# Global age-sex-specific fertility, mortality, healthy life expectancy (HALE), and population estimates in 204 countries and territories, 1950–2019: a comprehensive demographic analysis for the Global Burden of Disease Study 2019

**DOI:** 10.1016/S0140-6736(20)30977-6

**Published:** 2020-10-17

**Authors:** GBD 2019 Demographics Collaborators, Haidong Wang, Kaja M Abbas, Mitra Abbasifard, Mohsen Abbasi-Kangevari, Hedayat Abbastabar, Foad Abd-Allah, Ahmed Abdelalim, Hassan Abolhassani, Lucas Guimarães Abreu, Michael R M Abrigo, Abdelrahman I Abushouk, Maryam Adabi, Tim Adair, Oladimeji M Adebayo, Isaac Akinkunmi Adedeji, Victor Adekanmbi, Abiodun Moshood Adeoye, Olatunji O Adetokunboh, Shailesh M Advani, Ashkan Afshin, Mohammad Aghaali, Anurag Agrawal, Keivan Ahmadi, Hamid Ahmadieh, Muktar Beshir Ahmed, Ziyad Al-Aly, Khurshid Alam, Tahiya Alam, Fahad Mashhour Alanezi, Turki M Alanzi, Jacqueline Elizabeth Alcalde-Rabanal, Muhammad Ali, Gianfranco Alicandro, Mehran Alijanzadeh, Cyrus Alinia, Vahid Alipour, Hesam Alizade, Syed Mohamed Aljunid, Peter Allebeck, Majid Abdulrahman Hamad Almadi, Amir Almasi-Hashiani, Hesham M Al-Mekhlafi, Khalid A Altirkawi, Arwa Khalid Alumran, Nelson Alvis-Guzman, Mostafa Amini-Rarani, Arya Aminorroaya, Arianna Maever L Amit, Robert Ancuceanu, Catalina Liliana Andrei, Sofia Androudi, Colin Angus, Mina Anjomshoa, Fereshteh Ansari, Iman Ansari, Alireza Ansari-Moghaddam, Carl Abelardo T Antonio, Catherine M Antony, Davood Anvari, Seth Christopher Yaw Appiah, Jalal Arabloo, Morteza Arab-Zozani, Aleksandr Y Aravkin, Olatunde Aremu, Johan Ärnlöv, Krishna K Aryal, Ali A Asadi-Pooya, Samaneh Asgari, Mohammad Asghari Jafarabadi, Madhu Sudhan Atteraya, Marcel Ausloos, Leticia Avila-Burgos, Euripide Frinel Gbenato Arthur Avokpaho, Beatriz Paulina Ayala Quintanilla, Getinet Ayano, Martin Amogre Ayanore, Ghasem Azarian, Ebrahim Babaee, Ashish D Badiye, Eleni Bagli, Mohammad Amin Bahrami, Ahad Bakhtiari, Shelly Balassyano, Maciej Banach, Palash Chandra Banik, Suzanne Lyn Barker-Collo, Till Winfried Bärnighausen, Akbar Barzegar, Sanjay Basu, Bernhard T Baune, Mohsen Bayati, Gholamreza Bazmandegan, Neeraj Bedi, Michellr L Bell, Derrick A Bennett, Isabela M Bensenor, Kidanemaryam Berhe, Adam E Berman, Gregory J Bertolacci, Reshmi Bhageerathy, Neeraj Bhala, Krittika Bhattacharyya, Zulfiqar A Bhutta, Ali Bijani, Antonio Biondi, Donal Bisanzio, Catherine Bisignano, Raaj Kishore Biswas, Tone Bjørge, Somayeh Bohlouli, Mehdi Bohluli, Srinivasa Rao Rao Bolla, Antonio Maria Borzì, Shiva Borzouei, Oliver J Brady, Dejana Braithwaite, Michael Brauer, Andrey Nikolaevich Briko, Nikolay Ivanovich Briko, Blair R Bumgarner, Sharath Burugina Nagaraja, Zahid A Butt, Florentino Luciano Caetano dos Santos, Tianji Cai, Charlton SKH Callender, Luis LA Alberto Cámera, Ismael R Campos-Nonato, Rosario Cárdenas, Giulia Carreras, Juan J Carrero, Felix Carvalho, Joao Mauricio Castaldelli-Maia, Giulio Castelpietra, Franz Castro, Ferrán Catalá-López, Christopher R Cederroth, Ester Cerin, Vijay Kumar Chattu, Ken Lee Chin, Dinh-Toi Chu, Liliana G Ciobanu, Massimo Cirillo, Haley Comfort, Vera Marisa Costa, Richard G Cowden, Elizabeth A Cromwell, Andrew J Croneberger, Matthew Cunningham, Saad M A Dahlawi, Giovanni Damiani, Emanuele D'Amico, Lalit Dandona, Rakhi Dandona, Paul I Dargan, Aso Mohammad Darwesh, Ahmad Daryani, Rajat Das Gupta, José das Neves, Kairat Davletov, Diego De Leo, Edgar Denova-Gutiérrez, Kebede Deribe, Nikolaos Dervenis, Rupak Desai, Govinda Prasad Dhungana, Diana Dias da Silva, Daniel Diaz, Ilse N Dippenaar, Shirin Djalalinia, Hoa Thi Do, Klara Dokova, David Teye Doku, Fariba Dorostkar, Chirag P Doshi, Leila Doshmangir, Kerrie E Doyle, Eleonora Dubljanin, Andre Rodrigues Duraes, David Edvardsson, Andem Effiong, Iman El Sayed, Maha El Tantawi, Iffat Elbarazi, Shaimaa I El-Jaafary, Mohammad Hassan Emamian, Sharareh Eskandarieh, Firooz Esmaeilzadeh, Kara Estep, Mohammad Farahmand, Anwar Faraj, Mohammad Fareed, Roghiyeh Faridnia, Andre Faro, Farshad Farzadfar, Nazir Fattahi, Ali Akbar Fazaeli, Mehdi Fazlzadeh, Valery L Feigin, Seyed-Mohammad Fereshtehnejad, Eduarda Fernandes, Manuela L Ferreira, Irina Filip, Florian Fischer, Carsten Flohr, Nataliya A Foigt, Morenike Oluwatoyin Folayan, Artem Alekseevich Fomenkov, Marisa Freitas, Takeshi Fukumoto, John E Fuller, João M Furtado, Mohamed M Gad, Emmanuela Gakidou, Silvano Gallus, Abiyu Mekonnen Gebrehiwot, Ketema Bizuwork Gebremedhin, Peter W Gething, Farhad Ghamari, Ahmad Ghashghaee, Asadollah Gholamian, Syed Amir Gilani, Mojgan Gitimoghaddam, Ekaterina Vladimirovna Glushkova, Elena V Gnedovskaya, Sameer Vali Gopalani, Alessandra C Goulart, Harish Chander Gugnani, Yuming Guo, Rajeev Gupta, Subodh Sharan Gupta, Juanita A Haagsma, Arvin Haj-Mirzaian, Arya Haj-Mirzaian, Iman Halvaei, Randah R Hamadeh, Kanaan Hamagharib Abdullah, Chieh Han, Demelash Woldeyohannes Handiso, Graeme J Hankey, Hamidreza Haririan, Josep Maria Haro, Ahmed I Hasaballah, Soheil Hassanipour, Hadi Hassankhani, Simon I Hay, Behzad Heibati, Reza Heidari-Soureshjani, Kiana Henny, Nathaniel J Henry, Claudiu Herteliu, Fatemeh Heydarpour, Michael K Hole, Praveen Hoogar, H Dean Hosgood, Naznin Hossain, Mehdi Hosseinzadeh, Mihaela Hostiuc, Sorin Hostiuc, Mowafa Househ, Damian G Hoy, Guoqing Hu, Tanvir M Huda, Segun Emmanuel Ibitoye, Kevin S Ikuta, Olayinka Stephen Ilesanmi, Irena M Ilic, Milena D Ilic, Mohammad Hasan Imani-Nasab, MdMohaimenul Islam, Hiroyasu Iso, Chinwe Juliana Iwu, Jalil Jaafari, Kathryn H Jacobsen, Deepa Jahagirdar, Nader Jahanmehr, Amir Jalali, Farzad Jalilian, Spencer L James, Hosna Janjani, Ensiyeh Jenabi, Ravi Prakash Jha, Vivekanand Jha, John S Ji, Jost B Jonas, Farahnaz Joukar, Jacek Jerzy Jozwiak, Mikk Jürisson, Zubair Kabir, Hamed Kalani, Leila R Kalankesh, Zahra Kamiab, Tanuj Kanchan, Neeti Kapoor, André Karch, Salah Eddin Karimi, Seyed Asaad Karimi, Nicholas J Kassebaum, Srinivasa Vittal Katikireddi, Norito Kawakami, Gbenga A Kayode, Peter Njenga Keiyoro, Cathleen Keller, Yousef Saleh Khader, Nauman Khalid, Ejaz Ahmad Khan, Maseer Khan, Young-Ho Khang, Amir M Khater, Mona M Khater, Salman Khazaei, Habibolah Khazaie, Mohammad Taghi Khodayari, Jagdish Khubchandani, Neda Kianipour, Cho-il Kim, Young-Eun Kim, Yun Jin Kim, Yohannes Kinfu, Adnan Kisa, Sezer Kisa, Katarzyna Kissimova-Skarbek, Mika Kivimäki, Hamidreza Komaki, Jacek A Kopec, Soewarta Kosen, Parvaiz A Koul, Ai Koyanagi, Michael A Kravchenko, Kewal Krishan, Kris J Krohn, Barthelemy Kuate Defo, G Anil Kumar, Manasi Kumar, Pushpendra Kumar, Vivek Kumar, Dian Kusuma, Hmwe Hmwe Kyu, Carlo La Vecchia, Ben Lacey, Dharmesh Kumar Lal, Ratilal Lalloo, Faris Hasan Lami, Sonia Lansky, Samantha Leigh Larson, Anders O Larsson, Savita Lasrado, Zohra S Lassi, Jeffrey V Lazarus, Paul H Lee, Shaun Wen Huey Lee, Andrew T Leever, Kate E LeGrand, Matilde Leonardi, Shanshan Li, Lee-Ling Lim, Stephen S Lim, Shai Linn, Rakesh Lodha, Giancarlo Logroscino, Alan D Lopez, Platon D Lopukhov, Paulo A Lotufo, Rafael Lozano, Alton Lu, Raimundas Lunevicius, Mohammed Madadin, Emilie R Maddison, Hassan Magdy Abd El Razek, Muhammed Magdy Abd El Razek, Phetole Walter Mahasha, Mokhtar Mahdavi Mahdavi, Reza Malekzadeh, Abdullah A Mamun, Navid Manafi, Fariborz Mansour-Ghanaei, Borhan Mansouri, Mohammad Ali Mansournia, Chabila Christopher Mapoma, Santi Martini, Francisco Rogerlândio Martins-Melo, Anthony Masaka, Claudia I Mastrogiacomo, Manu Raj Mathur, Erin A May, Colm McAlinden, John J McGrath, Martin McKee, Man Mohan Mehndiratta, Fereshteh Mehri, Kala M Mehta, Wahengbam Bigyananda Meitei, Peter T N Memiah, Walter Mendoza, Ritesh G Menezes, Endalkachew Worku Mengesha, George A Mensah, Atte Meretoja, Tuomo J Meretoja, Tomislav Mestrovic, Irmina Maria Michalek, Kebadnew Mulatu Mihretie, Ted R Miller, Edward J Mills, George J Milne, Erkin M Mirrakhimov, Hamed Mirzaei, Maryam Mirzaei, Mehdi Mirzaei-Alavijeh, Awoke Temesgen Misganaw, Babak Moazen, Masoud Moghadaszadeh, Efat Mohamadi, Dara K Mohammad, Yousef Mohammad, Naser Mohammad Gholi Mezerji, Abolfazl Mohammadbeigi, Abdollah Mohammadian-Hafshejani, Reza Mohammadpourhodki, Hussen Mohammed, Shafiu Mohammed, Farnam Mohebi, Mohammad A Mohseni Bandpei, Amin Mokari, Ali H Mokdad, Natalie C Momen, Lorenzo Monasta, Meghan D Mooney, Ghobad Moradi, Masoud Moradi, Mohammad Moradi-Joo, Maziar Moradi-Lakeh, Rahmatollah Moradzadeh, Paula Moraga, Ilais Moreno Velásquez, Joana Morgado-da-Costa, Shane Douglas Morrison, Jonathan F Mosser, Simin Mouodi, Seyyed Meysam Mousavi, Amin Mousavi Khaneghah, Ulrich Otto Mueller, Kamarul Imran Musa, Saravanan Muthupandian, Behnam Nabavizadeh, Mehdi Naderi, Ahamarshan Jayaraman Nagarajan, Mohsen Naghavi, Behshad Naghshtabrizi, Gurudatta Naik, Farid Najafi, Vinay Nangia, Jobert Richie Nansseu, Duduzile Edith Ndwandwe, Ionut Negoi, Ruxandra Irina Negoi, Josephine W Ngunjiri, Huong Lan Thi Nguyen, Trang Huyen Nguyen, Yeshambel T Nigatu, Rajan Nikbakhsh, Amin Reza Nikpoor, Molly R Nixon, Chukwudi A Nnaji, Shuhei Nomura, Jean Jacques Noubiap, Soraya Nouraei Motlagh, Christoph Nowak, Adrian Oţoiu, Christopher M Odell, In-Hwan Oh, Morteza Oladnabi, Andrew T Olagunju, Bolajoko Olubukunola Olusanya, Jacob Olusegun Olusanya, Ahmed Omar Bali, Kanyin L Ong, Obinna E Onwujekwe, Alberto Ortiz, Nikita Otstavnov, Stanislav S Otstavnov, Simon Øverland, Mayowa O Owolabi, Mahesh P A, Jagadish Rao Padubidri, Keyvan Pakshir, Raffaele Palladino, Adrian Pana, Songhomitra Panda-Jonas, James Park, Deepak Kumar Pasupula, Jenil R Patel, Sangram Kishor Patel, George C Patton, Katherine R Paulson, Hamidreza Pazoki Toroudi, Spencer A Pease, Amy E Peden, Veincent Christian Filipino Pepito, Emmanuel K Peprah, Alexandre Pereira, David M Pereira, Norberto Perico, David M Pigott, Thomas Pilgrim, Tessa M Pilz, Michael A Piradov, Meghdad Pirsaheb, Khem Narayan Pokhrel, Maarten J Postma, Hadi Pourjafar, Farshad Pourmalek, Akram Pourshams, Anna Poznańska, Sergio I Prada, Sanjay Prakash, Liliana Preotescu, Zahiruddin Quazi Syed, Mohammad Rabiee, Navid Rabiee, Amir Radfar, Alireza Rafiei, Alberto Raggi, Muhammad Aziz Rahman, Ali Rajabpour-Sanati, Pradhum Ram, Chhabi Lal Ranabhat, Sowmya J Rao, Davide Rasella, Vahid Rashedi, Prateek Rastogi, Priya Rathi, Lal Rawal, Giuseppe Remuzzi, Vishnu Renjith, Andre M N Renzaho, Serge Resnikoff, Nima Rezaei, Mohammad sadegh Rezai, Aziz Rezapour, Jennifer Rickard, Leonardo Roever, Luca Ronfani, Gholamreza Roshandel, Morteza Rostamian, Enrico Rubagotti, Godfrey M Rwegerera, Siamak Sabour, Basema Saddik, Ehsan Sadeghi, Masoumeh Sadeghi, Sahar Saeedi Moghaddam, Yahya Safari, Sare Safi, Saeid Safiri, Rajesh Sagar, Amirhossein Sahebkar, Mohammad Ali Sahraian, S Mohammad Sajadi, Mohammad Reza Salahshoor, Joseph S Salama, Payman Salamati, Marwa R Rashad Salem, Yahya Salimi, Joshua A Salomon, Inbal Salz, Zainab Samad, Abdallah M Samy, Juan Sanabria, Milena M Santric-Milicevic, Sivan Yegnanarayana Iyer Saraswathy, Benn Sartorius, Arash Sarveazad, Brijesh Sathian, Thirunavukkarasu Sathish, Davide Sattin, Mete Saylan, Lauren E Schaeffer, Silvia Schiavolin, David C Schwebel, Falk Schwendicke, Mario Sekerija, Anbissa Muleta Senbeta, Subramanian Senthilkumaran, Sadaf G Sepanlou, Edson Serván-Mori, Mahsima Shabani, Saeed Shahabi, Mohammad Shahbaz, Amira A Shaheen, Masood Ali Shaikh, Ali S Shalash, Mehran Shams-Beyranvand, MohammadBagher Shamsi, Morteza Shamsizadeh, Mohammed Shannawaz, Kiomars Sharafi, Zeinab Sharafi, Fablina Sharara, Rajesh Sharma, David H Shaw, Aziz Sheikh, Jae Il Shin, Rahman Shiri, Mark G Shrime, Kerem Shuval, Soraya Siabani, Inga Dora Sigfusdottir, Rannveig Sigurvinsdottir, Diego Augusto Santos Silva, Biagio Simonetti, Kyle E Simpson, Jasvinder A Singh, Eirini Skiadaresi, Valentin Yurievich Skryabin, Amin Soheili, Anton Sokhan, Reed J D Sorensen, Joan B Soriano, Muluken Bekele Sorrie, Ireneous N Soyiri, Emma Elizabeth Spurlock, Chandrashekhar T Sreeramareddy, Leo Stockfelt, Mark A Stokes, Jacob L Stubbs, Agus Sudaryanto, Mu'awiyyah Babale Sufiyan, Rizwan Suliankatchi Abdulkader, Bryan L Sykes, Rafael Tabarés-Seisdedos, Karen M Tabb, Santosh Kumar Tadakamadla, Amir Taherkhani, Muming Tang, Nuno Taveira, Heather Jean Taylor, Whitney L Teagle, Arash Tehrani-Banihashemi, Berhane Fseha Teklehaimanot, Zemenu Tadesse Tessema, Kavumpurathu Raman Thankappan, Nihal Thomas, Amanda G Thrift, Mariya Vladimirovna Titova, Hamid Reza Tohidinik, Marcello Tonelli, Roman Topor-Madry, Fotis Topouzis, Marcos Roberto Roberto Tovani-Palone, Eugenio Traini, Bach Xuan Tran, Ravensara Travillian, Sergi Trias-Llimós, Thomas Clement Truelsen, Lorainne Tudor Car, Bhaskaran Unnikrishnan, Era Upadhyay, Marco Vacante, Alireza Vakilian, Pascual R Valdez, Alessandro Valli, Constantine Vardavas, Tommi Juhani Vasankari, Ana Maria Nogales Vasconcelos, Yasser Vasseghian, Yousef Veisani, Narayanaswamy Venketasubramanian, Simone Vidale, Francesco S Violante, Vasily Vlassov, Stein Emil Vollset, Theo Vos, Isidora S Vujcic, Ana Vukovic, Rade Vukovic, Yasir Waheed, Mitchell Taylor Wallin, Magdalene K Walters, Hongbo Wang, Yuan-Pang Wang, Stefanie Watson, Jingkai Wei, Jordan Weiss, Girmay Teklay Weldesamuel, Andrea Werdecker, Ronny Westerman, Harvey A Whiteford, Taweewat Wiangkham, Kirsten E Wiens, Tissa Wijeratne, Charles Shey Wiysonge, Bogdan Wojtyniak, Charles D A Wolfe, Adam Belay Wondmieneh, Eve E Wool, Ai-Min Wu, Junjie Wu, Gelin Xu, Tomohide Yamada, Kazumasa Yamagishi, Yuichiro Yano, Sanni Yaya, Vahid Yazdi-Feyzabadi, Jamal A Yearwood, Tomas Y Yeheyis, Christopher Sabo Yilgwan, Paul Yip, Naohiro Yonemoto, Seok-Jun Yoon, Javad Yoosefi Lebni, Hunter W York, Mustafa Z Younis, Theodore Patrick Younker, Zabihollah Yousefi, Taraneh Yousefinezhadi, Abdilahi Yousuf Yousuf, Hasan Yusefzadeh, Telma Zahirian Moghadam, Josefina Zakzuk, Sojib Bin Zaman, Mohammad Zamani, Maryam Zamanian, Hamed Zandian, Zhi-Jiang Zhang, Peng Zheng, Maigeng Zhou, Arash Ziapour, Christopher J L Murray

## Abstract

**Background:**

Accurate and up-to-date assessment of demographic metrics is crucial for understanding a wide range of social, economic, and public health issues that affect populations worldwide. The Global Burden of Diseases, Injuries, and Risk Factors Study (GBD) 2019 produced updated and comprehensive demographic assessments of the key indicators of fertility, mortality, migration, and population for 204 countries and territories and selected subnational locations from 1950 to 2019.

**Methods:**

8078 country-years of vital registration and sample registration data, 938 surveys, 349 censuses, and 238 other sources were identified and used to estimate age-specific fertility. Spatiotemporal Gaussian process regression (ST-GPR) was used to generate age-specific fertility rates for 5-year age groups between ages 15 and 49 years. With extensions to age groups 10–14 and 50–54 years, the total fertility rate (TFR) was then aggregated using the estimated age-specific fertility between ages 10 and 54 years. 7417 sources were used for under-5 mortality estimation and 7355 for adult mortality. ST-GPR was used to synthesise data sources after correction for known biases. Adult mortality was measured as the probability of death between ages 15 and 60 years based on vital registration, sample registration, and sibling histories, and was also estimated using ST-GPR. HIV-free life tables were then estimated using estimates of under-5 and adult mortality rates using a relational model life table system created for GBD, which closely tracks observed age-specific mortality rates from complete vital registration when available. Independent estimates of HIV-specific mortality generated by an epidemiological analysis of HIV prevalence surveys and antenatal clinic serosurveillance and other sources were incorporated into the estimates in countries with large epidemics. Annual and single-year age estimates of net migration and population for each country and territory were generated using a Bayesian hierarchical cohort component model that analysed estimated age-specific fertility and mortality rates along with 1250 censuses and 747 population registry years. We classified location-years into seven categories on the basis of the natural rate of increase in population (calculated by subtracting the crude death rate from the crude birth rate) and the net migration rate. We computed healthy life expectancy (HALE) using years lived with disability (YLDs) per capita, life tables, and standard demographic methods. Uncertainty was propagated throughout the demographic estimation process, including fertility, mortality, and population, with 1000 draw-level estimates produced for each metric.

**Findings:**

The global TFR decreased from 2·72 (95% uncertainty interval [UI] 2·66–2·79) in 2000 to 2·31 (2·17–2·46) in 2019. Global annual livebirths increased from 134·5 million (131·5–137·8) in 2000 to a peak of 139·6 million (133·0–146·9) in 2016. Global livebirths then declined to 135·3 million (127·2–144·1) in 2019. Of the 204 countries and territories included in this study, in 2019, 102 had a TFR lower than 2·1, which is considered a good approximation of replacement-level fertility. All countries in sub-Saharan Africa had TFRs above replacement level in 2019 and accounted for 27·1% (95% UI 26·4–27·8) of global livebirths. Global life expectancy at birth increased from 67·2 years (95% UI 66·8–67·6) in 2000 to 73·5 years (72·8–74·3) in 2019. The total number of deaths increased from 50·7 million (49·5–51·9) in 2000 to 56·5 million (53·7–59·2) in 2019. Under-5 deaths declined from 9·6 million (9·1–10·3) in 2000 to 5·0 million (4·3–6·0) in 2019. Global population increased by 25·7%, from 6·2 billion (6·0–6·3) in 2000 to 7·7 billion (7·5–8·0) in 2019. In 2019, 34 countries had negative natural rates of increase; in 17 of these, the population declined because immigration was not sufficient to counteract the negative rate of decline. Globally, HALE increased from 58·6 years (56·1–60·8) in 2000 to 63·5 years (60·8–66·1) in 2019. HALE increased in 202 of 204 countries and territories between 2000 and 2019.

**Interpretation:**

Over the past 20 years, fertility rates have been dropping steadily and life expectancy has been increasing, with few exceptions. Much of this change follows historical patterns linking social and economic determinants, such as those captured by the GBD Socio-demographic Index, with demographic outcomes. More recently, several countries have experienced a combination of low fertility and stagnating improvement in mortality rates, pushing more populations into the late stages of the demographic transition. Tracking demographic change and the emergence of new patterns will be essential for global health monitoring.

**Funding:**

Bill & Melinda Gates Foundation.

## Introduction

Age-specific mortality rates are a crucial dimension of population health. Fertility rates and population size and composition also have profound effects on the challenges faced by health systems. With rising mean age, for example, diseases such as dementia are a greater burden on individuals, families, and health providers. Assessing the trends in key demographic indicators is a core challenge for global health surveillance. Trends in age-specific mortality rates can also provide important evidence on where new diseases are emerging or adverse risk factor trends are having an impact. Understanding what demographic trends are expected on the basis of improvements in educational attainment and increased income per capita, or where the observed trends diverge from expected, can also help to identify national success stories in reducing mortality rates that could be useful for other countries to learn from.

A variety of sources are available on fertility, mortality, population, and migration, but they vary widely in the quality and completeness of registration. National statistical offices report on demographic indicators using a variety of different data-collection practices, estimation methods, and reporting intervals.^1^ The Organisation for Economic Co-operation and Development^2^ and the EU^3^ produce demographic estimates for selected locations. WHO generates mortality estimates for all of its member states, but not estimates of population and fertility.^4^ A wider array of demographic estimates is produced for 228 countries and areas by the US Census Bureau International Division, but only a small set of countries are updated each year.^5^ The UN Population Division produces biannual fertility, mortality, migration, and population estimates for 235 countries or areas for 5-year age groups in every 5-year period starting in 1950.^6^ Although these sources provide a diversity of estimates, they do not use a standardised set of statistical methods across all locations. None of these estimates is compliant with the Guidelines on Accurate and Transparent Health Estimates Reporting (GATHER). In particular, they do not make their statistical code available, provide details on why some sources are used and others are not, report how primary data are adjusted, or estimate uncertainty.

**Research in context**

**Evidence before this study**

Many national statistical offices report demographic estimates, but the UN Population Division of the Department of Economic and Social Affairs and the Global Burden of Diseases, Injuries, and Risk Factors Study (GBD) produce comprehensive and regularly updated demographic assessments for all or most countries and territories. Since 1951, the UN has produced estimates of some fertility, mortality, migration, and population metrics for every 5-year period and for each 5-year age group starting in 1950. Updated estimates are produced biannually with forecasts up to the year 2100 in more recent iterations. Other institutions such as the US Census Bureau, WHO, the Organisation for Economic Co-operation and Development, and the EU generate estimates less regularly or for either selected demographic metrics or locations. Since 2010, GBD has published estimates of age-specific mortality for single calendar years from 1950 onwards. In 2017, GBD began to produce comprehensive and internally consistent estimates of fertility, mortality, migration, and population by sex and age for each calendar year since 1950 at the national level and for selected subnational locations. Of all these estimates, only those from GBD are compliant with the Guidelines on Accurate and Transparent Health Estimates Reporting.

**Added value of this study**

GBD 2019 has produced comprehensive and comparable assessments of key demographic indicators, generating estimates for a total of 990 locations at the most detailed level. GBD 2019 improved demographic estimation from the GBD 2017 cycle in six ways. First, additional sources of data were incorporated. For fertility, we added 150 surveys, 561 vital registration years, 61 censuses, and 11 other sources; for population, 60 censuses and 290 years of population registry data; and for mortality, 116 surveys, 244 vital registration years, 32 censuses, and 47 other sources. Second, GBD 2019 expanded its assessment of population health to include all WHO member states, adding nine national-level units to the GBD location hierarchy. Third, for GBD 2019, estimates have been made more consistent and stable across estimation cycles, including using a GBD standard location list for estimating regression fixed effects. This ensured that our estimates were derived from relationships extrapolated from locations with more robust data. Fourth, we made improvements to key demographic modelling steps, including enhanced methods for estimating the completeness of vital registration systems by adding two new methods of evaluating completeness using the Bayesian analytical framework developed for population estimation in GBD 2017. Fifth, we improved the vetting mechanism for age patterns of mortality by using machine vision, a form of machine learning. Sixth, we took advantage of the comprehensive nature of this study of fertility, mortality, migration, and population to revise the taxonomy of the demographic transition. Many countries have moved into the post-transition phase of the demographic transition.

**Implications of all the available evidence**

In 2019, with half of countries and territories with below-replacement fertility, and 34 with negative natural rates of increase, challenges associated with the late stages of the demographic transition such as the declining size of workforces and ageing populations are becoming real policy issues. The global health community needs to simultaneously address supporting continued global health improvement in developing nations and helping to manage the new policy challenges emerging from the latter stages of the demographic transition.

Despite limitations, these various sources have quantified the profound demographic shifts that have been underway, especially since 1950. Demographers broadly characterise these shifts using the construct of the demographic transition.^7^, ^8^ The classical formulation of demographic transition theory says countries go from a state of high mortality and high fertility with a very young age structure to a state of low fertility and low mortality with a much older age structure. Economists have proposed a demographic dividend, which implies that after a decline in fertility, the share of the population in the working adult age groups will increase for a period, and thus decrease the dependency ratio, make available more resources and capital for investment, and with appropriate national policy interventions stimulate faster economic growth.^9^ The stage of the demographic transition can have important social, economic, and geopolitical effects.^10^, ^11^, ^12^, ^13^ Demographic changes underway suggest that there are varied routes of the demographic transition; in particular, countries might enter a stage of sustained below-replacement fertility and experience inverted age-structures with more people in older 5-year age groups than younger 5-year age groups. There is no intrinsic or biological reason that individual female's fertility choices will necessarily lead to a state of replacement fertility. Sustained population decline with profound fiscal, economic, social, and geopolitical consequences is possible. Understanding where countries are in the demographic transition is important for broader health and social policy.

In this study, we present the 2019 revision of demographic estimates for the Global Burden of Diseases, Injuries, and Risk Factors Study (GBD). This incorporates newly released census, survey, vital registration, and sample registration data. Methods innovations based on critical feedback of GBD 2017^14^, ^15^ in the published literature, from the GBD Independent Advisory Committee, and across the extensive GBD collaborative network have been incorporated.

The present study aims to produce up-to-date estimates of fertility, mortality, migration, and population by age and sex for 204 countries and territories and selected subnational locations for each calendar year from 1950 to 2019. We generated estimates for a total of 990 locations at the most detailed level. To better characterise where countries are in the demographic transition, we have developed a seven-category taxonomy.

## Methods

### Overview

The GBD estimation strategy for fertility, mortality, and population is designed to work with the diversity of data sources and potential biases in data available for each of these demographic components and to use replicable statistical code for data synthesis. The analysis can be divided into seven main steps: age-specific fertility estimation, under-5 mortality estimation, adult mortality estimation, age-specific mortality estimation using a relational model life table system, HIV adjustments, accounting for fatal discontinuities such as wars or natural disasters, and population estimation. For each component, it is useful to think of the data available, the data processing steps required to account for known biases, and the data synthesis stage, which deals with the challenges of both missing measurements in given location-years and the common problem of different measurements disagreeing with each other.

For GBD 2019, we instituted the GBD standard location list, which consists of all national-level locations as well as subnational locations in the UK, India, China, and the USA. In each modelling step, effects of the covariates were derived from empirical data observed from standard locations. This ensured that our estimates were derived from robust relationships extrapolated from locations with more robust empirical data, thus ensuring long-term stability in our estimates.

Below, we provide a high-level description of each analytical component, with an emphasis on new steps and other updates for GBD 2019. Methods used in the GBD demographic estimation process have been described extensively in previous publications,^14^, ^15^, ^16^, ^17^, ^18^ and additional detail on estimation for the 2019 cycle is available in appendix 1.

This study complies with GATHER;^19^ a completed GATHER checklist is available in appendix 1. Analyses used Python version 3.6.2 and 3.6.8, Stata versions 13 and 15, and R versions 3.4.2 and 3.5.0.

### Geographical units, age groups, and time periods

We produced estimates from 1950 to 2019 for 204 countries and territories that were grouped into 21 regions and seven super-regions. For GBD 2019, nine countries and territories (Cook Islands, Monaco, San Marino, Nauru, Niue, Palau, Saint Kitts and Nevis, Tokelau, and Tuvalu) were added, such that the GBD location hierarchy now includes all WHO member states. GBD 2019 includes subnational analyses for Italy, Nigeria, Pakistan, the Philippines, and Poland, and 16 countries previously estimated at subnational levels (Brazil, China, Ethiopia, India, Indonesia, Iran, Japan, Kenya, Mexico, New Zealand, Norway, Russia, South Africa, Sweden, the UK, and the USA). All subnational analyses are at the first level of administrative organisation within each country except for New Zealand (by Māori ethnicity), Sweden (by Stockholm and non-Stockholm), the UK (by local government authorities), Kenya (by district and province), and the Philippines (by province). For the demographic analyses, we seek to make the most of rich demographic data, more readily available and robust at aggregate level, and increase the precision of estimates at the aggregate level by running the modelling process at both the most detailed level and at the aggregate level (whether national, subnational, or both national and subnational). In this publication, we present subnational estimates for Brazil, India, Indonesia, Japan, Kenya, Mexico, Sweden, the UK, and the USA; given space constraints, these results are presented in appendix 2.

Following previous GBD studies, mortality and population are estimated for 23 age groups: early neonatal (0–6 days), late neonatal (7–27 days), post-neonatal (28–365 days), 1–4 years, 5–9 years, every 5-year age group up to 95 years, and 95 years and older. Age-specific fertility is estimated for 5-year age groups between ages 10 years and 54 years.

### Fertility estimation

Age-specific fertility estimation largely followed the analytical steps used in GBD 2017 (appendix 1 figure S3).^15^ We systematically searched government websites, statistical annuals, and demographic compendia for data on registered births by age of mother, total registered births, and complete and summary birth histories in censuses and surveys. We identified 439 complete birth histories and 628 summary birth histories from 938 surveys, 349 censuses, and 238 other sources. We also used 8078 location-years of national-level vital registration and sample registration data. Compared with GBD 2017, GBD 2019 incorporated 222 additional sources composed of 150 surveys, 61 censuses, and 11 other sources, as well as 561 additional location-years of vital registration (appendix 1 tables S10, S11). We used spatiotemporal Gaussian process regression (ST-GPR) to model age-specific fertility rates for 5-year age groups between ages 15 and 49 years in each location from 1950 to 2019. Educational attainment among females by age was included as a covariate, and the estimated age-specific fertility rate for the age group 20–24 years was included as a covariate for all other ages. Appendix 1 (section 3) includes model details. The model includes source-specific random effects: after a reference source was selected for each location, any other sources were adjusted on the basis of the difference in the random effects between the reference source and the source of interest. To be able to incorporate data on total births and summary birth histories, we first modelled age-specific fertility with vital registration data and complete birth history data to generate a first-round estimate of age-specific fertility. These first-round results were used to incorporate total birth and summary birth history data in a second final round of estimation for each location using the same analytical process described above (appendix 1 section 3). We then used these age-specific fertility estimates to extrapolate fertility estimates to age groups 10–14 years and 50–54 years.

### Under-5 mortality estimation

GBD 2019 estimation of under-5 mortality rate (U5MR) follows the analytical framework for mortality analysis used since GBD 2015.^14^, ^17^, ^18^ Across mortality estimation, we added 116 surveys, 244 vital registration years, 32 censuses, and 47 other sources for GBD 2019 (appendix 1 tables S3, S4). 7417 sources were used for under-5 mortality estimation. We systematically identified vital registration data on under-5 mortality and mortality for the early neonatal, late neonatal, post neonatal, and 1–4-year age groups; in total, GBD 2019 used 28 016 location-years of data, including 330 additional location-years of national data and 3736 additional location-years of subnational data compared with GBD 2017 (appendix 1 table S5). We also identified 481 surveys with complete birth histories, of which 21 are new for GBD 2019. 1081 sources on summary birth histories were also used, 127 of which are new for GBD 2019. To convert the ratio of children ever surviving to children ever born by age of mother to an estimate of U5MR, we used updated and validated methods.^20^ Next, we estimated U5MR without fatal discontinuities using ST-GPR. Education, HIV, and lag-distributed income were included as covariates. Appendix 1 (section 2) provides details on the model structure for U5MR. We similarly estimated mortality rates for the more detailed age groups younger than 5 years, and constrained these estimates to equal U5MR.

### Adult mortality estimation

7355 sources were used in adult mortality estimation. National-level data from 7000 location-years of vital registration and 322 location-years of sample vital registration were used as inputs to the estimation process for adult mortality rate, defined as the probability of death between ages 15 and 60 years. We also used 66 sources of household deaths, 102 censuses, and 133 surveys. Additionally, 161 sources of sibling history data were analysed using published methods that correct for various biases inherent in such data.^20^ The completeness of vital registration data was evaluated using death distribution methods (DDMs). To enhance the performance of classic DDMs, especially in settings with migration and age misreporting, we used five different methods to assess completeness, three of which—the generalised growth balance method (GGB), the synthetic extinct generations (SEG) method, and a combined method (GGB-SEG)^16^—had been previously used. Two new methods were added based on a modification of the Bayesian hierarchical cohort component model for population projection (BCCMP). The GBD 2019 BCCMP DDMs can take input data on registered death, fertility rate, and census population while considering the uncertainty associated with each input datapoint. Out-of-sample validity testing as detailed in appendix 1 (section 2) shows that the two BCCMP DDMs, one of which simultaneously estimates the age pattern of migration as well, outperform the traditional GGB, SEG, and GGB-SEG methods.

Additionally, through extensive validation, we have chosen optimum age trims for all five of the DDMs used here. Here, age trim means the range of ages from which inference on completeness of a vital registration system is drawn.

DDM results are used in a data synthesis step where completeness in U5MR—defined as the ratio between observed U5MR from vital registration and other U5MR sources and those estimated in step 1 of U5MR synthesis (appendix 1 section 2.2.6)—is used as a covariate to help to arrive at time series estimates of completeness together with the DDM points derived using the methods described above. Adult mortality data were synthesised using education, lag-distributed income, HIV crude death rate for ages 15–59 years, and U5MR as covariates in a non-linear mixed-effects model that helps to provide a prior for the ST-GPR model (appendix 1 section 2.3.4). Because of the way that independent estimates of HIV mortality rates based on epidemiological data on prevalence are used below, the models developed for U5MR and adult mortality are used to also generate a counterfactual estimate of U5MR and adult mortality in the absence of HIV.

### HIV-free life table estimation

Estimates of HIV-free U5MR and adult mortality are then used with a model life table system to generate HIV-free age-specific mortality rates. Since the 1960s,^21^ demographic estimation has routinely made use of model life tables that embody observed relationships between levels of age-specific mortality. For example, the UN Population Division makes extensive use of the UN Model Life Tables based on 72 observed life tables in their estimation process.^22^ For GBD 2019, we used the GBD relational model life table system. Details on the GBD relational model life table with a flexible standard life table selection process can be found in appendix 1 (section 2). GBD 2019 used a machine vision model to improve the screening process of empirical life tables used in the model life table stage. This model life table system is now based on 11 139 empirical life tables from 1950 to 2019; the GBD model life table system outperforms other life table systems such as Coale and Demeny,^21^ UN Model Life Tables,^22^ and others,^23^ in cross-validation exercises.^24^ A crucial component of this model life table analysis is how older-age mortality is estimated, especially over age 90 years (appendix 1 section 2).

### HIV adjustment

HIV mortality rates have been estimated as part of GBD using HIV prevalence survey data, antenatal clinic serosurveillance, and vital registration data. Estimation and Projection Package Age-Sex Model (EPP-ASM)^25^ was used to estimate HIV deaths in high-burden countries. This model fits possible transmission rates to observed prevalence data to determine the most likely epidemic time series at the age-sex-specific level. In the remaining locations, we used Spectrum. This model is a natural history progression model that generates mortality rates from input incidence and prevalence curves, along with assumptions about intervention scale-up and local variation in epidemiology (appendix 1 section 2).

### Fatal discontinuities

Fatal discontinuities or shocks are events that are stochastic in nature, and that cannot be modelled because they do not have a predictable time trend. Demographic estimation of age-specific mortality does not account for fatal discontinuities. Fatal discontinuity causes largely consist of natural disasters and conflicts. Input data for fatal discontinuities are compiled from a range of sources, including country vital registration data, international databases that capture several cause-specific fatal discontinuities, and supplemental data in the presence of known issues with data quality. The international databases used in GBD 2019 are Uppsala Conflict Data Program, International Institute for Strategic Studies, Armed Conflict Location & Event Data Project, Global Terrorism Database, the Chicago Project on Security and Threats Suicide Attack Database, and Amnesty International. A Twitter scrape was used to identify supplemental input data for missing fatal discontinuities. The total number of location-years from vital registration for fatal discontinuities in GBD 2019 is 1822, and the total number of other sources reporting unique events is 253.

Most data on fatal discontinuities are for both sexes and all ages combined. We drew on the cause of death research in GBD,^26^ which disaggregated these data by using observed global sex and age patterns of mortality rate due to specific causes of death that are considered fatal discontinuities. Details on their method can be found elsewhere.^27^ The sex-redistributed and age-redistributed fatal discontinuities by cause were then aggregated by age and sex and added to the estimated number of deaths from the previous step. These are the final all-cause mortality envelopes by location, year, sex, and age. Finally, we recalculated abridged life tables for each location, year, and sex combination to reflect the impact of fatal discontinuities (appendix 1 section 2). Full life tables by single year of age are then generated using the with-fatal-discontinuities abridged life tables.

### Population estimation

We identified 1250 censuses and 747 location-years of population registry data, of which 60 censuses and 290 location-years are new compared with GBD 2017 (appendix 1 table S12). A Bayesian hierarchical cohort component model for population projection developed by Wheldon and colleagues^28^ and improved by Murray and colleagues^29^ was used to estimate an age-specific 1950 baseline population and age-specific net migration consistent with our estimates of age-specific fertility and age-specific mortality and available census and registry data. The estimated 1950–2019 age-specific fertility, mortality, net migration, and 1950 baseline population were then used to produce fully consistent age-specific population estimates. The Bayesian model prior for net migration included information from estimates of refugee movements from the UN High Commissioner for Refugees^30^ and migration data for select countries, mainly in the EU and Gulf States. Details of the population model can be found in appendix 1 (section 5).

### Estimation of healthy life expectancy

Healthy life expectancy (HALE) is an essential measurement of years of life spent in good health. It serves as a summary metric for both the age-specific mortality and morbidity for a given population in a calendar year. We followed the analytical methods used to generate HALE in the GBD 2017 cycle.^31^ We calculated the Pearson's correlation coefficient between the Socio-demographic Index (SDI) and HALE.

### Age-specific mortality and life expectancy expected on the basis of SDI

To explore the role of broader social, economic, and demographic conditions associated with the levels and trend of mortality at the population level, we analysed the relationship between log mortality rates and SDI using MR-BRT (meta-regression-Bayesian regularised trimmed), a meta-regression program (appendix 1 section 6). SDI is a composite indicator of a country's lag-distributed income per capita, average years of schooling, and the total fertility rate (TFR) in females under the age of 25 years. MR-BRT defines a linear mixed-effects model with a B-spline specification for the relationship between outcomes of interest and SDI. We used a cubic spline with five knots between 0 and 1, with left-most and right-most spline segments enforced to be linear, and with slopes matching adjacent interior segments.^32^ To ensure that the results were not sensitive to the choice of spline knots, we used a model ensemble over 50 cubic spline models, as described above. For each model, interior knot placement was randomly generated to be between 0·1 and 0·8, with minimum interknot distance of 0·1. The final predictions were obtained using the ensemble aggregate over these 50 models. This model was performed separately for each GBD age-sex group. Expected mortality rates for each age-sex group were used to estimate expected life expectancy. A similar analysis was done for age-specific fertility rates and the TFR. Age-specific expected rates of fertility were raked to the expected TFR.

### Stages of the demographic transition

Demographic transition is a general theory about the transition from high mortality and high fertility to low mortality and low fertility. Various stages of the demographic transition have been proposed.^7^, ^8^, ^10^, ^33^ To help to elucidate key demographic trends, we defined seven categories of demographic transition based on five stages: before transition, early transition, mid-transition, late transition, and post transition. The first three categories map to more traditional notions of demographic change, identifying stages of demographic change on the basis of declines in crude birth rate and crude death rate, and changes in the natural rate of increase in population (calculated as crude birth rate minus crude death rate). In the first stage, before transition, both crude birth rate and crude death rate are high and there is no sustained decline in either. The early transition stage is where crude death rate has started to decline, yet the natural rate of increase in population has not achieved 3·0% per year. The mid-transition stage is where both crude birth rate and crude death rate are experiencing sustained decline, and the maximum annual natural rate of increase has achieved 3·0%. Towards the end of this stage, while crude birth rate is still in decline, the improvement in crude death rate has slowed down. The remaining four categories regard the late-transition and post-transition stages. The late-transition stage sees further decline in the natural rate of increase as fertility continues to decline and the improvement in crude death rate is attenuated. At the end of this stage in the demographic transition, we see a crossover of crude birth rate and crude death rate where the natural rate of increase in population becomes negative. In the final post-transition stage, countries see the crossover of crude birth rate and crude death rate, which makes the natural rate of population growth negative. In this stage, both crude birth and death rates are substantially lower than those in the early stages of the demographic transition. For these last two stages of demographic transition, where the natural rate of increase slows down considerably and then becomes negative, it is important to examine the level and trend of net migration, which is the difference between immigration rate and emigration rate. Based on whether net migration rate is positive (net immigration) or negative (net emigration), we disaggregate these two stages into four groups.

### Uncertainty analysis

Uncertainty has been propagated throughout the analytical process. ST-GPR for U5MR and adult mortality rate generated 1000 draws of U5MR and adult mortality rate for every location, year, and sex combination included in GBD, together with the same number of draws for crude death rate due to HIV estimates. These draw-level inputs helped to generate 1000 draws of all-cause mortality estimates together with draw-level estimates of fatal discontinuity. Mean estimates and 95% uncertainty intervals (UIs; the 25th and 975th estimates among the 1000 draws) were generated using the draw-level estimates. A similar ST-GPR process also generated means and UIs for our fertility estimates. The estimated uncertainty of fertility and mortality estimates were useful inputs for the BCCMP model used in GBD 2019 to estimate population.

### Role of the funding source

The funders of the study had no role in study design, data collection, data analysis, data interpretation, or the writing of the report. The corresponding author had full access to the data in the study and final responsibility for the decision to submit for publication.

## Results

### Fertility

Global TFR in 1950 was 4·97 (95% UI 4·79 to 5·16; table 1). There has been a continuous decline in TFR since then. TFR was 4·40 (4·28 to 4·51) in 1970, 3·18 (3·11 to 3·25) in 1990, 2·72 (2·66 to 2·79) in 2000, and 2·31 (2·17 to 2·46) in 2019, roughly 10% higher than the replacement level of 2·1. While TFR has decreased over the past seven decades, global annual livebirths increased. In 1950, global annual livebirths totalled 95·9 million (92·6 to 99·4). By 2000, annual livebirths had reached 134·5 million (131·5 to 137·8), and in 2016, the peak of annual livebirths was reached at 139·6 million (133·0 to 146·9). Between 2017 and 2019, global livebirths declined at an annualised rate of 0·6% (−0·2 to 1·4) per year. The estimated total number of livebirths in 2019 was 135·3 million (127·2 to 144·1; table 1).

**Table 1:** The 2019 population; annualised rate of change in population (2010–19); total fertility rate and livebirths (1950, 1980, and 2019); and 2019 net reproductive rate, globally and for GBD regions, super-regions, countries, and territories

		**Population in 2019 (thousands)**			**Annualised rate of change in population, 2010–19**	**Total fertility rate**			**Livebirths (thousands)**			**Net reproductive rate, 2019**
		All ages	15–64 years	<5 years		1950	1980	2019	1950	1980	2019	
**Global**		**7 737 464·6 (7 482 639·9 to 7 992 501·5)**	**5 055 473·0 (4 879 934·2 to 5 232 218·8)**	**662 842·7 (643 879·2 to 681 974·5)**	**1·1% (1·0 to 1·3)**	**4·97 (4·79 to 5·16)**	**3·82 (3·74 to 3·90)**	**2·31 (2·17 to 2·46)**	**95 940·2 (92 550·3 to 99 388·8)**	**130 420·3 (127 720·9 to 132 974·6)**	**135 350·0 (127 167·1 to 144 081·2)**	**1·1 (1·0 to 1·1)**
**Central Europe, eastern Europe, and central Asia**		**417 725·1 (396 014·3 to 440 103·3)**	**277 648·1 (263 234·8 to 292 468·6)**	**27 561·1 (25961·0 to 29 081·7)**	**0·2% (−0·1 to 0·4)**	**3·07 (3·00 to 3·15)**	**2·26 (2·23 to 2·29)**	**1·84 (1·66 to 2·06)**	**7593·5 (7417·7 to 7782·2)**	**7173·5 (7094·2 to 7257·0)**	**5206·6 (4672·8 to 5811·1)**	**0·9 (0·8 to 1·0)**
Central Asia		93 530·8 (85 150·4 to 102 488·4)	61 608·6 (56 096·7 to 67 506·2)	9572·4 (8656·9 to 10 530·7)	1·4% (1·1 to 1·7)	4·80 (4·59 to 5·01)	3·86 (3·75 to 3·98)	2·47 (2·28 to 2·66)	1083·0 (1036·1 to 1128·5)	1756·2 (1706·6 to 1806·9)	1895·5 (1755·2 to 2040·6)	1·2 (1·1 to 1·2)
	Armenia	3019·7 (2651·9 to 3385·9)	2045·7 (1796·5 to 2293·8)	204·5 (179·6 to 229·3)	−0·3% (−0·9 to 0·1)	4·65 (4·35 to 4·95)	2·88 (2·73 to 3·02)	1·74 (1·56 to 1·91)	54·6 (51·4 to 57·8)	89·2 (84·7 to 93·2)	38·4 (34·5 to 42·5)	0·8 (0·7 to 0·9)
	Azerbaijan	10 278·7 (8953·5 to 11 640·1)	7360·2 (6411·3 to 8335·0)	759·7 (661·8 to 860·4)	1·1% (0·6 to 1·7)	5·07 (4·74 to 5·39)	3·48 (3·33 to 3·64)	1·84 (1·59 to 2·12)	123·4 (115·5 to 131·4)	176·7 (168·8 to 184·5)	153·5 (133·2 to 176·4)	0·8 (0·7 to 0·9)
	Georgia	3664·8 (3306·2 to 4043·3)	2371·0 (2139·1 to 2616·0)	246·8 (222·7 to 272·3)	−0·9% (−1·0 to −0·8)	2·73 (2·48 to 3·01)	2·24 (2·11 to 2·37)	2·01 (1·73 to 2·32)	86·5 (78·7 to 95·0)	92·6 (87·4 to 97·9)	46·0 (39·8 to 53·1)	1·0 (0·8 to 1·1)
	Kazakhstan	18 392·1 (16 794·1 to 19 921·6)	11 998·3 (10 955·9 to 12 996·1)	1842·4 (1682·3 to 1995·6)	1·4% (0·5 to 2·2)	4·07 (3·88 to 4·26)	3·01 (2·91 to 3·10)	2·45 (2·23 to 2·67)	257·9 (245·5 to 270·3)	368·7 (358·2 to 379·7)	350·6 (320·5 to 381·5)	1·2 (1·1 to 1·3)
	Kyrgyzstan	6535·5 (5697·8 to 7315·2)	4133·8 (3603·9 to 4627·0)	752·3 (655·9 to 842·0)	1·7% (0·9 to 2·2)	4·31 (4·05 to 4·57)	4·21 (4·03 to 4·40)	2·61 (2·36 to 2·89)	58·5 (54·9 to 61·9)	113·5 (108·4 to 118·5)	143·9 (130·5 to 158·5)	1·2 (1·1 to 1·4)
	Mongolia	3387·6 (2977·5 to 3795·4)	2239·3 (1968·2 to 2508·8)	394·1 (346·4 to 441·6)	2·0% (1·3 to 2·5)	5·27 (4·92 to 5·59)	5·92 (5·71 to 6·12)	3·02 (2·66 to 3·40)	31·7 (29·7 to 33·7)	63·3 (61·2 to 65·4)	83·1 (73·5 to 93·4)	1·4 (1·3 to 1·6)
	Tajikistan	9492·4 (8213·9 to 10 674·8)	5957·2 (5154·9 to 6699·3)	1205·5 (1043·1 to 1355·7)	2·2% (1·3 to 2·8)	7·24 (6·93 to 7·54)	6·12 (5·95 to 6·30)	3·07 (2·79 to 3·40)	92·5 (88·7 to 96·2)	171·4 (166·5 to 176·5)	2 53·0 (229·8 to 279·9)	1·4 (1·3 to 1·6)
	Turkmenistan	5083·1 (4614·0 to 5544·9)	3302·2 (2997·5 to 3602·1)	550·7 (499·9 to 600·7)	1·2% (1·0 to 1·3)	5·24 (5·03 to 5·44)	5·27 (5·13 to 5·40)	2·92 (2·65 to 3·22)	53·2 (51·0 to 55·4)	108·2 (105·3 to 111·0)	113·1 (103·2 to 124·4)	1·4 (1·2 to 1·5)
	Uzbekistan	33 677·1 (25 411·0 to 42 319·4)	22 201·0 (16 751·7 to 27 898·2)	3616·4 (2728·7 to 4544·4)	1·6% (1·0 to 2·1)	6·25 (5·84 to 6·66)	4·70 (4·51 to 4·90)	2·44 (2·17 to 2·74)	324·7 (304·7 to 345·0)	572·6 (552·4 to 593·0)	713·9 (635·4 to 799·1)	1·1 (1·0 to 1·3)
Central Europe		114 223·6 (109 875·9 to 118 673·0)	75 341·3 (72 457·6 to 78 285·8)	5652·2 (5439·4 to 5872·4)	−0·3% (−0·6 to 0·0)	3·19 (3·10 to 3·28)	2·19 (2·16 to 2·21)	1·49 (1·31 to 1·70)	2299·3 (2240·9 to 2362·4)	2053·0 (2032·5 to 2075·8)	1069·3 (940·4 to 1218·7)	0·7 (0·6 to 0·8)
	Albania	2720·4 (2418·3 to 3021·8)	1847·2 (1642·1 to 2052·0)	162·8 (144·8 to 180·9)	−0·7% (−1·1 to −0·3)	6·15 (5·89 to 6·38)	3·46 (3·28 to 3·64)	1·94 (1·73 to 2·18)	50·2 (48·1 to 52·1)	73·4 (69·9 to 77·0)	37·4 (33·5 to 41·9)	0·9 (0·8 to 1·0)
	Bosnia and Herzegovina	3300·0 (2949·6 to 3649·2)	2259·3 (2019·4 to 2498·4)	146·4 (130·8 to 161·9)	−1·5% (−1·7 to −1·3)	3·91 (3·44 to 4·42)	2·19 (1·97 to 2·42)	1·25 (1·14 to 1·37)	90·8 (79·9 to 102·7)	77·5 (69·8 to 85·8)	26·1 (23·8 to 28·5)	0·6 (0·5 to 0·7)
	Bulgaria	6934·6 (6360·0 to 7553·9)	4454·0 (4084·9 to 4851·7)	313·8 (287·8 to 341·8)	−0·8% (−1·7 to 0·1)	2·75 (2·73 to 2·76)	2·06 (2·05 to 2·07)	1·56 (1·44 to 1·70)	166·8 (166·0 to 167·7)	127·3 (126·6 to 128·0)	60·1 (55·4 to 65·2)	0·7 (0·7 to 0·8)
	Croatia	4247·9 (3748·4 to 4764·2)	2775·2 (2448·8 to 3112·5)	185·6 (163·7 to 208·1)	−0·3% (−0·9 to 0·2)	2·91 (2·89 to 2·92)	1·82 (1·81 to 1·83)	1·34 (1·18 to 1·52)	91·3 (90·8 to 91·8)	67·8 (67·4 to 68·3)	35·2 (31·0 to 39·9)	0·6 (0·6 to 0·7)
	Czech Republic	10 643·5 (9779·1 to 11 500·1)	6793·8 (6242·0 to 7340·5)	568·3 (522·1 to 614·0)	0·2% (−0·7 to 1·0)	2·83 (2·82 to 2·84)	2·06 (2·05 to 2·07)	1·71 (1·52 to 1·91)	188·9 (188·1 to 189·8)	149·5 (148·8 to 150·2)	108·9 (97·4 to 121·9)	0·8 (0·7 to 0·9)
	Hungary	9674·4 (8515·5 to 10 789·0)	6346·6 (5586·4 to 7077·8)	439·9 (387·2 to 490·6)	−0·3% (−0·9 to 0·1)	2·59 (2·58 to 2·60)	1·90 (1·89 to 1·91)	1·41 (1·24 to 1·61)	196·3 (195·4 to 197·2)	148·8 (147·9 to 149·6)	82·2 (72·3 to 93·6)	0·7 (0·6 to 0·8)
	Montenegro	620·3 (545·9 to 695·6)	418·7 (368·5 to 469·5)	34·6 (30·5 to 38·8)	−0·2% (−0·7 to 0·3)	4·19 (3·87 to 4·52)	2·20 (2·06 to 2·35)	1·60 (1·49 to 1·74)	12·3 (11·4 to 13·3)	10·5 (9·8 to 11·2)	6·6 (6·1 to 7·1)	0·8 (0·7 to 0·8)
	North Macedonia	2152·7 (1785·5 to 2527·6)	1513·9 (1255·6 to 1777·5)	113·9 (94·5 to 133·8)	0·2% (−0·4 to 0·7)	3·98 (3·53 to 4·47)	2·45 (2·29 to 2·62)	1·44 (1·30 to 1·60)	39·5 (35·2 to 44·2)	39·8 (37·3 to 42·5)	22·3 (20·1 to 24·5)	0·7 (0·6 to 0·8)
	Poland	38 434·4 (35 379·0 to 41 364·9)	25 714·1 (23 669·9 to 27 674·7)	1908·6 (1756·8 to 2054·1)	0·0% (−0·8 to 0·8)	3·48 (3·37 to 3·59)	2·21 (2·16 to 2·27)	1·39 (1·20 to 1·61)	730·2 (709·4 to 752·8)	678·1 (662·8 to 695·5)	363·0 (313·9 to 420·0)	0·7 (0·6 to 0·8)
	Romania	19 237·1 (17 030·1 to 21 542·5)	12 510·7 (11 075·4 to 14 010·0)	940·0 (832·2 to 1052·7)	−0·8% (−1·3 to −0·4)	3·06 (2·83 to 3·31)	2·36 (2·35 to 2·37)	1·59 (1·35 to 1·87)	418·8 (387·1 to 455·0)	398·8 (397·4 to 400·1)	173·3 (146·9 to 204·3)	0·8 (0·6 to 0·9)
	Serbia	8746·8 (7829·8 to 9730·6)	5662·8 (5069·1 to 6299·7)	452·2 (404·8 to 503·0)	−0·3% (−0·7 to 0·1)	3·27 (3·25 to 3·29)	2·21 (2·20 to 2·22)	1·43 (1·21 to 1·69)	182·7 (181·4 to 184·0)	157·1 (156·4 to 157·9)	79·9 (67·6 to 94·5)	0·7 (0·6 to 0·8)
	Slovakia	5437·2 (4969·9 to 5923·6)	3700·4 (3382·3 to 4031·4)	285·3 (260·8 to 310·8)	0·0% (−0·9 to 0·9)	3·63 (3·61 to 3·65)	2·31 (2·30 to 2·32)	1·53 (1·35 to 1·73)	99·6 (99·0 to 100·1)	94·7 (94·2 to 95·2)	55·5 (49·0 to 62·9)	0·7 (0·7 to 0·8)
	Slovenia	2074·3 (1914·4 to 2243·2)	1344·7 (1241·1 to 1454·2)	100·8 (93·1 to 109·0)	0·2% (−0·6 to 1·0)	2·79 (2·48 to 3·17)	2·01 (1·96 to 2·06)	1·55 (1·36 to 1·78)	31·8 (28·2 to 36·0)	29·5 (28·8 to 30·3)	18·8 (16·4 to 21·6)	0·8 (0·7 to 0·9)
Eastern Europe		209 970·7 (189 853·2 to 228 336·8)	140 698·2 (127 218·3 to 152 944·5)	12 336·5 (11 115·2 to 13 473·5)	−0·1% (−0·6 to 0·3)	2·77 (2·70 to 2·84)	1·91 (1·88 to 1·93)	1·63 (1·40 to 1·89)	4211·2 (4107·3 to 4325·8)	3364·2 (3327·8 to 3401·9)	2241·8 (1935·0 to 2596·5)	0·8 (0·7 to 0·9)
	Belarus	9500·8 (8345·4 to 10 677·7)	6413·3 (5633·4 to 7207·8)	563·7 (495·2 to 633·6)	−0·2% (−0·8 to 0·3)	3·03 (2·91 to 3·16)	2·00 (1·93 to 2·07)	1·66 (1·39 to 1·98)	194·2 (186·6 to 201·8)	156·5 (151·1 to 161·8)	102·3 (86·0 to 121·5)	0·8 (0·7 to 0·9)
	Estonia	1312·4 (1204·4 to 1415·5)	835·5 (766·8 to 901·2)	69·7 (64·0 to 75·2)	−0·2% (−1·0 to 0·6)	2·29 (2·26 to 2·31)	2·05 (2·03 to 2·07)	1·57 (1·38 to 1·78)	20·1 (19·9 to 20·3)	22·5 (22·3 to 22·7)	13·2 (11·7 to 15·0)	0·8 (0·7 to 0·9)
	Latvia	1915·3 (1760·2 to 2071·4)	1219·4 (1120·7 to 1318·8)	103·9 (95·5 to 112·4)	−1·1% (−2·0 to −0·3)	1·96 (1·92 to 2·00)	1·89 (1·87 to 1·91)	1·63 (1·44 to 1·85)	32·7 (32·0 to 33·3)	35·6 (35·3 to 36·0)	19·2 (17·0 to 21·7)	0·8 (0·7 to 0·9)
	Lithuania	2794·2 (2574·8 to 3026·0)	1823·2 (1680·0 to 1974·4)	143·1 (131·9 to 155·0)	−1·1% (−1·2 to −1·0)	2·96 (2·79 to 3·17)	1·95 (1·94 to 1·96)	1·54 (1·37 to 1·74)	57·9 (54·5 to 62·0)	49·9 (49·6 to 50·3)	27·0 (24·0 to 30·4)	0·7 (0·7 to 0·8)
	Moldova	3688·2 (3095·7 to 4327·4)	2582·7 (2167·7 to 3030·3)	173·4 (145·5 to 203·4)	−0·6% (−1·2 to 0·1)	3·89 (3·69 to 4·08)	2·52 (2·40 to 2·66)	1·25 (1·09 to 1·43)	85·4 (80·9 to 89·9)	88·9 (84·2 to 93·7)	32·1 (28·2 to 36·5)	0·6 (0·5 to 0·7)
	Russia	146 717·4 (128 850·2 to 165 171·8)	97 916·2 (85 992·0 to 110 232·3)	9139·0 (8026·0 to 10 288·5)	0·1% (−0·6 to 0·7)	2·89 (2·88 to 2·91)	1·87 (1·86 to 1·87)	1·72 (1·49 to 1·98)	2962·2 (2941·9 to 2981·7)	2251·7 (2245·3 to 2258·8)	1660·8 (1441·5 to 1913·3)	0·8 (0·7 to 0·9)
	Ukraine	44 042·4 (35 745·5 to 52 268·0)	29 907·8 (24 273·7 to 35 493·6)	2143·7 (1739·9 to 2544·1)	−0·6% (−1·4 to 0·1)	2·34 (2·11 to 2·62)	1·94 (1·85 to 2·02)	1·38 (1·16 to 1·62)	858·6 (775·8 to 957·7)	759·1 (727·4 to 792·3)	387·2 (327·0 to 458·0)	0·7 (0·6 to 0·8)
**High income**		**1 083 976·1 (1 036 700·3 to 1 131 810·4)**	**700 212·4 (669 195·0 to 731 848·0)**	**56 941·9 (54 278·6 to 59 734·0)**	**0·5% (0·3 to 0·7)**	**2·84 (2·80 to 2·87)**	**1·87 (1·86 to 1·88)**	**1·63 (1·49 to 1·80)**	**13 588·8 (13 426·7 to 13 752·3)**	**12 482·3 (12 409·5 to 12 555·5)**	**11 186·1 (10 206·9 to 12 315·2)**	**0·8 (0·7 to 0·9)**
Australasia		29 063·8 (26 953·5 to 31 370·1)	18 813·5 (17 444·8 to 20 307·4)	1819·2 (1687·7 to 1963·4)	1·3% (1·2 to 1·5)	3·13 (3·11 to 3·15)	1·94 (1·93 to 1·94)	1·83 (1·64 to 2·03)	252·3 (250·9 to 253·7)	279·3 (278·2 to 280·3)	370·9 (334·0 to 412·2)	0·9 (0·8 to 1·0)
	Australia	24 568·1 (22 510·1 to 26 779·2)	15 960·0 (14 623·0 to 17 396·3)	1525·6 (1397·8 to 1662·9)	1·4% (1·3 to 1·6)	3·04 (3·03 to 3·06)	1·92 (1·91 to 1·93)	1·78 (1·61 to 1·98)	202·1 (201·1 to 203·0)	228·0 (226·9 to 229·0)	311·4 (281·1 to 345·2)	0·9 (0·8 to 1·0)
	New Zealand	4495·7 (4005·5 to 4968·1)	2853·6 (2542·4 to 3153·4)	293·7 (261·6 to 324·5)	0·6% (0·3 to 0·7)	3·52 (3·45 to 3·59)	1·99 (1·98 to 2·00)	2·08 (1·85 to 2·35)	50·2 (49·2 to 51·3)	51·3 (50·9 to 51·6)	59·5 (52·8 to 67·1)	1·0 (0·9 to 1·1)
High-income Asia Pacific		187 291·2 (173 225·9 to 200 835·0)	119 112·0 (110 488·9 to 127 556·8)	7286·4 (6748·7 to 7803·2)	0·1% (−0·0 to 0·4)	3·74 (3·62 to 3·87)	1·92 (1·86 to 1·97)	1·29 (1·20 to 1·40)	3069·5 (2957·6 to 3180·8)	2445·5 (2376·5 to 2513·3)	1376·6 (1276·3 to 1489·7)	0·6 (0·6 to 0·7)
	Brunei	437·1 (382·0 to 491·7)	323·1 (282·3 to 363·4)	31·5 (27·5 to 35·5)	1·2% (0·6 to 1·7)	6·98 (6·75 to 7·20)	3·85 (3·69 to 4·01)	1·71 (1·47 to 1·95)	3·1 (3·0 to 3·2)	5·8 (5·5 to 6·0)	6·6 (5·7 to 7·5)	0·8 (0·7 to 0·9)
	Japan	127 788·4 (115 774·1 to 139 878·5)	75 832·6 (68 703·1 to 83 007·1)	4791·1 (4340·7 to 5244·4)	−0·2% (−0·5 to 0·1)	3·31 (3·18 to 3·44)	1·68 (1·63 to 1·74)	1·34 (1·22 to 1·48)	2217·7 (2121·9 to 2314·2)	1574·7 (1527·4 to 1623·0)	900·4 (822·4 to 990·1)	0·7 (0·6 to 0·7)
	Singapore	5667·5 (5233·1 to 6058·7)	4207·5 (3885·1 to 4498·0)	289·3 (267·1 to 309·2)	1·2% (1·1 to 1·3)	5·68 (5·50 to 5·84)	1·81 (1·70 to 1·93)	1·16 (0·92 to 1·46)	45·0 (43·4 to 46·4)	43·1 (40·1 to 46·3)	57·1 (45·7 to 71·0)	0·6 (0·4 to 0·7)
	South Korea	53 398·3 (48 441·0 to 58 407·1)	38 748·8 (35 151·5 to 42 383·5)	2174·5 (1972·7 to 2378·5)	0·9% (0·6 to 1·1)	5·67 (5·27 to 6·07)	2·49 (2·33 to 2·64)	1·22 (1·09 to 1·38)	803·7 (750·5 to 858·0)	821·9 (768·9 to 874·7)	412·5 (369·2 to 466·3)	0·6 (0·5 to 0·7)
High-income North America		364 560·6 (323 053·2 to 406 080·4)	238 207·0 (211 083·2 to 265 338·3)	20 984·0 (18 568·5 to 23 383·5)	0·7% (0·0 to 1·2)	3·10 (3·09 to 3·11)	1·79 (1·79 to 1·80)	1·73 (1·62 to 1·85)	4015·0 (4001·9 to 4028·2)	3974·8 (3967·0 to 3984·4)	4200·8 (3932·7 to 4495·2)	0·8 (0·8 to 0·9)
	Canada	36 519·8 (33 331·5 to 39 599·8)	23 836·8 (21 755·7 to 25 847·1)	1925·4 (1757·3 to 2087·7)	0·9% (0·8 to 1·1)	3·30 (3·29 to 3·31)	1·65 (1·65 to 1·66)	1·56 (1·46 to 1·68)	361·8 (360·7 to 362·9)	358·8 (357·6 to 360·1)	373·3 (347·5 to 401·7)	0·8 (0·7 to 0·8)
	Greenland	56·2 (51·5 to 60·8)	39·3 (36·1 to 42·6)	4·0 (3·7 to 4·3)	−0·1% (−1·0 to 0·7)	5·70 (5·48 to 5·91)	2·33 (2·26 to 2·39)	1·95 (1·74 to 2·22)	1·0 (1·0 to 1·0)	1·0 (0·9 to 1·0)	0·8 (0·7 to 0·9)	0·9 (0·8 to 1·0)
	USA	327 978·7 (285 959·3 to 369 324·2)	214 327·1 (186 868·3 to 241 345·4)	19 054·3 (16 613·1 to 21 456·3)	0·6% (−0·1 to 1·3)	3·09 (3·08 to 3·10)	1·80 (1·80 to 1·81)	1·75 (1·63 to 1·87)	3652·1 (3639·4 to 3664·9)	3614·9 (3607·4 to 3623·9)	3826·7 (3584·3 to 4092·5)	0·8 (0·8 to 0·9)
Southern Latin America		66 753·1 (61 104·2 to 72 982·8)	44 129·1 (40 432·9 to 48 217·7)	4854·3 (4421·3 to 5327·8)	1·0% (0·5 to 1·4)	3·25 (3·16 to 3·34)	2·94 (2·92 to 2·95)	1·90 (1·60 to 2·27)	685·8 (668·5 to 704·8)	977·6 (973·6 to 982·7)	971·9 (817·9 to 1158·6)	0·9 (0·8 to 1·1)
	Argentina	45 115·3 (39 507·2 to 51 073·4)	29 488·6 (25 823·0 to 33 383·0)	3465·2 (3034·4 to 3922·8)	1·0% (0·3 to 1·7)	3·03 (2·93 to 3·15)	3·17 (3·16 to 3·19)	2·00 (1·65 to 2·42)	443·1 (428·5 to 459·4)	683·2 (680·1 to 686·8)	698·8 (578·3 to 846·1)	1·0 (0·8 to 1·2)
	Chile	18 198·4 (16 753·5 to 19 617·2)	12 420·8 (11 434·7 to 13 389·2)	1158·6 (1066·6 to 1248·9)	1·0% (0·7 to 1·4)	4·23 (4·19 to 4·28)	2·47 (2·46 to 2·48)	1·65 (1·46 to 1·88)	196·9 (194·5 to 199·3)	240·2 (239·1 to 241·6)	226·7 (200·7 to 257·4)	0·8 (0·7 to 0·9)
	Uruguay	3436·1 (3031·2 to 3877·0)	2217·4 (1956·1 to 2501·9)	230·3 (203·2 to 259·9)	0·2% (−0·3 to 0·8)	2·49 (2·34 to 2·65)	2·55 (2·47 to 2·62)	1·90 (1·59 to 2·26)	45·8 (43·0 to 48·9)	54·1 (52·5 to 55·9)	46·3 (38·8 to 55·0)	0·9 (0·8 to 1·1)
Western Europe		436 307·4 (422 667·7 to 450 260·4)	279 950·8 (271 212·8 to 288 867·6)	21 997·9 (21 292·6 to 22 709·6)	0·4% (0·1 to 0·6)	2·37 (2·33 to 2·40)	1·78 (1·78 to 1·78)	1·59 (1·43 to 1·77)	5566·2 (5491·3 to 5645·4)	4805·1 (4794·8 to 4816·0)	4265·9 (3836·0 to 4761·0)	0·8 (0·7 to 0·9)
	Andorra	83·1 (76·2 to 89·7)	60·1 (55·1 to 64·9)	2·7 (2·5 to 2·9)	−0·1% (−1·0 to 0·7)	2·65 (2·06 to 3·35)	1·56 (1·46 to 1·67)	1·13 (1·00 to 1·26)	0·1 (0·1 to 0·2)	0·5 (0·5 to 0·6)	0·6 (0·6 to 0·7)	0·5 (0·5 to 0·6)
	Austria	8916·2 (8169·6 to 9666·5)	5946·1 (5448·2 to 6446·4)	440·8 (403·9 to 477·9)	0·7% (−0·2 to 1·5)	2·05 (2·02 to 2·08)	1·67 (1·66 to 1·68)	1·50 (1·40 to 1·61)	105·0 (103·5 to 106·6)	91·5 (91·0 to 92·0)	87·7 (82·3 to 93·7)	0·7 (0·7 to 0·8)
	Belgium	11 419·2 (10 536·9 to 12 318·0)	7311·7 (6746·8 to 7887·2)	618·7 (570·9 to 667·4)	0·5% (−0·3 to 1·3)	2·29 (2·28 to 2·30)	1·69 (1·68 to 1·69)	1·67 (1·46 to 1·91)	142·7 (142·0 to 143·5)	122·9 (122·2 to 123·5)	121·6 (106·3 to 138·9)	0·8 (0·7 to 0·9)
	Cyprus	1313·5 (1162·1 to 1476·2)	916·2 (810·6 to 1029·7)	74·4 (65·8 to 83·6)	1·7% (1·3 to 2·2)	3·94 (3·77 to 4·11)	2·42 (2·36 to 2·49)	1·34 (1·13 to 1·58)	13·8 (13·3 to 14·4)	13·4 (13·1 to 13·8)	15·2 (12·9 to 17·9)	0·6 (0·5 to 0·8)
	Denmark	5802·7 (5330·0 to 6262·2)	3701·7 (3400·2 to 3994·8)	308·0 (282·9 to 332·4)	0·5% (−0·3 to 1·3)	2·55 (2·45 to 2·66)	1·49 (1·45 to 1·53)	1·76 (1·55 to 2·00)	78·4 (75·1 to 81·7)	55·3 (53·7 to 56·7)	62·8 (55·4 to 71·2)	0·9 (0·8 to 1·0)
	Finland	5534·1 (5086·5 to 5992·0)	3419·5 (3142·9 to 3702·4)	261·4 (240·3 to 283·0)	0·3% (−0·5 to 1·1)	3·07 (3·06 to 3·09)	1·64 (1·63 to 1·65)	1·48 (1·35 to 1·62)	95·4 (94·8 to 95·9)	63·5 (63·1 to 63·9)	49·9 (45·6 to 54·6)	0·7 (0·7 to 0·8)
	France	66 204·3 (60 093·8 to 72 433·7)	41 089·9 (37 297·4 to 44 956·2)	3650·4 (3313·5 to 3993·9)	0·4% (0·1 to 0·7)	2·80 (2·78 to 2·81)	1·92 (1·91 to 1·92)	1·80 (1·63 to 1·99)	840·7 (837·7 to 843·7)	803·7 (801·3 to 806·1)	718·7 (650·9 to 794·1)	0·9 (0·8 to 1·0)
	Germany	84 914·1 (77 688·6 to 92 219·5)	55 164·1 (50 470·1 to 59 910·0)	3923·7 (3589·8 to 4261·2)	0·4% (−0·5 to 1·2)	2·01 (1·93 to 2·08)	1·47 (1·47 to 1·48)	1·43 (1·31 to 1·56)	1059·1 (1020·3 to 1099·5)	833·1 (830·7 to 835·4)	740·3 (681·9 to 806·3)	0·7 (0·6 to 0·8)
	Greece	10 337·2 (9070·6 to 11 489·3)	6589·1 (5781·8 to 7323·5)	452·3 (396·8 to 502·7)	−0·8% (−1·4 to −0·3)	2·53 (2·49 to 2·57)	2·08 (2·07 to 2·09)	1·40 (1·24 to 1·60)	155·5 (153·0 to 158·1)	143·4 (142·5 to 144·3)	86·0 (76·3 to 97·9)	0·7 (0·6 to 0·8)
	Iceland	344·9 (316·9 to 373·2)	225·8 (207·5 to 244·4)	21·2 (19·4 to 22·9)	0·9% (0·0 to 1·7)	3·78 (3·67 to 3·90)	2·40 (2·36 to 2·45)	1·81 (1·56 to 2·12)	4·0 (3·9 to 4·1)	4·4 (4·3 to 4·4)	4·3 (3·7 to 5·0)	0·9 (0·8 to 1·0)
	Ireland	4910·4 (4483·8 to 5355·9)	3184·7 (2908·0 to 3473·7)	317·6 (290·0 to 346·4)	0·7% (0·6 to 0·8)	3·19 (3·06 to 3·33)	3·06 (2·99 to 3·12)	1·78 (1·53 to 2·08)	64·1 (61·4 to 66·9)	72·0 (70·4 to 73·4)	60·8 (52·0 to 70·9)	0·9 (0·7 to 1·0)
	Israel	9309·6 (8164·7 to 10 550·9)	5602·8 (4913·8 to 6349·9)	946·4 (830·0 to 1072·6)	1·9% (1·4 to 2·4)	3·84 (3·69 to 4·00)	3·14 (3·12 to 3·16)	3·11 (2·58 to 3·70)	47·6 (45·8 to 49·6)	92·9 (92·2 to 93·6)	192·6 (160·1 to 229·0)	1·5 (1·2 to 1·8)
	Italy	60 313·2 (55 356·1 to 64 983·9)	38 578·5 (35 407·7 to 41 566·1)	2359·7 (2165·8 to 2542·4)	0·0% (−0·9 to 0·7)	2·44 (2·42 to 2·45)	1·63 (1·62 to 1·63)	1·30 (1·19 to 1·44)	882·2 (877·1 to 886·9)	635·8 (632·7 to 639·1)	439·7 (401·5 to 485·6)	0·6 (0·6 to 0·7)
	Luxembourg	618·6 (568·1 to 666·4)	429·5 (394·5 to 462·7)	32·4 (29·8 to 34·9)	2·3% (1·4 to 3·0)	1·93 (1·89 to 1·97)	1·50 (1·47 to 1·53)	1·40 (1·24 to 1·59)	4·4 (4·3 to 4·5)	4·2 (4·1 to 4·2)	6·4 (5·7 to 7·3)	0·7 (0·6 to 0·8)
	Malta	439·2 (389·2 to 489·6)	282·2 (250·1 to 314·6)	21·8 (19·3 to 24·3)	0·4% (−0·1 to 0·8)	4·07 (3·91 to 4·25)	1·98 (1·95 to 2·01)	1·47 (1·25 to 1·74)	9·8 (9·4 to 10·3)	5·7 (5·7 to 5·9)	4·2 (3·6 to 5·0)	0·7 (0·6 to 0·8)
	Monaco	37·6 (34·3 to 40·8)	23·3 (21·2 to 25·2)	1·6 (1·5 to 1·8)	0·6% (0·4 to 0·8)	2·80 (2·41 to 3·25)	1·79 (1·51 to 2·09)	1·48 (1·24 to 1·79)	0·4 (0·3 to 0·5)	0·3 (0·3 to 0·4)	0·3 (0·2 to 0·3)	0·7 (0·6 to 0·9)
	Netherlands	17 156·8 (15 675·2 to 18 613·3)	11 101·0 (10 142·4 to 12 043·5)	879·8 (803·8 to 954·4)	0·4% (−0·5 to 1·2)	3·08 (3·07 to 3·10)	1·60 (1·59 to 1·61)	1·69 (1·45 to 1·96)	228·1 (226·8 to 229·4)	179·6 (178·2 to 181·1)	177·6 (152·8 to 206·4)	0·8 (0·7 to 0·9)
	Norway	5348·8 (4936·7 to 5754·8)	3488·0 (3219·3 to 3752·7)	294·2 (271·6 to 316·6)	1·0% (0·3 to 1·8)	2·51 (2·49 to 2·53)	1·71 (1·70 to 1·72)	1·59 (1·45 to 1·76)	61·9 (61·4 to 62·3)	50·7 (50·4 to 51·1)	56·7 (51·4 to 62·6)	0·8 (0·7 to 0·9)
	Portugal	10 651·3 (9433·2 to 11 909·0)	6912·3 (6121·8 to 7728·6)	415·1 (367·7 to 464·2)	−0·2% (−0·7 to 0·3)	3·03 (2·89 to 3·17)	2·13 (2·07 to 2·18)	1·25 (1·06 to 1·48)	205·0 (195·5 to 214·8)	154·0 (150·0 to 158·1)	79·6 (67·0 to 94·4)	0·6 (0·5 to 0·7)
	San Marino	33·1 (28·9 to 37·2)	21·7 (18·9 to 24·4)	1·6 (1·4 to 1·8)	0·7% (−0·0 to 1·3)	2·23 (1·89 to 2·62)	1·58 (1·33 to 1·85)	1·44 (1·20 to 1·74)	0·3 (0·2 to 0·3)	0·2 (0·2 to 0·3)	0·3 (0·3 to 0·4)	0·7 (0·6 to 0·8)
	Spain	46 021·2 (42 088·0 to 49 981·5)	30 244·7 (27 659·8 to 32 847·4)	2013·4 (1841·3 to 2186·6)	−0·2% (−1·1 to 0·6)	2·40 (2·38 to 2·41)	2·12 (2·11 to 2·14)	1·31 (1·16 to 1·49)	545·1 (541·0 to 549·2)	544·9 (541·4 to 548·6)	369·2 (329·4 to 418·8)	0·6 (0·6 to 0·7)
	Sweden	10 222·5 (9312·3 to 11 127·5)	6337·4 (5773·1 to 6898·4)	595·7 (542·7 to 648·5)	0·9% (−0·0 to 1·8)	2·26 (2·25 to 2·27)	1·67 (1·66 to 1·68)	1·78 (1·66 to 1·90)	113·4 (112·8 to 114·0)	96·2 (95·6 to 96·7)	117·4 (109·9 to 125·6)	0·9 (0·8 to 0·9)
	Switzerland	8775·2 (8021·7 to 9564·6)	5829·5 (5328·9 to 6353·9)	446·6 (408·3 to 486·8)	1·1% (0·2 to 1·9)	2·35 (2·34 to 2·37)	1·53 (1·52 to 1·54)	1·48 (1·37 to 1·60)	83·2 (82·7 to 83·8)	73·4 (73·0 to 73·8)	88·3 (82·1 to 95·2)	0·7 (0·7 to 0·8)
	UK	67 220·4 (60 468·7 to 73 925·4)	43 247·1 (38 906·0 to 47 560·3)	3899·2 (3500·5 to 4292·3)	0·6% (0·2 to 1·0)	2·19 (2·14 to 2·25)	1·87 (1·86 to 1·87)	1·73 (1·55 to 1·93)	822·0 (802·1 to 845·8)	759·7 (757·9 to 761·5)	782·1 (700·7 to 875·3)	0·8 (0·7 to 0·9)
**Latin America and Caribbean**		**584 378·2 (550 808·2 to 616 150·2)**	**389 534·9 (366 772·0 to 410 991·0)**	**48 074·1 (45 533·0 to 50 539·5)**	**1·1% (0·8 to 1·3)**	**6·05 (5·76 to 6·34)**	**4·27 (4·16 to 4·37)**	**2·07 (1·89 to 2·25)**	**6504·1 (6209·5 to 6799·1)**	**10 773·7 (10 520·3 to 11 028·0)**	**9793·7 (8950·1 to 10 685·6)**	**1·0 (0·9 to 1·1)**
Andean Latin America		63 595·5 (59 801·9 to 67 247·4)	40 733·9 (38 317·7 to 43 086·2)	6334·9 (5962·3 to 6690·4)	1·8% (1·6 to 2·0)	7·10 (6·79 to 7·42)	5·48 (5·32 to 5·64)	2·61 (2·23 to 3·03)	719·7 (686·9 to 753·9)	1231·6 (1193·9 to 1270·6)	1329·8 (1139·7 to 1543·9)	1·2 (1·1 to 1·4)
	Bolivia	12 011·7 (10 641·7 to 13 418·2)	7356·5 (6517·4 to 8217·8)	1511·7 (1339·3 to 1688·7)	1·8% (1·4 to 2·2)	7·56 (7·27 to 7·86)	6·05 (5·85 to 6·22)	3·44 (2·98 to 3·94)	165·6 (159·1 to 172·4)	229·7 (222·9 to 236·4)	326·9 (283·8 to 374·2)	1·6 (1·4 to 1·8)
	Ecuador	17 588·4 (15 403·9 to 19 749·9)	11 264·8 (9865·7 to 12 649·1)	1709·9 (1497·5 to 1920·0)	1·8% (1·1 to 2·4)	6·60 (6·28 to 6·96)	4·93 (4·79 to 5·07)	2·40 (2·05 to 2·80)	161·5 (153·4 to 170·2)	293·2 (285·0 to 301·4)	349·1 (298·8 to 406·2)	1·1 (1·0 to 1·3)
	Peru	33 995·4 (31 120·1 to 36 626·2)	22 112·6 (20 242·4 to 23 823·9)	3113·3 (2850·0 to 3354·3)	1·8% (1·7 to 1·8)	7·13 (6·81 to 7·45)	5·56 (5·33 to 5·78)	2·42 (2·06 to 2·83)	392·6 (374·6 to 411·3)	708·7 (677·4 to 739·6)	653·8 (557·1 to 763·9)	1·2 (1·0 to 1·3)
	Caribbean	47 167·0 (44 197·4 to 50 167·4)	30 885·7 (28 957·8 to 32 810·6)	3950·2 (3633·9 to 4285·3)	0·8% (0·5 to 1·1)	4·94 (4·78 to 5·11)	3·35 (3·27 to 3·43)	2·23 (2·03 to 2·46)	687·9 (666·0 to 710·1)	820·3 (800·5 to 838·7)	819·0 (743·2 to 901·7)	1·0 (0·9 to 1·1)
	Antigua and Barbuda	88·5 (77·6 to 98·9)	63·3 (55·5 to 70·7)	5·1 (4·5 to 5·7)	0·3% (−0·3 to 0·7)	4·72 (4·46 to 4·98)	2·69 (2·62 to 2·76)	1·41 (1·17 to 1·68)	1·7 (1·6 to 1·8)	1·4 (1·4 to 1·5)	1·0 (0·8 to 1·2)	0·7 (0·6 to 0·8)
	The Bahamas	376·9 (330·4 to 424·7)	267·1 (234·1 to 300·9)	21·8 (19·1 to 24·5)	0·7% (0·0 to 1·3)	4·01 (3·75 to 4·27)	2·66 (2·61 to 2·70)	1·33 (1·10 to 1·62)	2·6 (2·5 to 2·8)	5·0 (4·9 to 5·1)	4·1 (3·3 to 4·9)	0·6 (0·5 to 0·8)
	Barbados	297·8 (263·6 to 334·6)	202·5 (179·3 to 227·6)	14·6 (12·9 to 16·4)	0·6% (0·0 to 1·2)	3·65 (3·37 to 3·92)	1·97 (1·88 to 2·06)	1·42 (1·17 to 1·72)	6·9 (6·4 to 7·5)	4·4 (4·2 to 4·6)	2·8 (2·3 to 3·4)	0·7 (0·6 to 0·8)
	Belize	410·1 (358·8 to 459·1)	267·1 (233·7 to 299·0)	37·5 (32·8 to 42·0)	2·4% (1·7 to 3·0)	5·81 (5·49 to 6·16)	5·25 (5·10 to 5·40)	2·07 (1·79 to 2·40)	3·0 (2·9 to 3·2)	5·4 (5·3 to 5·6)	7·6 (6·5 to 8·8)	1·0 (0·9 to 1·1)
	Bermuda	64·0 (58·3 to 69·7)	42·9 (39·1 to 46·7)	2·6 (2·4 to 2·9)	−0·2% (−0·5 to −0·1)	3·53 (3·32 to 3·74)	1·66 (1·59 to 1·73)	1·28 (1·08 to 1·52)	1·1 (1·0 to 1·2)	0·8 (0·8 to 0·9)	0·5 (0·4 to 0·6)	0·6 (0·5 to 0·7)
	Cuba	11 358·5 (10 094·7 to 12 738·8)	7822·1 (6951·8 to 8772·6)	562·7 (500·1 to 631·1)	−0·1% (−0·5 to 0·3)	3·39 (3·22 to 3·60)	1·54 (1·52 to 1·57)	1·46 (1·26 to 1·70)	154·9 (147·3 to 164·0)	129·8 (127·2 to 132·5)	104·3 (89·8 to 121·0)	0·7 (0·6 to 0·8)
	Dominica	68·7 (60·1 to 77·1)	46·1 (40·4 to 51·8)	4·2 (3·7 to 4·8)	−0·2% (−0·7 to 0·3)	5·63 (5·37 to 5·88)	3·35 (3·15 to 3·57)	1·66 (1·39 to 1·97)	2·2 (2·1 to 2·2)	1·8 (1·7 to 1·9)	0·8 (0·7 to 1·0)	0·8 (0·7 to 0·9)
	Dominican Republic	10 881·9 (9629·8 to 12 279·8)	7066·0 (6253·0 to 7973·7)	1094·5 (968·5 to 1235·1)	1·1% (0·6 to 1·8)	6·76 (6·39 to 7·16)	5·16 (4·93 to 5·40)	2·48 (2·13 to 2·88)	117·3 (111·3 to 123·8)	223·9 (213·6 to 234·2)	230·3 (198·3 to 266·3)	1·2 (1·0 to 1·4)
	Grenada	103·2 (90·7 to 115·5)	71·8 (63·0 to 80·3)	7·0 (6·2 to 7·8)	−0·4% (−0·9 to 0·1)	5·79 (5·59 to 5·99)	3·57 (3·35 to 3·77)	1·81 (1·50 to 2·17)	3·8 (3·7 to 3·9)	2·7 (2·5 to 2·9)	1·4 (1·2 to 1·7)	0·9 (0·7 to 1·0)
	Guyana	770·7 (683·8 to 857·1)	514·8 (456·7 to 572·4)	71·0 (63·0 to 78·9)	0·3% (−0·1 to 0·7)	6·64 (6·37 to 6·93)	3·96 (3·74 to 4·19)	2·10 (1·77 to 2·48)	20·8 (19·9 to 21·7)	26·9 (25·4 to 28·4)	14·4 (12·1 to 16·9)	1·0 (0·8 to 1·2)
	Haiti	12 402·1 (10 373·6 to 14 713·4)	7620·5 (6374·1 to 9040·7)	1523·3 (1274·2 to 1807·2)	2·0% (1·3 to 2·7)	6·48 (6·04 to 6·91)	5·56 (5·29 to 5·82)	3·11 (2·80 to 3·47)	171·1 (159·4 to 182·4)	212·9 (202·7 to 222·8)	333·3 (299·6 to 373·0)	1·4 (1·2 to 1·5)
	Jamaica	2810·8 (2482·6 to 3132·0)	1931·7 (1706·2 to 2152·5)	184·9 (163·3 to 206·1)	0·2% (−0·3 to 0·6)	4·09 (3·79 to 4·40)	3·18 (3·04 to 3·32)	1·48 (1·26 to 1·74)	50·5 (46·9 to 54·3)	56·5 (54·0 to 59·1)	35·8 (30·4 to 42·1)	0·7 (0·6 to 0·8)
	PuertoRico	3521·4 (3106·9 to 3986·0)	2284·4 (2015·5 to 2585·8)	131·6 (116·1 to 149·0)	−0·9% (−1·5 to −0·2)	5·24 (5·21 to 5·27)	2·60 (2·58 to 2·62)	1·10 (0·96 to 1·26)	85·2 (84·6 to 85·7)	71·3 (70·9 to 71·8)	25·3 (22·1 to 29·0)	0·5 (0·5 to 0·6)
	Saint Kitts and Nevis	59·5 (48·0 to 70·9)	42·8 (34·5 to 51·0)	3·6 (2·9 to 4·3)	1·0% (0·1 to 1·7)	5·63 (5·11 to 6·16)	3·12 (2·87 to 3·37)	1·52 (1·26 to 1·87)	2·7 (2·4 to 2·9)	1·1 (1·0 to 1·2)	0·7 (0·6 to 0·8)	0·7 (0·6 to 0·9)
	Saint Lucia	174·6 (153·6 to 195·0)	124·7 (109·7 to 139·2)	9·3 (8·1 to 10·3)	0·4% (−0·3 to 0·9)	5·43 (5·09 to 5·77)	4·71 (4·53 to 4·88)	1·31 (1·08 to 1·59)	3·2 (3·0 to 3·4)	4·2 (4·0 to 4·4)	1·8 (1·5 to 2·1)	0·6 (0·5 to 0·8)
	Saint Vincent and the Grenadines	113·1 (101·2 to 125·6)	76·6 (68·5 to 85·1)	7·7 (6·9 to 8·6)	0·2% (−0·2 to 0·5)	6·04 (5·83 to 6·25)	4·03 (3·81 to 4·24)	1·83 (1·53 to 2·18)	3·4 (3·3 to 3·5)	3·3 (3·1 to 3·5)	1·5 (1·3 to 1·8)	0·9 (0·7 to 1·0)
	Suriname	575·9 (512·2 to 648·6)	380·9 (338·8 to 429·0)	45·7 (40·7 to 51·5)	0·7% (0·4 to 1·2)	5·37 (5·21 to 5·51)	3·91 (3·76 to 4·05)	2·11 (1·78 to 2·48)	7·5 (7·3 to 7·7)	10·6 (10·1 to 10·9)	9·1 (7·7 to 10·7)	1·0 (0·8 to 1·2)
	Trinidad and Tobago	1387·5 (1226·3 to 1549·4)	948·9 (838·7 to 1059·7)	82·8 (73·2 to 92·5)	0·3% (−0·1 to 0·8)	4·75 (4·47 to 5·07)	3·10 (3·00 to 3·19)	1·53 (1·30 to 1·79)	24·4 (23·0 to 26·0)	29·2 (28·2 to 30·2)	15·3 (13·0 to 18·0)	0·7 (0·6 to 0·9)
	Virgin Islands	104·0 (91·2 to 116·7)	65·3 (57·2 to 73·2)	6·4 (5·6 to 7·2)	−0·5% (−1·1 to 0·2)	5·09 (4·83 to 5·33)	2·97 (2·80 to 3·15)	2·05 (1·74 to 2·40)	0·9 (0·9 to 1·0)	2·5 (2·3 to 2·6)	1·3 (1·1 to 1·5)	1·0 (0·8 to 1·2)
Central Latin America		250 020·4 (232 493·3 to 267 248·3)	164 770·6 (153 208·2 to 176 282·8)	21 656·4 (20 178·7 to 23 113·0)	1·0% (0·6 to 1·3)	6·15 (5·91 to 6·39)	4·79 (4·68 to 4·89)	2·13 (1·91 to 2·36)	2473·4 (2381·3 to 2563·4)	4916·6 (4800·8 to 5027·2)	4404·2 (3952·7 to 4878·9)	1·0 (0·9 to 1·1)
	Colombia	47 776·7 (44 174·3 to 51 539·5)	32 136·5 (29 713·4 to 34 667·6)	3821·5 (3533·3 to 4122·4)	0·7% (0·5 to 1·0)	6·15 (5·75 to 6·58)	3·94 (3·75 to 4·11)	2·08 (1·76 to 2·44)	537·1 (503·2 to 574·1)	891·3 (853·5 to 928·7)	804·6 (682·6 to 943·2)	1·0 (0·8 to 1·2)
	Costa Rica	4716·7 (4164·8 to 5271·5)	3220·8 (2843·8 to 3599·6)	339·7 (300·0 to 379·7)	0·8% (0·2 to 1·2)	6·05 (5·94 to 6·15)	3·60 (3·52 to 3·69)	1·67 (1·40 to 1·97)	38·1 (37·4 to 38·8)	69·7 (67·9 to 71·6)	66·3 (55·9 to 78·3)	0·8 (0·7 to 0·9)
	El Salvador	6256·1 (5393·1 to 7102·9)	4014·9 (3461·0 to 4558·2)	570·9 (492·1 to 648·1)	0·4% (−0·1 to 0·9)	6·41 (6·15 to 6·71)	4·76 (4·64 to 4·88)	2·01 (1·72 to 2·33)	96·5 (92·6 to 100·8)	166·0 (161·5 to 170·5)	112·0 (96·2 to 130·0)	1·0 (0·8 to 1·1)
	Guatemala	17 776·5 (14 652·1 to 20 926·0)	11 024·0 (9086·4 to 12 977·1)	1992·7 (1642·4 to 2345·7)	2·2% (1·4 to 2·8)	6·53 (6·34 to 6·73)	6·72 (6·64 to 6·81)	2·53 (2·14 to 2·97)	147·5 (142·9 to 152·1)	307·5 (303·7 to 311·4)	409·1 (347·8 to 478·7)	1·2 (1·0 to 1·4)
	Honduras	9814·4 (8823·0 to 10 833·4)	6027·8 (5418·9 to 6653·7)	1128·1 (1014·1 to 1245·2)	2·3% (2·1 to 2·5)	6·78 (6·43 to 7·11)	6·14 (5·99 to 6·29)	2·60 (2·29 to 2·96)	73·7 (70·4 to 76·9)	149·1 (145·5 to 152·7)	233·1 (206·9 to 264·0)	1·2 (1·1 to 1·4)
	Mexico	124 940·2 (108 607·1 to 140 630·3)	82 961·5 (72 116·2 to 93 379·9)	10 466·6 (9098·4 to 11 781·0)	1·0% (0·2 to 1·6)	6·21 (5·85 to 6·55)	5·09 (4·93 to 5·23)	2·04 (1·80 to 2·33)	1266·8 (1201·6 to 1331·2)	2662·4 (2574·7 to 2745·0)	2095·8 (1848·0 to 2393·4)	1·0 (0·9 to 1·1)
	Nicaragua	6510·4 (5514·4 to 7574·3)	4165·6 (3528·3 to 4846·3)	659·4 (558·5 to 767·2)	1·4% (0·7 to 2·0)	7·62 (7·29 to 7·94)	6·34 (6·14 to 6·53)	2·28 (2·04 to 2·57)	66·7 (64·2 to 69·1)	130·6 (126·9 to 133·9)	130·4 (115·9 to 147·5)	1·1 (1·0 to 1·2)
	Panama	4160·5 (3659·3 to 4679·2)	2653·6 (2333·9 to 2984·5)	386·3 (339·8 to 434·5)	1·9% (1·3 to 2·5)	4·08 (3·94 to 4·23)	3·44 (3·34 to 3·55)	2·38 (2·07 to 2·73)	26·6 (25·7 to 27·6)	52·6 (50·9 to 54·3)	75·9 (65·8 to 86·9)	1·1 (1·0 to 1·3)
	Venezuela	28 069·0 (24 769·4 to 31 423·8)	18 566·0 (16 383·5 to 20 785·0)	2291·2 (2021·9 to 2565·0)	0·1% (−0·5 to 0·6)	5·41 (5·24 to 5·57)	4·09 (4·00 to 4·19)	2·19 (1·85 to 2·57)	220·3 (213·0 to 227·1)	487·5 (475·5 to 498·9)	477·0 (404·3 to 558·4)	1·0 (0·9 to 1·2)
Tropical Latin America		223 595·3 (196 813·0 to 249 498·1)	153 144·8 (134 770·8 to 170 939·6)	16 132·6 (14 217·4 to 17 986·9)	1·0% (0·4 to 1·5)	6·08 (5·59 to 6·56)	3·69 (3·57 to 3·82)	1·80 (1·61 to 2·02)	2623·2 (2422·9 to 2820·0)	3805·2 (3695·7 to 3913·1)	3240·7 (2890·4 to 3639·8)	0·9 (0·8 to 1·0)
	Brazil	216 664·8 (189 879·2 to 242 502·4)	148 638·5 (130 262·8 to 166 363·9)	15 496·0 (13 580·3 to 17 344·0)	1·0% (0·3 to 1·5)	6·07 (5·57 to 6·56)	3·66 (3·54 to 3·79)	1·79 (1·60 to 2·02)	2558·1 (2358·2 to 2754·1)	3696·3 (3591·5 to 3802·8)	3115·1 (2766·4 to 3503·6)	0·9 (0·8 to 1·0)
	Paraguay	6930·5 (5699·2 to 8111·0)	4506·2 (3705·7 to 5273·8)	636·6 (523·5 to 745·0)	1·4% (0·7 to 1·9)	6·47 (6·08 to 6·90)	4·98 (4·81 to 5·17)	2·17 (1·85 to 2·53)	65·0 (61·1 to 69·2)	108·8 (105·1 to 112·4)	125·6 (107·6 to 146·1)	1·0 (0·9 to 1·2)
**North Africa and Middle East**		**608 713·6 (585 677·9 to 634 586·8)**	**400 862·6 (386 122·8 to 416 975·8)**	**59 719·9 (56 802·9 to 62 767·3)**	**1·6% (1·5 to 1·8)**	**6·88 (6·60 to 7·16)**	**6·34 (6·19 to 6·48)**	**2·50 (2·26 to 2·77)**	**5617·1 (5392·9 to 5838·0)**	**11 238·7 (10 969·5 to 11 490·4)**	**12 197·8 (11 052·0 to 13 533·2)**	**1·2 (1·1 to 1·3)**
Afghanistan		38 277·5 (26 161·8 to 50 468·6)	20 234·9 (13 830·1 to 26 679·6)	6645·1 (4541·8 to 8761·6)	3·2% (2·4 to 3·6)	7·27 (6·91 to 7·62)	7·21 (7·00 to 7·40)	5·39 (5·11 to 5·69)	372·1 (354·1 to 389·7)	524·6 (510·7 to 538·3)	1496·2 (1417·3 to 1580·5)	2·4 (2·3 to 2·5)
Algeria		41 847·3 (36 020·5 to 47 461·9)	27 484·4 (23 657·5 to 31 171·9)	4281·8 (3685·6 to 4856·3)	1·6% (0·8 to 2·1)	6·29 (5·90 to 6·67)	6·45 (6·25 to 6·63)	2·54 (2·27 to 2·86)	386·8 (362·7 to 411·1)	744·6 (718·2 to 768·0)	885·9 (794·9 to 993·0)	1·2 (1·1 to 1·3)
Bahrain		1442·7 (1254·8 to 1605·9)	1156·7 (1006·1 to 1287·6)	70·7 (61·5 to 78·7)	1·6% (0·8 to 2·1)	5·95 (5·48 to 6·41)	4·06 (3·80 to 4·31)	1·35 (1·11 to 1·66)	3·8 (3·6 to 4·1)	9·3 (8·6 to 9·9)	13·0 (10·7 to 16·0)	0·6 (0·5 to 0·8)
Egypt		99 069·6 (90 571·9 to 107 515·8)	61 855·8 (56 550·1 to 67 129·3)	10 825·2 (9896·7 to 11 748·1)	1·9% (1·8 to 2·0)	6·66 (6·45 to 6·86)	5·92 (5·76 to 6·06)	2·63 (2·34 to 2·91)	1045·7 (1011·3 to 1081·0)	1884·4 (1832·0 to 1933·5)	2107·7 (1879·6 to 2346·0)	1·2 (1·1 to 1·4)
Iran		84 297·9 (77 330·6 to 91 935·9)	58 348·2 (53 525·7 to 63 635·0)	7066·7 (6482·7 to 7707·0)	1·1% (0·9 to 1·3)	6·55 (6·14 to 6·96)	7·73 (7·64 to 7·81)	1·92 (1·61 to 2·31)	822·5 (773·9 to 870·5)	2082·9 (2056·8 to 2108·5)	1349·8 (1135·5 to 1623·2)	0·9 (0·8 to 1·1)
Iraq		42 119·5 (31 429·3 to 52 981·9)	26 718·3 (19 937·0 to 33 608·8)	4660·1 (3477·3 to 5861·9)	2·4% (1·9 to 2·8)	7·19 (6·88 to 7·48)	6·93 (6·67 to 7·16)	2·70 (2·33 to 3·15)	251·4 (241·4 to 260·9)	594·5 (573·5 to 614·2)	956·7 (832·7 to 1109·8)	1·3 (1·1 to 1·5)
Jordan		11 636·7 (10 588·3 to 12 678·9)	7466·3 (6793·6 to 8135·0)	1200·8 (1092·6 to 1308·3)	5·2% (5·1 to 5·3)	9·19 (9·14 to 9·22)	7·64 (7·50 to 7·77)	2·80 (2·44 to 3·25)	25·6 (25·5 to 25·7)	99·6 (97·5 to 101·8)	240·4 (209·7 to 277·7)	1·3 (1·2 to 1·5)
Kuwait		4426·6 (3926·5 to 4929·5)	3419·0 (3032·7 to 3807·5)	298·8 (265·1 to 332·8)	4·4% (3·9 to 4·8)	5·71 (5·26 to 6·16)	5·26 (5·05 to 5·45)	1·37 (1·13 to 1·68)	3·0 (2·7 to 3·2)	50·1 (48·3 to 51·8)	60·8 (50·3 to 74·5)	0·7 (0·5 to 0·8)
Lebanon		5177·1 (4455·3 to 5928·5)	3321·0 (2858·0 to 3803·1)	507·2 (436·5 to 580·8)	2·4% (1·8 to 3·0)	7·32 (6·96 to 7·68)	5·84 (5·47 to 6·20)	2·59 (2·23 to 3·03)	63·9 (60·8 to 66·9)	112·2 (105·0 to 119·2)	107·3 (92·9 to 124·7)	1·2 (1·1 to 1·4)
Libya		6735·5 (5705·4 to 7670·8)	4910·0 (4159·1 to 5591·8)	424·0 (359·1 to 482·8)	1·1% (0·3 to 1·7)	7·45 (7·20 to 7·69)	7·19 (6·97 to 7·40)	1·40 (1·17 to 1·69)	51·6 (50·0 to 53·1)	130·2 (125·9 to 134·3)	81·3 (68·1 to 98·5)	0·7 (0·6 to 0·8)
Morocco		35 952·2 (32 338·7 to 39 427·4)	24 182·7 (21 752·1 to 26 520·2)	3047·0 (2740·8 to 3341·6)	0·8% (0·7 to 0·8)	7·25 (6·92 to 7·58)	5·61 (5·32 to 5·89)	2·13 (1·81 to 2·49)	463·2 (441·1 to 486·4)	788·8 (746·5 to 829·9)	608·2 (516·7 to 713·2)	1·0 (0·9 to 1·2)
Oman		4584·0 (4209·6 to 4952·3)	3462·7 (3179·9 to 3740·9)	391·2 (359·2 to 422·6)	5·3% (5·1 to 5·5)	7·98 (7·74 to 8·20)	7·51 (7·26 to 7·76)	2·37 (2·10 to 2·64)	25·7 (24·9 to 26·5)	60·0 (57·9 to 62·1)	77·8 (69·2 to 86·8)	1·1 (1·0 to 1·3)
Palestine		4956·6 (4561·8 to 5329·7)	2939·8 (2705·6 to 3161·0)	623·6 (573·9 to 670·5)	2·0% (1·9 to 2·0)	7·95 (7·68 to 8·21)	7·35 (7·10 to 7·58)	2·99 (2·70 to 3·35)	39·5 (38·2 to 40·8)	69·3 (67·1 to 71·4)	125·2 (112·1 to 140·2)	1·4 (1·3 to 1·6)
Qatar		2864·5 (2595·4 to 3123·7)	2415·9 (2188·9 to 2634·5)	141·6 (128·3 to 154·4)	5·6% (5·3 to 5·8)	5·99 (5·54 to 6·46)	4·85 (4·59 to 5·10)	1·70 (1·45 to 1·97)	1·1 (1·0 to 1·1)	7·9 (7·5 to 8·3)	26·8 (23·1 to 30·9)	0·8 (0·7 to 0·9)
Saudi Arabia		35 732·0 (31 175·7 to 40 192·0)	27 759·4 (24 219·7 to 31 224·3)	2281·6 (1990·6 to 2566·3)	2·7% (2·0 to 3·3)	7·66 (7·41 to 7·91)	6·81 (6·47 to 7·17)	1·44 (1·28 to 1·62)	133·8 (128·6 to 138·8)	406·1 (384·3 to 430·4)	455·3 (403·8 to 510·7)	0·7 (0·6 to 0·8)
Sudan		40 808·4 (35 356·1 to 46 009·7)	23 669·7 (20 507·2 to 26 686·5)	5512·7 (4776·2 to 6215·4)	2·1% (1·4 to 2·6)	7·55 (7·19 to 7·90)	7·46 (7·16 to 7·73)	3·66 (3·20 to 4·21)	279·2 (266·5 to 291·3)	793·4 (764·4 to 820·3)	1206·7 (1051·7 to 1388·3)	1·7 (1·5 to 1·9)
Syria		14 491·2 (12 173·7 to 16 795·6)	9515·8 (7994·0 to 11 029·0)	1136·9 (955·1 to 1317·7)	−4·1% (−4·8 to −3·5)	8·16 (7·96 to 8·36)	7·34 (7·17 to 7·51)	2·26 (1·91 to 2·71)	177·6 (172·9 to 182·3)	412·4 (400·5 to 424·0)	231·9 (192·5 to 282·6)	1·1 (0·9 to 1·3)
Tunisia		11 571·6 (10 423·7 to 12 757·9)	7878·1 (7096·6 to 8685·7)	868·7 (782·5 to 957·7)	0·7% (0·6 to 0·9)	6·51 (6·10 to 6·91)	5·66 (5·50 to 5·82)	1·79 (1·53 to 2·11)	168·4 (157·8 to 178·8)	253·8 (245·1 to 262·7)	167·6 (143·7 to 197·8)	0·8 (0·7 to 1·0)
Turkey		81 359·7 (71 366·1 to 91 236·7)	58 155·6 (51 012·2 to 65 215·6)	4893·9 (4292·8 to 5488·0)	0·9% (0·4 to 1·4)	6·54 (6·10 to 6·99)	4·70 (4·45 to 4·95)	1·48 (1·29 to 1·71)	1055·8 (988·6 to 1123·1)	1640·7 (1550·4 to 1726·1)	981·7 (856·9 to 1135·2)	0·7 (0·6 to 0·8)
United Arab Emirates		9241·7 (7764·6 to 10 592·7)	7957·2 (6685·5 to 9120·5)	333·4 (280·1 to 382·2)	1·0% (0·3 to 1·5)	7·43 (7·08 to 7·75)	6·18 (5·99 to 6·36)	1·14 (0·98 to 1·33)	3·5 (3·4 to 3·7)	35·5 (34·5 to 36·5)	56·2 (48·1 to 66·3)	0·5 (0·5 to 0·6)
Yemen		31 502·9 (26 596·9 to 36 775·1)	17 603·7 (14 862·2 to 20 549·7)	4448·2 (3755·4 to 5192·6)	2·5% (1·9 to 3·1)	7·92 (7·65 to 8·18)	8·21 (8·09 to 8·32)	3·90 (3·46 to 4·41)	241·9 (233·6 to 249·8)	531·1 (522·7 to 539·3)	948·9 (838·1 to 1077·0)	1·8 (1·6 to 2·0)
**South Asia**		**1 805 200·3 (1 650 386·8 to 1 971 037·9)**	**1 174 895·9 (1 072 148·8 to 1 284 426·1)**	**164 404·5 (151 118·0 to 178 694·1)**	**1·4% (1·1 to 1·8)**	**6·18 (5·70 to 6·66)**	**5·21 (4·97 to 5·44)**	**2·21 (2·03 to 2·40)**	**20 216·6 (18 733·9 to 21 701·2)**	**33 101·1 (31 750·4 to 34 432·9)**	**33 438·5 (30 722·0 to 36 348·4)**	**1·0 (0·9 to 1·1)**
Bangladesh		159 259·8 (141 199·8 to 177 852·8)	104 364·9 (92 529·9 to 116 549·1)	13 745·9 (12 187·1 to 15 350·7)	1·1% (0·6 to 1·5)	7·81 (7·50 to 8·12)	5·52 (5·34 to 5·71)	1·82 (1·63 to 2·02)	2220·8 (2145·9 to 2289·5)	3265·3 (3160·1 to 3376·7)	2659·5 (2378·5 to 2945·6)	0·9 (0·8 to 0·9)
Bhutan		754·2 (697·0 to 815·4)	513·9 (474·9 to 555·6)	65·3 (60·4 to 70·6)	0·3% (0·1 to 0·6)	6·52 (6·08 to 6·97)	5·67 (5·25 to 6·12)	2·06 (1·76 to 2·42)	8·2 (7·7 to 8·8)	19·1 (17·9 to 20·4)	13·8 (11·8 to 16·1)	1·0 (0·8 to 1·1)
India		1 390 707·0 (1 237 773·4 to 1 558 771·7)	921 391·2 (820 067·4 to 1 032 739·8)	117 070·8 (104 196·7 to 131 218·6)	1·3% (0·9 to 1·8)	5·91 (5·40 to 6·42)	4·95 (4·70 to 5·20)	2·02 (1·82 to 2·22)	15 887·5 (14 589·5 to 17 194·3)	25 192·9 (24 001·9 to 26 356·9)	23 461·0 (21 145·4 to 25 772·6)	0·9 (0·8 to 1·0)
Nepal		30 416·4 (26 611·4 to 34 238·8)	19 373·6 (16 950·0 to 21 808·3)	2996·0 (2621·2 to 3372·5)	1·0% (0·4 to 1·6)	6·46 (5·99 to 6·93)	6·10 (5·80 to 6·40)	2·05 (1·83 to 2·33)	429·8 (400·1 to 459·1)	724·1 (690·3 to 757·1)	618·1 (546·5 to 704·2)	1·0 (0·8 to 1·1)
Pakistan		224 062·8 (207 077·3 to 241 657·4)	129 252·3 (119 454·1 to 139 401·8)	30 526·5 (28 212·4 to 32 923·6)	2·3% (1·9 to 2·7)	7·27 (6·88 to 7·64)	6·99 (6·75 to 7·20)	3·72 (3·31 to 4·20)	1670·3 (1589·4 to 1748·8)	3899·7 (3770·3 to 4020·5)	6686·0 (5942·0 to 7548·1)	1·6 (1·4 to 1·8)
**Southeast Asia, east Asia, and Oceania**		**2 159 262·0 (1 973 725·4 to 2 334 066·8)**	**1 521 841·5 (1 389 093·2 to 1 646 668·4)**	**140 474·2 (129 639·5 to 150 870·5)**	**0·6% (0·1 to 1·0)**	**6·04 (5·87 to 6·21)**	**3·42 (3·36 to 3·47)**	**1·67 (1·59 to 1·75)**	**33 486·0 (32 623·7 to 34 352·1)**	**37 910·4 (37 354·7 to 38 445·7)**	**26 800·3 (25 656·2 to 28 075·6)**	**0·8 (0·7 to 0·8)**
East Asia		1 472 203·5 (1 291 741·2 to 1 646 505·6)	1 054 890·1 (925 570·0 to 1 179 794·5)	84 138·8 (73 763·3 to 94 132·0)	0·4% (−0·3 to 1·0)	5·92 (5·82 to 6·02)	3·08 (3·02 to 3·14)	1·43 (1·39 to 1·48)	24 649·5 (24 277·1 to 25 036·8)	24 963·0 (24 514·5 to 25 393·0)	15 448·0 (14 999·4 to 15 903·6)	0·7 (0·6 to 0·7)
	China	1 422 350·4 (1 239 302·4 to 1 597 063·5)	1 019 223·9 (888 059·7 to 1 144 416·1)	81 490·9 (70 988·9 to 91 525·7)	0·4% (−0·3 to 1·0)	5·91 (5·82 to 6·00)	3·05 (2·99 to 3·11)	1·43 (1·39 to 1·48)	23 813·1 (23 464·2 to 24 175·5)	23 907·6 (23 459·9 to 24 329·2)	14 921·8 (14 467·4 to 15 355·4)	0·7 (0·6 to 0·7)
	North Korea	26 232·9 (22 628·9 to 29 910·5)	18 690·0 (16 122·3 to 21 310·2)	1665·4 (1436·6 to 1898·8)	0·3% (−0·4 to 0·9)	6·30 (5·97 to 6·65)	5·31 (4·95 to 5·69)	1·74 (1·51 to 1·99)	488·5 (464·2 to 513·1)	645·7 (599·7 to 695·1)	350·7 (304·4 to 400·0)	0·8 (0·7 to 0·9)
	Taiwan (province of China)	23 620·2 (21 658·6 to 25 443·2)	16 976·2 (15 566·3 to 18 286·4)	982·6 (901·0 to 1058·4)	0·2% (−0·7 to 0·9)	6·47 (6·44 to 6·49)	2·44 (2·43 to 2·44)	1·06 (0·94 to 1·20)	348·0 (346·8 to 349·1)	409·6 (408·2 to 411·2)	175·5 (154·7 to 199·4)	0·5 (0·4 to 0·6)
Oceania		13 276·4 (11 989·8 to 14 430·4)	7996·0 (7228·5 to 8685·5)	1850·7 (1664·4 to 2018·9)	2·4% (1·9 to 2·7)	6·49 (6·24 to 6·74)	5·05 (4·90 to 5·19)	4·01 (3·66 to 4·39)	117·9 (113·4 to 122·2)	181·6 (176·5 to 186·6)	415·8 (380·9 to 452·6)	1·8 (1·6 to 1·9)
	American Samoa	55·5 (48·4 to 62·8)	35·3 (30·7 to 39·9)	5·2 (4·5 to 5·9)	−0·3% (−1·1 to 0·3)	6·15 (5·90 to 6·42)	4·35 (4·09 to 4·61)	2·67 (2·31 to 3·08)	0·8 (0·8 to 0·9)	1·1 (1·1 to 1·2)	1·1 (0·9 to 1·2)	1·3 (1·1 to 1·4)
	Cook Islands	18·0 (16·6 to 19·6)	11·5 (10·6 to 12·5)	1·3 (1·2 to 1·5)	−0·4% (−0·5 to −0·3)	6·43 (5·98 to 6·87)	3·95 (3·58 to 4·33)	2·23 (1·97 to 2·56)	0·7 (0·6 to 0·7)	0·4 (0·4 to 0·5)	0·3 (0·2 to 0·3)	1·1 (0·9 to 1·2)
	Federated States of Micronesia	102·1 (89·8 to 114·4)	66·4 (58·4 to 74·4)	9·8 (8·6 to 10·9)	−0·4% (−1·0 to 0·1)	8·04 (7·92 to 8·15)	6·80 (6·57 to 6·99)	2·53 (2·16 to 2·99)	1·8 (1·8 to 1·9)	3·5 (3·4 to 3·6)	1·9 (1·7 to 2·3)	1·2 (1·0 to 1·4)
	Fiji	911·2 (838·8 to 984·2)	592·3 (545·2 to 639·7)	88·7 (81·7 to 95·8)	0·5% (0·4 to 0·6)	5·70 (5·38 to 6·04)	3·28 (3·08 to 3·48)	2·53 (2·19 to 2·91)	11·5 (10·9 to 12·3)	18·6 (17·4 to 19·8)	17·5 (15·2 to 20·2)	1·2 (1·0 to 1·3)
	Guam	170·6 (149·1 to 191·5)	108·9 (95·2 to 122·3)	15·9 (13·9 to 17·8)	0·4% (−0·3 to 1·0)	5·40 (5·19 to 5·63)	3·06 (3·00 to 3·13)	2·89 (2·63 to 3·16)	1·8 (1·7 to 1·8)	3·0 (2·9 to 3·0)	3·3 (3·0 to 3·6)	1·4 (1·2 to 1·5)
	Kiribati	118·6 (107·4 to 128·9)	72·6 (65·7 to 78·9)	14·6 (13·2 to 15·9)	1·3% (1·0 to 1·5)	6·87 (6·68 to 7·04)	4·85 (4·56 to 5·18)	3·09 (2·69 to 3·54)	1·5 (1·4 to 1·5)	2·3 (2·2 to 2·4)	3·0 (2·6 to 3·4)	1·4 (1·2 to 1·6)
	Marshall Islands	56·8 (49·8 to 63·4)	36·2 (31·7 to 40·4)	6·1 (5·3 to 6·8)	0·4% (−0·2 to 0·8)	6·94 (6·54 to 7·34)	5·67 (5·35 to 5·96)	2·74 (2·41 to 3·09)	0·6 (0·5 to 0·6)	1·4 (1·3 to 1·5)	1·2 (1·1 to 1·4)	1·3 (1·1 to 1·4)
	Nauru	10·6 (9·3 to 11·8)	6·5 (5·7 to 7·3)	1·4 (1·2 to 1·6)	0·1% (−0·4 to 0·7)	7·26 (6·92 to 7·60)	6·30 (5·94 to 6·66)	3·43 (2·98 to 3·91)	0·1 (0·1 to 0·1)	0·4 (0·4 to 0·4)	0·3 (0·3 to 0·3)	1·6 (1·4 to 1·8)
	Niue	1·7 (1·5 to 1·9)	1·1 (1·0 to 1·2)	0·1 (0·1 to 0·1)	0·4% (−0·1 to 0·9)	6·87 (6·49 to 7·24)	4·64 (4·17 to 5·13)	2·39 (2·01 to 2·86)	0·2 (0·2 to 0·2)	0·1 (0·1 to 0·1)	0·0 (0·0 to 0·0)	1·1 (0·9 to 1·3)
	Northern Mariana Islands	42·5 (37·2 to 47·9)	31·6 (27·7 to 35·6)	2·3 (2·0 to 2·6)	−2·8% (−3·4 to −2·2)	5·62 (5·13 to 6·12)	3·27 (2·97 to 3·61)	2·01 (1·72 to 2·34)	0·2 (0·2 to 0·2)	0·4 (0·4 to 0·5)	0·5 (0·4 to 0·6)	0·9 (0·8 to 1·1)
	Palau	18·0 (16·3 to 19·6)	13·2 (11·9 to 14·3)	1·0 (0·9 to 1·1)	−0·3% (−0·5 to −0·2)	5·95 (5·47 to 6·44)	3·44 (3·10 to 3·86)	1·93 (1·70 to 2·21)	0·3 (0·3 to 0·3)	0·3 (0·3 to 0·4)	0·2 (0·2 to 0·2)	0·9 (0·8 to 1·0)
	Papua New Guinea	9866·6 (8688·2 to 10 952·4)	5898·3 (5193·8 to 6547·4)	1448·8 (1275·8 to 1608·3)	2·9% (2·4 to 3·3)	6·61 (6·31 to 6·89)	5·45 (5·27 to 5·62)	4·27 (3·89 to 4·68)	80·0 (76·7 to 83·1)	117·9 (114·1 to 121·4)	331·9 (304·5 to 361·6)	1·9 (1·7 to 2·1)
	Samoa	211·4 (193·0 to 229·3)	127·0 (115·9 to 137·7)	21·0 (19·2 to 22·8)	1·5% (1·3 to 1·6)	5·76 (5·49 to 6·00)	3·87 (3·61 to 4·15)	2·38 (2·03 to 2·78)	3·5 (3·4 to 3·7)	3·8 (3·5 to 4·0)	3·5 (3·0 to 4·1)	1·1 (0·9 to 1·3)
	Solomon Islands	655·6 (570·7 to 742·6)	378·7 (329·6 to 428·9)	96·0 (83·6 to 108·8)	1·8% (1·2 to 2·5)	7·31 (7·03 to 7·57)	6·80 (6·56 to 7·04)	4·22 (3·83 to 4·66)	5·4 (5·2 to 5·5)	10·2 (9·8 to 10·6)	21·2 (19·2 to 23·3)	1·9 (1·7 to 2·1)
	Tokelau	1·4 (1·3 to 1·5)	0·8 (0·7 to 0·9)	0·2 (0·2 to 0·2)	1·4% (1·2 to 1·5)	7·82 (7·51 to 8·11)	6·81 (6·38 to 7·21)	4·13 (3·64 to 4·72)	0·1 (0·1 to 0·1)	0·1 (0·1 to 0·1)	0·0 (0·0 to 0·0)	1·9 (1·7 to 2·2)
	Tonga	102·4 (93·6 to 111·2)	59·8 (54·7 to 65·0)	12·1 (11·1 to 13·2)	−0·4% (−0·6 to −0·3)	5·63 (5·34 to 5·92)	4·48 (4·27 to 4·69)	3·17 (2·77 to 3·61)	1·8 (1·7 to 1·9)	2·8 (2·6 to 2·9)	2·3 (2·0 to 2·6)	1·5 (1·3 to 1·7)
	Tuvalu	11·8 (10·5 to 13·2)	7·5 (6·7 to 8·5)	1·1 (1·0 to 1·2)	1·1% (0·7 to 1·5)	6·12 (5·69 to 6·55)	4·75 (4·41 to 5·14)	2·51 (2·23 to 2·85)	0·2 (0·2 to 0·2)	0·3 (0·3 to 0·4)	0·2 (0·2 to 0·2)	1·2 (1·0 to 1·3)
	Vanuatu	294·6 (268·0 to 320·6)	170·6 (155·2 to 185·7)	37·6 (34·2 to 41·0)	2·0% (1·8 to 2·1)	6·53 (6·11 to 6·96)	5·65 (5·30 to 5·99)	3·22 (2·81 to 3·71)	2·0 (1·8 to 2·1)	4·7 (4·4 to 5·0)	7·6 (6·7 to 8·8)	1·5 (1·3 to 1·7)
Southeast Asia		673 782·0 (635 730·9 to 715 879·5)	458 955·4 (432 545·6 to 487 783·0)	54 484·7 (51 527·2 to 57 498·7)	1·0% (0·7 to 1·2)	6·41 (6·02 to 6·79)	4·30 (4·18 to 4·41)	2·04 (1·86 to 2·25)	8718·7 (8229·6 to 9194·4)	12 765·8 (12 465·8 to 13 058·8)	10 936·5 (9991·6 to 12 064·5)	1·0 (0·9 to 1·1)
	Cambodia	16 603·1 (14 206·2 to 18 867·7)	10 682·7 (9140·4 to 12 139·7)	1767·5 (1512·4 to 2008·6)	1·5% (0·7 to 2·0)	6·62 (6·19 to 7·05)	6·29 (6·09 to 6·50)	2·59 (2·34 to 2·90)	218·7 (204·6 to 232·6)	375·7 (365·2 to 386·1)	369·2 (333·7 to 413·0)	1·2 (1·1 to 1·3)
	Indonesia	259 465·8 (226 843·4 to 291 997·9)	178 967·5 (156 466·1 to 201 406·6)	19 706·4 (17 228·7 to 22 177·2)	0·8% (0·1 to 1·4)	6·36 (5·96 to 6·74)	4·30 (4·19 to 4·41)	1·86 (1·68 to 2·08)	3701·3 (3507·3 to 3887·8)	5211·1 (5087·6 to 5330·1)	3837·8 (3457·9 to 4287·3)	0·9 (0·8 to 1·0)
	Laos	7158·2 (6469·6 to 7826·1)	4602·3 (4159·5 to 5031·7)	801·5 (724·4 to 876·3)	1·3% (1·3 to 1·4)	6·60 (6·15 to 7·04)	5·91 (5·67 to 6·14)	2·76 (2·56 to 2·98)	83·8 (78·3 to 89·2)	144·0 (139·2 to 148·8)	173·7 (160·5 to 187·8)	1·3 (1·2 to 1·4)
	Malaysia	31 301·4 (27 339·3 to 35 190·5)	21 462·0 (18 745·3 to 24 128·6)	2567·8 (2242·7 to 2886·8)	1·2% (0·4 to 1·7)	5·94 (5·45 to 6·44)	3·58 (3·56 to 3·59)	2·08 (1·87 to 2·30)	265·7 (245·0 to 286·5)	379·9 (378·3 to 381·5)	538·7 (485·7 to 595·6)	1·0 (0·9 to 1·1)
	Maldives	498·4 (448·5 to 546·4)	368·7 (331·8 to 404·2)	41·2 (37·1 to 45·1)	3·7% (3·6 to 3·7)	5·12 (4·78 to 5·45)	6·47 (6·29 to 6·64)	2·32 (2·02 to 2·69)	2·8 (2·6 to 2·9)	6·8 (6·6 to 7·0)	8·5 (7·4 to 9·9)	1·1 (1·0 to 1·3)
	Mauritius	1276·7 (1113·7 to 1442·9)	910·7 (794·4 to 1029·3)	64·4 (56·1 to 72·7)	0·1% (−0·6 to 0·6)	6·36 (6·18 to 6·57)	2·66 (2·63 to 2·69)	1·38 (1·20 to 1·57)	23·4 (22·7 to 24·2)	23·8 (23·6 to 24·0)	12·8 (11·2 to 14·6)	0·7 (0·6 to 0·8)
	Myanmar	54 676·9 (48 907·8 to 60 236·3)	36 169·5 (32 353·2 to 39 847·2)	5018·7 (4489·1 to 5529·0)	0·8% (0·7 to 0·9)	6·51 (6·08 to 6·94)	5·25 (4·88 to 5·59)	2·35 (2·07 to 2·66)	903·7 (844·4 to 963·1)	1304·9 (1218·1 to 1388·8)	1054·9 (930·5 to 1193·8)	1·1 (1·0 to 1·2)
	Philippines	112 142·8 (101 580·9 to 121 868·8)	70 858·0 (64 184·4 to 77 003·5)	12 654·3 (11 462·5 to 13 751·8)	1·6% (1·3 to 1·9)	6·88 (6·50 to 7·26)	5·16 (4·98 to 5·33)	2·98 (2·67 to 3·31)	983·1 (930·0 to 1035·6)	1943·3 (1876·6 to 2011·5)	2666·1 (2390·3 to 2962·9)	1·4 (1·2 to 1·5)
	Seychelles	102·1 (89·5 to 114·5)	72·0 (63·1 to 80·8)	7·5 (6·5 to 8·4)	1·0% (0·3 to 1·6)	4·21 (3·89 to 4·57)	3·67 (3·53 to 3·81)	2·15 (1·89 to 2·45)	1·1 (1·0 to 1·2)	1·7 (1·6 to 1·8)	1·5 (1·3 to 1·7)	1·0 (0·9 to 1·2)
	Sri Lanka	21 854·5 (19 445·1 to 24 140·0)	14 511·9 (12 912·0 to 16 029·6)	1545·6 (1375·2 to 1707·3)	0·7% (0·3 to 1·0)	5·20 (4·97 to 5·46)	3·28 (3·11 to 3·45)	1·83 (1·55 to 2·16)	306·4 (292·9 to 321·8)	410·7 (388·4 to 432·3)	295·6 (249·8 to 349·0)	0·9 (0·7 to 1·0)
	Thailand	70 111·6 (61 329·0 to 78 909·5)	50 703·4 (44 352·0 to 57 065·9)	3125·3 (2733·8 to 3517·4)	0·4% (−0·3 to 1·0)	6·78 (6·38 to 7·17)	3·25 (3·10 to 3·39)	1·20 (1·02 to 1·41)	984·2 (925·5 to 1042·1)	1231·1 (1166·1 to 1297·0)	584·0 (493·0 to 691·2)	0·6 (0·5 to 0·7)
	Timor-Leste	1334·8 (1208·8 to 1447·4)	759·6 (687·9 to 823·6)	175·9 (159·3 to 190·7)	2·1% (1·7 to 2·3)	7·19 (6·92 to 7·48)	6·52 (6·32 to 6·70)	4·06 (3·60 to 4·54)	23·1 (22·1 to 24·1)	28·1 (27·2 to 29·0)	38·9 (34·5 to 43·7)	1·9 (1·7 to 2·1)
	Vietnam	96 372·9 (83 066·4 to 109 007·0)	68 286·0 (58 857·5 to 77 238·0)	6937·4 (5979·6 to 7846·9)	0·8% (0·0 to 1·4)	6·19 (5·77 to 6·63)	3·92 (3·75 to 4·11)	1·68 (1·51 to 1·88)	1208·8 (1128·0 to 1291·1)	1687·1 (1620·1 to 1754·1)	1340·4 (1213·5 to 1498·6)	0·8 (0·7 to 0·9)
**Sub-Saharan Africa**		**1 078 209·3 (1 036 018·4 to 1 119 304·1)**	**590 477·6 (567 469·7 to 612 781·8)**	**165 667·0 (159 058·5 to 171 945·1)**	**2·6% (2·5 to 2·7)**	**7·03 (6·67 to 7·38)**	**6·79 (6·58 to 6·99)**	**4·39 (4·12 to 4·71)**	**8934·2 (8474·2 to 9368·6)**	**17 740·7 (17 216·5 to 18 236·3)**	**36 726·9 (34 252·4 to 39 559·0)**	**1·9 (1·8 to 2·1)**
Central sub-Saharan Africa		131 544·6 (105 155·5 to 156 302·3)	71 070·7 (56 705·7 to 84 558·2)	20 700·4 (16 610·1 to 24 537·3)	2·8% (2·3 to 3·2)	7·37 (7·10 to 7·61)	7·32 (7·07 to 7·54)	4·59 (4·28 to 4·90)	1016·1 (979·1 to 1050·3)	2117·9 (2045·9 to 2183·3)	4464·2 (4147·8 to 4782·4)	2·1 (1·9 to 2·2)
	Angola	30 138·5 (27 054·8 to 33 116·6)	15 450·0 (13 869·2 to 16 976·7)	5147·2 (4620·5 to 5655·8)	3·6% (3·5 to 3·6)	7·16 (6·89 to 7·41)	7·43 (7·19 to 7·65)	4·97 (4·50 to 5·43)	247·6 (238·9 to 256·9)	380·4 (367·3 to 393·1)	1104·4 (999·0 to 1214·1)	2·2 (2·0 to 2·4)
	Central African Republic	5299·9 (4459·3 to 6192·6)	2942·2 (2475·6 to 3437·8)	841·4 (708·0 to 983·2)	1·5% (0·8 to 2·1)	6·17 (5·80 to 6·53)	6·47 (6·09 to 6·82)	4·62 (4·24 to 5·03)	63·0 (59·4 to 66·4)	110·6 (104·5 to 116·6)	197·5 (182·0 to 213·0)	1·9 (1·8 to 2·0)
	Congo (Brazzaville)	5265·8 (4507·1 to 6008·8)	3107·9 (2660·1 to 3546·4)	695·3 (595·1 to 793·4)	2·5% (1·7 to 3·1)	7·15 (6·74 to 7·52)	6·77 (6·53 to 7·00)	3·47 (3·19 to 3·77)	43·5 (41·0 to 45·8)	83·4 (80·5 to 86·3)	145·1 (133·1 to 157·6)	1·6 (1·5 to 1·7)
	Democratic Republic of the Congo	87 670·4 (61 748·6 to 112 590·4)	47 655·3 (33 564·9 to 61 201·1)	13 629·5 (9599·6 to 17 503·6)	2·7% (1·8 to 3·3)	7·69 (7·36 to 7·99)	7·41 (7·14 to 7·66)	4·60 (4·20 to 5·00)	633·0 (604·7 to 659·0)	1494·5 (1437·7 to 1546·8)	2935·8 (2666·3 to 3206·1)	2·1 (1·9 to 2·3)
	Equatorial Guinea	1419·8 (1290·6 to 1552·9)	822·1 (747·3 to 899·1)	185·0 (168·2 to 202·4)	3·4% (3·3 to 3·5)	7·98 (7·68 to 8·26)	7·81 (7·55 to 8·05)	3·49 (3·04 to 4·00)	10·9 (10·5 to 11·2)	16·3 (15·7 to 16·8)	38·5 (33·5 to 44·2)	1·6 (1·4 to 1·8)
	Gabon	1750·0 (1566·3 to 1939·2)	1093·3 (978·5 to 1211·5)	202·0 (180·8 to 223·9)	1·7% (1·4 to 1·9)	6·39 (5·90 to 6·87)	6·51 (6·18 to 6·83)	2·82 (2·42 to 3·29)	18·2 (16·8 to 19·5)	32·6 (30·9 to 34·2)	42·9 (36·9 to 50·1)	1·3 (1·1 to 1·5)
Eastern sub-Saharan Africa		411 777·3 (390 580·5 to 431 677·4)	223 851·0 (212 444·8 to 234 781·9)	64 138·4 (60 907·4 to 67 174·0)	2·6% (2·4 to 2·8)	7·20 (6·84 to 7·54)	7·08 (6·88 to 7·25)	4·41 (4·11 to 4·76)	3417·6 (3250·6 to 3575·8)	7085·4 (6900·8 to 7258·6)	14 162·8 (13 111·2 to 15 323·9)	2·0 (1·9 to 2·1)
	Burundi	11 934·4 (10 304·6 to 13 532·1)	6235·2 (5383·7 to 7069·9)	2079·0 (1795·1 to 2357·3)	3·2% (2·4 to 3·7)	7·58 (7·30 to 7·85)	7·23 (7·07 to 7·38)	5·63 (5·32 to 5·97)	130·9 (125·5 to 136·0)	223·3 (217·6 to 228·5)	466·2 (436·5 to 499·1)	2·5 (2·4 to 2·6)
	Comoros	714·4 (593·3 to 837·2)	443·1 (368·0 to 519·3)	78·1 (64·9 to 91·5)	1·2% (0·4 to 1·7)	4·73 (4·27 to 5·19)	7·20 (6·94 to 7·42)	3·02 (2·57 to 3·51)	5·7 (5·1 to 6·3)	16·9 (16·3 to 17·5)	16·8 (14·4 to 19·5)	1·4 (1·2 to 1·6)
	Djibouti	1202·8 (1050·5 to 1362·7)	744·6 (650·3 to 843·6)	159·2 (139·0 to 180·3)	2·9% (2·3 to 3·5)	7·48 (7·12 to 7·84)	7·39 (7·10 to 7·65)	3·71 (3·20 to 4·27)	3·3 (3·1 to 3·4)	14·8 (14·2 to 15·3)	35·5 (30·8 to 40·6)	1·7 (1·4 to 1·9)
	Eritrea	6711·2 (4780·6 to 8595·7)	3910·1 (2785·3 to 5008·1)	929·8 (662·4 to 1190·9)	1·9% (1·3 to 2·3)	7·29 (6·92 to 7·63)	6·91 (6·60 to 7·19)	3·90 (3·42 to 4·45)	57·5 (54·4 to 60·4)	122·4 (116·5 to 128·0)	199·2 (174·0 to 228·0)	1·8 (1·6 to 2·0)
	Ethiopia	107 591·2 (92 024·3 to 122 776·2)	58 479·1 (50 018·0 to 66 732·6)	16 679·7 (14 266·4 to 19 033·8)	2·5% (1·8 to 3·1)	7·04 (6·63 to 7·44)	7·04 (6·76 to 7·30)	4·47 (4·14 to 4·84)	911·5 (857·1 to 962·1)	1811·1 (1736·6 to 1880·3)	3674·6 (3394·8 to 4002·6)	2·0 (1·9 to 2·2)
	Kenya	50 227·7 (43 651·1 to 56 751·2)	29 558·9 (25 688·6 to 33 398·0)	6416·0 (5575·9 to 7249·4)	2·3% (1·7 to 2·9)	7·97 (7·68 to 8·23)	7·11 (6·90 to 7·31)	3·12 (2·70 to 3·63)	319·2 (308·1 to 329·4)	813·5 (790·2 to 835·1)	1342·7 (1166·7 to 1554·7)	1·4 (1·2 to 1·7)
	Madagascar	26 690·3 (20 373·8 to 32 844·3)	15 098·8 (11 525·5 to 18 580·1)	3939·2 (3006·9 to 4847·4)	2·6% (1·7 to 3·0)	6·70 (6·44 to 6·98)	7·05 (6·82 to 7·26)	3·99 (3·49 to 4·54)	219·9 (211·1 to 229·5)	442·3 (428·8 to 454·6)	861·8 (756·9 to 973·3)	1·8 (1·6 to 2·0)
	Malawi	18 442·2 (17 149·8 to 19 745·2)	10 021·0 (9318·8 to 10 729·0)	2586·0 (2404·8 to 2768·7)	2·8% (2·7 to 3·0)	6·90 (6·46 to 7·30)	6·91 (6·69 to 7·11)	3·65 (3·33 to 4·02)	152·7 (143·3 to 161·5)	307·2 (297·4 to 316·2)	545·1 (498·5 to 600·7)	1·7 (1·5 to 1·8)
	Mozambique	29 528·0 (27 057·9 to 31 808·3)	14 994·9 (13 740·5 to 16 152·9)	5105·0 (4678·0 to 5499·3)	2·8% (2·7 to 2·9)	6·75 (6·28 to 7·19)	6·76 (6·51 to 7·00)	4·89 (4·53 to 5·28)	327·5 (305·5 to 348·2)	600·2 (580·5 to 619·5)	1124·7 (1045·3 to 1206·2)	2·1 (2·0 to 2·3)
	Rwanda	12 688·1 (11 344·3 to 14 076·2)	7422·6 (6636·4 to 8234·6)	1650·4 (1475·6 to 1831·0)	2·3% (1·9 to 2·6)	7·84 (7·61 to 8·05)	7·33 (7·23 to 7·43)	3·53 (3·24 to 3·86)	147·7 (143·1 to 152·1)	259·5 (255·3 to 263·5)	352·3 (321·2 to 387·9)	1·6 (1·5 to 1·8)
	Somalia	20 343·1 (15 201·4 to 25 703·6)	10 403·7 (7774·2 to 13 145·1)	3732·0 (2788·8 to 4715·4)	3·6% (3·1 to 4·1)	7·59 (7·28 to 7·88)	7·55 (7·27 to 7·81)	6·36 (5·92 to 6·83)	105·2 (100·3 to 109·7)	316·0 (303·4 to 327·7)	871·2 (805·6 to 941·2)	2·7 (2·5 to 2·8)
	South Sudan	9283·0 (8050·8 to 10 613·5)	4861·4 (4216·2 to 5558·2)	1516·4 (1315·1 to 1733·7)	−0·1% (−0·7 to 0·6)	5·89 (5·38 to 6·40)	6·17 (5·83 to 6·51)	5·51 (5·13 to 5·92)	105·3 (96·3 to 114·0)	203·7 (192·7 to 215·4)	364·1 (339·4 to 393·2)	2·4 (2·3 to 2·5)
	Tanzania	56 736·1 (50 495·5 to 63 230·4)	30 373·8 (27 032·9 to 33 850·6)	9307·3 (8283·5 to 10 372·6)	2·6% (2·2 to 3·0)	7·25 (6·86 to 7·62)	6·98 (6·72 to 7·21)	4·67 (4·36 to 5·05)	461·6 (437·3 to 484·0)	944·9 (907·7 to 979·5)	2087·5 (1926·2 to 2266·0)	2·1 (2·0 to 2·3)
	Uganda	41 117·9 (37 023·7 to 44 955·5)	21 067·6 (18 969·9 to 23 033·9)	7089·7 (6383·8 to 7751·5)	2·6% (2·5 to 2·6)	7·69 (7·34 to 7·99)	7·58 (7·41 to 7·74)	5·00 (4·73 to 5·29)	333·4 (319·8 to 345·8)	702·9 (688·1 to 717·0)	1593·7 (1505·2 to 1695·3)	2·3 (2·2 to 2·4)
	Zambia	18 237·7 (15 885·9 to 20 473·3)	10 057·1 (8760·2 to 11 289·9)	2819·4 (2455·8 to 3165·0)	3·2% (2·4 to 3·8)	7·81 (7·49 to 8·10)	7·34 (7·12 to 7·54)	4·12 (3·74 to 4·54)	135·0 (129·7 to 139·9)	302·7 (293·2 to 311·4)	615·9 (556·3 to 684·1)	1·9 (1·7 to 2·0)
Southern sub-Saharan Africa		78 574·8 (71 923·5 to 85 743·8)	50 599·6 (46 173·2 to 55 304·0)	8096·4 (7450·5 to 8792·0)	1·1% (0·8 to 1·5)	6·31 (5·82 to 6·79)	4·51 (4·21 to 4·81)	2·48 (2·23 to 2·77)	803·4 (742·0 to 863·0)	1427·7 (1330·4 to 1520·8)	1670·8 (1504·6 to 1864·3)	1·1 (1·0 to 1·2)
	Botswana	2338·7 (2084·7 to 2607·8)	1539·1 (1372·0 to 1716·2)	236·5 (210·8 to 263·7)	1·7% (1·3 to 2·2)	6·66 (6·22 to 7·09)	5·08 (4·71 to 5·45)	2·29 (2·00 to 2·64)	18·3 (17·1 to 19·6)	35·0 (32·3 to 37·6)	48·2 (42·0 to 55·5)	1·0 (0·9 to 1·2)
	eSwatini	1142·1 (1051·1 to 1230·3)	689·7 (634·7 to 742·9)	140·6 (129·4 to 151·5)	0·7% (0·6 to 0·8)	7·04 (6·67 to 7·38)	6·13 (5·86 to 6·40)	2·91 (2·55 to 3·35)	12·2 (11·6 to 12·7)	27·4 (26·2 to 28·6)	30·0 (26·4 to 34·1)	1·3 (1·1 to 1·5)
	Lesotho	2091·6 (1910·3 to 2273·3)	1334·8 (1219·2 to 1450·8)	218·4 (199·5 to 237·4)	0·7% (0·6 to 0·7)	5·67 (5·14 to 6·21)	4·99 (4·48 to 5·54)	2·56 (2·20 to 2·94)	26·0 (23·6 to 28·4)	52·3 (47·1 to 57·8)	46·9 (40·5 to 53·8)	1·1 (0·9 to 1·2)
	Namibia	2403·1 (2112·7 to 2685·1)	1456·4 (1280·4 to 1627·3)	296·8 (260·9 to 331·6)	1·4% (0·8 to 1·8)	7·13 (6·72 to 7·51)	5·57 (5·25 to 5·89)	3·08 (2·79 to 3·44)	21·9 (20·6 to 23·1)	39·7 (37·5 to 41·9)	62·8 (56·9 to 70·2)	1·4 (1·3 to 1·6)
	South Africa	55 588·4 (49 169·7 to 62 724·7)	37 046·7 (32 769·0 to 41 802·7)	5094·5 (4506·3 to 5748·5)	1·0% (0·5 to 1·6)	6·08 (5·56 to 6·59)	3·92 (3·56 to 4·27)	2·17 (1·92 to 2·47)	585·6 (536·8 to 633·5)	922·1 (833·5 to 1007·1)	1035·0 (917·7 to 1172·5)	1·0 (0·9 to 1·1)
	Zimbabwe	15 010·9 (13 317·2 to 16 650·7)	8532·8 (7570·0 to 9464·9)	2109·6 (1871·5 to 2340·0)	1·6% (1·1 to 1·9)	7·41 (7·04 to 7·76)	6·74 (6·57 to 6·91)	3·50 (3·24 to 3·82)	139·4 (132·4 to 145·8)	351·1 (343·5 to 359·0)	448·0 (413·1 to 489·5)	1·6 (1·5 to 1·7)
Western sub-Saharan Africa		456 312·7 (432 022·5 to 478 778·4)	244 956·4 (231 698·7 to 256 902·5)	72 731·9 (68 976·8 to 76 211·5)	2·9% (2·8 to 3·0)	6·98 (6·61 to 7·32)	7·06 (6·84 to 7·27)	4·71 (4·41 to 5·05)	3697·0 (3504·1 to 3879·6)	7109·7 (6883·8 to 7316·6)	16 429·1 (15 275·3 to 17 702·8)	2·0 (1·9 to 2·1)
	Benin	12 665·8 (11 316·9 to 13 983·2)	6556·4 (5858·2 to 7238·4)	2228·1 (1990·8 to 2459·8)	3·3% (3·1 to 3·5)	6·95 (6·51 to 7·36)	7·20 (6·94 to 7·44)	5·24 (4·90 to 5·61)	99·5 (93·3 to 105·3)	175·3 (169·6 to 180·7)	505·2 (474·8 to 541·2)	2·3 (2·2 to 2·4)
	Burkina Faso	22 691·8 (19 383·3 to 26 173·3)	11 654·5 (9955·2 to 13 442·6)	4045·0 (3455·2 to 4665·6)	3·2% (2·5 to 3·8)	6·32 (5·83 to 6·80)	7·14 (6·92 to 7·36)	5·56 (5·21 to 5·95)	185·5 (171·7 to 198·9)	348·6 (338·6 to 358·3)	937·3 (873·5 to 1007·7)	2·4 (2·2 to 2·5)
	Cameroon	29 101·9 (24 783·0 to 33 604·8)	16 244·6 (13 833·8 to 18 758·2)	4207·1 (3582·7 to 4858·1)	3·1% (2·5 to 3·7)	6·32 (5·83 to 6·80)	6·53 (6·31 to 6·74)	3·81 (3·36 to 4·33)	211·9 (195·8 to 227·3)	381·6 (369·7 to 392·9)	903·8 (797·3 to 1027·7)	1·7 (1·5 to 1·9)
	Cape Verde	563·6 (494·6 to 632·1)	372·3 (326·7 to 417·6)	53·2 (46·6 to 59·6)	1·0% (0·4 to 1·6)	5·27 (5·00 to 5·52)	4·96 (4·81 to 5·11)	2·30 (1·92 to 2·73)	7·1 (6·7 to 7·5)	10·4 (10·0 to 10·7)	10·8 (9·1 to 12·8)	1·1 (0·9 to 1·3)
	Chad	16 398·9 (14 327·4 to 18 680·1)	7686·2 (6715·3 to 8755·4)	3332·7 (2911·7 to 3796·3)	3·7% (3·0 to 4·4)	7·40 (7·00 to 7·77)	7·61 (7·39 to 7·83)	6·87 (6·58 to 7·19)	129·5 (122·8 to 135·6)	243·2 (237·0 to 248·9)	787·6 (753·5 to 823·7)	2·9 (2·8 to 3·0)
	Côte d'Ivoire	26 171·5 (23 573·1 to 28 873·0)	14 803·6 (13 333·8 to 16 331·6)	4012·0 (3613·7 to 4426·1)	2·1% (2·0 to 2·3)	6·56 (6·08 to 7·01)	6·80 (6·56 to 7·03)	4·35 (3·99 to 4·78)	122·4 (113·6 to 130·7)	397·7 (383·7 to 410·8)	892·3 (813·1 to 981·6)	1·9 (1·8 to 2·1)
	The Gambia	2245·9 (2028·0 to 2476·2)	1248·4 (1127·3 to 1376·5)	329·6 (297·6 to 363·4)	2·6% (2·5 to 2·8)	6·60 (6·14 to 7·04)	7·13 (6·78 to 7·46)	4·03 (3·54 to 4·59)	11·3 (10·5 to 12·1)	35·6 (33·7 to 37·3)	72·0 (63·0 to 82·3)	1·8 (1·6 to 2·1)
	Ghana	31 536·2 (27 445·3 to 35 185·8)	19 074·6 (16 600·2 to 21 282·0)	3970·6 (3455·6 to 4430·1)	2·4% (1·7 to 2·9)	5·87 (5·44 to 6·33)	6·84 (6·64 to 7·04)	3·16 (2·72 to 3·66)	219·3 (201·1 to 238·8)	560·8 (541·9 to 578·8)	854·9 (734·9 to 987·7)	1·4 (1·2 to 1·6)
	Guinea	12 643·1 (11 365·9 to 13 951·9)	6508·0 (5850·6 to 7181·7)	2143·4 (1926·9 to 2365·3)	2·6% (2·5 to 2·7)	7·19 (6·86 to 7·52)	6·97 (6·70 to 7·20)	4·73 (4·36 to 5·08)	129·7 (123·8 to 135·0)	251·9 (242·9 to 260·3)	477·3 (440·9 to 512·5)	2·0 (1·9 to 2·1)
	Guinea-Bissau	1901·2 (1666·3 to 2146·2)	1058·4 (927·7 to 1194·8)	287·4 (251·9 to 324·5)	2·2% (1·6 to 2·8)	7·24 (6·93 to 7·53)	6·23 (5·87 to 6·59)	4·02 (3·57 to 4·49)	31·7 (30·3 to 33·0)	37·7 (35·4 to 39·9)	62·8 (55·4 to 70·6)	1·8 (1·6 to 2·0)
	Liberia	4789·9 (4131·0 to 5420·9)	2783·4 (2400·5 to 3150·0)	633·3 (546·2 to 716·8)	1·8% (1·1 to 2·3)	6·59 (6·13 to 7·04)	6·81 (6·55 to 7·06)	3·49 (3·04 to 4·02)	42·9 (39·9 to 45·8)	98·3 (94·4 to 101·7)	135·5 (118·0 to 156·2)	1·5 (1·4 to 1·8)
	Mali	21 917·5 (19 126·1 to 24 868·4)	10 965·8 (9569·2 to 12 442·2)	4064·3 (3546·7 to 4611·6)	3·6% (3·0 to 4·1)	7·33 (6·94 to 7·69)	7·58 (7·39 to 7·75)	6·04 (5·70 to 6·41)	185·0 (175·0 to 194·1)	389·6 (379·9 to 398·1)	961·1 (902·4 to 1023·6)	2·5 (2·4 to 2·6)
	Mauritania	4014·3 (3561·9 to 4442·0)	2239·2 (1986·9 to 2477·8)	531·2 (471·4 to 587·8)	2·1% (1·8 to 2·3)	6·24 (5·76 to 6·71)	6·89 (6·62 to 7·12)	3·59 (3·26 to 3·99)	31·1 (28·7 to 33·5)	72·3 (69·6 to 74·7)	109·0 (98·1 to 122·0)	1·6 (1·5 to 1·8)
	Niger	23 295·4 (20 797·7 to 25 931·6)	10 738·1 (9586·8 to 11 953·4)	4852·9 (4332·5 to 5402·1)	3·8% (3·5 to 4·2)	7·33 (6·94 to 7·70)	7·98 (7·79 to 8·15)	7·44 (7·20 to 7·69)	140·4 (133·2 to 147·1)	349·7 (342·4 to 356·5)	1142·8 (1101·5 to 1186·0)	3·1 (3·1 to 3·2)
	Nigeria	214 823·8 (193 132·5 to 236 573·6)	115 220·2 (103 586·2 to 126 885·7)	33 521·6 (30 136·9 to 36 915·5)	2·8% (2·7 to 2·9)	7·32 (6·95 to 7·66)	7·12 (6·83 to 7·38)	4·68 (4·36 to 5·06)	1863·4 (1767·1 to 1951·9)	3199·5 (3055·2 to 3329·8)	7587·2 (7020·7 to 8238·2)	2·0 (1·9 to 2·1)
	São Tomé and Príncipe	205·4 (182·4 to 229·1)	123·7 (109·9 to 138·0)	23·3 (20·7 to 26·0)	1·8% (1·4 to 2·2)	6·26 (5·95 to 6·60)	6·36 (6·21 to 6·50)	2·87 (2·45 to 3·33)	2·4 (2·3 to 2·6)	3·8 (3·7 to 3·9)	4·7 (4·0 to 5·4)	1·4 (1·2 to 1·6)
	Senegal	15 134·1 (13 504·0 to 16 852·5)	8468·5 (7556·3 to 9430·0)	2150·6 (1919·0 to 2394·8)	2·1% (1·9 to 2·3)	6·93 (6·49 to 7·34)	7·21 (7·04 to 7·39)	4·11 (3·79 to 4·48)	130·6 (122·4 to 138·4)	294·4 (287·7 to 300·9)	466·5 (430·4 to 510·2)	1·9 (1·7 to 2·0)
	Sierra Leone	8284·8 (7526·3 to 9080·2)	4679·2 (4250·8 to 5128·4)	1229·7 (1117·1 to 1347·7)	2·9% (2·9 to 3·0)	5·63 (5·09 to 6·18)	5·84 (5·46 to 6·19)	4·07 (3·72 to 4·48)	79·4 (72·1 to 86·7)	130·7 (122·0 to 138·4)	280·7 (256·5 to 309·4)	1·7 (1·6 to 1·9)
	Togo	7921·5 (6943·0 to 8897·9)	4527·8 (3968·5 to 5085·9)	1114·9 (977·1 to 1252·3)	2·4% (1·7 to 2·9)	7·21 (6·84 to 7·55)	6·99 (6·80 to 7·16)	3·78 (3·47 to 4·12)	73·6 (69·7 to 77·2)	128·5 (125·2 to 131·6)	237·5 (219·7 to 258·9)	1·7 (1·6 to 1·8)

Data in parentheses are 95% uncertainty intervals. Super-regions, regions, and countries are listed in alphabetical order. GBD=Global Burden of Diseases, Injuries, and Risk Factors Study.

The distribution of livebirths among GBD regions has also changed considerably. In 1950, east Asia accounted for 24·6 million (95% UI 24·3–25·0) livebirths or more than a quarter (25·7%, 25·2–26·3) of all livebirths globally (table 1). The share of livebirths from east Asia has been in sharp decline since then: 22·1% (21·5–22·6) of global livebirths in 1970, 16·2% (15·8–16·5) in 1990, and 11·4% (10·7–12·1) in 2019. Since 1969, south Asia has been the region with the most annual livebirths, consistently accounting for around a quarter of global livebirths (24·7%, 23·5–25·8 in 2019). The share of livebirths from sub-Saharan Africa increased from 23·7% (23·3–24·1) in 1990 to 27·1% (26·4–27·8) in 2019.

**Figure 1:**
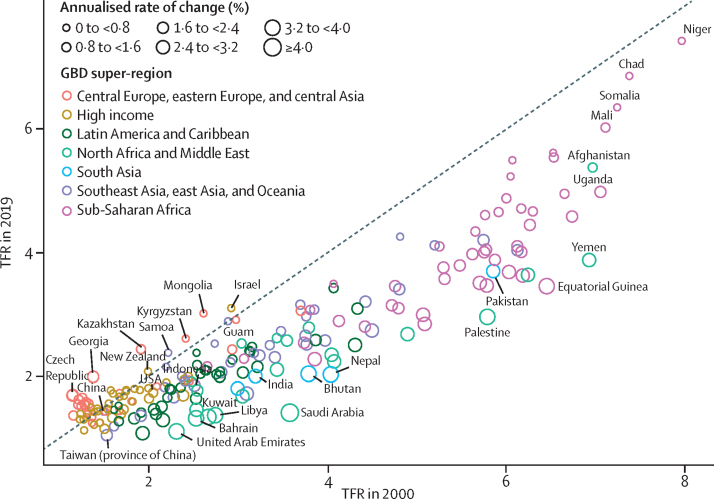
TFR by country or territory, 2000 and 2019 Each point represents the TFR for a country or territory in 2000 and 2019. The size of the point indicates the absolute annualised rate of change in total fertility rate between 2000 and 2019. Points above the diagonal line show countries or territories that have seen an increase in TFR between 2000 and 2019, whereas those below the diagonal had a decline in TFR between 2000 and 2019. GBD=Global Burden of Diseases, Injuries, and Risk Factors Study. TFR=total fertility rate.

179 (88%) of 204 countries and territories saw a decrease in TFR between 2010 and 2019 (figure 1). Saudi Arabia, Kuwait, Puerto Rico, Palestine, and Yemen experienced the fastest annualised decline at a rate of at least 3·6% per year. 57 countries had a more than 2·0% annual decline in TFR, including India, Pakistan, Bangladesh, and Zambia. Declines in TFR have accelerated in the majority of countries, with 128 (63%) experiencing a larger decline in 2010–19 compared with the preceding decade.

The result of these accelerated declines in fertility is that by 2019, half of all countries and territories (102 out of 204) had reached below-replacement-level TFR, with 43 of these having reached an ultra-low TFR of 1·5 or lower. China, for example, has experienced sustained decline in TFR since 1950 (table 1; appendix 2 table S8). After reaching a local minimum in 2010 of 1·23 (95% UI 1·19–1·27), China's TFR started to increase again, especially for females in the older maternal ages who already had one child. The peak TFR in this period of fertility increase was reached in 2016 at 1·65 (1·61–1·68). Since then, there has been a decrease of 13·0% (10·0–16·2) in TFR over a period of just 3 years. In the past four decades, countries such as Belgium, Czech Republic, Germany, Poland, and Slovakia reached the lowest TFR in their history and then subsequently experienced increases in TFR. These increases are difficult to interpret because TFR, as a period measurement of fertility level, is affected by both levels of and changes in age-specific fertility rates from different maternal birth cohorts in that year. This means TFR can fluctuate for a long period even if cohort completed fertility, a measurement of cumulative fertility by cohort, remains stable. In 2019, 102 countries had TFR over 2·1. This includes all countries in the sub-Saharan Africa super-region and at least one country in every other super-region, such as Papua New Guinea, Solomon Islands, Pakistan, Afghanistan, Yemen, Bolivia, Haiti, Israel, Tajikistan, and Mongolia.

### Mortality

The global number of deaths has steadily increased in the past seven decades, excluding spikes due to fatal discontinuities from causes such as famines in China in the early 1960s and from HIV/AIDS in the 1990s and 2000s. Global deaths increased from 43·6 million (95% UI 40·2–47·5) in 1950 to 50·7 million (49·5 −51·9) in 2000 and to 56·5 million (53·7–59·2) in 2019 (table 2).

**Table 2:** Under-5 mortality rate, rate of change in under-5 mortality (2010–19), probability of death between ages 15 and 60 years and life expectancy at birth (by sex), HALE, total number of deaths, and total number of deaths among children under 5 years, globally and for GBD regions, super-regions, countries, and territories, 2019

		**Under-5 mortality**		**Probability of death between ages 15 and 60 years, 2019**		**Life expectancy at birth in 2019 (years)**			**HALE in 2019 (years)**	**Total deaths in 2019 (thousands)**	**Total deaths among children younger than 5 years in 2019 (thousands)**
		Mortality rate in 2019 (deaths per 1000)	Annualised rate of change, 2010–19	Female	Male	Female	Male	Both sexes			
**Global**		**37·1 (33·2 to 41·7)**	**−4·0% (−5·1 to −2·9)**	**0·10 (0·09 to 0·11)**	**0·16 (0·15 to 0·17)**	**76·1 (75·4 to 76·9)**	**71·0 (70·1 to 71·9)**	**73·5 (72·8 to 74·3)**	**63·5 (60·8 to 66·1)**	**56 496·5 (53 714·0 to 59 172·5)**	**5045·4 (4273·0 to 6017·7)**
**Central Europe, eastern Europe, and central Asia**		**11·5 (10·4 to 12·8)**	**−3·8% (−4·8 to −2·8)**	**0·10 (0·09 to 0·11)**	**0·24 (0·22 to 0·26)**	**77·7 (76·8 to 78·6)**	**69·1 (67·9 to 70·3)**	**73·4 (72·5 to 74·3)**	**63·0 (60·2 to 65·3)**	**4741·2 (4436·2 to 5047·8)**	**61·1 (52·2 to 72·1)**
Central Asia		20·7 (18·3 to 23·7)	−4·4% (−5·6 to −3·1)	0·11 (0·10 to 0·13)	0·22 (0·20 to 0·24)	74·0 (73·0 to 75·0)	67·4 (66·2 to 68·6)	70·7 (69·6 to 71·8)	61·8 (59·4 to 64·2)	637·4 (587·4 to 696·5)	39·7 (33·2 to 48·4)
	Armenia	11·4 (9·6 to 13·7)	−4·2% (−5·9 to −2·3)	0·06 (0·05 to 0·08)	0·16 (0·14 to 0·19)	78·8 (77·5 to 80·2)	72·5 (70·8 to 74·3)	75·7 (74·2 to 77·3)	66·6 (63·9 to 69·1)	28·0 (24·4 to 32·0)	0·5 (0·4 to 0·6)
	Azerbaijan	27·6 (23·2 to 33·4)	−3·6% (−5·3 to −1·7)	0·09 (0·07 to 0·11)	0·18 (0·15 to 0·22)	73·7 (72·2 to 75·1)	68·3 (65·8 to 70·6)	71·0 (69·3 to 72·4)	63·0 (60·2 to 65·6)	75·1 (67·1 to 85·4)	4·3 (3·4 to 5·4)
	Georgia	10·3 (8·6 to 12·5)	−5·2% (−7·0 to −3·2)	0·08 (0·07 to 0·10)	0·24 (0·20 to 0·27)	78·1 (76·6 to 79·6)	68·8 (67·0 to 70·7)	73·4 (71·7 to 75·2)	64·4 (61·7 to 67·0)	49·4 (43·3 to 56·1)	0·5 (0·4 to 0·6)
	Kazakhstan	12·1 (10·1 to 14·7)	−6·4% (−8·2 to −4·5)	0·11 (0·09 to 0·12)	0·24 (0·22 to 0·27)	76·0 (74·8 to 77·2)	67·7 (66·2 to 69·2)	71·9 (70·5 to 73·3)	63·1 (60·3 to 65·8)	139·5 (124·9 to 155·3)	4·3 (3·4 to 5·5)
	Kyrgyzstan	17·4 (15·9 to 19·0)	−5·1% (−6·1 to −4·1)	0·09 (0·08 to 0·10)	0·20 (0·18 to 0·23)	76·9 (75·8 to 78·0)	69·8 (68·5 to 71·2)	73·4 (72·2 to 74·7)	64·9 (62·1 to 67·4)	34·7 (31·4 to 38·2)	2·5 (2·2 to 2·9)
	Mongolia	17·0 (14·5 to 20·4)	−6·3% (−8·1 to −4·6)	0·15 (0·12 to 0·20)	0·33 (0·27 to 0·41)	72·7 (69·8 to 75·3)	63·8 (60·5 to 66·9)	68·1 (64·9 to 71·0)	60·4 (57·0 to 63·7)	24·9 (20·0 to 31·1)	1·4 (1·2 to 1·8)
	Tajikistan	32·1 (27·5 to 37·3)	−3·0% (−4·6 to −1·3)	0·12 (0·09 to 0·15)	0·17 (0·14 to 0·21)	71·7 (69·8 to 73·5)	67·6 (65·5 to 69·5)	69·6 (67·6 to 71·4)	61·8 (59·0 to 64·5)	48·7 (41·3 to 57·9)	8·1 (6·5 to 10·0)
	Turkmenistan	25·2 (21·4 to 29·6)	−4·9% (−6·6 to −3·1)	0·13 (0·10 to 0·16)	0·24 (0·20 to 0·29)	74·4 (71·8 to 76·7)	67·4 (64·7 to 70·0)	70·8 (68·1 to 73·3)	62·7 (59·6 to 65·8)	33·6 (27·9 to 40·9)	2·9 (2·4 to 3·5)
	Uzbekistan	21·2 (17·8 to 25·7)	−4·3% (−6·1 to −2·4)	0·13 (0·11 to 0·16)	0·23 (0·19 to 0·27)	71·0 (69·5 to 72·4)	65·9 (64·2 to 67·6)	68·4 (66·9 to 70·0)	60·9 (58·3 to 63·2)	203·6 (175·7 to 235·4)	15·2 (12·4 to 19·2)
Central Europe		5·1 (4·5 to 5·6)	−4·2% (−5·4 to −3·0)	0·07 (0·06 to 0·08)	0·16 (0·14 to 0·18)	80·4 (79·2 to 81·5)	73·7 (72·2 to 75·2)	77·0 (75·7 to 78·3)	65·6 (62·6 to 68·1)	1372·2 (1227·5 to 1518·4)	5·5 (4·5 to 6·8)
	Albania	11·9 (9·9 to 14·4)	−3·5% (−5·3 to −1·5)	0·05 (0·03 to 0·06)	0·09 (0·07 to 0·12)	81·4 (79·3 to 83·5)	75·8 (73·2 to 78·5)	78·5 (76·1 to 81·0)	68·7 (65·3 to 71·9)	22·7 (17·9 to 28·3)	0·5 (0·3 to 0·6)
	Bosnia and Herzegovina	5·4 (4·6 to 6·4)	−2·7% (−4·7 to −0·7)	0·06 (0·05 to 0·08)	0·12 (0·10 to 0·16)	79·3 (77·4 to 81·2)	74·6 (72·1 to 76·9)	77·0 (74·7 to 79·1)	66·9 (63·5 to 70·0)	37·4 (30·7 to 45·3)	0·1 (0·1 to 0·2)
	Bulgaria	7·3 (6·2 to 8·6)	−4·7% (−6·5 to −2·9)	0·10 (0·08 to 0·12)	0·21 (0·17 to 0·26)	77·0 (74·9 to 79·0)	69·9 (67·3 to 72·5)	73·3 (70·9 to 75·7)	64·6 (61·3 to 67·5)	124·2 (103·5 to 148·3)	0·4 (0·4 to 0·6)
	Croatia	4·0 (3·3 to 4·7)	−3·1% (−5·2 to −1·1)	0·05 (0·04 to 0·06)	0·12 (0·09 to 0·15)	81·7 (79·9 to 83·5)	75·6 (73·2 to 77·9)	78·7 (76·5 to 80·8)	68·1 (64·8 to 71·4)	52·3 (43·2 to 63·0)	0·1 (0·1 to 0·2)
	Czech Republic	2·6 (2·3 to 3·1)	−3·0% (−4·8 to −1·2)	0·05 (0·04 to 0·06)	0·11 (0·09 to 0·13)	82·3 (80·7 to 83·9)	76·6 (74·5 to 78·6)	79·5 (77·6 to 81·3)	68·5 (65·0 to 71·6)	113·8 (96·6 to 133·9)	0·3 (0·2 to 0·4)
	Hungary	4·0 (3·5 to 4·6)	−4·2% (−5·8 to −2·5)	0·08 (0·06 to 0·10)	0·17 (0·14 to 0·20)	79·8 (78·0 to 81·6)	73·2 (71·1 to 75·3)	76·6 (74·6 to 78·6)	66·7 (63·5 to 69·7)	128·9 (109·9 to 150·9)	0·3 (0·3 to 0·4)
	Montenegro	3·7 (3·2 to 4·4)	−7·3% (−9·1 to −5·3)	0·08 (0·07 to 0·09)	0·14 (0·12 to 0·17)	78·7 (77·5 to 79·8)	73·1 (71·2 to 74·9)	75·9 (74·2 to 77·4)	66·4 (63·4 to 69·2)	6·8 (5·9 to 7·8)	0·0 (0·0 to 0·0)
	North Macedonia	8·5 (7·2 to 10·0)	−1·2% (−3·1 to 0·8)	0·08 (0·06 to 0·10)	0·14 (0·11 to 0·18)	76·4 (74·6 to 78·2)	72·5 (70·2 to 74·7)	74·4 (72·3 to 76·4)	65·4 (62·3 to 68·3)	24·0 (19·9 to 29·0)	0·2 (0·2 to 0·2)
	Poland	3·8 (3·3 to 4·5)	−4·6% (−6·4 to −2·8)	0·06 (0·05 to 0·08)	0·16 (0·12 to 0·20)	81·9 (80·0 to 83·9)	74·2 (71·6 to 76·8)	78·1 (76·3 to 79·9)	68·0 (64·7 to 71·0)	406·3 (350·6 to 465·4)	1·4 (1·1 to 1·9)
	Romania	8·0 (7·0 to 9·3)	−4·0% (−5·5 to −2·4)	0·08 (0·06 to 0·10)	0·19 (0·16 to 0·22)	79·2 (77·5 to 80·9)	72·0 (69·8 to 74·1)	75·5 (73·5 to 77·5)	66·3 (63·3 to 69·1)	262·8 (224·7 to 305·1)	1·4 (1·2 to 1·7)
	Serbia	4·1 (3·5 to 4·9)	−5·7% (−7·5 to −3·7)	0·08 (0·06 to 0·10)	0·14 (0·11 to 0·18)	77·9 (76·1 to 79·6)	73·5 (71·2 to 75·7)	75·7 (73·6 to 77·7)	66·3 (63·1 to 69·2)	117·6 (98·1 to 140·6)	0·3 (0·3 to 0·4)
	Slovakia	5·3 (4·5 to 6·3)	−2·7% (−4·5 to −0·8)	0·06 (0·05 to 0·08)	0·14 (0·11 to 0·18)	80·8 (78·8 to 82·7)	74·3 (71·7 to 76·8)	77·6 (75·2 to 79·9)	67·5 (64·0 to 70·7)	54·5 (44·7 to 66·2)	0·3 (0·2 to 0·4)
	Slovenia	2·0 (1·7 to 2·3)	−5·6% (−7·4 to −3·7)	0·04 (0·03 to 0·06)	0·10 (0·08 to 0·13)	84·6 (82·4 to 86·6)	78·2 (75·5 to 80·5)	81·4 (78·9 to 83·7)	70·1 (66·5 to 73·5)	20·8 (17·0 to 25·9)	0·0 (0·0 to 0·0)
Eastern Europe		6·9 (6·3 to 7·5)	−3·8% (−4·8 to −2·8)	0·11 (0·09 to 0·12)	0·29 (0·25 to 0·32)	77·6 (76·1 to 78·9)	67·3 (65·5 to 69·1)	72·5 (71·3 to 73·7)	62·0 (59·1 to 64·4)	2731·5 (2523·9 to 2972·0)	15·9 (13·3 to 18·6)
	Belarus	5·3 (4·4 to 6·5)	−4·9% (−6·9 to −3·0)	0·08 (0·06 to 0·11)	0·24 (0·19 to 0·30)	78·9 (76·4 to 81·1)	68·9 (65·8 to 71·8)	74·0 (71·0 to 76·7)	65·0 (61·5 to 68·2)	121·8 (99·7 to 150·5)	0·6 (0·4 to 0·7)
	Estonia	2·6 (2·3 to 3·1)	−6·3% (−8·2 to −4·3)	0·07 (0·05 to 0·08)	0·17 (0·13 to 0·21)	81·8 (79·8 to 83·7)	74·0 (71·3 to 76·7)	78·0 (75·6 to 80·5)	68·0 (64·6 to 71·1)	15·9 (13·1 to 19·1)	0·0 (0·0 to 0·0)
	Latvia	4·2 (3·6 to 4·9)	−6·9% (−8·5 to −5·0)	0·08 (0·06 to 0·11)	0·21 (0·17 to 0·26)	79·9 (77·3 to 82·2)	71·5 (68·8 to 74·2)	75·9 (73·8 to 77·7)	66·2 (63·1 to 69·1)	27·4 (24·0 to 31·9)	0·1 (0·1 to 0·1)
	Lithuania	4·0 (3·5 to 4·6)	−4·6% (−6·1 to −2·9)	0·08 (0·06 to 0·09)	0·21 (0·17 to 0·25)	80·7 (79·0 to 82·3)	71·5 (69·2 to 73·8)	76·2 (74·1 to 78·3)	66·5 (63·3 to 69·4)	38·5 (32·8 to 44·8)	0·1 (0·1 to 0·1)
	Moldova	12·2 (10·2 to 14·7)	−3·2% (−5·0 to −1·2)	0·09 (0·07 to 0·10)	0·22 (0·20 to 0·24)	78·2 (77·0 to 79·4)	70·2 (68·8 to 71·7)	74·3 (73·0 to 75·7)	65·0 (62·2 to 67·7)	41·0 (37·0 to 45·4)	0·4 (0·3 to 0·5)
	Russia	6·5 (5·7 to 7·4)	−4·2% (−5·6 to −2·8)	0·11 (0·09 to 0·13)	0·27 (0·23 to 0·32)	77·8 (75·9 to 79·6)	68·0 (65·6 to 70·4)	73·0 (71·3 to 74·5)	63·7 (60·5 to 66·2)	1788·3 (1603·2 to 1997·9)	11·2 (9·2 to 13·4)
	Ukraine	8·8 (7·8 to 9·7)	−1·9% (−3·1 to −0·7)	0·11 (0·09 to 0·14)	0·34 (0·29 to 0·40)	78·3 (76·5 to 80·1)	69·0 (66·6 to 71·4)	73·8 (72·2 to 75·4)	61·7 (58·7 to 64·5)	698·7 (615·5 to 796·9)	3·5 (2·9 to 4·3)
**High income**		**4·9 (4·8 to 5·1)**	**−2·1% (−2·5 to −1·7)**	**0·06 (0·05 to 0·06)**	**0·10 (0·10 to 0·10)**	**83·8 (83·8 to 83·9)**	**78·8 (78·7 to 78·9)**	**81·3 (81·2 to 81·4)**	**67·4 (64·2 to 70·4)**	**9951·5 (9886·3 to 10 019·8)**	**55·6 (49·7 to 62·6)**
Australasia		3·7 (3·5 to 3·9)	−3·7% (−4·3 to −3·0)	0·05 (0·04 to 0·05)	0·08 (0·08 to 0·08)	84·8 (84·6 to 84·9)	80·7 (80·5 to 80·9)	82·7 (82·5 to 82·9)	68·2 (64·7 to 71·2)	205·4 (202·2 to 208·6)	1·4 (1·2 to 1·6)
	Australia	3·6 (3·4 to 3·8)	−3·5% (−4·0 to −2·9)	0·04 (0·04 to 0·05)	0·08 (0·07 to 0·08)	85·0 (84·8 to 85·1)	80·8 (80·6 to 81·0)	82·9 (82·7 to 83·1)	70·0 (66·4 to 73·1)	170·9 (167·8 to 174·1)	1·1 (1·0 to 1·3)
	New Zealand	4·5 (4·2 to 5·0)	−4·2% (−5·3 to −3·1)	0·05 (0·05 to 0·05)	0·08 (0·08 to 0·08)	83·6 (83·4 to 83·8)	79·9 (79·6 to 80·1)	81·8 (81·6 to 82·0)	69·4 (65·9 to 72·4)	34·5 (34·0 to 35·0)	0·3 (0·2 to 0·3)
High-income Asia Pacific		2·6 (2·5 to 2·7)	−3·0% (−3·4 to −2·6)	0·03 (0·03 to 0·04)	0·07 (0·07 to 0·07)	87·3 (87·2 to 87·4)	81·5 (81·3 to 81·6)	84·4 (84·3 to 84·6)	71·2 (68·2 to 73·8)	1743·7 (1726·6 to 1761·1)	3·7 (3·4 to 4·0)
	Brunei	9·2 (7·7 to 10·9)	−0·2% (−2·4 to 2·0)	0·09 (0·08 to 0·11)	0·13 (0·11 to 0·15)	76·4 (75·4 to 77·4)	72·3 (70·9 to 73·7)	74·4 (73·2 to 75·4)	65·2 (62·5 to 67·7)	1·9 (1·7 to 2·1)	0·1 (0·0 to 0·1)
	Japan	2·4 (2·4 to 2·5)	−2·9% (−3·3 to −2·6)	0·03 (0·03 to 0·04)	0·07 (0·06 to 0·07)	87·7 (87·5 to 87·8)	81·9 (81·7 to 82·0)	84·8 (84·7 to 84·9)	73·3 (70·2 to 76·0)	1400·0 (1387·1 to 1412·9)	2·2 (2·1 to 2·4)
	Singapore	1·8 (1·6 to 2·1)	−3·5% (−5·1 to −1·8)	0·03 (0·03 to 0·03)	0·05 (0·05 to 0·05)	86·7 (86·5 to 86·9)	82·9 (82·7 to 83·2)	84·9 (84·6 to 85·1)	73·9 (70·9 to 76·4)	23·2 (22·7 to 23·8)	0·1 (0·1 to 0·1)
	South Korea	3·0 (2·8 to 3·3)	−3·3% (−4·2 to −2·4)	0·03 (0·03 to 0·04)	0·08 (0·08 to 0·08)	85·6 (85·4 to 85·9)	80·0 (79·7 to 80·4)	82·9 (82·6 to 83·3)	72·0 (68·9 to 74·6)	318·6 (307·8 to 330·1)	1·3 (1·1 to 1·5)
High-income North America		6·3 (6·2 to 6·5)	−1·4% (−1·6 to −1·1)	0·08 (0·08 to 0·08)	0·13 (0·13 to 0·13)	81·8 (81·7 to 81·9)	76·7 (76·6 to 76·9)	79·3 (79·2 to 79·3)	64·2 (60·8 to 67·4)	3235·1 (3212·2 to 3258·5)	26·6 (24·6 to 28·7)
	Canada	4·9 (4·7 to 5·1)	−1·8% (−2·3 to −1·2)	0·05 (0·05 to 0·05)	0·08 (0·08 to 0·08)	84·3 (84·1 to 84·4)	80·0 (79·9 to 80·2)	82·2 (82·0 to 82·3)	70·1 (66·9 to 73·0)	288·2 (284·8 to 291·8)	1·8 (1·6 to 2·0)
	Greenland	9·5 (6·9 to 12·9)	−2·9% (−6·7 to 0·9)	0·13 (0·10 to 0·15)	0·20 (0·16 to 0·25)	76·3 (73·9 to 78·4)	71·1 (68·6 to 73·7)	73·3 (70·9 to 75·8)	62·8 (59·4 to 66·1)	0·5 (0·4 to 0·6)	0·0 (0·0 to 0·0)
	USA	6·5 (6·3 to 6·6)	−1·3% (−1·6 to −1·1)	0·08 (0·08 to 0·08)	0·14 (0·13 to 0·14)	81·5 (81·4 to 81·6)	76·4 (76·2 to 76·5)	78·9 (78·8 to 79·0)	65·2 (61·6 to 68·5)	2946·5 (2924·3 to 2968·8)	24·7 (23·0 to 26·7)
Southern Latin America		9·6 (9·1 to 10·2)	−3·2% (−3·8 to −2·5)	0·07 (0·07 to 0·07)	0·13 (0·13 to 0·14)	80·4 (80·2 to 80·7)	74·6 (74·3 to 74·9)	77·6 (77·3 to 77·8)	66·0 (63·1 to 68·5)	495·8 (485·2 to 507·3)	9·4 (7·6 to 11·6)
	Argentina	10·6 (10·3 to 11·0)	−3·2% (−3·6 to −2·8)	0·08 (0·08 to 0·08)	0·14 (0·14 to 0·15)	79·6 (79·4 to 79·8)	73·5 (73·3 to 73·8)	76·6 (76·3 to 76·8)	66·7 (63·8 to 69·2)	348·8 (342·1 to 356·2)	7·4 (6·1 to 9·1)
	Chile	6·8 (5·9 to 8·0)	−3·1% (−4·8 to −1·3)	0·05 (0·05 to 0·06)	0·11 (0·10 to 0·11)	82·6 (82·2 to 82·9)	77·7 (77·3 to 78·1)	80·2 (79·8 to 80·5)	69·0 (65·8 to 71·7)	113·1 (110·1 to 116·4)	1·6 (1·2 to 2·0)
	Uruguay	8·3 (7·3 to 9·5)	−3·9% (−5·6 to −2·0)	0·07 (0·07 to 0·08)	0·14 (0·14 to 0·15)	80·8 (80·5 to 81·1)	74·1 (73·6 to 74·5)	77·5 (77·1 to 77·9)	67·3 (64·3 to 69·8)	33·8 (33·0 to 34·8)	0·4 (0·3 to 0·5)
Western Europe		3·4 (3·3 to 3·6)	−2·4% (−2·9 to −2·0)	0·04 (0·04 to 0·05)	0·08 (0·08 to 0·08)	84·4 (84·3 to 84·5)	79·8 (79·6 to 79·9)	82·1 (82·0 to 82·2)	68·5 (65·3 to 71·4)	4271·5 (4231·5 to 4313·0)	14·7 (12·9 to 16·8)
	Andorra	1·8 (1·5 to 2·1)	−5·0% (−7·1 to −3·0)	0·05 (0·04 to 0·06)	0·09 (0·07 to 0·11)	84·9 (82·6 to 87·4)	79·8 (77·6 to 82·0)	82·2 (79·9 to 84·5)	70·6 (67·0 to 73·9)	0·6 (0·5 to 0·8)	0·0 (0·0 to 0·0)
	Austria	3·2 (3·0 to 3·4)	−3·7% (−4·6 to −2·9)	0·04 (0·04 to 0·04)	0·08 (0·08 to 0·08)	84·5 (84·3 to 84·6)	79·8 (79·6 to 80·0)	82·2 (82·0 to 82·3)	70·4 (67·0 to 73·3)	82·5 (81·2 to 83·9)	0·3 (0·3 to 0·3)
	Belgium	3·5 (3·3 to 3·7)	−2·7% (−3·4 to −1·9)	0·05 (0·05 to 0·05)	0·09 (0·08 to 0·09)	83·8 (83·6 to 84·0)	79·0 (78·7 to 79·2)	81·4 (81·2 to 81·6)	69·5 (66·1 to 72·5)	114·1 (111·9 to 116·4)	0·4 (0·4 to 0·5)
	Cyprus	3·2 (2·5 to 3·9)	−1·7% (−4·6 to 1·0)	0·04 (0·03 to 0·04)	0·08 (0·07 to 0·09)	82·8 (82·1 to 83·4)	78·9 (78·0 to 79·9)	80·8 (80·0 to 81·6)	69·8 (66·7 to 72·8)	8·7 (8·0 to 9·5)	0·0 (0·0 to 0·1)
	Denmark	3·5 (3·2 to 3·8)	−1·6% (−2·7 to −0·5)	0·05 (0·05 to 0·05)	0·08 (0·08 to 0·08)	83·1 (82·8 to 83·4)	79·2 (78·8 to 79·5)	81·1 (80·8 to 81·4)	69·7 (66·5 to 72·5)	55·4 (54·0 to 56·9)	0·2 (0·2 to 0·3)
	Finland	2·2 (2·0 to 2·4)	−3·4% (−4·5 to −2·2)	0·04 (0·04 to 0·05)	0·09 (0·09 to 0·10)	84·7 (84·4 to 85·0)	79·1 (78·7 to 79·5)	81·9 (81·5 to 82·2)	70·1 (66·8 to 73·0)	56·1 (54·5 to 57·9)	0·1 (0·1 to 0·1)
	France	3·6 (3·4 to 3·8)	−1·7% (−2·4 to −1·1)	0·05 (0·05 to 0·05)	0·09 (0·09 to 0·10)	85·7 (85·6 to 85·9)	79·9 (79·7 to 80·1)	82·9 (82·7 to 83·1)	71·2 (67·9 to 74·1)	603·3 (593·8 to 613·2)	2·6 (2·3 to 3·0)
	Germany	3·3 (3·2 to 3·5)	−2·4% (−2·8 to −1·9)	0·05 (0·05 to 0·05)	0·09 (0·09 to 0·09)	83·5 (83·3 to 83·6)	78·9 (78·7 to 79·1)	81·2 (81·0 to 81·4)	69·5 (66·3 to 72·5)	959·9 (945·8 to 976·7)	2·5 (2·2 to 2·7)
	Greece	3·9 (3·5 to 4·2)	−1·0% (−2·1 to 0·1)	0·05 (0·04 to 0·05)	0·10 (0·10 to 0·10)	83·3 (83·1 to 83·5)	78·5 (78·2 to 78·8)	80·9 (80·7 to 81·2)	69·7 (66·5 to 72·5)	128·7 (126·1 to 131·4)	0·3 (0·3 to 0·4)
	Iceland	2·3 (1·6 to 3·2)	−1·6% (−5·8 to 2·8)	0·04 (0·03 to 0·04)	0·07 (0·06 to 0·07)	86·9 (86·1 to 87·6)	81·5 (80·5 to 82·3)	84·1 (83·2 to 84·9)	71·9 (68·7 to 74·8)	2·1 (2·0 to 2·3)	0·0 (0·0 to 0·0)
	Ireland	3·3 (3·0 to 3·5)	−3·1% (−4·1 to −2·1)	0·04 (0·04 to 0·05)	0·07 (0·07 to 0·07)	83·9 (83·6 to 84·2)	80·2 (79·8 to 80·5)	82·0 (81·7 to 82·4)	70·1 (66·8 to 73·1)	32·4 (31·4 to 33·4)	0·2 (0·2 to 0·2)
	Israel	3·2 (3·1 to 3·3)	−4·0% (−4·5 to −3·4)	0·04 (0·04 to 0·04)	0·07 (0·06 to 0·07)	84·6 (84·4 to 84·8)	81·3 (81·0 to 81·5)	83·0 (82·7 to 83·2)	71·5 (68·4 to 74·4)	47·9 (46·8 to 49·2)	0·6 (0·5 to 0·7)
	Italy	3·0 (2·9 to 3·1)	−3·0% (−3·5 to −2·6)	0·04 (0·04 to 0·04)	0·07 (0·07 to 0·07)	85·3 (85·2 to 85·4)	80·8 (80·7 to 80·9)	83·1 (83·0 to 83·2)	71·0 (67·6 to 74·0)	642·3 (636·8 to 647·8)	1·3 (1·2 to 1·5)
	Luxembourg	2·4 (1·7 to 3·4)	−1·7% (−5·8 to 2·6)	0·04 (0·04 to 0·05)	0·07 (0·06 to 0·08)	85·0 (84·0 to 85·9)	80·8 (79·6 to 81·9)	82·9 (81·8 to 83·9)	70·7 (67·2 to 73·7)	4·1 (3·8 to 4·6)	0·0 (0·0 to 0·0)
	Malta	5·7 (4·5 to 7·2)	−1·8% (−4·7 to 1·2)	0·04 (0·03 to 0·04)	0·07 (0·06 to 0·07)	84·9 (84·0 to 85·9)	80·2 (79·2 to 81·1)	82·6 (81·6 to 83·5)	70·7 (67·5 to 73·7)	3·8 (3·5 to 4·1)	0·0 (0·0 to 0·0)
	Monaco	2·6 (2·2 to 3·1)	−2·5% (−4·5 to −0·5)	0·06 (0·05 to 0·08)	0·10 (0·08 to 0·13)	82·3 (80·5 to 84·6)	77·9 (76·3 to 79·7)	80·1 (78·3 to 82·1)	68·9 (65·4 to 72·2)	0·5 (0·4 to 0·6)	0·0 (0·0 to 0·0)
	Netherlands	3·7 (3·5 to 3·9)	−2·1% (−2·7 to −1·5)	0·05 (0·05 to 0·05)	0·06 (0·06 to 0·07)	83·4 (83·2 to 83·6)	80·0 (79·8 to 80·3)	81·7 (81·5 to 82·0)	70·4 (67·3 to 73·2)	157·0 (153·6 to 160·7)	0·7 (0·6 to 0·8)
	Norway	2·5 (2·3 to 2·7)	−3·1% (−3·9 to −2·2)	0·04 (0·04 to 0·04)	0·06 (0·06 to 0·06)	84·7 (84·4 to 84·9)	81·1 (80·9 to 81·3)	82·9 (82·7 to 83·1)	70·6 (67·2 to 73·6)	41·4 (40·7 to 42·0)	0·1 (0·1 to 0·2)
	Portugal	2·8 (2·6 to 3·1)	−2·9% (−3·9 to −1·8)	0·04 (0·04 to 0·04)	0·10 (0·10 to 0·10)	84·5 (84·3 to 84·7)	78·7 (78·5 to 79·0)	81·7 (81·5 to 82·0)	70·0 (66·7 to 72·9)	116·4 (114·0 to 118·9)	0·2 (0·2 to 0·3)
	San Marino	3·6 (3·0 to 4·4)	−1·4% (−3·2 to 0·5)	0·05 (0·03 to 0·07)	0·08 (0·05 to 0·12)	84·3 (80·7 to 87·6)	80·0 (76·4 to 83·9)	82·2 (78·5 to 85·8)	70·6 (66·3 to 74·5)	0·3 (0·2 to 0·4)	0·0 (0·0 to 0·0)
	Spain	3·0 (2·9 to 3·1)	−3·1% (−3·5 to −2·6)	0·04 (0·04 to 0·04)	0·08 (0·07 to 0·08)	85·7 (85·6 to 85·9)	80·4 (80·1 to 80·6)	83·1 (82·9 to 83·3)	71·3 (68·0 to 74·2)	428·6 (421·7 to 435·9)	1·1 (1·0 to 1·3)
	Sweden	2·6 (2·4 to 2·8)	−1·9% (−2·9 to −0·8)	0·04 (0·04 to 0·04)	0·06 (0·06 to 0·06)	84·6 (84·4 to 84·7)	81·1 (80·9 to 81·3)	82·8 (82·7 to 83·0)	71·1 (67·9 to 74·0)	93·8 (92·7 to 95·0)	0·3 (0·3 to 0·3)
	Switzerland	3·7 (3·5 to 3·9)	−2·2% (−3·0 to −1·4)	0·04 (0·03 to 0·04)	0·06 (0·06 to 0·06)	85·8 (85·6 to 86·0)	82·0 (81·8 to 82·3)	84·0 (83·7 to 84·2)	71·7 (68·3 to 74·7)	69·8 (68·4 to 71·4)	0·3 (0·3 to 0·4)
	UK	4·1 (4·0 to 4·2)	−2·4% (−2·8 to −2·0)	0·05 (0·05 to 0·05)	0·08 (0·08 to 0·08)	82·9 (82·8 to 83·0)	79·2 (79·1 to 79·4)	81·1 (81·0 to 81·2)	68·9 (65·5 to 71·9)	621·8 (616·8 to 627·2)	3·2 (2·8 to 3·7)
**Latin America and Caribbean**		**19·0 (16·2 to 22·3)**	**−4·7% (−6·2 to −3·1)**	**0·09 (0·08 to 0·10)**	**0·17 (0·16 to 0·19)**	**79·0 (78·0 to 80·0)**	**72·9 (71·5 to 74·3)**	**76·0 (74·8 to 77·1)**	**64·7 (61·7 to 67·2)**	**3565·5 (3298·9 to 3863·5)**	**186·9 (149·0 to 231·4)**
Andean Latin America		18·6 (16·5 to 21·1)	−4·4% (−5·8 to −2·9)	0·08 (0·07 to 0·10)	0·12 (0·10 to 0·15)	79·1 (77·1 to 81·0)	75·9 (73·6 to 78·3)	77·5 (75·3 to 79·6)	66·5 (63·5 to 69·4)	320·9 (269·6 to 381·0)	24·9 (19·2 to 31·9)
	Bolivia	29·5 (25·2 to 35·1)	−4·4% (−6·1 to −2·6)	0·13 (0·10 to 0·16)	0·15 (0·11 to 0·19)	72·9 (71·0 to 74·8)	71·1 (68·8 to 73·7)	72·0 (69·9 to 74·2)	63·0 (59·9 to 66·0)	75·9 (63·5 to 88·8)	9·6 (7·8 to 11·8)
	Ecuador	15·1 (12·7 to 18·1)	−4·6% (−7·1 to −2·0)	0·09 (0·07 to 0·11)	0·15 (0·12 to 0·19)	78·7 (76·4 to 80·6)	74·2 (71·2 to 76·8)	76·4 (73·8 to 78·7)	66·7 (63·2 to 69·8)	92·5 (76·6 to 113·5)	5·3 (3·8 to 7·2)
	Peru	15·1 (12·6 to 18·1)	−4·6% (−6·8 to −2·3)	0·07 (0·05 to 0·09)	0·10 (0·08 to 0·13)	81·8 (79·0 to 84·4)	78·7 (75·3 to 82·0)	80·2 (77·1 to 83·2)	69·5 (65·8 to 72·9)	152·4 (118·7 to 194·5)	10·0 (7·1 to 13·8)
Caribbean		38·8 (33·2 to 45·3)	−7·4% (−8·9 to −5·7)	0·13 (0·11 to 0·15)	0·18 (0·16 to 0·21)	75·1 (73·3 to 76·8)	70·2 (68·1 to 72·4)	72·6 (70·7 to 74·6)	63·0 (60·1 to 65·8)	366·4 (323·3 to 412·5)	32·0 (26·0 to 39·0)
	Antigua and Barbuda	10·3 (8·6 to 12·3)	−1·8% (−3·6 to 0·2)	0·09 (0·07 to 0·10)	0·13 (0·11 to 0·15)	77·9 (76·7 to 79·1)	74·6 (72·9 to 76·3)	76·3 (74·8 to 77·7)	66·5 (63·3 to 69·4)	0·6 (0·5 to 0·7)	0·0 (0·0 to 0·0)
	The Bahamas	11·7 (9·9 to 14·1)	−1·3% (−3·1 to 0·5)	0·13 (0·11 to 0·16)	0·24 (0·20 to 0·28)	76·9 (74·5 to 79·0)	69·9 (67·1 to 72·4)	73·4 (70·7 to 75·7)	64·1 (60·7 to 67·2)	2·7 (2·3 to 3·3)	0·0 (0·0 to 0·1)
	Barbados	12·1 (10·2 to 14·5)	−1·4% (−3·3 to 0·6)	0·09 (0·08 to 0·11)	0·13 (0·11 to 0·16)	77·8 (76·0 to 79·6)	74·5 (72·4 to 76·6)	76·2 (74·3 to 78·1)	66·6 (63·4 to 69·5)	3·1 (2·7 to 3·5)	0·0 (0·0 to 0·0)
	Belize	15·4 (13·0 to 18·6)	−2·8% (−4·6 to −0·9)	0·12 (0·10 to 0·13)	0·21 (0·19 to 0·24)	78·0 (76·7 to 79·2)	71·3 (69·6 to 73·0)	74·5 (72·9 to 76·0)	64·8 (61·9 to 67·5)	2·0 (1·8 to 2·2)	0·1 (0·1 to 0·1)
	Bermuda	4·7 (3·9 to 5·5)	−1·5% (−3·4 to 0·4)	0·04 (0·04 to 0·05)	0·10 (0·09 to 0·12)	85·3 (83·5 to 86·7)	77·5 (75·7 to 79·0)	81·3 (79·5 to 82·8)	70·8 (67·5 to 73·7)	0·6 (0·5 to 0·7)	0·0 (0·0 to 0·0)
	Cuba	4·7 (4·1 to 5·4)	−2·3% (−4·0 to −0·7)	0·07 (0·06 to 0·08)	0·12 (0·10 to 0·15)	81·1 (79·3 to 82·9)	76·3 (74·1 to 78·5)	78·7 (76·6 to 80·7)	68·2 (65·0 to 71·2)	106·0 (89·7 to 124·4)	0·5 (0·4 to 0·6)
	Dominica	26·0 (21·8 to 31·0)	1·4% (−0·6 to 3·3)	0·11 (0·09 to 0·13)	0·18 (0·15 to 0·22)	75·0 (72·8 to 76·9)	70·0 (67·3 to 72·4)	72·3 (69·8 to 74·6)	63·0 (59·7 to 66·0)	0·7 (0·6 to 0·9)	0·0 (0·0 to 0·0)
	Dominican Republic	25·2 (21·1 to 30·2)	−2·5% (−4·9 to −0·1)	0·11 (0·09 to 0·14)	0·20 (0·16 to 0·25)	76·7 (74·1 to 79·1)	70·0 (66·6 to 73·3)	73·2 (70·1 to 76·1)	64·1 (60·5 to 67·5)	70·5 (57·3 to 87·1)	5·8 (4·2 to 7·9)
	Grenada	13·8 (11·6 to 16·5)	−1·1% (−2·9 to 0·8)	0·11 (0·10 to 0·13)	0·17 (0·15 to 0·19)	75·8 (74·8 to 76·8)	71·0 (70·1 to 72·0)	73·3 (72·3 to 74·3)	63·9 (61·0 to 66·5)	0·8 (0·8 to 0·9)	0·0 (0·0 to 0·0)
	Guyana	23·1 (19·4 to 27·4)	−3·4% (−5·3 to −1·3)	0·19 (0·15 to 0·24)	0·32 (0·26 to 0·38)	70·8 (67·7 to 73·7)	64·1 (60·7 to 67·6)	67·3 (64·0 to 70·6)	58·3 (54·7 to 61·8)	6·7 (5·3 to 8·3)	0·3 (0·2 to 0·5)
	Haiti	68·3 (58·7 to 79·5)	−9·7% (−11·3 to −8·1)	0·25 (0·20 to 0·31)	0·26 (0·20 to 0·33)	64·6 (61·8 to 67·4)	62·9 (59·8 to 66·0)	63·8 (60·8 to 66·7)	55·4 (52·0 to 58·6)	99·7 (82·0 to 121·5)	22·6 (18·8 to 27·0)
	Jamaica	15·7 (13·1 to 18·7)	−1·6% (−3·6 to 0·4)	0·11 (0·09 to 0·14)	0·13 (0·11 to 0·17)	78·0 (75·5 to 80·4)	74·6 (71·9 to 77·2)	76·2 (73·6 to 78·8)	66·2 (62·8 to 69·4)	19·7 (16·3 to 23·6)	0·6 (0·4 to 0·8)
	PuertoRico	7·7 (6·5 to 9·0)	−1·6% (−3·4 to 0·4)	0·06 (0·05 to 0·08)	0·14 (0·11 to 0·18)	83·7 (81·4 to 86·0)	77·2 (74·1 to 80·2)	80·5 (77·8 to 83·2)	69·1 (65·5 to 72·4)	32·9 (26·8 to 40·3)	0·2 (0·1 to 0·3)
	Saint Kitts and Nevis	15·3 (13·1 to 17·9)	−1·2% (−3·0 to 0·7)	0·11 (0·09 to 0·13)	0·22 (0·19 to 0·26)	75·8 (74·0 to 77·2)	68·9 (67·1 to 70·2)	72·1 (70·3 to 73·5)	63·0 (60·1 to 65·7)	0·5 (0·5 to 0·6)	0·0 (0·0 to 0·0)
	Saint Lucia	14·6 (12·2 to 17·4)	−2·3% (−4·0 to −0·3)	0·10 (0·08 to 0·12)	0·17 (0·15 to 0·20)	78·4 (76·7 to 80·1)	72·1 (70·1 to 74·0)	75·1 (73·2 to 77·0)	65·1 (61·8 to 68·1)	1·4 (1·2 to 1·6)	0·0 (0·0 to 0·0)
	Saint Vincent and the Grenadines	15·2 (12·8 to 18·2)	−2·9% (−4·9 to −0·9)	0·13 (0·11 to 0·15)	0·20 (0·18 to 0·23)	75·3 (73·7 to 76·7)	70·9 (69·2 to 72·5)	72·9 (71·3 to 74·5)	63·6 (60·5 to 66·4)	1·0 (0·9 to 1·1)	0·0 (0·0 to 0·0)
	Suriname	25·7 (21·6 to 30·6)	−2·8% (−4·7 to −1·0)	0·13 (0·11 to 0·15)	0·21 (0·18 to 0·24)	75·7 (73·7 to 77·5)	69·8 (67·3 to 72·3)	72·7 (70·4 to 74·9)	62·7 (59·4 to 65·7)	4·4 (3·8 to 5·1)	0·2 (0·2 to 0·3)
	Trinidad and Tobago	15·2 (12·7 to 18·2)	−1·9% (−4·2 to 0·4)	0·11 (0·08 to 0·14)	0·18 (0·14 to 0·23)	77·9 (74·8 to 80·9)	72·1 (68·4 to 75·7)	74·9 (71·4 to 78·3)	64·9 (61·2 to 68·5)	11·8 (9·2 to 15·0)	0·2 (0·2 to 0·3)
	Virgin Islands	6·8 (5·7 to 8·0)	−2·9% (−4·7 to −1·0)	0·08 (0·07 to 0·10)	0·22 (0·18 to 0·27)	79·1 (77·5 to 80·5)	69·9 (67·9 to 71·8)	74·3 (72·6 to 76·1)	65·1 (62·1 to 67·9)	1·3 (1·1 to 1·4)	0·0 (0·0 to 0·0)
Central Latin America		14·8 (12·4 to 17·5)	−3·8% (−5·6 to −2·0)	0·09 (0·07 to 0·10)	0·17 (0·15 to 0·20)	79·4 (77·8 to 80·9)	73·4 (71·2 to 75·4)	76·4 (74·5 to 78·1)	64·9 (61·8 to 67·5)	1433·0 (1259·7 to 1628·2)	65·4 (50·7 to 83·7)
	Colombia	12·6 (10·6 to 15·0)	−4·4% (−6·4 to −2·3)	0·06 (0·05 to 0·08)	0·12 (0·10 to 0·16)	82·9 (80·4 to 85·2)	77·5 (74·1 to 80·7)	80·2 (77·3 to 83·0)	69·0 (65·5 to 72·4)	246·7 (197·9 to 306·1)	10·3 (7·4 to 13·9)
	Costa Rica	7·9 (6·8 to 9·3)	−3·4% (−5·3 to −1·6)	0·06 (0·05 to 0·08)	0·13 (0·10 to 0·16)	83·0 (80·8 to 85·2)	77·1 (74·1 to 80·0)	80·1 (77·4 to 82·7)	69·0 (65·4 to 72·3)	24·4 (19·7 to 30·1)	0·5 (0·4 to 0·7)
	El Salvador	10·9 (9·2 to 13·1)	−5·1% (−7·2 to −3·1)	0·09 (0·07 to 0·12)	0·23 (0·18 to 0·29)	79·9 (77·0 to 82·7)	71·1 (67·3 to 74·9)	75·7 (72·3 to 79·1)	65·2 (61·5 to 68·8)	40·2 (31·8 to 50·2)	1·2 (0·9 to 1·7)
	Guatemala	21·7 (18·3 to 25·8)	−4·4% (−6·5 to −2·3)	0·13 (0·10 to 0·16)	0·23 (0·18 to 0·29)	75·5 (72·8 to 78·0)	69·5 (66·1 to 72·8)	72·6 (69·5 to 75·5)	62·9 (59·2 to 66·3)	94·8 (75·9 to 117·7)	8·9 (6·4 to 12·1)
	Honduras	17·2 (14·4 to 20·5)	−4·4% (−6·3 to −2·3)	0·15 (0·11 to 0·20)	0·17 (0·13 to 0·23)	73·3 (70·6 to 75·3)	70·8 (68·3 to 72·6)	72·1 (69·5 to 73·9)	63·0 (59·6 to 65·8)	52·6 (46·2 to 62·4)	4·0 (3·1 to 4·9)
	Mexico	14·4 (12·2 to 17·1)	−3·8% (−5·8 to −1·8)	0·09 (0·08 to 0·10)	0·19 (0·17 to 0·21)	78·6 (77·5 to 79·9)	72·6 (71·1 to 74·3)	75·6 (74·6 to 76·7)	65·3 (62·1 to 68·0)	738·4 (654·7 to 827·7)	30·3 (24·2 to 37·5)
	Nicaragua	14·4 (12·1 to 17·1)	−4·4% (−6·2 to −2·6)	0·08 (0·06 to 0·10)	0·14 (0·12 to 0·18)	78·4 (77·1 to 79·7)	72·4 (70·4 to 74·6)	75·4 (73·7 to 77·2)	65·7 (62·6 to 68·6)	29·2 (24·9 to 33·6)	1·9 (1·5 to 2·3)
	Panama	14·5 (12·1 to 17·3)	−2·5% (−4·8 to −0·1)	0·07 (0·05 to 0·09)	0·12 (0·09 to 0·15)	82·7 (80·2 to 85·1)	77·5 (74·3 to 80·6)	80·0 (77·2 to 82·8)	68·8 (65·2 to 72·1)	19·8 (15·9 to 24·6)	1·1 (0·8 to 1·5)
	Venezuela	14·8 (12·4 to 17·6)	−2·3% (−4·2 to −0·4)	0·10 (0·07 to 0·13)	0·19 (0·15 to 0·25)	78·9 (75·9 to 81·7)	71·4 (67·7 to 75·0)	75·0 (71·6 to 78·4)	65·2 (61·5 to 68·7)	186·9 (146·8 to 237·0)	7·2 (5·2 to 9·9)
Tropical Latin America		19·8 (16·7 to 23·4)	−4·2% (−5·9 to −2·4)	0·09 (0·08 to 0·09)	0·18 (0·17 to 0·19)	79·4 (78·9 to 79·9)	72·4 (71·7 to 73·0)	75·9 (75·4 to 76·3)	64·3 (61·4 to 66·9)	1445·2 (1406·6 to 1485·4)	64·6 (51·3 to 79·2)
	Brazil	20·0 (16·9 to 23·7)	−4·2% (−5·9 to −2·4)	0·09 (0·08 to 0·09)	0·18 (0·17 to 0·19)	79·4 (78·9 to 79·8)	72·3 (71·7 to 72·9)	75·8 (75·4 to 76·3)	65·0 (62·0 to 67·6)	1411·0 (1375·4 to 1448·3)	62·8 (49·9 to 77·0)
	Paraguay	14·0 (11·8 to 16·6)	−4·3% (−6·3 to −2·2)	0·09 (0·07 to 0·11)	0·16 (0·12 to 0·20)	79·7 (77·0 to 82·3)	73·8 (70·5 to 77·0)	76·6 (73·6 to 79·6)	66·2 (62·5 to 69·6)	34·2 (27·1 to 42·7)	1·8 (1·3 to 2·4)
**North Africa and Middle East**		**24·4 (22·3 to 26·7)**	**−4·3% (−5·2 to −3·4)**	**0·10 (0·09 to 0·11)**	**0·15 (0·13 to 0·17)**	**75·5 (74·3 to 76·6)**	**72·3 (70·9 to 73·6)**	**73·8 (72·5 to 75·0)**	**63·3 (60·5 to 65·9)**	**3099·5 (2812·9 to 3411·8)**	**300·0 (255·2 to 353·3)**
Afghanistan		55·3 (47·9 to 63·5)	−3·9% (−5·4 to −2·3)	0·29 (0·23 to 0·35)	0·26 (0·21 to 0·33)	63·2 (60·6 to 65·8)	63·5 (60·9 to 66·0)	63·3 (60·7 to 65·9)	54·1 (50·7 to 57·2)	251·4 (214·6 to 292·6)	81·4 (67·9 to 97·2)
Algeria		19·5 (17·0 to 22·4)	−3·9% (−5·4 to −2·2)	0·08 (0·07 to 0·10)	0·10 (0·08 to 0·12)	76·8 (75·4 to 78·2)	75·6 (73·7 to 77·5)	76·2 (74·5 to 77·8)	66·3 (63·1 to 69·0)	201·1 (172·2 to 234·2)	17·3 (14·5 to 20·5)
Bahrain		6·5 (5·8 to 7·4)	−2·5% (−4·0 to −1·1)	0·06 (0·05 to 0·07)	0·07 (0·06 to 0·09)	77·6 (76·2 to 78·9)	76·6 (74·9 to 78·3)	77·0 (75·5 to 78·6)	66·6 (63·4 to 69·4)	4·3 (3·6 to 5·1)	0·1 (0·1 to 0·1)
Egypt		15·3 (12·8 to 18·3)	−6·1% (−8·6 to −3·7)	0·12 (0·10 to 0·16)	0·20 (0·16 to 0·25)	71·9 (69·6 to 74·1)	70·1 (67·1 to 73·1)	71·0 (68·3 to 73·6)	62·4 (59·2 to 65·5)	561·6 (448·0 to 697·8)	32·6 (24·6 to 42·6)
Iran		11·1 (10·2 to 12·0)	−9·6% (−11·3 to −8·1)	0·06 (0·06 to 0·06)	0·12 (0·11 to 0·12)	79·6 (79·2 to 79·9)	76·1 (75·6 to 76·5)	77·8 (77·5 to 78·0)	66·7 (63·6 to 69·5)	391·1 (381·5 to 402·1)	15·2 (12·7 to 18·4)
Iraq		15·7 (13·2 to 18·9)	−6·9% (−8·7 to −5·1)	0·11 (0·08 to 0·15)	0·17 (0·13 to 0·22)	75·9 (73·7 to 77·9)	71·0 (68·6 to 73·7)	73·3 (71·0 to 75·7)	63·3 (60·1 to 66·3)	179·6 (148·1 to 214·1)	15·0 (11·0 to 20·7)
Jordan		15·3 (13·0 to 18·3)	−1·9% (−3·6 to 0·1)	0·06 (0·05 to 0·07)	0·09 (0·07 to 0·11)	79·1 (77·1 to 80·9)	77·5 (75·2 to 79·7)	78·2 (76·6 to 79·7)	67·8 (64·7 to 70·7)	32·3 (28·0 to 37·6)	3·6 (2·7 to 5·0)
Kuwait		9·2 (7·8 to 10·8)	−2·4% (−4·4 to −0·4)	0·03 (0·03 to 0·04)	0·08 (0·06 to 0·09)	84·7 (83·2 to 86·1)	79·5 (77·4 to 81·6)	81·5 (79·8 to 83·0)	69·9 (66·5 to 72·8)	10·0 (8·7 to 11·6)	0·6 (0·4 to 0·7)
Lebanon		9·0 (7·5 to 11·0)	−4·2% (−6·0 to −2·4)	0·08 (0·06 to 0·09)	0·13 (0·11 to 0·15)	79·3 (78·1 to 80·4)	74·0 (72·6 to 75·6)	76·6 (75·3 to 78·0)	65·9 (62·7 to 68·8)	33·9 (30·7 to 37·1)	1·0 (0·7 to 1·4)
Libya		13·3 (11·3 to 15·7)	−2·7% (−4·4 to −0·8)	0·10 (0·08 to 0·13)	0·14 (0·12 to 0·18)	77·6 (75·4 to 79·6)	74·5 (72·0 to 76·8)	75·9 (73·6 to 78·1)	65·1 (61·7 to 68·4)	31·7 (26·5 to 38·3)	1·1 (0·9 to 1·4)
Morocco		17·9 (15·0 to 21·4)	−5·9% (−7·7 to −4·0)	0·12 (0·09 to 0·16)	0·14 (0·11 to 0·19)	74·7 (72·8 to 76·7)	72·0 (70·2 to 74·6)	73·3 (71·5 to 75·6)	63·8 (60·6 to 66·9)	228·1 (187·8 to 261·3)	11·1 (7·9 to 15·4)
Oman		10·4 (9·4 to 11·4)	−1·0% (−2·1 to 0·2)	0·08 (0·07 to 0·09)	0·10 (0·09 to 0·12)	75·4 (74·4 to 76·3)	73·0 (71·8 to 74·1)	74·0 (73·2 to 74·8)	64·7 (62·0 to 67·1)	12·4 (11·4 to 13·4)	0·8 (0·7 to 0·9)
Palestine		12·4 (10·5 to 14·9)	−6·0% (−7·7 to −4·2)	0·08 (0·07 to 0·09)	0·12 (0·11 to 0·14)	76·5 (75·3 to 77·6)	73·2 (71·9 to 74·5)	74·8 (73·5 to 76·1)	64·4 (61·2 to 67·1)	16·6 (14·8 to 18·6)	1·6 (1·2 to 2·1)
Qatar		8·0 (6·7 to 9·7)	−3·6% (−5·4 to −1·7)	0·05 (0·04 to 0·07)	0·07 (0·05 to 0·09)	75·3 (74·0 to 76·6)	76·7 (74·8 to 78·6)	76·3 (74·5 to 78·0)	66·3 (63·1 to 69·1)	4·4 (3·5 to 5·5)	0·2 (0·2 to 0·3)
Saudi Arabia		5·7 (4·8 to 7·0)	−7·6% (−9·5 to −5·6)	0·12 (0·10 to 0·15)	0·17 (0·14 to 0·21)	76·4 (74·2 to 78·6)	73·3 (71·5 to 75·3)	74·5 (72·5 to 76·6)	64·4 (61·2 to 67·3)	128·5 (105·3 to 156·2)	2·6 (2·1 to 3·3)
Sudan		41·9 (35·7 to 50·0)	−5·1% (−6·8 to −3·3)	0·13 (0·10 to 0·17)	0·17 (0·13 to 0·22)	72·3 (70·1 to 74·0)	69·0 (66·4 to 71·4)	70·5 (68·1 to 72·6)	61·3 (58·1 to 64·2)	202·2 (173·6 to 235·4)	50·7 (37·3 to 68·6)
Syria		13·6 (11·7 to 15·8)	−0·4% (−2·0 to 1·3)	0·09 (0·07 to 0·12)	0·15 (0·12 to 0·19)	75·3 (73·1 to 77·3)	72·4 (69·6 to 75·1)	73·9 (71·3 to 76·2)	63·9 (60·2 to 67·1)	84·4 (67·8 to 106·2)	3·2 (2·6 to 4·0)
Tunisia		11·3 (9·5 to 13·5)	−5·7% (−7·5 to −3·8)	0·06 (0·05 to 0·08)	0·11 (0·08 to 0·14)	80·0 (77·6 to 82·4)	75·5 (72·5 to 78·5)	77·7 (74·9 to 80·4)	67·3 (63·6 to 70·6)	67·6 (53·1 to 85·4)	1·9 (1·5 to 2·4)
Turkey		15·4 (12·9 to 18·4)	−4·8% (−6·8 to −2·6)	0·05 (0·04 to 0·06)	0·10 (0·08 to 0·13)	80·6 (79·0 to 82·3)	76·2 (74·1 to 78·3)	78·4 (76·5 to 80·3)	67·6 (64·4 to 70·7)	454·7 (382·1 to 536·6)	15·1 (12·1 to 18·7)
United Arab Emirates		5·0 (4·2 to 6·0)	−3·5% (−5·3 to −1·8)	0·10 (0·08 to 0·13)	0·15 (0·12 to 0·19)	76·5 (74·4 to 78·4)	72·9 (70·6 to 75·0)	73·9 (71·7 to 76·0)	64·3 (61·2 to 67·2)	29·1 (22·5 to 37·3)	0·3 (0·2 to 0·4)
Yemen		46·7 (40·2 to 54·4)	−2·5% (−4·0 to −0·9)	0·16 (0·12 to 0·21)	0·23 (0·18 to 0·29)	69·9 (67·3 to 72·0)	65·7 (62·9 to 68·1)	67·7 (65·0 to 70·0)	58·5 (55·0 to 61·5)	174·5 (148·0 to 209·9)	44·2 (36·7 to 53·1)
**South Asia**		**40·5 (36·0 to 46·0)**	**−4·6% (−6·0 to −3·0)**	**0·14 (0·12 to 0·16)**	**0·19 (0·16 to 0·22)**	**71·7 (70·1 to 73·4)**	**69·1 (67·3 to 71·0)**	**70·4 (69·2 to 71·7)**	**60·7 (57·7 to 63·5)**	**11 939·1 (10 903·9 to 13 016·7)**	**1357·5 (1143·5 to 1612·4)**
Bangladesh		29·2 (24·9 to 34·2)	−6·5% (−8·1 to −4·9)	0·10 (0·08 to 0·12)	0·13 (0·11 to 0·16)	75·9 (73·9 to 77·9)	73·4 (71·1 to 75·6)	74·6 (72·4 to 76·7)	64·4 (61·2 to 67·3)	849·6 (712·9 to 1005·6)	77·9 (63·0 to 94·2)
Bhutan		31·5 (26·7 to 36·9)	−4·7% (−6·4 to −2·8)	0·10 (0·08 to 0·13)	0·13 (0·10 to 0·17)	74·3 (72·3 to 76·4)	72·2 (69·7 to 74·8)	73·2 (70·9 to 75·6)	63·1 (59·8 to 66·2)	4·3 (3·5 to 5·0)	0·4 (0·3 to 0·6)
India		35·8 (30·2 to 43·0)	−5·4% (−7·5 to −3·3)	0·14 (0·11 to 0·16)	0·19 (0·16 to 0·23)	72·1 (70·2 to 74·0)	69·5 (67·3 to 71·8)	70·8 (69·3 to 72·2)	60·5 (57·4 to 63·3)	9392·1 (8430·3 to 10 426·1)	840·9 (692·3 to 1037·9)
Nepal		29·1 (24·8 to 34·1)	−6·2% (−7·7 to −4·5)	0·13 (0·10 to 0·16)	0·18 (0·15 to 0·22)	73·0 (71·3 to 74·8)	69·2 (67·4 to 71·5)	71·1 (69·4 to 73·2)	61·5 (58·5 to 64·3)	193·3 (165·4 to 216·2)	18·0 (14·7 to 21·8)
Pakistan		63·3 (53·9 to 74·3)	−2·3% (−4·1 to −0·3)	0·20 (0·15 to 0·26)	0·22 (0·17 to 0·29)	66·8 (63·9 to 69·5)	65·0 (61·9 to 68·0)	65·9 (63·8 to 67·8)	57·4 (54·5 to 60·2)	1499·9 (1328·7 to 1695·2)	420·3 (346·4 to 507·3)
**Southeast Asia, east Asia, and Oceania**		**14·8 (13·2 to 16·5)**	**−5·4% (−6·4 to −4·5)**	**0·07 (0·06 to 0·08)**	**0·14 (0·12 to 0·16)**	**79·2 (77·9 to 80·4)**	**73·2 (71·8 to 74·7)**	**76·1 (75·1 to 77·1)**	**65·8 (63·3 to 68·1)**	**15 551·0 (14 134·9 to 16 985·4)**	**405·4 (349·0 to 467·0)**
East Asia		8·6 (7·5 to 9·8)	−7·0% (−8·6 to −5·4)	0·06 (0·04 to 0·07)	0·12 (0·09 to 0·14)	80·7 (79·1 to 82·4)	74·7 (72·8 to 76·6)	77·6 (76·3 to 78·9)	67·0 (64·4 to 69·3)	11 075·9 (9731·6 to 12 488·1)	136·0 (118·6 to 155·4)
	China	8·5 (7·5 to 9·7)	−7·1% (−8·7 to −5·4)	0·05 (0·04 to 0·07)	0·12 (0·09 to 0·14)	80·8 (79·1 to 82·5)	74·7 (72·8 to 76·7)	77·6 (76·3 to 79·0)	68·4 (65·8 to 70·9)	10 653·4 (9311·0 to 12 069·8)	130·7 (114·0 to 149·0)
	North Korea	12·6 (10·5 to 15·4)	−6·8% (−8·6 to −4·9)	0·11 (0·09 to 0·15)	0·20 (0·16 to 0·25)	76·2 (73·9 to 78·0)	69·8 (67·9 to 71·6)	73·1 (71·0 to 75·1)	64·9 (62·1 to 67·4)	237·1 (207·4 to 274·0)	4·5 (3·5 to 5·7)
	Taiwan (province of China)	4·5 (3·8 to 5·4)	−1·2% (−3·0 to 0·9)	0·05 (0·04 to 0·07)	0·13 (0·10 to 0·17)	83·6 (81·3 to 85·7)	77·1 (74·1 to 79·9)	80·3 (77·5 to 82·7)	70·3 (67·0 to 73·5)	185·4 (150·6 to 231·6)	0·8 (0·7 to 1·0)
Oceania		48·4 (40·7 to 57·5)	−2·0% (−3·7 to −0·3)	0·22 (0·18 to 0·28)	0·29 (0·24 to 0·36)	67·1 (64·0 to 69·9)	63·1 (60·0 to 66·1)	64·9 (61·8 to 67·9)	57·1 (53·8 to 60·2)	92·6 (76·2 to 112·8)	19·9 (15·8 to 25·0)
	American Samoa	10·2 (8·7 to 12·0)	−2·1% (−3·8 to −0·2)	0·14 (0·12 to 0·17)	0·22 (0·18 to 0·27)	74·6 (72·4 to 76·6)	69·9 (67·8 to 71·9)	72·2 (70·0 to 74·2)	62·5 (59·3 to 65·4)	0·4 (0·3 to 0·4)	0·0 (0·0 to 0·0)
	Cook Islands	2·5 (2·1 to 2·9)	−8·2% (−10·0 to −6·2)	0·09 (0·08 to 0·12)	0·20 (0·17 to 0·24)	79·0 (76·8 to 81·0)	72·2 (70·0 to 74·1)	75·4 (73·3 to 77·3)	65·2 (62·0 to 68·2)	0·2 (0·1 to 0·2)	0·0 (0·0 to 0·0)
	Federated States of Micronesia	14·5 (12·1 to 17·4)	−3·8% (−5·8 to −1·8)	0·26 (0·21 to 0·32)	0·40 (0·33 to 0·46)	67·1 (64·4 to 69·7)	61·5 (58·8 to 64·4)	64·1 (61·4 to 66·9)	56·7 (53·7 to 59·6)	0·9 (0·8 to 1·1)	0·0 (0·0 to 0·0)
	Fiji	21·7 (18·1 to 25·9)	−1·4% (−3·3 to 0·3)	0·18 (0·14 to 0·23)	0·25 (0·20 to 0·31)	70·6 (67·8 to 73·4)	66·3 (63·4 to 69·1)	68·4 (65·5 to 71·2)	59·6 (56·2 to 62·8)	7·4 (6·0 to 9·1)	0·4 (0·3 to 0·5)
	Guam	13·3 (11·6 to 15·3)	−0·1% (−1·7 to 1·5)	0·10 (0·08 to 0·12)	0·19 (0·16 to 0·22)	79·7 (77·7 to 81·5)	73·5 (71·3 to 75·7)	76·4 (74·3 to 78·5)	66·2 (63·0 to 69·1)	1·1 (1·0 to 1·3)	0·0 (0·0 to 0·1)
	Kiribati	33·5 (28·1 to 40·0)	−3·5% (−5·3 to −1·5)	0·30 (0·24 to 0·36)	0·46 (0·39 to 0·54)	63·9 (61·3 to 66·6)	57·6 (55·0 to 60·5)	60·8 (58·0 to 63·5)	53·5 (50·6 to 56·4)	1·2 (1·0 to 1·4)	0·1 (0·1 to 0·1)
	Marshall Islands	19·3 (16·2 to 23·4)	−3·6% (−5·3 to −1·8)	0·25 (0·20 to 0·32)	0·33 (0·27 to 0·40)	67·3 (64·2 to 70·2)	63·9 (60·4 to 67·0)	65·5 (62·2 to 68·5)	57·4 (53·8 to 60·7)	0·4 (0·3 to 0·5)	0·0 (0·0 to 0·0)
	Nauru	23·2 (19·5 to 28·1)	−5·7% (−7·5 to −3·9)	0·26 (0·20 to 0·32)	0·41 (0·34 to 0·48)	66·7 (63·6 to 69·5)	60·3 (57·5 to 63·2)	63·4 (61·1 to 65·6)	56·1 (53·3 to 58·7)	0·1 (0·1 to 0·1)	0·0 (0·0 to 0·0)
	Niue	18·6 (15·5 to 22·2)	−2·0% (−4·0 to −0·1)	0·14 (0·11 to 0·19)	0·24 (0·18 to 0·29)	73·6 (70·6 to 76·2)	67·8 (65·2 to 70·2)	70·6 (67·8 to 73·1)	61·3 (57·9 to 64·4)	0·0 (0·0 to 0·0)	0·0 (0·0 to 0·0)
	Northern Mariana Islands	9·1 (7·7 to 10·7)	1·0% (−0·7 to 3·0)	0·12 (0·10 to 0·15)	0·21 (0·17 to 0·25)	76·3 (74·1 to 78·1)	70·2 (68·5 to 71·9)	72·9 (71·1 to 74·7)	63·8 (60·9 to 66·4)	0·4 (0·3 to 0·4)	0·0 (0·0 to 0·0)
	Palau	14·0 (11·7 to 17·0)	−2·3% (−4·1 to −0·4)	0·18 (0·14 to 0·23)	0·32 (0·26 to 0·39)	71·2 (68·6 to 74·1)	64·8 (61·8 to 67·6)	67·5 (64·7 to 70·4)	59·2 (56·0 to 62·3)	0·2 (0·2 to 0·3)	0·0 (0·0 to 0·0)
	Papua New Guinea	53·8 (45·3 to 63·9)	−2·2% (−4·0 to −0·5)	0·22 (0·17 to 0·29)	0·29 (0·22 to 0·37)	66·8 (63·1 to 70·1)	63·0 (59·3 to 66·4)	64·7 (61·1 to 68·0)	56·3 (52·5 to 60·0)	69·8 (55·8 to 87·3)	17·6 (14·0 to 22·0)
	Samoa	13·2 (11·1 to 15·9)	−3·4% (−5·3 to −1·5)	0·18 (0·14 to 0·23)	0·23 (0·18 to 0·28)	71·9 (69·0 to 74·7)	69·2 (66·4 to 71·5)	70·5 (67·6 to 73·1)	61·8 (58·5 to 64·8)	1·4 (1·1 to 1·7)	0·0 (0·0 to 0·1)
	Solomon Islands	24·6 (20·6 to 29·4)	−3·5% (−5·5 to −1·6)	0·39 (0·32 to 0·46)	0·48 (0·41 to 0·56)	61·2 (58·5 to 63·7)	57·4 (54·9 to 60·1)	59·2 (56·6 to 61·8)	52·7 (50·0 to 55·5)	6·2 (5·2 to 7·4)	0·5 (0·4 to 0·6)
	Tokelau	7·7 (6·4 to 9·3)	−4·6% (−6·5 to −2·7)	0·20 (0·15 to 0·25)	0·17 (0·13 to 0·22)	71·3 (68·1 to 74·2)	73·3 (70·4 to 75·6)	72·2 (69·1 to 74·9)	63·1 (59·7 to 66·2)	0·0 (0·0 to 0·0)	0·0 (0·0 to 0·0)
	Tonga	13·6 (11·4 to 16·3)	−3·2% (−5·1 to −1·3)	0·12 (0·09 to 0·16)	0·20 (0·16 to 0·25)	76·4 (73·7 to 78·8)	69·8 (67·3 to 72·3)	73·0 (70·4 to 75·4)	63·8 (60·5 to 66·8)	0·7 (0·6 to 0·8)	0·0 (0·0 to 0·0)
	Tuvalu	13·7 (11·4 to 16·7)	−5·2% (−7·0 to −3·4)	0·21 (0·16 to 0·27)	0·29 (0·23 to 0·37)	70·0 (66·6 to 73·1)	66·1 (62·4 to 69·3)	67·9 (64·4 to 71·1)	59·8 (56·1 to 63·2)	0·1 (0·1 to 0·1)	0·0 (0·0 to 0·0)
	Vanuatu	24·4 (20·5 to 29·2)	−2·7% (−4·6 to −0·9)	0·22 (0·17 to 0·29)	0·34 (0·27 to 0·43)	68·5 (65·3 to 71·4)	62·9 (59·5 to 65·8)	65·5 (62·2 to 68·3)	57·6 (54·2 to 60·7)	2·2 (1·8 to 2·8)	0·2 (0·1 to 0·2)
Southeast Asia		22·6 (20·1 to 25·6)	−4·1% (−5·4 to −2·8)	0·11 (0·09 to 0·12)	0·18 (0·16 to 0·21)	75·9 (74·6 to 77·2)	70·1 (68·7 to 71·6)	72·9 (71·8 to 74·2)	63·1 (60·6 to 65·5)	4382·5 (4008·3 to 4743·2)	249·6 (208·7 to 296·5)
	Cambodia	31·3 (26·5 to 37·5)	−5·0% (−6·7 to −3·2)	0·14 (0·11 to 0·17)	0·22 (0·18 to 0·27)	72·6 (70·9 to 74·6)	67·0 (65·0 to 69·4)	69·9 (68·0 to 72·2)	61·2 (58·2 to 63·8)	110·9 (93·2 to 126·1)	11·6 (8·9 to 15·3)
	Indonesia	25·5 (21·6 to 30·2)	−4·1% (−6·0 to −2·1)	0·13 (0·10 to 0·16)	0·17 (0·14 to 0·22)	73·5 (71·6 to 75·6)	69·4 (67·2 to 71·6)	71·4 (70·0 to 73·0)	62·5 (59·8 to 64·9)	1705·9 (1480·2 to 1887·0)	98·9 (80·6 to 120·7)
	Laos	40·9 (35·0 to 47·5)	−5·8% (−7·4 to −4·0)	0·15 (0·11 to 0·18)	0·21 (0·17 to 0·26)	71·4 (69·1 to 73·8)	66·7 (64·0 to 69·6)	69·0 (66·4 to 71·6)	60·6 (57·6 to 63·6)	44·5 (36·6 to 53·0)	7·1 (5·7 to 8·9)
	Malaysia	6·4 (5·4 to 7·6)	−1·5% (−3·3 to 0·4)	0·09 (0·07 to 0·11)	0·16 (0·13 to 0·20)	77·4 (75·4 to 79·4)	72·9 (70·3 to 75·5)	75·0 (72·7 to 77·4)	65·5 (62·4 to 68·6)	175·9 (145·0 to 212·7)	3·4 (2·8 to 4·2)
	Maldives	16·2 (13·7 to 19·4)	−2·4% (−4·2 to −0·5)	0·05 (0·04 to 0·06)	0·08 (0·07 to 0·09)	80·5 (79·1 to 82·0)	78·0 (76·5 to 79·6)	79·1 (77·6 to 80·6)	68·9 (65·9 to 71·6)	1·5 (1·3 to 1·7)	0·1 (0·1 to 0·2)
	Mauritius	12·6 (10·8 to 14·7)	−1·4% (−3·3 to 0·4)	0·09 (0·07 to 0·11)	0·18 (0·15 to 0·21)	78·5 (76·4 to 80·6)	72·5 (69·9 to 75·0)	75·5 (73·1 to 77·8)	65·0 (61·6 to 68·2)	10·7 (8·9 to 12·8)	0·2 (0·1 to 0·2)
	Myanmar	40·3 (34·3 to 47·1)	−5·5% (−7·3 to −3·7)	0·13 (0·10 to 0·16)	0·23 (0·18 to 0·27)	72·6 (70·5 to 74·4)	66·2 (63·5 to 68·6)	69·5 (66·9 to 71·5)	60·8 (57·7 to 63·7)	420·9 (367·0 to 494·4)	42·8 (32·1 to 56·3)
	Philippines	22·6 (19·1 to 26·7)	−3·1% (−5·1 to −1·1)	0·12 (0·09 to 0·15)	0·21 (0·17 to 0·27)	75·1 (72·6 to 77·5)	68·8 (65·9 to 71·9)	71·8 (69·9 to 73·9)	62·6 (59·7 to 65·3)	638·8 (548·4 to 736·3)	60·0 (47·8 to 73·2)
	Seychelles	11·5 (9·9 to 13·4)	−2·0% (−3·7 to −0·1)	0·10 (0·08 to 0·11)	0·20 (0·18 to 0·23)	77·4 (76·0 to 78·8)	70·3 (68·7 to 71·8)	73·6 (72·4 to 74·7)	64·3 (61·7 to 66·8)	0·8 (0·7 to 0·9)	0·0 (0·0 to 0·0)
	Sri Lanka	7·6 (6·4 to 9·1)	−6·4% (−8·3 to −4·4)	0·06 (0·05 to 0·08)	0·15 (0·11 to 0·19)	80·2 (77·7 to 82·6)	74·3 (71·1 to 77·4)	77·3 (74·4 to 80·1)	66·8 (63·3 to 70·2)	135·6 (105·3 to 172·6)	2·3 (1·6 to 3·1)
	Thailand	7·6 (6·8 to 8·5)	−2·8% (−4·1 to −1·7)	0·08 (0·06 to 0·10)	0·17 (0·14 to 0·22)	81·9 (79·4 to 84·5)	74·9 (71·5 to 78·5)	78·4 (75·3 to 81·5)	68·0 (64·4 to 71·3)	497·5 (389·3 to 628·0)	4·6 (3·8 to 5·5)
	Timor-Leste	32·1 (27·2 to 38·5)	−3·3% (−5·0 to −1·4)	0·14 (0·11 to 0·17)	0·18 (0·14 to 0·21)	72·4 (70·8 to 74·3)	69·0 (67·2 to 71·3)	70·6 (69·0 to 72·7)	61·6 (58·7 to 64·4)	7·8 (6·7 to 8·6)	1·2 (1·0 to 1·5)
	Vietnam	12·4 (10·5 to 15·0)	−4·2% (−5·9 to −2·3)	0·07 (0·06 to 0·10)	0·19 (0·15 to 0·23)	78·8 (77·3 to 80·6)	70·2 (68·5 to 72·3)	74·5 (72·8 to 76·4)	65·7 (62·8 to 68·3)	631·8 (538·1 to 714·1)	17·1 (13·0 to 22·7)
**Sub-Saharan Africa**		**74·1 (65·3 to 85·2)**	**−4·0% (−5·3 to −2·8)**	**0·20 (0·18 to 0·23)**	**0·27 (0·25 to 0·30)**	**66·8 (65·1 to 68·1)**	**62·2 (60·4 to 63·7)**	**64·5 (62·8 to 65·9)**	**57·4 (54·8 to 59·8)**	**7648·7 (6919·1 to 8577·3)**	**2678·8 (2219·8 to 3252·5)**
Central sub-Saharan Africa		58·8 (51·7 to 67·5)	−6·4% (−7·8 to −5·1)	0·22 (0·18 to 0·26)	0·29 (0·25 to 0·34)	66·8 (64·4 to 69·1)	62·1 (59·7 to 64·5)	64·4 (62·0 to 66·8)	56·6 (53·5 to 59·5)	871·9 (747·9 to 1011·8)	259·7 (222·4 to 310·0)
	Angola	54·2 (46·4 to 62·9)	−6·6% (−8·2 to −5·0)	0·22 (0·18 to 0·27)	0·29 (0·24 to 0·34)	67·5 (64·6 to 70·1)	62·6 (59·7 to 65·5)	65·1 (62·2 to 67·8)	56·7 (53·4 to 59·9)	184·9 (154·0 to 219·1)	58·8 (48·1 to 70·8)
	Central African Republic	123·5 (105·4 to 146·5)	−2·8% (−4·3 to −1·2)	0·38 (0·31 to 0·46)	0·53 (0·45 to 0·61)	55·8 (51·6 to 59·9)	49·3 (45·2 to 52·9)	52·3 (48·2 to 56·2)	45·7 (41·7 to 49·4)	67·8 (54·9 to 84·4)	24·0 (19·2 to 30·0)
	Congo (Brazzaville)	39·5 (33·7 to 46·1)	−5·4% (−7·1 to −3·7)	0·26 (0·21 to 0·31)	0·27 (0·22 to 0·33)	66·4 (63·3 to 69·2)	64·5 (61·6 to 67·0)	65·4 (62·5 to 68·1)	57·0 (53·7 to 60·2)	35·7 (30·0 to 43·0)	5·8 (4·8 to 6·9)
	Democratic Republic of the Congo	57·9 (49·3 to 69·1)	−6·8% (−8·6 to −5·2)	0·20 (0·16 to 0·25)	0·28 (0·23 to 0·33)	67·4 (65·0 to 69·7)	62·7 (60·3 to 65·2)	65·0 (62·7 to 67·5)	56·3 (53·1 to 59·3)	564·1 (479·8 to 658·6)	168·5 (140·9 to 204·9)
	Equatorial Guinea	38·1 (31·9 to 46·3)	−5·4% (−7·1 to −3·4)	0·26 (0·19 to 0·35)	0·28 (0·22 to 0·36)	67·1 (62·8 to 71·3)	64·5 (60·9 to 68·1)	66·0 (62·1 to 69·9)	57·2 (53·1 to 60·9)	7·6 (5·8 to 9·8)	1·5 (1·1 to 2·0)
	Gabon	30·1 (25·3 to 36·4)	−6·0% (−7·8 to −4·1)	0·19 (0·15 to 0·24)	0·27 (0·22 to 0·33)	71·1 (68·5 to 73·6)	64·8 (62·6 to 67·3)	67·8 (65·5 to 70·3)	58·8 (55·6 to 61·8)	11·8 (9·9 to 13·6)	1·3 (0·9 to 1·8)
Eastern sub-Saharan Africa		58·3 (50·5 to 68·1)	−4·6% (−5·9 to −3·2)	0·20 (0·18 to 0·22)	0·27 (0·25 to 0·30)	68·1 (66·6 to 69·3)	63·3 (61·7 to 64·7)	65·6 (64·1 to 66·9)	58·1 (55·6 to 60·5)	2585·8 (2337·9 to 2893·7)	814·2 (657·8 to 1011·6)
	Burundi	65·4 (55·7 to 77·8)	−4·6% (−6·3 to −2·9)	0·21 (0·17 to 0·27)	0·29 (0·23 to 0·36)	66·3 (63·1 to 69·4)	61·7 (58·1 to 65·1)	63·8 (60·3 to 67·0)	55·4 (51·8 to 58·9)	83·5 (67·5 to 103·2)	29·7 (23·8 to 37·4)
	Comoros	49·8 (42·1 to 59·9)	−4·5% (−6·3 to −2·7)	0·16 (0·12 to 0·20)	0·19 (0·15 to 0·24)	70·0 (67·7 to 71·9)	67·4 (64·9 to 69·6)	68·7 (66·4 to 70·8)	60·2 (57·2 to 63·0)	5·0 (4·4 to 5·8)	0·8 (0·7 to 1·0)
	Djibouti	47·0 (39·4 to 56·9)	−4·4% (−6·1 to −2·5)	0·20 (0·15 to 0·26)	0·25 (0·19 to 0·32)	68·9 (65·2 to 72·0)	65·2 (61·5 to 68·4)	66·8 (63·2 to 70·0)	58·9 (55·1 to 62·2)	7·6 (6·1 to 9·5)	1·7 (1·3 to 2·1)
	Eritrea	47·5 (39·8 to 57·5)	−4·4% (−6·2 to −2·5)	0·23 (0·18 to 0·30)	0·34 (0·27 to 0·42)	66·7 (62·7 to 70·0)	60·9 (57·0 to 64·1)	63·8 (59·9 to 67·1)	55·8 (52·0 to 59·3)	43·4 (34·1 to 56·6)	9·4 (6·9 to 12·9)
	Ethiopia	52·4 (44·7 to 62·4)	−5·8% (−7·6 to −4·1)	0·15 (0·13 to 0·18)	0·19 (0·16 to 0·23)	70·8 (69·2 to 72·3)	67·1 (65·1 to 69·2)	68·8 (67·5 to 70·2)	60·1 (57·4 to 62·6)	560·0 (506·1 to 622·0)	190·2 (149·8 to 242·6)
	Kenya	40·6 (34·6 to 47·7)	−3·9% (−5·5 to −2·3)	0·22 (0·18 to 0·25)	0·29 (0·25 to 0·33)	69·0 (66·9 to 71·2)	64·2 (62·3 to 66·3)	66·5 (65·1 to 67·9)	58·3 (55·5 to 60·7)	294·7 (268·8 to 322·6)	54·1 (43·7 to 65·7)
	Madagascar	56·6 (48·1 to 67·7)	−3·8% (−5·4 to −2·0)	0·21 (0·17 to 0·27)	0·25 (0·19 to 0·30)	66·7 (64·1 to 69·3)	64·3 (61·6 to 67·2)	65·5 (62·9 to 68·2)	57·5 (54·5 to 60·5)	164·2 (135·3 to 196·9)	48·2 (39·1 to 59·0)
	Malawi	59·1 (50·6 to 70·0)	−5·2% (−6·8 to −3·4)	0·20 (0·17 to 0·24)	0·31 (0·26 to 0·36)	68·2 (66·0 to 70·1)	61·4 (59·1 to 63·6)	64·7 (62·4 to 66·8)	56·5 (53·5 to 59·3)	116·7 (101·2 to 135·1)	31·8 (24·9 to 40·8)
	Mozambique	69·4 (59·0 to 82·8)	−4·8% (−6·5 to −3·1)	0·31 (0·25 to 0·37)	0·47 (0·40 to 0·53)	62·2 (59·3 to 65·1)	54·8 (52·1 to 57·3)	58·4 (55·7 to 60·9)	50·7 (47·8 to 53·7)	264·8 (228·3 to 307·6)	76·5 (62·8 to 93·8)
	Rwanda	46·6 (39·6 to 55·8)	−5·0% (−6·7 to −3·2)	0·16 (0·13 to 0·19)	0·22 (0·18 to 0·26)	70·7 (68·6 to 72·4)	66·3 (64·0 to 68·4)	68·6 (66·4 to 70·5)	59·8 (56·7 to 62·5)	68·6 (59·7 to 79·5)	16·3 (12·6 to 21·2)
	Somalia	95·4 (80·8 to 114·1)	−4·1% (−5·6 to −2·4)	0·28 (0·22 to 0·35)	0·38 (0·31 to 0·47)	61·3 (57·1 to 64·8)	55·8 (51·4 to 59·6)	58·5 (54·2 to 62·3)	51·4 (47·5 to 55·1)	184·2 (147·1 to 235·8)	80·6 (62·9 to 103·7)
	South Sudan	92·6 (78·9 to 108·0)	−2·7% (−4·3 to −1·0)	0·19 (0·14 to 0·25)	0·25 (0·19 to 0·32)	66·2 (63·0 to 69·0)	61·7 (58·3 to 65·0)	63·7 (60·4 to 66·9)	54·5 (50·9 to 57·9)	72·7 (61·0 to 87·5)	33·1 (26·8 to 40·6)
	Tanzania	57·1 (48·9 to 67·7)	−3·8% (−5·5 to −2·1)	0·17 (0·15 to 0·21)	0·23 (0·20 to 0·28)	69·1 (67·4 to 70·8)	65·3 (63·1 to 67·3)	67·2 (65·2 to 69·0)	58·6 (55·8 to 61·3)	354·4 (312·2 to 408·3)	117·9 (93·2 to 149·9)
	Uganda	58·4 (50·4 to 68·7)	−4·6% (−6·2 to −2·9)	0·17 (0·14 to 0·20)	0·28 (0·23 to 0·33)	69·8 (67·8 to 71·7)	62·7 (60·4 to 64·8)	66·2 (64·1 to 68·2)	57·7 (54·7 to 60·5)	242·8 (212·6 to 279·7)	91·7 (75·0 to 112·5)
	Zambia	51·8 (44·0 to 61·8)	−5·0% (−6·6 to −3·2)	0·25 (0·21 to 0·30)	0·36 (0·30 to 0·41)	66·4 (63·8 to 68·5)	60·4 (57·9 to 62·8)	63·2 (60·7 to 65·4)	55·0 (52·0 to 57·9)	123·4 (106·8 to 144·3)	31·5 (24·2 to 41·3)
Southern sub-Saharan Africa		42·0 (36·3 to 49·3)	−3·9% (−5·3 to −2·4)	0·27 (0·25 to 0·30)	0·39 (0·36 to 0·42)	67·1 (65·7 to 68·3)	60·5 (59·0 to 61·8)	63·8 (62·5 to 64·9)	55·6 (53·1 to 58·0)	733·0 (688·3 to 788·1)	70·7 (56·3 to 89·8)
	Botswana	41·3 (34·7 to 49·9)	−2·8% (−4·4 to −0·8)	0·29 (0·23 to 0·35)	0·42 (0·35 to 0·50)	65·6 (62·2 to 68·7)	59·0 (55·8 to 62·3)	62·2 (59·0 to 65·3)	54·0 (50·6 to 57·3)	21·2 (17·5 to 25·9)	2·0 (1·5 to 2·8)
	eSwatini	47·3 (40·3 to 55·3)	−5·4% (−7·0 to −3·6)	0·32 (0·26 to 0·39)	0·55 (0·48 to 0·63)	63·6 (60·2 to 67·5)	53·5 (50·3 to 56·4)	58·3 (55·0 to 61·5)	50·6 (47·3 to 53·8)	11·6 (9·5 to 14·1)	1·4 (1·2 to 1·8)
	Lesotho	64·4 (54·9 to 75·6)	−3·1% (−4·8 to −1·5)	0·49 (0·41 to 0·57)	0·66 (0·59 to 0·72)	55·4 (51·7 to 59·3)	48·6 (46·1 to 51·1)	51·8 (49·1 to 54·8)	45·2 (42·4 to 48·0)	32·5 (27·3 to 38·4)	3·0 (2·4 to 3·7)
	Namibia	35·1 (29·6 to 42·2)	−4·1% (−5·8 to −2·2)	0·22 (0·18 to 0·28)	0·37 (0·30 to 0·44)	69·3 (65·9 to 72·3)	61·3 (58·2 to 64·4)	65·2 (62·0 to 68·2)	56·7 (53·2 to 60·2)	18·9 (15·7 to 23·0)	2·2 (1·7 to 2·9)
	South Africa	36·9 (31·6 to 43·6)	−4·1% (−5·8 to −2·4)	0·26 (0·23 to 0·29)	0·37 (0·34 to 0·40)	68·3 (66·9 to 69·5)	61·9 (60·3 to 63·2)	65·1 (63·9 to 66·1)	56·2 (53·7 to 58·6)	522·4 (495·1 to 555·5)	38·5 (30·2 to 49·4)
	Zimbabwe	52·5 (45·0 to 62·0)	−3·7% (−5·3 to −2·0)	0·30 (0·25 to 0·35)	0·42 (0·36 to 0·48)	64·0 (61·5 to 66·6)	57·9 (55·5 to 60·2)	61·0 (58·6 to 63·4)	53·5 (50·6 to 56·3)	126·5 (109·7 to 147·1)	23·6 (18·6 to 29·9)
Western sub-Saharan Africa		95·3 (84·7 to 108·5)	−3·4% (−4·6 to −2·1)	0·19 (0·16 to 0·22)	0·24 (0·21 to 0·27)	65·7 (63·6 to 67·6)	62·0 (59·8 to 64·0)	63·8 (61·9 to 65·6)	57·5 (54·8 to 60·1)	3458·1 (3068·6 to 3944·2)	1534·2 (1278·2 to 1855·2)
	Benin	85·0 (73·8 to 99·3)	−3·2% (−4·8 to −1·5)	0·18 (0·14 to 0·23)	0·24 (0·19 to 0·31)	66·9 (63·3 to 69·8)	62·2 (58·0 to 65·6)	64·5 (60·6 to 67·7)	56·6 (52·7 to 60·1)	93·1 (75·8 to 115·4)	42·0 (34·3 to 51·5)
	Burkina Faso	108·8 (93·0 to 129·0)	−2·8% (−4·3 to −1·1)	0·19 (0·16 to 0·23)	0·28 (0·24 to 0·33)	64·3 (62·0 to 66·3)	59·1 (56·5 to 61·4)	61·7 (59·2 to 63·9)	54·1 (51·1 to 56·8)	201·8 (176·5 to 235·5)	98·8 (78·3 to 124·7)
	Cameroon	71·7 (61·2 to 84·0)	−4·0% (−5·6 to −2·4)	0·24 (0·19 to 0·29)	0·30 (0·24 to 0·36)	65·5 (62·7 to 68·1)	61·4 (58·7 to 64·1)	63·4 (60·6 to 66·0)	55·4 (52·2 to 58·5)	207·3 (173·0 to 247·4)	64·0 (52·5 to 77·2)
	Cape Verde	17·1 (14·7 to 19·8)	−5·2% (−6·9 to −3·3)	0·08 (0·06 to 0·10)	0·19 (0·16 to 0·23)	78·0 (76·6 to 79·1)	69·7 (68·2 to 71·1)	73·7 (72·2 to 75·1)	64·5 (61·8 to 67·1)	3·5 (3·2 to 3·8)	0·2 (0·1 to 0·3)
	Chad	112·9 (97·0 to 133·3)	−3·0% (−4·6 to −1·4)	0·23 (0·18 to 0·28)	0·28 (0·23 to 0·33)	62·2 (59·7 to 64·6)	58·9 (56·0 to 61·6)	60·4 (57·7 to 63·0)	52·8 (49·6 to 55·7)	156·6 (136·5 to 182·1)	85·6 (70·4 to 104·8)
	Côte d'Ivoire	73·3 (62·8 to 85·5)	−4·3% (−5·8 to −2·7)	0·20 (0·16 to 0·24)	0·27 (0·23 to 0·32)	67·0 (64·5 to 69·2)	62·0 (59·5 to 64·4)	64·3 (61·9 to 66·5)	56·1 (53·1 to 59·0)	182·4 (158·6 to 210·2)	64·8 (54·3 to 77·1)
	The Gambia	37·8 (31·9 to 45·5)	−5·0% (−6·8 to −3·2)	0·21 (0·16 to 0·25)	0·27 (0·22 to 0·33)	68·9 (66·5 to 71·0)	64·8 (62·5 to 67·1)	66·7 (64·4 to 68·9)	58·3 (55·2 to 61·1)	13·5 (11·8 to 15·6)	2·7 (2·0 to 3·7)
	Ghana	52·2 (44·9 to 60·7)	−4·3% (−6·0 to −2·6)	0·19 (0·16 to 0·22)	0·27 (0·23 to 0·32)	69·0 (66·9 to 70·9)	63·6 (61·4 to 65·6)	66·3 (64·2 to 68·3)	58·2 (55·3 to 60·7)	208·2 (180·7 to 239·1)	44·3 (33·3 to 58·4)
	Guinea	97·1 (83·8 to 111·9)	−3·4% (−5·0 to −1·8)	0·24 (0·20 to 0·29)	0·28 (0·23 to 0·34)	62·8 (59·6 to 66·1)	59·8 (56·4 to 63·2)	61·2 (57·9 to 64·6)	53·8 (50·4 to 57·2)	114·3 (93·7 to 138·0)	45·4 (36·4 to 55·8)
	Guinea-Bissau	71·2 (60·7 to 83·0)	−4·4% (−6·1 to −2·7)	0·26 (0·21 to 0·32)	0·37 (0·31 to 0·44)	64·0 (60·7 to 67·1)	58·1 (54·8 to 61·2)	61·0 (57·7 to 64·1)	53·6 (50·3 to 56·7)	14·8 (12·4 to 17·9)	4·4 (3·5 to 5·4)
	Liberia	60·9 (51·9 to 72·6)	−4·2% (−5·8 to −2·6)	0·21 (0·17 to 0·25)	0·23 (0·18 to 0·27)	66·9 (64·4 to 69·2)	65·4 (62·8 to 67·8)	66·1 (63·5 to 68·5)	56·8 (53·5 to 59·8)	29·7 (25·4 to 35·2)	8·3 (6·1 to 11·2)
	Mali	118·5 (102·6 to 138·4)	−2·8% (−4·3 to −1·2)	0·21 (0·17 to 0·27)	0·22 (0·18 to 0·27)	62·7 (59·0 to 65·6)	61·0 (56·8 to 64·7)	61·8 (57·9 to 65·2)	54·2 (50·3 to 57·7)	201·4 (165·3 to 248·6)	109·6 (89·5 to 135·3)
	Mauritania	42·8 (36·6 to 50·8)	−4·6% (−6·2 to −2·8)	0·15 (0·12 to 0·20)	0·15 (0·11 to 0·19)	71·0 (68·1 to 73·1)	70·6 (67·5 to 73·0)	70·8 (67·8 to 73·1)	62·1 (58·6 to 65·2)	21·0 (17·6 to 25·8)	4·7 (3·6 to 6·1)
	Niger	110·7 (94·5 to 131·5)	−3·0% (−4·5 to −1·3)	0·19 (0·15 to 0·24)	0·23 (0·18 to 0·29)	63·8 (60·1 to 66·9)	61·2 (57·0 to 64·9)	62·5 (58·5 to 65·9)	55·0 (51·1 to 58·5)	202·6 (166·1 to 249·4)	120·2 (99·5 to 146·5)
	Nigeria	103·7 (90·6 to 120·3)	−3·6% (−5·1 to −2·0)	0·17 (0·13 to 0·23)	0·21 (0·17 to 0·28)	65·9 (62·9 to 68·7)	62·8 (59·5 to 65·8)	64·3 (62·2 to 66·6)	55·5 (52·5 to 58·3)	1593·2 (1391·4 to 1830·8)	773·1 (627·7 to 956·7)
	São Tomé and Príncipe	25·4 (21·4 to 30·0)	−5·0% (−6·6 to −3·3)	0·14 (0·11 to 0·18)	0·18 (0·14 to 0·22)	72·5 (70·6 to 74·4)	69·5 (67·5 to 71·7)	70·9 (69·0 to 73·1)	62·2 (59·3 to 65·0)	1·0 (0·8 to 1·2)	0·1 (0·1 to 0·2)
	Senegal	49·3 (42·8 to 57·7)	−4·1% (−5·6 to −2·4)	0·16 (0·13 to 0·20)	0·20 (0·17 to 0·25)	70·1 (68·1 to 71·8)	66·8 (64·6 to 68·8)	68·5 (66·3 to 70·3)	59·8 (56·7 to 62·5)	89·9 (79·0 to 104·1)	22·8 (18·4 to 28·6)
	Sierra Leone	102·1 (88·6 to 117·1)	−4·5% (−6·1 to −2·9)	0·23 (0·19 to 0·28)	0·26 (0·21 to 0·31)	63·1 (59·8 to 66·0)	60·6 (57·0 to 64·0)	61·8 (58·4 to 65·0)	54·0 (50·4 to 57·4)	70·1 (58·2 to 84·6)	28·1 (22·9 to 34·3)
	Togo	63·2 (53·9 to 75·3)	−4·3% (−6·0 to −2·5)	0·19 (0·15 to 0·24)	0·29 (0·23 to 0·34)	67·9 (65·5 to 70·1)	62·0 (59·5 to 64·5)	65·0 (62·5 to 67·4)	56·9 (53·9 to 59·9)	53·6 (45·6 to 63·0)	15·0 (12·0 to 19·0)

Data in the parentheses are 95% uncertainty intervals. Super-regions, regions, and countries are listed in alphabetical order. HALE=healthy life expectancy. GBD=Global Burden of Diseases, Injuries, and Risk Factors Study.

**Figure 2:**
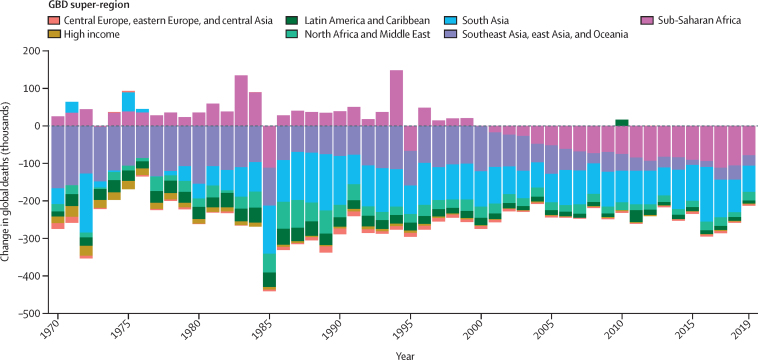
Annual change in under-5 deaths by GBD super-region, 1970–2019 The annual change in under-5 deaths is defined as the simple difference between deaths in the under-5 age group from the current year and the year before. Different colours show the annual changes in under-5 deaths from the different GBD super-regions. The height of the bar indicates the number of deaths. GBD=Global Burden of Diseases, Injuries, and Risk Factors Study.

The global number of under-5 deaths decreased from 19·9 million (95% UI 17·2–23·2) in 1950 to 16·3 million (15·1–17·6) in 1970. Further decline is evident in 2000 at 9·6 million (9·1–10·3) and 5·0 million (4·3–6·0) in 2019 (table 2). When considering changes by GBD super-region, north Africa and the Middle East; Latin America and the Caribbean; central Europe, eastern Europe, and central Asia; and the high-income super-region, while experiencing consistent decreases, have contributed relatively little to the global decline in child deaths from 1970 to 2019 (figure 2). Most of the decline was initially driven by southeast Asia, east Asia, and Oceania, and then by south Asia; since 2001, sub-Saharan Africa has contributed to an increasingly larger proportion of the decline in deaths, to a level similar to south Asia (figure 2). In 2019, decline in under-5 deaths from sub-Saharan Africa accounted for 36·5% (20·2–49·6) of the global decline, higher than that of south Asia, at 31·9% (8·2–45·9) in the same year. By contrast, since 2000, the contribution of southeast Asia, east Asia, and Oceania has gradually declined such that this super-region contributed only to 13·6% (8·8–22·0) of the global decline in 2019.

**Figure 3:**
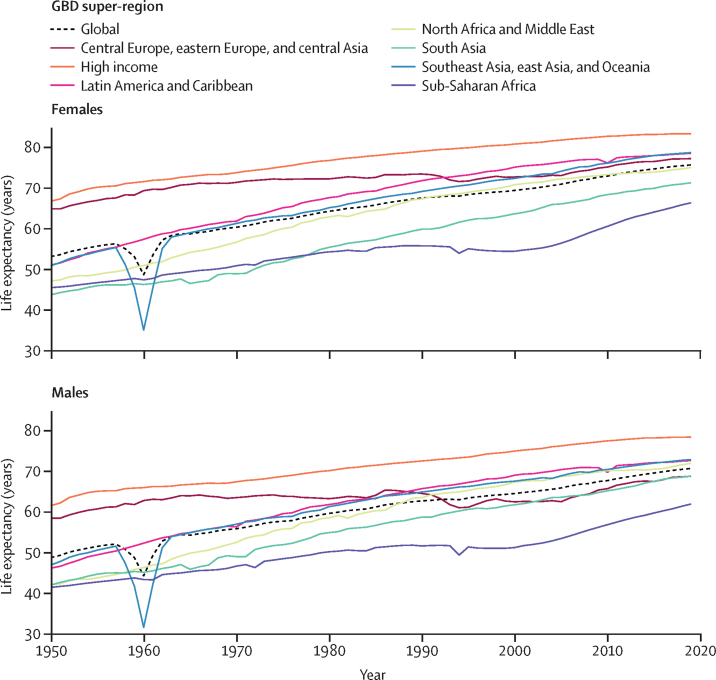
Life expectancy at birth by sex and GBD super-region, 1950–2019 Each line shows life expectancy at birth globally or for a GBD super-region, indicated by colour, between 1950 and 2019. GBD=Global Burden of Diseases, Injuries, and Risk Factors Study.

Global life expectancy at birth increased from 51·1 years (95% UI 49·4–52·7) in 1950 to 65·4 years (65·0–65·8) in 1990, 67·2 years (66·8–67·6) in 2000, and 73·5 years (72·8–74·3) in 2019 (table 2; appendix 2 table S2A). The difference in life expectancy at birth between females and males increased from 4·5 years (3·5–5·5) in 1950 to 5·4 years (4·9–5·8) in 2012, then decreased to 5·1 years (4·3–6·0) in 2019 (figure 3; table 2). Five of the seven super-regions saw life expectancy at birth improve continuously in the past seven decades (figure 3). Southeast Asia, east Asia, and Oceania; north Africa and the Middle East; Latin America and the Caribbean; and, to a lesser extent, south Asia (particularly among females) exhibit some convergence toward the life expectancy of the high-income super-region, although inequalities persist. The other two super-regions show markedly different temporal patterns. Sub-Saharan Africa experienced declines in the 1980s, 1990s, and early 2000s, but accelerated improvements since then. While life expectancy in central Europe, eastern Europe, and central Asia in the 1950s and 1960s was not much lower than in the high-income super-region, the central Europe, eastern Europe, and central Asia super-region saw relatively little improvement up until the mid-2000s when life expectancy began to increase (figure 3).

**Figure 4:**
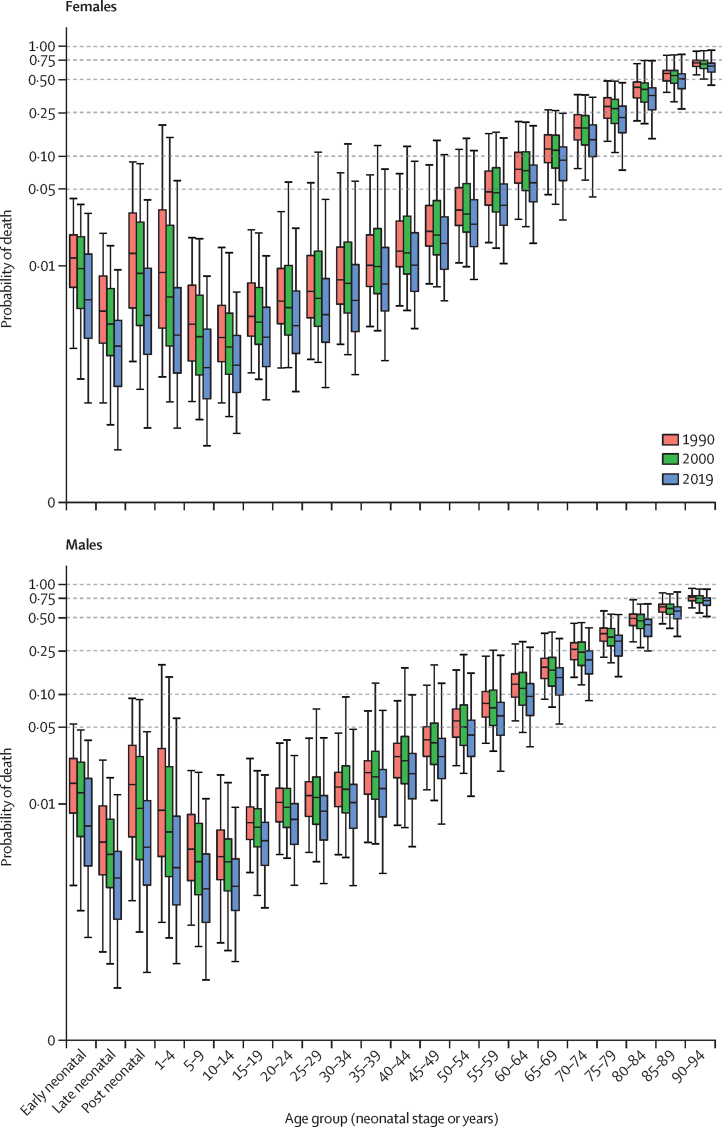
Distribution of probability of death by age, in 1990, 2000, and 2019 The graph shows the distribution of probability of death by age group for the years 1990, 2000, and 2019, calculated for the 204 countries and territories included in this study and plotted in a natural logarithmic scale. The boxes indicate the middle 50% of the distribution (75th and 25th percentiles) and the mean (horizontal bar in the box), while the whiskers indicate the middle 95% of the distribution (97·5th and 2·5th percentiles).

When considering the distribution of country-level age-specific probability of death for both sexes, all age groups younger than 20 years have seen a narrowing of the distribution among the years 1990, 2000, and 2019, as shown by the decreasing width of the middle 95% of age-specific probability of death, indicating decreasing disparities between countries (figure 4). Between 2000 and 2019, however, age groups between 20–24 years and 70–74 years have seen decreases in the width of the middle 95% while the widths increased during the same time period for age groups older than 70–74 for both sexes. Increases in the width of the total range, however, occurred in all age groups older than 20 years between 1990 and 2000. For age groups older than 75 years, the increase in the width of the middle 95% of the age-specific probability of death is much more pronounced during the past three decades.

While there have been improvements in survival across the age spectrum in the past seven decades, this has not been universal. Increases in mortality, especially among adult age groups, have been observed in countries severely affected by the HIV/AIDS epidemic in the 1990s and early 2000s. Fatal discontinuities due to events such as civil conflict and natural disasters have led to sharp increases in mortality, too. In more recent years, similar increases in mortality have been seen among young adult age groups in the USA, Sweden, the UK, Norway, New Zealand, Malta, Israel, Iceland, Greece, Brunei, Belgium, and Argentina.

### HALE

Global HALE increased from 56·9 years (95% UI 54·4–59·1) in 1990 to 58·6 years (56·1–60·8) in 2000, 61·3 years (58·7–63·6) in 2010, and 63·5 years (60·8–66·1) in 2019. Between 2000 and 2019, HALE increased in 202 of 204 countries and territories. HALE expanded in every region of the world between 1990 and 2019, with the largest increases occurring in eastern sub-Saharan Africa. Seven of the 15 countries in eastern sub-Saharan Africa had an increase in HALE at birth greater than 10 years, with an average annual improvement of at least 0·3 years between 1990 and 2019 (appendix 2 table S7). There is a strong correlation between SDI and HALE at birth and at age 65 years over this period. The correlation coefficient between HALE at birth and SDI is 0·85 based on data for years 1990, 2010, and 2019. The correlation coefficient is 0·70 for age 65 years. Similar to the improvement in life expectancy at birth, 200 of the 204 countries and territories analysed have seen improvement in HALE at birth for both sexes between 1990 and 2019. The four countries that have experienced declines in HALE at birth are Lesotho, eSwatini, Zimbabwe, and Uzbekistan. In 21 countries, HALE at birth increased by more than 10 years between 1990 and 2019. Among them, Ethiopia, Rwanda, Eritrea, and Uganda had the largest improvement in HALE at birth, between 15·7 and 19·1 years. Among the 21 countries with the biggest gains in HALE at birth between 1990 and 2019, 20 of them are from the low and low-middle SDI quintiles. During this same time period, 187 countries had improvements in HALE at age 65 years. Among the 11 countries with more than a 3-year increase in HALE at age 65 years between 1990 and 2019, only four countries—Maldives, Ethiopia, Bangladesh, and Rwanda—are from the low and low-middle SDI quintiles. While the correlation between SDI and HALE is strong, the changes in SDI and changes in HALE between 1990 and 2019 are much more modest. The correlation between changes in HALE at birth and change in SDI is 0·34. The similar correlation for change in HALE at age 65 years and changes in SDI during the same period is much weaker at about 0·06.

**Figure 5:**
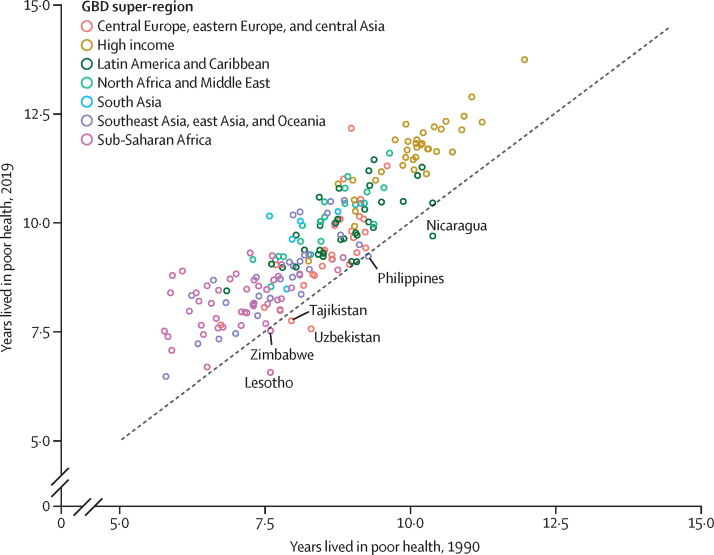
Years lived in poor health in 1990 and 2019 The scatter plot shows years lived in poor health, calculated by subtracting HALE from life expectancy at birth, for 1990 and 2019. Datapoints are coloured by GBD super-region. GBD=Global Burden of Diseases, Injuries, and Risk Factors Study. HALE=healthy life expectancy.

When considering years lived in poor health, as measured by the difference between life expectancy at birth and HALE at birth, only six countries—Lesotho, Uzbekistan, Nicaragua, Tajikistan, the Philippines, and Zimbabwe—have seen a decrease between 1990 and 2019 (figure 5). The rest of the 204 countries and territories have all seen numbers of years spent in poor health increase, even though both life expectancy at birth and HALE at birth have improved in the same period.

### Observed versus expected life expectancy based on SDI

**Figure 6:**
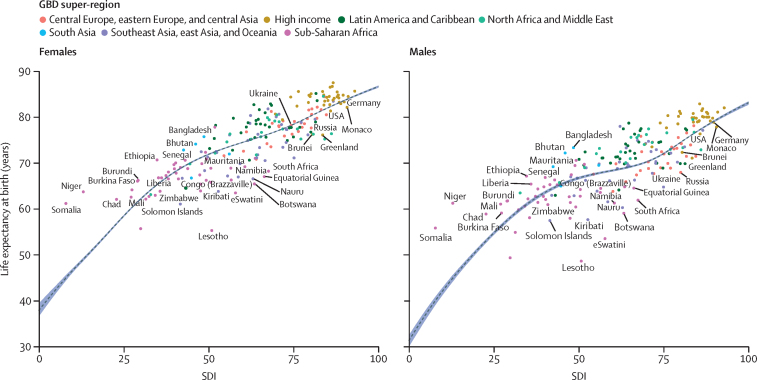
Life expectancy at birth and fit of expected value based on SDI, 2019 The scatter plot shows estimated life expectancy at birth in years for 204 countries and territories coloured by super-region for 2019. The dashed black line represents life expectancy that is predicted on the basis of SDI, with 95% uncertainty intervals shaded. GBD=Global Burden of Diseases, Injuries, and Risk Factors Study. SDI=Socio-demographic Index.

Figure 6 shows for each country in 2019 the observed life expectancy at birth versus expected life expectancy—ie, comparing GBD estimates for each location with life expectancy anticipated on the basis of SDI. For males, both the high-income and Latin America and Caribbean super-regions had the highest proportion of countries that have achieved higher life expectancy than expected. For the high-income super-region, 31 (86%) of 36 countries had male life expectancy at birth higher than expected. Spain, Portugal, Israel, Italy, Singapore, and Malta had estimated male life expectancy at birth more than 5 years higher than what would be expected based on SDI. The five countries in this super-region that had male life expectancy at birth lower than expected are Brunei, Greenland, Monaco, the USA, and Germany. For the USA, the difference between male life expectancy at birth and that based on its level of SDI in 2019 was −1·1 years. The difference was −3·4 years for Brunei, −1·5 years for Greenland, −1·5 years for Monaco, and −0·3 years for Germany. For sub-Saharan Africa, 18 (39%) of 46 countries had male life expectancy at birth higher than the expected value based on SDI. All 18 of these countries were in either eastern or western sub-Saharan Africa. The ten countries in sub-Saharan Africa with the biggest positive difference between observed and expected male life expectancy at birth are Somalia (15·1 years), Niger (13·6 years), Ethiopia (6·4 years), Mali (5·5 years), Chad (5·2 years), Burundi (5·7 years), Burkina Faso (4·0 years), Mauritania (3·8 years), Senegal (3·7 years), and Liberia (3·2 years). Ten countries in the sub-Saharan Africa super-region had male life expectancy at birth more than 5 years lower than what SDI in the same year would predict: Lesotho (−18·4 years), eSwatini (−14·7 years), Botswana (−10·0 years), Zimbabwe (−8·3 years), South Africa (−8·0 years), Namibia (−7·5 years), Central African Republic (−7·1 years), Zambia (−6·5 years), Equatorial Guinea (−5·6 years), and Cameroon (−5·2 years). For males, countries in sub-Saharan Africa accounted for seven of the ten countries with the largest negative difference between life expectancy at birth in 2019 and the expected values based on SDI. The three countries outside of sub-Saharan Africa are Kiribati (−9·7 years), Nauru (−8·5 years), and Russia (−7·1 years).

The high-income GBD super-region also had the most countries proportionally (80·6%) and in absolute terms (n=29) with higher female life expectancy at birth than expected based on SDI. Of the 19 such countries in sub-Saharan Africa, 18 are either in eastern or western sub-Saharan Africa. One country from central sub-Saharan Africa, Democratic Republic of the Congo, was included in this list and southern sub-Saharan Africa had no countries with a higher female life expectancy than expected. Among the ten countries with the largest negative difference between estimated and expected life expectancy at birth for females in 2019, five are in southern sub-Saharan Africa (Lesotho [−16·8 years], eSwatini [−10·5 years], Botswana [−9·9 years], South Africa [−8·2 years], and Zimbabwe [−7·2 years]), three are in Oceania (Kiribati [−8·9 years], Nauru [−8·4 years], and Solomon Islands [−7·2 years]), and the rest are in central sub-Saharan Africa (Equatorial Guinea [−9·5 years] and Congo [Brazzaville; −7·5 years]).

**Figure 7:**
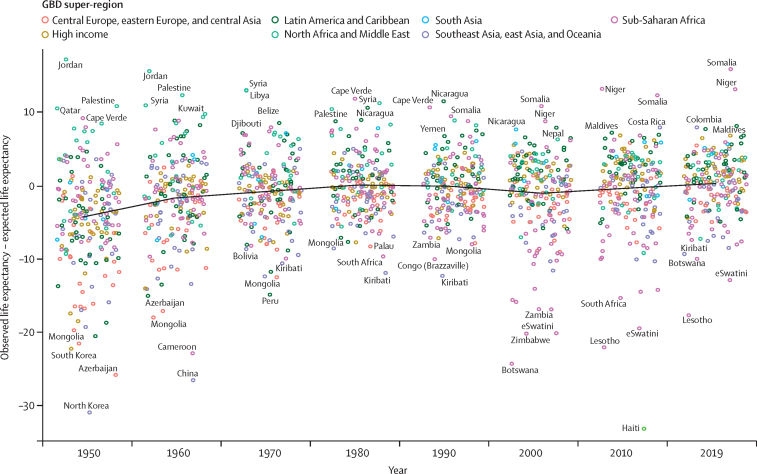
Difference between observed life expectancy and expected life expectancy by decade Datapoints show the difference between observed life expectancy and the expected life expectancy at birth based on SDI by country or territory for every tenth year between 1950 and 2010, and for 2019. The black line shows the mean difference by year over time. GBD=Global Burden of Diseases, Injuries, and Risk Factors Study. SDI=Socio-demographic Index.

Between 1950 and 1980, there was a general improvement in the mean difference between observed and expected life expectancy at birth for both sexes combined (figure 7). This improvement mainly levelled off between 1980 and 2000. Countries from the sub-Saharan Africa super-region and the central Europe, eastern Europe, and central Asia super-region have more often had lower observed life expectancy at birth than that expected. In the most recent years after 2000, however, countries from sub-Saharan Africa, including Niger and Somalia with higher life expectancy than expected, are among the outliers. Such a trend is visible when we examine the observed versus expected life expectancy at birth by SDI quintiles (table 3). For 2019, for example, the difference between observed and expected life expectancy at birth is positive across all SDI quintiles.

**Table 3:** Observed and expected life expectancy, globally and by SDI quintile, for 1990, 2000, 2010, and 2019

	**1990**			**2000**			**2010**			**2019**		
	Observed life expectancy	Expected life expectancy	Difference	Observed life expectancy	Expected life expectancy	Difference	Observed life expectancy	Expected life expectancy	Difference	Observed life expectancy	Expected life expectancy	Difference
**Global**	**67·2**	**65·1**	**2·1**	**67·2**	**67·2**	**0·0**	**70·6**	**69·6**	**1·0**	**73·5**	**71·7**	**1·8**
Low SDI	53·8	52·3	1·5	55·4	54·5	0·9	60·5	58·7	1·8	65·6	63·1	2·5
Low-middle SDI	59·7	59·4	0·3	62·4	63·4	−1·0	66·5	67·1	−0·6	69·7	69·7	0·0
Middle SDI	67·0	66·7	0·3	69·5	69·4	0·1	72·1	71·2	0·9	74·7	72·6	2·1
High-middle SDI	70·4	70·4	0·0	71·6	72·1	−0·5	75·0	73·9	1·1	77·5	75·2	2·3
High SDI	76·0	75·9	0·1	78·1	77·6	0·5	80·4	79·0	1·4	81·2	80·2	1·0

GBD=Global Burden of Diseases, Injuries, and Risk Factors Study. SDI=Socio-demographic Index.

### Population

**Figure 8:**
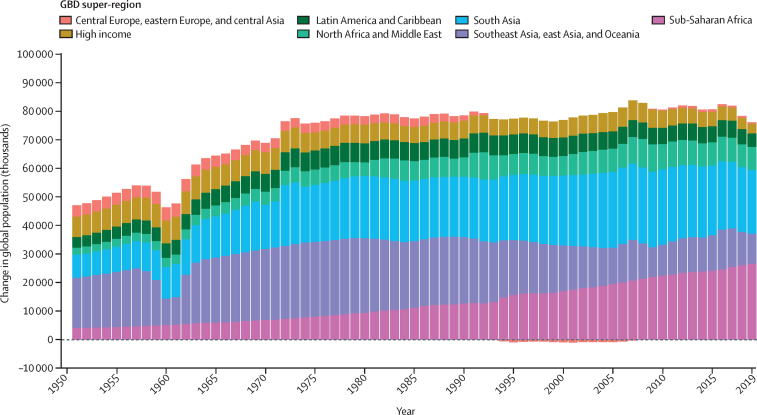
Annual change in global total population by GBD super-region, 1950–2019 The stacked bar chart shows the changes in global total population from year to year. The height of the bar (or, when there is negative change from a region, for example in the 1990s and 2000s in central Europe, eastern Europe, and central Asia, the difference between the height of the bar above zero and the height of the bar below zero) indicates the total annual change in global population. GBD=Global Burden of Diseases, Injuries, and Risk Factors Study.

Global population increased from 2·5 billion (95% UI 2·5 to 2·6) in 1950 to 5·3 billion (5·2 to 5·5) in 1990, to 6·2 billion (6·0 to 6·3) in 2000, and to 7·7 billion (7·5 to 8·0) in 2019 (appendix 2 table S6A). From 2000 to 2019 alone, that is an increase in population of 25·7%. The annual increment in population from 1950 to 1951 increased from 48·8 million (42·4 to 54·2) in 1951 to a peak of 86·9 million (73·2 to 102·5) from 2006 to 2007. Since then, the increase has slowed down. The annual increase in population in 2019 is estimated to have been 78·9 million (51·9 to 102·9). When considering the distribution of annual population change by super-region, the story at the global level is primarily about three super-regions: sub-Saharan Africa; southeast Asia, east Asia, and Oceania; and south Asia (figure 8). The contribution of sub-Saharan Africa to the annual increase in total global population has varied over time. It accounted for only 8·8% (7·1 to 10·4) of the total increase in global population in 1951. Its share of the global annual increase in population changed to 10·0% (8·6 to 11·6) in 1970, then rose to 16·0% (14·0 to 18·4) in 1990, and finally to 35·9% (25·8 to 53·6) in 2019. In contrast, the proportion of annual global population increase coming from the southeast Asia, east Asia, and Oceania super-region declined from 37·3% (28·5 to 43·4) in 1951 to just less than 11·7% (−26·2 to 27·7) in 2019. The proportion of annual global population increase from south Asia increased between 1953 and 1980, largely stabilised in the 1980s before continuing to increase in the 1990s, and has started to decline since 2005.

At the national level, from 1950 to 2019, the total population size declined in 48 of the 204 countries and territories included in this study, 20 of which are in the GBD super-region of central Europe, eastern Europe, and central Asia. Seven countries in the high-income super-region have experienced declines in total population: Greece, Greenland, Andorra, Japan, Portugal, Spain, and Italy. In 2019, 34 countries had negative natural rates of increase; 17 countries experienced negative net migration.

**Figure 9:**
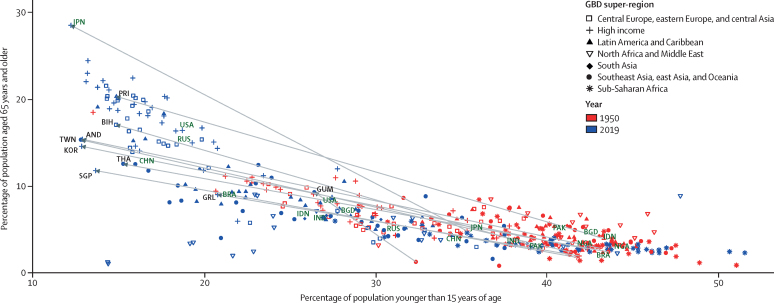
Percentage of the population younger than 15 years of age or aged 65 years and older, in 1950 and 2019 This figure shows the long-range changes in the distribution of population at the national level from 1950 to 2019. For reference, countries with the top ten population size in 2019 are shown in green. The grey lines connect the countries and territories with the most substantial changes between the two years of comparison. Codes within the graph are ISO 3 codes. GBD=Global Burden of Diseases, Injuries, and Risk Factors Study. AND=Andorra. BGD=Bangladesh. BIH=Bosnia and Herzegovina. BRA=Brazil. CHN=China. GRL=Greenland. GUM=Guam. IDN=Indonesia. IND=India. JPN=Japan. KOR=South Korea. NGA=Nigeria. PAK=Pakistan. PRI=Puerto Rico. RUS=Russia. SGP=Singapore. THA=Thailand. TWN=Taiwan (province of China).

There has been considerable change in the age structure of populations across the GBD regions (figure 9). For 174 of the 204 countries and territories, the proportion of the population in the under-15 age groups declined between 1950 and 2019. United Arab Emirates, Qatar, South Korea, Bahrain, Taiwan (province of China), Puerto Rico, Thailand, Andorra, Northern Mariana Islands, Singapore, Saudi Arabia, North Korea, and Bosnia and Herzegovina have had declines of more than 25 percentage points in the proportion of the population younger than 15 years during the past seven decades. Many countries in the high-income super-region as well as countries in other regions in Europe have had substantial increases in the proportion of population aged 65 years and older over the past seven decades, often by more than ten percentage points. In Japan, for example, the proportion of people aged 65 years and older increased by more than 480%, from 4·9% in 1950 to 28·4% in 2019. The 20 countries where the proportion of the population younger than 15 years has increased the most between 1950 and 2019 are nearly all in sub-Saharan Africa, with Afghanistan being the only exception. Similarly, the ten countries with the greatest decline in the proportion of population aged 65 years and older are all in sub-Saharan Africa and the north Africa and Middle East super-regions (appendix 2 figure S12).

### Demographic transition

Between 1950 and 2019, no country was found to be in the first stage of demographic transition—ie, before transition—which describes locations where both the crude birth rate and crude death rate are and have been high.

The early transition stage identifies locations where mortality has started to decline, yet crude birth rate is still relatively stable. By 1970, 17 countries were in this stage, with a high level of heterogeneity in the natural rate of increase among these countries. The natural rate of increase ranged from 2·6% per year in Burkina Faso to 3·8% in the Gambia. Among these 17 countries, TFR ranged from 6·2 (95% UI 5·8–6·5) in South Sudan to 8·0 (7·7–8·2) in Equatorial Guinea, almost four times replacement fertility. By 2019, there were no countries and territories in this stage.

The mid-transition stage consists of countries where crude birth rate has started sustained decline after a period of decline in crude death rate. In 1950, only 15 countries were in this group. The number of countries in this stage has quickly increased with the fast reduction in mortality since the 1950s. In 1970, 93 of 204 countries and territories were in the middle transition stage; between 1970 and 2019, this number decreased to 35.

In the late-transition stage, while crude birth rate continues to decline, crude death rate has stayed relatively stable. In 1970, 94 countries were in the late-transition stage: 46 with net emigration and 48 with net immigration. In 2019, 131 countries were in the late-transition stage, 81 of which had net emigration and 50 net immigration.

**Figure 10:**
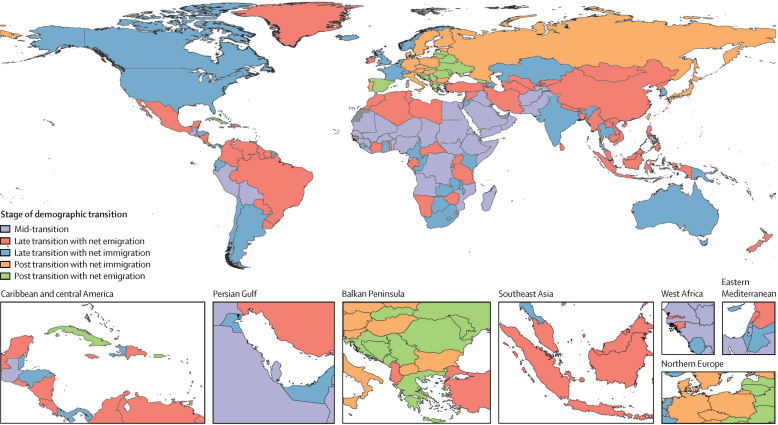
Stage of demographic transition by location, 2019 The map shows the distribution of locations across five stages of demographic transition in 2019. No countries or territories were in the before transition or early transition stages. Late demographic transition and post demographic transition locations are differentiated between those with net emigration and those with net immigration.

The final stage of the demographic transition comes after the crossover between crude birth rate and crude death rate where the natural rate of increase in population is negative. Before 1970, no country was in the post demographic transition stage. In 2000, 22 countries were in this stage. Among them, Belarus, Bulgaria, Croatia, Estonia, Georgia, Lithuania, Poland, Romania, and Ukraine belong to the post-demographic transition with net emigration group. Austria, Czech Republic, Denmark, Germany, Greece, Hungary, Italy, Luxembourg, Russia, and Sweden were in the post-transition with net immigration group. By 2019, the number of countries in this stage had increased to 38. 18 of these countries had a net emigration rate and 20 had a net immigration rate. Figure 10 shows the distribution of countries across these stages of demographic change for year 2019.

## Discussion

### Main findings

Our paper presents a comprehensive assessment of demographic changes in 204 countries and territories from 1950 to 2019, with a focus on the past two decades. There have been substantial changes in the demographics of most countries and territories since the turn of the millennium. Both life expectancy and HALE have expanded almost universally during our study period. While there have been impressive improvements in mortality reductions, recent slowdowns and reversals in improvements observed especially among adults indicate that progress is not guaranteed. We also see that in 2019, half the nations in the world had below-replacement fertility and 34 had a crude birth rate that was less than the crude death rate. 17 countries were in the precarious state of having a negative rate of population change and negative net migration. Demographic transition, which has largely been a story of faster or slower rates of change, is entering a new phase in many countries with high levels of development. Low fertility and rising average age might lead to inverted population pyramids, which will be fiscally and socially challenging.

### Global population health improvement

Our analysis shows that at the global level, all major mortality indicators and HALE have been improving. Rates of progress slowed from 1980 to 2000 but have since improved. Slower progress in these two decades can in part be traced to both the HIV epidemic and the mortality reversals in eastern Europe and central Asia. The strong correlation of SDI with age-specific mortality, life expectancy, and HALE suggests that the pace of improvement in key social, demographic, and economic conditions is a crucial factor determining global health progress. Average SDI has improved steadily in the last 50 years, but notably there has been faster progress for less developed countries in the past 20 years (appendix 1 table S16). This faster progress in the lower SDI quintiles has fuelled larger gains in life expectancy and HALE in these countries. While this study does not explore the causal pathways through which improvements in education,^34^, ^35^ income,^36^ and the status of women^37^, ^38^, ^39^ drive reductions in mortality, the strength of these associations and the myriad studies supporting causal pathways suggest that an important strategy for global health is to promote improvements in development as measured by key social and economic metrics including SDI. Strategies that increase educational attainment faster and improve the status of women might be particularly effective in stimulating health improvement.

### Implications for future progress

Improvement in mortality in the under-5 age group is an important achievement in global health in the past seven decades, and especially since the beginning of the UN Sustainable Development Goals (SDGs) era. Global under-5 deaths maintained a continuous decline despite increases in annual livebirths during the same period. The proportion of under-5 deaths globally that occur in sub-Saharan Africa has continuously increased in the past decades (figure 2). This shift towards contributing a higher proportion of global under-5 deaths, combined with higher TFR in the region compared with other regions, indicates that the pace of improvement in reducing under-5 deaths globally has and will experience further slowdowns due to such compositional shifts. This has important implications for further reducing deaths in the under-5 age groups, especially given that based on the trajectory of U5MR between 2010 and 2019, 37 of the 46 countries in sub-Saharan Africa will not be able to reduce the level of U5MR below the SDG target of 25 per 1000 livebirths by 2030 (appendix 2 table S5). Eliminating preventable under-5 deaths globally will require more strenuous efforts in the coming decades. In addition to changes in U5MR, changes in fertility, and the pace of that change across super-regions, have implications for achieving SDG goals. For instance, the trend in the fertility rate will have an important impact on the global population size in the future, which will have potentially large consequences for carbon production.^40^

Compared with the trend in child mortality, advances in reducing adult mortality have not been as continuous in every country in every past decade. Reversals in the global improvement in mortality have been observed in countries across the SDI spectrum. In the 1990s, countries in eastern and southern Africa, eastern Europe, and central Asia experienced increases in mortality (appendix 2 table S3A). In the past two decades, some countries in the high-income super-region have seen increases in the probability of death in adult age groups. In the USA, the increase in mortality, which was largely concentrated in adults between the ages of 20 and 50 years, was seen in at least 5 years of the most recent decade, with a similar pattern for both males and females. Such increases in adult mortality in the USA have been described as deaths of despair.^41^ This study also shows a similar pattern of increase in young adult mortality in the most recent two decades in other high-income countries, including Sweden, the UK, Norway, New Zealand, Malta, Israel, Iceland, Greece, Brunei, Belgium, and Argentina.

Identifying countries that have longer life expectancy than predicted by SDI can help to shed light on effective public health and health system policies. Exemplar countries include both locations where observed life expectancy at birth is much higher than expected based on SDI and those countries where life expectancy at birth is lower than expected. Our analysis shows that countries including Somalia, Niger, Ethiopia, Portugal, and Spain have achieved life expectancy at birth for both sexes in 2019 that is more than 5 years higher than the expected values based on SDI. Such countries come from many GBD regions, including regions with the lowest average life expectancy (such as eastern and western sub-Saharan Africa), as well as regions with high average life expectancy (such as western Europe). Further study comparing observed age-specific mortality rates and mortality rates predicted by SDI could lay the groundwork for research on programmes or policies responsible for such differences. Understanding broader demographic variation across countries and territories can also help to provide context for efforts to make further progress on child and adult mortality and other SDG-related goals.

### Stages of demographic transition

Our seven-category taxonomy of demographic transition is based on classical demographic transition theory but offers several refinements. First, we identify a post-transition stage where the natural rate of increase in population is negative. Classical theory postulated that societies would avoid sustained periods of negative natural rates of increase. Second, our taxonomy identifies emigration as a key factor affecting demographic conditions in late-transition and post-transition countries. Migration is a key element affecting the size of a population, so it is increasingly unrealistic to assume as zero, as in classical demographic analysis. This taxonomy can highlight discordant population profiles and provide an entry point for research into the economic, social, cultural, religious, and political factors that produce variation.

Our analysis shows that only 19% of all countries were in post transition in 2019. These 38 countries are mostly in Europe, including central, eastern, and western Europe. Post-transition countries that do not experience net emigration tend to reach their population peak roughly 35 years from when their net reproductive rate falls below 1·0 (35 years corresponds to the span of the active reproductive age group between ages 15 and 49 years). This underscores the point that attempting to increase fertility through policy interventions can only result in expansion in total population size and in the relative size of the working-age population in the long run. In the shorter term, it will create an exacerbated dependency ratio.

Countries in the two late-transition groups are still experiencing increasing population. They will probably move into the post-transition phase in the coming decades. Population increases should end soon, given that population momentum can be expected to end one to two generations after fertility reaches below-replacement level. Net emigration among the 81 late-transition countries in 2019 will contribute to slowing population growth. Such slowdowns, coupled with the further ageing of populations, have important implications for socioeconomic dynamics. Economists have pointed out potential links between a slow economic growth rate, ageing populations, and shrinking workforces.^42^, ^43^ Some middle SDI countries, however, have reaped the benefits of a demographic dividend^44^ in which a larger proportion of the working-age young adult population contributed to a high level of economic growth. High-income countries in the post-transition and late-transition stages have faced a variety of issues related to ageing populations and the declining size of the workforce. Innovative social and economic policies focused on migration represent one route for addressing labour force shortages. The Global Compact for Safe, Orderly and Regular Migration,^45^ prepared under the auspices of the UN, provides a useful framework for countries to rethink their migration policies in ways that facilitate mutually beneficial migration and ultimately mitigate the impediment of population ageing on economic growth.

The difficulty of expanding population through policy interventions is evident in recent country experiences. Pro-natalist policies^46^ have been proposed to change the level and trend of fertility, but such policies face substantial challenges once fertility decline has begun. A few Nordic countries including Sweden have implemented a variety of pro-natalist policies such as subsidised public day care and long paid leaves for both parents. Such policies might have contributed to the increase in TFR in Sweden in the 2000s, although TFR started to decline again in Sweden in the past decade. The Japanese Government has implemented similar pro-natalist policies since 1994, such as policies to provide more accessible child-care services; the TFR in Japan did start to improve in the early 2000s.^47^ Other countries have seen unsatisfactory results from pro-natalist policies. In an effort to increase fertility after its TFR fell far below replacement level, the Chinese Government reversed its longstanding family planning policy by first allowing a second child for couples from single-child families, then by implementing the two-child family planning policy.^48^ Our observations suggest the impact of such policy changes has been short lived. TFR reached a local peak in 2016, largely coming from a higher fertility rate among females in older reproductive age groups. Steep fertility decline soon followed, however. Annual livebirths in China declined from 18·2 million (95% UI 17·8–18·6) in 2016 to 15·3 million (14·9–15·7) in 2018, with further decline as shown in our model for year 2019. The one-child policy played an important role in the decline of fertility in China, but its role should not be exaggerated. Economic growth and improvement in the schooling and labour force participation rate of women in China all contributed to the decline in fertility and maintain fertility well below replacement level over the past two decades.

The 35 countries in mid-transition are primarily in sub-Saharan Africa and north Africa and the Middle East. In these locations, socioeconomic, political, and religious influences on both fertility and mortality have contributed to demographic profiles that are quite different from the post-transition group.^49^, ^50^ The presence of the 102 countries and territories where TFR is above replacement level in the mid-transition and late-transition groups underscores the persistence of above-replacement-level fertility coupled with a delay in the realisation of lowest observed mortality.

While there is movement between categories over time, such changes in demographic factors are difficult to steer. Fertility offers a good example. This study shows temporal increases in TFR in some countries after relatively long periods of decline. Yet, it is far from certain that fertility will increase or converge in the long term. Patterns in cohort fertility indicate otherwise. Cohort-completed fertility by age 50 years (CCF50) is a much better measure than TFR of the true level of fertility because measuring fertility by birth cohort controls for general shifts in the timing of childbearing. When we look at CCF50, temporal rebounding in fertility is much less common. Since the 1940 birth cohort, CCF50 has peaked and begun to decline in all countries and territories (1969 represents the last birth cohort for which we have complete age-specific fertility data between ages 15 and 50 years). The strong relationship between fertility behaviour, socioeconomic indicators of education, and met need for contraception also suggest fertility is unlikely to rebound and converge toward replacement levels in the long run. Yet, replacement fertility is not constant at 2·1, as usually implied in the literature. Replacement level fertility is realised when the net reproduction rate is 1·0, meaning a female is producing one daughter if she is subject to the current age pattern of mortality and fertility. As it is affected by both the sex ratio at birth and the mortality rate, the replacement level fertility varies over time and by location. Based on our current estimates of fertility, mortality, and sex ratio at birth, in 2019 replacement level fertility varied from 2·05 to 2·44.

### Comparisons between GBD demographic estimates and other demographic estimates

There are considerable differences between GBD and other demographic studies in the methods used to synthesise input data from diverse sources and to process data. The correlation between our U5MR estimates and those from UNICEF^51^—another major source for global U5MR estimates—is high, with a correlation coefficient of 0·98 between the two sets of estimates for the period between 1970 and 2019. At the global level, the most recent UNICEF estimates put global U5MR in 2018 at 38·6 deaths per 1000 livebirths; for the same year, our estimate is 38·5 deaths (95% UI 34·7–43·0). The estimates of global under-5 deaths for 2018 are also similar, with both UNICEF and GBD reporting 5·3 million deaths in that year. There are differences for estimated U5MR at the country level, however. Although the mean relative difference at the country level between our study and UNICEF's most recent estimates is only 4·2%, differences ranged from −62·4% to 198·6%. This reflects how raw data are processed, and the different data synthesis methods that have been used. For adult mortality rate, the correlation between our study and UN Population Division estimates in the most recent World Population Prospects (WPP 2019)^52^ is still relatively high at 0·89. Relative differences in country-level adult mortality rate estimates, however, range from −68·4% for males in the US Virgin Islands in 2017 to 160·5% for males in Iran in 1982. In 15·6% of available country-years, the absolute relative difference between WPP 2019 and this study is more than 30%. Countries where the differences are largest tend to be those that have non-complete vital registration systems, and those where our study has employed indirect estimates of adult mortality rate based on sibling survival data from surveys. The difference in both U5MR and adult mortality rate is reflected in the difference in life expectancy at birth estimates from our study and those from WPP 2019. The maximum absolute difference in life expectancy estimates can be as high as 29 years, especially in country-years that have sparse vital registration data and those years affected by major fatal discontinuities.

The difference between the TFR estimates from GBD 2019 and those from WPP 2019 is much smaller. Among 2618 country-years between 1952 and 2017 (WPP 2019 only provides estimates for every 5-year period), only 6·0% (157 of 2618) have an absolute relative difference higher than 20%. There are an additional 467 country-years where the absolute relative difference is between 10% and 20%. Countries where GBD 2019 and WPP 2019 have the biggest relative differences are Libya, North Korea, and Samoa, where the empirical data on fertility are sparse.

### Limitations

This study had several limitations. First, our computational resources did not allow us to propagate uncertainty for some covariates throughout our analytical process. Uncertainty from model estimation at each stage and the model life tables along with key demographics inputs and outputs such as U5MR and age-specific mortality rates are included, but uncertainties for some covariates such as lag-distributed income and education were not. A similar limitation applies to covariates used in other analytical steps, such as completeness in the under-5 age group in the completeness synthesis model, which does not account for measurement error.

Second, our migration estimates could be improved. We made an effort to strengthen our population estimation model by integrating information on the overall level and age pattern of migration, especially for countries experiencing large-scale net immigration or emigration. A standalone migration model that estimates age-specific and sex-specific immigration and emigration is necessary to have more consistent estimates among all key components of the demographic balancing equation: fertility, mortality, migration, and population.

Third, this study faced a range of limitations due to the availability of high quality data or use of lower quality data. Sparsity of census data in certain locations and the lag between censuses affected our completeness estimation for vital and sample registration systems, and for household death recall in censuses and surveys. The mortality analysis for the adult age groups in locations without a vital registration system relied heavily on indirect estimates of adult mortality based on sibling survival data, household death recall from censuses and surveys, and covariates in data-sparse periods. Nationally representative sample registration or disease surveillance systems should be set up in locations where these systems do not exist. The availability and quality of migration data are generally poor compared with data on mortality and fertility. This is true even for high-income countries that generally keep more accurate accounts of immigration, although that is less the case for emigration. Information on migration is also scarce in censuses and surveys. When migration data are collected, questionnaires tend to be non-standard, which makes cross-country comparison and integration of information on migration from different sources difficult. This is especially a problem for low SDI countries that are both a source and destination of most migration in recent years. As the most influential international survey, only about 25% of all Demographic and Health Surveys include any questions that can be used to infer information on migration. Country governments and international organisations should improve data collection on migration, which is crucial for the accurate assessment of populations over time.

Lastly, when calculating data variance, we have assumed a binomial distribution. There might be overdispersion in some of the data we used, leading to inaccurate estimates of data variance. We have not tested such models. Given the sampling errors of the mortality and fertility rate data we have used from vital registration and civil registration systems are probably much smaller than the non-sampling errors, we do not anticipate such a change will have a sizeable impact on our estimates. We will consider testing such assumptions in future rounds of GBD.

### Future directions

This study suggests several directions for future research. As stated above, a separate assessment of the volume, flow, and age pattern of migration and integration of that assessment into the GBD demographic estimation process would be valuable.

Further integration of the components in the demographic analysis is another avenue for future work. Our Bayesian hierarchical cohort component model for population projection takes as inputs age-specific mortality and fertility estimated by other components of the GBD demographic estimation process to estimate age-specific population. It will be worth exploring whether estimates will be improved by allowing this model to generate not only age-specific population but also posterior age-specific fertility and mortality estimates.

Increasing the frequency, quality, and coverage of censuses and post-enumeration surveys in many locations would produce better data that would ultimately contribute to more accurate demographic estimates. Post-enumeration surveys are an important tool to gauge the accuracy of censuses, yet information on post-enumeration surveys is scarce. Among the 1250 censuses we included in our analysis, only 161 came with information on census coverage from the corresponding post-enumeration survey.

Future research could use our taxonomy to assess the consequences of demographic variation across countries and territories. Relating variation in demographic transitions to variation in epidemiological transitions would be one direction. Another possibility would be to research in more detail the movement of countries between categories.

### Conclusion

The most recent decade continued the trend of general progress in reducing global fertility and global mortality. While the majority of countries are following this pattern of progress, there is evidence that the world is nearing a demographic inflection point. Half of the 204 countries and territories in our analysis had below-replacement fertility in 2019. Nearly one in five had entered the post-transition stage where the natural rate of increase was negative, and nearly half of them faced the twofold challenge of a negative natural increase in population and net emigration. These demographic shifts, combined with the trend toward stagnation in or reversals of mortality improvements in some high SDI countries, highlight that continued declines in mortality are not a guarantee. Additionally, sustained low fertility in the setting of slowing or reversing mortality could hasten the number of countries entering the challenging post-transition phase.

Correspondence to: Dr Haidong Wang, Institute for Health Metrics and Evaluation, University of Washington, Seattle, WA 98195, USA haidong@uw.edu

## Data sharing

To download the data used in these analyses, please visit the Global Health Data Exchange GBD 2019 website.
