# Supplementary material for: Global age-sex-specific fertility, mortality, healthy life expectancy (HALE), and population estimates in 204 countries and territories, 1950–2019: a comprehensive demographic analysis for the Global Burden of Disease Study 2019
Source: Lancet. 2020 Oct 17;396(10258):1160–203. doi: 10.1016/S0140-6736(20)30977-6 (PMC7566045; doi:10.1016/S0140-6736(20)30977-6)
Supplement: Supplementary appendix 2 [file mmc2.pdf]

THE LANCET

Supplementary appendix 2

This appendix formed part of the original submission and has been peer reviewed. We post it as supplied by the authors.

Supplement to: GBD 2019 Demographics Collaborators. Global age-sex-specific fertility, mortality, healthy life expectancy (HALE), and population estimates in 204 countries and territories, 1950–2019: a comprehensive demographic analysis for the Global Burden of Disease Study 2019. *Lancet* 2020; **396:** 1160–203.

Appendix 2: Supplementary results appendix for "Global, regional, and national age-sex-specific fertility, mortality, and population estimates, 1950–2019: a comprehensive demographic analysis for the Global Burden of Disease Study 2019"

This supplement provides additional figures and more detailed results for Demographics results.

These results are available in an interactive data downloading tool in the Global Health Data Exchange (GHDx). The tool contains the complete set of results from all GBD 2019 papers; however, specialised tables from the papers will be available as separate entries in the GHDx. Access to the results and tables through the GHDx will be made publically available upon publication.

Figures

Supplemental Results Figure 1: Most recent year of available vital registration……………………………………….4

Supplemental Results Figure 2: Comparison of estimates of under−5 mortality from GBD 2019 and IGME 2018…….5

Supplemental Results Figure 3: Comparison of life expectancy at birth by location in GBD 2017 vs GBD 2019……….6

Supplemental Results Figure 4: Comparison of life expectancy at birth for countries in GBD 2019 vs WPP 2019, both sexes combined, for every five years from 1950 to 2015……………………………………………………7

Supplemental Results Figure 5a: Distance to Life Expectancy at birth from the GBD-YLL-LT, 2019, Males..22

Supplemental Results Figure 5b: Distance to Life Expectancy at birth from the GBD-YLL-LT, 2019, Females………23

Supplemental Results Figure 6: Correlation of Baseline nqx and the annualized rate of change in every age group by decade………..24

Supplemental Results Figure 7: Number of deaths over time by age group, by sex, 1950-2019……………25

Supplemental Results Figure 8: Death Structure by SDI in 2019…………………………………………………………26

Supplemental Results Figure 9: Percent difference between GBD 2019 and UN WPP population estimates, by location and sex, all ages, for 1950, 1975, 2000, 2015, and 2019……………………………………27

Supplemental Results Figure 10: Percent difference between GBD 2019 and UN WPP population estimates, by location and sex, ages 5-9, 45-49, 70-74, for 2019…………………………………………………………28

Supplemental Results Figure 11: Population predictions by SDI…………………………………………………………29

Supplemental Results Figure 12: Population pyramids for females and males, by age and by location for 1950, 1975, 2000 and 2019………………………………………………………………………………………………………30

Supplemental Results Figure 13: Age-specific fertility fitted trend lines by location, 1950-2019………...234

Tables

Supplemental Results Table 1a: Live births (in thousands) by location, both sexes combined, 1950, 1960, 1970, 1980, 1990, 2000, 2010, 2019……2264

Supplemental Results Table 1b: Live births (in thousands) by location, males, 1950, 1960, 1970, 1980, 1990, 2000, 2010, 2019……2277

Supplemental Results Table 1c: Live births (in thousands) by location, females, 1950, 1960, 1970, 1980, 1990, 2000, 2010, 2019……2290

Supplemental Results Table 2a: Estimates of life expectancy at birth by location, both sexes combined, 1950, 1960, 1970, 1980, 1990, 2000, 2010, 2019……2303

Supplemental Results Table 2b: Estimates of life expectancy at birth by location, males, 1950, 1960, 1970, 1980, 1990, 2000, 2010, 2019……2322

Supplemental Results Table 2c: Estimates of life expectancy at birth by location, females, 1950, 1960, 1970, 1980, 1990, 2000, 2010, 2019……2340

Supplemental Results Table 3a: Age-standardised mortality rates by location, both sexes combined, 1950, 1960, 1970, 1980, 1990, 2000, 2010, 2019……2359

Supplemental Results Table 3b: Age-standardised mortality rates by location, males, 1950, 1960, 1970, 1980, 1990, 2000, 2010, 2019……2369

Supplemental Results Table 3c: Age-standardised mortality rates by location, females, 1950, 1960, 1970, 1980, 1990, 2000, 2010, 2019……2378

Supplemental Results Table 4a: Under-5 mortality rates by location, both sexes combined, 1950, 1960, 1970, 1980, 1990, 2000, 2010, 2019……2387

Supplemental Results Table 4b: Under-5 mortality rates by location, males, 1950, 1960, 1970, 1980, 1990, 2000, 2010, 2019……2401

Supplemental Results Table 4c: Under-5 mortality rates by location, females, 1950, 1960, 1970, 1980, 1990, 2000, 2010, 2019……2415

Supplemental Results Table 5: Under-5 death numbers by location, both sexes combined, 1950, 1960, 1970, 1980, 1990, 2000, 2010, 2019……2429

Supplemental Results Table 6a: Population (in thousands) by location, both sexes combined, 1950, 1960, 1970, 1980, 1990, 2000, 2010, 2019……2441

Supplemental Results Table 6b: Population (in thousands) by location, males, 1950, 1960, 1970, 1980, 1990, 2000, 2010, 2019……2453

Supplemental Results Table 6c: Population (in thousands) by location, females, 1950, 1960, 1970, 1980, 1990, 2000, 2010, 2019……2465

Supplemental Results Table 7: Healthy life expectancy (HALE) by location, both sexes combined, 1990, 2000, 2019, and difference between 1990 and 2019……………………………………………………………………..2477

Supplemental Results Table 8: Replacement Level Fertility by country, 2019……...............................2500

Supplemental Results Figure 1a: Most recent year of available vital registration

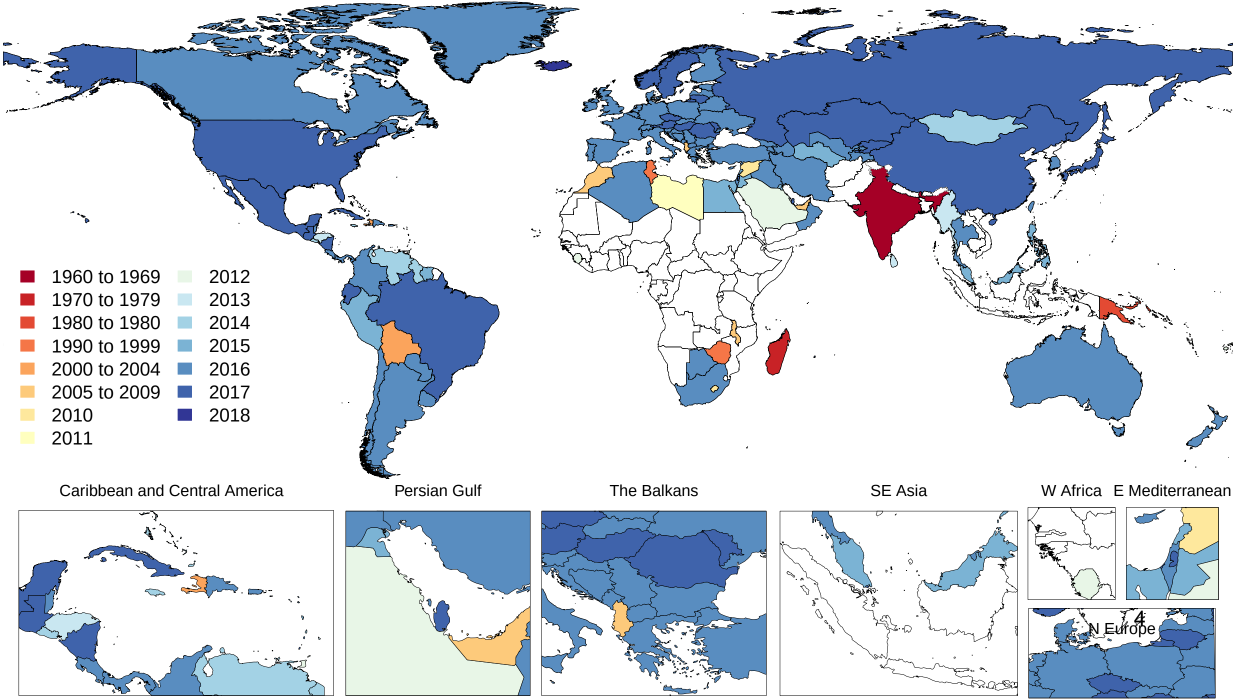

Ratio of vital registration data to estimated with shock death numbers

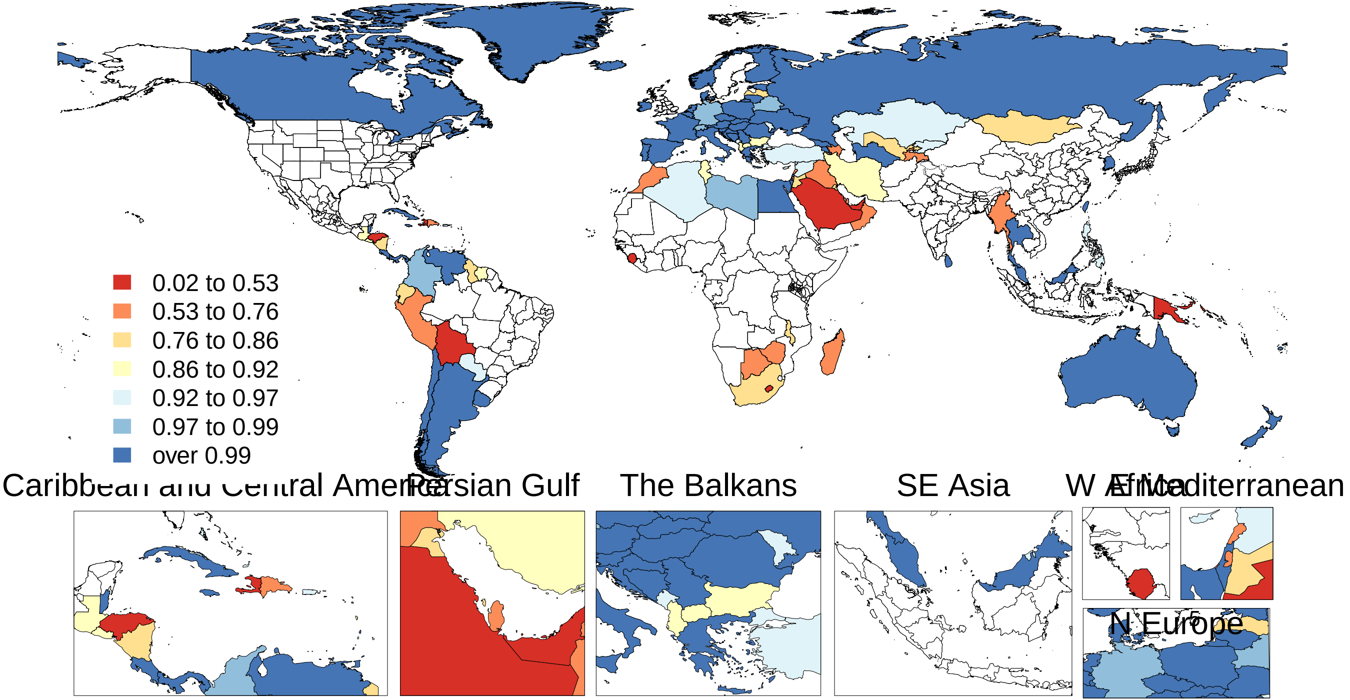

Supplemental Results Figure 2: Comparison of estimates of under 5 mortality from GBD 2019 and IGME

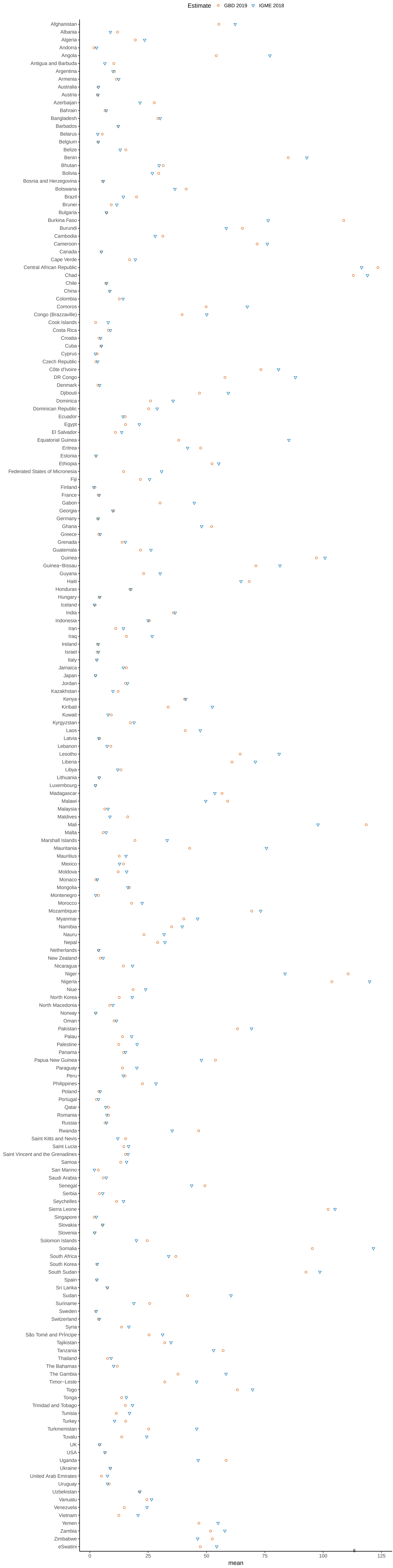

Supplemental Results Figure 3: Comparison of life expectancy at birth by location in GBD 2017 vs GBD 2019

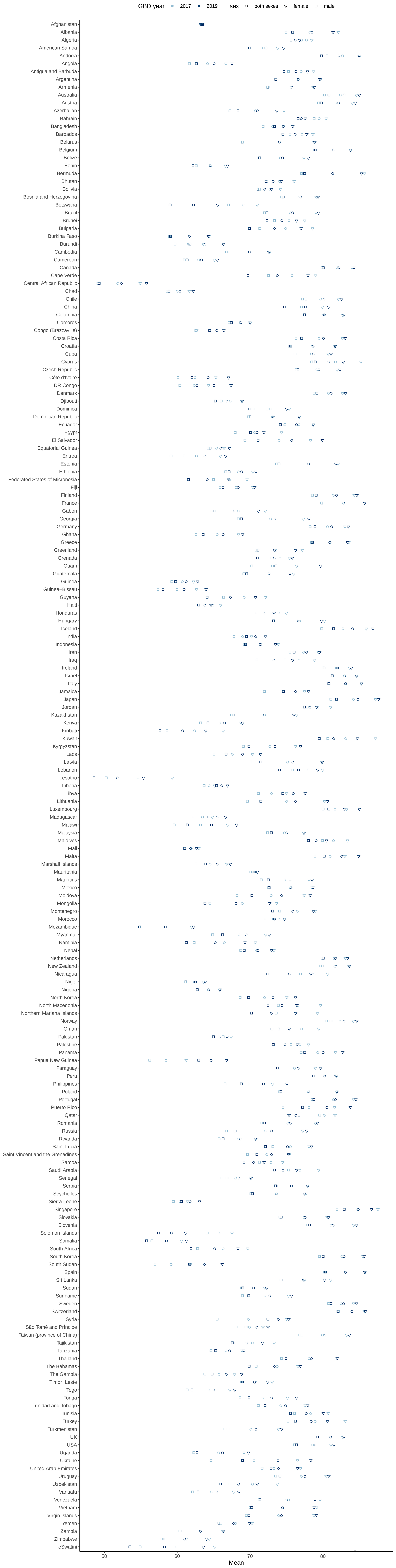

Supplemental Results Figure 4: Comparison of life expectancy at birth for countries in GBD 2019 vs WPP 2019 for 1950, both sexes combined

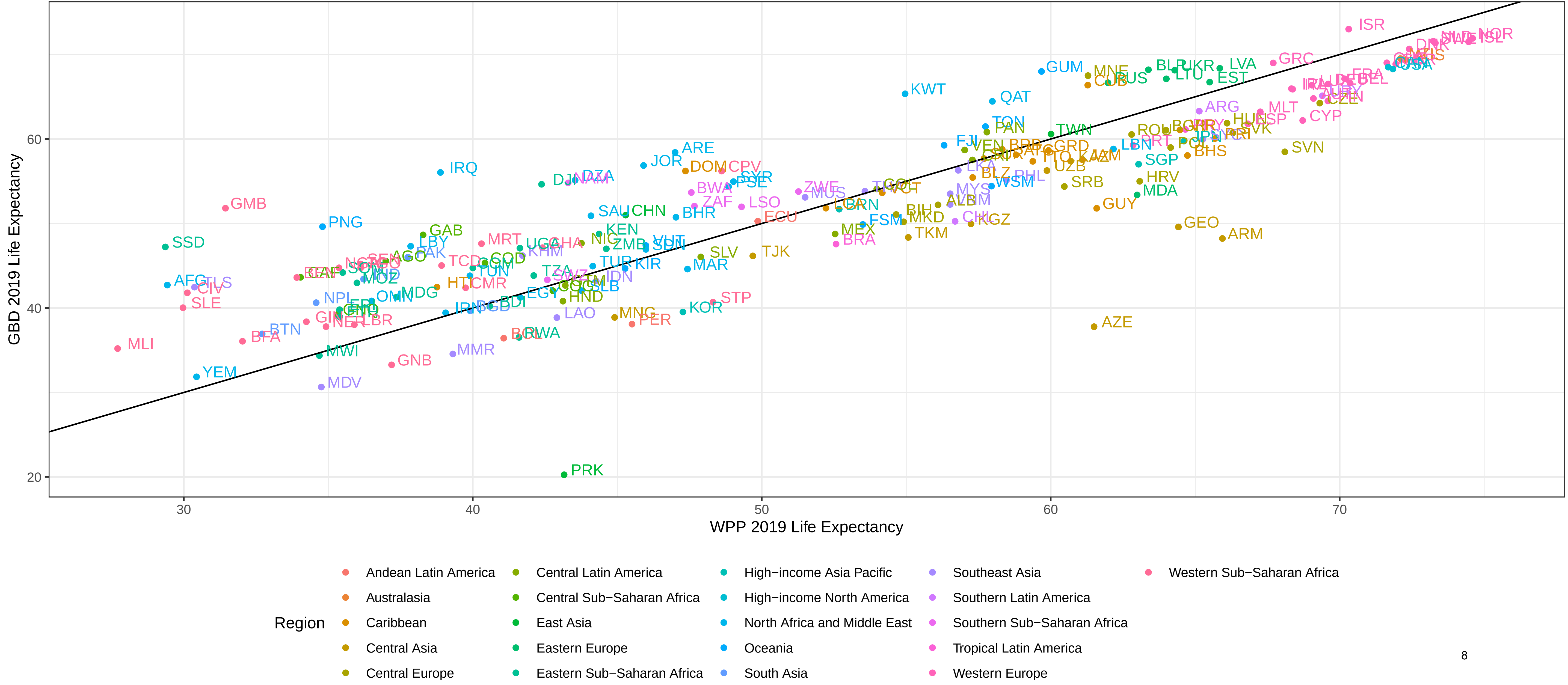

Supplemental Results Figure 4: Comparison of life expectancy at birth for countries in GBD 2019 vs WPP 2019 for 1955, both sexes combined

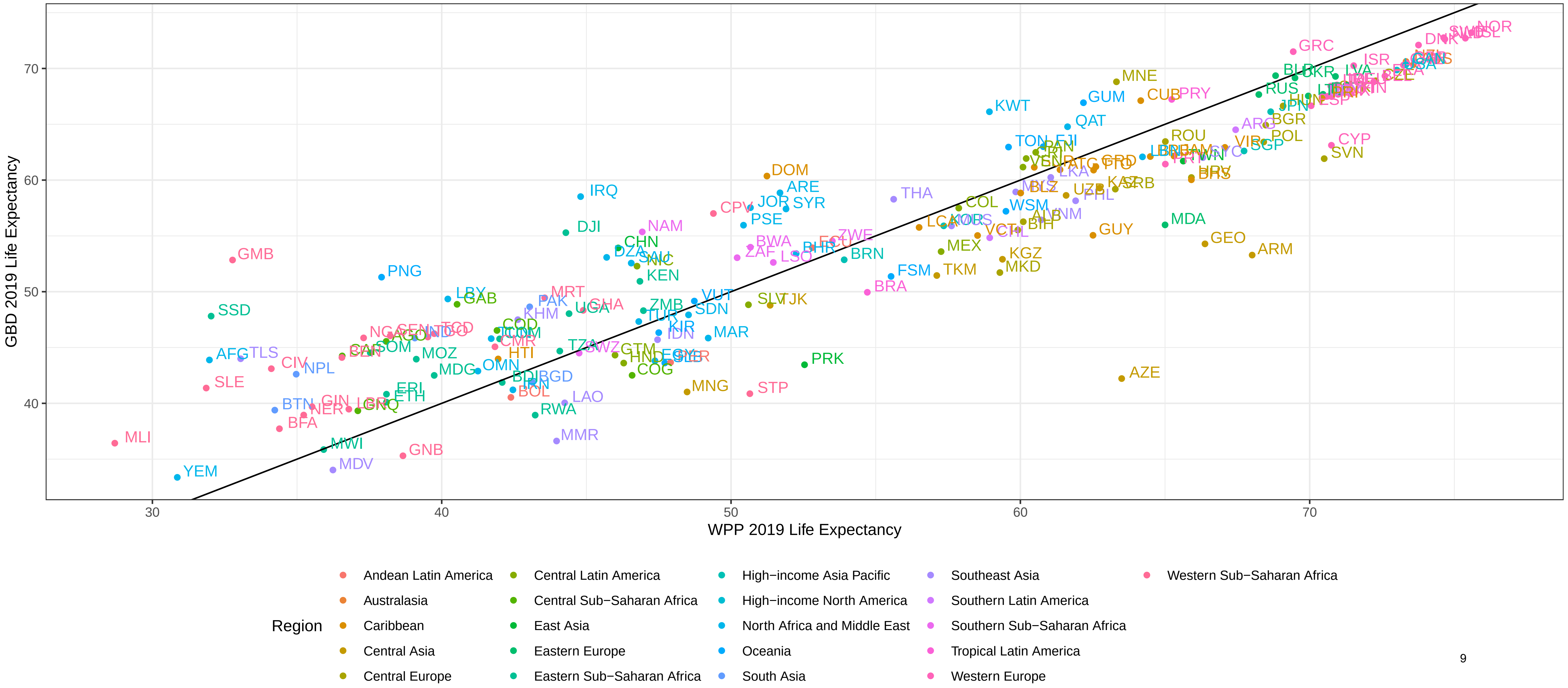

Supplemental Results Figure 4: Comparison of life expectancy at birth for countries in GBD 2019 vs WPP 2019 for 1960, both sexes combined

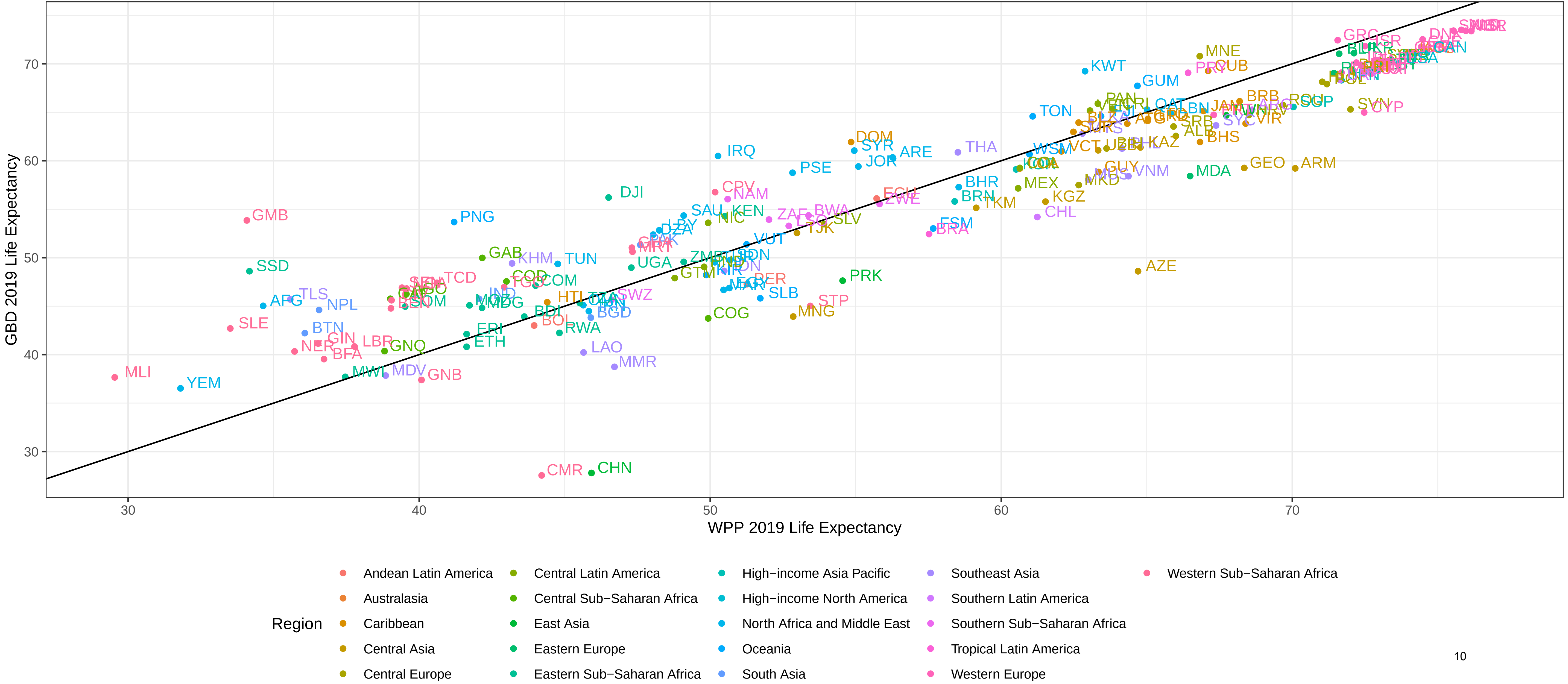

Supplemental Results Figure 4: Comparison of life expectancy at birth for countries in GBD 2019 vs WPP 2019 for 1965, both sexes combined

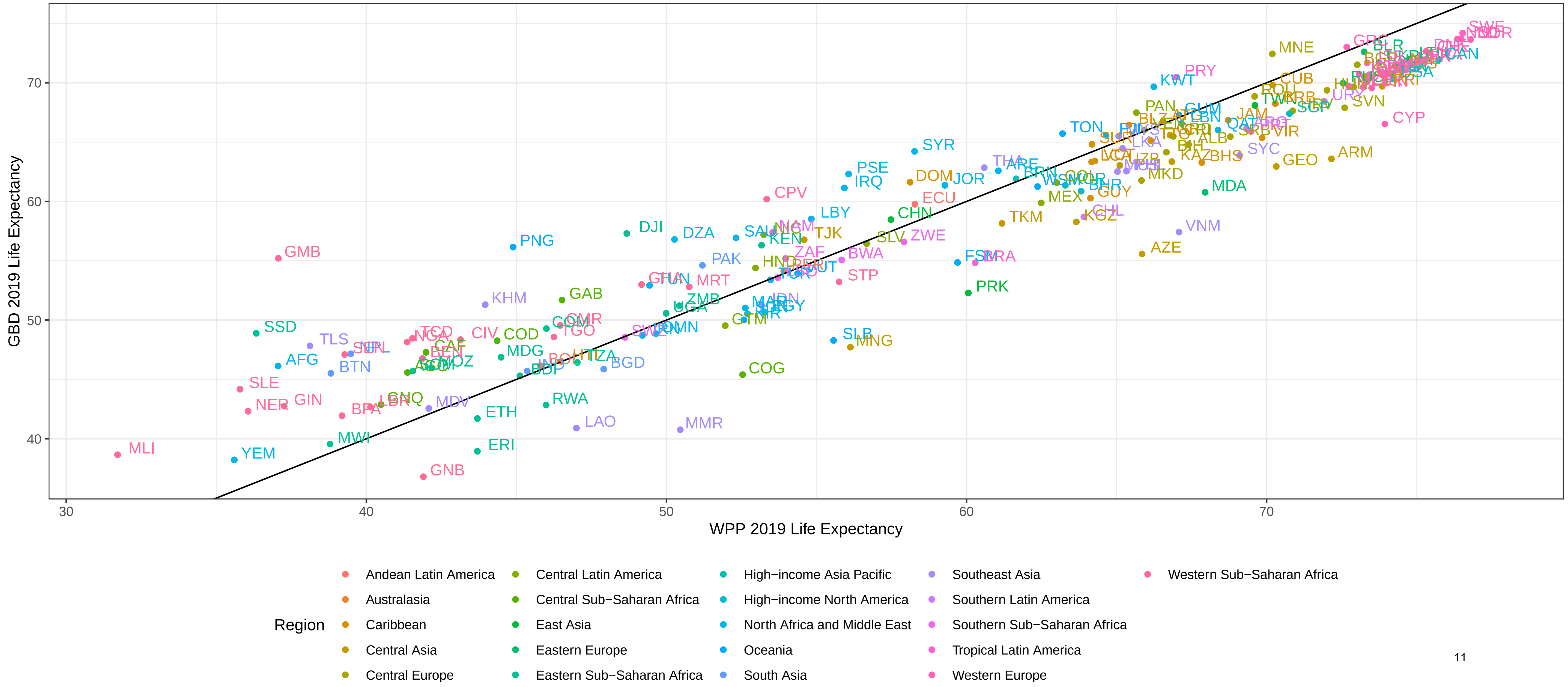

Supplemental Results Figure 4: Comparison of life expectancy at birth for countries in GBD 2019 vs WPP 2019 for 1970, both sexes combined

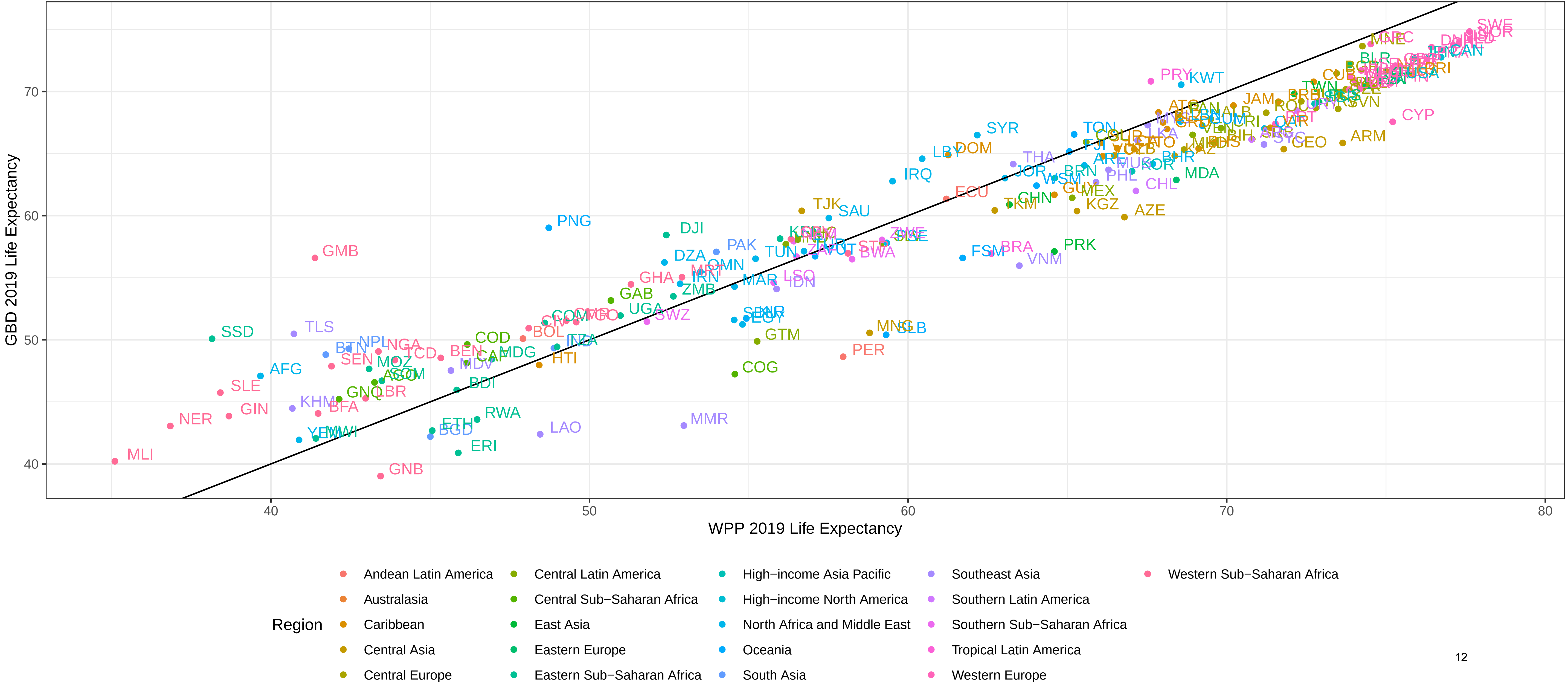

Supplemental Results Figure 4: Comparison of life expectancy at birth for countries in GBD 2019 vs WPP 2019 for 1975, both sexes combined

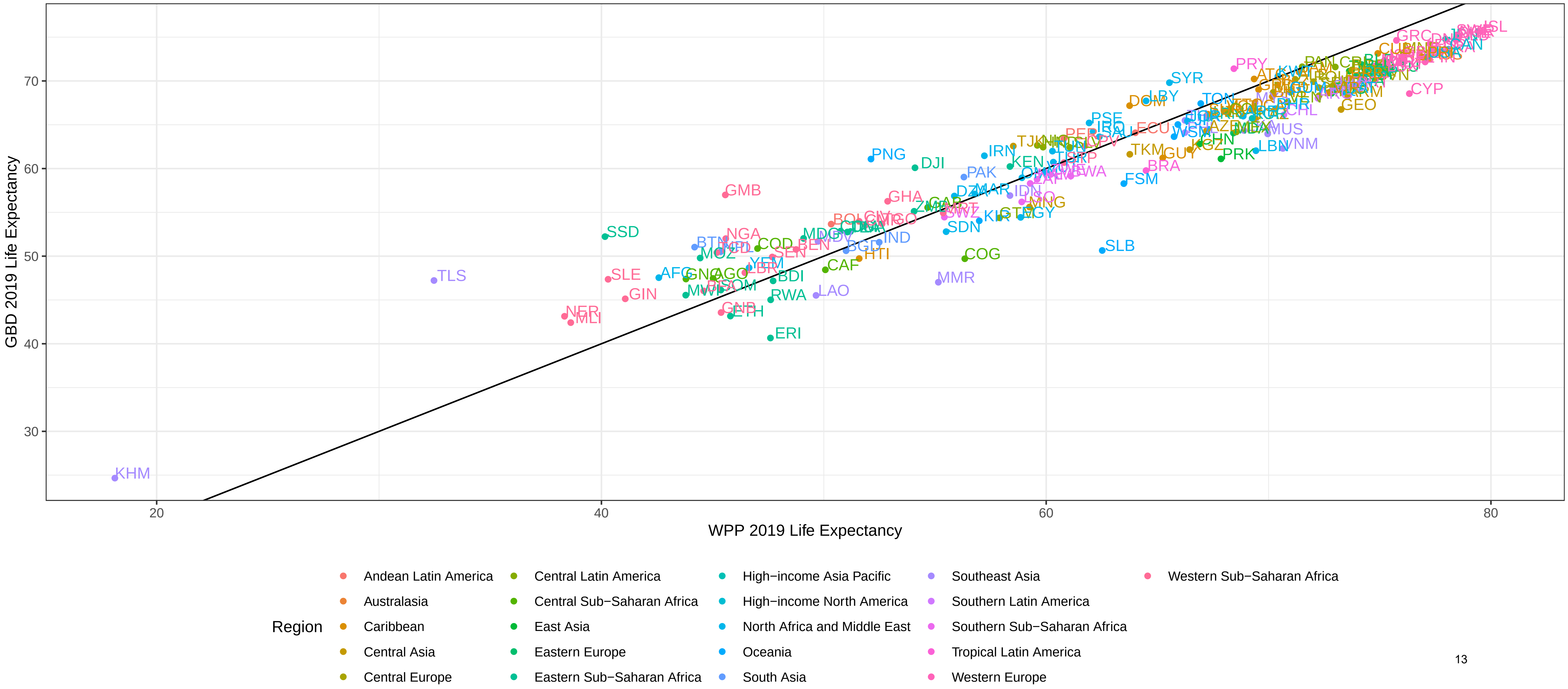

Supplemental Results Figure 4: Comparison of life expectancy at birth for countries in GBD 2019 vs WPP 2019 for 1980, both sexes combined

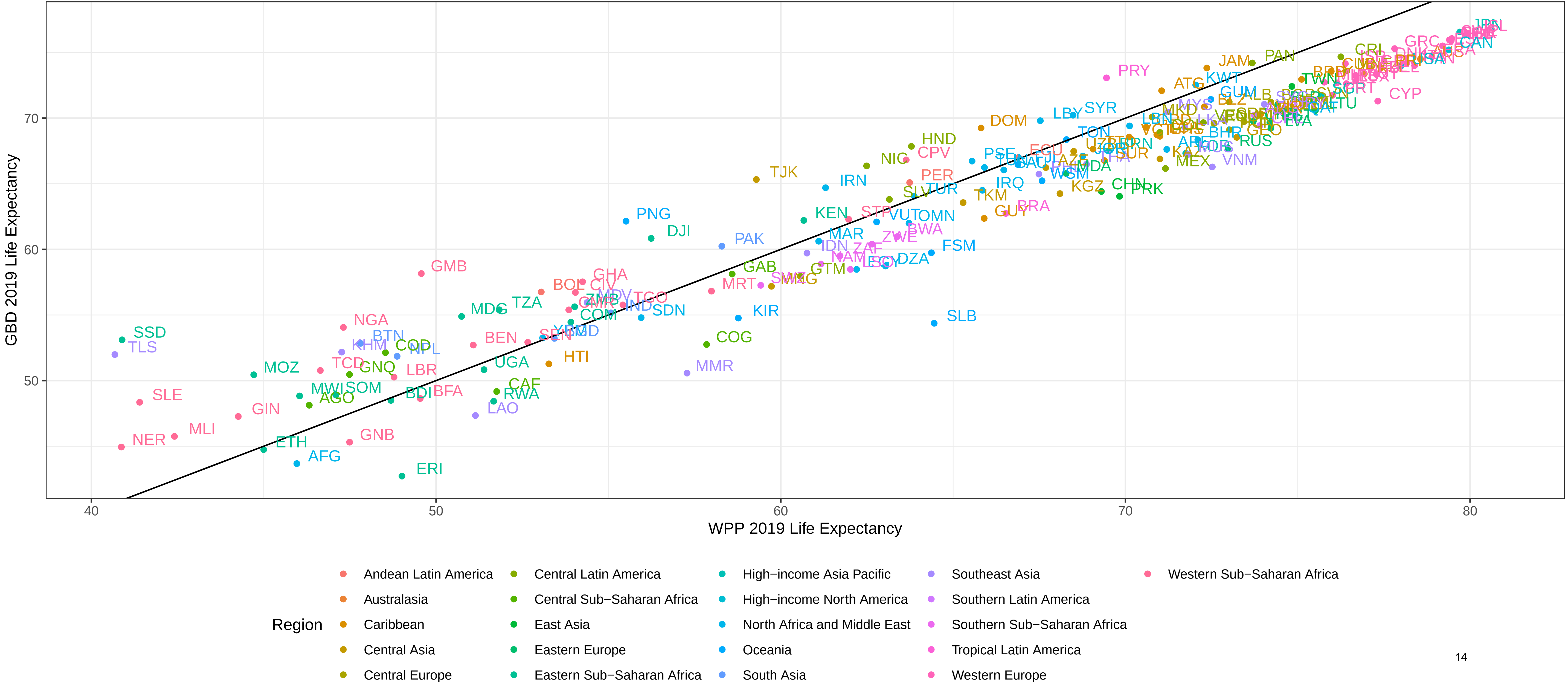

Supplemental Results Figure 4: Comparison of life expectancy at birth for countries in GBD 2019 vs WPP 2019 for 1985, both sexes combined

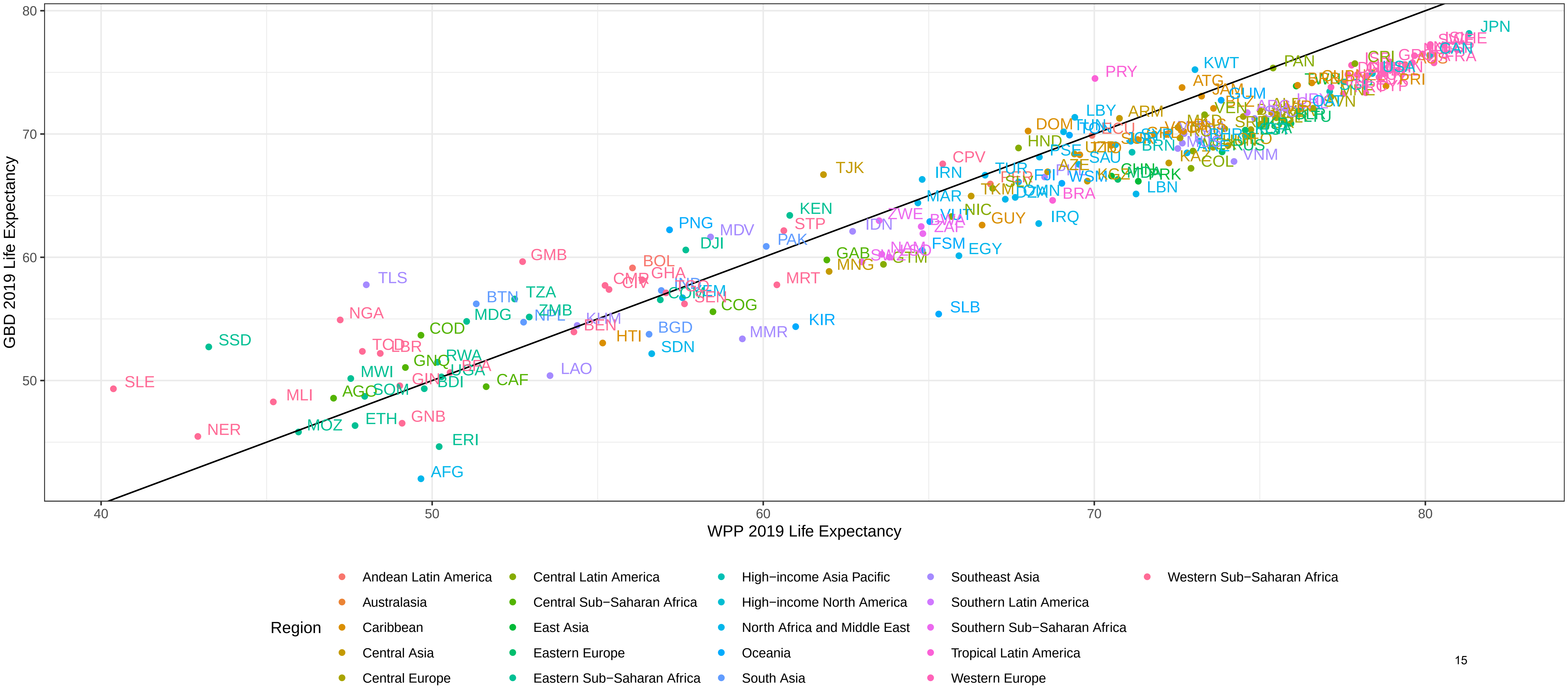

Supplemental Results Figure 4: Comparison of life expectancy at birth for countries in GBD 2019 vs WPP 2019 for 1990, both sexes combined

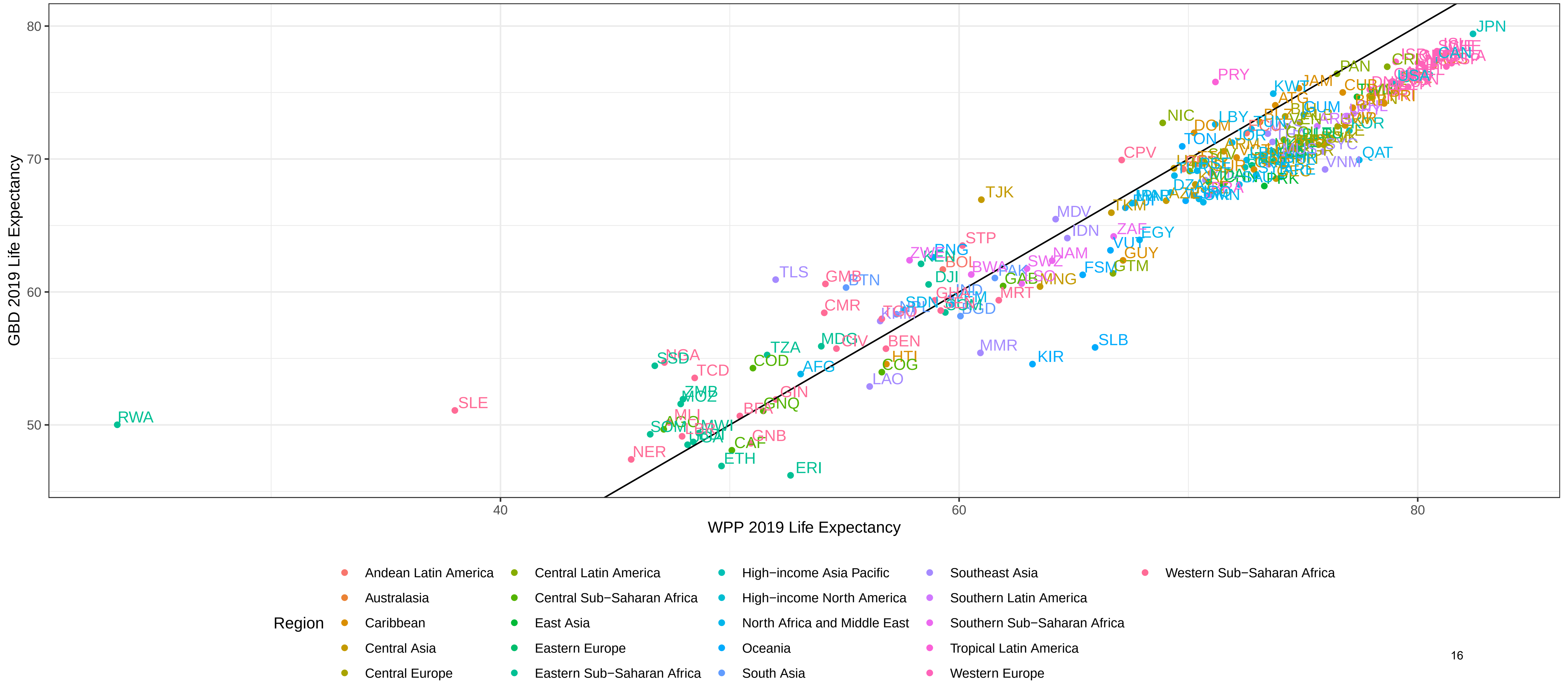

Supplemental Results Figure 4: Comparison of life expectancy at birth for countries in GBD 2019 vs WPP 2019 for 1995, both sexes combined

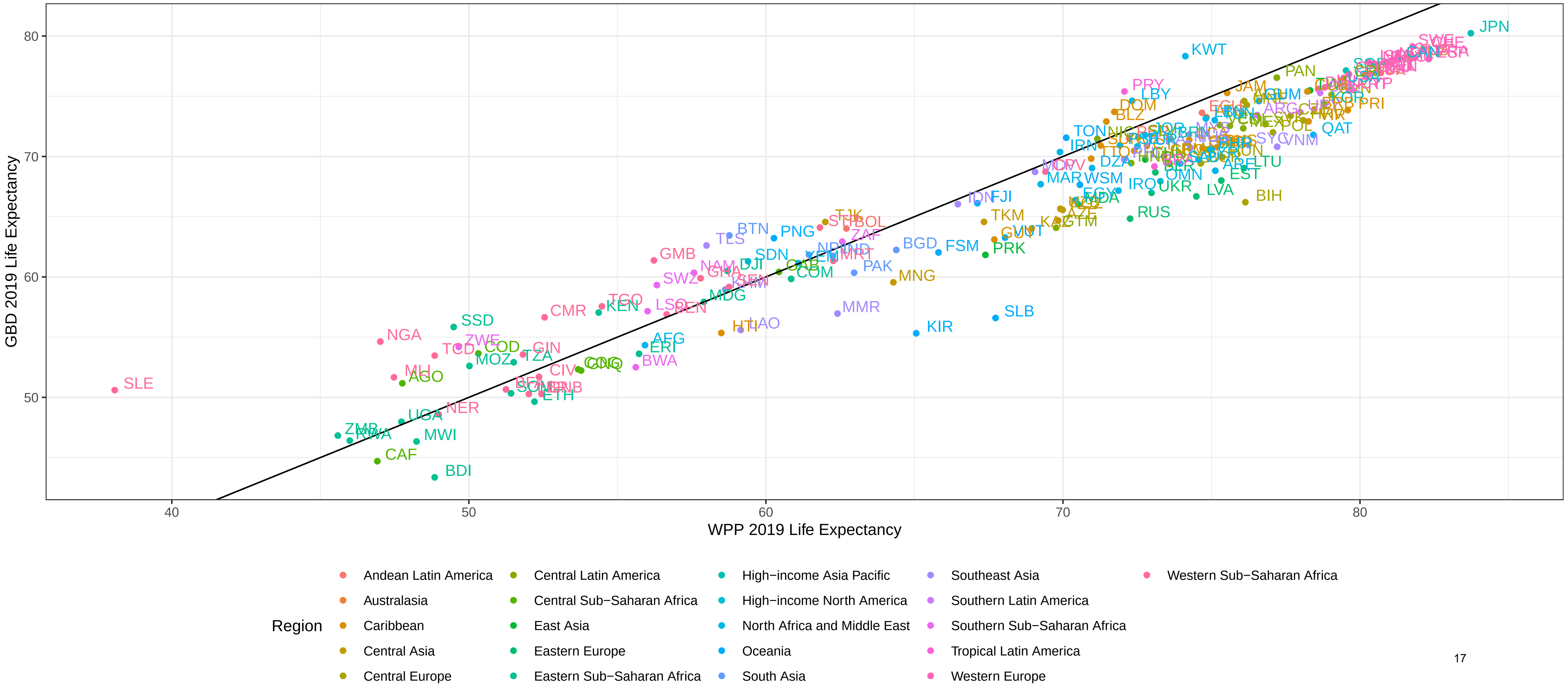

Supplemental Results Figure 4: Comparison of life expectancy at birth for countries in GBD 2019 vs WPP 2019 for 2000, both sexes combined

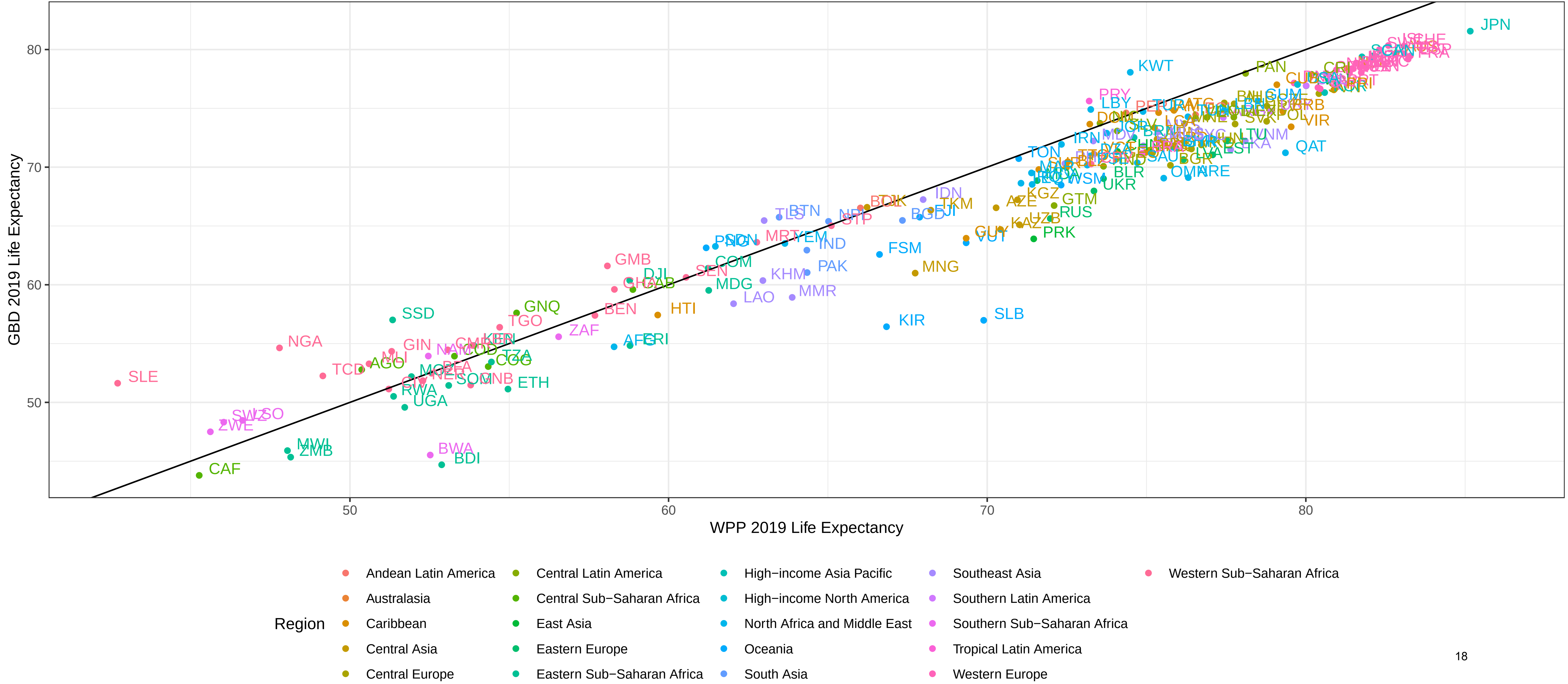

Supplemental Results Figure 4: Comparison of life expectancy at birth for countries in GBD 2019 vs WPP 2019 for 2005, both sexes combined

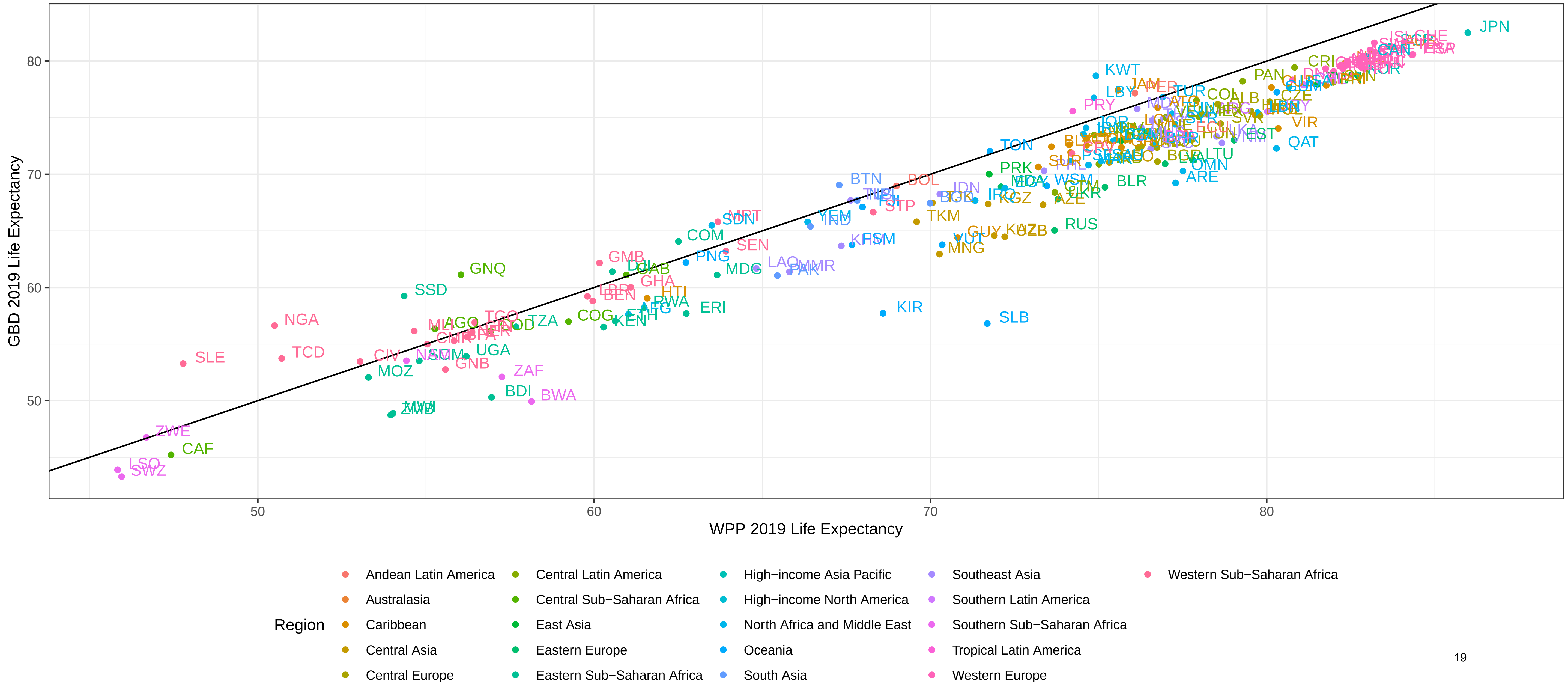

Supplemental Results Figure 4: Comparison of life expectancy at birth for countries in GBD 2019 vs WPP 2019 for 2010, both sexes combined

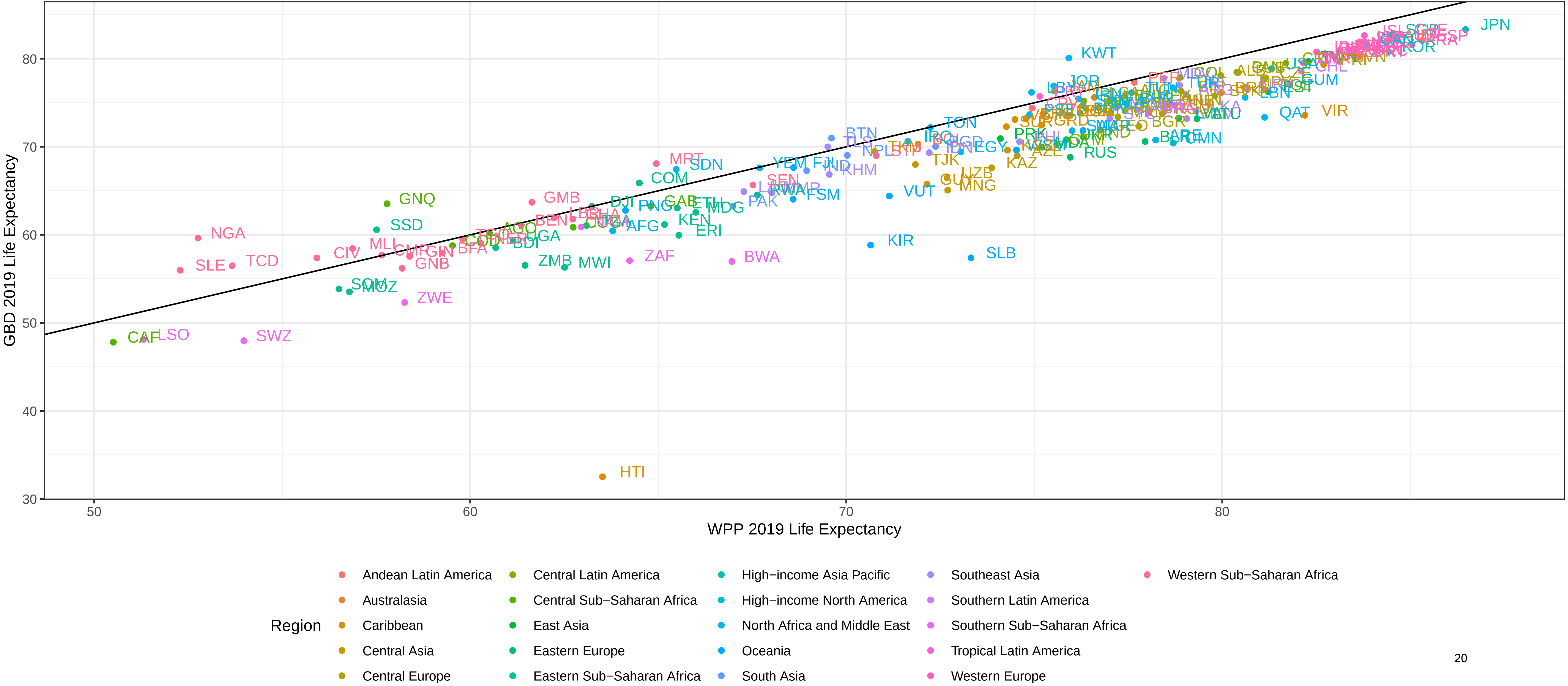

Supplemental Results Figure 4: Comparison of life expectancy at birth for countries in GBD 2019 vs WPP 2019 for 2015, both sexes combined

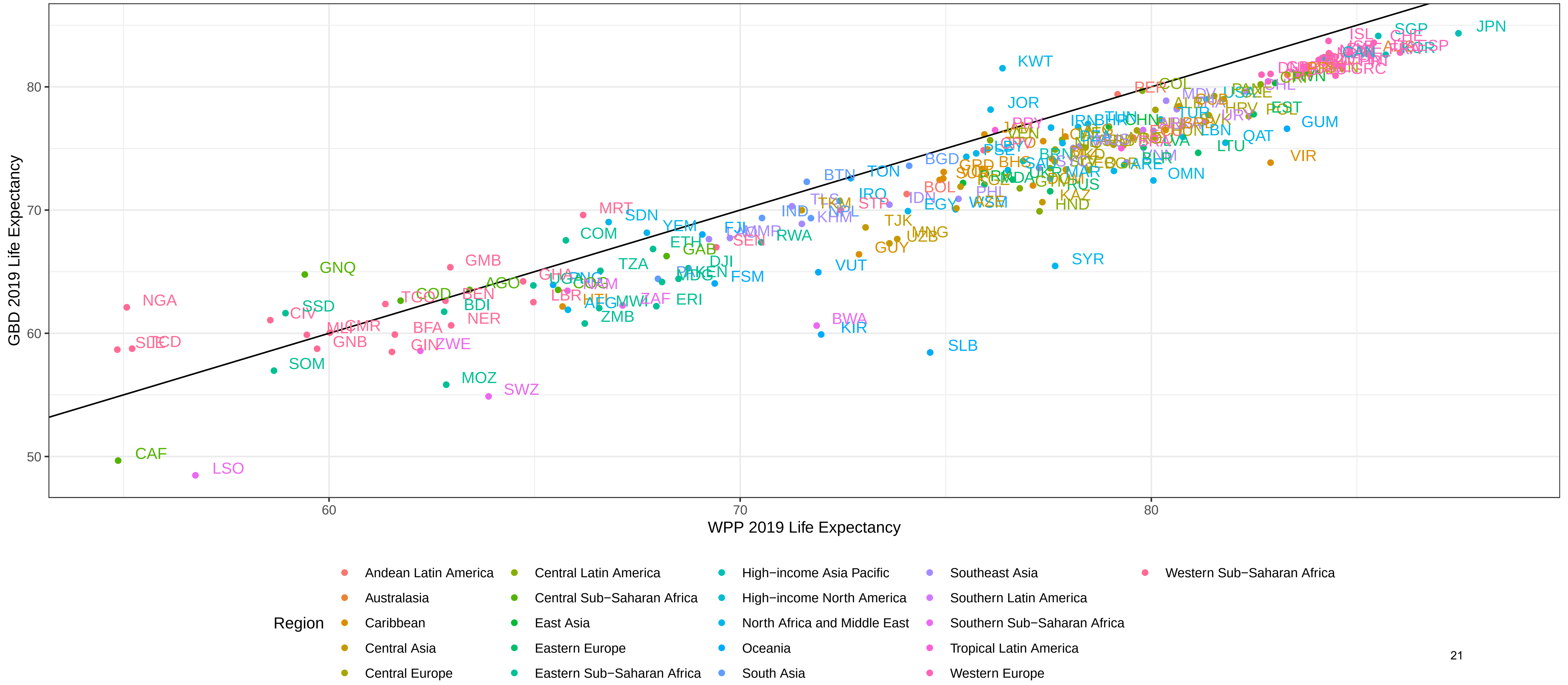

Figure 5a: Distance to Life Expectancy at Birth from the GBD−YLL−LT, 2019,Males

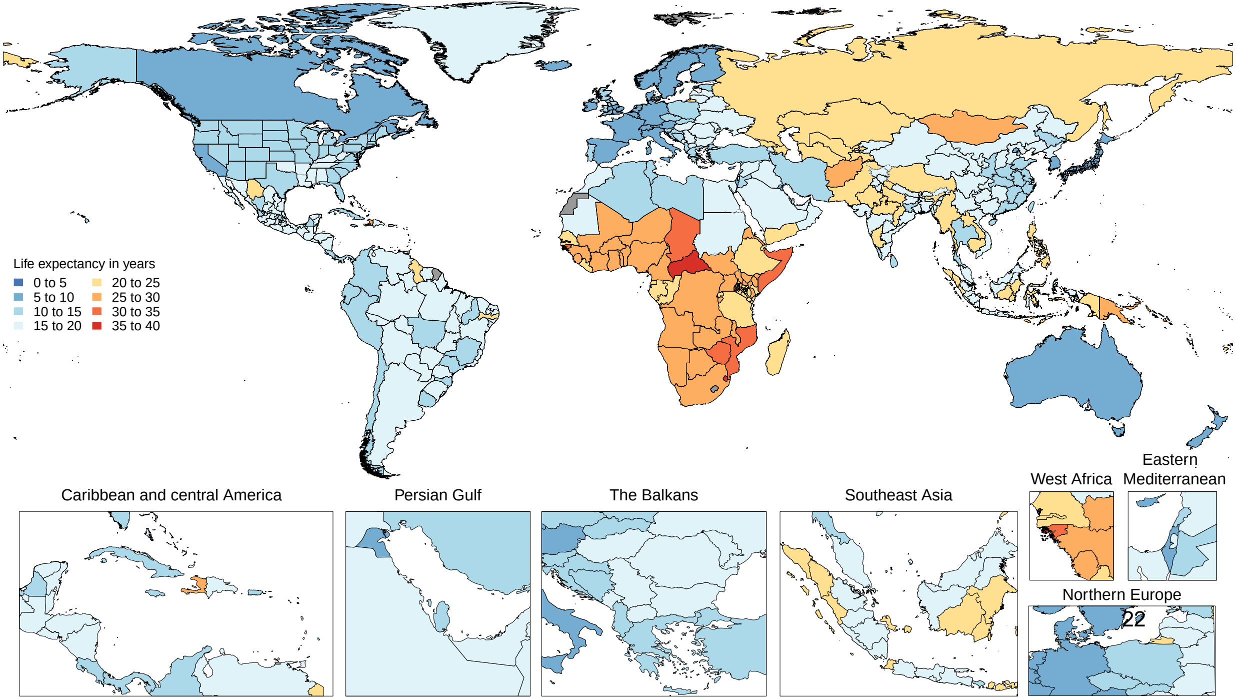

Figure 5b: Distance to Life Expectancy at Birth from the GBD−YLL−LT, 2019,Females

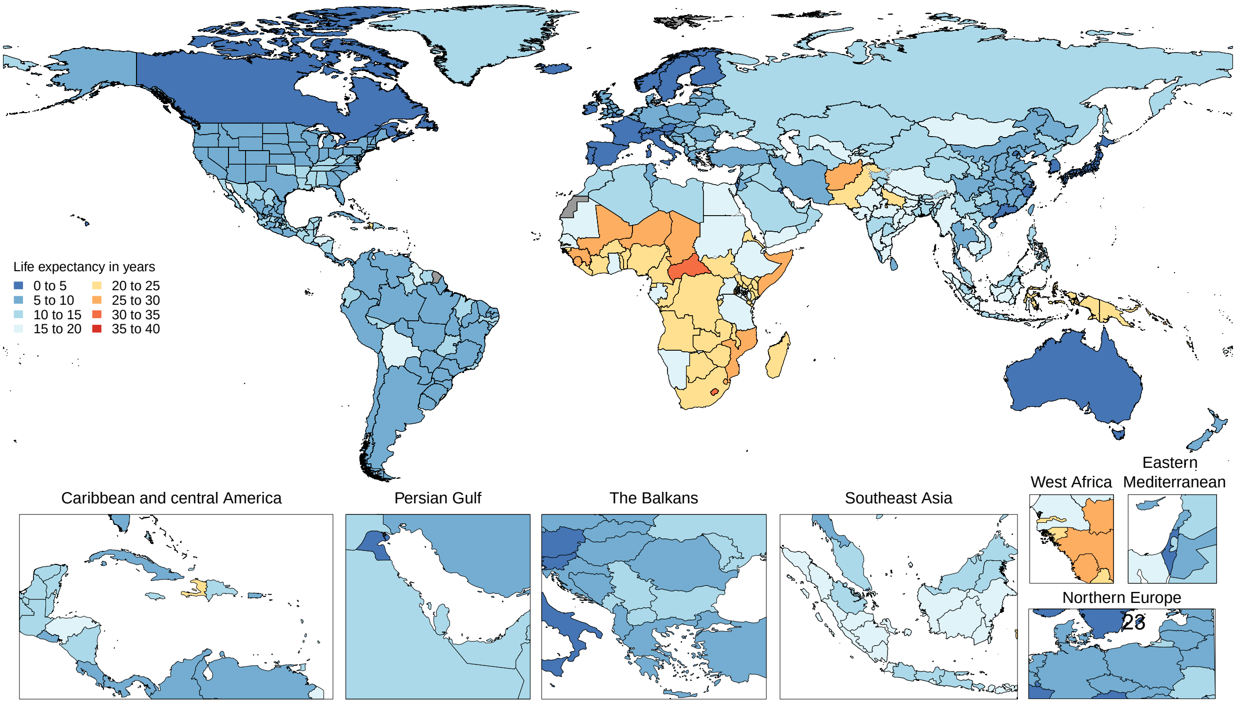

Supplemental Results Figure 6: Corelation of Baseline nqx and the ARC in every decade per age group by decade

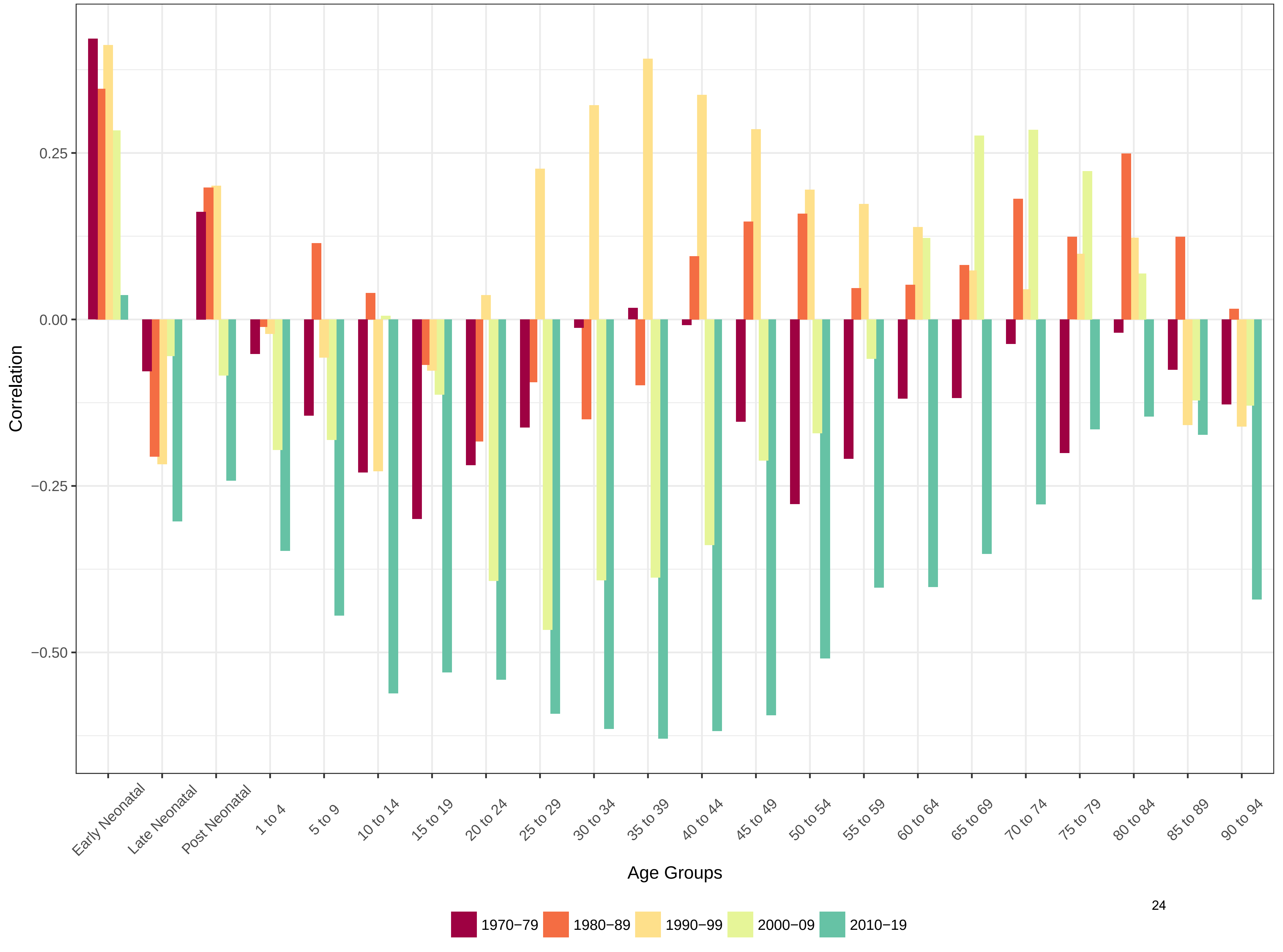

SR Figure 7: Number of deaths over time by age group, 1950−2019

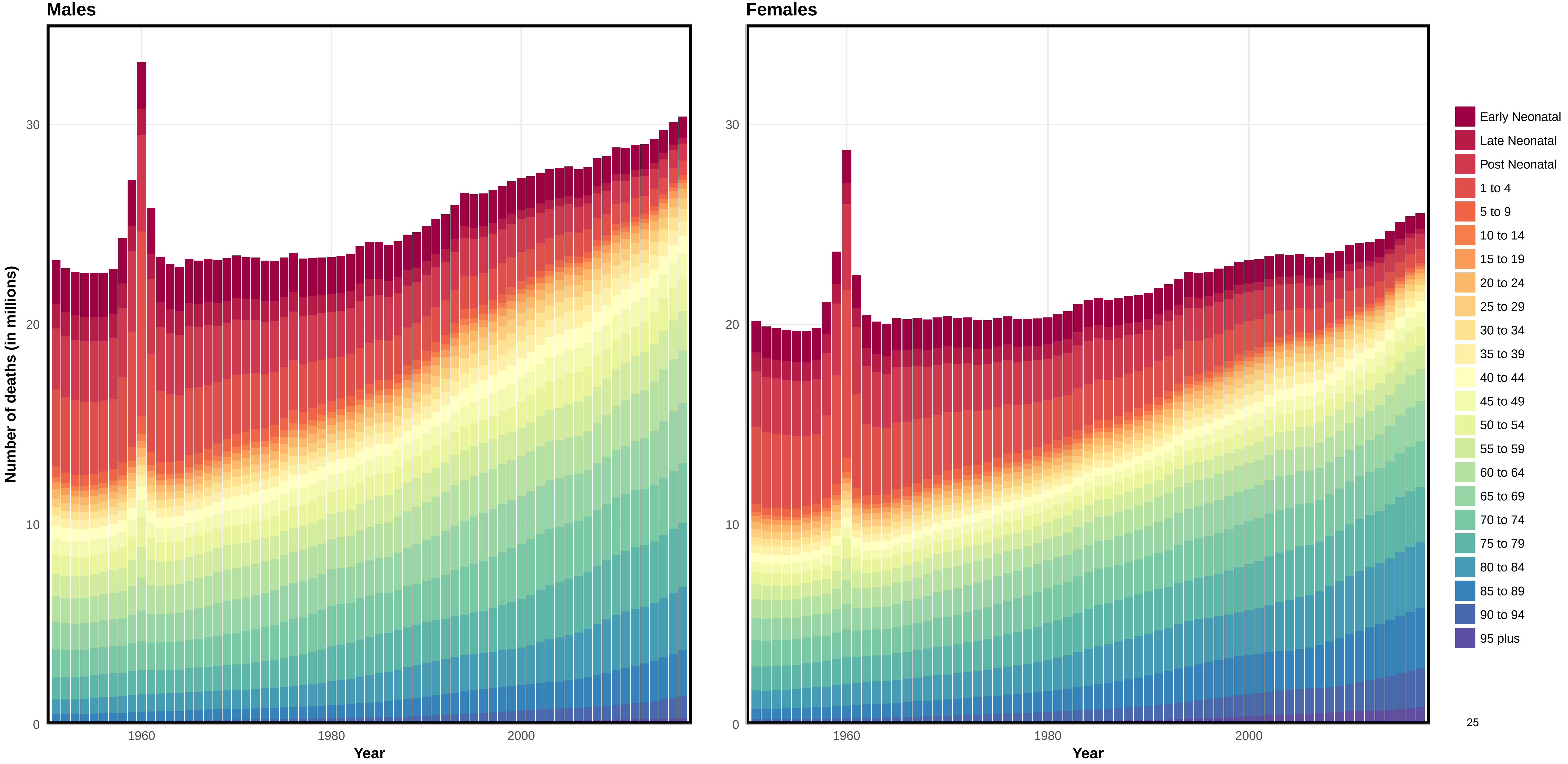

Supplemental Results Figure 8: Death Structure by SDI in 2019

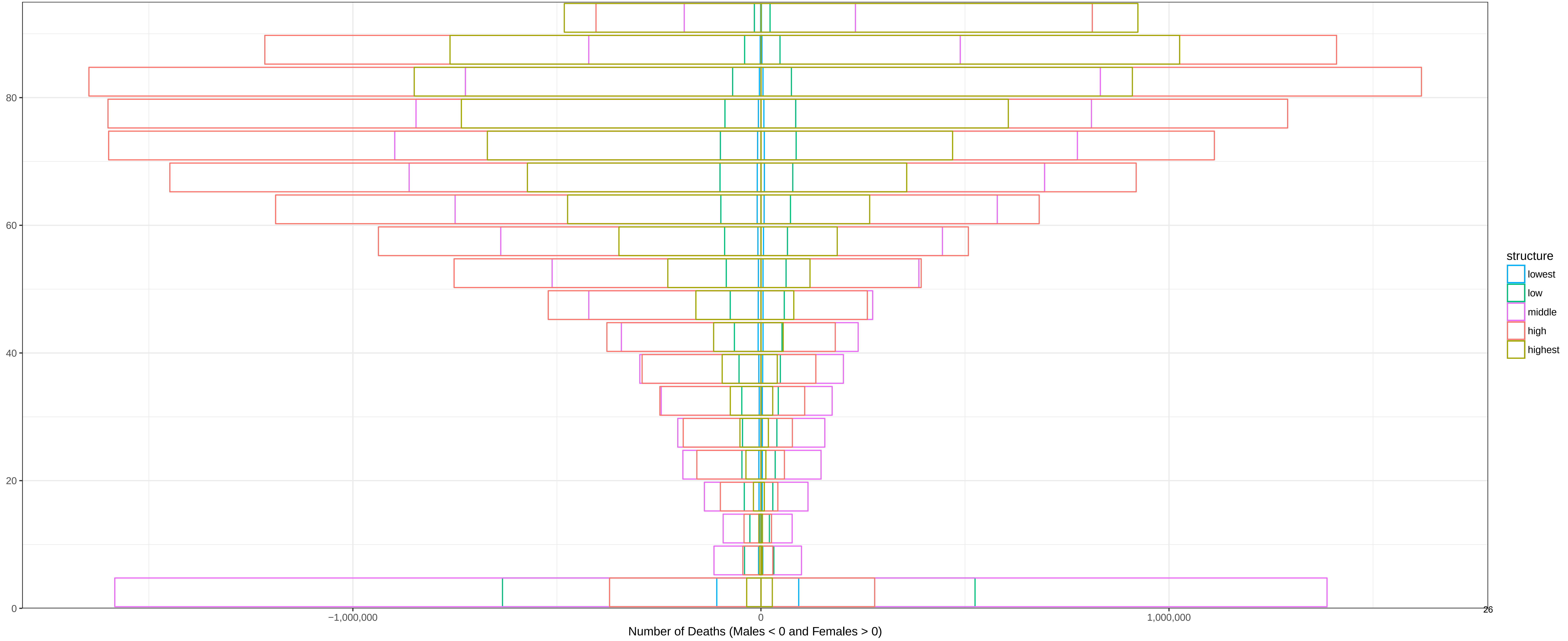

Supplemental Results Figure 9: Death Structure by SDI in 2019

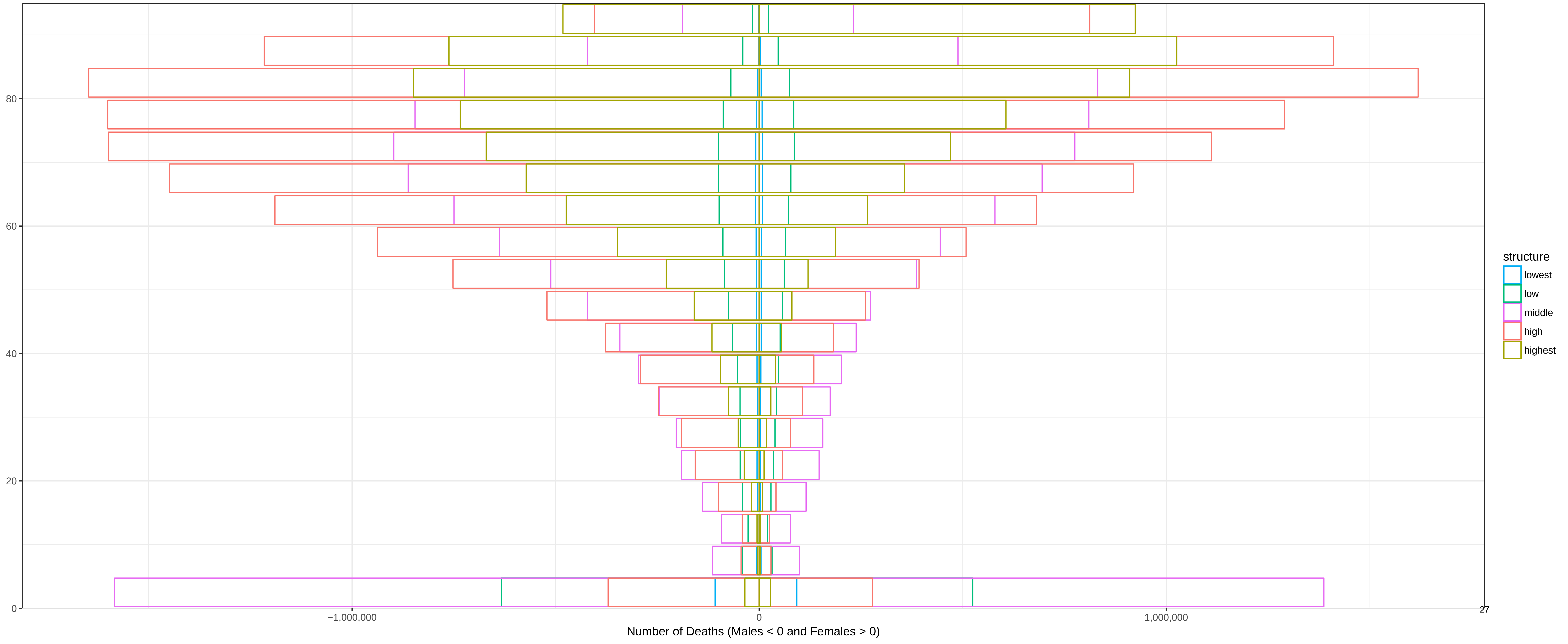

Supplemental Results Figure 10: Percent difference between GBD 2019 population estimates and UN WPP estimates, by location, ages 5−9, 45−49, 70−74, by sex, 2019

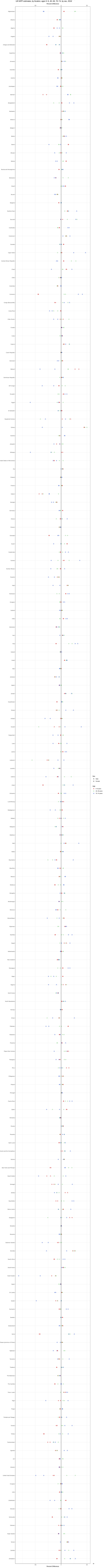

Supplemental Results Figure 11: Population Structure by SDI in 2019

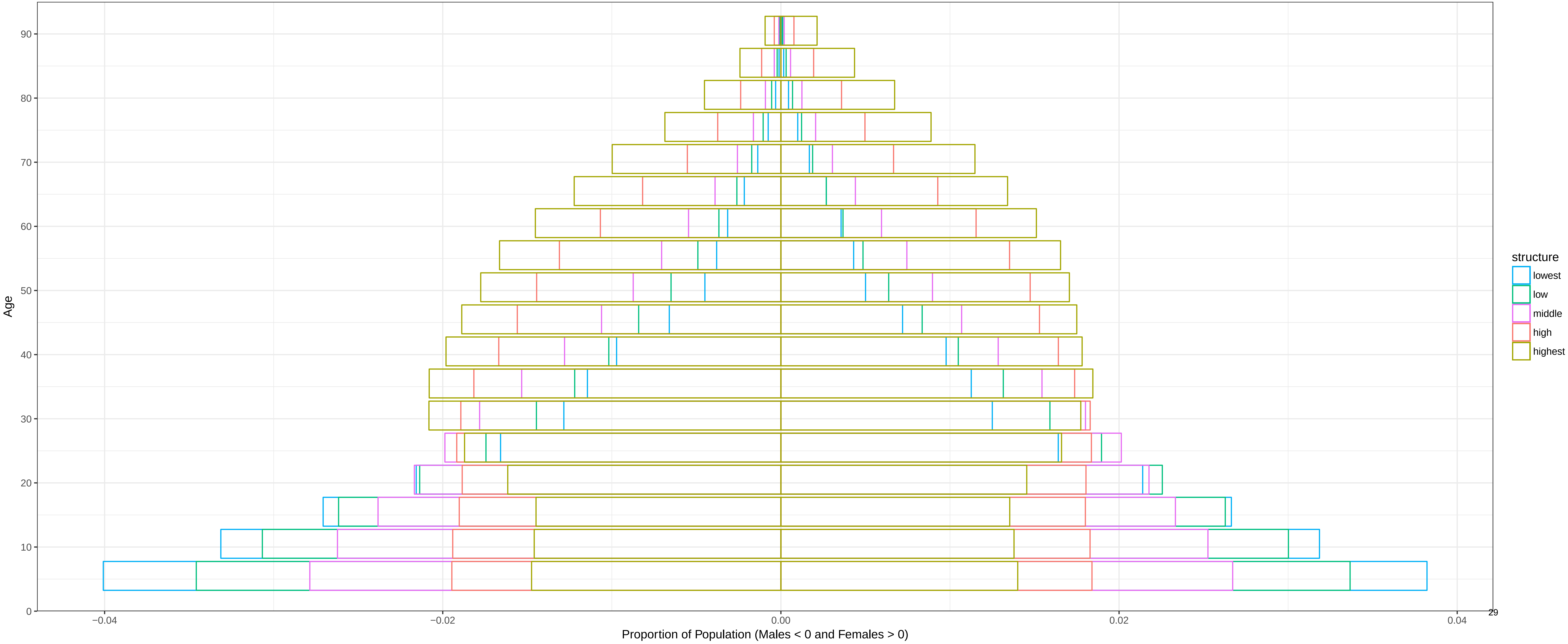

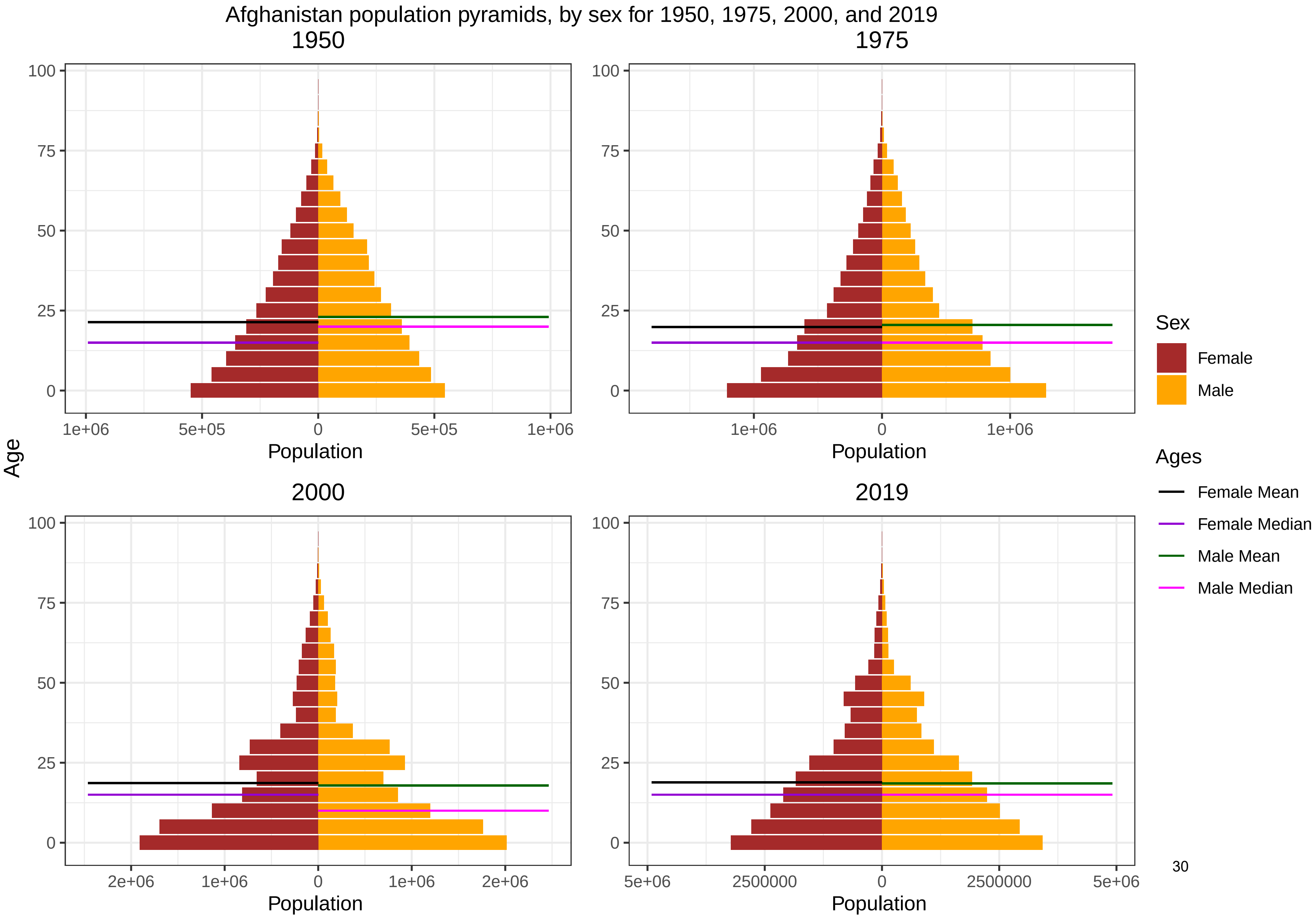
Afghanistan population pyramids, by sex for 1950, 1975, 2000, and 2019
1950
1975
2000
2019
Age
Population
Sex
Female
Male
Ages
Female Mean
Female Median
Male Mean
Male Median

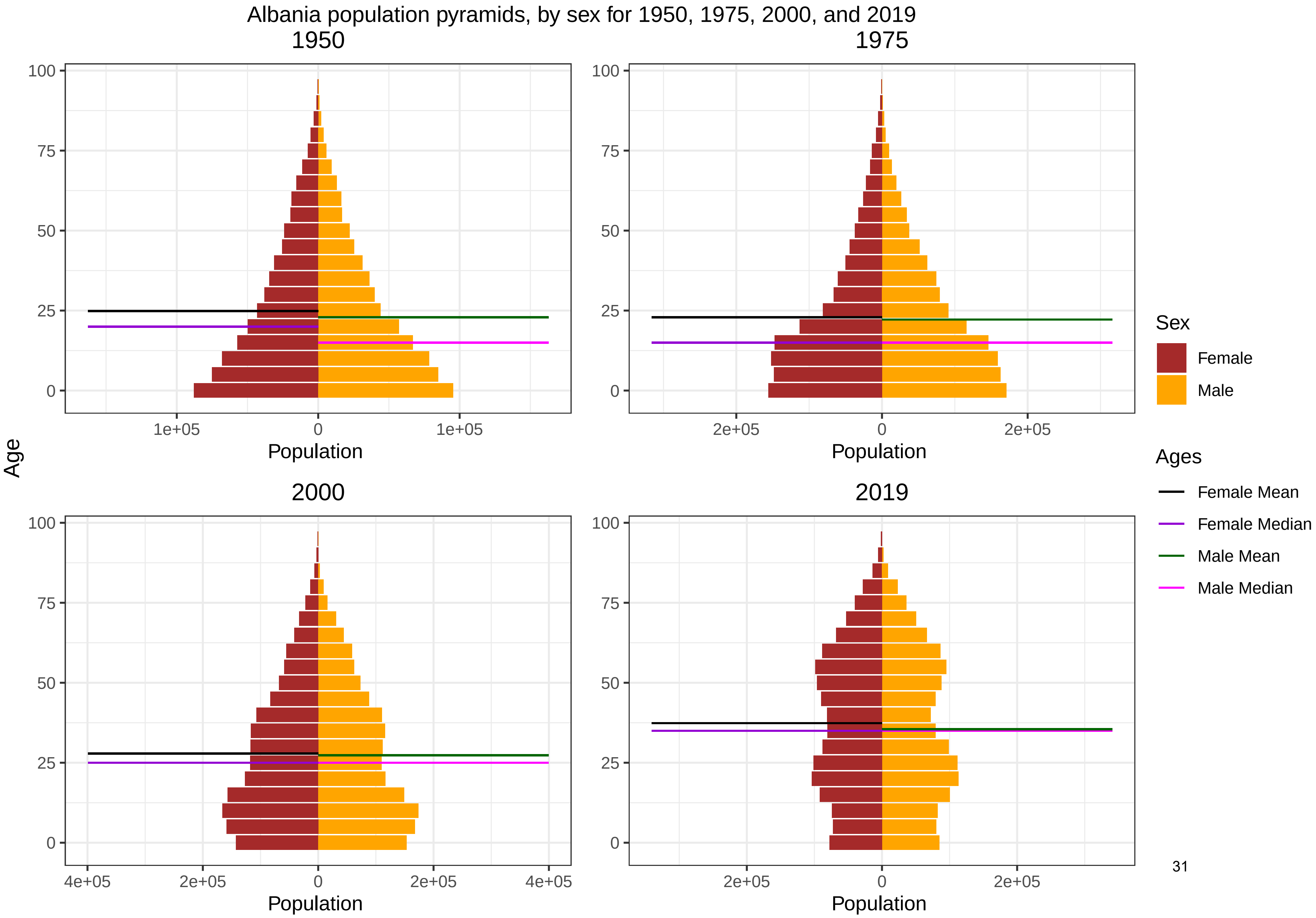

Albania population pyramids, by sex for 1950, 1975, 2000, and 2019
1950
1975
2000
2019
Age
Population
Sex
Female
Male
Ages
Female Mean
Female Median
Male Mean
Male Median

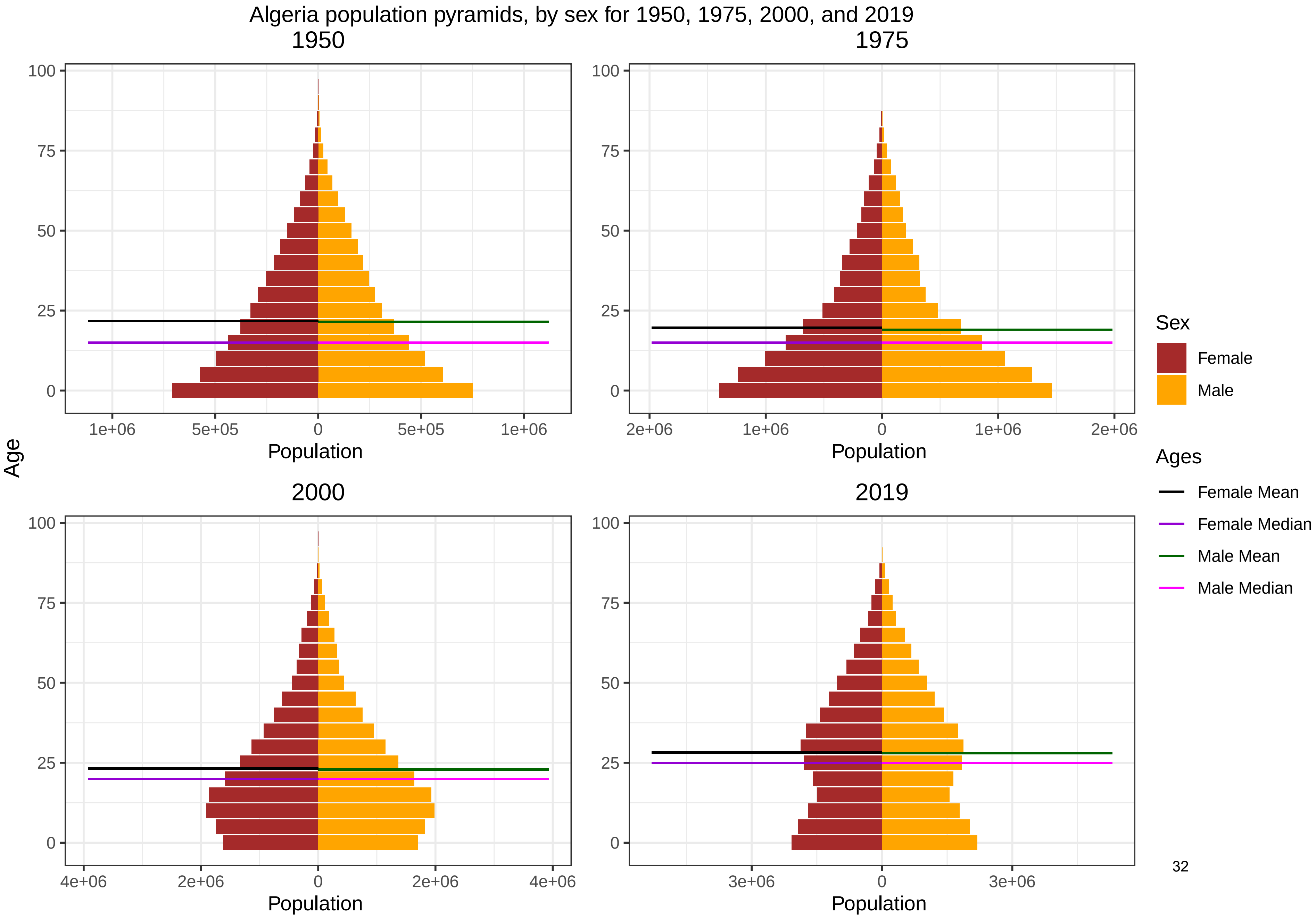
Algeria population pyramids, by sex for 1950, 1975, 2000, and 2019
1950
1975
2000
2019
Age
Population
Sex
Female
Male
Ages
Female Mean
Female Median
Male Mean
Male Median
100
75
50
25
0
1e+06
5e+05
0
5e+05
1e+06
2e+06
1e+06
0
1e+06
2e+06
4e+06
2e+06
0
2e+06
4e+06
3e+06
0
3e+06

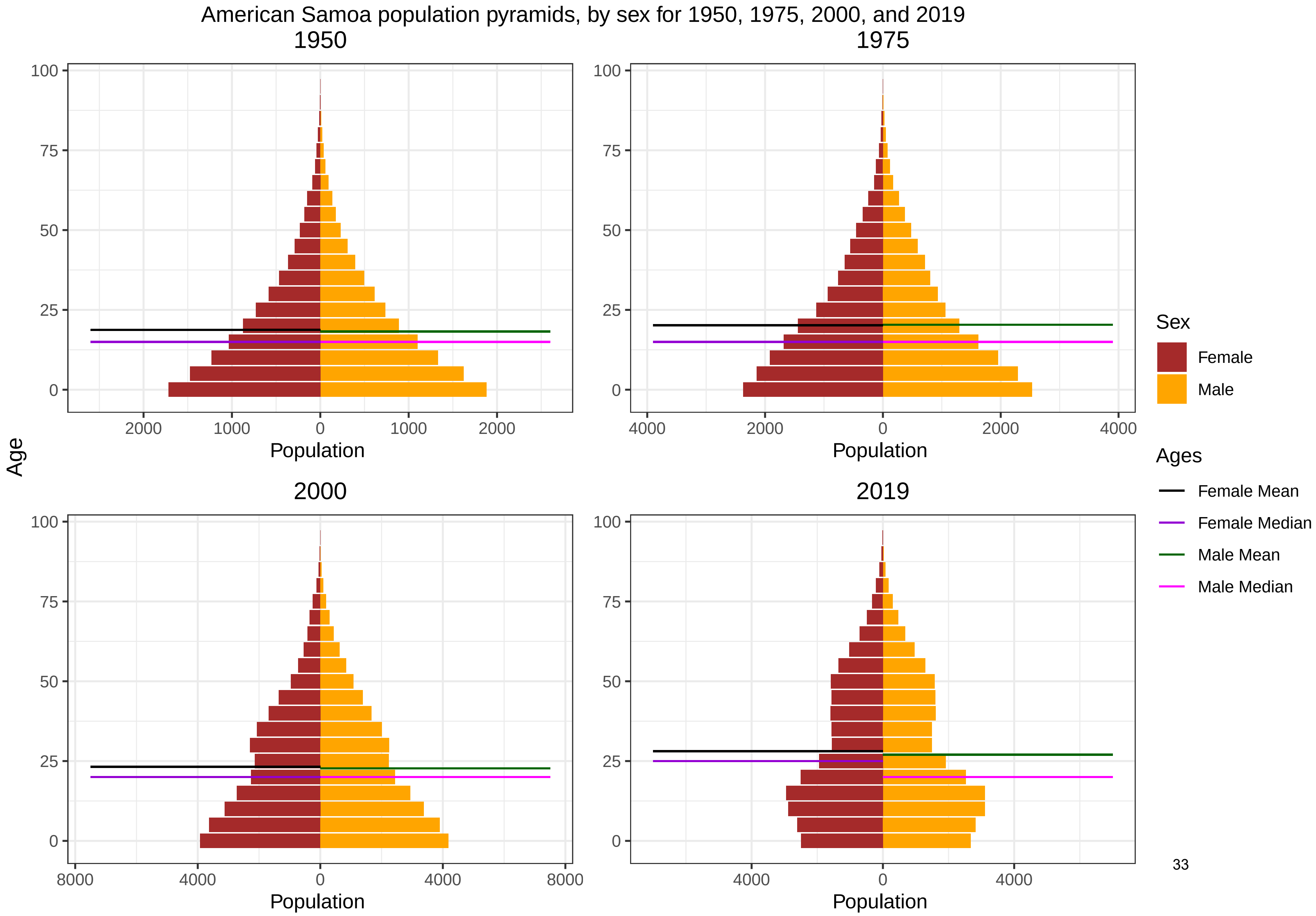
American Samoa population pyramids, by sex for 1950, 1975, 2000, and 2019
1950
1975
2000
2019
Age
Population
100
75
50
25
0
2000
1000
0
1000
2000
4000
2000
0
2000
4000
8000
4000
0
4000
8000
4000
0
4000
Sex
Female
Male
Ages
Female Mean
Female Median
Male Mean
Male Median

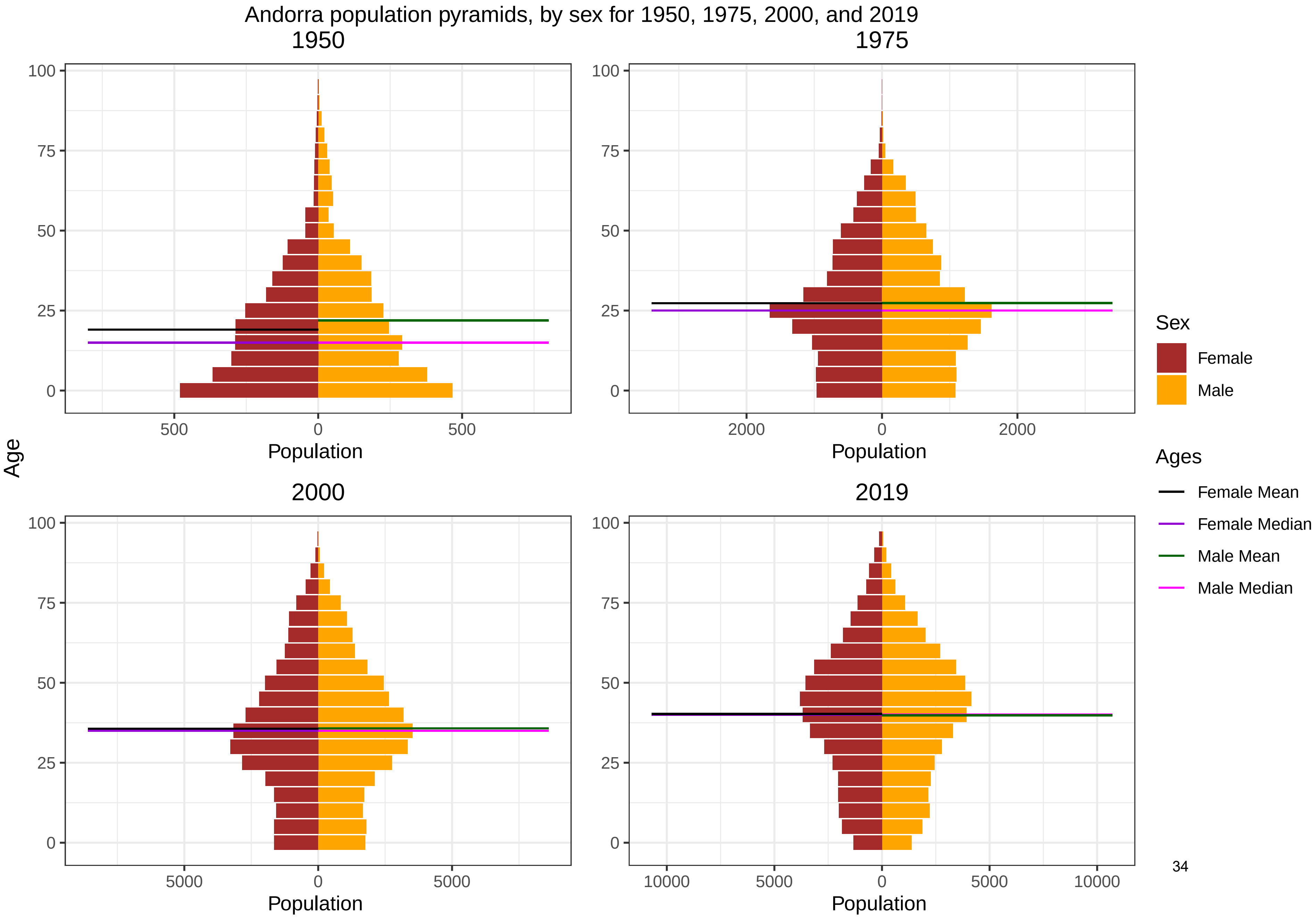
Andorra population pyramids, by sex for 1950, 1975, 2000, and 2019
1950
1975
2000
2019
Age
Population
Sex
Female
Male
Ages
Female Mean
Female Median
Male Mean
Male Median

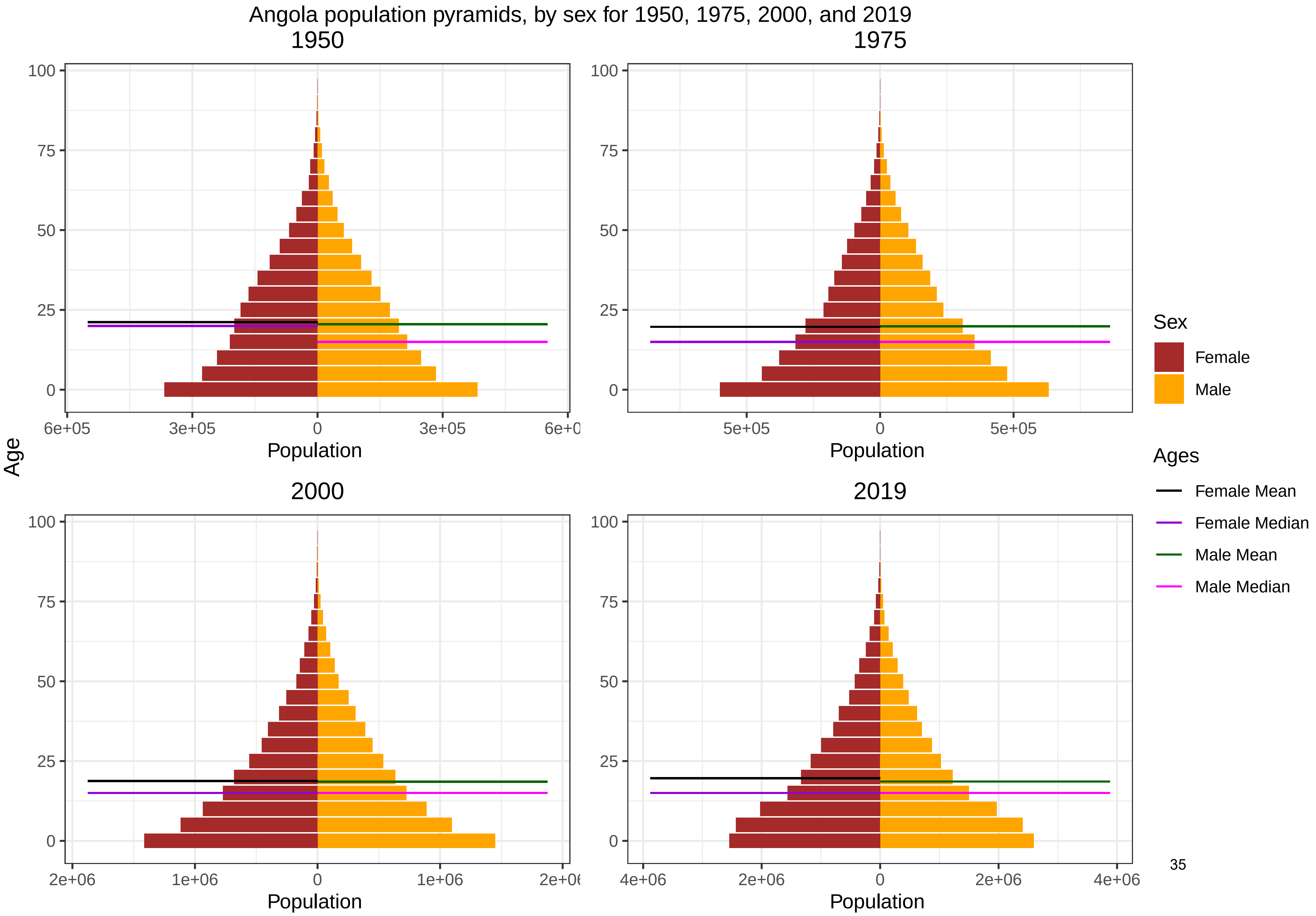

Angola population pyramids, by sex for 1950, 1975, 2000, and 2019
1950
1975
2000
2019
Age
Population
100
75
50
25
0
6e+05
3e+05
0
3e+05
5e+05
0
5e+05
2e+06
1e+06
0
1e+06
4e+06
2e+06
0
2e+06
4e+06
Sex
Female
Male
Ages
Female Mean
Female Median
Male Mean
Male Median

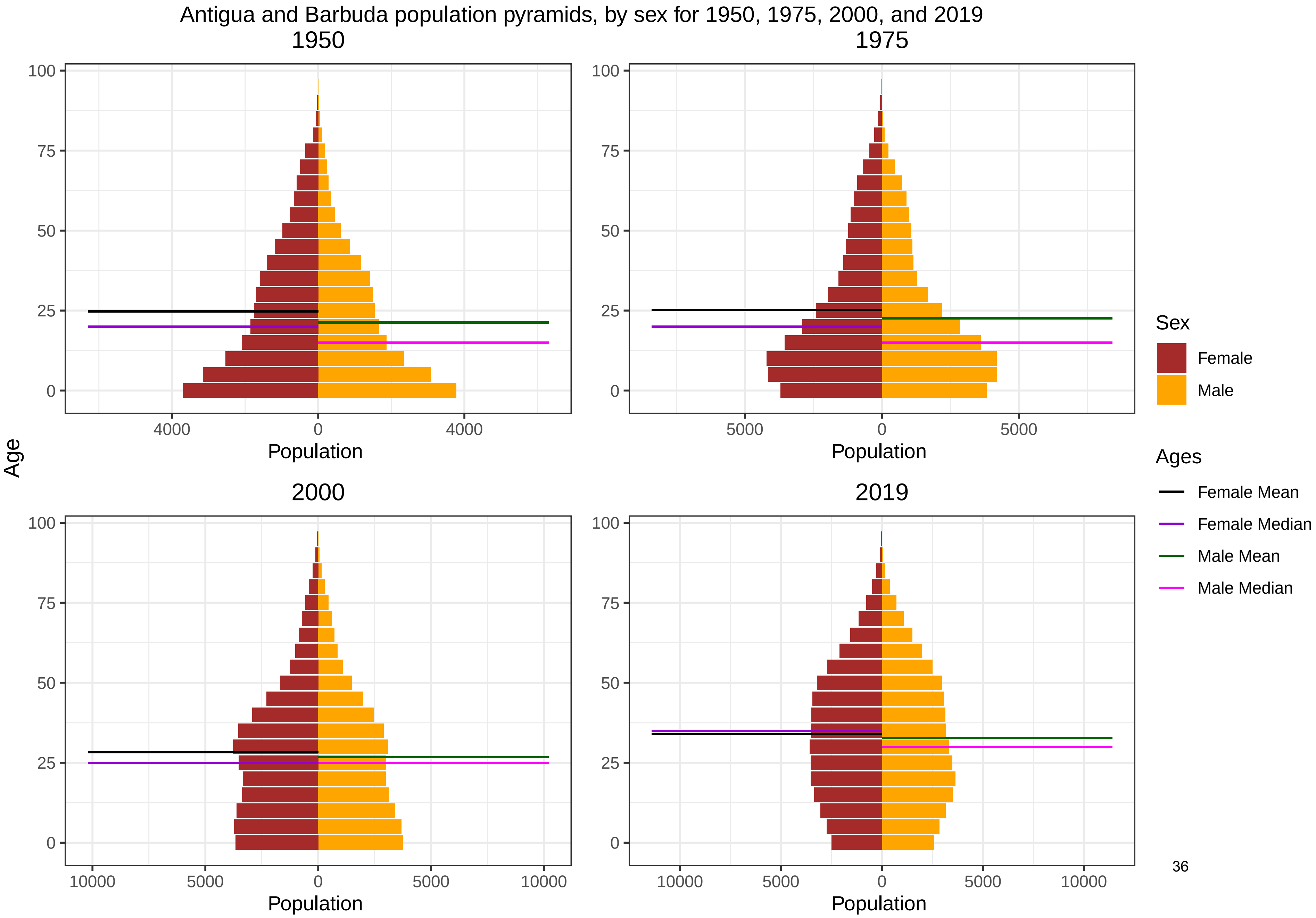
Antigua and Barbuda population pyramids, by sex for 1950, 1975, 2000, and 2019
1950
1975
2000
2019
Age
Population
Sex
Female
Male
Ages
Female Mean
Female Median
Male Mean
Male Median

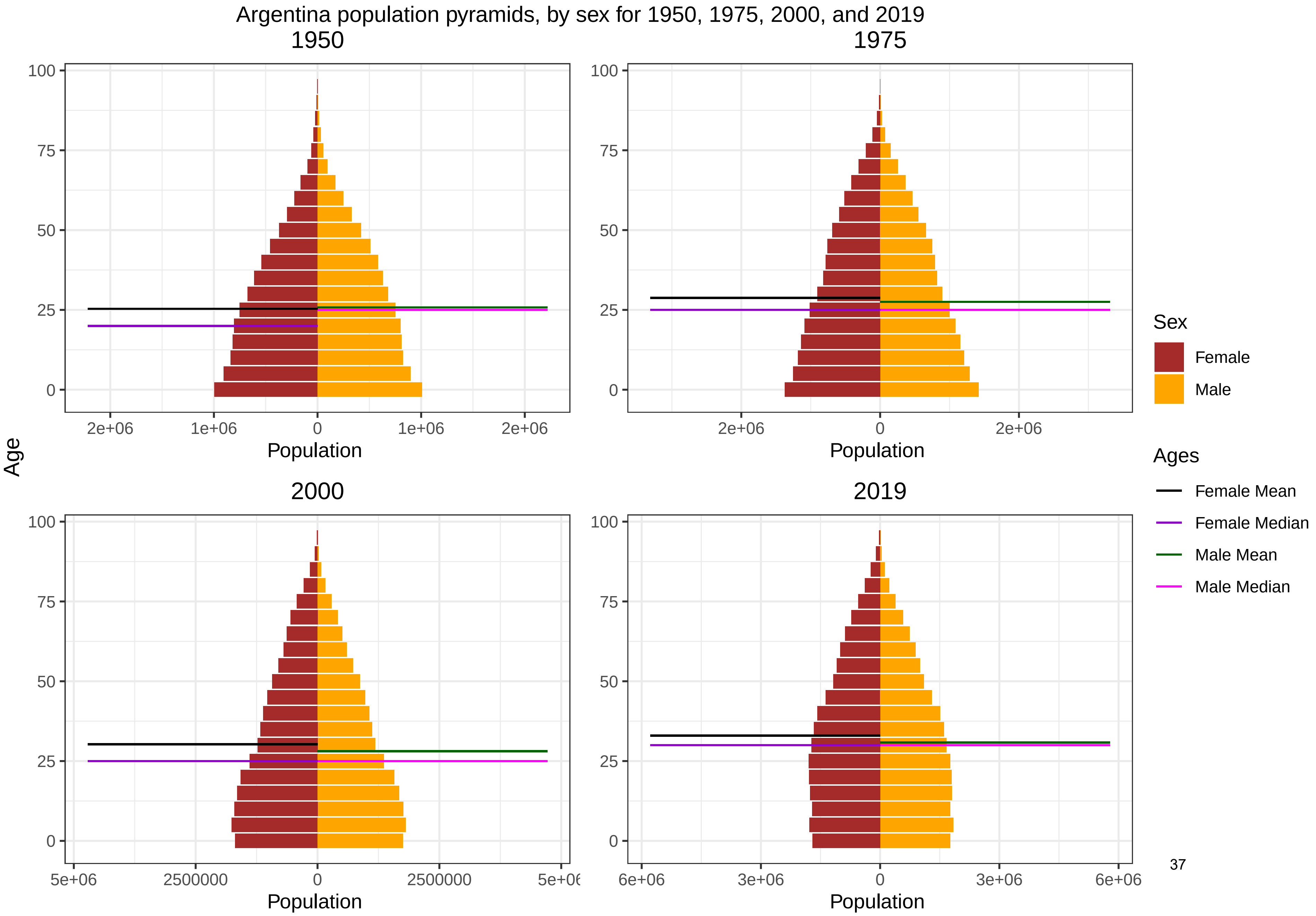
Argentina population pyramids, by sex for 1950, 1975, 2000, and 2019
1950
1975
2000
2019
Age
Population
Sex
Female
Male
Ages
Female Mean
Female Median
Male Mean
Male Median
100
75
50
25
0
2e+06
1e+06
0
2500000
5e+06
3e+06
6e+06

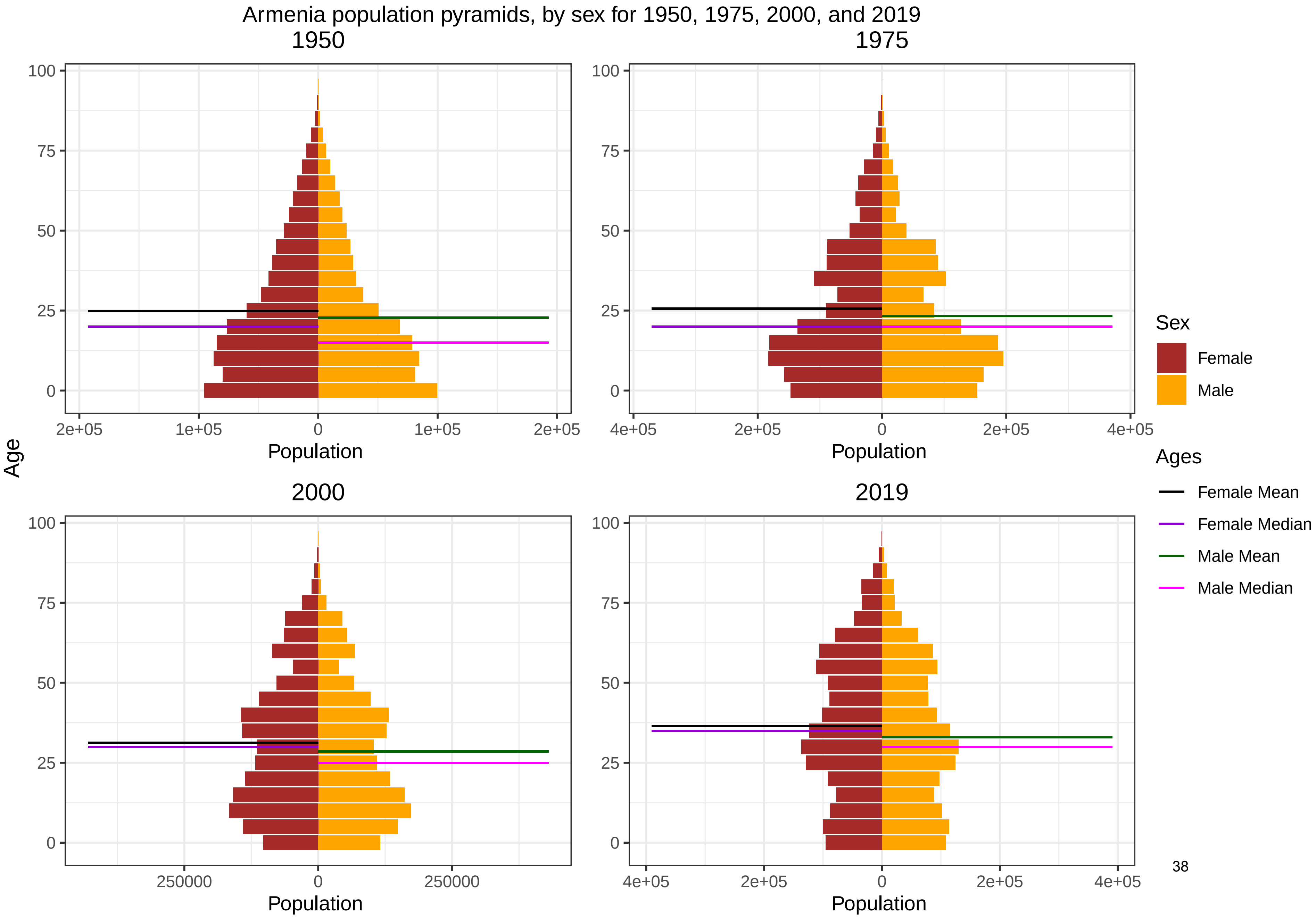

Armenia population pyramids, by sex for 1950, 1975, 2000, and 2019
1950
1975
2000
2019
Age
Population
Sex
Female
Male
Ages
Female Mean
Female Median
Male Mean
Male Median

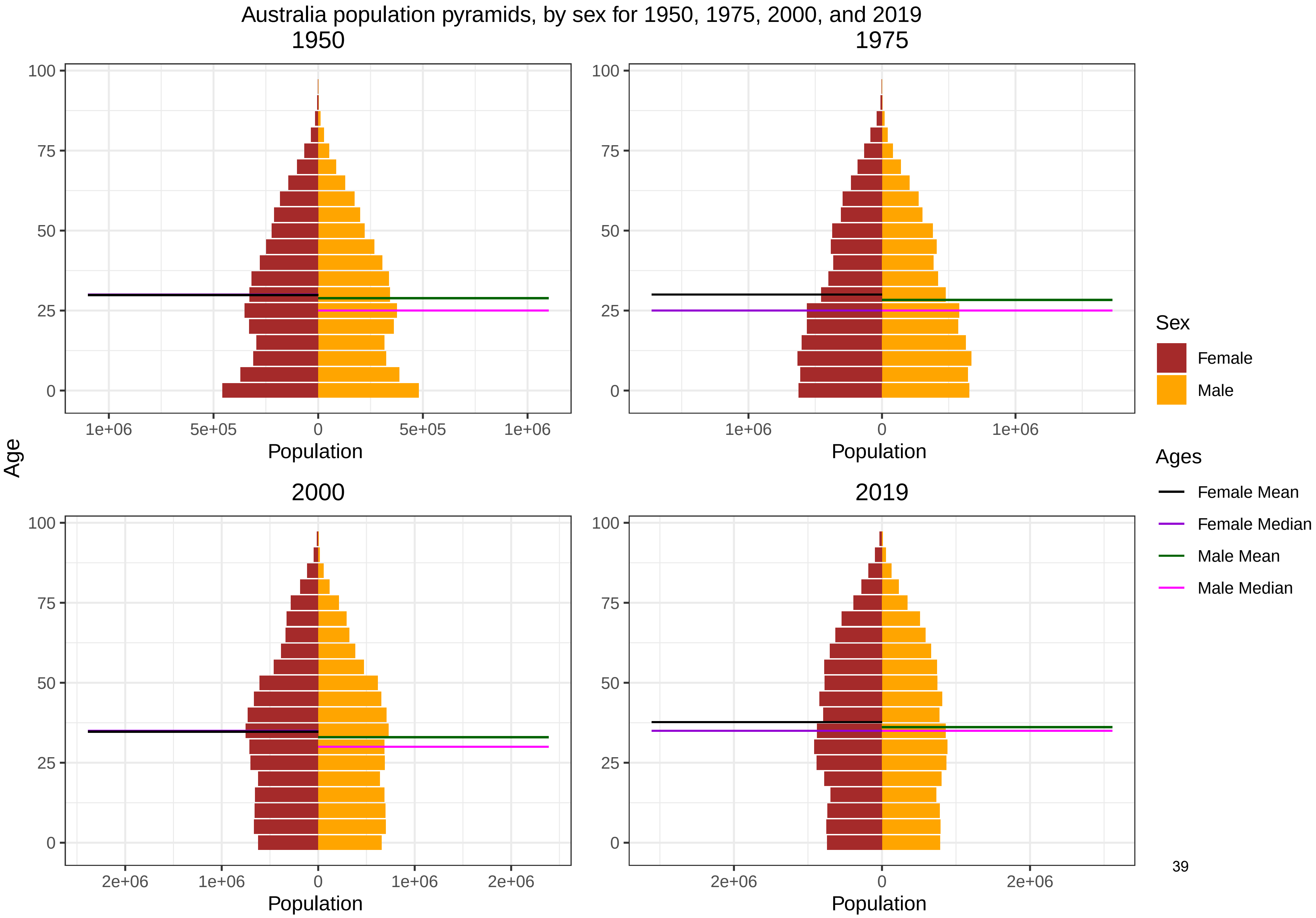

Australia population pyramids, by sex for 1950, 1975, 2000, and 2019
1950
1975
2000
2019
Age
Population
Sex
Female
Male
Ages
Female Mean
Female Median
Male Mean
Male Median

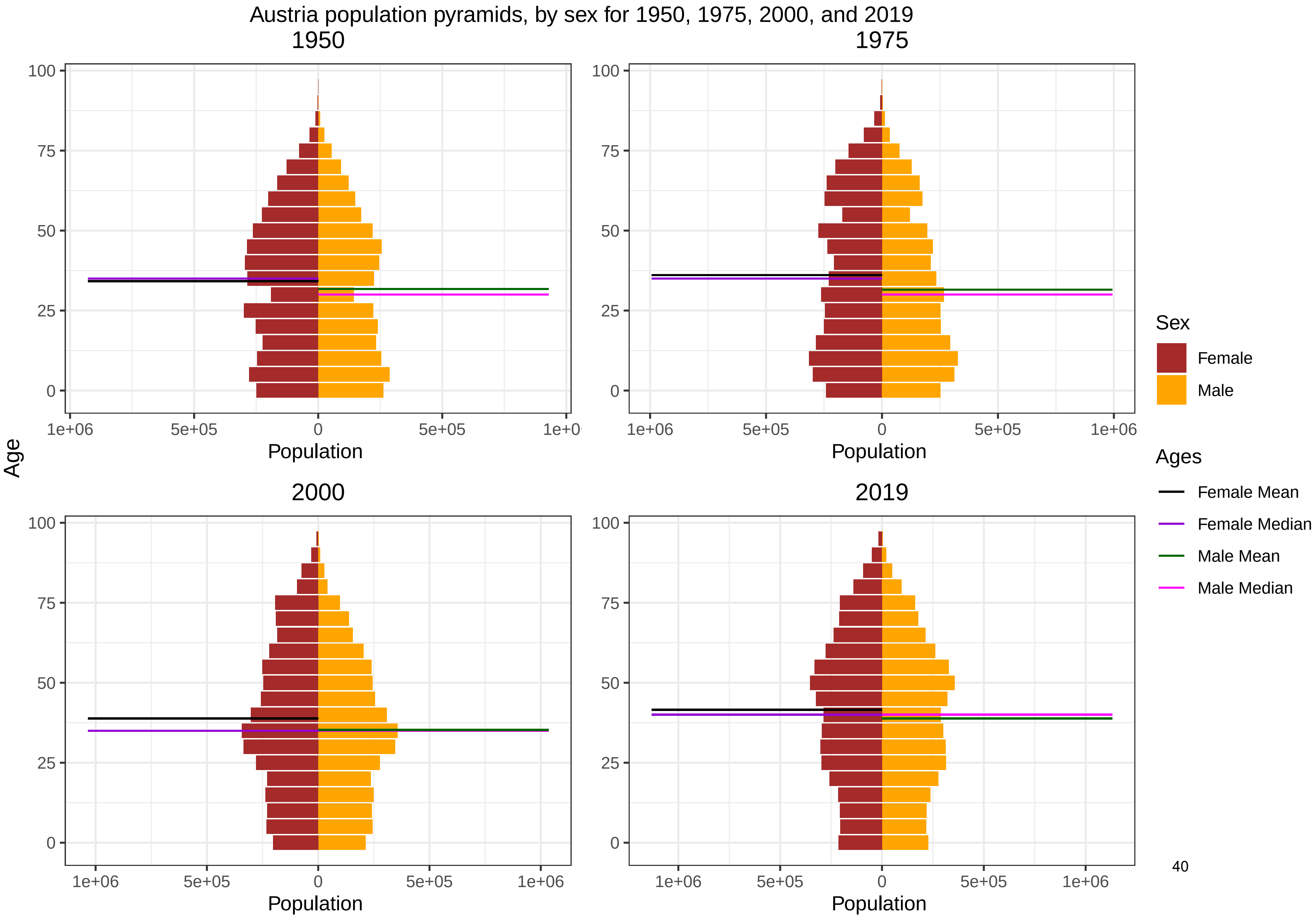
Austria population pyramids, by sex for 1950, 1975, 2000, and 2019
1950
1975
2000
2019
Age
Population
100
75
50
25
0
1e+06
5e+05
0
5e+05
1e+06
Sex
Female
Male
Ages
Female Mean
Female Median
Male Mean
Male Median

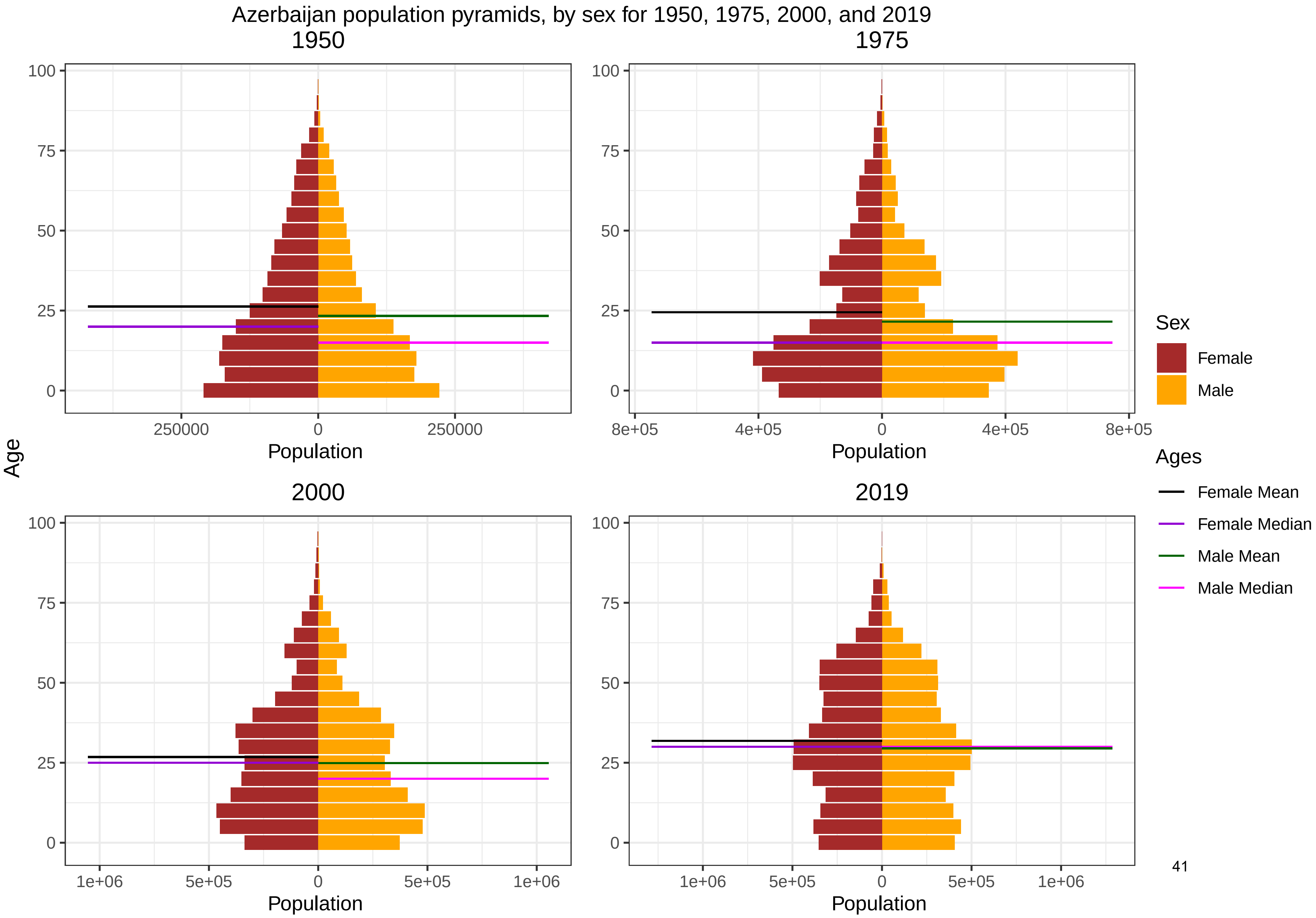

Azerbaijan population pyramids, by sex for 1950, 1975, 2000, and 2019
1950
1975
2000
2019
Age
Population
100
75
50
25
0
250000
0
250000
8e+05
4e+05
0
4e+05
8e+05
1e+06
5e+05
0
5e+05
1e+06
Sex
Female
Male
Ages
Female Mean
Female Median
Male Mean
Male Median

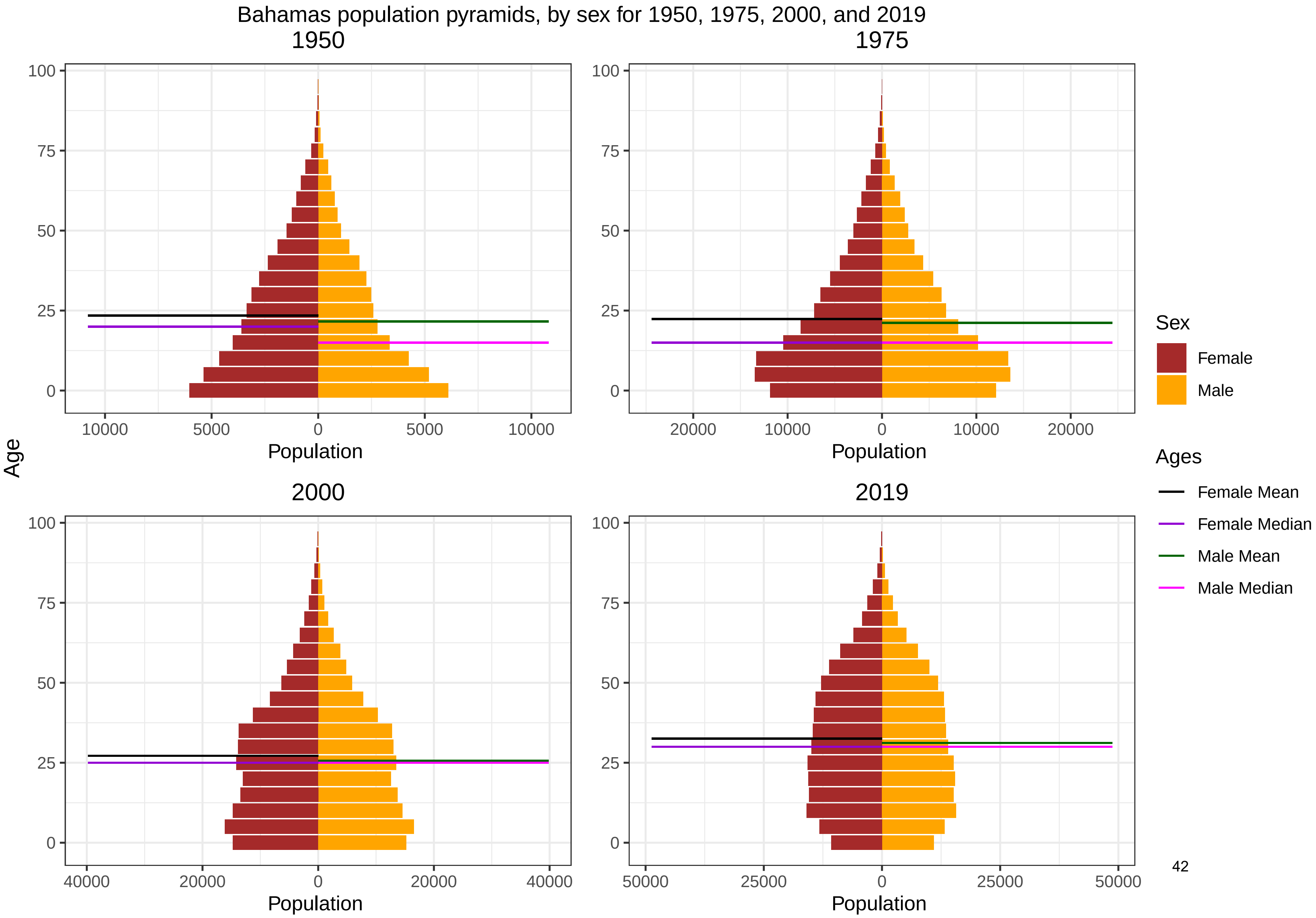
Bahamas population pyramids, by sex for 1950, 1975, 2000, and 2019
1950
1975
2000
2019
Age
Population
Sex
Female
Male
Ages
Female Mean
Female Median
Male Mean
Male Median

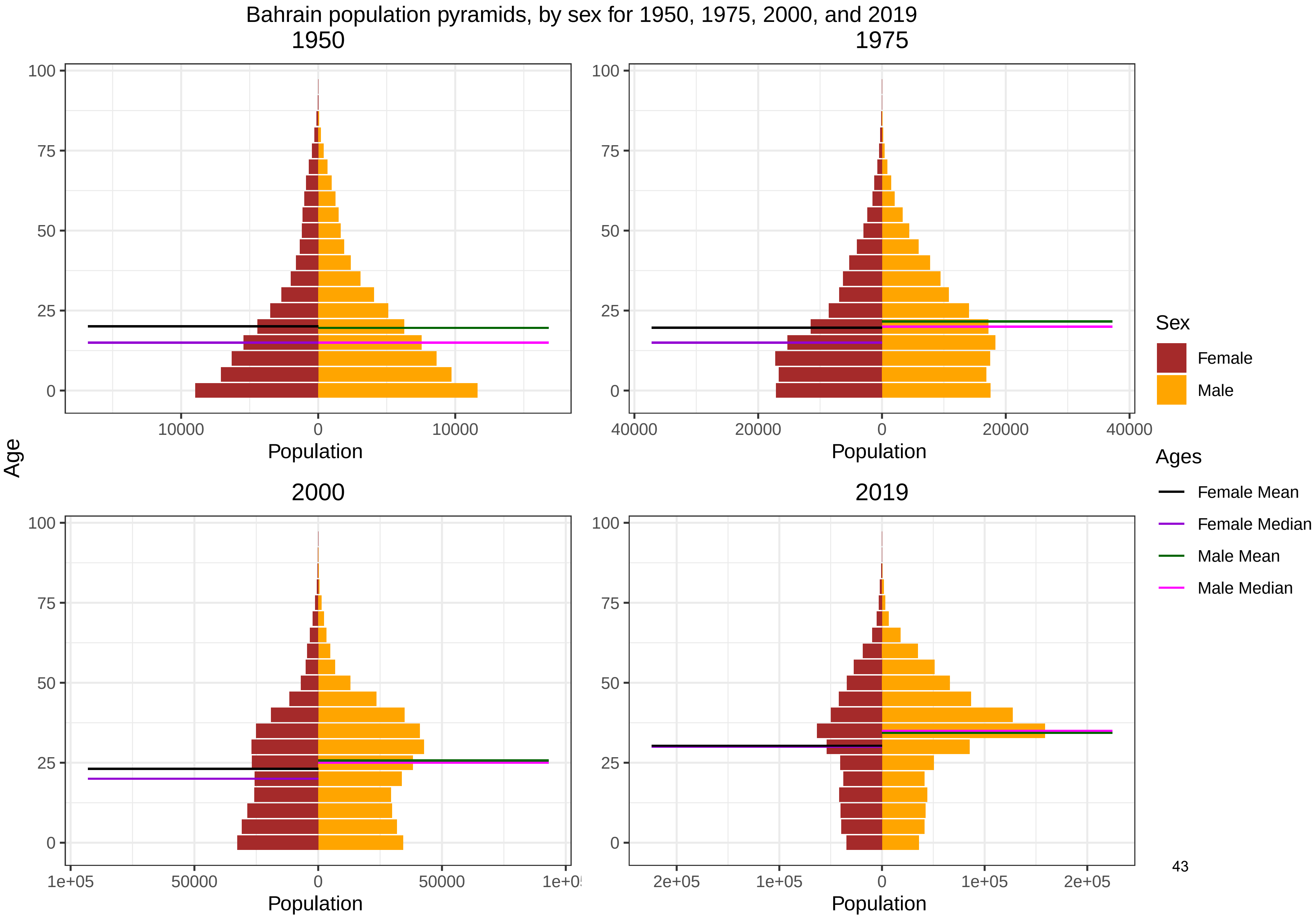

Bahrain population pyramids, by sex for 1950, 1975, 2000, and 2019
1950
1975
2000
2019
Age
Population
Sex
Female
Male
Ages
Female Mean
Female Median
Male Mean
Male Median

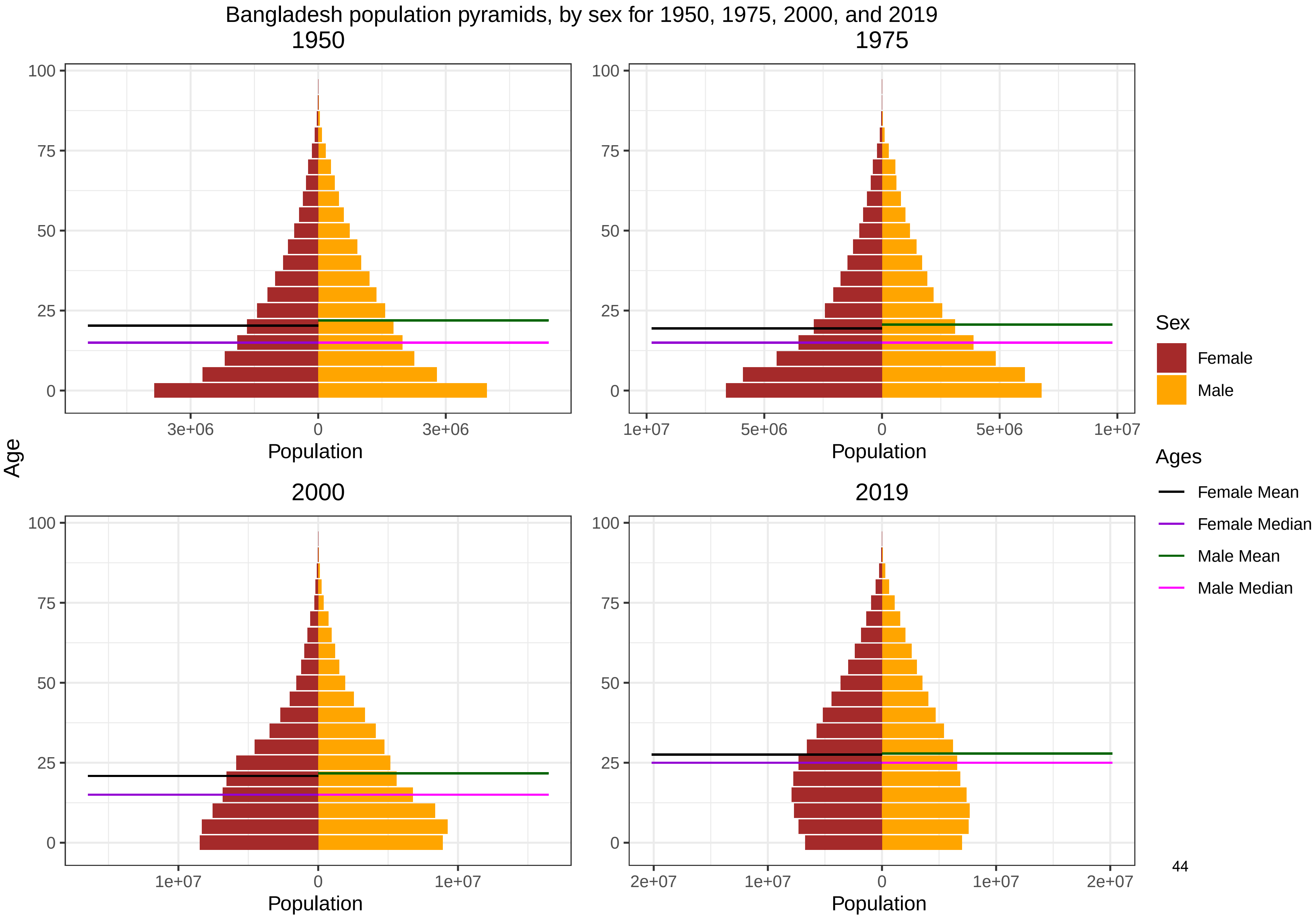
Bangladesh population pyramids, by sex for 1950, 1975, 2000, and 2019
1950
1975
2000
2019
Age
Population
100
75
50
25
0
3e+06
0
3e+06
1e+07
5e+06
0
5e+06
1e+07
1e+07
0
1e+07
2e+07
1e+07
0
1e+07
2e+07
Sex
Female
Male
Ages
Female Mean
Female Median
Male Mean
Male Median

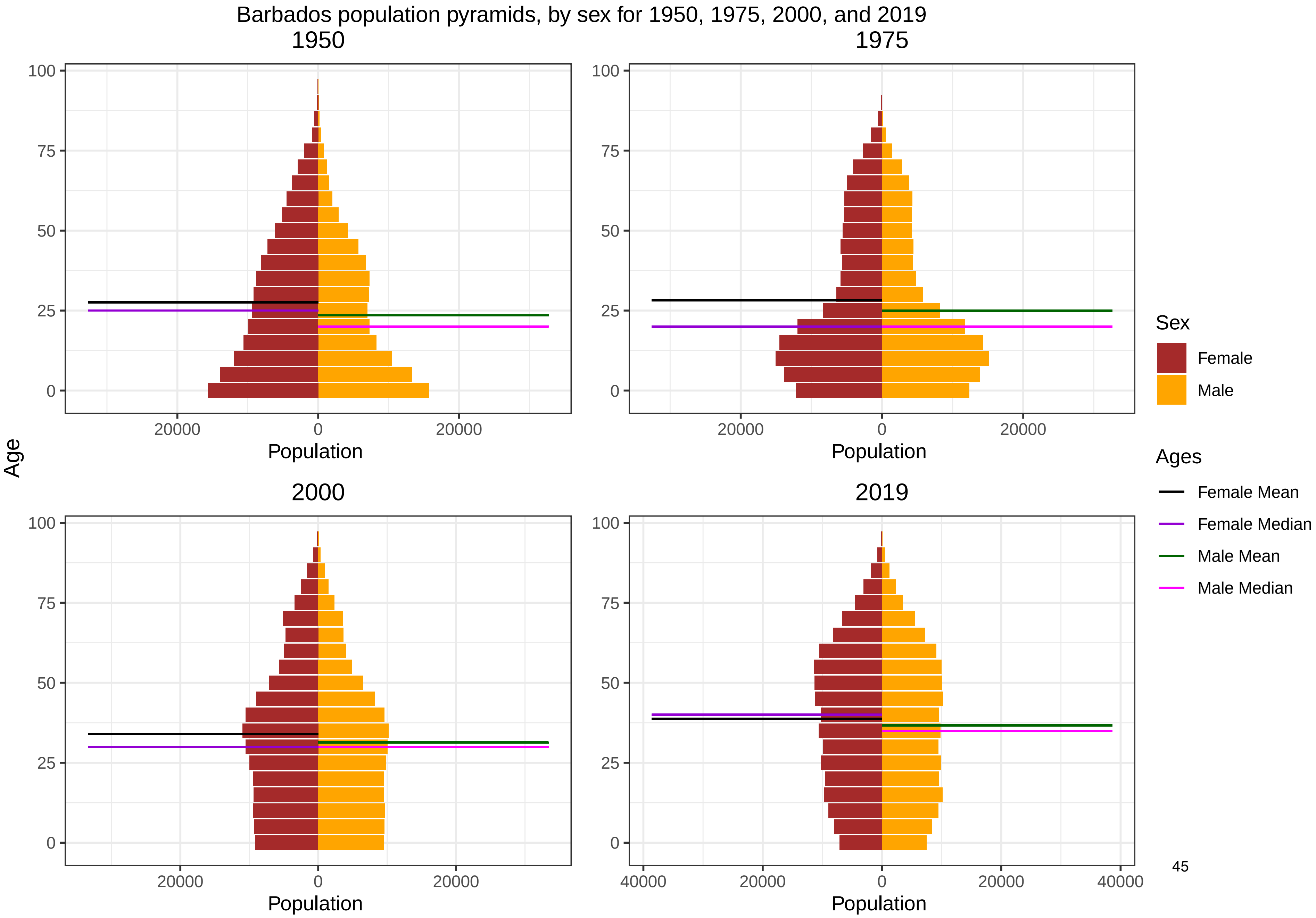

Barbados population pyramids, by sex for 1950, 1975, 2000, and 2019
1950
1975
2000
2019
Age
Population
Sex
Female
Male
Ages
Female Mean
Female Median
Male Mean
Male Median

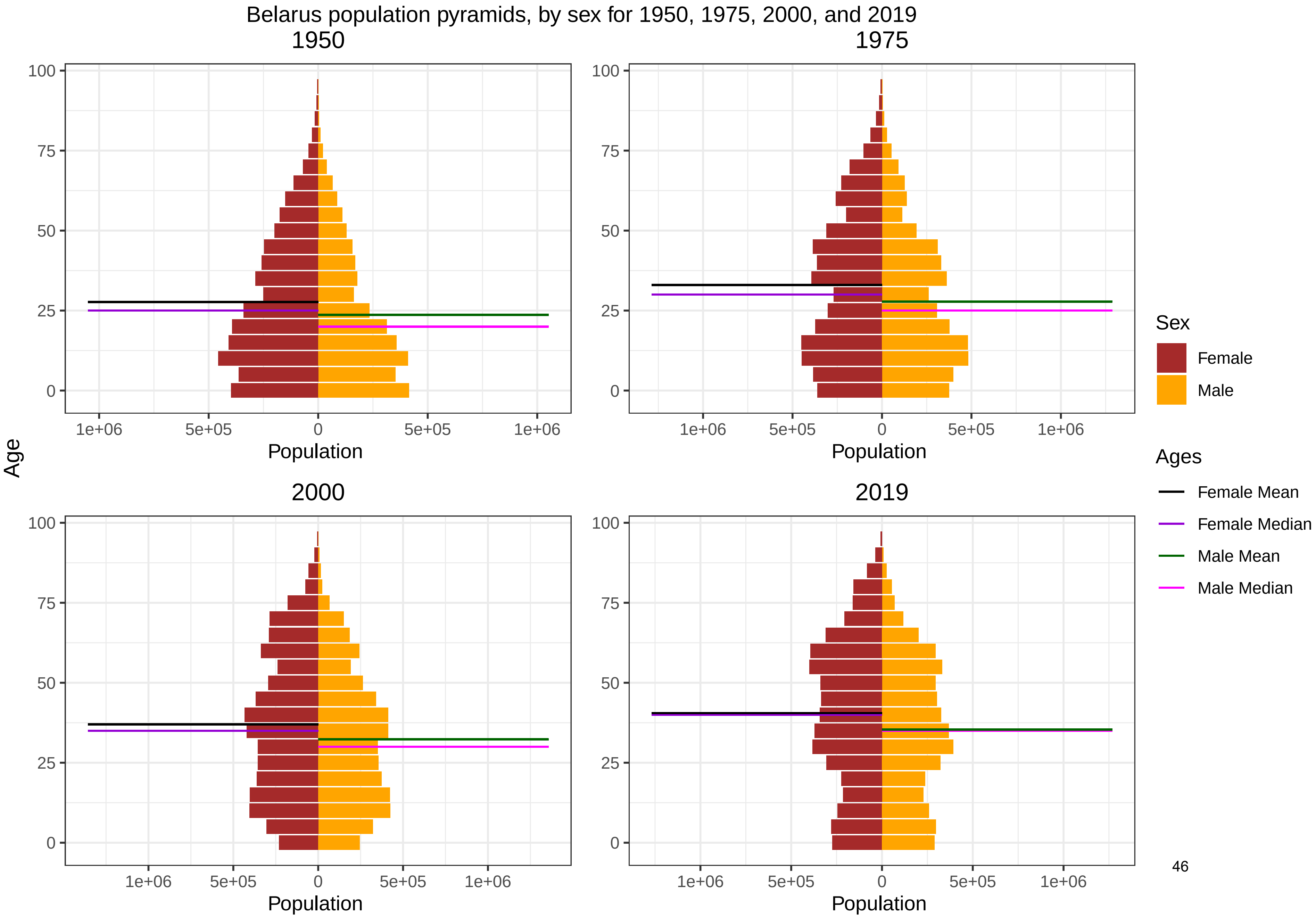

Belarus population pyramids, by sex for 1950, 1975, 2000, and 2019
1950
1975
2000
2019
Age
Population
100
75
50
25
0
1e+06
5e+05
0
5e+05
1e+06
Sex
Female
Male
Ages
Female Mean
Female Median
Male Mean
Male Median

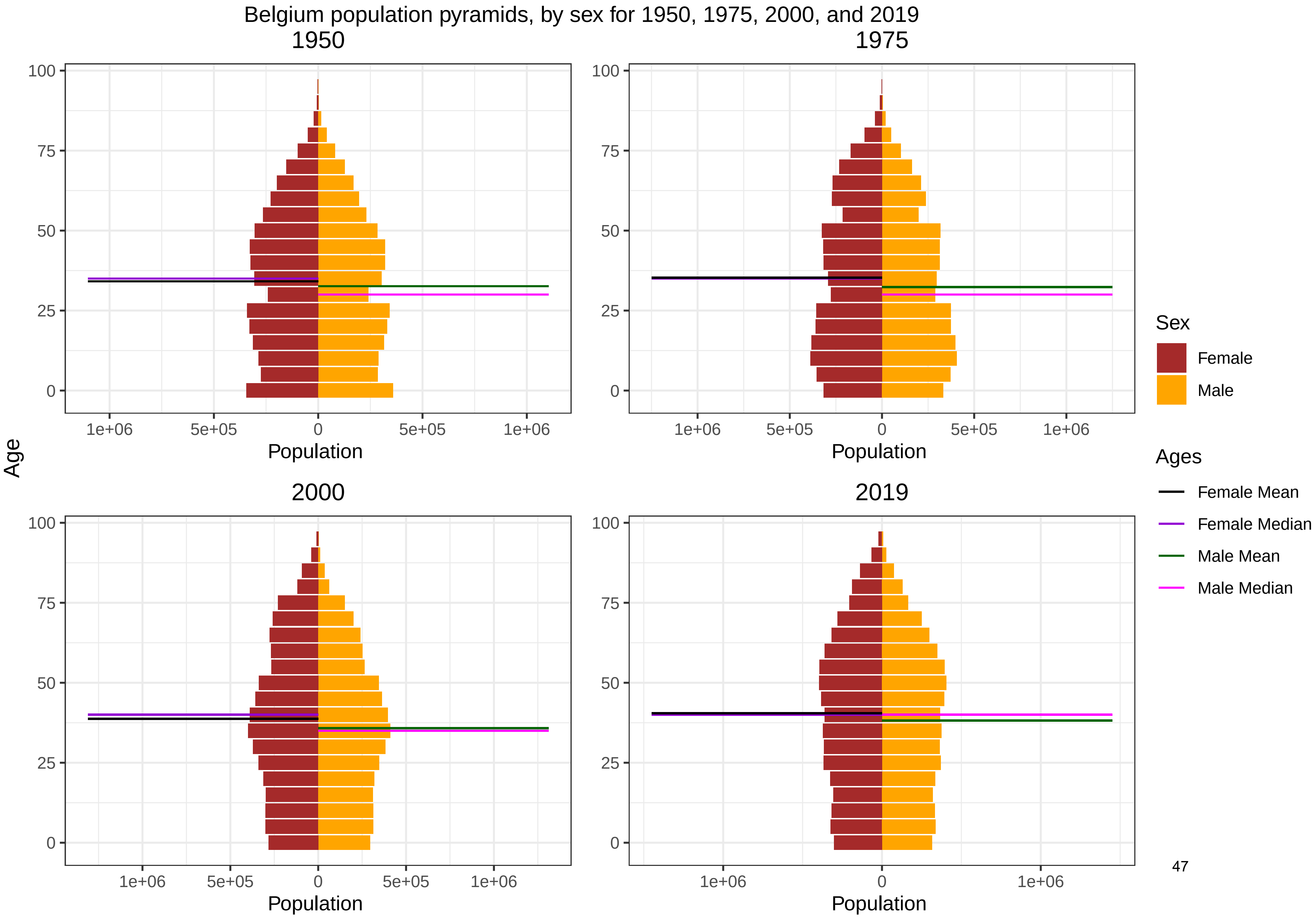
Belgium population pyramids, by sex for 1950, 1975, 2000, and 2019
1950
1975
2000
2019
Age
Population
100
75
50
25
0
1e+06
5e+05
0
5e+05
1e+06
Sex
Female
Male
Ages
Female Mean
Female Median
Male Mean
Male Median

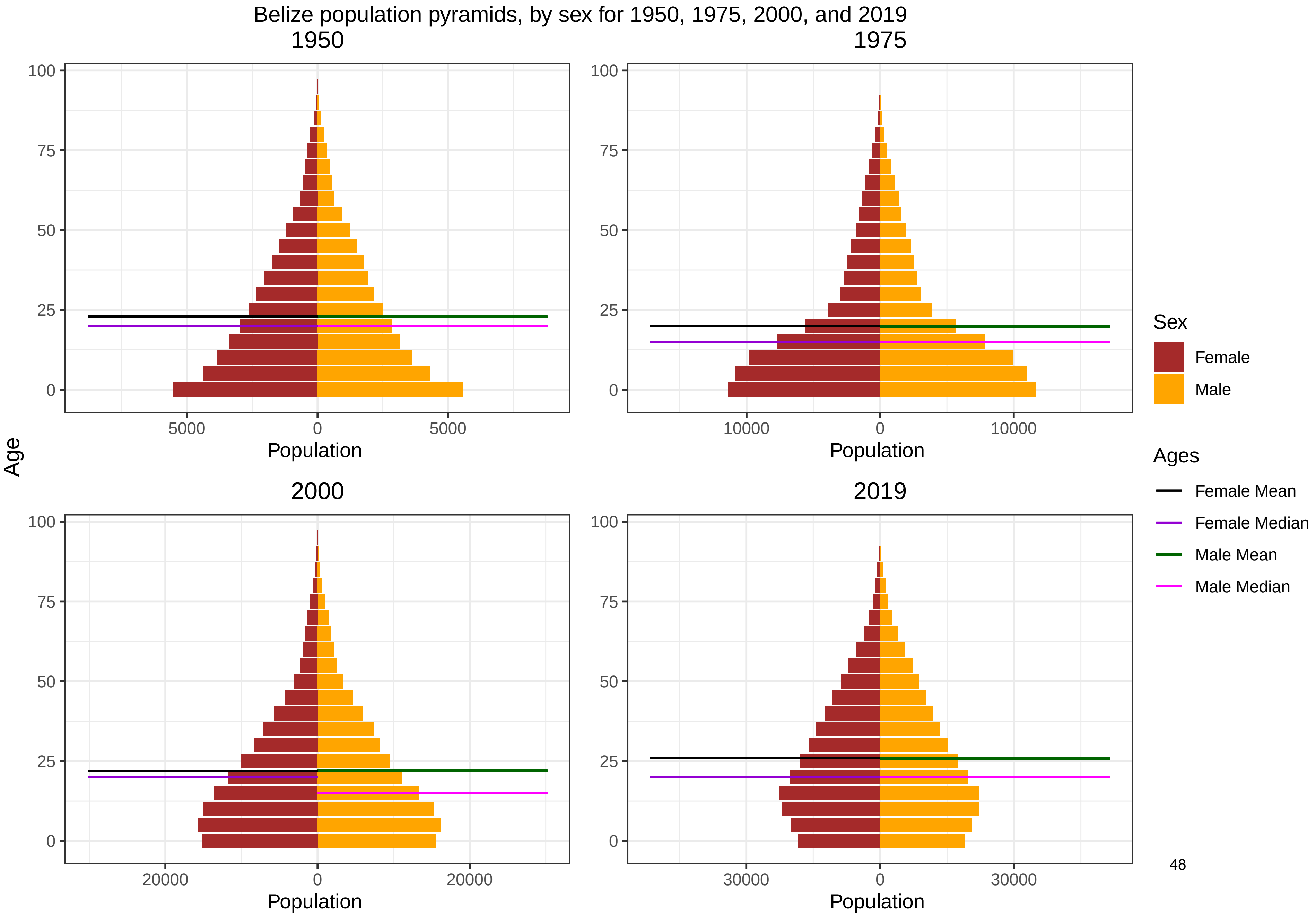
Belize population pyramids, by sex for 1950, 1975, 2000, and 2019
1950
1975
2000
2019
Age
Population
Sex
Female
Male
Ages
Female Mean
Female Median
Male Mean
Male Median

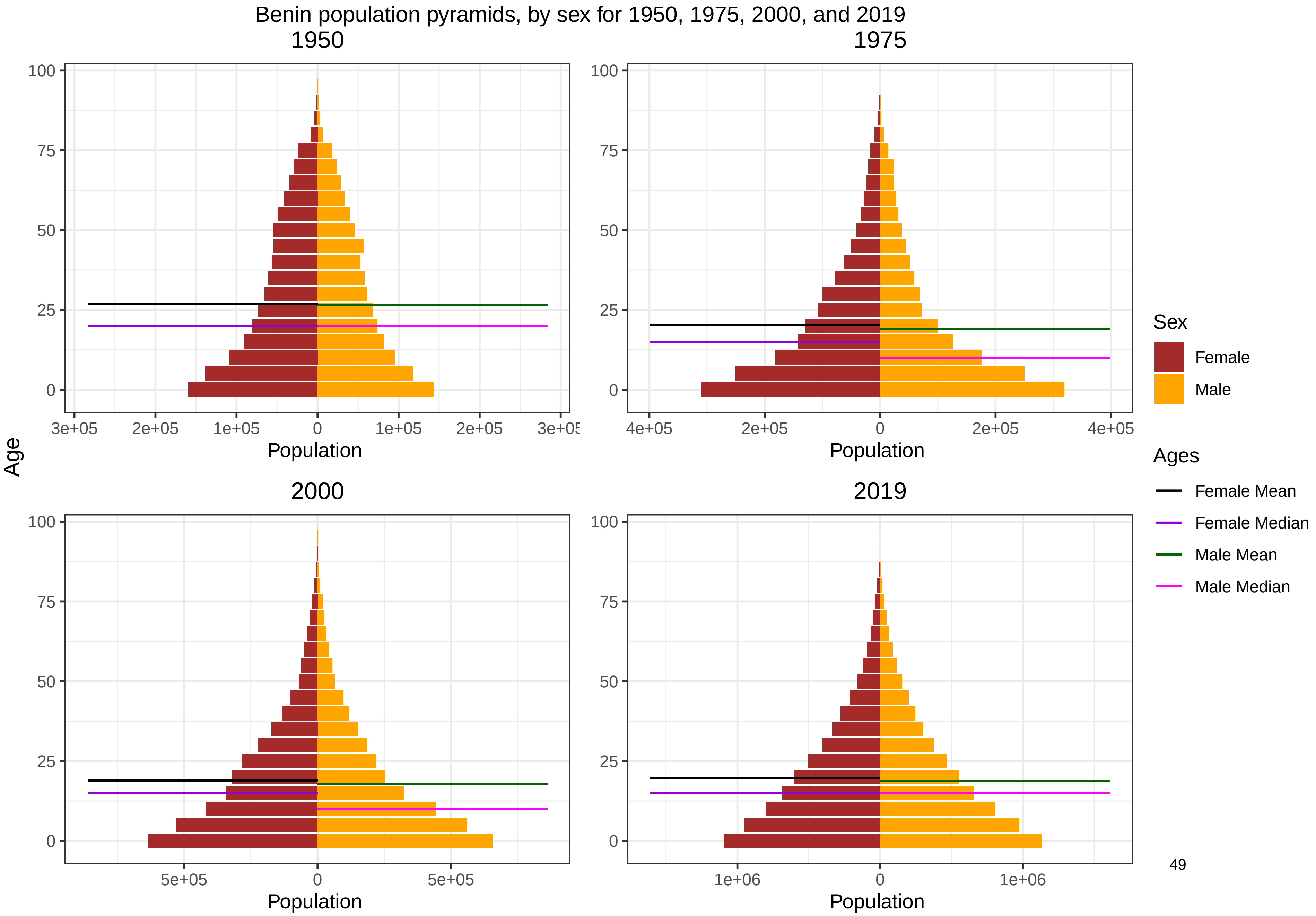
Benin population pyramids, by sex for 1950, 1975, 2000, and 2019
1950
1975
2000
2019
Age
Population
Sex
Female
Male
Ages
Female Mean
Female Median
Male Mean
Male Median

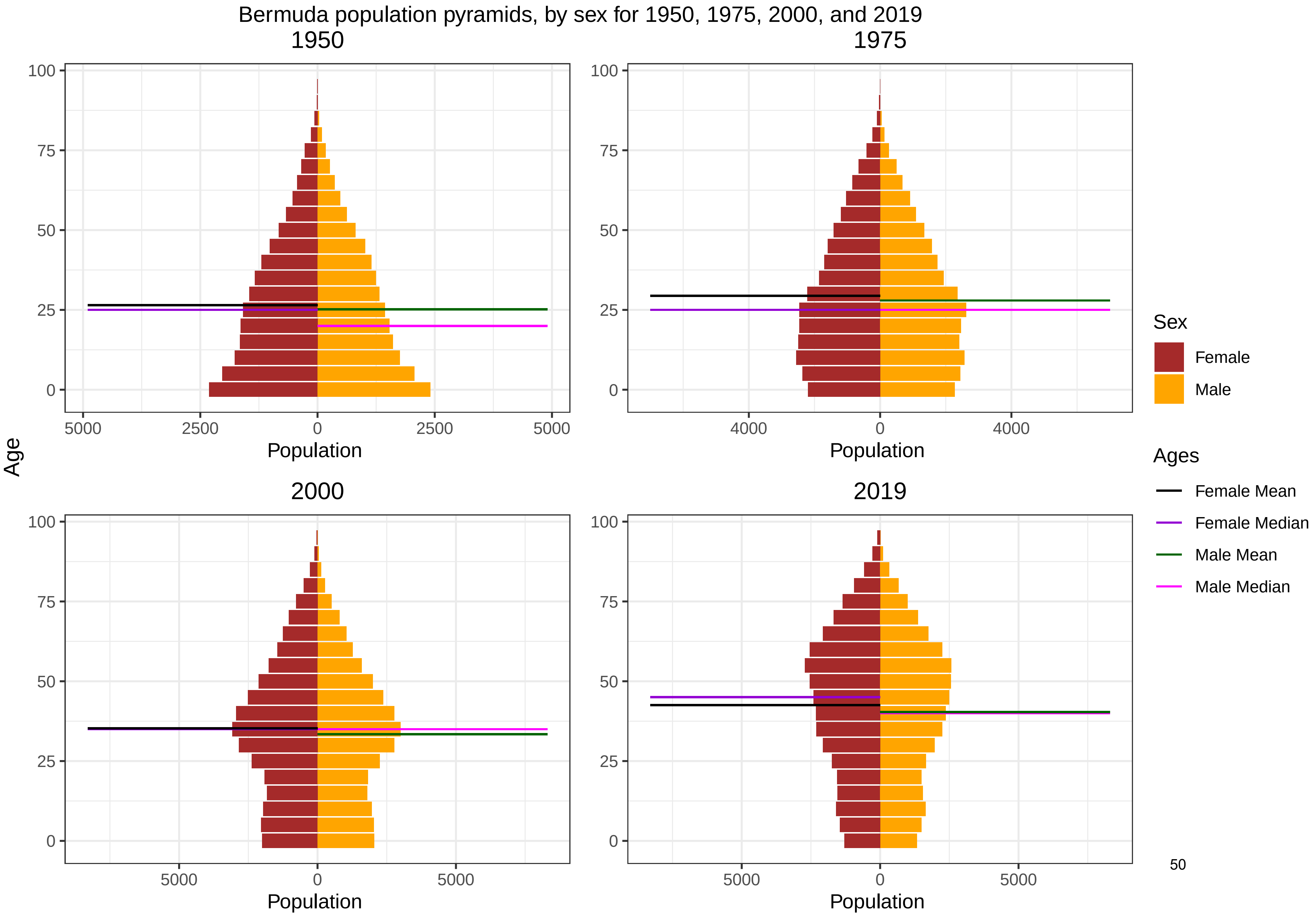

Bermuda population pyramids, by sex for 1950, 1975, 2000, and 2019
1950
1975
2000
2019
Age
Population
Sex
Female
Male
Ages
Female Mean
Female Median
Male Mean
Male Median

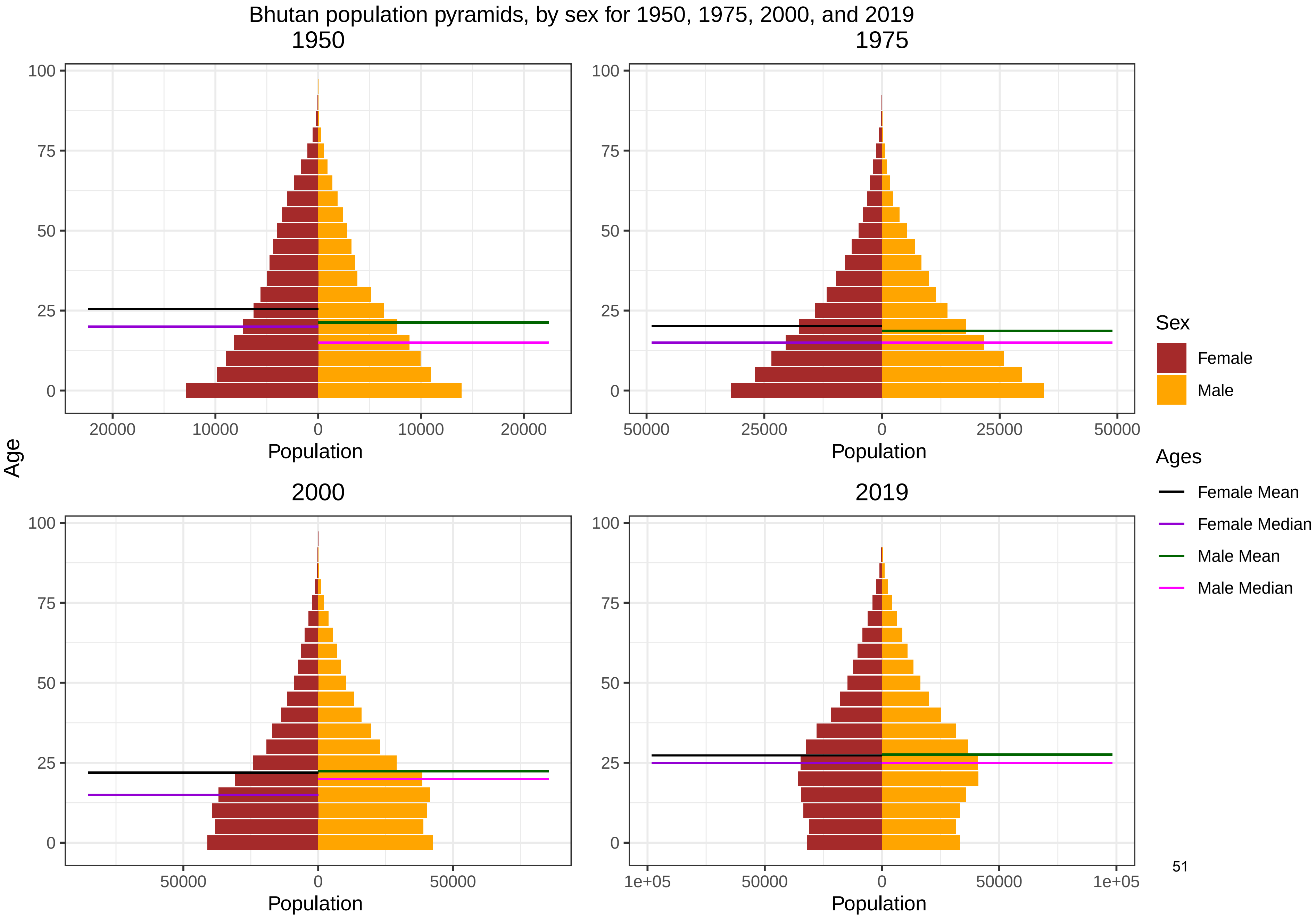
Bhutan population pyramids, by sex for 1950, 1975, 2000, and 2019
1950
1975
2000
2019
Age
Population
100
75
50
25
0
20000
10000
0
10000
20000
50000
25000
0
25000
50000
50000
0
50000
1e+05
50000
0
50000
1e+05
Sex
Female
Male
Ages
Female Mean
Female Median
Male Mean
Male Median

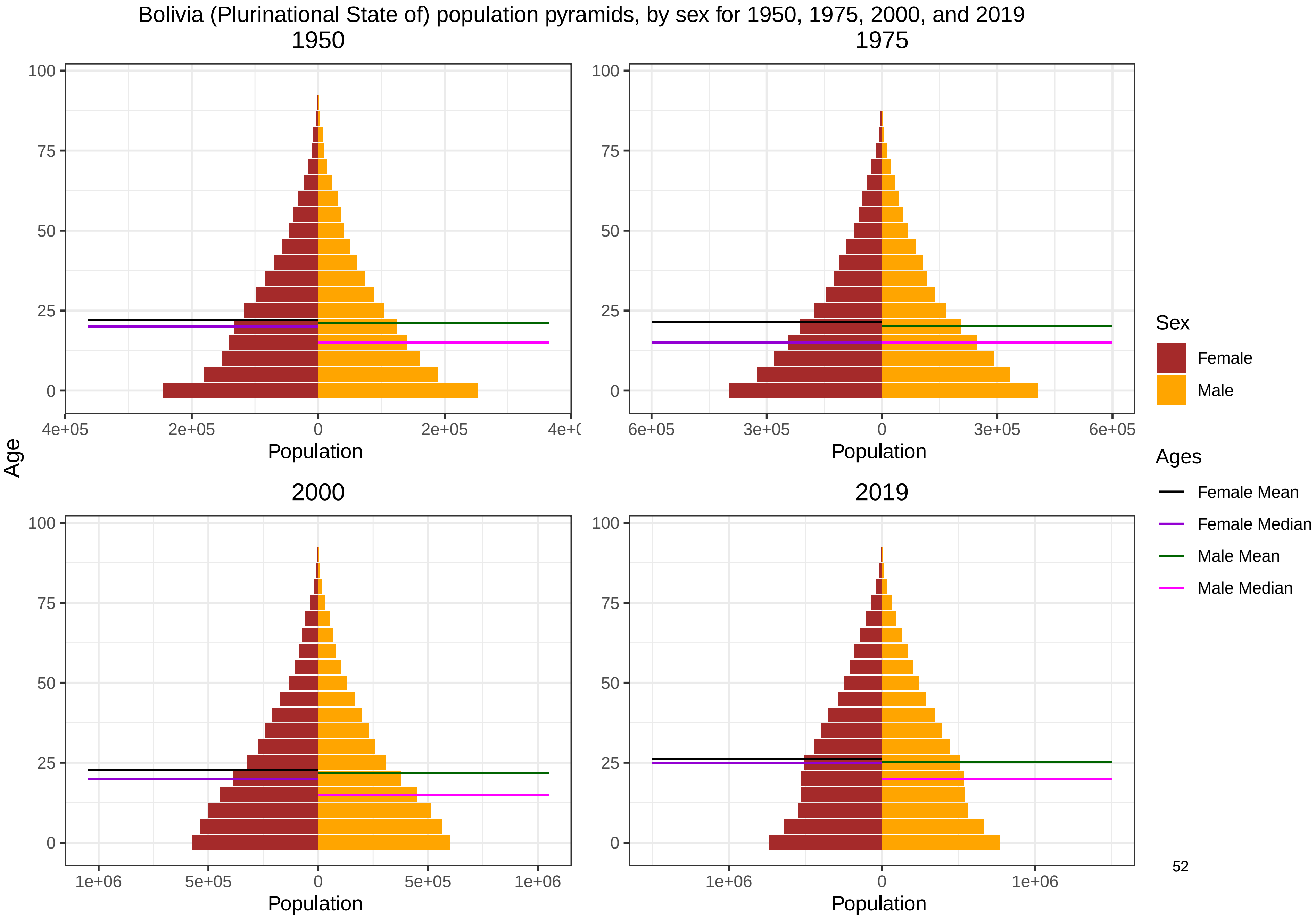
Bolivia (Plurinational State of) population pyramids, by sex for 1950, 1975, 2000, and 2019
1950
1975
2000
2019
Age
Population
Sex
Female
Male
Ages
Female Mean
Female Median
Male Mean
Male Median

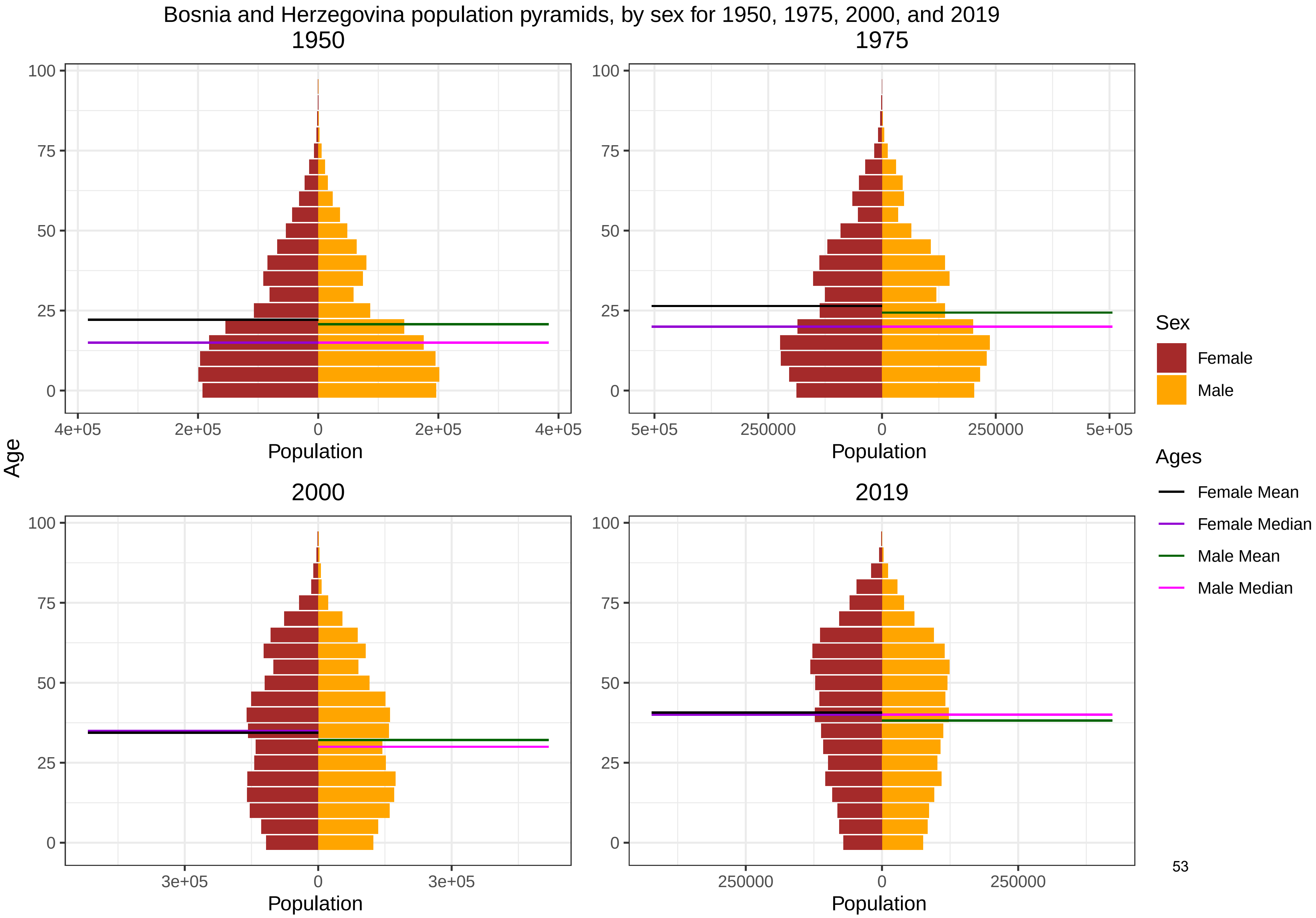
Bosnia and Herzegovina population pyramids, by sex for 1950, 1975, 2000, and 2019
1950
1975
2000
2019
Age
Population
Sex
Female
Male
Ages
Female Mean
Female Median
Male Mean
Male Median
4e+05
2e+05
0
250000
5e+05
3e+05
100
75
50
25

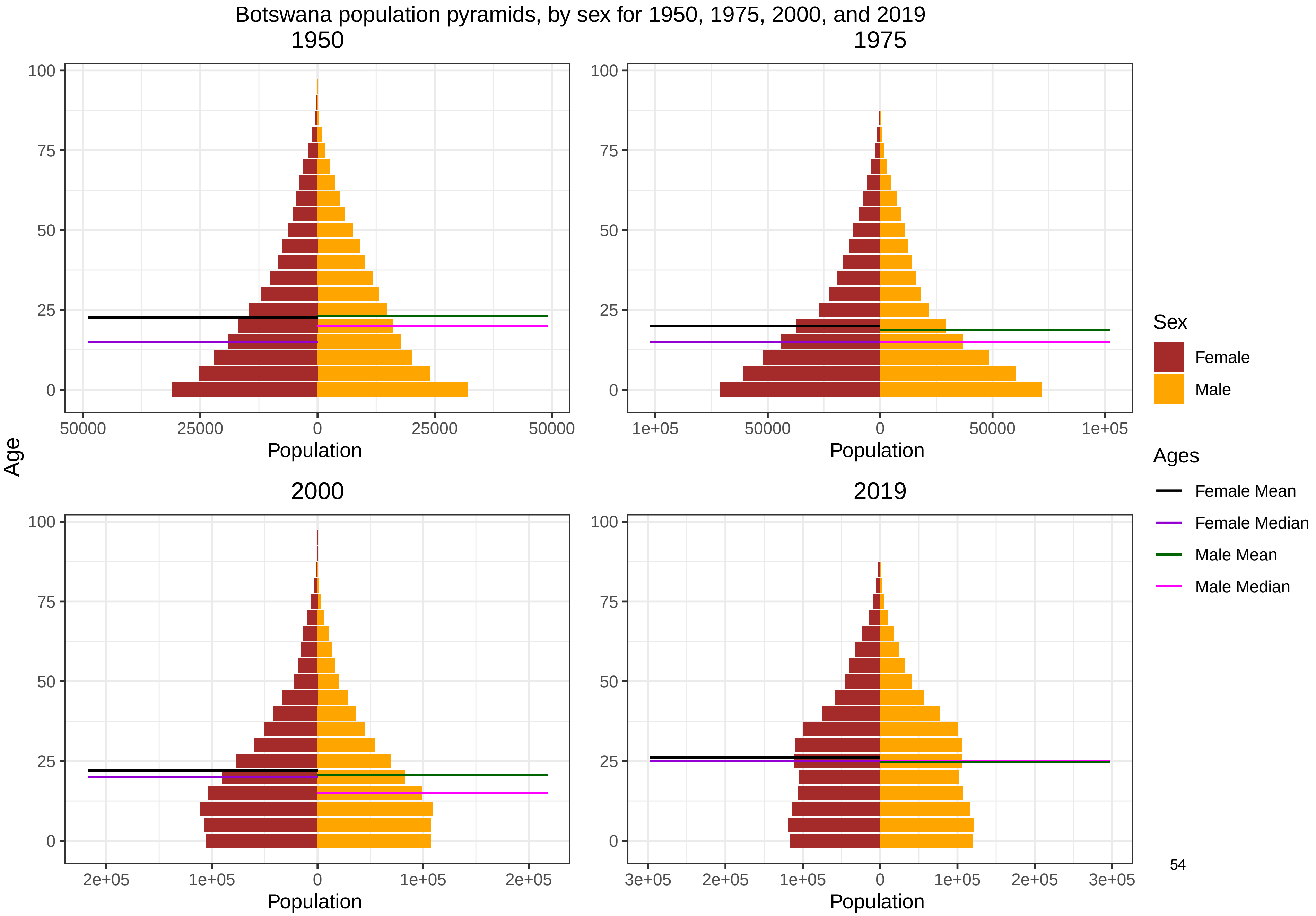
Botswana population pyramids, by sex for 1950, 1975, 2000, and 2019
1950
1975
2000
2019
Age
Population
Sex
Female
Male
Ages
Female Mean
Female Median
Male Mean
Male Median

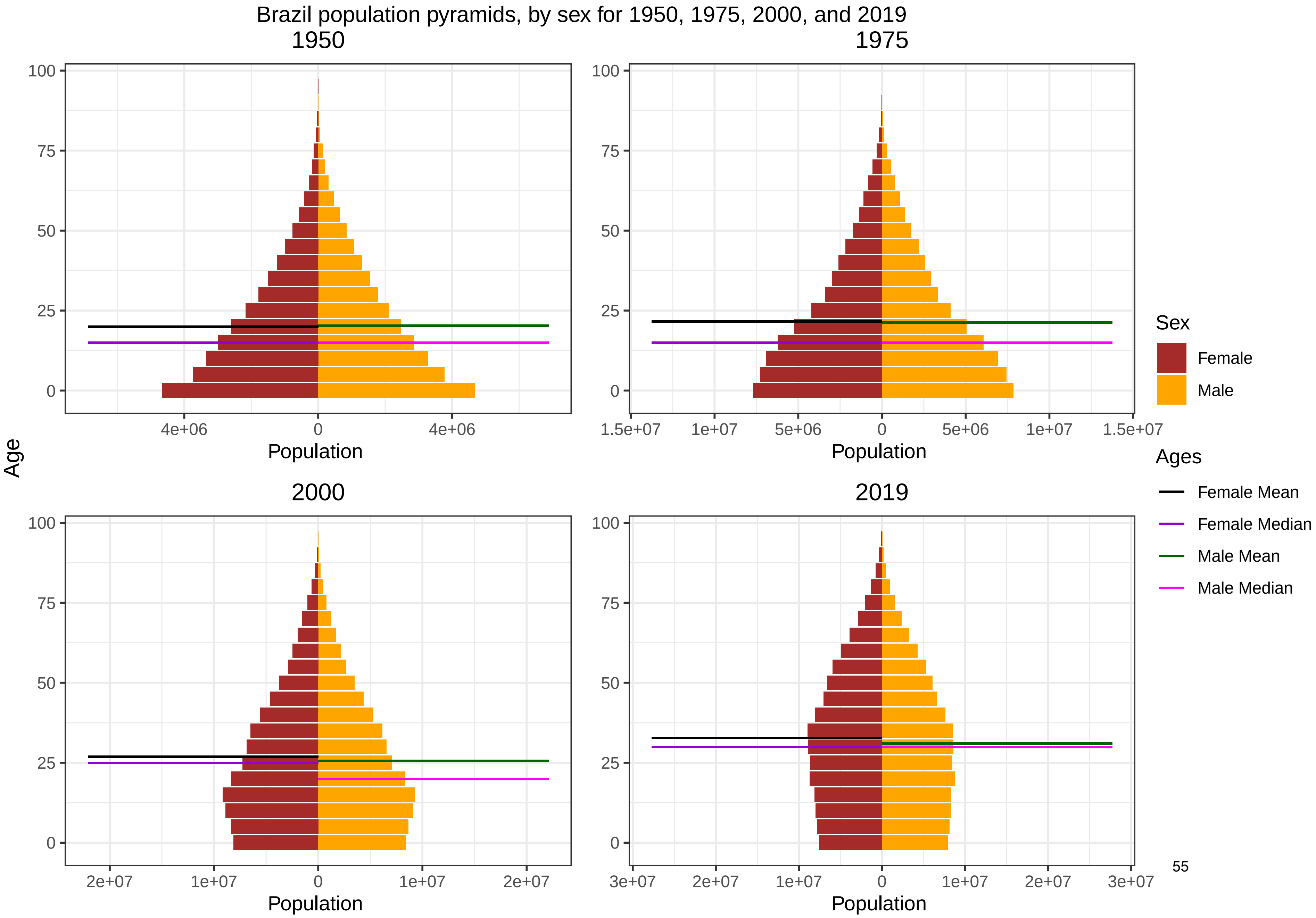
Brazil population pyramids, by sex for 1950, 1975, 2000, and 2019
1950
1975
2000
2019
Age
Population
100
75
50
25
0
4e+06
0
4e+06
1.5e+07
1e+07
5e+06
5e+06
1e+07
1.5e+07
2e+07
1e+07
1e+07
2e+07
3e+07
2e+07
1e+07
1e+07
2e+07
3e+07
Sex
Female
Male
Ages
Female Mean
Female Median
Male Mean
Male Median

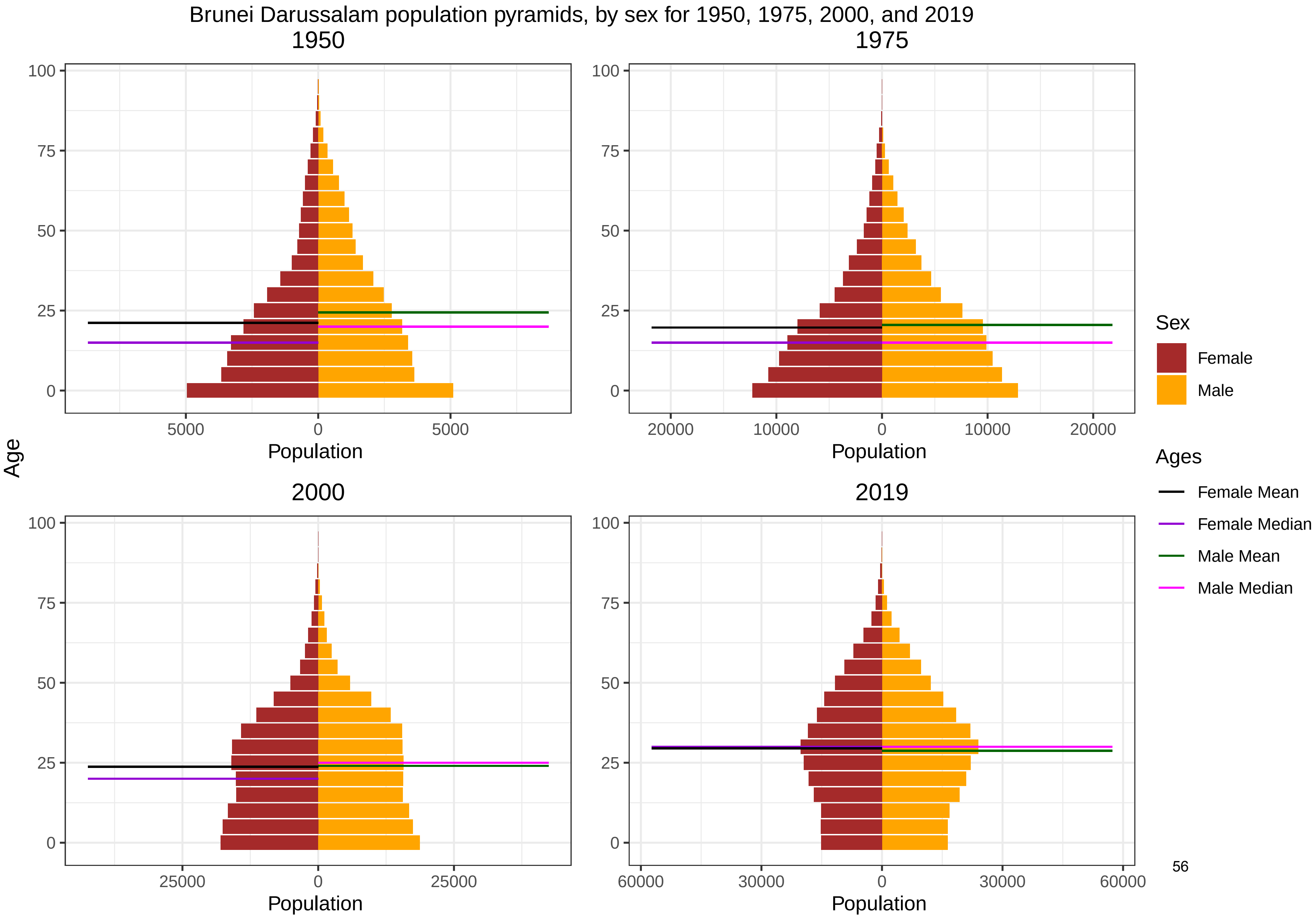

Brunei Darussalam population pyramids, by sex for 1950, 1975, 2000, and 2019
1950
1975
2000
2019
Age
Population
Sex
Female
Male
Ages
Female Mean
Female Median
Male Mean
Male Median

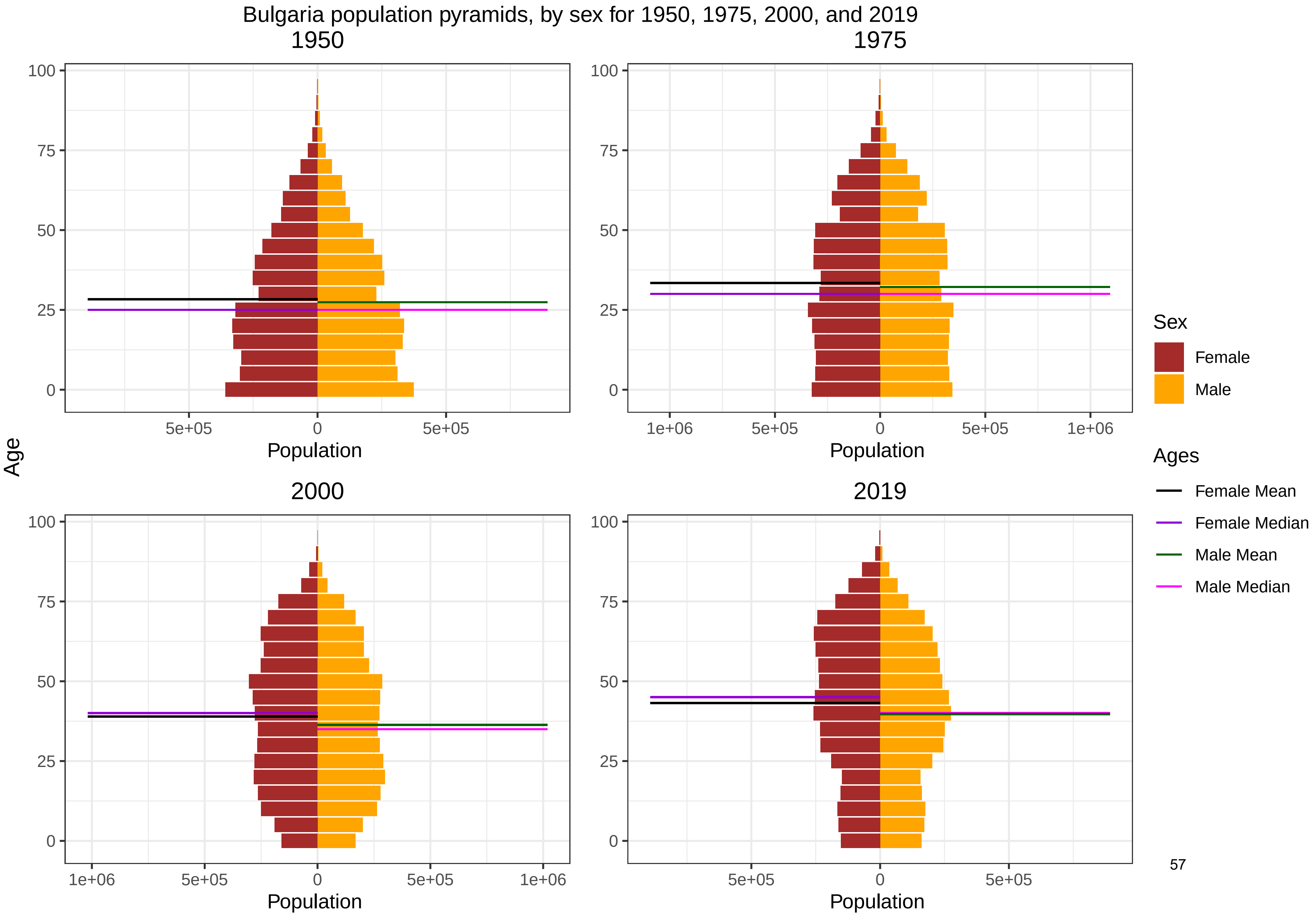

Bulgaria population pyramids, by sex for 1950, 1975, 2000, and 2019
1950
1975
2000
2019
Age
Population
100
75
50
25
0
5e+05
0
5e+05
1e+06
Sex
Female
Male
Ages
Female Mean
Female Median
Male Mean
Male Median

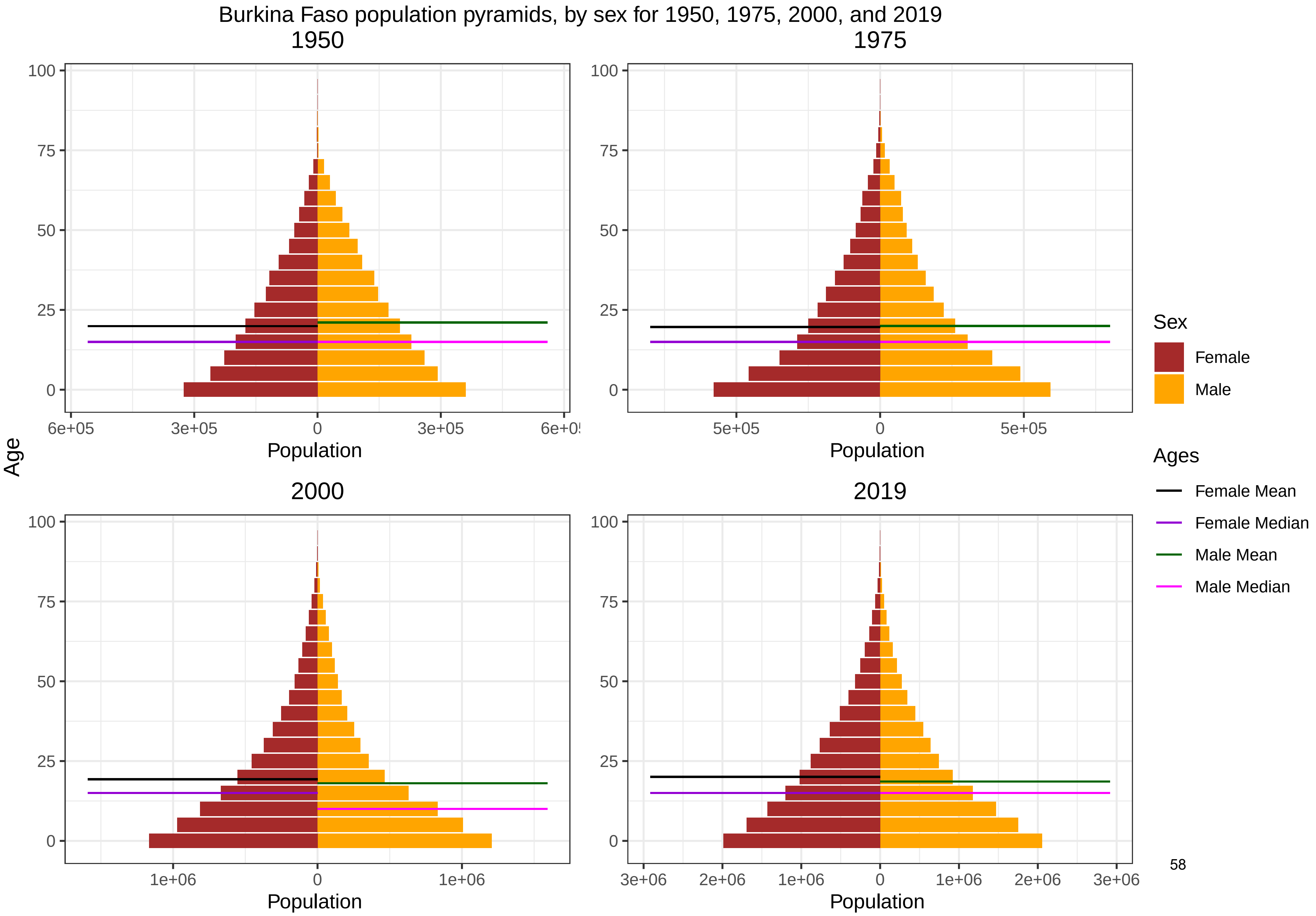
Burkina Faso population pyramids, by sex for 1950, 1975, 2000, and 2019
1950
1975
2000
2019
Age
Population
Sex
Female
Male
Ages
Female Mean
Female Median
Male Mean
Male Median

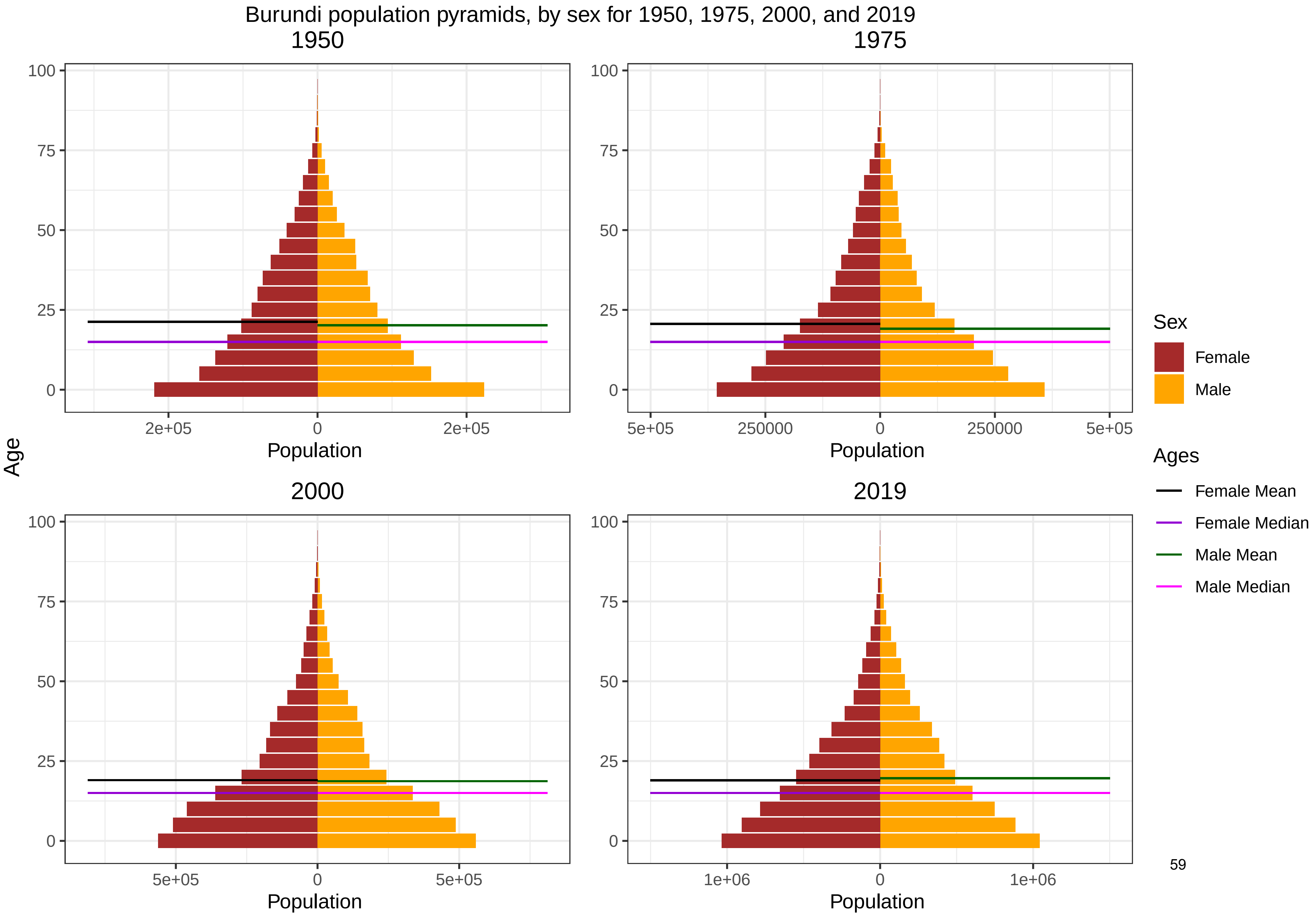

Burundi population pyramids, by sex for 1950, 1975, 2000, and 2019
1950
1975
2000
2019
Age
Population
Sex
Female
Male
Ages
Female Mean
Female Median
Male Mean
Male Median

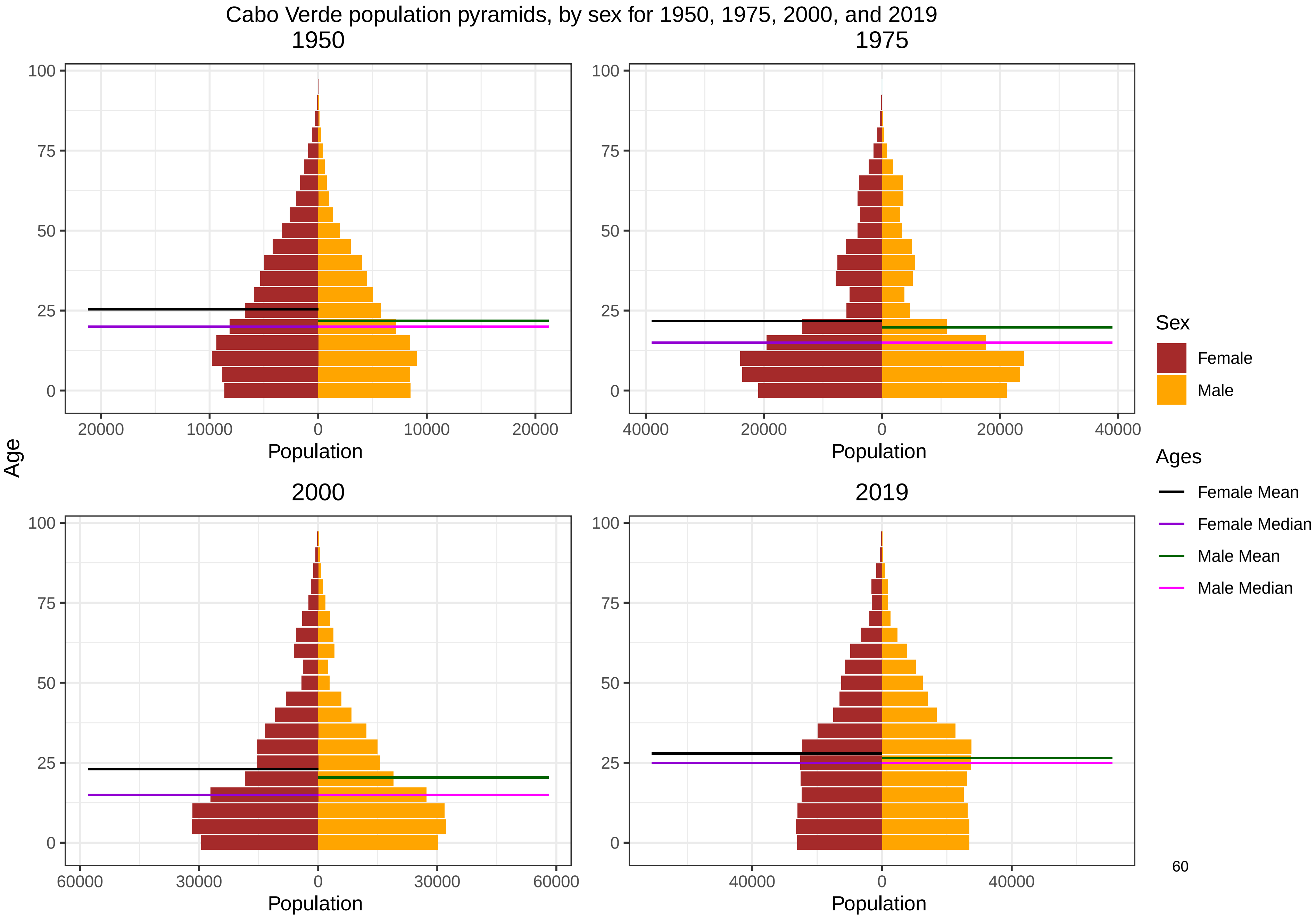
Cabo Verde population pyramids, by sex for 1950, 1975, 2000, and 2019
1950
1975
2000
2019
Age
Population
100
75
50
25
0
20000
10000
0
10000
20000
40000
20000
0
20000
40000
60000
30000
0
30000
60000
40000
0
40000
Sex
Female
Male
Ages
Female Mean
Female Median
Male Mean
Male Median

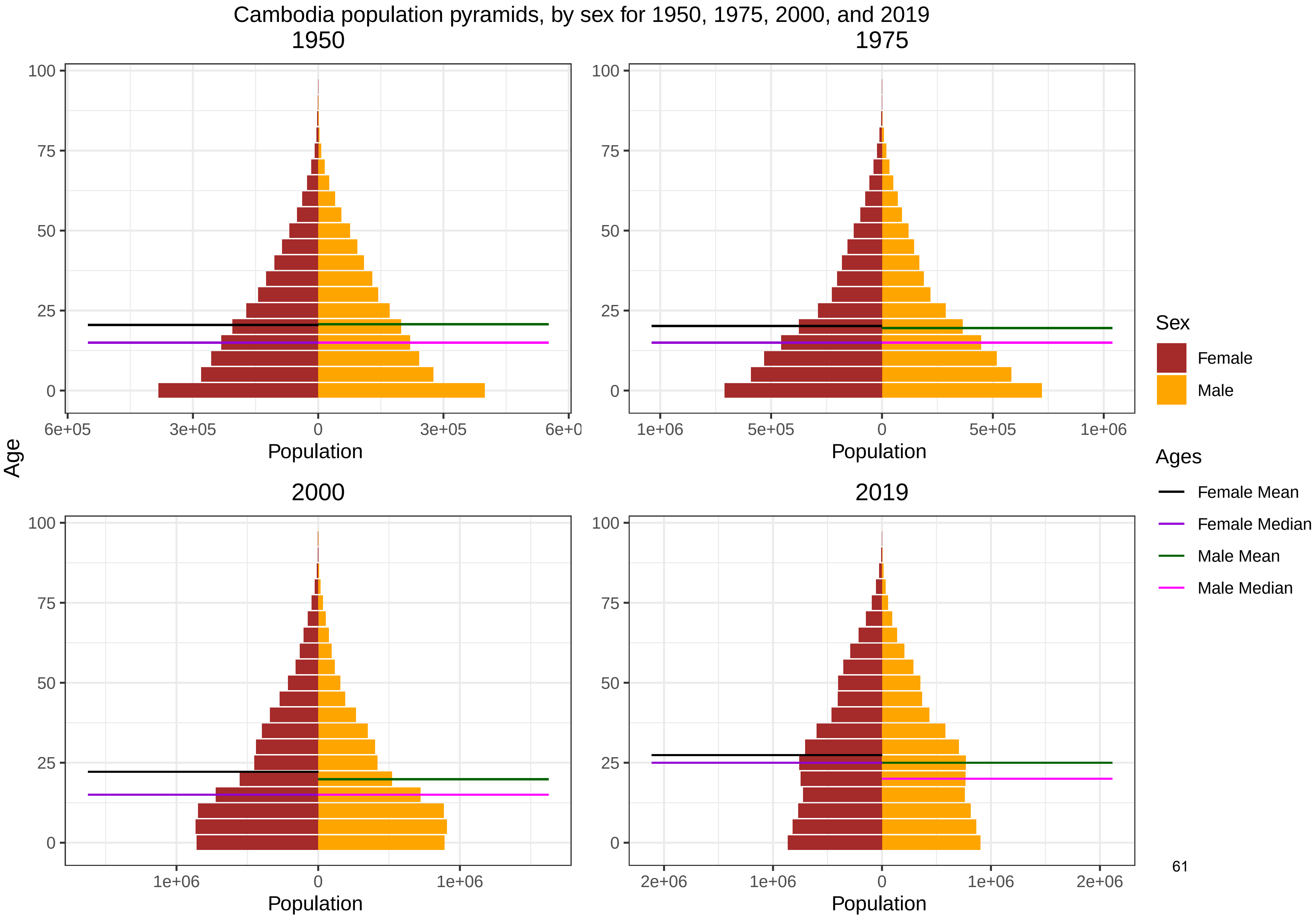
Cambodia population pyramids, by sex for 1950, 1975, 2000, and 2019
1950
1975
2000
2019
Age
Population
0
25
50
75
100
6e+05
3e+05
0
3e+05
1e+06
5e+05
5e+05
1e+06
2e+06
Sex
Female
Male
Ages
Female Mean
Female Median
Male Mean
Male Median

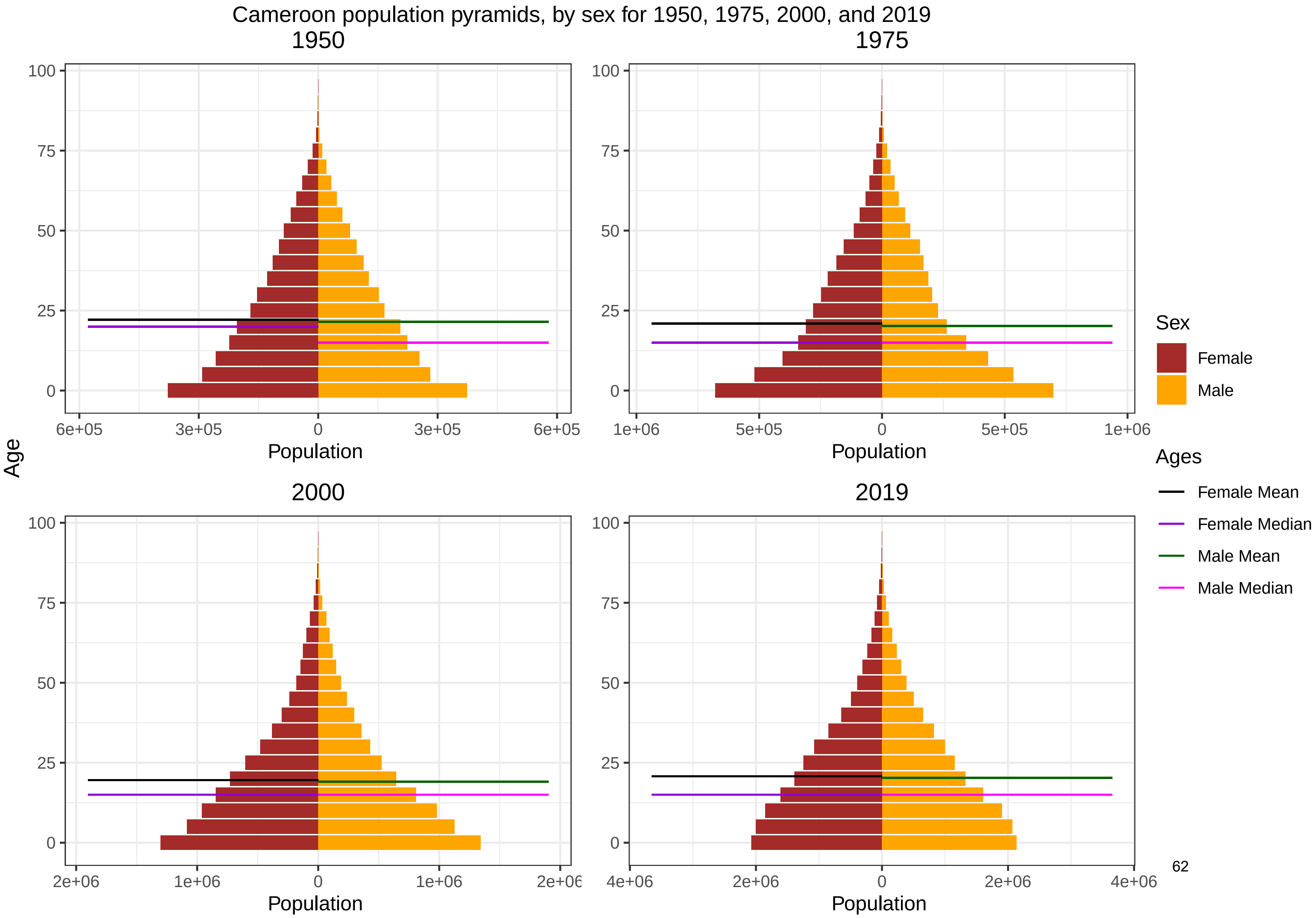
Cameroon population pyramids, by sex for 1950, 1975, 2000, and 2019
1950
1975
2000
2019
Age
Population
Sex
Female
Male
Ages
Female Mean
Female Median
Male Mean
Male Median
100
75
50
25
0
6e+05
3e+05
0
3e+05
6e+05
1e+06
5e+05
5e+05
1e+06
2e+06
1e+06
1e+06
2e+06
4e+06
2e+06
2e+06
4e+06

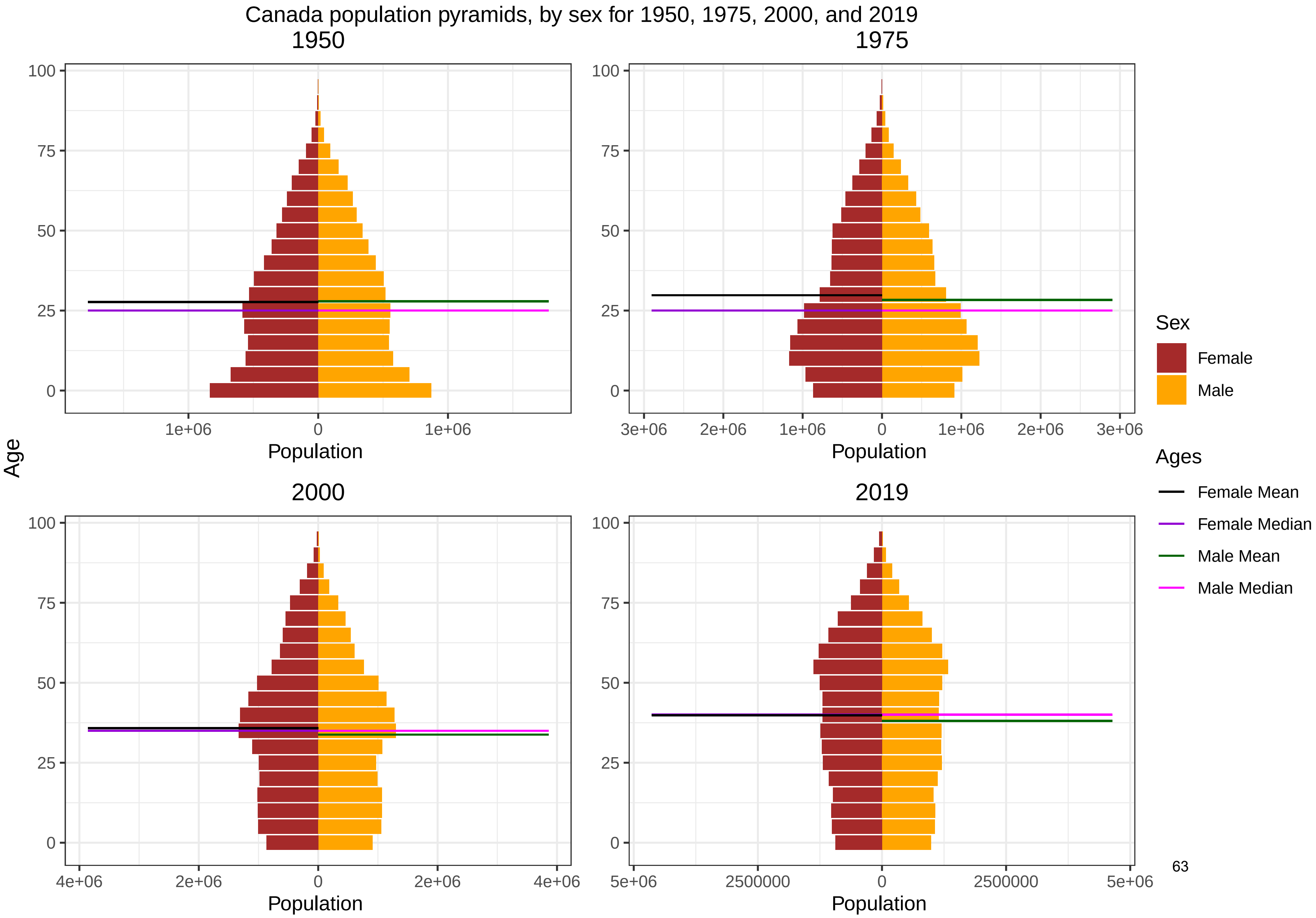

Canada population pyramids, by sex for 1950, 1975, 2000, and 2019
1950
1975
2000
2019
Age
Population
100
75
50
25
0
1e+06
0
1e+06
3e+06
2e+06
1e+06
0
1e+06
2e+06
3e+06
4e+06
2e+06
0
2e+06
4e+06
5e+06
2500000
0
2500000
5e+06
Sex
Female
Male
Ages
Female Mean
Female Median
Male Mean
Male Median

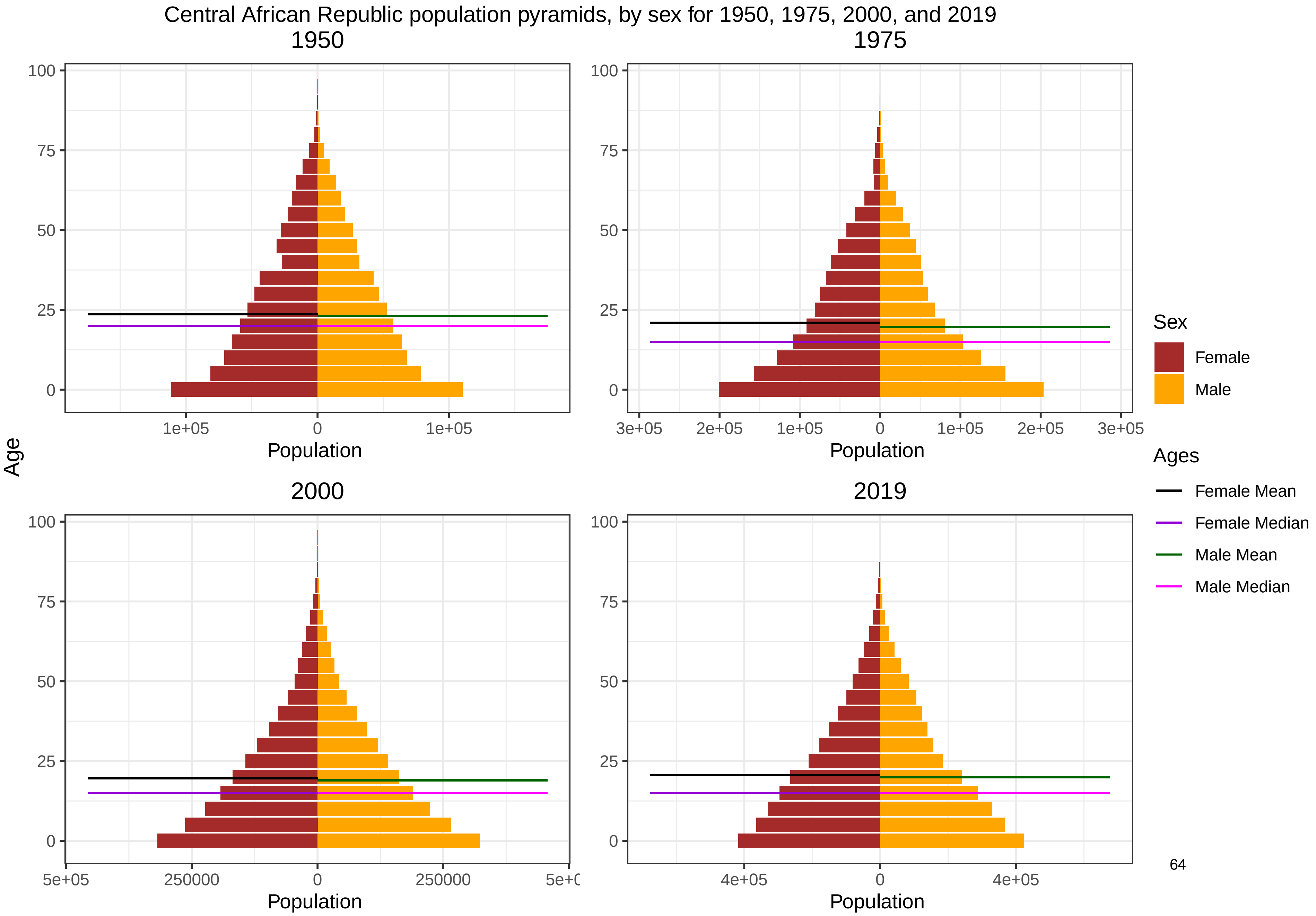
Central African Republic population pyramids, by sex for 1950, 1975, 2000, and 2019
1950
1975
2000
2019
Age
Population
Sex
Female
Male
Ages
Female Mean
Female Median
Male Mean
Male Median

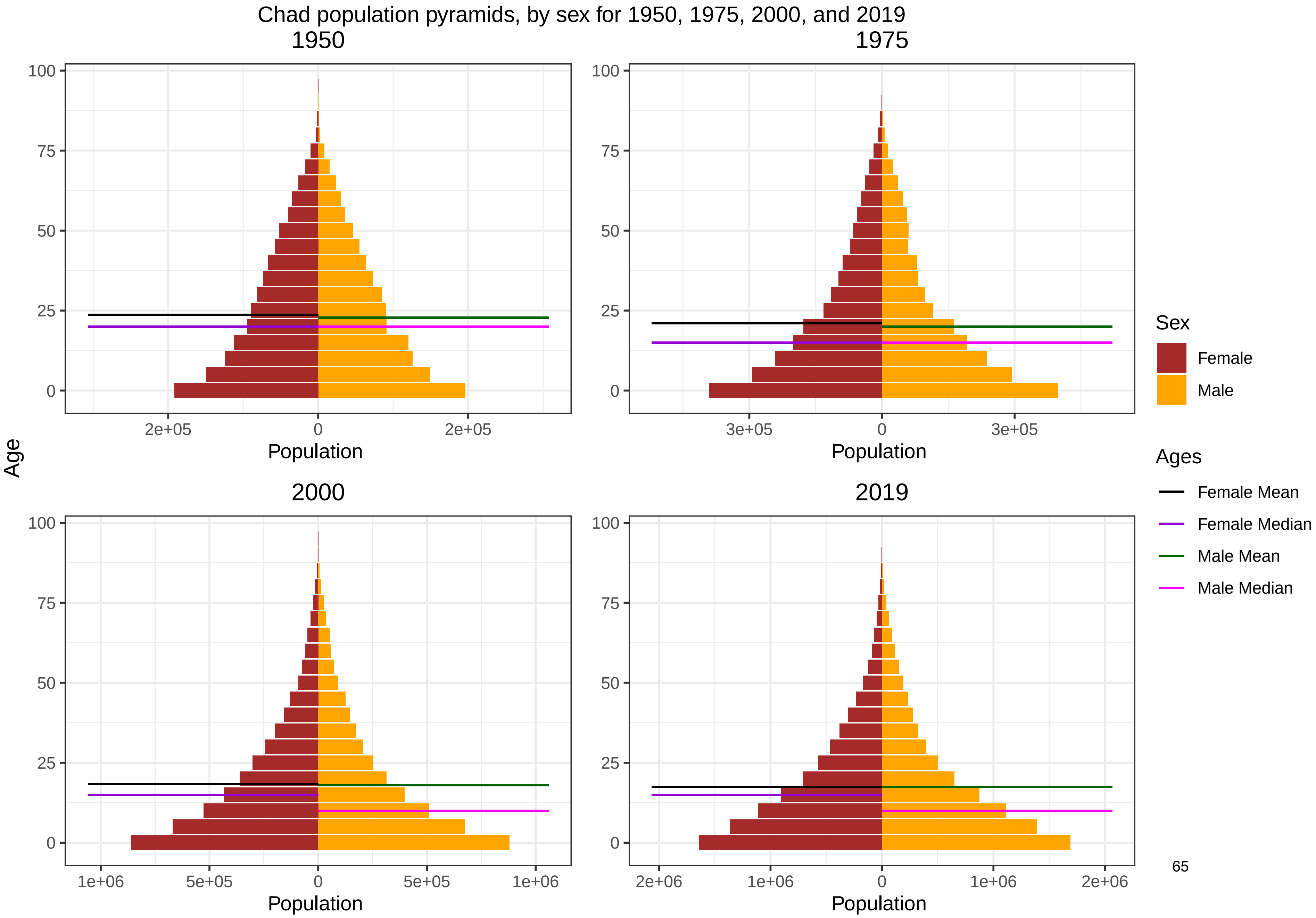
Chad population pyramids, by sex for 1950, 1975, 2000, and 2019
1950
1975
2000
2019
Age
Population
100
75
50
25
0
2e+05
0
2e+05
3e+05
0
3e+05
1e+06
5e+05
0
5e+05
1e+06
2e+06
1e+06
0
1e+06
2e+06
Sex
Female
Male
Ages
Female Mean
Female Median
Male Mean
Male Median

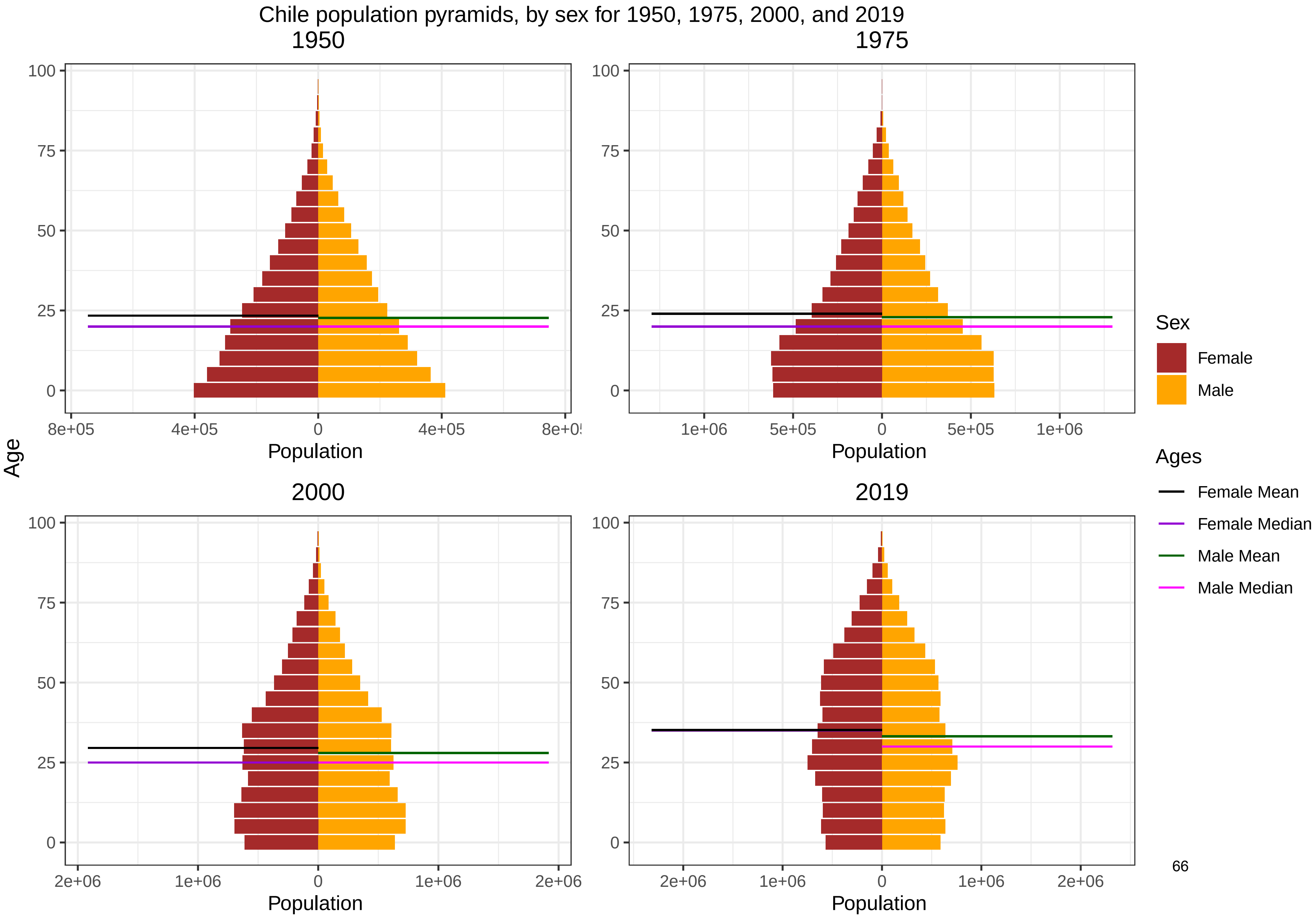

Chile population pyramids, by sex for 1950, 1975, 2000, and 2019
1950
1975
2000
2019
Age
Population
100
75
50
25
0
8e+05
4e+05
0
1e+06
5e+05
2e+06
Sex
Female
Male
Ages
Female Mean
Female Median
Male Mean
Male Median

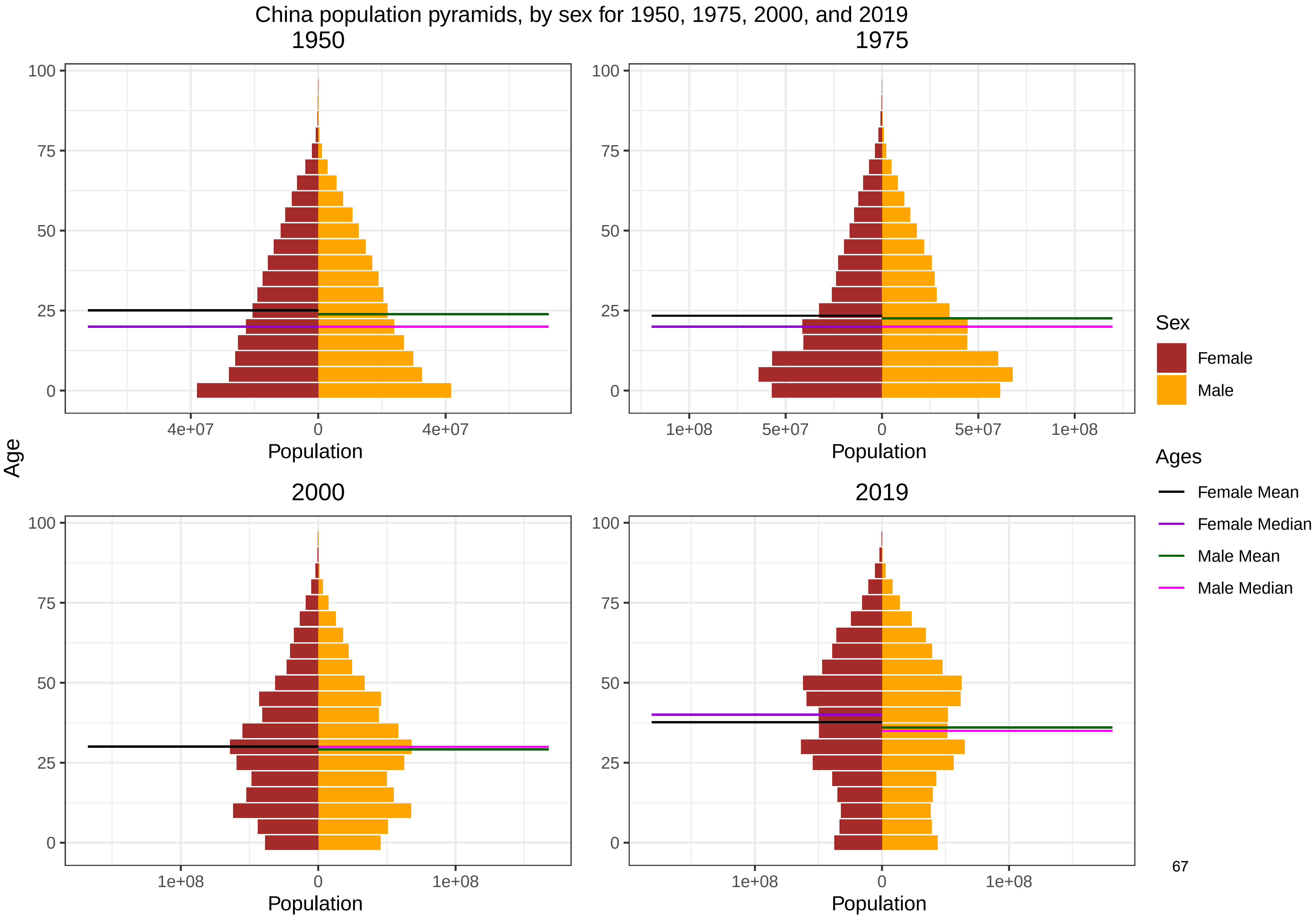
China population pyramids, by sex for 1950, 1975, 2000, and 2019
1950
1975
2000
2019
Age
Population
100
75
50
25
0
4e+07
0
4e+07
1e+08
5e+07
0
5e+07
1e+08
1e+08
0
1e+08
Sex
Female
Male
Ages
Female Mean
Female Median
Male Mean
Male Median

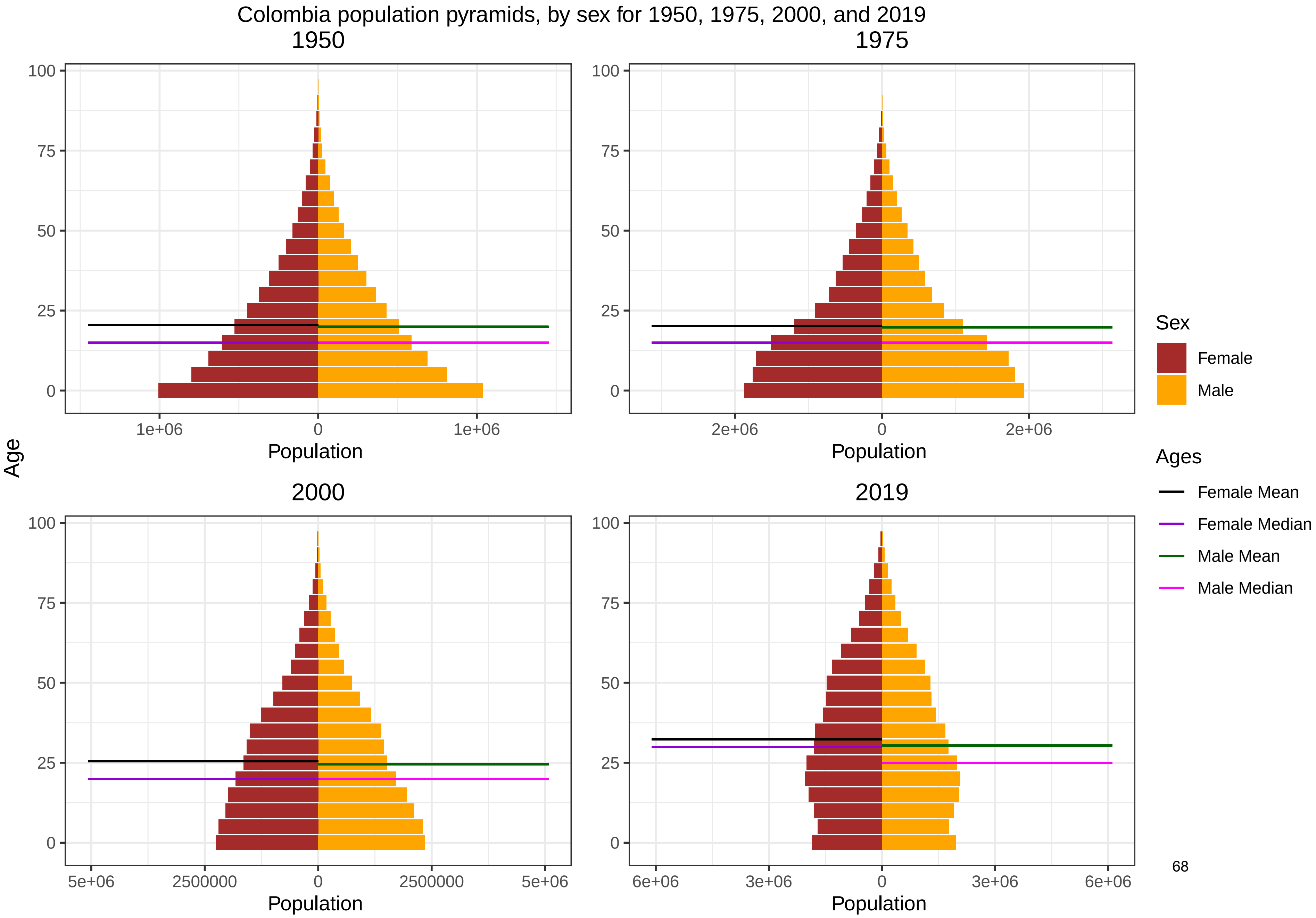

Colombia population pyramids, by sex for 1950, 1975, 2000, and 2019
1950
1975
2000
2019
Age
Population
Sex
Female
Male
Ages
Female Mean
Female Median
Male Mean
Male Median

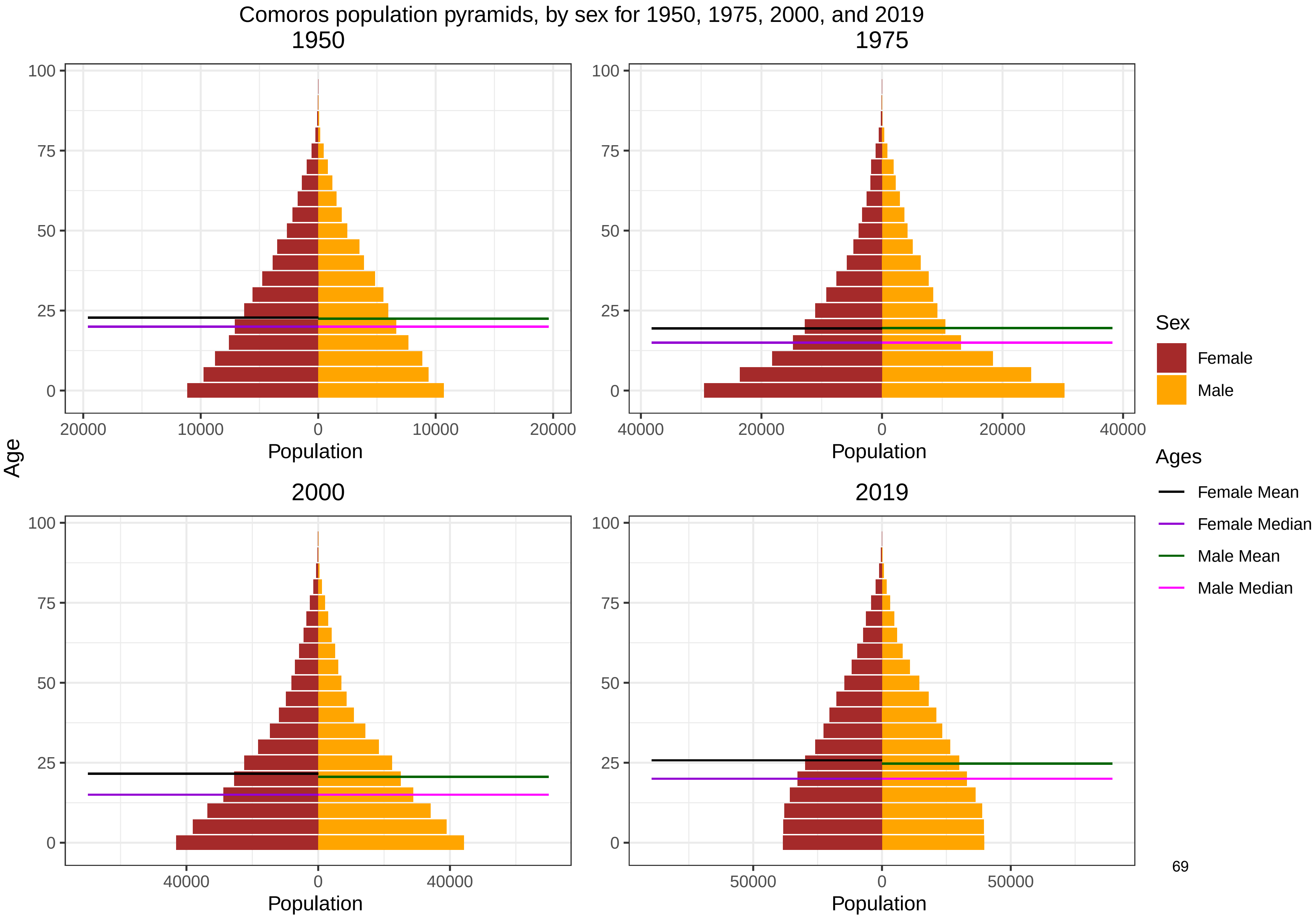
Comoros population pyramids, by sex for 1950, 1975, 2000, and 2019
1950
1975
2000
2019
Age
Population
Sex
Female
Male
Ages
Female Mean
Female Median
Male Mean
Male Median
100
75
50
25
0
20000
10000
0
10000
20000
40000
20000
0
20000
40000
40000
0
40000
50000
0
50000

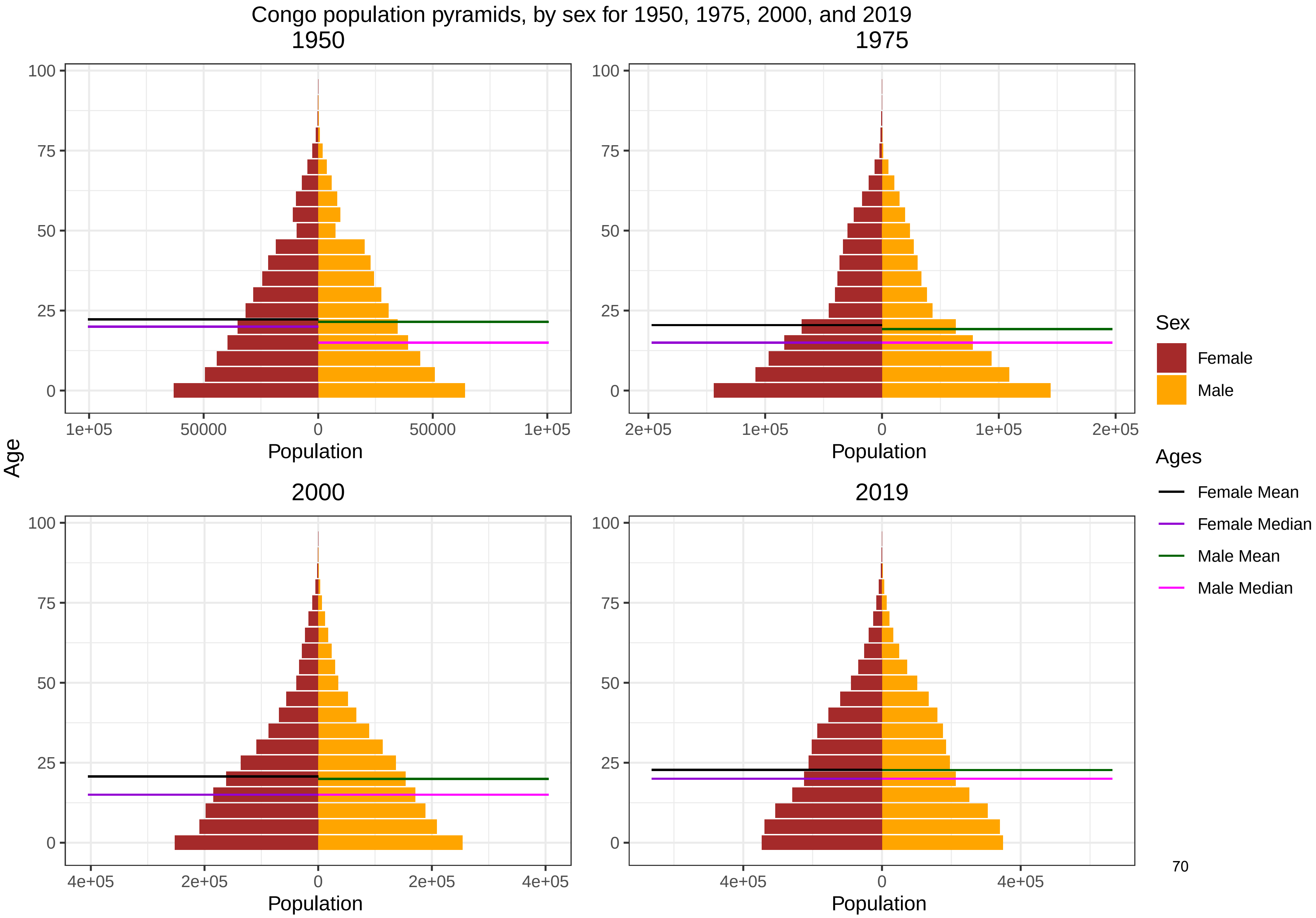

Congo population pyramids, by sex for 1950, 1975, 2000, and 2019
1950
1975
2000
2019
Age
Population
100
75
50
25
0
1e+05
50000
0
50000
1e+05
2e+05
1e+05
0
1e+05
2e+05
4e+05
2e+05
0
2e+05
4e+05
4e+05
0
4e+05
Sex
Female
Male
Ages
Female Mean
Female Median
Male Mean
Male Median

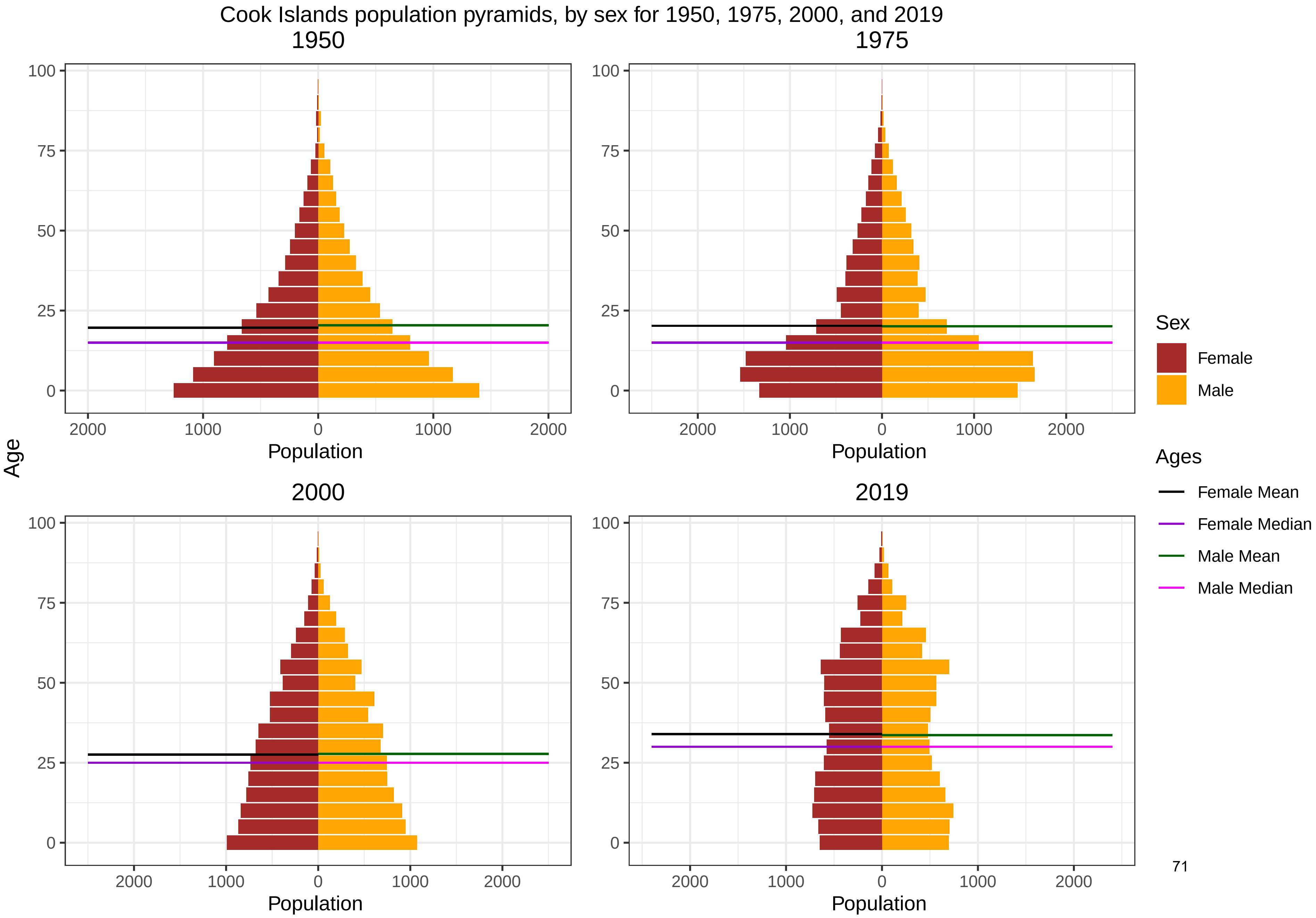

Cook Islands population pyramids, by sex for 1950, 1975, 2000, and 2019
1950
1975
2000
2019
Age
Population
Sex
Female
Male
Ages
Female Mean
Female Median
Male Mean
Male Median

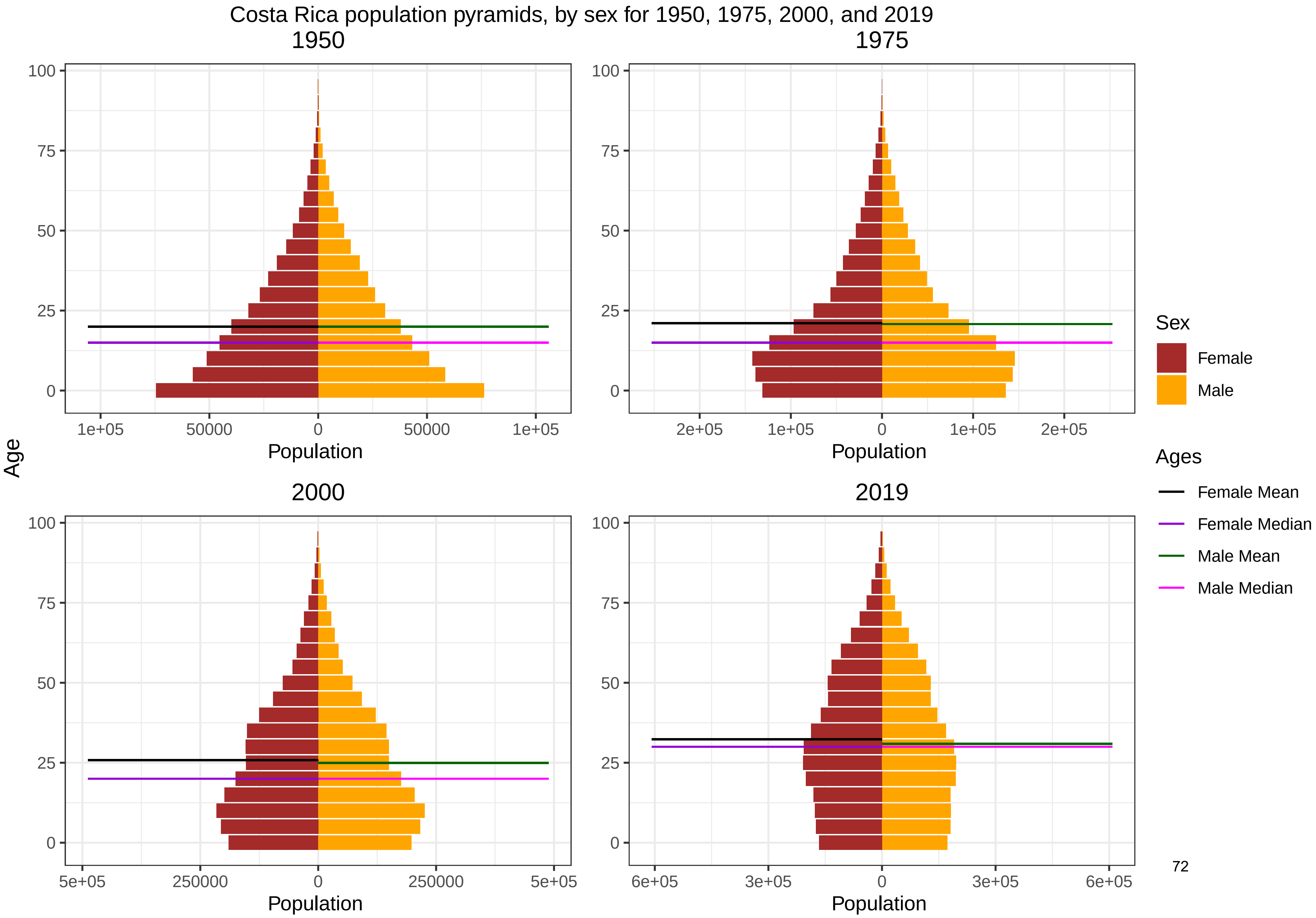

Costa Rica population pyramids, by sex for 1950, 1975, 2000, and 2019
1950
1975
2000
2019
Age
Population
100
75
50
25
0
1e+05
50000
0
50000
1e+05
2e+05
1e+05
0
1e+05
2e+05
5e+05
250000
0
250000
5e+05
6e+05
3e+05
0
3e+05
6e+05
Sex
Female
Male
Ages
Female Mean
Female Median
Male Mean
Male Median

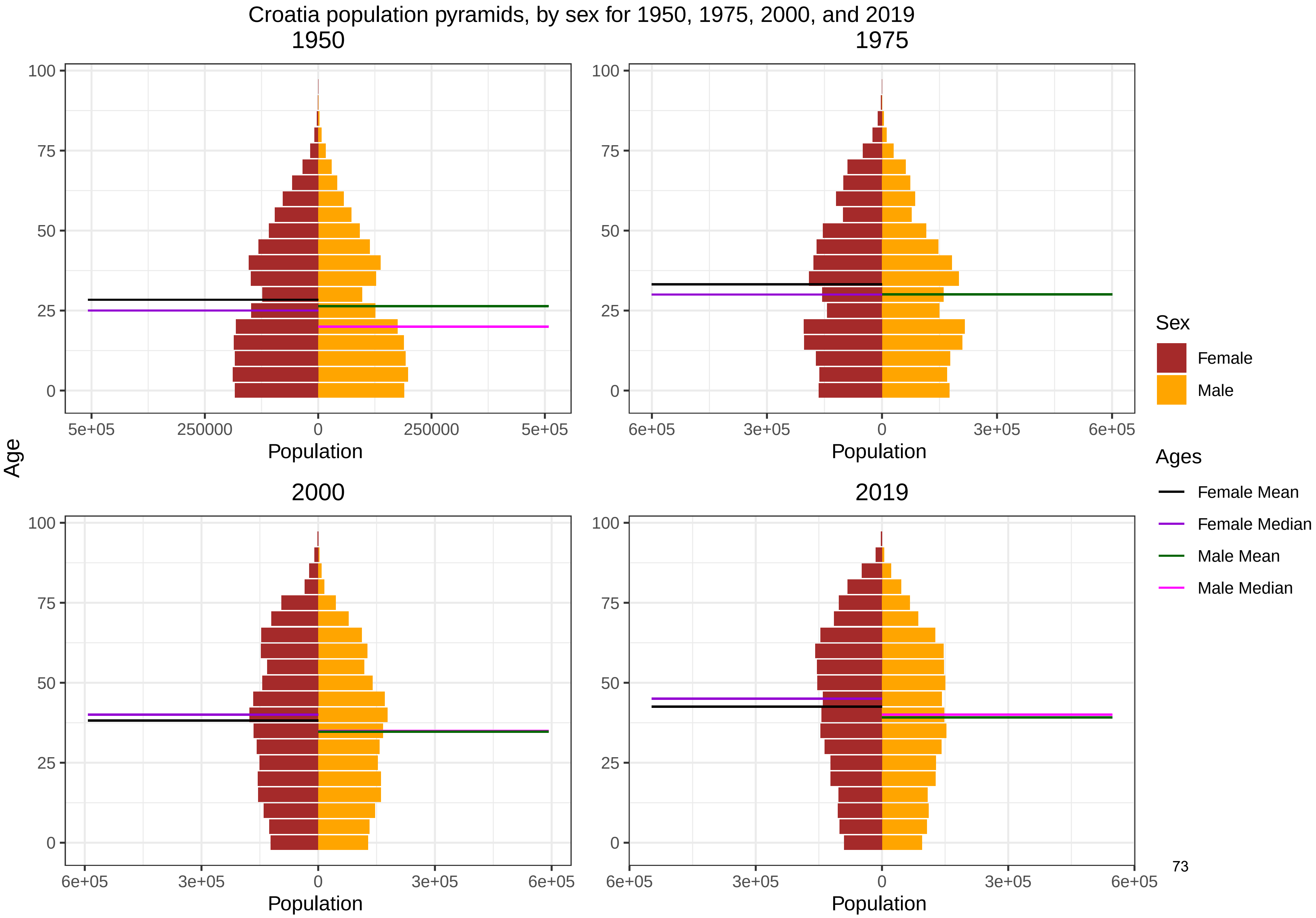

Croatia population pyramids, by sex for 1950, 1975, 2000, and 2019
1950
1975
2000
2019
Age
Population
100
75
50
25
0
5e+05
250000
0
250000
5e+05
6e+05
3e+05
0
3e+05
6e+05
Sex
Female
Male
Ages
Female Mean
Female Median
Male Mean
Male Median

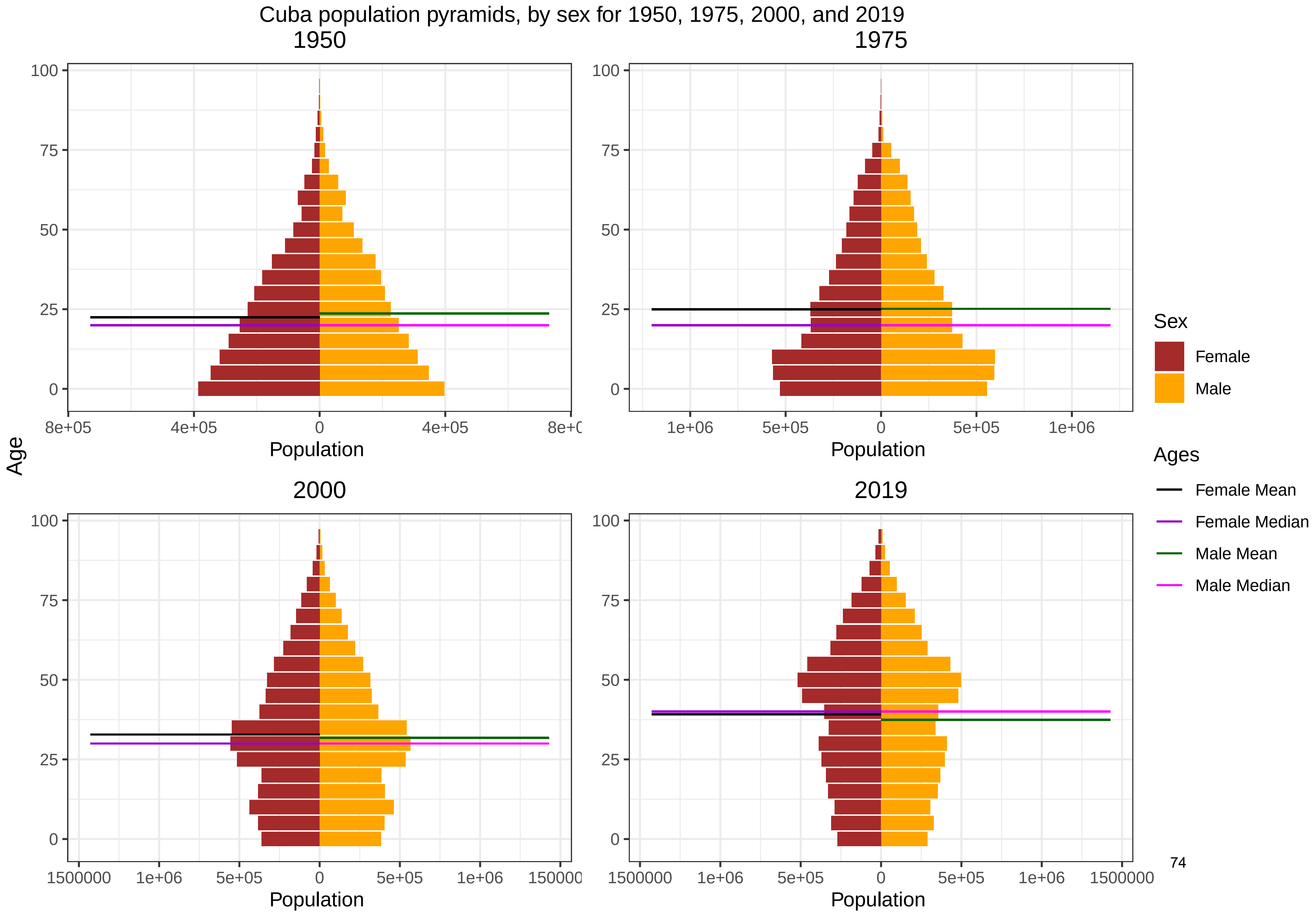

Cuba population pyramids, by sex for 1950, 1975, 2000, and 2019
1950
1975
2000
2019
Age
Population
Sex
Female
Male
Ages
Female Mean
Female Median
Male Mean
Male Median

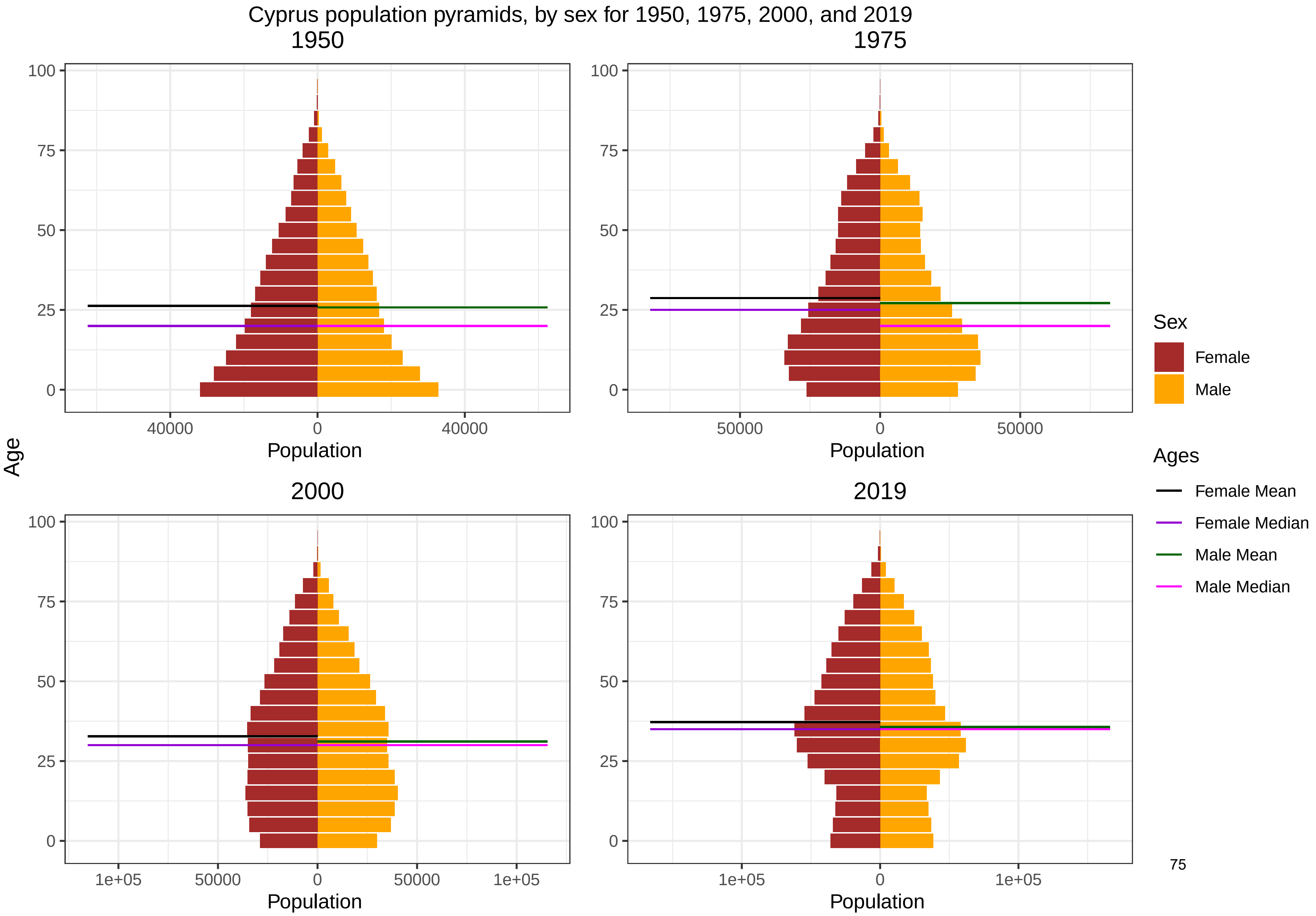

Cyprus population pyramids, by sex for 1950, 1975, 2000, and 2019
1950
1975
2000
2019
Age
Population
Sex
Female
Male
Ages
Female Mean
Female Median
Male Mean
Male Median

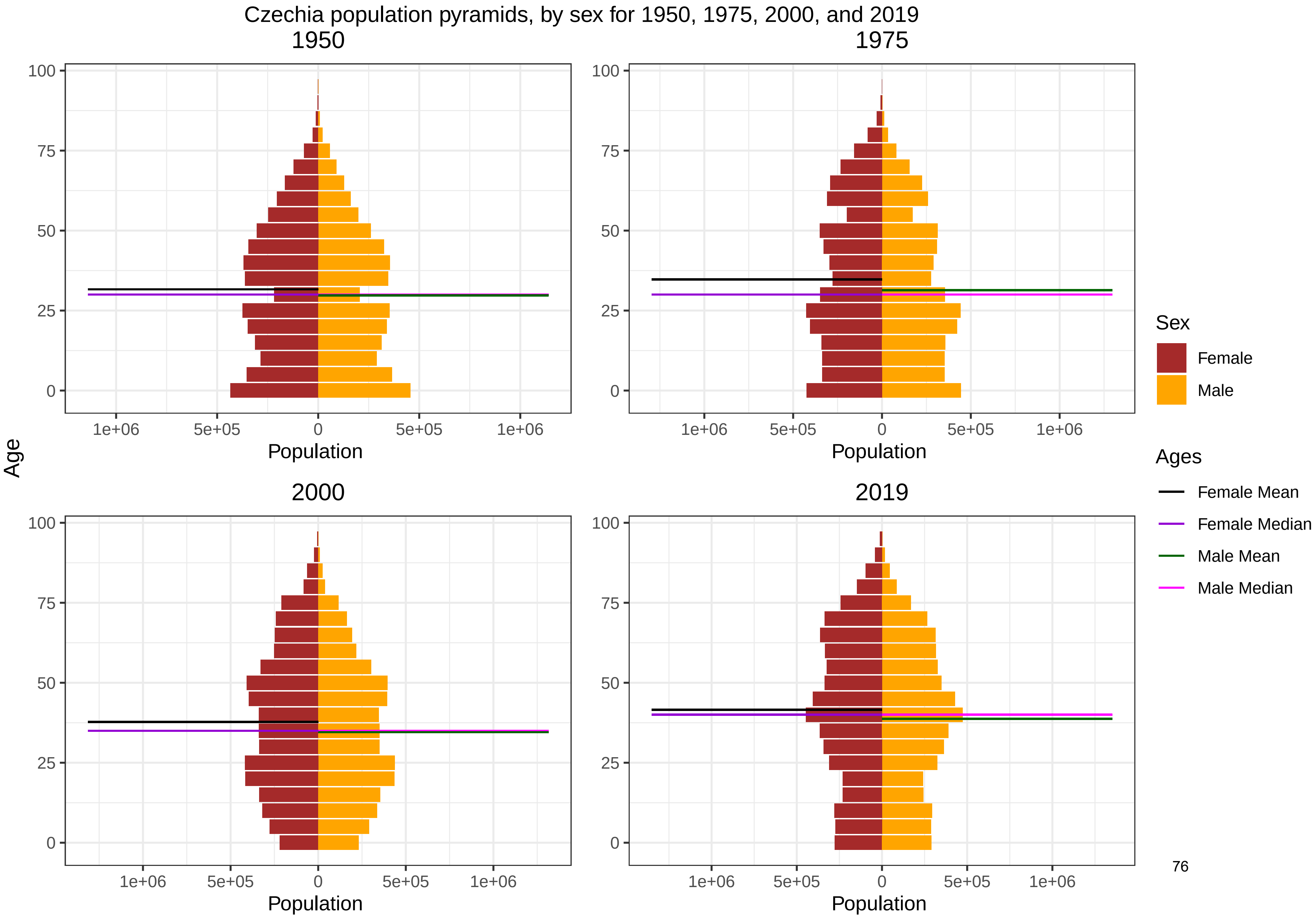

Czechia population pyramids, by sex for 1950, 1975, 2000, and 2019
1950
1975
2000
2019
Age
Population
100
75
50
25
0
1e+06
5e+05
0
5e+05
1e+06
Sex
Female
Male
Ages
Female Mean
Female Median
Male Mean
Male Median

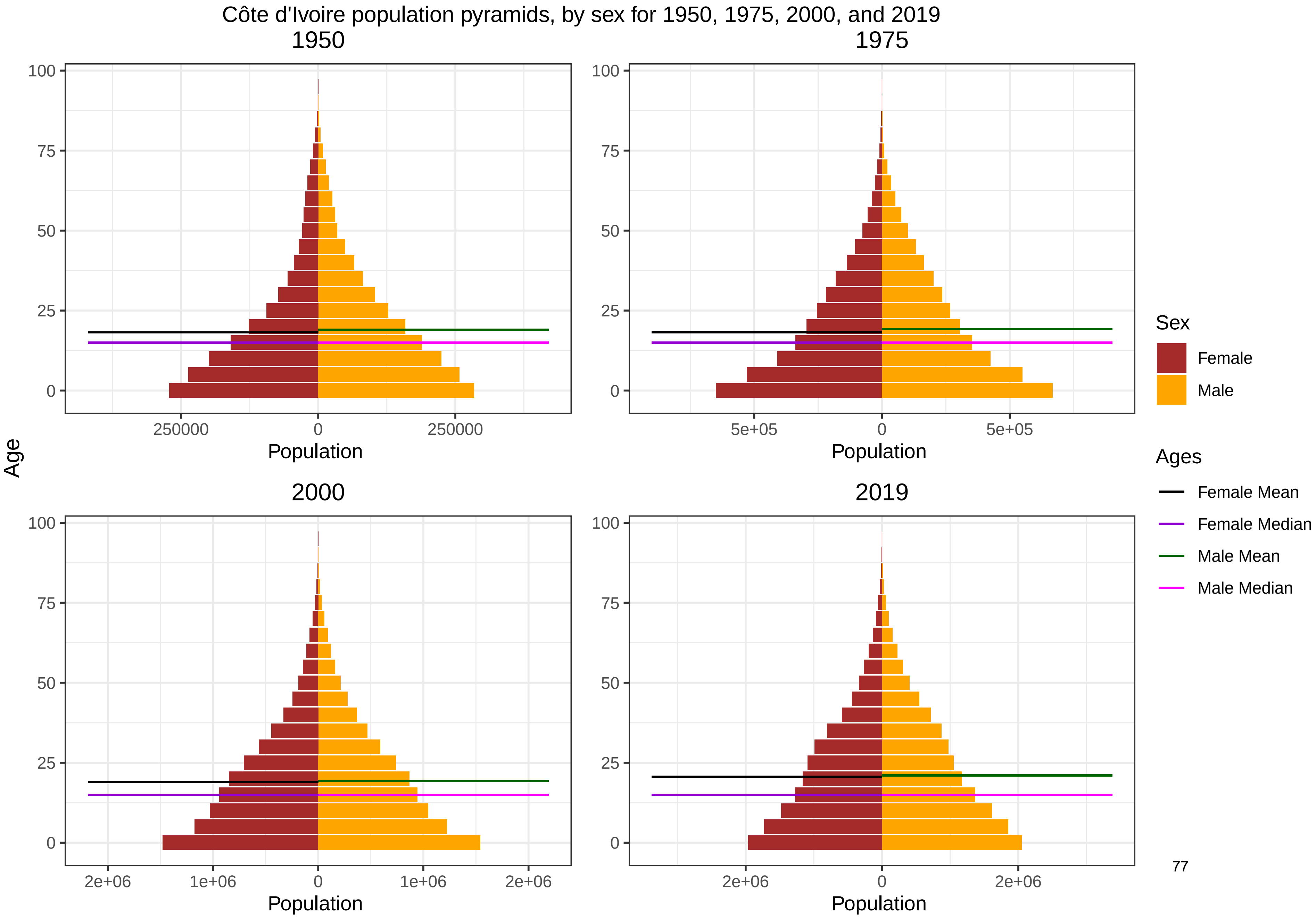
Côte d'Ivoire population pyramids, by sex for 1950, 1975, 2000, and 2019
1950
1975
2000
2019
Age
Population
100
75
50
25
0
250000
0
250000
5e+05
0
5e+05
2e+06
1e+06
0
1e+06
2e+06
Sex
Female
Male
Ages
Female Mean
Female Median
Male Mean
Male Median

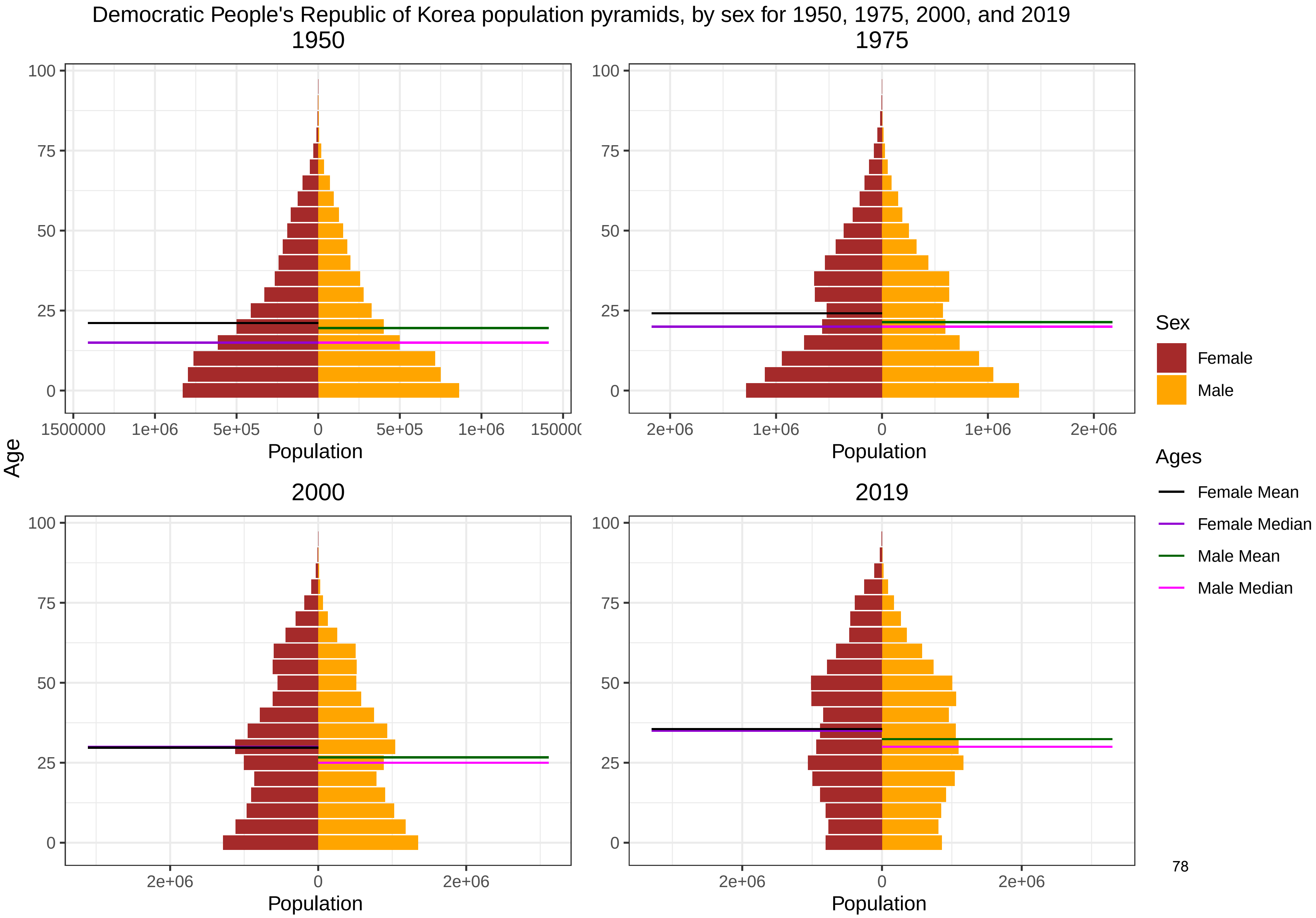
Democratic People's Republic of Korea population pyramids, by sex for 1950, 1975, 2000, and 2019
1950
1975
2000
2019
Age
Population
Sex
Female
Male
Ages
Female Mean
Female Median
Male Mean
Male Median

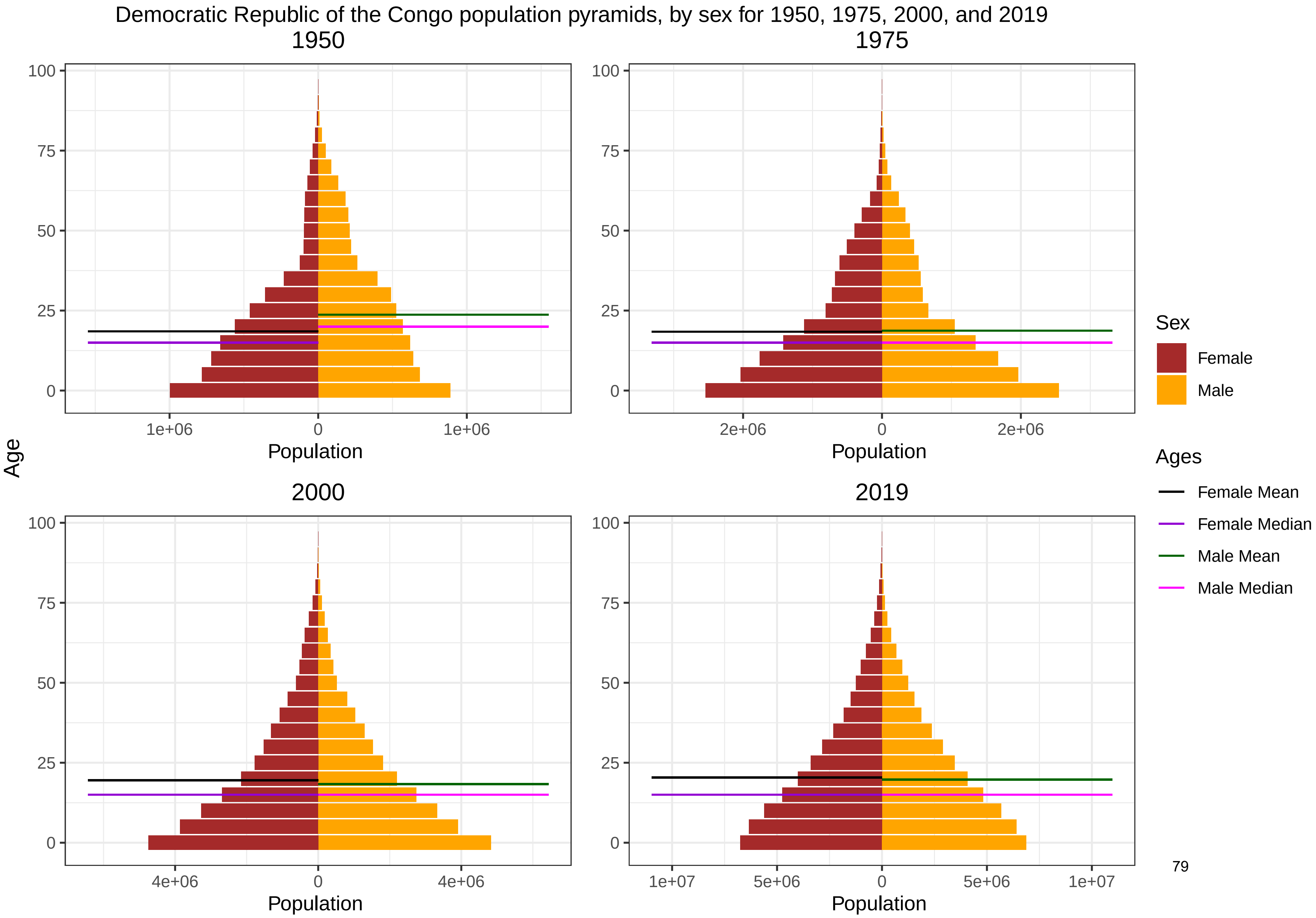

Democratic Republic of the Congo population pyramids, by sex for 1950, 1975, 2000, and 2019
1950
1975
2000
2019
Age
Population
100
75
50
25
0
1e+06
0
1e+06
2e+06
0
2e+06
4e+06
0
4e+06
1e+07
5e+06
0
5e+06
1e+07
Sex
Female
Male
Ages
Female Mean
Female Median
Male Mean
Male Median

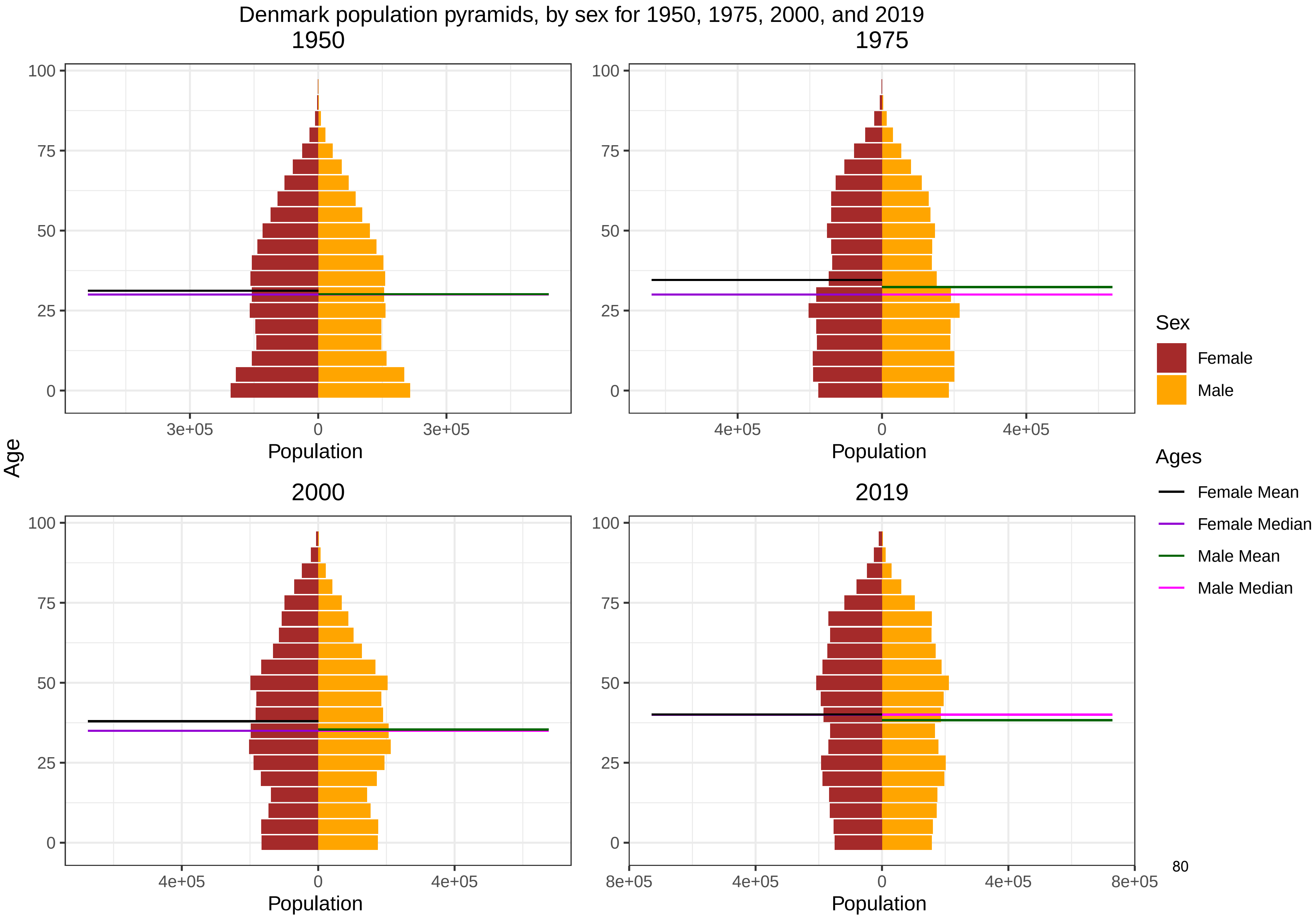

Denmark population pyramids, by sex for 1950, 1975, 2000, and 2019
1950
1975
2000
2019
Age
Population
100
75
50
25
0
3e+05
0
3e+05
4e+05
0
4e+05
8e+05
4e+05
0
4e+05
8e+05
Sex
Female
Male
Ages
Female Mean
Female Median
Male Mean
Male Median

80

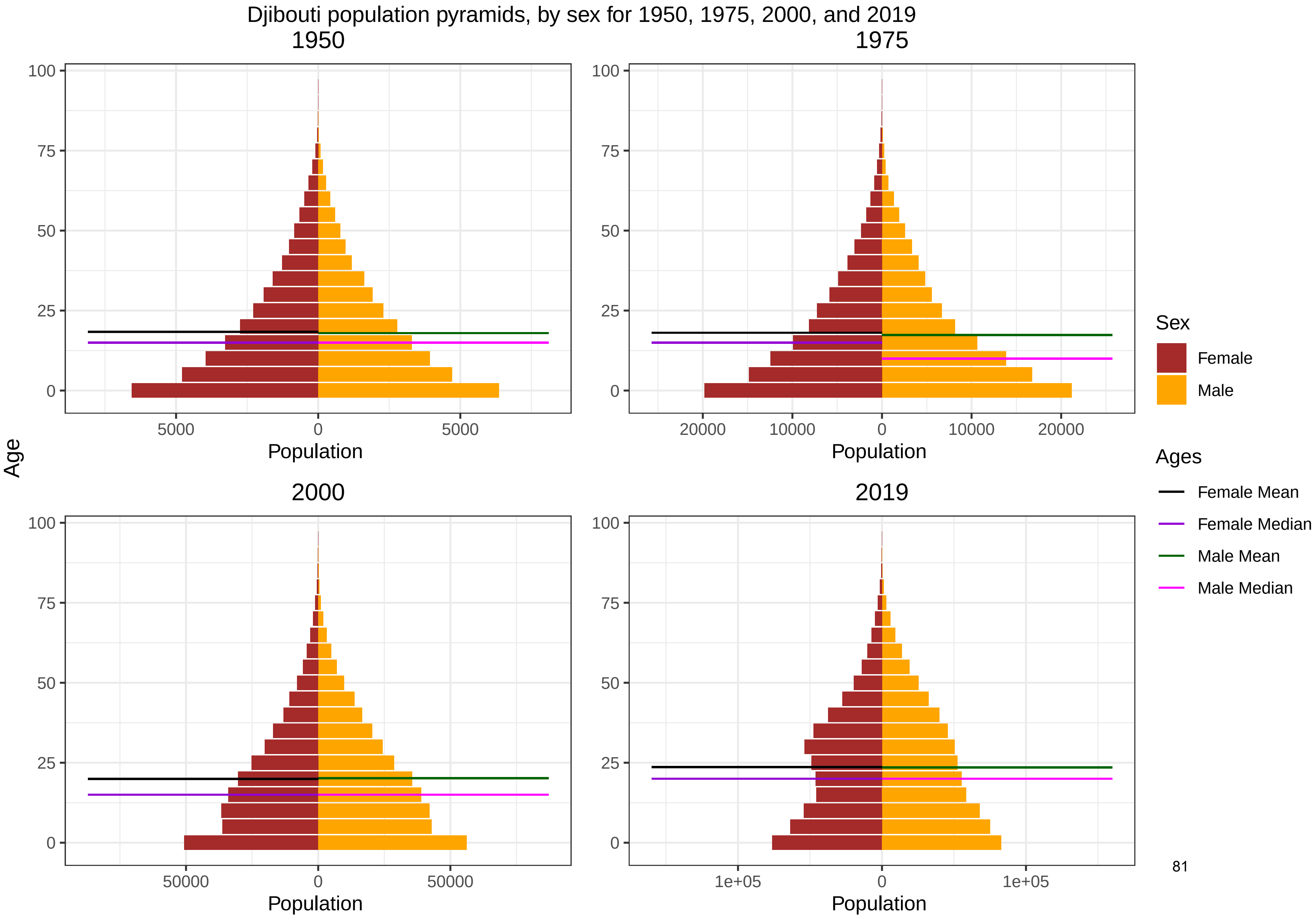
Djibouti population pyramids, by sex for 1950, 1975, 2000, and 2019
1950
1975
2000
2019
Age
Population
100
75
50
25
0
5000
0
5000
20000
10000
0
10000
20000
50000
0
50000
1e+05
0
1e+05
Sex
Female
Male
Ages
Female Mean
Female Median
Male Mean
Male Median

Dominica population pyramids, by sex for 1950, 1975, 2000, and 2019

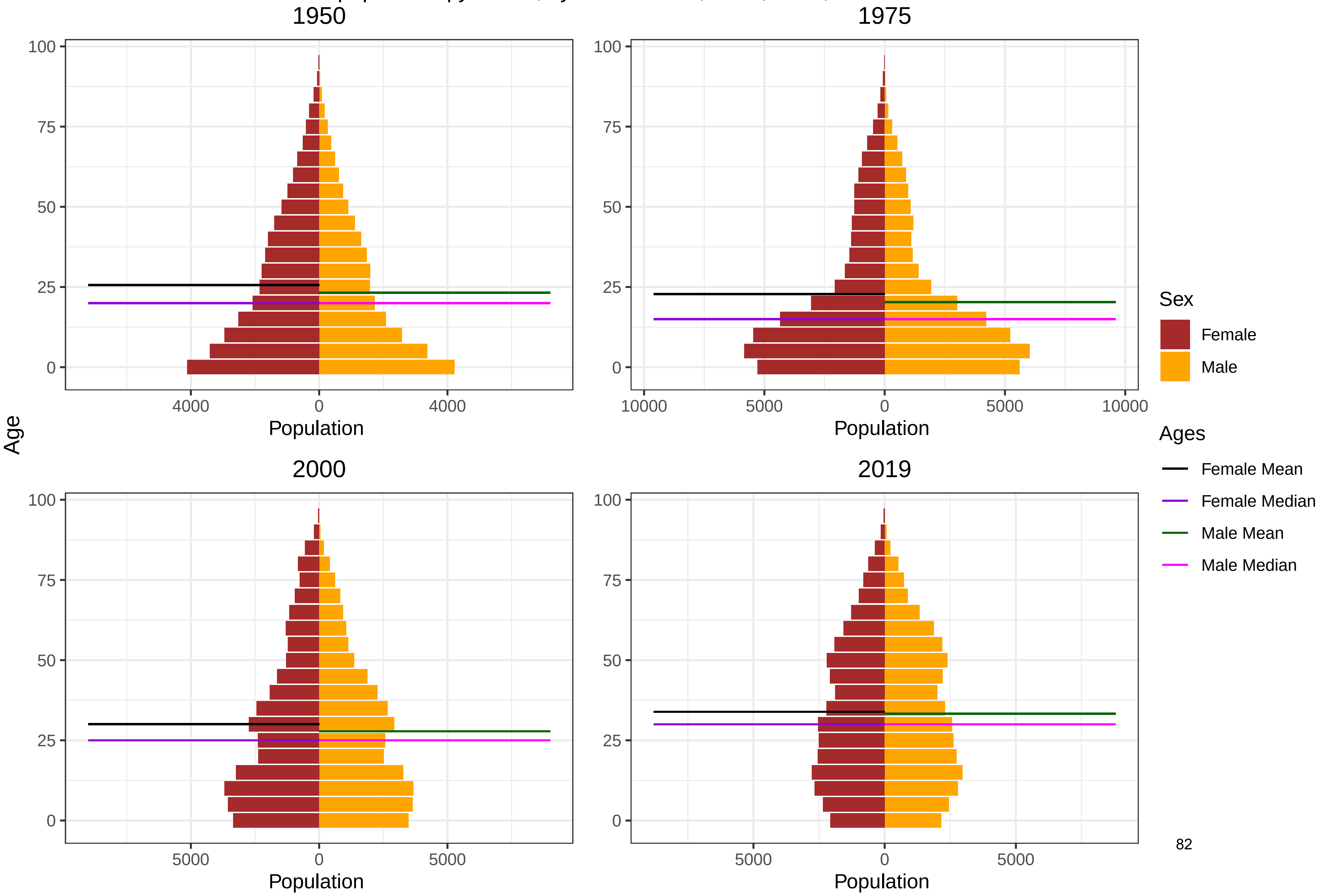

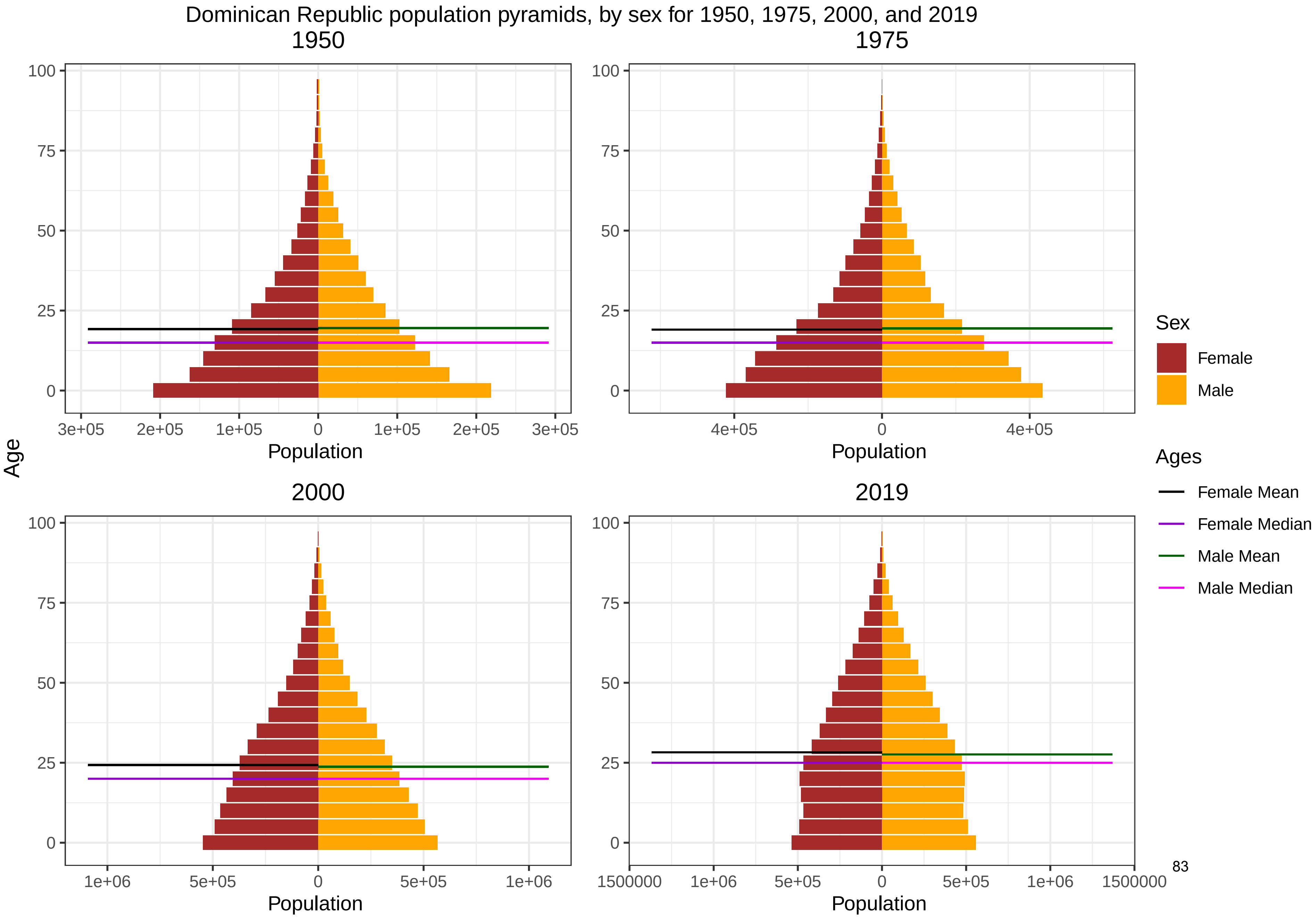

Dominican Republic population pyramids, by sex for 1950, 1975, 2000, and 2019
1950
1975
2000
2019
Age
Population
100
75
50
25
0
3e+05
2e+05
1e+05
0
4e+05
5e+05
1e+06
1500000
Sex
Female
Male
Ages
Female Mean
Female Median
Male Mean
Male Median

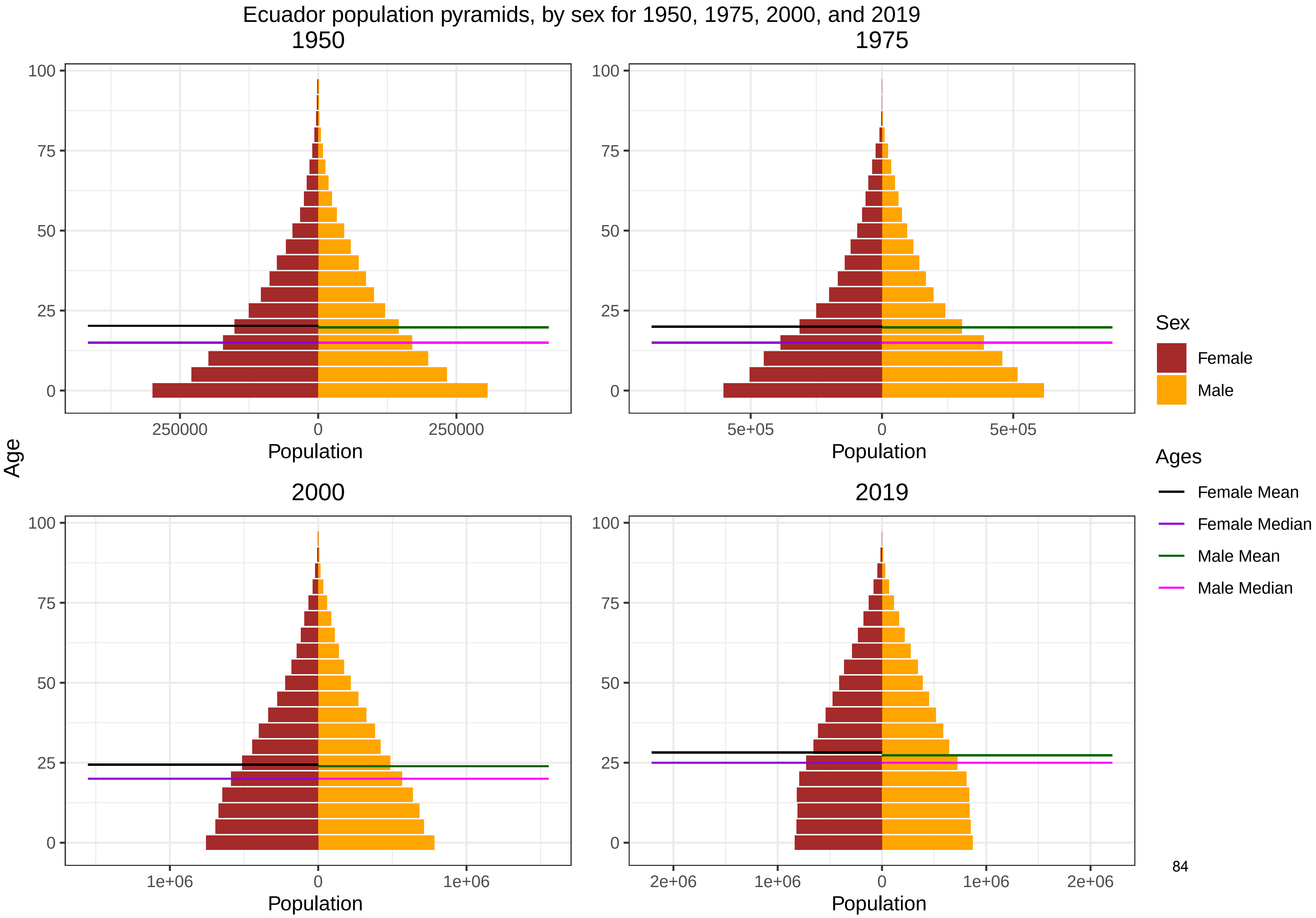
Ecuador population pyramids, by sex for 1950, 1975, 2000, and 2019
1950
1975
2000
2019
Age
Population
100
75
50
25
0
250000
0
250000
5e+05
0
5e+05
1e+06
0
1e+06
2e+06
1e+06
0
1e+06
2e+06
Sex
Female
Male
Ages
Female Mean
Female Median
Male Mean
Male Median

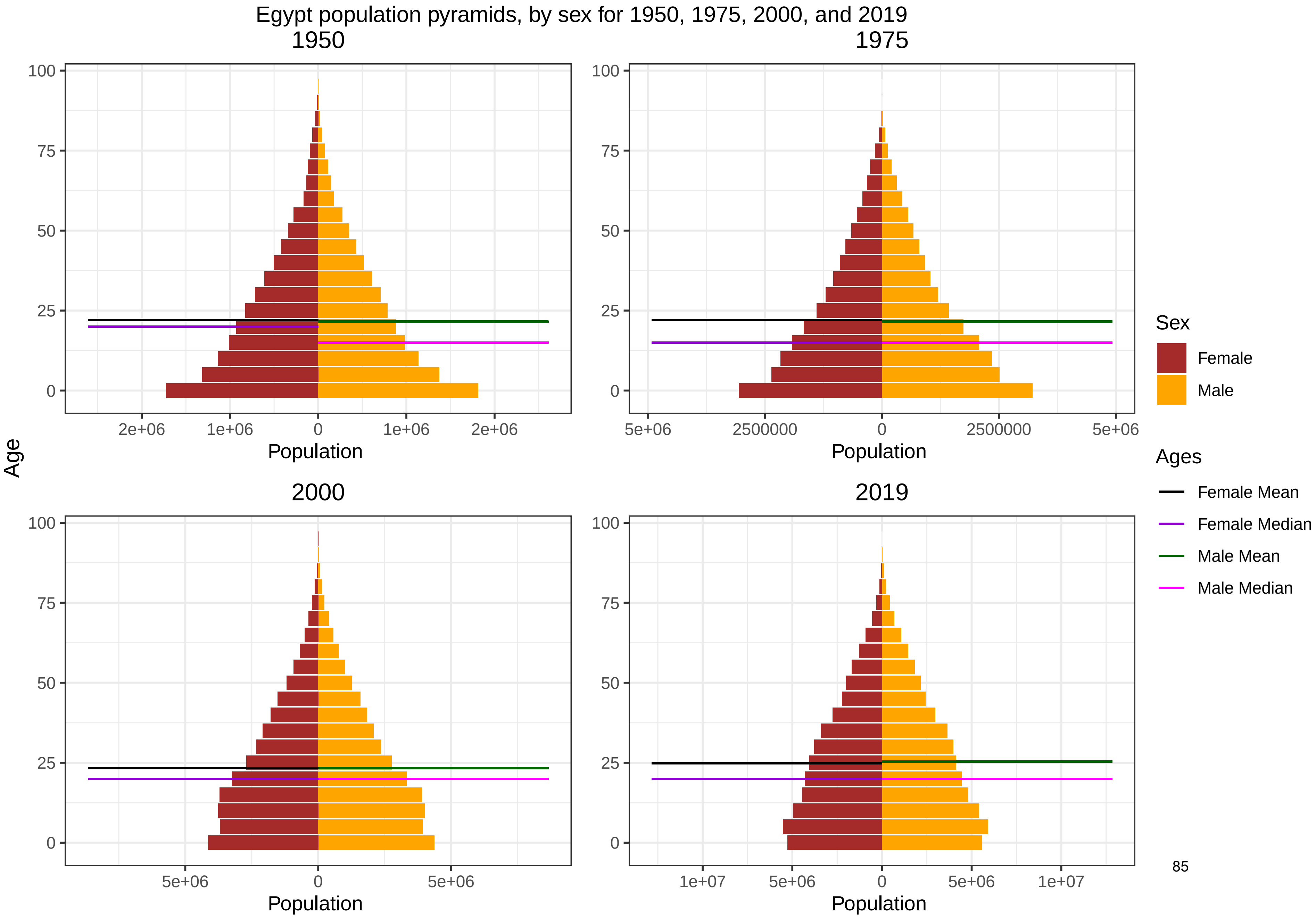
Egypt population pyramids, by sex for 1950, 1975, 2000, and 2019
1950
1975
2000
2019
Age
Population
100
75
50
25
0
2e+06
1e+06
0
2500000
5e+06
1e+07
Sex
Female
Male
Ages
Female Mean
Female Median
Male Mean
Male Median

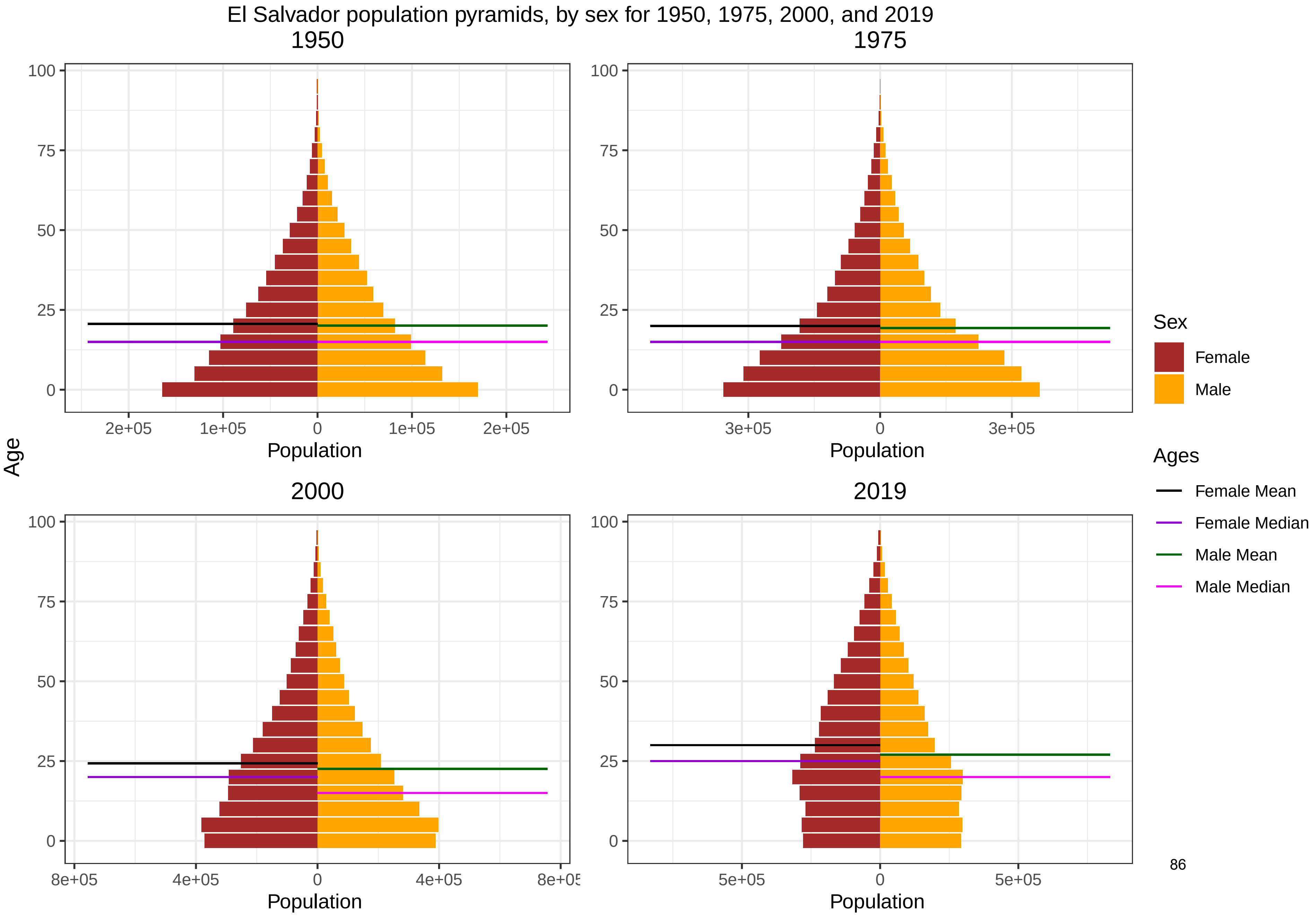

El Salvador population pyramids, by sex for 1950, 1975, 2000, and 2019
1950
1975
2000
2019
100
75
50
25
0
2e+05
1e+05
0
1e+05
2e+05
3e+05
0
3e+05
8e+05
4e+05
0
4e+05
8e+05
5e+05
0
5e+05
Population
Age
Sex
Female
Male
Ages
Female Mean
Female Median
Male Mean
Male Median

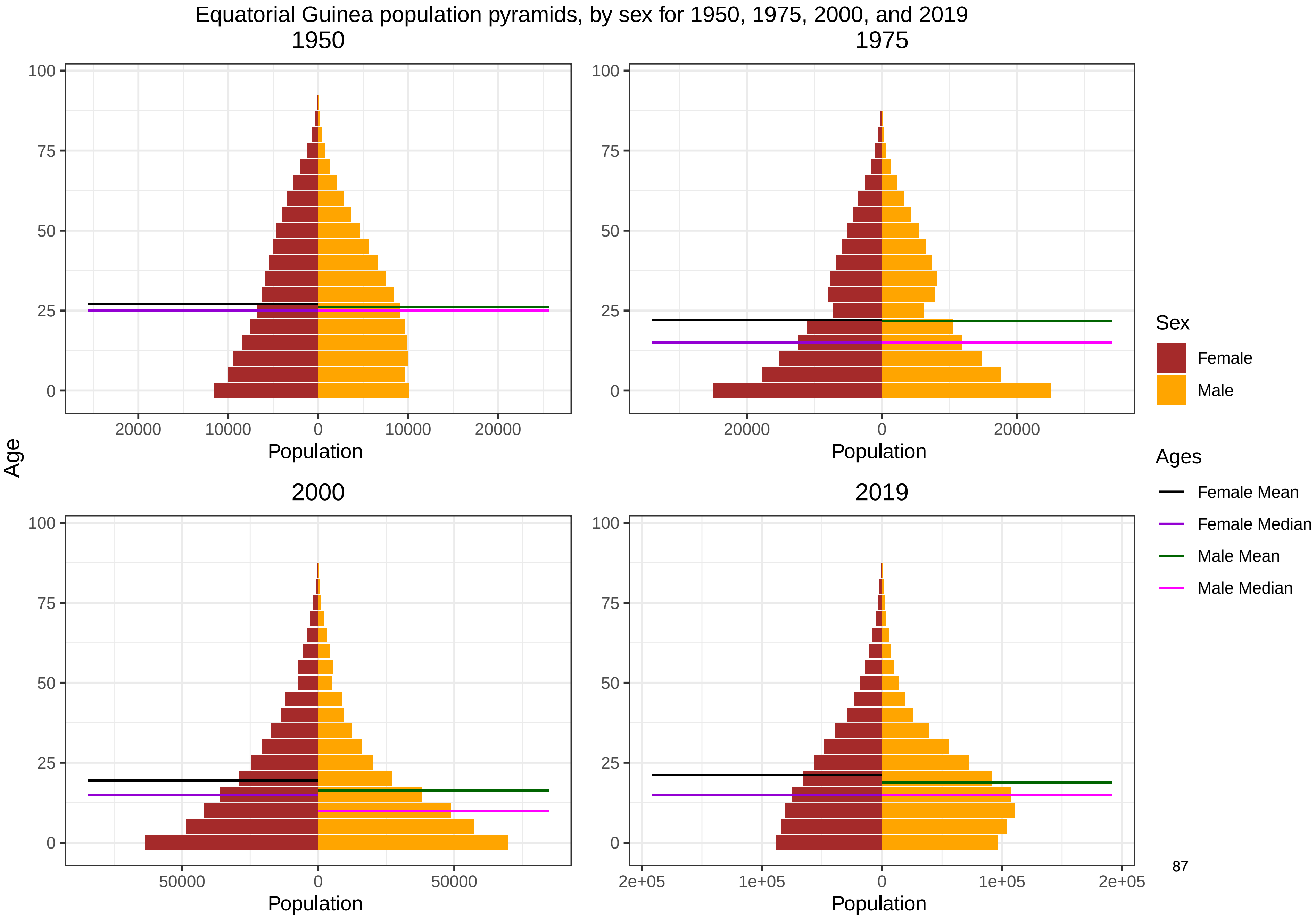

Equatorial Guinea population pyramids, by sex for 1950, 1975, 2000, and 2019
1950
1975
2000
2019
Age
Population
Sex
Female
Male
Ages
Female Mean
Female Median
Male Mean
Male Median

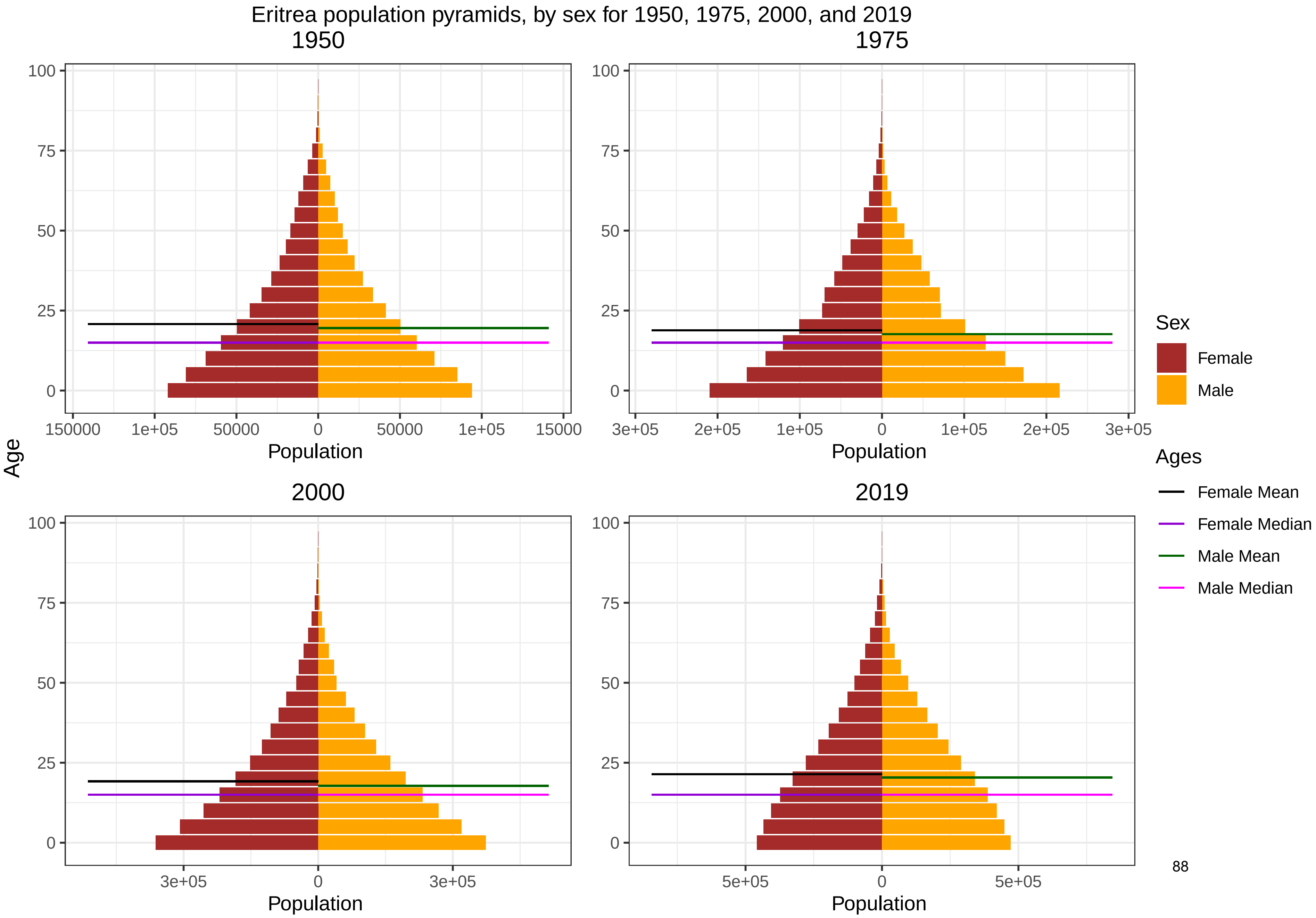
Eritrea population pyramids, by sex for 1950, 1975, 2000, and 2019
1950
1975
2000
2019
Age
Population
100
75
50
25
0
150000
1e+05
50000
0
50000
1e+05
15000
3e+05
2e+05
1e+05
0
1e+05
2e+05
3e+05
3e+05
0
3e+05
5e+05
0
5e+05
Sex
Female
Male
Ages
Female Mean
Female Median
Male Mean
Male Median

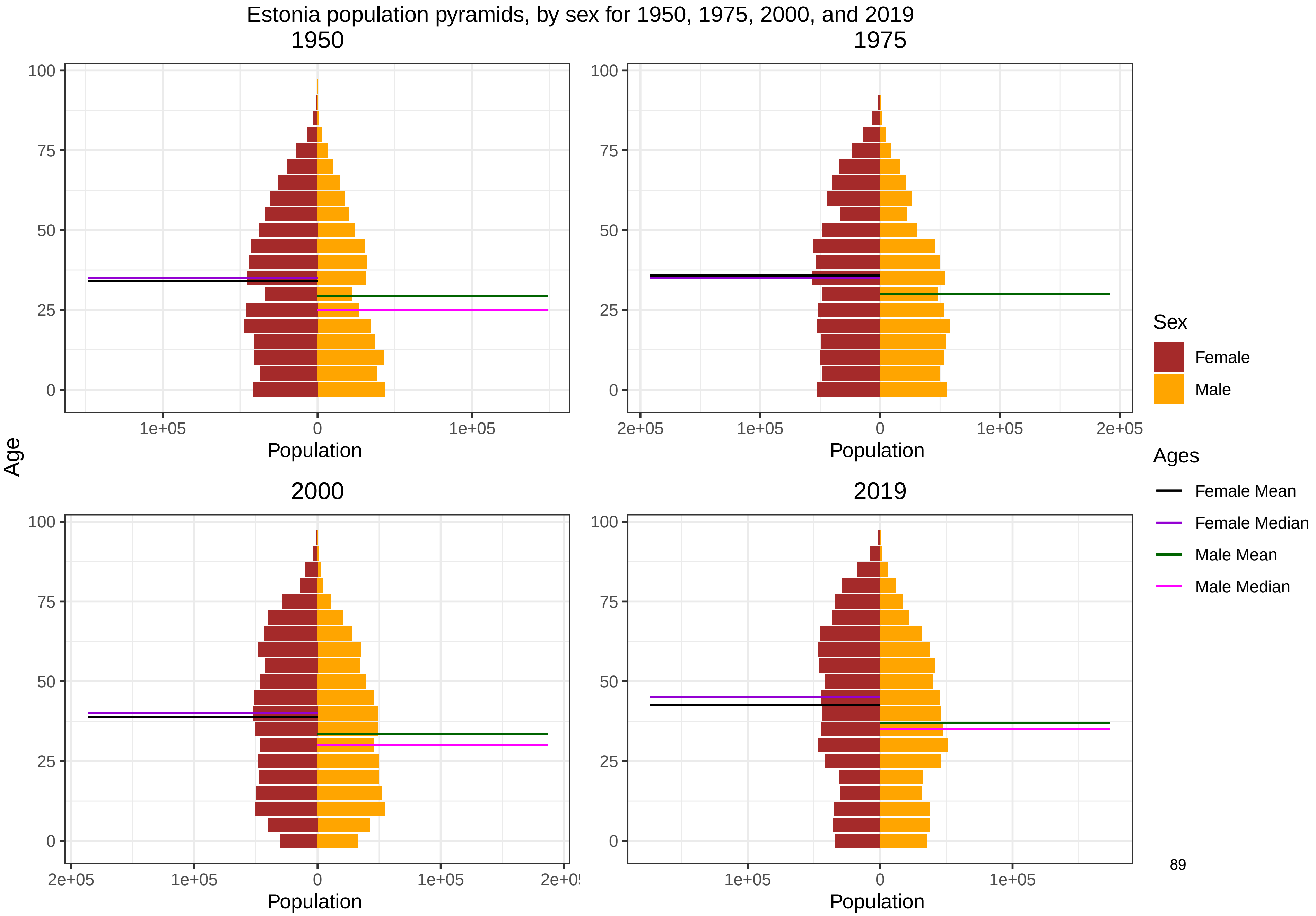

Estonia population pyramids, by sex for 1950, 1975, 2000, and 2019
1950
1975
2000
2019
100
75
50
25
0
1e+05
2e+05
Population
Age
Sex
Female
Male
Ages
Female Mean
Female Median
Male Mean
Male Median

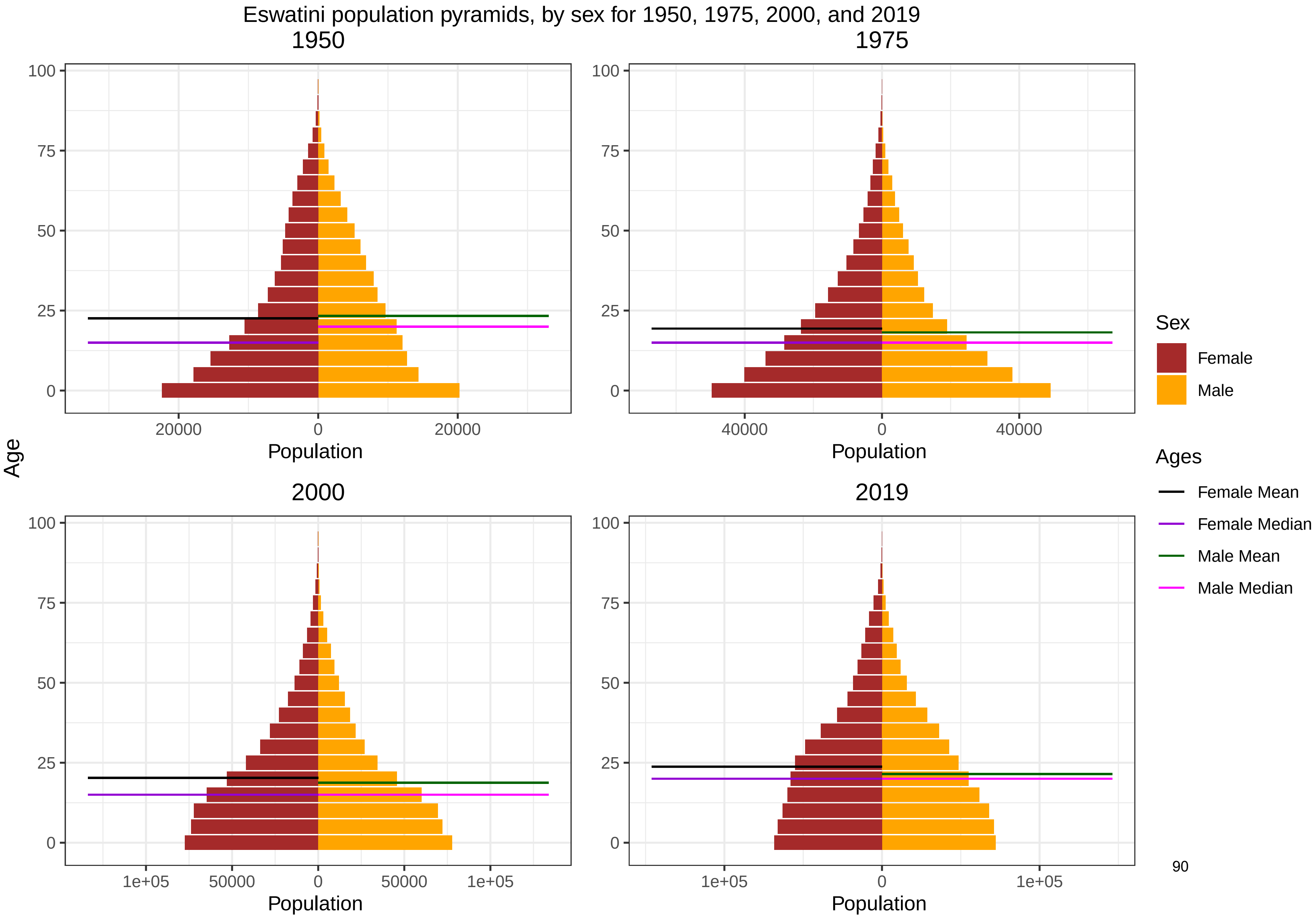
Eswatini population pyramids, by sex for 1950, 1975, 2000, and 2019
1950
1975
2000
2019
Age
Population
Sex
Female
Male
Ages
Female Mean
Female Median
Male Mean
Male Median

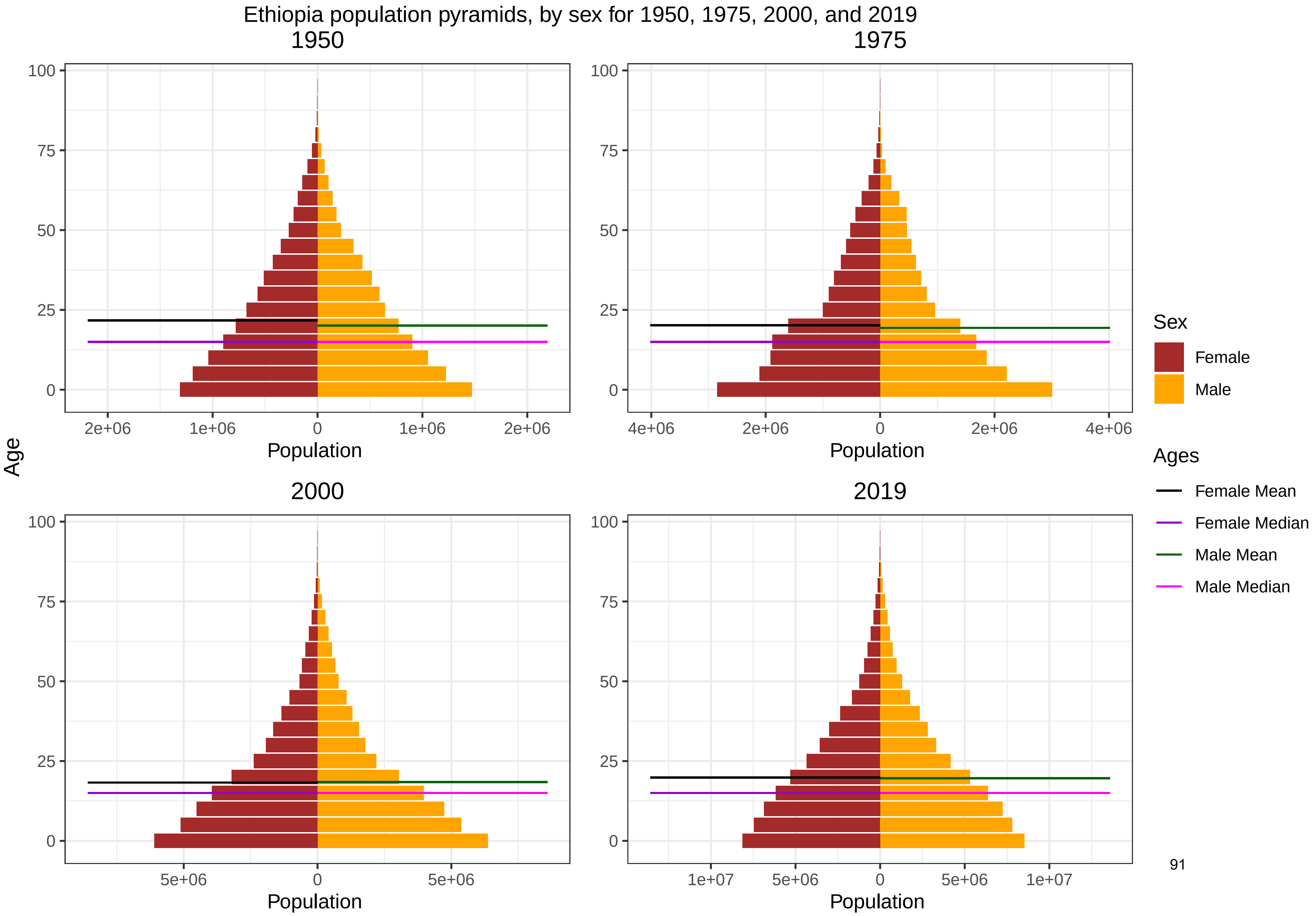
Ethiopia population pyramids, by sex for 1950, 1975, 2000, and 2019
1950
1975
2000
2019
Age
Population
100
75
50
25
0
2e+06
1e+06
0
4e+06
5e+06
1e+07
Sex
Female
Male
Ages
Female Mean
Female Median
Male Mean
Male Median

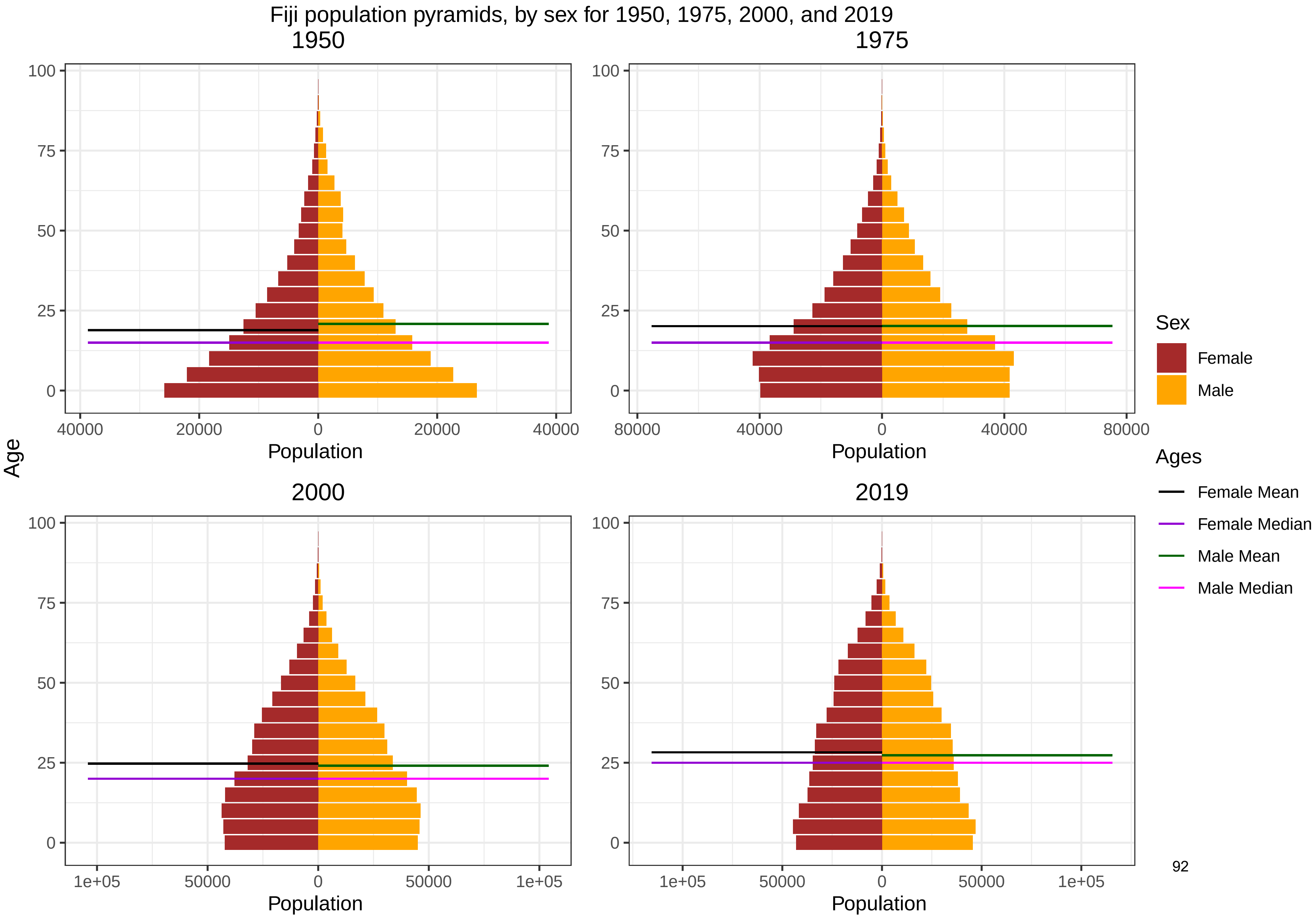
Fiji population pyramids, by sex for 1950, 1975, 2000, and 2019
1950
1975
2000
2019
Age
Population
100
75
50
25
0
40000
20000
0
20000
40000
80000
40000
0
40000
80000
1e+05
50000
0
50000
1e+05
Sex
Female
Male
Ages
Female Mean
Female Median
Male Mean
Male Median

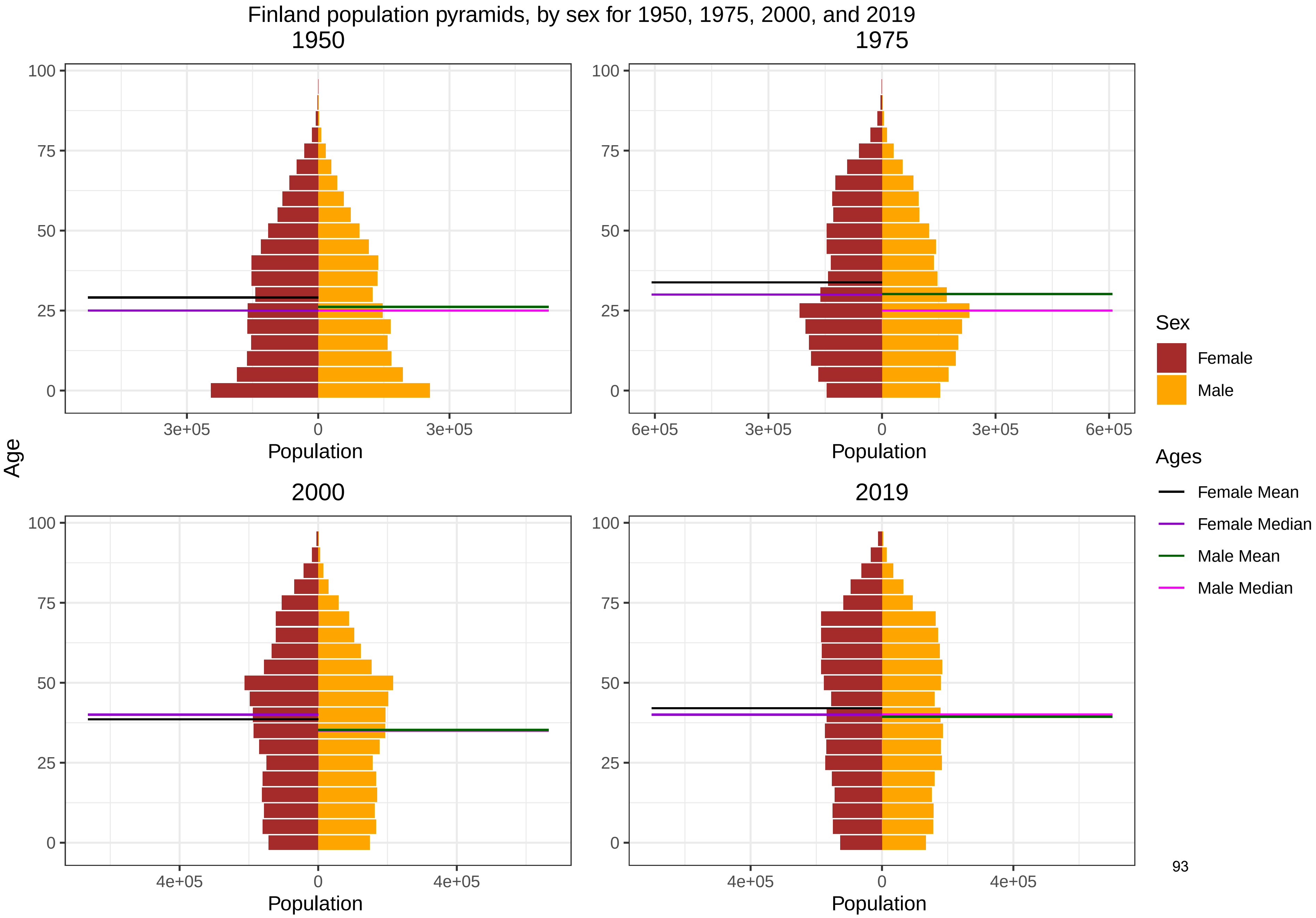
Finland population pyramids, by sex for 1950, 1975, 2000, and 2019
1950
1975
2000
2019
Age
Population
100
75
50
25
0
3e+05
0
3e+05
6e+05
4e+05
Sex
Female
Male
Ages
Female Mean
Female Median
Male Mean
Male Median

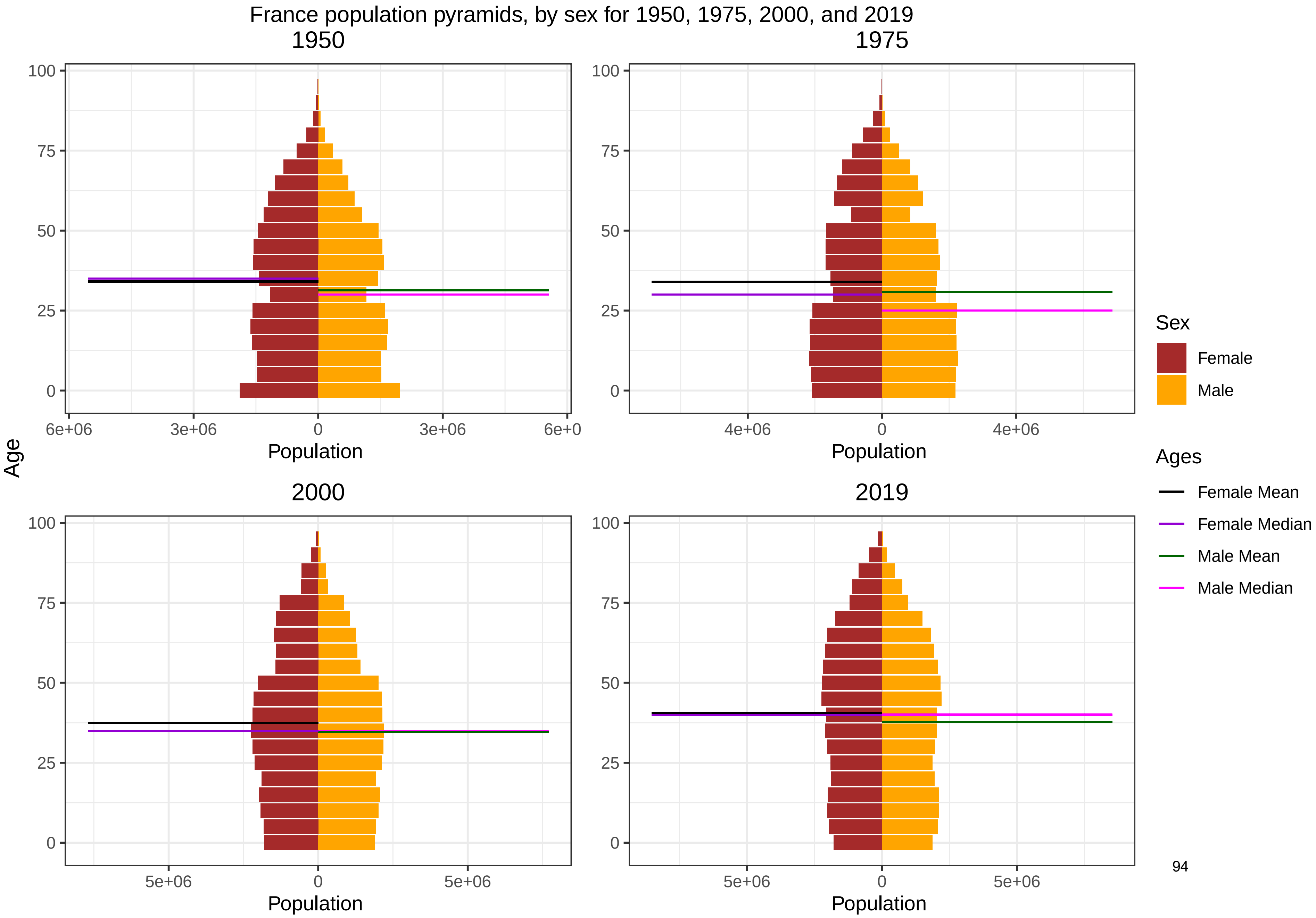

France population pyramids, by sex for 1950, 1975, 2000, and 2019
1950
1975
2000
2019
Age
Population
0
25
50
75
100
6e+06
3e+06
3e+06
6e+0
4e+06
4e+06
5e+06
5e+06
Sex
Female
Male
Ages
Female Mean
Female Median
Male Mean
Male Median

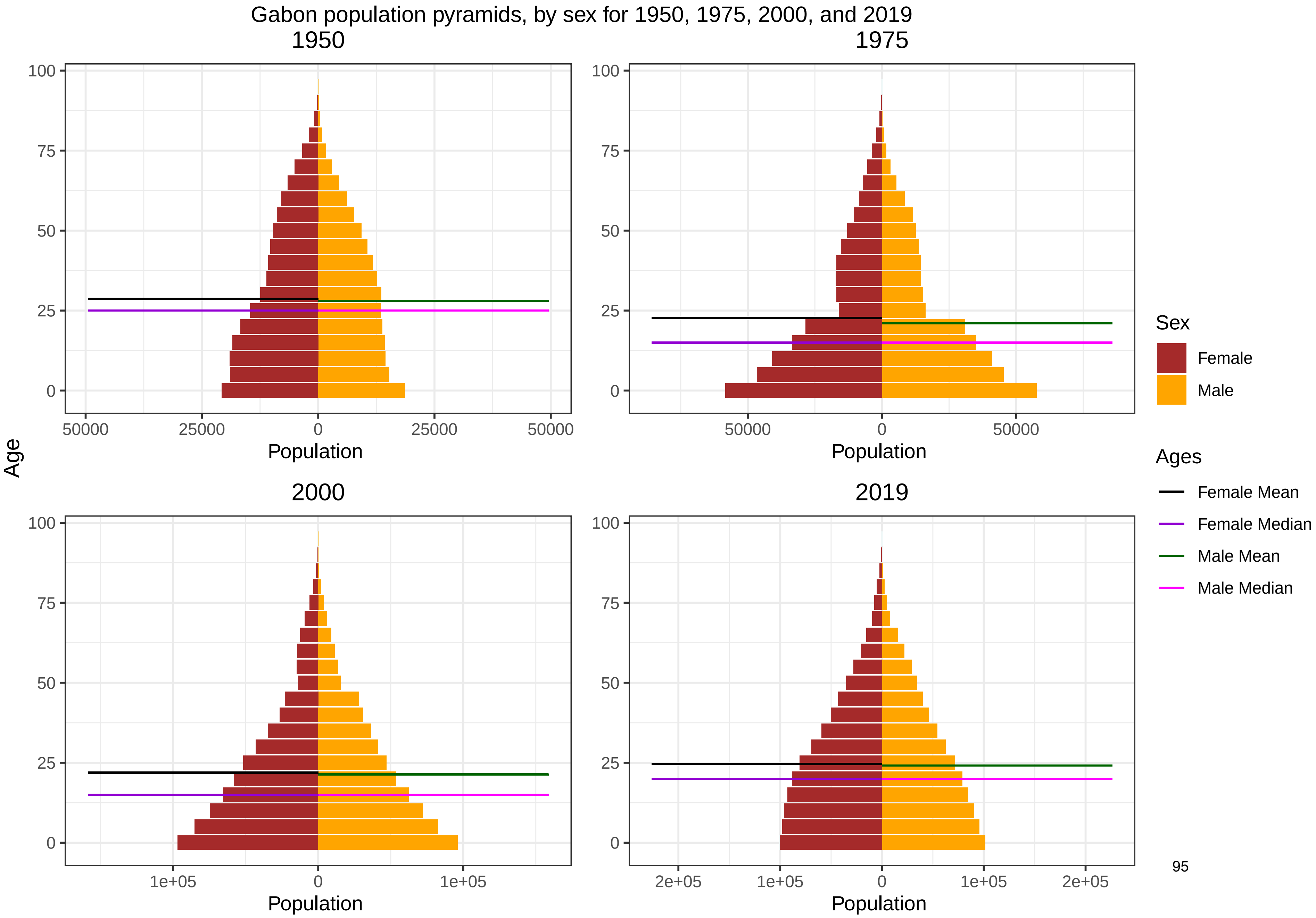
Gabon population pyramids, by sex for 1950, 1975, 2000, and 2019
1950
1975
2000
2019
Age
Population
100
75
50
25
0
50000
25000
0
25000
50000
1e+05
2e+05
Sex
Female
Male
Ages
Female Mean
Female Median
Male Mean
Male Median

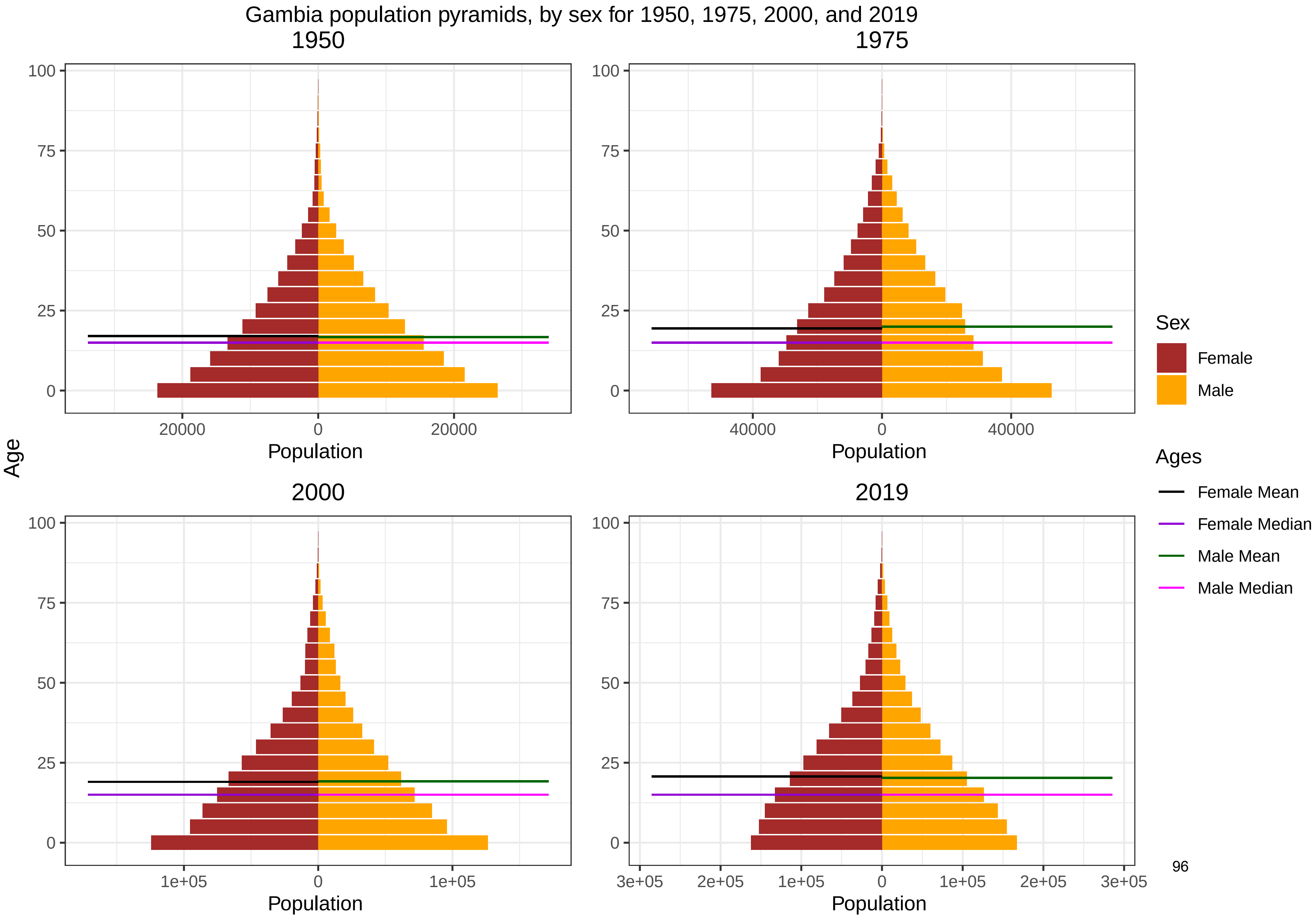
Gambia population pyramids, by sex for 1950, 1975, 2000, and 2019
1950
1975
2000
2019
Age
Population
Sex
Female
Male
Ages
Female Mean
Female Median
Male Mean
Male Median

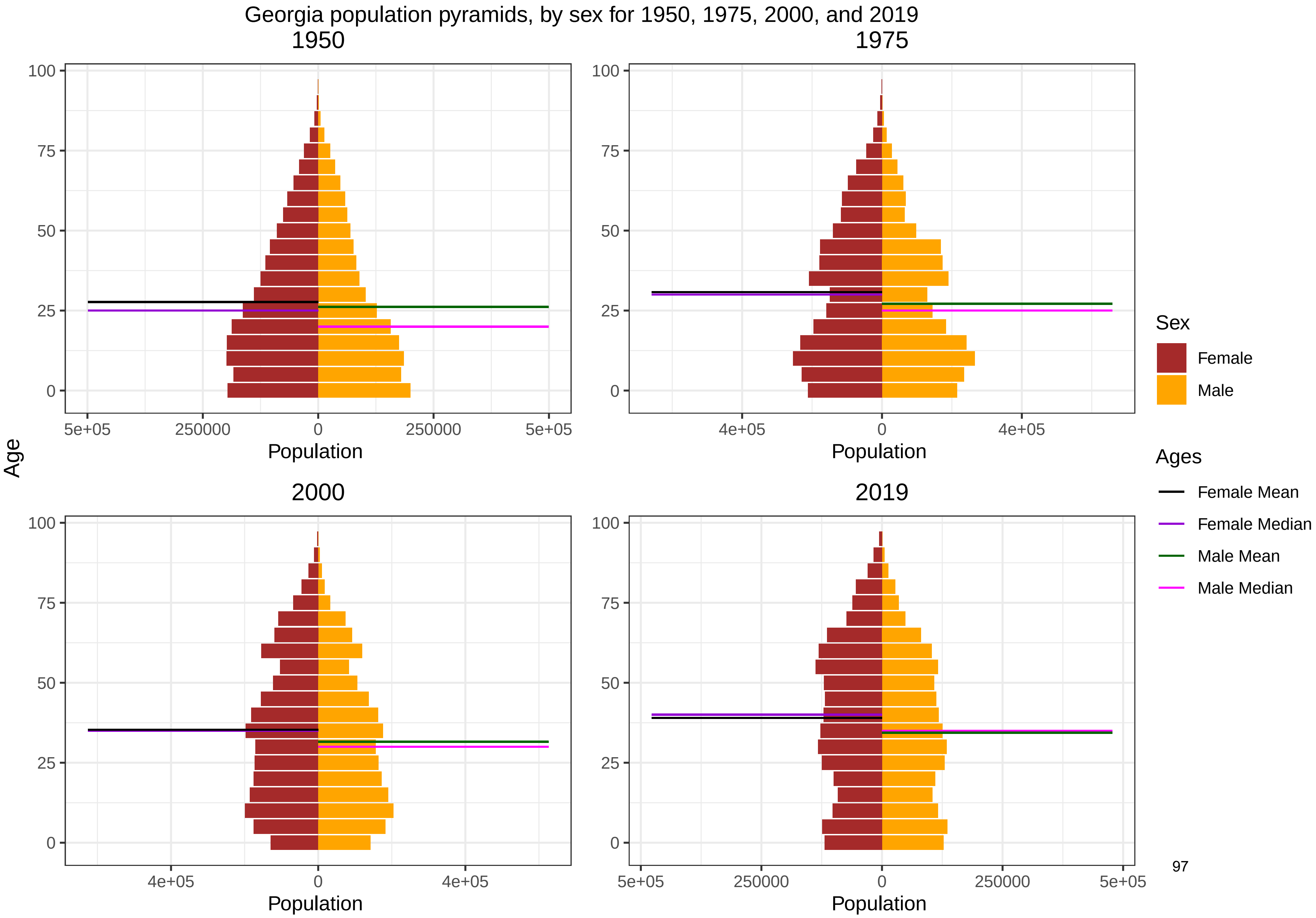
Georgia population pyramids, by sex for 1950, 1975, 2000, and 2019
1950
1975
2000
2019
Age
Population
0
25
50
75
100
5e+05
250000
4e+05
Sex
Female
Male
Ages
Female Mean
Female Median
Male Mean
Male Median

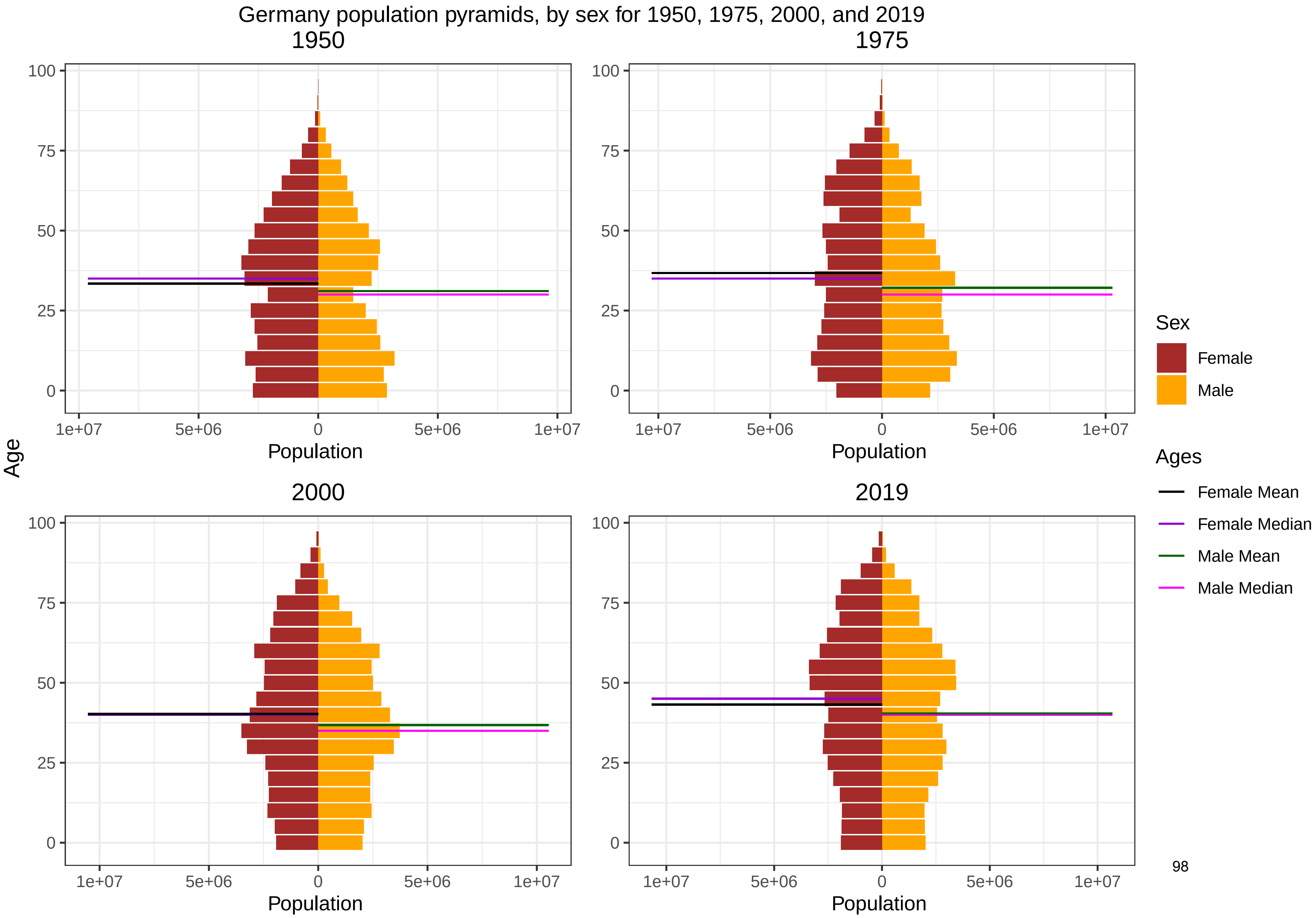
Germany population pyramids, by sex for 1950, 1975, 2000, and 2019
1950
1975
2000
2019
Age
Population
100
75
50
25
0
1e+07
5e+06
0
5e+06
1e+07
Sex
Female
Male
Ages
Female Mean
Female Median
Male Mean
Male Median

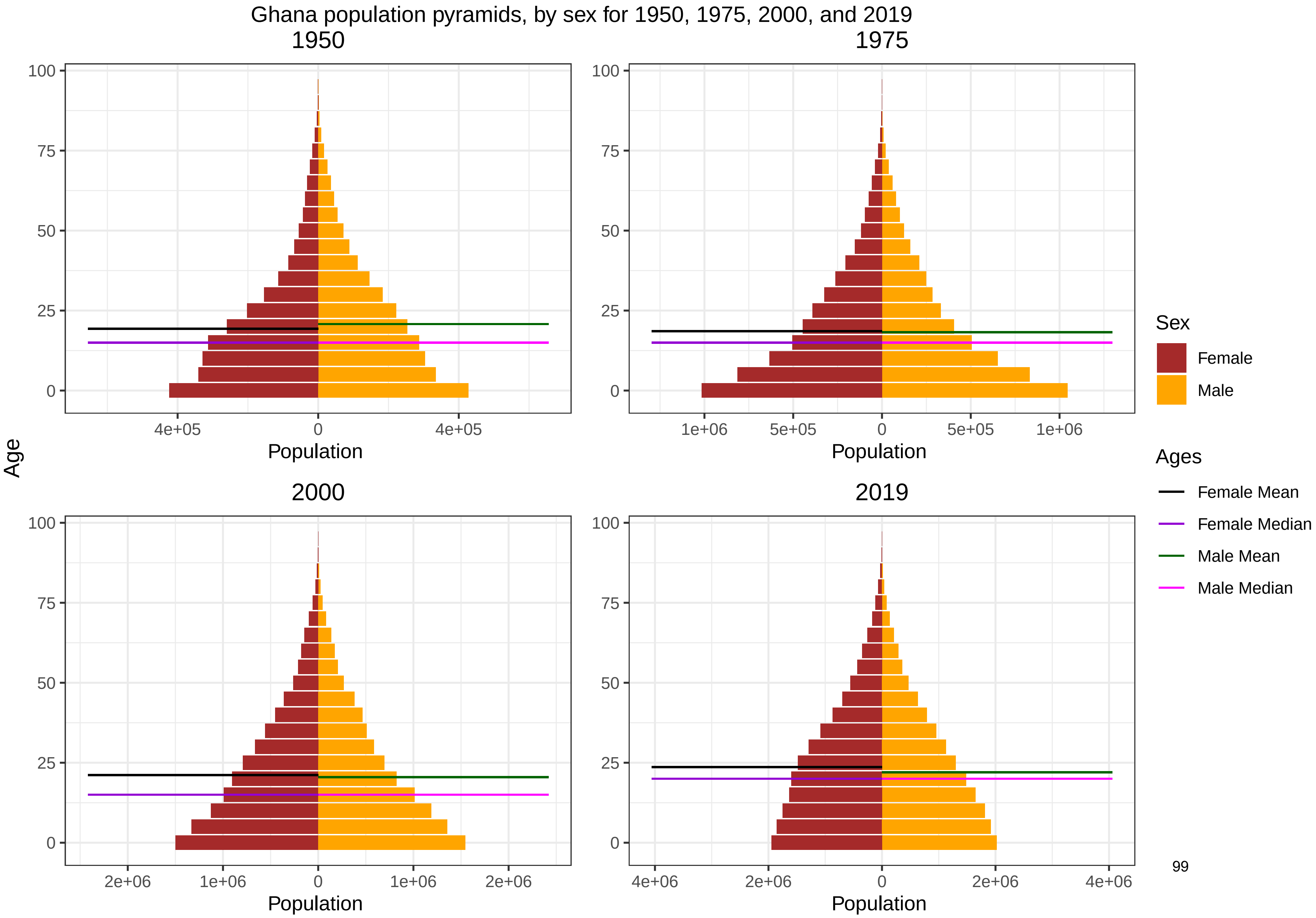
Ghana population pyramids, by sex for 1950, 1975, 2000, and 2019
1950
1975
2000
2019
Age
Population
0
25
50
75
100
4e+05
0
4e+05
1e+06
5e+05
0
5e+05
1e+06
2e+06
1e+06
0
1e+06
2e+06
4e+06
2e+06
0
2e+06
4e+06
Sex
Female
Male
Ages
Female Mean
Female Median
Male Mean
Male Median

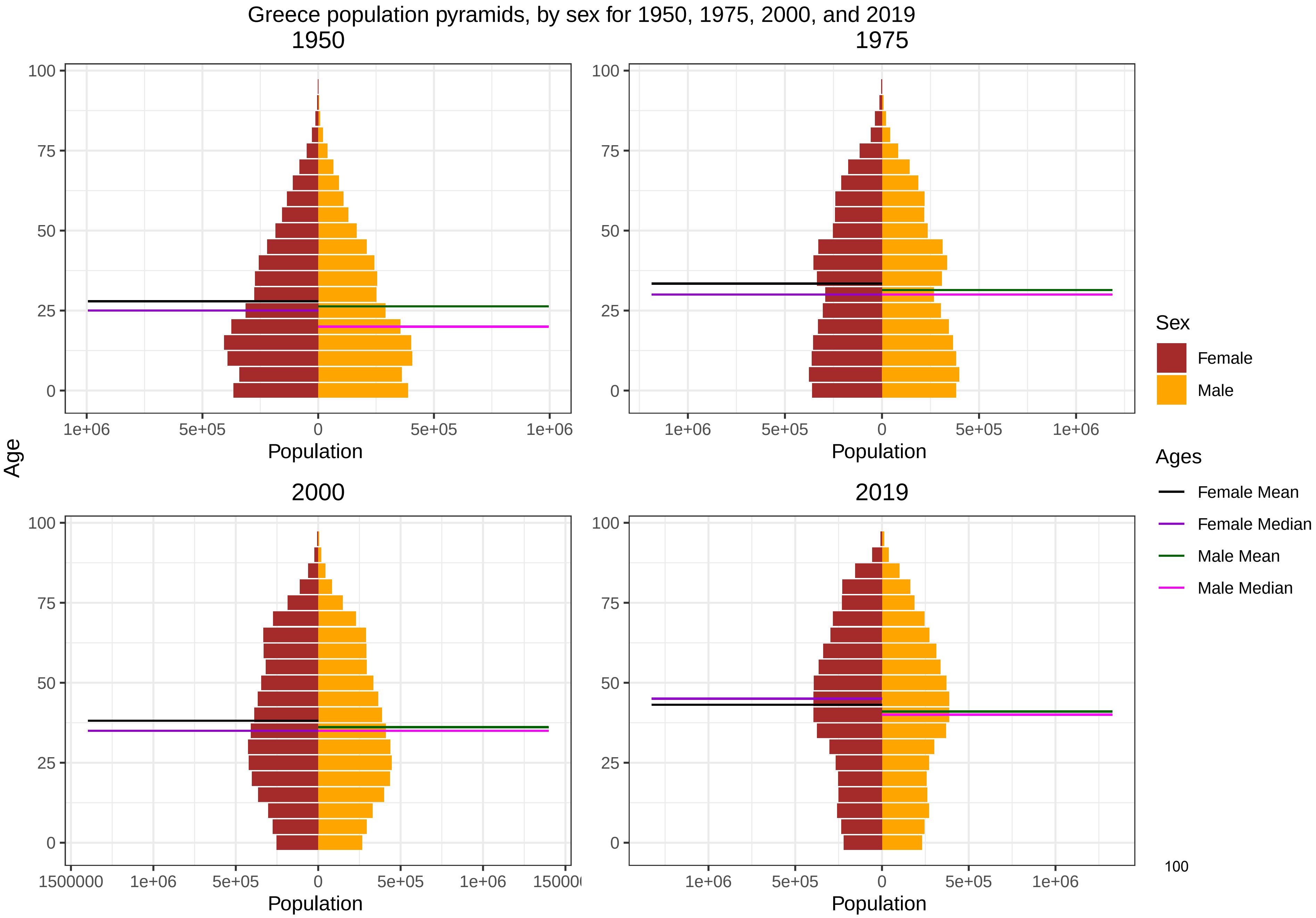
Greece population pyramids, by sex for 1950, 1975, 2000, and 2019
1950
1975
2000
2019
Age
Population
Sex
Female
Male
Ages
Female Mean
Female Median
Male Mean
Male Median
100

Greenland population pyramids, by sex for 1950, 1975, 2000, and 2019

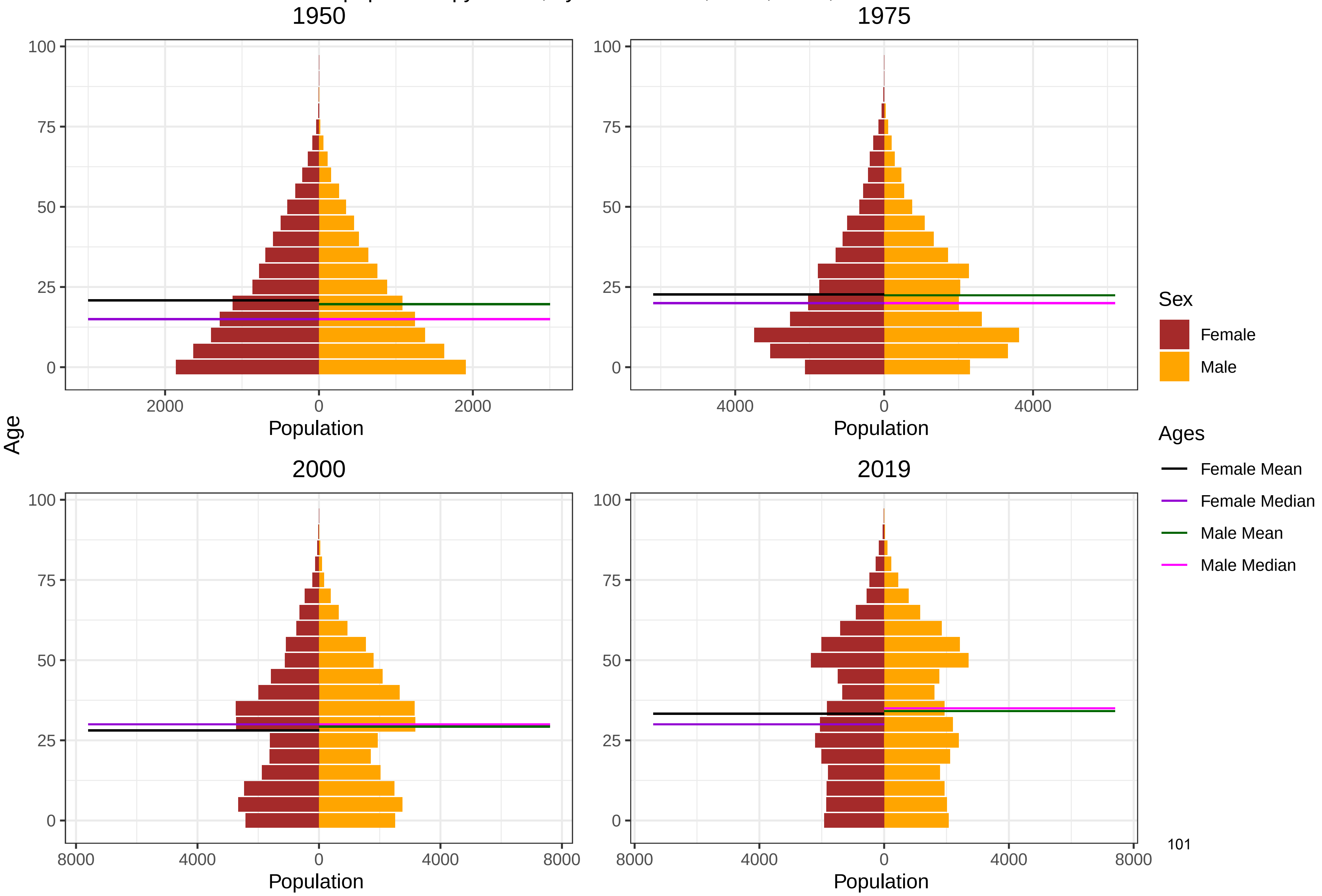

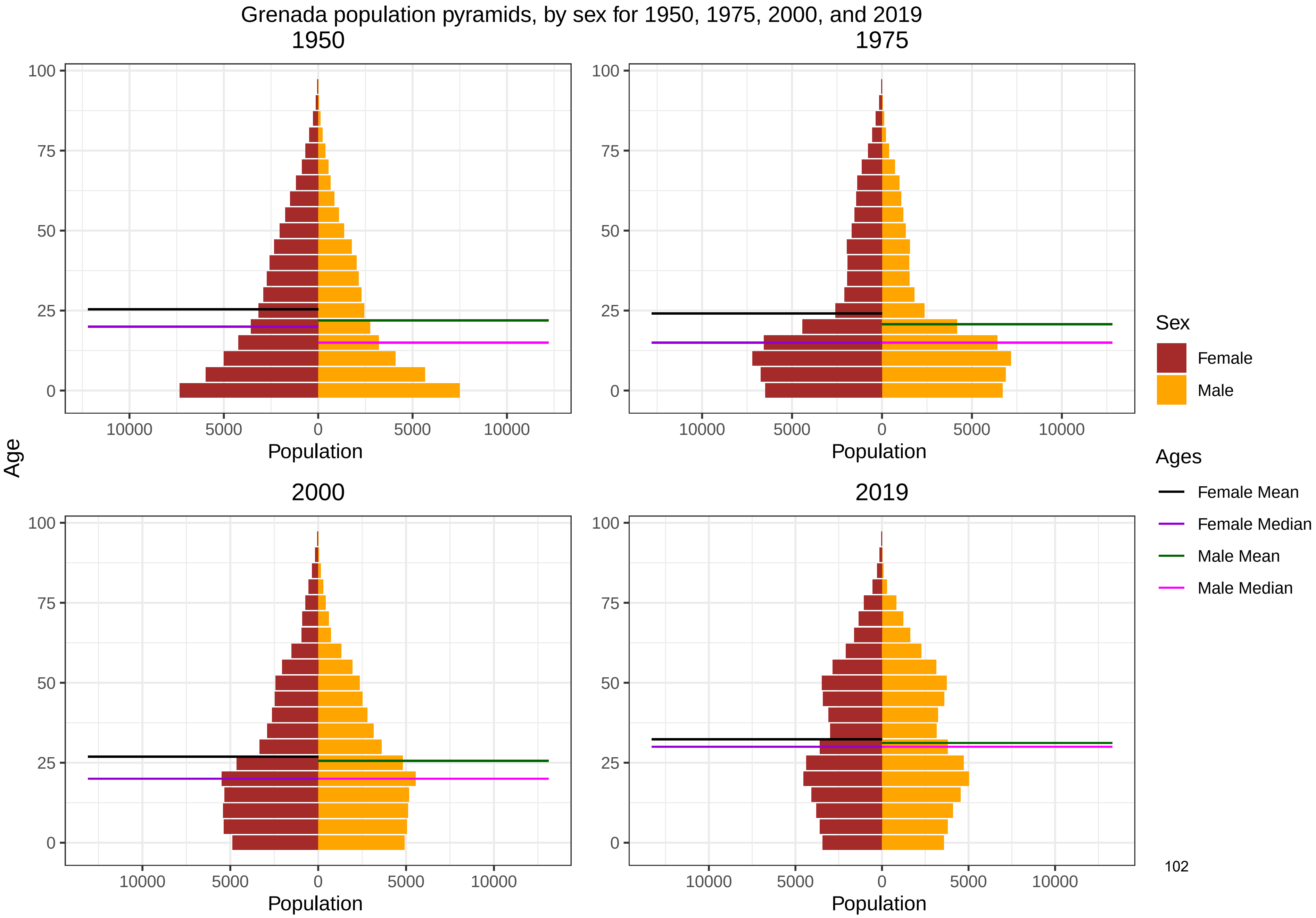
Grenada population pyramids, by sex for 1950, 1975, 2000, and 2019
1950
1975
2000
2019
Age
Population
100
75
50
25
0
10000
5000
0
5000
10000
Sex
Female
Male
Ages
Female Mean
Female Median
Male Mean
Male Median

Guam population pyramids, by sex for 1950, 1975, 2000, and 2019

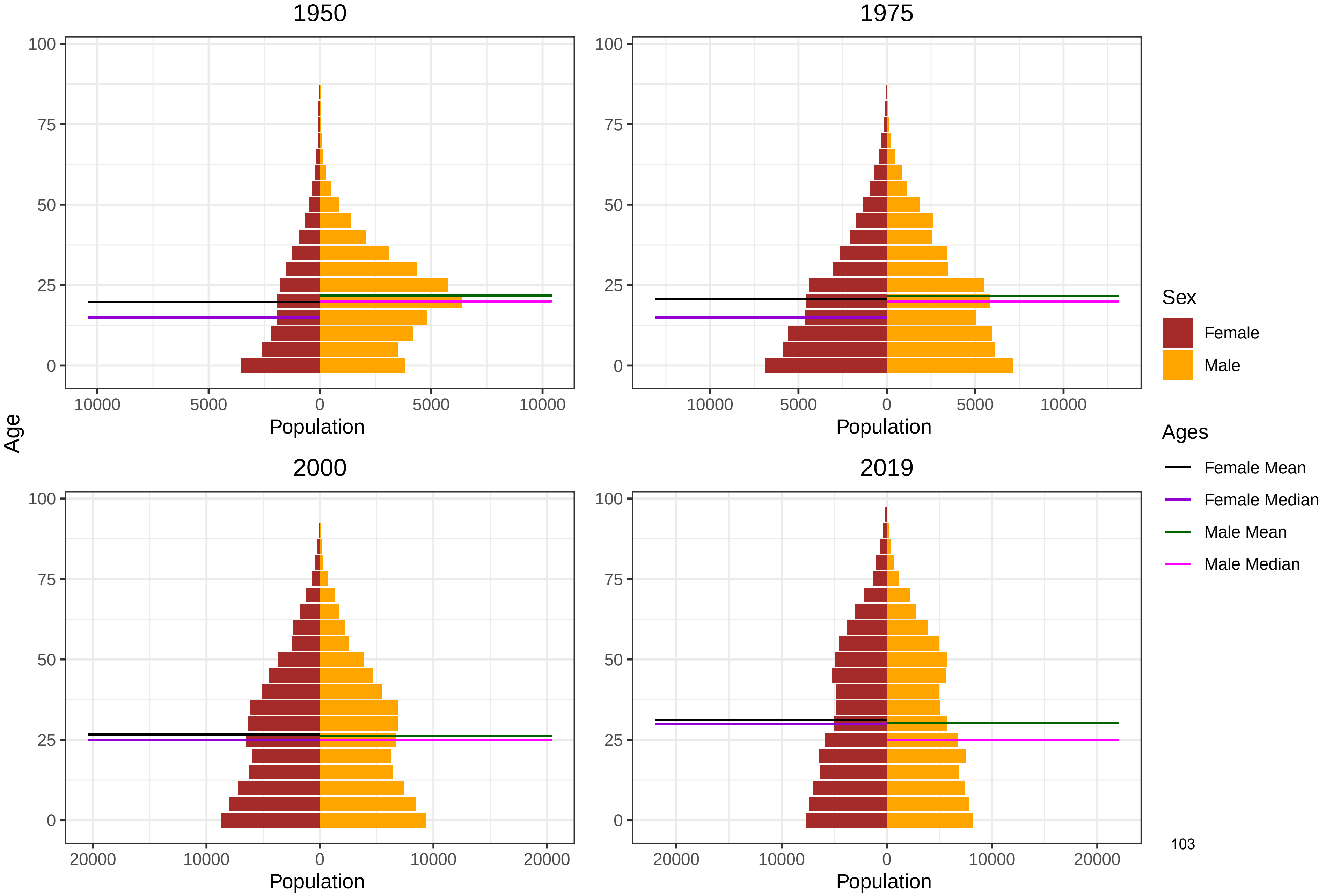

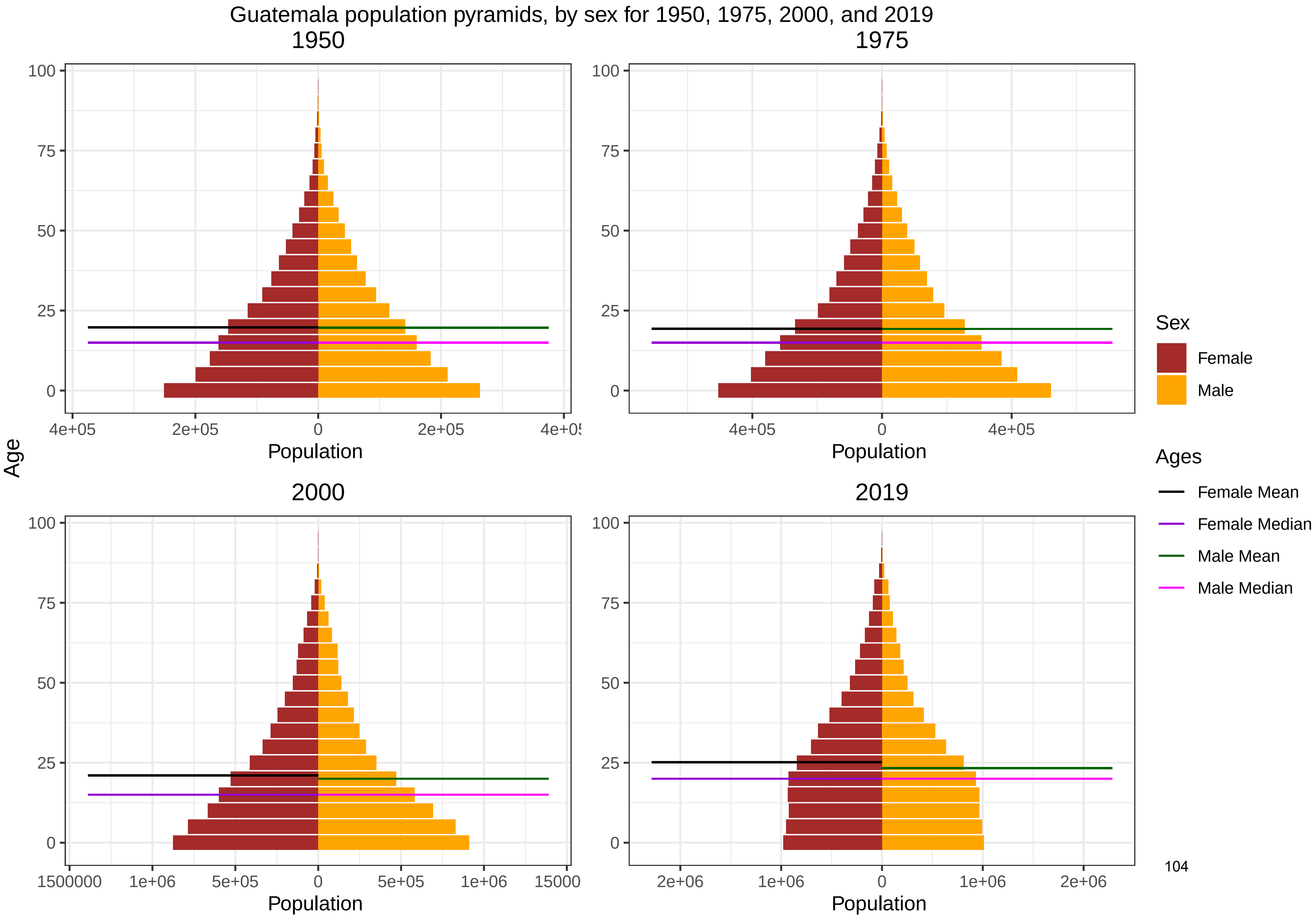

Guatemala population pyramids, by sex for 1950, 1975, 2000, and 2019
1950
1975
2000
2019
Age
Population
100
75
50
25
0
4e+05
2e+05
0
2e+05
4e+05
1e+06
5e+05
1500000
15000
2e+06
Sex
Female
Male
Ages
Female Mean
Female Median
Male Mean
Male Median

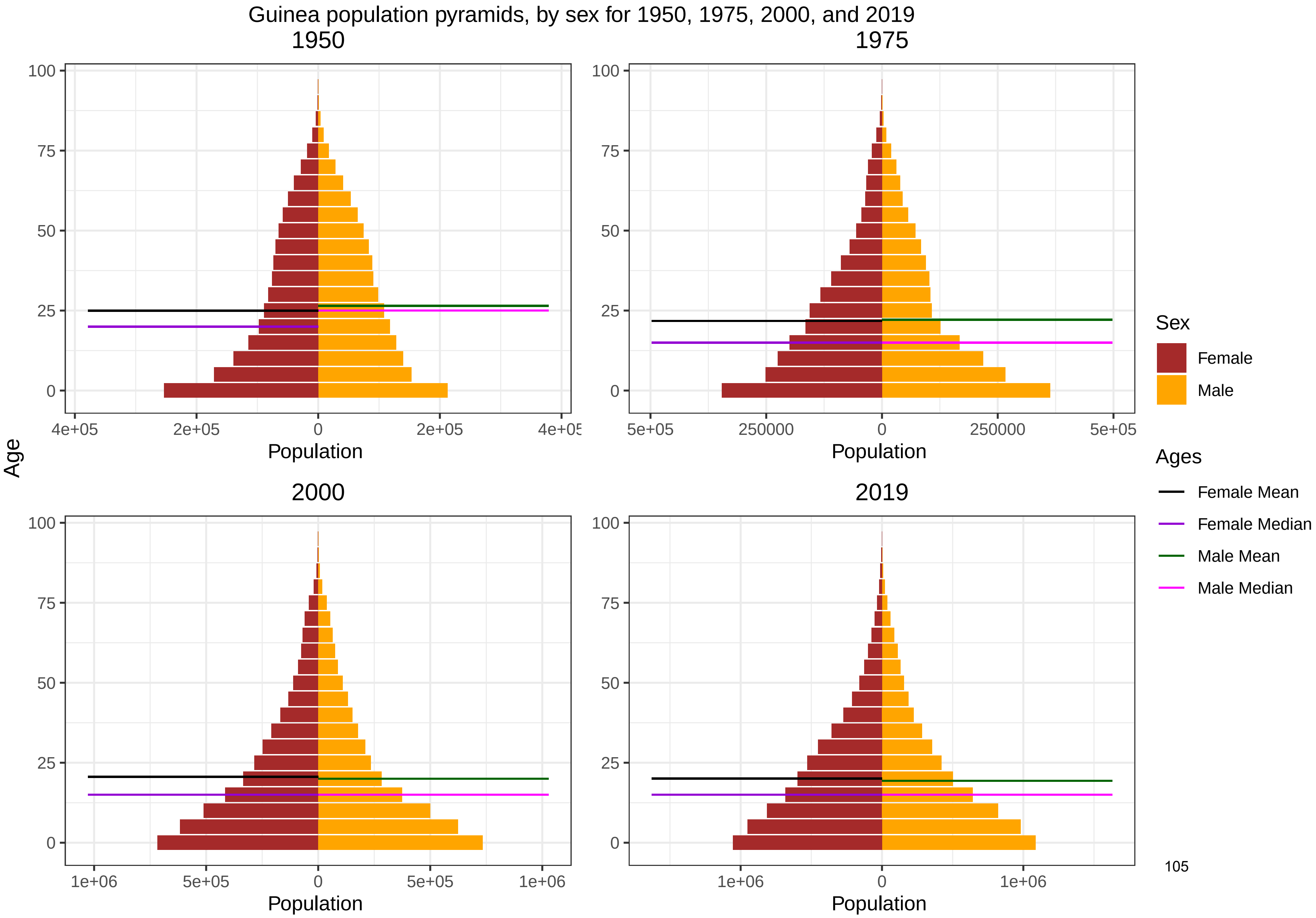

Guinea population pyramids, by sex for 1950, 1975, 2000, and 2019
1950
1975
2000
2019
Age
Population
100
75
50
25
0
4e+05
2e+05
0
2e+05
4e+05
5e+05
250000
0
250000
5e+05
1e+06
5e+05
0
5e+05
1e+06
1e+06
0
1e+06
Sex
Female
Male
Ages
Female Mean
Female Median
Male Mean
Male Median

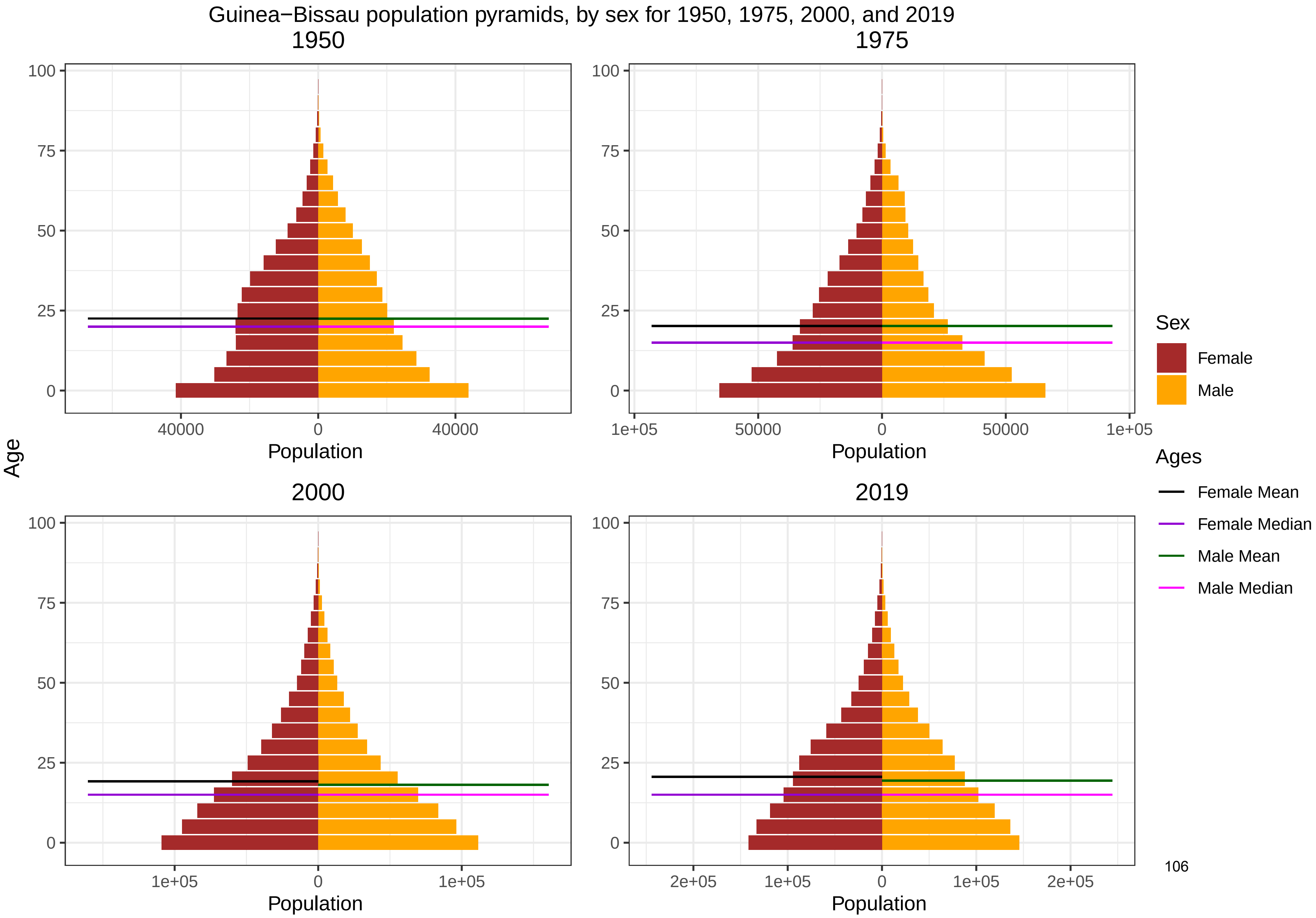
Guinea−Bissau population pyramids, by sex for 1950, 1975, 2000, and 2019
1950
1975
2000
2019
Age
Population
Sex
Female
Male
Ages
Female Mean
Female Median
Male Mean
Male Median

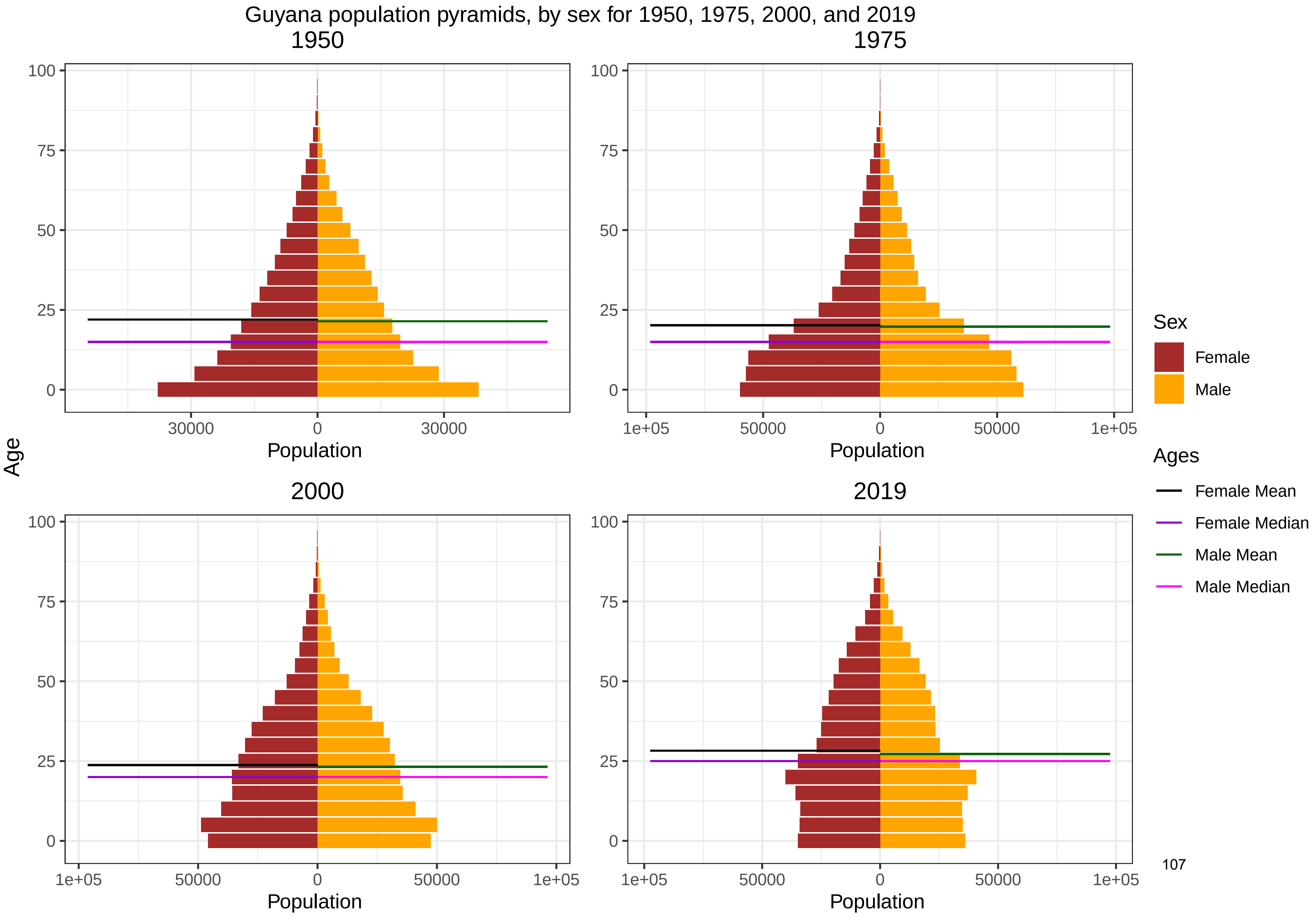

Guyana population pyramids, by sex for 1950, 1975, 2000, and 2019
1950
1975
2000
2019
Age
Population
Sex
Female
Male
Ages
Female Mean
Female Median
Male Mean
Male Median

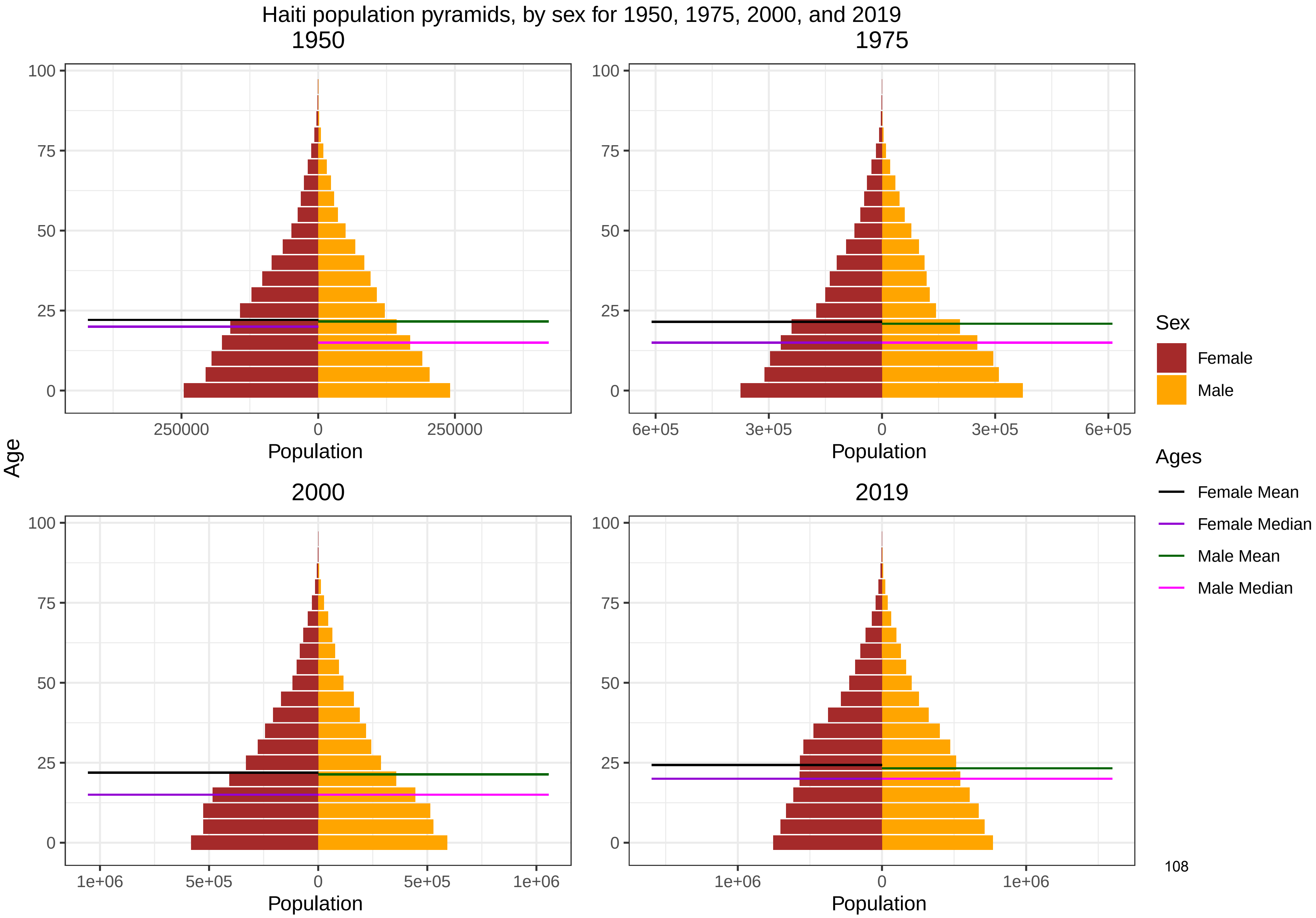
Haiti population pyramids, by sex for 1950, 1975, 2000, and 2019
1950
1975
2000
2019
Age
Population
0
25
50
75
100
250000
6e+05
3e+05
5e+05
1e+06
Sex
Female
Male
Ages
Female Mean
Female Median
Male Mean
Male Median

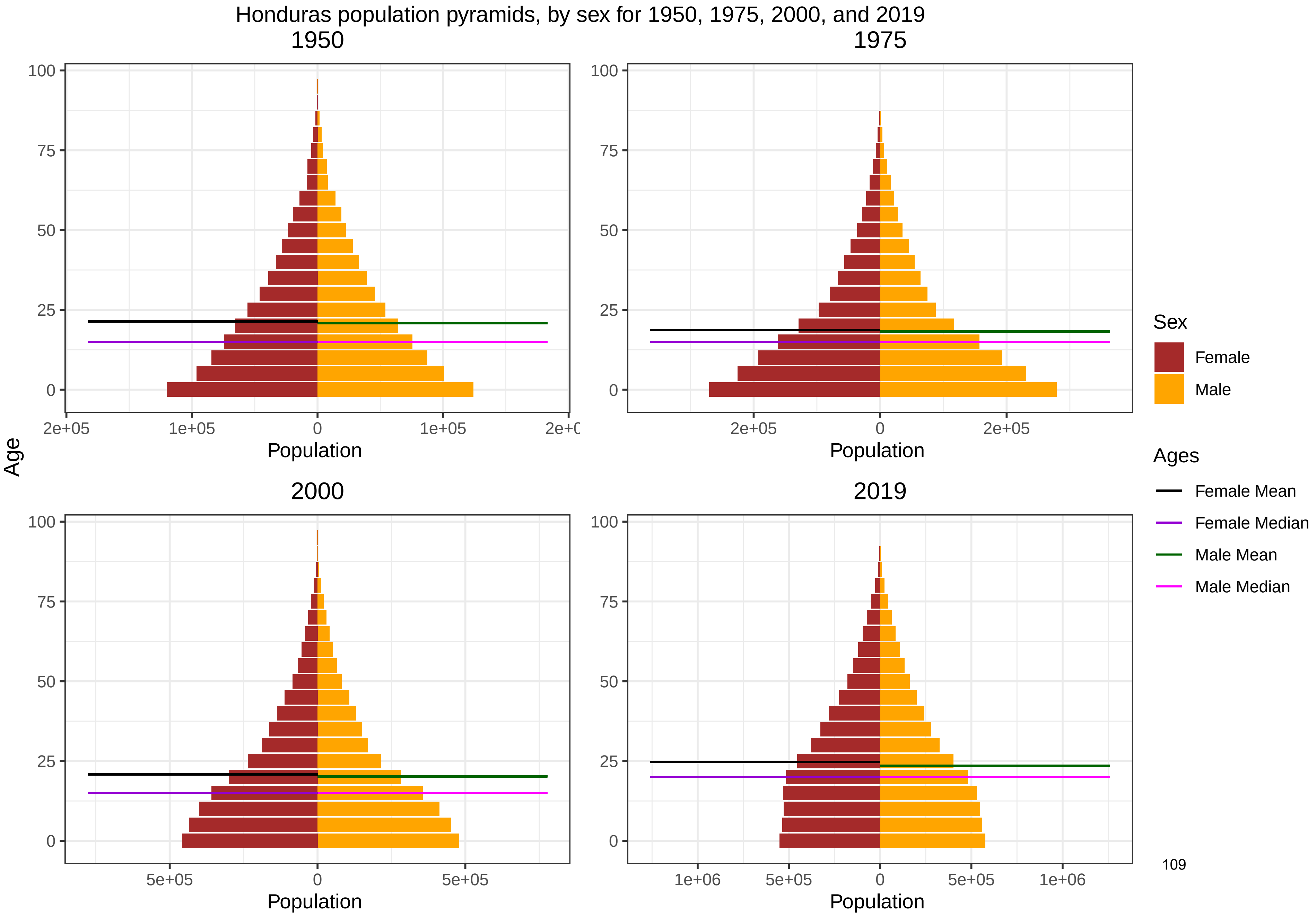

Honduras population pyramids, by sex for 1950, 1975, 2000, and 2019
1950
1975
2000
2019
Age
Population
Sex
Female
Male
Ages
Female Mean
Female Median
Male Mean
Male Median

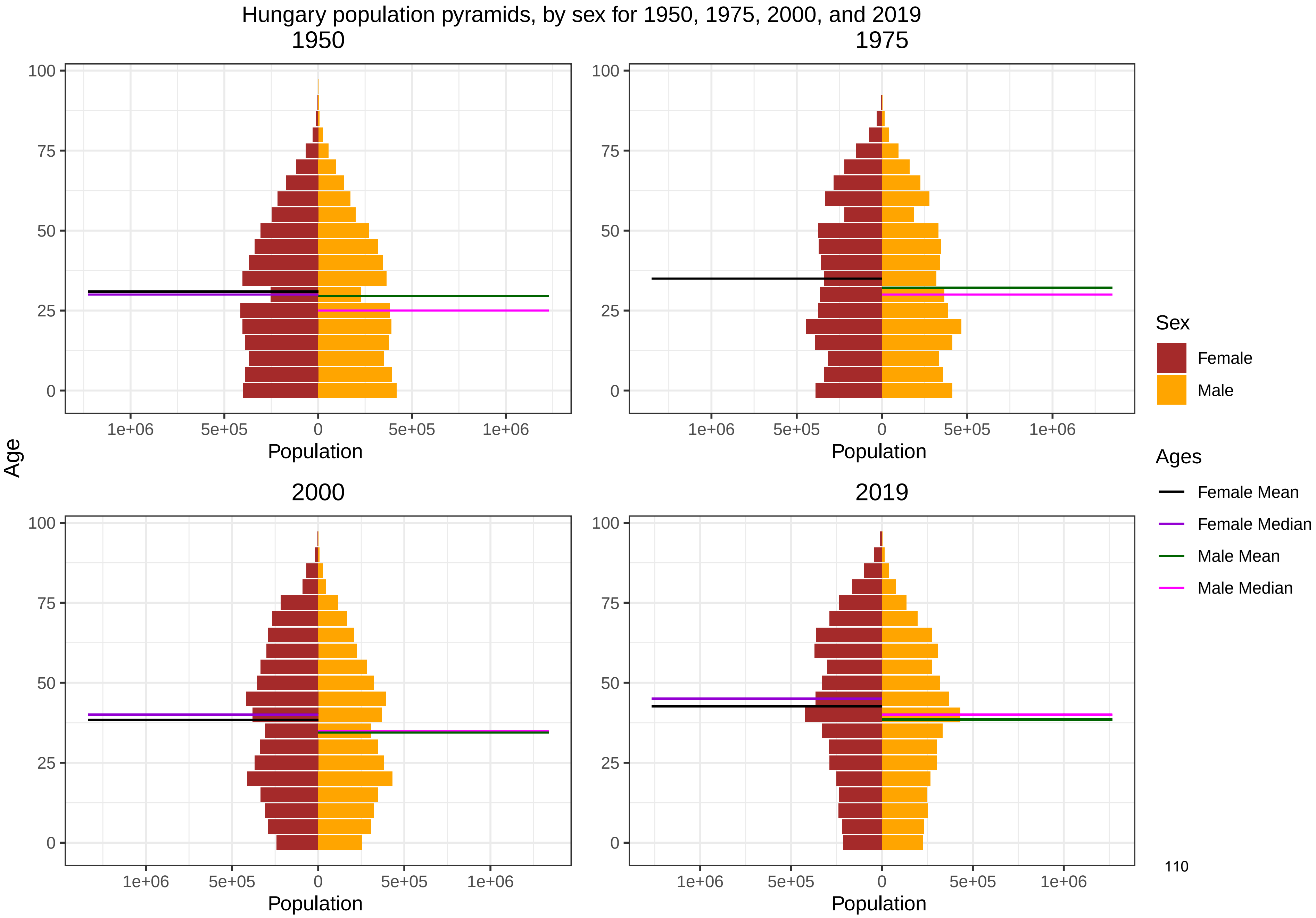
Hungary population pyramids, by sex for 1950, 1975, 2000, and 2019
1950
1975
2000
2019
Age
Population
100
75
50
25
0
1e+06
5e+05
0
5e+05
1e+06
Sex
Female
Male
Ages
Female Mean
Female Median
Male Mean
Male Median

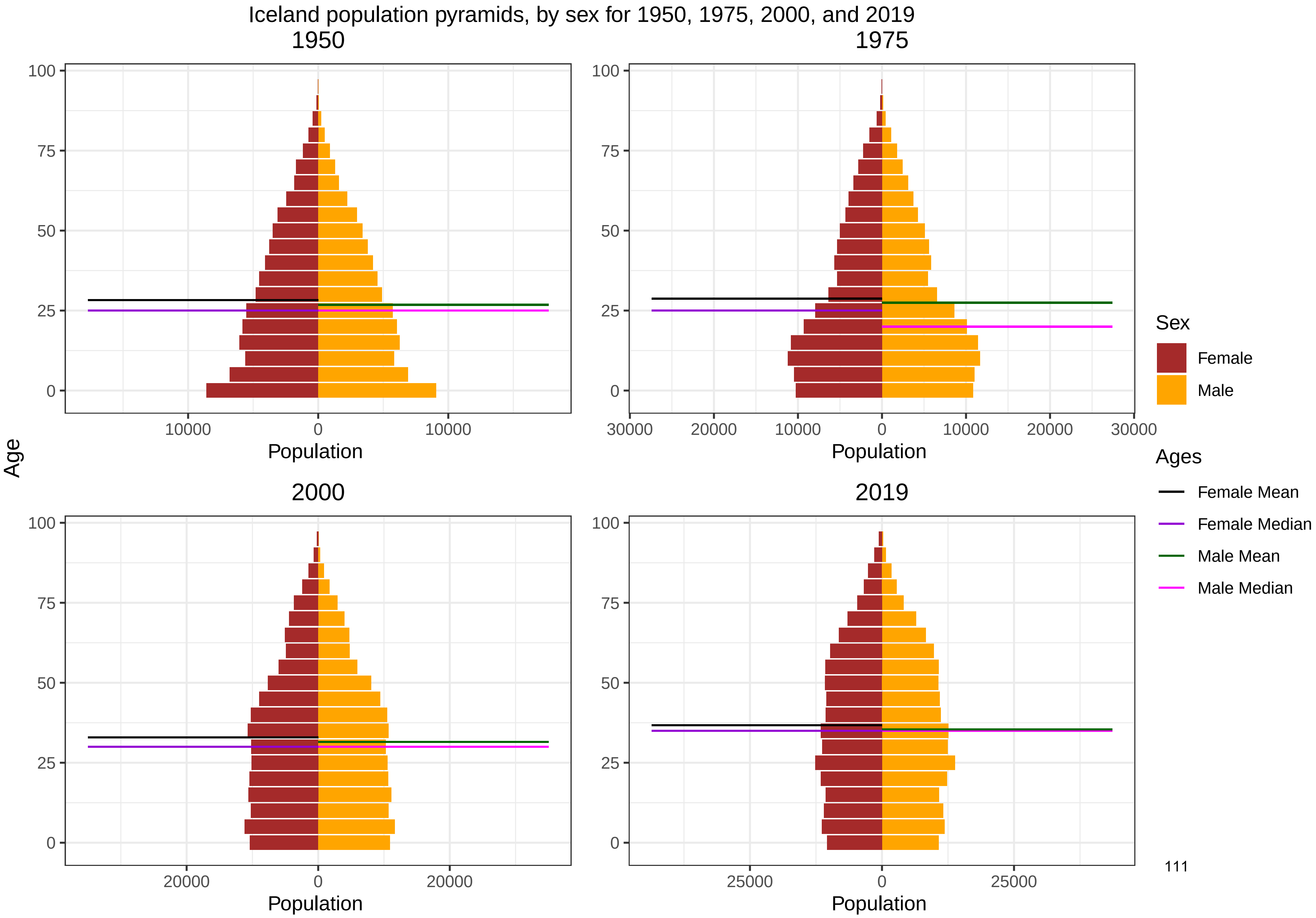

Iceland population pyramids, by sex for 1950, 1975, 2000, and 2019
1950
1975
2000
2019
Age
Population
Sex
Female
Male
Ages
Female Mean
Female Median
Male Mean
Male Median

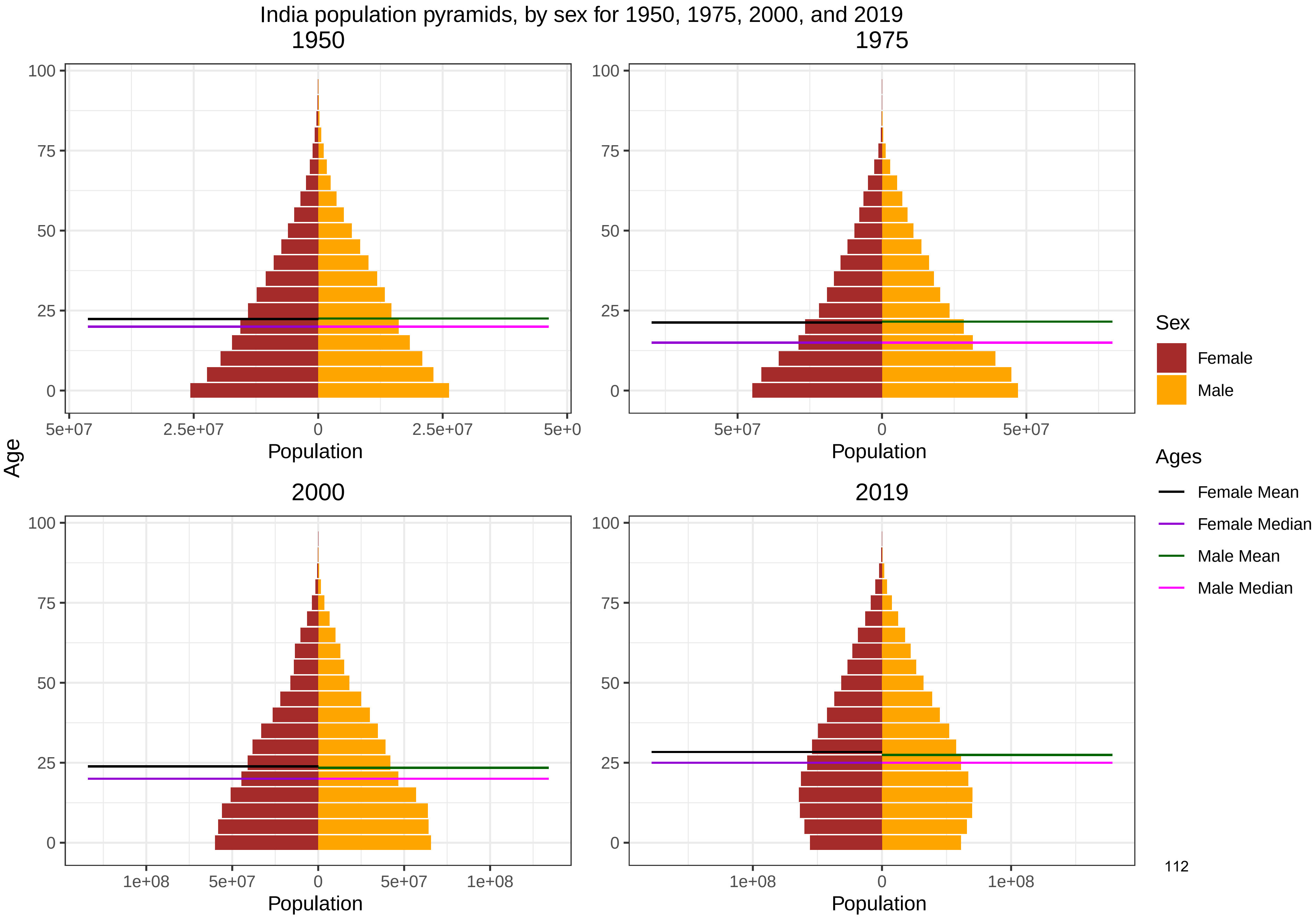
India population pyramids, by sex for 1950, 1975, 2000, and 2019
1950
1975
2000
2019
Age
Population
100
75
50
25
0
5e+07
2.5e+07
0
2.5e+07
5e+0
5e+07
0
5e+07
1e+08
5e+07
0
5e+07
1e+08
1e+08
0
1e+08
Sex
Female
Male
Ages
Female Mean
Female Median
Male Mean
Male Median

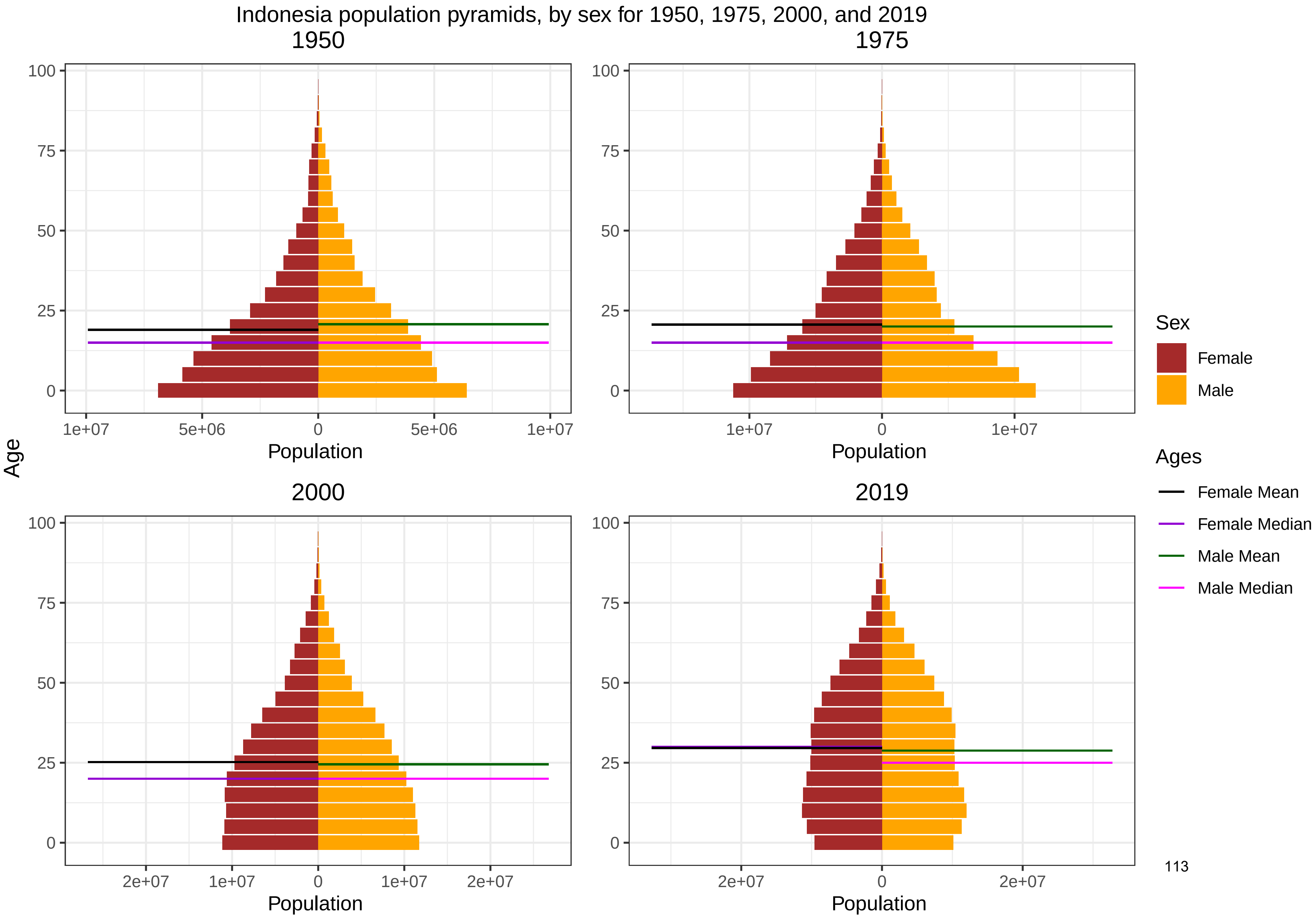

Indonesia population pyramids, by sex for 1950, 1975, 2000, and 2019
1950
1975
2000
2019
Age
Population
Sex
Female
Male
Ages
Female Mean
Female Median
Male Mean
Male Median

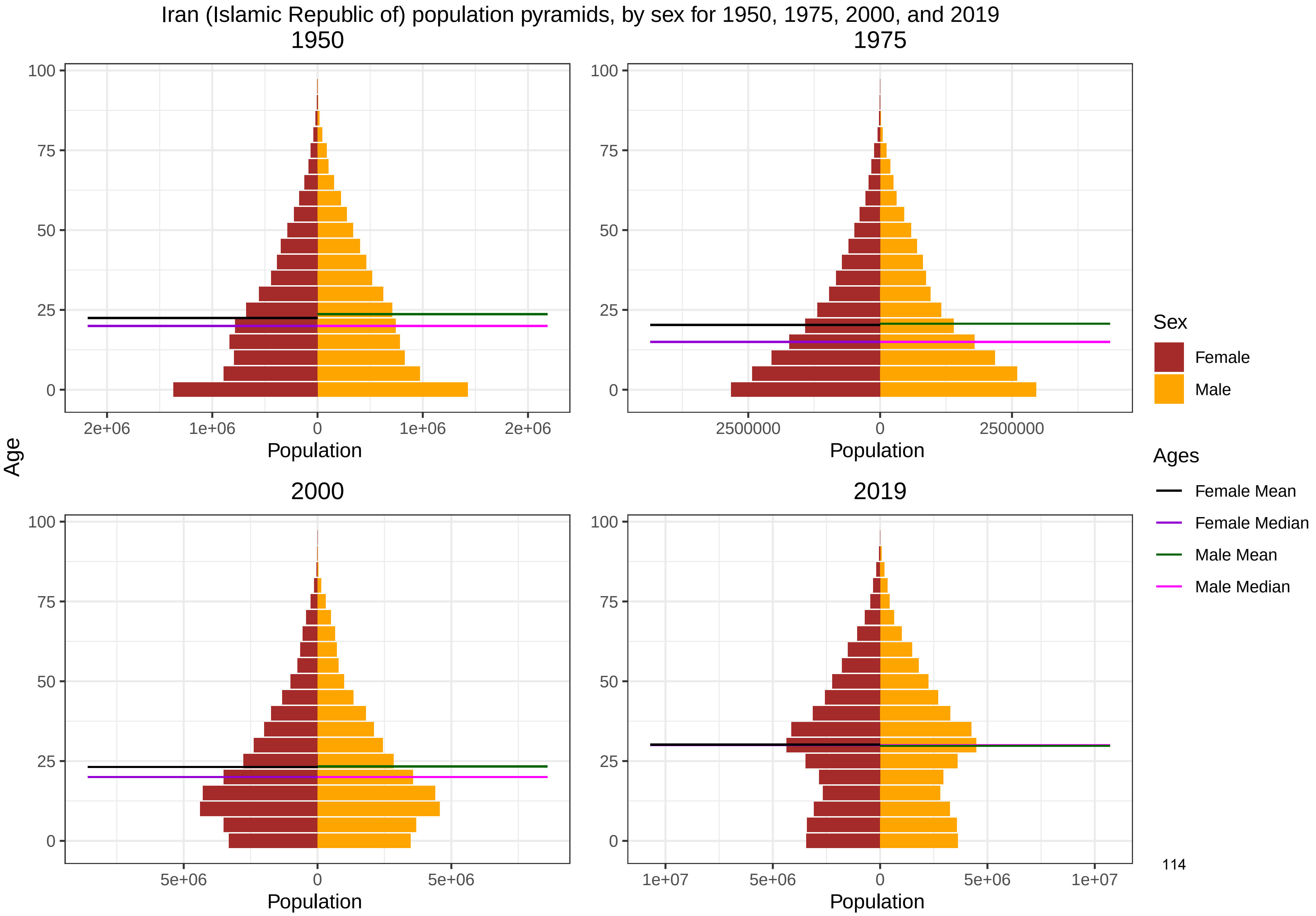

Iran (Islamic Republic of) population pyramids, by sex for 1950, 1975, 2000, and 2019
1950
1975
2000
2019
Age
Population
100
75
50
25
0
2e+06
1e+06
0
1e+06
2e+06
2500000
0
2500000
5e+06
0
5e+06
1e+07
5e+06
0
5e+06
1e+07
Sex
Female
Male
Ages
Female Mean
Female Median
Male Mean
Male Median

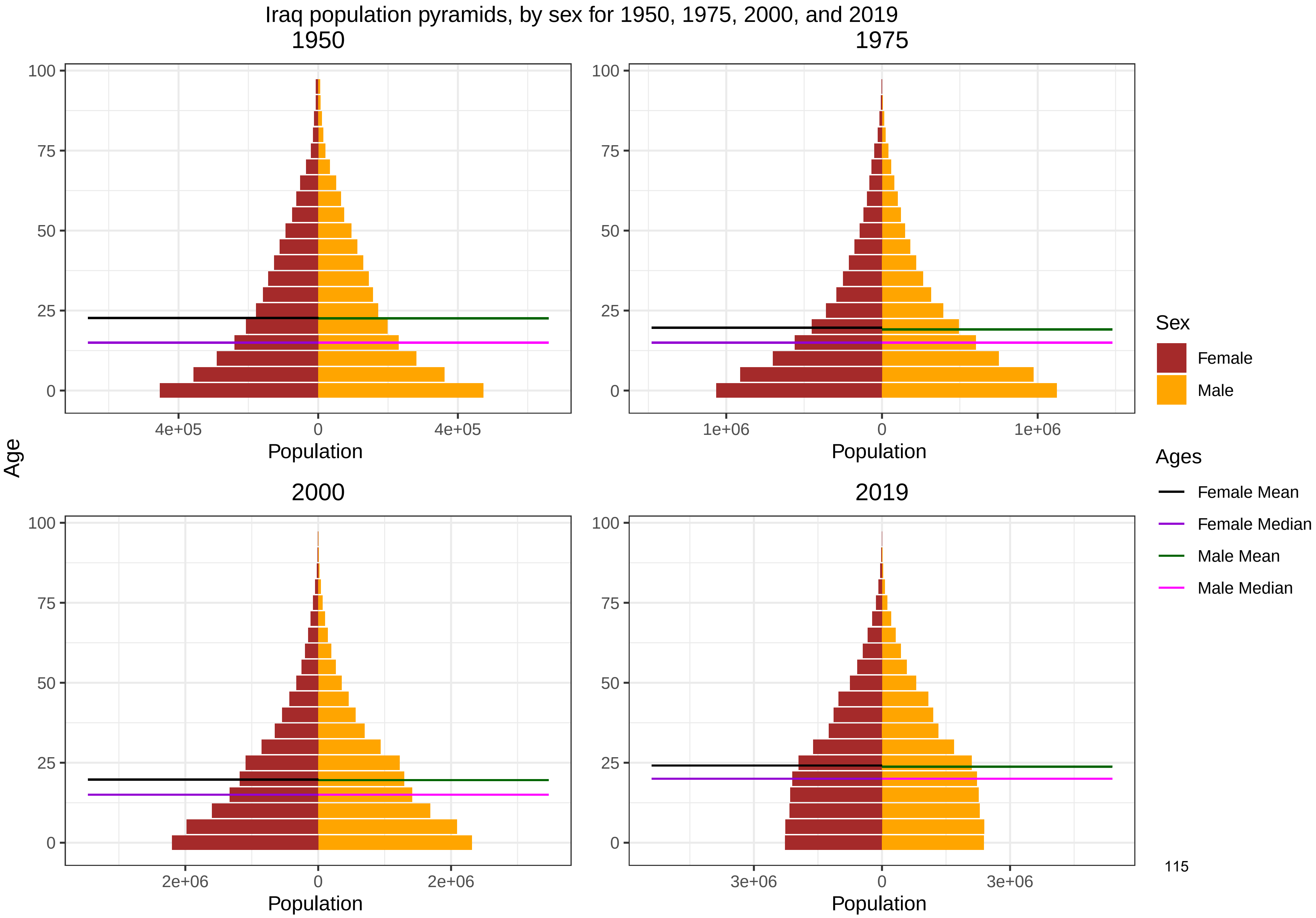
Iraq population pyramids, by sex for 1950, 1975, 2000, and 2019
1950
1975
2000
2019
Age
Population
100
75
50
25
0
4e+05
0
4e+05
1e+06
0
1e+06
2e+06
0
2e+06
3e+06
0
3e+06
Sex
Female
Male
Ages
Female Mean
Female Median
Male Mean
Male Median

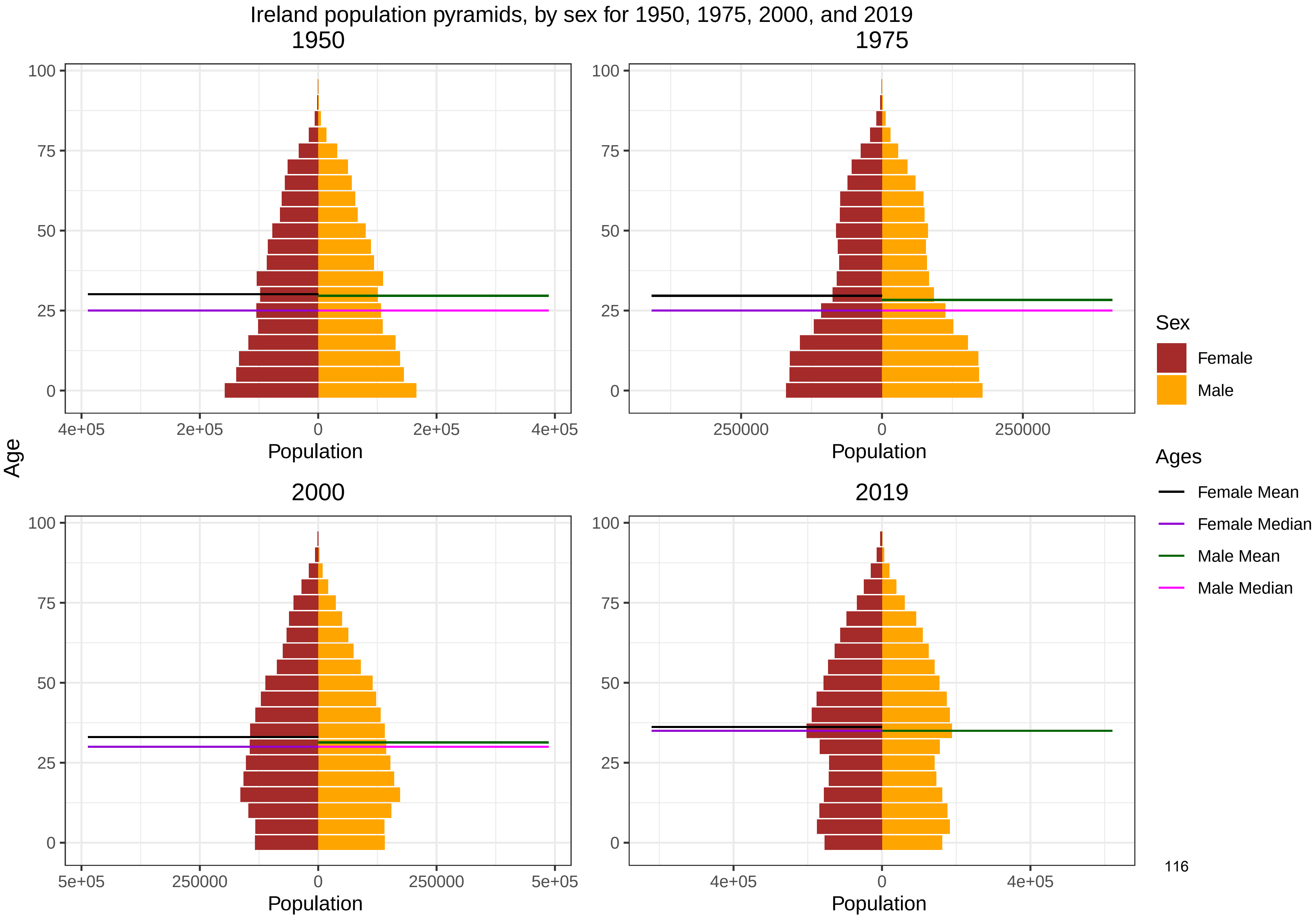
Ireland population pyramids, by sex for 1950, 1975, 2000, and 2019
1950
1975
2000
2019
Age
Population
100
75
50
25
0
4e+05
2e+05
0
250000
5e+05
Sex
Female
Male
Ages
Female Mean
Female Median
Male Mean
Male Median

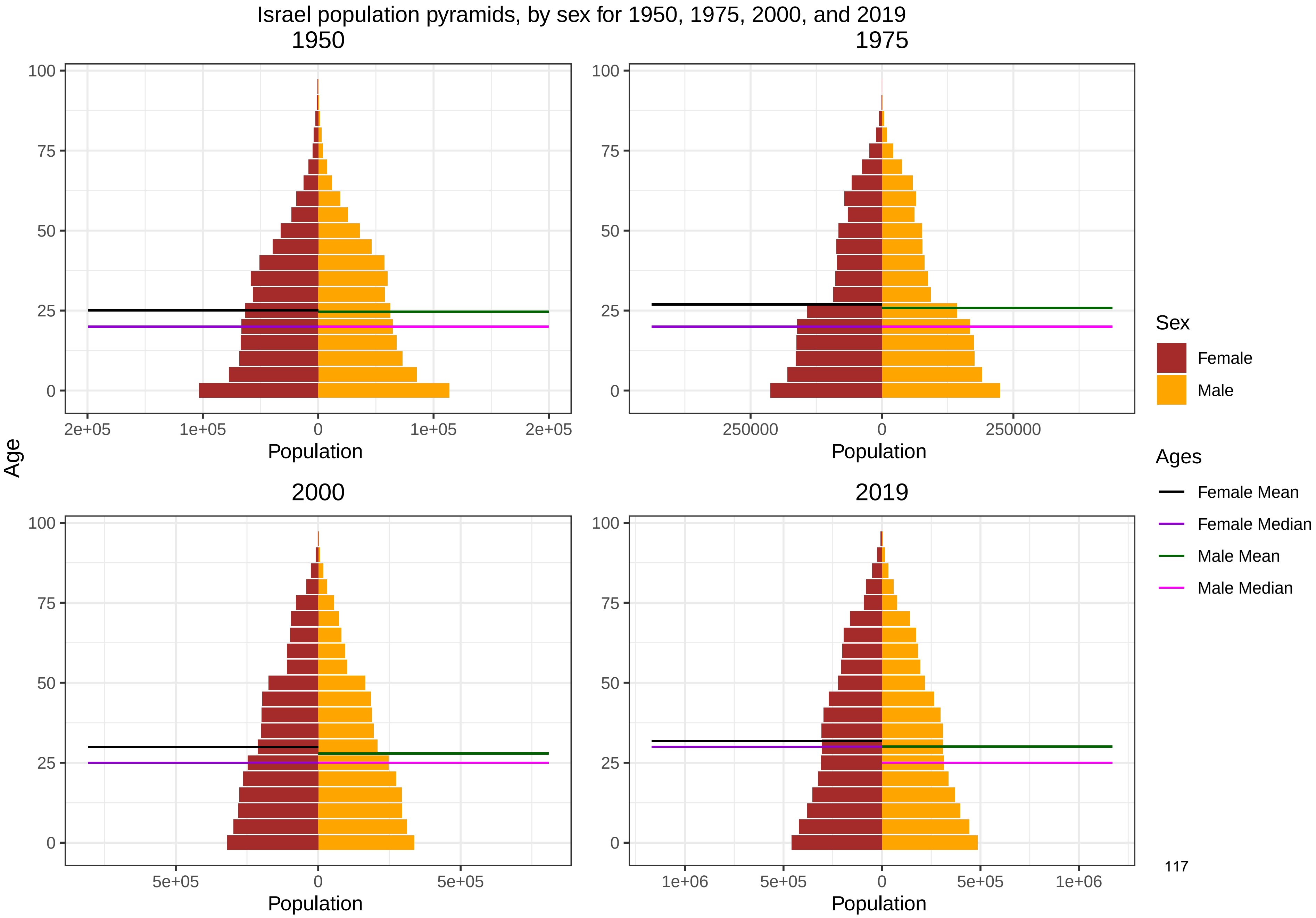

Israel population pyramids, by sex for 1950, 1975, 2000, and 2019
1950
1975
2000
2019
Age
Population
Sex
Female
Male
Ages
Female Mean
Female Median
Male Mean
Male Median

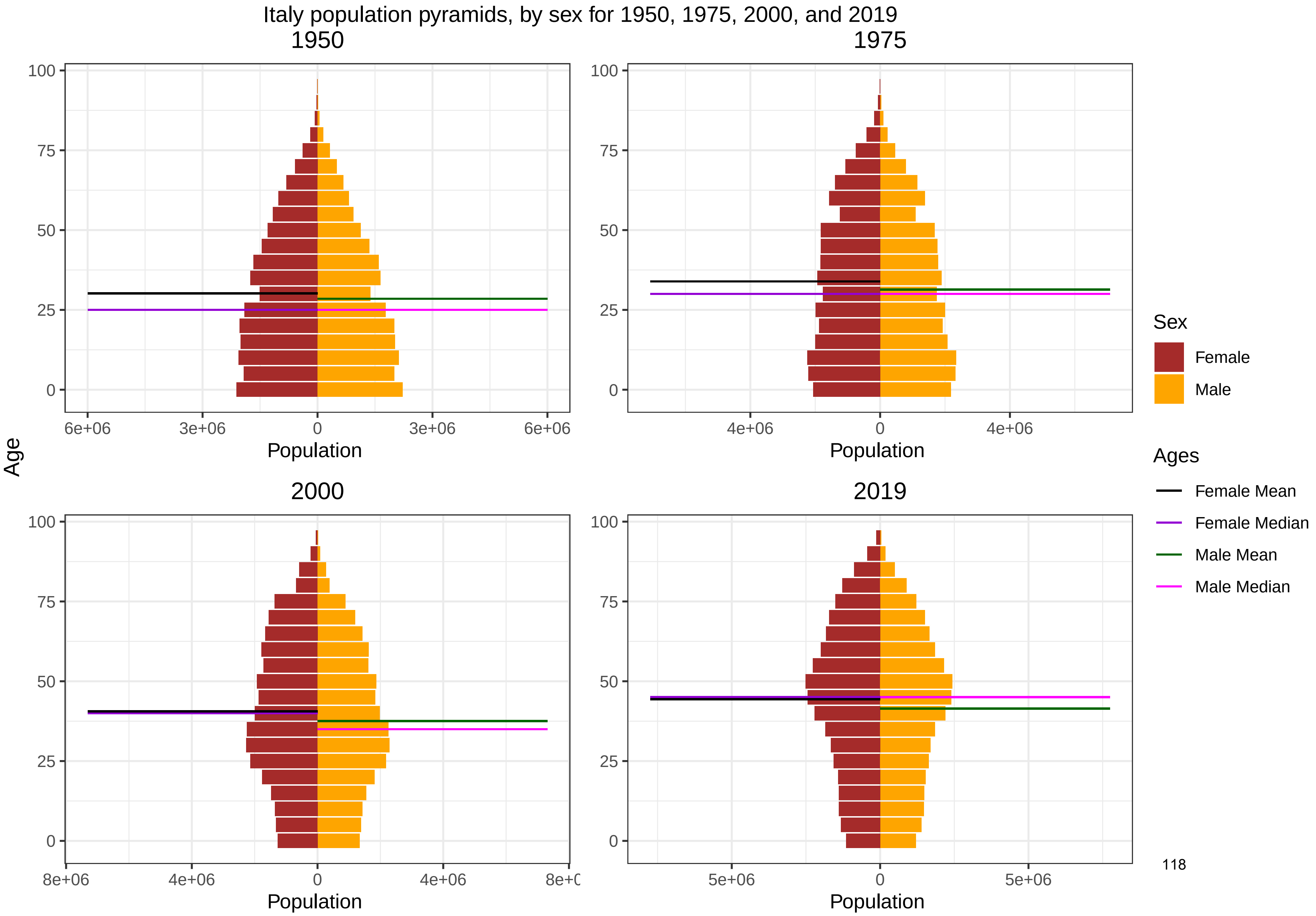
Italy population pyramids, by sex for 1950, 1975, 2000, and 2019
1950
1975
2000
2019
Age
Population
0
25
50
75
100
6e+06
3e+06
0
4e+06
8e+06
5e+06
Sex
Female
Male
Ages
Female Mean
Female Median
Male Mean
Male Median

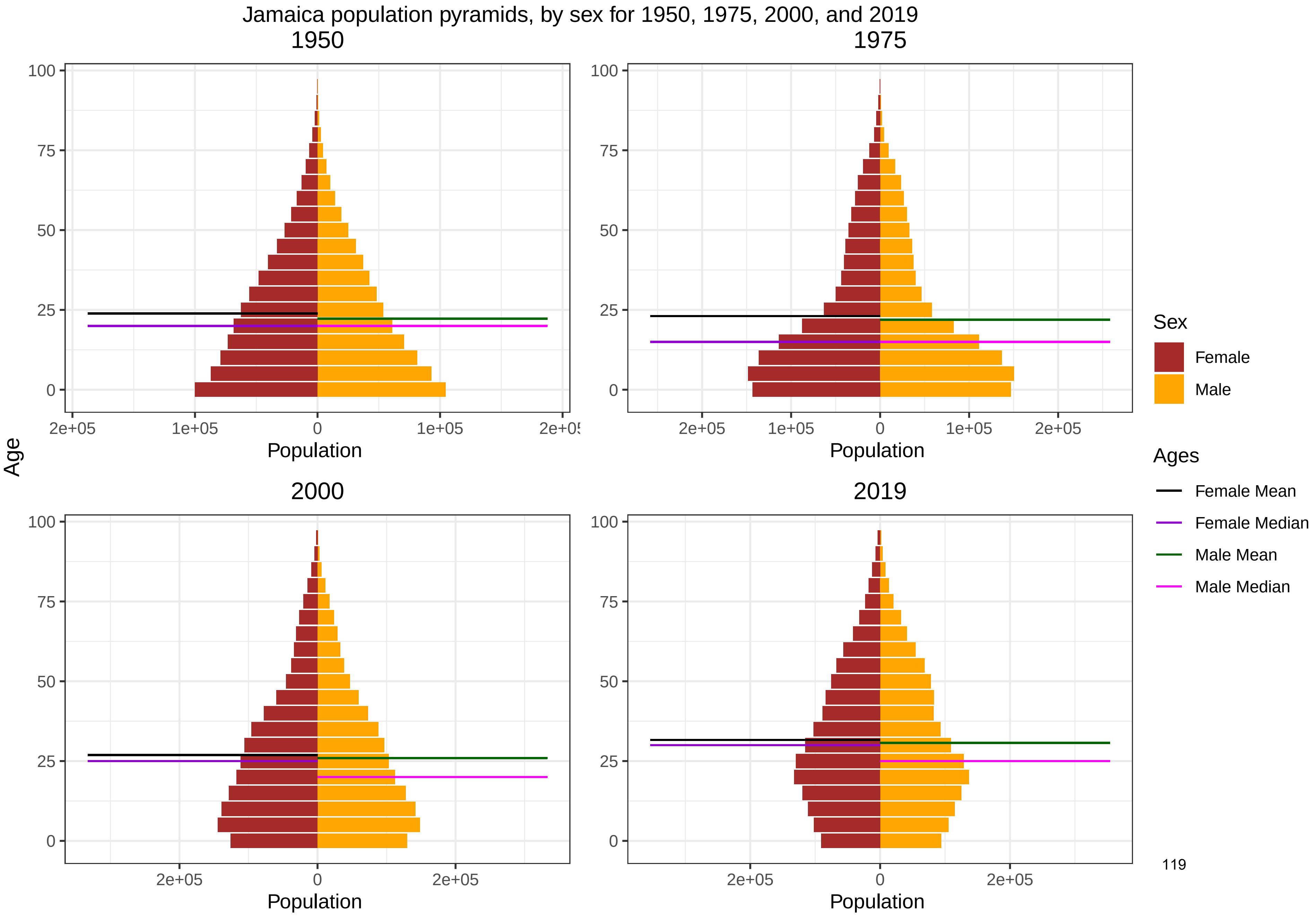
Jamaica population pyramids, by sex for 1950, 1975, 2000, and 2019
1950
1975
2000
2019
Age
Population
Sex
Female
Male
Ages
Female Mean
Female Median
Male Mean
Male Median

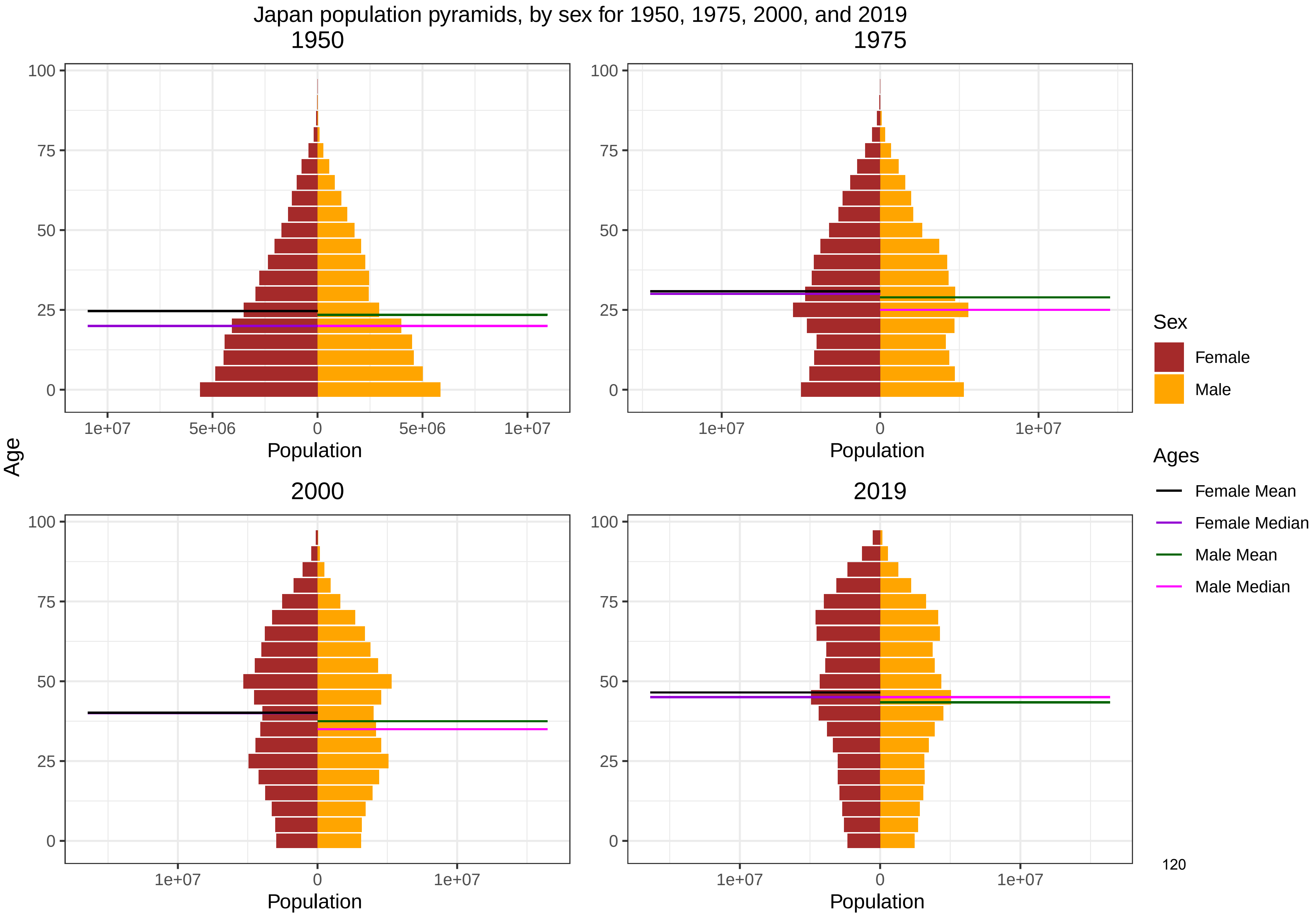
Japan population pyramids, by sex for 1950, 1975, 2000, and 2019
1950
1975
2000
2019
Age
Population
0
25
50
75
100
1e+07
5e+06
0
5e+06
1e+07
Sex
Female
Male
Ages
Female Mean
Female Median
Male Mean
Male Median

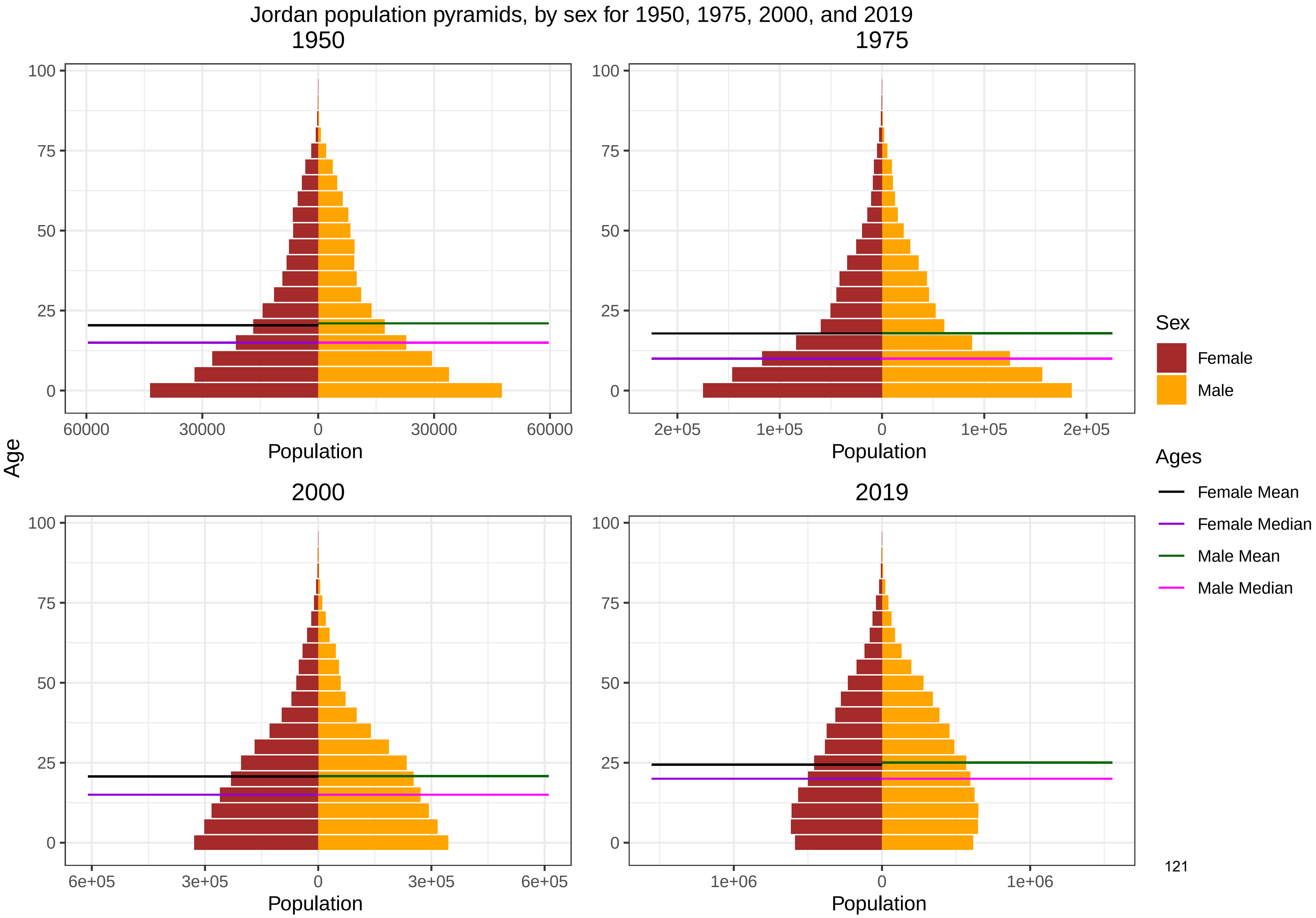

Jordan population pyramids, by sex for 1950, 1975, 2000, and 2019
1950
1975
2000
2019
Age
Population
100
75
50
25
0
60000
30000
0
30000
60000
2e+05
1e+05
0
1e+05
2e+05
6e+05
3e+05
0
3e+05
6e+05
1e+06
0
1e+06
Sex
Female
Male
Ages
Female Mean
Female Median
Male Mean
Male Median

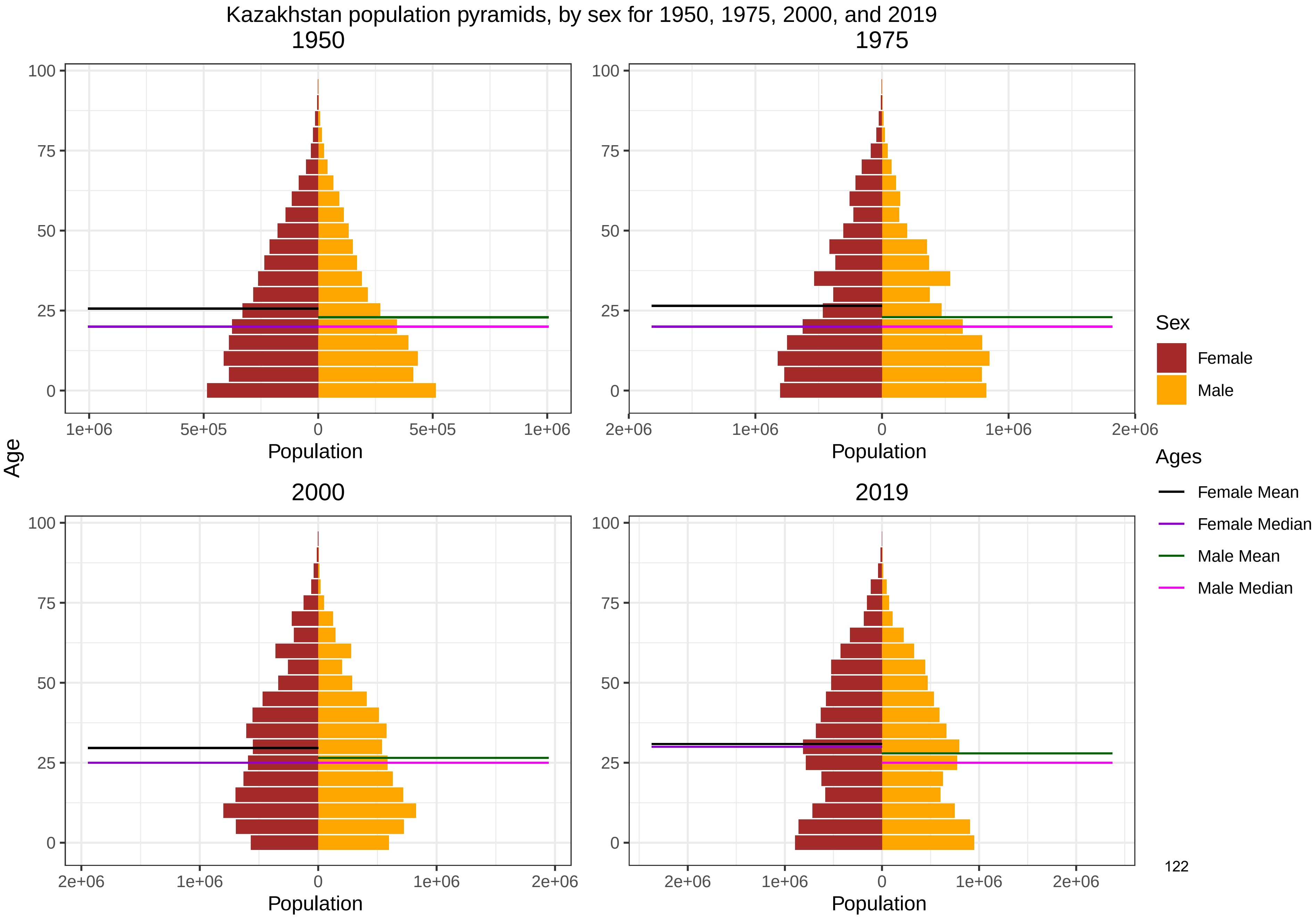
Kazakhstan population pyramids, by sex for 1950, 1975, 2000, and 2019
1950
1975
2000
2019
Age
Population
Sex
Female
Male
Ages
Female Mean
Female Median
Male Mean
Male Median

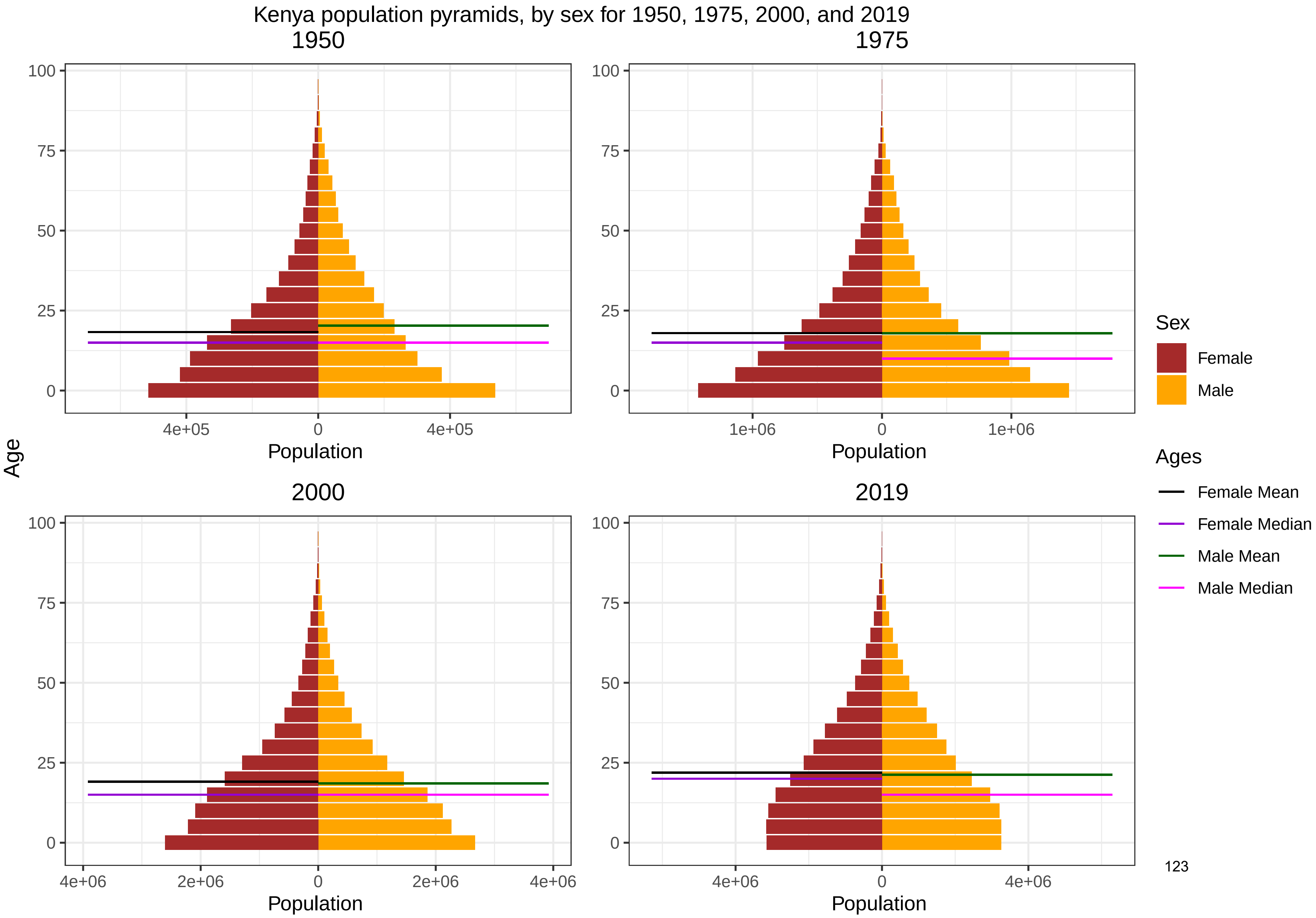

Kenya population pyramids, by sex for 1950, 1975, 2000, and 2019
1950
1975
2000
2019
Age
Population
100
75
50
25
0
4e+05
0
4e+05
1e+06
0
1e+06
4e+06
2e+06
0
2e+06
4e+06
4e+06
0
4e+06
Sex
Female
Male
Ages
Female Mean
Female Median
Male Mean
Male Median

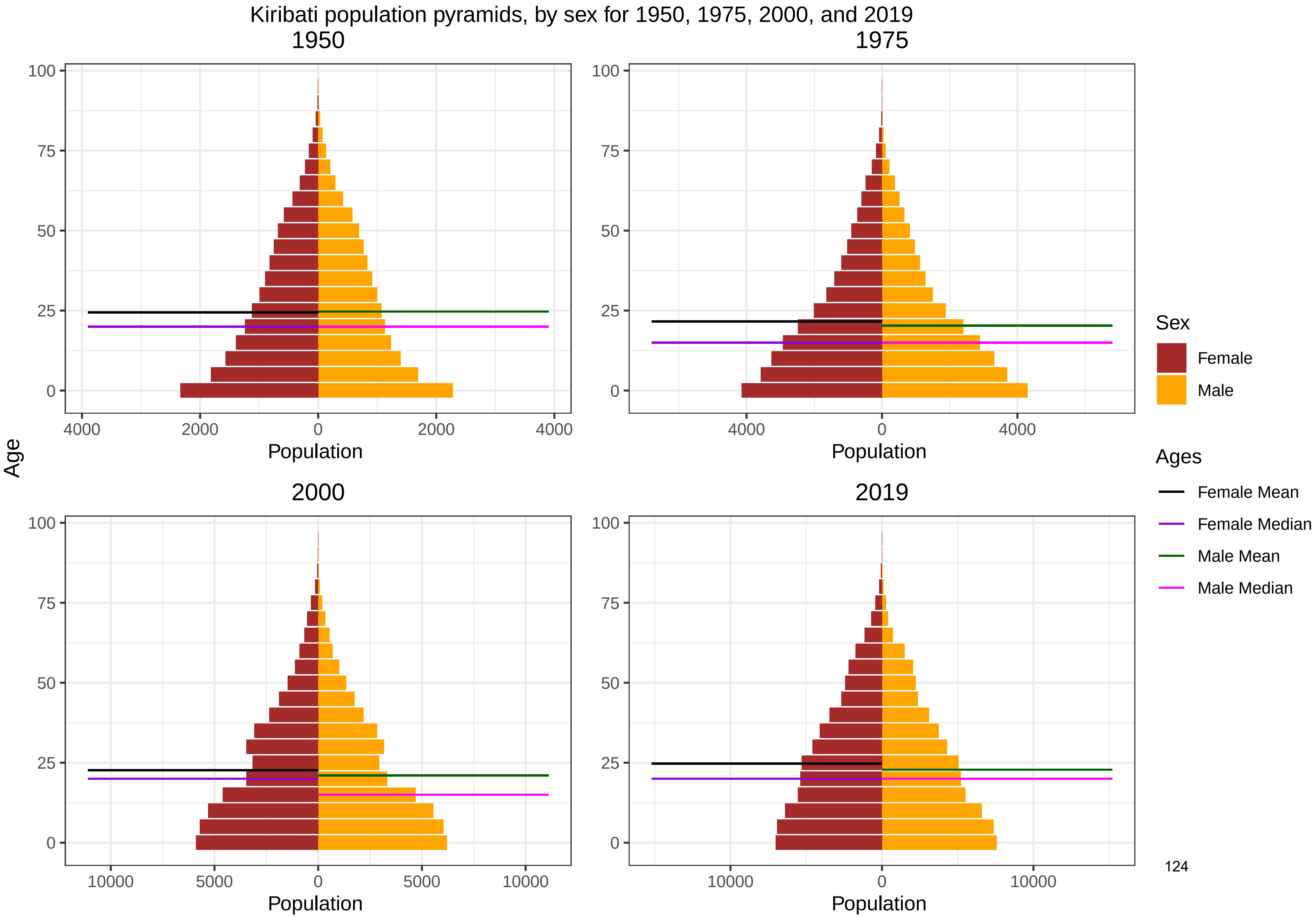
Kiribati population pyramids, by sex for 1950, 1975, 2000, and 2019
1950
1975
2000
2019
Age
Population
Sex
Female
Male
Ages
Female Mean
Female Median
Male Mean
Male Median

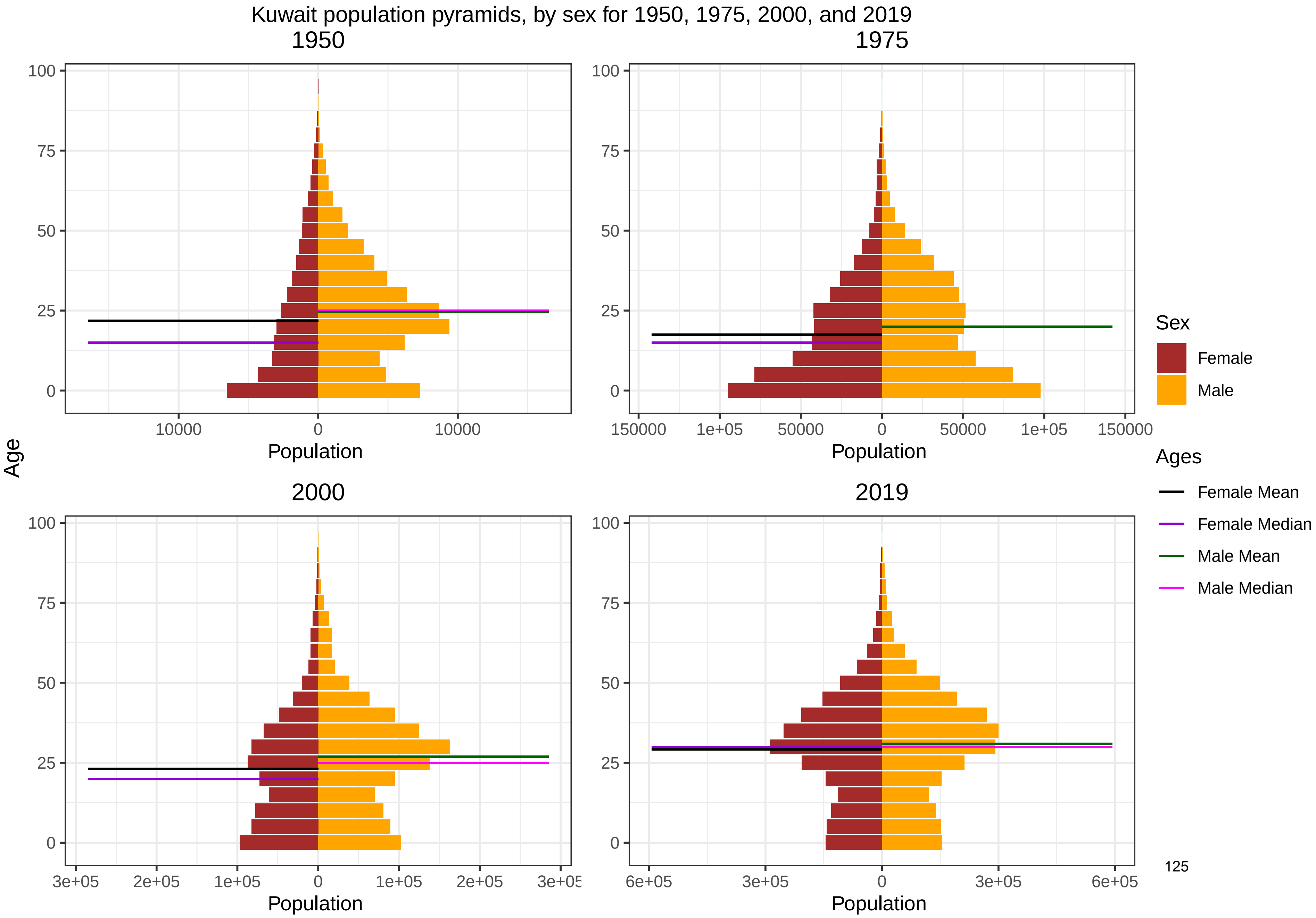
Kuwait population pyramids, by sex for 1950, 1975, 2000, and 2019
1950
1975
2000
2019
Age
Population
Sex
Female
Male
Ages
Female Mean
Female Median
Male Mean
Male Median

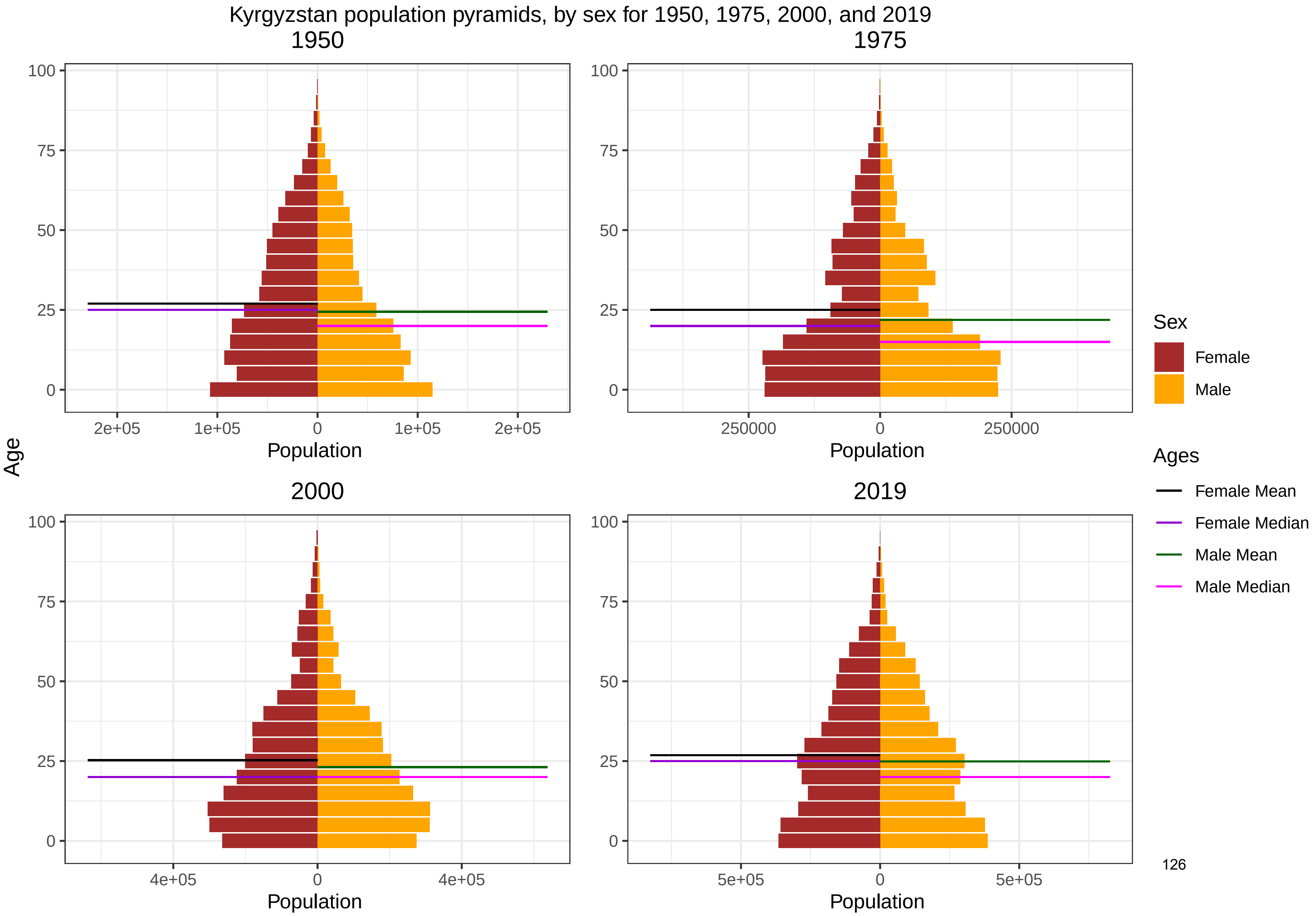
Kyrgyzstan population pyramids, by sex for 1950, 1975, 2000, and 2019
1950
1975
2000
2019
Age
Population
100
75
50
25
0
2e+05
1e+05
0
1e+05
2e+05
250000
0
250000
4e+05
0
4e+05
5e+05
0
5e+05
Sex
Female
Male
Ages
Female Mean
Female Median
Male Mean
Male Median

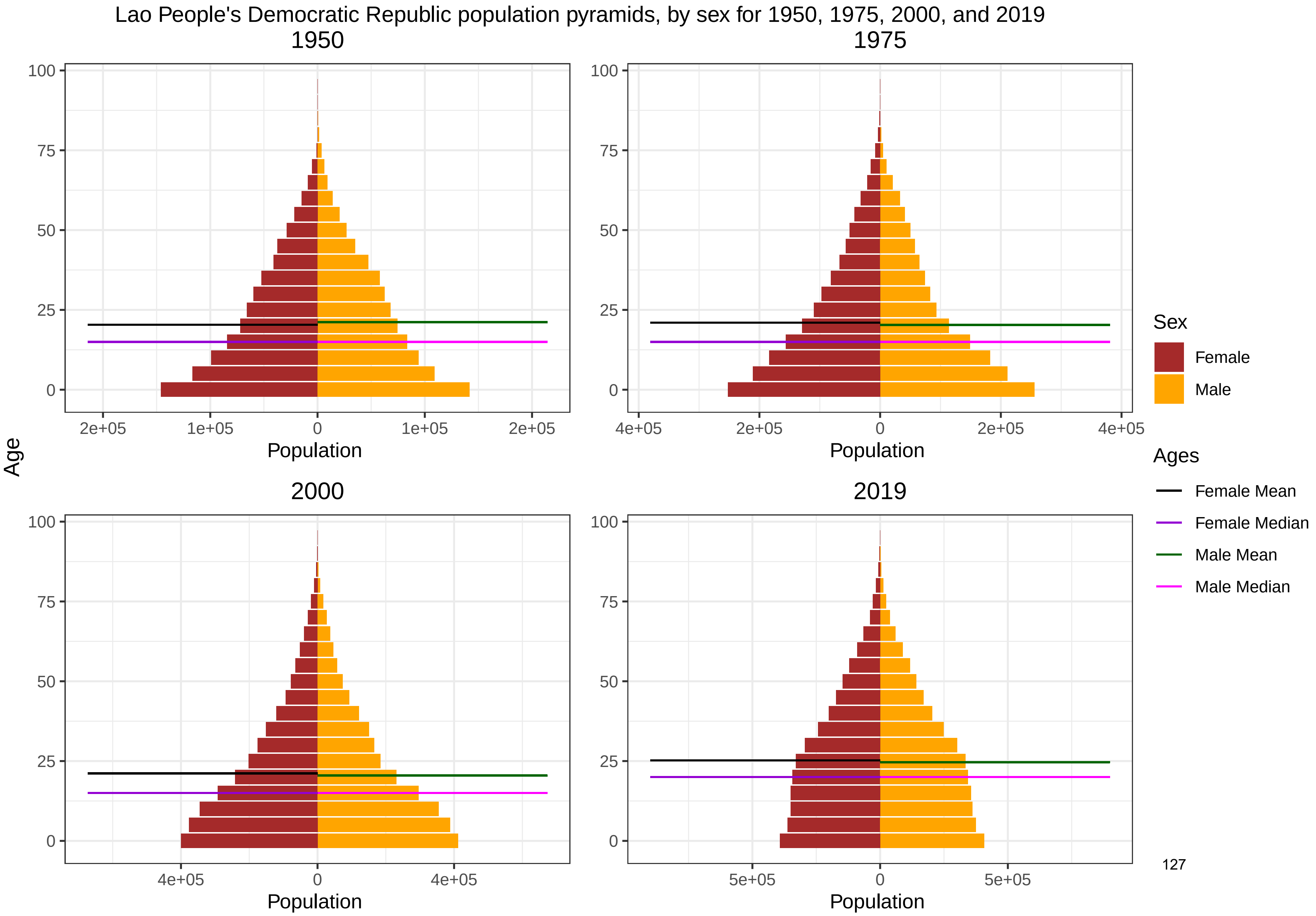

Lao People's Democratic Republic population pyramids, by sex for 1950, 1975, 2000, and 2019
1950
1975
2000
2019
Age
Population
100
75
50
25
0
2e+05
1e+05
0
1e+05
2e+05
4e+05
2e+05
0
2e+05
4e+05
4e+05
0
4e+05
5e+05
0
5e+05
Sex
Female
Male
Ages
Female Mean
Female Median
Male Mean
Male Median

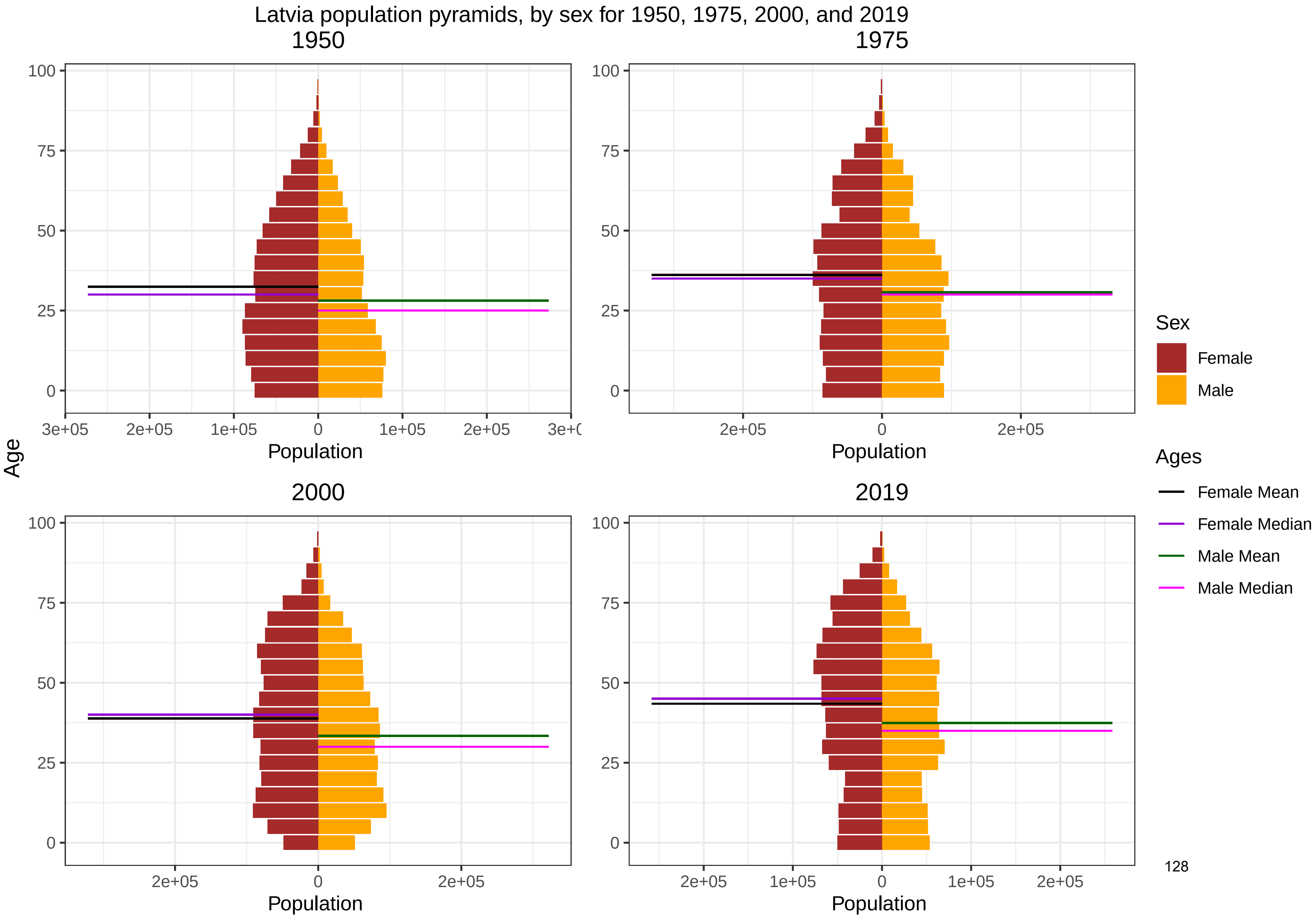

Latvia population pyramids, by sex for 1950, 1975, 2000, and 2019
1950
1975
2000
2019
Age
Population
100
75
50
25
0
3e+05
2e+05
1e+05
0
1e+05
2e+05
Sex
Female
Male
Ages
Female Mean
Female Median
Male Mean
Male Median

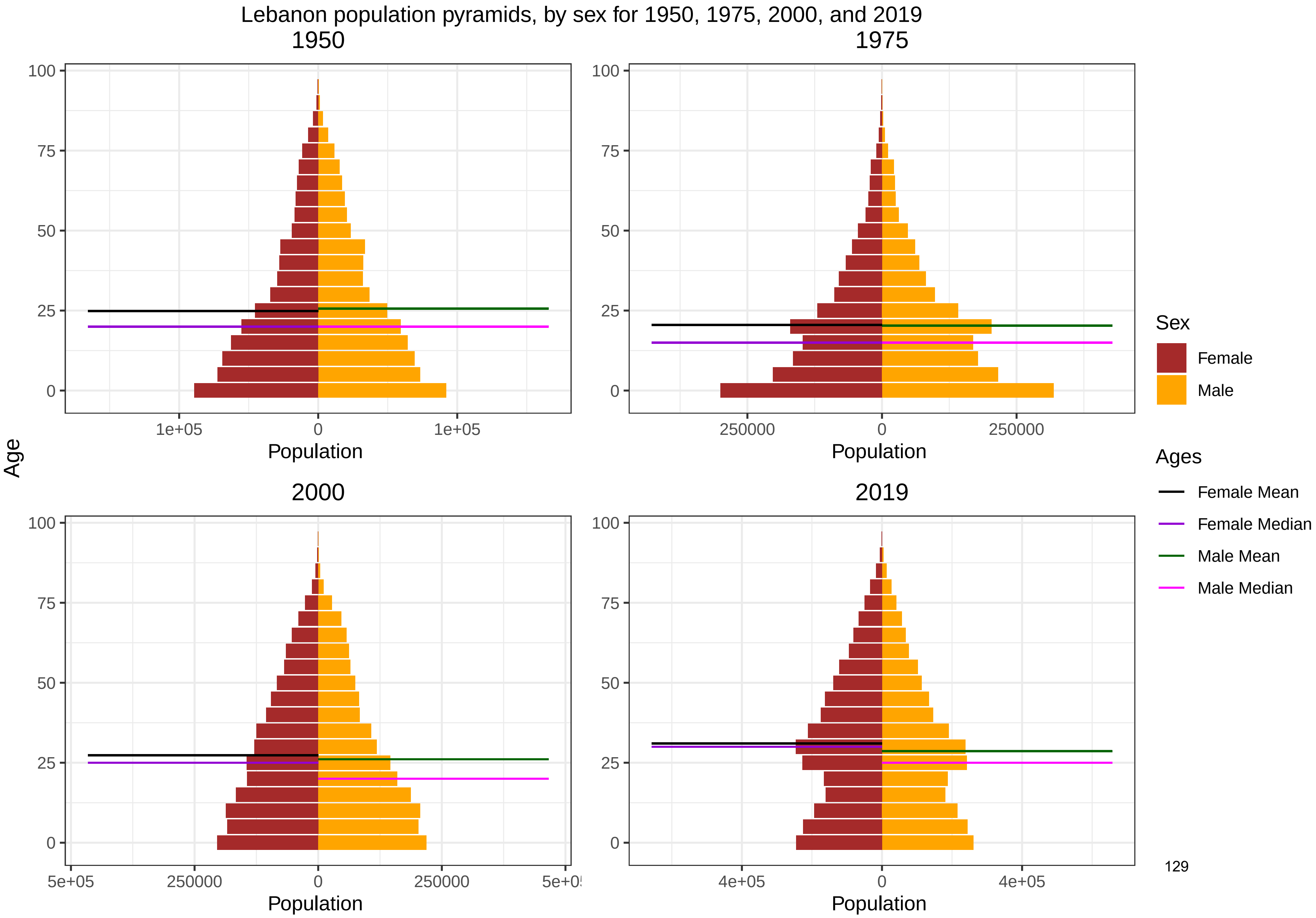
Lebanon population pyramids, by sex for 1950, 1975, 2000, and 2019
1950
1975
2000
2019
Age
Population
0
25
50
75
100
1e+05
250000
5e+05
4e+05
Sex
Female
Male
Ages
Female Mean
Female Median
Male Mean
Male Median

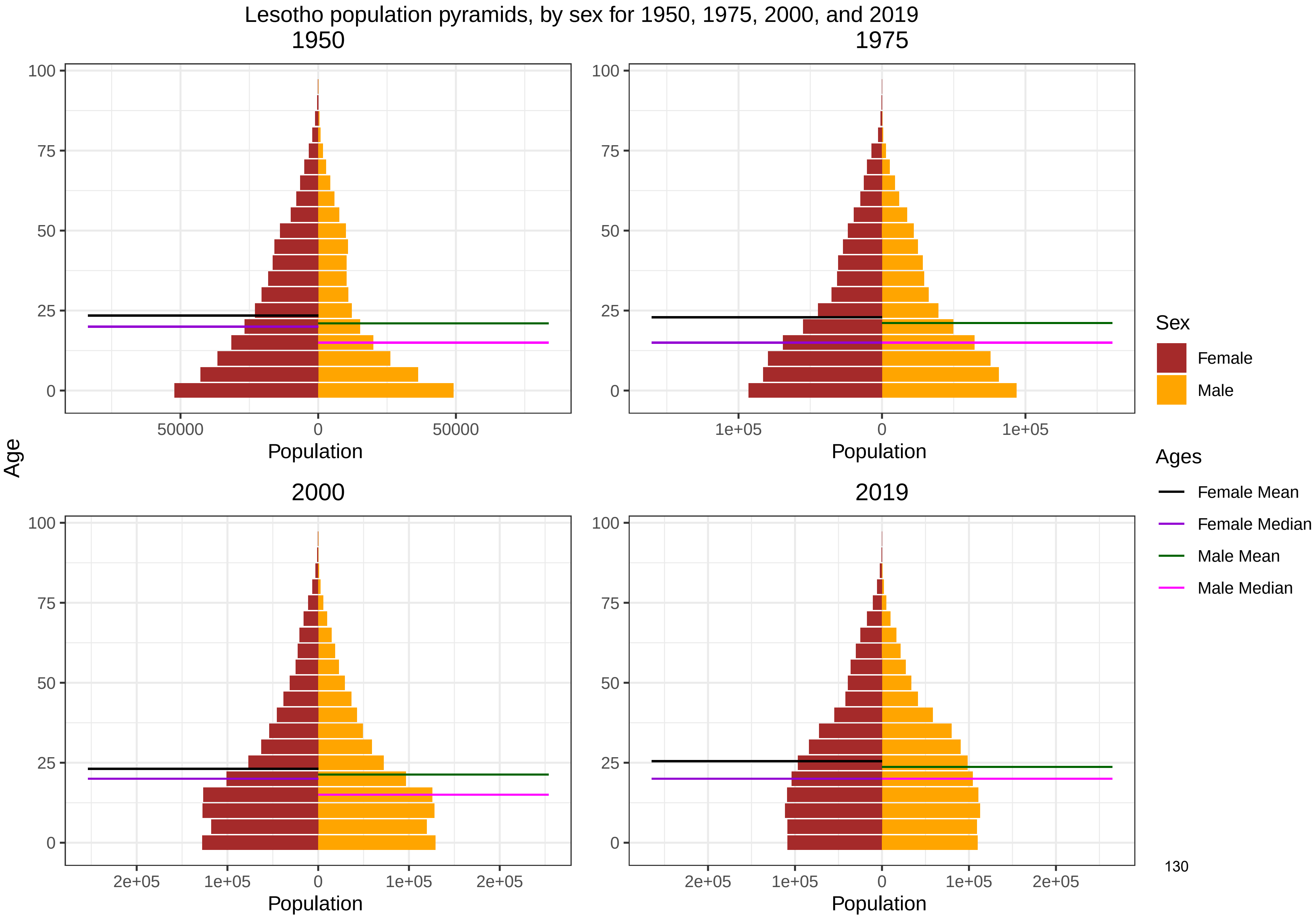
Lesotho population pyramids, by sex for 1950, 1975, 2000, and 2019
1950
1975
2000
2019
Age
Population
Sex
Female
Male
Ages
Female Mean
Female Median
Male Mean
Male Median

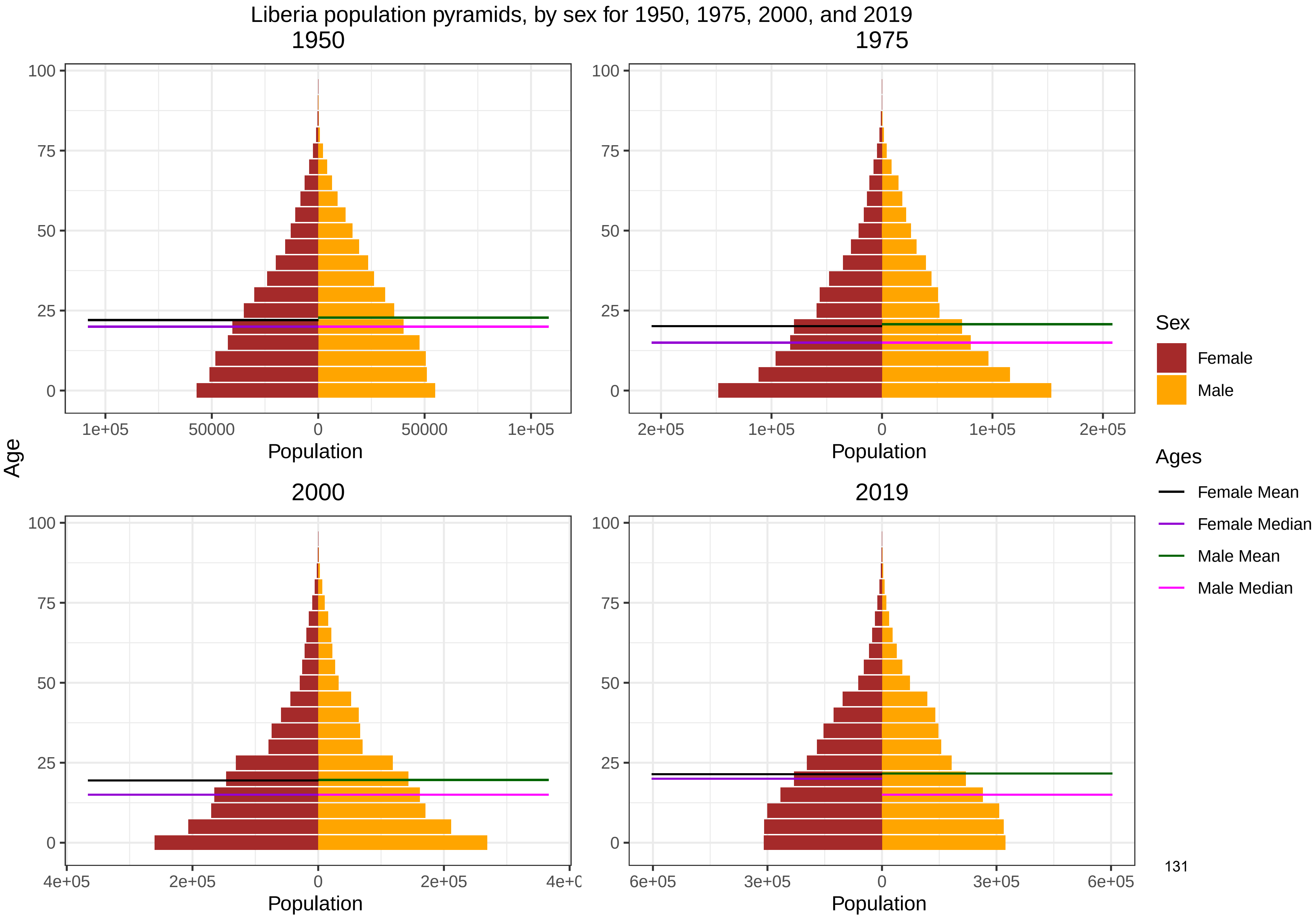
Liberia population pyramids, by sex for 1950, 1975, 2000, and 2019
1950
1975
2000
2019
Age
Population
Sex
Female
Male
Ages
Female Mean
Female Median
Male Mean
Male Median

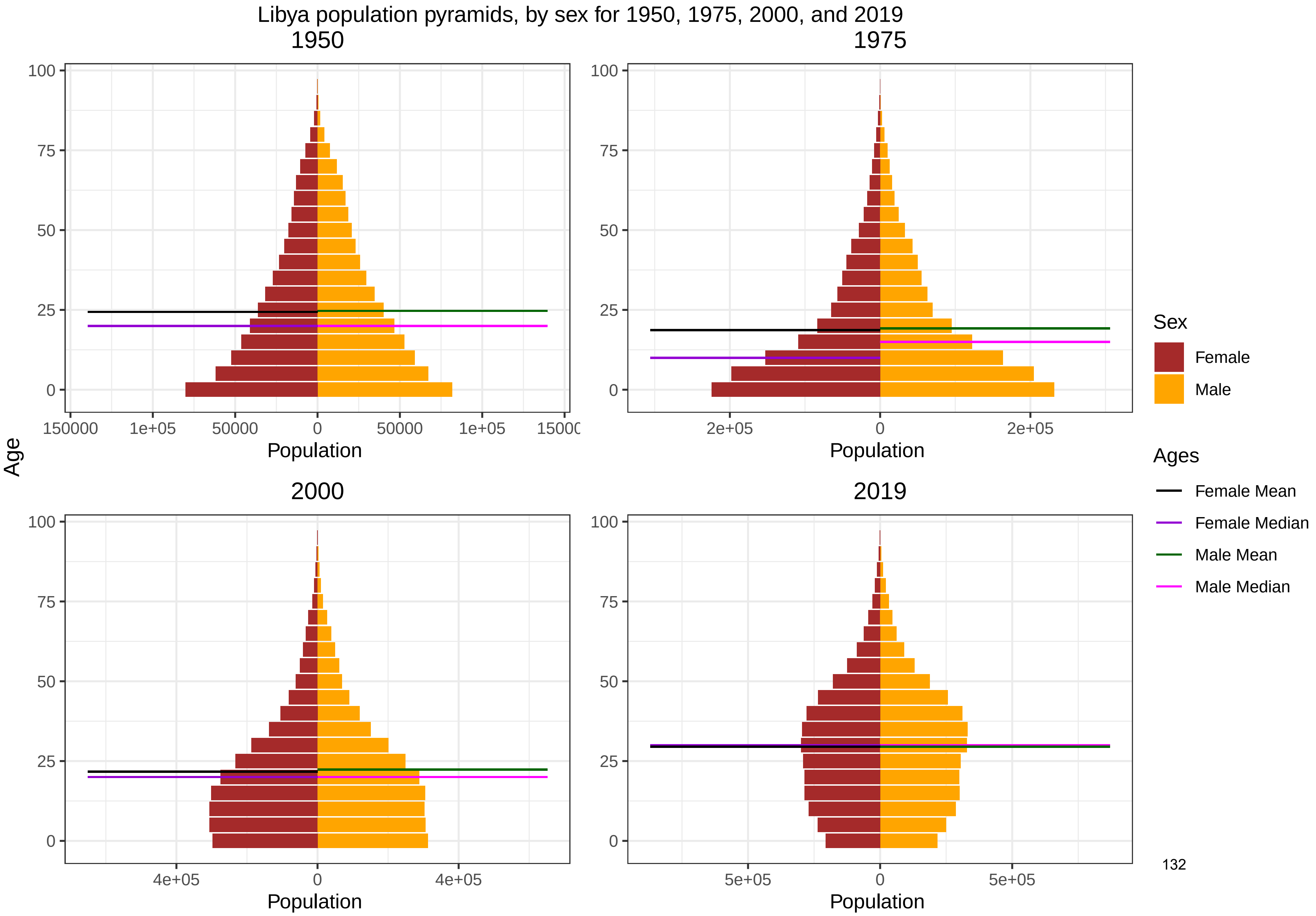
Libya population pyramids, by sex for 1950, 1975, 2000, and 2019
1950
1975
2000
2019
Age
Population
Sex
Female
Male
Ages
Female Mean
Female Median
Male Mean
Male Median

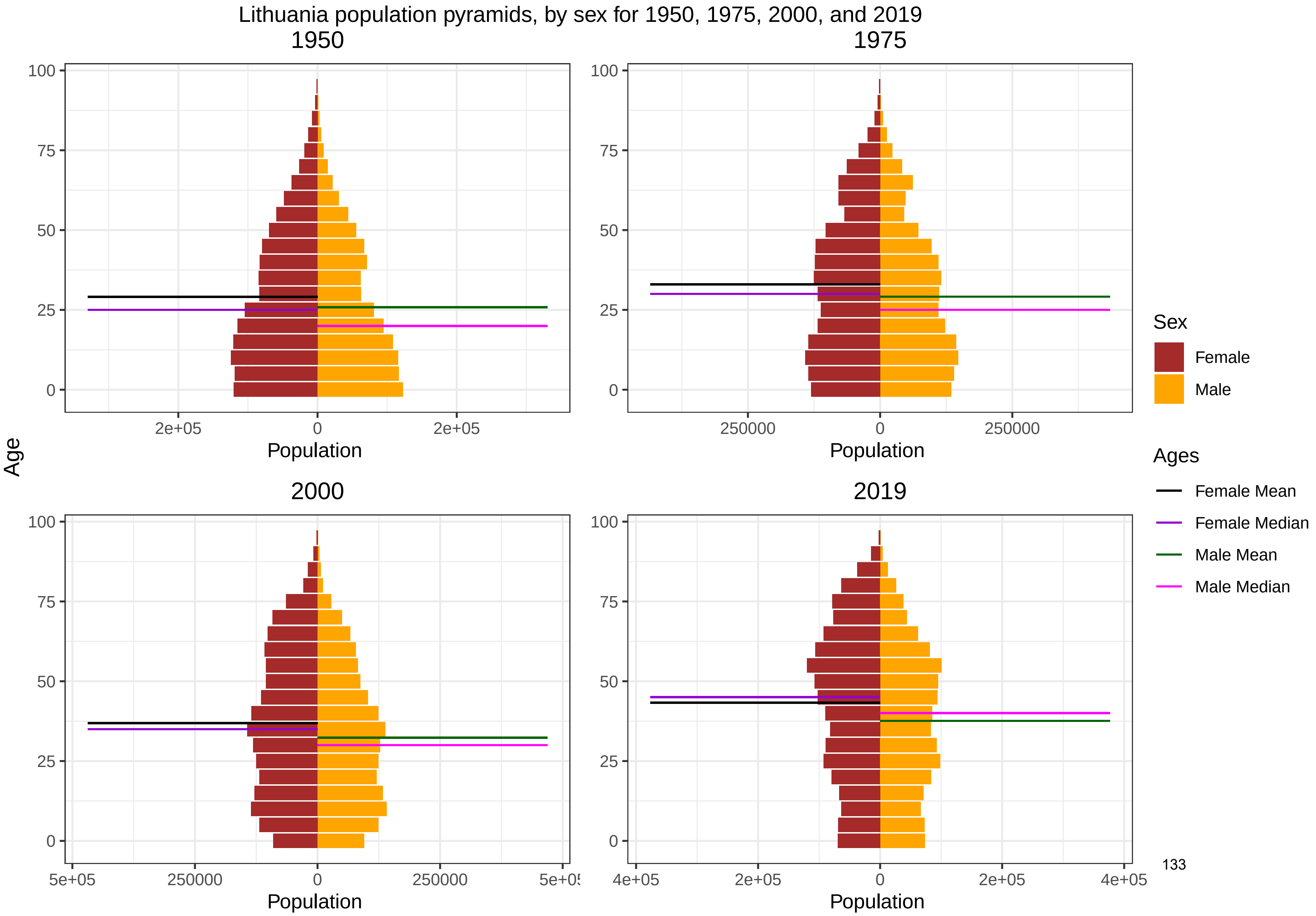

Lithuania population pyramids, by sex for 1950, 1975, 2000, and 2019
1950
1975
2000
2019
Age
Population
2e+05
0
250000
5e+05
4e+05
100
75
50
25
Sex
Female
Male
Ages
Female Mean
Female Median
Male Mean
Male Median

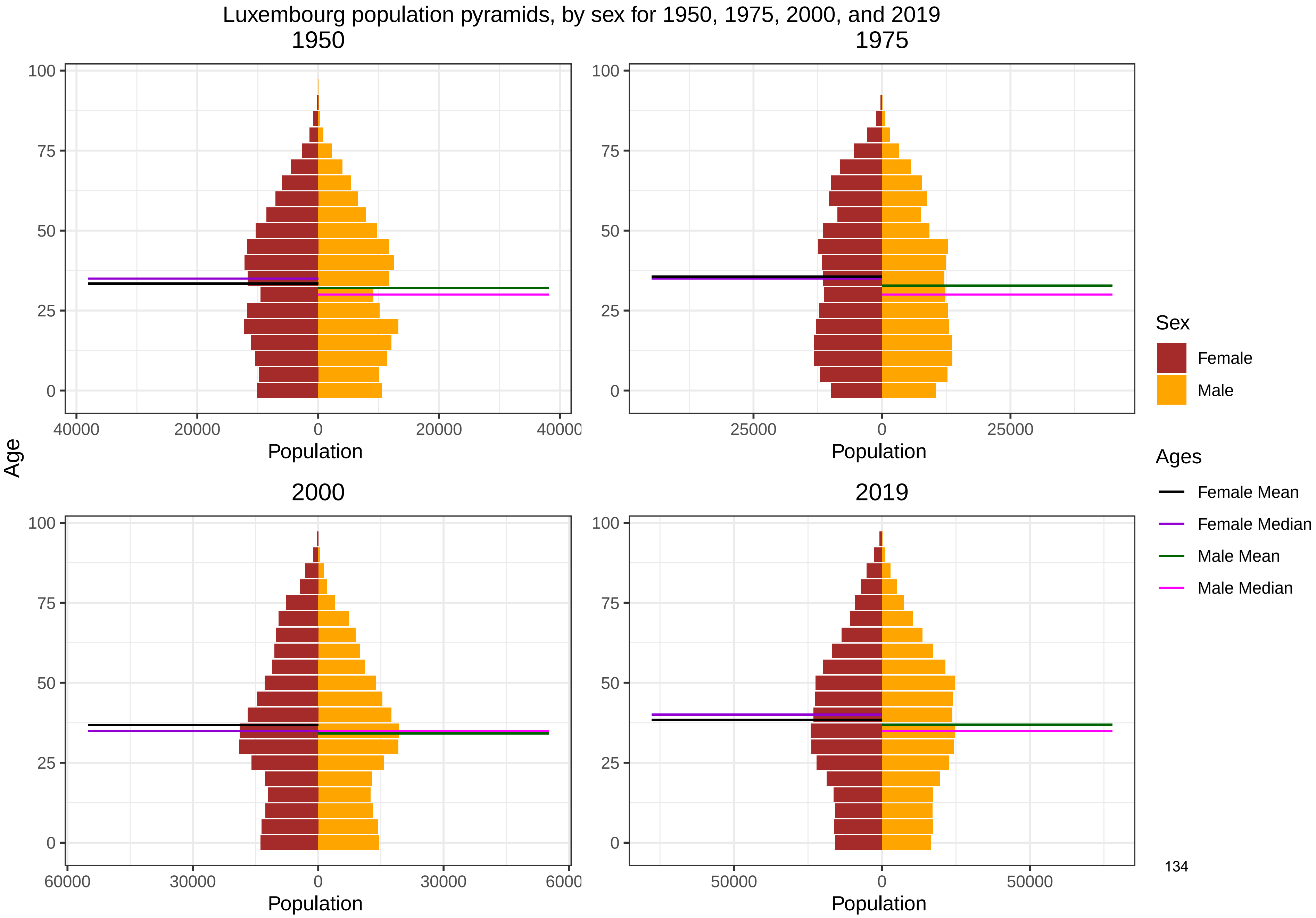
Luxembourg population pyramids, by sex for 1950, 1975, 2000, and 2019
1950
1975
2000
2019
Age
Population
Sex
Female
Male
Ages
Female Mean
Female Median
Male Mean
Male Median

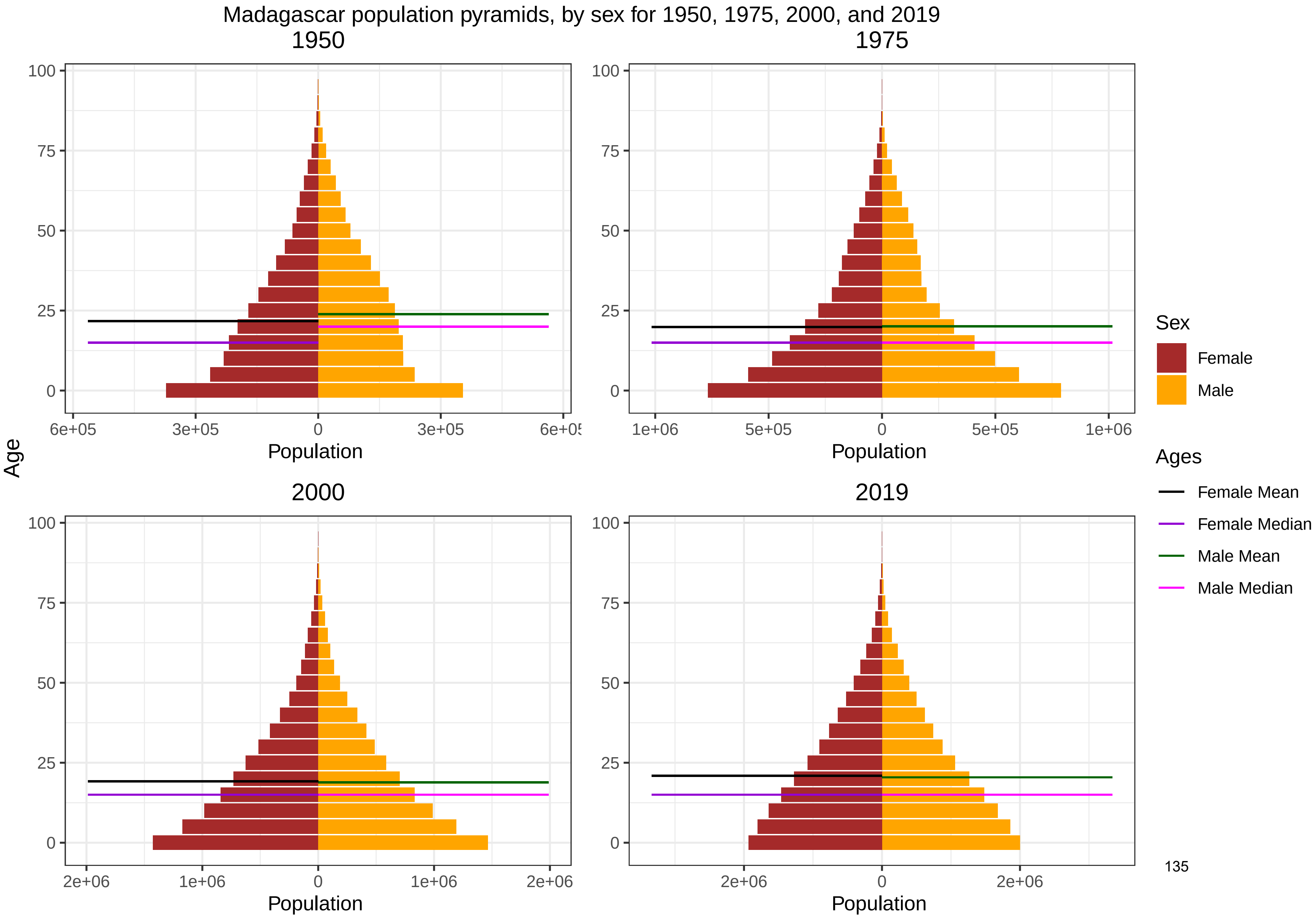
Madagascar population pyramids, by sex for 1950, 1975, 2000, and 2019
1950
1975
2000
2019
Age
Population
100
75
50
25
0
6e+05
3e+05
1e+06
5e+05
2e+06
1e+06
Sex
Female
Male
Ages
Female Mean
Female Median
Male Mean
Male Median

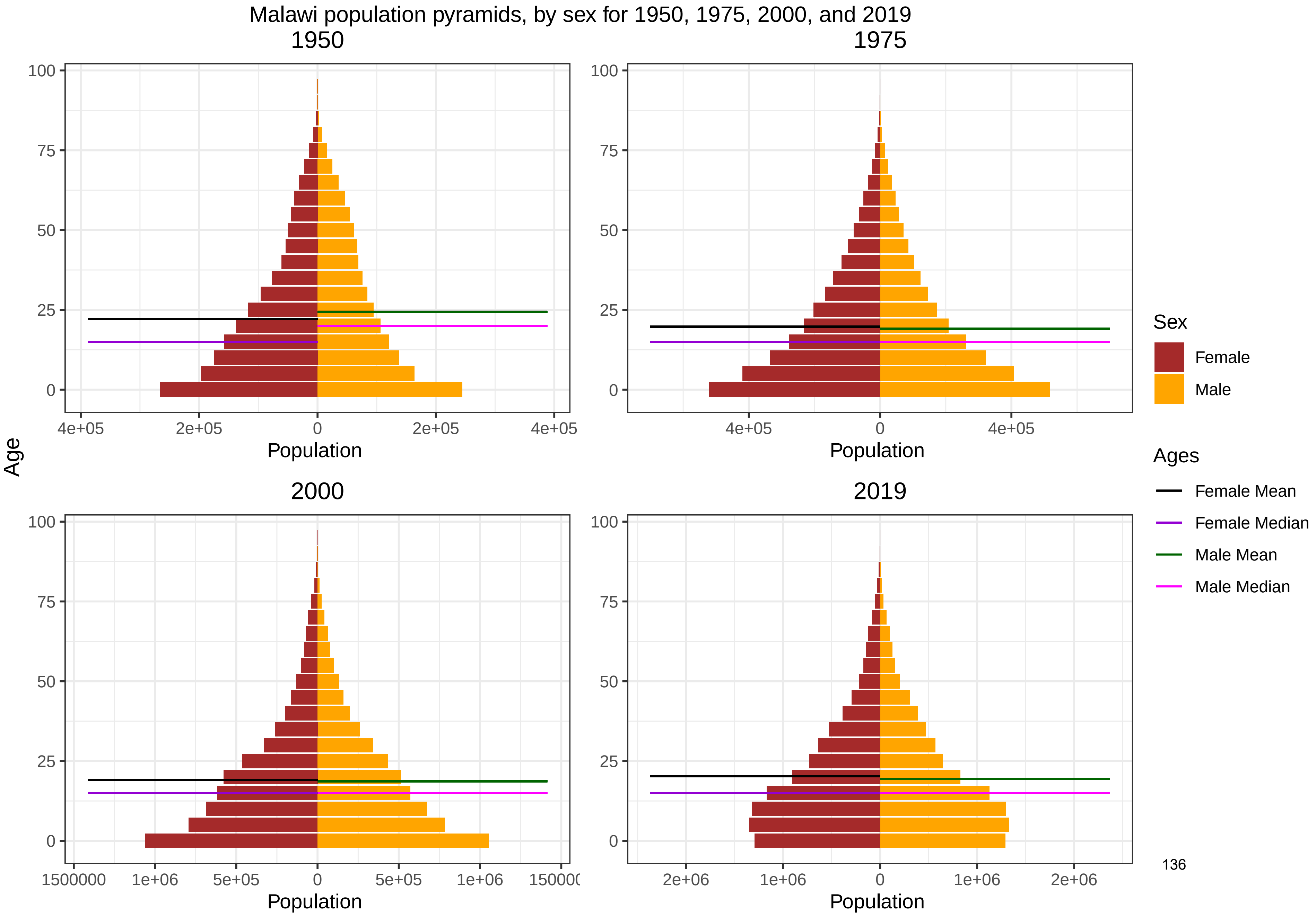

Malawi population pyramids, by sex for 1950, 1975, 2000, and 2019
1950
1975
2000
2019
Age
Population
Sex
Female
Male
Ages
Female Mean
Female Median
Male Mean
Male Median

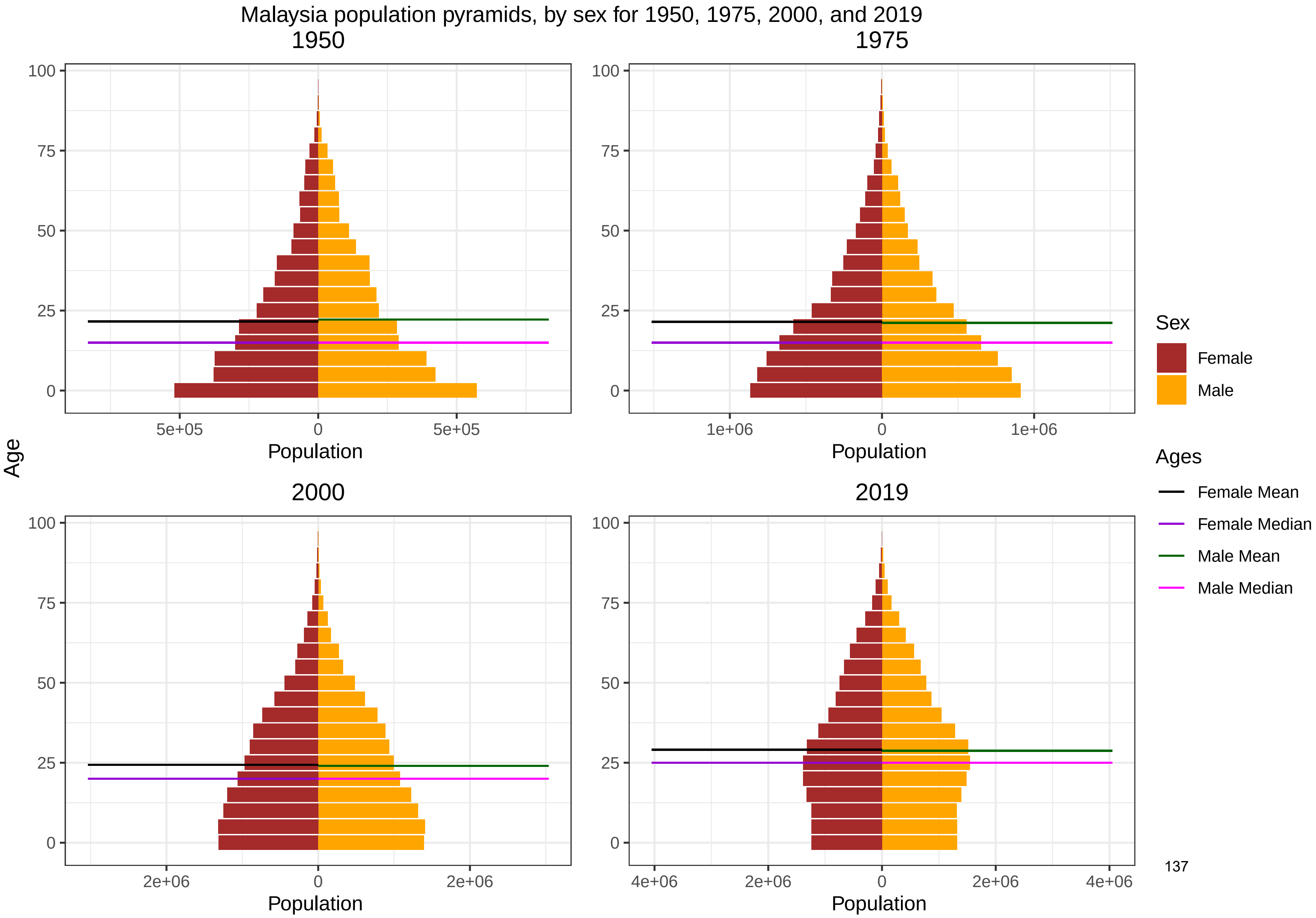
Malaysia population pyramids, by sex for 1950, 1975, 2000, and 2019
1950
1975
2000
2019
Age
Population
5e+05
0
1e+06
2e+06
4e+06
100
75
50
25
Sex
Female
Male
Ages
Female Mean
Female Median
Male Mean
Male Median

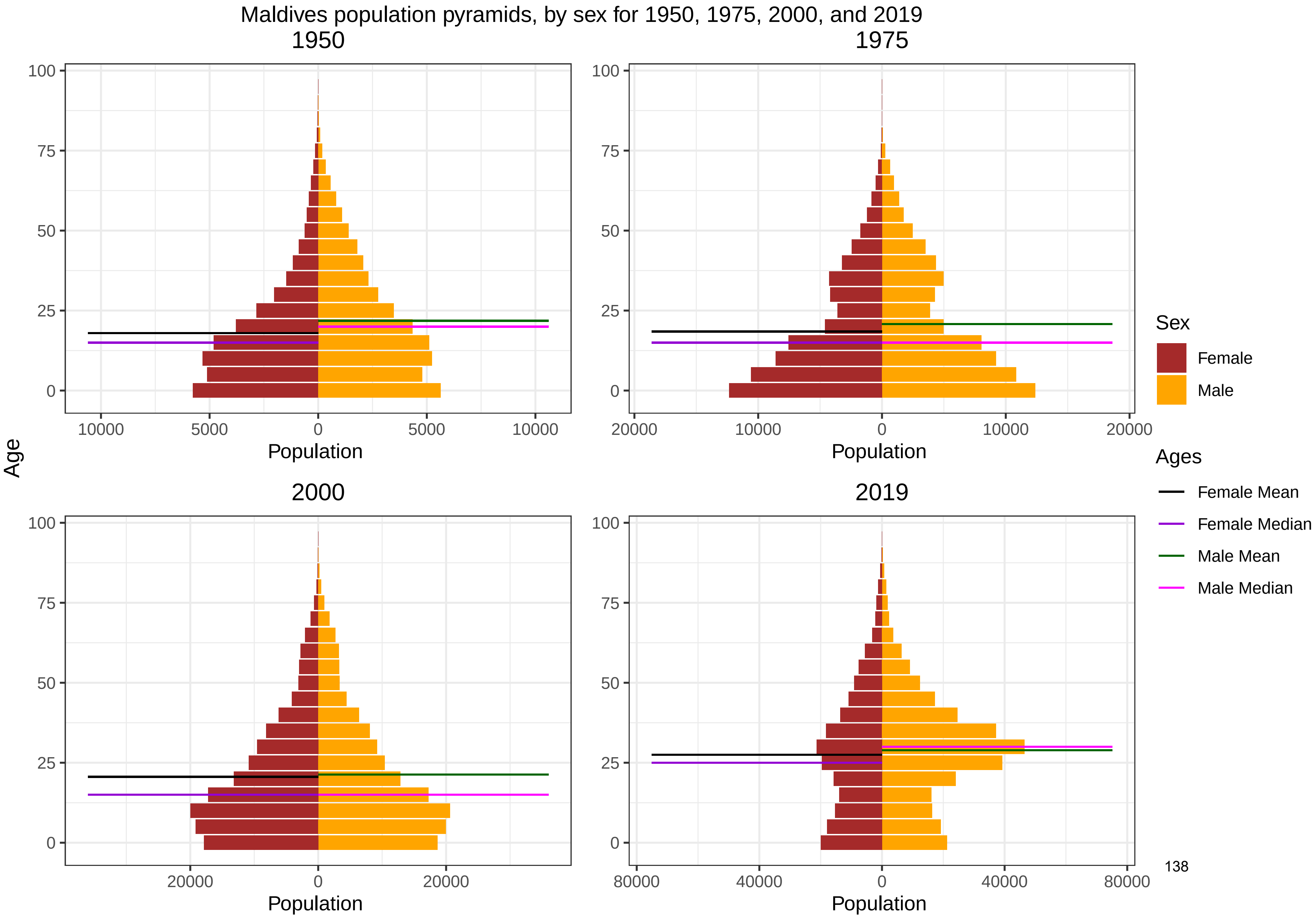

Maldives population pyramids, by sex for 1950, 1975, 2000, and 2019
1950
1975
2000
2019
Age
Population
Sex
Female
Male
Ages
Female Mean
Female Median
Male Mean
Male Median

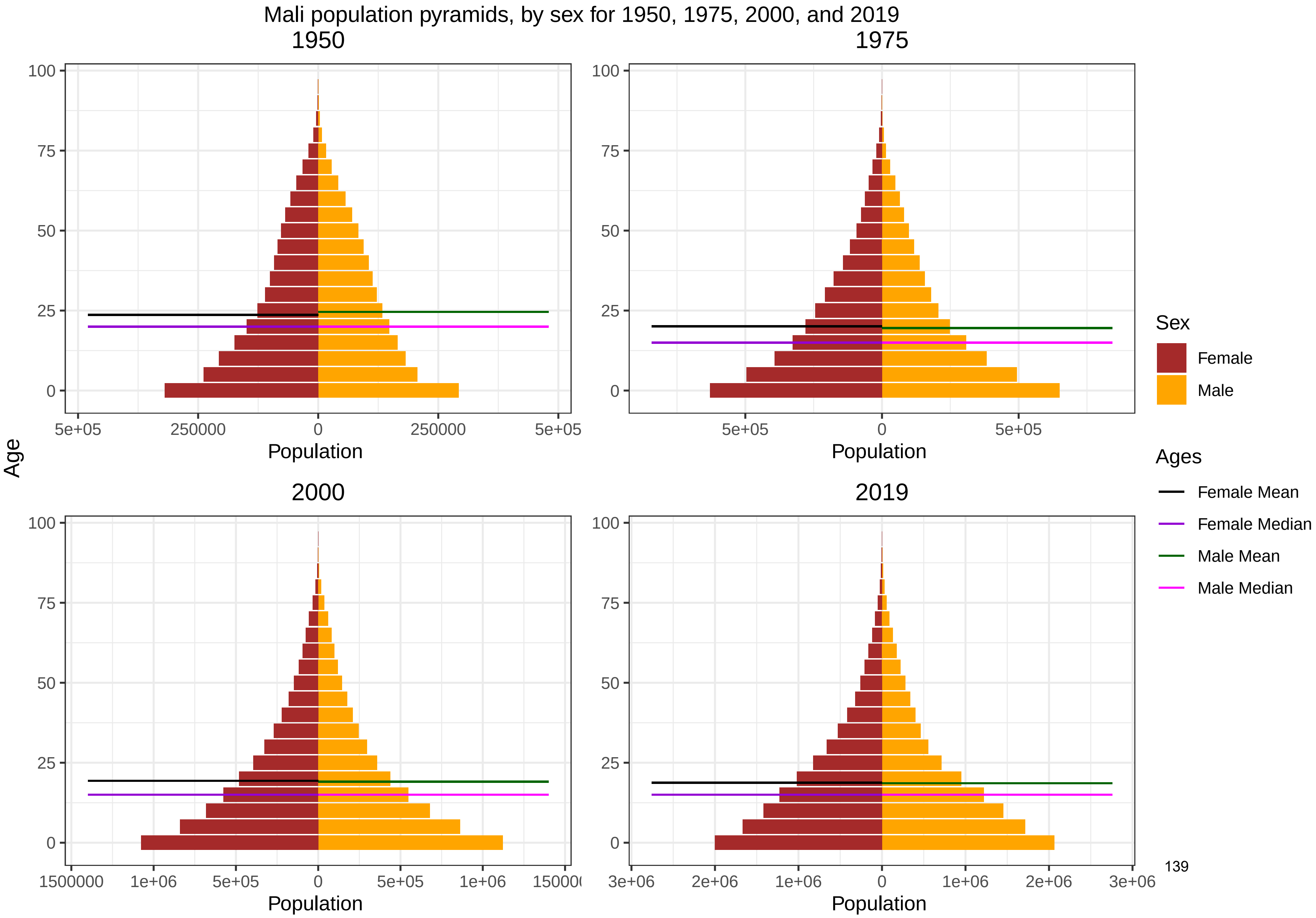

Mali population pyramids, by sex for 1950, 1975, 2000, and 2019
1950
1975
2000
2019
Age
Population
100
75
50
25
0
5e+05
250000
0
250000
5e+05
1500000
1e+06
5e+05
1e+06
150000
3e+06
2e+06
Sex
Female
Male
Ages
Female Mean
Female Median
Male Mean
Male Median

Malta population pyramids, by sex for 1950, 1975, 2000, and 2019

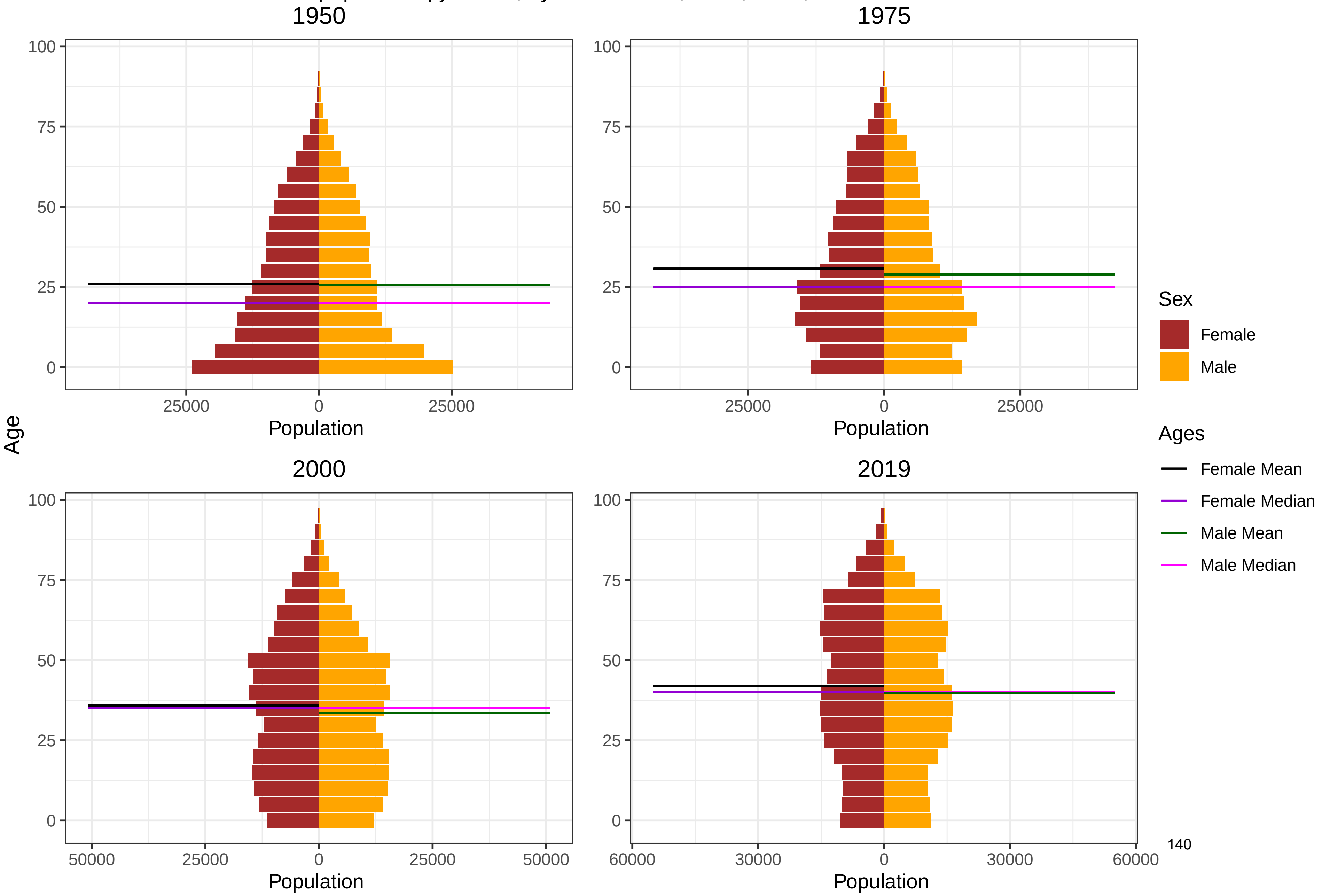

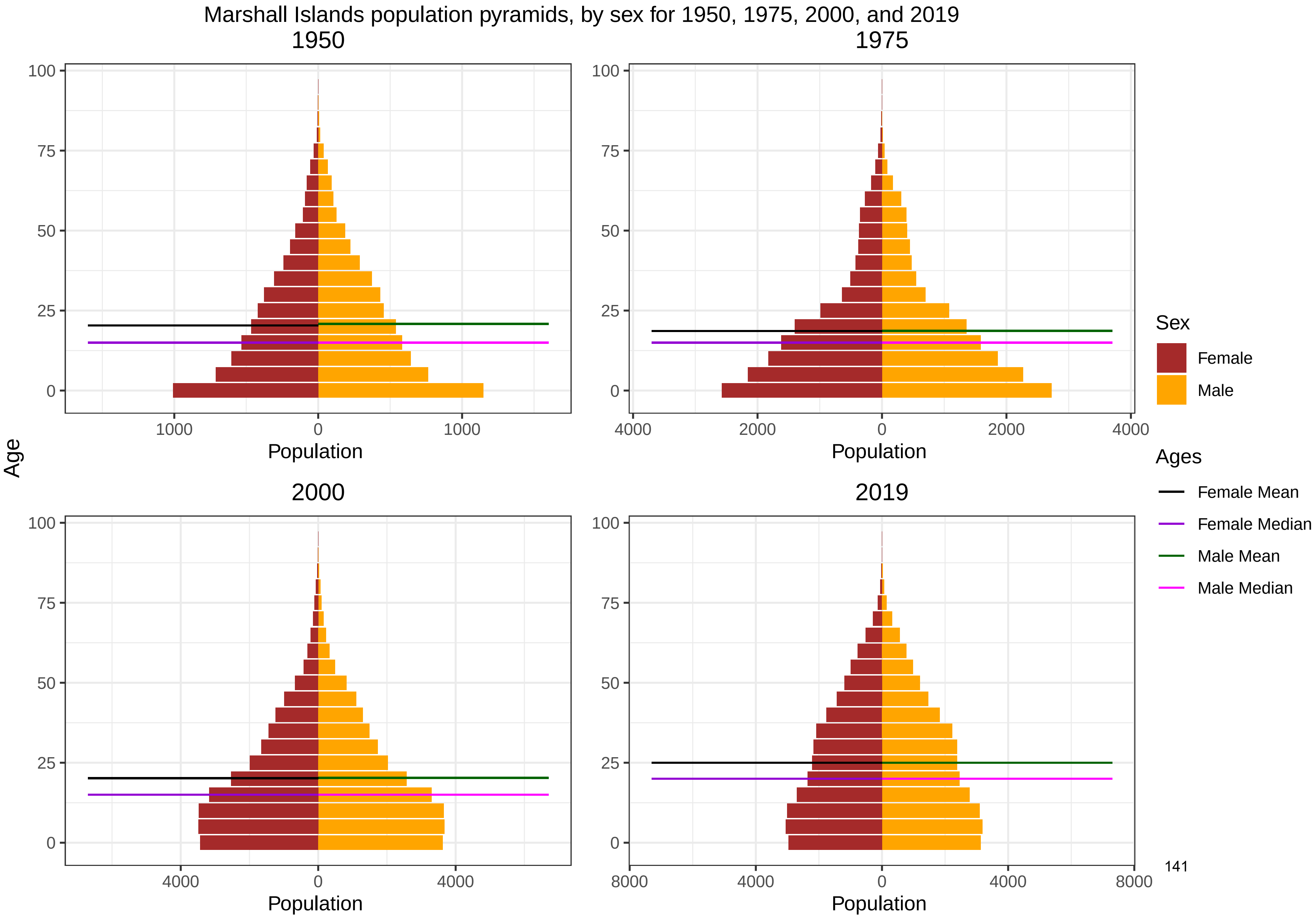
Marshall Islands population pyramids, by sex for 1950, 1975, 2000, and 2019
1950
1975
2000
2019
Age
Population
100
75
50
25
0
1000
0
1000
4000
2000
0
2000
4000
4000
0
4000
8000
4000
0
4000
8000
Sex
Female
Male
Ages
Female Mean
Female Median
Male Mean
Male Median

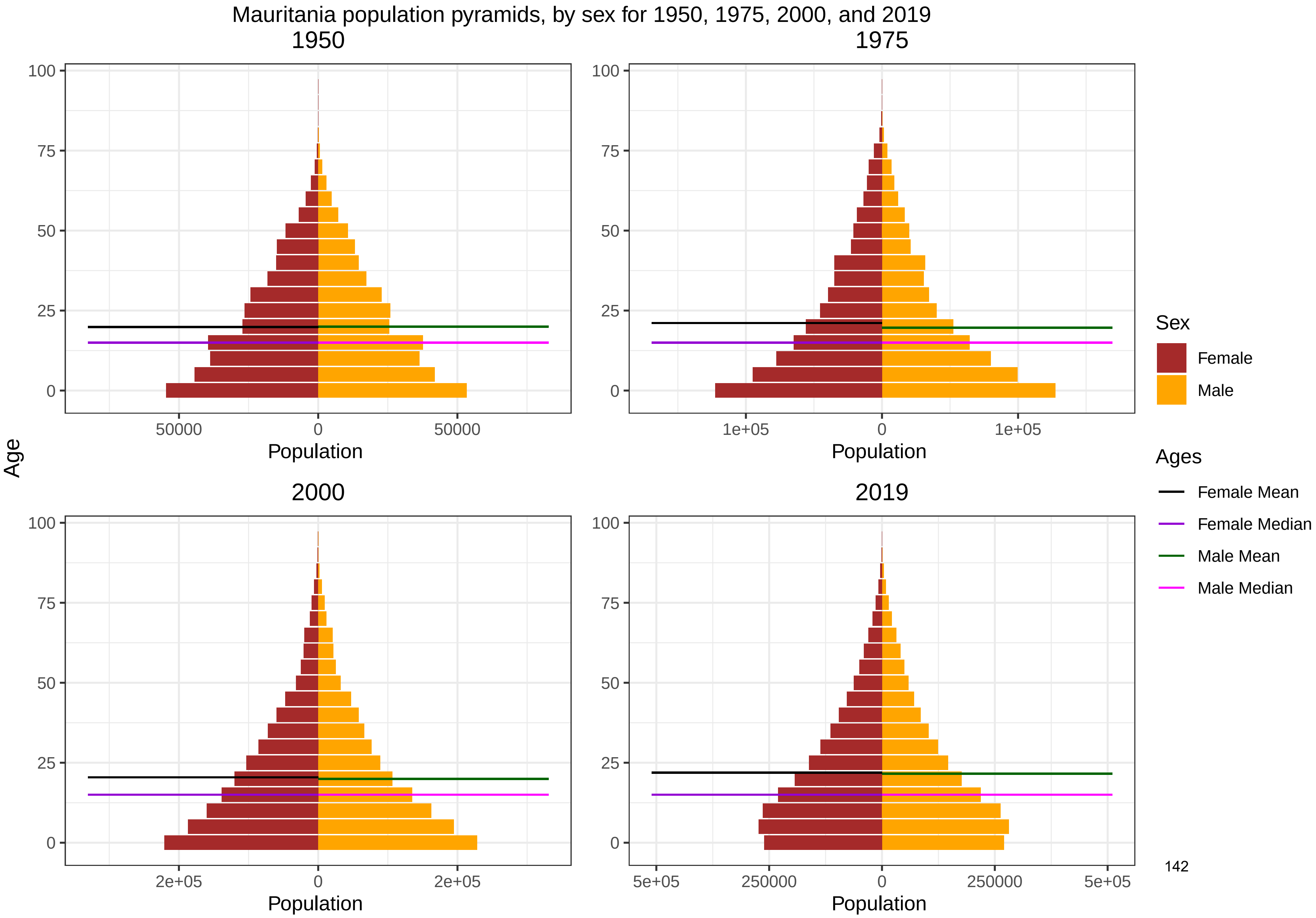

Mauritania population pyramids, by sex for 1950, 1975, 2000, and 2019
1950
1975
2000
2019
Age
Population
100
75
50
25
0
50000
0
50000
1e+05
0
1e+05
2e+05
0
2e+05
5e+05
250000
0
250000
5e+05
Sex
Female
Male
Ages
Female Mean
Female Median
Male Mean
Male Median

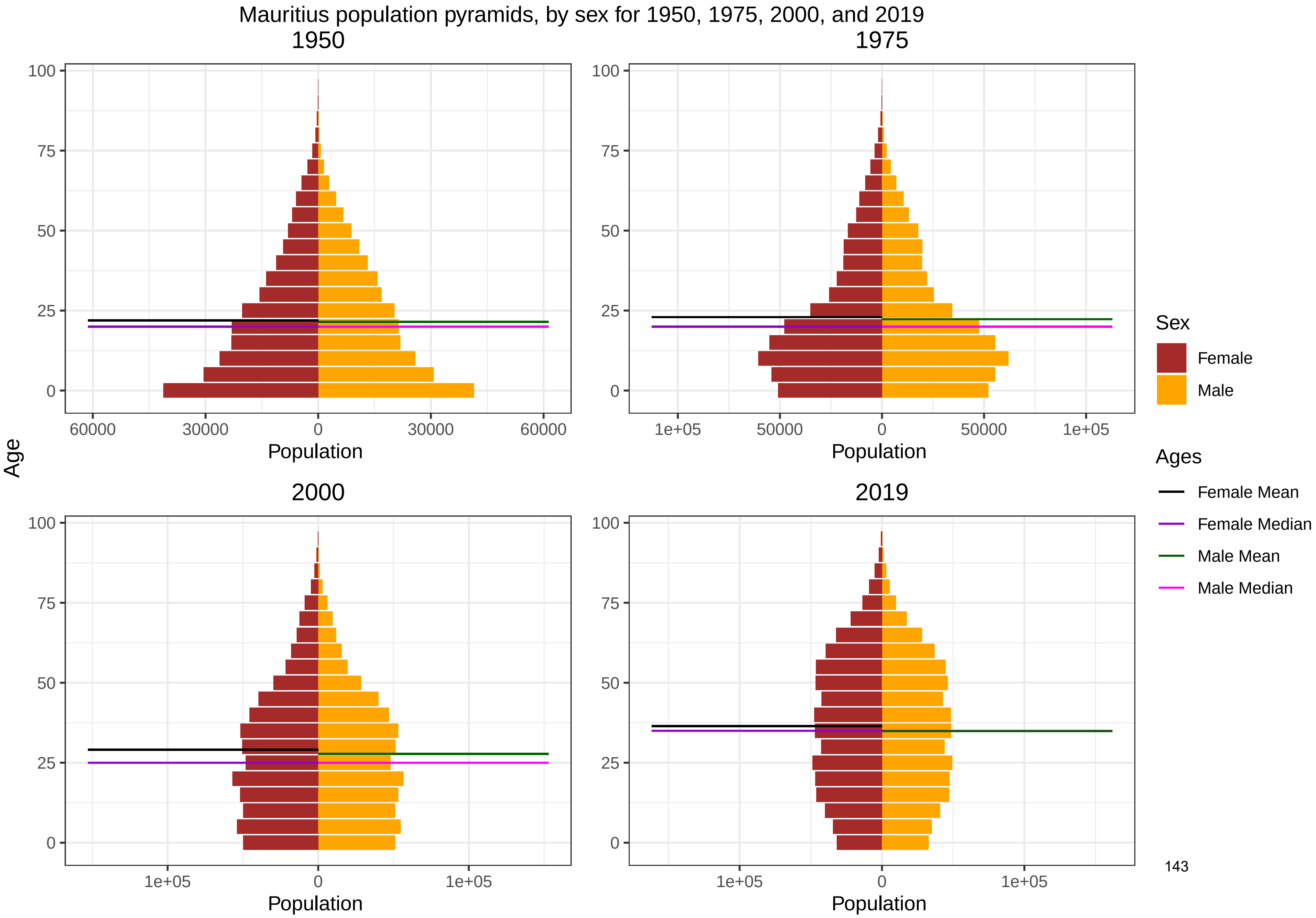

Mauritius population pyramids, by sex for 1950, 1975, 2000, and 2019
1950
1975
2000
2019
Age
Population
60000
30000
0
1e+05
50000
100
75
50
25
Sex
Female
Male
Ages
Female Mean
Female Median
Male Mean
Male Median

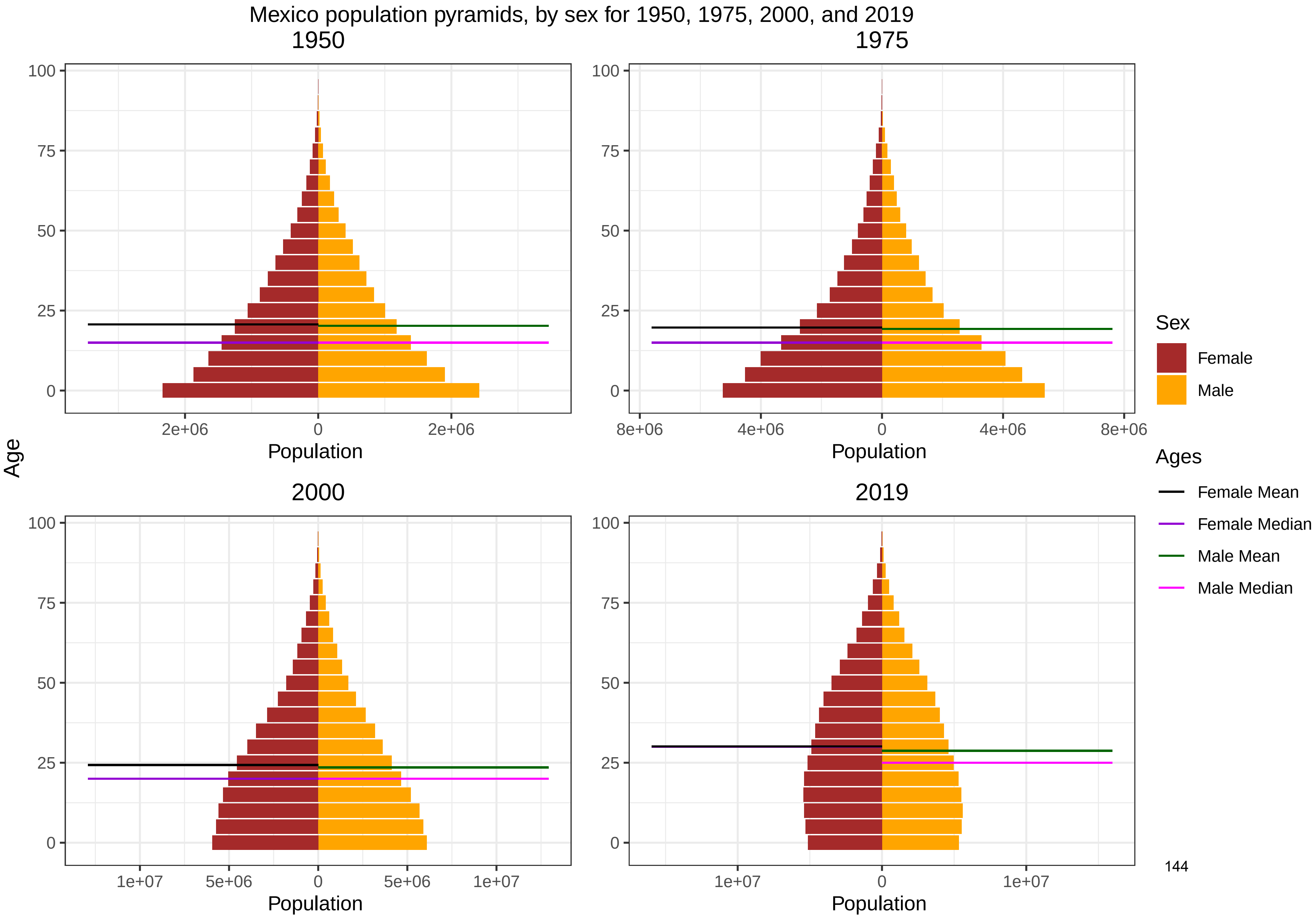

Mexico population pyramids, by sex for 1950, 1975, 2000, and 2019
1950
1975
2000
2019
Age
Population
2e+06
0
8e+06
4e+06
1e+07
5e+06
100
75
50
25
Sex
Female
Male
Ages
Female Mean
Female Median
Male Mean
Male Median

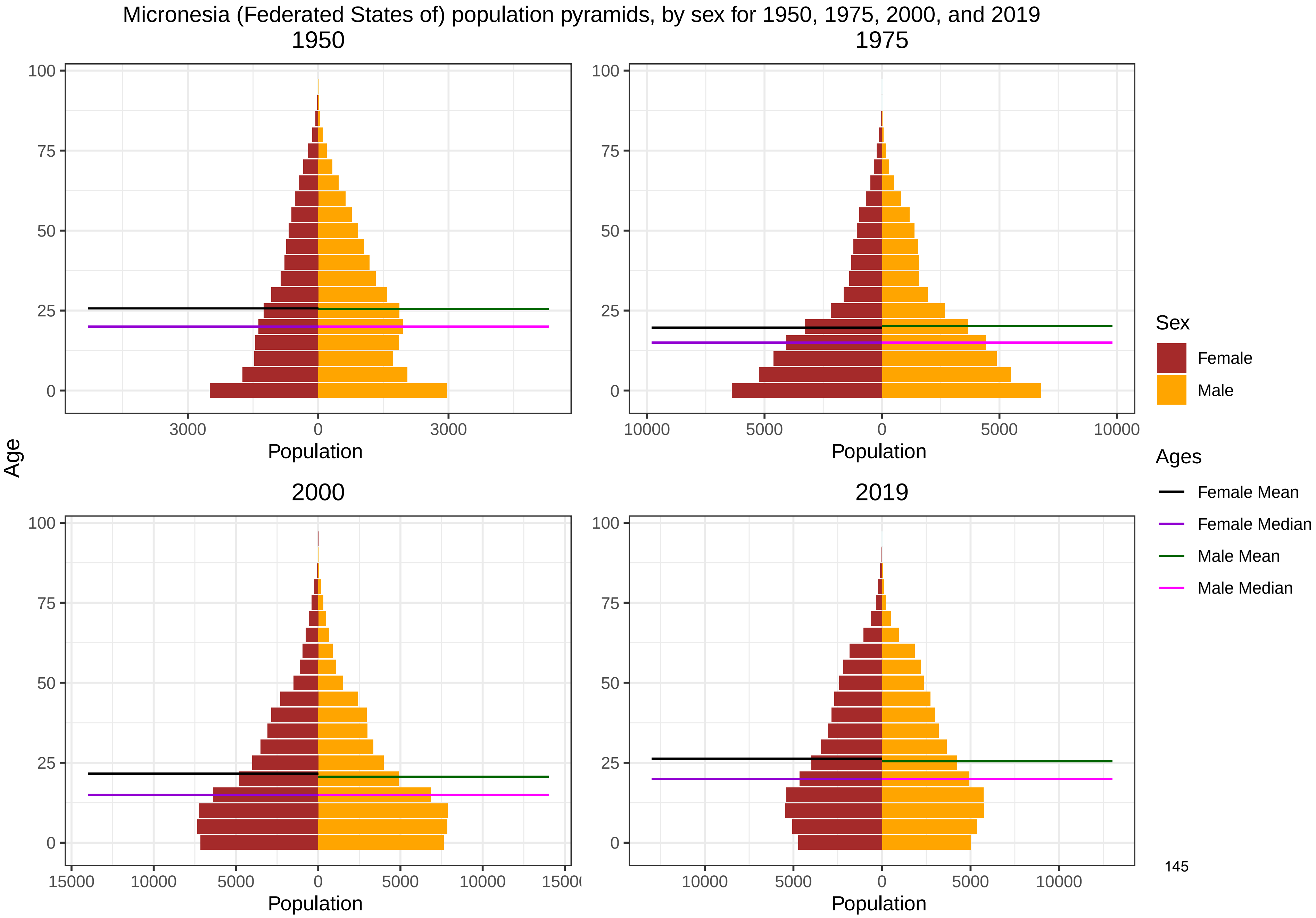
Micronesia (Federated States of) population pyramids, by sex for 1950, 1975, 2000, and 2019
1950
1975
2000
2019
Age
Population
0
25
50
75
100
3000
0
3000
10000
5000
0
5000
10000
15000
10000
5000
0
5000
10000
Sex
Female
Male
Ages
Female Mean
Female Median
Male Mean
Male Median

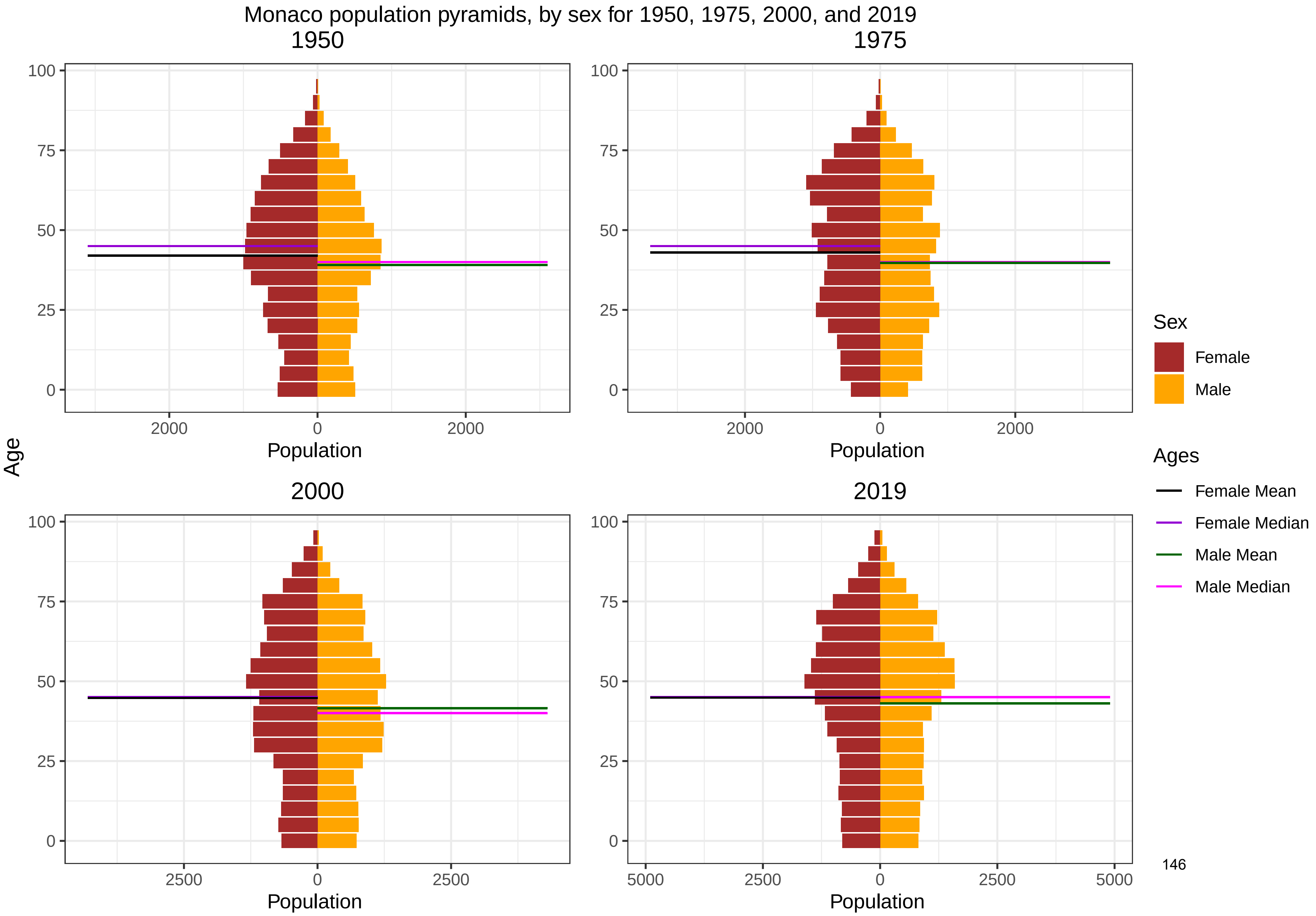

Monaco population pyramids, by sex for 1950, 1975, 2000, and 2019
1950
1975
2000
2019
Age
Population
Sex
Female
Male
Ages
Female Mean
Female Median
Male Mean
Male Median

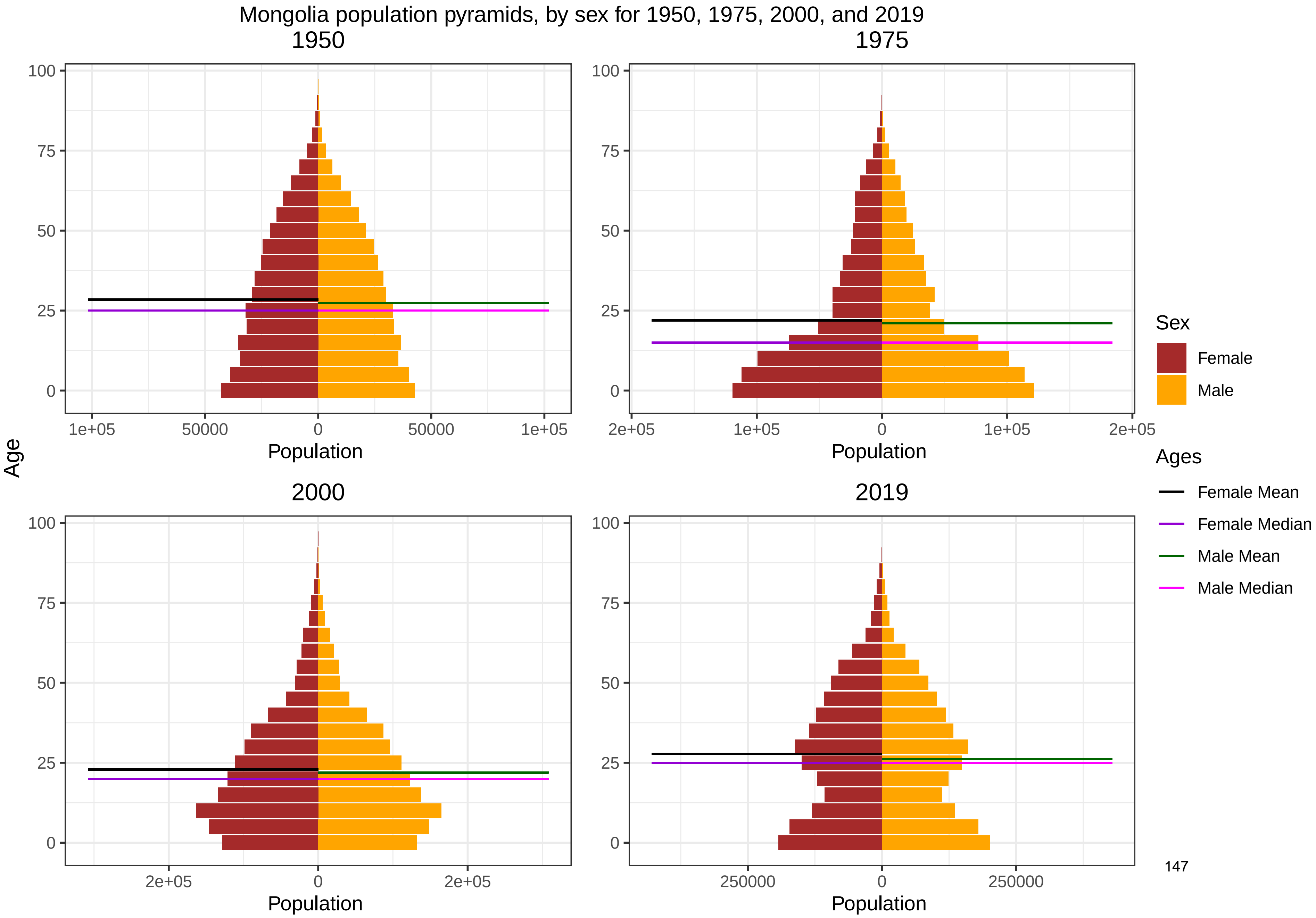
Mongolia population pyramids, by sex for 1950, 1975, 2000, and 2019
1950
1975
2000
2019
Age
Population
100
75
50
25
0
1e+05
50000
0
50000
1e+05
2e+05
1e+05
0
1e+05
2e+05
2e+05
0
2e+05
250000
0
250000
Sex
Female
Male
Ages
Female Mean
Female Median
Male Mean
Male Median

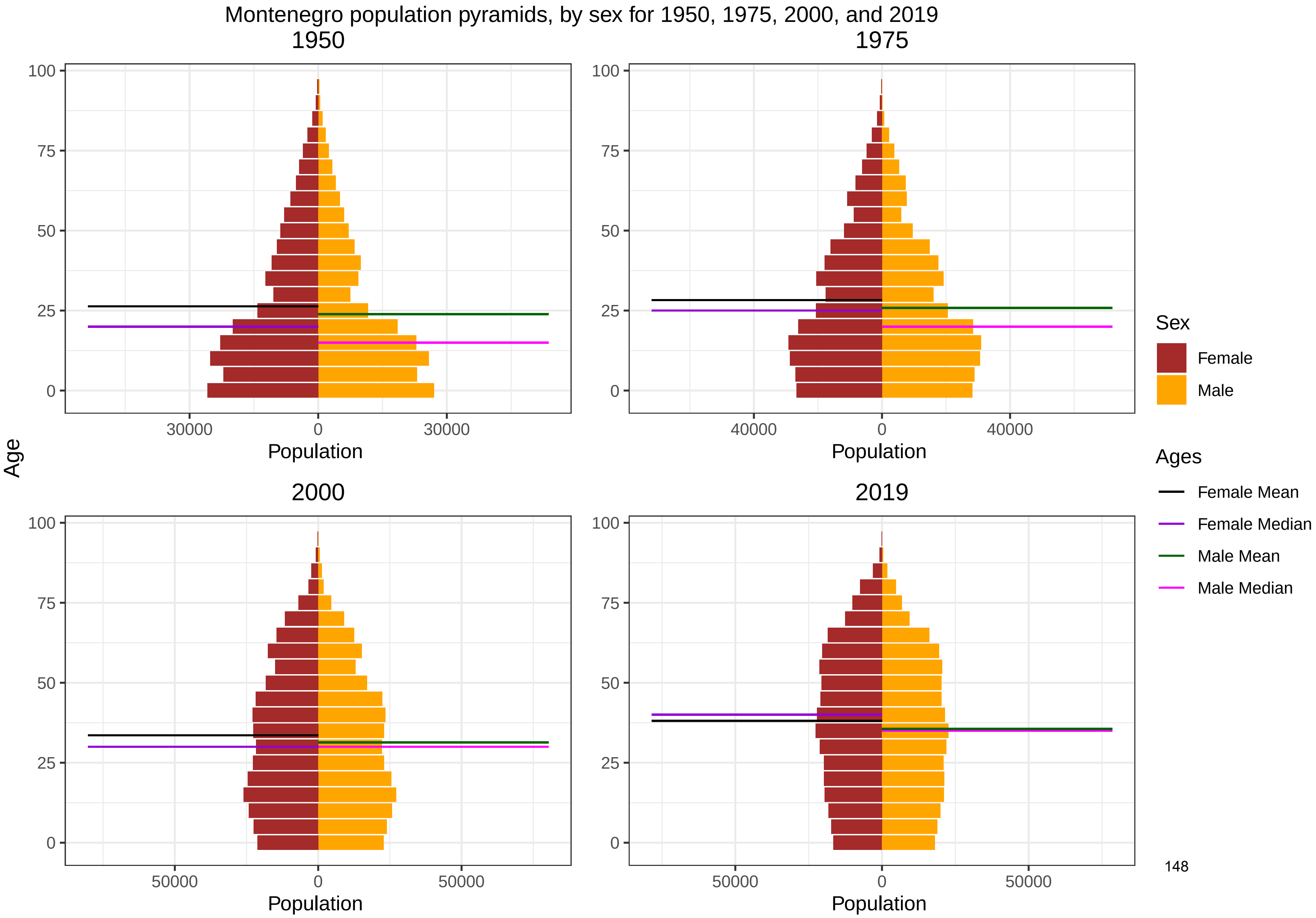
Montenegro population pyramids, by sex for 1950, 1975, 2000, and 2019
1950
1975
2000
2019
Age
Population
100
75
50
25
0
30000
0
30000
40000
0
40000
50000
0
50000
Sex
Female
Male
Ages
Female Mean
Female Median
Male Mean
Male Median

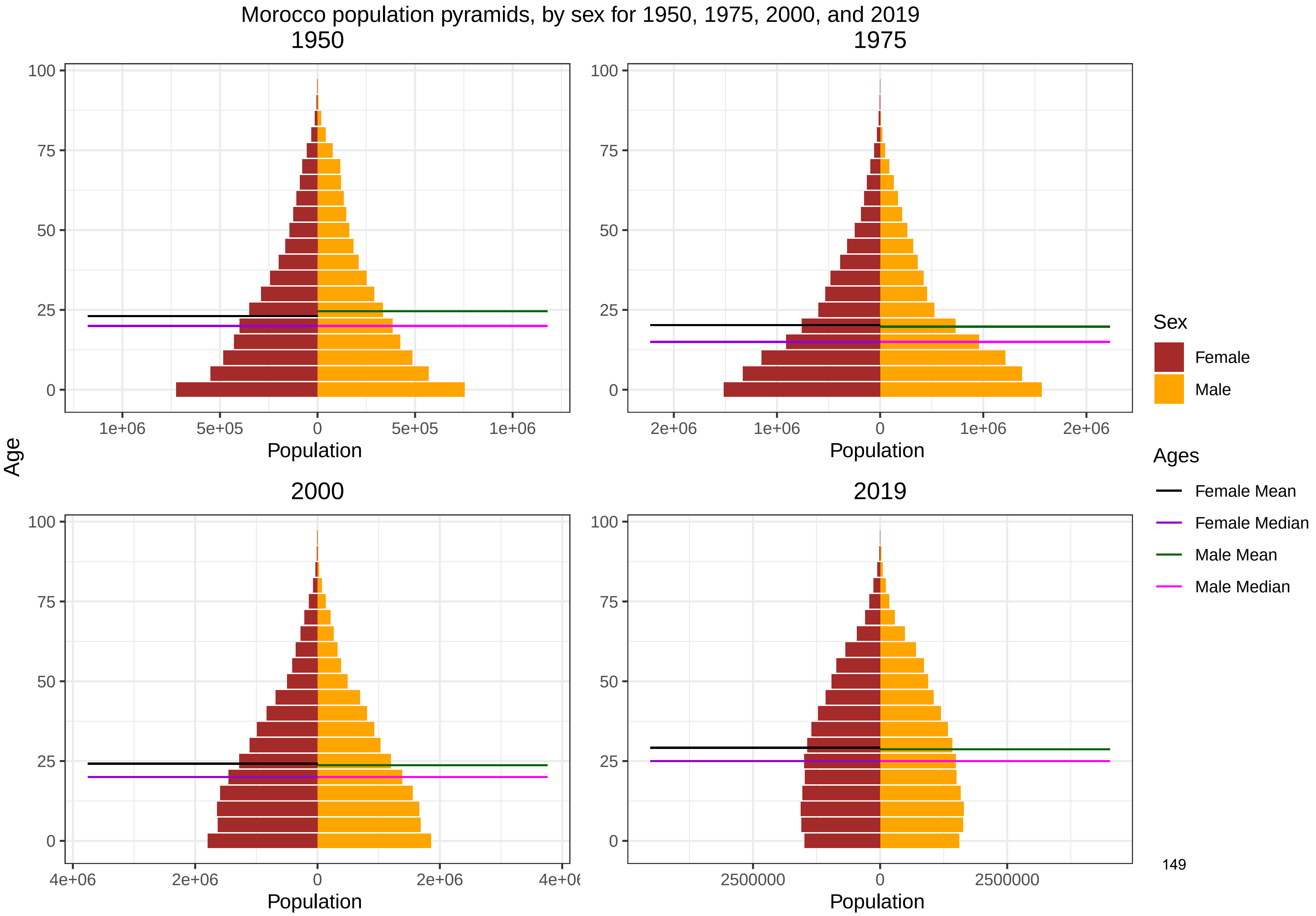
Morocco population pyramids, by sex for 1950, 1975, 2000, and 2019
1950
1975
2000
2019
Age
Population
100
75
50
25
0
1e+06
5e+05
0
5e+05
1e+06
2e+06
1e+06
0
1e+06
2e+06
4e+06
2e+06
0
2e+06
2500000
0
2500000
Sex
Female
Male
Ages
Female Mean
Female Median
Male Mean
Male Median

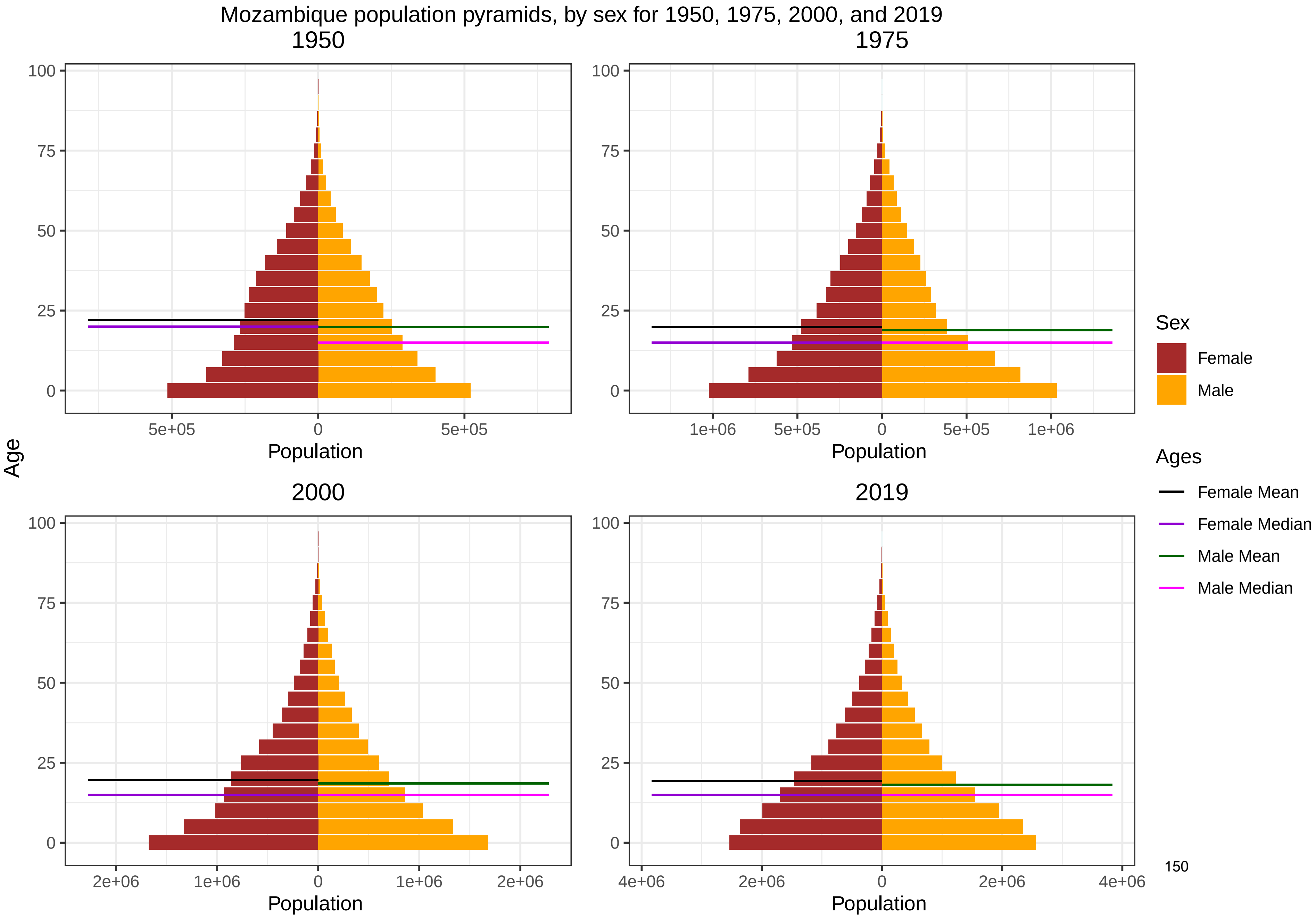
Mozambique population pyramids, by sex for 1950, 1975, 2000, and 2019
1950
1975
2000
2019
Age
Population
0
25
50
75
100
5e+05
1e+06
2e+06
4e+06
Sex
Female
Male
Ages
Female Mean
Female Median
Male Mean
Male Median

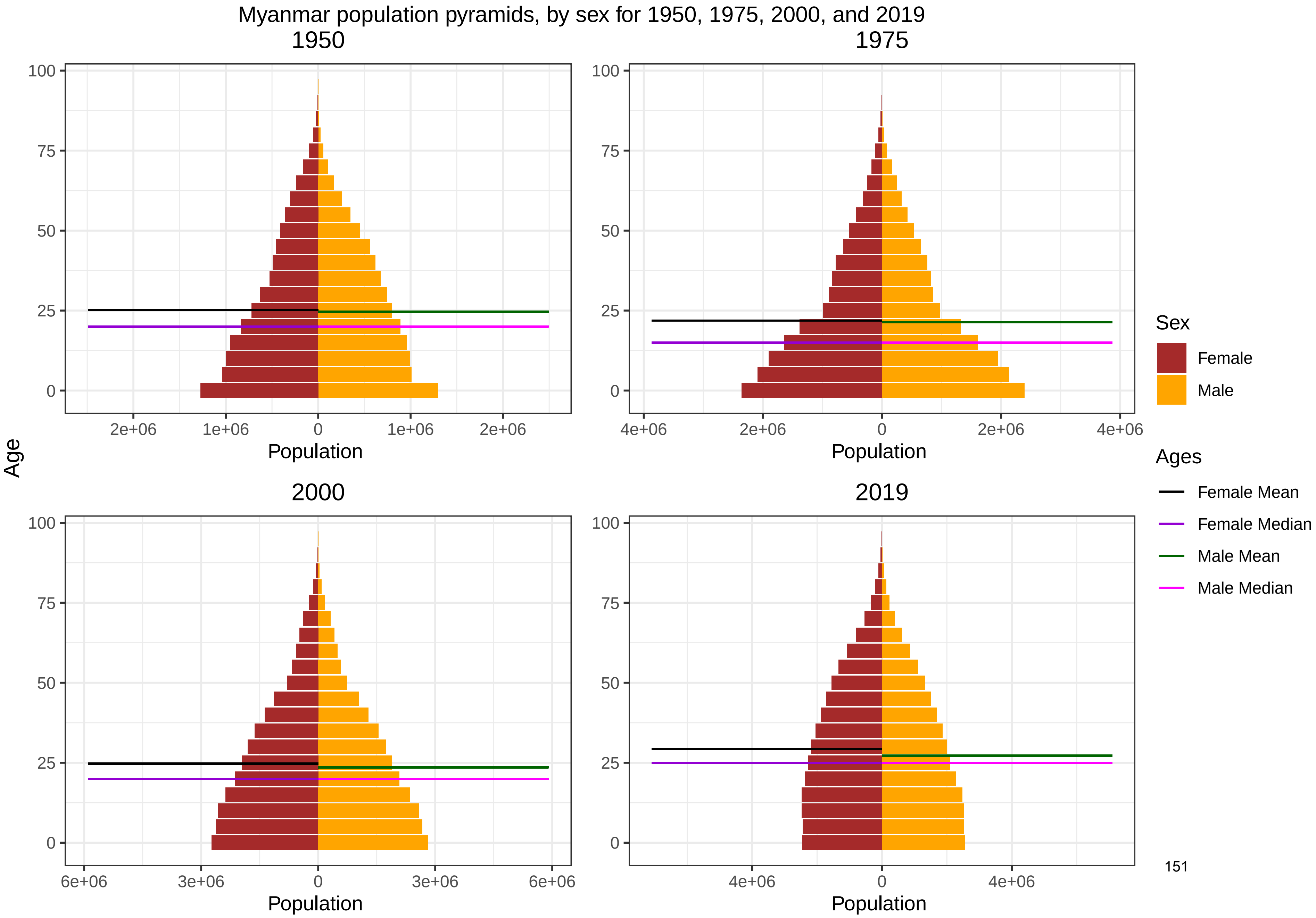

Myanmar population pyramids, by sex for 1950, 1975, 2000, and 2019
1950
1975
2000
2019
Age
Population
Sex
Female
Male
Ages
Female Mean
Female Median
Male Mean
Male Median

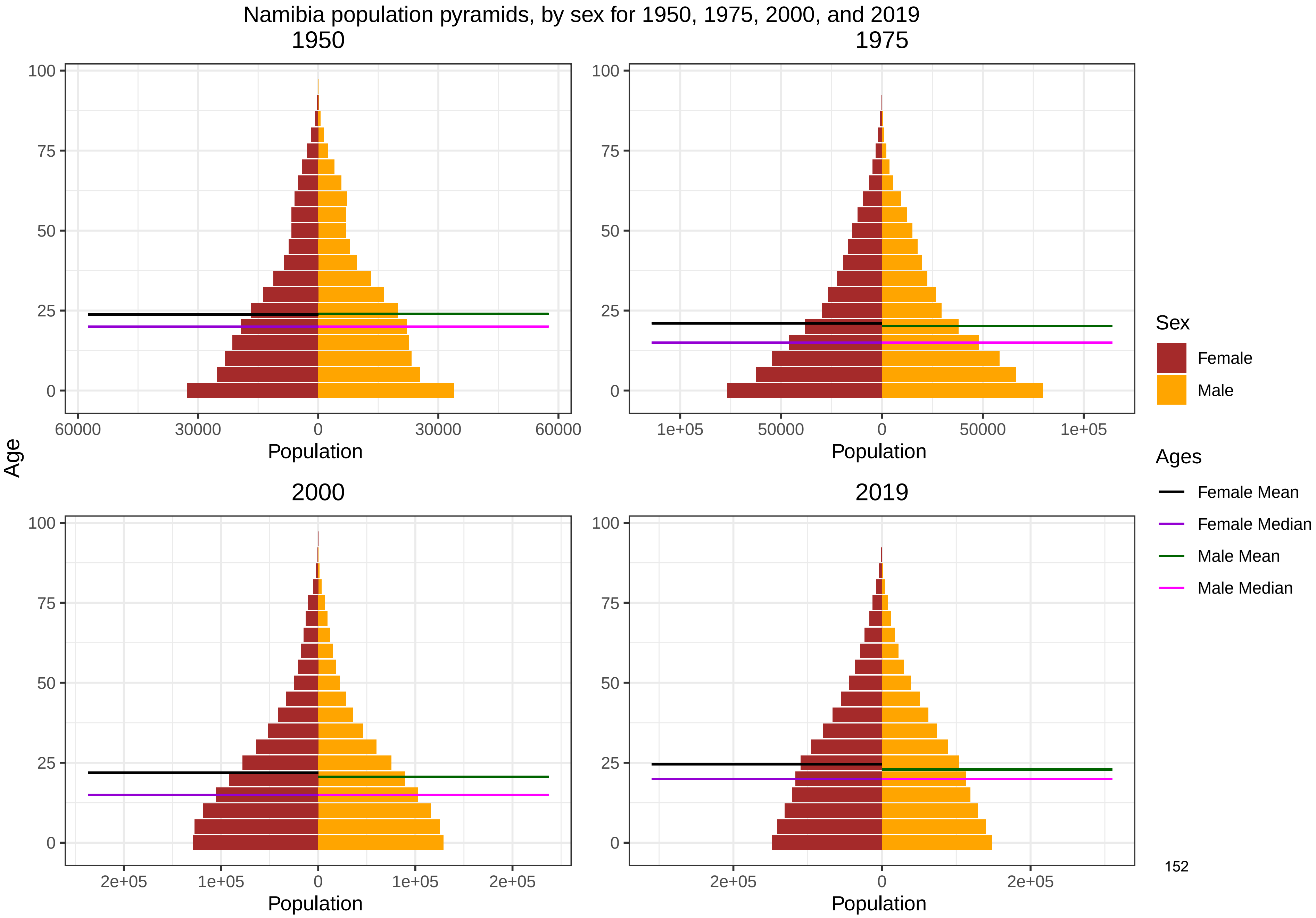

Namibia population pyramids, by sex for 1950, 1975, 2000, and 2019
1950
1975
2000
2019
Age
Population
Sex
Female
Male
Ages
Female Mean
Female Median
Male Mean
Male Median

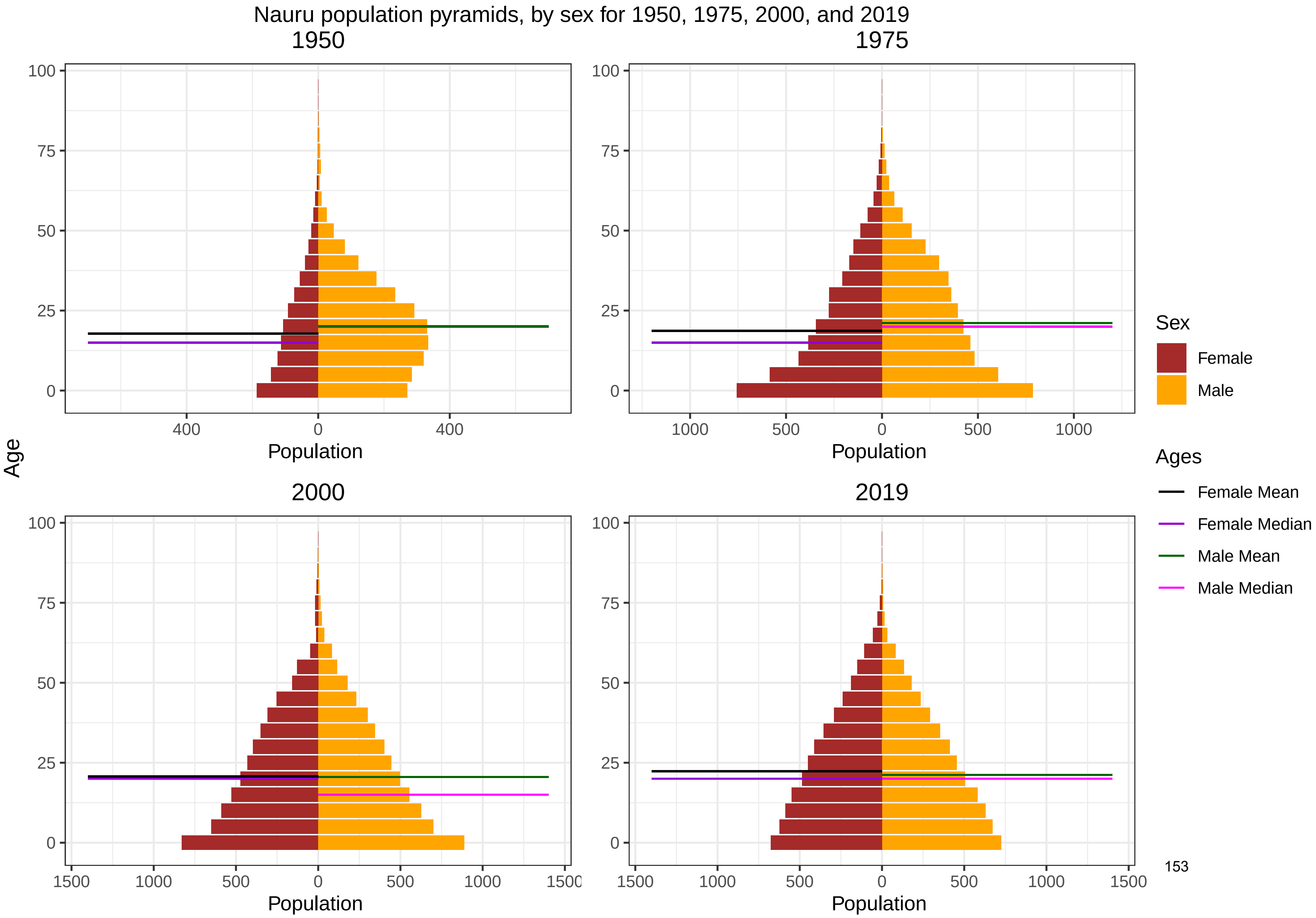
Nauru population pyramids, by sex for 1950, 1975, 2000, and 2019
1950
1975
2000
2019
Age
Population
Sex
Female
Male
Ages
Female Mean
Female Median
Male Mean
Male Median

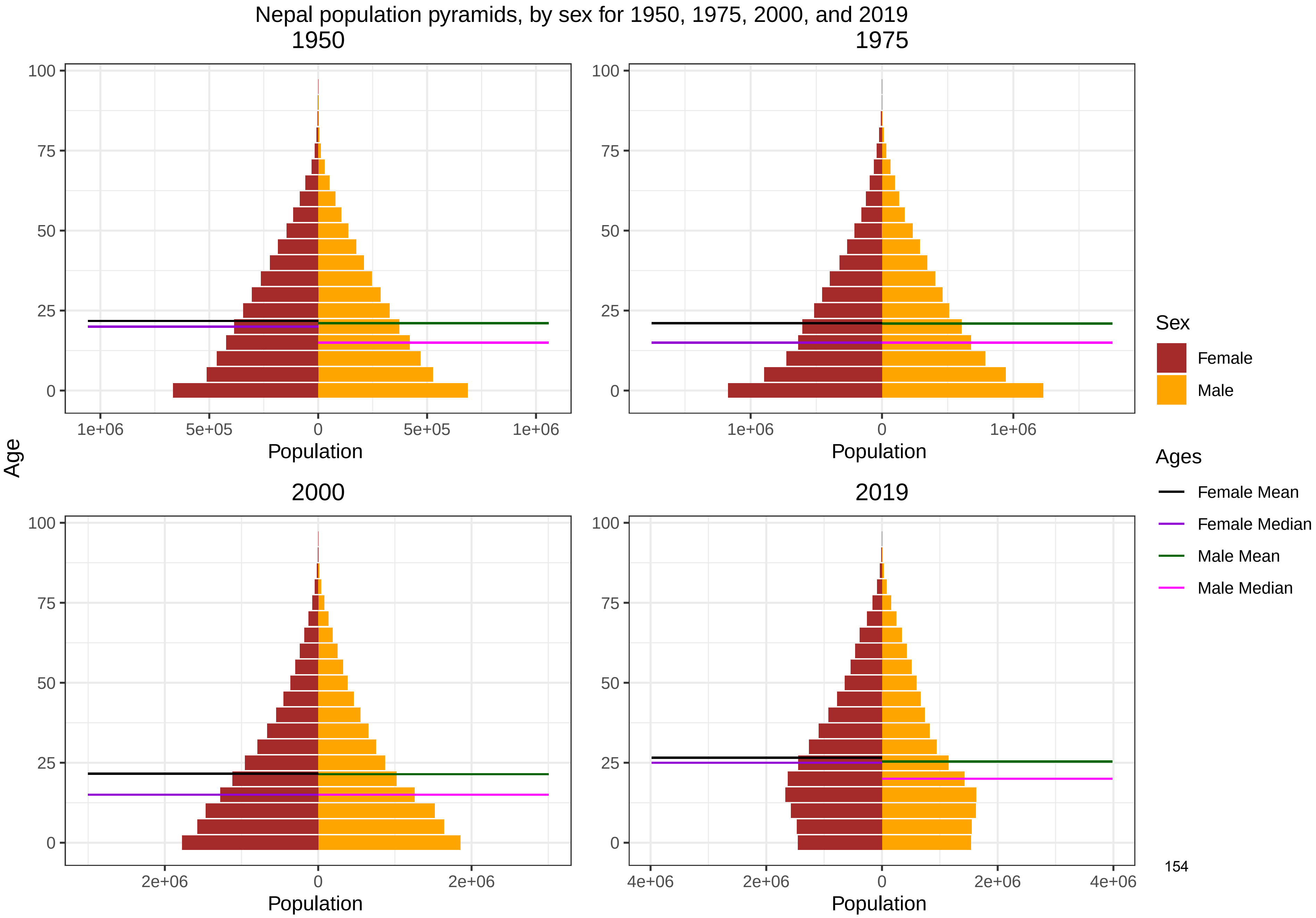

Nepal population pyramids, by sex for 1950, 1975, 2000, and 2019
1950
1975
2000
2019
Age
Population
100
75
50
25
0
1e+06
5e+05
0
5e+05
1e+06
2e+06
4e+06
Sex
Female
Male
Ages
Female Mean
Female Median
Male Mean
Male Median

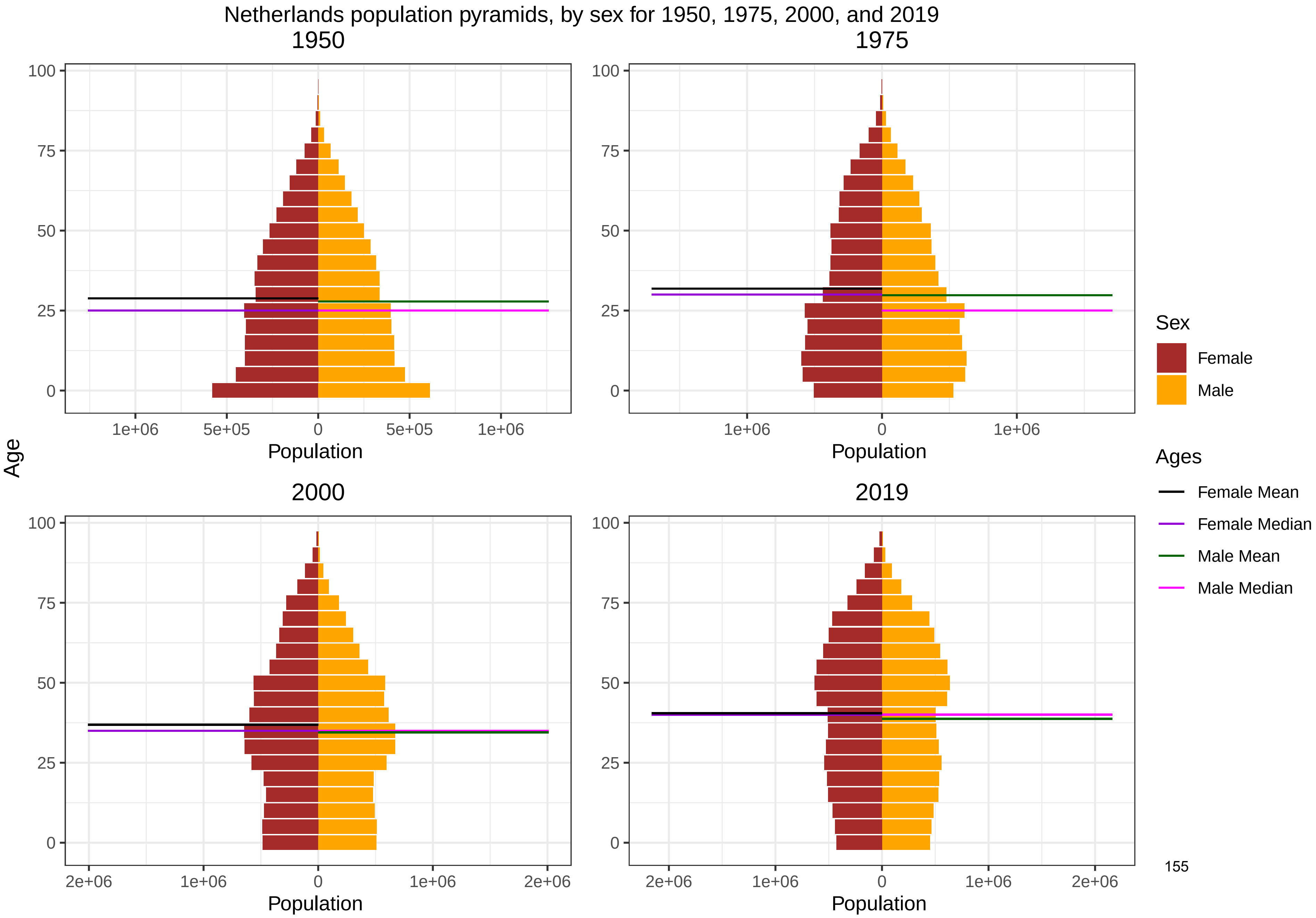
Netherlands population pyramids, by sex for 1950, 1975, 2000, and 2019
1950
1975
2000
2019
Age
Population
1e+06
5e+05
0
2e+06
Sex
Female
Male
Ages
Female Mean
Female Median
Male Mean
Male Median

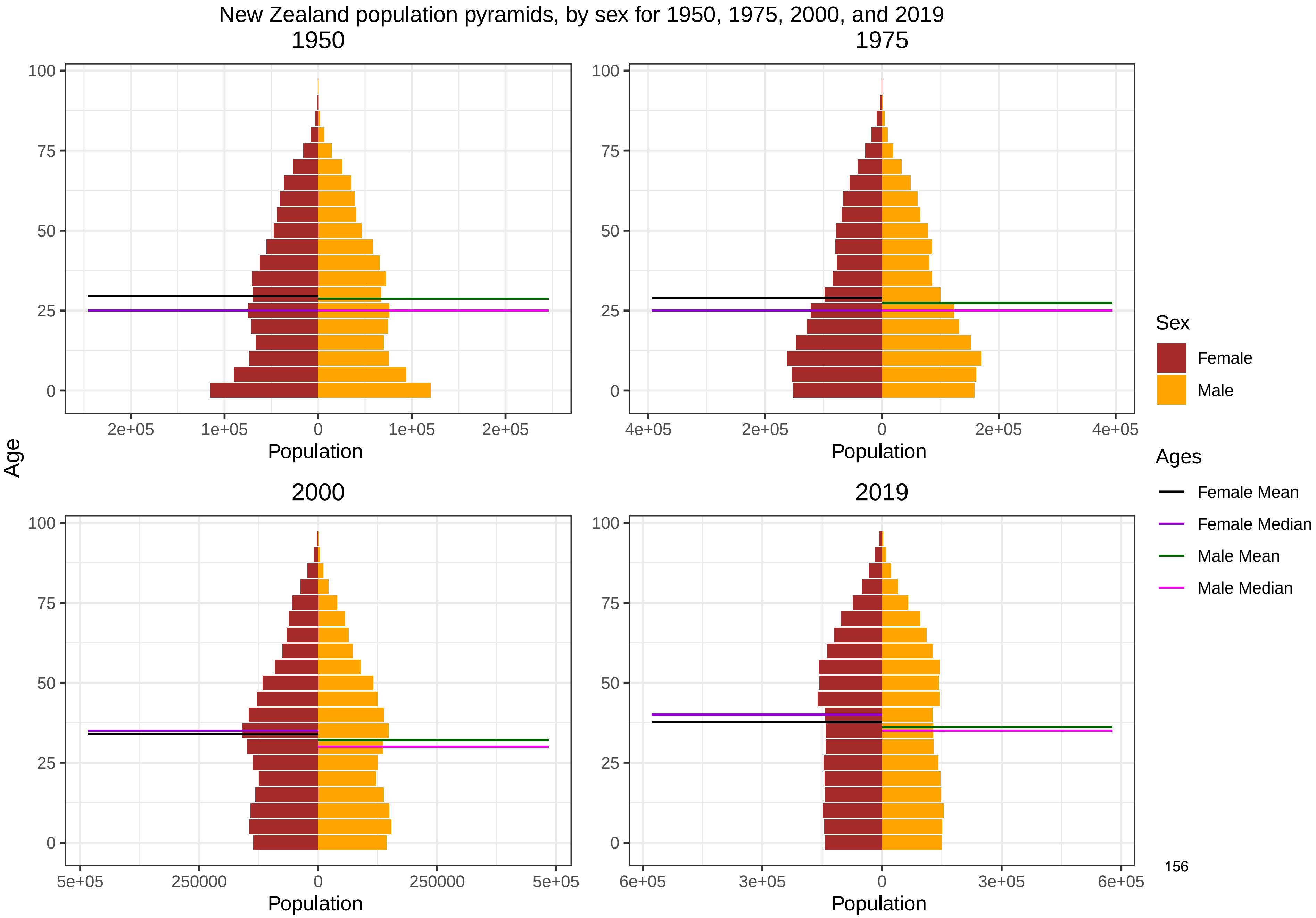
New Zealand population pyramids, by sex for 1950, 1975, 2000, and 2019
1950
1975
2000
2019
Age
Population
Sex
Female
Male
Ages
Female Mean
Female Median
Male Mean
Male Median
100
75
50
25
0
2e+05
1e+05
4e+05
5e+05
250000
6e+05
3e+05

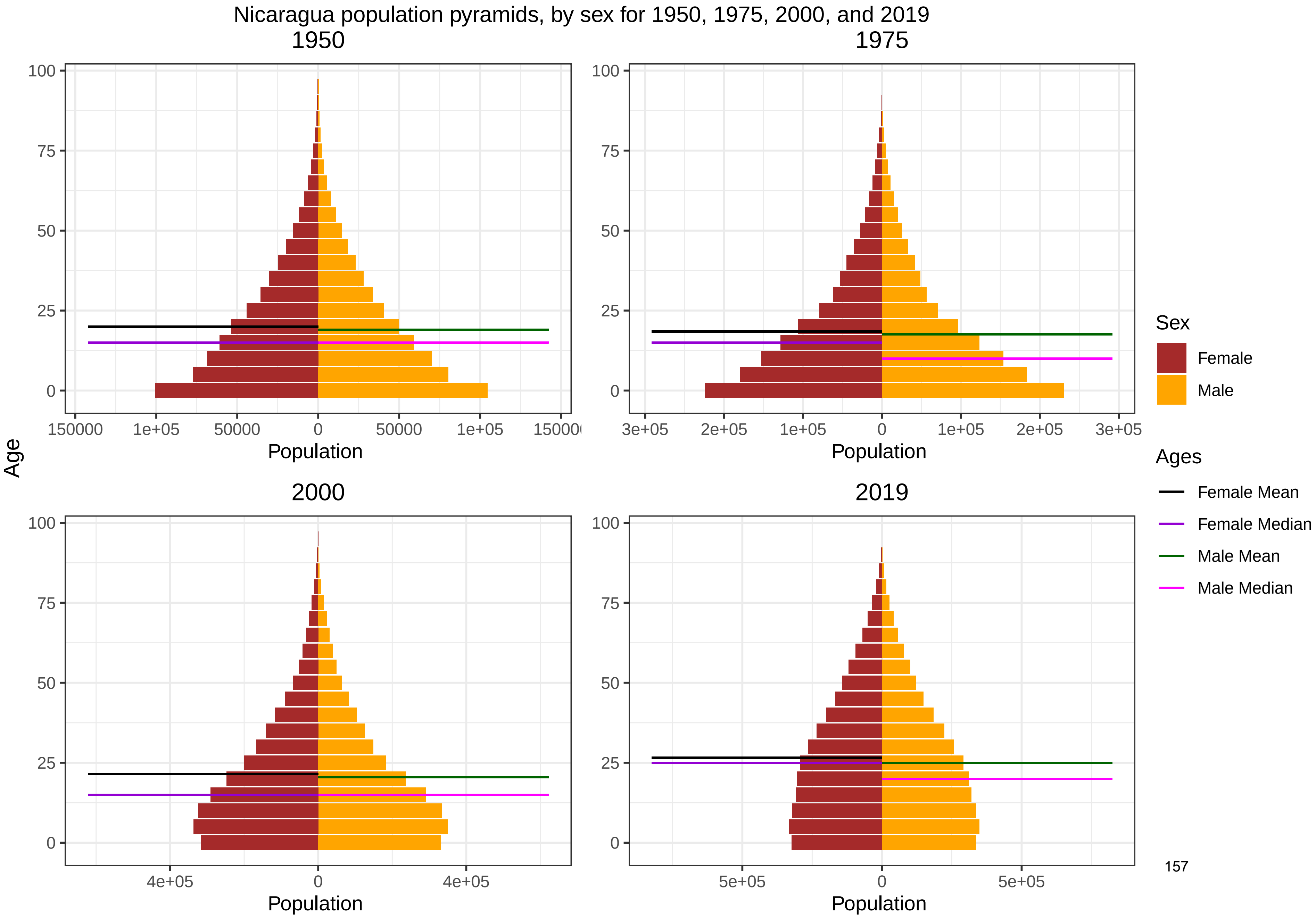
Nicaragua population pyramids, by sex for 1950, 1975, 2000, and 2019
1950
1975
2000
2019
Age
Population
100
75
50
25
0
150000
1e+05
50000
0
3e+05
2e+05
4e+05
5e+05
Sex
Female
Male
Ages
Female Mean
Female Median
Male Mean
Male Median

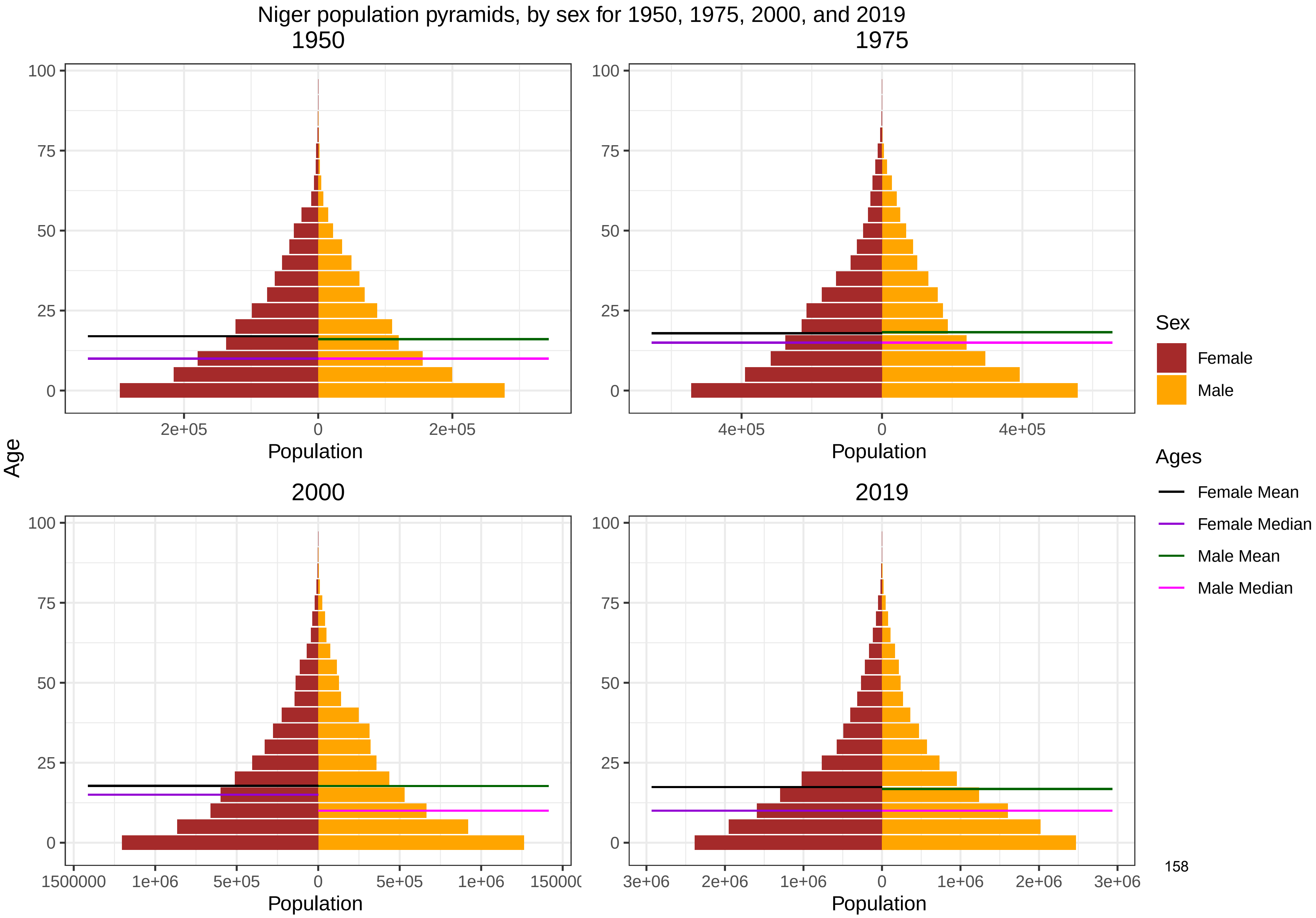
Niger population pyramids, by sex for 1950, 1975, 2000, and 2019
1950
1975
2000
2019
Age
Population
100
75
50
25
0
2e+05
0
2e+05
4e+05
0
4e+05
1500000
1e+06
5e+05
0
5e+05
1e+06
150000
3e+06
2e+06
1e+06
0
1e+06
2e+06
3e+06
Sex
Female
Male
Ages
Female Mean
Female Median
Male Mean
Male Median

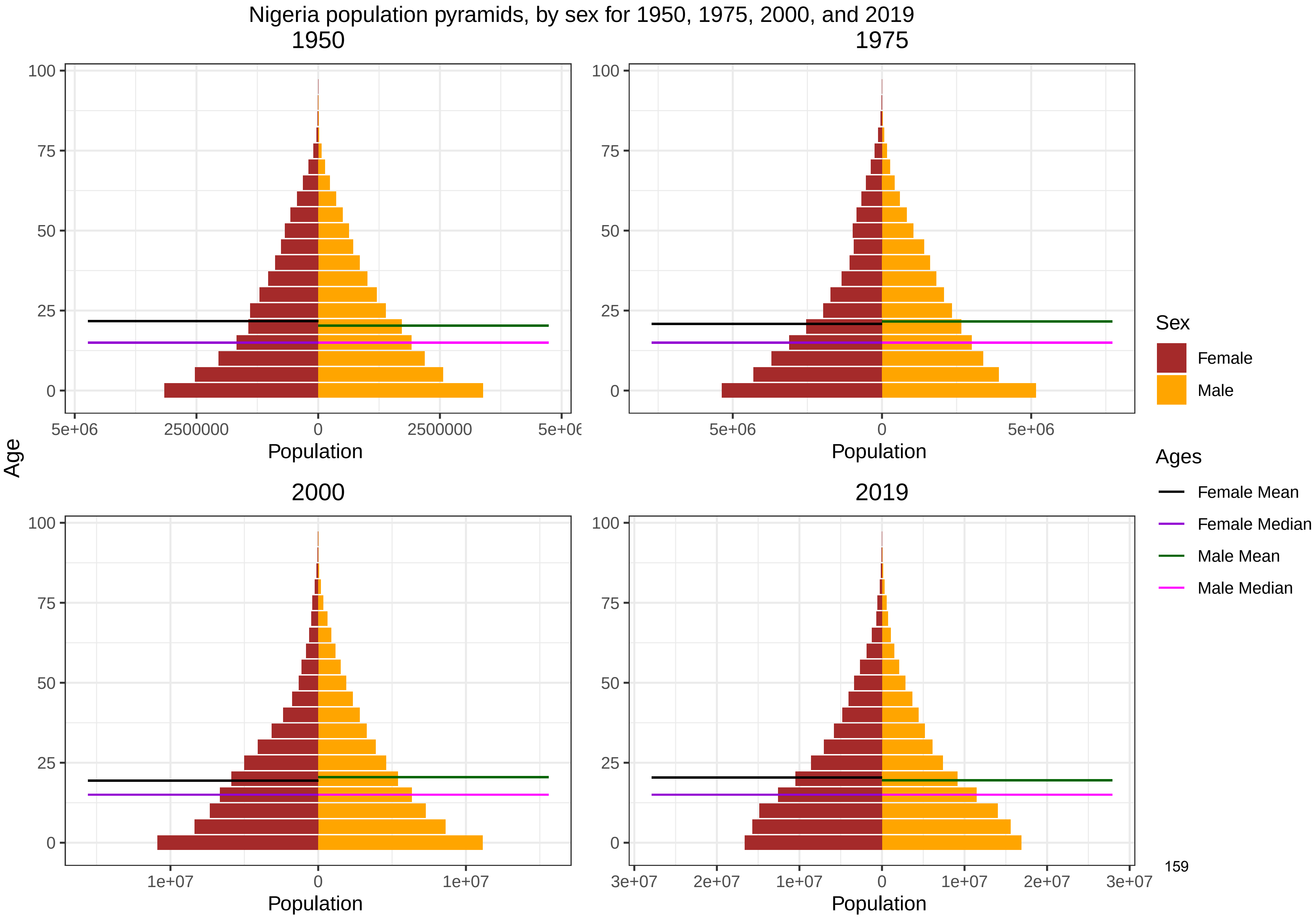
Nigeria population pyramids, by sex for 1950, 1975, 2000, and 2019
1950
1975
2000
2019
Age
Population
100
75
50
25
0
5e+06
2500000
0
2500000
5e+06
1e+07
2e+07
3e+07
Sex
Female
Male
Ages
Female Mean
Female Median
Male Mean
Male Median

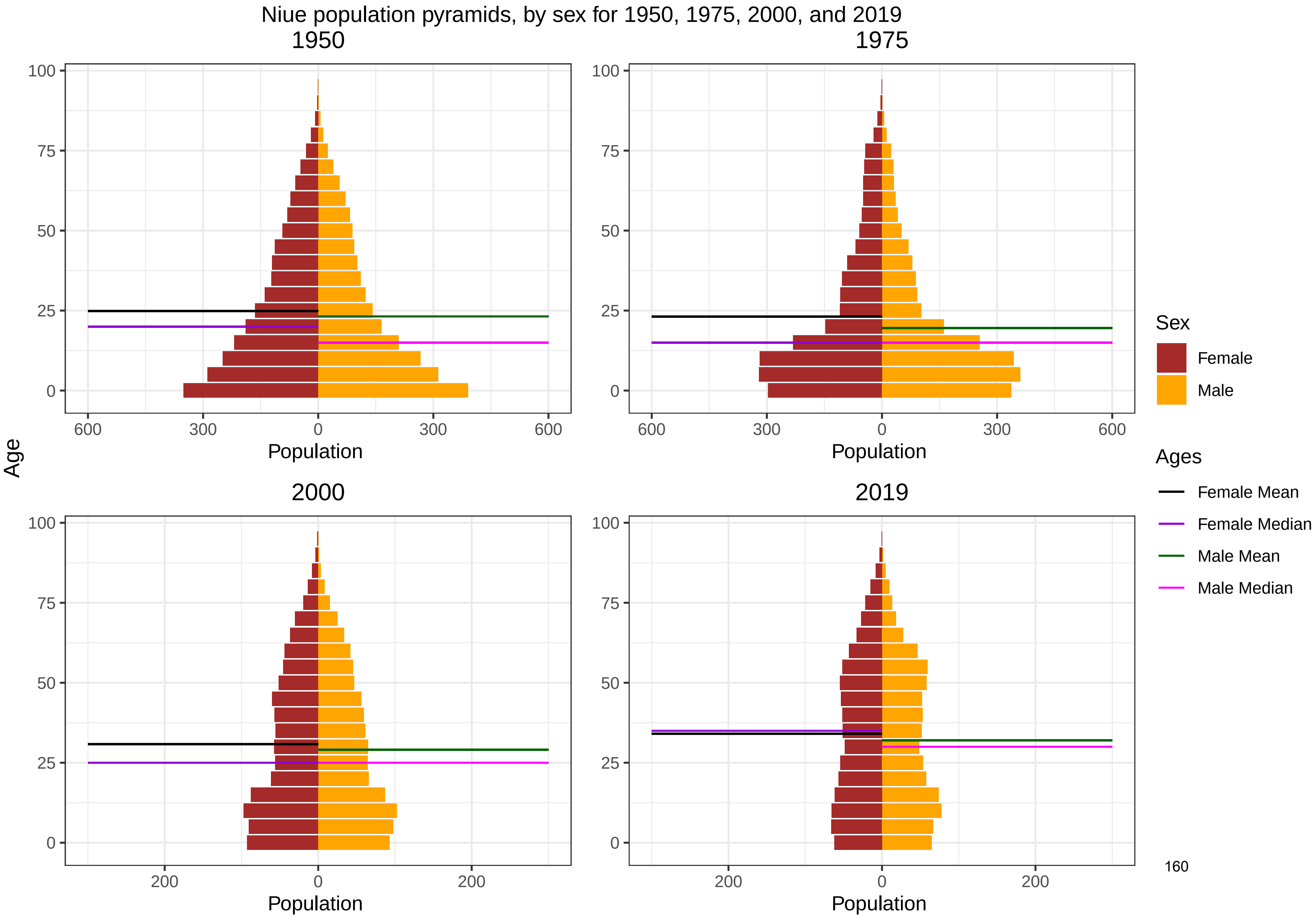
Niue population pyramids, by sex for 1950, 1975, 2000, and 2019
1950
1975
2000
2019
Age
Population
Sex
Female
Male
Ages
Female Mean
Female Median
Male Mean
Male Median

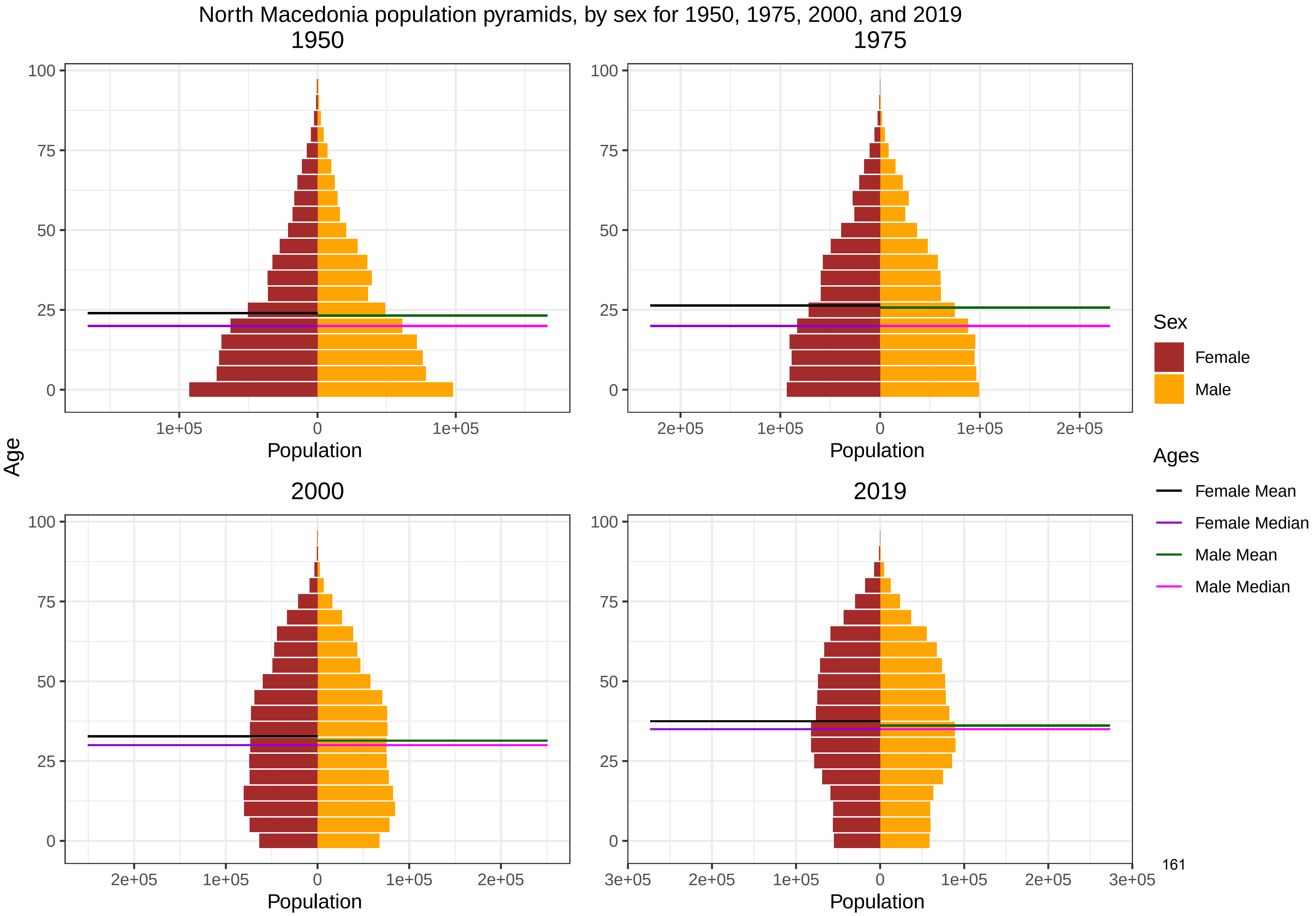
North Macedonia population pyramids, by sex for 1950, 1975, 2000, and 2019
1950
1975
2000
2019
Age
Population
Sex
Female
Male
Ages
Female Mean
Female Median
Male Mean
Male Median

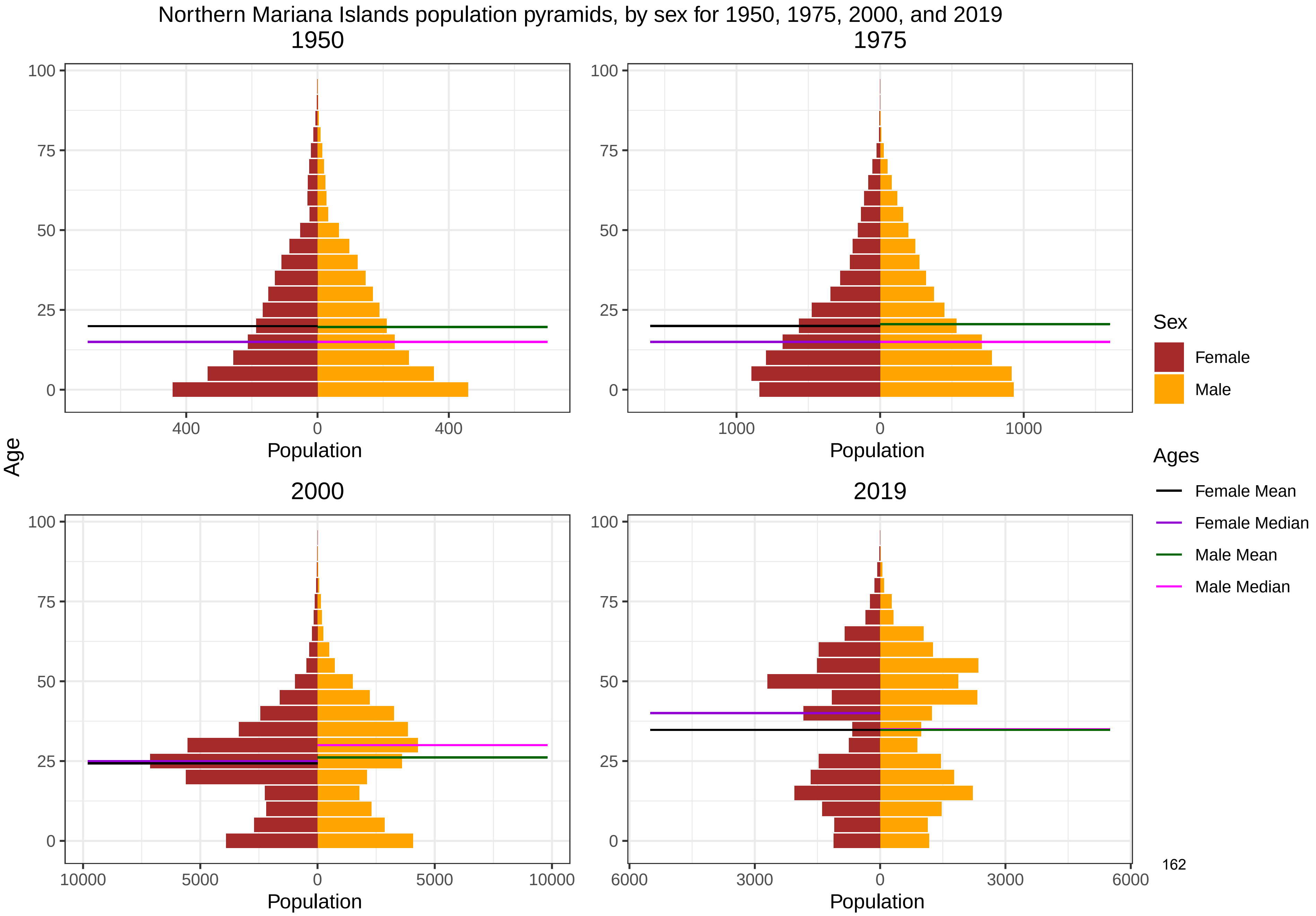
Northern Mariana Islands population pyramids, by sex for 1950, 1975, 2000, and 2019
1950
1975
2000
2019
Age
Population
Sex
Female
Male
Ages
Female Mean
Female Median
Male Mean
Male Median

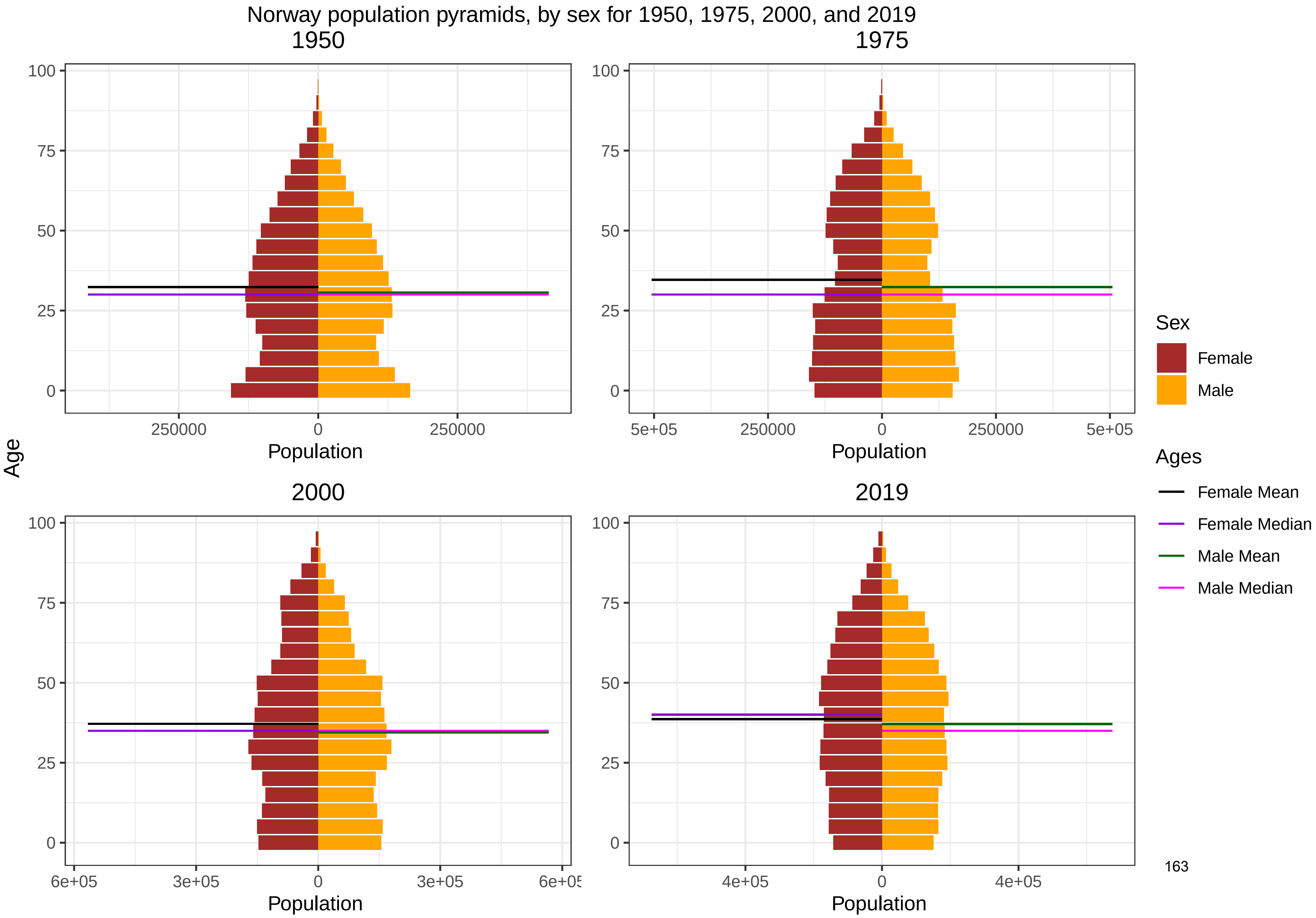
Norway population pyramids, by sex for 1950, 1975, 2000, and 2019
1950
1975
2000
2019
Age
Population
Sex
Female
Male
Ages
Female Mean
Female Median
Male Mean
Male Median

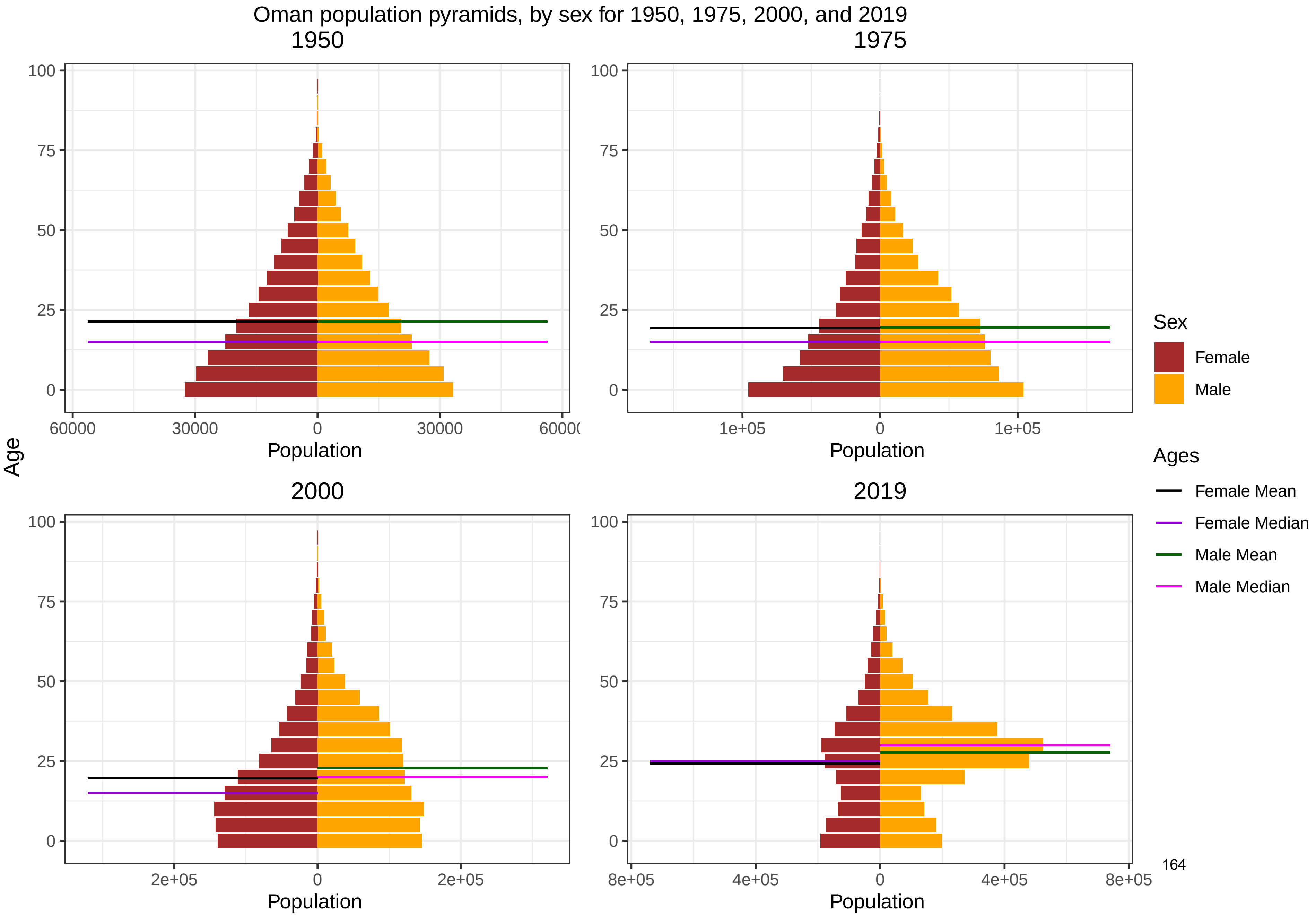

Oman population pyramids, by sex for 1950, 1975, 2000, and 2019
1950
1975
2000
2019
Age
Population
Sex
Female
Male
Ages
Female Mean
Female Median
Male Mean
Male Median

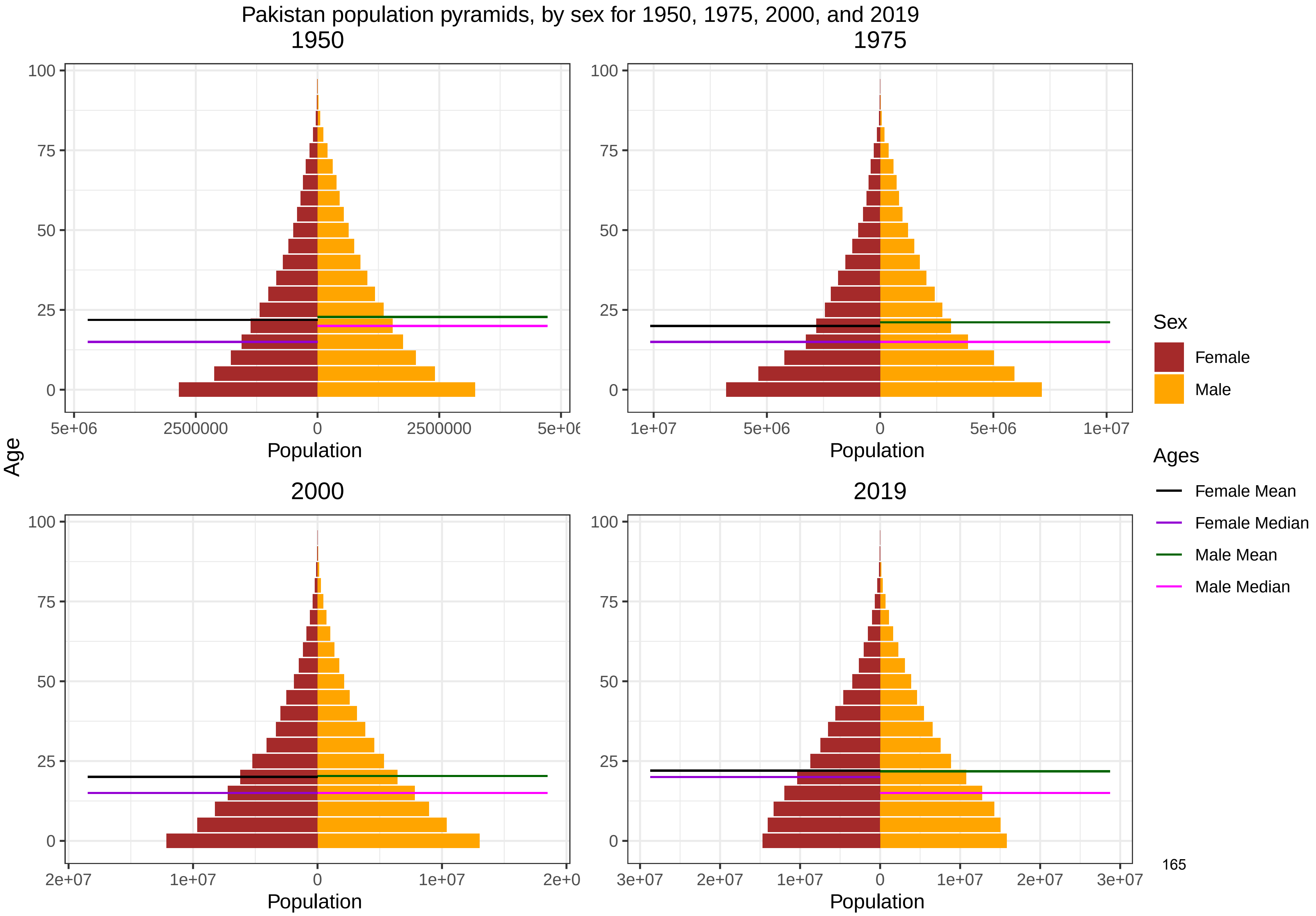

Pakistan population pyramids, by sex for 1950, 1975, 2000, and 2019
1950
1975
2000
2019
Age
Population
Sex
Female
Male
Ages
Female Mean
Female Median
Male Mean
Male Median

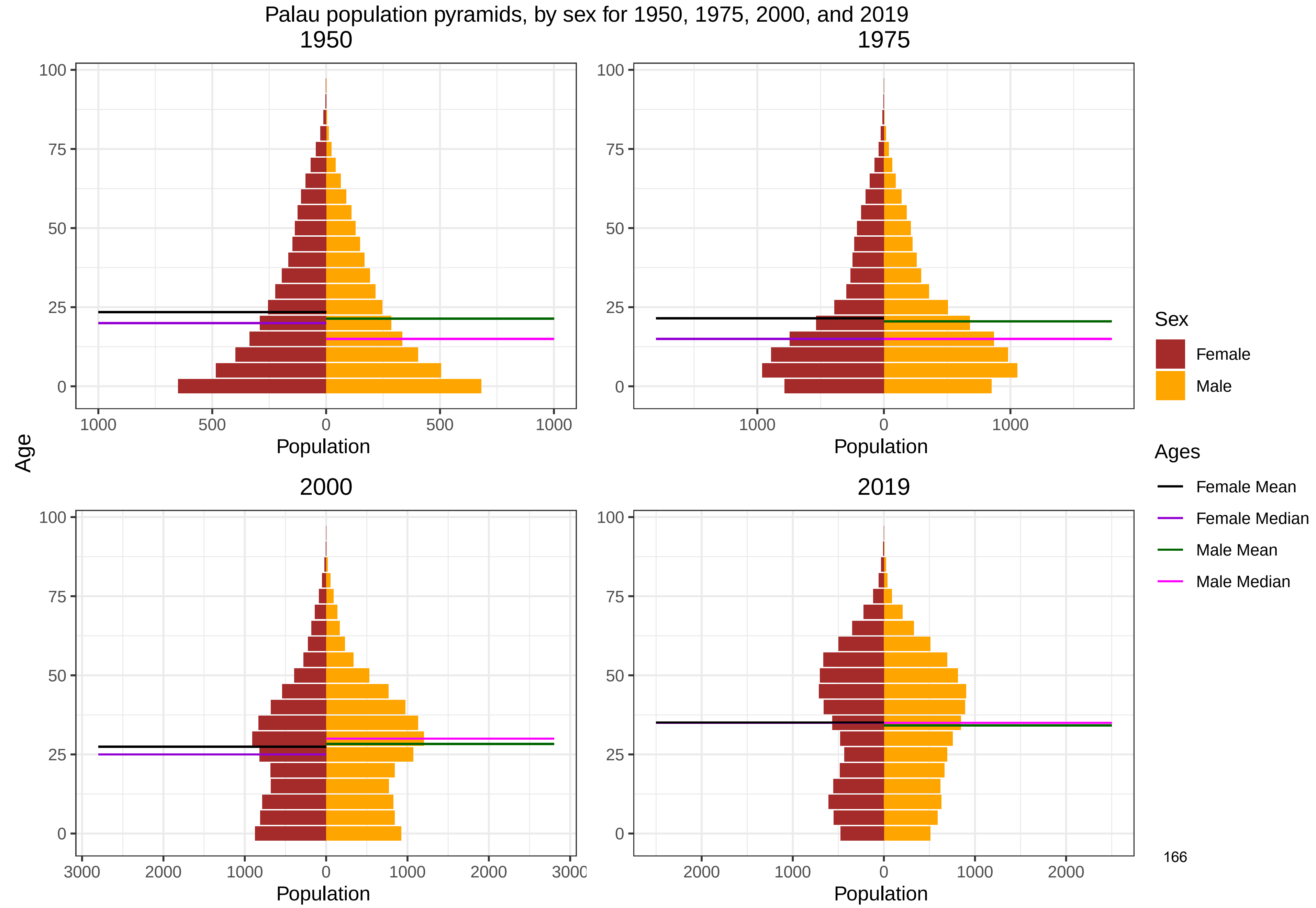
Palau population pyramids, by sex for 1950, 1975, 2000, and 2019
1950
1975
2000
2019
Age
Population
Sex
Female
Male
Ages
Female Mean
Female Median
Male Mean
Male Median

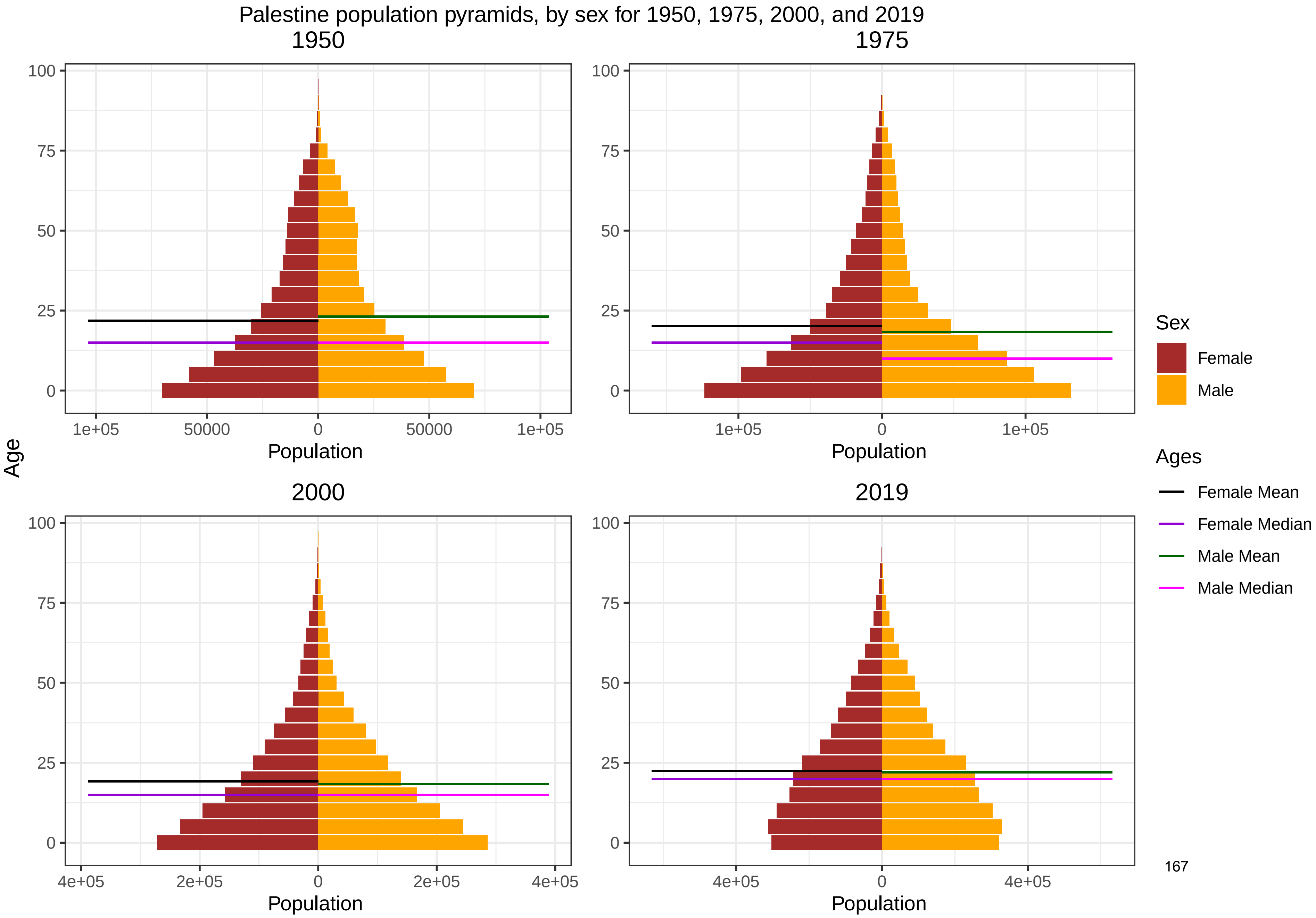

Palestine population pyramids, by sex for 1950, 1975, 2000, and 2019
1950
1975
2000
2019
Age
Population
0
25
50
75
100
1e+05
50000
0
50000
1e+05
2e+05
4e+05
Sex
Female
Male
Ages
Female Mean
Female Median
Male Mean
Male Median

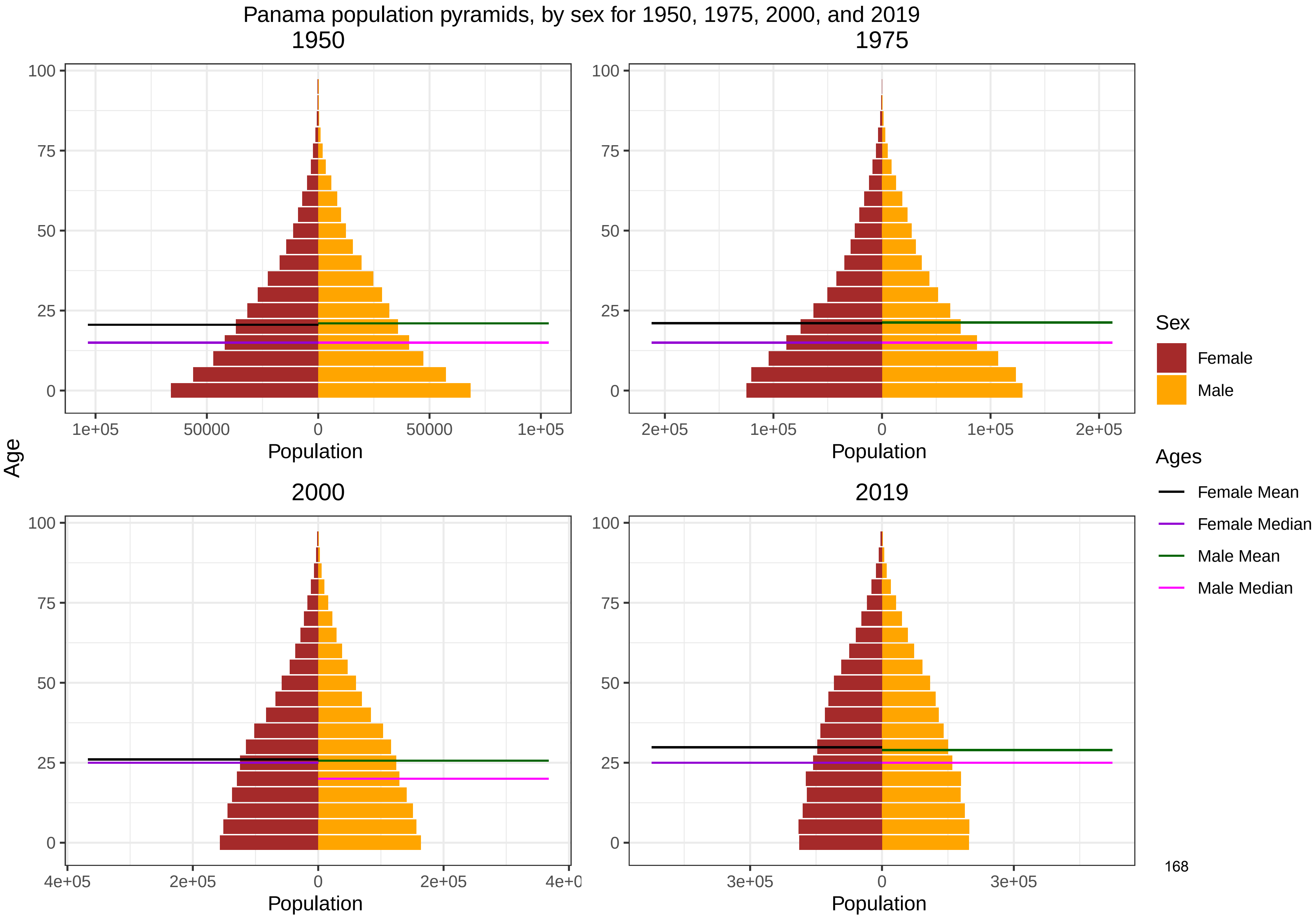
Panama population pyramids, by sex for 1950, 1975, 2000, and 2019
1950
1975
2000
2019
Age
Population
Sex
Female
Male
Ages
Female Mean
Female Median
Male Mean
Male Median

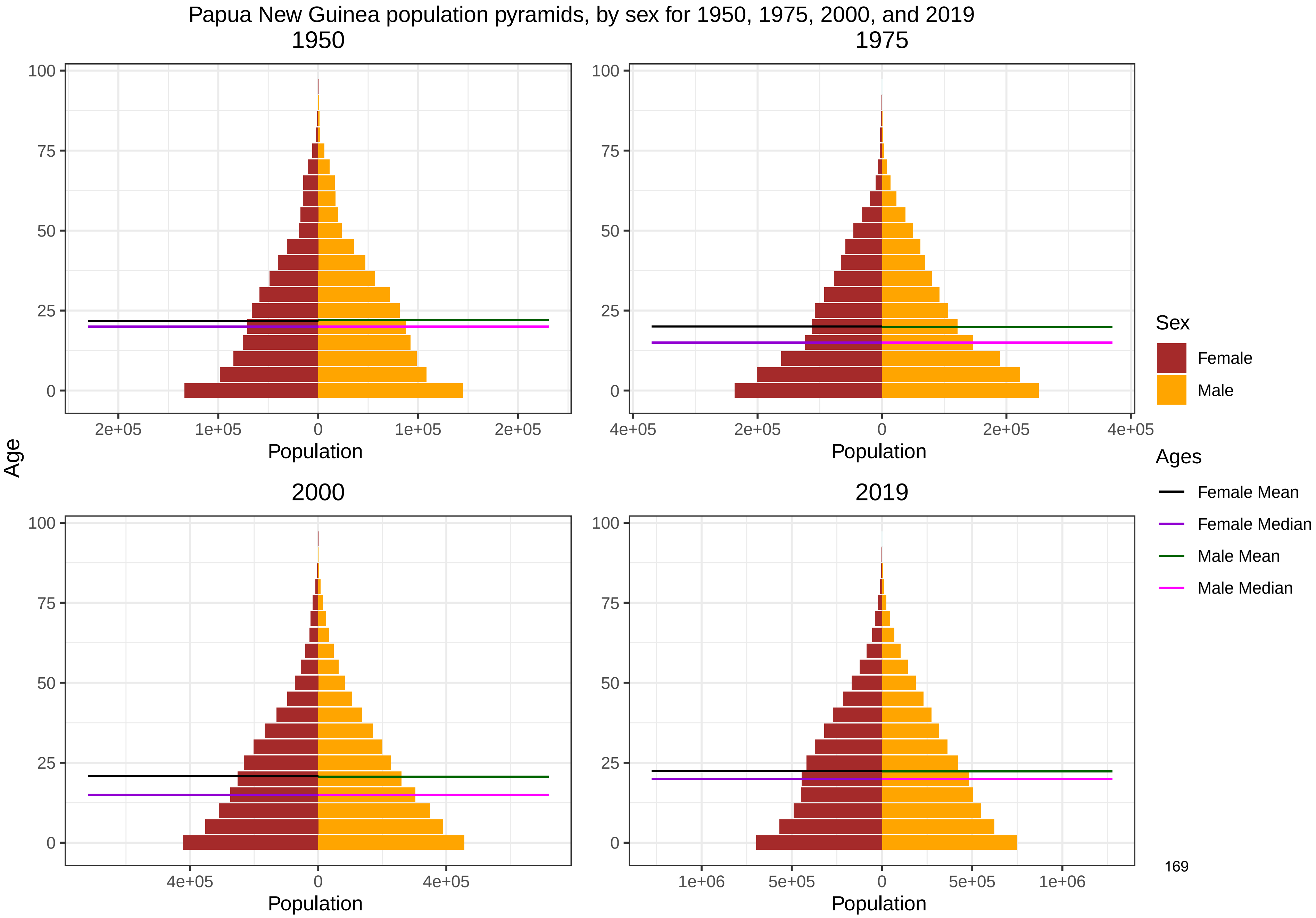
Papua New Guinea population pyramids, by sex for 1950, 1975, 2000, and 2019
1950
1975
2000
2019
Age
Population
Sex
Female
Male
Ages
Female Mean
Female Median
Male Mean
Male Median

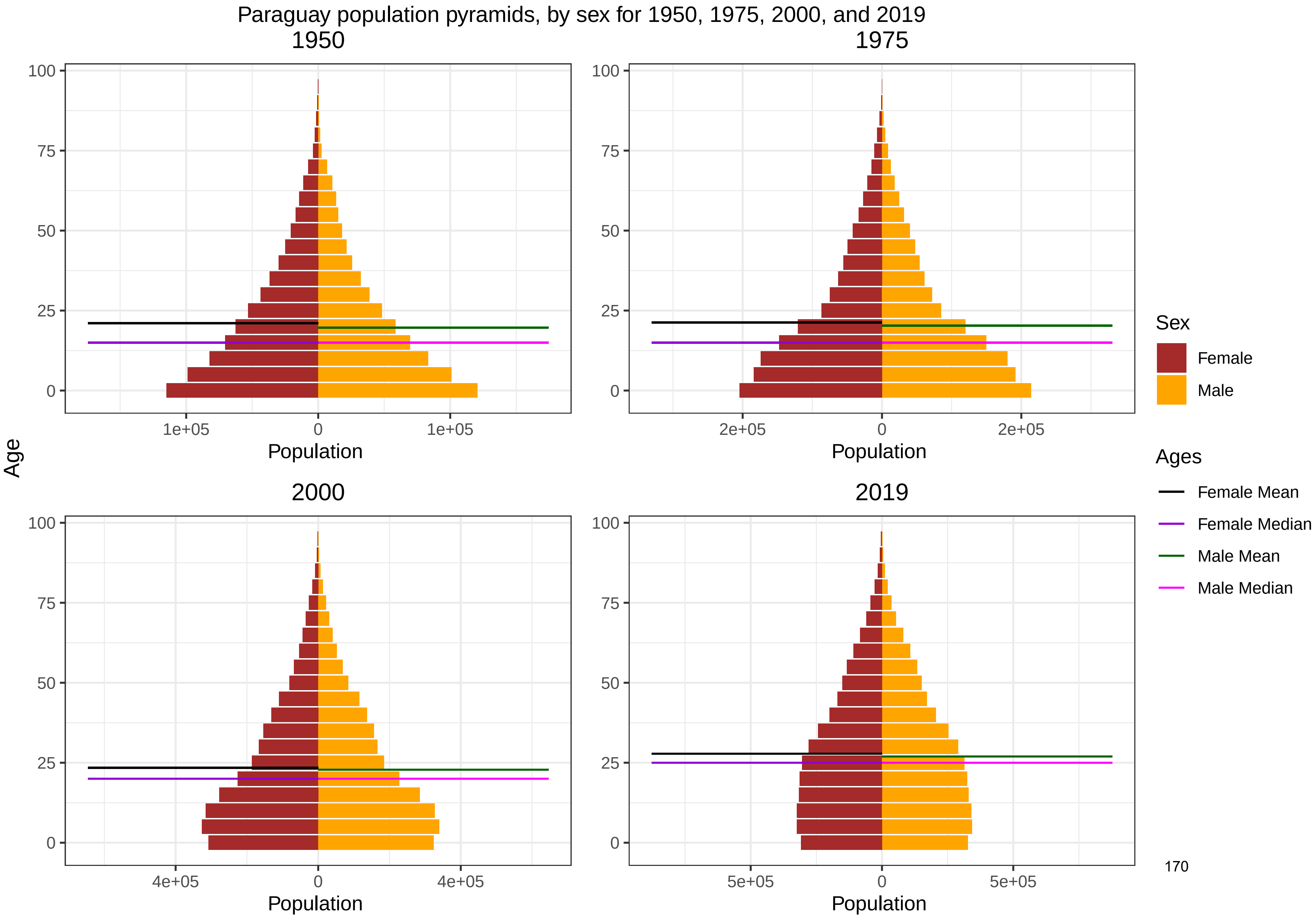
Paraguay population pyramids, by sex for 1950, 1975, 2000, and 2019
1950
1975
2000
2019
Age
Population
100
75
50
25
0
1e+05
0
1e+05
2e+05
0
2e+05
4e+05
0
4e+05
5e+05
0
5e+05
Sex
Female
Male
Ages
Female Mean
Female Median
Male Mean
Male Median

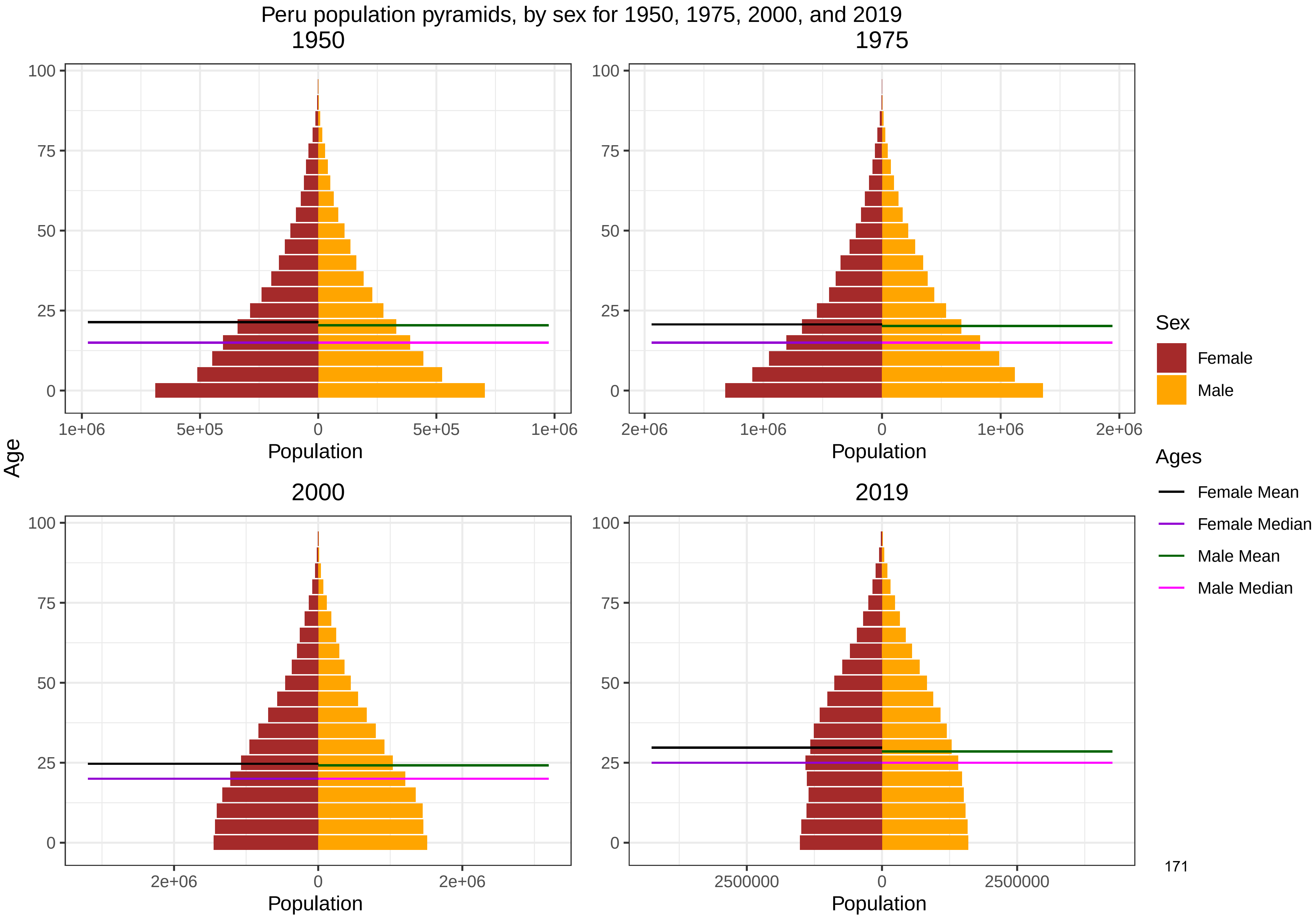
Peru population pyramids, by sex for 1950, 1975, 2000, and 2019
1950
1975
2000
2019
Age
Population
100
75
50
25
0
1e+06
5e+05
0
5e+05
1e+06
2e+06
1e+06
0
1e+06
2e+06
2e+06
0
2e+06
2500000
0
2500000
Sex
Female
Male
Ages
Female Mean
Female Median
Male Mean
Male Median

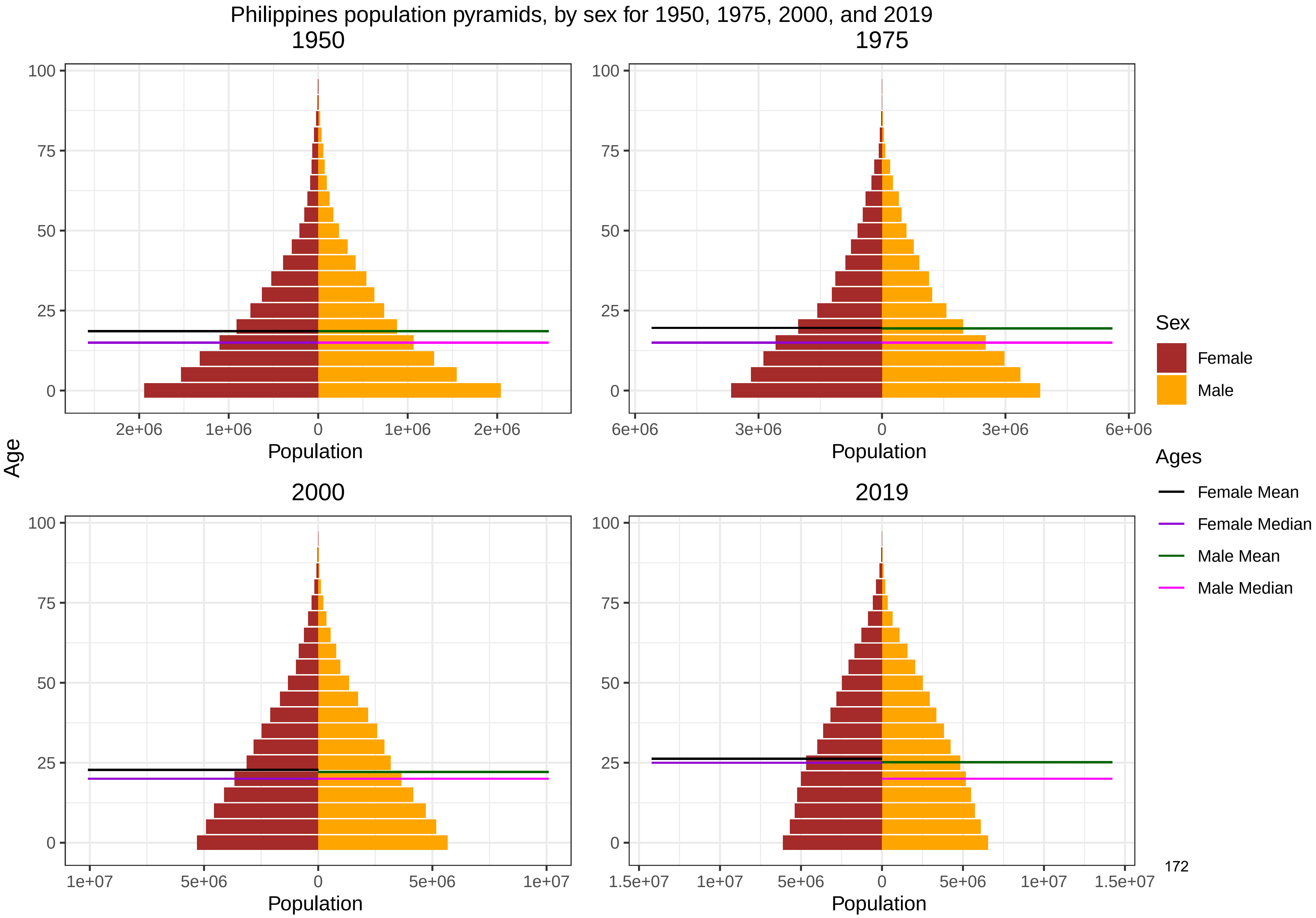

Philippines population pyramids, by sex for 1950, 1975, 2000, and 2019
1950
1975
2000
2019
Age
Population
Sex
Female
Male
Ages
Female Mean
Female Median
Male Mean
Male Median

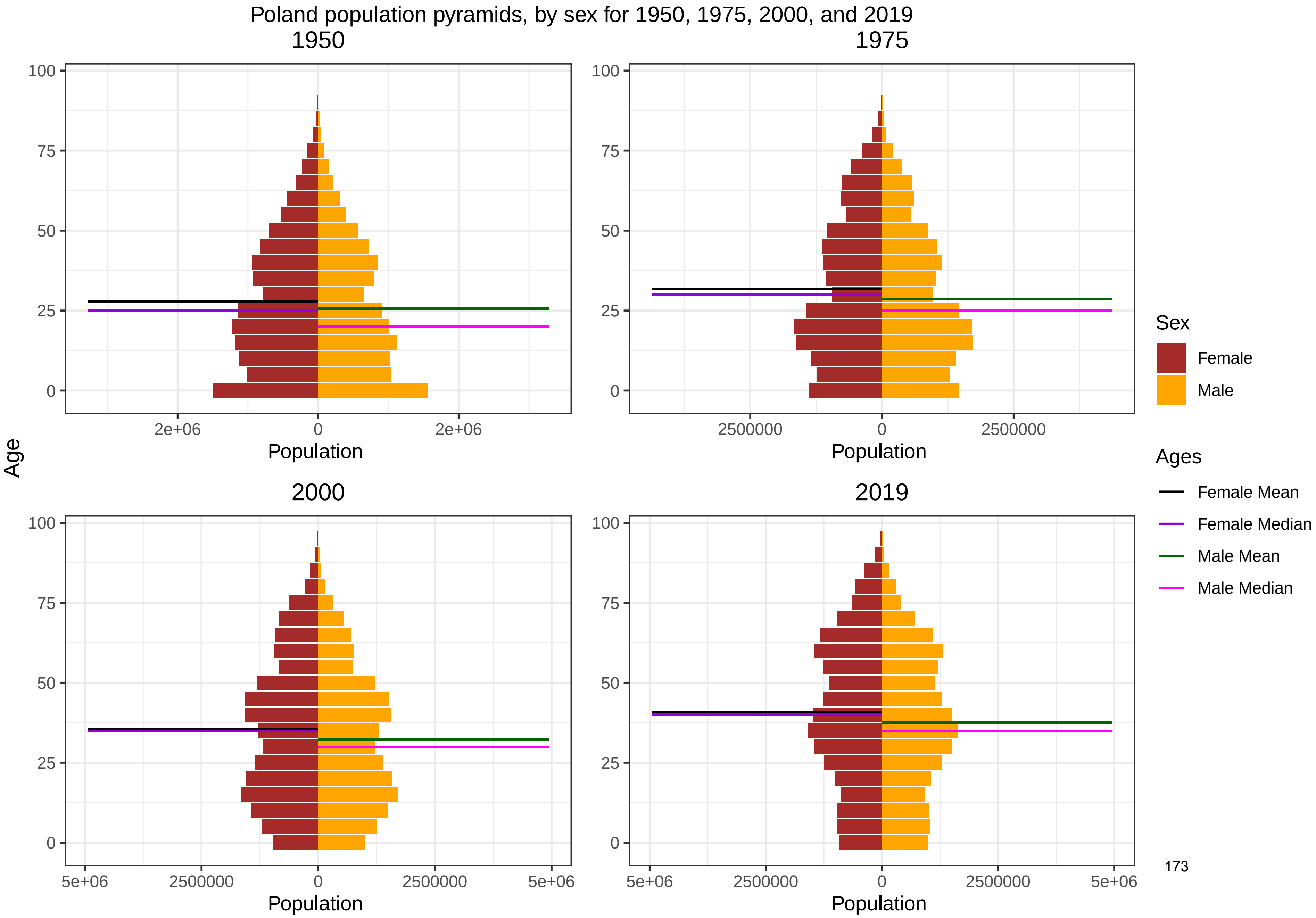

Poland population pyramids, by sex for 1950, 1975, 2000, and 2019
1950
1975
2000
2019
Age
Population
Sex
Female
Male
Ages
Female Mean
Female Median
Male Mean
Male Median

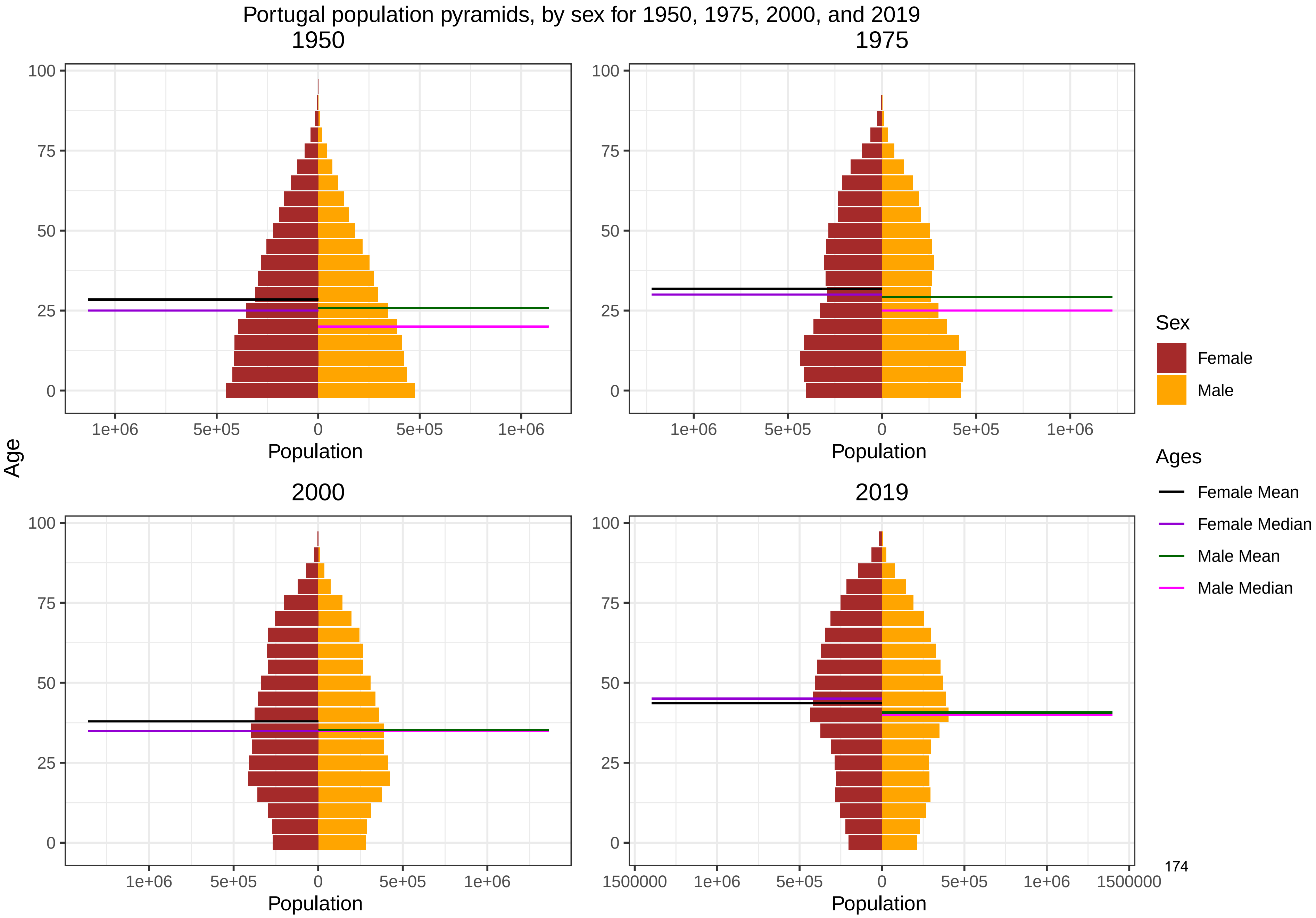

Portugal population pyramids, by sex for 1950, 1975, 2000, and 2019
1950
1975
2000
2019
Age
Population
1e+06
5e+05
0
1500000
100
75
50
25
Sex
Female
Male
Ages
Female Mean
Female Median
Male Mean
Male Median

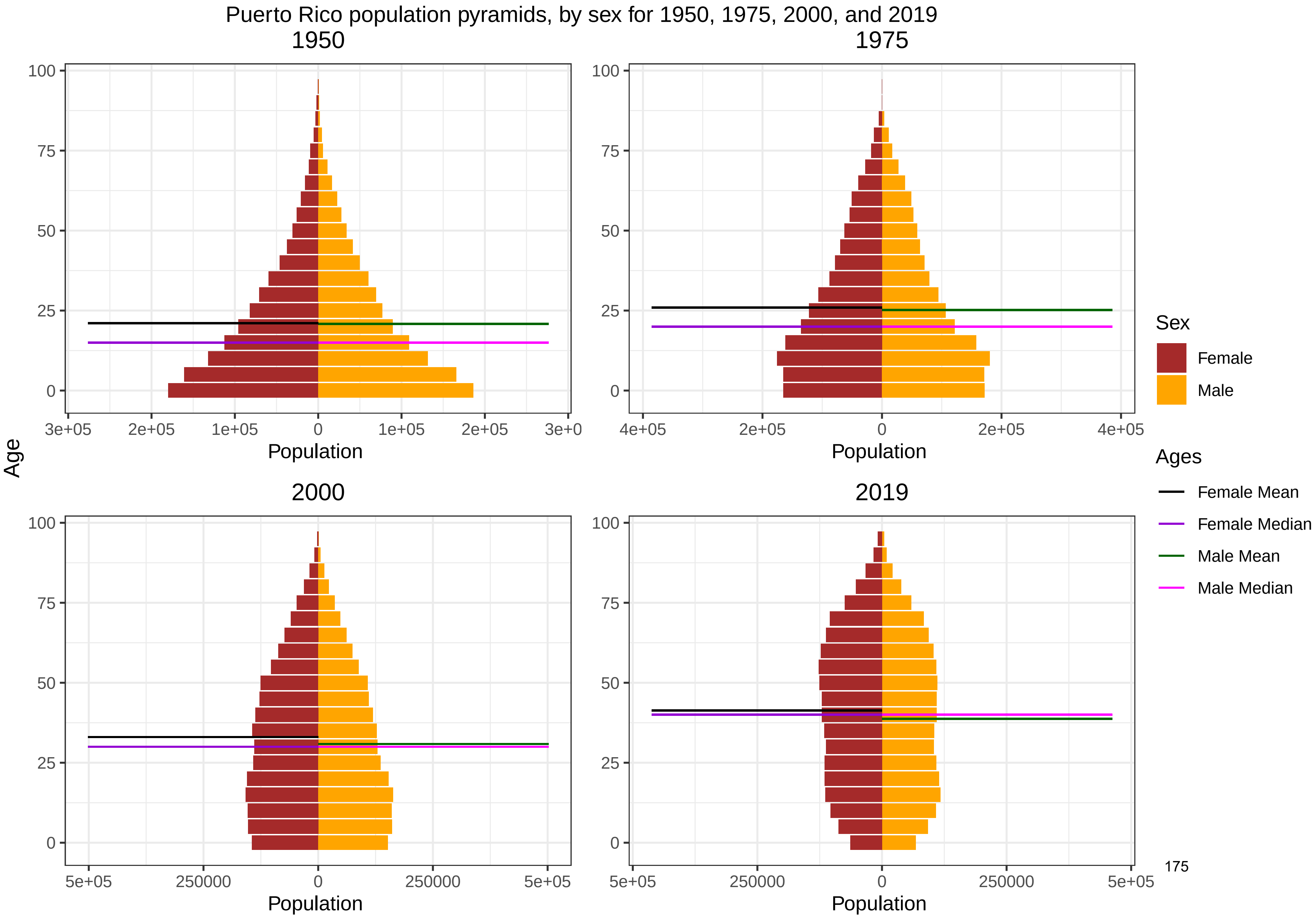

Puerto Rico population pyramids, by sex for 1950, 1975, 2000, and 2019
1950
1975
2000
2019
Age
Population
Sex
Female
Male
Ages
Female Mean
Female Median
Male Mean
Male Median

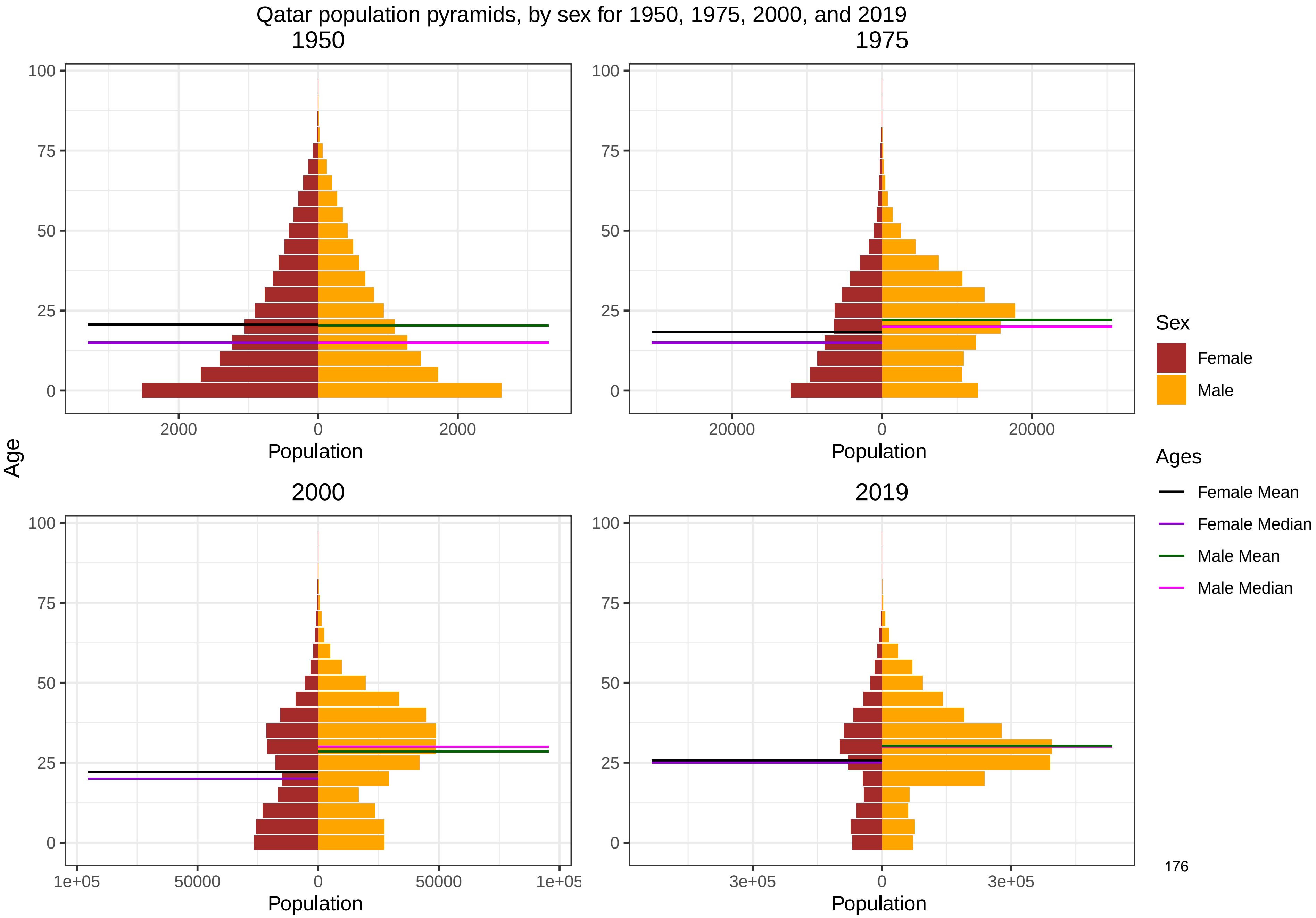
Qatar population pyramids, by sex for 1950, 1975, 2000, and 2019
1950
1975
2000
2019
Age
Population
0
25
50
75
100
2000
20000
50000
1e+05
3e+05
Sex
Female
Male
Ages
Female Mean
Female Median
Male Mean
Male Median

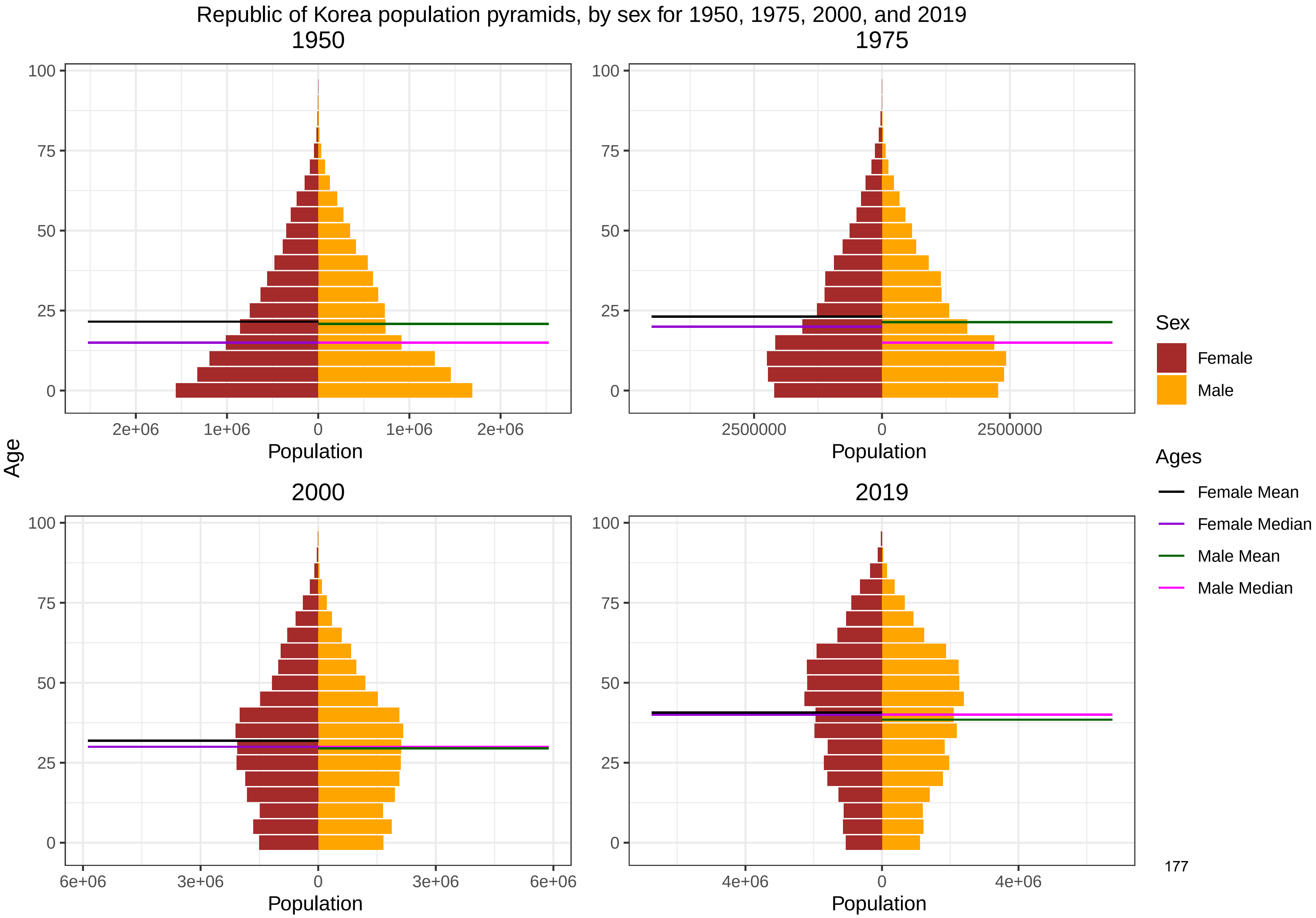
Republic of Korea population pyramids, by sex for 1950, 1975, 2000, and 2019
1950
1975
2000
2019
Age
Population
Sex
Female
Male
Ages
Female Mean
Female Median
Male Mean
Male Median

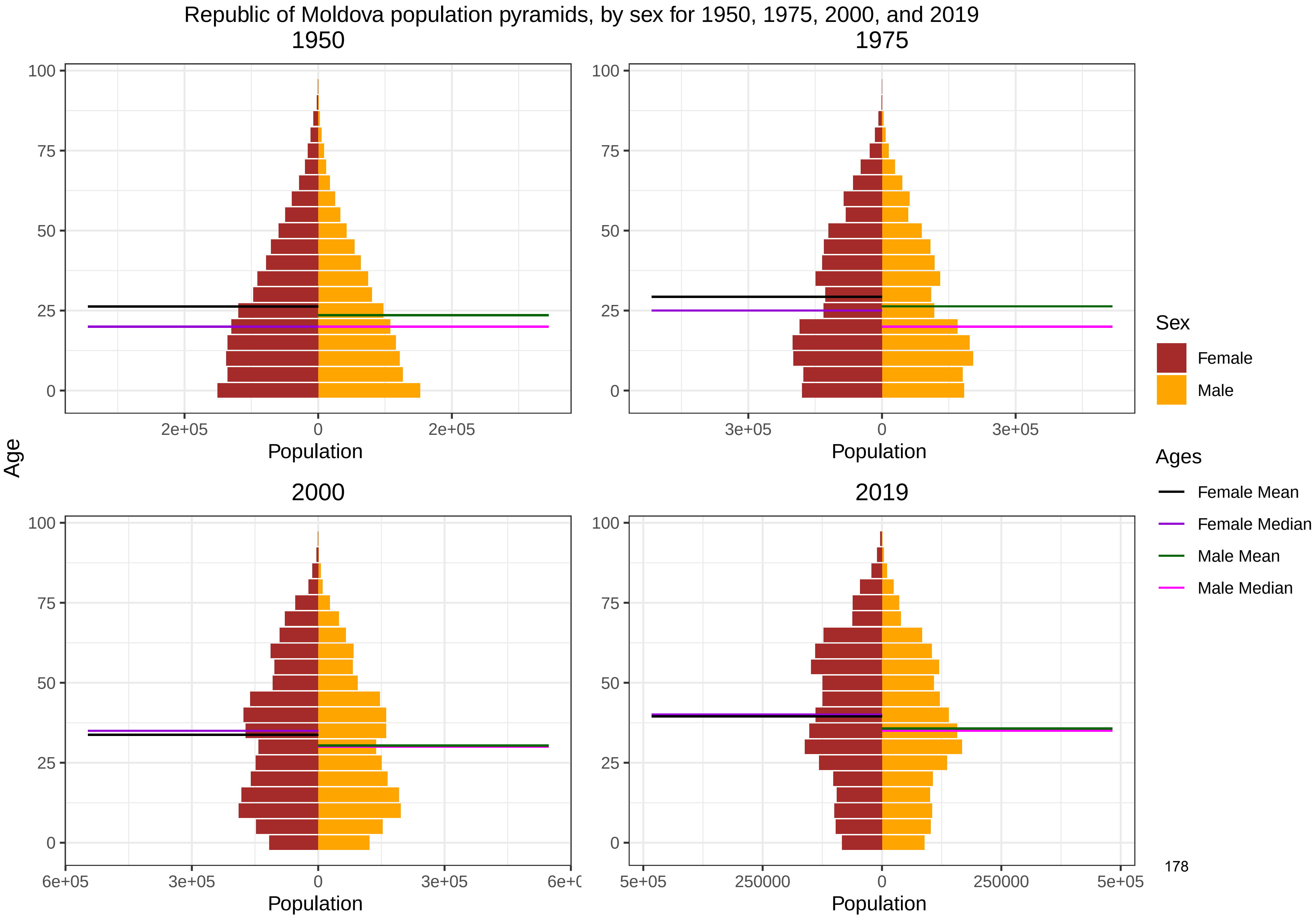

Republic of Moldova population pyramids, by sex for 1950, 1975, 2000, and 2019
1950
1975
2000
2019
Age
Population
2e+05
0
3e+05
6e+05
250000
5e+05
100
75
50
25
Sex
Female
Male
Ages
Female Mean
Female Median
Male Mean
Male Median

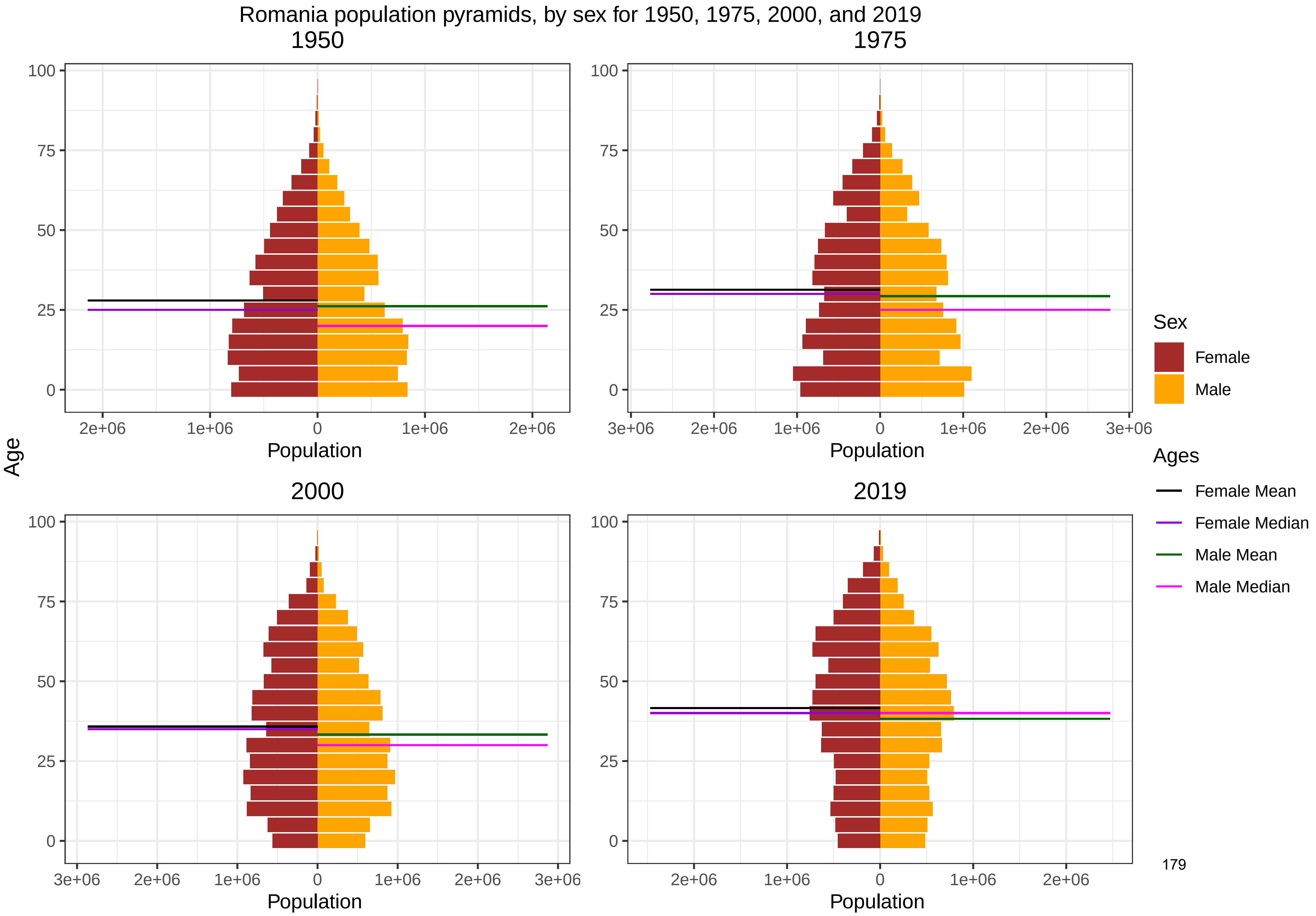

Romania population pyramids, by sex for 1950, 1975, 2000, and 2019
1950
1975
2000
2019
Age
Population
Sex
Female
Male
Ages
Female Mean
Female Median
Male Mean
Male Median

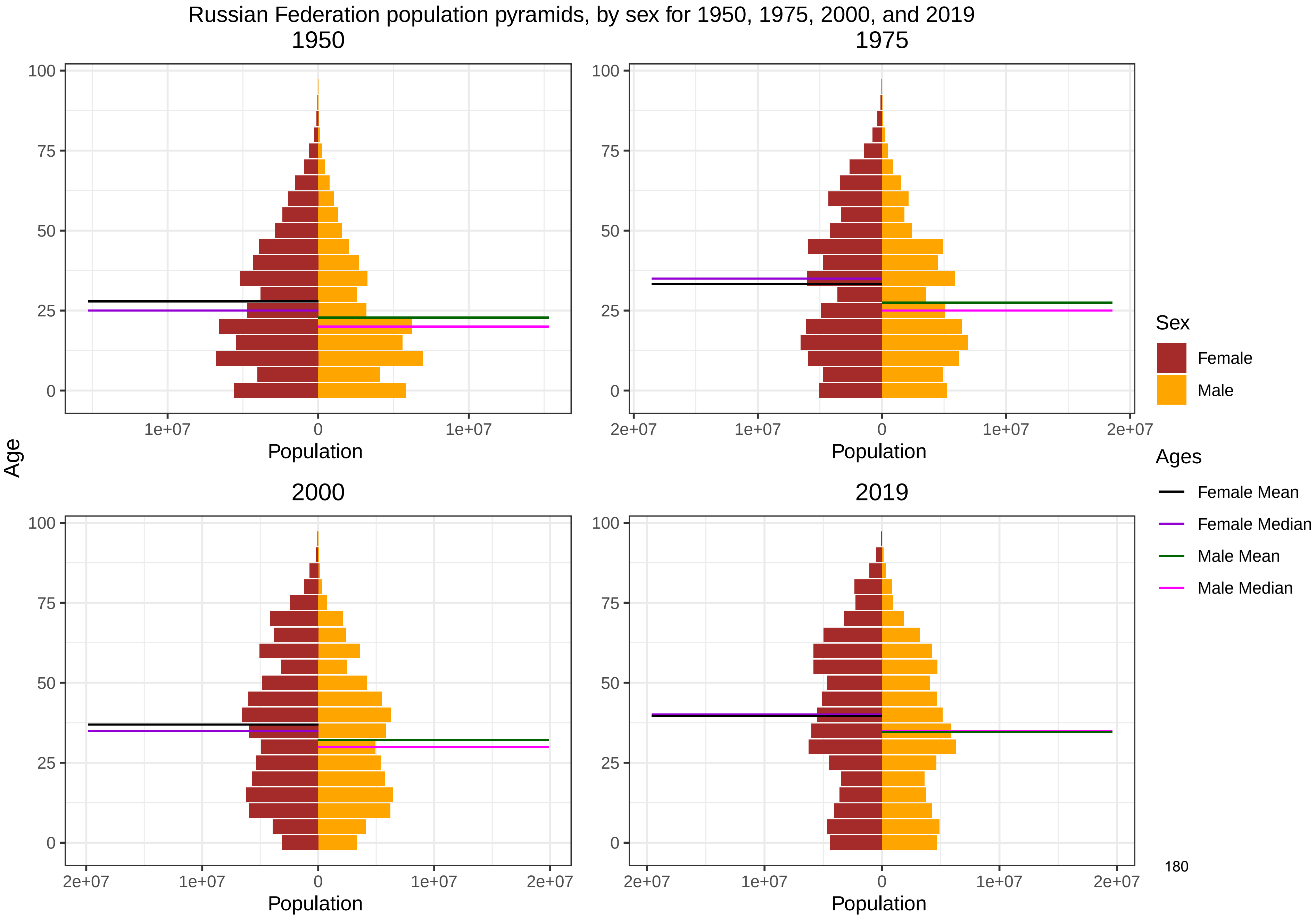

Russian Federation population pyramids, by sex for 1950, 1975, 2000, and 2019
1950
1975
2000
2019
Age
Population
100
75
50
25
0
1e+07
0
2e+07
Sex
Female
Male
Ages
Female Mean
Female Median
Male Mean
Male Median

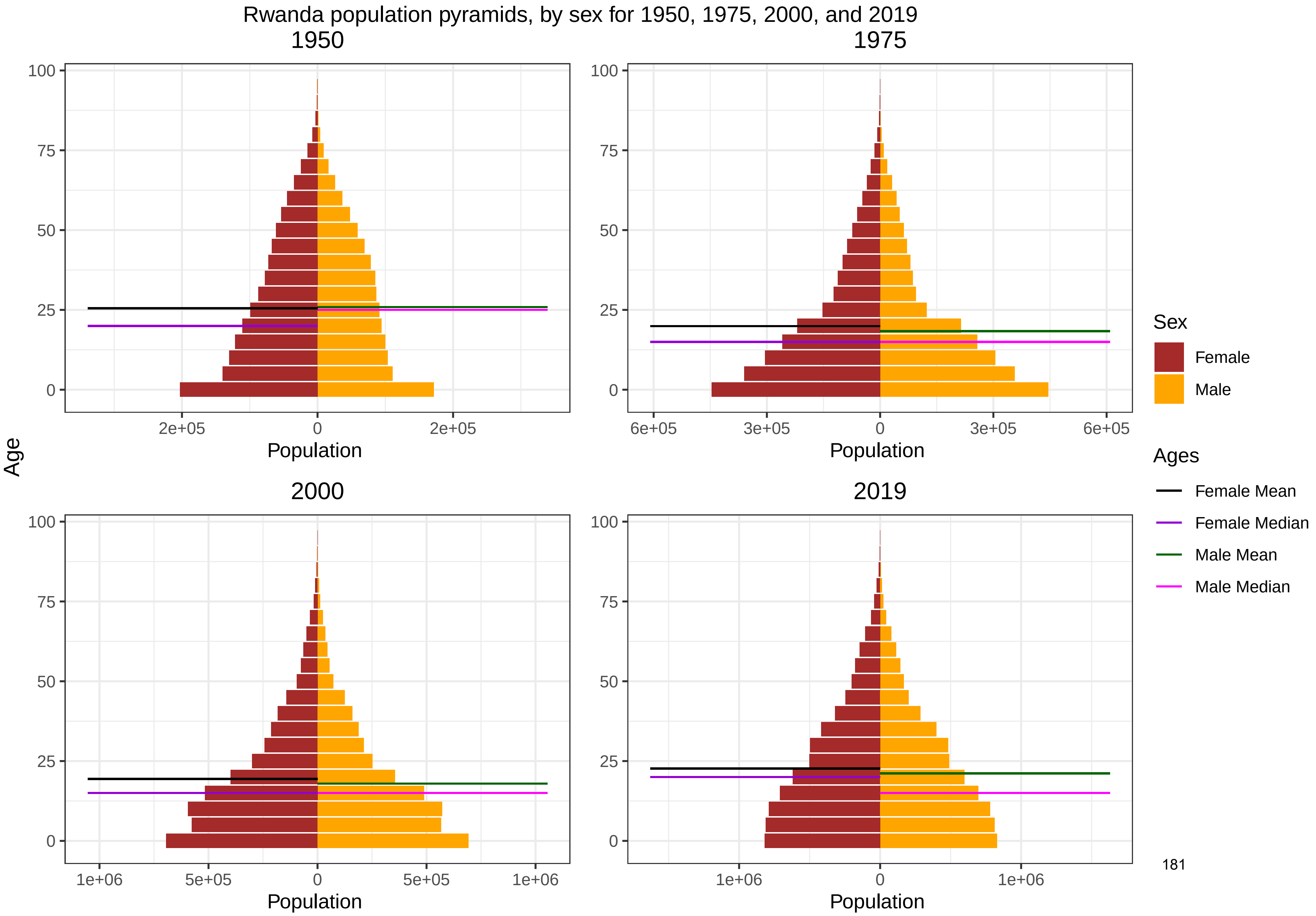

Rwanda population pyramids, by sex for 1950, 1975, 2000, and 2019
1950
1975
2000
2019
Age
Population
Sex
Female
Male
Ages
Female Mean
Female Median
Male Mean
Male Median

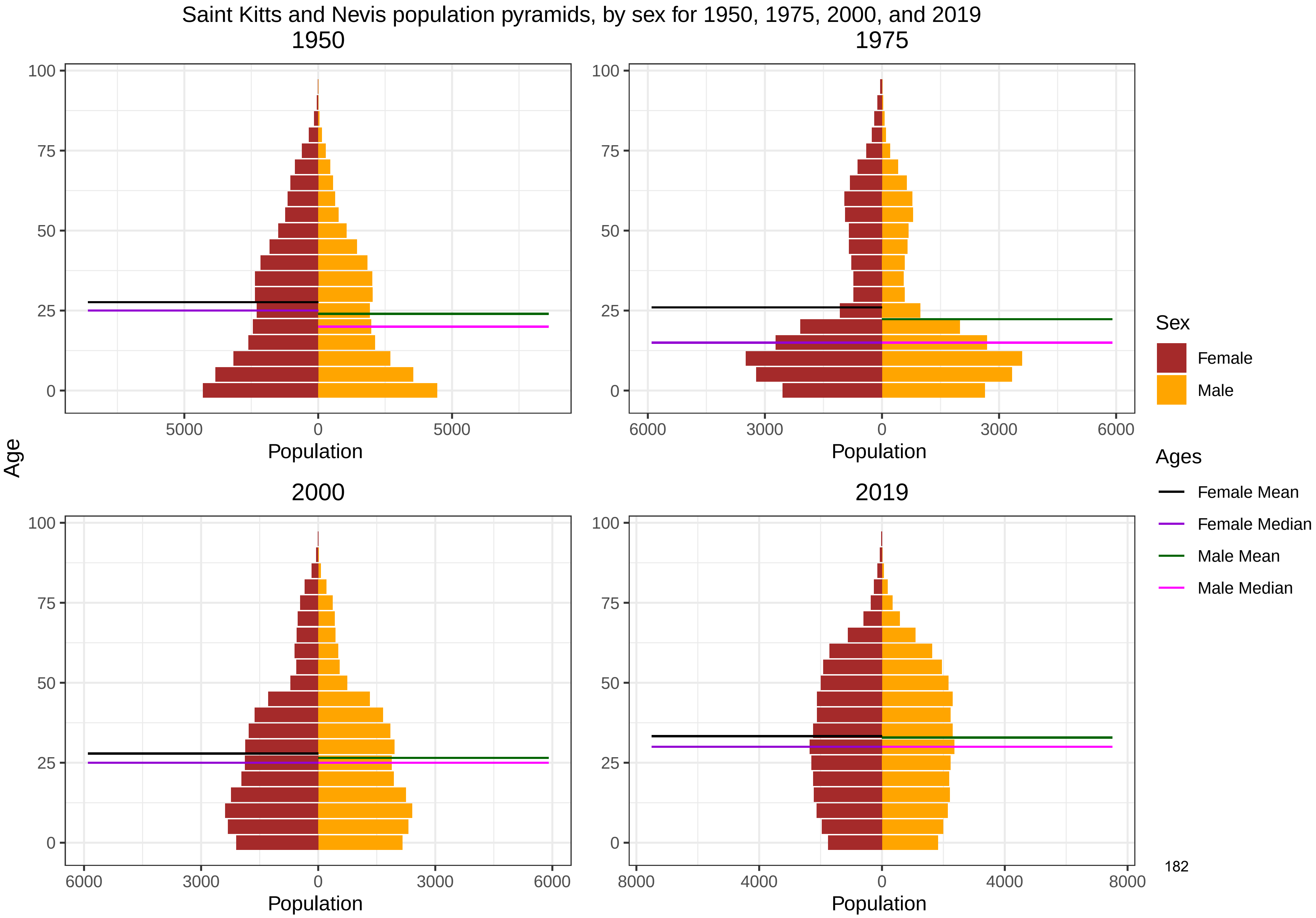
Saint Kitts and Nevis population pyramids, by sex for 1950, 1975, 2000, and 2019
1950
1975
2000
2019
Age
Population
Sex
Female
Male
Ages
Female Mean
Female Median
Male Mean
Male Median

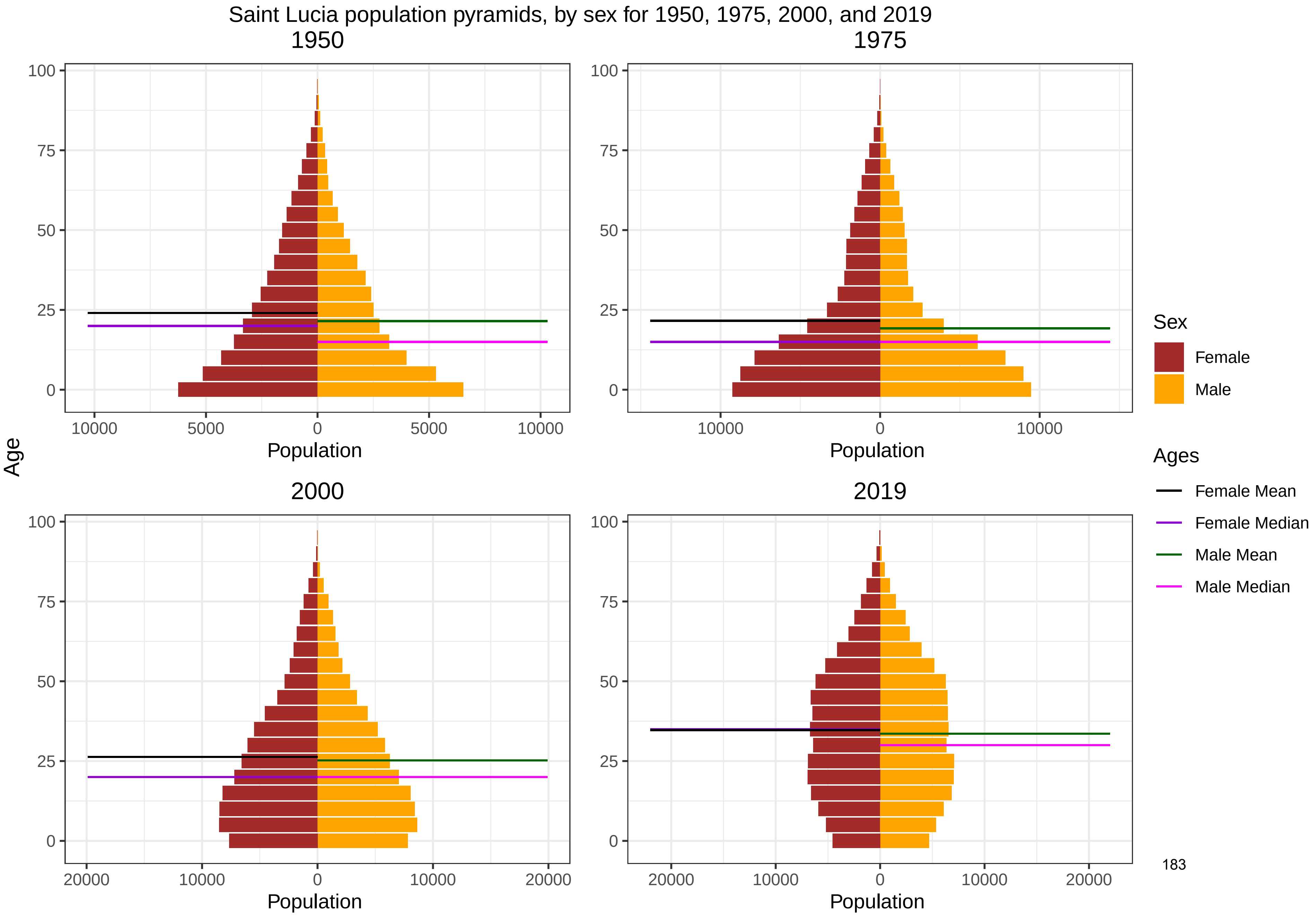
Saint Lucia population pyramids, by sex for 1950, 1975, 2000, and 2019
1950
1975
2000
2019
Age
Population
Sex
Female
Male
Ages
Female Mean
Female Median
Male Mean
Male Median

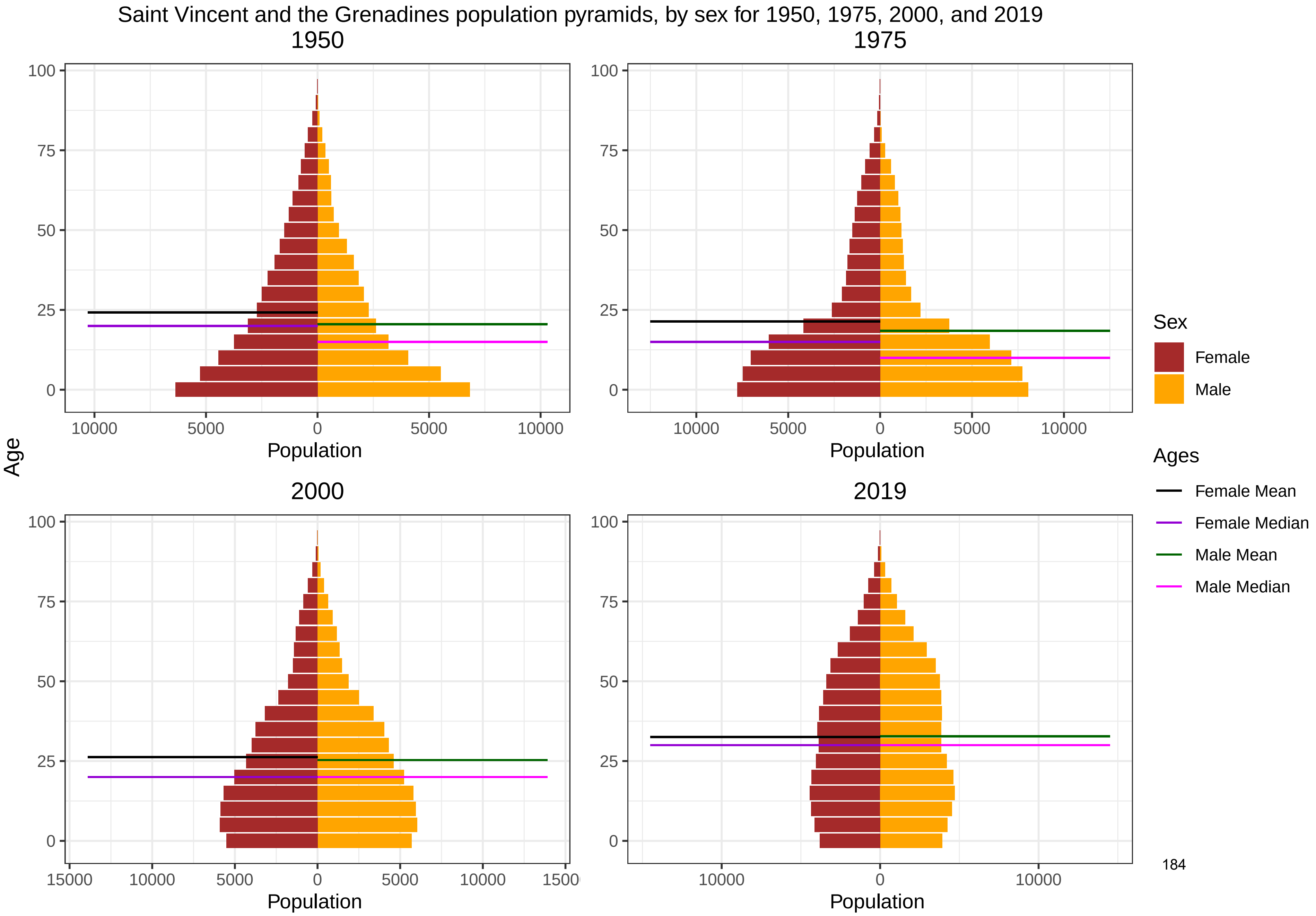

Saint Vincent and the Grenadines population pyramids, by sex for 1950, 1975, 2000, and 2019
1950
1975
2000
2019
Age
Population
Sex
Female
Male
Ages
Female Mean
Female Median
Male Mean
Male Median

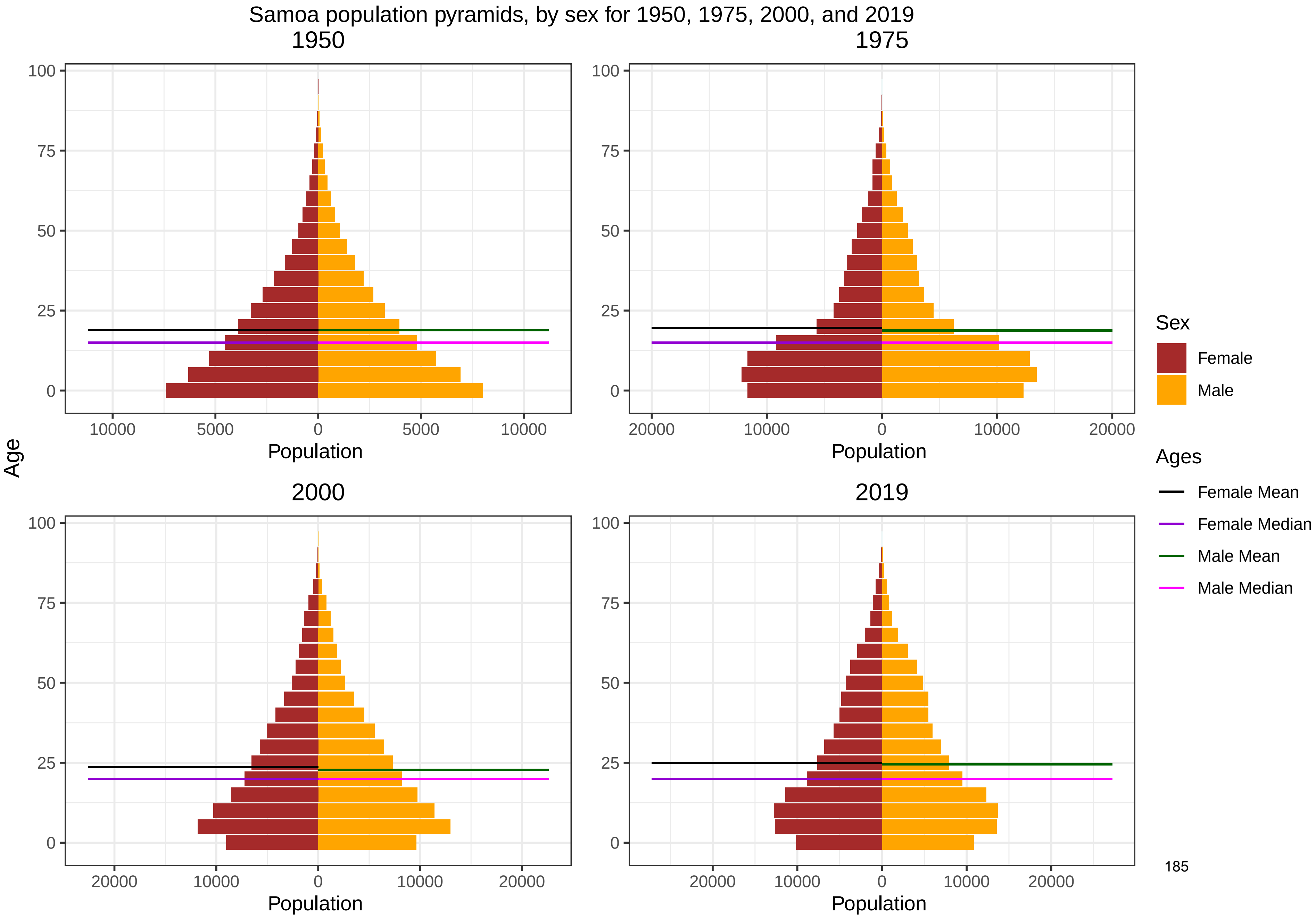

Samoa population pyramids, by sex for 1950, 1975, 2000, and 2019
1950
1975
2000
2019
Age
Population
Sex
Female
Male
Ages
Female Mean
Female Median
Male Mean
Male Median

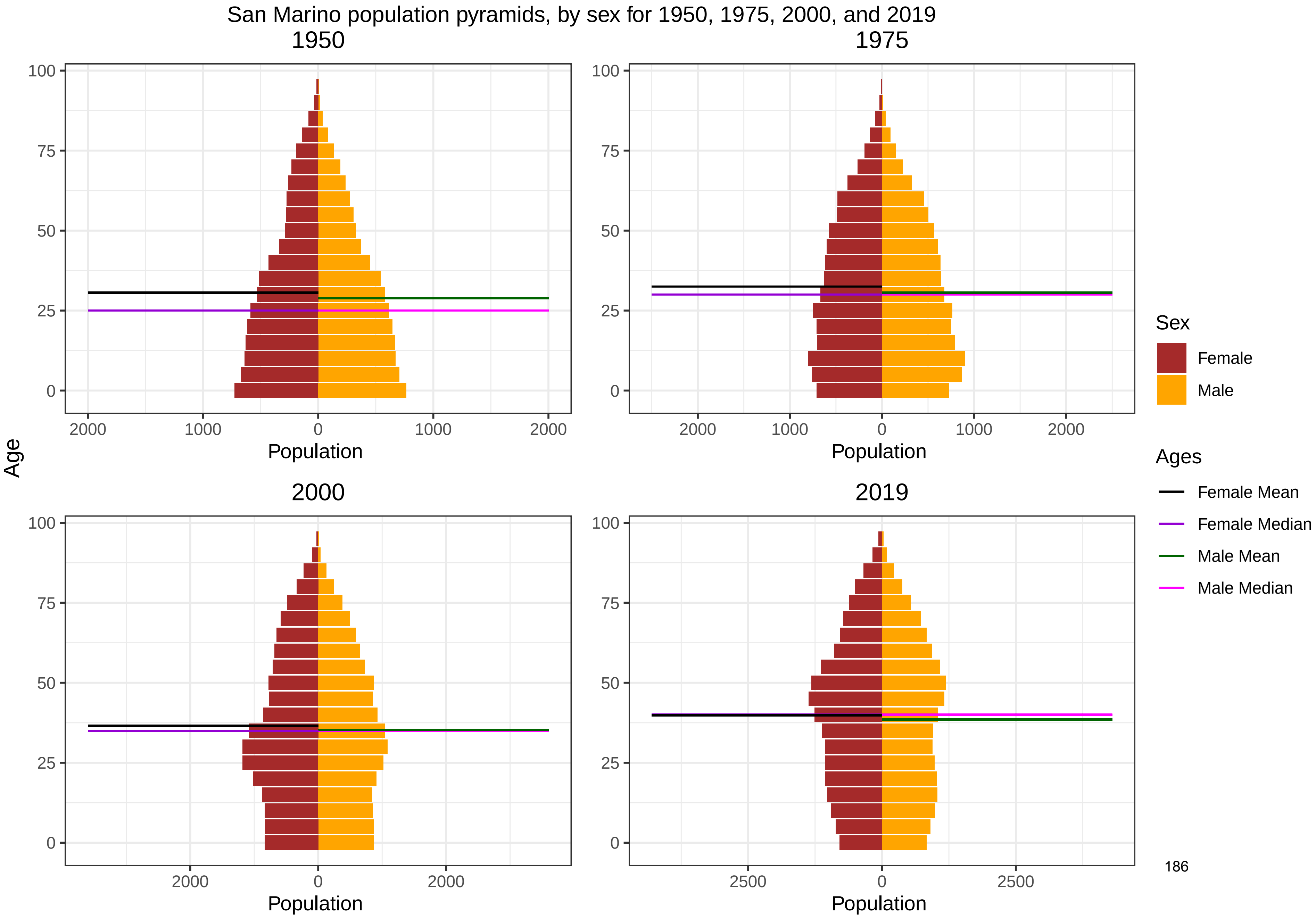

San Marino population pyramids, by sex for 1950, 1975, 2000, and 2019
1950
1975
2000
2019
Age
Population
100
75
50
25
0
2000
1000
0
2500
Sex
Female
Male
Ages
Female Mean
Female Median
Male Mean
Male Median

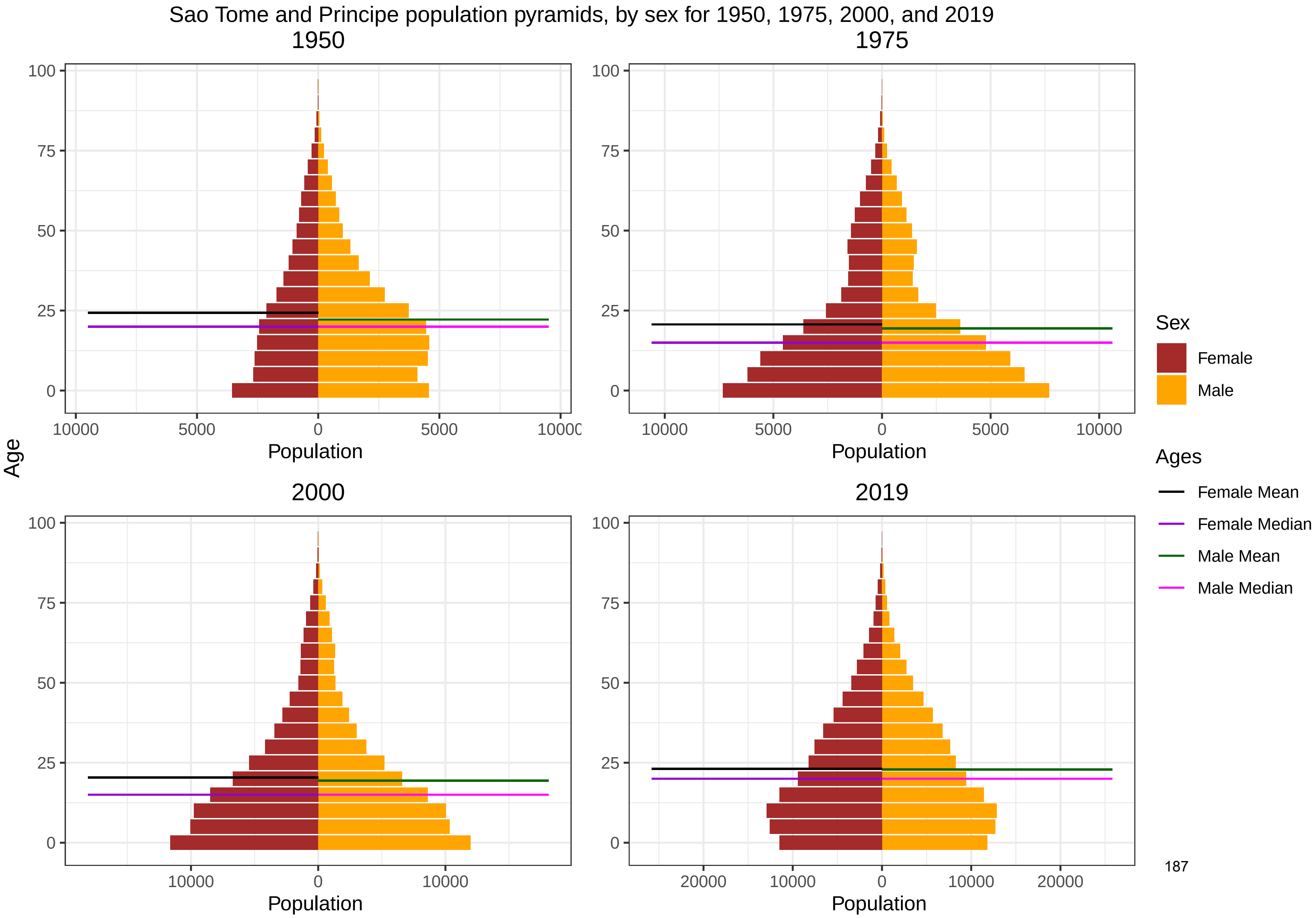
Sao Tome and Principe population pyramids, by sex for 1950, 1975, 2000, and 2019
1950
1975
2000
2019
Age
Population
Sex
Female
Male
Ages
Female Mean
Female Median
Male Mean
Male Median

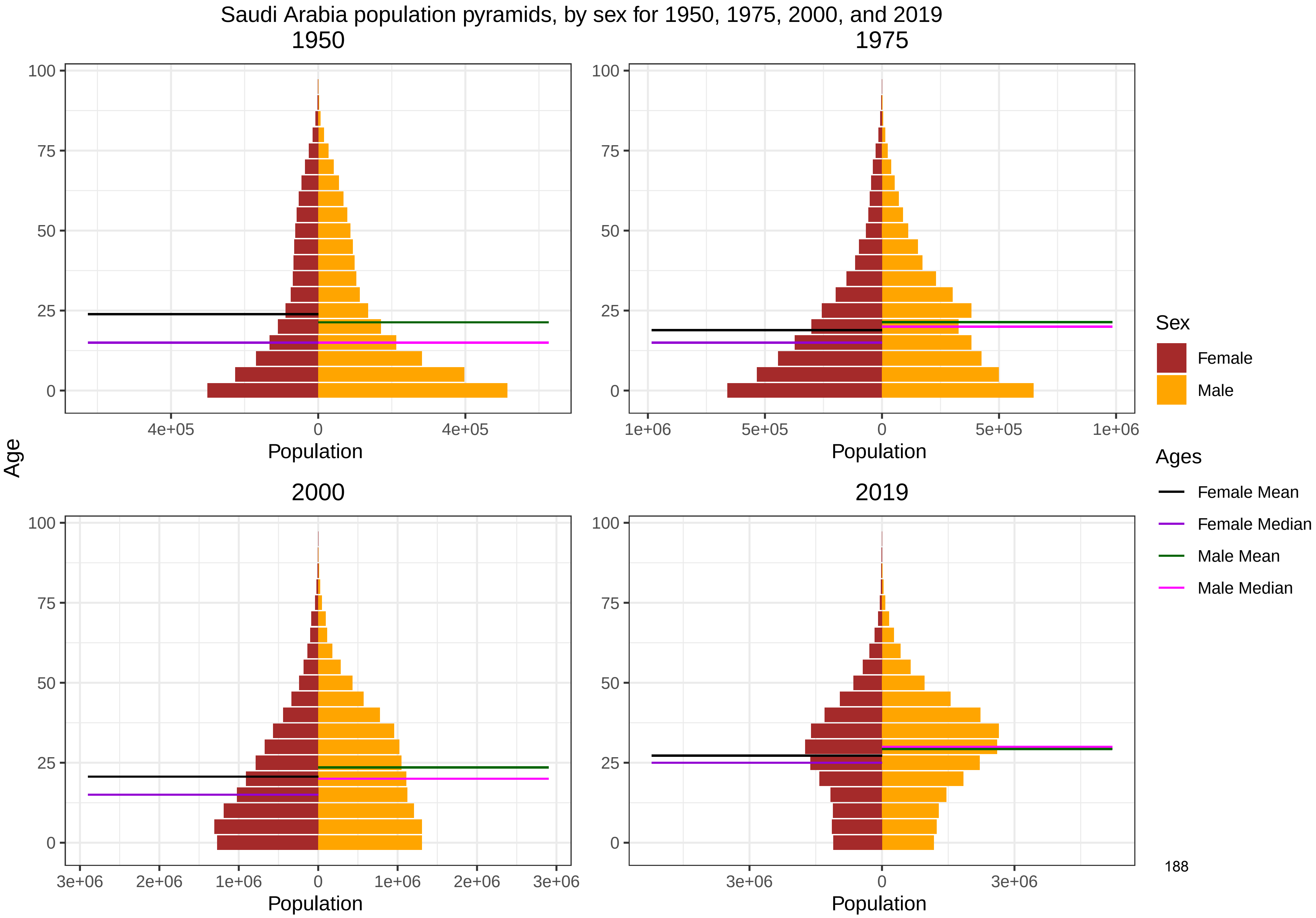

Saudi Arabia population pyramids, by sex for 1950, 1975, 2000, and 2019
1950
1975
2000
2019
Age
Population
Sex
Female
Male
Ages
Female Mean
Female Median
Male Mean
Male Median

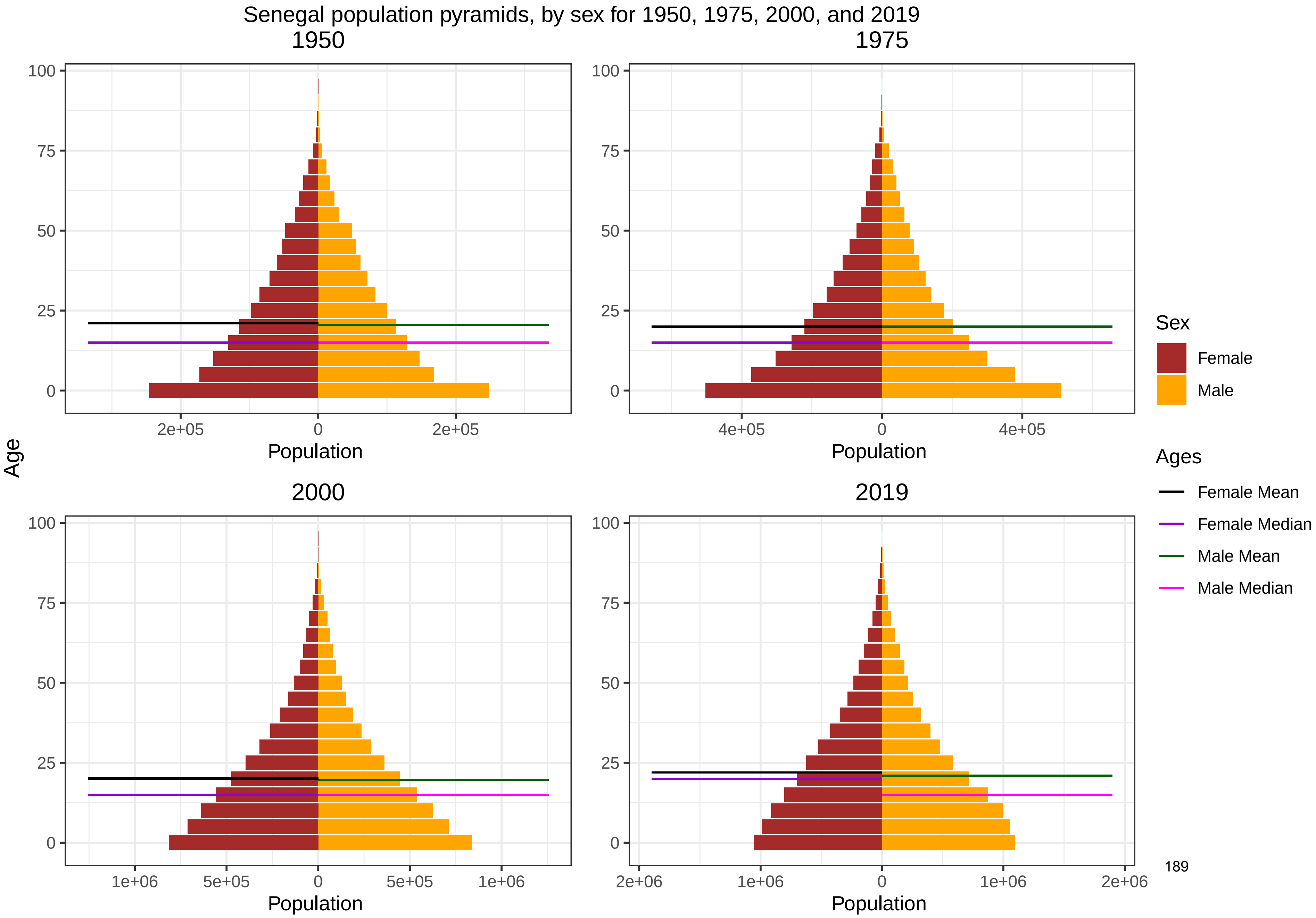
Senegal population pyramids, by sex for 1950, 1975, 2000, and 2019
1950
1975
2000
2019
Age
Population
Sex
Female
Male
Ages
Female Mean
Female Median
Male Mean
Male Median

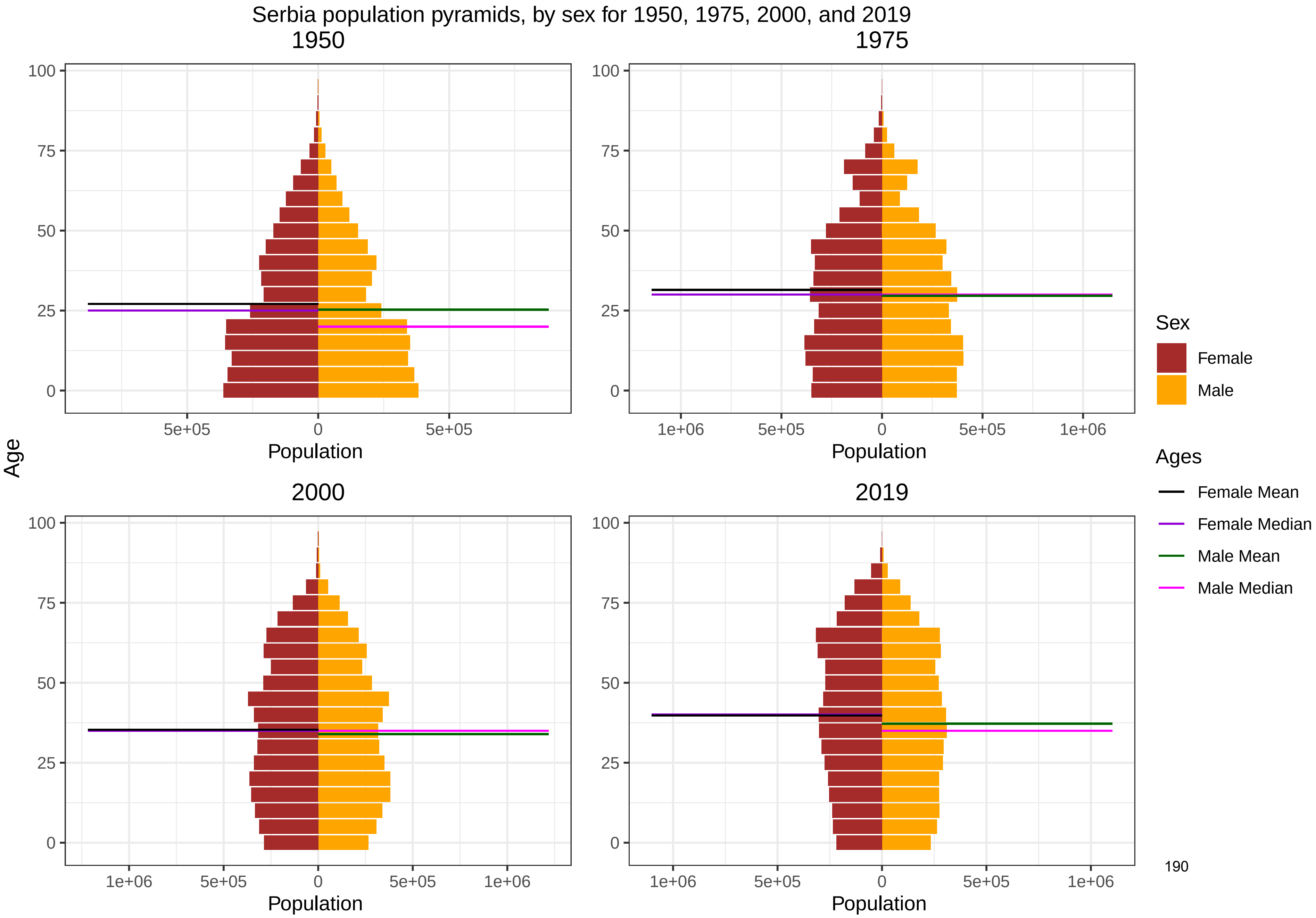

Serbia population pyramids, by sex for 1950, 1975, 2000, and 2019
1950
1975
2000
2019
Age
Population
Sex
Female
Male
Ages
Female Mean
Female Median
Male Mean
Male Median

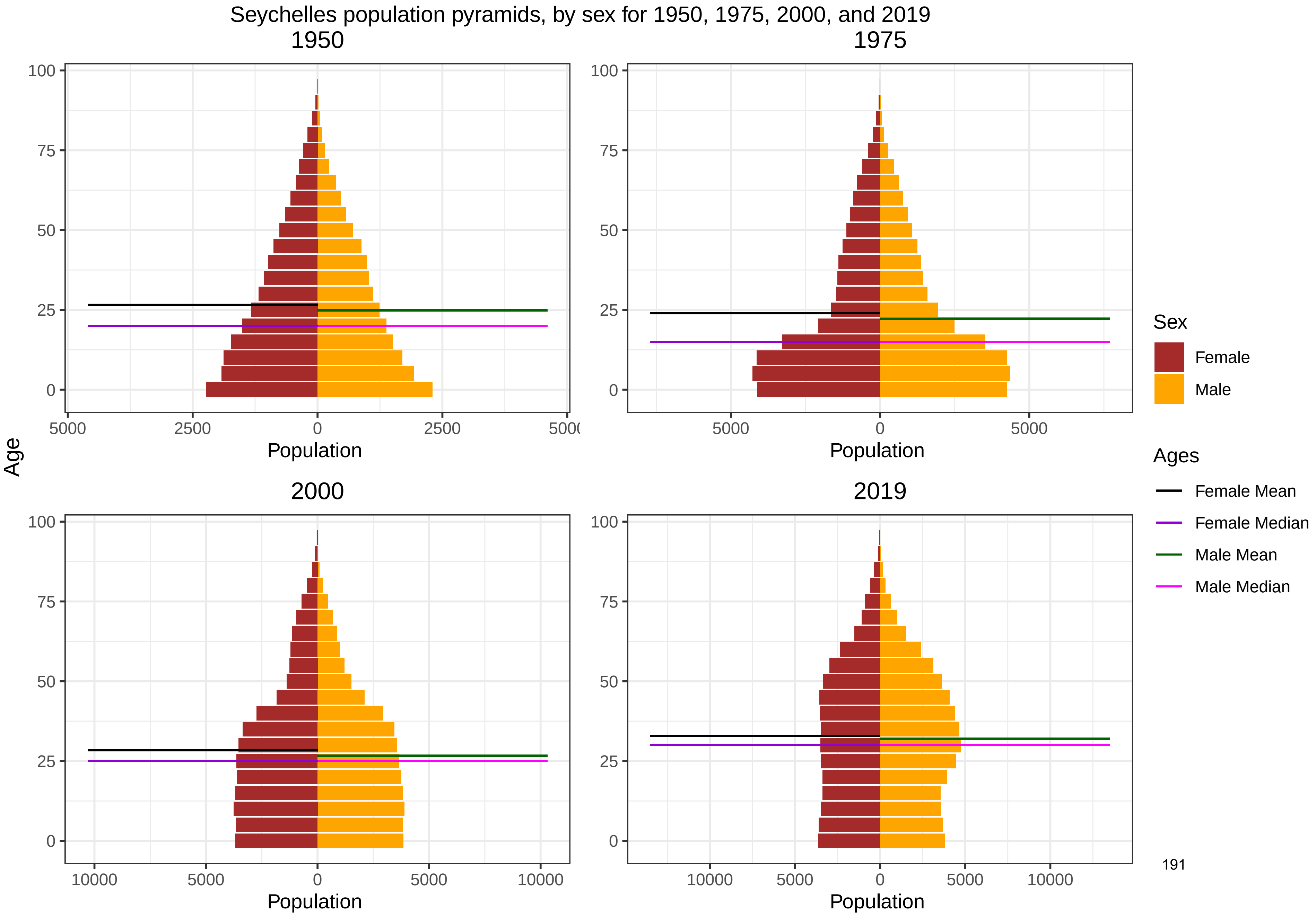
Seychelles population pyramids, by sex for 1950, 1975, 2000, and 2019
1950
1975
2000
2019
Age
Population
Sex
Female
Male
Ages
Female Mean
Female Median
Male Mean
Male Median

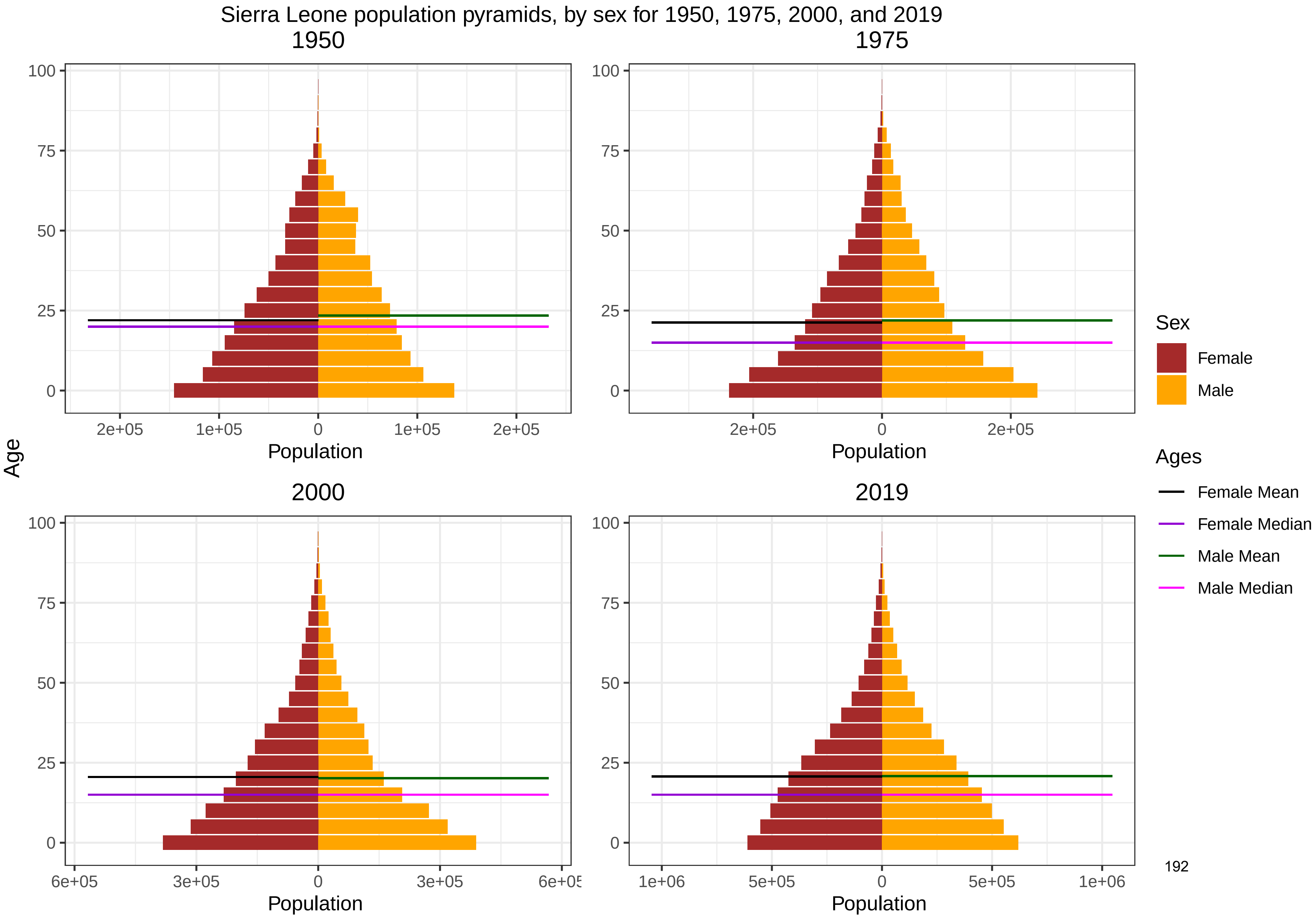

Sierra Leone population pyramids, by sex for 1950, 1975, 2000, and 2019
1950
1975
2000
2019
Age
Population
Sex
Female
Male
Ages
Female Mean
Female Median
Male Mean
Male Median

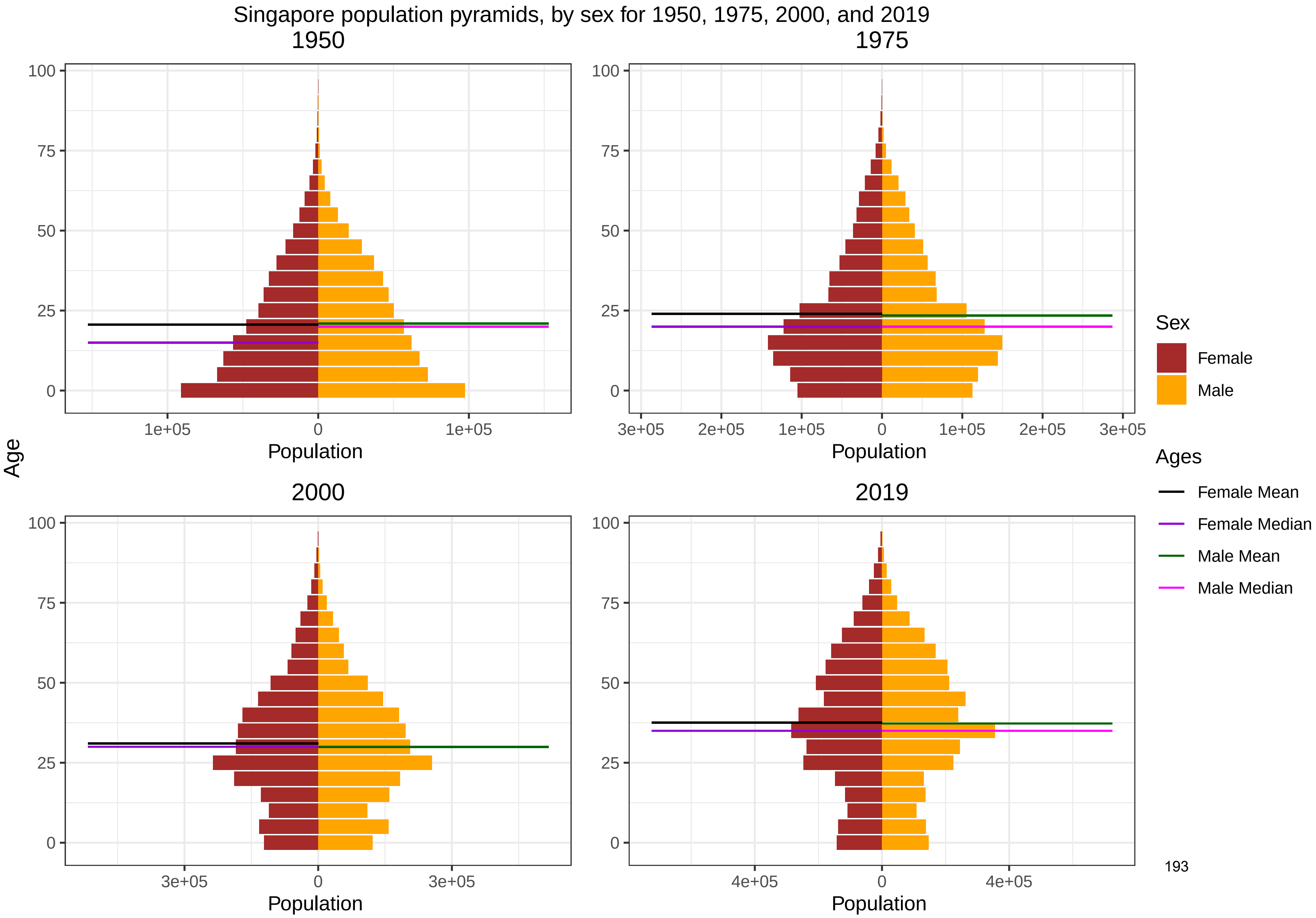

Singapore population pyramids, by sex for 1950, 1975, 2000, and 2019
1950
1975
2000
2019
Age
Population
0
25
50
75
100
1e+05
2e+05
3e+05
4e+05
Sex
Female
Male
Ages
Female Mean
Female Median
Male Mean
Male Median

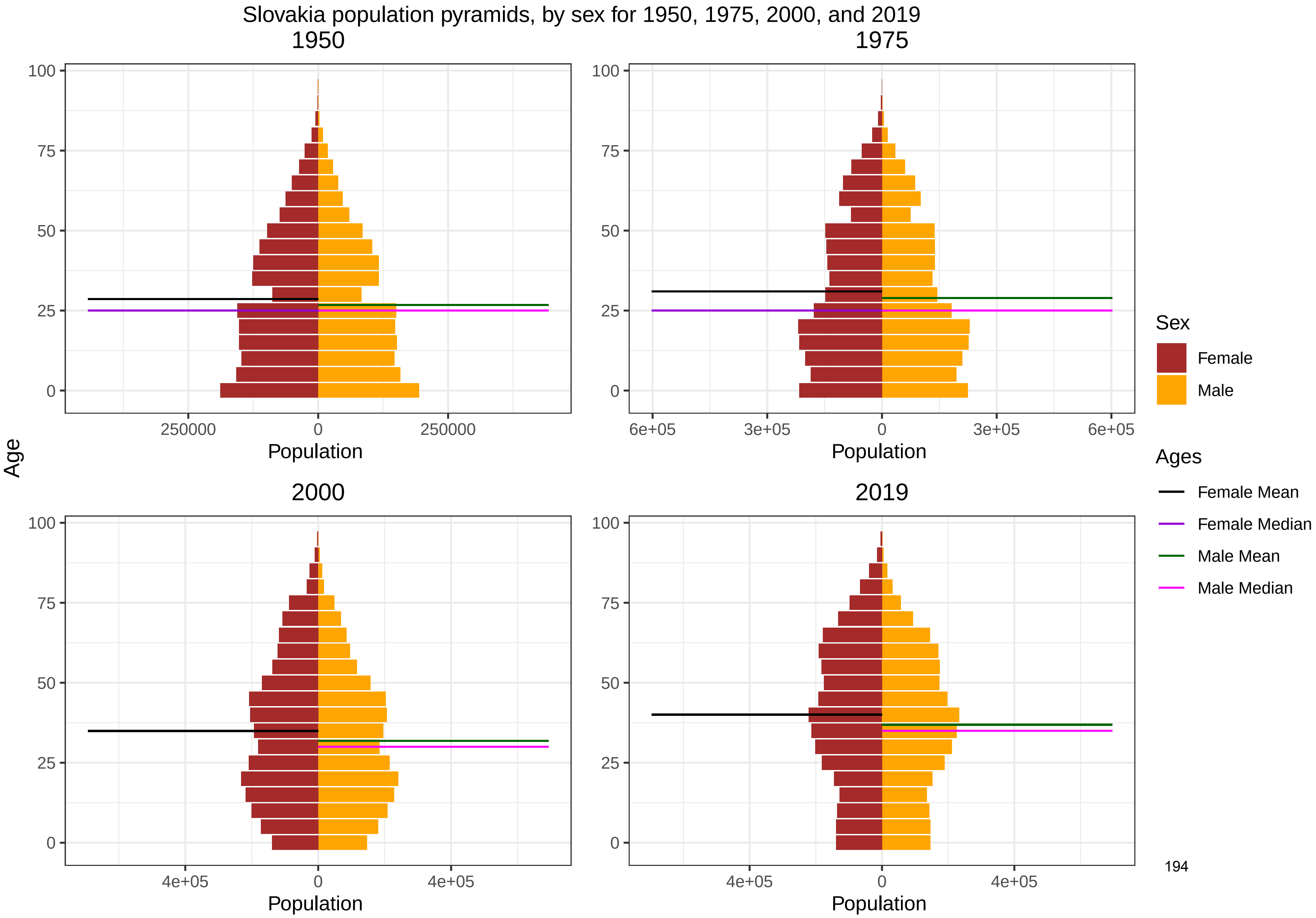

Slovakia population pyramids, by sex for 1950, 1975, 2000, and 2019
1950
1975
2000
2019
Age
Population
Sex
Female
Male
Ages
Female Mean
Female Median
Male Mean
Male Median

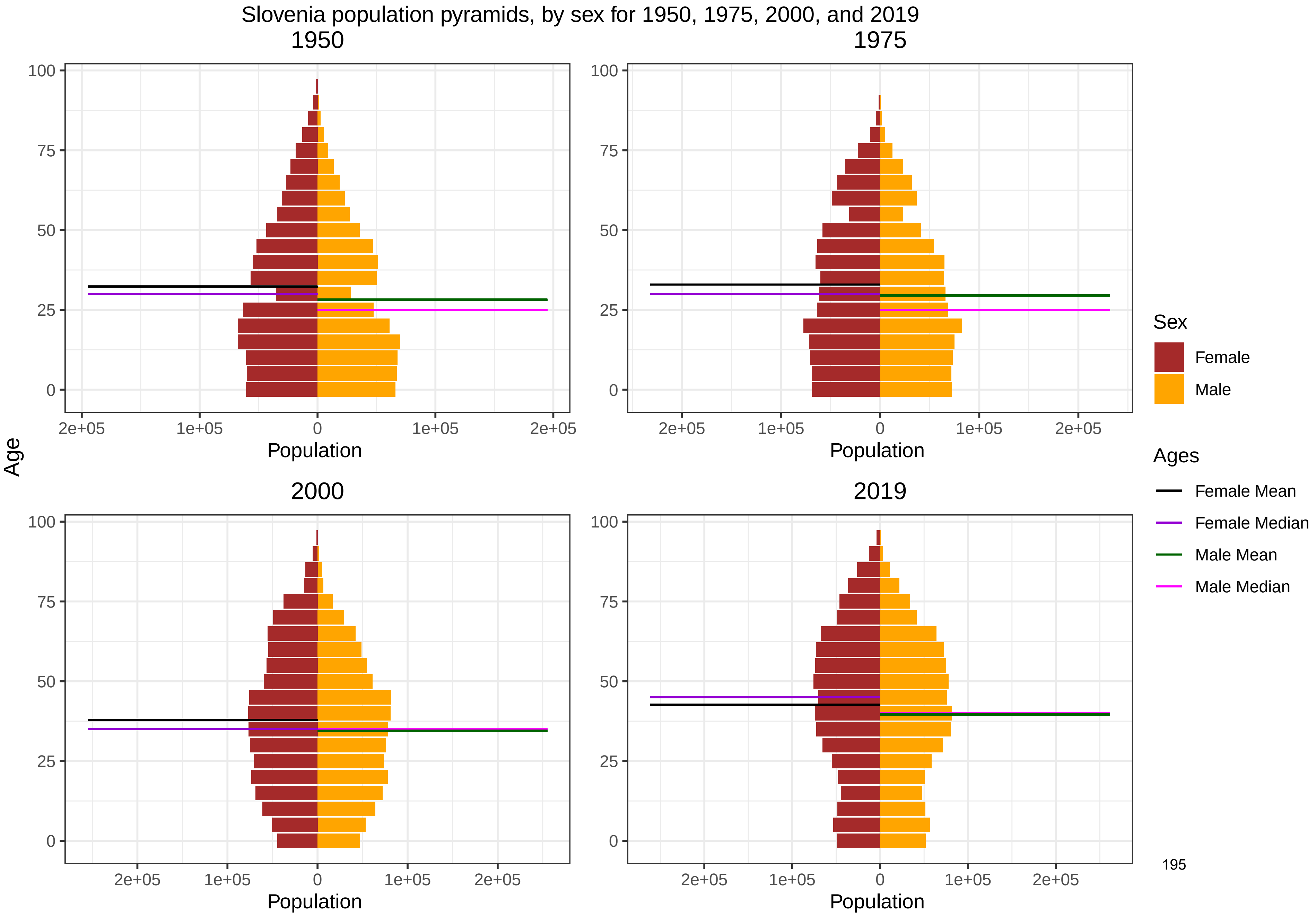
Slovenia population pyramids, by sex for 1950, 1975, 2000, and 2019
1950
1975
2000
2019
Age
Population
100
75
50
25
0
2e+05
1e+05
0
1e+05
2e+05
Sex
Female
Male
Ages
Female Mean
Female Median
Male Mean
Male Median

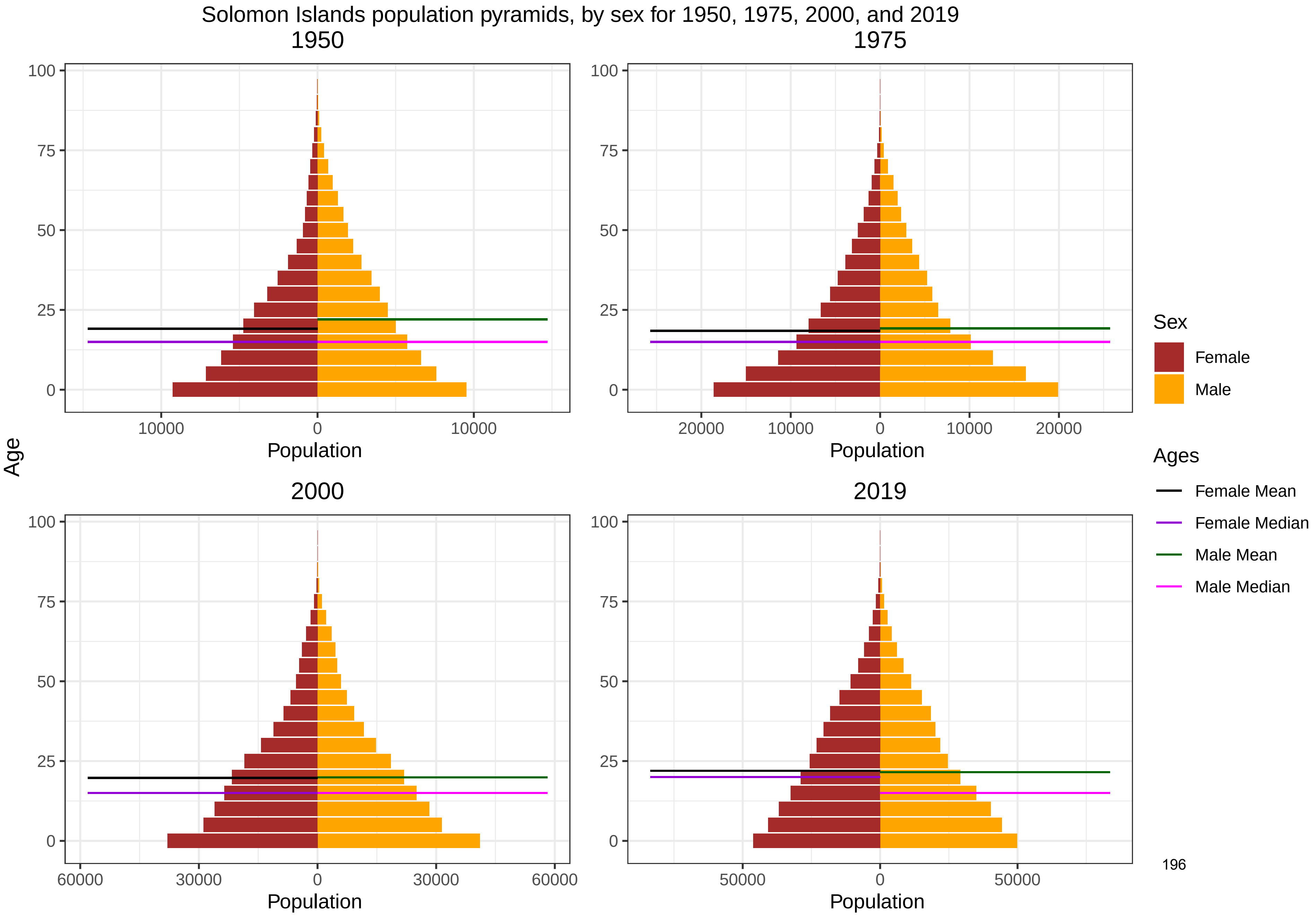
Solomon Islands population pyramids, by sex for 1950, 1975, 2000, and 2019
1950
1975
2000
2019
Age
Population
100
75
50
25
0
10000
0
20000
30000
60000
50000
Sex
Female
Male
Ages
Female Mean
Female Median
Male Mean
Male Median

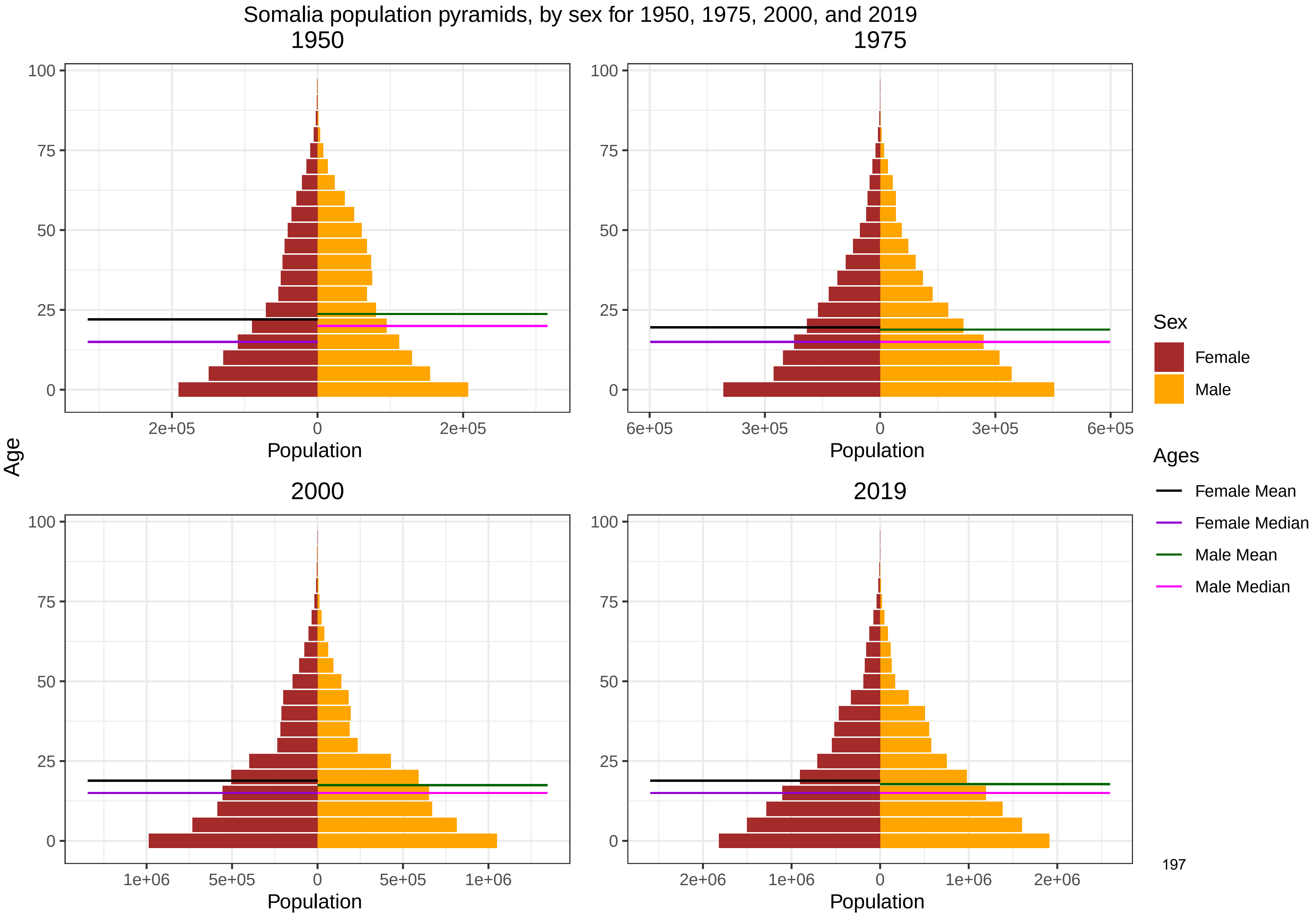
Somalia population pyramids, by sex for 1950, 1975, 2000, and 2019
1950
1975
2000
2019
Age
Population
Sex
Female
Male
Ages
Female Mean
Female Median
Male Mean
Male Median

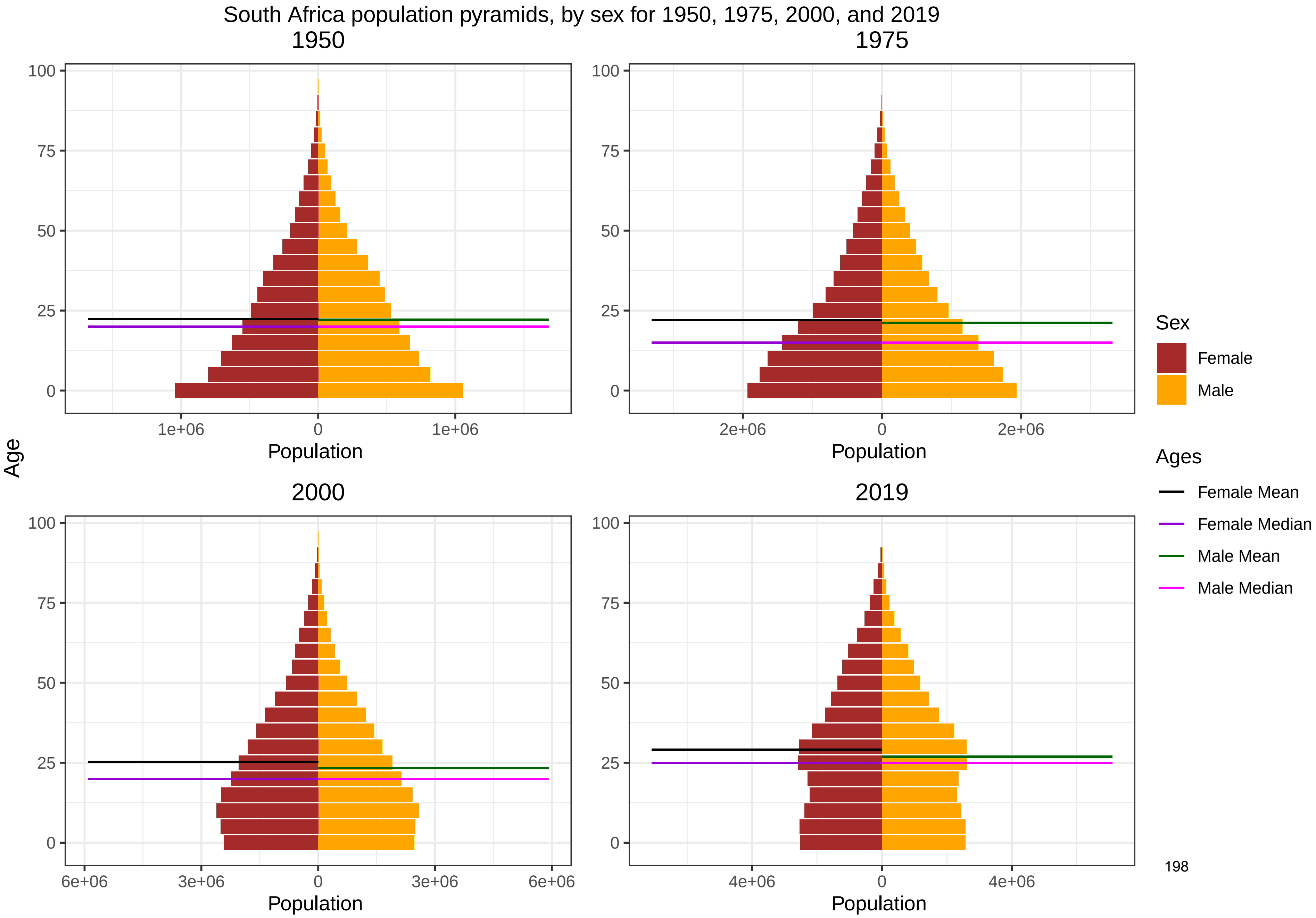
South Africa population pyramids, by sex for 1950, 1975, 2000, and 2019
1950
1975
2000
2019
Age
Population
100
75
50
25
0
1e+06
0
1e+06
2e+06
0
2e+06
6e+06
3e+06
0
3e+06
6e+06
4e+06
0
4e+06
Sex
Female
Male
Ages
Female Mean
Female Median
Male Mean
Male Median

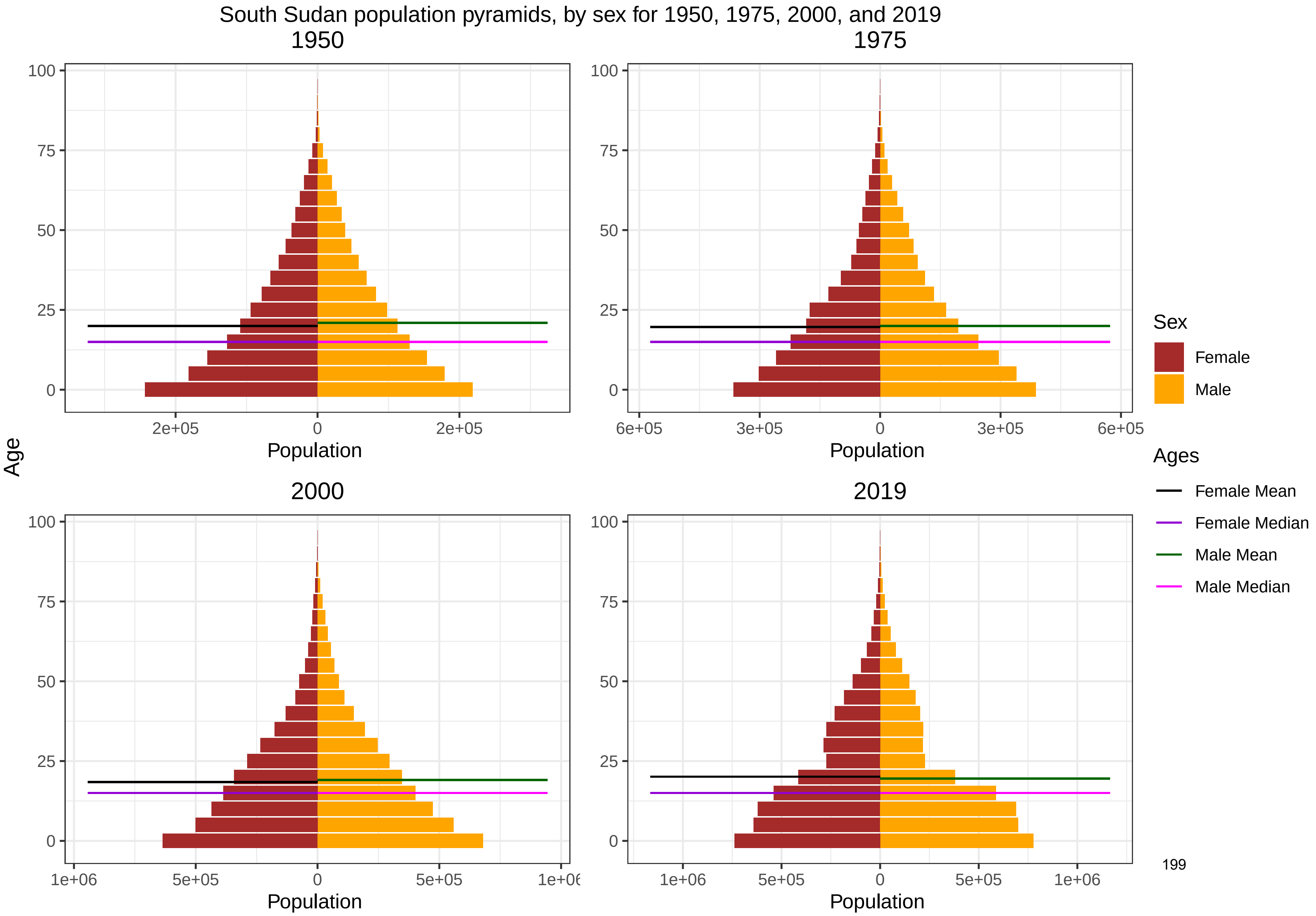

South Sudan population pyramids, by sex for 1950, 1975, 2000, and 2019
1950
1975
2000
2019
Age
Population
100
75
50
25
0
2e+05
0
2e+05
6e+05
3e+05
0
3e+05
6e+05
1e+06
5e+05
0
5e+05
1e+06
Sex
Female
Male
Ages
Female Mean
Female Median
Male Mean
Male Median

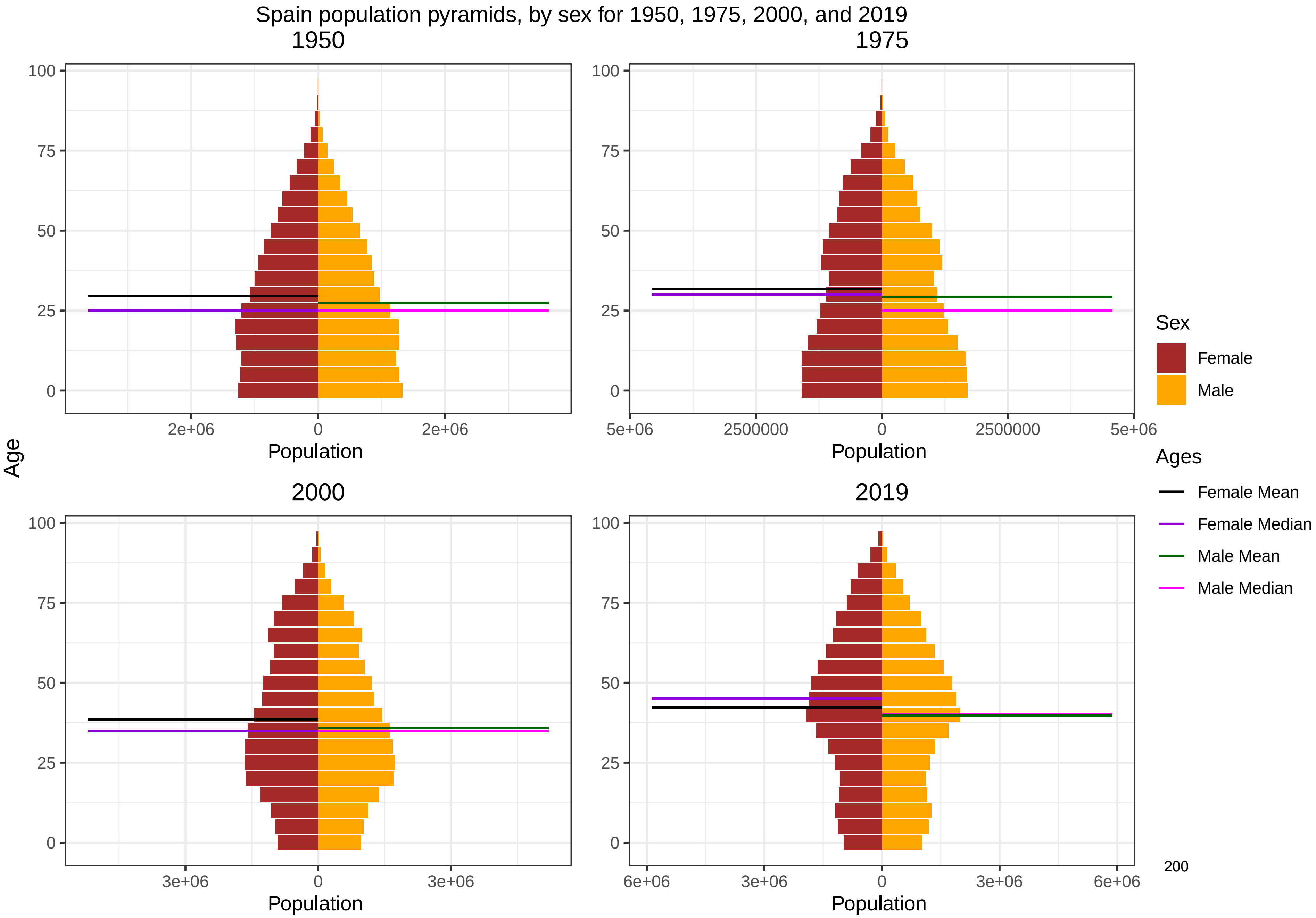

Spain population pyramids, by sex for 1950, 1975, 2000, and 2019
1950
1975
2000
2019
Age
Population
Sex
Female
Male
Ages
Female Mean
Female Median
Male Mean
Male Median

200

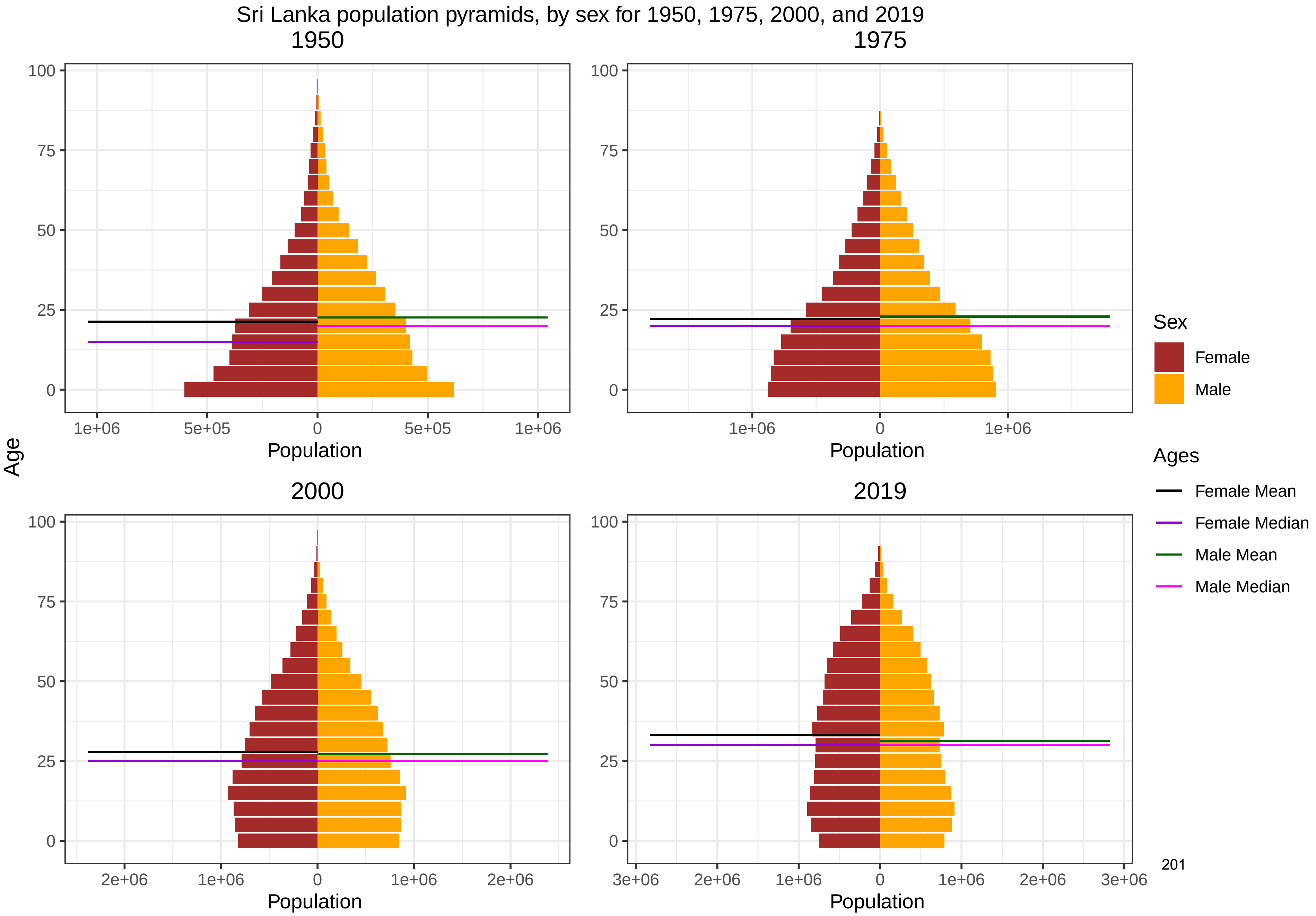

Sri Lanka population pyramids, by sex for 1950, 1975, 2000, and 2019
1950
1975
2000
2019
Age
Population
100
75
50
25
0
1e+06
5e+05
0
5e+05
1e+06
2e+06
3e+06
Sex
Female
Male
Ages
Female Mean
Female Median
Male Mean
Male Median

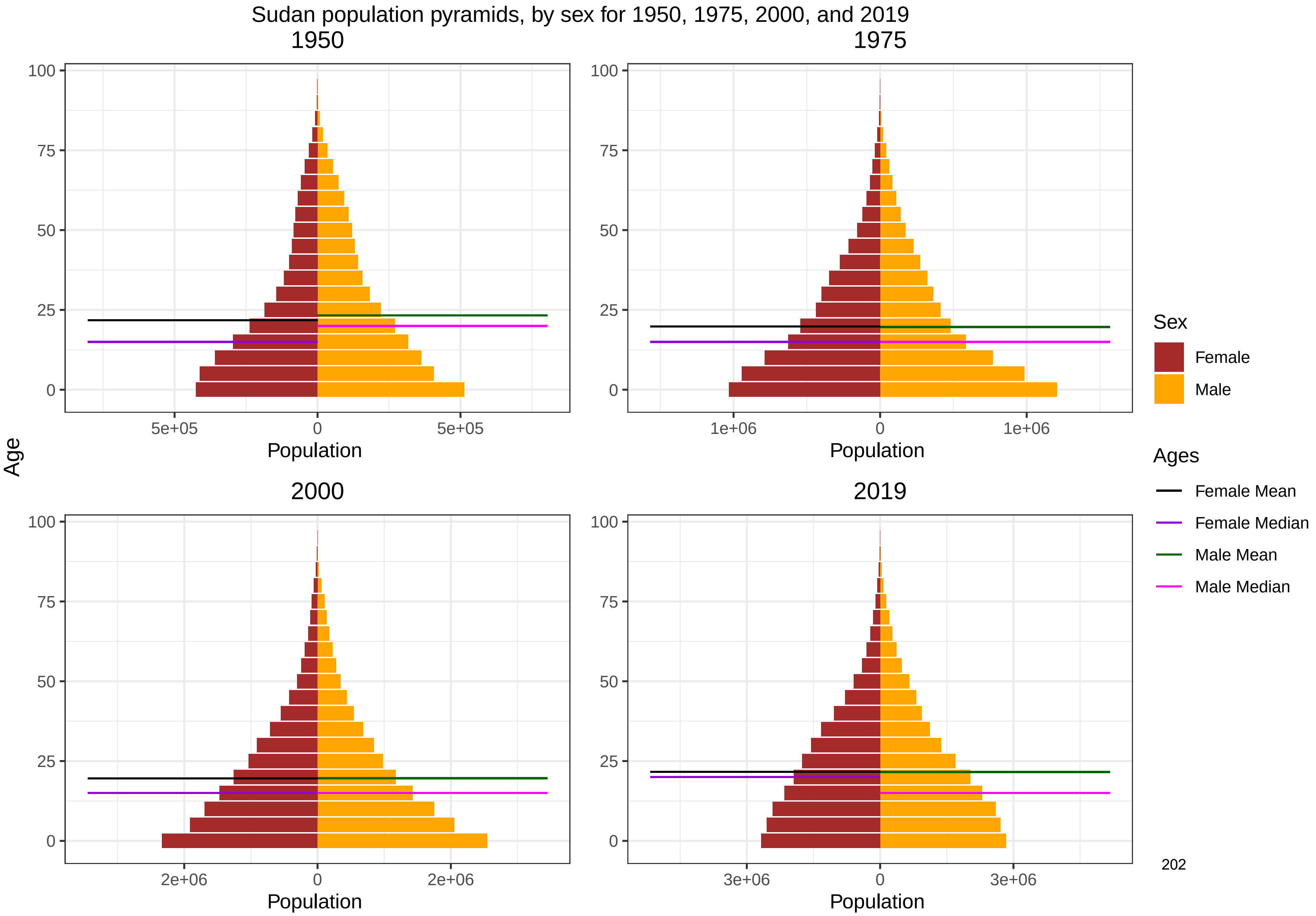

Sudan population pyramids, by sex for 1950, 1975, 2000, and 2019
1950
1975
2000
2019
Age
Population
100
75
50
25
0
5e+05
0
5e+05
1e+06
0
1e+06
2e+06
0
2e+06
3e+06
0
3e+06
Sex
Female
Male
Ages
Female Mean
Female Median
Male Mean
Male Median

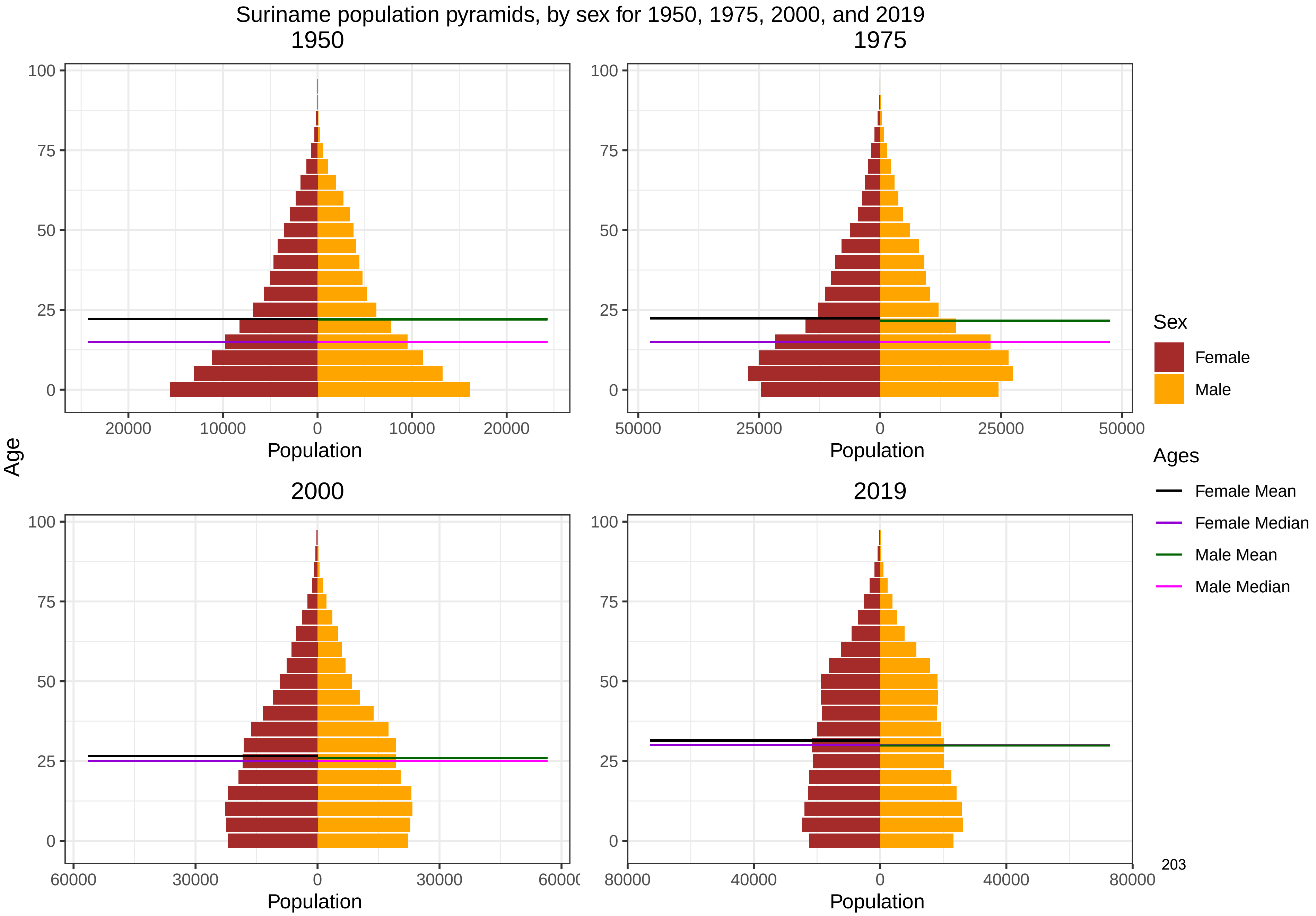

Suriname population pyramids, by sex for 1950, 1975, 2000, and 2019
1950
1975
2000
2019
Age
Population
100
75
50
25
0
20000
10000
0
10000
20000
50000
25000
0
25000
50000
60000
30000
0
30000
60000
80000
40000
0
40000
80000
Sex
Female
Male
Ages
Female Mean
Female Median
Male Mean
Male Median

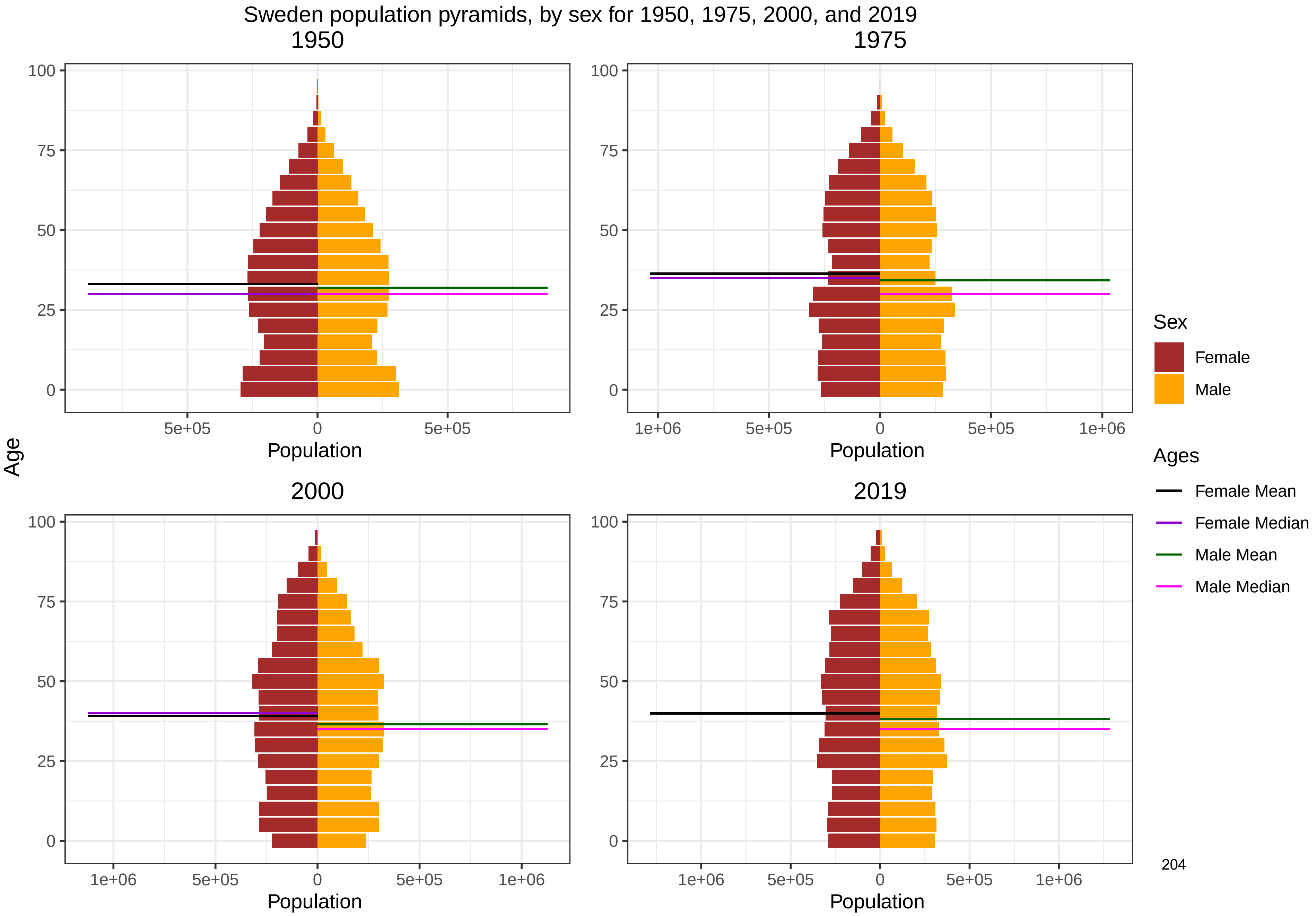

Sweden population pyramids, by sex for 1950, 1975, 2000, and 2019
1950
1975
2000
2019
Age
Population
0
25
50
75
100
5e+05
1e+06
Sex
Female
Male
Ages
Female Mean
Female Median
Male Mean
Male Median

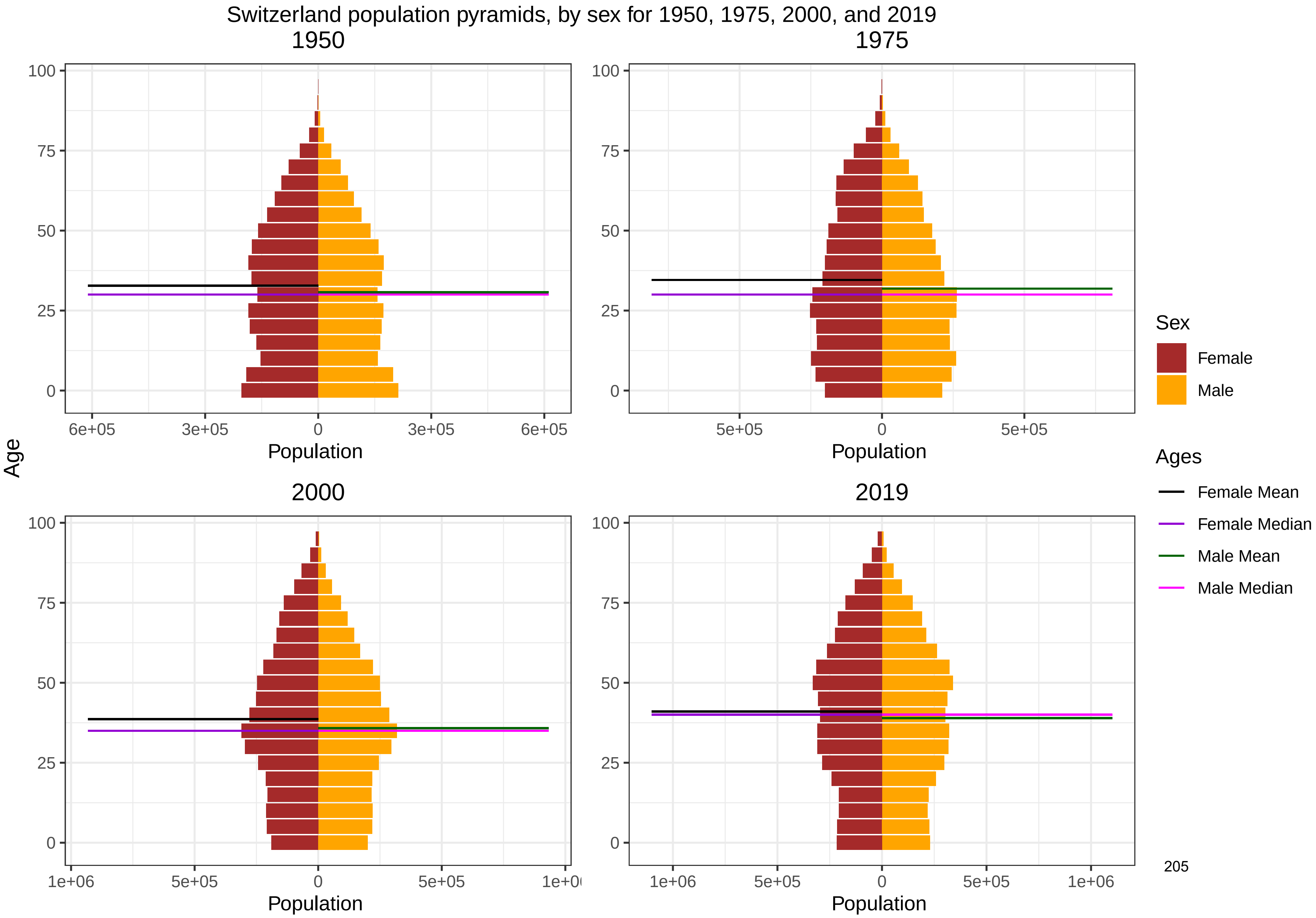

Switzerland population pyramids, by sex for 1950, 1975, 2000, and 2019
1950
1975
2000
2019
Age
Population
Sex
Female
Male
Ages
Female Mean
Female Median
Male Mean
Male Median

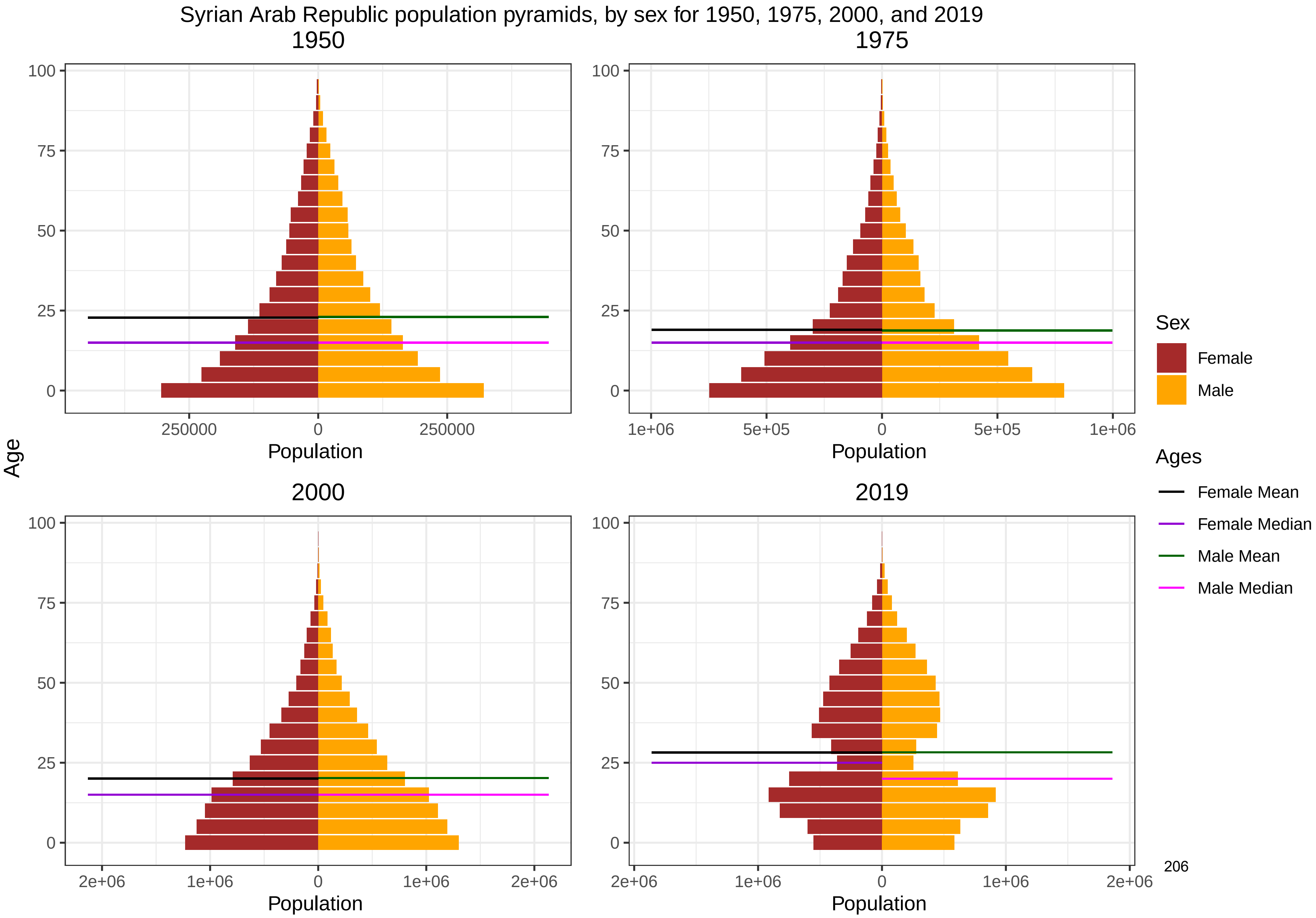

Syrian Arab Republic population pyramids, by sex for 1950, 1975, 2000, and 2019
1950
1975
2000
2019
Age
Population
Sex
Female
Male
Ages
Female Mean
Female Median
Male Mean
Male Median

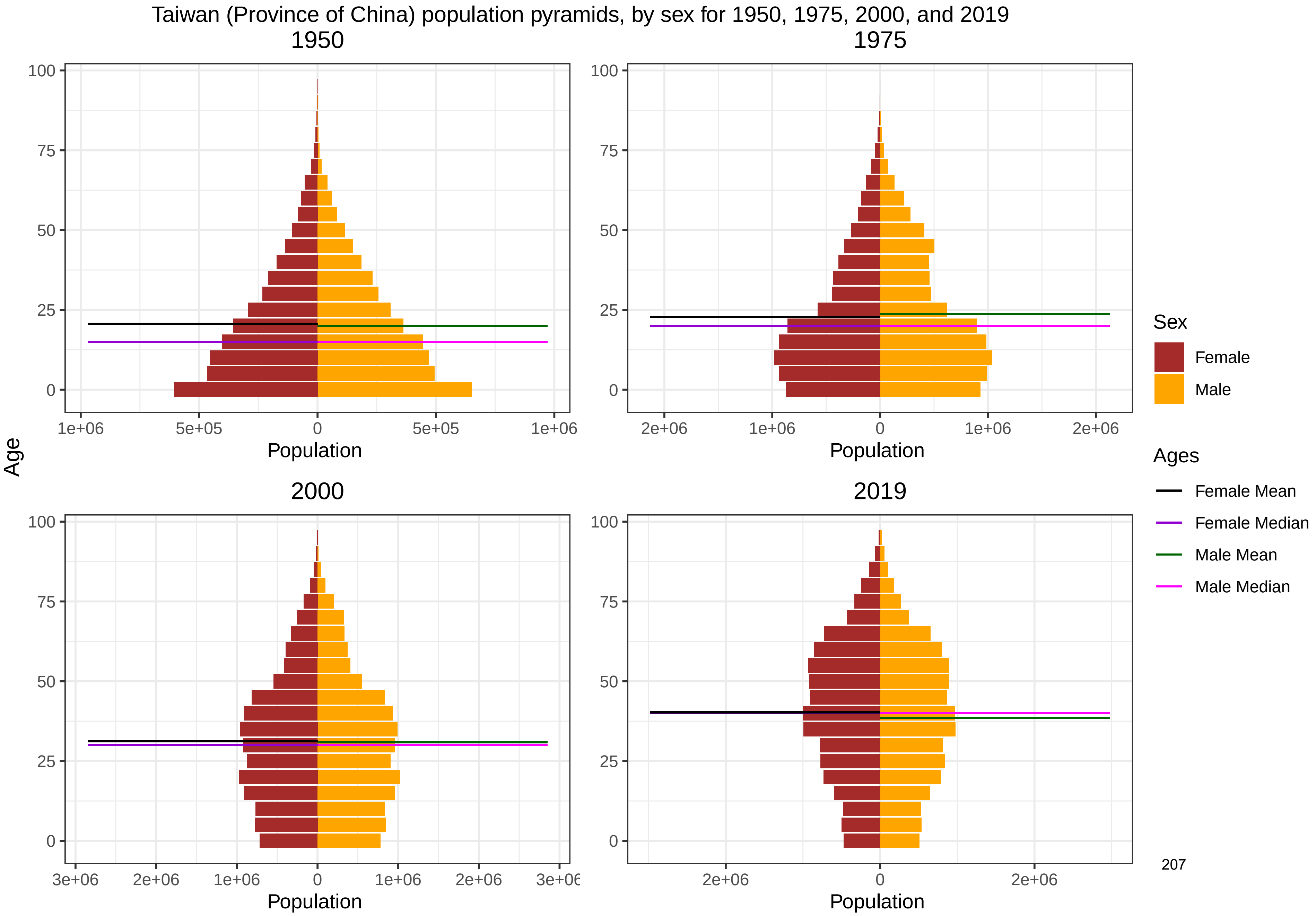

Taiwan (Province of China) population pyramids, by sex for 1950, 1975, 2000, and 2019
1950
1975
2000
2019
Age
Population
Sex
Female
Male
Ages
Female Mean
Female Median
Male Mean
Male Median

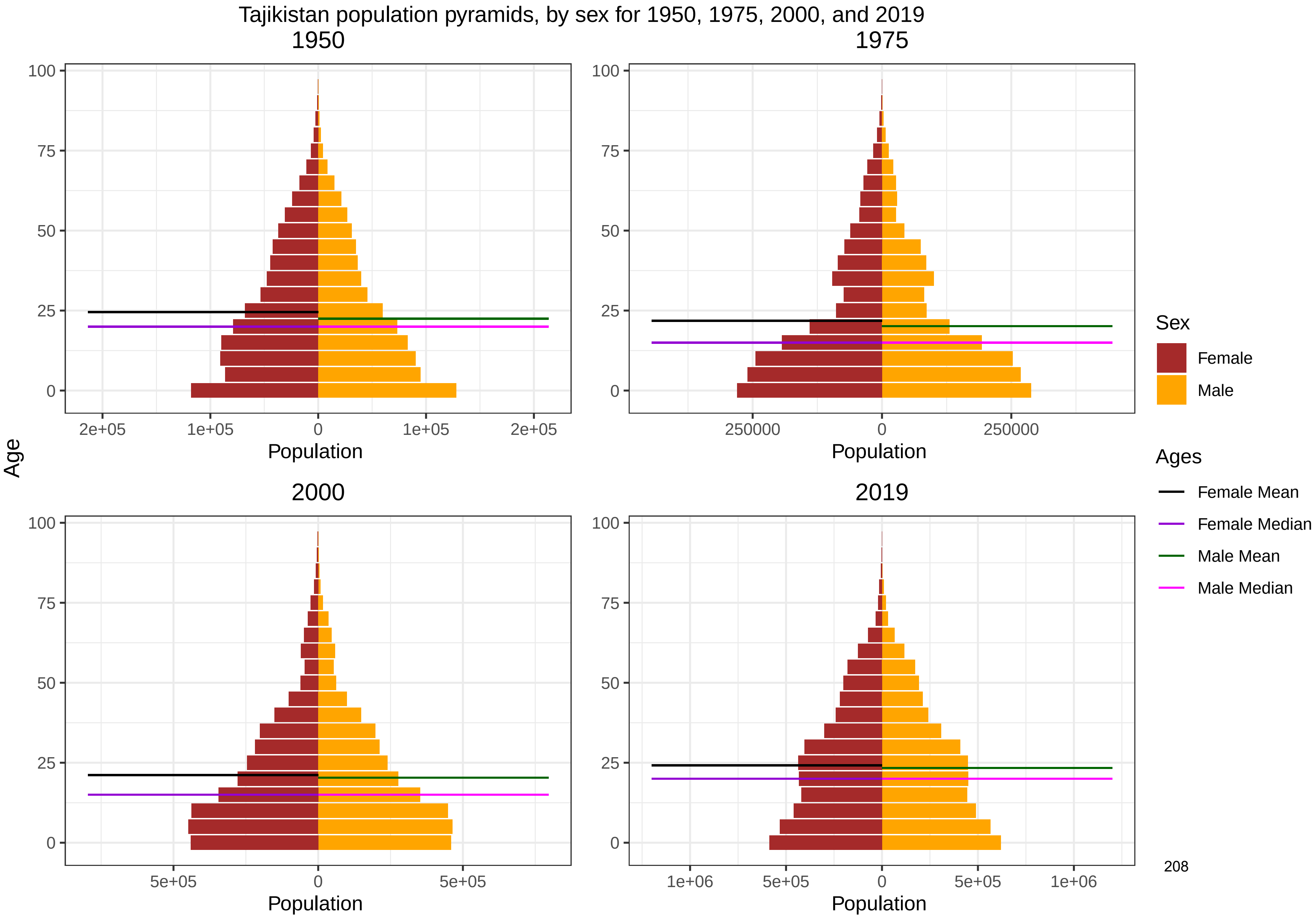
Tajikistan population pyramids, by sex for 1950, 1975, 2000, and 2019
1950
1975
2000
2019
Age
Population
0
25
50
75
100
2e+05
1e+05
250000
5e+05
1e+06
Sex
Female
Male
Ages
Female Mean
Female Median
Male Mean
Male Median

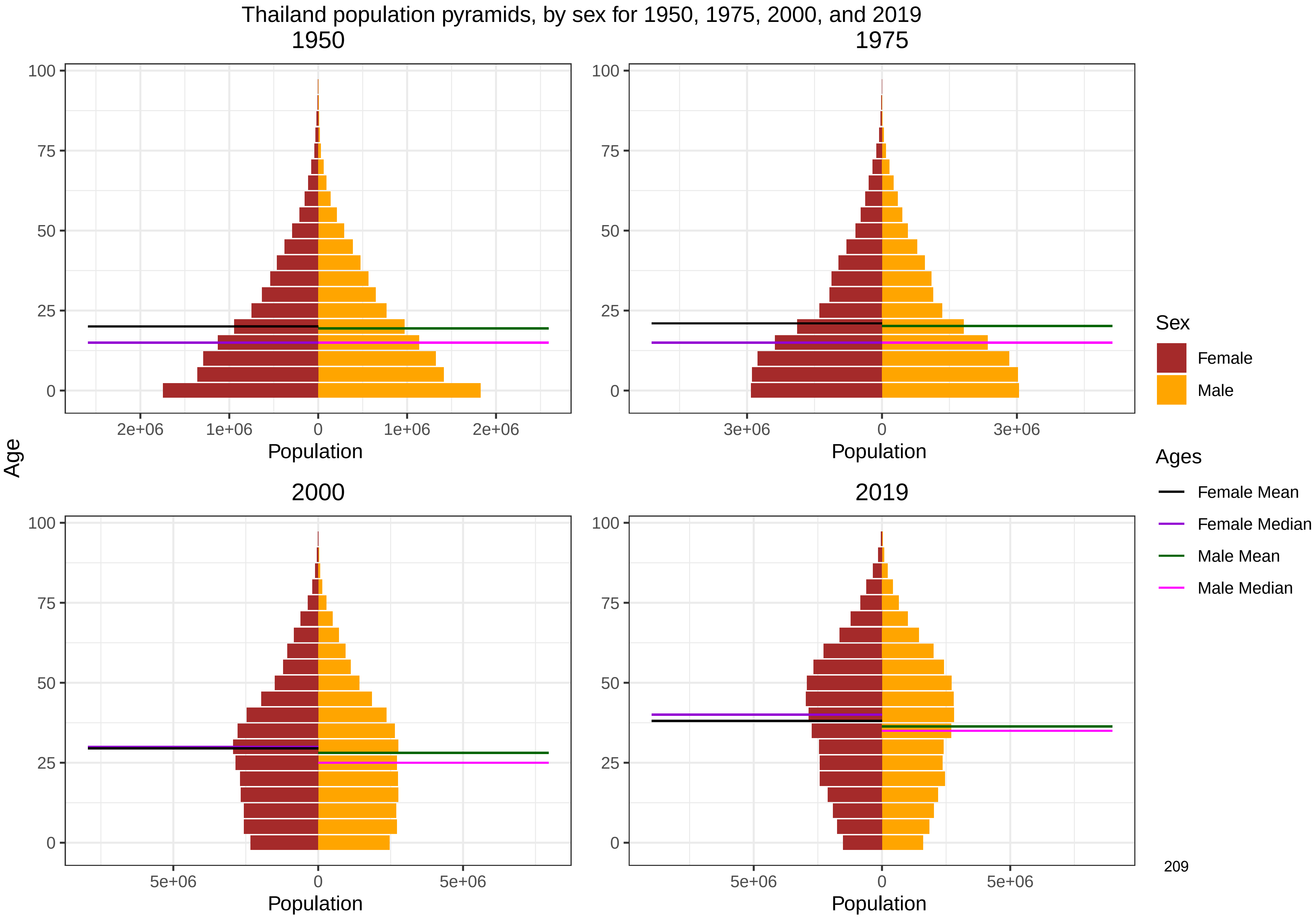

Thailand population pyramids, by sex for 1950, 1975, 2000, and 2019
1950
1975
2000
2019
Age
Population
100
75
50
25
0
2e+06
1e+06
0
3e+06
5e+06
Sex
Female
Male
Ages
Female Mean
Female Median
Male Mean
Male Median

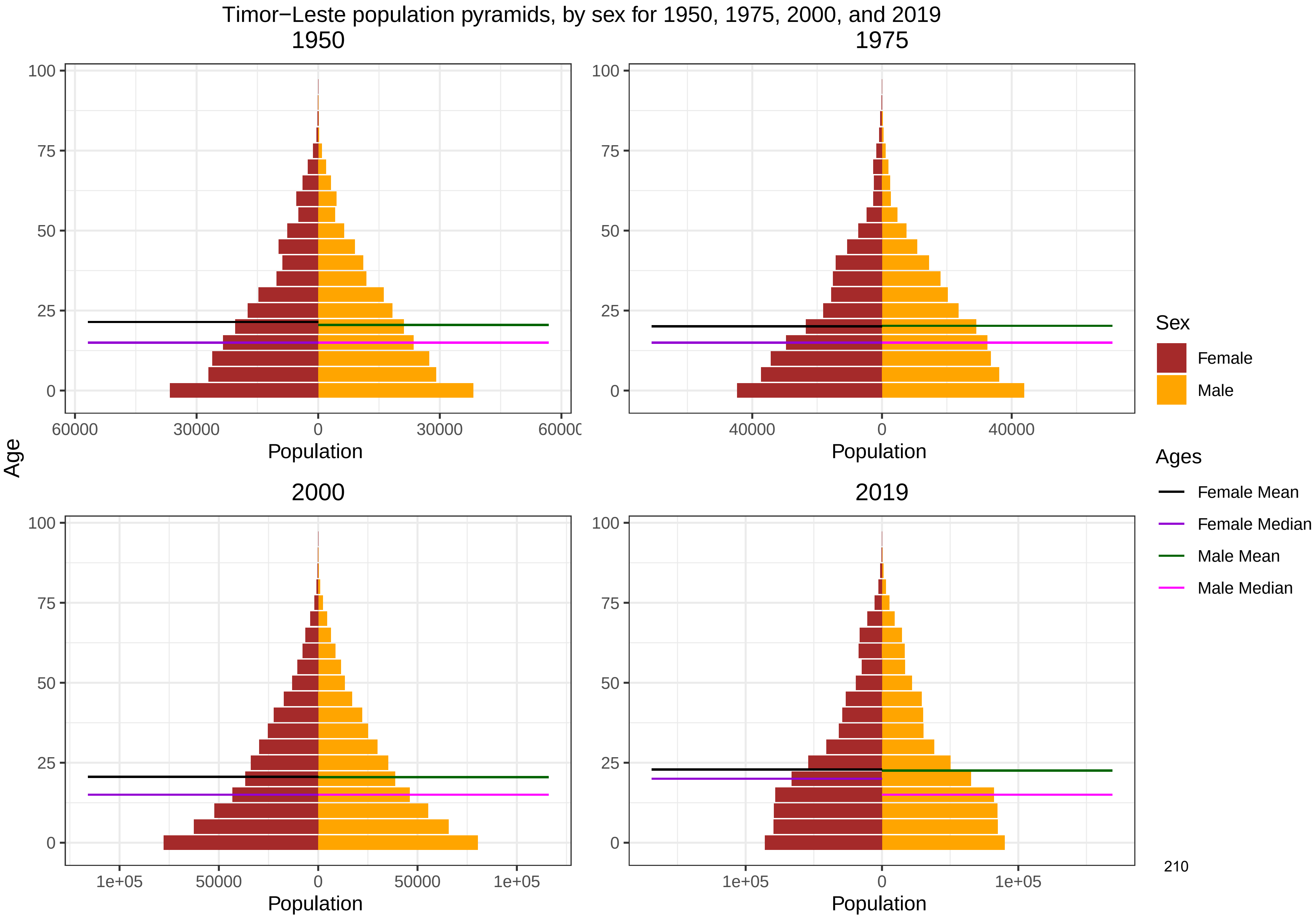

Timor−Leste population pyramids, by sex for 1950, 1975, 2000, and 2019
1950
1975
2000
2019
Age
Population
Sex
Female
Male
Ages
Female Mean
Female Median
Male Mean
Male Median

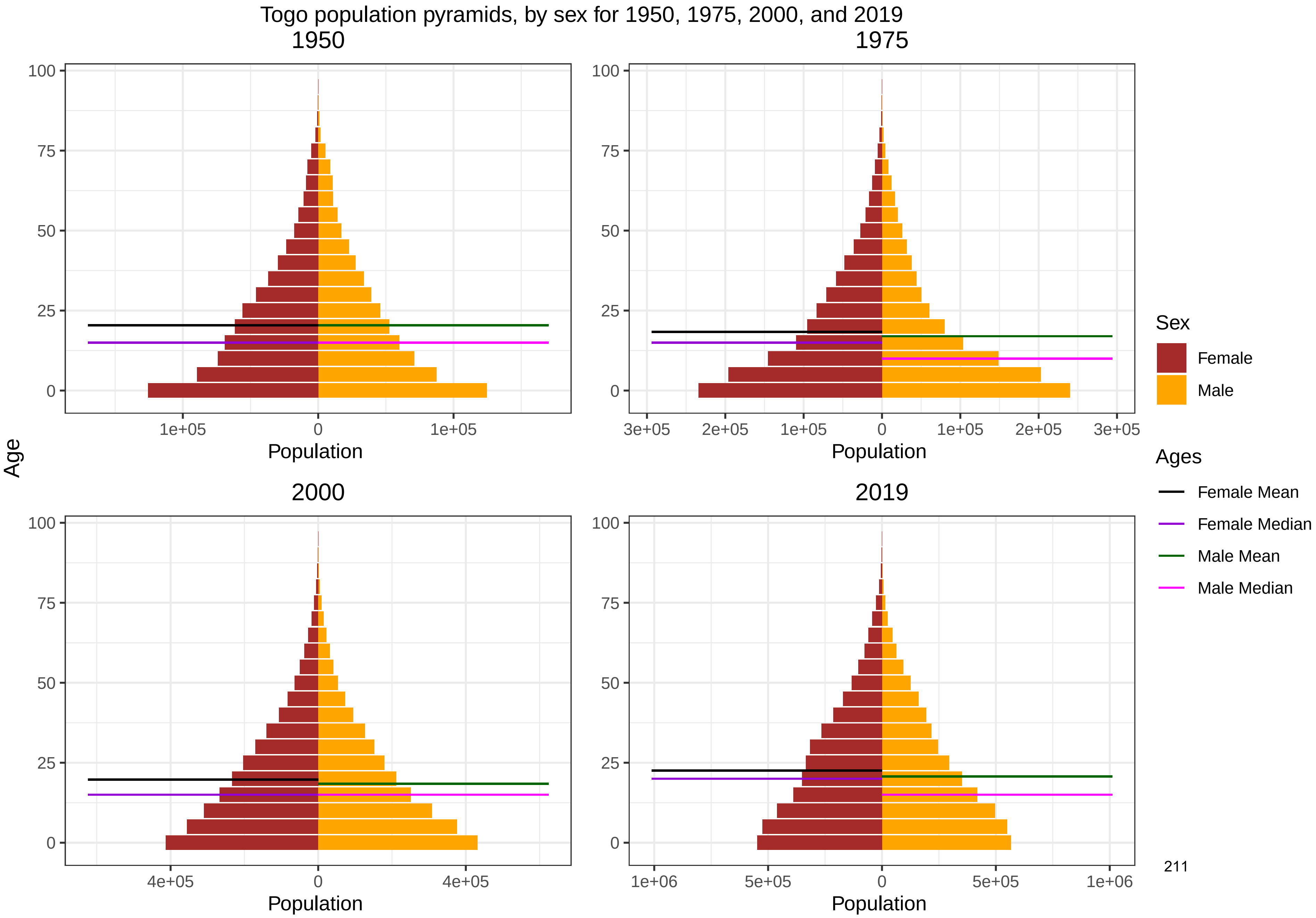

Togo population pyramids, by sex for 1950, 1975, 2000, and 2019
1950
1975
2000
2019
Age
Population
100
75
50
25
0
1e+05
0
1e+05
3e+05
2e+05
1e+05
0
1e+05
2e+05
3e+05
4e+05
0
4e+05
1e+06
5e+05
0
5e+05
1e+06
Sex
Female
Male
Ages
Female Mean
Female Median
Male Mean
Male Median

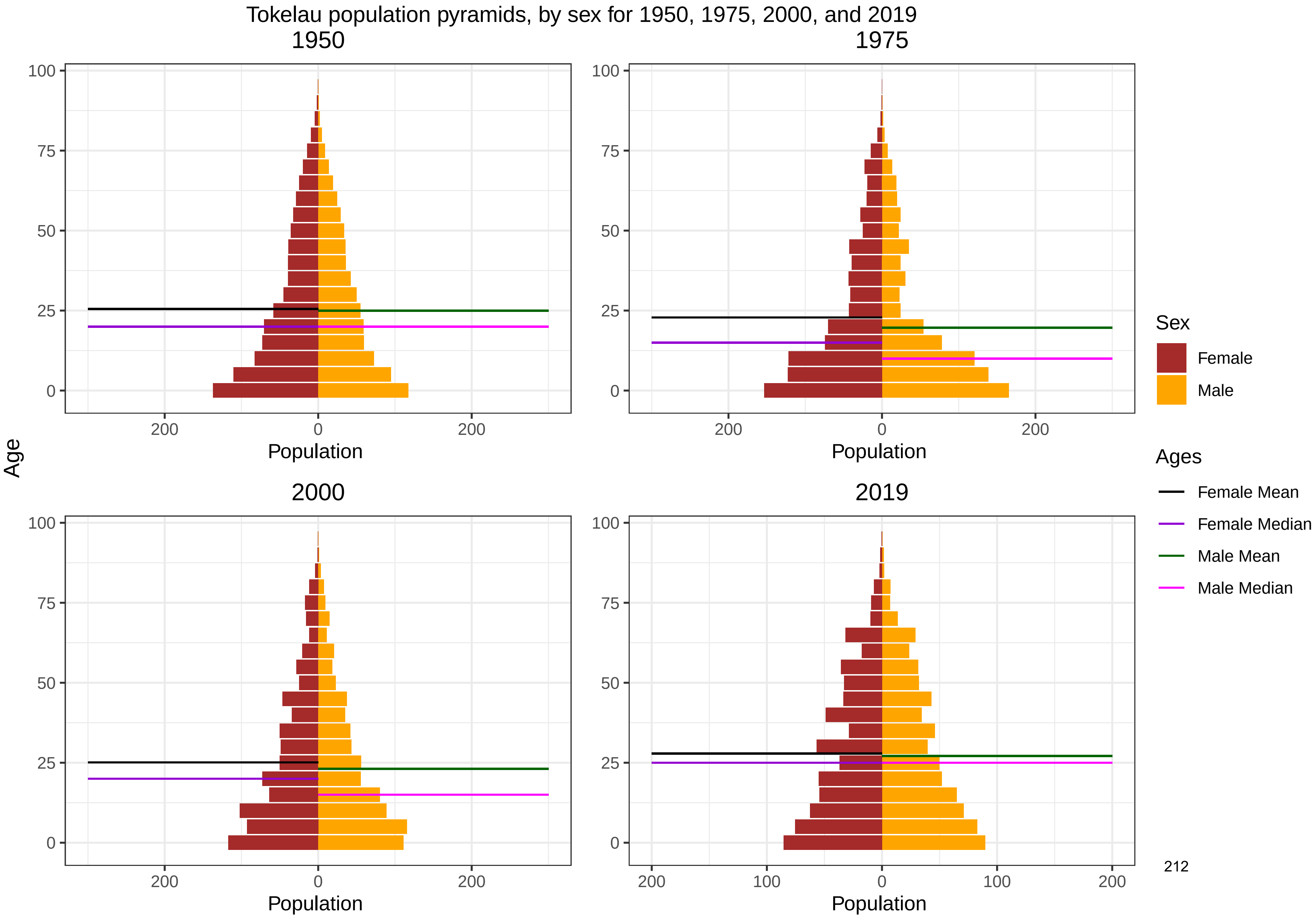

Tokelau population pyramids, by sex for 1950, 1975, 2000, and 2019
1950
1975
2000
2019
Age
Population
0
25
50
75
100
200
100
Sex
Female
Male
Ages
Female Mean
Female Median
Male Mean
Male Median

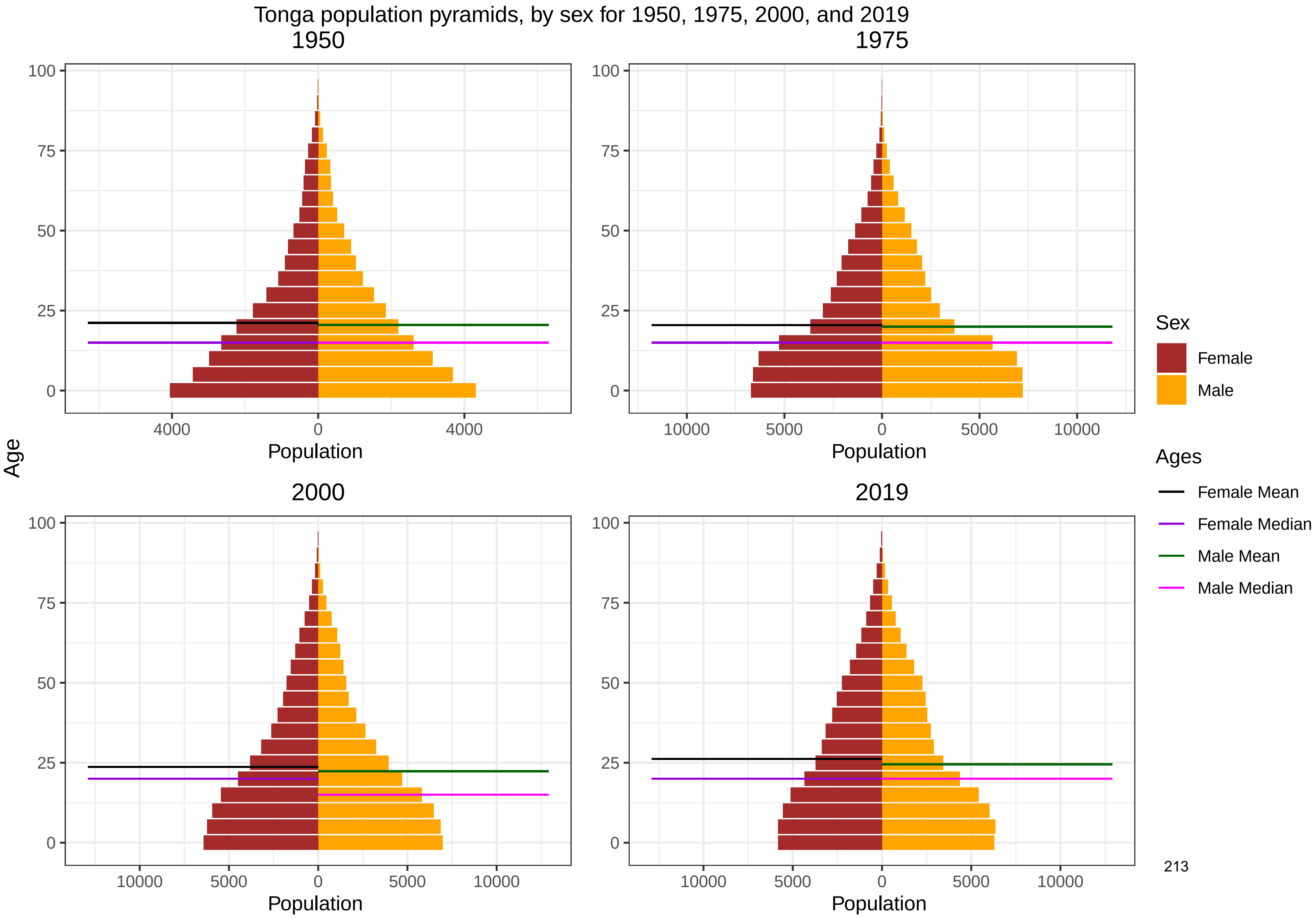
Tonga population pyramids, by sex for 1950, 1975, 2000, and 2019
1950
1975
2000
2019
Age
Population
Sex
Female
Male
Ages
Female Mean
Female Median
Male Mean
Male Median

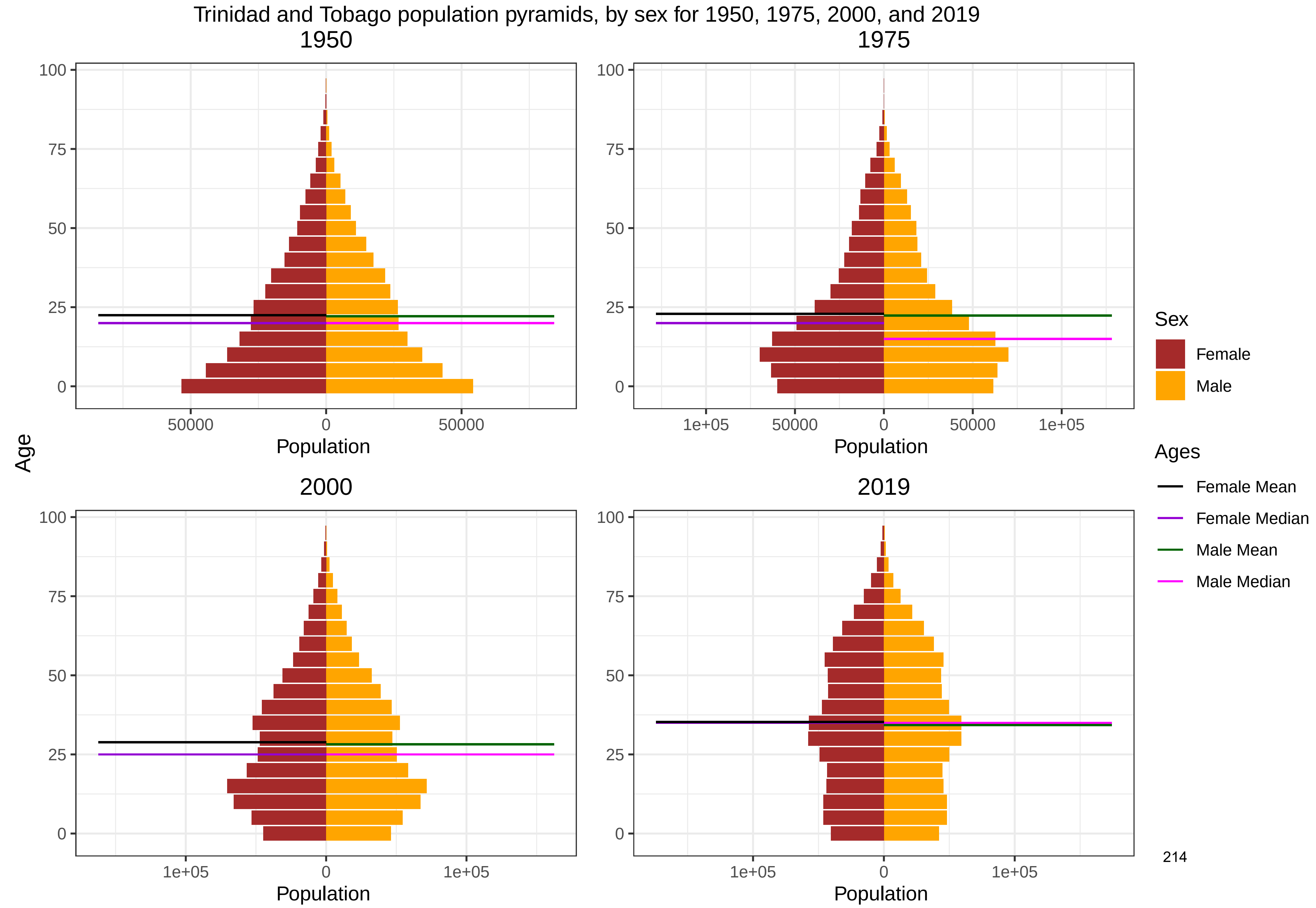

Trinidad and Tobago population pyramids, by sex for 1950, 1975, 2000, and 2019
1950
1975
2000
2019
Age
Population
Sex
Female
Male
Ages
Female Mean
Female Median
Male Mean
Male Median

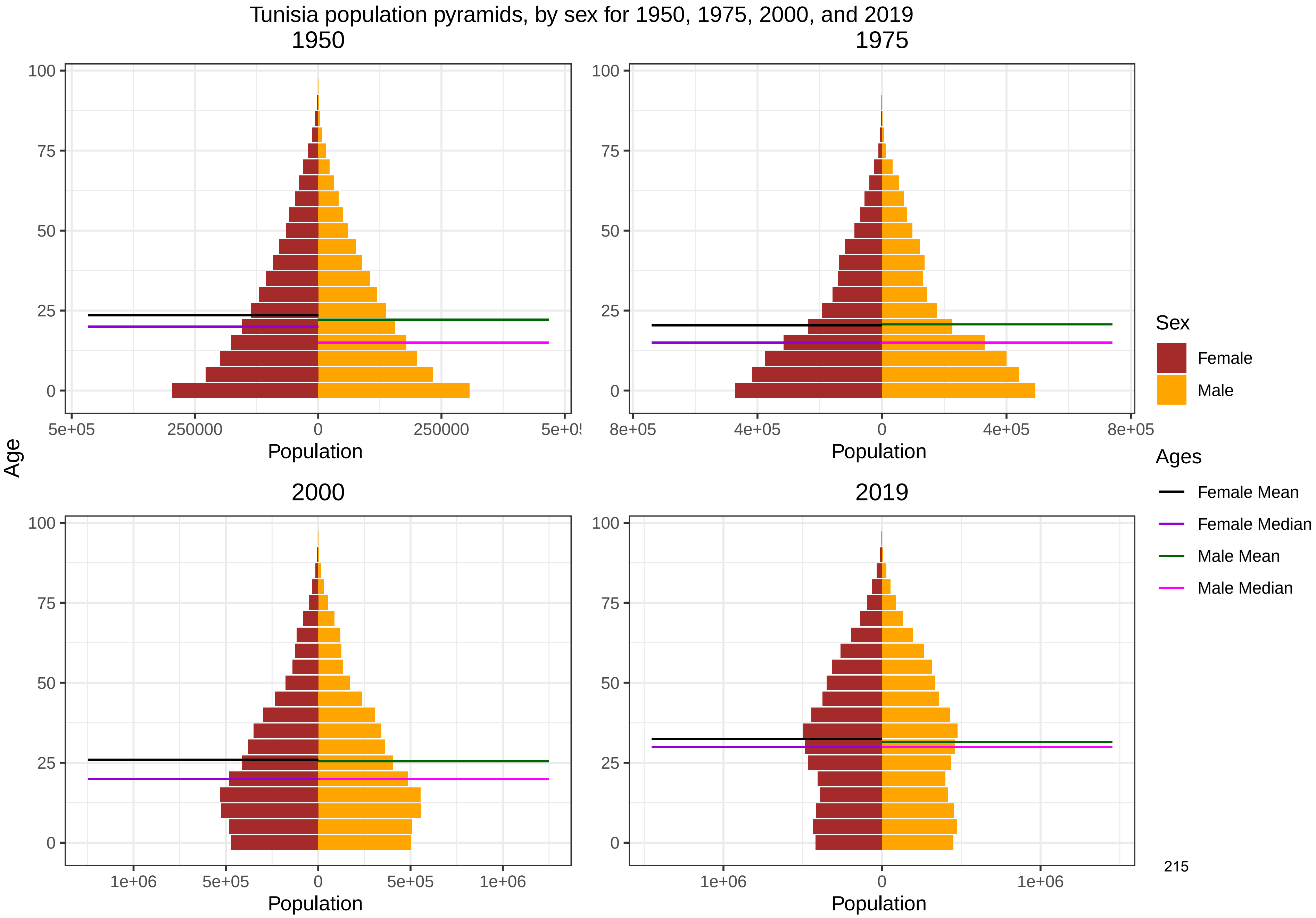

Tunisia population pyramids, by sex for 1950, 1975, 2000, and 2019
1950
1975
2000
2019
Age
Population
100
75
50
25
0
5e+05
250000
0
250000
8e+05
4e+05
0
4e+05
8e+05
1e+06
5e+05
0
5e+05
1e+06
Sex
Female
Male
Ages
Female Mean
Female Median
Male Mean
Male Median

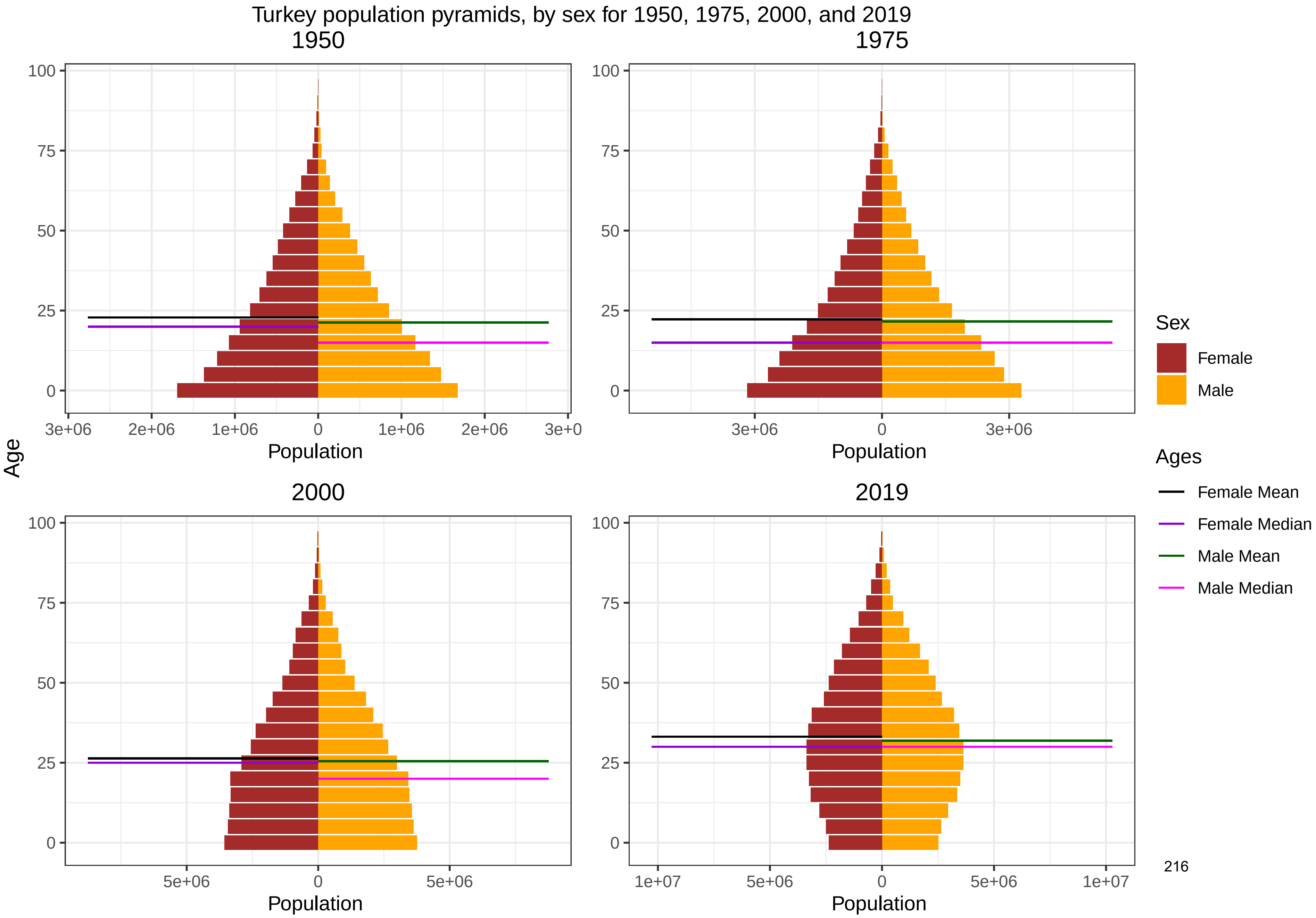

Turkey population pyramids, by sex for 1950, 1975, 2000, and 2019
1950
1975
2000
2019
Age
Population
100
75
50
25
0
3e+06
2e+06
1e+06
0
1e+06
2e+06
3e+0
3e+06
5e+06
1e+07
Sex
Female
Male
Ages
Female Mean
Female Median
Male Mean
Male Median

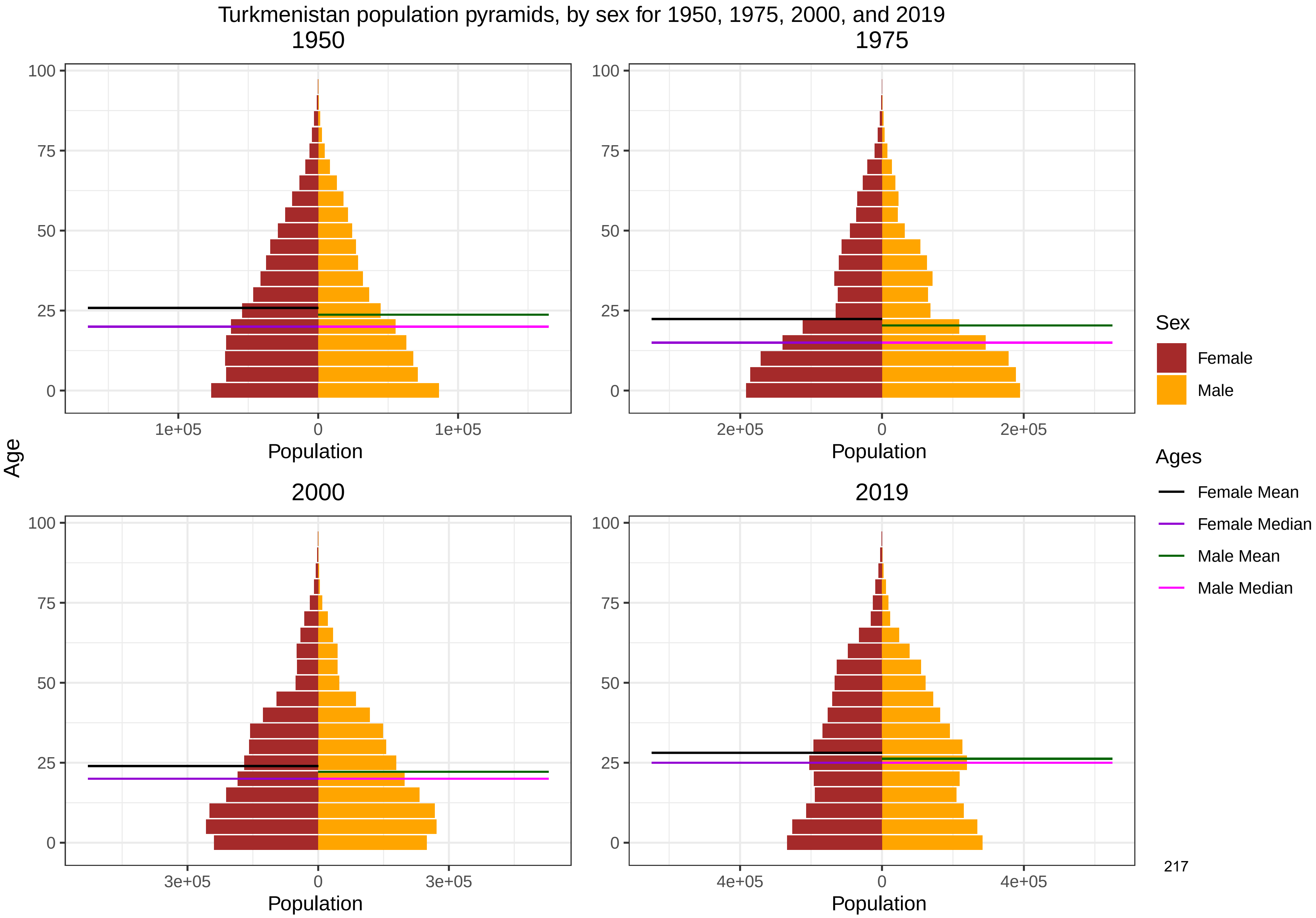

Turkmenistan population pyramids, by sex for 1950, 1975, 2000, and 2019
1950
1975
2000
2019
Age
Population
100
75
50
25
0
1e+05
2e+05
3e+05
4e+05
Sex
Female
Male
Ages
Female Mean
Female Median
Male Mean
Male Median

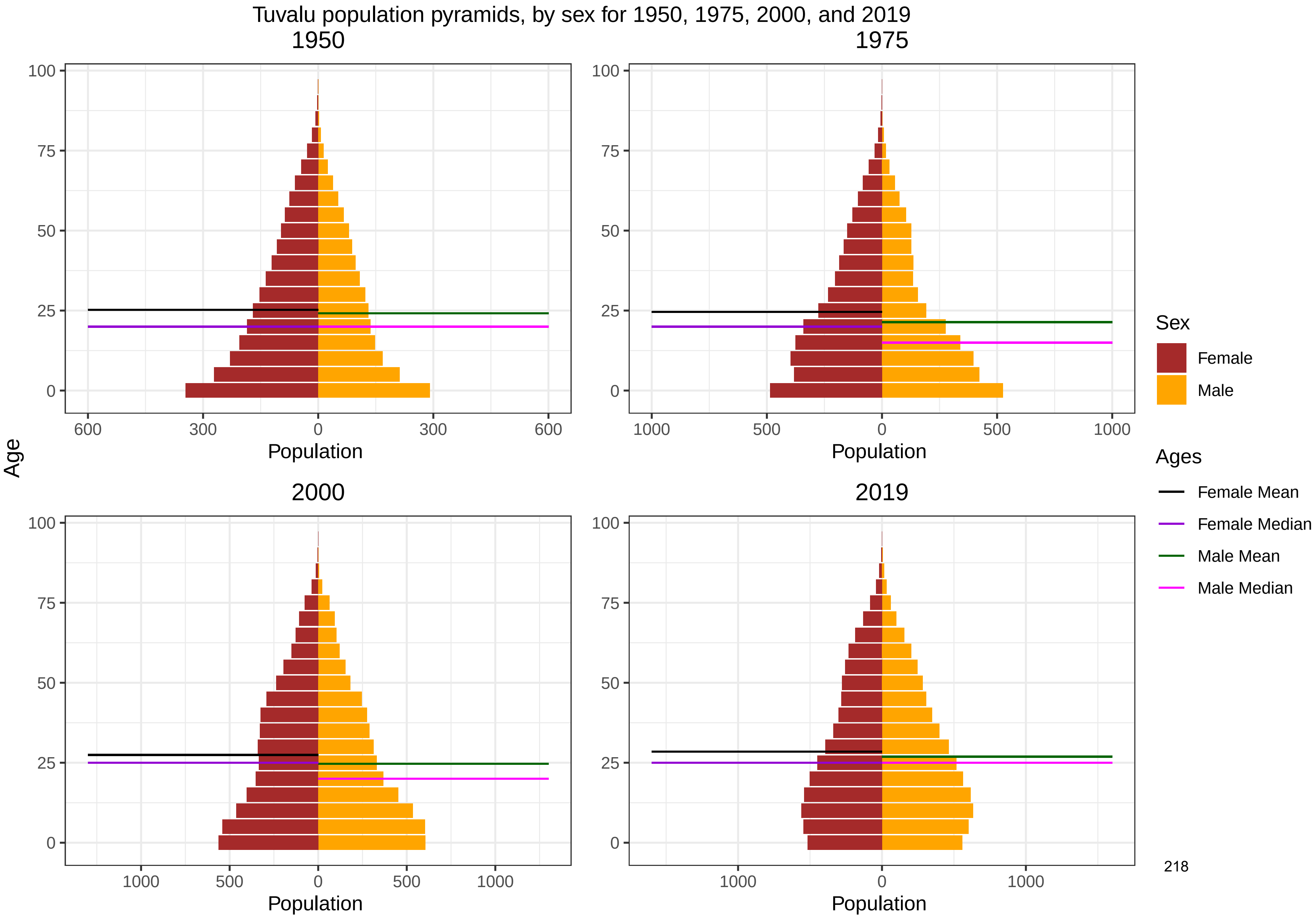

Tuvalu population pyramids, by sex for 1950, 1975, 2000, and 2019
1950
1975
2000
2019
Age
Population
Sex
Female
Male
Ages
Female Mean
Female Median
Male Mean
Male Median

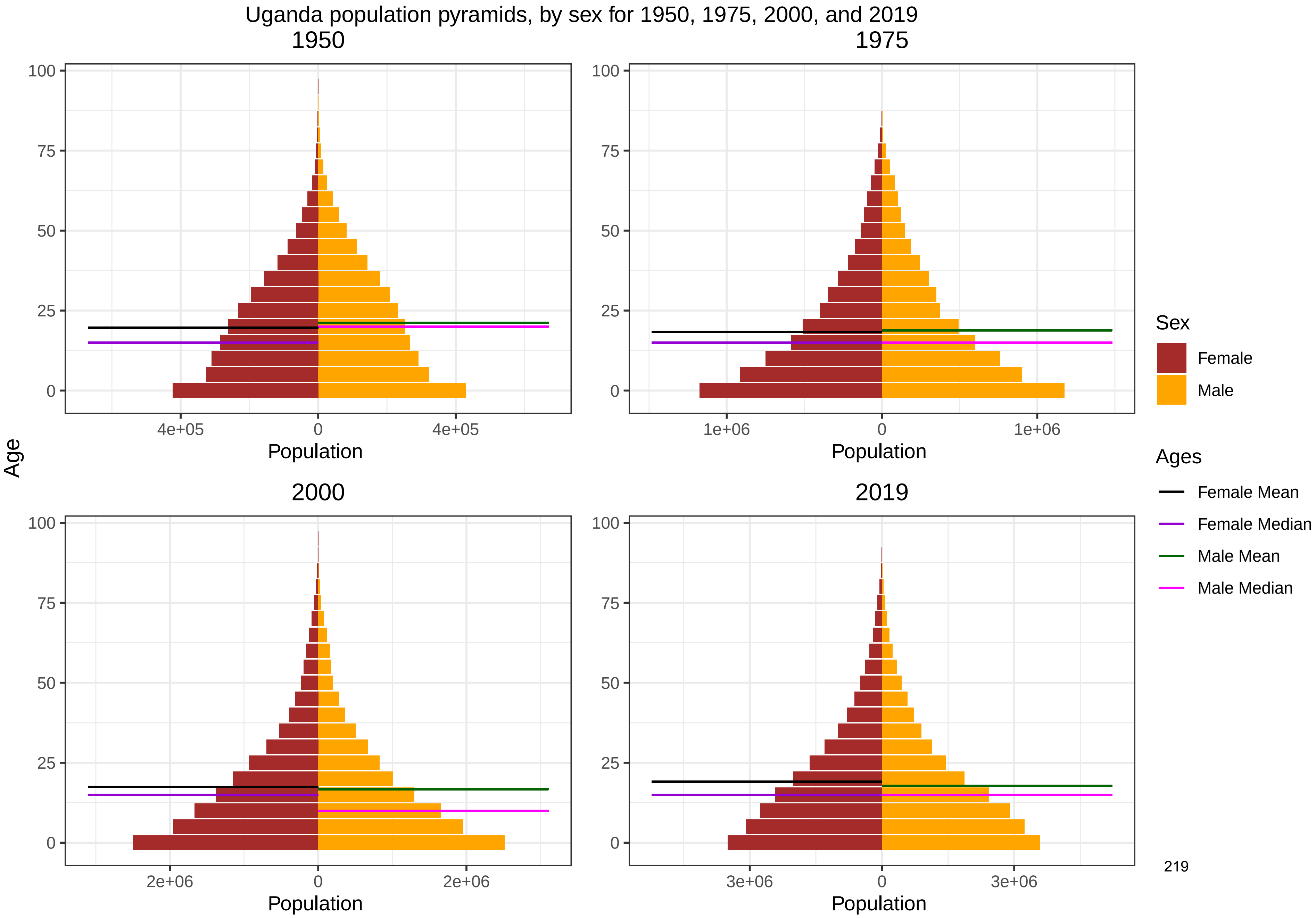

Uganda population pyramids, by sex for 1950, 1975, 2000, and 2019
1950
1975
2000
2019
Age
Population
100
75
50
25
0
4e+05
1e+06
2e+06
3e+06
Sex
Female
Male
Ages
Female Mean
Female Median
Male Mean
Male Median

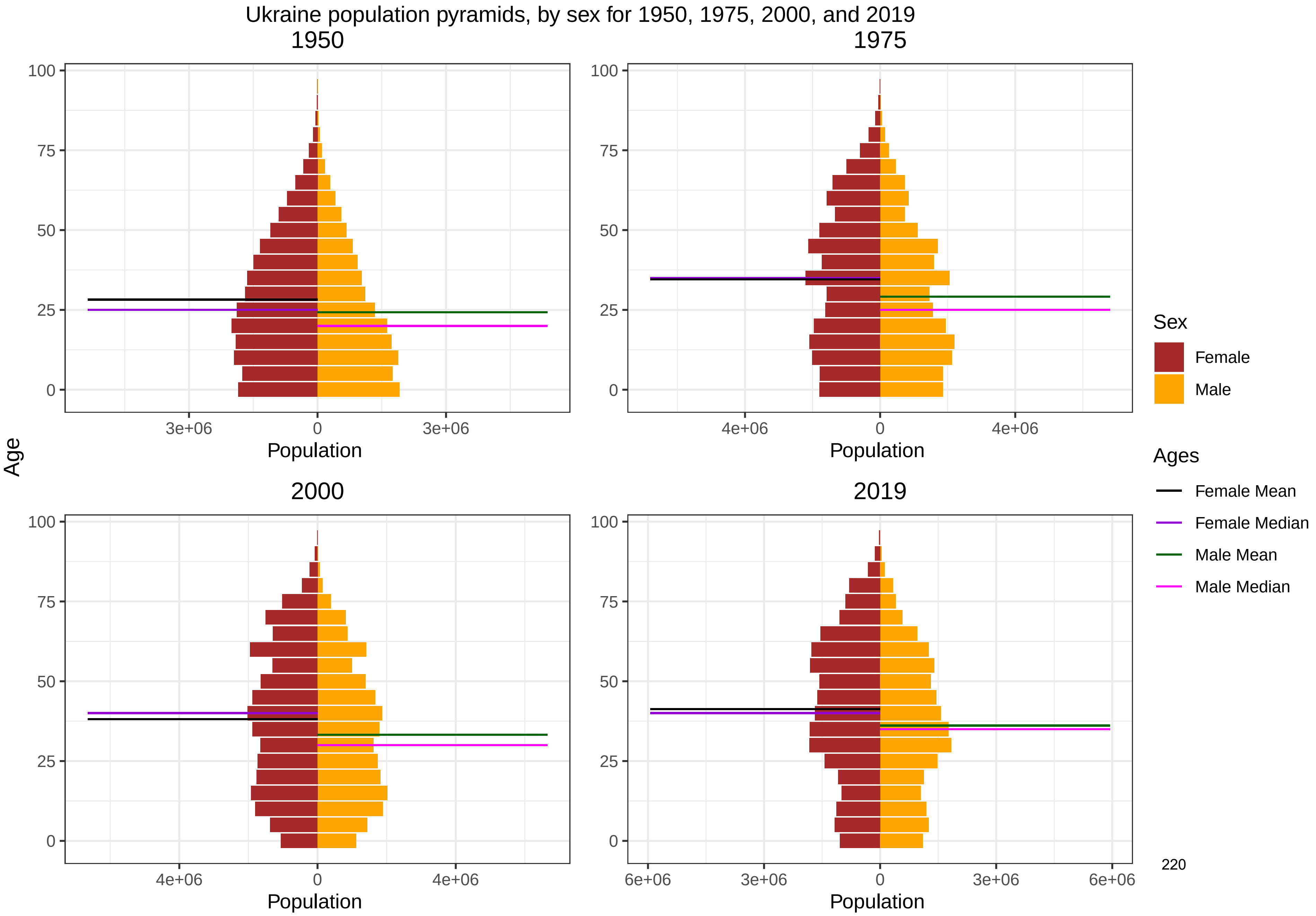
Ukraine population pyramids, by sex for 1950, 1975, 2000, and 2019
1950
1975
2000
2019
Age
Population
0
25
50
75
100
3e+06
4e+06
6e+06
Sex
Female
Male
Ages
Female Mean
Female Median
Male Mean
Male Median

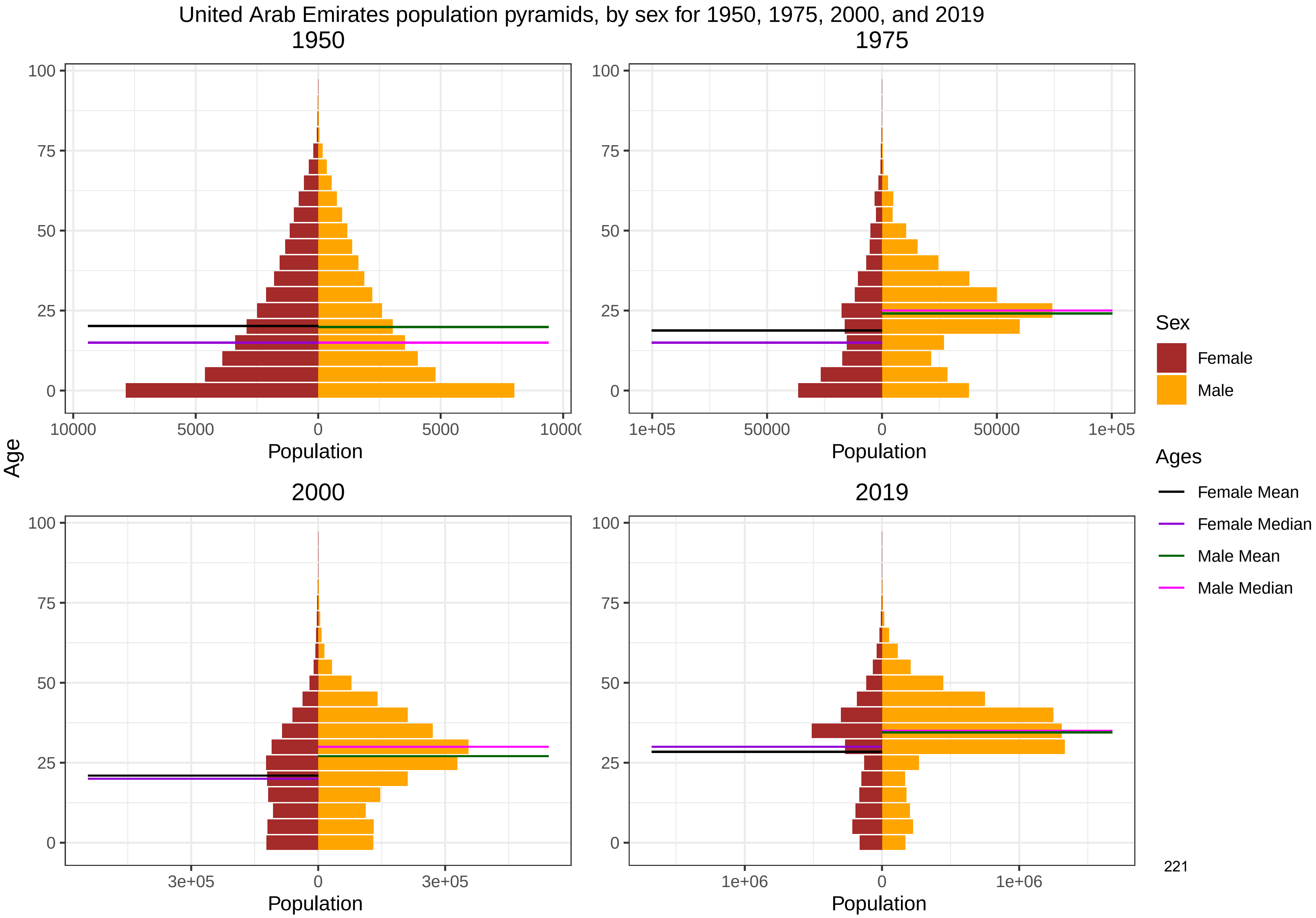

United Arab Emirates population pyramids, by sex for 1950, 1975, 2000, and 2019
1950
1975
2000
2019
Age
Population
10000
5000
0
5000
1e+05
50000
0
50000
1e+05
3e+05
0
3e+05
1e+06
0
1e+06
100
75
50
25
0
Sex
Female
Male
Ages
Female Mean
Female Median
Male Mean
Male Median

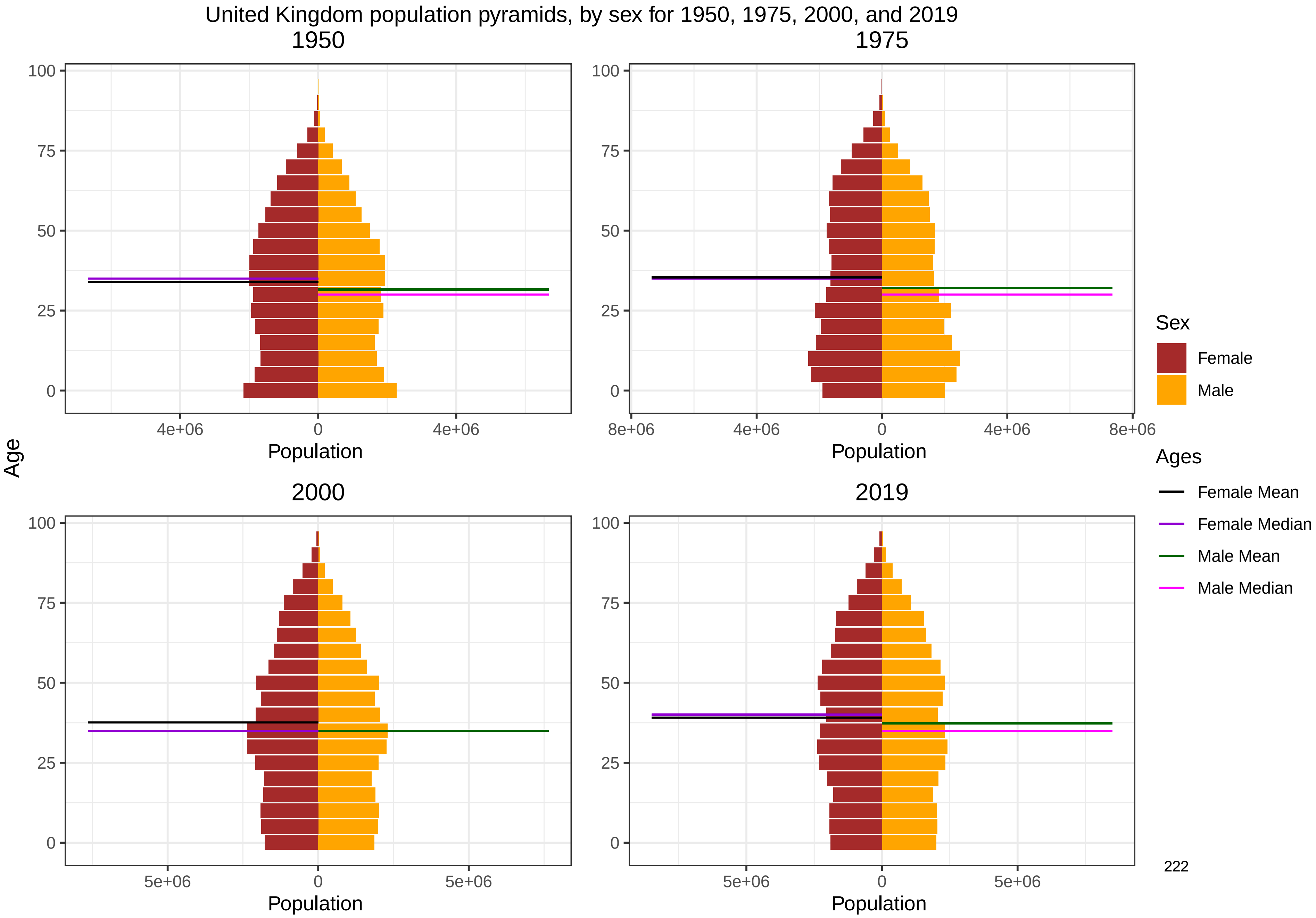
United Kingdom population pyramids, by sex for 1950, 1975, 2000, and 2019
1950
1975
2000
2019
Age
Population
100
75
50
25
0
4e+06
0
4e+06
8e+06
5e+06
Sex
Female
Male
Ages
Female Mean
Female Median
Male Mean
Male Median

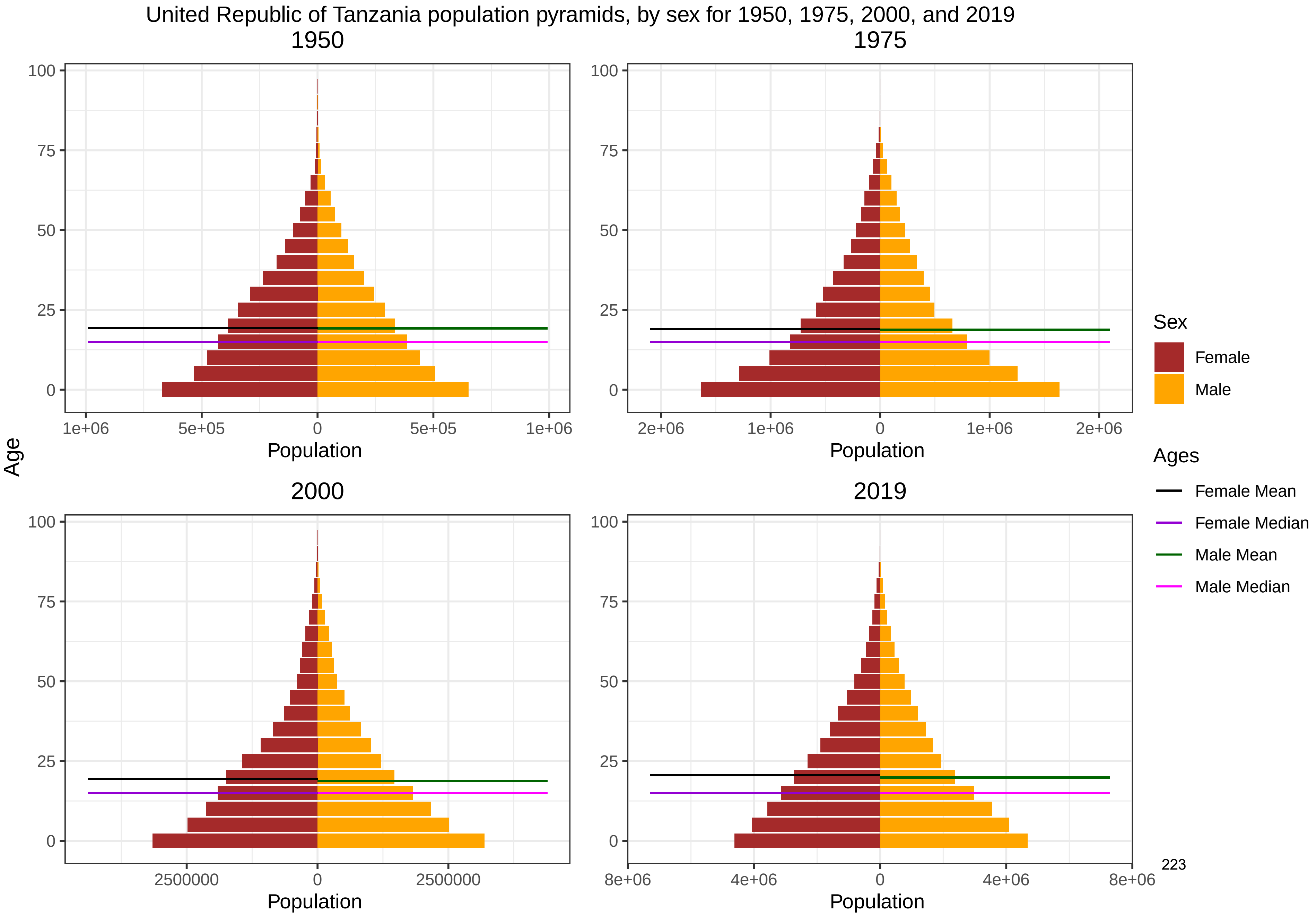
United Republic of Tanzania population pyramids, by sex for 1950, 1975, 2000, and 2019
1950
1975
2000
2019
Age
Population
Sex
Female
Male
Ages
Female Mean
Female Median
Male Mean
Male Median
100
75
50
25
0
1e+06
5e+05
2e+06
2500000
8e+06
4e+06

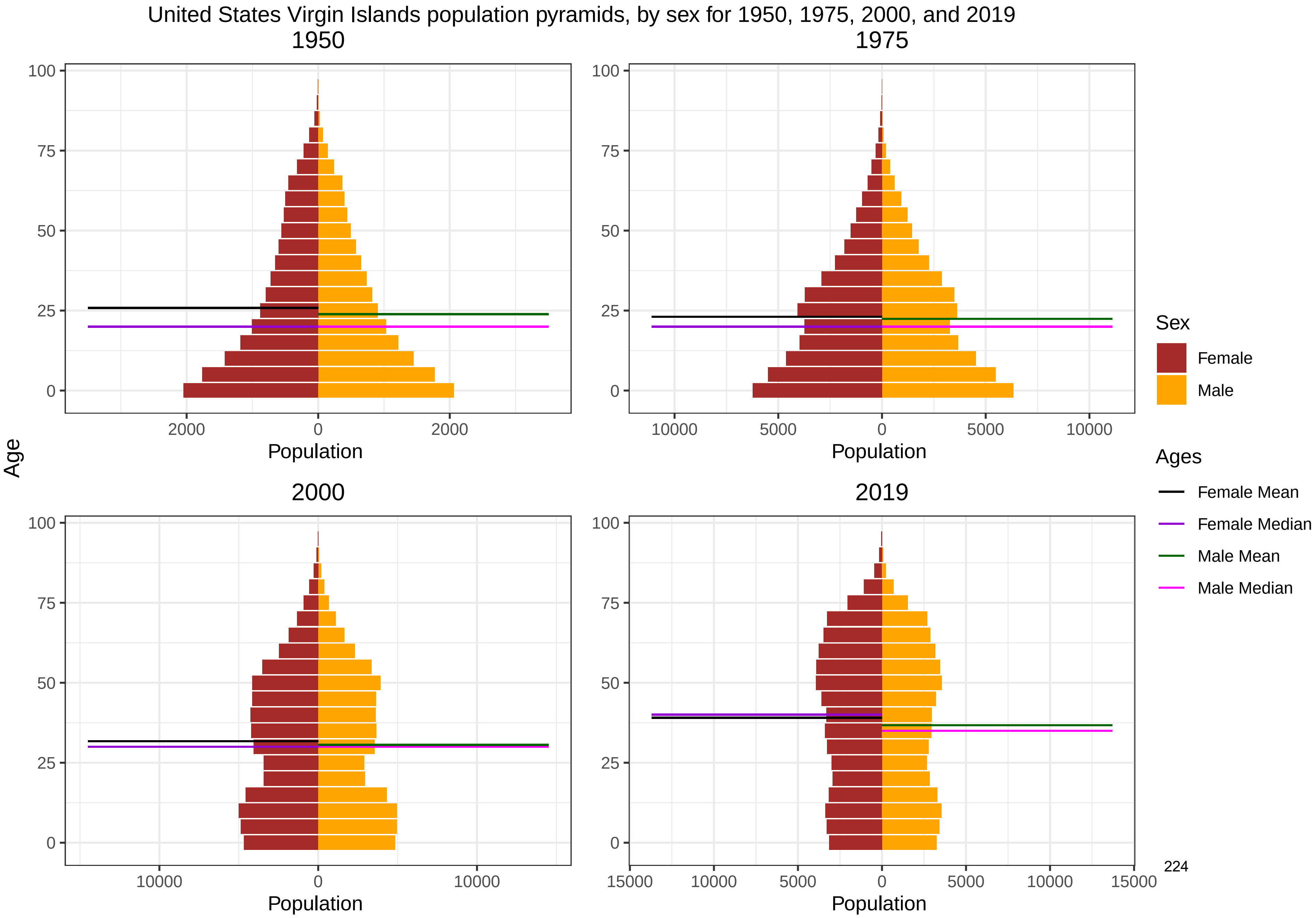
United States Virgin Islands population pyramids, by sex for 1950, 1975, 2000, and 2019
1950
1975
2000
2019
Age
Population
Sex
Female
Male
Ages
Female Mean
Female Median
Male Mean
Male Median

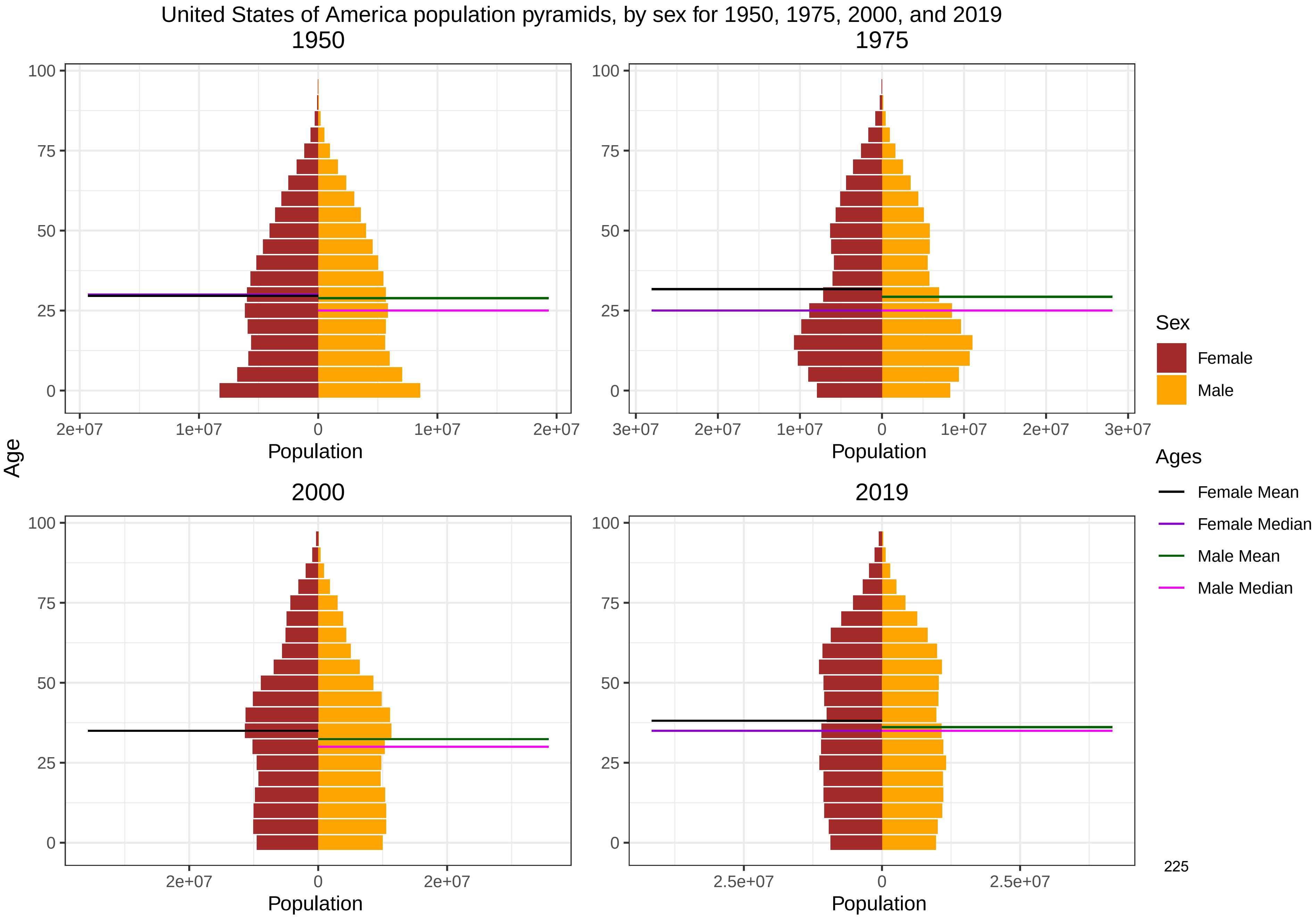
United States of America population pyramids, by sex for 1950, 1975, 2000, and 2019
1950
1975
2000
2019
Age
Population
100
75
50
25
0
2e+07
1e+07
0
3e+07
2.5e+07
Sex
Female
Male
Ages
Female Mean
Female Median
Male Mean
Male Median

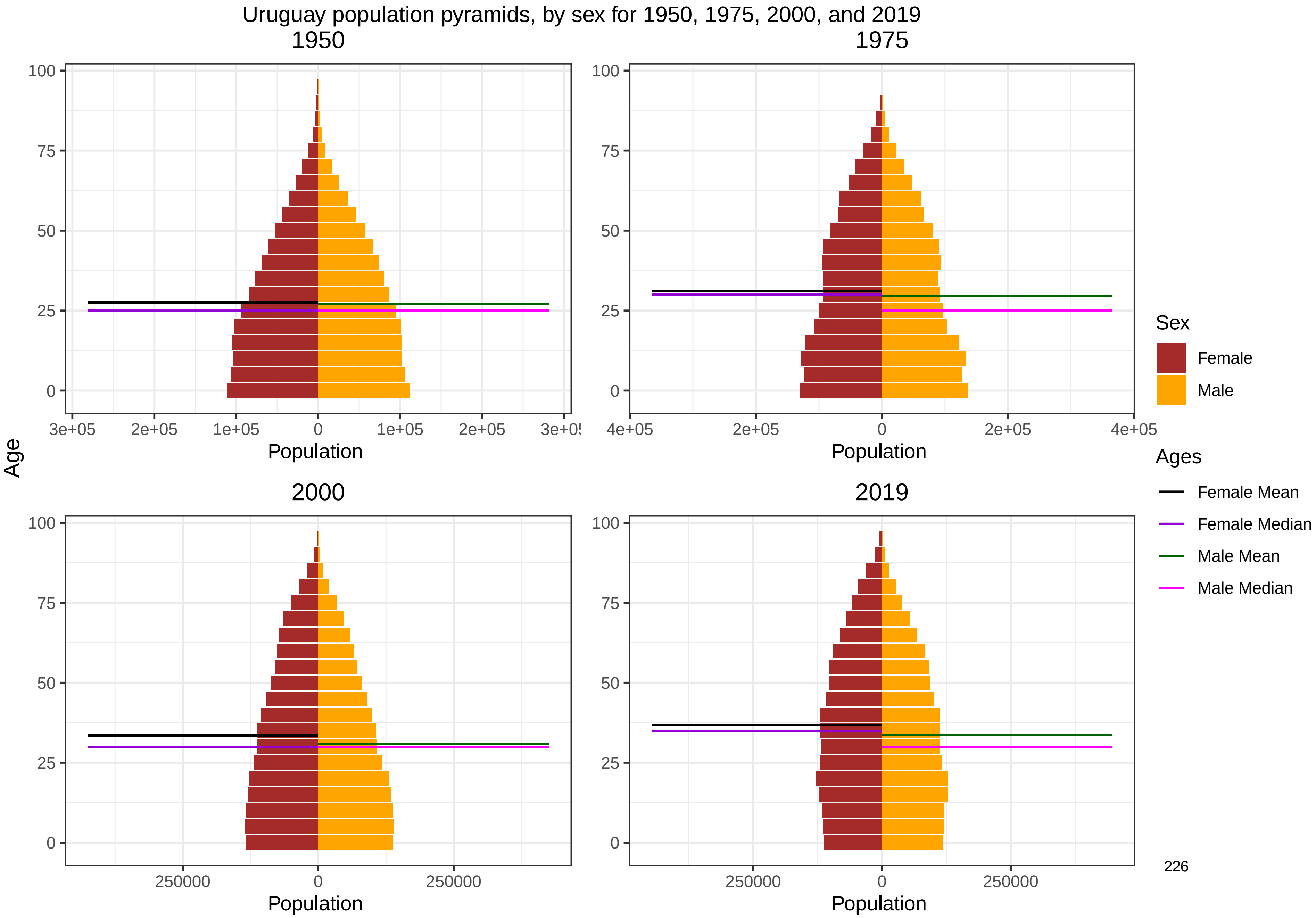
Uruguay population pyramids, by sex for 1950, 1975, 2000, and 2019
1950
1975
2000
2019
Age
Population
Sex
Female
Male
Ages
Female Mean
Female Median
Male Mean
Male Median

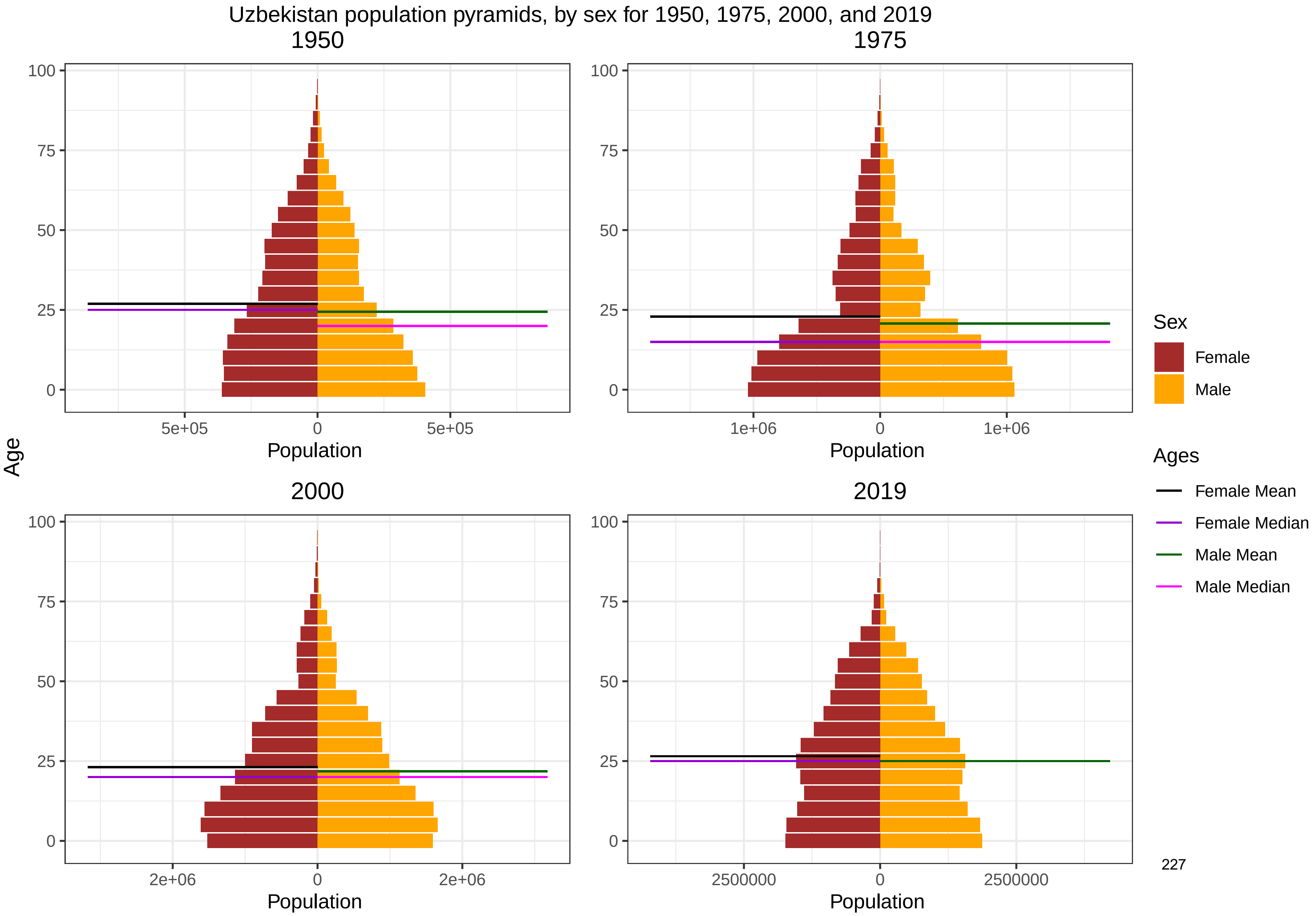

Uzbekistan population pyramids, by sex for 1950, 1975, 2000, and 2019
1950
1975
2000
2019
Age
Population
100
75
50
25
0
5e+05
0
5e+05
1e+06
0
1e+06
2e+06
0
2e+06
2500000
0
2500000
Sex
Female
Male
Ages
Female Mean
Female Median
Male Mean
Male Median

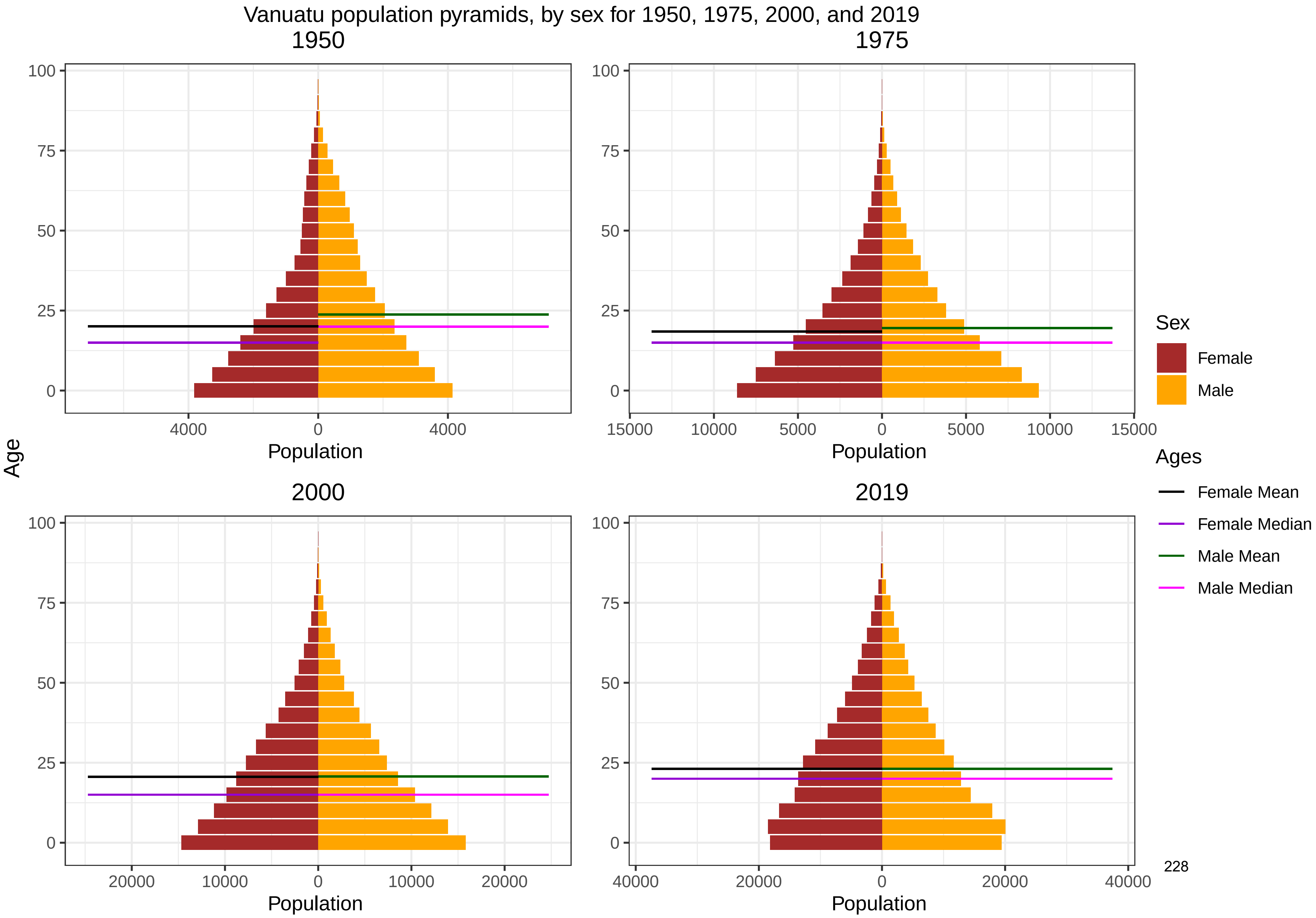

Vanuatu population pyramids, by sex for 1950, 1975, 2000, and 2019
1950
1975
2000
2019
Age
Population
Sex
Female
Male
Ages
Female Mean
Female Median
Male Mean
Male Median

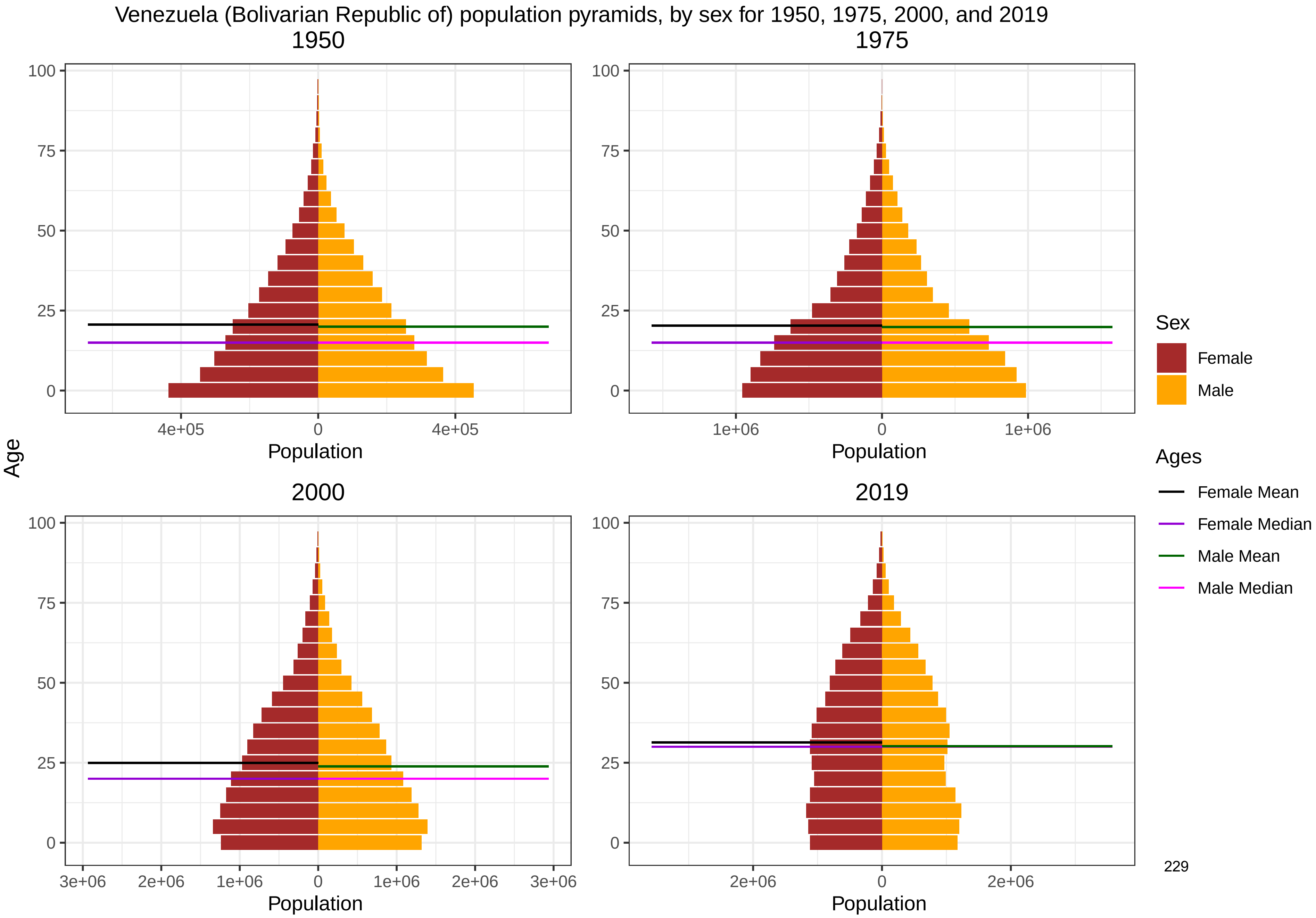

Venezuela (Bolivarian Republic of) population pyramids, by sex for 1950, 1975, 2000, and 2019
1950
1975
2000
2019
Age
Population
0
25
50
75
100
4e+05
1e+06
2e+06
3e+06
Sex
Female
Male
Ages
Female Mean
Female Median
Male Mean
Male Median

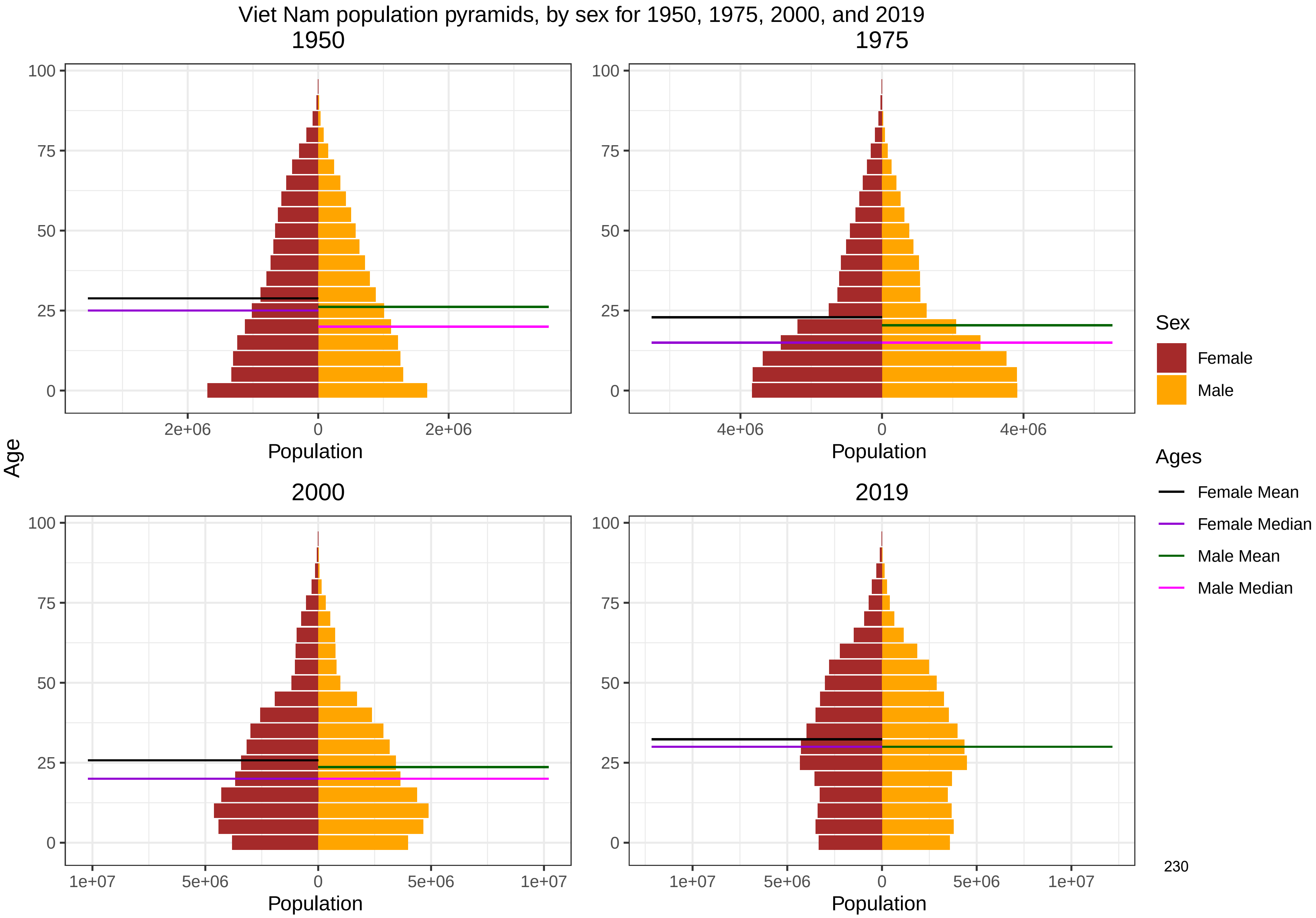

Viet Nam population pyramids, by sex for 1950, 1975, 2000, and 2019
1950
1975
2000
2019
Age
Population
2e+06
0
4e+06
1e+07
5e+06
100
75
50
25
0
Sex
Female
Male
Ages
Female Mean
Female Median
Male Mean
Male Median

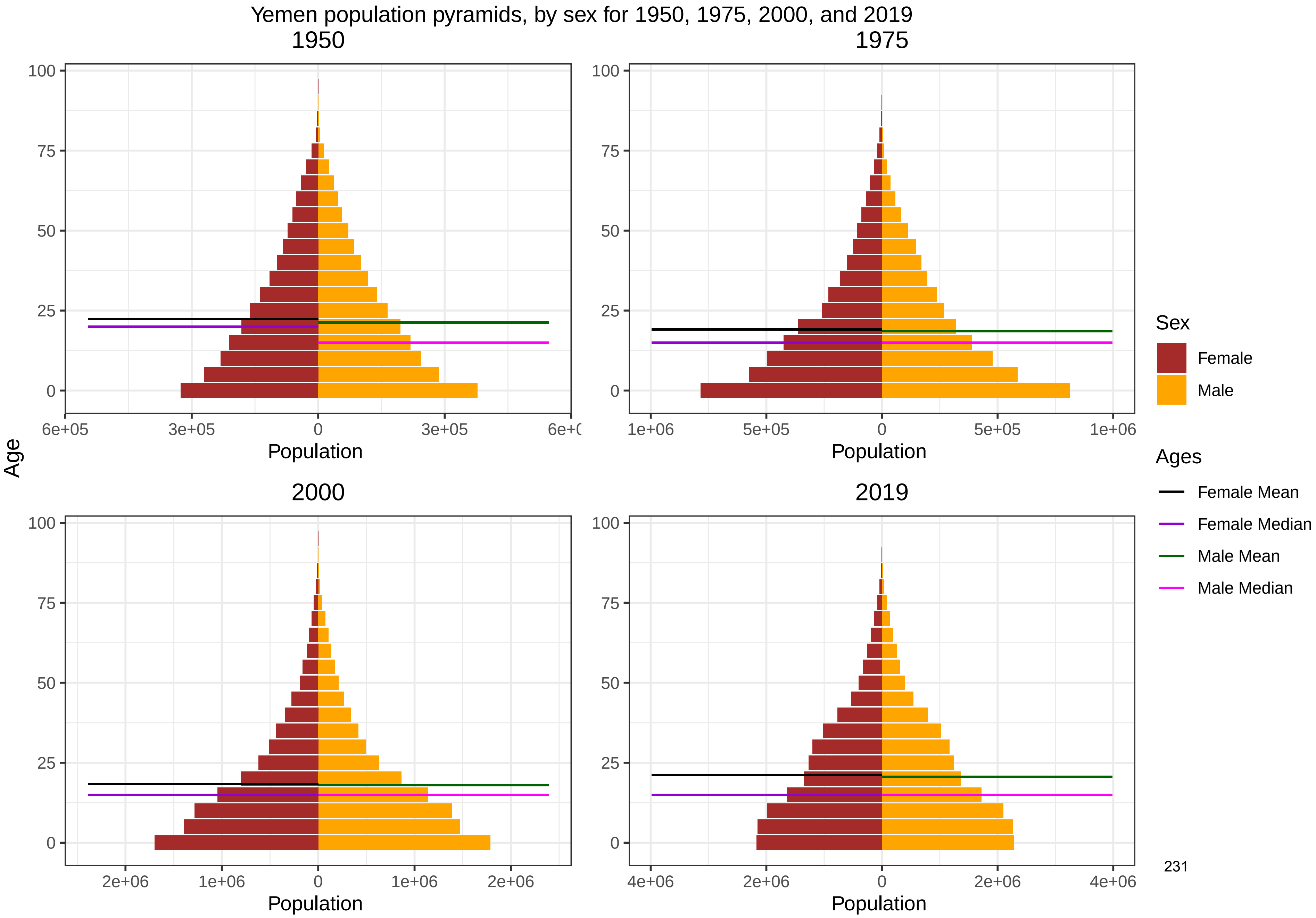

Yemen population pyramids, by sex for 1950, 1975, 2000, and 2019
1950
1975
2000
2019
Age
Population
100
75
50
25
0
6e+05
3e+05
0
3e+05
6e+0
1e+06
5e+05
5e+05
1e+06
2e+06
1e+06
1e+06
2e+06
4e+06
2e+06
2e+06
4e+06
Sex
Female
Male
Ages
Female Mean
Female Median
Male Mean
Male Median

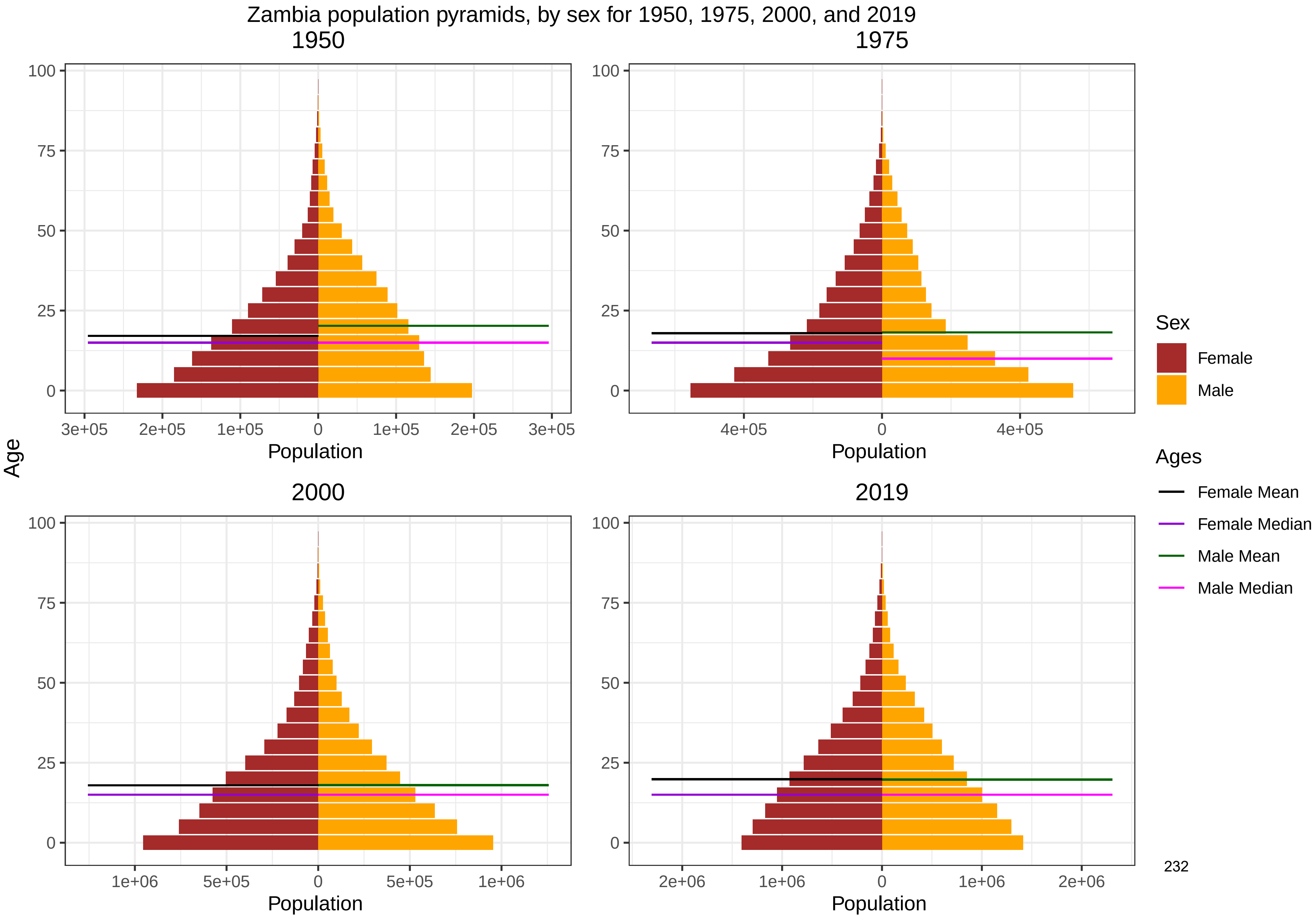
Zambia population pyramids, by sex for 1950, 1975, 2000, and 2019
1950
1975
2000
2019
Age
Population
Sex
Female
Male
Ages
Female Mean
Female Median
Male Mean
Male Median

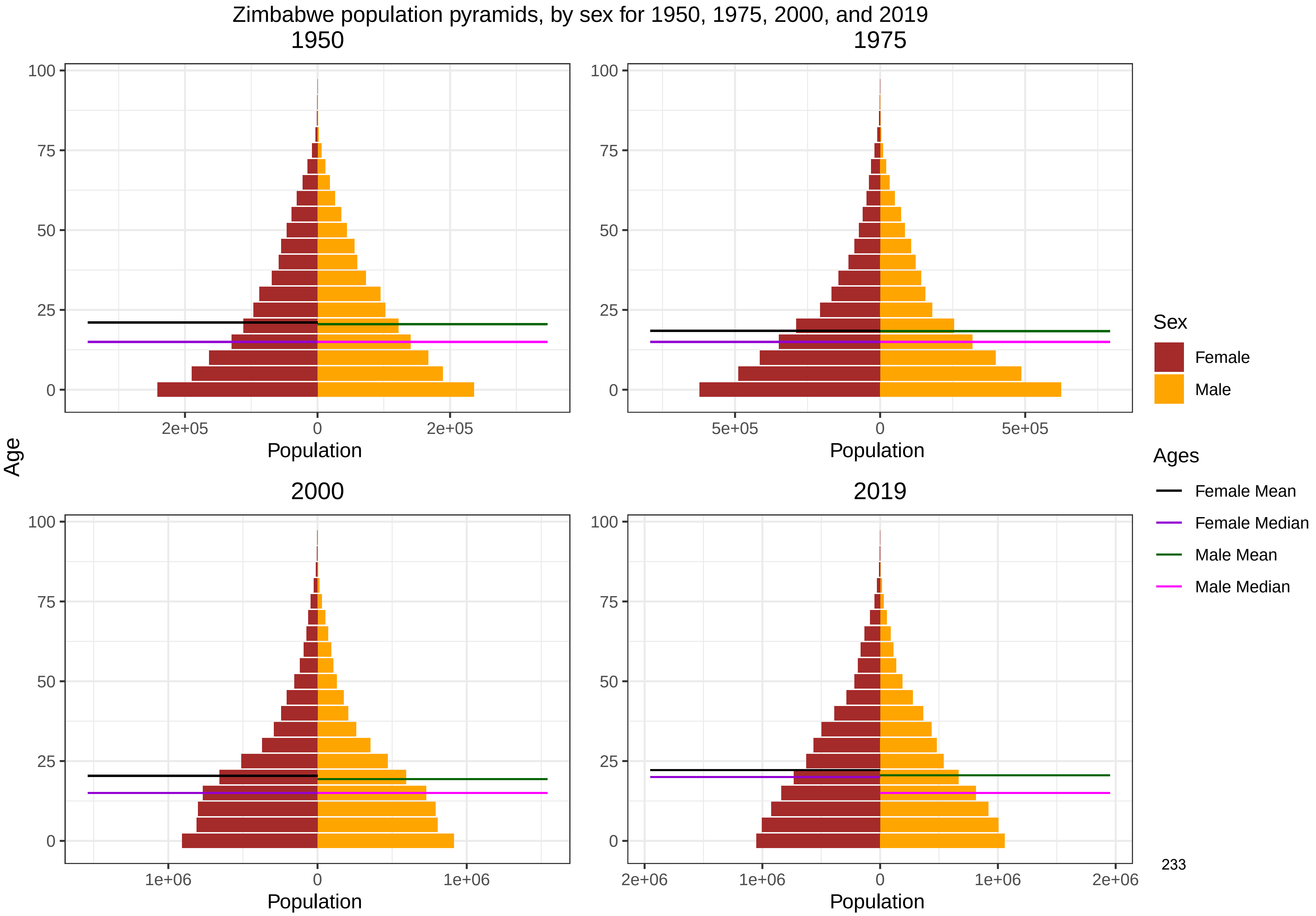

Zimbabwe population pyramids, by sex for 1950, 1975, 2000, and 2019
1950
1975
2000
2019
Age
Population
100
75
50
25
0
2e+05
0
2e+05
5e+05
0
5e+05
1e+06
0
1e+06
2e+06
1e+06
0
1e+06
2e+06
Sex
Female
Male
Ages
Female Mean
Female Median
Male Mean
Male Median

Age−specific fertility fitted trend lines, 1950−2019: Afghanistan, Ages: 10−14
Data points enclosed with a diamond are reference sources

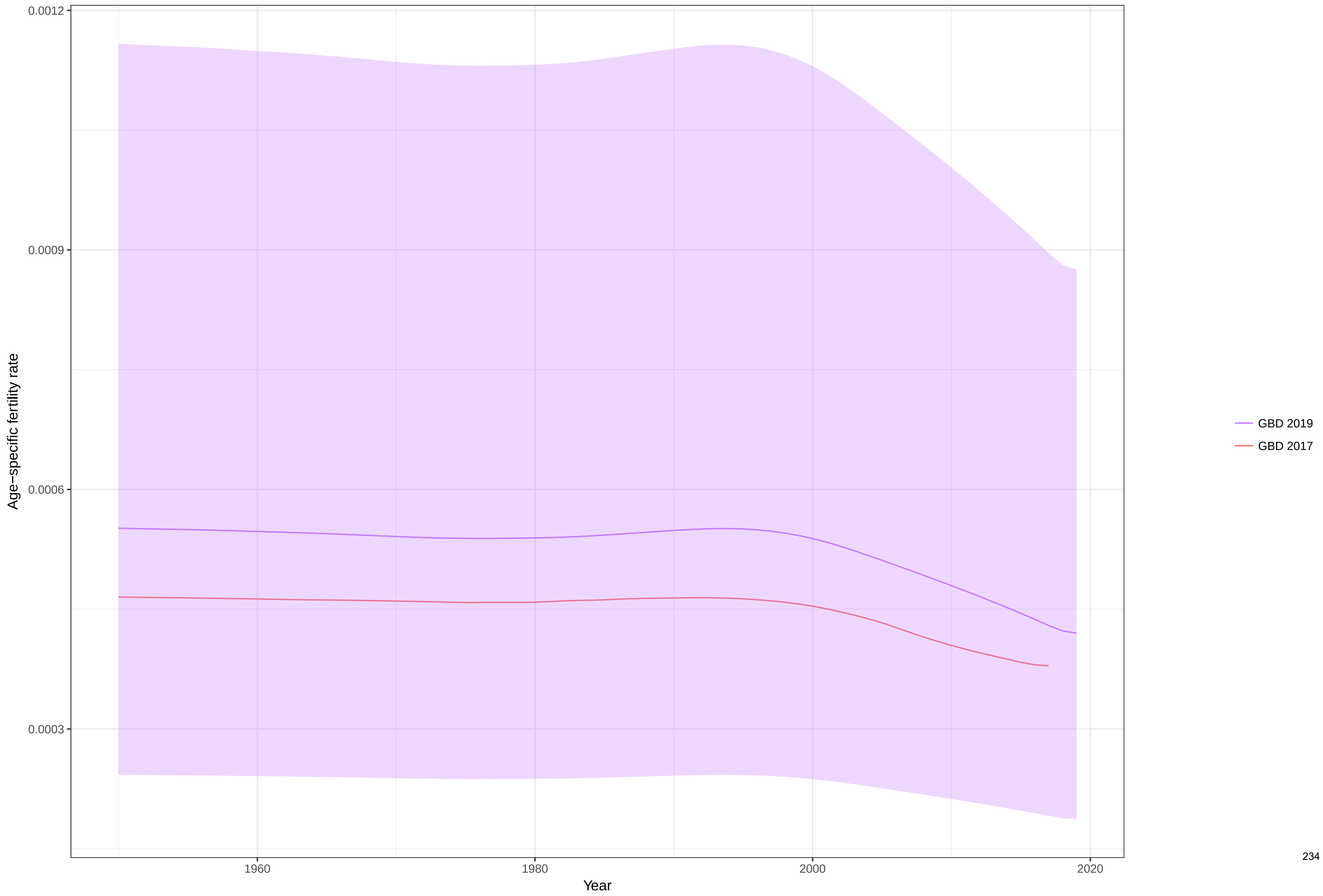

Age−specific fertility fitted trend lines, 1950−2019: Afghanistan, Ages: 15−19
Data points enclosed with a diamond are reference sources

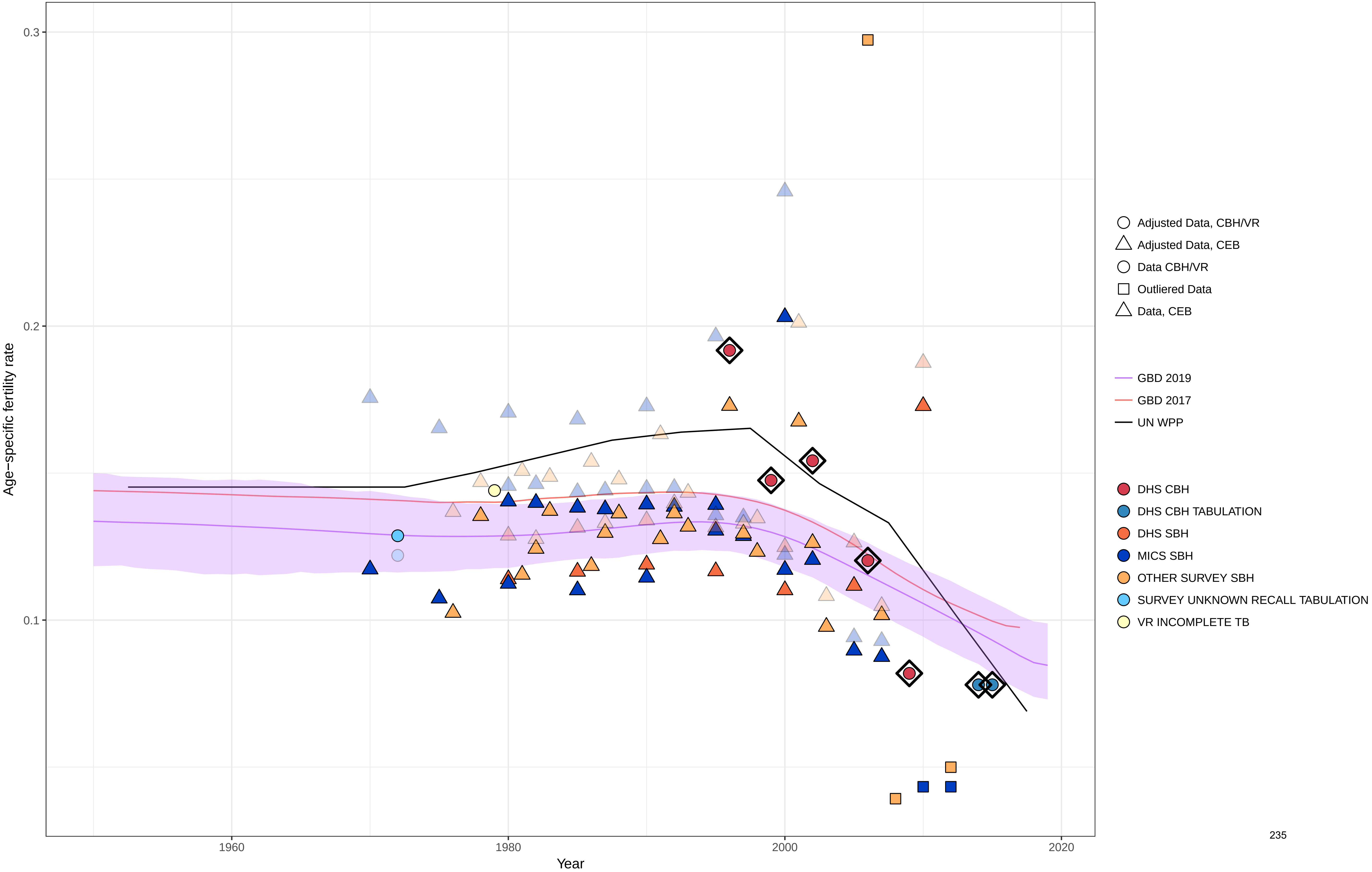

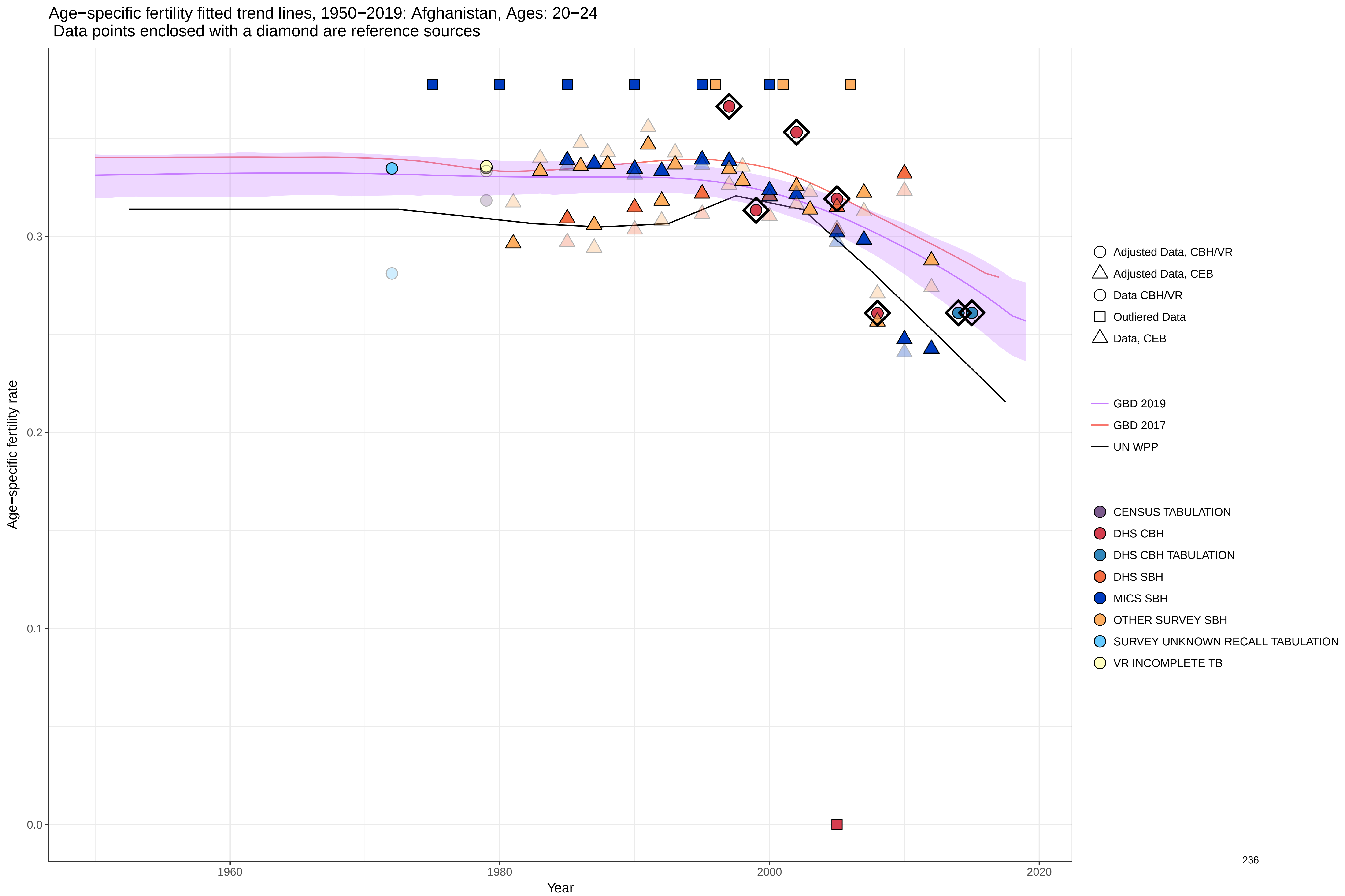
Age−specific fertility fitted trend lines, 1950−2019: Afghanistan, Ages: 20−24
Data points enclosed with a diamond are reference sources
Age−specific fertility rate
Year
0.0
0.1
0.2
0.3
1960
1980
2000
2020
Adjusted Data, CBH/VR
Adjusted Data, CEB
Data CBH/VR
Outliered Data
Data, CEB
GBD 2019
GBD 2017
UN WPP
CENSUS TABULATION
DHS CBH
DHS CBH TABULATION
DHS SBH
MICS SBH
OTHER SURVEY SBH
SURVEY UNKNOWN RECALL TABULATION
VR INCOMPLETE TB

Age−specific fertility fitted trend lines, 1950−2019: Afghanistan, Ages: 25−29
Data points enclosed with a diamond are reference sources

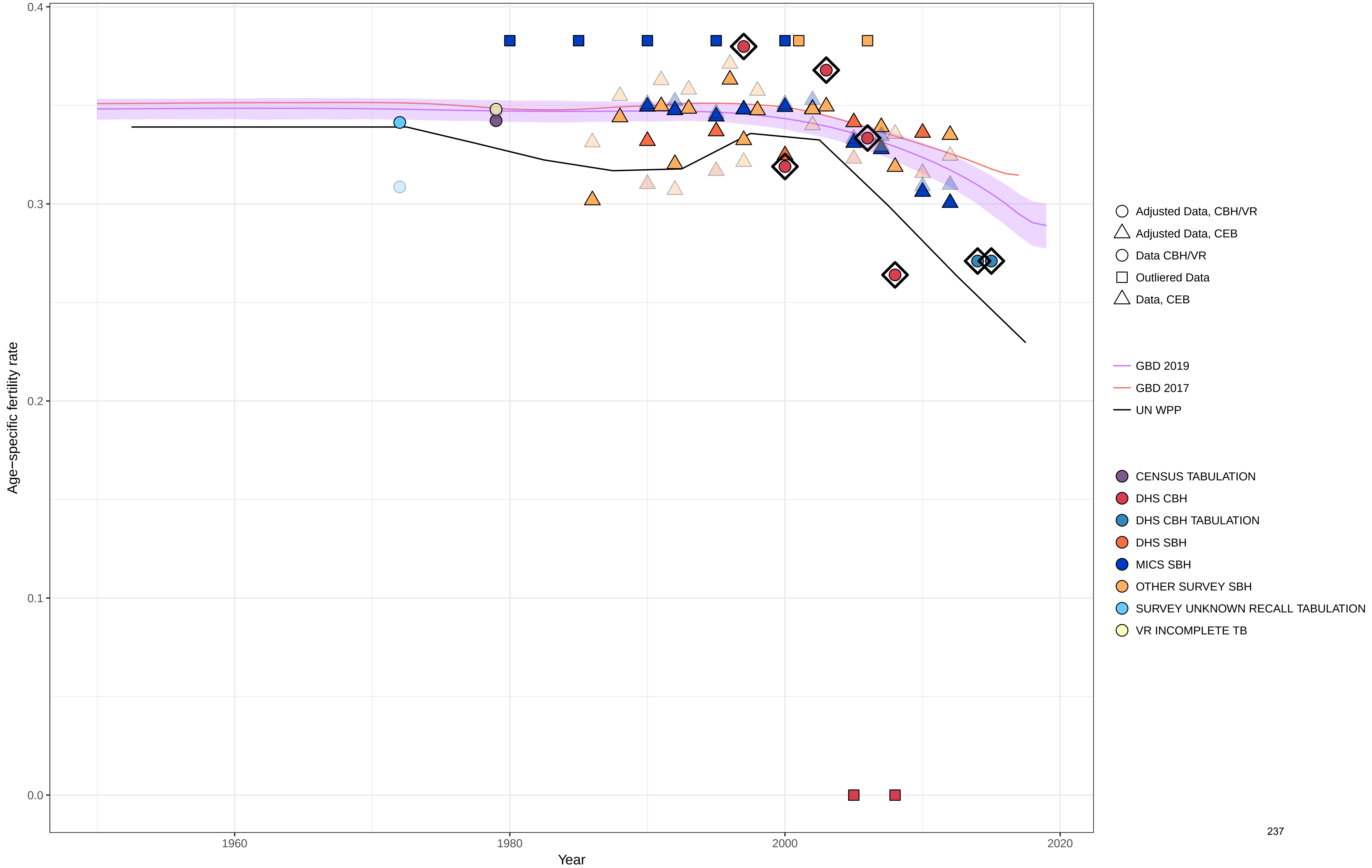

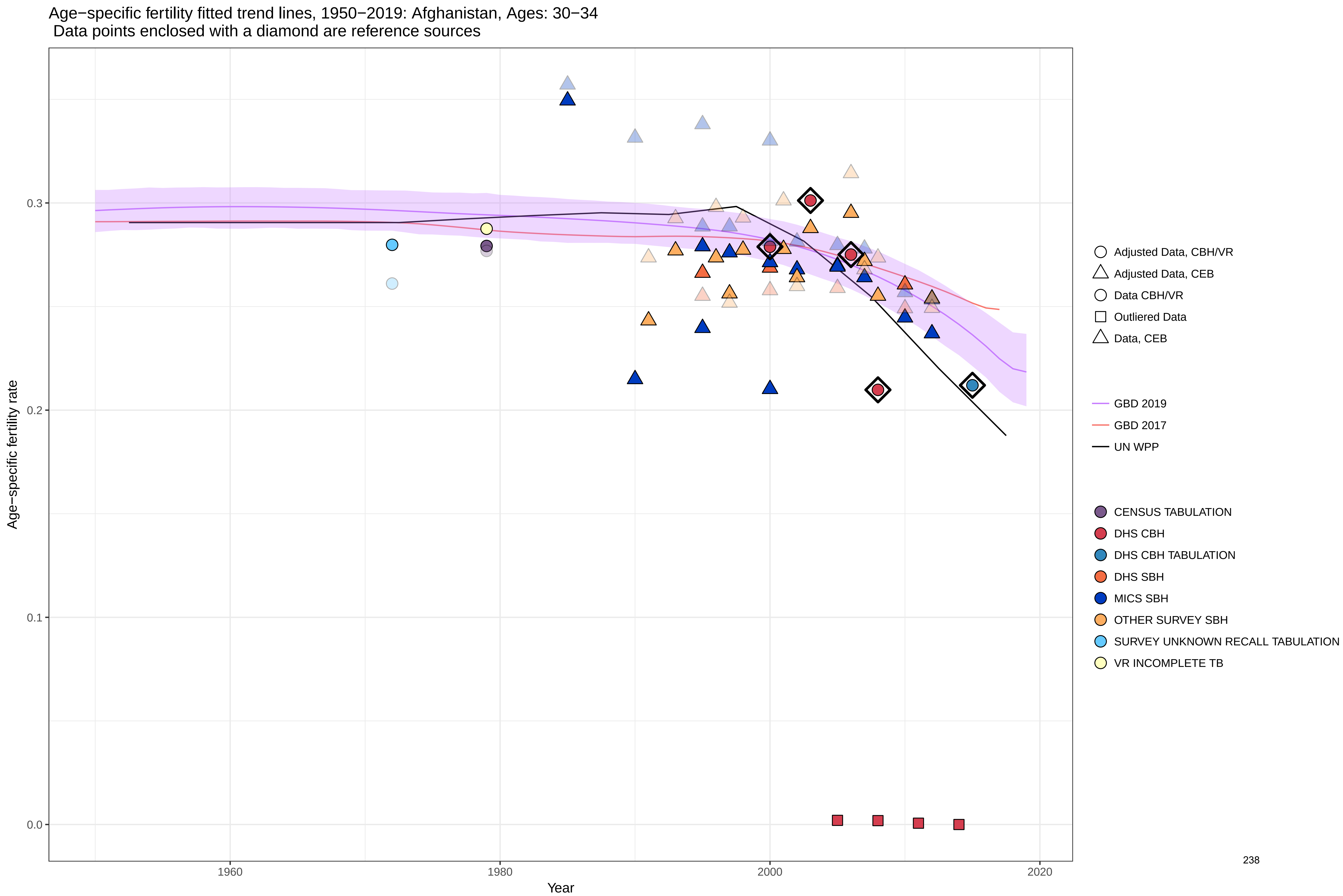

Age−specific fertility fitted trend lines, 1950−2019: Afghanistan, Ages: 30−34
Data points enclosed with a diamond are reference sources
Age−specific fertility rate
Year
0.0
0.1
0.2
0.3
1960
1980
2000
2020
Adjusted Data, CBH/VR
Adjusted Data, CEB
Data CBH/VR
Outliered Data
Data, CEB
GBD 2019
GBD 2017
UN WPP
CENSUS TABULATION
DHS CBH
DHS CBH TABULATION
DHS SBH
MICS SBH
OTHER SURVEY SBH
SURVEY UNKNOWN RECALL TABULATION
VR INCOMPLETE TB

Age−specific fertility fitted trend lines, 1950−2019: Afghanistan, Ages: 35−39
Data points enclosed with a diamond are reference sources

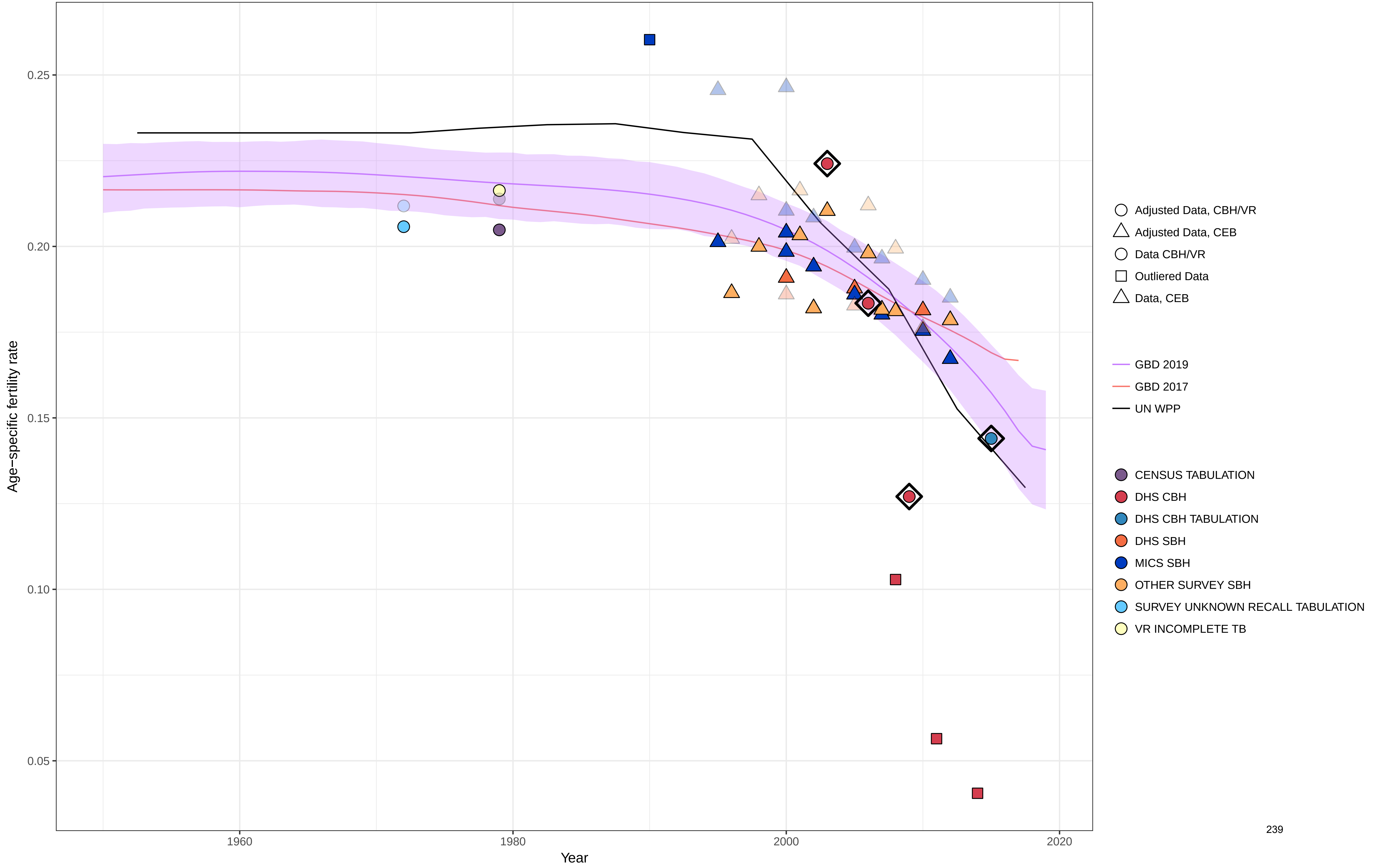

Age−specific fertility fitted trend lines, 1950−2019: Afghanistan, Ages: 40−44
Data points enclosed with a diamond are reference sources

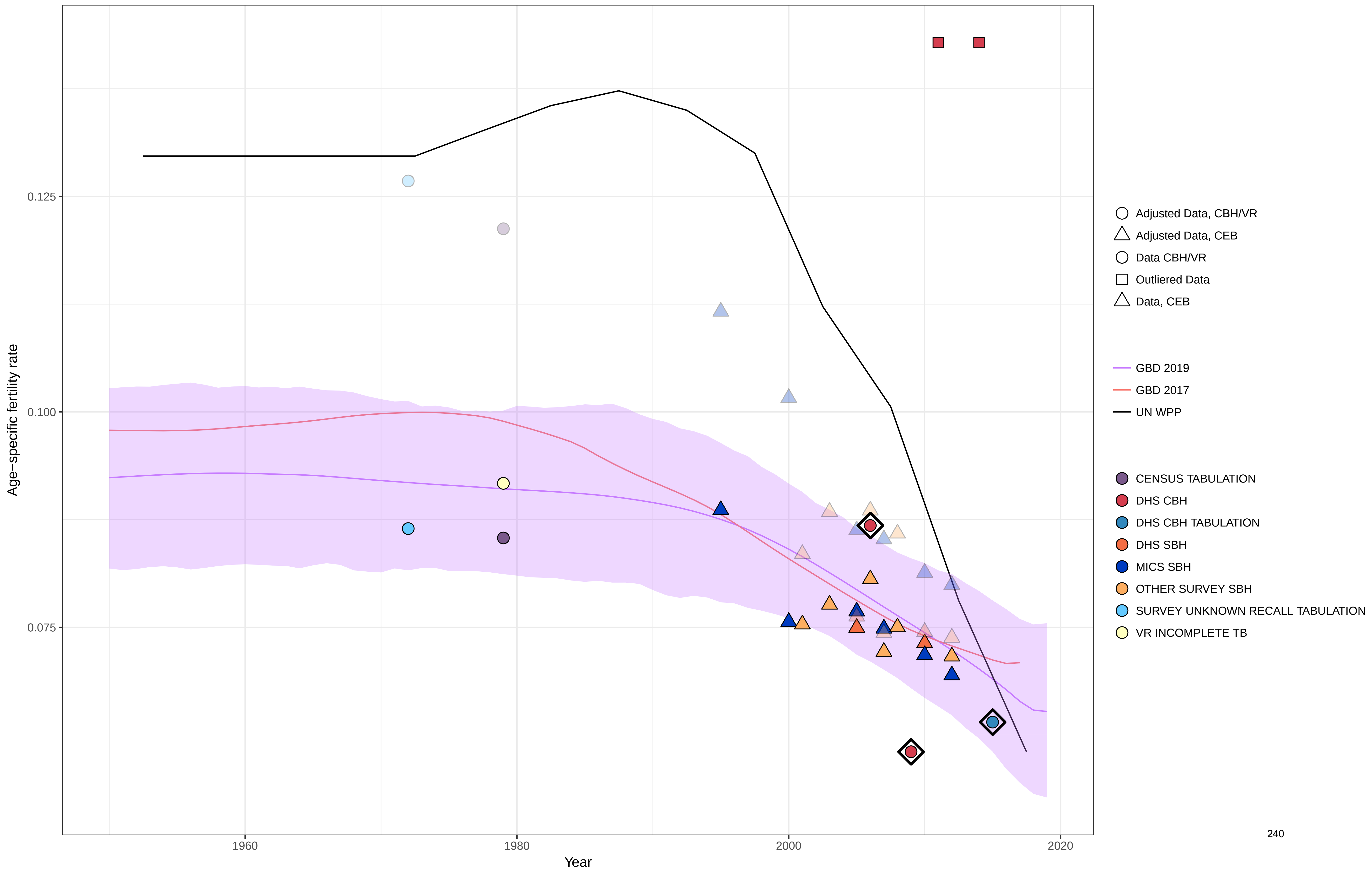

Age−specific fertility fitted trend lines, 1950−2019: Afghanistan, Ages: 45−49
Data points enclosed with a diamond are reference sources

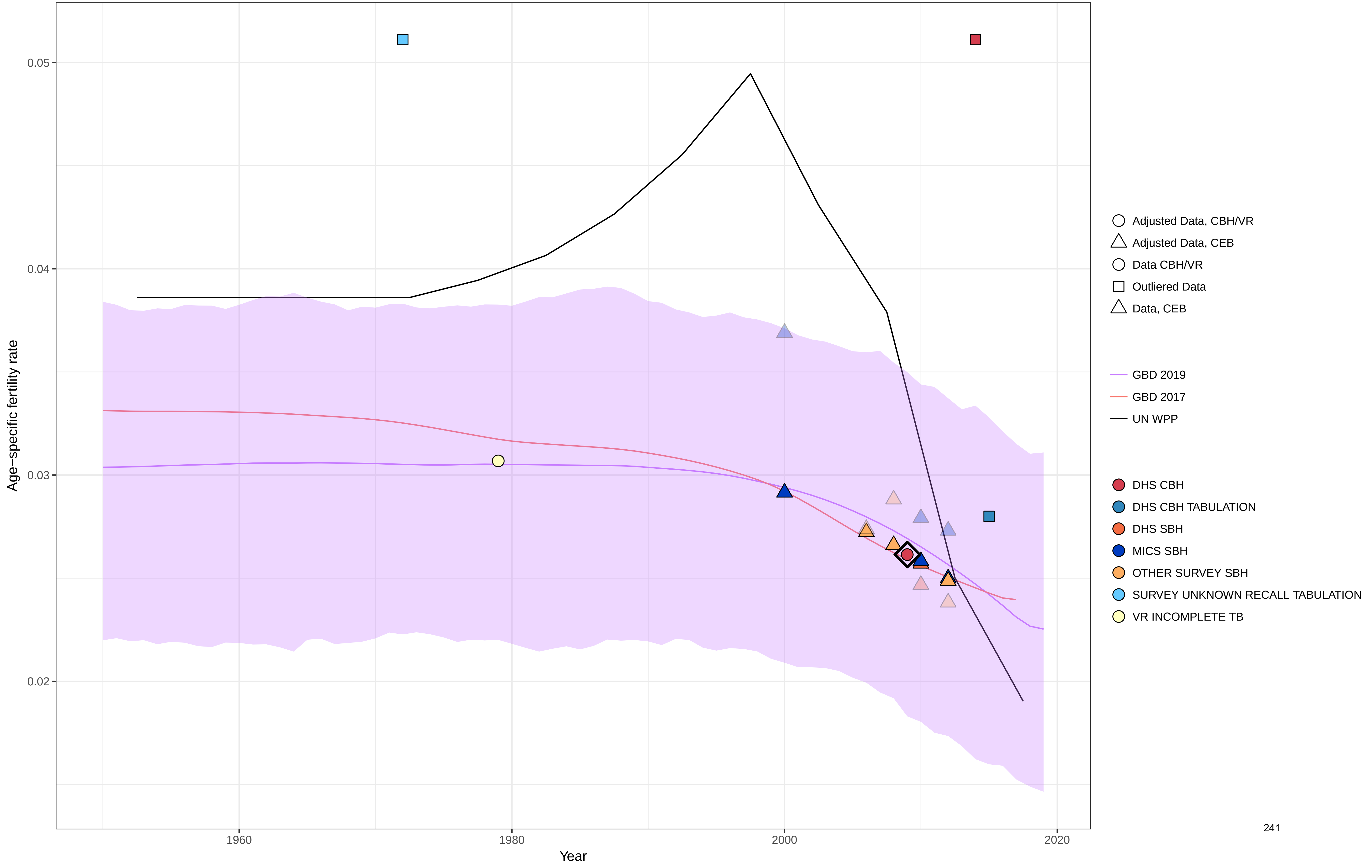

Age−specific fertility fitted trend lines, 1950−2019: Afghanistan, Ages: 50−54
Data points enclosed with a diamond are reference sources

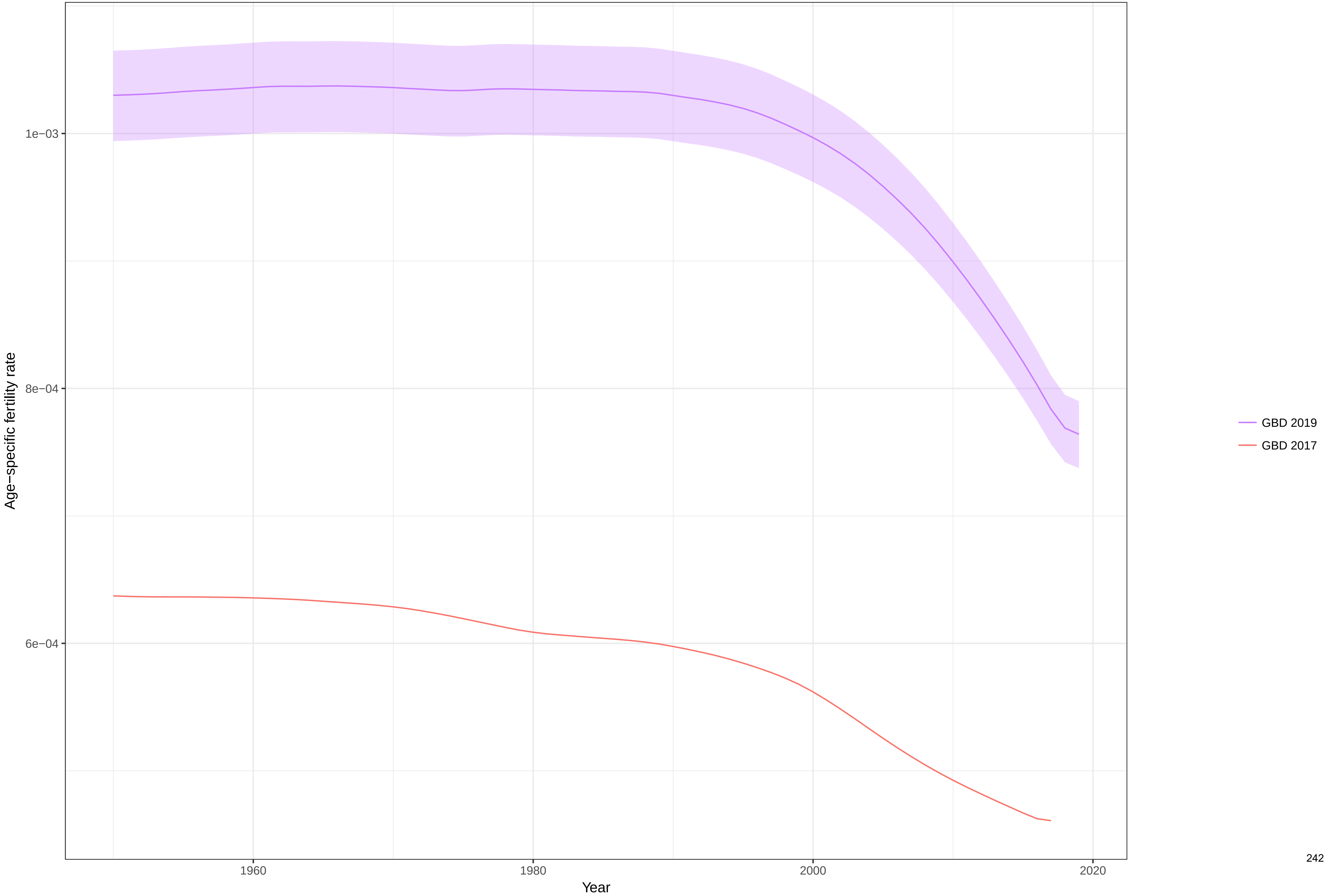

Total fertility rate fitted trend lines, 1950−2019: Afghanistan TFR
Data points enclosed with a diamond are reference sources

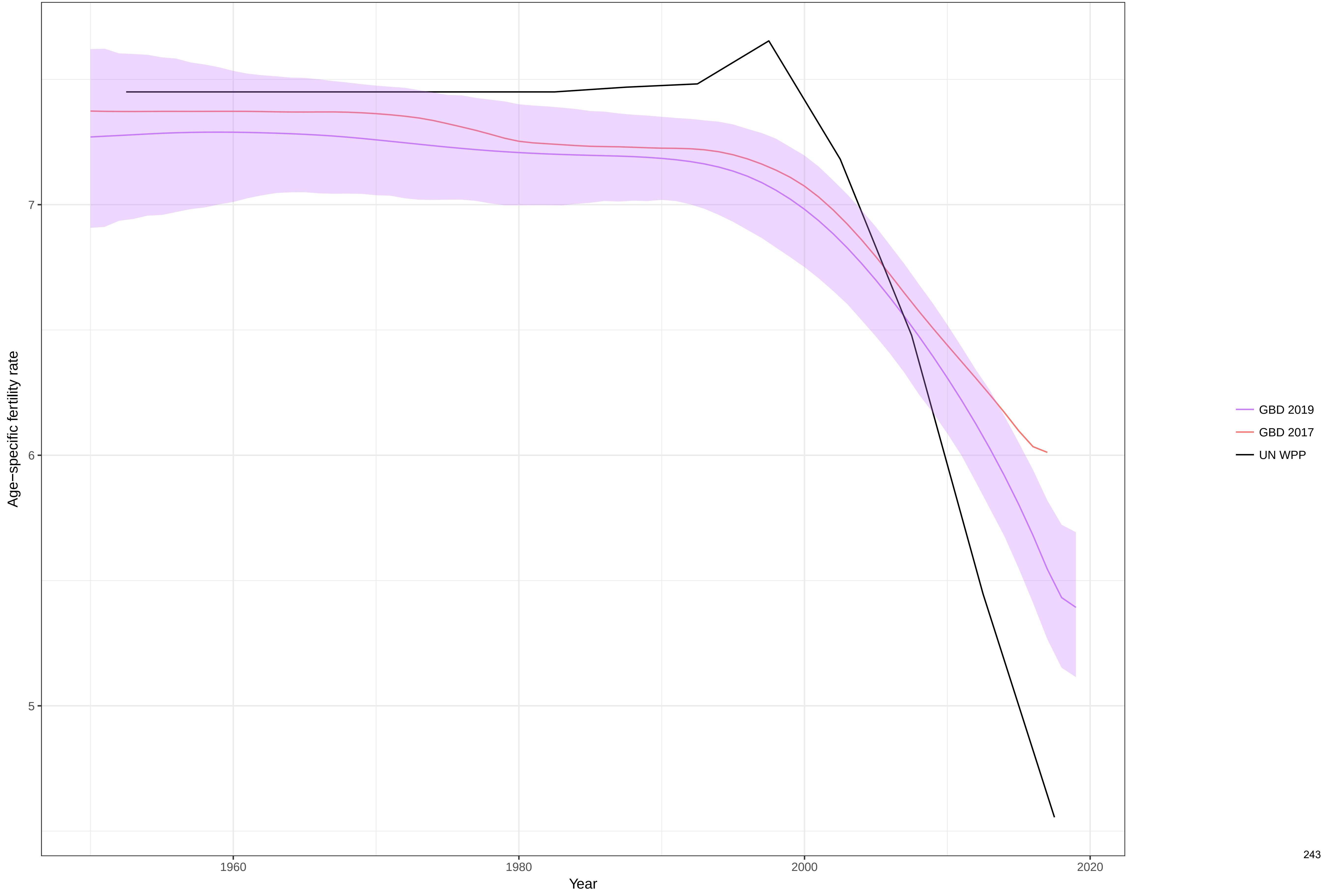

Age−specific fertility fitted trend lines, 1950−2019: Angola, Ages: 10−14
Data points enclosed with a diamond are reference sources

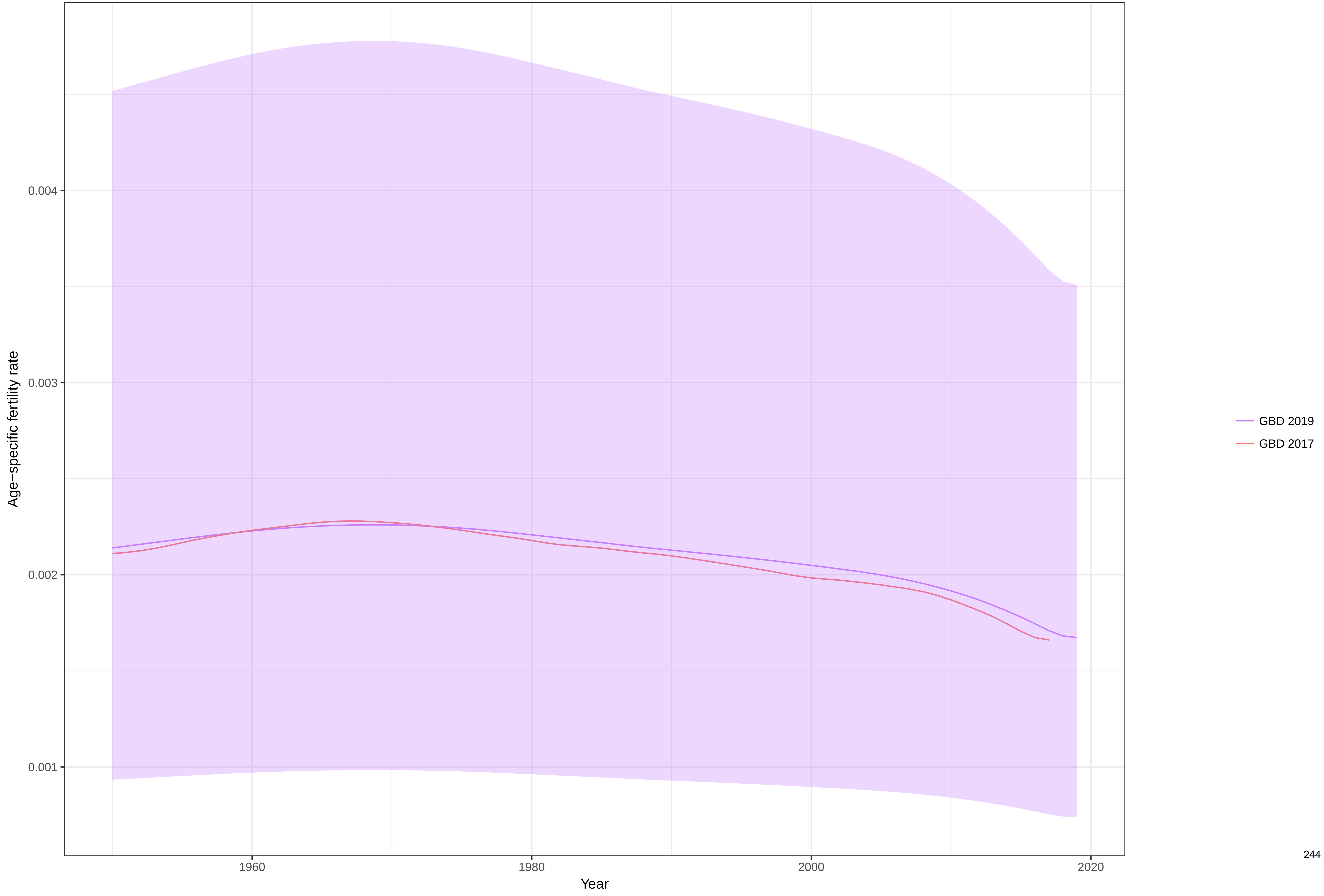

Age−specific fertility fitted trend lines, 1950−2019: Angola, Ages: 15−19
Data points enclosed with a diamond are reference sources

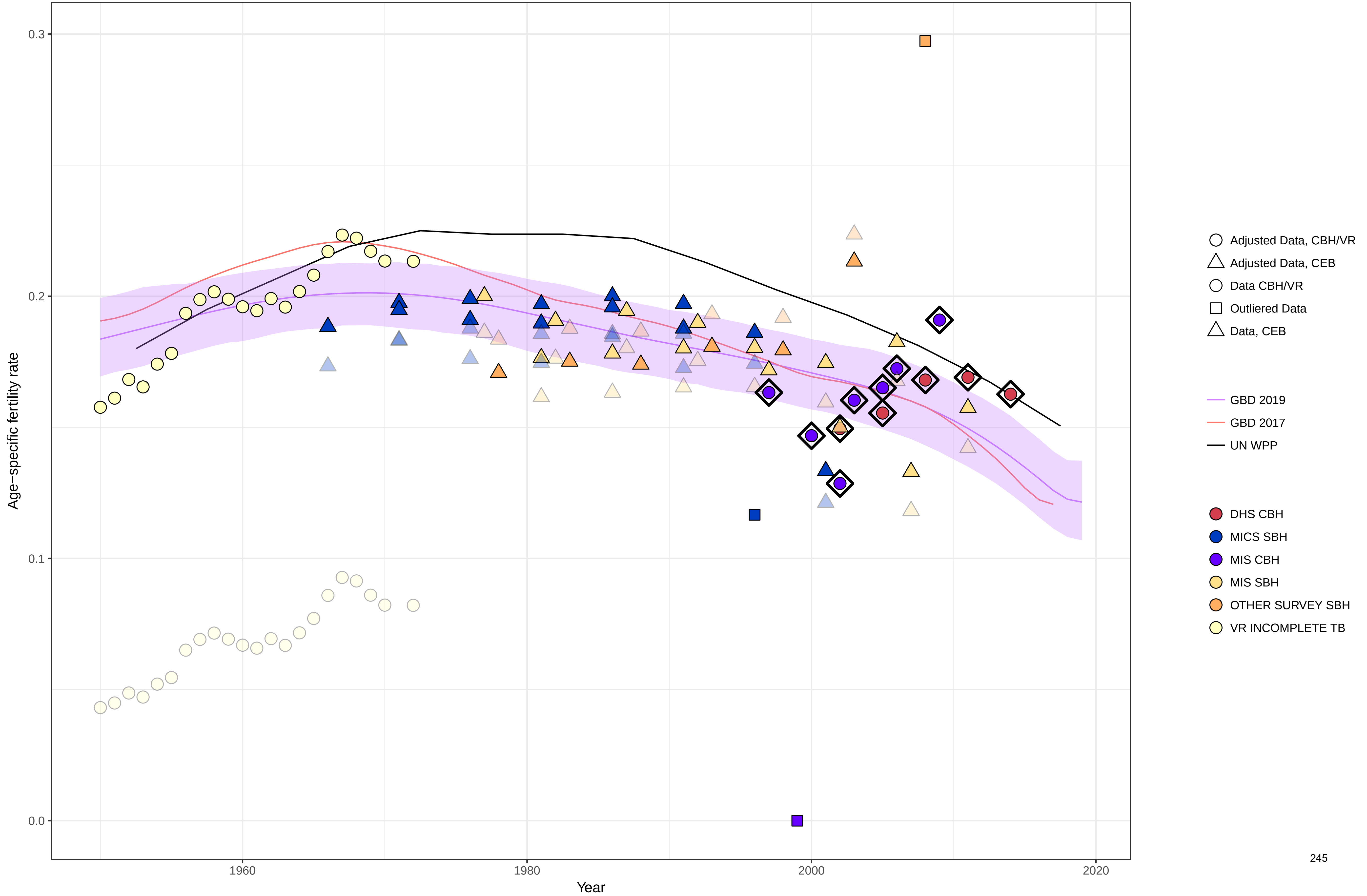

Age−specific fertility fitted trend lines, 1950−2019: Angola, Ages: 20−24
Data points enclosed with a diamond are reference sources

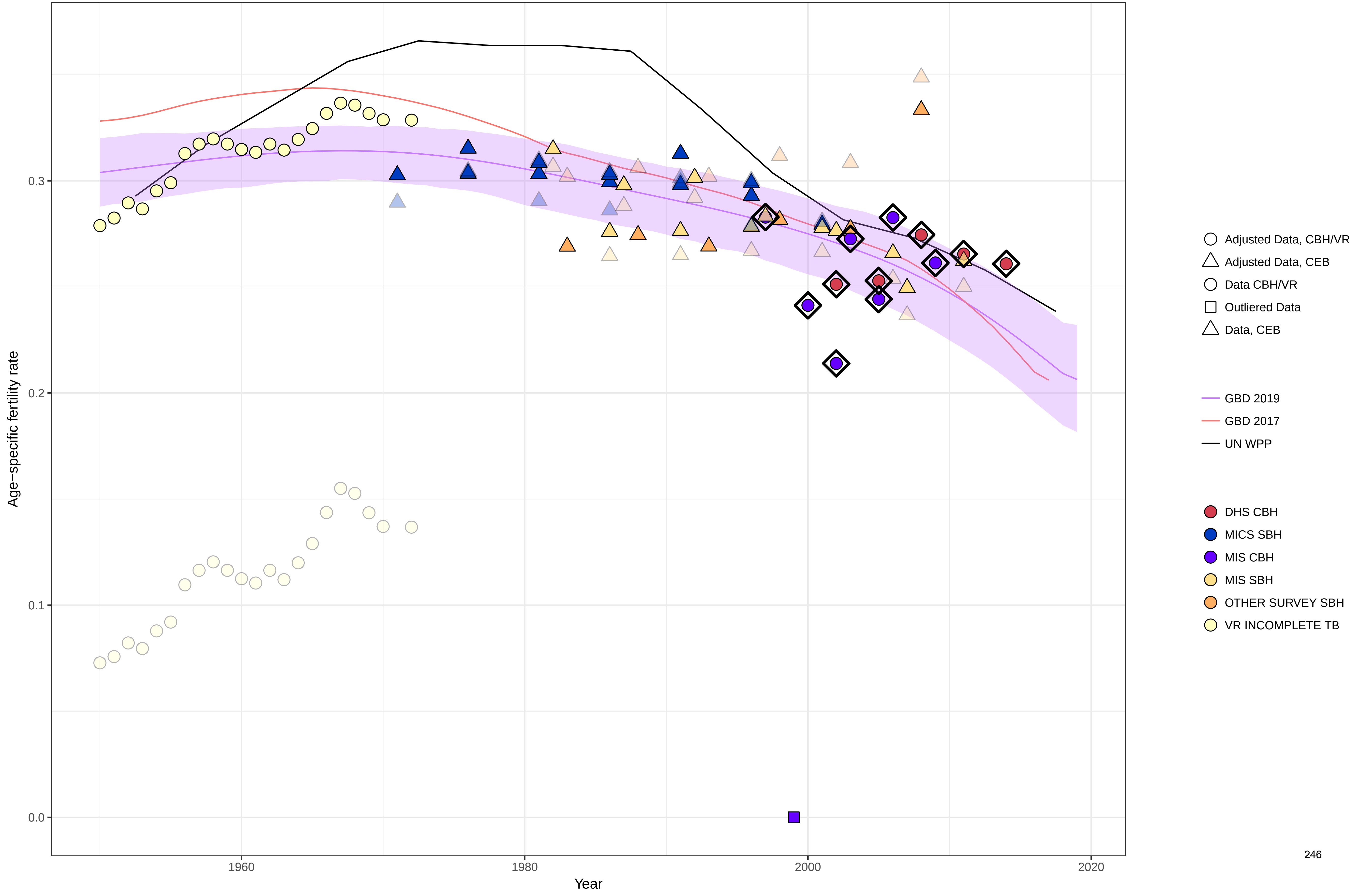

Age−specific fertility fitted trend lines, 1950−2019: Angola, Ages: 25−29
Data points enclosed with a diamond are reference sources

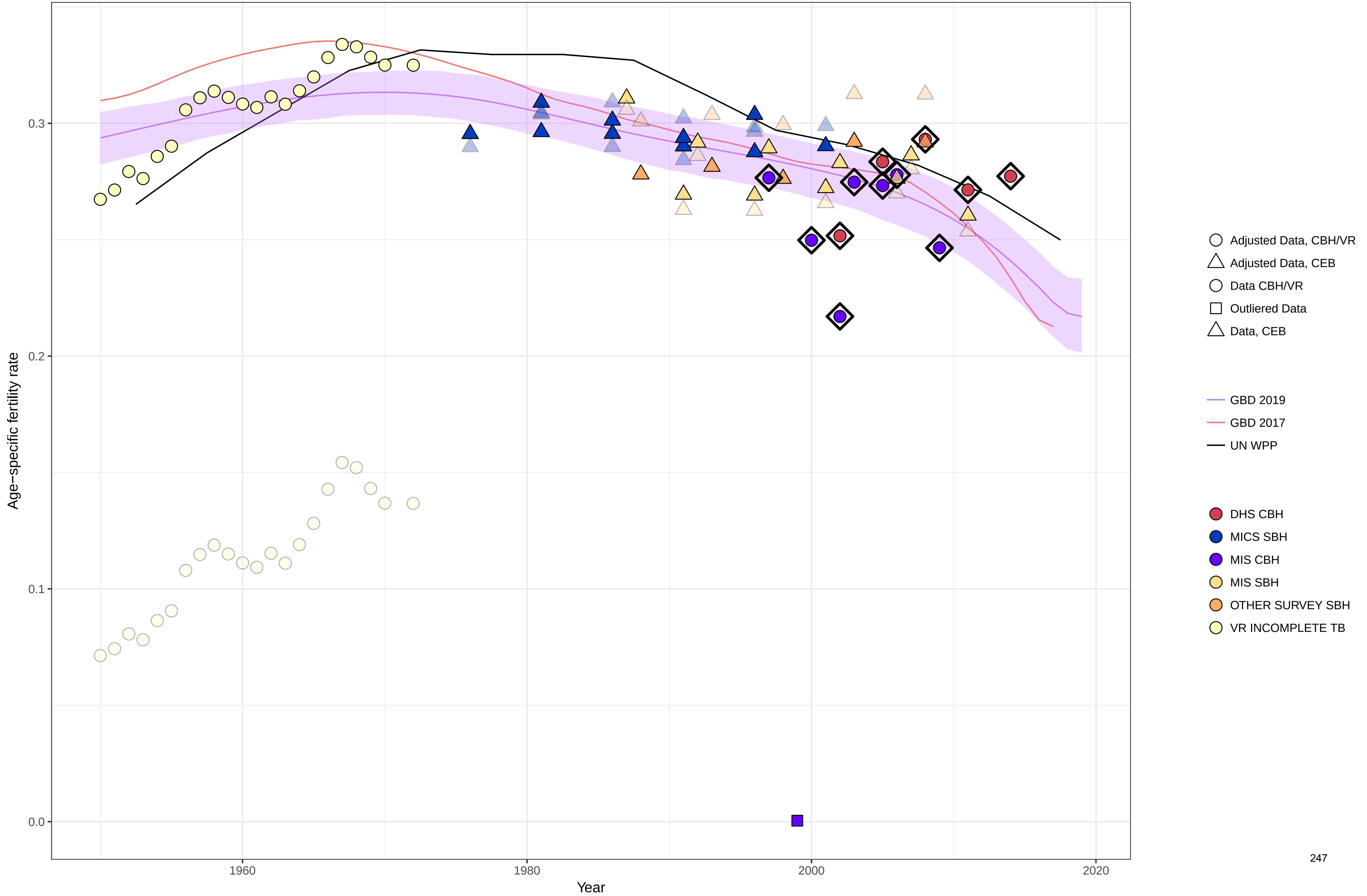

Age−specific fertility fitted trend lines, 1950−2019: Angola, Ages: 30−34
Data points enclosed with a diamond are reference sources

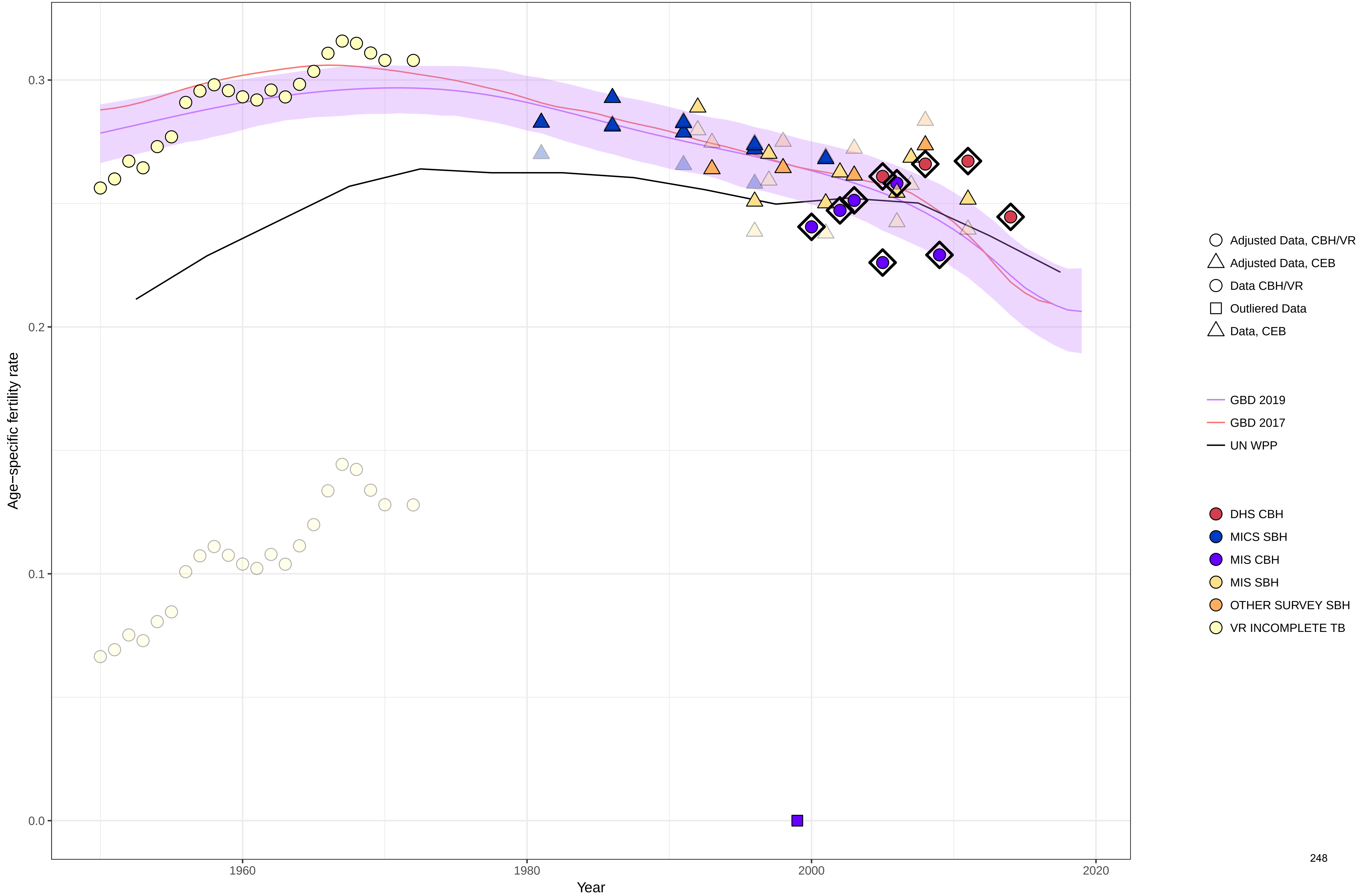

Age−specific fertility fitted trend lines, 1950−2019: Angola, Ages: 35−39
Data points enclosed with a diamond are reference sources

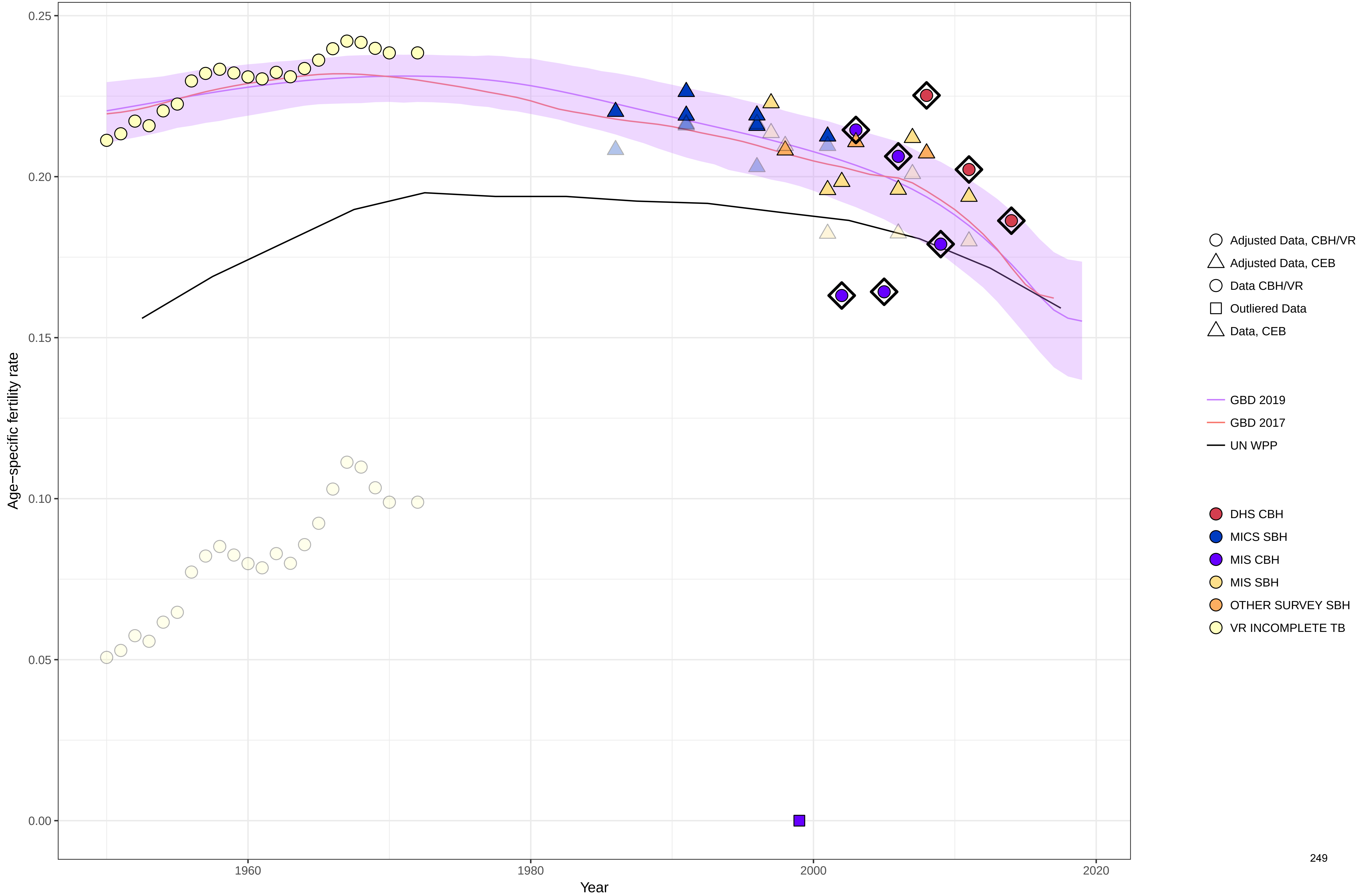

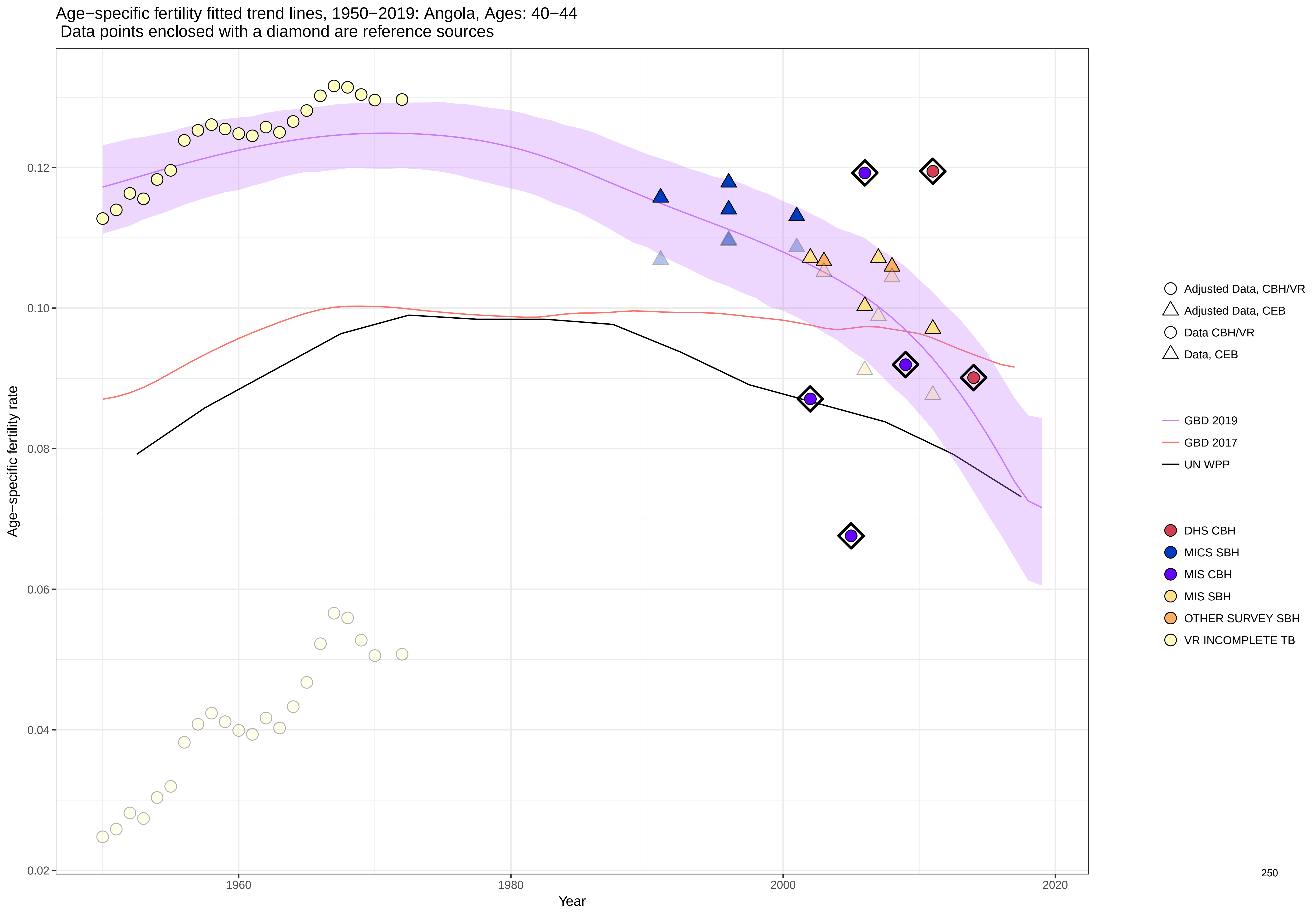

Age−specific fertility fitted trend lines, 1950−2019: Angola, Ages: 40−44
Data points enclosed with a diamond are reference sources
Age−specific fertility rate
0.12
0.10
0.08
0.06
0.04
0.02
1960
1980
2000
2020
Year
Adjusted Data, CBH/VR
Adjusted Data, CEB
Data CBH/VR
Data, CEB
GBD 2019
GBD 2017
UN WPP
DHS CBH
MICS SBH
MIS CBH
MIS SBH
OTHER SURVEY SBH
VR INCOMPLETE TB

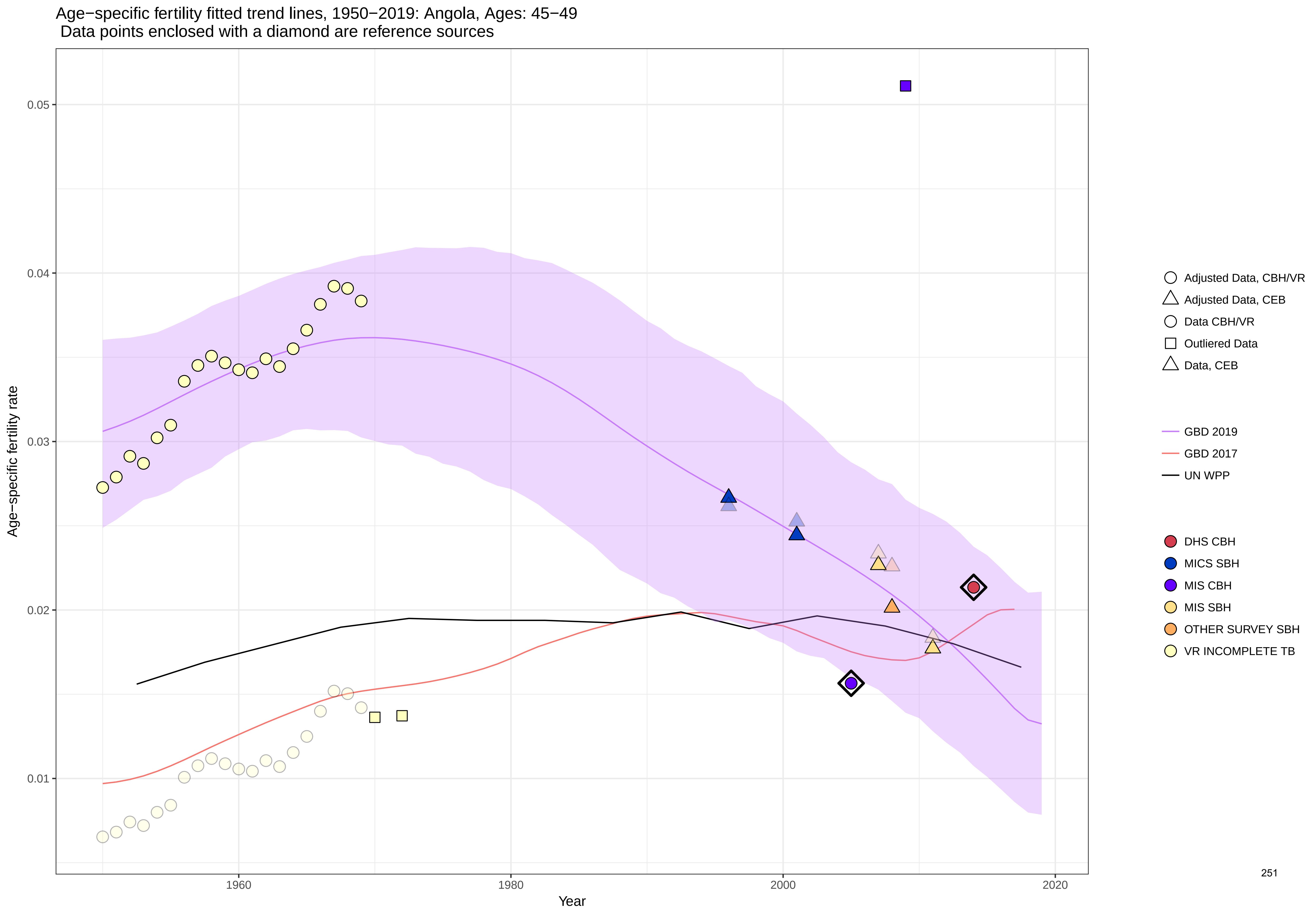

Age−specific fertility fitted trend lines, 1950−2019: Angola, Ages: 45−49
Data points enclosed with a diamond are reference sources
Age−specific fertility rate
0.05
0.04
0.03
0.02
0.01
1960
1980
2000
2020
Year
Adjusted Data, CBH/VR
Adjusted Data, CEB
Data CBH/VR
Outliered Data
Data, CEB
GBD 2019
GBD 2017
UN WPP
DHS CBH
MICS SBH
MIS CBH
MIS SBH
OTHER SURVEY SBH
VR INCOMPLETE TB

Age−specific fertility fitted trend lines, 1950−2019: Angola, Ages: 50−54
Data points enclosed with a diamond are reference sources

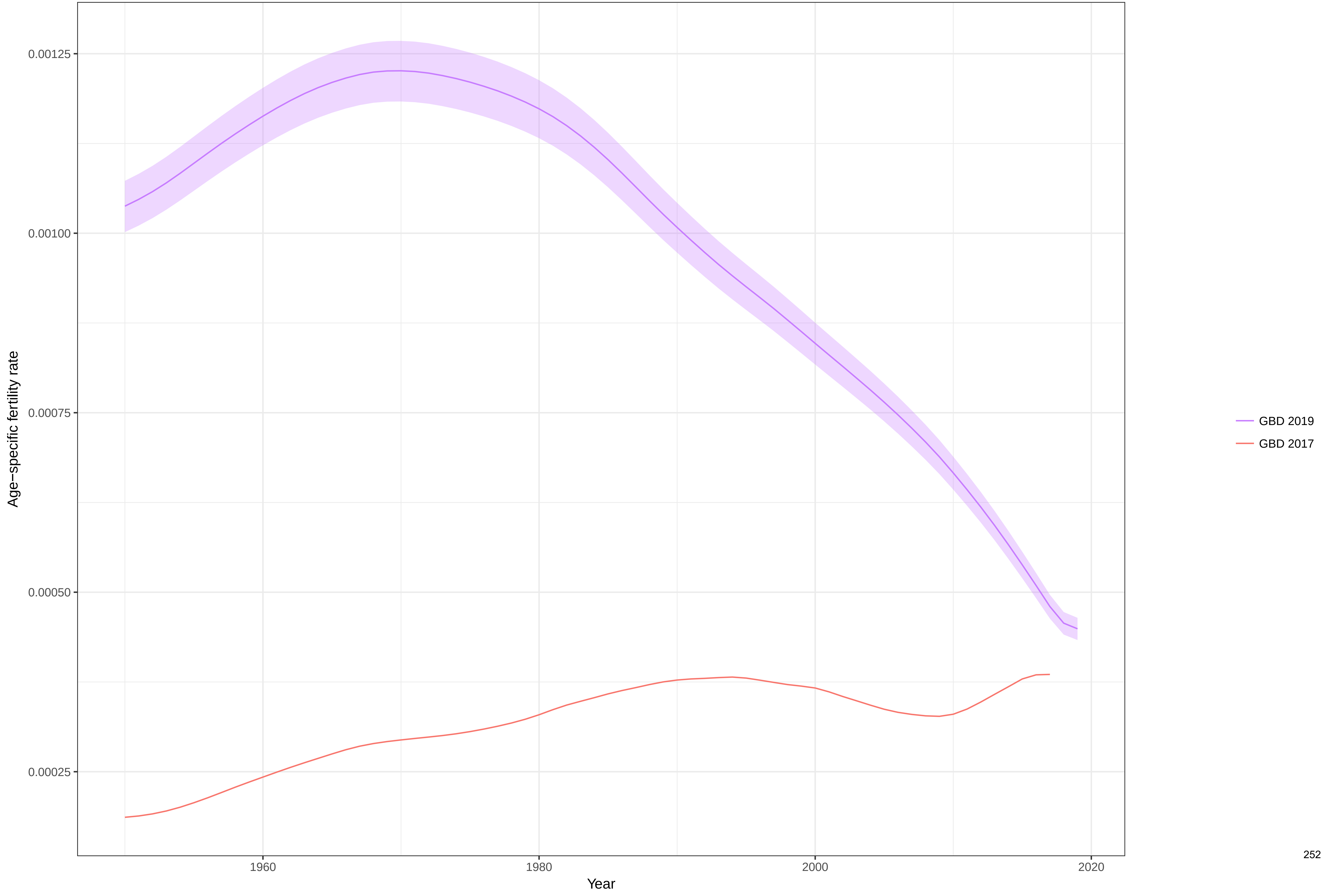

Total fertility rate fitted trend lines, 1950−2019: Angola TFR
Data points enclosed with a diamond are reference sources

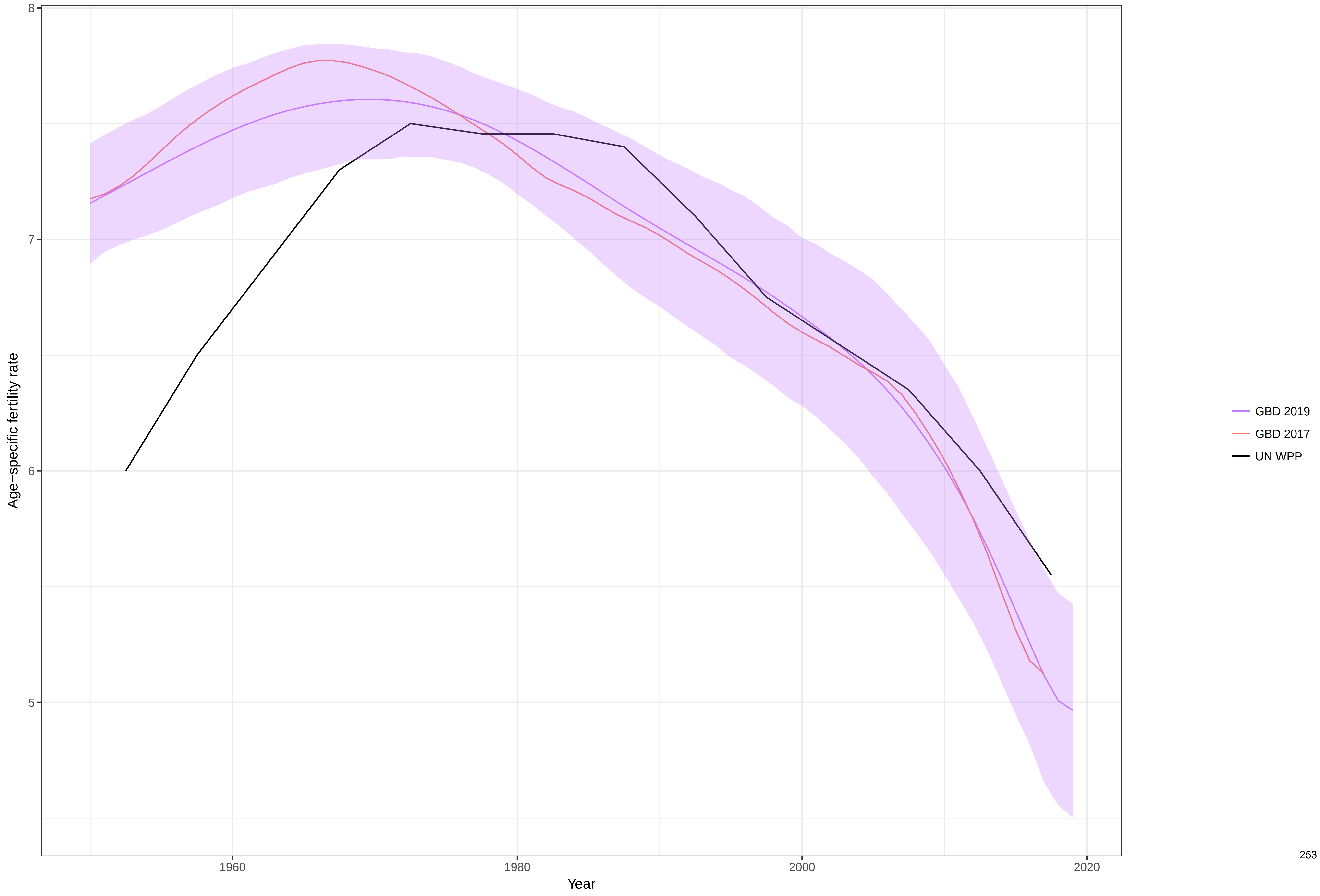

Age−specific fertility fitted trend lines, 1950−2019: Albania, Ages: 10−14
Data points enclosed with a diamond are reference sources

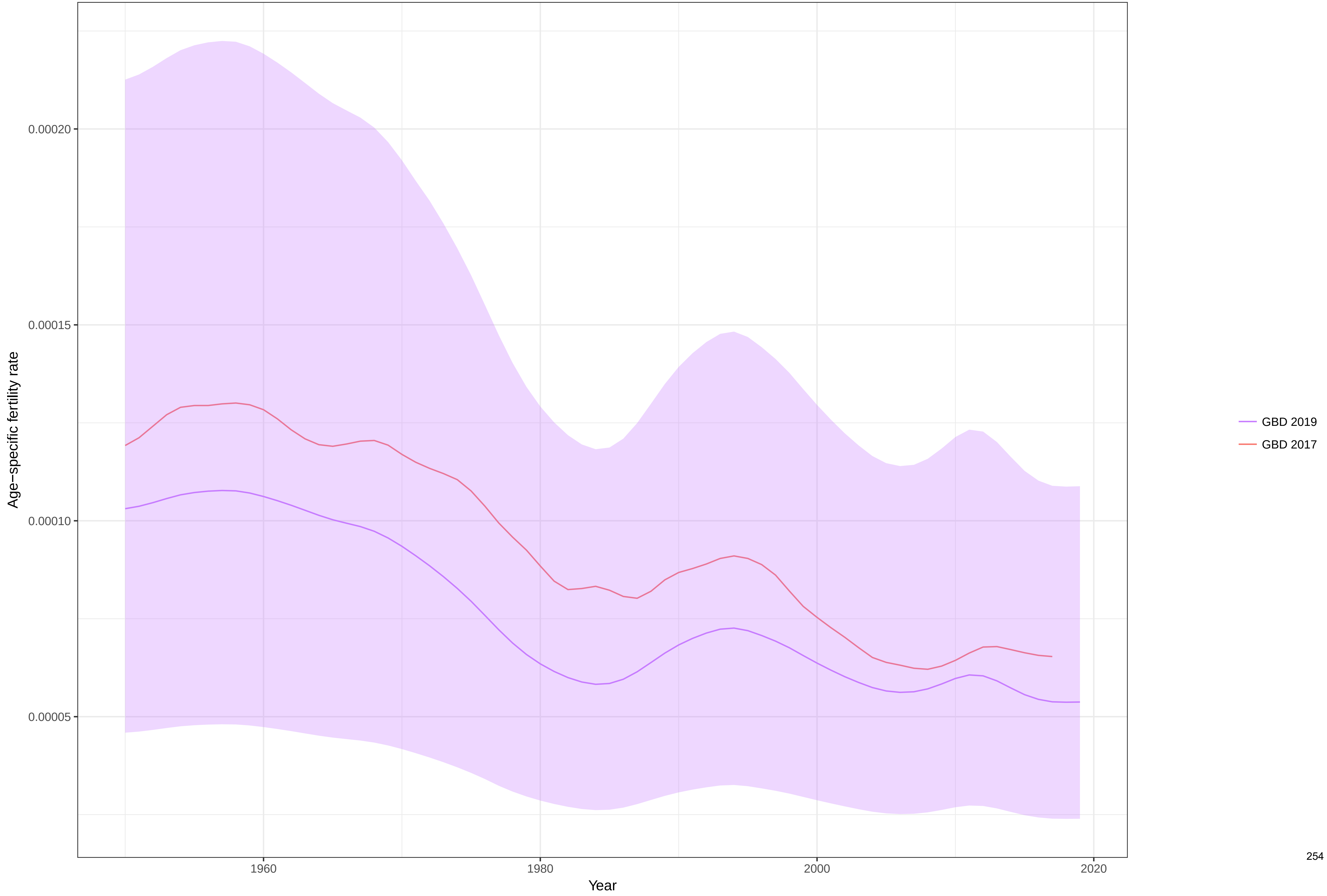

Age−specific fertility fitted trend lines, 1950−2019: Albania, Ages: 15−19
Data points enclosed with a diamond are reference sources

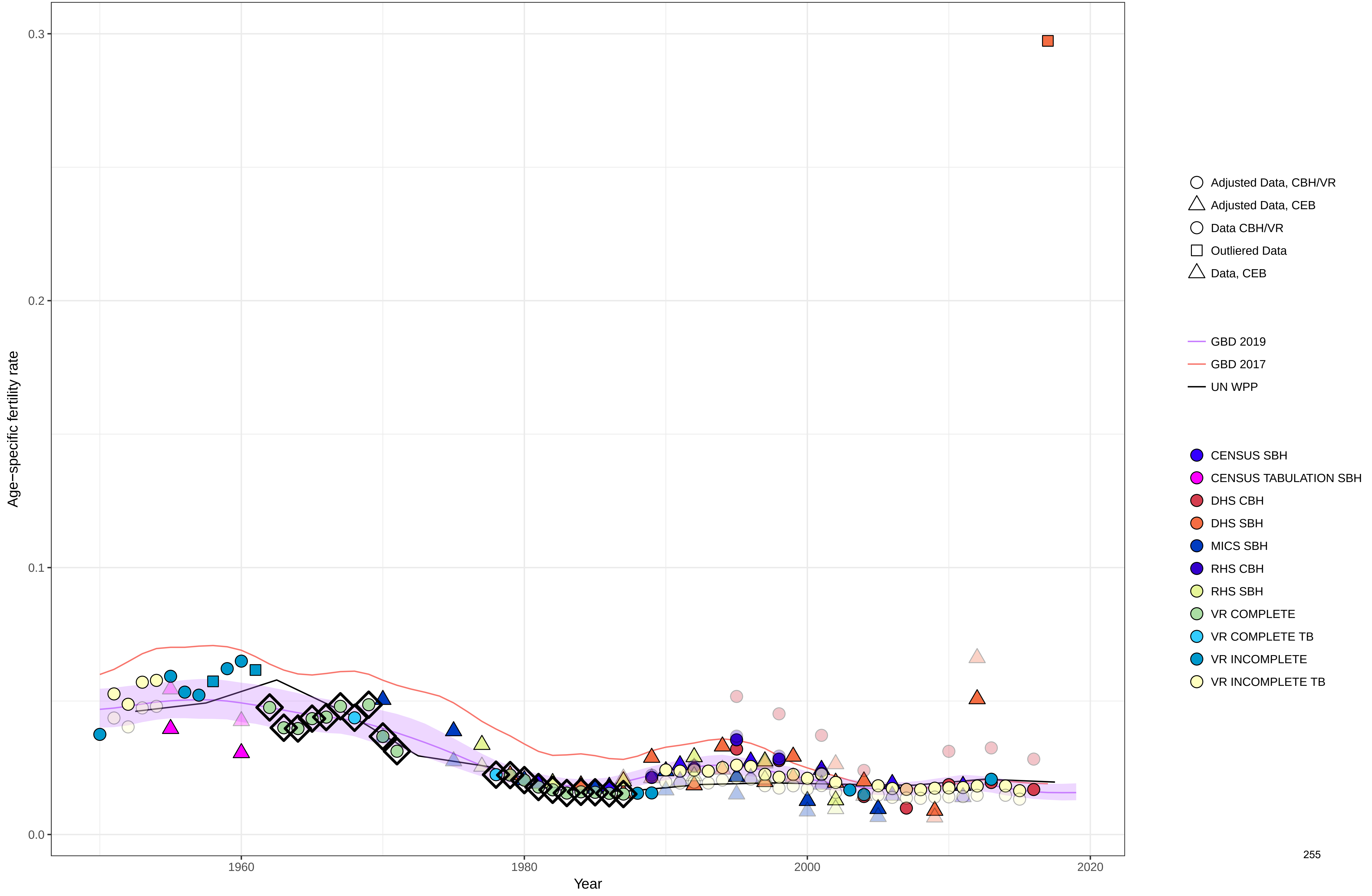

Age−specific fertility fitted trend lines, 1950−2019: Albania, Ages: 20−24
Data points enclosed with a diamond are reference sources

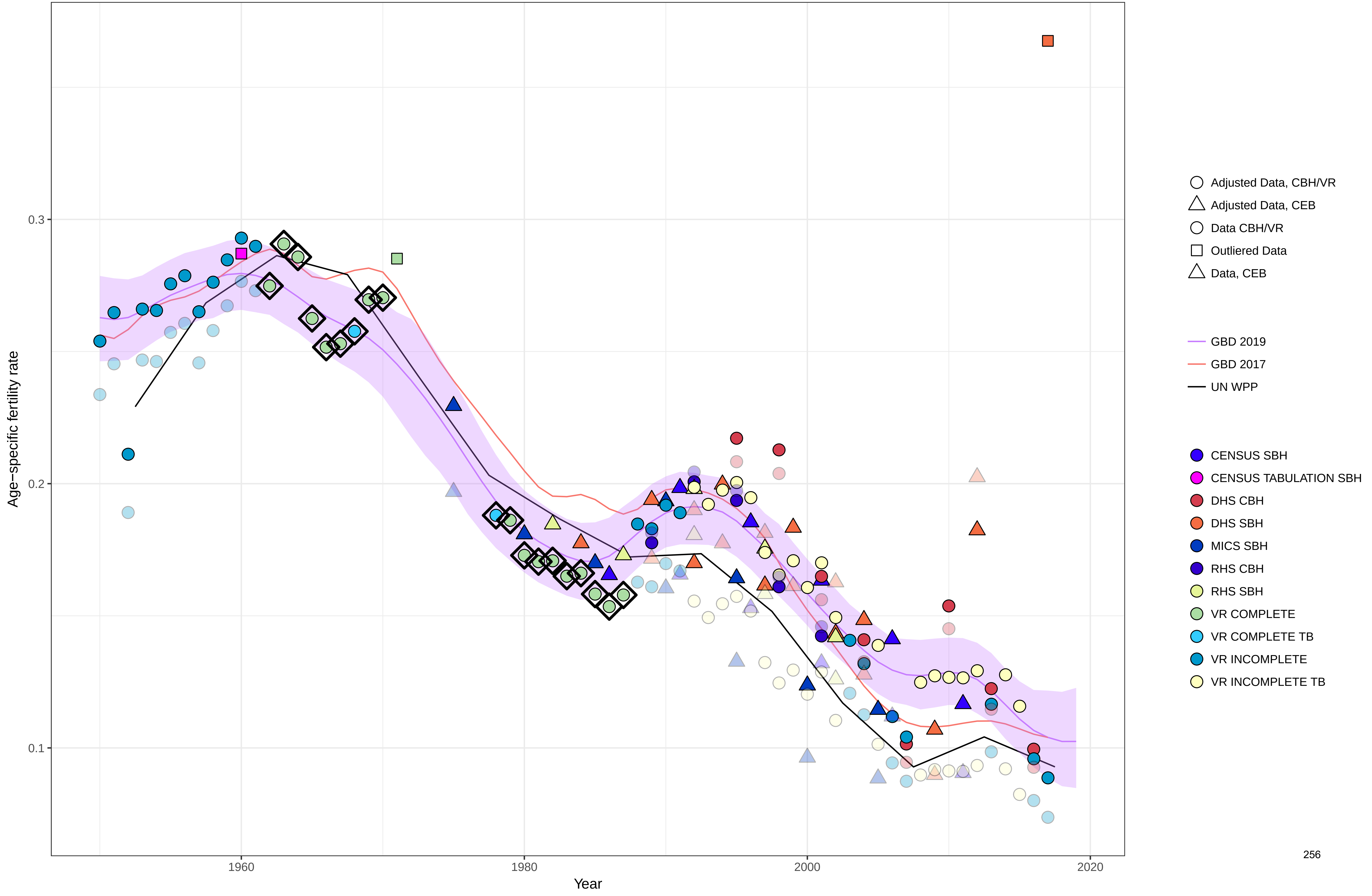

Age−specific fertility fitted trend lines, 1950−2019: Albania, Ages: 25−29
Data points enclosed with a diamond are reference sources

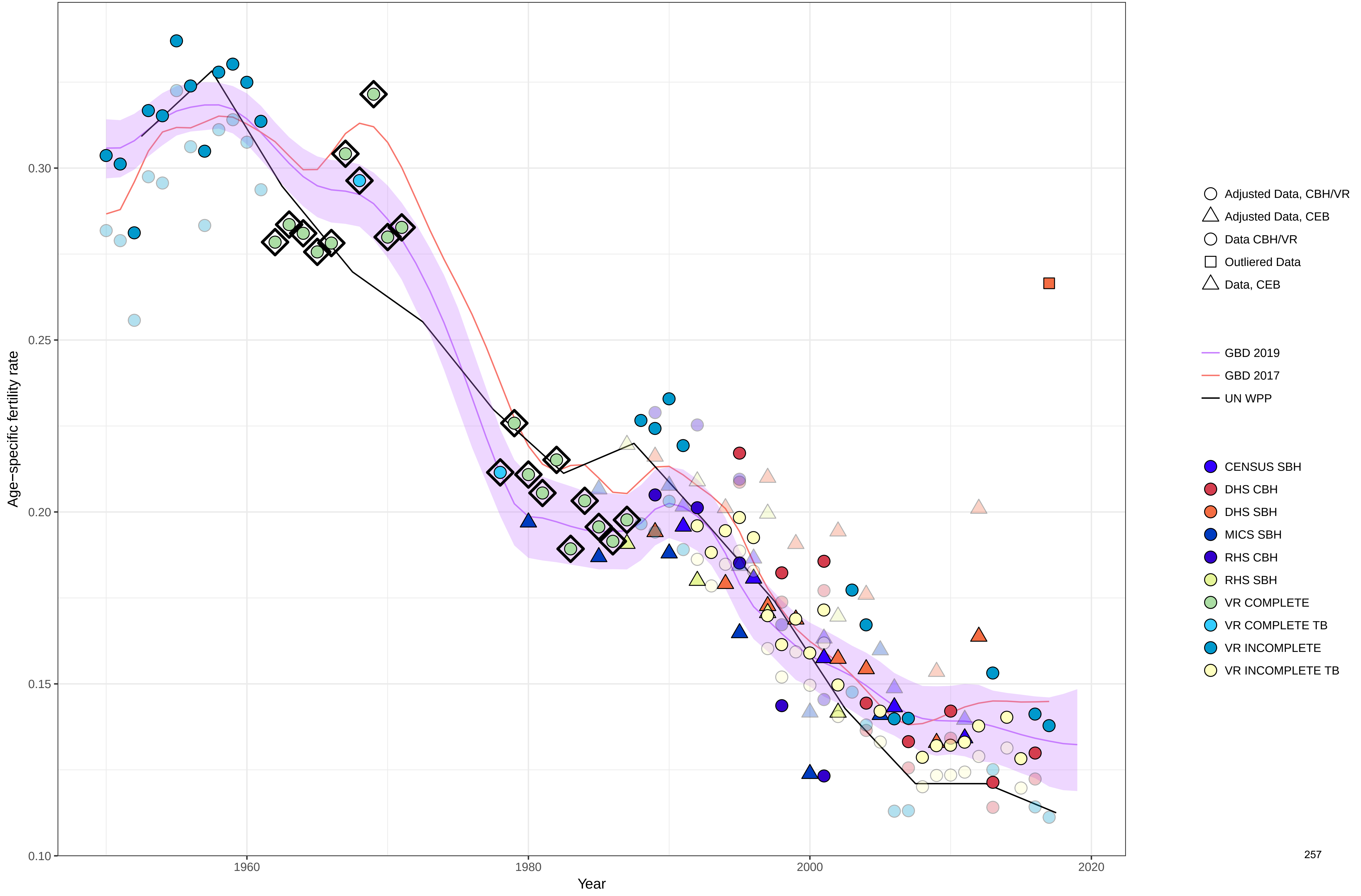

Age−specific fertility fitted trend lines, 1950−2019: Albania, Ages: 30−34
Data points enclosed with a diamond are reference sources

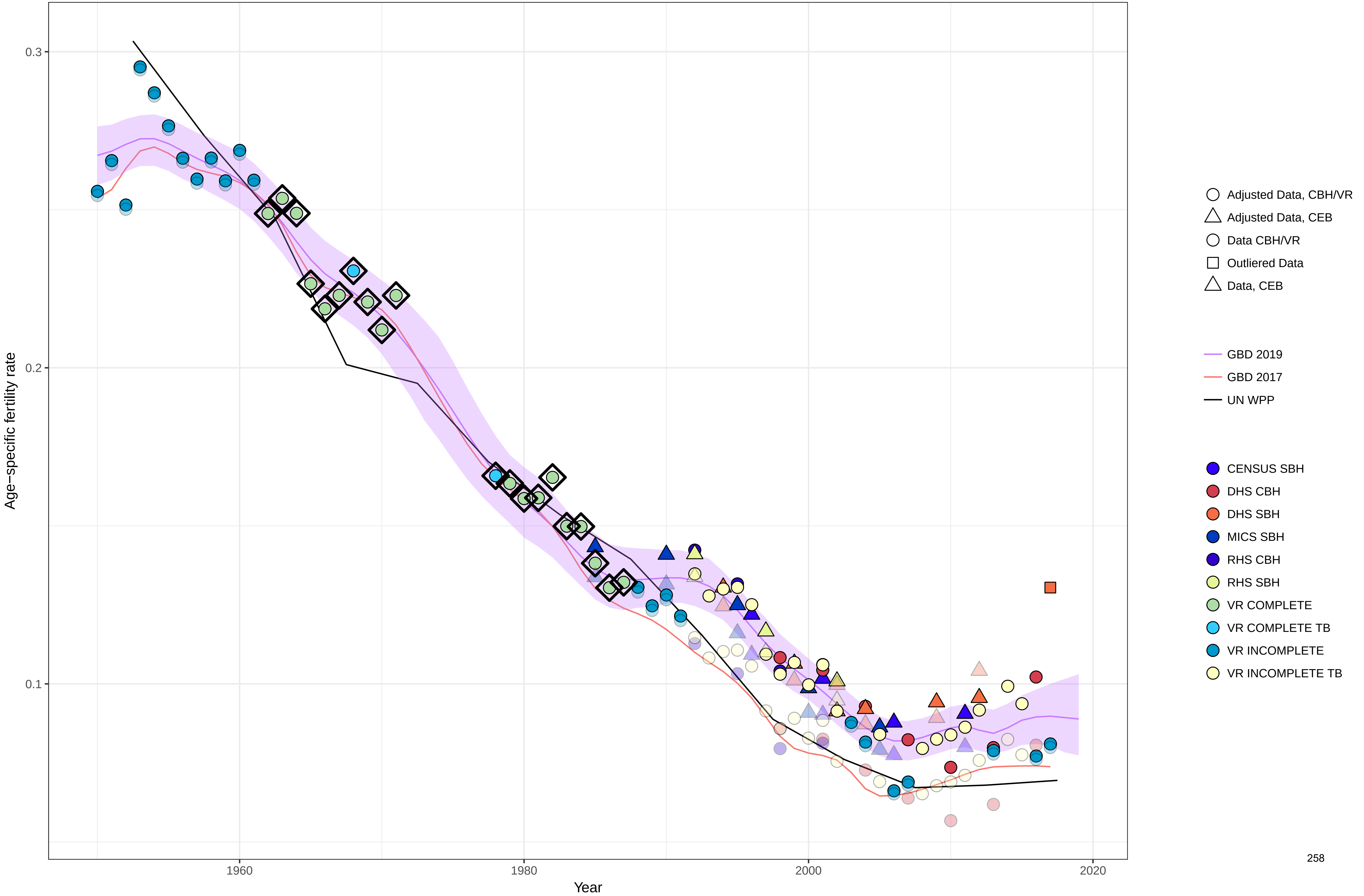

Age−specific fertility fitted trend lines, 1950−2019: Albania, Ages: 35−39
Data points enclosed with a diamond are reference sources

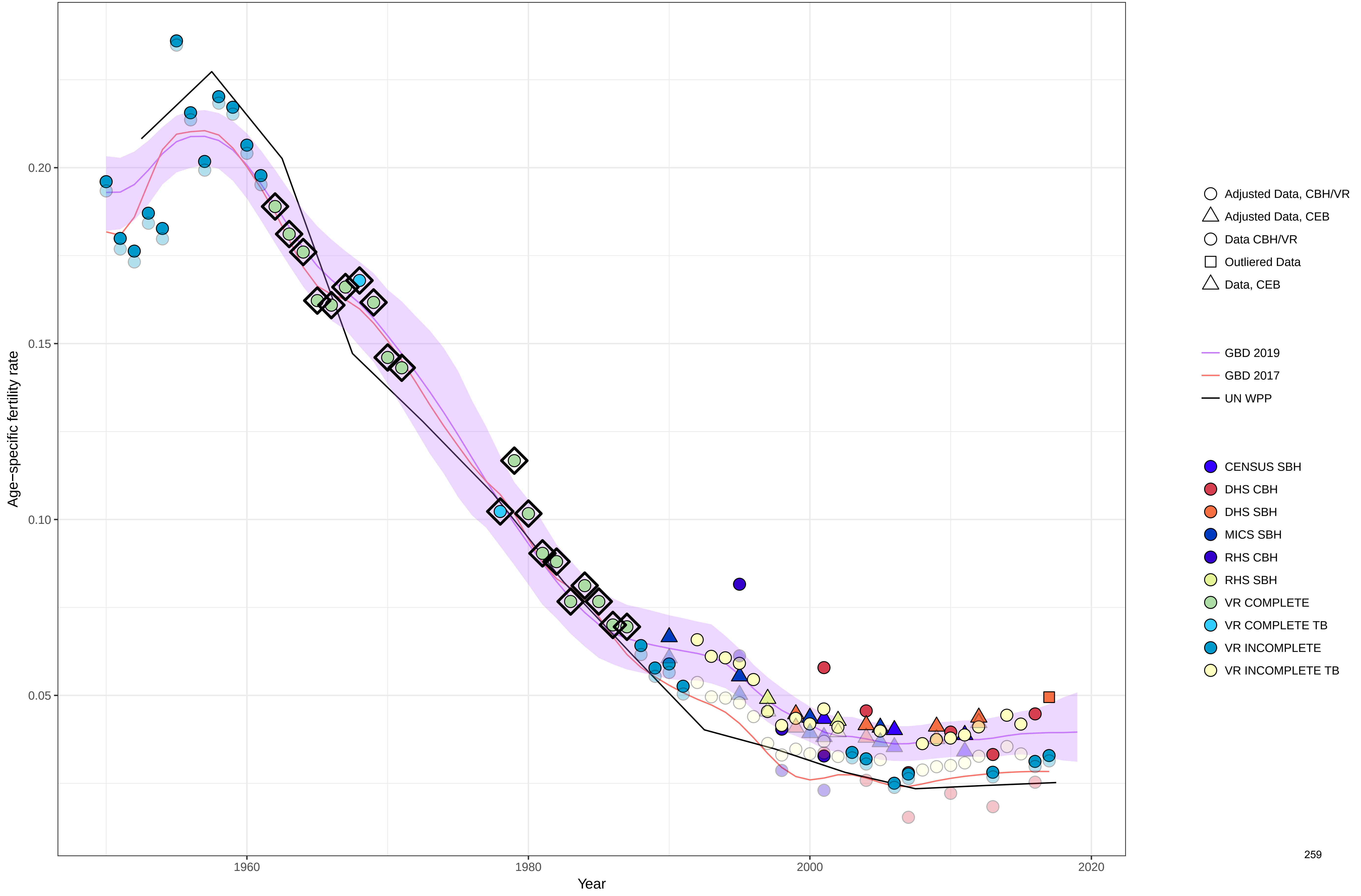

Age−specific fertility fitted trend lines, 1950−2019: Albania, Ages: 40−44
Data points enclosed with a diamond are reference sources

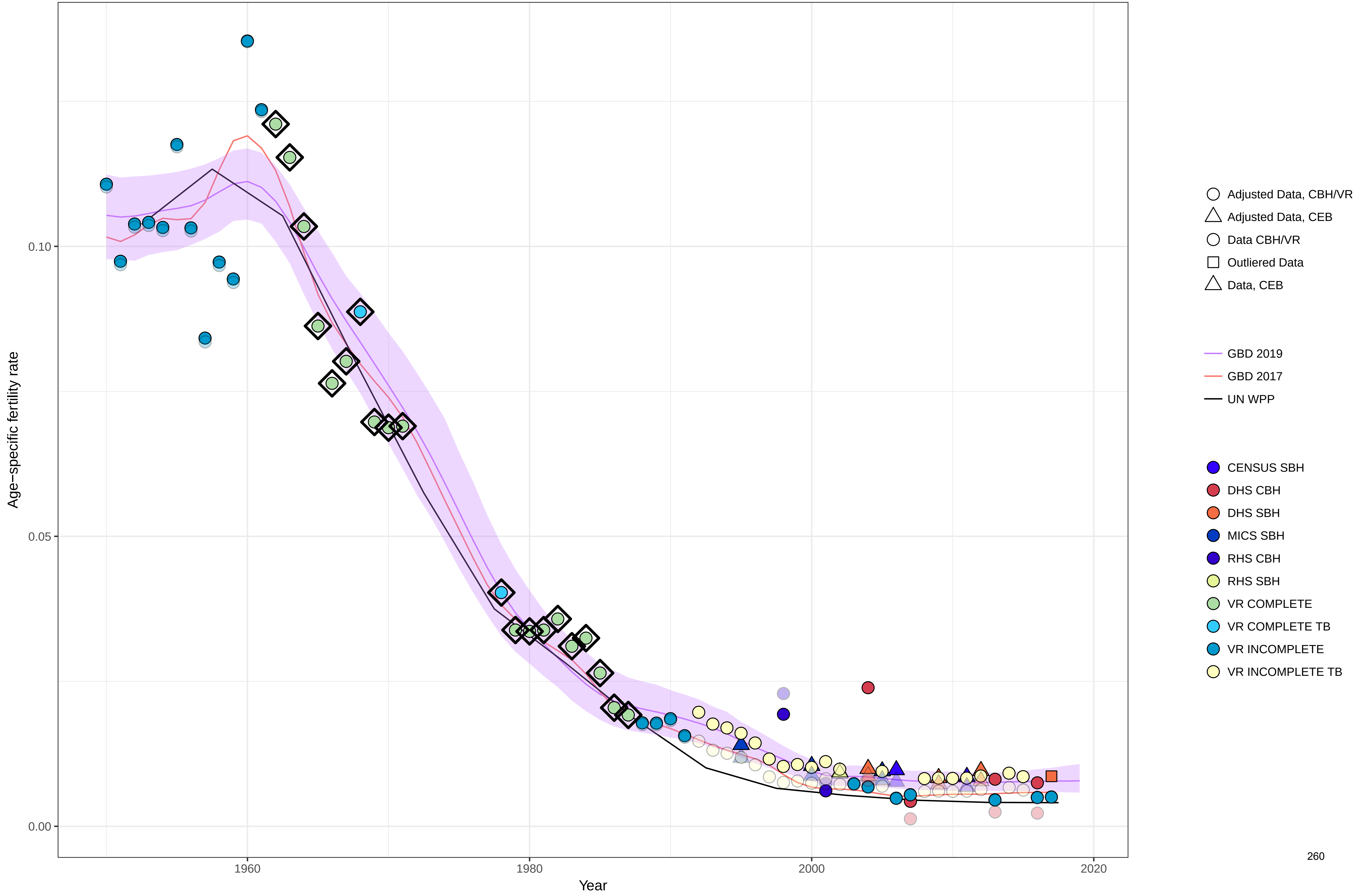

Age−specific fertility fitted trend lines, 1950−2019: Albania, Ages: 45−49
Data points enclosed with a diamond are reference sources

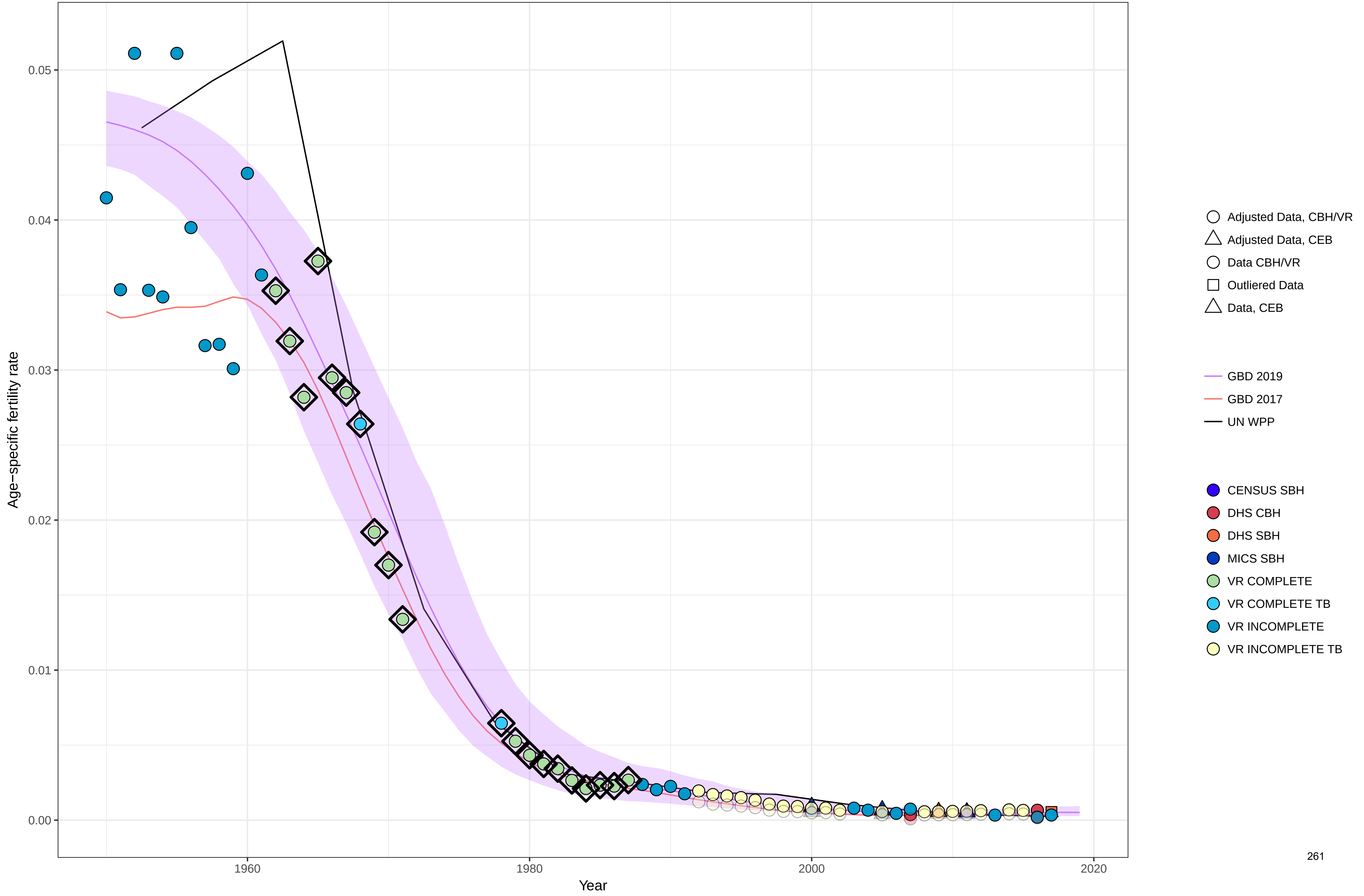

Age−specific fertility fitted trend lines, 1950−2019: Albania, Ages: 50−54
Data points enclosed with a diamond are reference sources

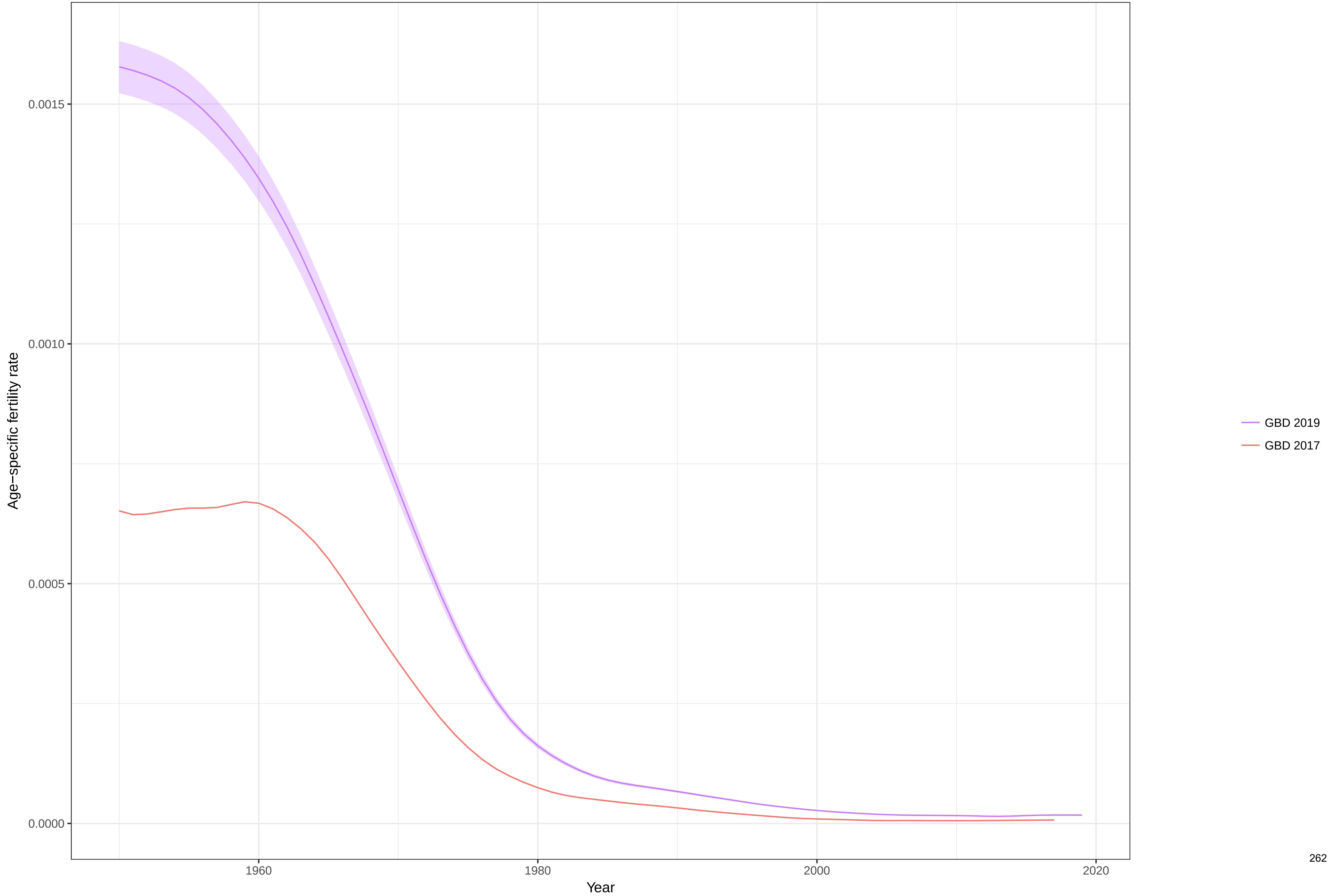

Total fertility rate fitted trend lines, 1950−2019: Albania TFR
Data points enclosed with a diamond are reference sources

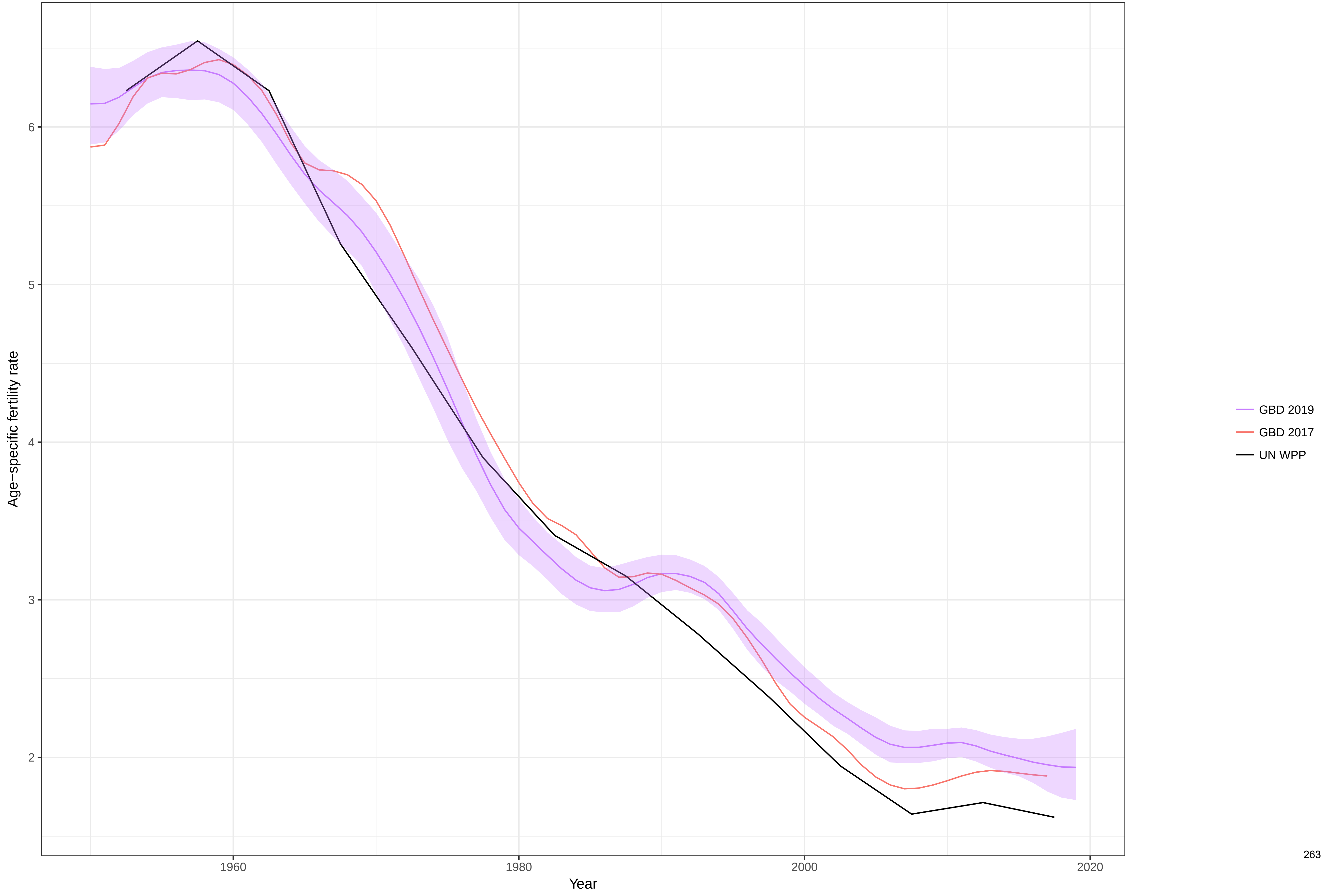

Age−specific fertility fitted trend lines, 1950−2019: Andorra, Ages: 10−14
Data points enclosed with a diamond are reference sources

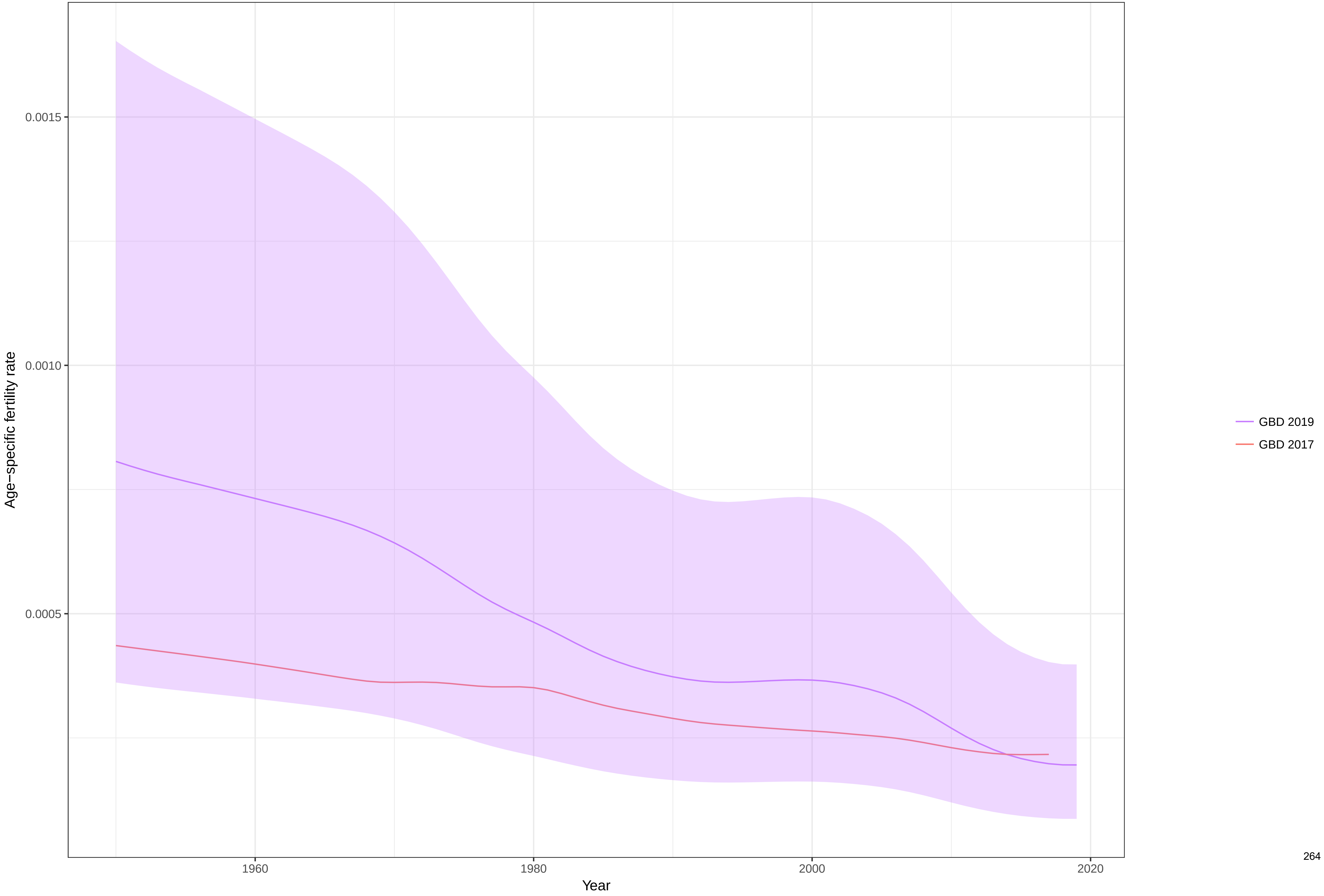

Age−specific fertility fitted trend lines, 1950−2019: Andorra, Ages: 15−19
Data points enclosed with a diamond are reference sources

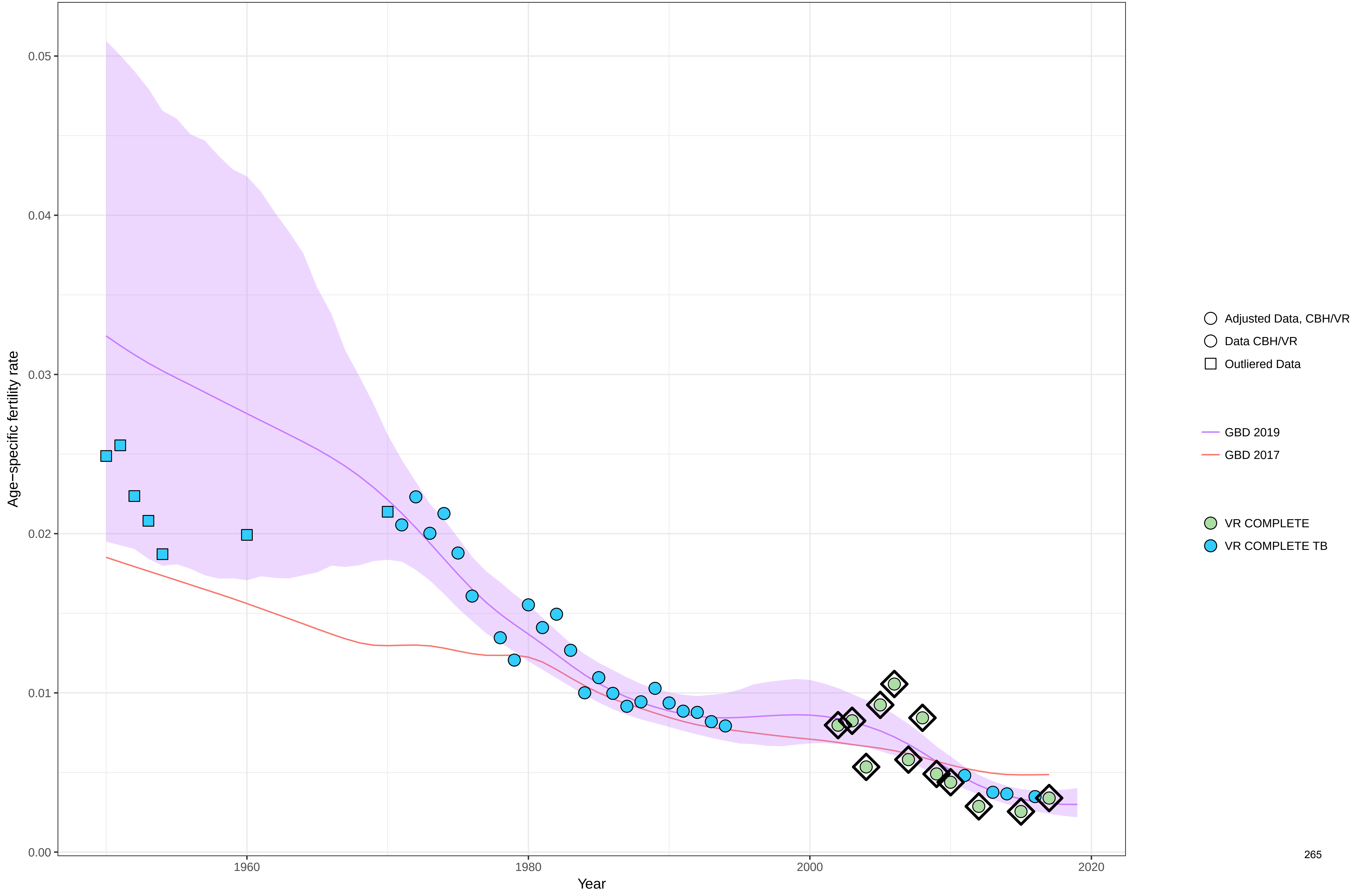

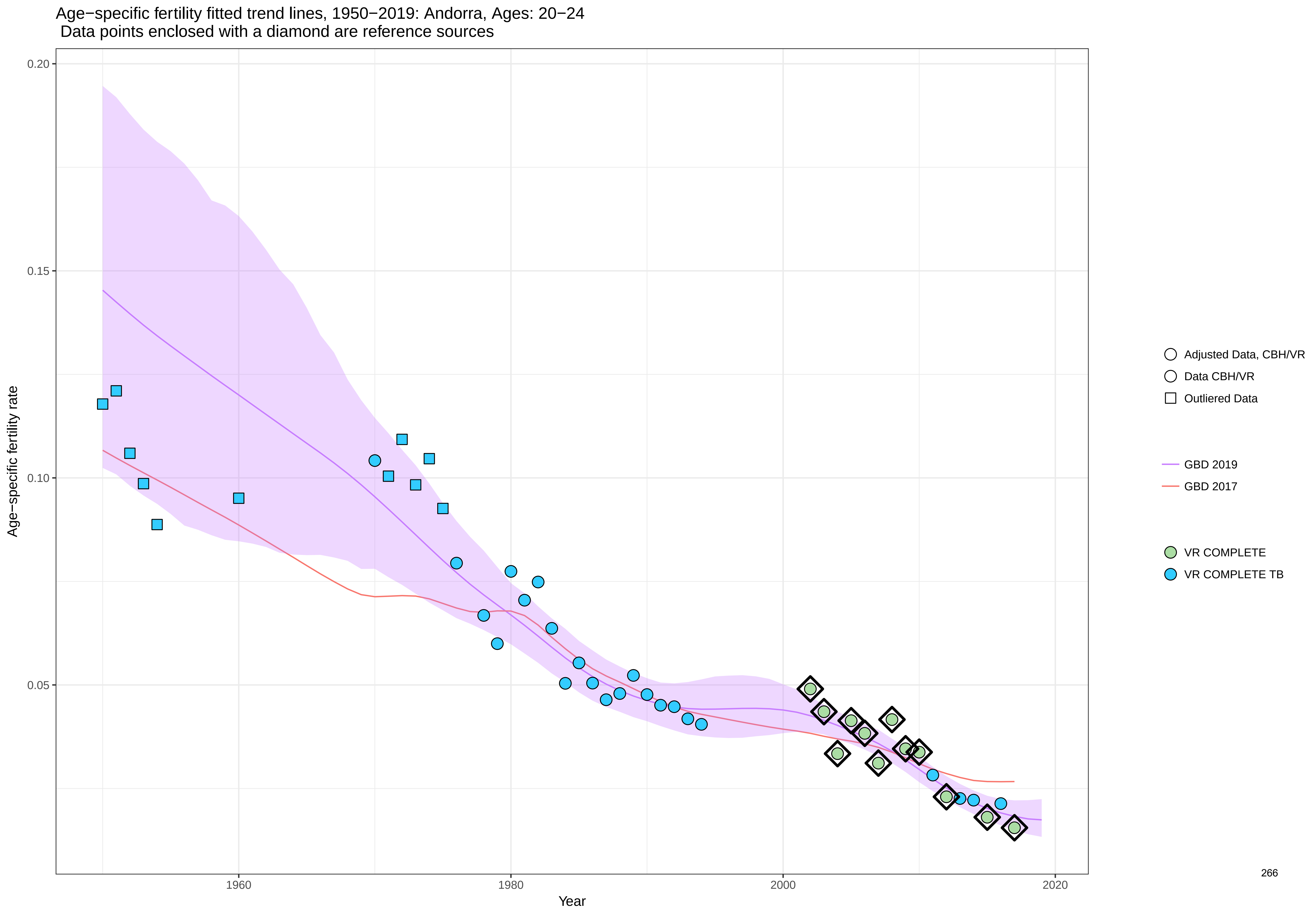
Age−specific fertility fitted trend lines, 1950−2019: Andorra, Ages: 20−24
Data points enclosed with a diamond are reference sources
Age−specific fertility rate
Year
0.20
0.15
0.10
0.05
1960
1980
2000
2020
Adjusted Data, CBH/VR
Data CBH/VR
Outliered Data
GBD 2019
GBD 2017
VR COMPLETE
VR COMPLETE TB

Age−specific fertility fitted trend lines, 1950−2019: Andorra, Ages: 25−29
Data points enclosed with a diamond are reference sources

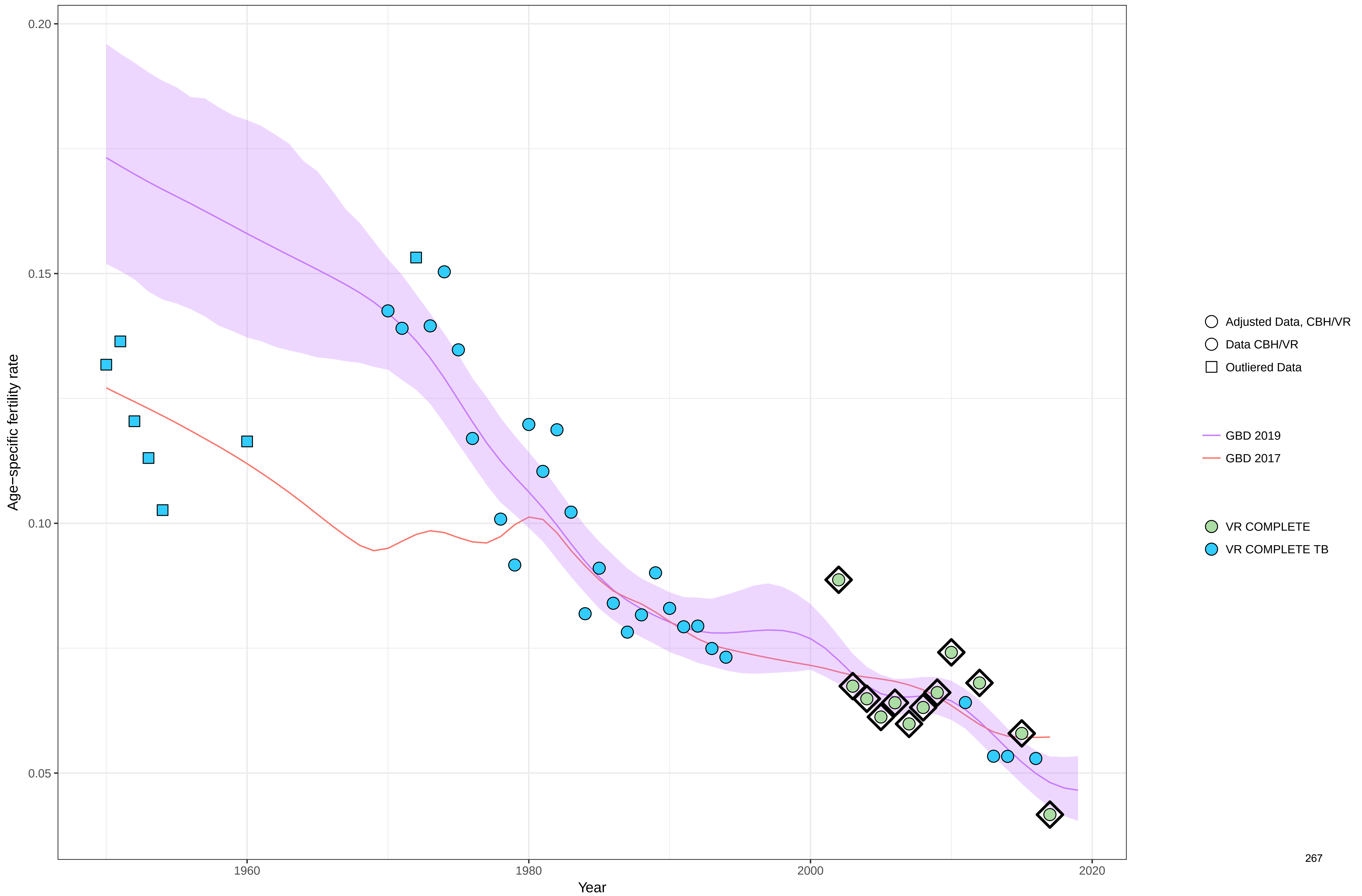

Age−specific fertility fitted trend lines, 1950−2019: Andorra, Ages: 30−34
Data points enclosed with a diamond are reference sources

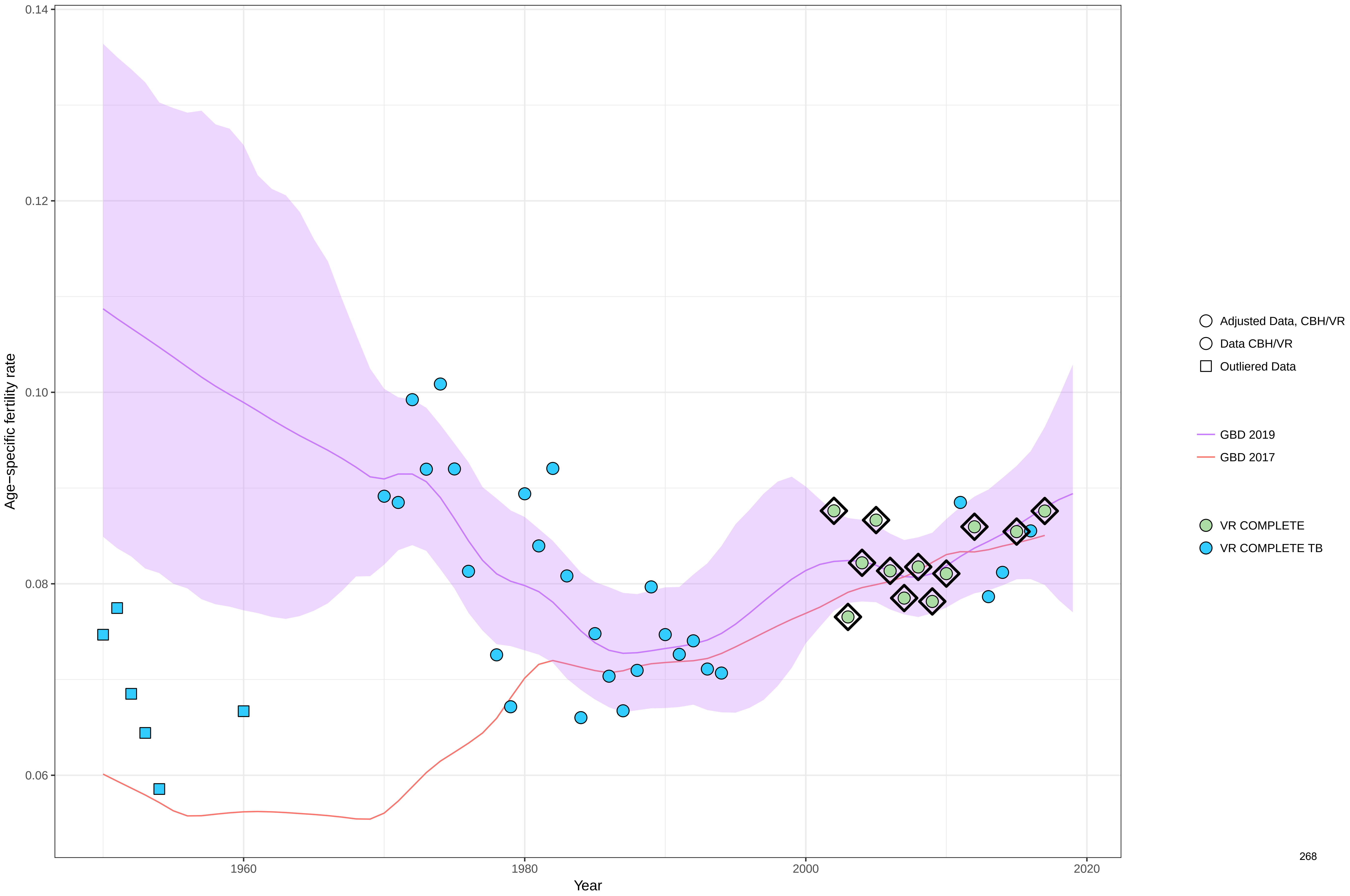

Age−specific fertility fitted trend lines, 1950−2019: Andorra, Ages: 35−39
Data points enclosed with a diamond are reference sources

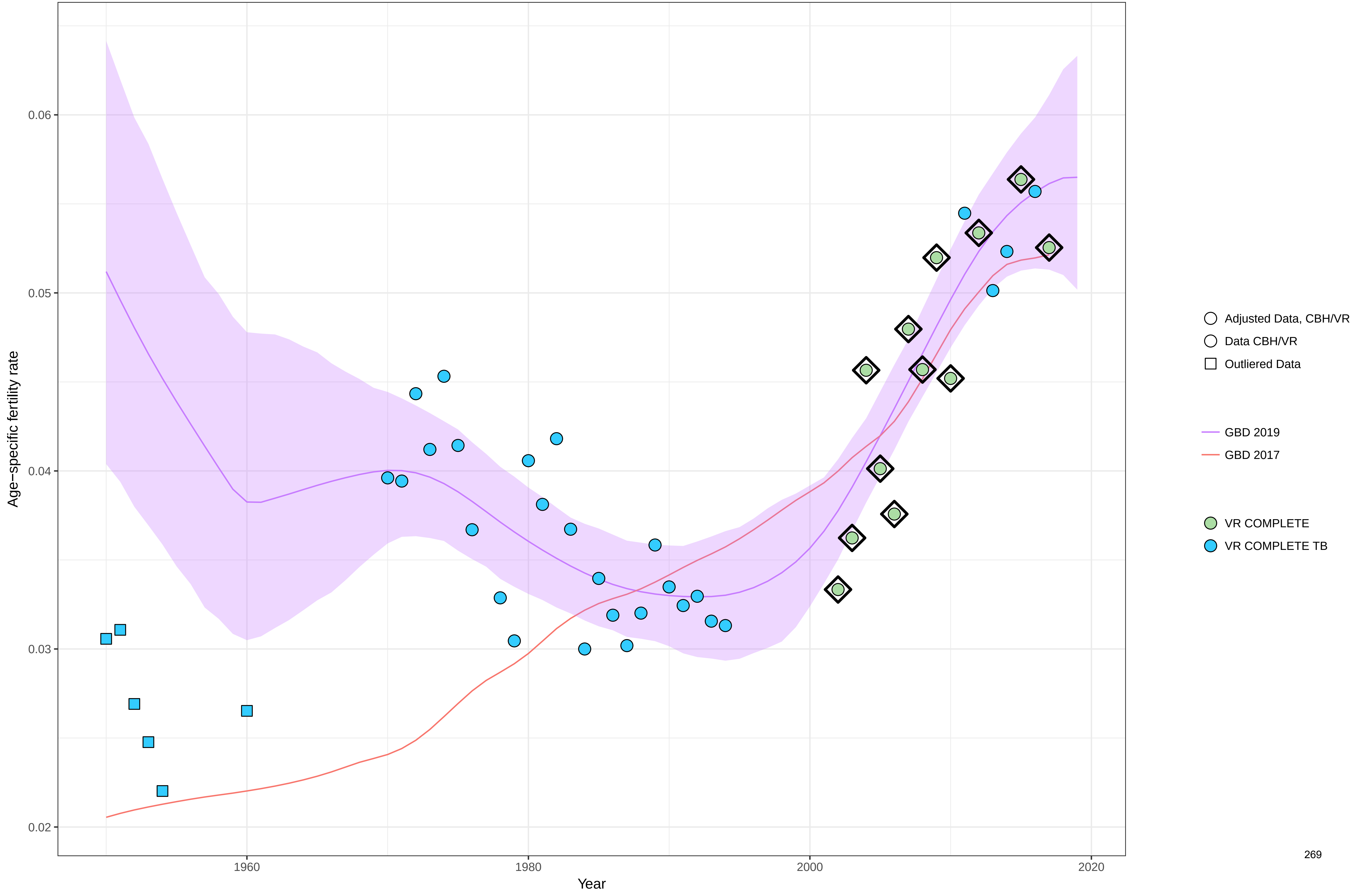

Age−specific fertility fitted trend lines, 1950−2019: Andorra, Ages: 40−44
Data points enclosed with a diamond are reference sources

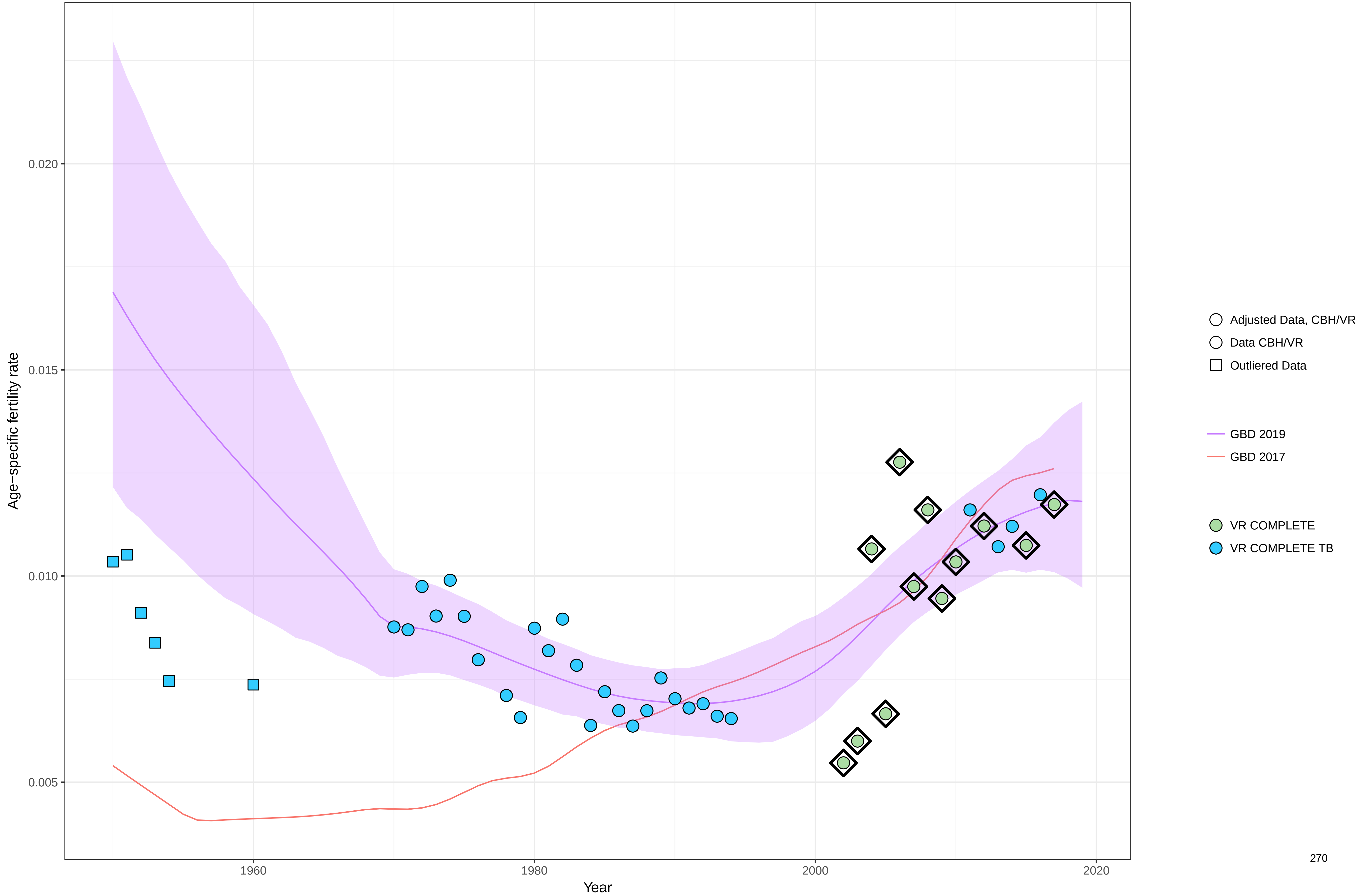

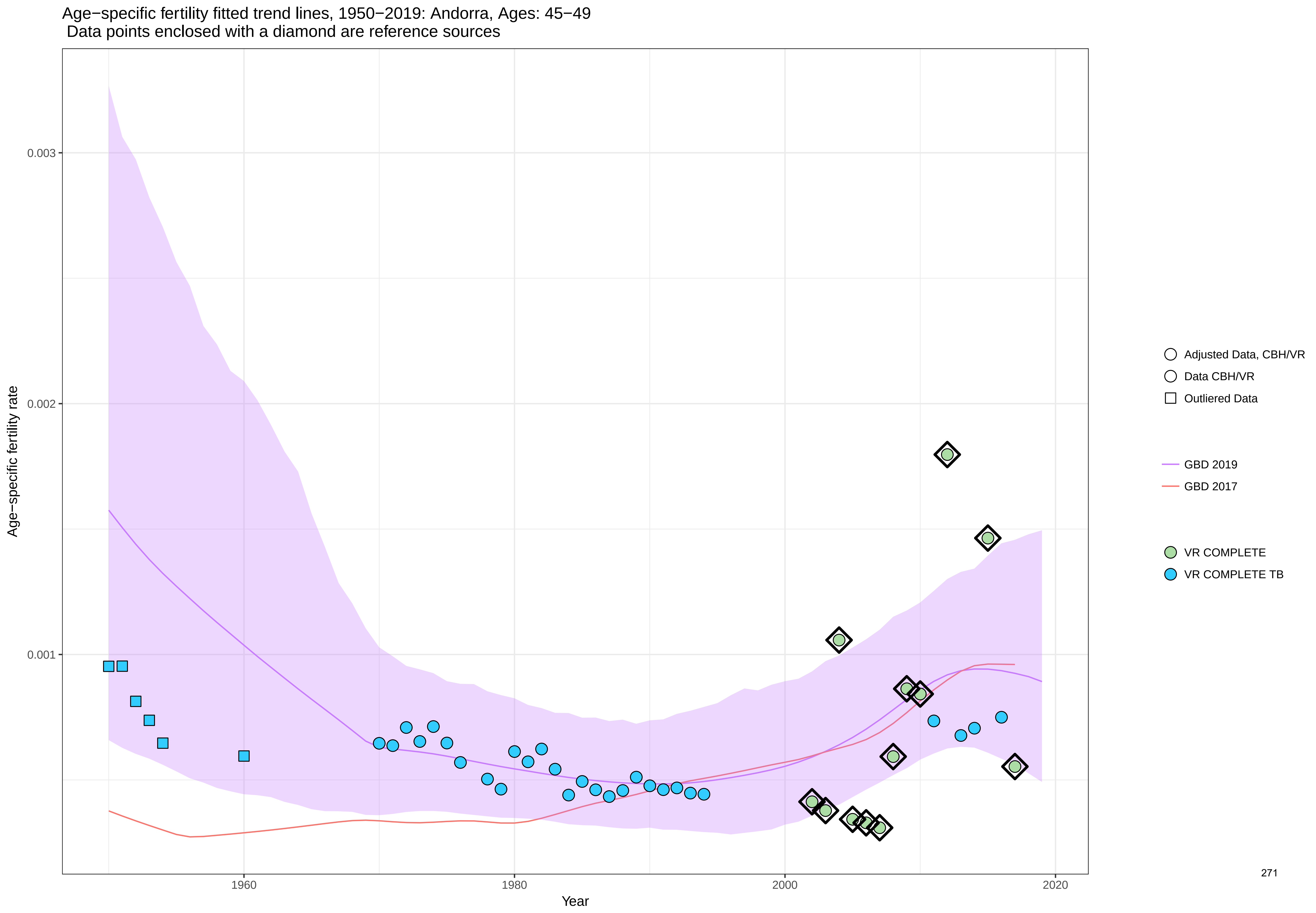

Age−specific fertility fitted trend lines, 1950−2019: Andorra, Ages: 45−49
Data points enclosed with a diamond are reference sources
0.003
0.002
0.001
Age−specific fertility rate
1960
1980
2000
2020
Year
Adjusted Data, CBH/VR
Data CBH/VR
Outliered Data
GBD 2019
GBD 2017
VR COMPLETE
VR COMPLETE TB

Age−specific fertility fitted trend lines, 1950−2019: Andorra, Ages: 50−54
Data points enclosed with a diamond are reference sources

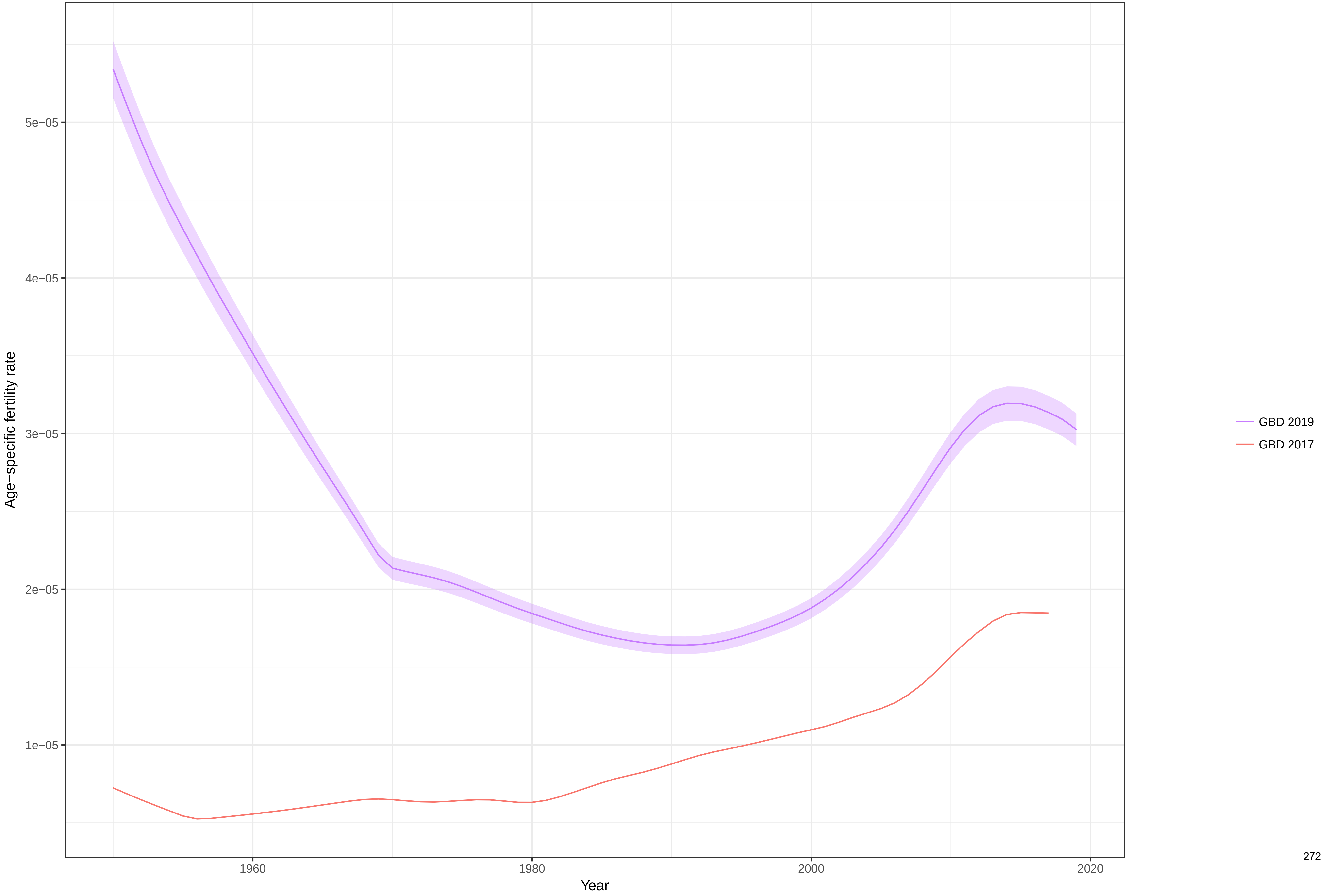

Total fertility rate fitted trend lines, 1950−2019: Andorra TFR
Data points enclosed with a diamond are reference sources

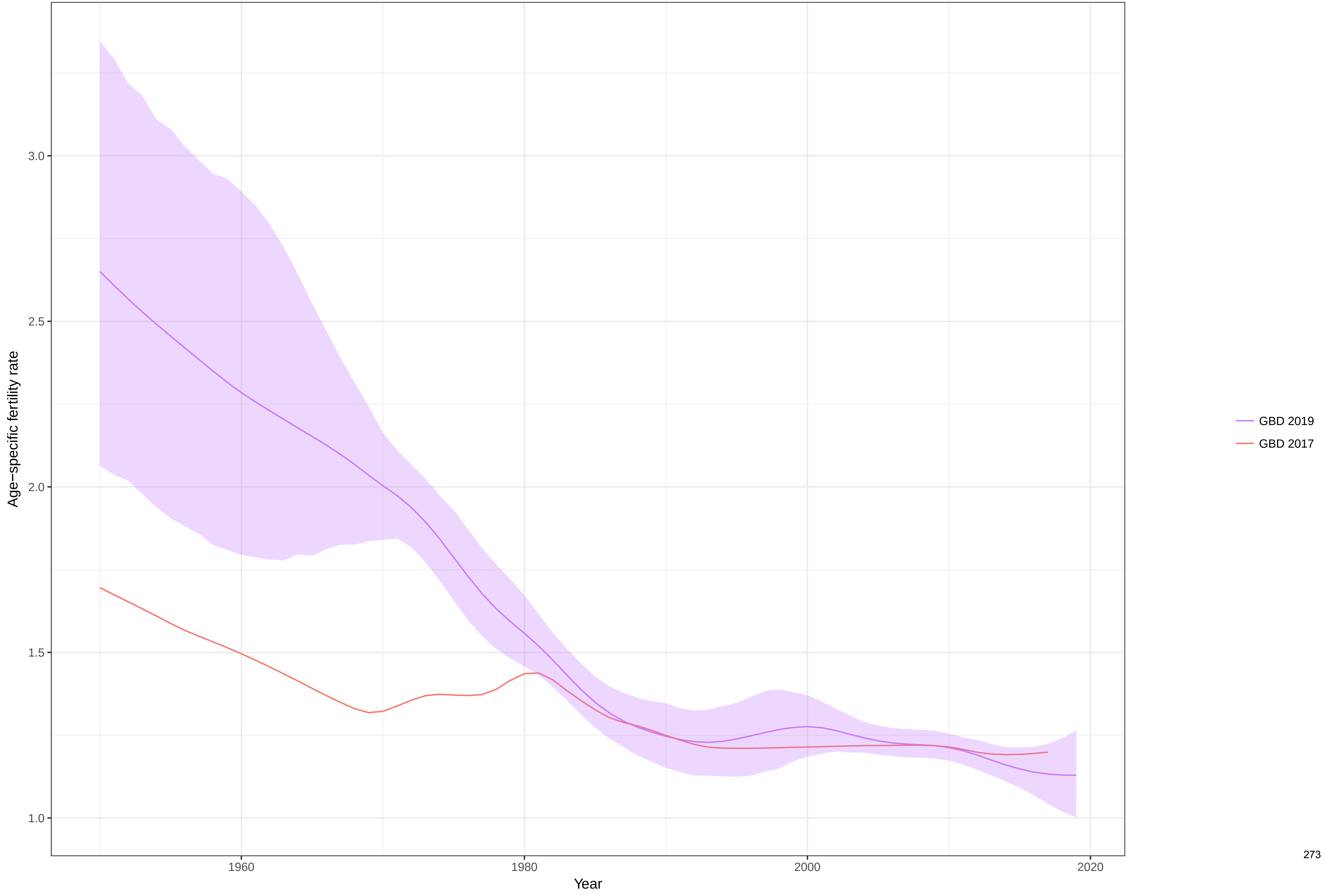

Age−specific fertility fitted trend lines, 1950−2019: United Arab Emirates, Ages: 10−14
Data points enclosed with a diamond are reference sources

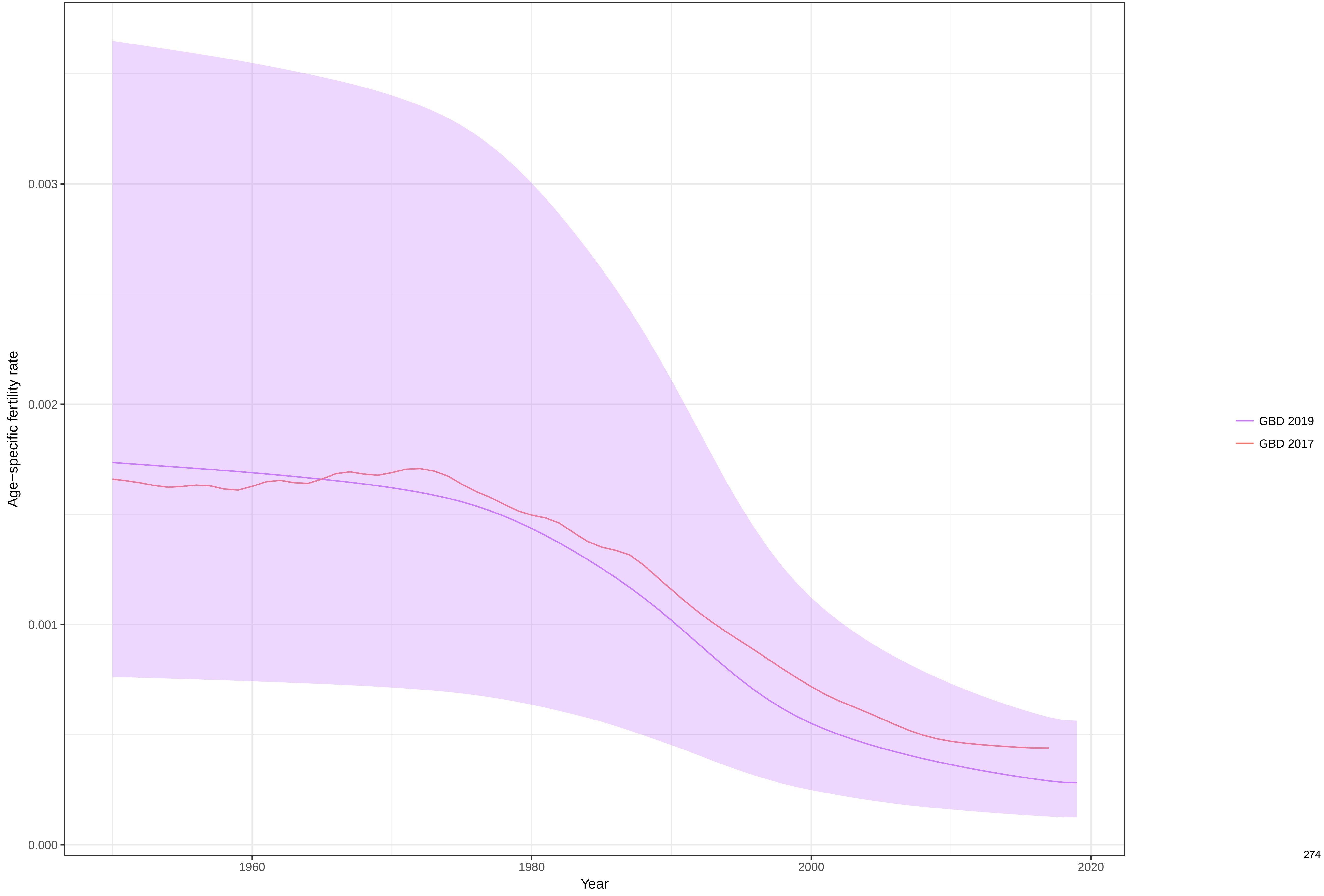

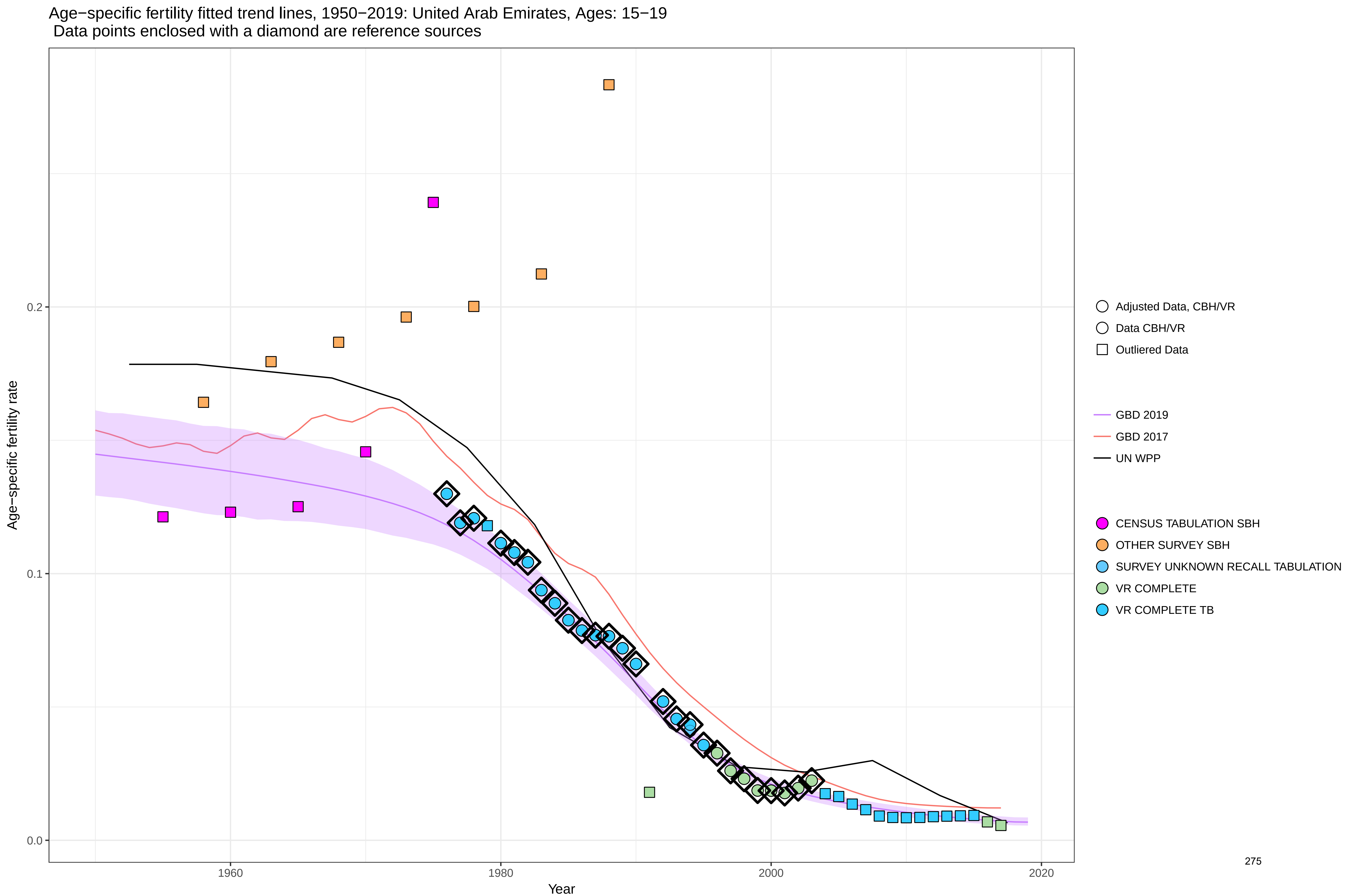

Age−specific fertility fitted trend lines, 1950−2019: United Arab Emirates, Ages: 15−19
Data points enclosed with a diamond are reference sources
Age−specific fertility rate
0.0
0.1
0.2
1960
1980
2000
2020
Year
Adjusted Data, CBH/VR
Data CBH/VR
Outliered Data
GBD 2019
GBD 2017
UN WPP
CENSUS TABULATION SBH
OTHER SURVEY SBH
SURVEY UNKNOWN RECALL TABULATION
VR COMPLETE
VR COMPLETE TB

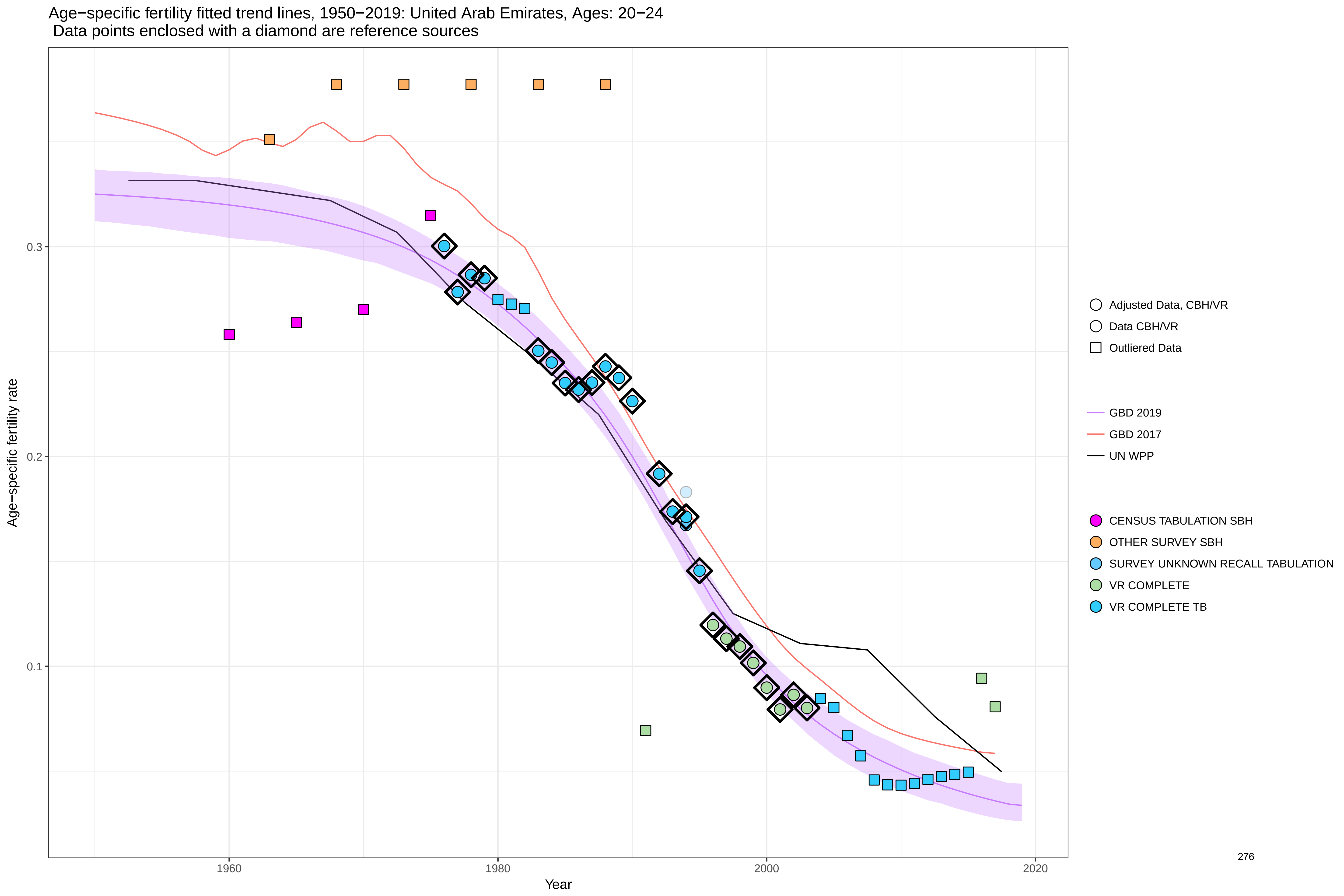
Age−specific fertility fitted trend lines, 1950−2019: United Arab Emirates, Ages: 20−24
Data points enclosed with a diamond are reference sources
Age−specific fertility rate
Year
0.1
0.2
0.3
1960
1980
2000
2020
Adjusted Data, CBH/VR
Data CBH/VR
Outliered Data
GBD 2019
GBD 2017
UN WPP
CENSUS TABULATION SBH
OTHER SURVEY SBH
SURVEY UNKNOWN RECALL TABULATION
VR COMPLETE
VR COMPLETE TB

Age−specific fertility fitted trend lines, 1950−2019: United Arab Emirates, Ages: 25−29
Data points enclosed with a diamond are reference sources

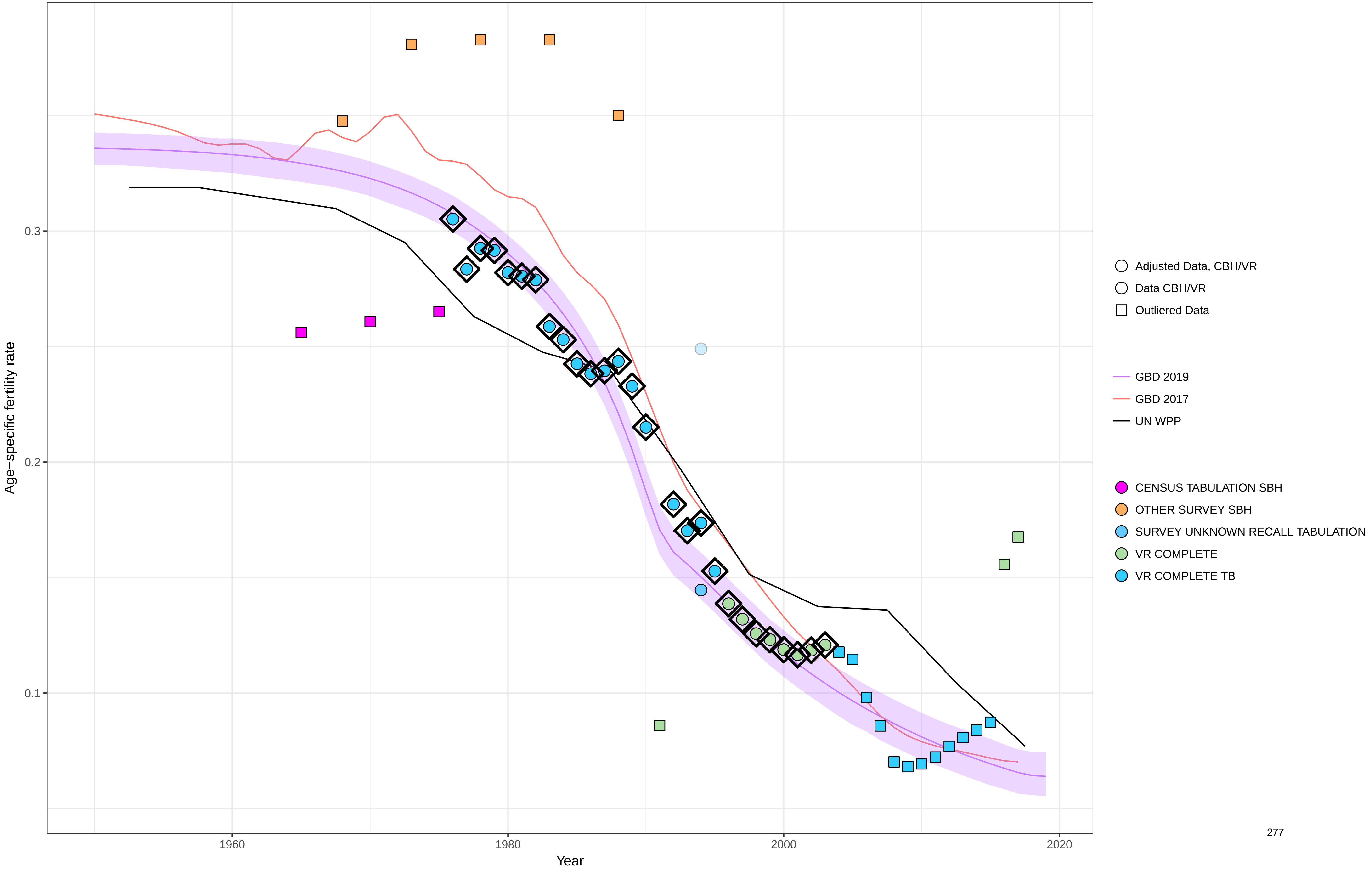

Age−specific fertility fitted trend lines, 1950−2019: United Arab Emirates, Ages: 30−34
Data points enclosed with a diamond are reference sources

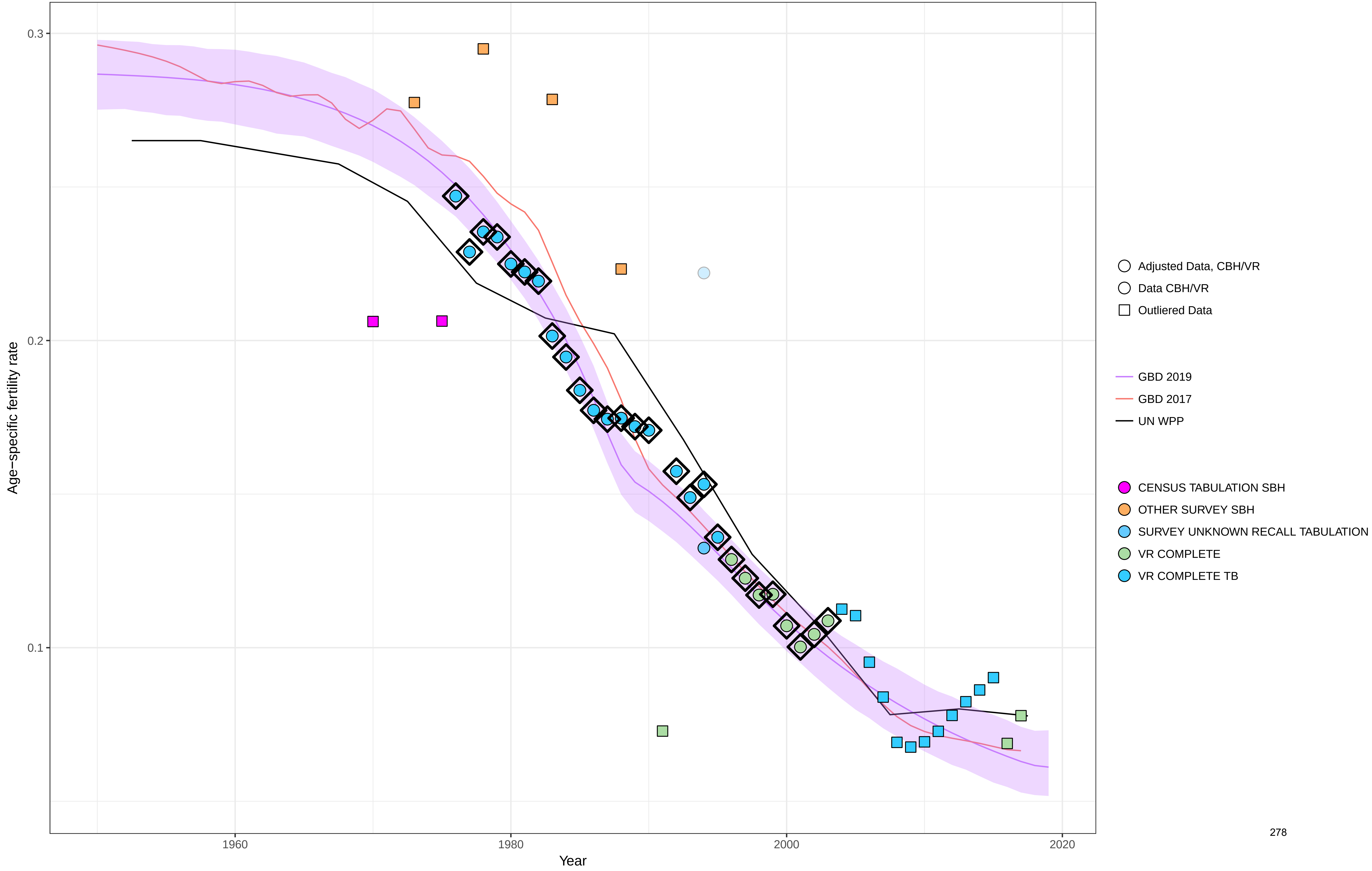

Age−specific fertility fitted trend lines, 1950−2019: United Arab Emirates, Ages: 35−39
Data points enclosed with a diamond are reference sources

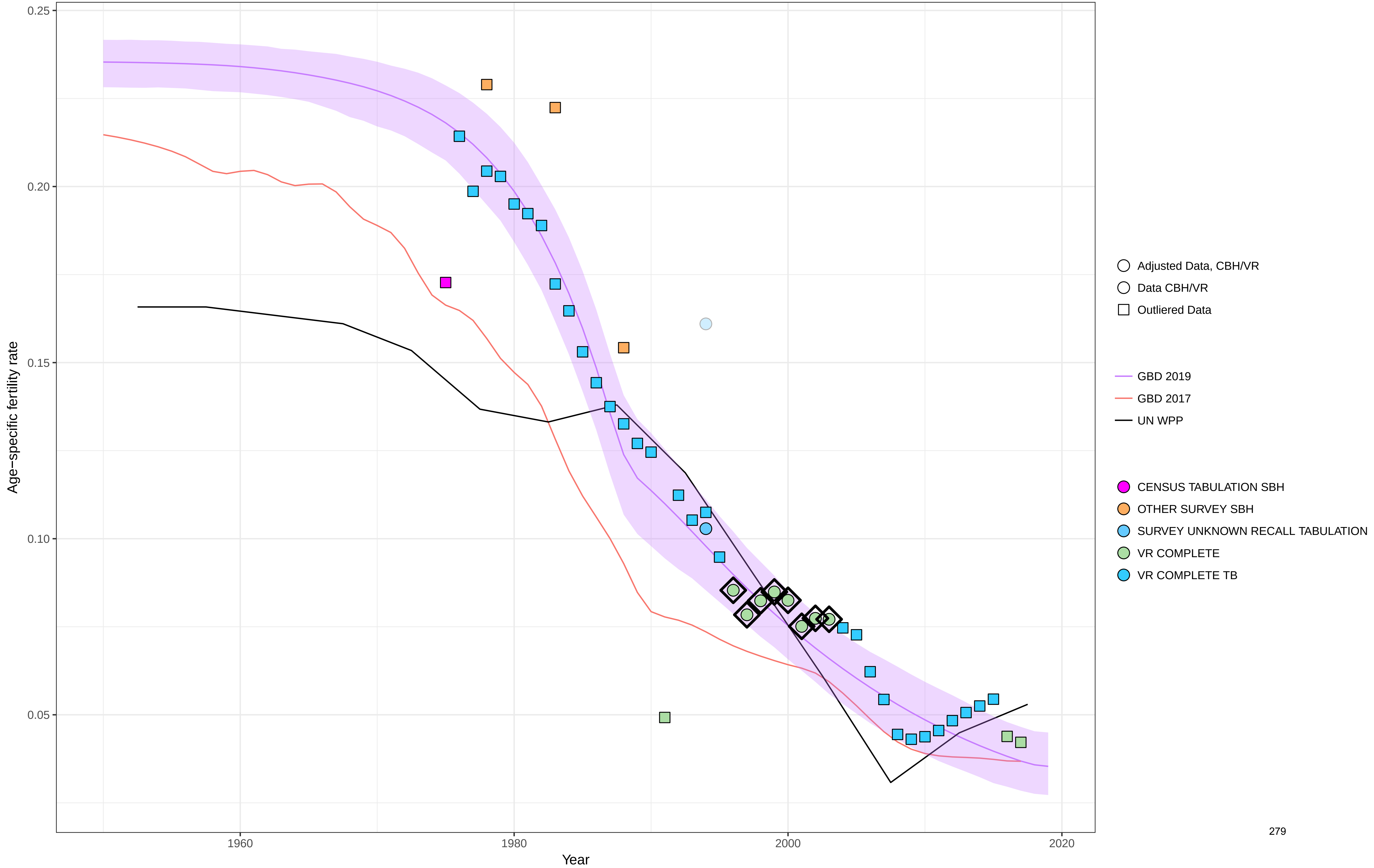

Age−specific fertility fitted trend lines, 1950−2019: United Arab Emirates, Ages: 40−44
Data points enclosed with a diamond are reference sources

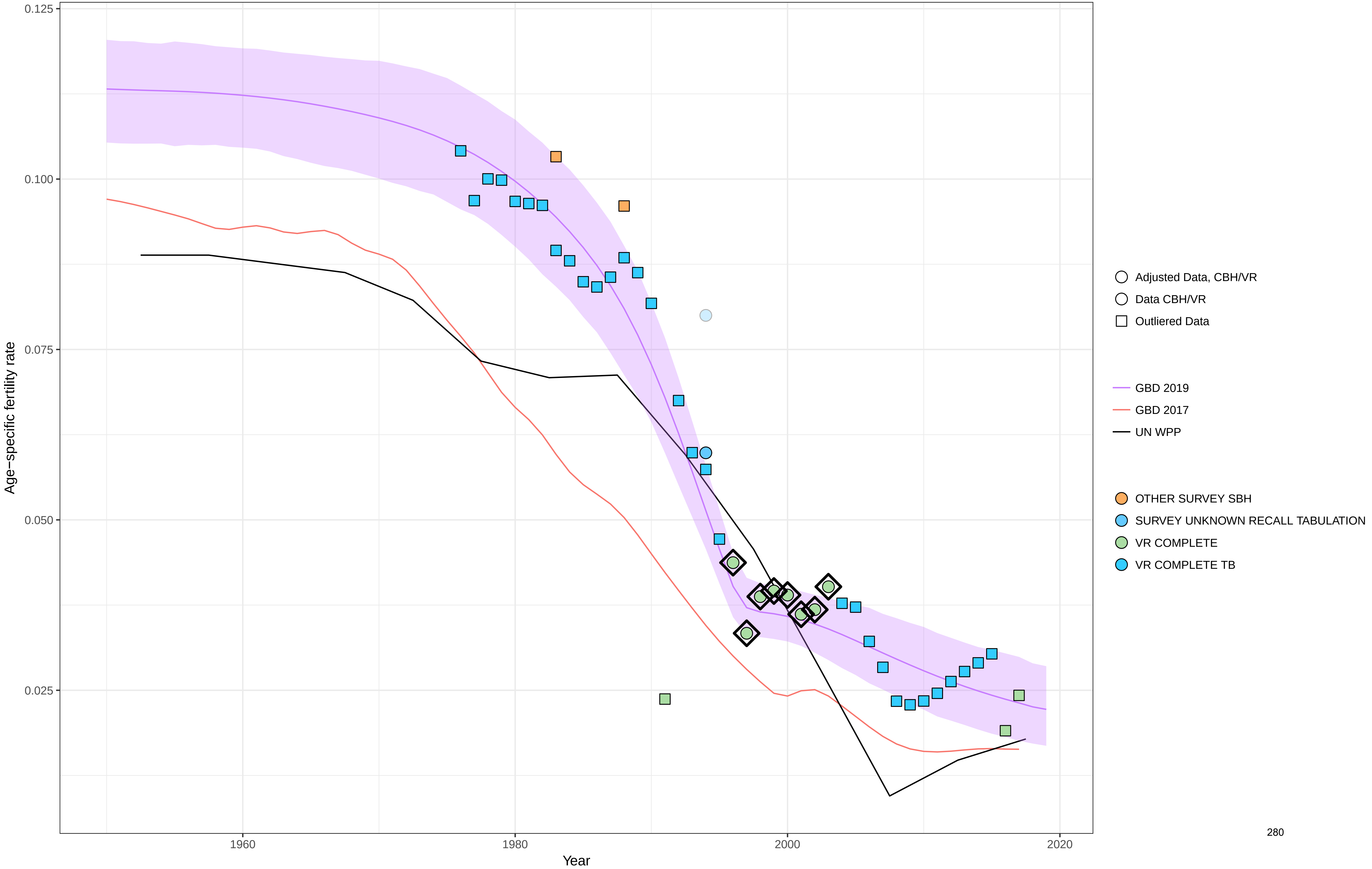

Age−specific fertility fitted trend lines, 1950−2019: United Arab Emirates, Ages: 45−49
Data points enclosed with a diamond are reference sources

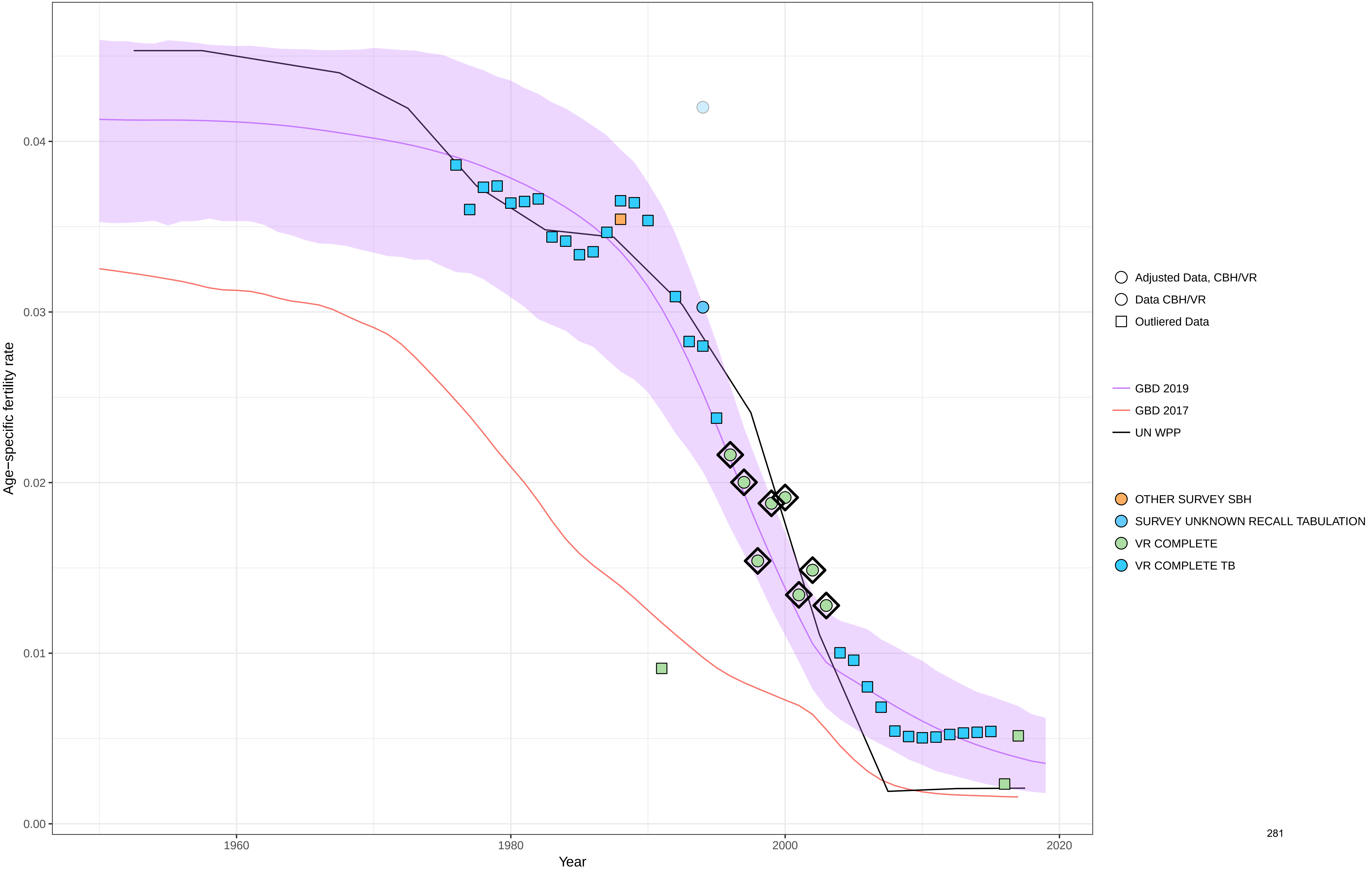

Age−specific fertility fitted trend lines, 1950−2019: United Arab Emirates, Ages: 50−54
Data points enclosed with a diamond are reference sources

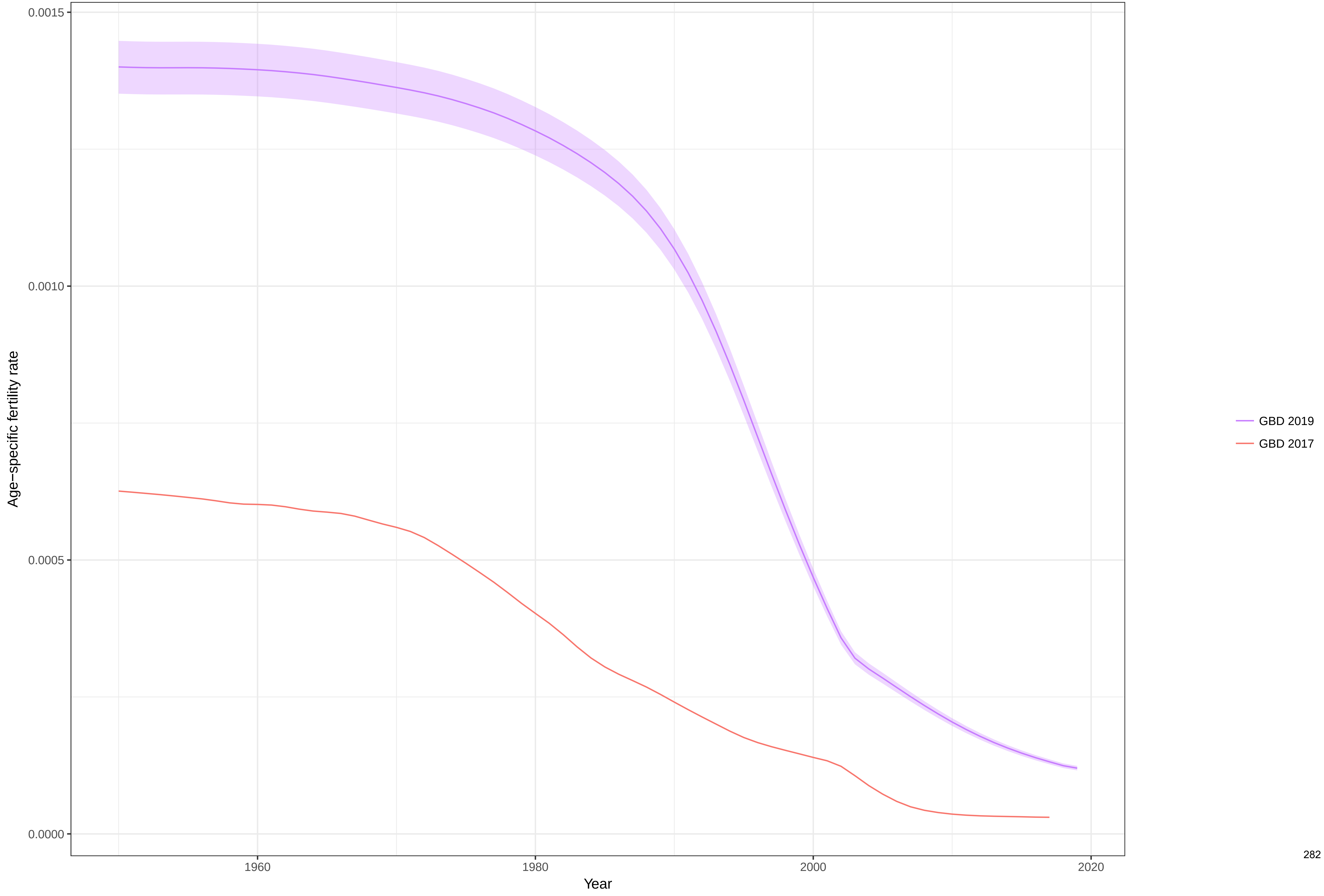

Total fertility rate fitted trend lines, 1950−2019: United Arab Emirates TFR
Data points enclosed with a diamond are reference sources

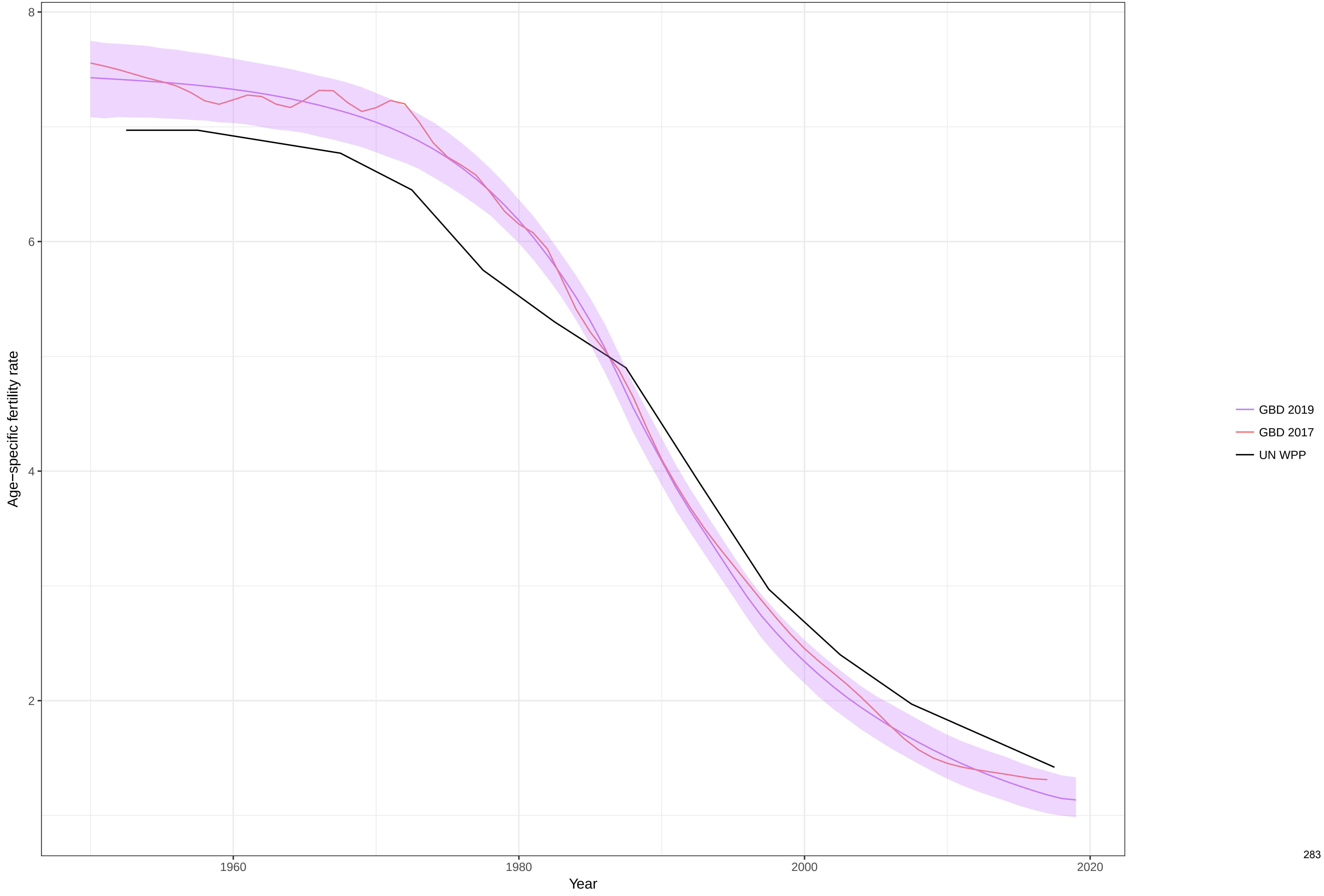

Age−specific fertility fitted trend lines, 1950−2019: Argentina, Ages: 10−14
Data points enclosed with a diamond are reference sources

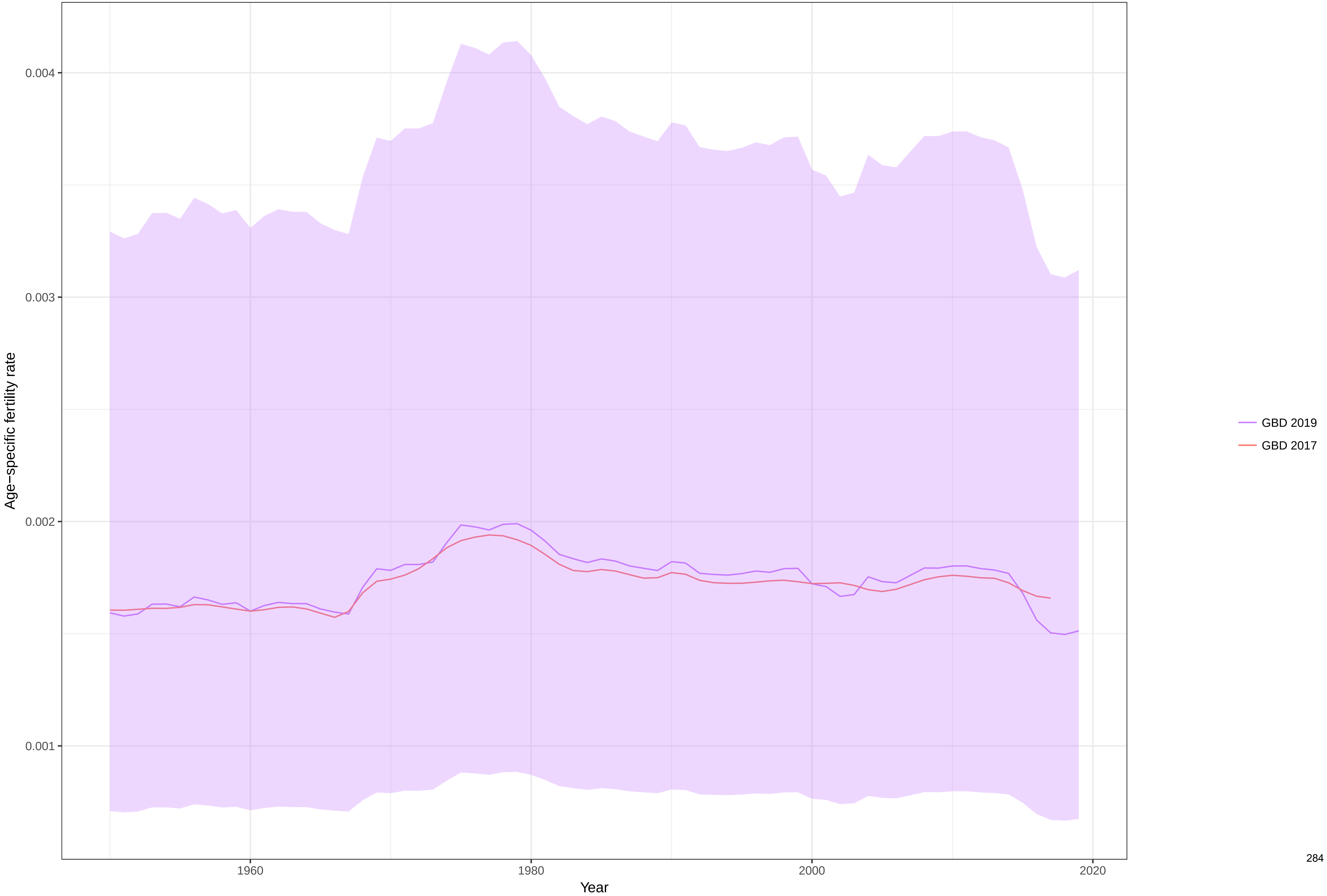

Age−specific fertility fitted trend lines, 1950−2019: Argentina, Ages: 15−19
Data points enclosed with a diamond are reference sources

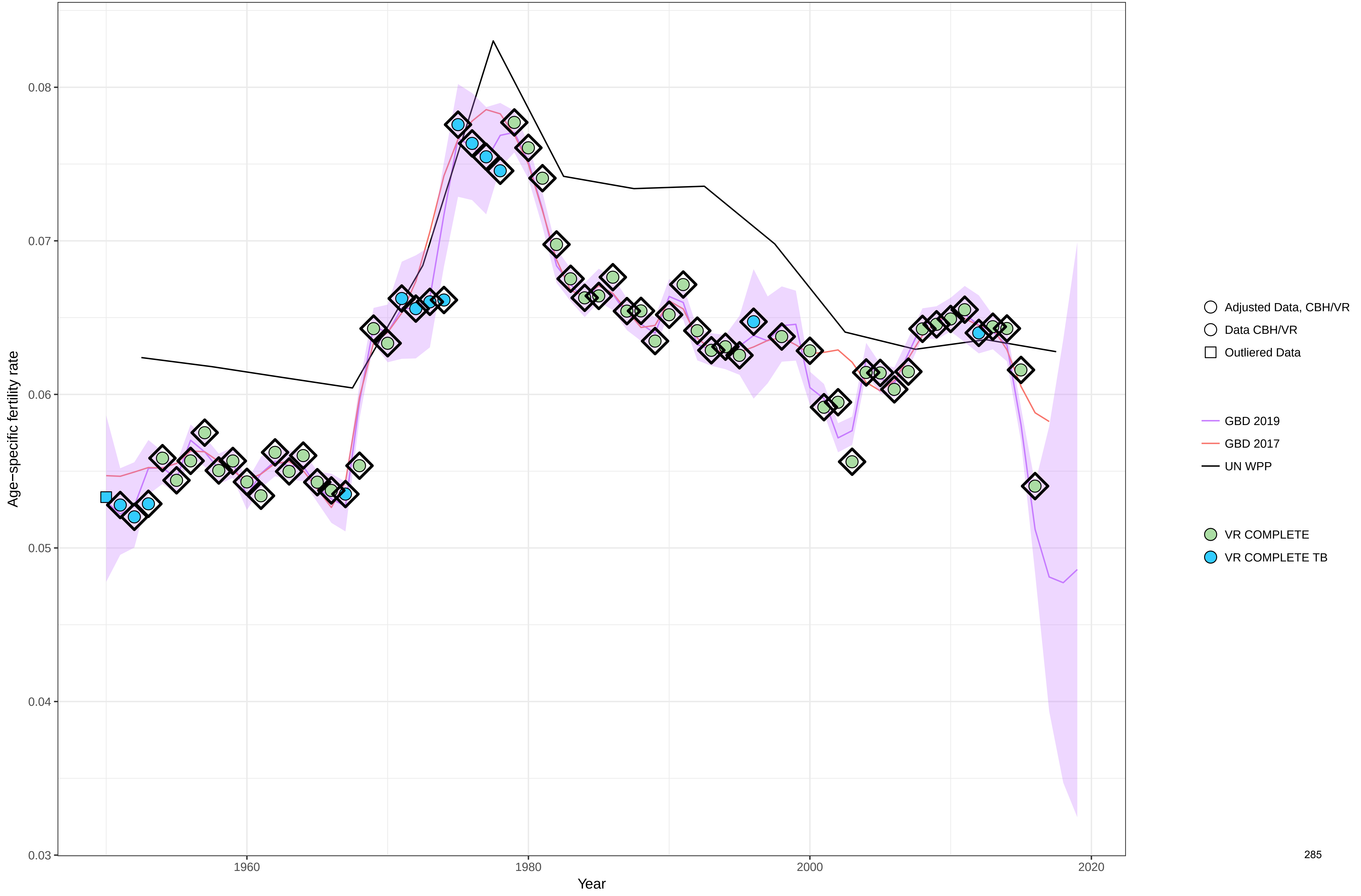

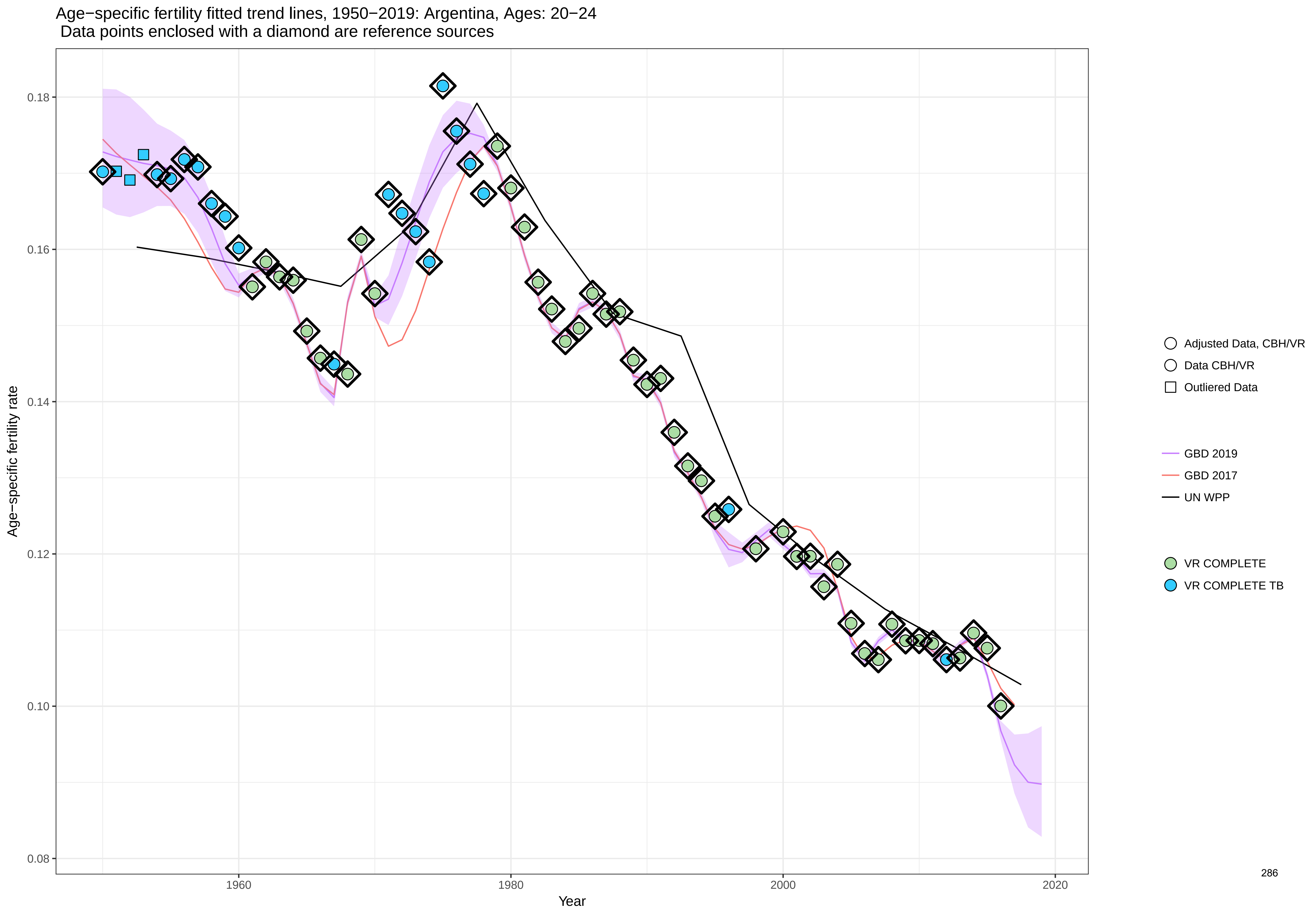
Age−specific fertility fitted trend lines, 1950−2019: Argentina, Ages: 20−24
Data points enclosed with a diamond are reference sources
Age−specific fertility rate
Year
0.08
0.10
0.12
0.14
0.16
0.18
1960
1980
2000
2020
Adjusted Data, CBH/VR
Data CBH/VR
Outliered Data
GBD 2019
GBD 2017
UN WPP
VR COMPLETE
VR COMPLETE TB

Age−specific fertility fitted trend lines, 1950−2019: Argentina, Ages: 25−29
Data points enclosed with a diamond are reference sources

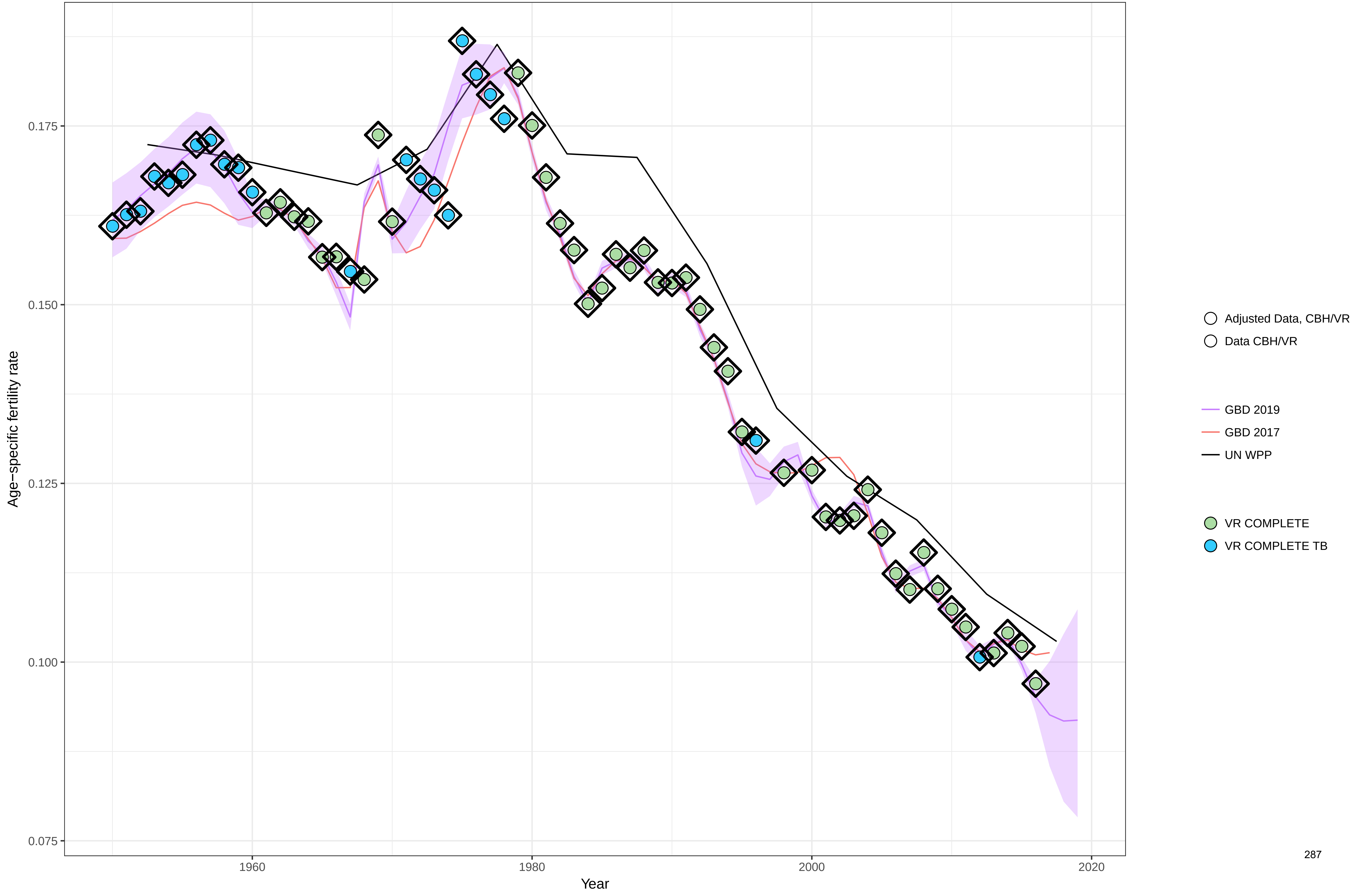

Age−specific fertility fitted trend lines, 1950−2019: Argentina, Ages: 30−34
Data points enclosed with a diamond are reference sources

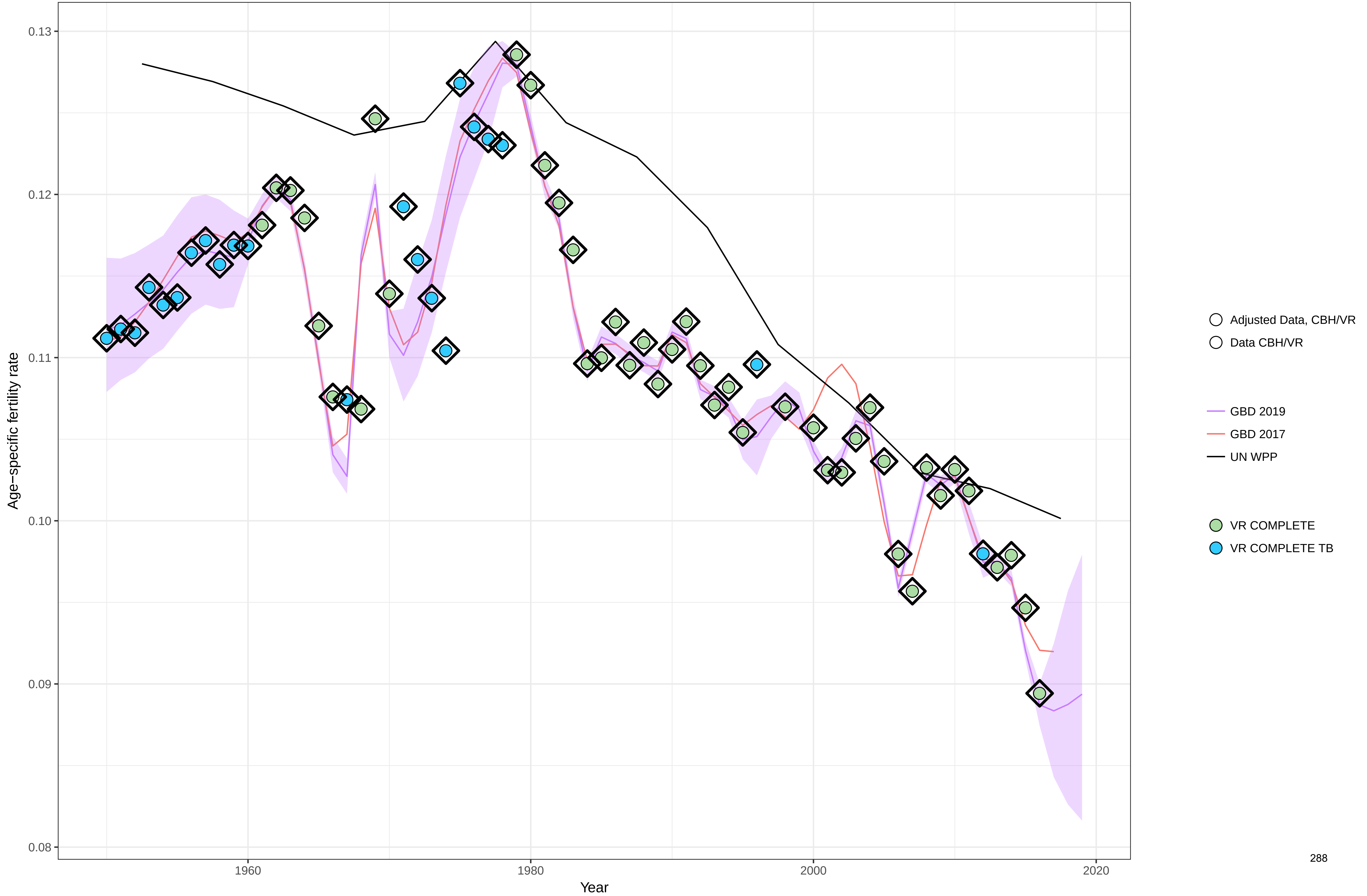

Age−specific fertility fitted trend lines, 1950−2019: Argentina, Ages: 35−39
Data points enclosed with a diamond are reference sources

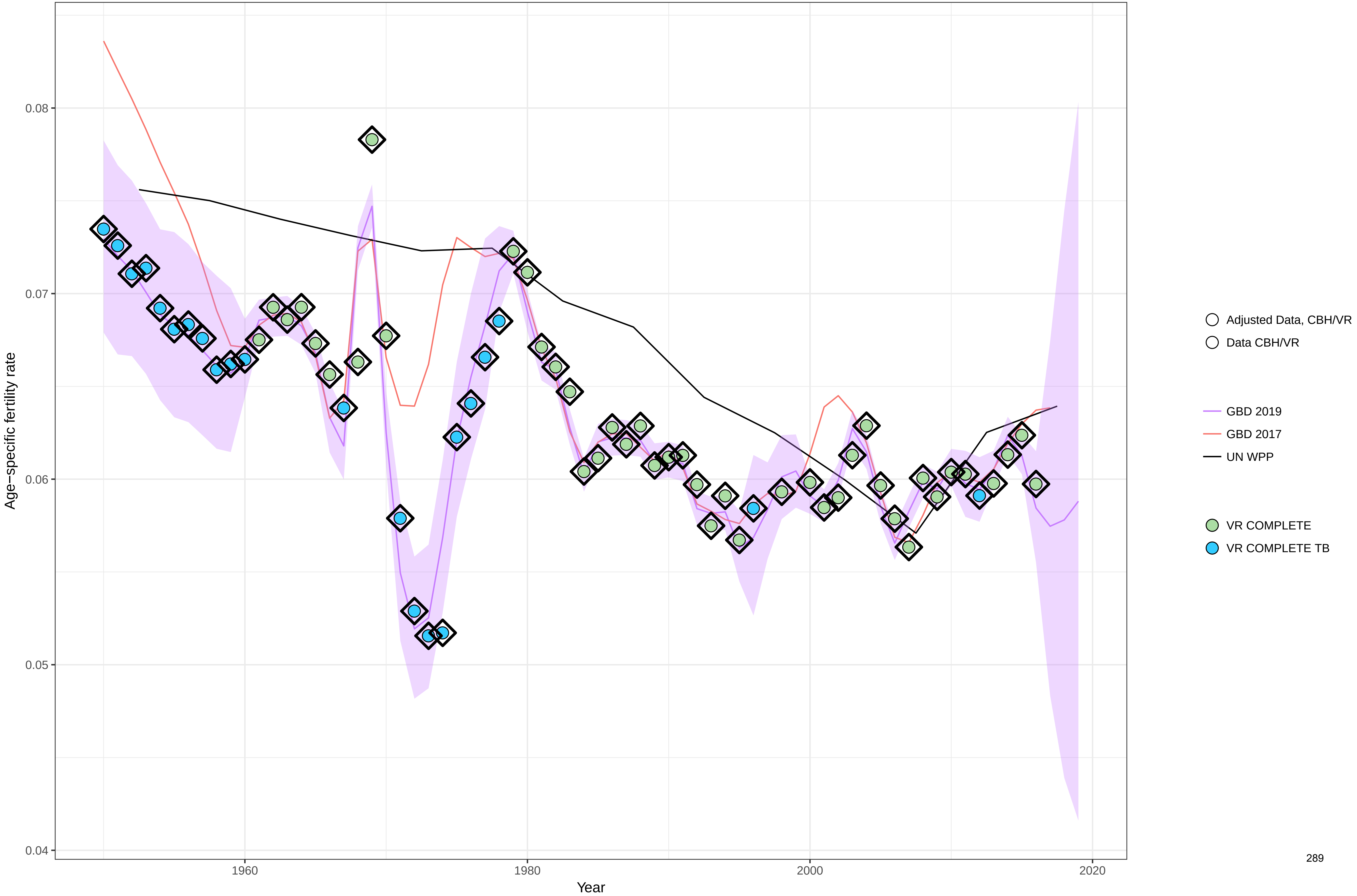

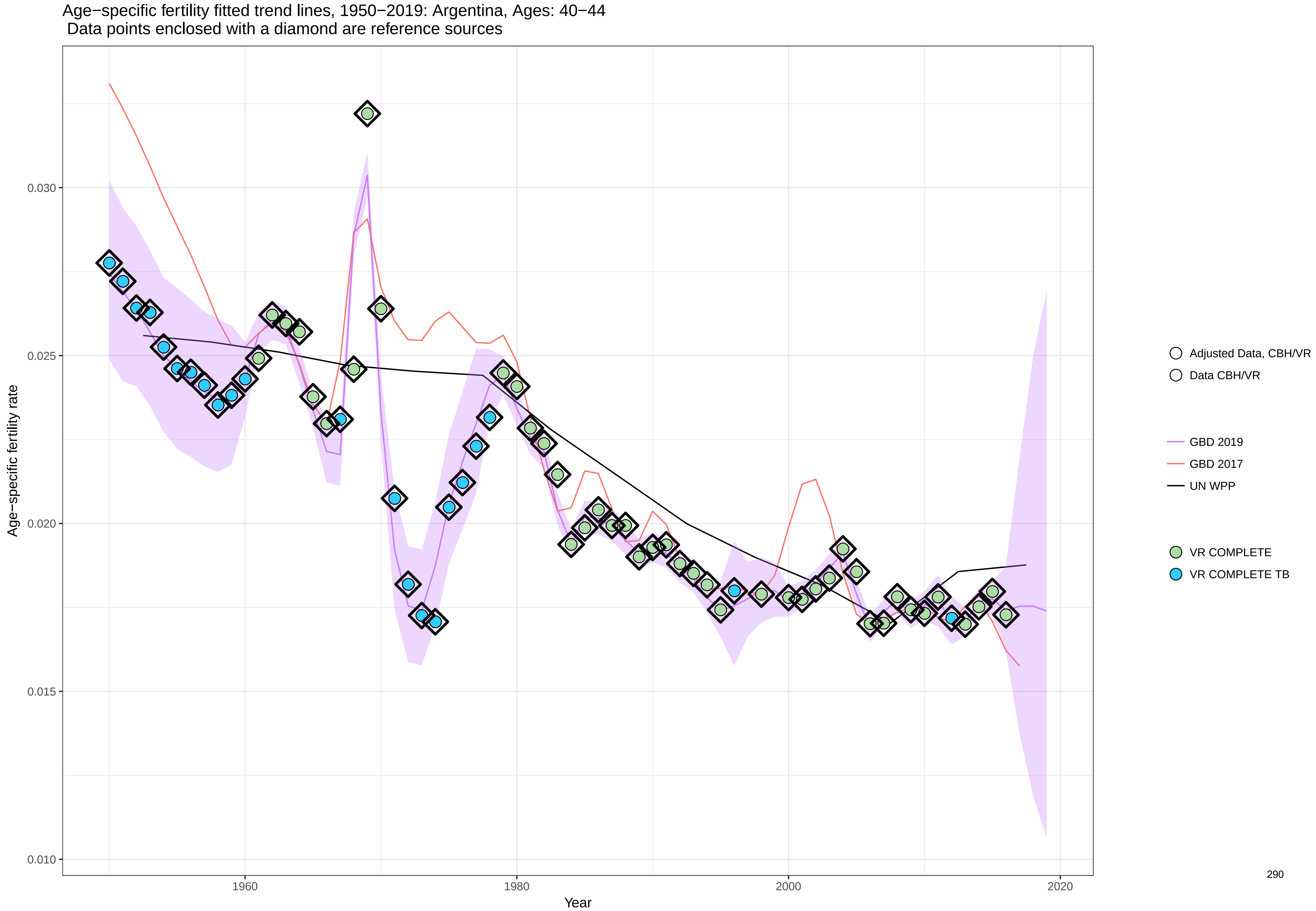
Age−specific fertility fitted trend lines, 1950−2019: Argentina, Ages: 40−44
Data points enclosed with a diamond are reference sources
Age−specific fertility rate
0.030
0.025
0.020
0.015
0.010
1960
1980
2000
2020
Year
Adjusted Data, CBH/VR
Data CBH/VR
GBD 2019
GBD 2017
UN WPP
VR COMPLETE
VR COMPLETE TB

Age−specific fertility fitted trend lines, 1950−2019: Argentina, Ages: 45−49
Data points enclosed with a diamond are reference sources

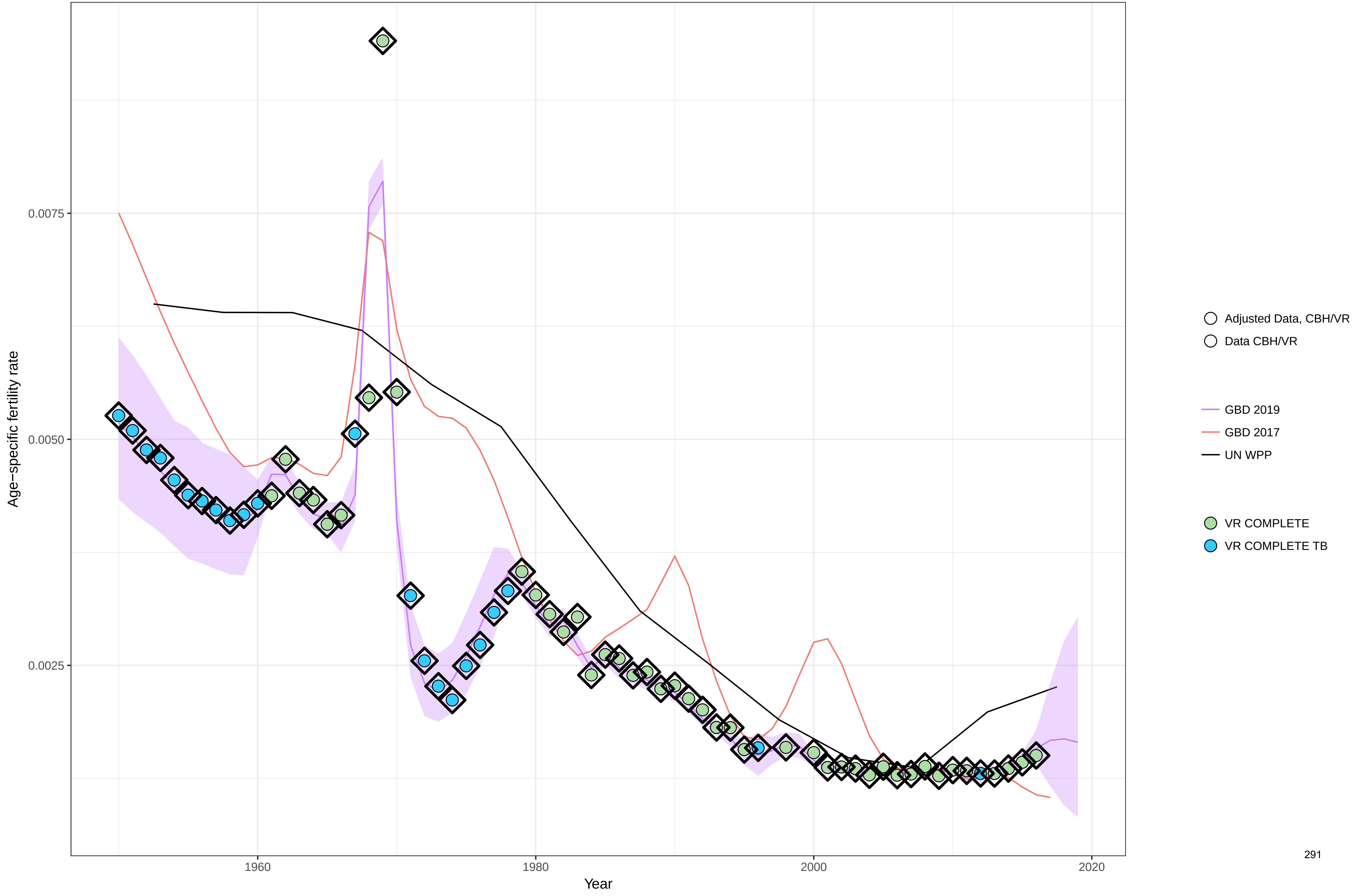

Age−specific fertility fitted trend lines, 1950−2019: Argentina, Ages: 50−54
Data points enclosed with a diamond are reference sources

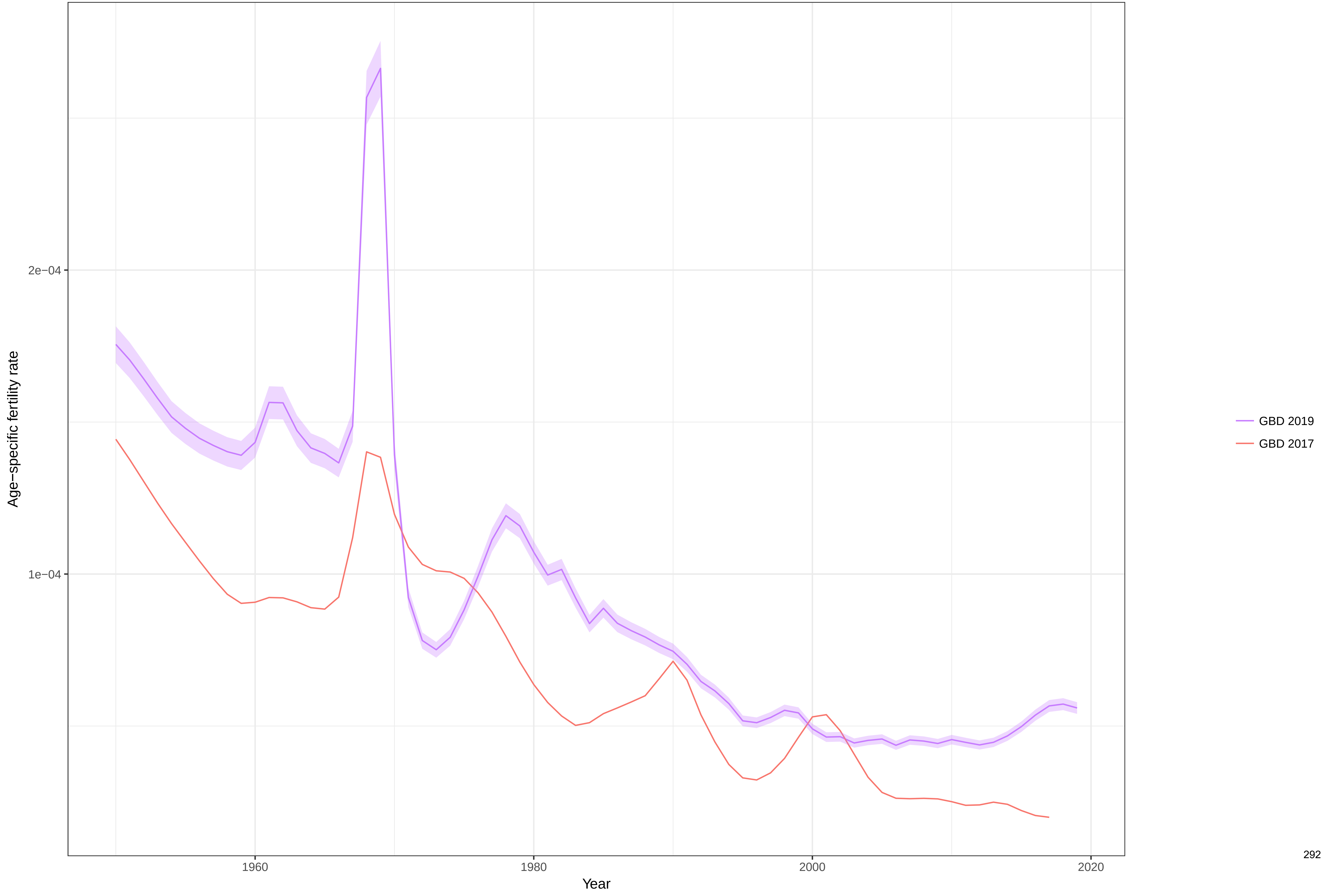

Total fertility rate fitted trend lines, 1950−2019: Argentina TFR
Data points enclosed with a diamond are reference sources

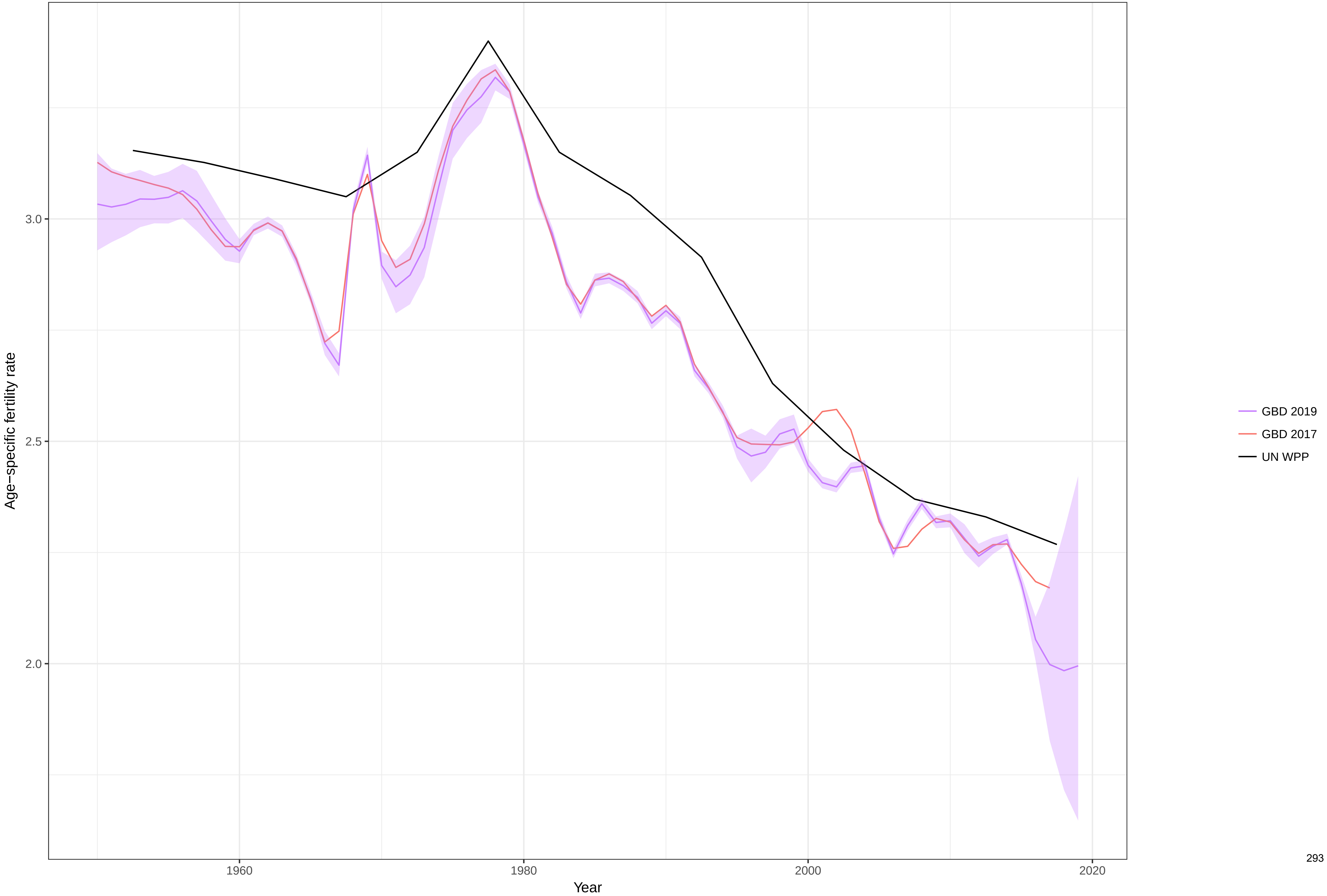

Age−specific fertility fitted trend lines, 1950−2019: Armenia, Ages: 10−14
Data points enclosed with a diamond are reference sources

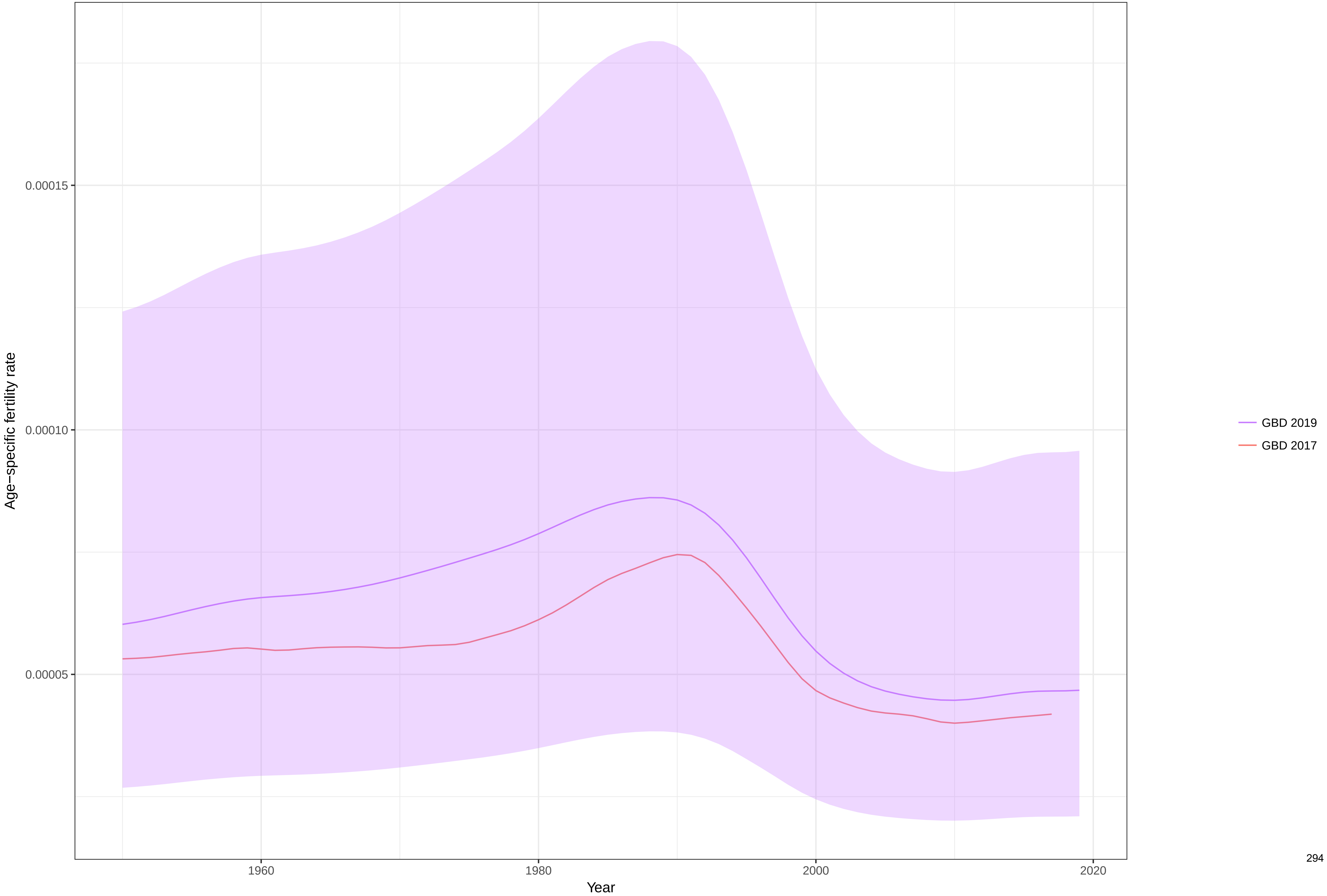

Age−specific fertility fitted trend lines, 1950−2019: Armenia, Ages: 15−19
Data points enclosed with a diamond are reference sources

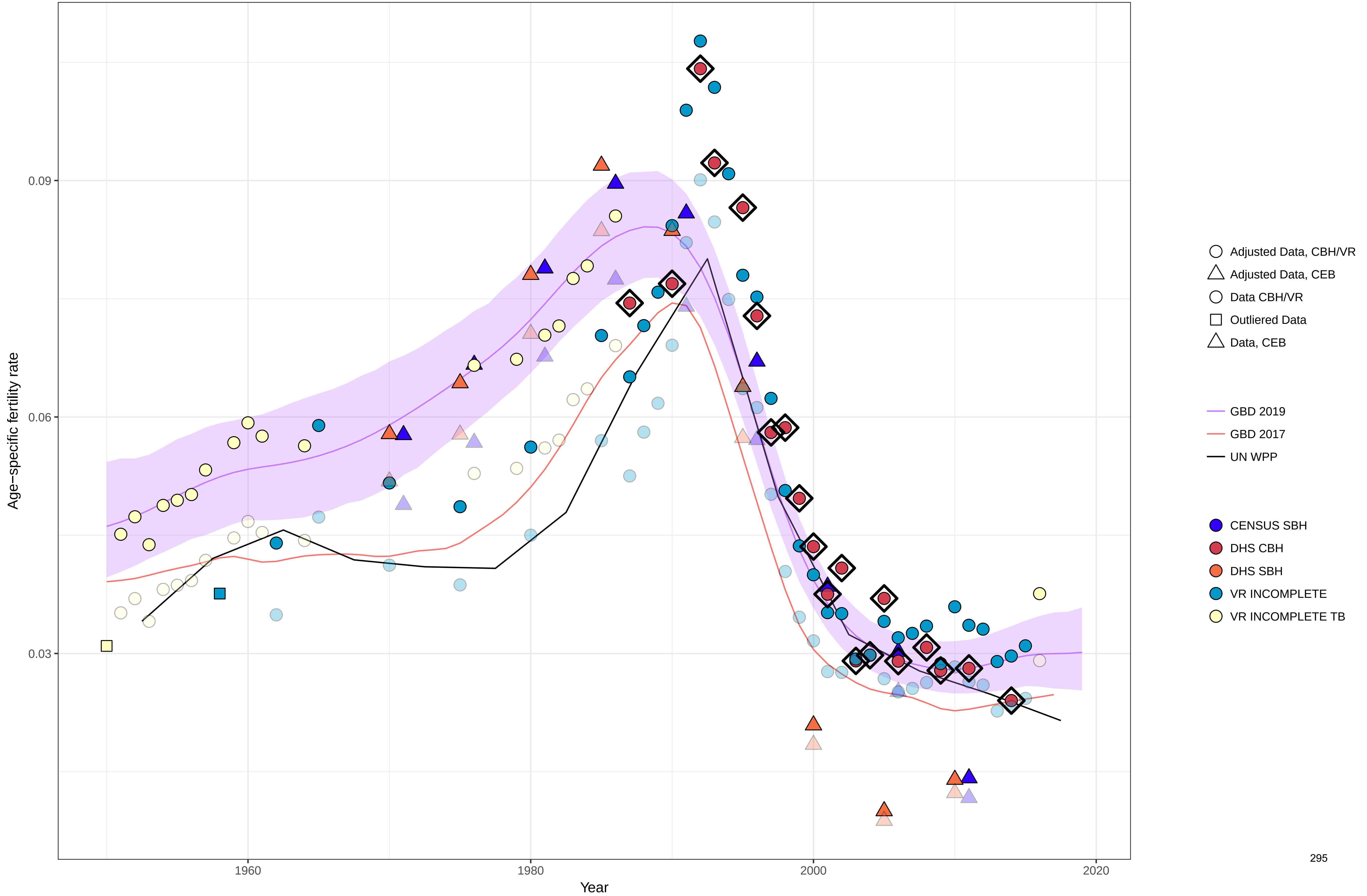

Age−specific fertility fitted trend lines, 1950−2019: Armenia, Ages: 20−24
Data points enclosed with a diamond are reference sources

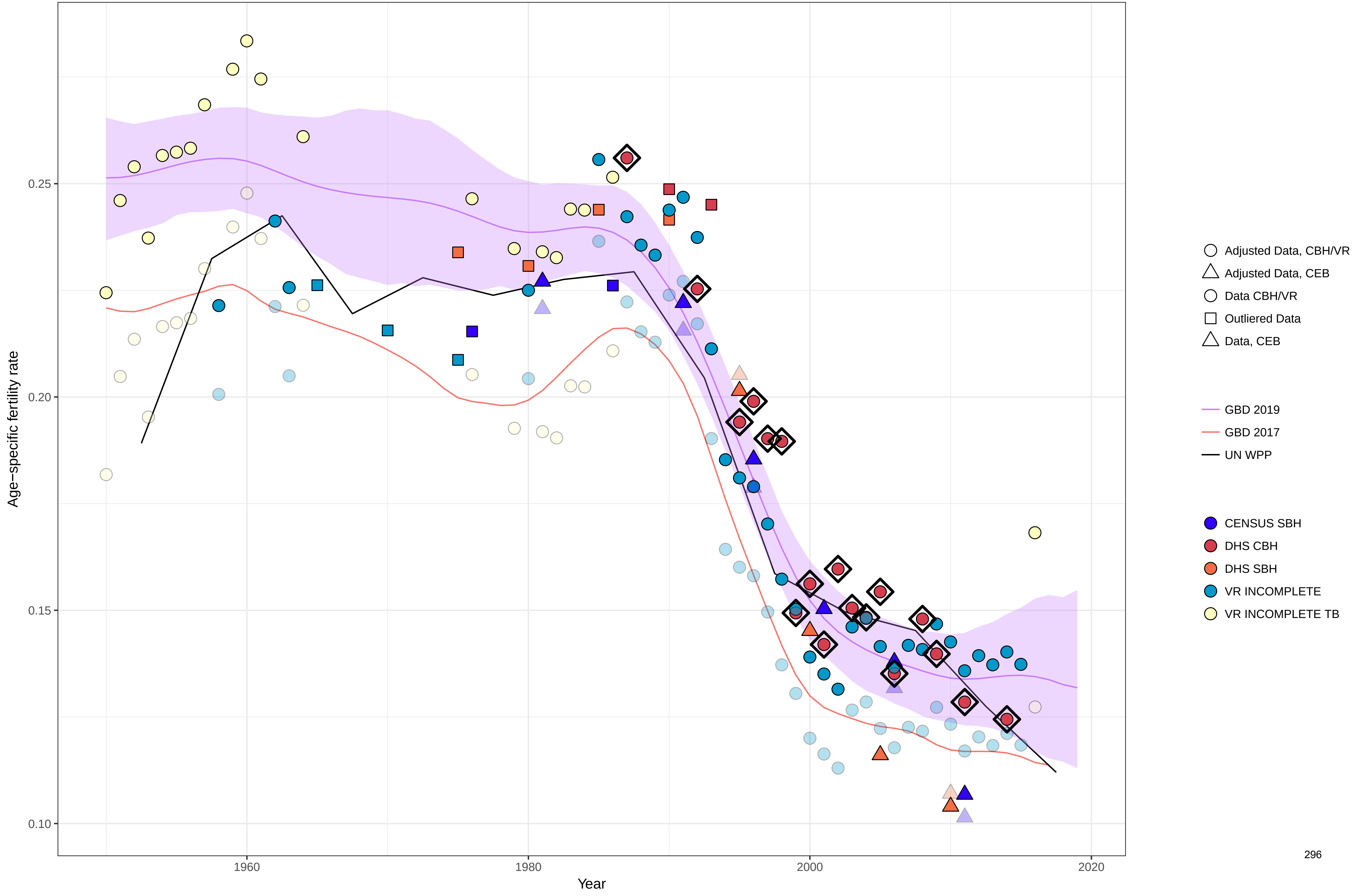

Age−specific fertility fitted trend lines, 1950−2019: Armenia, Ages: 25−29
Data points enclosed with a diamond are reference sources

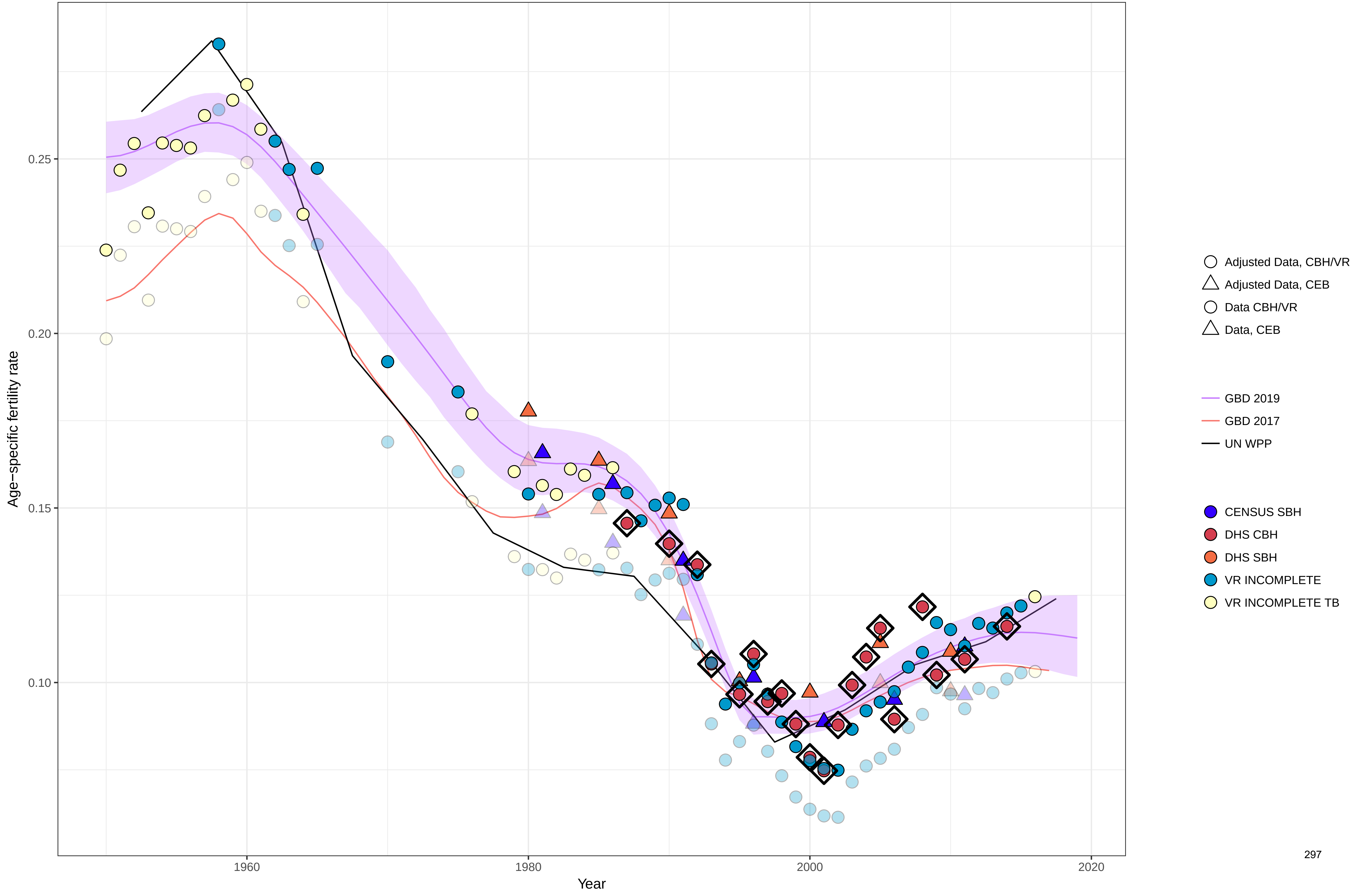

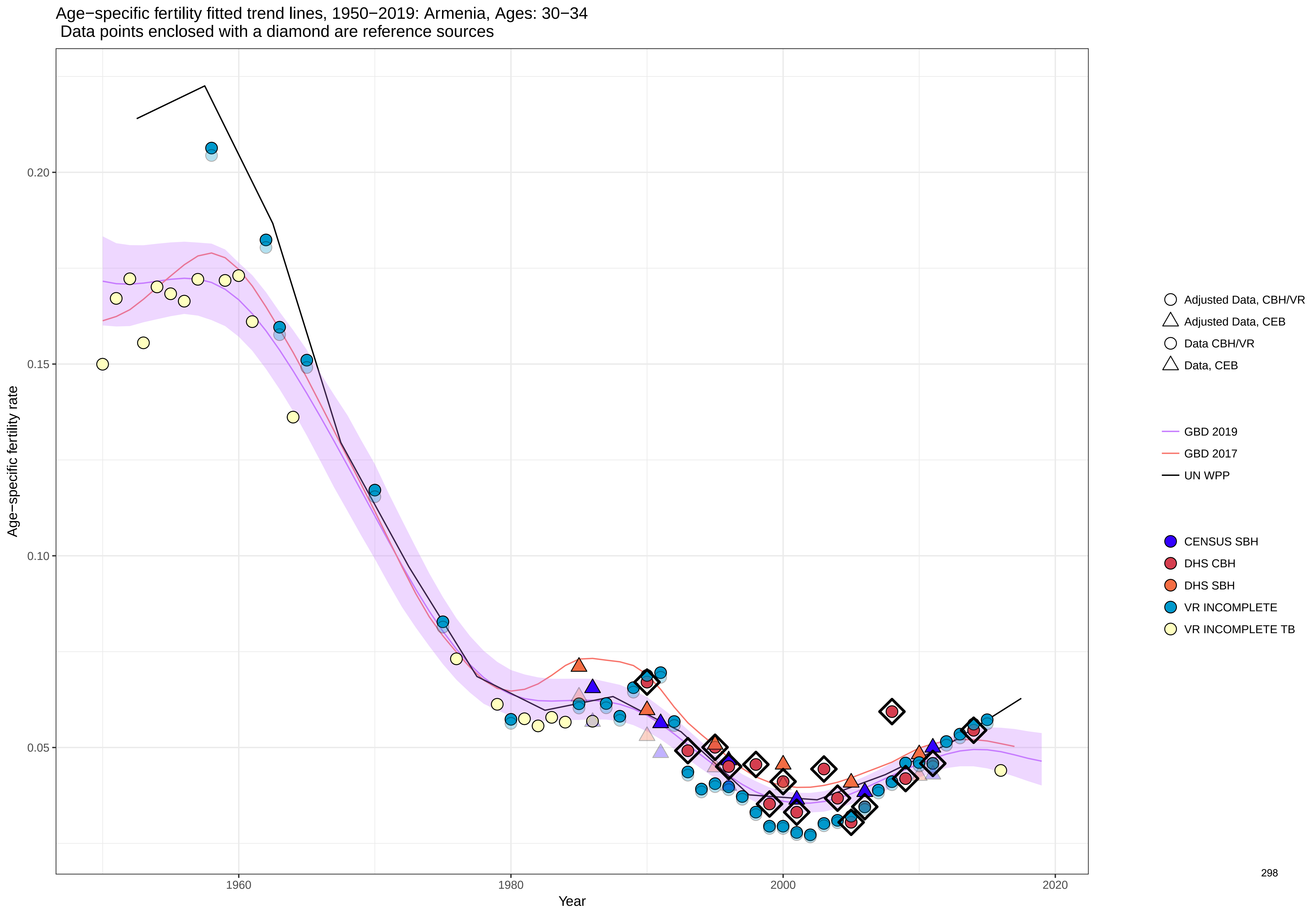

Age−specific fertility fitted trend lines, 1950−2019: Armenia, Ages: 30−34
Data points enclosed with a diamond are reference sources
Age−specific fertility rate
Year
0.20
0.15
0.10
0.05
1960
1980
2000
2020
Adjusted Data, CBH/VR
Adjusted Data, CEB
Data CBH/VR
Data, CEB
GBD 2019
GBD 2017
UN WPP
CENSUS SBH
DHS CBH
DHS SBH
VR INCOMPLETE
VR INCOMPLETE TB

Age−specific fertility fitted trend lines, 1950−2019: Armenia, Ages: 35−39
Data points enclosed with a diamond are reference sources

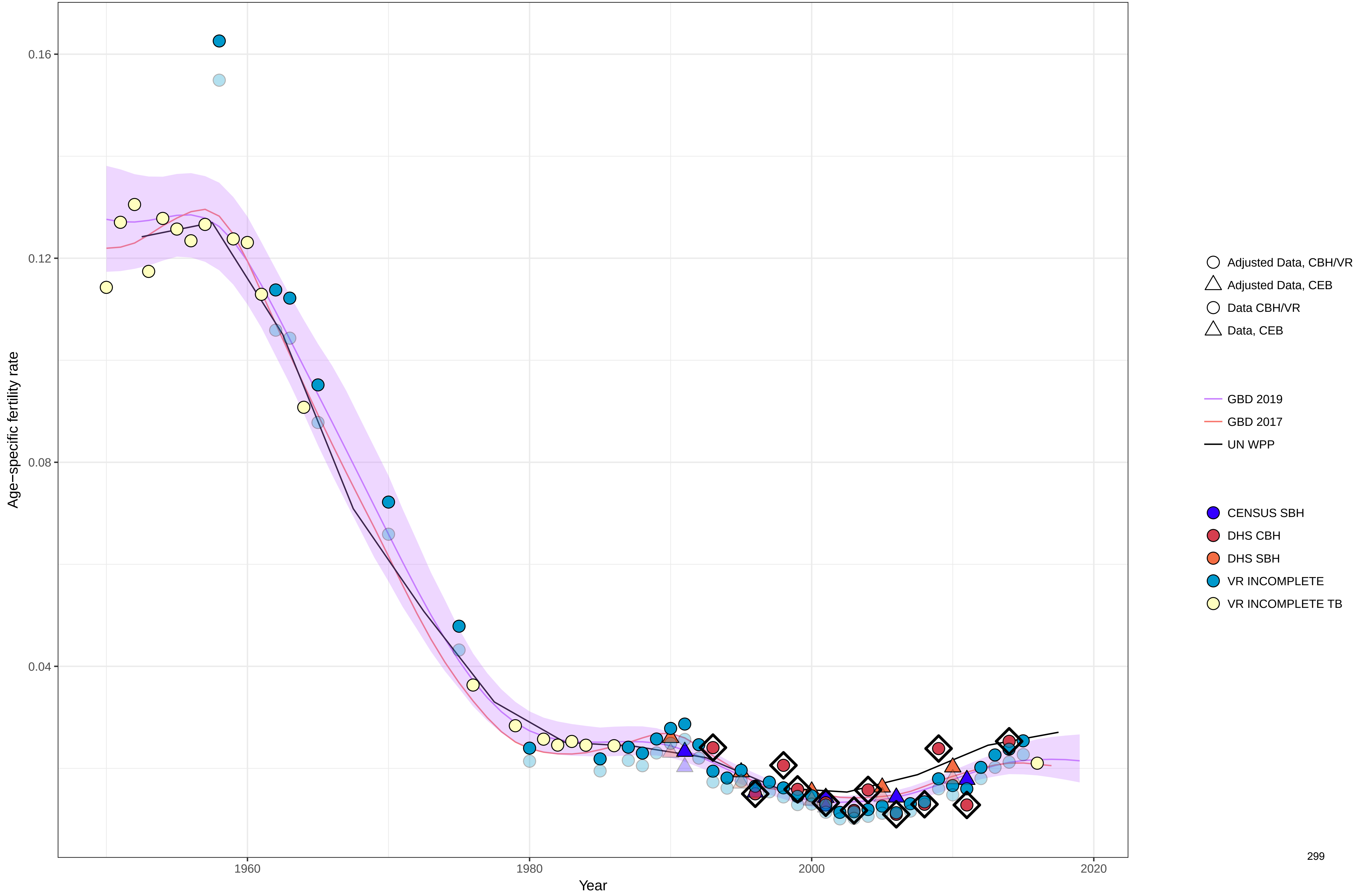

Age−specific fertility fitted trend lines, 1950−2019: Armenia, Ages: 40−44
Data points enclosed with a diamond are reference sources

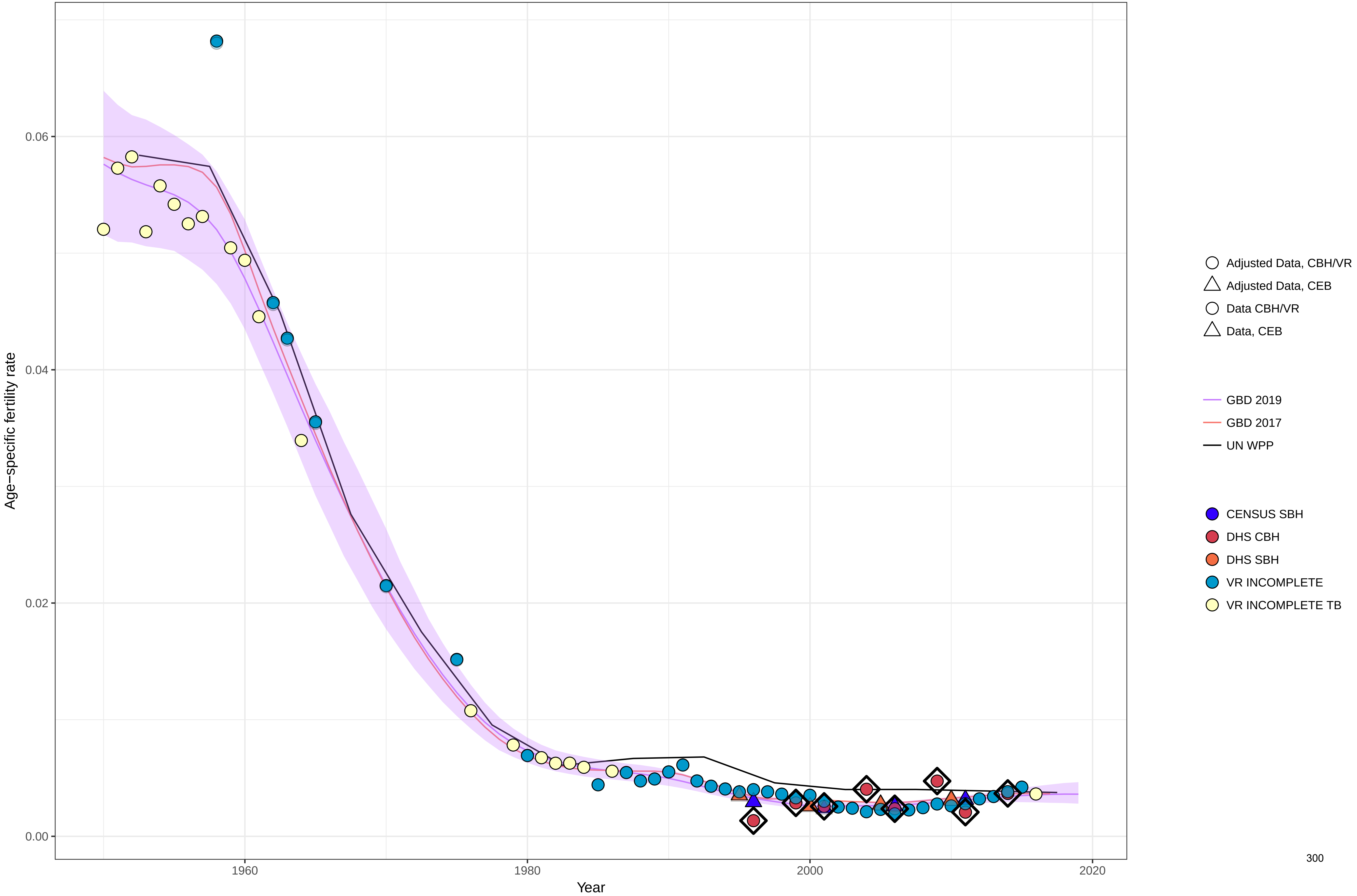

Age−specific fertility fitted trend lines, 1950−2019: Armenia, Ages: 45−49
Data points enclosed with a diamond are reference sources

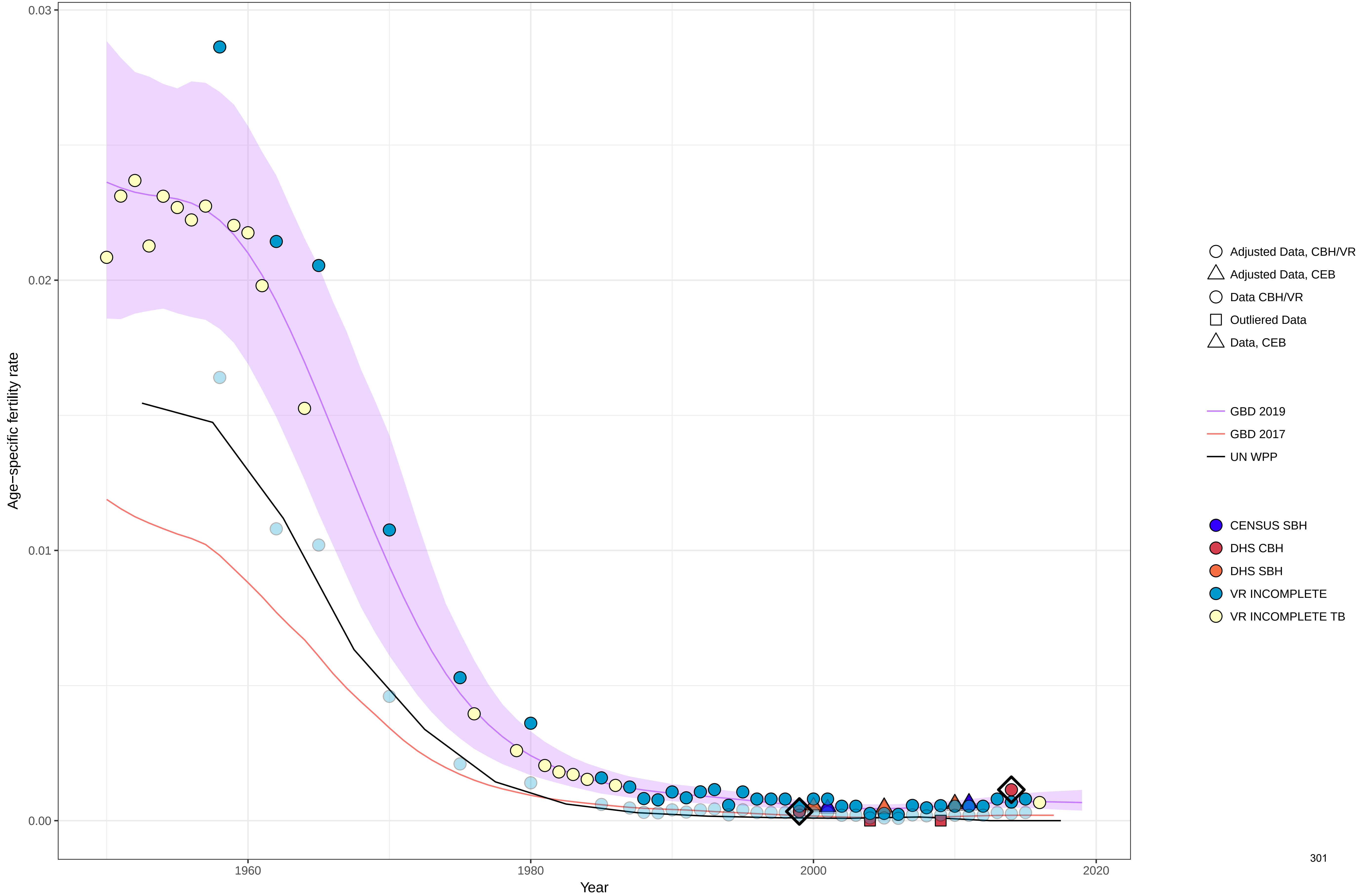

Age−specific fertility fitted trend lines, 1950−2019: Armenia, Ages: 50−54
Data points enclosed with a diamond are reference sources

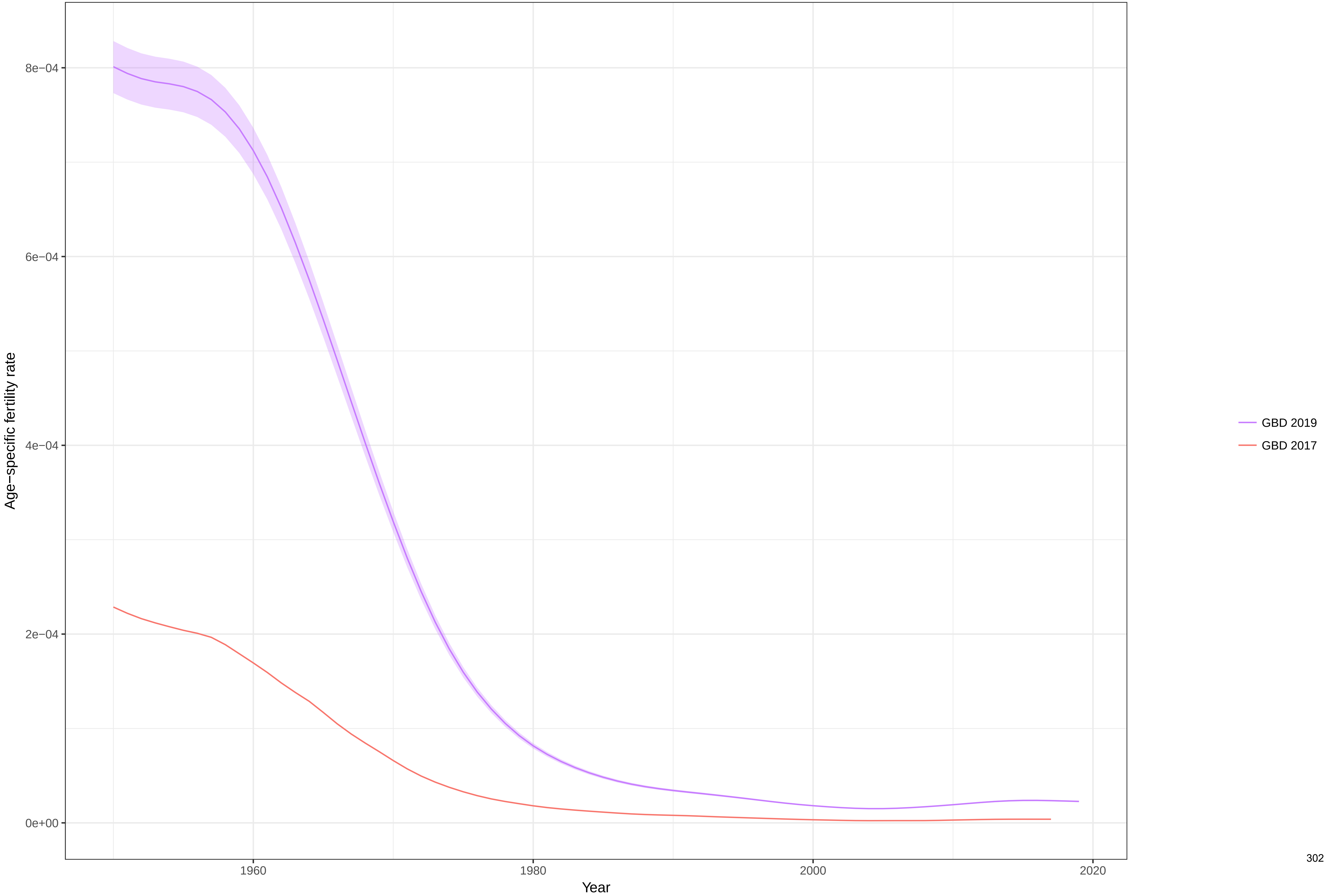

Total fertility rate fitted trend lines, 1950−2019: Armenia TFR
Data points enclosed with a diamond are reference sources

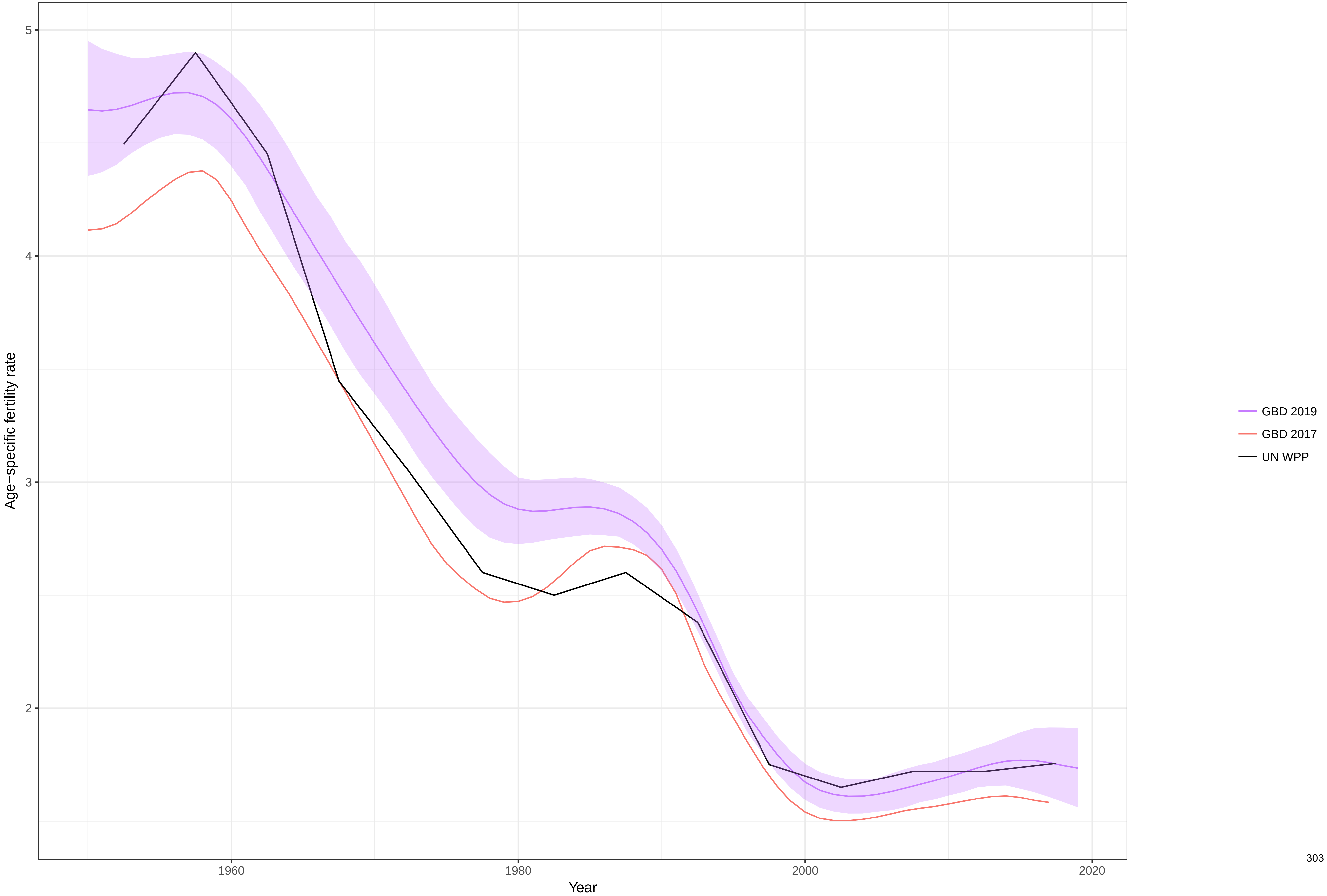

Age−specific fertility fitted trend lines, 1950−2019: American Samoa, Ages: 10−14
Data points enclosed with a diamond are reference sources

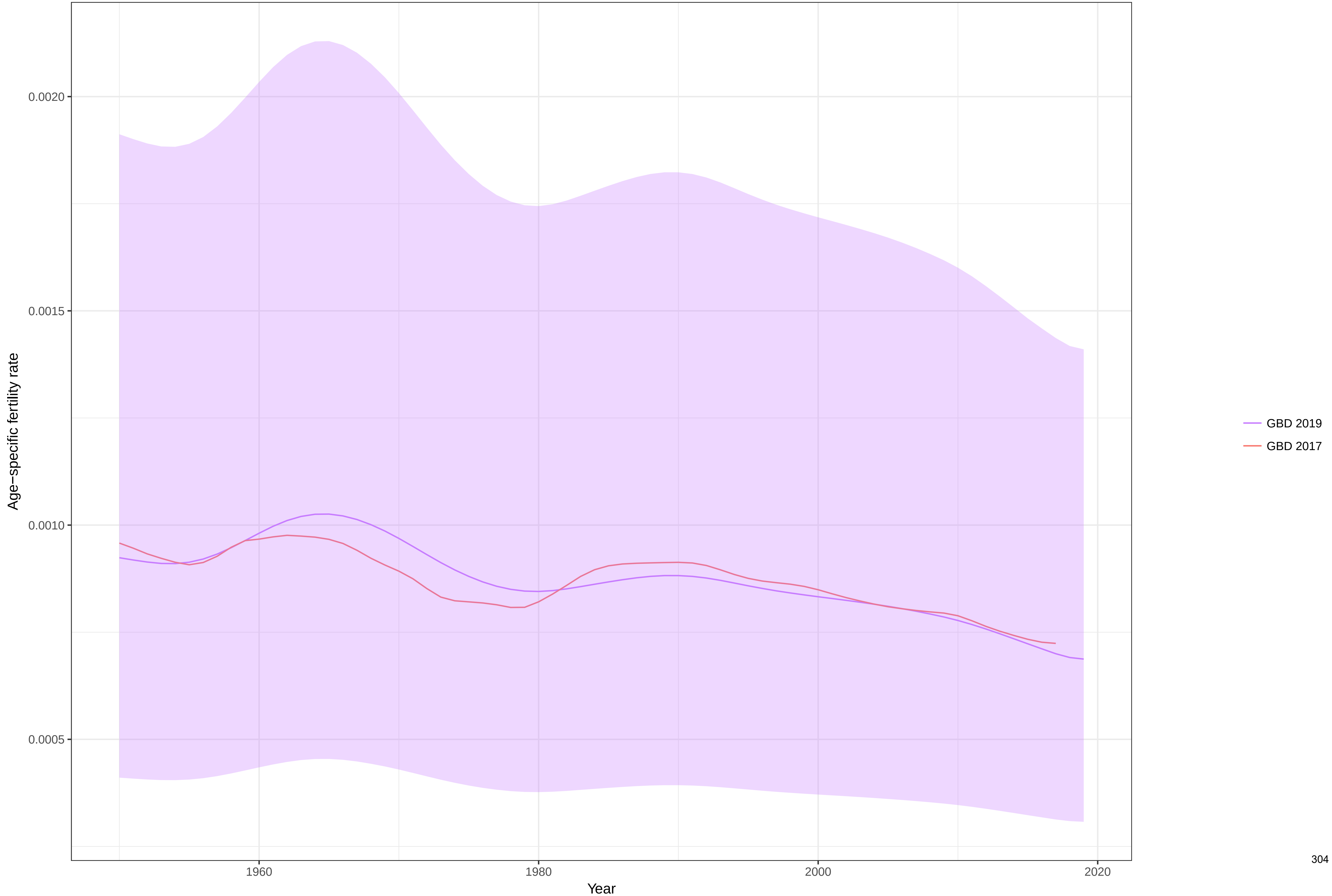

Age−specific fertility fitted trend lines, 1950−2019: American Samoa, Ages: 15−19
Data points enclosed with a diamond are reference sources

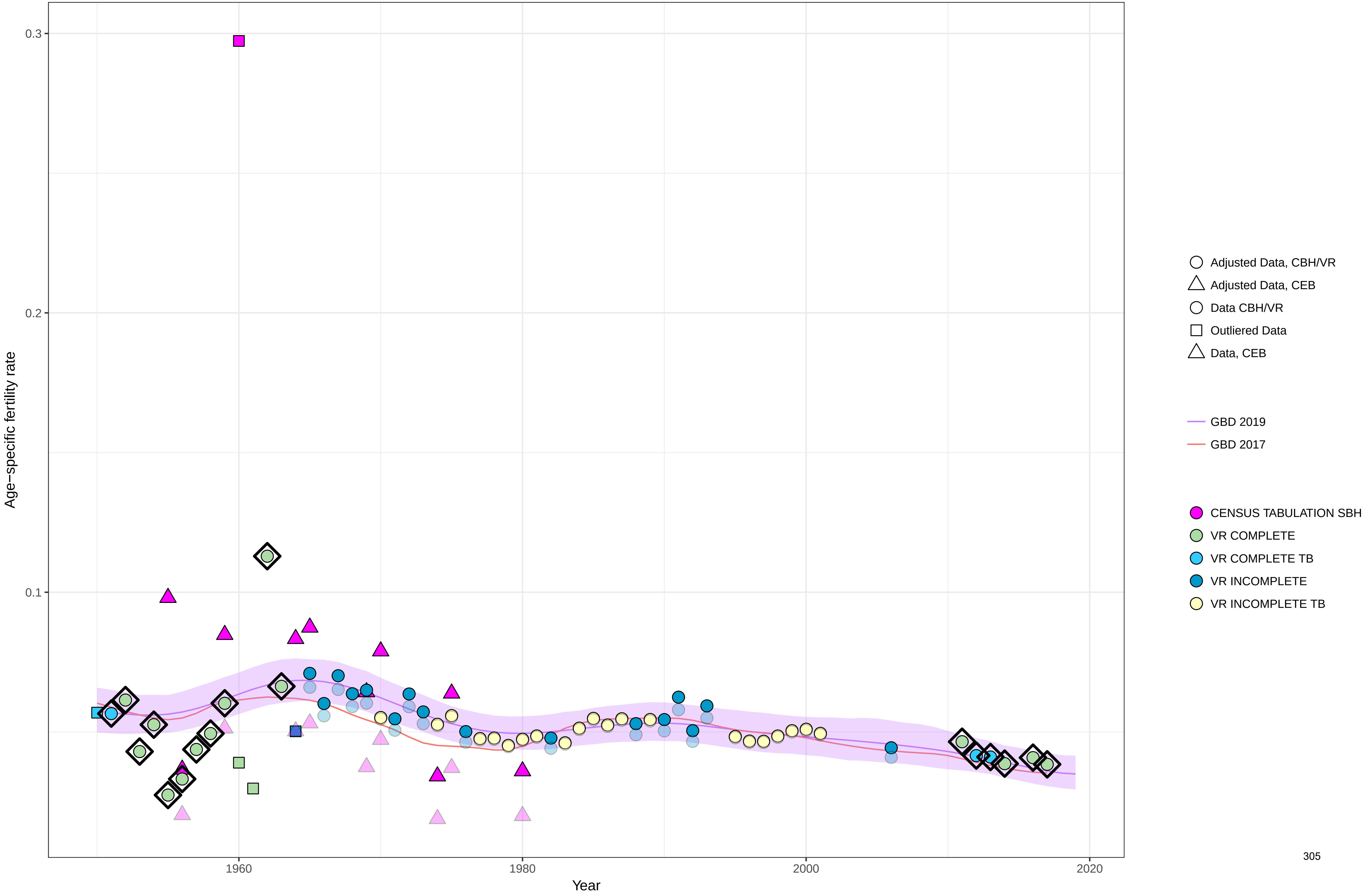

Age−specific fertility fitted trend lines, 1950−2019: American Samoa, Ages: 20−24
Data points enclosed with a diamond are reference sources

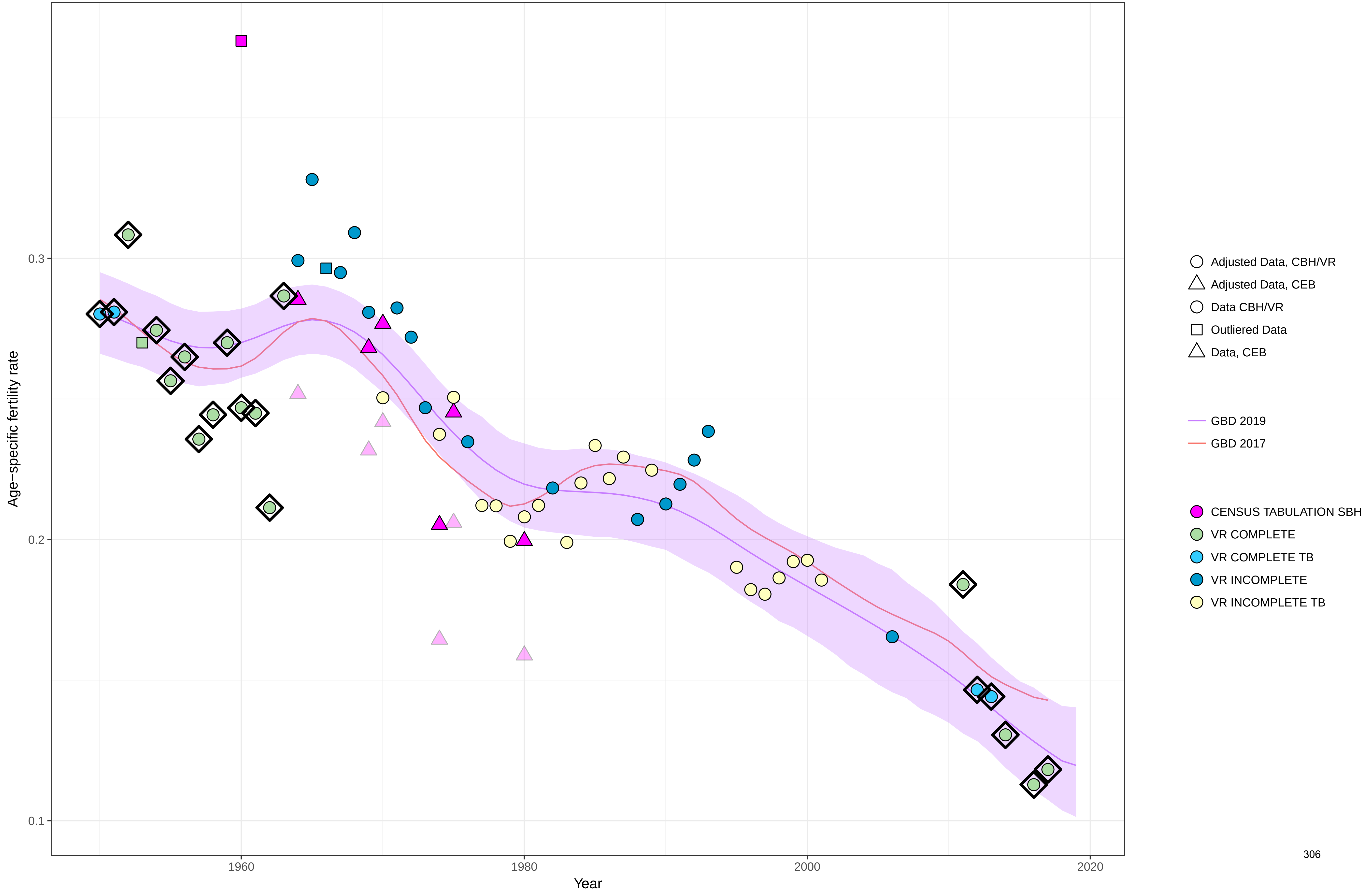

Age−specific fertility fitted trend lines, 1950−2019: American Samoa, Ages: 25−29
Data points enclosed with a diamond are reference sources

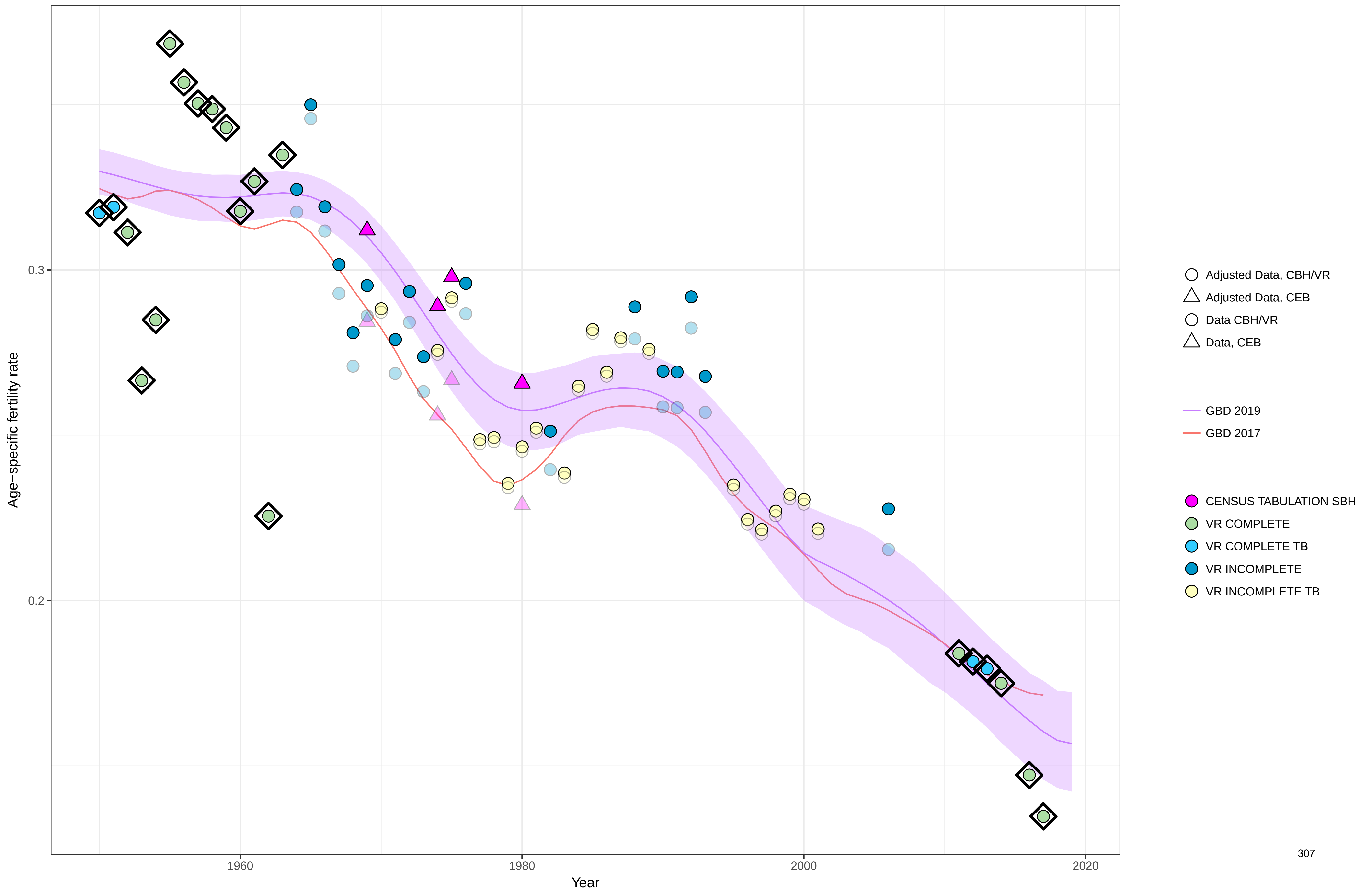

Age−specific fertility fitted trend lines, 1950−2019: American Samoa, Ages: 30−34
Data points enclosed with a diamond are reference sources

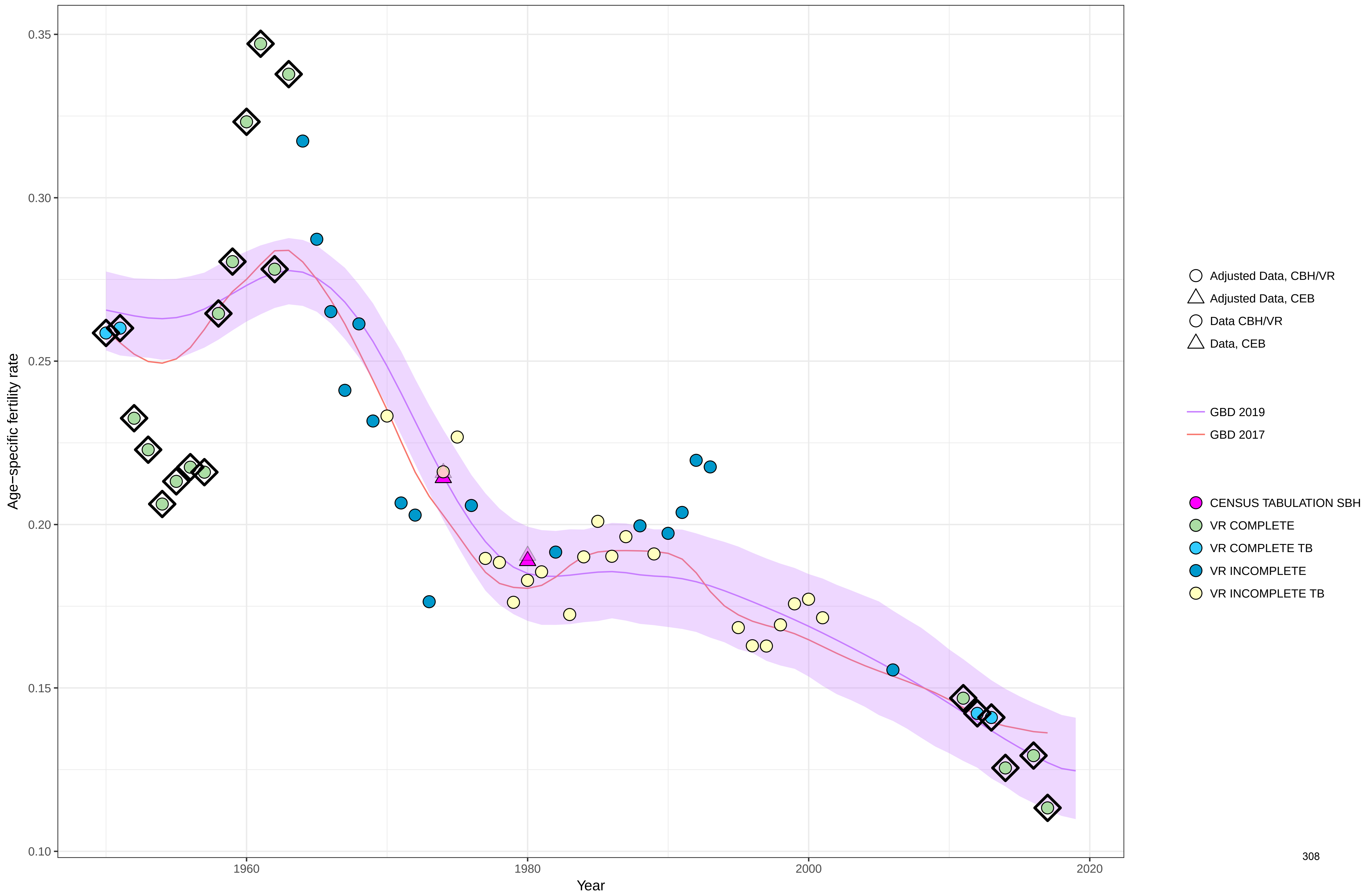

Age−specific fertility fitted trend lines, 1950−2019: American Samoa, Ages: 35−39
Data points enclosed with a diamond are reference sources

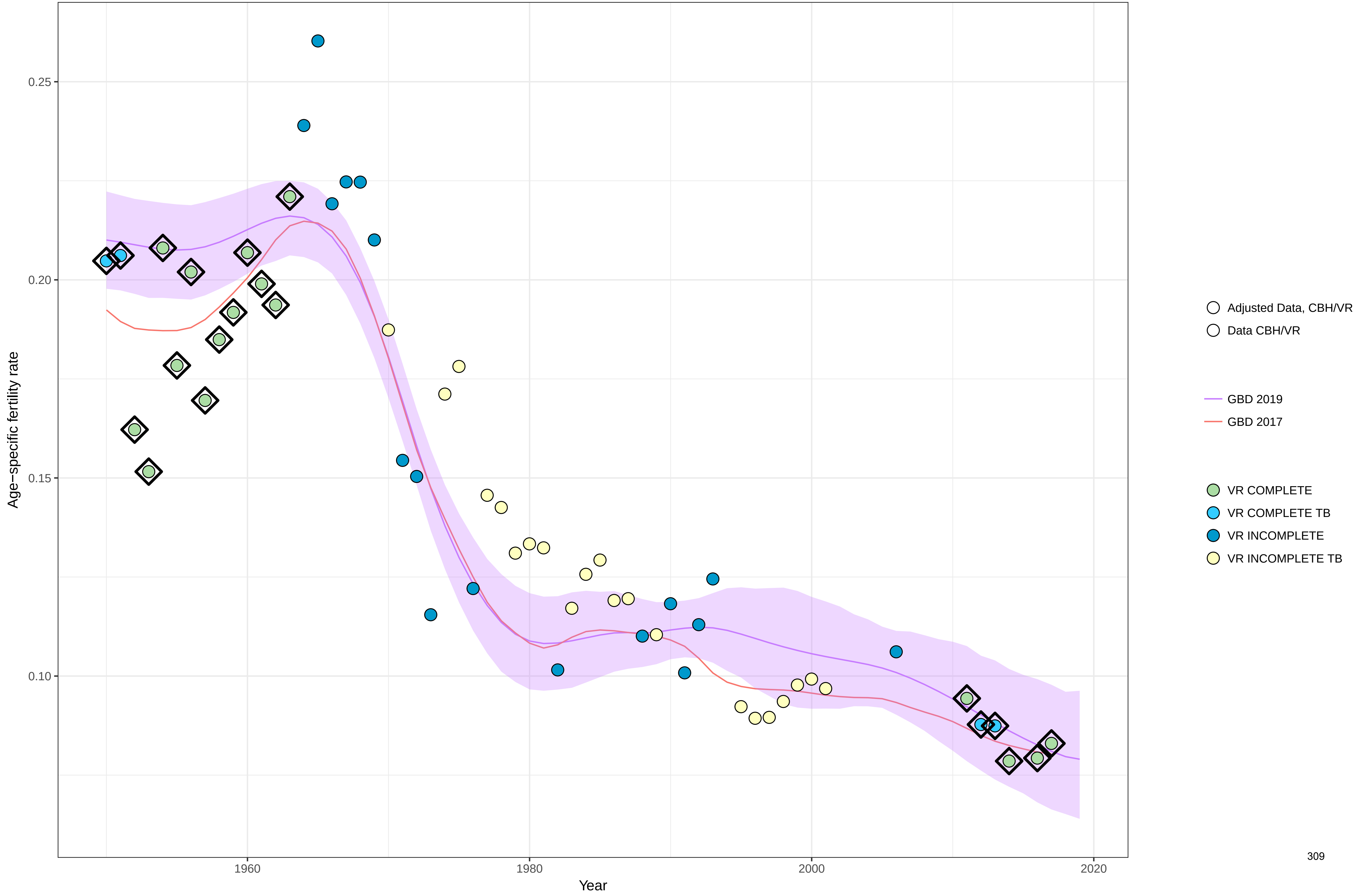

Age−specific fertility fitted trend lines, 1950−2019: American Samoa, Ages: 40−44
Data points enclosed with a diamond are reference sources

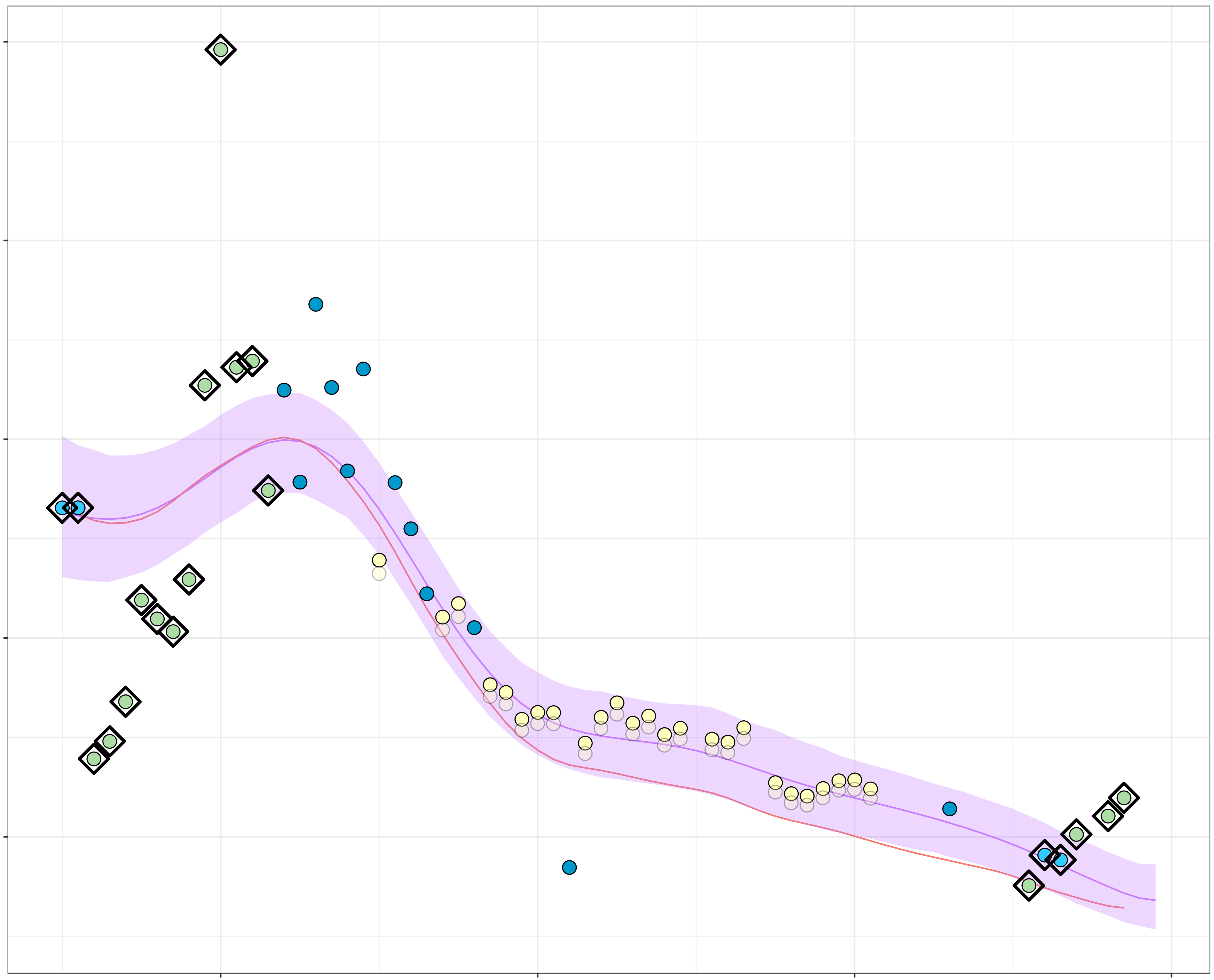

Age−specific fertility fitted trend lines, 1950−2019: American Samoa, Ages: 45−49
Data points enclosed with a diamond are reference sources

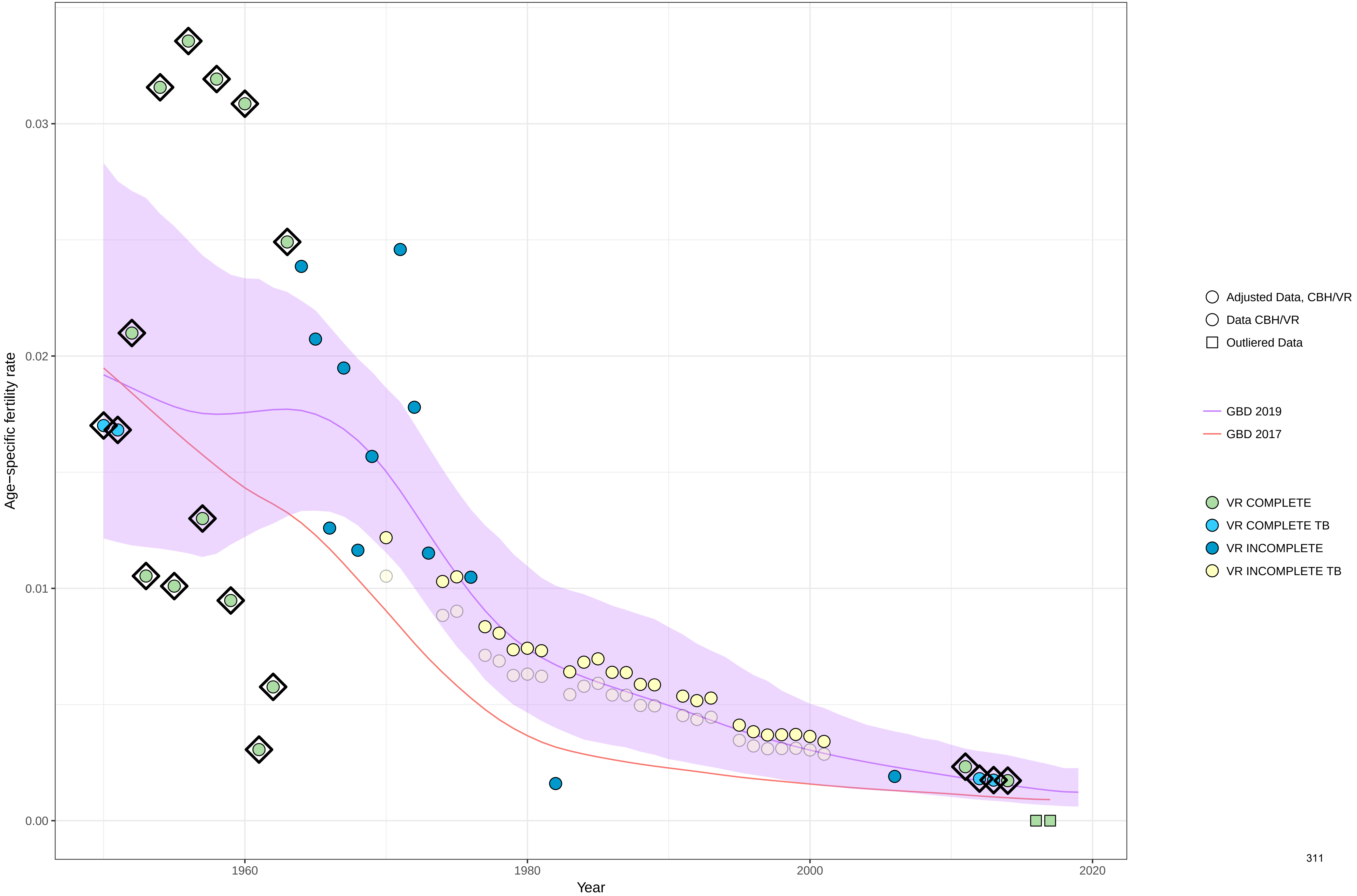

Age−specific fertility fitted trend lines, 1950−2019: American Samoa, Ages: 50−54
Data points enclosed with a diamond are reference sources

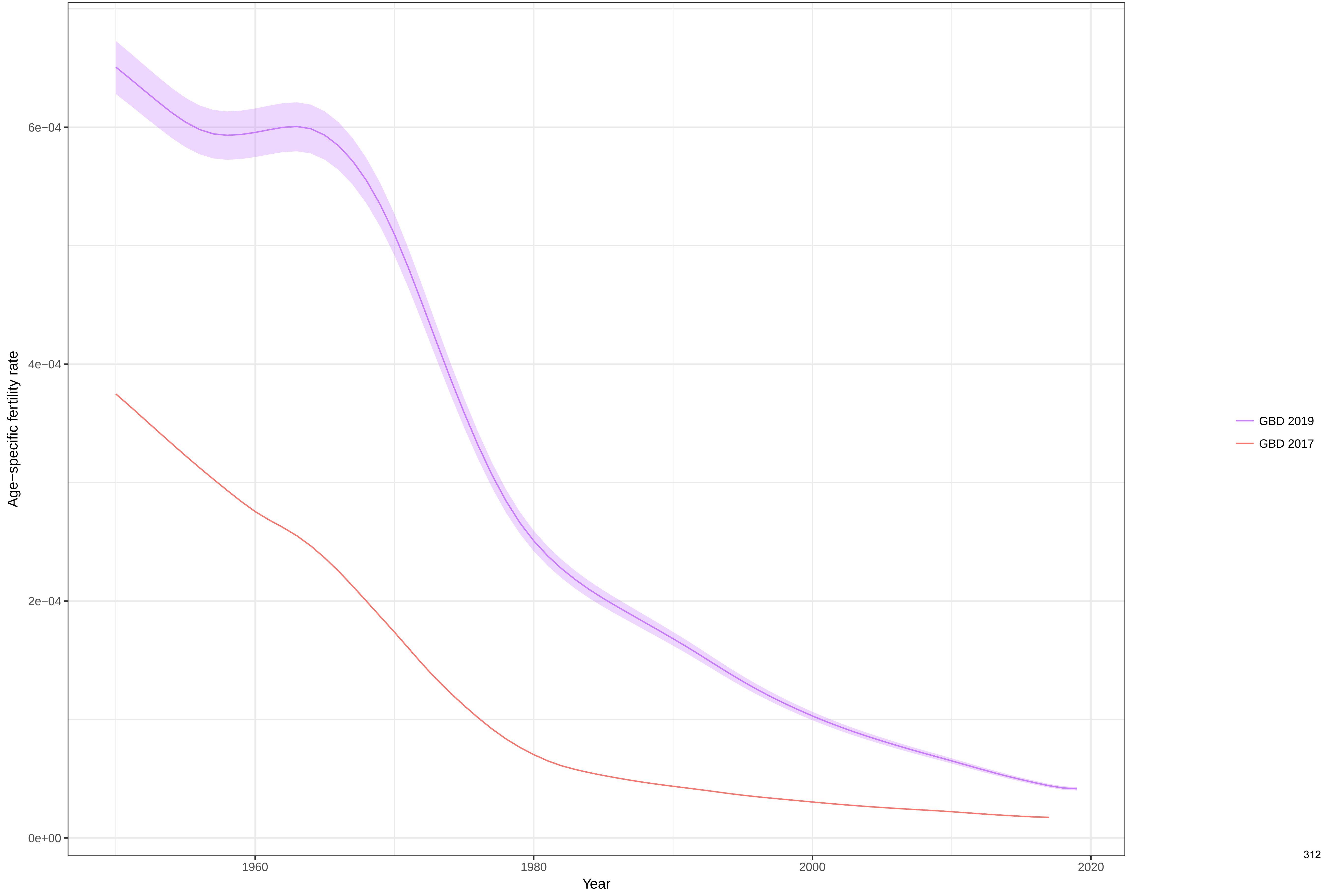

Total fertility rate fitted trend lines, 1950−2019: American Samoa TFR
Data points enclosed with a diamond are reference sources

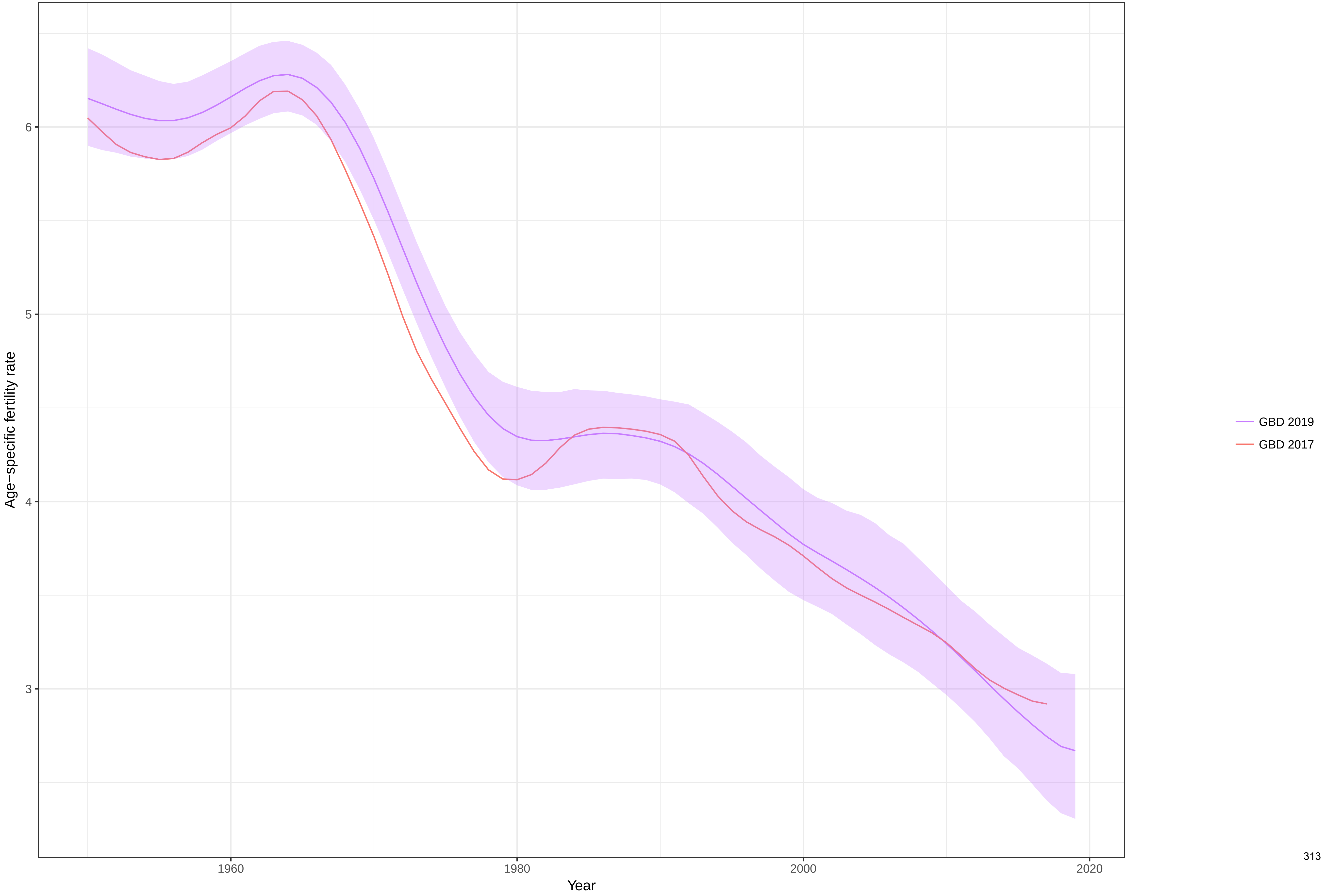

Age−specific fertility fitted trend lines, 1950−2019: Antigua and Barbuda, Ages: 10−14
Data points enclosed with a diamond are reference sources

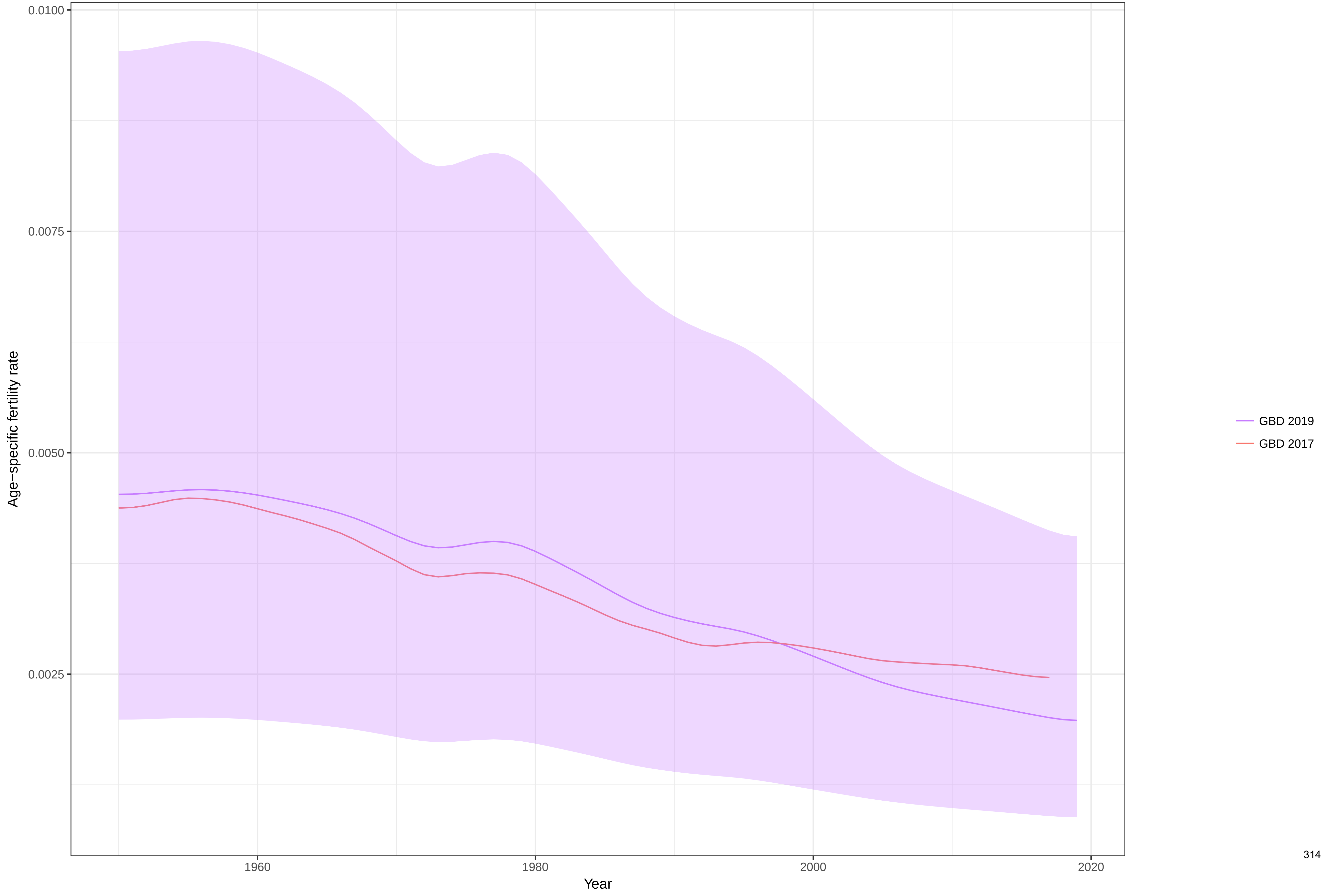

Age−specific fertility fitted trend lines, 1950−2019: Antigua and Barbuda, Ages: 15−19
Data points enclosed with a diamond are reference sources

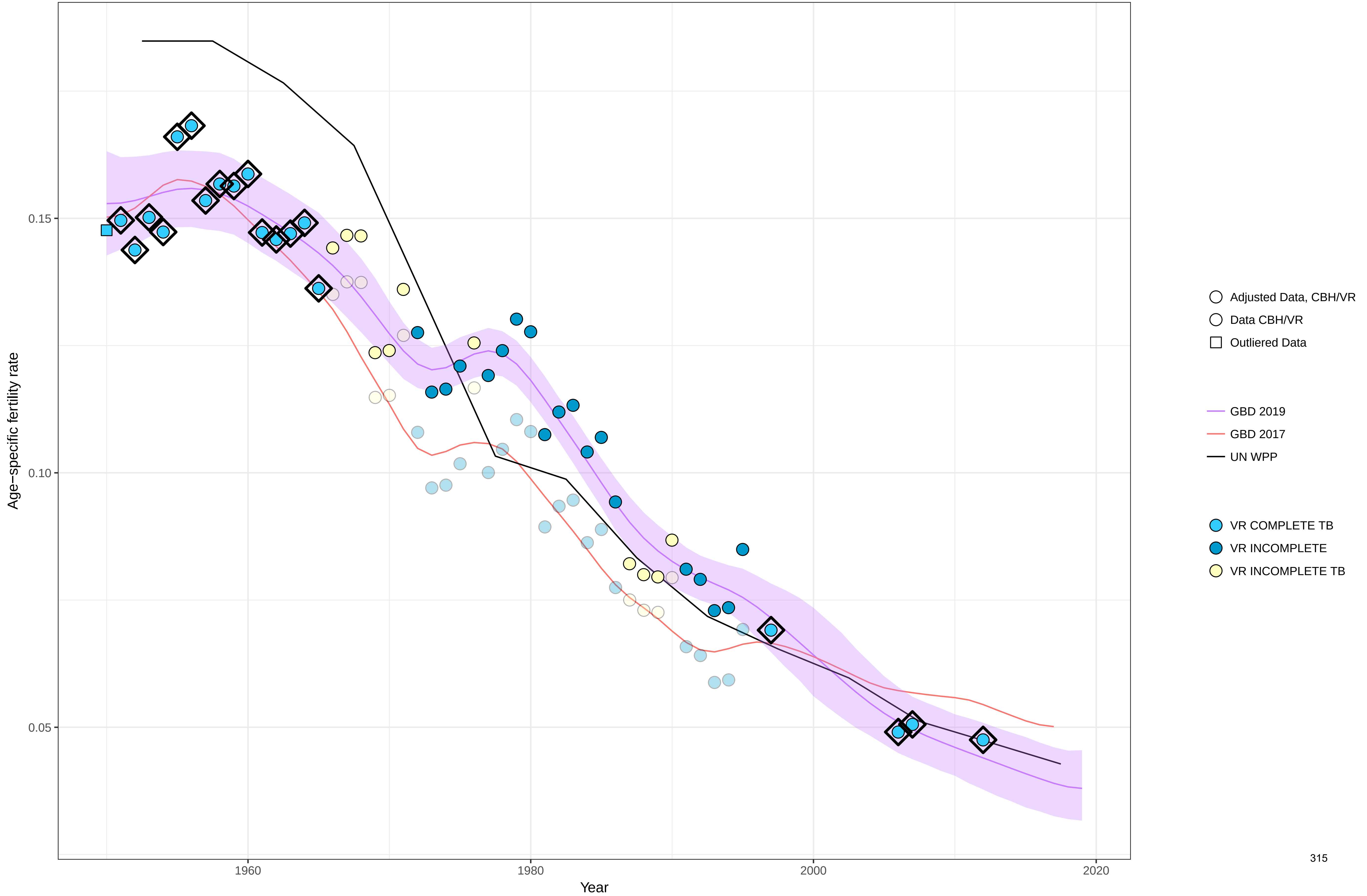

Age−specific fertility fitted trend lines, 1950−2019: Antigua and Barbuda, Ages: 20−24
Data points enclosed with a diamond are reference sources

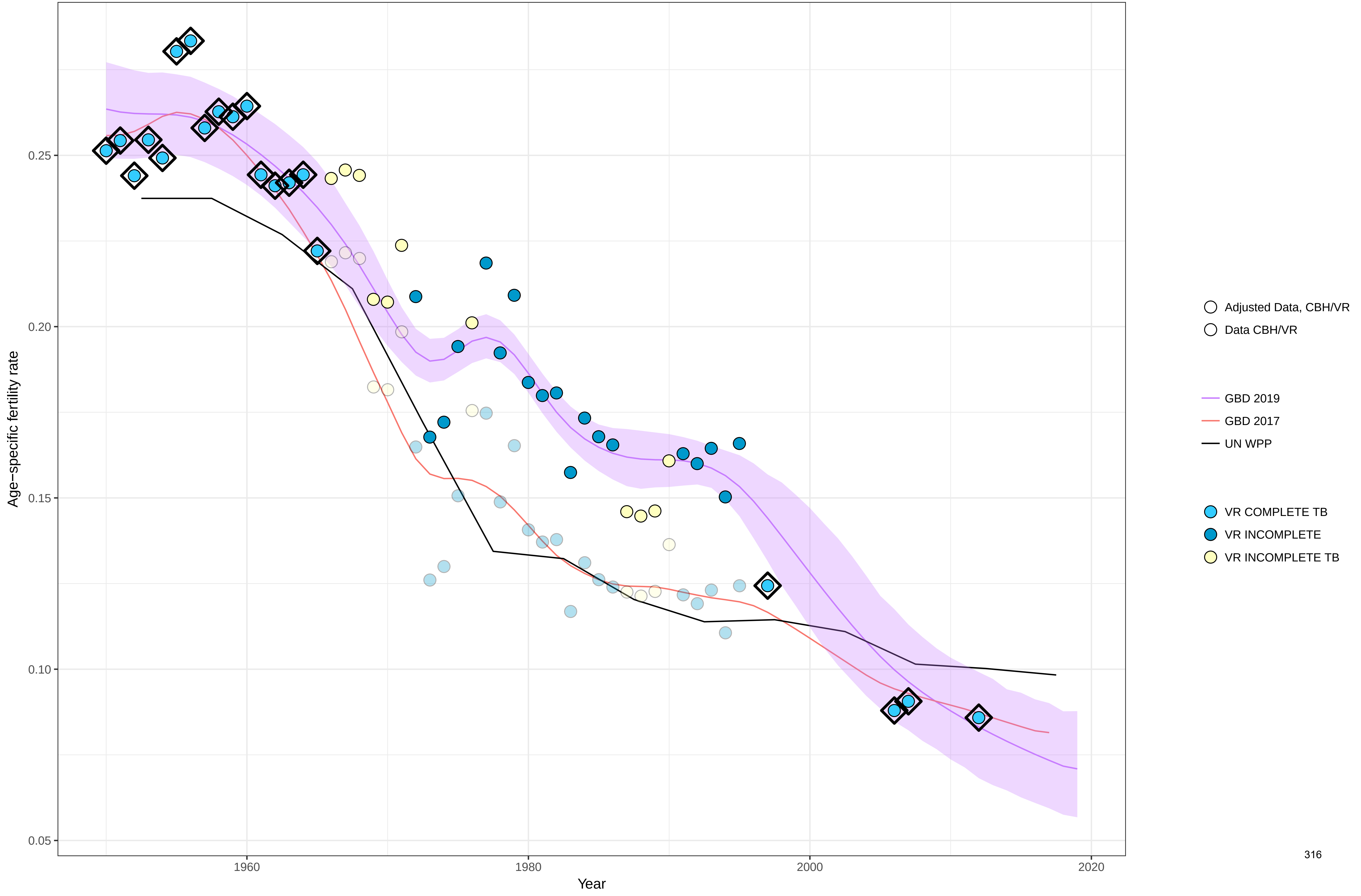

Age−specific fertility fitted trend lines, 1950−2019: Antigua and Barbuda, Ages: 25−29
Data points enclosed with a diamond are reference sources

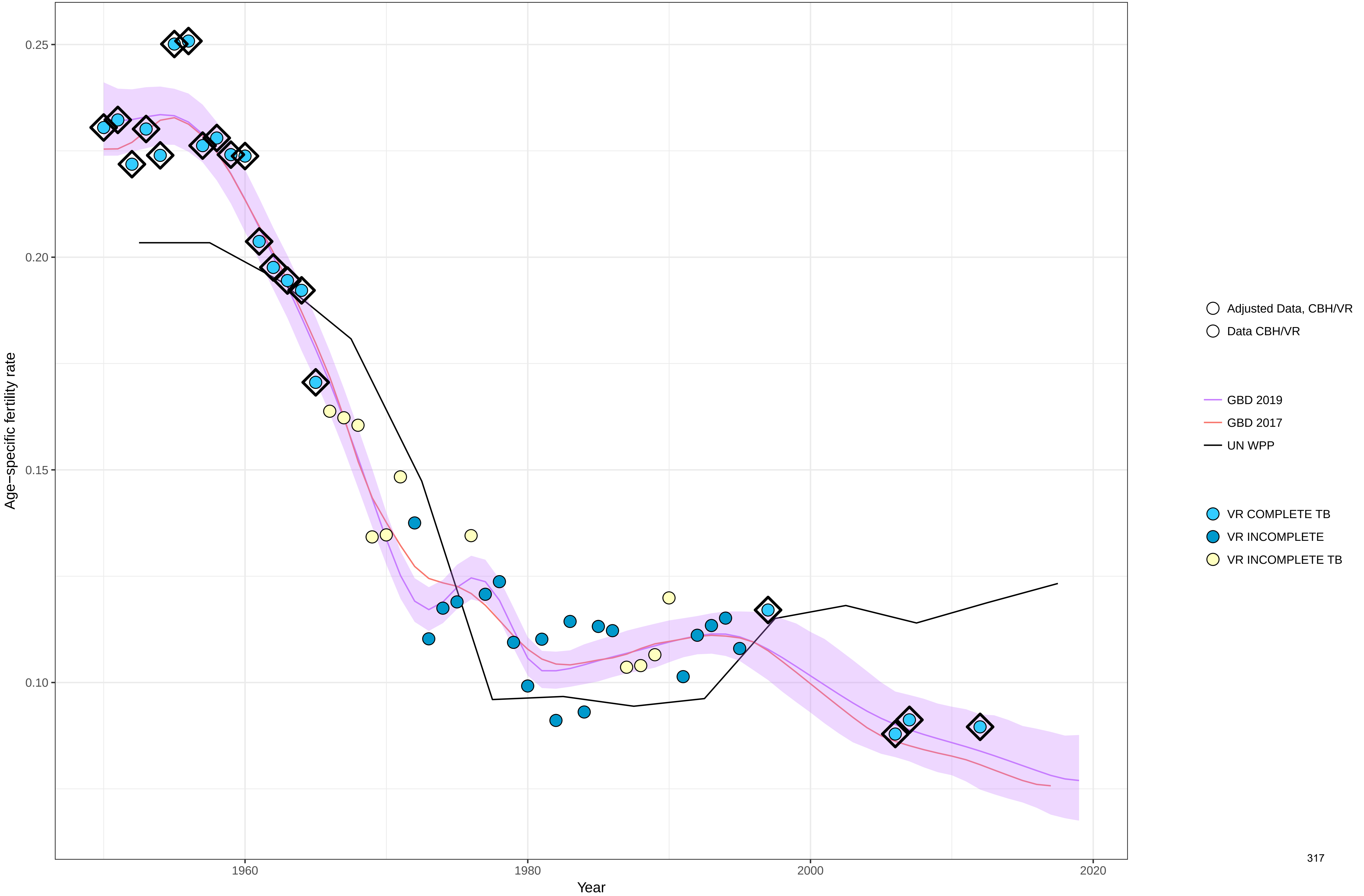

Age−specific fertility fitted trend lines, 1950−2019: Antigua and Barbuda, Ages: 30−34
Data points enclosed with a diamond are reference sources

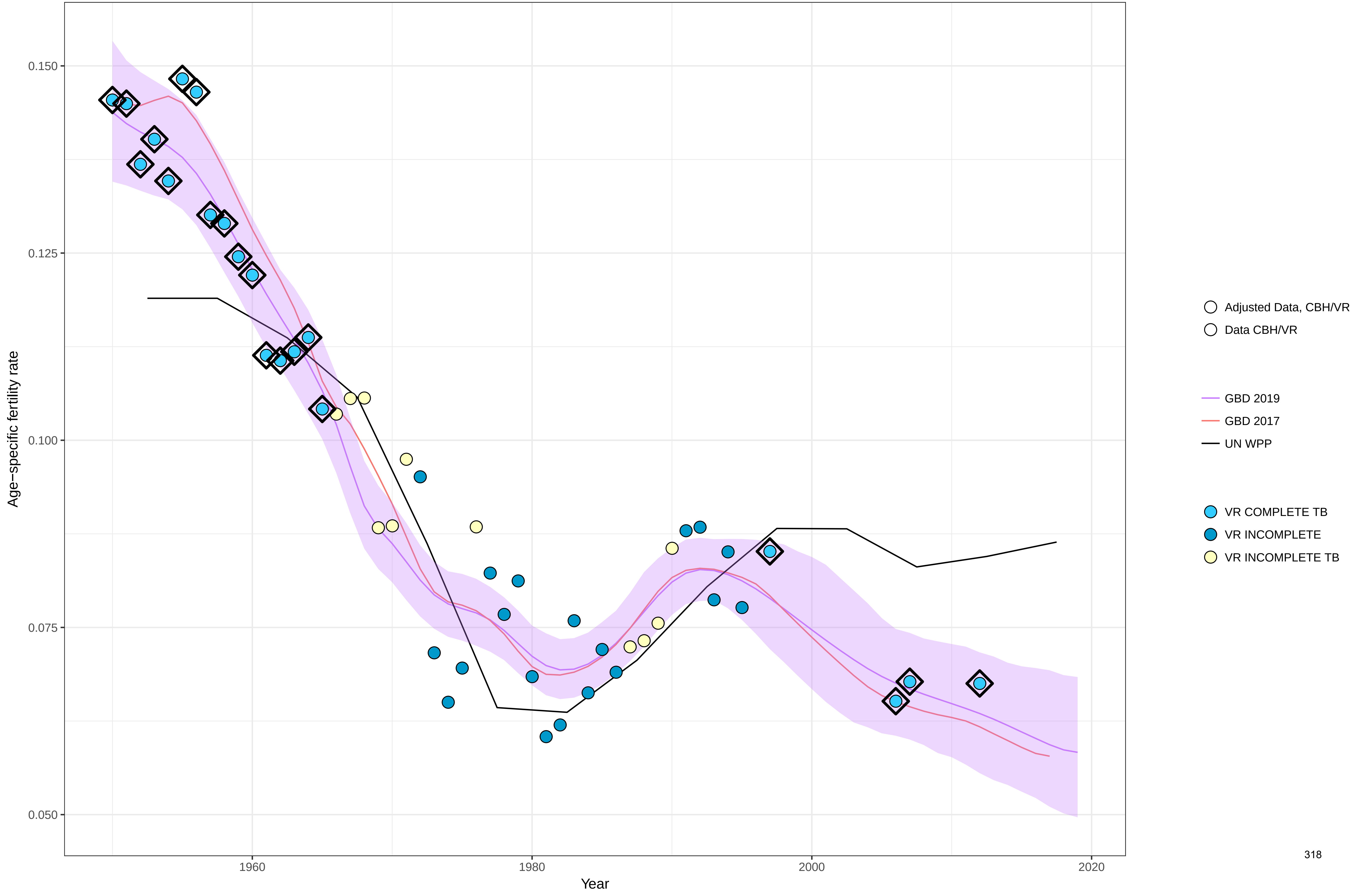

Age−specific fertility fitted trend lines, 1950−2019: Antigua and Barbuda, Ages: 35−39
Data points enclosed with a diamond are reference sources

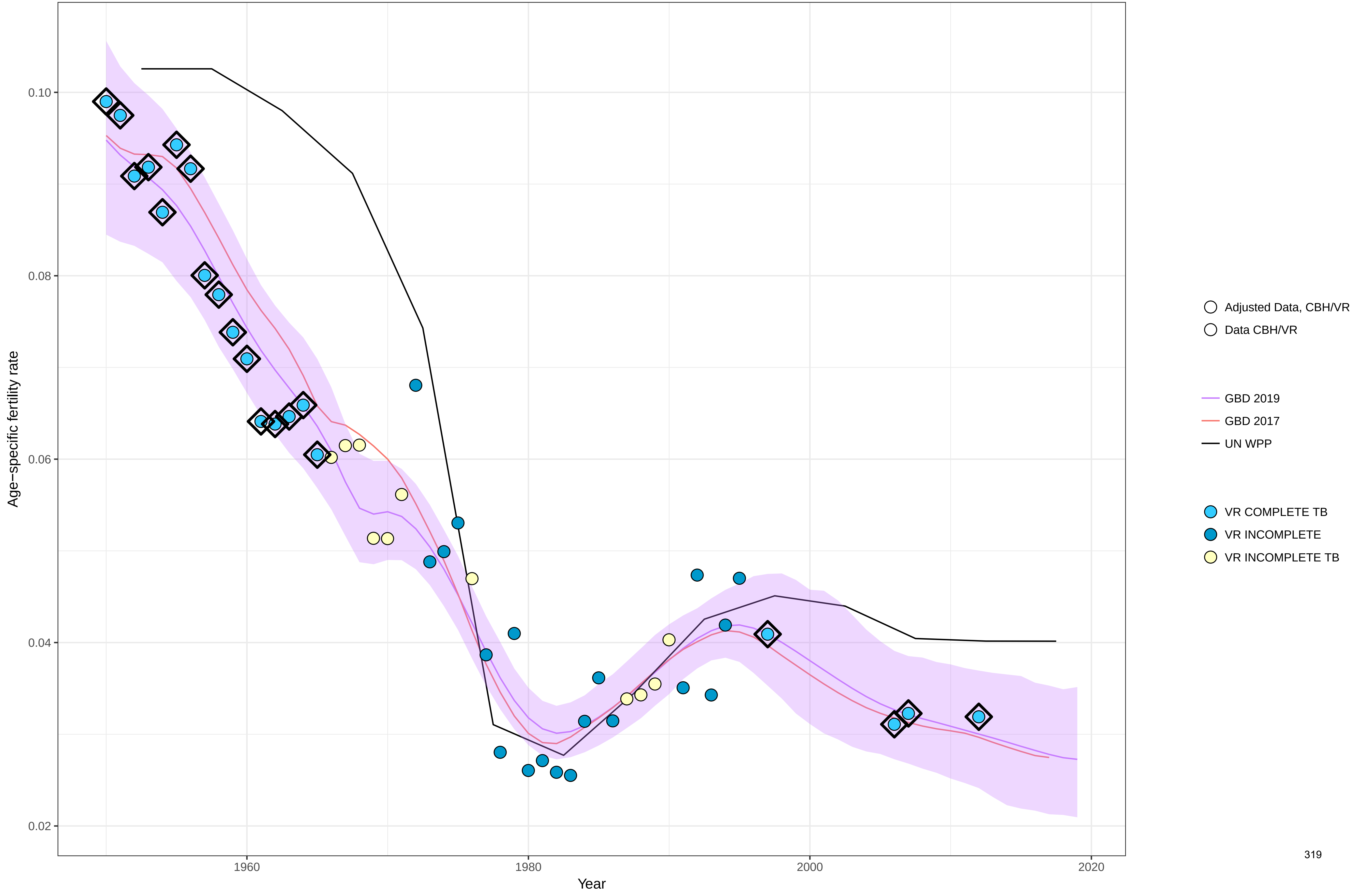

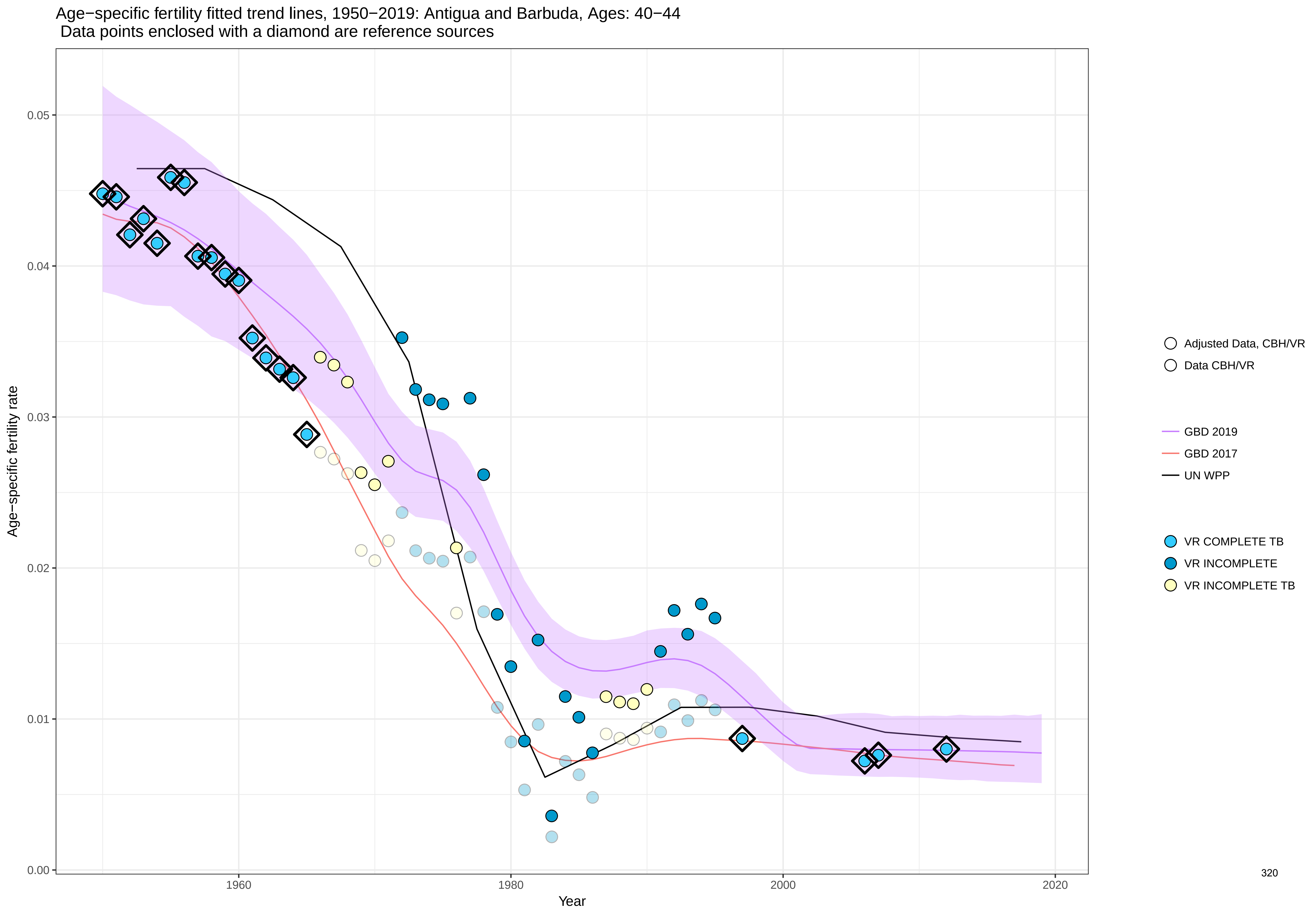
Age−specific fertility fitted trend lines, 1950−2019: Antigua and Barbuda, Ages: 40−44
Data points enclosed with a diamond are reference sources
Age−specific fertility rate
Year
0.00
0.01
0.02
0.03
0.04
0.05
1960
1980
2000
2020
Adjusted Data, CBH/VR
Data CBH/VR
GBD 2019
GBD 2017
UN WPP
VR COMPLETE TB
VR INCOMPLETE
VR INCOMPLETE TB

Age−specific fertility fitted trend lines, 1950−2019: Antigua and Barbuda, Ages: 45−49
Data points enclosed with a diamond are reference sources

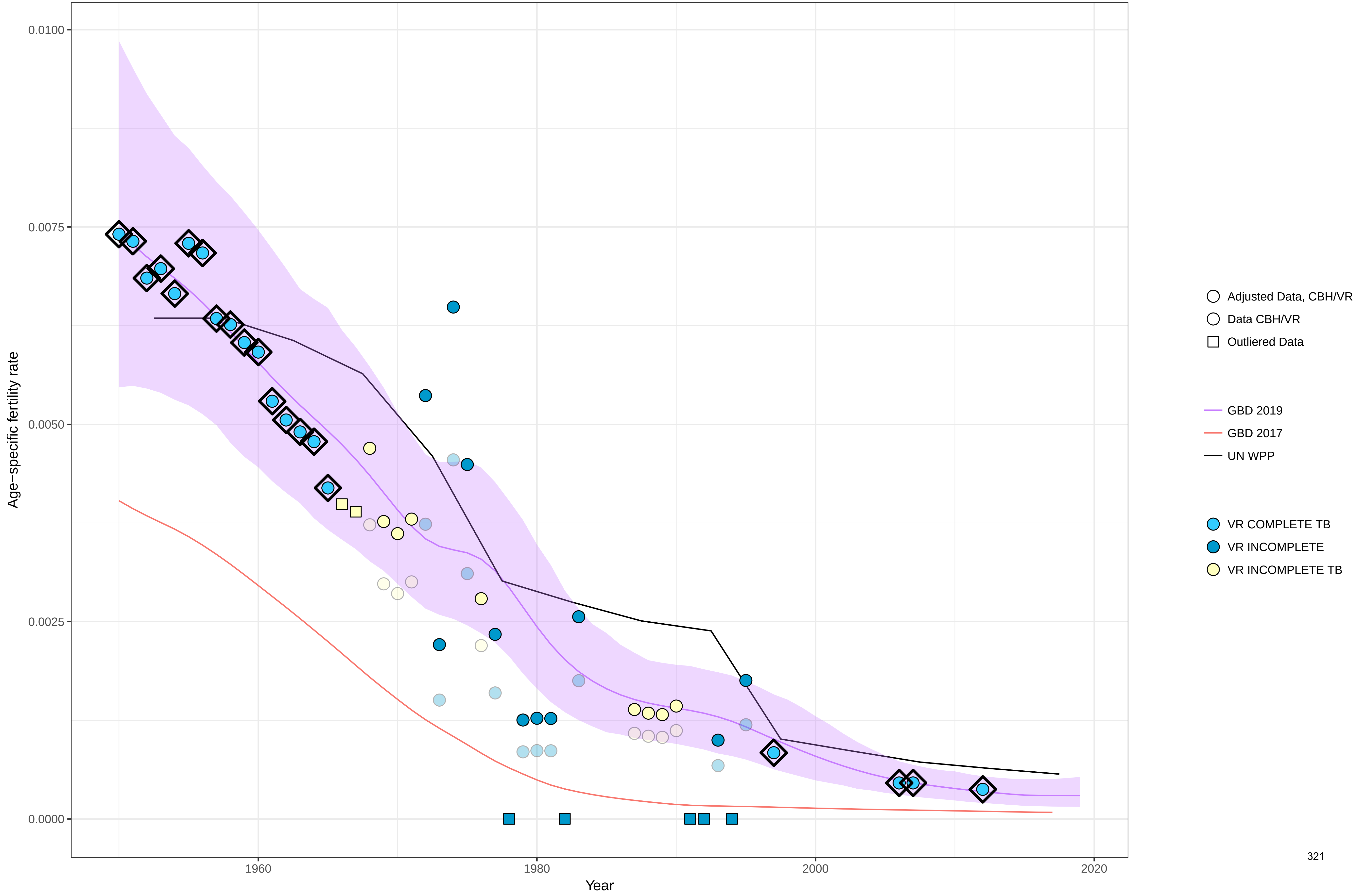

Age−specific fertility fitted trend lines, 1950−2019: Antigua and Barbuda, Ages: 50−54
Data points enclosed with a diamond are reference sources

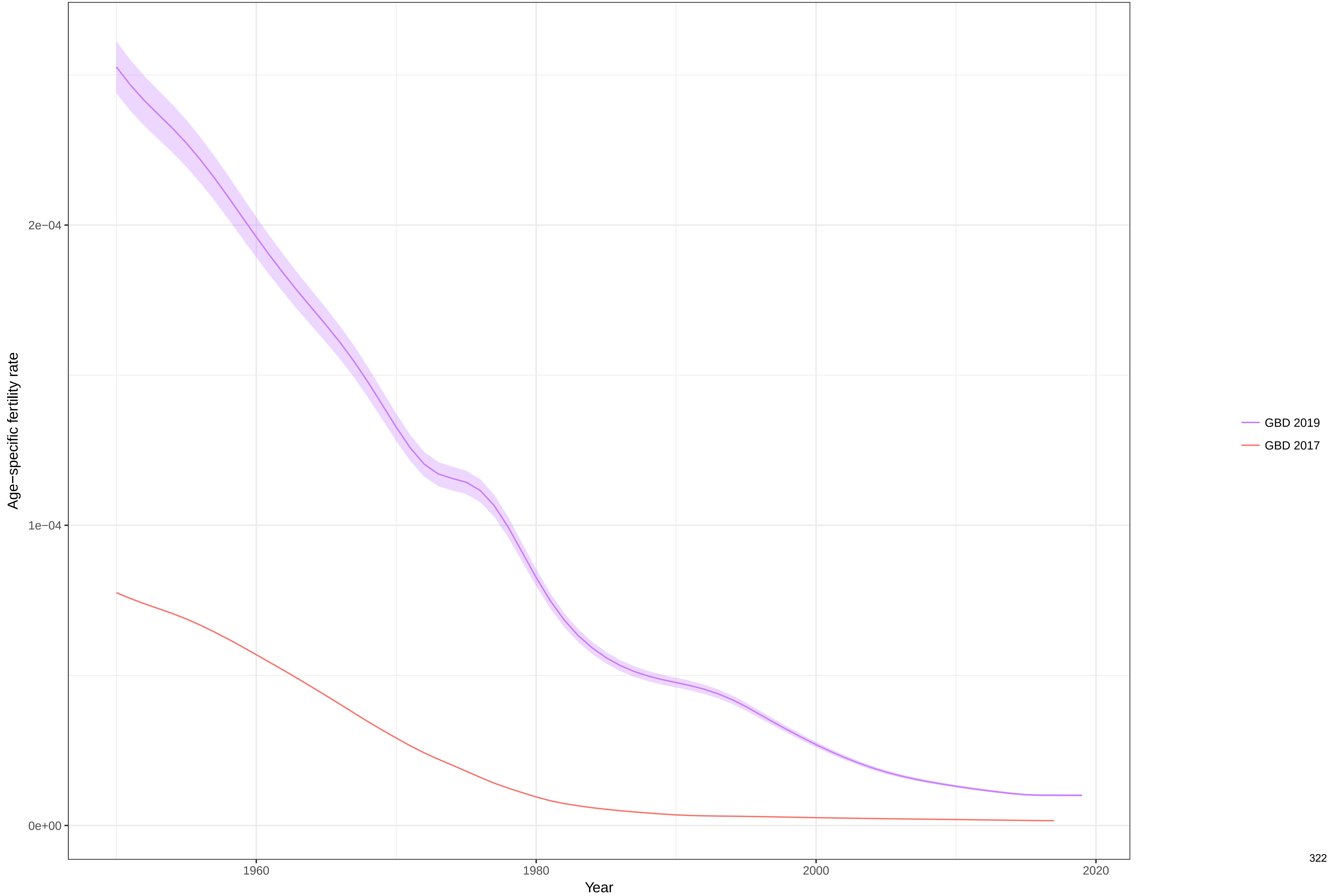

Total fertility rate fitted trend lines, 1950−2019: Antigua and Barbuda TFR
Data points enclosed with a diamond are reference sources

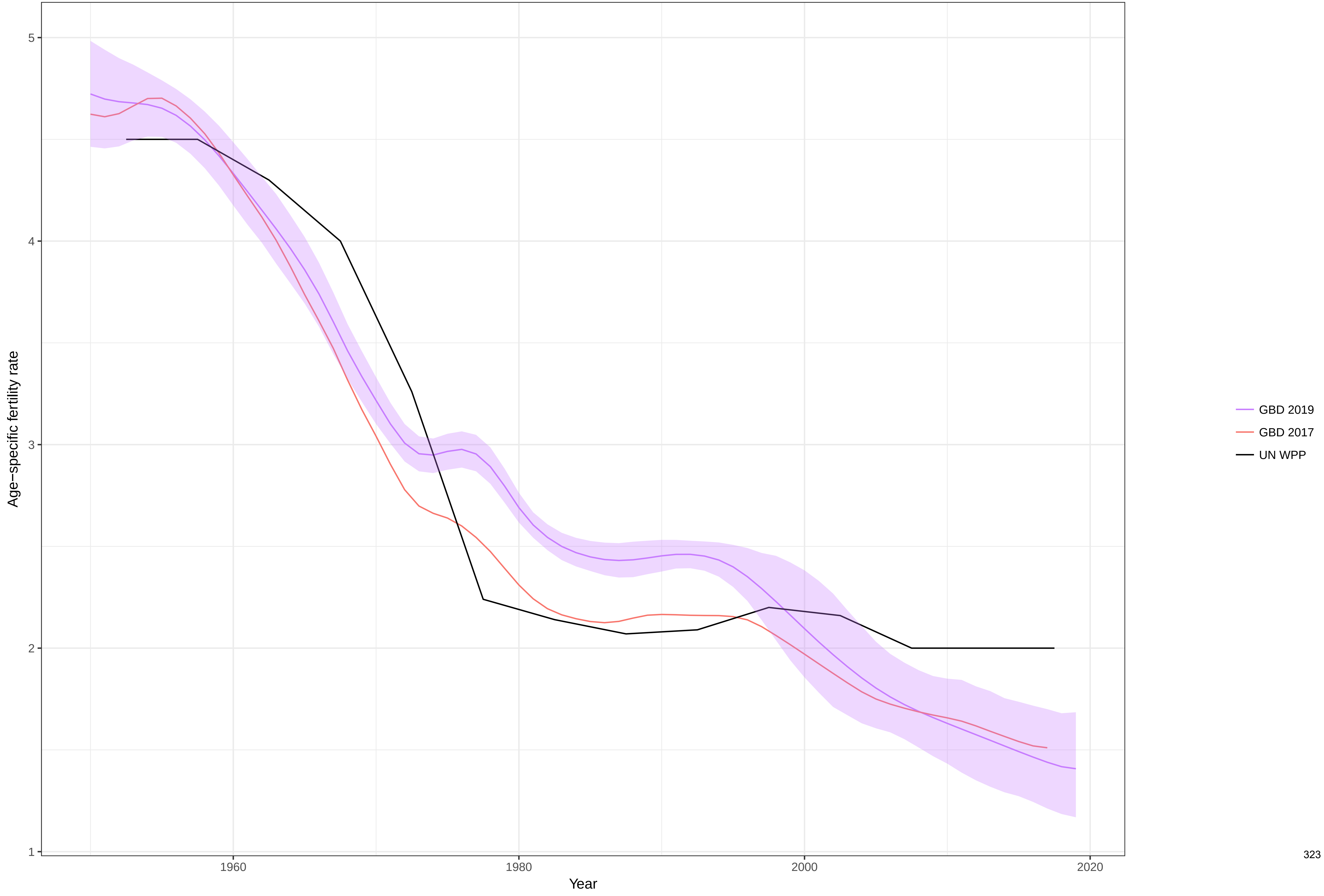

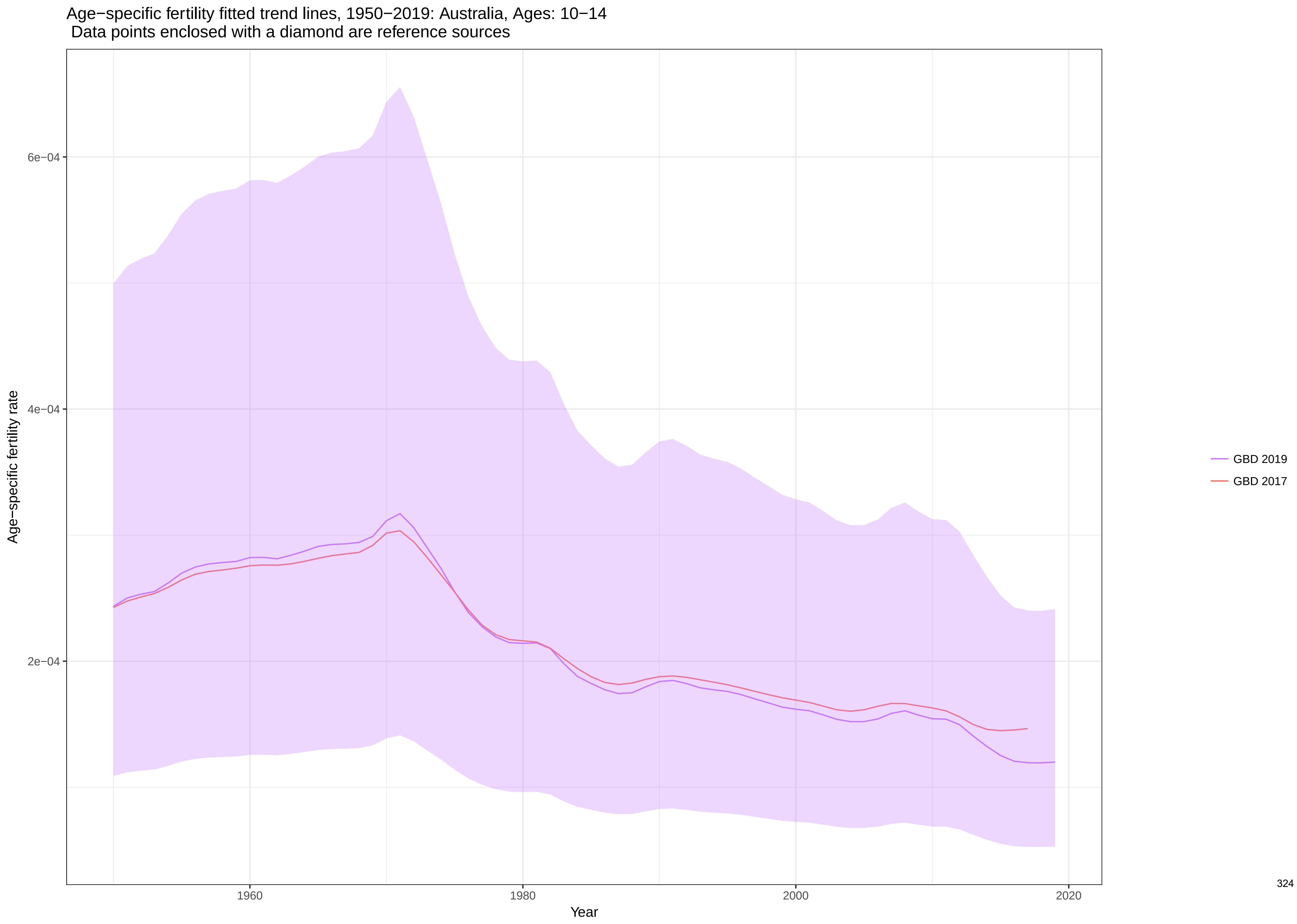
Age−specific fertility fitted trend lines, 1950−2019: Australia, Ages: 10−14
Data points enclosed with a diamond are reference sources
Age−specific fertility rate
6e−04
4e−04
2e−04
1960
1980
2000
2020
Year
GBD 2019
GBD 2017

Age−specific fertility fitted trend lines, 1950−2019: Australia, Ages: 15−19
Data points enclosed with a diamond are reference sources

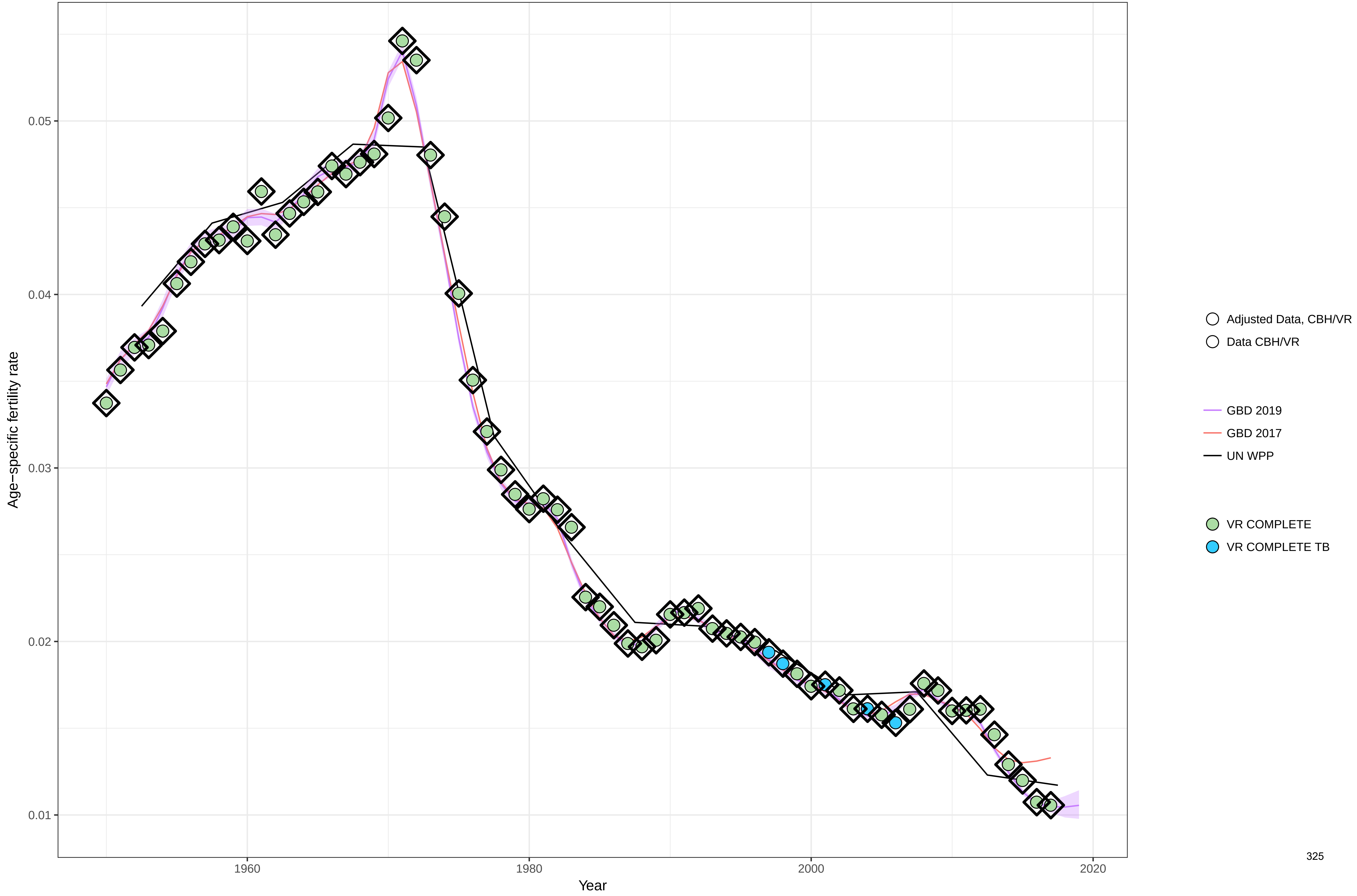

Age−specific fertility fitted trend lines, 1950−2019: Australia, Ages: 20−24
Data points enclosed with a diamond are reference sources

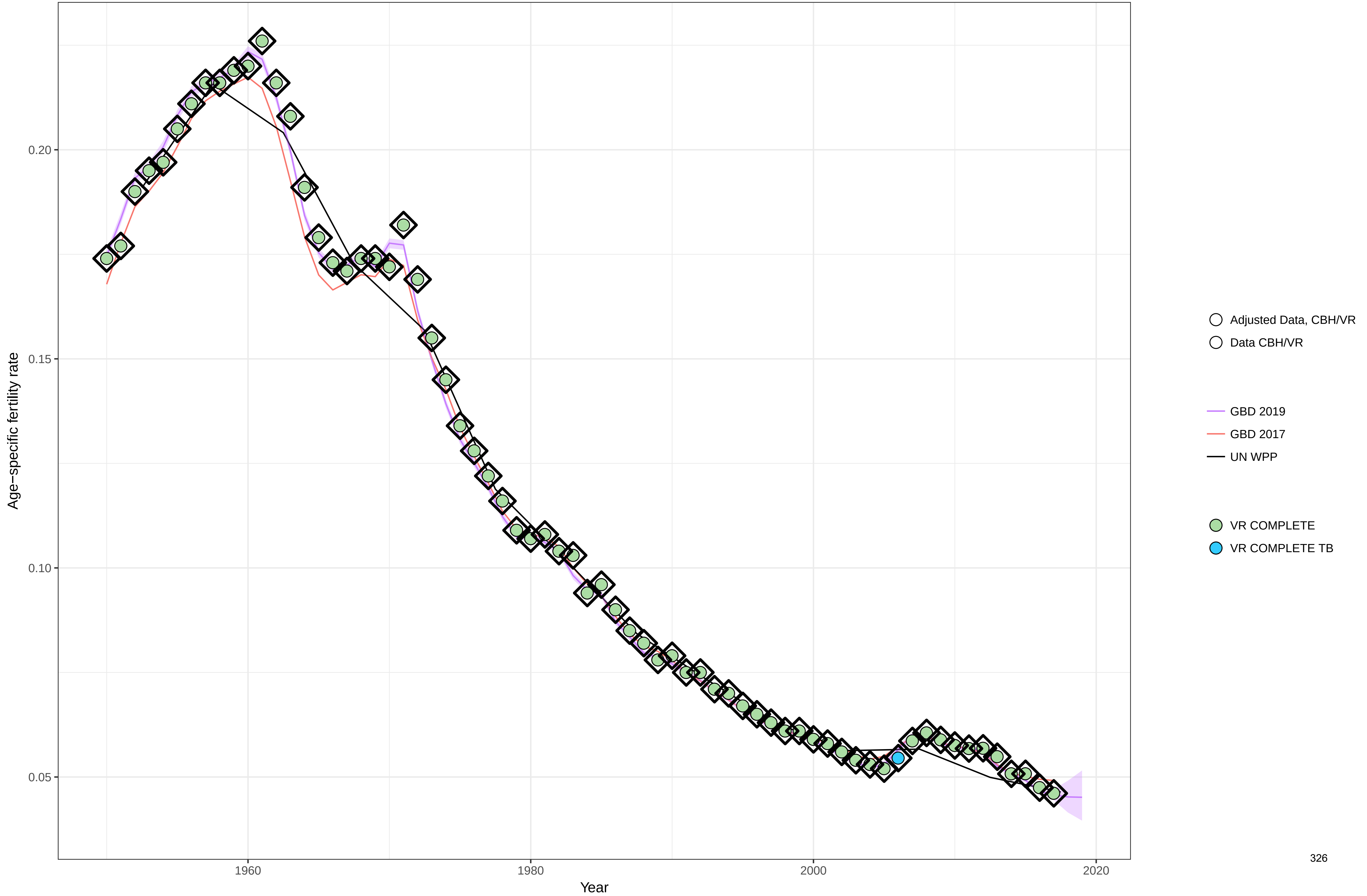

Age−specific fertility fitted trend lines, 1950−2019: Australia, Ages: 25−29
Data points enclosed with a diamond are reference sources

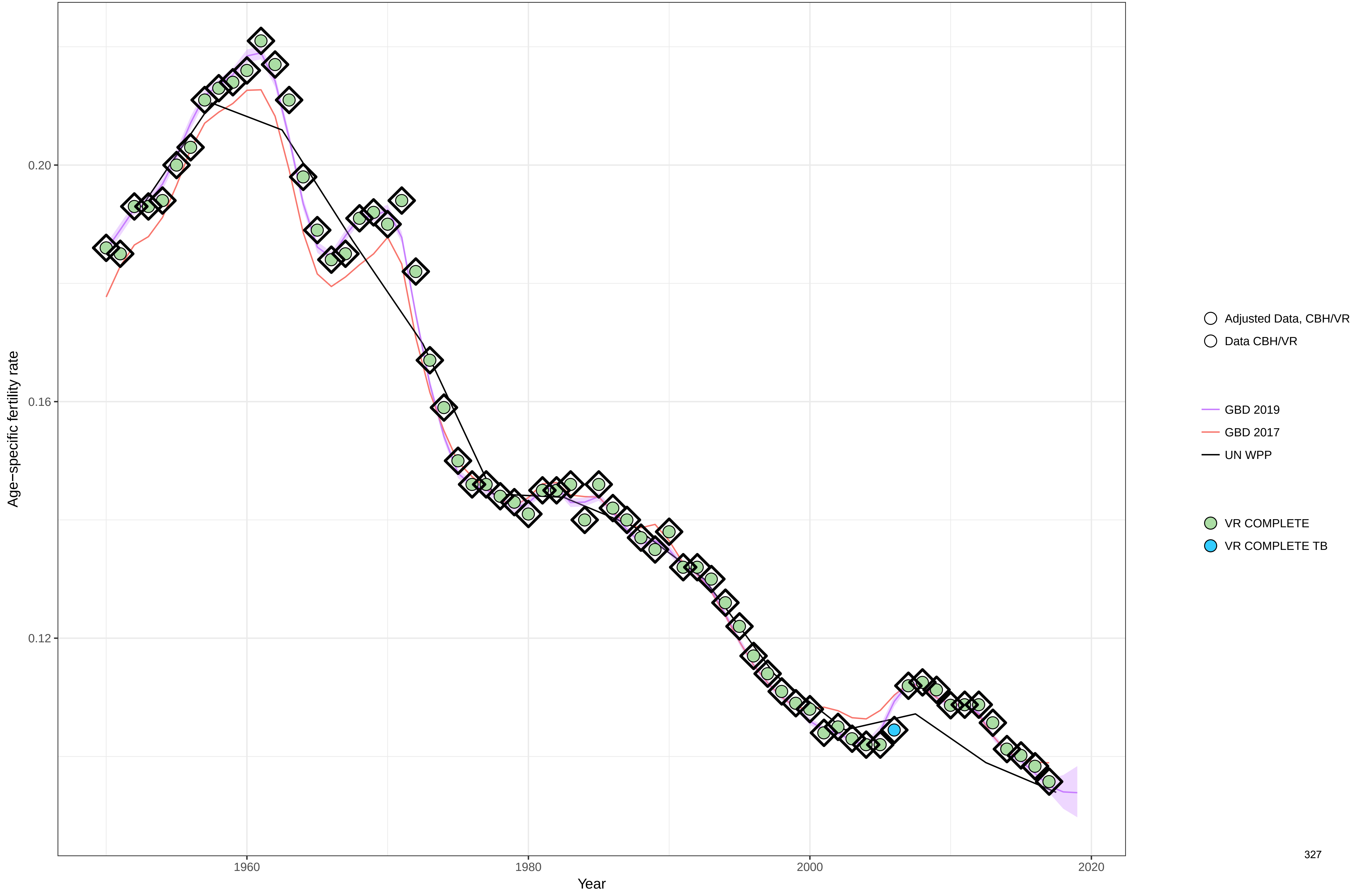

Age−specific fertility fitted trend lines, 1950−2019: Australia, Ages: 30−34
Data points enclosed with a diamond are reference sources

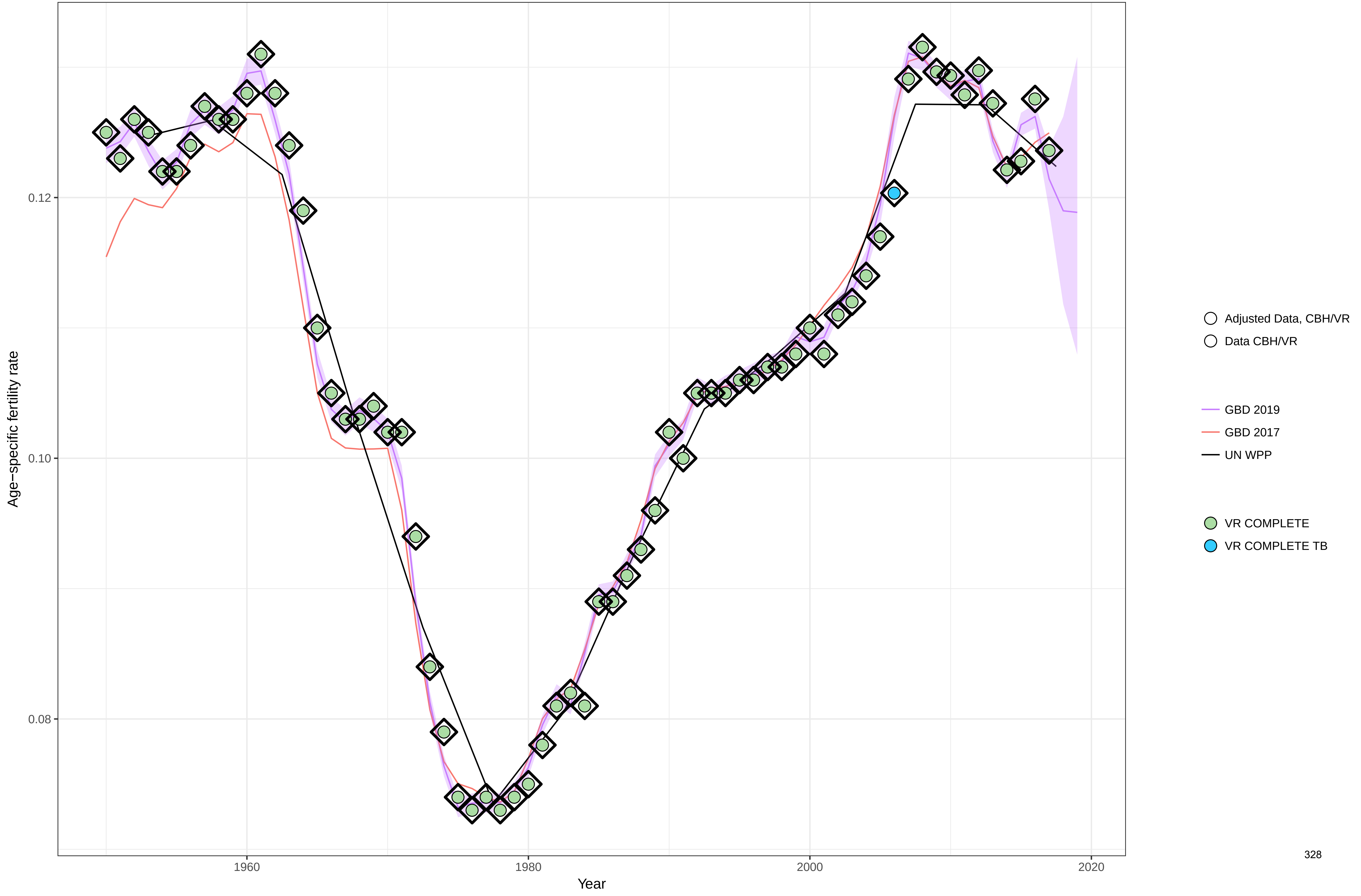

Age−specific fertility fitted trend lines, 1950−2019: Australia, Ages: 35−39
Data points enclosed with a diamond are reference sources

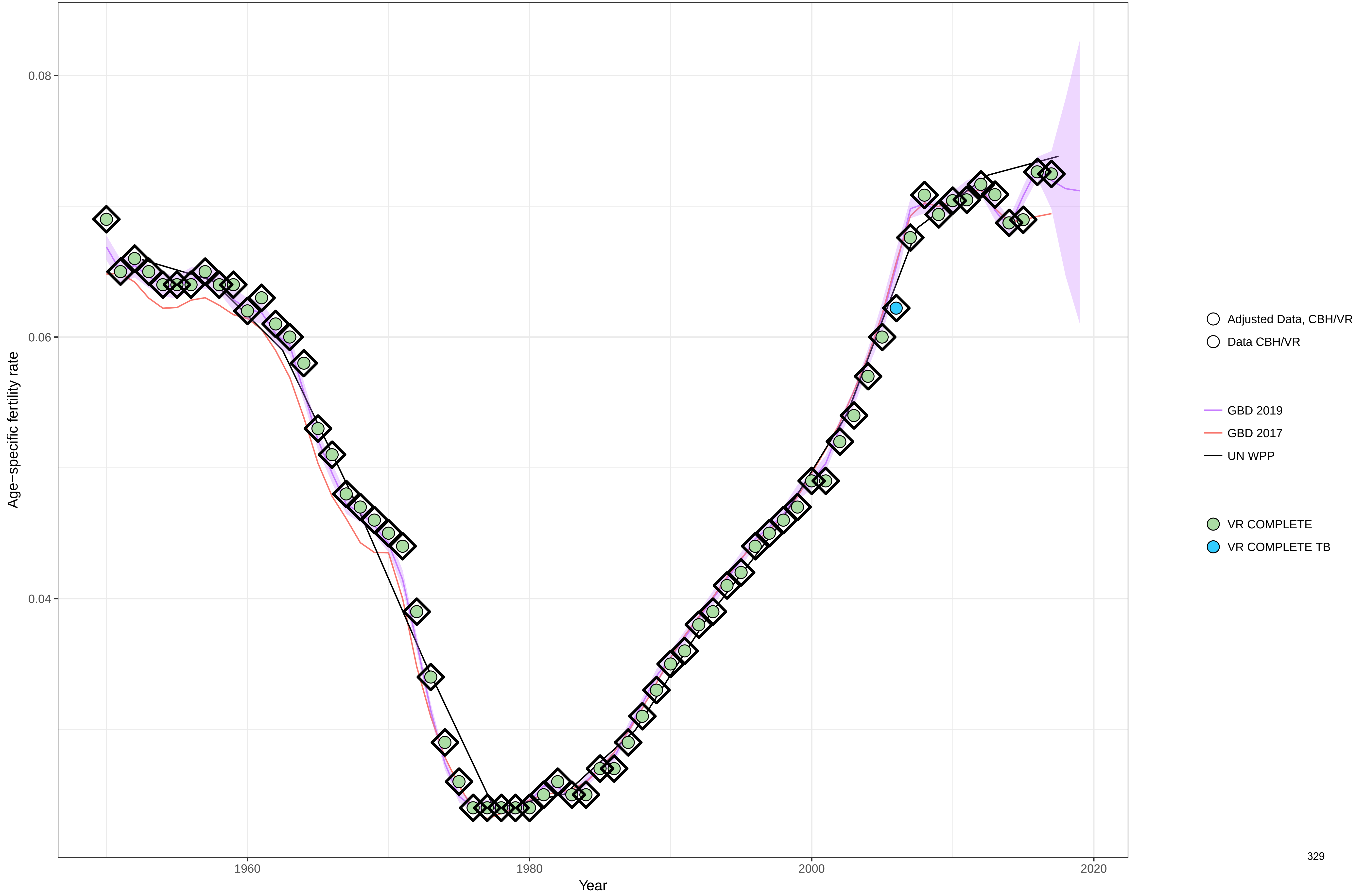

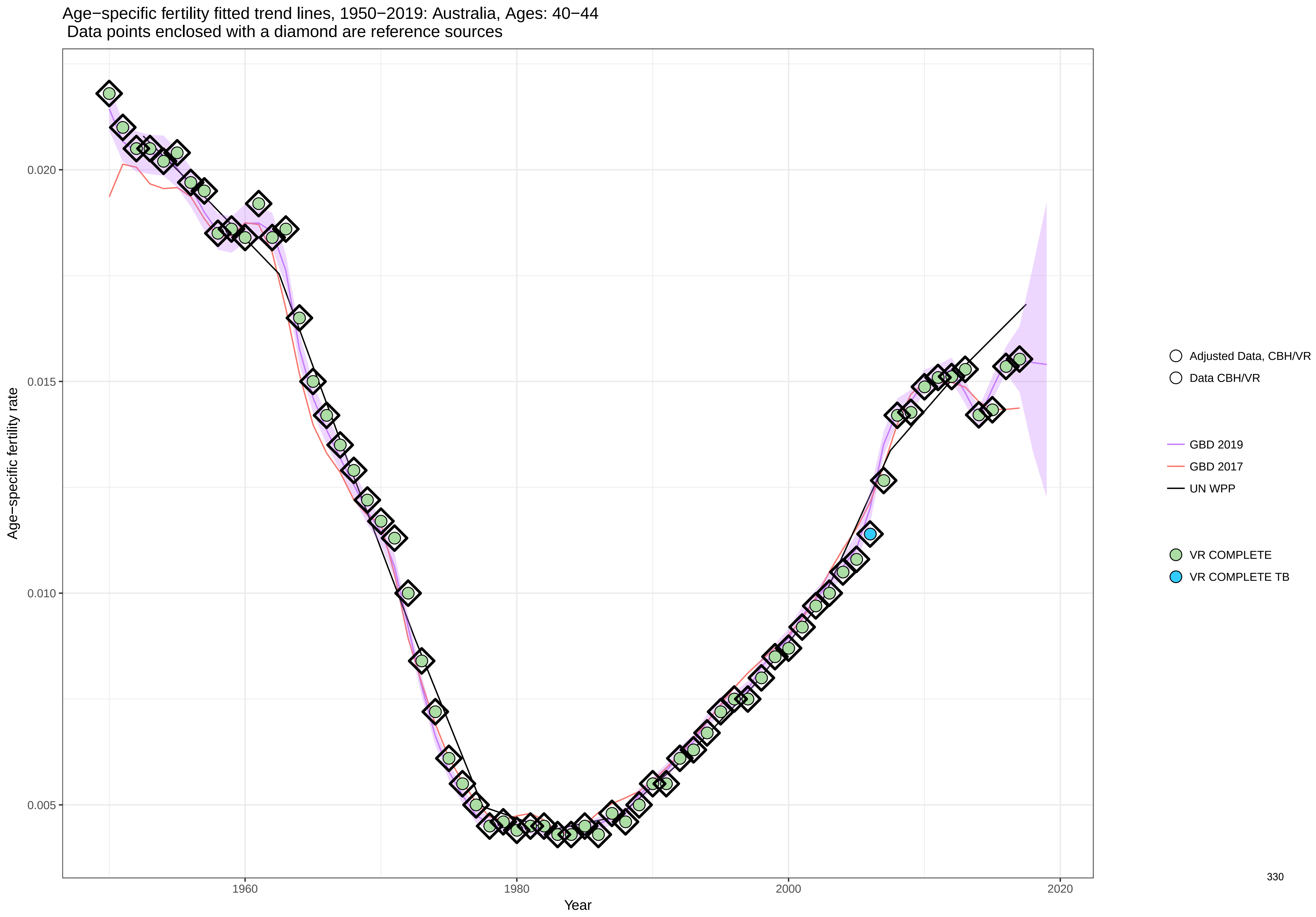
Age−specific fertility fitted trend lines, 1950−2019: Australia, Ages: 40−44
Data points enclosed with a diamond are reference sources
Age−specific fertility rate
0.020
0.015
0.010
0.005
1960
1980
2000
2020
Year
Adjusted Data, CBH/VR
Data CBH/VR
GBD 2019
GBD 2017
UN WPP
VR COMPLETE
VR COMPLETE TB

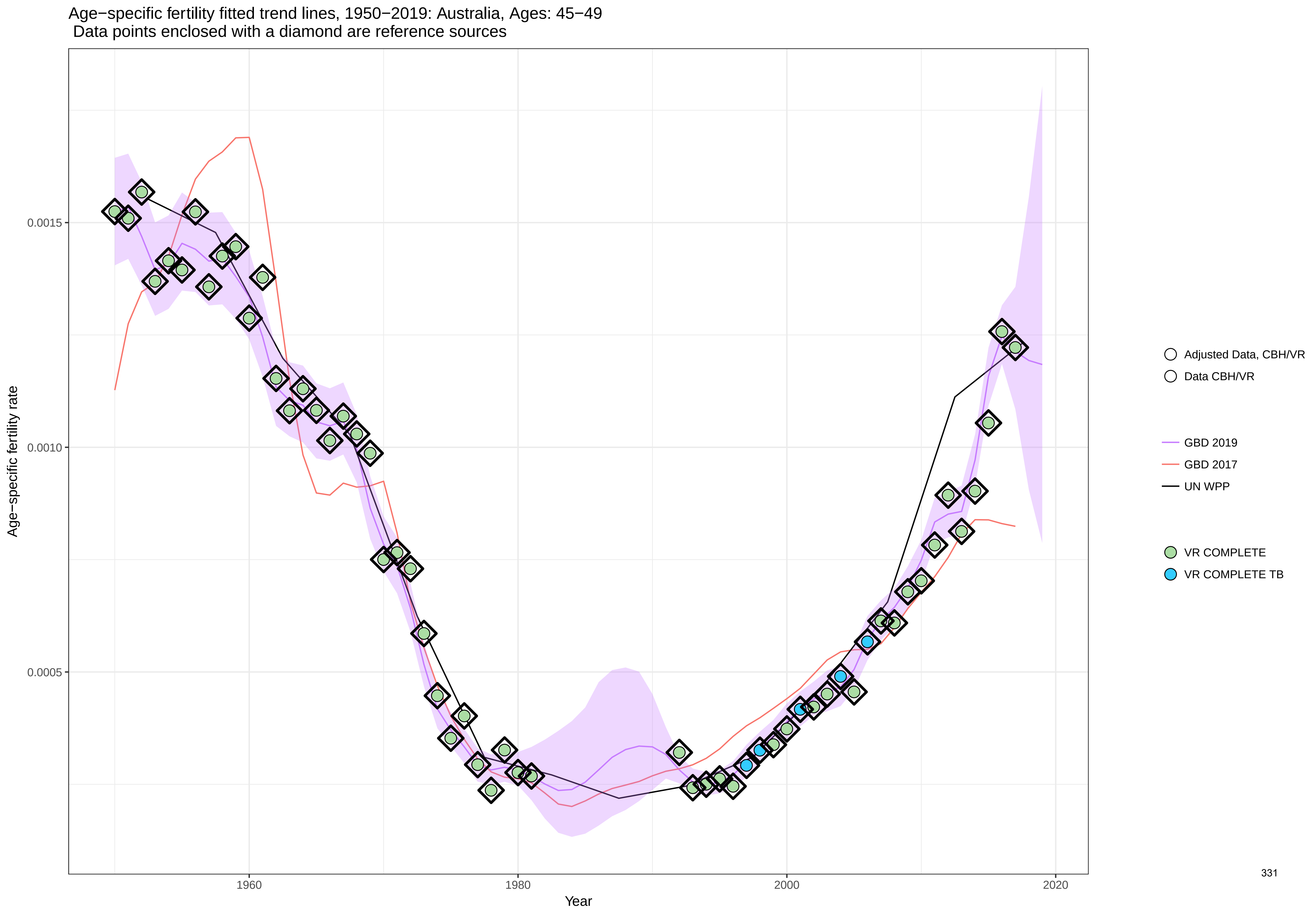

Age−specific fertility fitted trend lines, 1950−2019: Australia, Ages: 45−49
Data points enclosed with a diamond are reference sources
Age−specific fertility rate
0.0015
0.0010
0.0005
1960
1980
2000
2020
Year
Adjusted Data, CBH/VR
Data CBH/VR
GBD 2019
GBD 2017
UN WPP
VR COMPLETE
VR COMPLETE TB

Age−specific fertility fitted trend lines, 1950−2019: Australia, Ages: 50−54
Data points enclosed with a diamond are reference sources

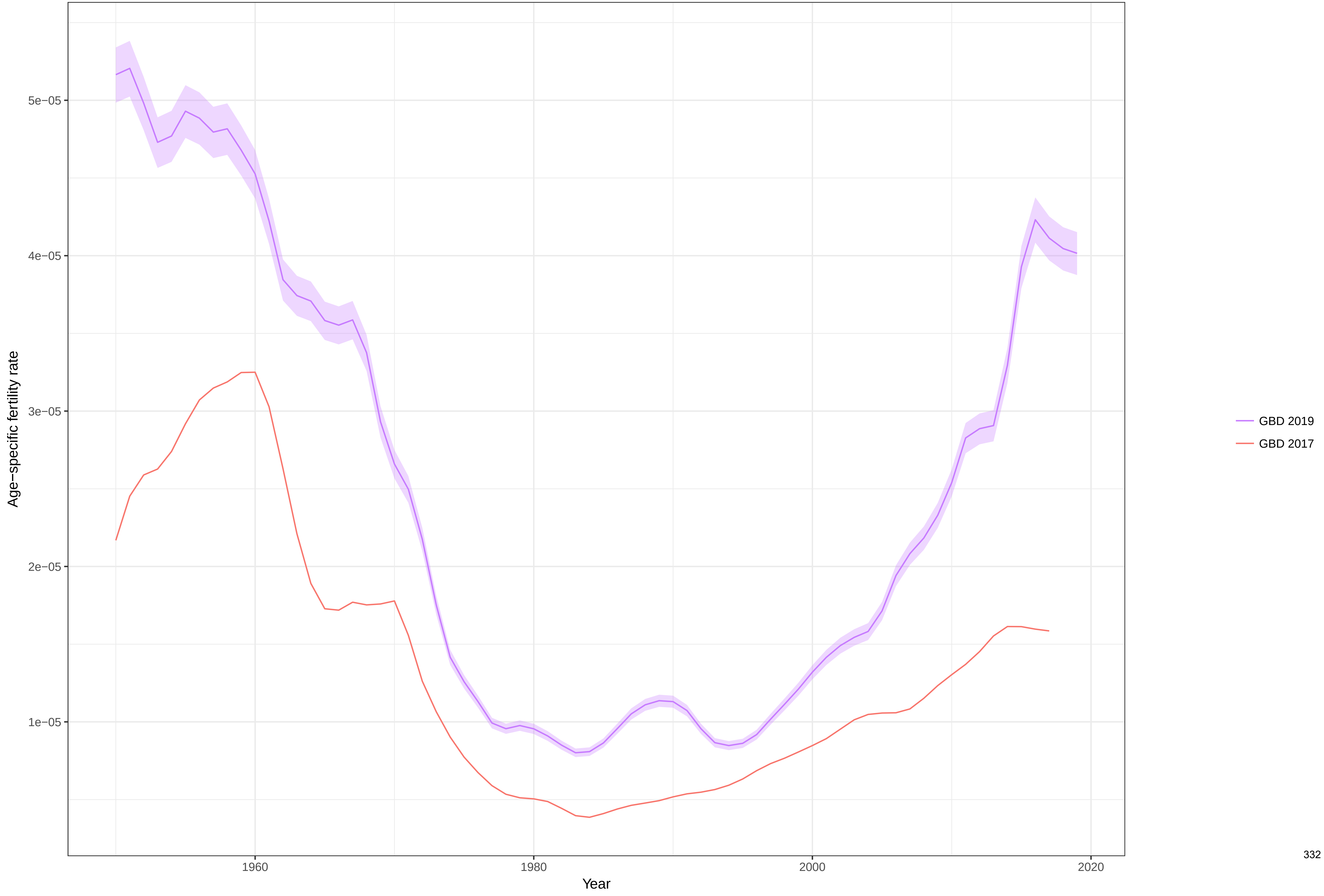

Total fertility rate fitted trend lines, 1950−2019: Australia TFR
Data points enclosed with a diamond are reference sources

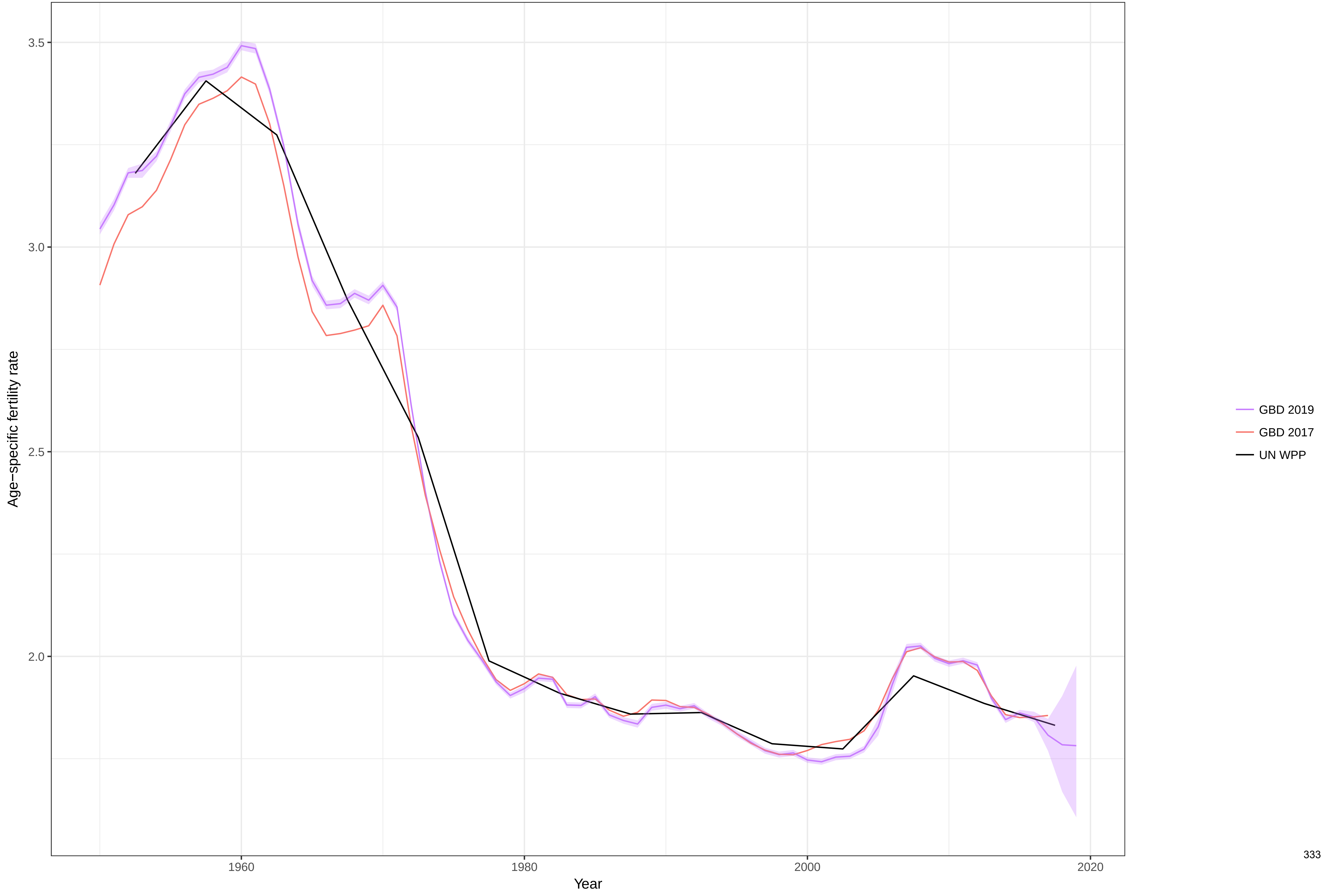

Age−specific fertility fitted trend lines, 1950−2019: Austria, Ages: 10−14
Data points enclosed with a diamond are reference sources

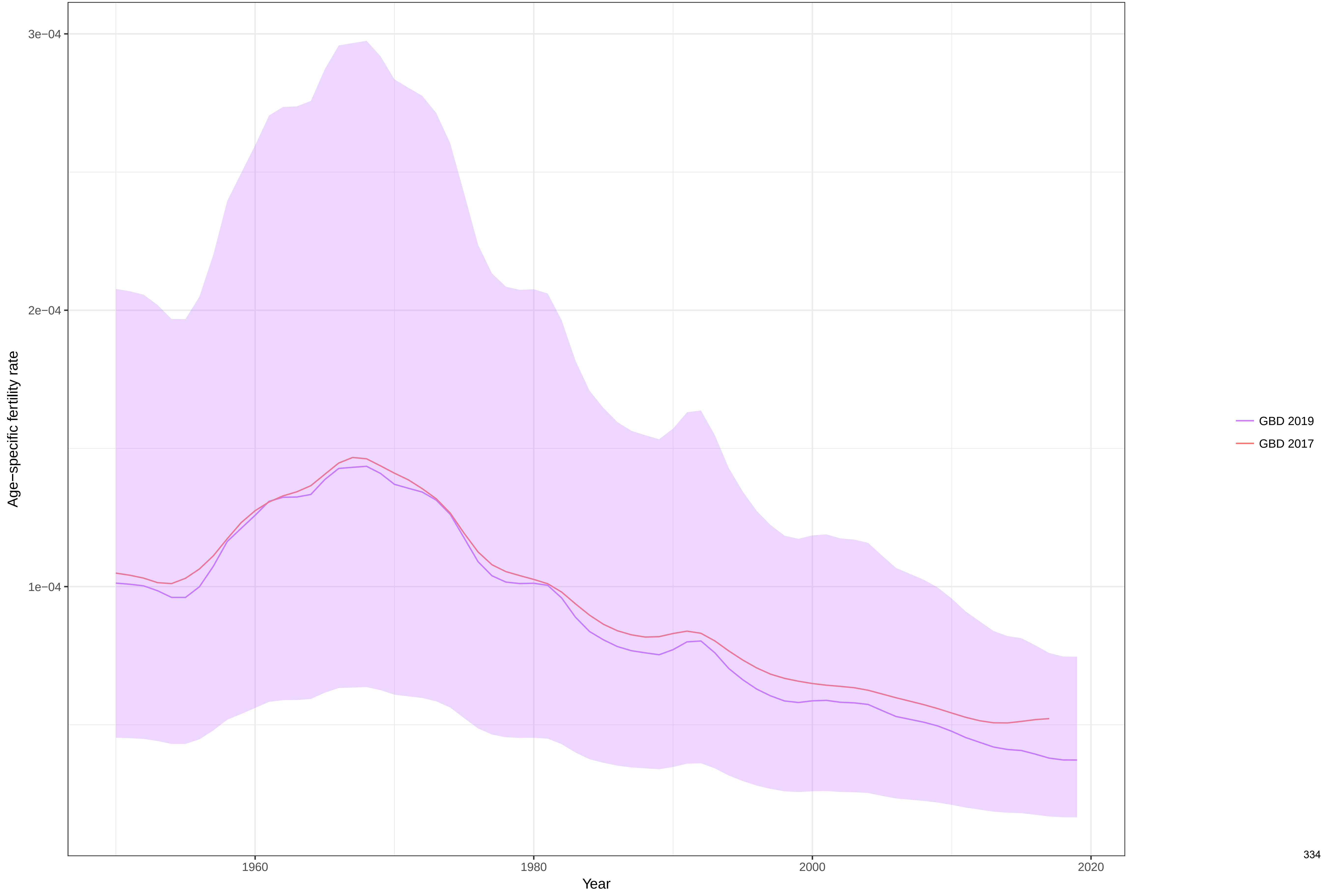

Age−specific fertility fitted trend lines, 1950−2019: Austria, Ages: 15−19
Data points enclosed with a diamond are reference sources

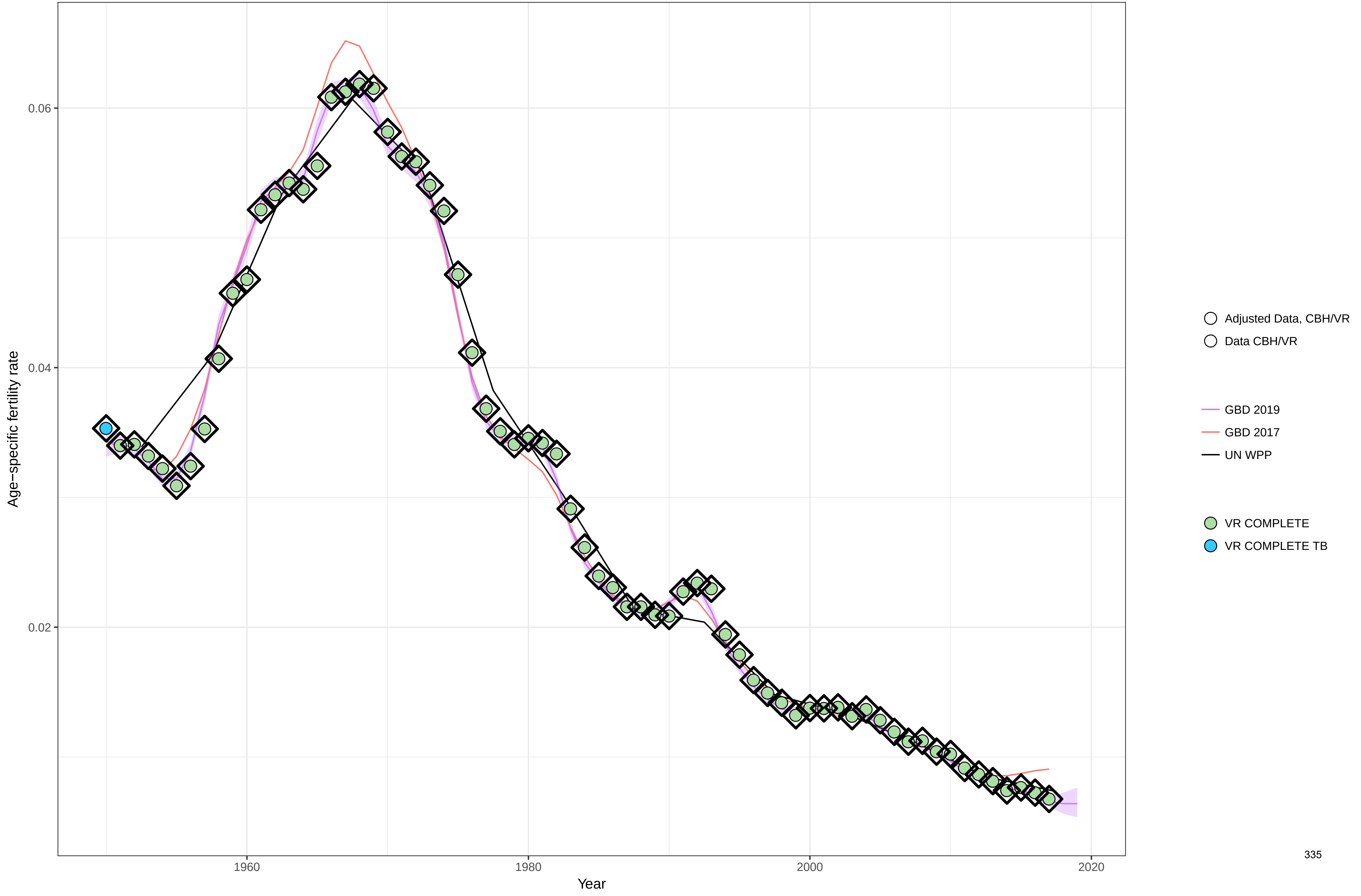

Age−specific fertility fitted trend lines, 1950−2019: Austria, Ages: 20−24
Data points enclosed with a diamond are reference sources

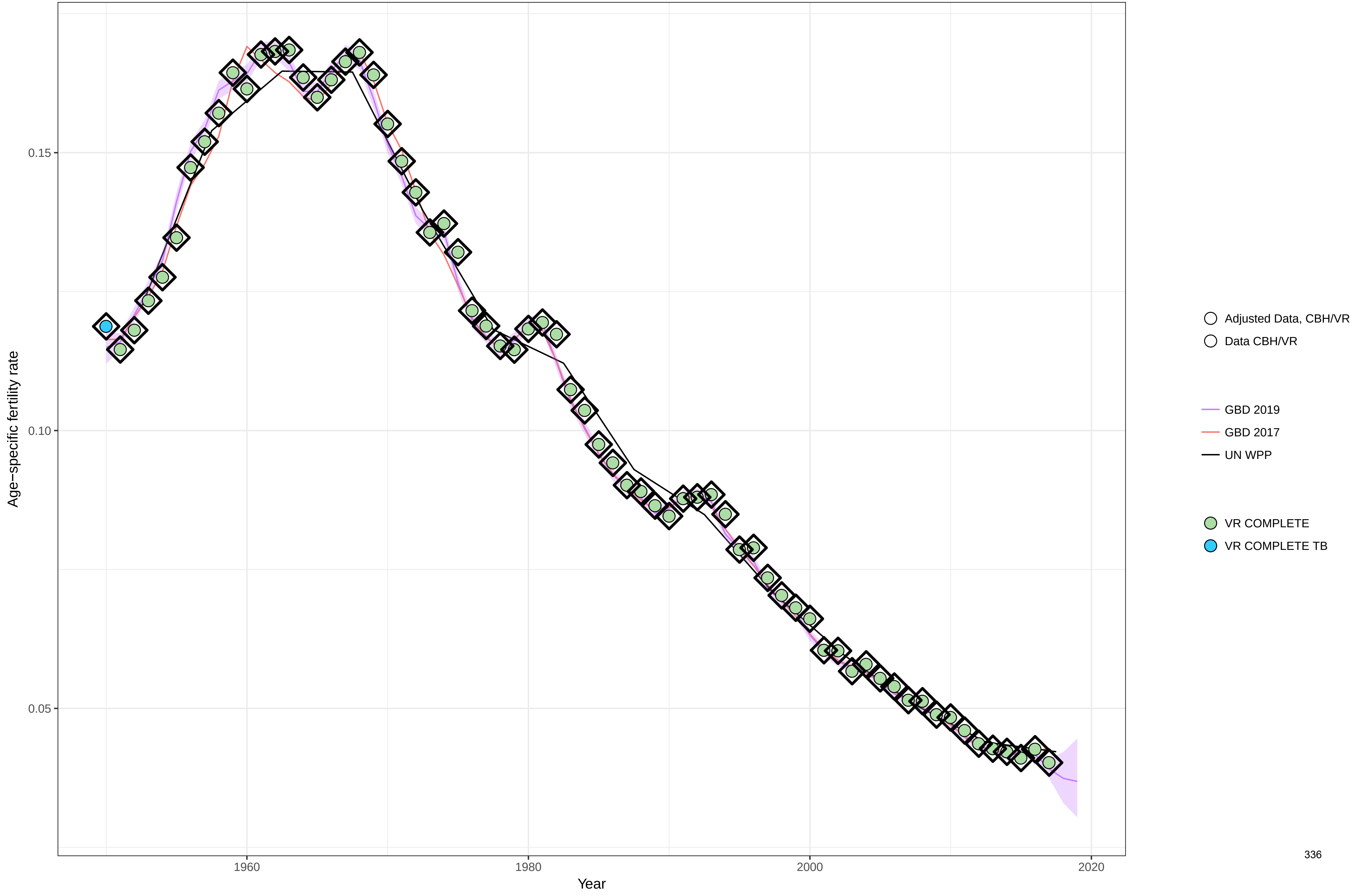

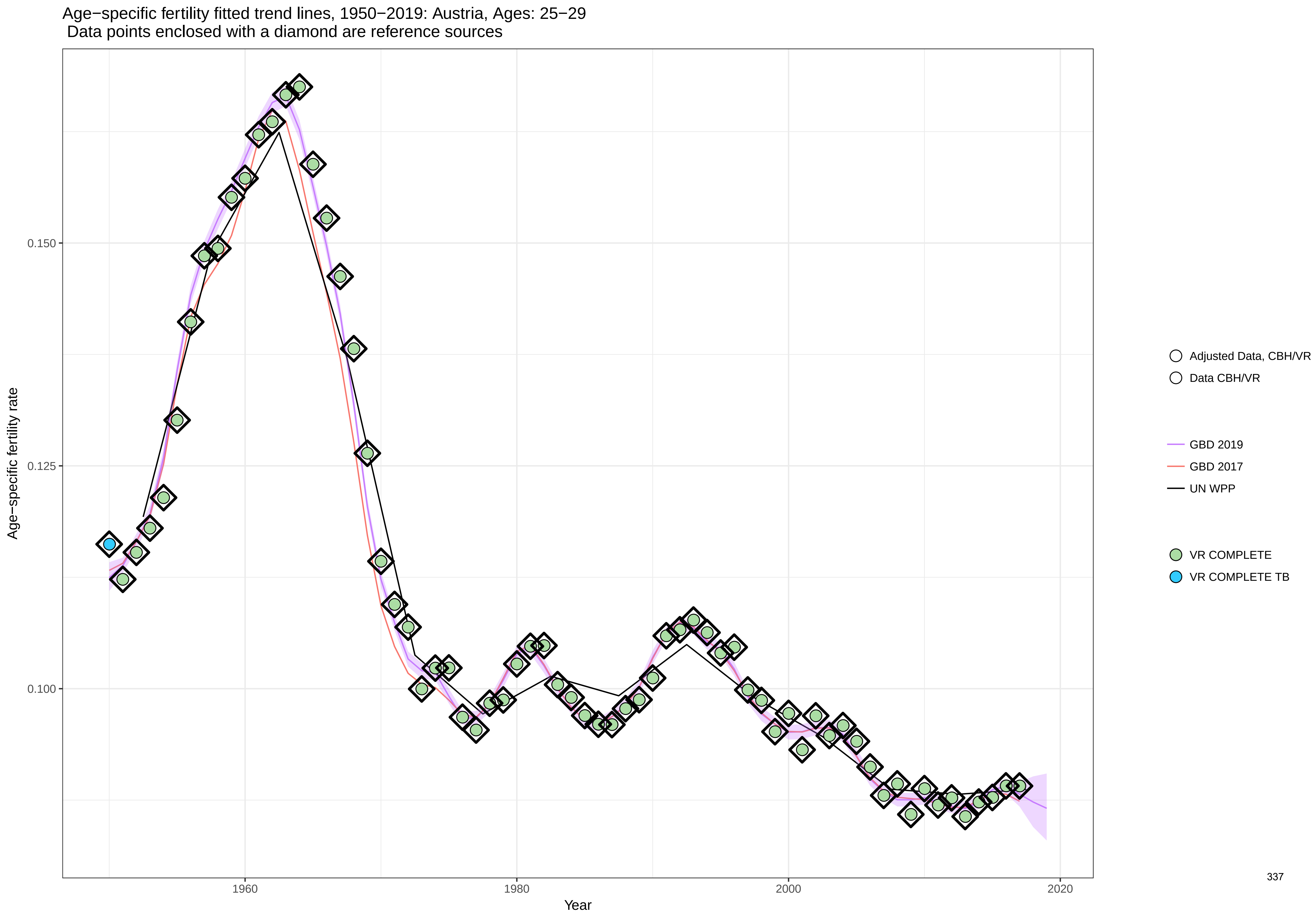

Age−specific fertility fitted trend lines, 1950−2019: Austria, Ages: 25−29
Data points enclosed with a diamond are reference sources
Age−specific fertility rate
0.150
0.125
0.100
1960
1980
2000
2020
Year
Adjusted Data, CBH/VR
Data CBH/VR
GBD 2019
GBD 2017
UN WPP
VR COMPLETE
VR COMPLETE TB

Age−specific fertility fitted trend lines, 1950−2019: Austria, Ages: 30−34
Data points enclosed with a diamond are reference sources

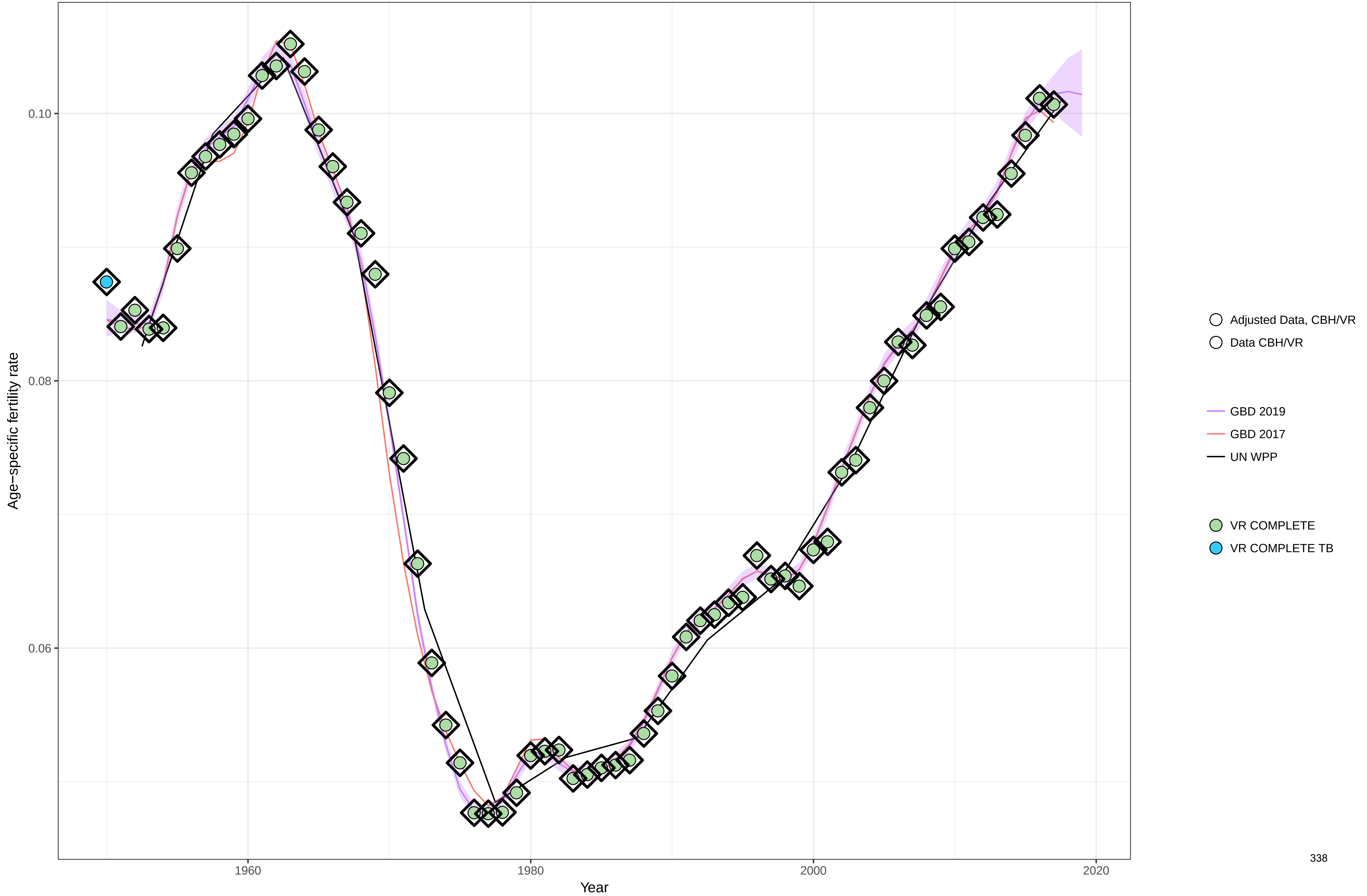

Age−specific fertility fitted trend lines, 1950−2019: Austria, Ages: 35−39
Data points enclosed with a diamond are reference sources

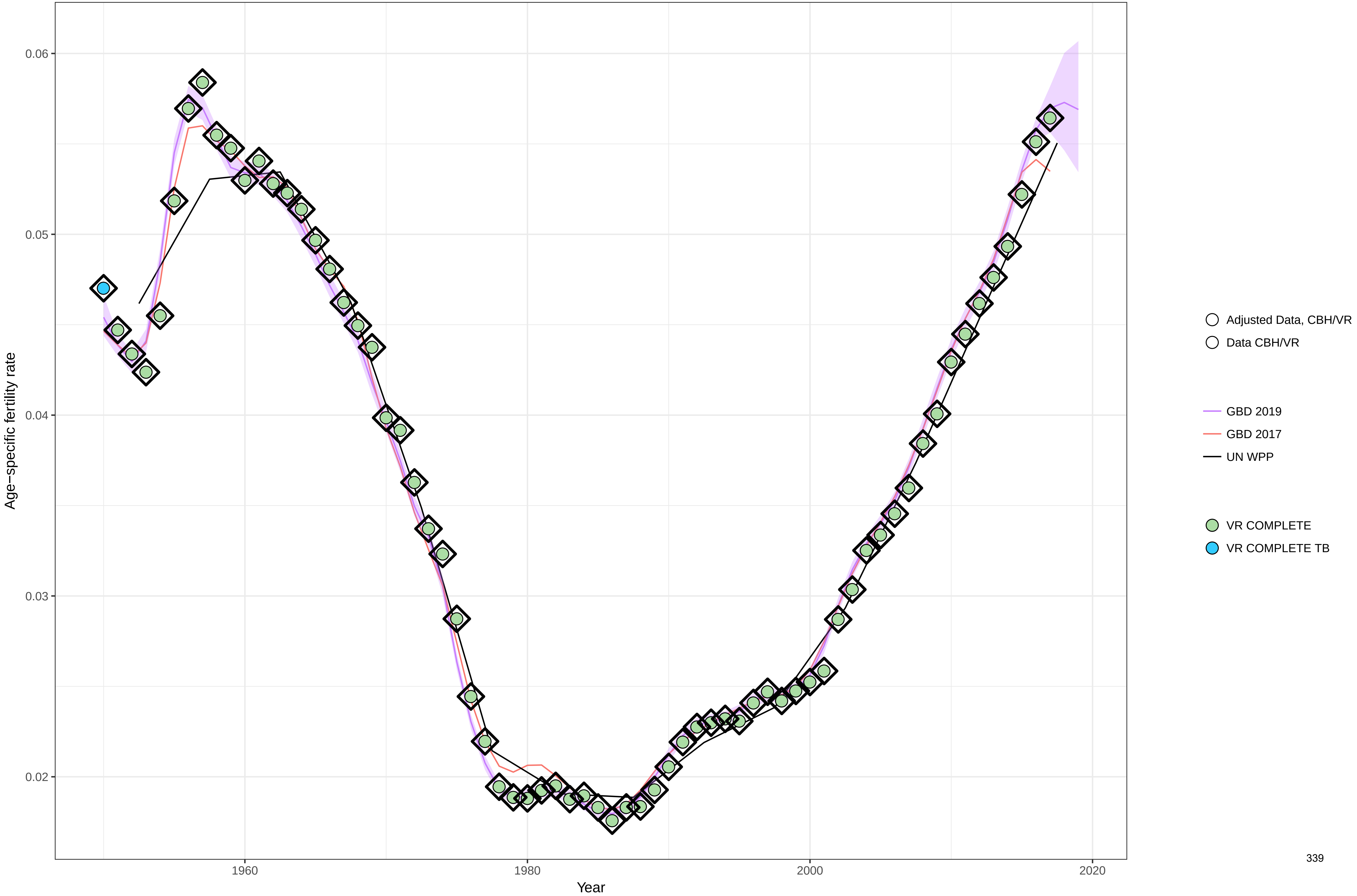

Age−specific fertility fitted trend lines, 1950−2019: Austria, Ages: 40−44
Data points enclosed with a diamond are reference sources

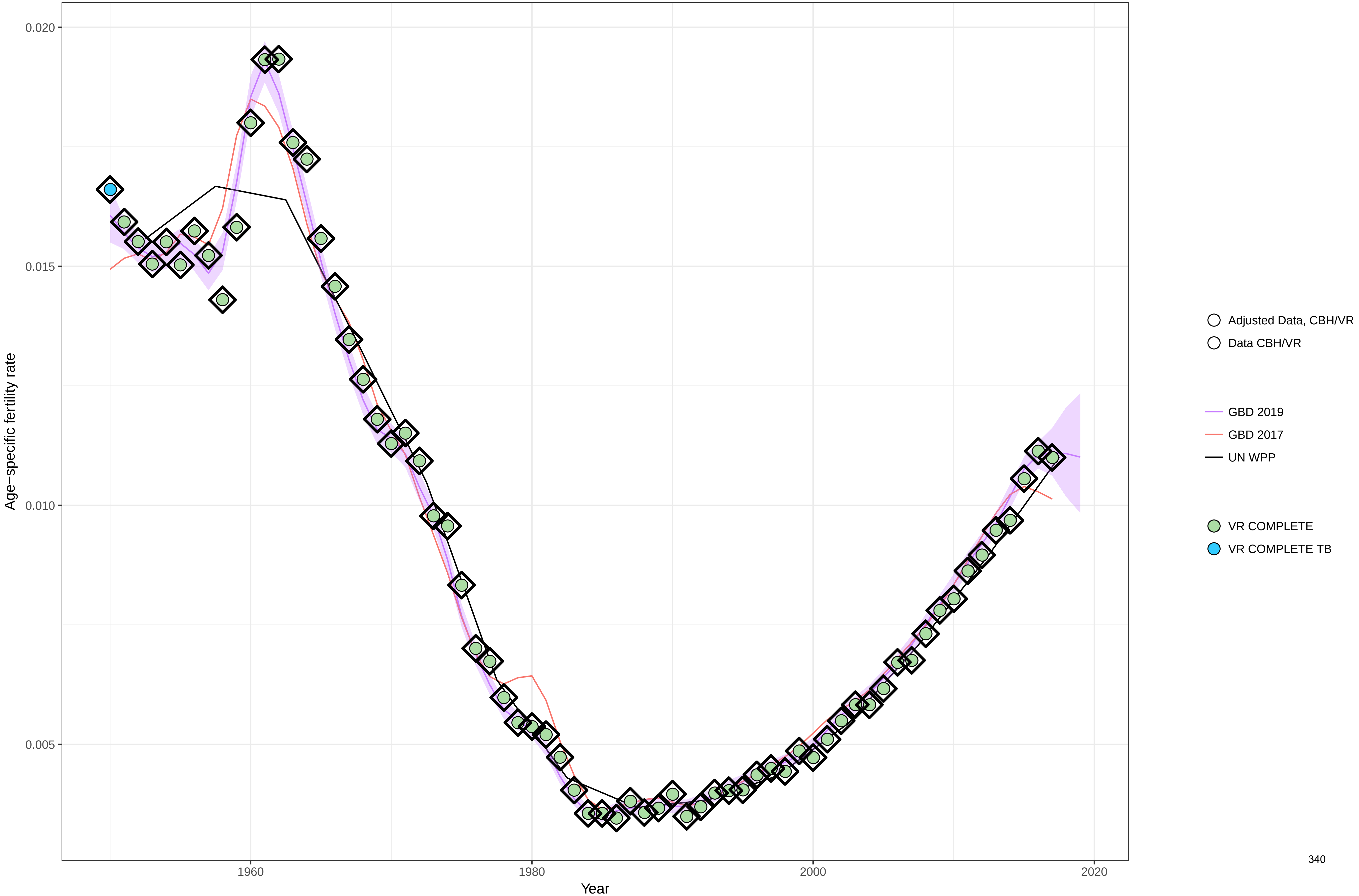

Age−specific fertility fitted trend lines, 1950−2019: Austria, Ages: 45−49
Data points enclosed with a diamond are reference sources

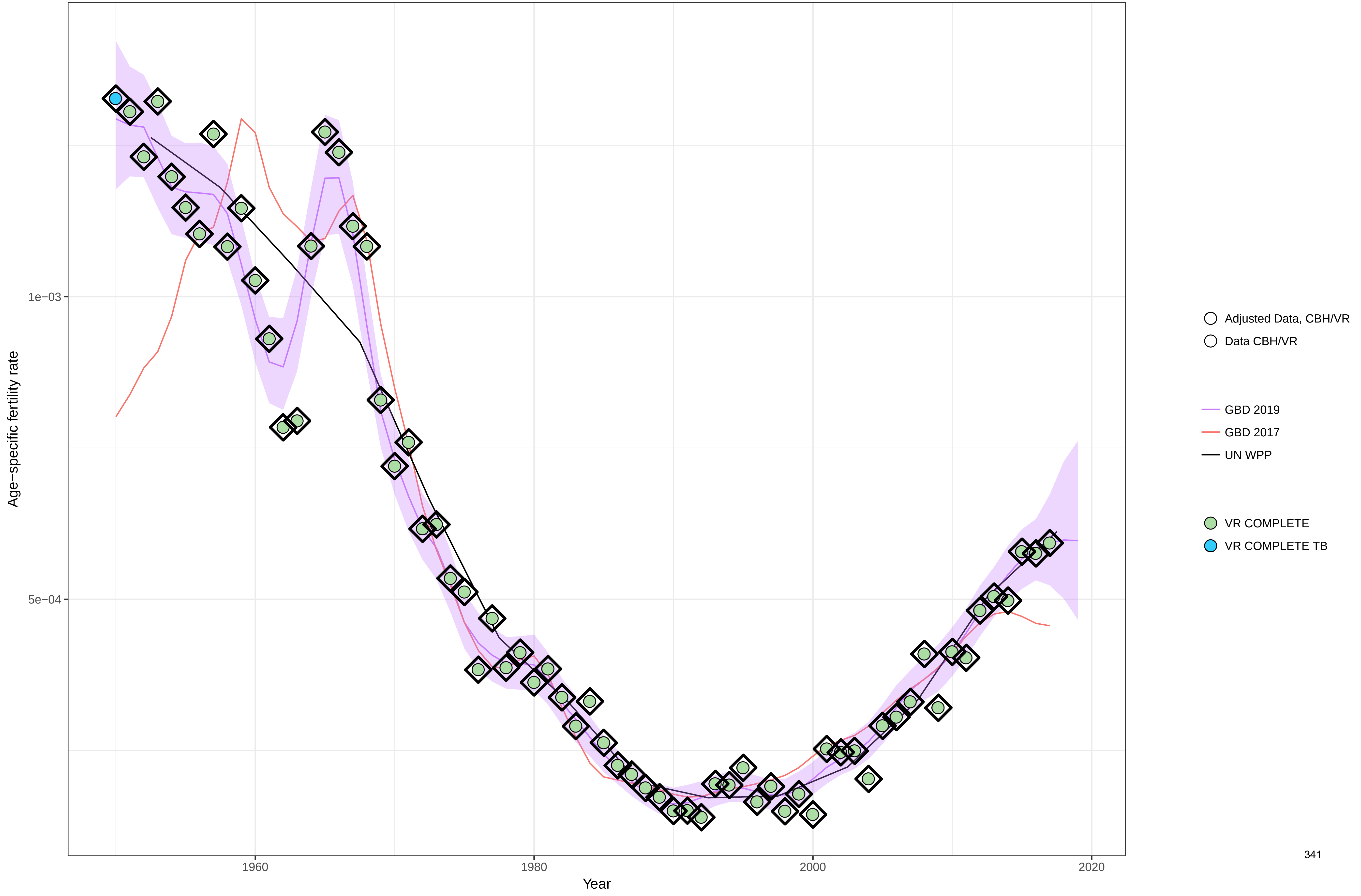

Age−specific fertility fitted trend lines, 1950−2019: Austria, Ages: 50−54
Data points enclosed with a diamond are reference sources

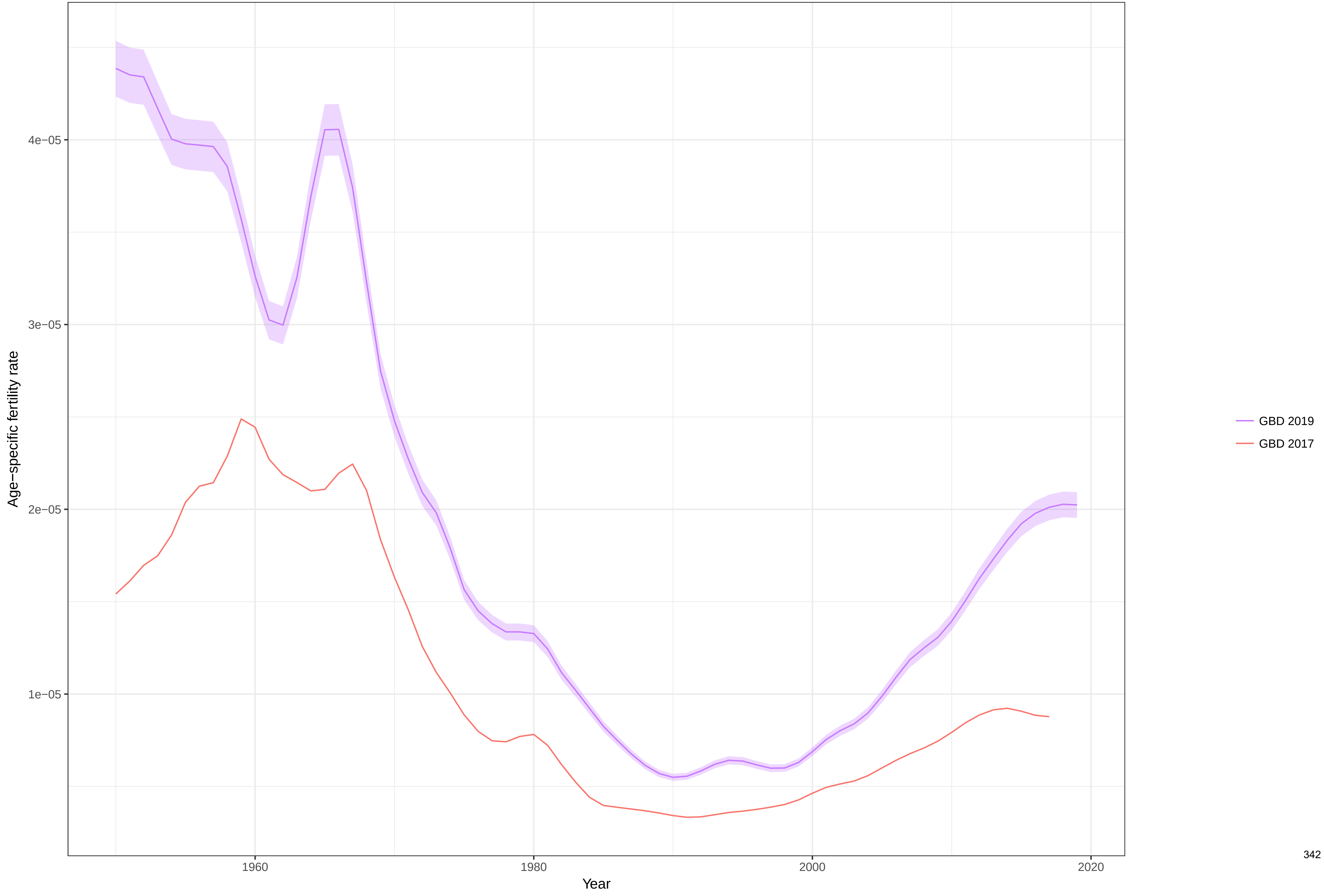

Total fertility rate fitted trend lines, 1950−2019: Austria TFR
Data points enclosed with a diamond are reference sources

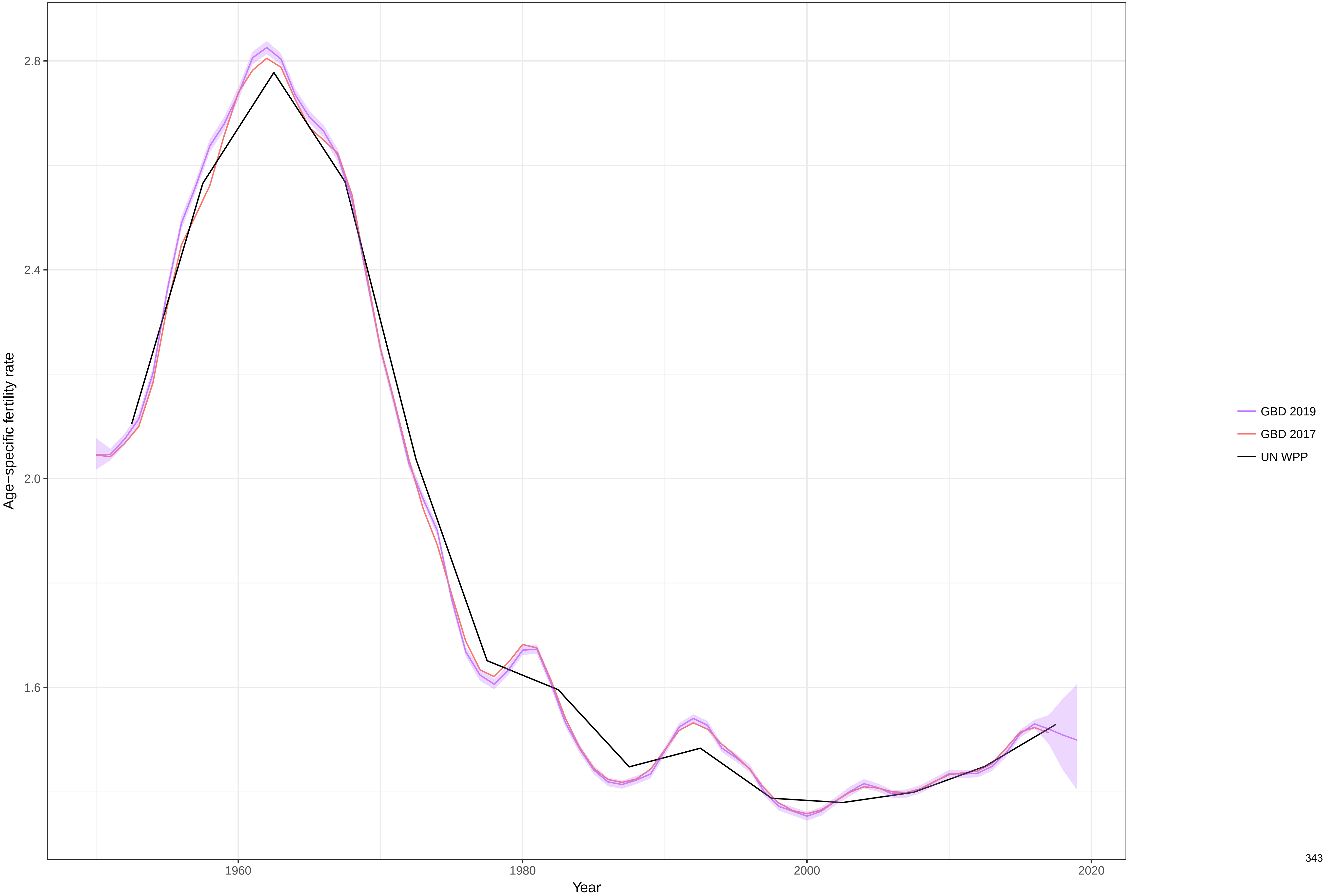

Age−specific fertility fitted trend lines, 1950−2019: Azerbaijan, Ages: 10−14
Data points enclosed with a diamond are reference sources

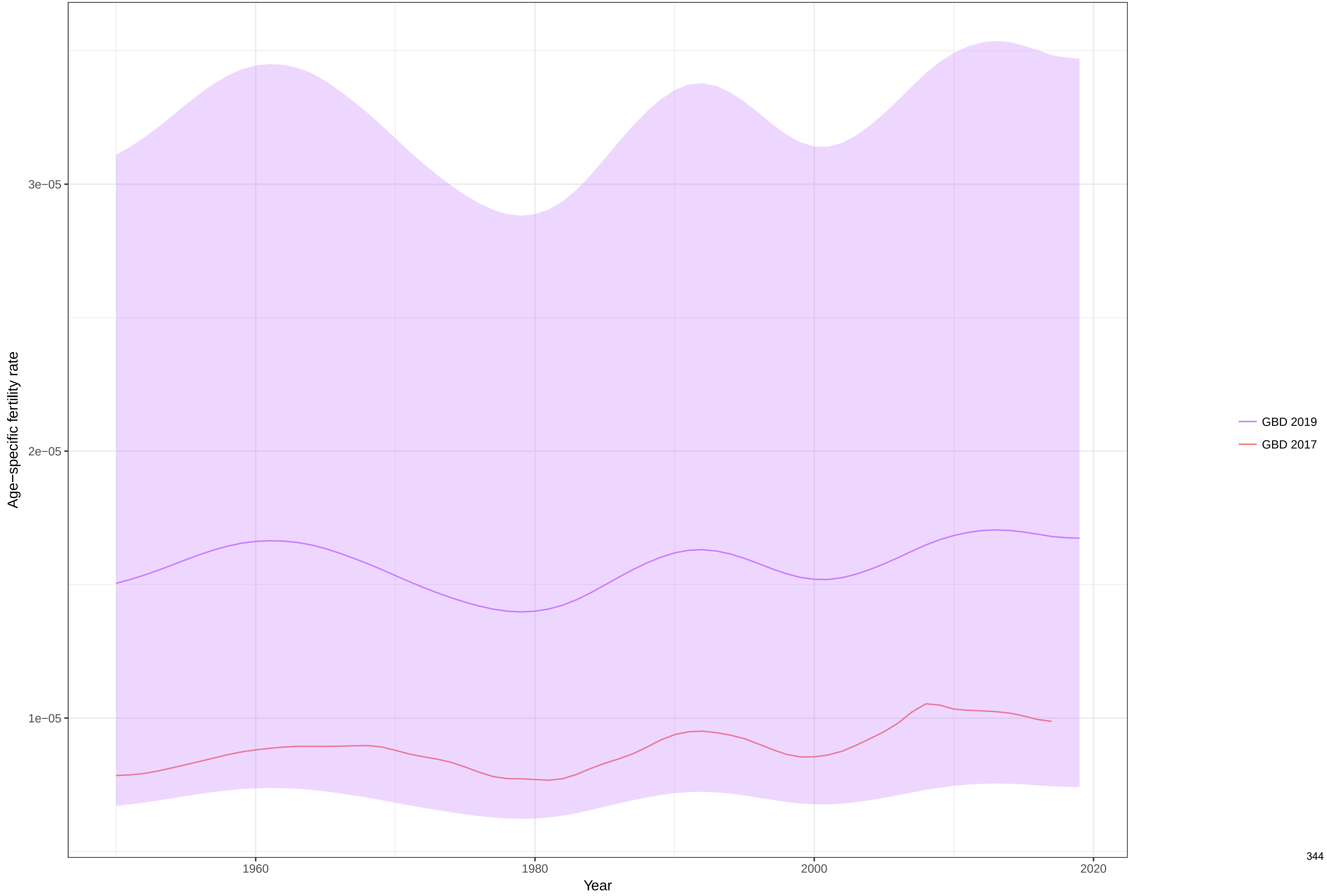

Age−specific fertility fitted trend lines, 1950−2019: Azerbaijan, Ages: 15−19
Data points enclosed with a diamond are reference sources

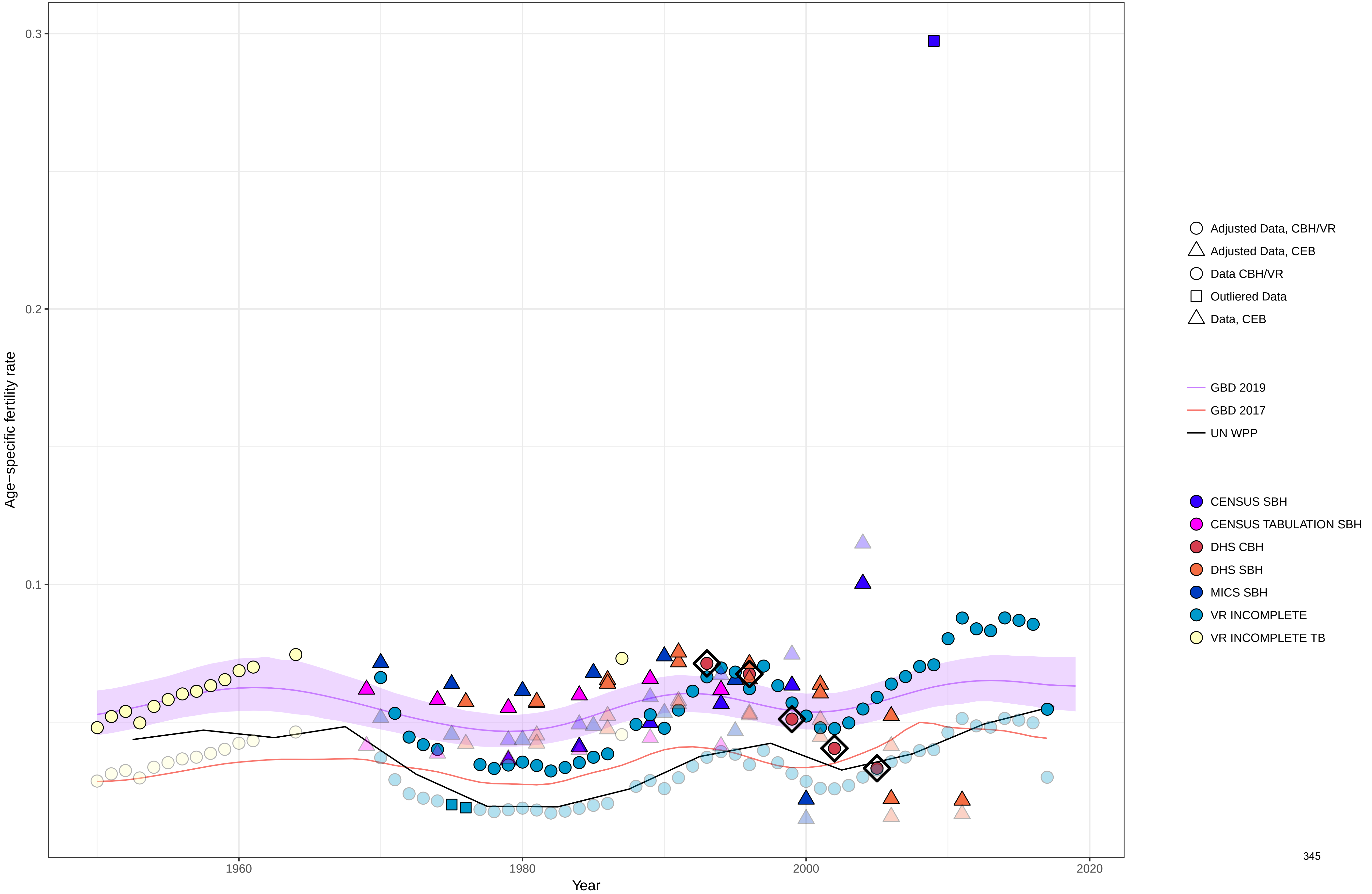

Age−specific fertility fitted trend lines, 1950−2019: Azerbaijan, Ages: 20−24
Data points enclosed with a diamond are reference sources

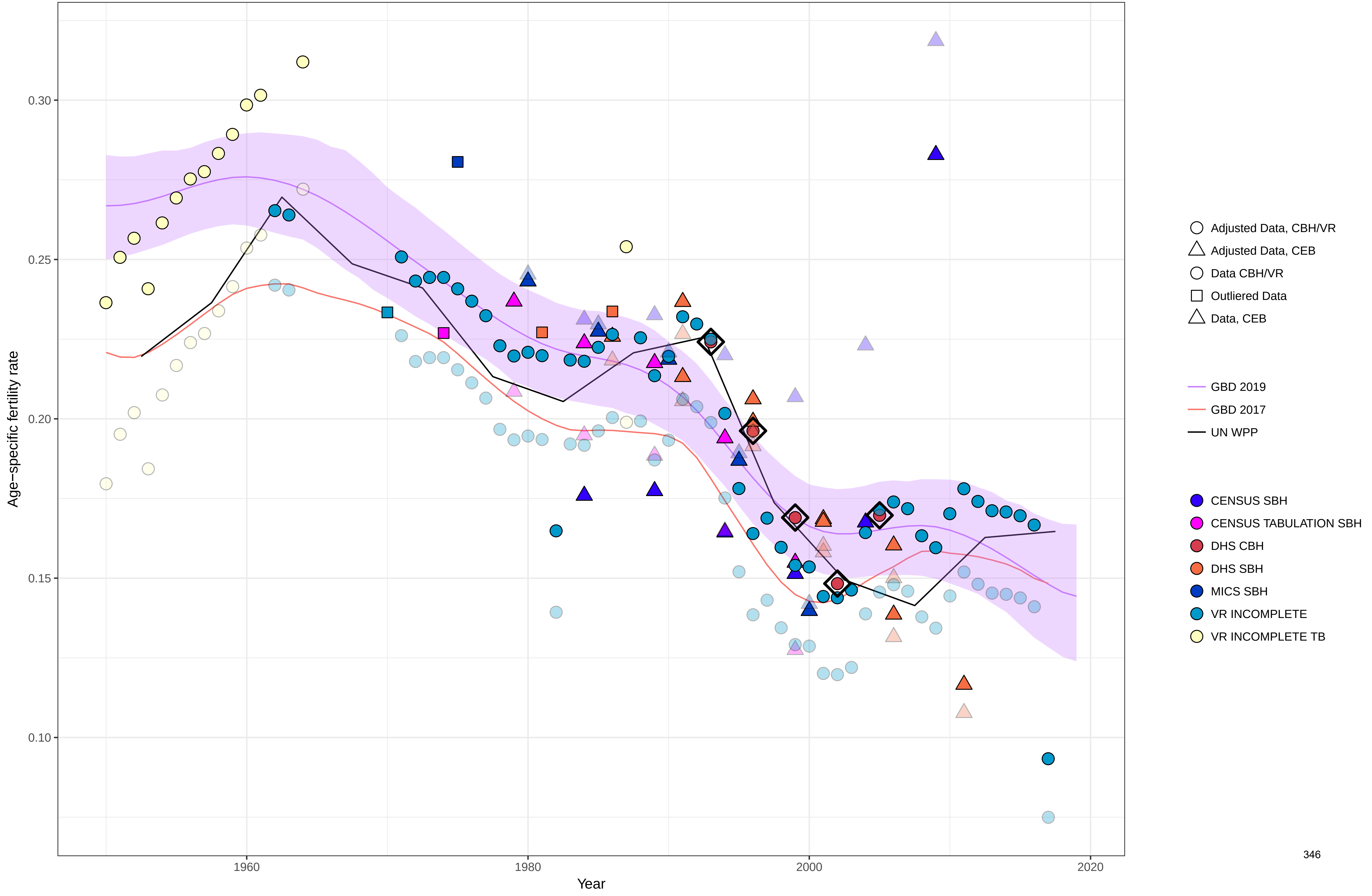

Age−specific fertility fitted trend lines, 1950−2019: Azerbaijan, Ages: 25−29
Data points enclosed with a diamond are reference sources

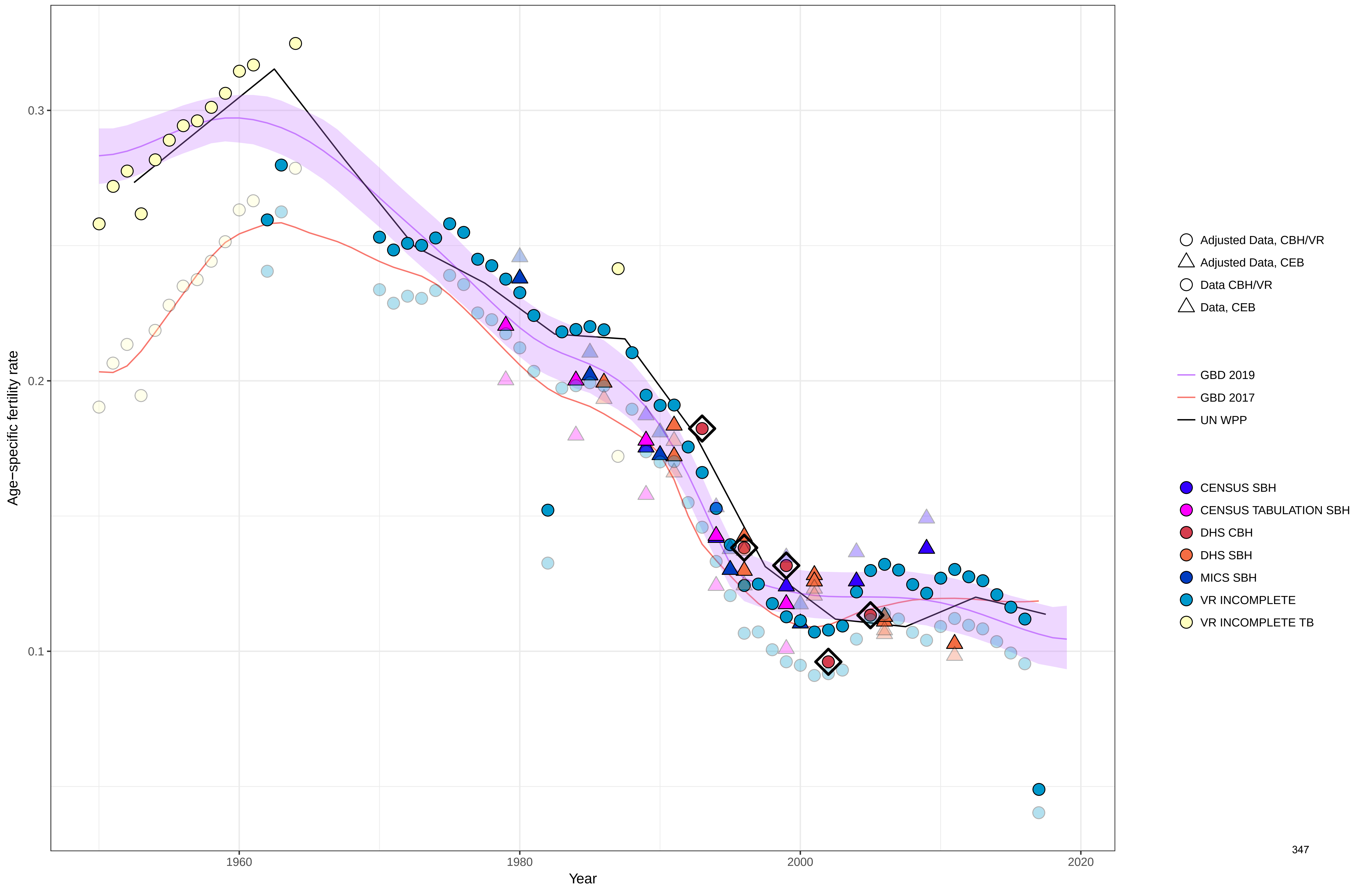

Age−specific fertility fitted trend lines, 1950−2019: Azerbaijan, Ages: 30−34
Data points enclosed with a diamond are reference sources

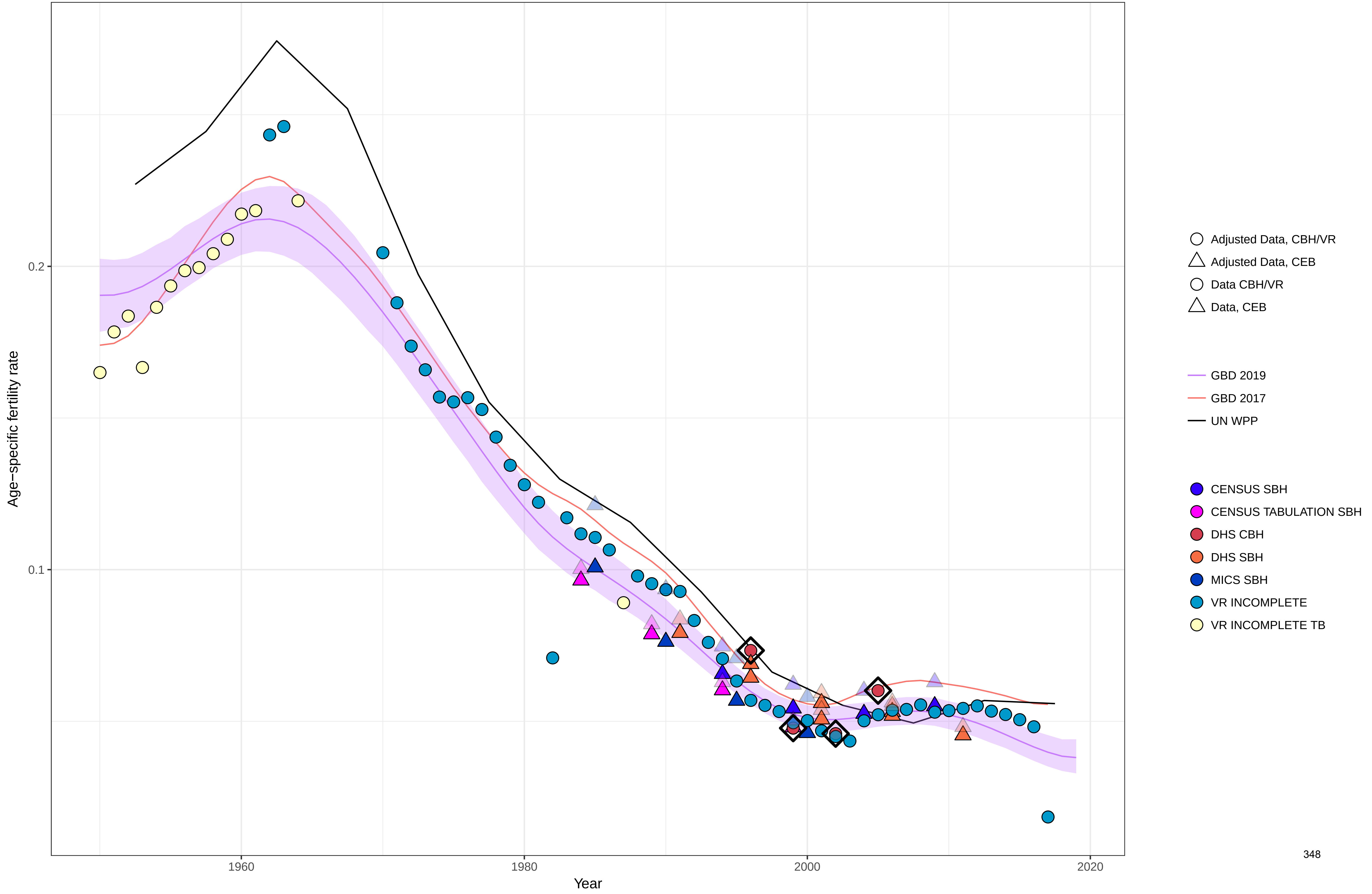

Age−specific fertility fitted trend lines, 1950−2019: Azerbaijan, Ages: 35−39
Data points enclosed with a diamond are reference sources

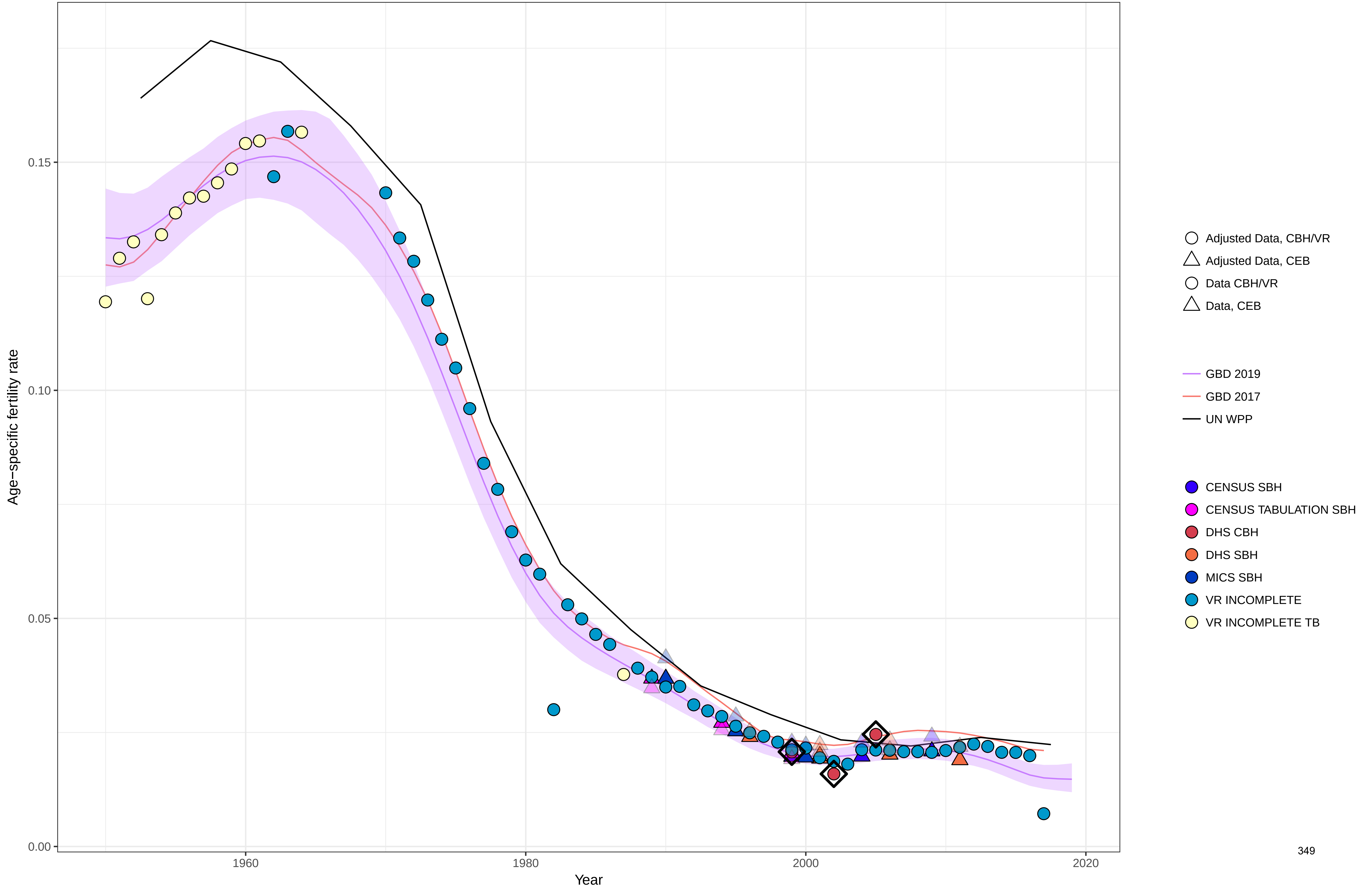

Age−specific fertility fitted trend lines, 1950−2019: Azerbaijan, Ages: 40−44
Data points enclosed with a diamond are reference sources

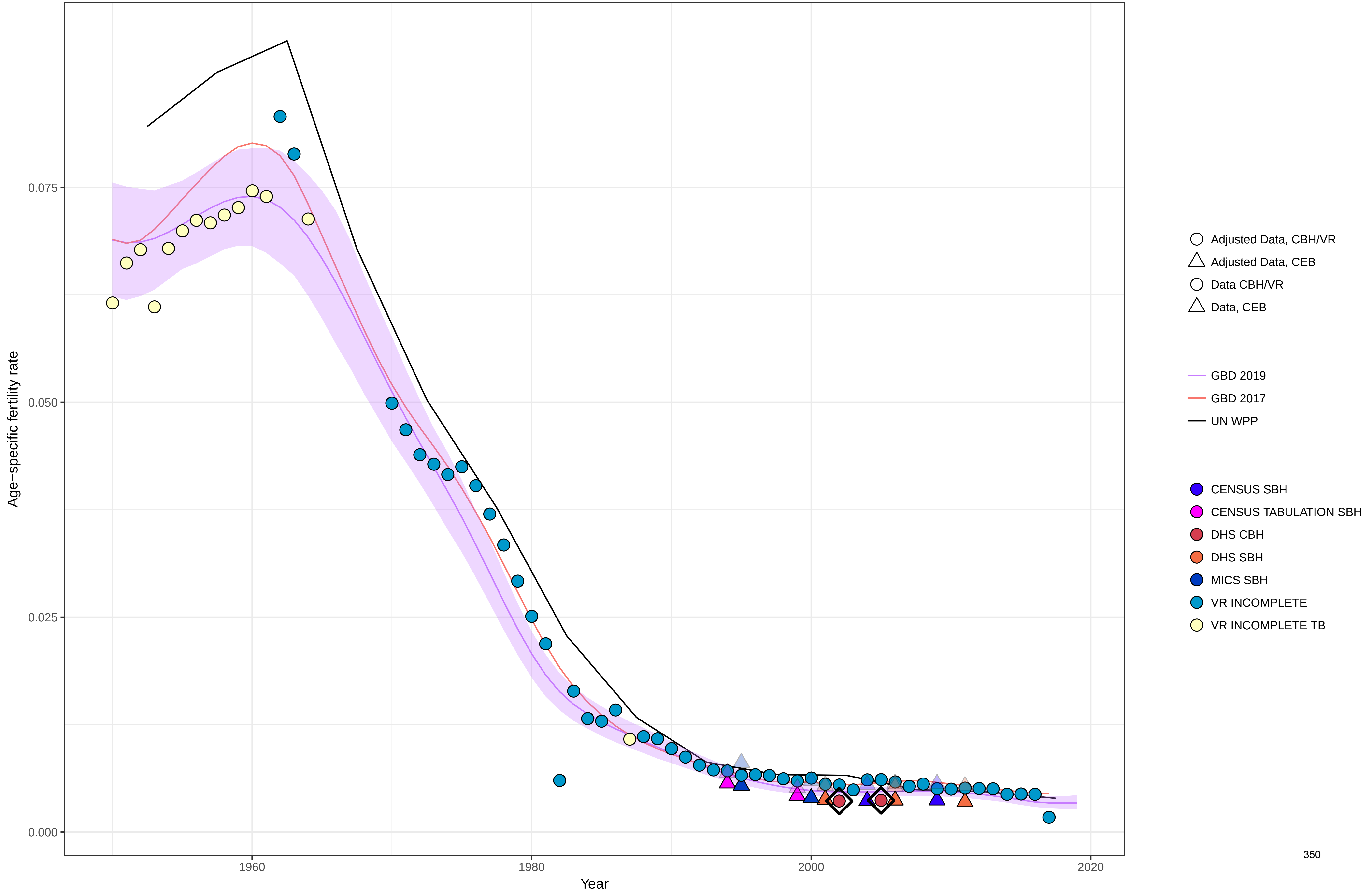

Age−specific fertility fitted trend lines, 1950−2019: Azerbaijan, Ages: 45−49
Data points enclosed with a diamond are reference sources

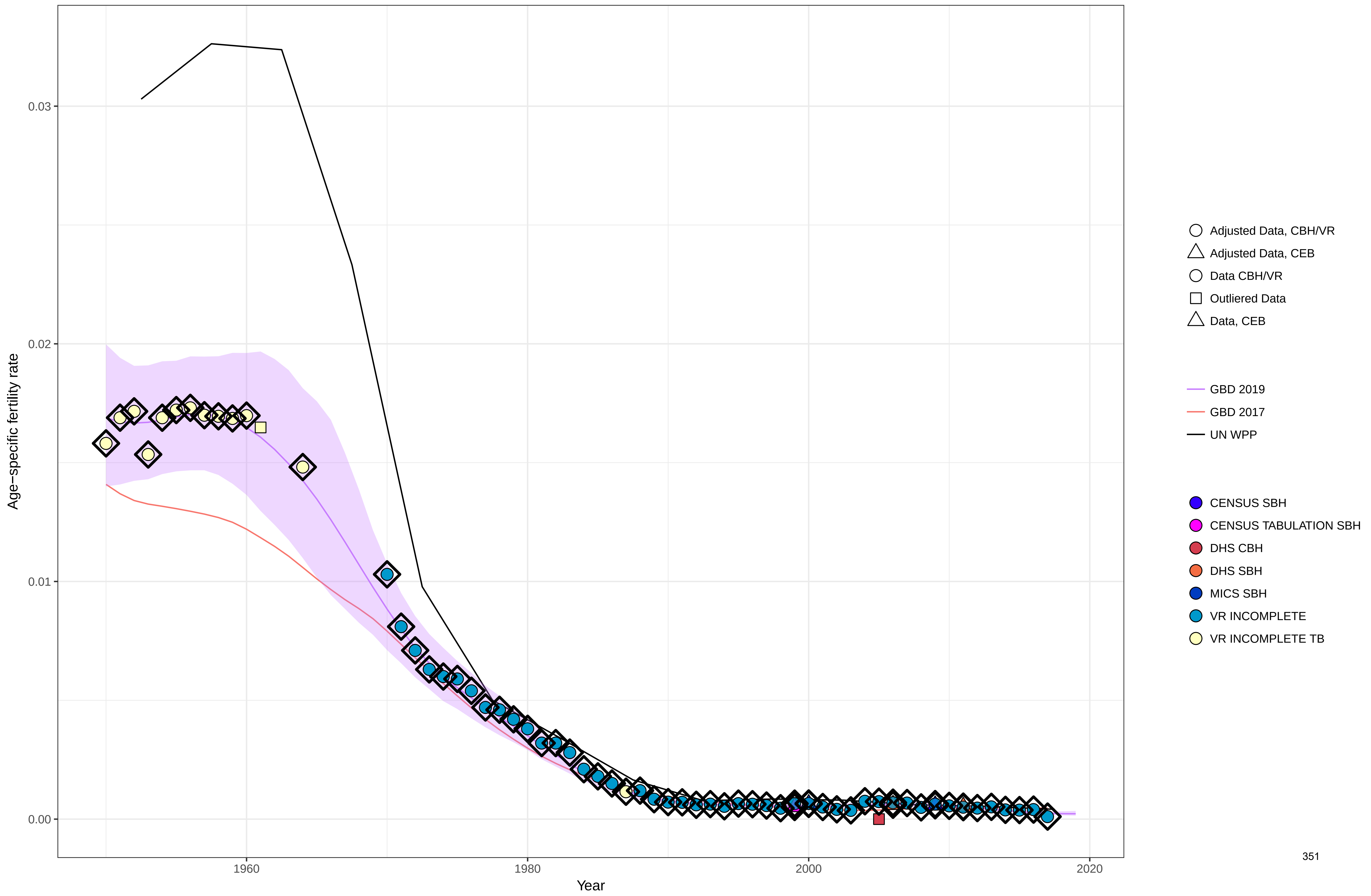

Age−specific fertility fitted trend lines, 1950−2019: Azerbaijan, Ages: 50−54
Data points enclosed with a diamond are reference sources

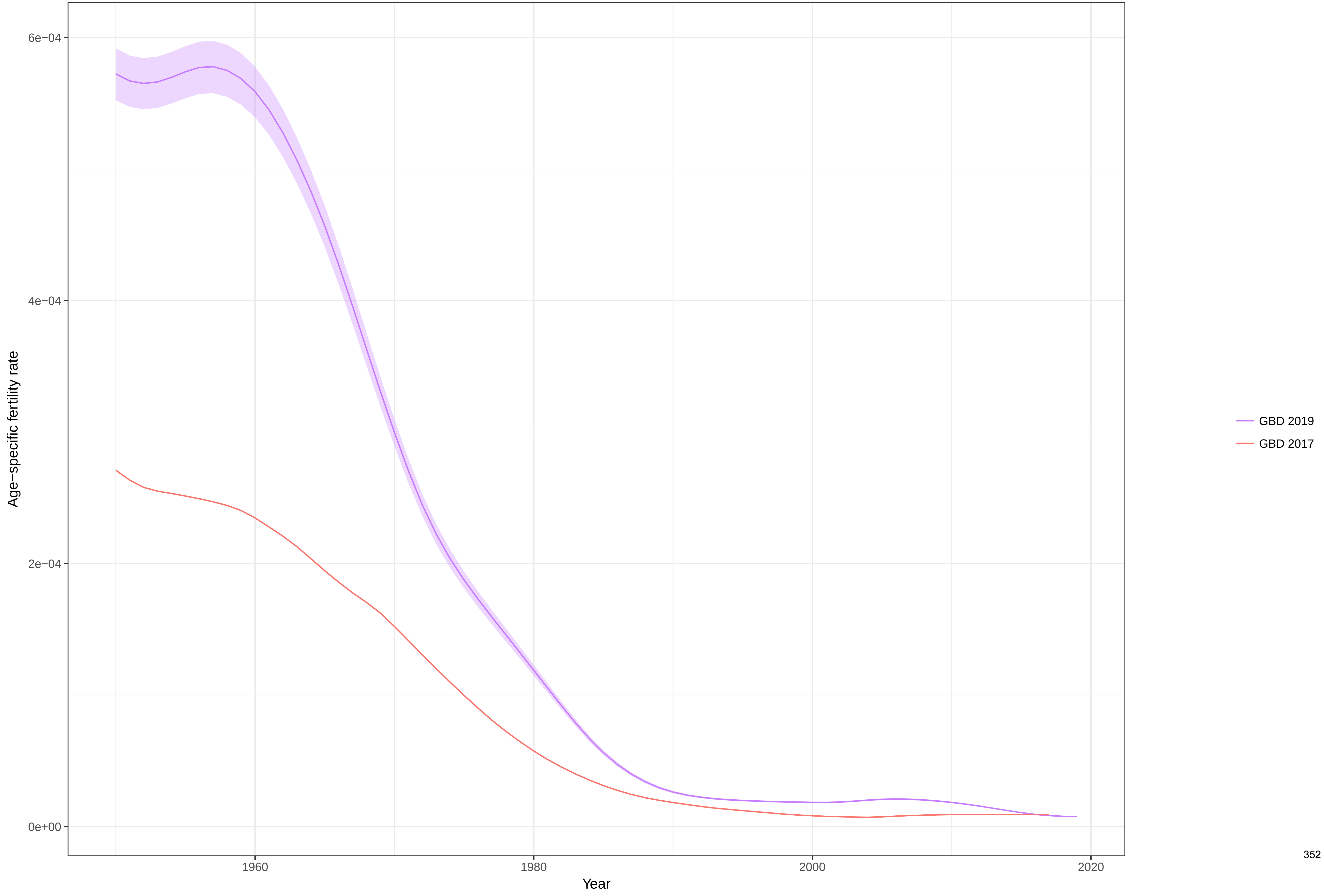

Total fertility rate fitted trend lines, 1950−2019: Azerbaijan TFR
Data points enclosed with a diamond are reference sources

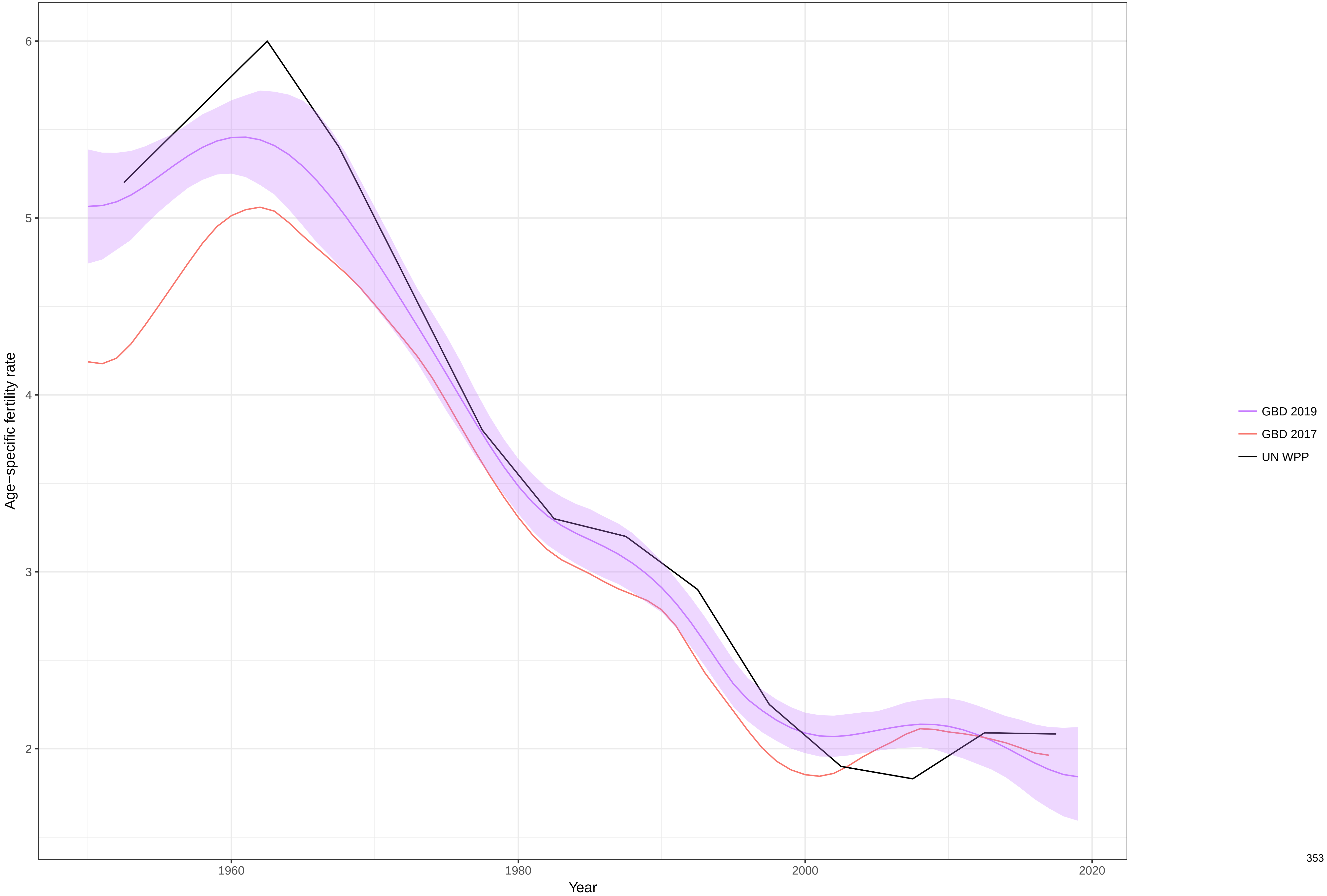

Age−specific fertility fitted trend lines, 1950−2019: Burundi, Ages: 10−14
Data points enclosed with a diamond are reference sources

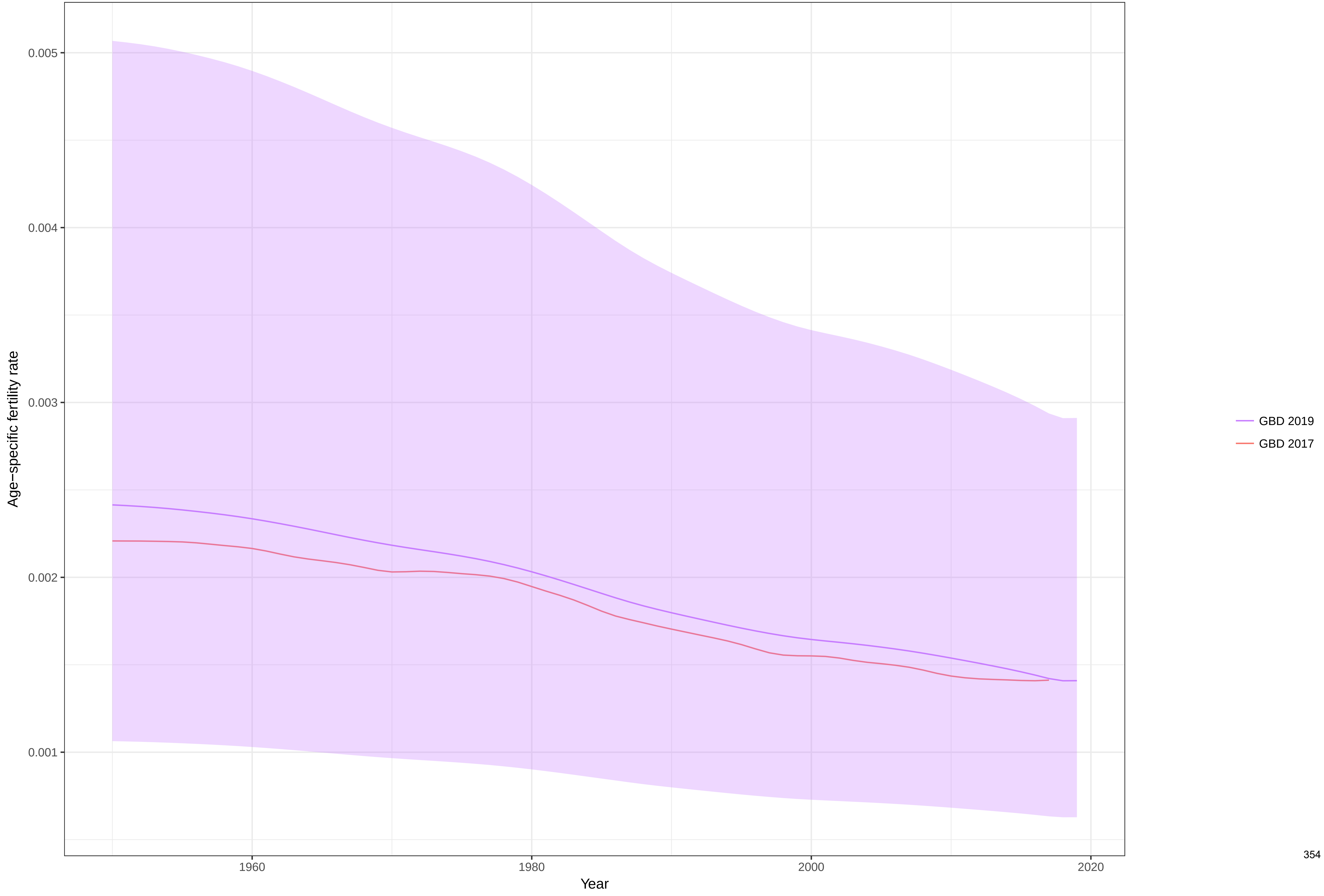

Age−specific fertility fitted trend lines, 1950−2019: Burundi, Ages: 15−19
Data points enclosed with a diamond are reference sources

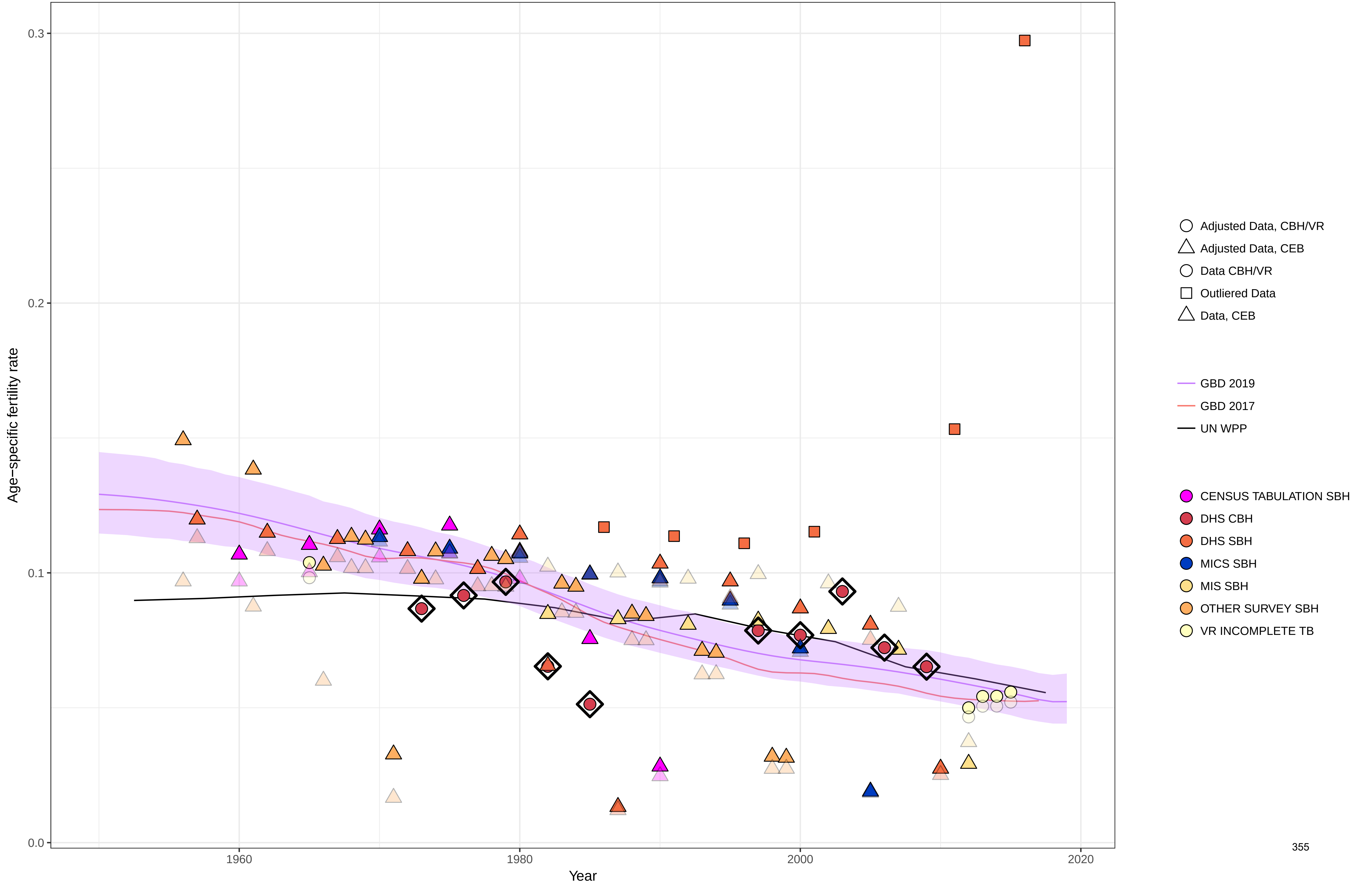

Age−specific fertility fitted trend lines, 1950−2019: Burundi, Ages: 20−24
Data points enclosed with a diamond are reference sources

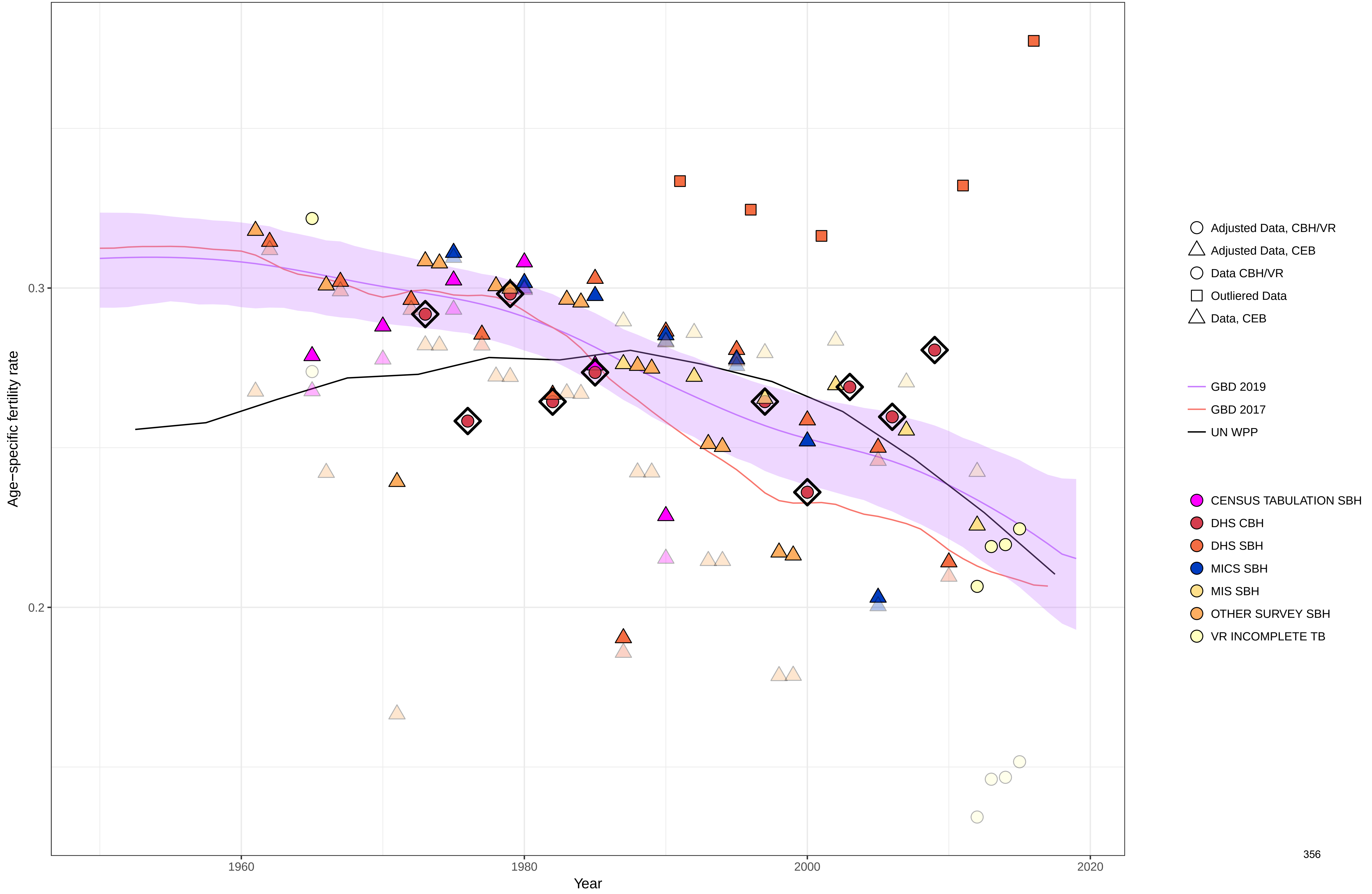

Age−specific fertility fitted trend lines, 1950−2019: Burundi, Ages: 25−29
Data points enclosed with a diamond are reference sources

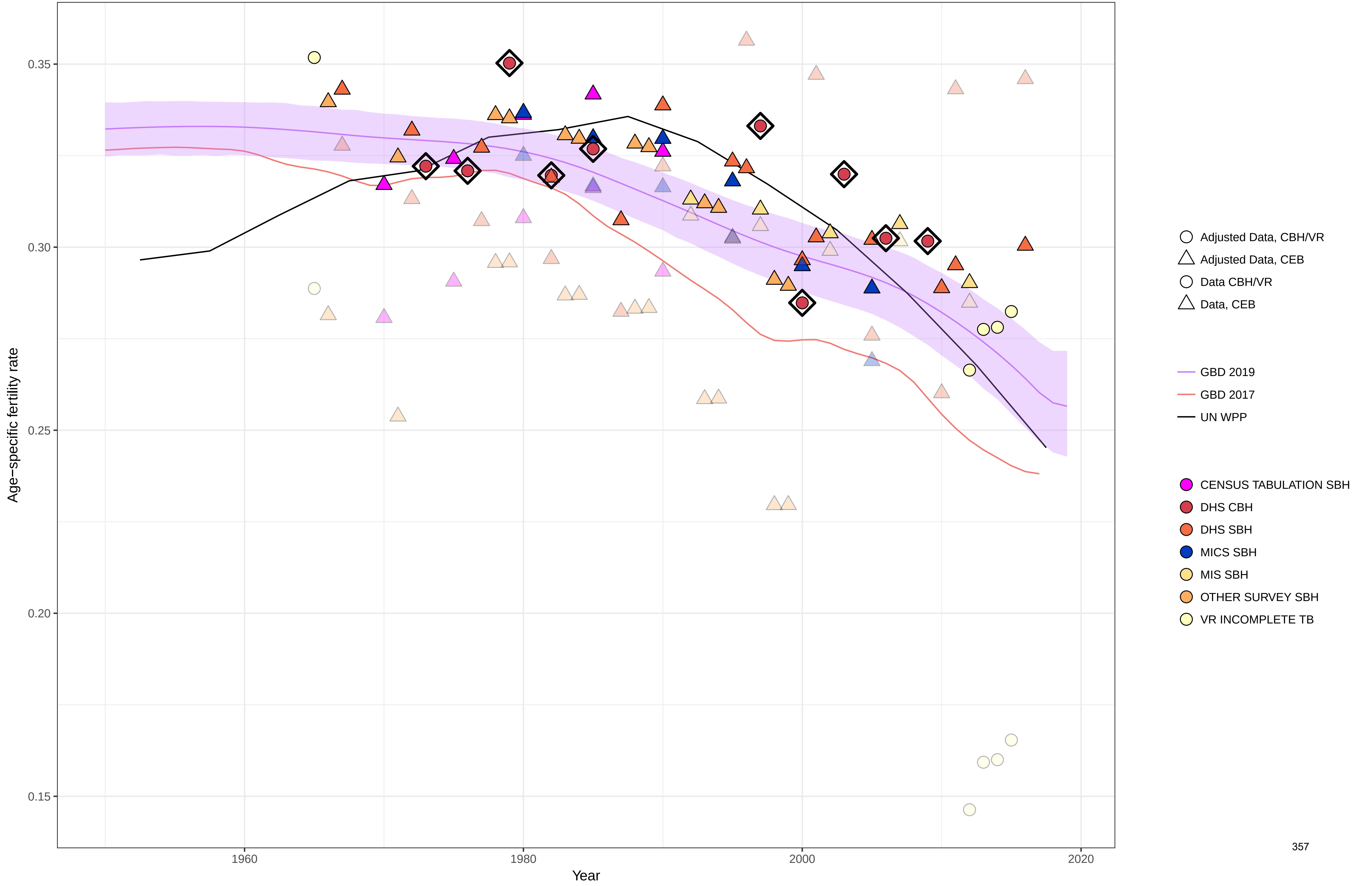

Age−specific fertility fitted trend lines, 1950−2019: Burundi, Ages: 30−34
Data points enclosed with a diamond are reference sources

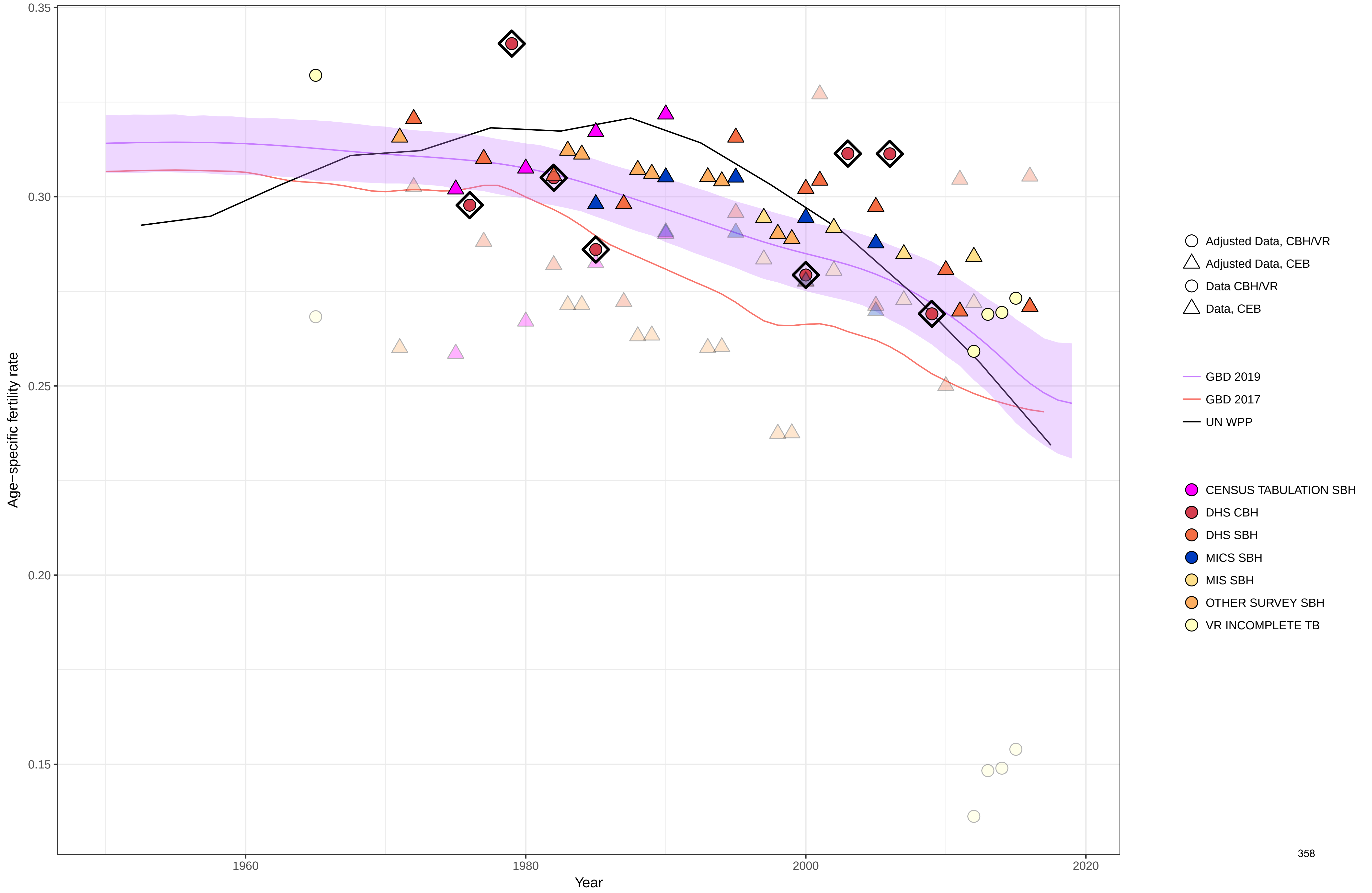

Age−specific fertility fitted trend lines, 1950−2019: Burundi, Ages: 35−39
Data points enclosed with a diamond are reference sources

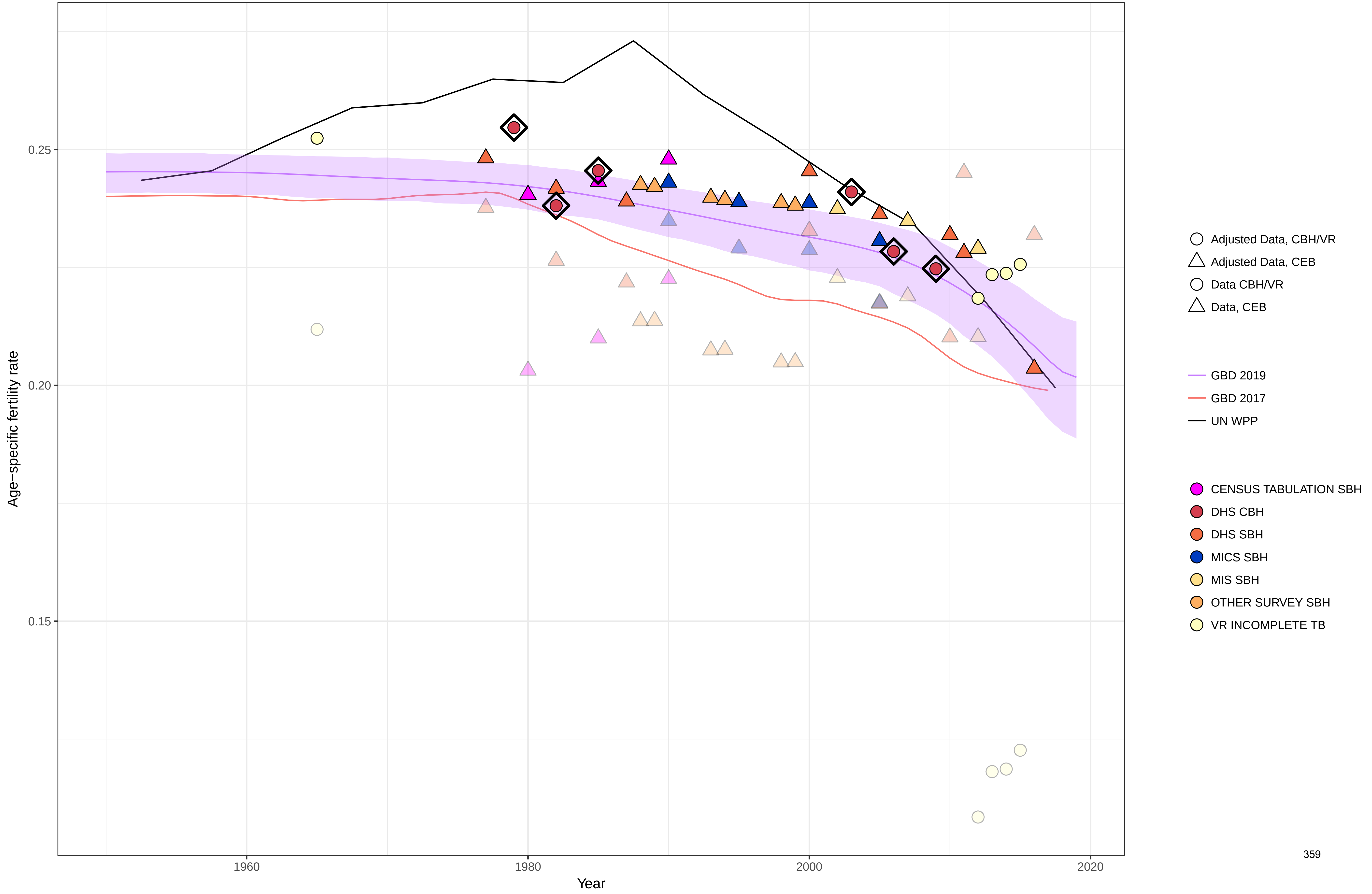

Age−specific fertility fitted trend lines, 1950−2019: Burundi, Ages: 40−44
Data points enclosed with a diamond are reference sources

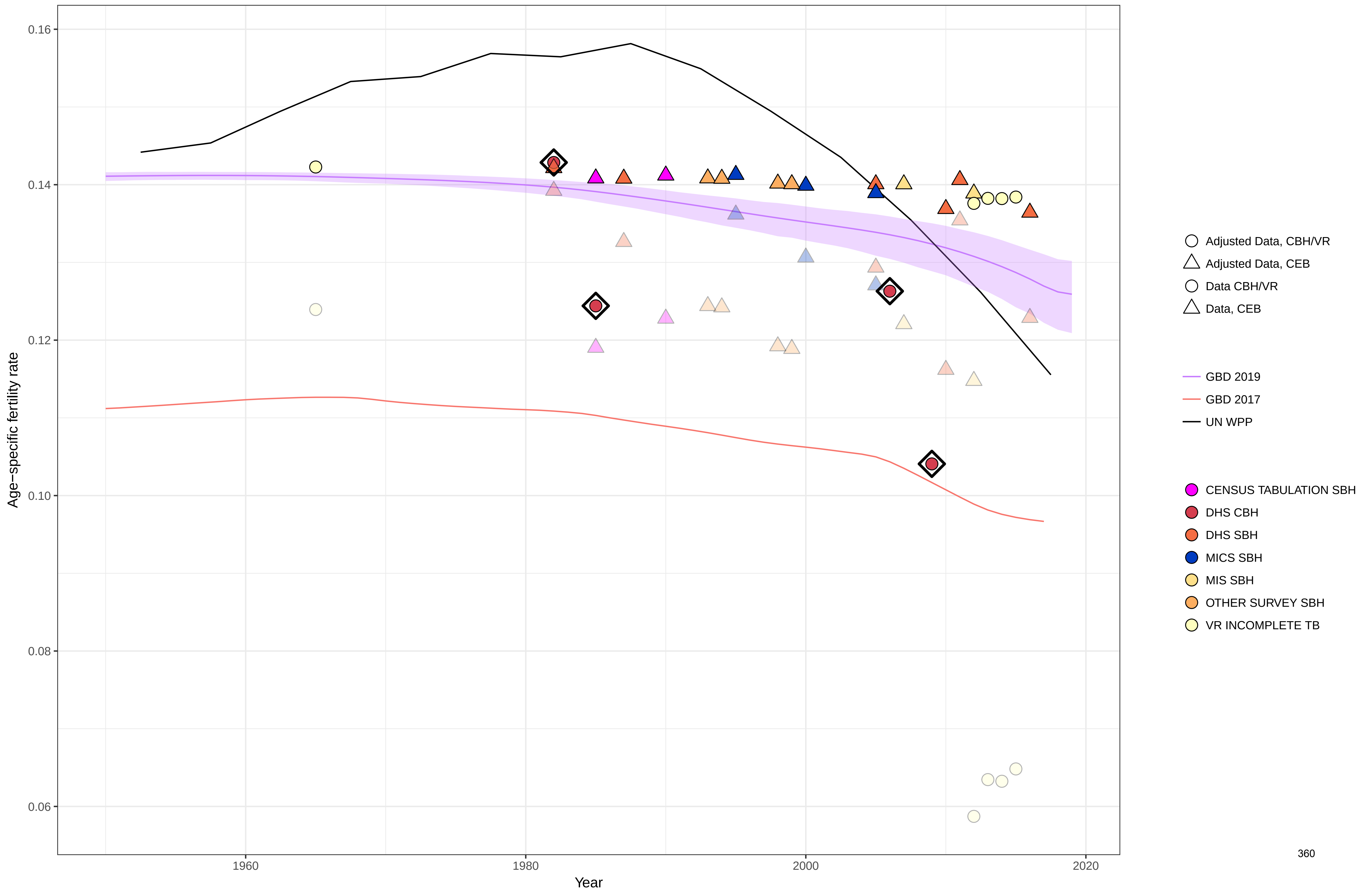

Age−specific fertility fitted trend lines, 1950−2019: Burundi, Ages: 45−49
Data points enclosed with a diamond are reference sources

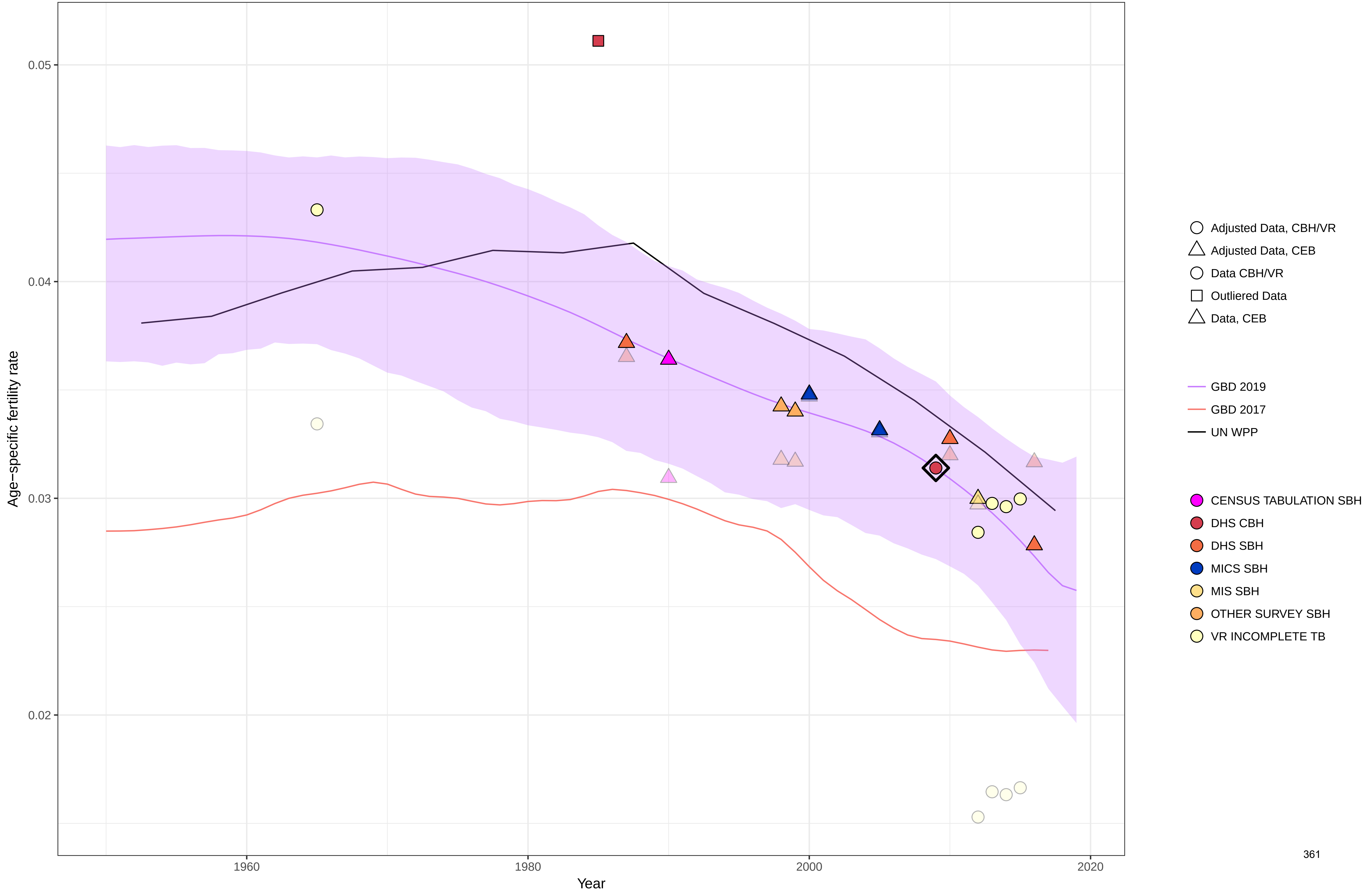

Age−specific fertility fitted trend lines, 1950−2019: Burundi, Ages: 50−54
Data points enclosed with a diamond are reference sources

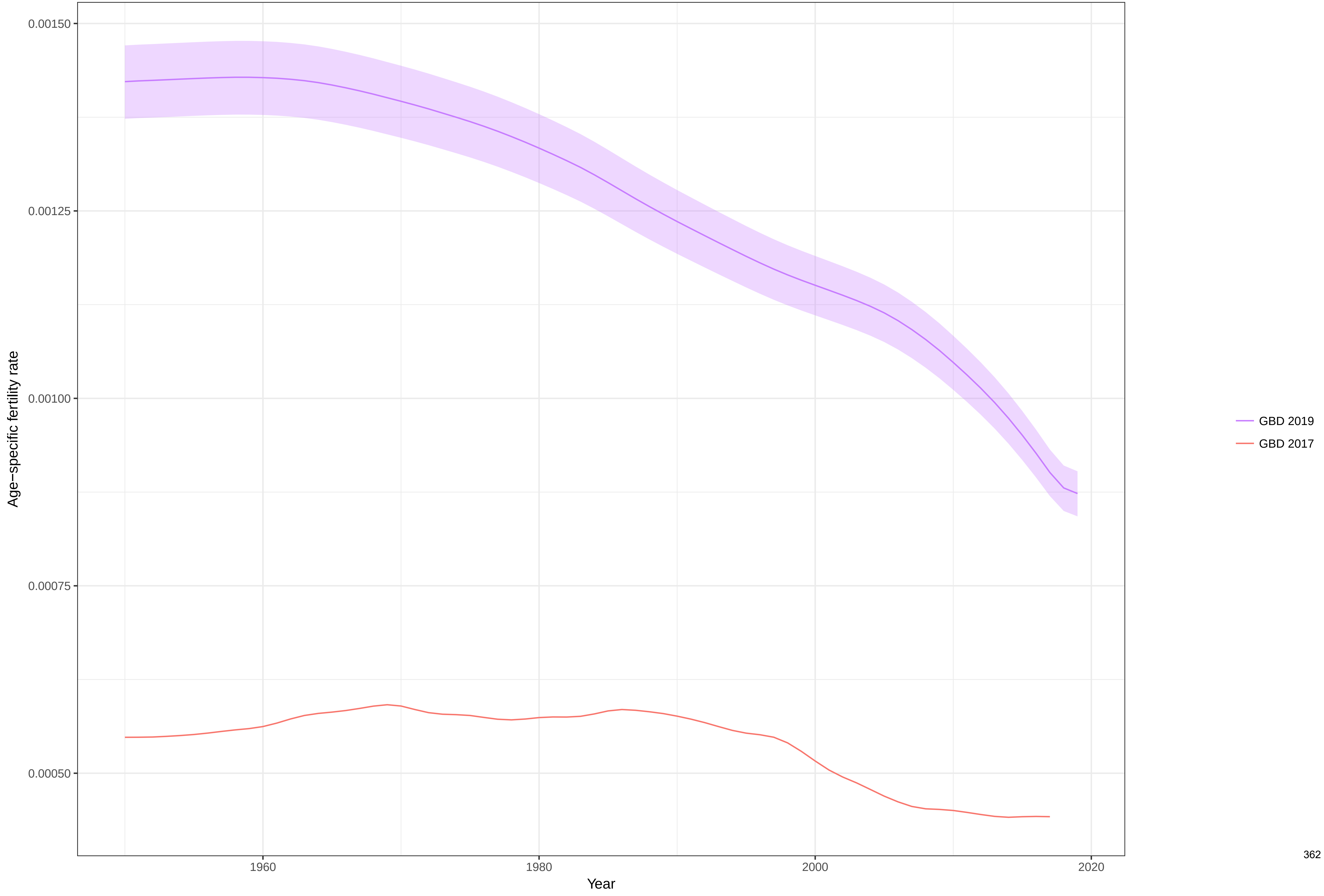

Total fertility rate fitted trend lines, 1950−2019: Burundi TFR
Data points enclosed with a diamond are reference sources

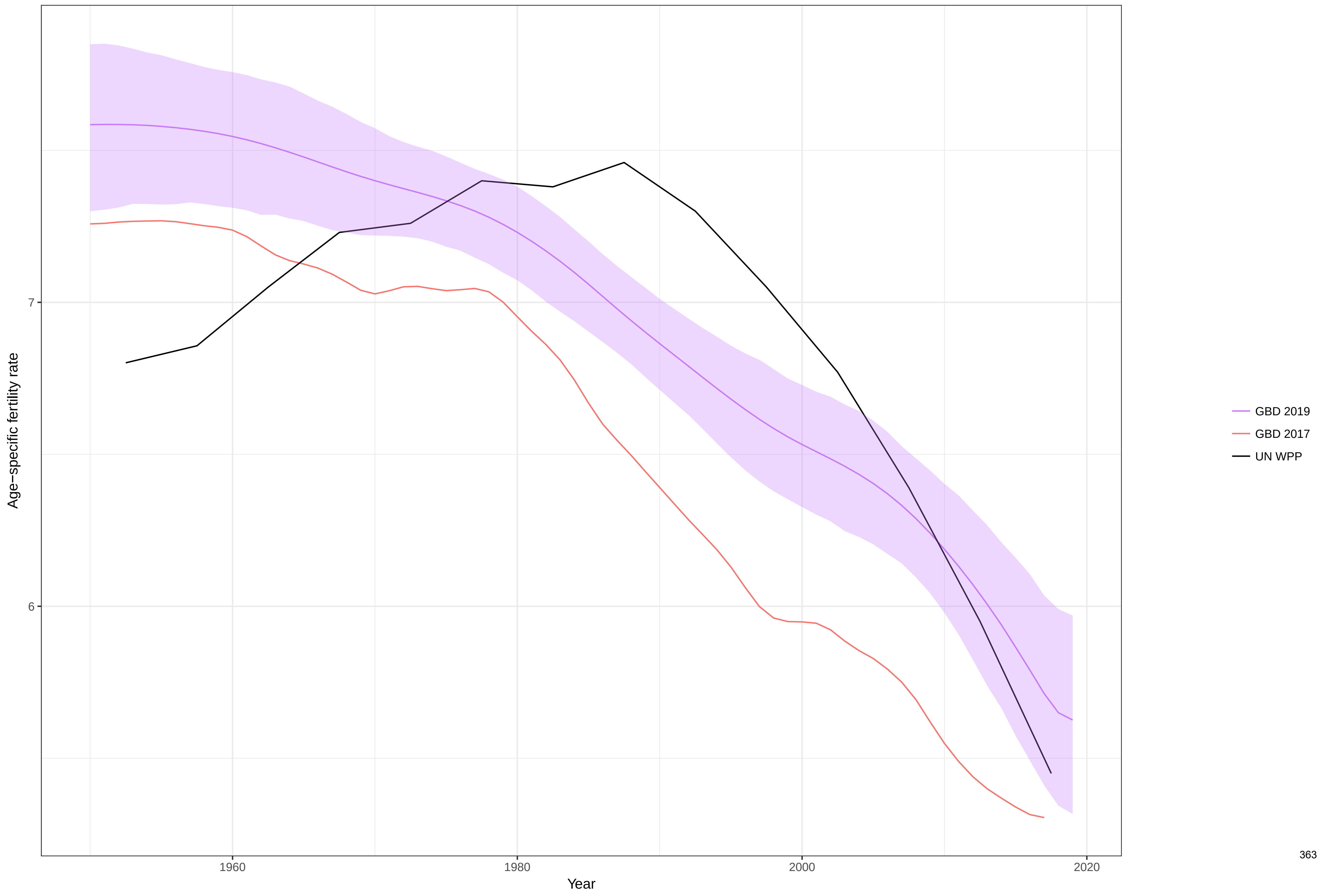

Age−specific fertility fitted trend lines, 1950−2019: Belgium, Ages: 10−14
Data points enclosed with a diamond are reference sources

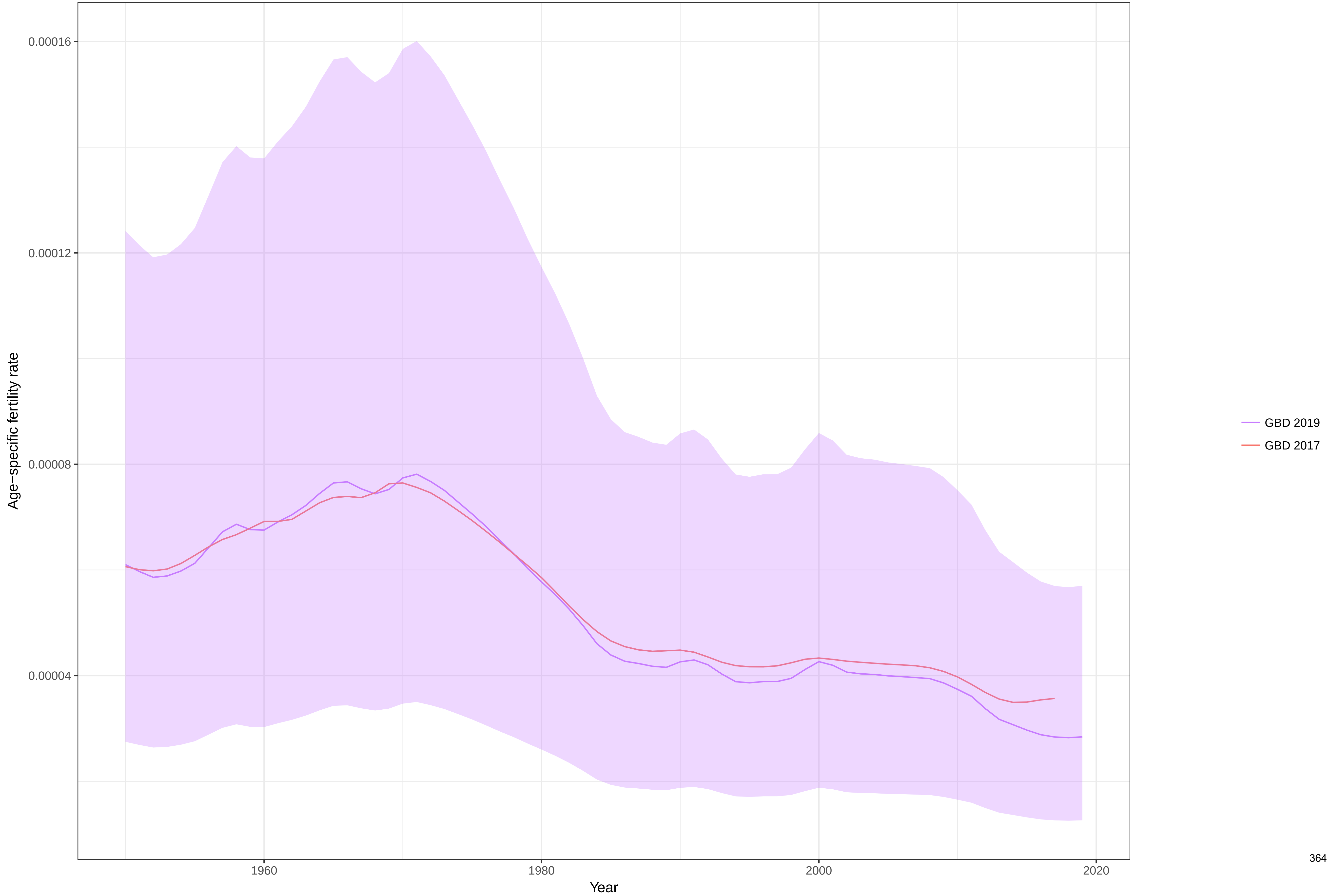

Age−specific fertility fitted trend lines, 1950−2019: Belgium, Ages: 15−19
Data points enclosed with a diamond are reference sources

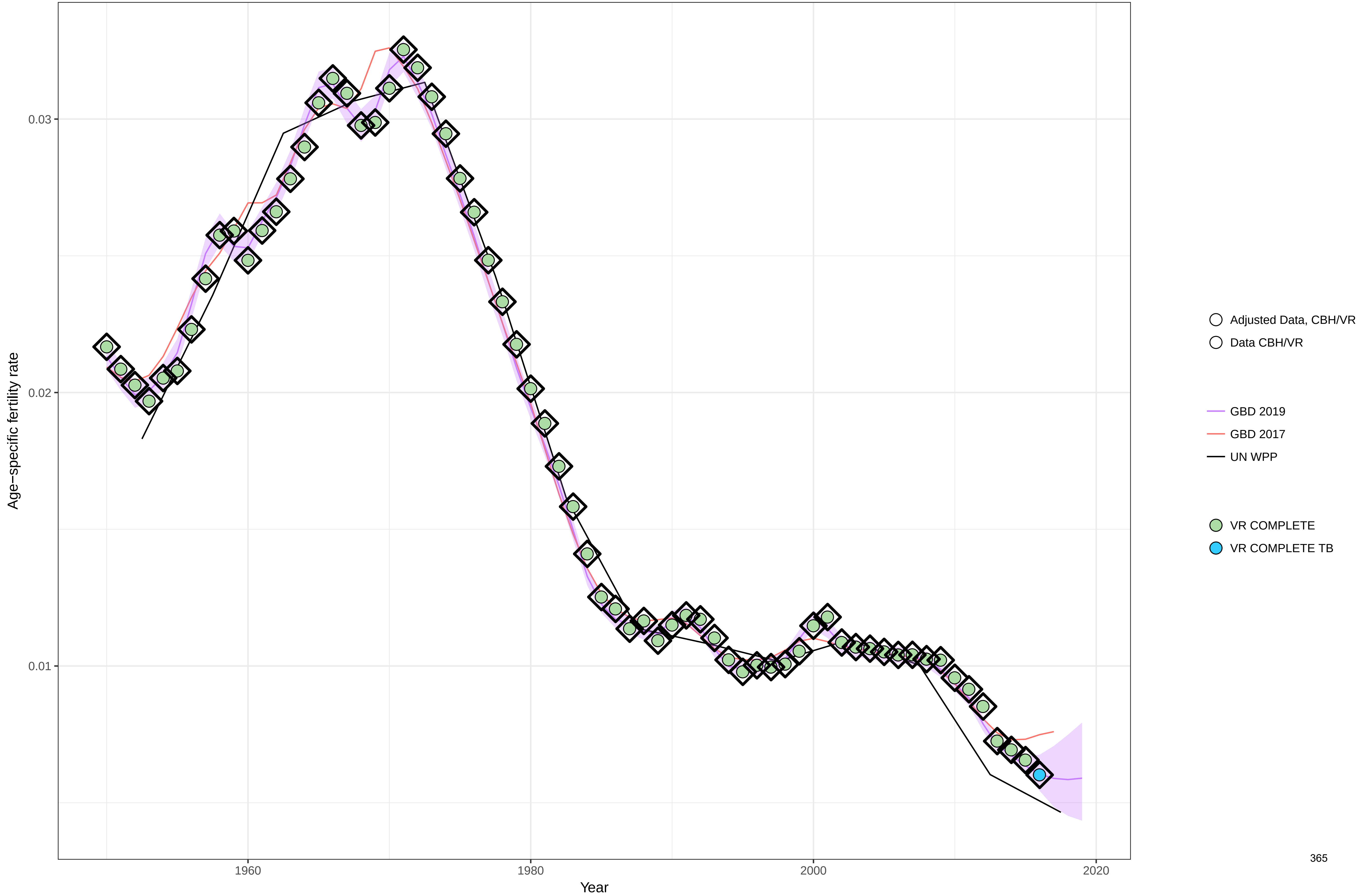

Age−specific fertility fitted trend lines, 1950−2019: Belgium, Ages: 20−24
Data points enclosed with a diamond are reference sources

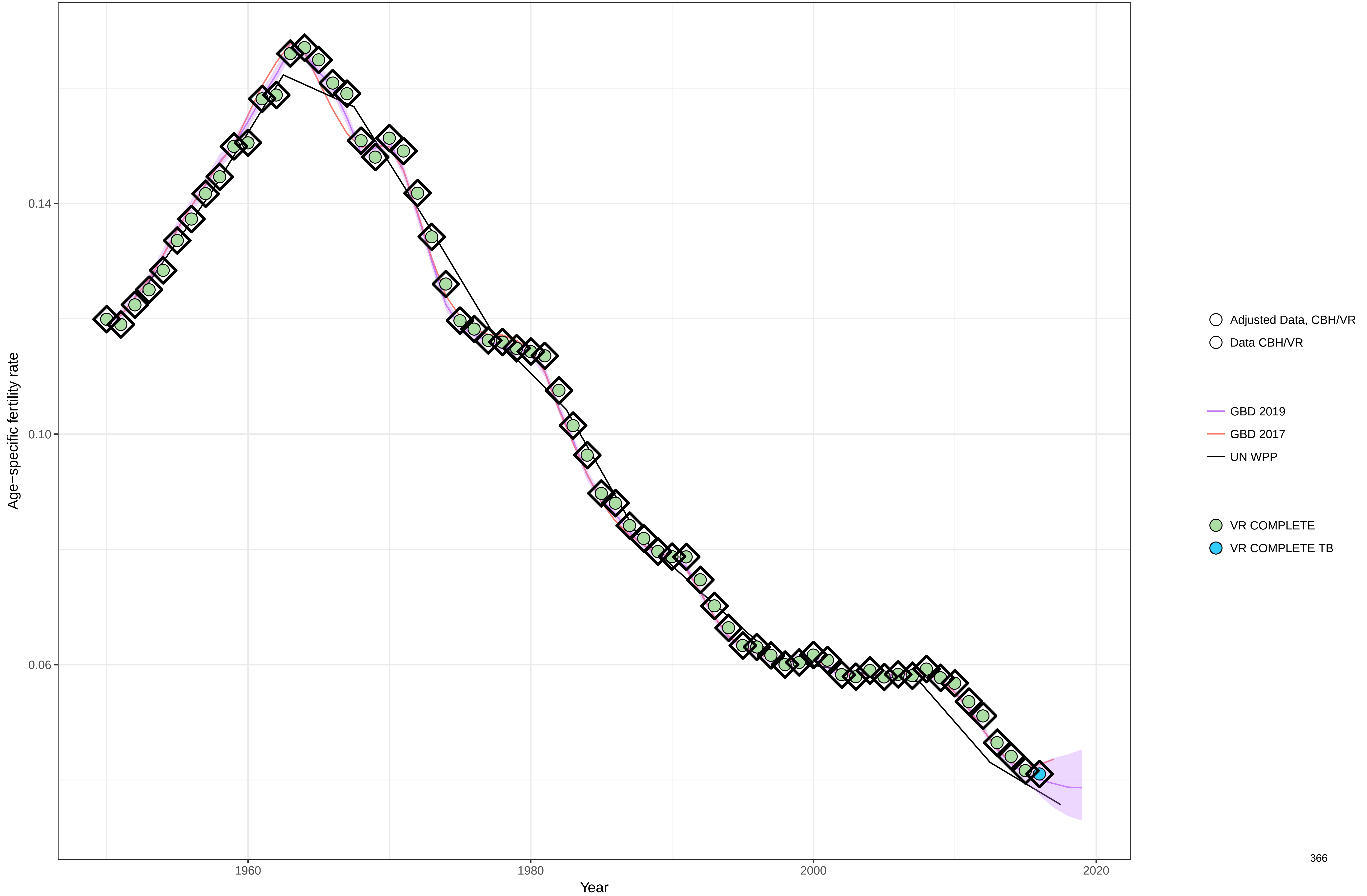

Age−specific fertility fitted trend lines, 1950−2019: Belgium, Ages: 25−29
Data points enclosed with a diamond are reference sources

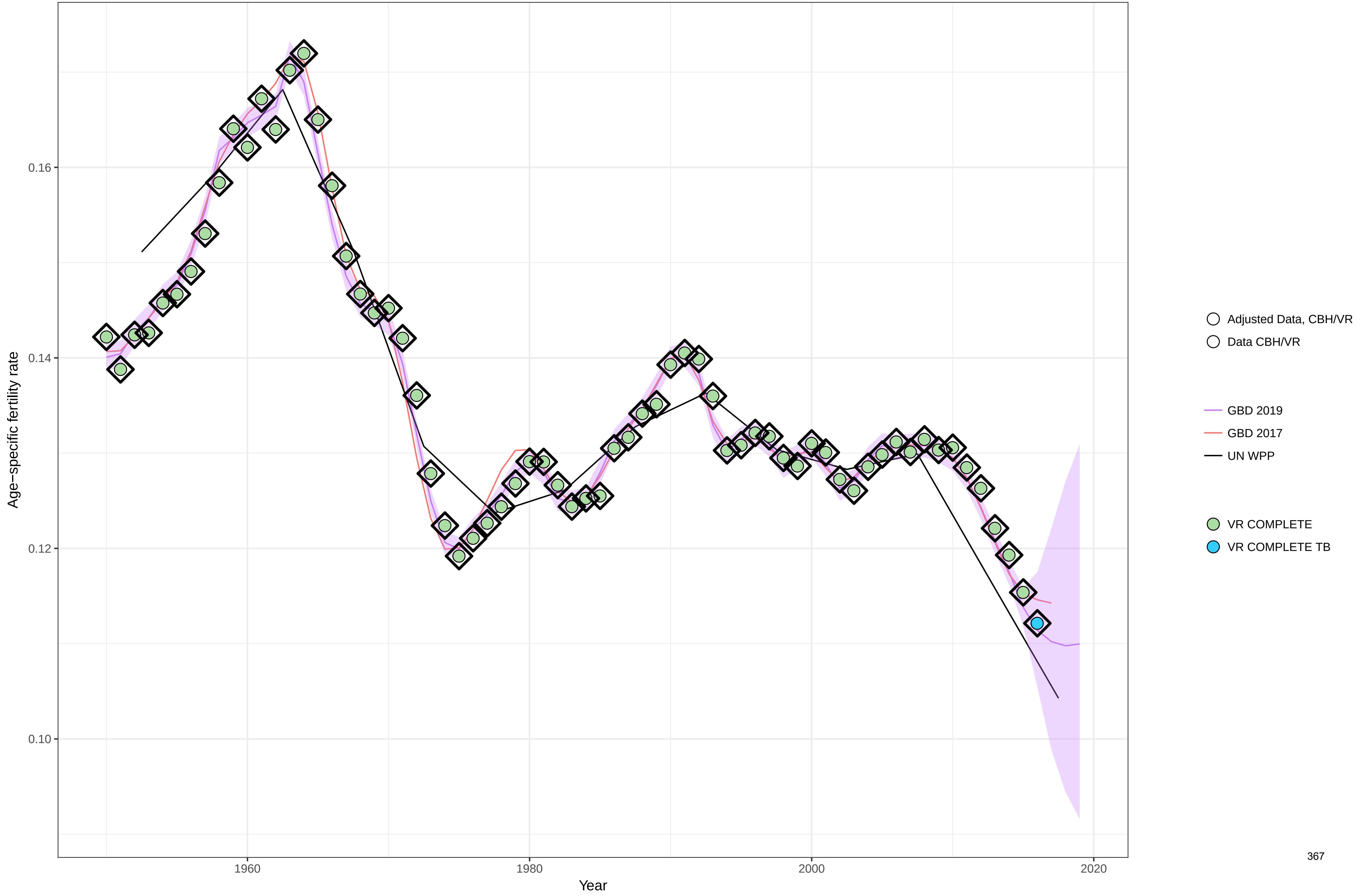

Age−specific fertility fitted trend lines, 1950−2019: Belgium, Ages: 30−34
Data points enclosed with a diamond are reference sources

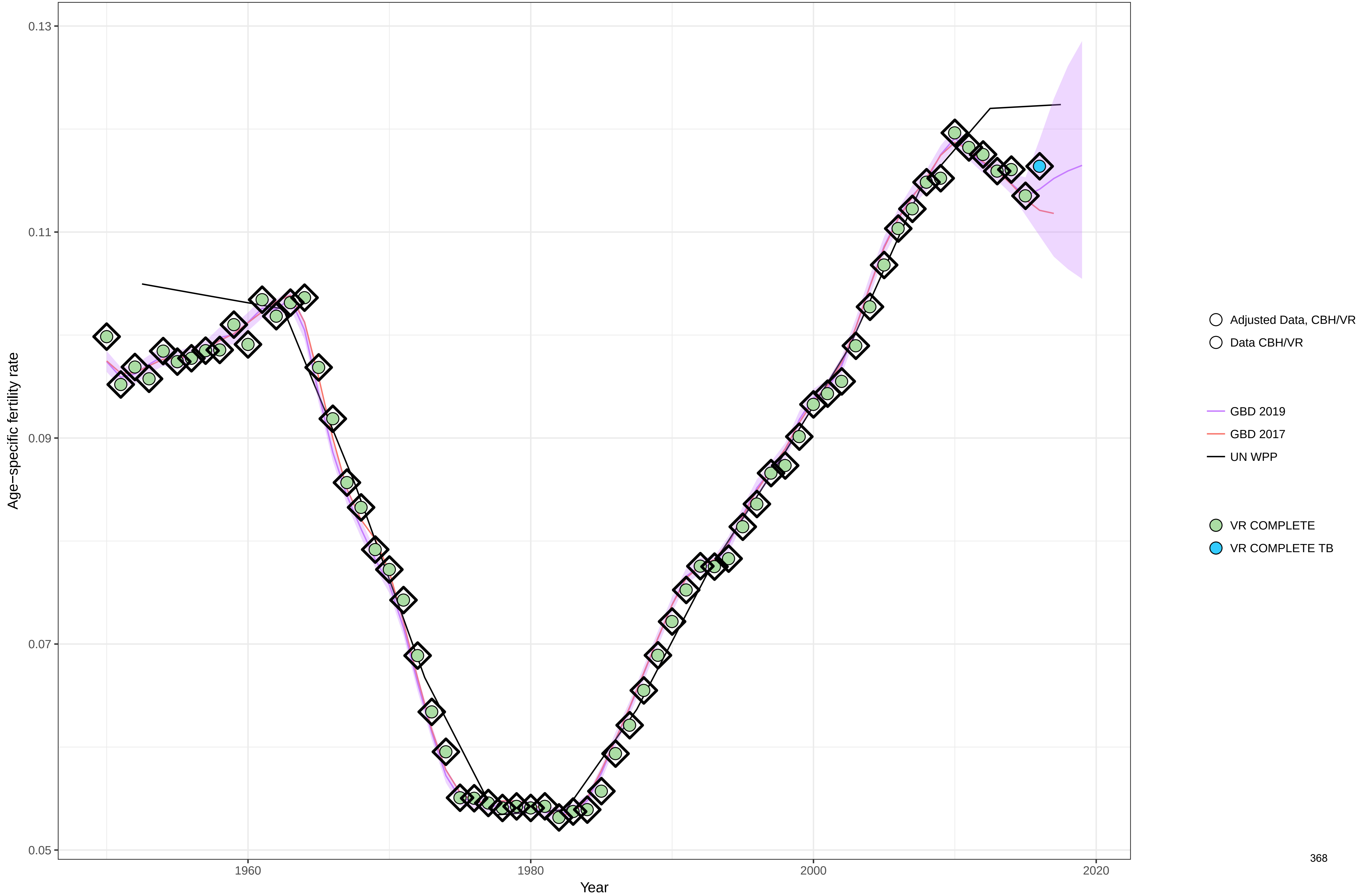

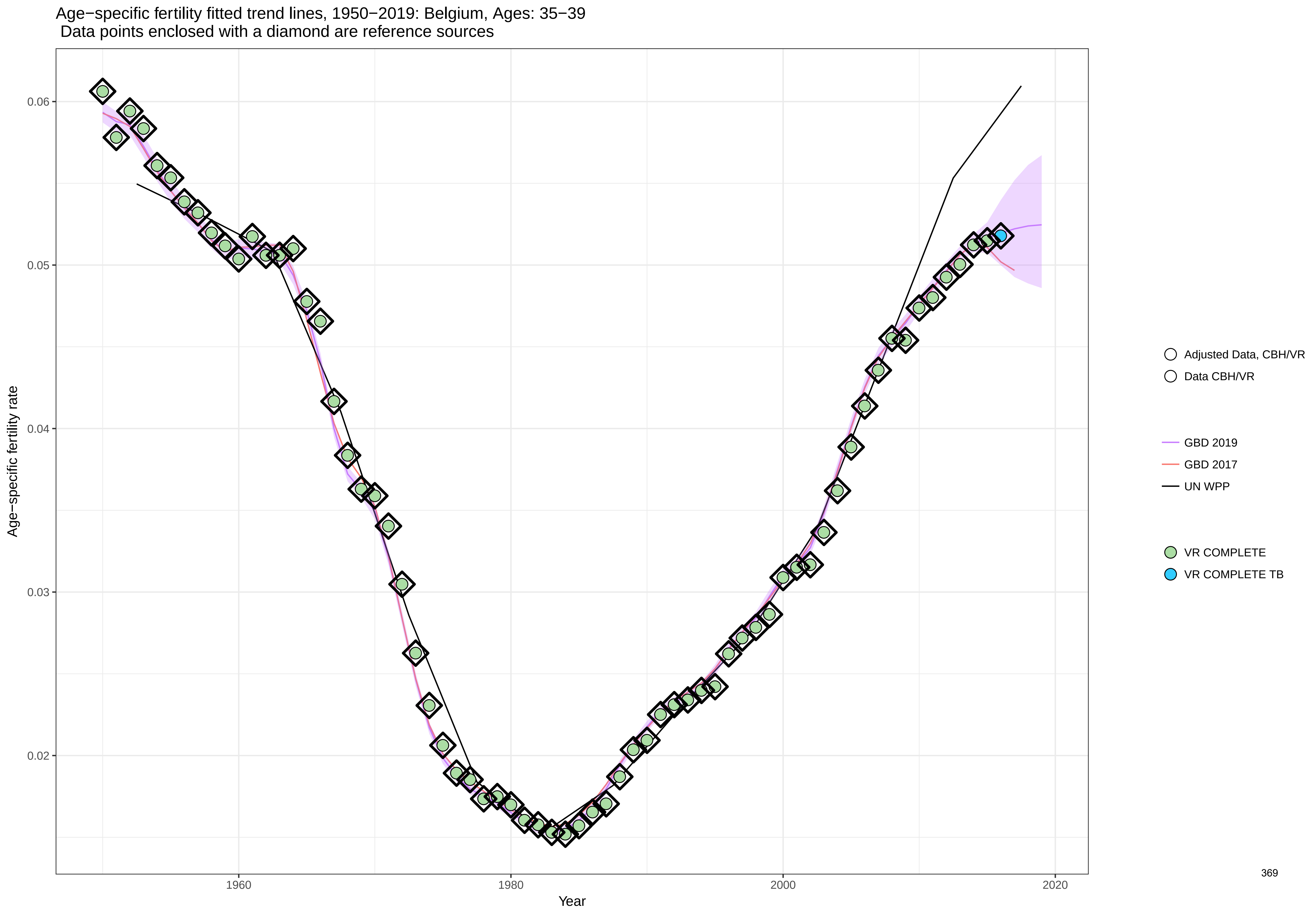

Age−specific fertility fitted trend lines, 1950−2019: Belgium, Ages: 35−39
Data points enclosed with a diamond are reference sources
0.06
0.05
0.04
0.03
0.02
Age−specific fertility rate
1960
1980
2000
2020
Year
Adjusted Data, CBH/VR
Data CBH/VR
GBD 2019
GBD 2017
UN WPP
VR COMPLETE
VR COMPLETE TB

Age−specific fertility fitted trend lines, 1950−2019: Belgium, Ages: 40−44
Data points enclosed with a diamond are reference sources

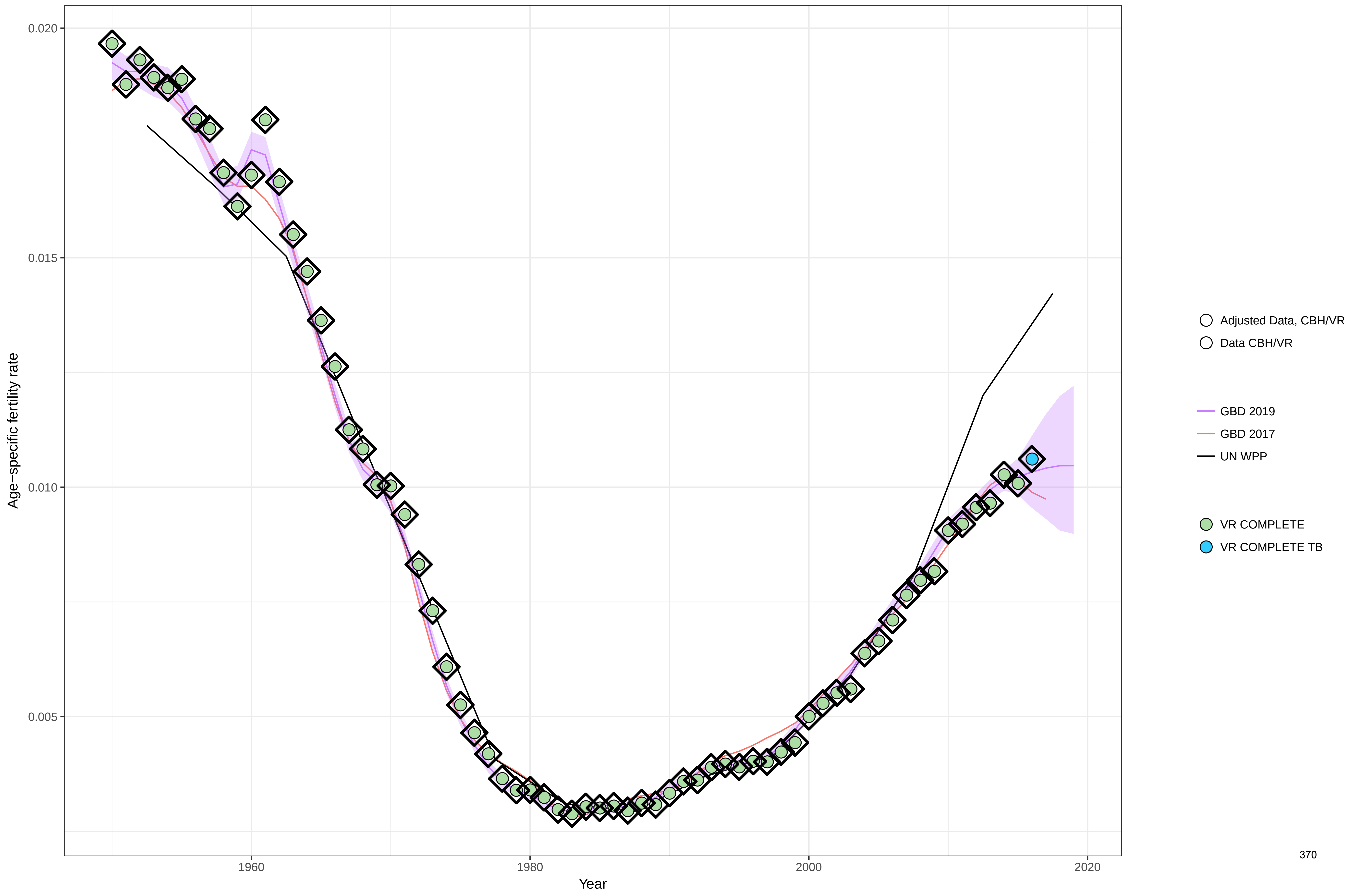

Age−specific fertility fitted trend lines, 1950−2019: Belgium, Ages: 45−49
Data points enclosed with a diamond are reference sources

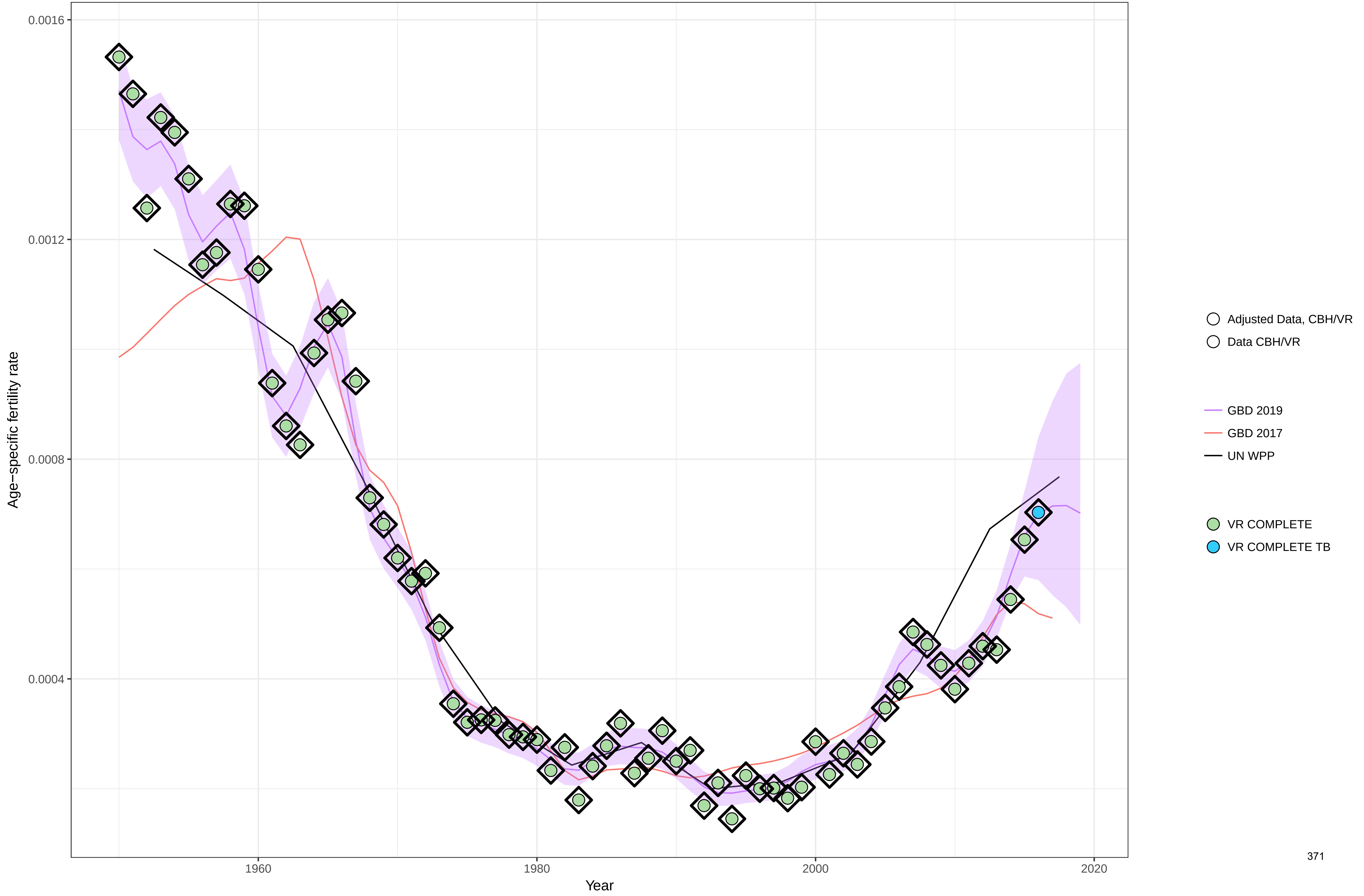

Age−specific fertility fitted trend lines, 1950−2019: Belgium, Ages: 50−54
Data points enclosed with a diamond are reference sources

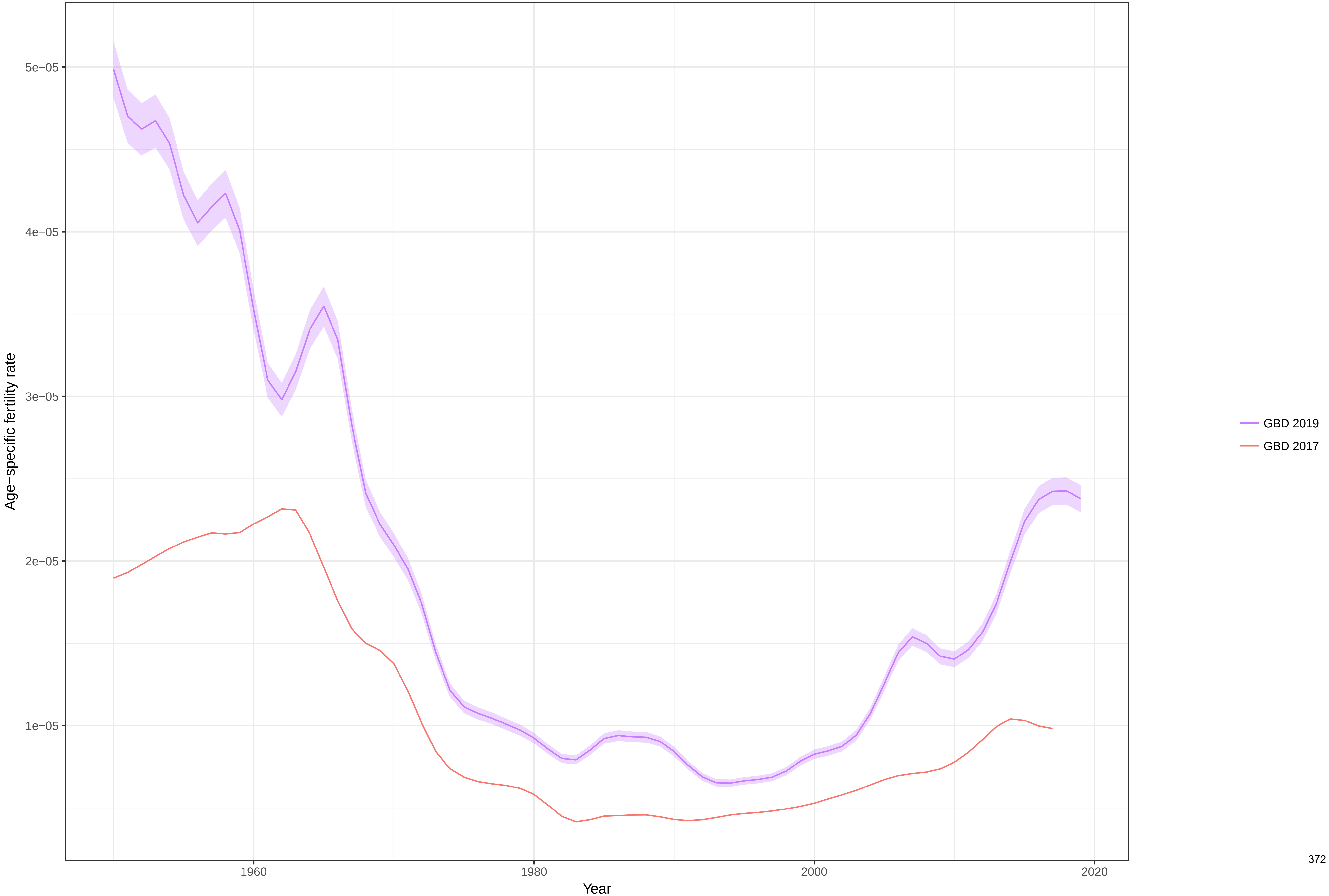

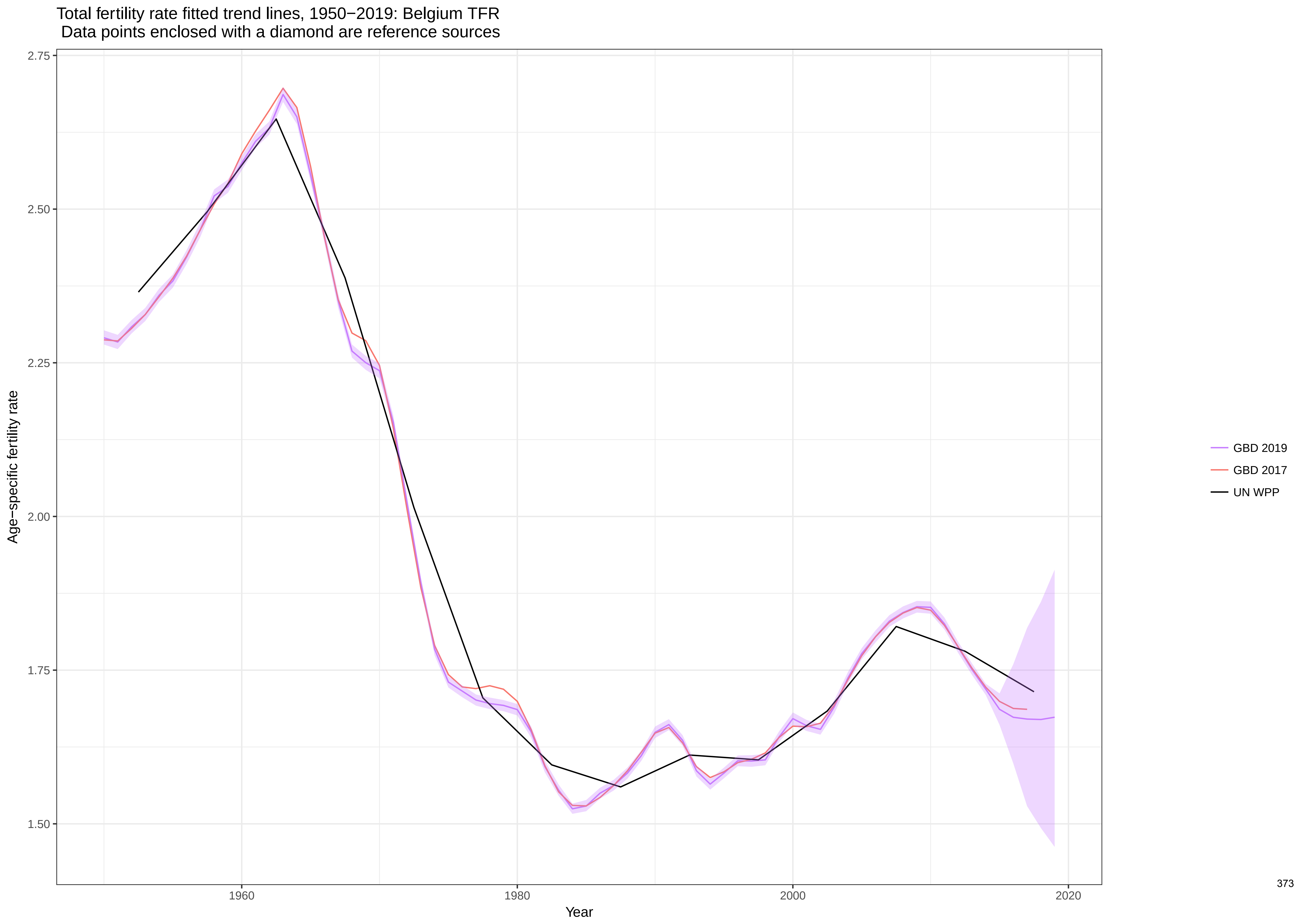
Total fertility rate fitted trend lines, 1950−2019: Belgium TFR
Data points enclosed with a diamond are reference sources
2.75
2.50
2.25
2.00
1.75
1.50
Age−specific fertility rate
1960
1980
2000
2020
Year
GBD 2019
GBD 2017
UN WPP

Age−specific fertility fitted trend lines, 1950−2019: Benin, Ages: 10−14
Data points enclosed with a diamond are reference sources

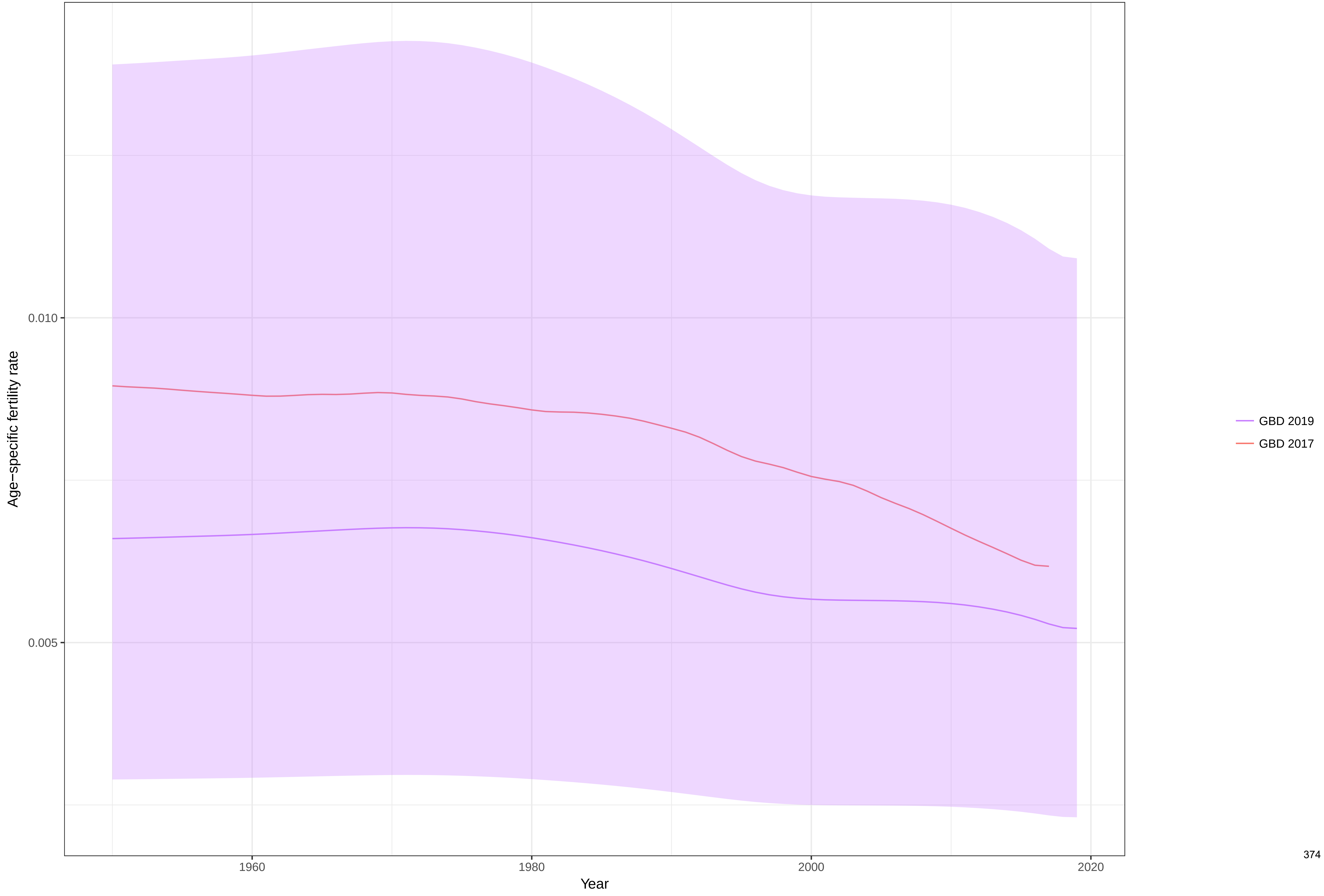

Age−specific fertility fitted trend lines, 1950−2019: Benin, Ages: 15−19
Data points enclosed with a diamond are reference sources

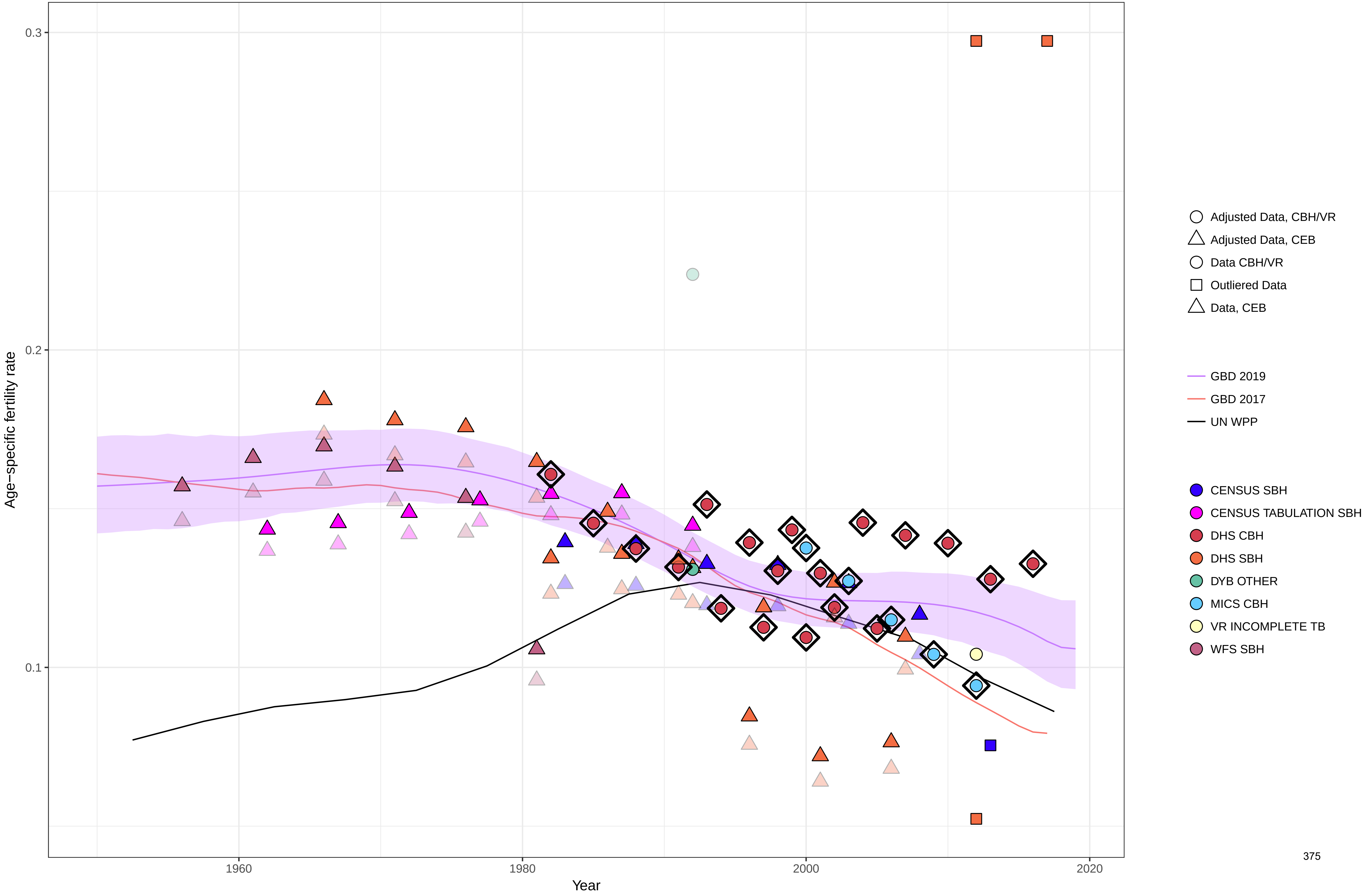

Age−specific fertility fitted trend lines, 1950−2019: Benin, Ages: 20−24
Data points enclosed with a diamond are reference sources

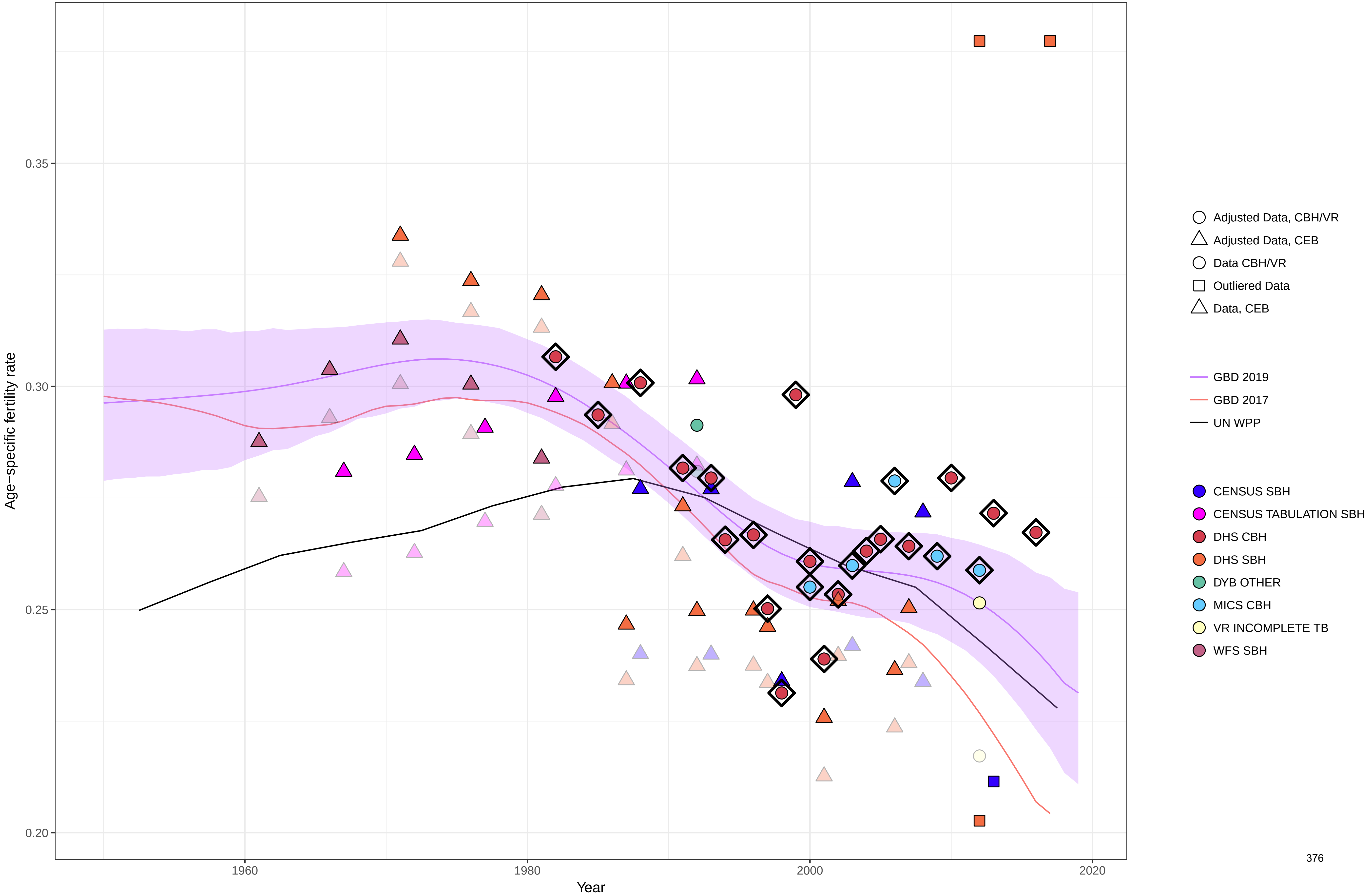

Age−specific fertility fitted trend lines, 1950−2019: Benin, Ages: 25−29
Data points enclosed with a diamond are reference sources

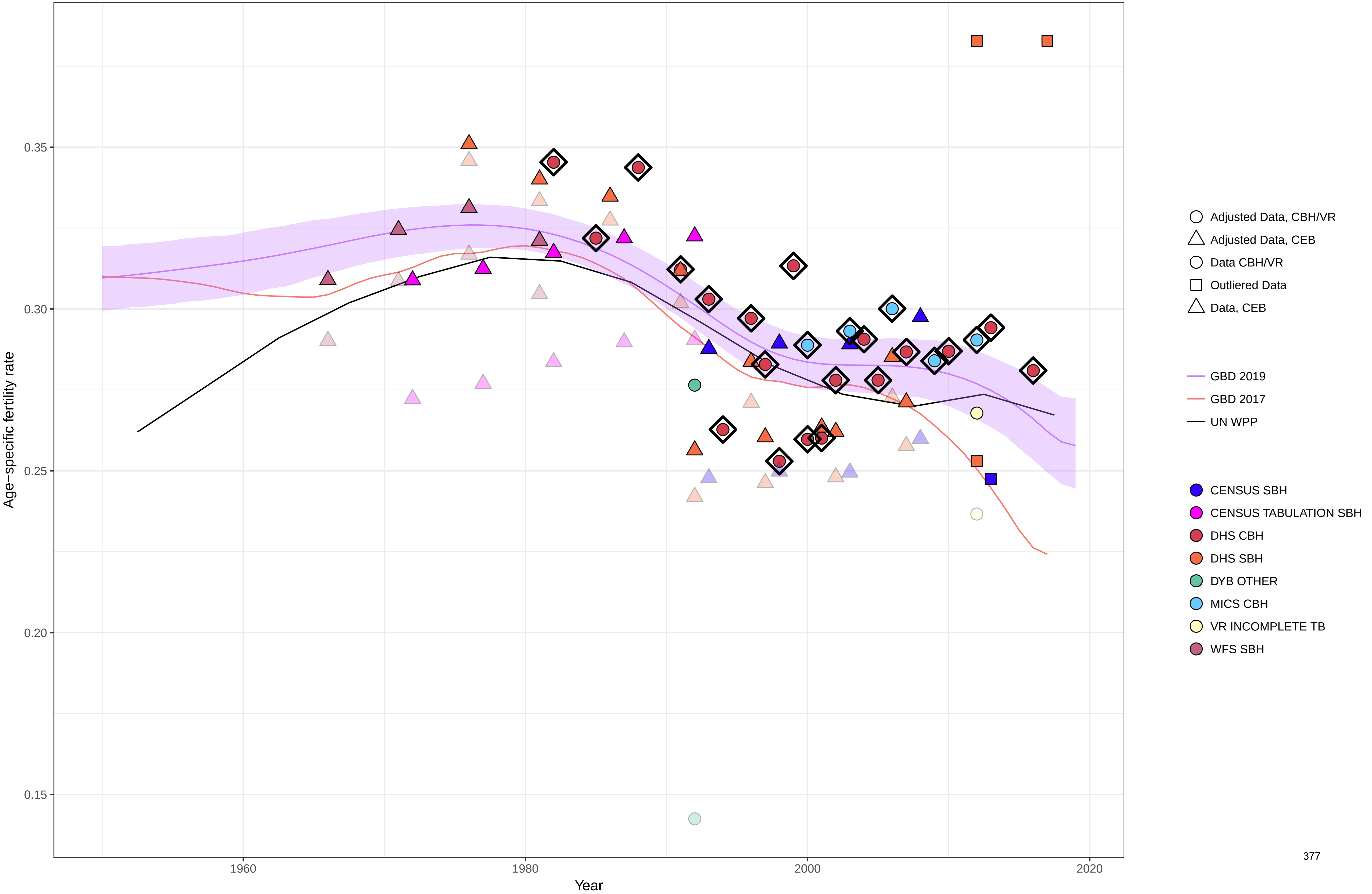

Age−specific fertility fitted trend lines, 1950−2019: Benin, Ages: 30−34
Data points enclosed with a diamond are reference sources

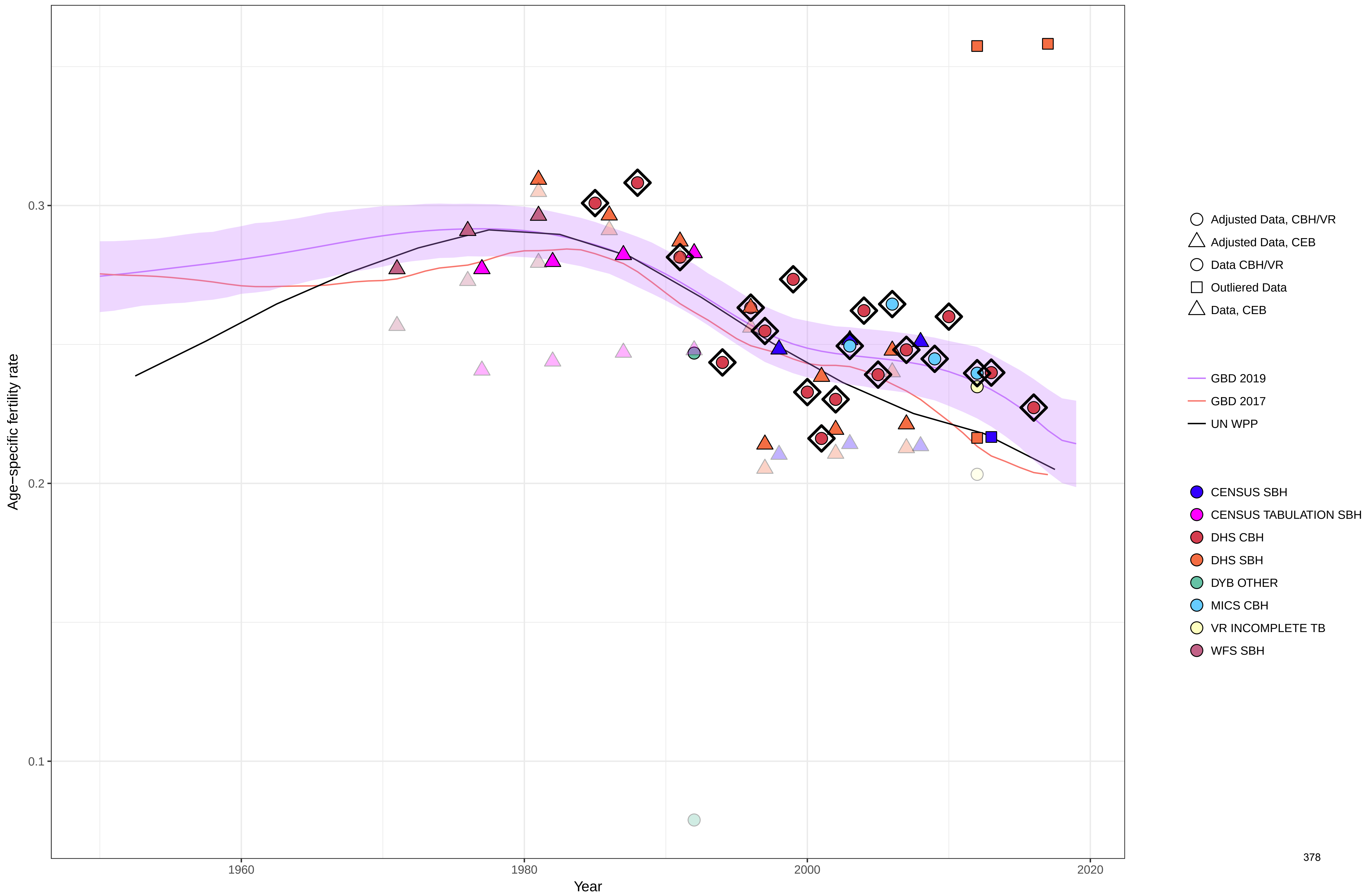

Age−specific fertility fitted trend lines, 1950−2019: Benin, Ages: 35−39
Data points enclosed with a diamond are reference sources

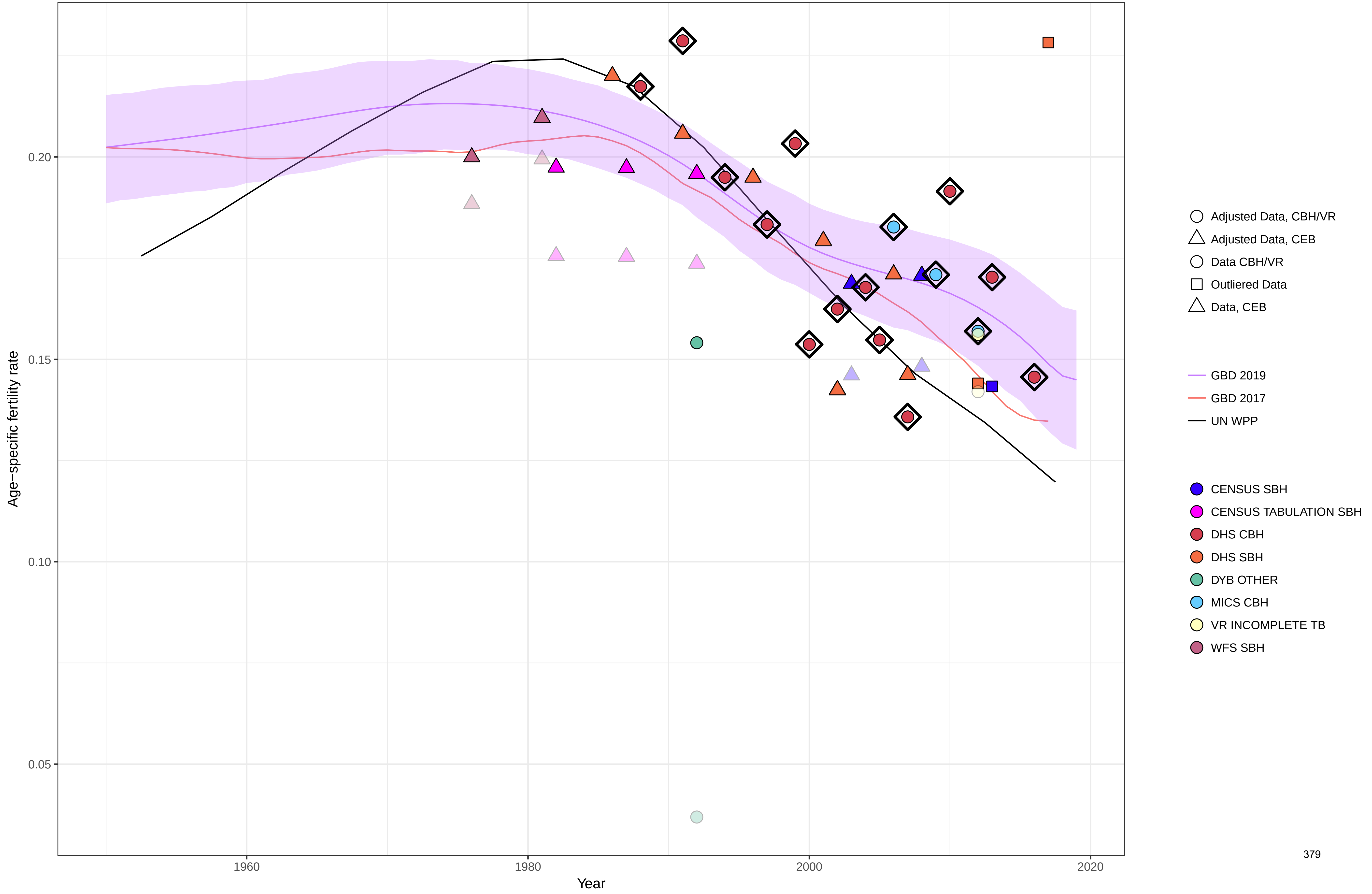

Age−specific fertility fitted trend lines, 1950−2019: Benin, Ages: 40−44
Data points enclosed with a diamond are reference sources

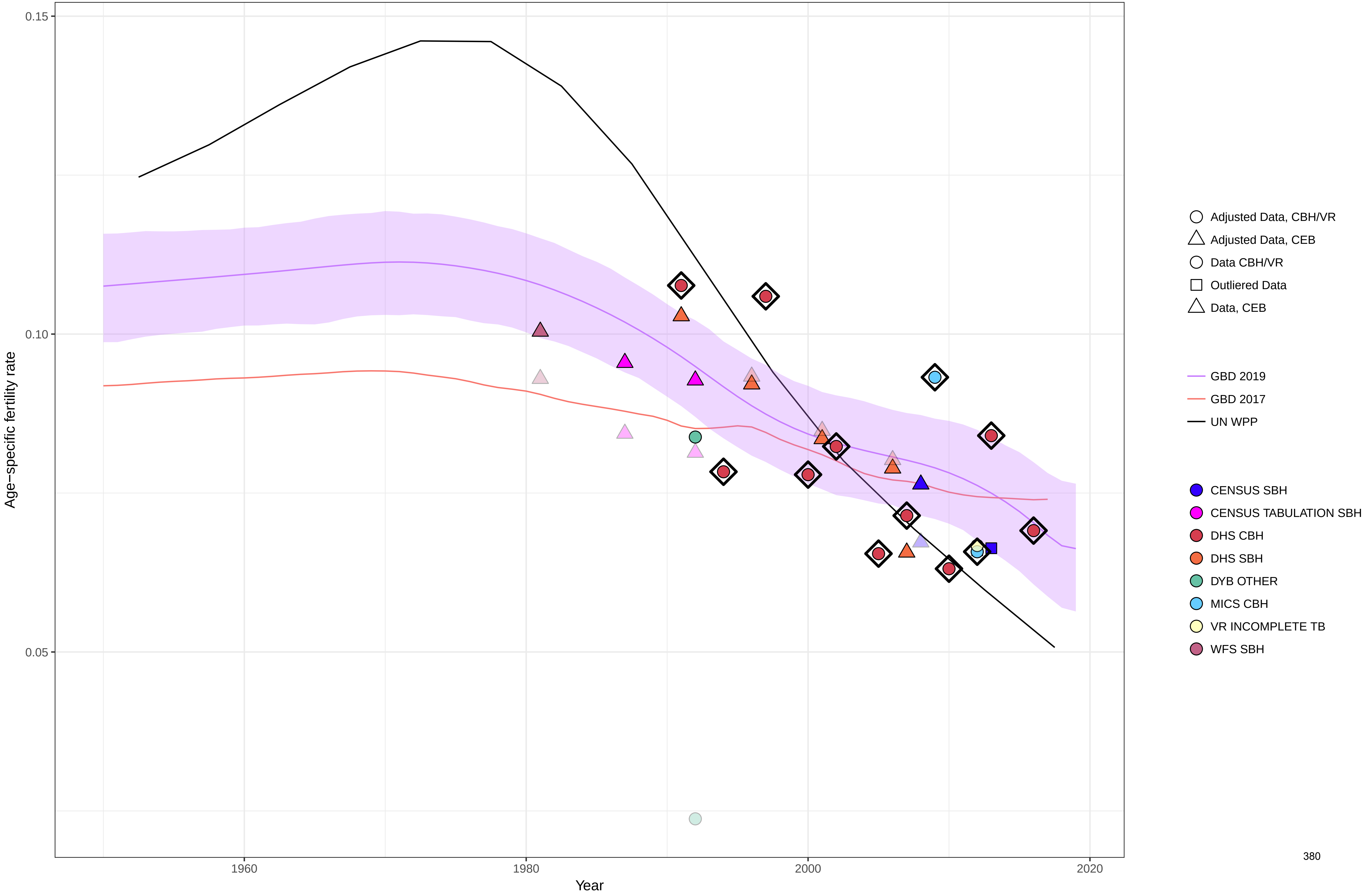

Age−specific fertility fitted trend lines, 1950−2019: Benin, Ages: 45−49
Data points enclosed with a diamond are reference sources

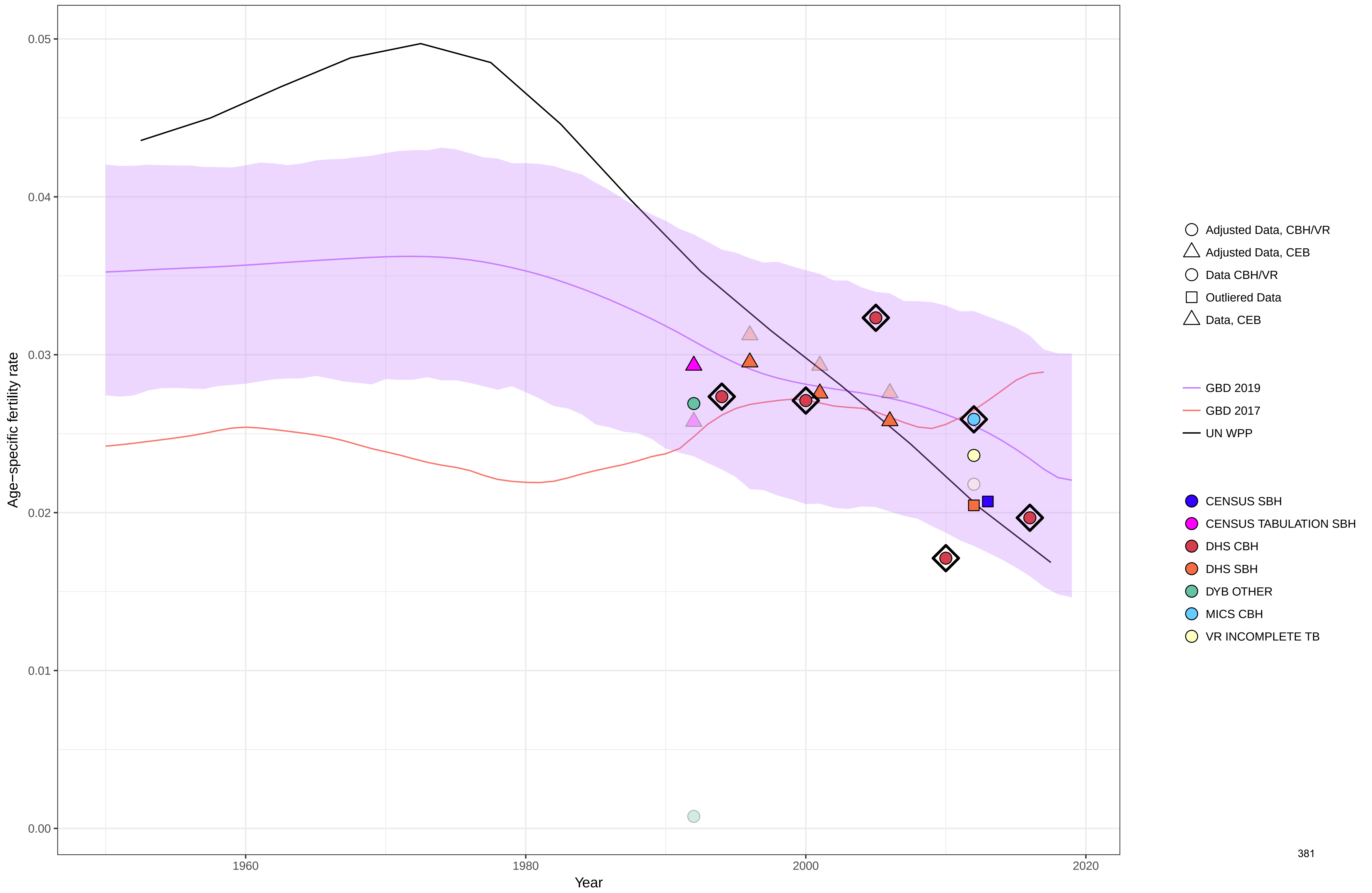

Age−specific fertility fitted trend lines, 1950−2019: Benin, Ages: 50−54
Data points enclosed with a diamond are reference sources

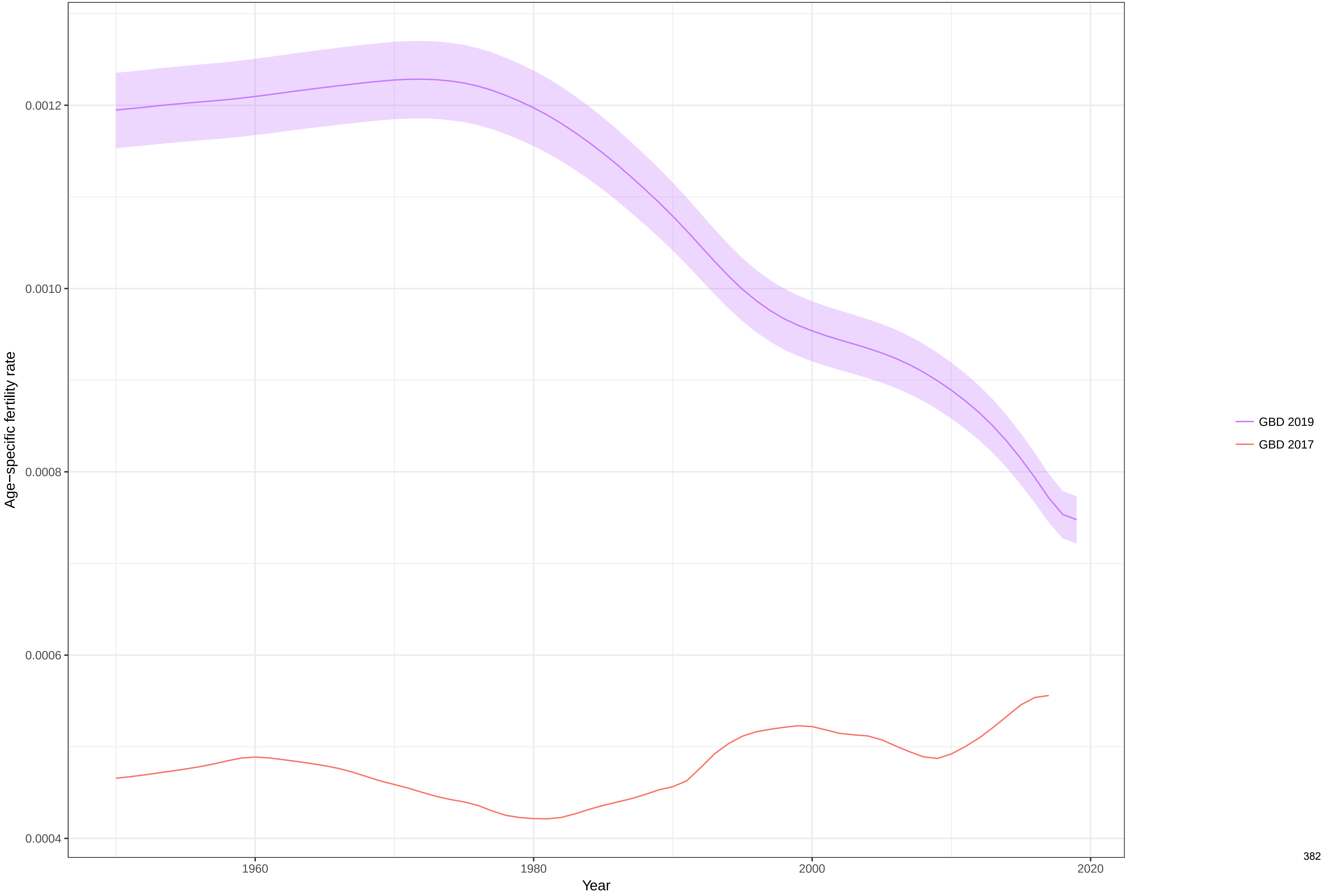

Total fertility rate fitted trend lines, 1950−2019: Benin TFR
Data points enclosed with a diamond are reference sources

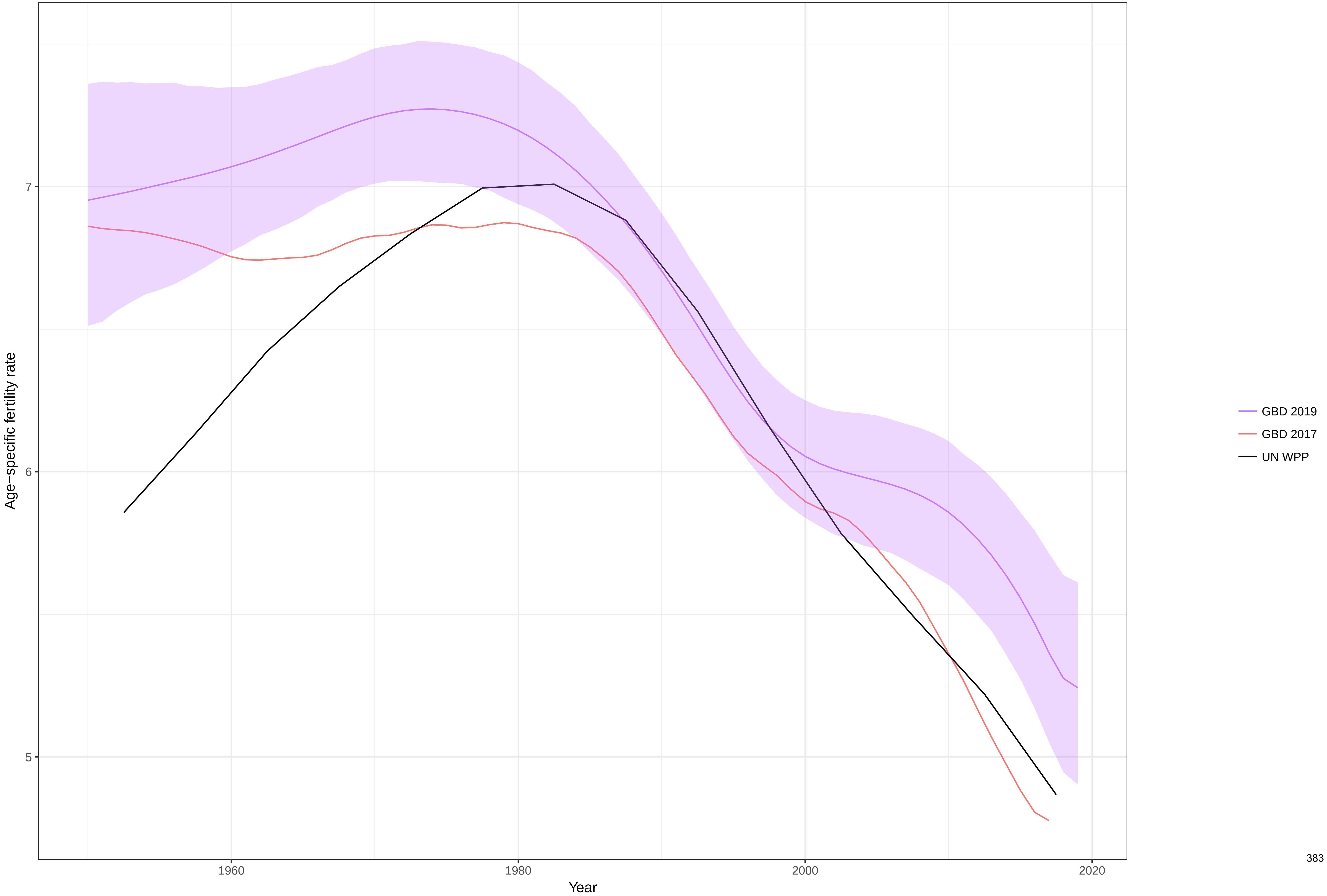

Age−specific fertility fitted trend lines, 1950−2019: Burkina Faso, Ages: 10−14
Data points enclosed with a diamond are reference sources

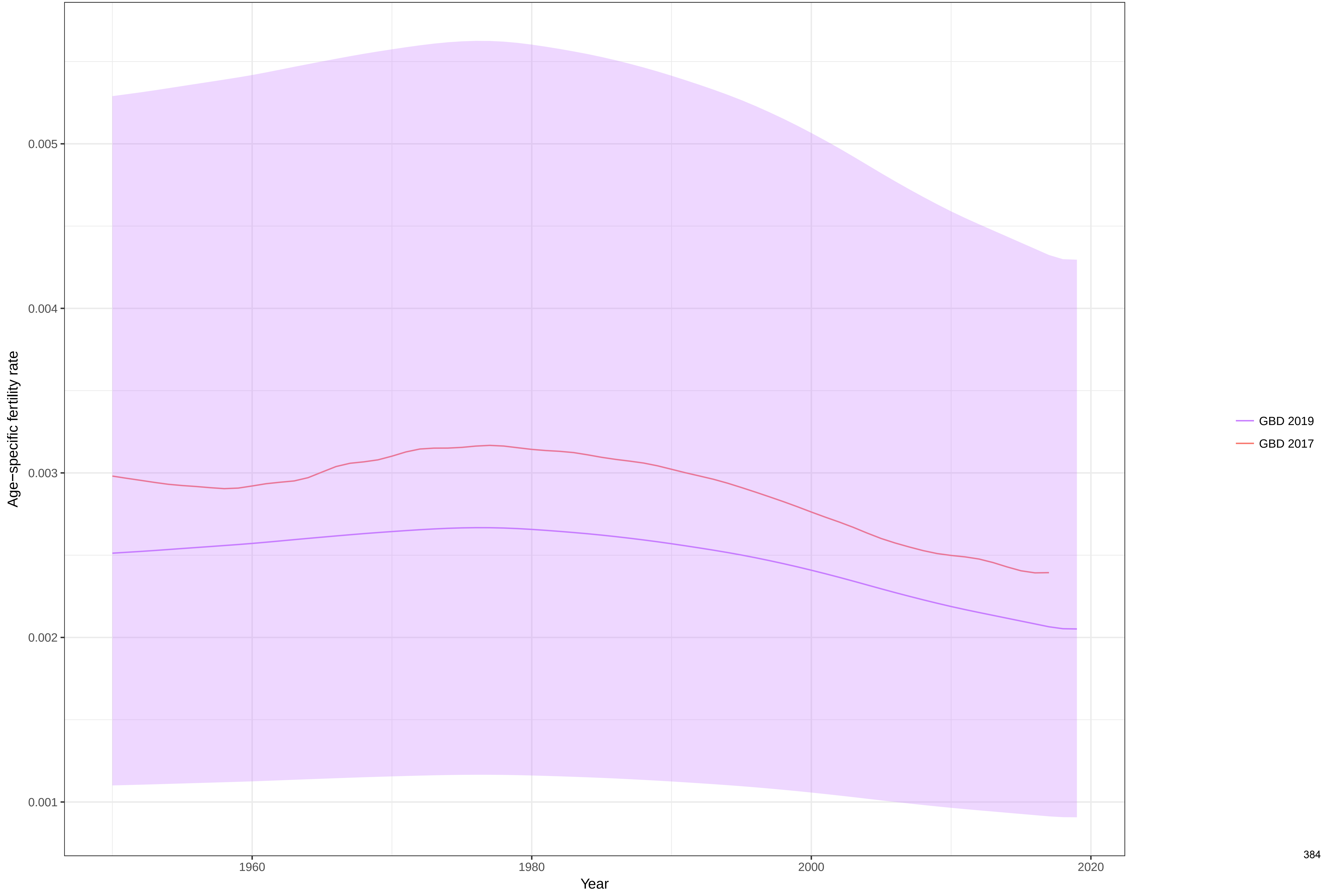

Age−specific fertility fitted trend lines, 1950−2019: Burkina Faso, Ages: 15−19
Data points enclosed with a diamond are reference sources

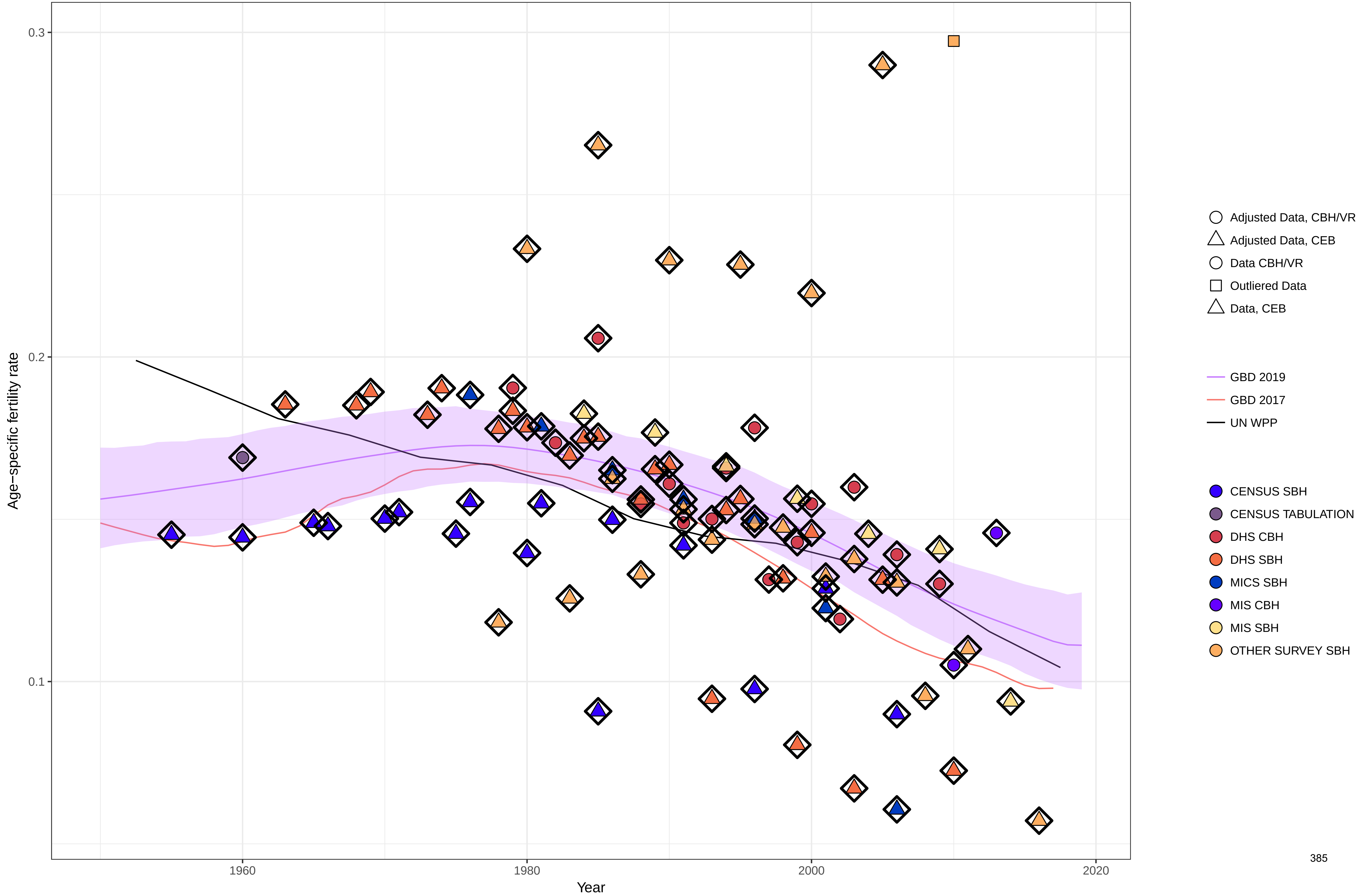

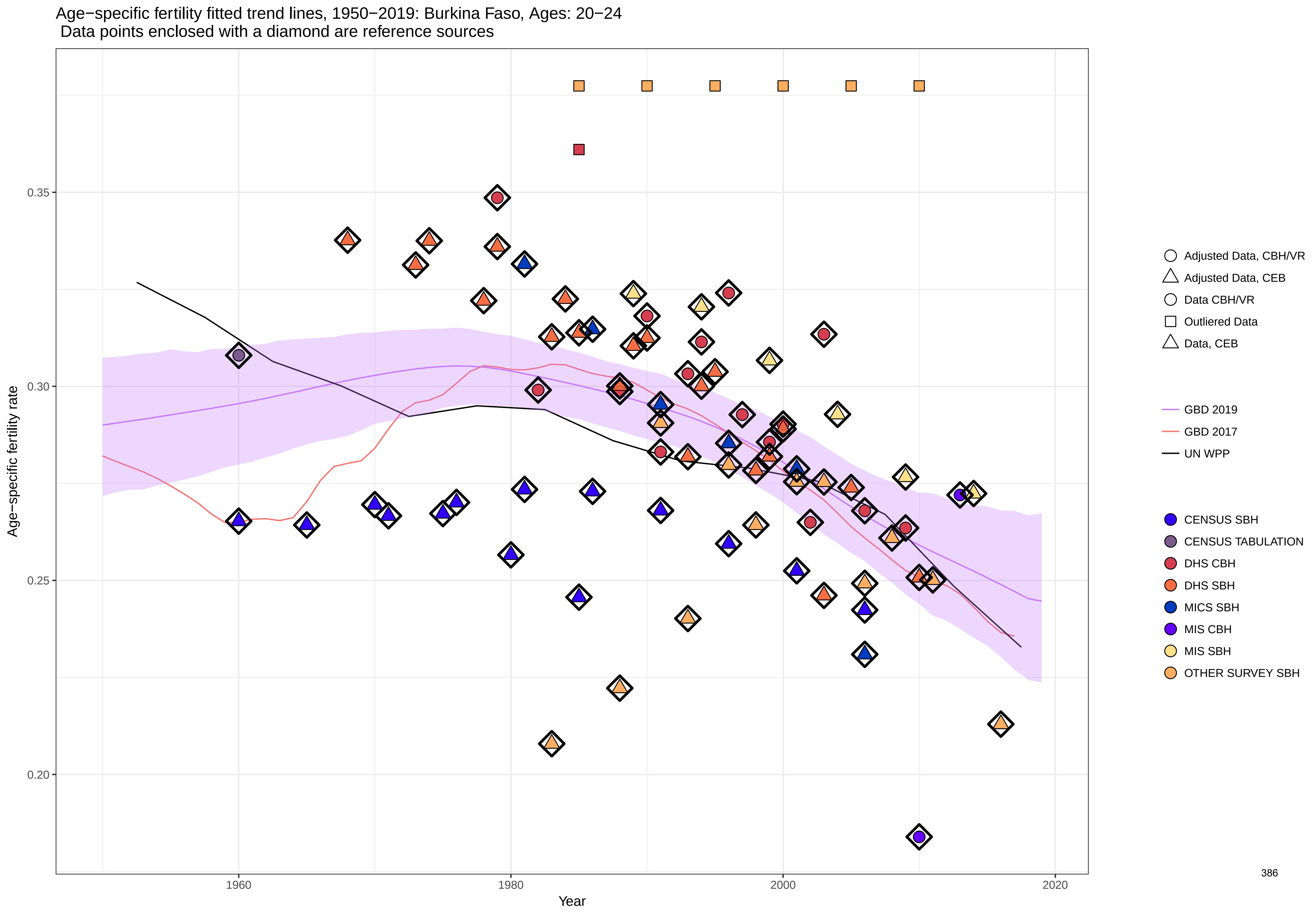

Age−specific fertility fitted trend lines, 1950−2019: Burkina Faso, Ages: 20−24
Data points enclosed with a diamond are reference sources
Age−specific fertility rate
0.35
0.30
0.25
0.20
Year
1960
1980
2000
2020
Adjusted Data, CBH/VR
Adjusted Data, CEB
Data CBH/VR
Outliered Data
Data, CEB
GBD 2019
GBD 2017
UN WPP
CENSUS SBH
CENSUS TABULATION
DHS CBH
DHS SBH
MICS SBH
MIS CBH
MIS SBH
OTHER SURVEY SBH

Age−specific fertility fitted trend lines, 1950−2019: Burkina Faso, Ages: 25−29
Data points enclosed with a diamond are reference sources

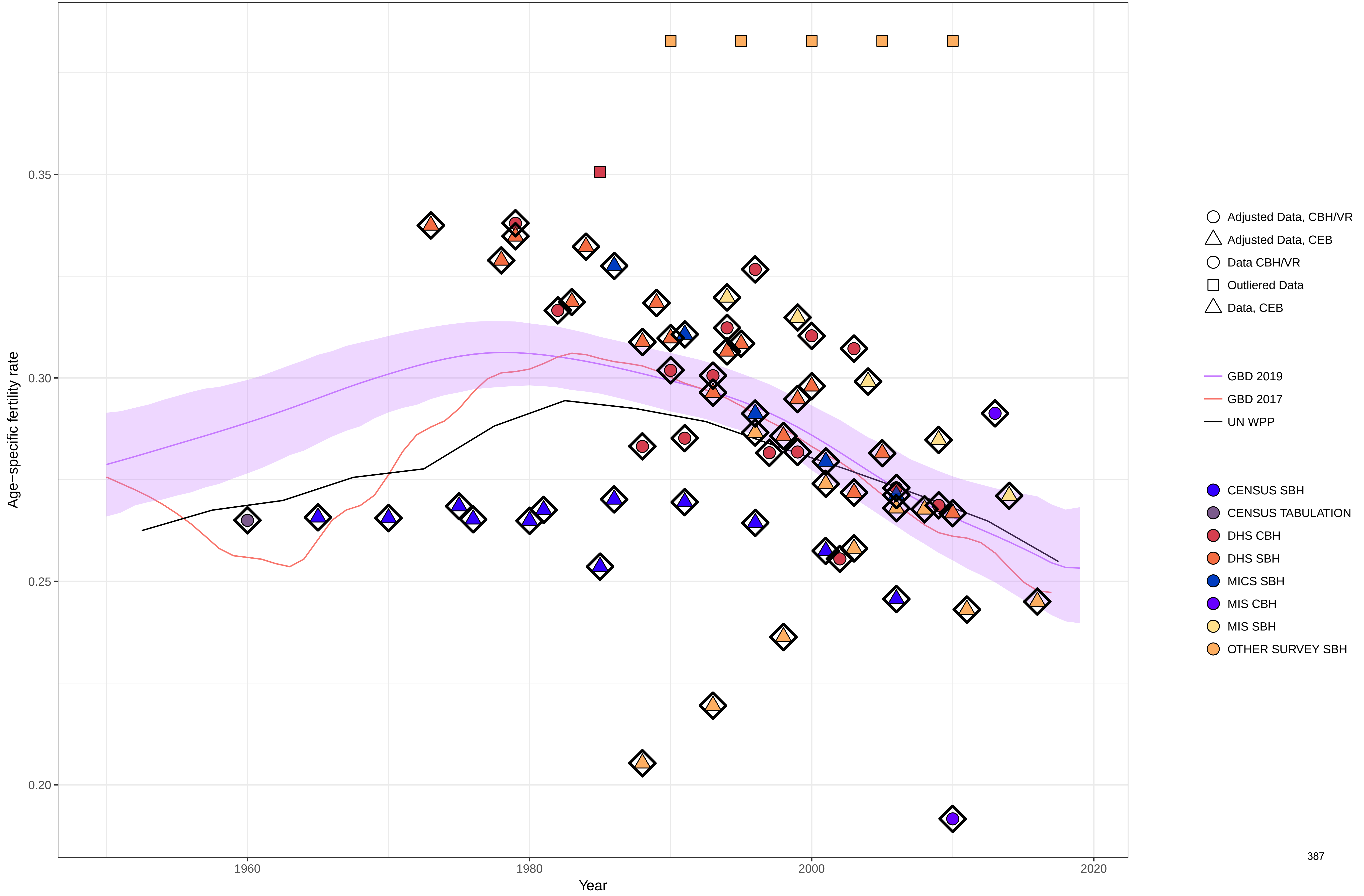

Age−specific fertility fitted trend lines, 1950−2019: Burkina Faso, Ages: 30−34
Data points enclosed with a diamond are reference sources

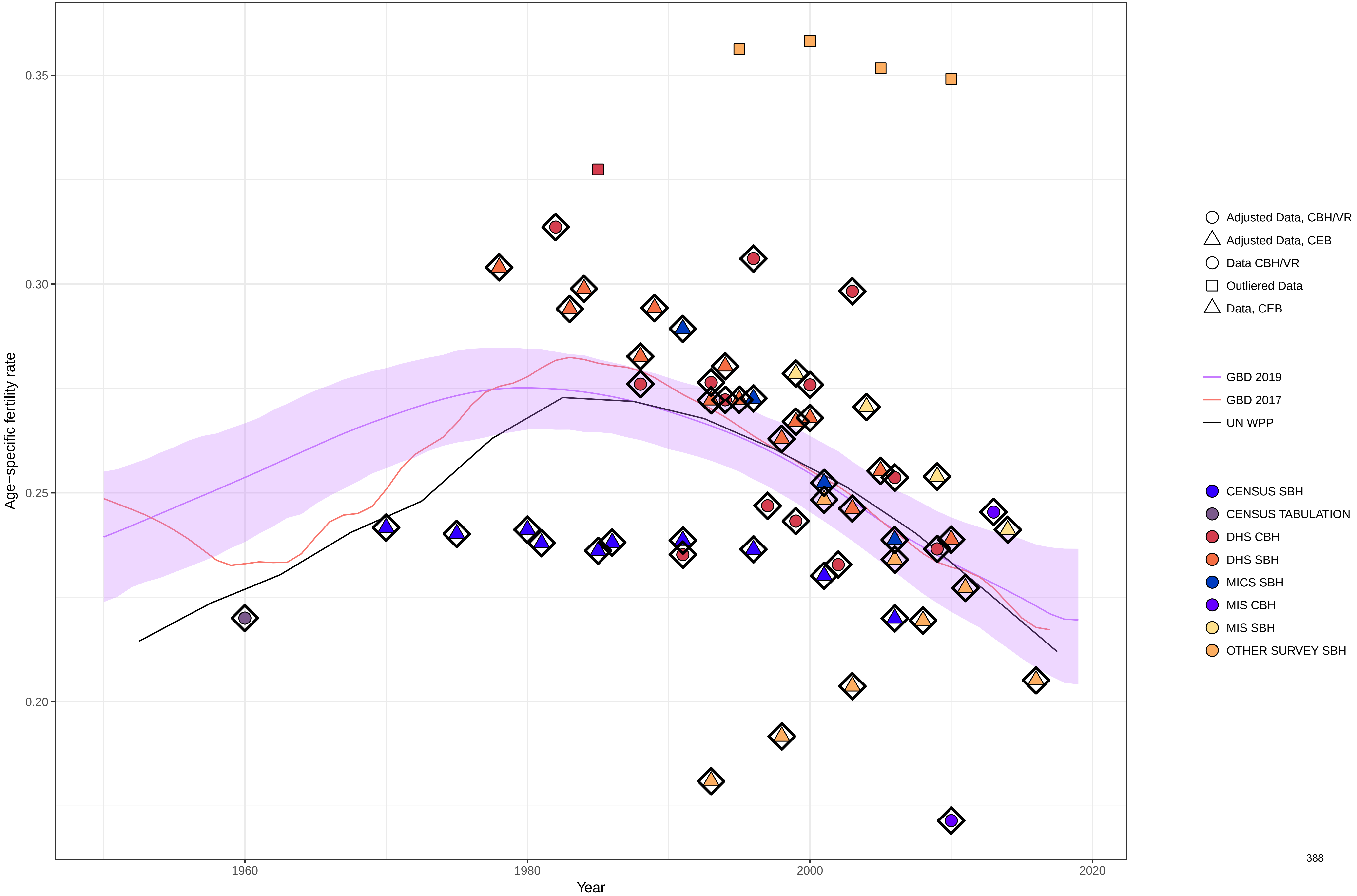

Age−specific fertility fitted trend lines, 1950−2019: Burkina Faso, Ages: 35−39
Data points enclosed with a diamond are reference sources

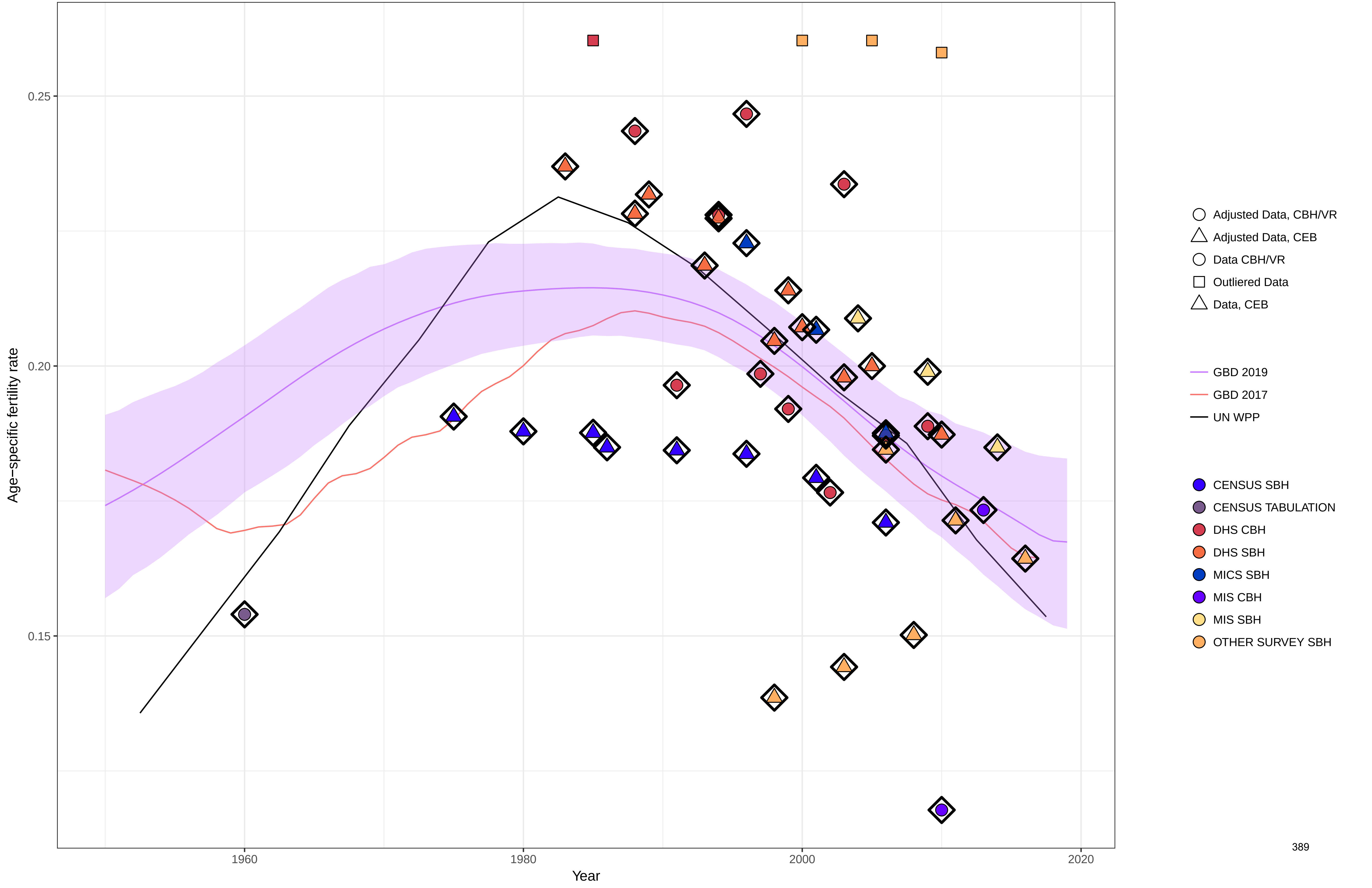

Age−specific fertility fitted trend lines, 1950−2019: Burkina Faso, Ages: 40−44
Data points enclosed with a diamond are reference sources

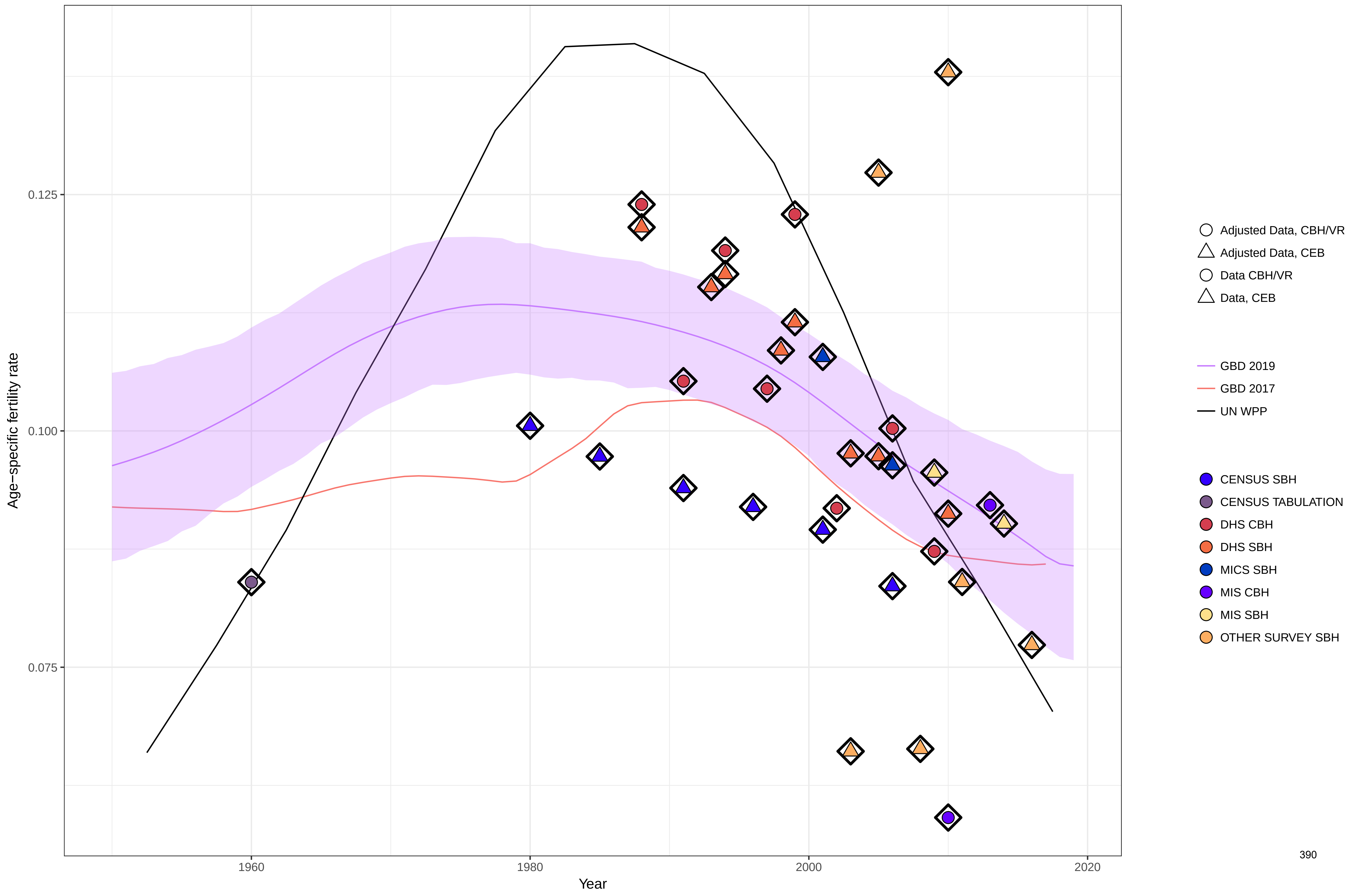

Age−specific fertility fitted trend lines, 1950−2019: Burkina Faso, Ages: 45−49
Data points enclosed with a diamond are reference sources

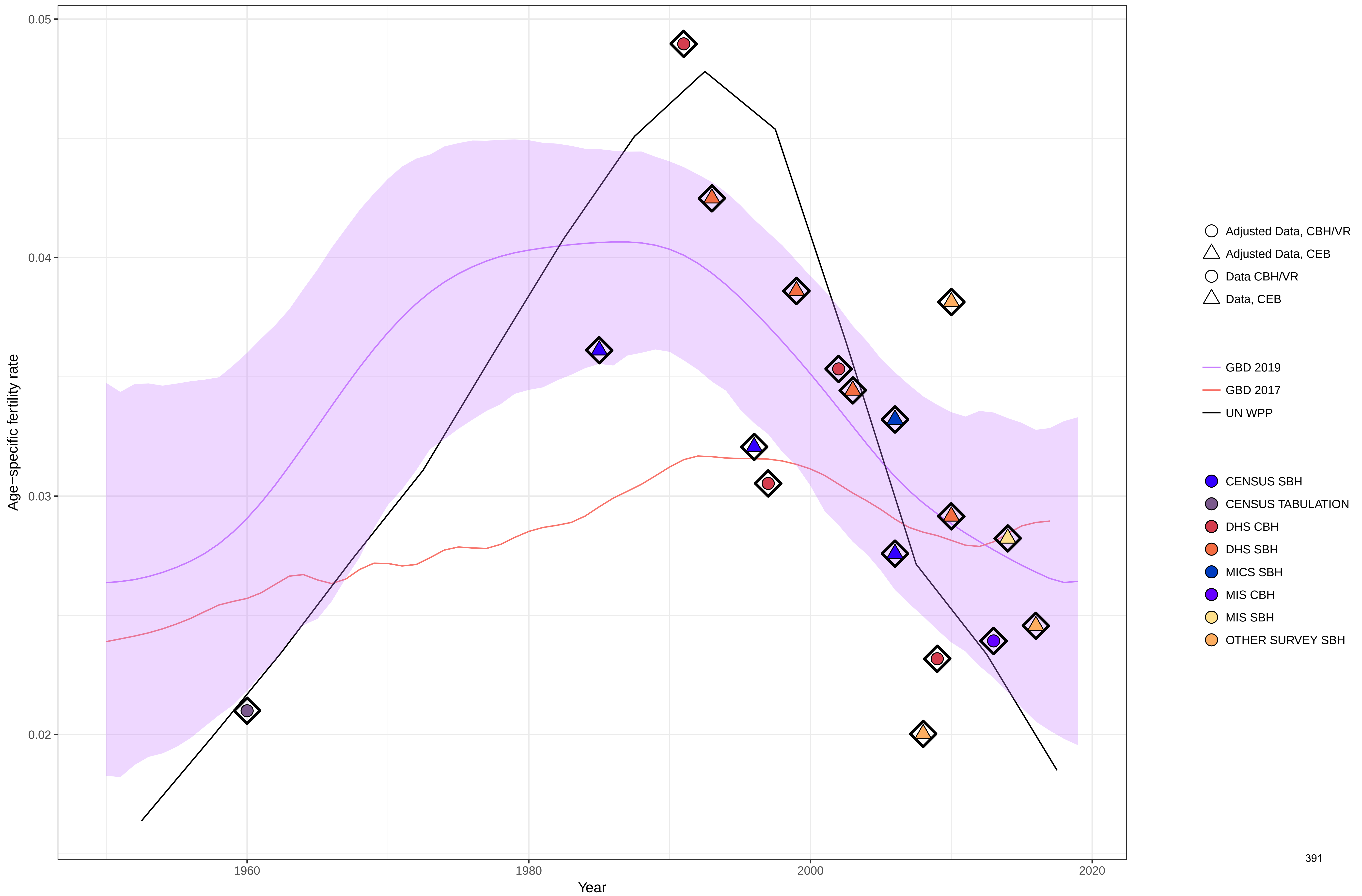

Age−specific fertility fitted trend lines, 1950−2019: Burkina Faso, Ages: 50−54
Data points enclosed with a diamond are reference sources

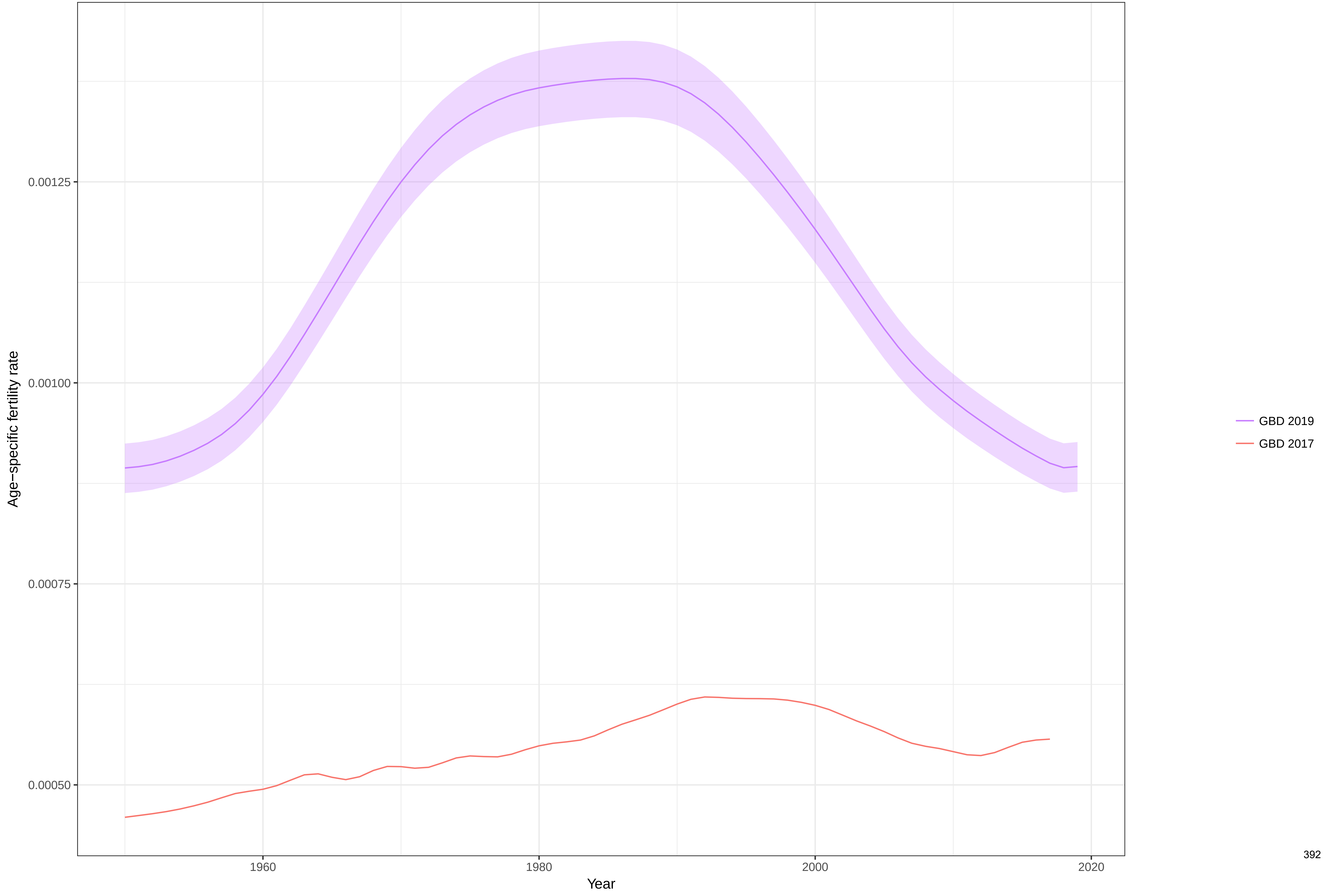

Total fertility rate fitted trend lines, 1950−2019: Burkina Faso TFR
Data points enclosed with a diamond are reference sources

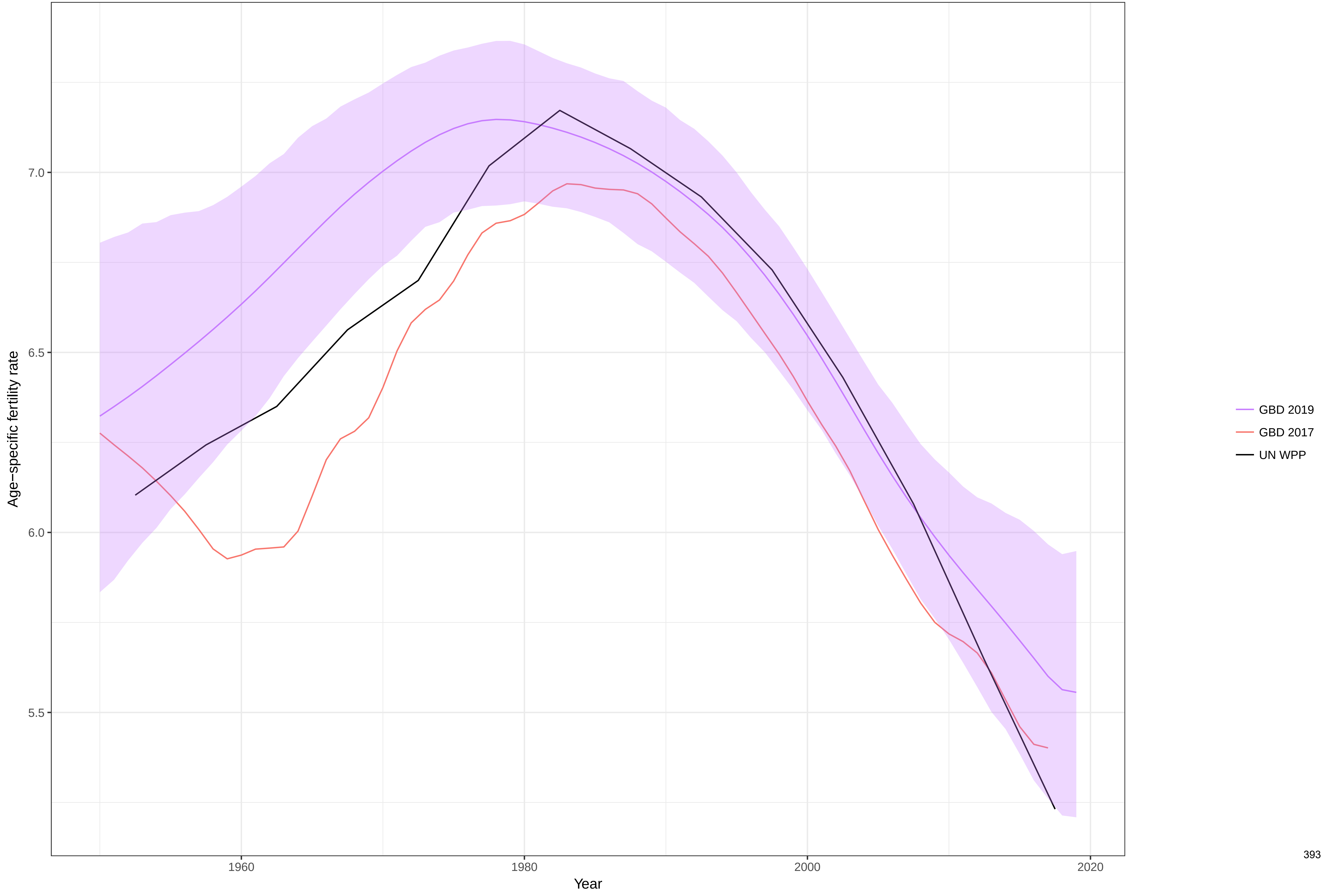

Age−specific fertility fitted trend lines, 1950−2019: Bangladesh, Ages: 10−14
Data points enclosed with a diamond are reference sources

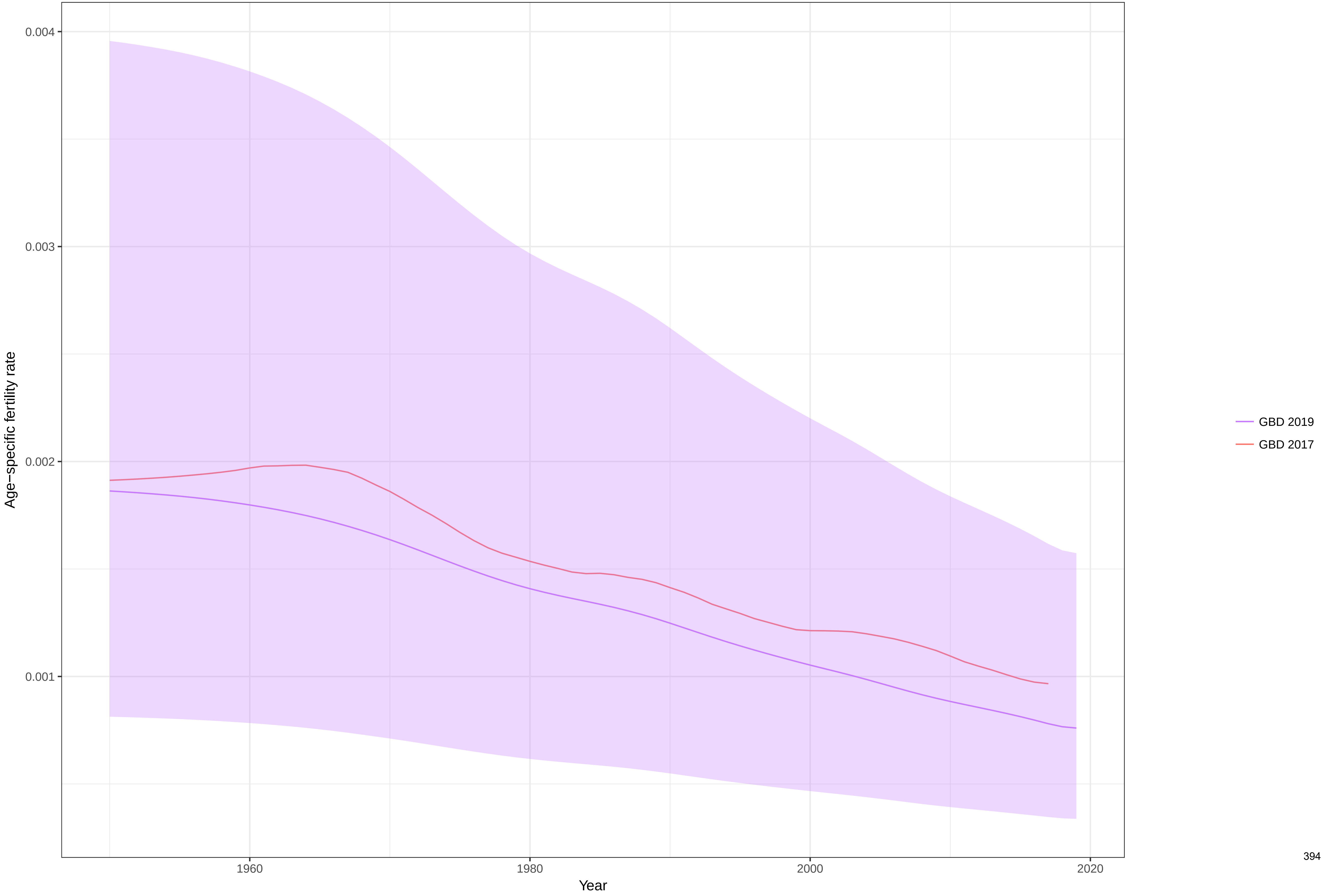

Age−specific fertility fitted trend lines, 1950−2019: Bangladesh, Ages: 15−19
Data points enclosed with a diamond are reference sources

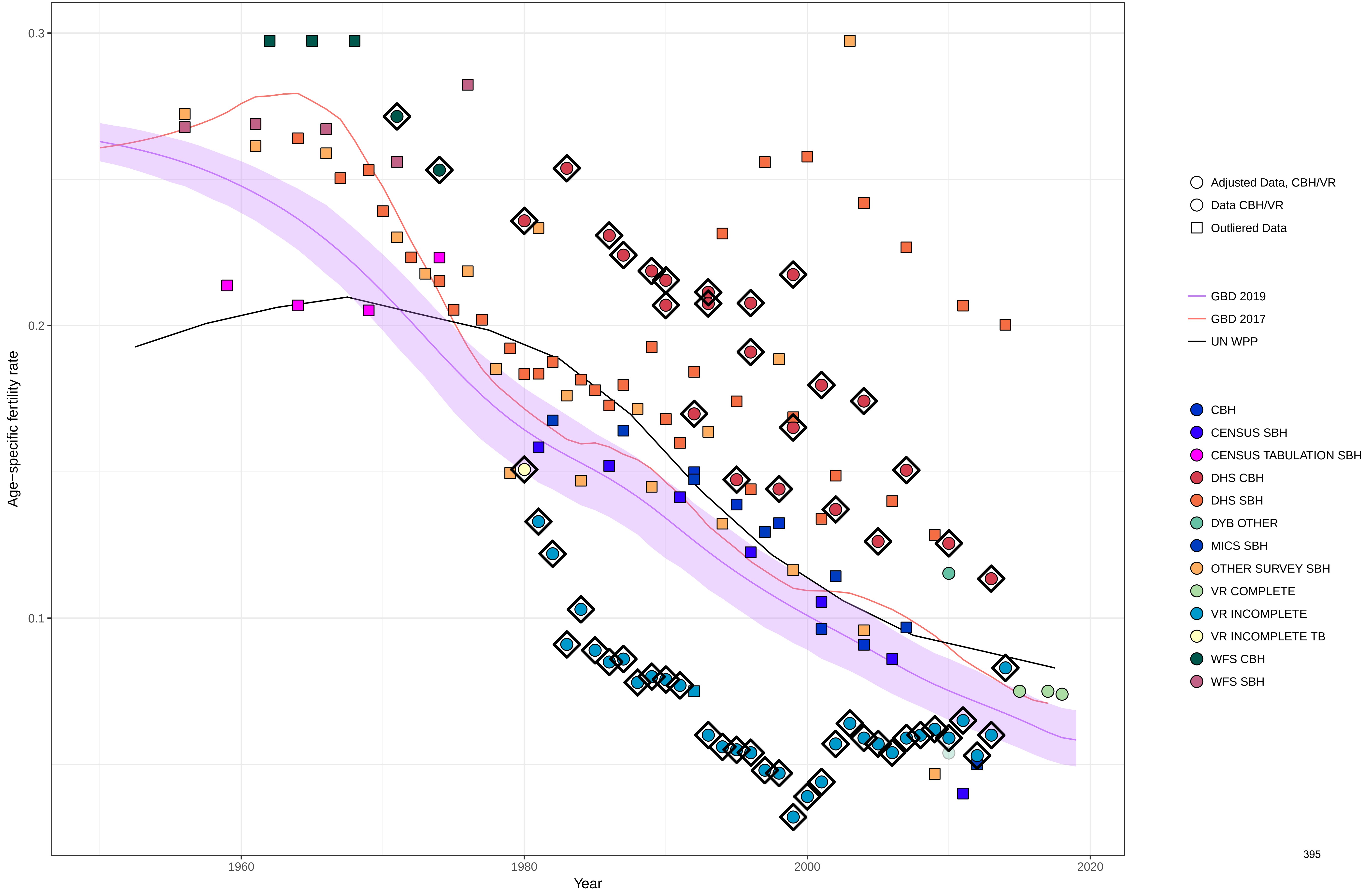

Age−specific fertility fitted trend lines, 1950−2019: Bangladesh, Ages: 20−24
Data points enclosed with a diamond are reference sources

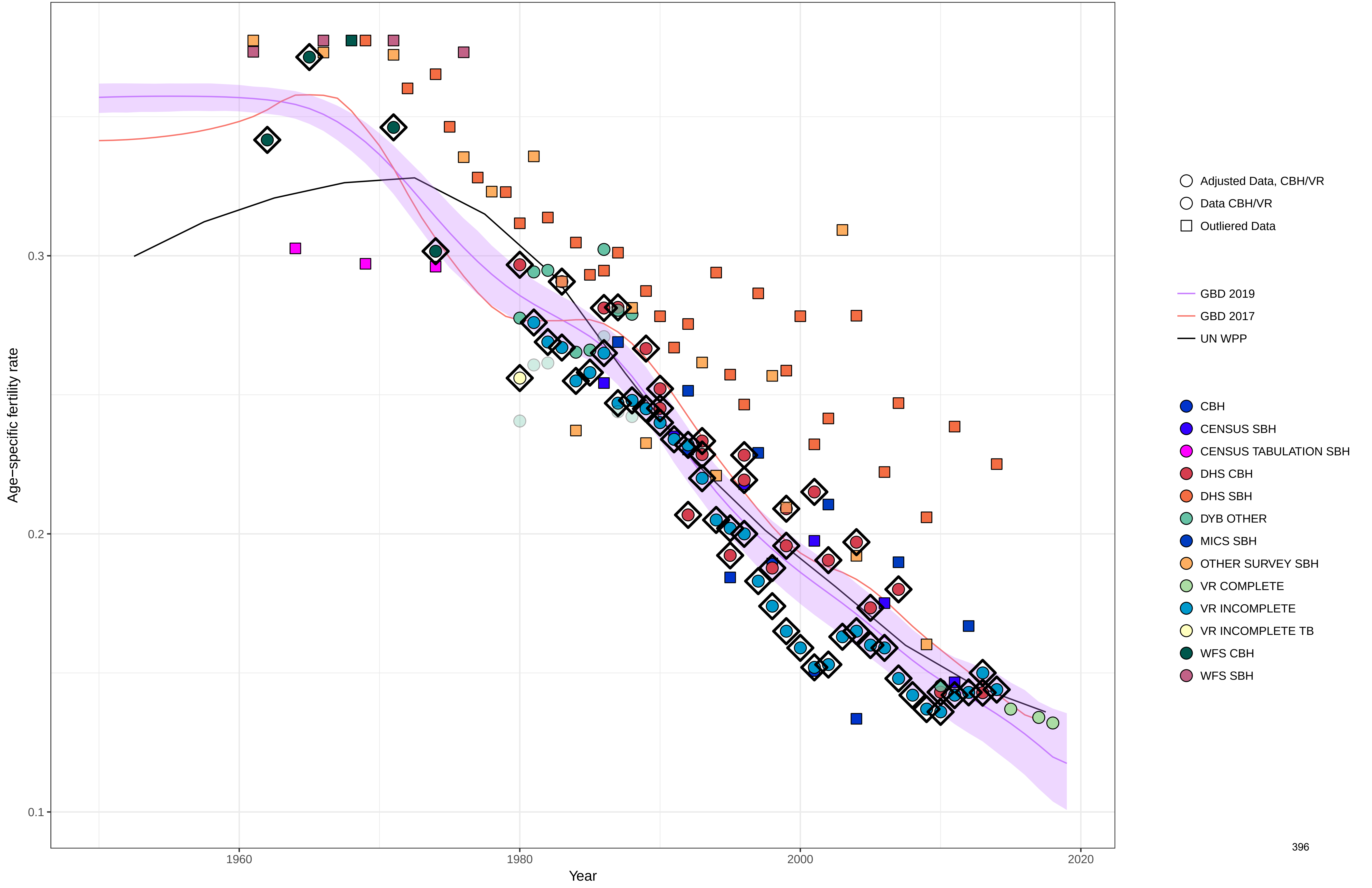

Age−specific fertility fitted trend lines, 1950−2019: Bangladesh, Ages: 25−29
Data points enclosed with a diamond are reference sources

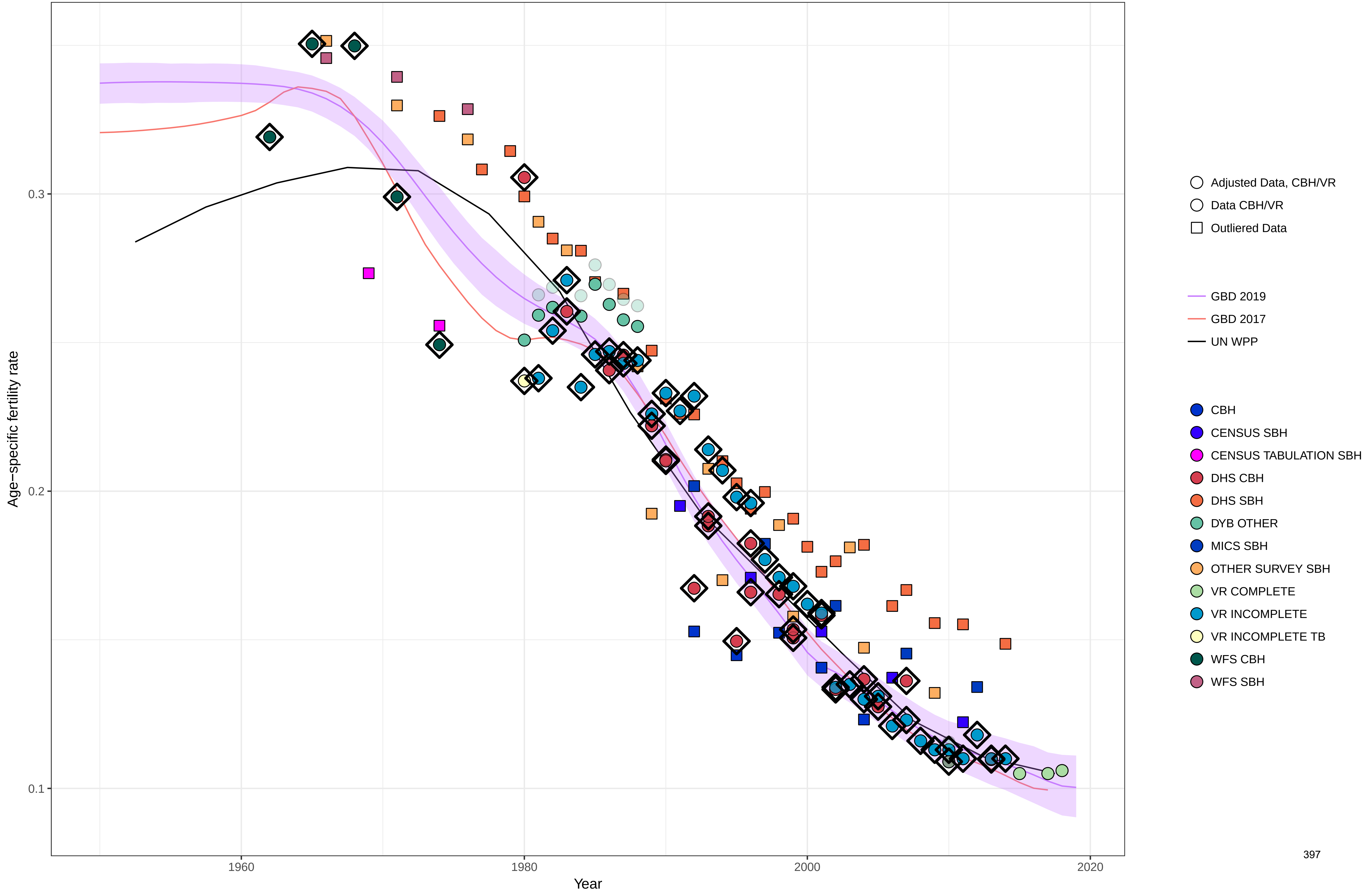

Age−specific fertility fitted trend lines, 1950−2019: Bangladesh, Ages: 30−34
Data points enclosed with a diamond are reference sources

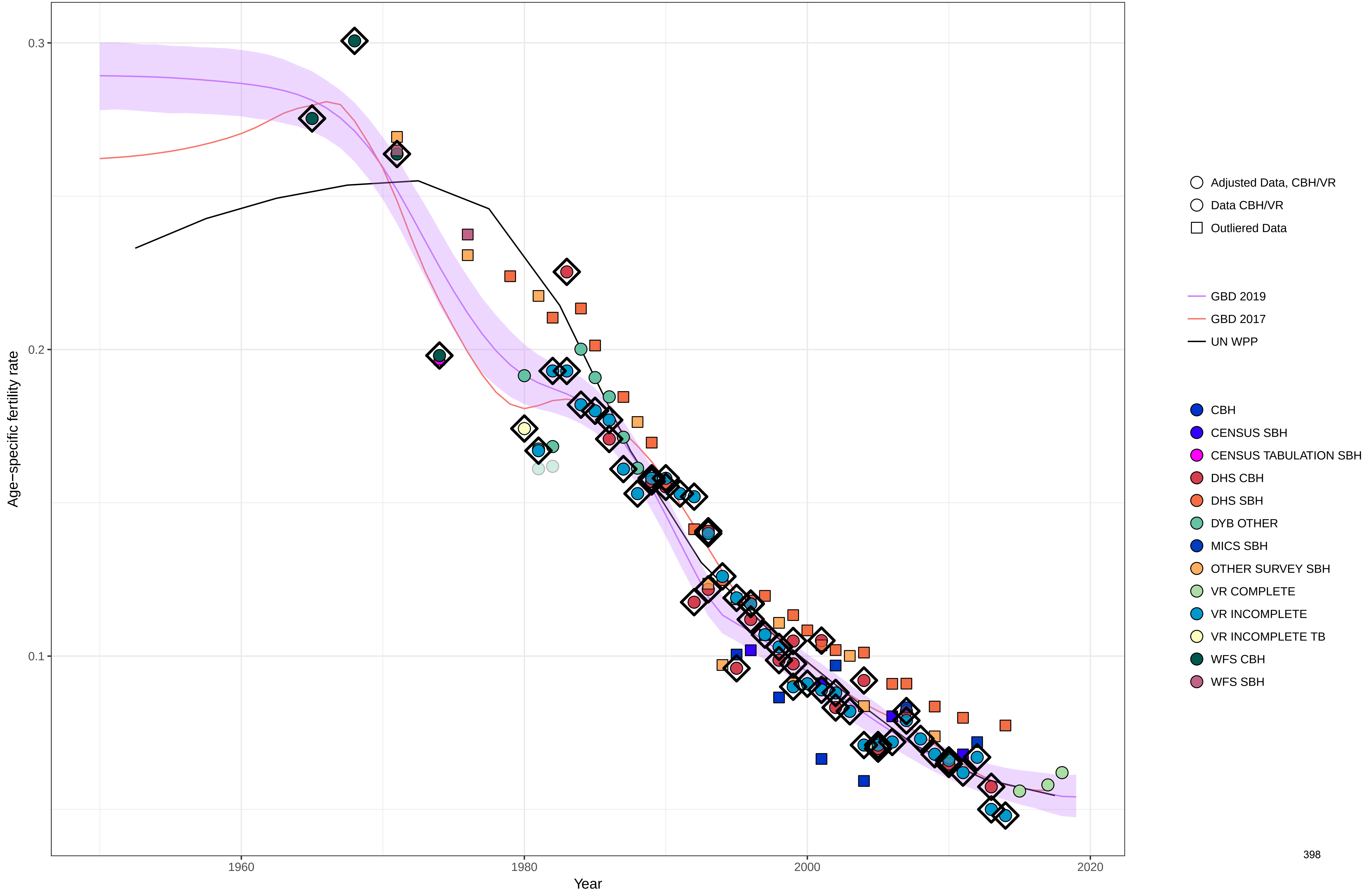

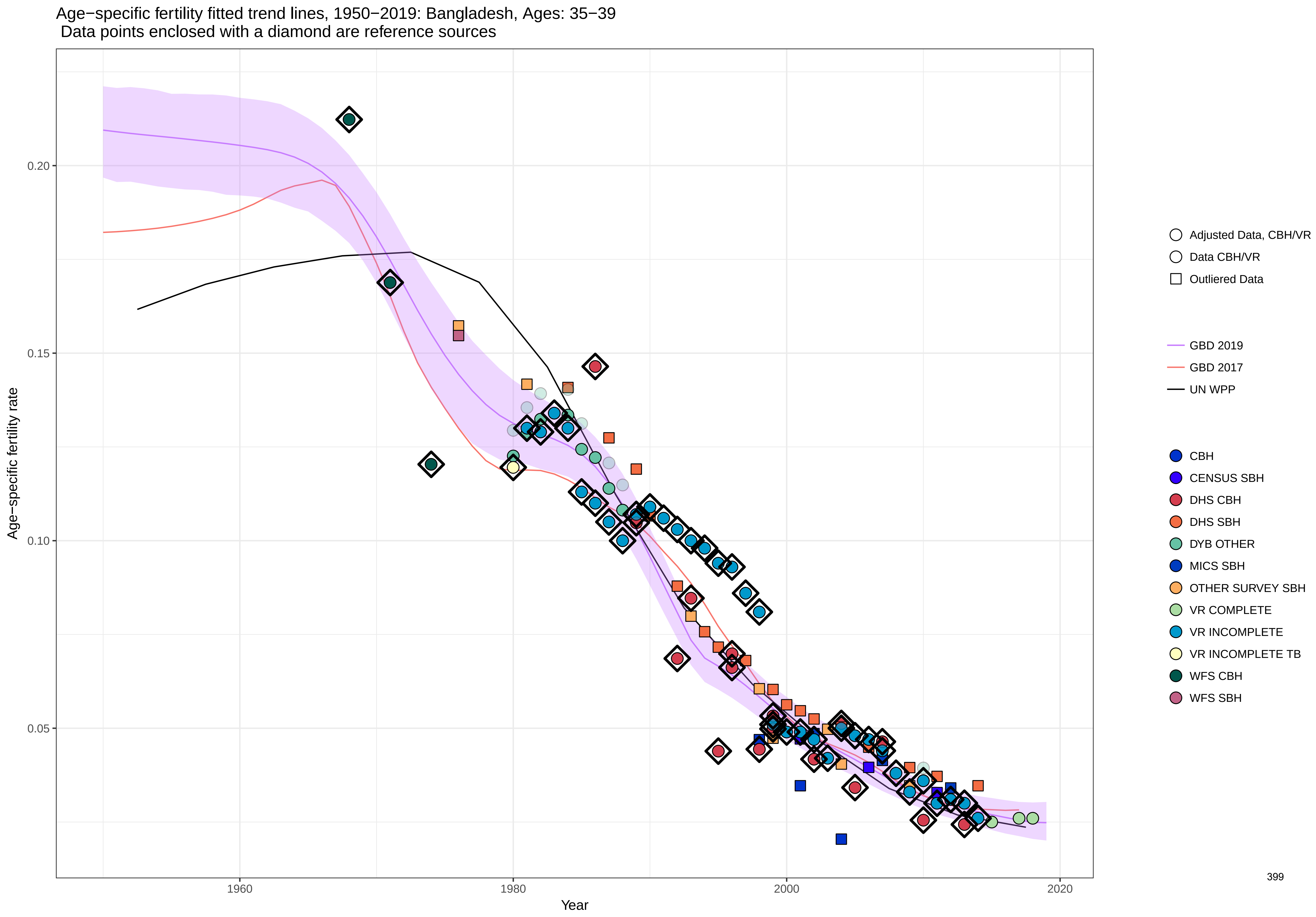

Age−specific fertility fitted trend lines, 1950−2019: Bangladesh, Ages: 35−39
Data points enclosed with a diamond are reference sources
Age−specific fertility rate
0.20
0.15
0.10
0.05
1960
1980
2000
2020
Year
Adjusted Data, CBH/VR
Data CBH/VR
Outliered Data
GBD 2019
GBD 2017
UN WPP
CBH
CENSUS SBH
DHS CBH
DHS SBH
DYB OTHER
MICS SBH
OTHER SURVEY SBH
VR COMPLETE
VR INCOMPLETE
VR INCOMPLETE TB
WFS CBH
WFS SBH

Age−specific fertility fitted trend lines, 1950−2019: Bangladesh, Ages: 40−44
Data points enclosed with a diamond are reference sources

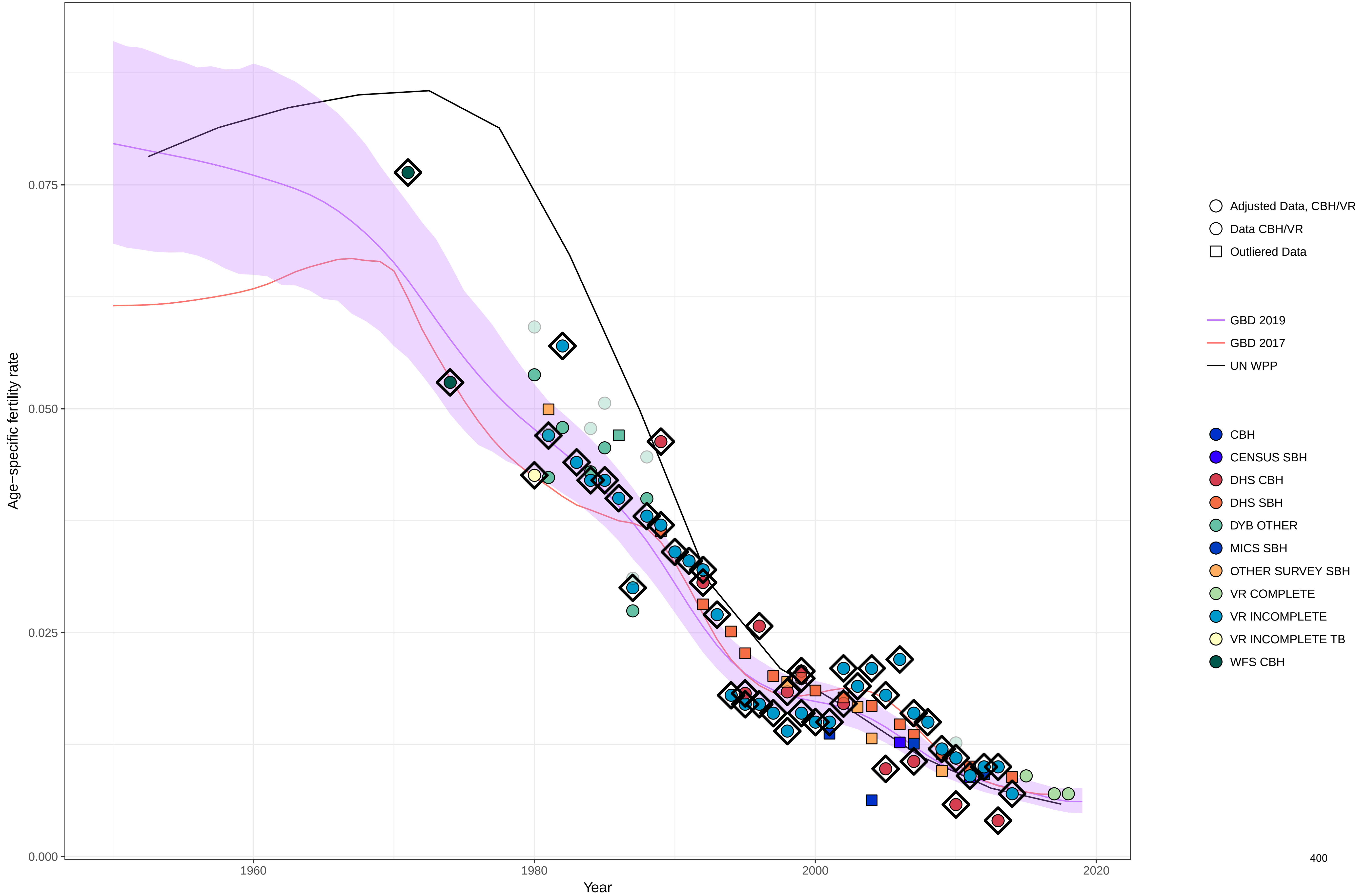

Age−specific fertility fitted trend lines, 1950−2019: Bangladesh, Ages: 45−49
Data points enclosed with a diamond are reference sources

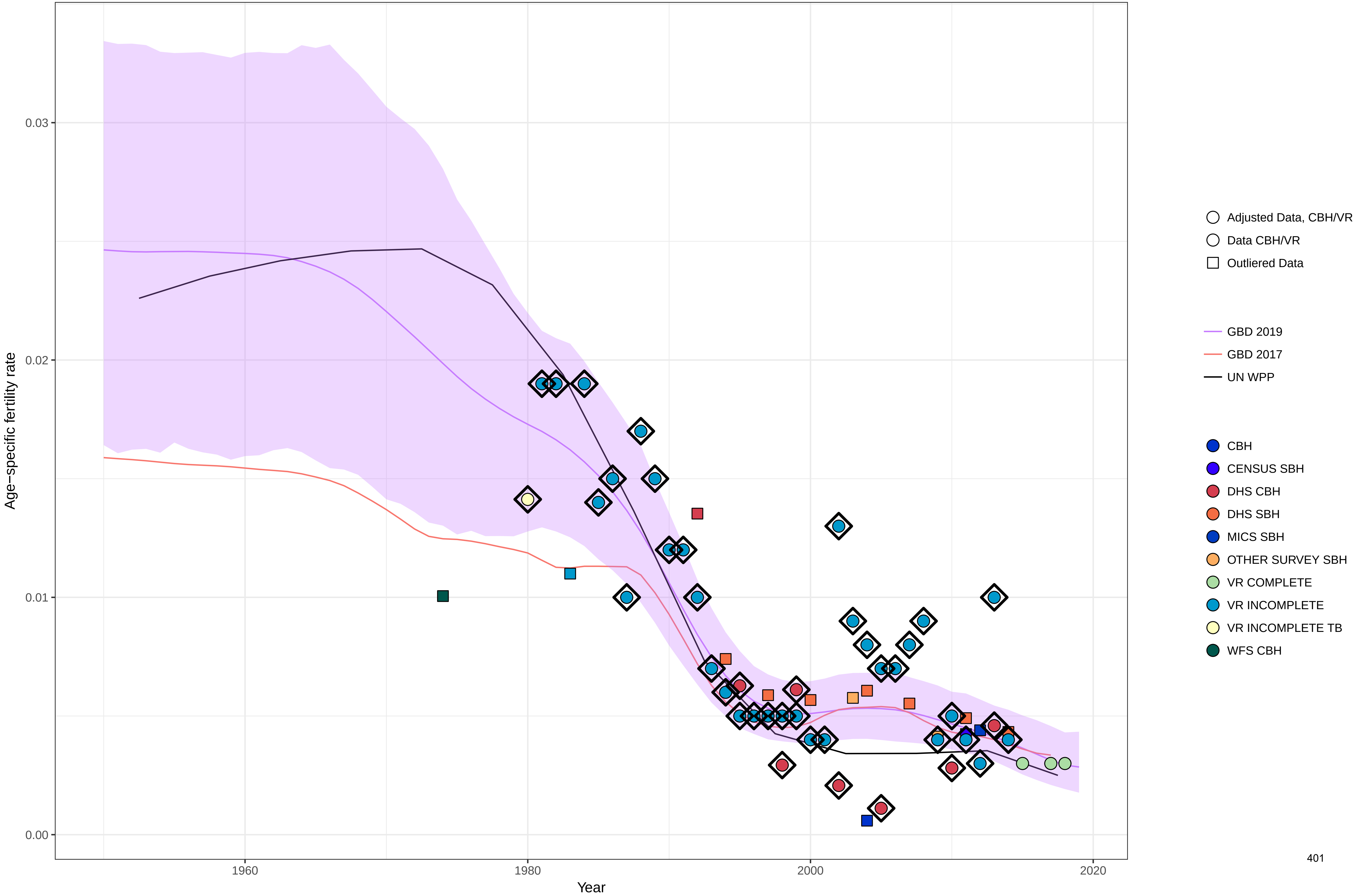

Age−specific fertility fitted trend lines, 1950−2019: Bangladesh, Ages: 50−54
Data points enclosed with a diamond are reference sources

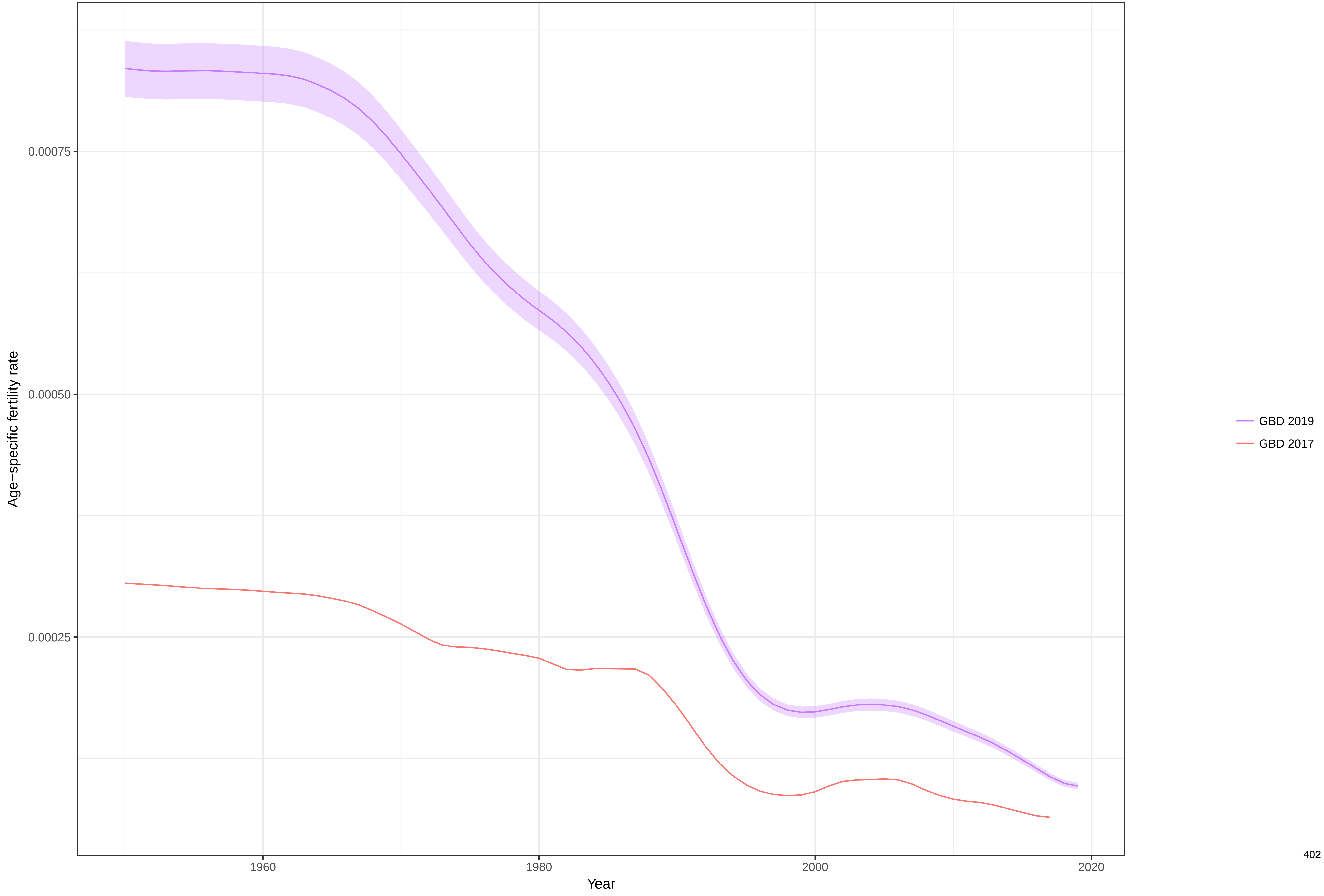

Total fertility rate fitted trend lines, 1950−2019: Bangladesh TFR
Data points enclosed with a diamond are reference sources

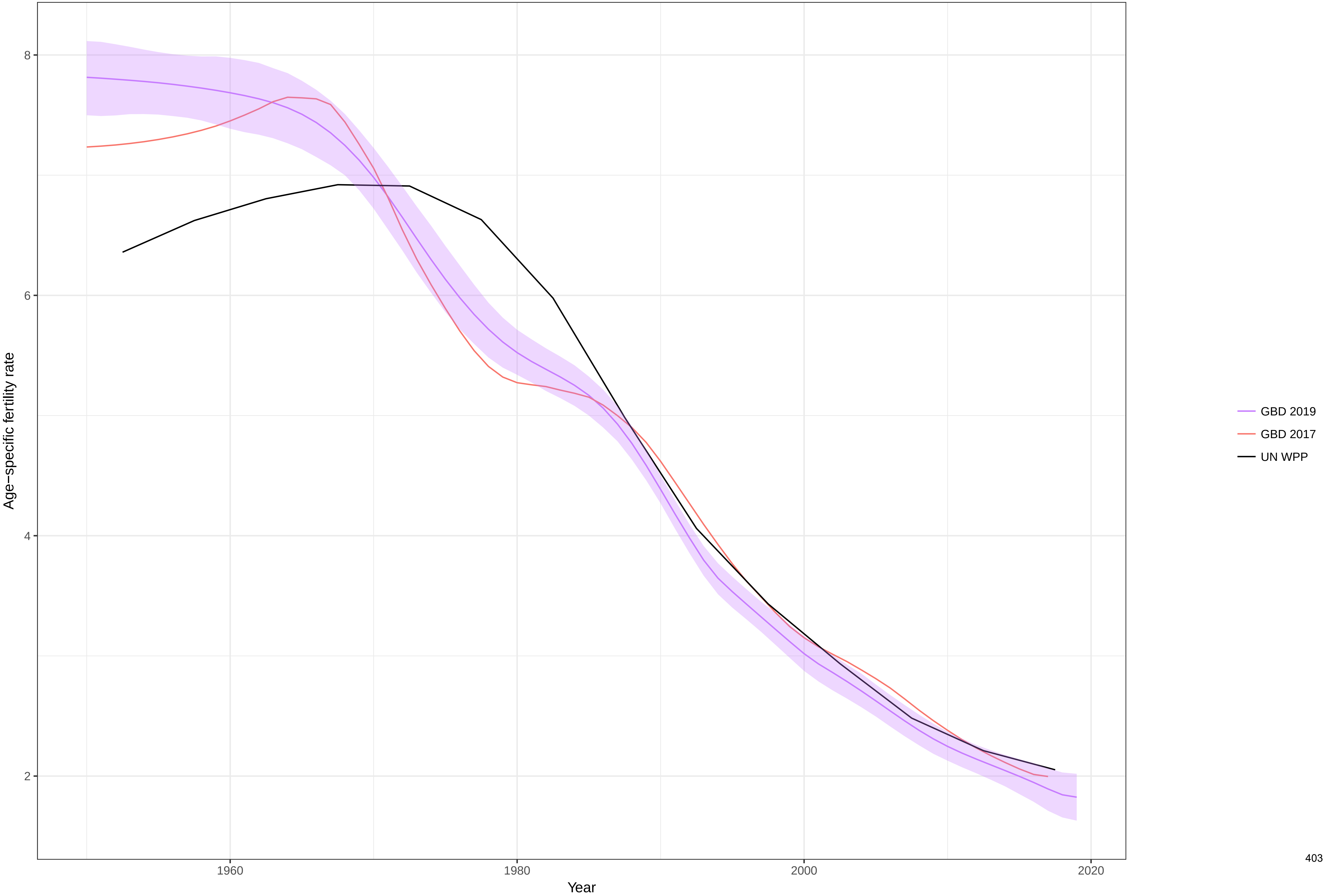

Age−specific fertility fitted trend lines, 1950−2019: Bulgaria, Ages: 10−14
Data points enclosed with a diamond are reference sources

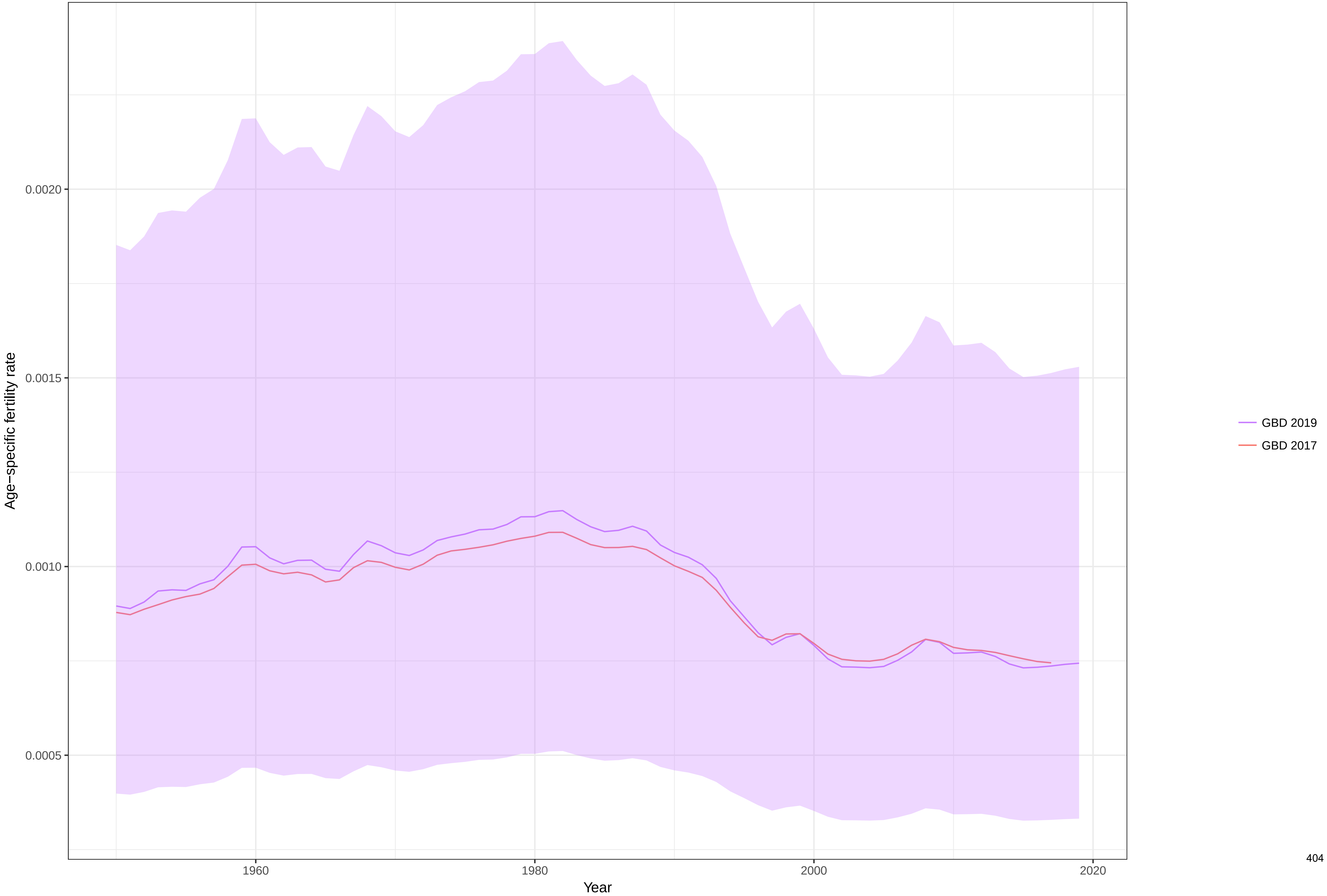

Age−specific fertility fitted trend lines, 1950−2019: Bulgaria, Ages: 15−19
Data points enclosed with a diamond are reference sources

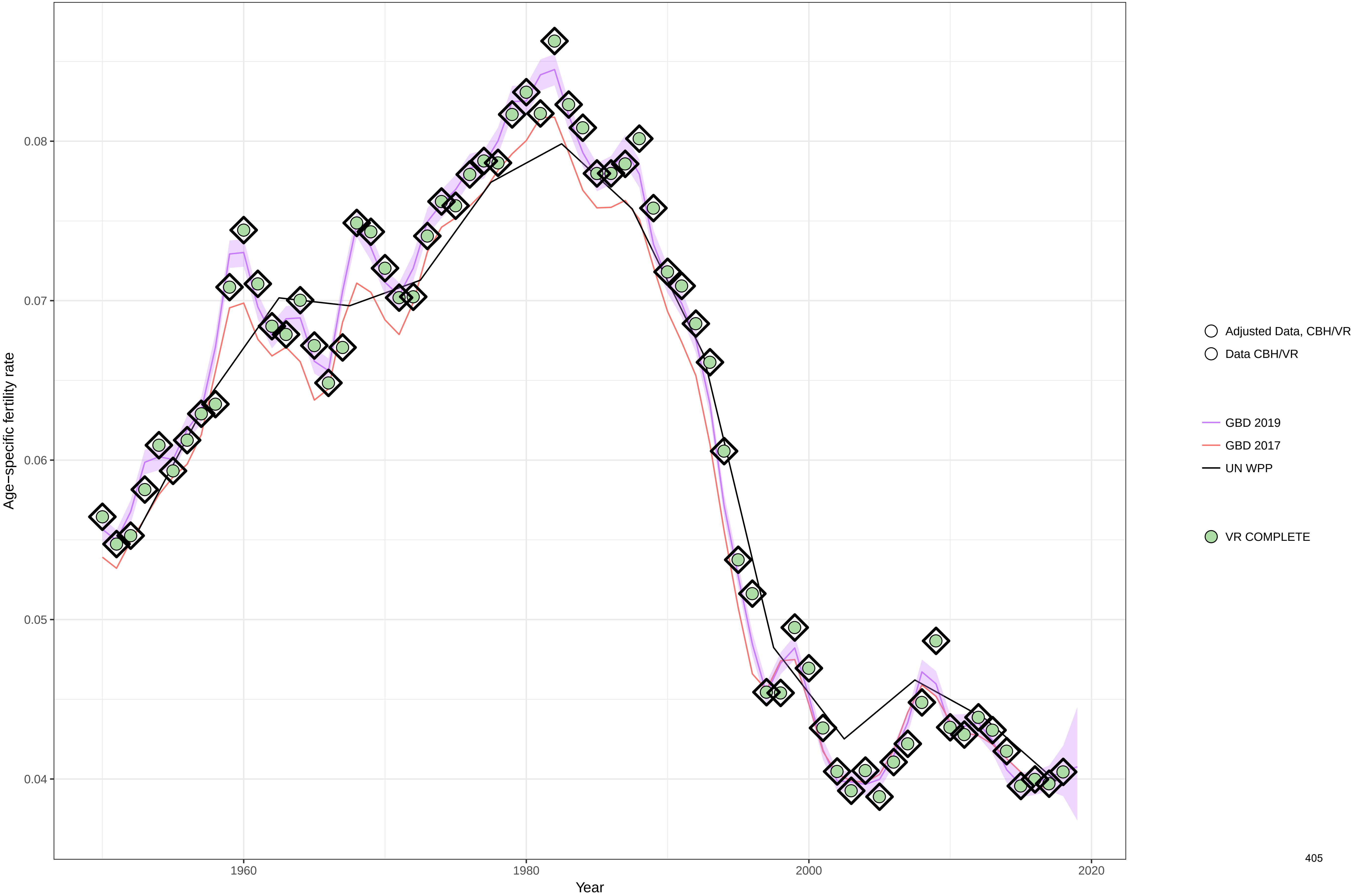

Age−specific fertility fitted trend lines, 1950−2019: Bulgaria, Ages: 20−24
Data points enclosed with a diamond are reference sources

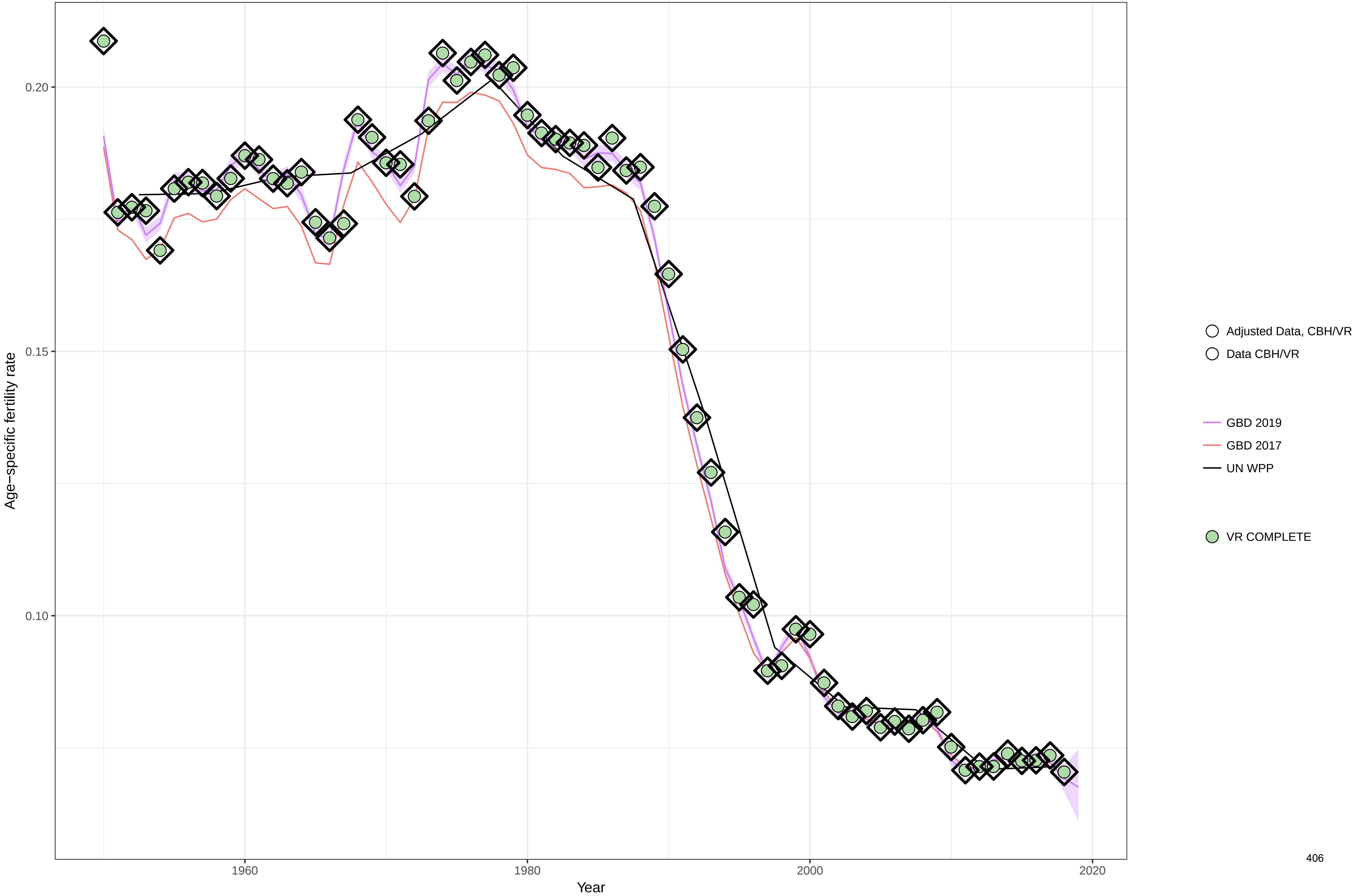

Age−specific fertility fitted trend lines, 1950−2019: Bulgaria, Ages: 25−29
Data points enclosed with a diamond are reference sources

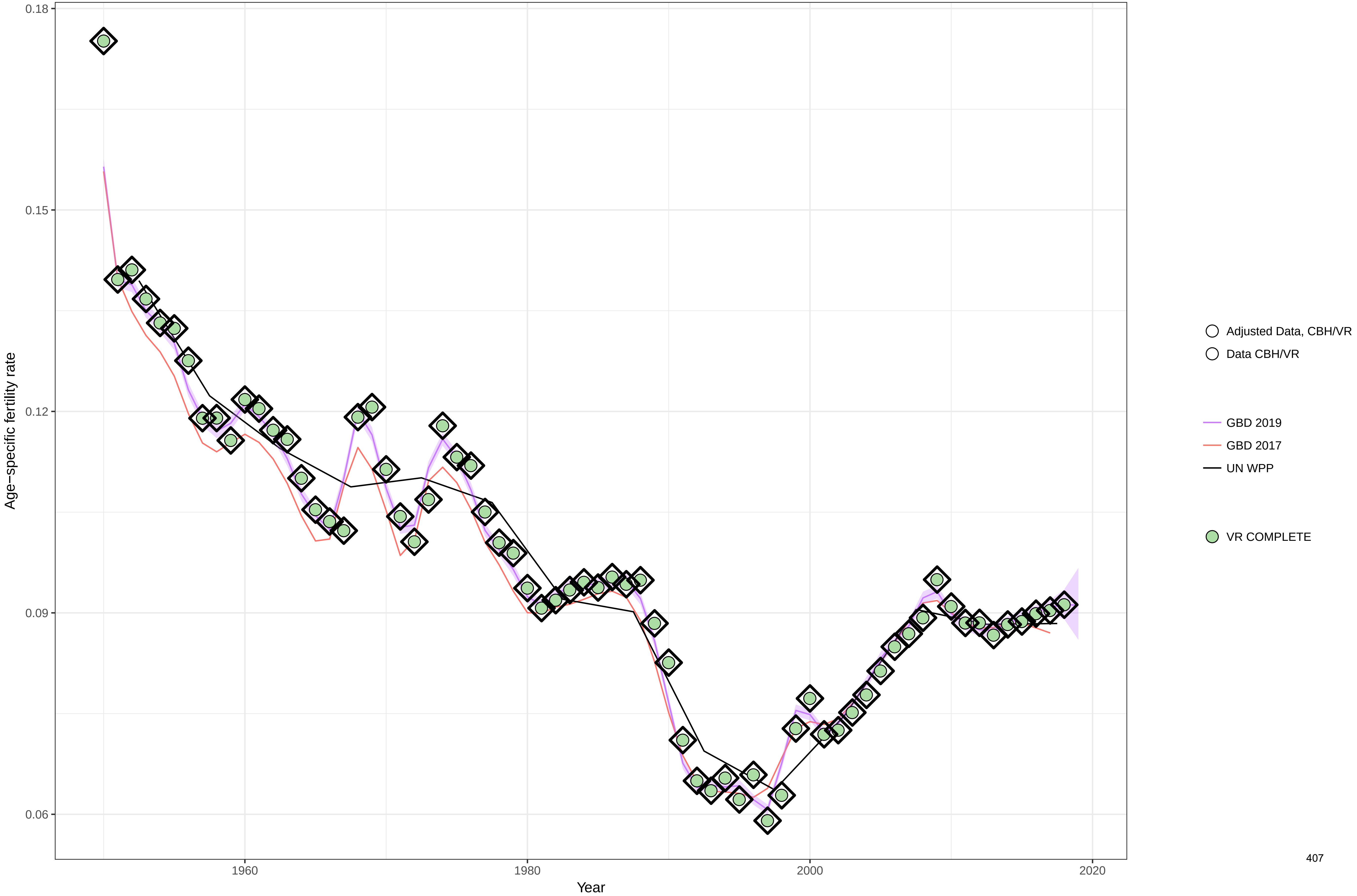

Age−specific fertility fitted trend lines, 1950−2019: Bulgaria, Ages: 30−34
Data points enclosed with a diamond are reference sources

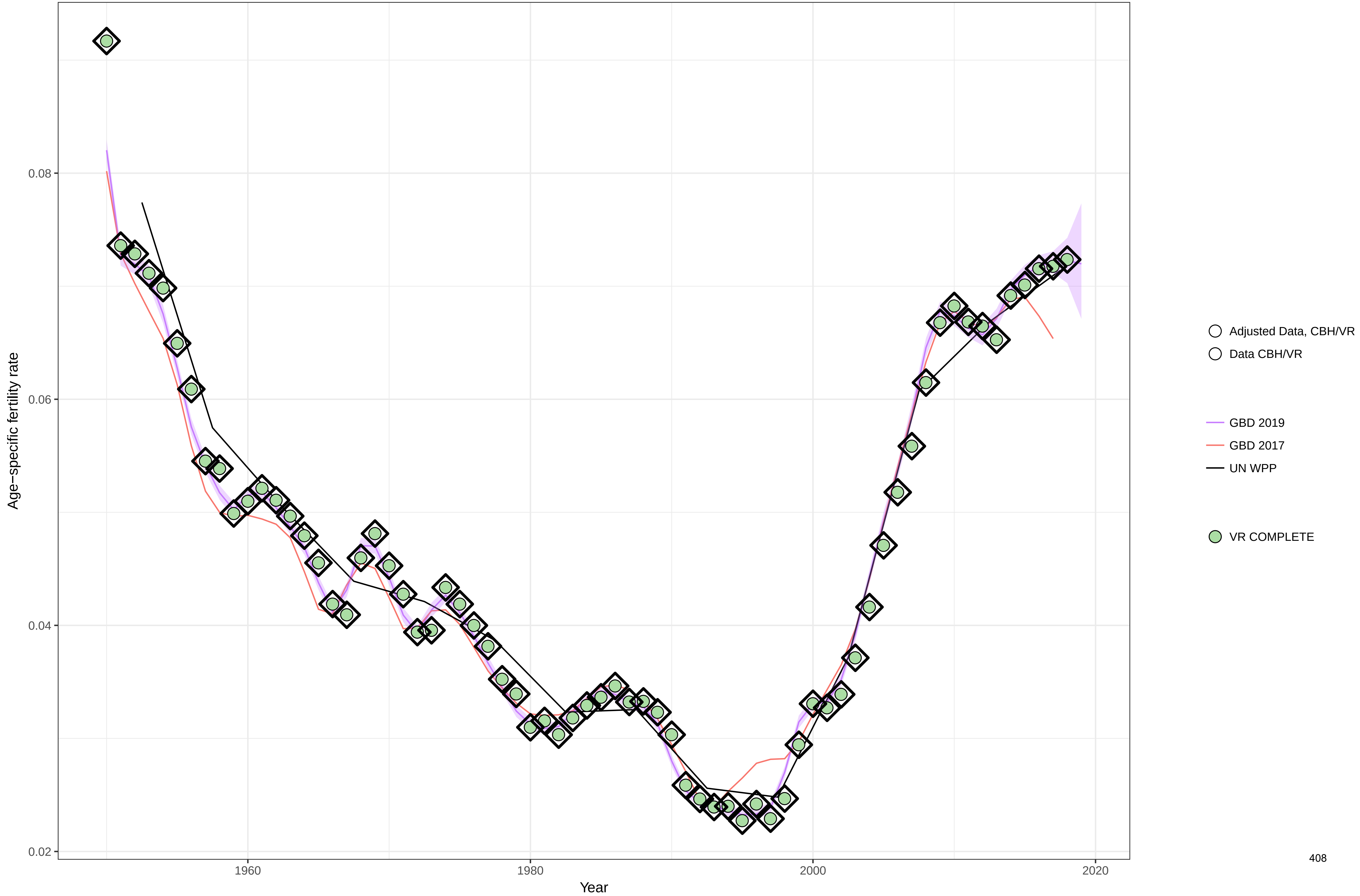

Age−specific fertility fitted trend lines, 1950−2019: Bulgaria, Ages: 35−39
Data points enclosed with a diamond are reference sources

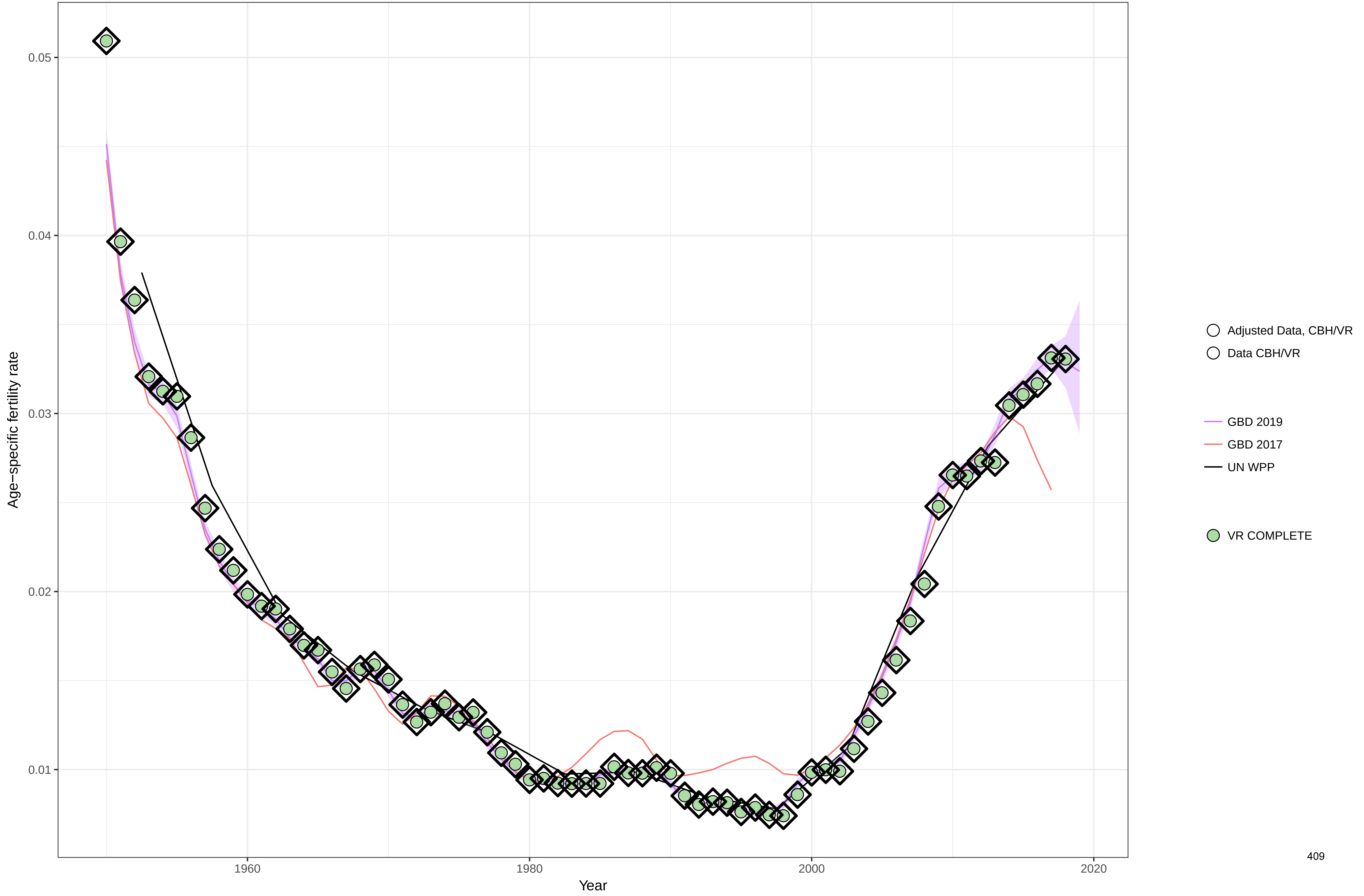

Age−specific fertility fitted trend lines, 1950−2019: Bulgaria, Ages: 40−44
Data points enclosed with a diamond are reference sources

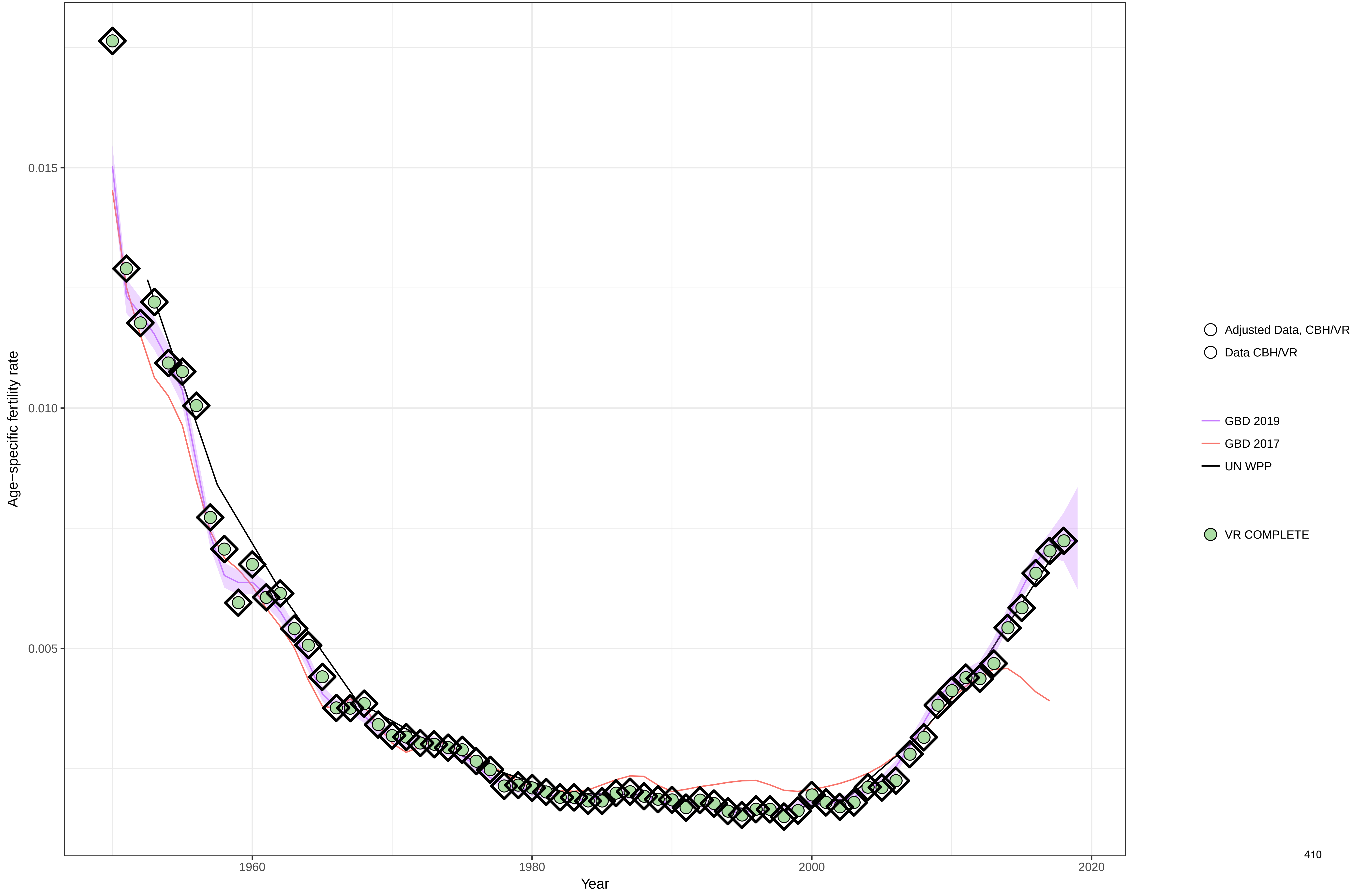

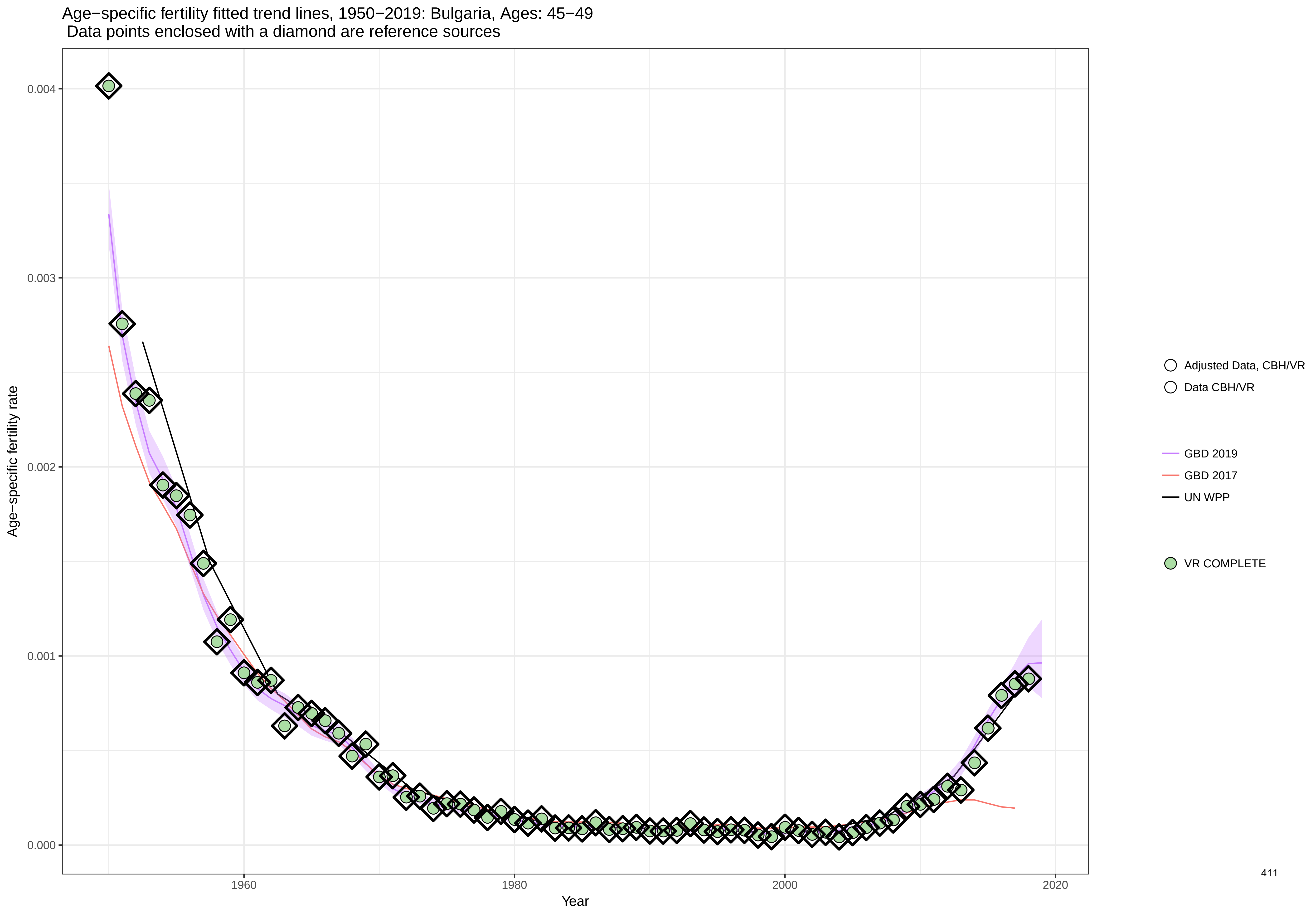
Age−specific fertility fitted trend lines, 1950−2019: Bulgaria, Ages: 45−49
Data points enclosed with a diamond are reference sources
0.004
0.003
0.002
0.001
0.000
Age−specific fertility rate
1960
1980
2000
2020
Year
Adjusted Data, CBH/VR
Data CBH/VR
GBD 2019
GBD 2017
UN WPP
VR COMPLETE

Age−specific fertility fitted trend lines, 1950−2019: Bulgaria, Ages: 50−54
Data points enclosed with a diamond are reference sources

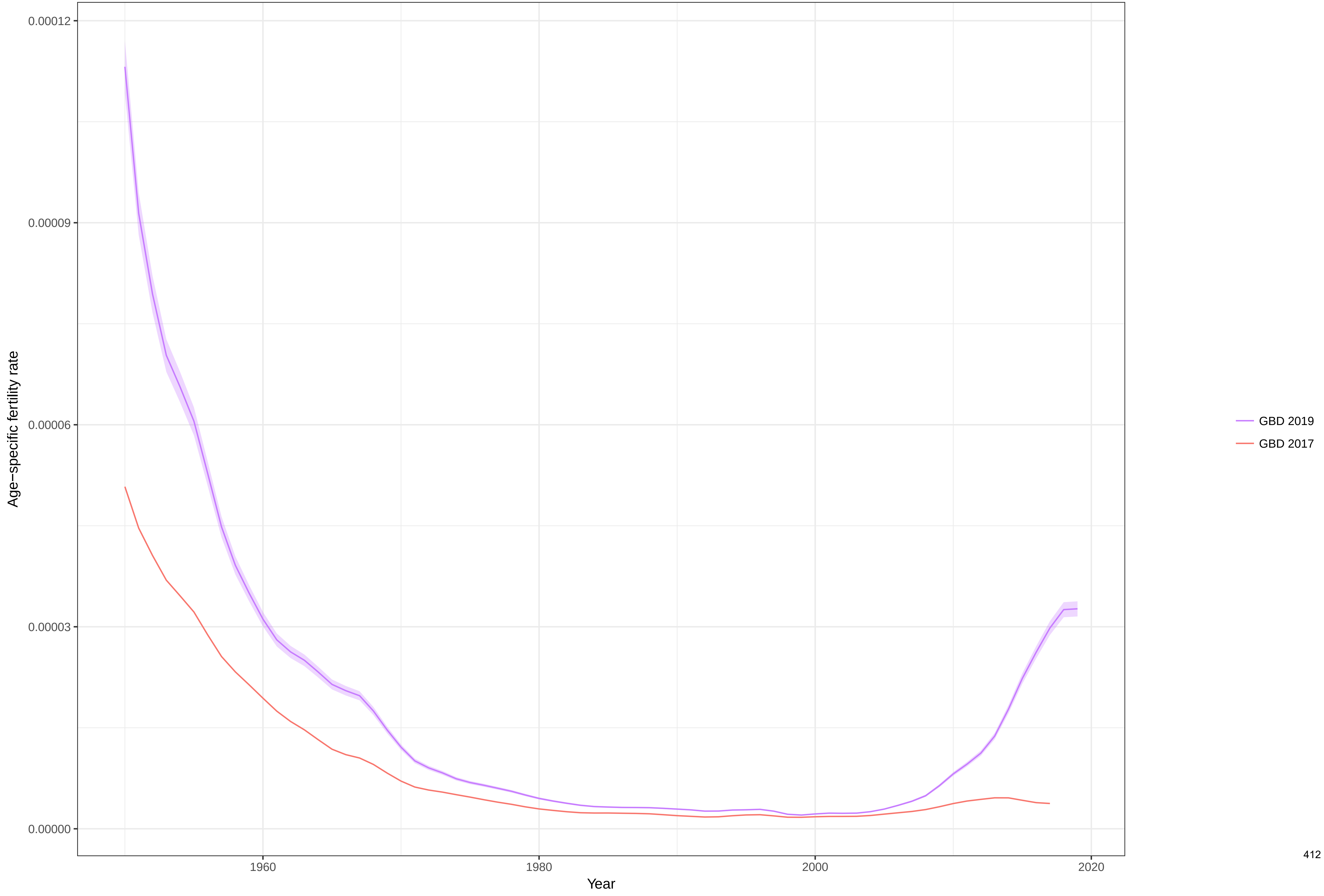

Total fertility rate fitted trend lines, 1950−2019: Bulgaria TFR
Data points enclosed with a diamond are reference sources

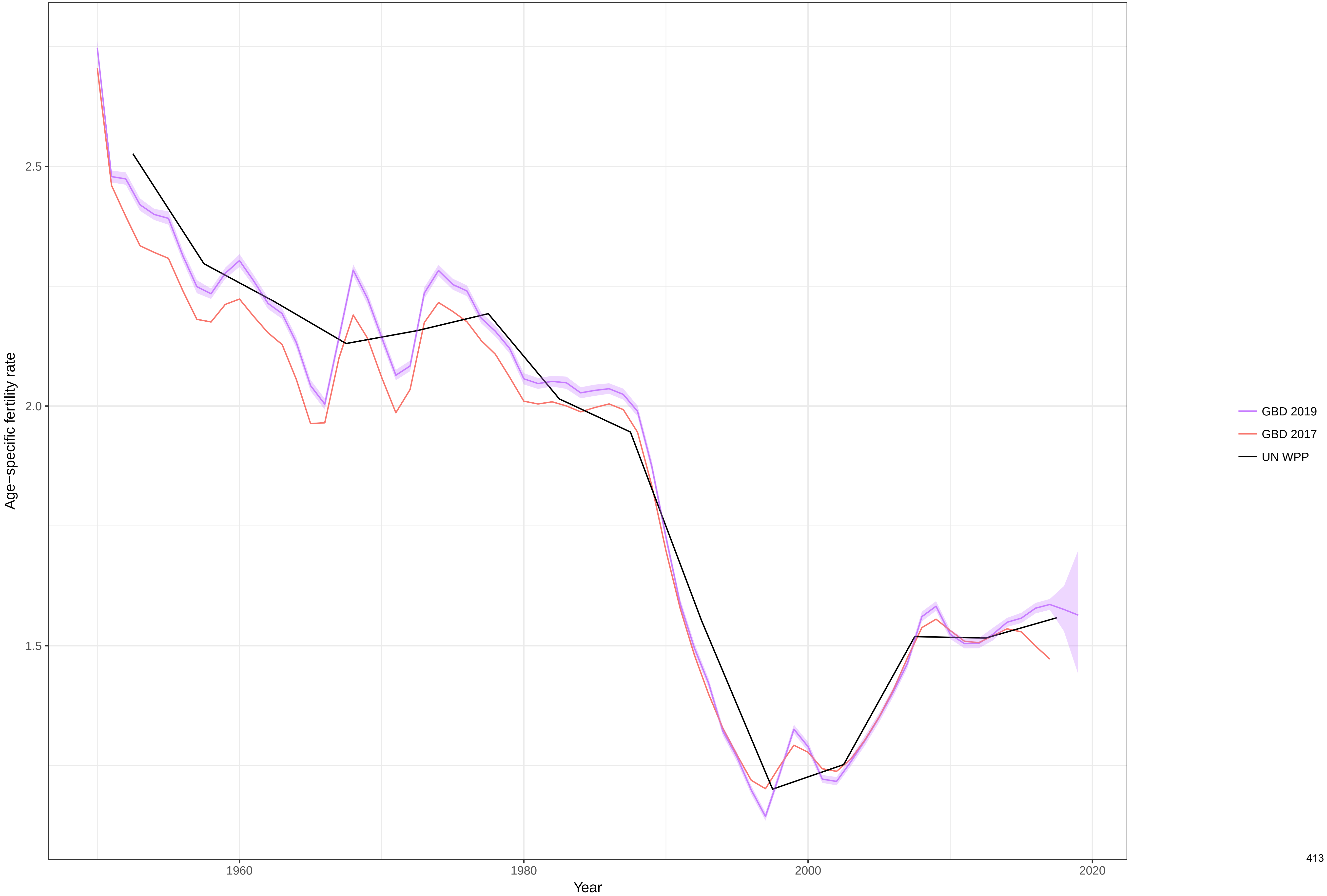

Age−specific fertility fitted trend lines, 1950−2019: Bahrain, Ages: 10−14
Data points enclosed with a diamond are reference sources

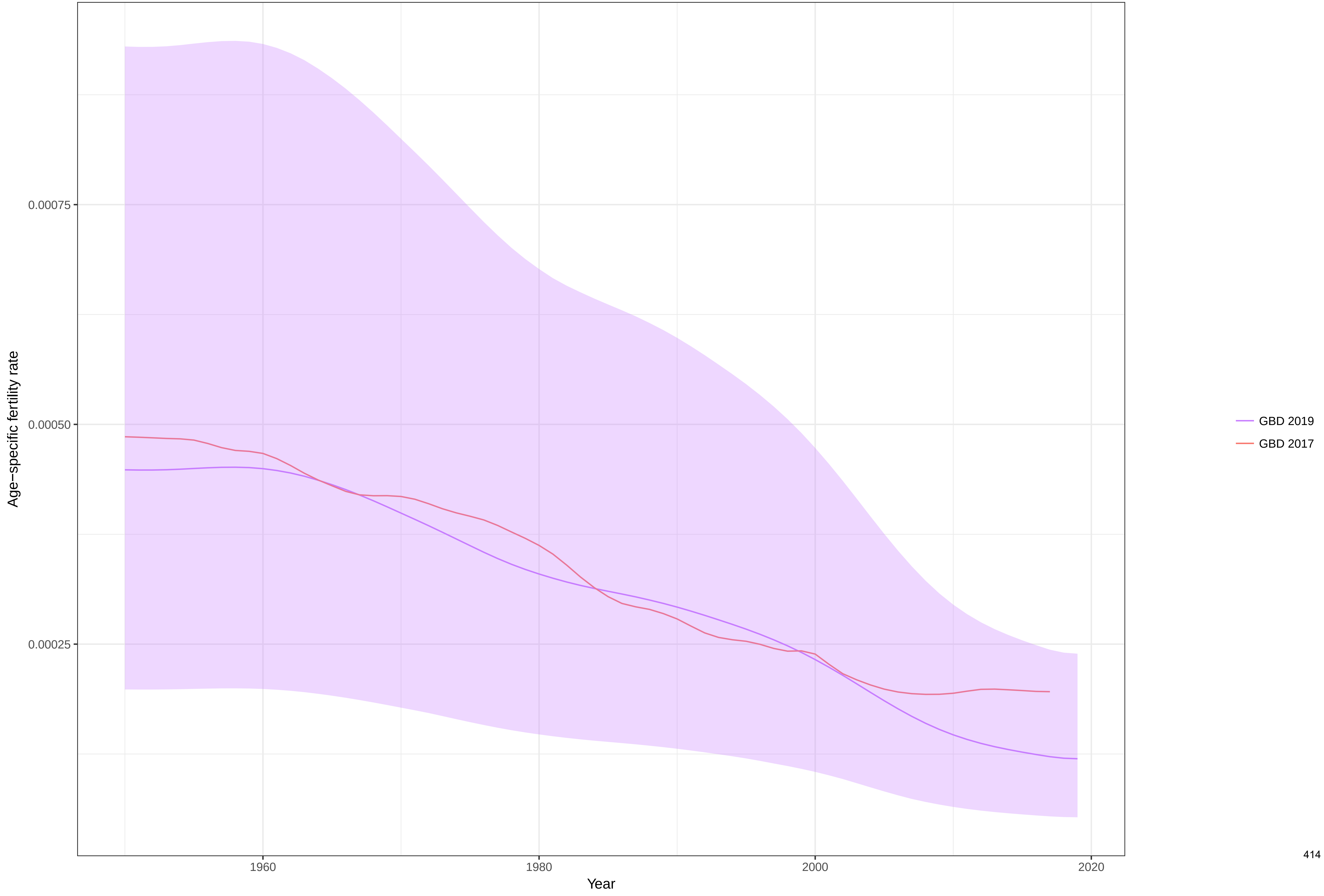

Age−specific fertility fitted trend lines, 1950−2019: Bahrain, Ages: 15−19
Data points enclosed with a diamond are reference sources

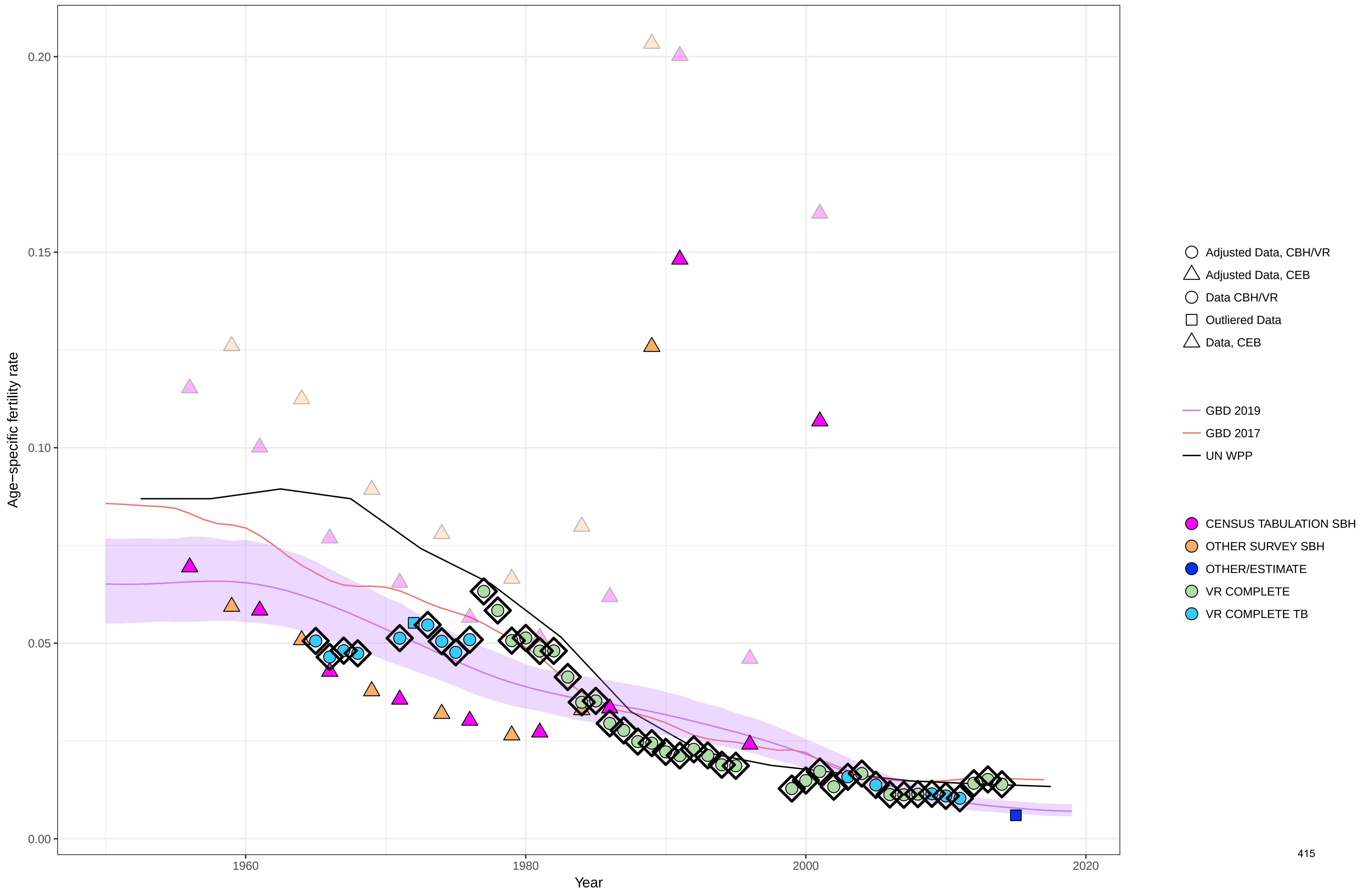

Age−specific fertility fitted trend lines, 1950−2019: Bahrain, Ages: 20−24
Data points enclosed with a diamond are reference sources

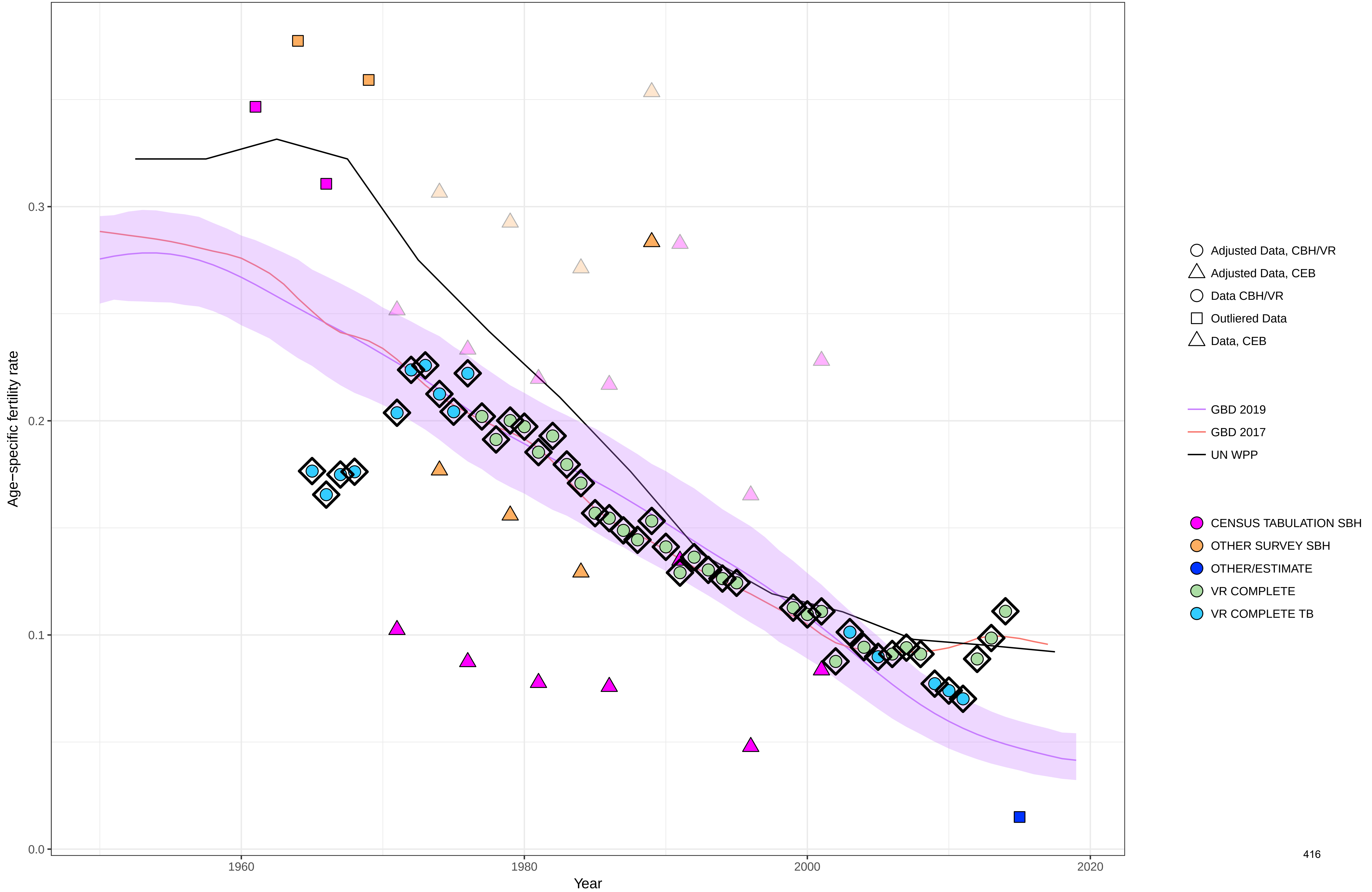

Age−specific fertility fitted trend lines, 1950−2019: Bahrain, Ages: 25−29
Data points enclosed with a diamond are reference sources

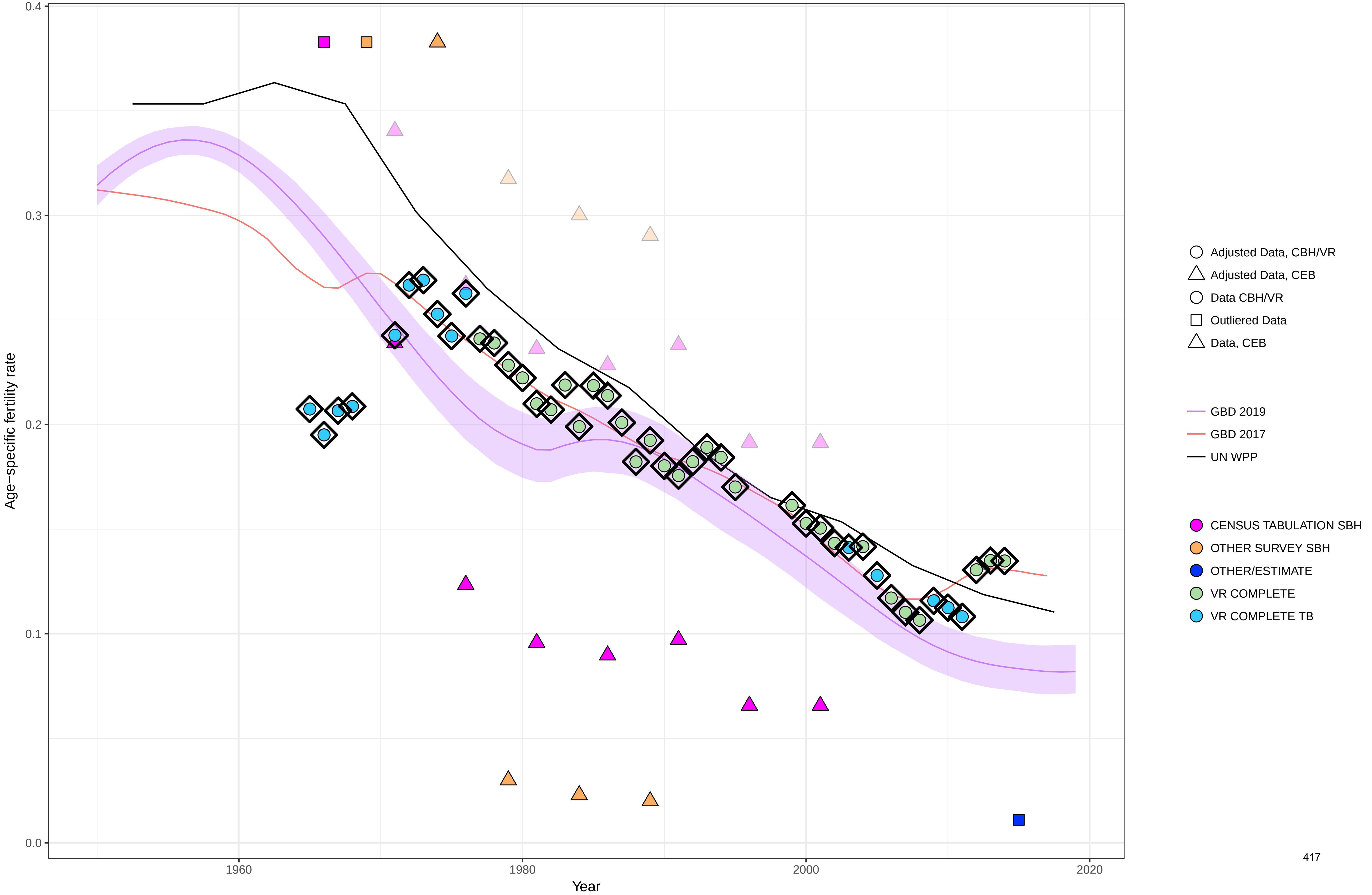

Age−specific fertility fitted trend lines, 1950−2019: Bahrain, Ages: 30−34
Data points enclosed with a diamond are reference sources

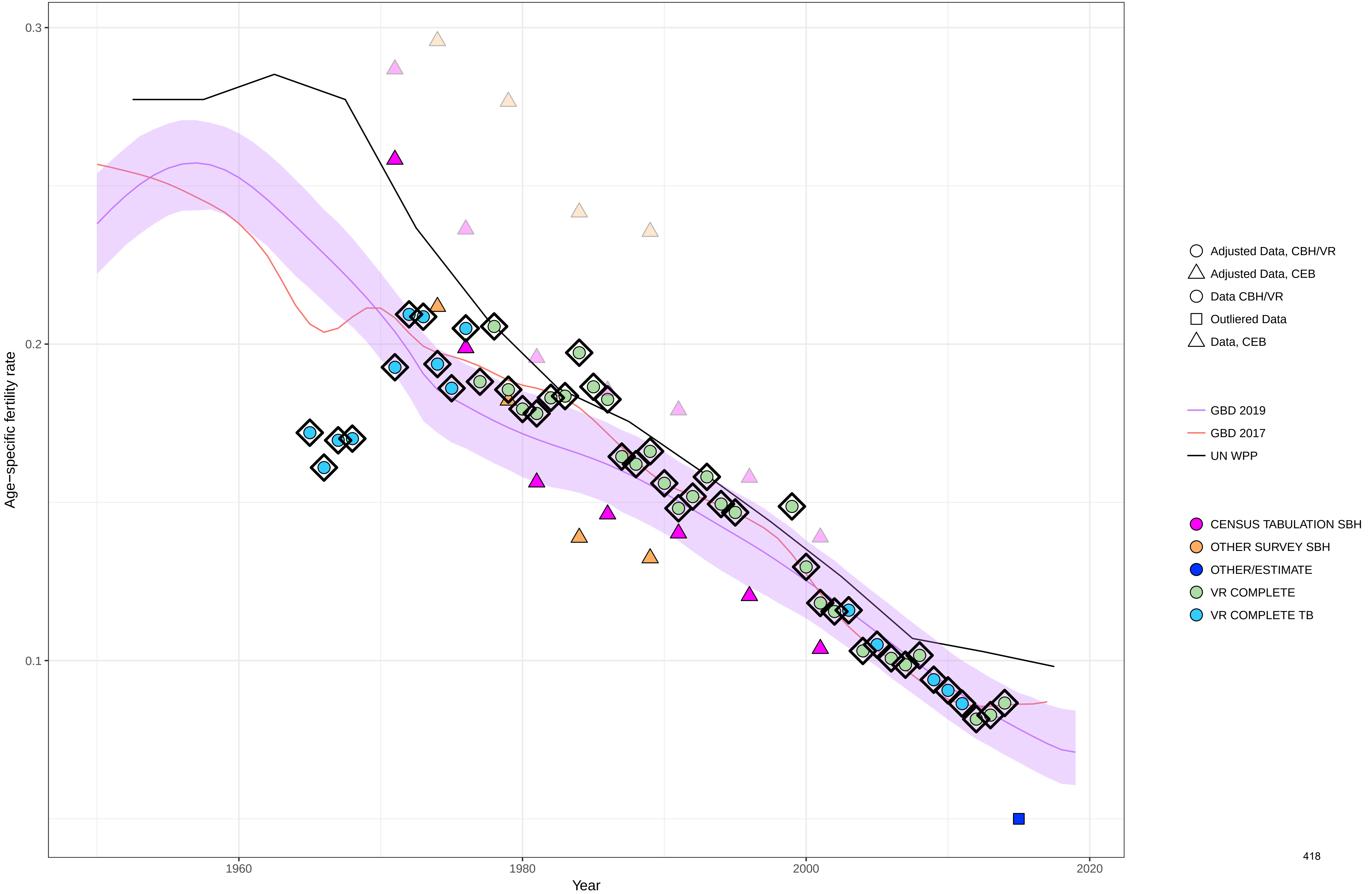

Age−specific fertility fitted trend lines, 1950−2019: Bahrain, Ages: 35−39
Data points enclosed with a diamond are reference sources

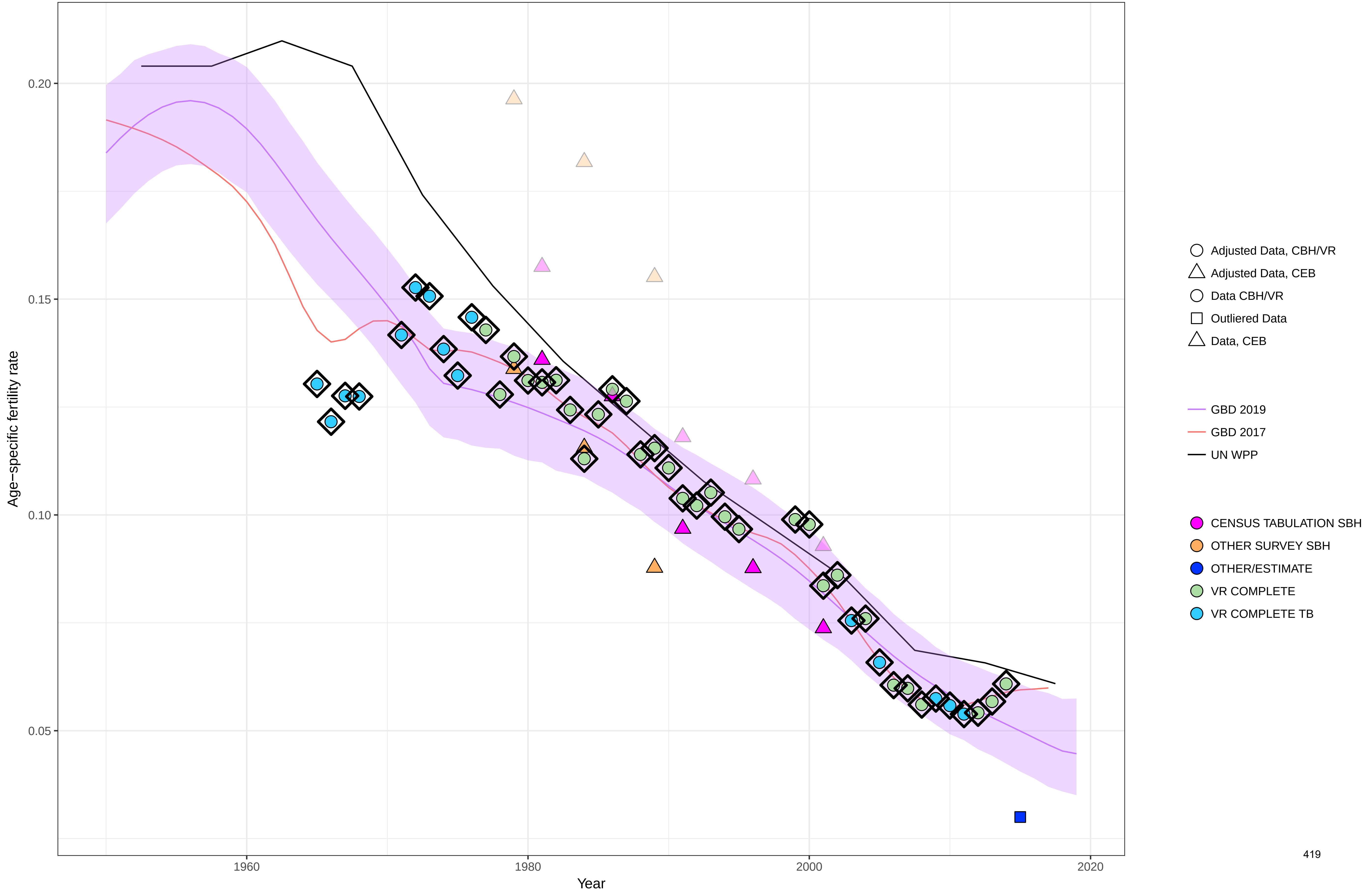

Age−specific fertility fitted trend lines, 1950−2019: Bahrain, Ages: 40−44
Data points enclosed with a diamond are reference sources

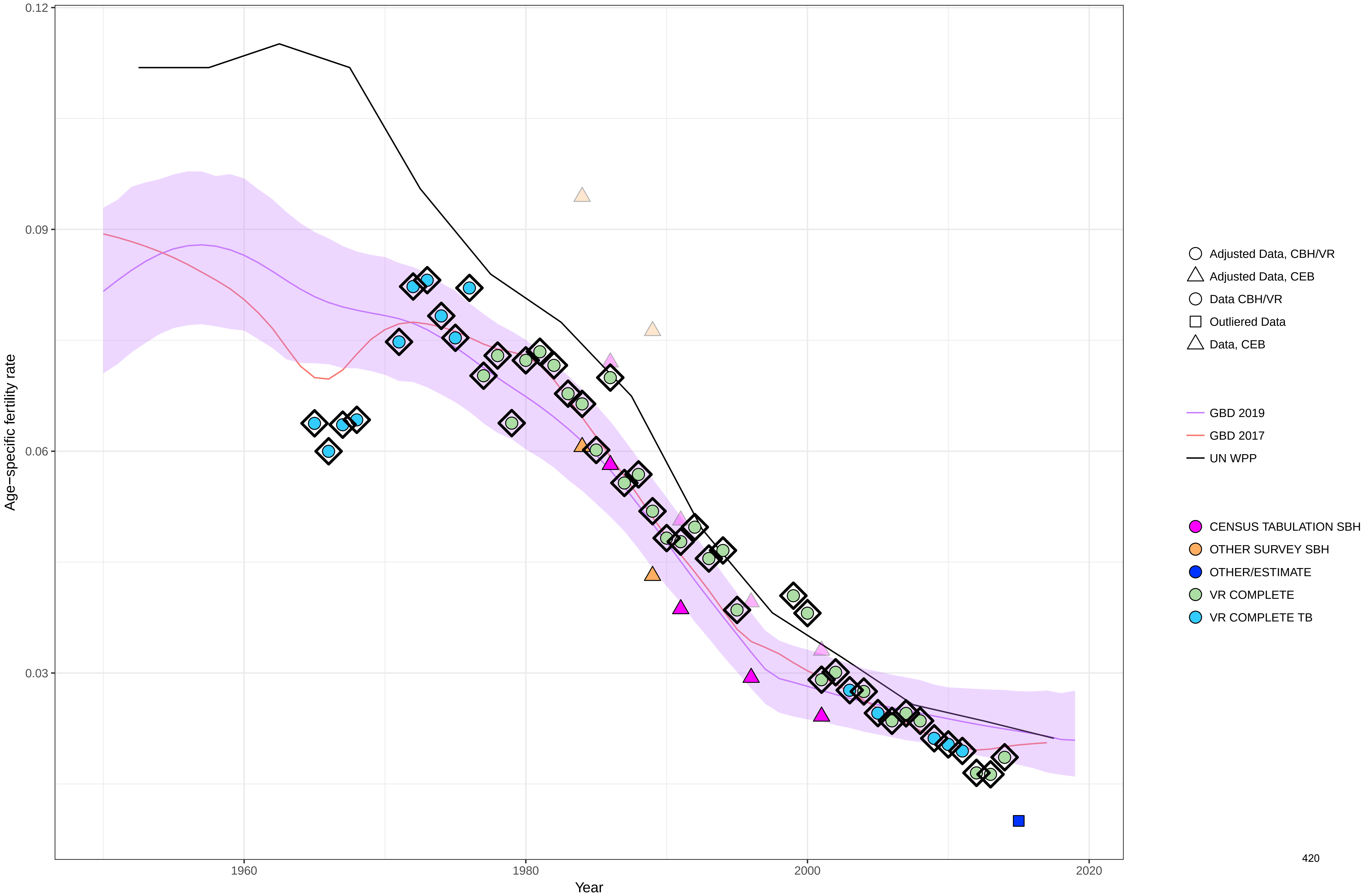

Age−specific fertility fitted trend lines, 1950−2019: Bahrain, Ages: 45−49
Data points enclosed with a diamond are reference sources

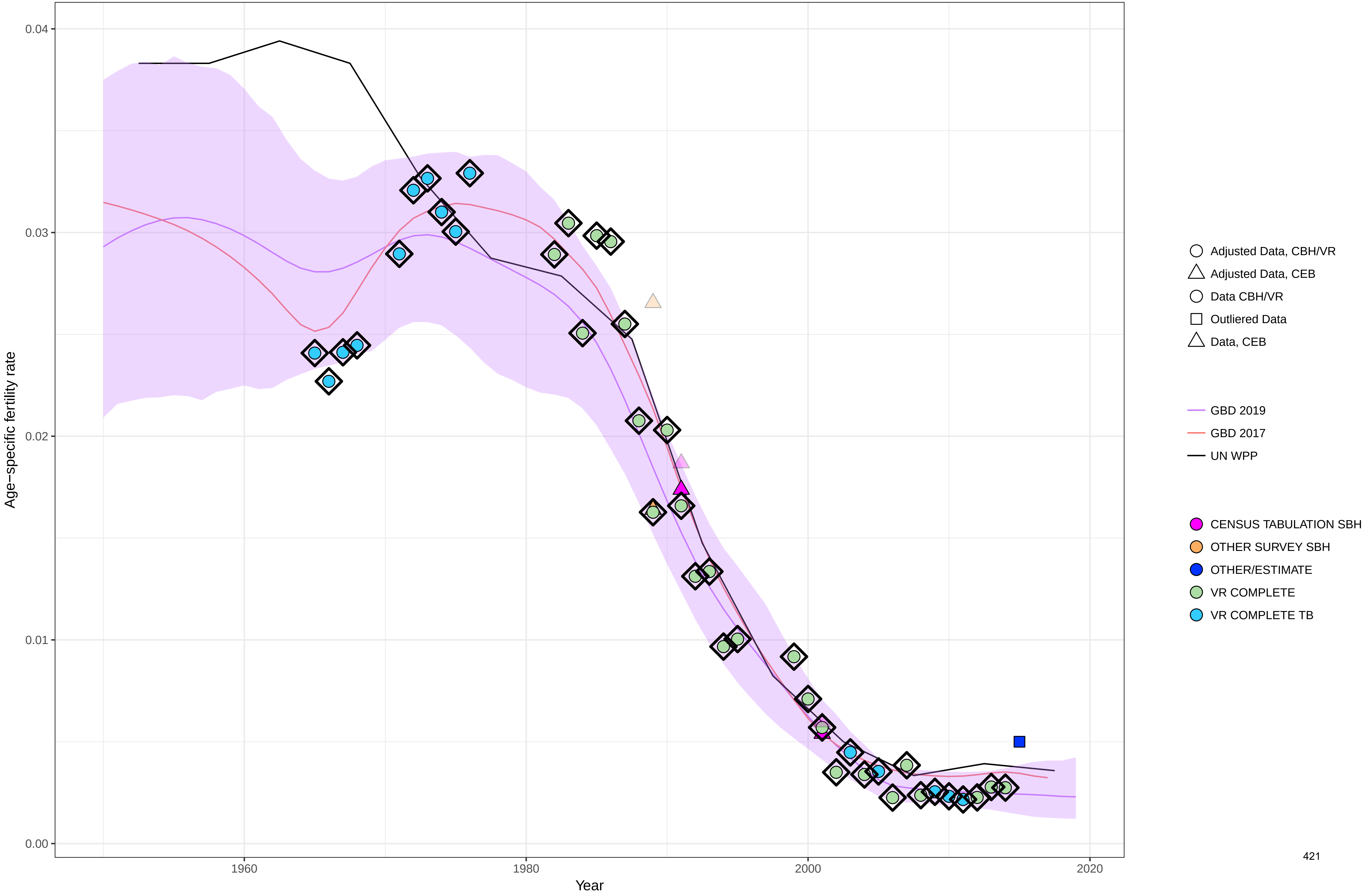

Age−specific fertility fitted trend lines, 1950−2019: Bahrain, Ages: 50−54
Data points enclosed with a diamond are reference sources

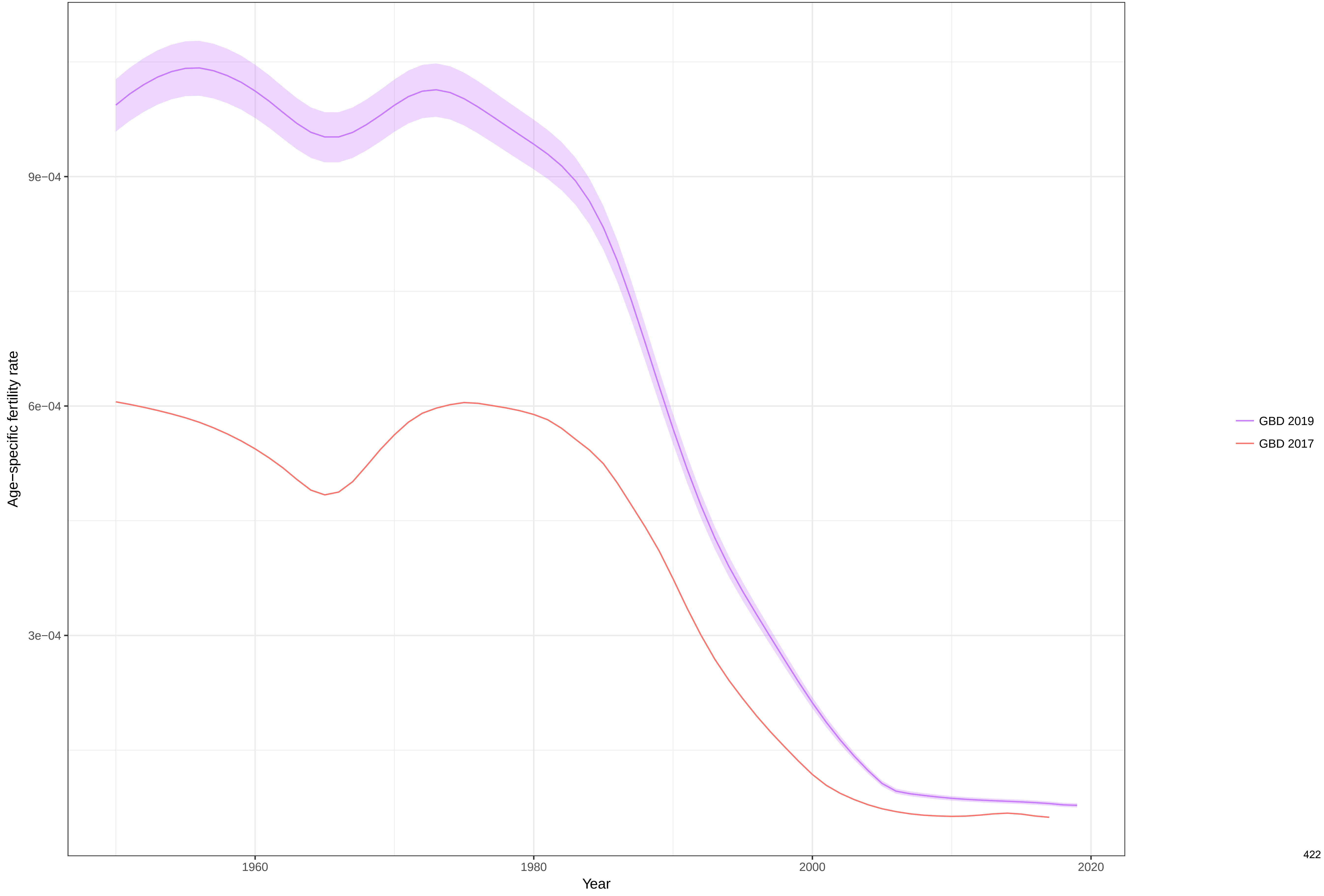

Total fertility rate fitted trend lines, 1950−2019: Bahrain TFR
Data points enclosed with a diamond are reference sources

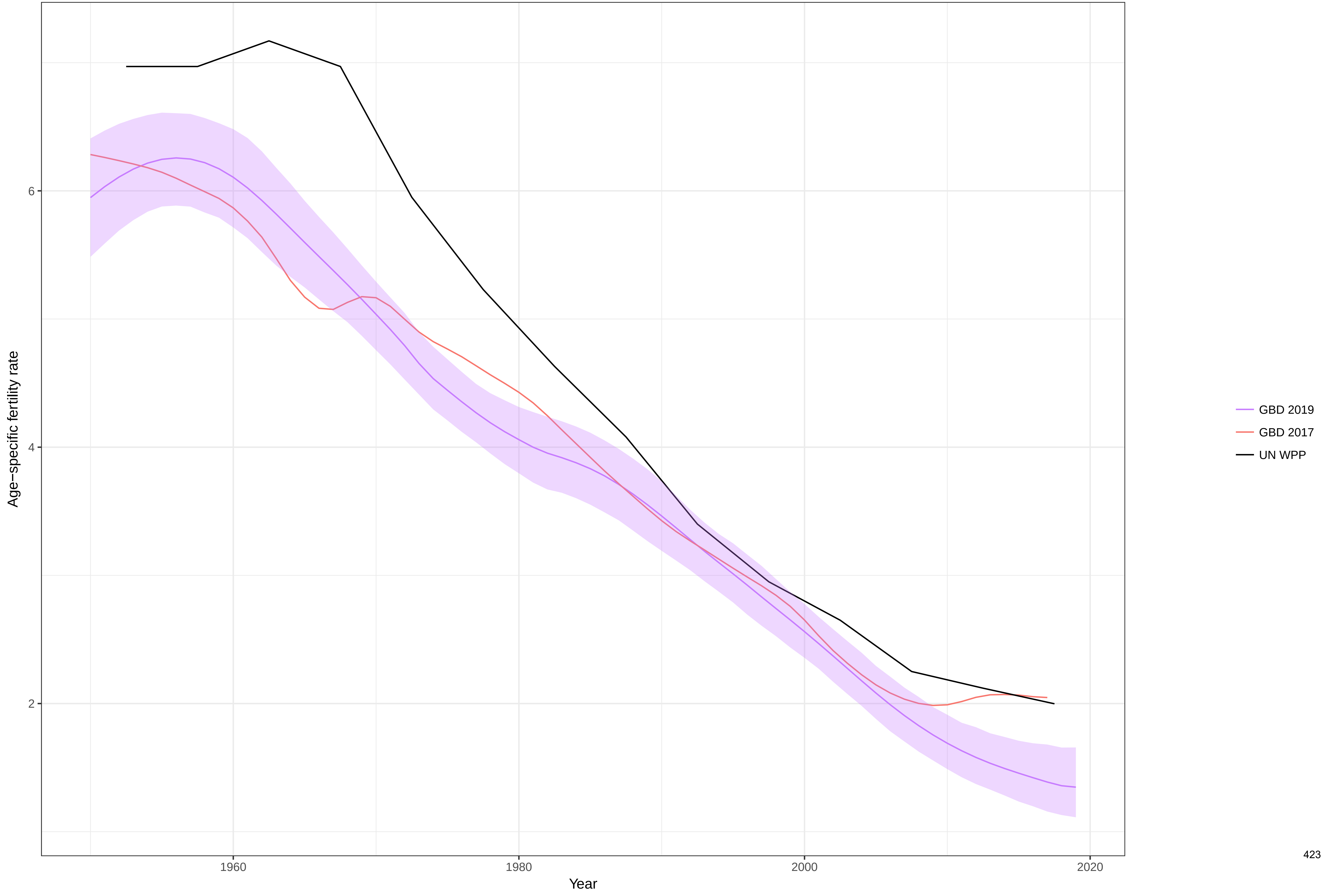

Age−specific fertility fitted trend lines, 1950−2019: Bahamas, Ages: 10−14
Data points enclosed with a diamond are reference sources

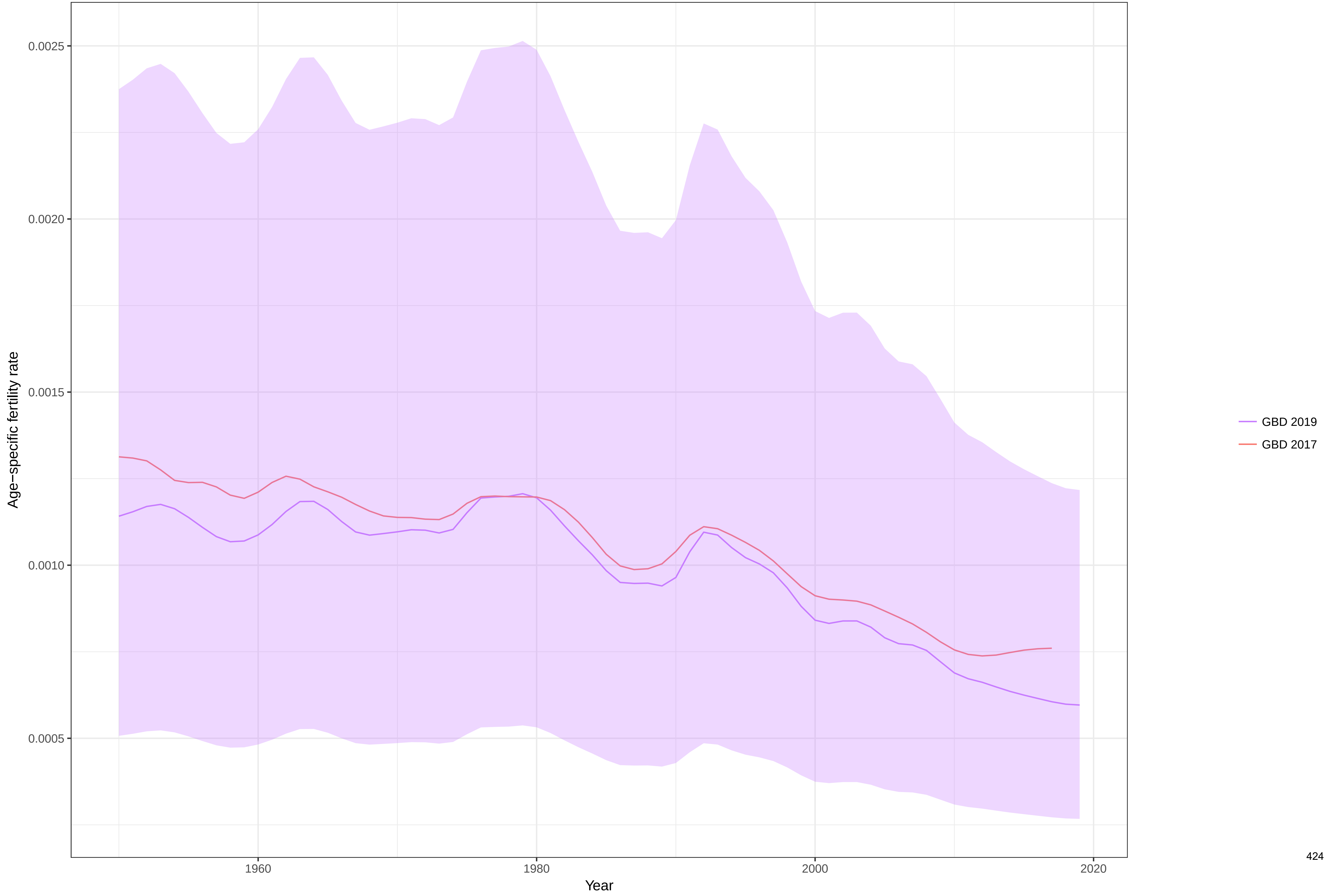

Age−specific fertility fitted trend lines, 1950−2019: Bahamas, Ages: 15−19
Data points enclosed with a diamond are reference sources

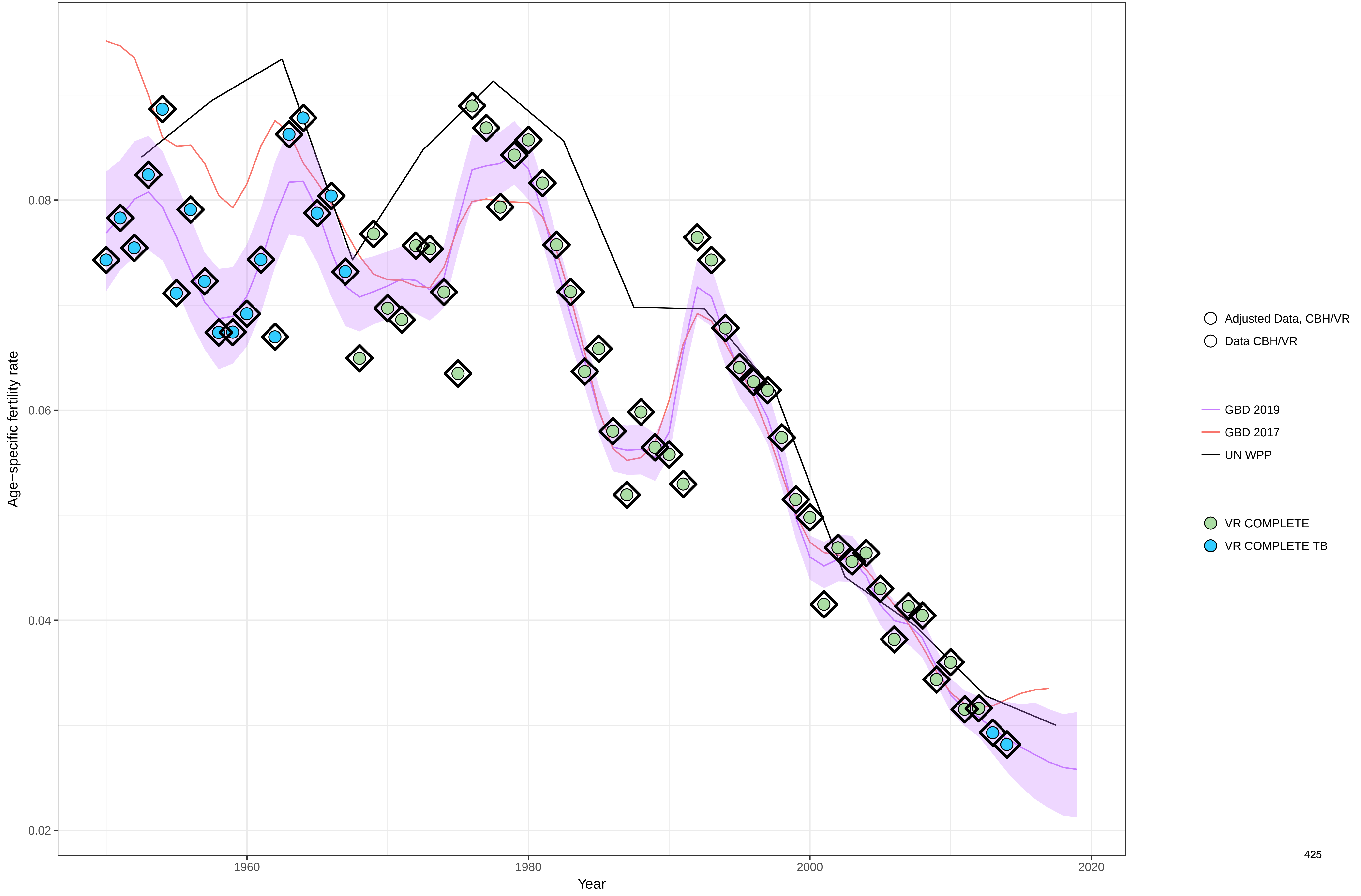

Age−specific fertility fitted trend lines, 1950−2019: Bahamas, Ages: 20−24
Data points enclosed with a diamond are reference sources

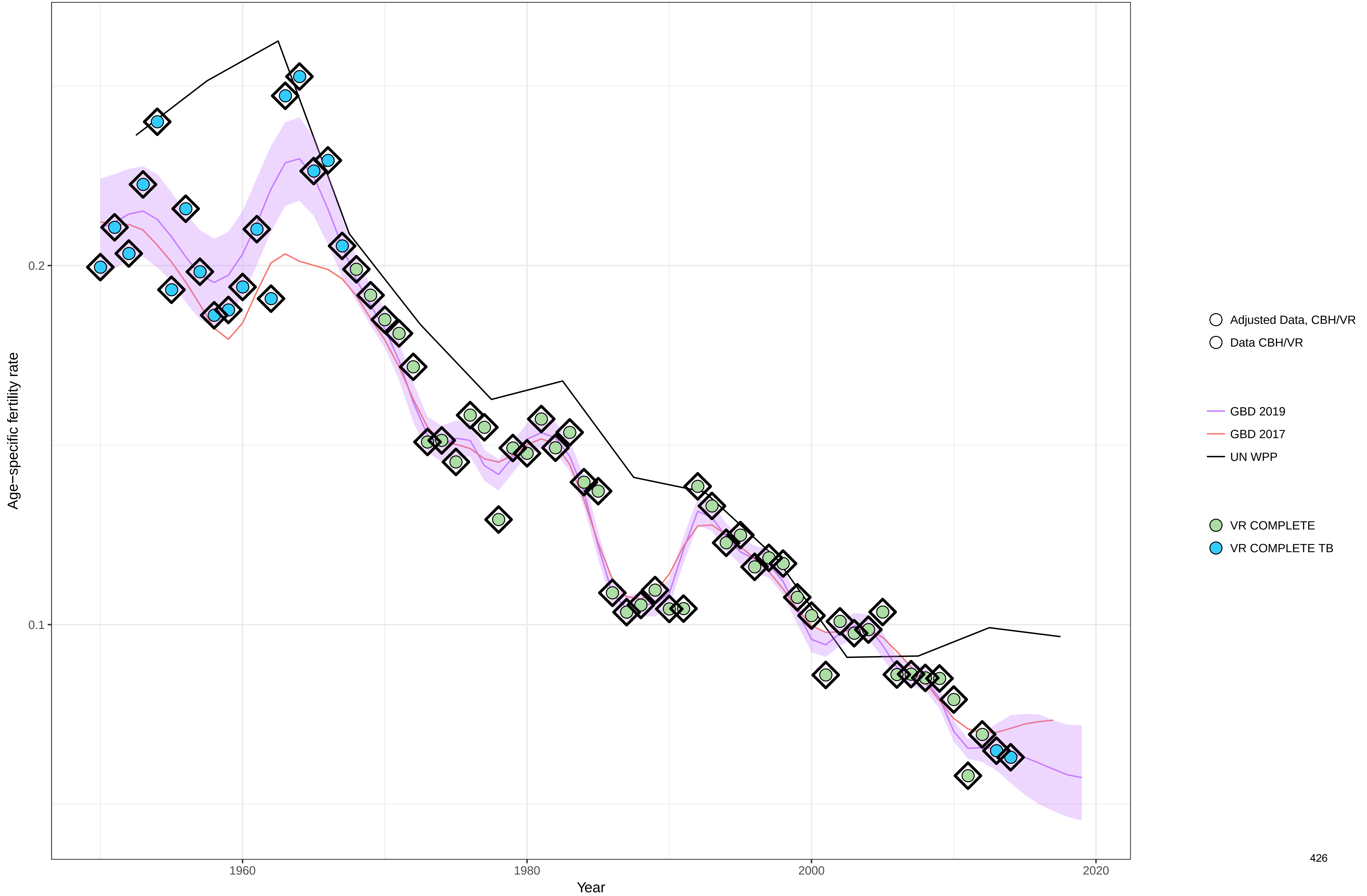

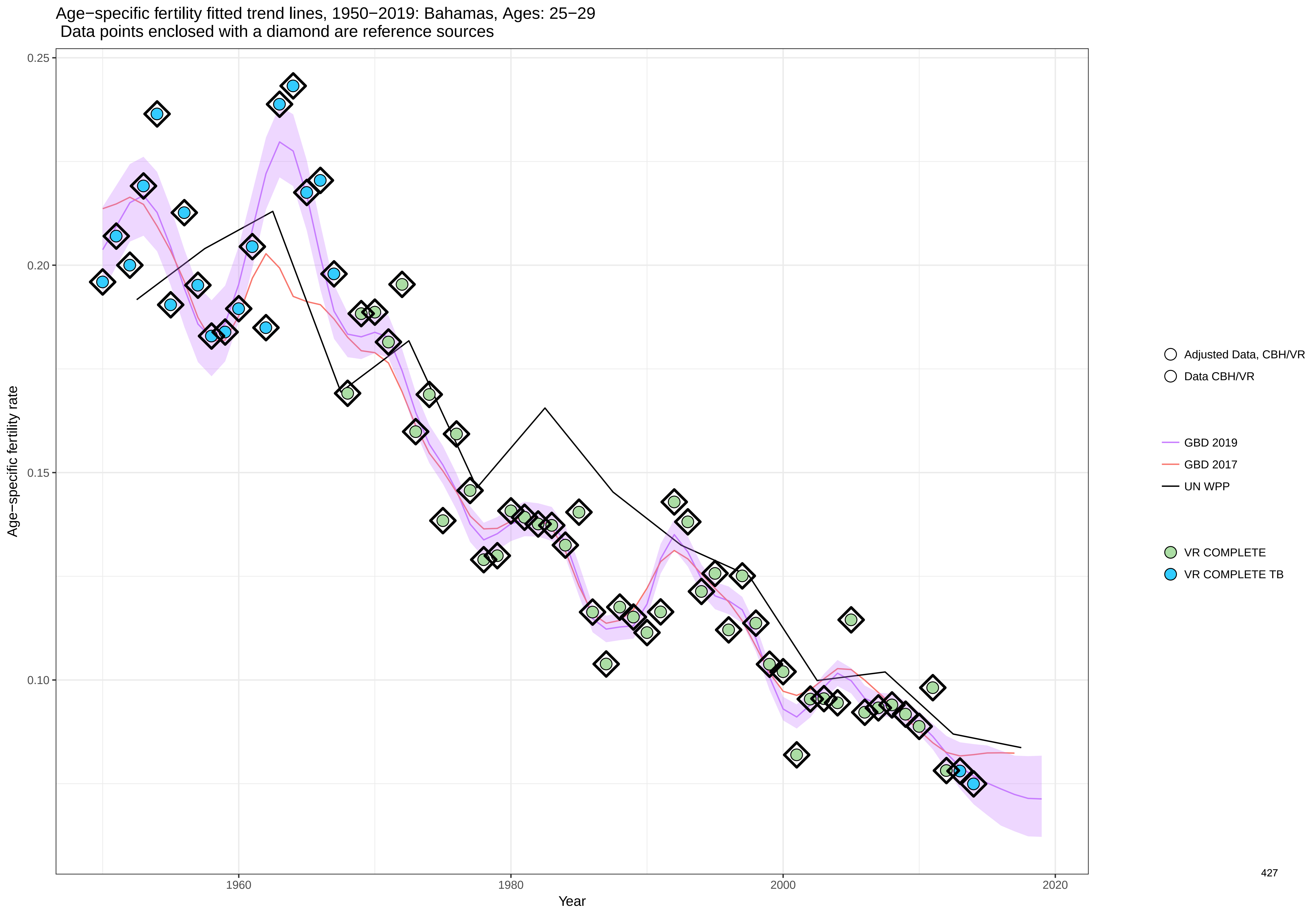

Age−specific fertility fitted trend lines, 1950−2019: Bahamas, Ages: 25−29
Data points enclosed with a diamond are reference sources
0.25
0.20
0.15
0.10
Age−specific fertility rate
1960
1980
2000
2020
Year
Adjusted Data, CBH/VR
Data CBH/VR
GBD 2019
GBD 2017
UN WPP
VR COMPLETE
VR COMPLETE TB

Age−specific fertility fitted trend lines, 1950−2019: Bahamas, Ages: 30−34
Data points enclosed with a diamond are reference sources

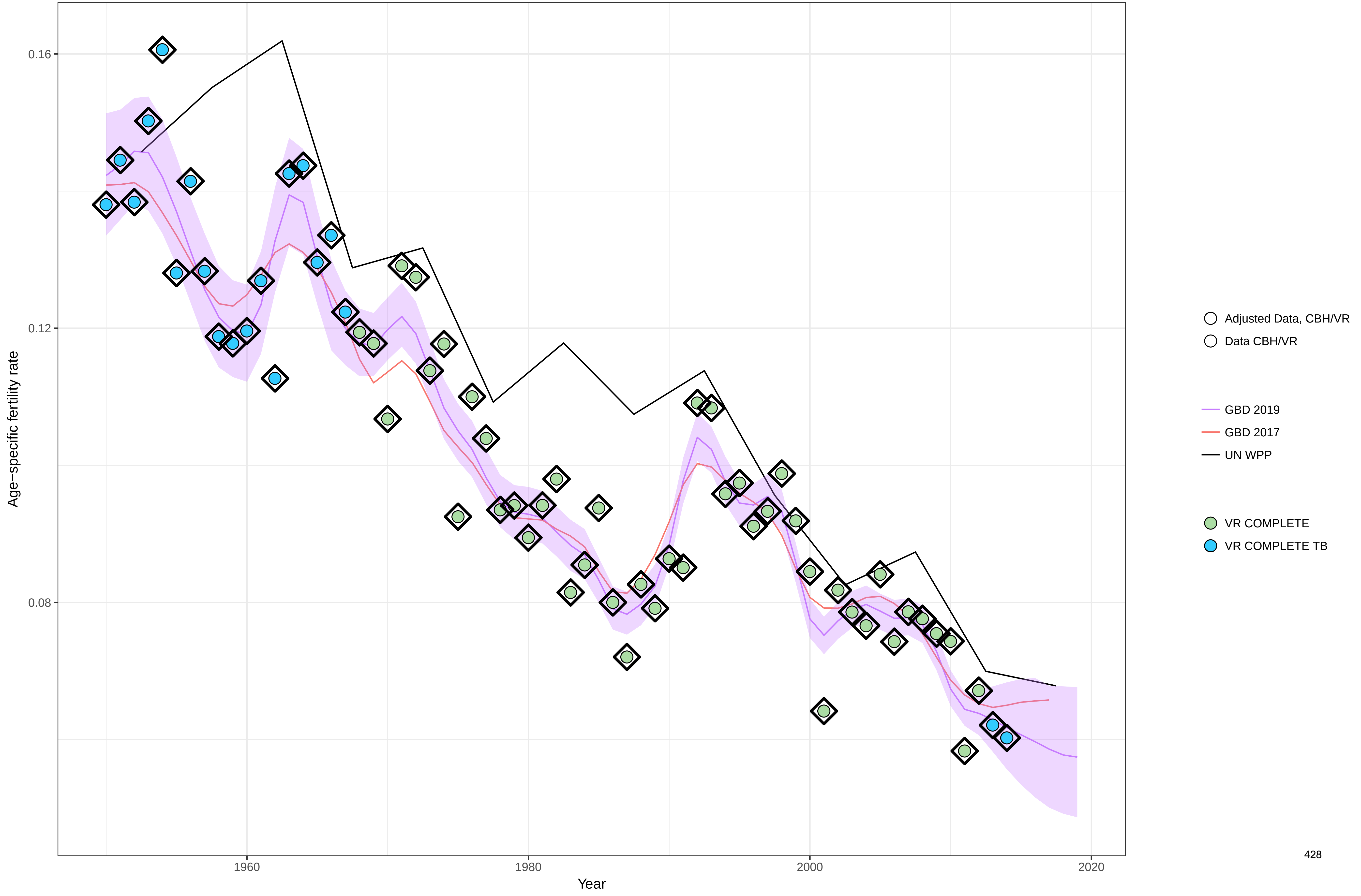

Age−specific fertility fitted trend lines, 1950−2019: Bahamas, Ages: 35−39
Data points enclosed with a diamond are reference sources

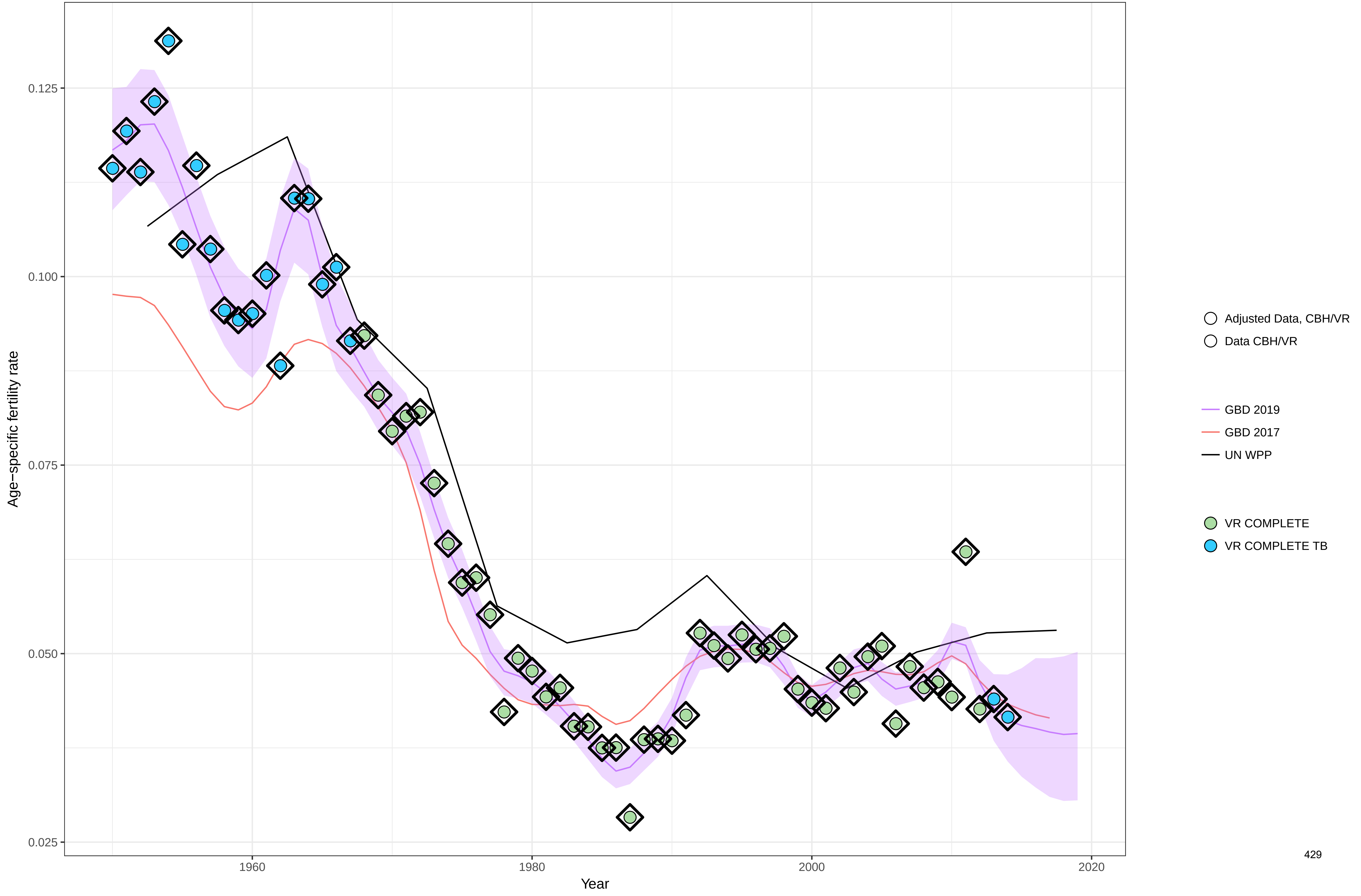

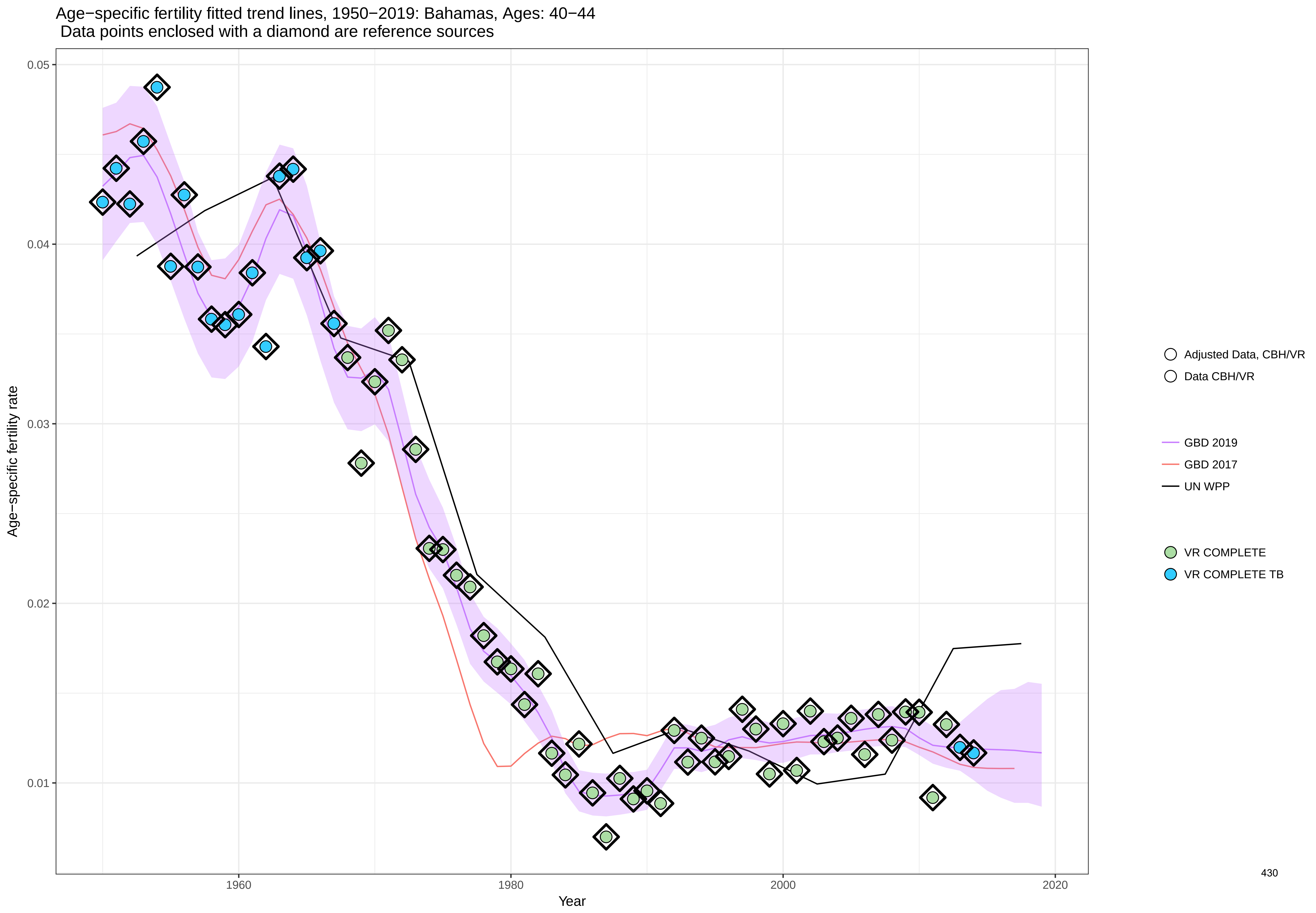
Age−specific fertility fitted trend lines, 1950−2019: Bahamas, Ages: 40−44
Data points enclosed with a diamond are reference sources
Age−specific fertility rate
0.05
0.04
0.03
0.02
0.01
1960
1980
2000
2020
Year
Adjusted Data, CBH/VR
Data CBH/VR
GBD 2019
GBD 2017
UN WPP
VR COMPLETE
VR COMPLETE TB

Age−specific fertility fitted trend lines, 1950−2019: Bahamas, Ages: 45−49
Data points enclosed with a diamond are reference sources

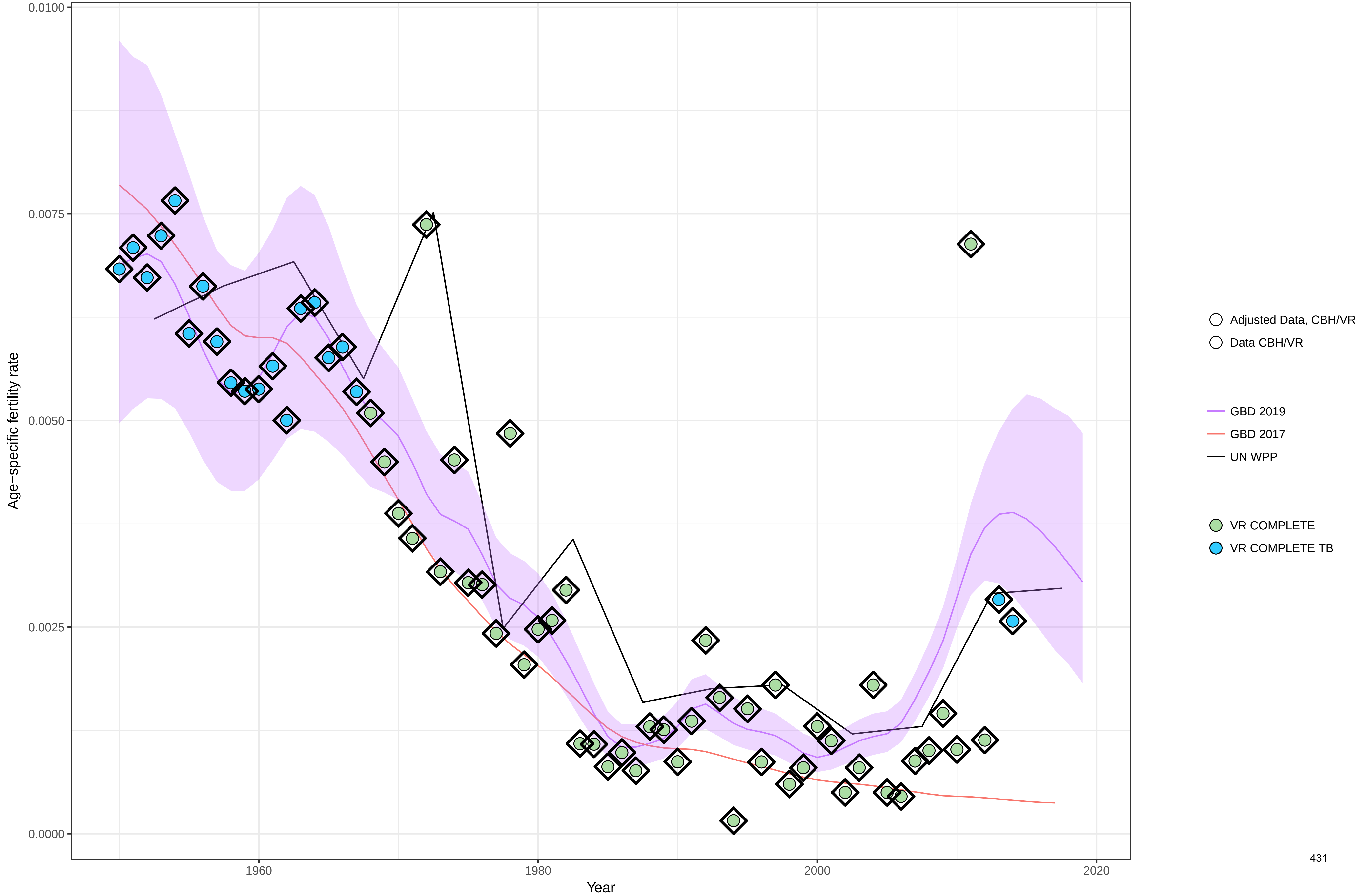

Age−specific fertility fitted trend lines, 1950−2019: Bahamas, Ages: 50−54
Data points enclosed with a diamond are reference sources

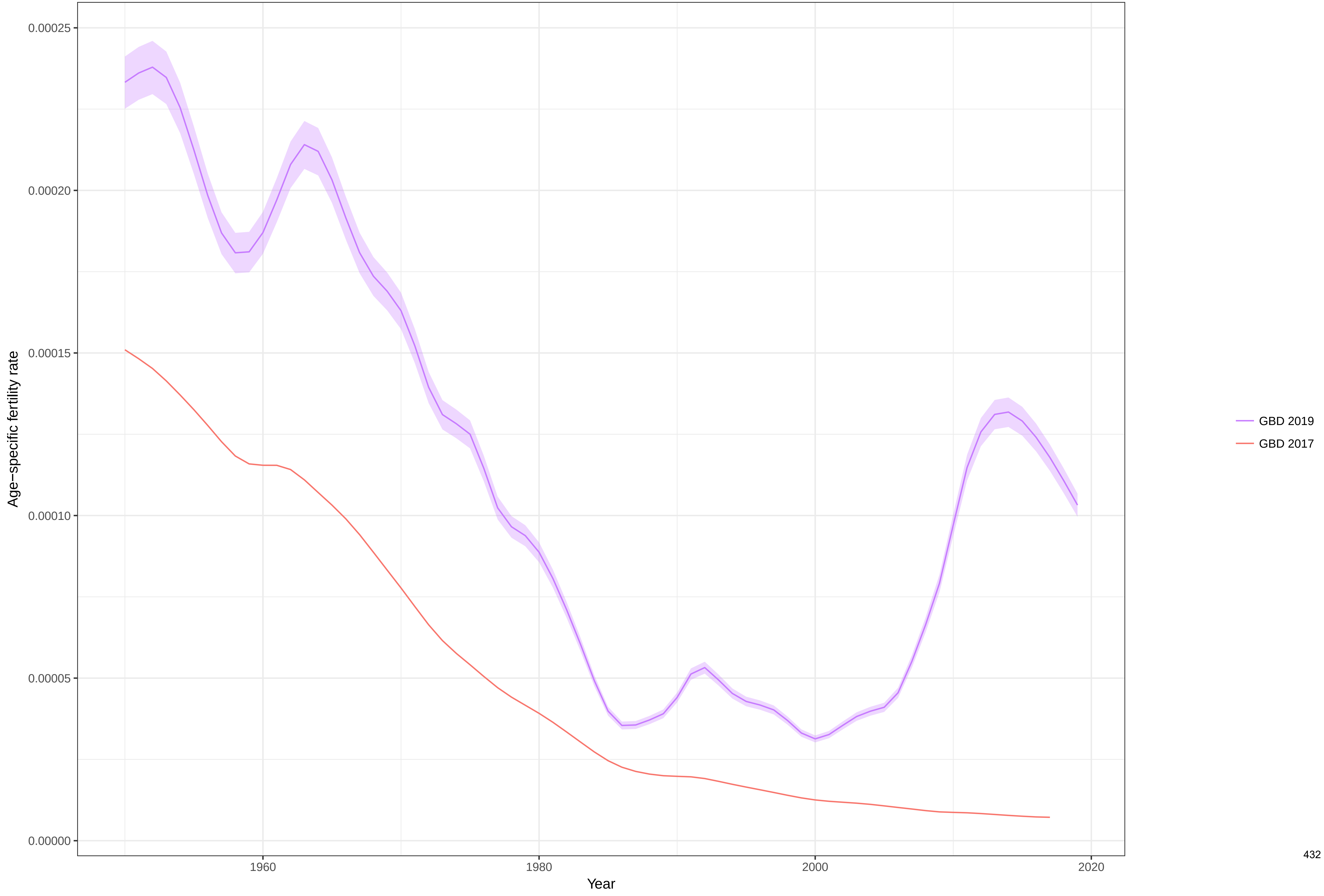

Total fertility rate fitted trend lines, 1950−2019: Bahamas TFR
Data points enclosed with a diamond are reference sources

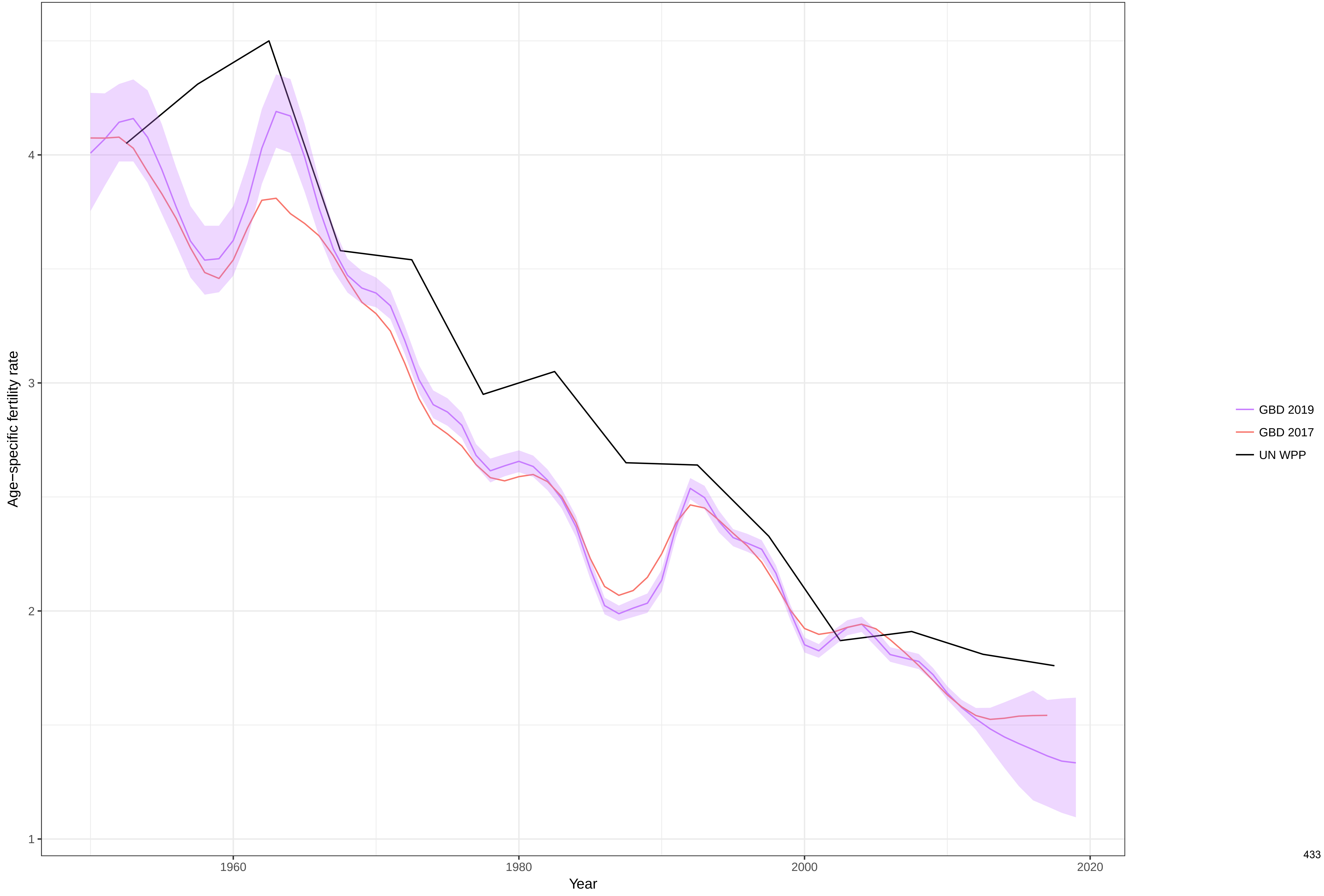

Age−specific fertility fitted trend lines, 1950−2019: Bosnia and Herzegovina, Ages: 10−14
Data points enclosed with a diamond are reference sources

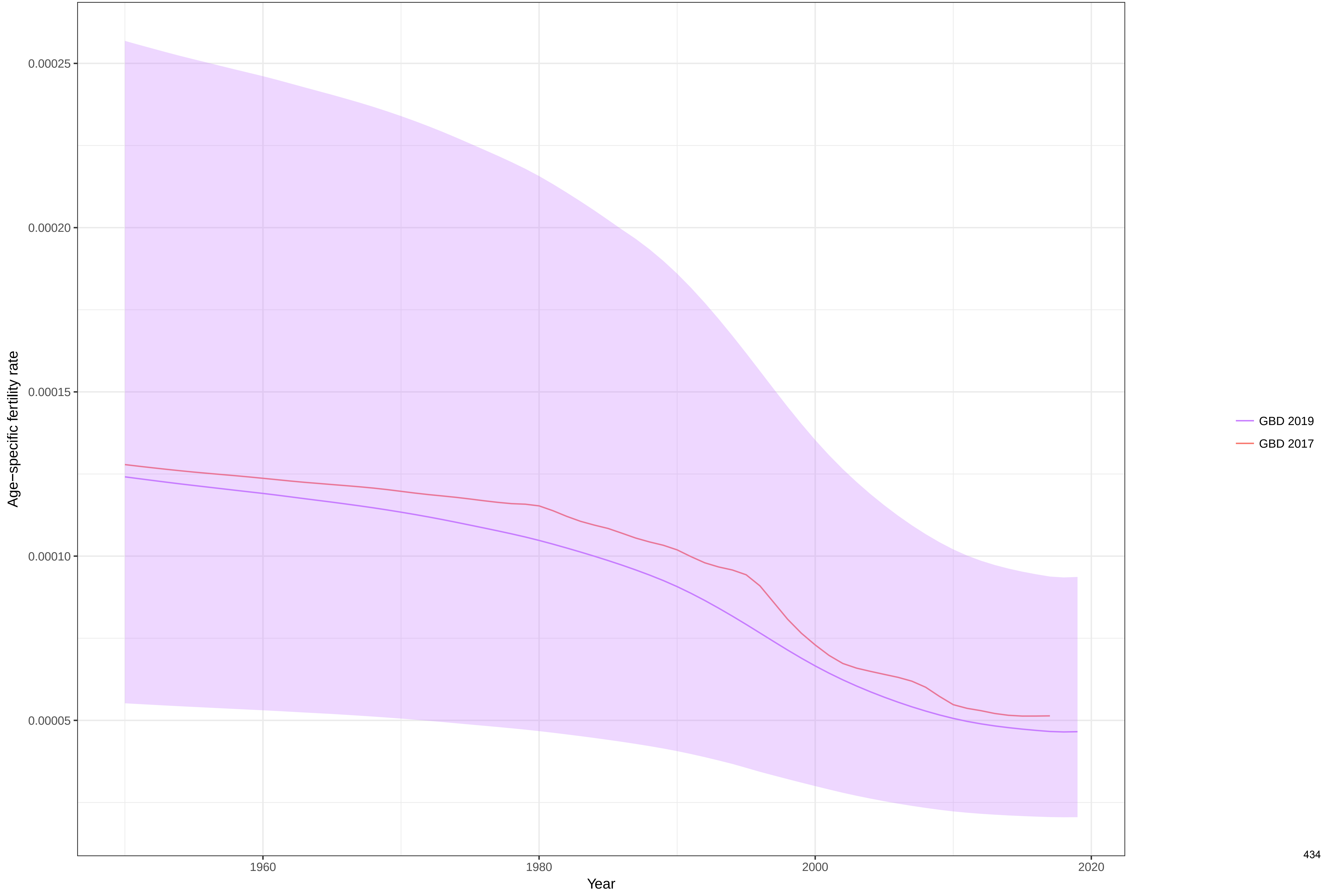

Age−specific fertility fitted trend lines, 1950−2019: Bosnia and Herzegovina, Ages: 15−19
Data points enclosed with a diamond are reference sources

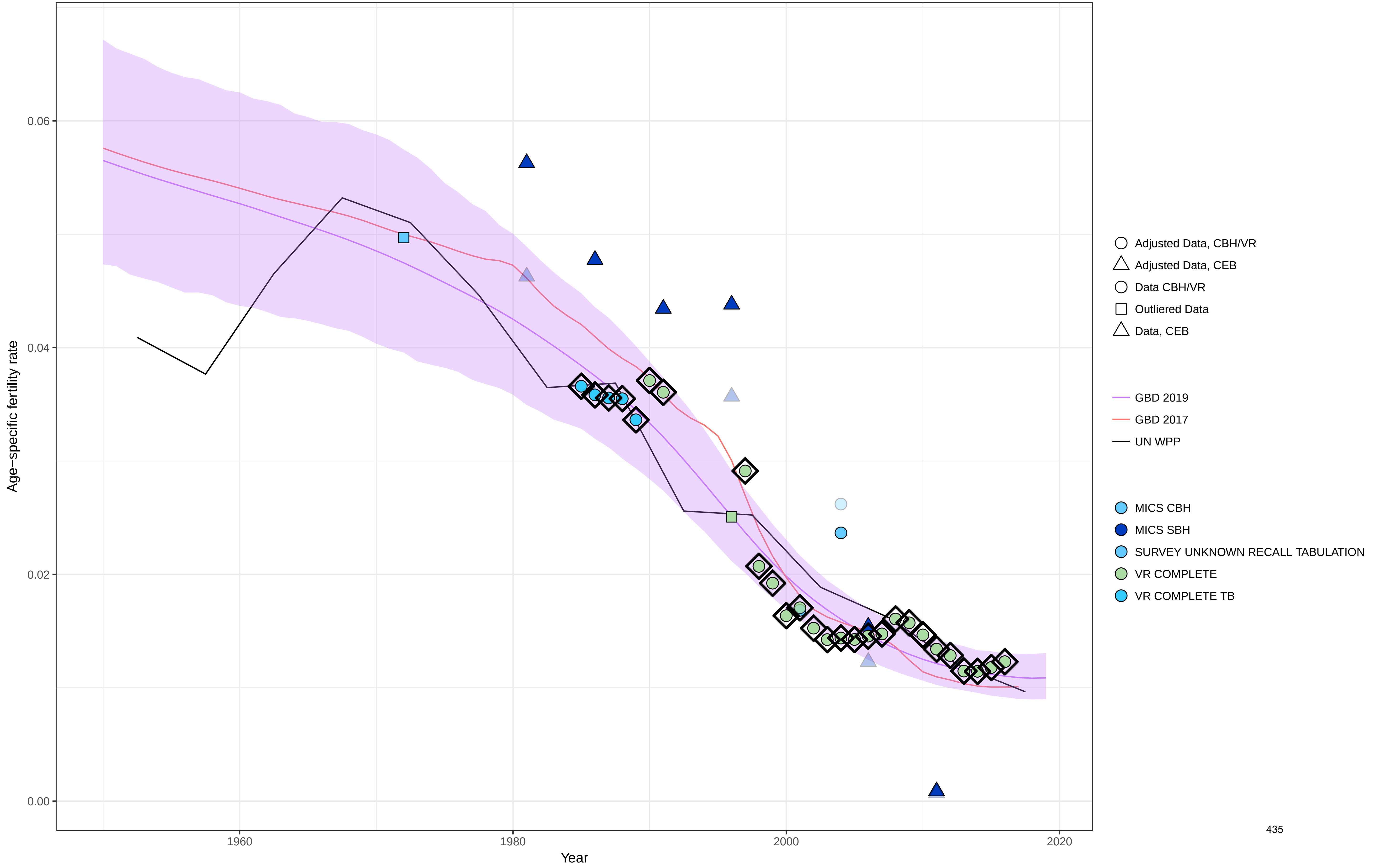

Age−specific fertility fitted trend lines, 1950−2019: Bosnia and Herzegovina, Ages: 20−24
Data points enclosed with a diamond are reference sources

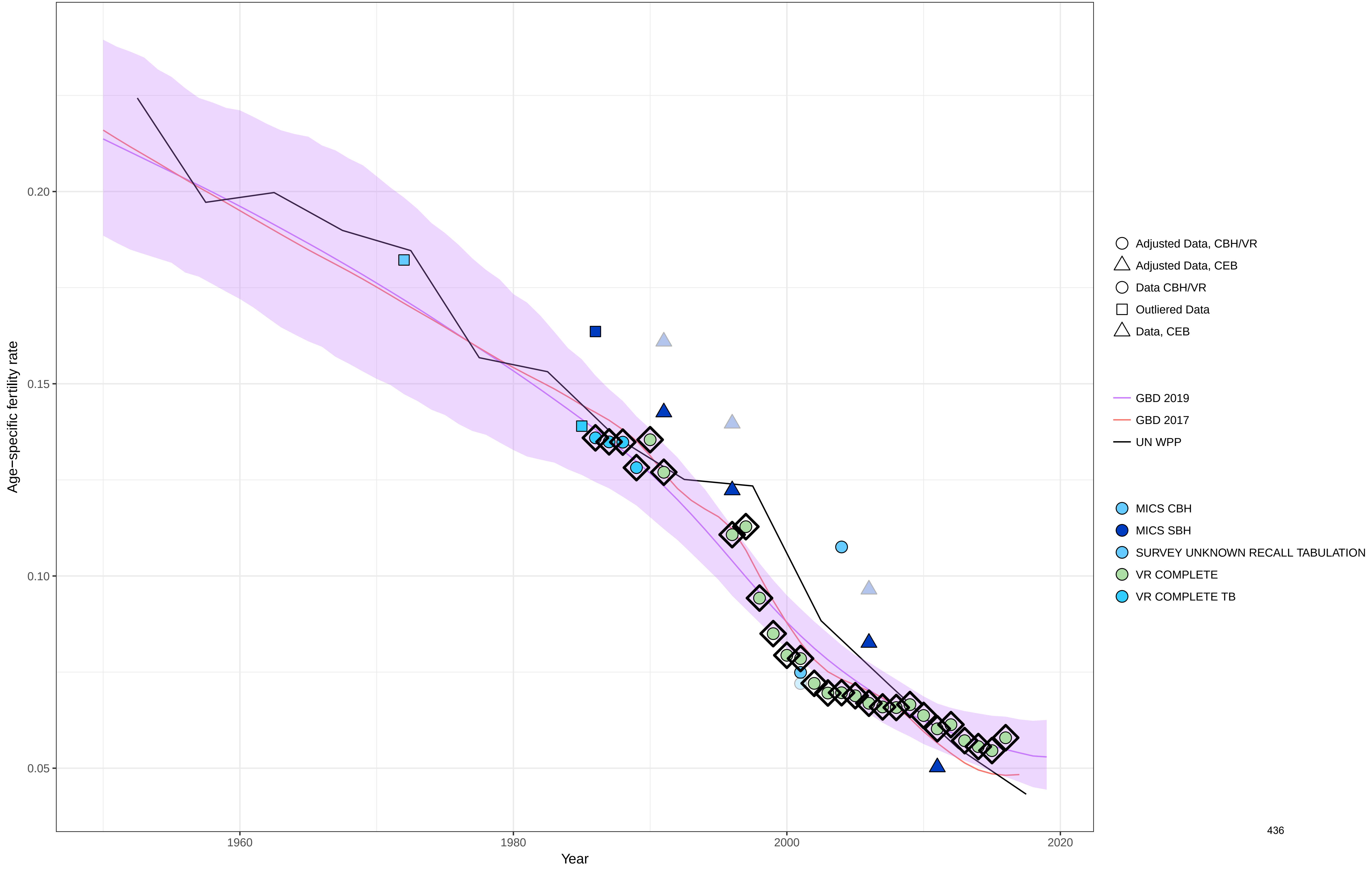

Age−specific fertility fitted trend lines, 1950−2019: Bosnia and Herzegovina, Ages: 25−29
Data points enclosed with a diamond are reference sources

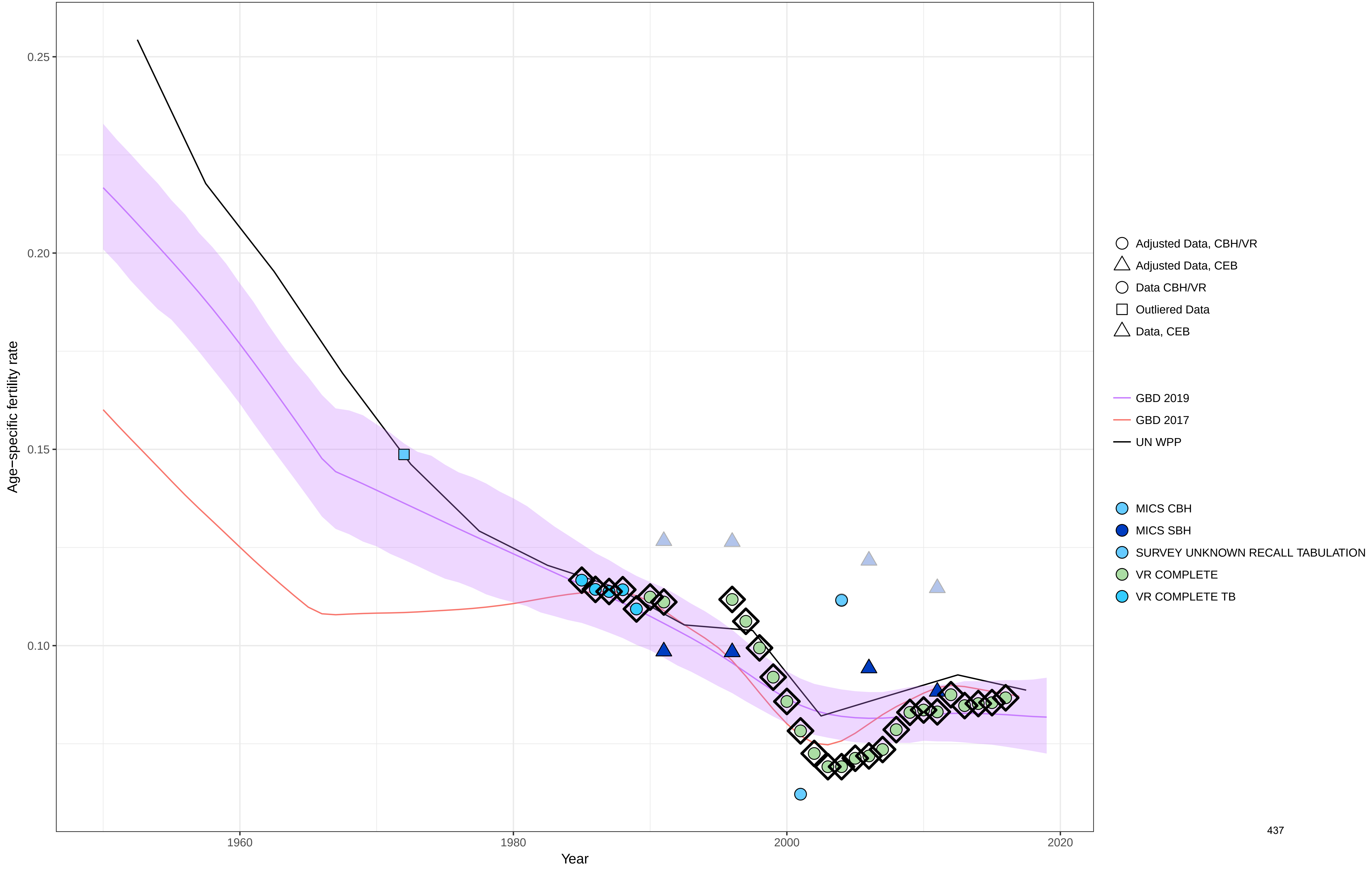

Age−specific fertility fitted trend lines, 1950−2019: Bosnia and Herzegovina, Ages: 30−34
Data points enclosed with a diamond are reference sources

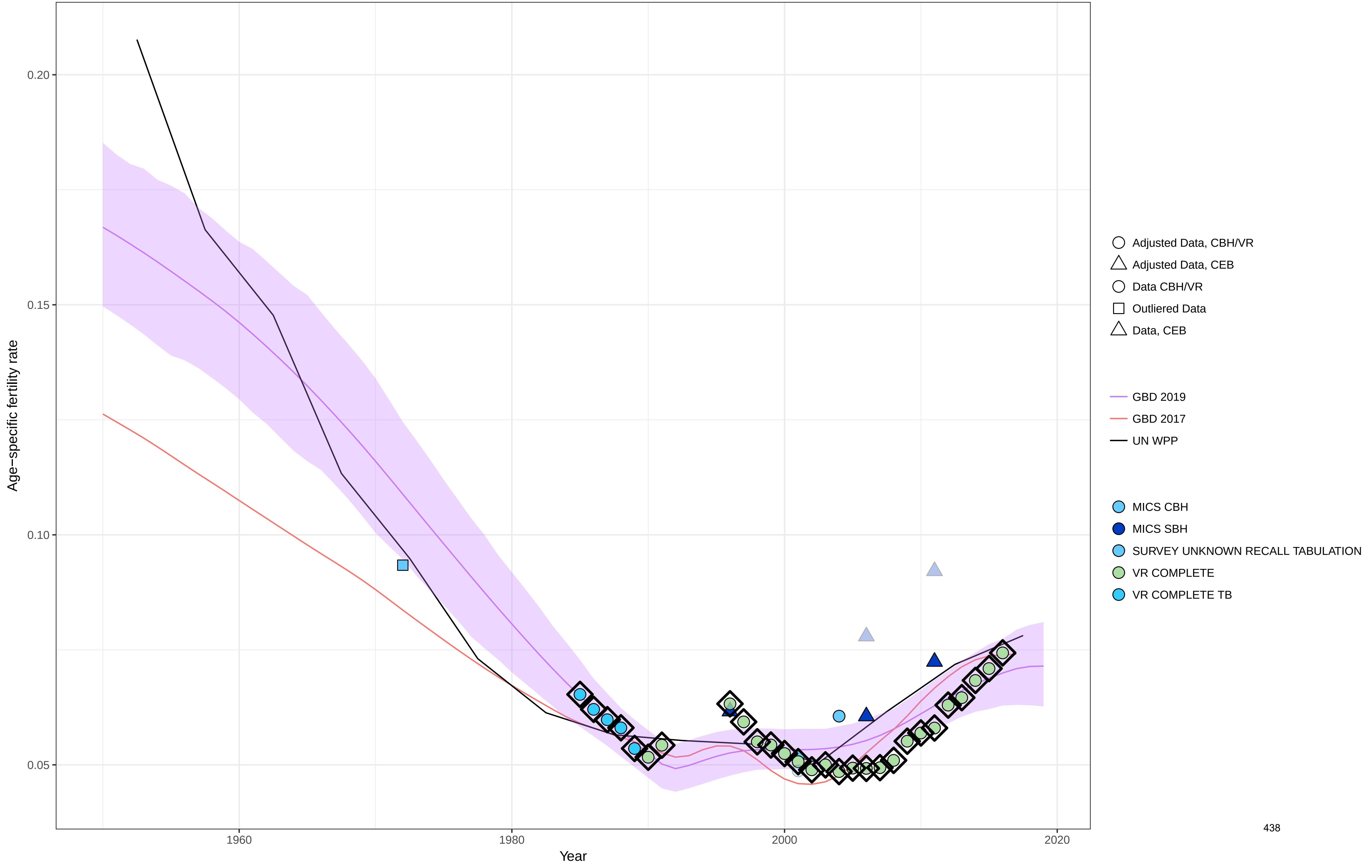

Age−specific fertility fitted trend lines, 1950−2019: Bosnia and Herzegovina, Ages: 35−39
Data points enclosed with a diamond are reference sources

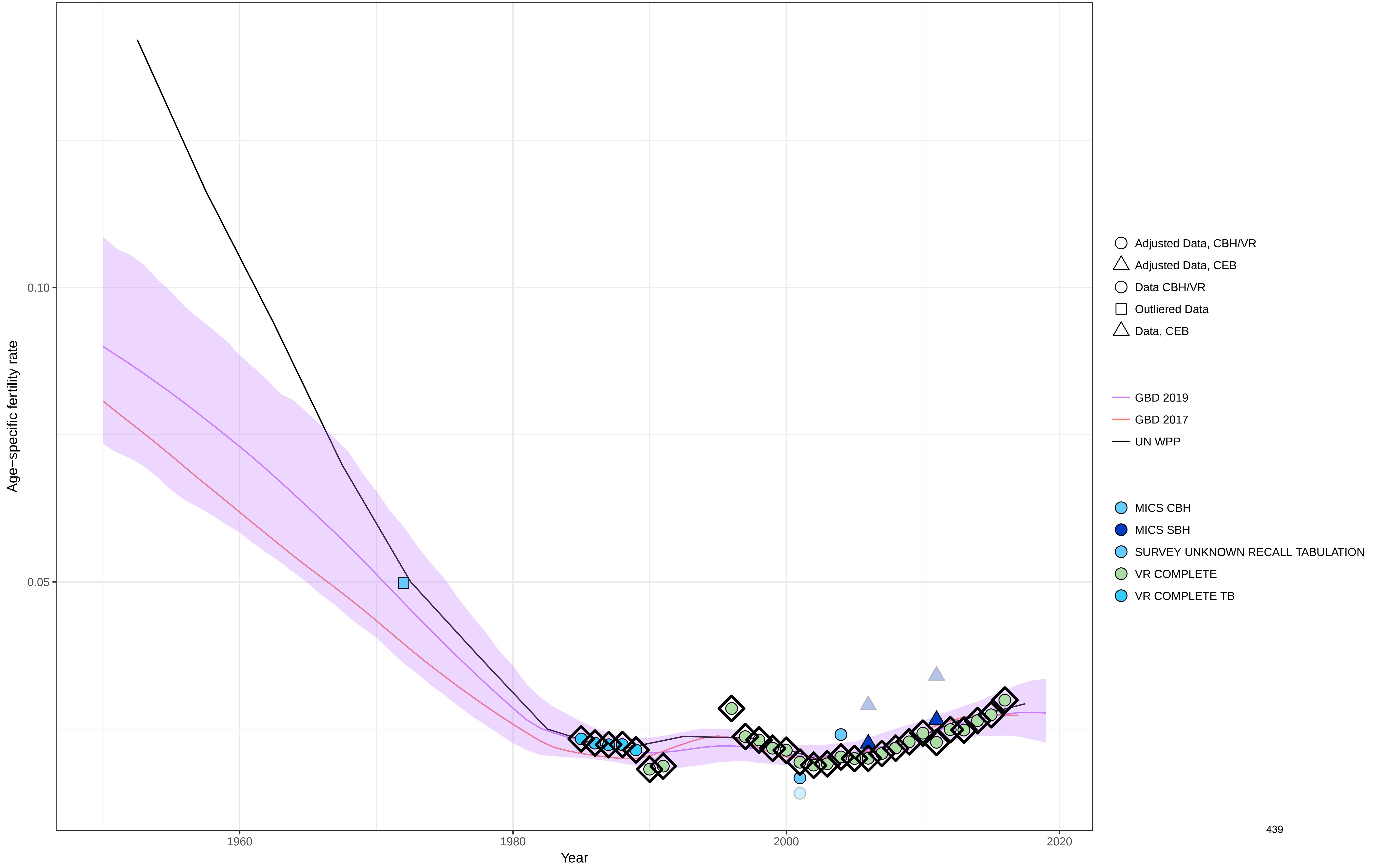

Age−specific fertility fitted trend lines, 1950−2019: Bosnia and Herzegovina, Ages: 40−44
Data points enclosed with a diamond are reference sources

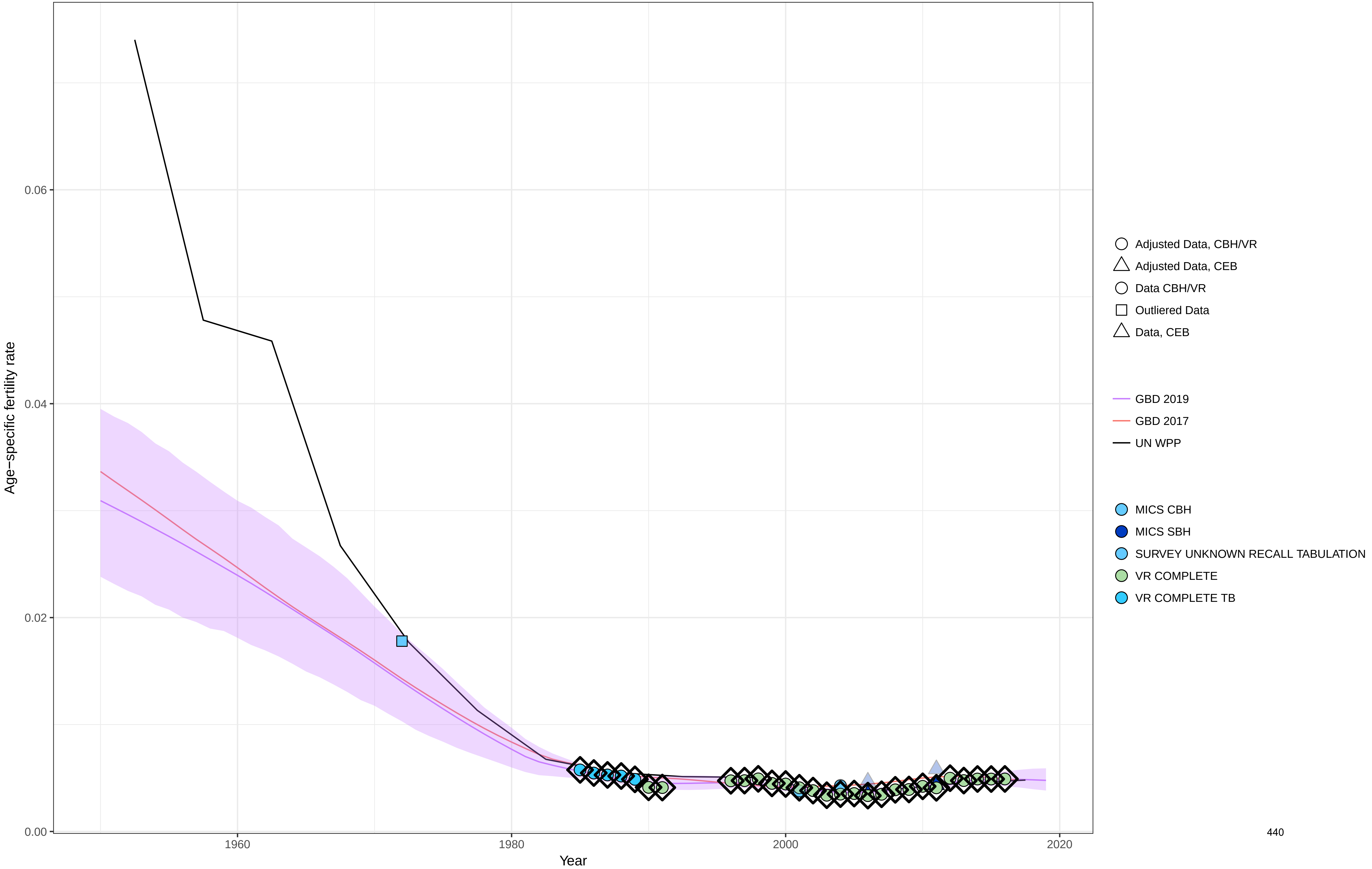

Age−specific fertility fitted trend lines, 1950−2019: Bosnia and Herzegovina, Ages: 45−49
Data points enclosed with a diamond are reference sources

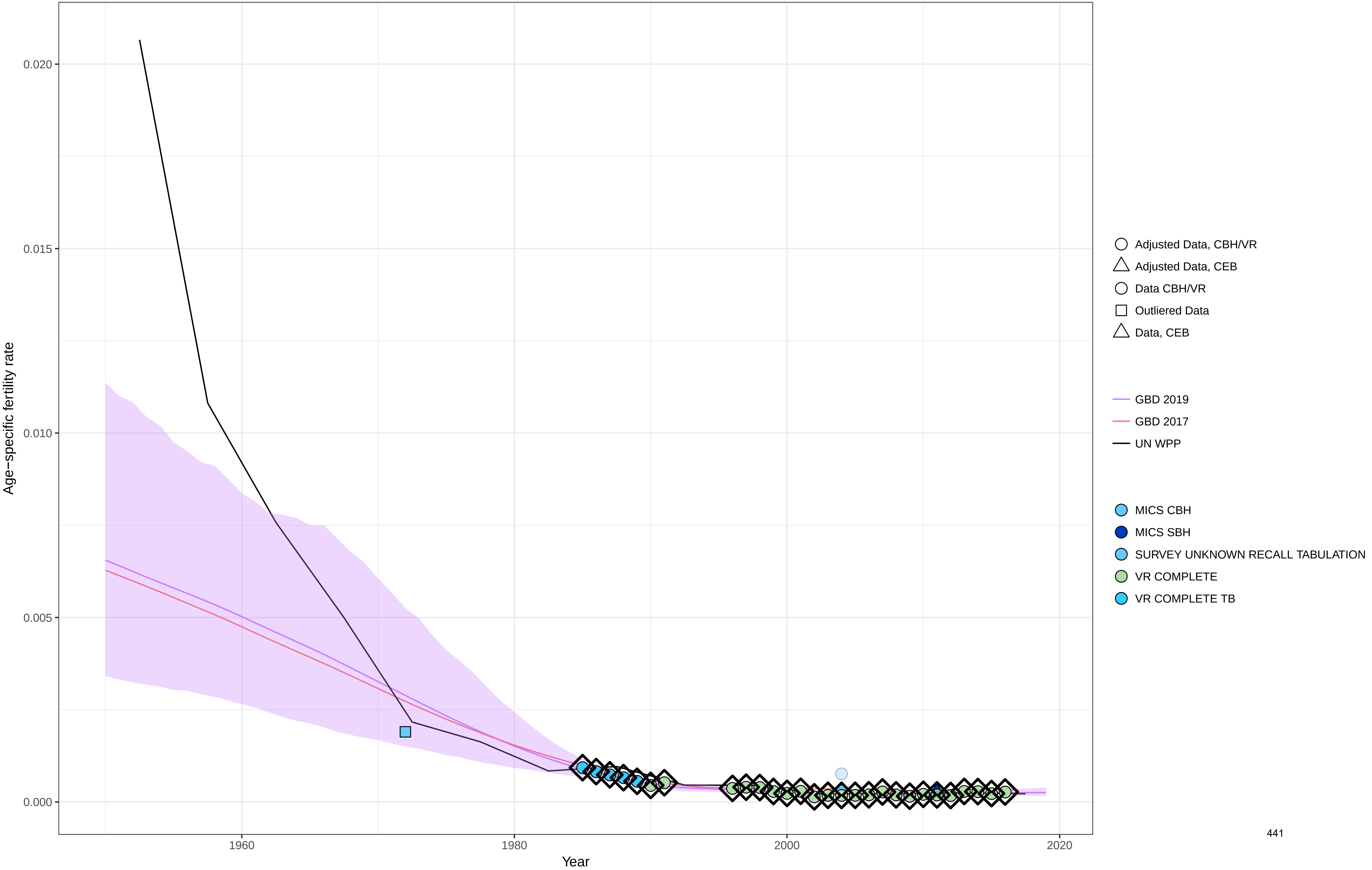

Age−specific fertility fitted trend lines, 1950−2019: Bosnia and Herzegovina, Ages: 50−54
Data points enclosed with a diamond are reference sources

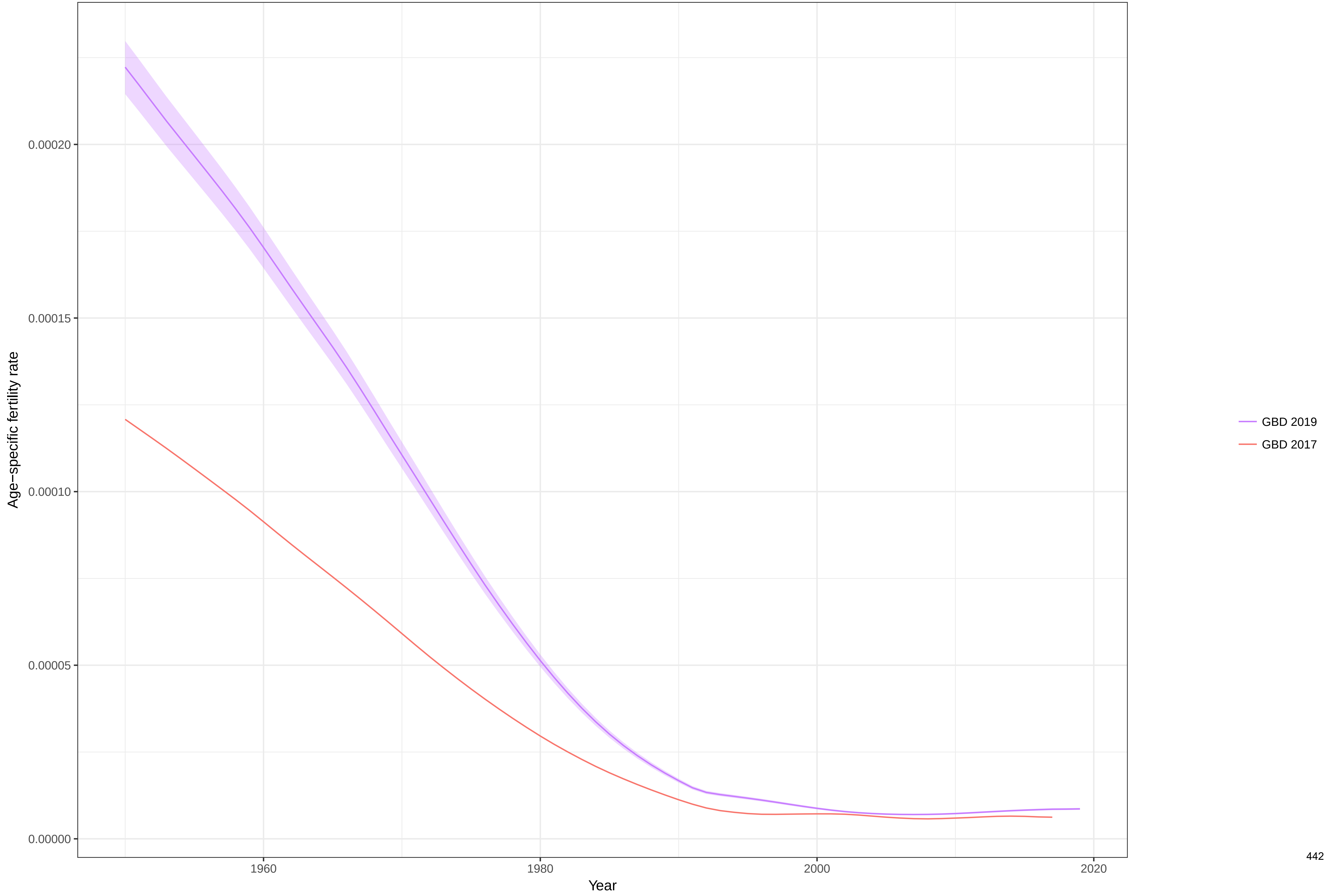

Total fertility rate fitted trend lines, 1950−2019: Bosnia and Herzegovina TFR
Data points enclosed with a diamond are reference sources

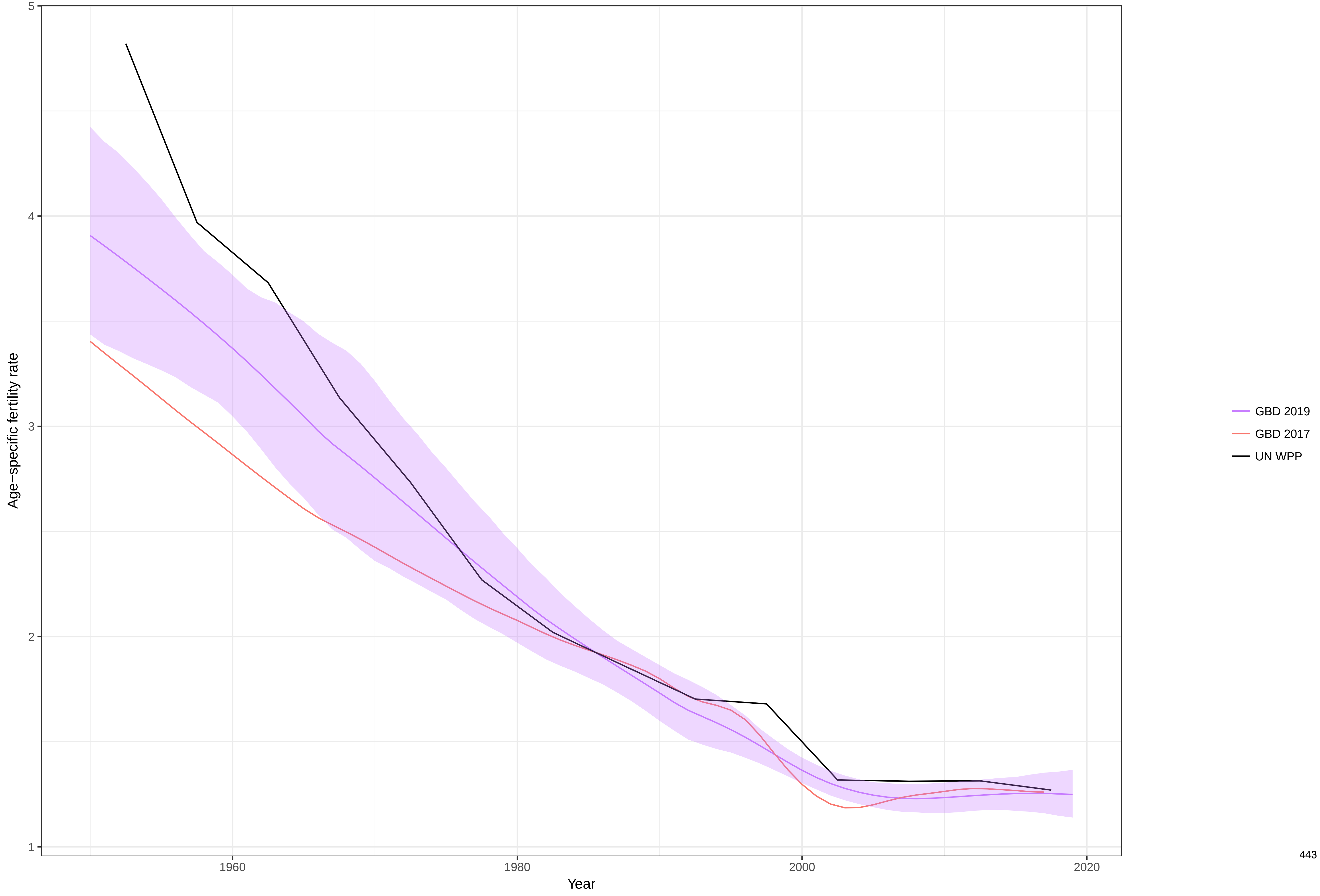

Age−specific fertility fitted trend lines, 1950−2019: Belarus, Ages: 10−14
Data points enclosed with a diamond are reference sources

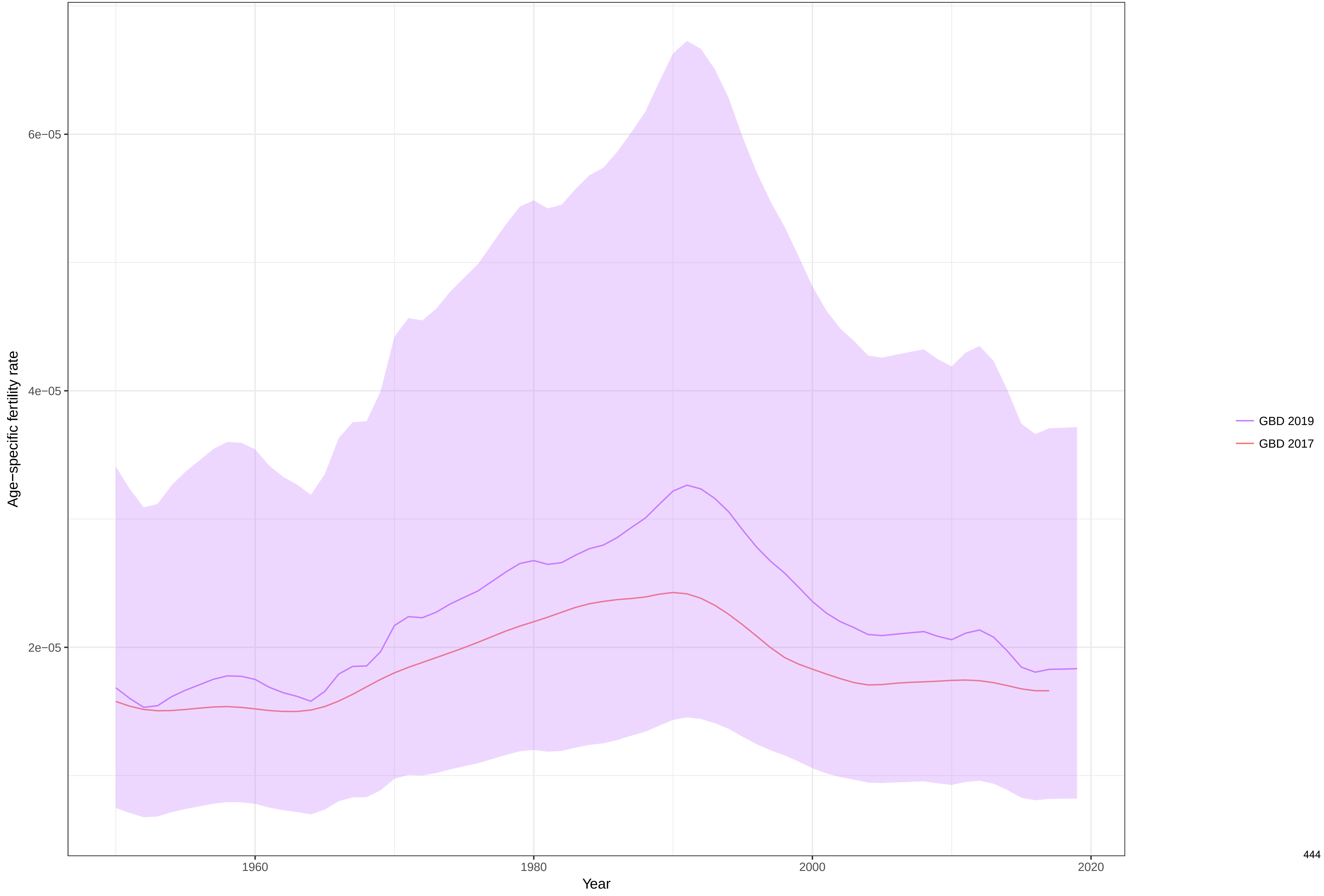

Age−specific fertility fitted trend lines, 1950−2019: Belarus, Ages: 15−19
Data points enclosed with a diamond are reference sources

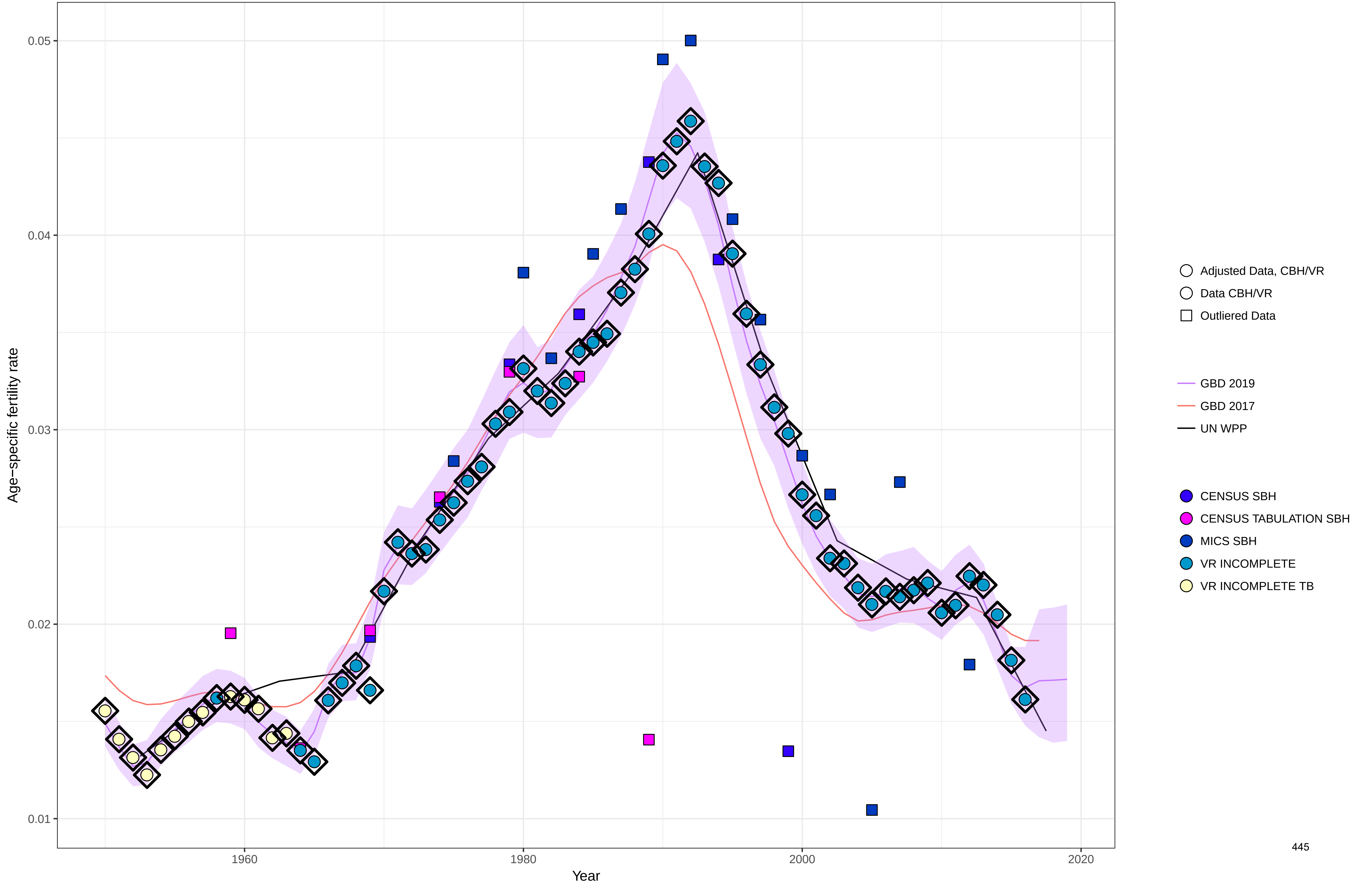

Age−specific fertility fitted trend lines, 1950−2019: Belarus, Ages: 20−24
Data points enclosed with a diamond are reference sources

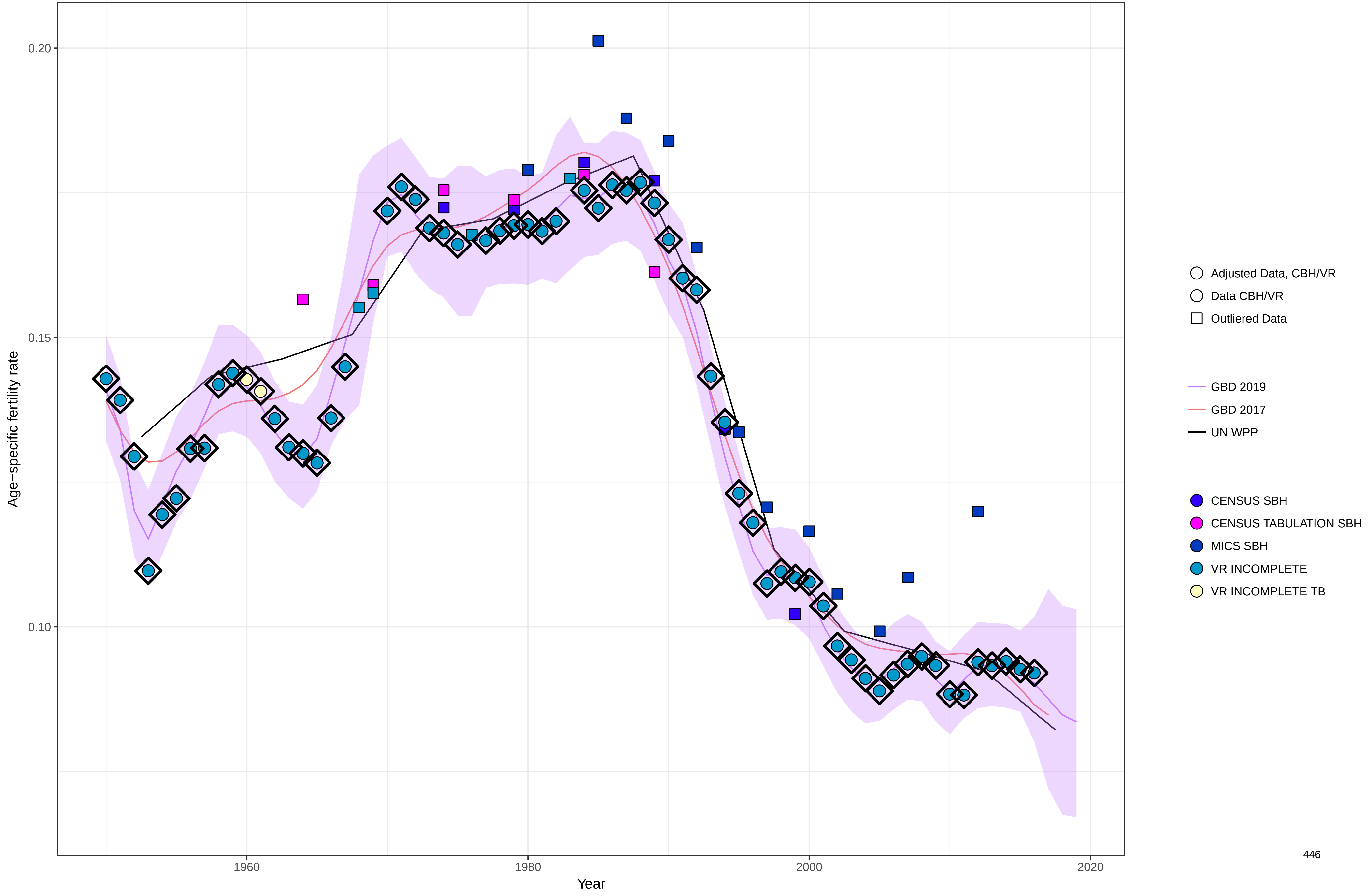

Age−specific fertility fitted trend lines, 1950−2019: Belarus, Ages: 25−29
Data points enclosed with a diamond are reference sources

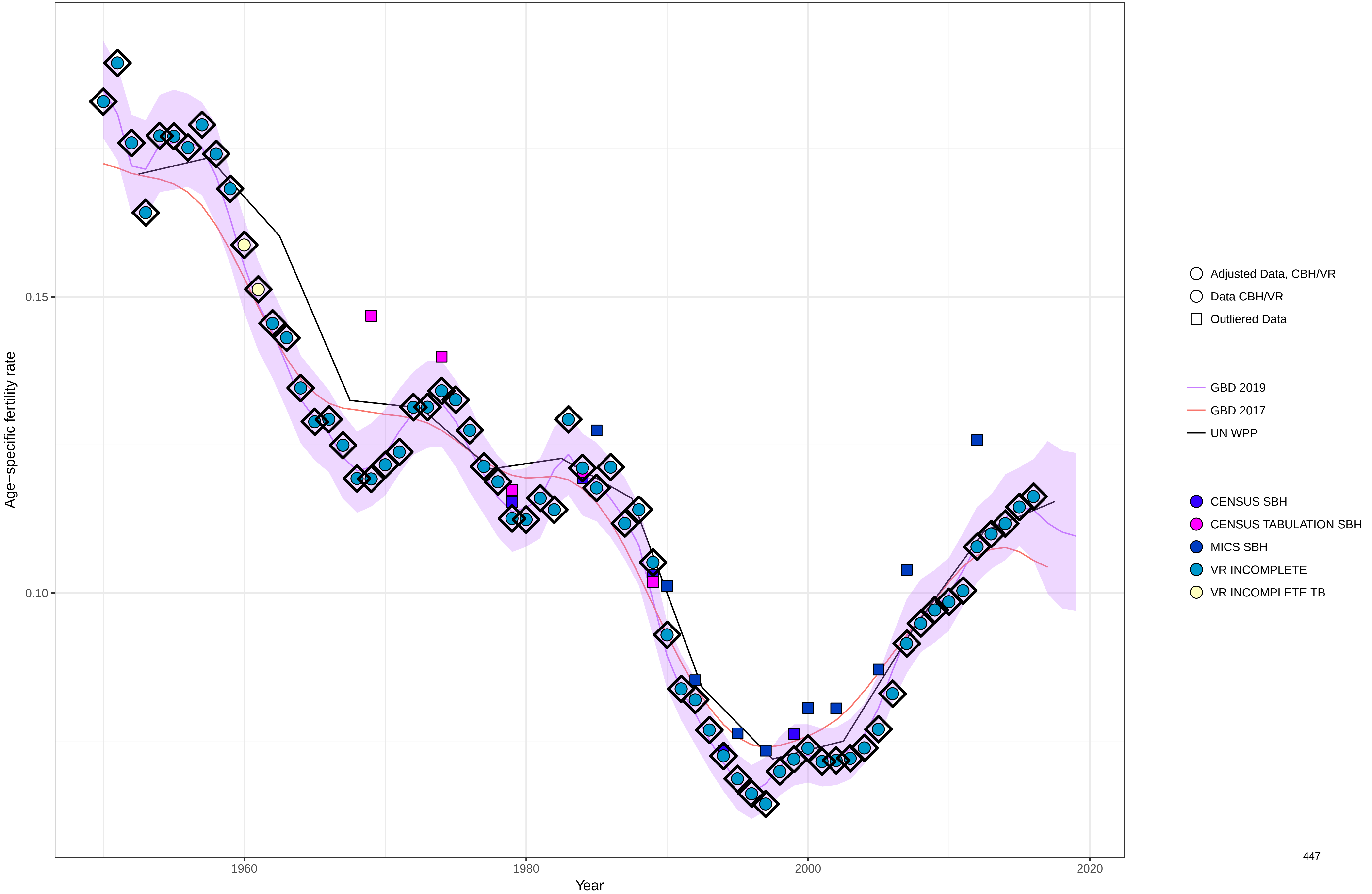

Age−specific fertility fitted trend lines, 1950−2019: Belarus, Ages: 30−34
Data points enclosed with a diamond are reference sources

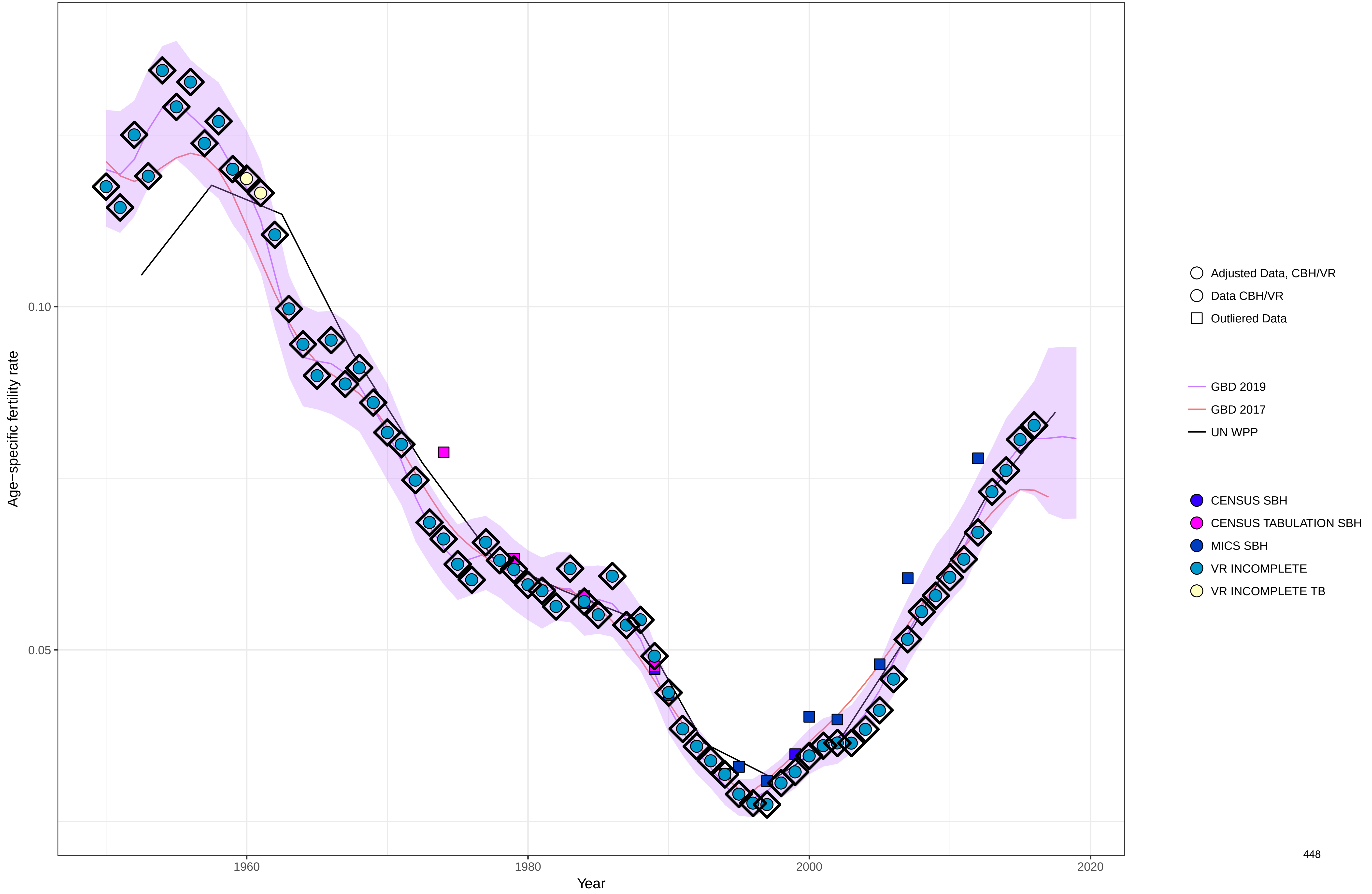

Age−specific fertility fitted trend lines, 1950−2019: Belarus, Ages: 35−39
Data points enclosed with a diamond are reference sources

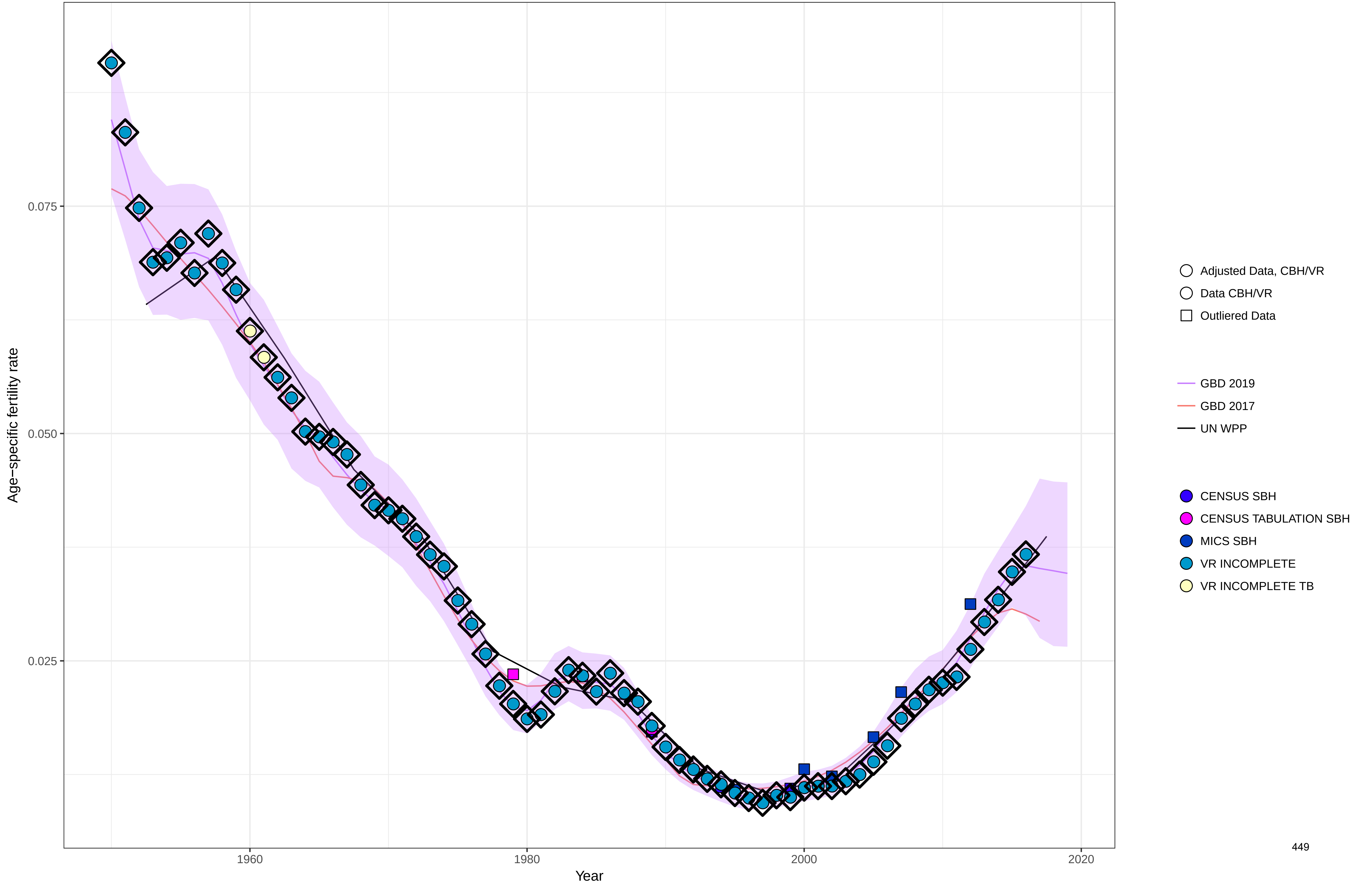

Age−specific fertility fitted trend lines, 1950−2019: Belarus, Ages: 40−44
Data points enclosed with a diamond are reference sources

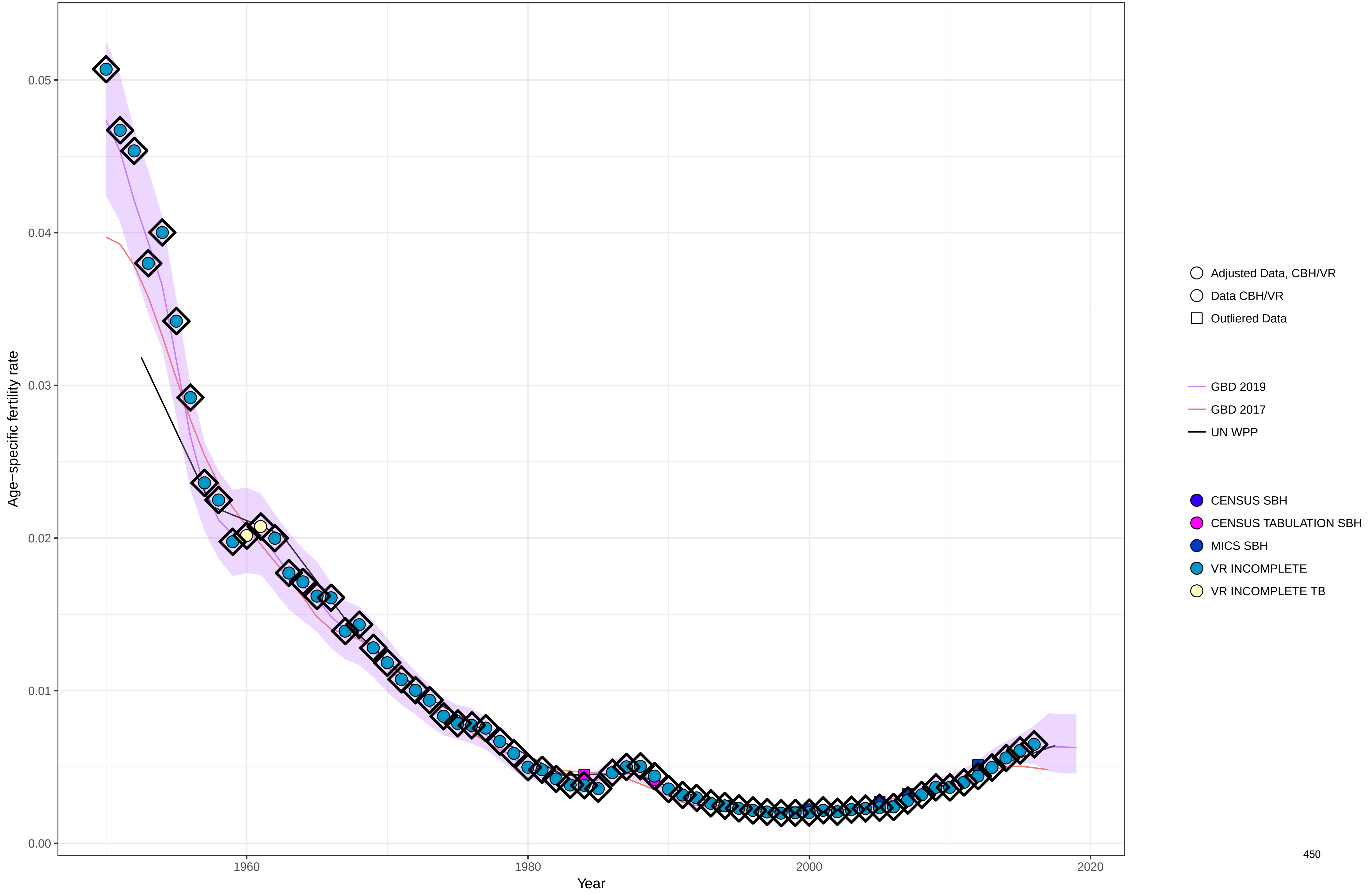

Age−specific fertility fitted trend lines, 1950−2019: Belarus, Ages: 45−49
Data points enclosed with a diamond are reference sources

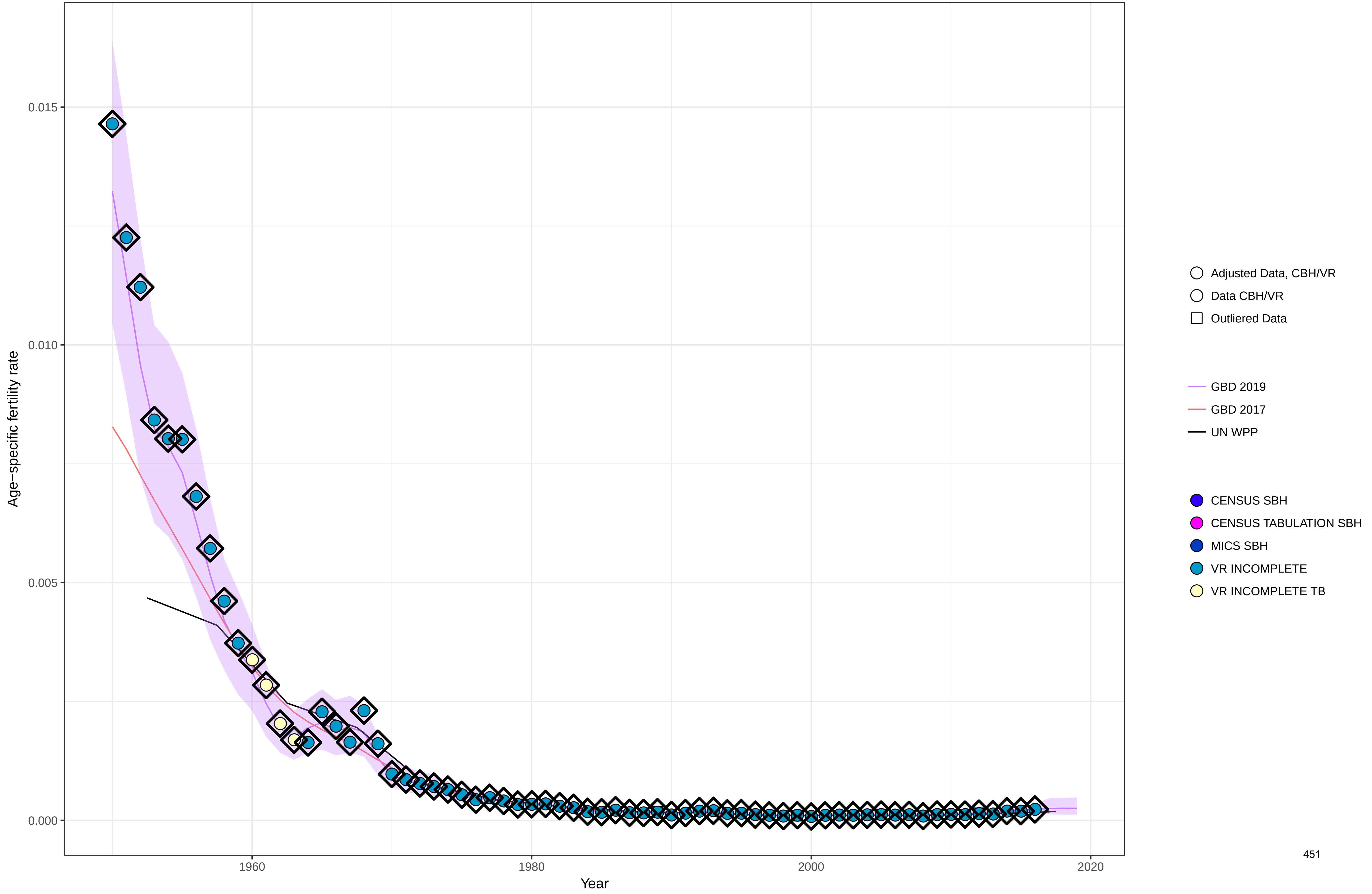

Age−specific fertility fitted trend lines, 1950−2019: Belarus, Ages: 50−54
Data points enclosed with a diamond are reference sources

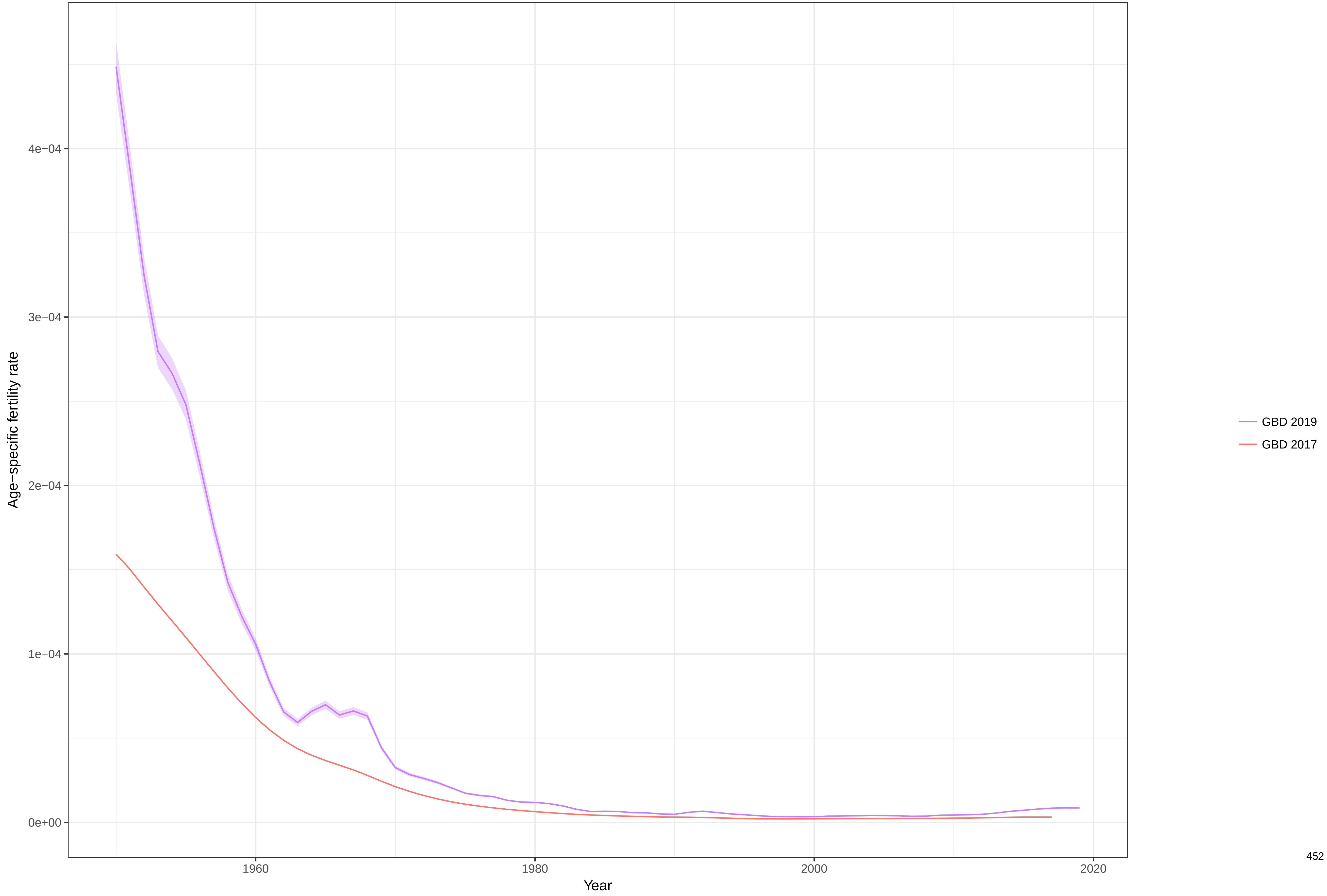

Total fertility rate fitted trend lines, 1950−2019: Belarus TFR
Data points enclosed with a diamond are reference sources

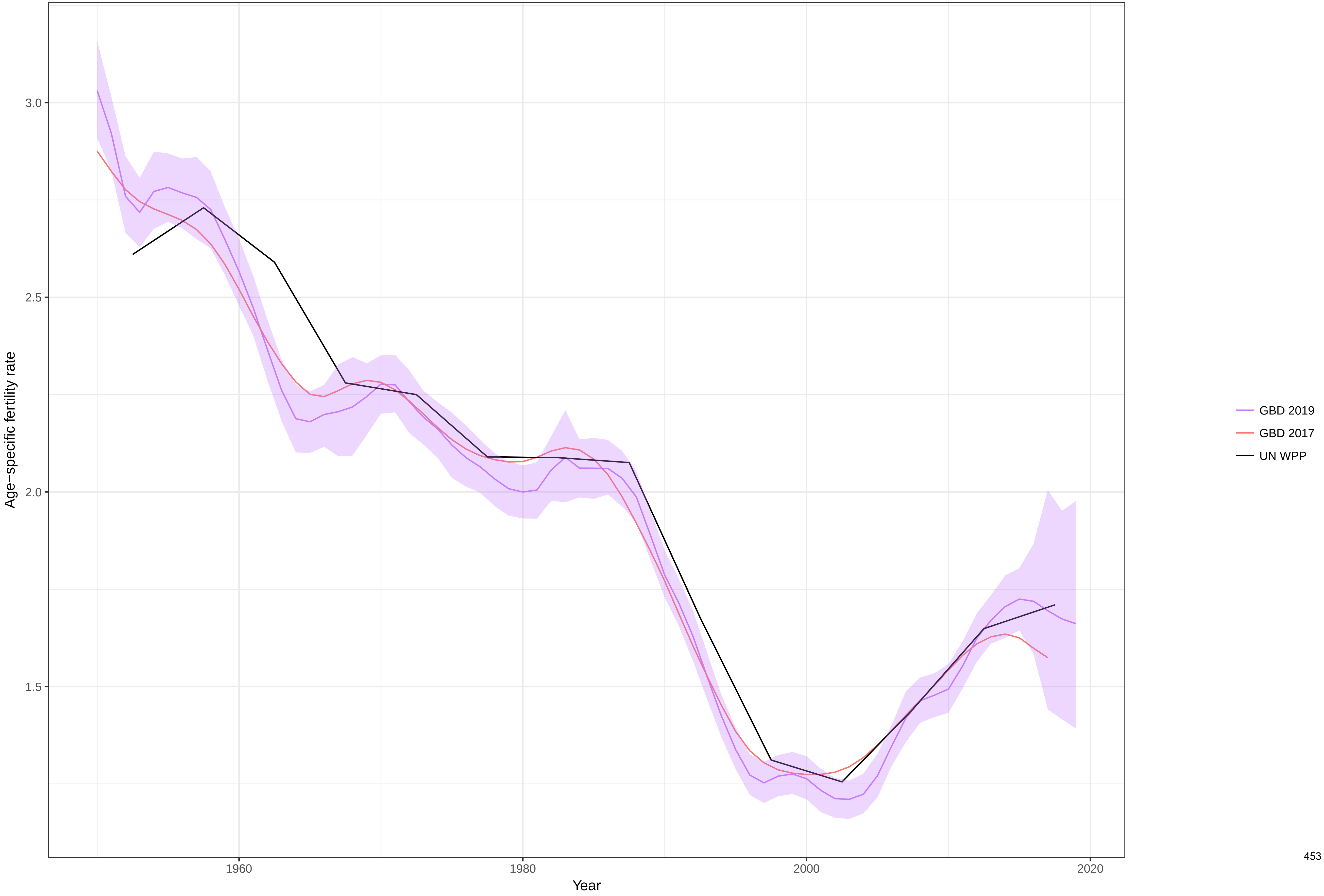

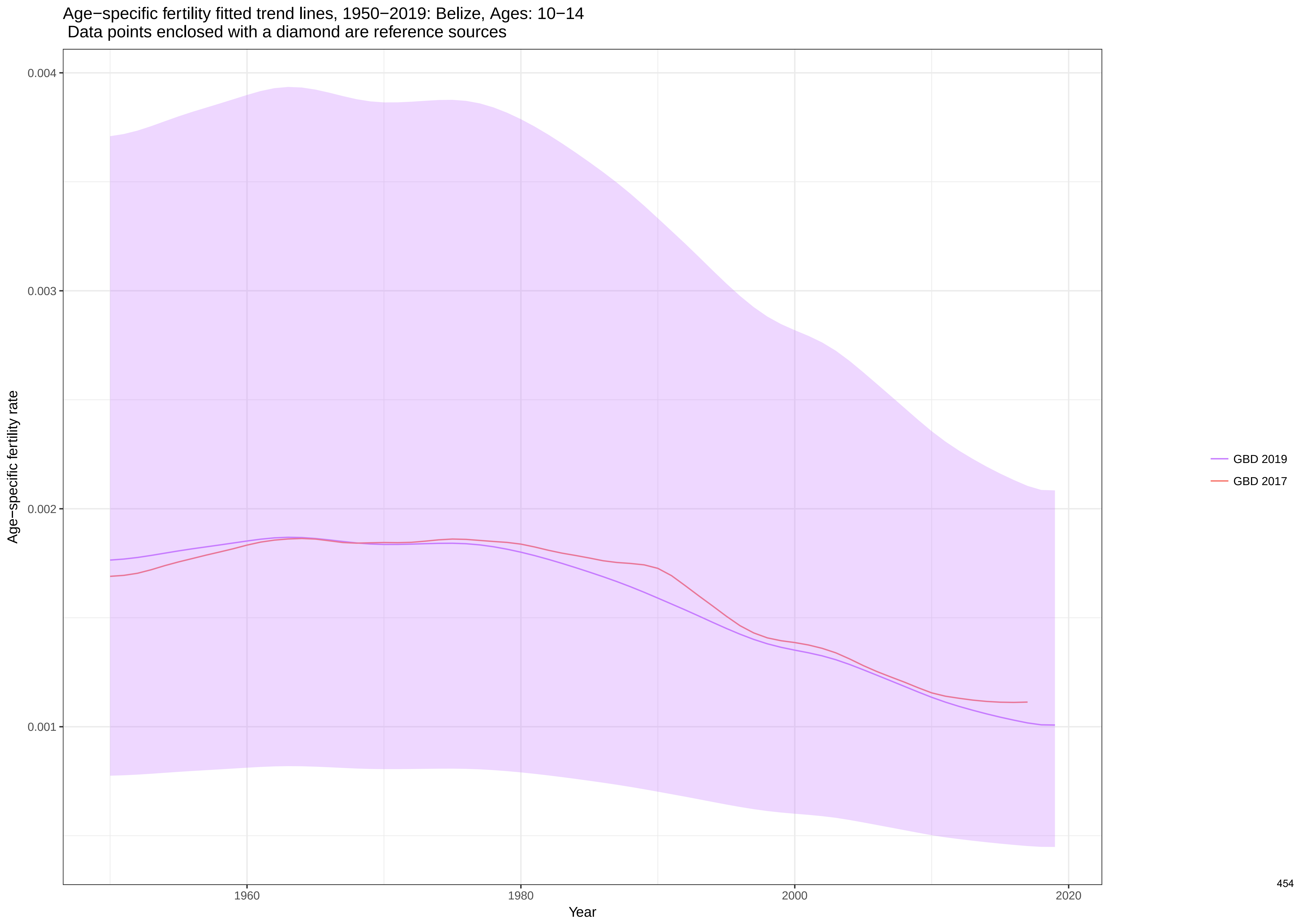

Age−specific fertility fitted trend lines, 1950−2019: Belize, Ages: 10−14
Data points enclosed with a diamond are reference sources
0.004
0.003
0.002
0.001
Age−specific fertility rate
1960
1980
2000
2020
Year
GBD 2019
GBD 2017

Age−specific fertility fitted trend lines, 1950−2019: Belize, Ages: 15−19
Data points enclosed with a diamond are reference sources

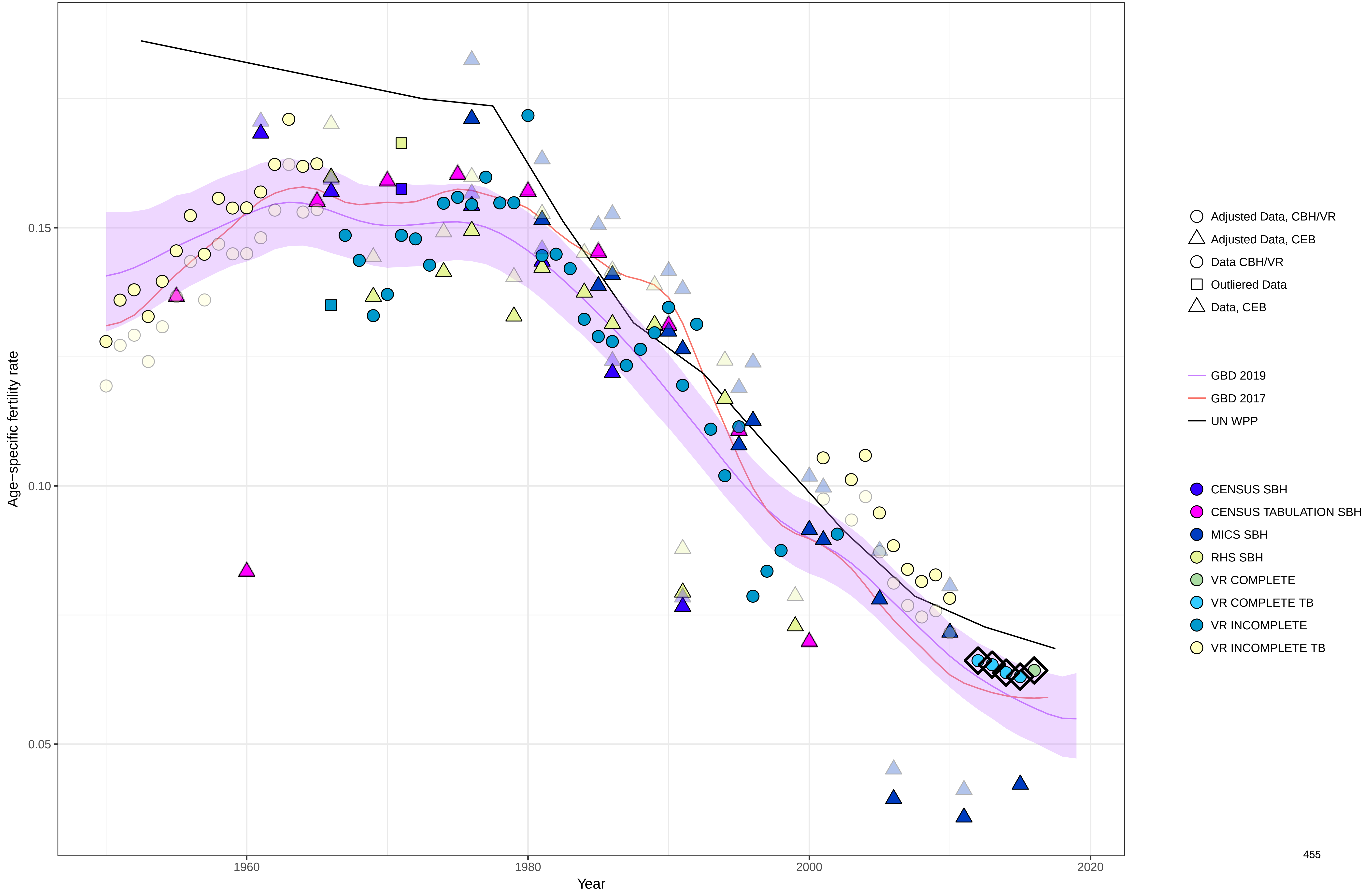

Age−specific fertility fitted trend lines, 1950−2019: Belize, Ages: 20−24
Data points enclosed with a diamond are reference sources

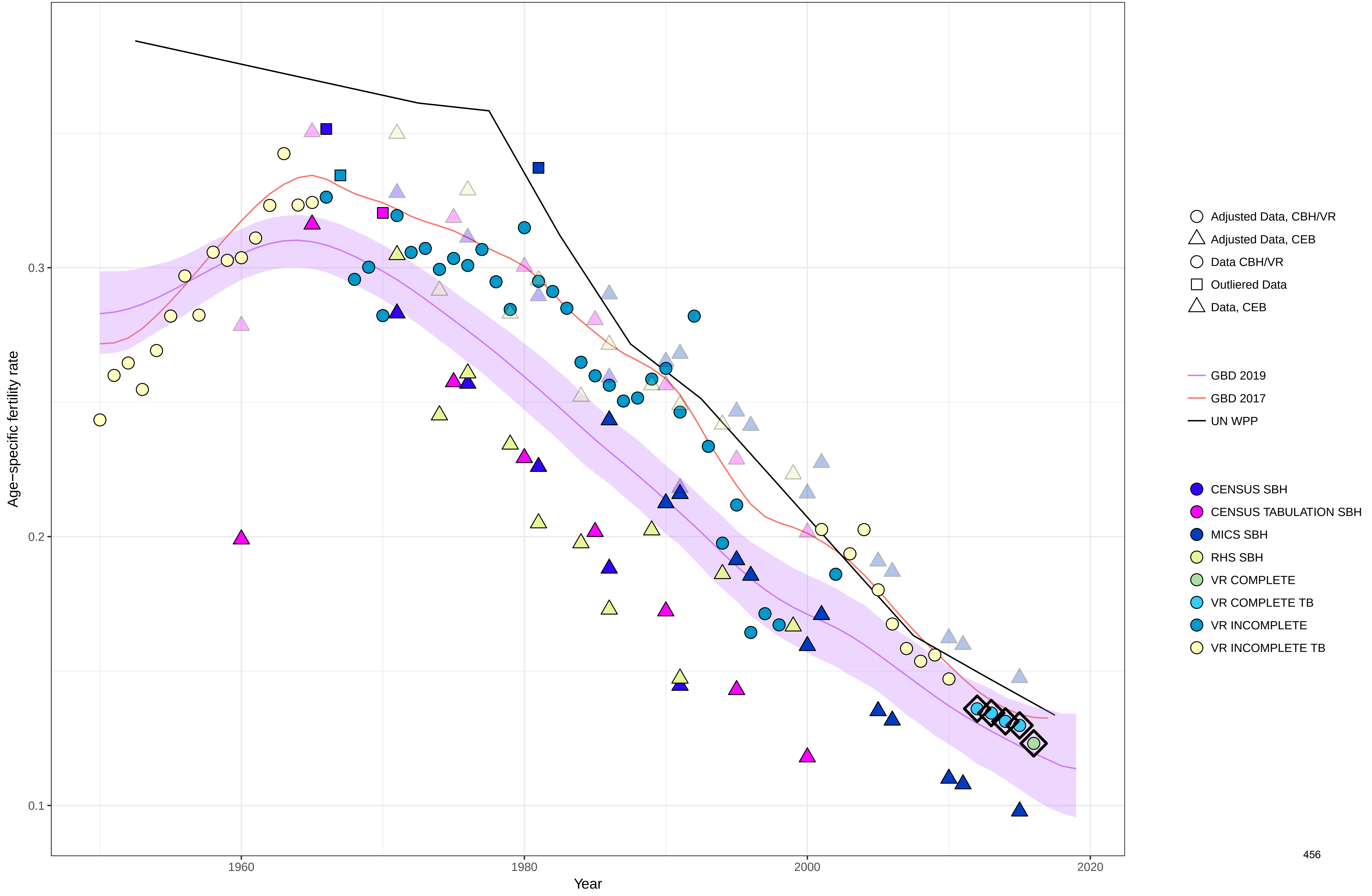

Age−specific fertility fitted trend lines, 1950−2019: Belize, Ages: 25−29
Data points enclosed with a diamond are reference sources

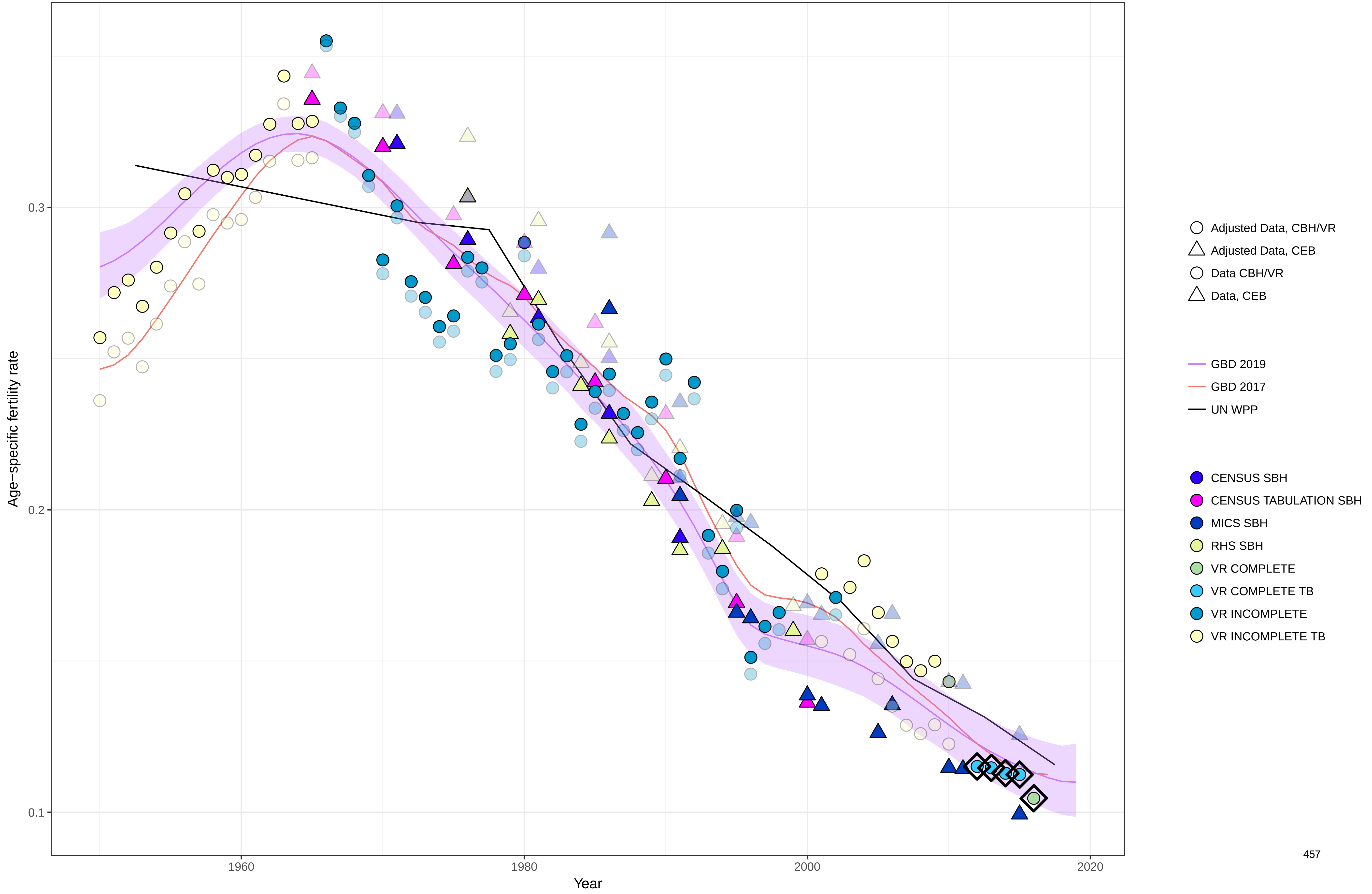

Age−specific fertility fitted trend lines, 1950−2019: Belize, Ages: 30−34
Data points enclosed with a diamond are reference sources

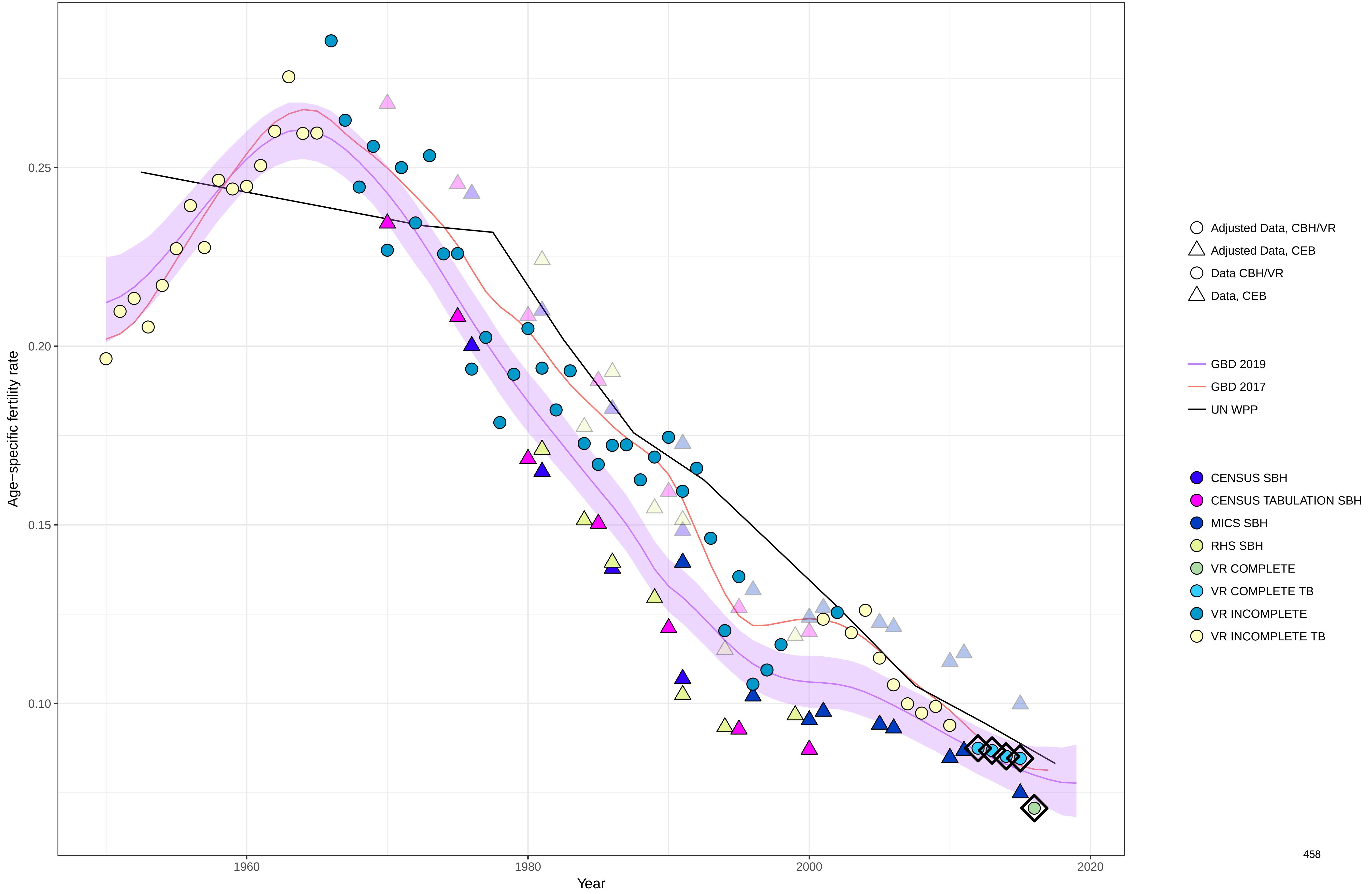

Age−specific fertility fitted trend lines, 1950−2019: Belize, Ages: 35−39
Data points enclosed with a diamond are reference sources

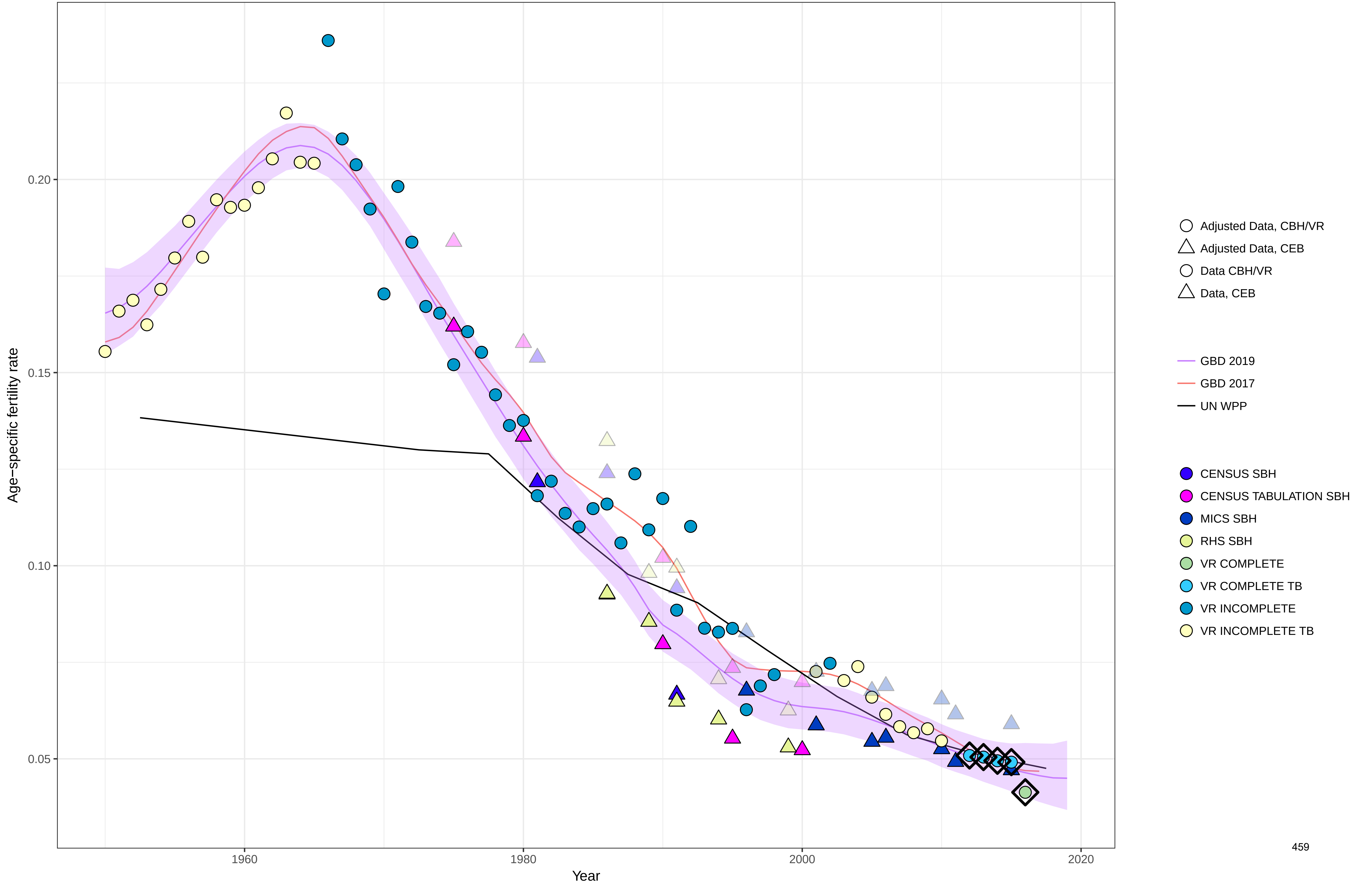

Age−specific fertility fitted trend lines, 1950−2019: Belize, Ages: 40−44
Data points enclosed with a diamond are reference sources

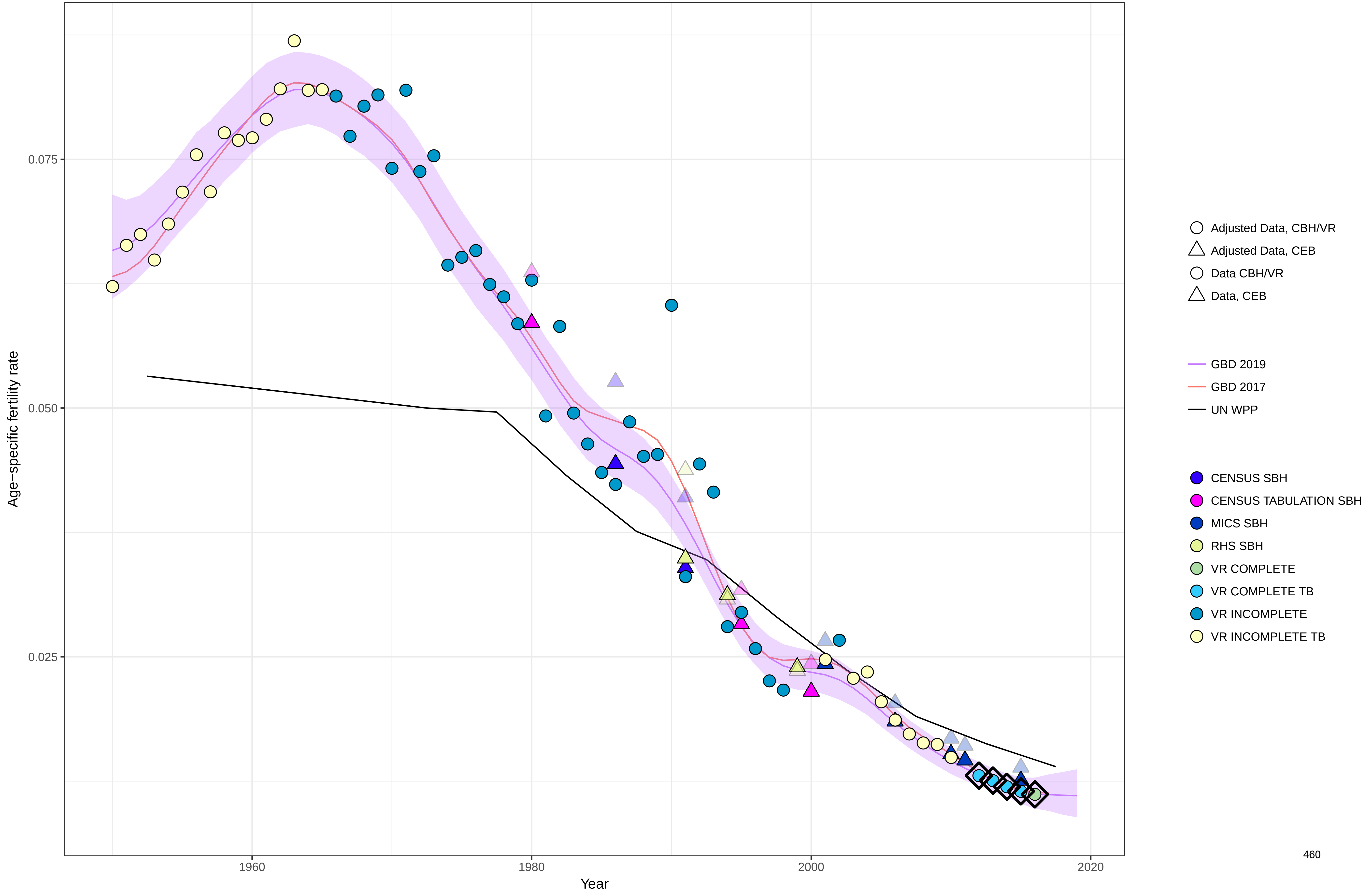

Age−specific fertility fitted trend lines, 1950−2019: Belize, Ages: 45−49
Data points enclosed with a diamond are reference sources

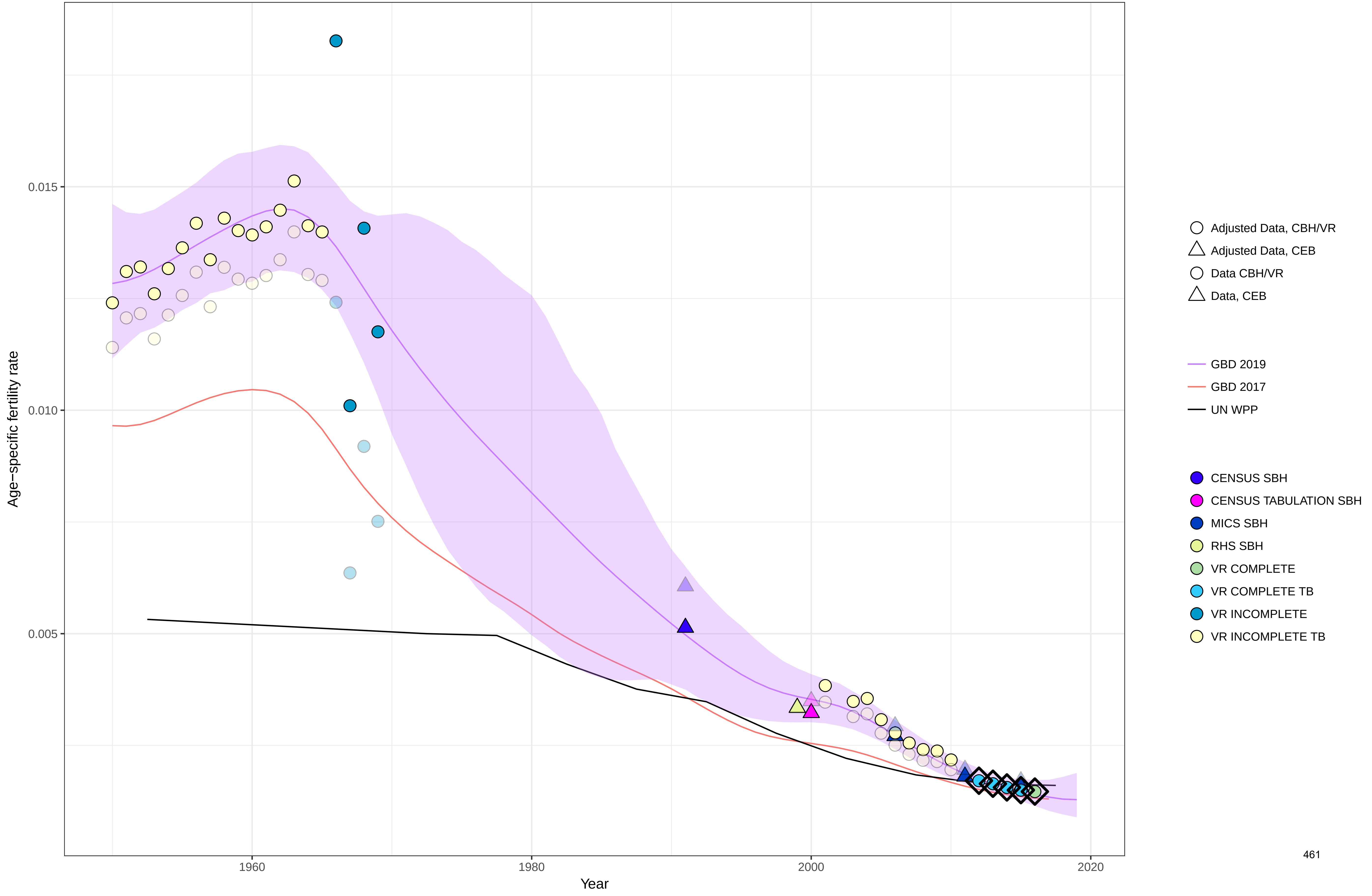

Age−specific fertility fitted trend lines, 1950−2019: Belize, Ages: 50−54
Data points enclosed with a diamond are reference sources

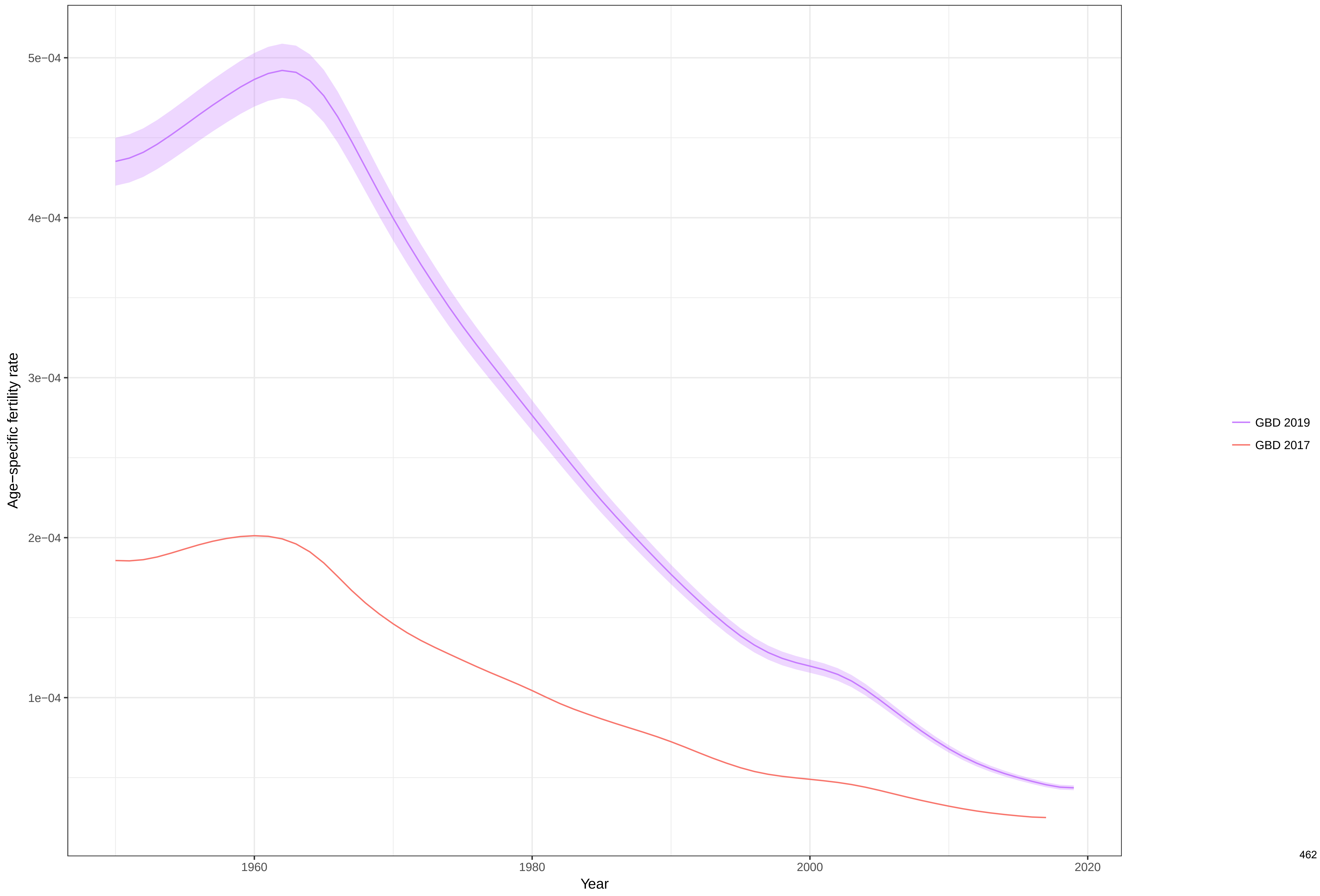

Total fertility rate fitted trend lines, 1950−2019: Belize TFR
Data points enclosed with a diamond are reference sources

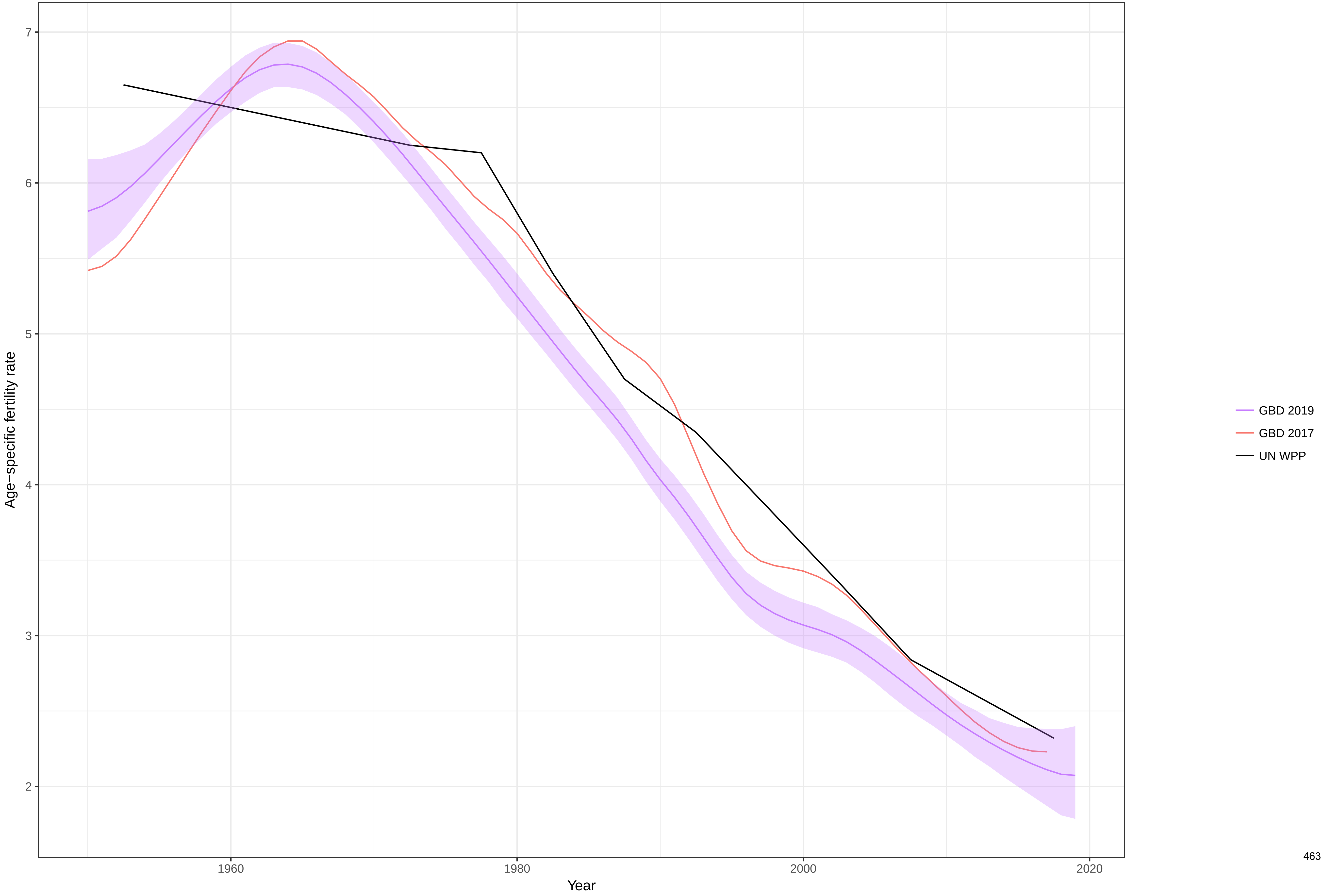

Age−specific fertility fitted trend lines, 1950−2019: Bermuda, Ages: 10−14
Data points enclosed with a diamond are reference sources

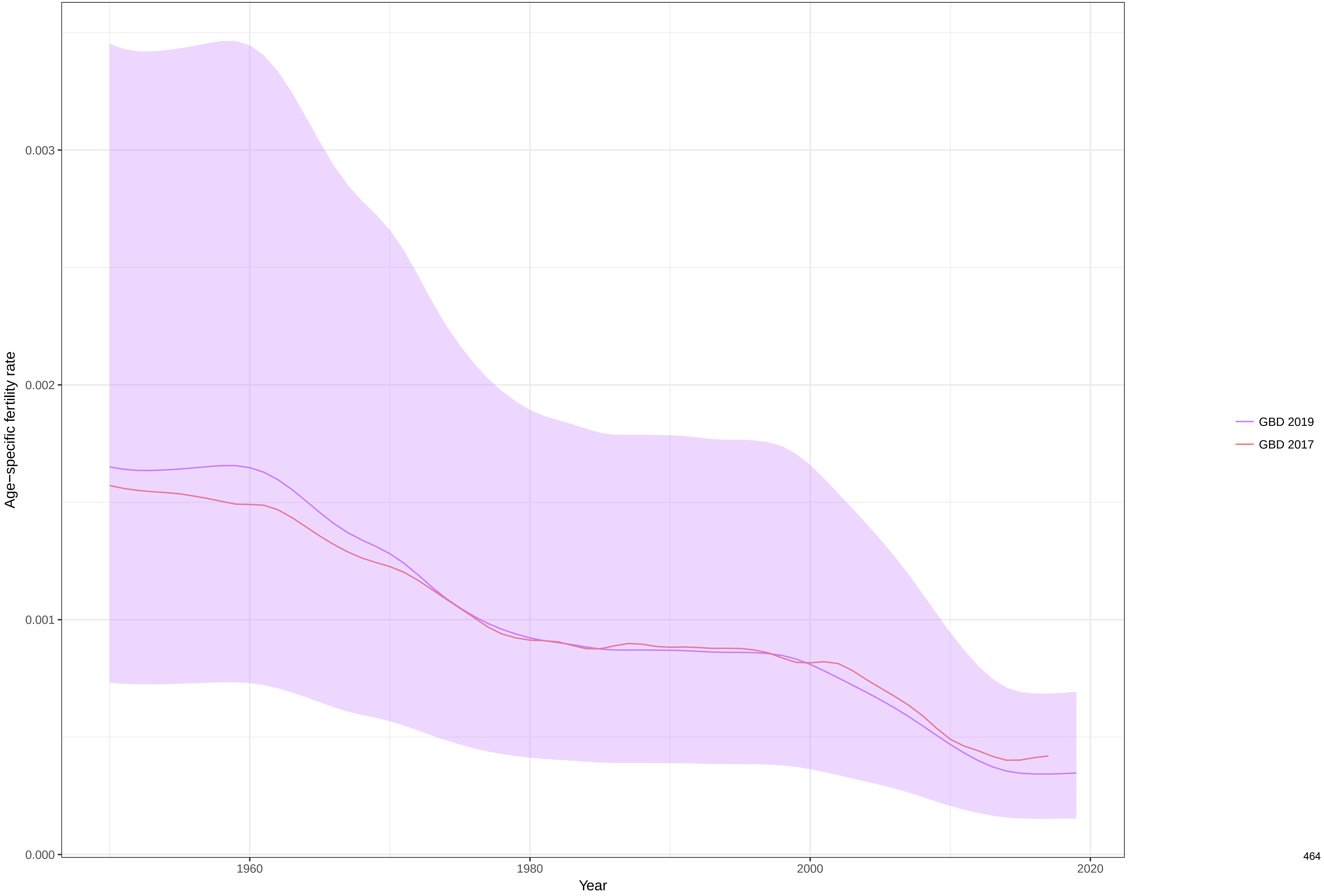

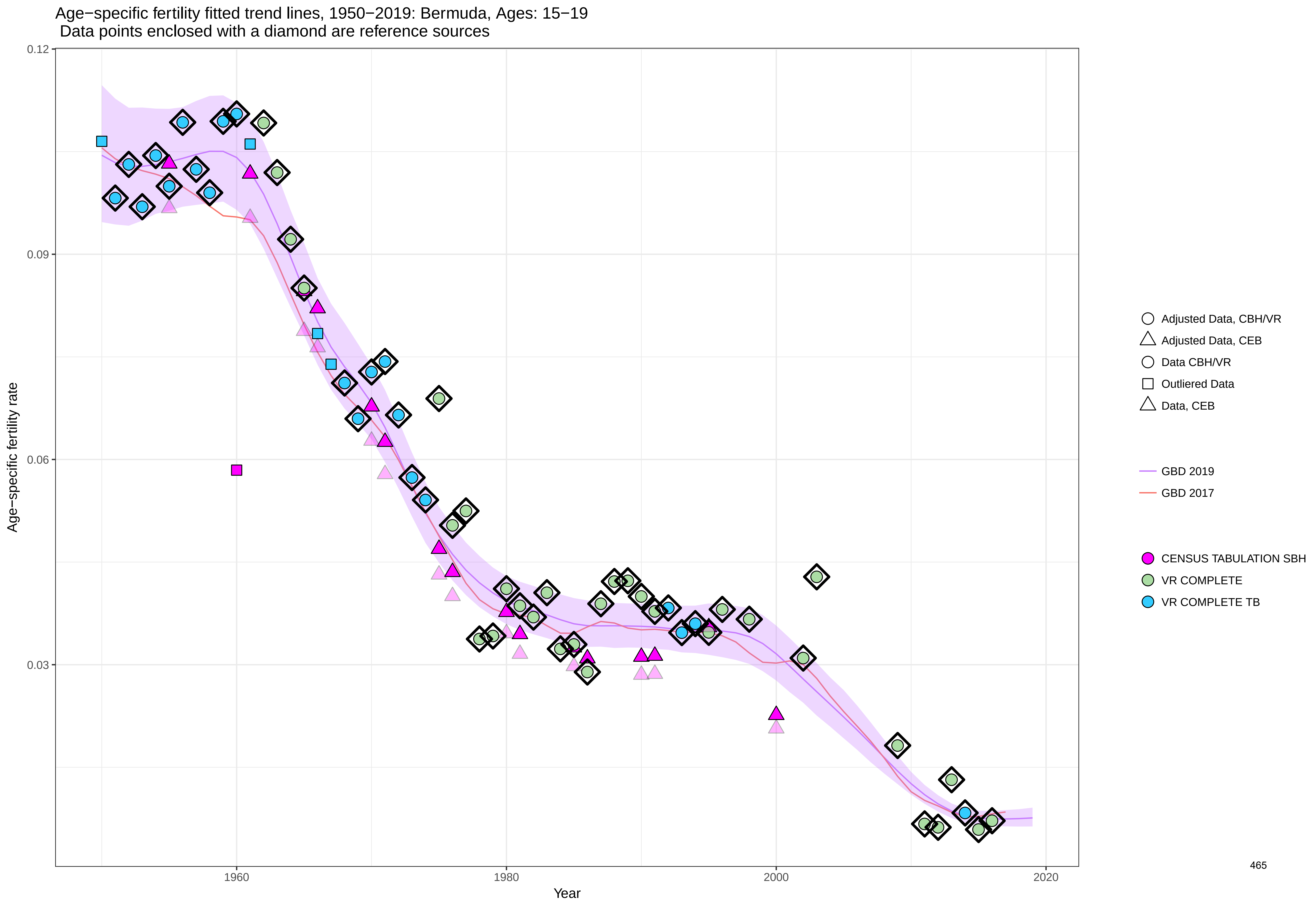
Age−specific fertility fitted trend lines, 1950−2019: Bermuda, Ages: 15−19
Data points enclosed with a diamond are reference sources
0.12
0.09
0.06
0.03
Age−specific fertility rate
1960
1980
2000
2020
Year
Adjusted Data, CBH/VR
Adjusted Data, CEB
Data CBH/VR
Outliered Data
Data, CEB
GBD 2019
GBD 2017
CENSUS TABULATION SBH
VR COMPLETE
VR COMPLETE TB

Age−specific fertility fitted trend lines, 1950−2019: Bermuda, Ages: 20−24
Data points enclosed with a diamond are reference sources

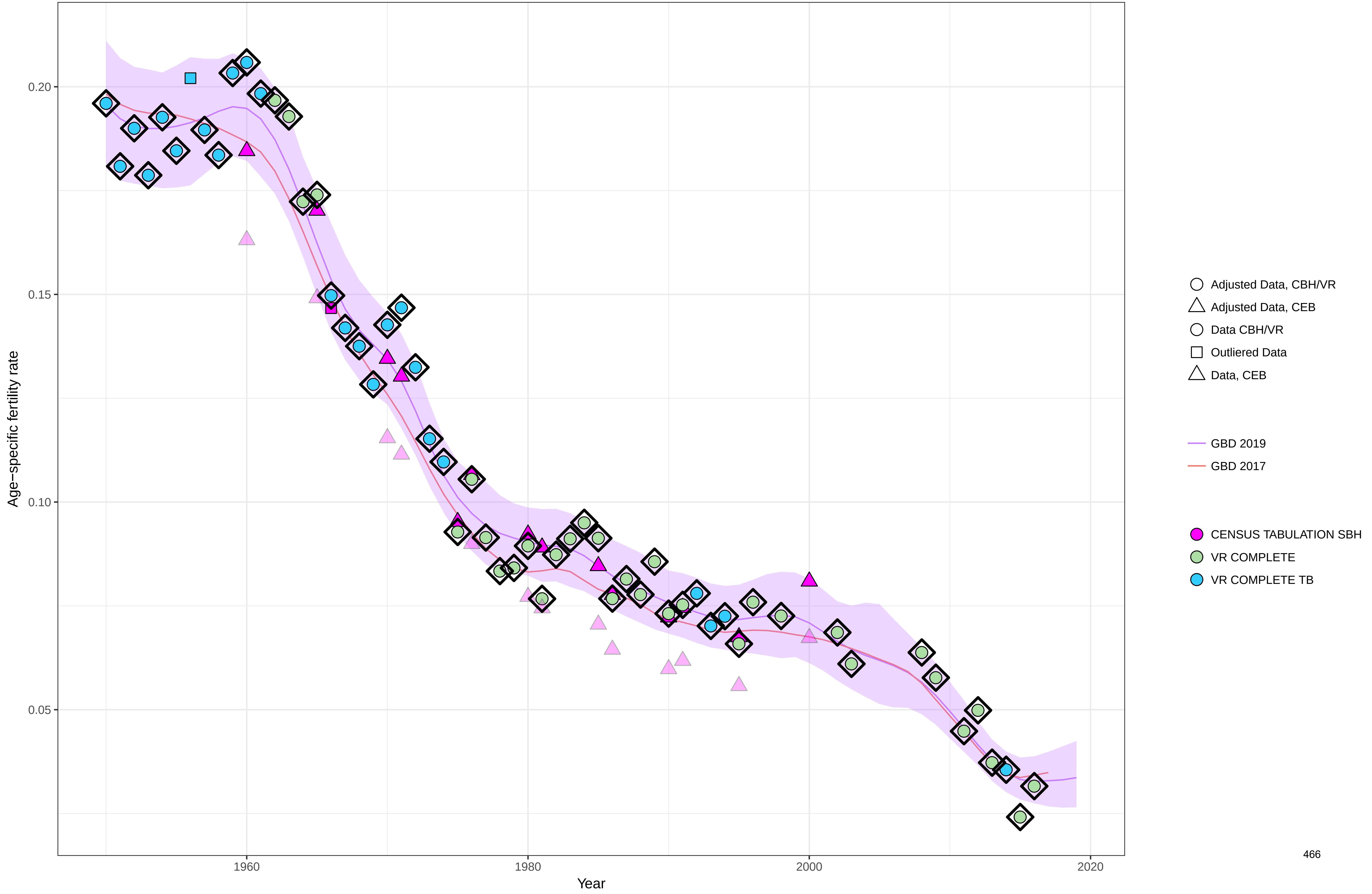

Age−specific fertility fitted trend lines, 1950−2019: Bermuda, Ages: 25−29
Data points enclosed with a diamond are reference sources

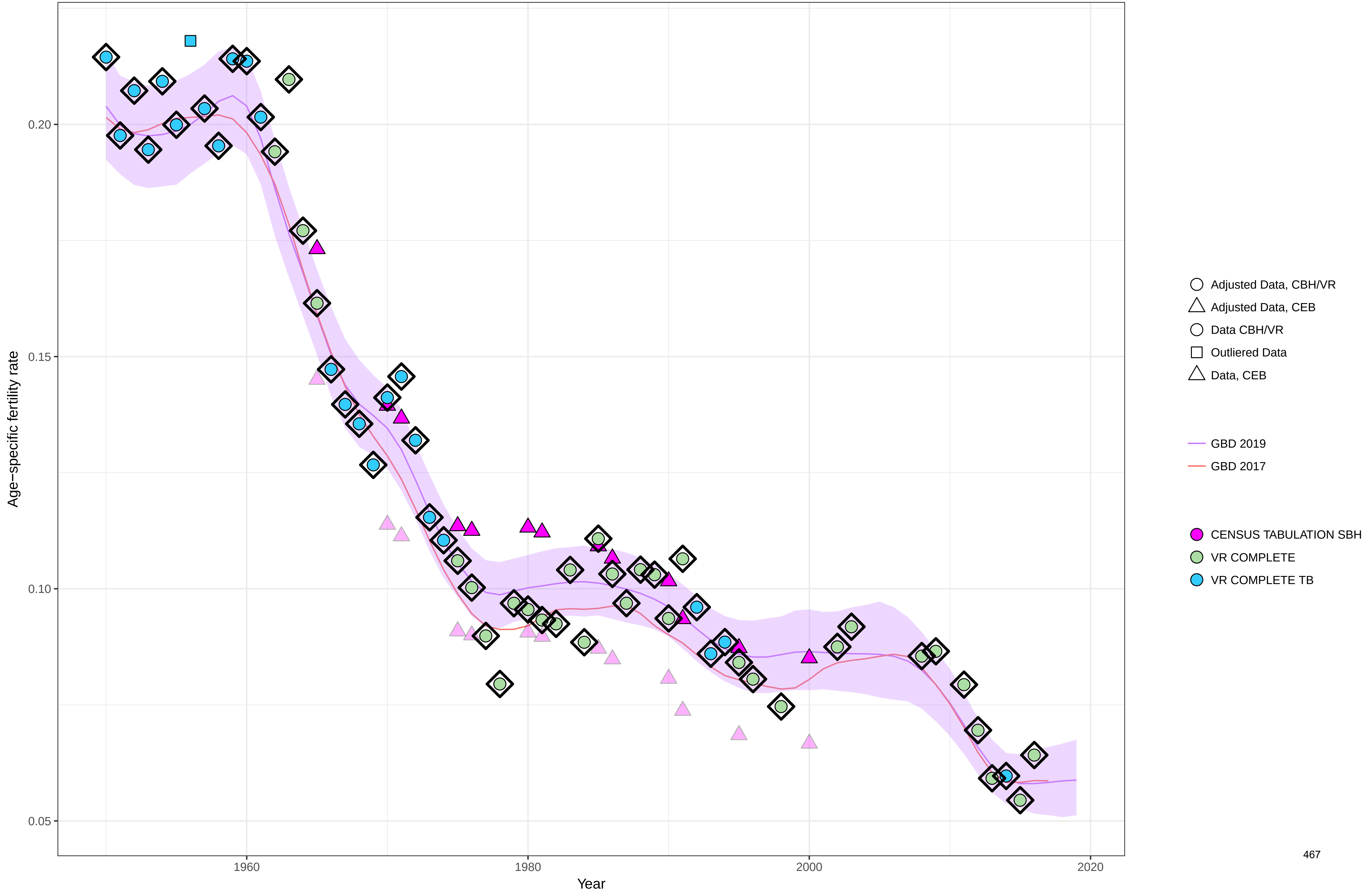

Age−specific fertility fitted trend lines, 1950−2019: Bermuda, Ages: 30−34
Data points enclosed with a diamond are reference sources

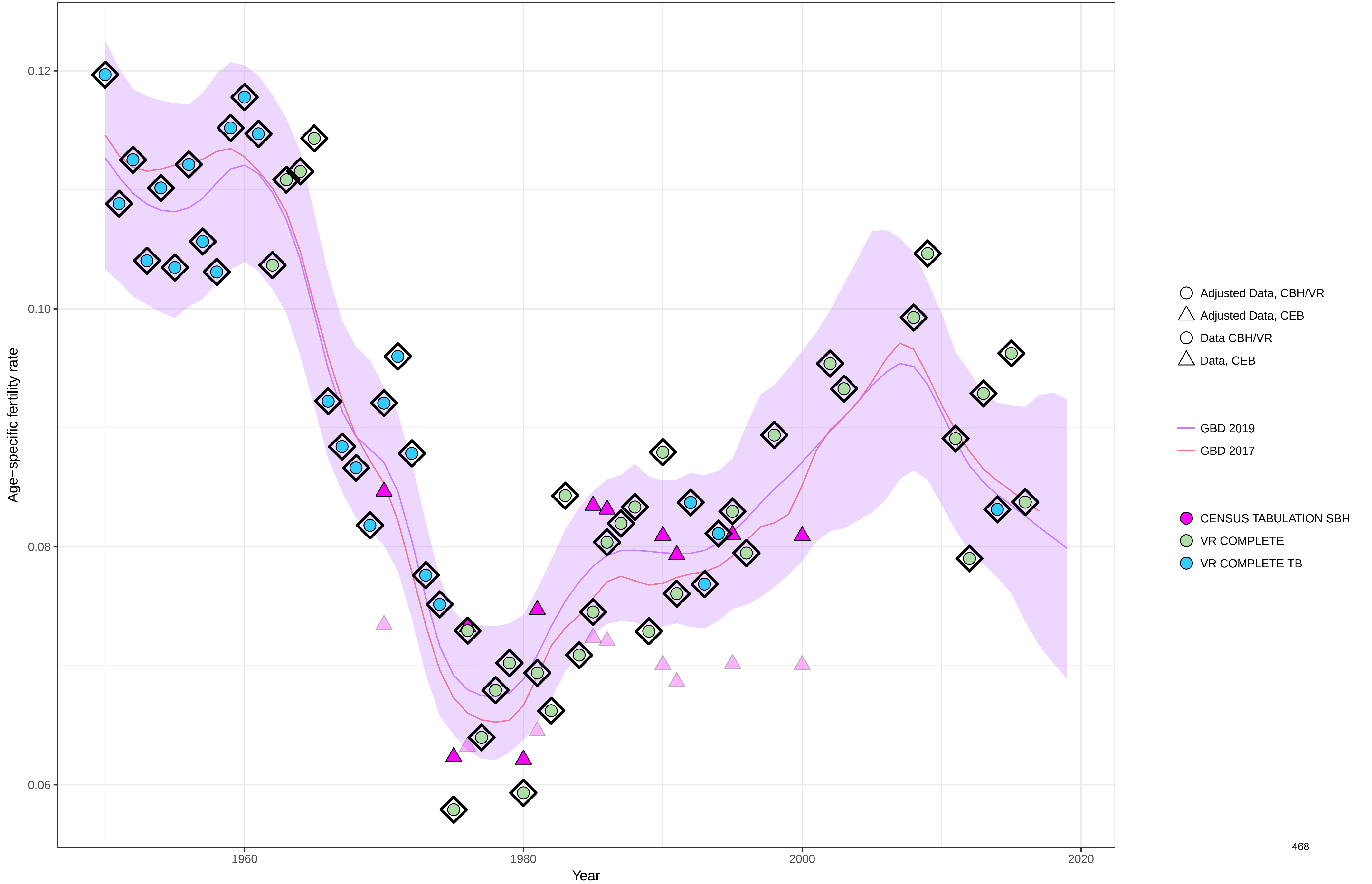

Age−specific fertility fitted trend lines, 1950−2019: Bermuda, Ages: 35−39
Data points enclosed with a diamond are reference sources

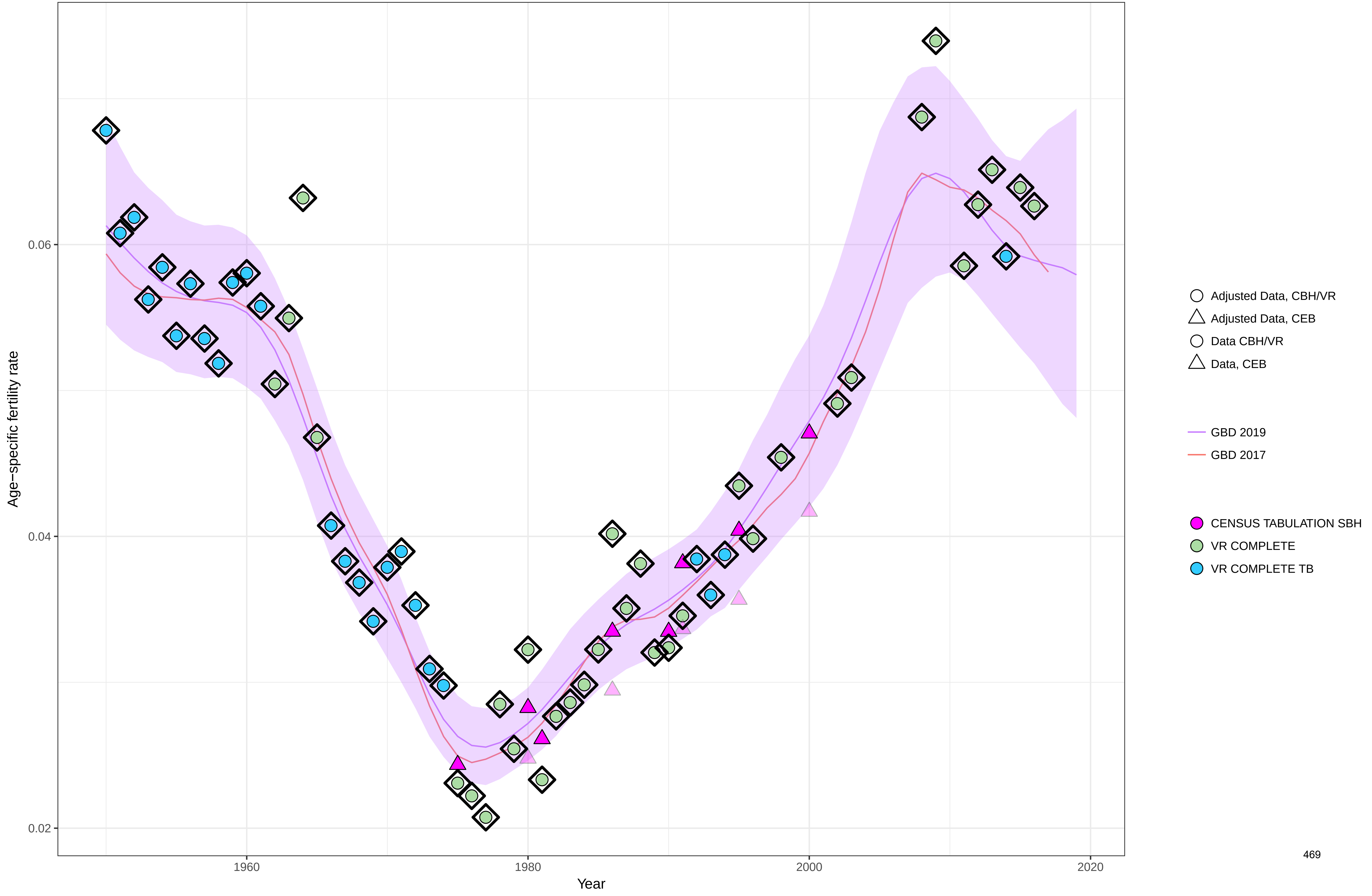

Age−specific fertility fitted trend lines, 1950−2019: Bermuda, Ages: 40−44
Data points enclosed with a diamond are reference sources

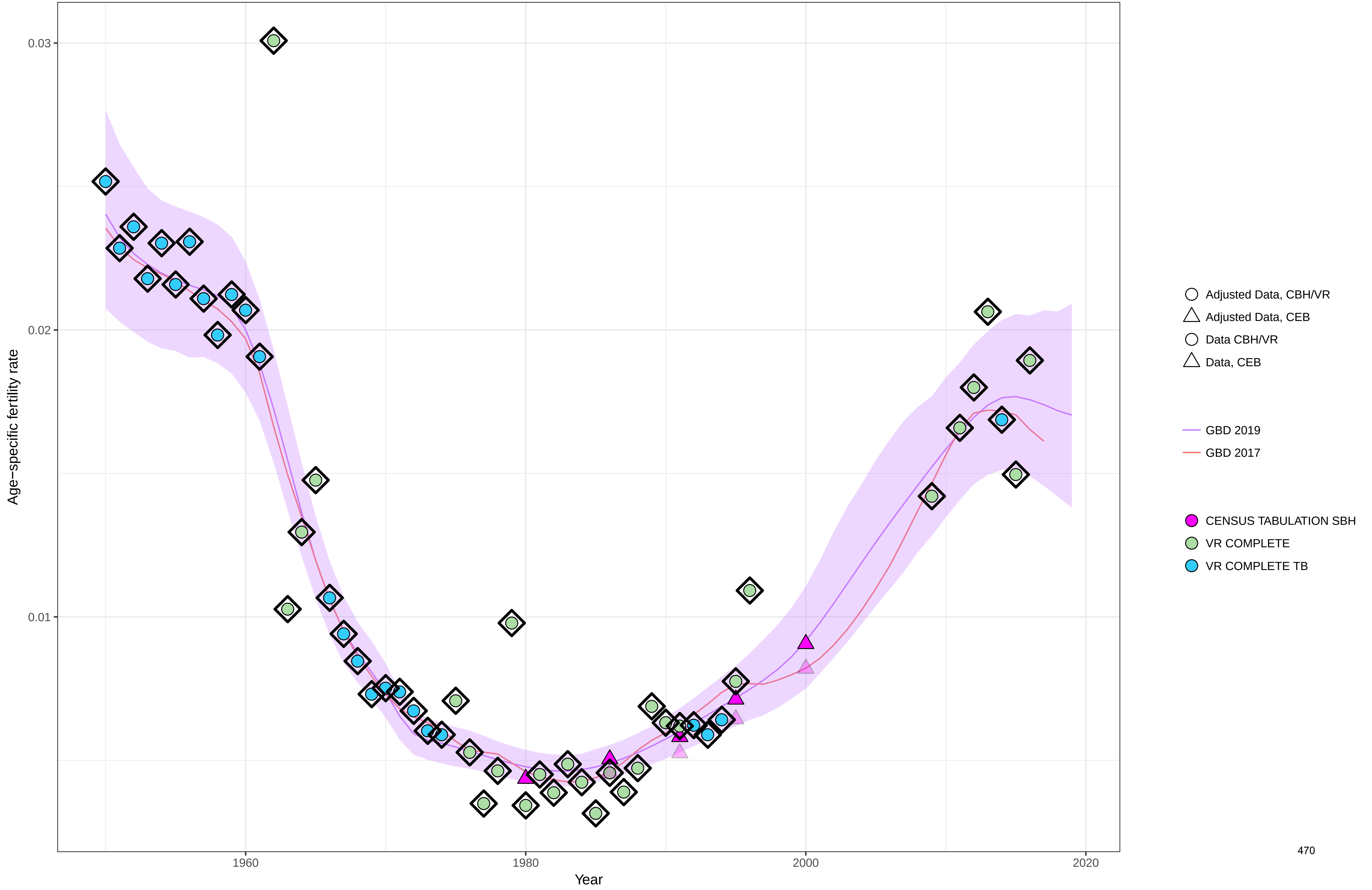

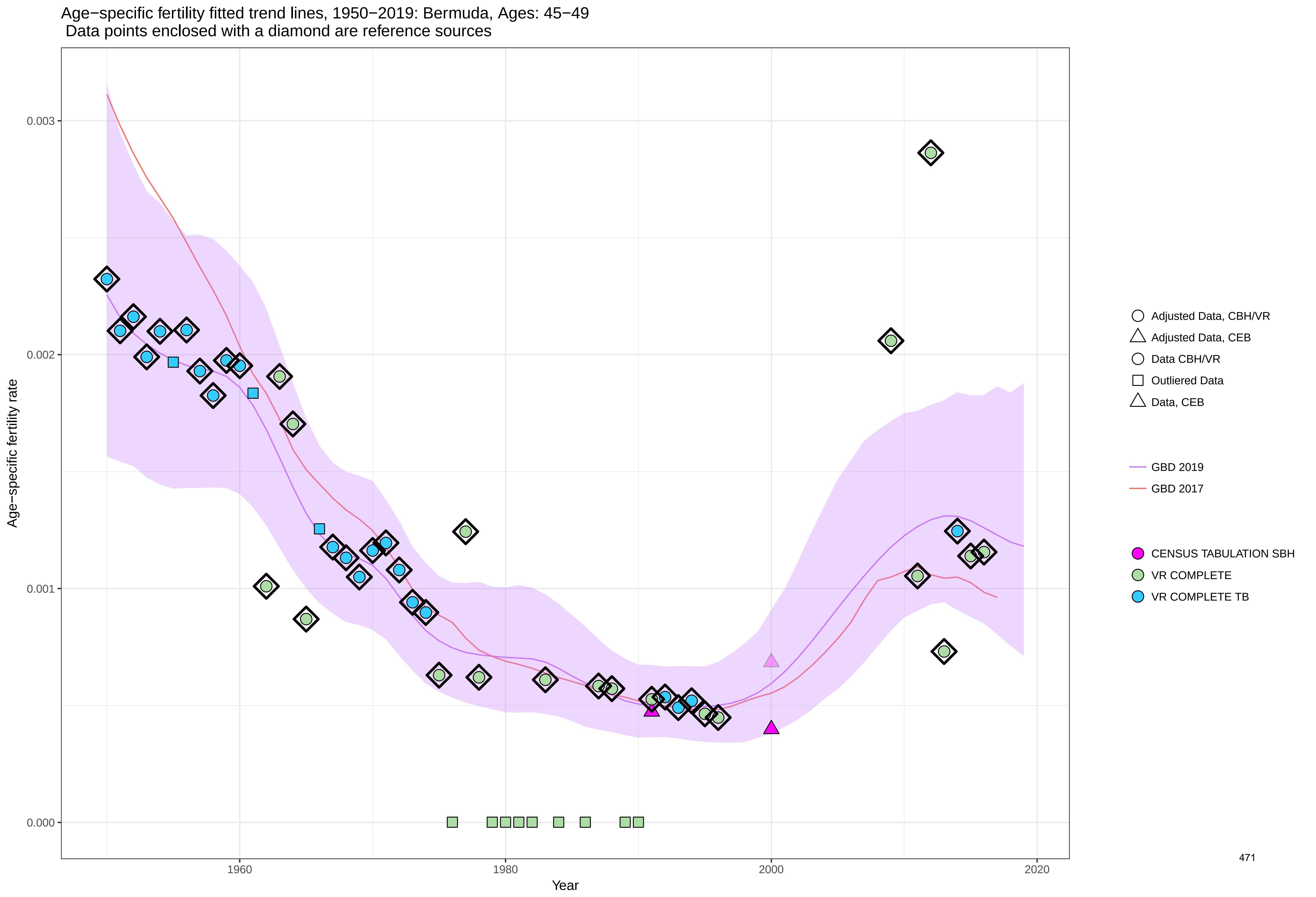

Age−specific fertility fitted trend lines, 1950−2019: Bermuda, Ages: 45−49
Data points enclosed with a diamond are reference sources
Age−specific fertility rate
Year
0.000
0.001
0.002
0.003
1960
1980
2000
2020
Adjusted Data, CBH/VR
Adjusted Data, CEB
Data CBH/VR
Outliered Data
Data, CEB
GBD 2019
GBD 2017
CENSUS TABULATION SBH
VR COMPLETE
VR COMPLETE TB

Age−specific fertility fitted trend lines, 1950−2019: Bermuda, Ages: 50−54
Data points enclosed with a diamond are reference sources

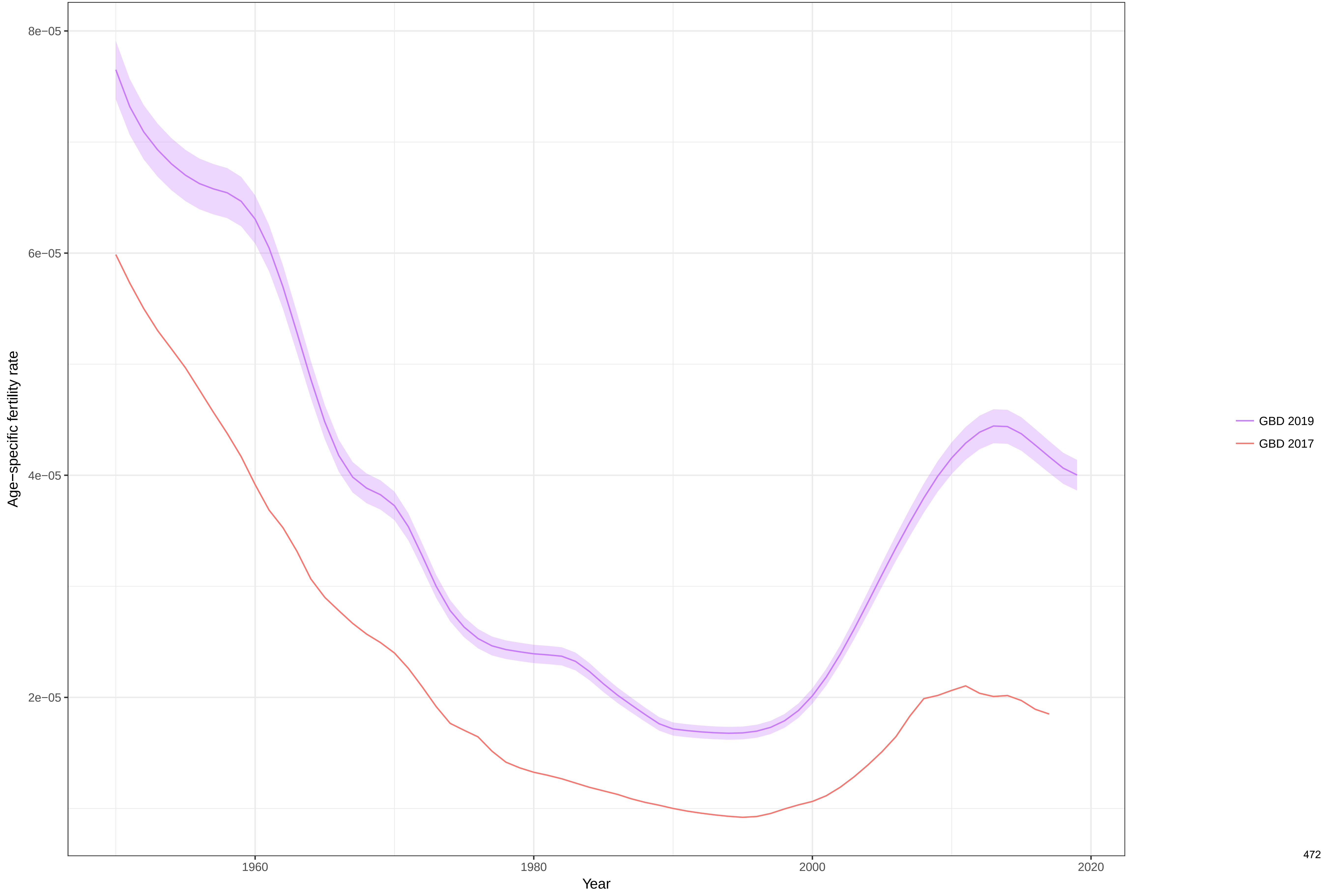

Total fertility rate fitted trend lines, 1950−2019: Bermuda TFR
Data points enclosed with a diamond are reference sources

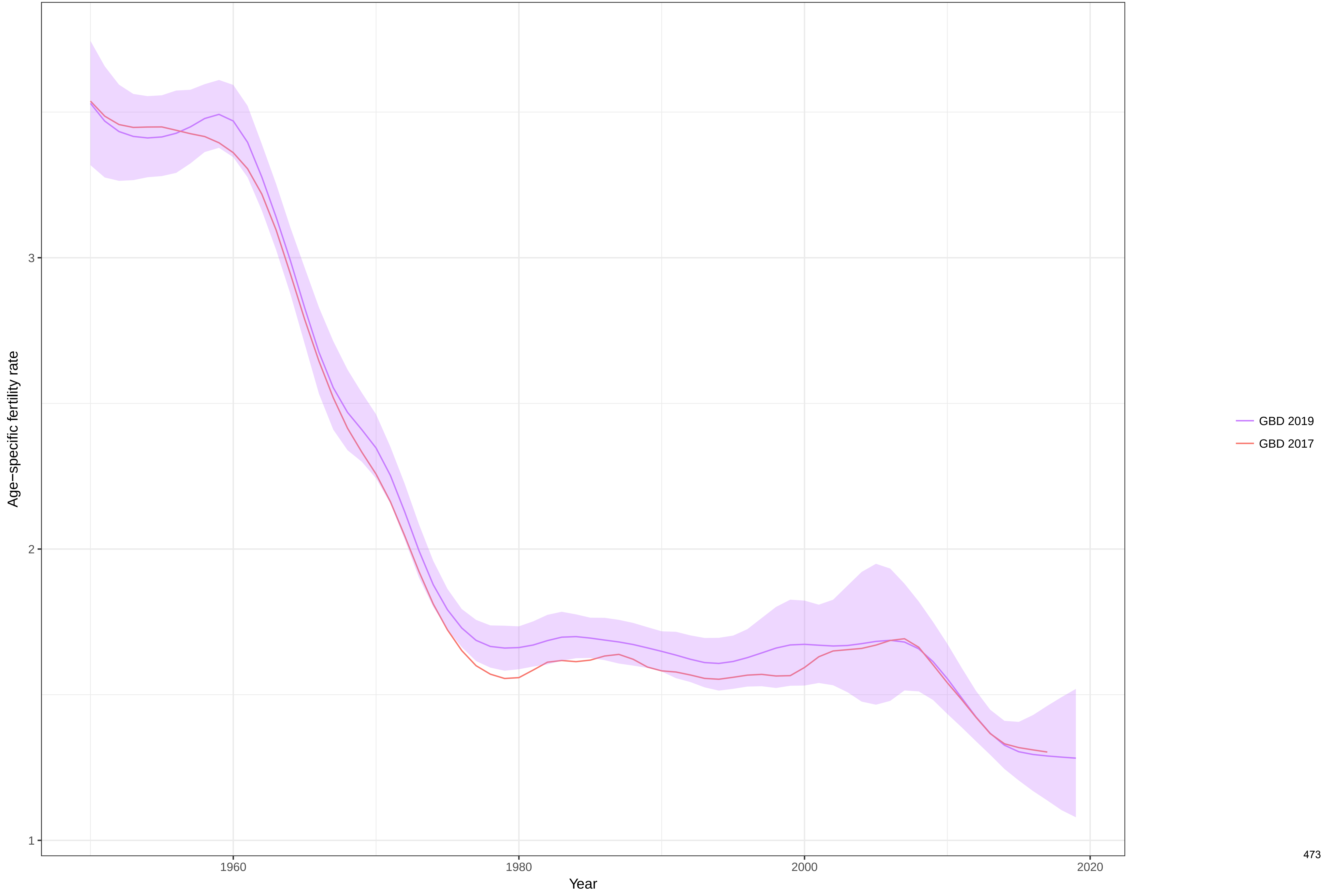

Age−specific fertility fitted trend lines, 1950−2019: Bolivia (Plurinational State of), Ages: 10−14
Data points enclosed with a diamond are reference sources

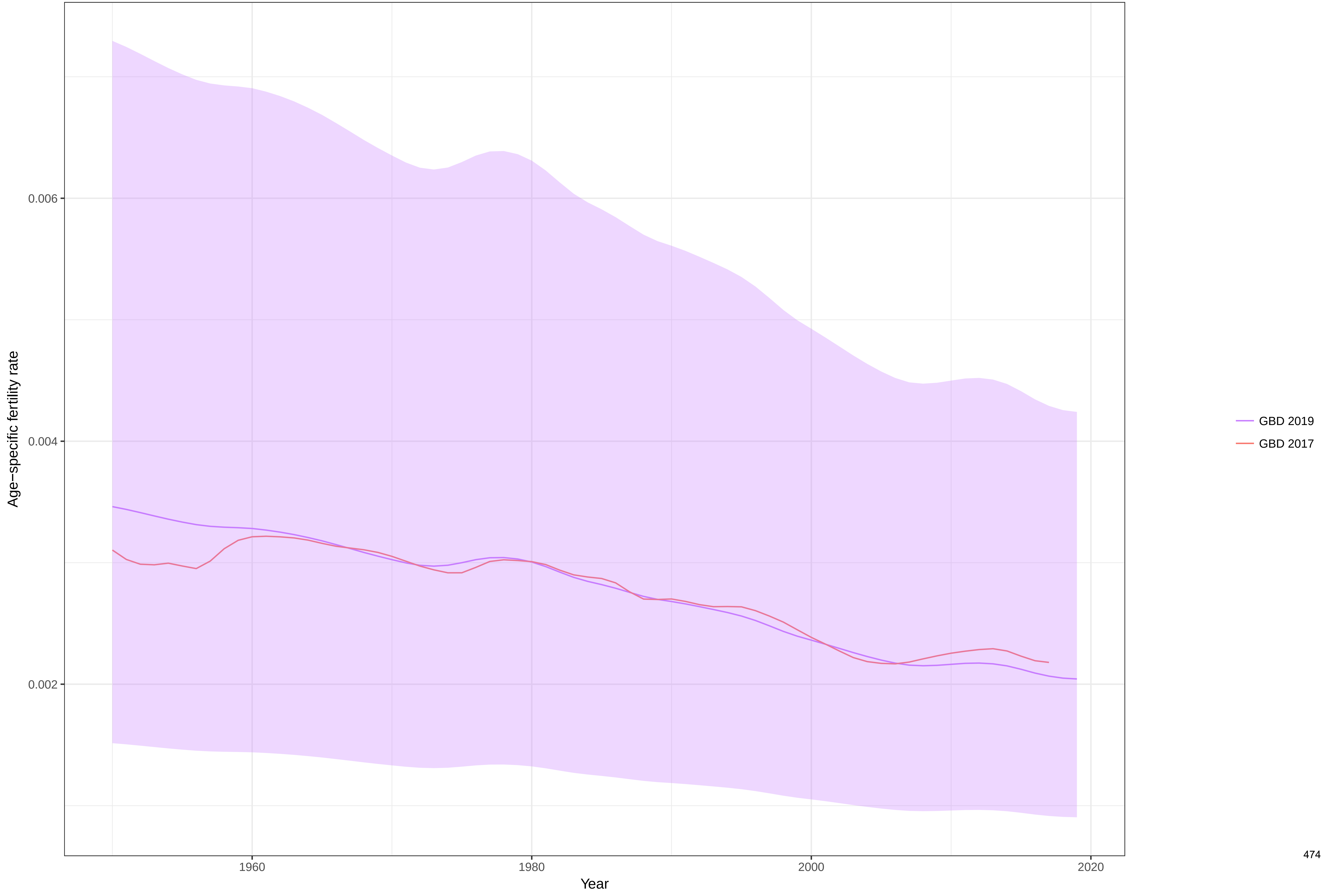

Age−specific fertility fitted trend lines, 1950−2019: Bolivia (Plurinational State of), Ages: 15−19
Data points enclosed with a diamond are reference sources

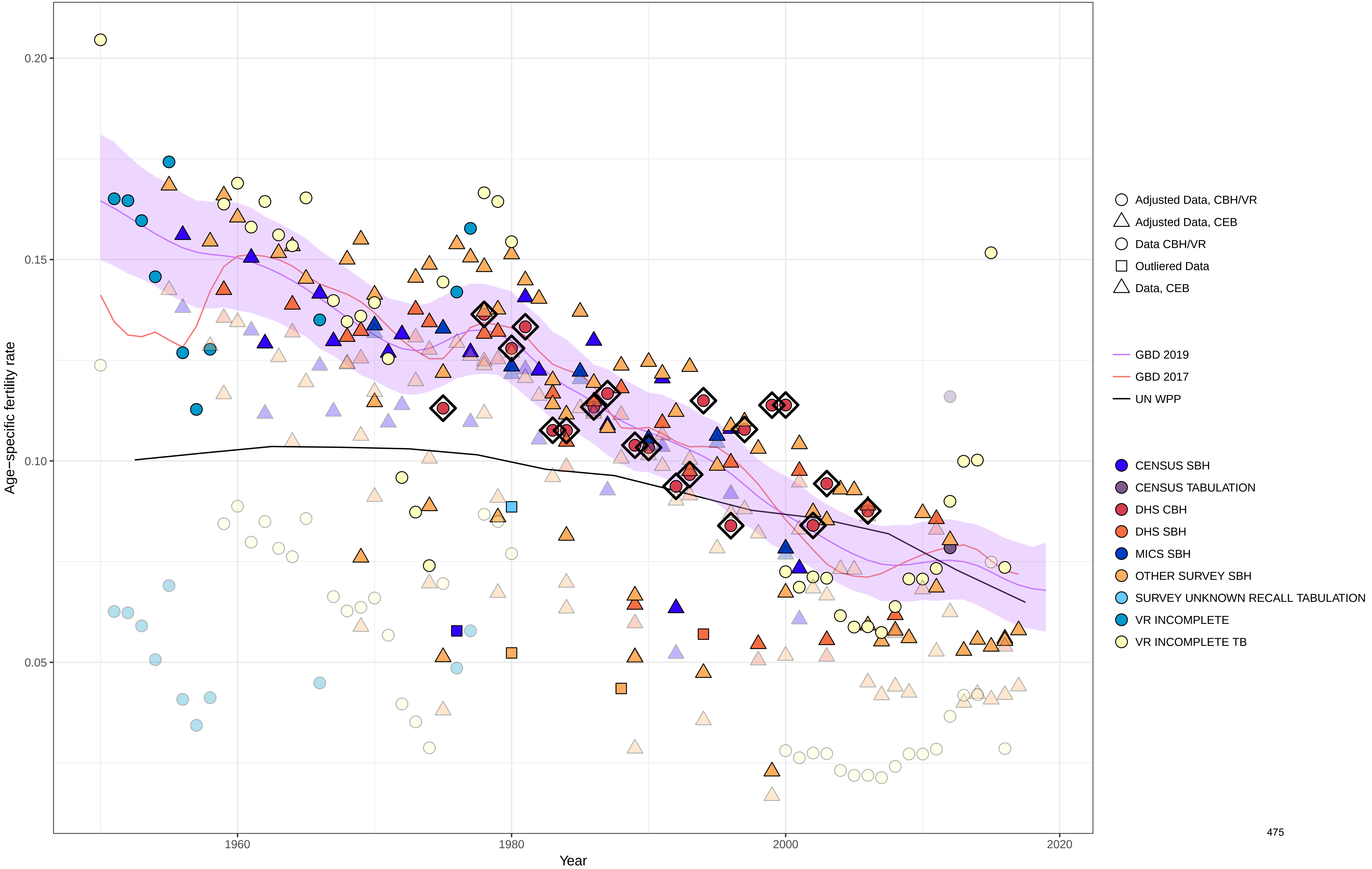

Age−specific fertility fitted trend lines, 1950−2019: Bolivia (Plurinational State of), Ages: 20−24
Data points enclosed with a diamond are reference sources

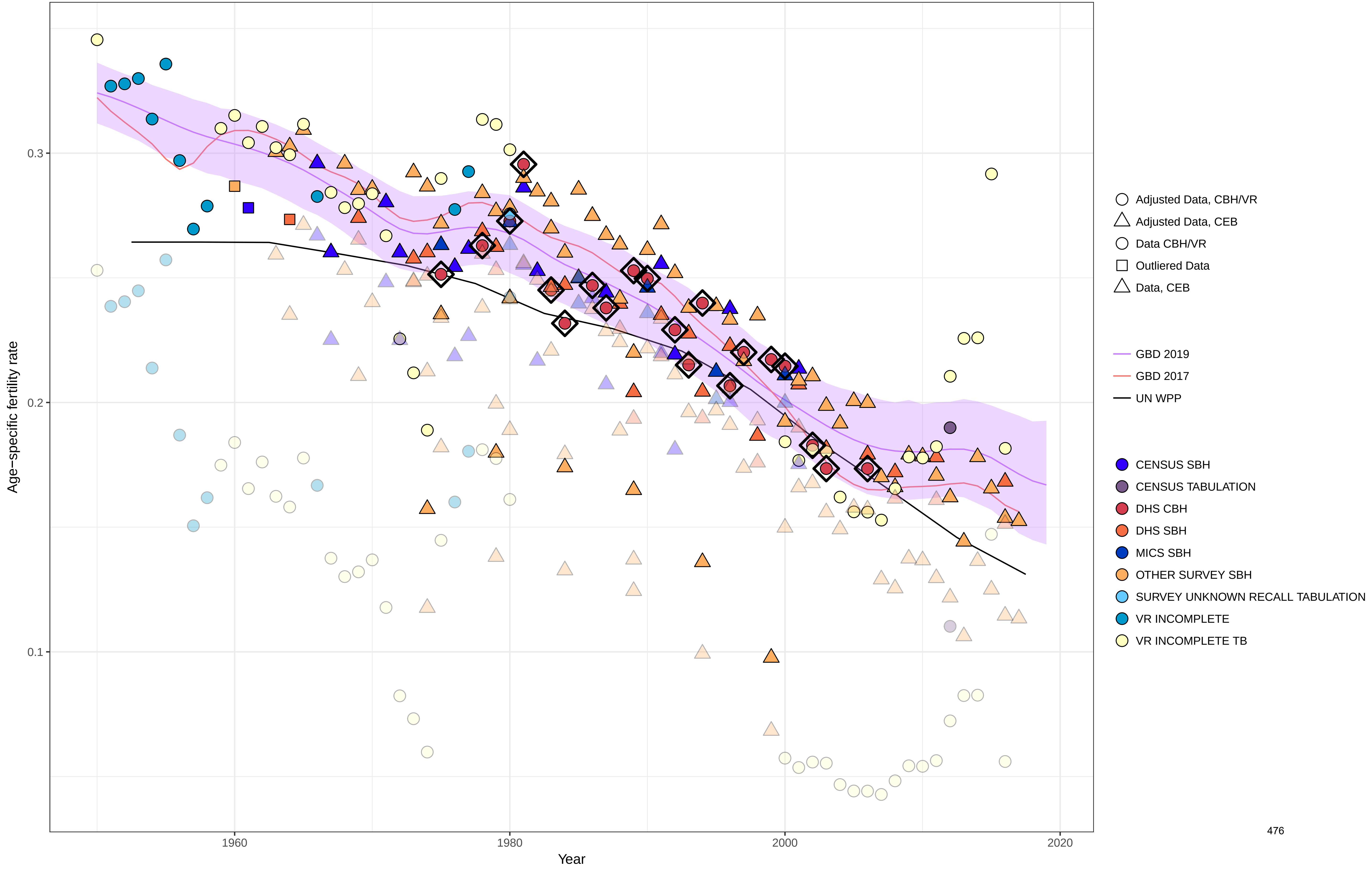

Age−specific fertility fitted trend lines, 1950−2019: Bolivia (Plurinational State of), Ages: 25−29
Data points enclosed with a diamond are reference sources

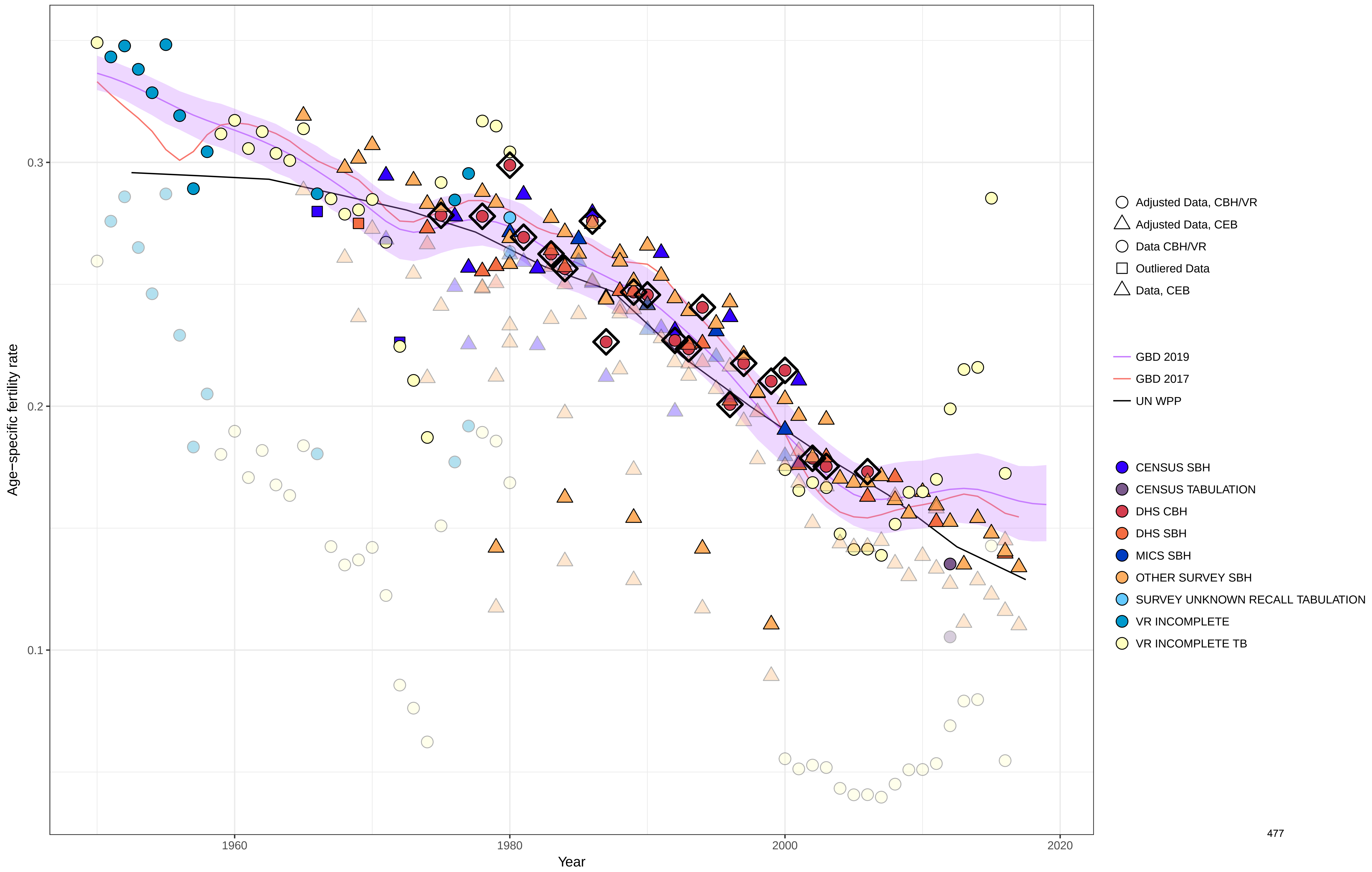

Age−specific fertility fitted trend lines, 1950−2019: Bolivia (Plurinational State of), Ages: 30−34
Data points enclosed with a diamond are reference sources

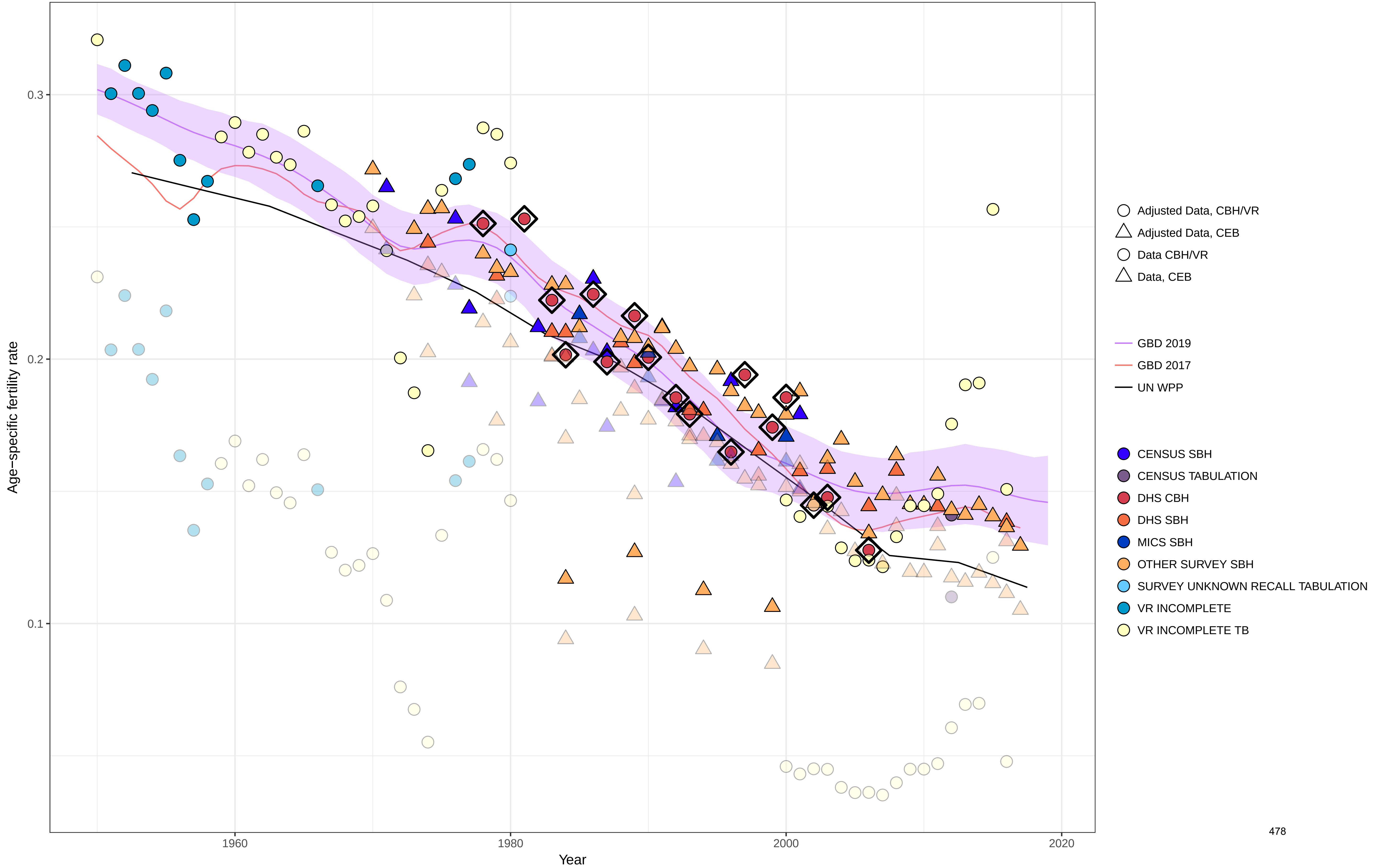

Age−specific fertility fitted trend lines, 1950−2019: Bolivia (Plurinational State of), Ages: 35−39
Data points enclosed with a diamond are reference sources

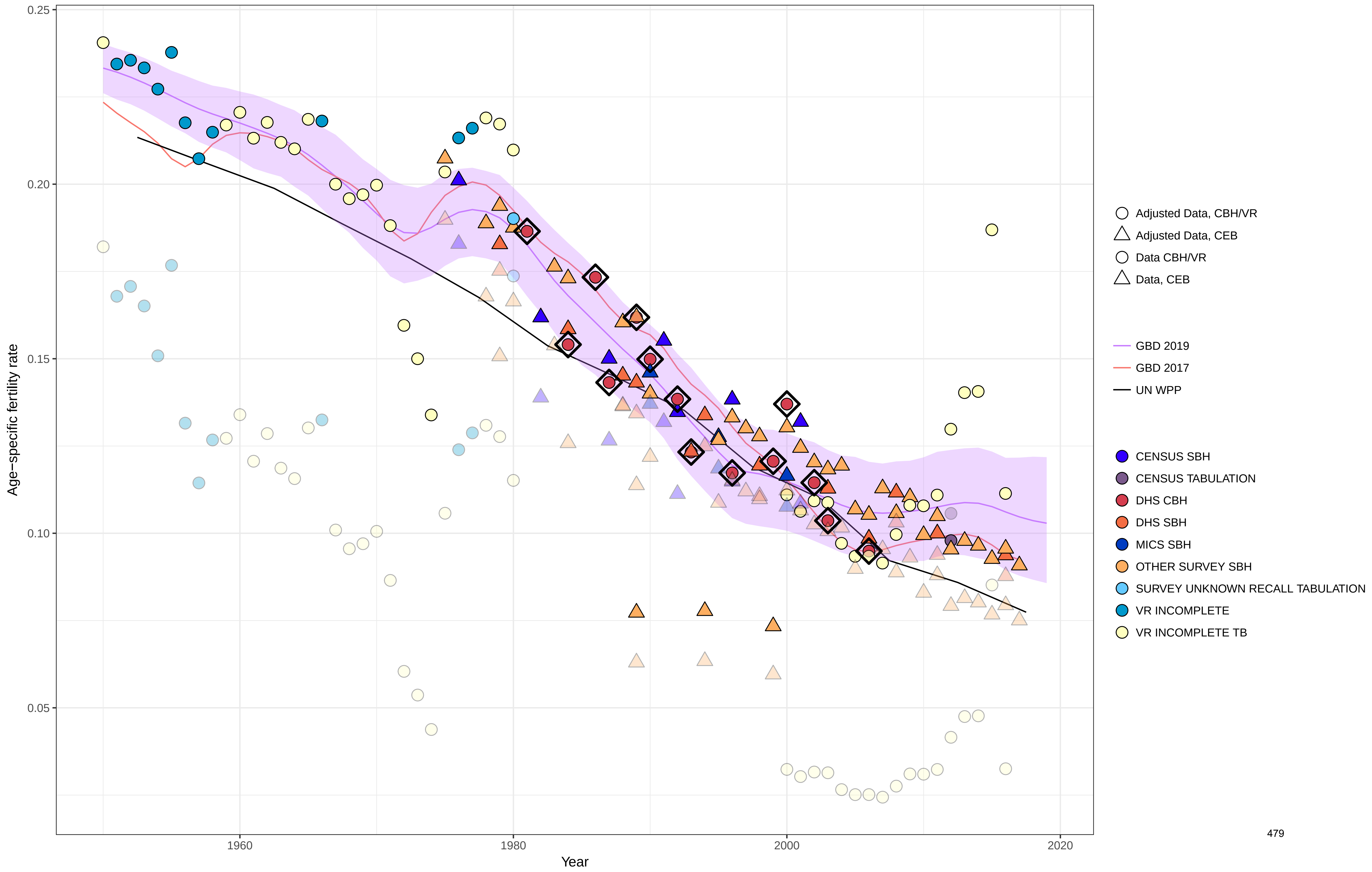

Age−specific fertility fitted trend lines, 1950−2019: Bolivia (Plurinational State of), Ages: 40−44
Data points enclosed with a diamond are reference sources

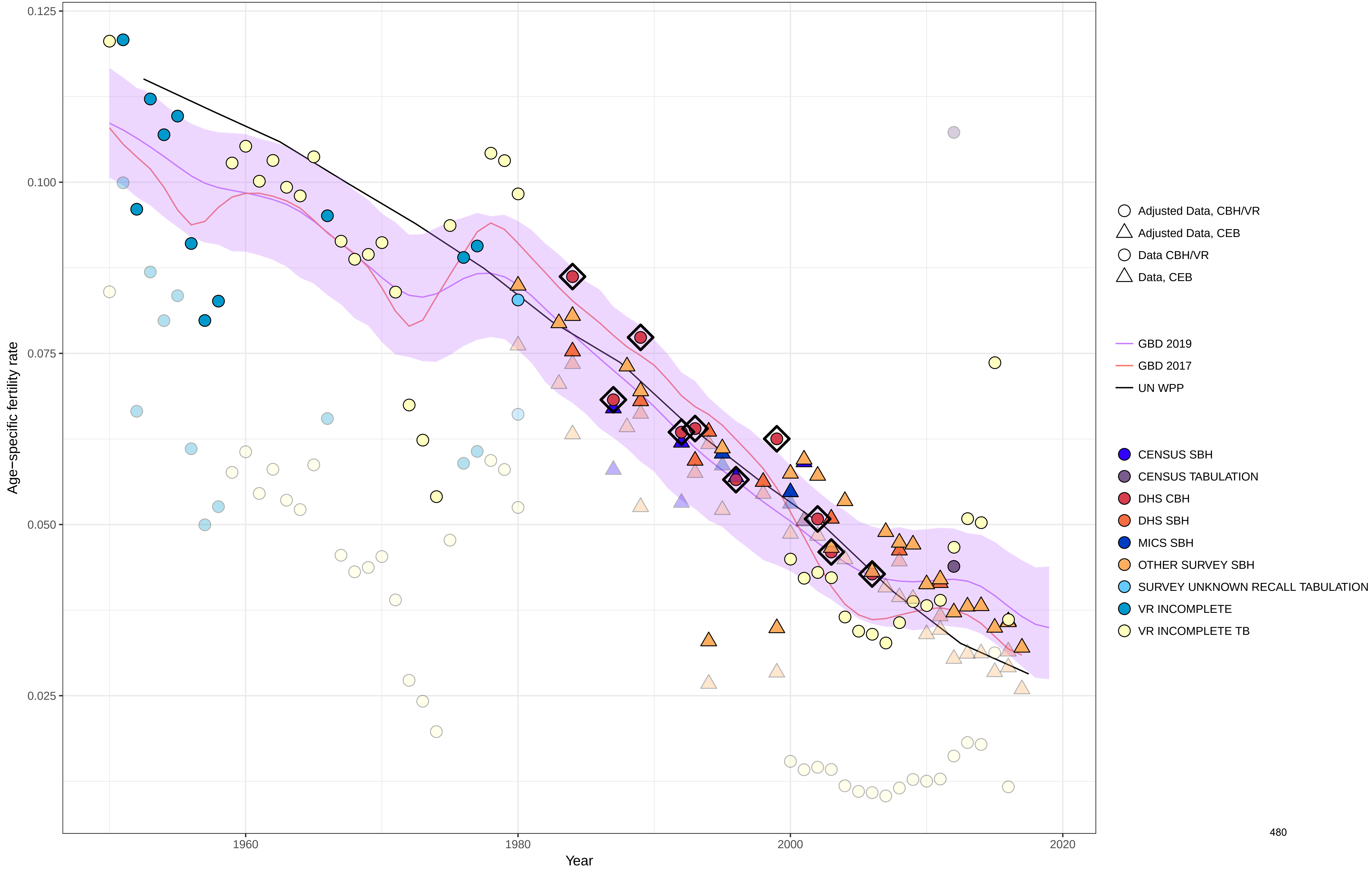

Age−specific fertility fitted trend lines, 1950−2019: Bolivia (Plurinational State of), Ages: 45−49
Data points enclosed with a diamond are reference sources

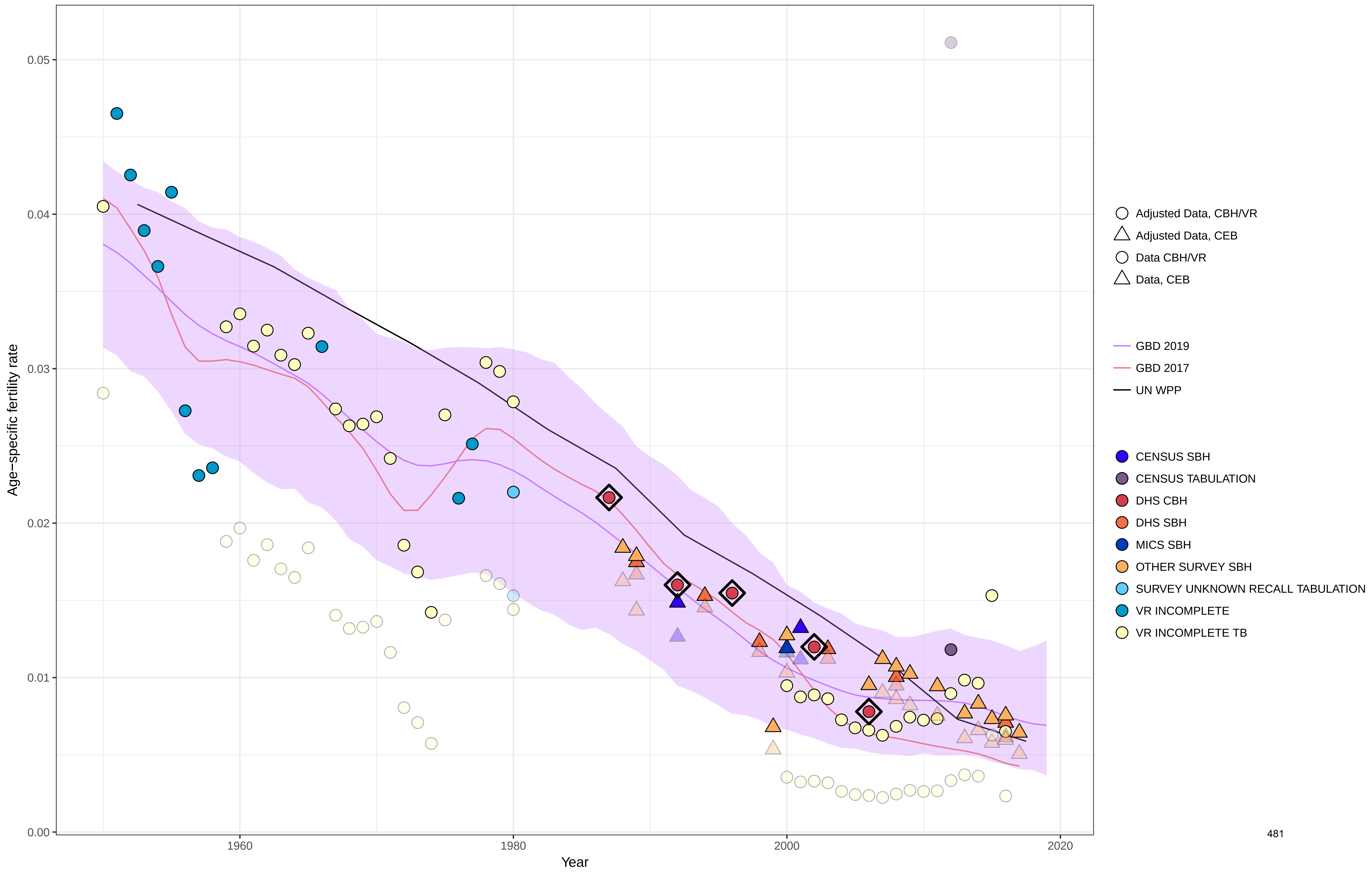

Age−specific fertility fitted trend lines, 1950−2019: Bolivia (Plurinational State of), Ages: 50−54
Data points enclosed with a diamond are reference sources

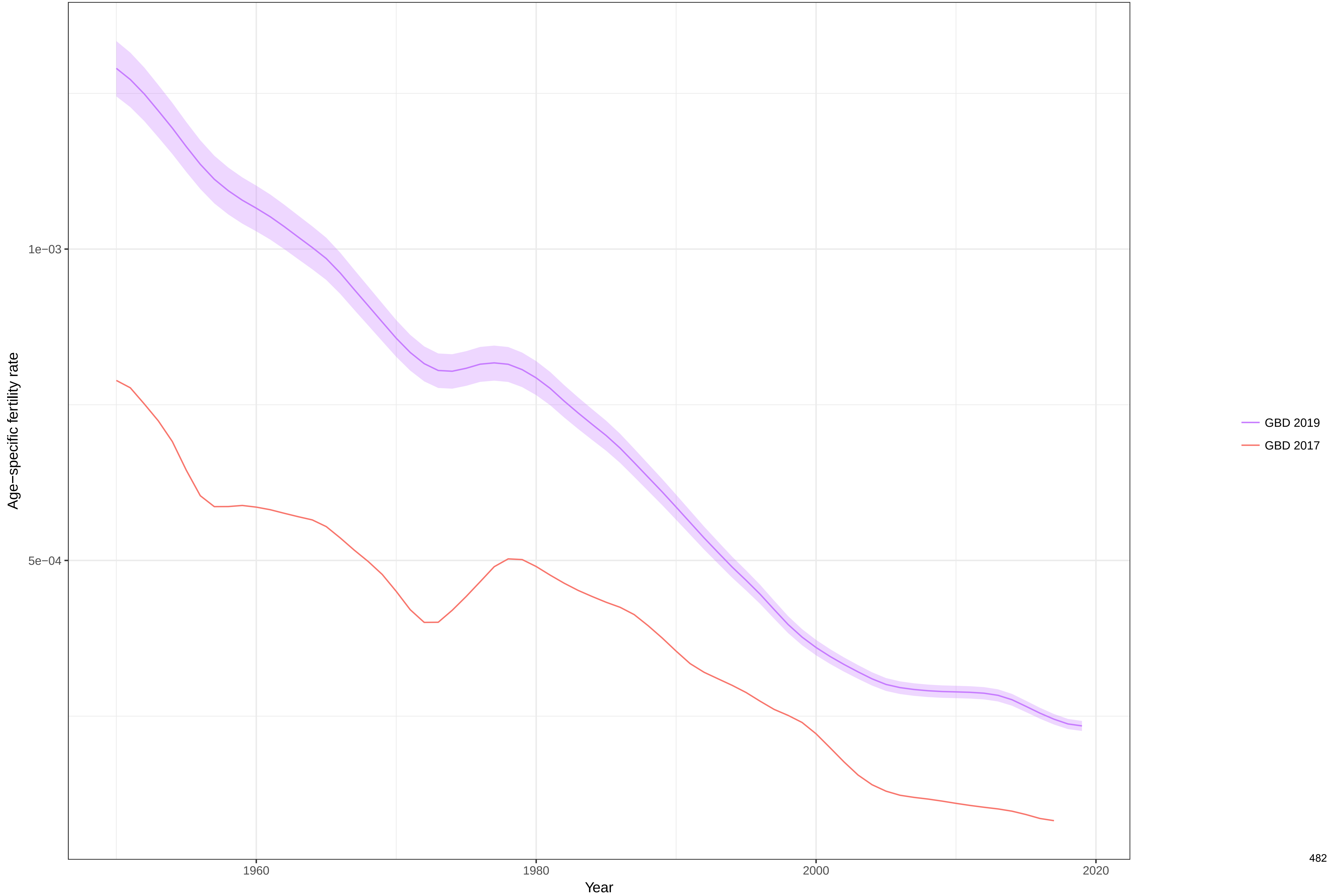

Total fertility rate fitted trend lines, 1950−2019: Bolivia (Plurinational State of) TFR
Data points enclosed with a diamond are reference sources

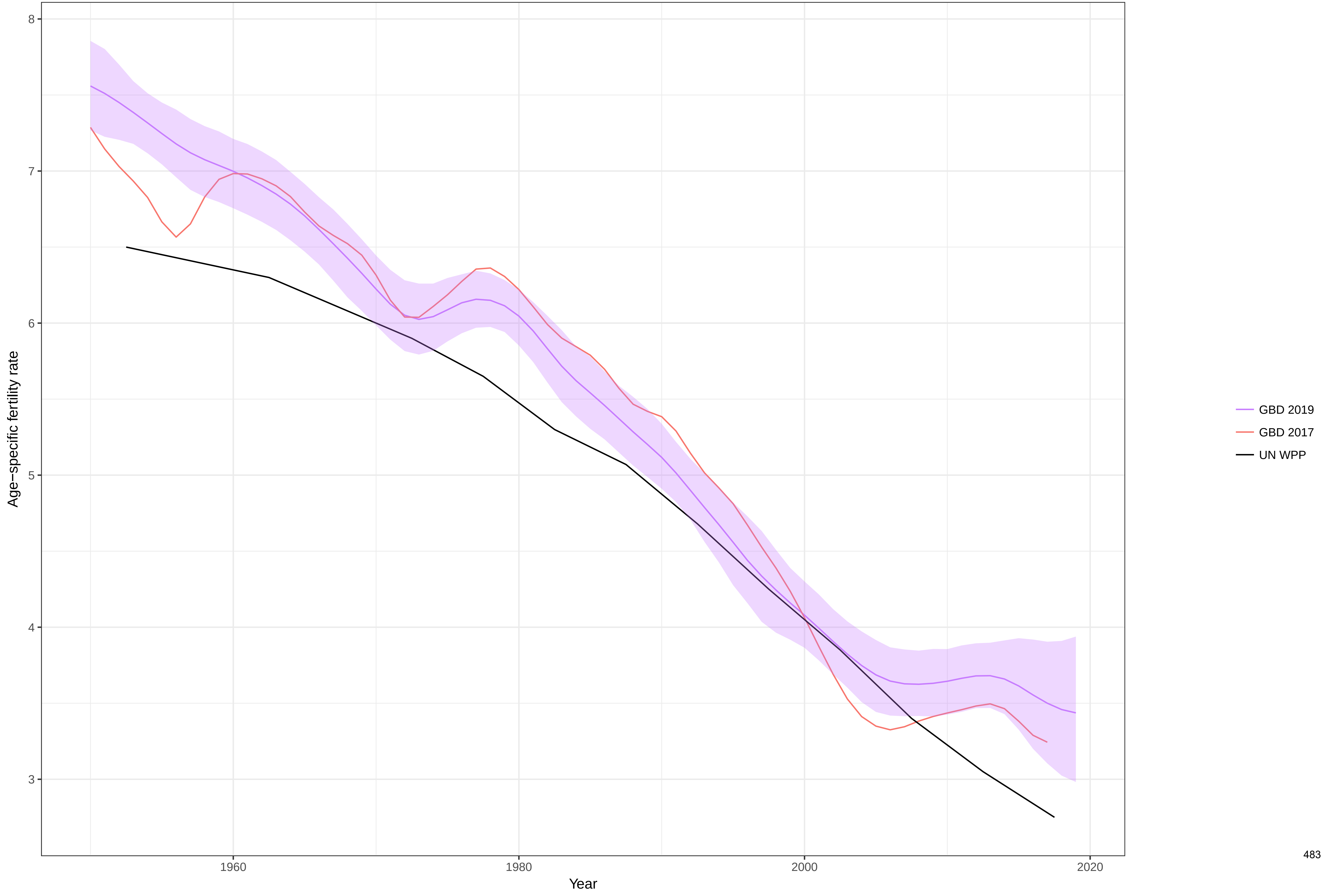

Age−specific fertility fitted trend lines, 1950−2019: Brazil, Ages: 10−14
Data points enclosed with a diamond are reference sources

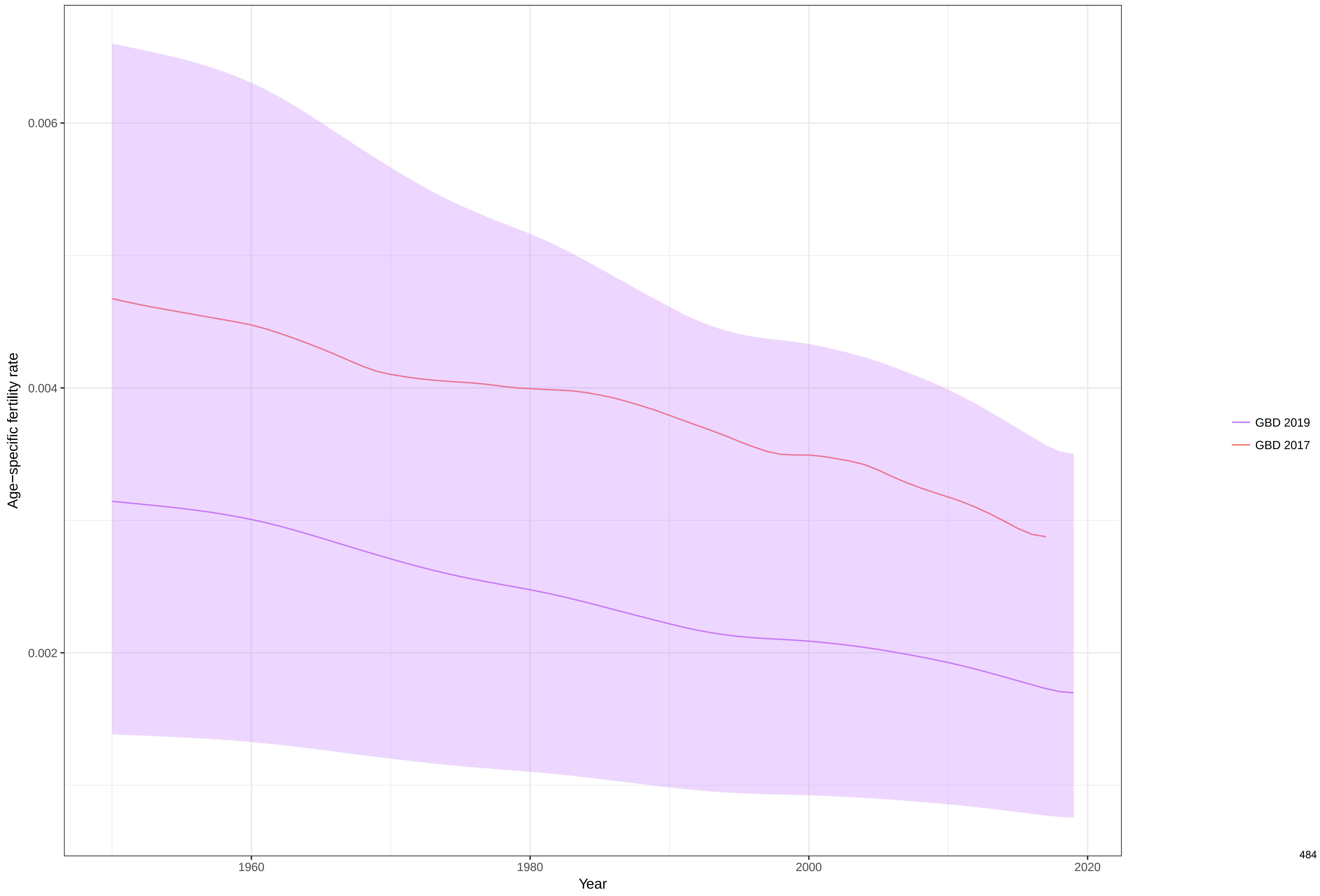

Age−specific fertility fitted trend lines, 1950−2019: Brazil, Ages: 15−19
Data points enclosed with a diamond are reference sources

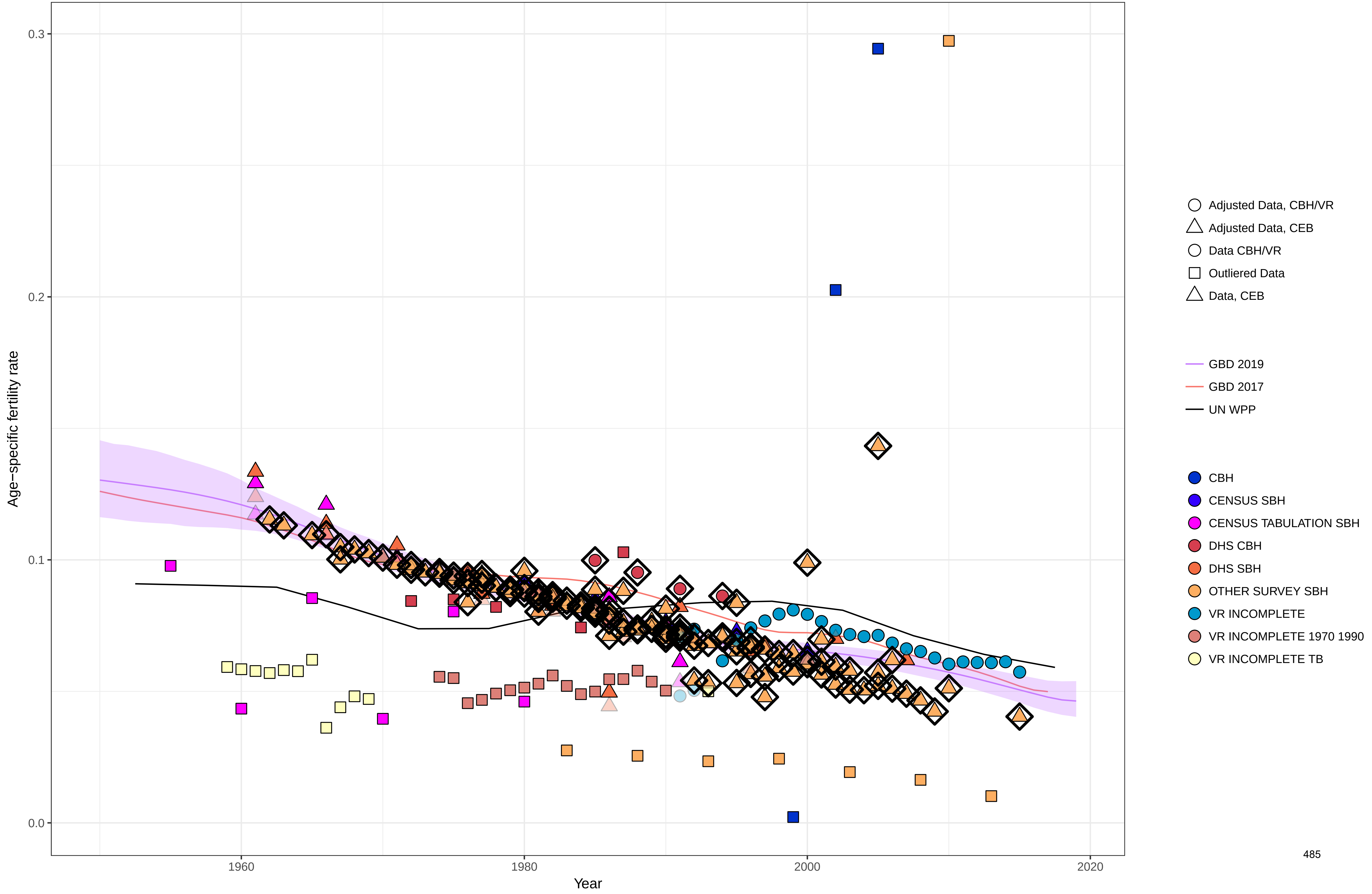

Age−specific fertility fitted trend lines, 1950−2019: Brazil, Ages: 20−24
Data points enclosed with a diamond are reference sources

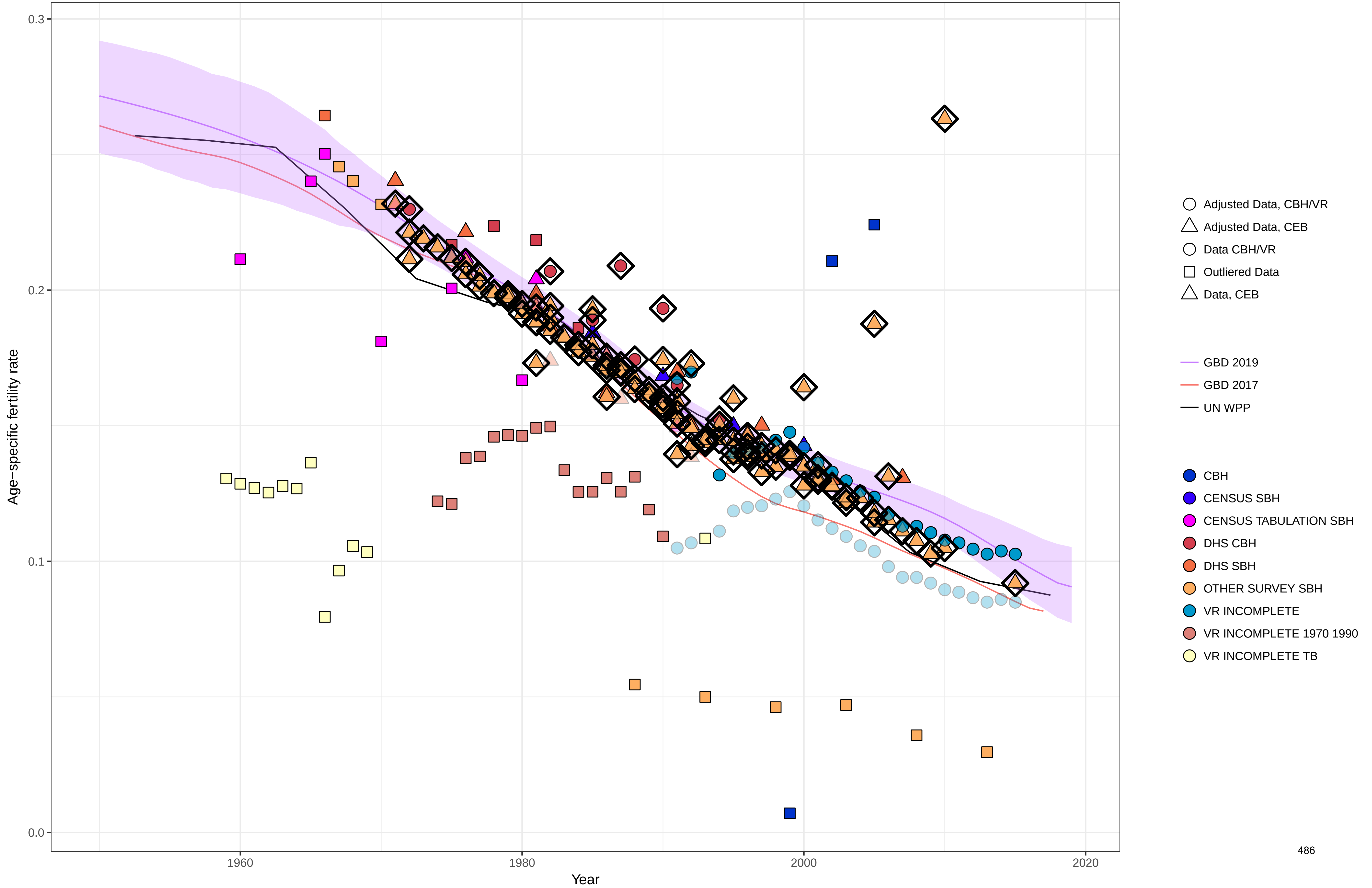

Age−specific fertility fitted trend lines, 1950−2019: Brazil, Ages: 25−29
Data points enclosed with a diamond are reference sources

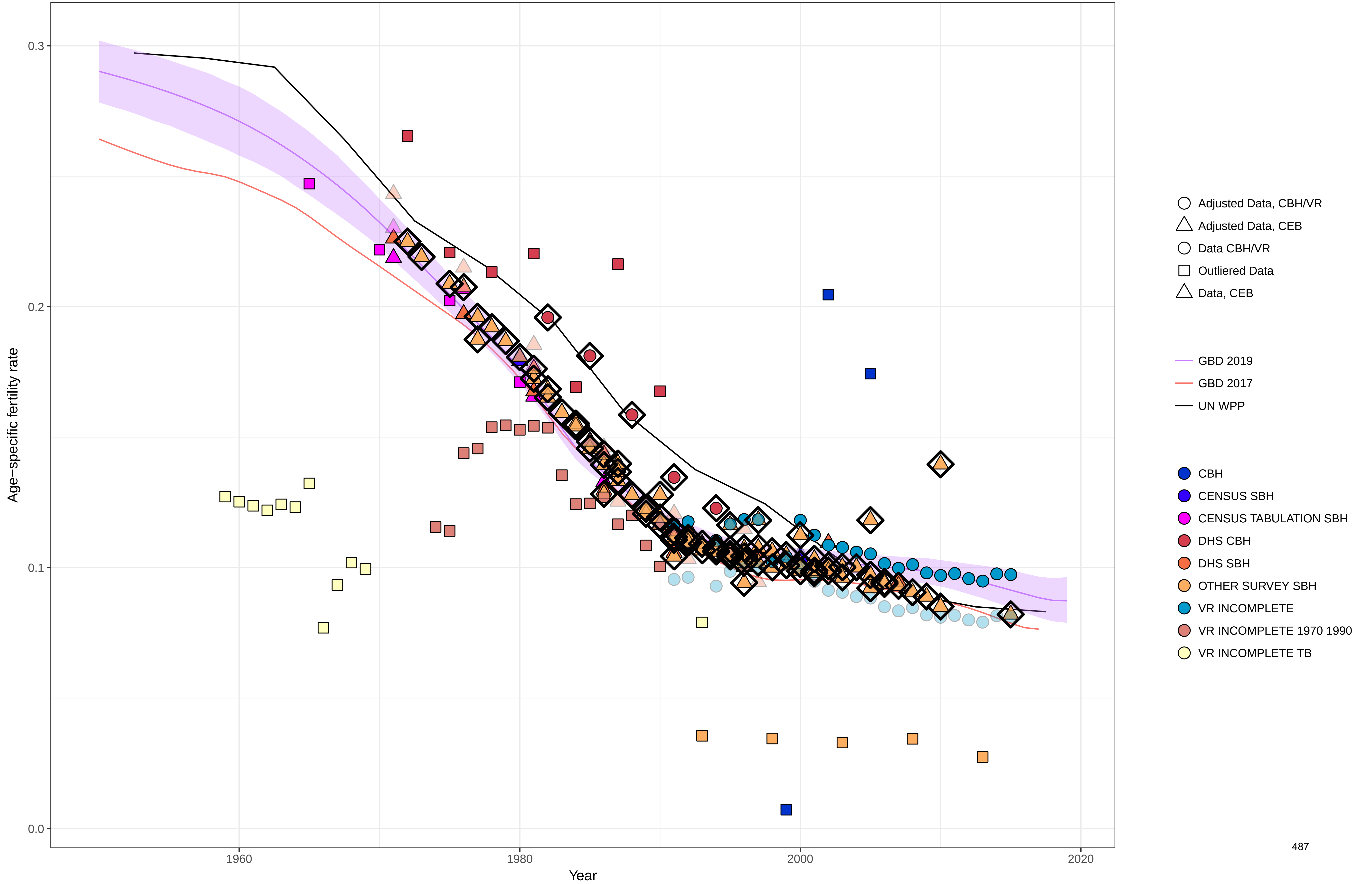

Age−specific fertility fitted trend lines, 1950−2019: Brazil, Ages: 30−34
Data points enclosed with a diamond are reference sources

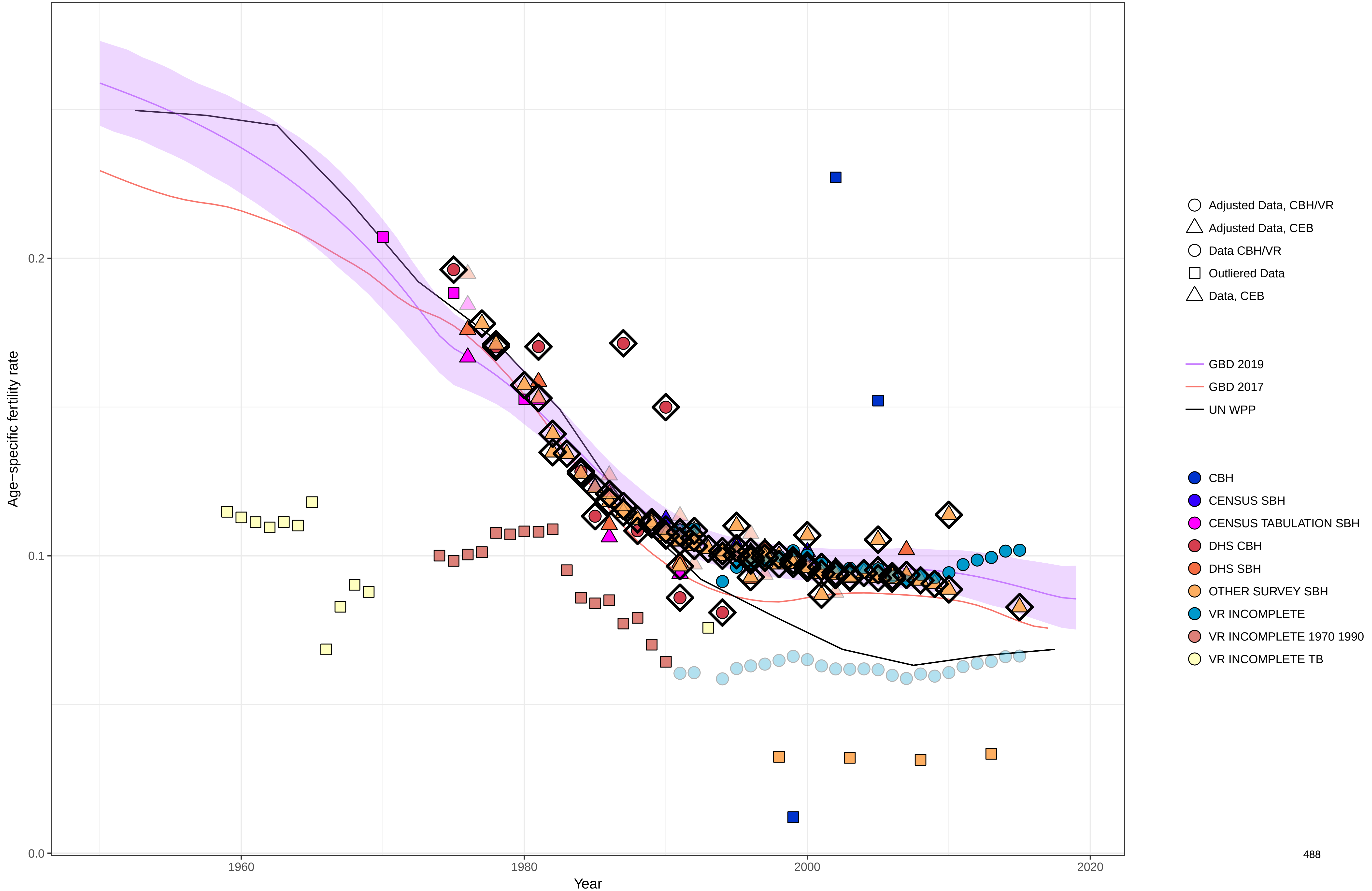

Age−specific fertility fitted trend lines, 1950−2019: Brazil, Ages: 35−39
Data points enclosed with a diamond are reference sources

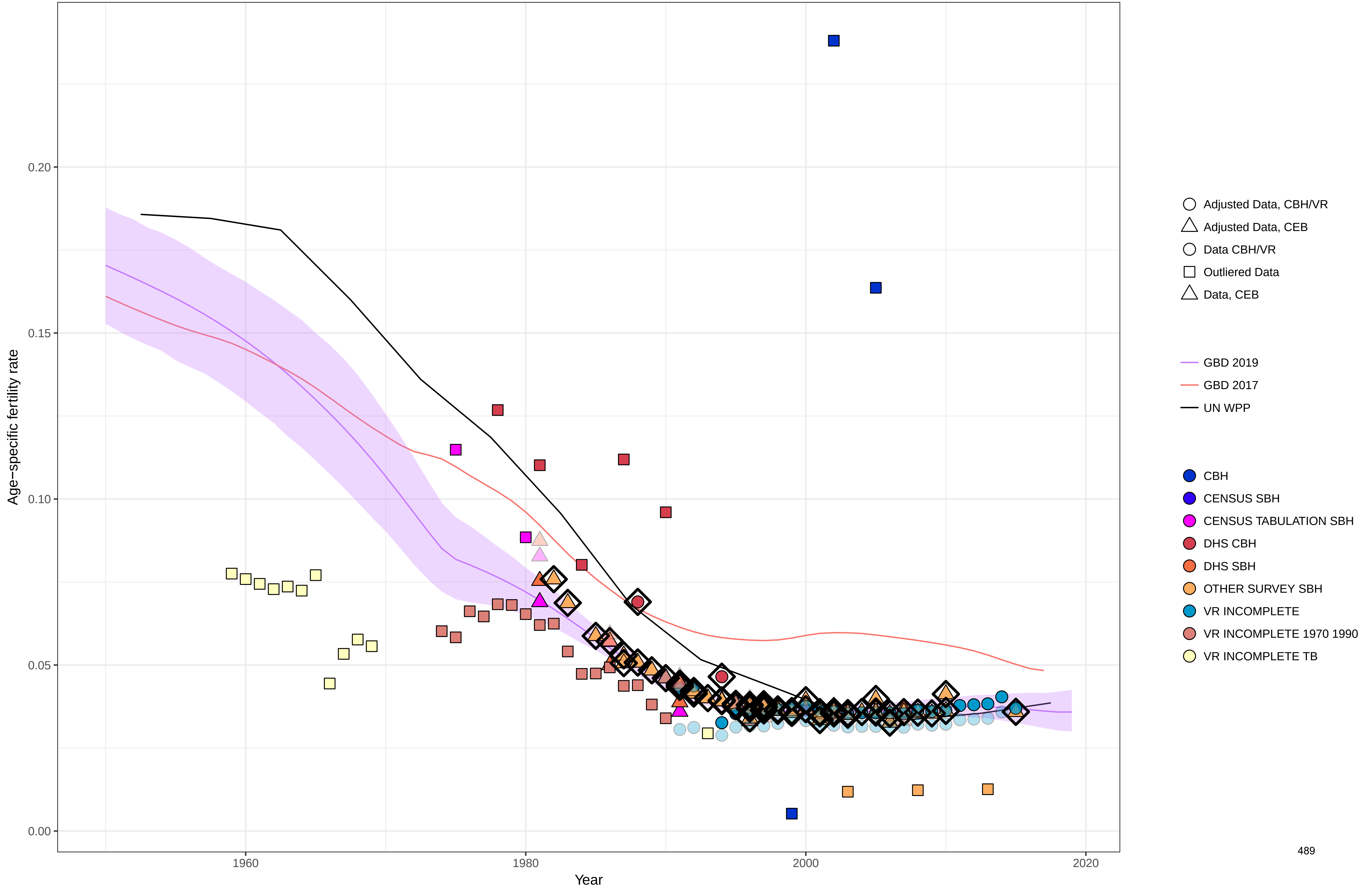

Age−specific fertility fitted trend lines, 1950−2019: Brazil, Ages: 40−44
Data points enclosed with a diamond are reference sources

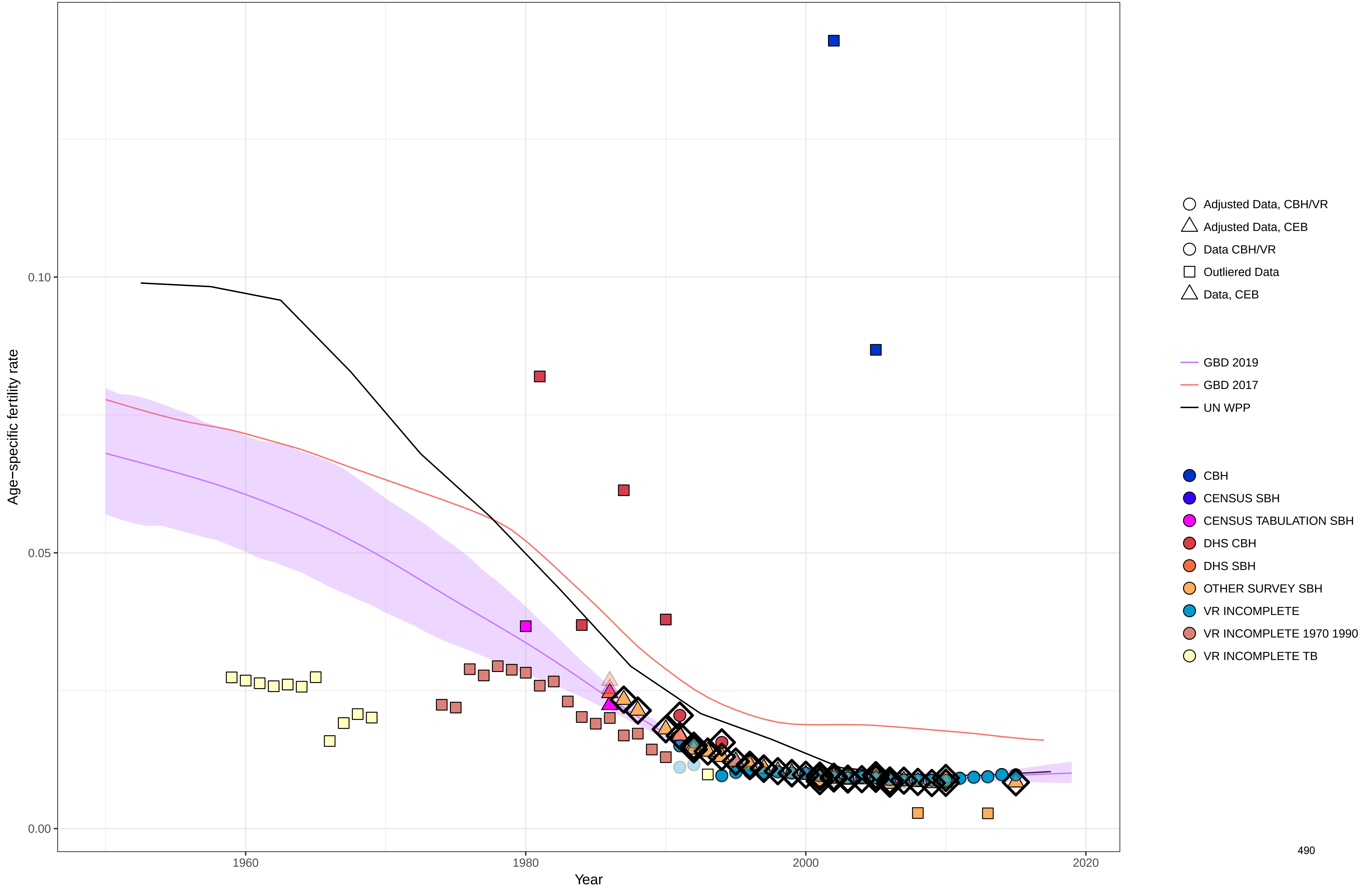

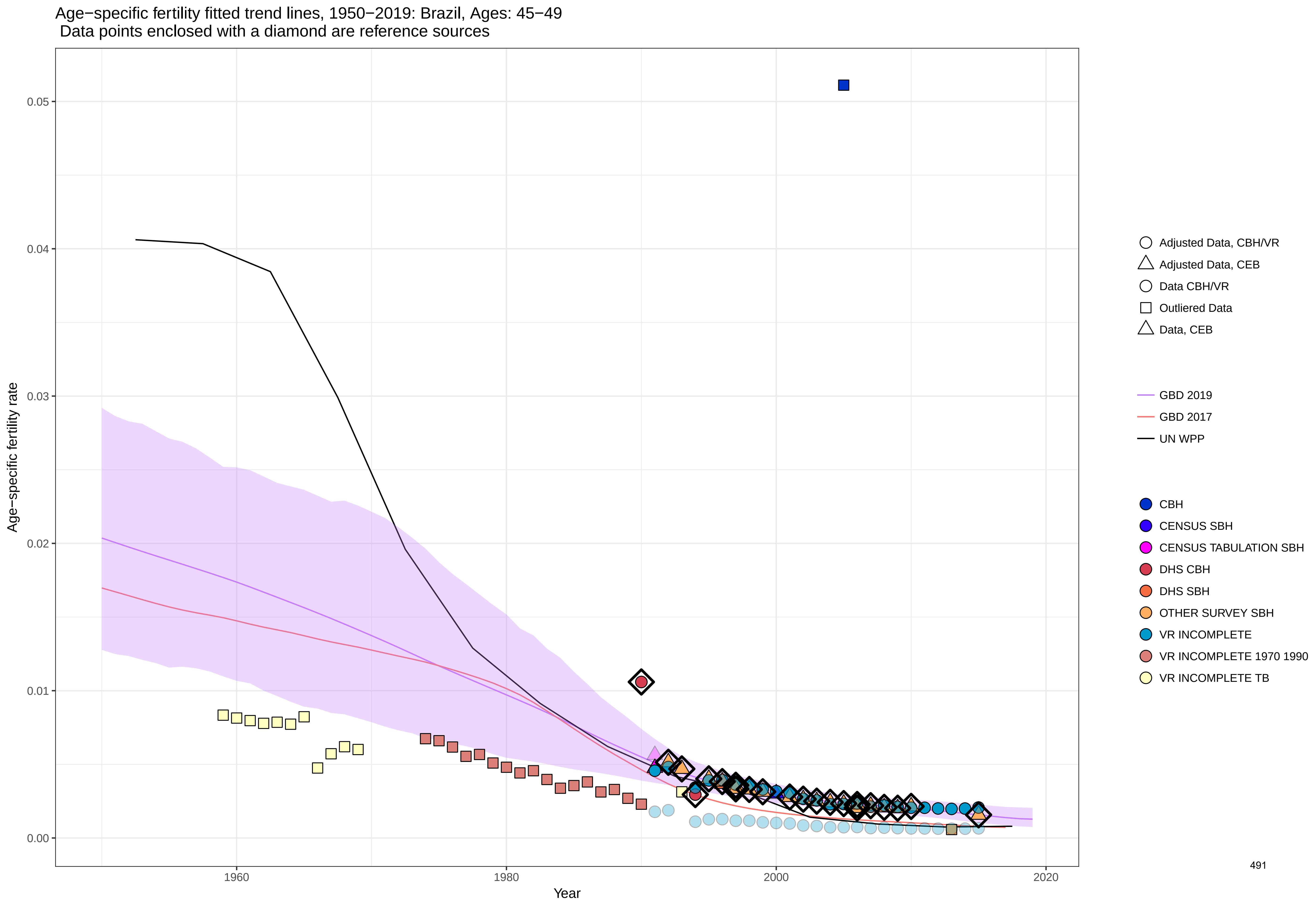

Age−specific fertility fitted trend lines, 1950−2019: Brazil, Ages: 45−49
Data points enclosed with a diamond are reference sources
Age−specific fertility rate
0.00
0.01
0.02
0.03
0.04
0.05
1960
1980
2000
2020
Year
Adjusted Data, CBH/VR
Adjusted Data, CEB
Data CBH/VR
Outliered Data
Data, CEB
GBD 2019
GBD 2017
UN WPP
CBH
CENSUS SBH
CENSUS TABULATION SBH
DHS CBH
DHS SBH
OTHER SURVEY SBH
VR INCOMPLETE
VR INCOMPLETE 1970 1990
VR INCOMPLETE TB

Age−specific fertility fitted trend lines, 1950−2019: Brazil, Ages: 50−54
Data points enclosed with a diamond are reference sources

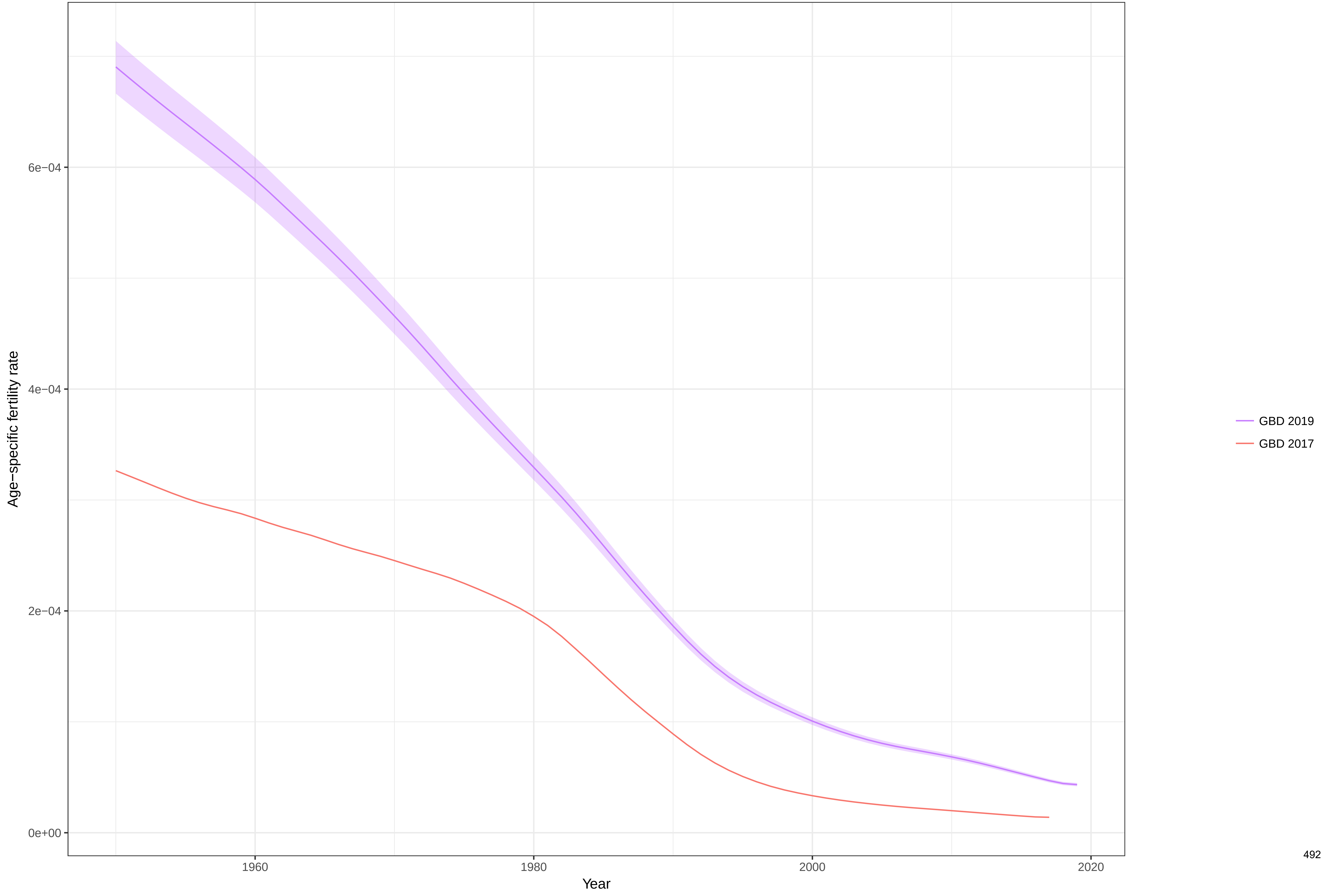

Total fertility rate fitted trend lines, 1950−2019: Brazil TFR
Data points enclosed with a diamond are reference sources

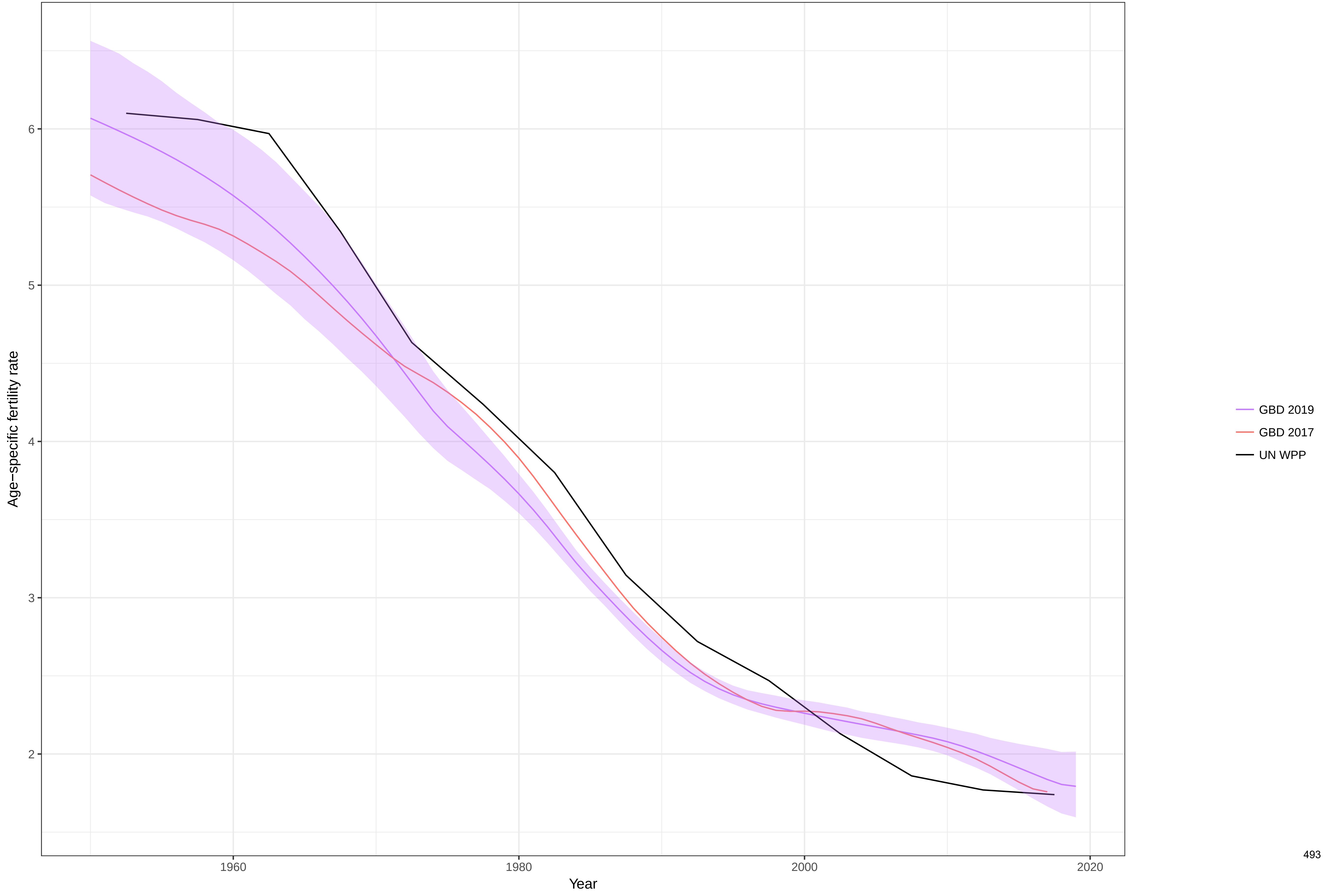

Age−specific fertility fitted trend lines, 1950−2019: Barbados, Ages: 10−14
 Data points enclosed with a diamond are reference sources

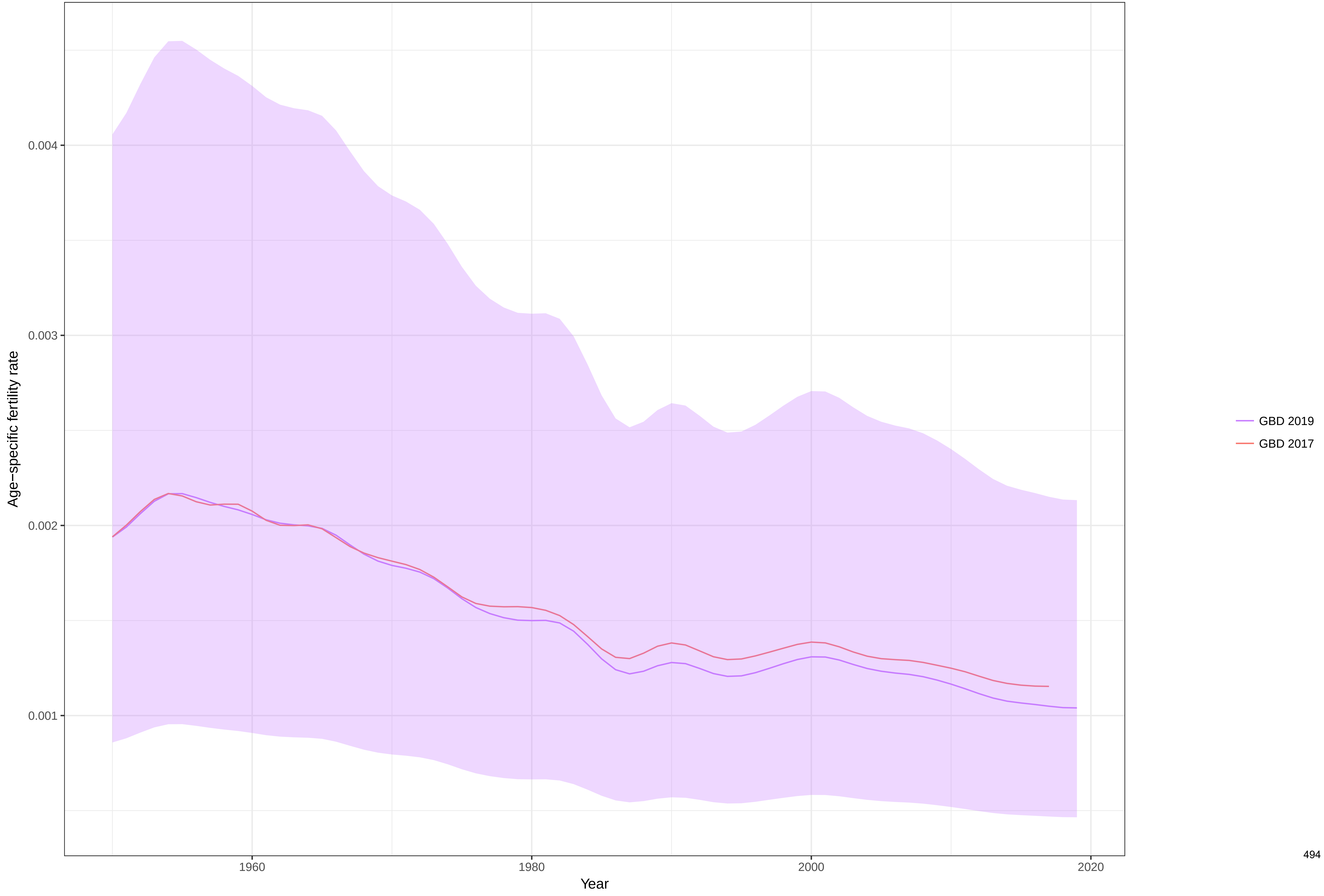

Age−specific fertility fitted trend lines, 1950−2019: Barbados, Ages: 15−19
Data points enclosed with a diamond are reference sources

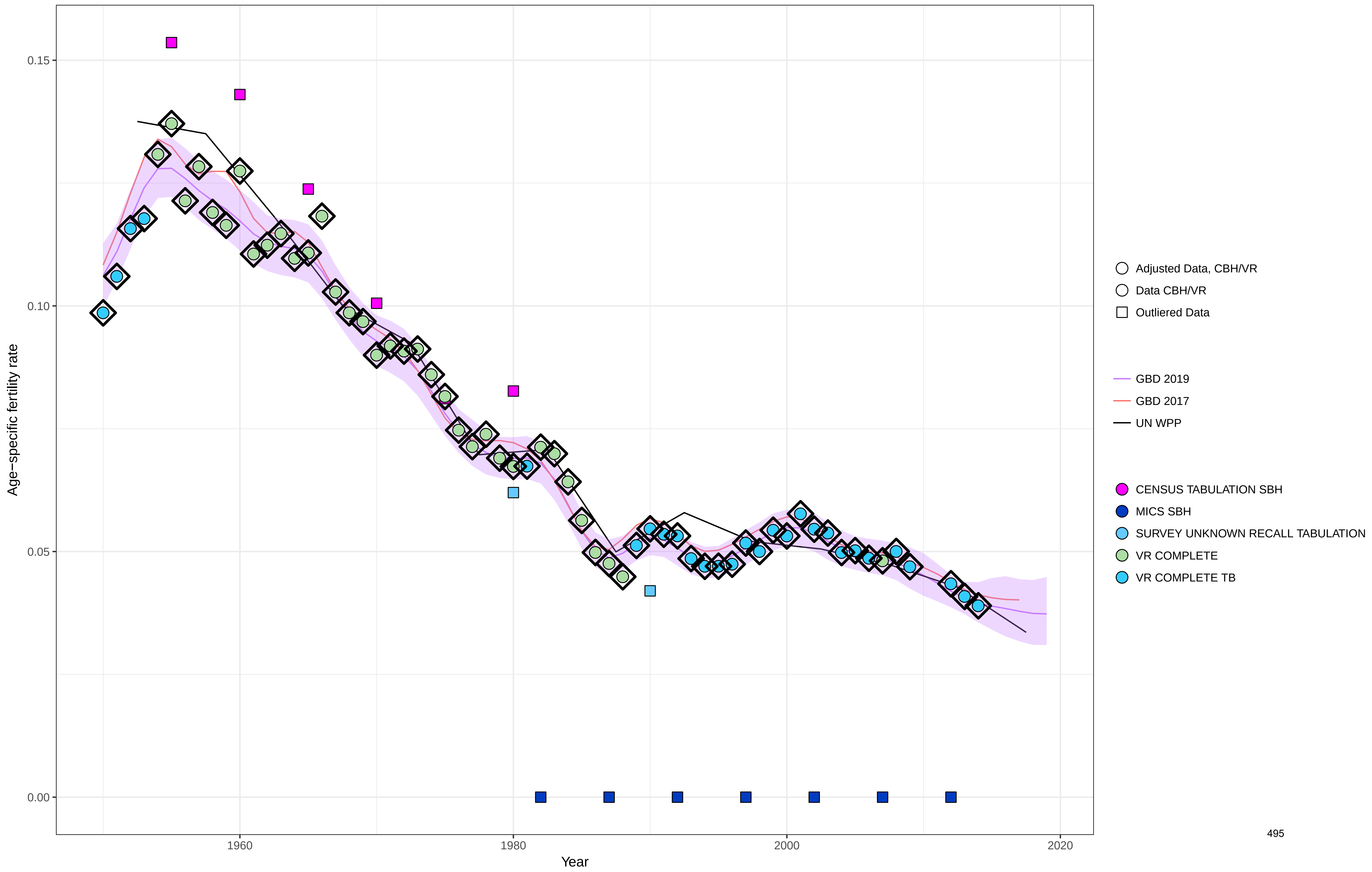

Age−specific fertility fitted trend lines, 1950−2019: Barbados, Ages: 20−24
Data points enclosed with a diamond are reference sources

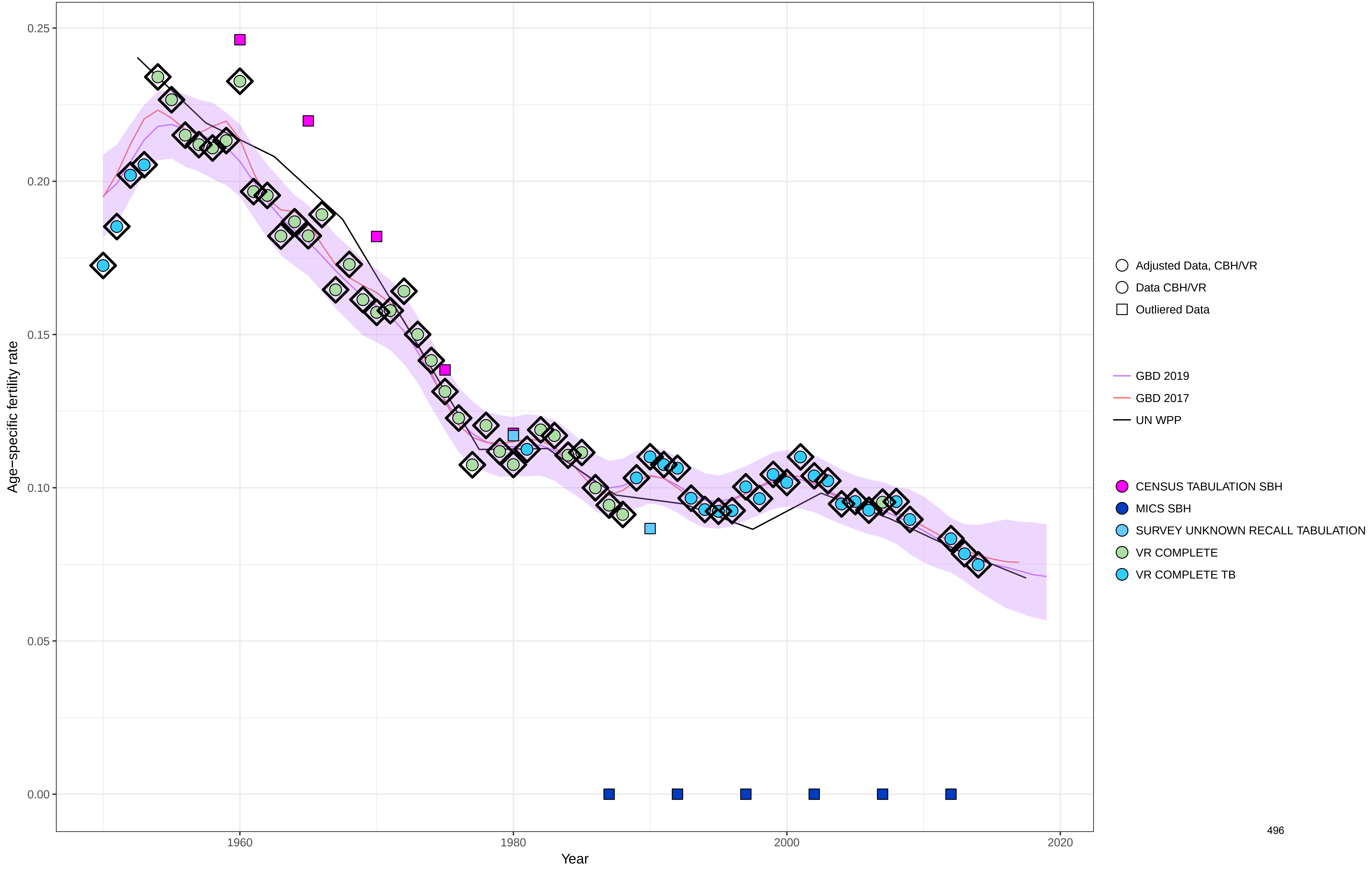

Age−specific fertility fitted trend lines, 1950−2019: Barbados, Ages: 25−29
Data points enclosed with a diamond are reference sources

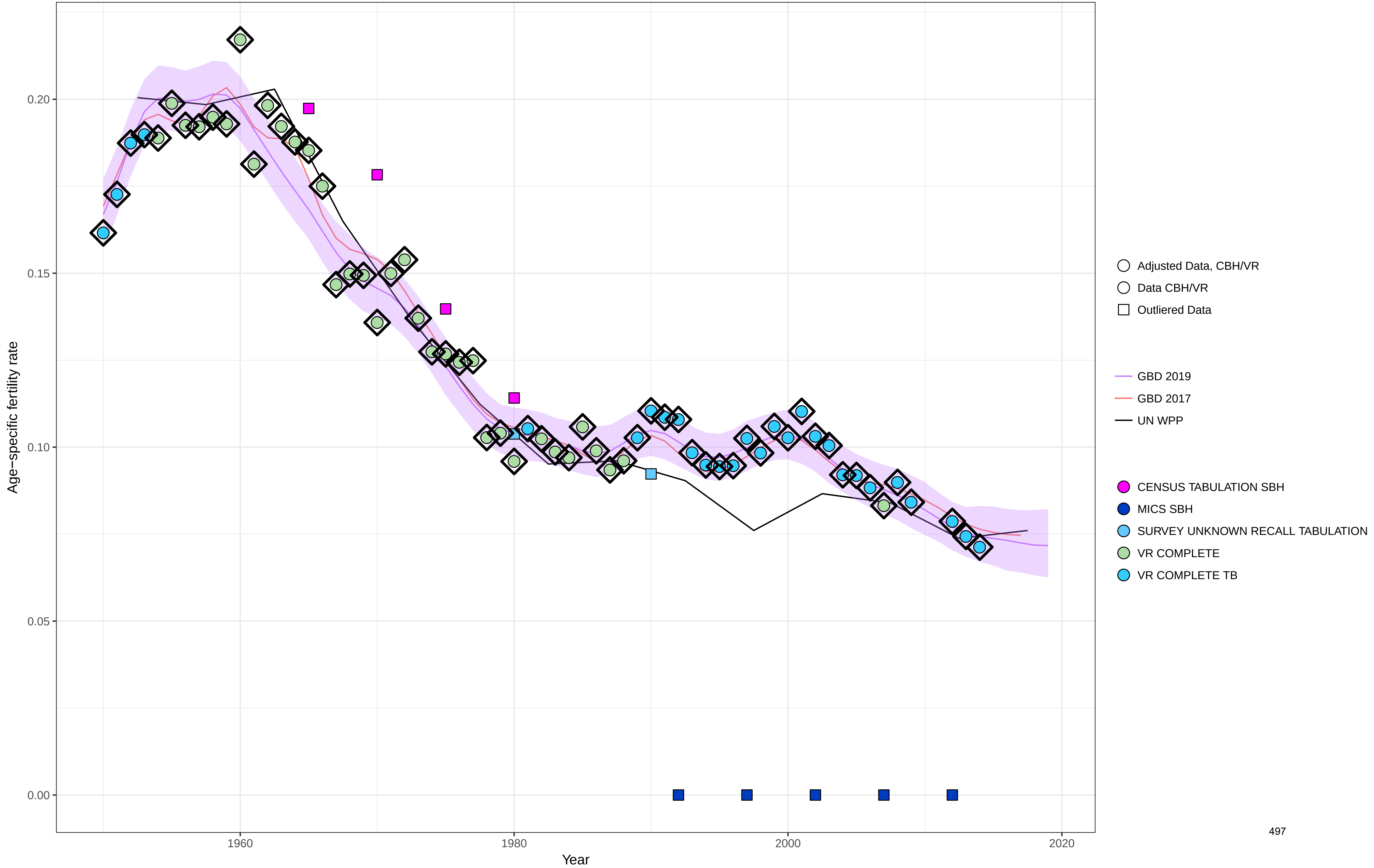

Age−specific fertility fitted trend lines, 1950−2019: Barbados, Ages: 30−34
Data points enclosed with a diamond are reference sources

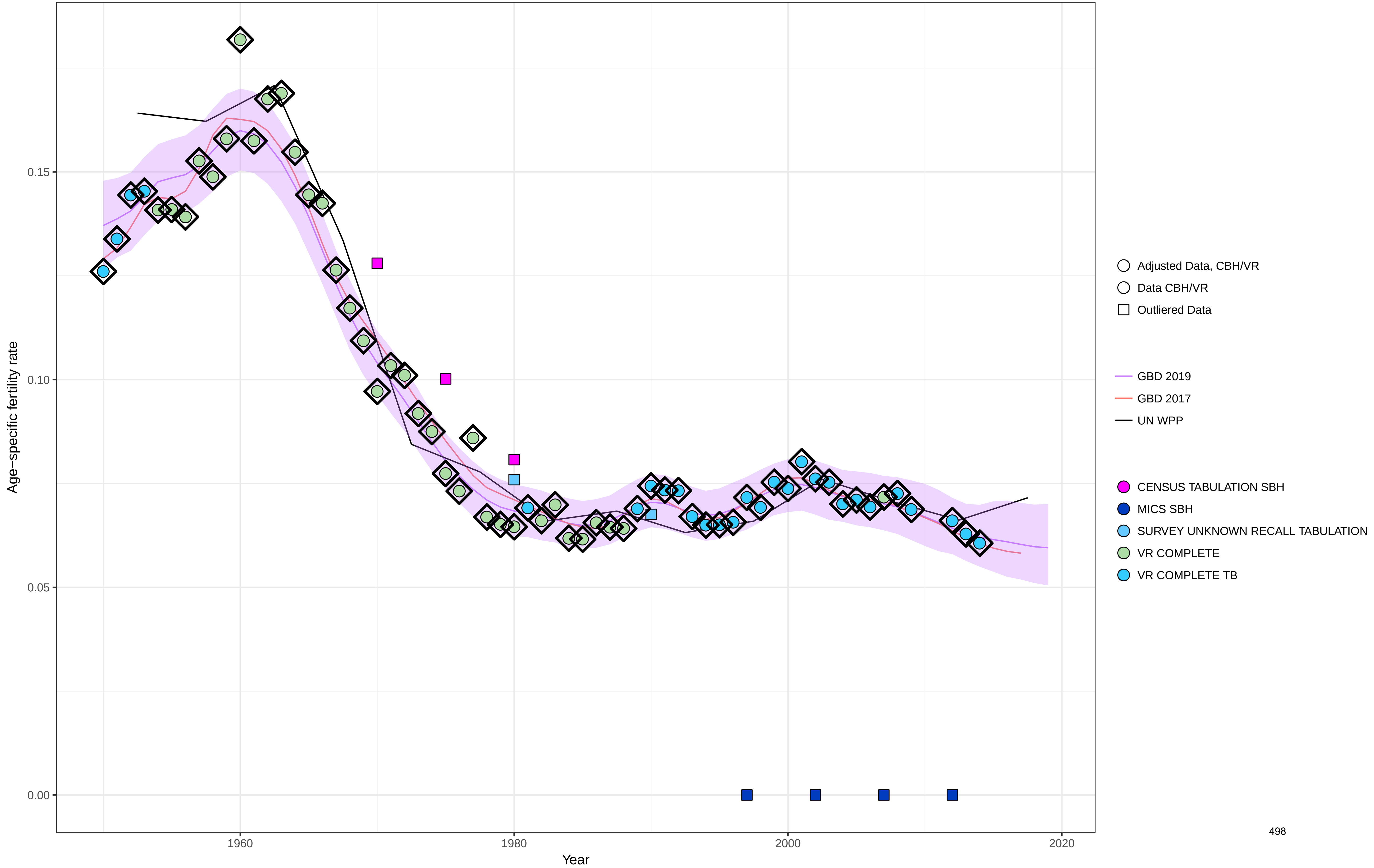

Age−specific fertility fitted trend lines, 1950−2019: Barbados, Ages: 35−39
Data points enclosed with a diamond are reference sources

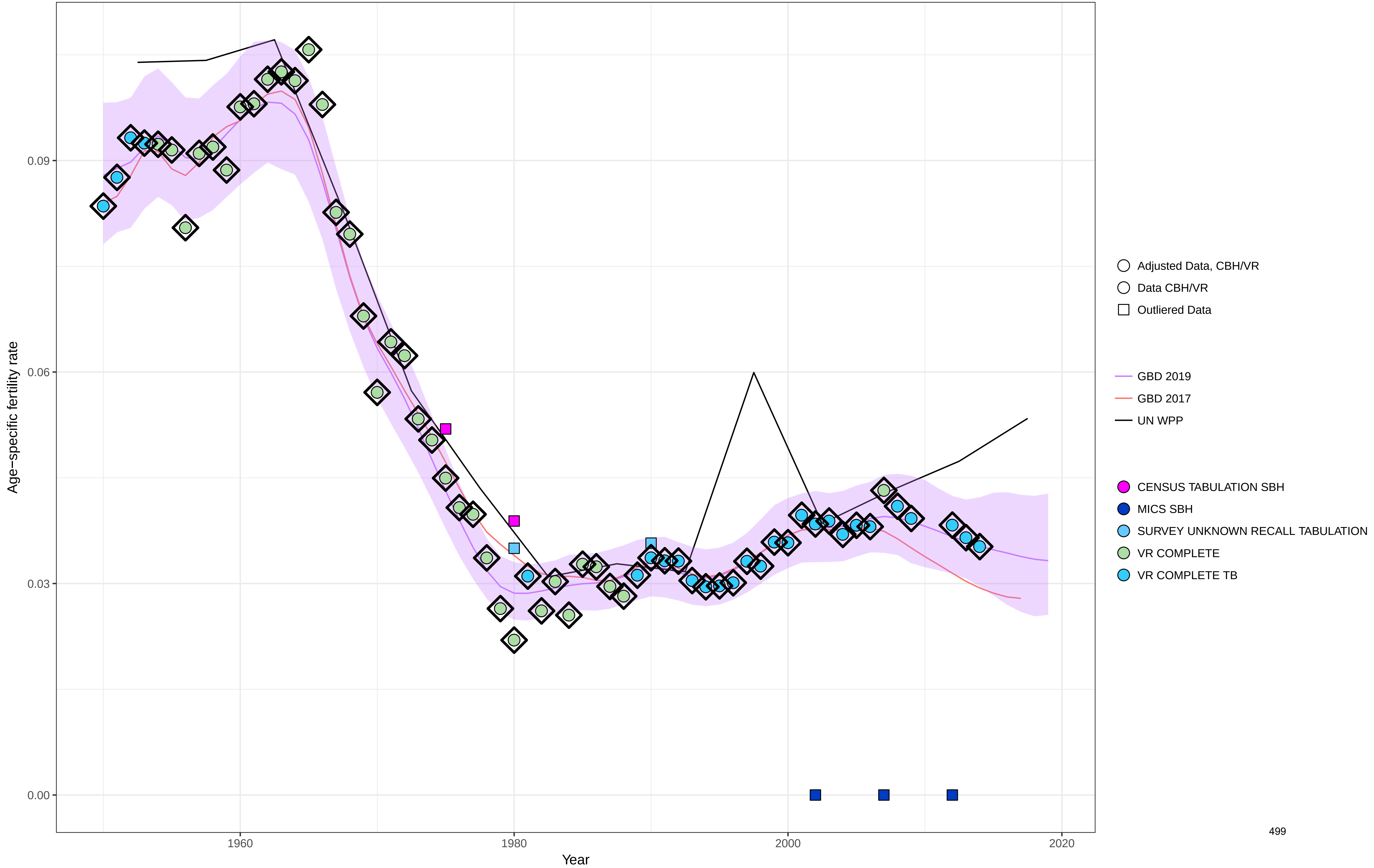

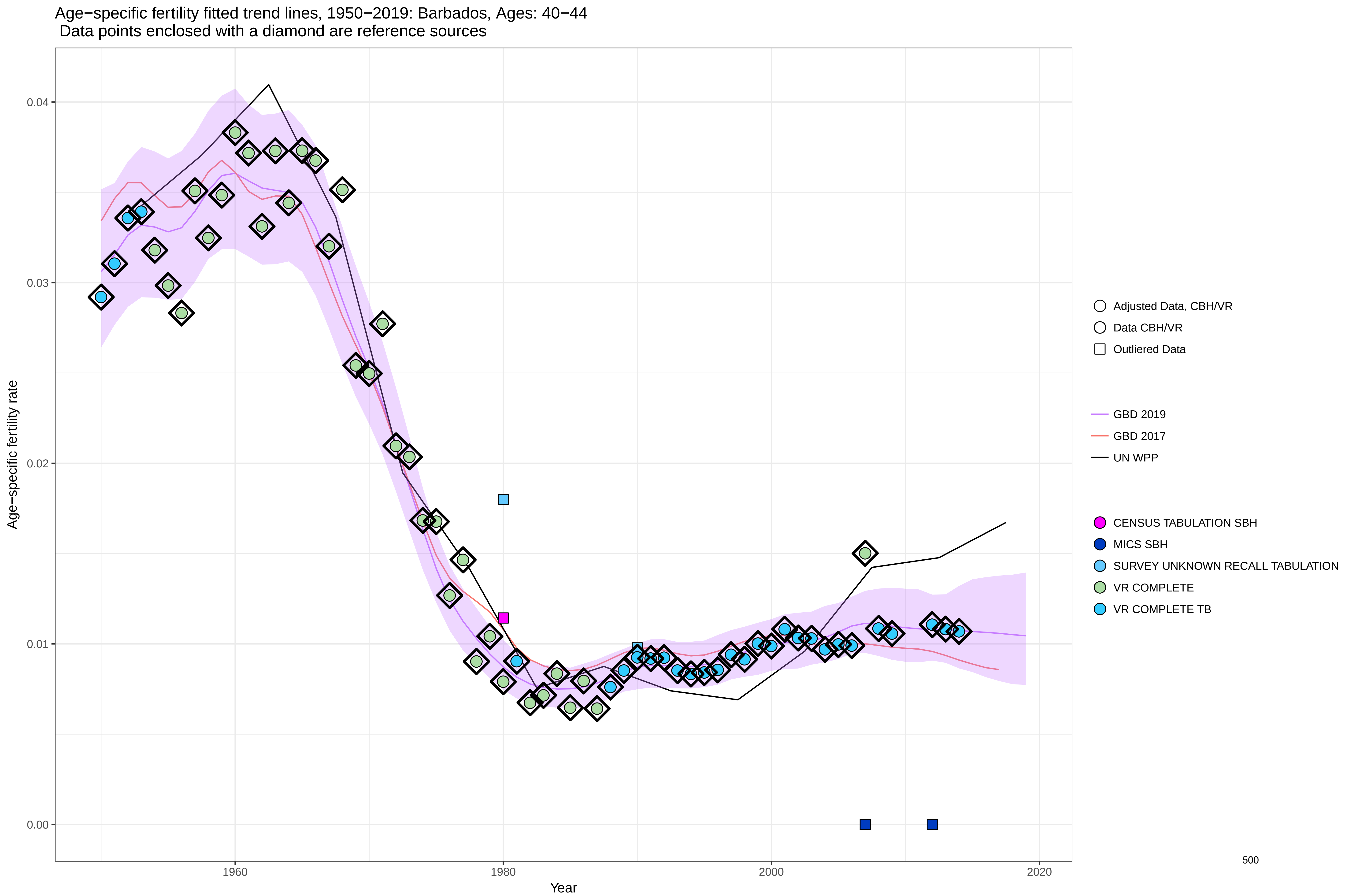

Age−specific fertility fitted trend lines, 1950−2019: Barbados, Ages: 40−44
Data points enclosed with a diamond are reference sources
Age−specific fertility rate
Year
0.00
0.01
0.02
0.03
0.04
1960
1980
2000
2020
Adjusted Data, CBH/VR
Data CBH/VR
Outliered Data
GBD 2019
GBD 2017
UN WPP
CENSUS TABULATION SBH
MICS SBH
SURVEY UNKNOWN RECALL TABULATION
VR COMPLETE
VR COMPLETE TB

500

Age−specific fertility fitted trend lines, 1950−2019: Barbados, Ages: 45−49
Data points enclosed with a diamond are reference sources

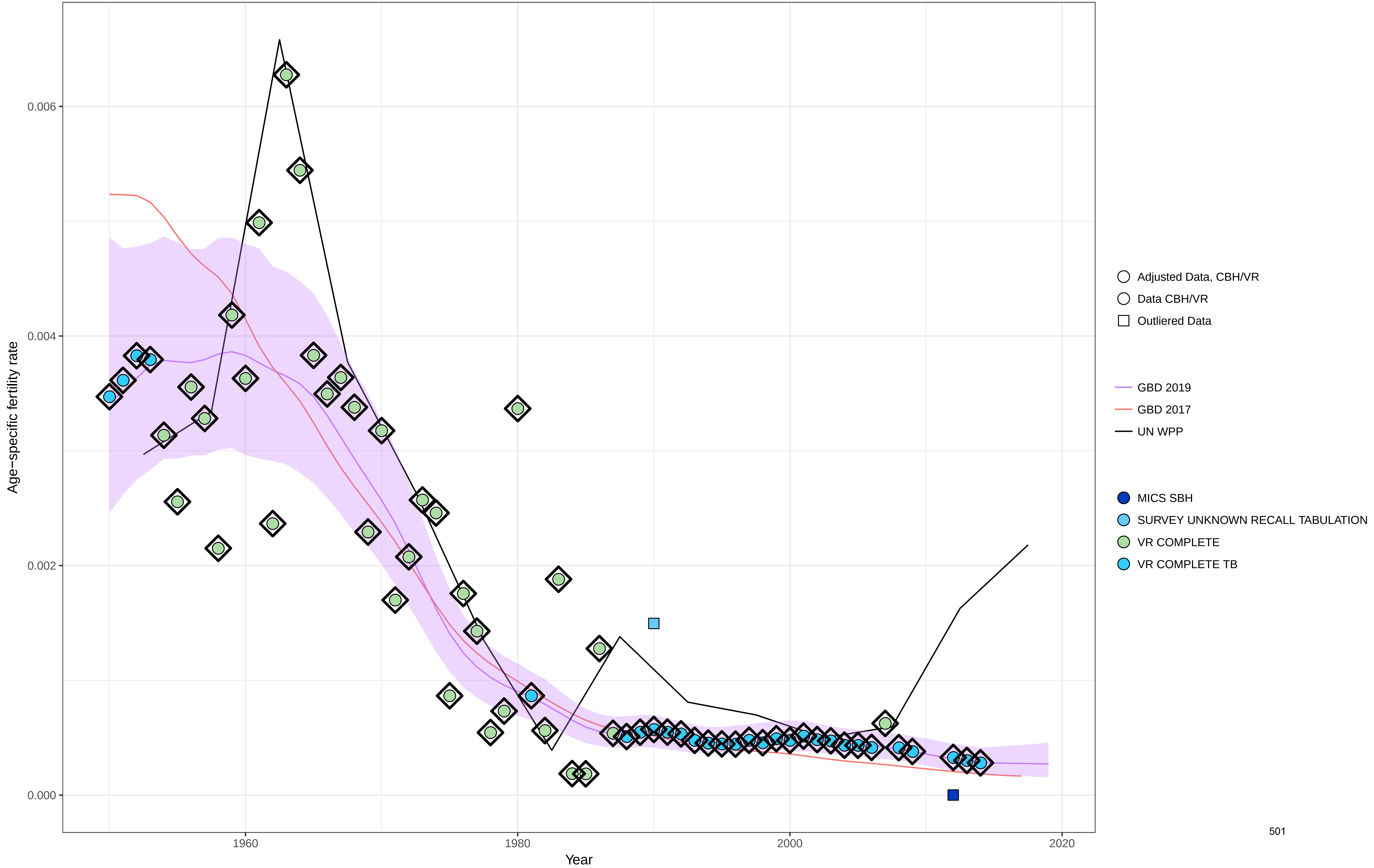

Age−specific fertility fitted trend lines, 1950−2019: Barbados, Ages: 50−54
Data points enclosed with a diamond are reference sources

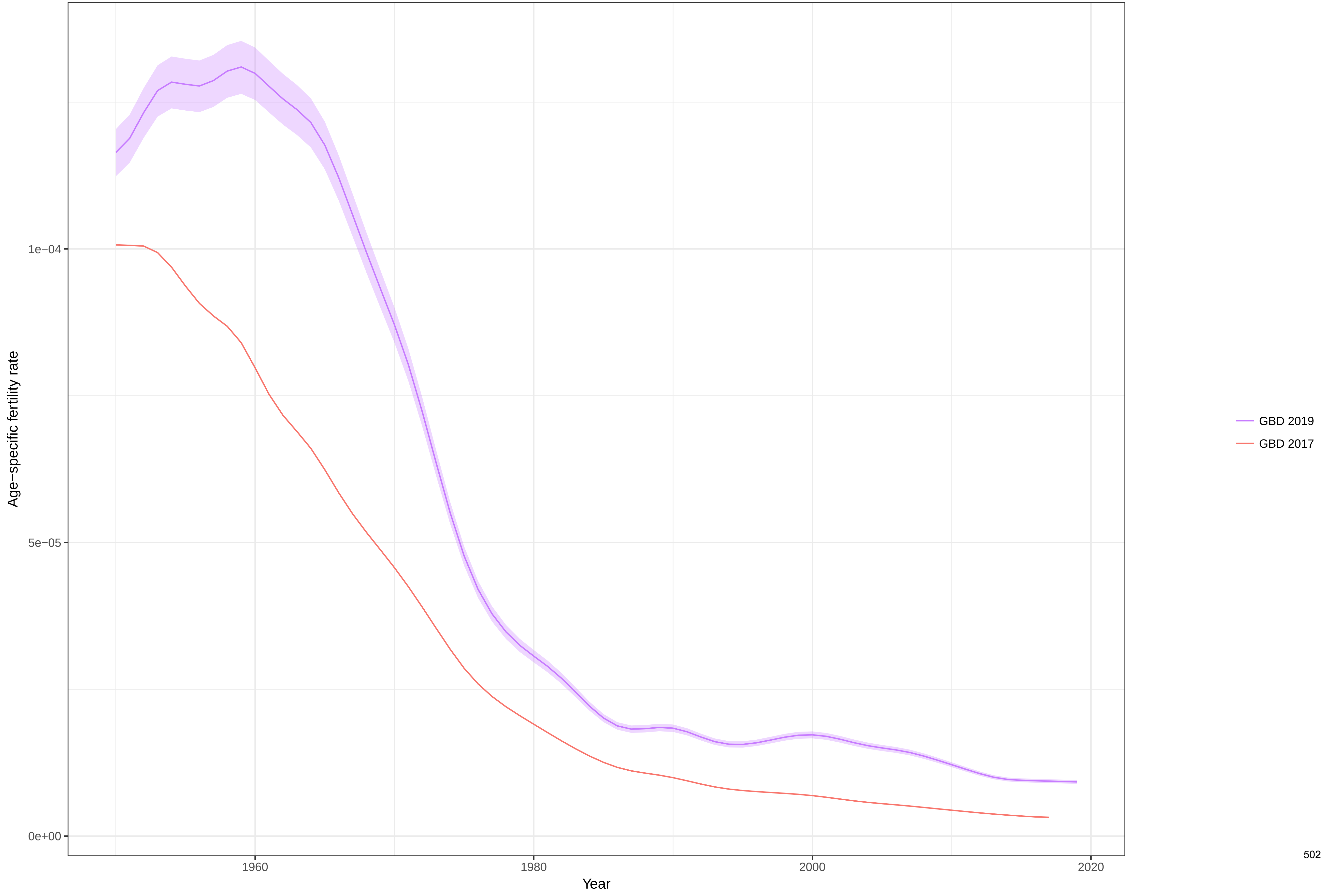

Total fertility rate fitted trend lines, 1950−2019: Barbados TFR
Data points enclosed with a diamond are reference sources

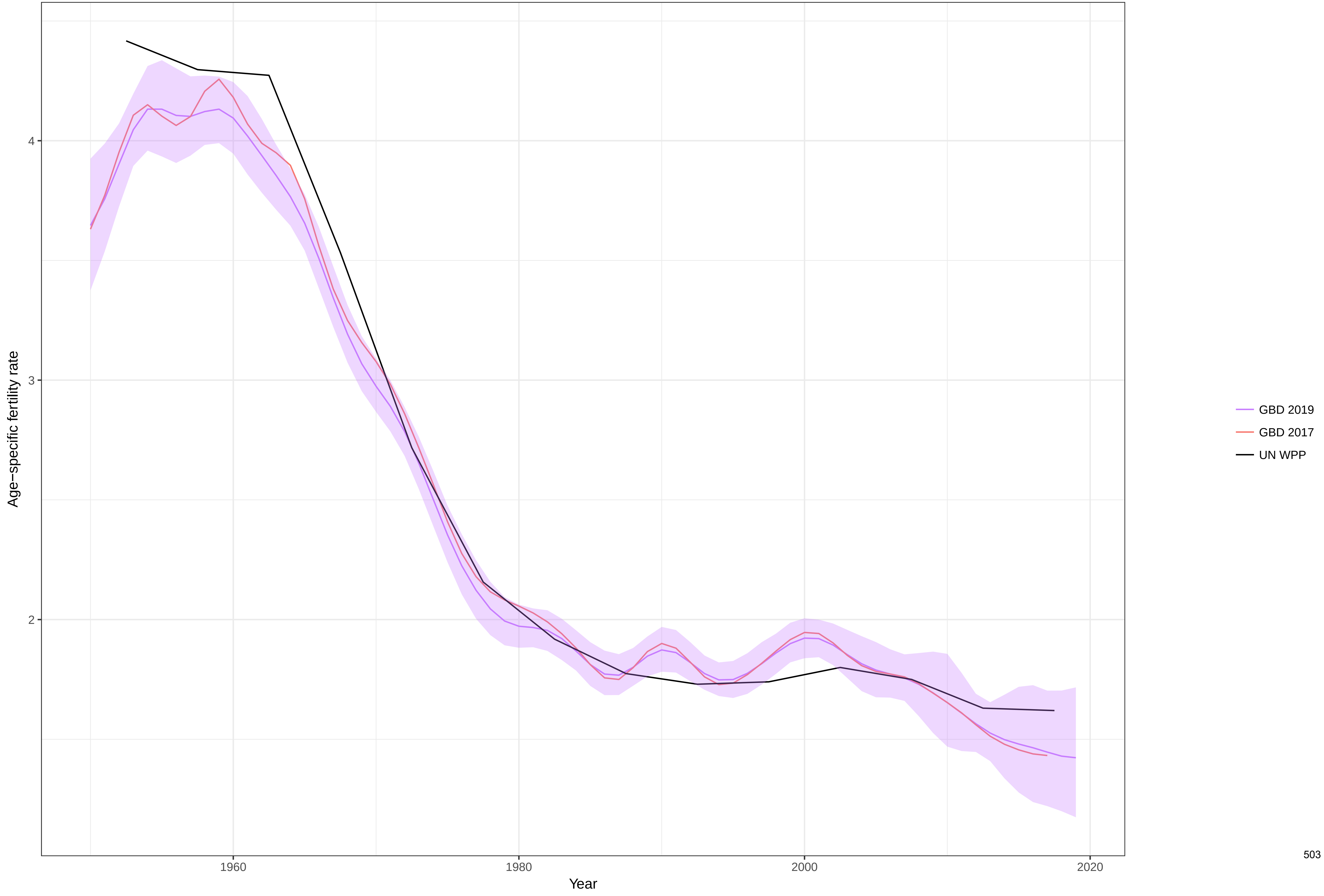

Age−specific fertility fitted trend lines, 1950−2019: Brunei Darussalam, Ages: 10−14
Data points enclosed with a diamond are reference sources

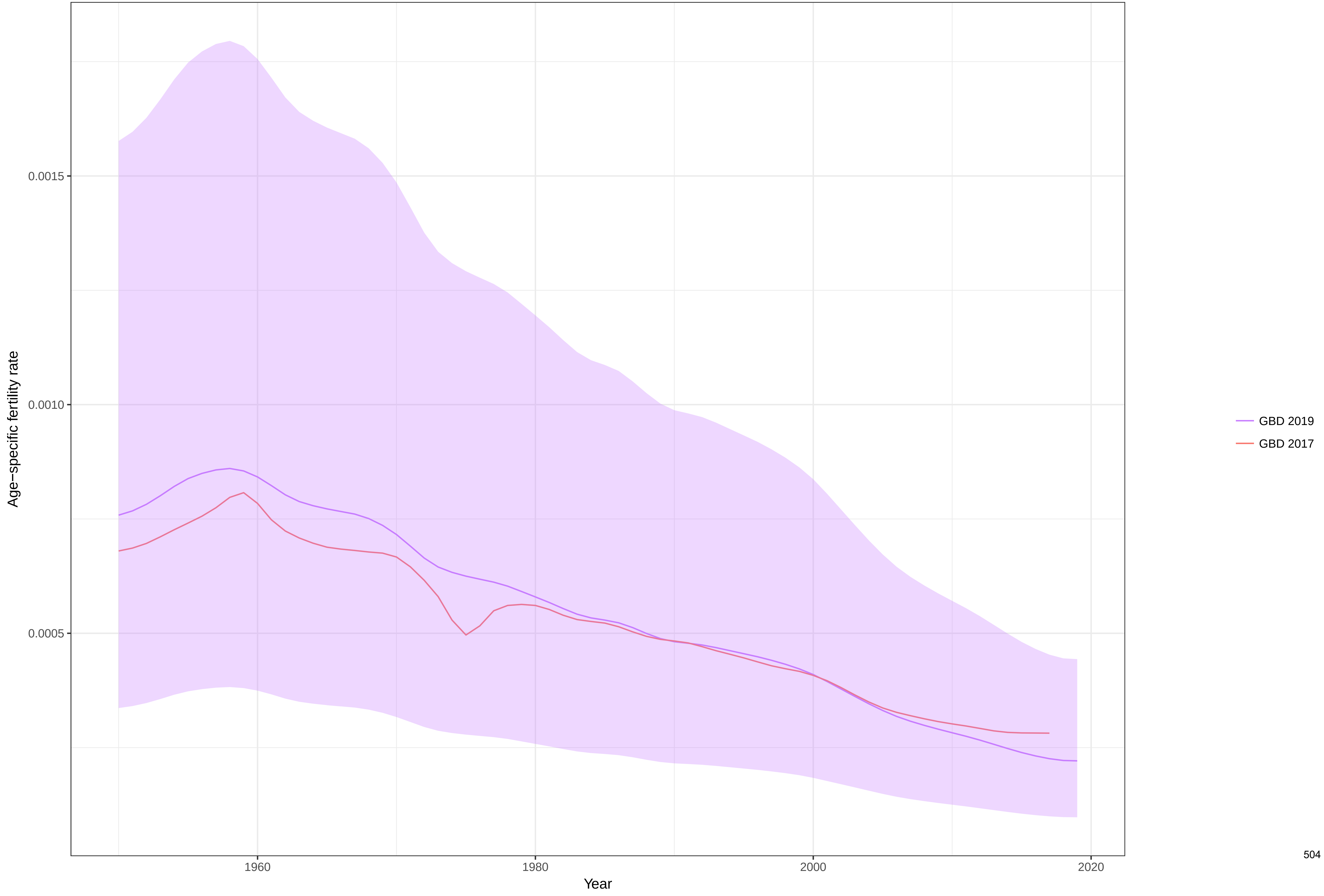

Age−specific fertility fitted trend lines, 1950−2019: Brunei Darussalam, Ages: 15−19
Data points enclosed with a diamond are reference sources

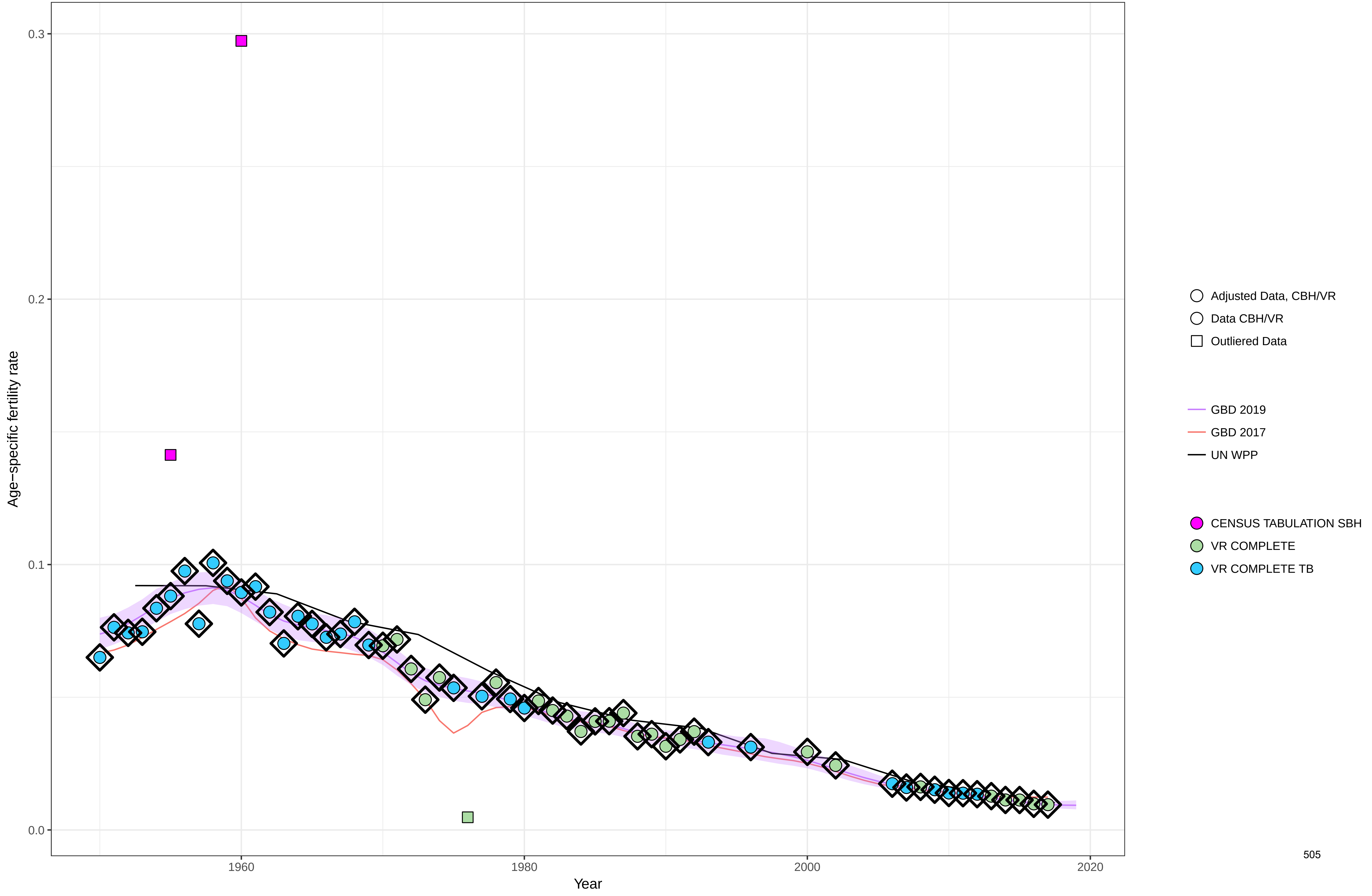

Age−specific fertility fitted trend lines, 1950−2019: Brunei Darussalam, Ages: 20−24
Data points enclosed with a diamond are reference sources

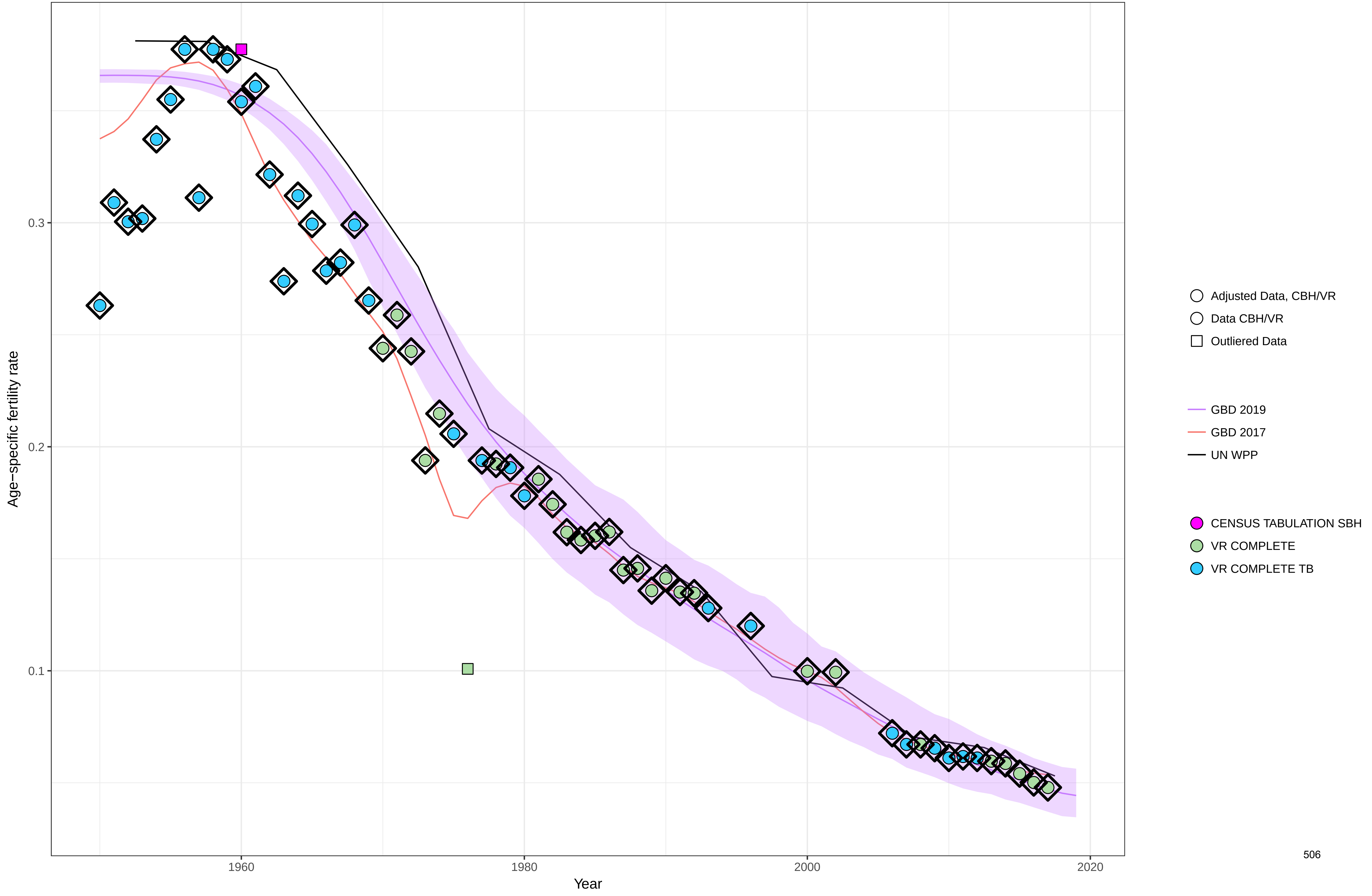

Age−specific fertility fitted trend lines, 1950−2019: Brunei Darussalam, Ages: 25−29
Data points enclosed with a diamond are reference sources

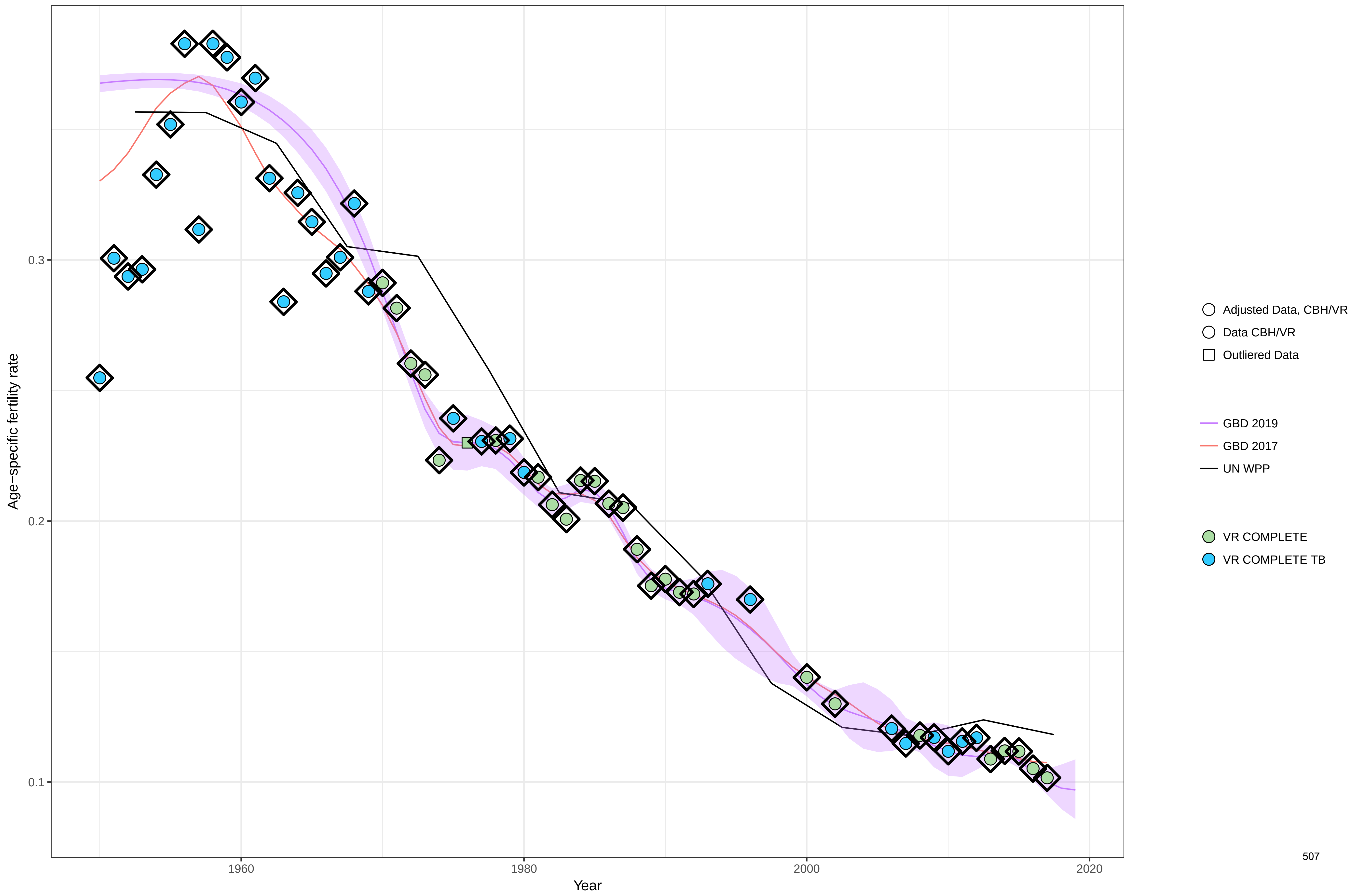

Age−specific fertility fitted trend lines, 1950−2019: Brunei Darussalam, Ages: 30−34
Data points enclosed with a diamond are reference sources

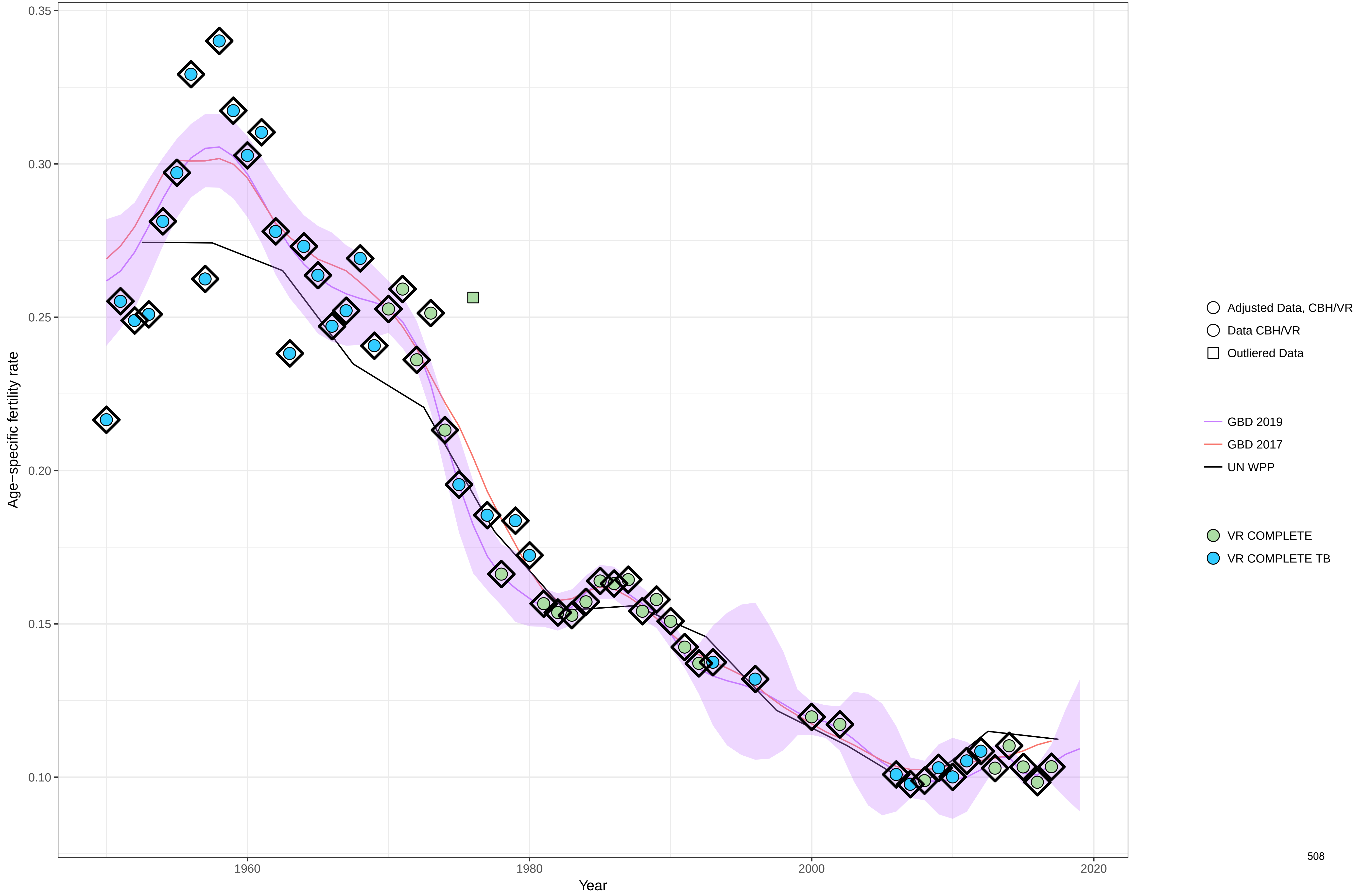

Age−specific fertility fitted trend lines, 1950−2019: Brunei Darussalam, Ages: 35−39
Data points enclosed with a diamond are reference sources

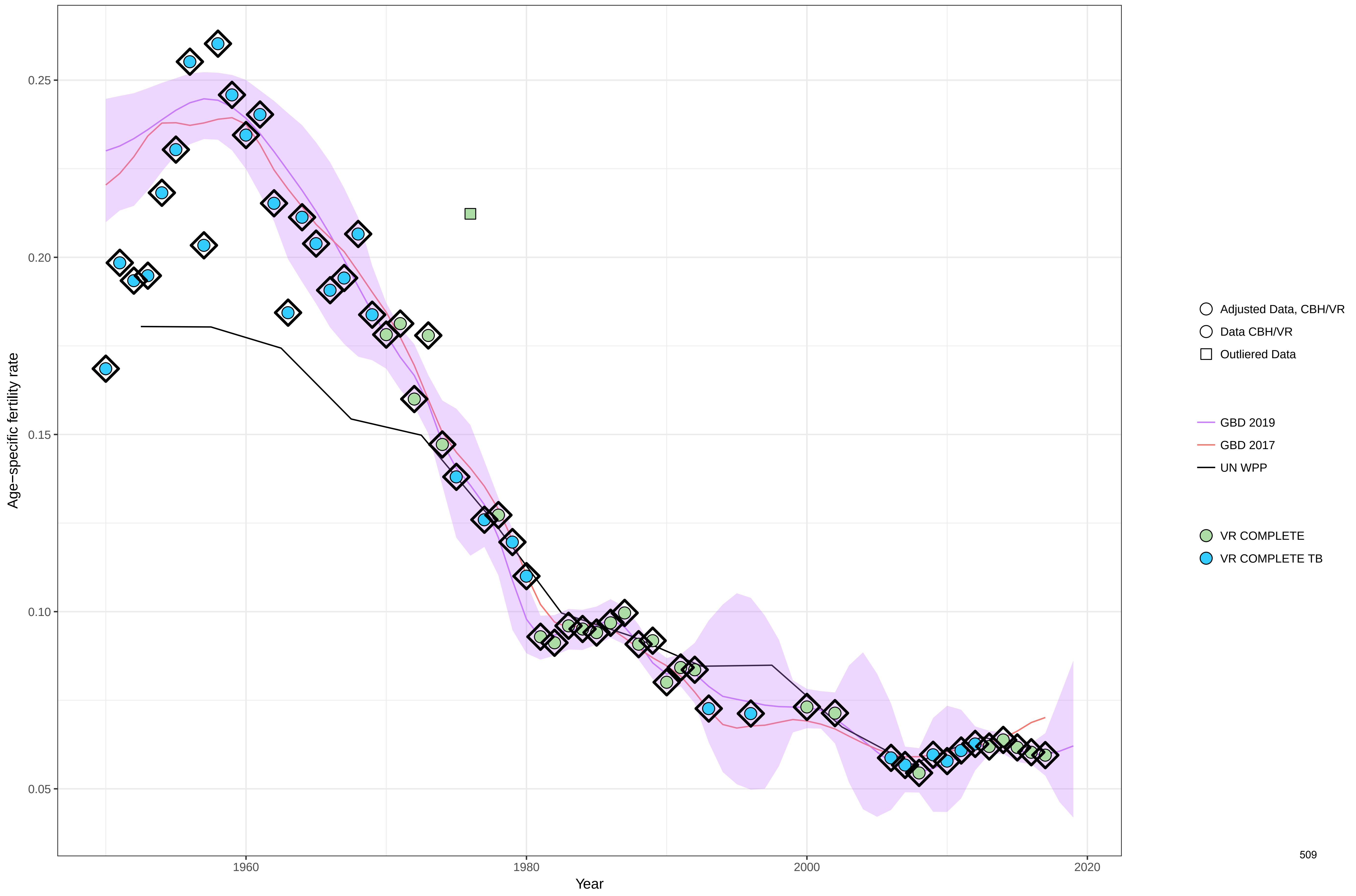

Age−specific fertility fitted trend lines, 1950−2019: Brunei Darussalam, Ages: 40−44
Data points enclosed with a diamond are reference sources

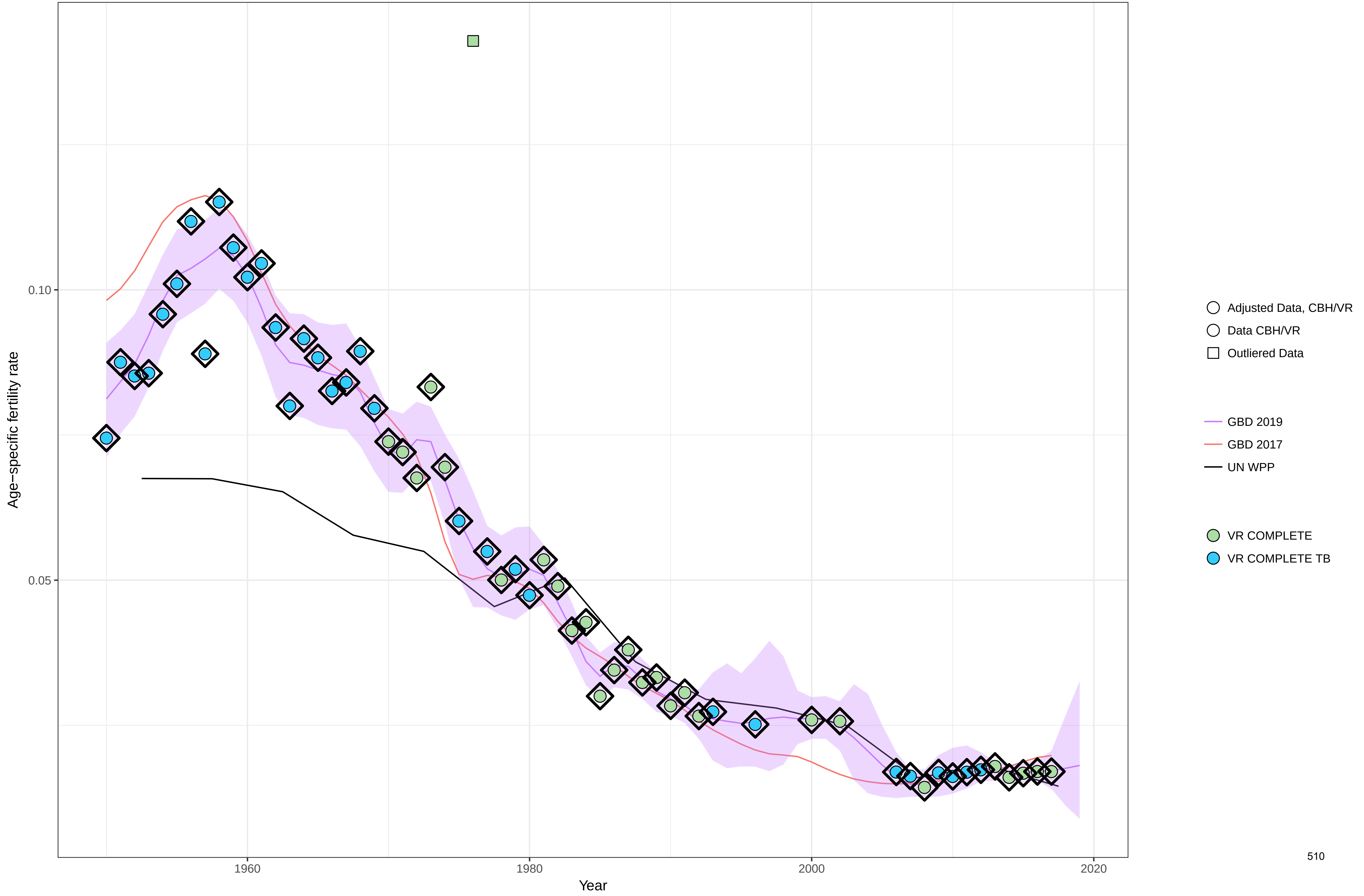

Age−specific fertility fitted trend lines, 1950−2019: Brunei Darussalam, Ages: 45−49
Data points enclosed with a diamond are reference sources

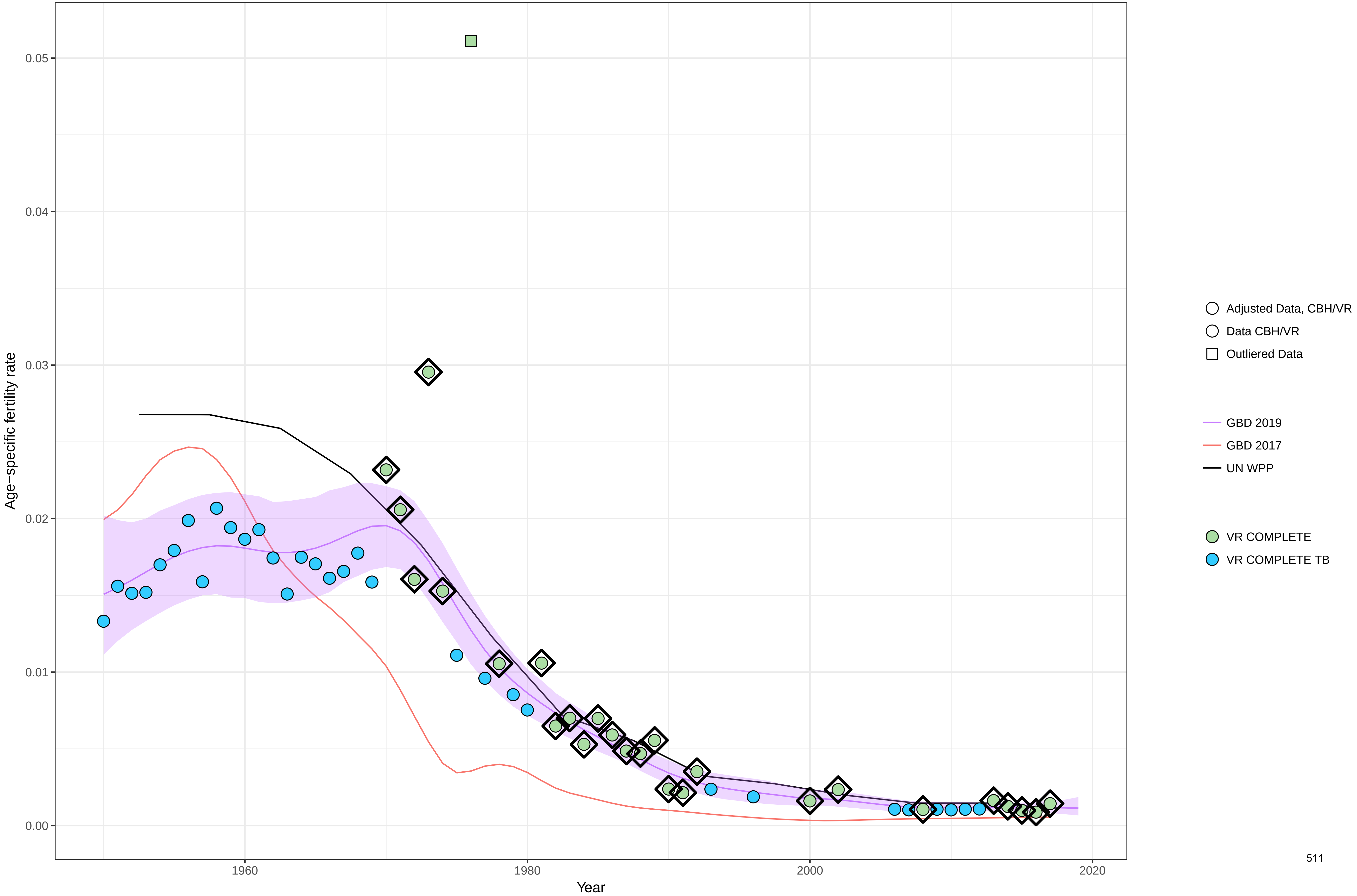

Age−specific fertility fitted trend lines, 1950−2019: Brunei Darussalam, Ages: 50−54
Data points enclosed with a diamond are reference sources

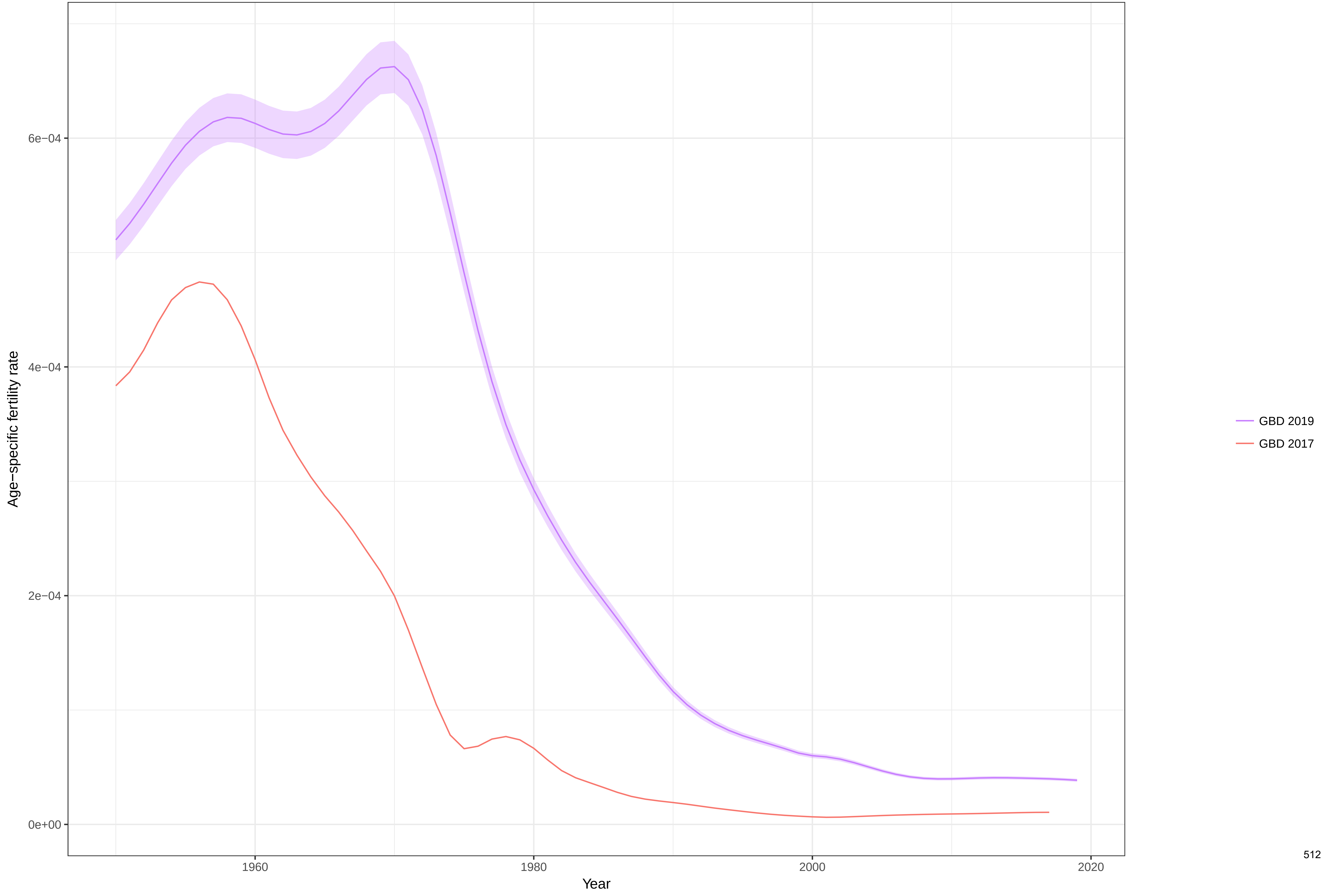

Total fertility rate fitted trend lines, 1950−2019: Brunei Darussalam TFR
Data points enclosed with a diamond are reference sources

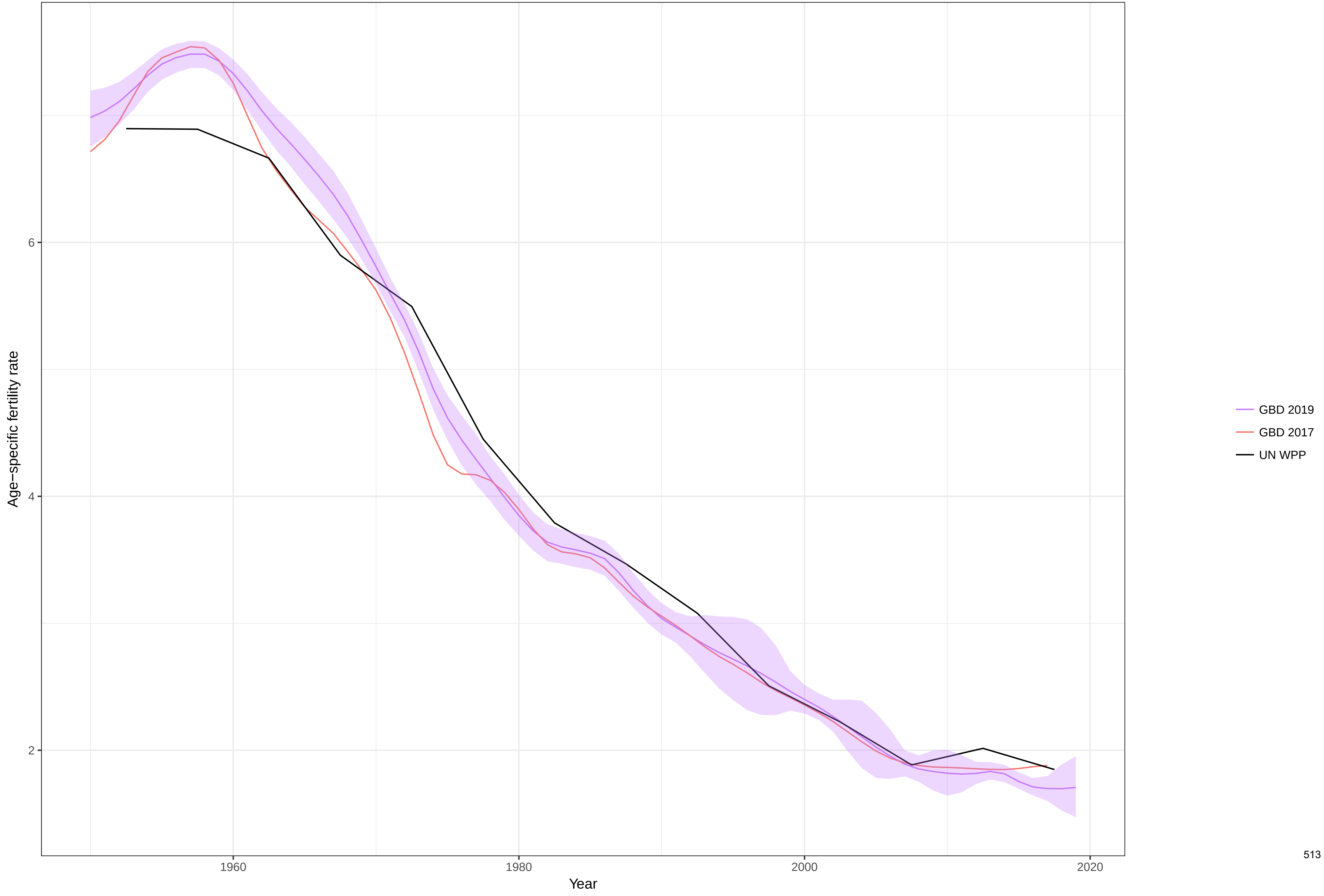

Age−specific fertility fitted trend lines, 1950−2019: Bhutan, Ages: 10−14
Data points enclosed with a diamond are reference sources

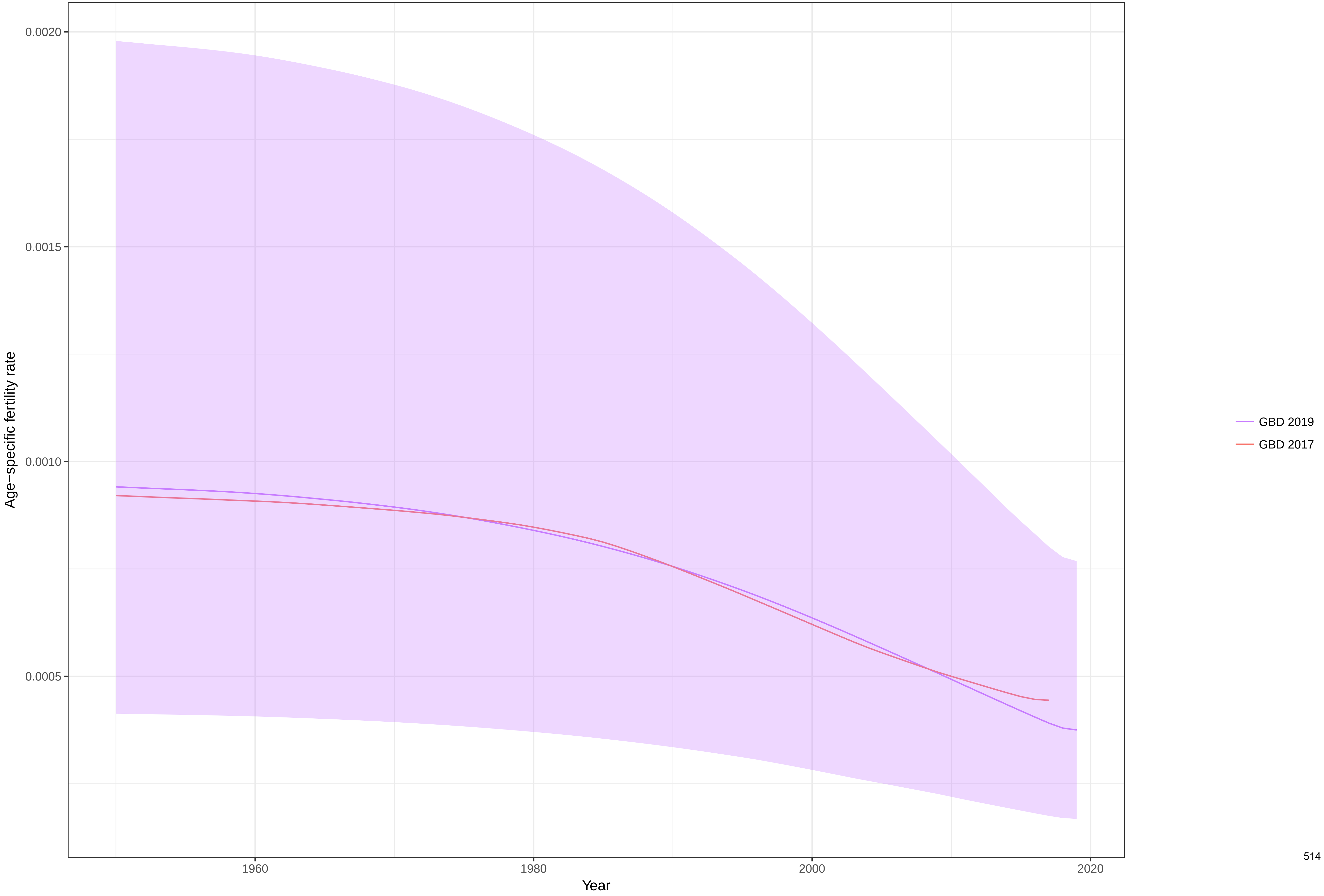

Age−specific fertility fitted trend lines, 1950−2019: Bhutan, Ages: 15−19
Data points enclosed with a diamond are reference sources

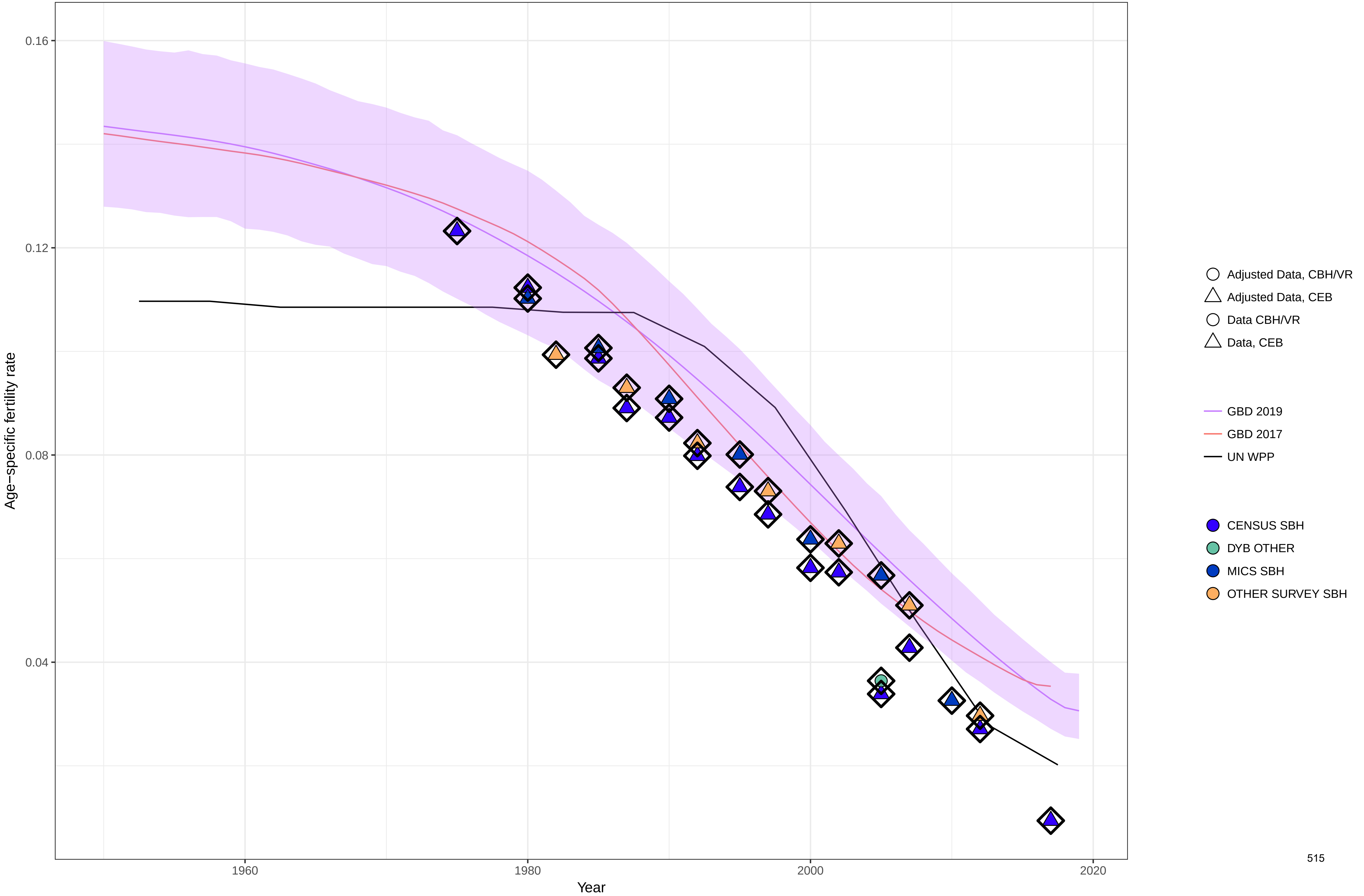

Age−specific fertility fitted trend lines, 1950−2019: Bhutan, Ages: 20−24
Data points enclosed with a diamond are reference sources

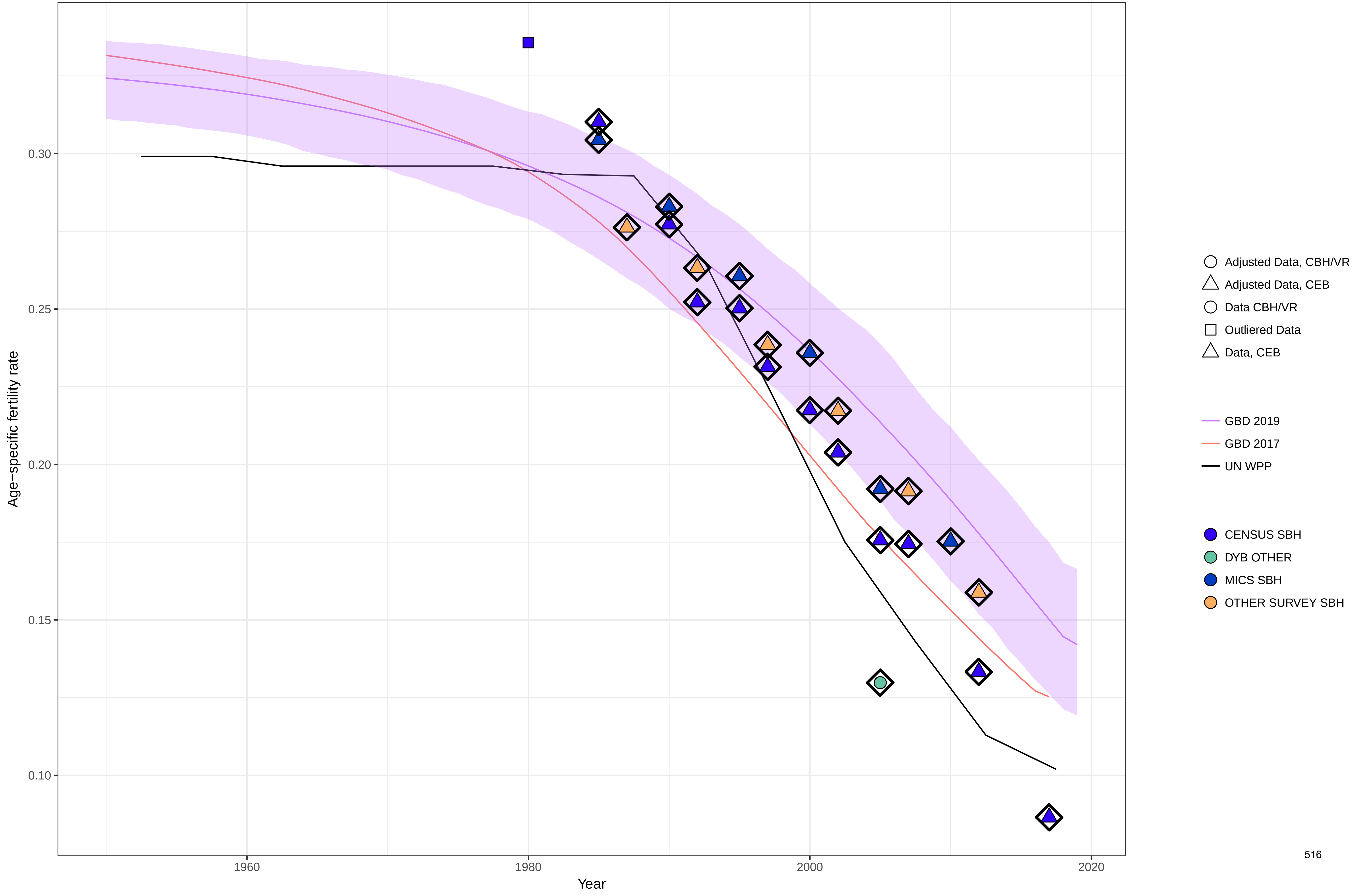

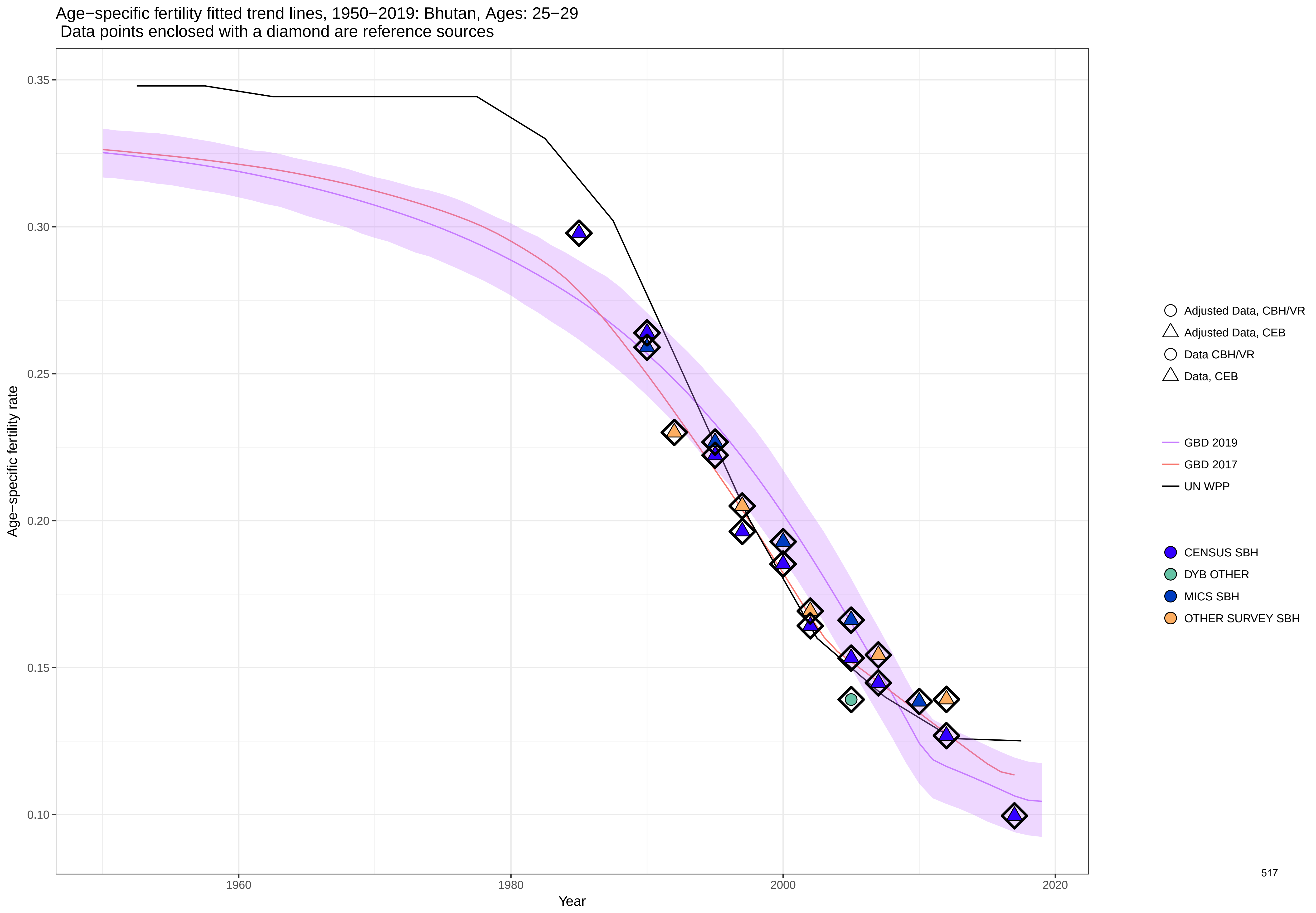

Age−specific fertility fitted trend lines, 1950−2019: Bhutan, Ages: 25−29
Data points enclosed with a diamond are reference sources
Age−specific fertility rate
Year
0.35
0.30
0.25
0.20
0.15
0.10
1960
1980
2000
2020
Adjusted Data, CBH/VR
Adjusted Data, CEB
Data CBH/VR
Data, CEB
GBD 2019
GBD 2017
UN WPP
CENSUS SBH
DYB OTHER
MICS SBH
OTHER SURVEY SBH

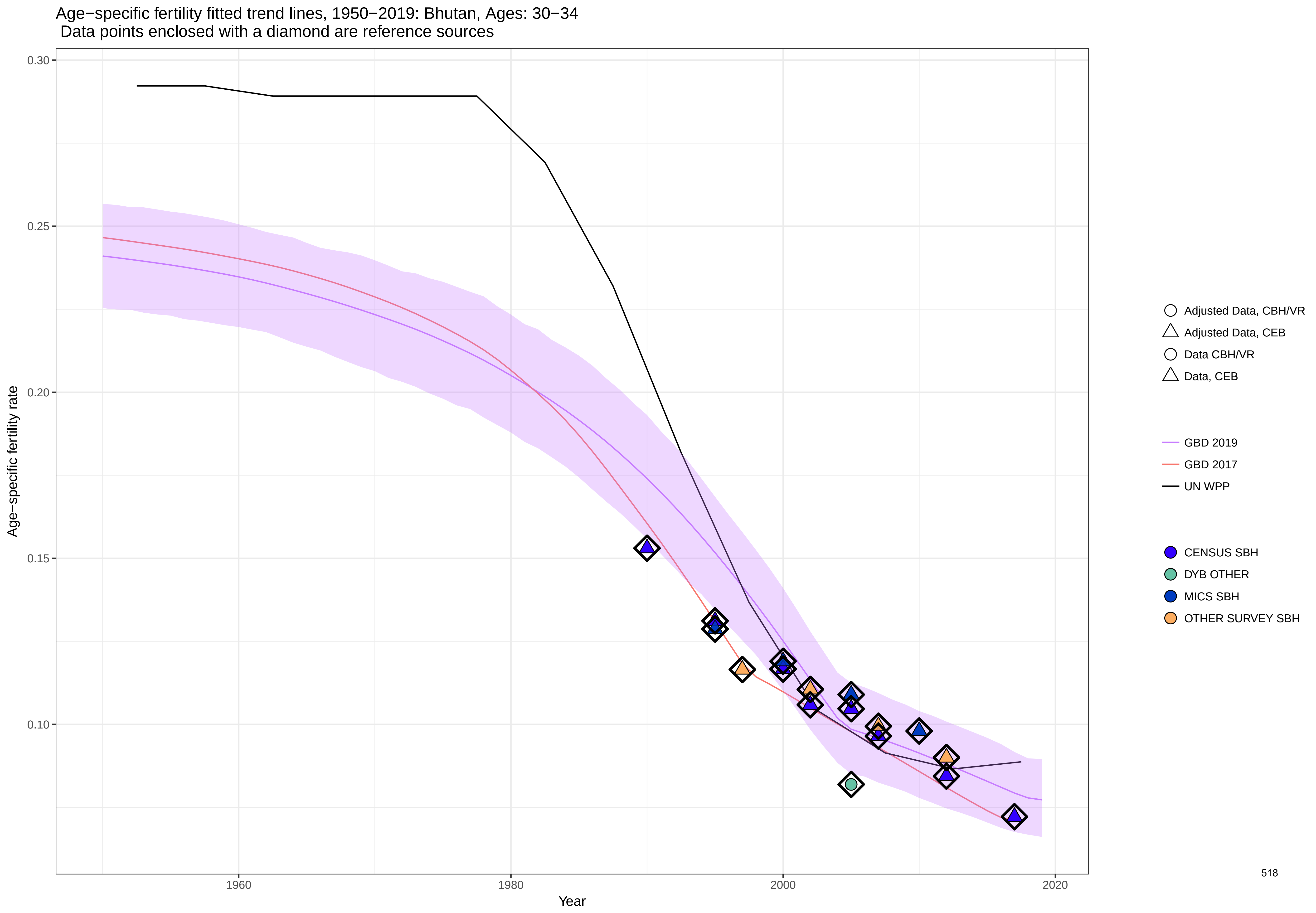
Age−specific fertility fitted trend lines, 1950−2019: Bhutan, Ages: 30−34
Data points enclosed with a diamond are reference sources
Age−specific fertility rate
Year
0.30
0.25
0.20
0.15
0.10
1960
1980
2000
2020
Adjusted Data, CBH/VR
Adjusted Data, CEB
Data CBH/VR
Data, CEB
GBD 2019
GBD 2017
UN WPP
CENSUS SBH
DYB OTHER
MICS SBH
OTHER SURVEY SBH

Age−specific fertility fitted trend lines, 1950−2019: Bhutan, Ages: 35−39
Data points enclosed with a diamond are reference sources

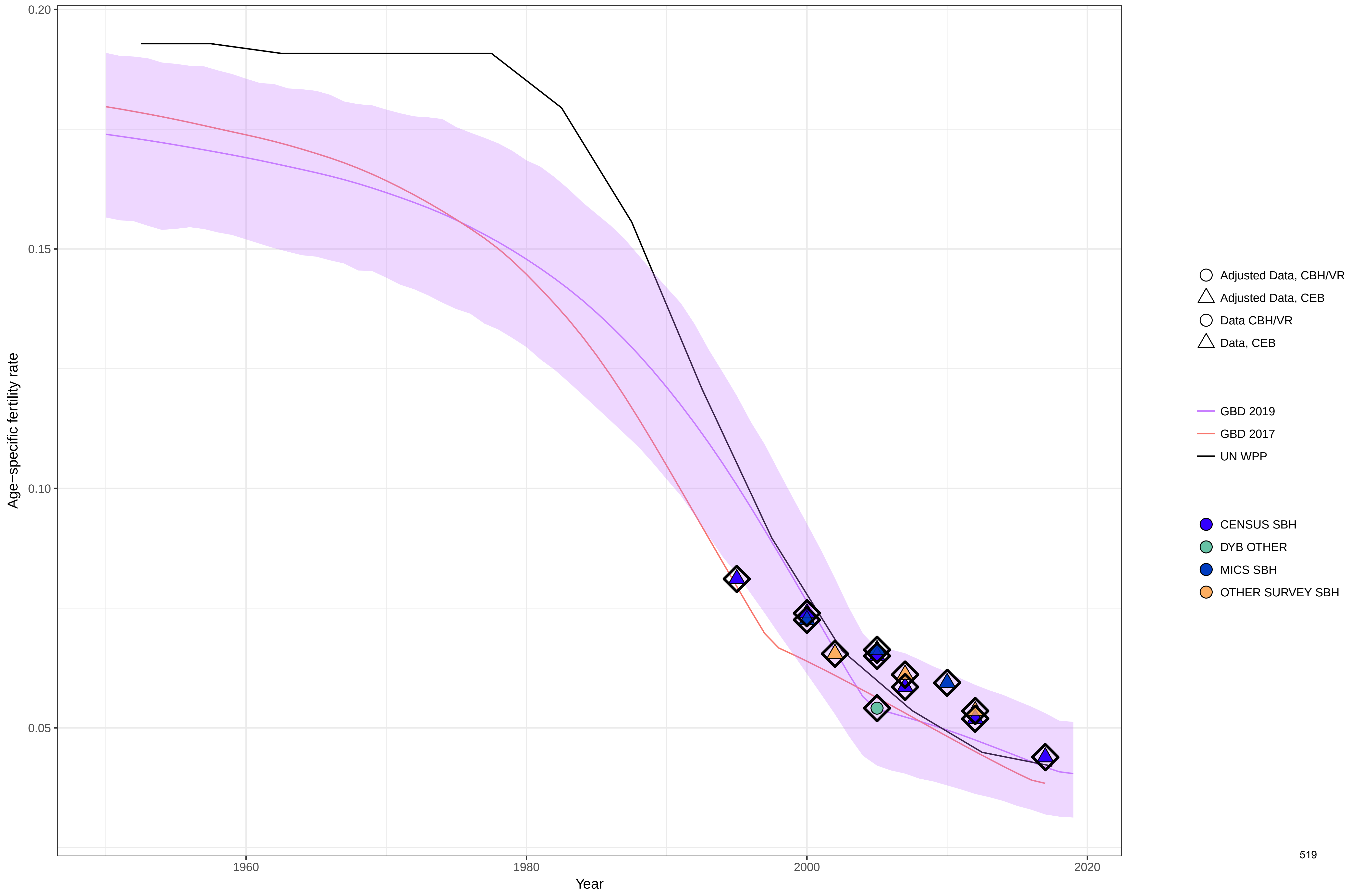

Age−specific fertility fitted trend lines, 1950−2019: Bhutan, Ages: 40−44
Data points enclosed with a diamond are reference sources

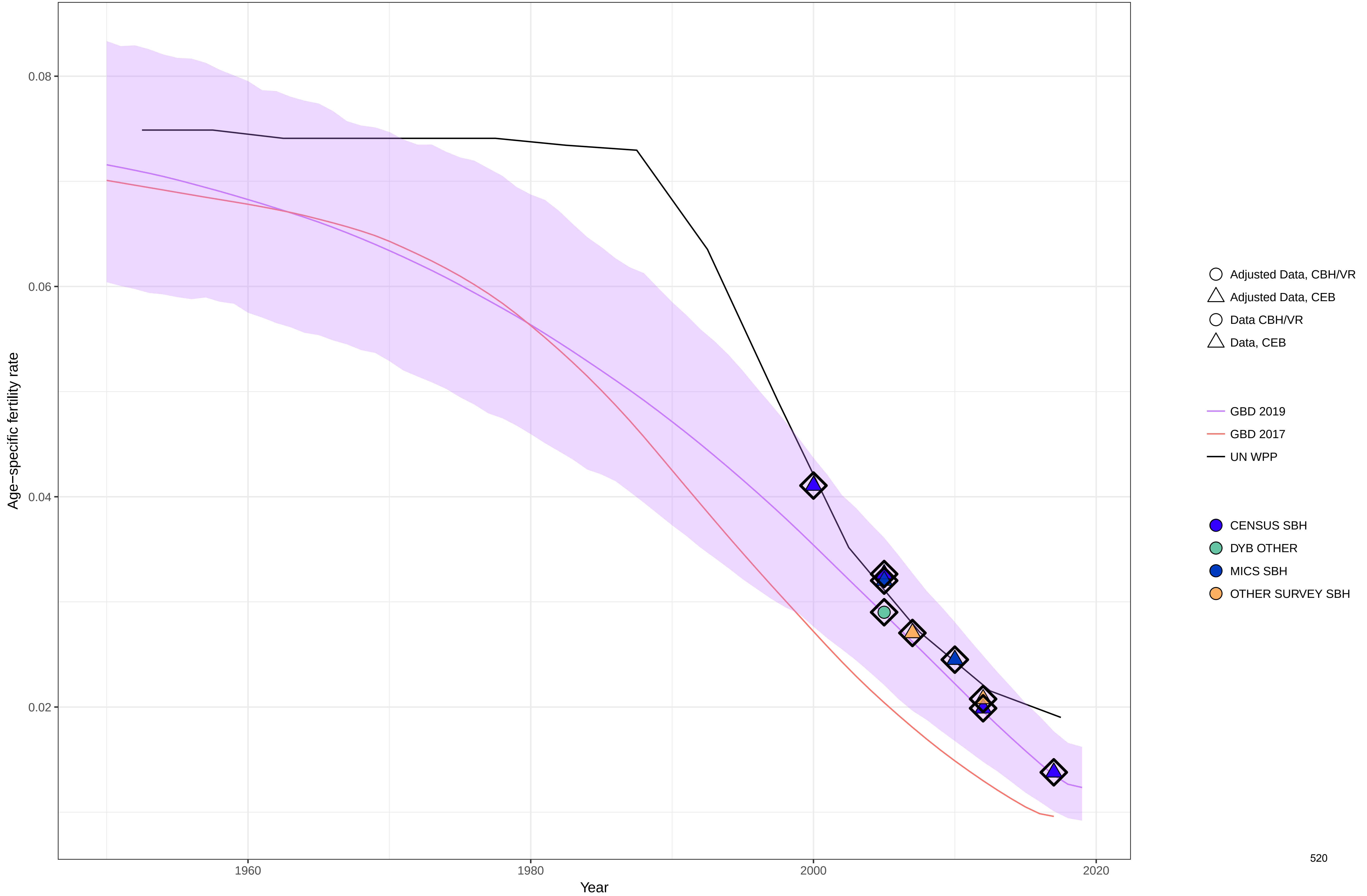

Age−specific fertility fitted trend lines, 1950−2019: Bhutan, Ages: 45−49
Data points enclosed with a diamond are reference sources

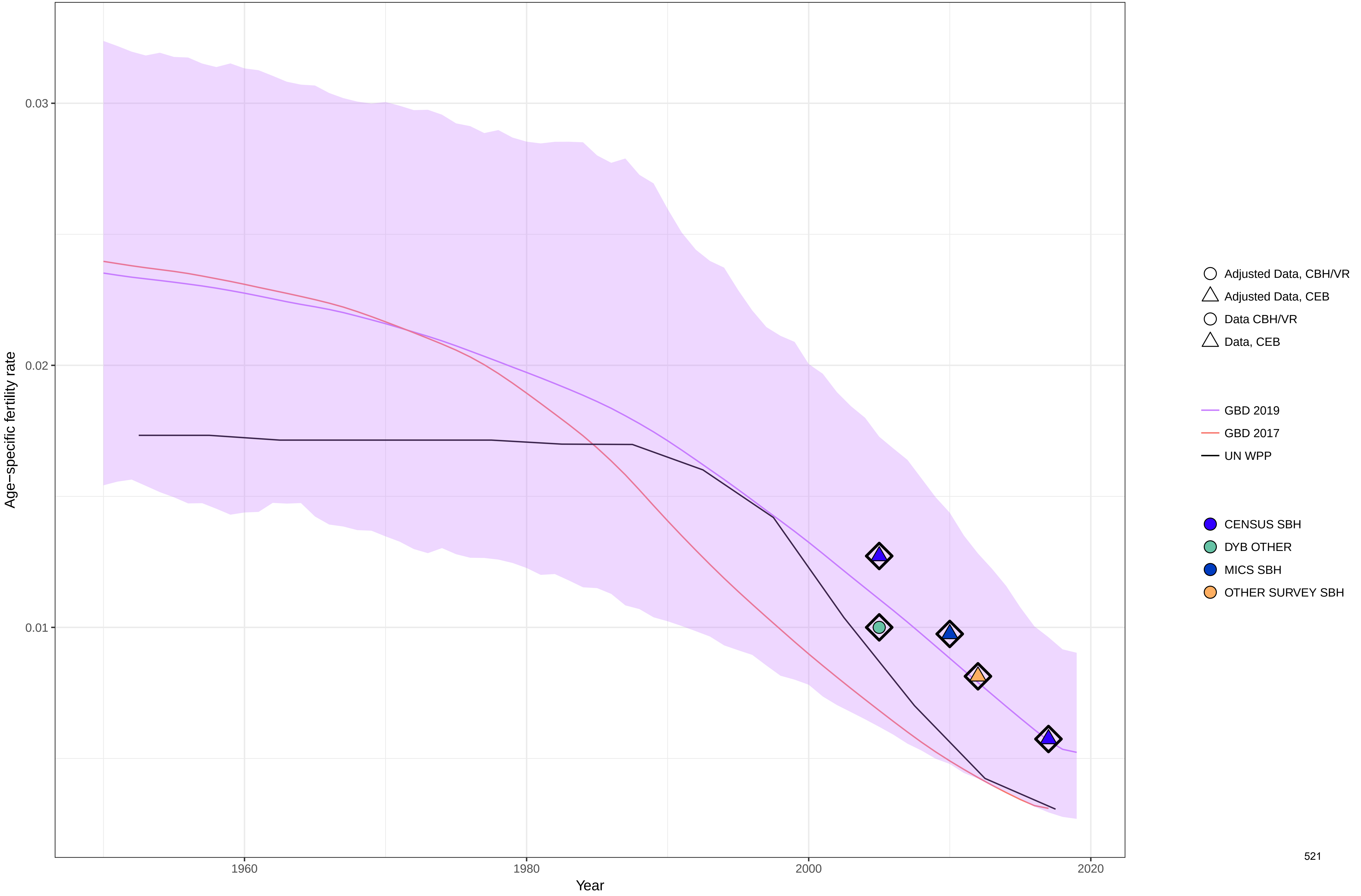

Age−specific fertility fitted trend lines, 1950−2019: Bhutan, Ages: 50−54
Data points enclosed with a diamond are reference sources

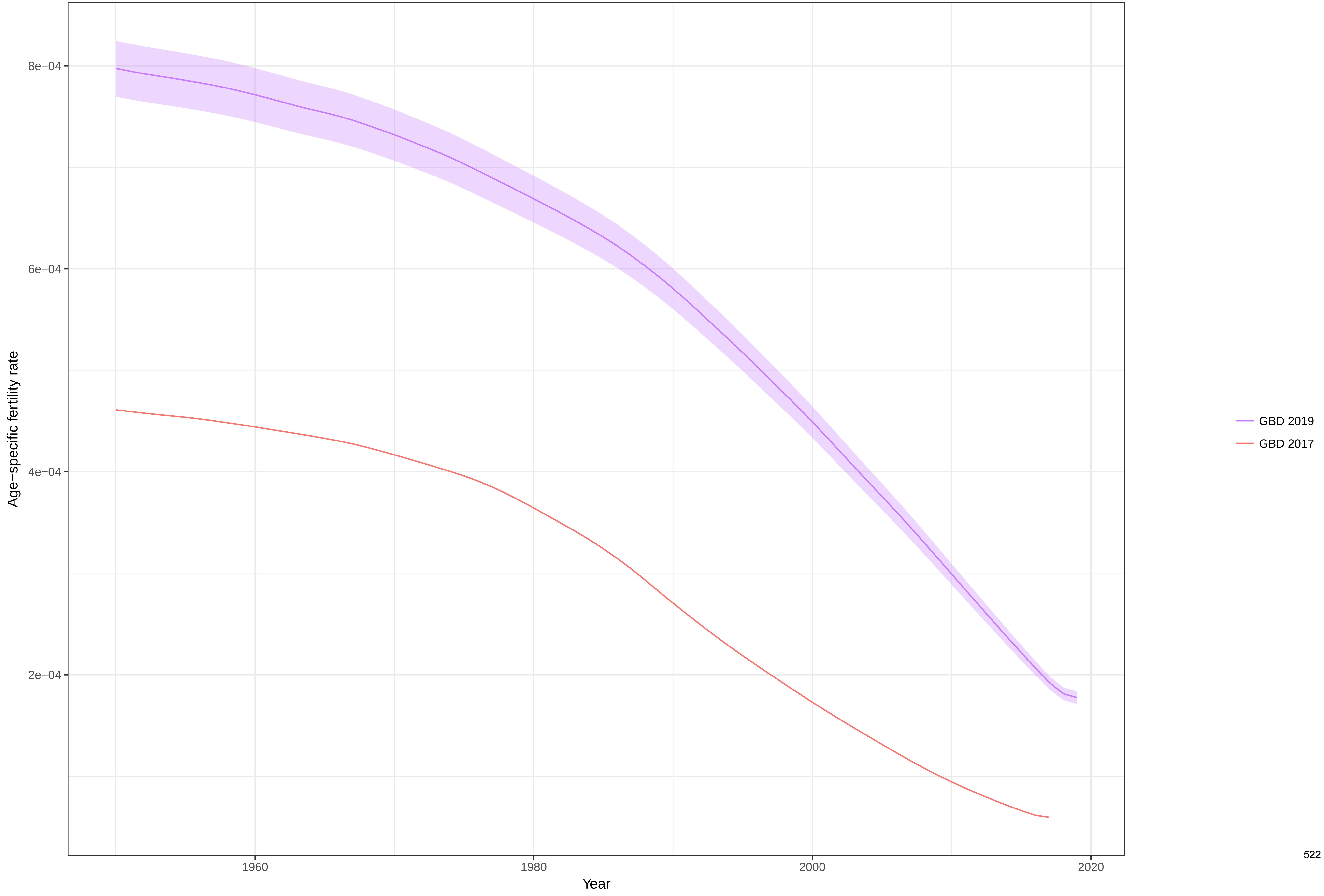

Total fertility rate fitted trend lines, 1950−2019: Bhutan TFR
Data points enclosed with a diamond are reference sources

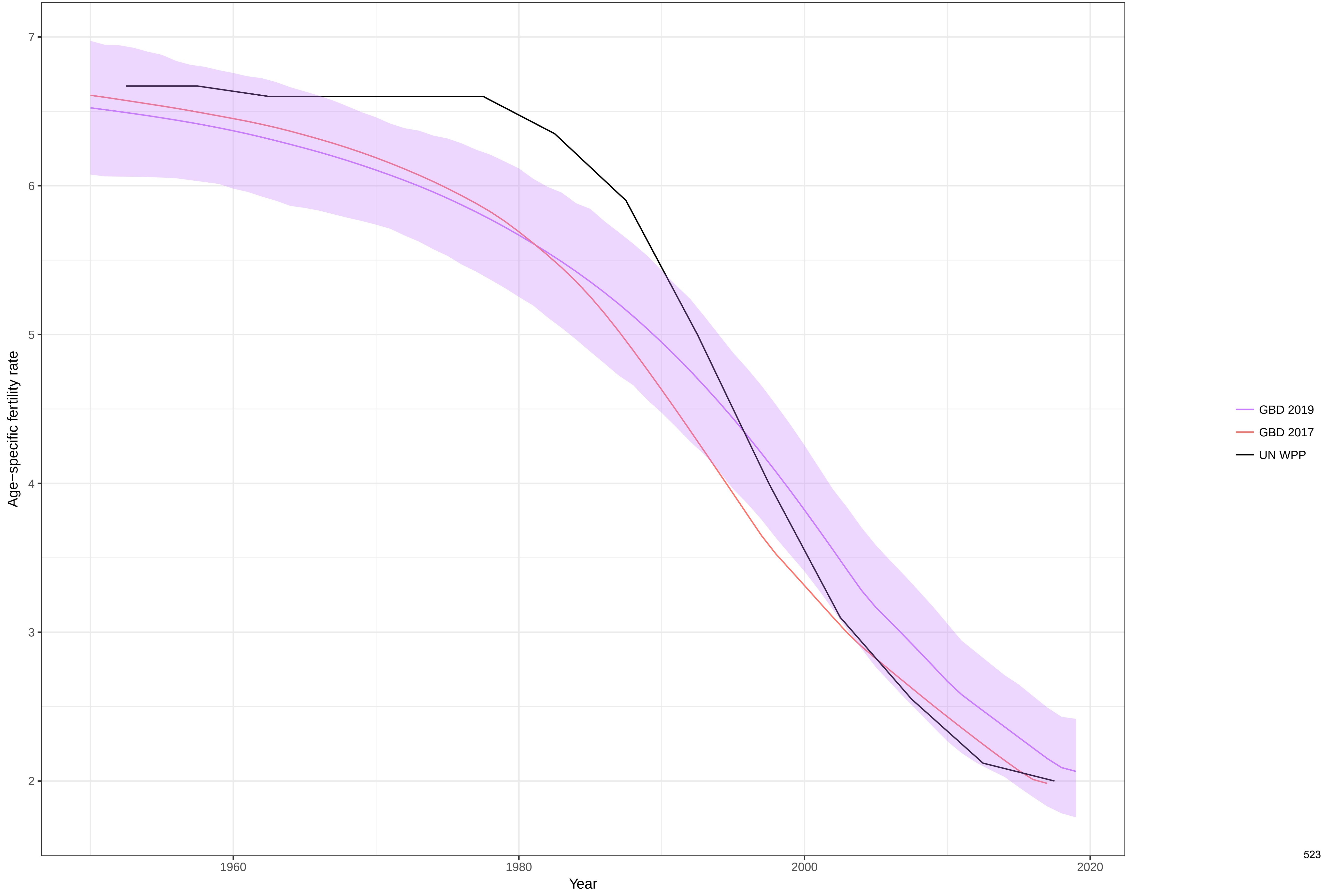

Age−specific fertility fitted trend lines, 1950−2019: Botswana, Ages: 10−14
Data points enclosed with a diamond are reference sources

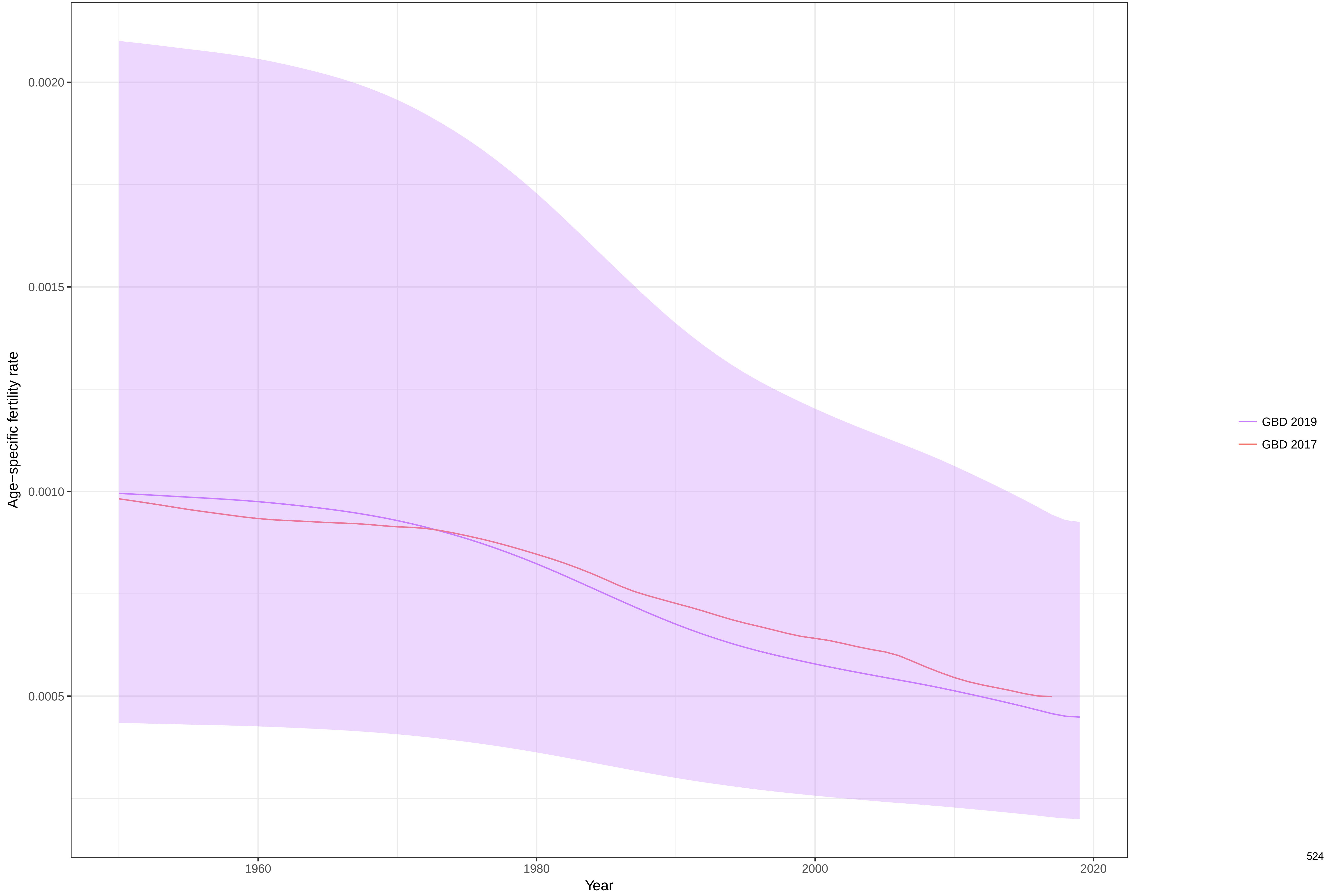

Age−specific fertility fitted trend lines, 1950−2019: Botswana, Ages: 15−19
Data points enclosed with a diamond are reference sources

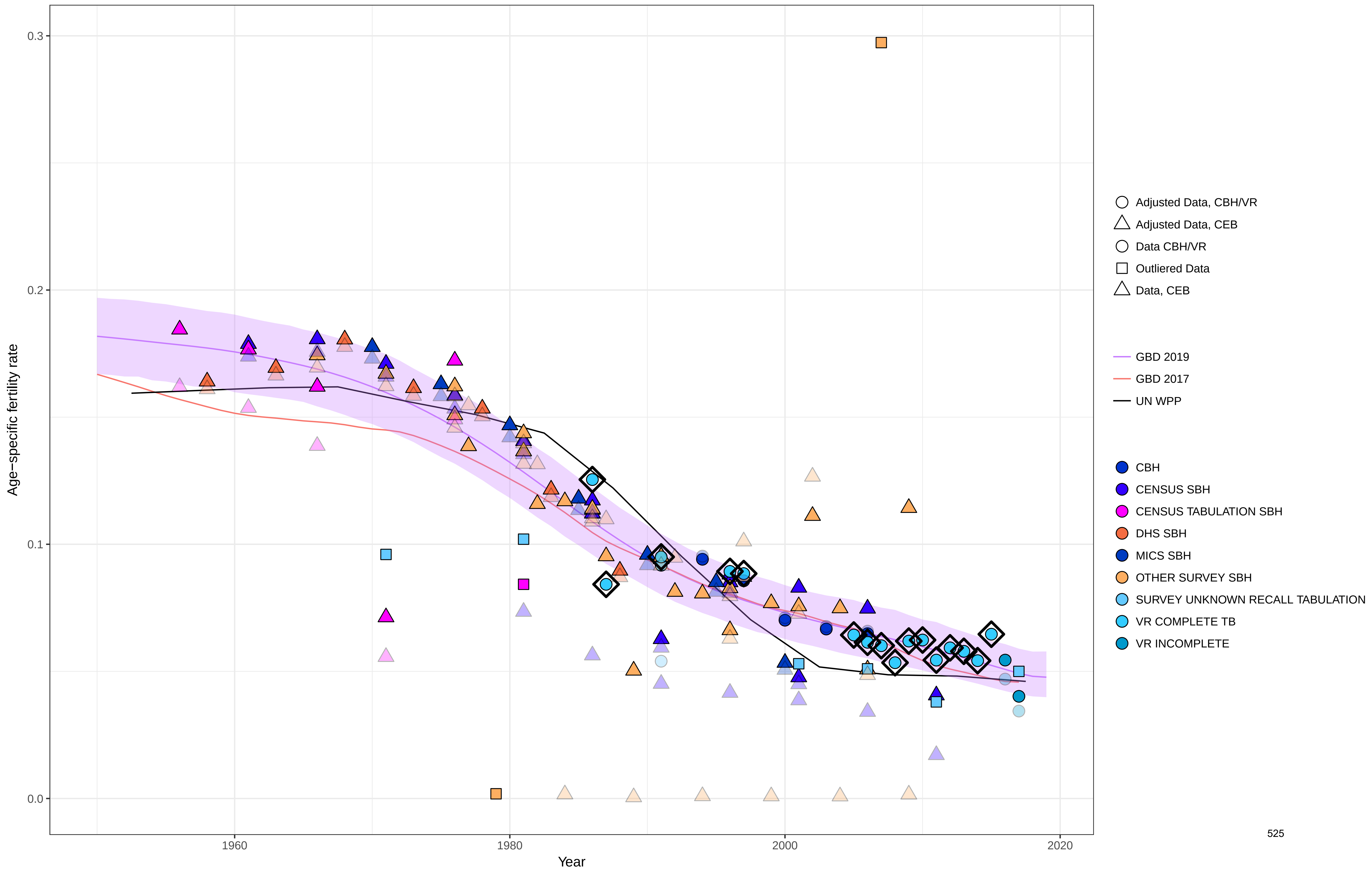

Age−specific fertility fitted trend lines, 1950−2019: Botswana, Ages: 20−24
Data points enclosed with a diamond are reference sources

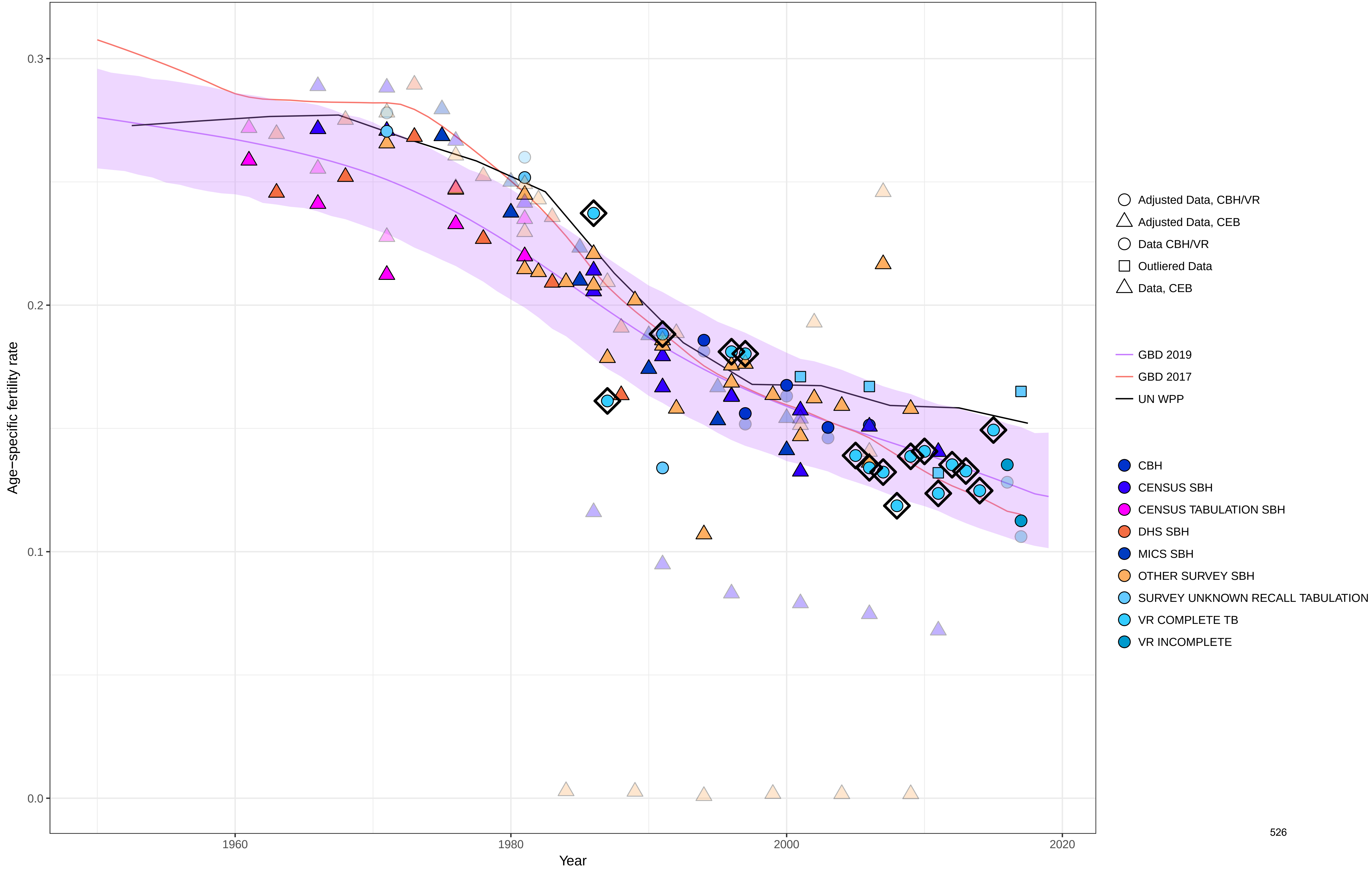

Age−specific fertility fitted trend lines, 1950−2019: Botswana, Ages: 25−29
Data points enclosed with a diamond are reference sources

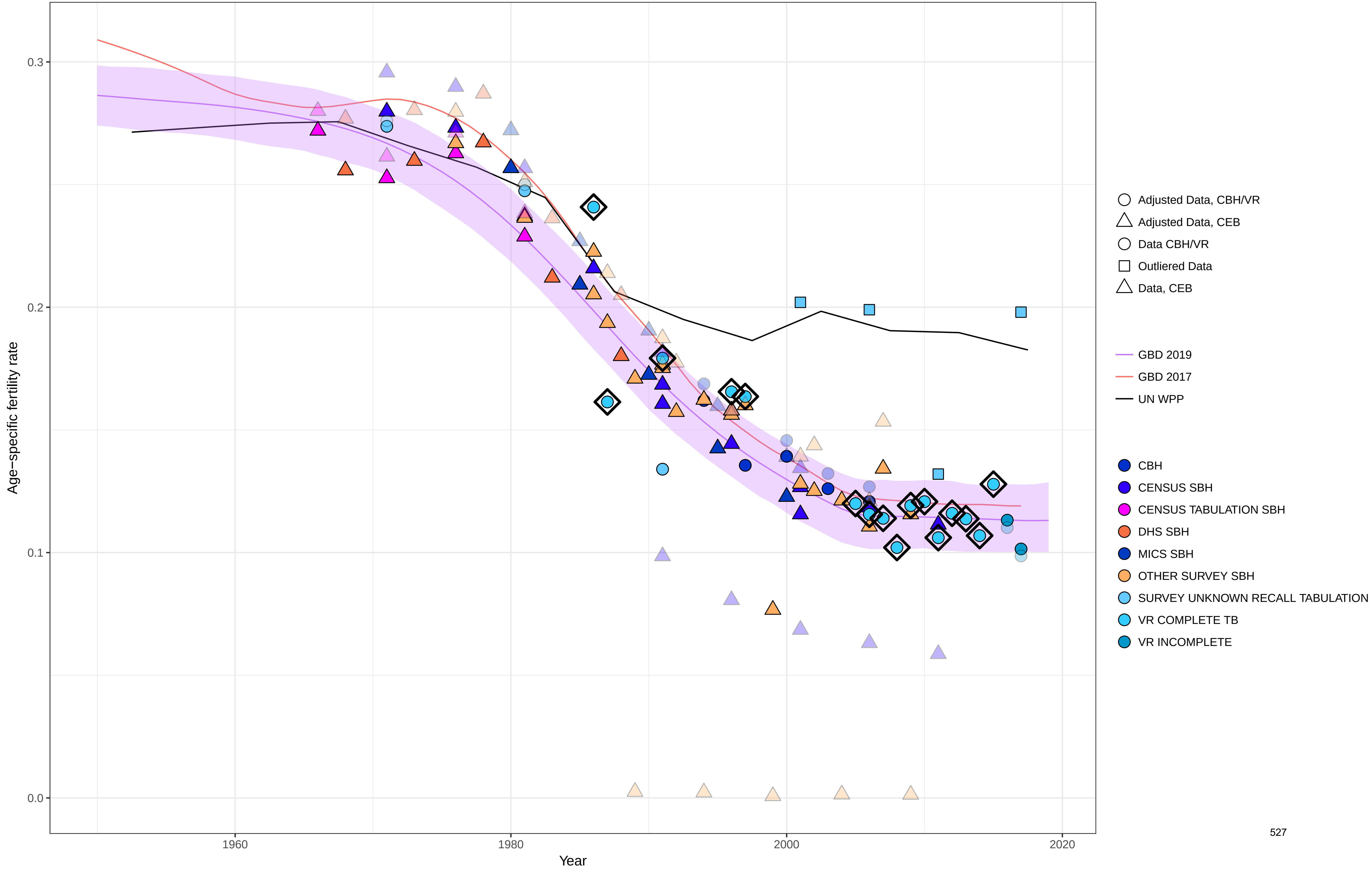

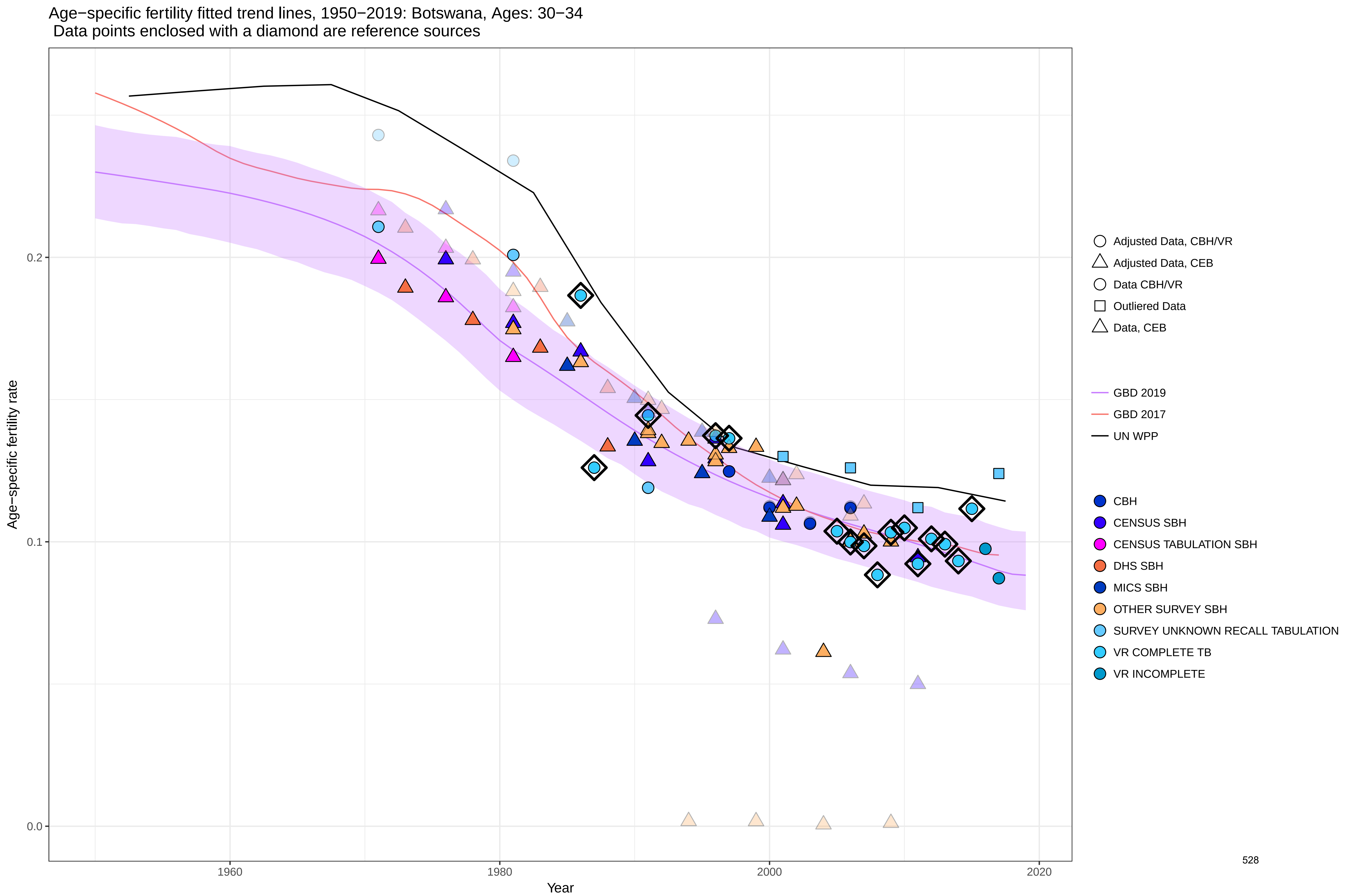
Age−specific fertility fitted trend lines, 1950−2019: Botswana, Ages: 30−34
Data points enclosed with a diamond are reference sources
Age−specific fertility rate
Year
0.0
0.1
0.2
1960
1980
2000
2020
Adjusted Data, CBH/VR
Adjusted Data, CEB
Data CBH/VR
Outliered Data
Data, CEB
GBD 2019
GBD 2017
UN WPP
CBH
CENSUS SBH
CENSUS TABULATION SBH
DHS SBH
MICS SBH
OTHER SURVEY SBH
SURVEY UNKNOWN RECALL TABULATION
VR COMPLETE TB
VR INCOMPLETE

Age−specific fertility fitted trend lines, 1950−2019: Botswana, Ages: 35−39
Data points enclosed with a diamond are reference sources

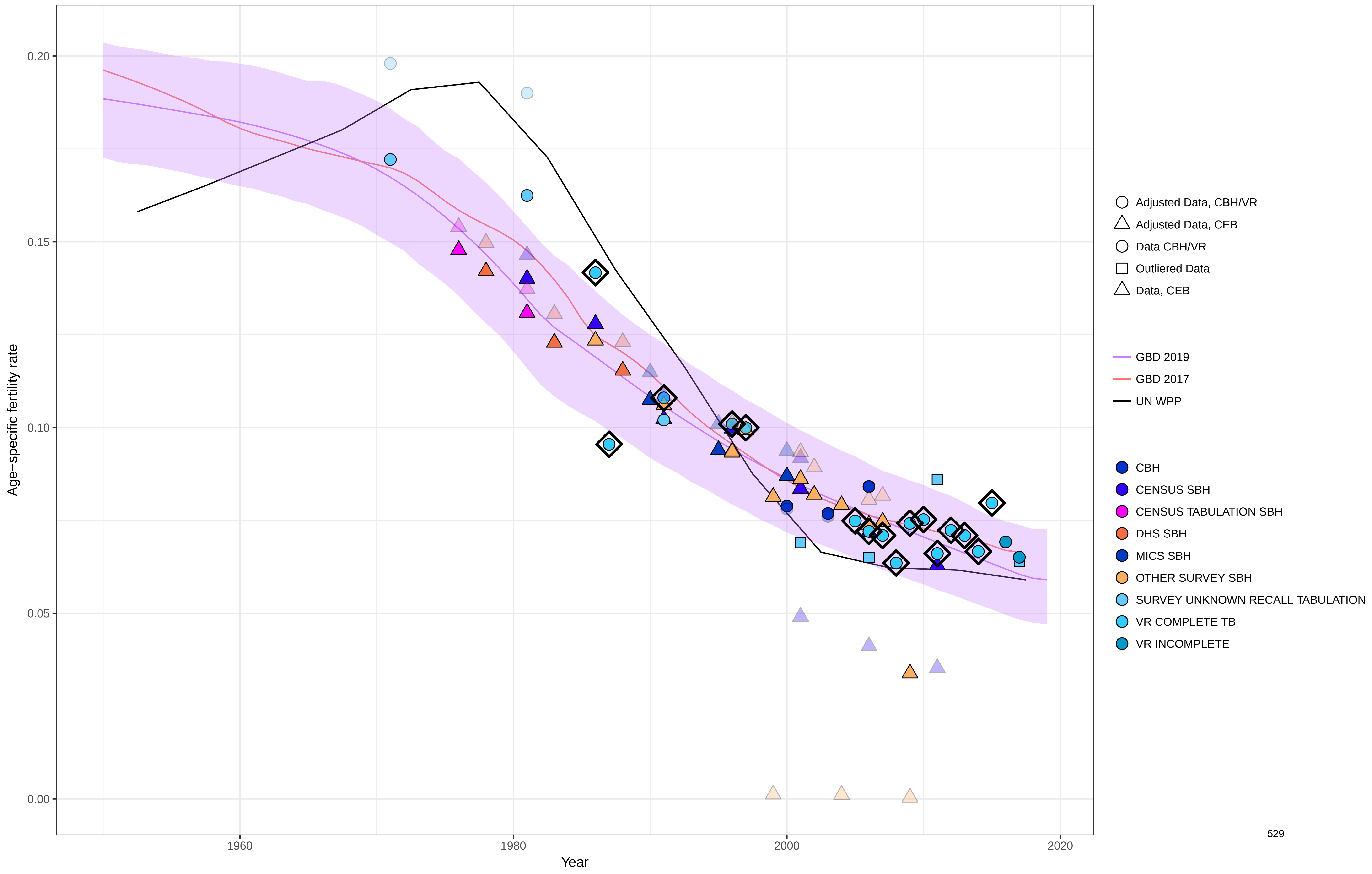

Age−specific fertility fitted trend lines, 1950−2019: Botswana, Ages: 40−44
Data points enclosed with a diamond are reference sources

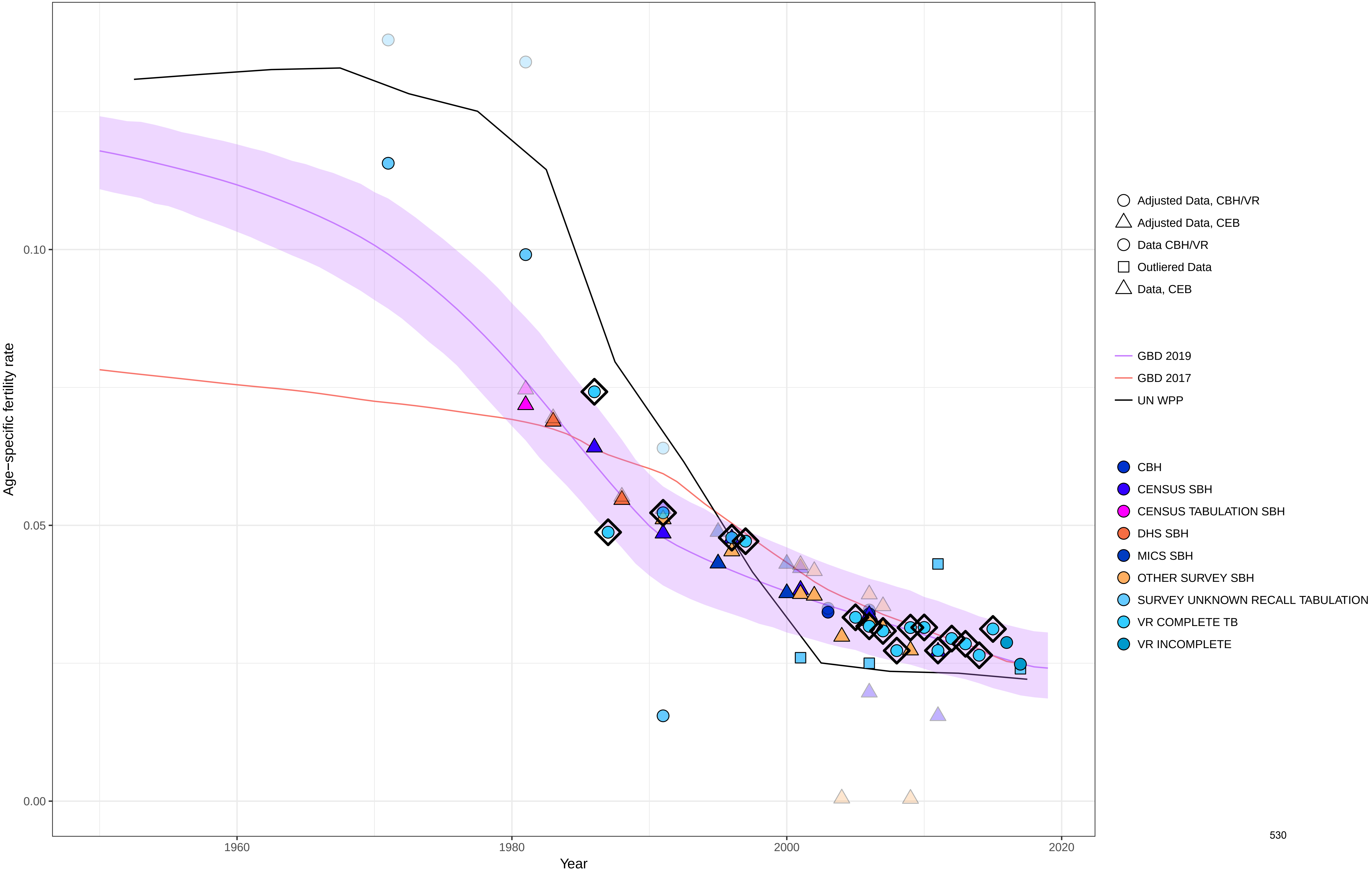

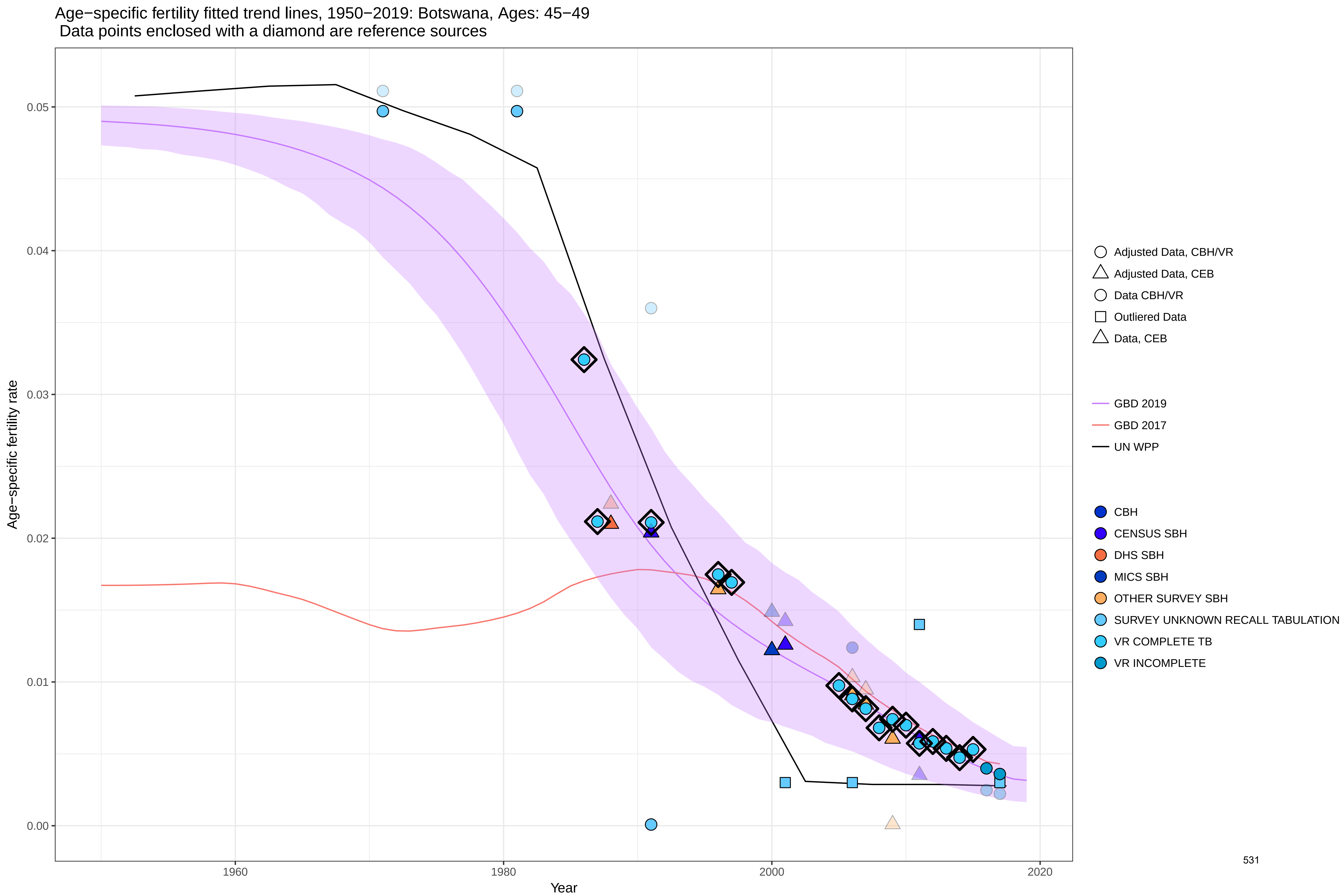
Age−specific fertility fitted trend lines, 1950−2019: Botswana, Ages: 45−49
Data points enclosed with a diamond are reference sources
Age−specific fertility rate
Year
0.00
0.01
0.02
0.03
0.04
0.05
1960
1980
2000
2020
Adjusted Data, CBH/VR
Adjusted Data, CEB
Data CBH/VR
Outliered Data
Data, CEB
GBD 2019
GBD 2017
UN WPP
CBH
CENSUS SBH
DHS SBH
MICS SBH
OTHER SURVEY SBH
SURVEY UNKNOWN RECALL TABULATION
VR COMPLETE TB
VR INCOMPLETE

Age−specific fertility fitted trend lines, 1950−2019: Botswana, Ages: 50−54
Data points enclosed with a diamond are reference sources

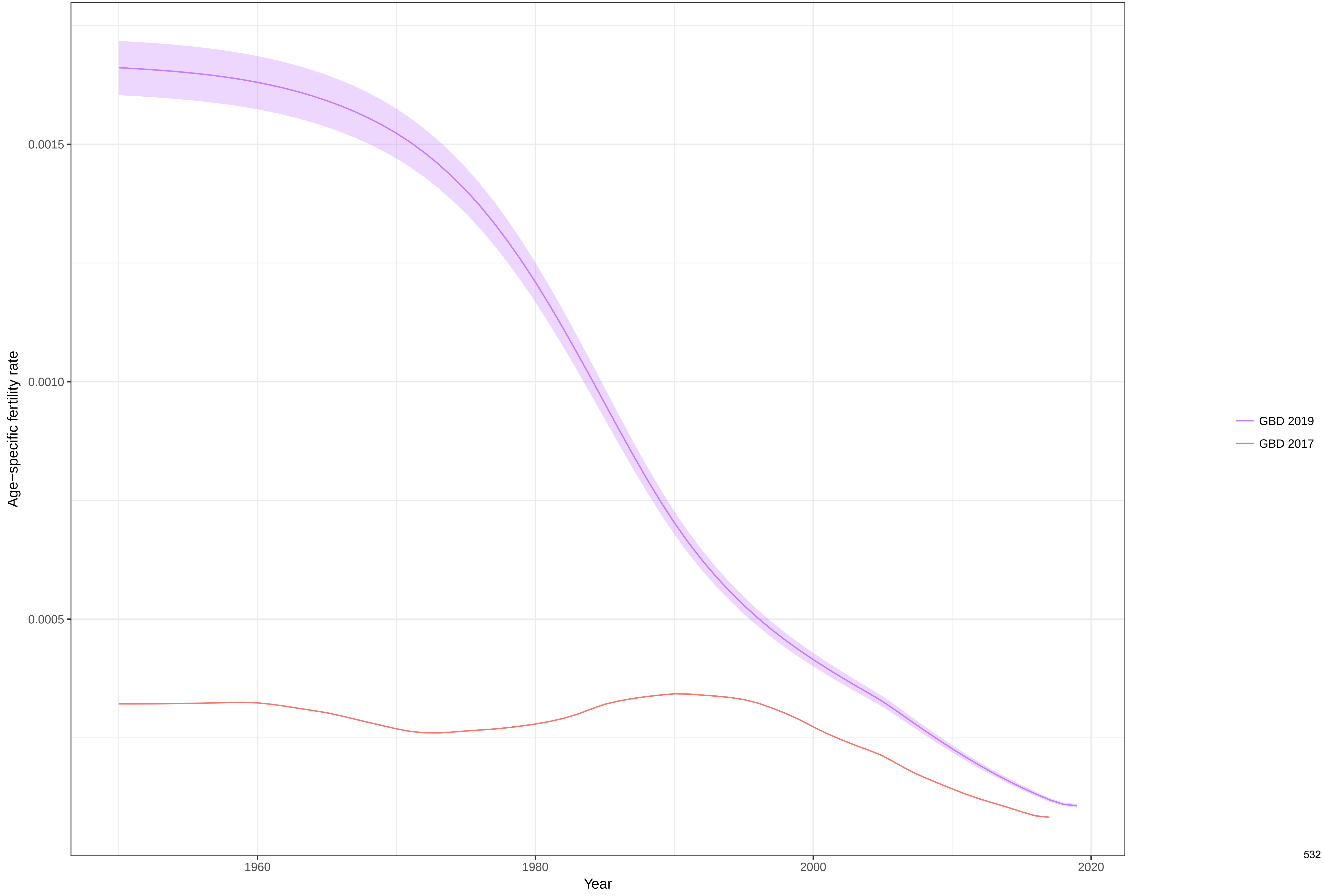

Total fertility rate fitted trend lines, 1950−2019: Botswana TFR
Data points enclosed with a diamond are reference sources

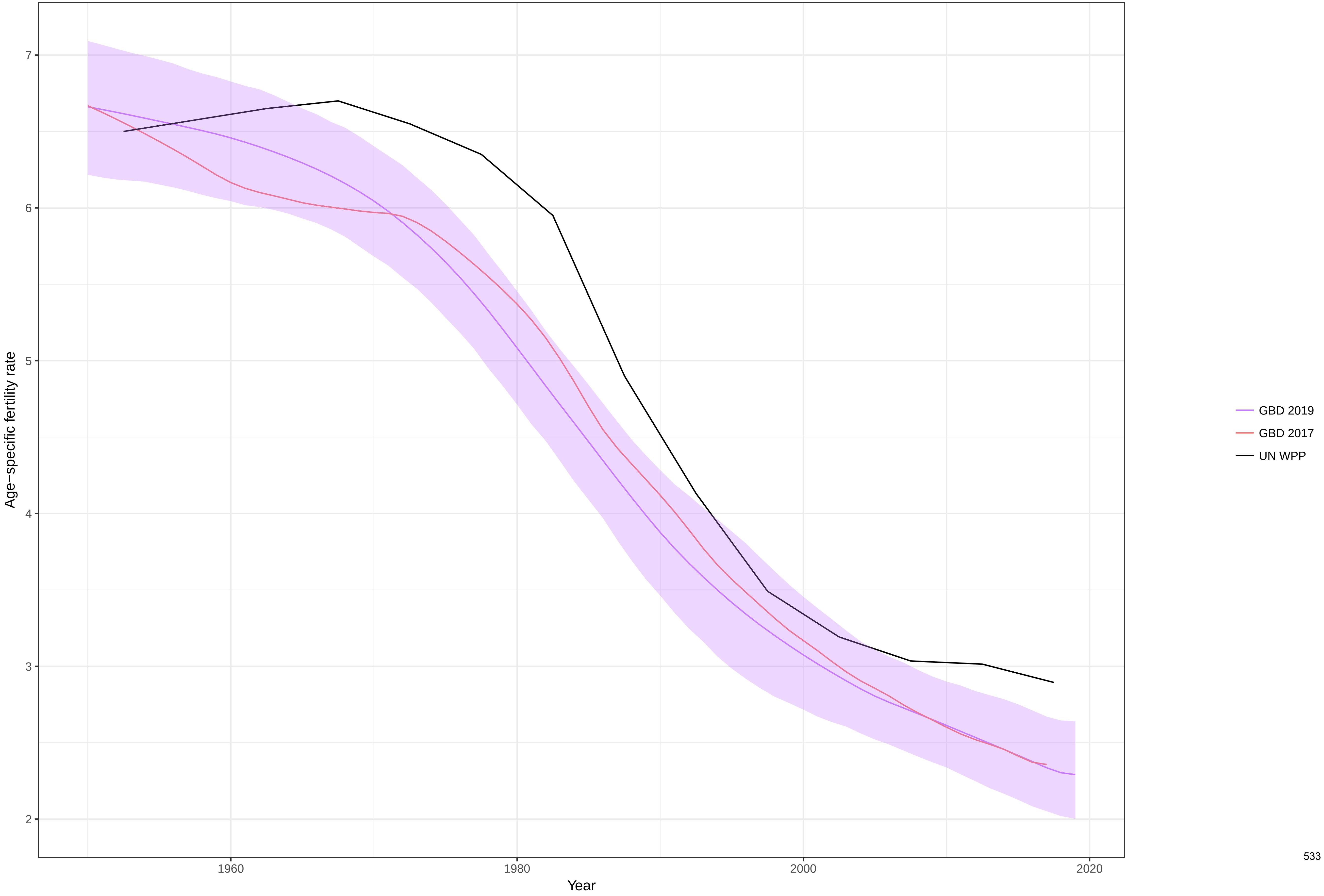

Age−specific fertility fitted trend lines, 1950−2019: Central African Republic, Ages: 10−14
Data points enclosed with a diamond are reference sources

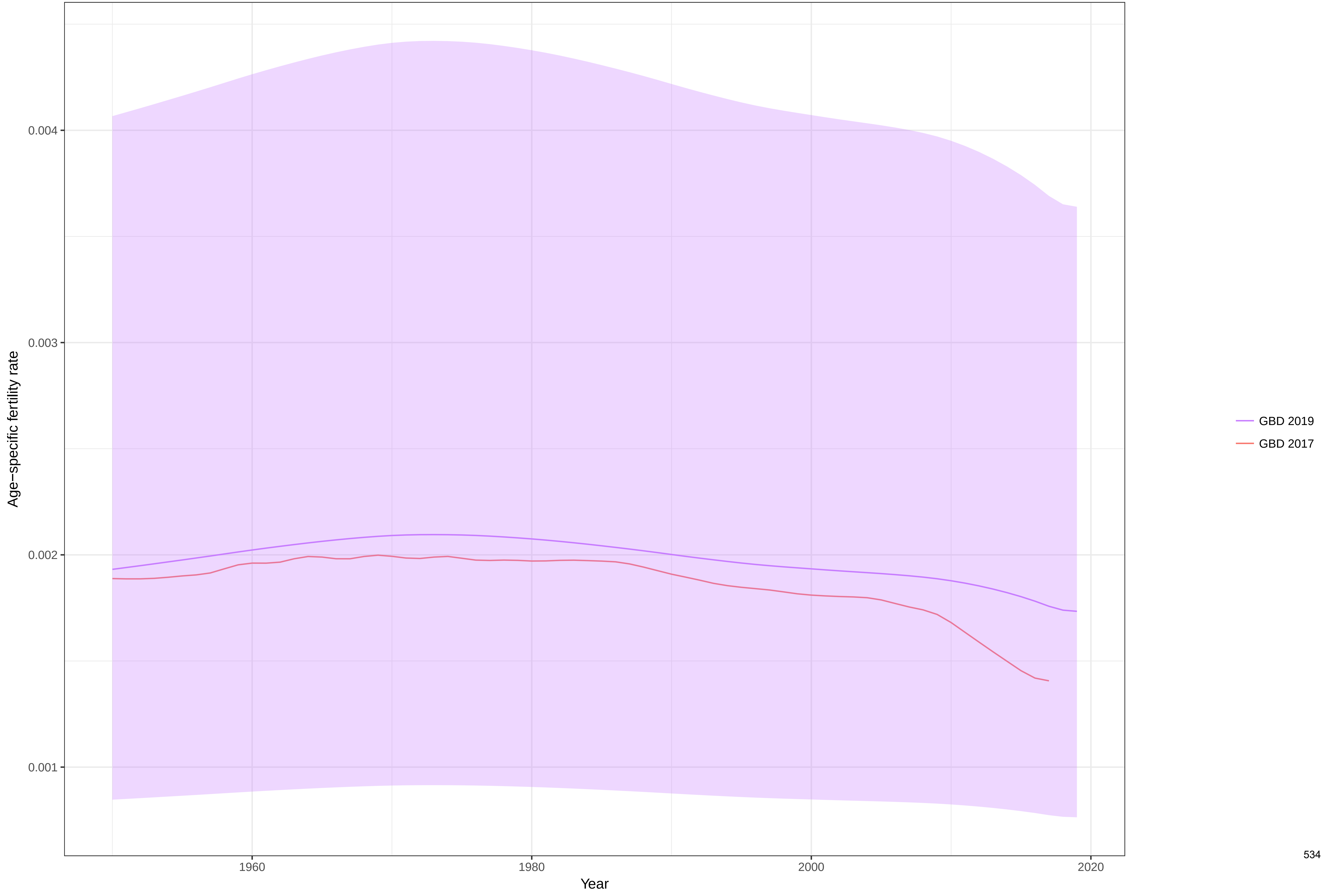

Age−specific fertility fitted trend lines, 1950−2019: Central African Republic, Ages: 15−19
Data points enclosed with a diamond are reference sources

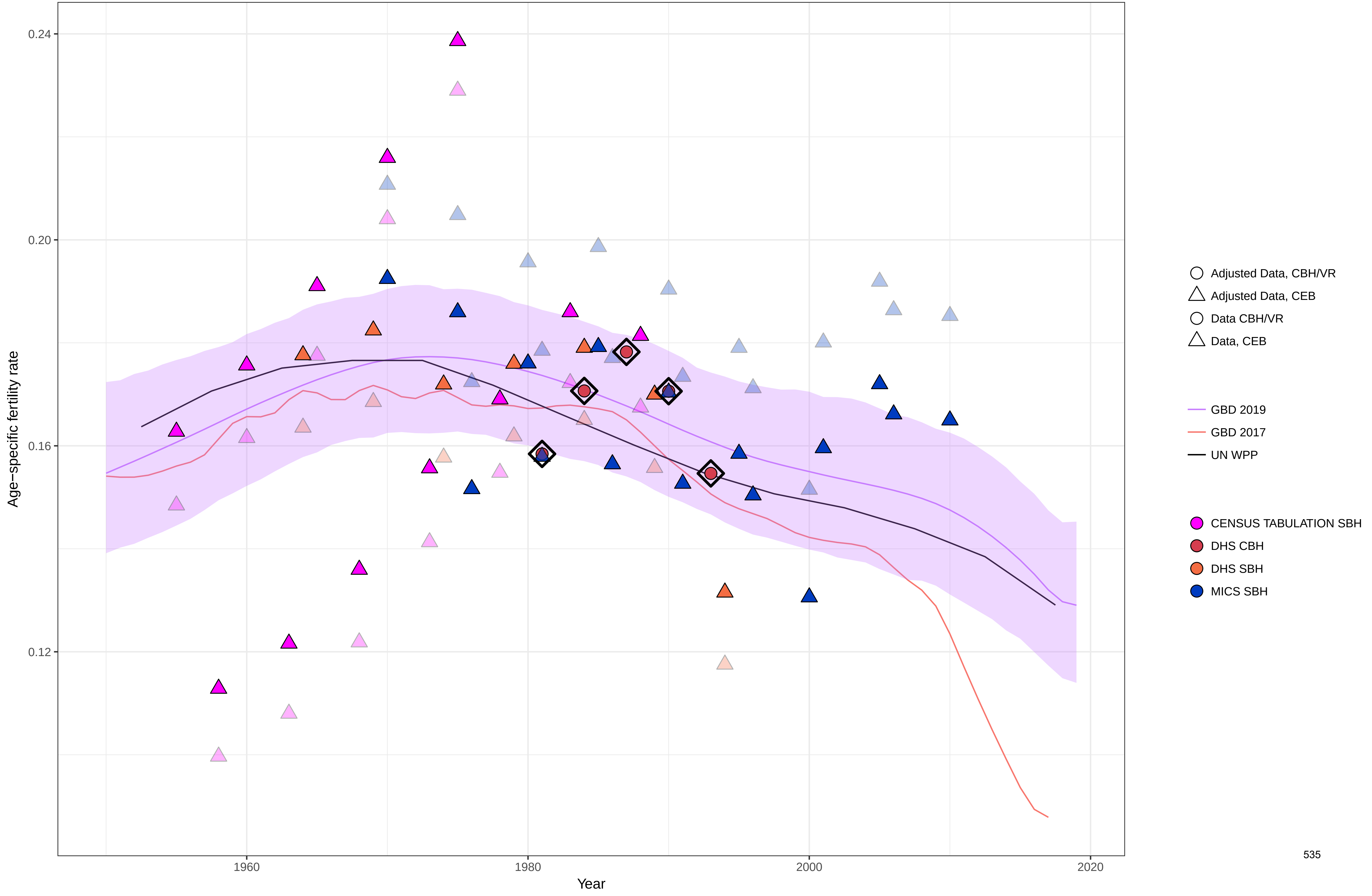

Age−specific fertility fitted trend lines, 1950−2019: Central African Republic, Ages: 20−24
Data points enclosed with a diamond are reference sources

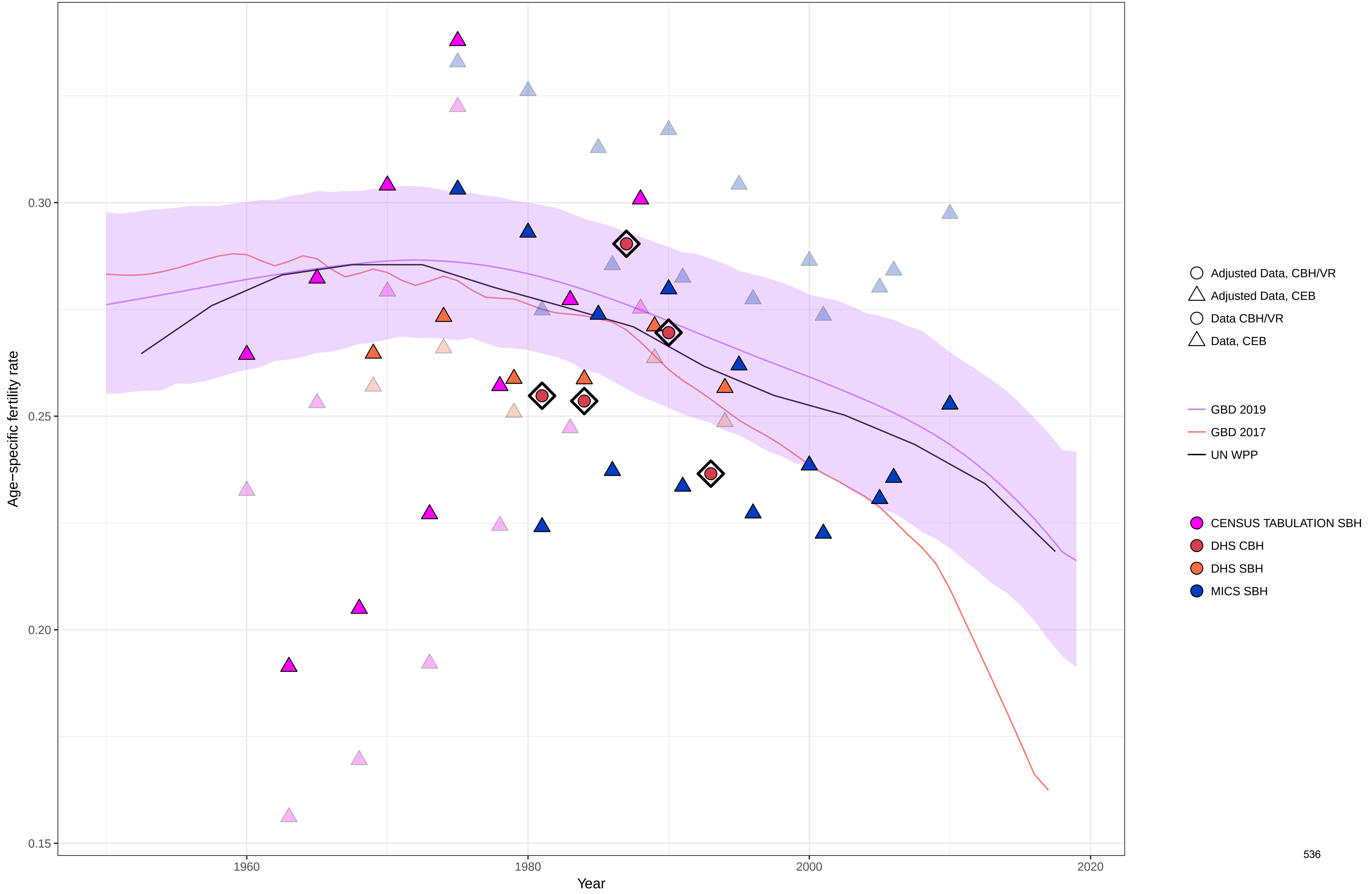

Age−specific fertility fitted trend lines, 1950−2019: Central African Republic, Ages: 25−29
Data points enclosed with a diamond are reference sources

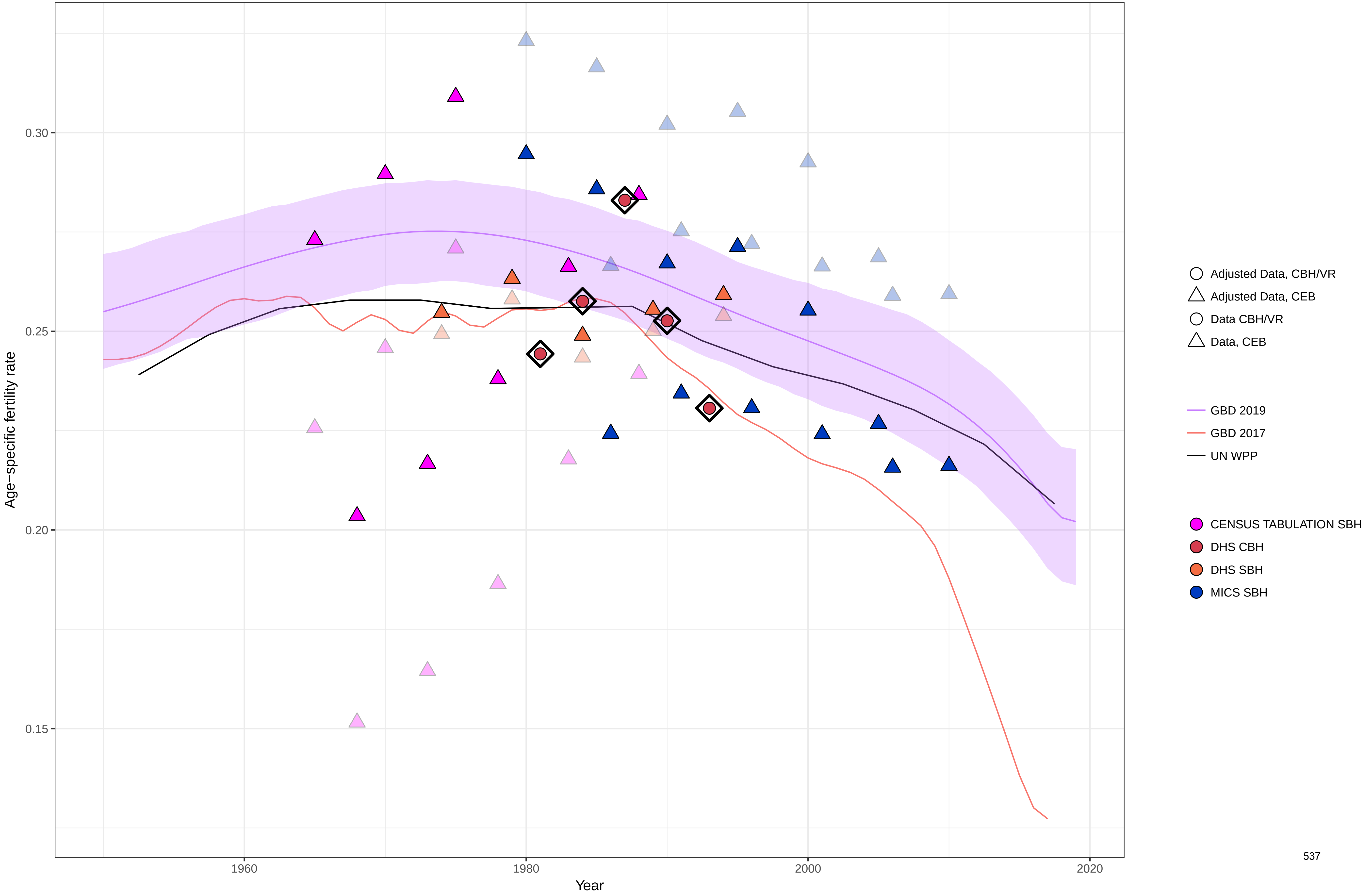

Age−specific fertility fitted trend lines, 1950−2019: Central African Republic, Ages: 30−34
Data points enclosed with a diamond are reference sources

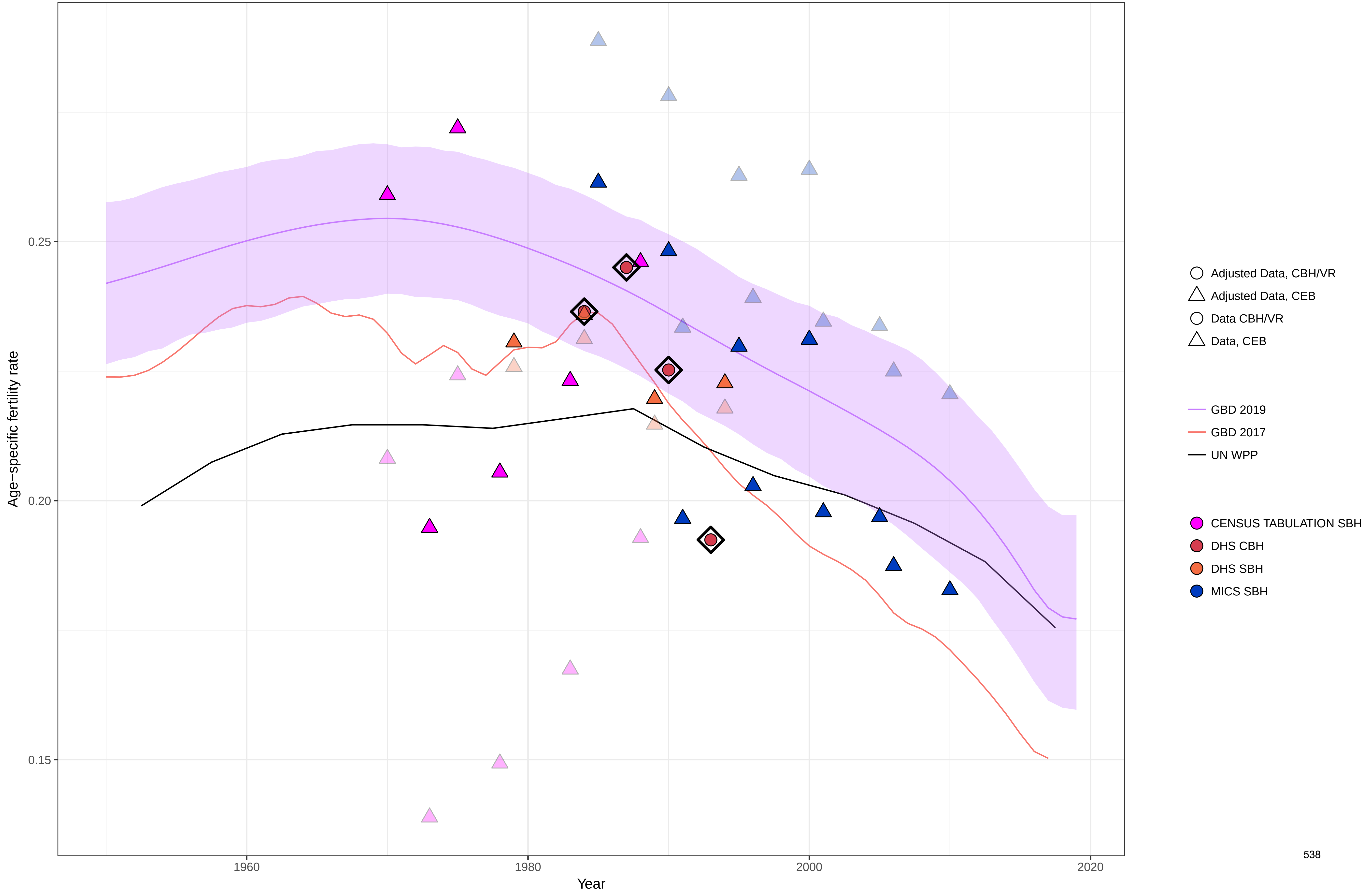

Age−specific fertility fitted trend lines, 1950−2019: Central African Republic, Ages: 35−39
Data points enclosed with a diamond are reference sources

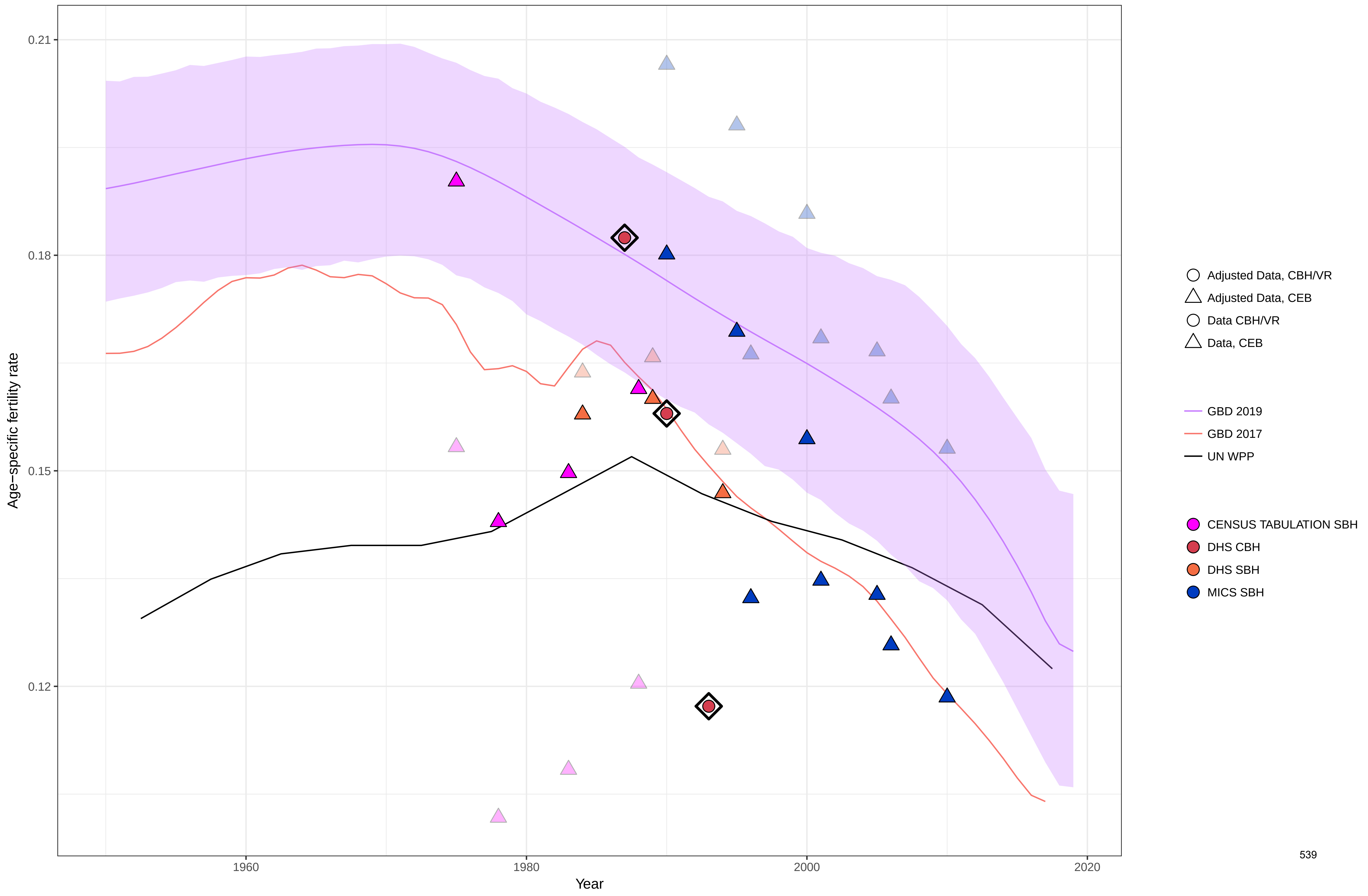

Age−specific fertility fitted trend lines, 1950−2019: Central African Republic, Ages: 40−44
Data points enclosed with a diamond are reference sources

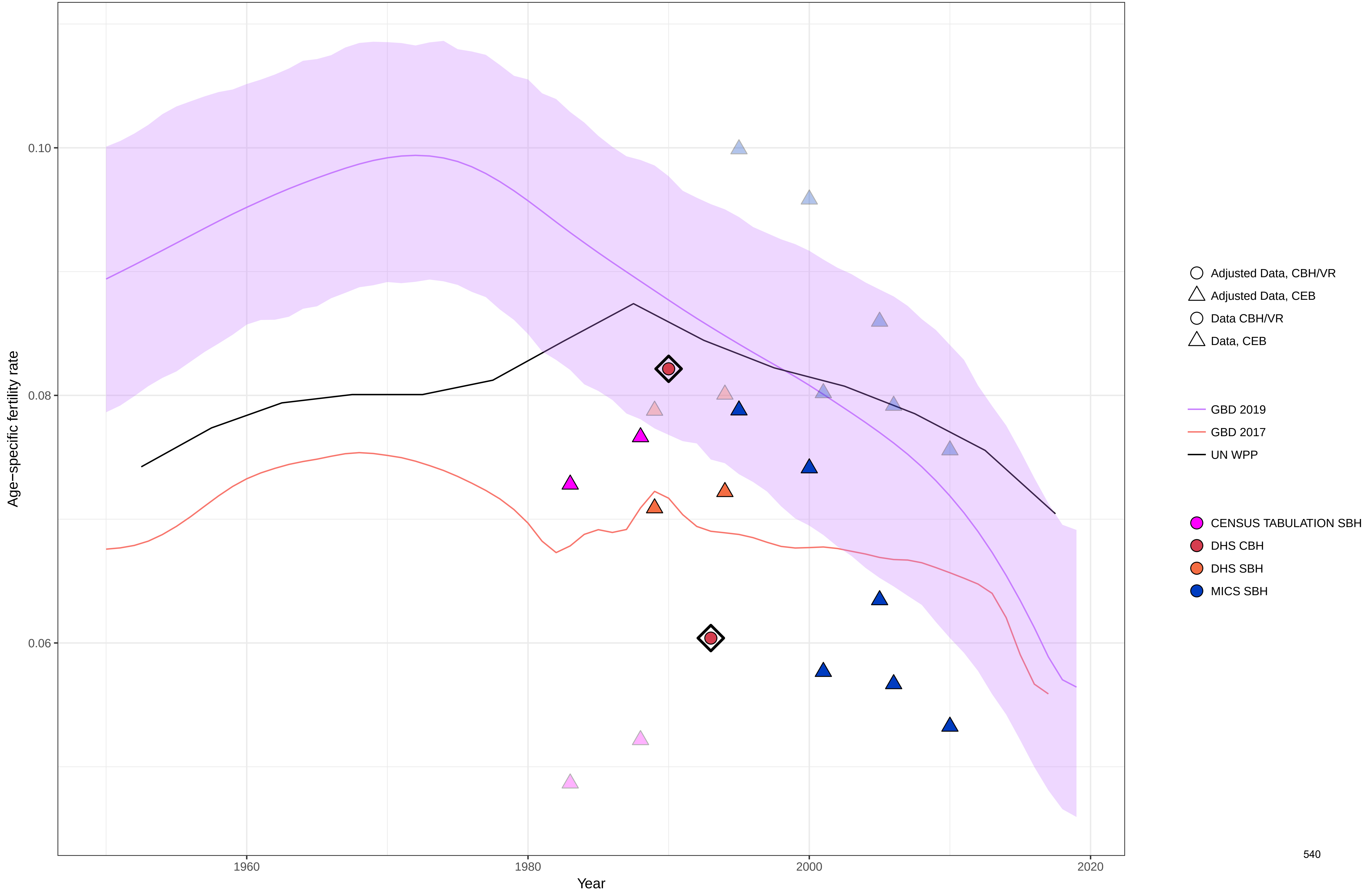

Age−specific fertility fitted trend lines, 1950−2019: Central African Republic, Ages: 45−49
Data points enclosed with a diamond are reference sources

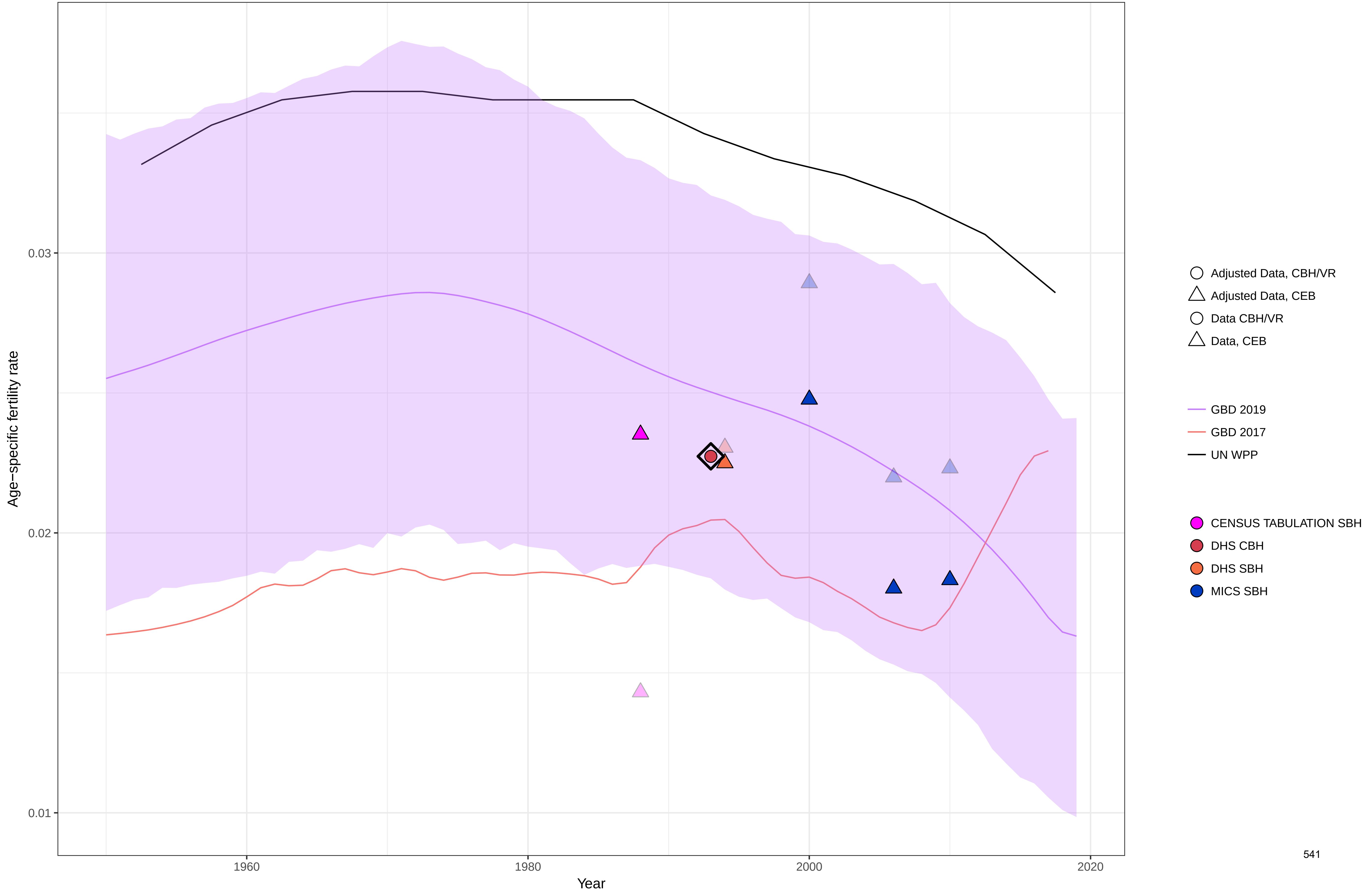

Age−specific fertility fitted trend lines, 1950−2019: Central African Republic, Ages: 50−54
Data points enclosed with a diamond are reference sources

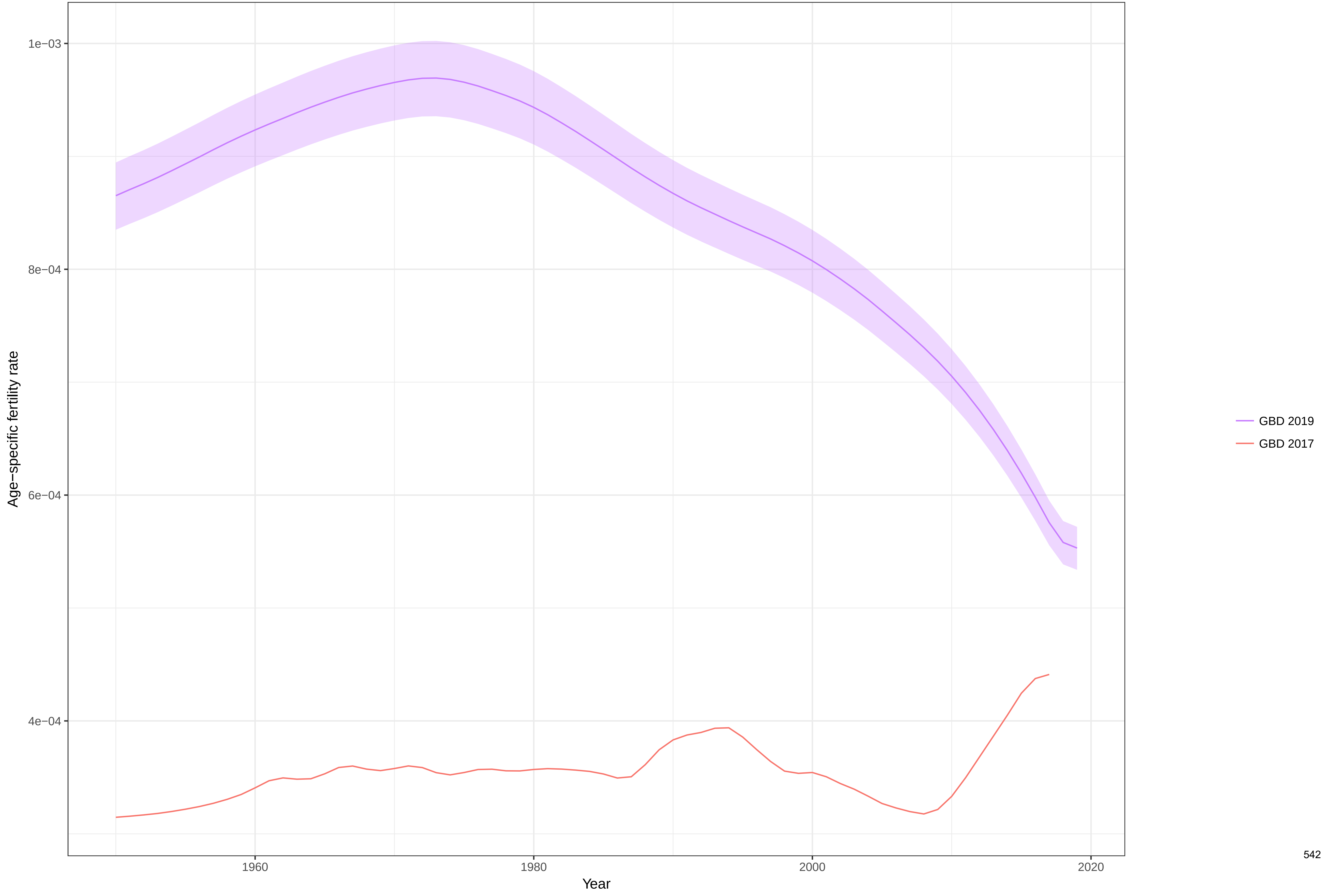

Total fertility rate fitted trend lines, 1950−2019: Central African Republic TFR
Data points enclosed with a diamond are reference sources

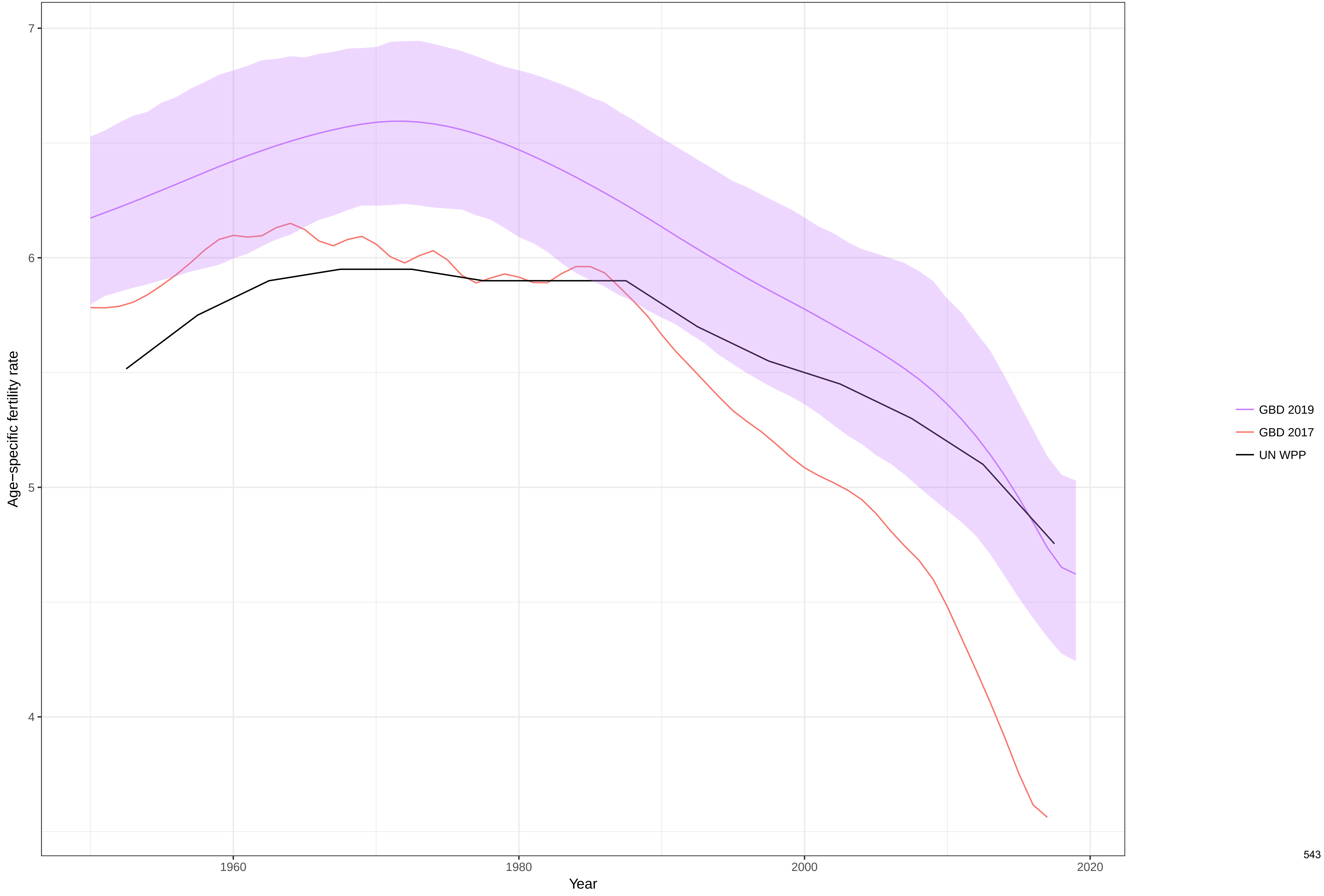

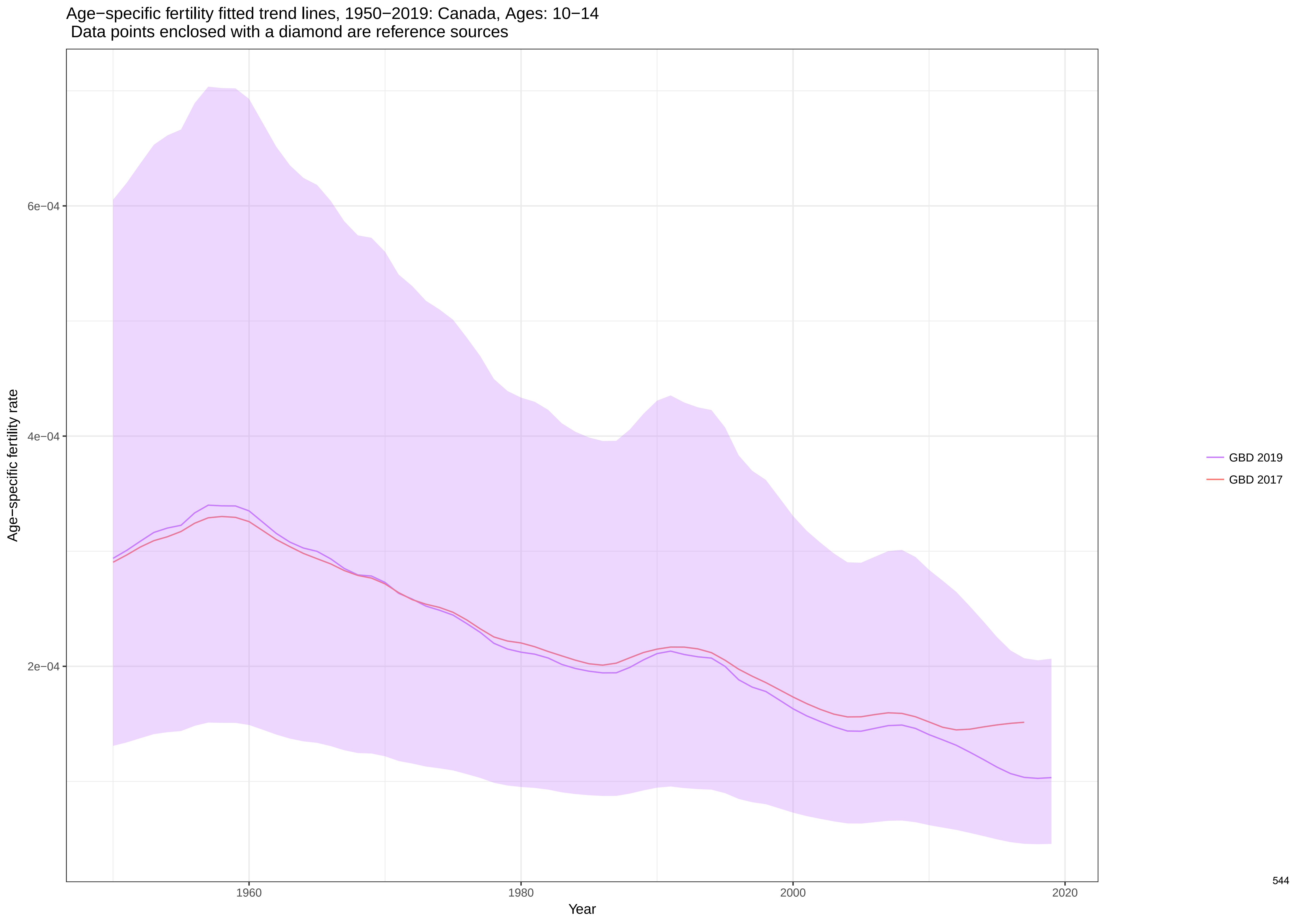
Age−specific fertility fitted trend lines, 1950−2019: Canada, Ages: 10−14
Data points enclosed with a diamond are reference sources
6e−04
4e−04
2e−04
Age−specific fertility rate
1960
1980
2000
2020
Year
GBD 2019
GBD 2017

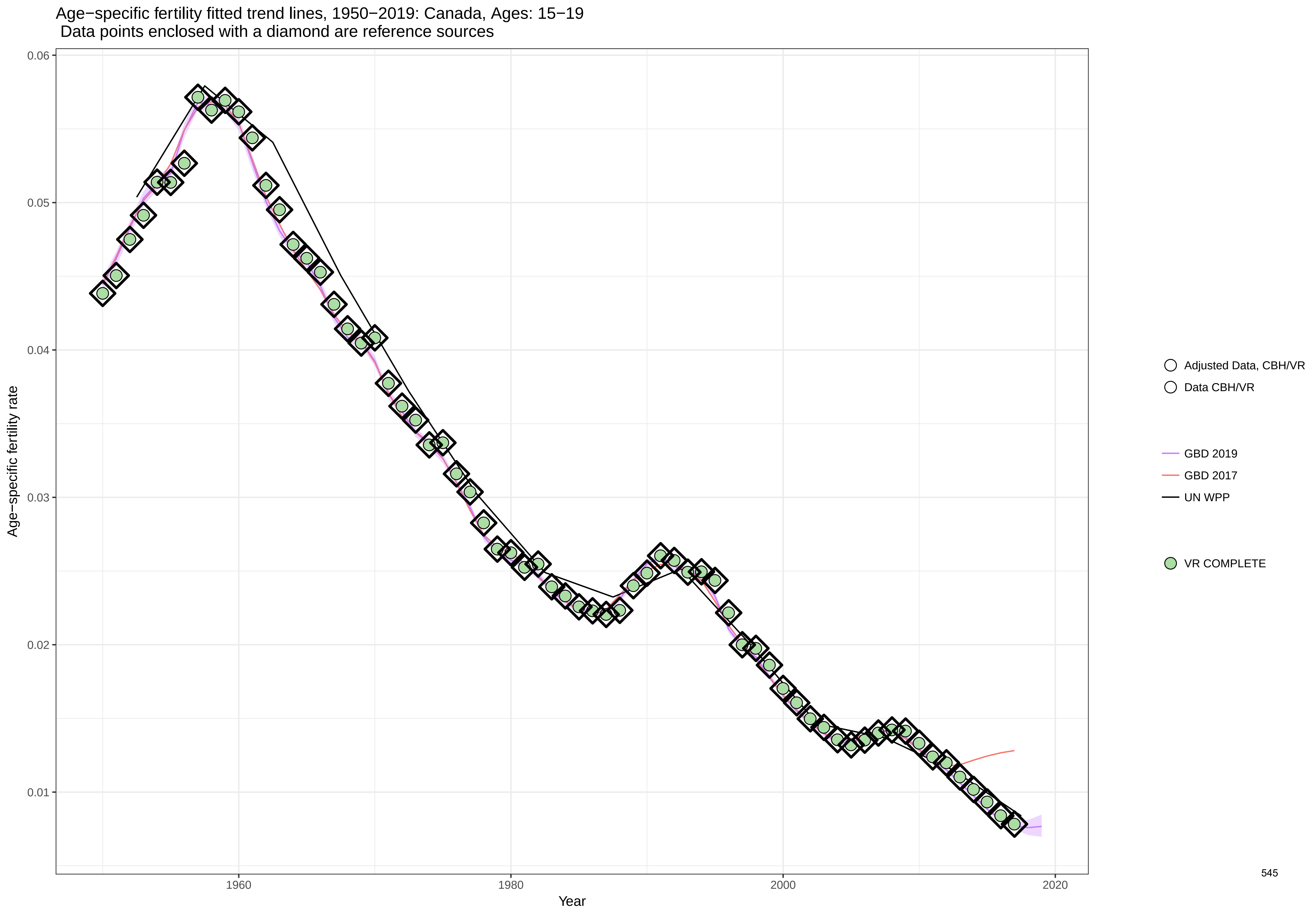
Age−specific fertility fitted trend lines, 1950−2019: Canada, Ages: 15−19
Data points enclosed with a diamond are reference sources
Age−specific fertility rate
Year
0.06
0.05
0.04
0.03
0.02
0.01
1960
1980
2000
2020
Adjusted Data, CBH/VR
Data CBH/VR
GBD 2019
GBD 2017
UN WPP
VR COMPLETE

Age−specific fertility fitted trend lines, 1950−2019: Canada, Ages: 20−24
Data points enclosed with a diamond are reference sources

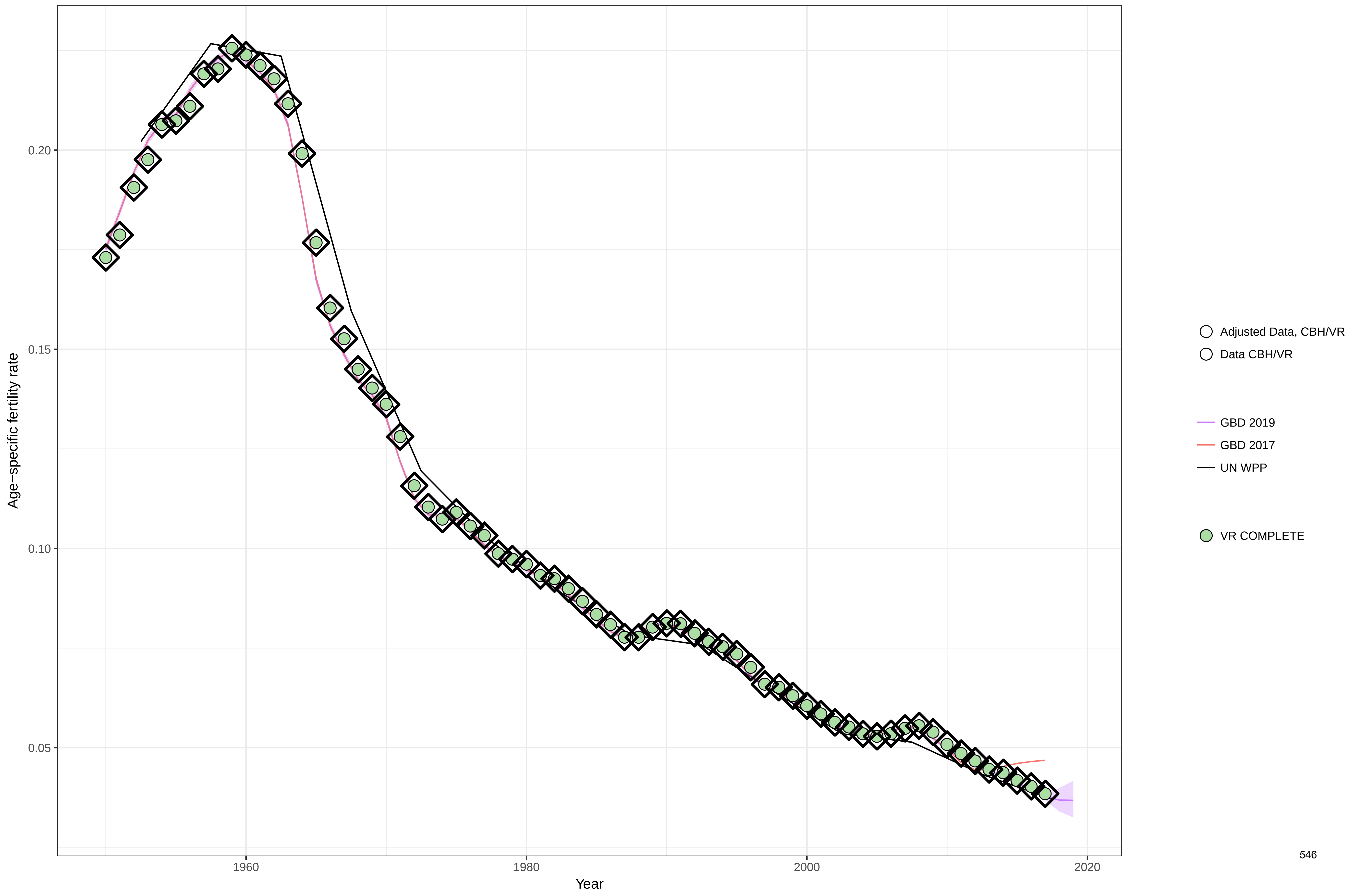

Age−specific fertility fitted trend lines, 1950−2019: Canada, Ages: 25−29
Data points enclosed with a diamond are reference sources

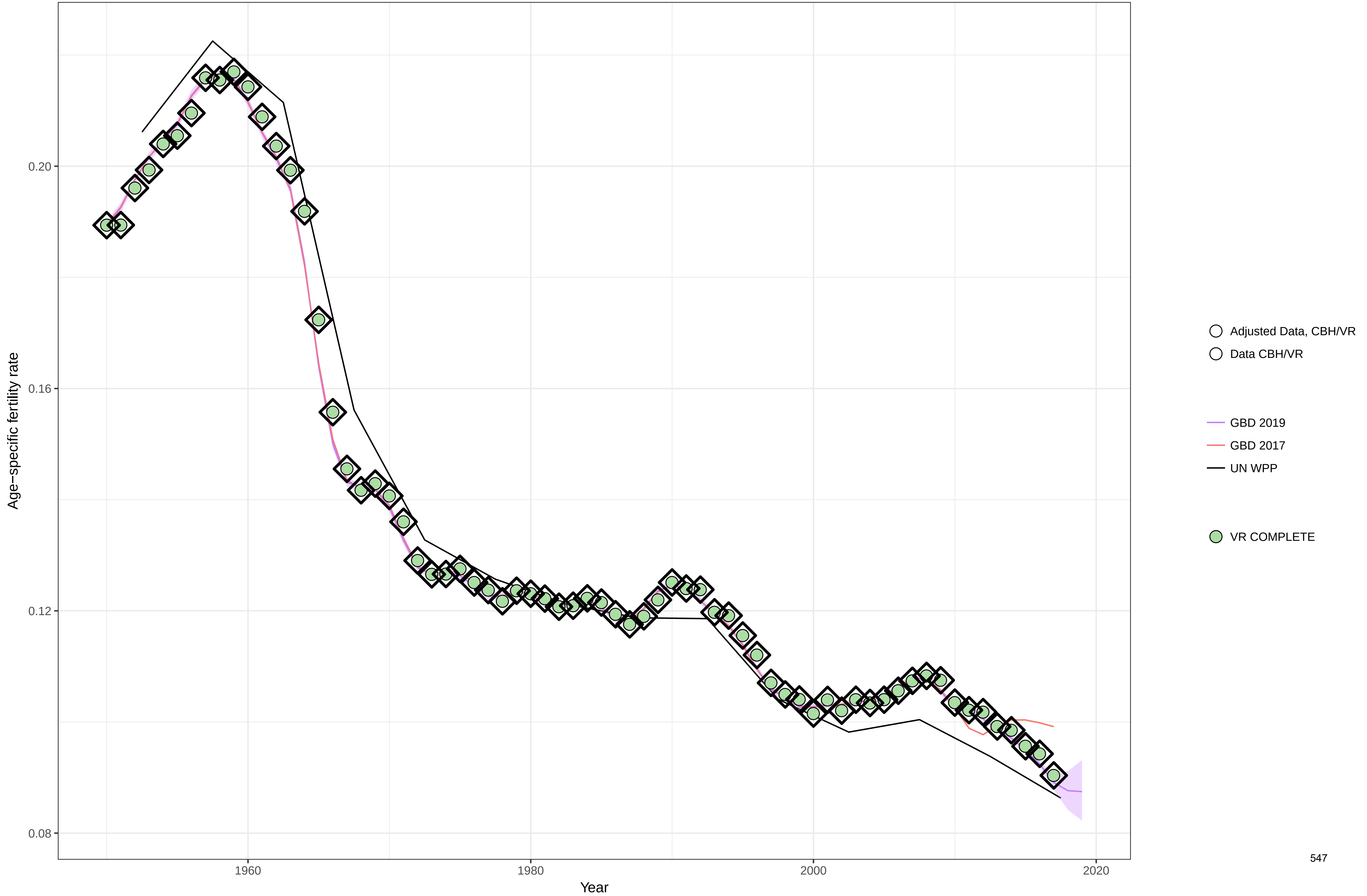

Age−specific fertility fitted trend lines, 1950−2019: Canada, Ages: 30−34
Data points enclosed with a diamond are reference sources

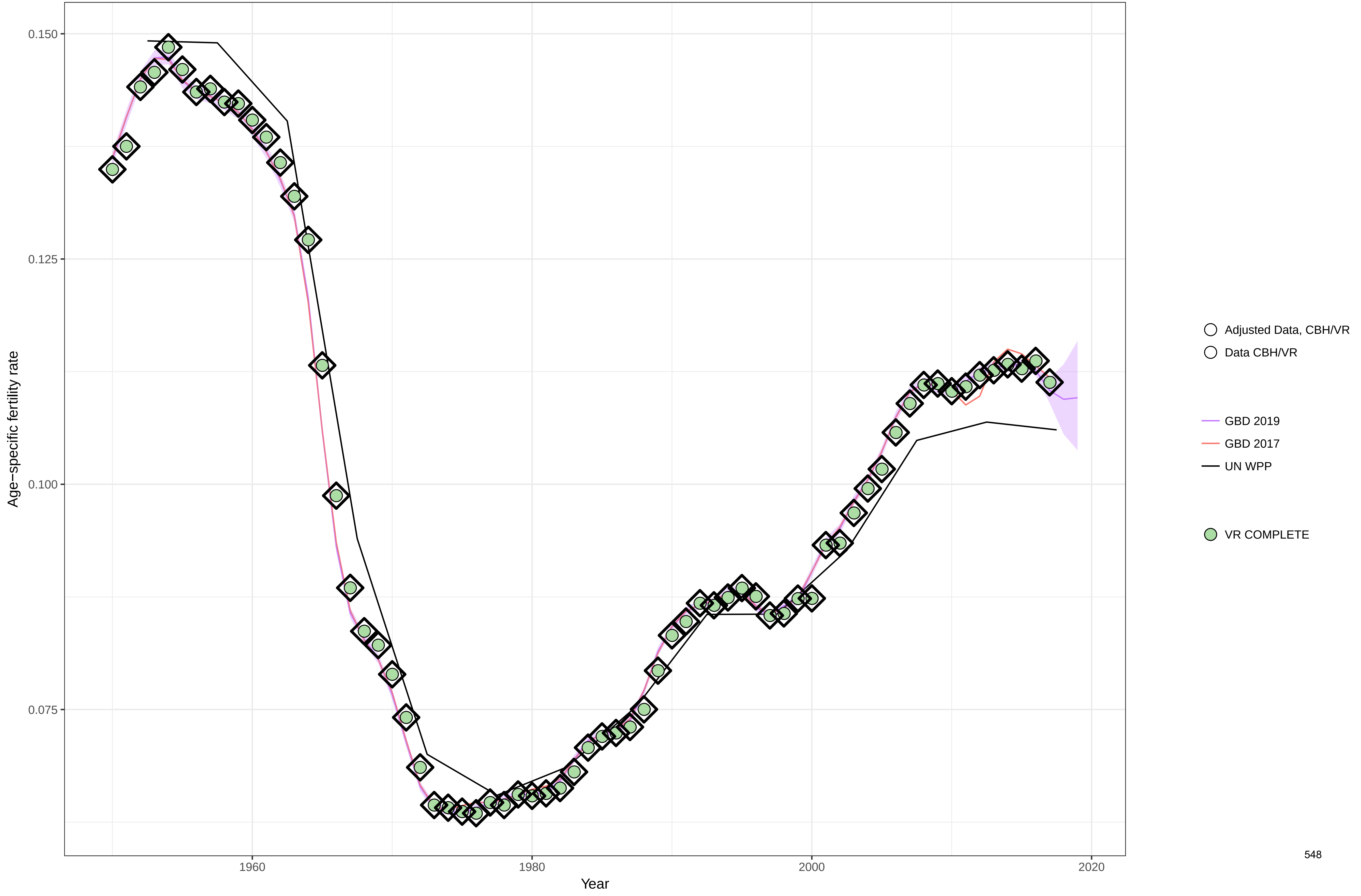

Age−specific fertility fitted trend lines, 1950−2019: Canada, Ages: 35−39
Data points enclosed with a diamond are reference sources

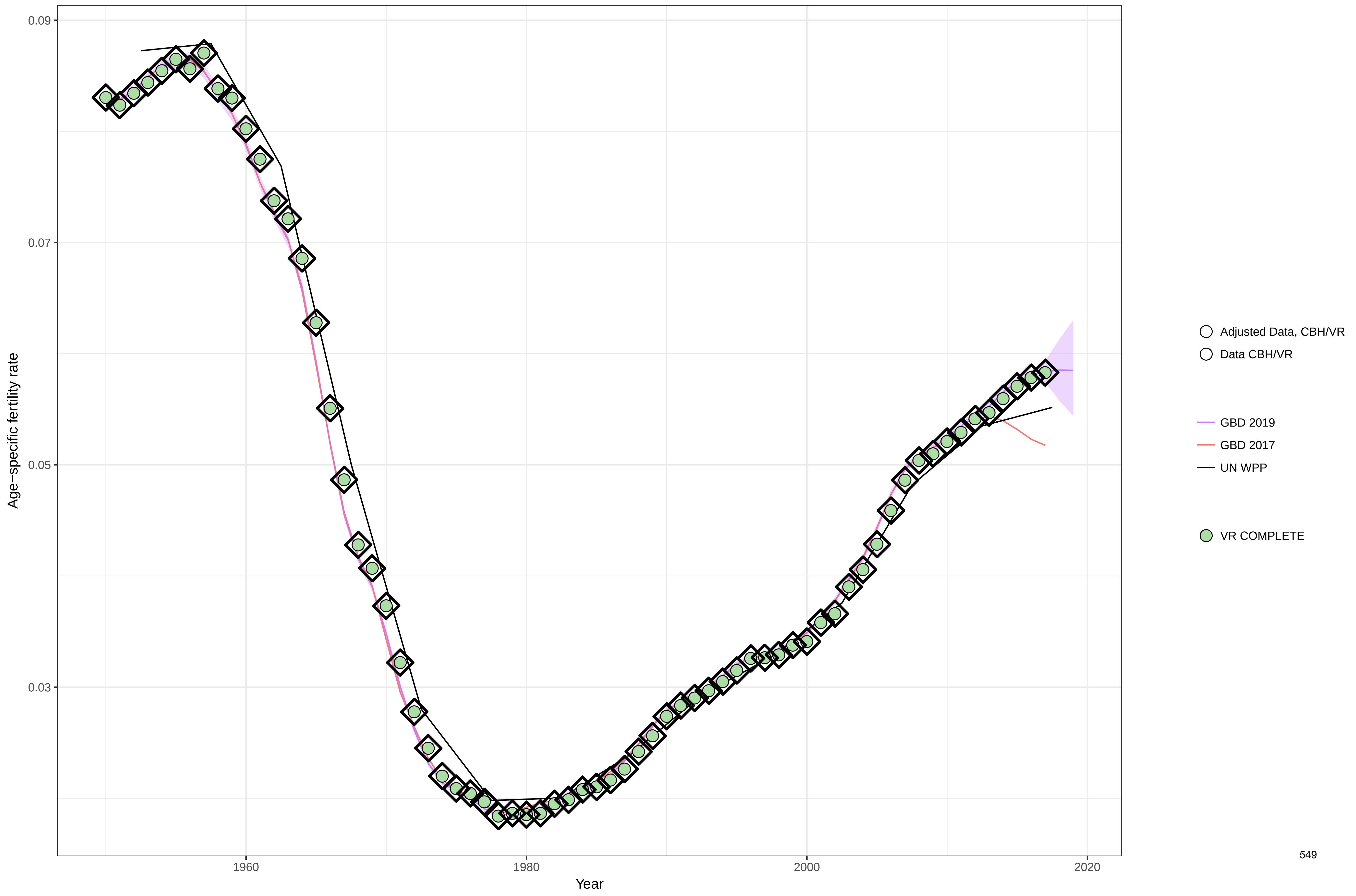

Age−specific fertility fitted trend lines, 1950−2019: Canada, Ages: 40−44
Data points enclosed with a diamond are reference sources

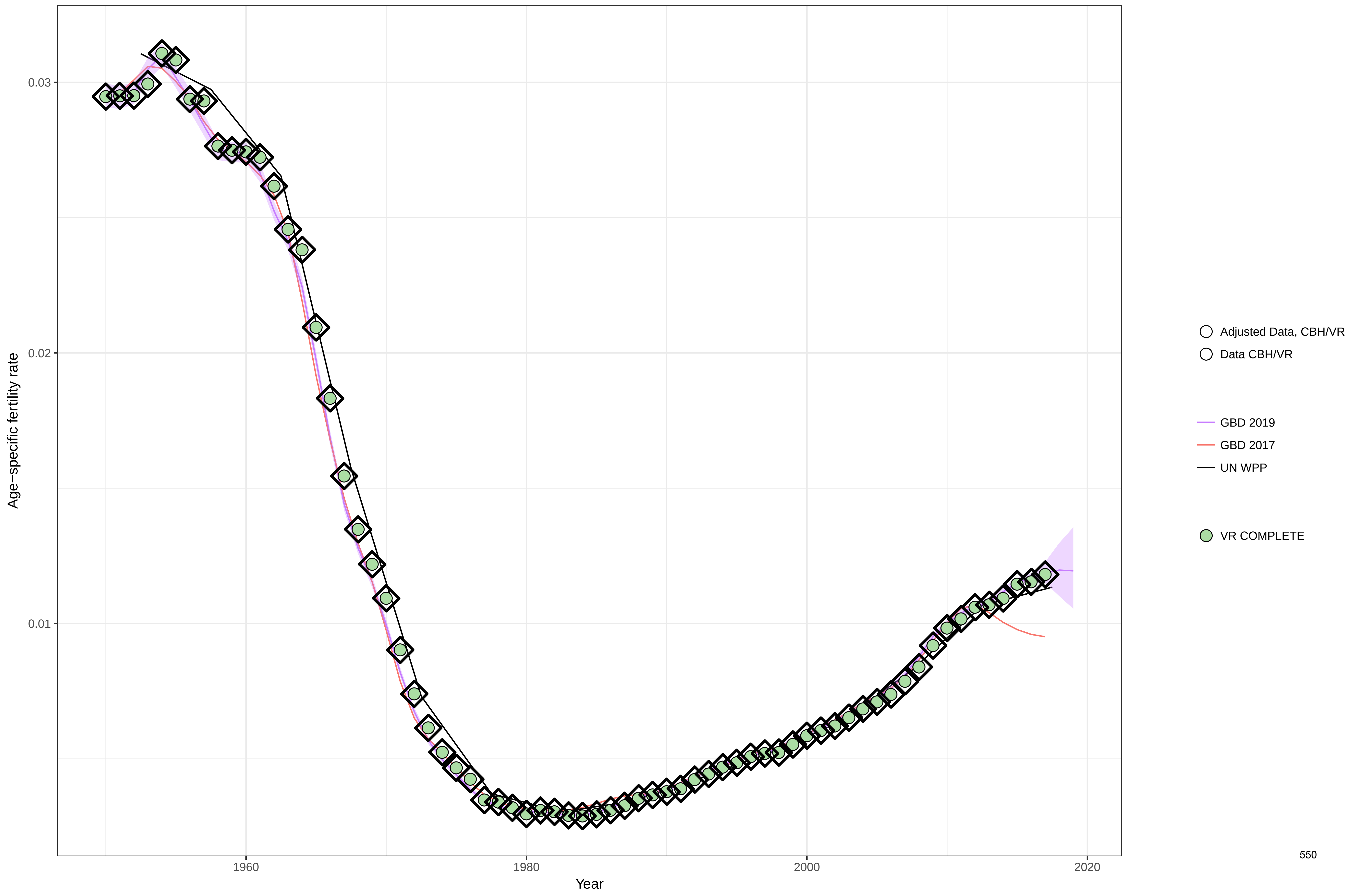

Age−specific fertility fitted trend lines, 1950−2019: Canada, Ages: 45−49
Data points enclosed with a diamond are reference sources

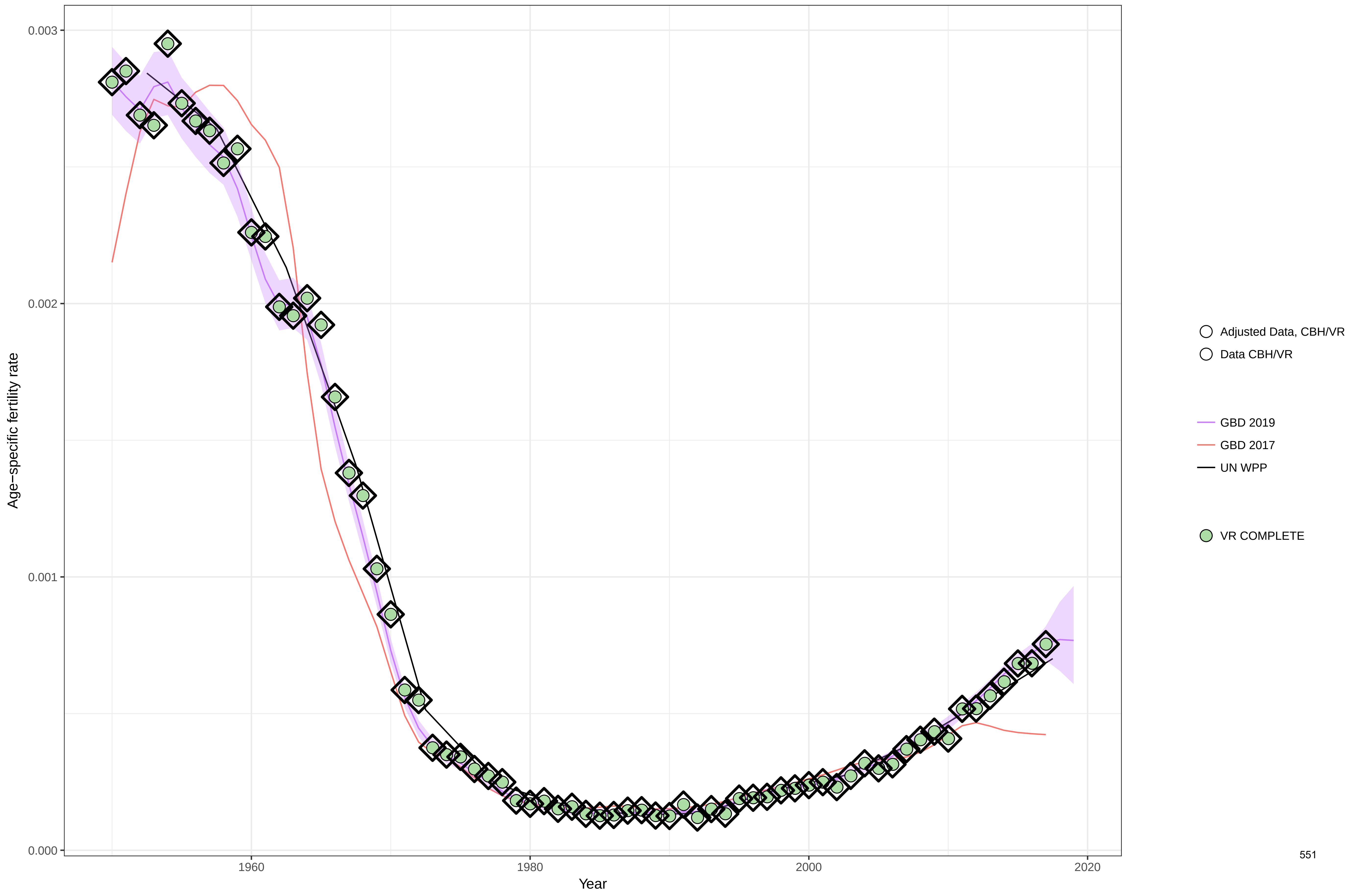

Age−specific fertility fitted trend lines, 1950−2019: Canada, Ages: 50−54
Data points enclosed with a diamond are reference sources

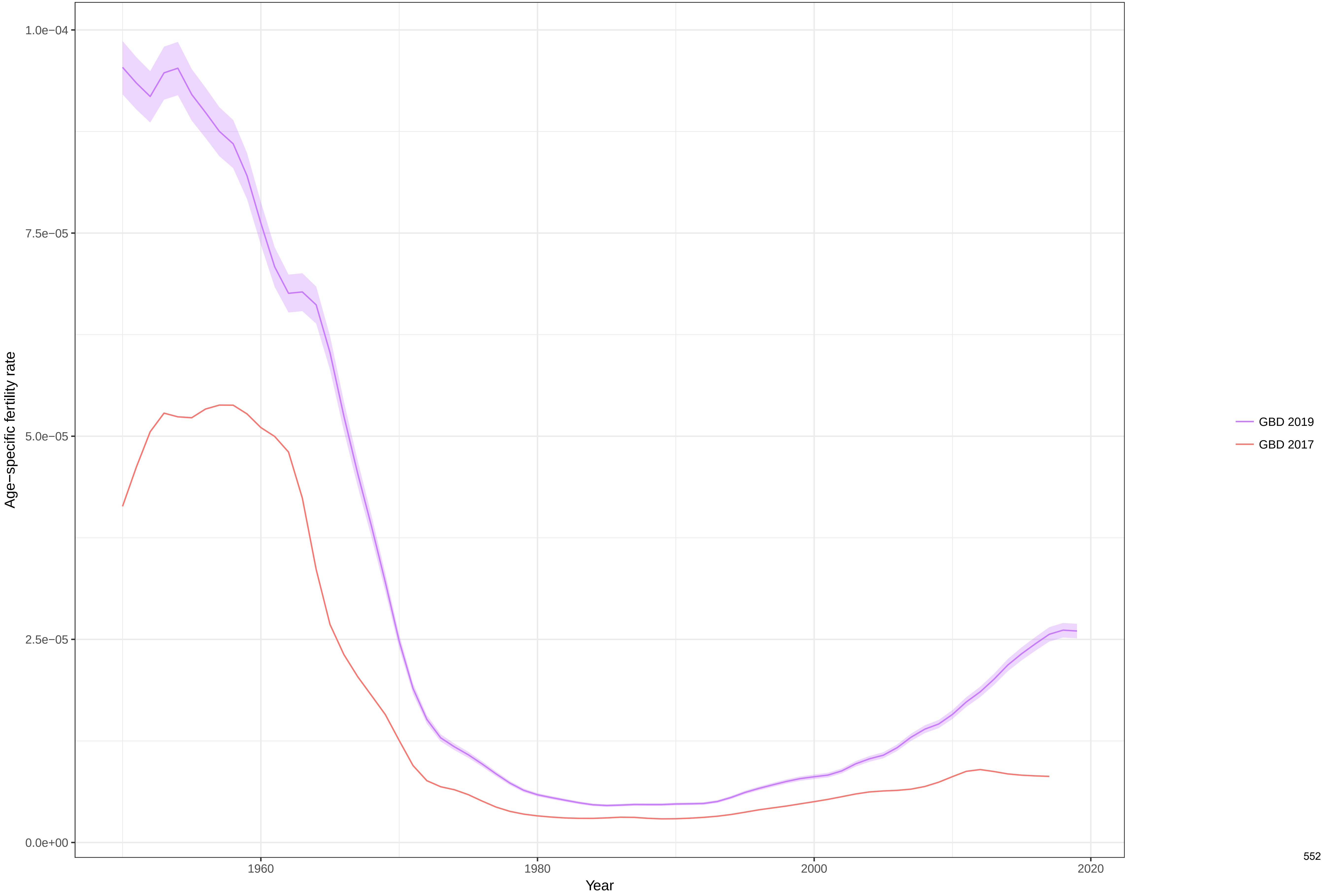

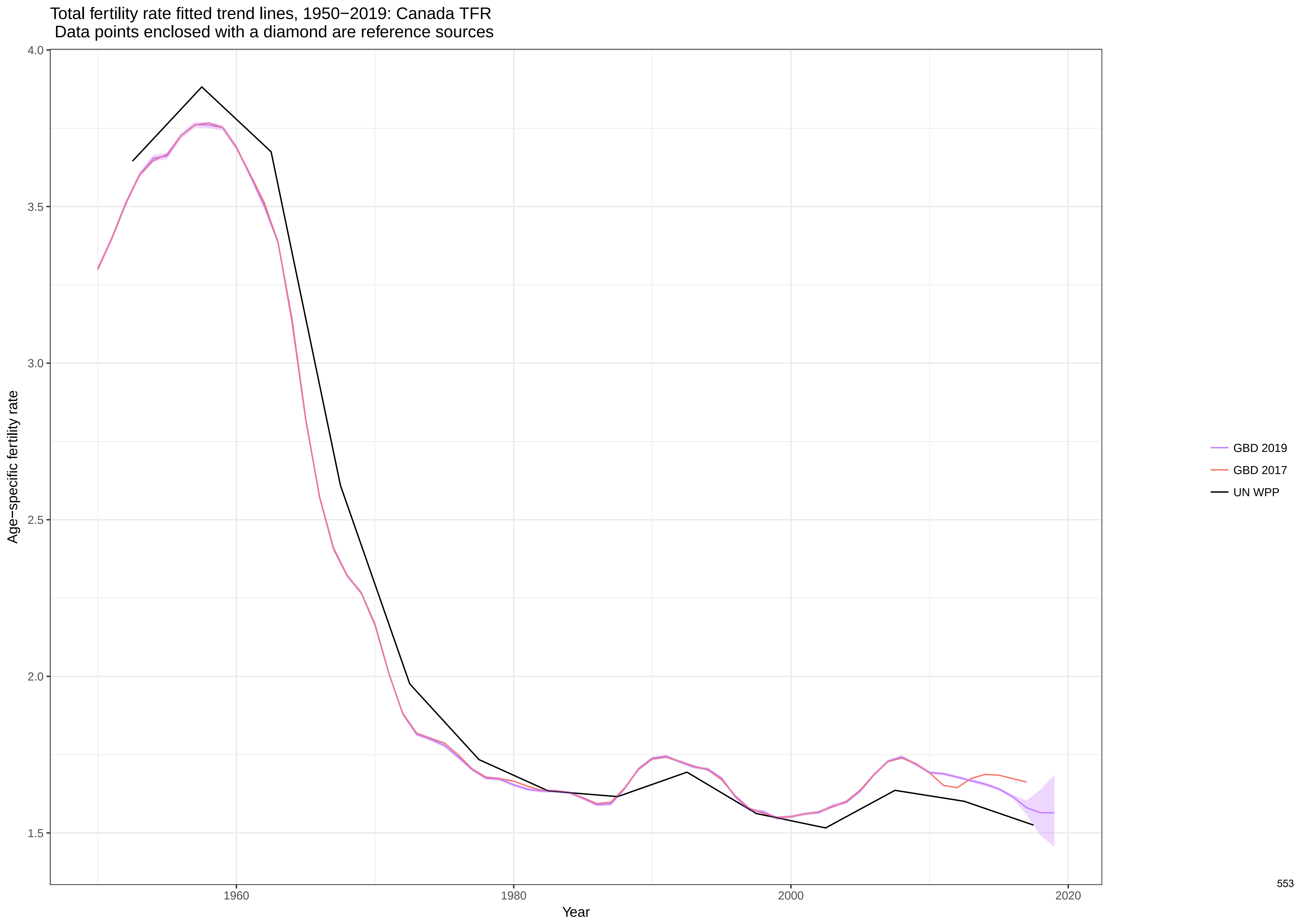
Total fertility rate fitted trend lines, 1950−2019: Canada TFR
Data points enclosed with a diamond are reference sources
4.0
3.5
3.0
2.5
2.0
1.5
Age−specific fertility rate
1960
1980
2000
2020
Year
GBD 2019
GBD 2017
UN WPP

Age−specific fertility fitted trend lines, 1950−2019: Switzerland, Ages: 10−14
Data points enclosed with a diamond are reference sources

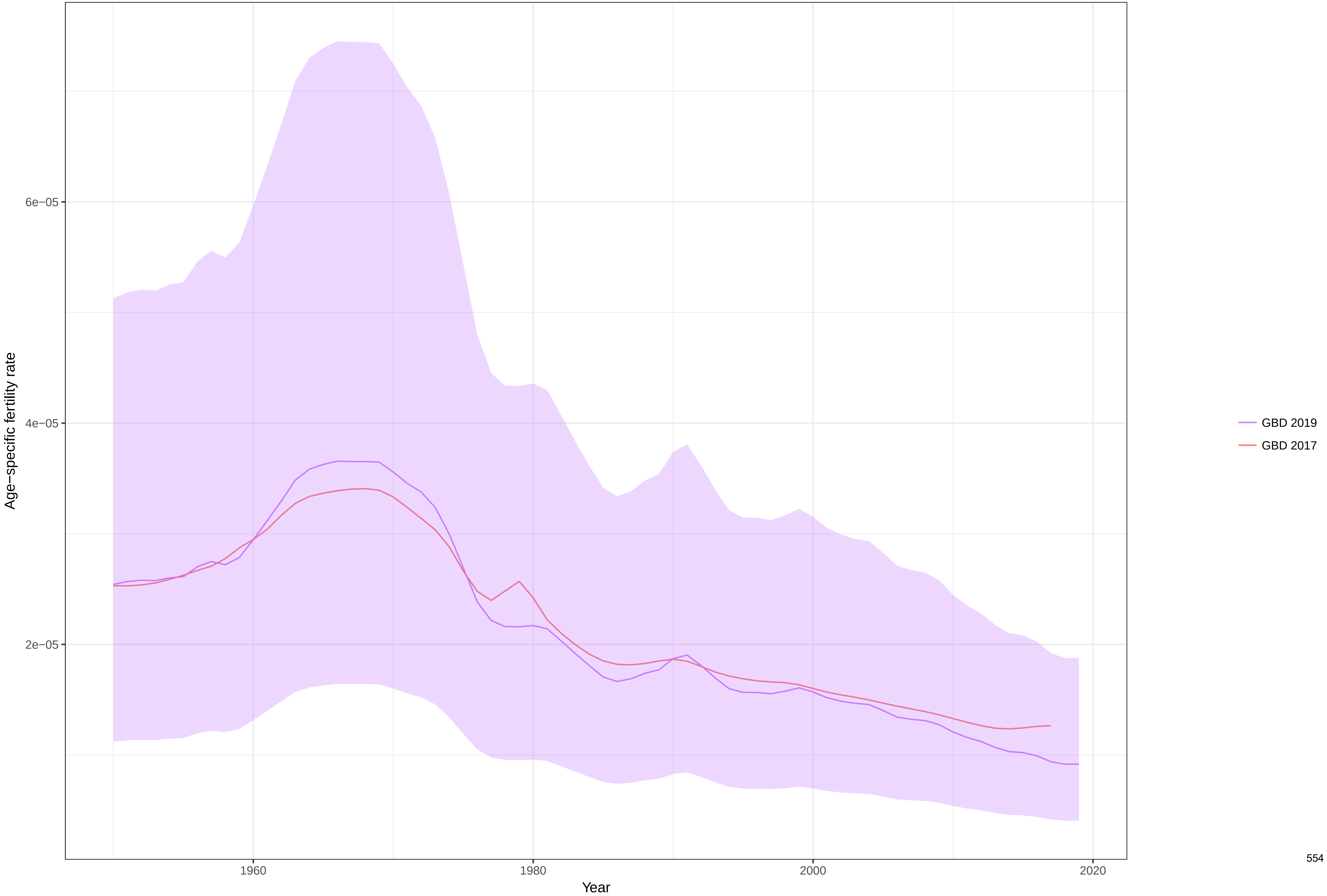

Age−specific fertility fitted trend lines, 1950−2019: Switzerland, Ages: 15−19
Data points enclosed with a diamond are reference sources

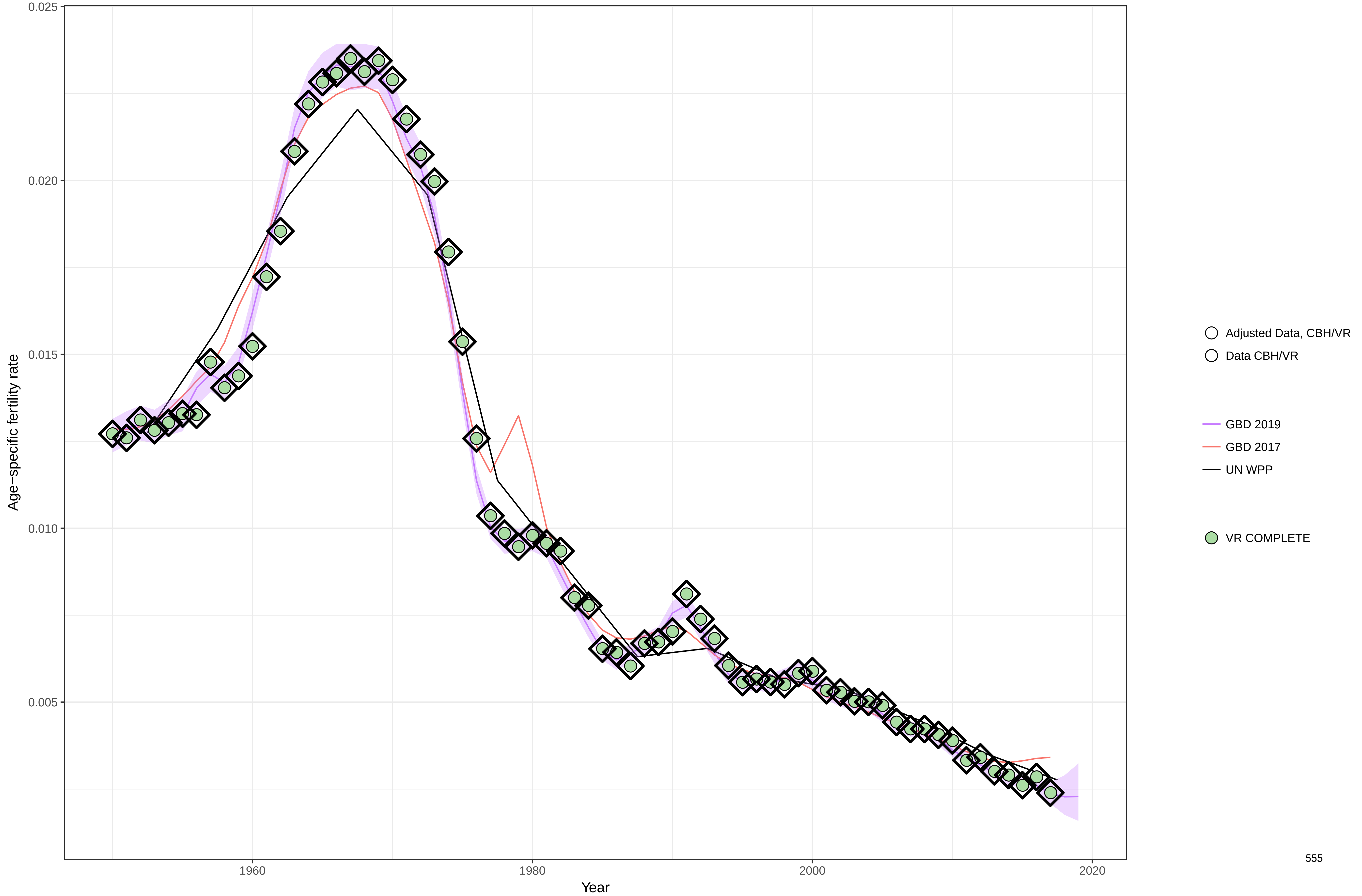

Age−specific fertility fitted trend lines, 1950−2019: Switzerland, Ages: 20−24
Data points enclosed with a diamond are reference sources

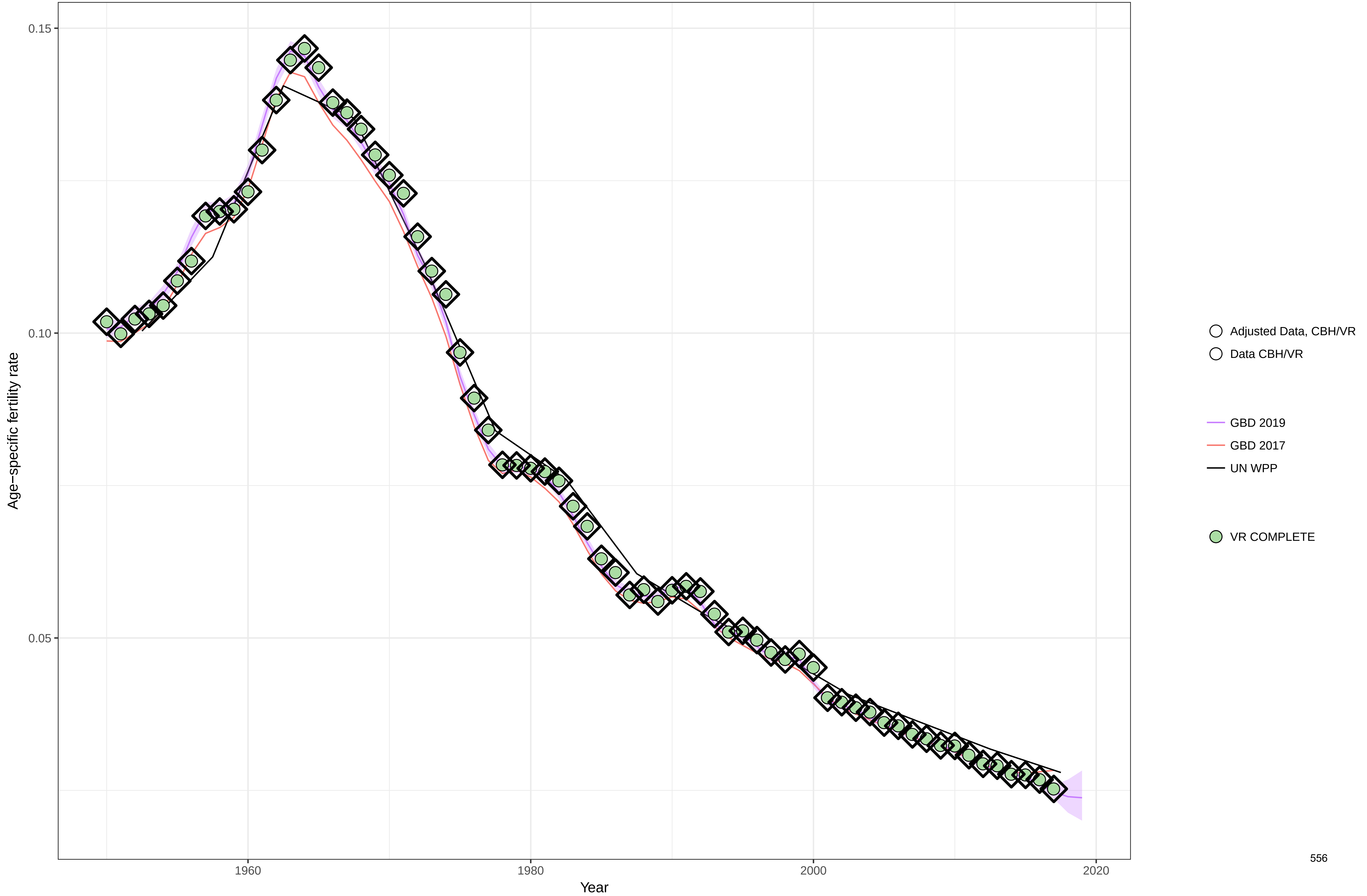

Age−specific fertility fitted trend lines, 1950−2019: Switzerland, Ages: 25−29
Data points enclosed with a diamond are reference sources

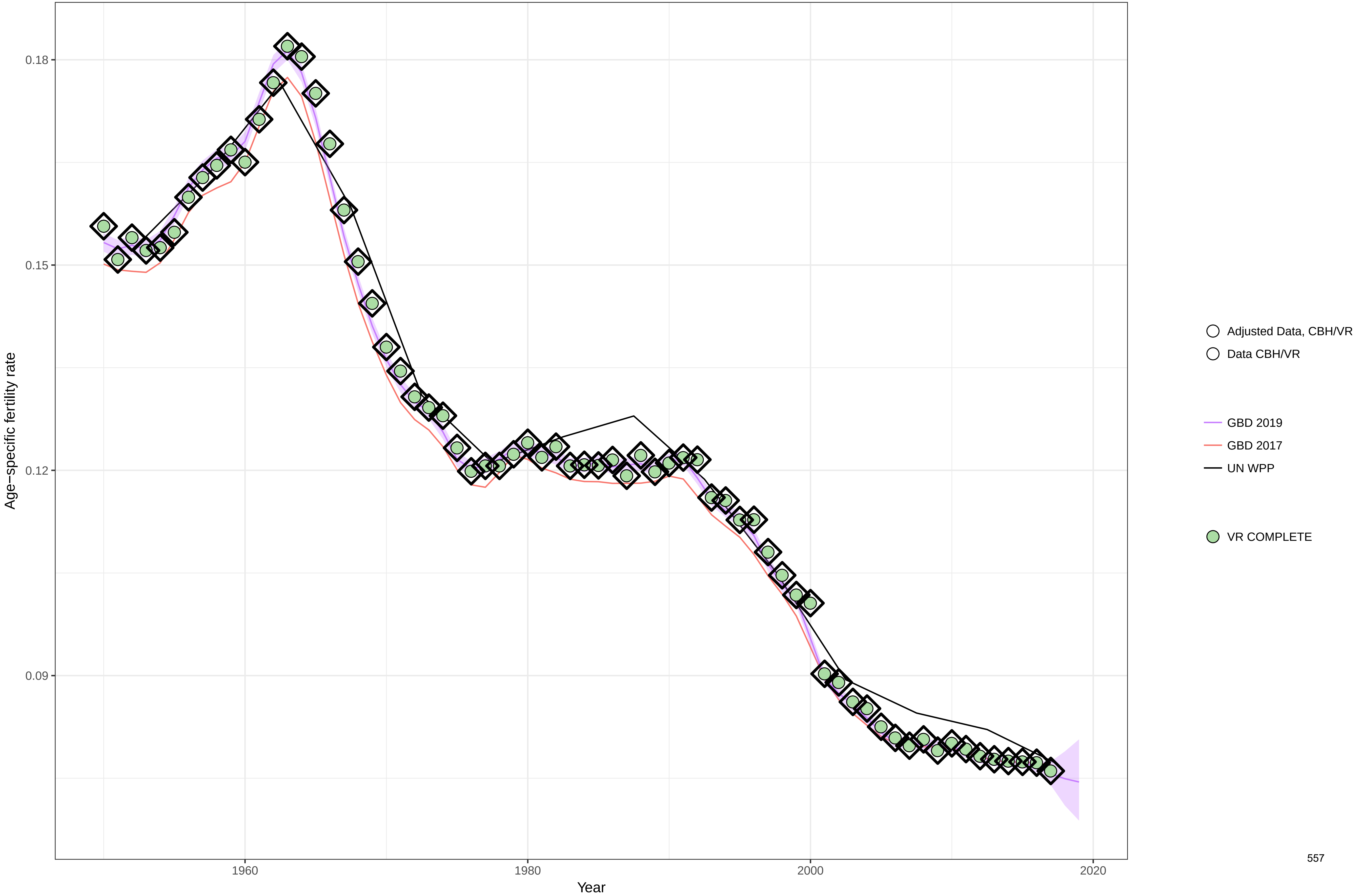

Age−specific fertility fitted trend lines, 1950−2019: Switzerland, Ages: 30−34
Data points enclosed with a diamond are reference sources

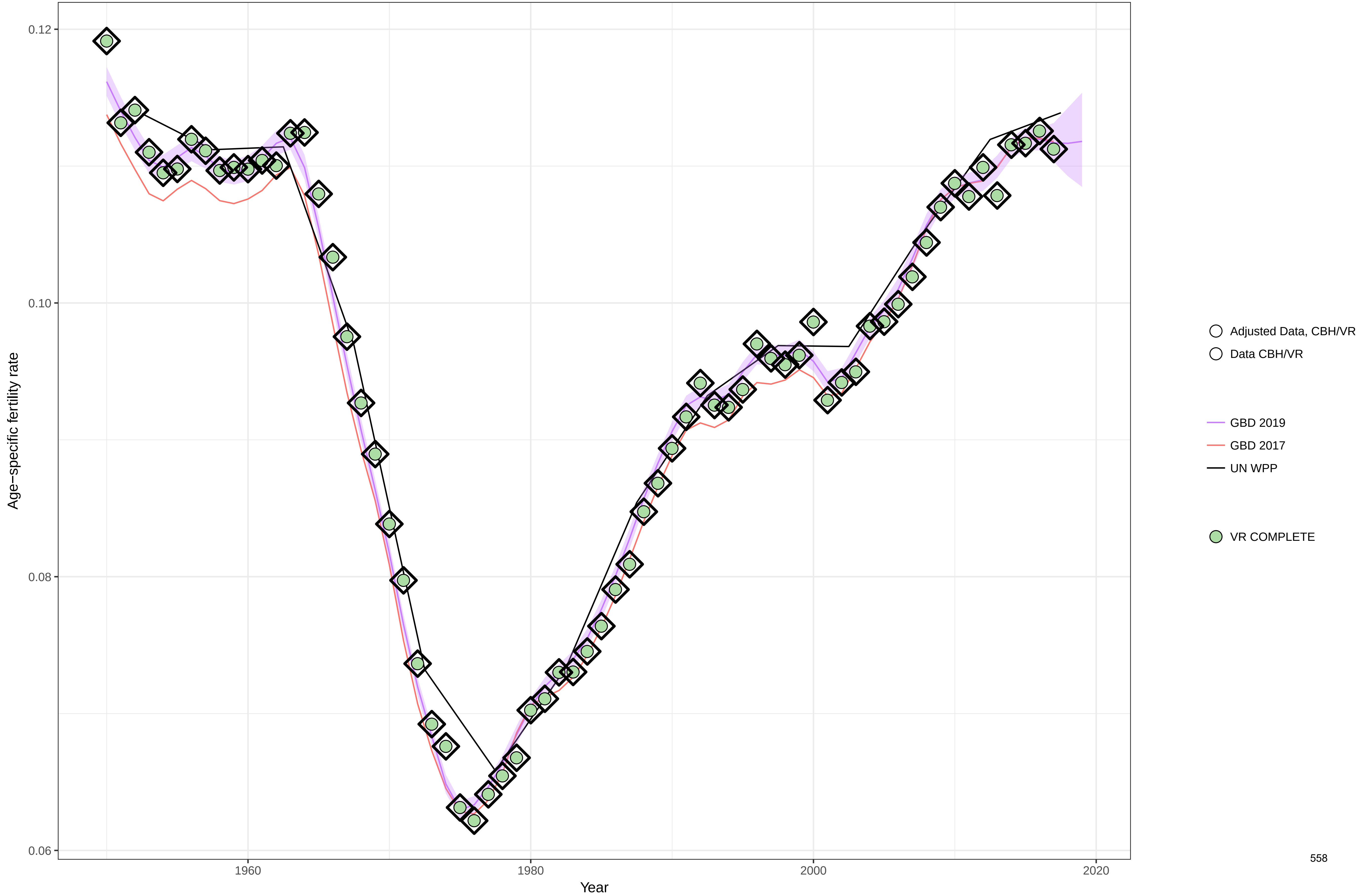

Age−specific fertility fitted trend lines, 1950−2019: Switzerland, Ages: 35−39
Data points enclosed with a diamond are reference sources

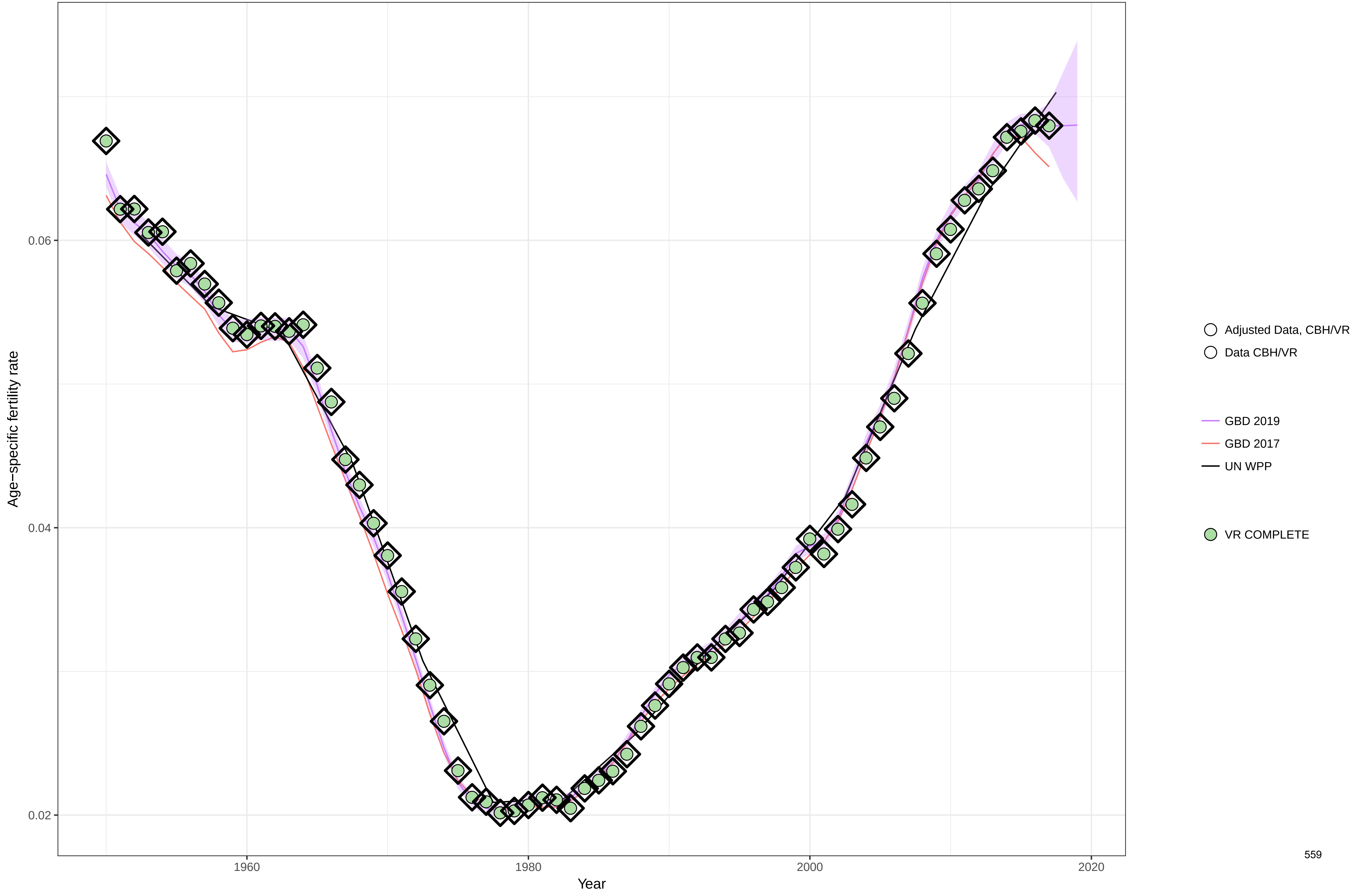

Age−specific fertility fitted trend lines, 1950−2019: Switzerland, Ages: 40−44
Data points enclosed with a diamond are reference sources

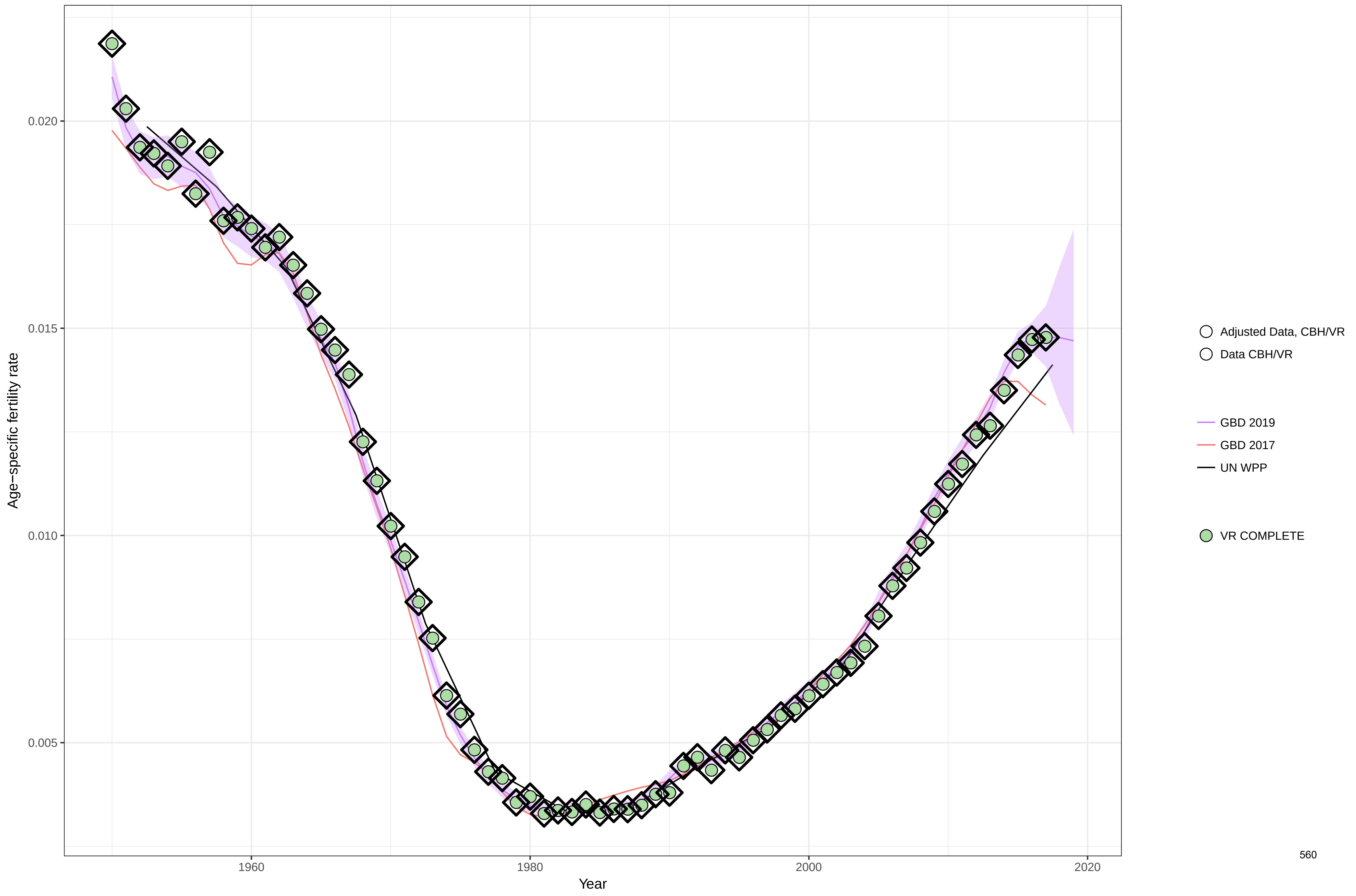

Age−specific fertility fitted trend lines, 1950−2019: Switzerland, Ages: 45−49
Data points enclosed with a diamond are reference sources

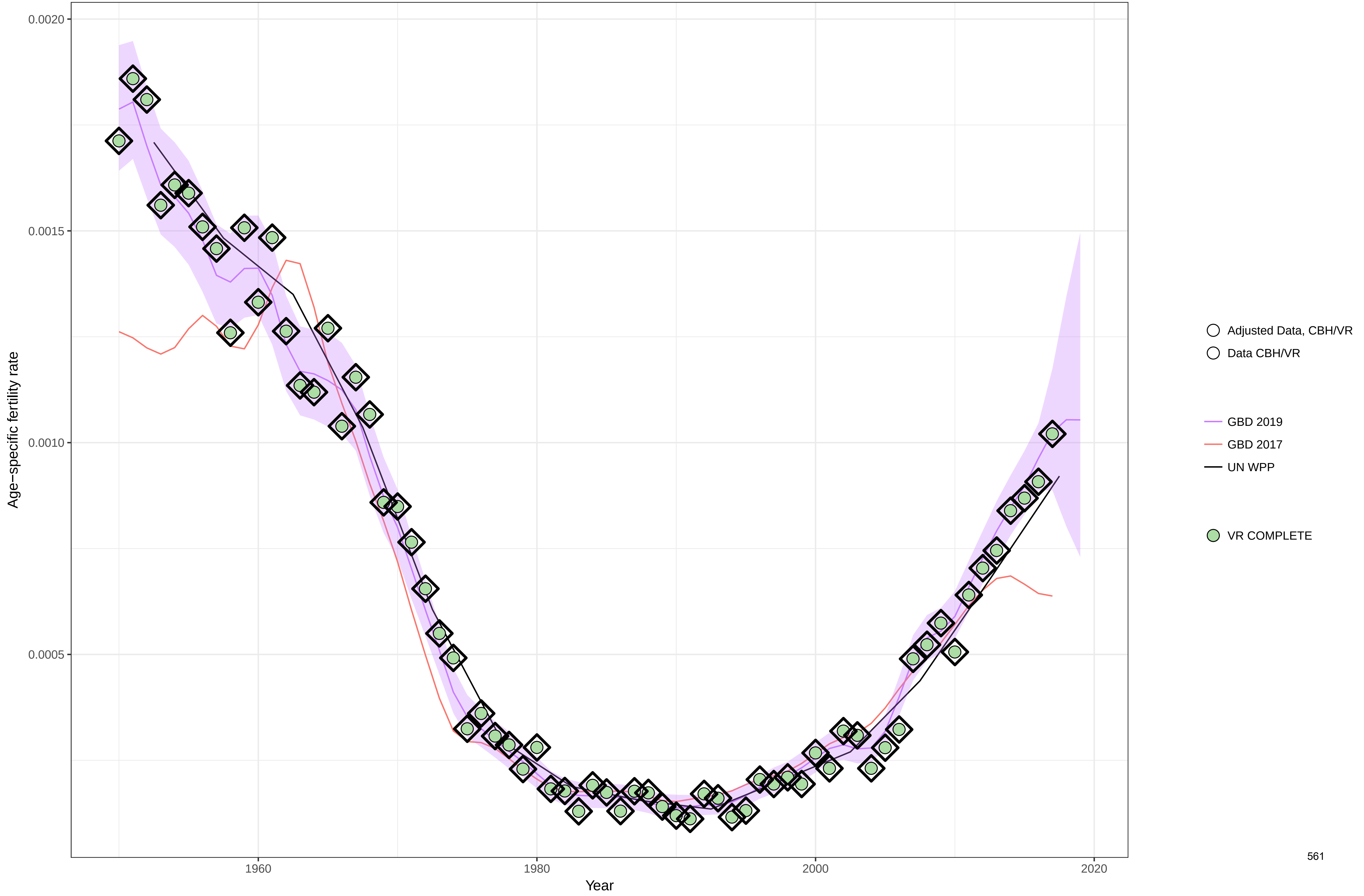

Age−specific fertility fitted trend lines, 1950−2019: Switzerland, Ages: 50−54
Data points enclosed with a diamond are reference sources

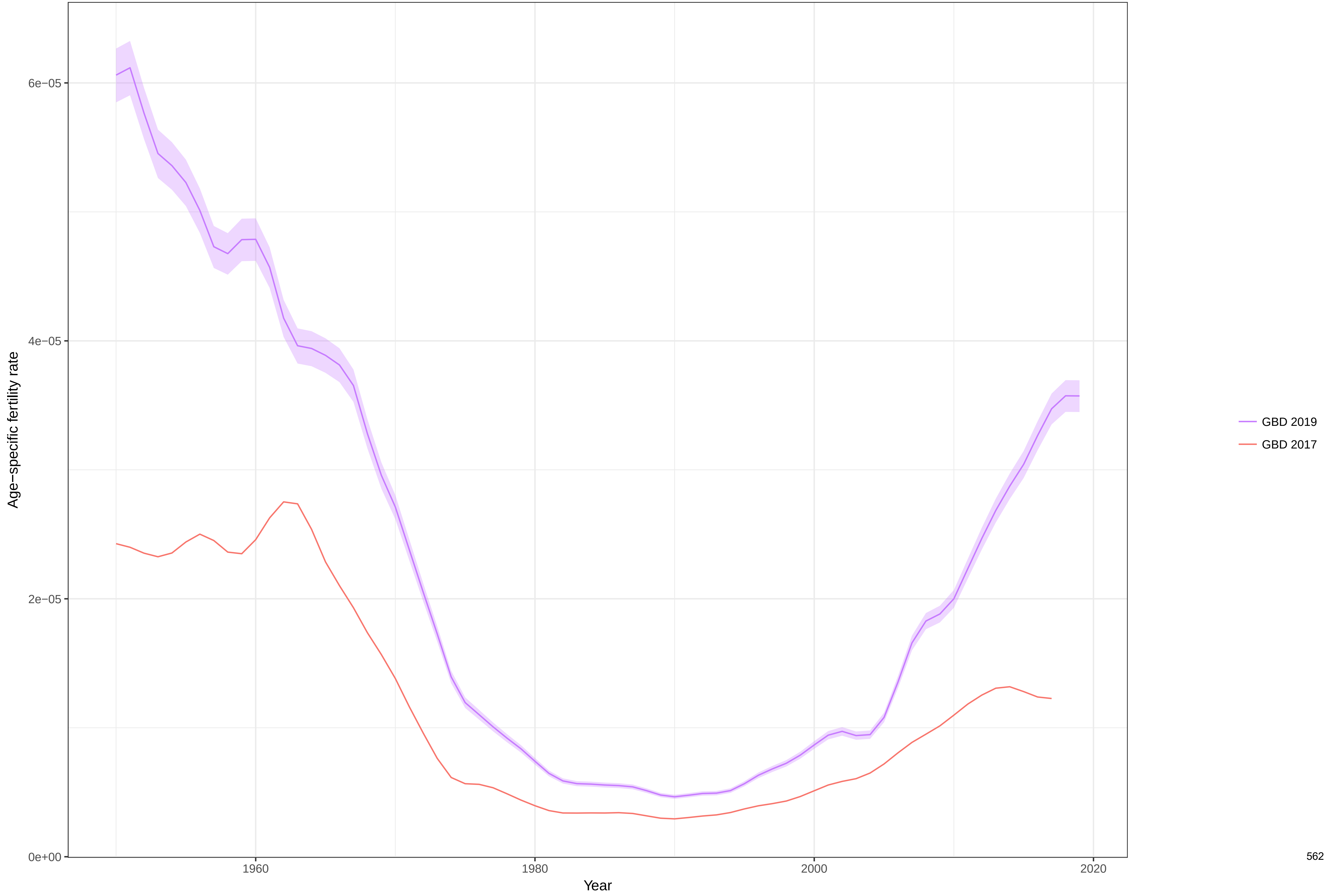

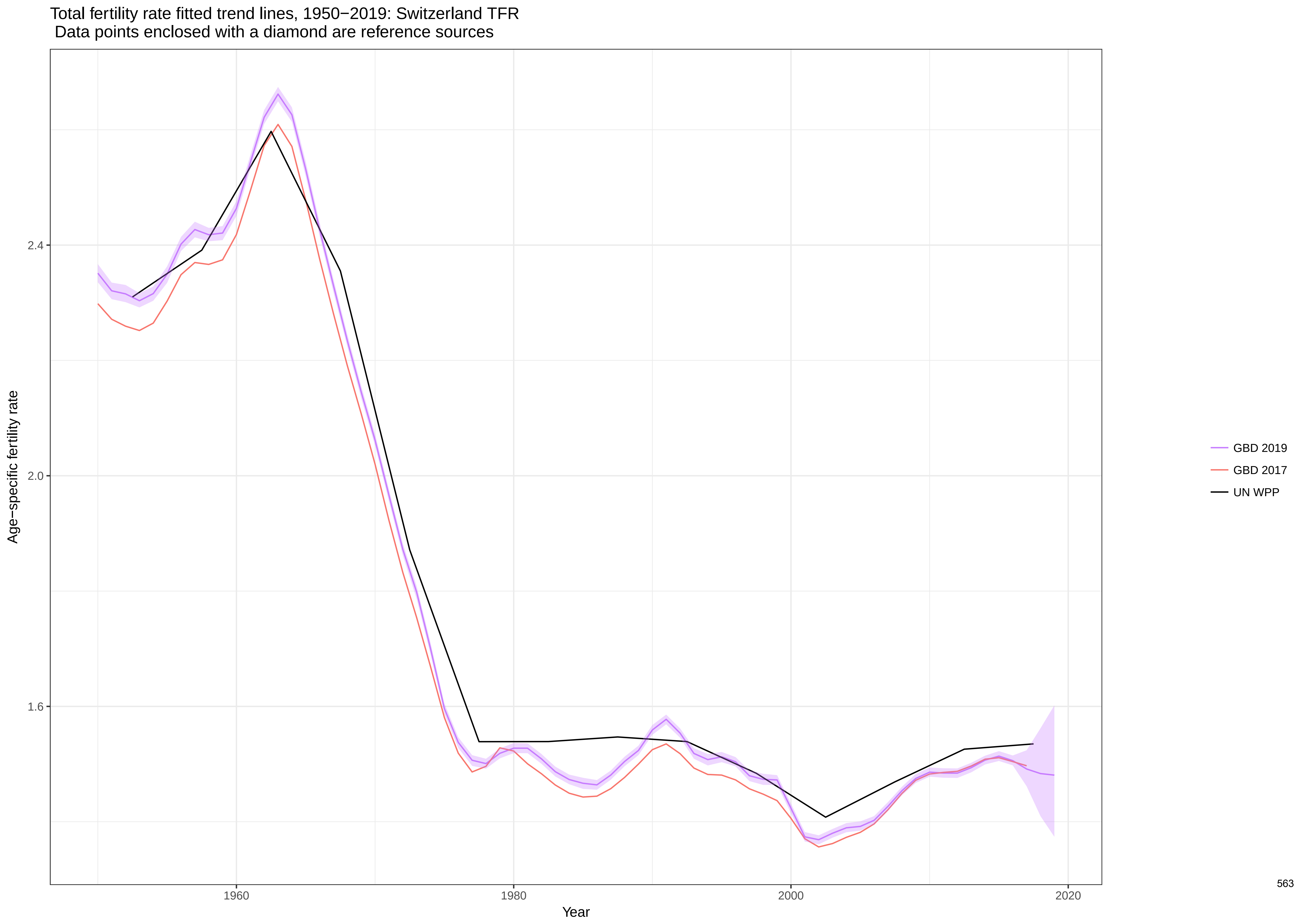

Total fertility rate fitted trend lines, 1950−2019: Switzerland TFR
Data points enclosed with a diamond are reference sources
Age−specific fertility rate
2.4
2.0
1.6
1960
1980
2000
2020
Year
GBD 2019
GBD 2017
UN WPP

Age−specific fertility fitted trend lines, 1950−2019: Chile, Ages: 10−14
Data points enclosed with a diamond are reference sources

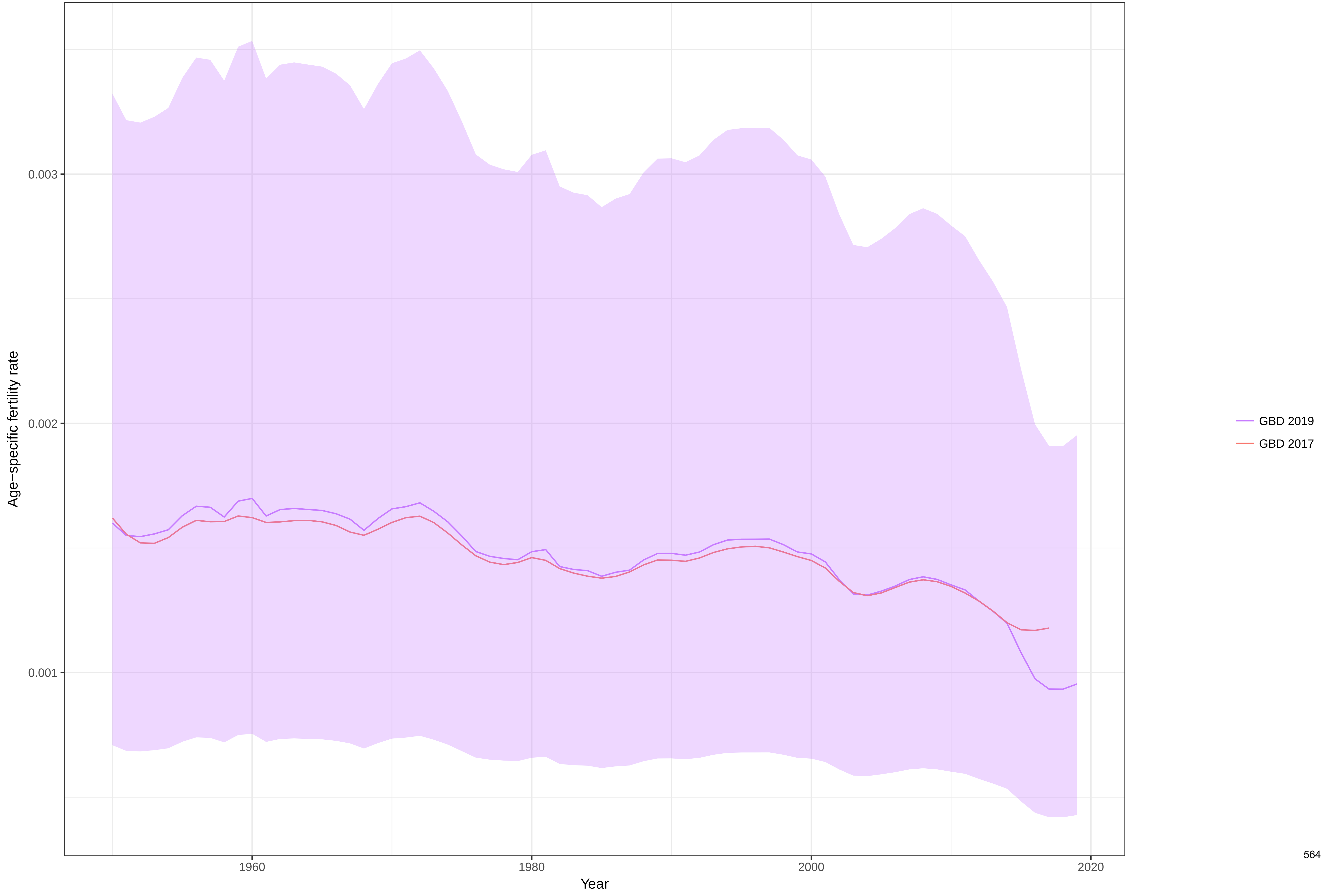

Age−specific fertility fitted trend lines, 1950−2019: Chile, Ages: 15−19
Data points enclosed with a diamond are reference sources

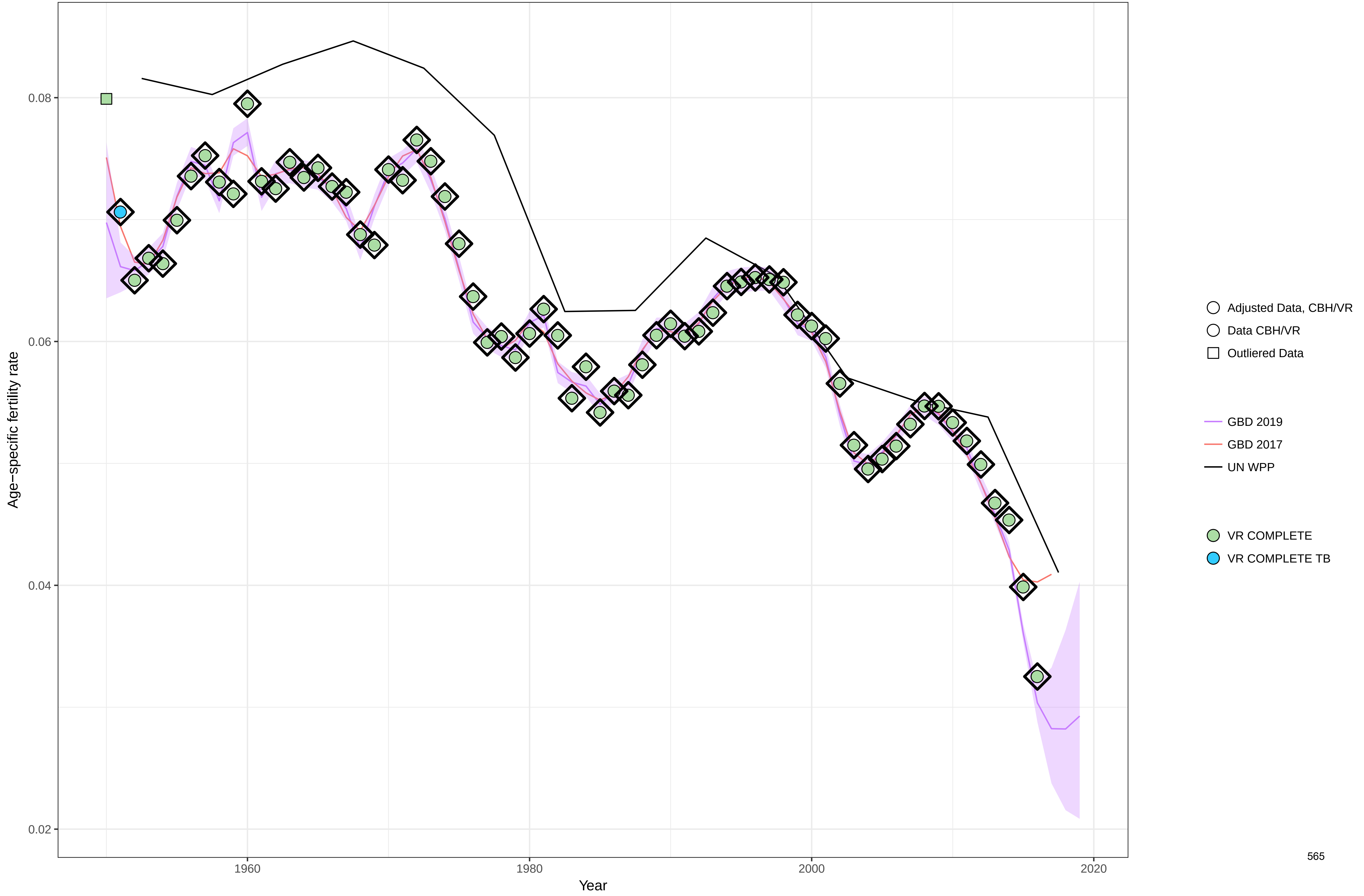

Age−specific fertility fitted trend lines, 1950−2019: Chile, Ages: 20−24
Data points enclosed with a diamond are reference sources

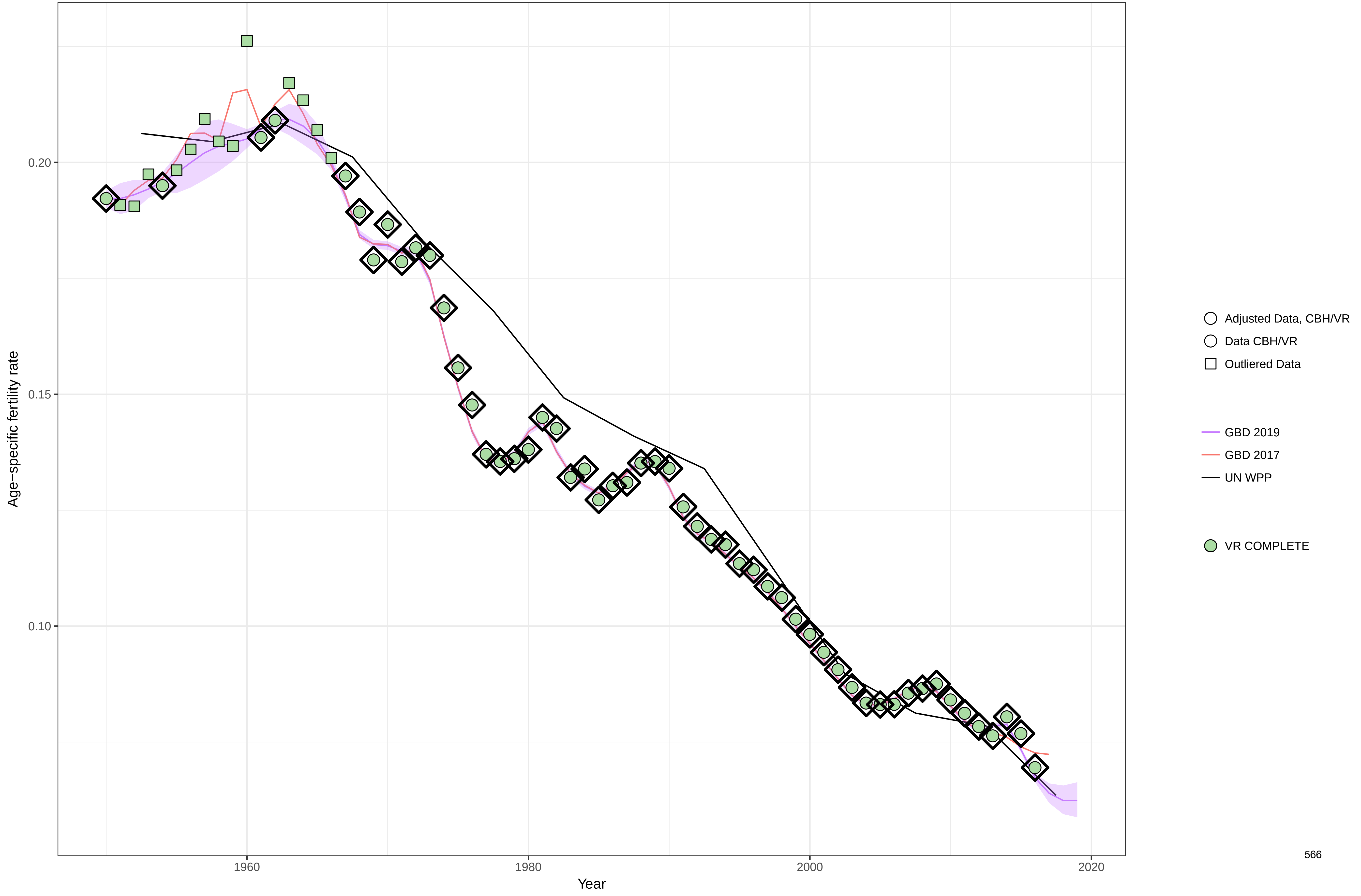

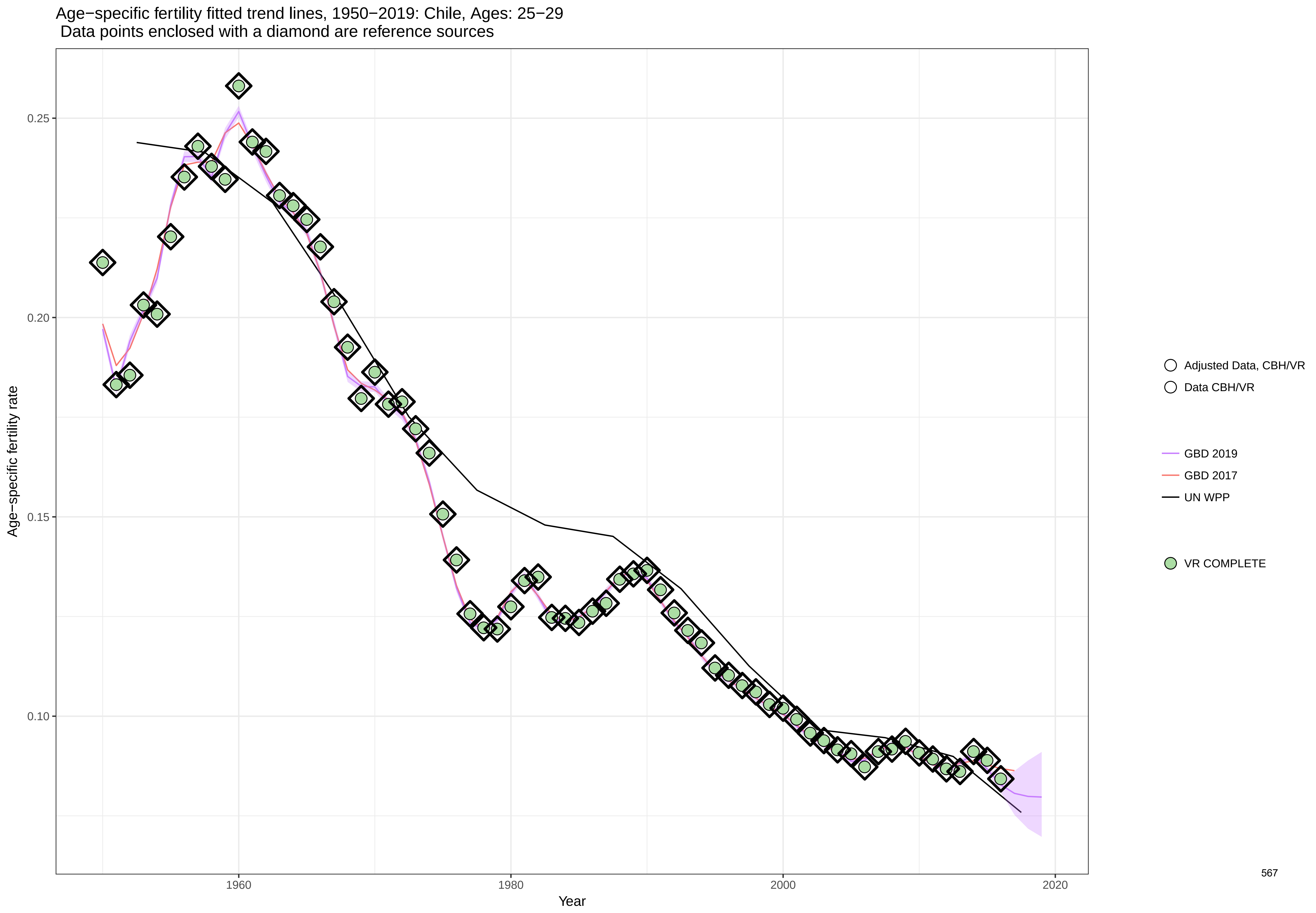
Age−specific fertility fitted trend lines, 1950−2019: Chile, Ages: 25−29
Data points enclosed with a diamond are reference sources
Age−specific fertility rate
0.25
0.20
0.15
0.10
1960
1980
2000
2020
Year
Adjusted Data, CBH/VR
Data CBH/VR
GBD 2019
GBD 2017
UN WPP
VR COMPLETE

Age−specific fertility fitted trend lines, 1950−2019: Chile, Ages: 30−34
Data points enclosed with a diamond are reference sources

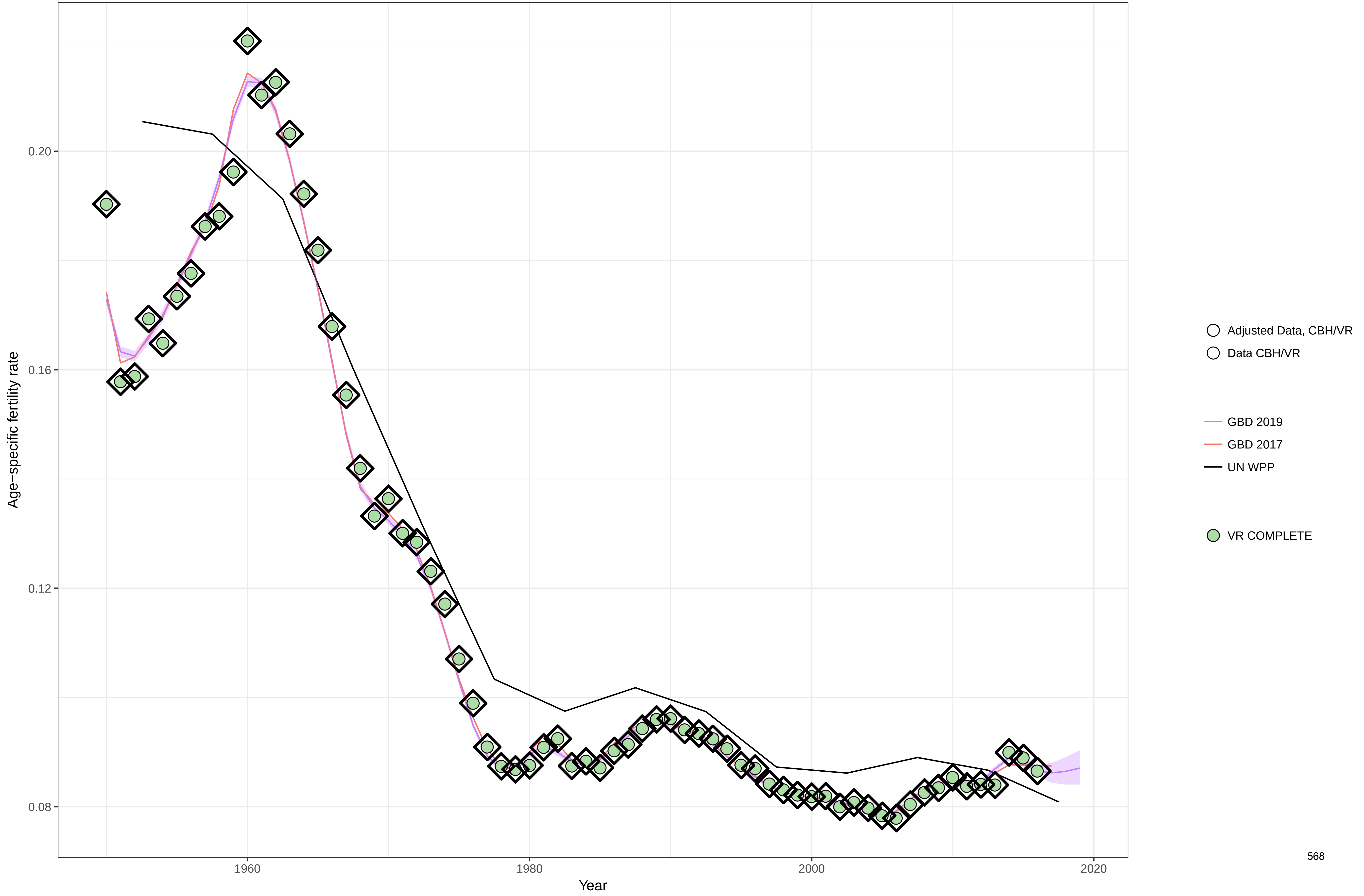

Age−specific fertility fitted trend lines, 1950−2019: Chile, Ages: 35−39
Data points enclosed with a diamond are reference sources

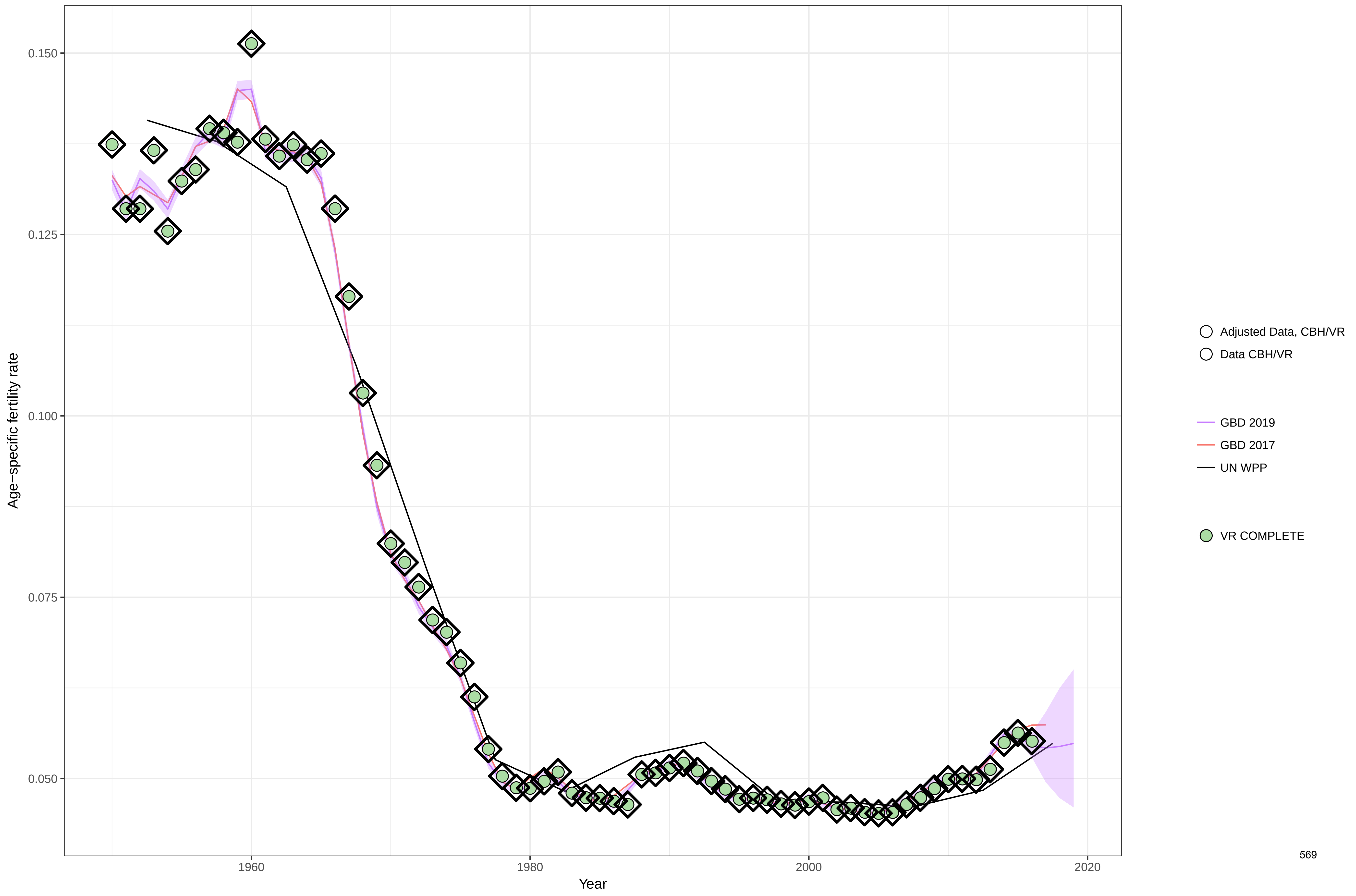

Age−specific fertility fitted trend lines, 1950−2019: Chile, Ages: 40−44
Data points enclosed with a diamond are reference sources

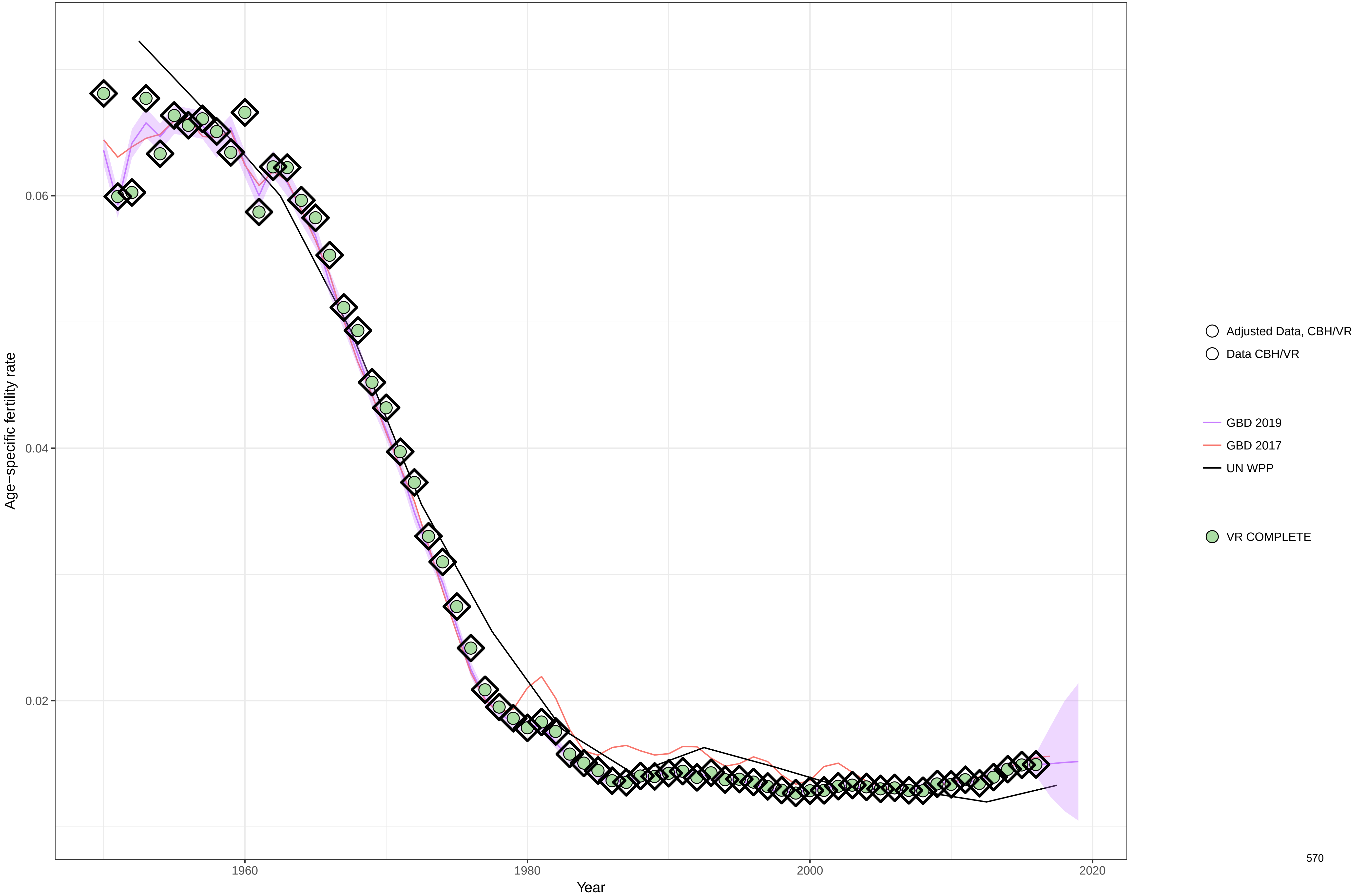

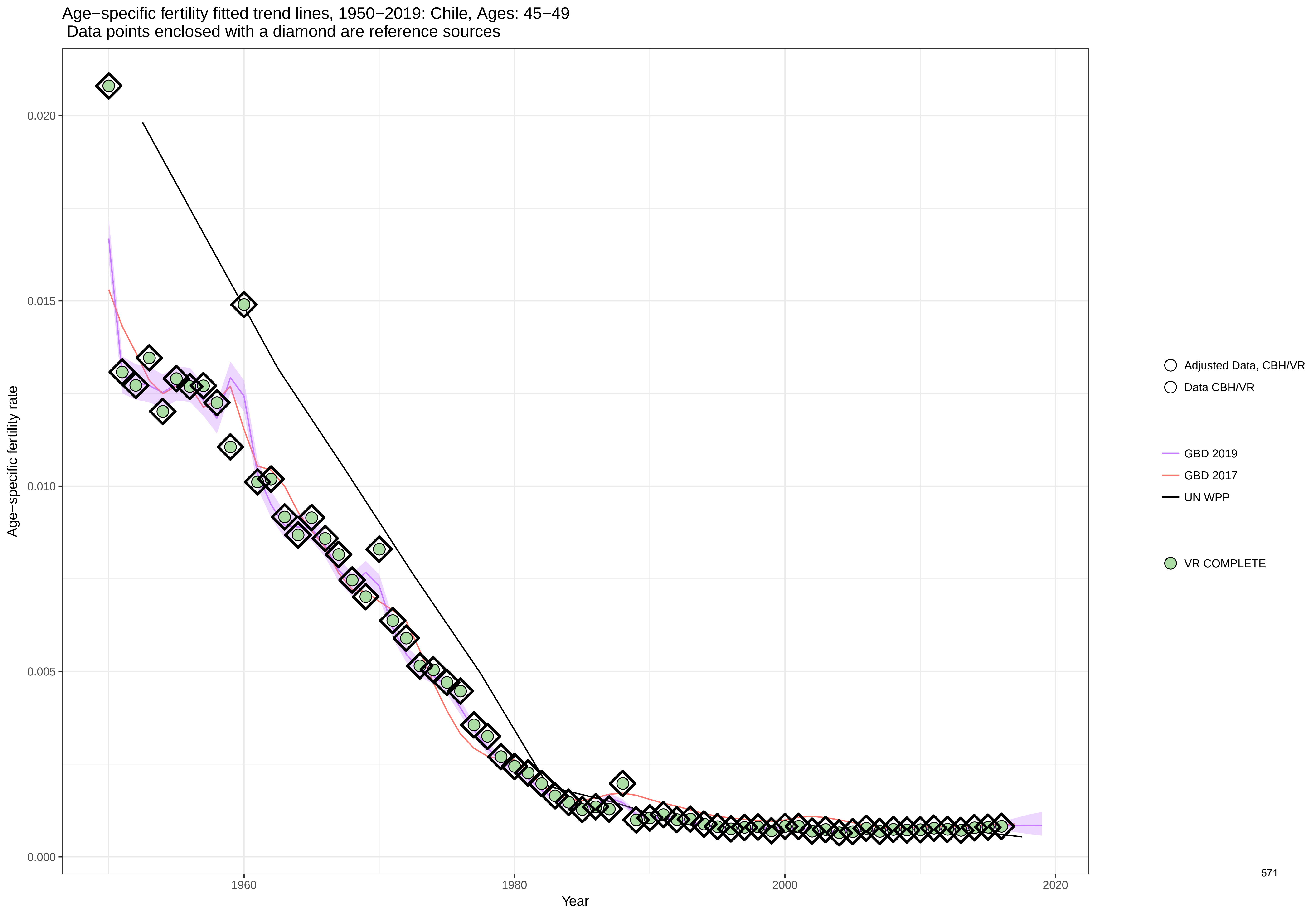

Age−specific fertility fitted trend lines, 1950−2019: Chile, Ages: 45−49
Data points enclosed with a diamond are reference sources
Age−specific fertility rate
0.000
0.005
0.010
0.015
0.020
1960
1980
2000
2020
Year
Adjusted Data, CBH/VR
Data CBH/VR
GBD 2019
GBD 2017
UN WPP
VR COMPLETE

Age−specific fertility fitted trend lines, 1950−2019: Chile, Ages: 50−54
Data points enclosed with a diamond are reference sources

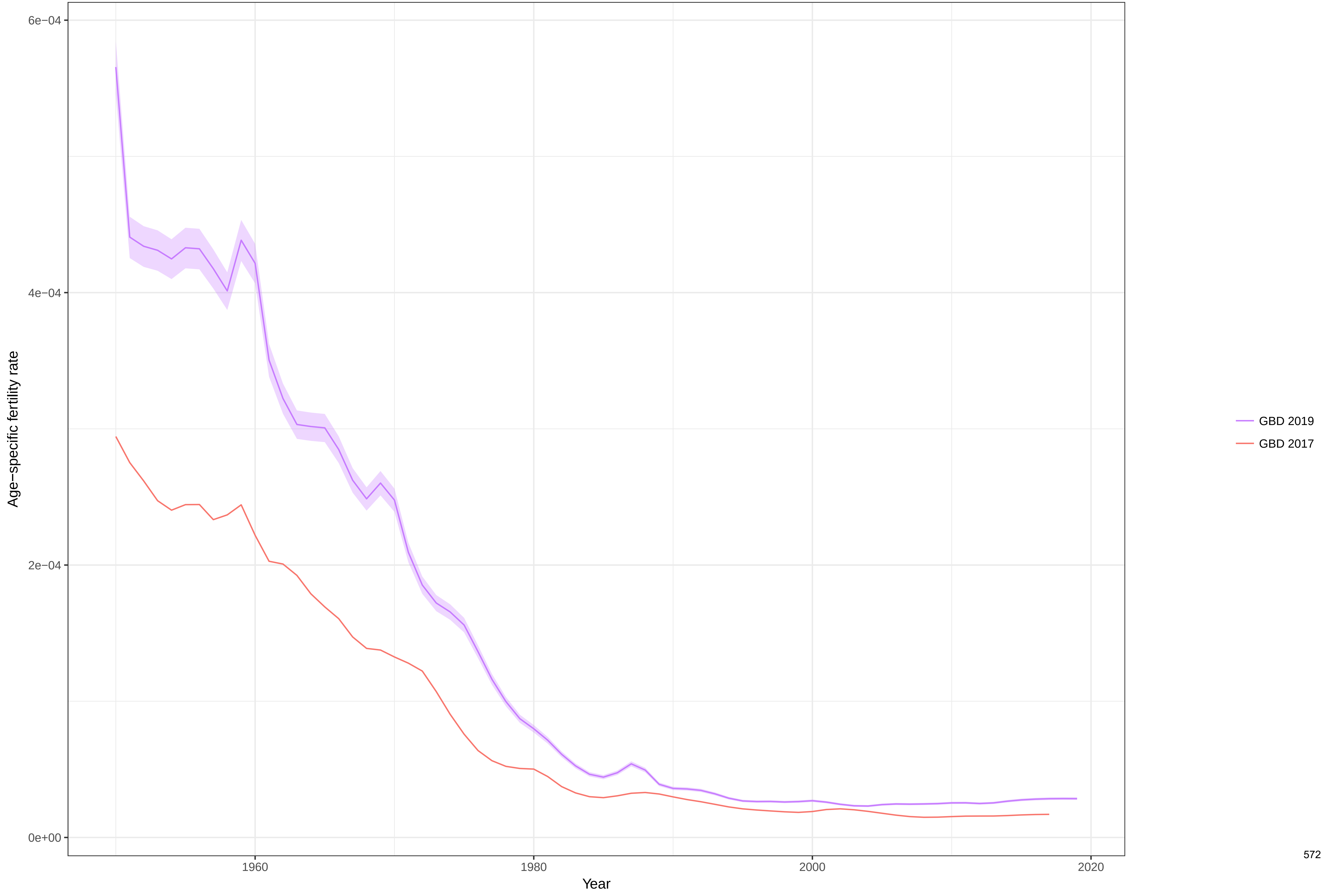

Total fertility rate fitted trend lines, 1950−2019: Chile TFR
Data points enclosed with a diamond are reference sources

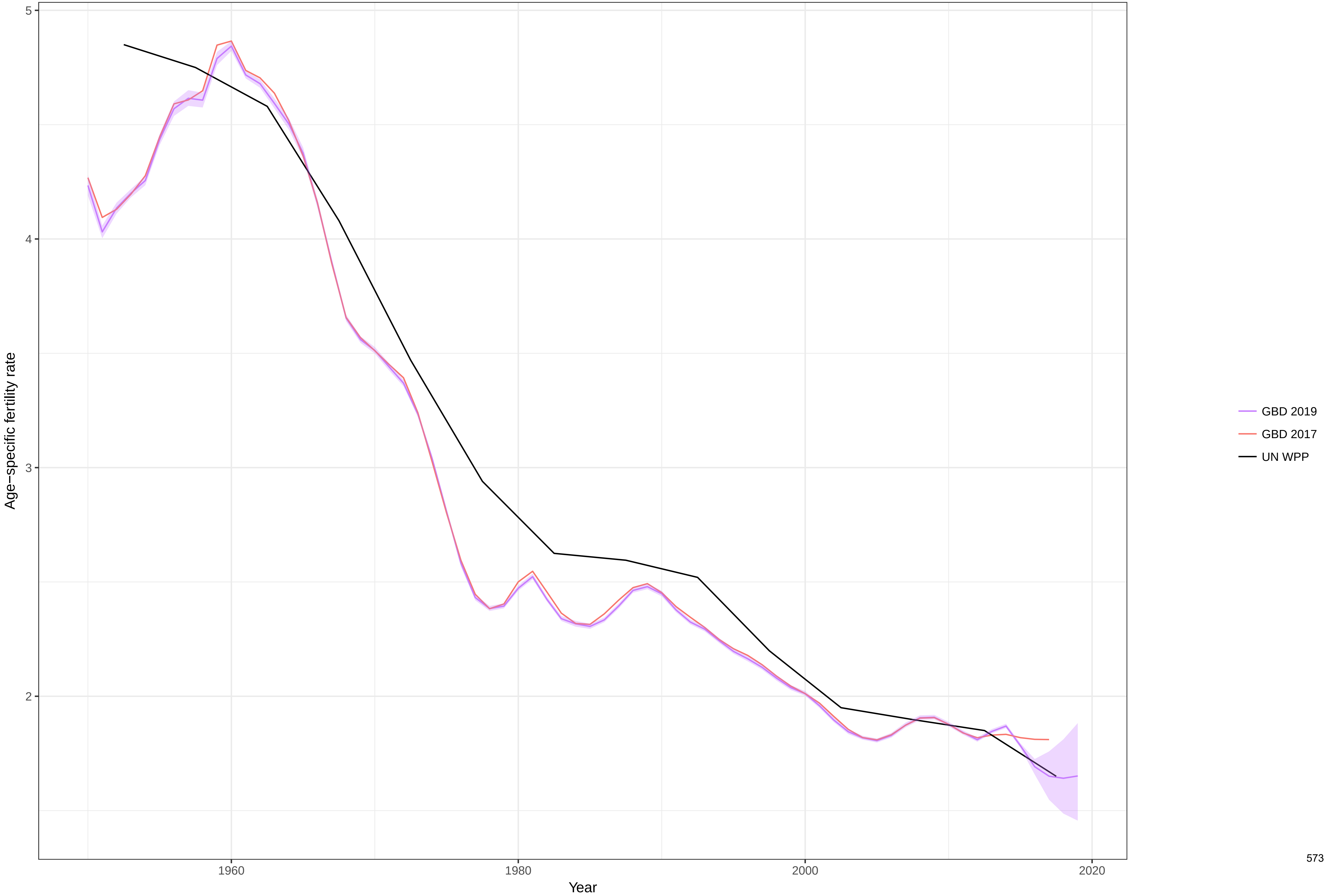

Age−specific fertility fitted trend lines, 1950−2019: Côte d'Ivoire, Ages: 10−14
Data points enclosed with a diamond are reference sources

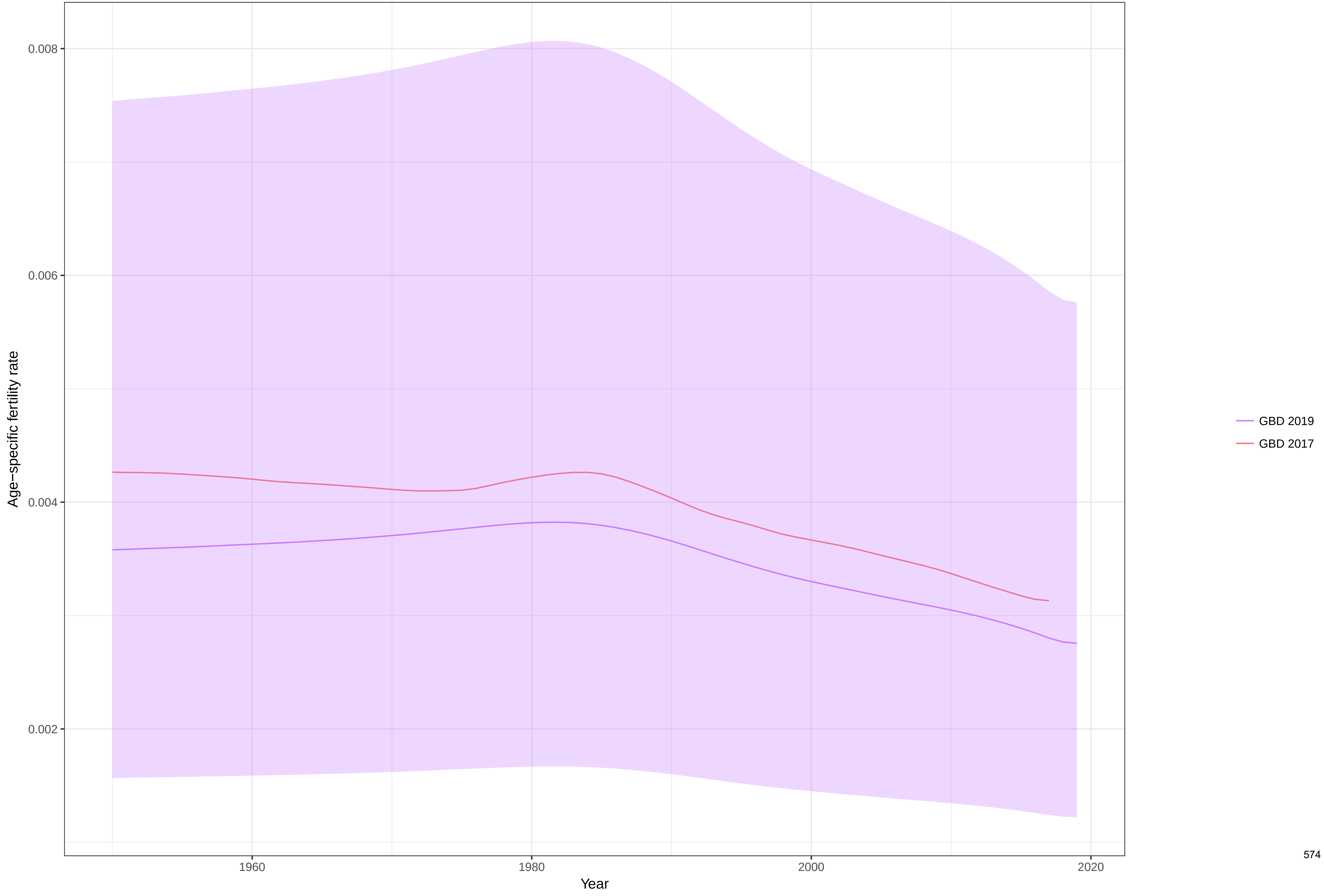

Age−specific fertility fitted trend lines, 1950−2019: Côte d'Ivoire, Ages: 15−19
Data points enclosed with a diamond are reference sources

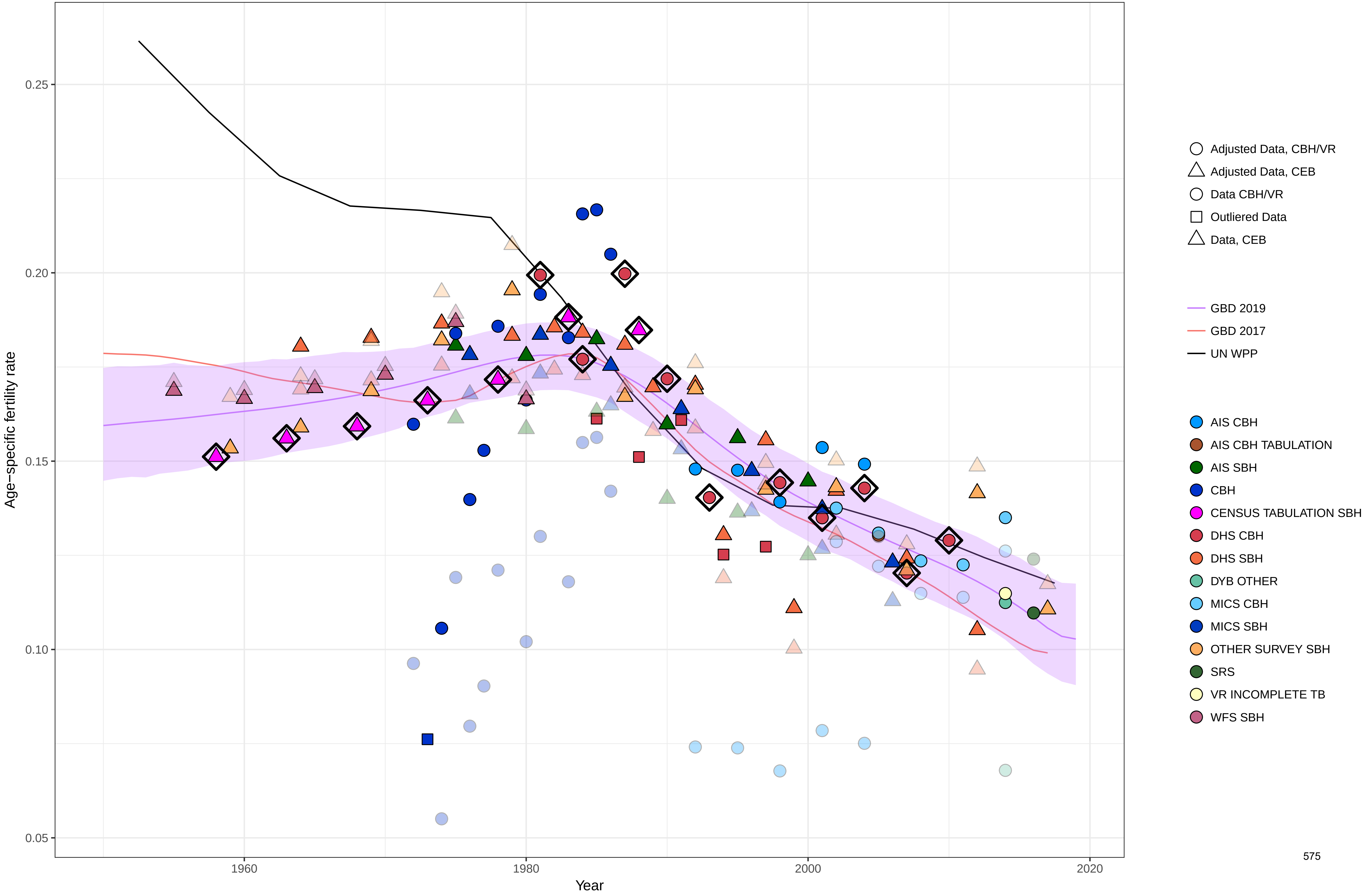

Age−specific fertility fitted trend lines, 1950−2019: Côte d'Ivoire, Ages: 20−24
Data points enclosed with a diamond are reference sources

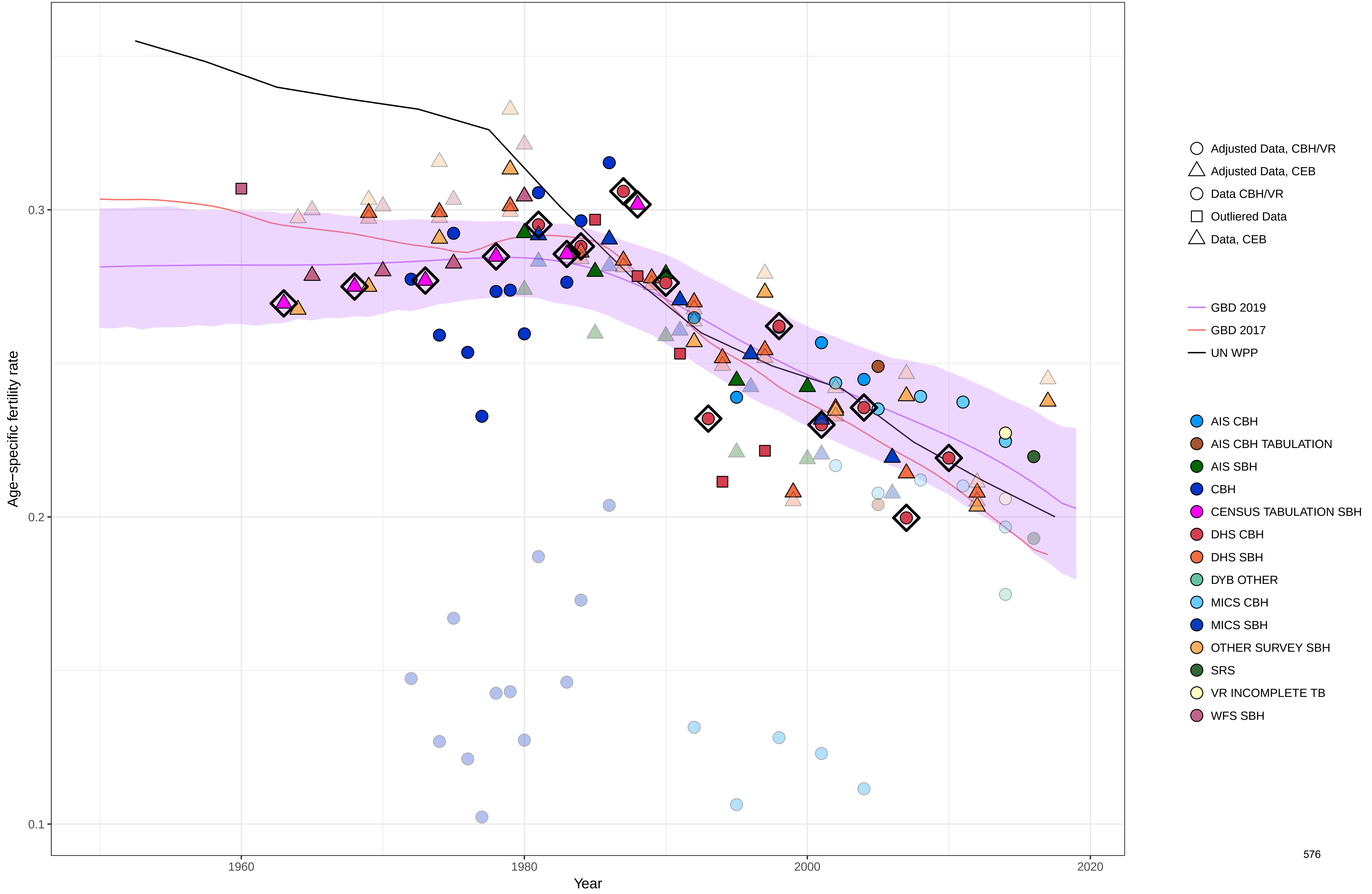

Age−specific fertility fitted trend lines, 1950−2019: Côte d'Ivoire, Ages: 25−29
Data points enclosed with a diamond are reference sources

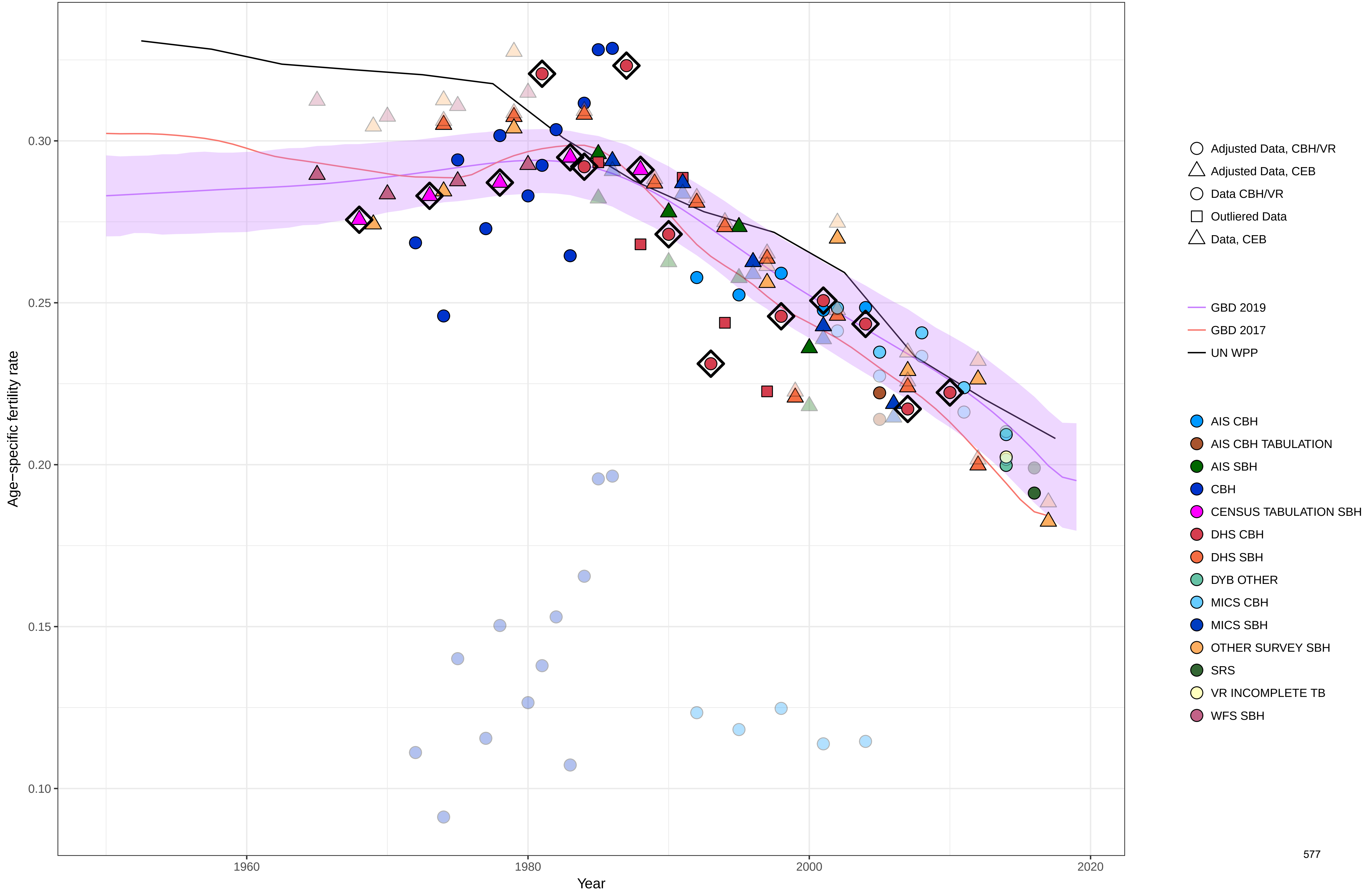

Age−specific fertility fitted trend lines, 1950−2019: Côte d'Ivoire, Ages: 30−34
Data points enclosed with a diamond are reference sources

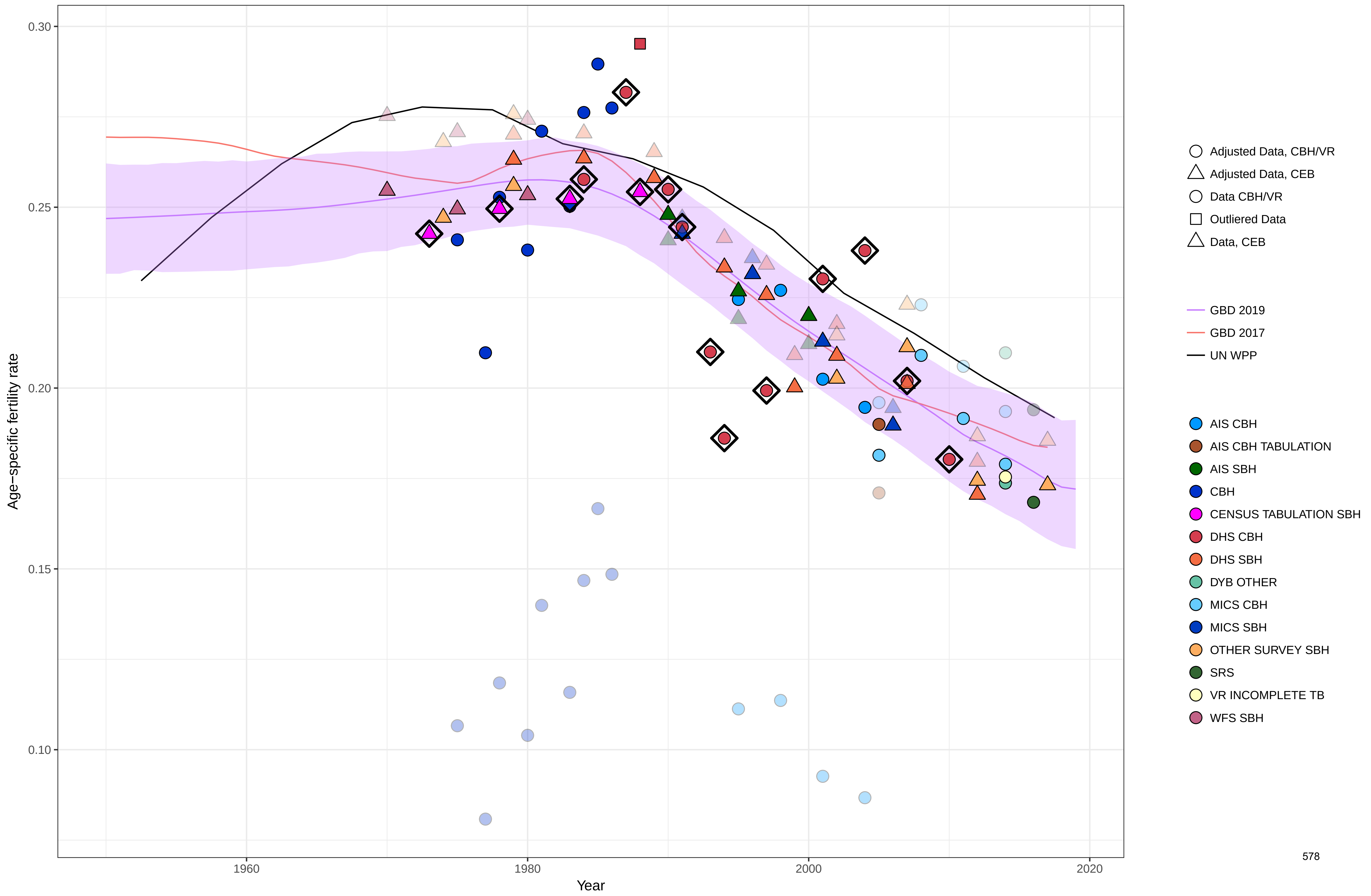

Age−specific fertility fitted trend lines, 1950−2019: Côte d'Ivoire, Ages: 35−39
Data points enclosed with a diamond are reference sources

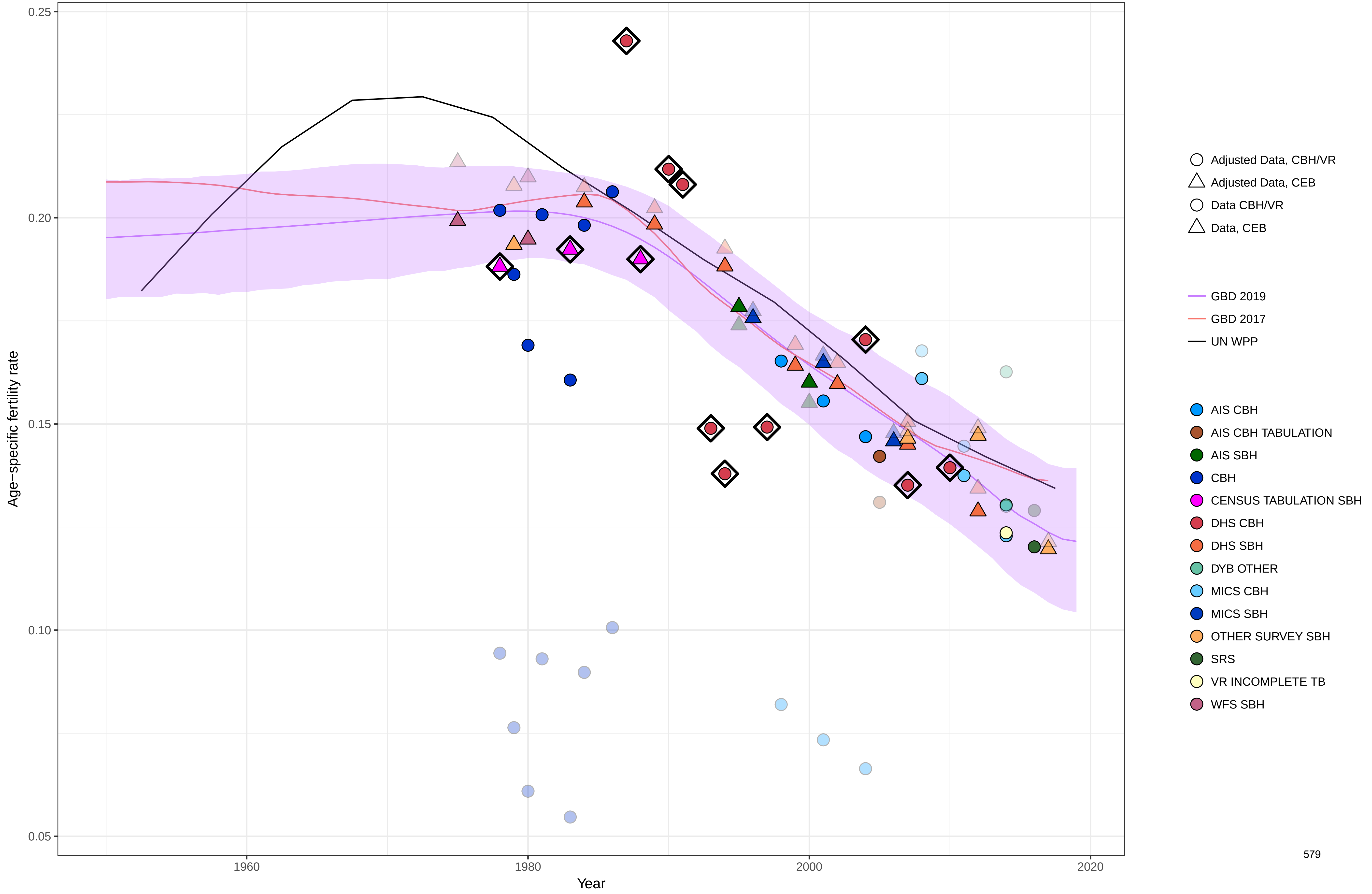

Age−specific fertility fitted trend lines, 1950−2019: Côte d'Ivoire, Ages: 40−44
Data points enclosed with a diamond are reference sources

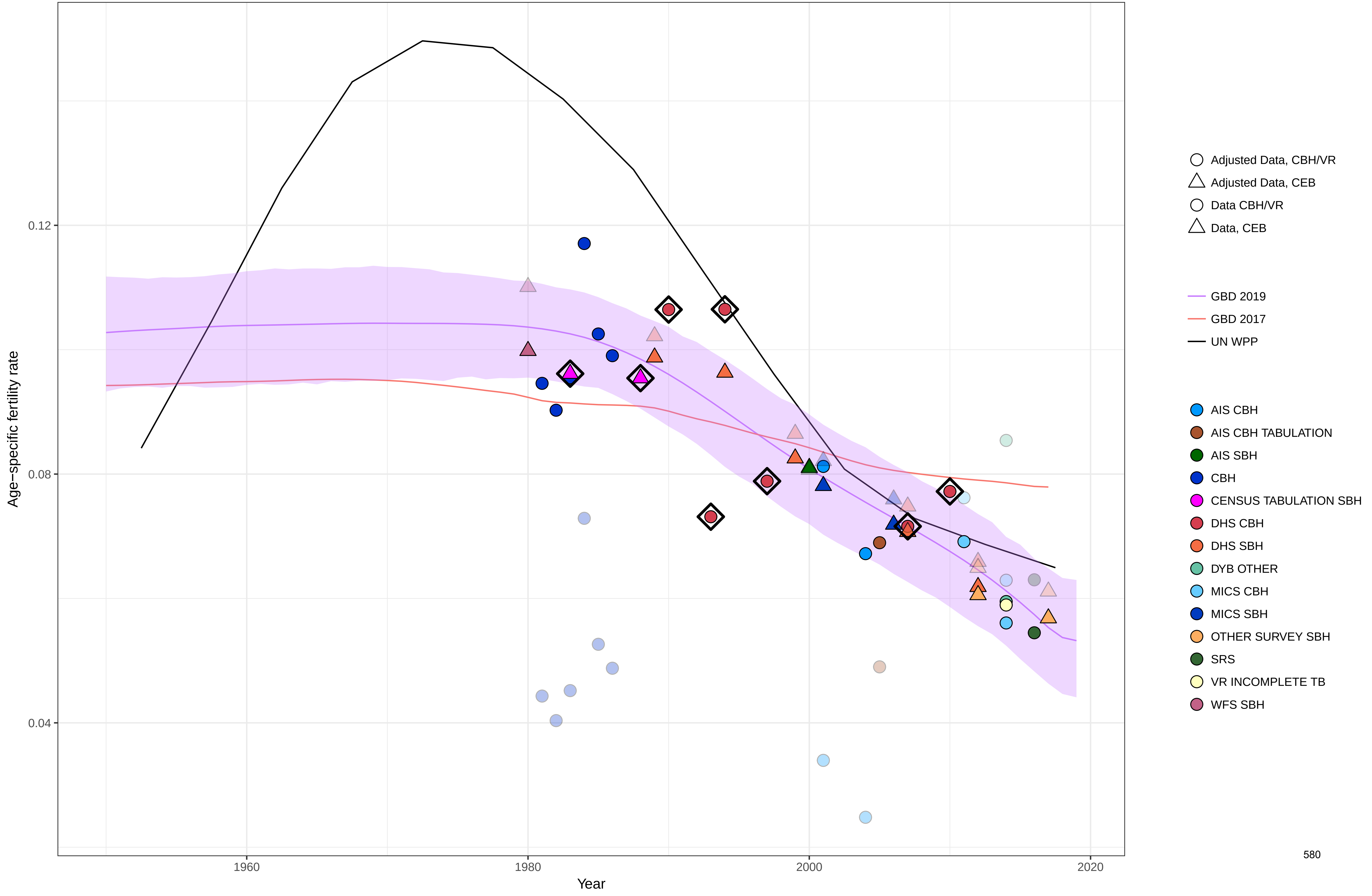

Age−specific fertility fitted trend lines, 1950−2019: Côte d'Ivoire, Ages: 45−49
Data points enclosed with a diamond are reference sources

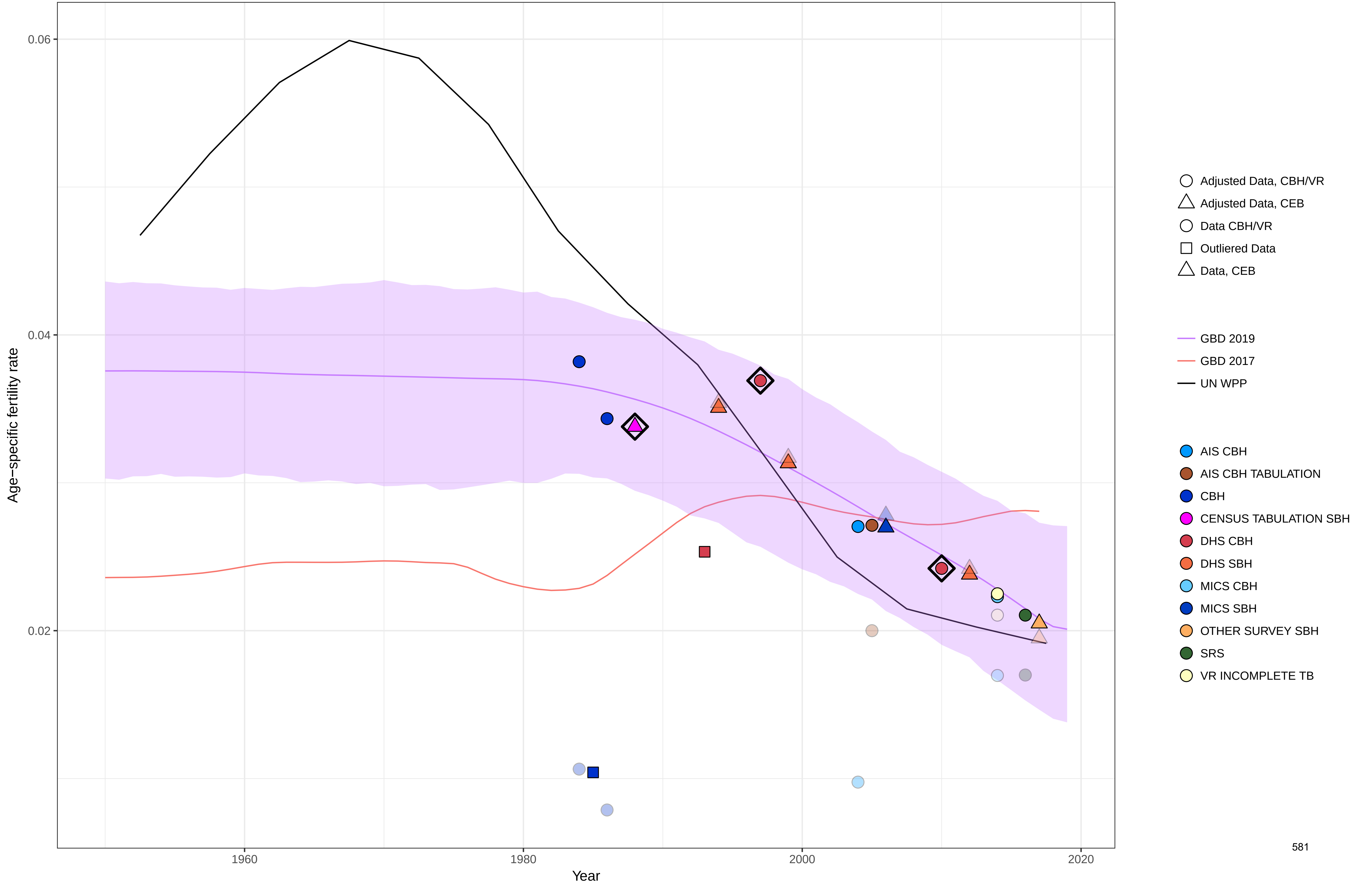

Age−specific fertility fitted trend lines, 1950−2019: Côte d'Ivoire, Ages: 50−54
Data points enclosed with a diamond are reference sources

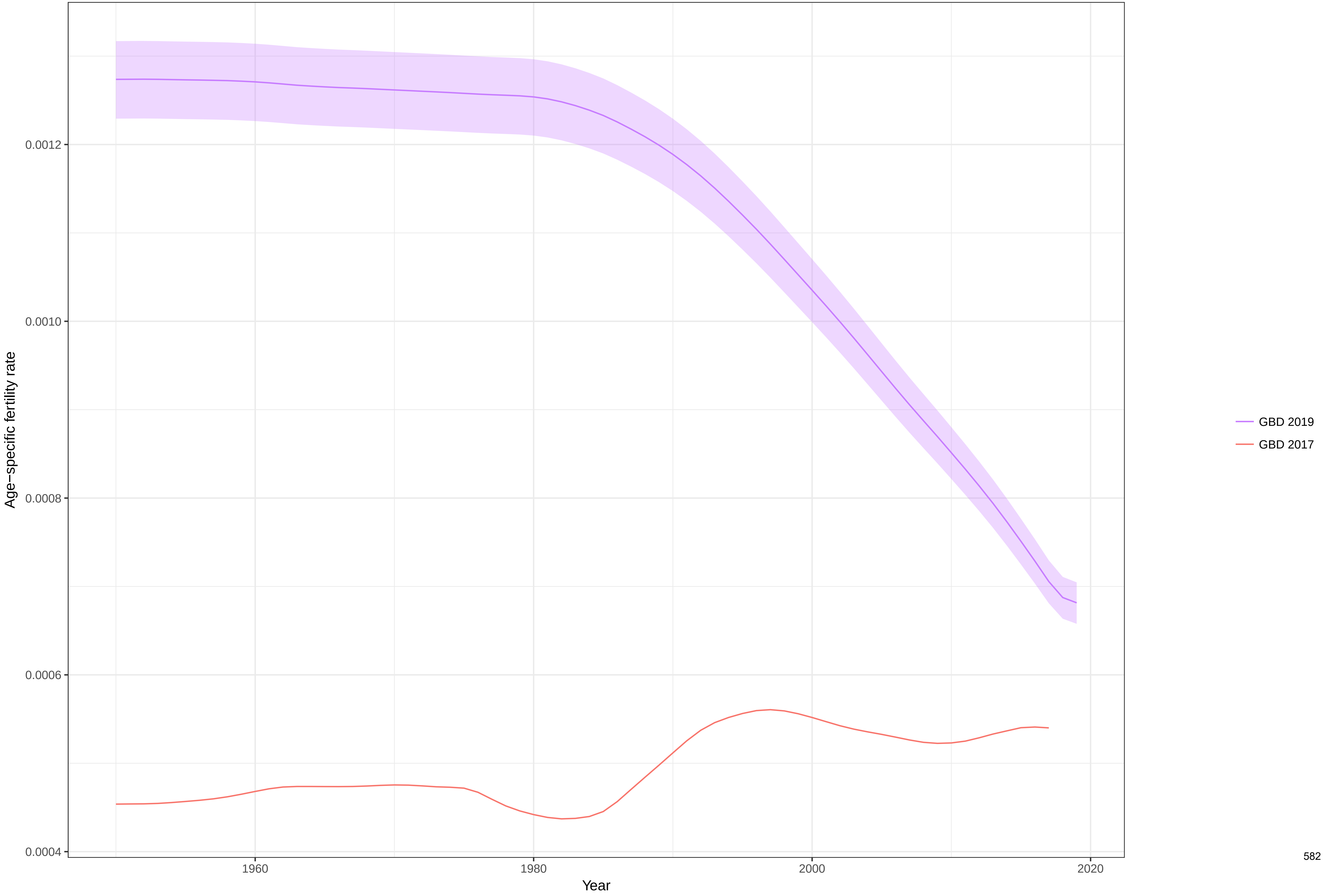

Total fertility rate fitted trend lines, 1950−2019: Côte d'Ivoire TFR
Data points enclosed with a diamond are reference sources

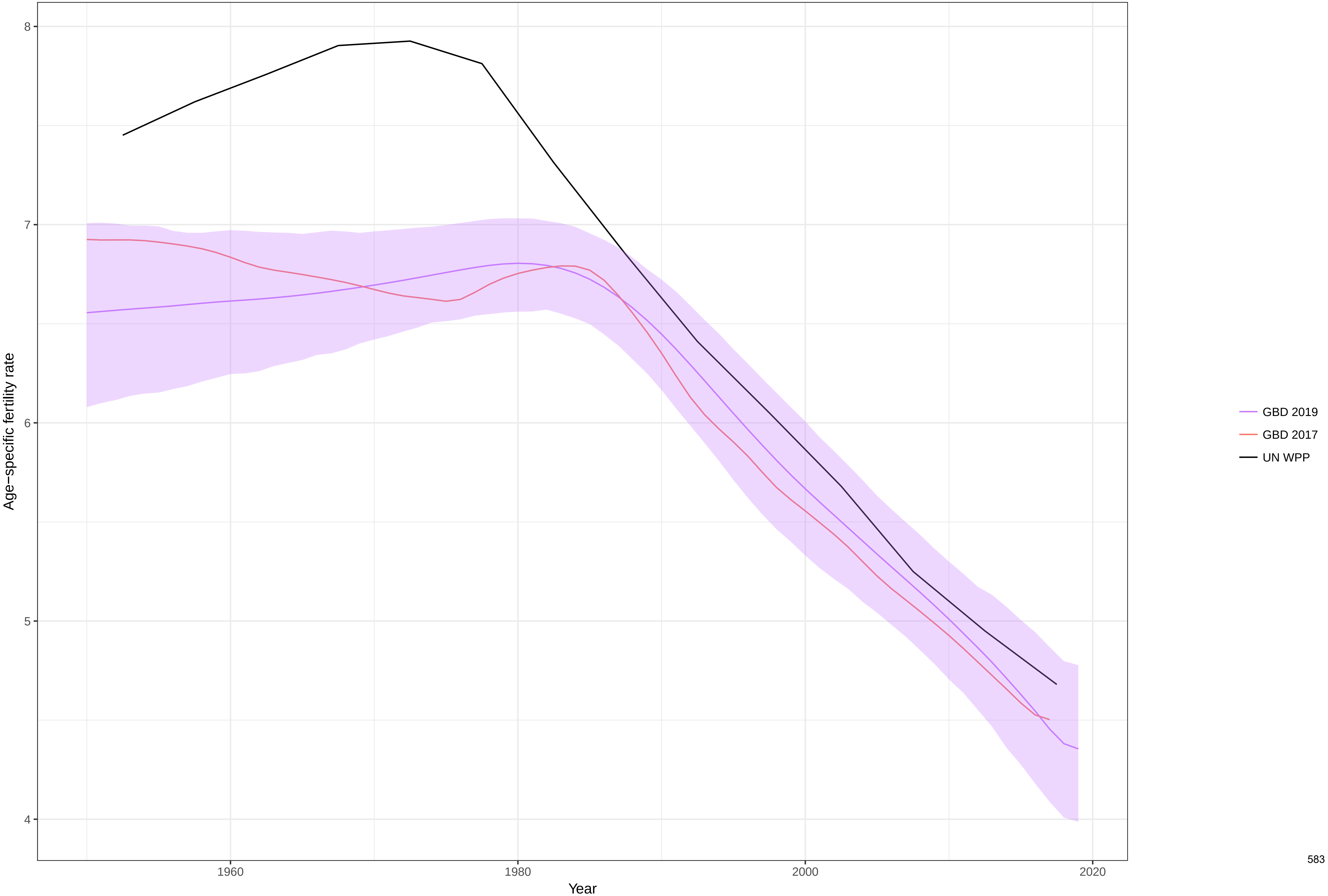

Age−specific fertility fitted trend lines, 1950−2019: Cameroon, Ages: 10−14
Data points enclosed with a diamond are reference sources

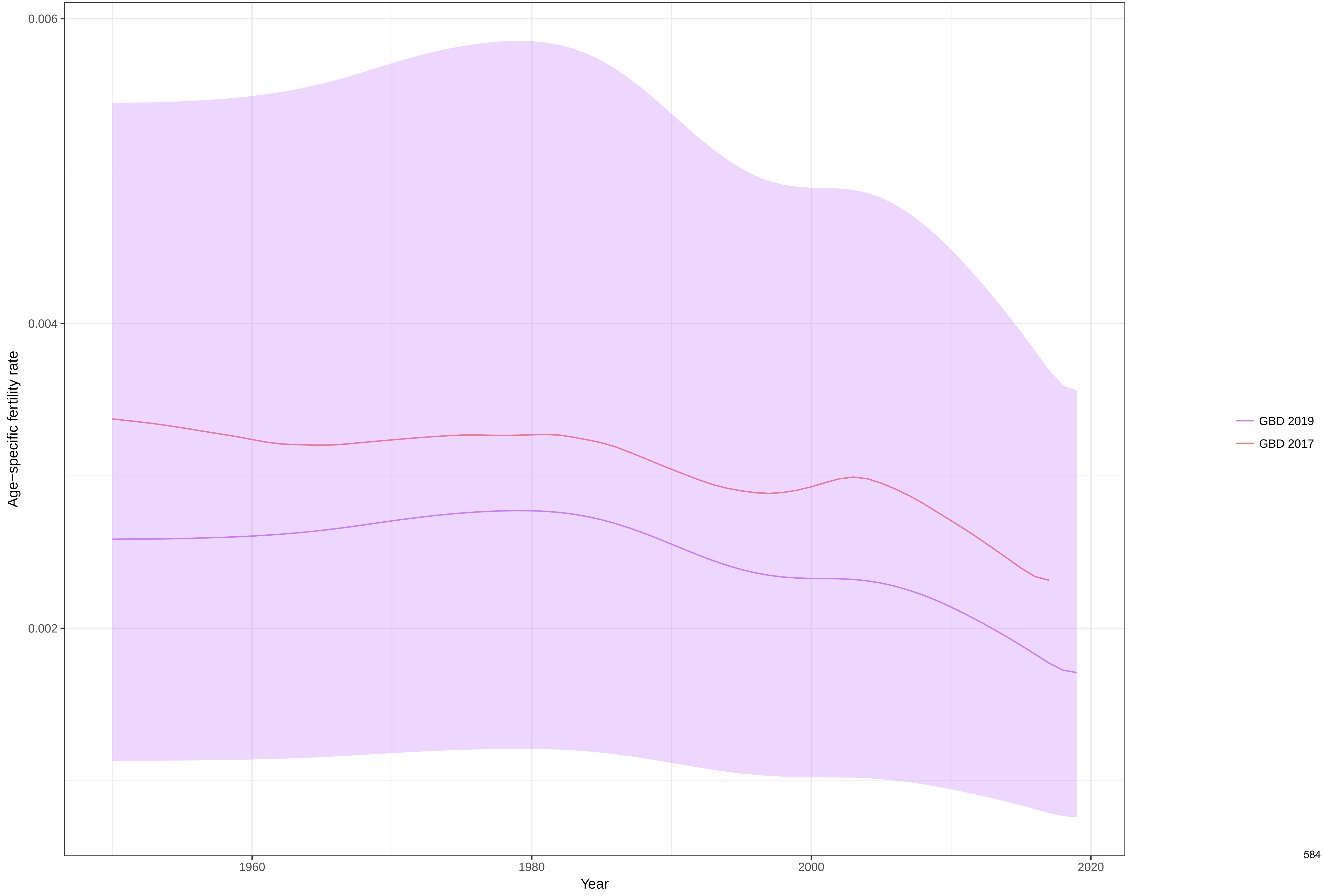

Age−specific fertility fitted trend lines, 1950−2019: Cameroon, Ages: 15−19
Data points enclosed with a diamond are reference sources

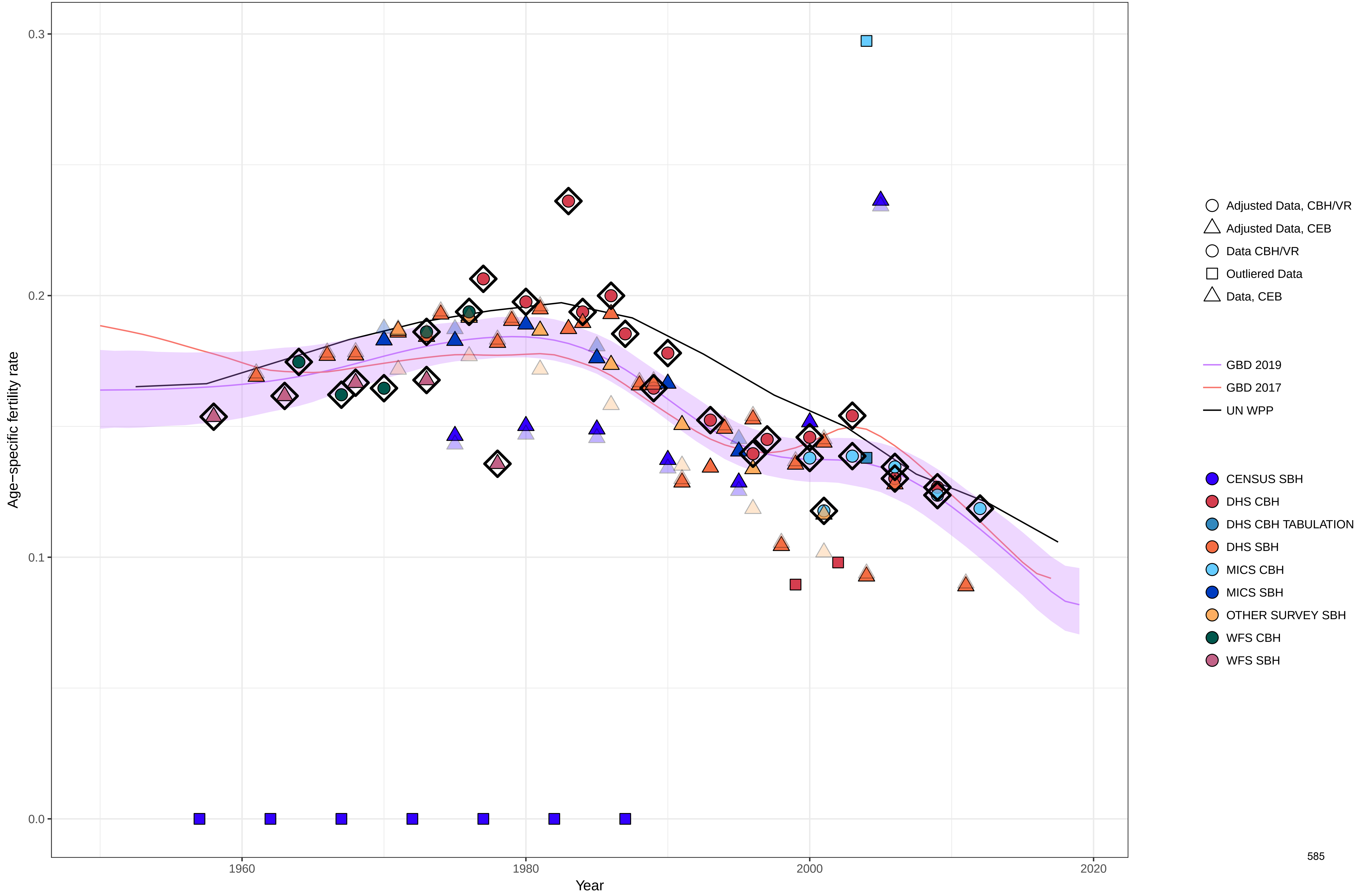

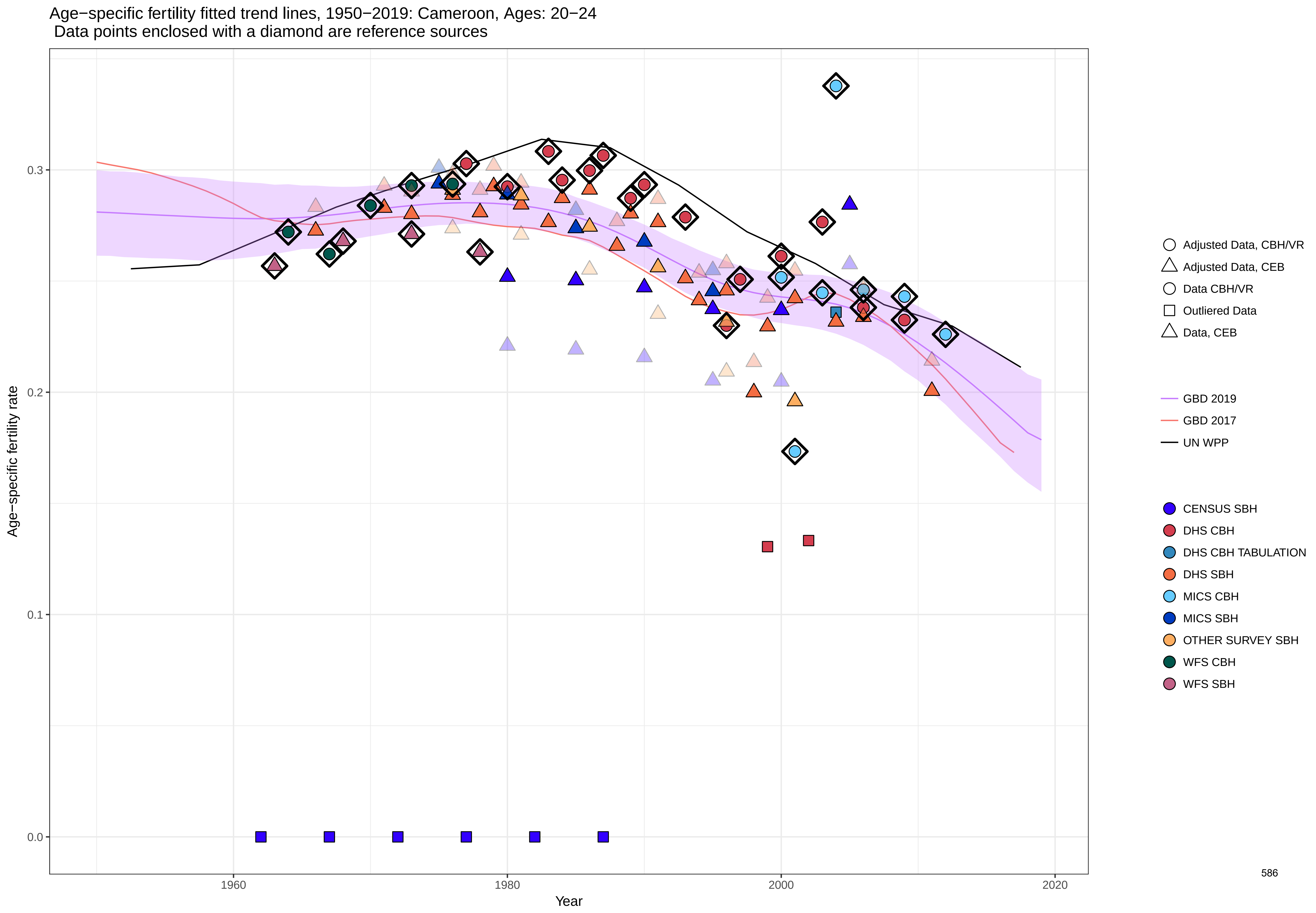
Age−specific fertility fitted trend lines, 1950−2019: Cameroon, Ages: 20−24
Data points enclosed with a diamond are reference sources
Age−specific fertility rate
Year
0.0
0.1
0.2
0.3
1960
1980
2000
2020
Adjusted Data, CBH/VR
Adjusted Data, CEB
Data CBH/VR
Outliered Data
Data, CEB
GBD 2019
GBD 2017
UN WPP
CENSUS SBH
DHS CBH
DHS CBH TABULATION
DHS SBH
MICS CBH
MICS SBH
OTHER SURVEY SBH
WFS CBH
WFS SBH

Age−specific fertility fitted trend lines, 1950−2019: Cameroon, Ages: 25−29
Data points enclosed with a diamond are reference sources

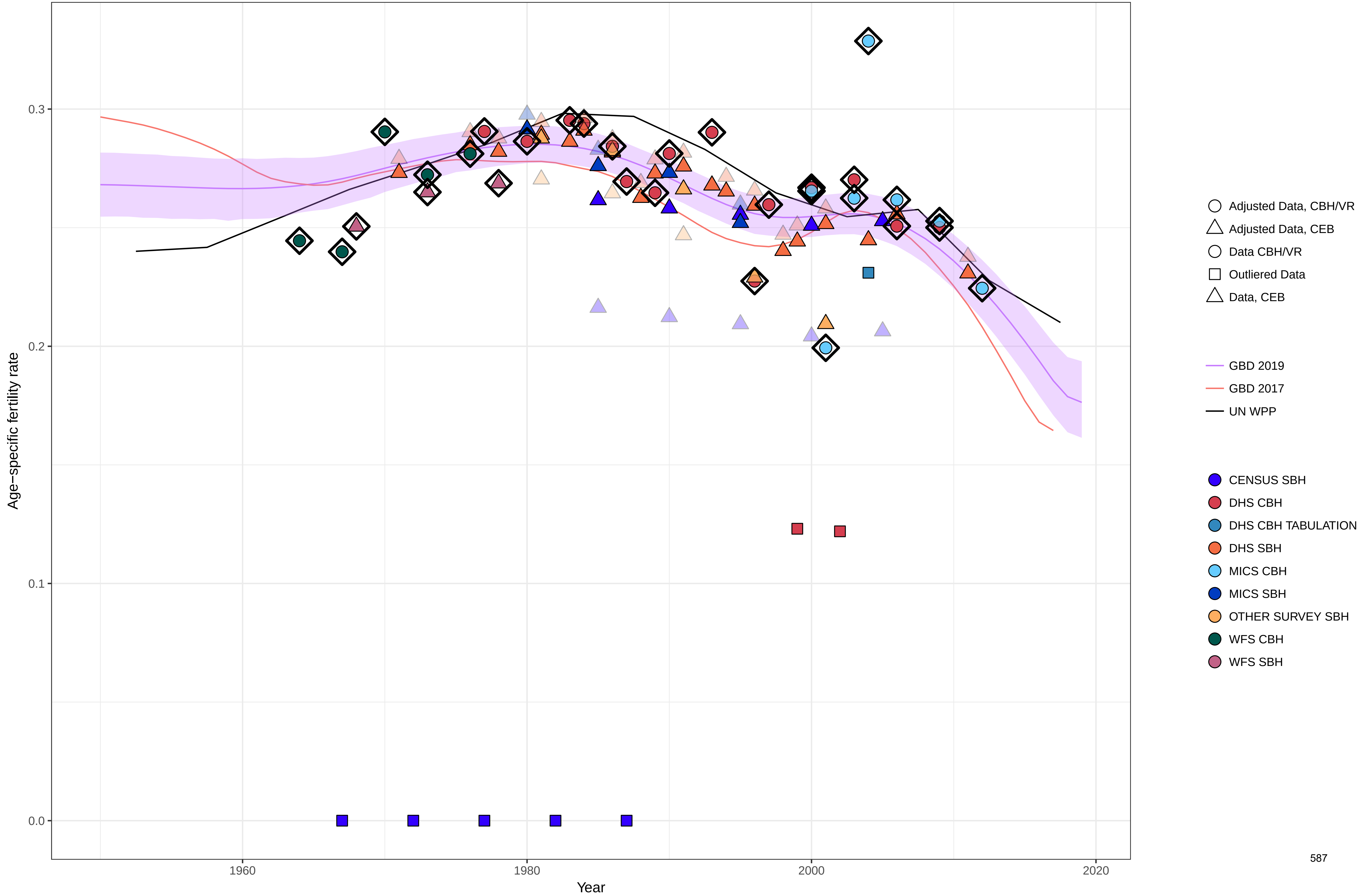

Age−specific fertility fitted trend lines, 1950−2019: Cameroon, Ages: 30−34
Data points enclosed with a diamond are reference sources

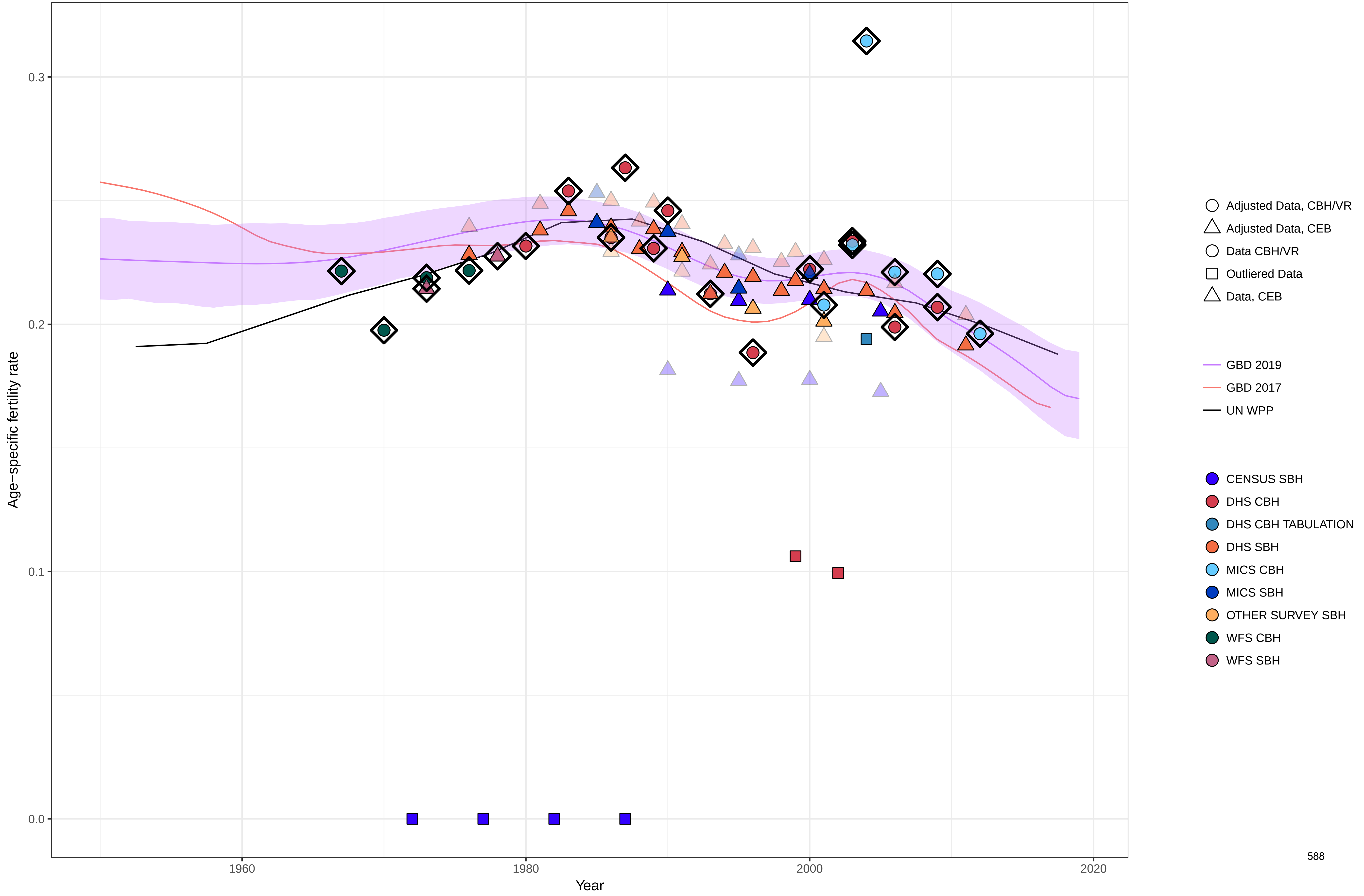

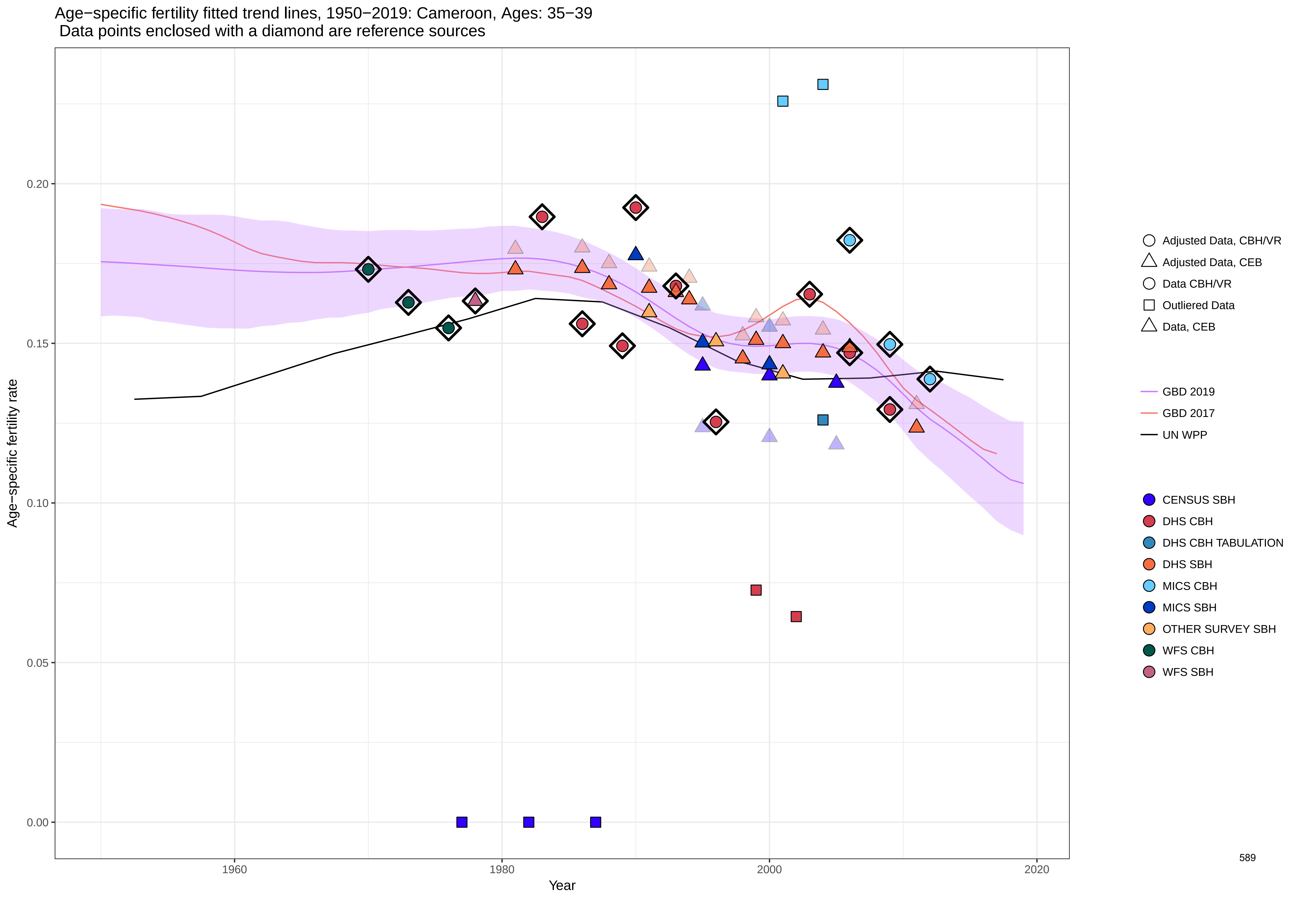

Age−specific fertility fitted trend lines, 1950−2019: Cameroon, Ages: 35−39
Data points enclosed with a diamond are reference sources
Age−specific fertility rate
Year
0.00
0.05
0.10
0.15
0.20
1960
1980
2000
2020
Adjusted Data, CBH/VR
Adjusted Data, CEB
Data CBH/VR
Outliered Data
Data, CEB
GBD 2019
GBD 2017
UN WPP
CENSUS SBH
DHS CBH
DHS CBH TABULATION
DHS SBH
MICS CBH
MICS SBH
OTHER SURVEY SBH
WFS CBH
WFS SBH

Age−specific fertility fitted trend lines, 1950−2019: Cameroon, Ages: 40−44
Data points enclosed with a diamond are reference sources

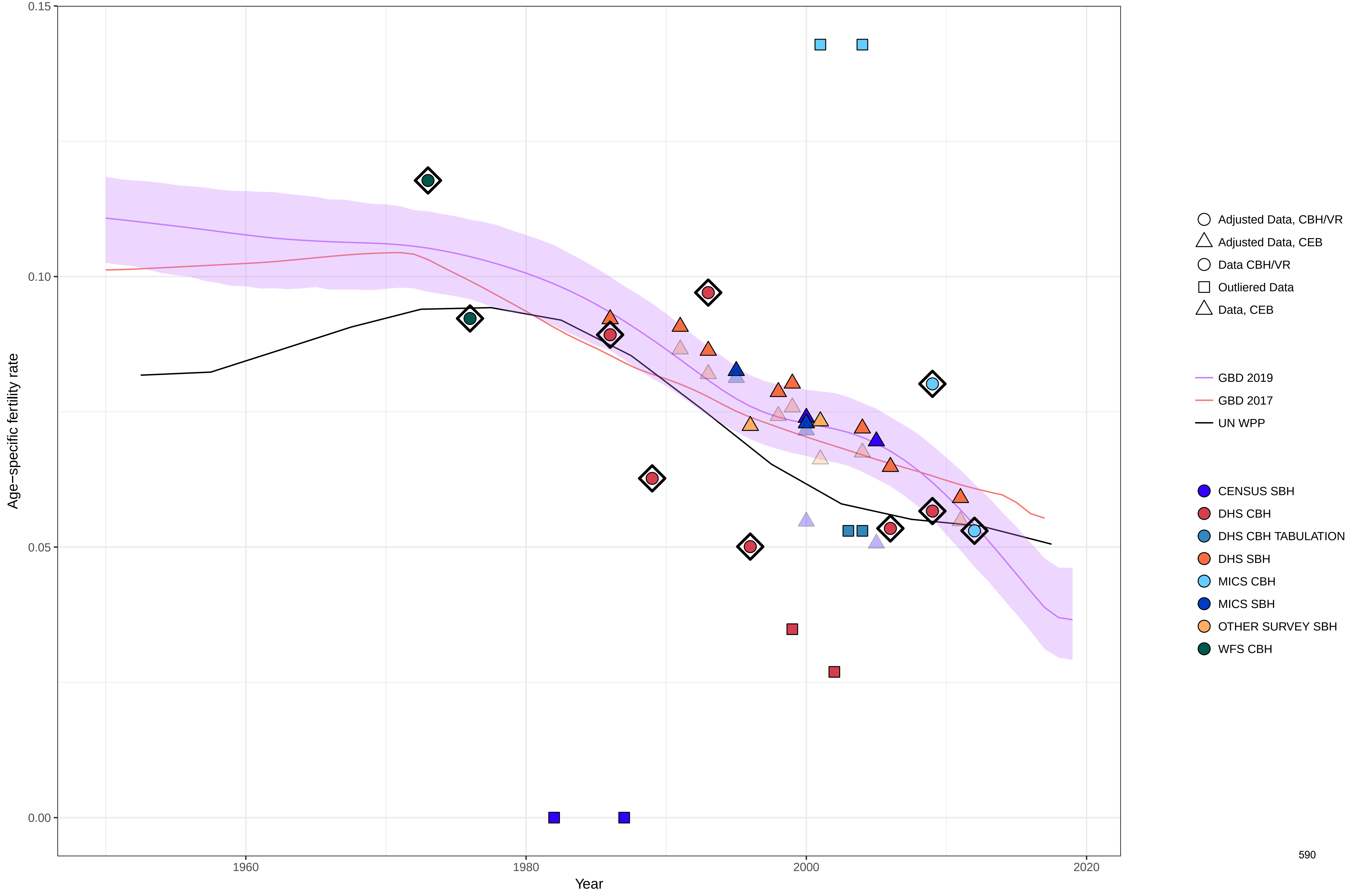

Age−specific fertility fitted trend lines, 1950−2019: Cameroon, Ages: 45−49
Data points enclosed with a diamond are reference sources

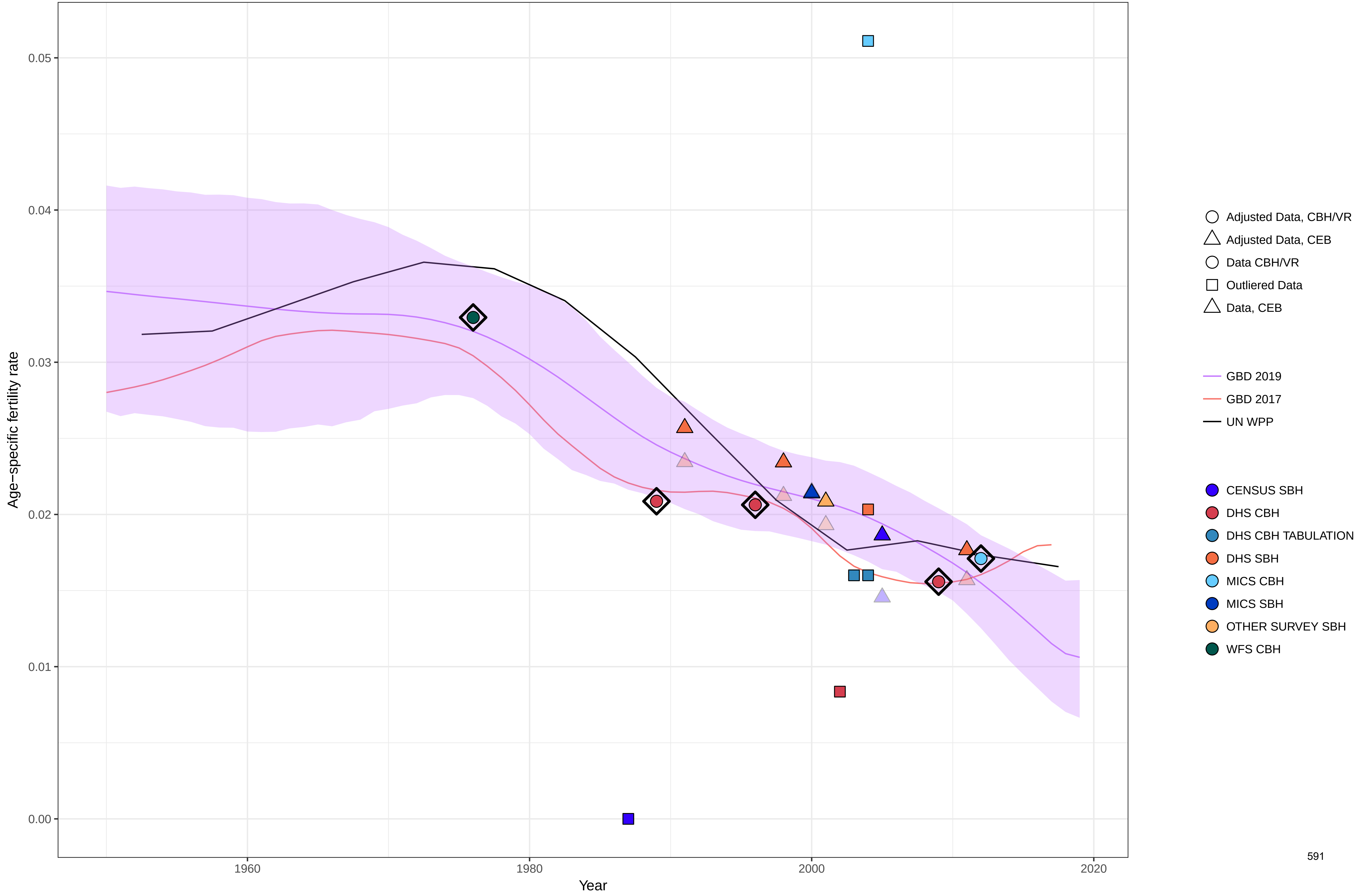

Age−specific fertility fitted trend lines, 1950−2019: Cameroon, Ages: 50−54
Data points enclosed with a diamond are reference sources

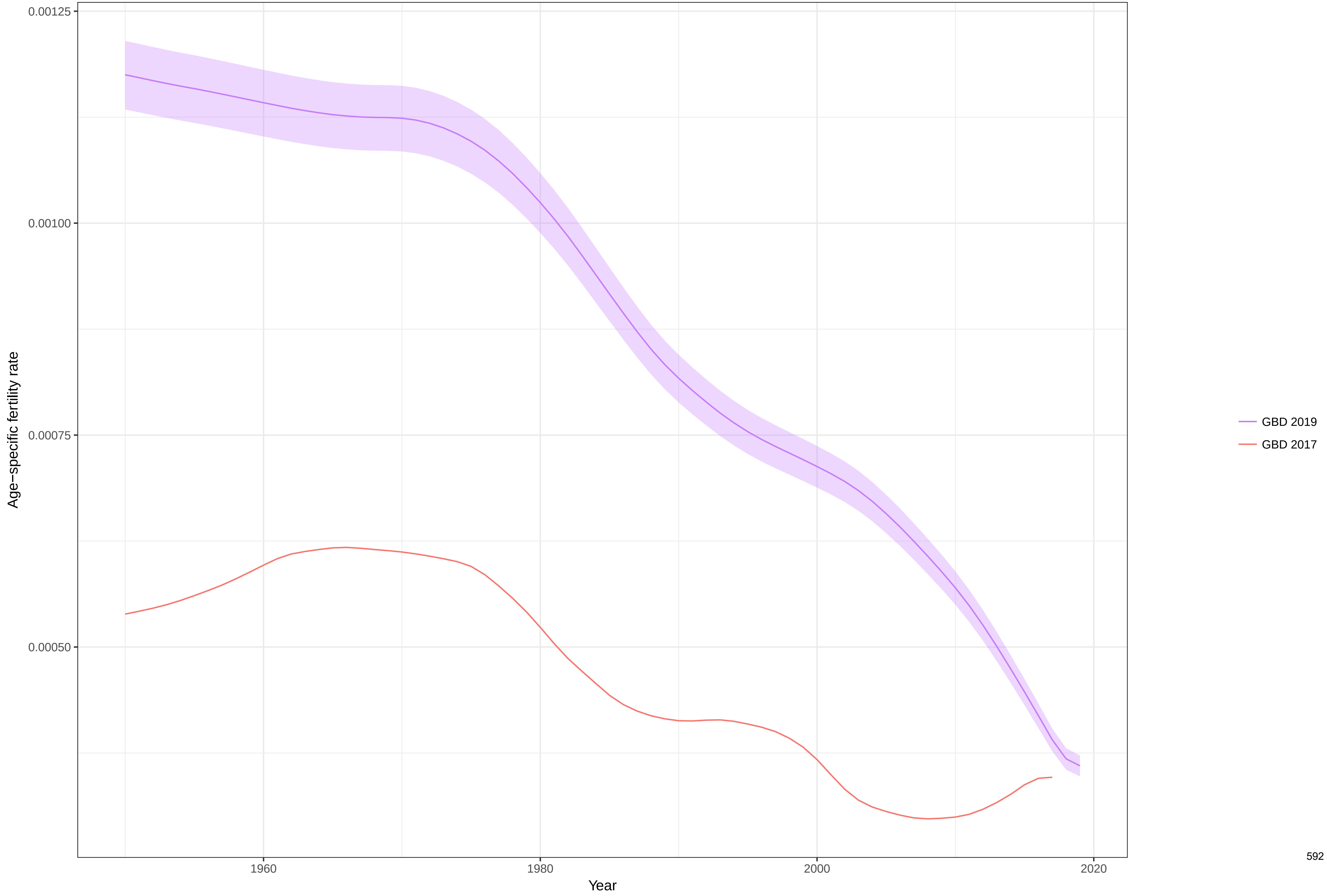

Total fertility rate fitted trend lines, 1950−2019: Cameroon TFR
Data points enclosed with a diamond are reference sources

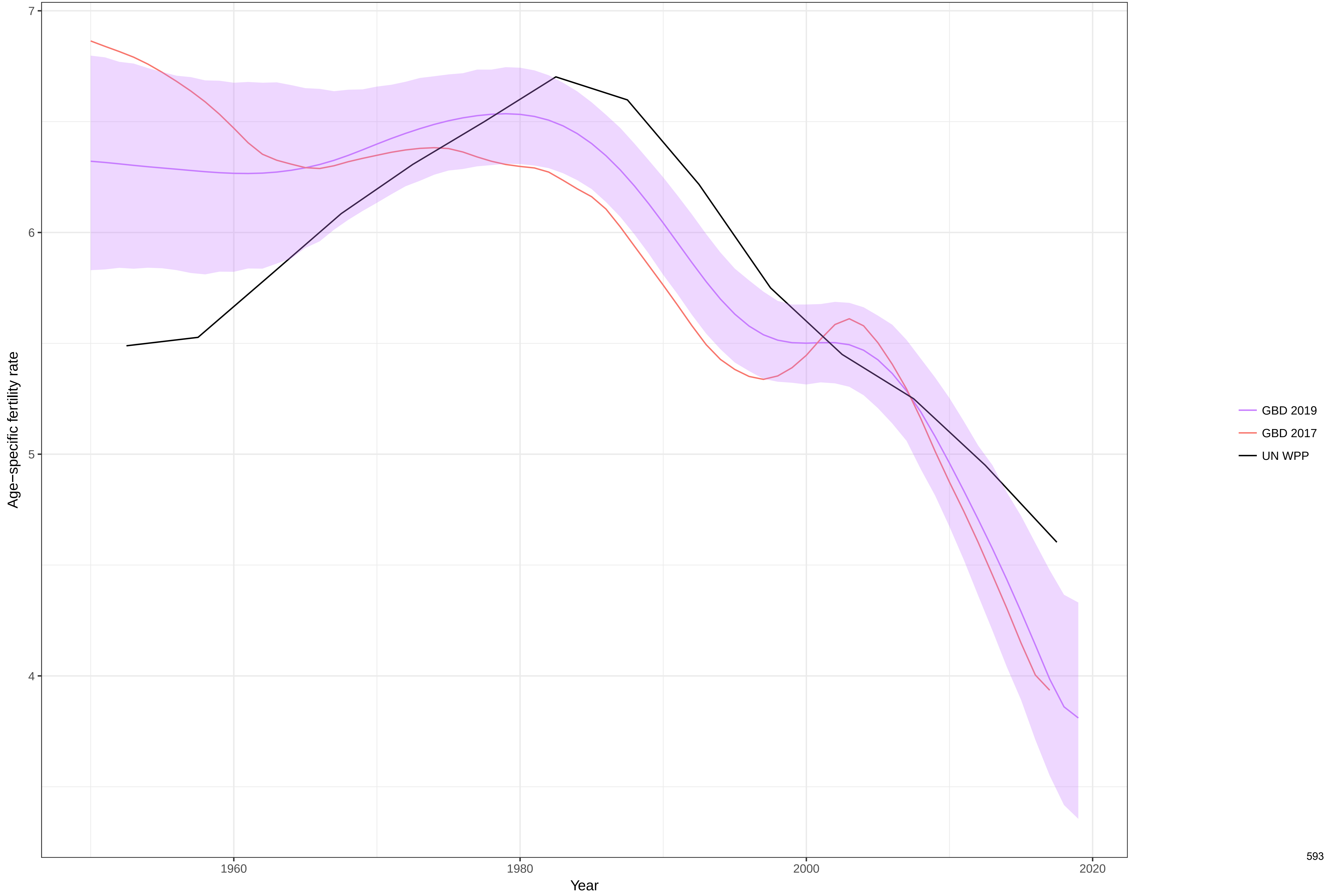

Age−specific fertility fitted trend lines, 1950−2019: Democratic Republic of the Congo, Ages: 10−14
Data points enclosed with a diamond are reference sources

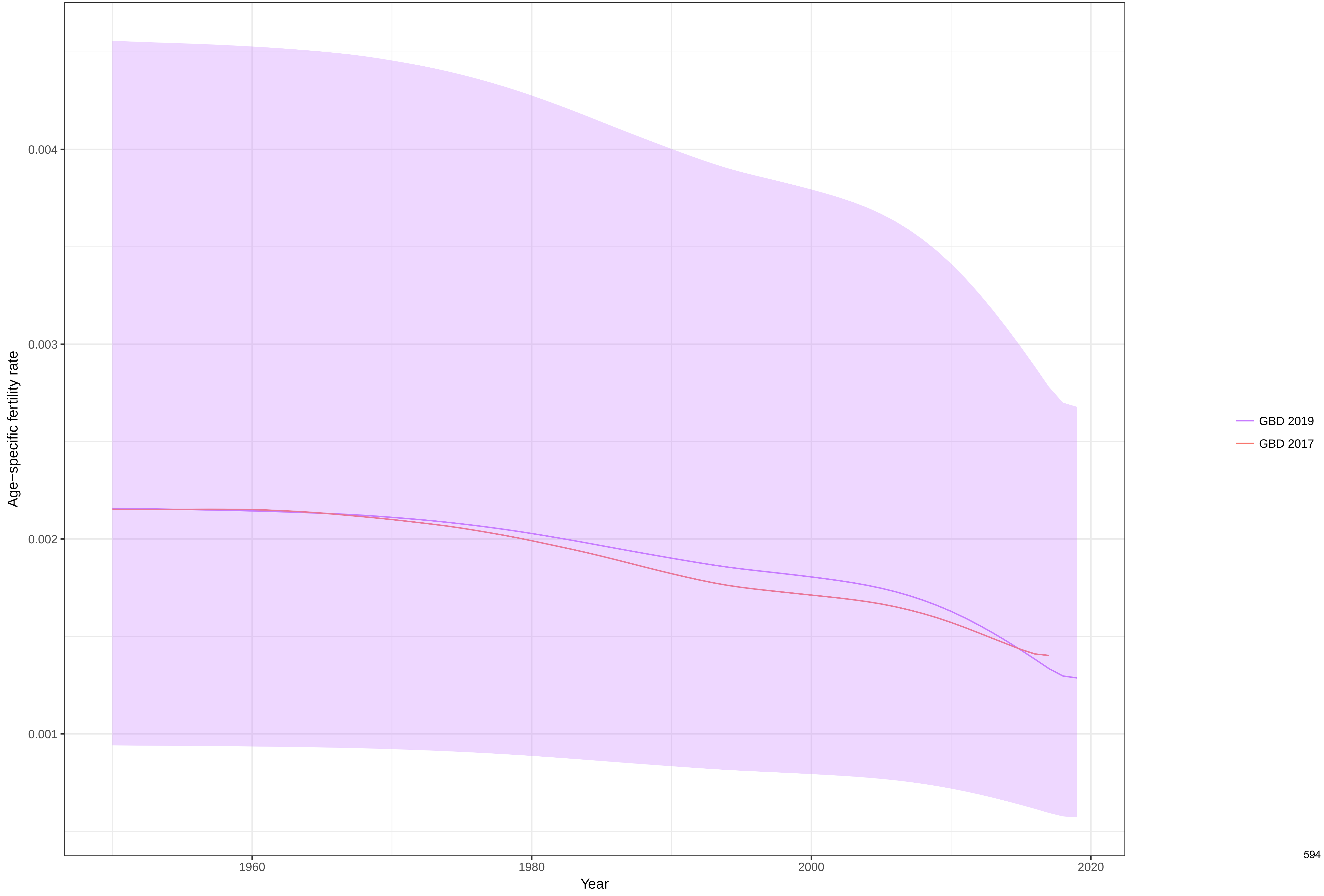

Age−specific fertility fitted trend lines, 1950−2019: Democratic Republic of the Congo, Ages: 15−19
Data points enclosed with a diamond are reference sources

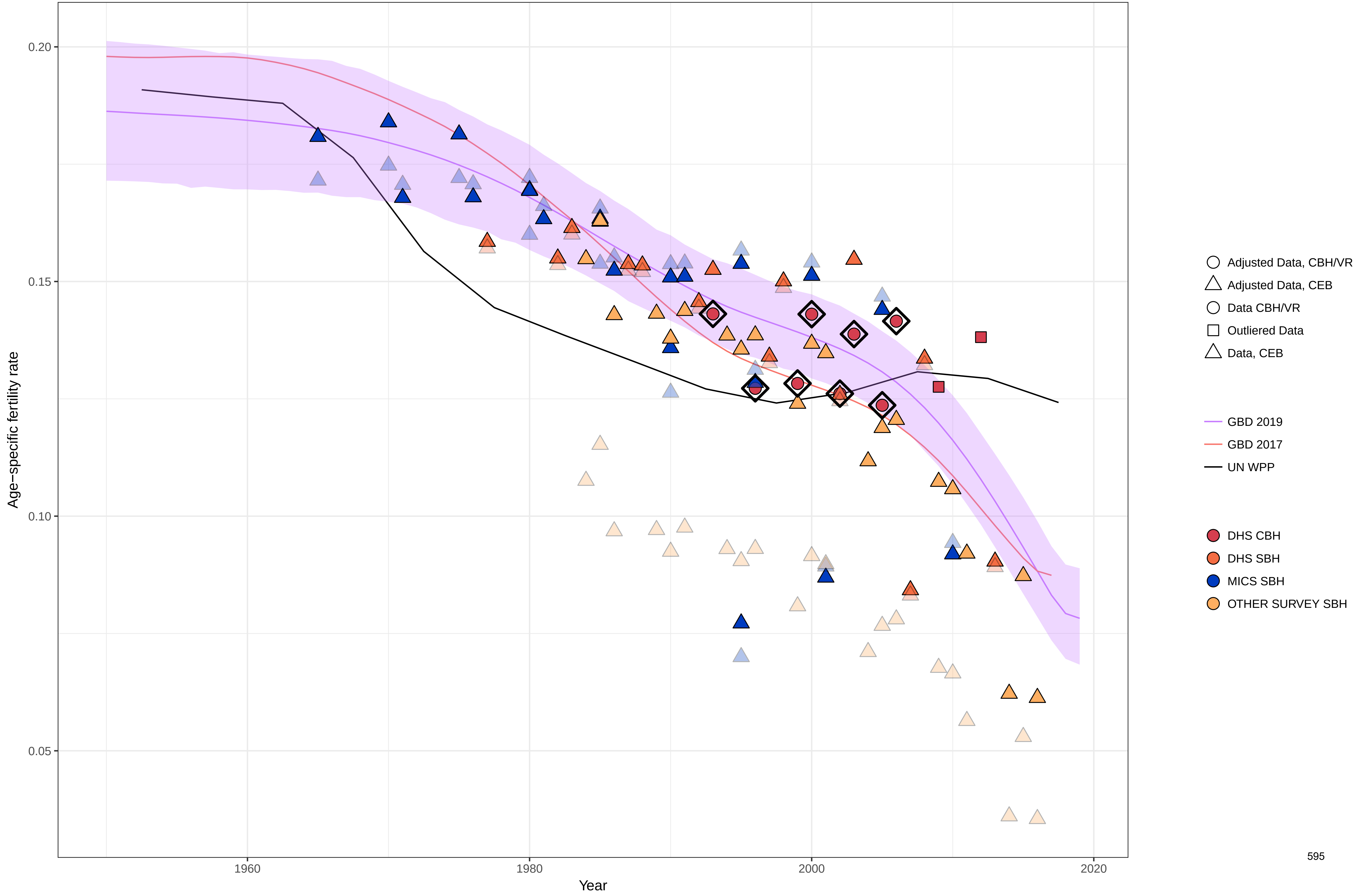

Age−specific fertility fitted trend lines, 1950−2019: Democratic Republic of the Congo, Ages: 20−24
Data points enclosed with a diamond are reference sources

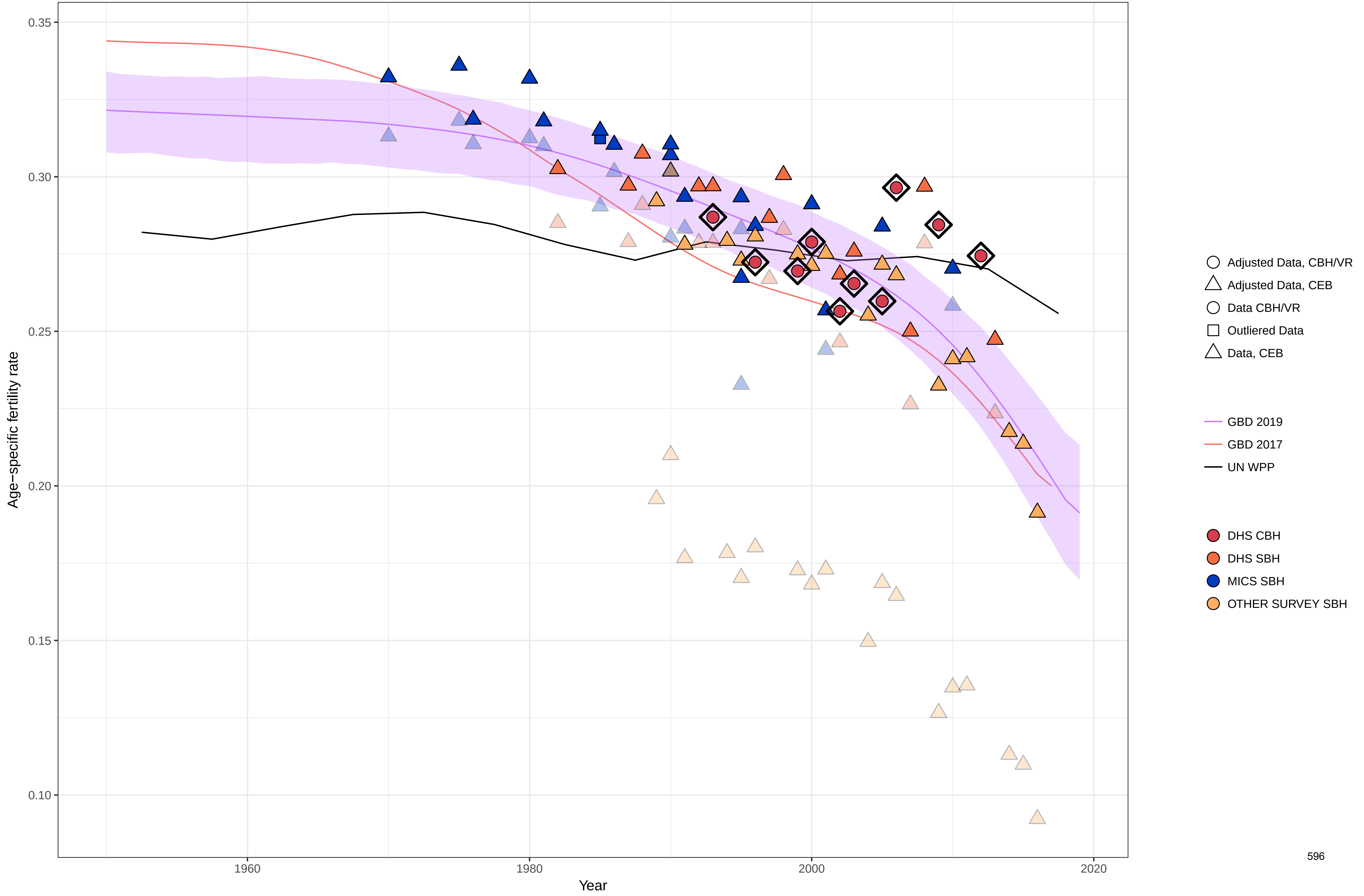

Age−specific fertility fitted trend lines, 1950−2019: Democratic Republic of the Congo, Ages: 25−29
Data points enclosed with a diamond are reference sources

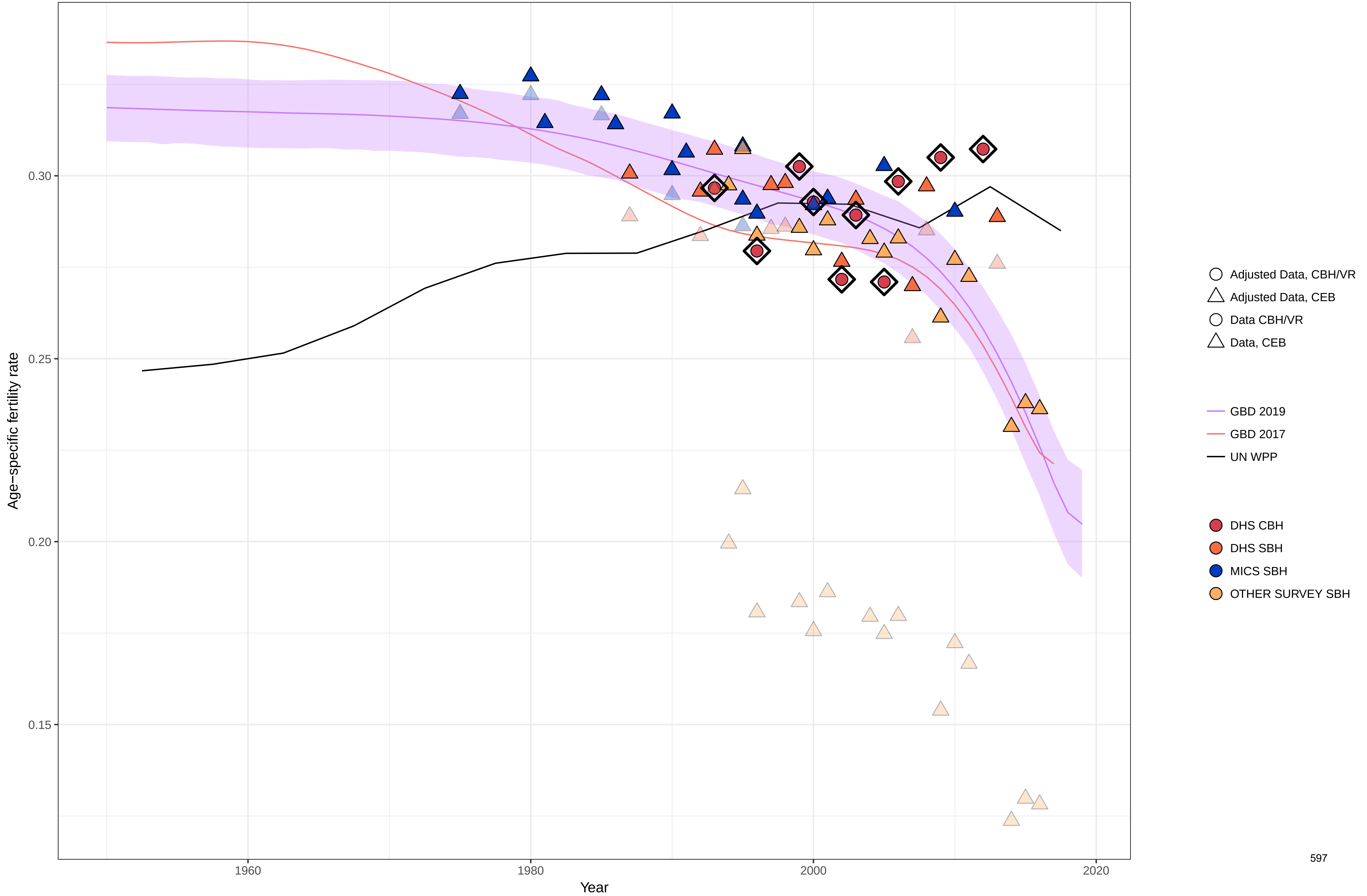

Age−specific fertility fitted trend lines, 1950−2019: Democratic Republic of the Congo, Ages: 30−34
Data points enclosed with a diamond are reference sources

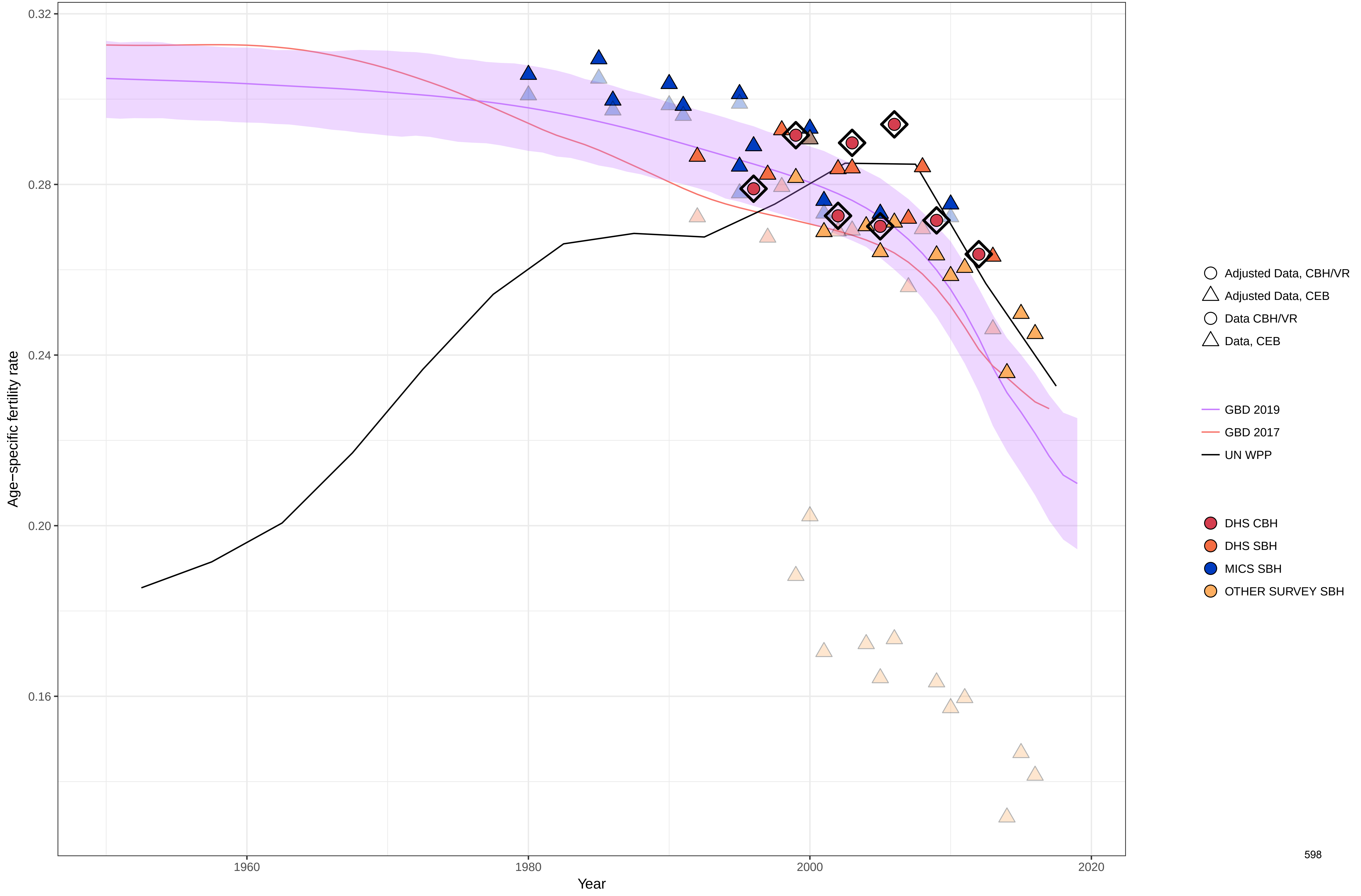

Age−specific fertility fitted trend lines, 1950−2019: Democratic Republic of the Congo, Ages: 35−39
Data points enclosed with a diamond are reference sources

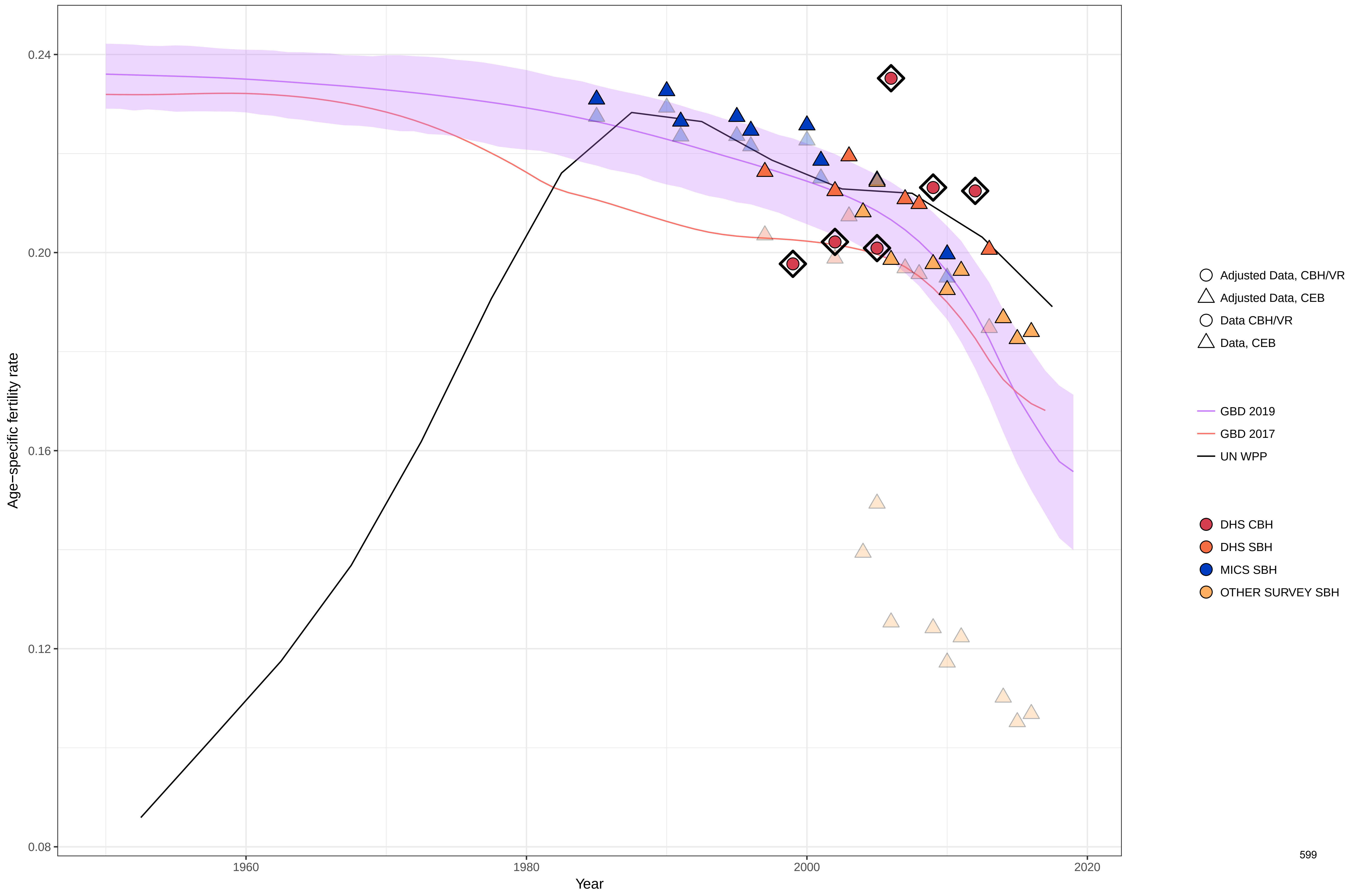

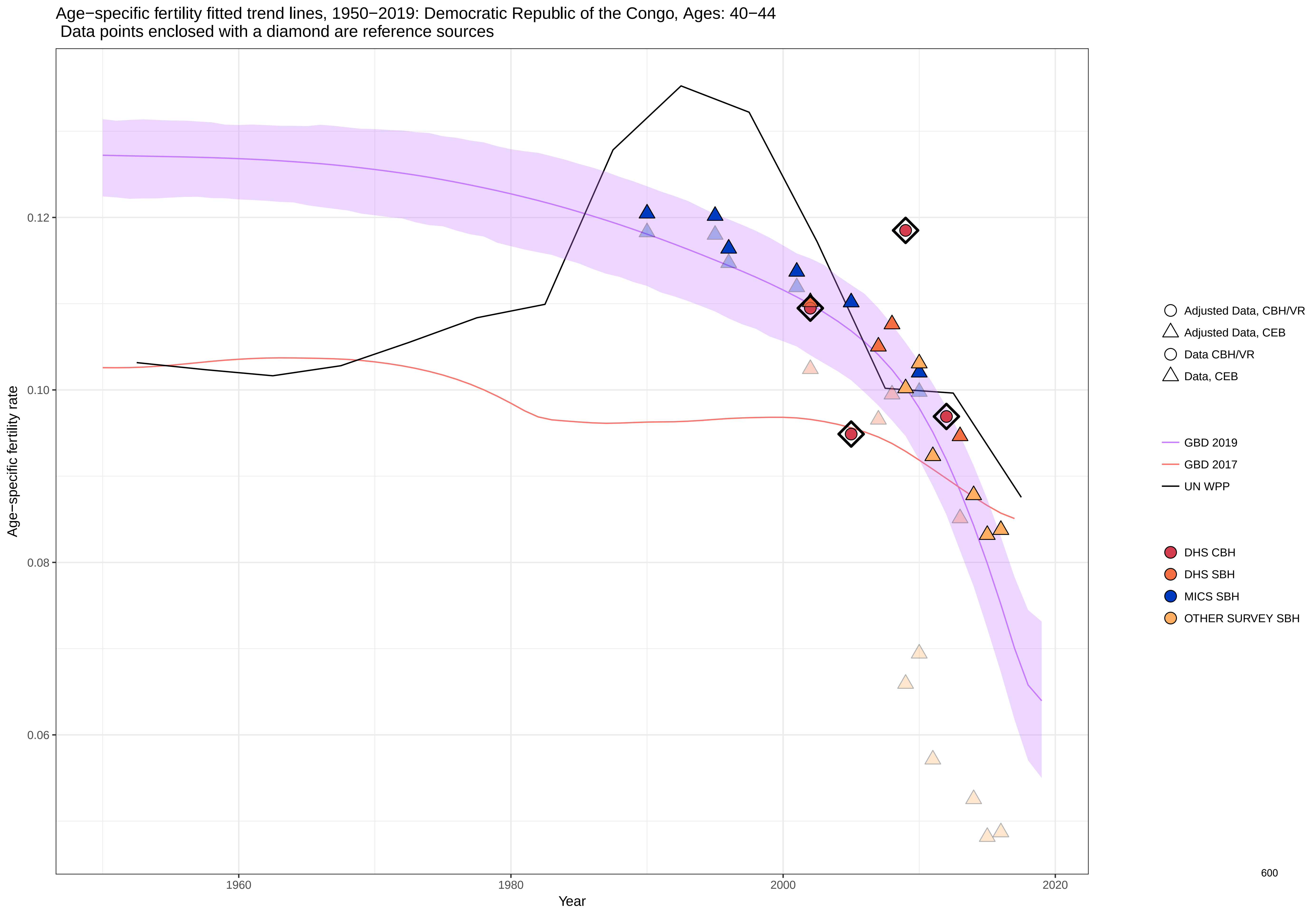
Age−specific fertility fitted trend lines, 1950−2019: Democratic Republic of the Congo, Ages: 40−44
Data points enclosed with a diamond are reference sources
Age−specific fertility rate
0.12
0.10
0.08
0.06
1960
1980
2000
2020
Year
Adjusted Data, CBH/VR
Adjusted Data, CEB
Data CBH/VR
Data, CEB
GBD 2019
GBD 2017
UN WPP
DHS CBH
DHS SBH
MICS SBH
OTHER SURVEY SBH

Age−specific fertility fitted trend lines, 1950−2019: Democratic Republic of the Congo, Ages: 45−49
Data points enclosed with a diamond are reference sources

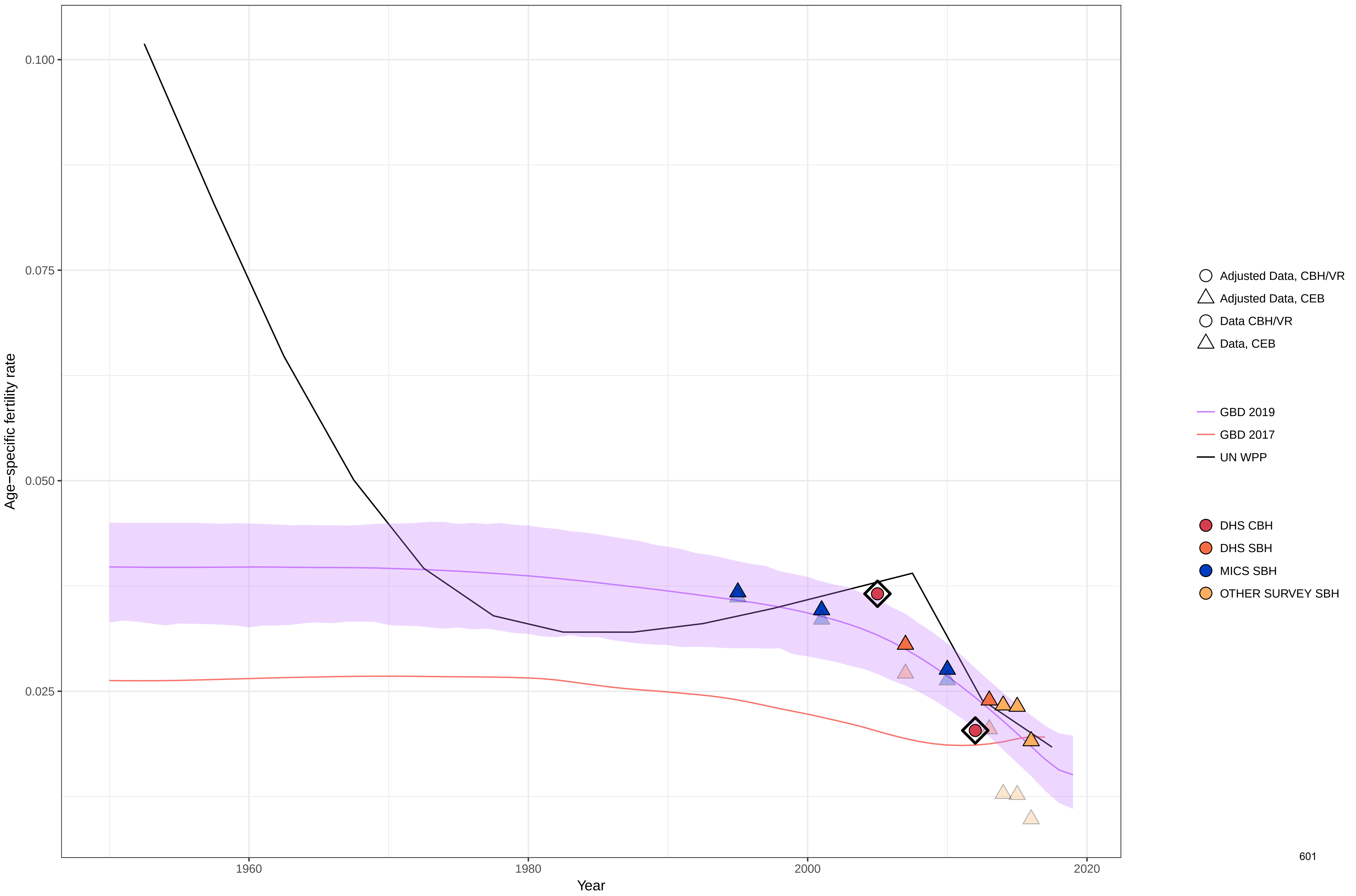

Age−specific fertility fitted trend lines, 1950−2019: Democratic Republic of the Congo, Ages: 50−54
Data points enclosed with a diamond are reference sources

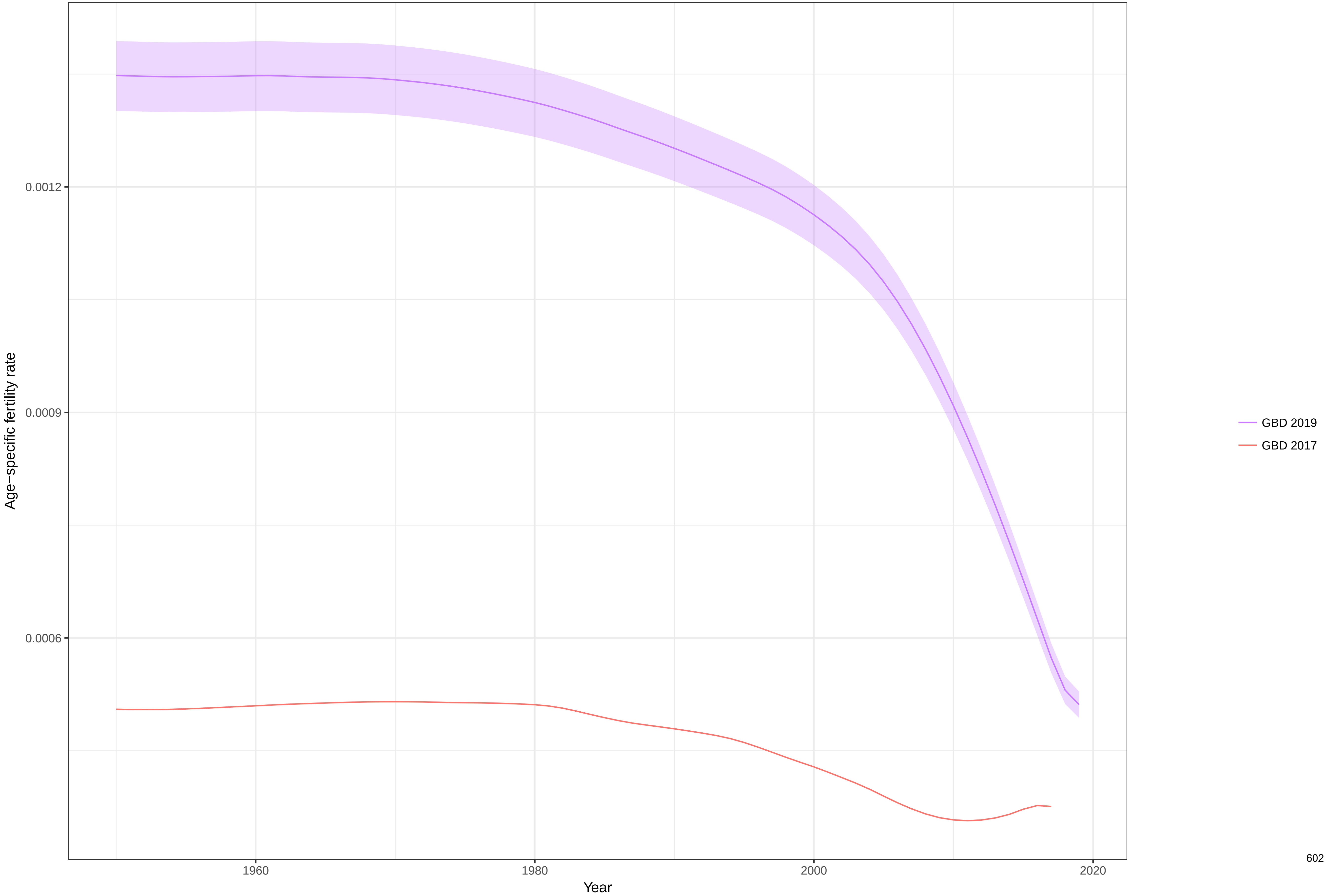

Total fertility rate fitted trend lines, 1950−2019: Democratic Republic of the Congo TFR
Data points enclosed with a diamond are reference sources

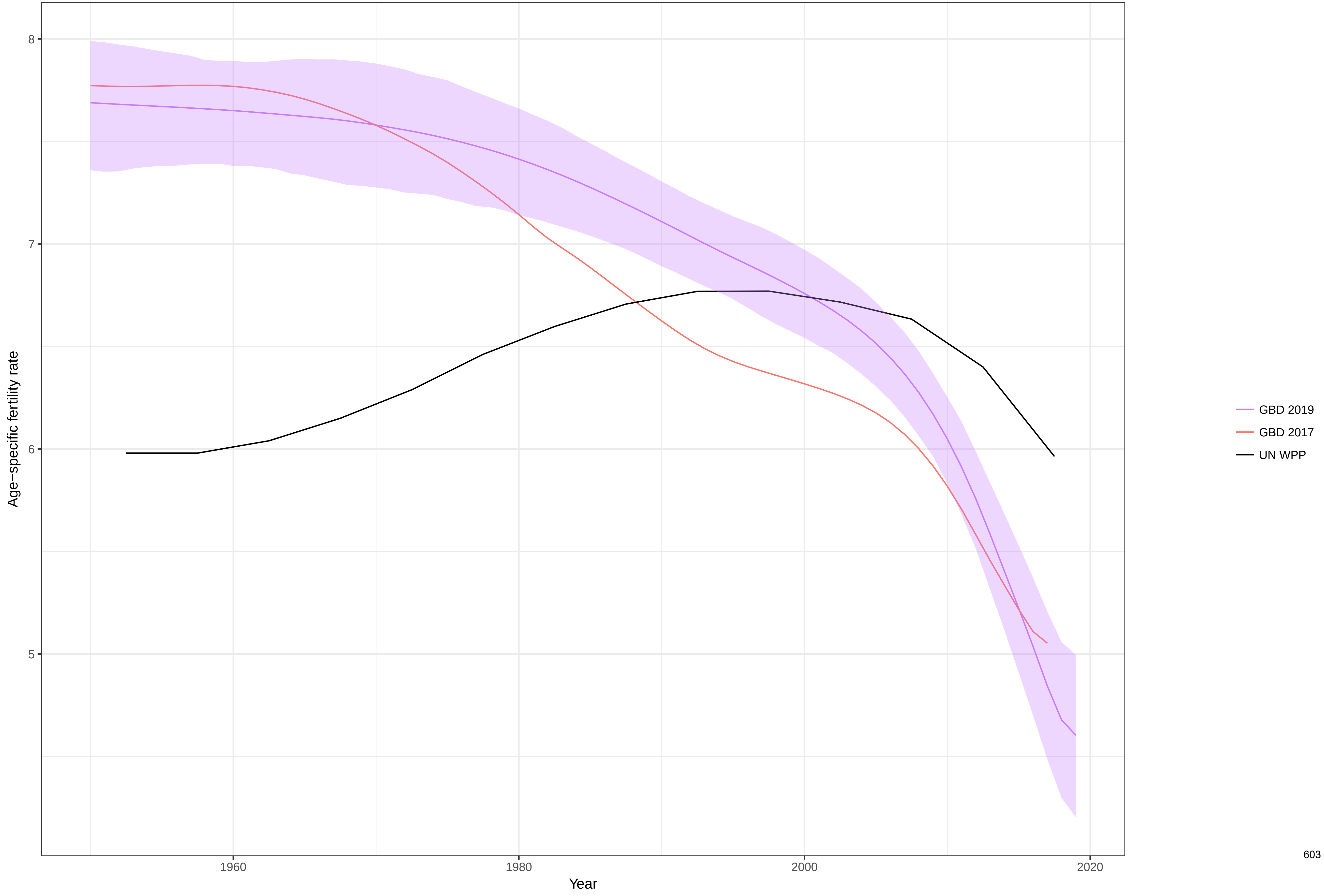

Age−specific fertility fitted trend lines, 1950−2019: Congo, Ages: 10−14
Data points enclosed with a diamond are reference sources

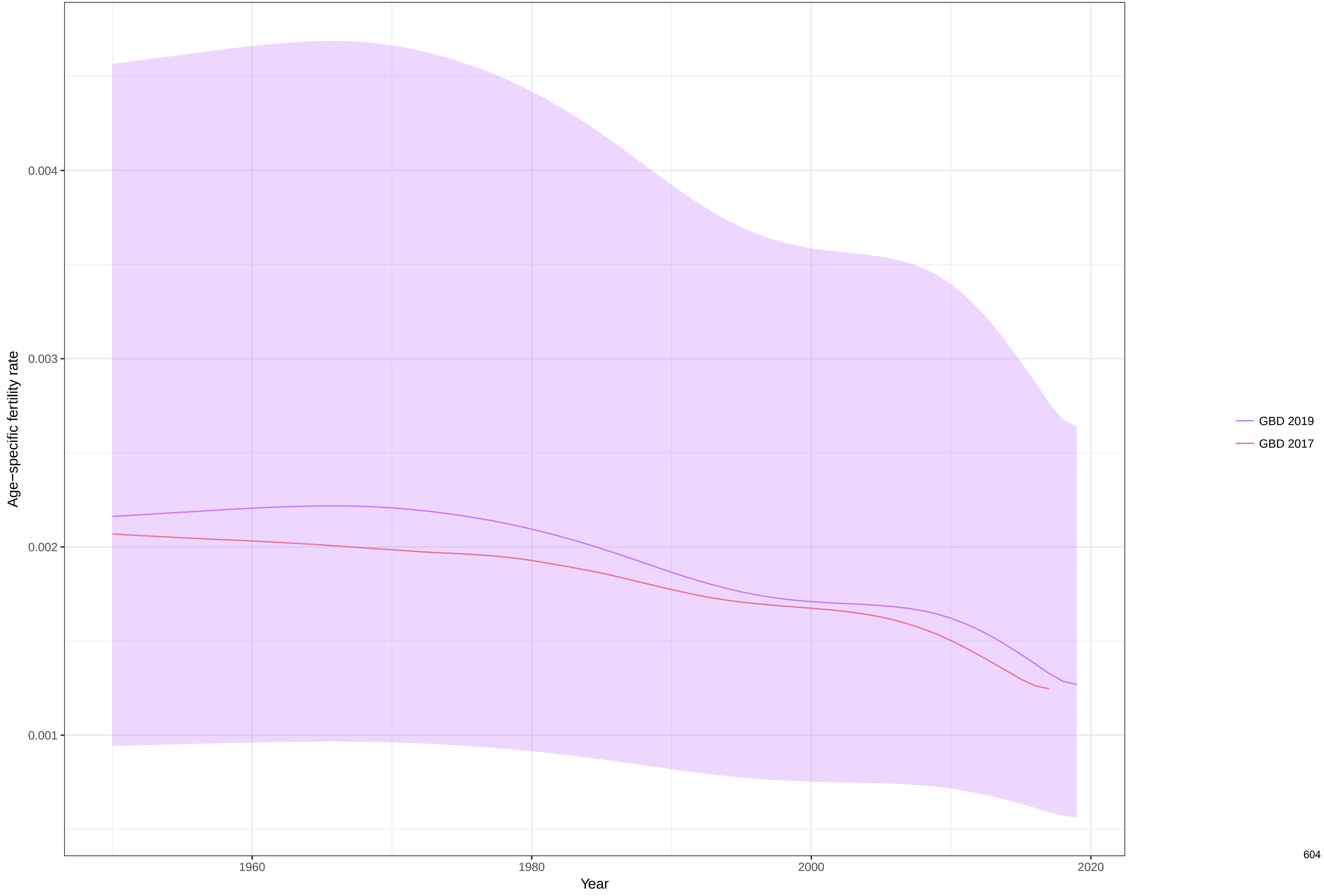

Age−specific fertility fitted trend lines, 1950−2019: Congo, Ages: 15−19
Data points enclosed with a diamond are reference sources

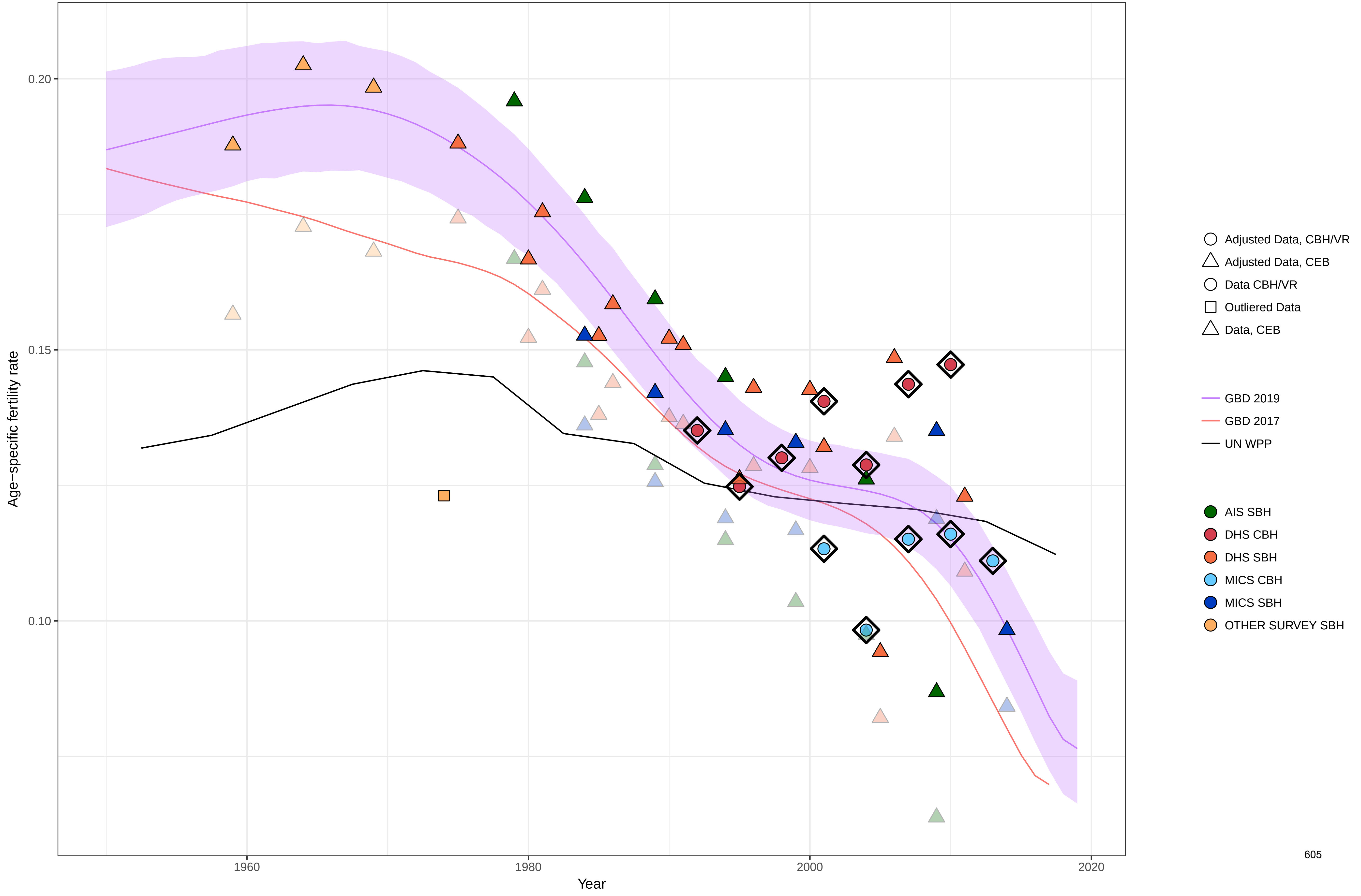

Age−specific fertility fitted trend lines, 1950−2019: Congo, Ages: 20−24
Data points enclosed with a diamond are reference sources

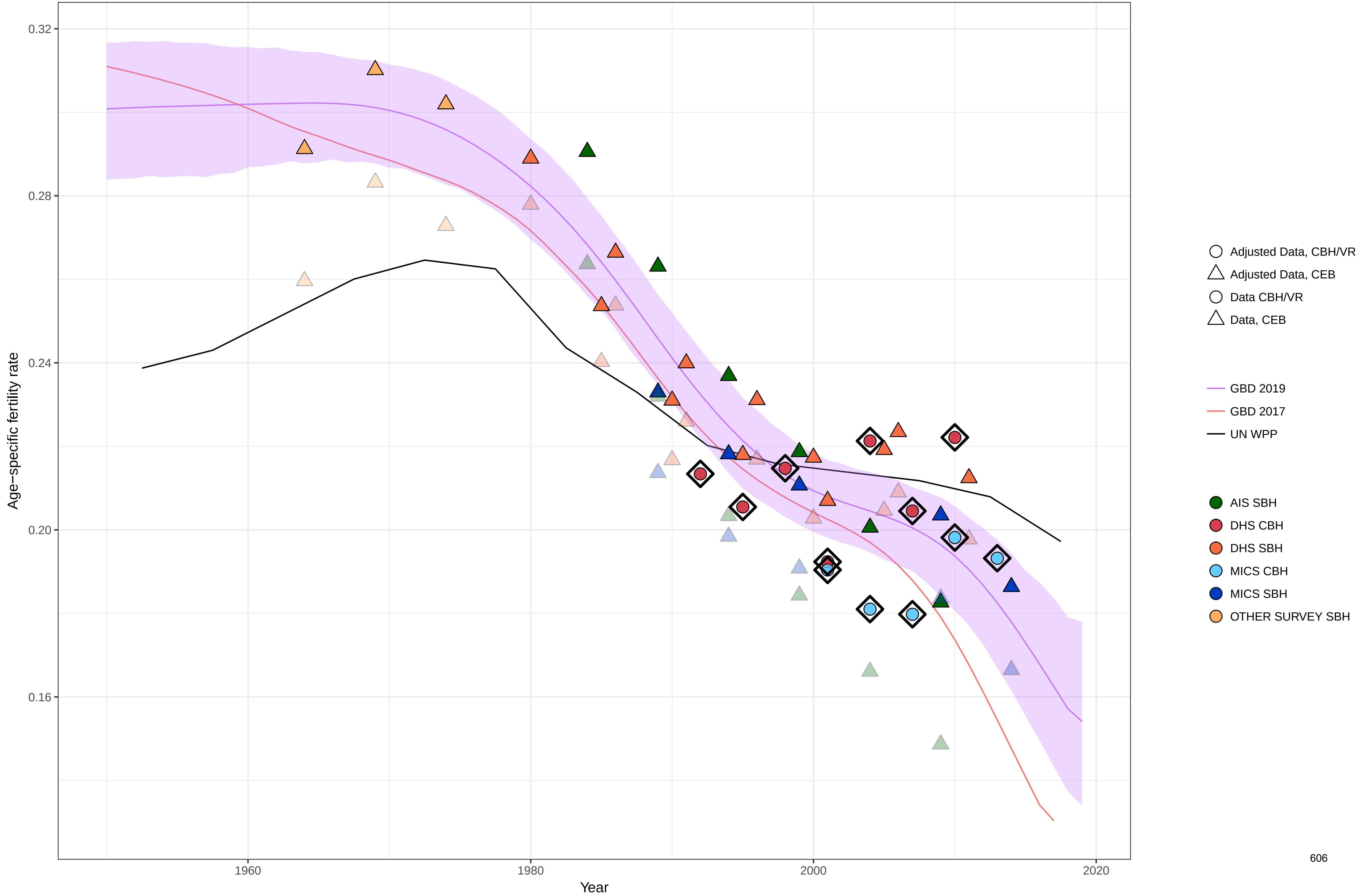

Age−specific fertility fitted trend lines, 1950−2019: Congo, Ages: 25−29
Data points enclosed with a diamond are reference sources

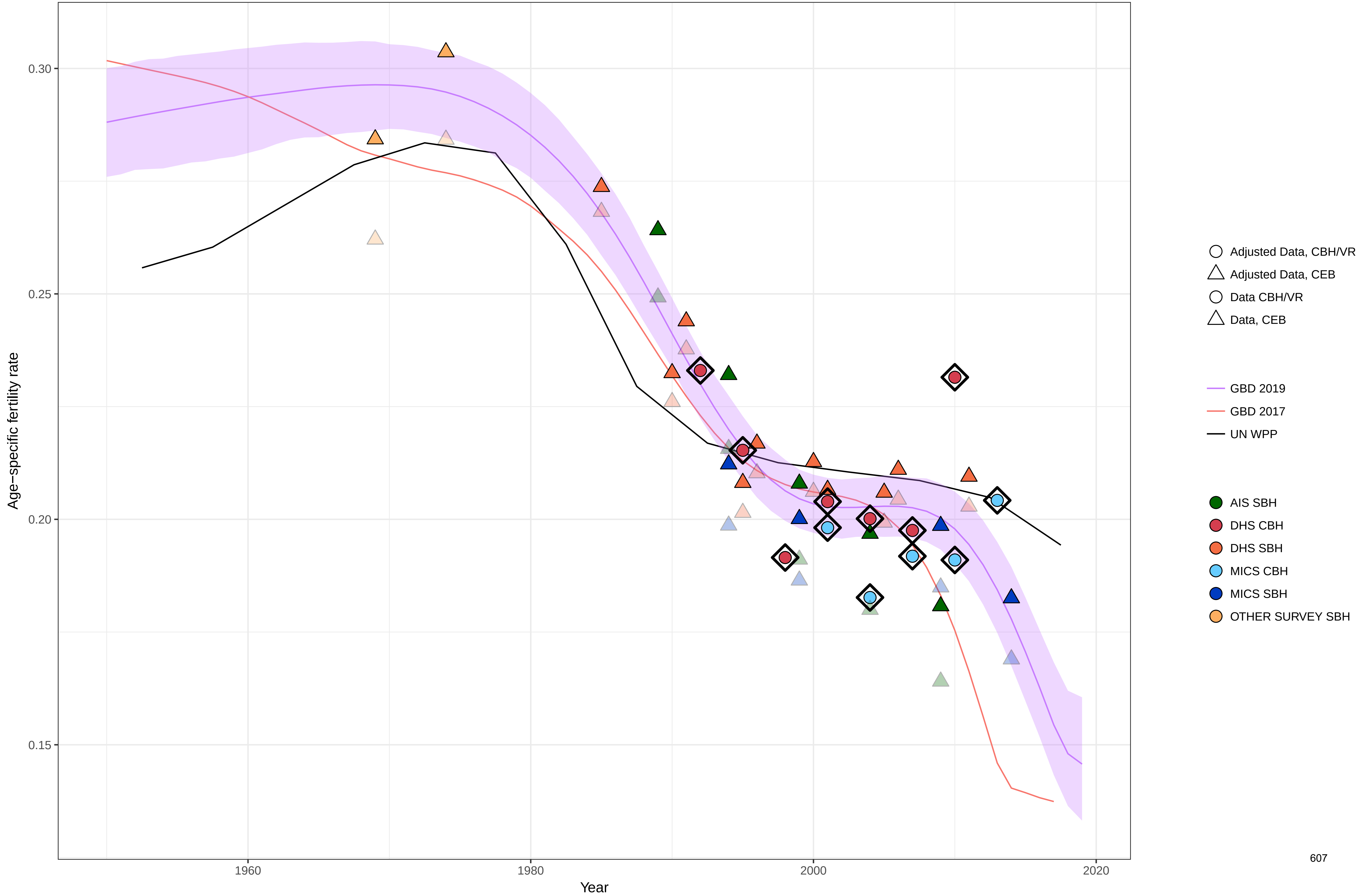

Age−specific fertility fitted trend lines, 1950−2019: Congo, Ages: 30−34
Data points enclosed with a diamond are reference sources

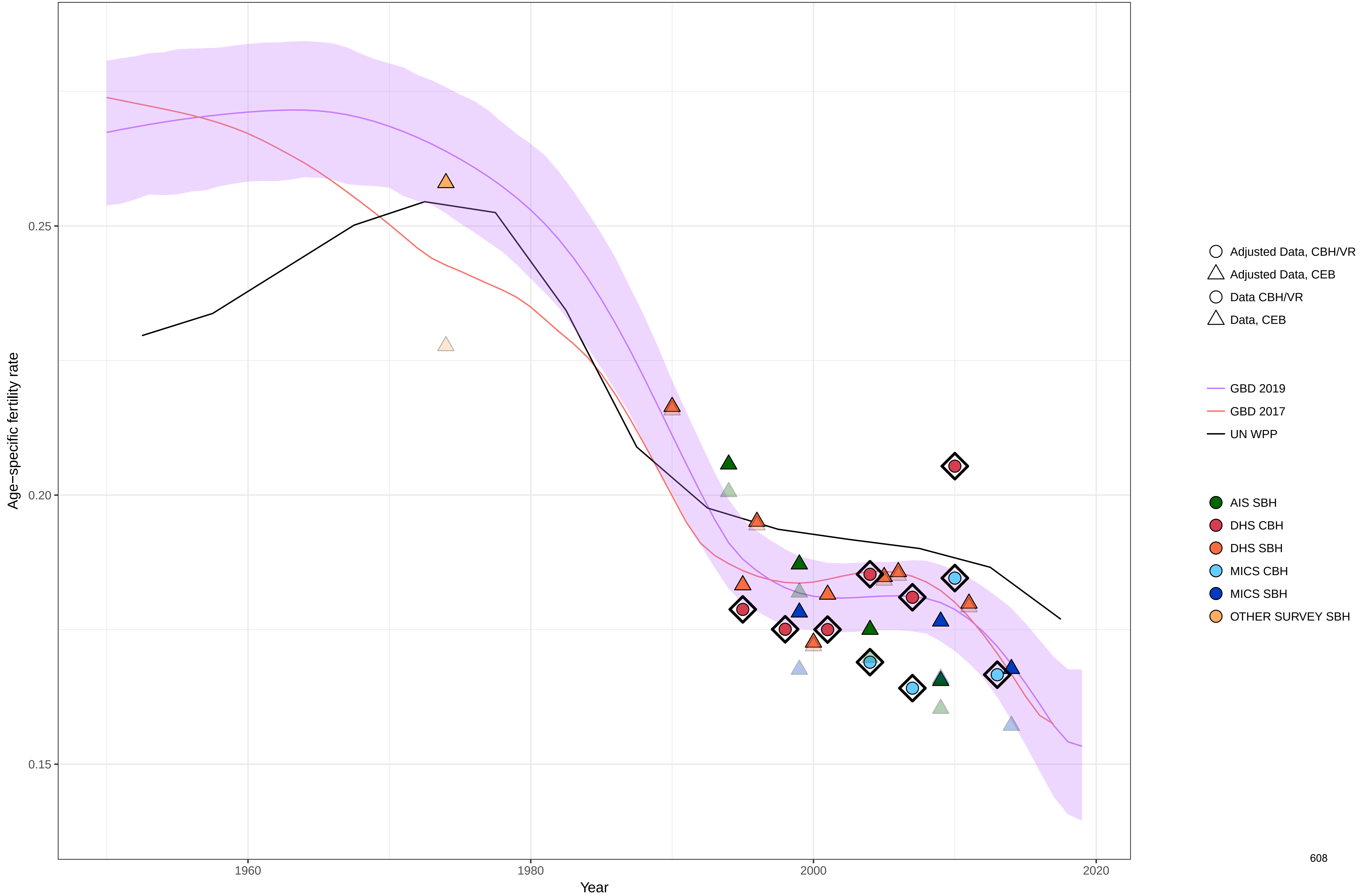

Age−specific fertility fitted trend lines, 1950−2019: Congo, Ages: 35−39
Data points enclosed with a diamond are reference sources

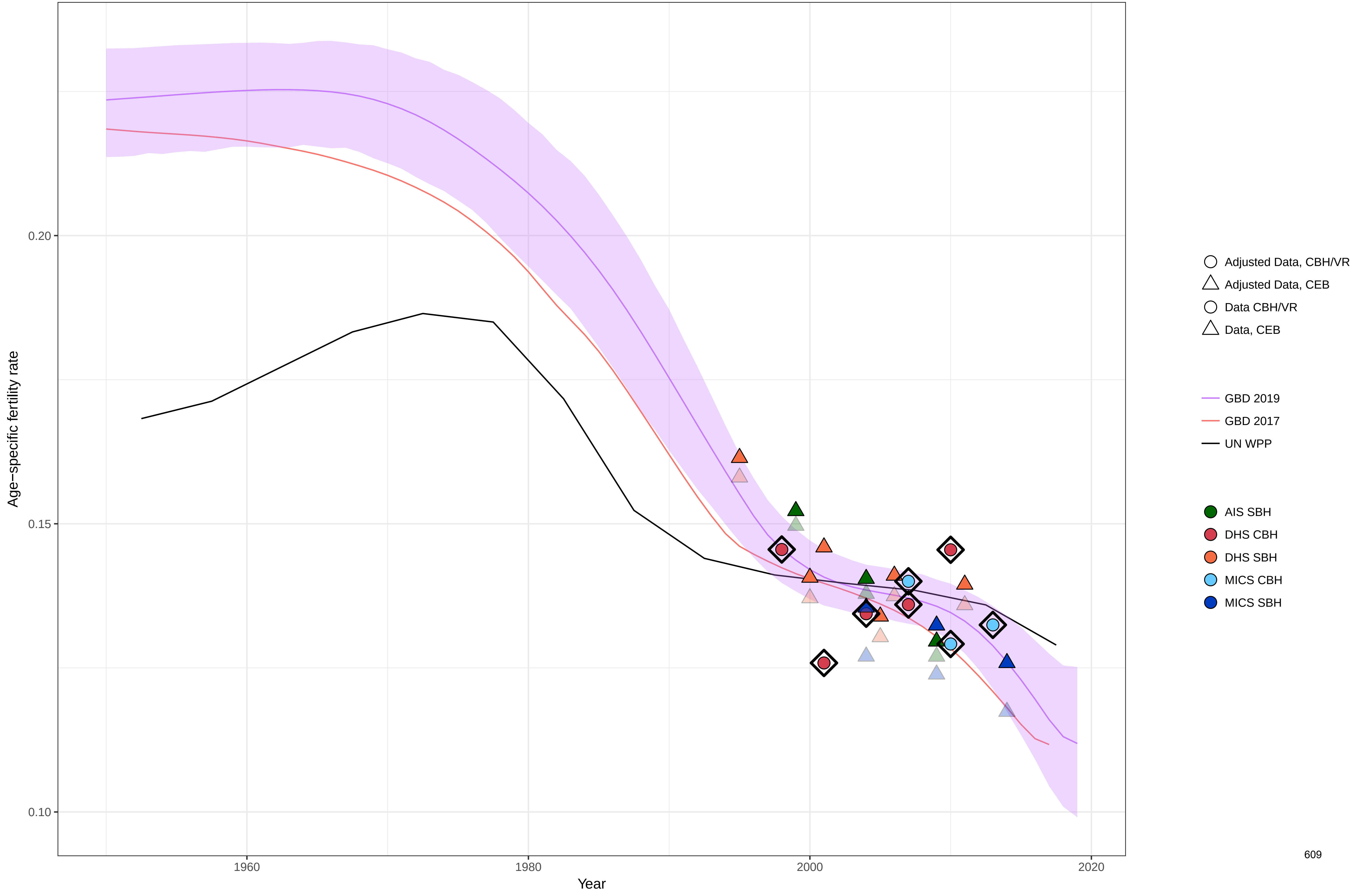

Age−specific fertility fitted trend lines, 1950−2019: Congo, Ages: 40−44
Data points enclosed with a diamond are reference sources

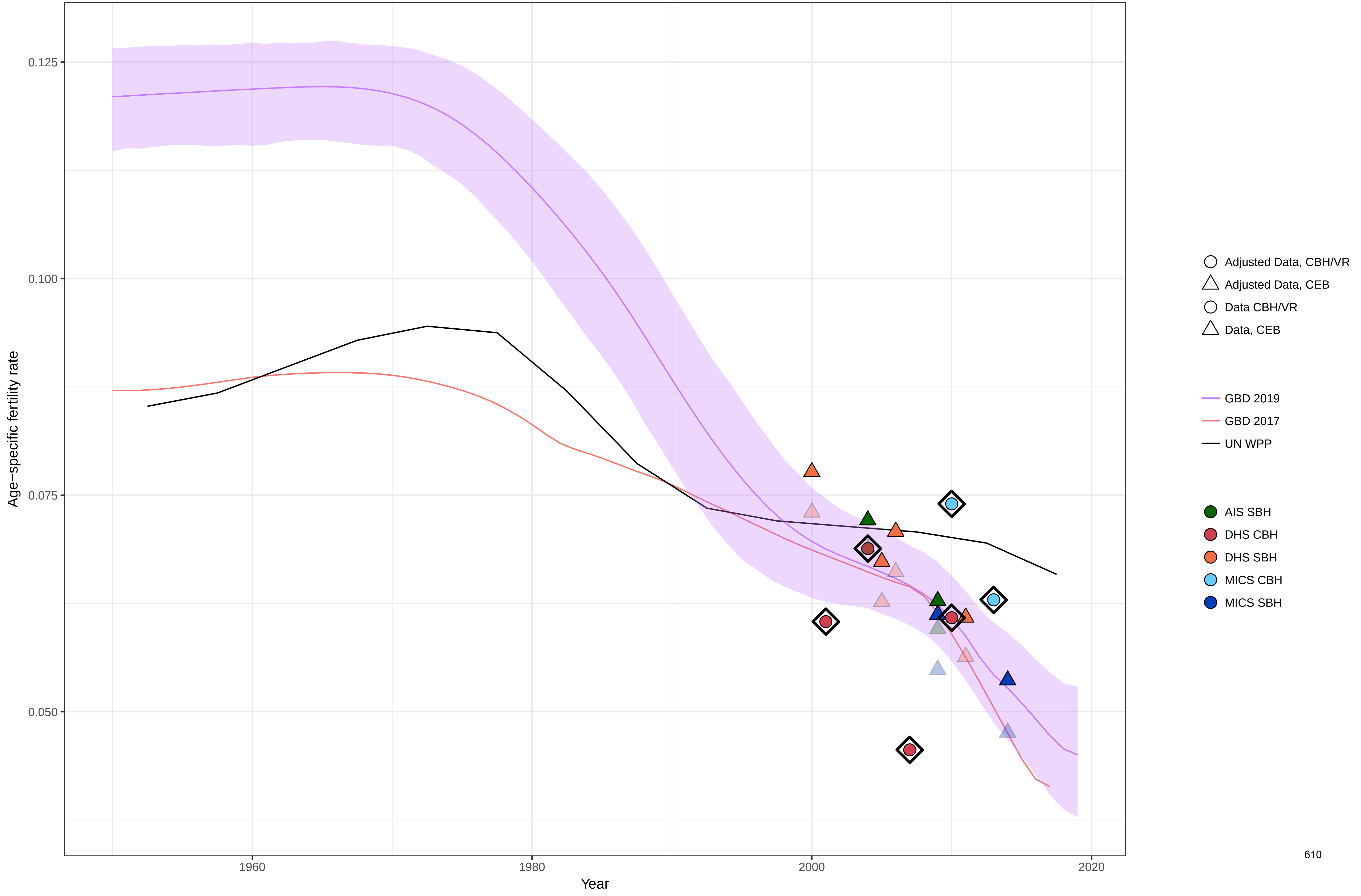

Age−specific fertility fitted trend lines, 1950−2019: Congo, Ages: 45−49
Data points enclosed with a diamond are reference sources

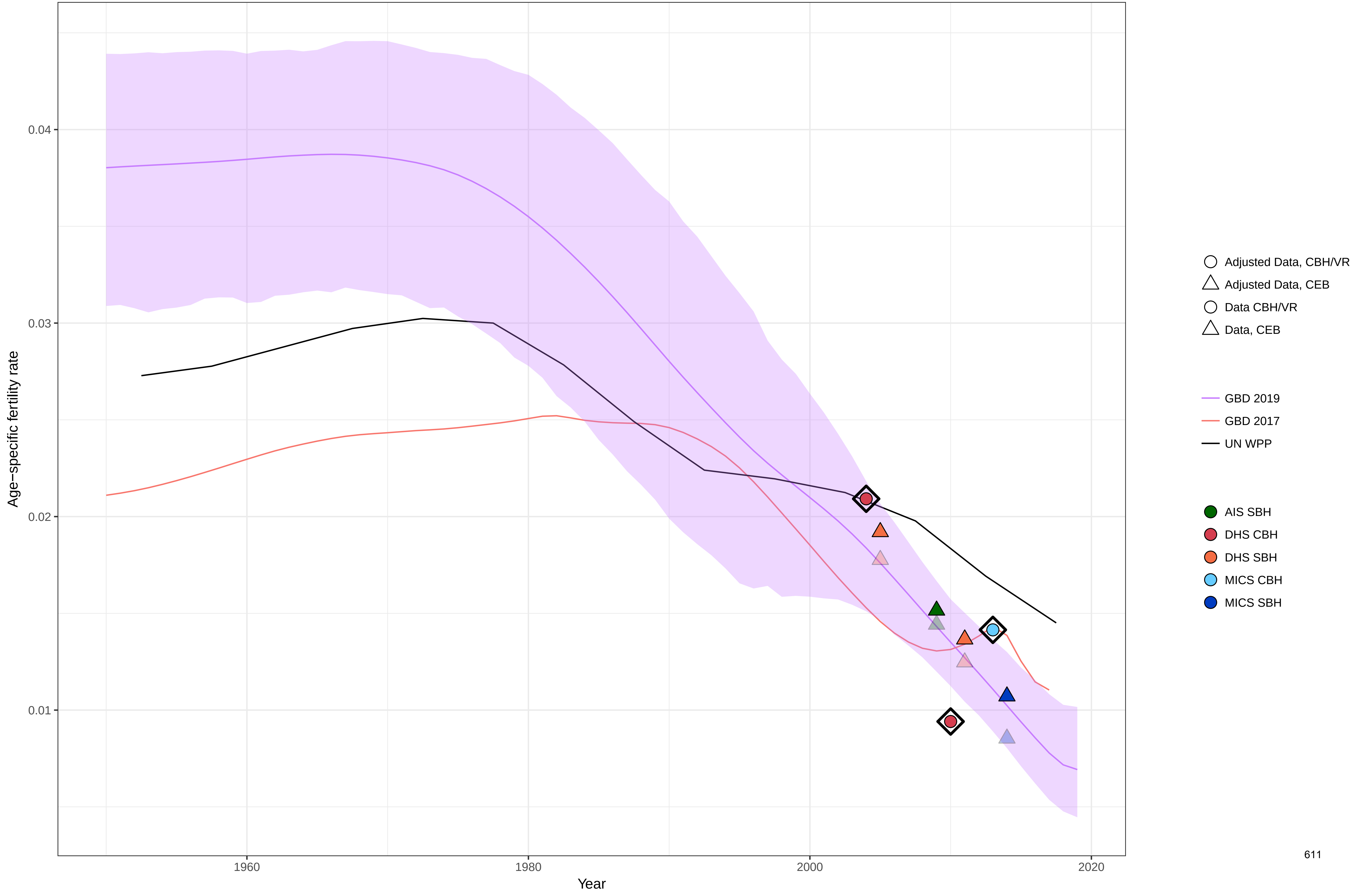

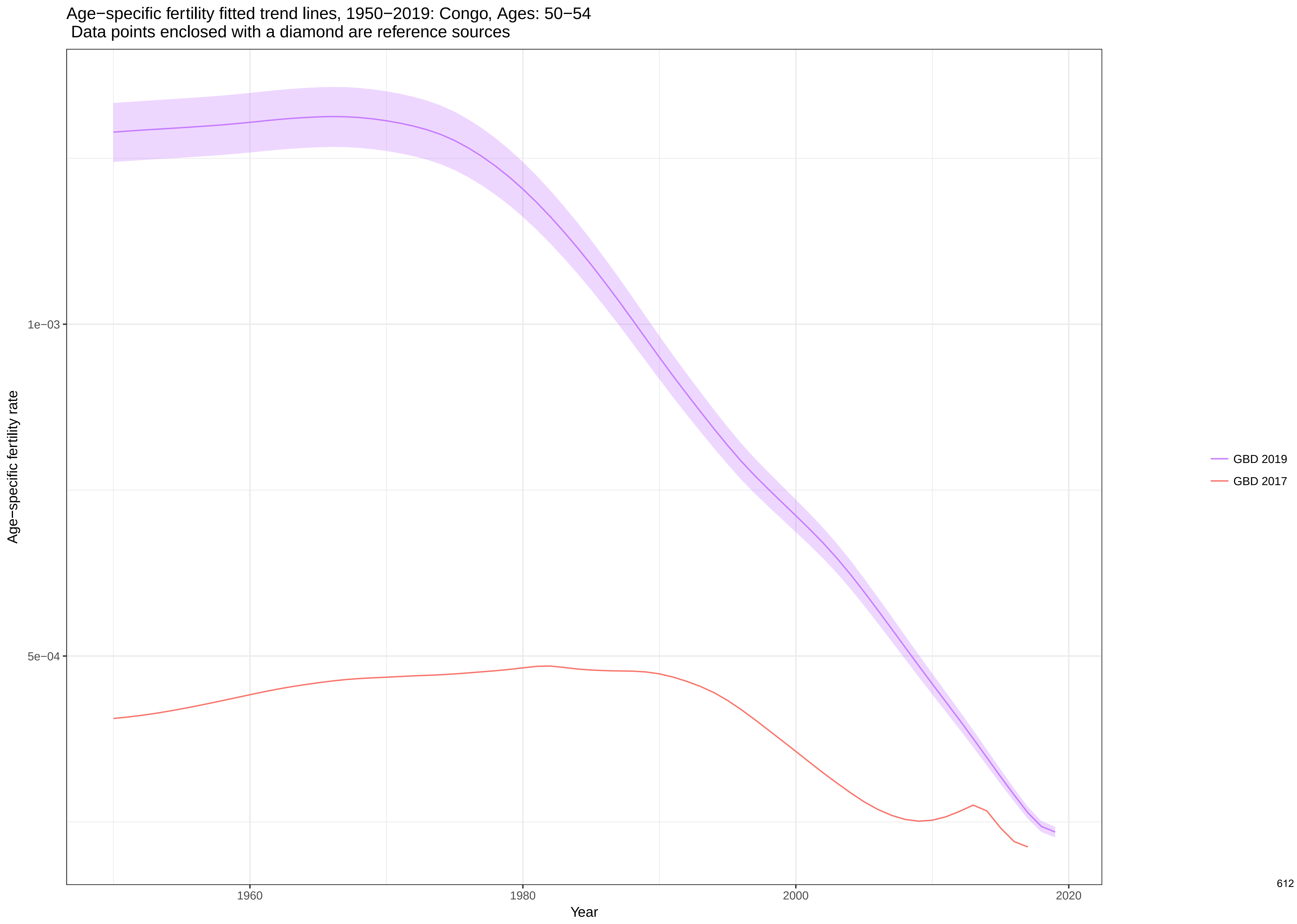

Age−specific fertility fitted trend lines, 1950−2019: Congo, Ages: 50−54
Data points enclosed with a diamond are reference sources
1e−03
5e−04
Age−specific fertility rate
1960
1980
2000
2020
Year
GBD 2019
GBD 2017

Total fertility rate fitted trend lines, 1950−2019: Congo TFR
Data points enclosed with a diamond are reference sources

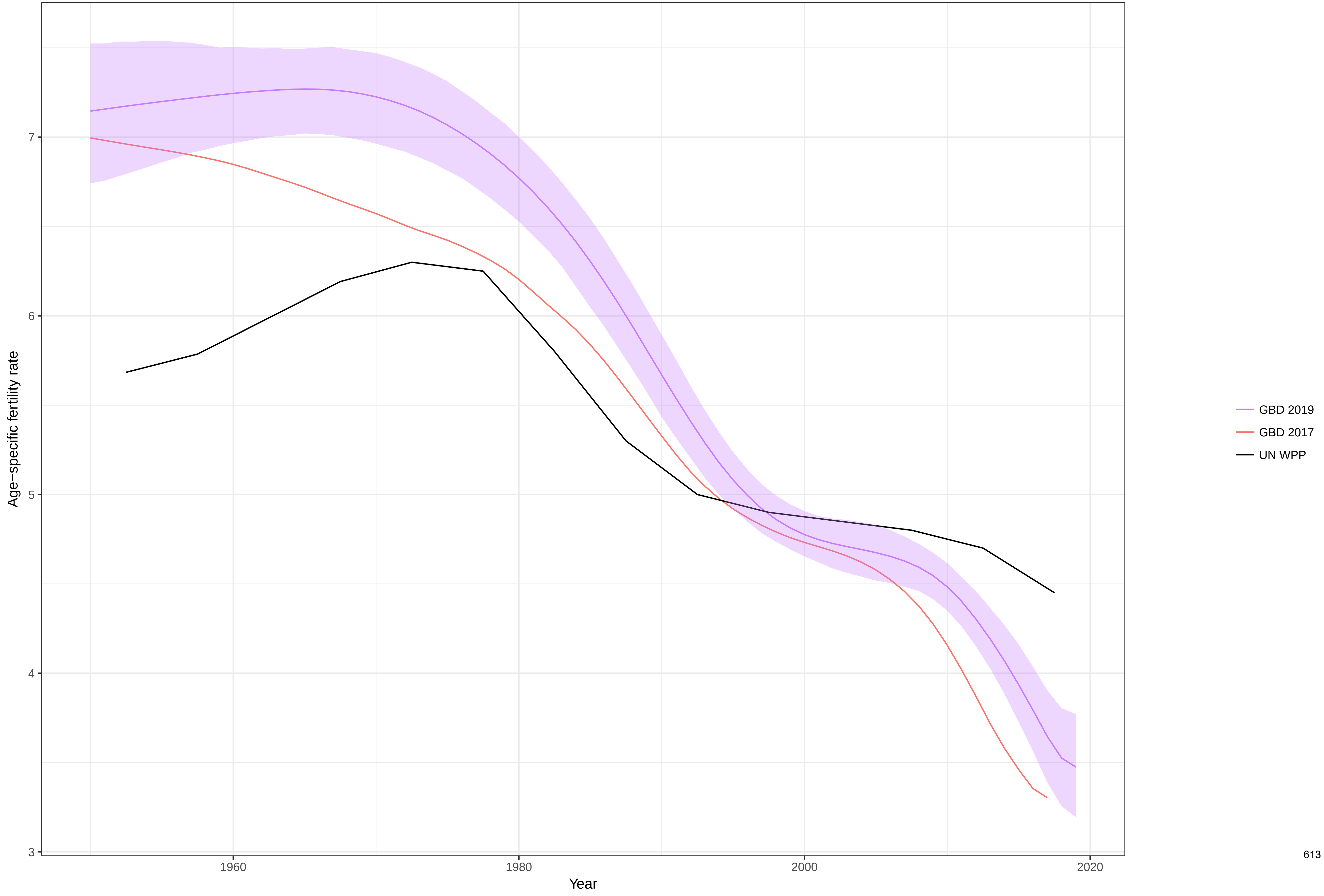

Age−specific fertility fitted trend lines, 1950−2019: Cook Islands, Ages: 10−14
Data points enclosed with a diamond are reference sources

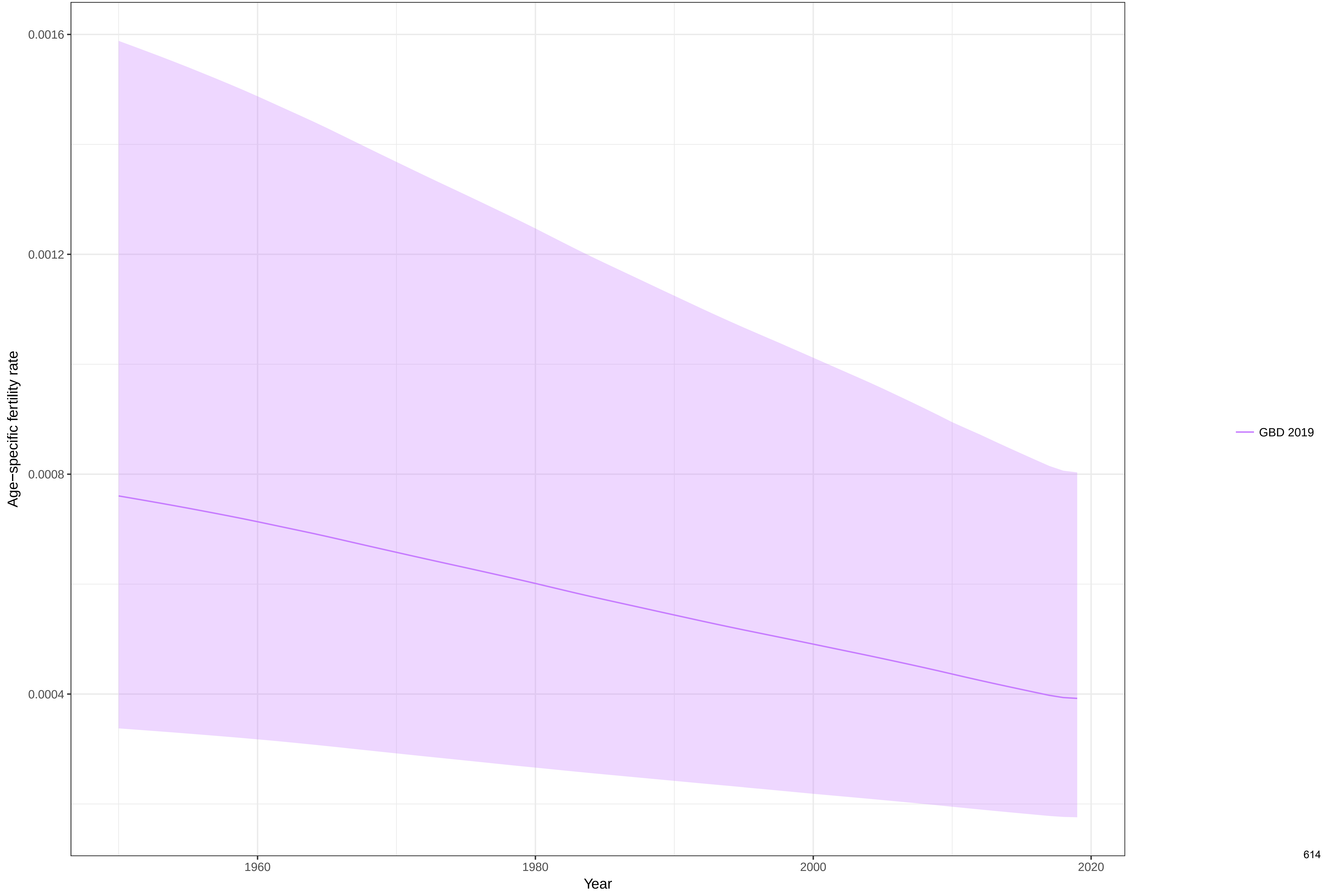

Age−specific fertility fitted trend lines, 1950−2019: Cook Islands, Ages: 15−19
Data points enclosed with a diamond are reference sources

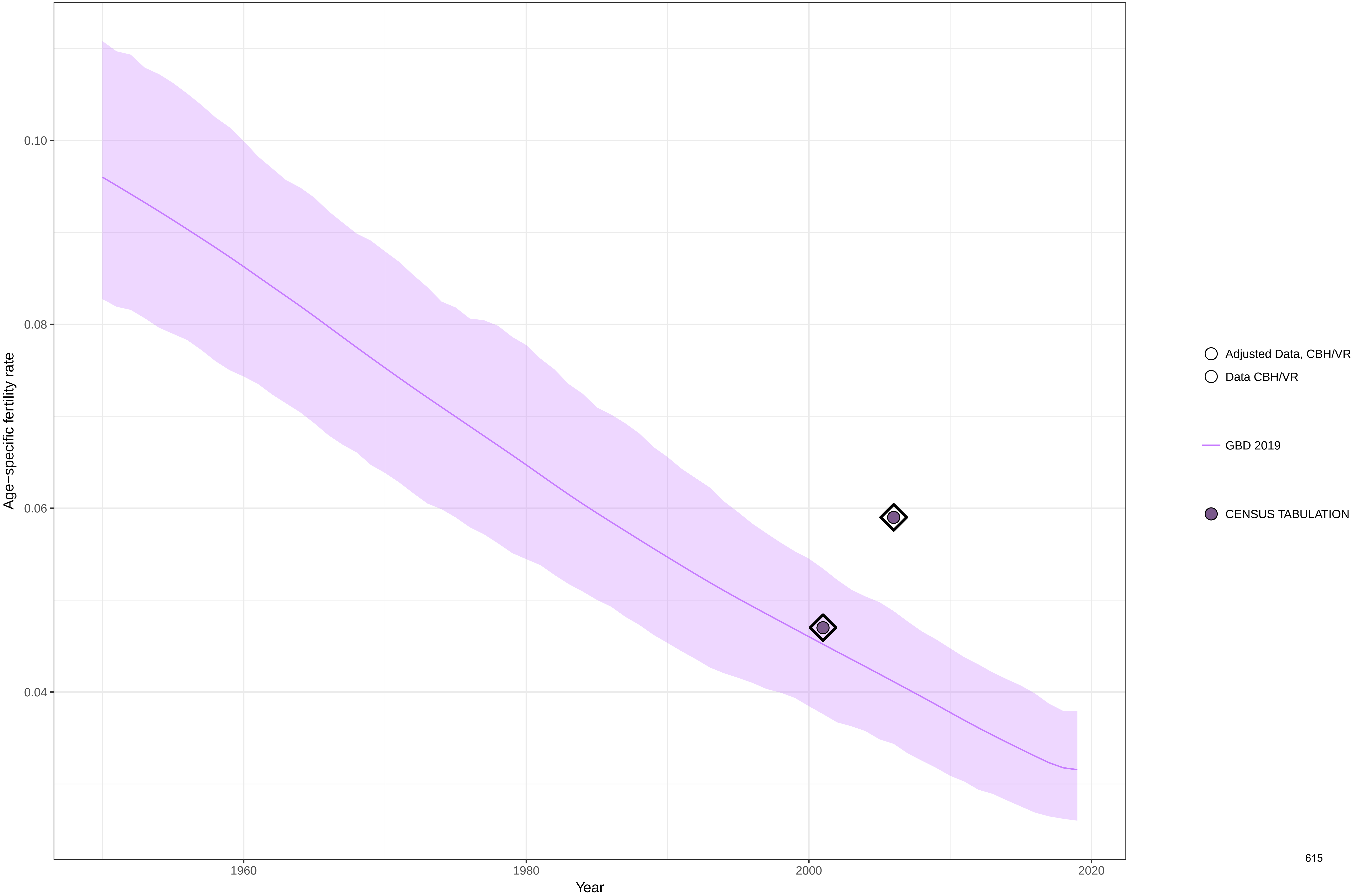

Age−specific fertility fitted trend lines, 1950−2019: Cook Islands, Ages: 20−24
Data points enclosed with a diamond are reference sources

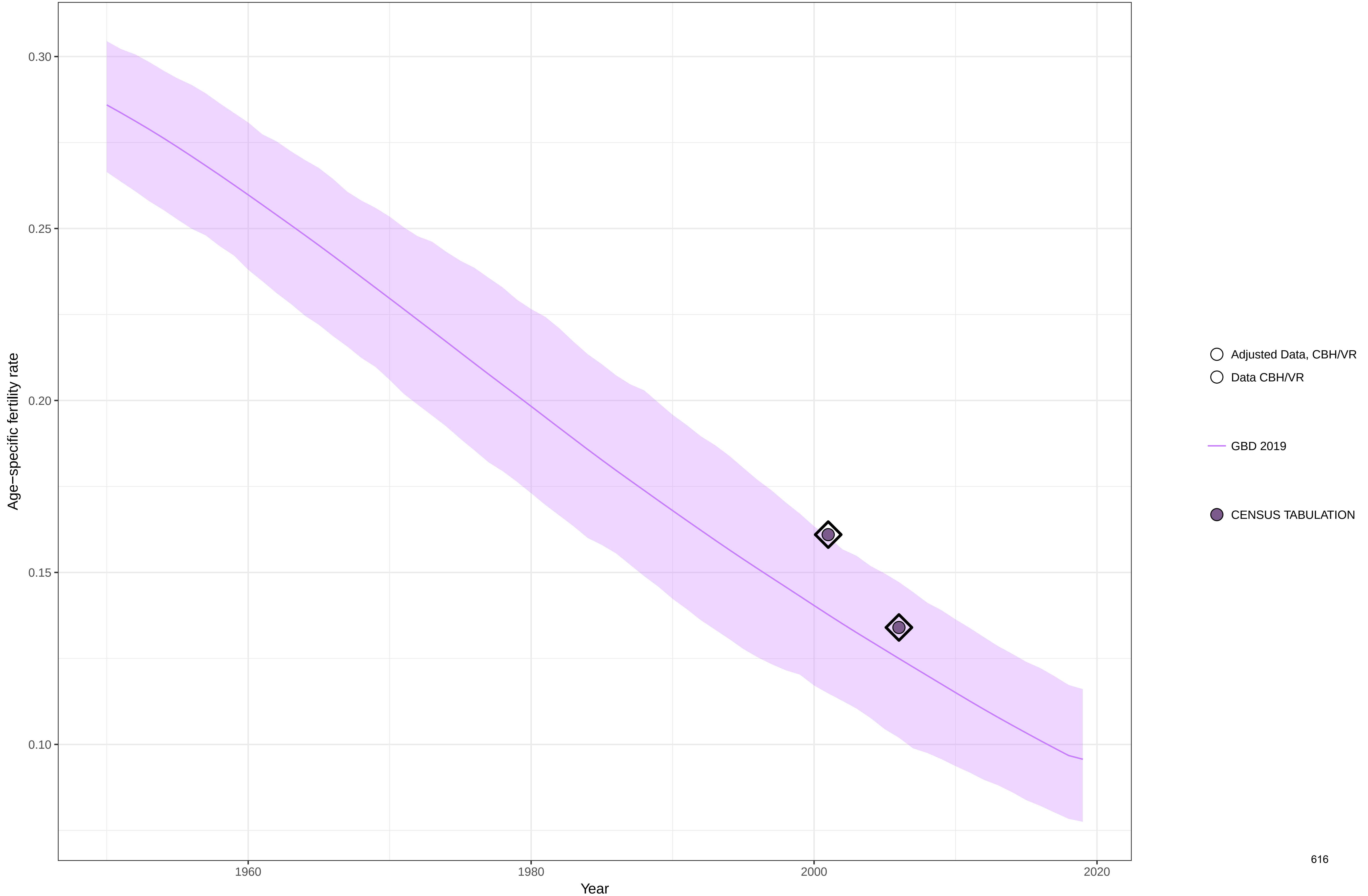

Age−specific fertility fitted trend lines, 1950−2019: Cook Islands, Ages: 25−29
Data points enclosed with a diamond are reference sources

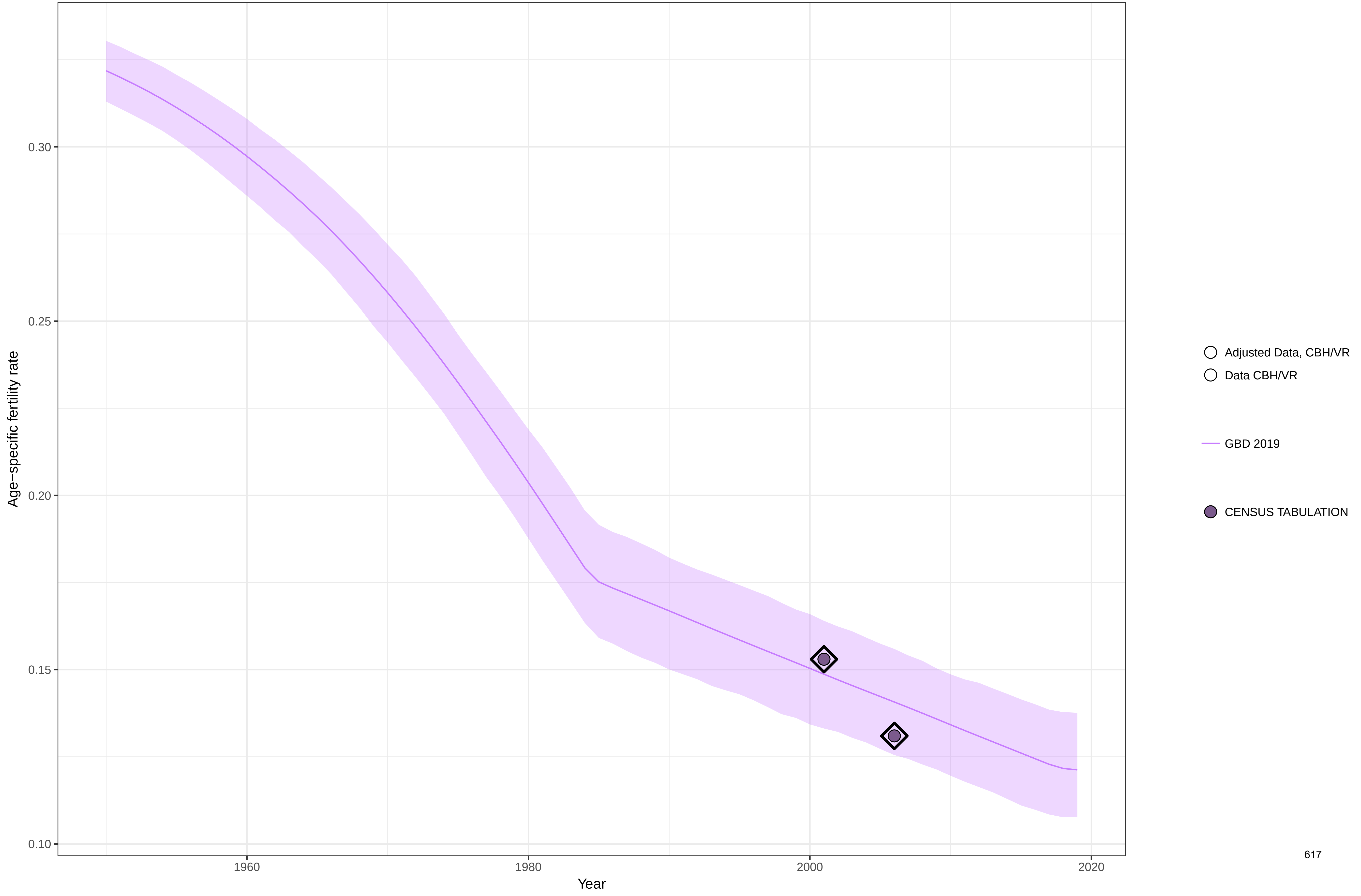

Age−specific fertility fitted trend lines, 1950−2019: Cook Islands, Ages: 30−34
Data points enclosed with a diamond are reference sources

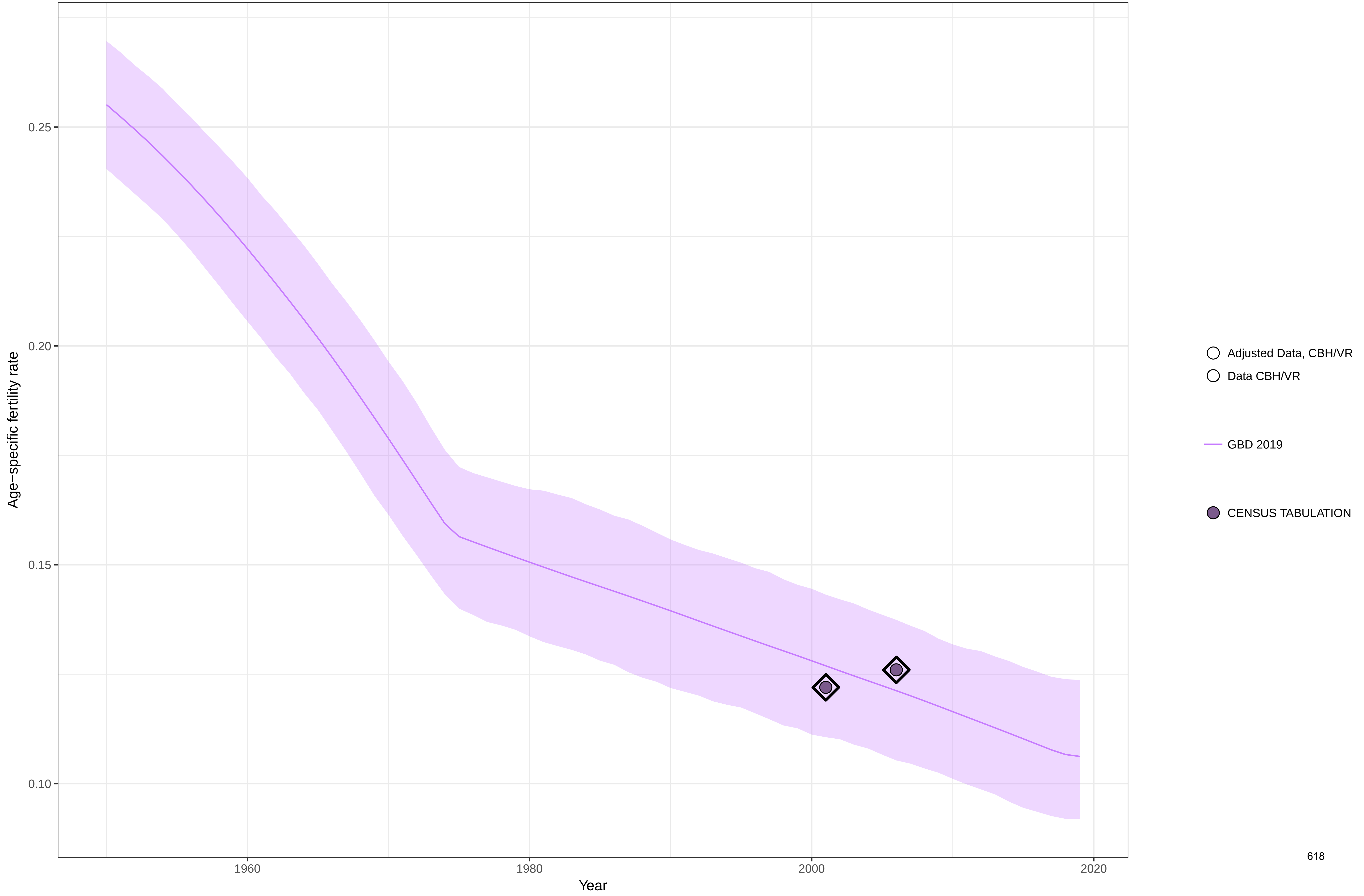

Age−specific fertility fitted trend lines, 1950−2019: Cook Islands, Ages: 35−39
Data points enclosed with a diamond are reference sources

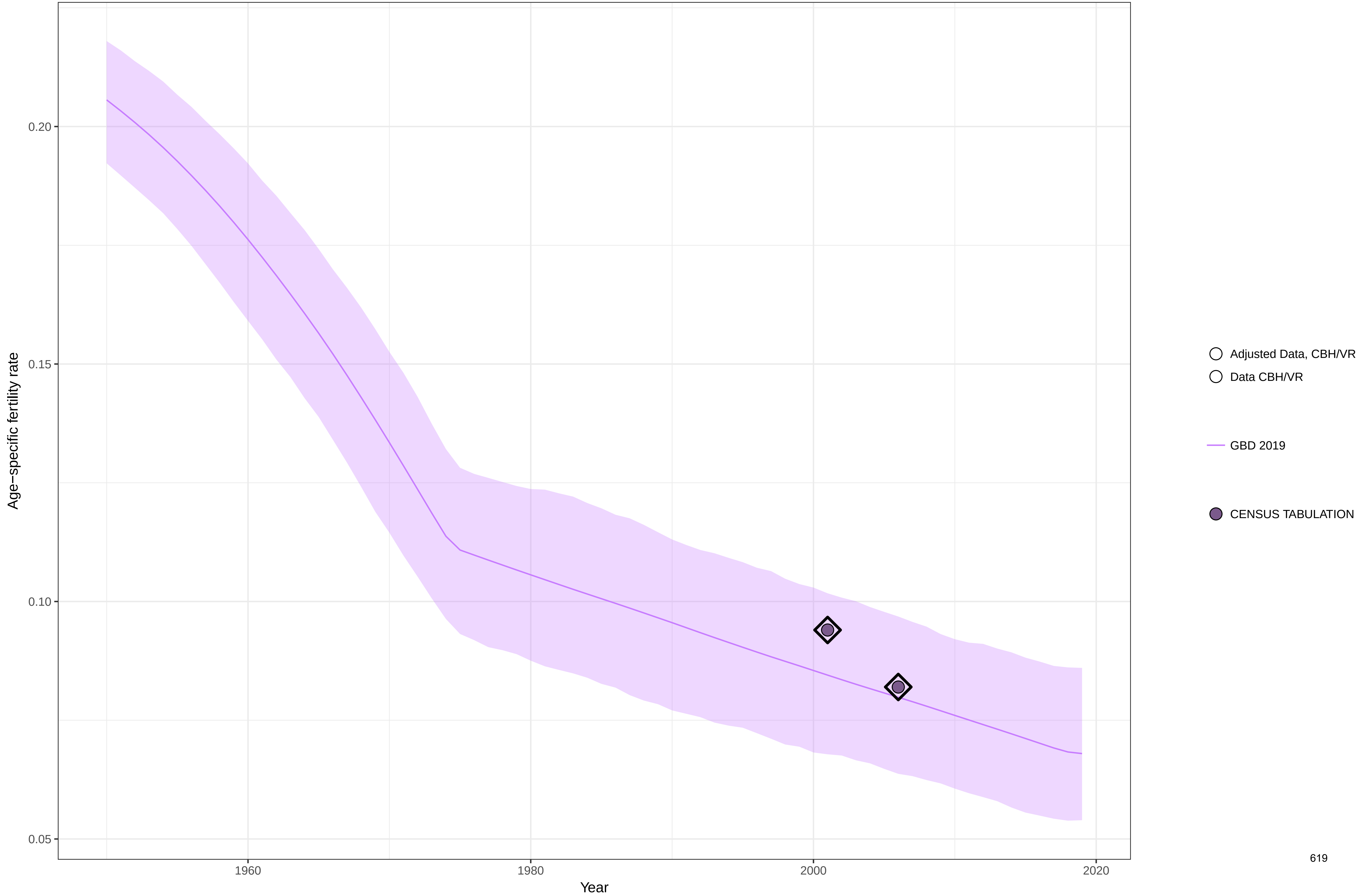

Age−specific fertility fitted trend lines, 1950−2019: Cook Islands, Ages: 40−44
Data points enclosed with a diamond are reference sources

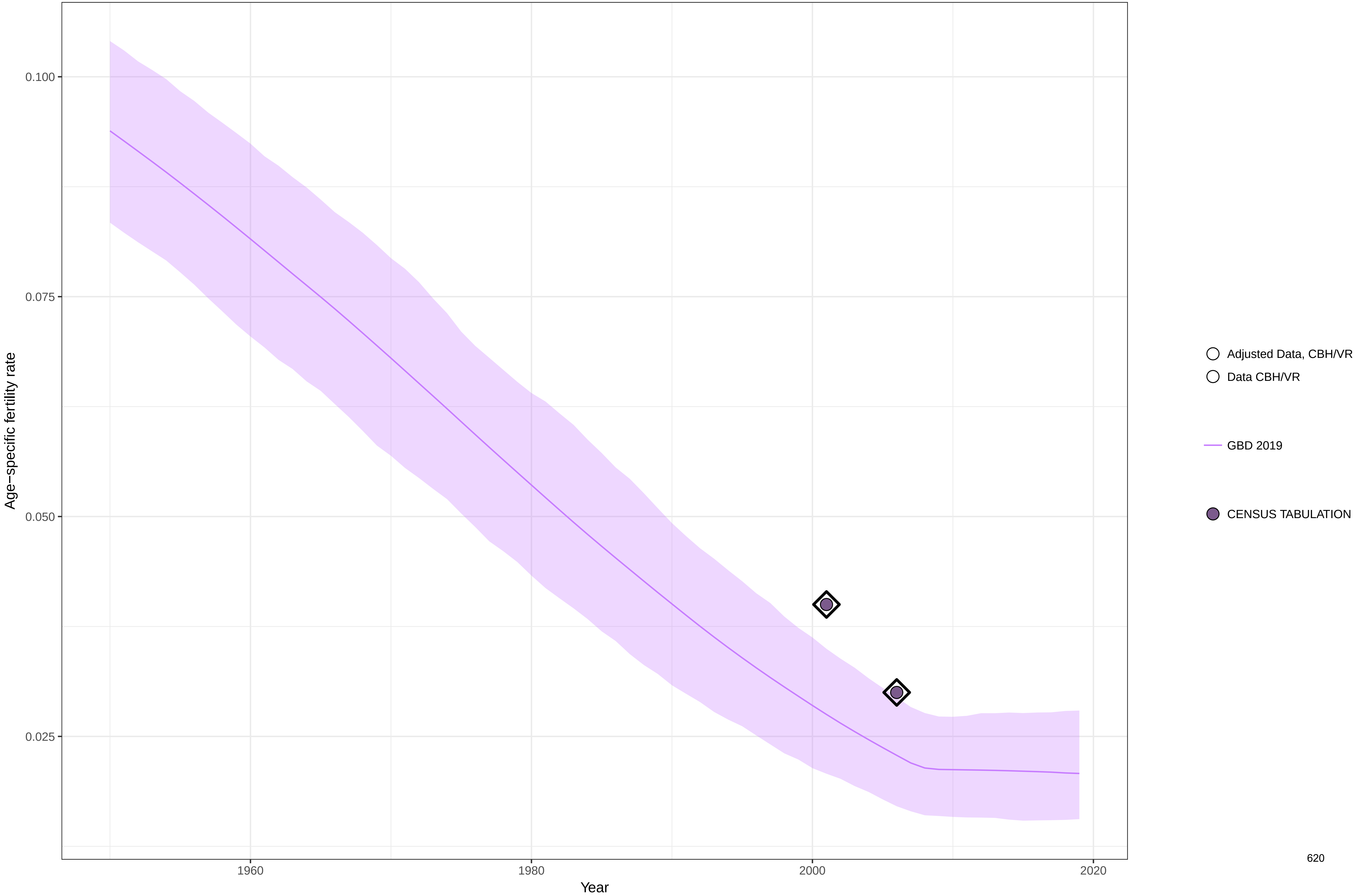

Age−specific fertility fitted trend lines, 1950−2019: Cook Islands, Ages: 45−49
Data points enclosed with a diamond are reference sources

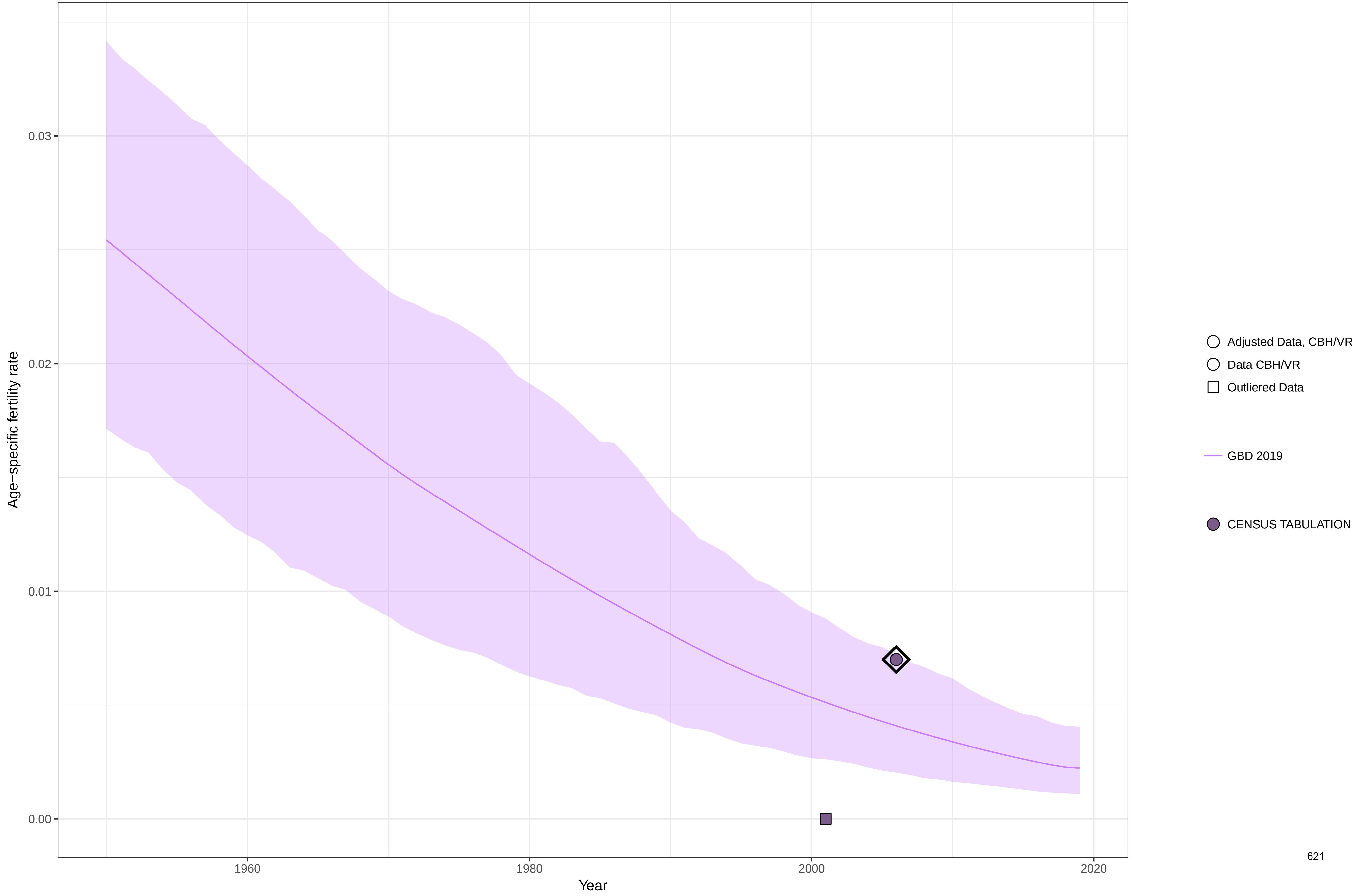

Age−specific fertility fitted trend lines, 1950−2019: Cook Islands, Ages: 50−54
Data points enclosed with a diamond are reference sources

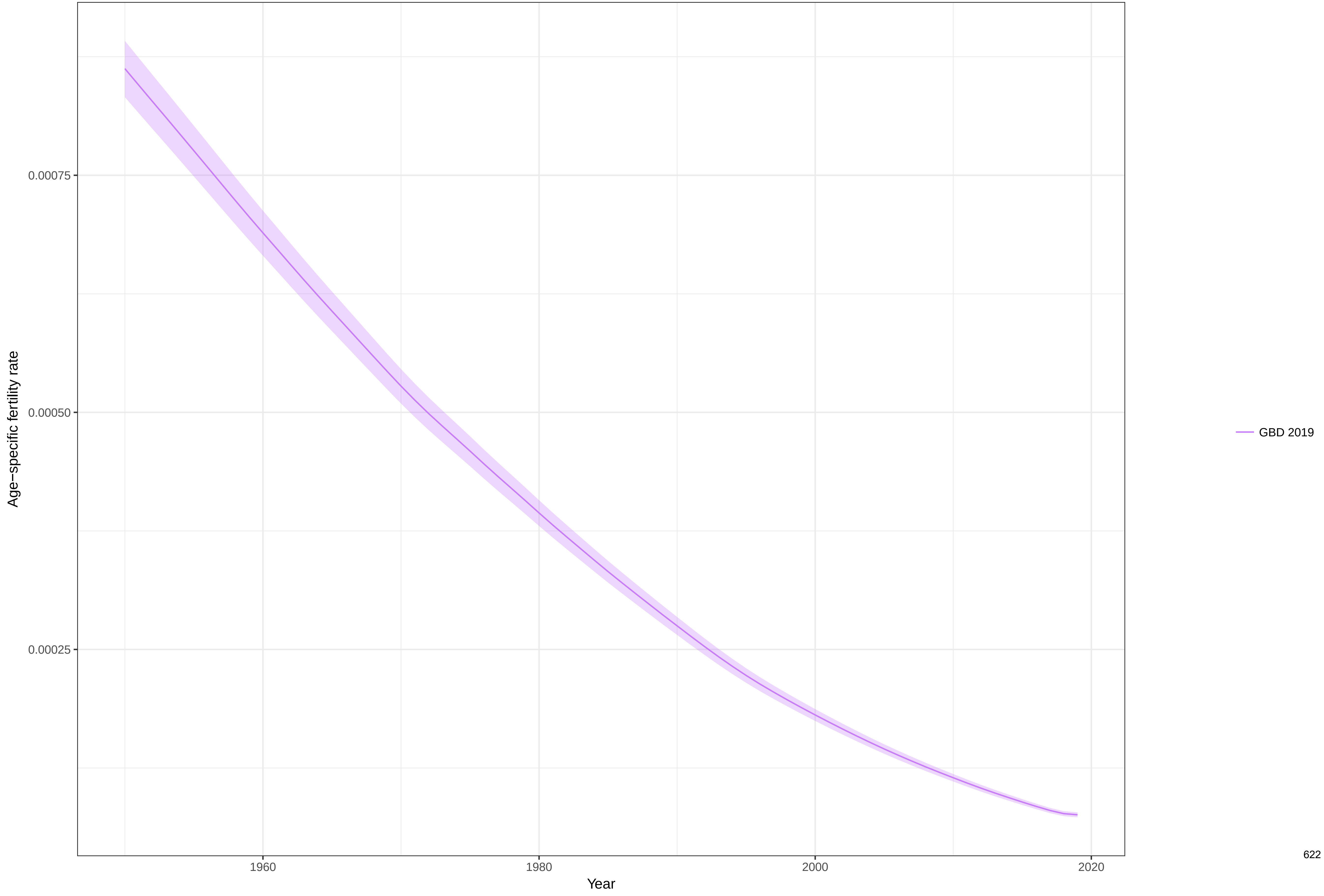

Total fertility rate fitted trend lines, 1950−2019: Cook Islands TFR
Data points enclosed with a diamond are reference sources

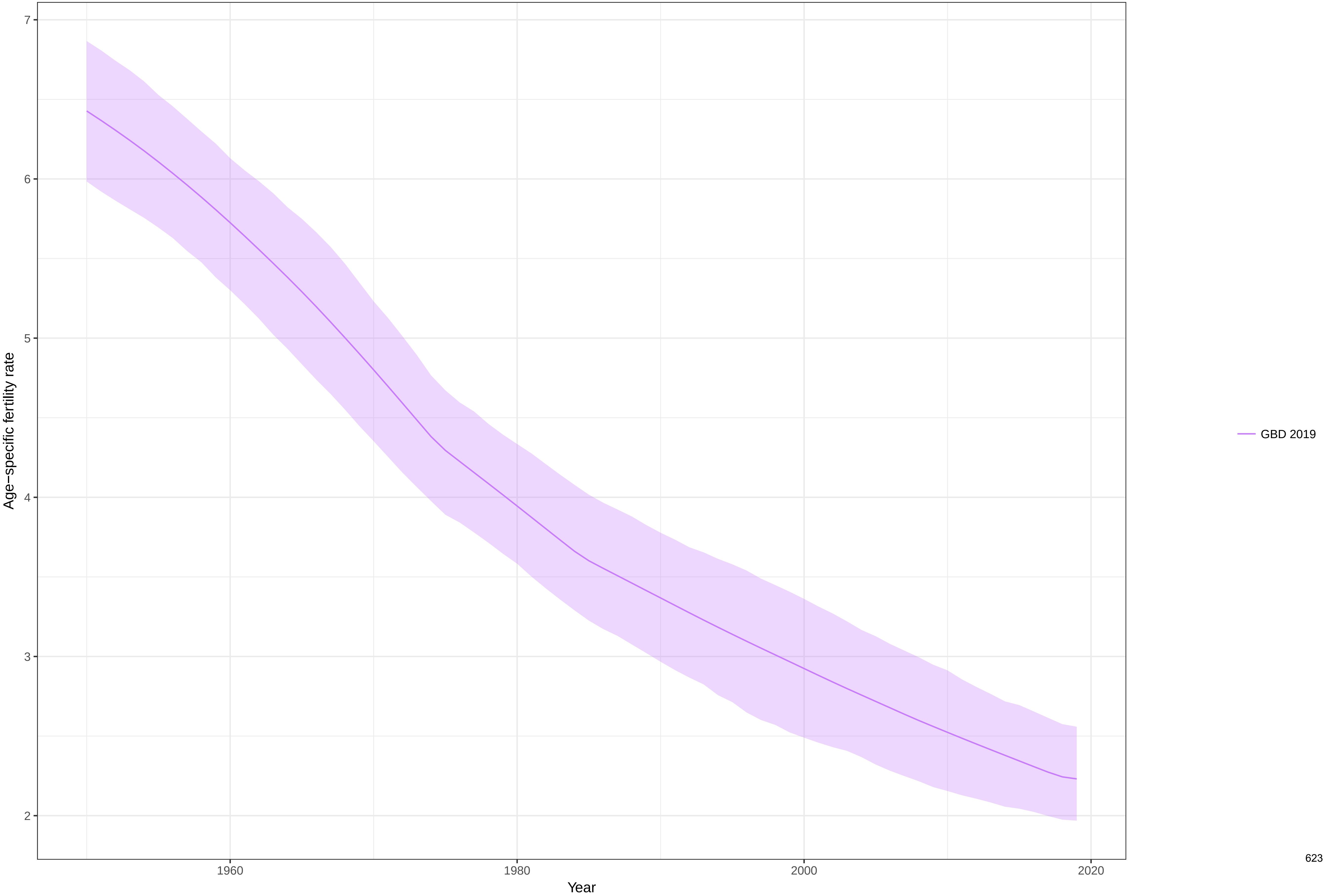

Age−specific fertility fitted trend lines, 1950−2019: Colombia, Ages: 10−14
Data points enclosed with a diamond are reference sources

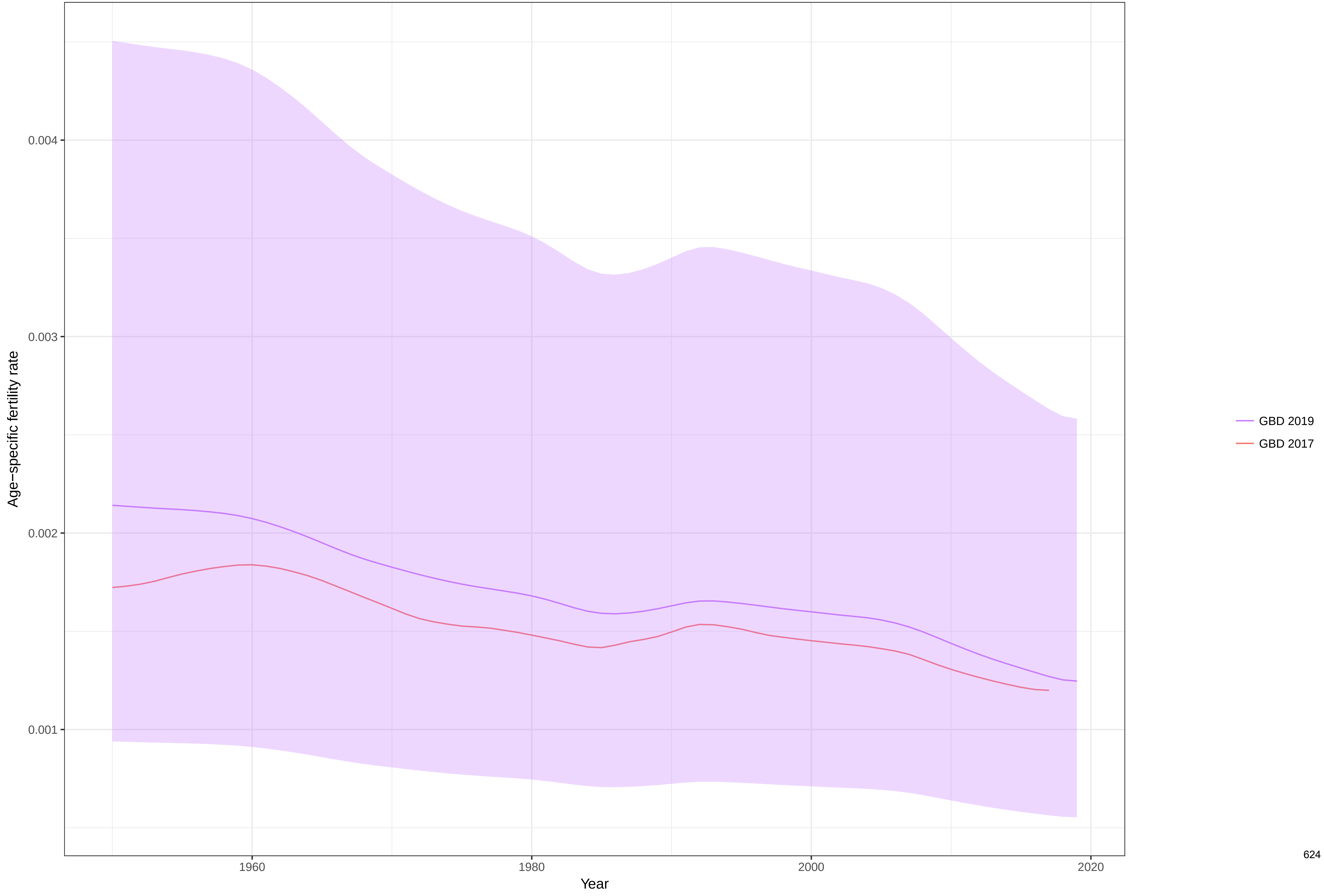

Age−specific fertility fitted trend lines, 1950−2019: Colombia, Ages: 15−19
Data points enclosed with a diamond are reference sources

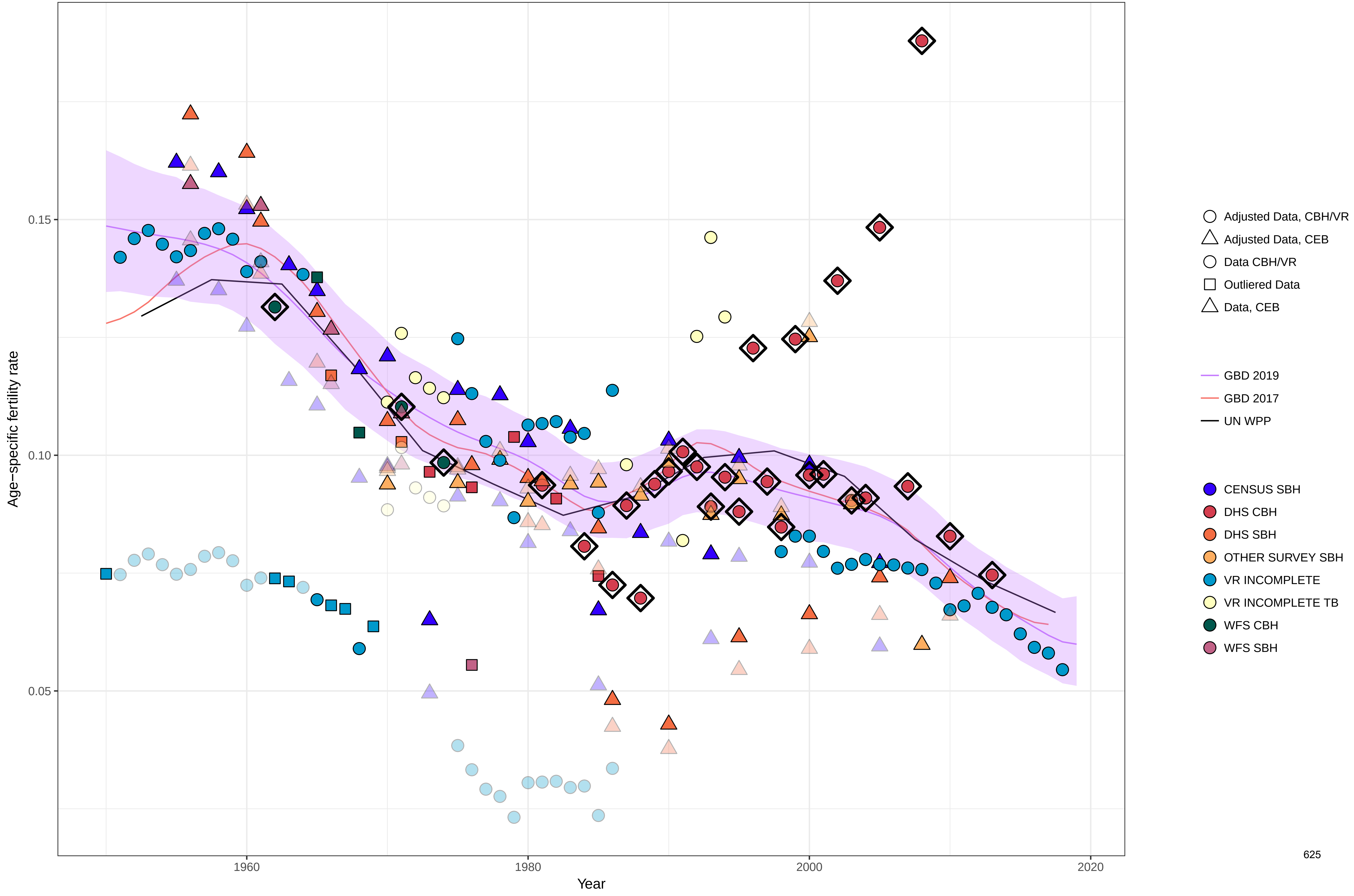

Age−specific fertility fitted trend lines, 1950−2019: Colombia, Ages: 20−24
Data points enclosed with a diamond are reference sources

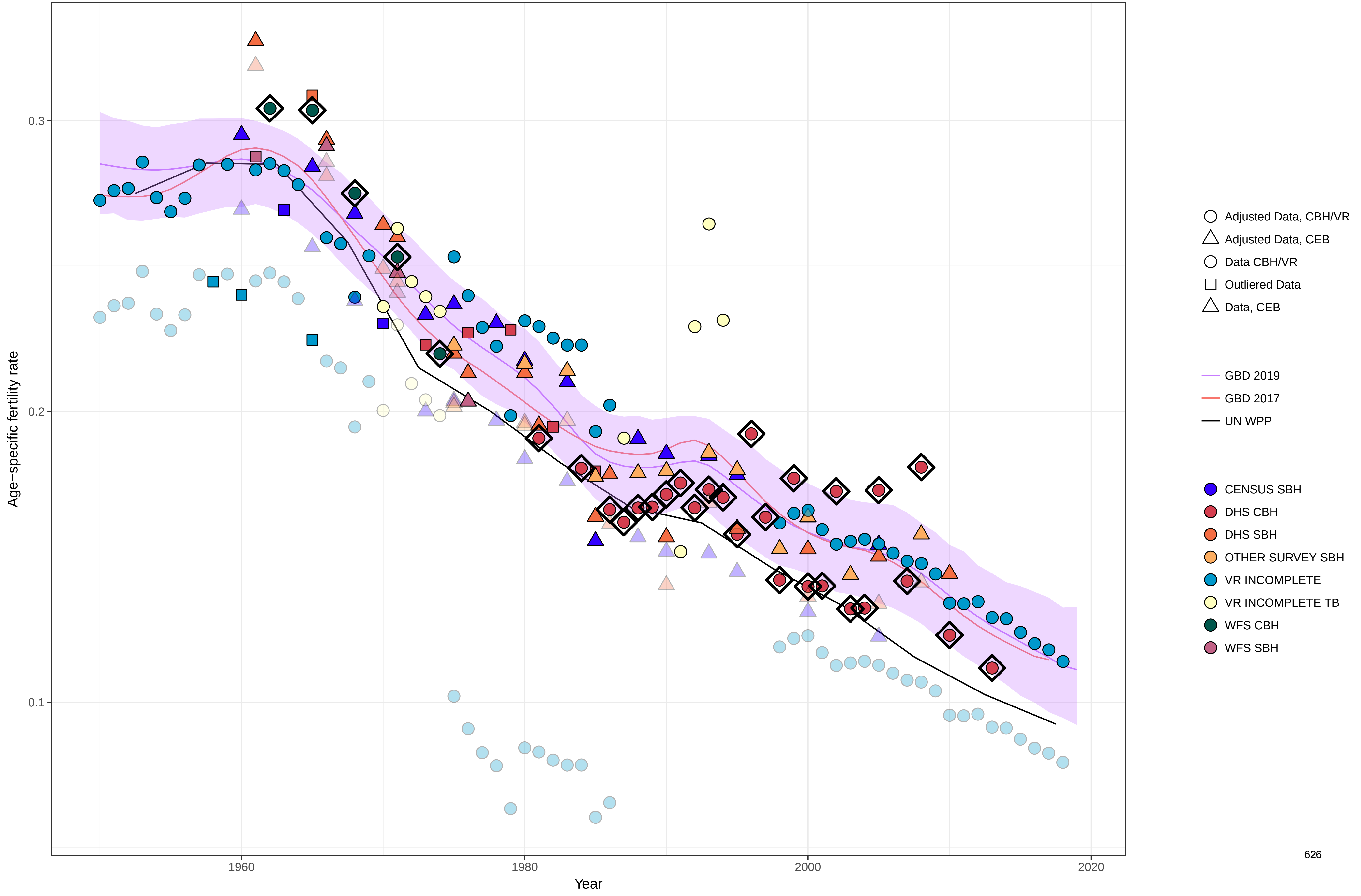

Age−specific fertility fitted trend lines, 1950−2019: Colombia, Ages: 25−29
Data points enclosed with a diamond are reference sources

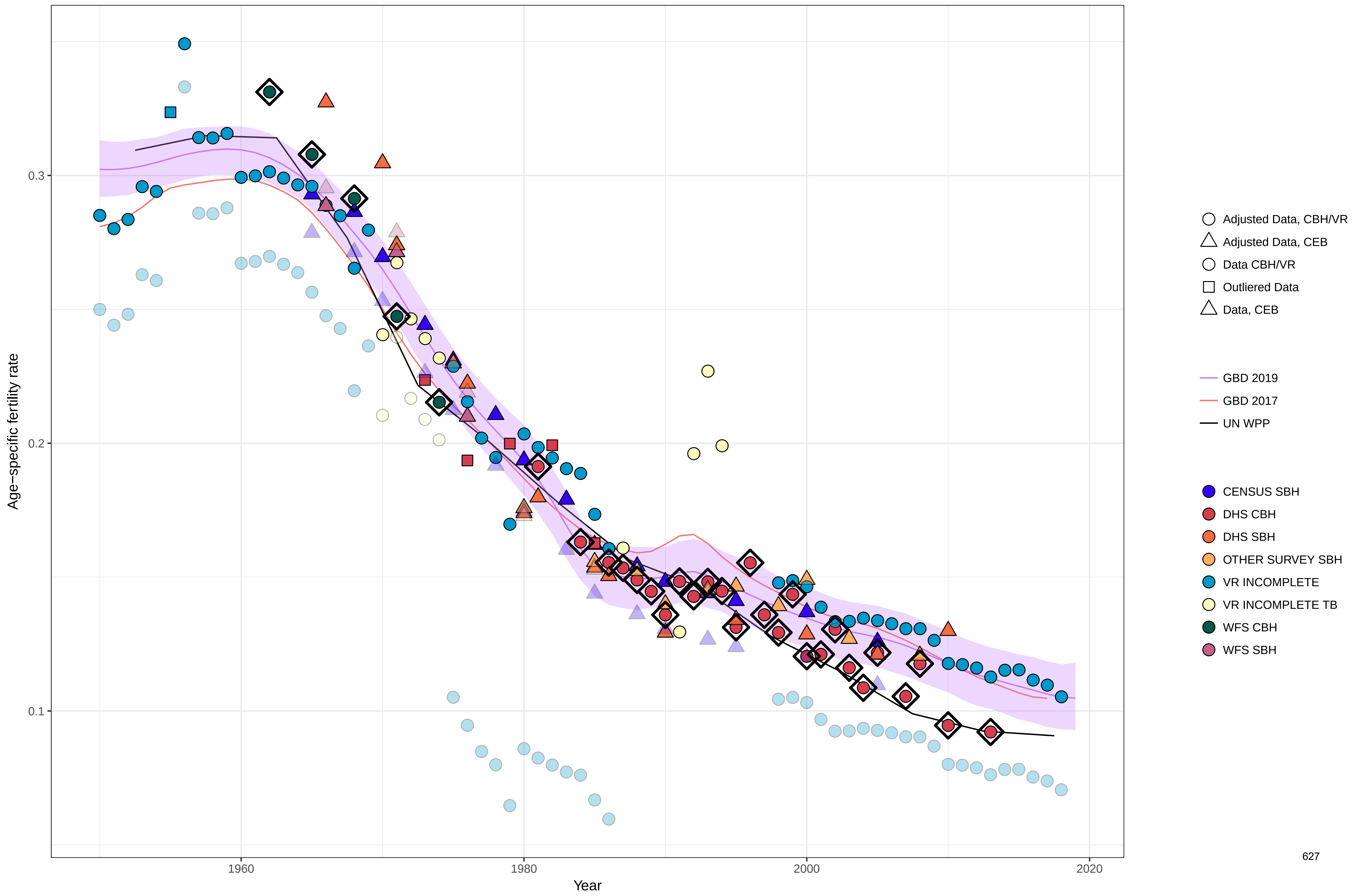

Age−specific fertility fitted trend lines, 1950−2019: Colombia, Ages: 30−34
Data points enclosed with a diamond are reference sources

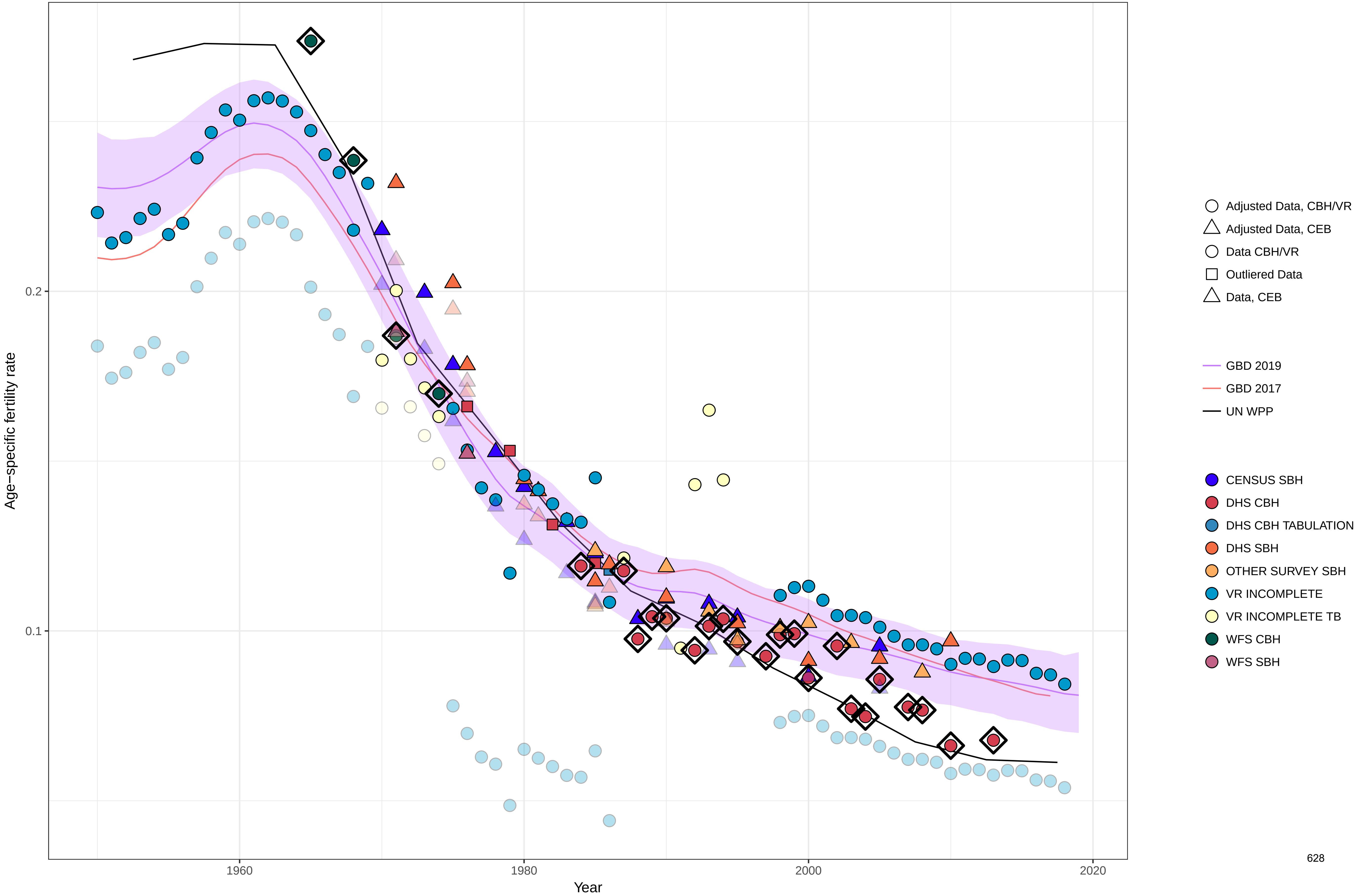

Age−specific fertility fitted trend lines, 1950−2019: Colombia, Ages: 35−39
Data points enclosed with a diamond are reference sources

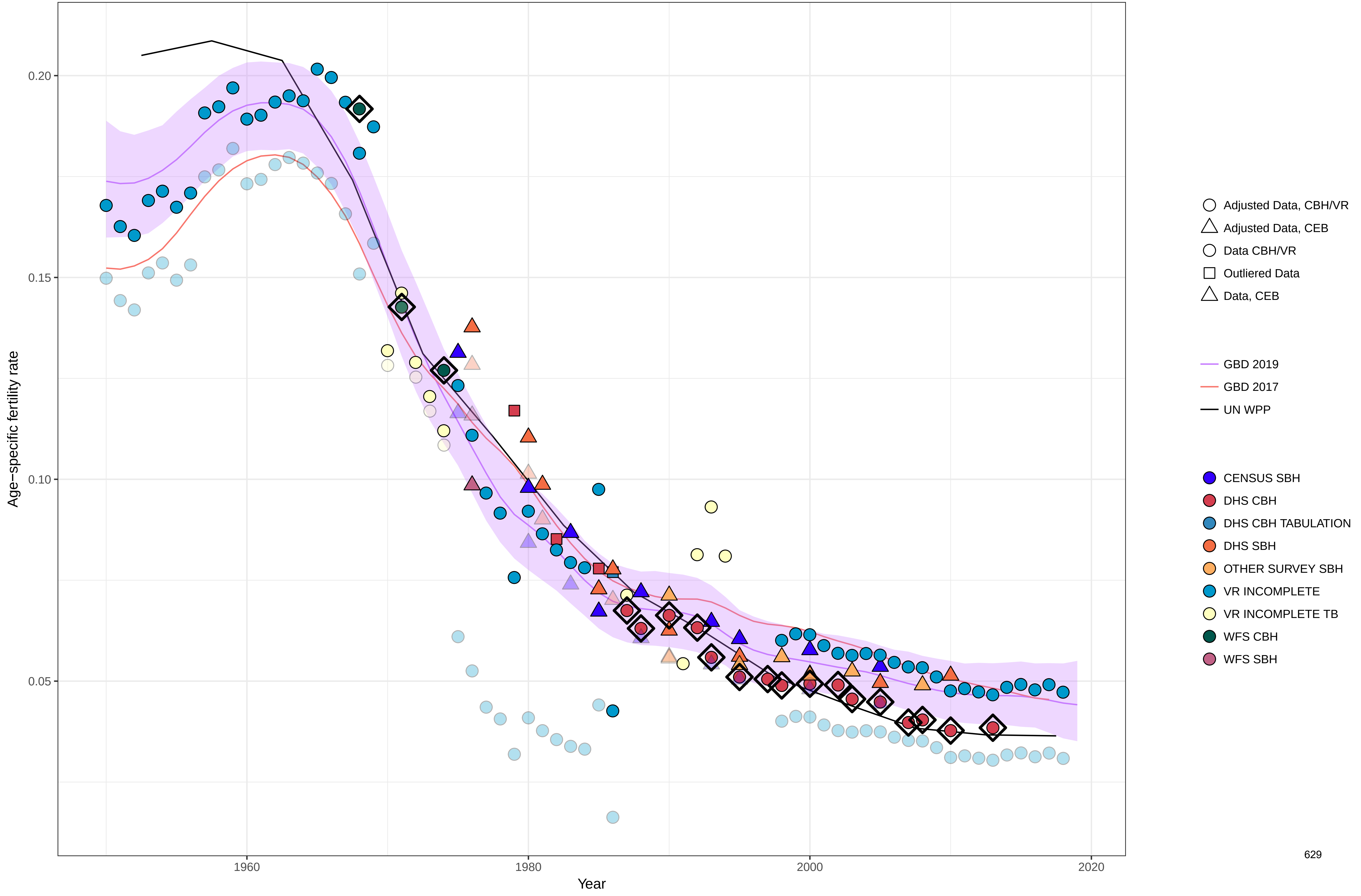

Age−specific fertility fitted trend lines, 1950−2019: Colombia, Ages: 40−44
Data points enclosed with a diamond are reference sources

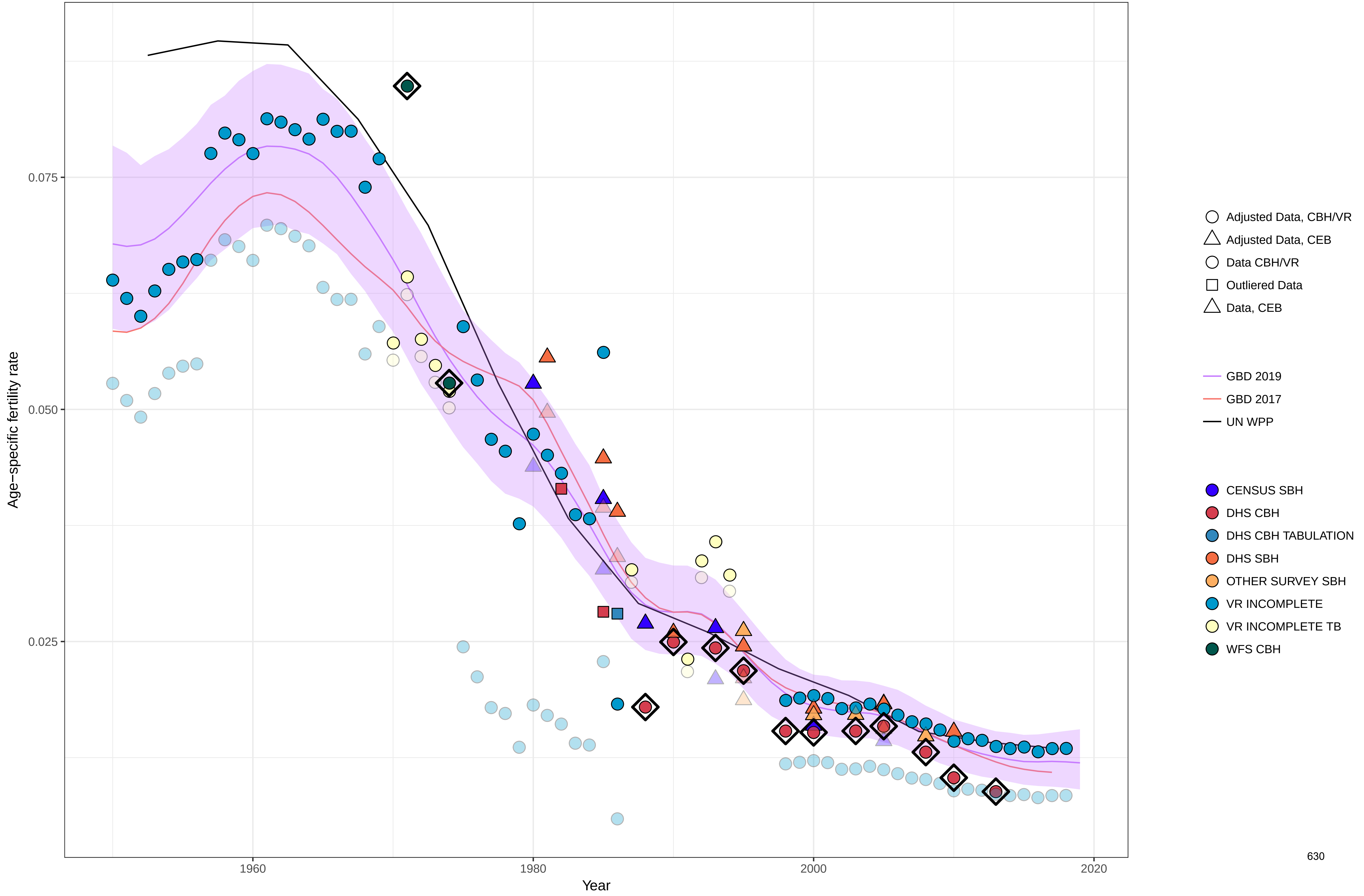

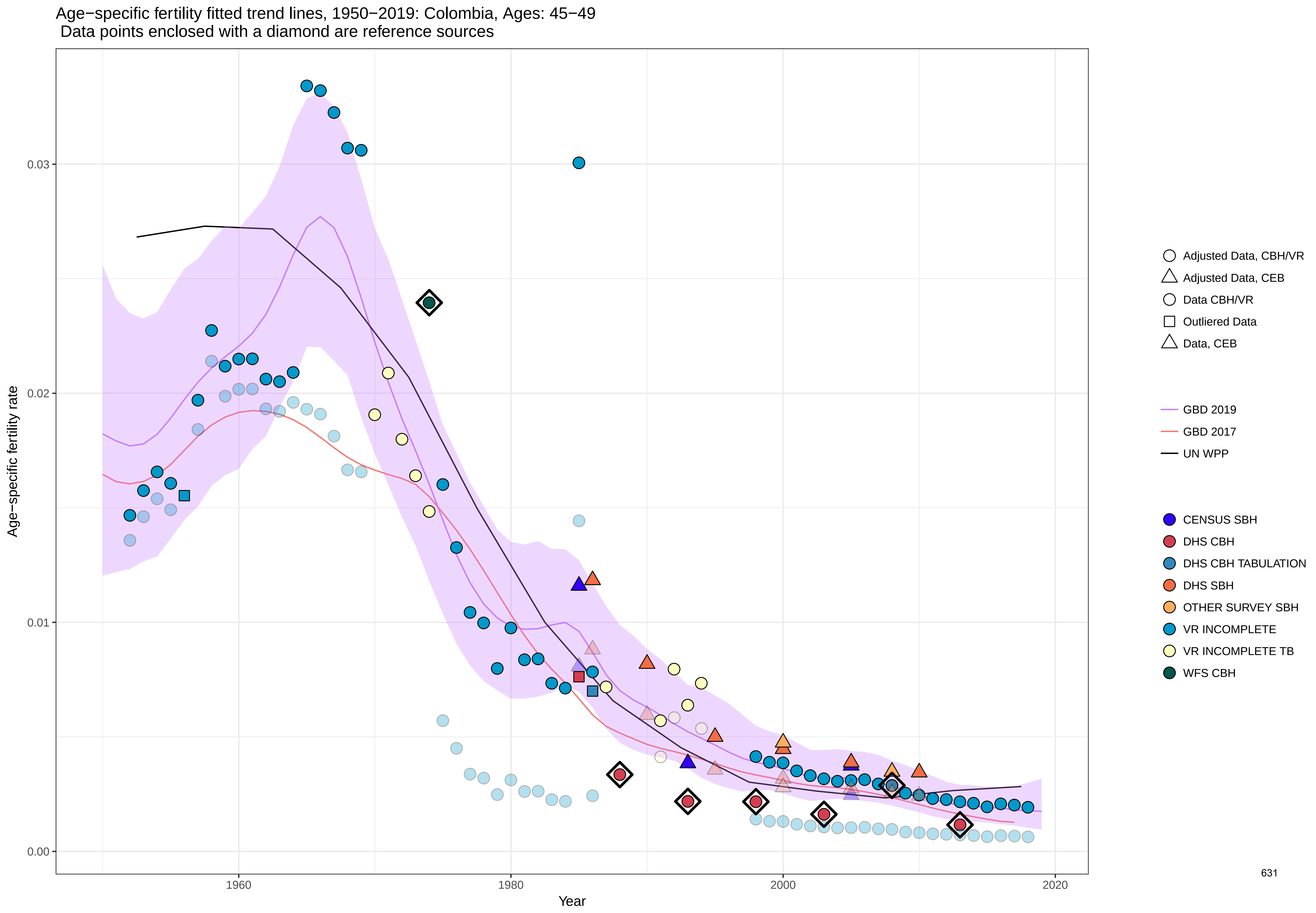
Age−specific fertility fitted trend lines, 1950−2019: Colombia, Ages: 45−49
Data points enclosed with a diamond are reference sources
Age−specific fertility rate
0.00
0.01
0.02
0.03
1960
1980
2000
2020
Year
Adjusted Data, CBH/VR
Adjusted Data, CEB
Data CBH/VR
Outliered Data
Data, CEB
GBD 2019
GBD 2017
UN WPP
CENSUS SBH
DHS CBH
DHS CBH TABULATION
DHS SBH
OTHER SURVEY SBH
VR INCOMPLETE
VR INCOMPLETE TB
WFS CBH

Age−specific fertility fitted trend lines, 1950−2019: Colombia, Ages: 50−54
Data points enclosed with a diamond are reference sources

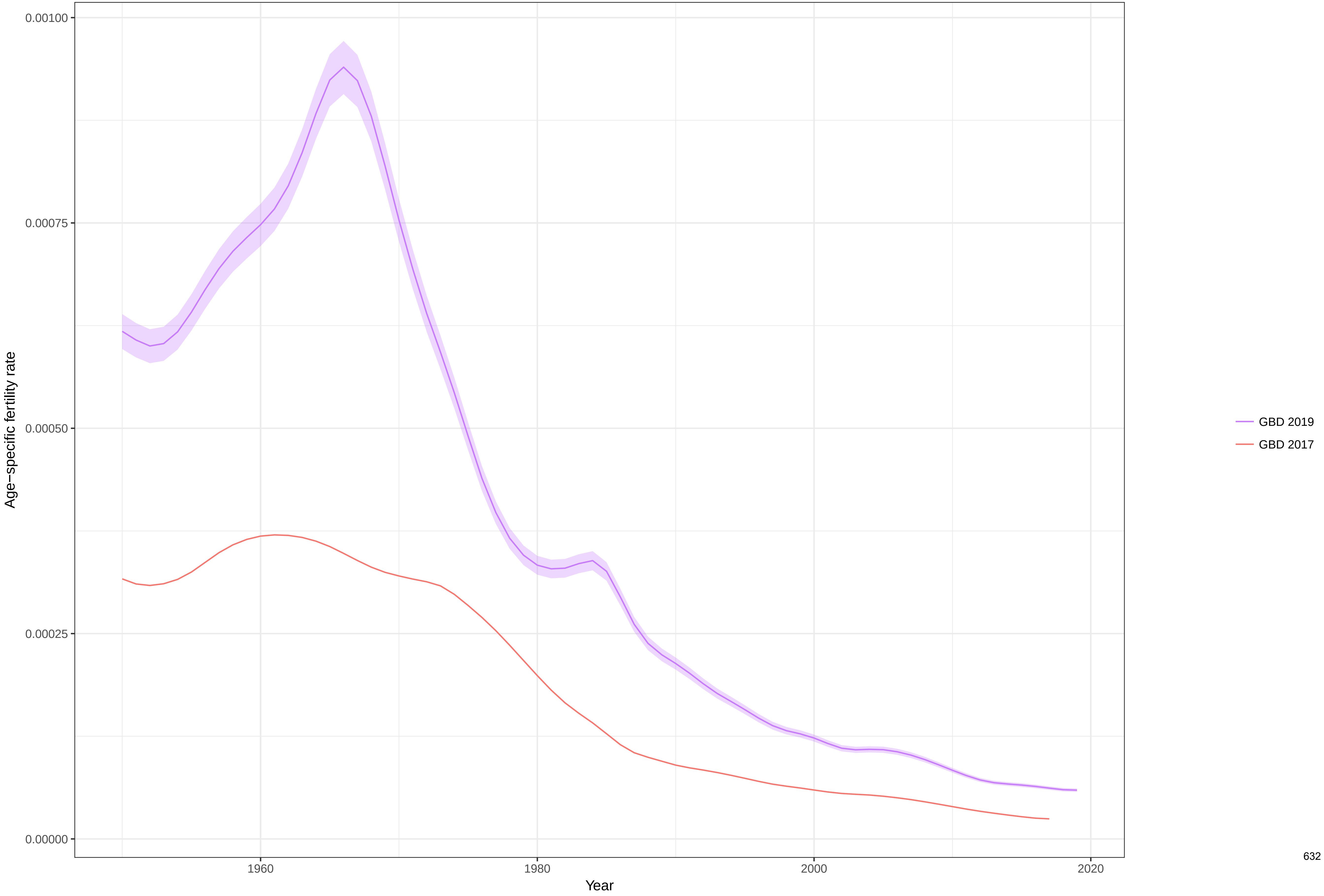

Total fertility rate fitted trend lines, 1950−2019: Colombia TFR
Data points enclosed with a diamond are reference sources

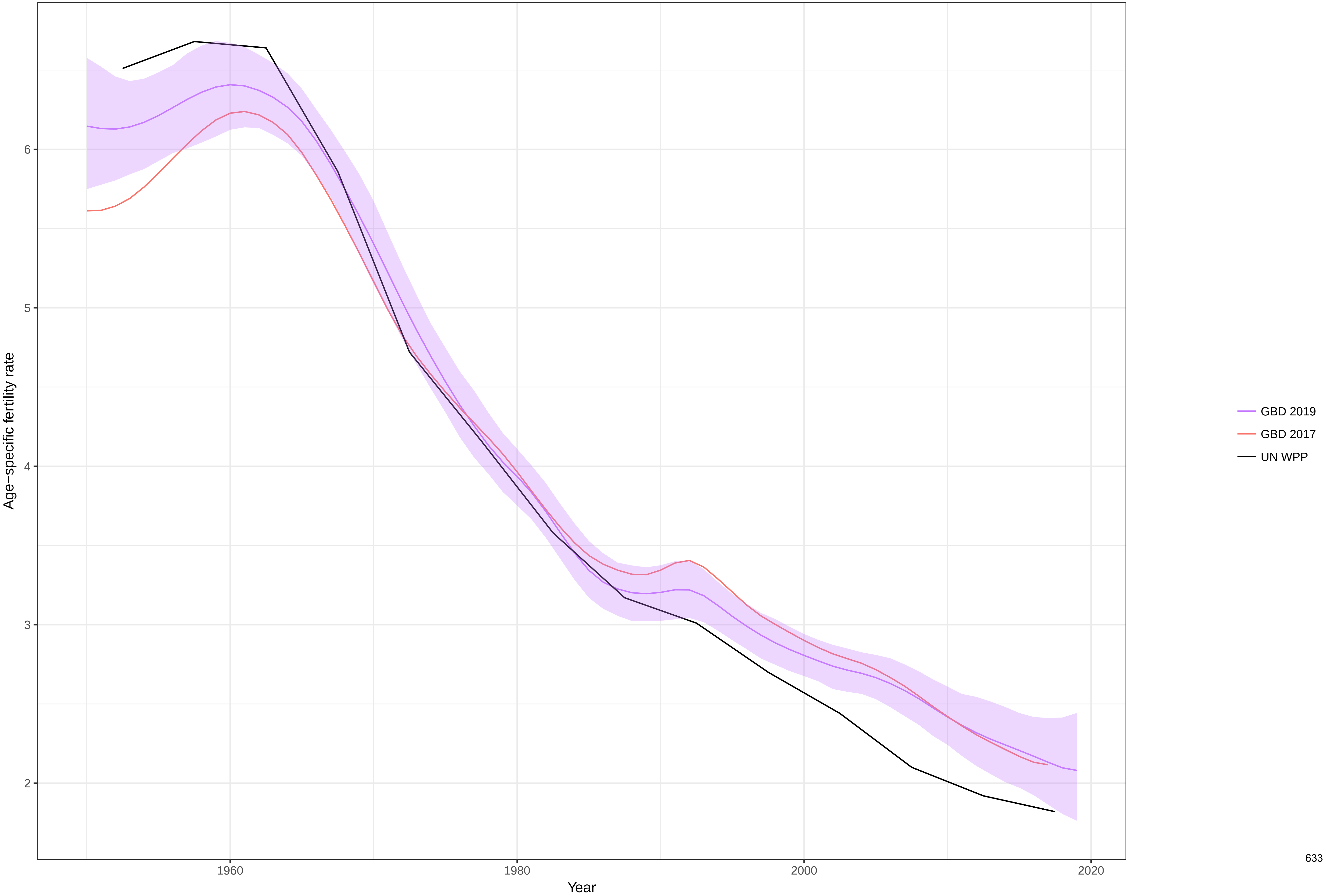

Age−specific fertility fitted trend lines, 1950−2019: Comoros, Ages: 10−14
Data points enclosed with a diamond are reference sources

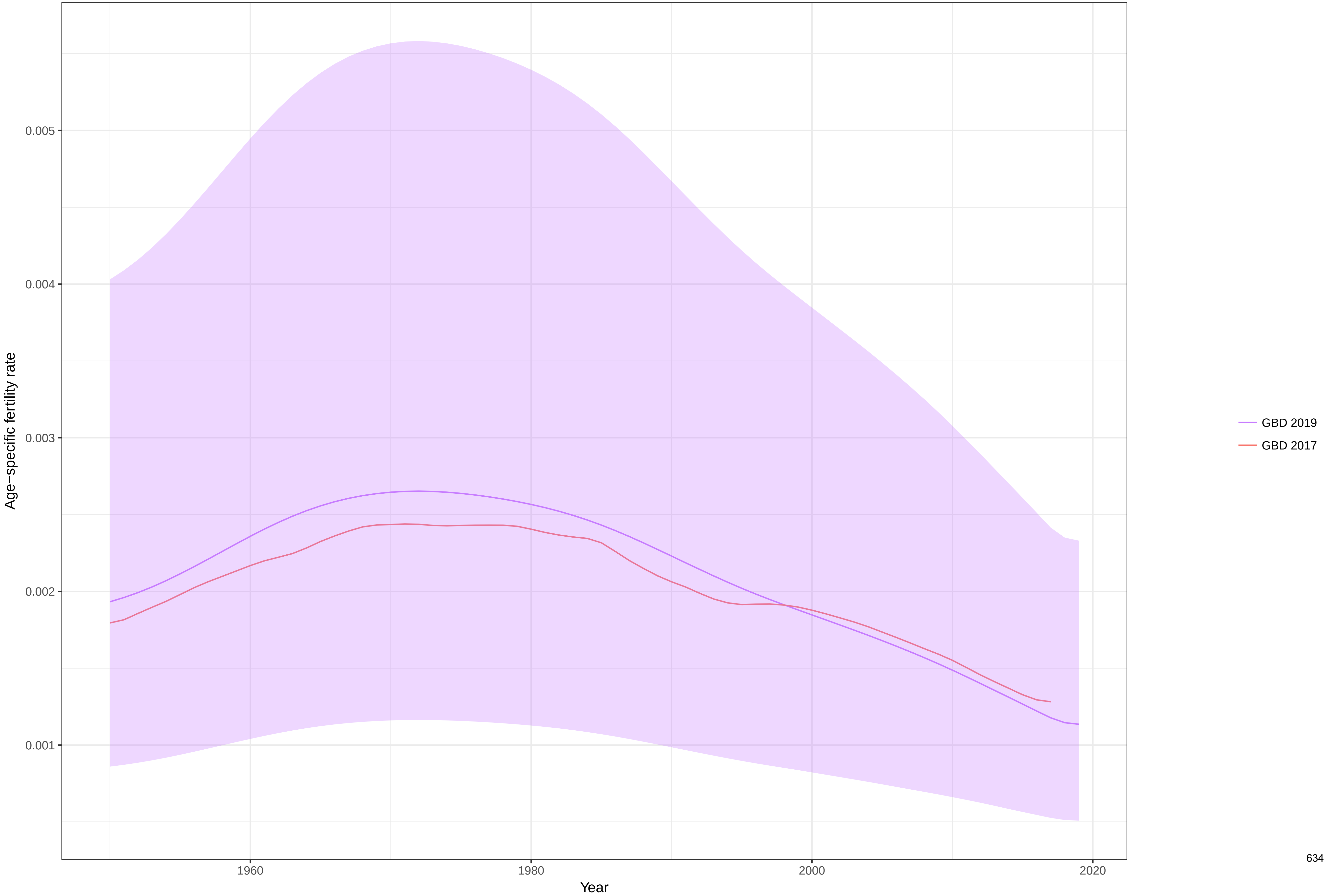

Age−specific fertility fitted trend lines, 1950−2019: Comoros, Ages: 15−19
Data points enclosed with a diamond are reference sources

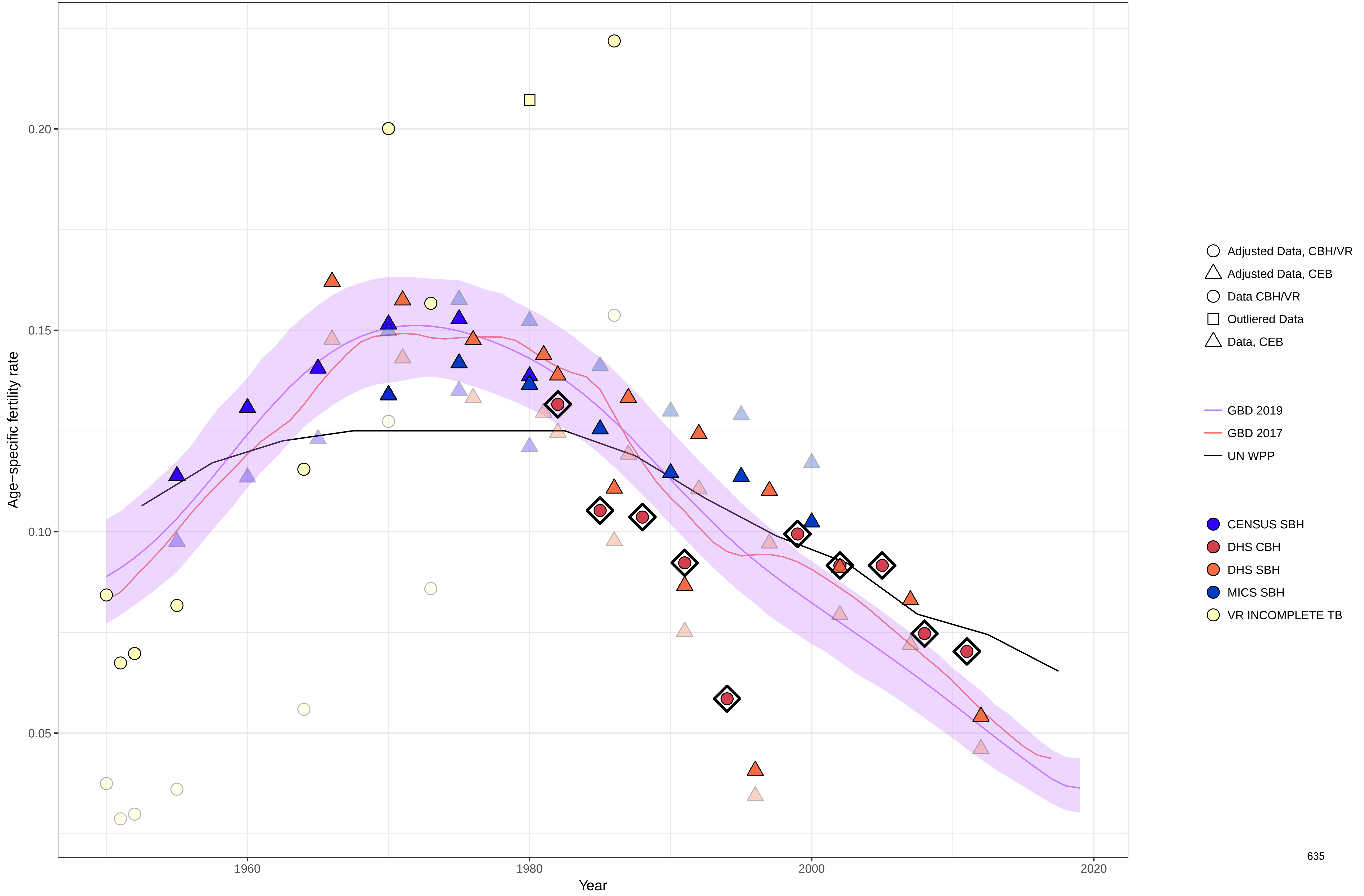

Age−specific fertility fitted trend lines, 1950−2019: Comoros, Ages: 20−24
Data points enclosed with a diamond are reference sources

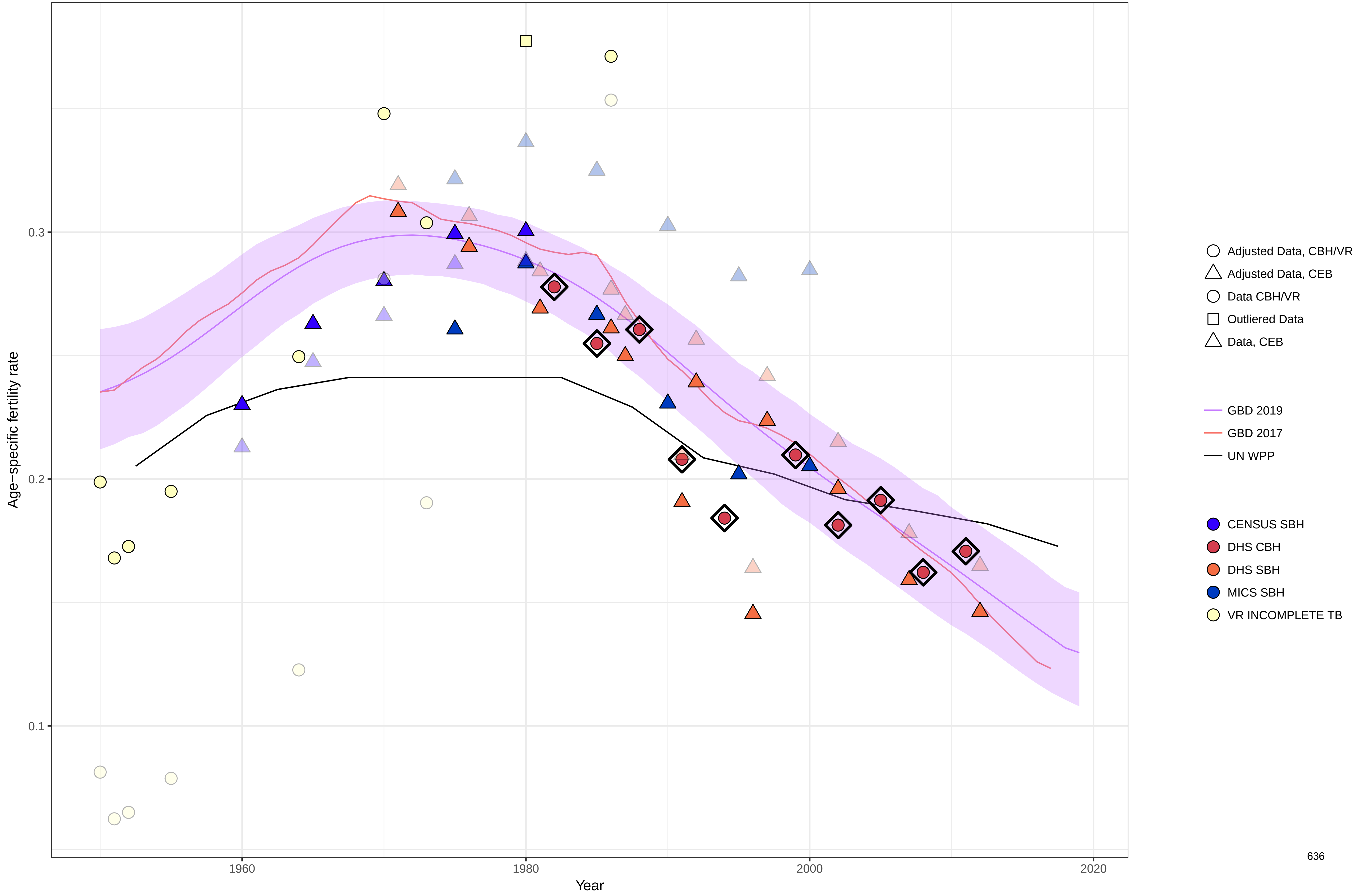

Age−specific fertility fitted trend lines, 1950−2019: Comoros, Ages: 25−29
Data points enclosed with a diamond are reference sources

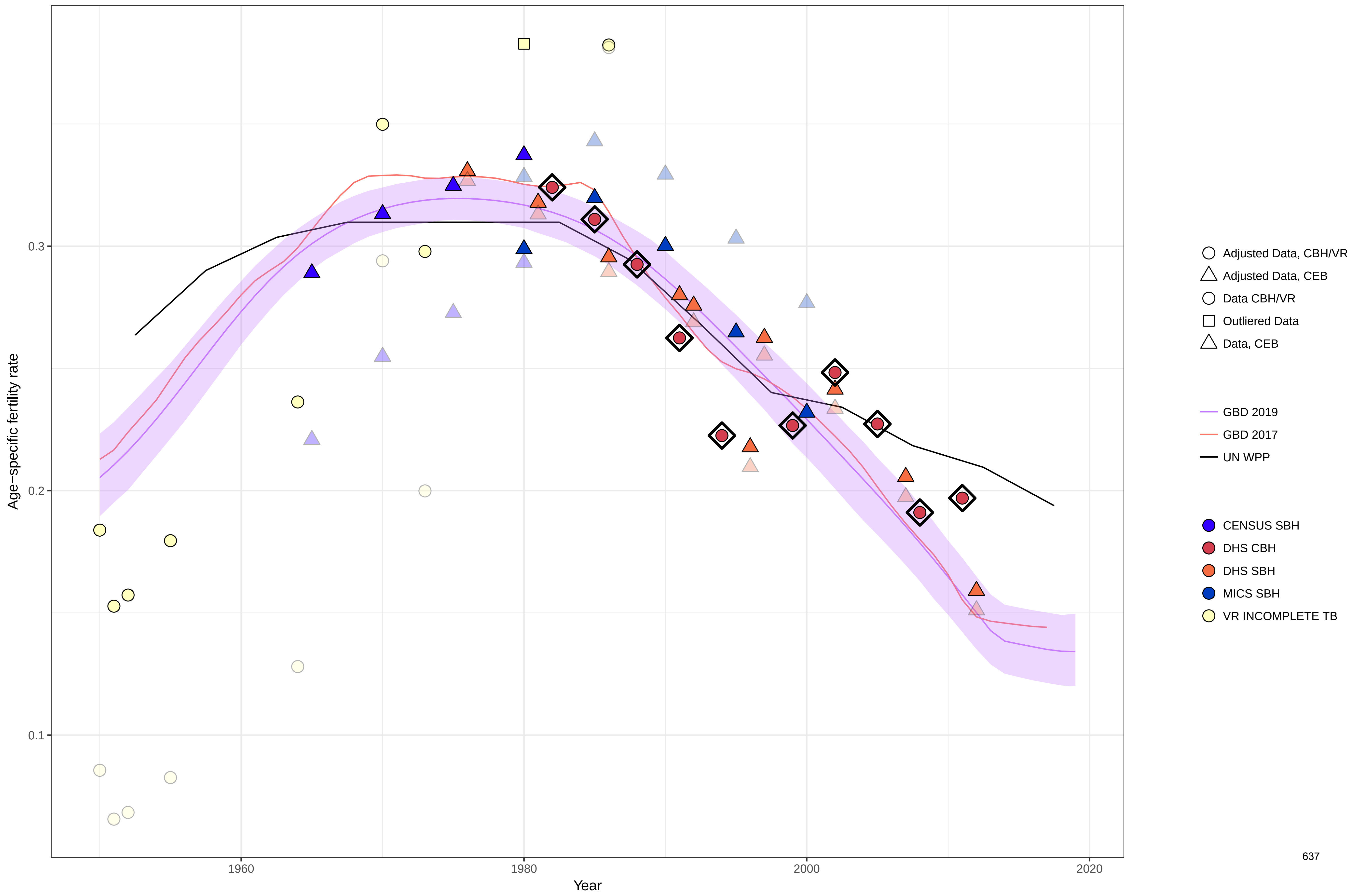

Age−specific fertility fitted trend lines, 1950−2019: Comoros, Ages: 30−34
Data points enclosed with a diamond are reference sources

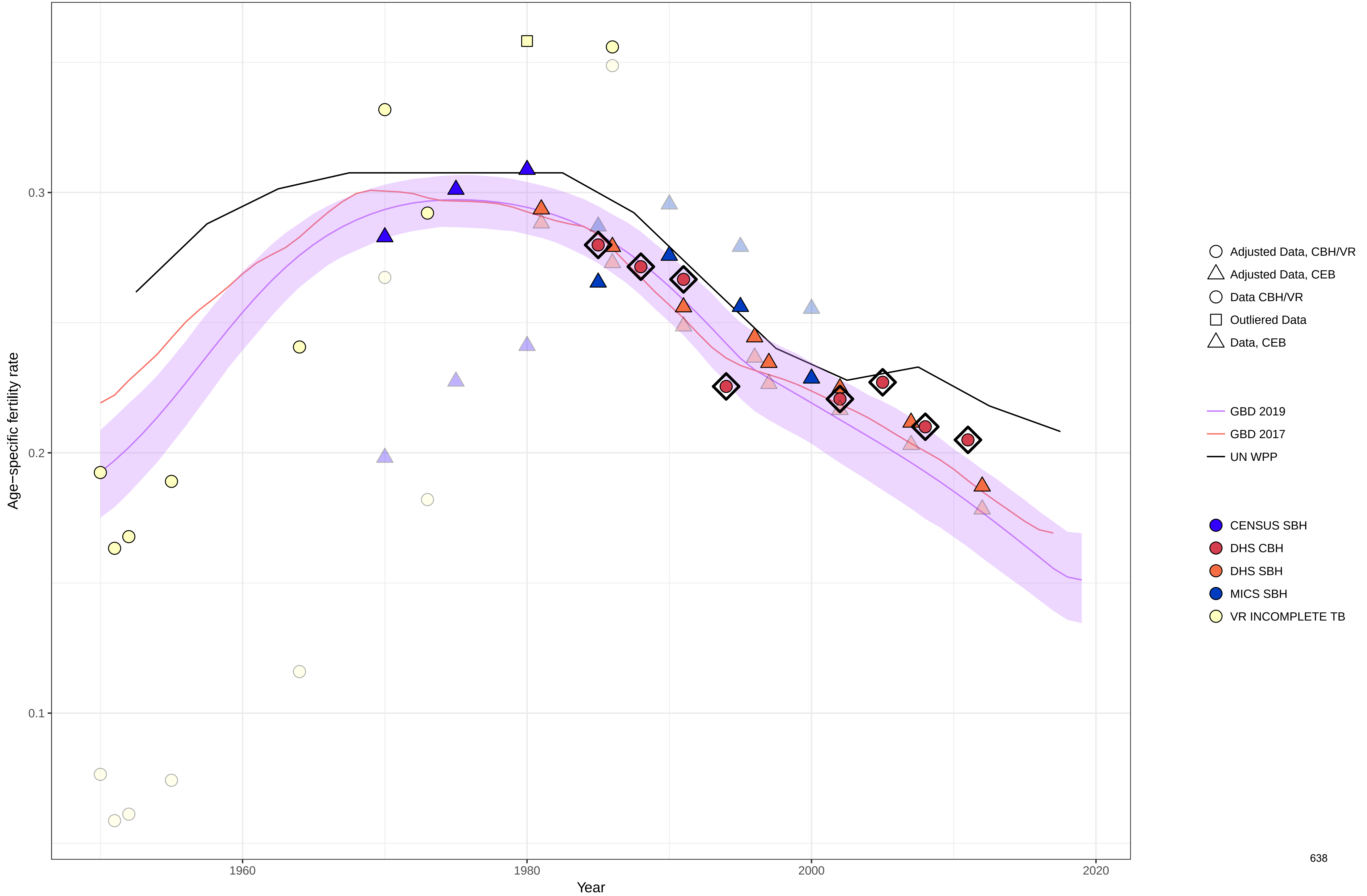

Age−specific fertility fitted trend lines, 1950−2019: Comoros, Ages: 35−39
Data points enclosed with a diamond are reference sources

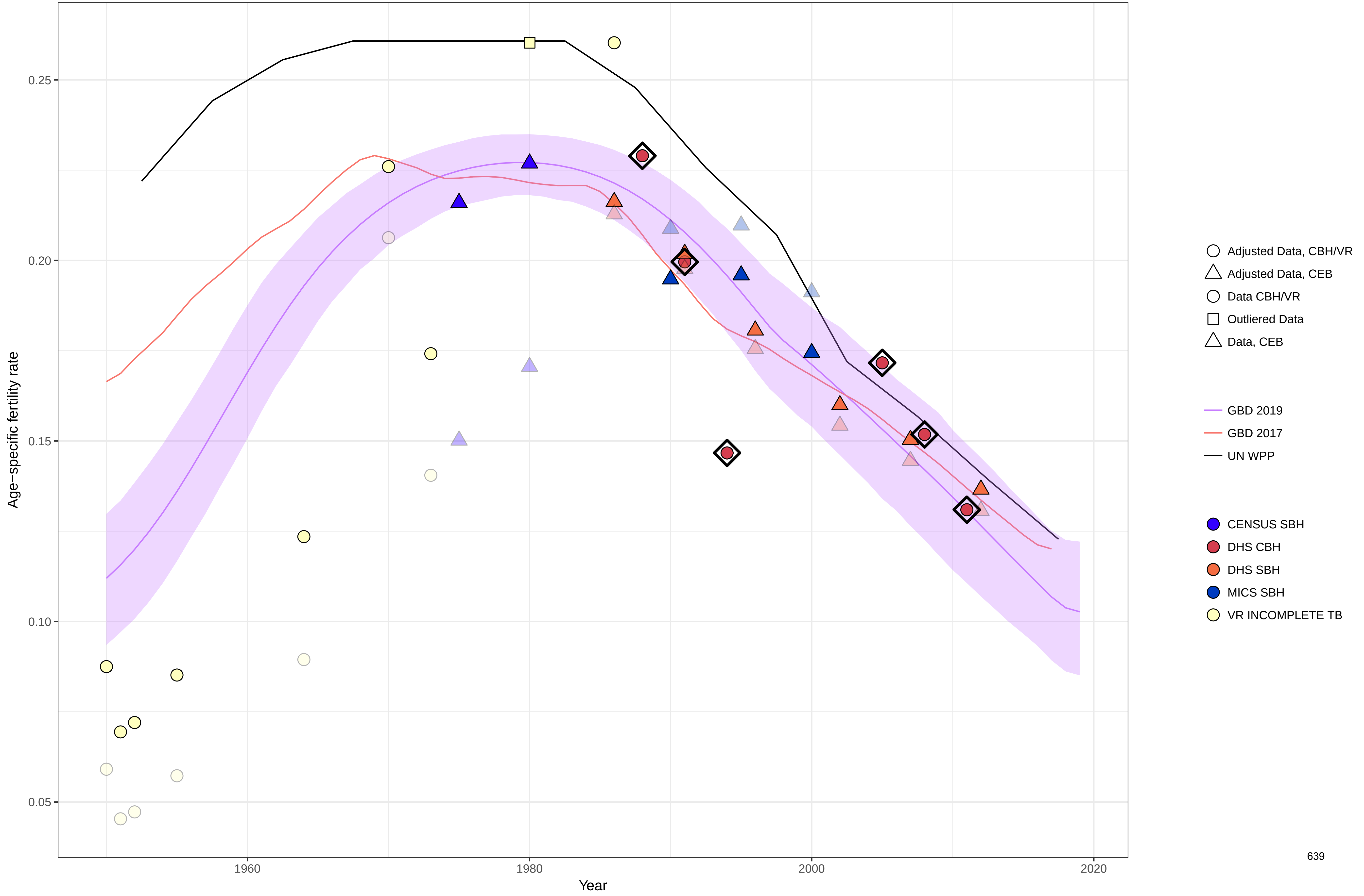

Age−specific fertility fitted trend lines, 1950−2019: Comoros, Ages: 40−44
Data points enclosed with a diamond are reference sources

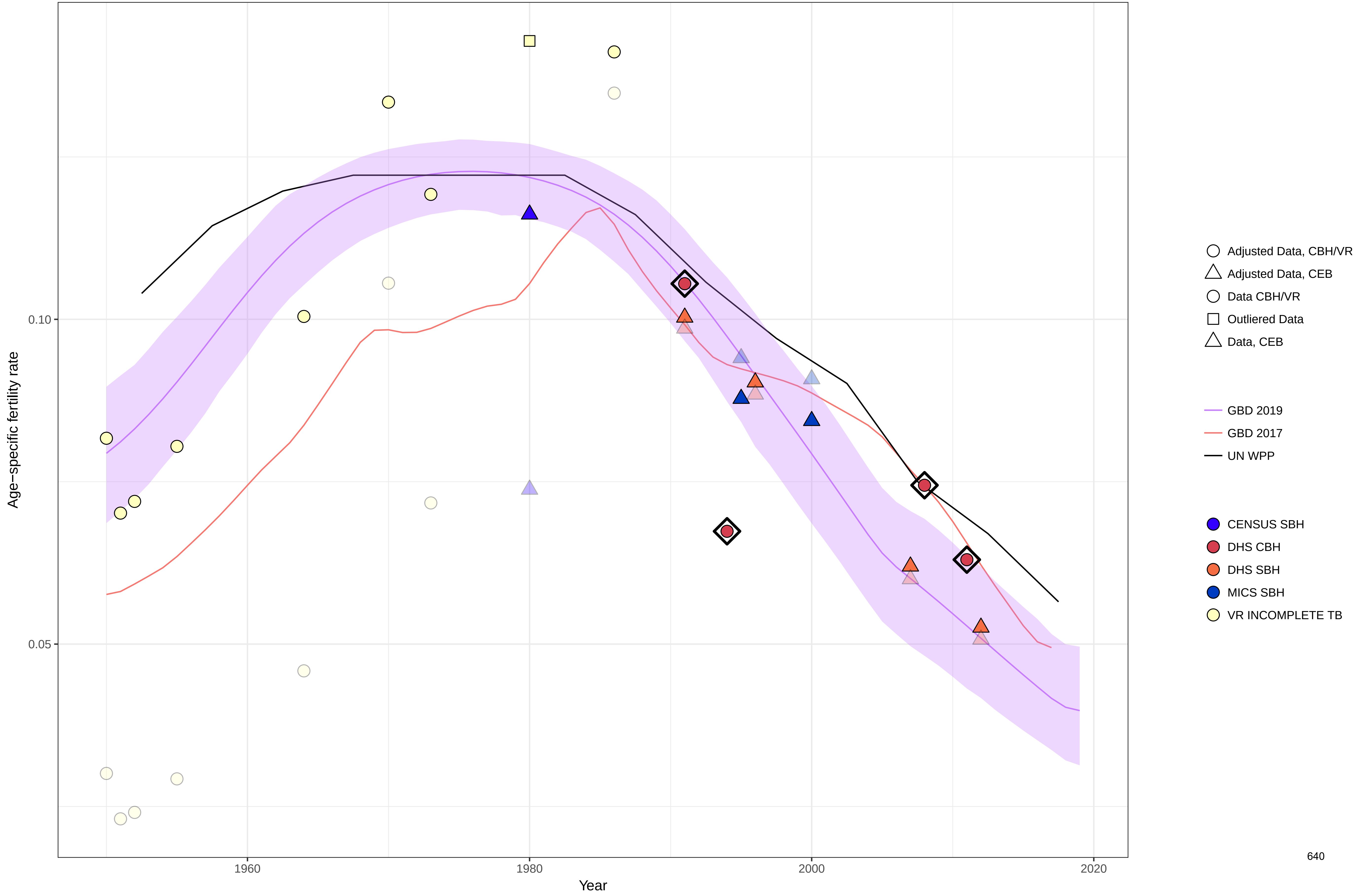

Age−specific fertility fitted trend lines, 1950−2019: Comoros, Ages: 45−49
Data points enclosed with a diamond are reference sources

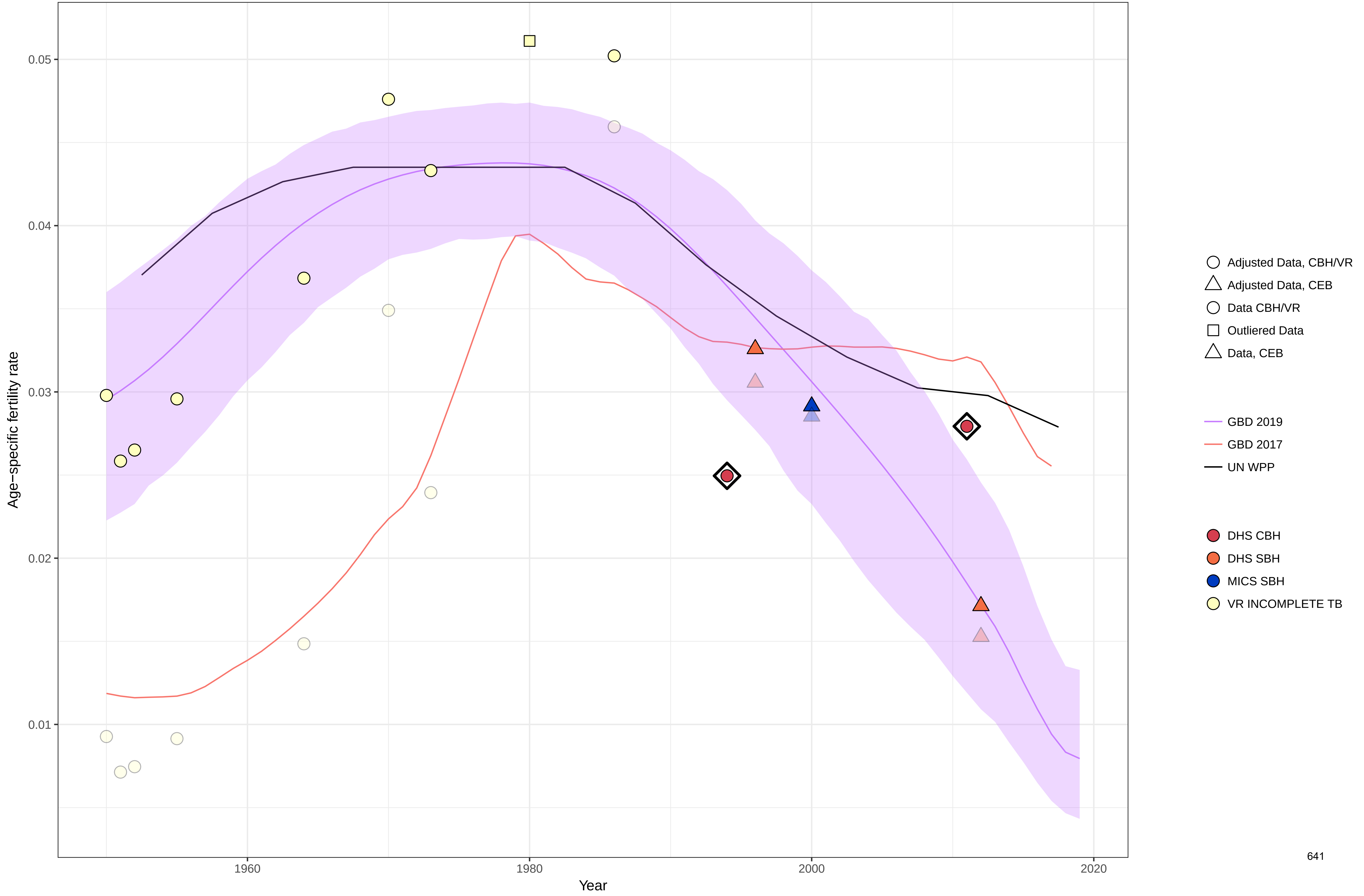

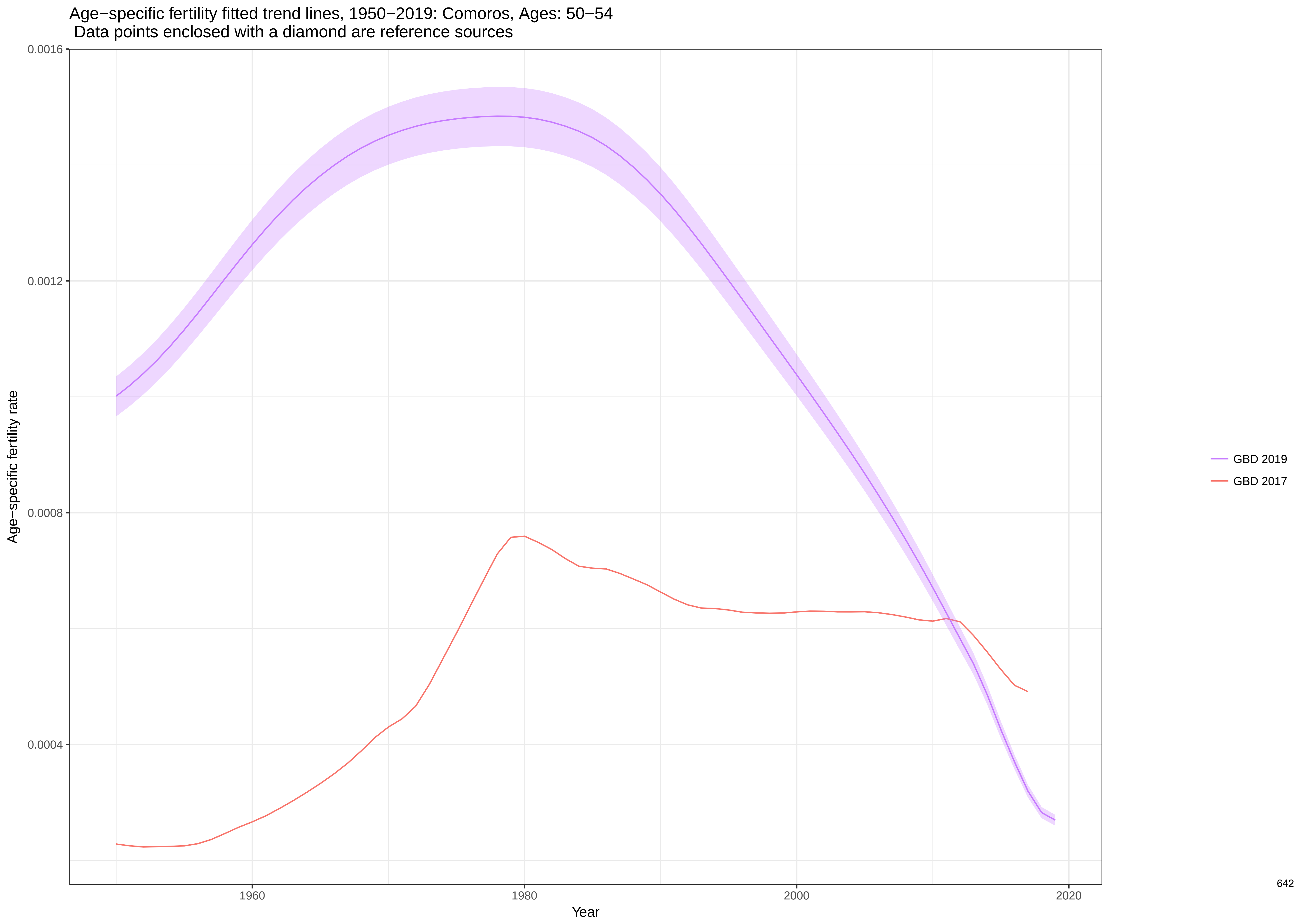

Age−specific fertility fitted trend lines, 1950−2019: Comoros, Ages: 50−54
Data points enclosed with a diamond are reference sources
0.0016
0.0012
0.0008
0.0004
Age−specific fertility rate
1960
1980
2000
2020
Year
GBD 2019
GBD 2017

Total fertility rate fitted trend lines, 1950−2019: Comoros TFR
Data points enclosed with a diamond are reference sources

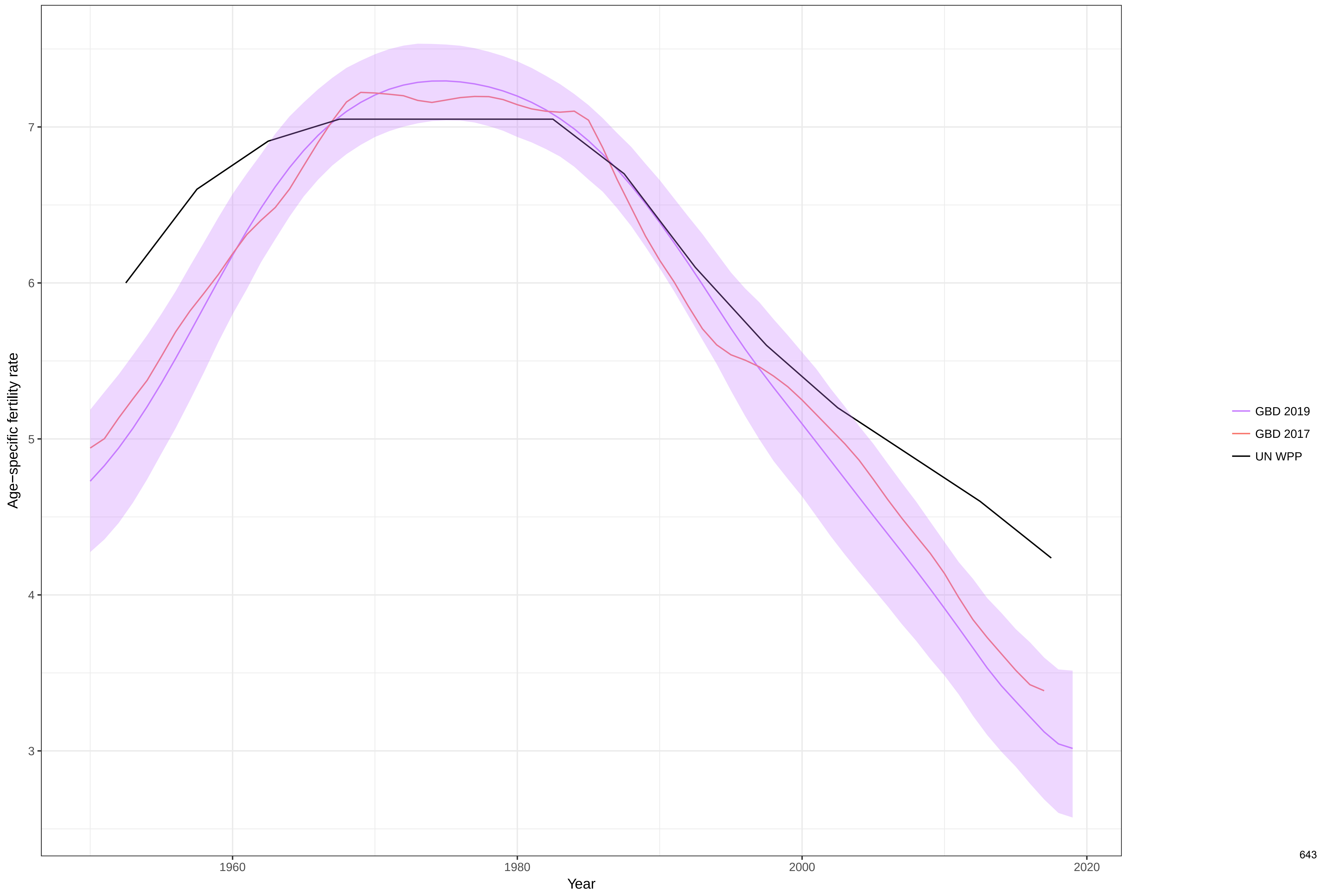

Age−specific fertility fitted trend lines, 1950−2019: Cabo Verde, Ages: 10−14
Data points enclosed with a diamond are reference sources

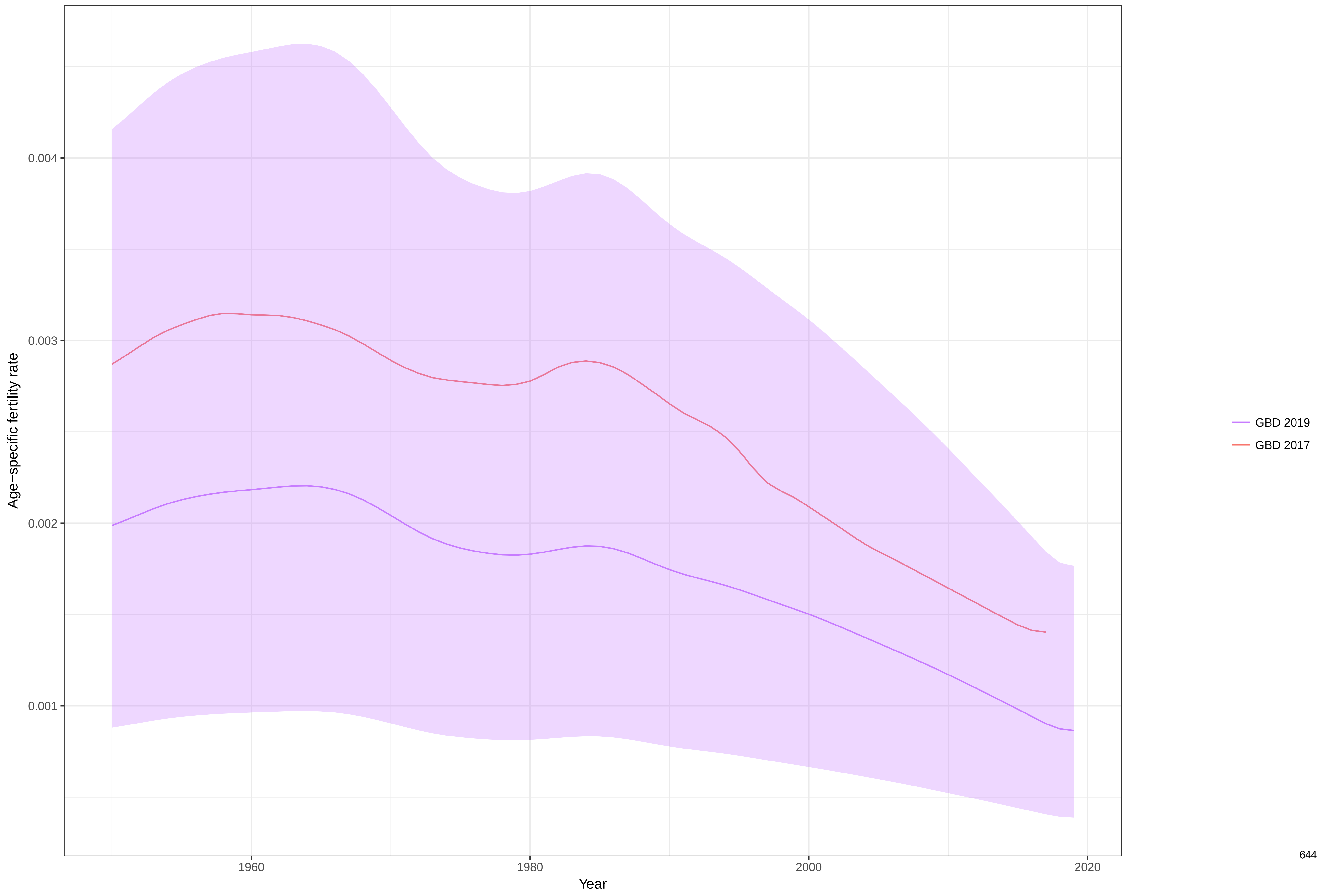

Age−specific fertility fitted trend lines, 1950−2019: Cabo Verde, Ages: 15−19
Data points enclosed with a diamond are reference sources

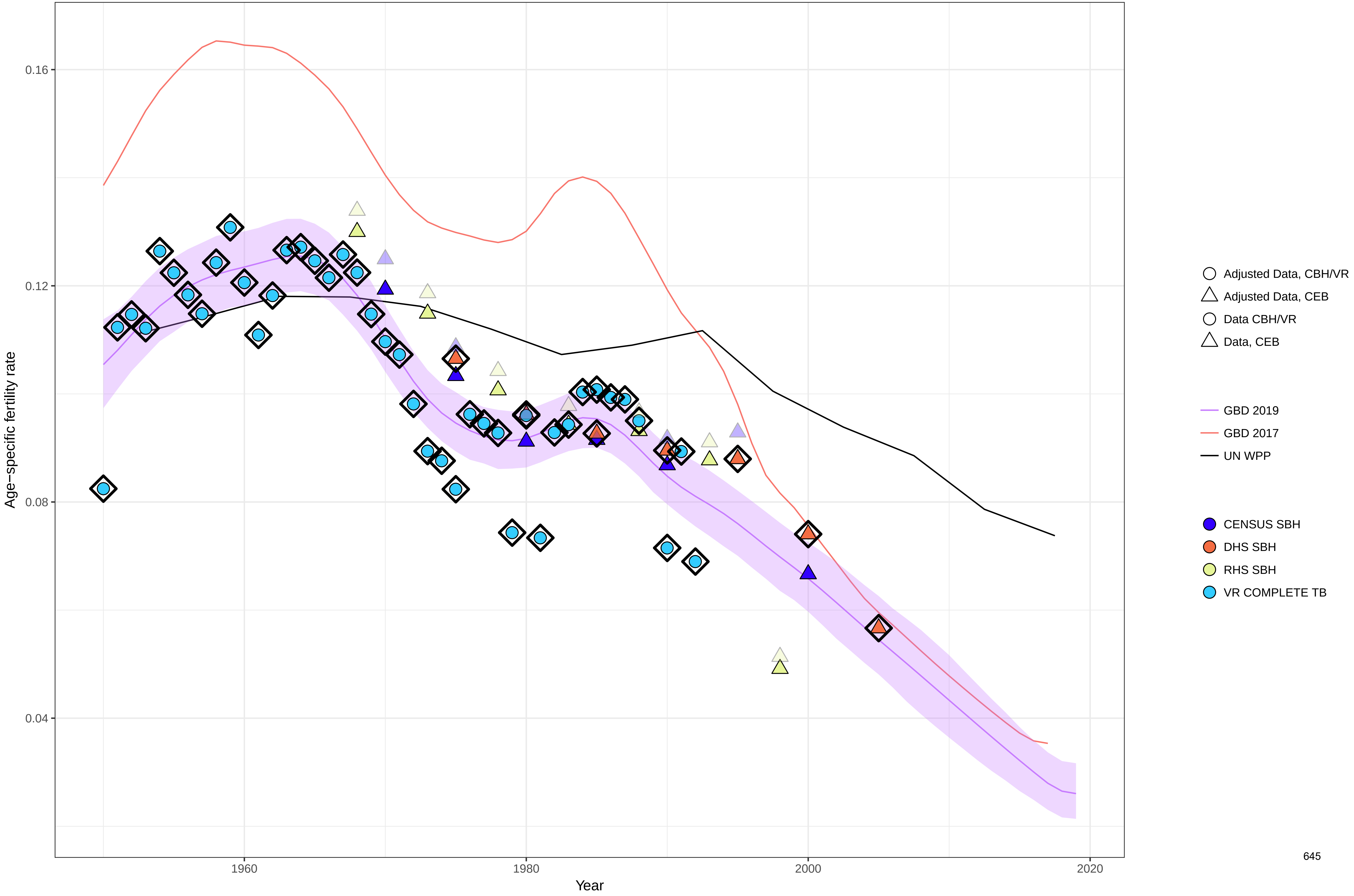

Age−specific fertility fitted trend lines, 1950−2019: Cabo Verde, Ages: 20−24
Data points enclosed with a diamond are reference sources

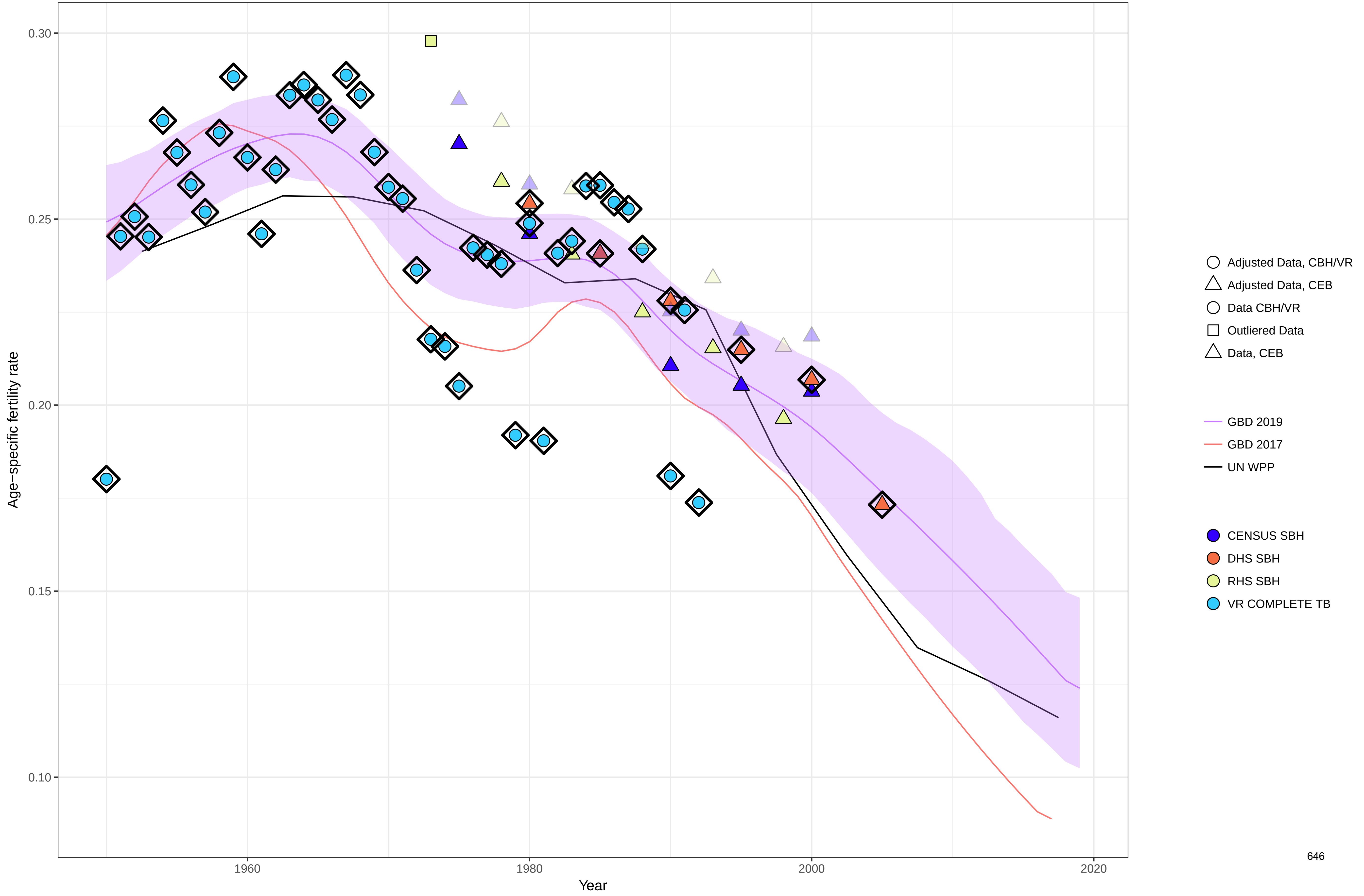

Age−specific fertility fitted trend lines, 1950−2019: Cabo Verde, Ages: 25−29
Data points enclosed with a diamond are reference sources

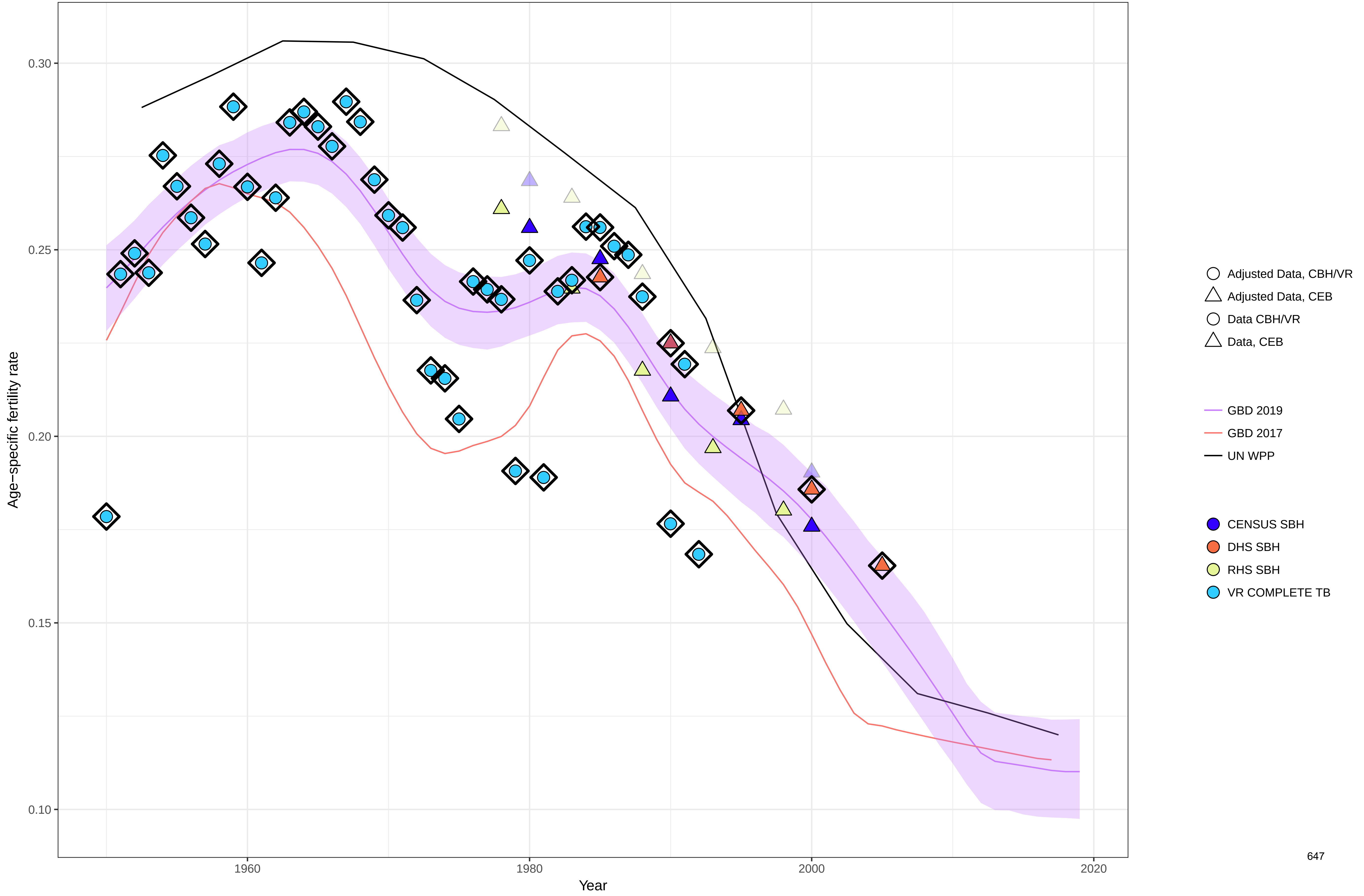

Age−specific fertility fitted trend lines, 1950−2019: Cabo Verde, Ages: 30−34
Data points enclosed with a diamond are reference sources

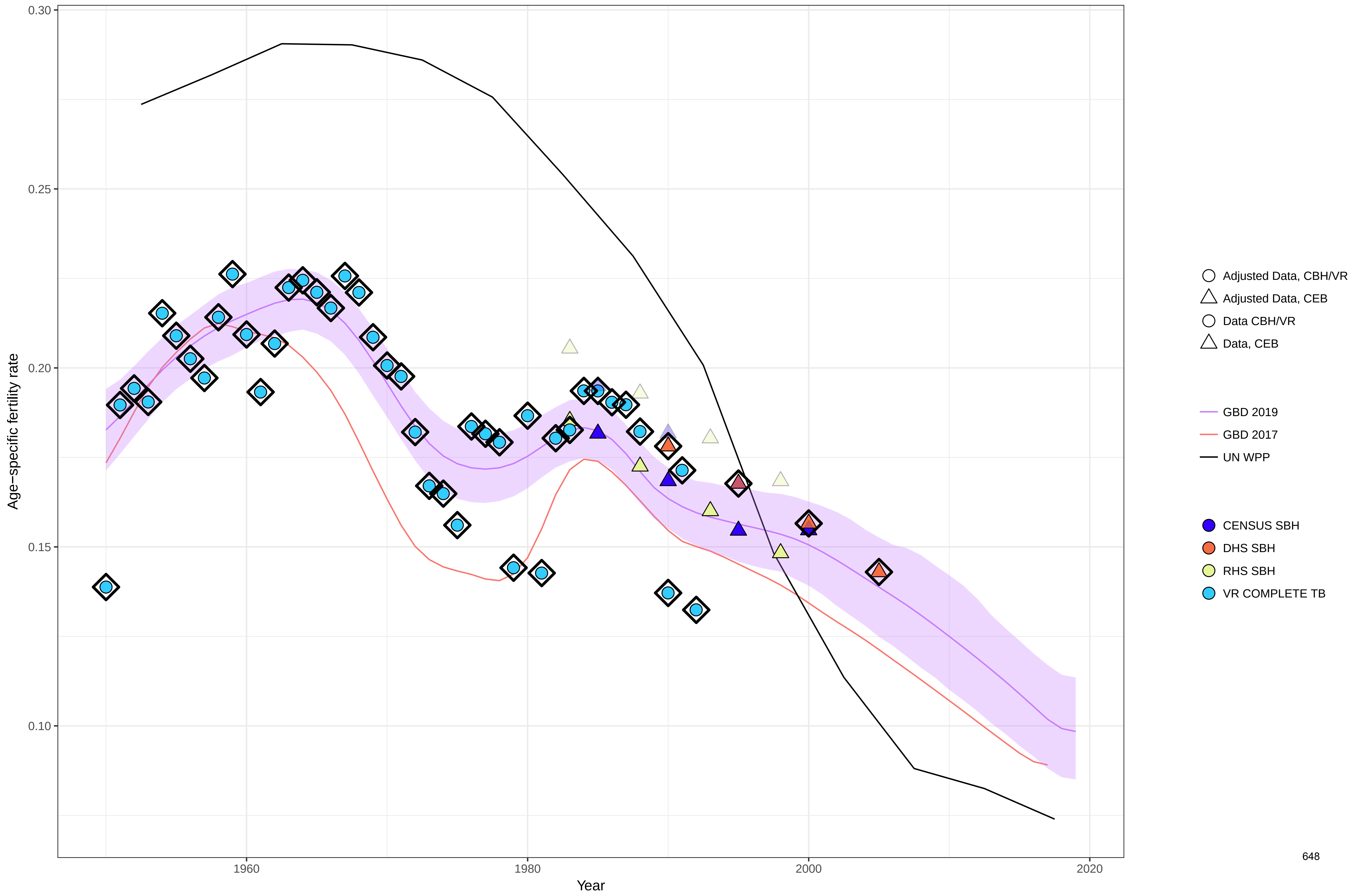

Age−specific fertility fitted trend lines, 1950−2019: Cabo Verde, Ages: 35−39
Data points enclosed with a diamond are reference sources

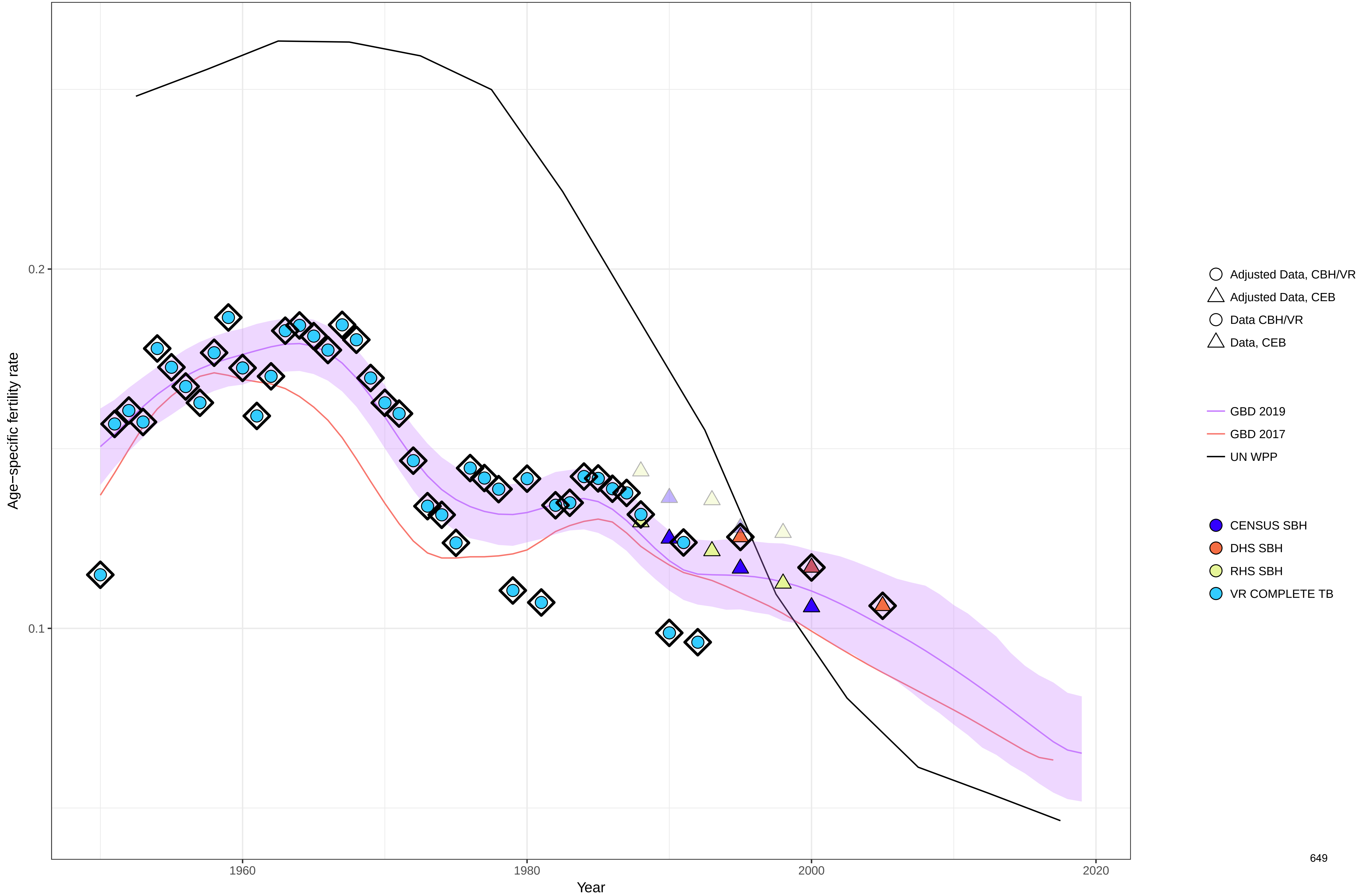

Age−specific fertility fitted trend lines, 1950−2019: Cabo Verde, Ages: 40−44
Data points enclosed with a diamond are reference sources

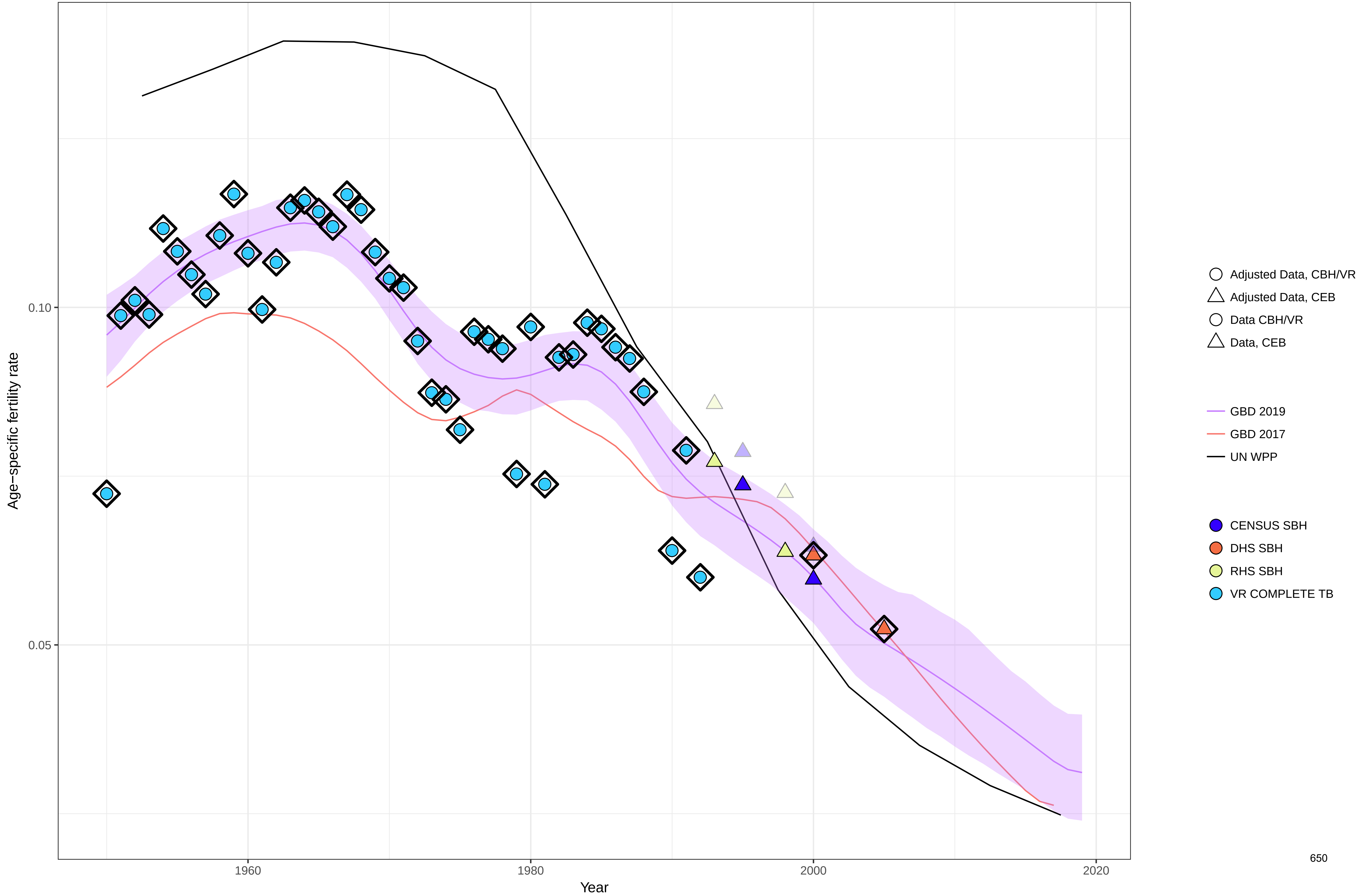

Age−specific fertility fitted trend lines, 1950−2019: Cabo Verde, Ages: 45−49
Data points enclosed with a diamond are reference sources

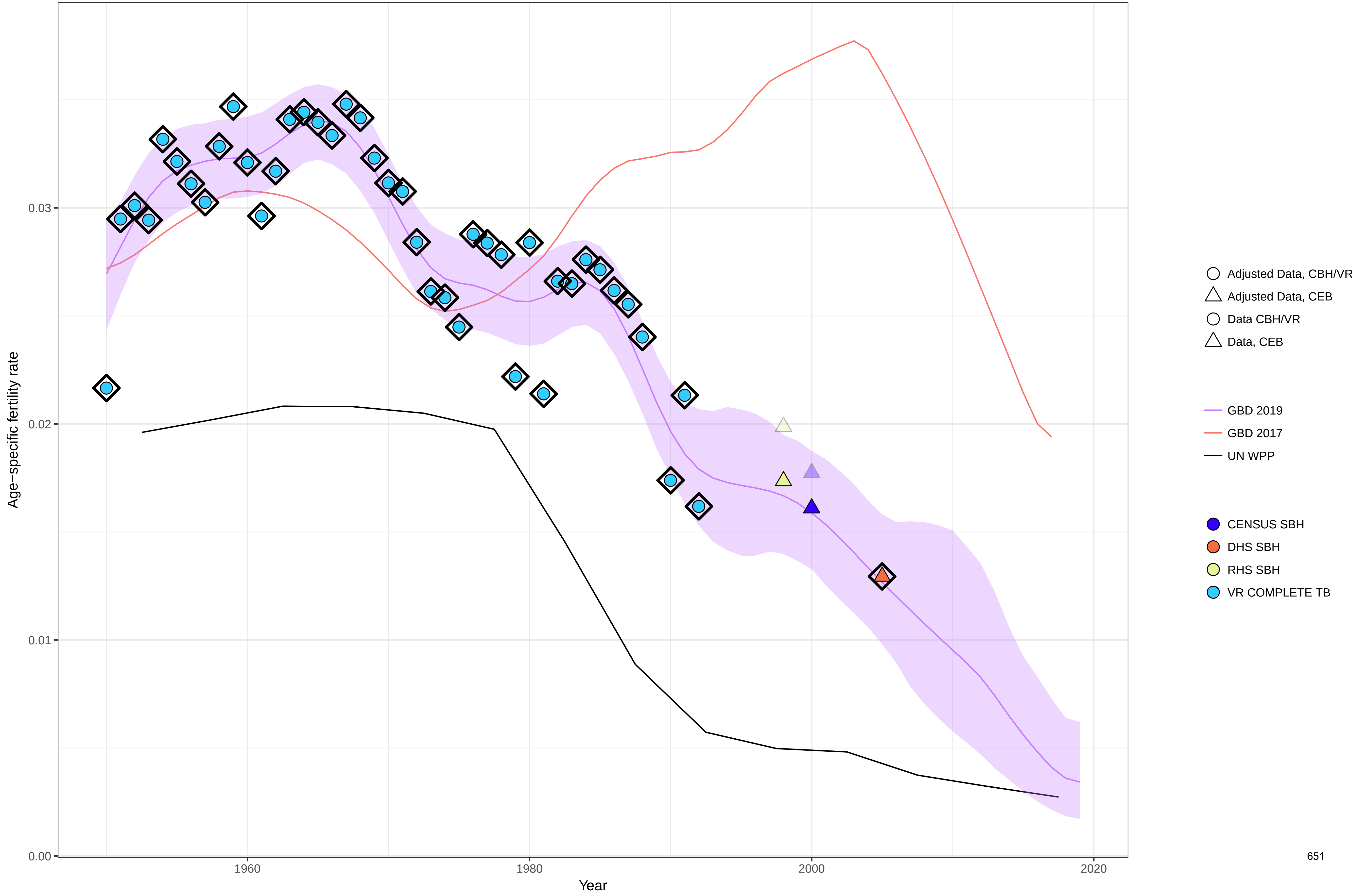

Age−specific fertility fitted trend lines, 1950−2019: Cabo Verde, Ages: 50−54
Data points enclosed with a diamond are reference sources

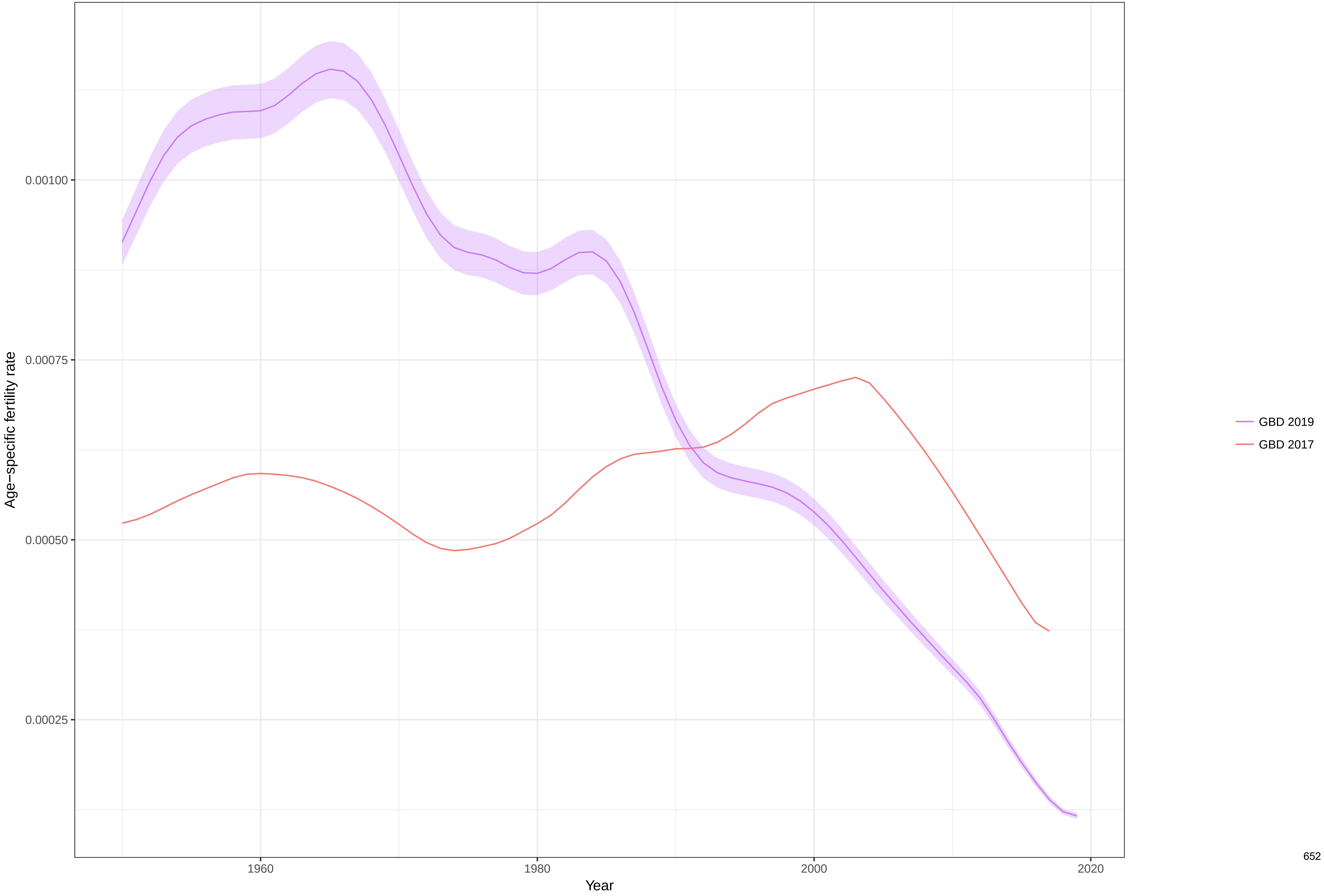

Total fertility rate fitted trend lines, 1950−2019: Cabo Verde TFR
Data points enclosed with a diamond are reference sources

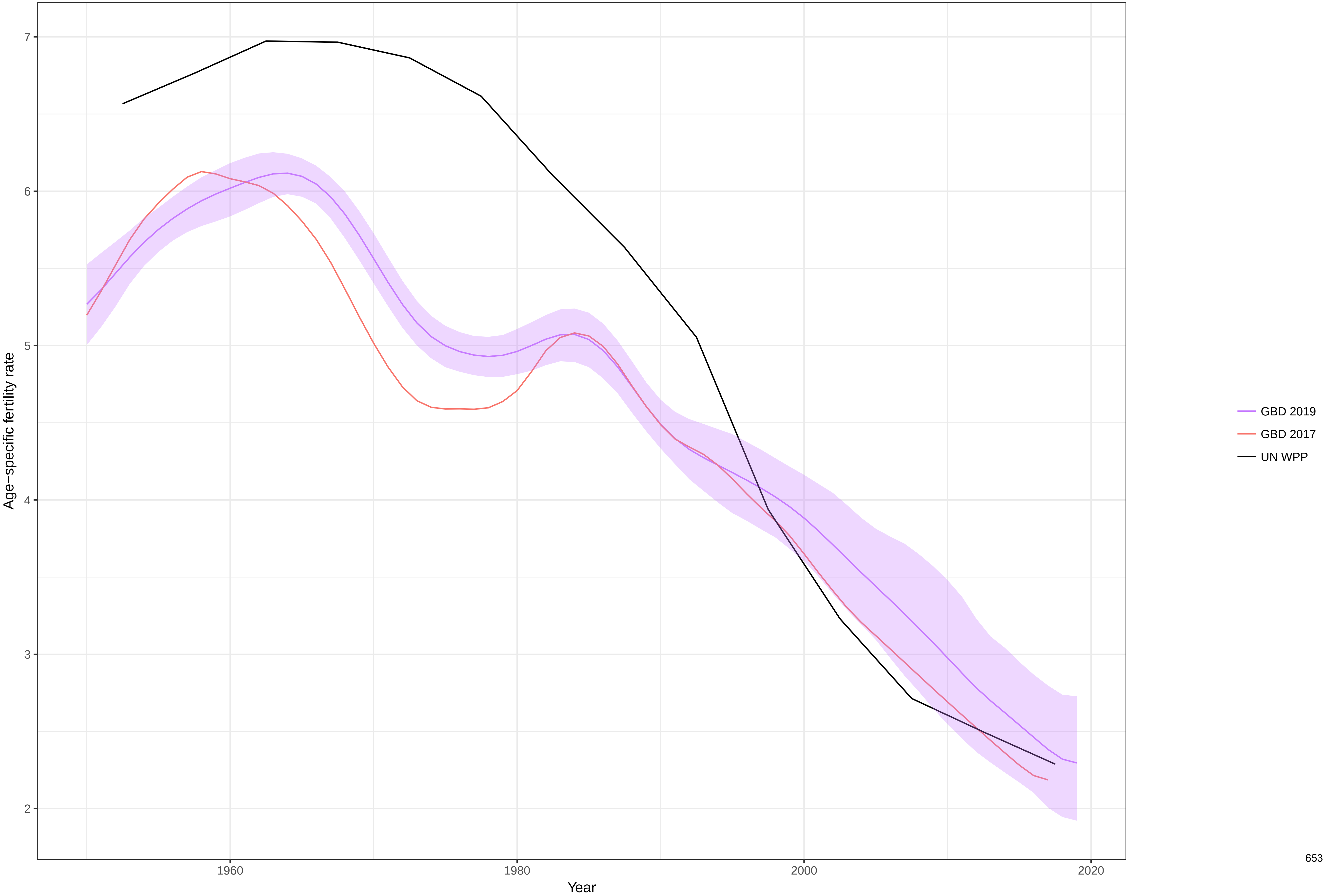

Age−specific fertility fitted trend lines, 1950−2019: Costa Rica, Ages: 10−14
Data points enclosed with a diamond are reference sources

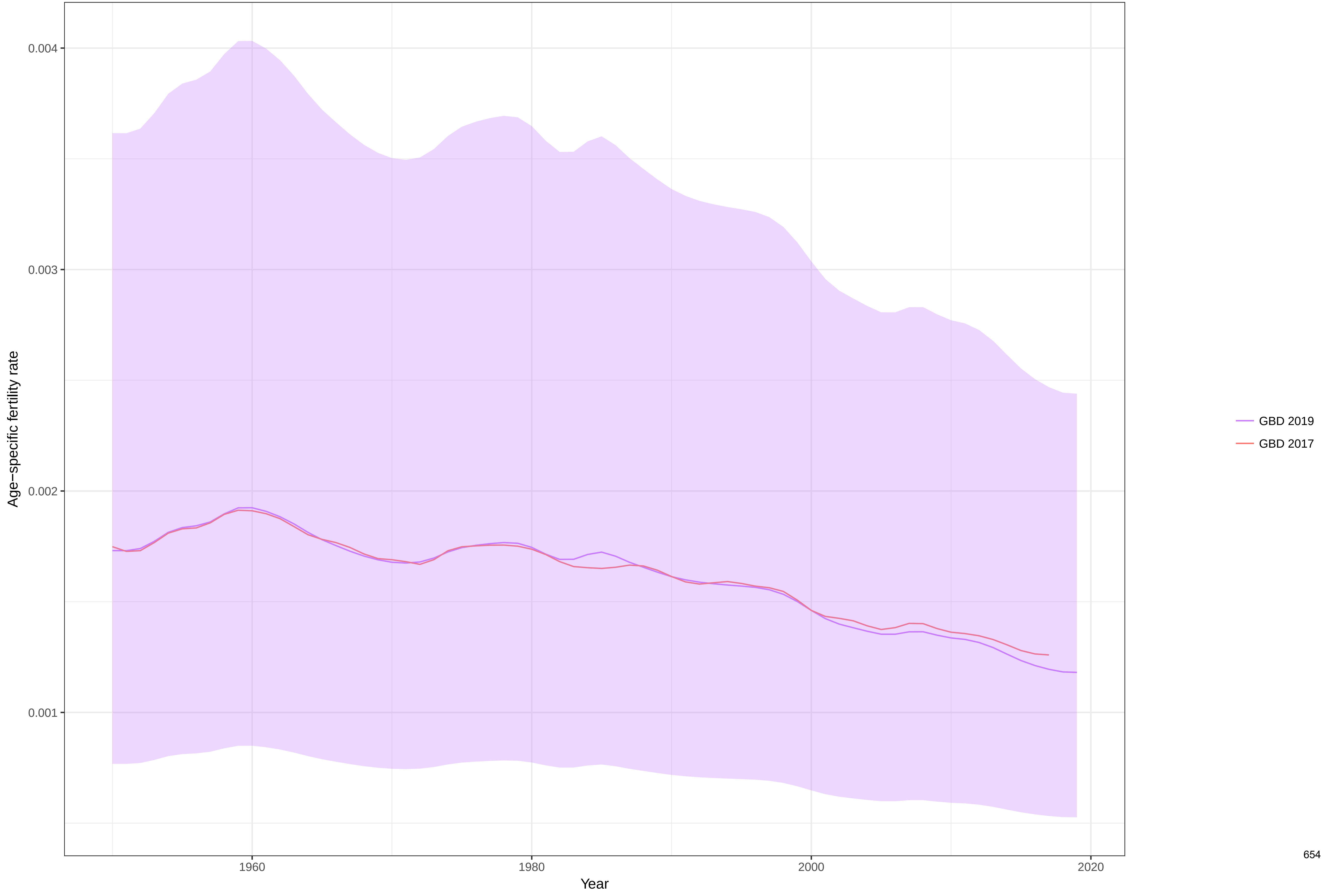

Age−specific fertility fitted trend lines, 1950−2019: Costa Rica, Ages: 15−19
Data points enclosed with a diamond are reference sources

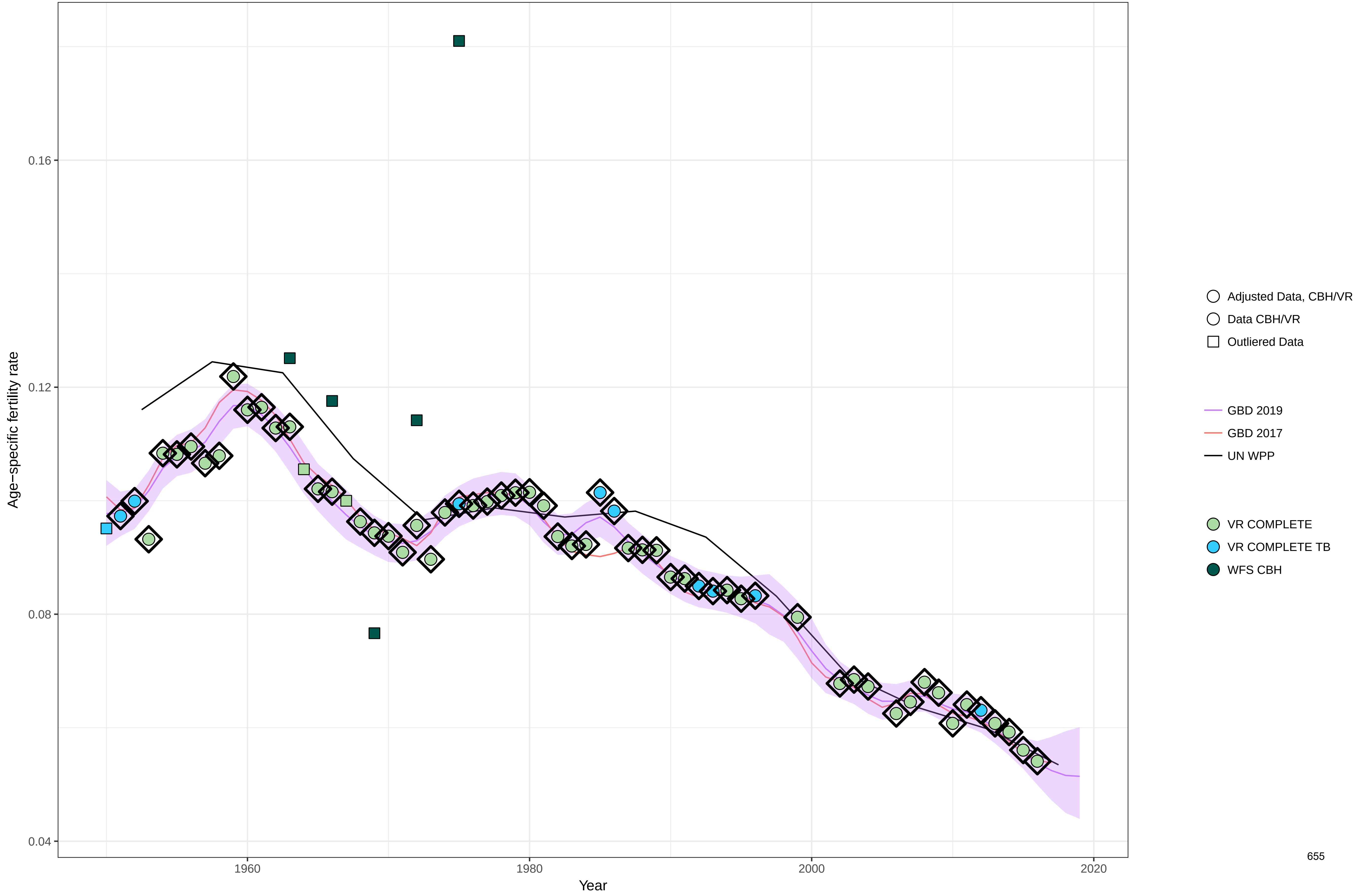

Age−specific fertility fitted trend lines, 1950−2019: Costa Rica, Ages: 20−24
Data points enclosed with a diamond are reference sources

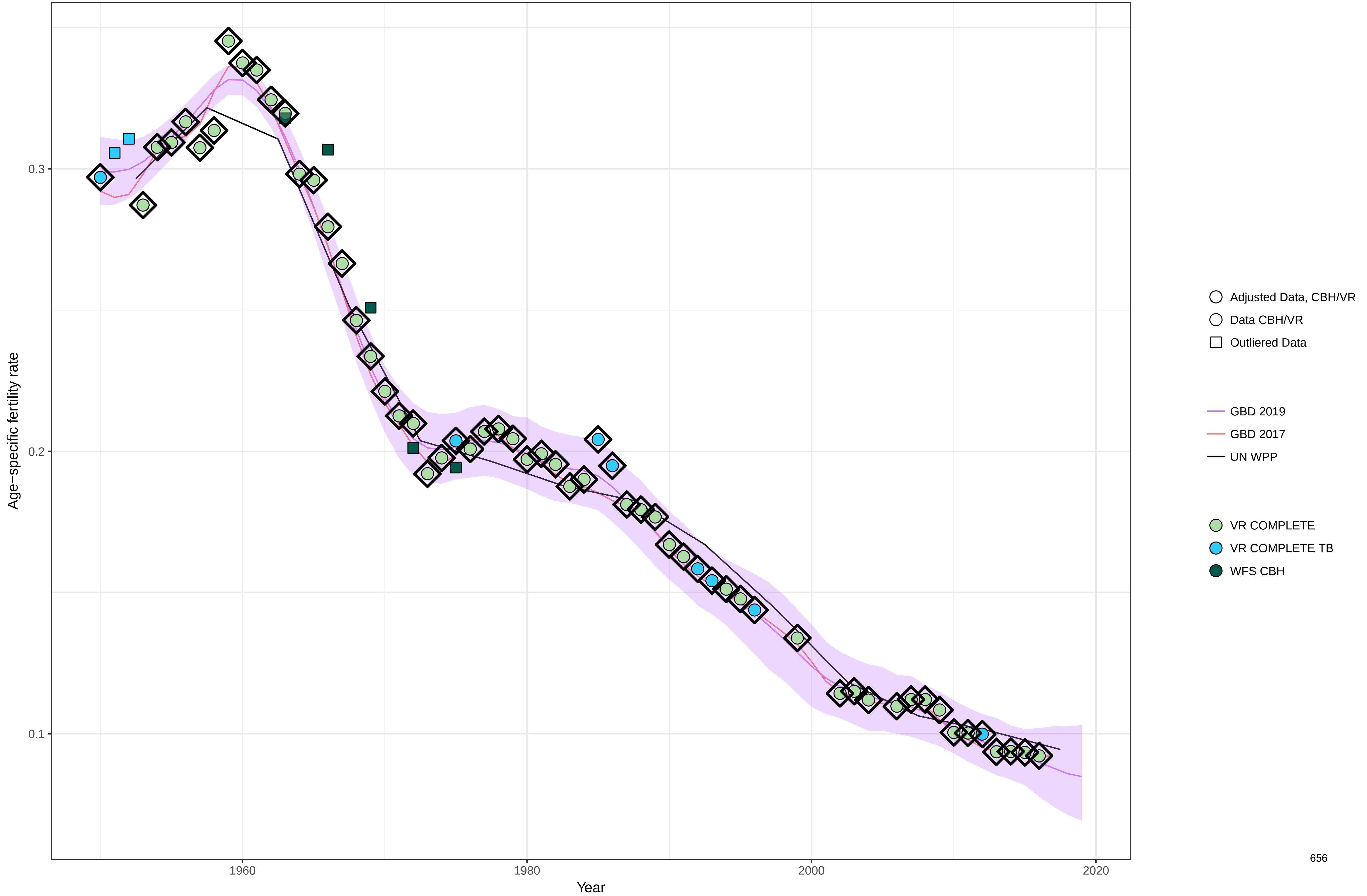

Age−specific fertility fitted trend lines, 1950−2019: Costa Rica, Ages: 25−29
Data points enclosed with a diamond are reference sources

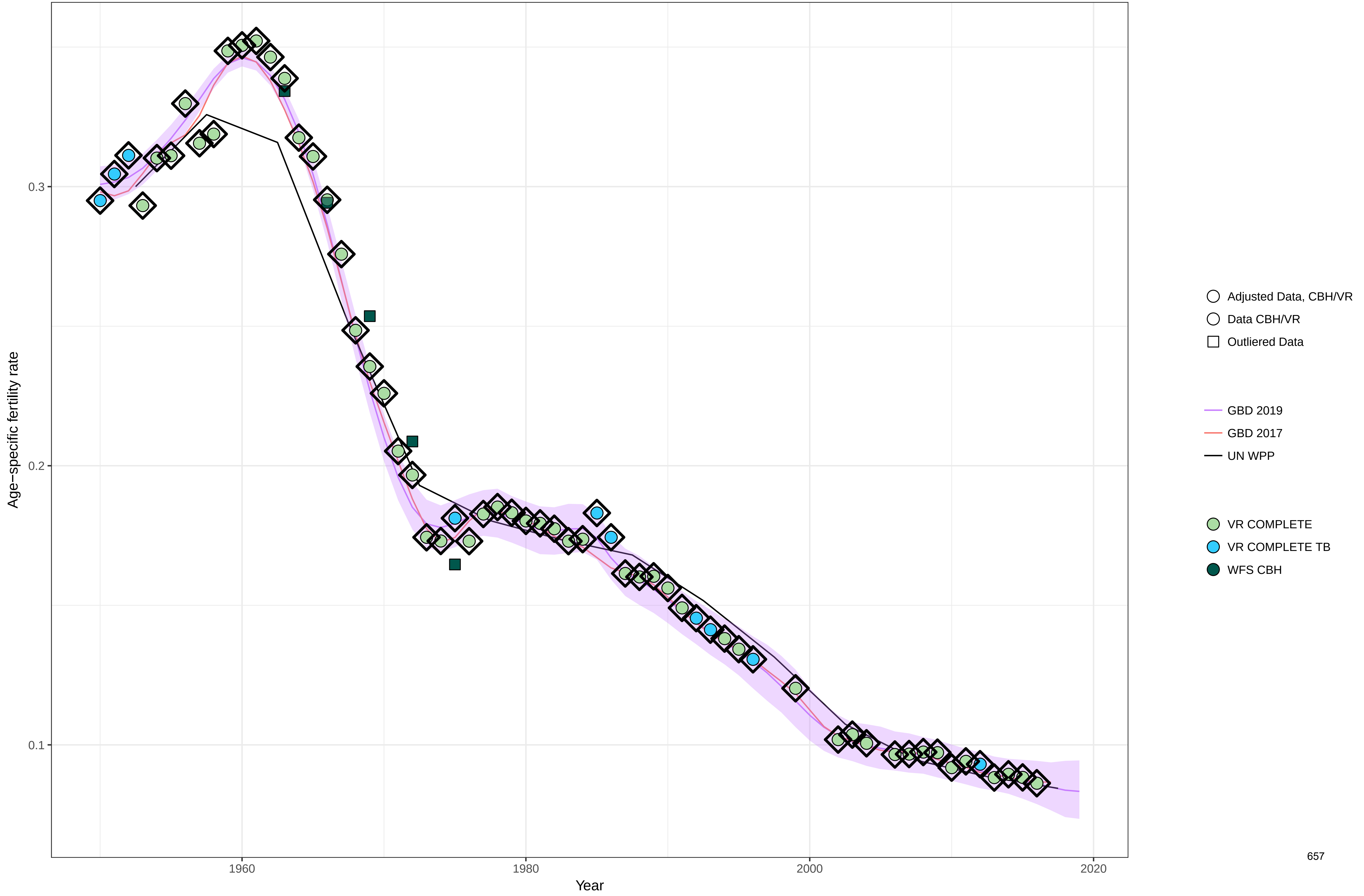

Age−specific fertility fitted trend lines, 1950−2019: Costa Rica, Ages: 30−34
Data points enclosed with a diamond are reference sources

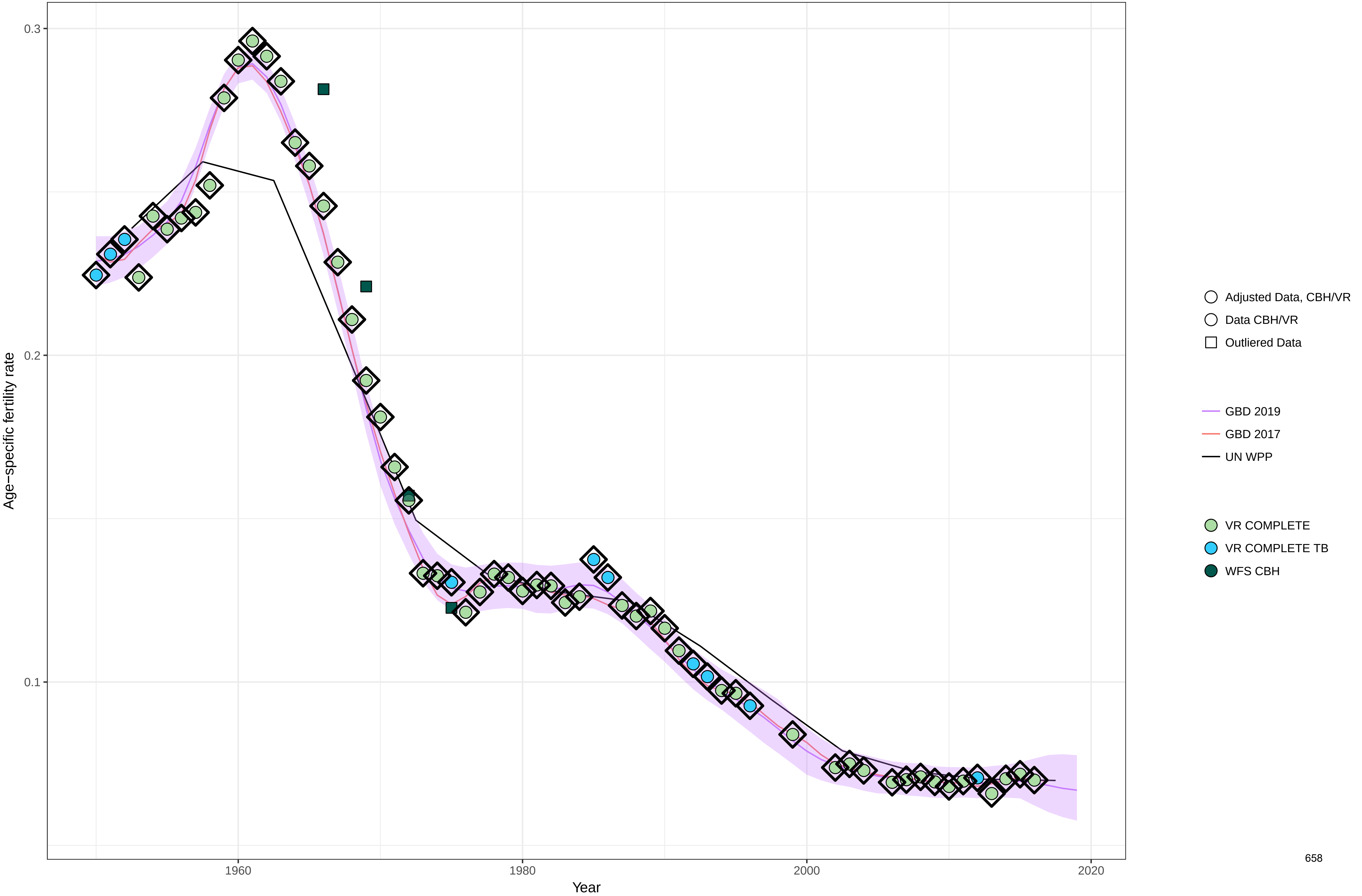

Age−specific fertility fitted trend lines, 1950−2019: Costa Rica, Ages: 35−39
Data points enclosed with a diamond are reference sources

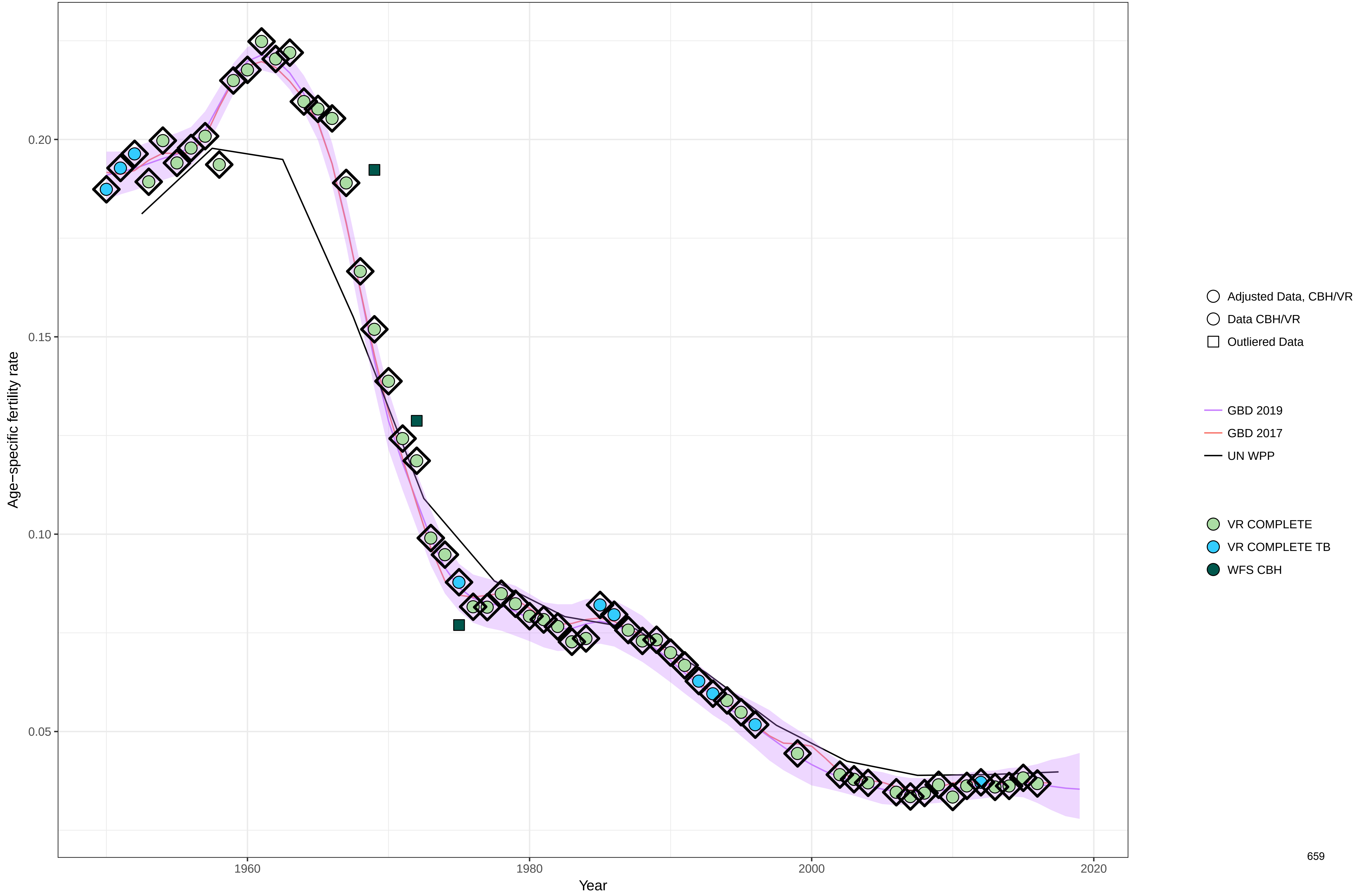

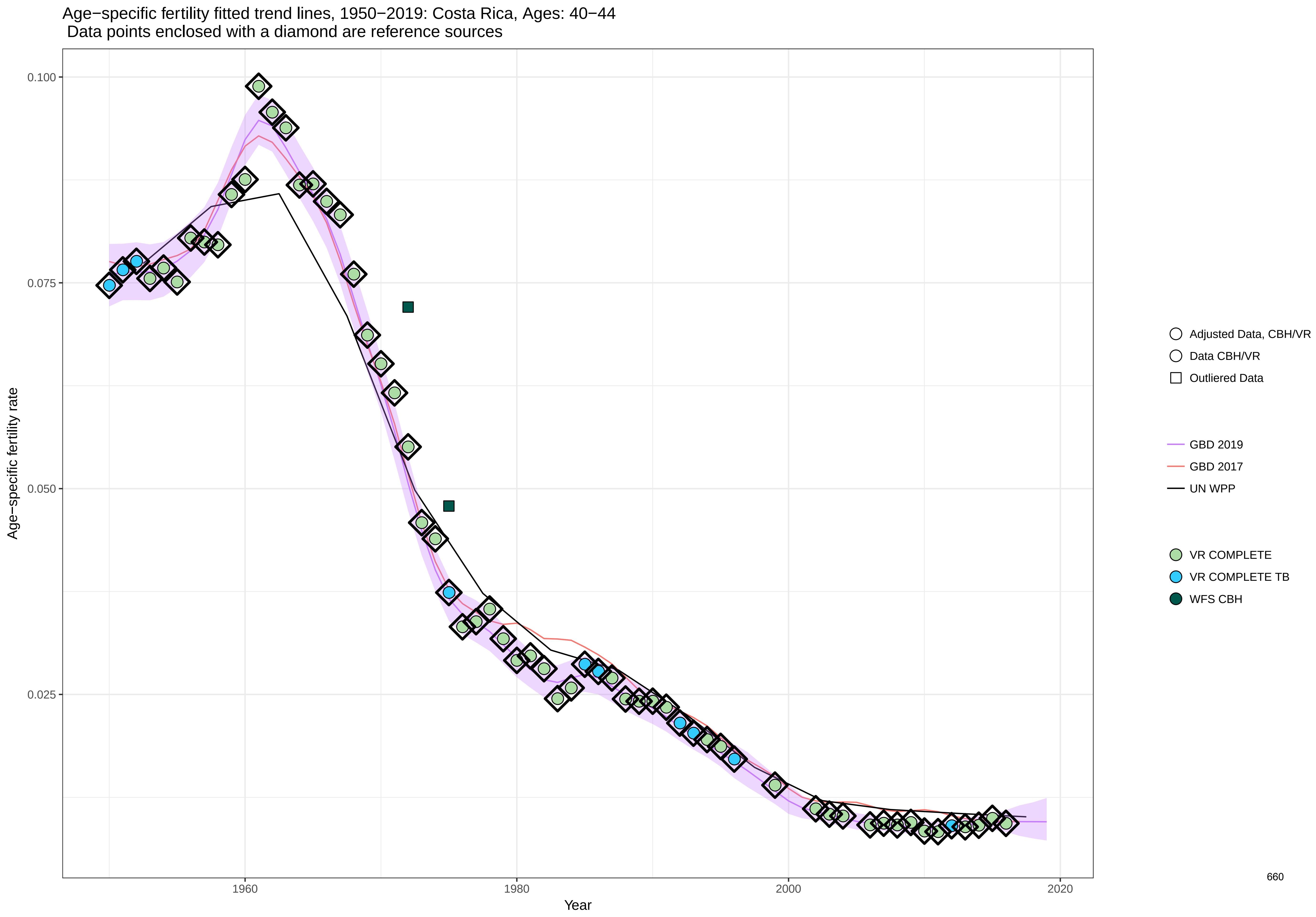
Age−specific fertility fitted trend lines, 1950−2019: Costa Rica, Ages: 40−44
Data points enclosed with a diamond are reference sources
Age−specific fertility rate
0.100
0.075
0.050
0.025
1960
1980
2000
2020
Year
Adjusted Data, CBH/VR
Data CBH/VR
Outliered Data
GBD 2019
GBD 2017
UN WPP
VR COMPLETE
VR COMPLETE TB
WFS CBH

Age−specific fertility fitted trend lines, 1950−2019: Costa Rica, Ages: 45−49
Data points enclosed with a diamond are reference sources

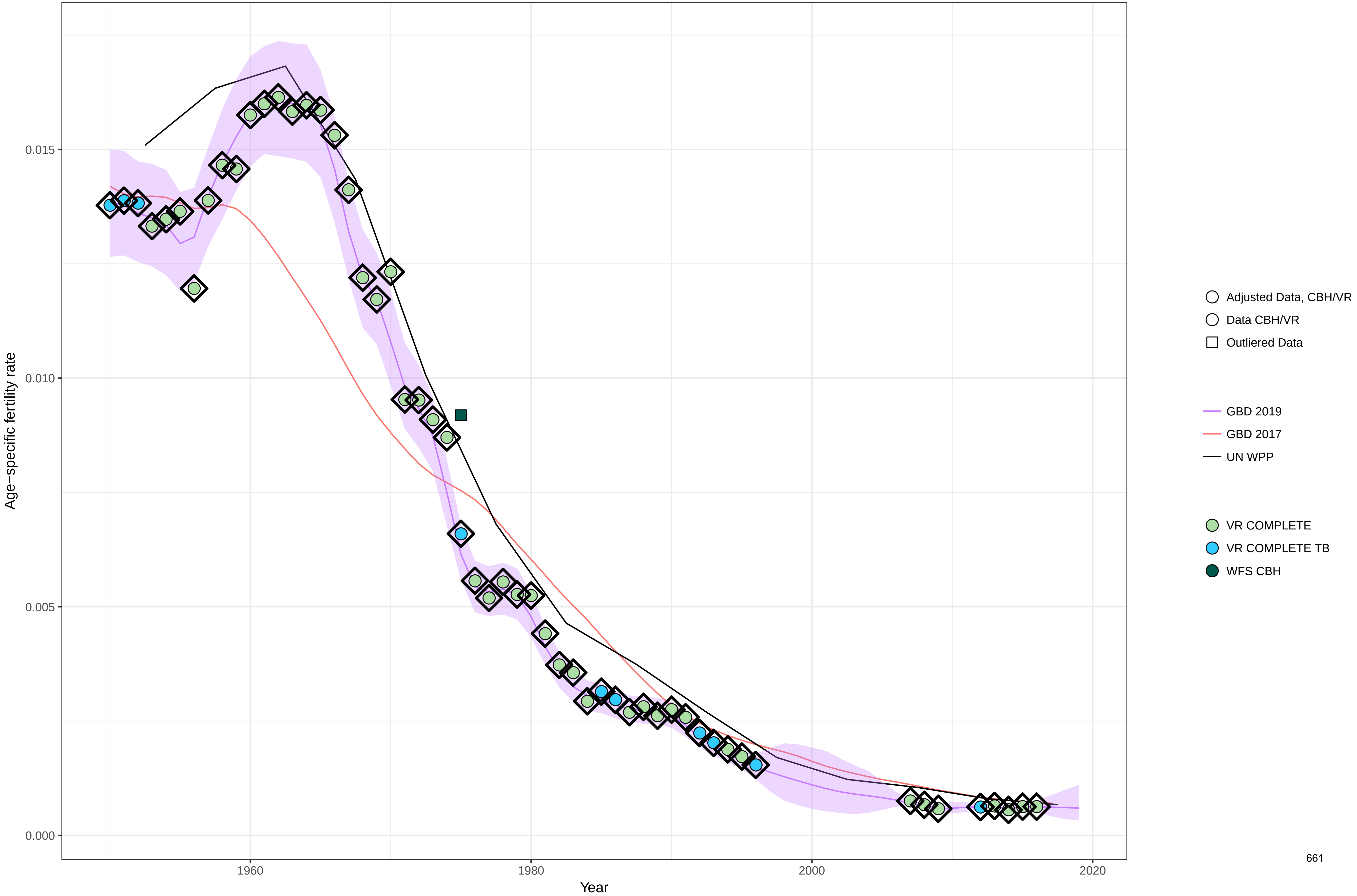

Age−specific fertility fitted trend lines, 1950−2019: Costa Rica, Ages: 50−54
Data points enclosed with a diamond are reference sources

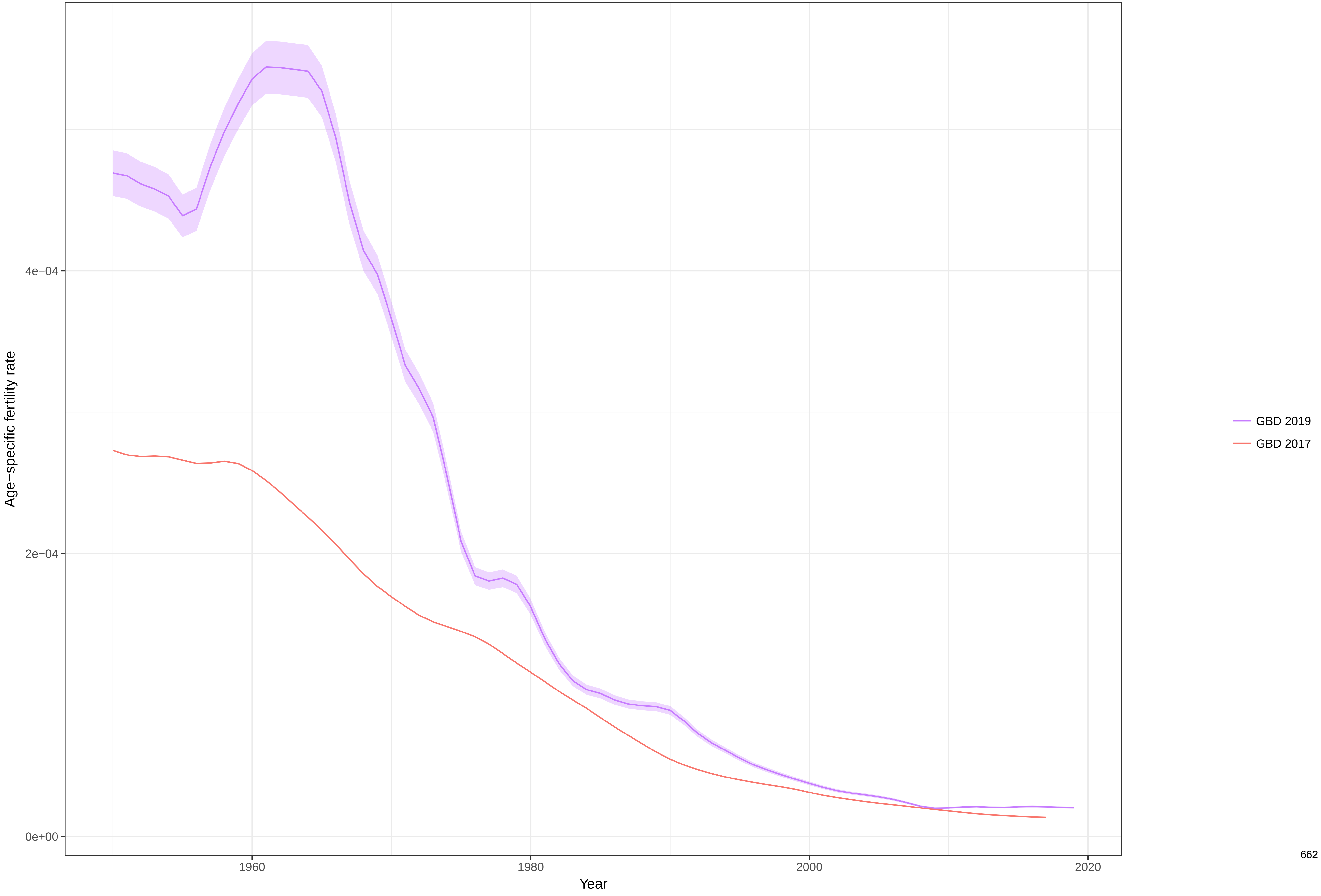

Total fertility rate fitted trend lines, 1950−2019: Costa Rica TFR
Data points enclosed with a diamond are reference sources

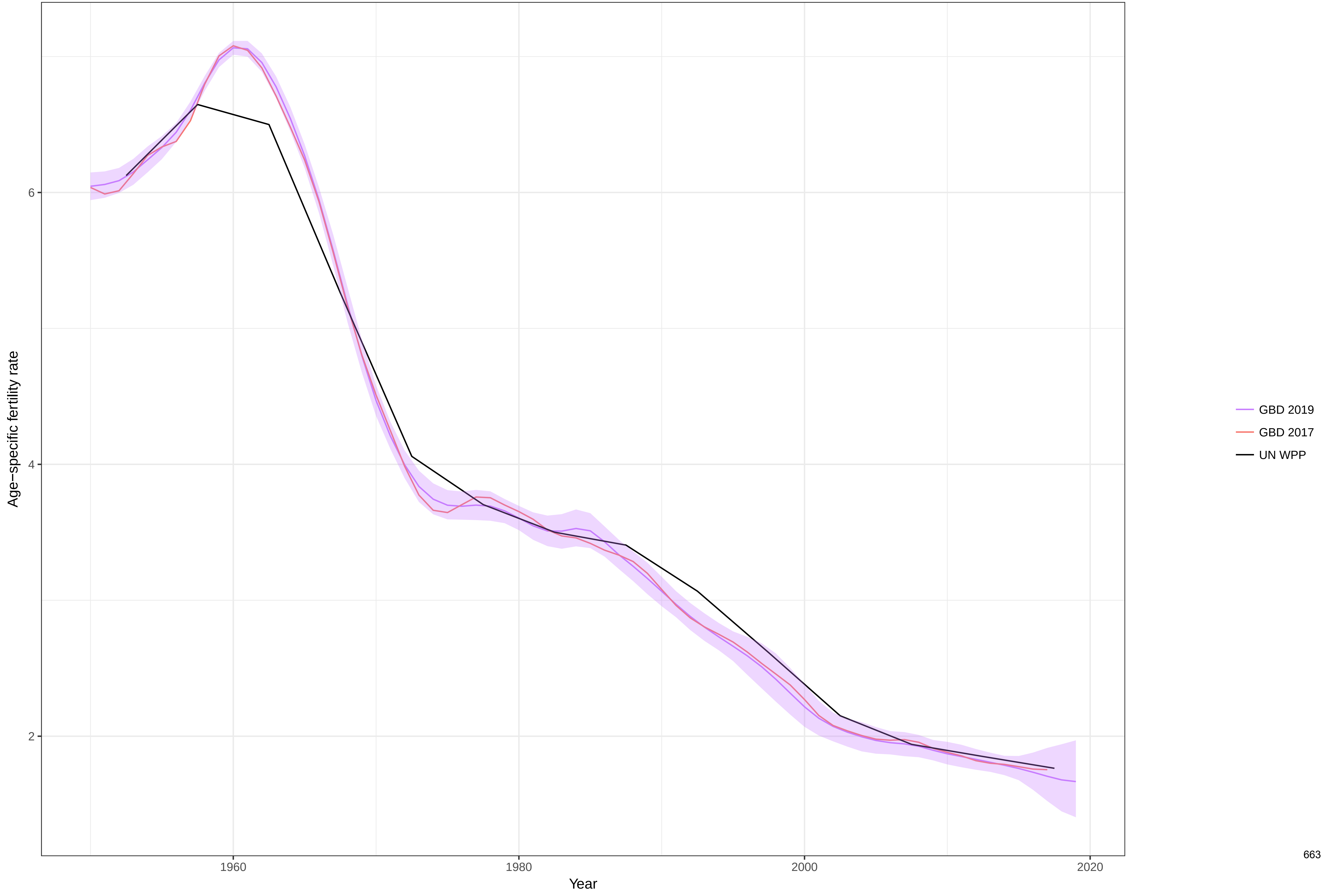

Age−specific fertility fitted trend lines, 1950−2019: Cuba, Ages: 10−14
Data points enclosed with a diamond are reference sources

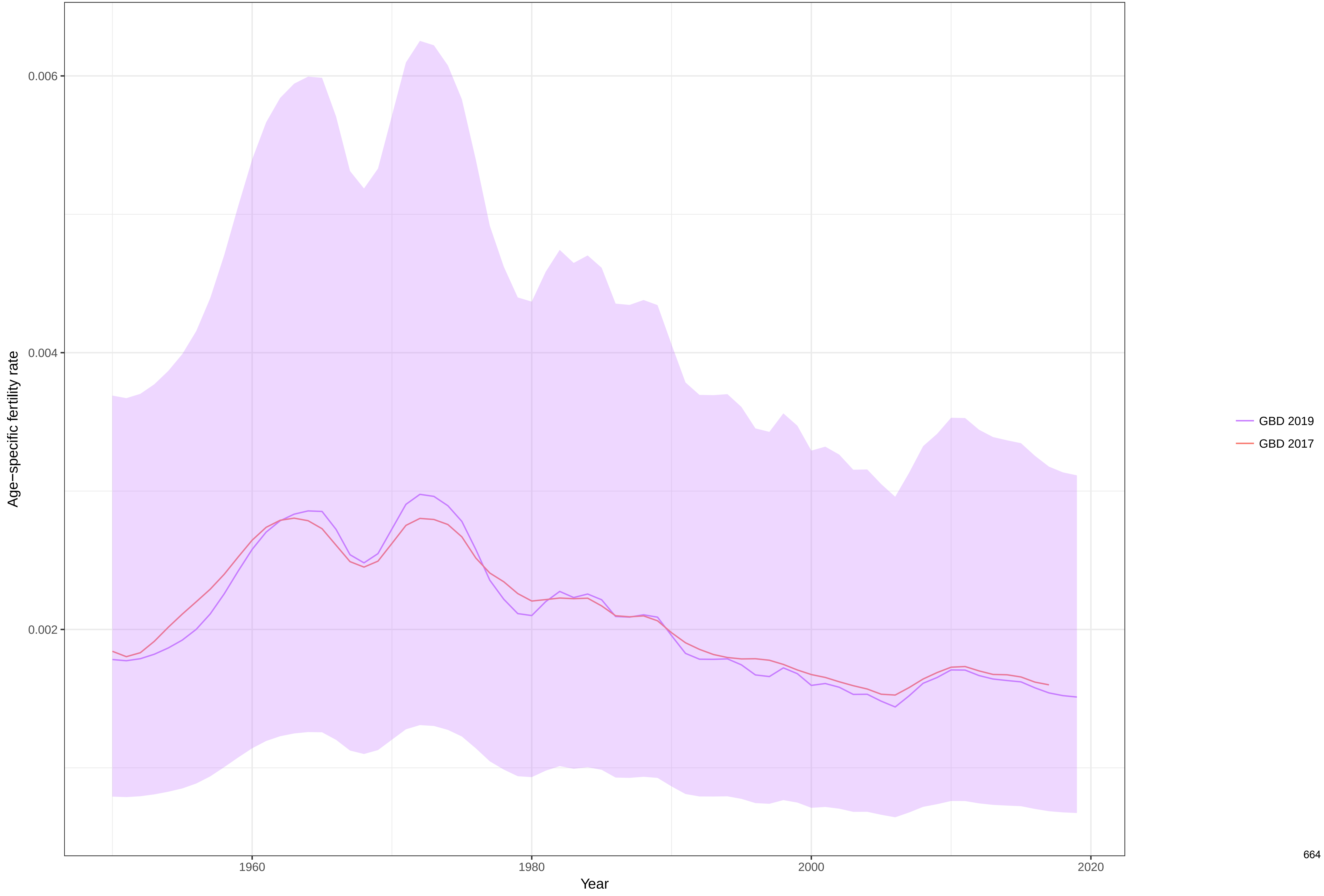

Age−specific fertility fitted trend lines, 1950−2019: Cuba, Ages: 15−19
Data points enclosed with a diamond are reference sources

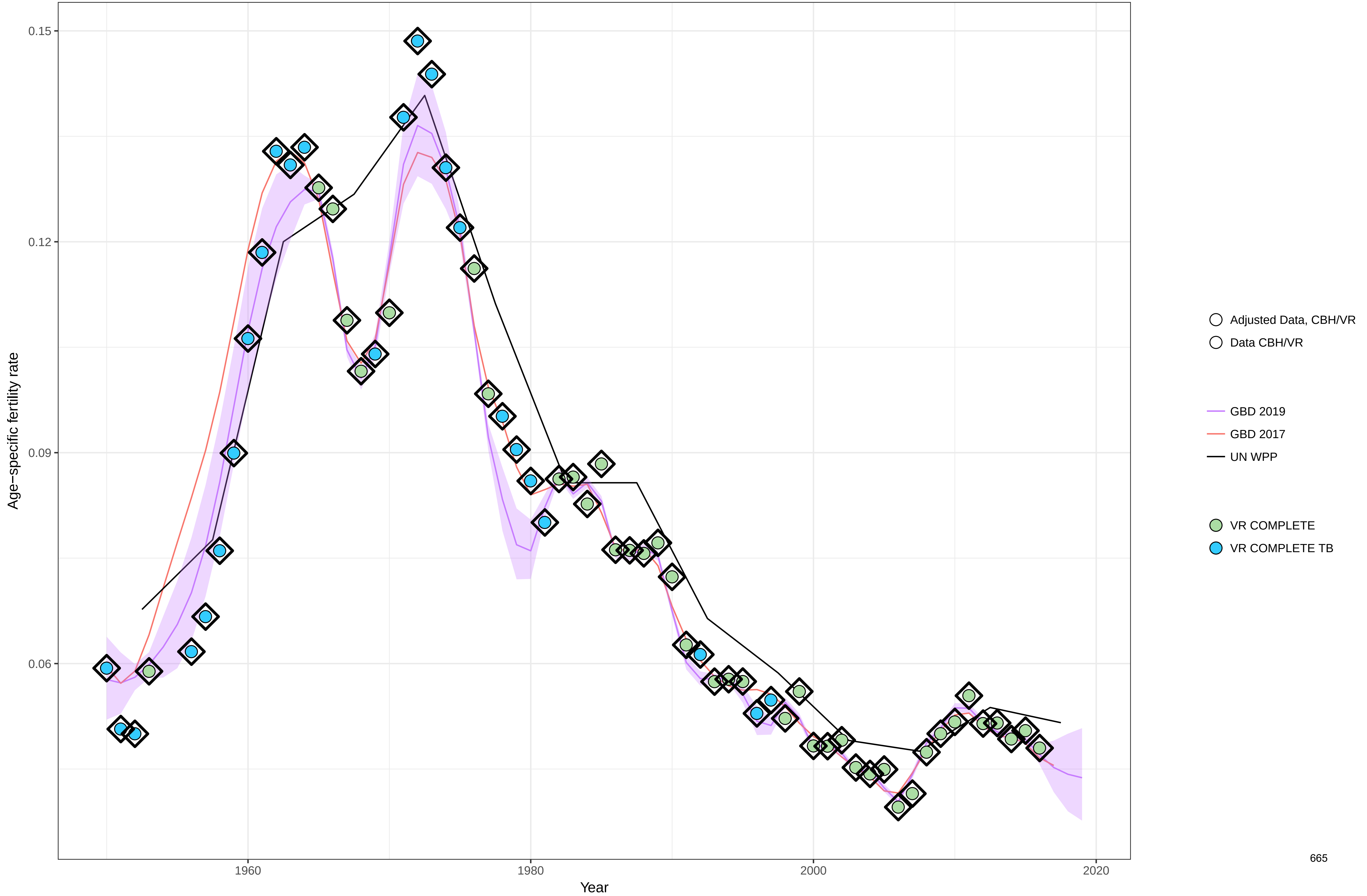

Age−specific fertility fitted trend lines, 1950−2019: Cuba, Ages: 20−24
Data points enclosed with a diamond are reference sources

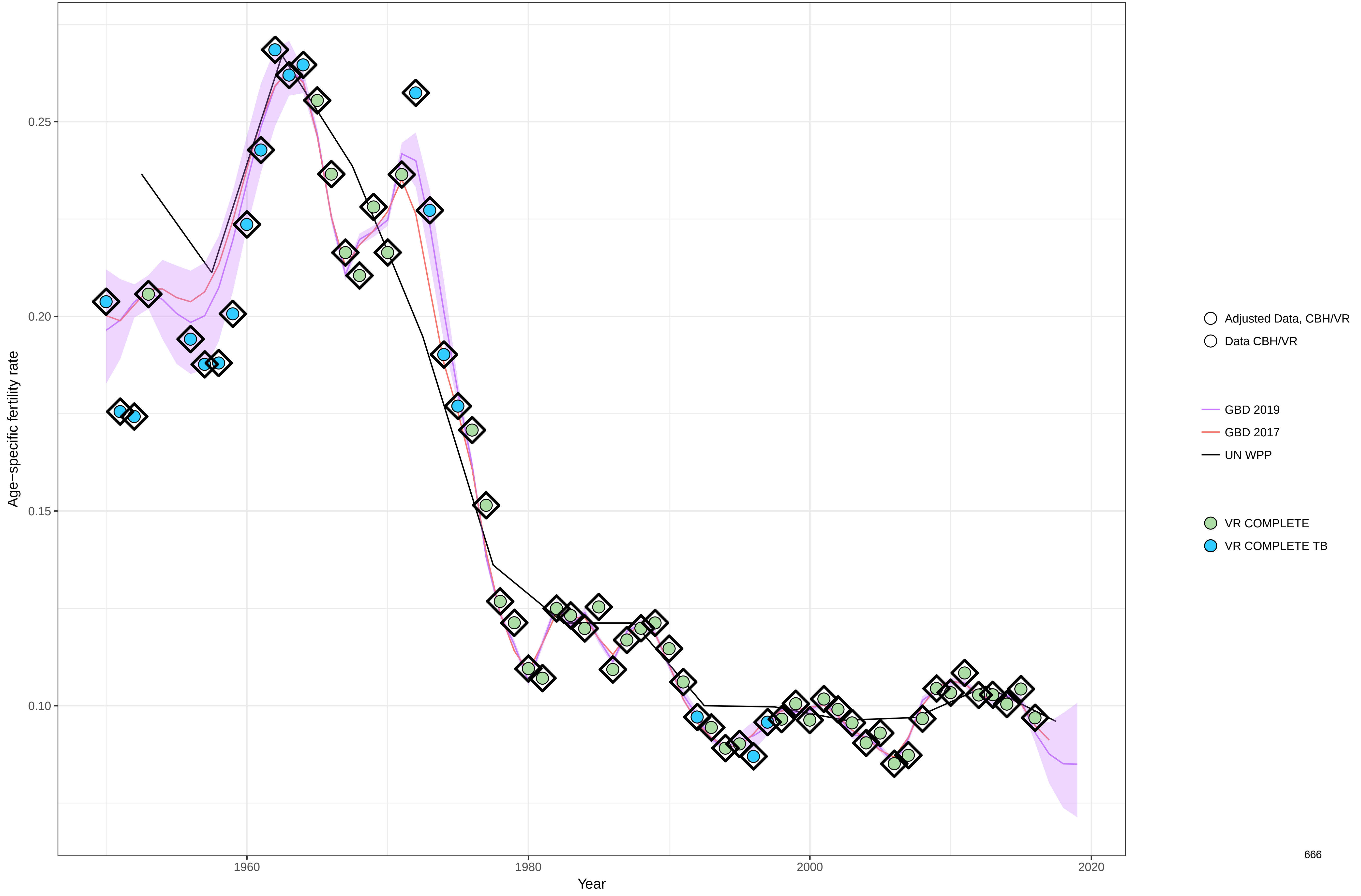

Age−specific fertility fitted trend lines, 1950−2019: Cuba, Ages: 25−29
Data points enclosed with a diamond are reference sources

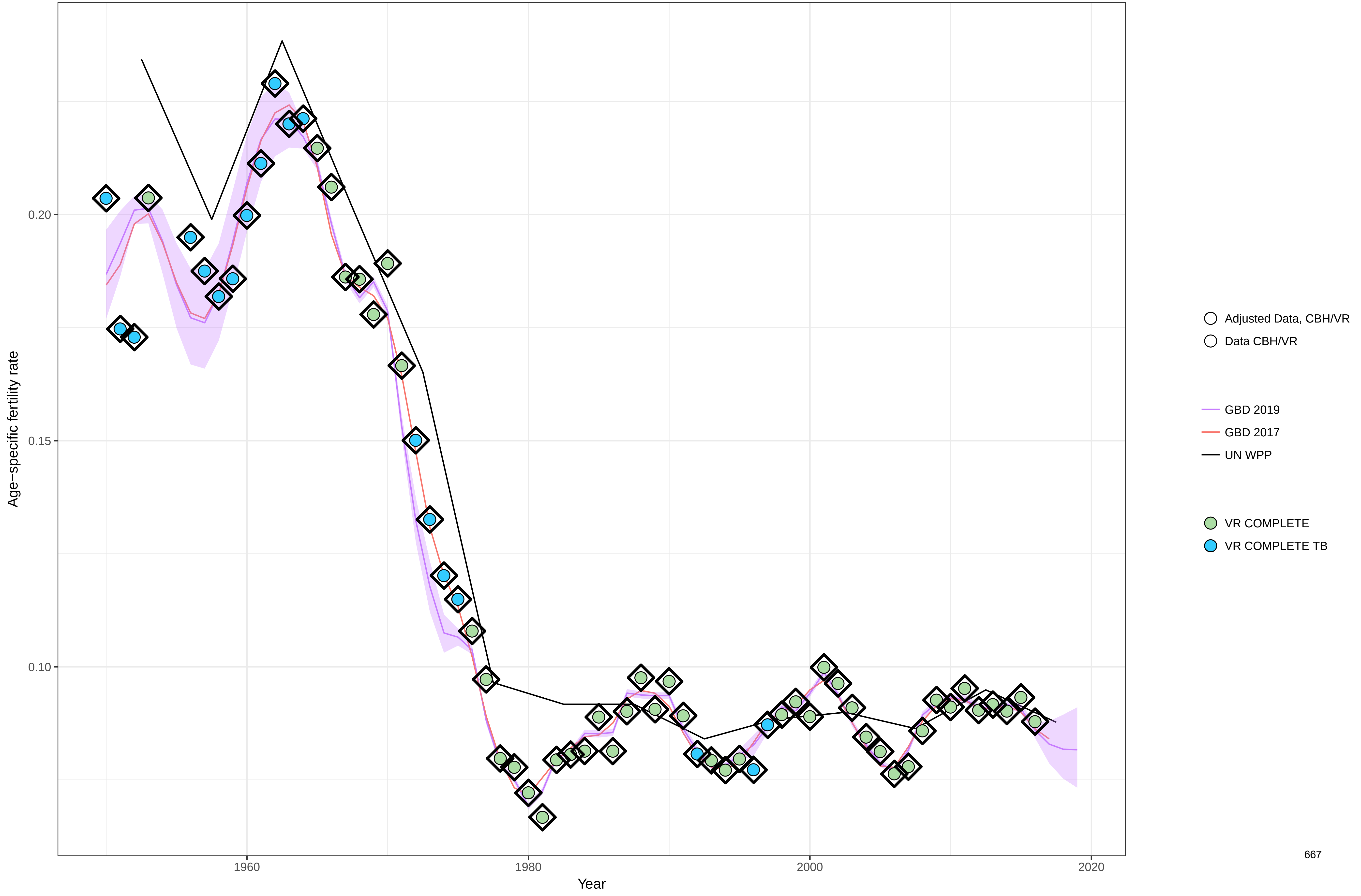

Age−specific fertility fitted trend lines, 1950−2019: Cuba, Ages: 30−34
Data points enclosed with a diamond are reference sources

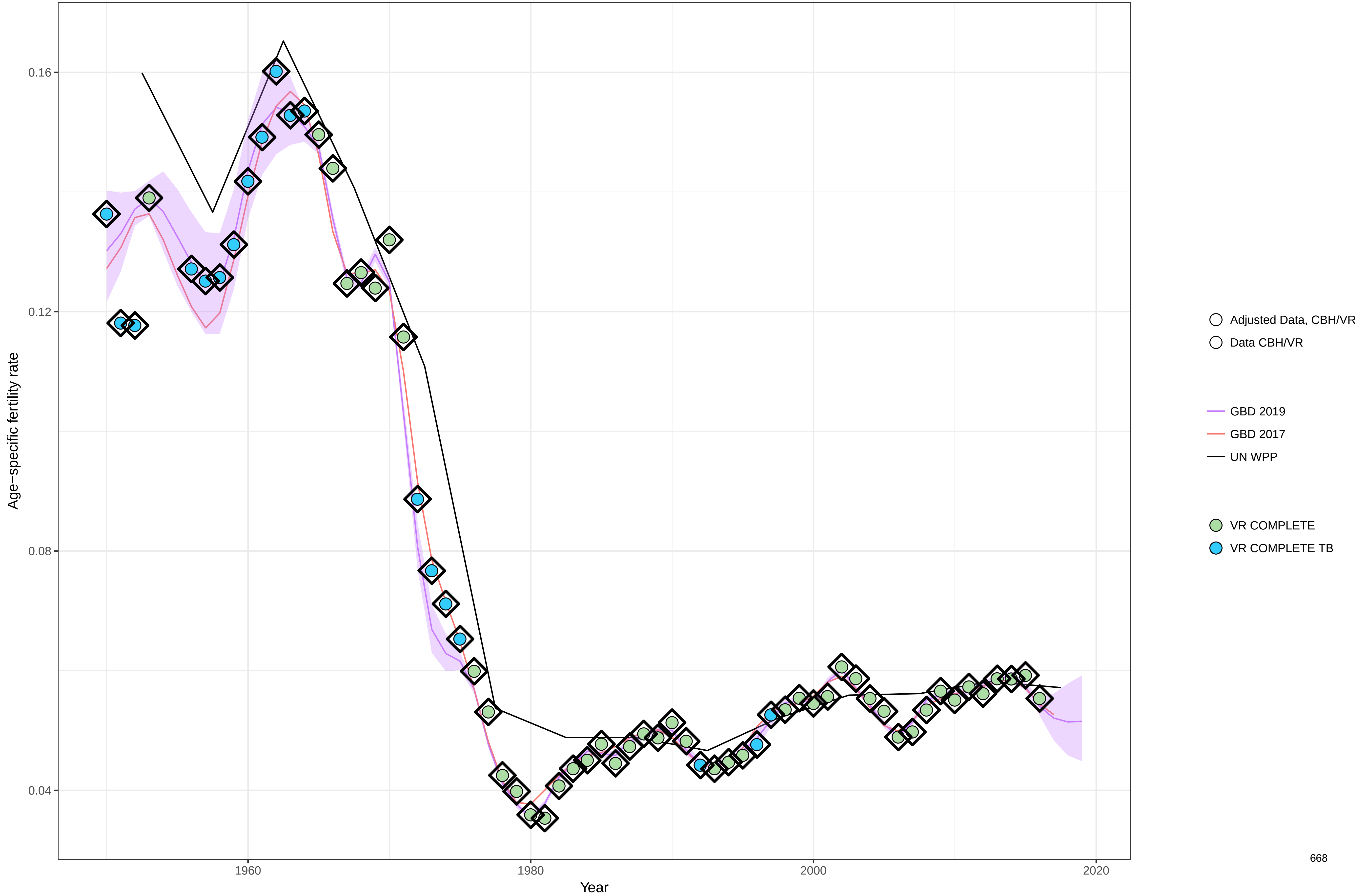

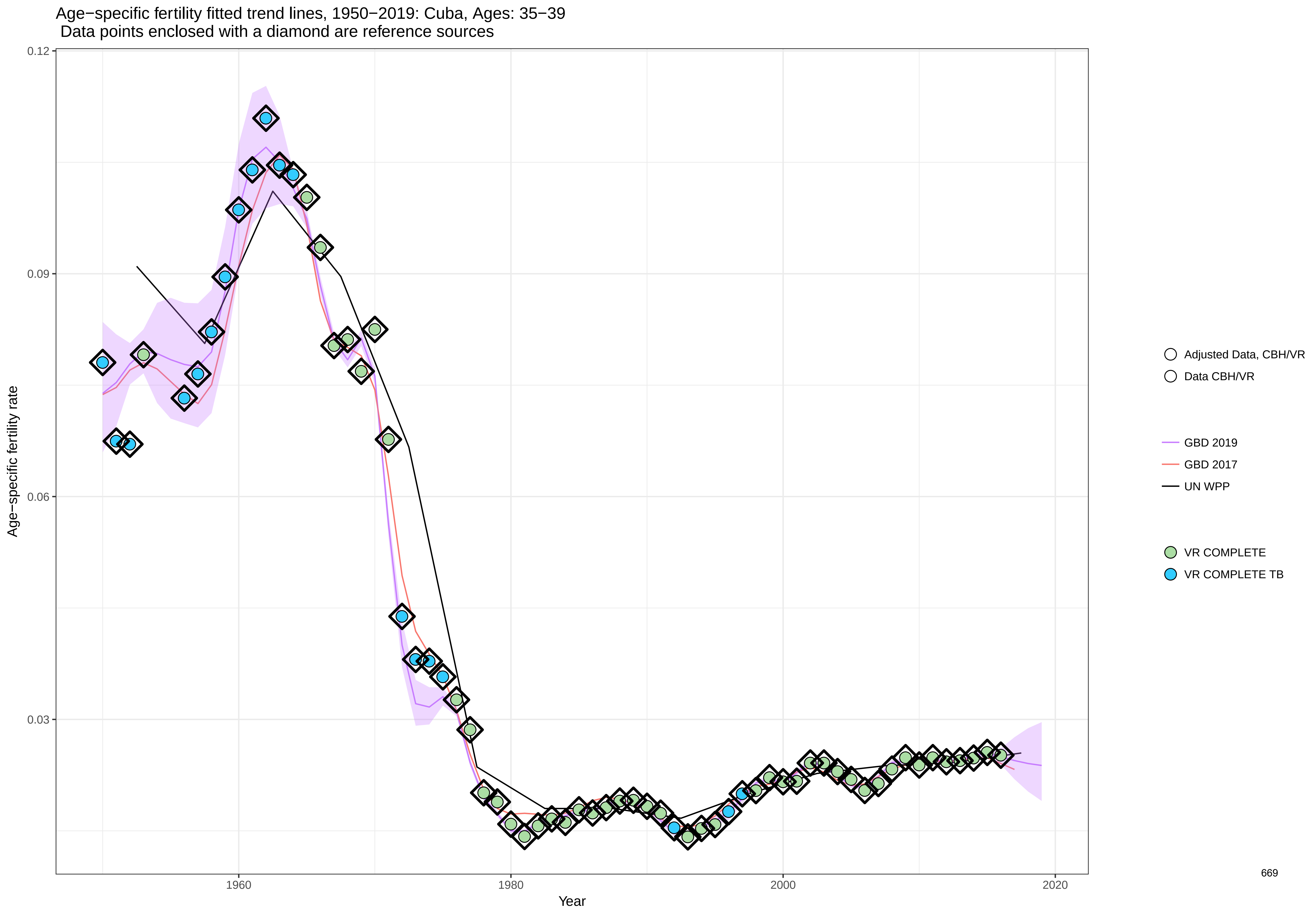

Age−specific fertility fitted trend lines, 1950−2019: Cuba, Ages: 35−39
Data points enclosed with a diamond are reference sources
0.12
0.09
0.06
0.03
Age−specific fertility rate
1960
1980
2000
2020
Year
Adjusted Data, CBH/VR
Data CBH/VR
GBD 2019
GBD 2017
UN WPP
VR COMPLETE
VR COMPLETE TB

Age−specific fertility fitted trend lines, 1950−2019: Cuba, Ages: 40−44
Data points enclosed with a diamond are reference sources

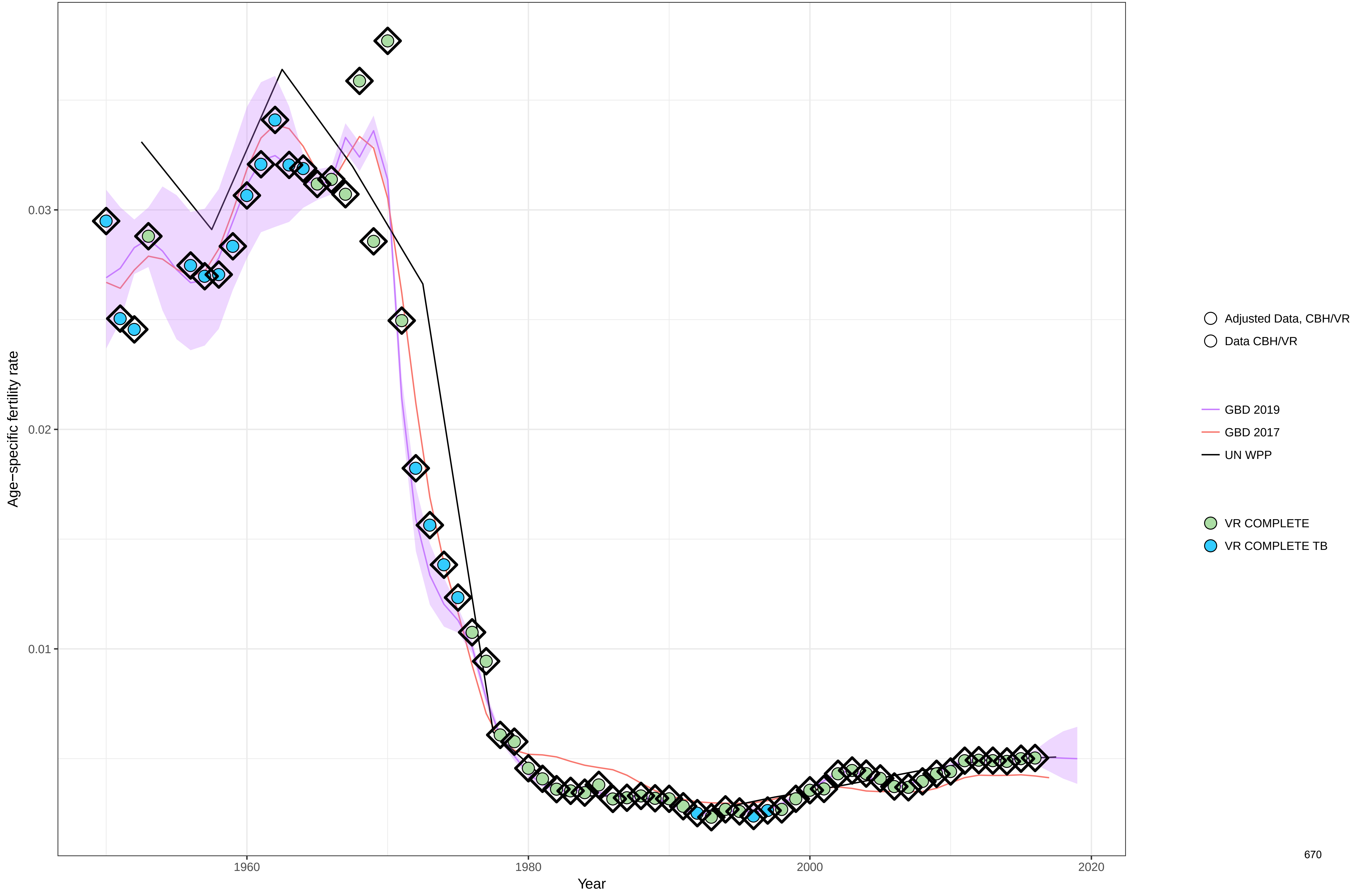

Age−specific fertility fitted trend lines, 1950−2019: Cuba, Ages: 45−49
Data points enclosed with a diamond are reference sources

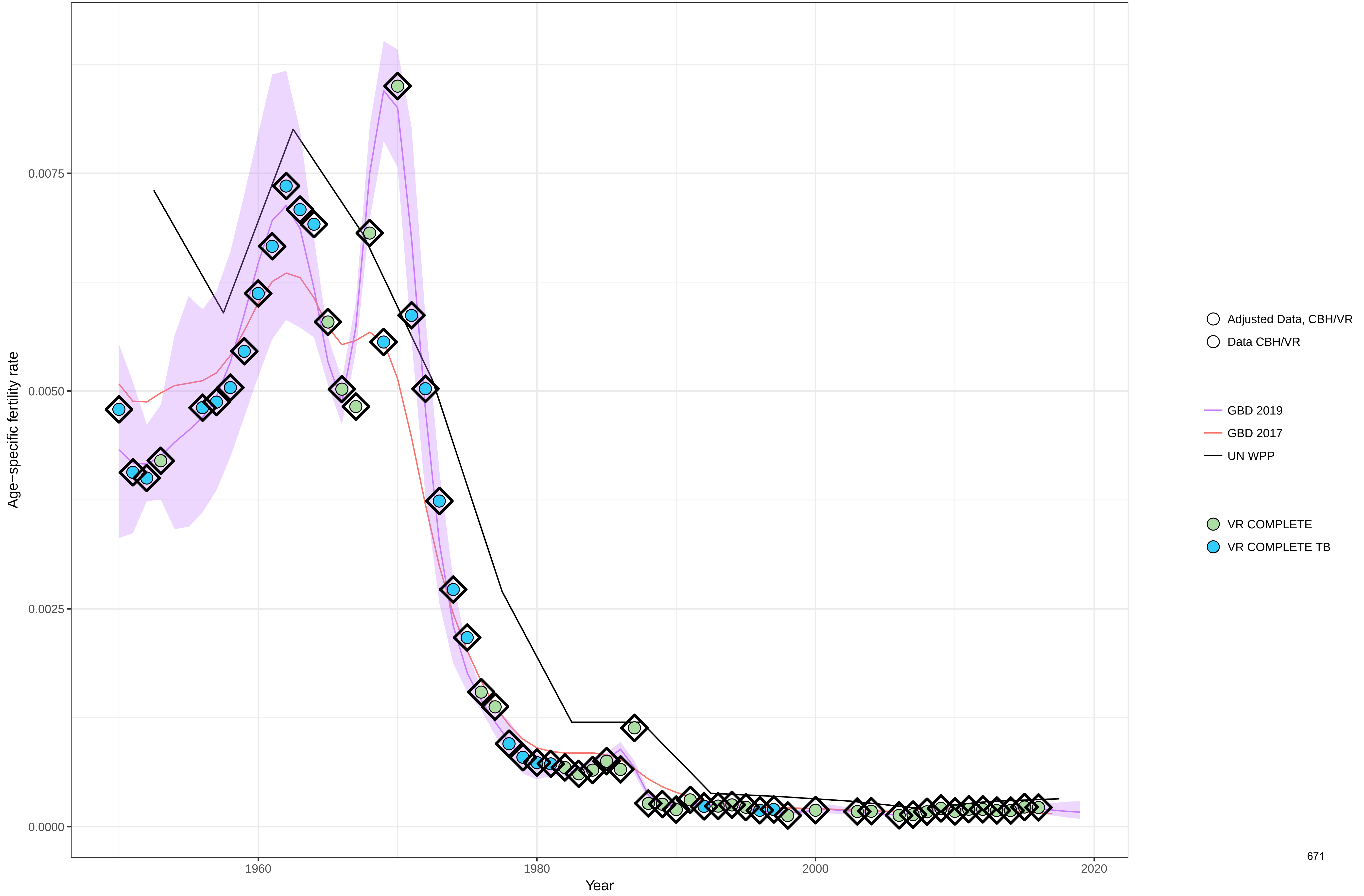

Age−specific fertility fitted trend lines, 1950−2019: Cuba, Ages: 50−54
Data points enclosed with a diamond are reference sources

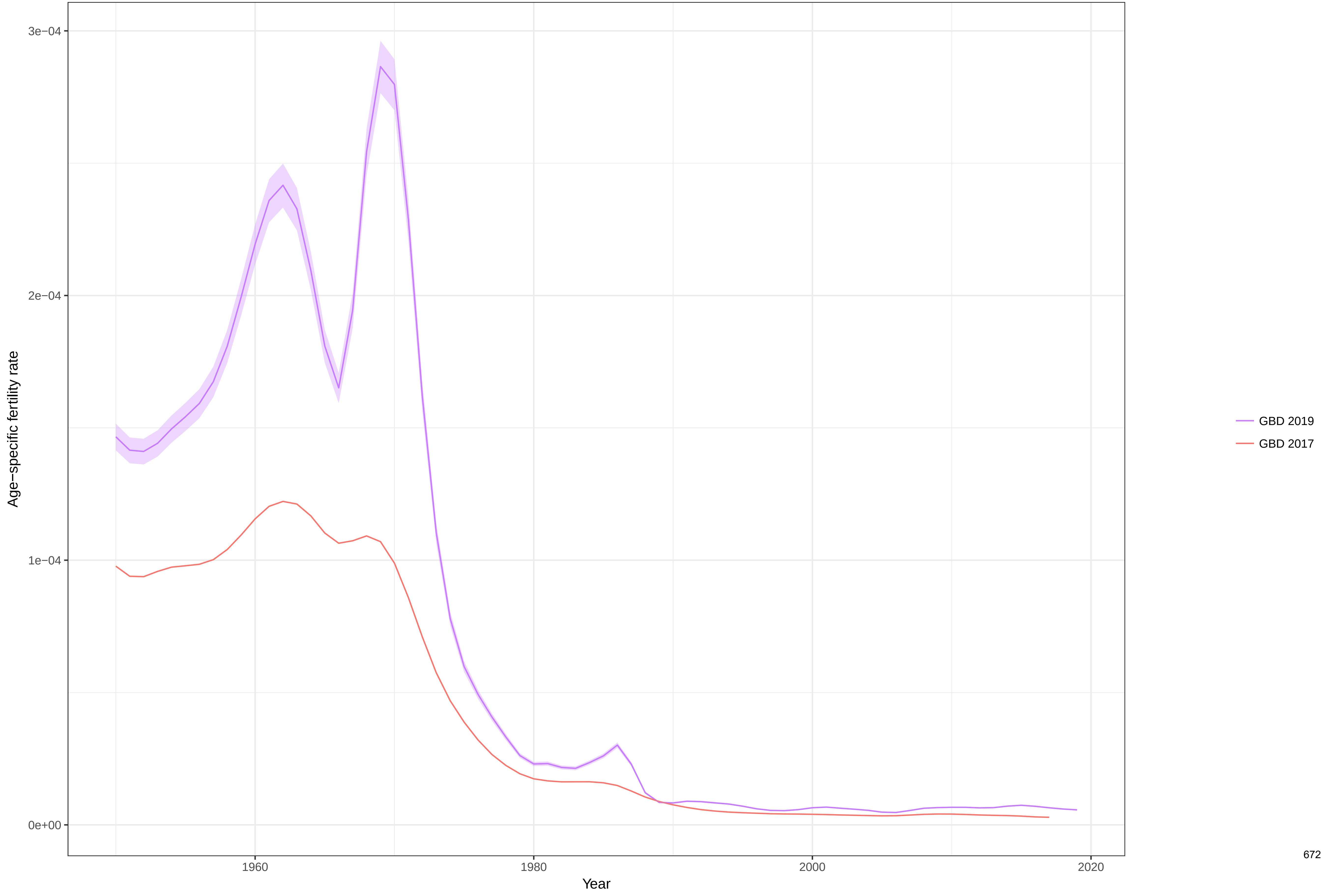

Total fertility rate fitted trend lines, 1950−2019: Cuba TFR
Data points enclosed with a diamond are reference sources

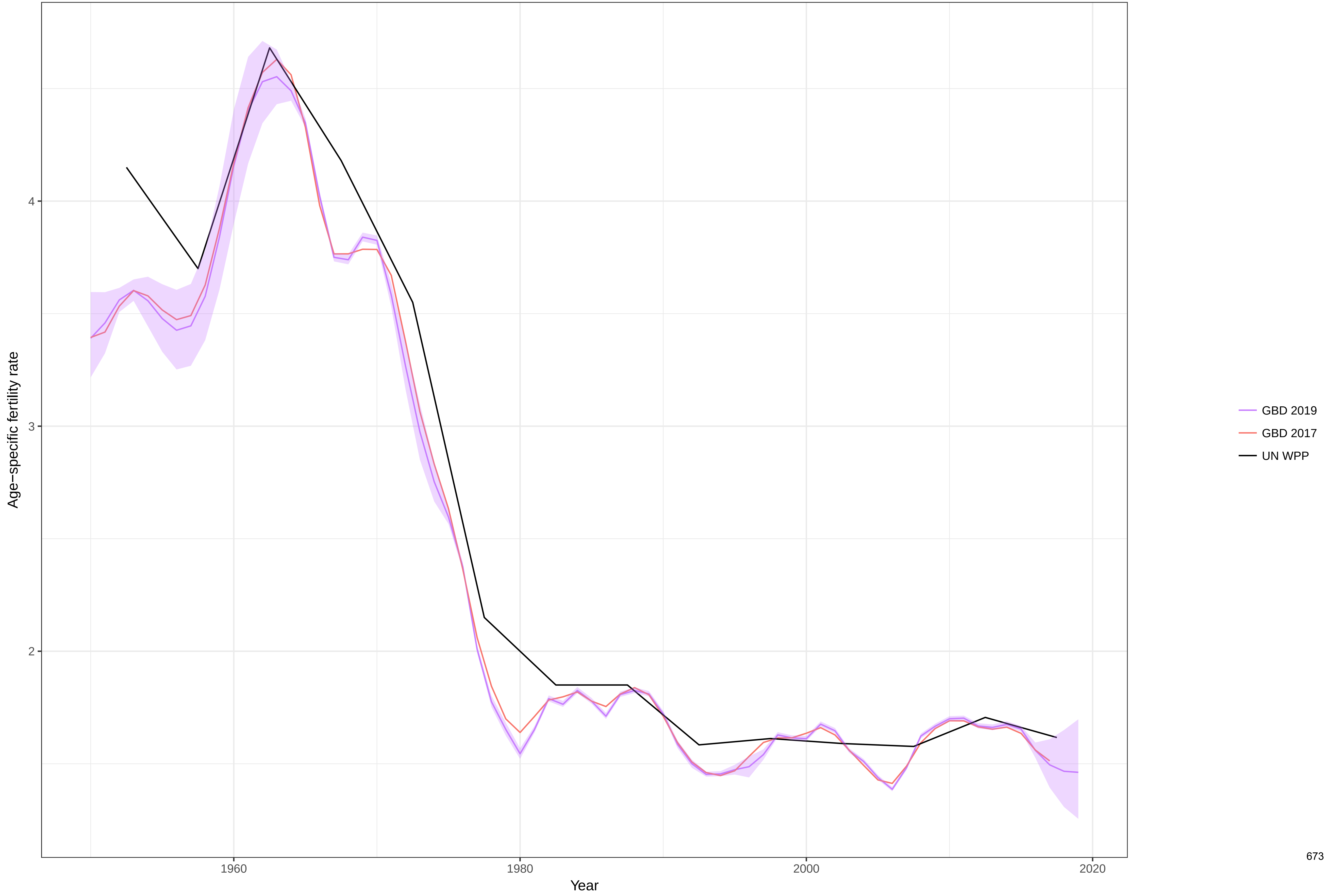

Age−specific fertility fitted trend lines, 1950−2019: Cyprus, Ages: 10−14
Data points enclosed with a diamond are reference sources

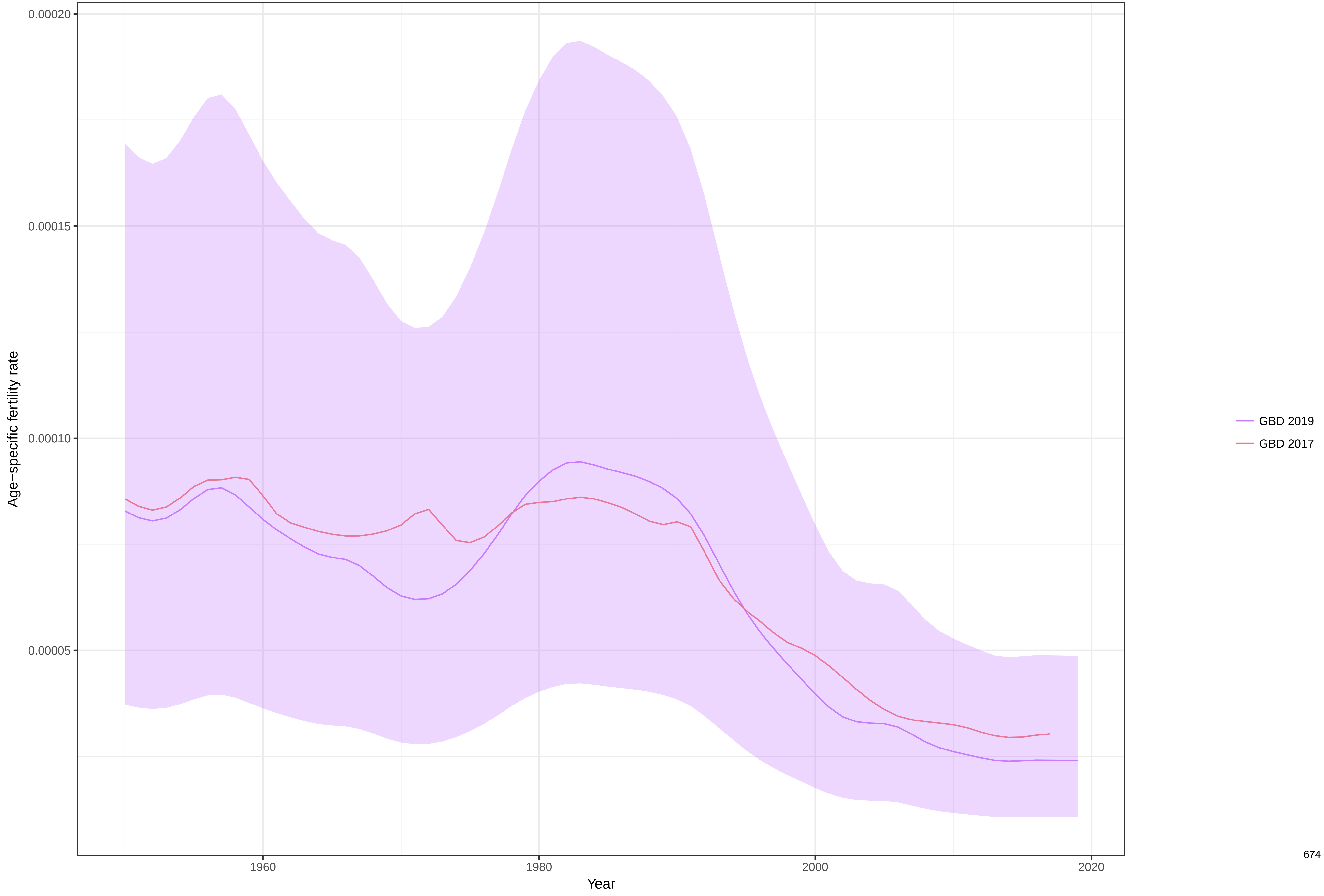

Age−specific fertility fitted trend lines, 1950−2019: Cyprus, Ages: 15−19
Data points enclosed with a diamond are reference sources

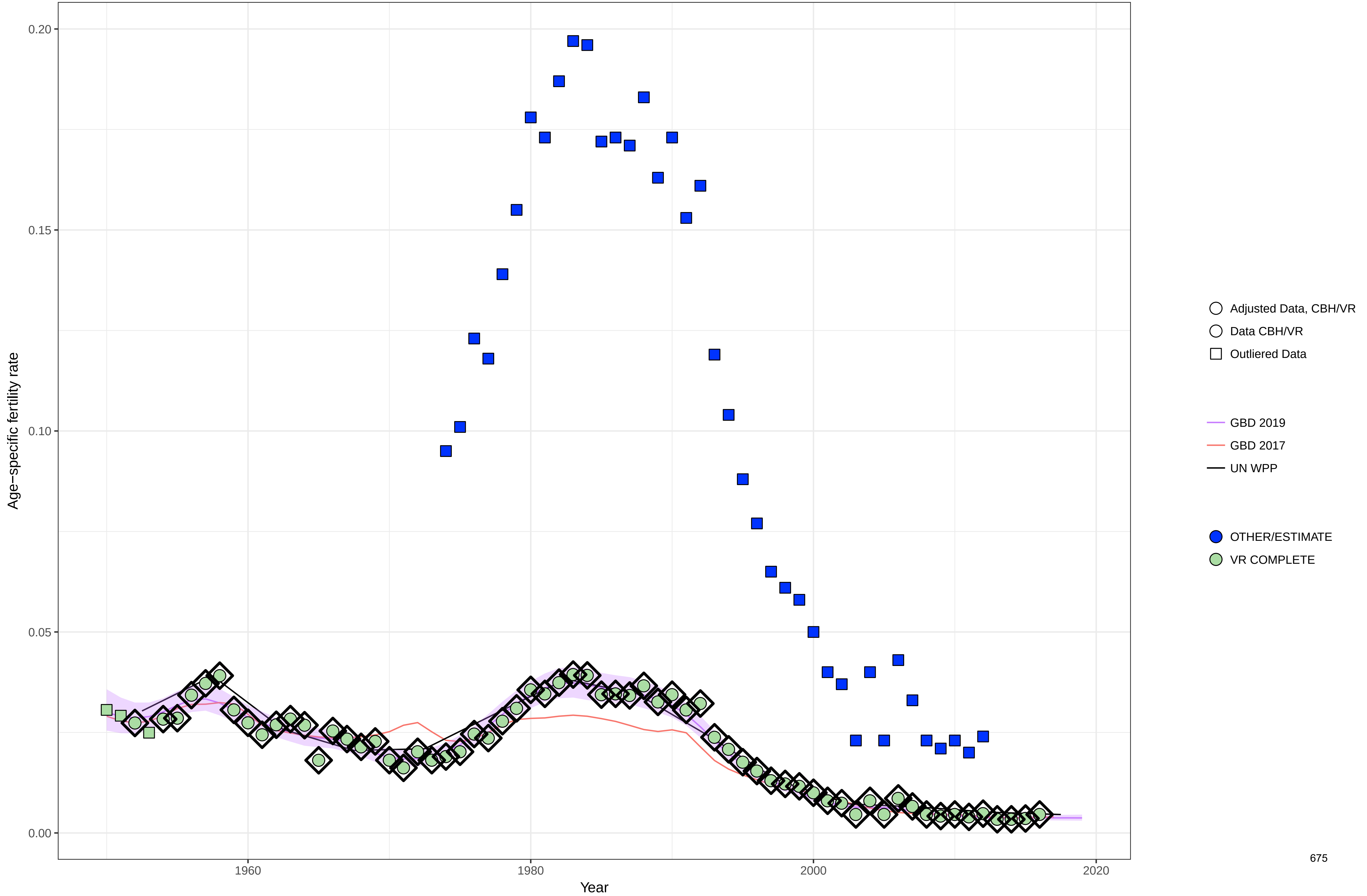

Age−specific fertility fitted trend lines, 1950−2019: Cyprus, Ages: 20−24
Data points enclosed with a diamond are reference sources

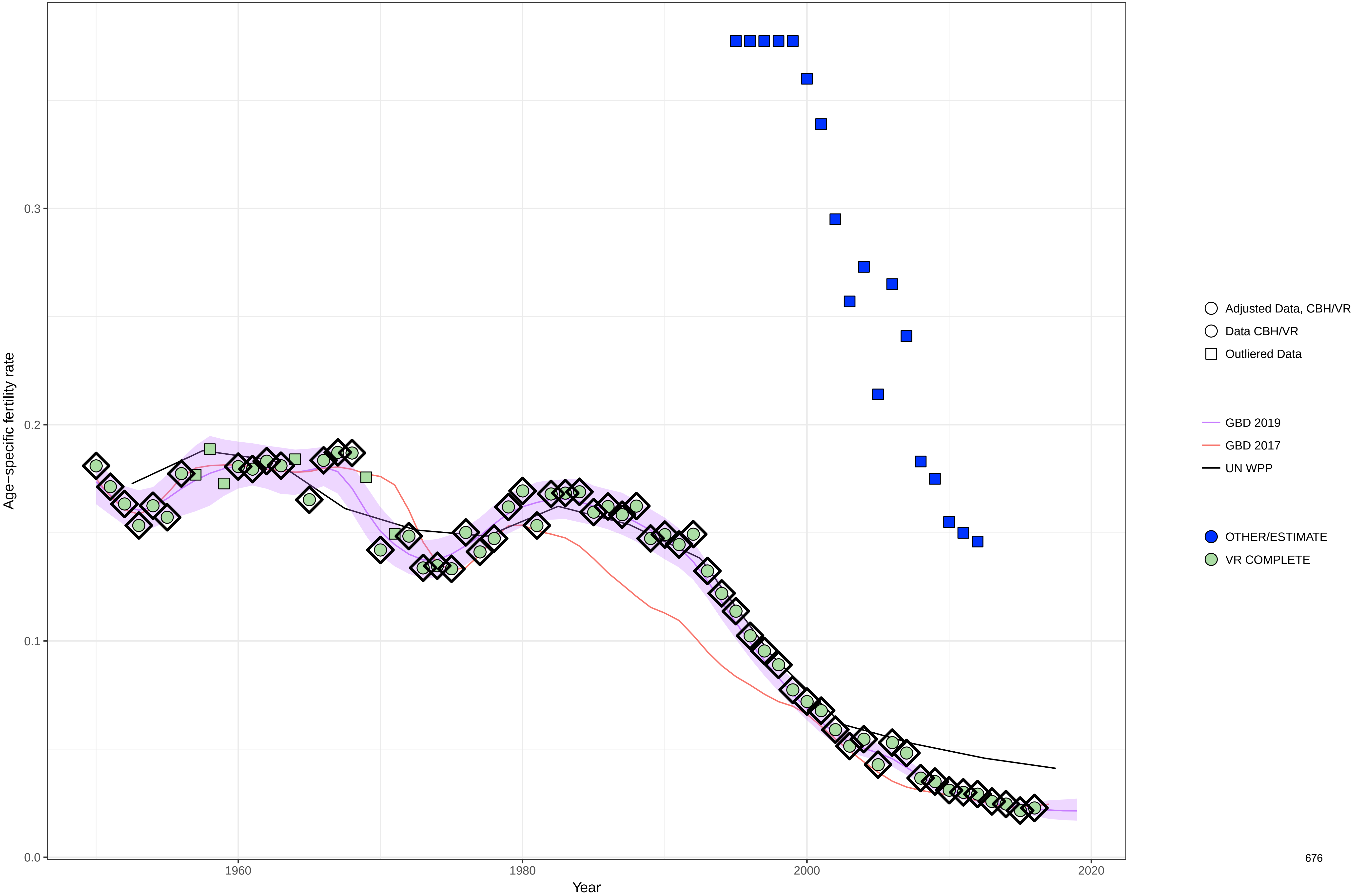

Age−specific fertility fitted trend lines, 1950−2019: Cyprus, Ages: 25−29
Data points enclosed with a diamond are reference sources

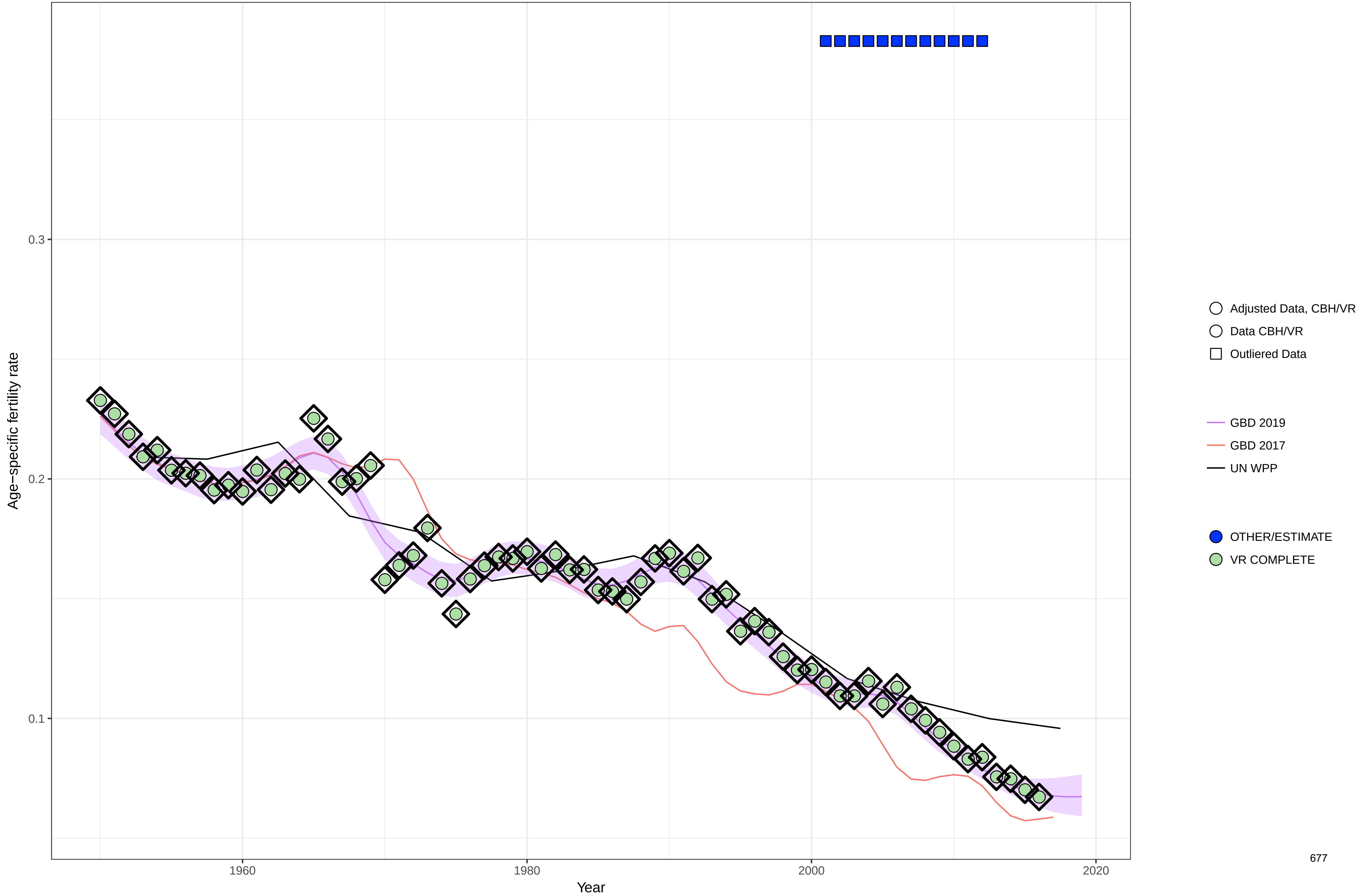

Age−specific fertility fitted trend lines, 1950−2019: Cyprus, Ages: 30−34
Data points enclosed with a diamond are reference sources

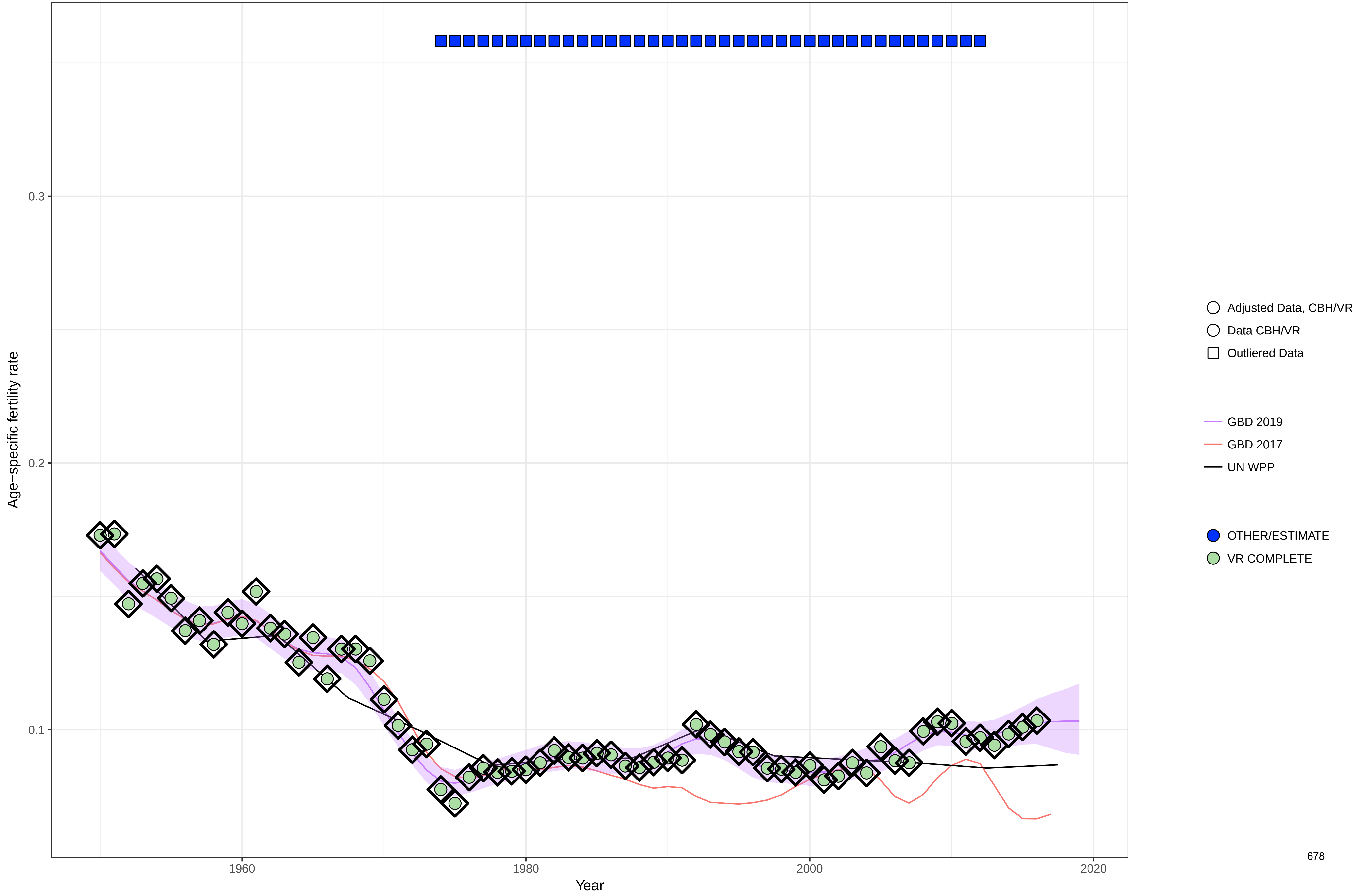

Age−specific fertility fitted trend lines, 1950−2019: Cyprus, Ages: 35−39
Data points enclosed with a diamond are reference sources

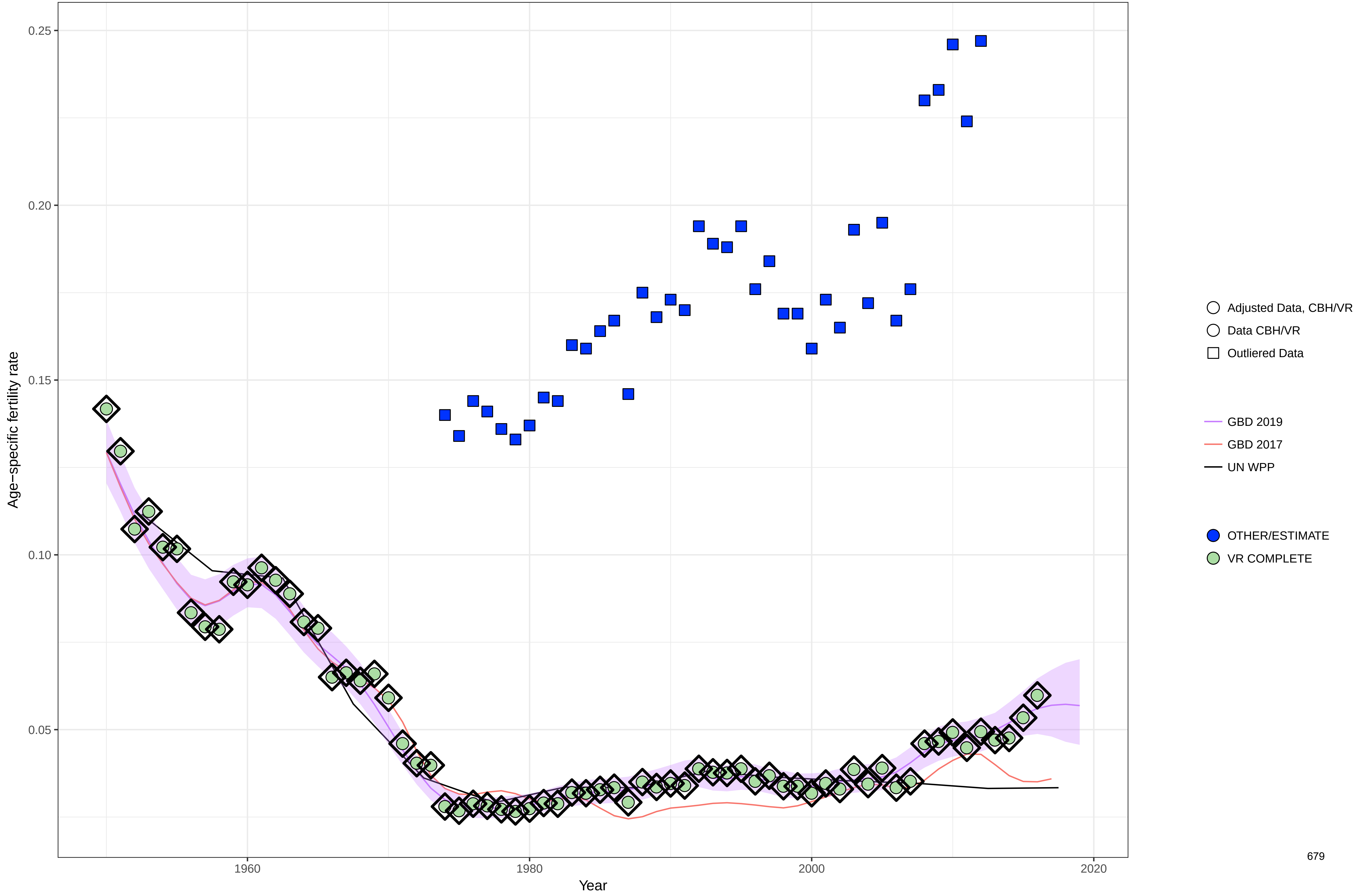

Age−specific fertility fitted trend lines, 1950−2019: Cyprus, Ages: 40−44
Data points enclosed with a diamond are reference sources

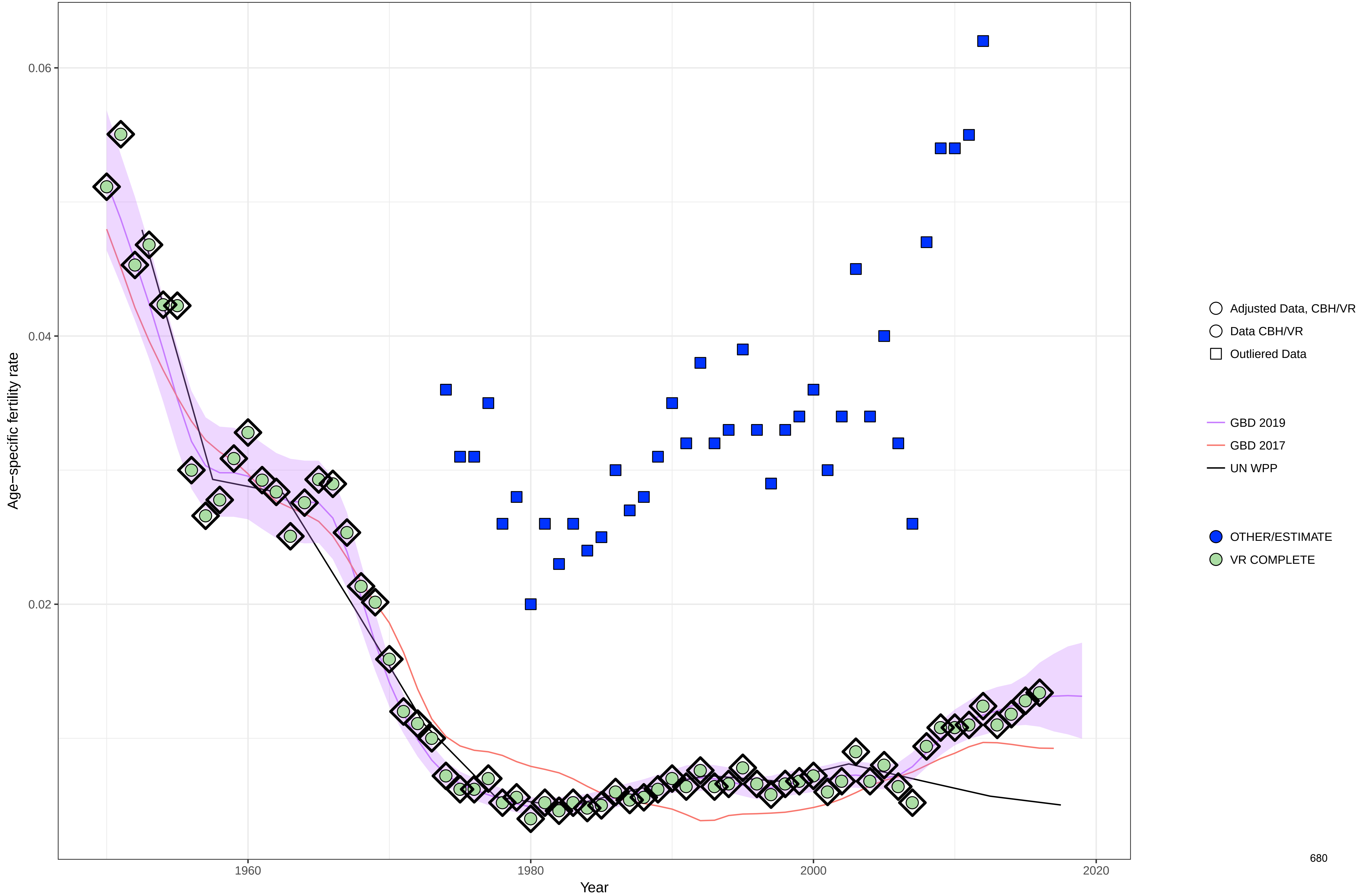

Age−specific fertility fitted trend lines, 1950−2019: Cyprus, Ages: 45−49
Data points enclosed with a diamond are reference sources

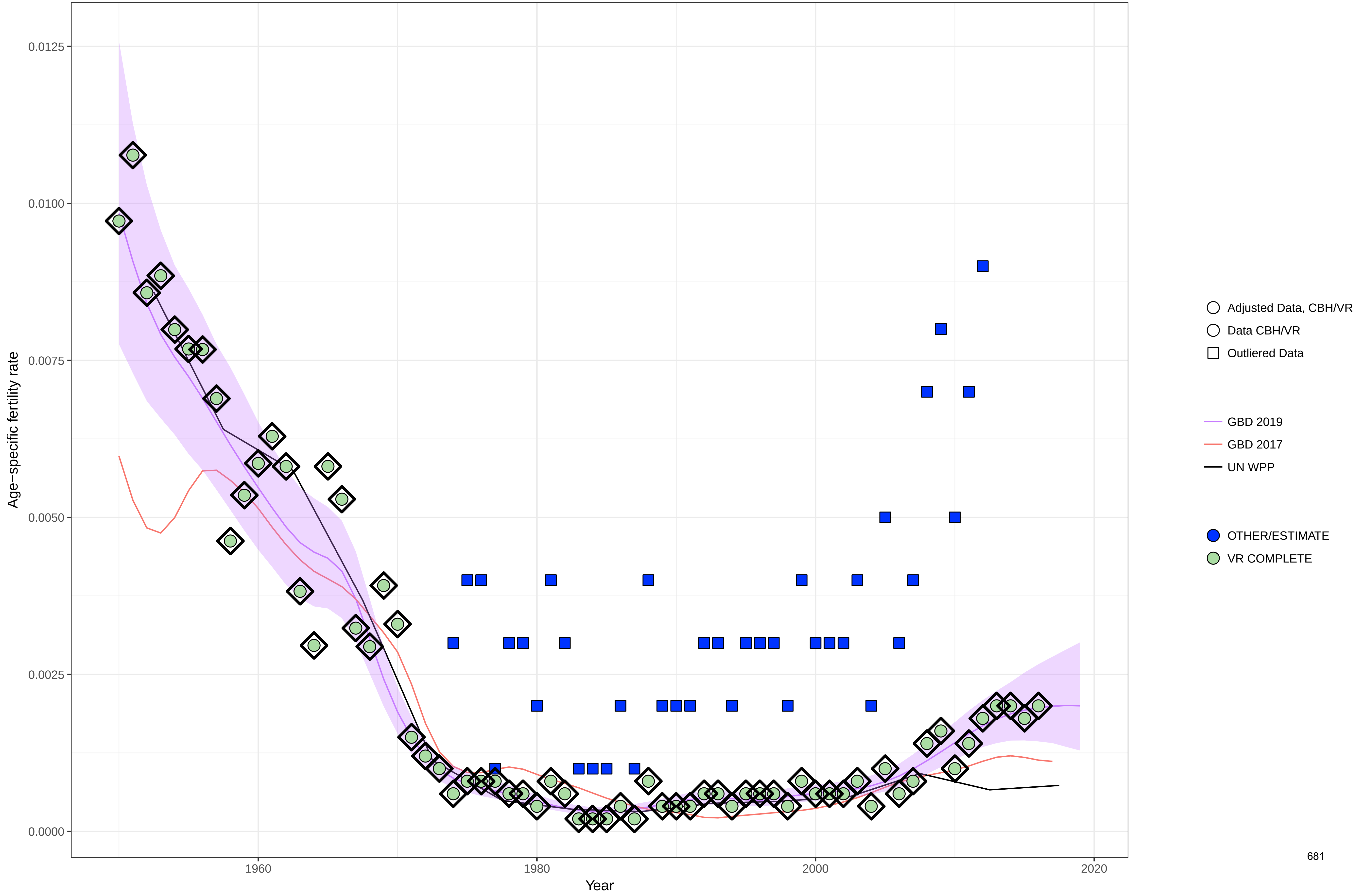

Age−specific fertility fitted trend lines, 1950−2019: Cyprus, Ages: 50−54
Data points enclosed with a diamond are reference sources

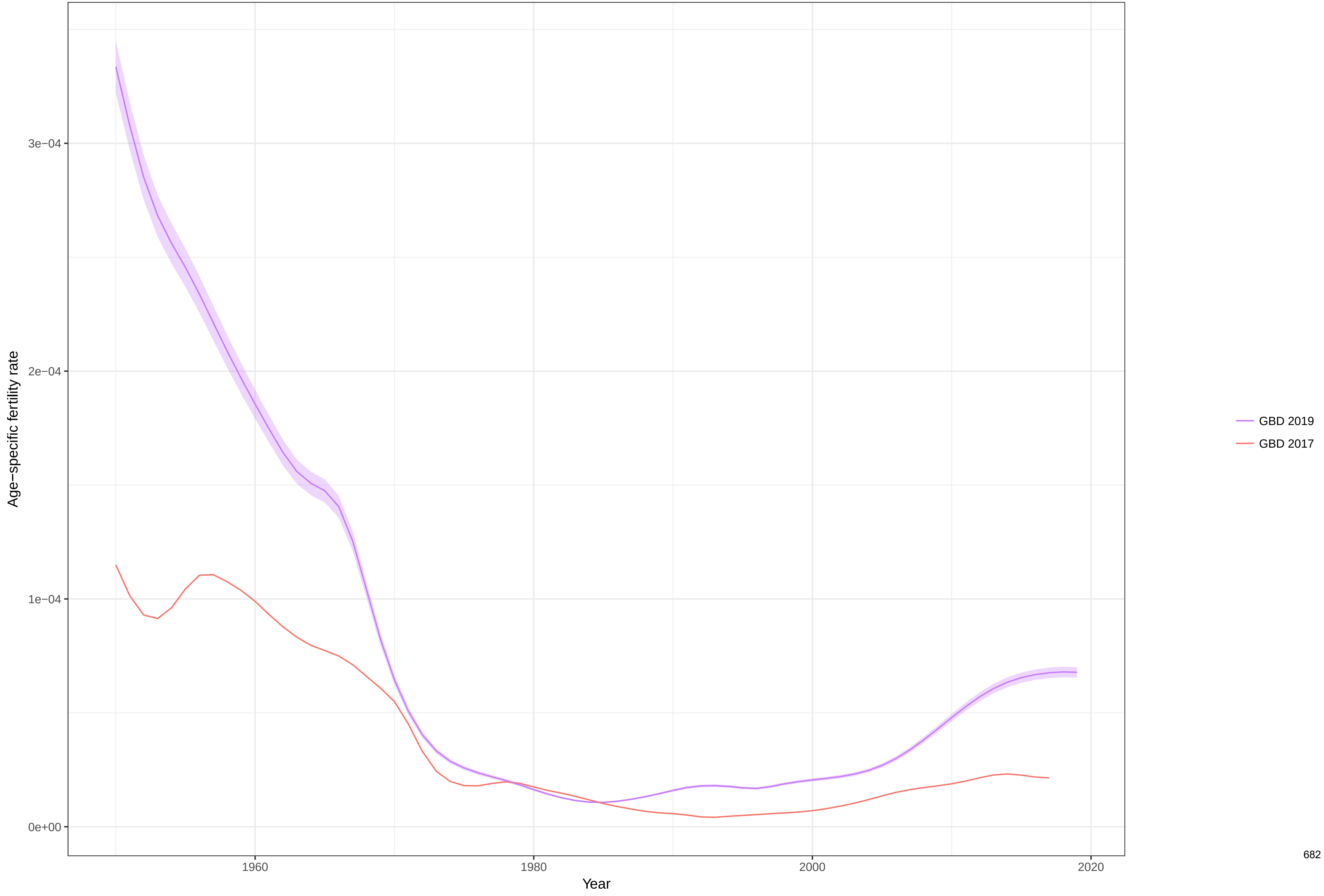

Total fertility rate fitted trend lines, 1950−2019: Cyprus TFR
Data points enclosed with a diamond are reference sources

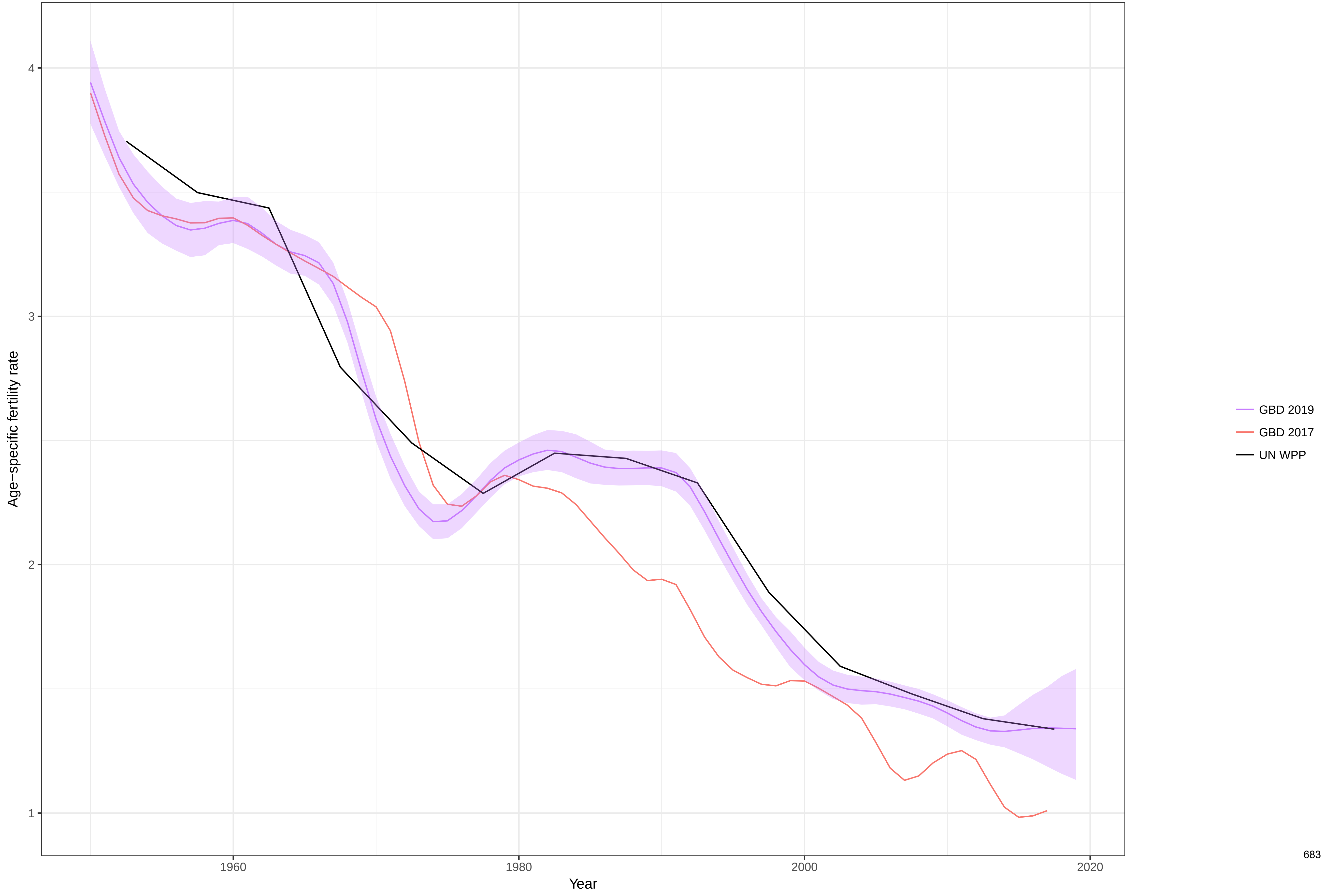

Age−specific fertility fitted trend lines, 1950−2019: Czechia, Ages: 10−14
Data points enclosed with a diamond are reference sources

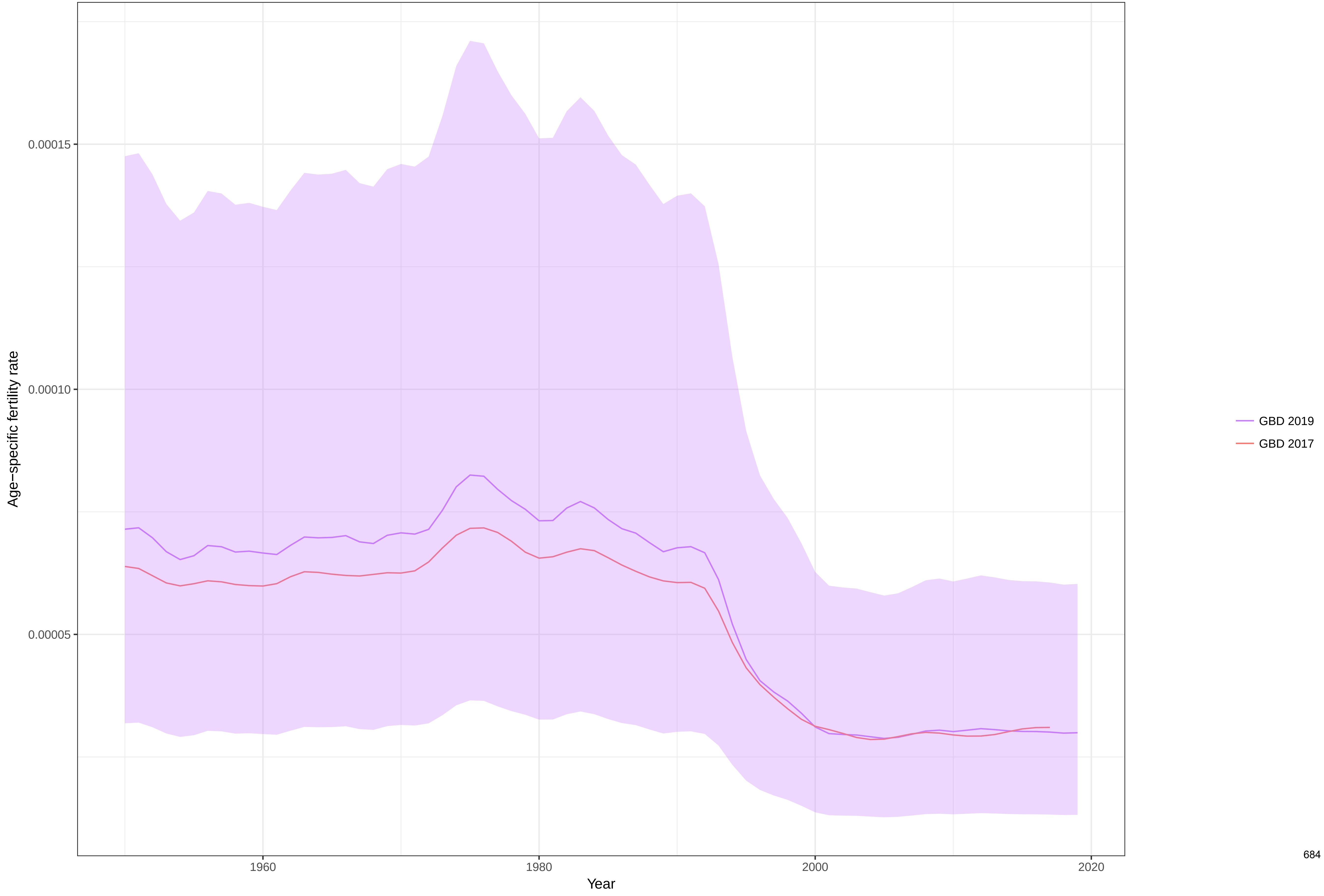

Age−specific fertility fitted trend lines, 1950−2019: Czechia, Ages: 15−19
Data points enclosed with a diamond are reference sources

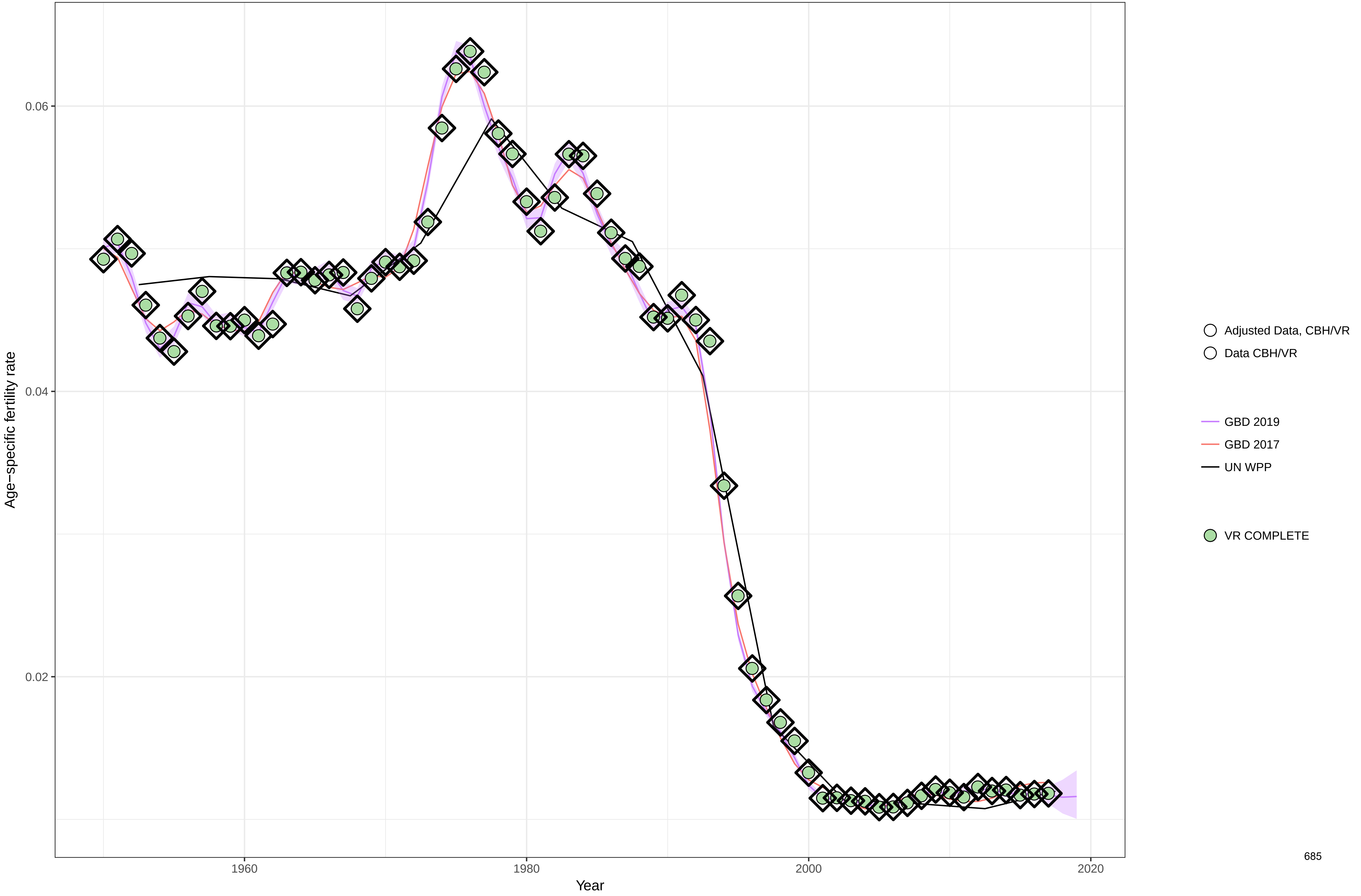

Age−specific fertility fitted trend lines, 1950−2019: Czechia, Ages: 20−24
Data points enclosed with a diamond are reference sources

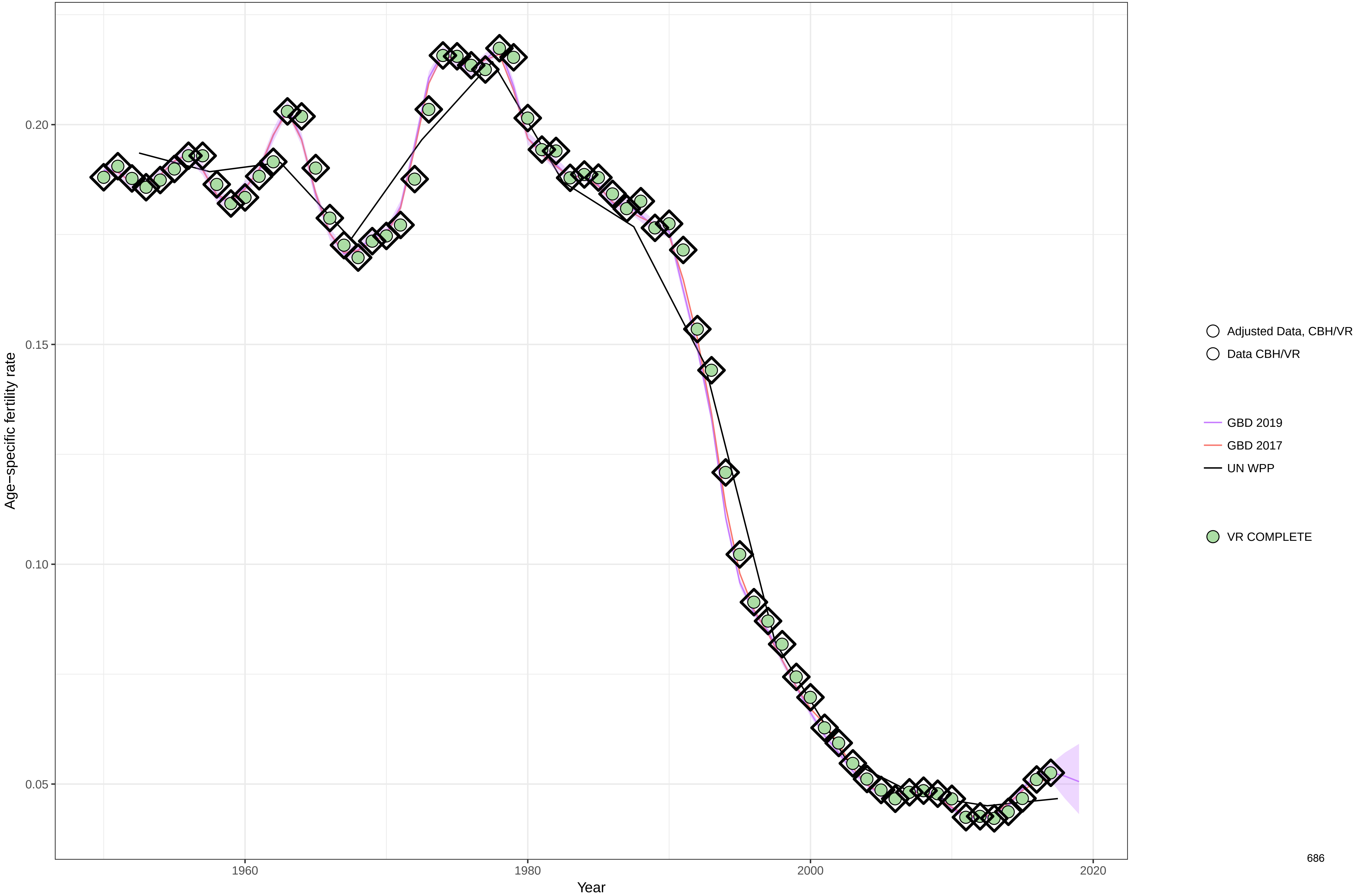

Age−specific fertility fitted trend lines, 1950−2019: Czechia, Ages: 25−29
Data points enclosed with a diamond are reference sources

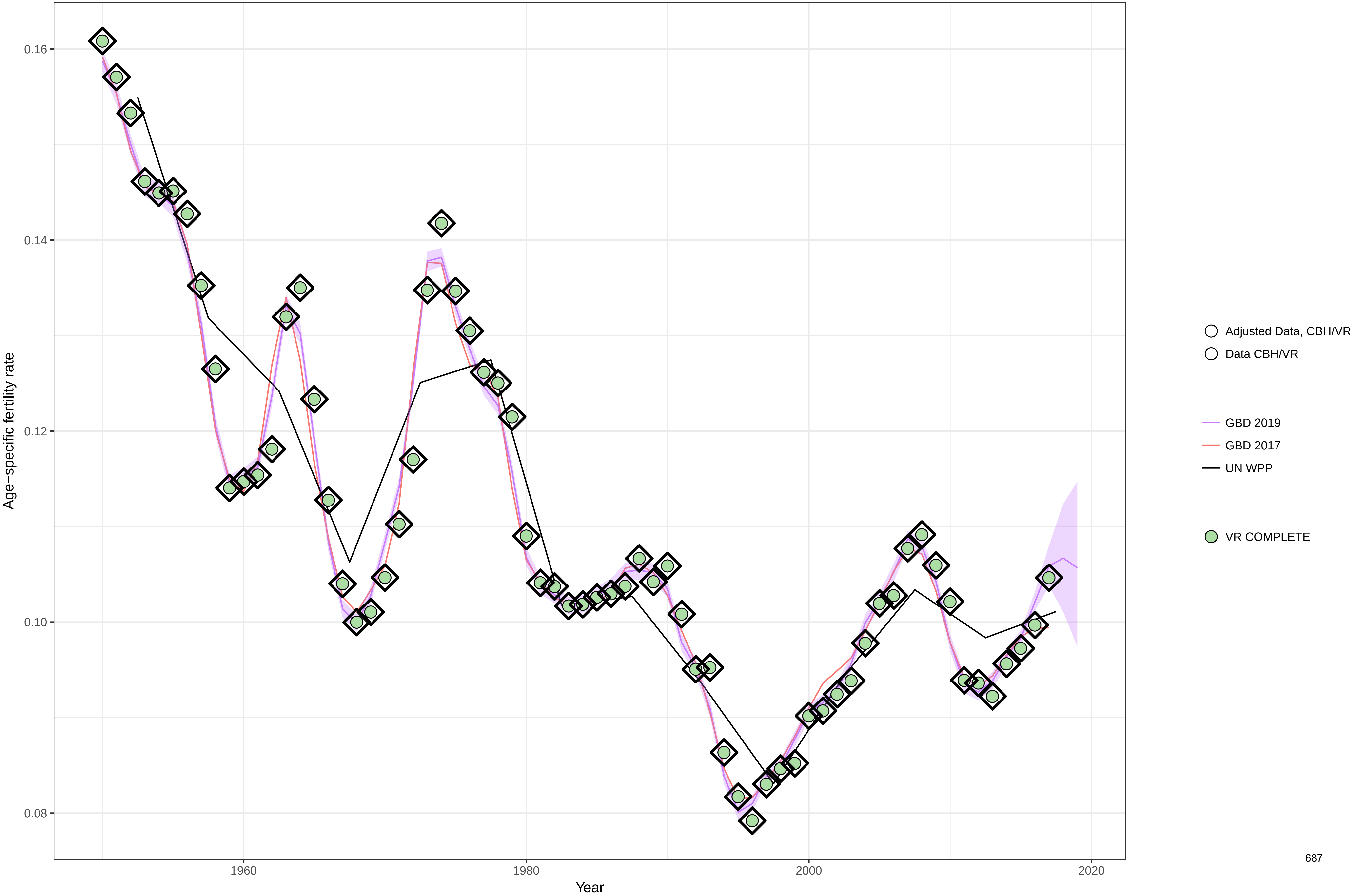

Age−specific fertility fitted trend lines, 1950−2019: Czechia, Ages: 30−34
Data points enclosed with a diamond are reference sources

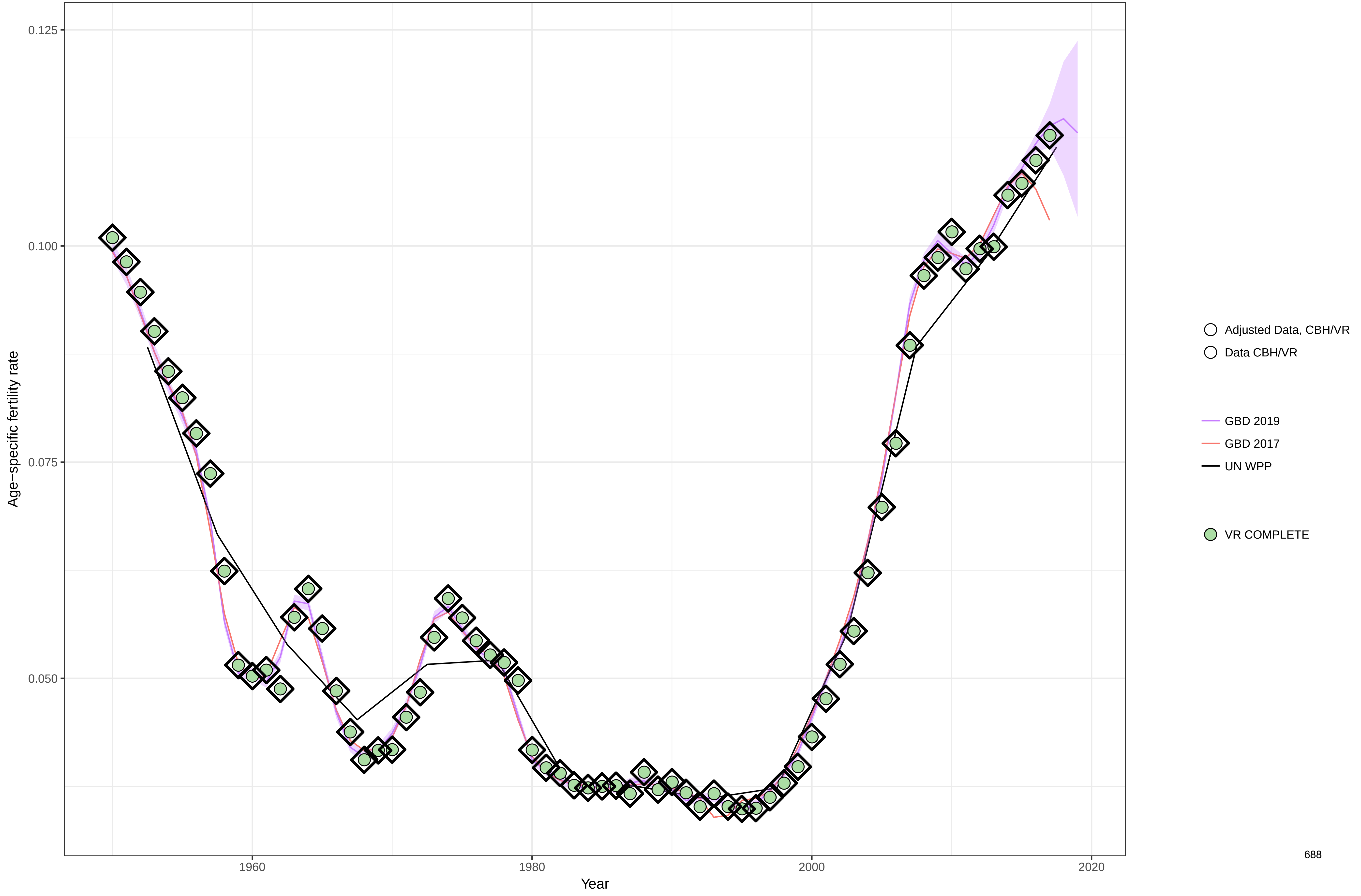

Age−specific fertility fitted trend lines, 1950−2019: Czechia, Ages: 35−39
Data points enclosed with a diamond are reference sources

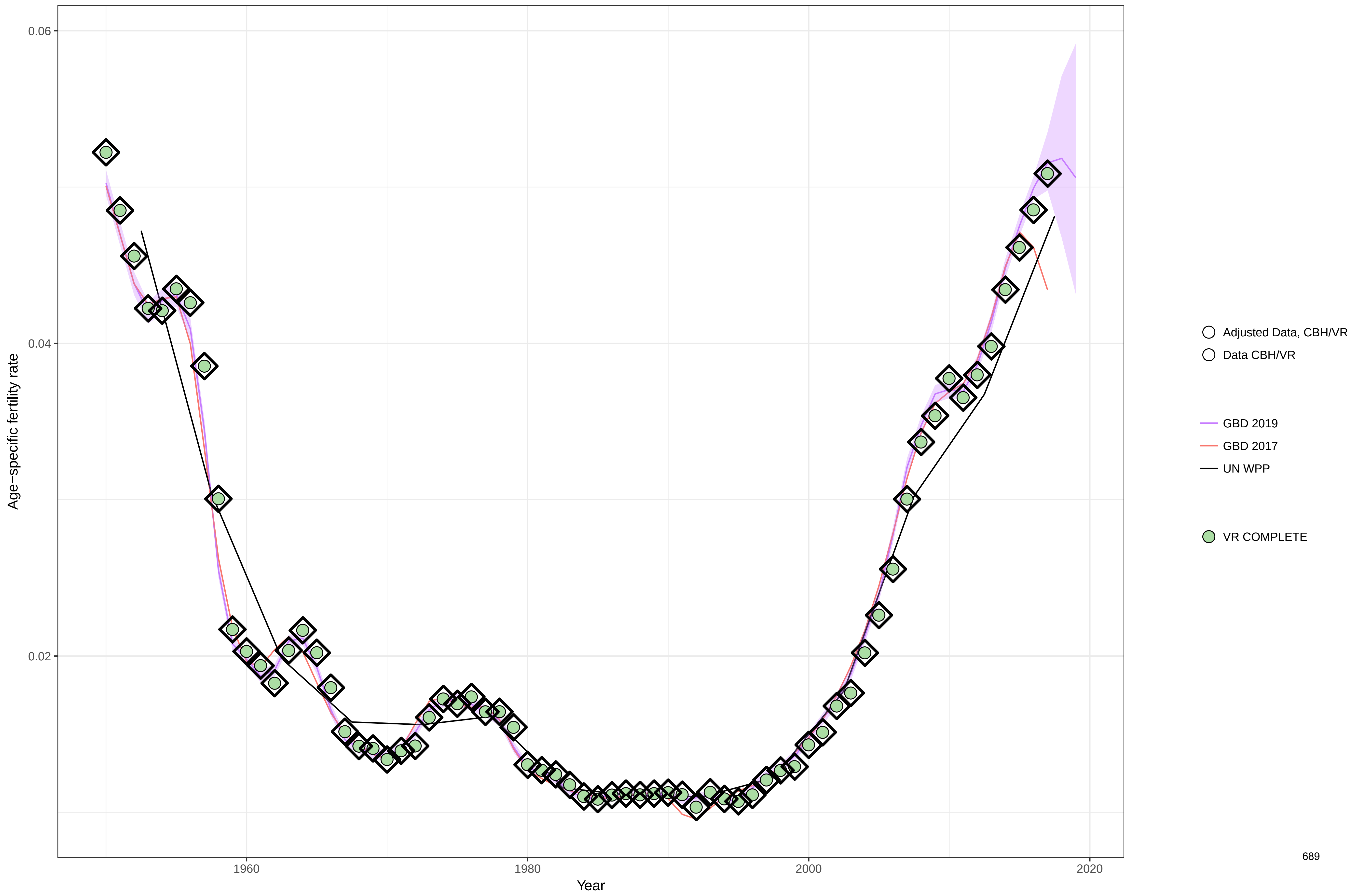

Age−specific fertility fitted trend lines, 1950−2019: Czechia, Ages: 40−44
Data points enclosed with a diamond are reference sources

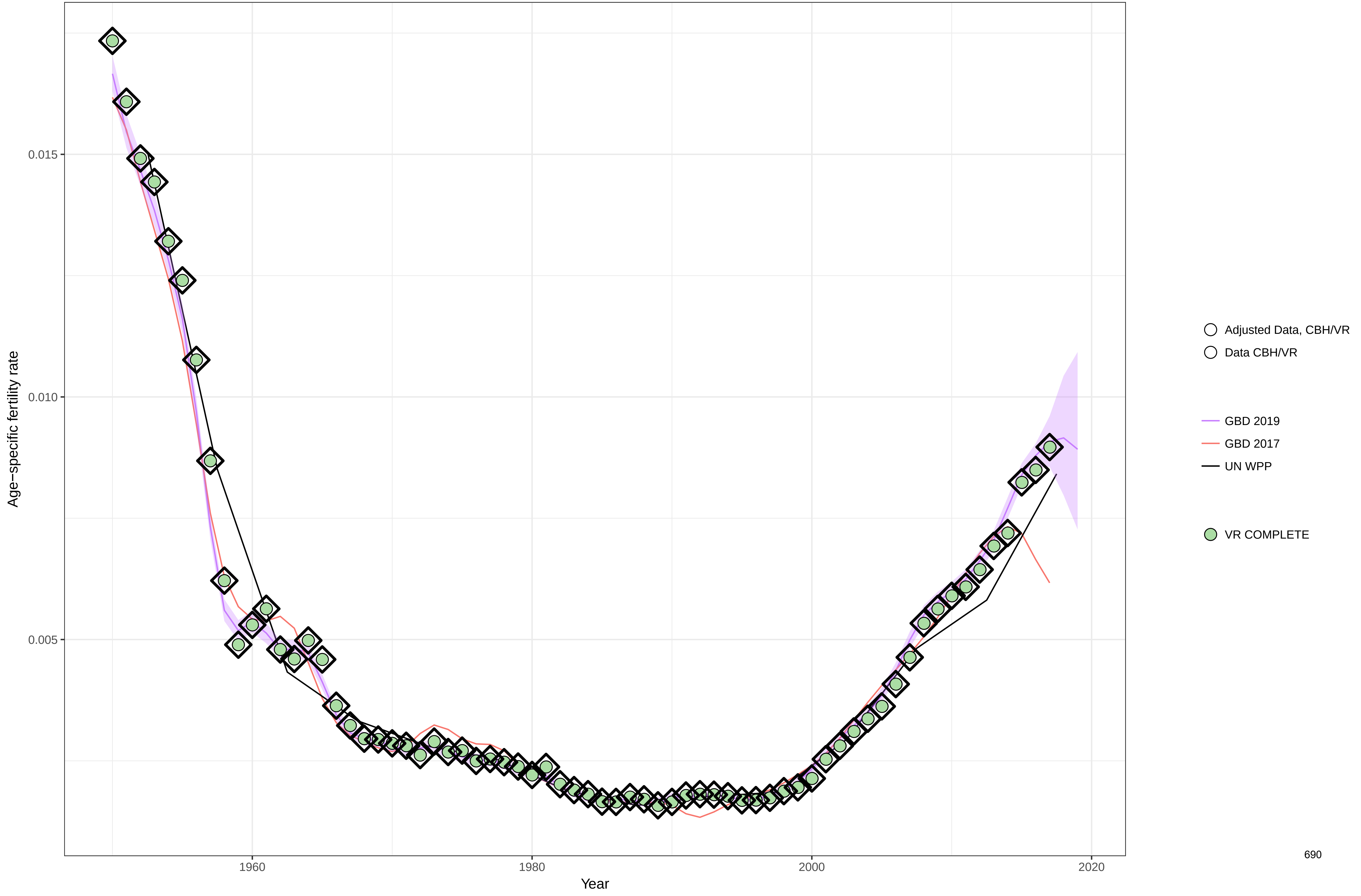

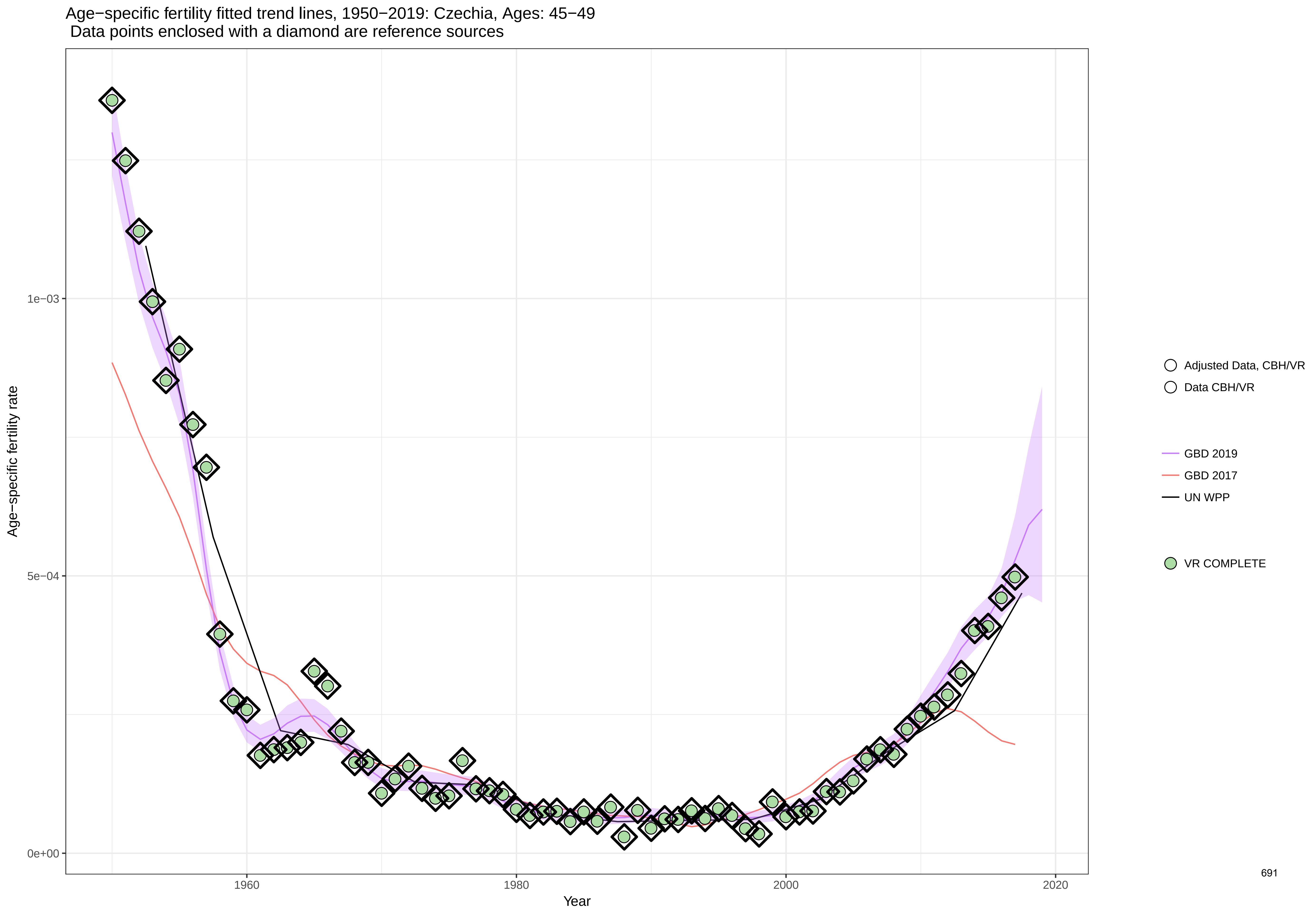

Age−specific fertility fitted trend lines, 1950−2019: Czechia, Ages: 45−49
Data points enclosed with a diamond are reference sources
Age−specific fertility rate
1e−03
5e−04
0e+00
1960
1980
2000
2020
Year
Adjusted Data, CBH/VR
Data CBH/VR
GBD 2019
GBD 2017
UN WPP
VR COMPLETE

Age−specific fertility fitted trend lines, 1950−2019: Czechia, Ages: 50−54
Data points enclosed with a diamond are reference sources

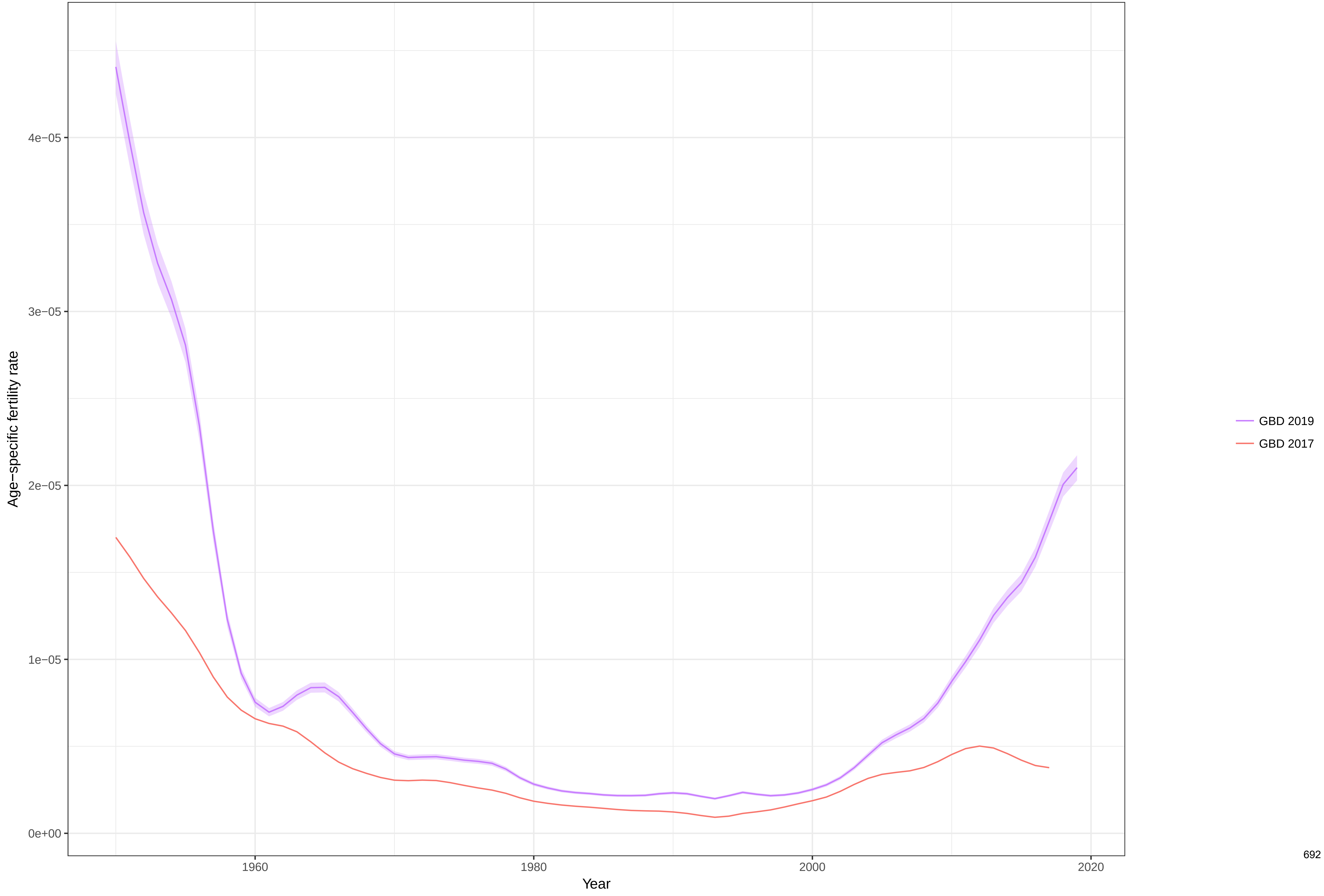

Total fertility rate fitted trend lines, 1950−2019: Czechia TFR
Data points enclosed with a diamond are reference sources

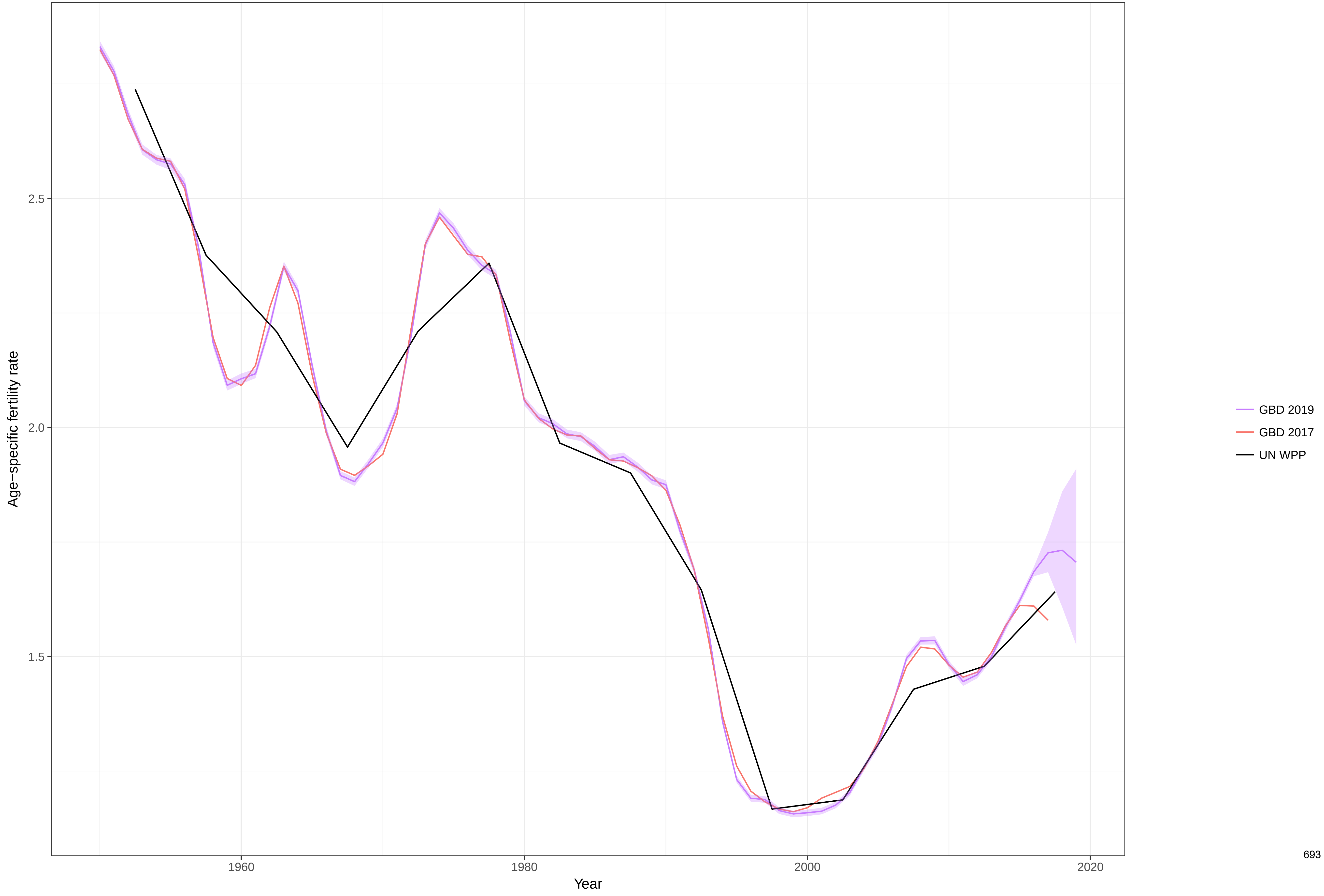

Age−specific fertility fitted trend lines, 1950−2019: Germany, Ages: 10−14
Data points enclosed with a diamond are reference sources

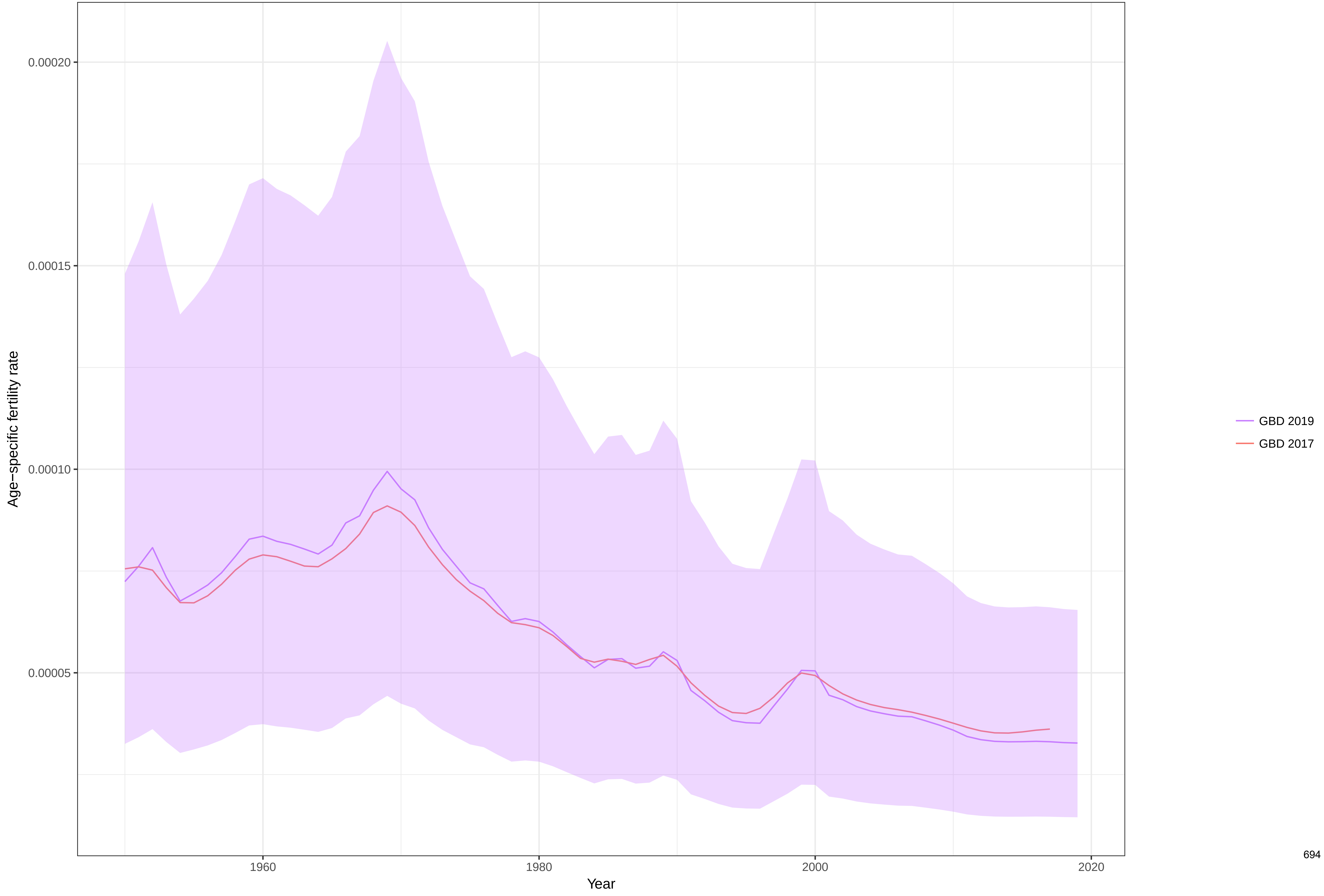

Age−specific fertility fitted trend lines, 1950−2019: Germany, Ages: 15−19
Data points enclosed with a diamond are reference sources

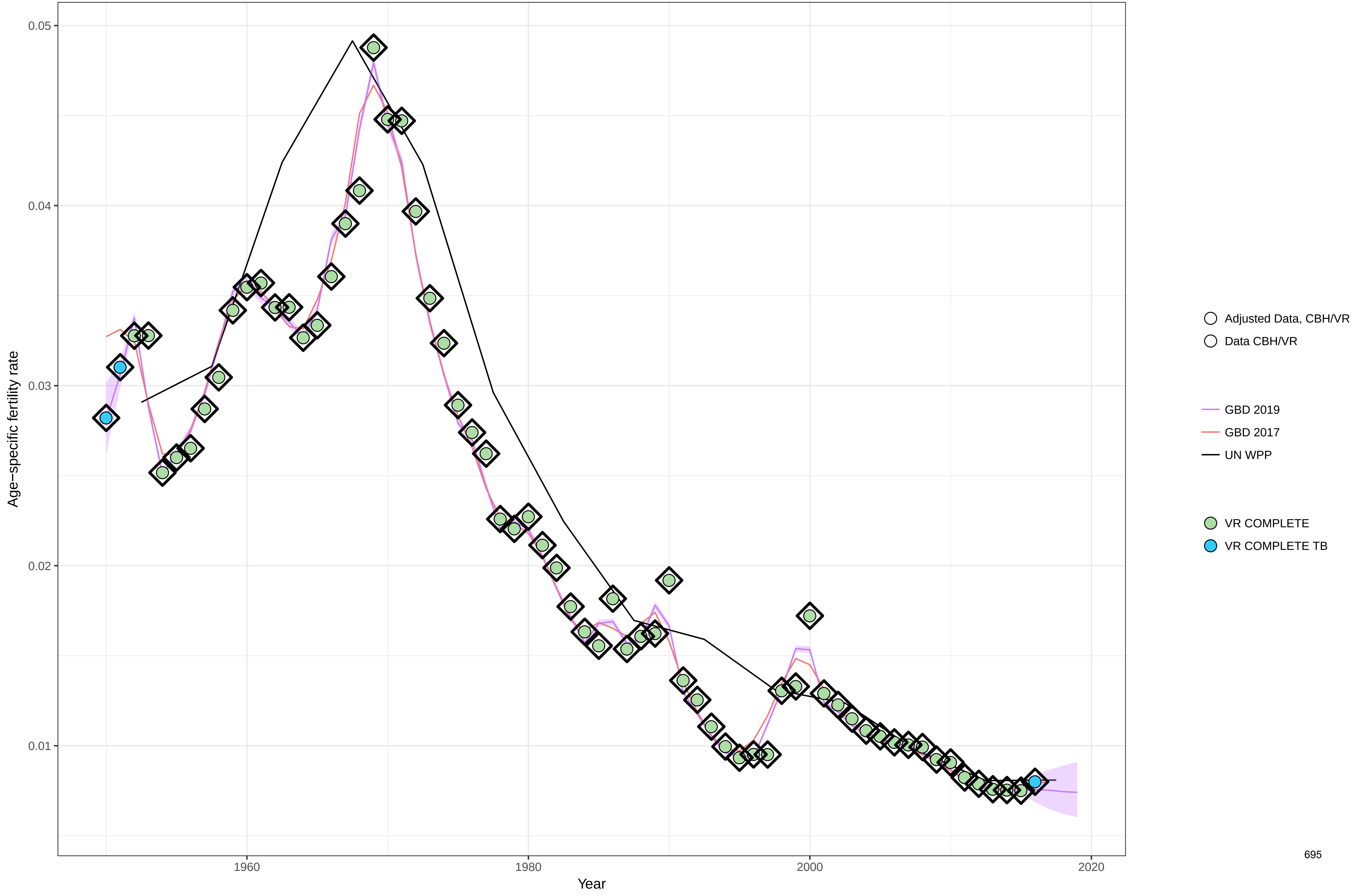

Age−specific fertility fitted trend lines, 1950−2019: Germany, Ages: 20−24
Data points enclosed with a diamond are reference sources

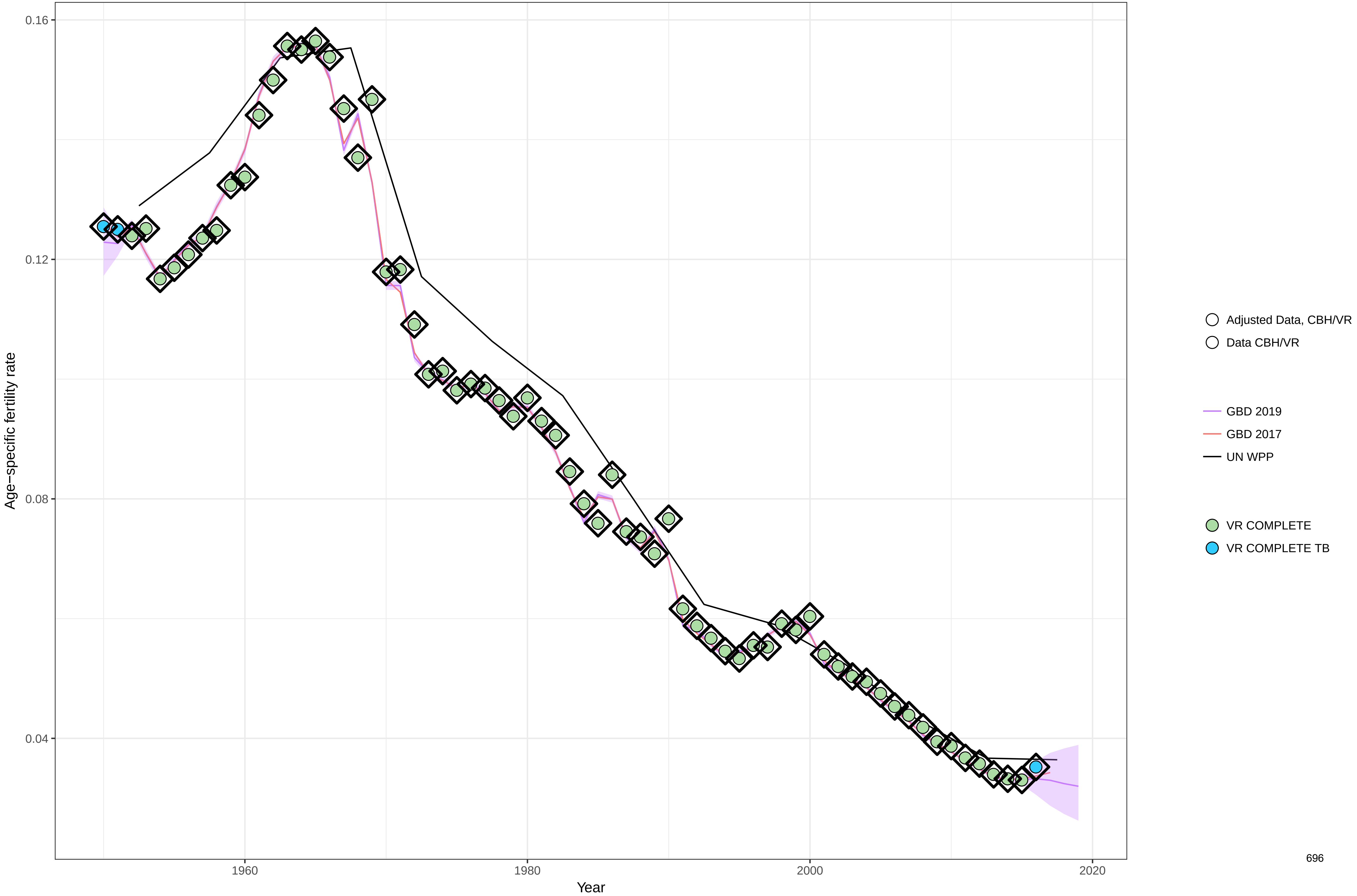

Age−specific fertility fitted trend lines, 1950−2019: Germany, Ages: 25−29
Data points enclosed with a diamond are reference sources

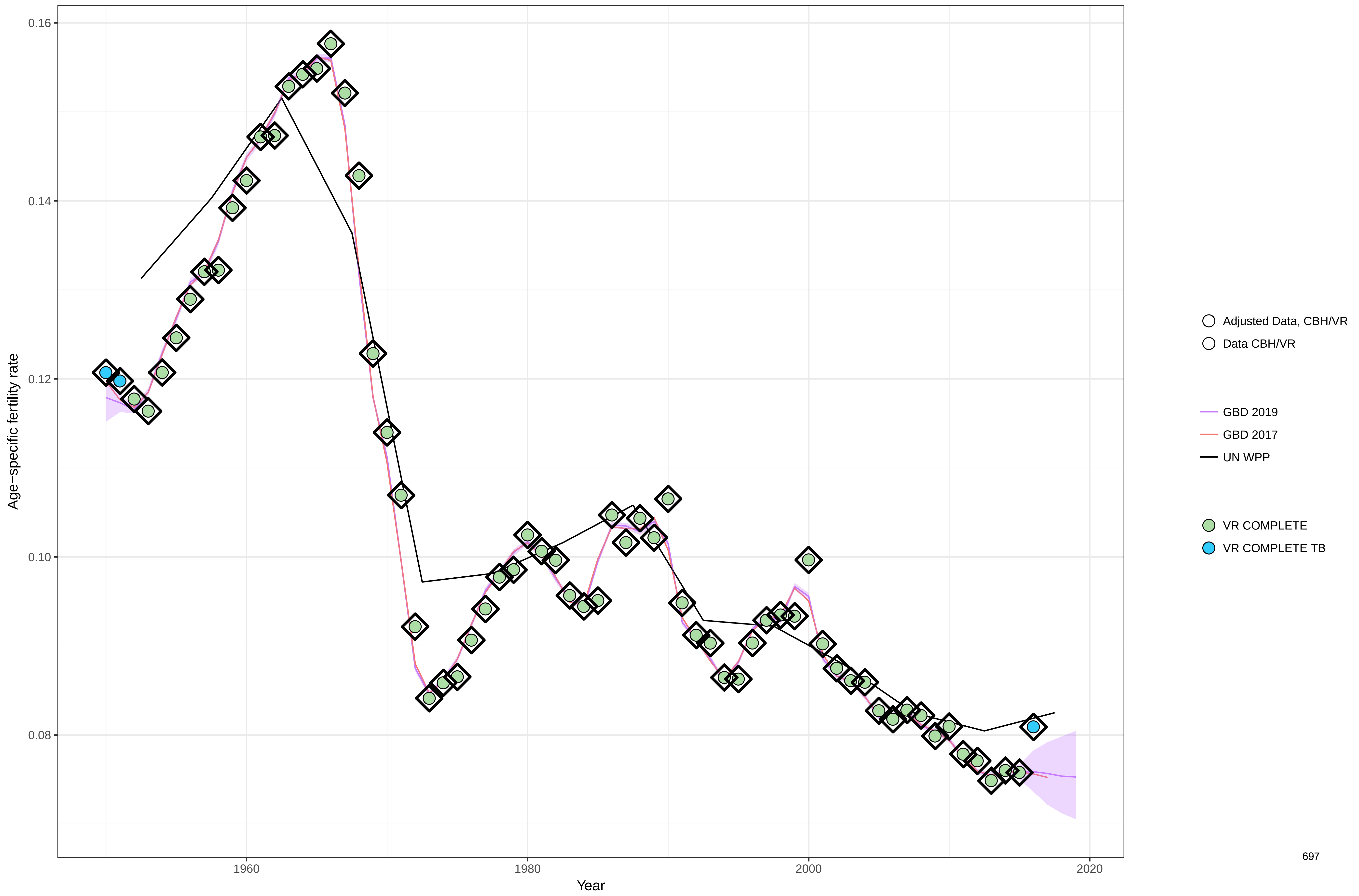

Age−specific fertility fitted trend lines, 1950−2019: Germany, Ages: 30−34
Data points enclosed with a diamond are reference sources

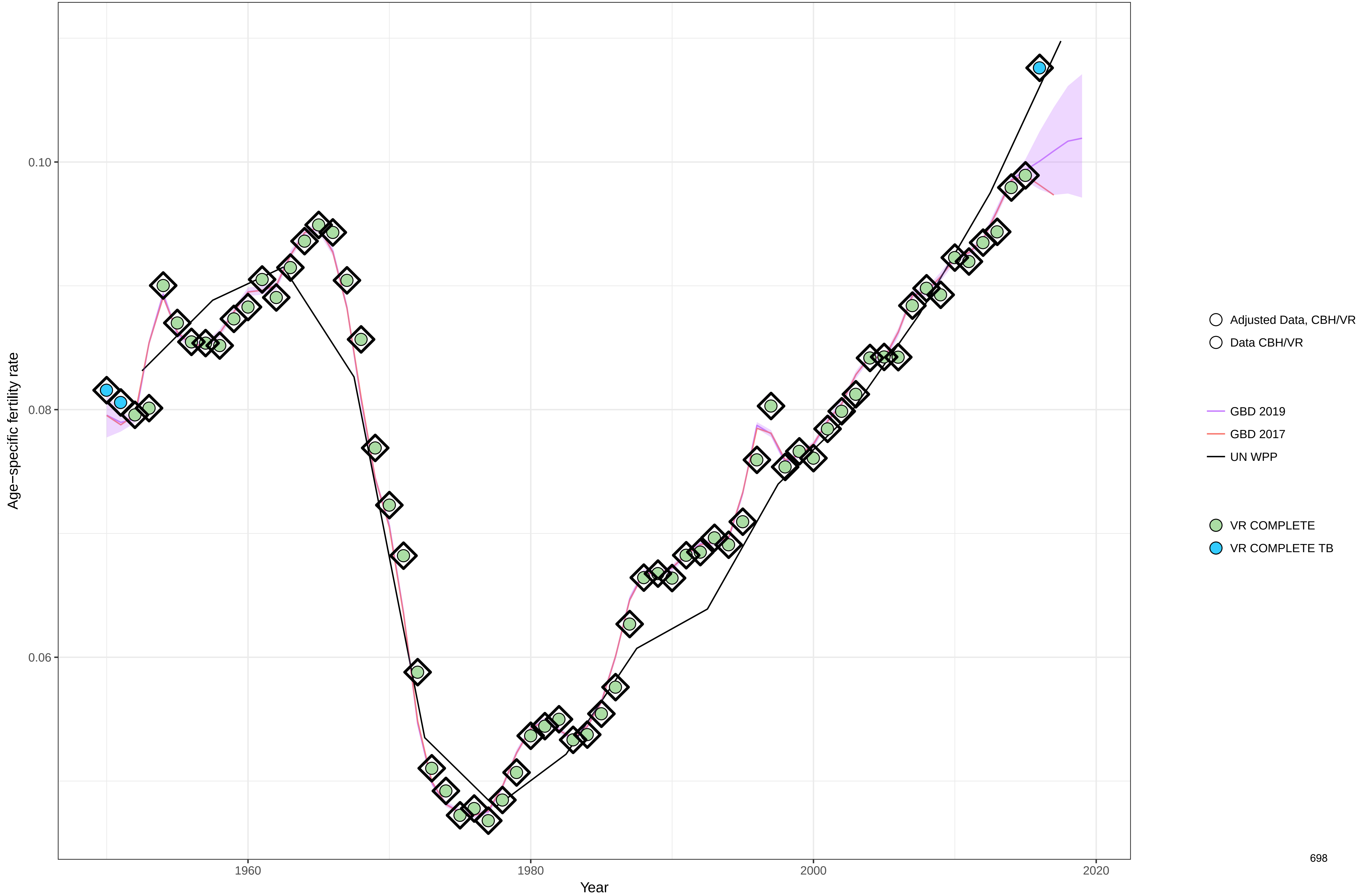

Age−specific fertility fitted trend lines, 1950−2019: Germany, Ages: 35−39
Data points enclosed with a diamond are reference sources

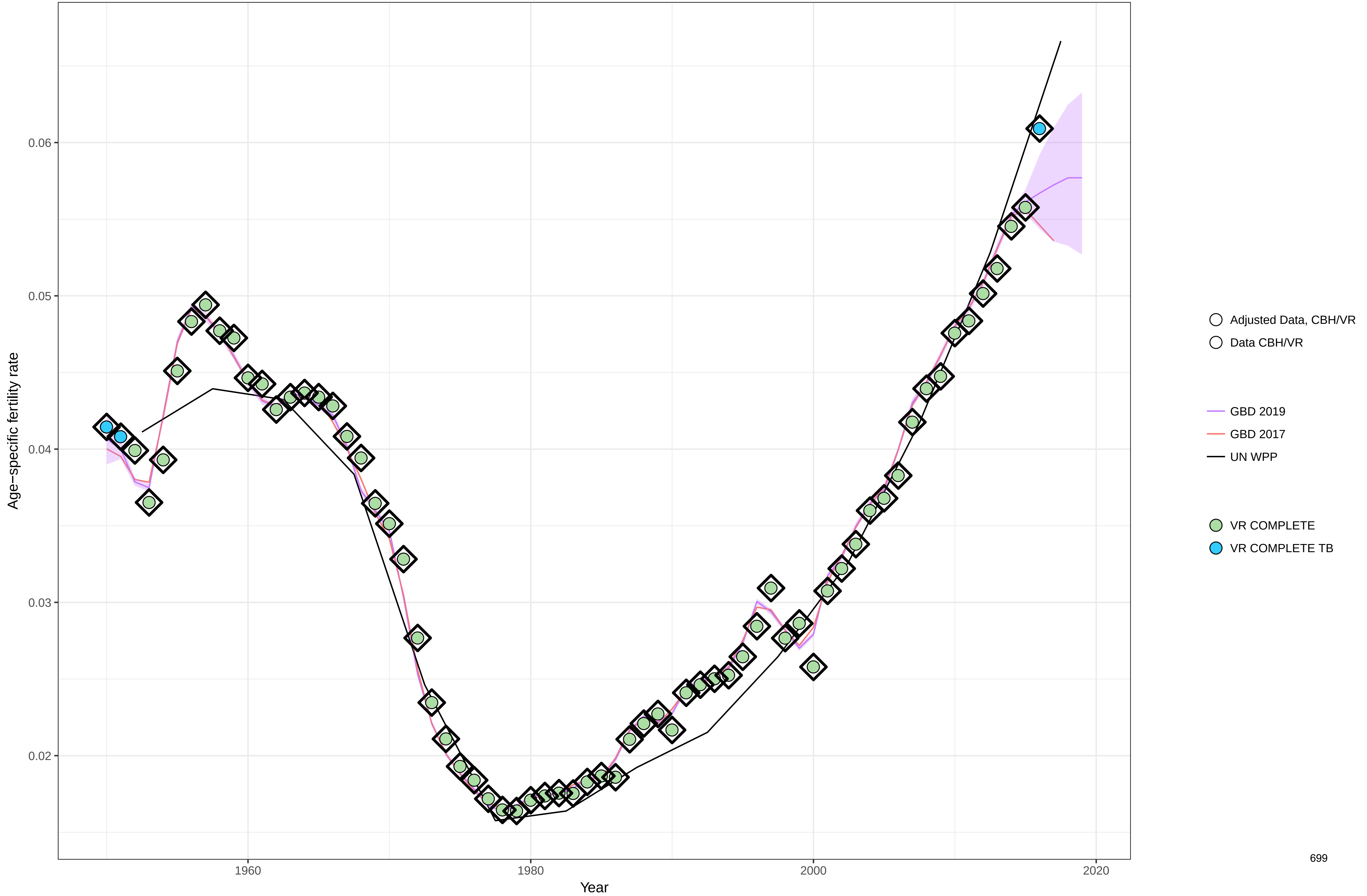

Age−specific fertility fitted trend lines, 1950−2019: Germany, Ages: 40−44
Data points enclosed with a diamond are reference sources

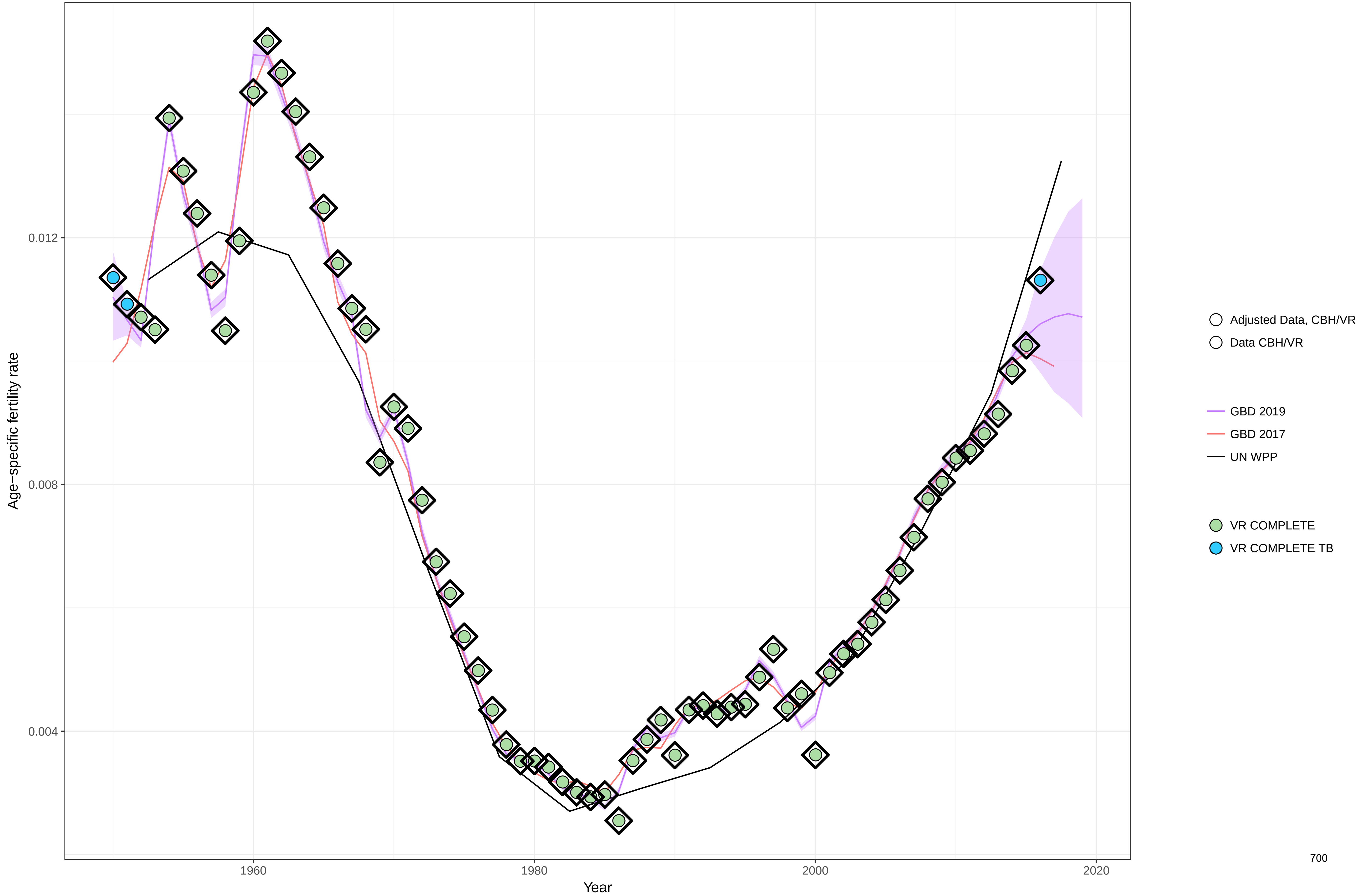

Age−specific fertility fitted trend lines, 1950−2019: Germany, Ages: 45−49
Data points enclosed with a diamond are reference sources

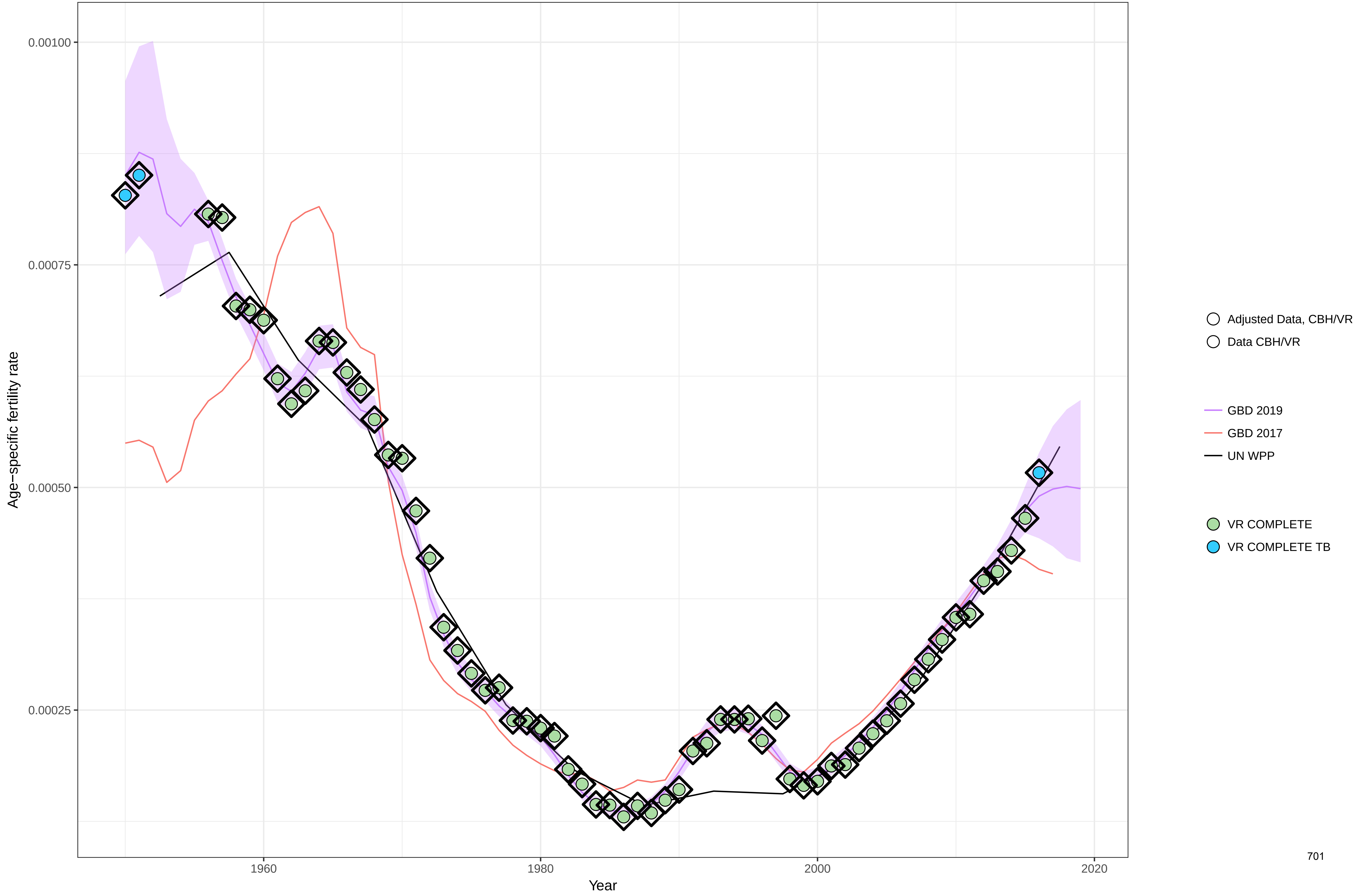

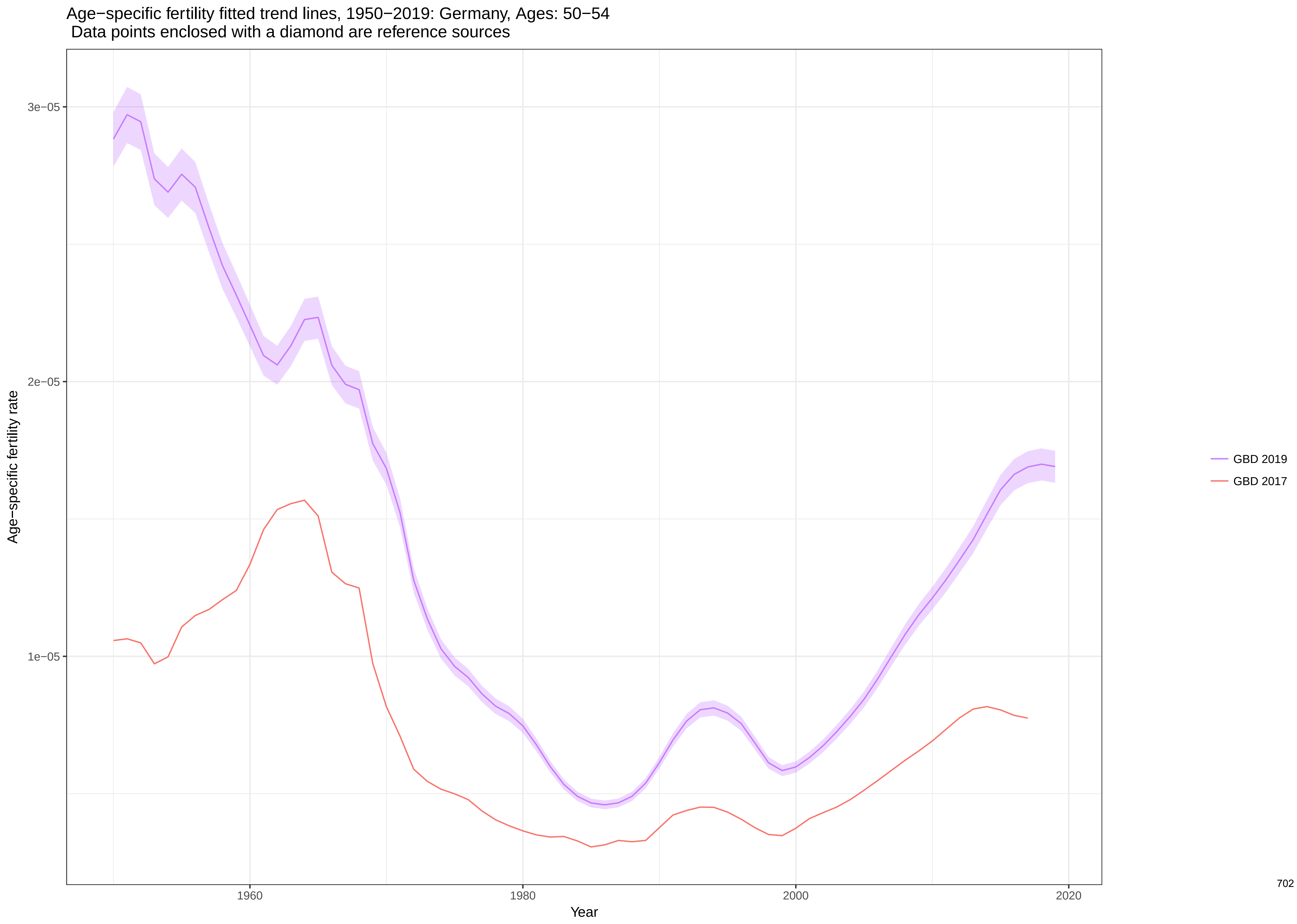
Age−specific fertility fitted trend lines, 1950−2019: Germany, Ages: 50−54
Data points enclosed with a diamond are reference sources
3e−05
2e−05
1e−05
1960
1980
2000
2020
Year
Age−specific fertility rate
GBD 2019
GBD 2017

Total fertility rate fitted trend lines, 1950−2019: Germany TFR
Data points enclosed with a diamond are reference sources

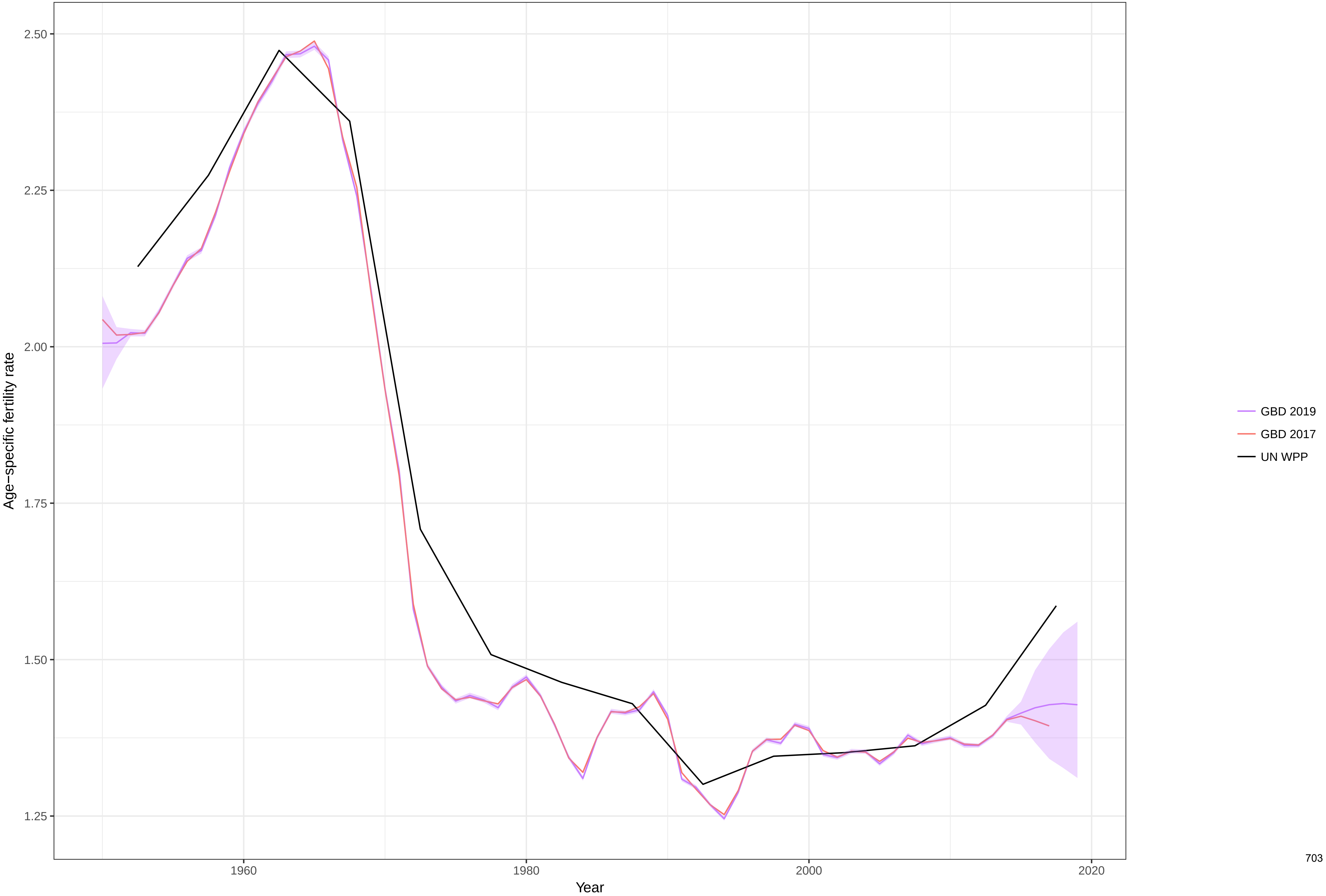

Age−specific fertility fitted trend lines, 1950−2019: Djibouti, Ages: 10−14
Data points enclosed with a diamond are reference sources

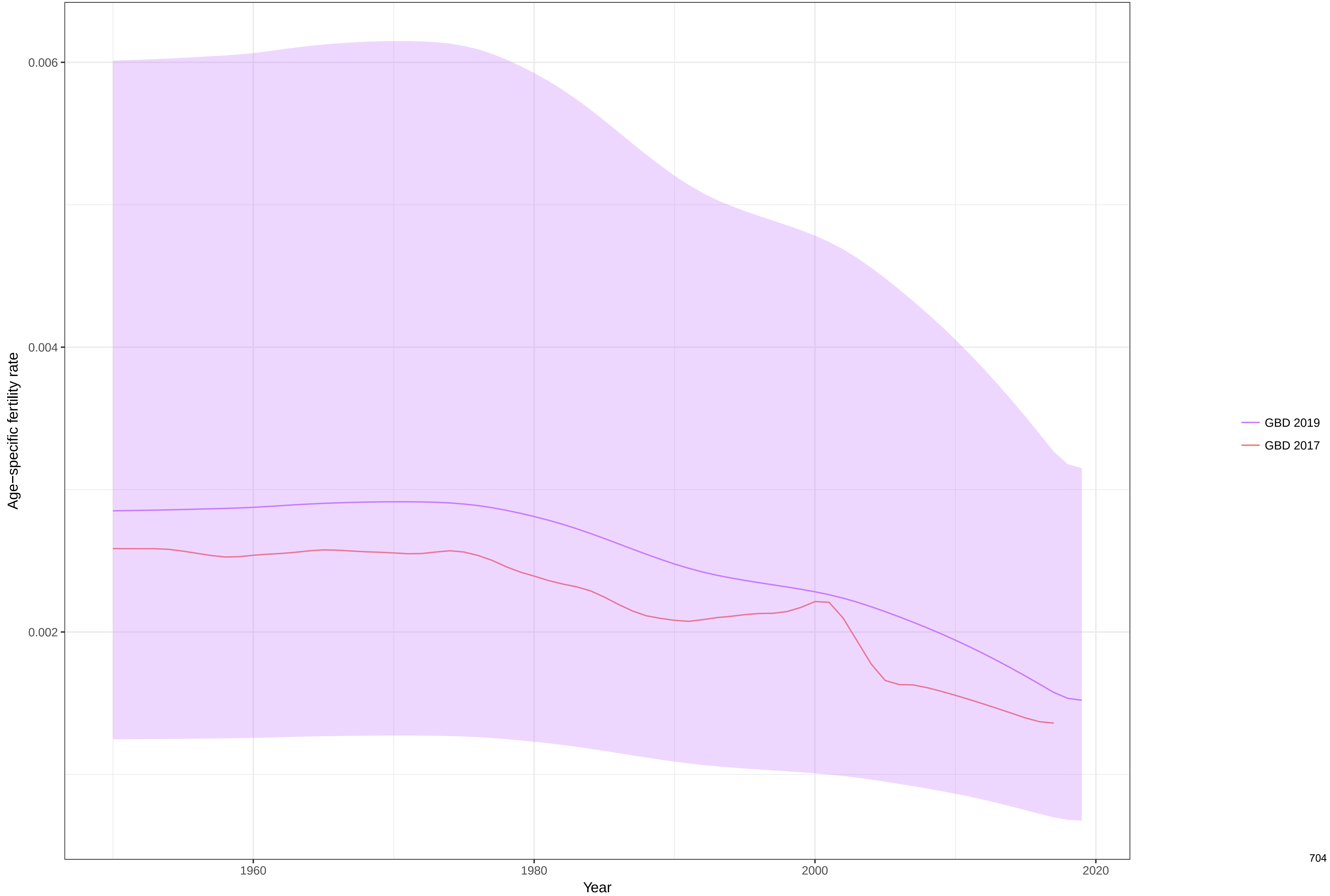

Age−specific fertility fitted trend lines, 1950−2019: Djibouti, Ages: 15−19
Data points enclosed with a diamond are reference sources

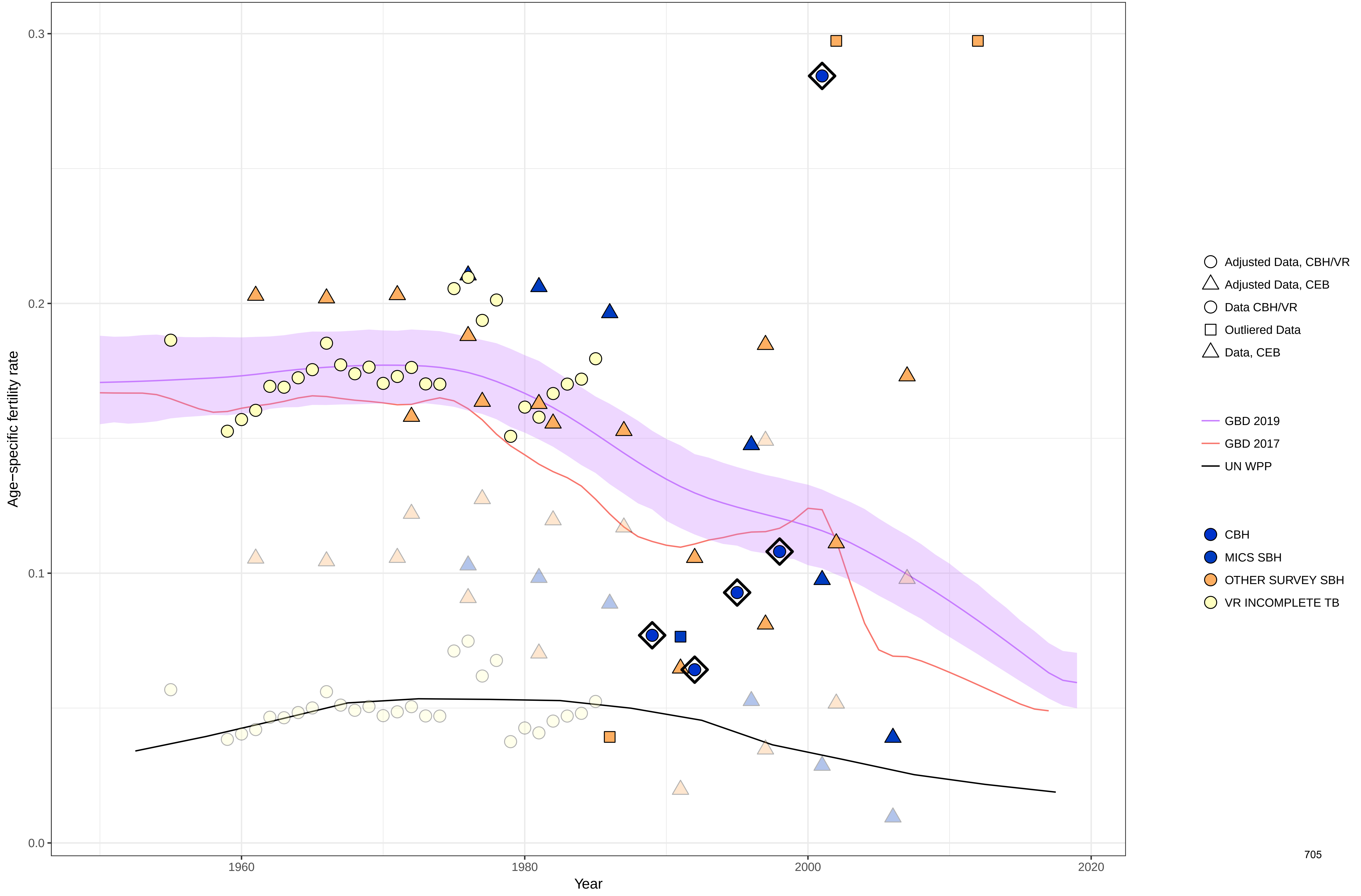

Age−specific fertility fitted trend lines, 1950−2019: Djibouti, Ages: 20−24
Data points enclosed with a diamond are reference sources

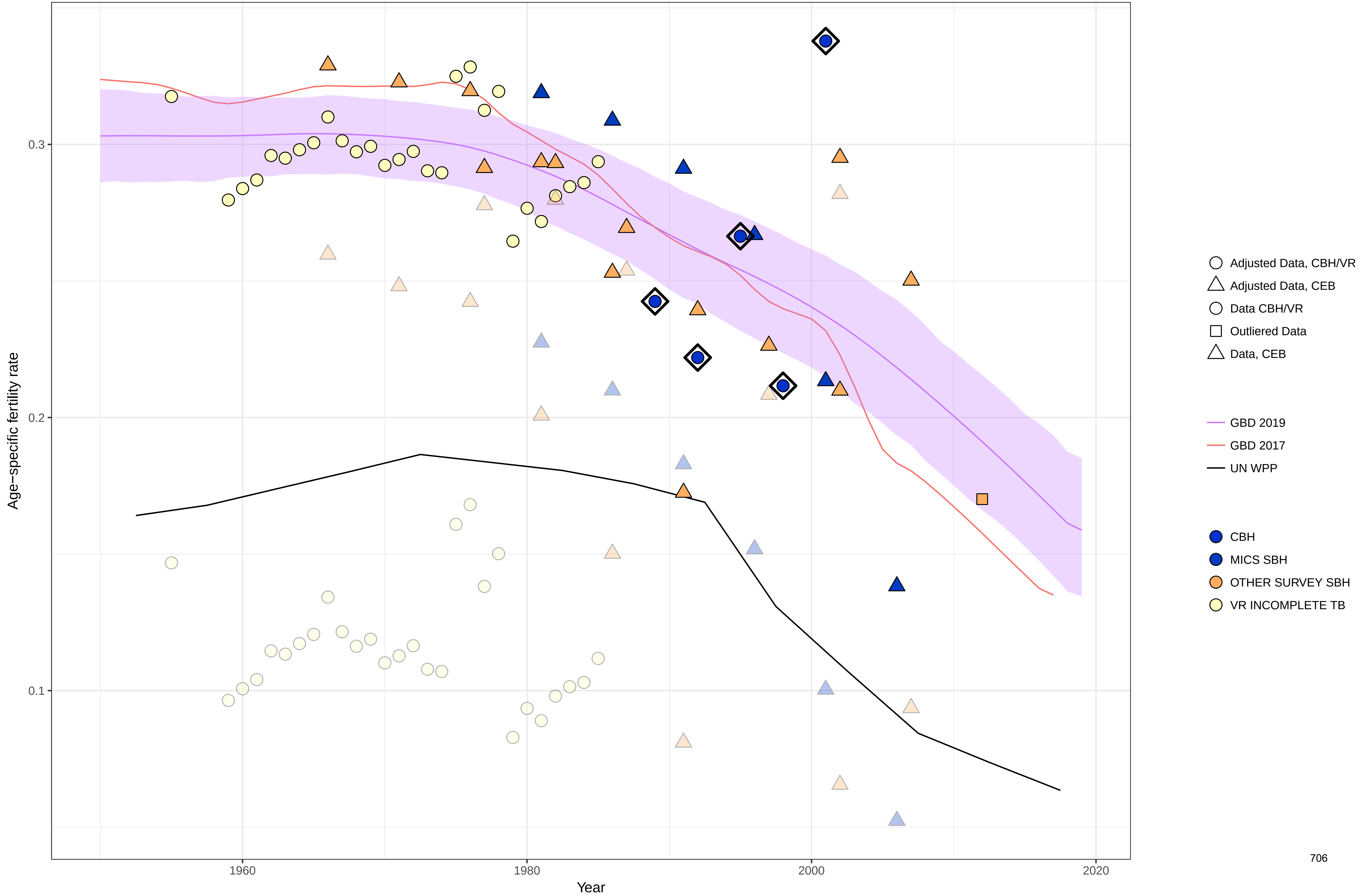

Age−specific fertility fitted trend lines, 1950−2019: Djibouti, Ages: 25−29
Data points enclosed with a diamond are reference sources

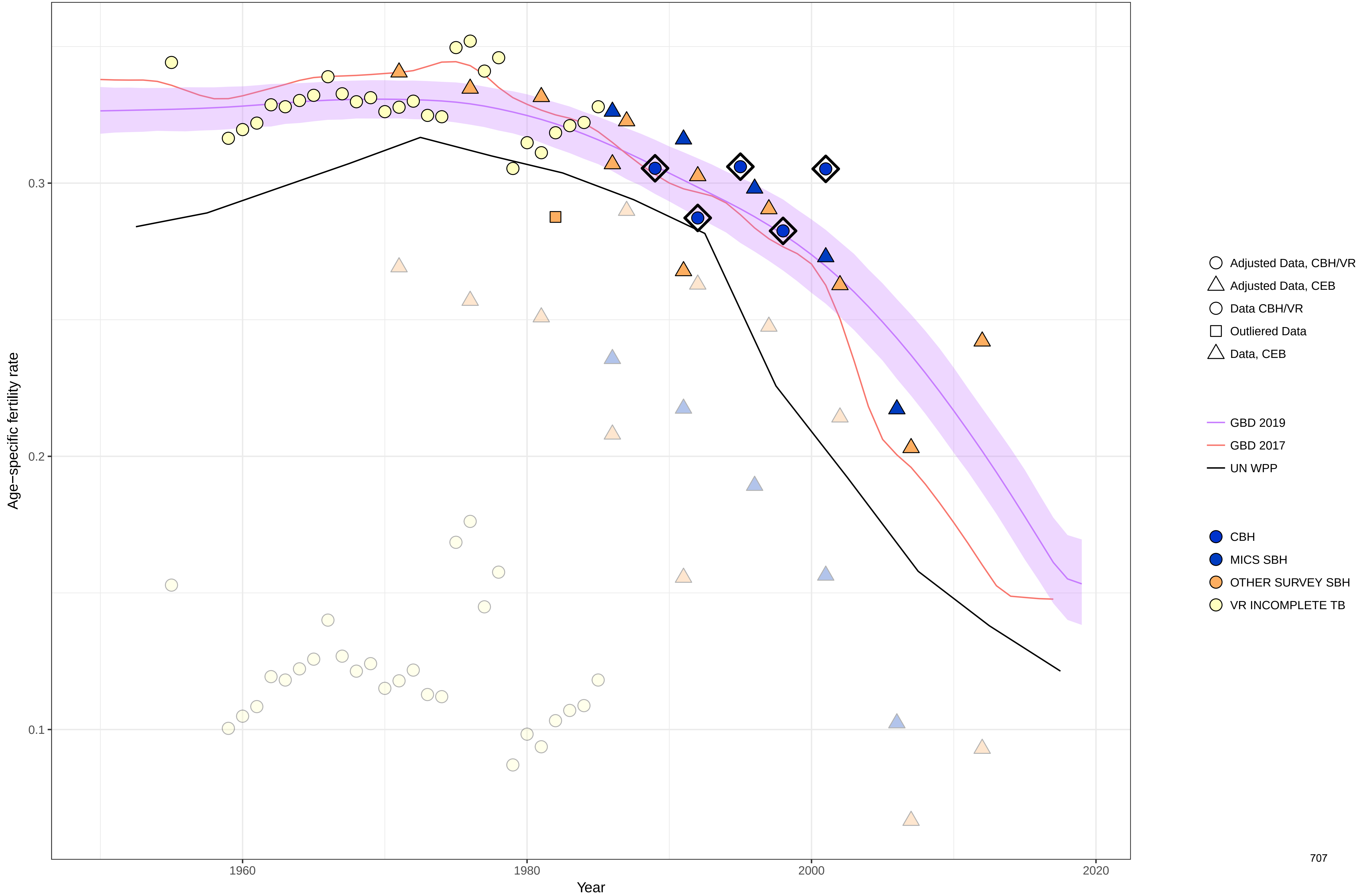

Age−specific fertility fitted trend lines, 1950−2019: Djibouti, Ages: 30−34
Data points enclosed with a diamond are reference sources

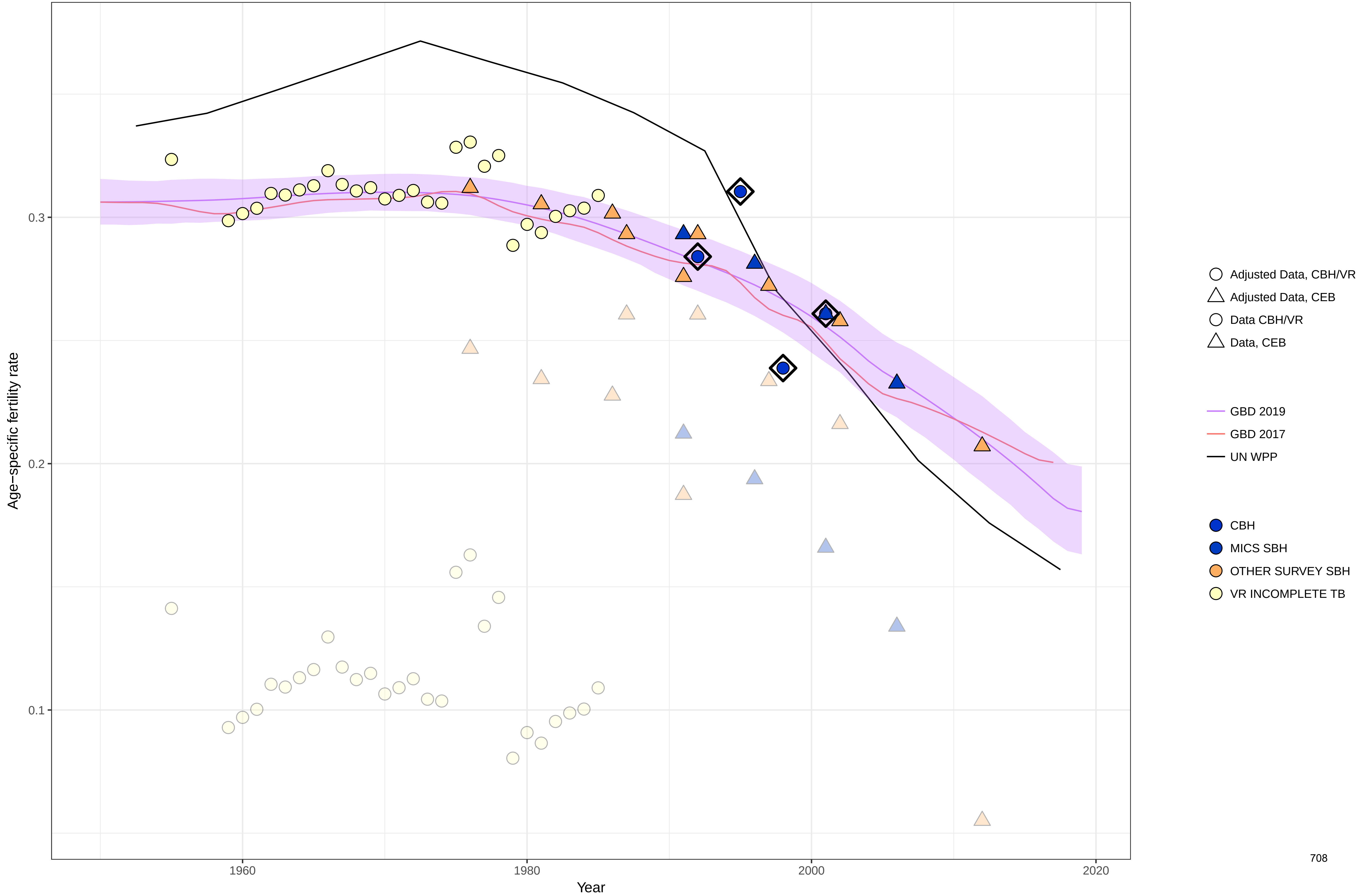

Age−specific fertility fitted trend lines, 1950−2019: Djibouti, Ages: 35−39
Data points enclosed with a diamond are reference sources

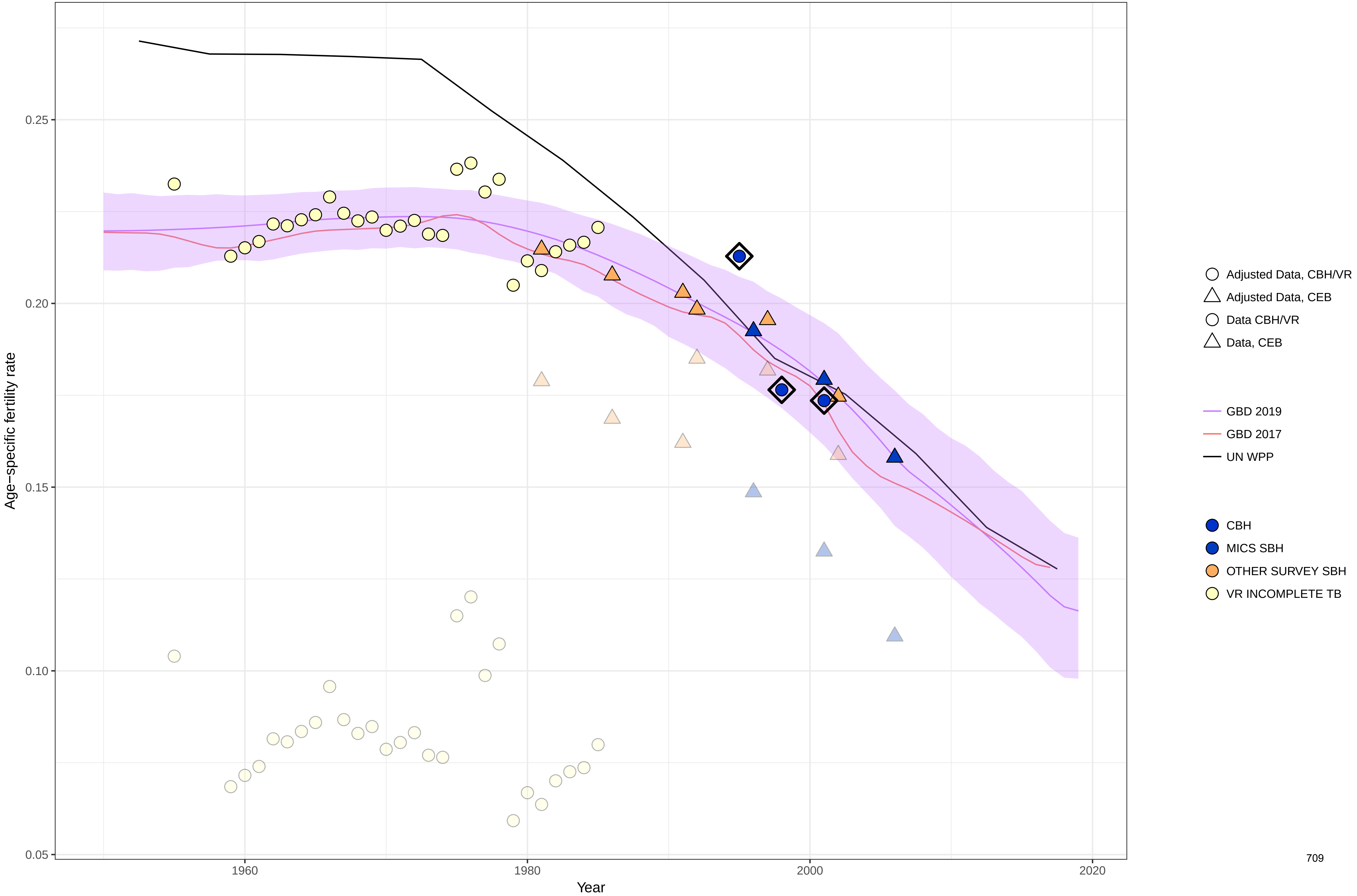

Age−specific fertility fitted trend lines, 1950−2019: Djibouti, Ages: 40−44
Data points enclosed with a diamond are reference sources

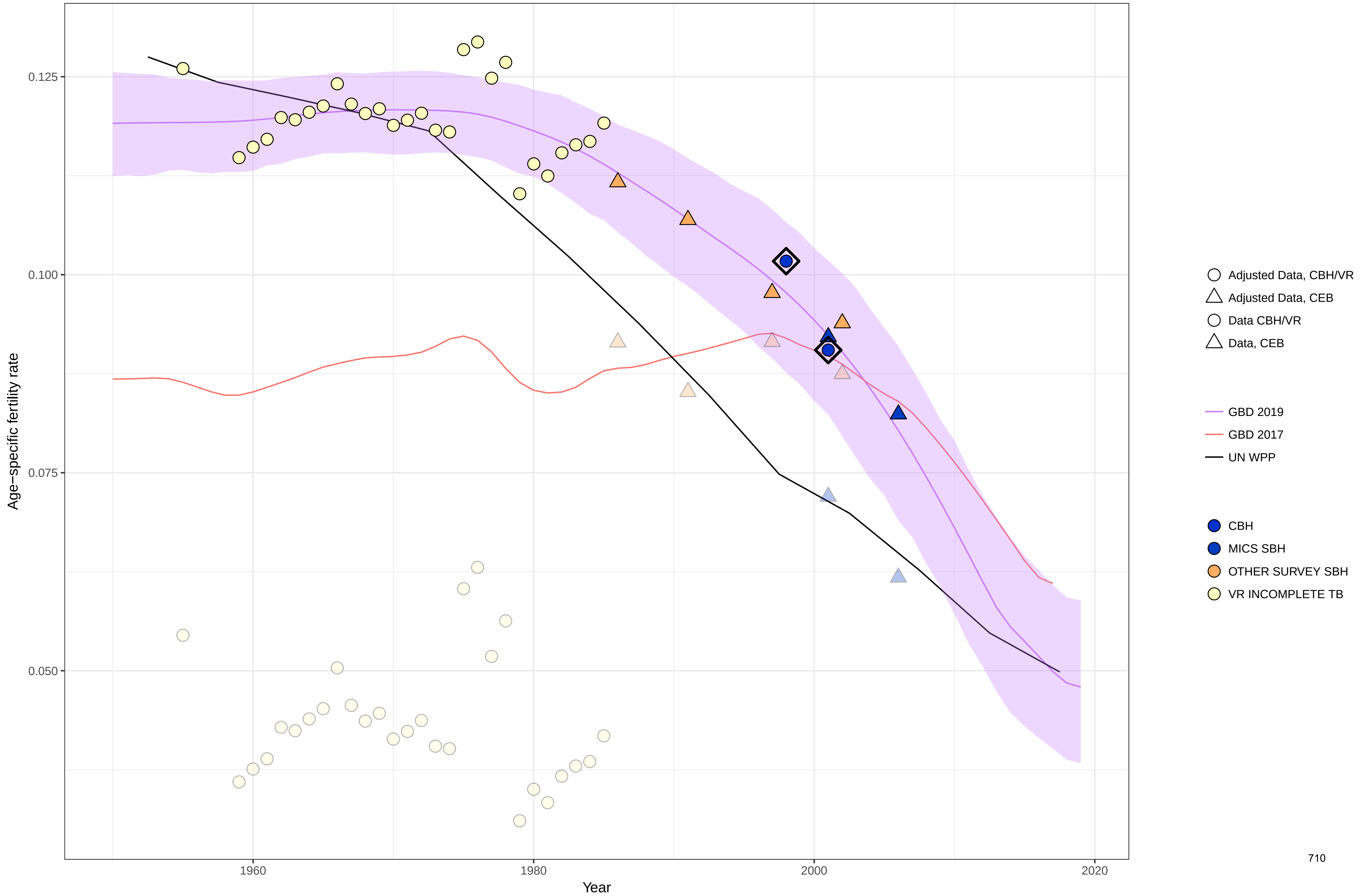

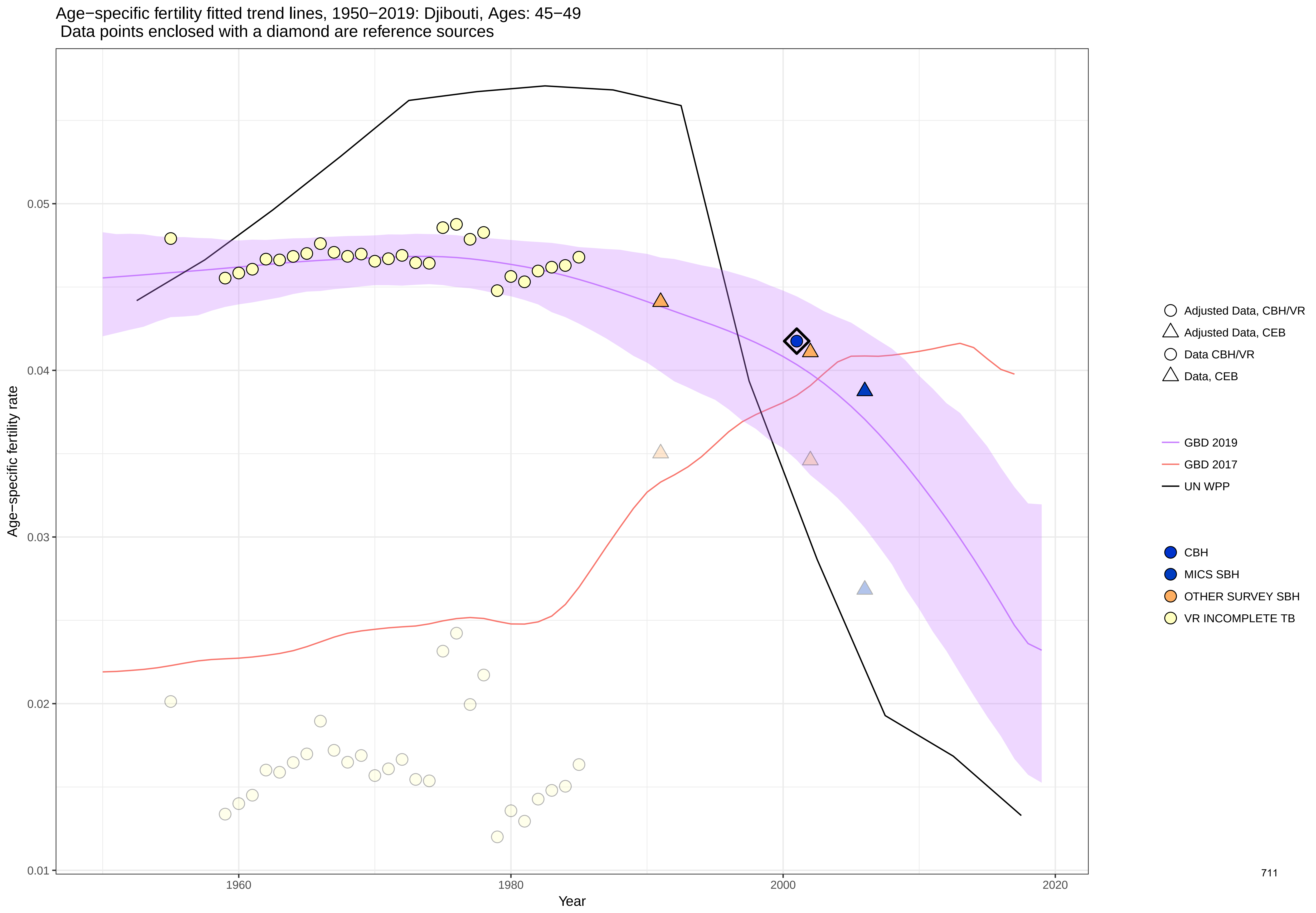
Age−specific fertility fitted trend lines, 1950−2019: Djibouti, Ages: 45−49
Data points enclosed with a diamond are reference sources
Age−specific fertility rate
Year
0.01
0.02
0.03
0.04
0.05
1960
1980
2000
2020
Adjusted Data, CBH/VR
Adjusted Data, CEB
Data CBH/VR
Data, CEB
GBD 2019
GBD 2017
UN WPP
CBH
MICS SBH
OTHER SURVEY SBH
VR INCOMPLETE TB

Age−specific fertility fitted trend lines, 1950−2019: Djibouti, Ages: 50−54
Data points enclosed with a diamond are reference sources

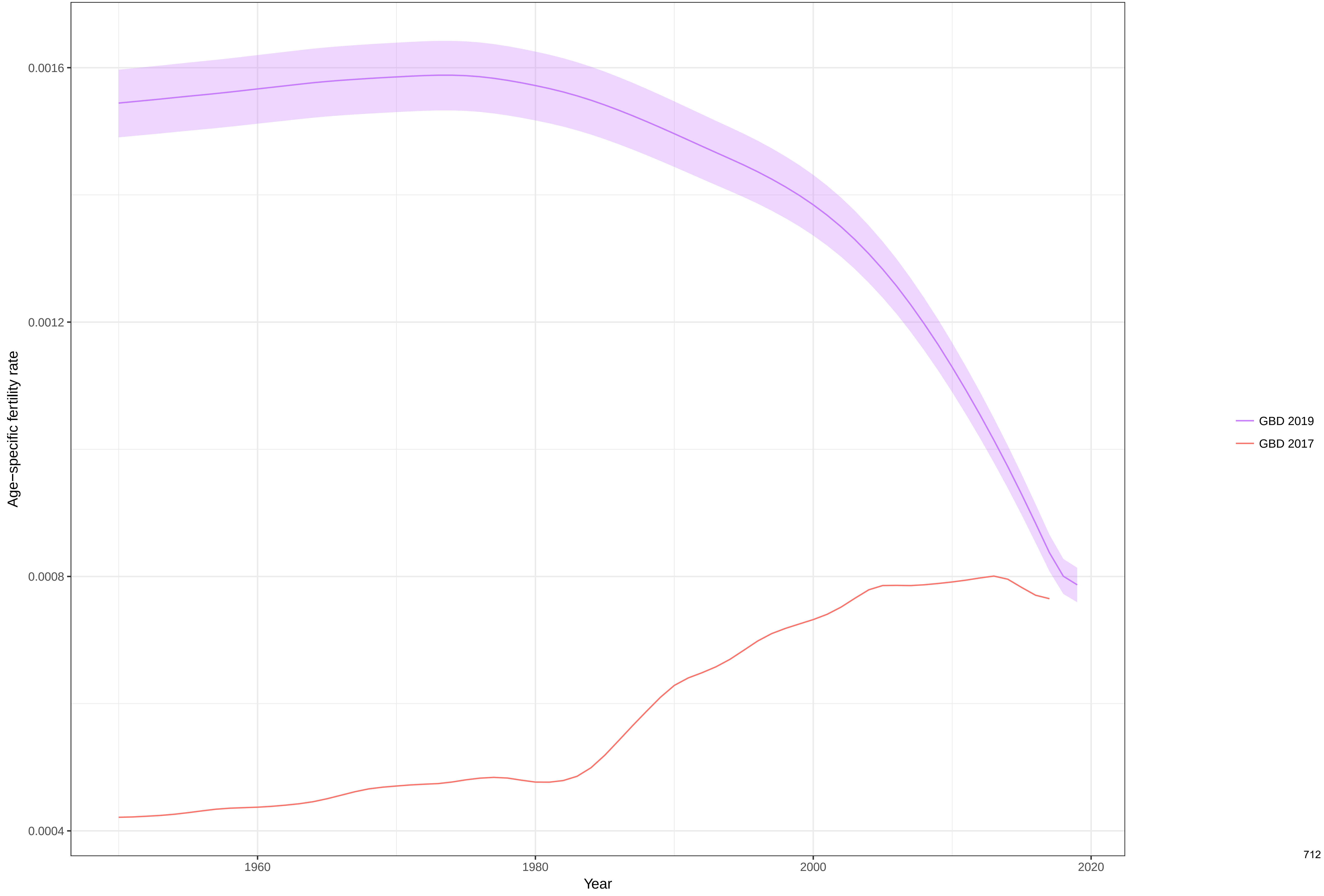

Total fertility rate fitted trend lines, 1950−2019: Djibouti TFR
Data points enclosed with a diamond are reference sources

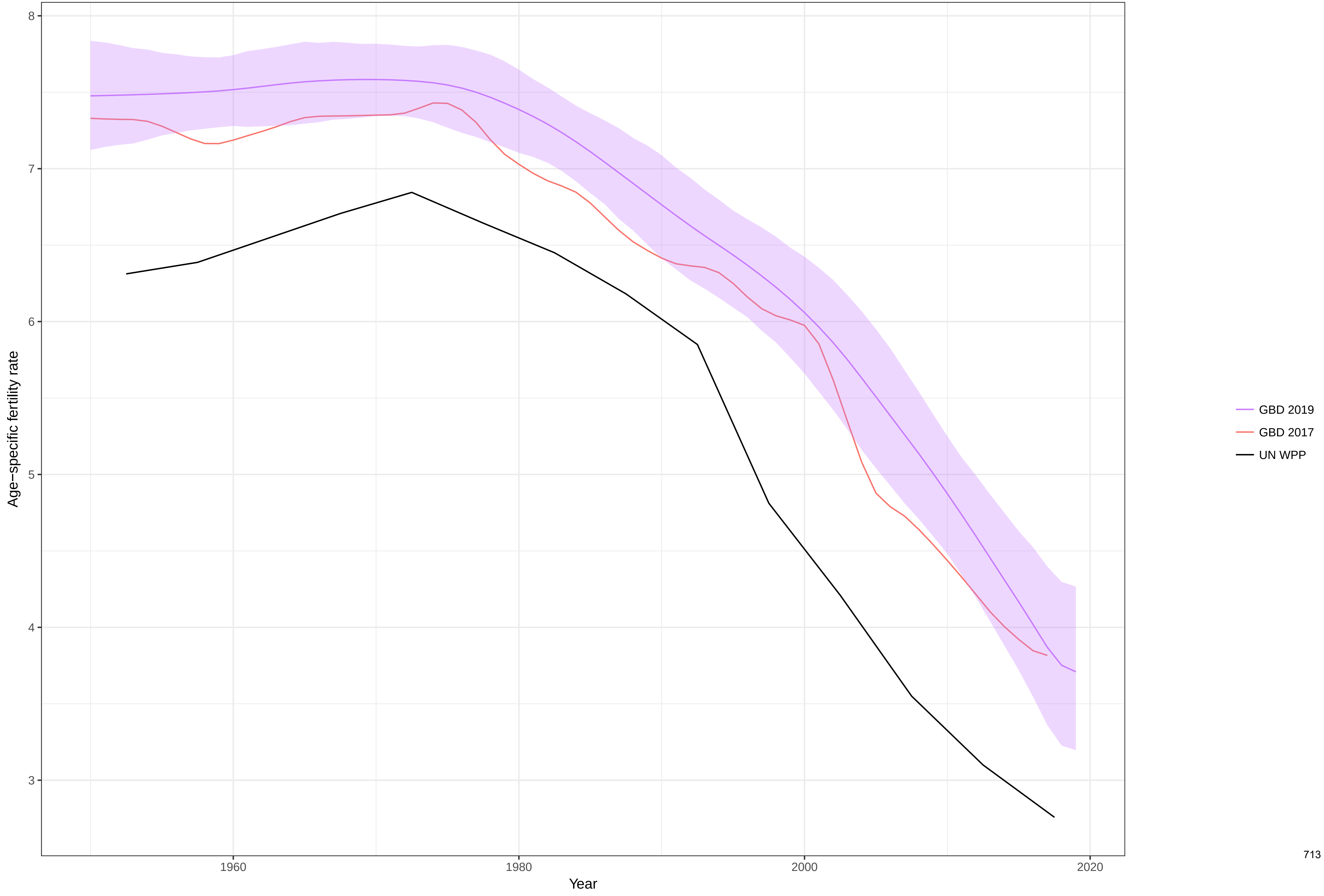

Age−specific fertility fitted trend lines, 1950−2019: Dominica, Ages: 10−14
Data points enclosed with a diamond are reference sources

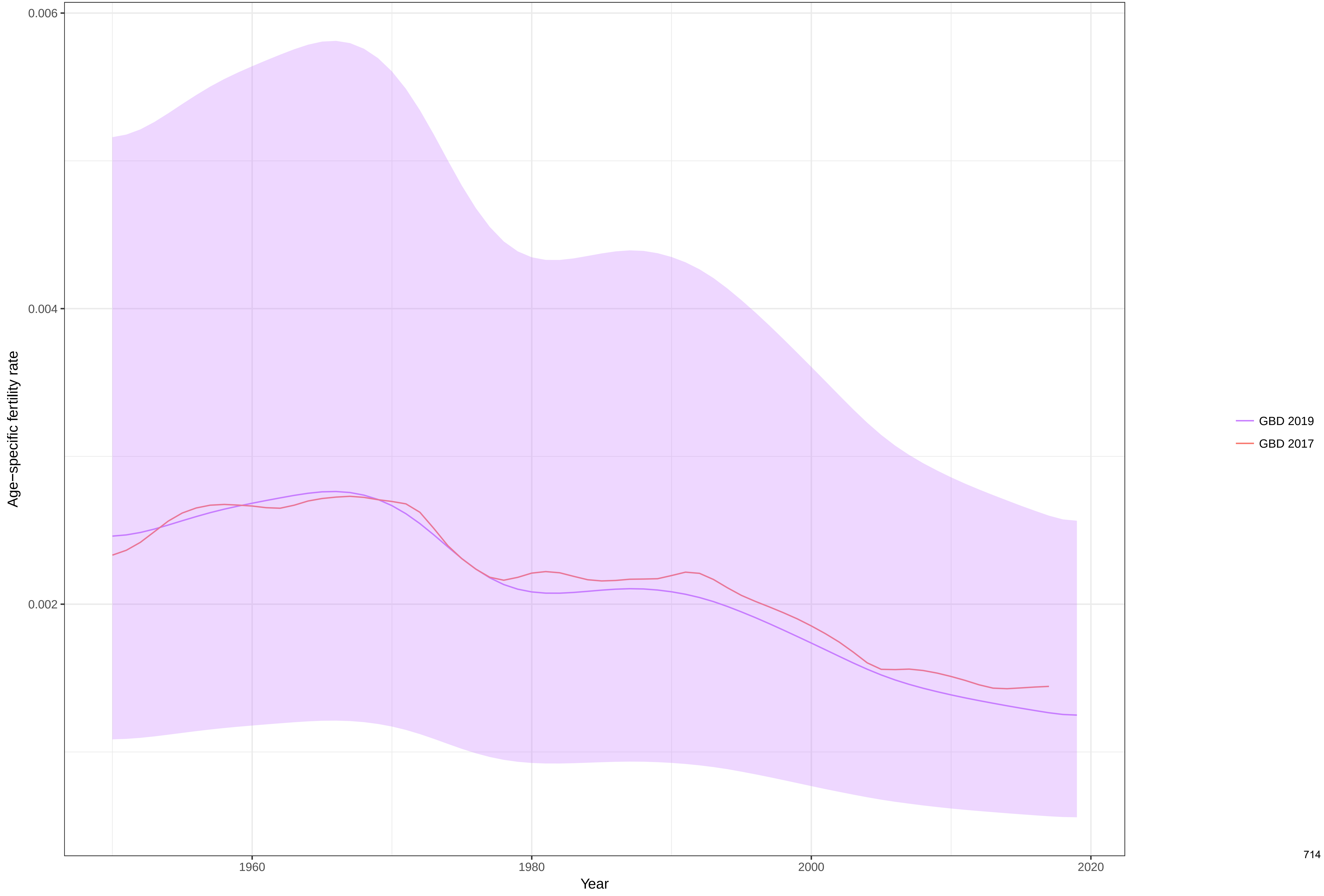

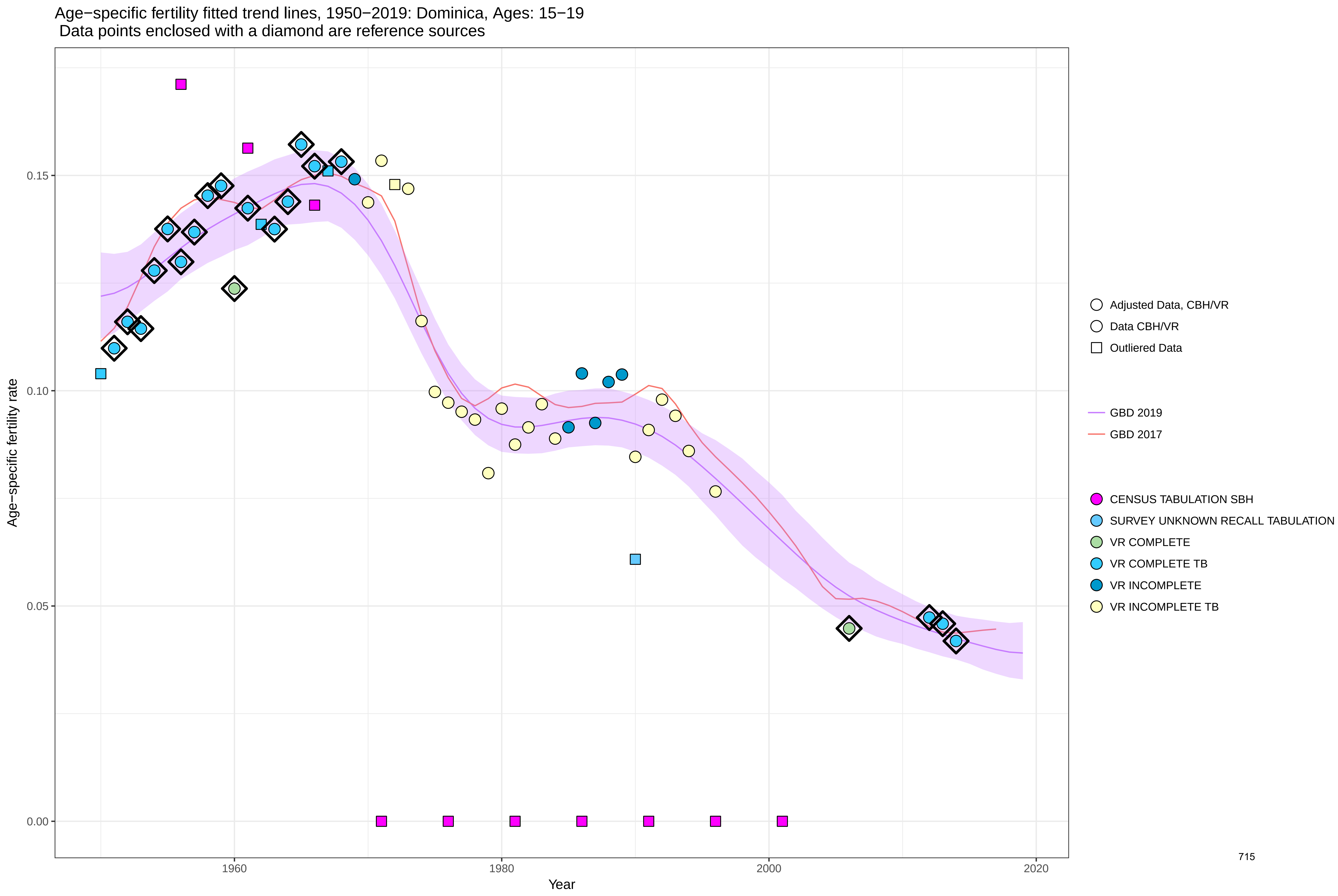

Age−specific fertility fitted trend lines, 1950−2019: Dominica, Ages: 15−19
Data points enclosed with a diamond are reference sources
Age−specific fertility rate
Year
0.00
0.05
0.10
0.15
1960
1980
2000
2020
Adjusted Data, CBH/VR
Data CBH/VR
Outliered Data
GBD 2019
GBD 2017
CENSUS TABULATION SBH
SURVEY UNKNOWN RECALL TABULATION
VR COMPLETE
VR COMPLETE TB
VR INCOMPLETE
VR INCOMPLETE TB

Age−specific fertility fitted trend lines, 1950−2019: Dominica, Ages: 20−24
Data points enclosed with a diamond are reference sources

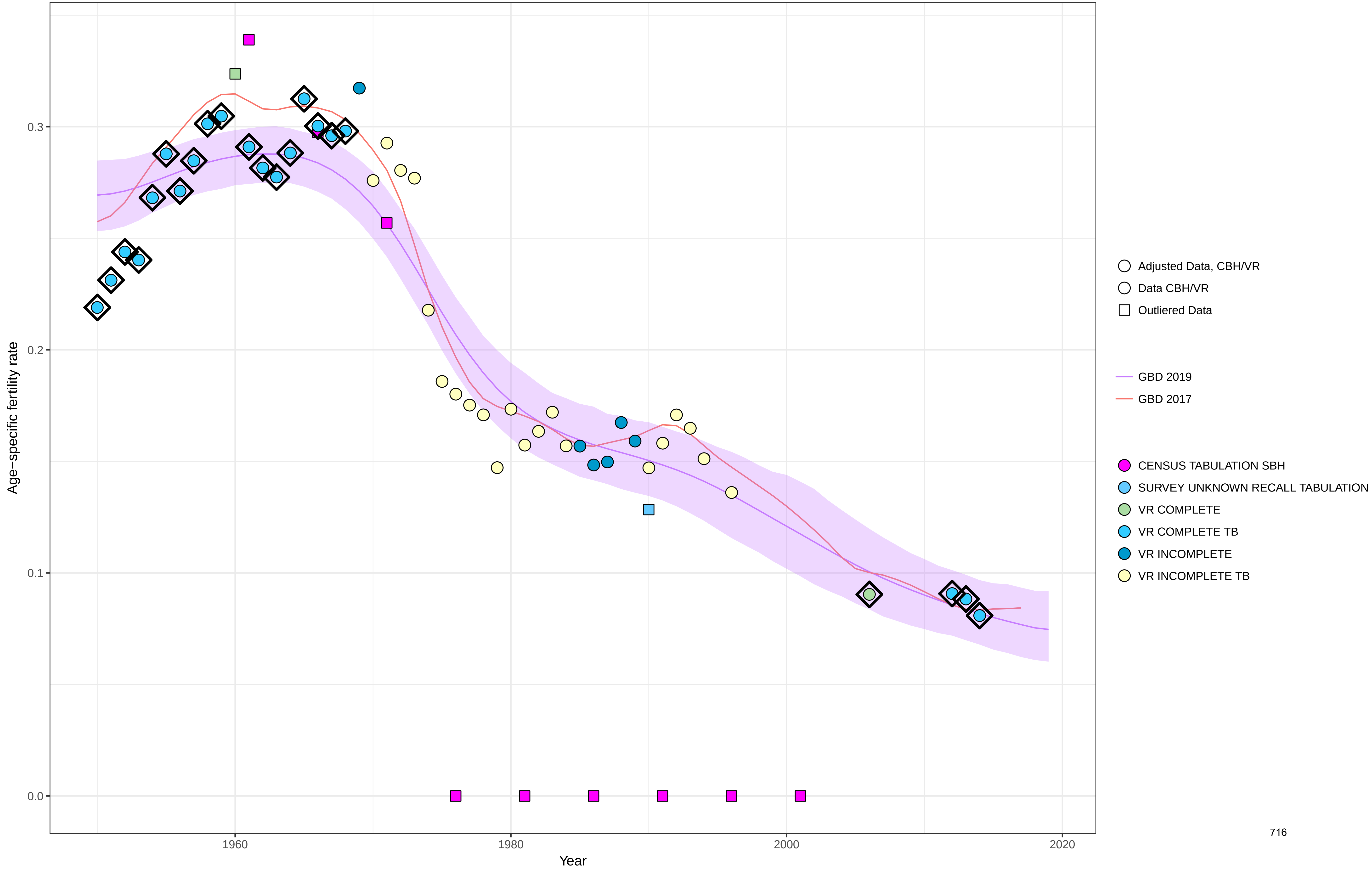

Age−specific fertility fitted trend lines, 1950−2019: Dominica, Ages: 25−29
Data points enclosed with a diamond are reference sources

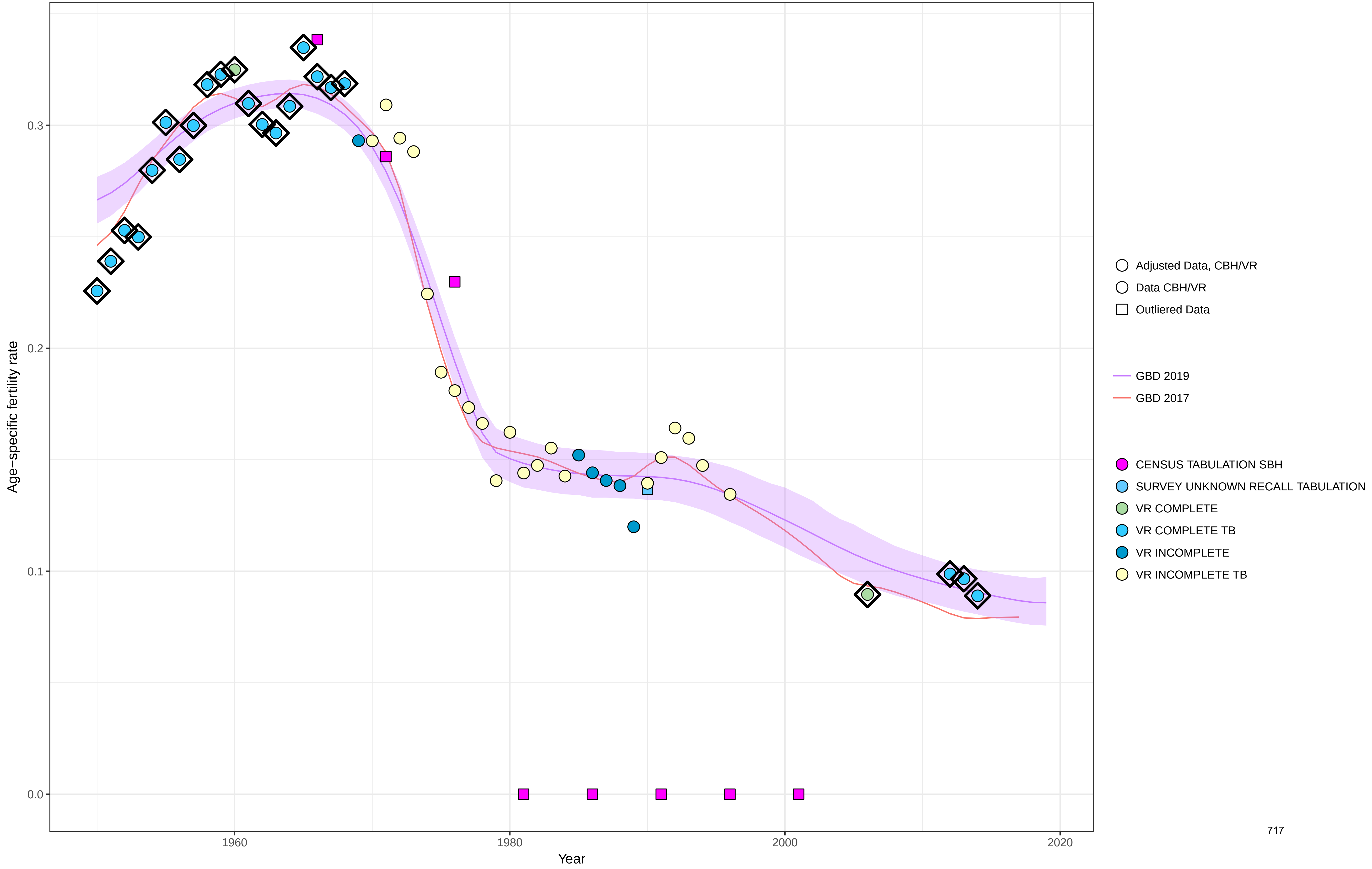

Age−specific fertility fitted trend lines, 1950−2019: Dominica, Ages: 30−34
Data points enclosed with a diamond are reference sources

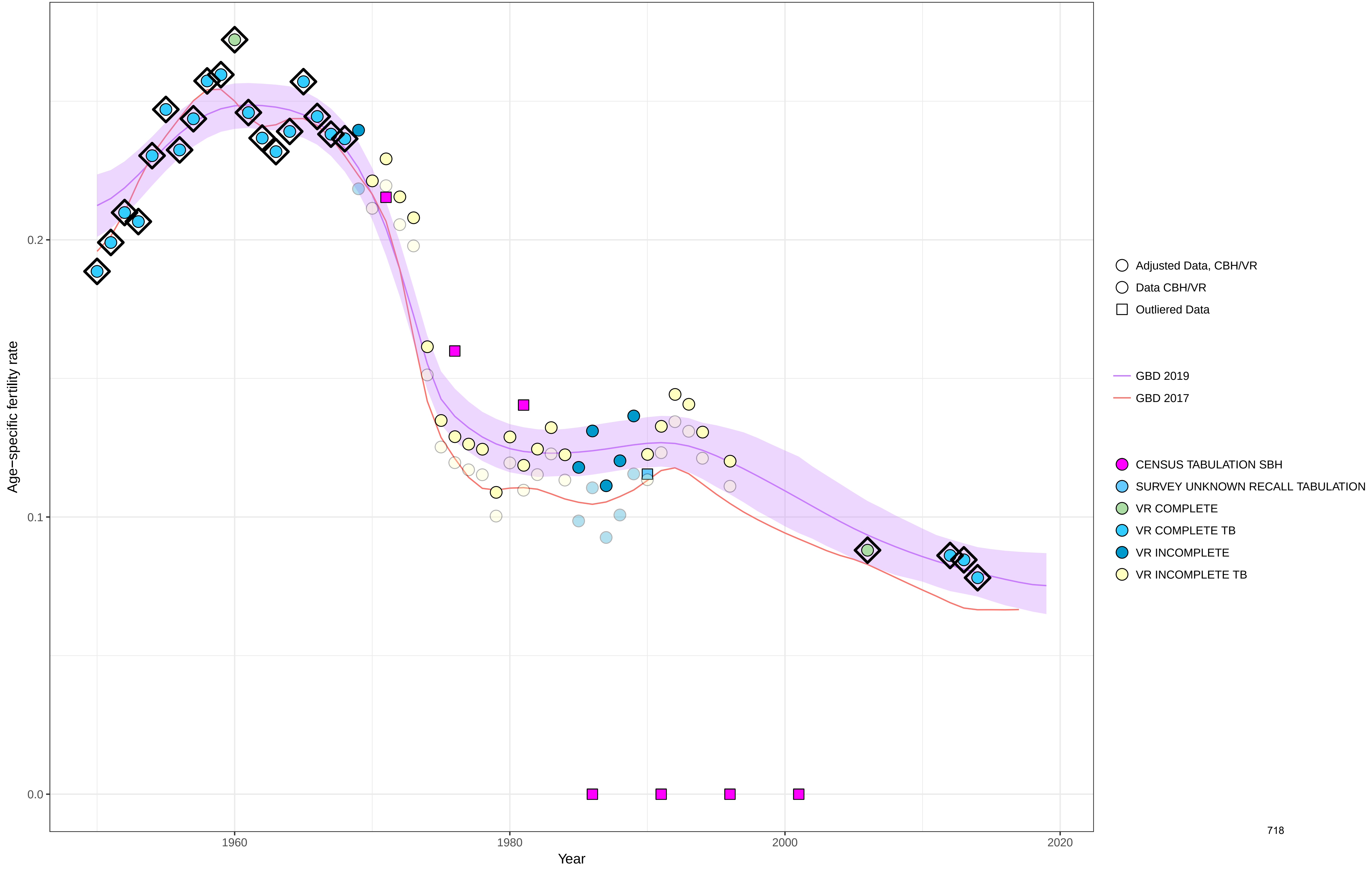

Age−specific fertility fitted trend lines, 1950−2019: Dominica, Ages: 35−39
Data points enclosed with a diamond are reference sources

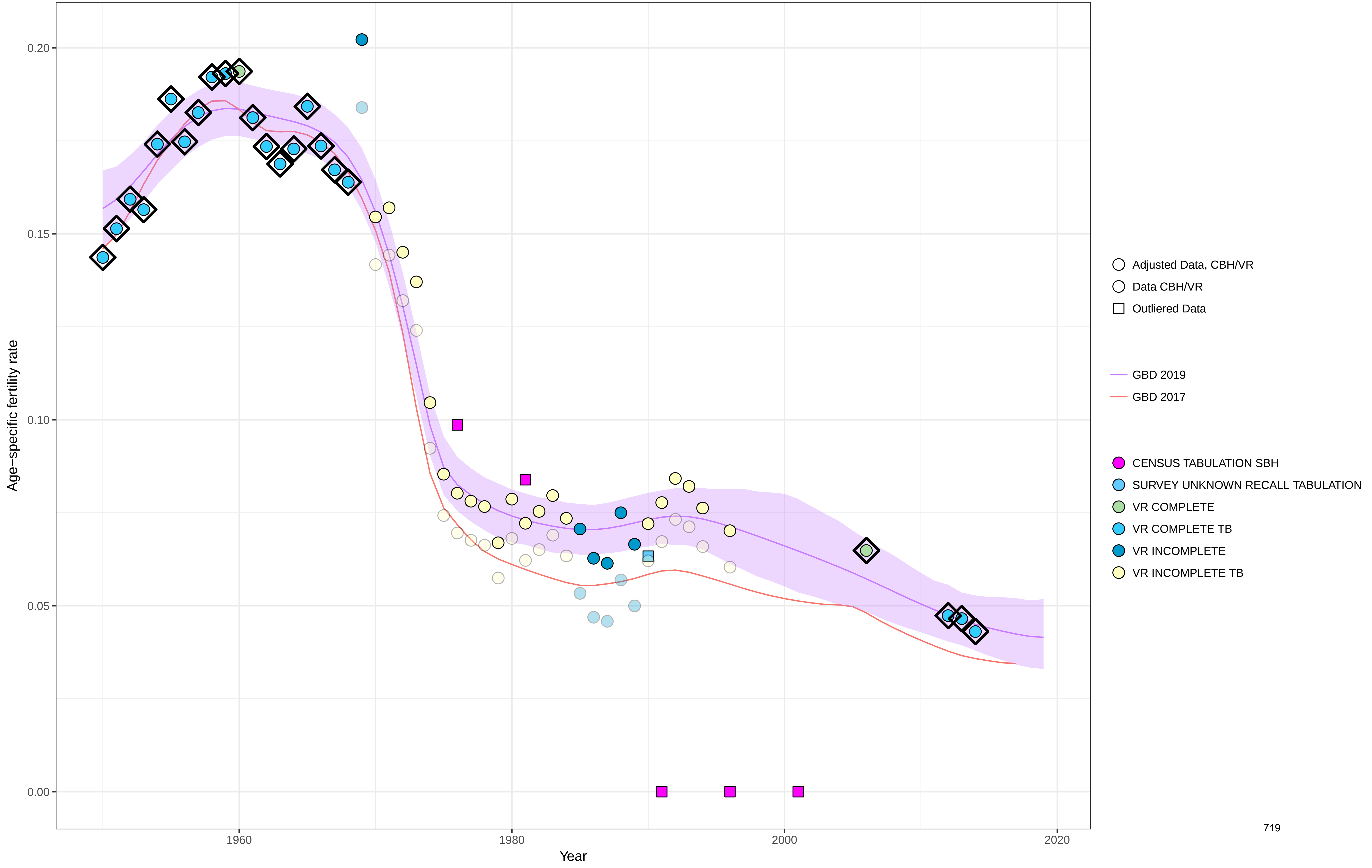

Age−specific fertility fitted trend lines, 1950−2019: Dominica, Ages: 40−44
Data points enclosed with a diamond are reference sources

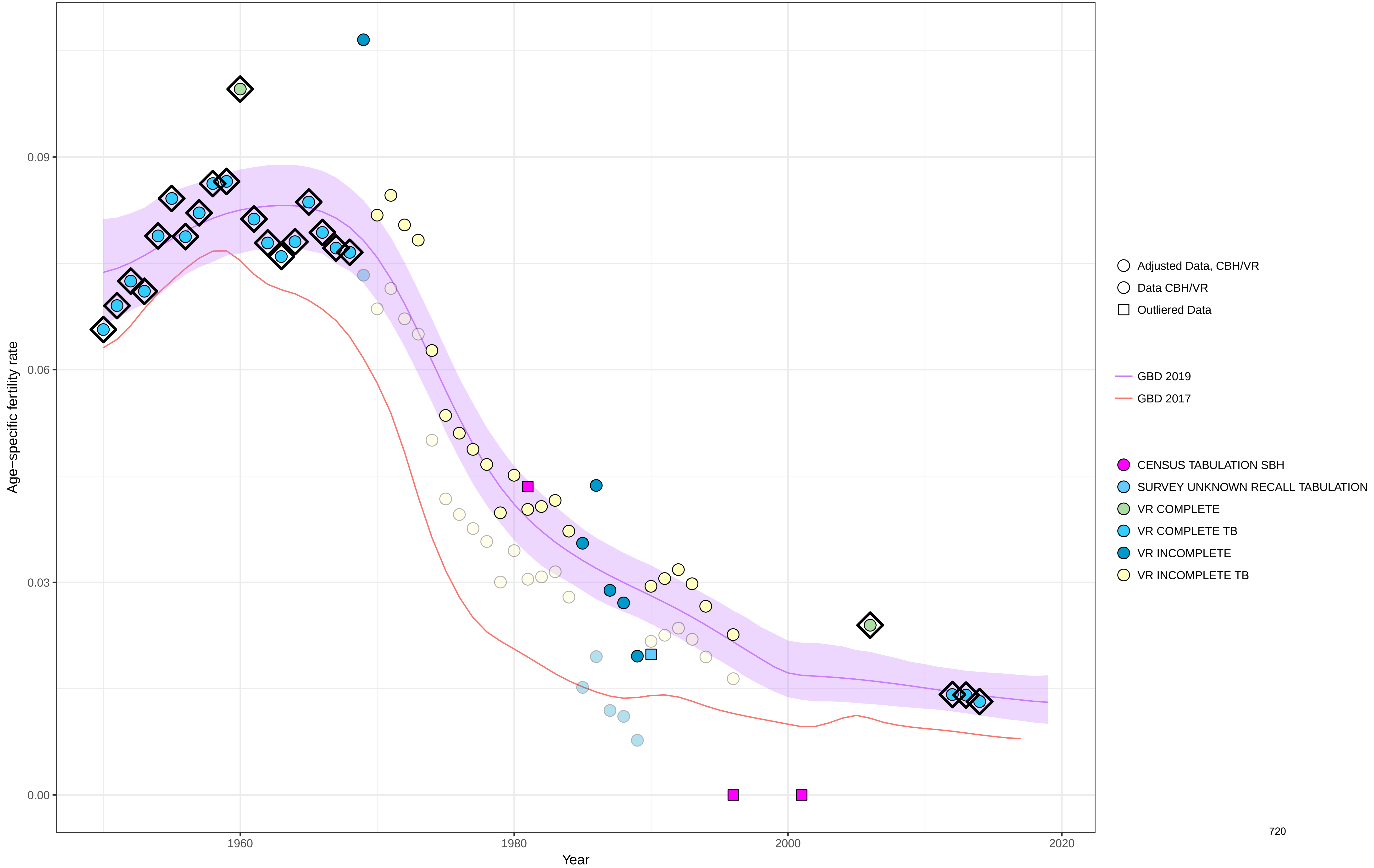

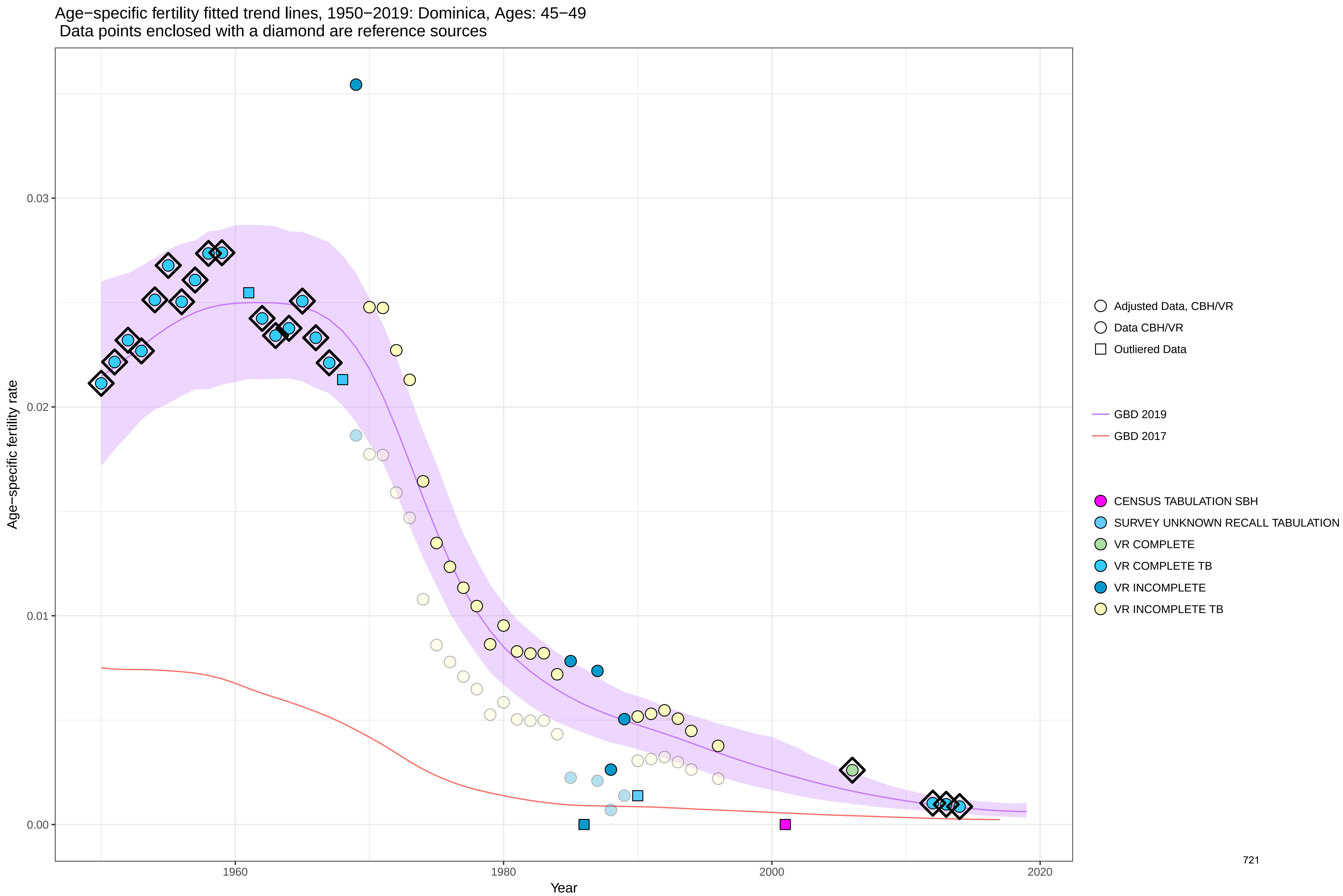
Age−specific fertility fitted trend lines, 1950−2019: Dominica, Ages: 45−49
Data points enclosed with a diamond are reference sources
Age−specific fertility rate
0.00
0.01
0.02
0.03
1960
1980
2000
2020
Year
Adjusted Data, CBH/VR
Data CBH/VR
Outliered Data
GBD 2019
GBD 2017
CENSUS TABULATION SBH
SURVEY UNKNOWN RECALL TABULATION
VR COMPLETE
VR COMPLETE TB
VR INCOMPLETE
VR INCOMPLETE TB

Age−specific fertility fitted trend lines, 1950−2019: Dominica, Ages: 50−54
Data points enclosed with a diamond are reference sources

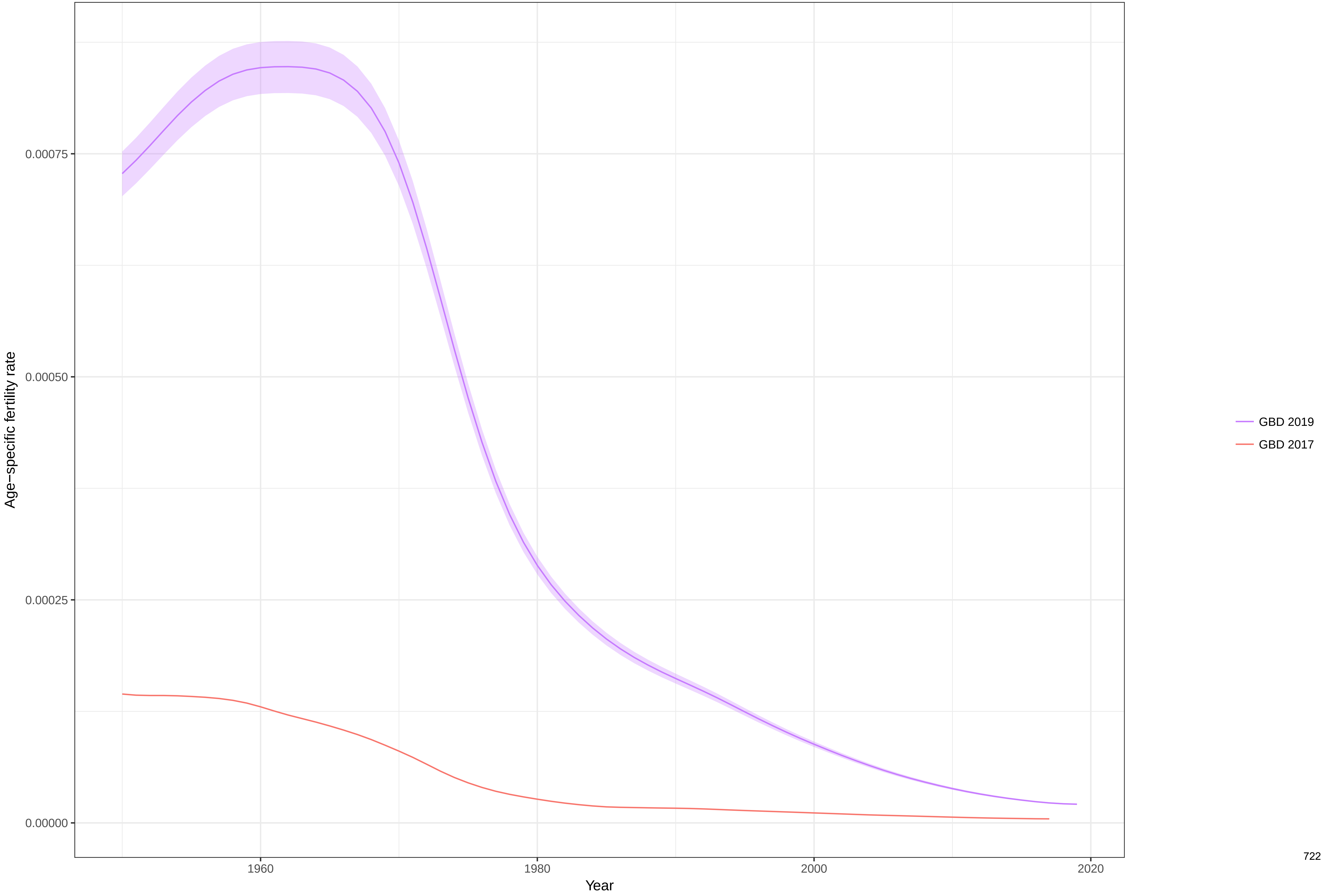

Total fertility rate fitted trend lines, 1950−2019: Dominica TFR
Data points enclosed with a diamond are reference sources

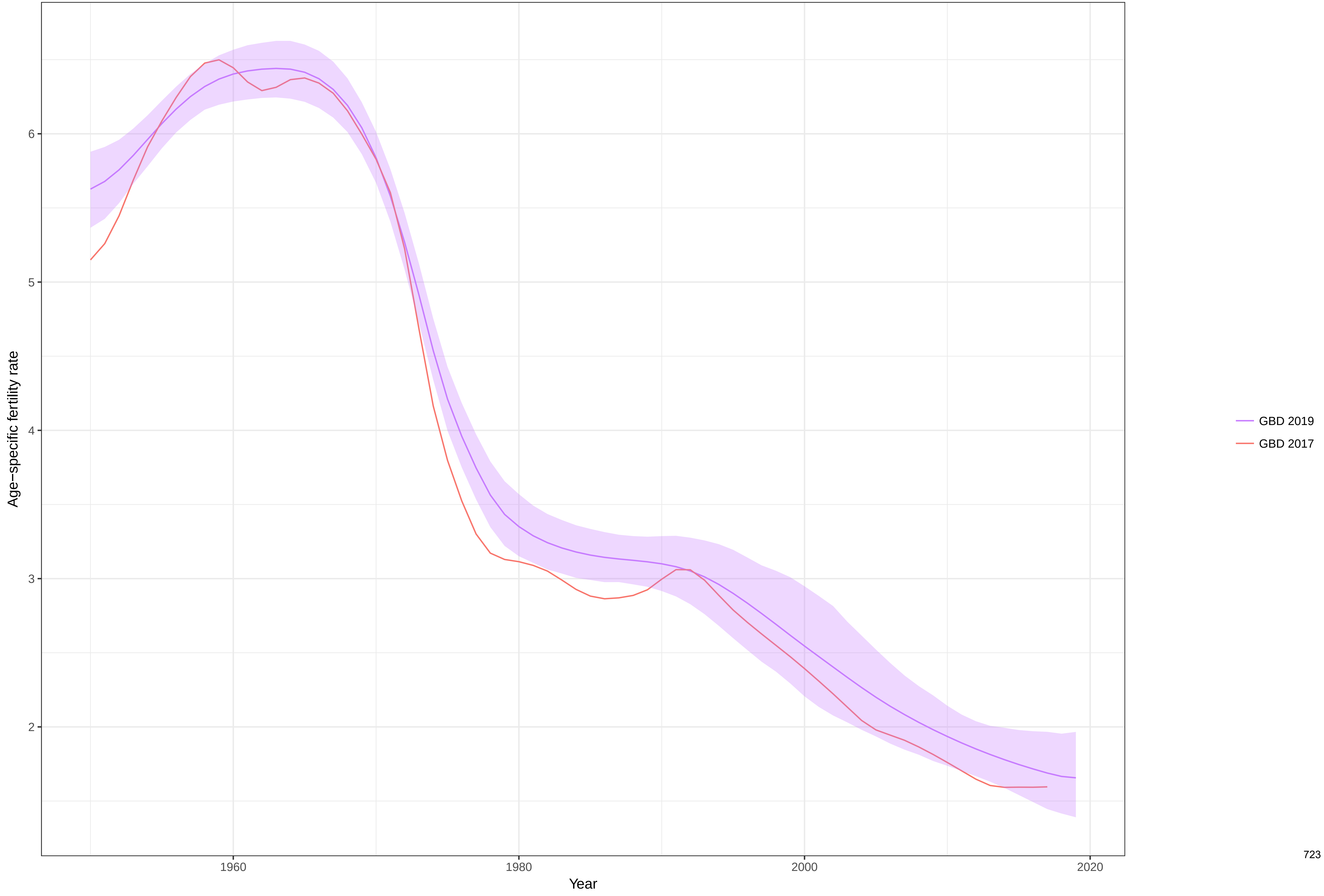

Age−specific fertility fitted trend lines, 1950−2019: Denmark, Ages: 10−14
Data points enclosed with a diamond are reference sources

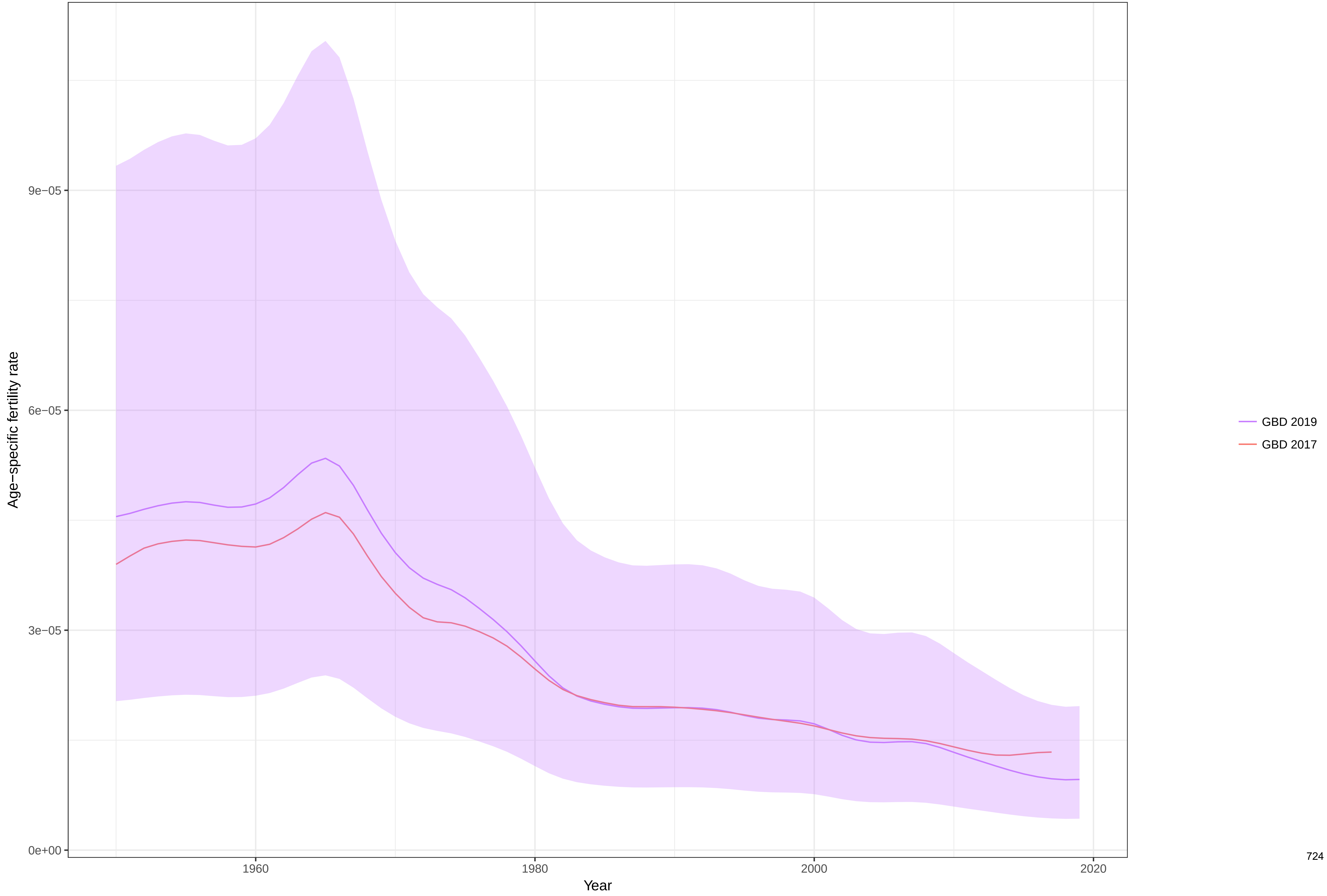

Age−specific fertility fitted trend lines, 1950−2019: Denmark, Ages: 15−19
Data points enclosed with a diamond are reference sources

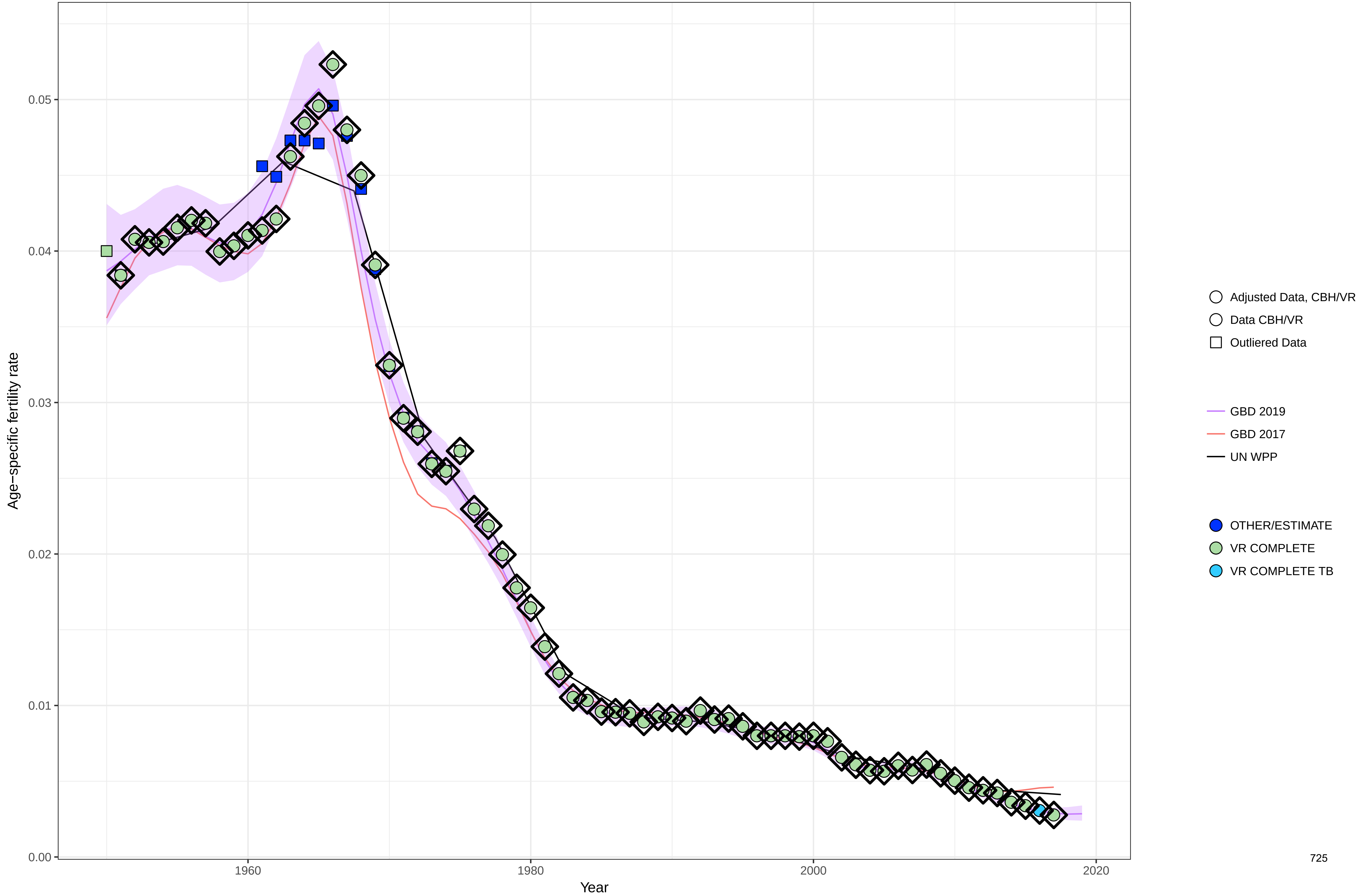

Age−specific fertility fitted trend lines, 1950−2019: Denmark, Ages: 20−24
Data points enclosed with a diamond are reference sources

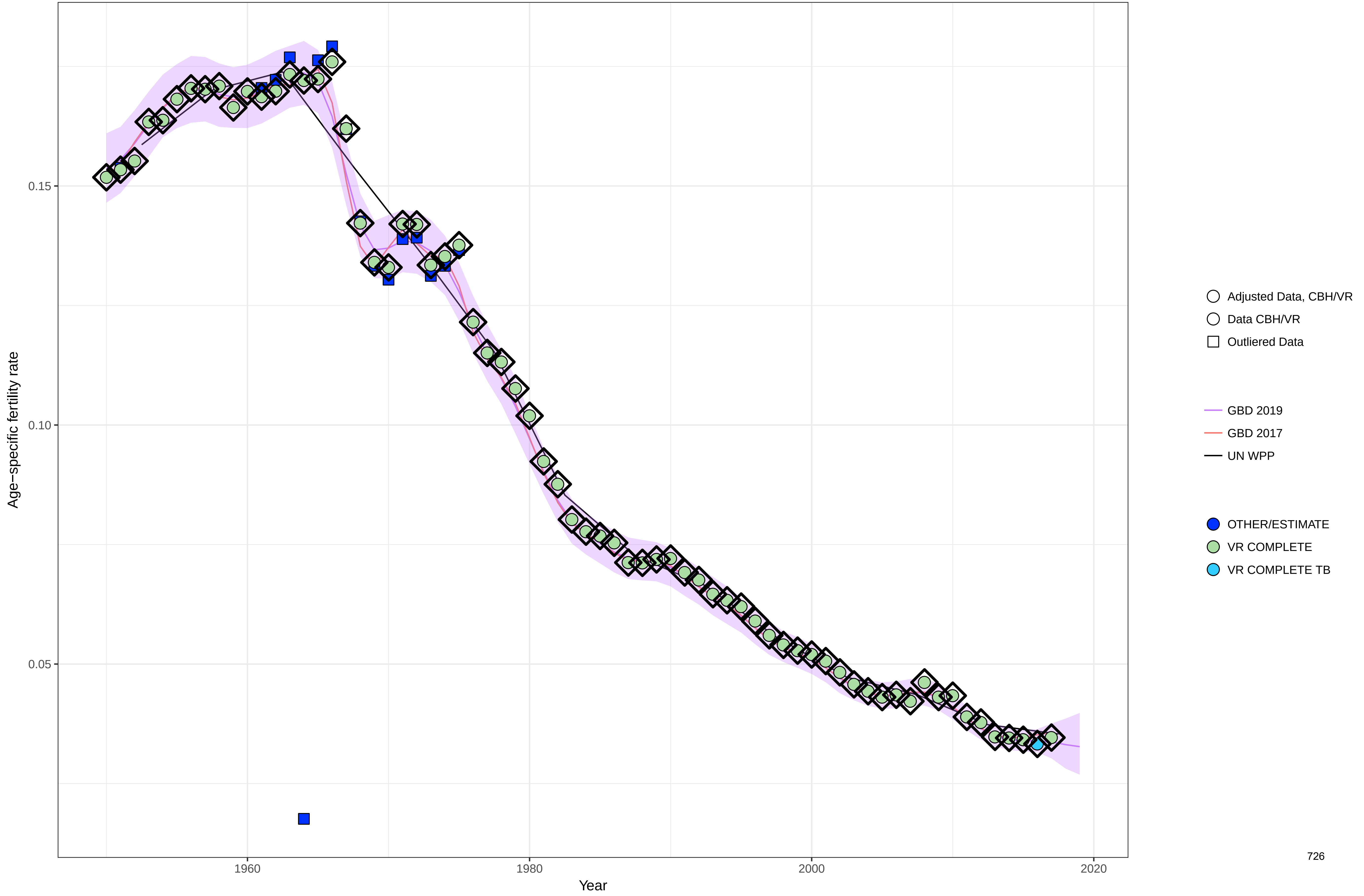

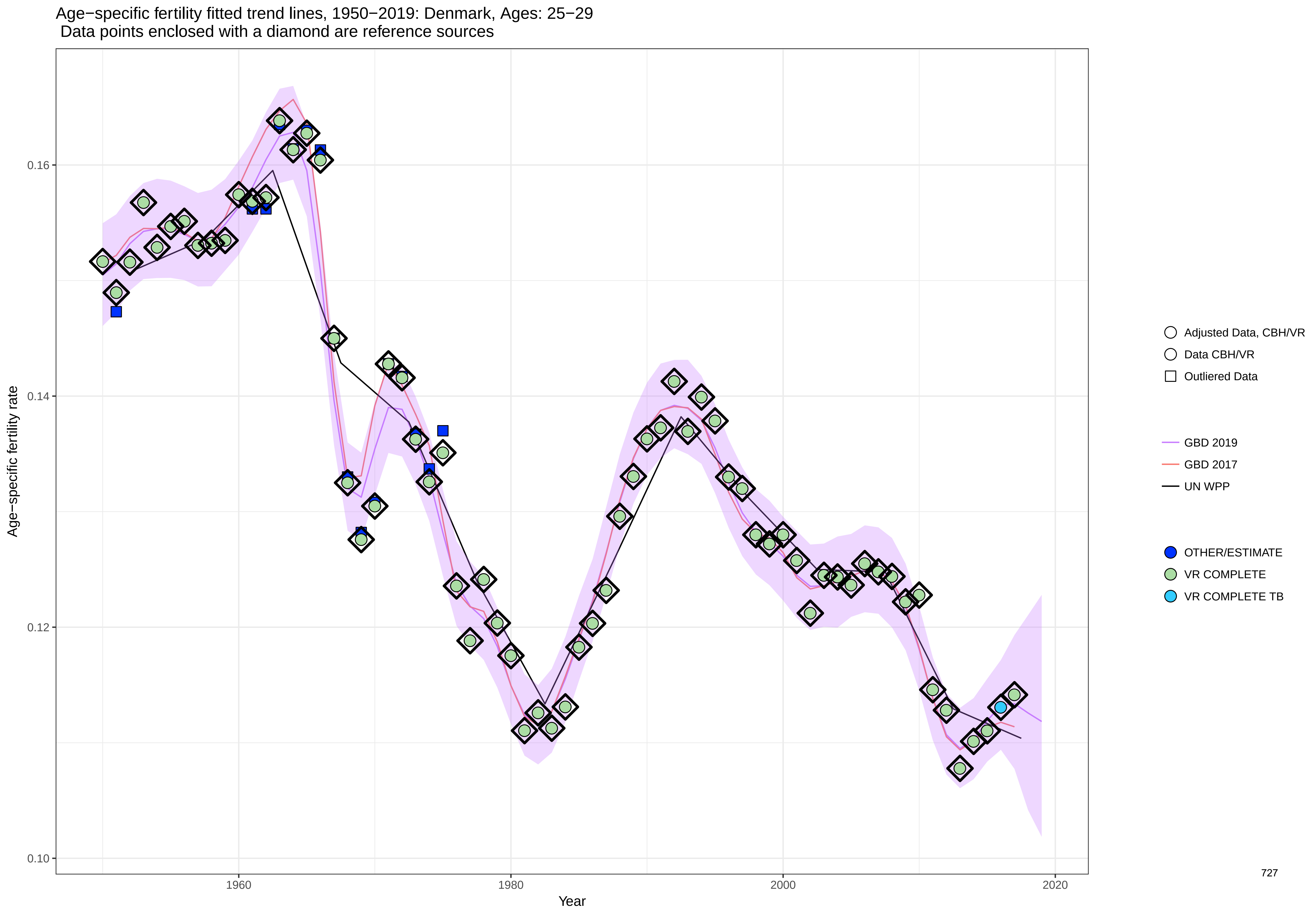

Age−specific fertility fitted trend lines, 1950−2019: Denmark, Ages: 25−29
Data points enclosed with a diamond are reference sources
Age−specific fertility rate
0.16
0.14
0.12
0.10
1960
1980
2000
2020
Year
Adjusted Data, CBH/VR
Data CBH/VR
Outliered Data
GBD 2019
GBD 2017
UN WPP
OTHER/ESTIMATE
VR COMPLETE
VR COMPLETE TB

Age−specific fertility fitted trend lines, 1950−2019: Denmark, Ages: 30−34
Data points enclosed with a diamond are reference sources

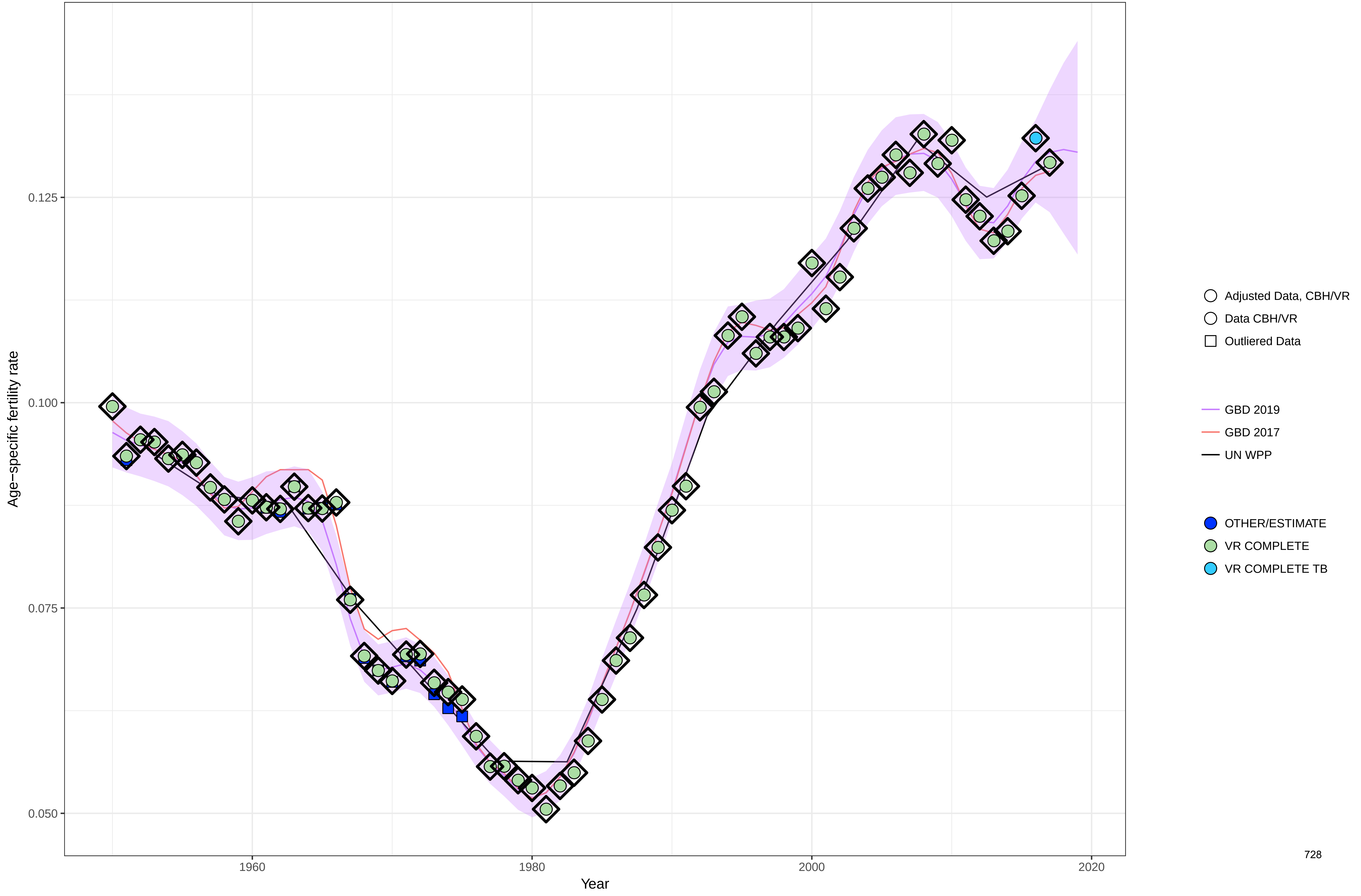

Age−specific fertility fitted trend lines, 1950−2019: Denmark, Ages: 35−39
Data points enclosed with a diamond are reference sources

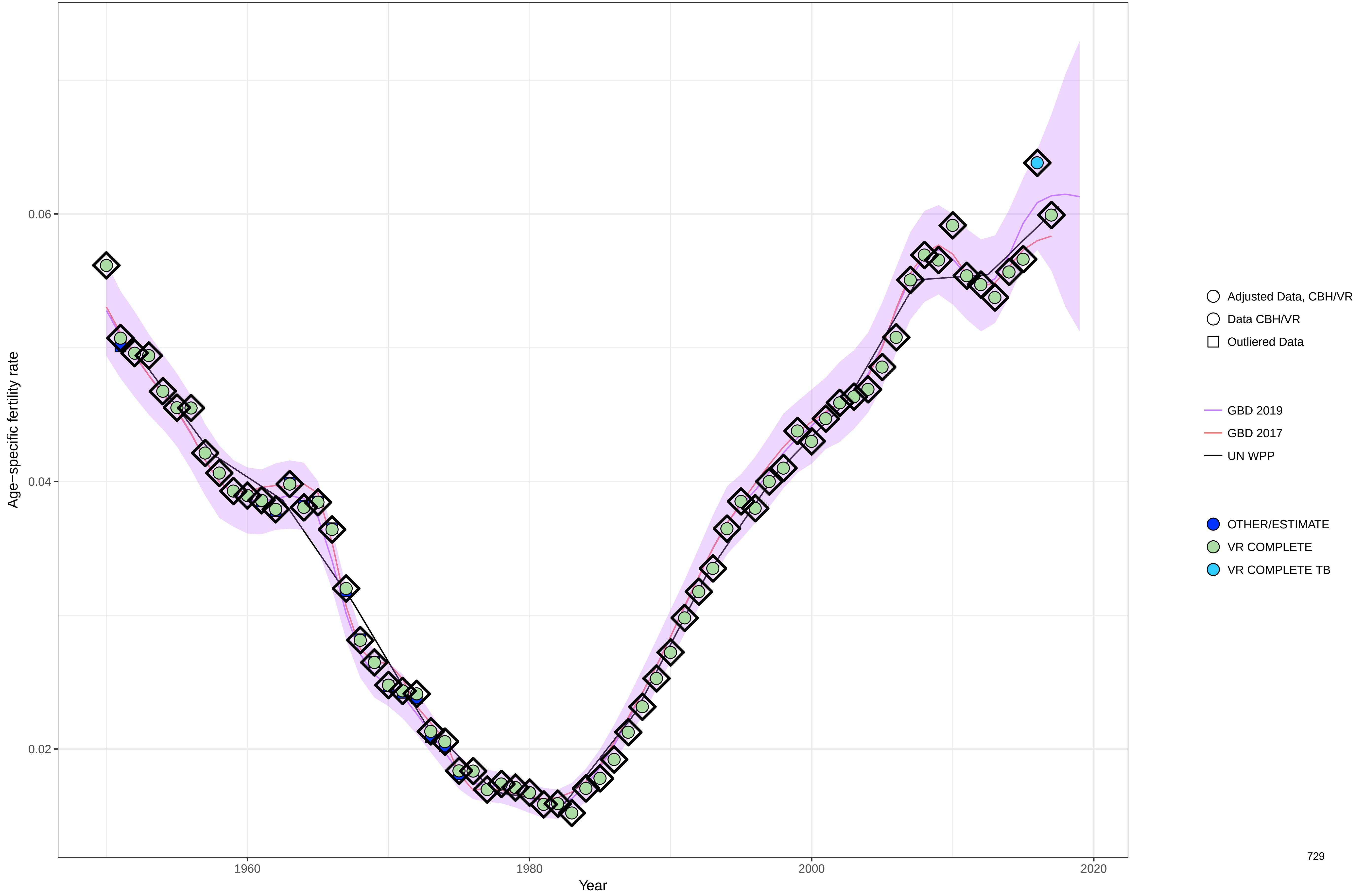

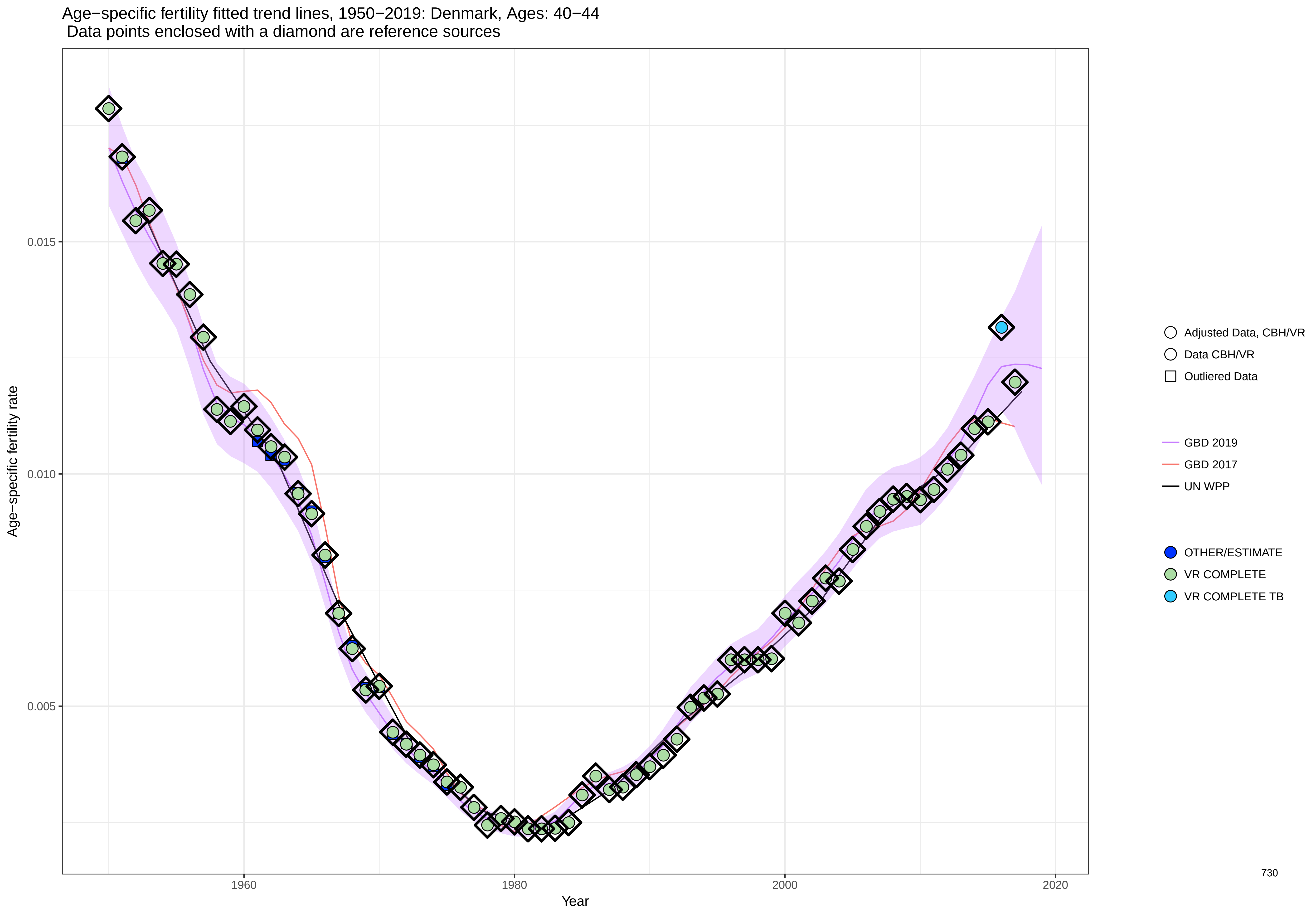
Age−specific fertility fitted trend lines, 1950−2019: Denmark, Ages: 40−44
Data points enclosed with a diamond are reference sources
Age−specific fertility rate
0.015
0.010
0.005
Year
1960
1980
2000
2020
Adjusted Data, CBH/VR
Data CBH/VR
Outliered Data
GBD 2019
GBD 2017
UN WPP
OTHER/ESTIMATE
VR COMPLETE
VR COMPLETE TB

Age−specific fertility fitted trend lines, 1950−2019: Denmark, Ages: 45−49
Data points enclosed with a diamond are reference sources

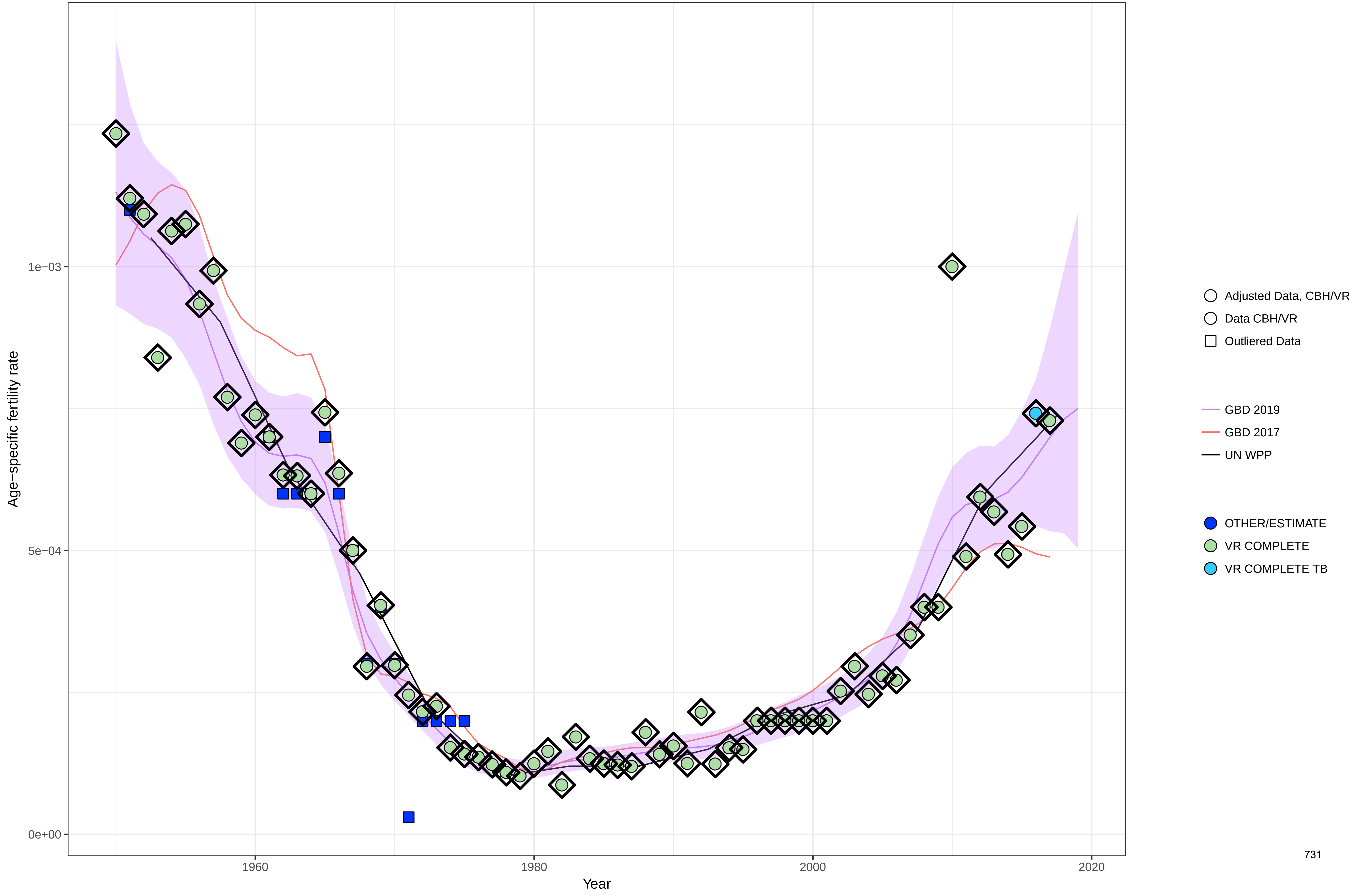

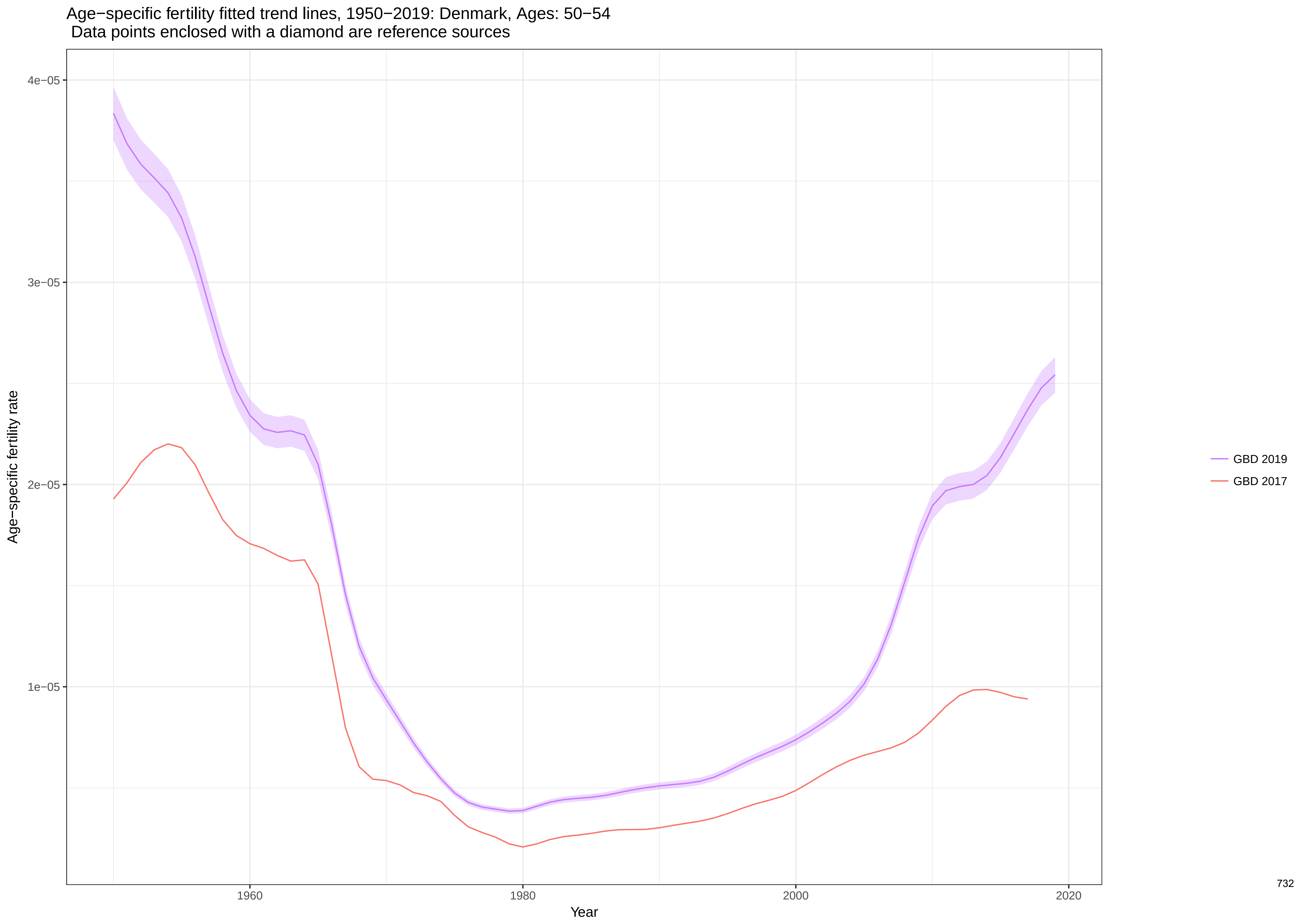
Age−specific fertility fitted trend lines, 1950−2019: Denmark, Ages: 50−54
Data points enclosed with a diamond are reference sources
4e−05
3e−05
2e−05
1e−05
Age−specific fertility rate
1960
1980
2000
2020
Year
GBD 2019
GBD 2017

Total fertility rate fitted trend lines, 1950−2019: Denmark TFR
Data points enclosed with a diamond are reference sources

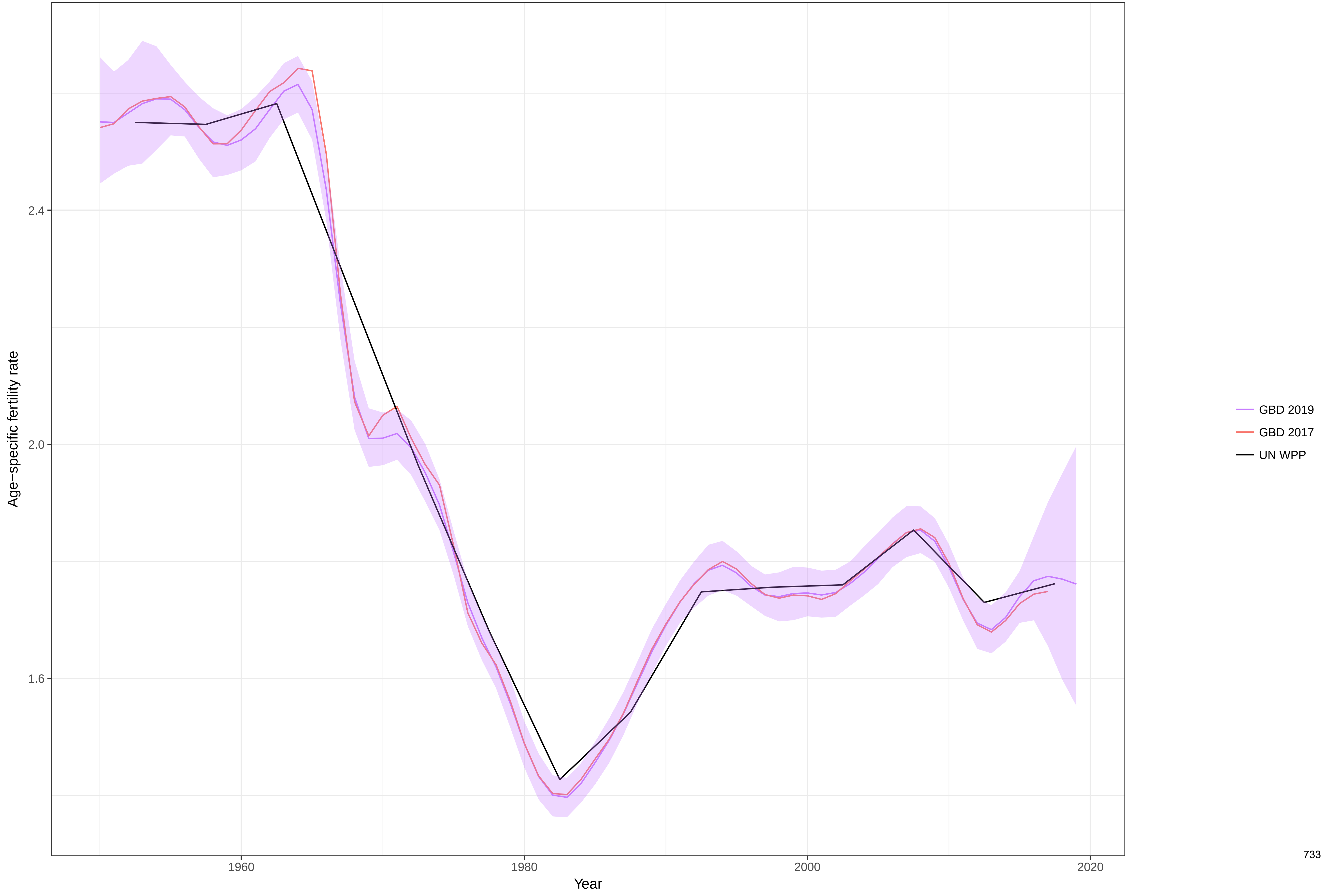

Age−specific fertility fitted trend lines, 1950−2019: Dominican Republic, Ages: 10−14
Data points enclosed with a diamond are reference sources

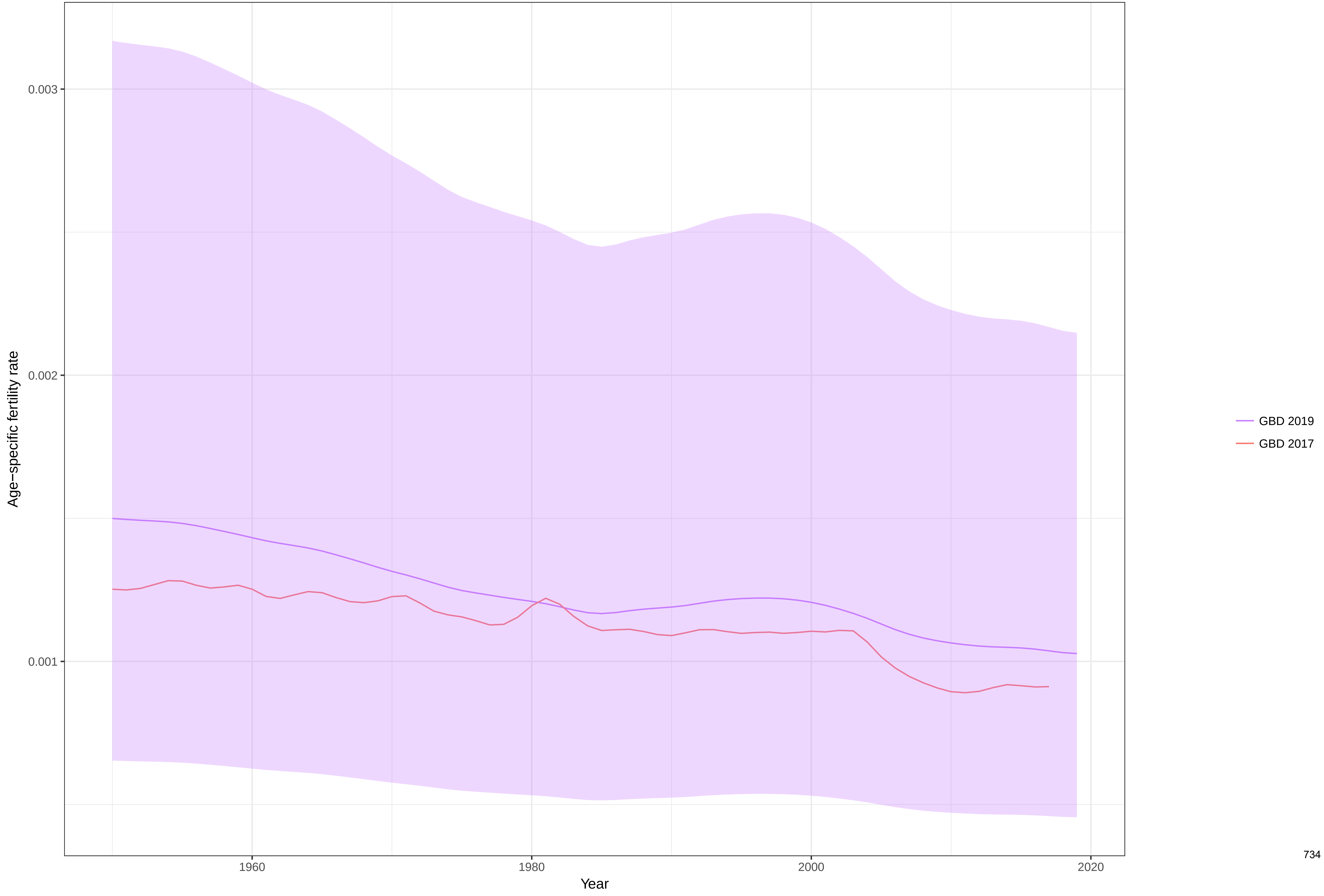

Age−specific fertility fitted trend lines, 1950−2019: Dominican Republic, Ages: 15−19
Data points enclosed with a diamond are reference sources

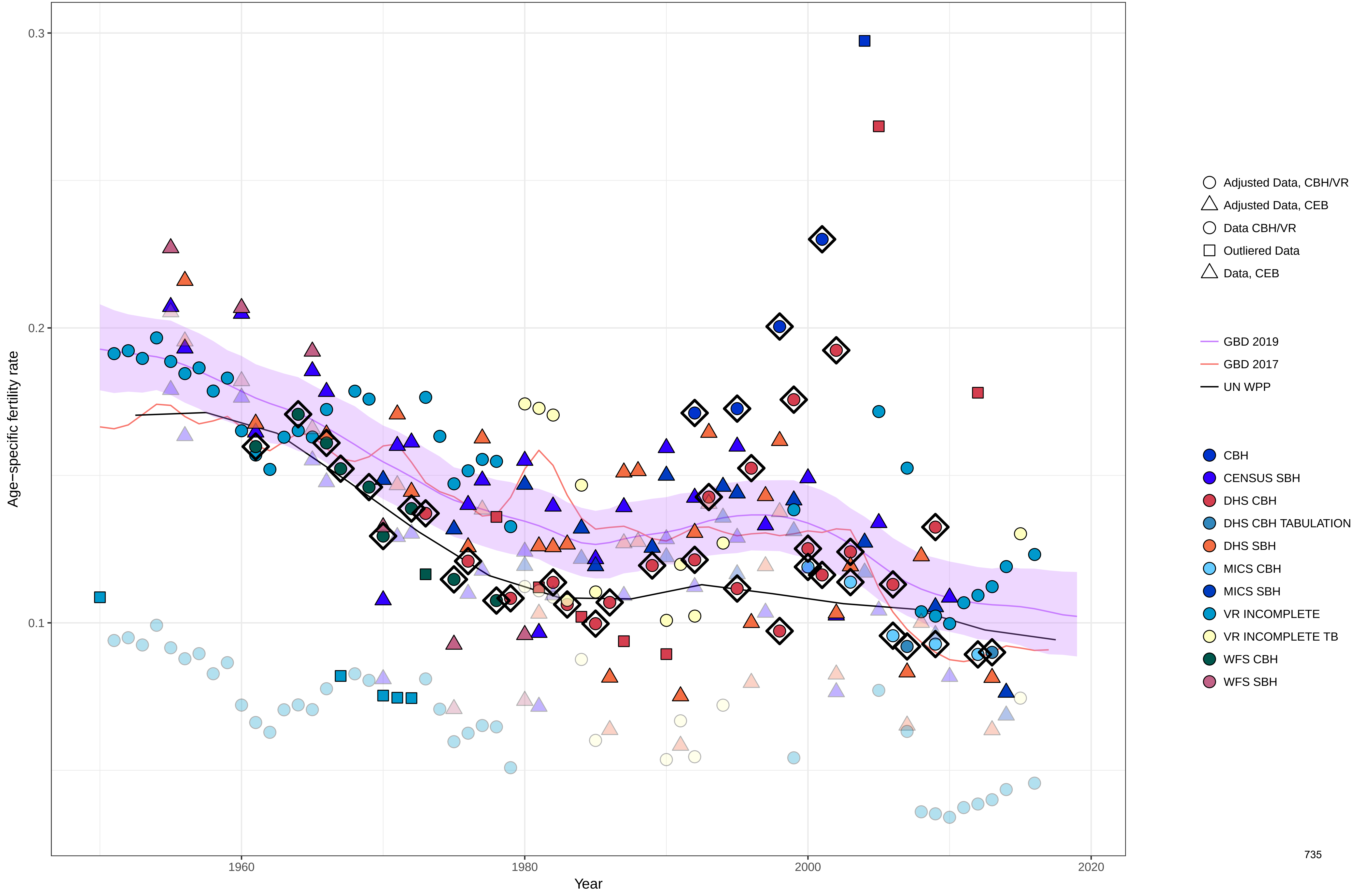

Age−specific fertility fitted trend lines, 1950−2019: Dominican Republic, Ages: 20−24
Data points enclosed with a diamond are reference sources

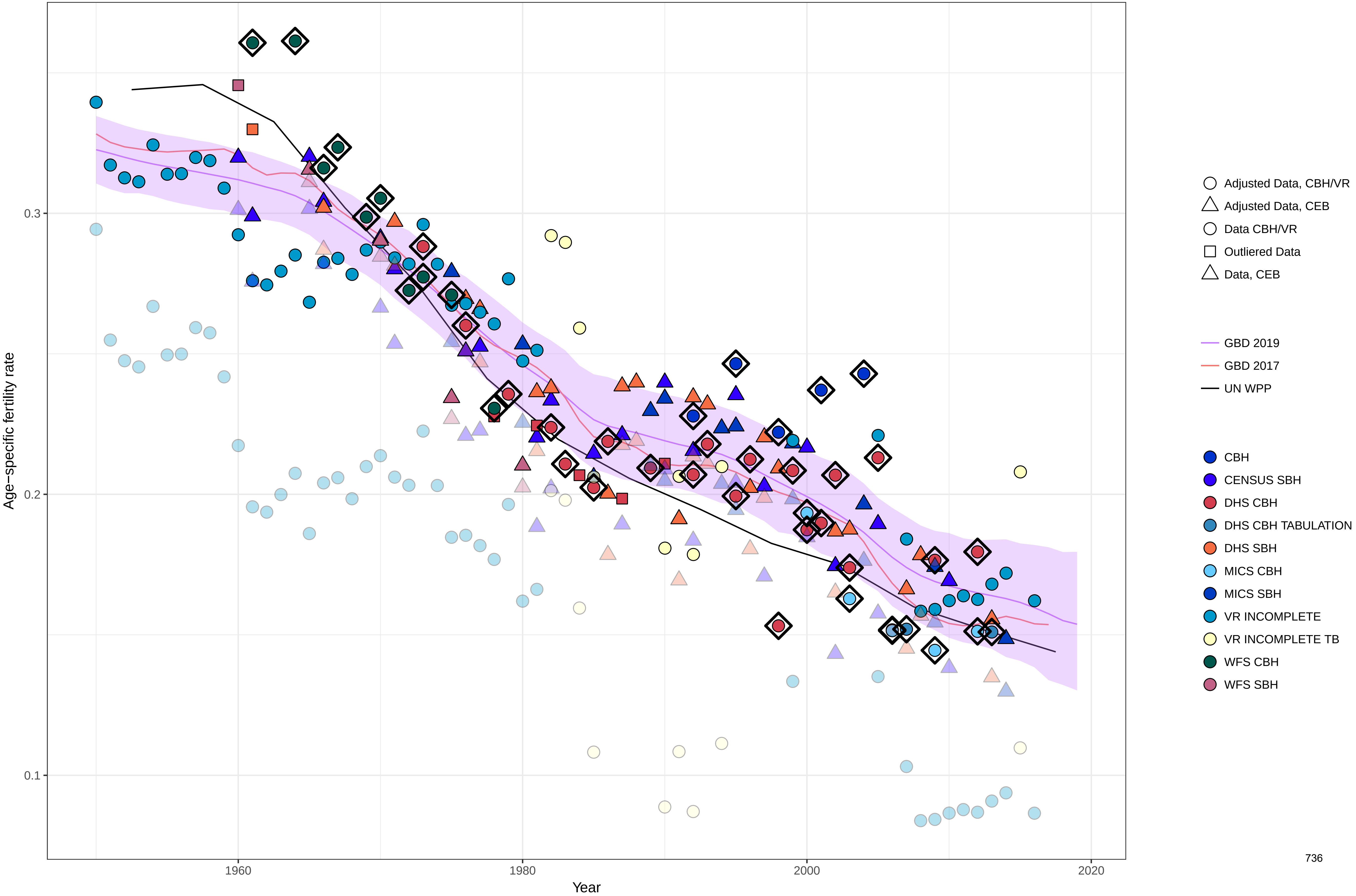

Age−specific fertility fitted trend lines, 1950−2019: Dominican Republic, Ages: 25−29
Data points enclosed with a diamond are reference sources

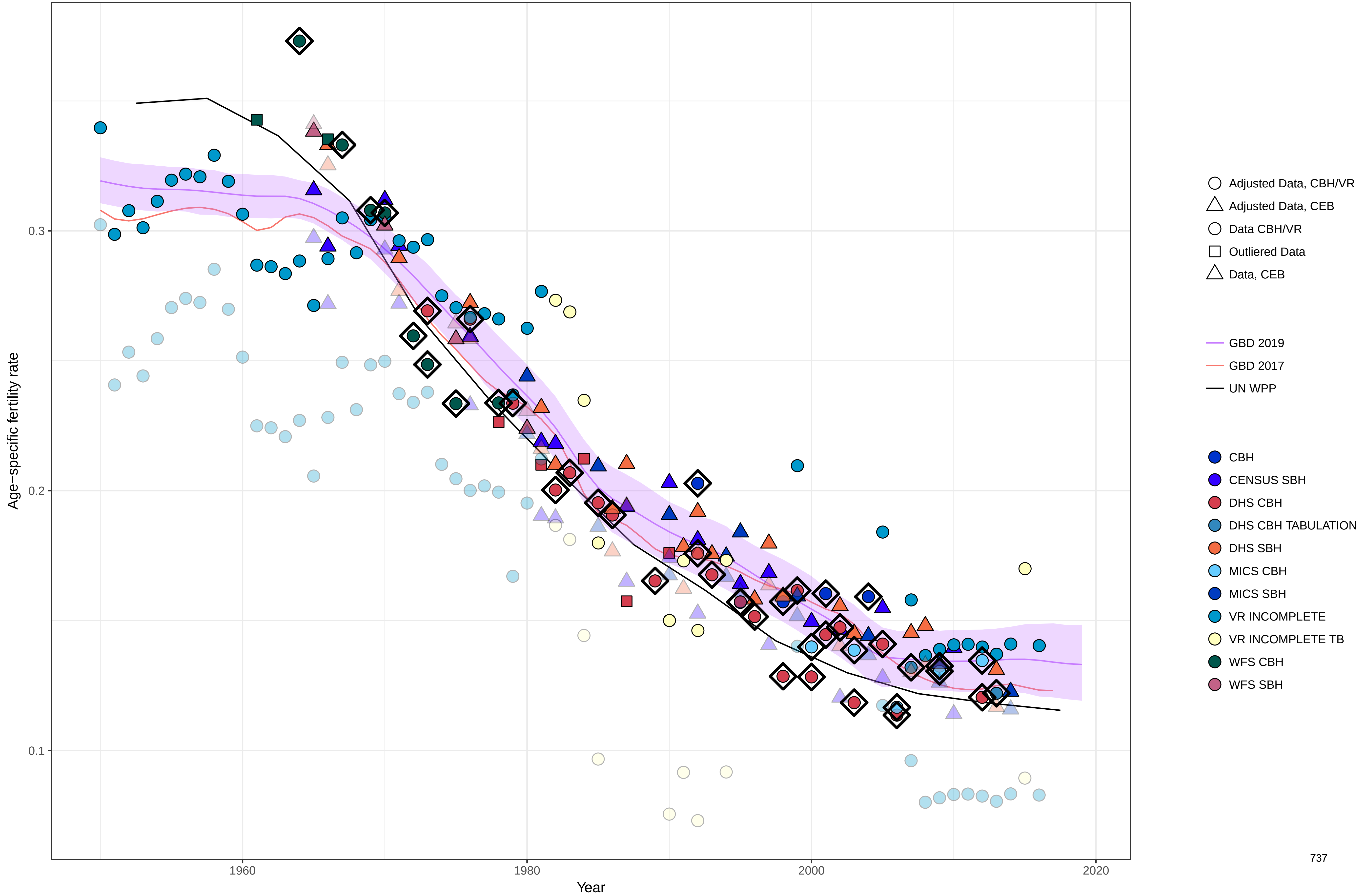

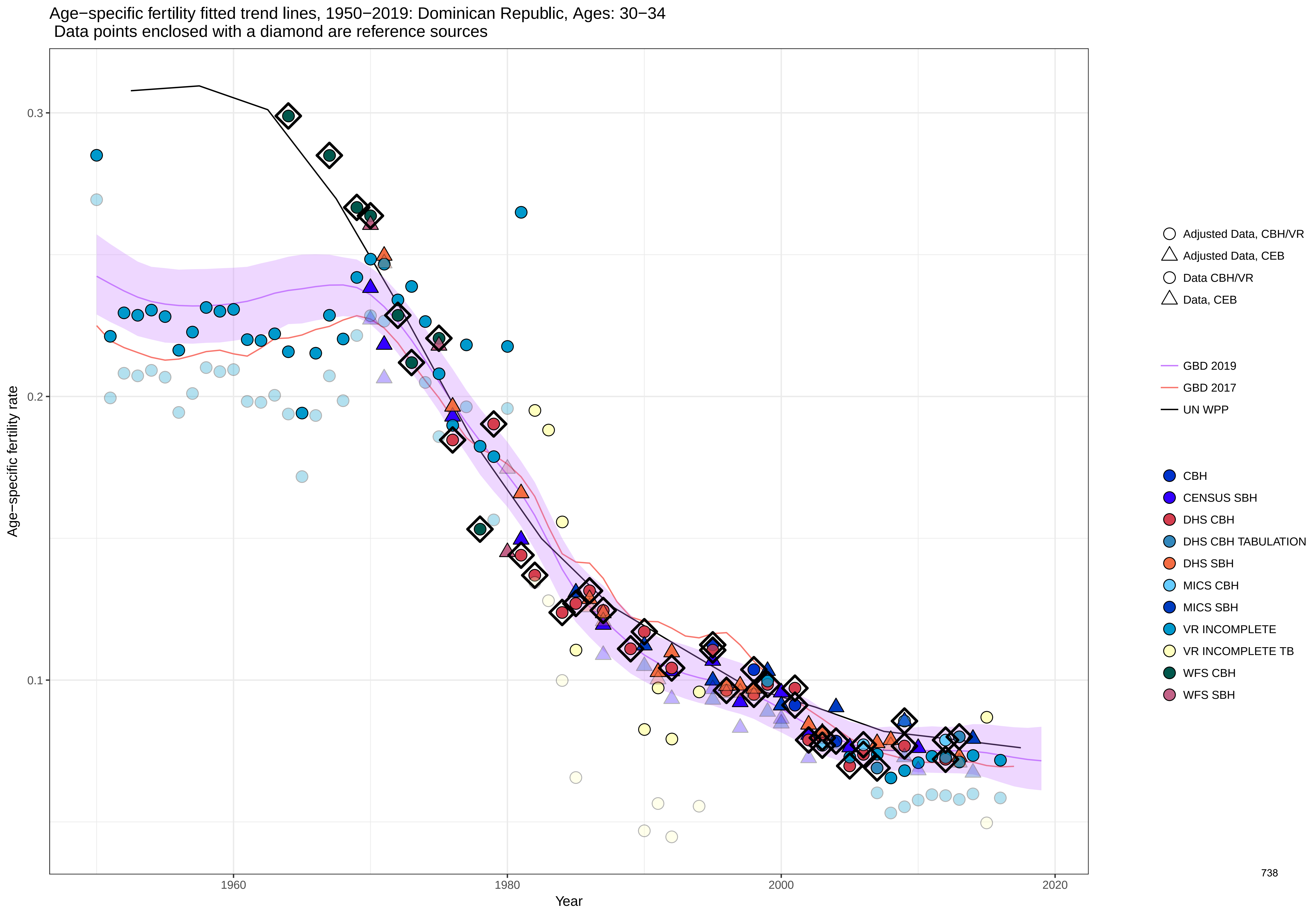

Age−specific fertility fitted trend lines, 1950−2019: Dominican Republic, Ages: 30−34
Data points enclosed with a diamond are reference sources
0.3
0.2
0.1
1960
1980
2000
2020
Year
Age−specific fertility rate
Adjusted Data, CBH/VR
Adjusted Data, CEB
Data CBH/VR
Data, CEB
GBD 2019
GBD 2017
UN WPP
CBH
CENSUS SBH
DHS CBH
DHS CBH TABULATION
DHS SBH
MICS CBH
MICS SBH
VR INCOMPLETE
VR INCOMPLETE TB
WFS CBH
WFS SBH

Age−specific fertility fitted trend lines, 1950−2019: Dominican Republic, Ages: 35−39
Data points enclosed with a diamond are reference sources

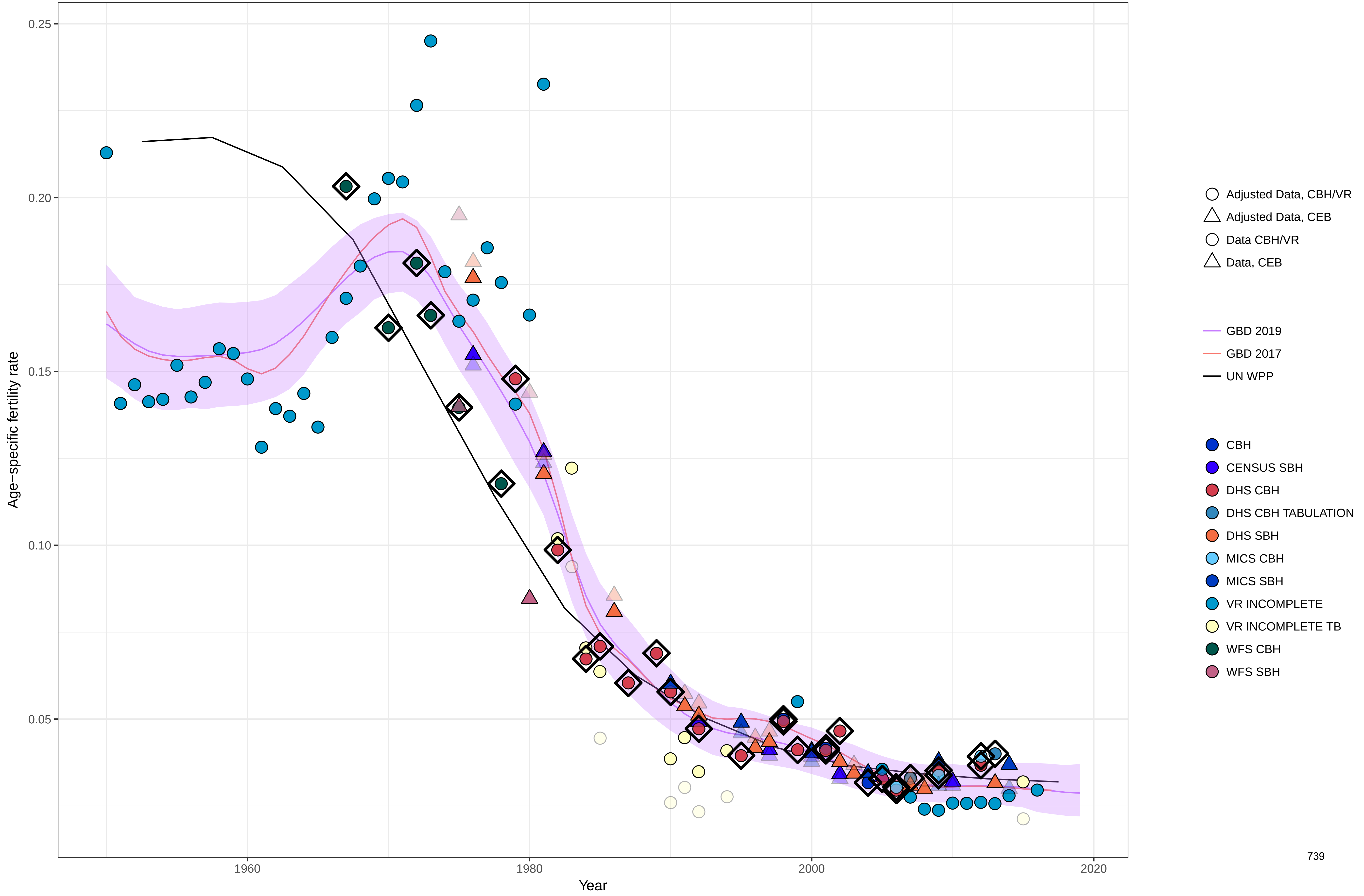

Age−specific fertility fitted trend lines, 1950−2019: Dominican Republic, Ages: 40−44
Data points enclosed with a diamond are reference sources

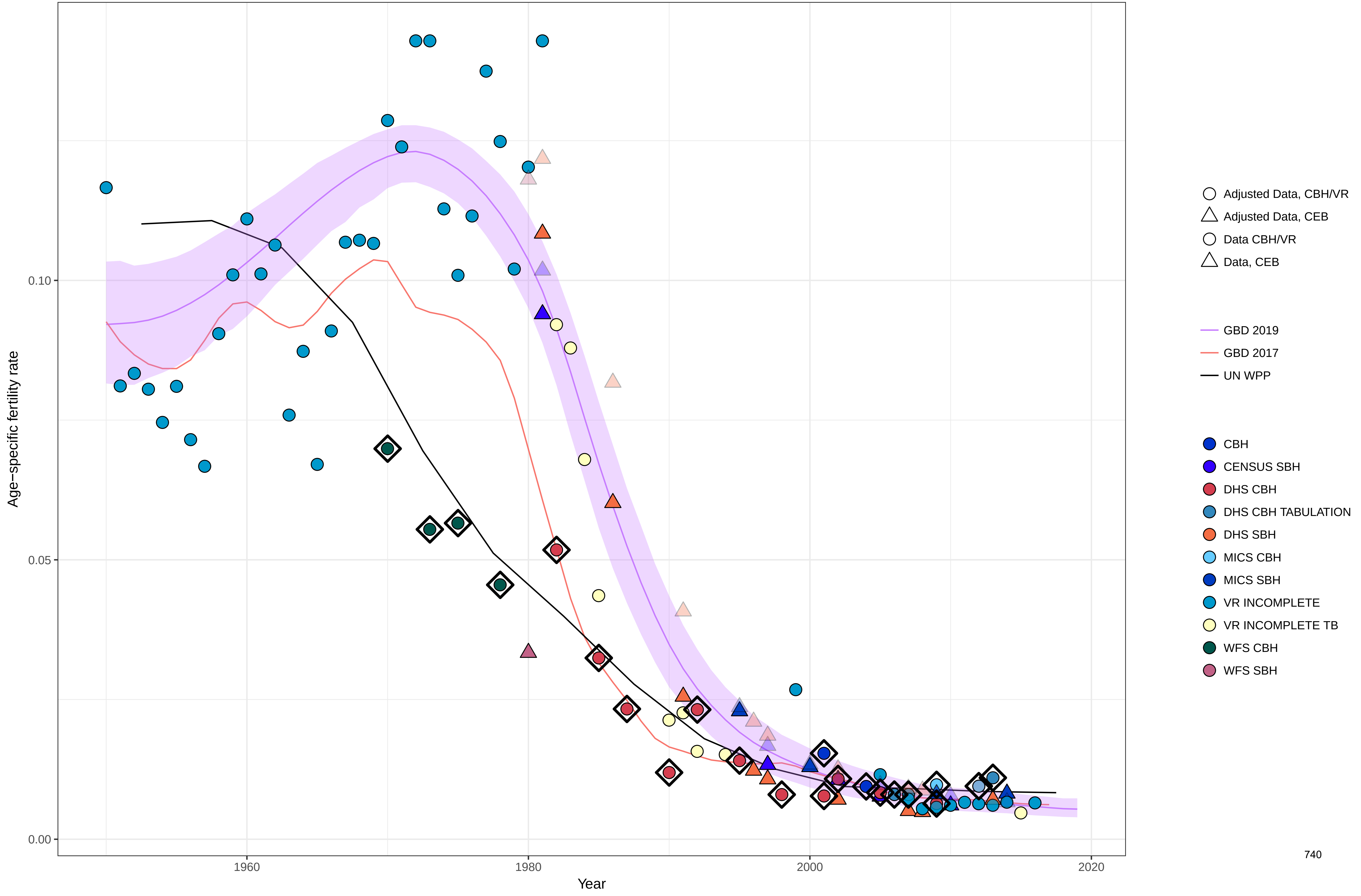

Age−specific fertility fitted trend lines, 1950−2019: Dominican Republic, Ages: 45−49
Data points enclosed with a diamond are reference sources

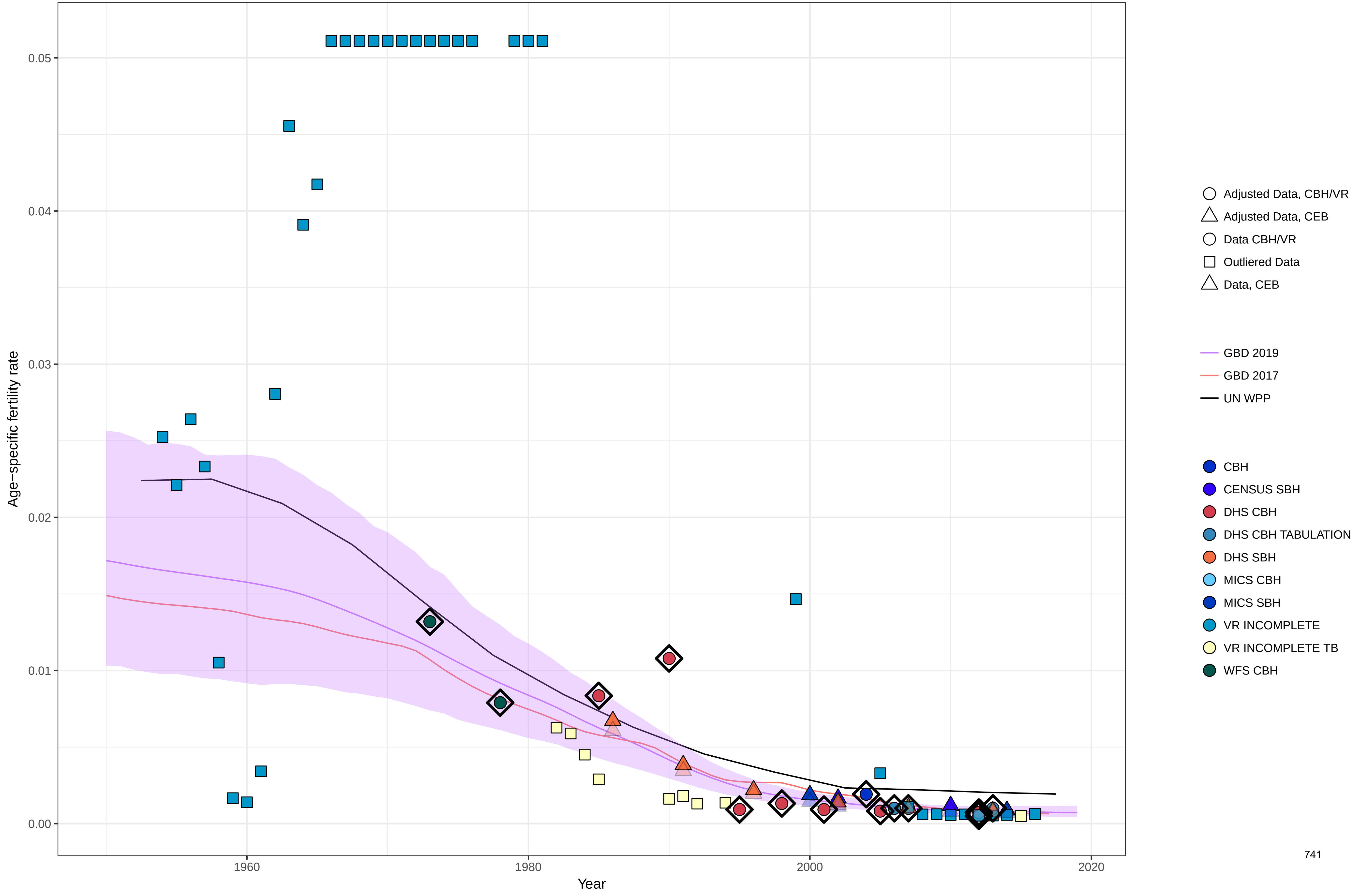

Age−specific fertility fitted trend lines, 1950−2019: Dominican Republic, Ages: 50−54
Data points enclosed with a diamond are reference sources

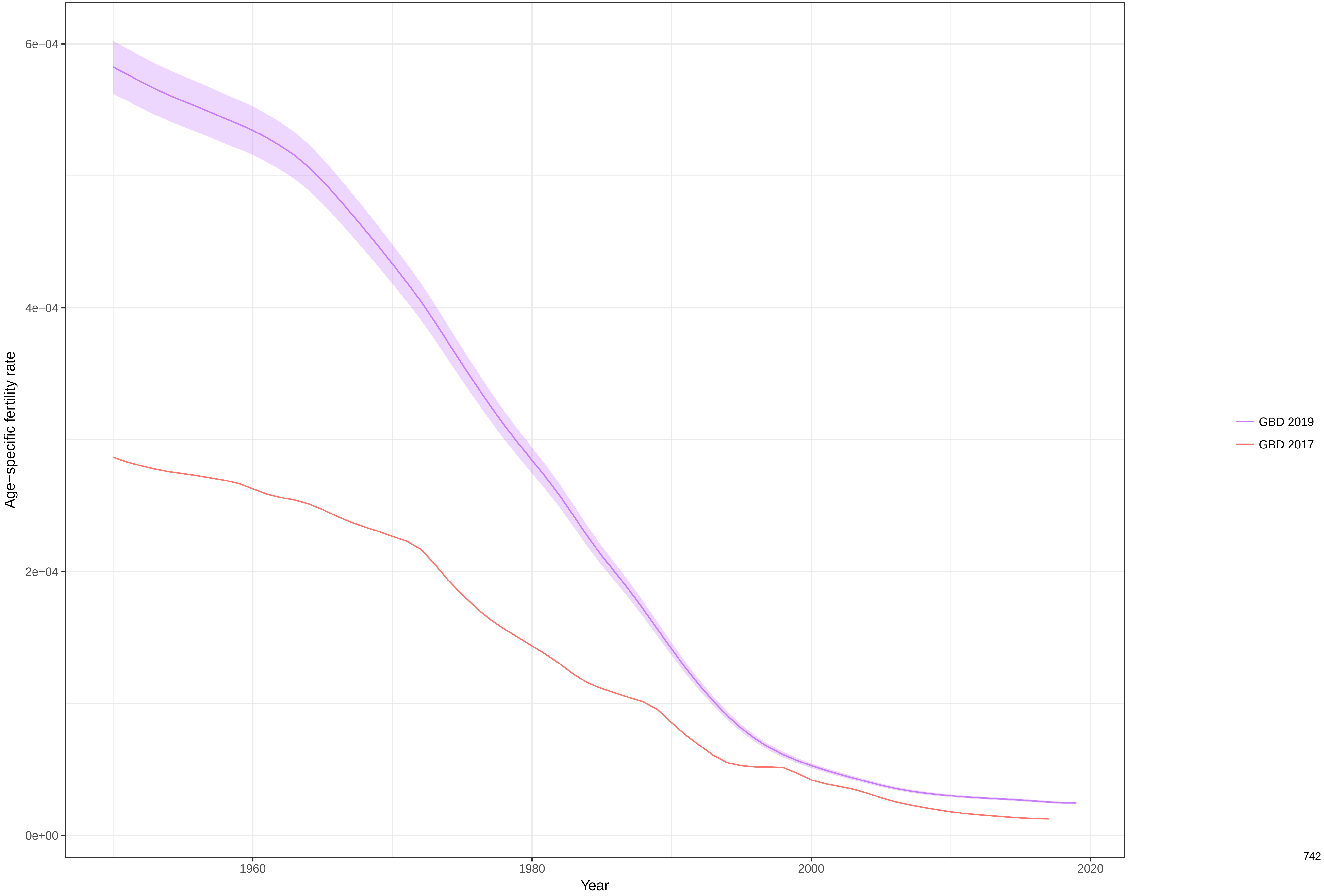

Total fertility rate fitted trend lines, 1950−2019: Dominican Republic TFR
Data points enclosed with a diamond are reference sources

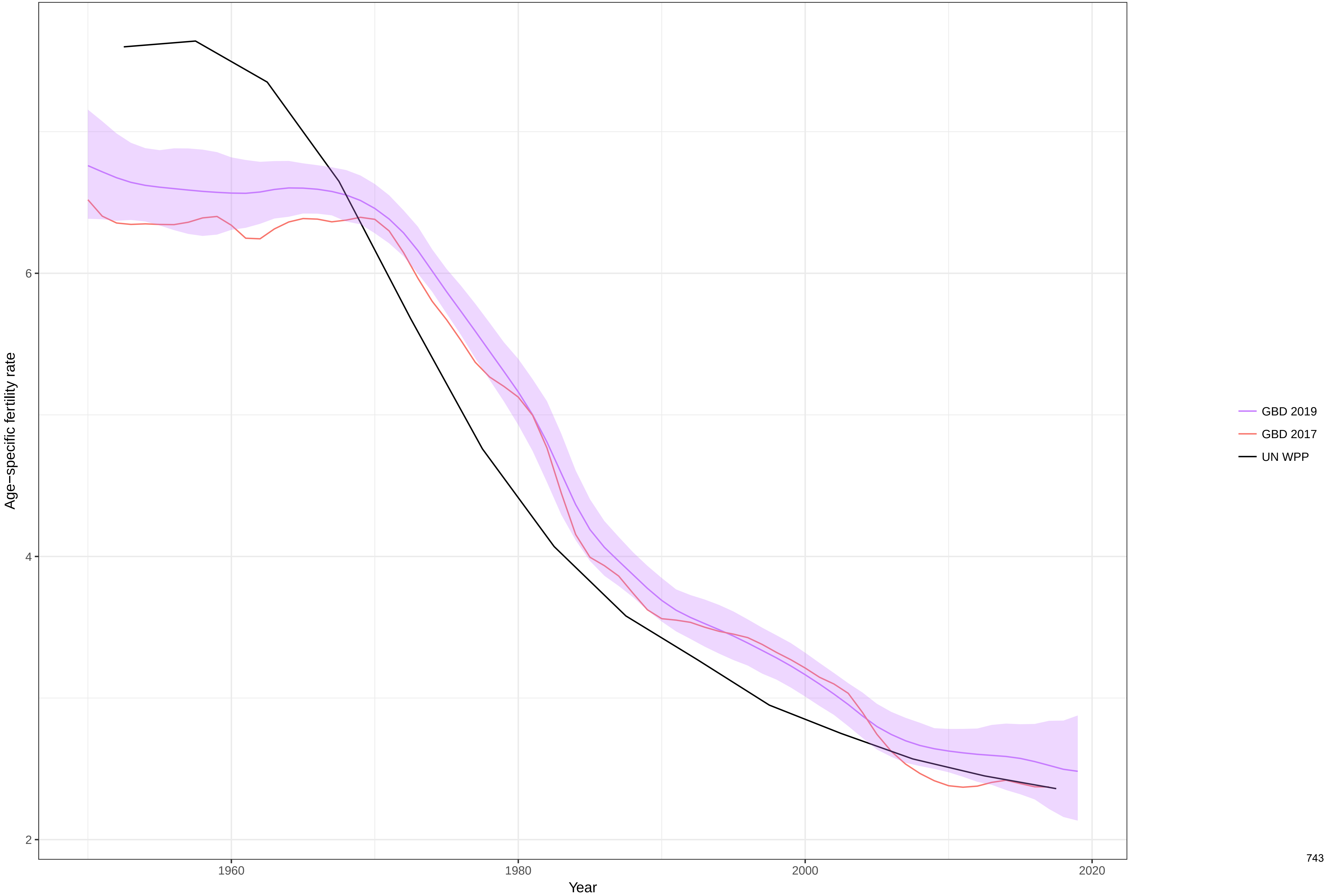

Age−specific fertility fitted trend lines, 1950−2019: Algeria, Ages: 10−14
Data points enclosed with a diamond are reference sources

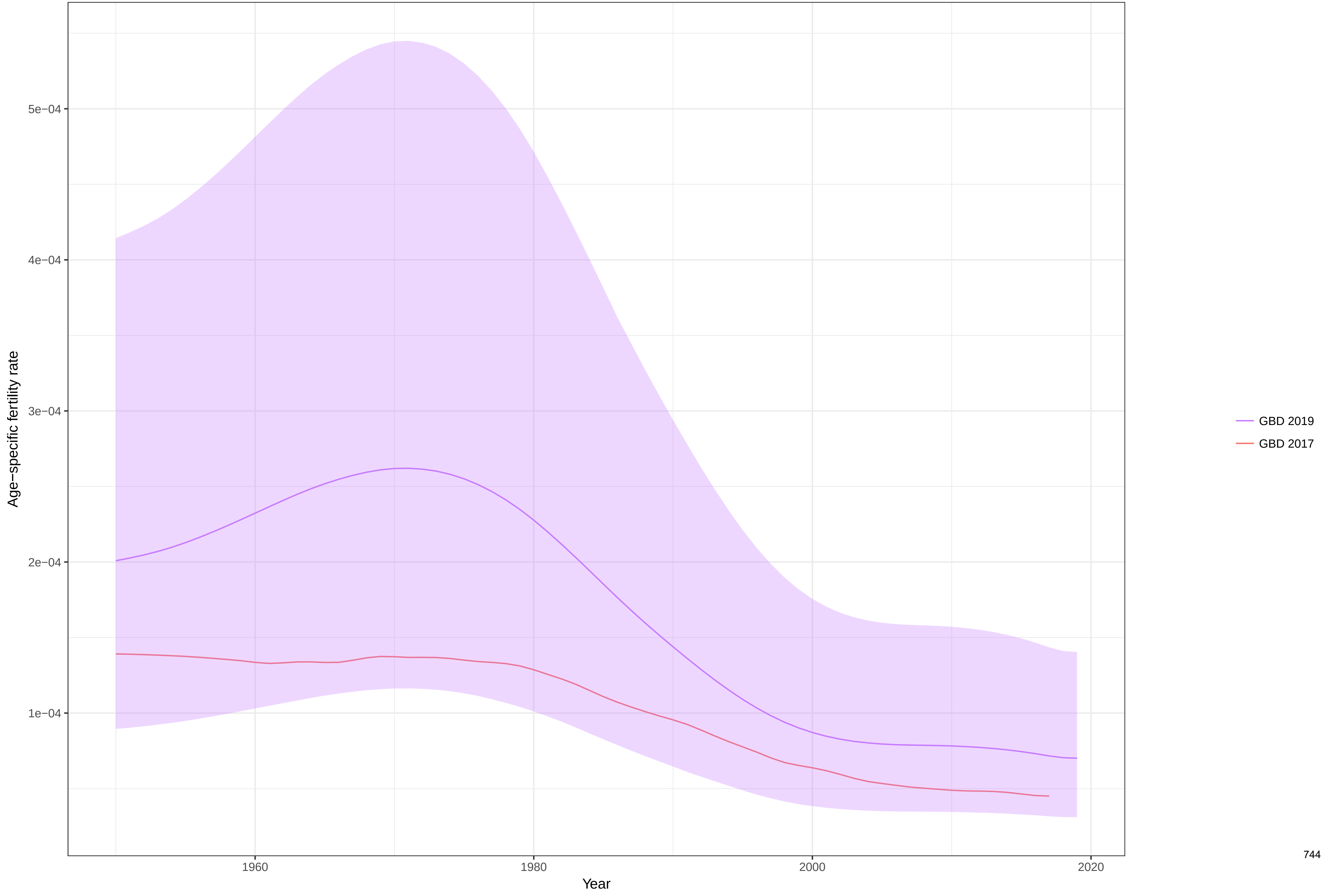

Age−specific fertility fitted trend lines, 1950−2019: Algeria, Ages: 15−19
Data points enclosed with a diamond are reference sources

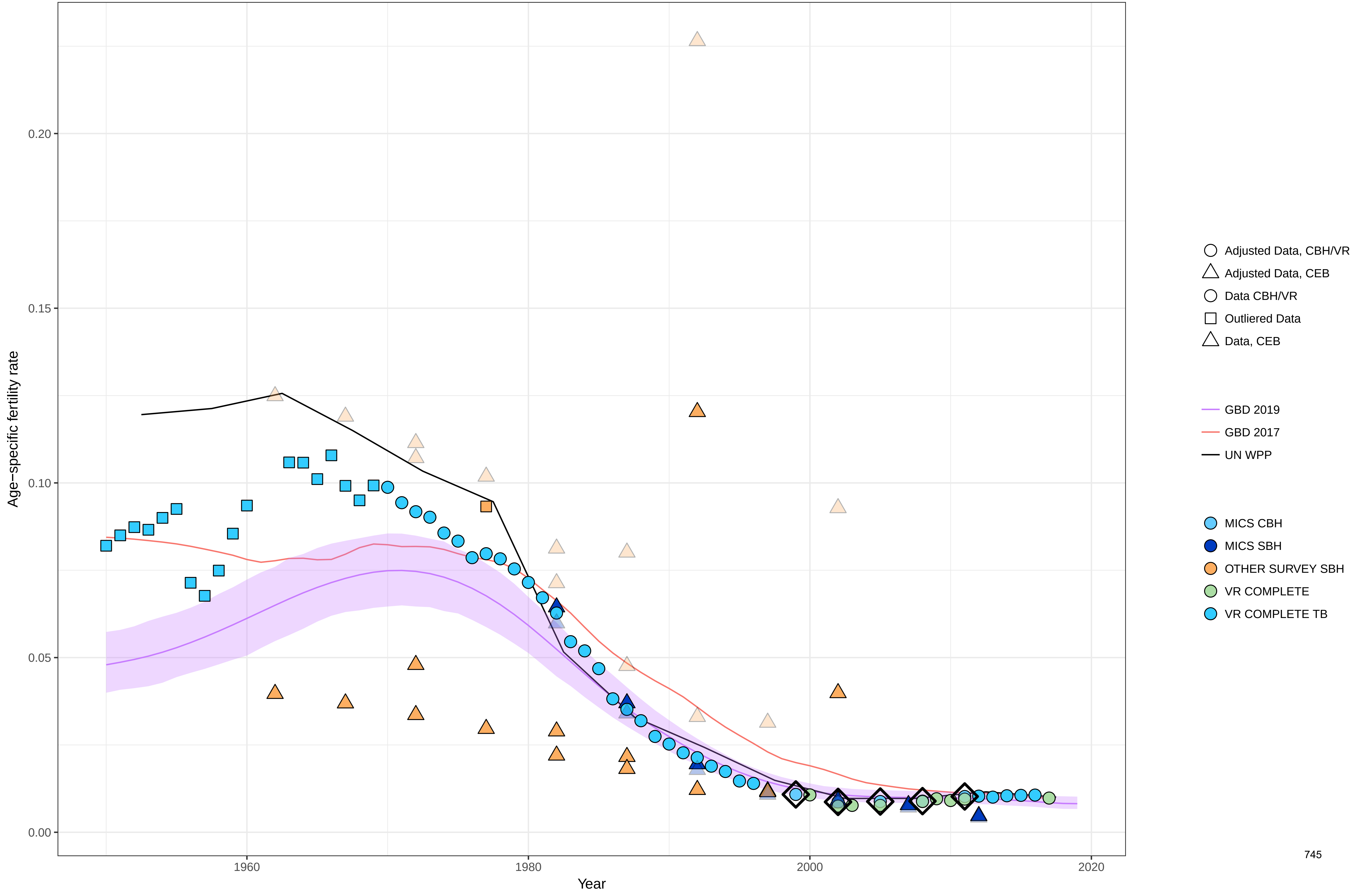

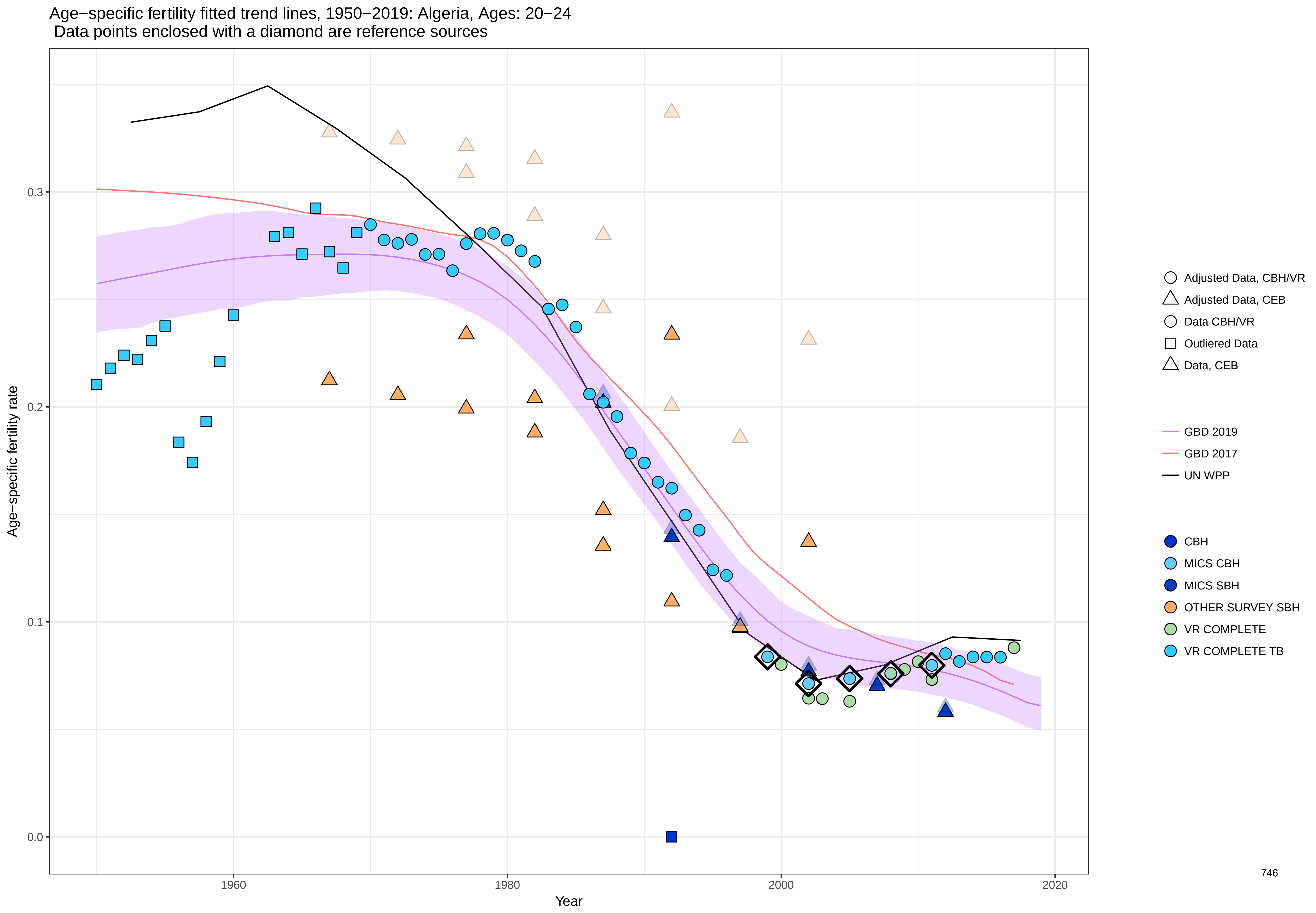
Age−specific fertility fitted trend lines, 1950−2019: Algeria, Ages: 20−24
Data points enclosed with a diamond are reference sources
Age−specific fertility rate
Year
0.0
0.1
0.2
0.3
1960
1980
2000
2020
Adjusted Data, CBH/VR
Adjusted Data, CEB
Data CBH/VR
Outliered Data
Data, CEB
GBD 2019
GBD 2017
UN WPP
CBH
MICS CBH
MICS SBH
OTHER SURVEY SBH
VR COMPLETE
VR COMPLETE TB

Age−specific fertility fitted trend lines, 1950−2019: Algeria, Ages: 25−29
Data points enclosed with a diamond are reference sources

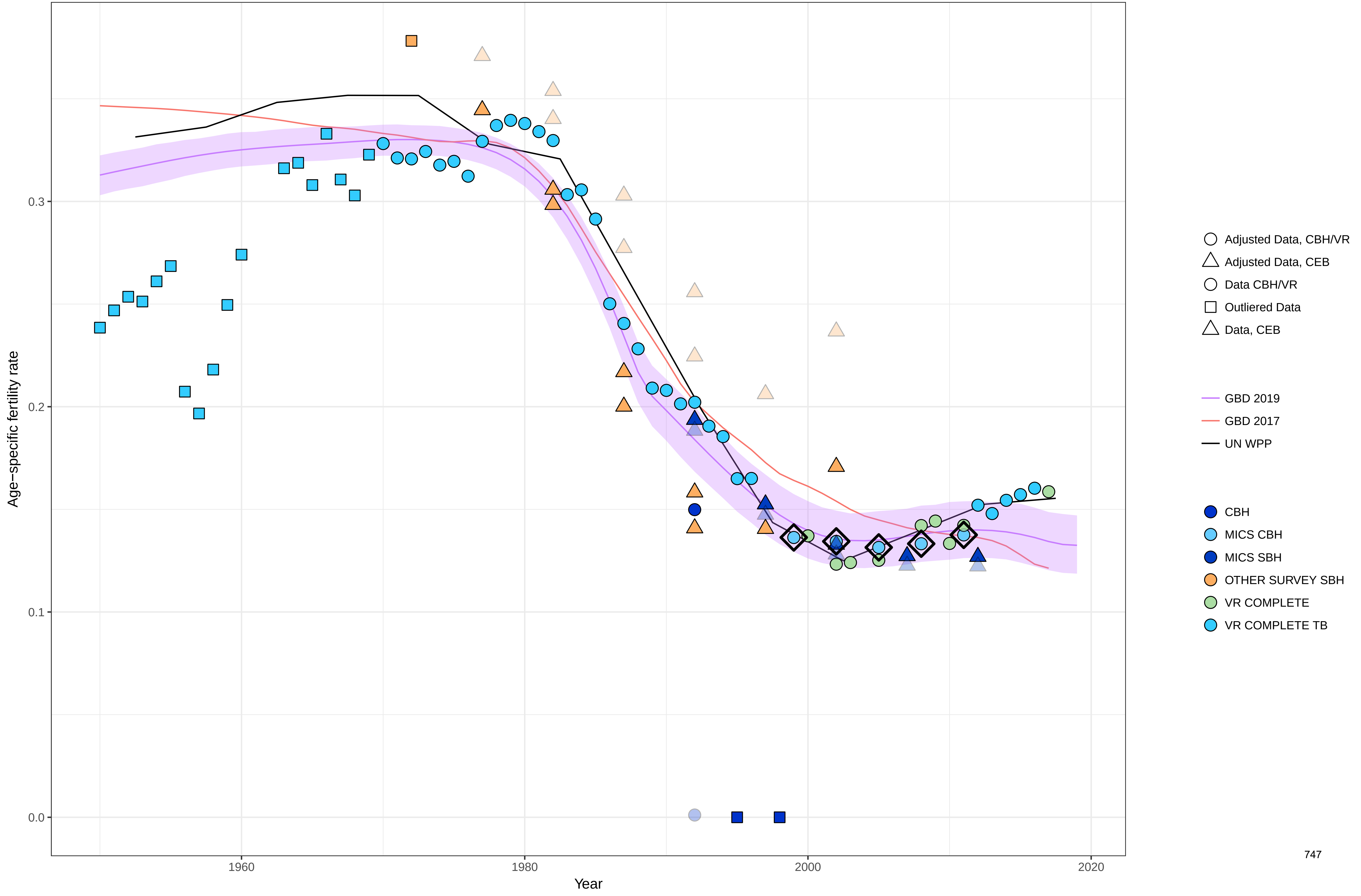

Age−specific fertility fitted trend lines, 1950−2019: Algeria, Ages: 30−34
Data points enclosed with a diamond are reference sources

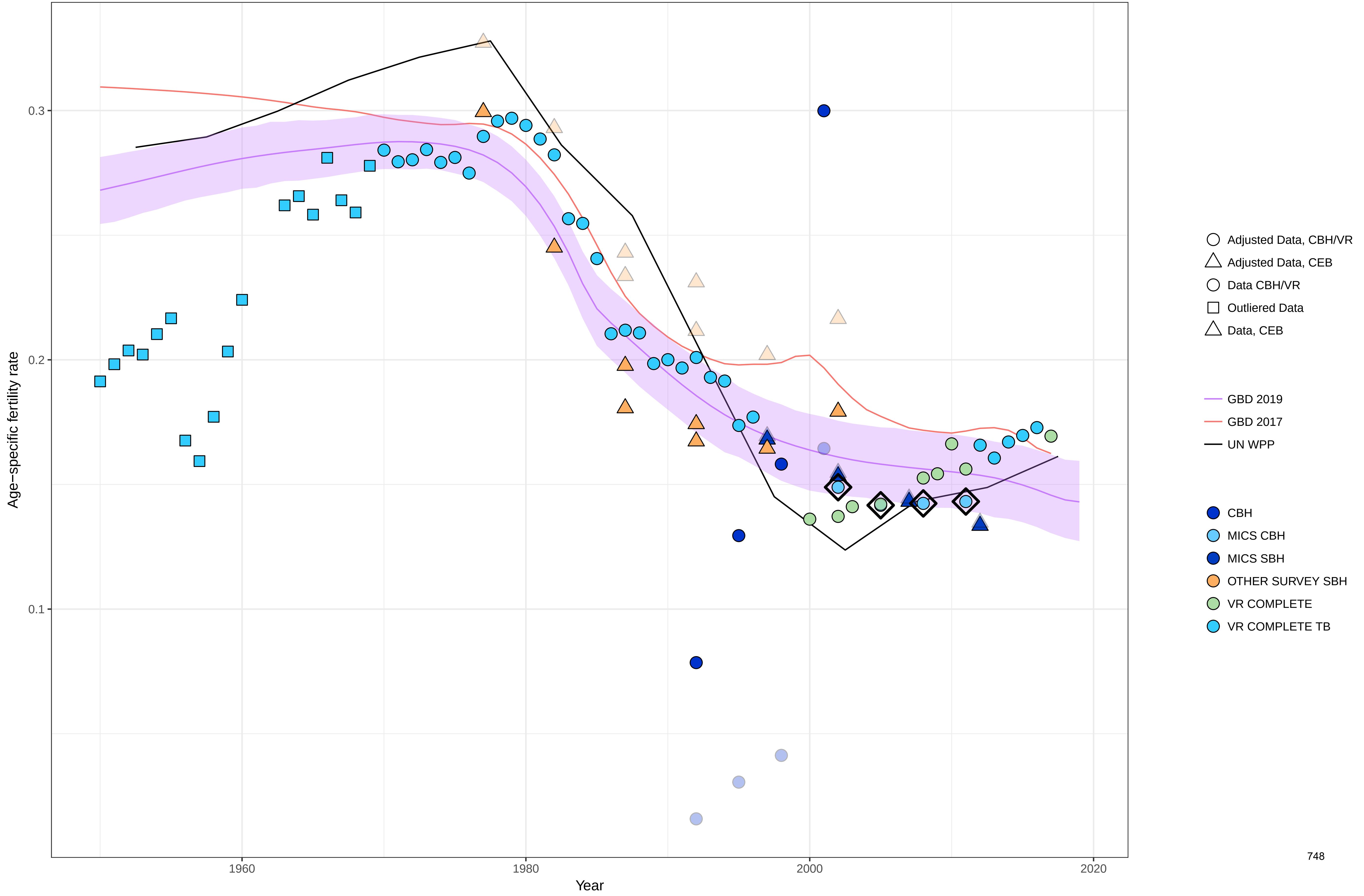

Age−specific fertility fitted trend lines, 1950−2019: Algeria, Ages: 35−39
Data points enclosed with a diamond are reference sources

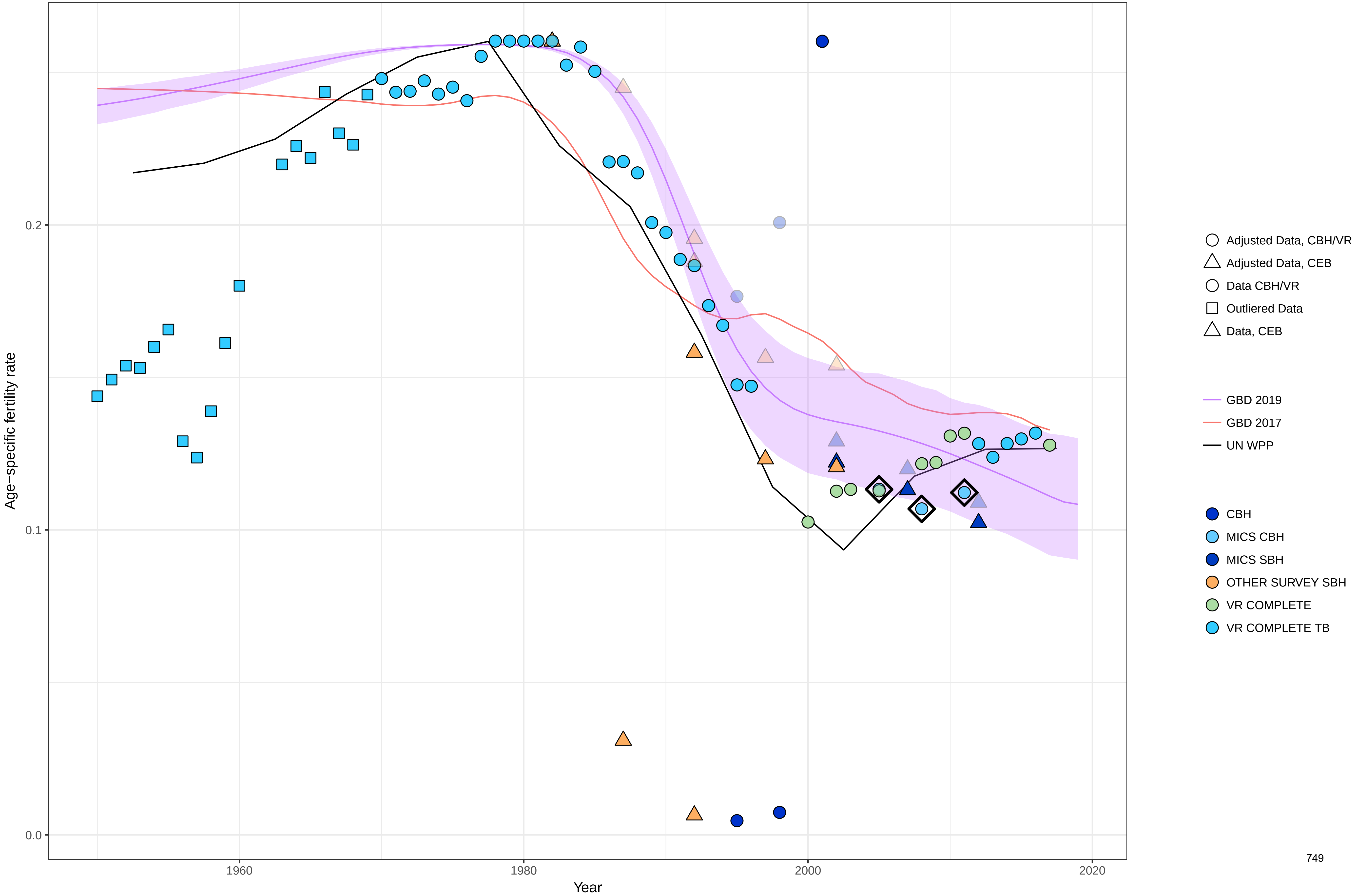

Age−specific fertility fitted trend lines, 1950−2019: Algeria, Ages: 40−44
Data points enclosed with a diamond are reference sources

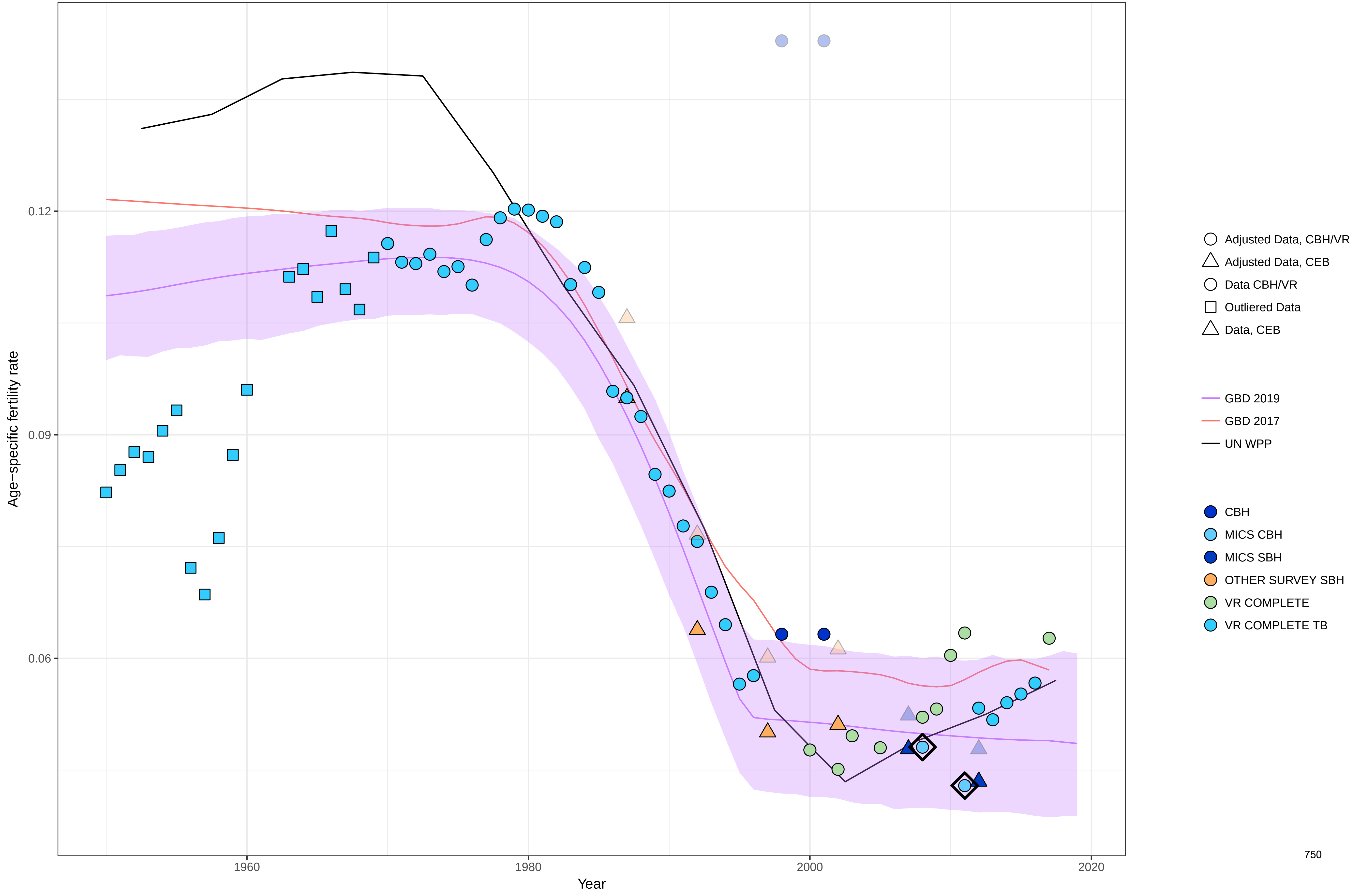

Age−specific fertility fitted trend lines, 1950−2019: Algeria, Ages: 45−49
Data points enclosed with a diamond are reference sources

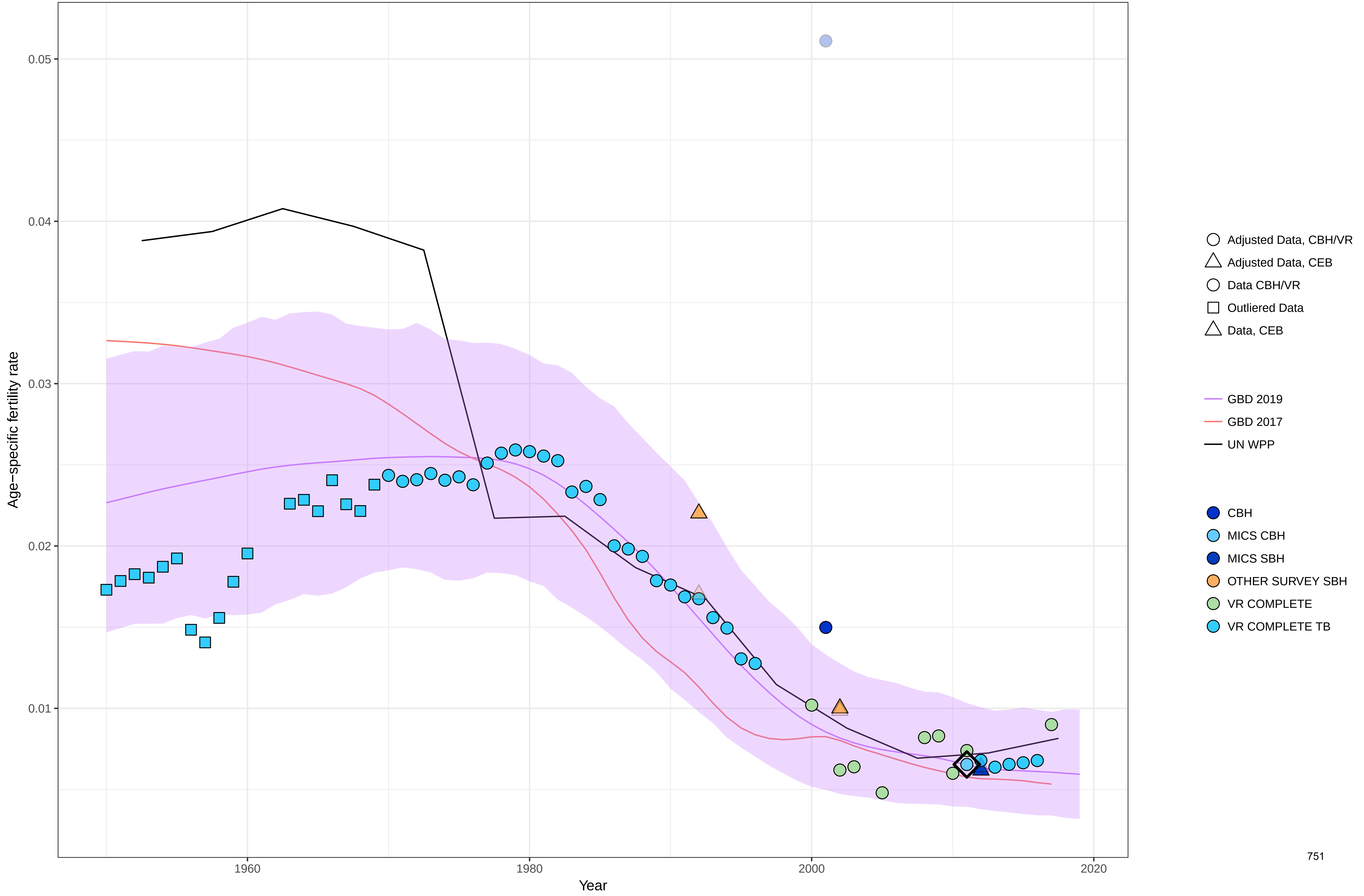

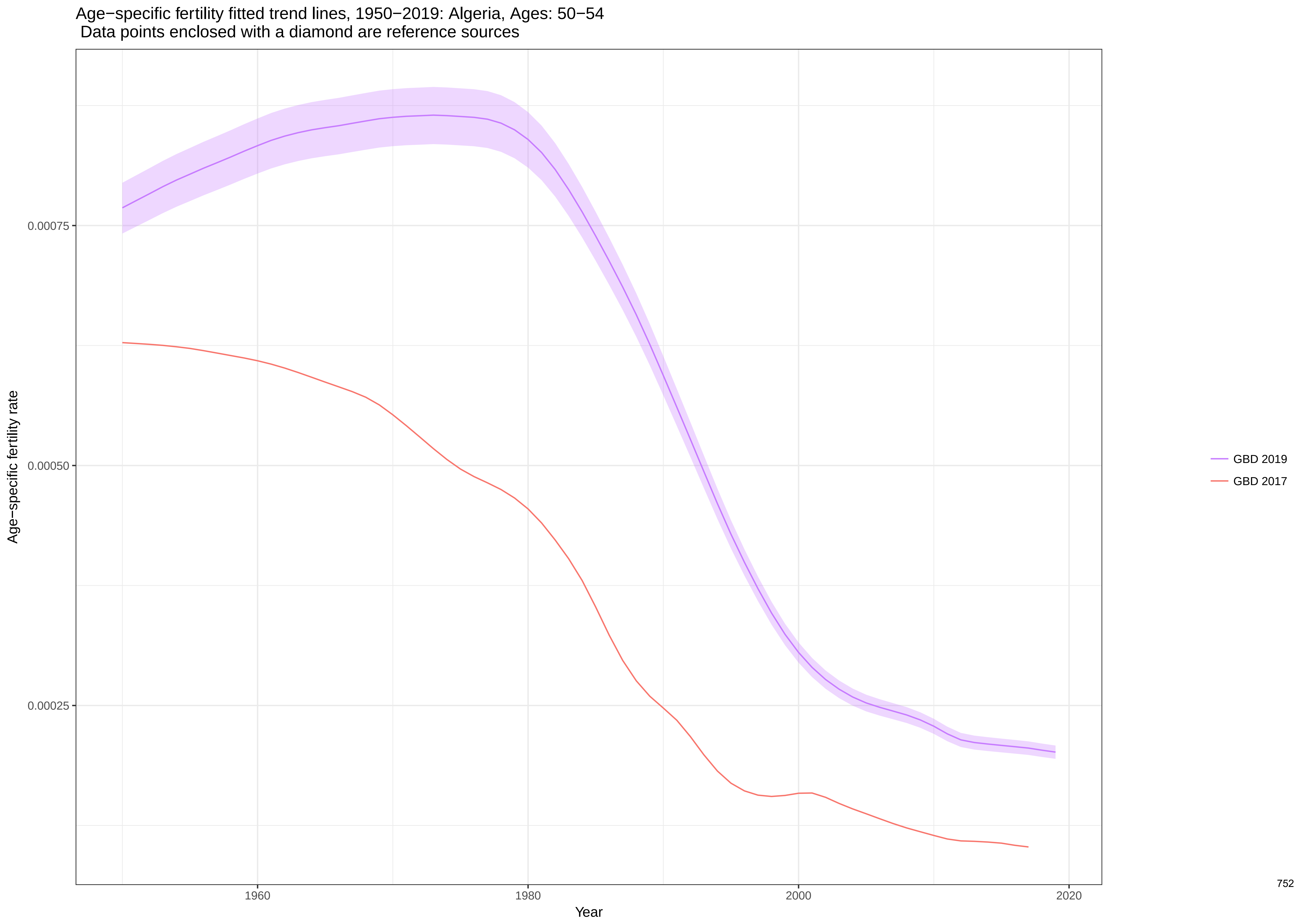
Age−specific fertility fitted trend lines, 1950−2019: Algeria, Ages: 50−54
Data points enclosed with a diamond are reference sources
Age−specific fertility rate
0.00075
0.00050
0.00025
1960
1980
2000
2020
Year
GBD 2019
GBD 2017

Total fertility rate fitted trend lines, 1950−2019: Algeria TFR
Data points enclosed with a diamond are reference sources

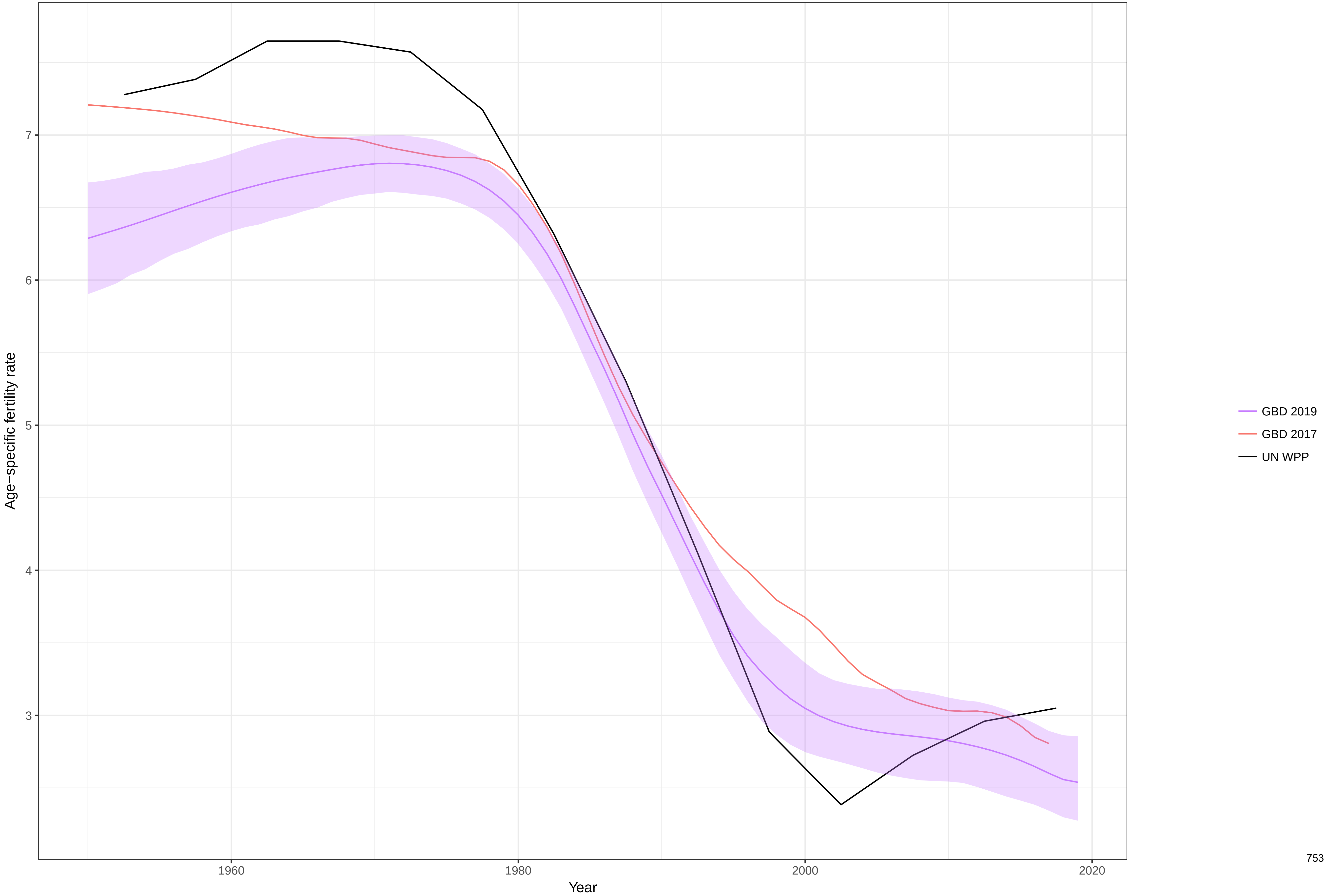

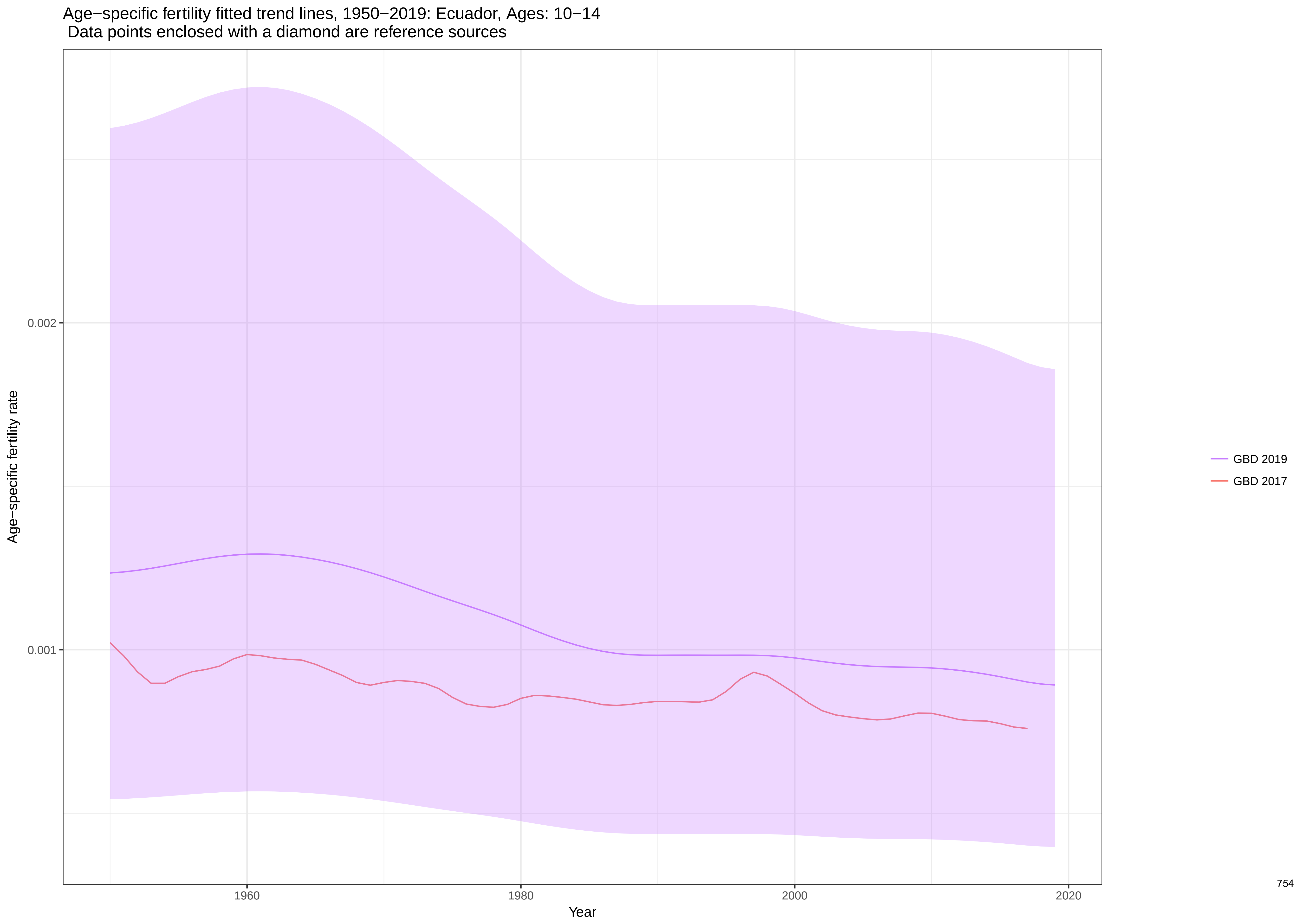
Age−specific fertility fitted trend lines, 1950−2019: Ecuador, Ages: 10−14
Data points enclosed with a diamond are reference sources
Age−specific fertility rate
0.002
0.001
1960
1980
2000
2020
Year
GBD 2019
GBD 2017

Age−specific fertility fitted trend lines, 1950−2019: Ecuador, Ages: 15−19
Data points enclosed with a diamond are reference sources

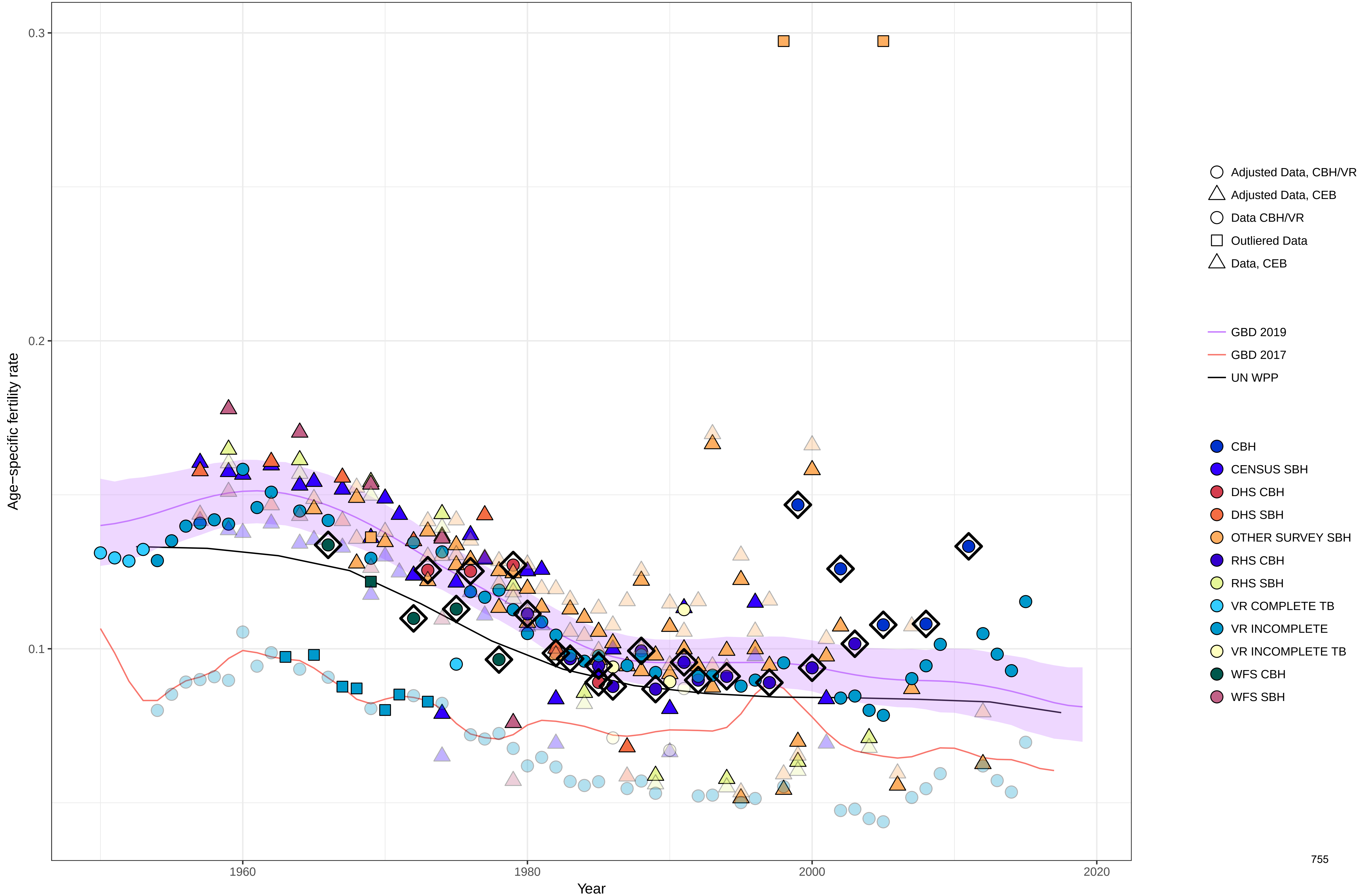

Age−specific fertility fitted trend lines, 1950−2019: Ecuador, Ages: 20−24
Data points enclosed with a diamond are reference sources

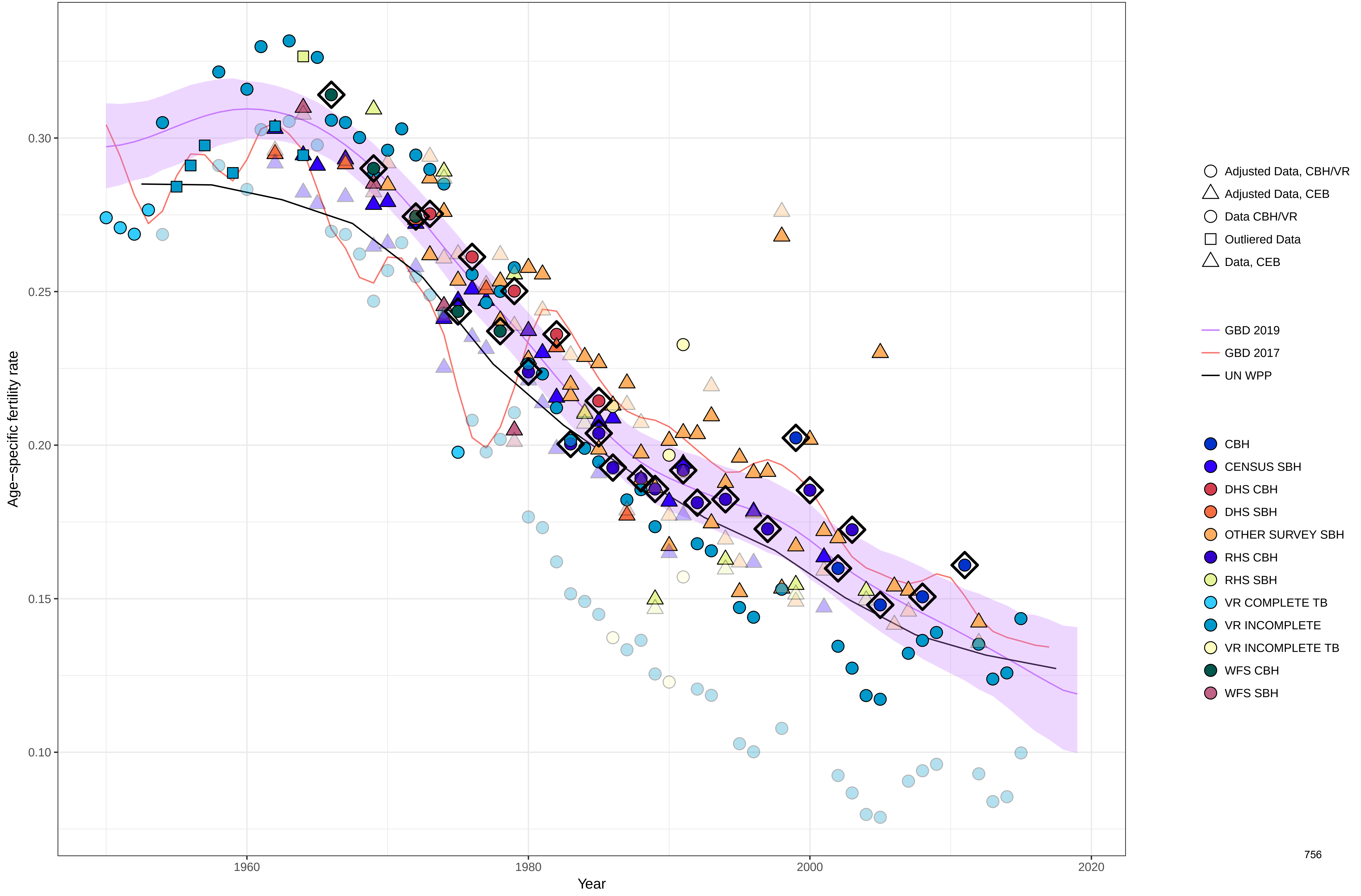

Age−specific fertility fitted trend lines, 1950−2019: Ecuador, Ages: 25−29
Data points enclosed with a diamond are reference sources

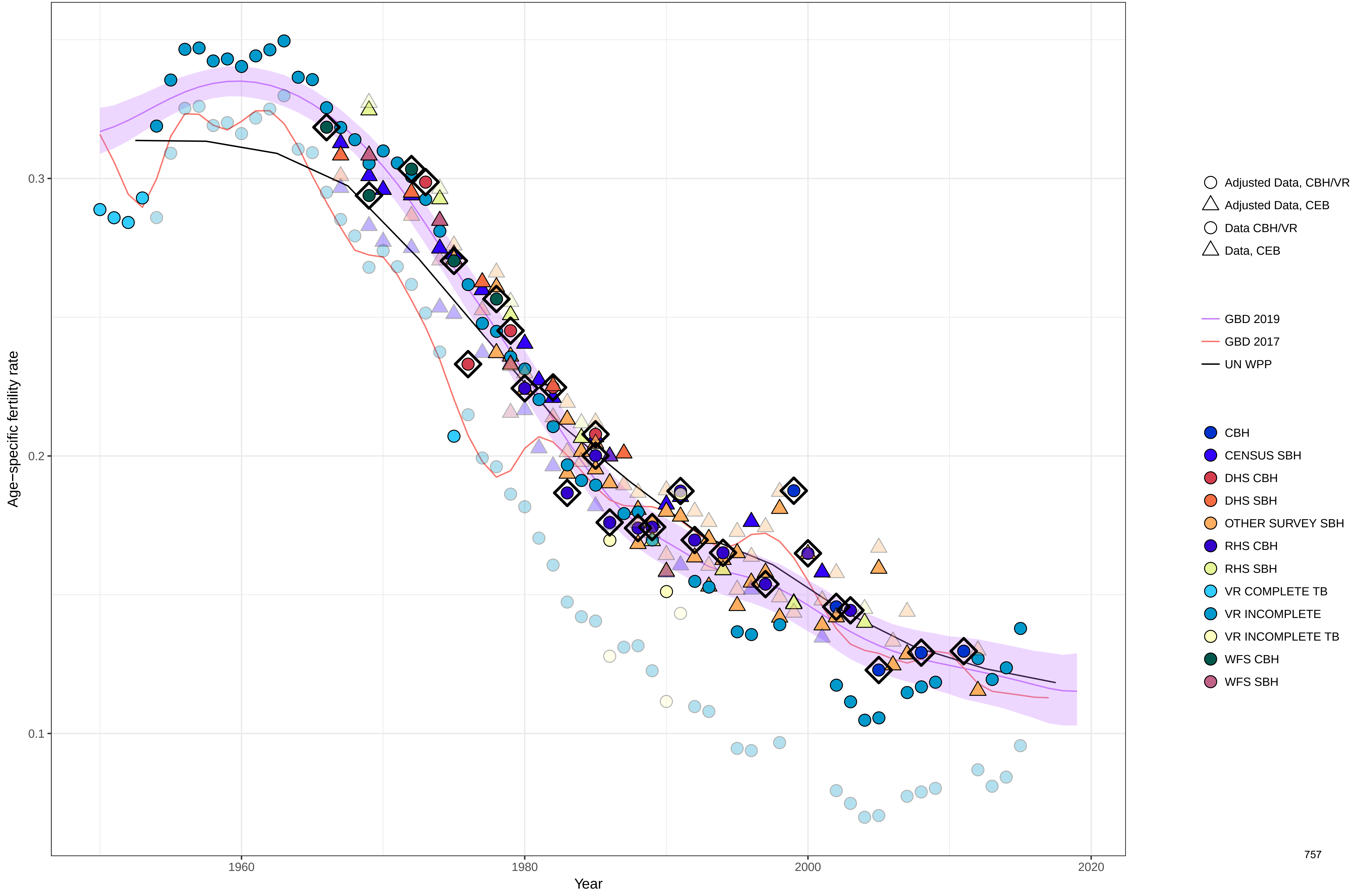

Age−specific fertility fitted trend lines, 1950−2019: Ecuador, Ages: 30−34
Data points enclosed with a diamond are reference sources

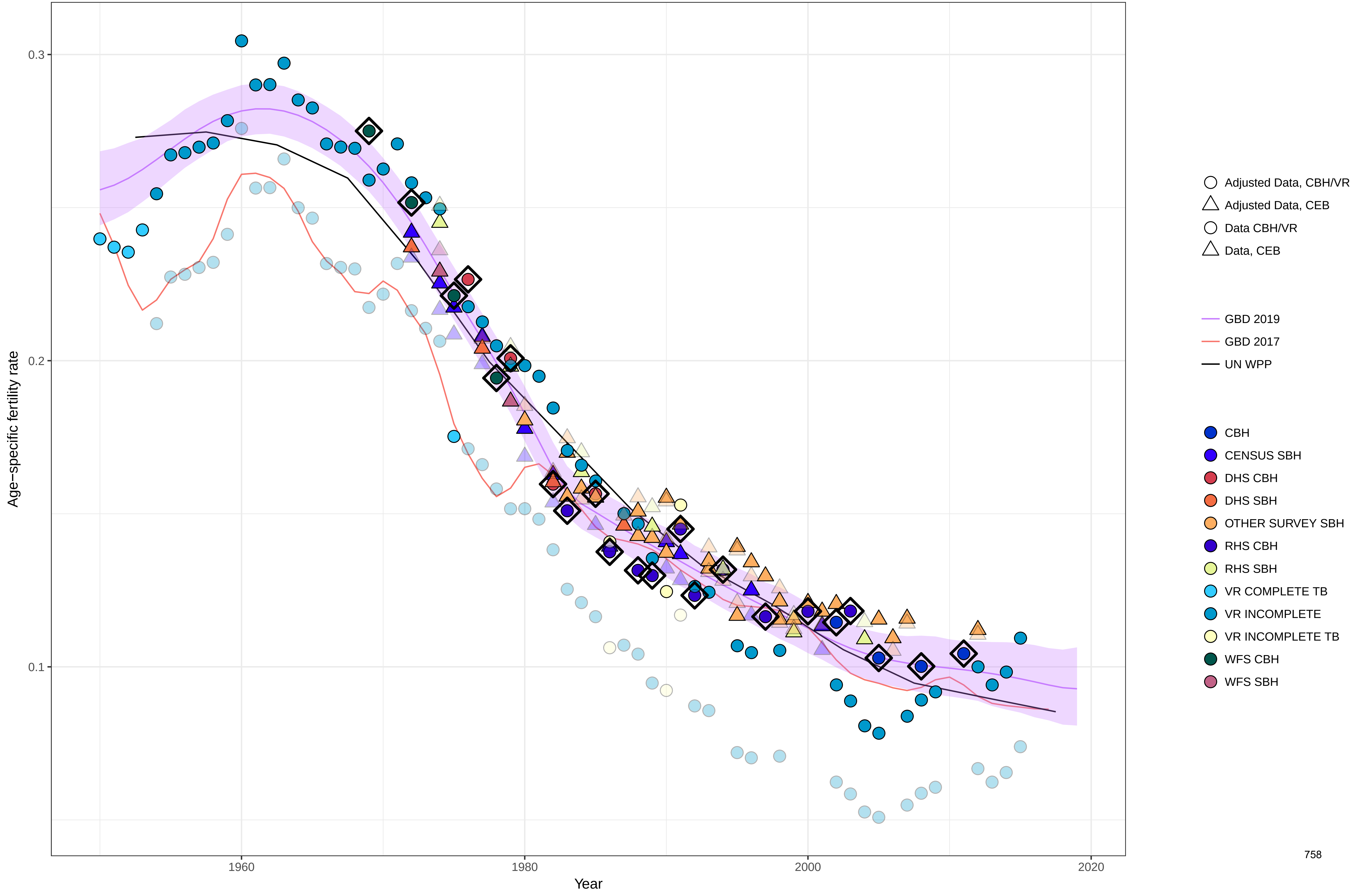

Age−specific fertility fitted trend lines, 1950−2019: Ecuador, Ages: 35−39
Data points enclosed with a diamond are reference sources

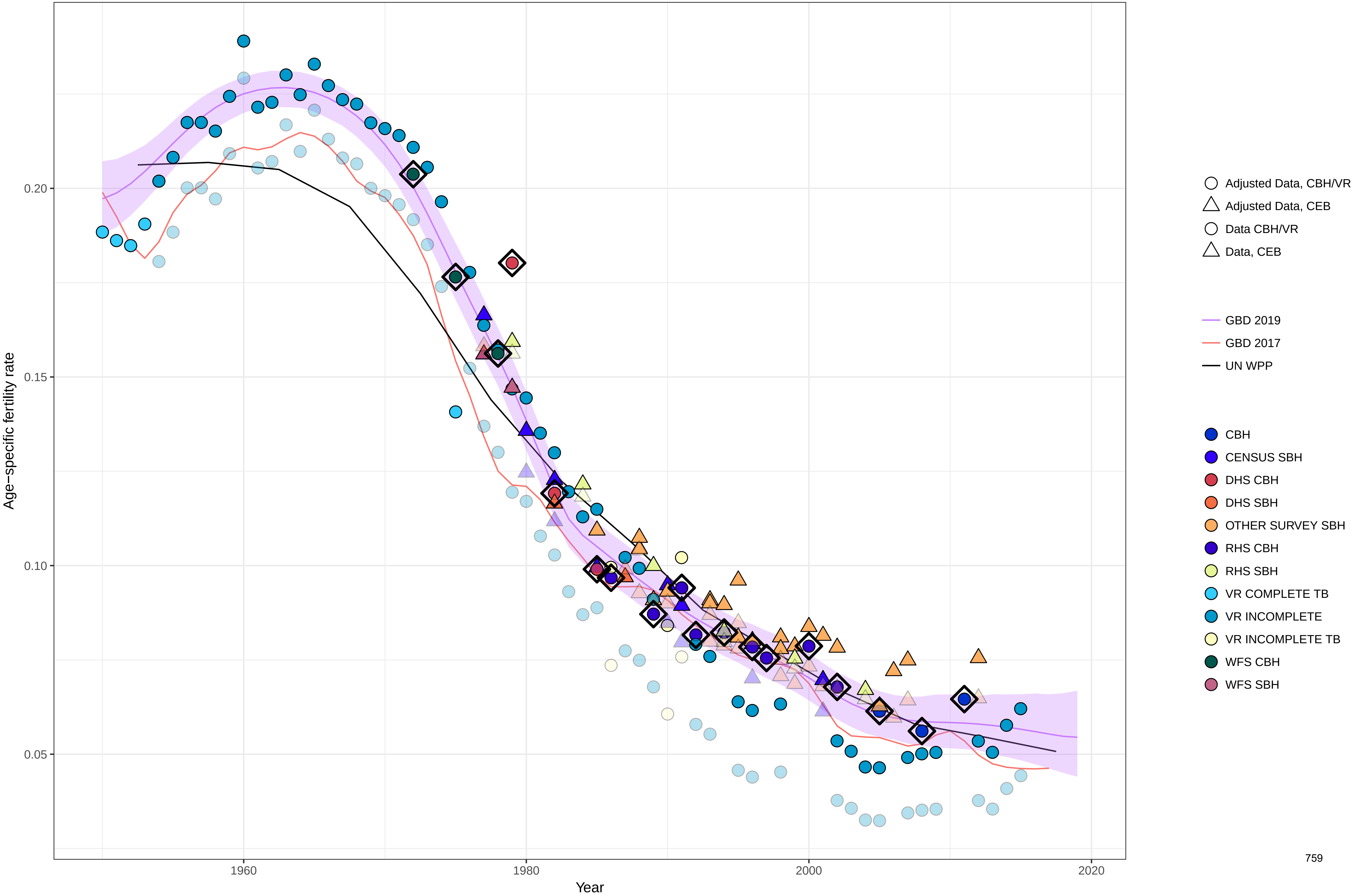

Age−specific fertility fitted trend lines, 1950−2019: Ecuador, Ages: 40−44
Data points enclosed with a diamond are reference sources

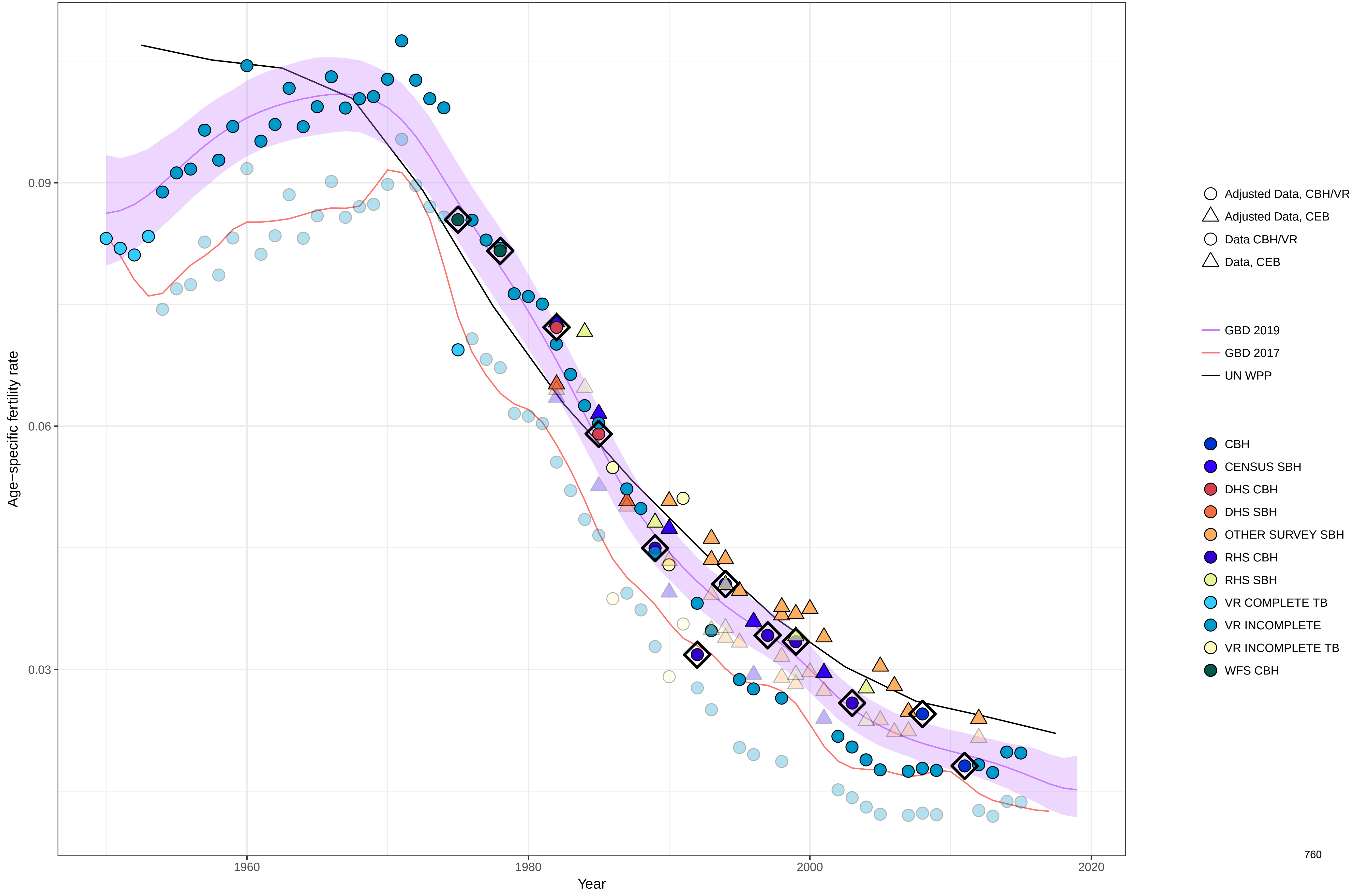

Age−specific fertility fitted trend lines, 1950−2019: Ecuador, Ages: 45−49
Data points enclosed with a diamond are reference sources

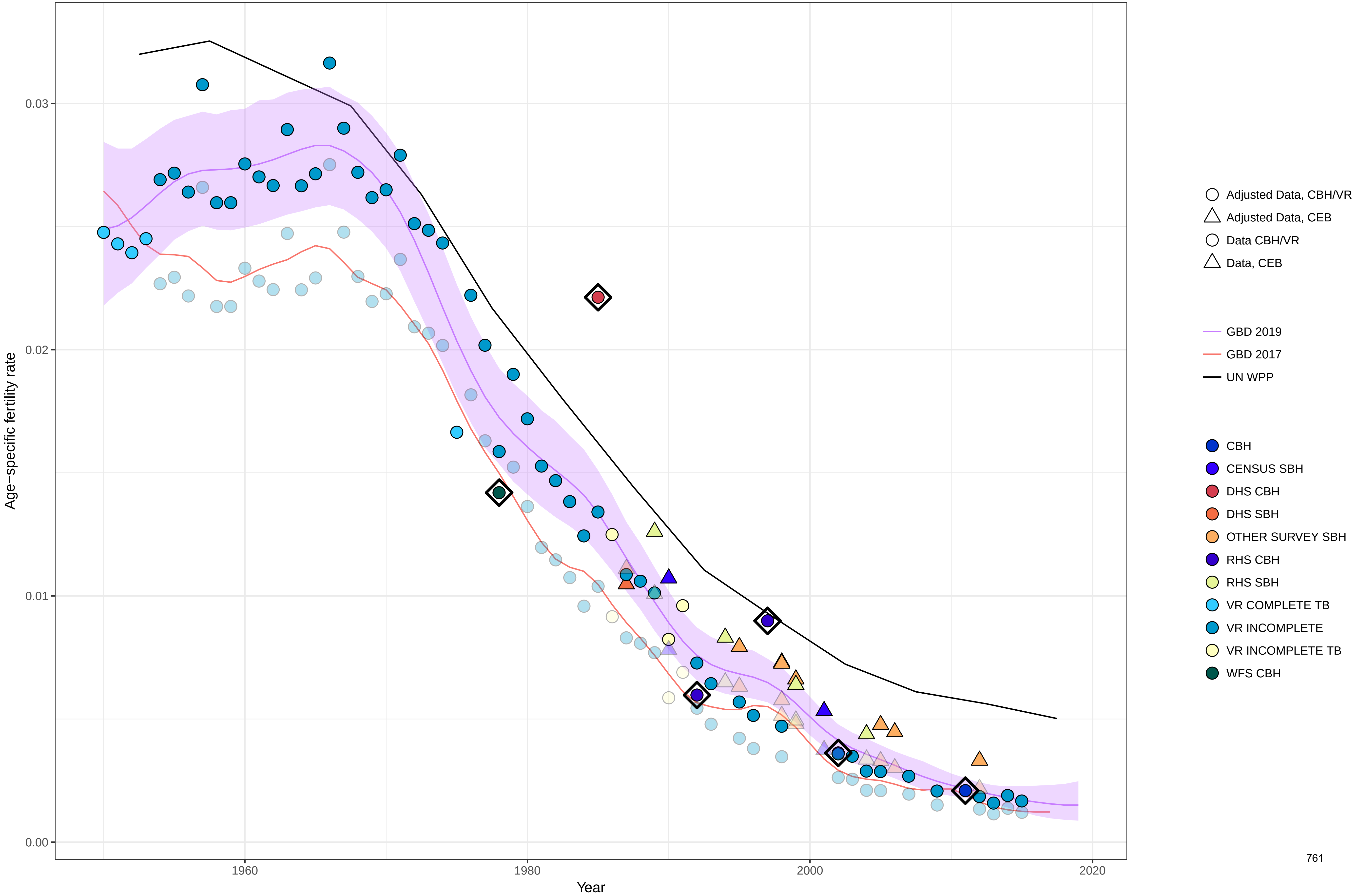

Age−specific fertility fitted trend lines, 1950−2019: Ecuador, Ages: 50−54
Data points enclosed with a diamond are reference sources

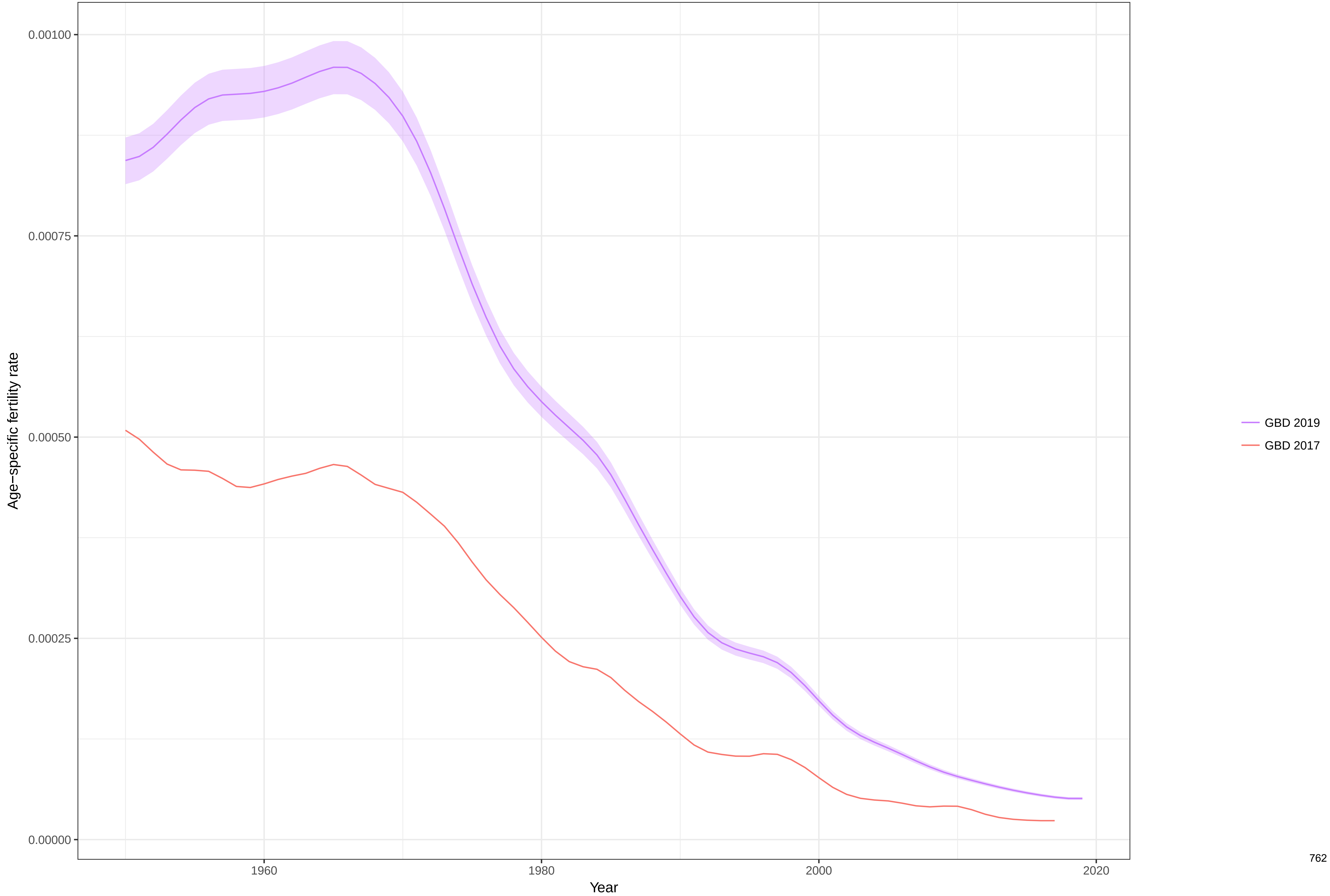

Total fertility rate fitted trend lines, 1950−2019: Ecuador TFR
Data points enclosed with a diamond are reference sources

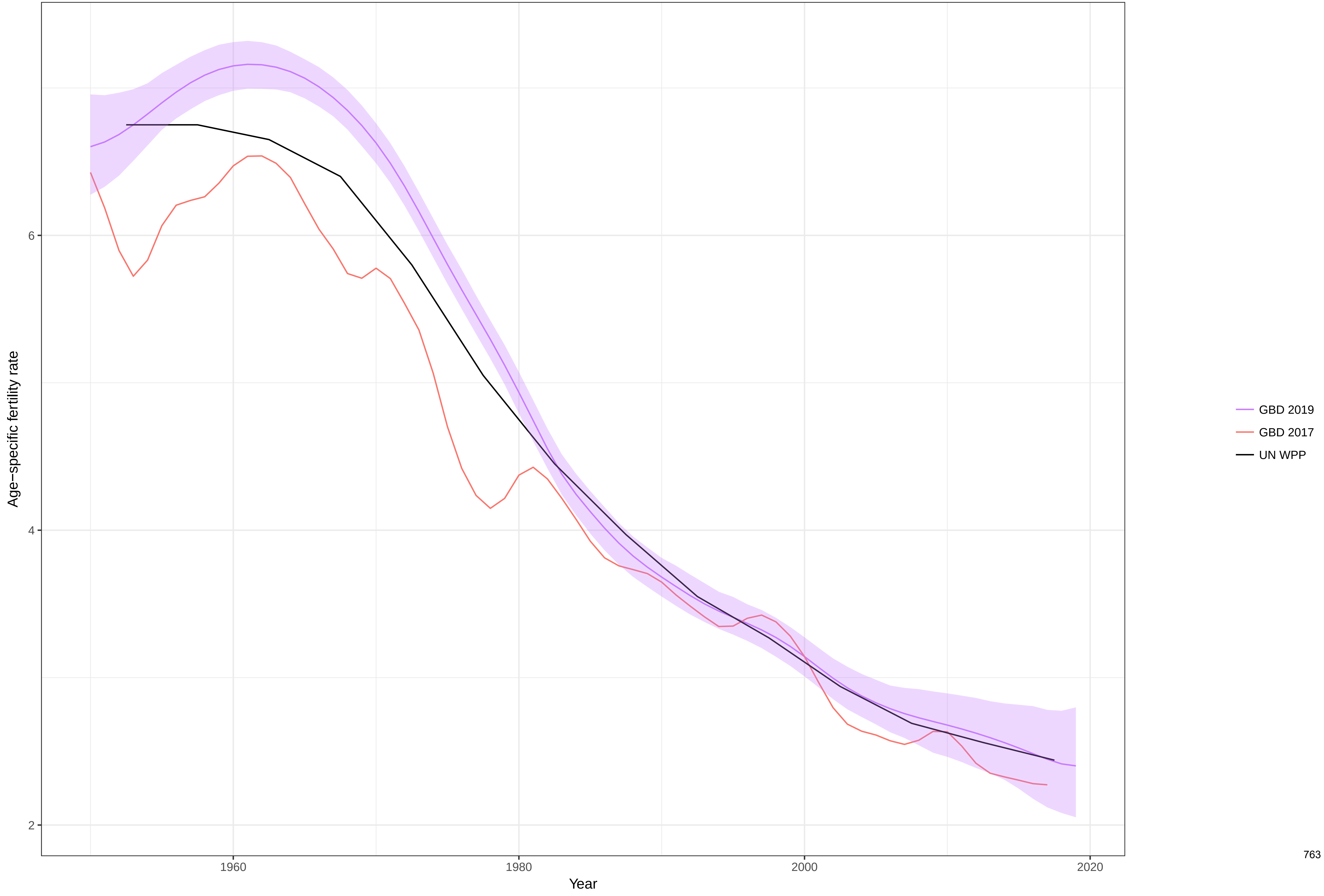

Age−specific fertility fitted trend lines, 1950−2019: Egypt, Ages: 10−14
Data points enclosed with a diamond are reference sources

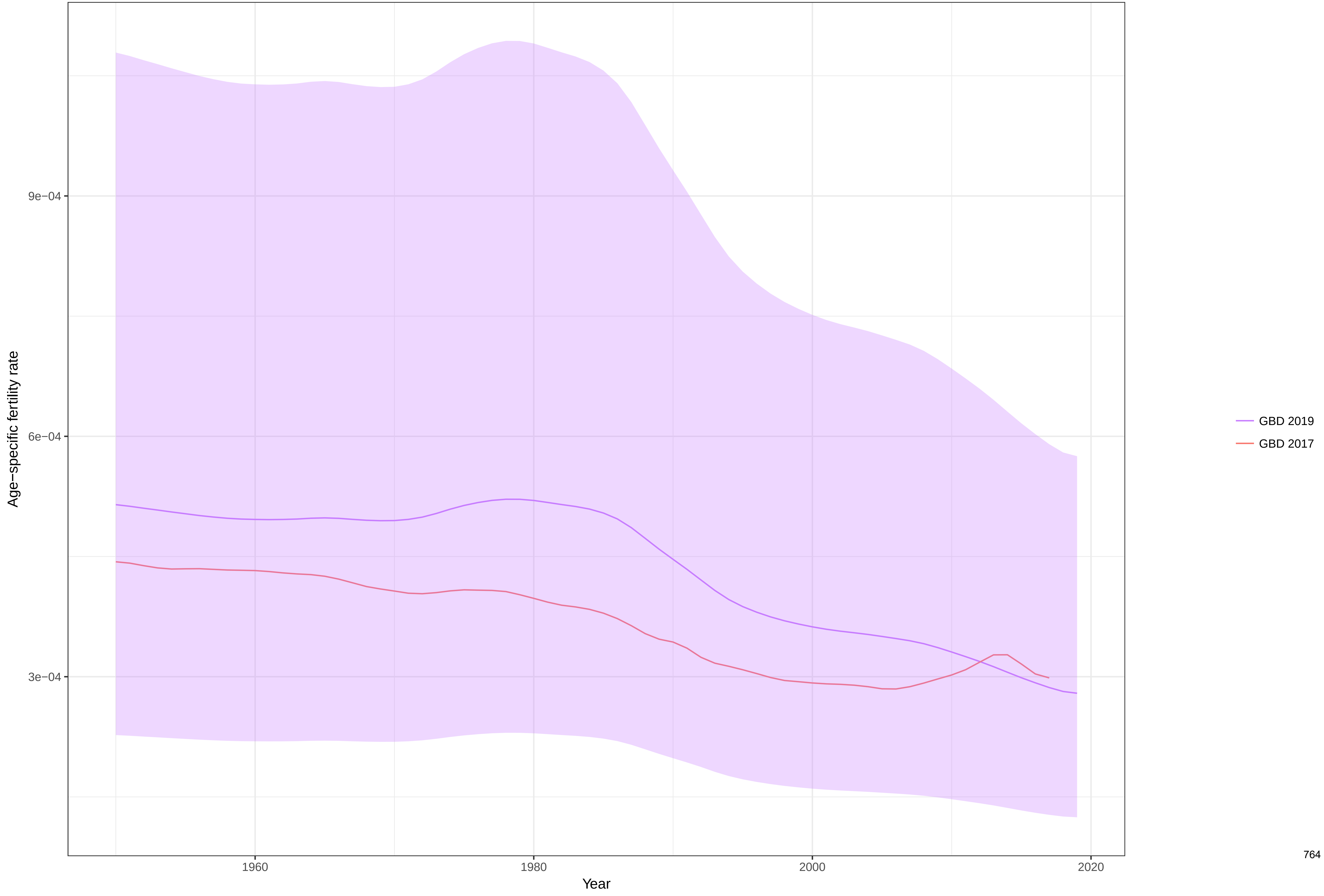

Age−specific fertility fitted trend lines, 1950−2019: Egypt, Ages: 15−19
Data points enclosed with a diamond are reference sources

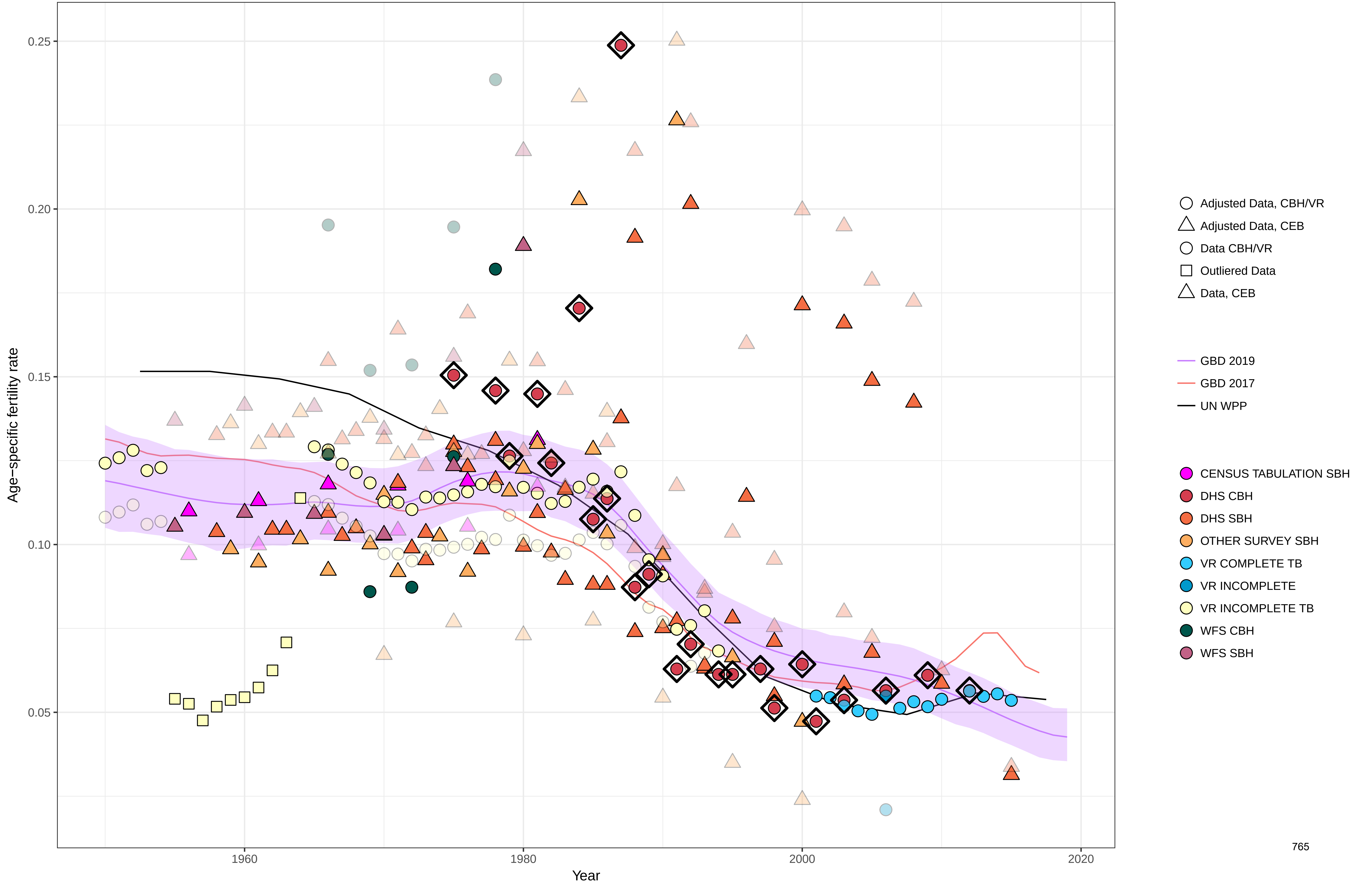

Age−specific fertility fitted trend lines, 1950−2019: Egypt, Ages: 20−24
Data points enclosed with a diamond are reference sources

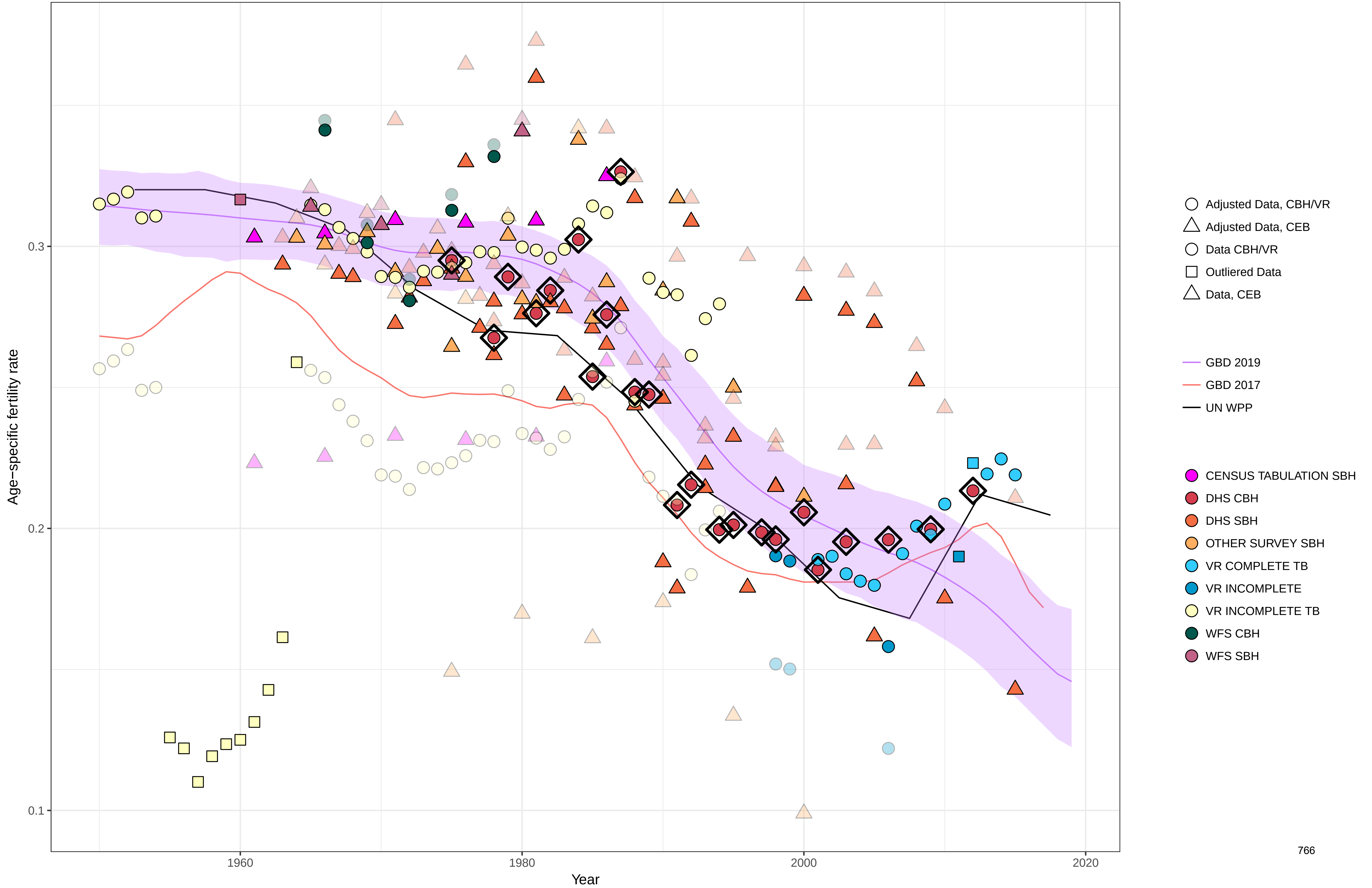

Age−specific fertility fitted trend lines, 1950−2019: Egypt, Ages: 25−29
Data points enclosed with a diamond are reference sources

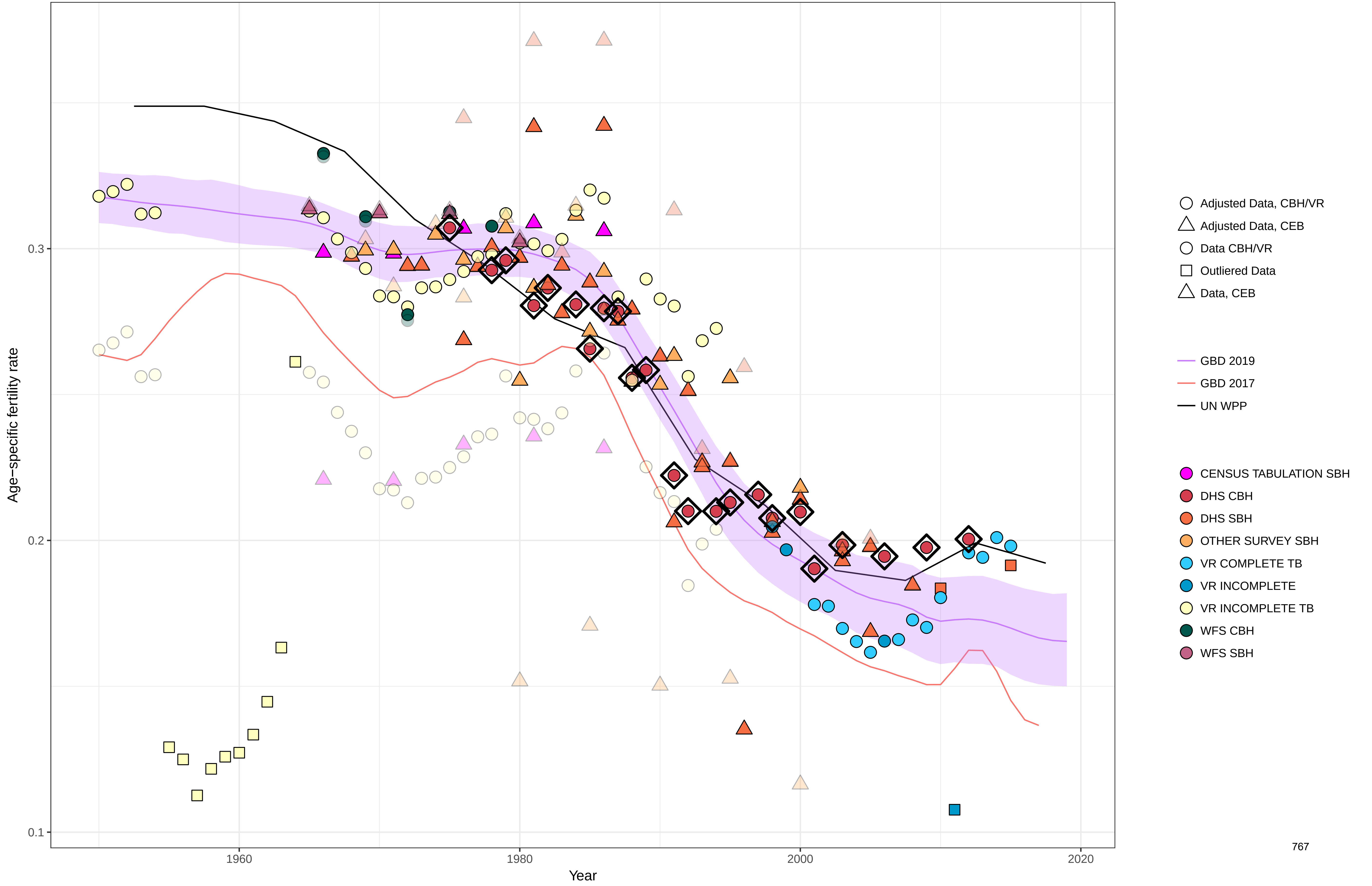

Age−specific fertility fitted trend lines, 1950−2019: Egypt, Ages: 30−34
Data points enclosed with a diamond are reference sources

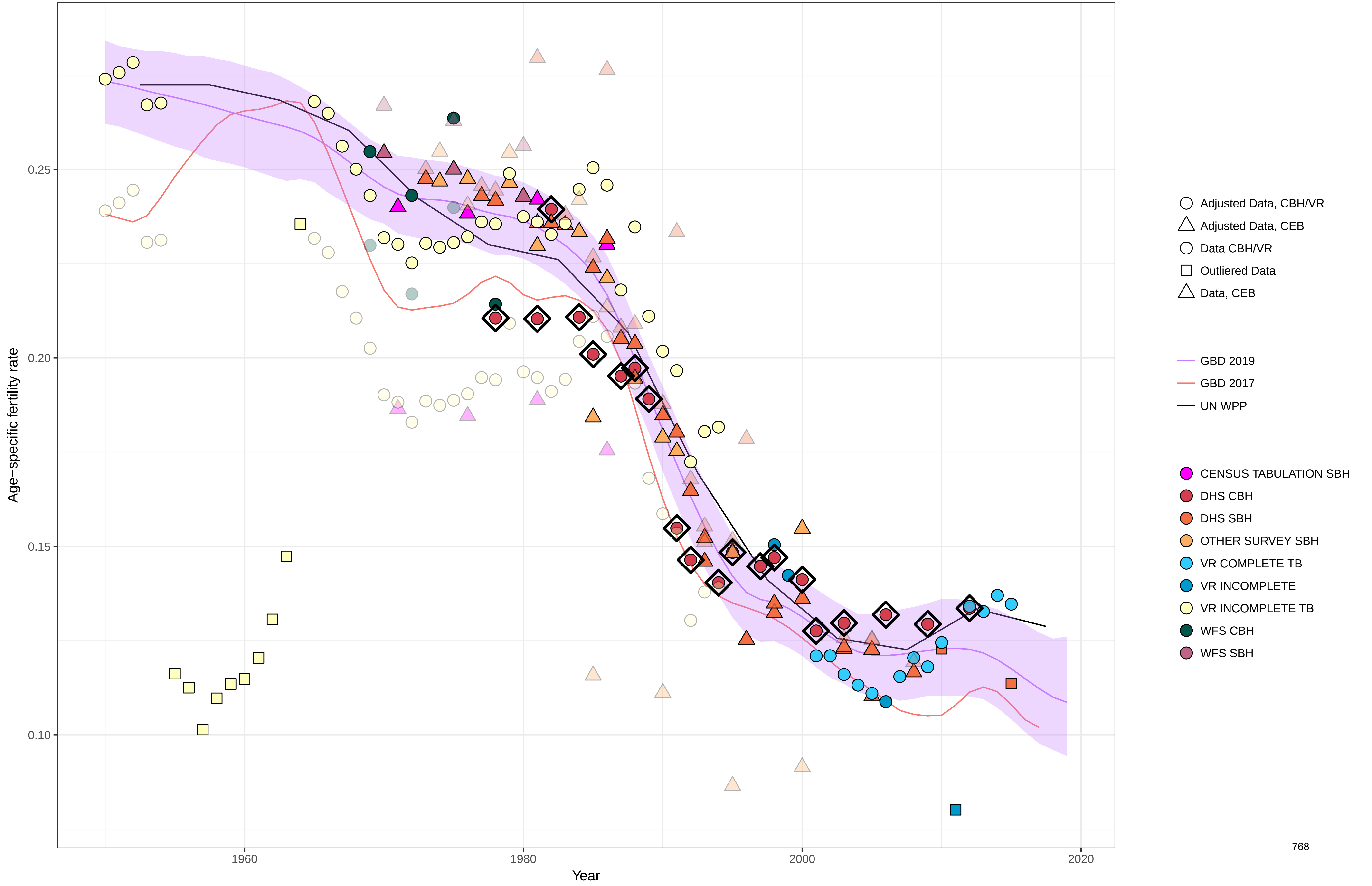

Age−specific fertility fitted trend lines, 1950−2019: Egypt, Ages: 35−39
Data points enclosed with a diamond are reference sources

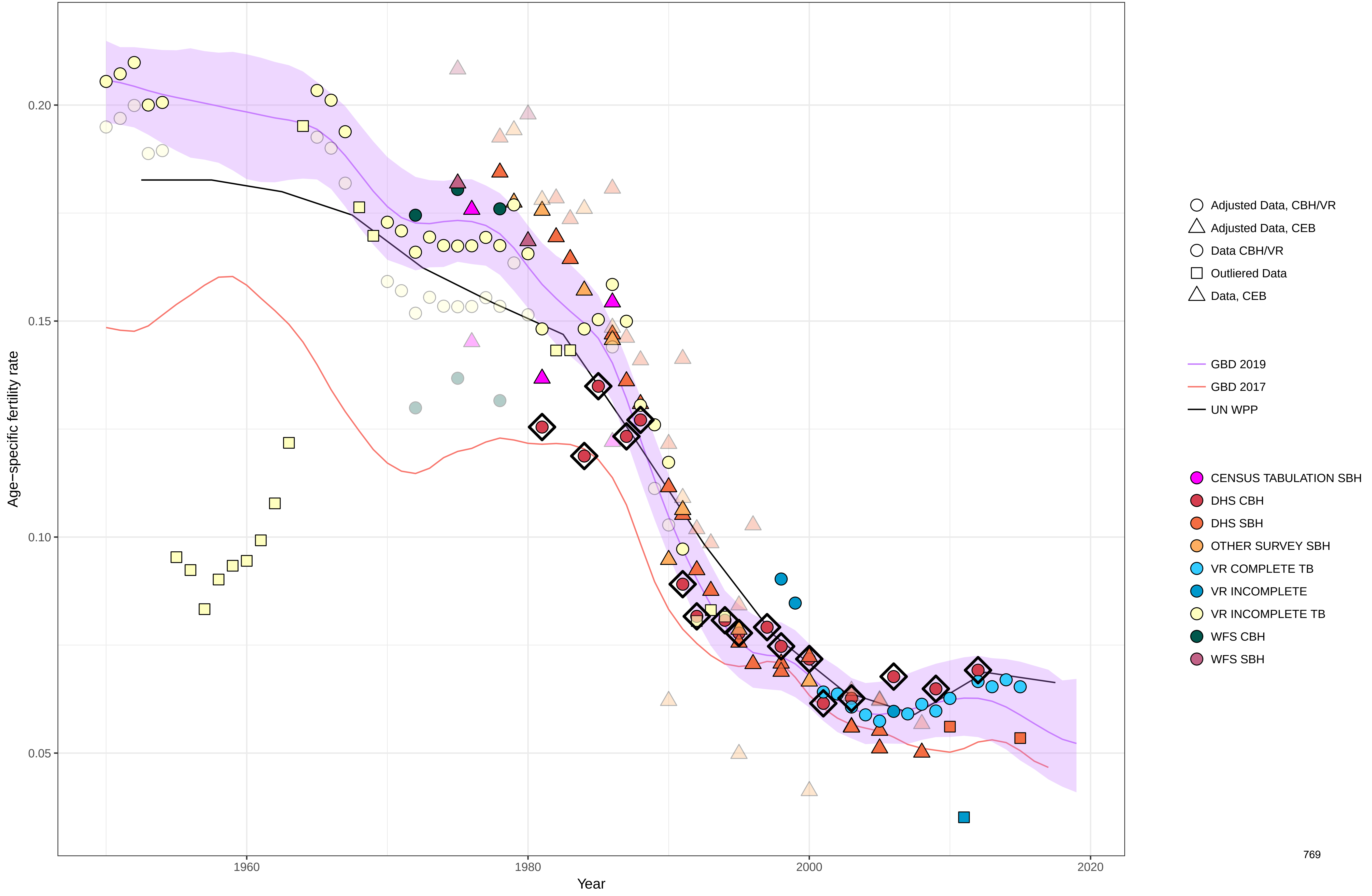

Age−specific fertility fitted trend lines, 1950−2019: Egypt, Ages: 40−44
Data points enclosed with a diamond are reference sources

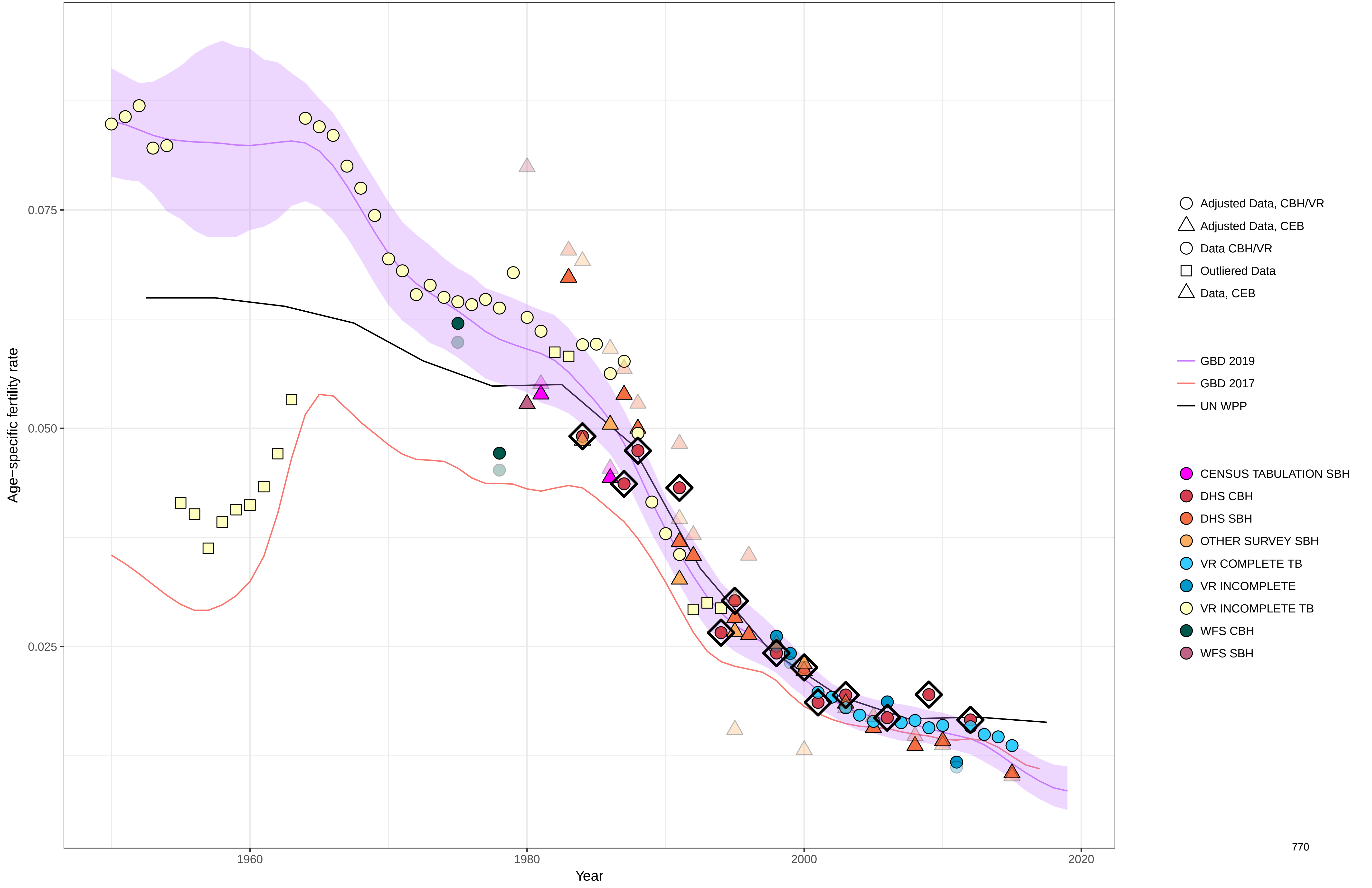

Age−specific fertility fitted trend lines, 1950−2019: Egypt, Ages: 45−49
Data points enclosed with a diamond are reference sources

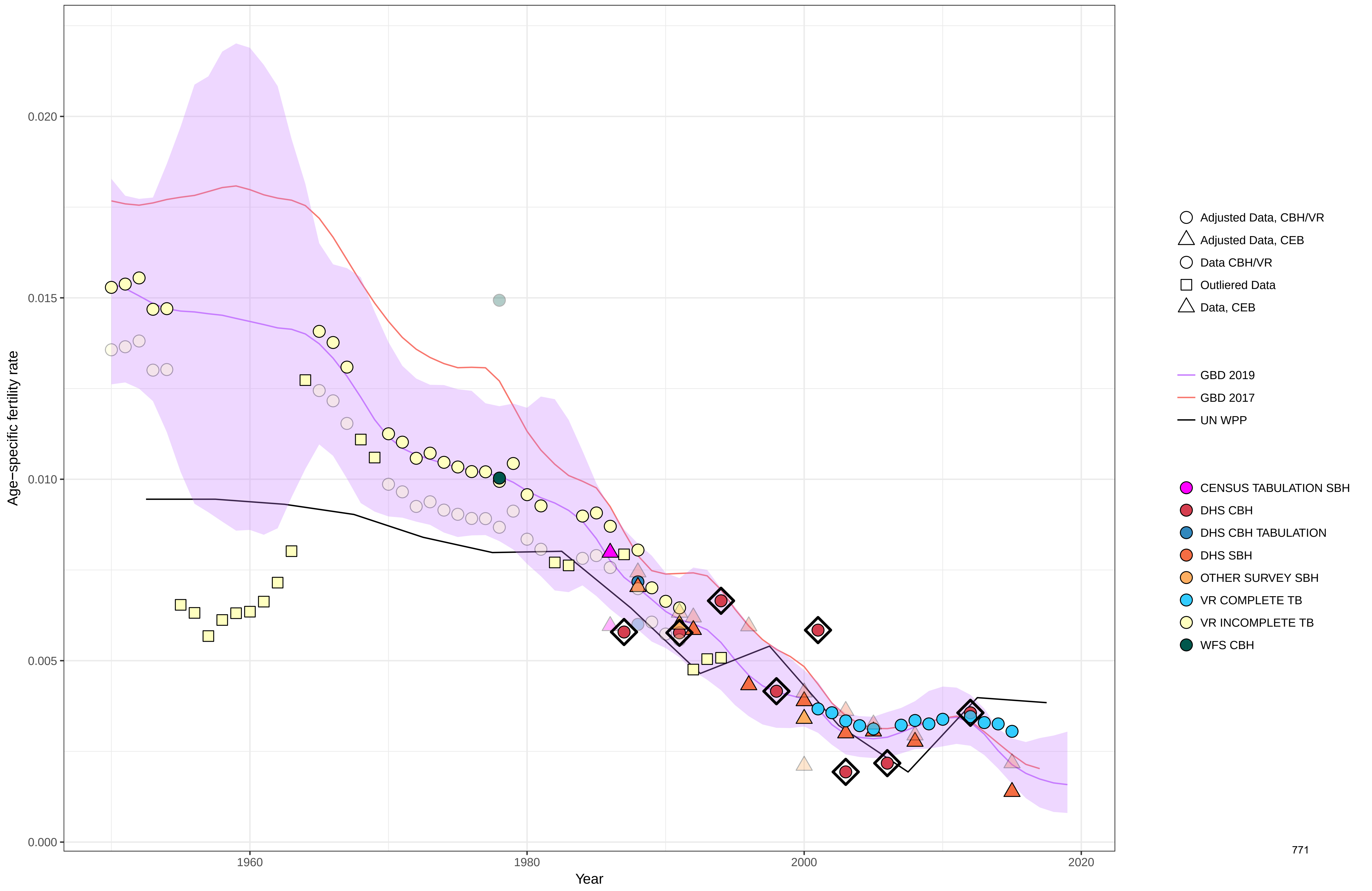

Age−specific fertility fitted trend lines, 1950−2019: Egypt, Ages: 50−54
Data points enclosed with a diamond are reference sources

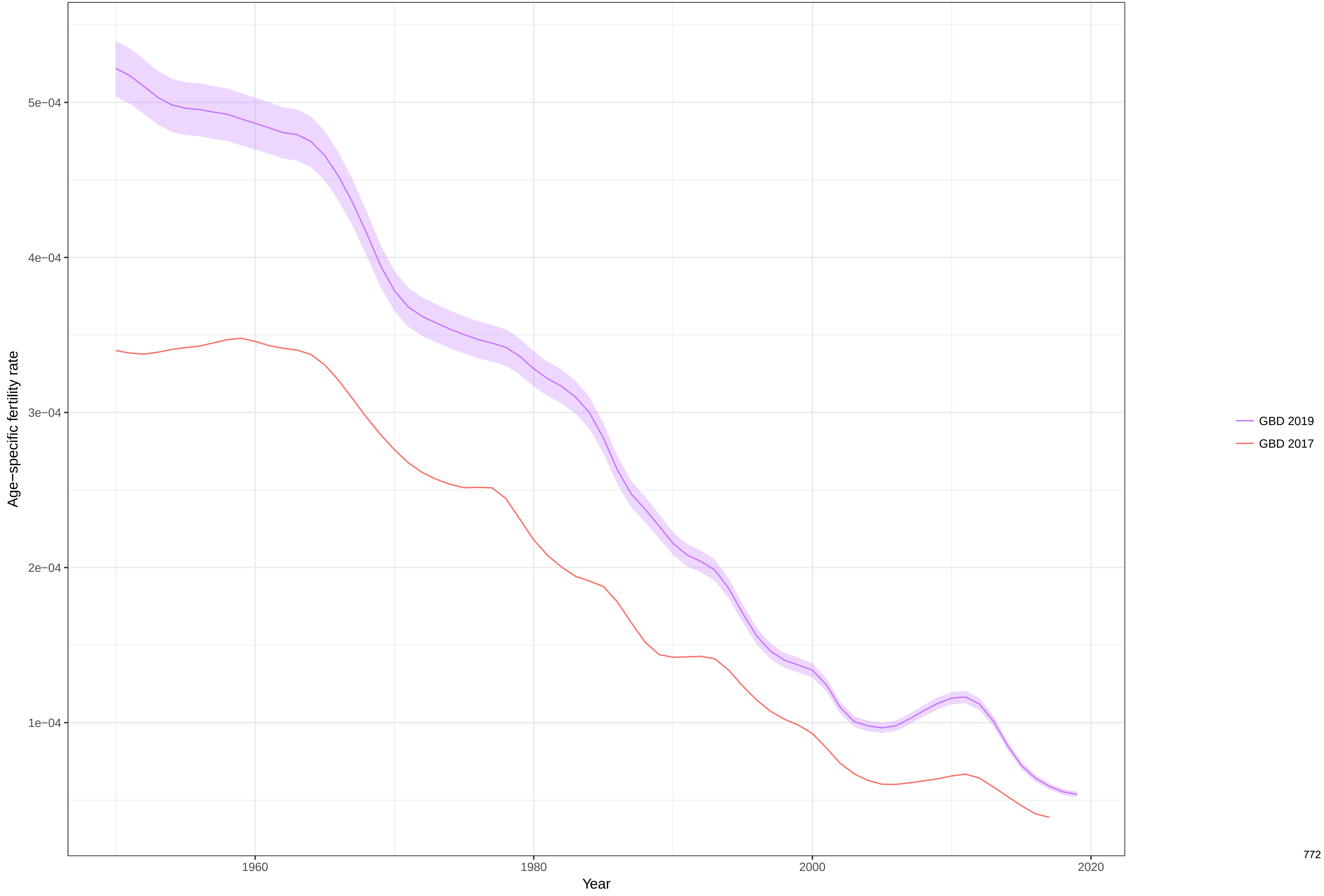

Total fertility rate fitted trend lines, 1950−2019: Egypt TFR
Data points enclosed with a diamond are reference sources

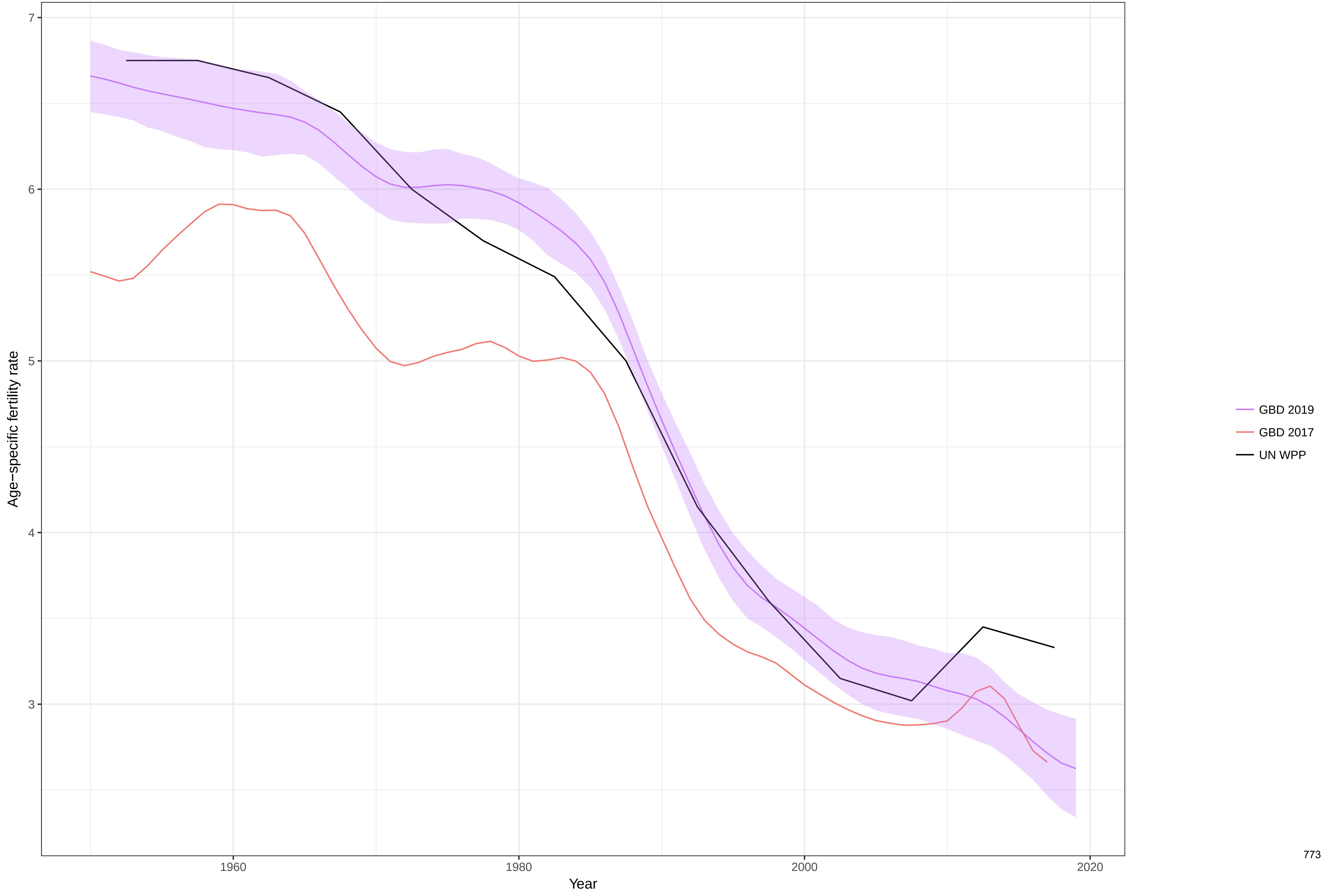

Age−specific fertility fitted trend lines, 1950−2019: Eritrea, Ages: 10−14
Data points enclosed with a diamond are reference sources

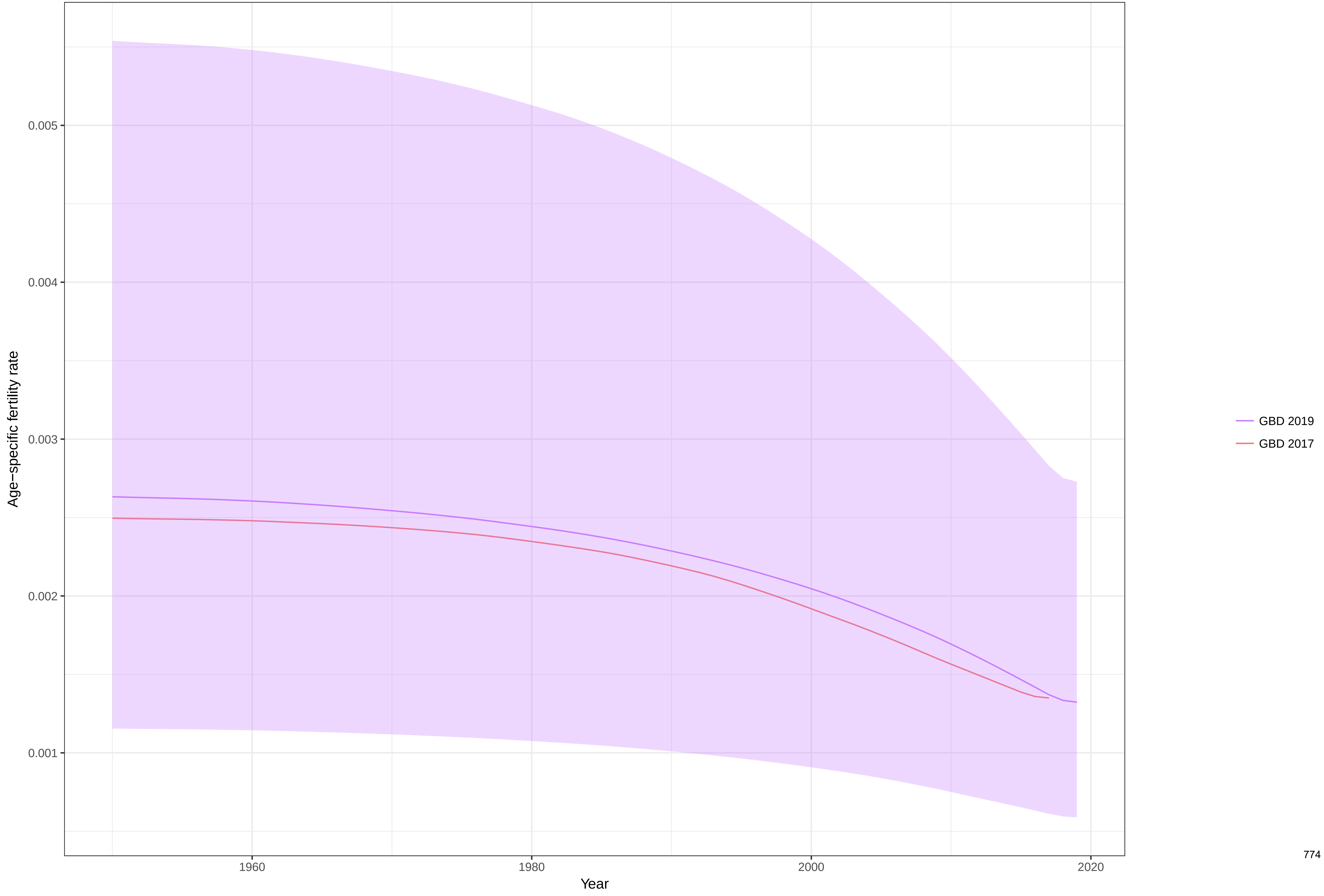

Age−specific fertility fitted trend lines, 1950−2019: Eritrea, Ages: 15−19
Data points enclosed with a diamond are reference sources

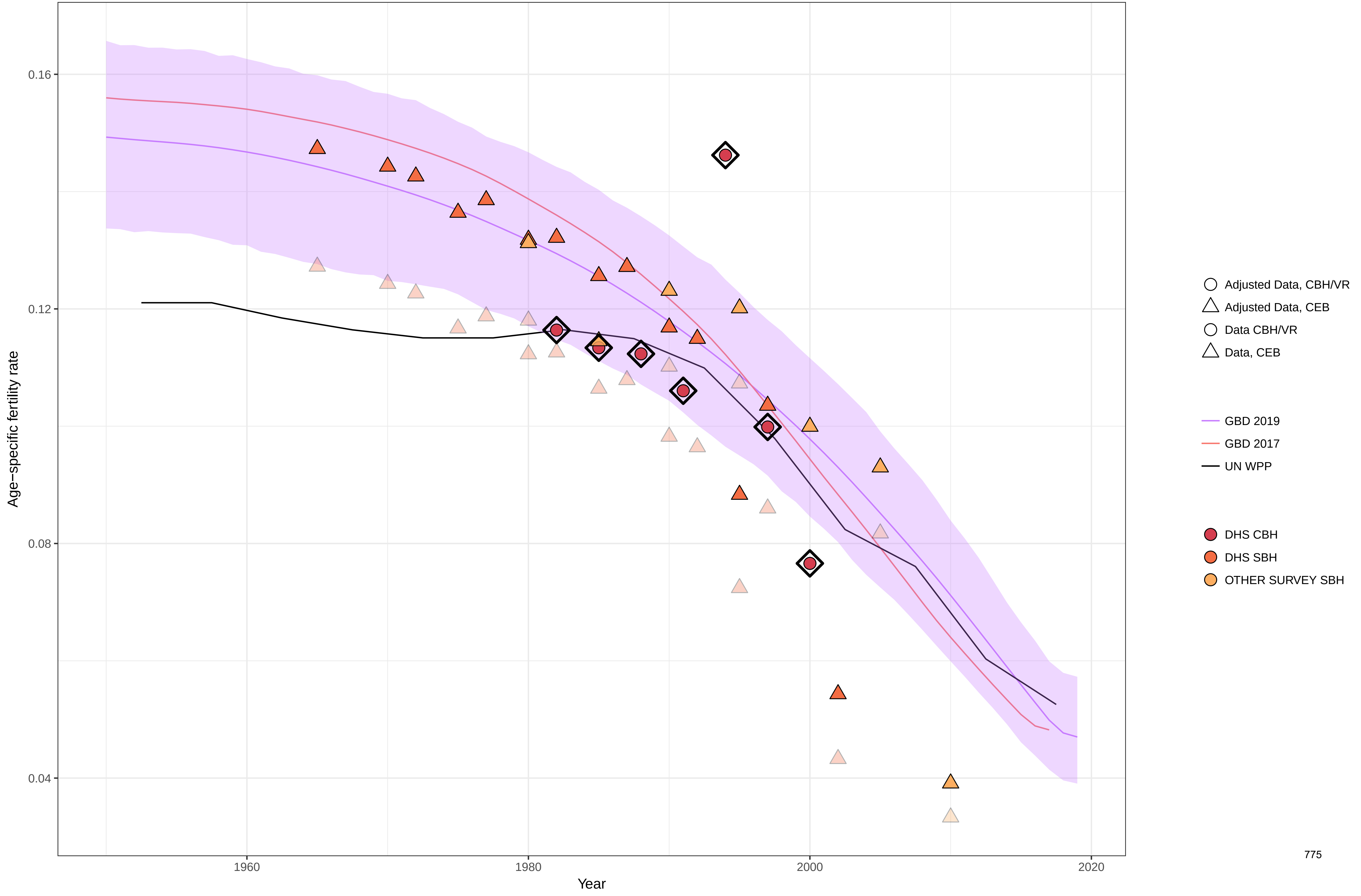

Age−specific fertility fitted trend lines, 1950−2019: Eritrea, Ages: 20−24
Data points enclosed with a diamond are reference sources

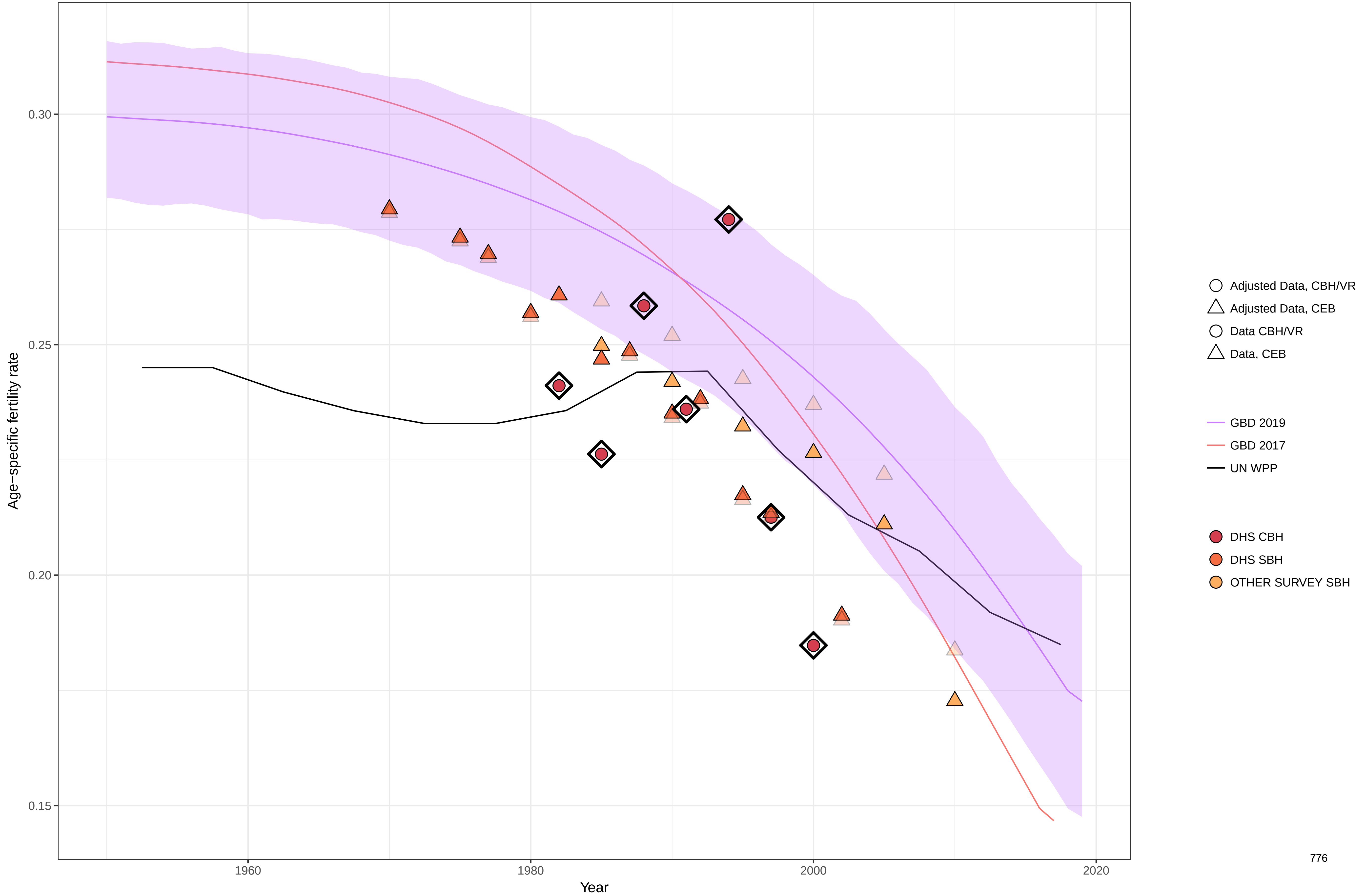

Age−specific fertility fitted trend lines, 1950−2019: Eritrea, Ages: 25−29
Data points enclosed with a diamond are reference sources

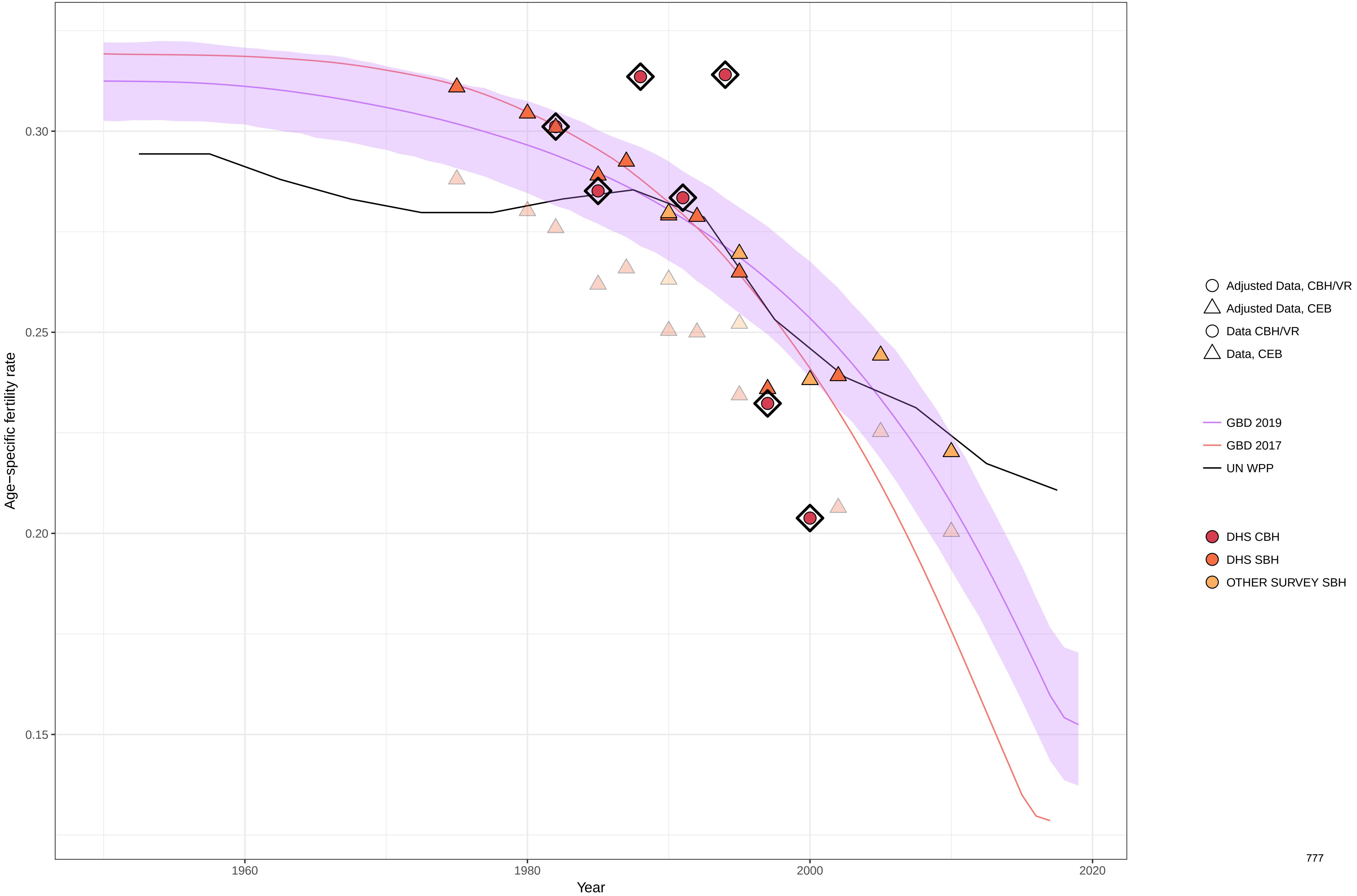

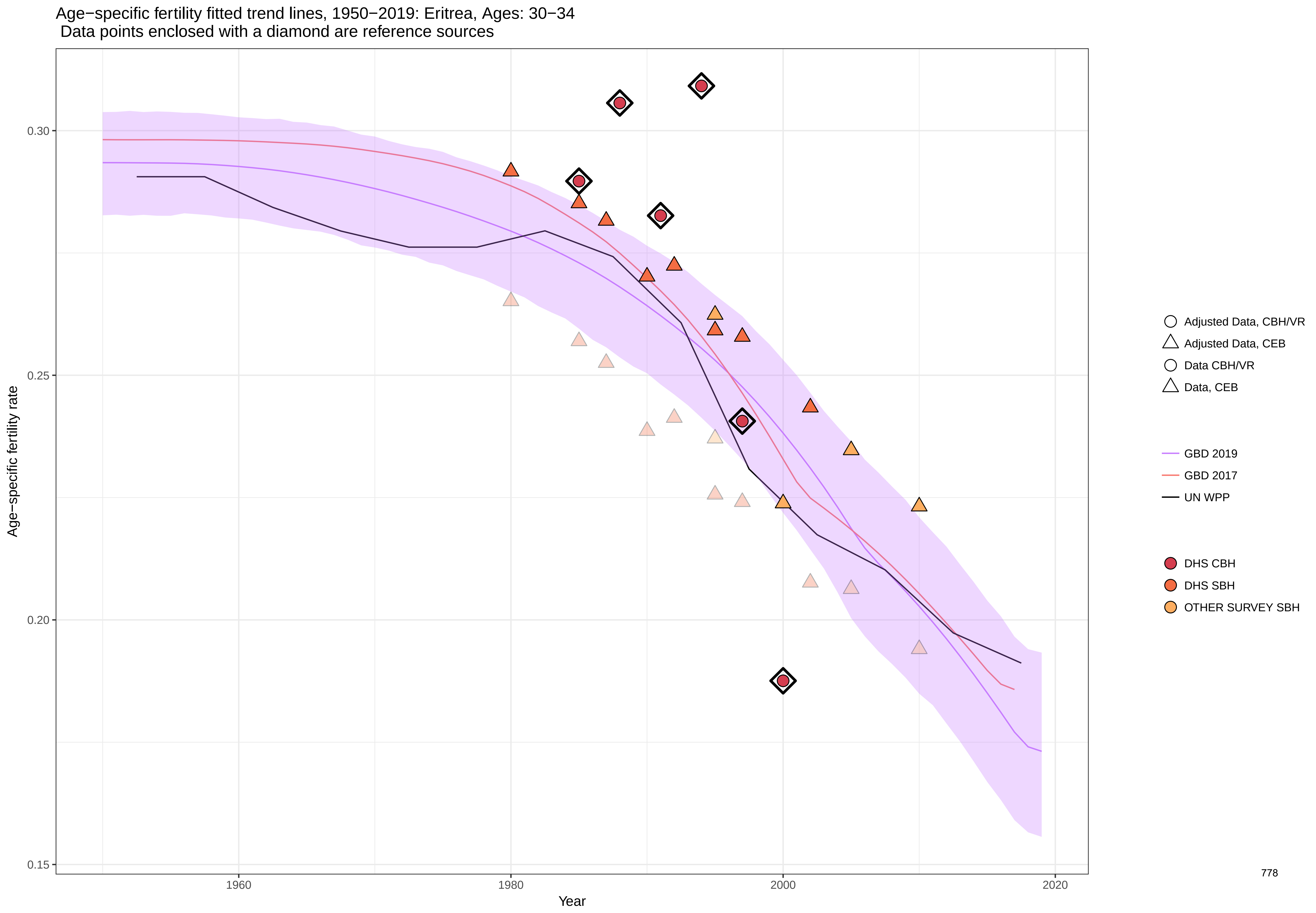

Age−specific fertility fitted trend lines, 1950−2019: Eritrea, Ages: 30−34
Data points enclosed with a diamond are reference sources
Age−specific fertility rate
0.30
0.25
0.20
0.15
1960
1980
2000
2020
Year
Adjusted Data, CBH/VR
Adjusted Data, CEB
Data CBH/VR
Data, CEB
GBD 2019
GBD 2017
UN WPP
DHS CBH
DHS SBH
OTHER SURVEY SBH

Age−specific fertility fitted trend lines, 1950−2019: Eritrea, Ages: 35−39
Data points enclosed with a diamond are reference sources

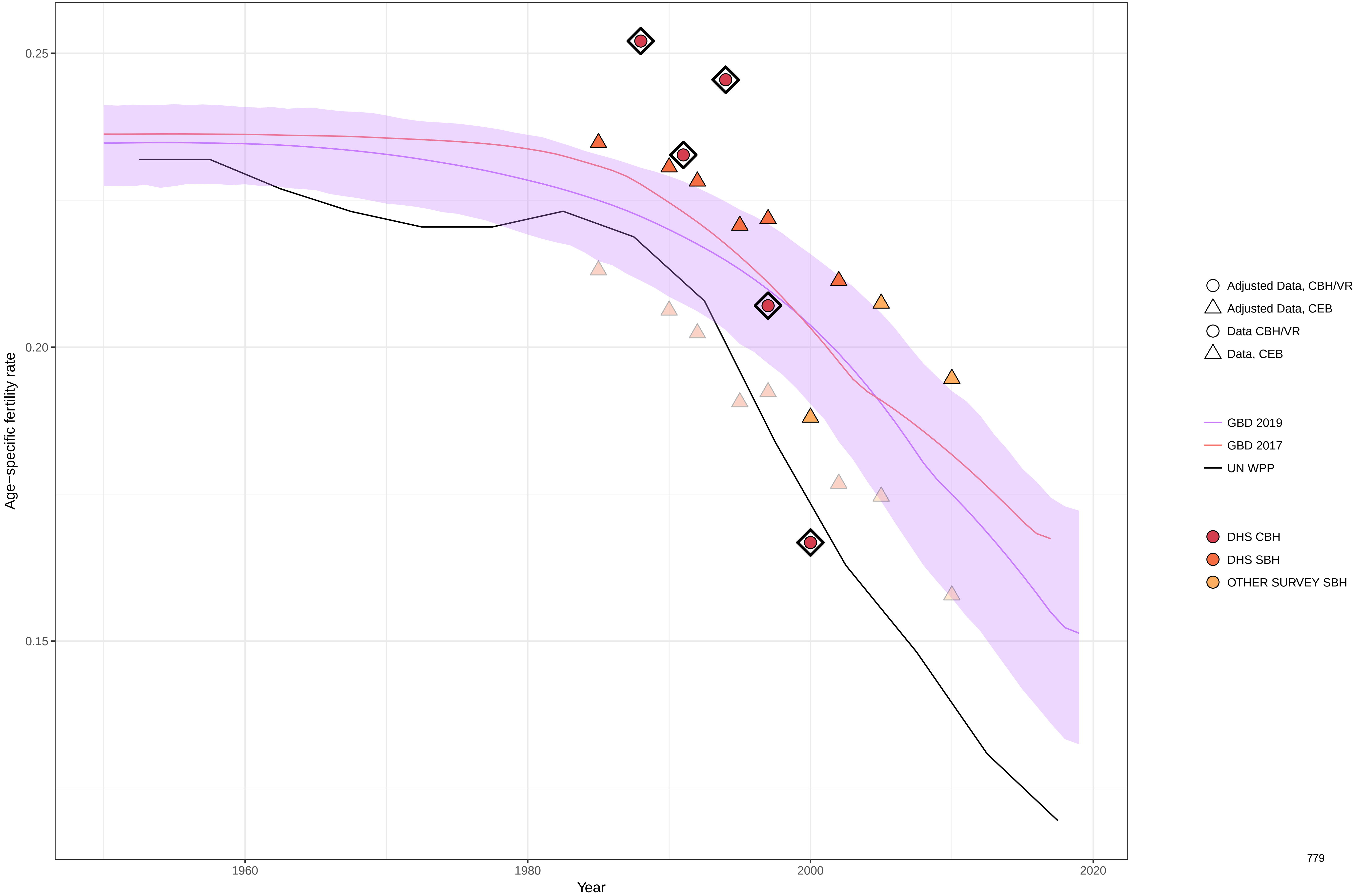

Age−specific fertility fitted trend lines, 1950−2019: Eritrea, Ages: 40−44
Data points enclosed with a diamond are reference sources

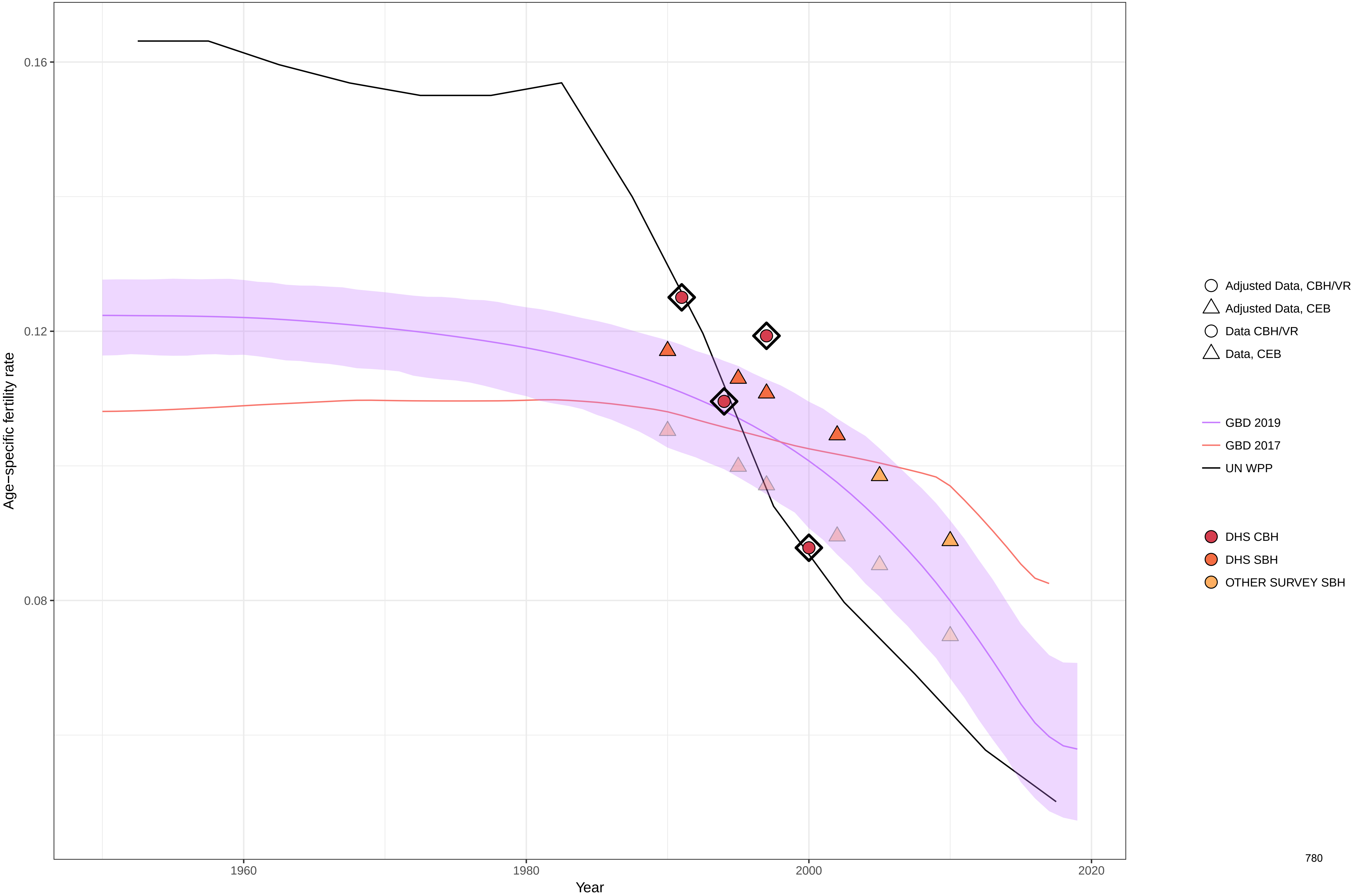

Age−specific fertility fitted trend lines, 1950−2019: Eritrea, Ages: 45−49
Data points enclosed with a diamond are reference sources

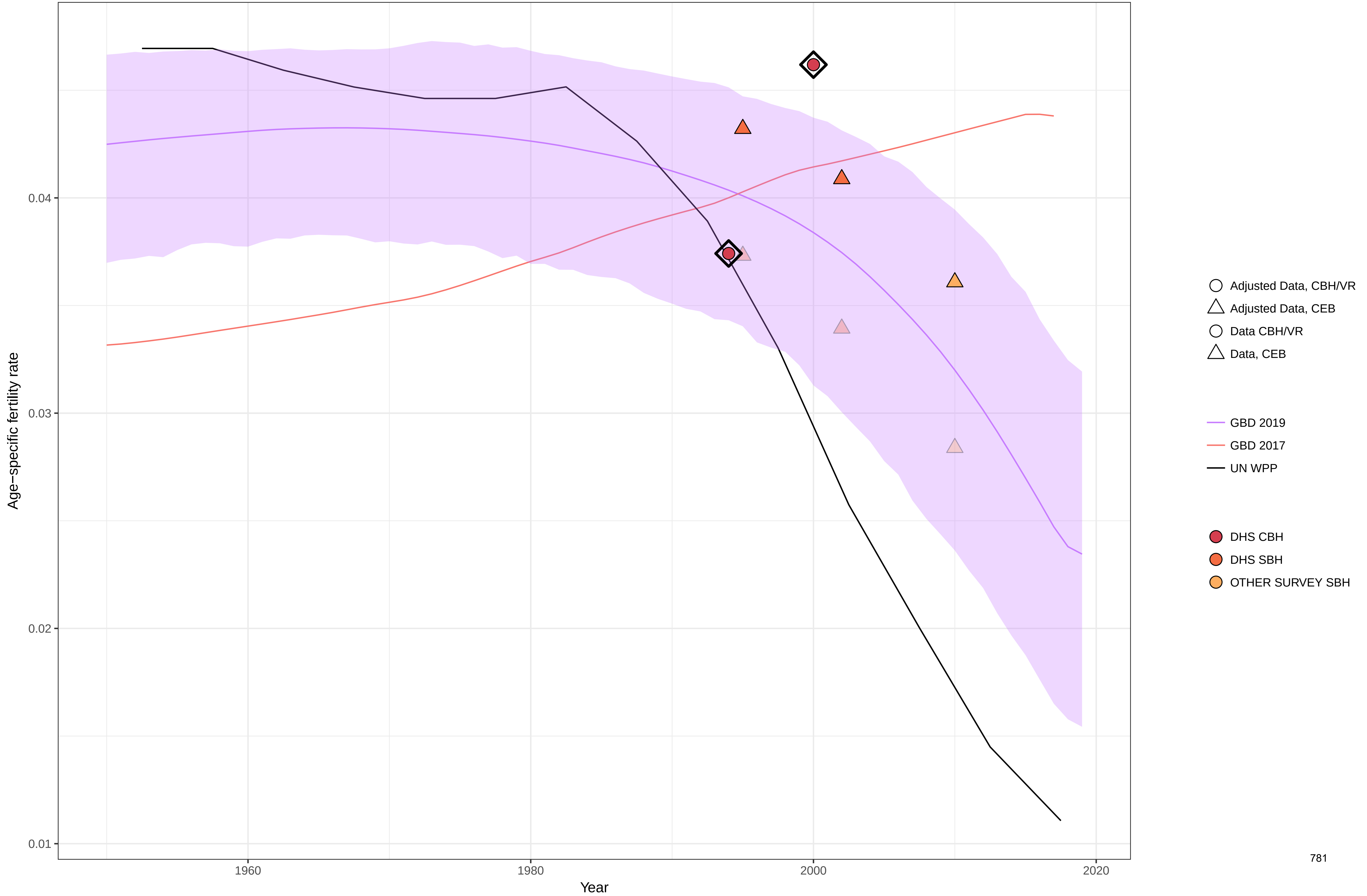

Age−specific fertility fitted trend lines, 1950−2019: Eritrea, Ages: 50−54
Data points enclosed with a diamond are reference sources

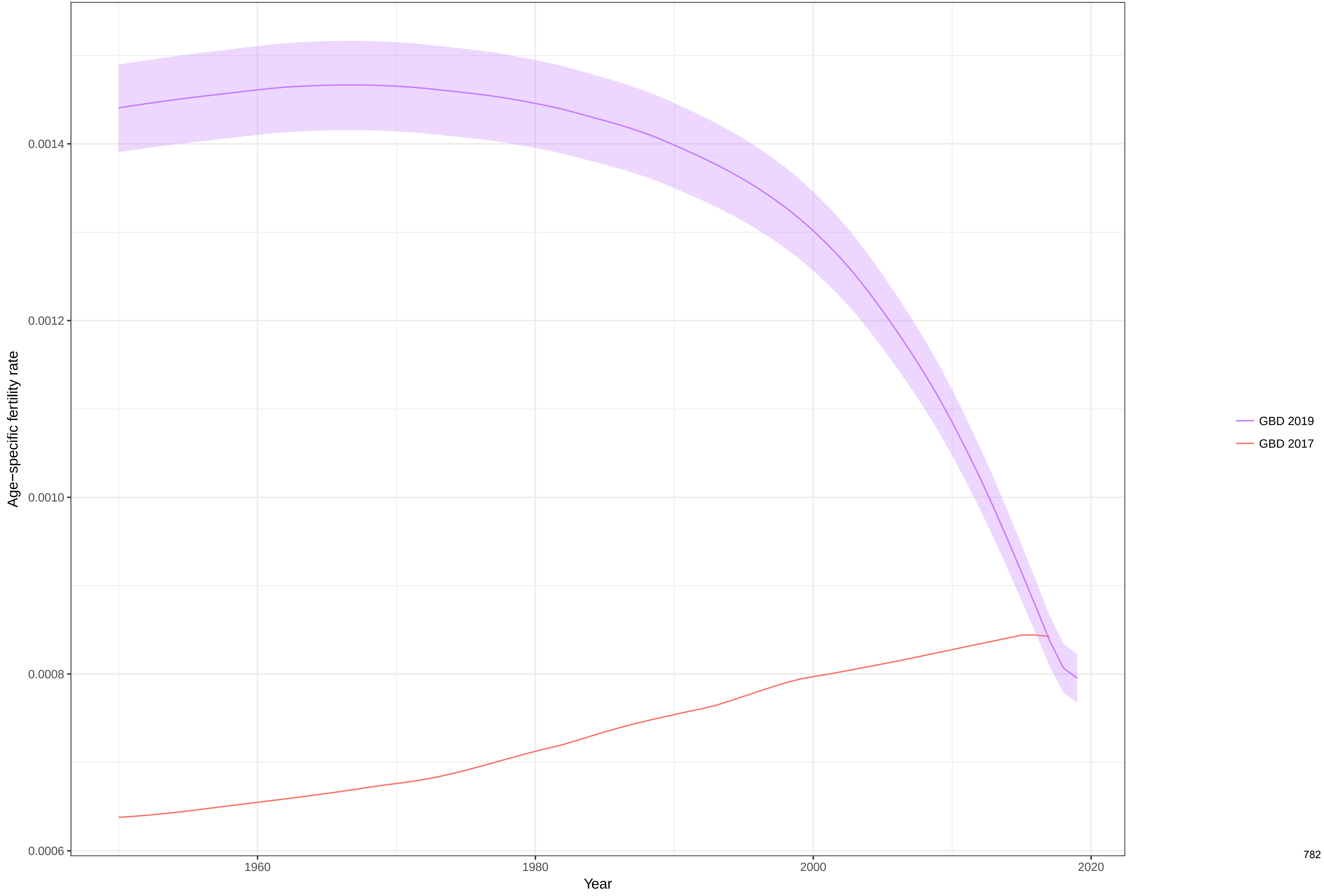

Total fertility rate fitted trend lines, 1950−2019: Eritrea TFR
Data points enclosed with a diamond are reference sources

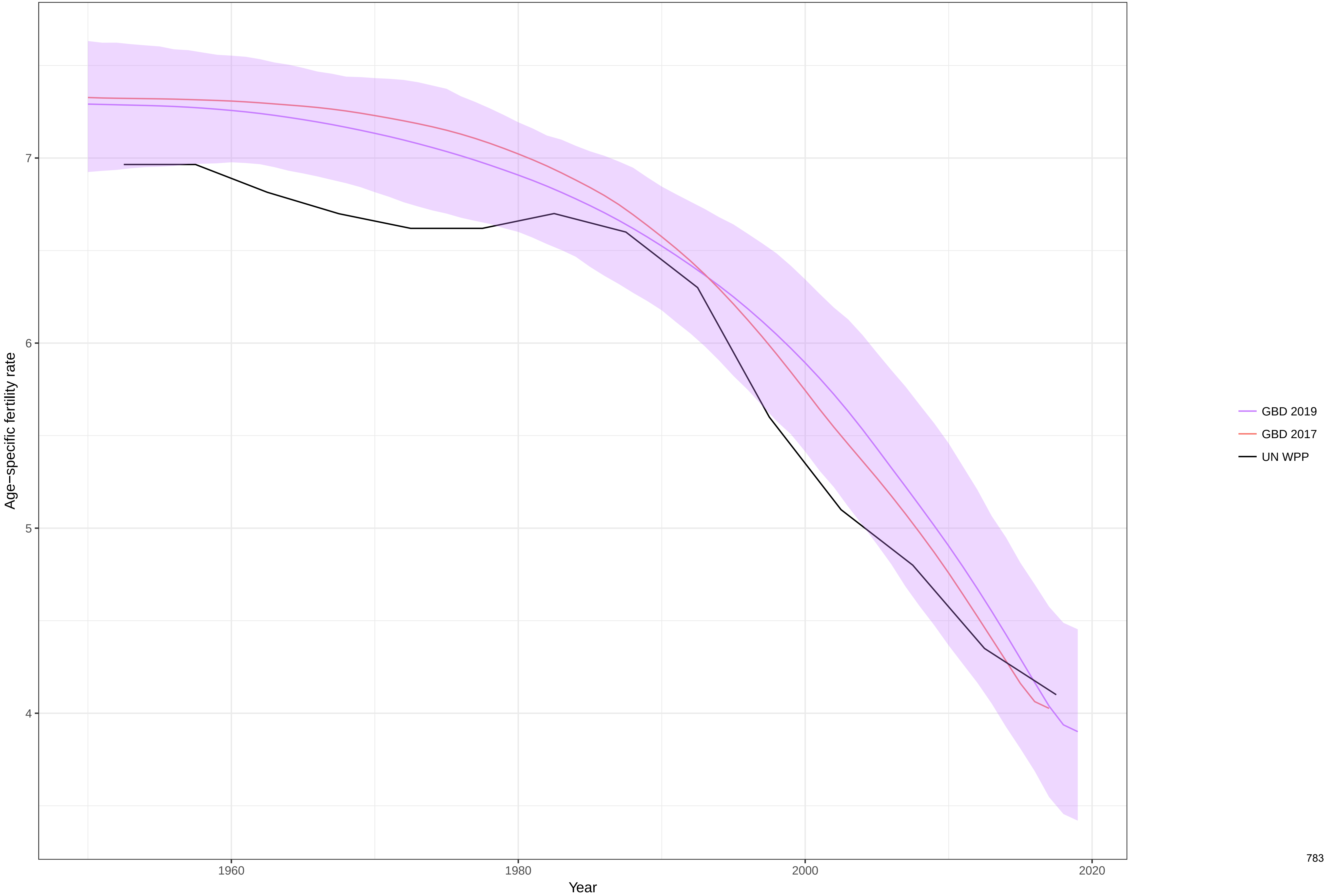

Age−specific fertility fitted trend lines, 1950−2019: Spain, Ages: 10−14
Data points enclosed with a diamond are reference sources

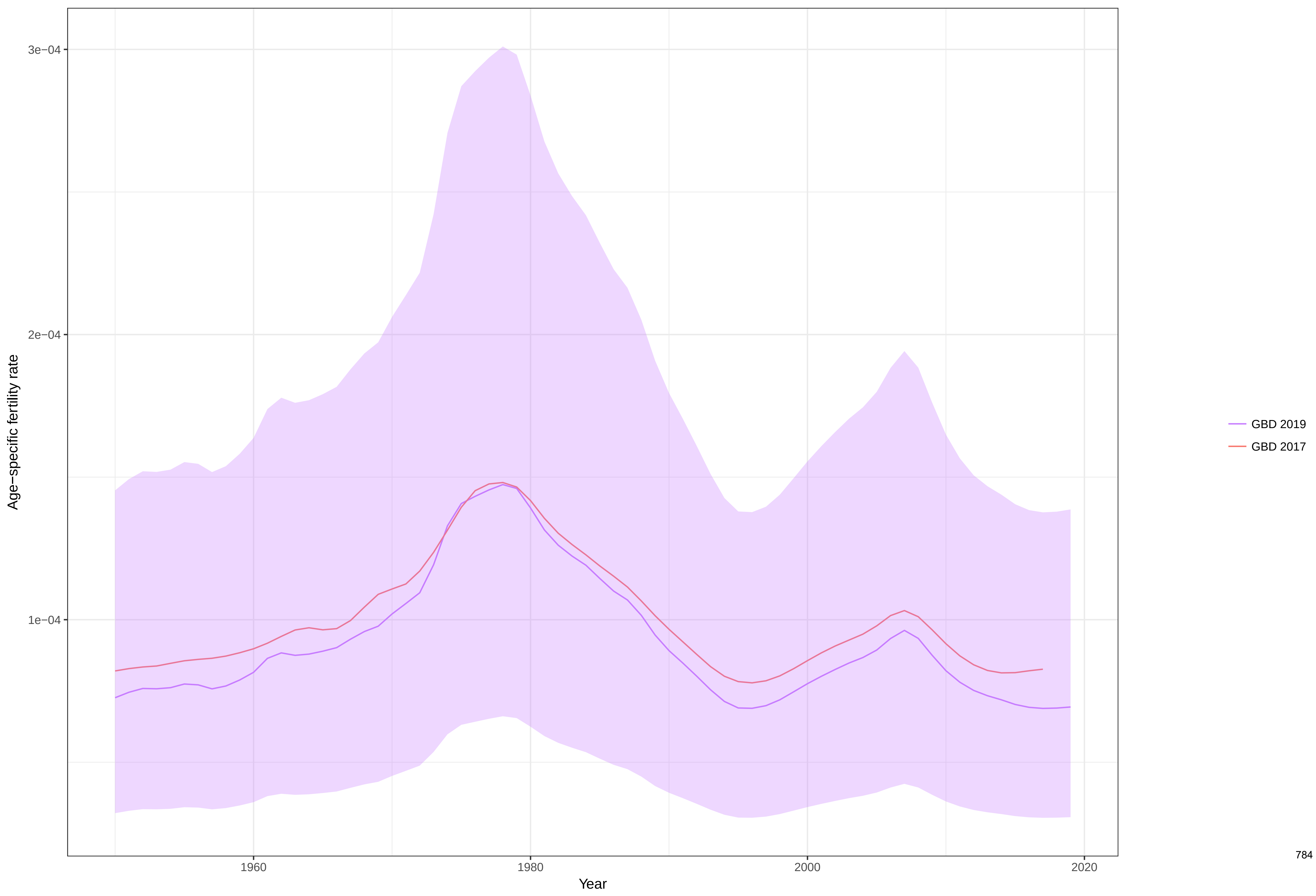

Age−specific fertility fitted trend lines, 1950−2019: Spain, Ages: 15−19
Data points enclosed with a diamond are reference sources

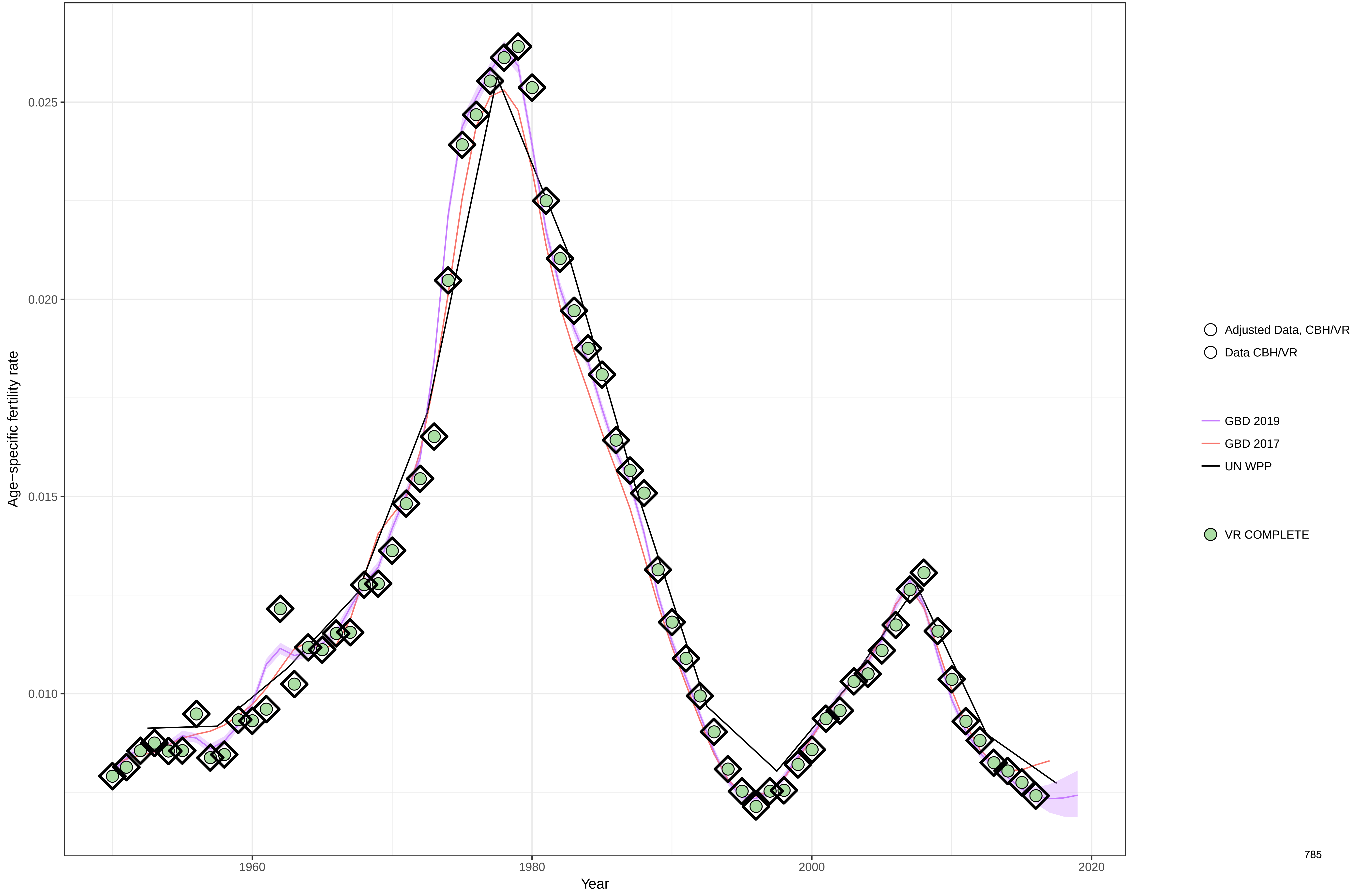

Age−specific fertility fitted trend lines, 1950−2019: Spain, Ages: 20−24
Data points enclosed with a diamond are reference sources

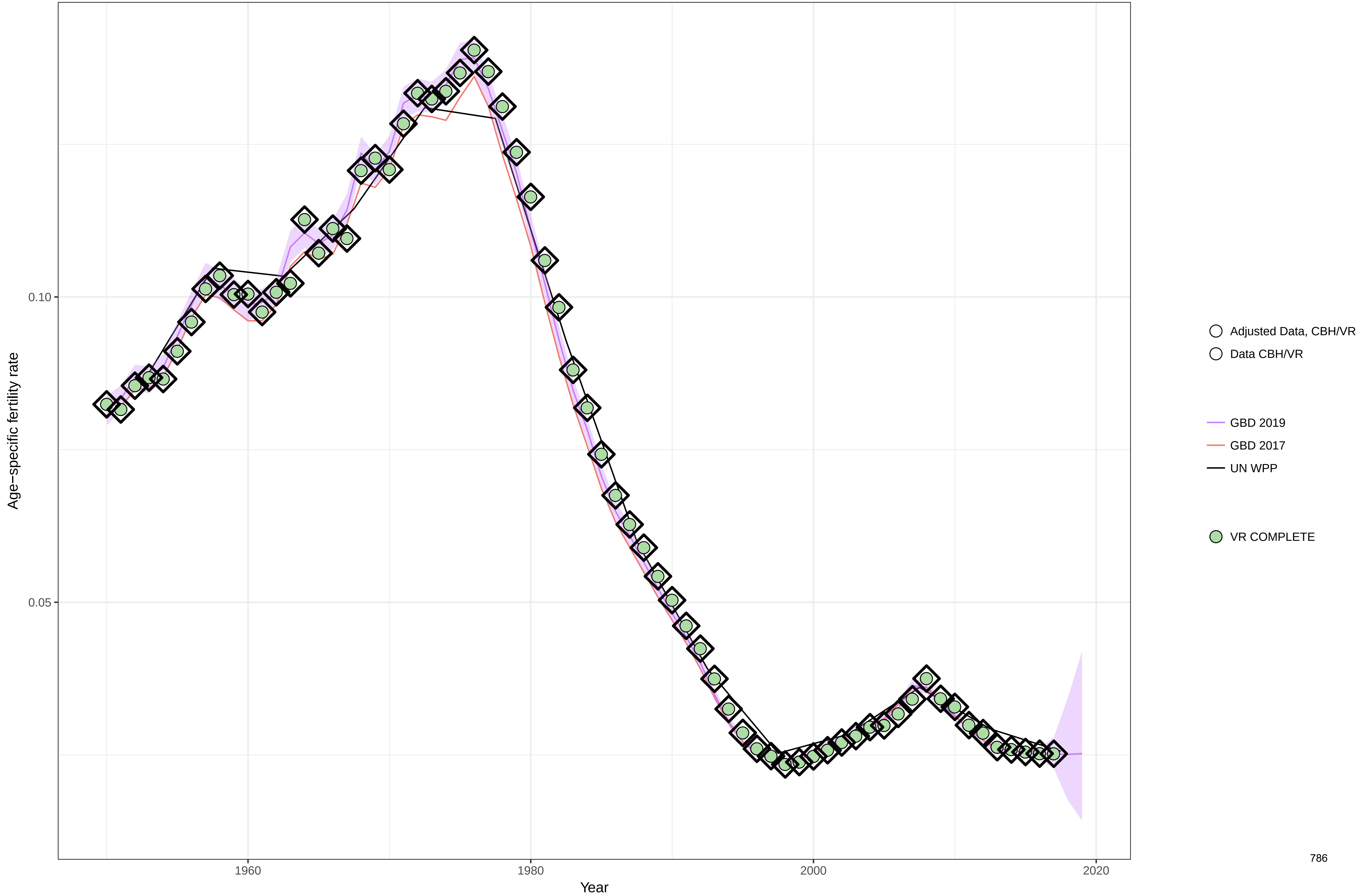

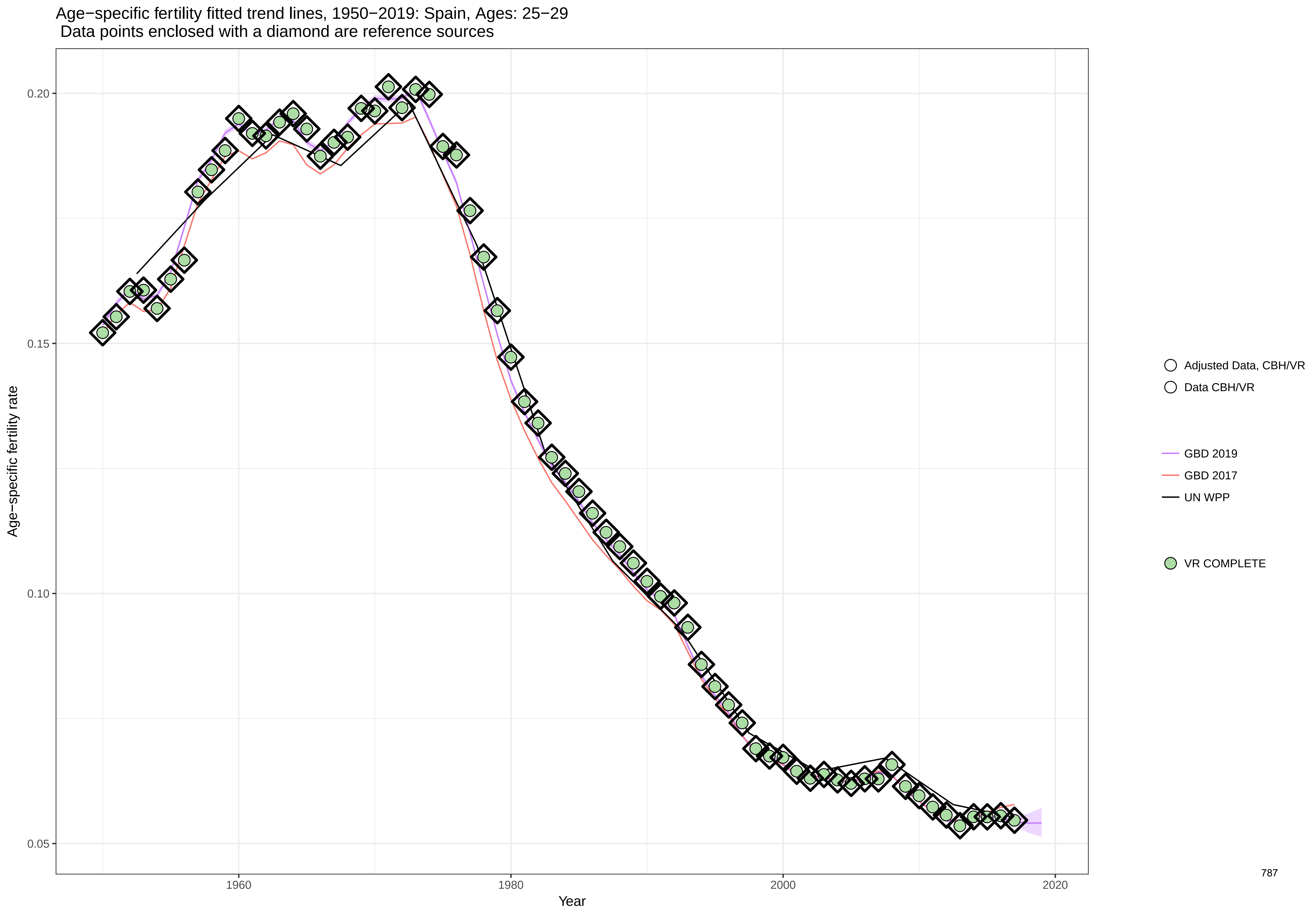
Age−specific fertility fitted trend lines, 1950−2019: Spain, Ages: 25−29
Data points enclosed with a diamond are reference sources
Age−specific fertility rate
0.20
0.15
0.10
0.05
1960
1980
2000
2020
Year
Adjusted Data, CBH/VR
Data CBH/VR
GBD 2019
GBD 2017
UN WPP
VR COMPLETE

Age−specific fertility fitted trend lines, 1950−2019: Spain, Ages: 30−34
Data points enclosed with a diamond are reference sources

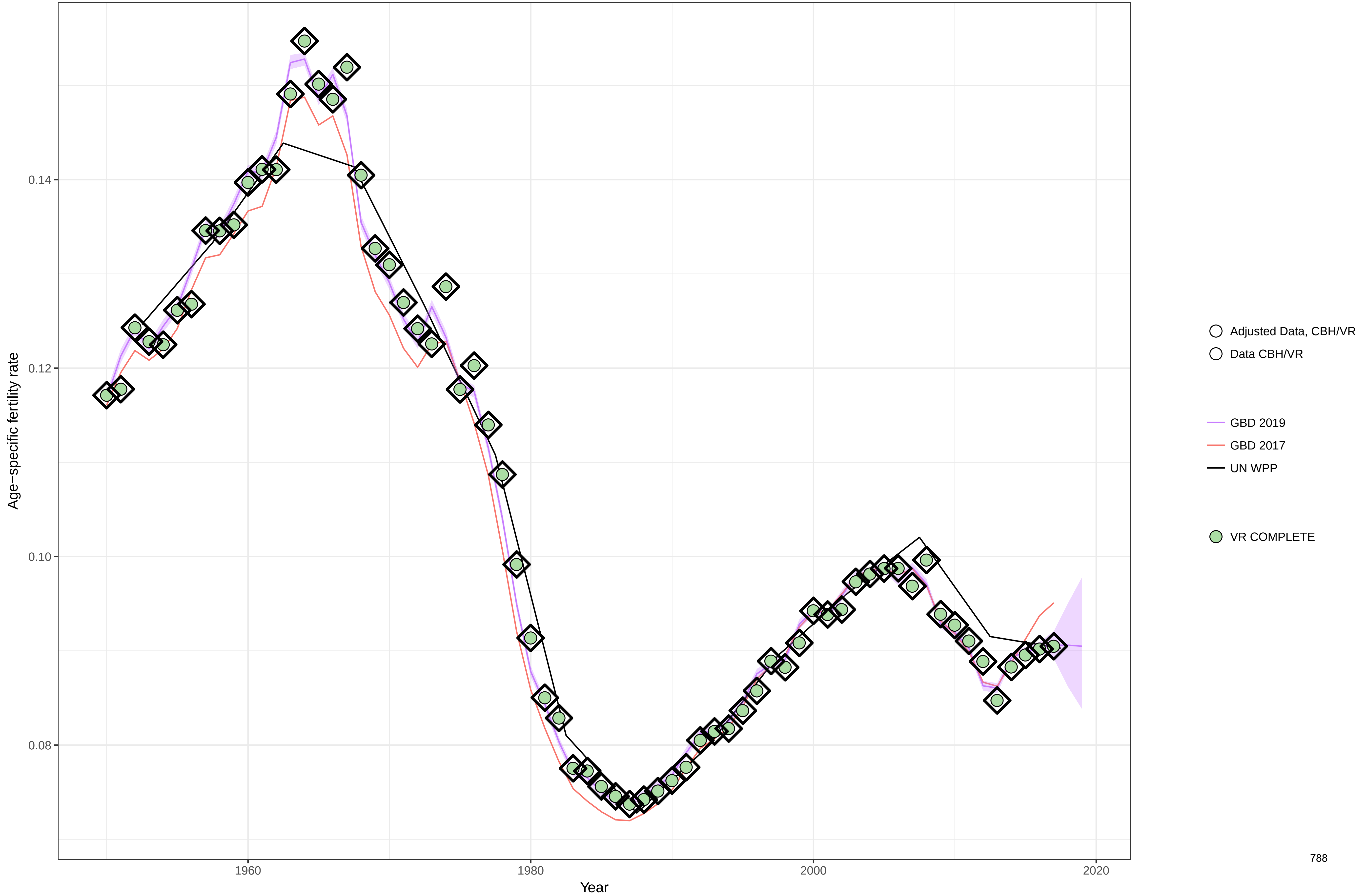

Age−specific fertility fitted trend lines, 1950−2019: Spain, Ages: 35−39
Data points enclosed with a diamond are reference sources

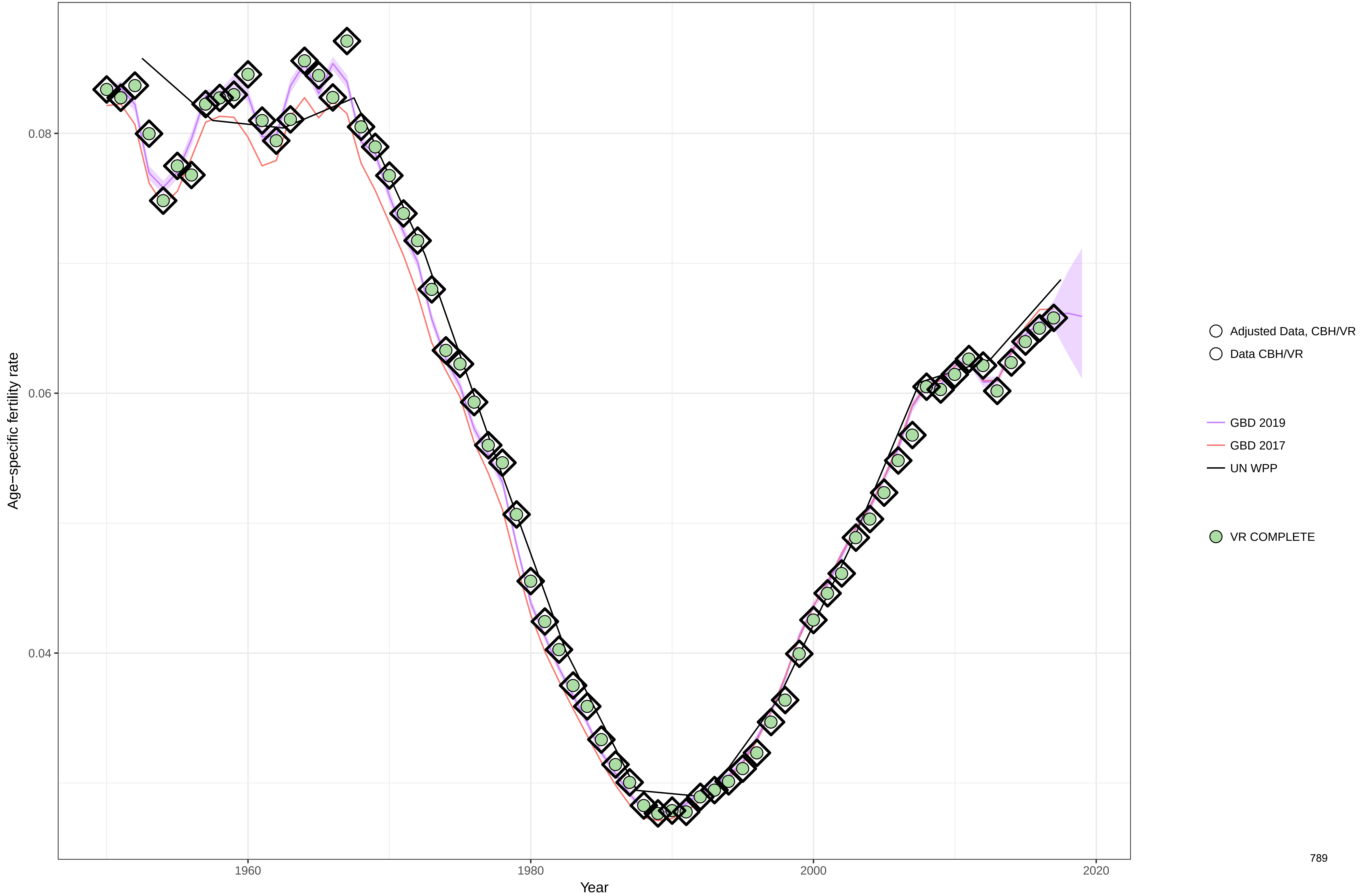

Age−specific fertility fitted trend lines, 1950−2019: Spain, Ages: 40−44
Data points enclosed with a diamond are reference sources

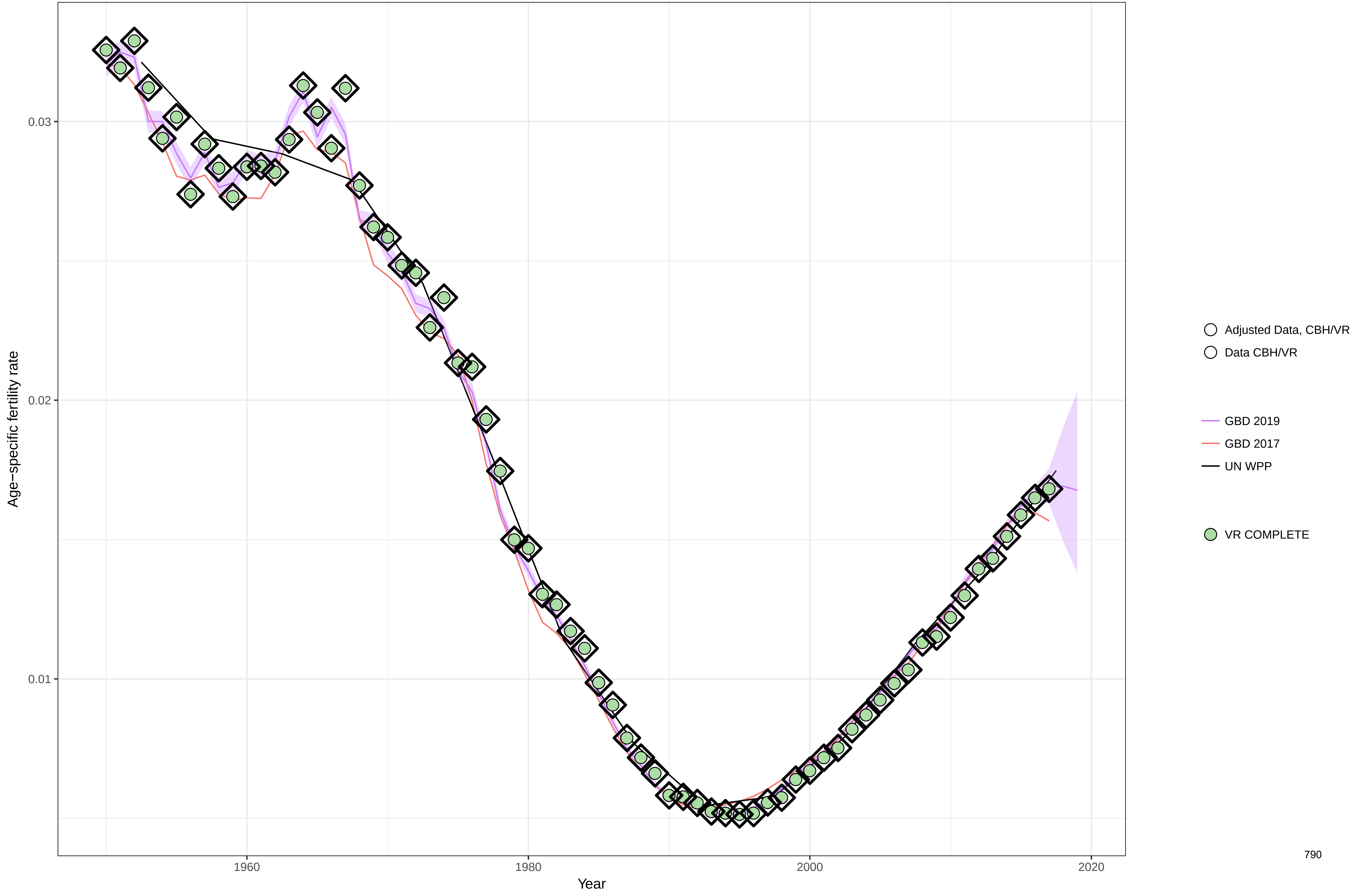

Age−specific fertility fitted trend lines, 1950−2019: Spain, Ages: 45−49
Data points enclosed with a diamond are reference sources

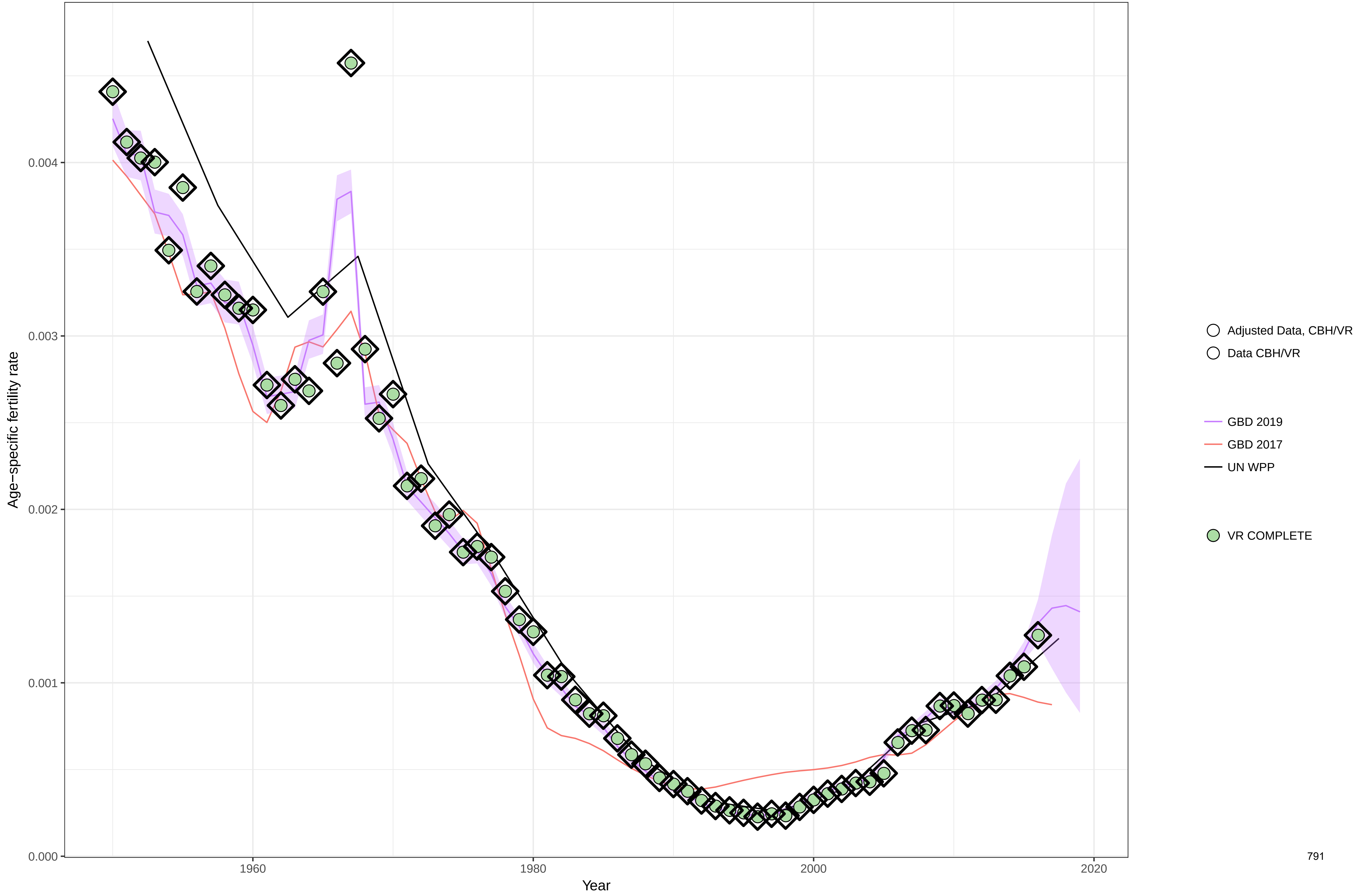

Age−specific fertility fitted trend lines, 1950−2019: Spain, Ages: 50−54
Data points enclosed with a diamond are reference sources

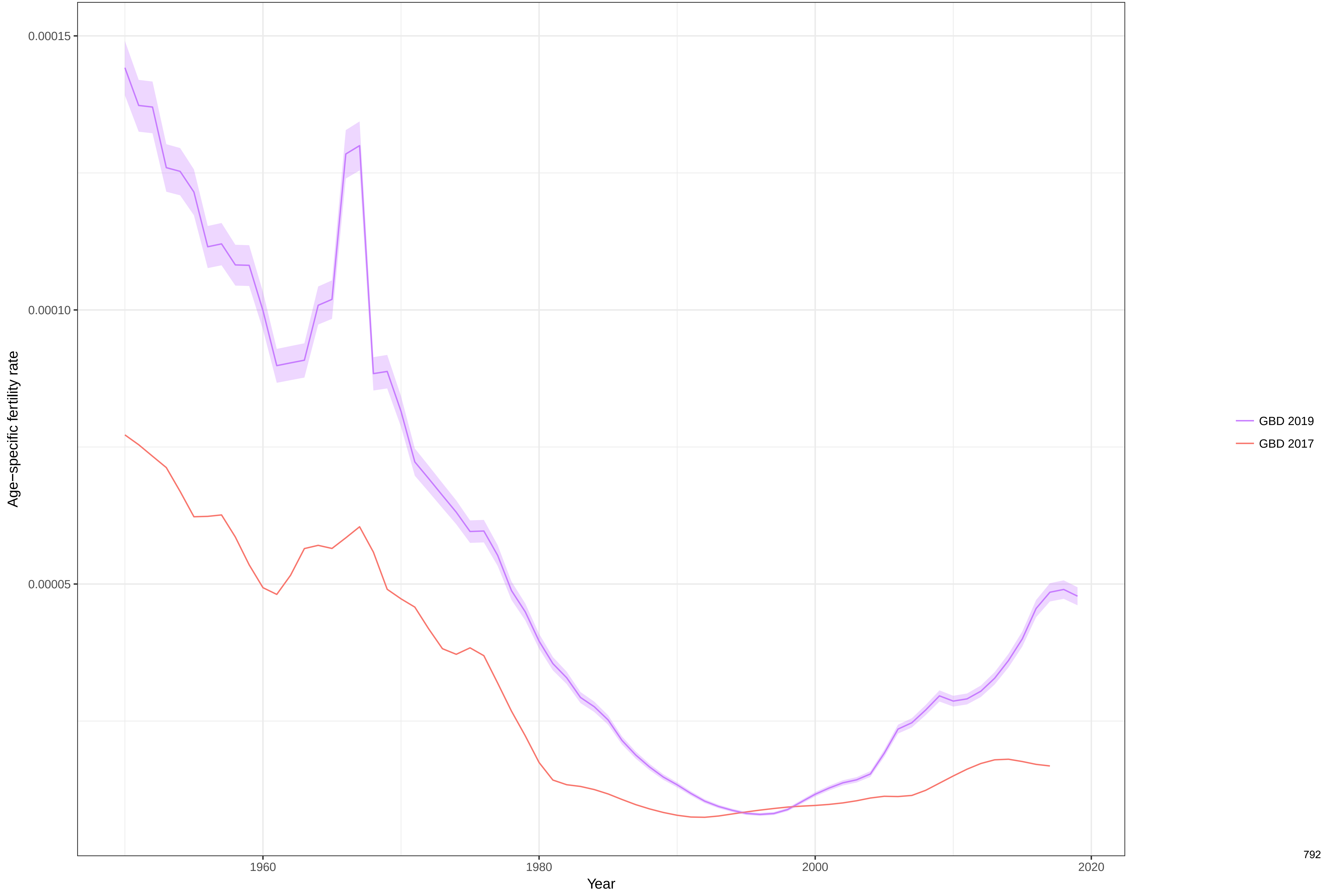

Total fertility rate fitted trend lines, 1950−2019: Spain TFR
Data points enclosed with a diamond are reference sources

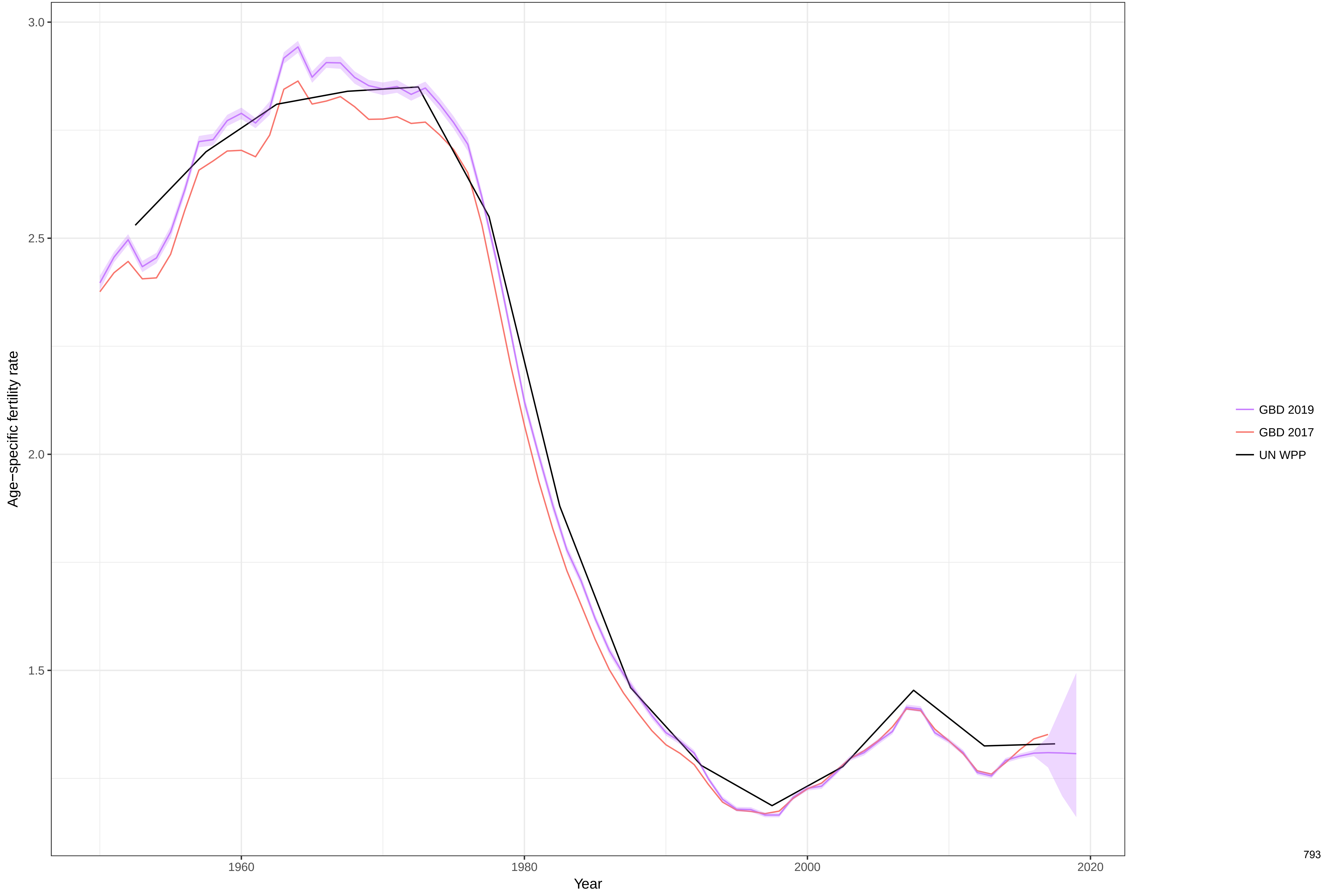

Age−specific fertility fitted trend lines, 1950−2019: Estonia, Ages: 10−14
Data points enclosed with a diamond are reference sources

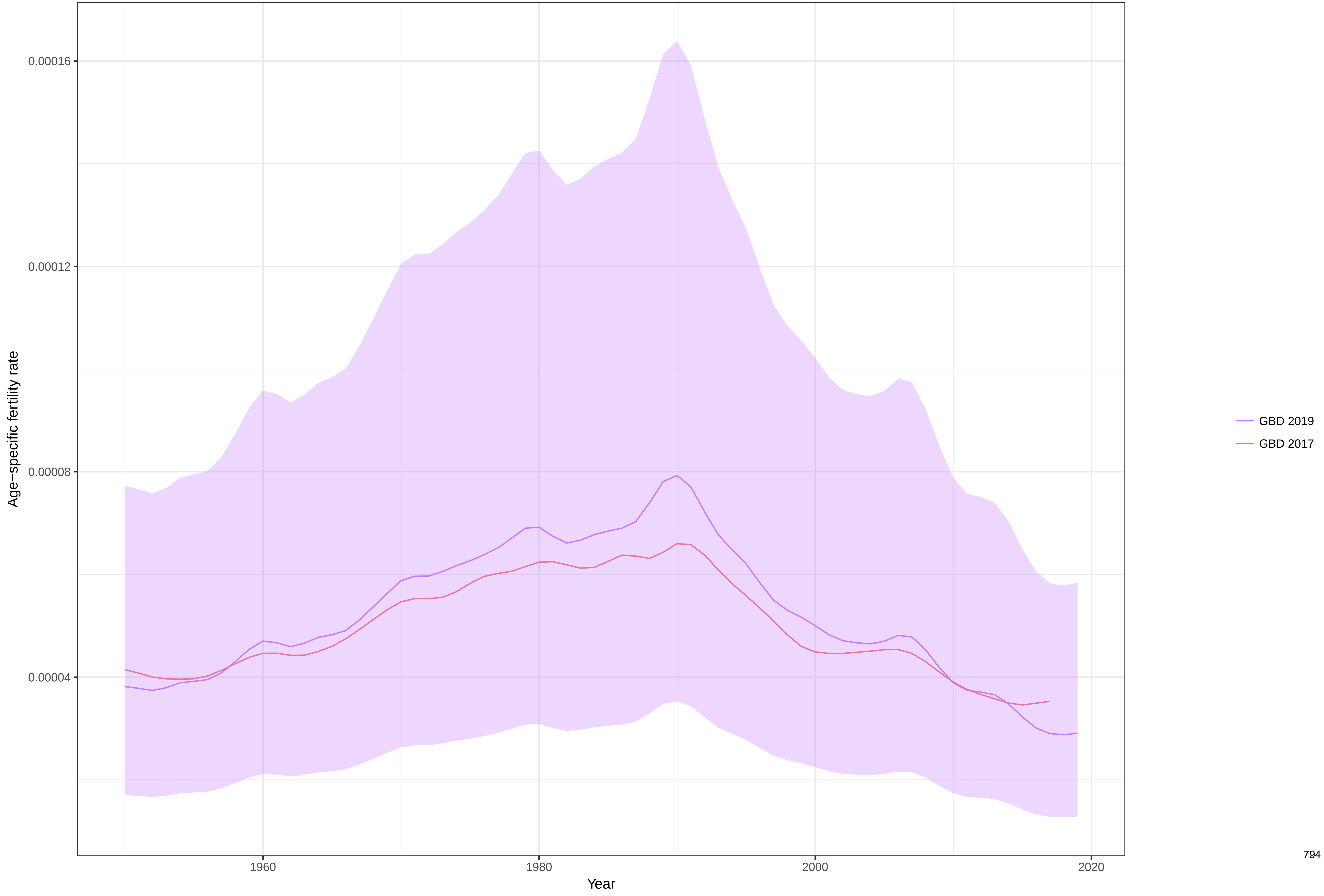

Age−specific fertility fitted trend lines, 1950−2019: Estonia, Ages: 15−19
Data points enclosed with a diamond are reference sources

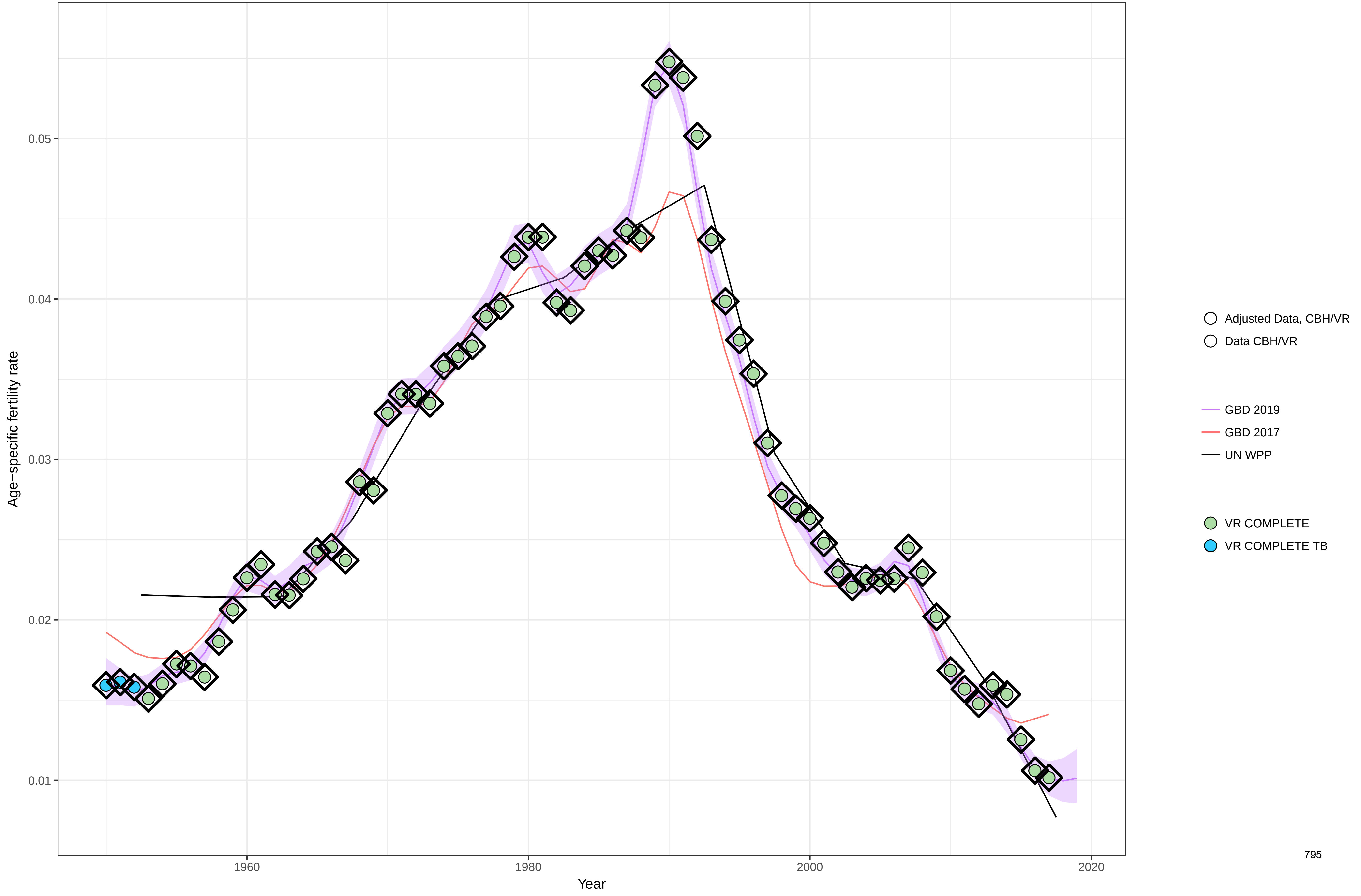

Age−specific fertility fitted trend lines, 1950−2019: Estonia, Ages: 20−24
Data points enclosed with a diamond are reference sources

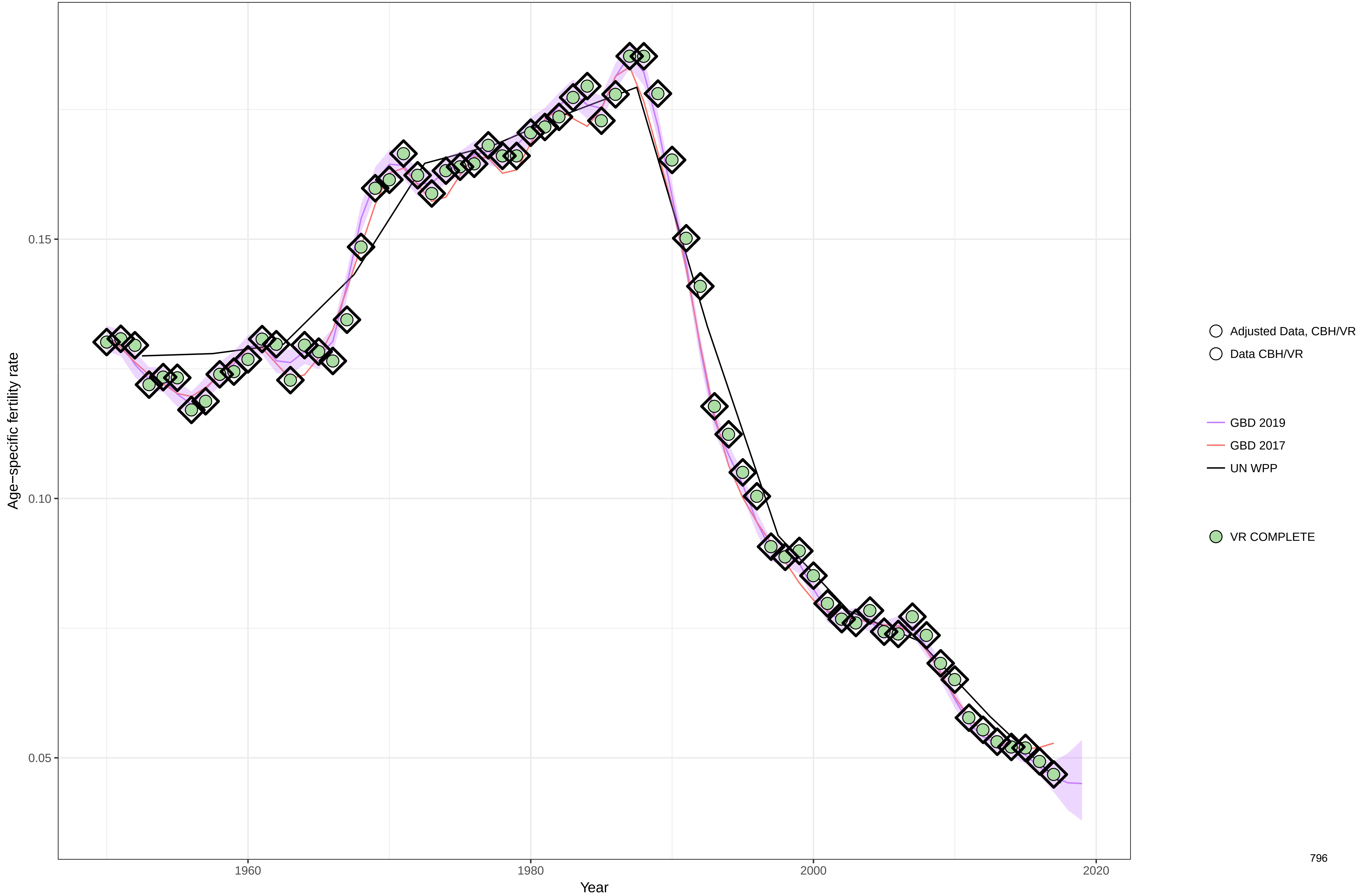

Age−specific fertility fitted trend lines, 1950−2019: Estonia, Ages: 25−29
Data points enclosed with a diamond are reference sources

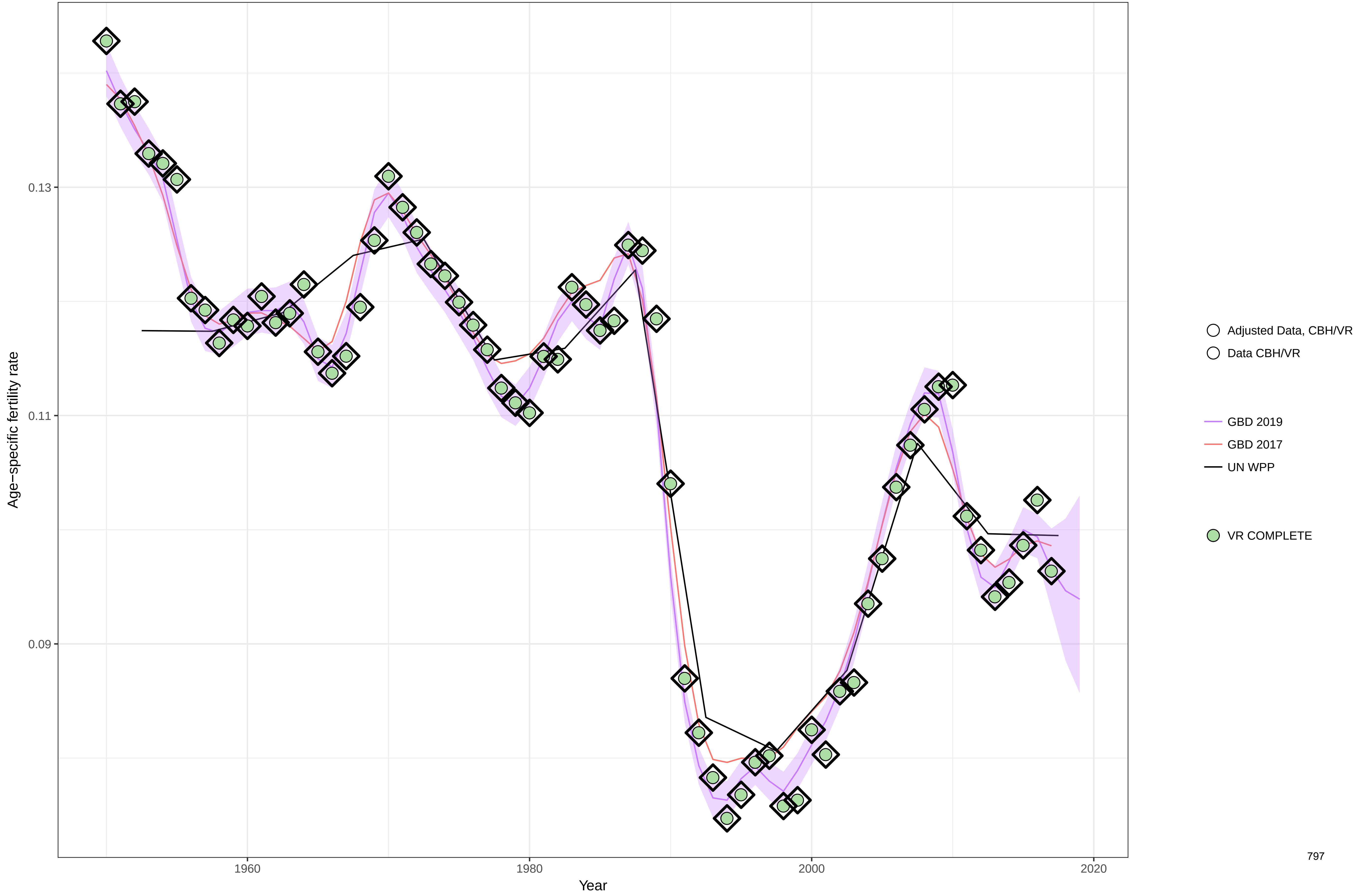

Age−specific fertility fitted trend lines, 1950−2019: Estonia, Ages: 30−34
Data points enclosed with a diamond are reference sources

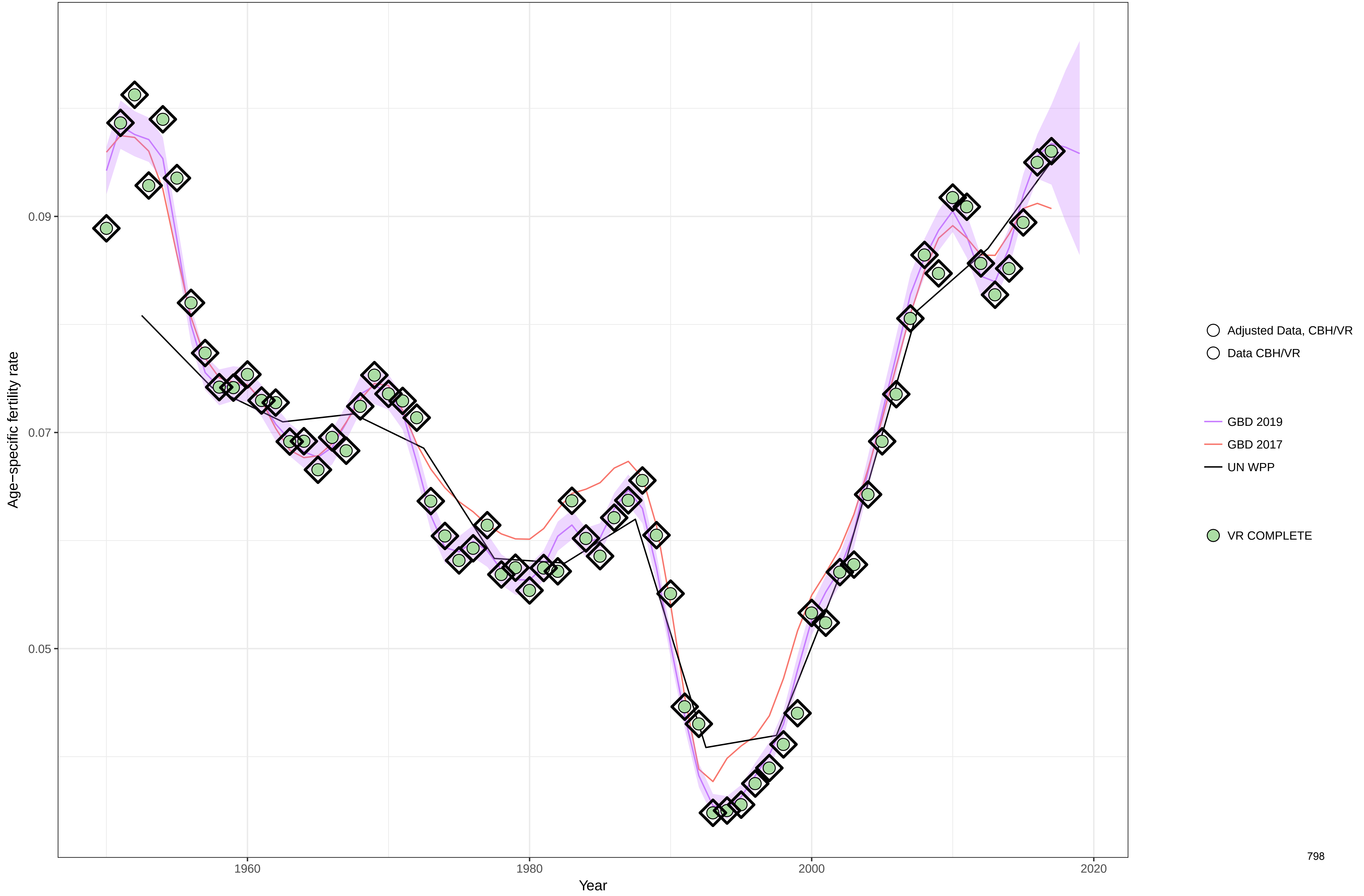

Age−specific fertility fitted trend lines, 1950−2019: Estonia, Ages: 35−39
Data points enclosed with a diamond are reference sources

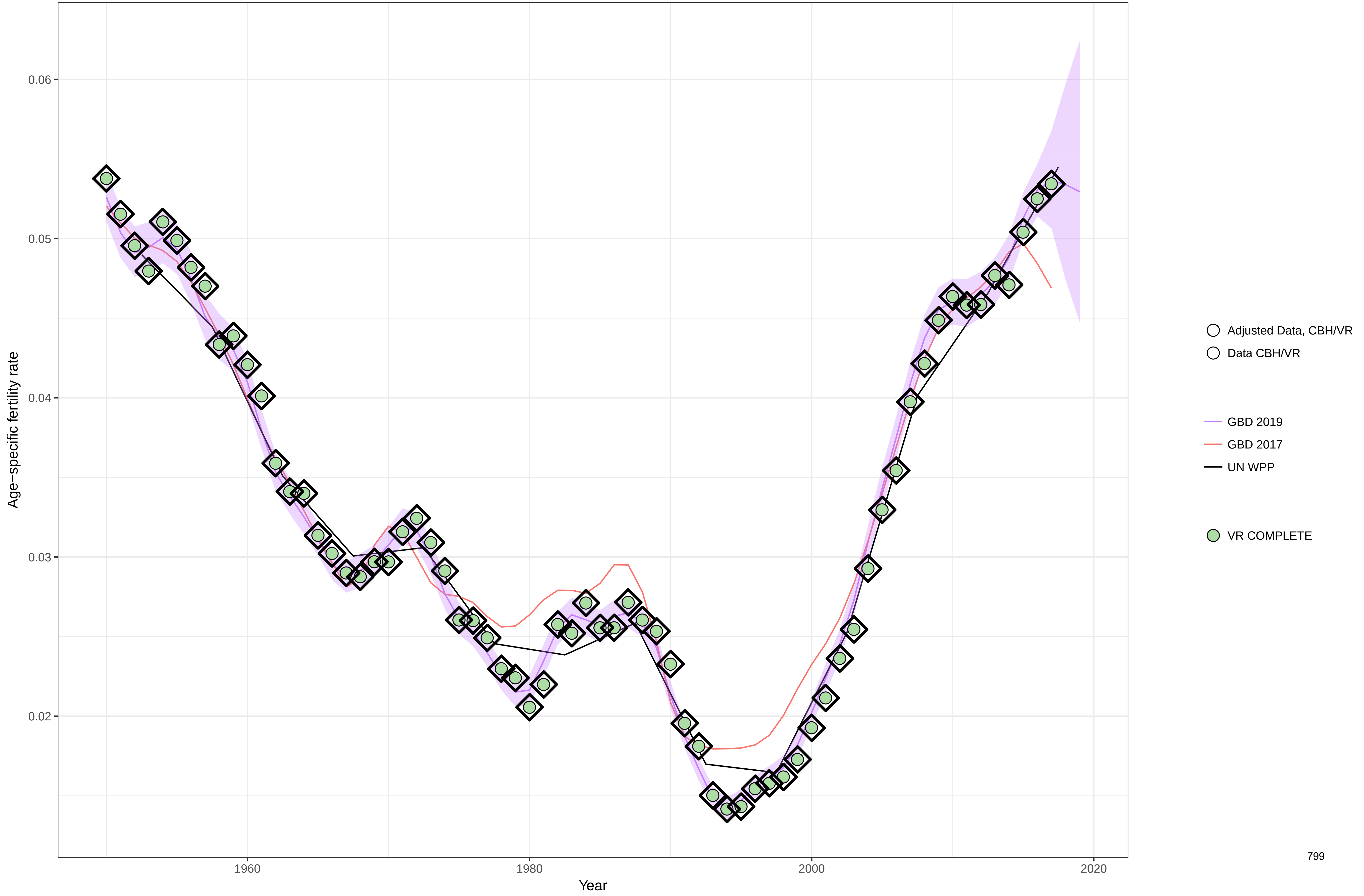

Age−specific fertility fitted trend lines, 1950−2019: Estonia, Ages: 40−44
Data points enclosed with a diamond are reference sources

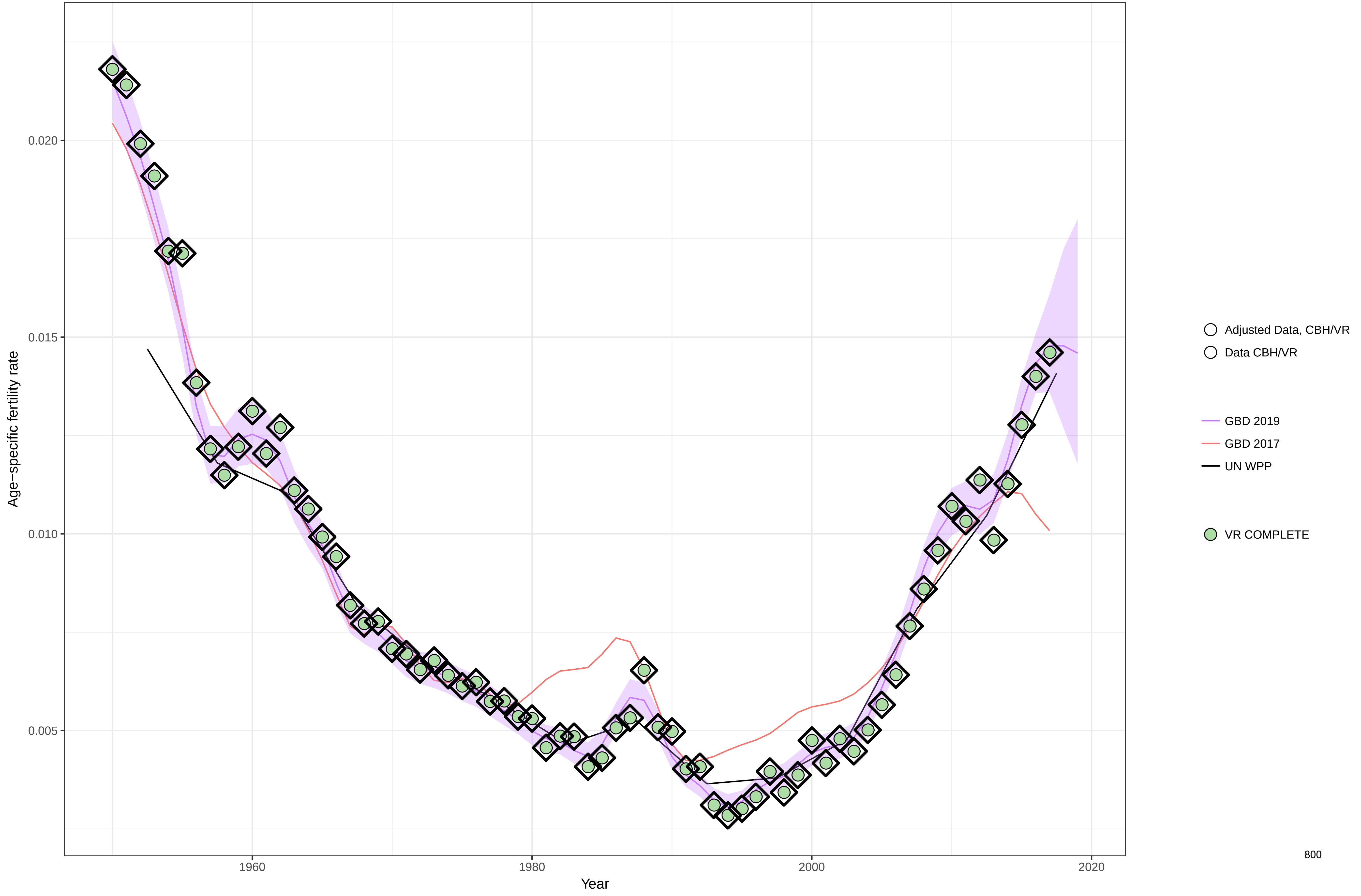

Age−specific fertility fitted trend lines, 1950−2019: Estonia, Ages: 45−49
Data points enclosed with a diamond are reference sources

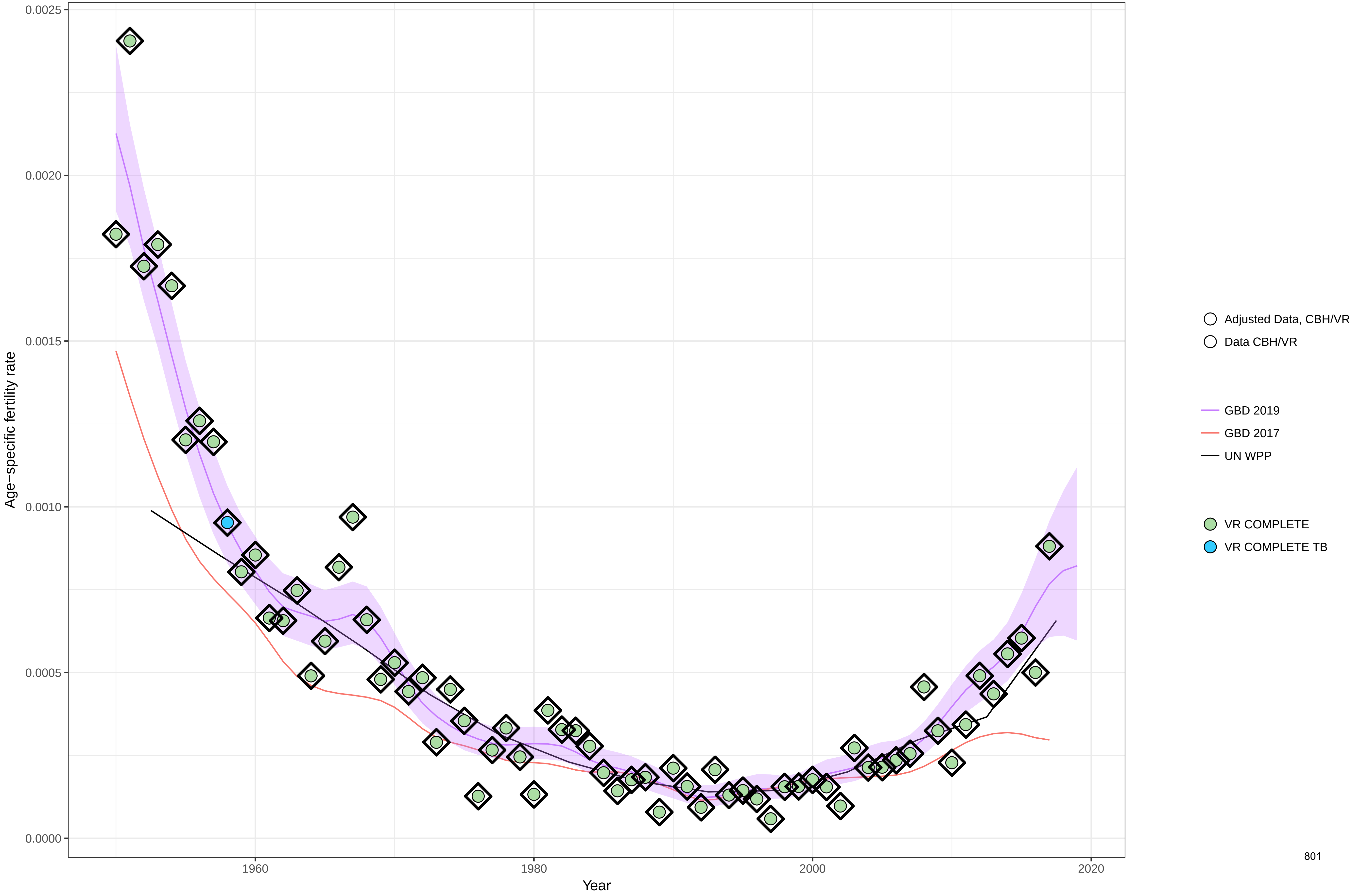

Age−specific fertility fitted trend lines, 1950−2019: Estonia, Ages: 50−54
Data points enclosed with a diamond are reference sources

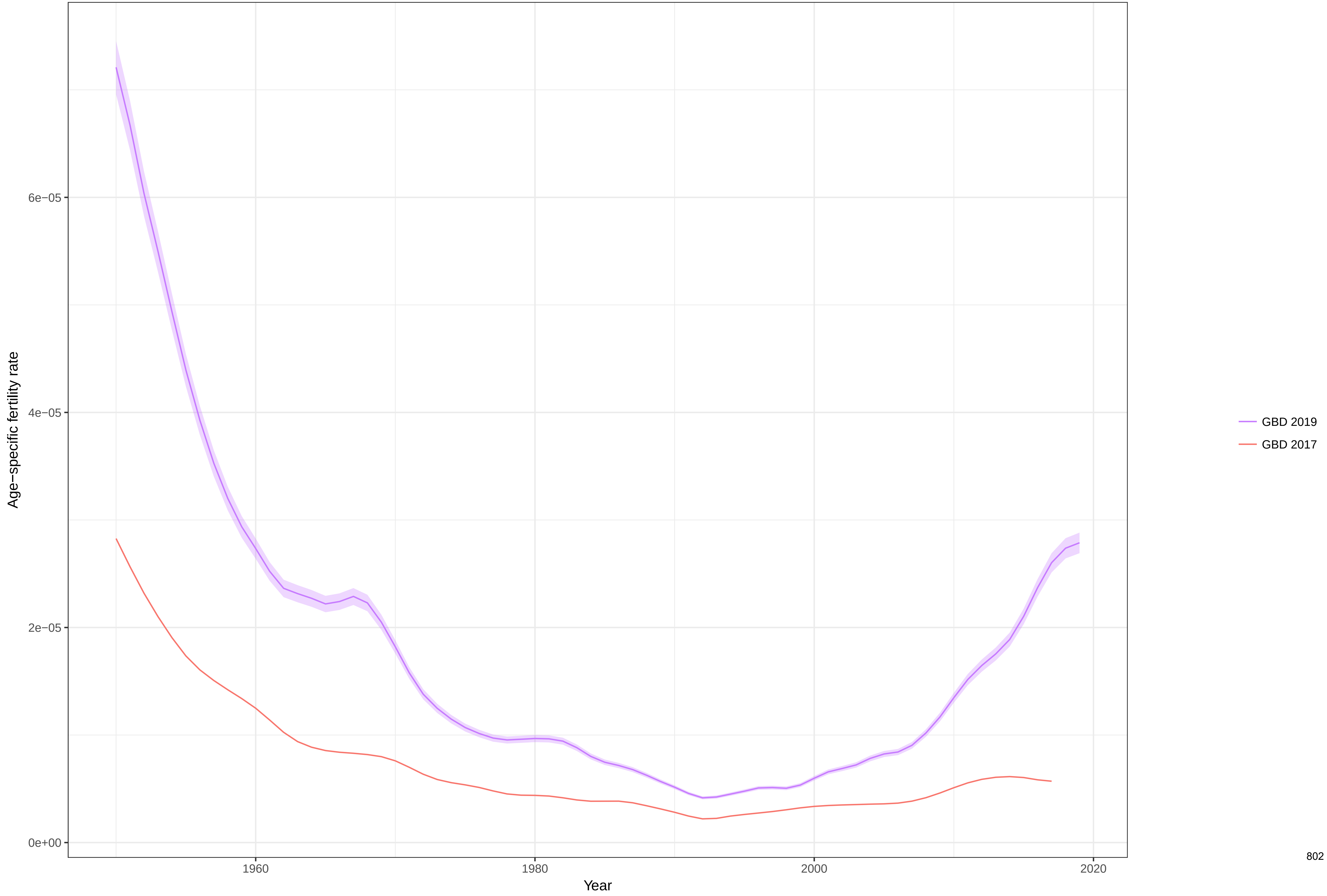

Total fertility rate fitted trend lines, 1950−2019: Estonia TFR
Data points enclosed with a diamond are reference sources

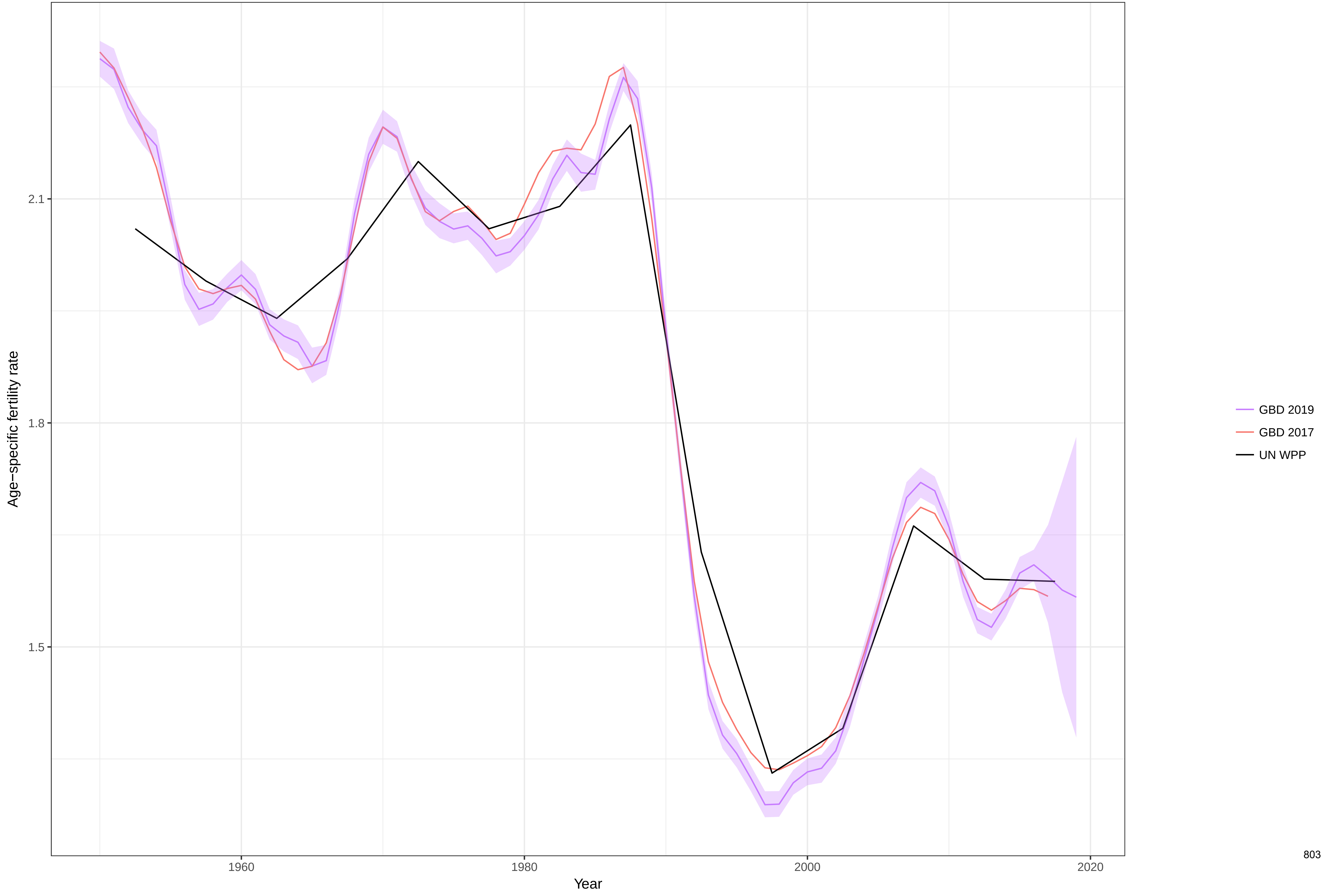

Age−specific fertility fitted trend lines, 1950−2019: Ethiopia, Ages: 10−14
Data points enclosed with a diamond are reference sources

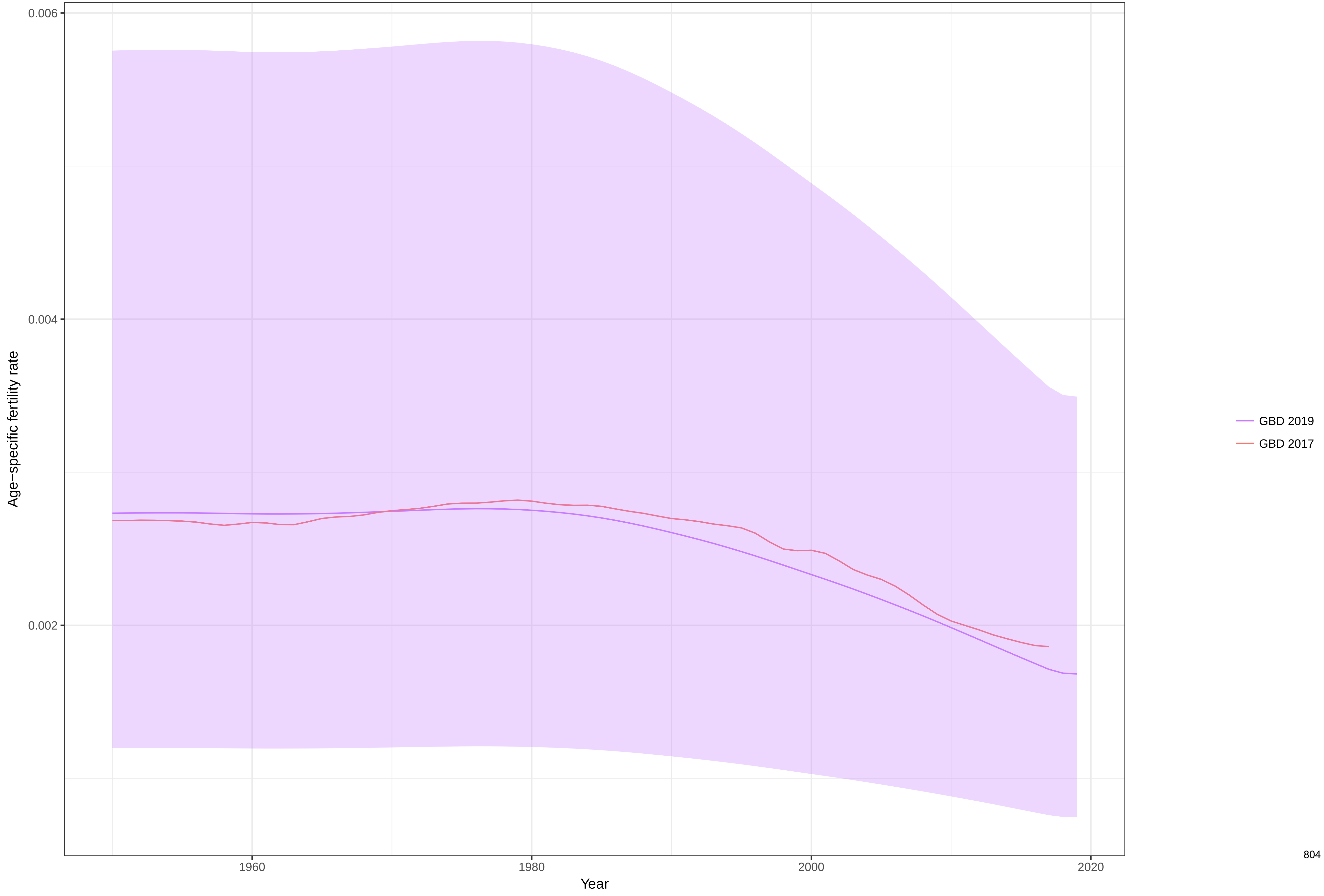

Age−specific fertility fitted trend lines, 1950−2019: Ethiopia, Ages: 15−19
Data points enclosed with a diamond are reference sources

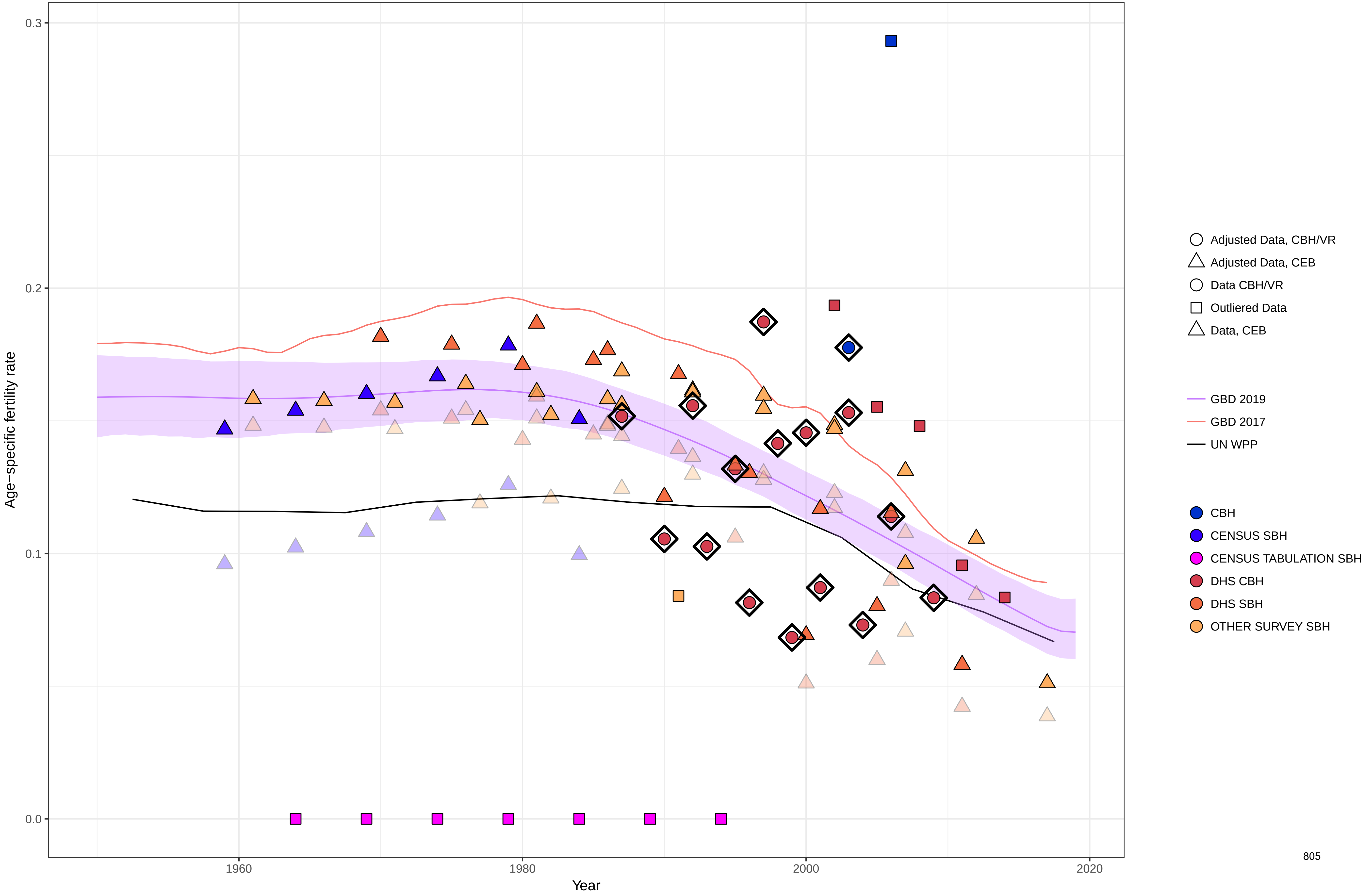

Age−specific fertility fitted trend lines, 1950−2019: Ethiopia, Ages: 20−24
Data points enclosed with a diamond are reference sources

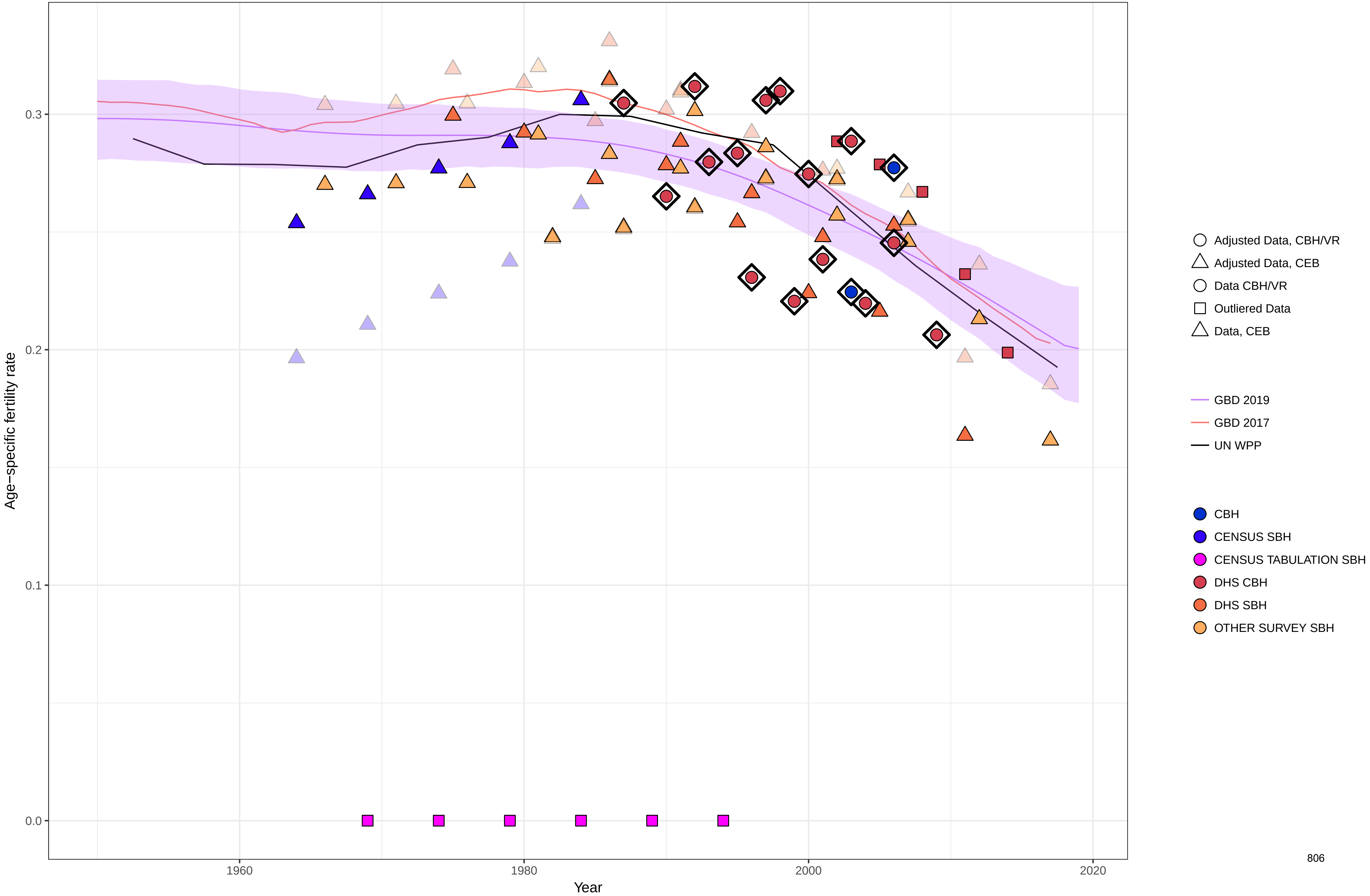

Age−specific fertility fitted trend lines, 1950−2019: Ethiopia, Ages: 25−29
Data points enclosed with a diamond are reference sources

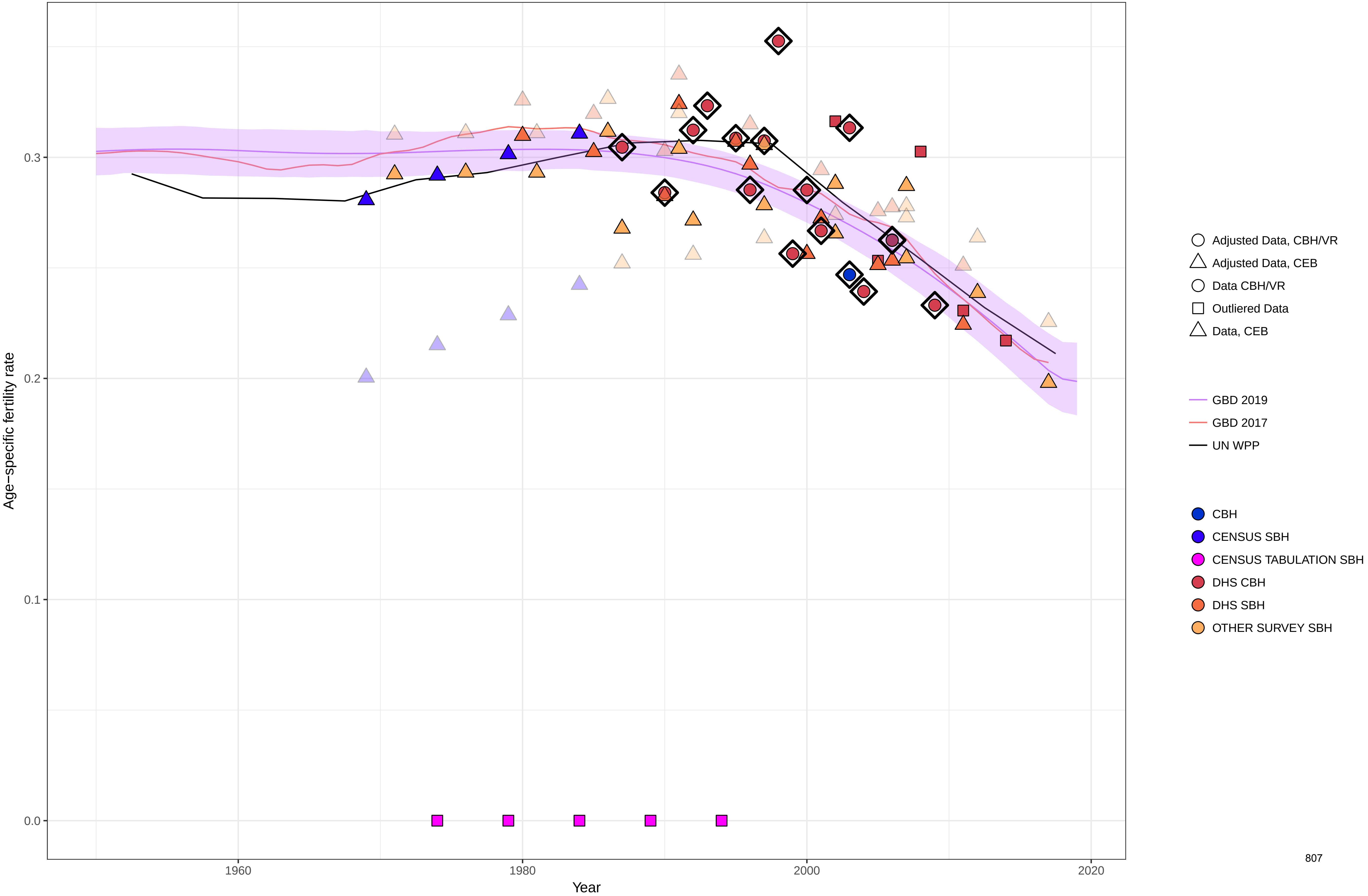

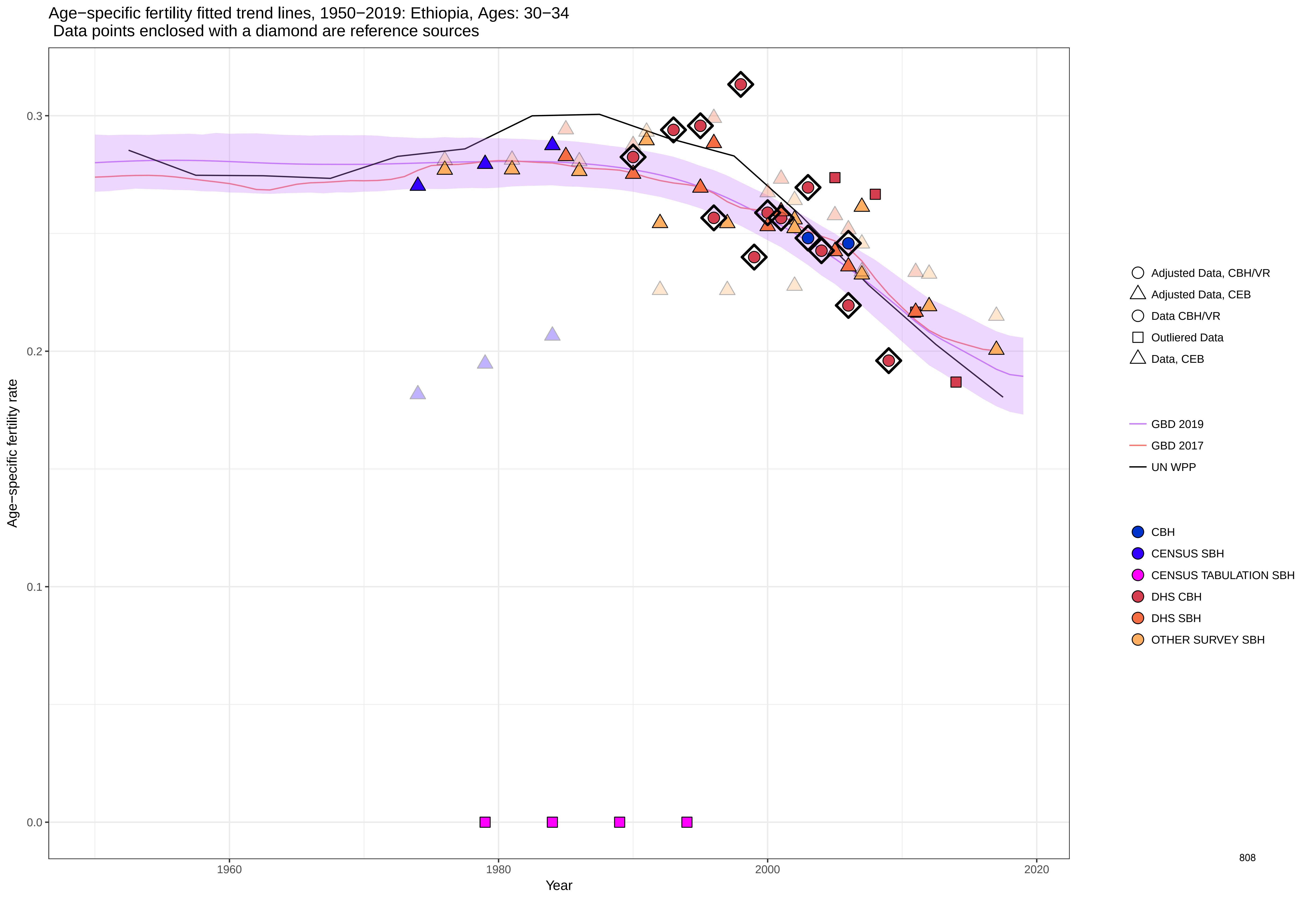

Age−specific fertility fitted trend lines, 1950−2019: Ethiopia, Ages: 30−34
Data points enclosed with a diamond are reference sources
Age−specific fertility rate
0.0
0.1
0.2
0.3
Year
1960
1980
2000
2020
Adjusted Data, CBH/VR
Adjusted Data, CEB
Data CBH/VR
Outliered Data
Data, CEB
GBD 2019
GBD 2017
UN WPP
CBH
CENSUS SBH
CENSUS TABULATION SBH
DHS CBH
DHS SBH
OTHER SURVEY SBH

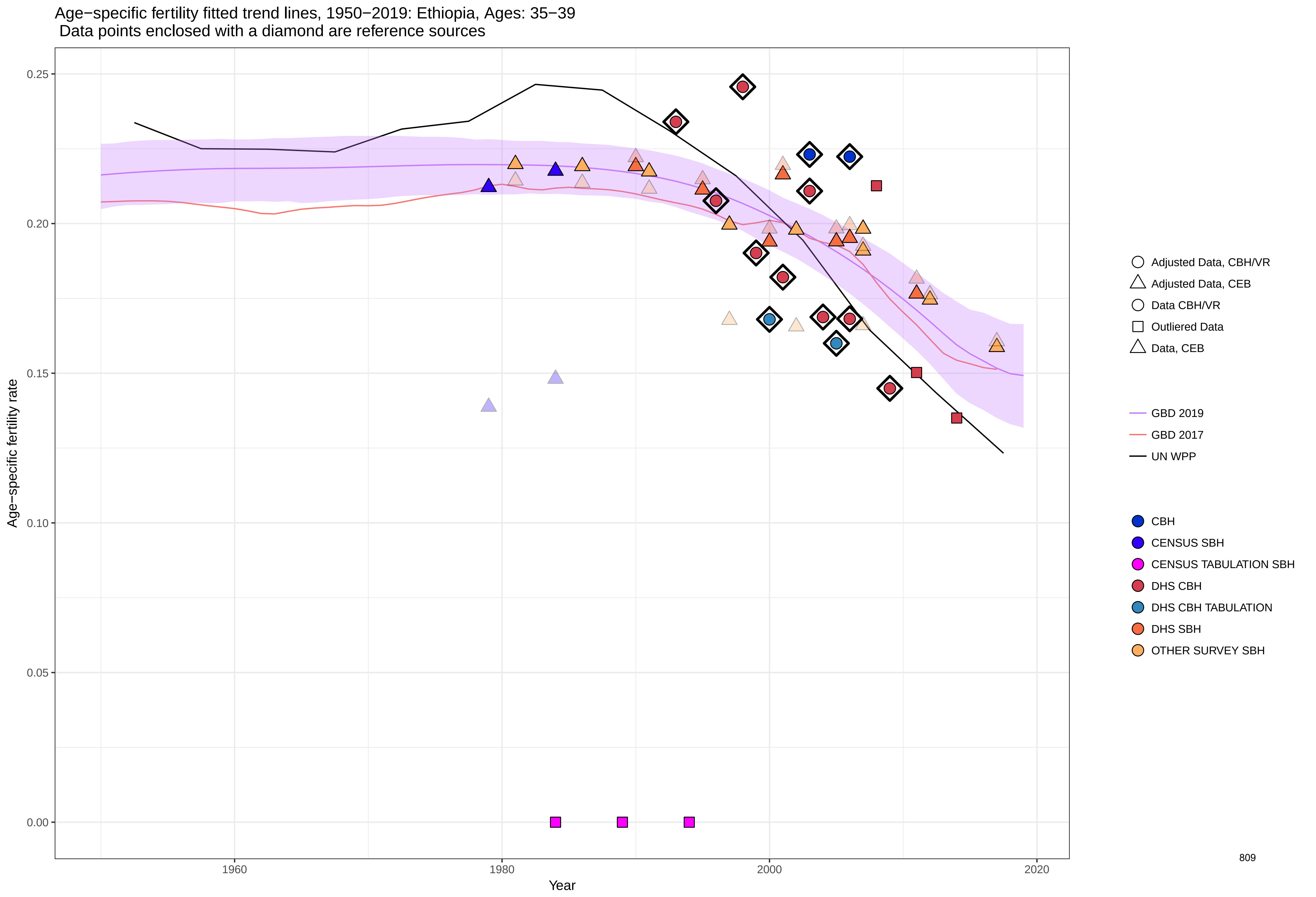

Age−specific fertility fitted trend lines, 1950−2019: Ethiopia, Ages: 35−39
Data points enclosed with a diamond are reference sources
Age−specific fertility rate
Year
0.00
0.05
0.10
0.15
0.20
0.25
1960
1980
2000
2020
Adjusted Data, CBH/VR
Adjusted Data, CEB
Data CBH/VR
Outliered Data
Data, CEB
GBD 2019
GBD 2017
UN WPP
CBH
CENSUS SBH
CENSUS TABULATION SBH
DHS CBH
DHS CBH TABULATION
DHS SBH
OTHER SURVEY SBH

Age−specific fertility fitted trend lines, 1950−2019: Ethiopia, Ages: 40−44
Data points enclosed with a diamond are reference sources

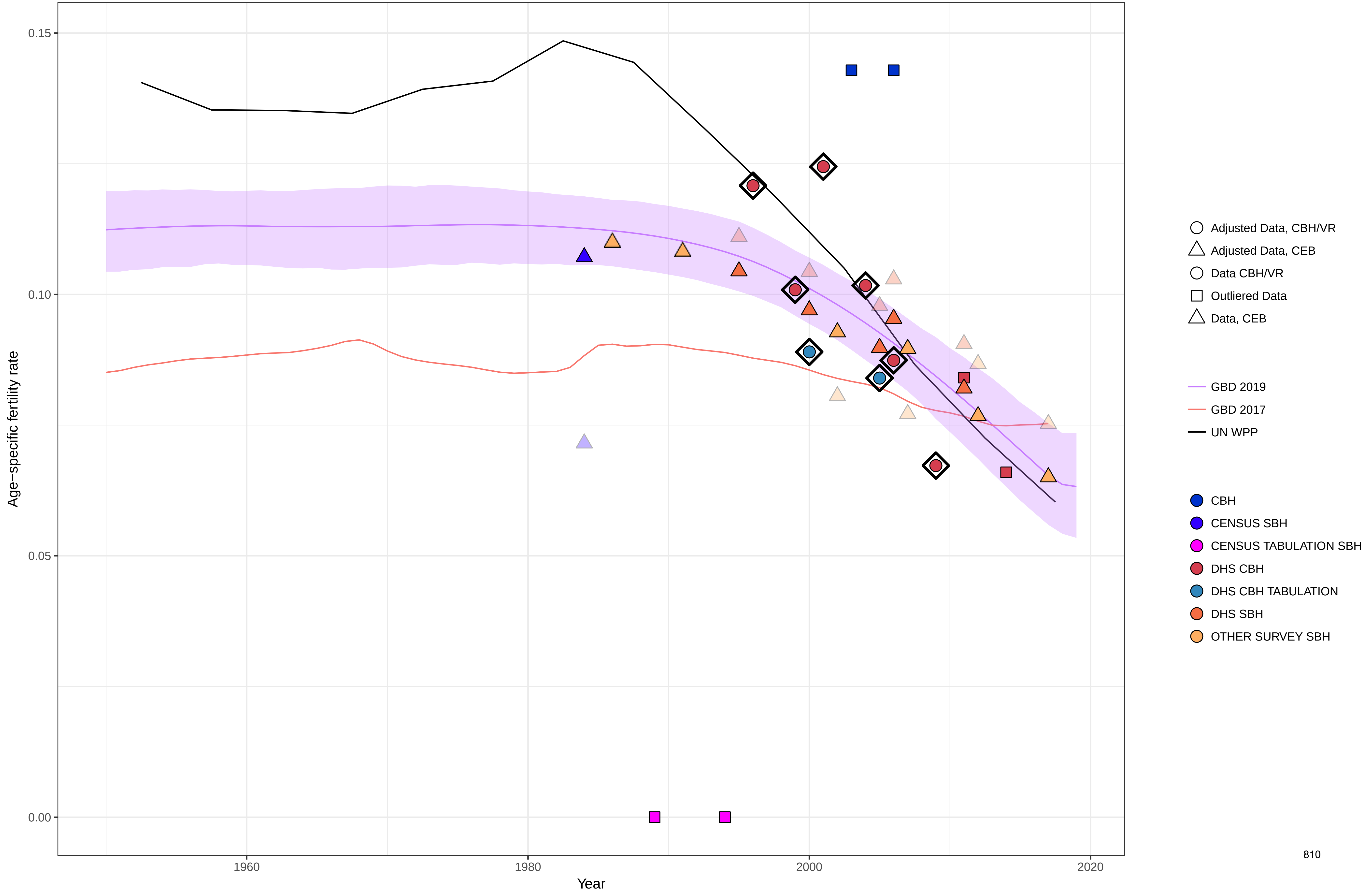

Age−specific fertility fitted trend lines, 1950−2019: Ethiopia, Ages: 45−49
Data points enclosed with a diamond are reference sources

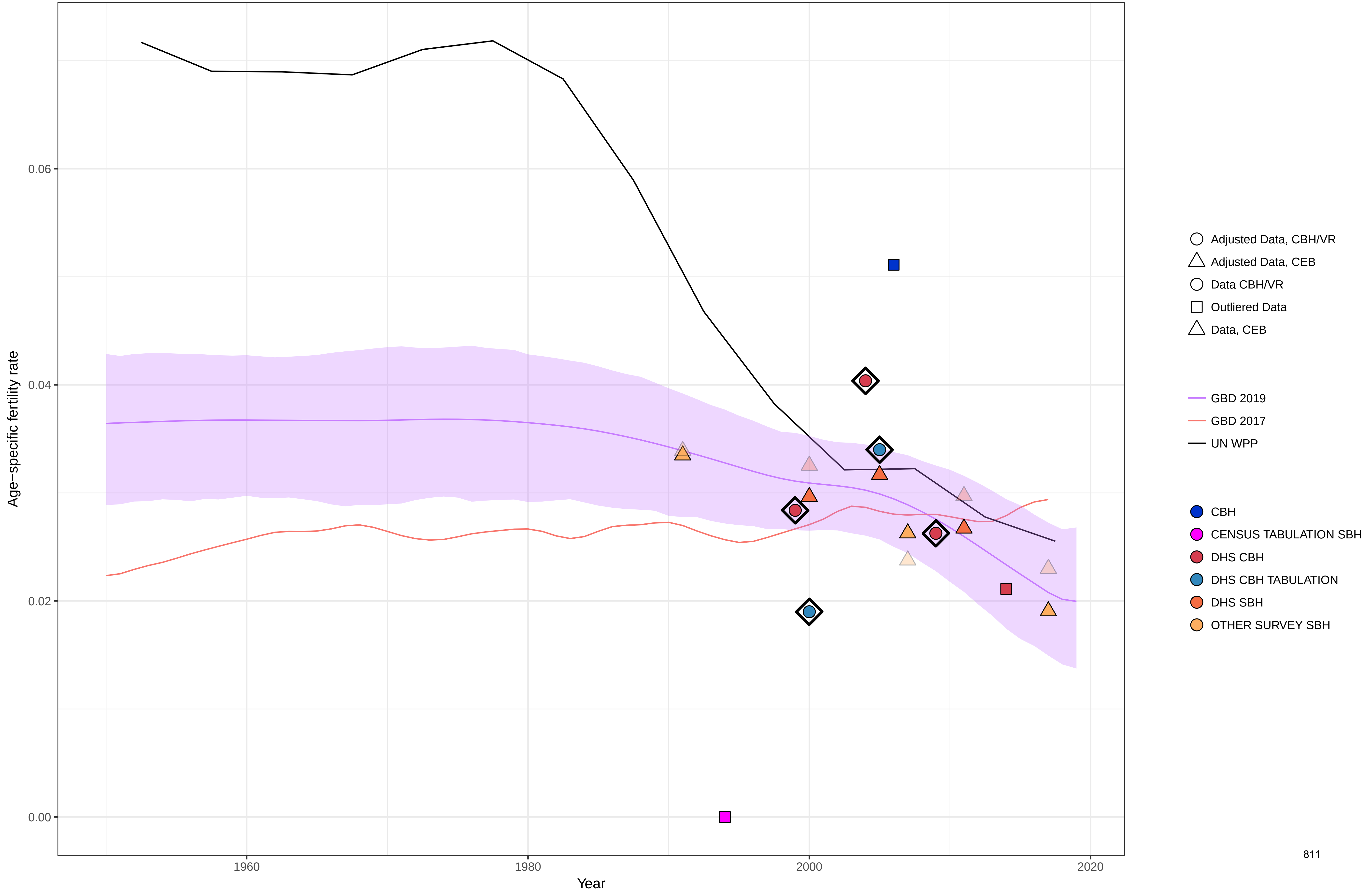

Age−specific fertility fitted trend lines, 1950−2019: Ethiopia, Ages: 50−54
Data points enclosed with a diamond are reference sources

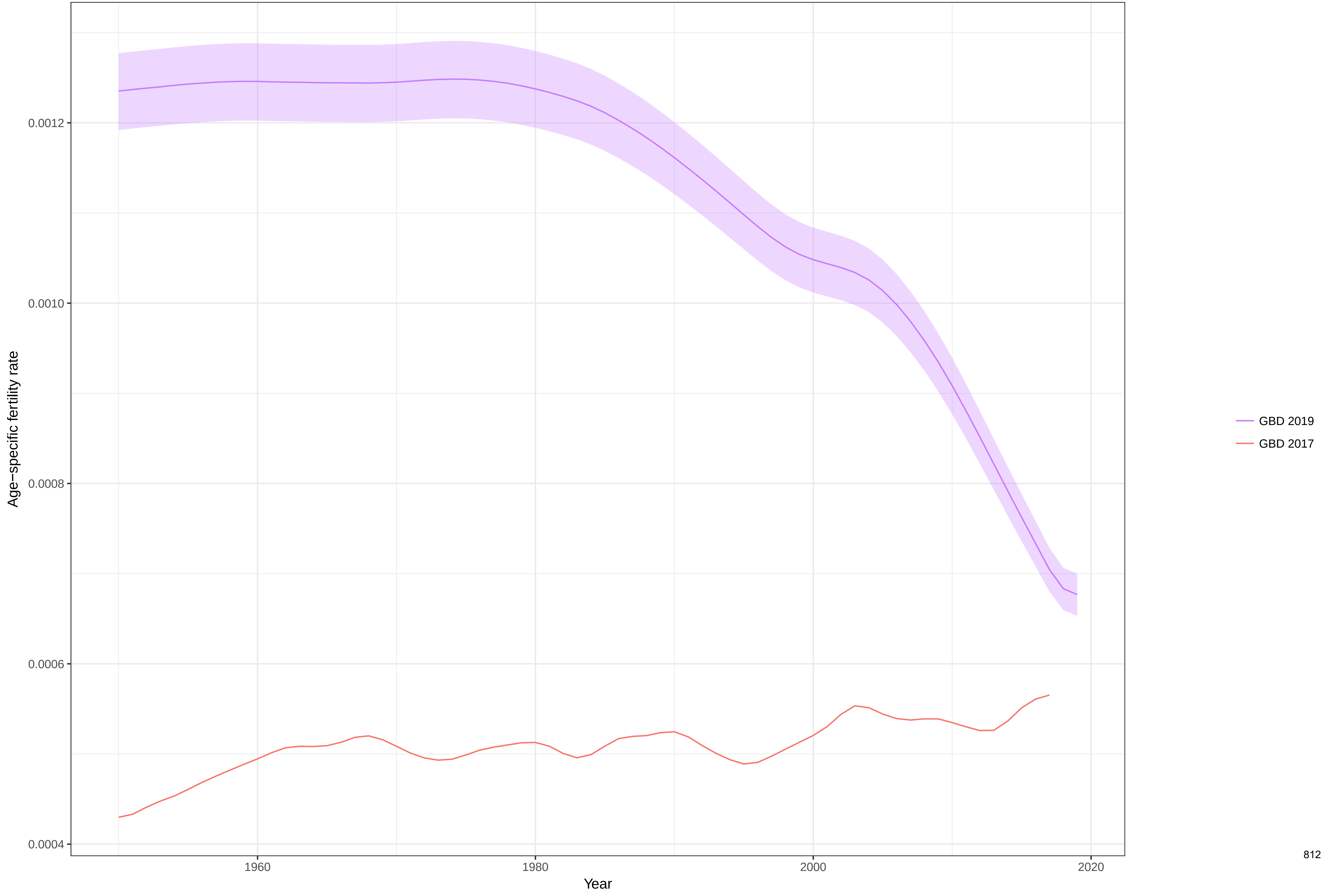

Total fertility rate fitted trend lines, 1950−2019: Ethiopia TFR
Data points enclosed with a diamond are reference sources

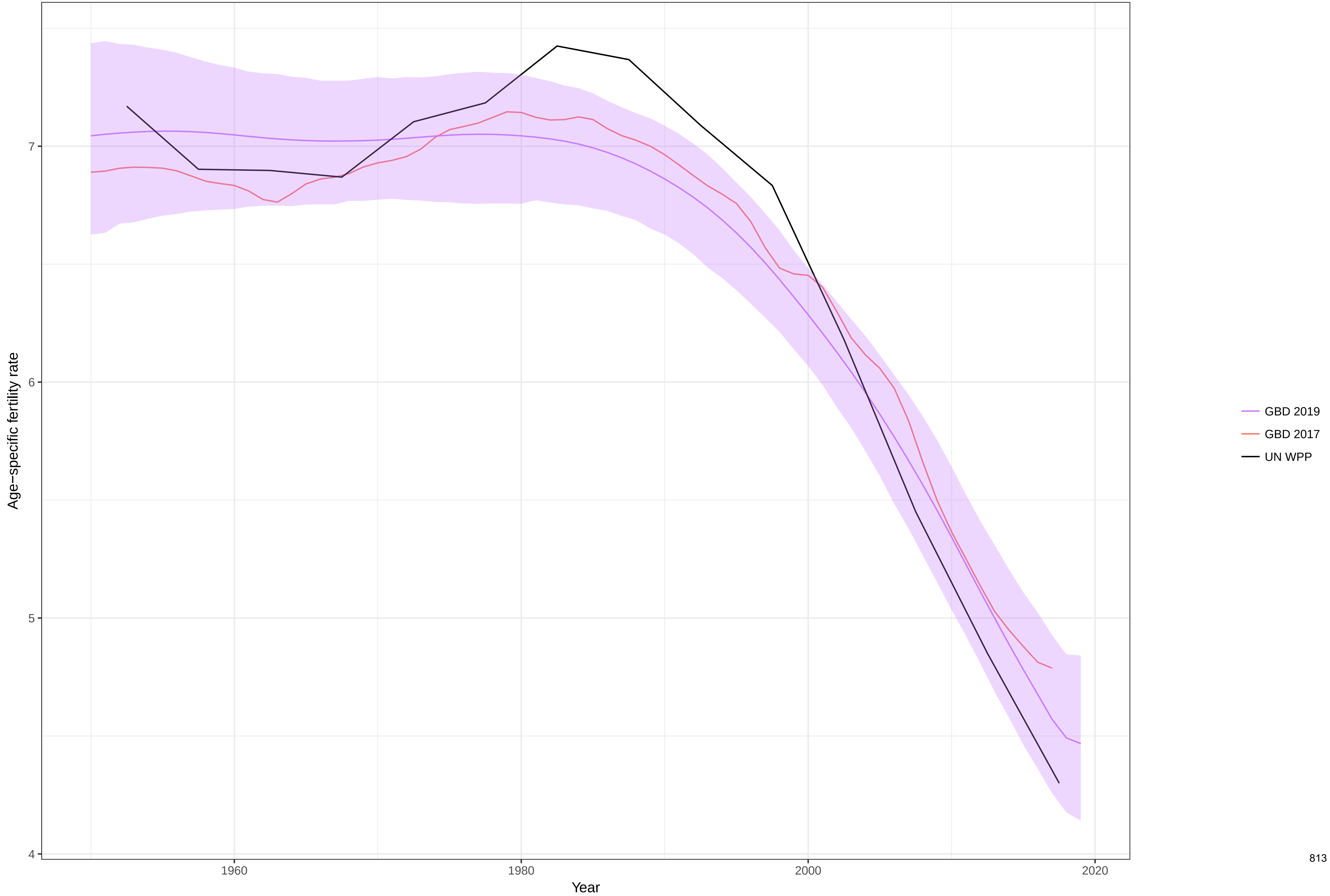

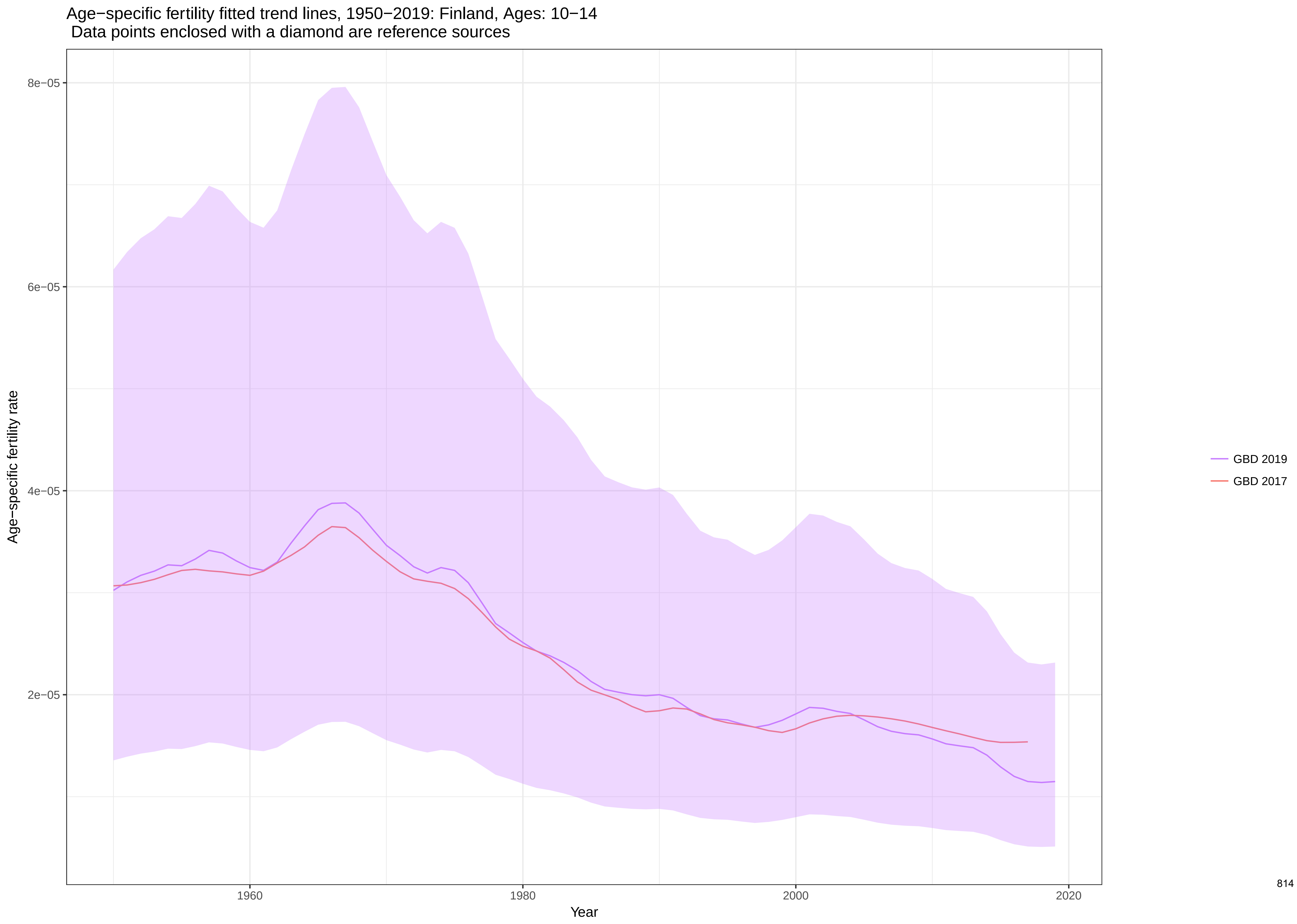
Age−specific fertility fitted trend lines, 1950−2019: Finland, Ages: 10−14
Data points enclosed with a diamond are reference sources
8e−05
6e−05
4e−05
2e−05
Age−specific fertility rate
1960
1980
2000
2020
Year
GBD 2019
GBD 2017

Age−specific fertility fitted trend lines, 1950−2019: Finland, Ages: 15−19
Data points enclosed with a diamond are reference sources

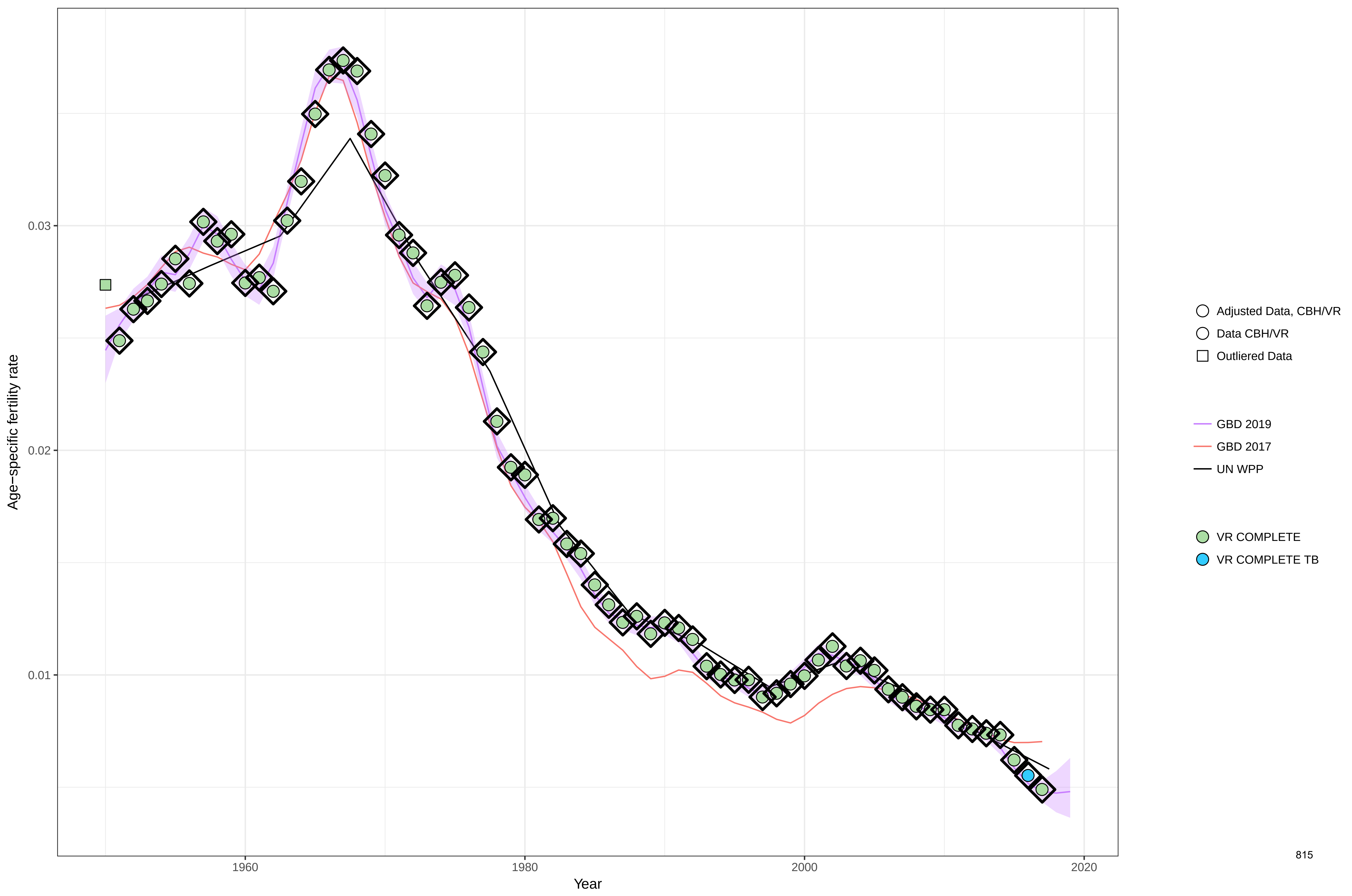

Age−specific fertility fitted trend lines, 1950−2019: Finland, Ages: 20−24
Data points enclosed with a diamond are reference sources

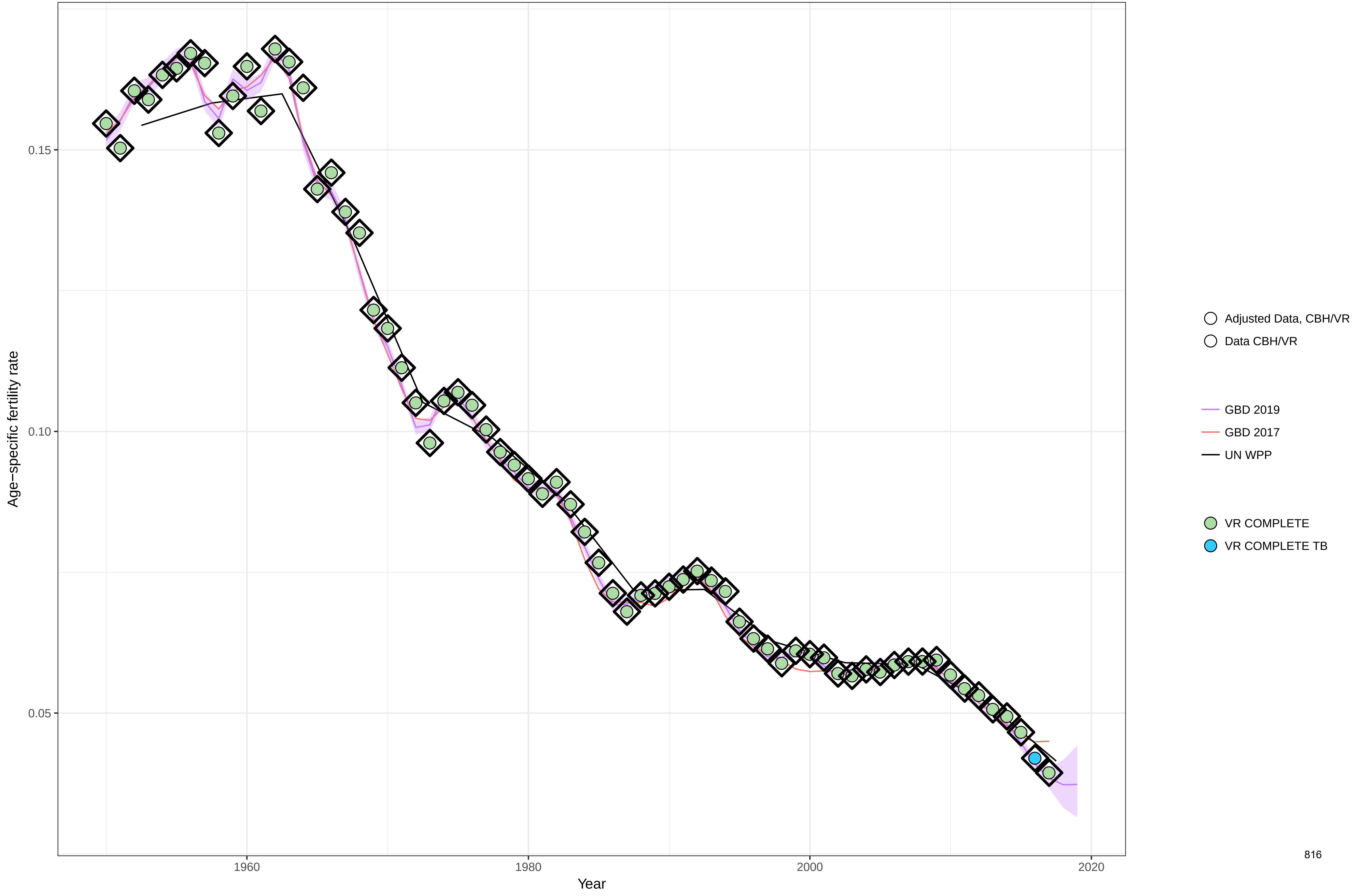

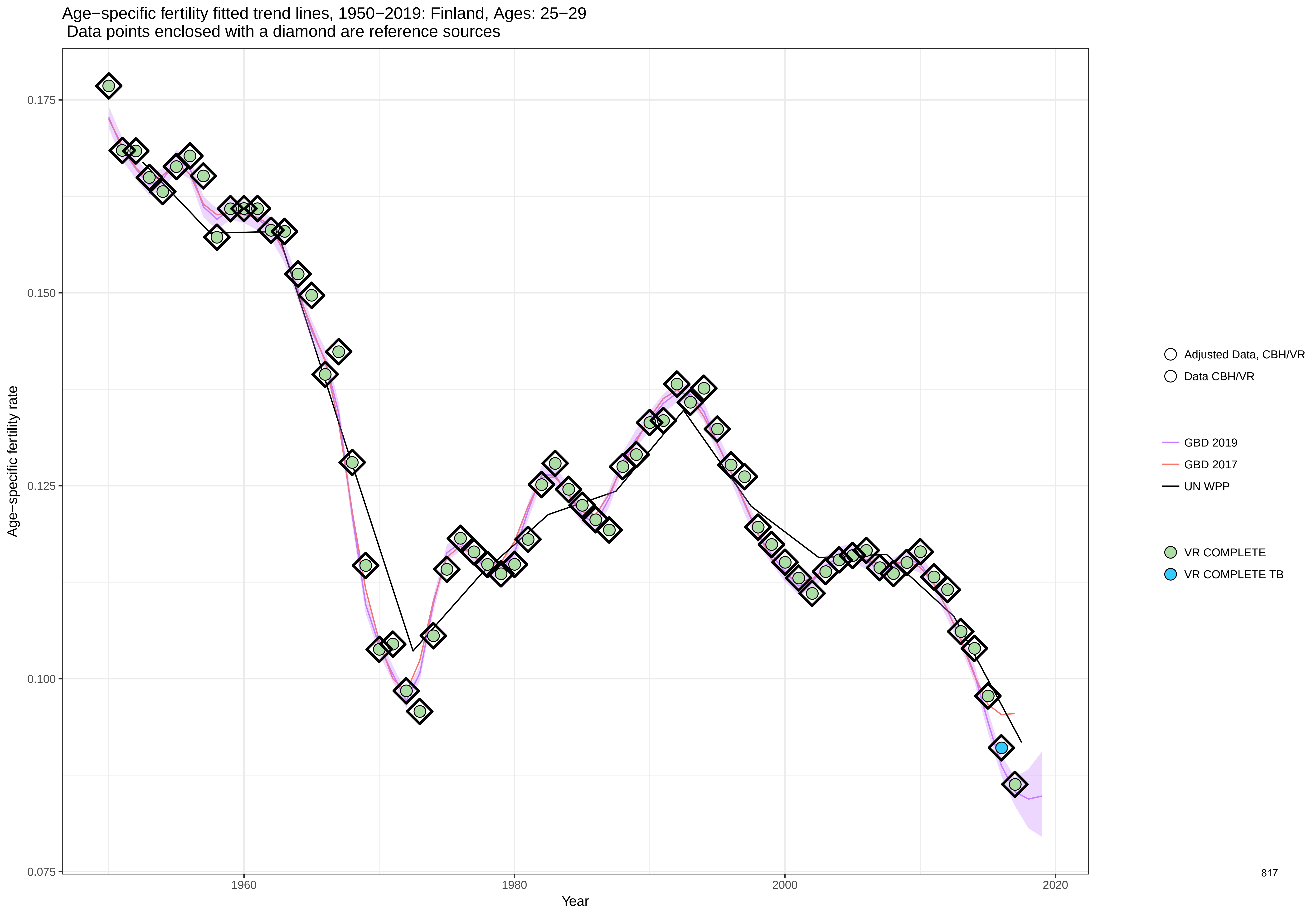
Age−specific fertility fitted trend lines, 1950−2019: Finland, Ages: 25−29
Data points enclosed with a diamond are reference sources
Age−specific fertility rate
0.175
0.150
0.125
0.100
0.075
1960
1980
2000
2020
Year
Adjusted Data, CBH/VR
Data CBH/VR
GBD 2019
GBD 2017
UN WPP
VR COMPLETE
VR COMPLETE TB

Age−specific fertility fitted trend lines, 1950−2019: Finland, Ages: 30−34
Data points enclosed with a diamond are reference sources

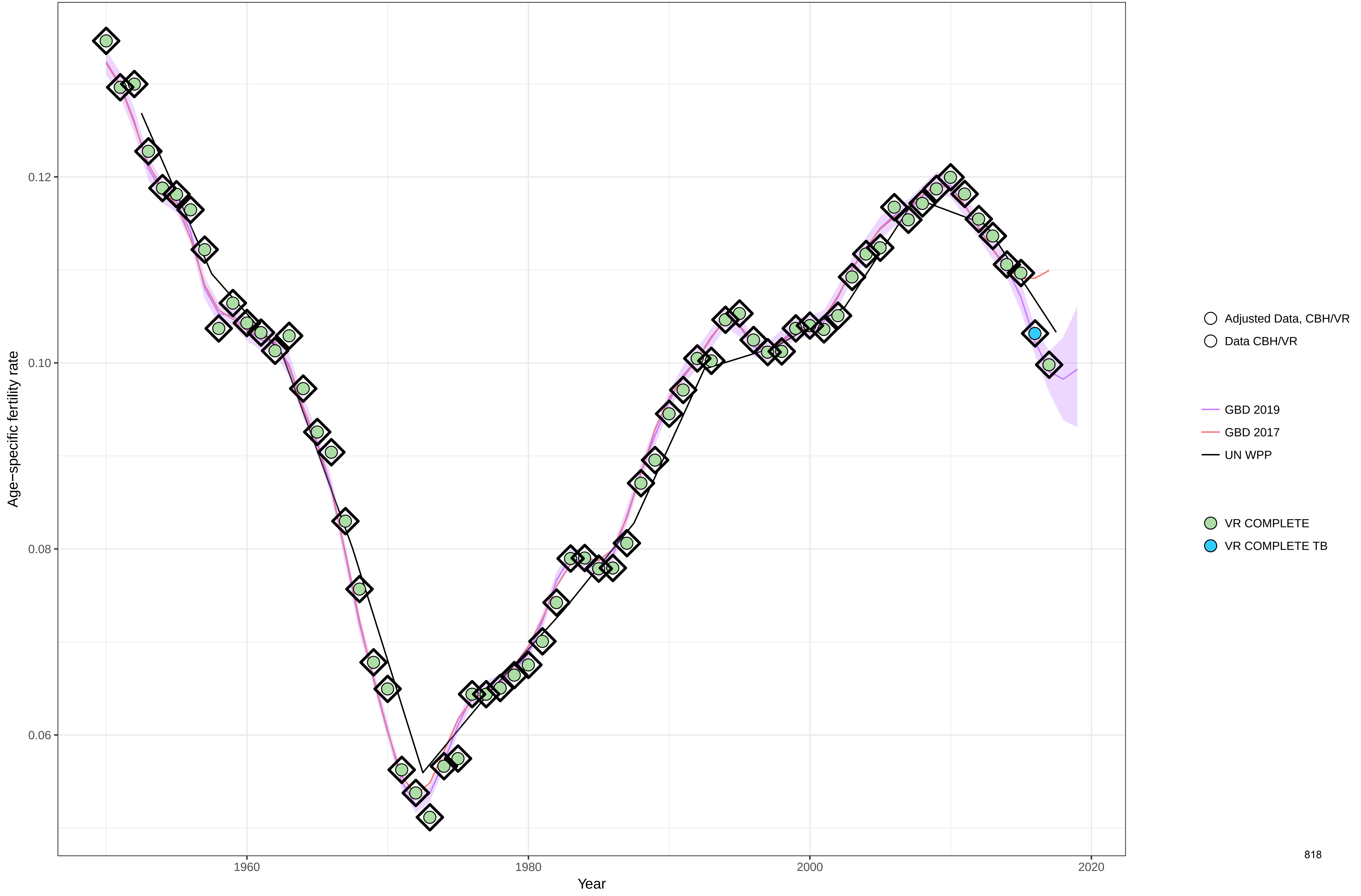

Age−specific fertility fitted trend lines, 1950−2019: Finland, Ages: 35−39
Data points enclosed with a diamond are reference sources

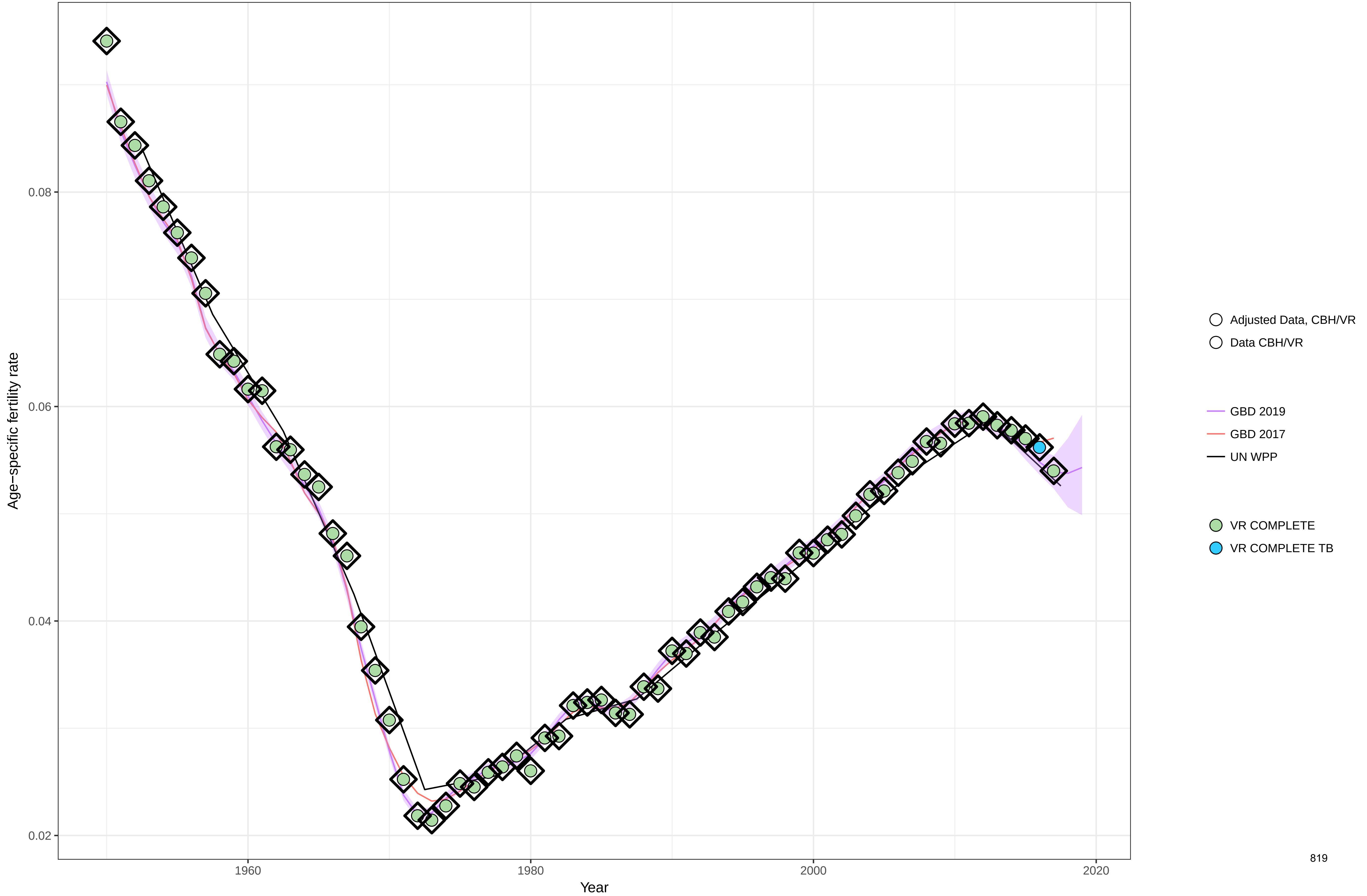

Age−specific fertility fitted trend lines, 1950−2019: Finland, Ages: 40−44
Data points enclosed with a diamond are reference sources

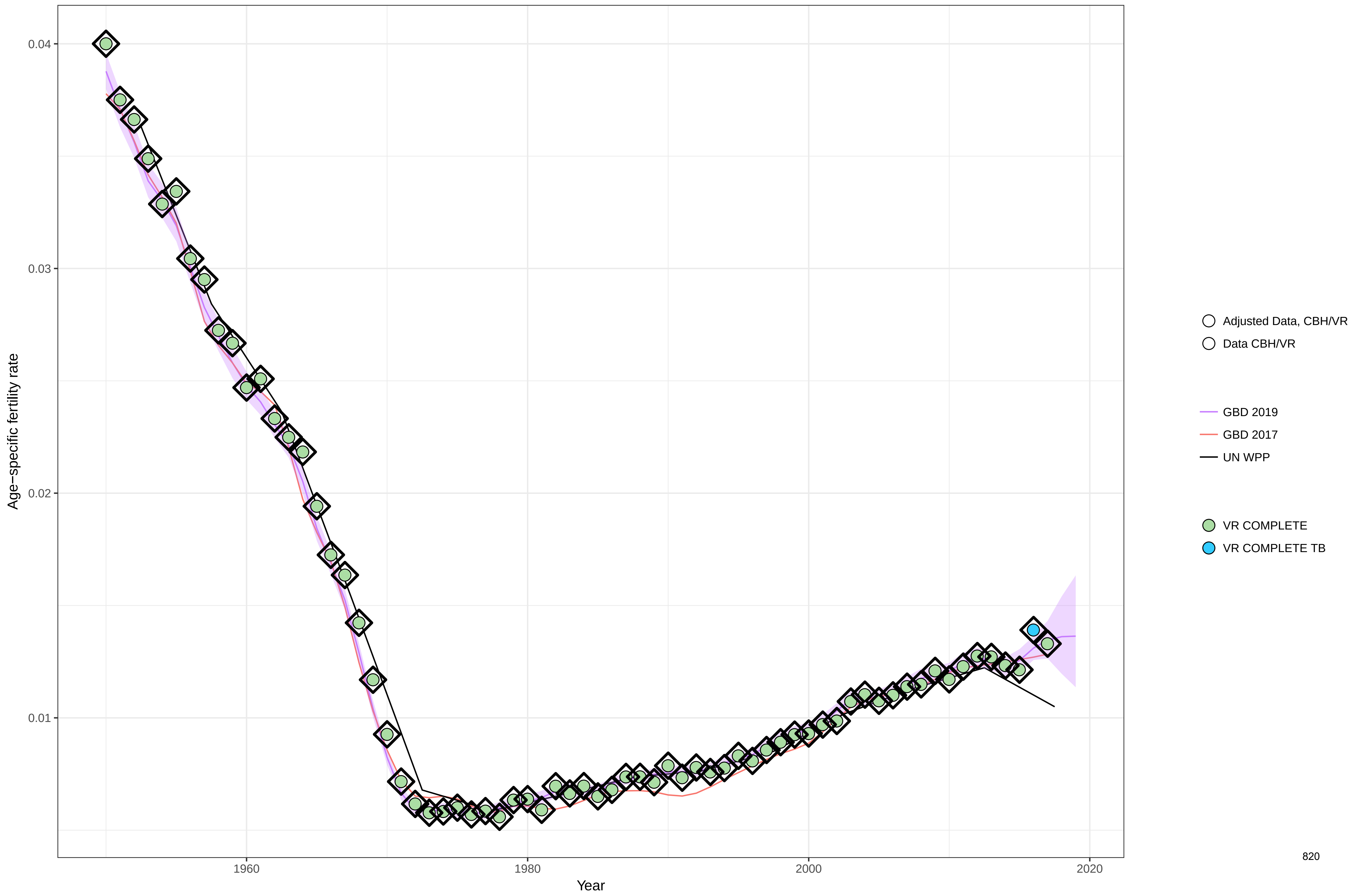

Age−specific fertility fitted trend lines, 1950−2019: Finland, Ages: 45−49
Data points enclosed with a diamond are reference sources

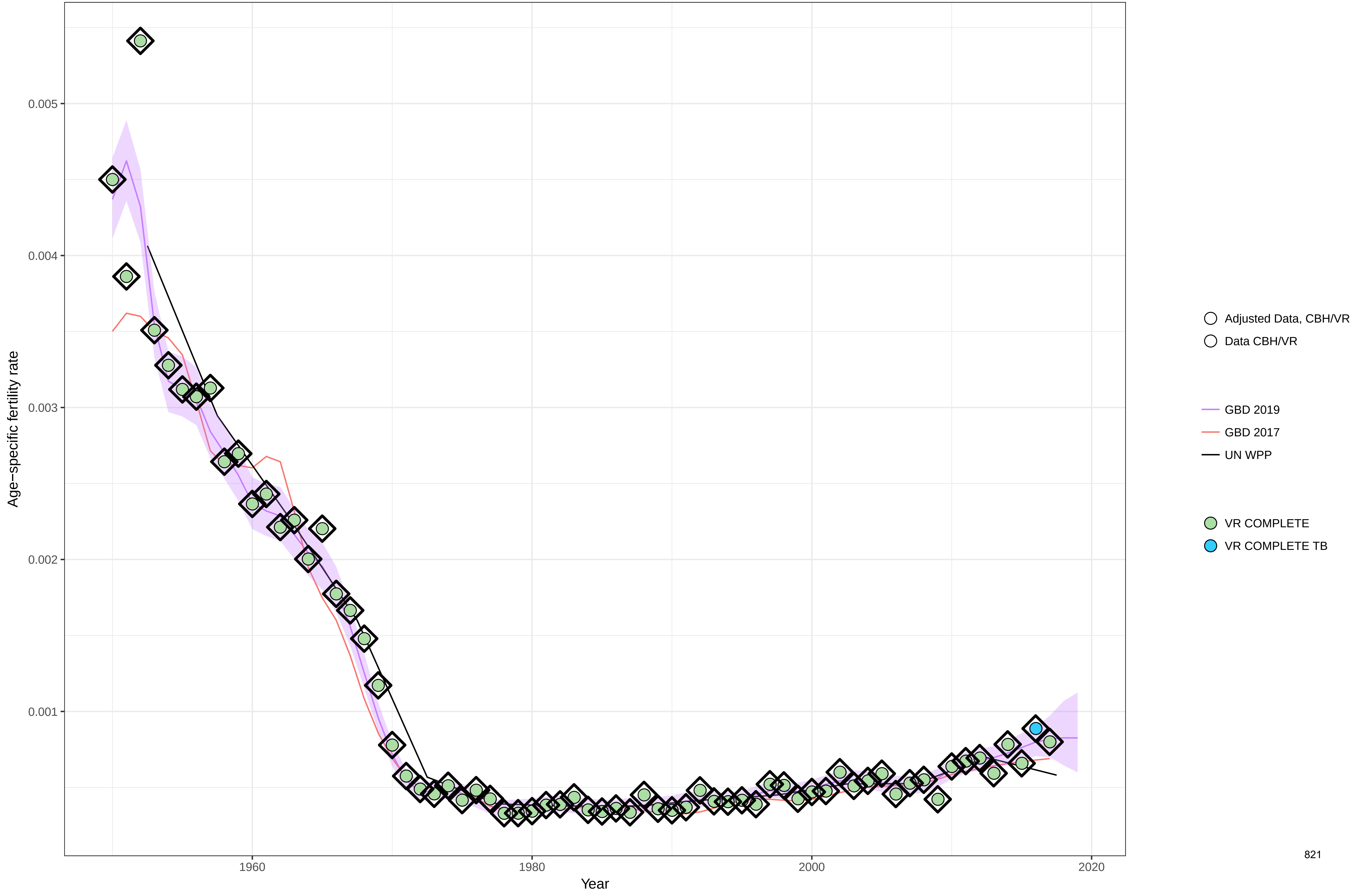

Age−specific fertility fitted trend lines, 1950−2019: Finland, Ages: 50−54
Data points enclosed with a diamond are reference sources

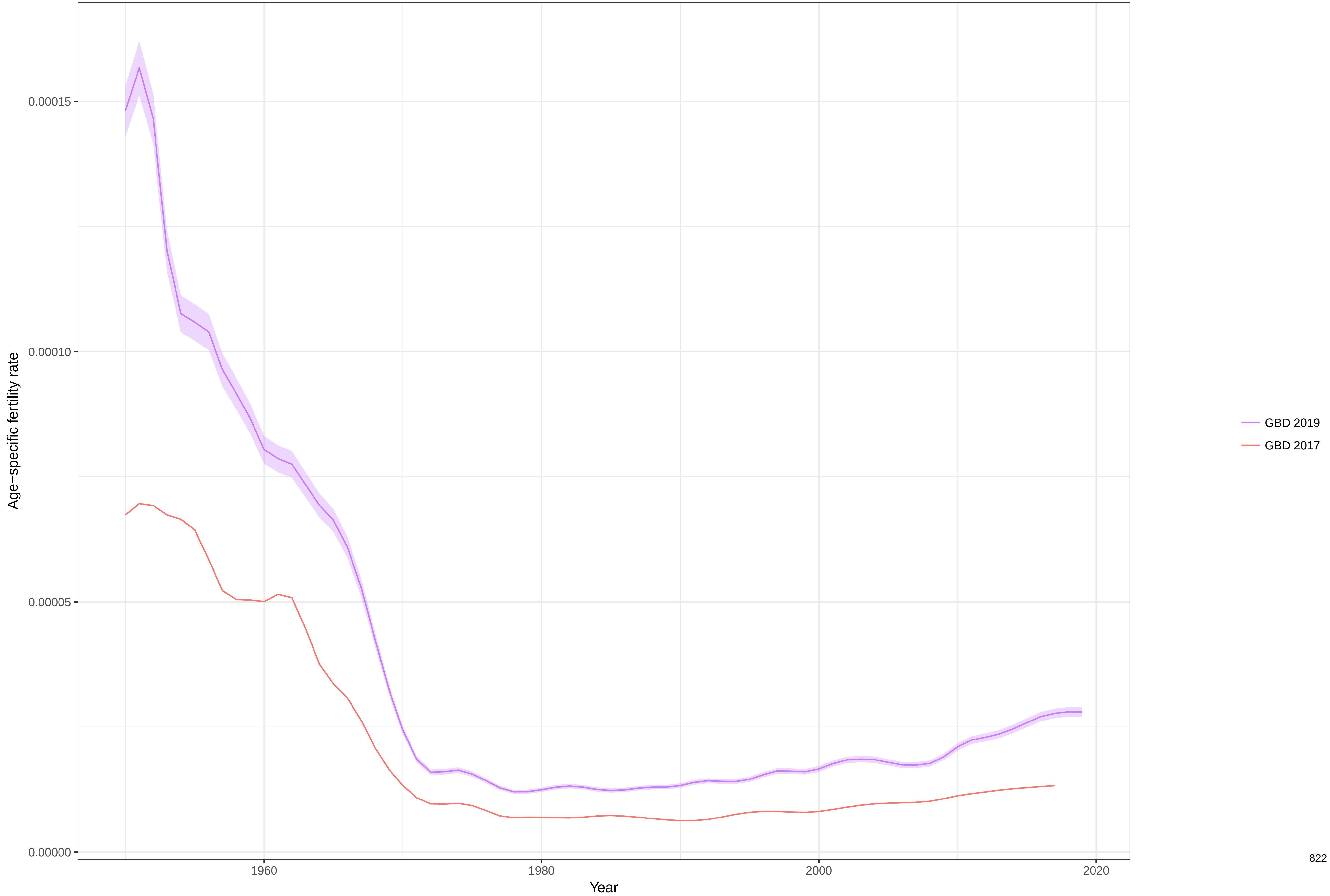

Total fertility rate fitted trend lines, 1950−2019: Finland TFR
Data points enclosed with a diamond are reference sources

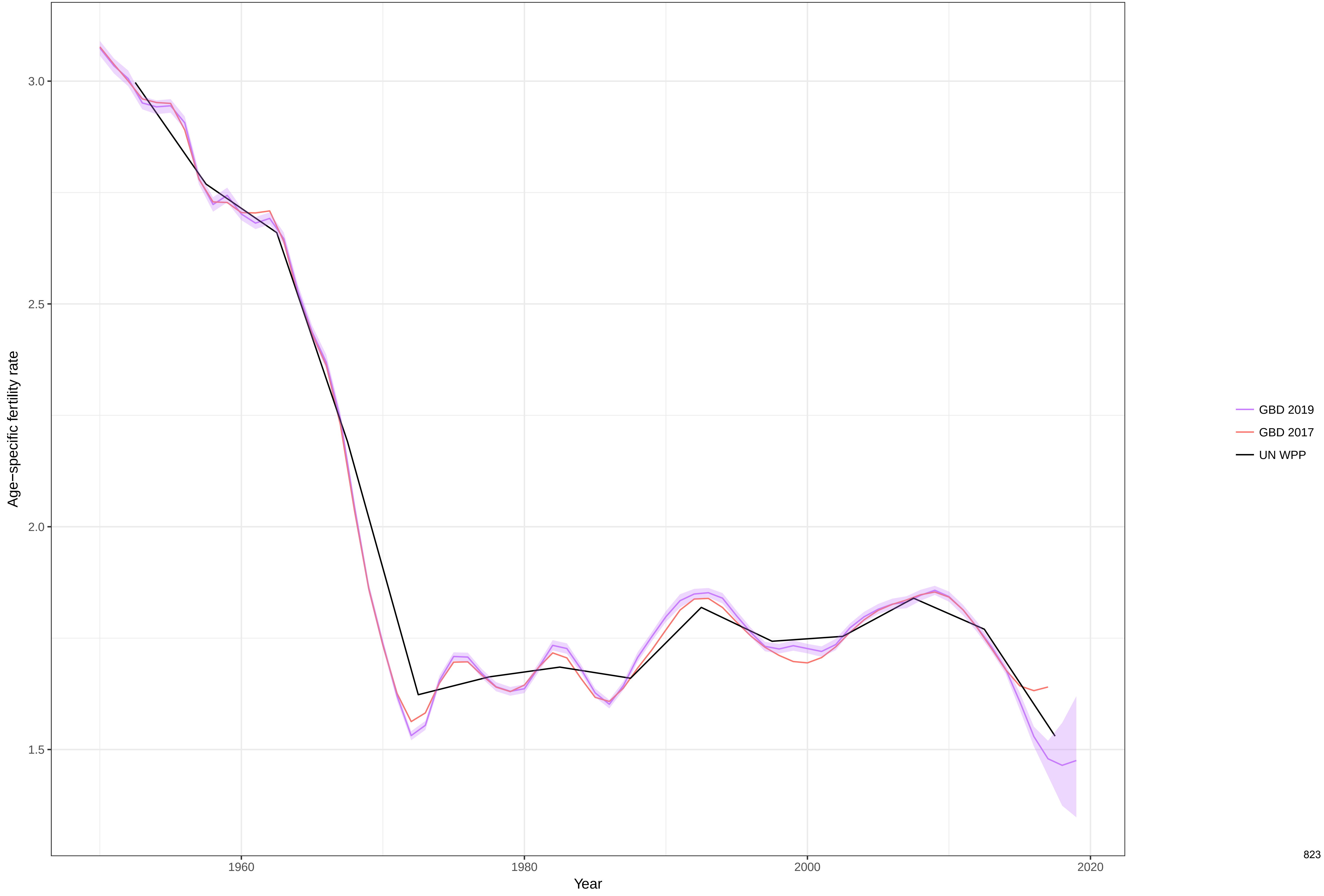

Age−specific fertility fitted trend lines, 1950−2019: Fiji, Ages: 10−14
Data points enclosed with a diamond are reference sources

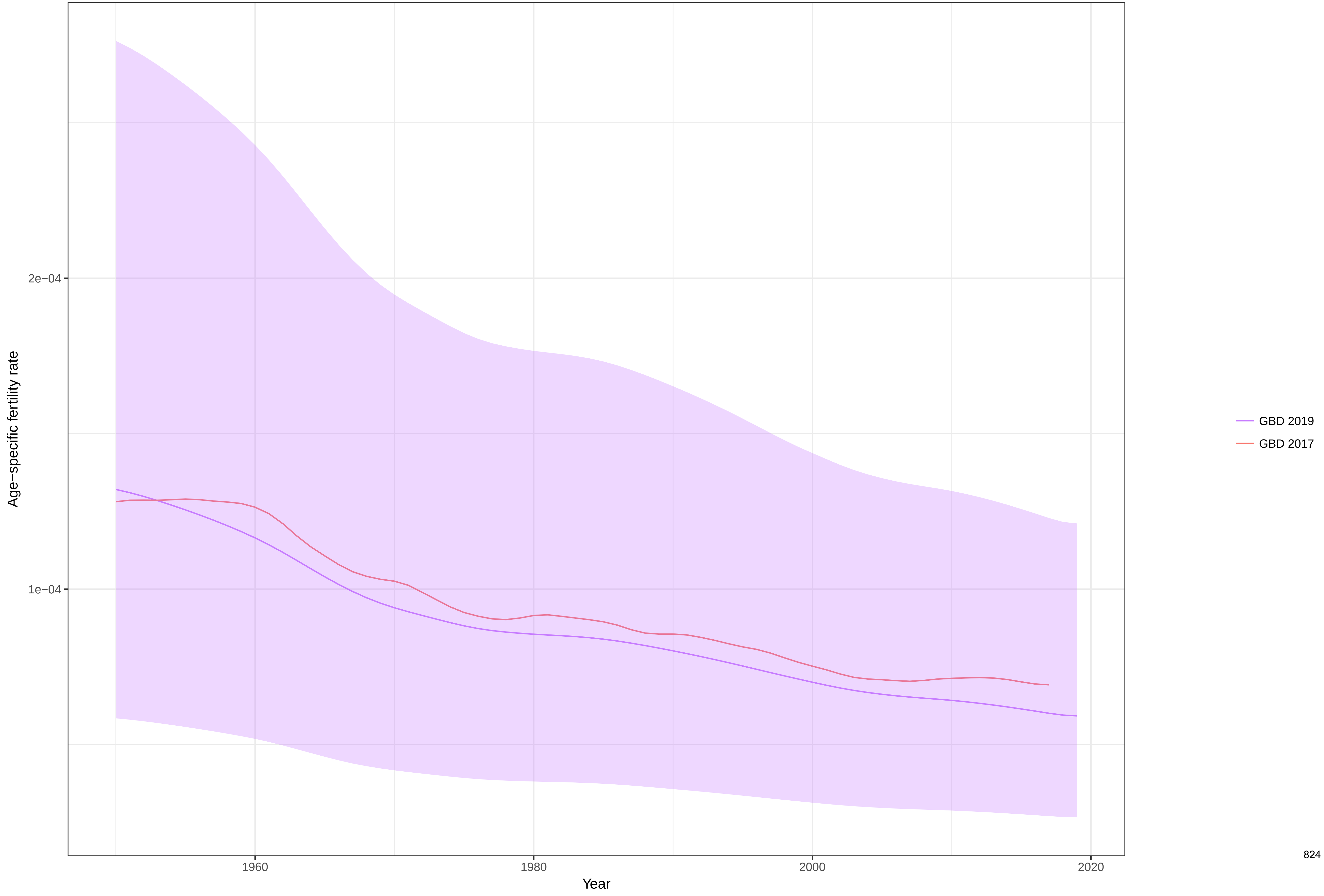

Age−specific fertility fitted trend lines, 1950−2019: Fiji, Ages: 15−19
Data points enclosed with a diamond are reference sources

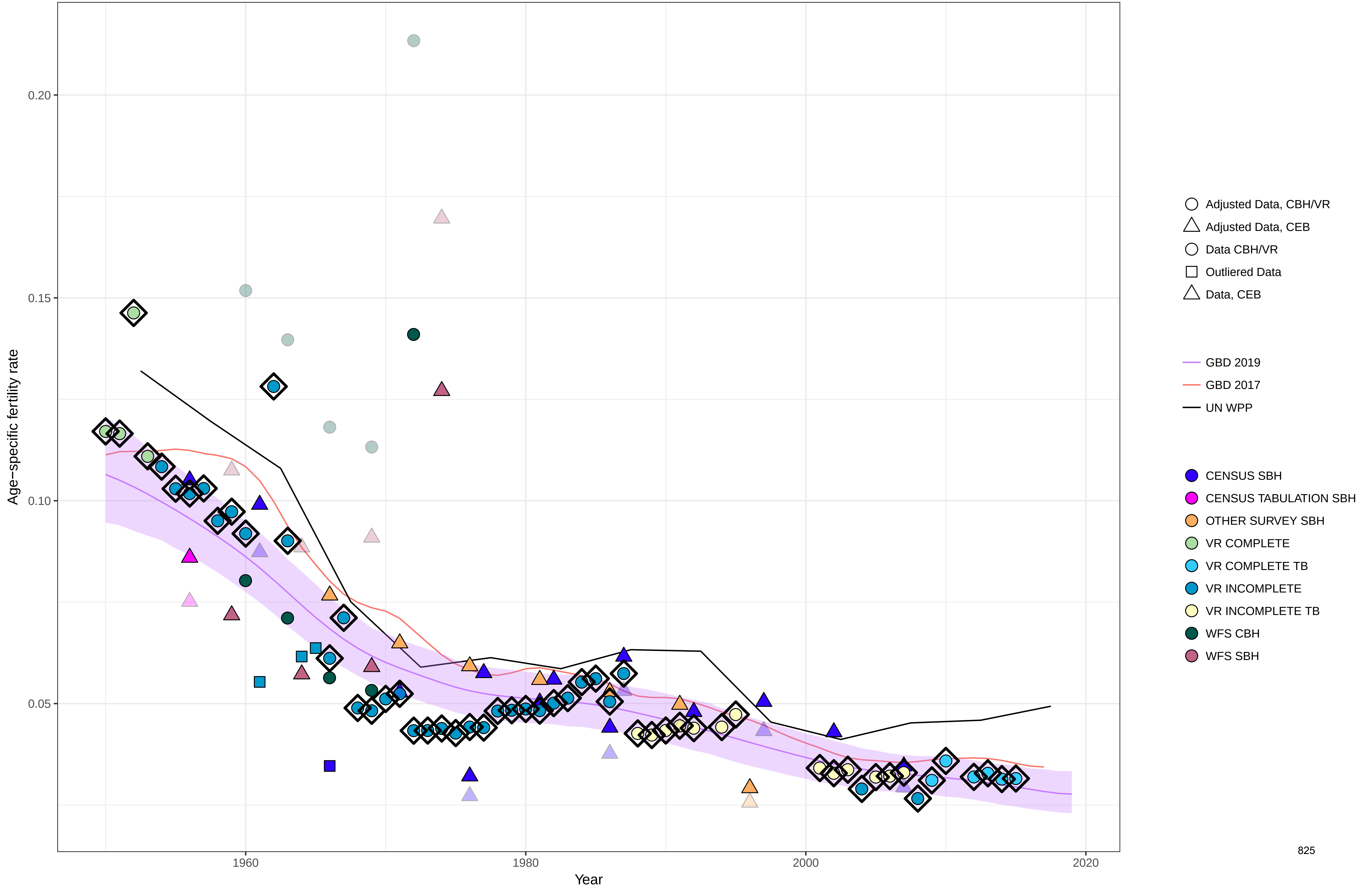

Age−specific fertility fitted trend lines, 1950−2019: Fiji, Ages: 20−24
Data points enclosed with a diamond are reference sources

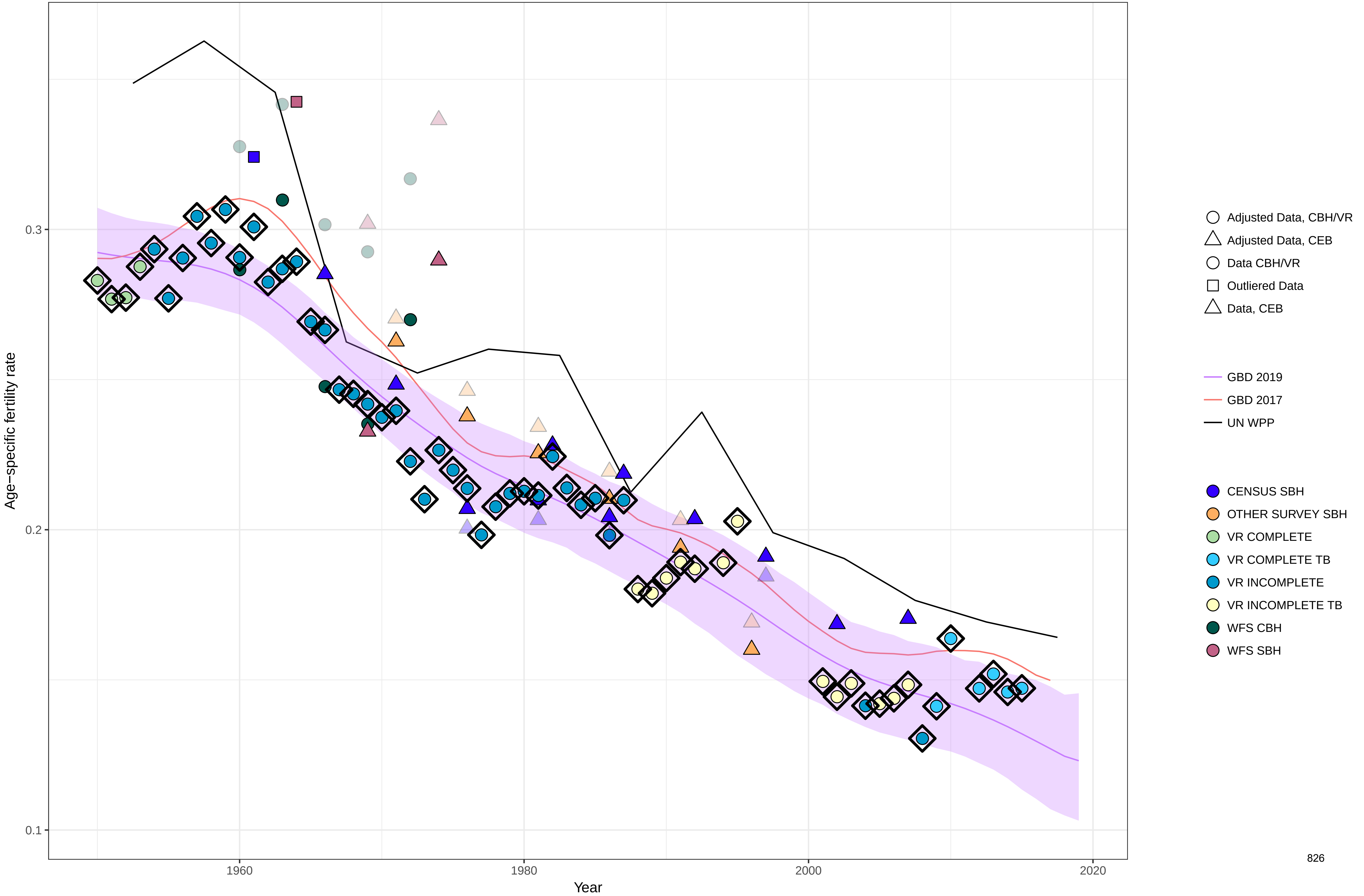

Age−specific fertility fitted trend lines, 1950−2019: Fiji, Ages: 25−29
Data points enclosed with a diamond are reference sources

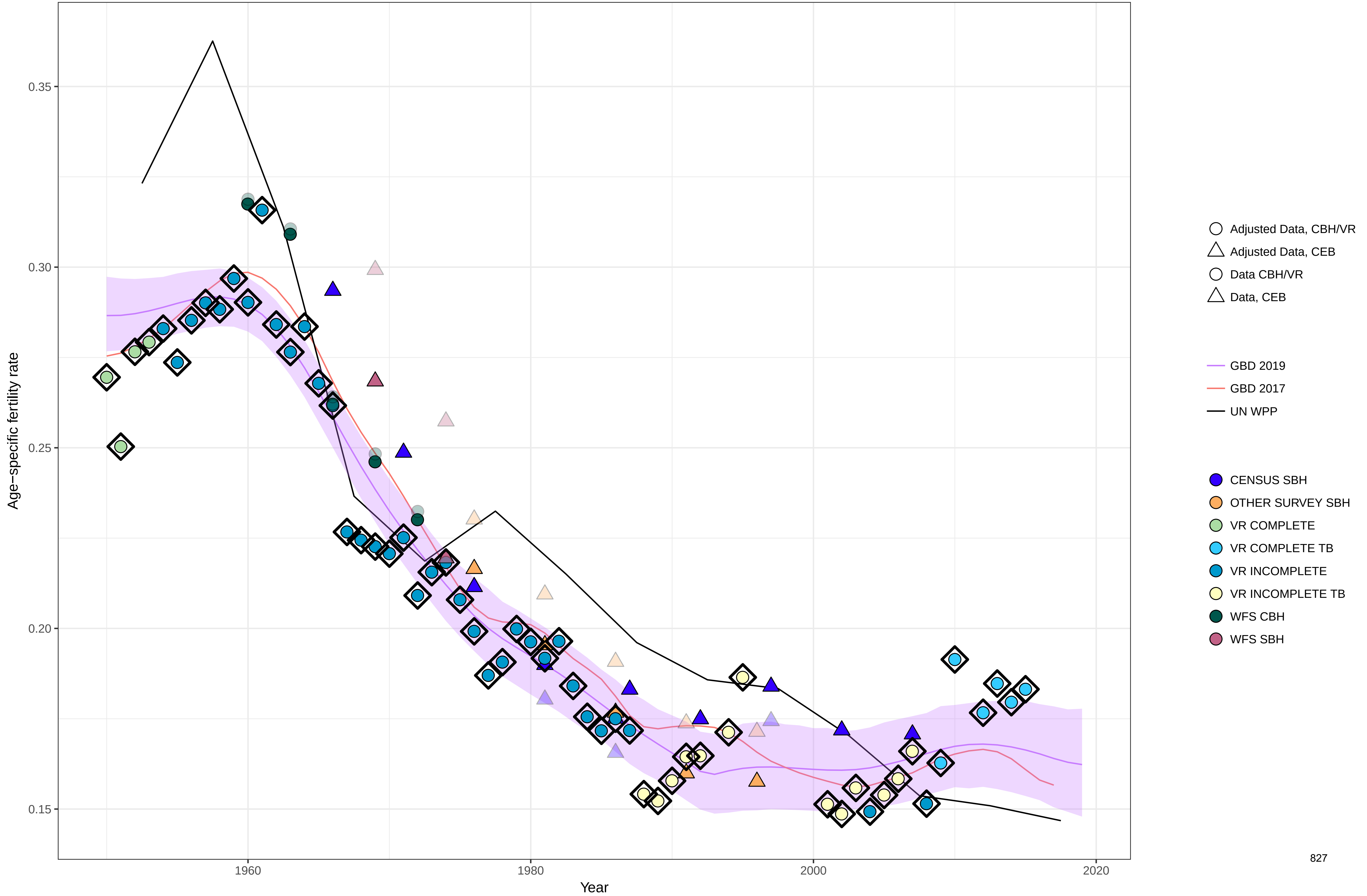

Age−specific fertility fitted trend lines, 1950−2019: Fiji, Ages: 30−34
Data points enclosed with a diamond are reference sources

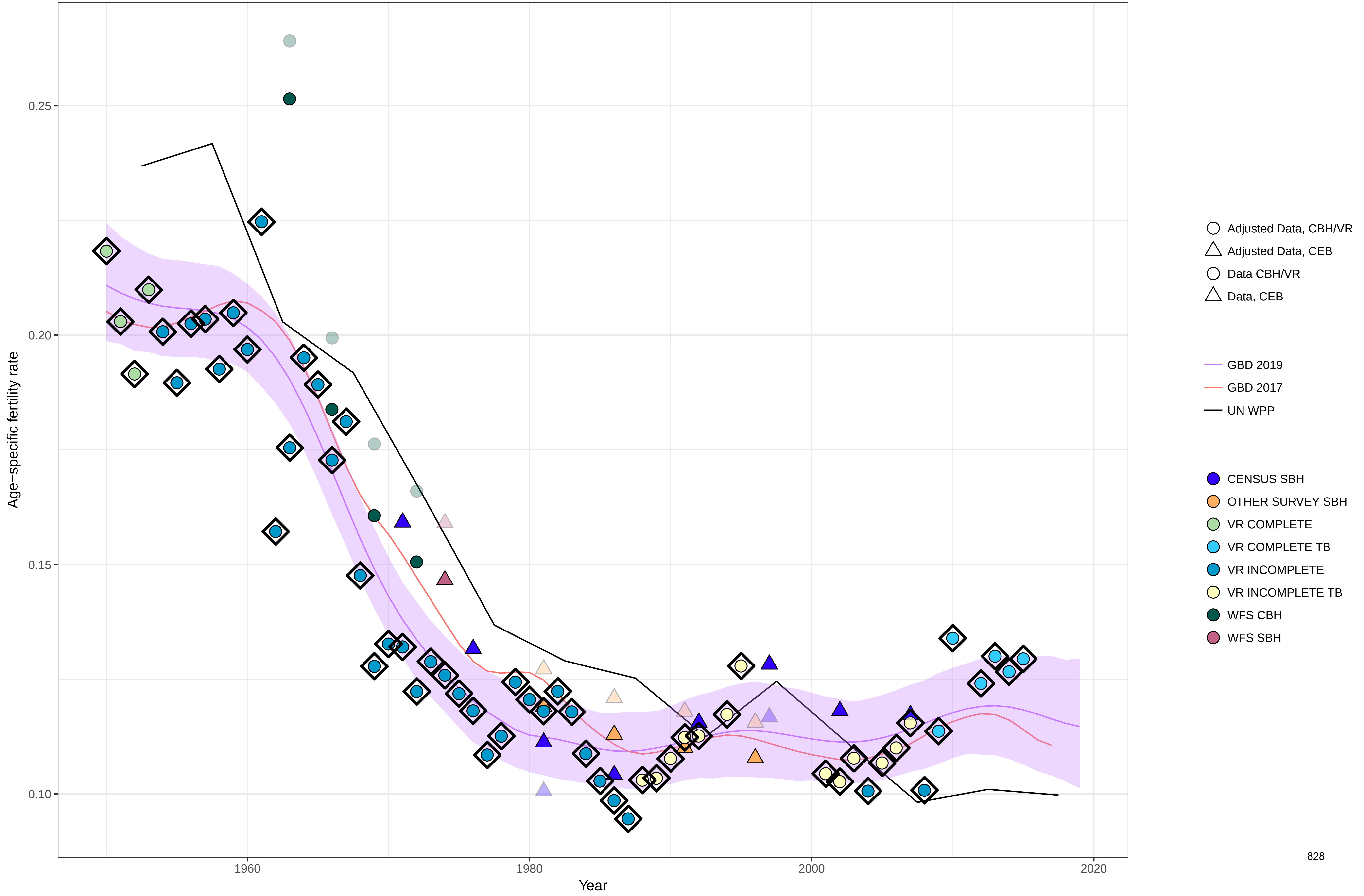

Age−specific fertility fitted trend lines, 1950−2019: Fiji, Ages: 35−39
Data points enclosed with a diamond are reference sources

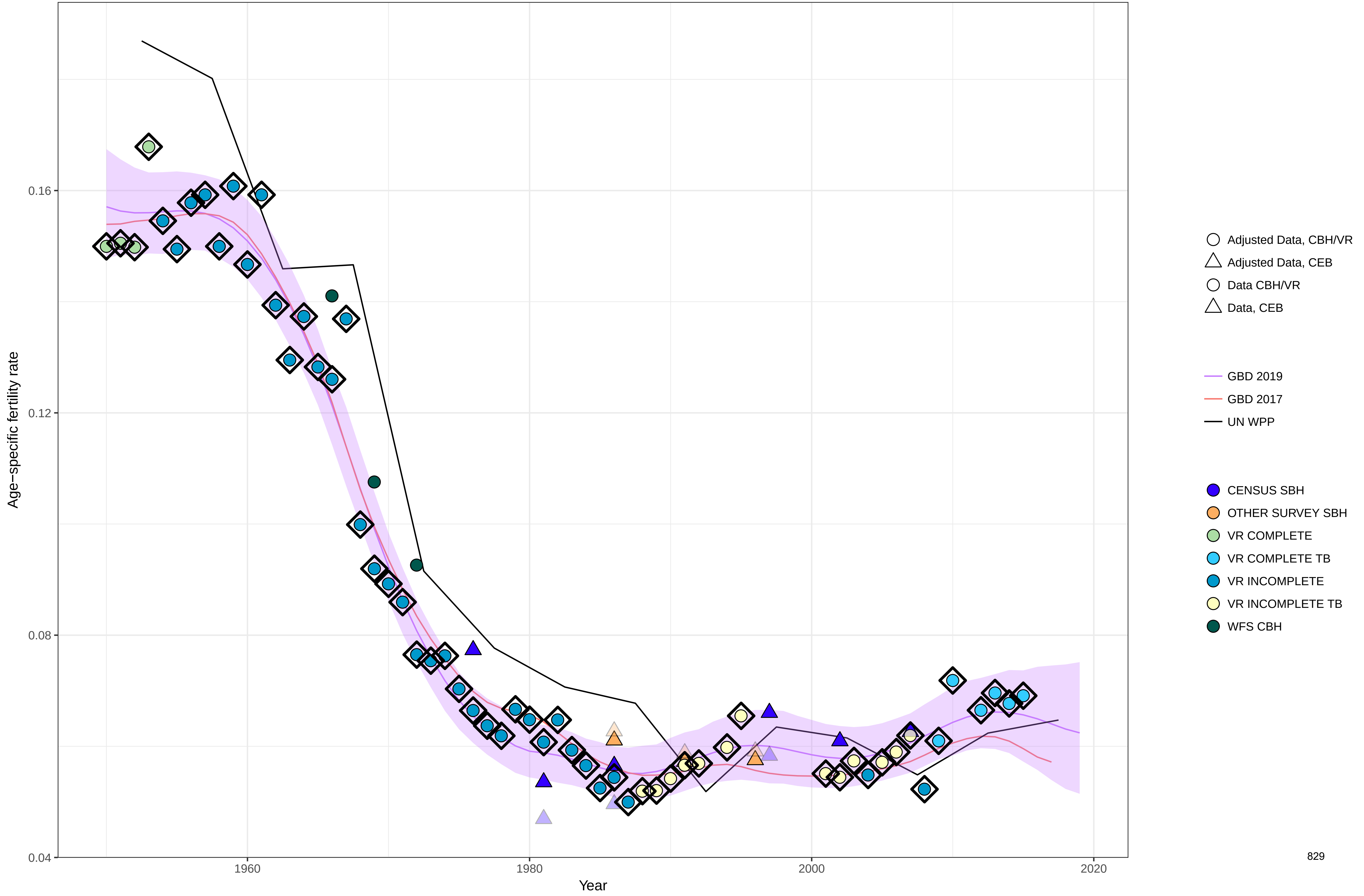

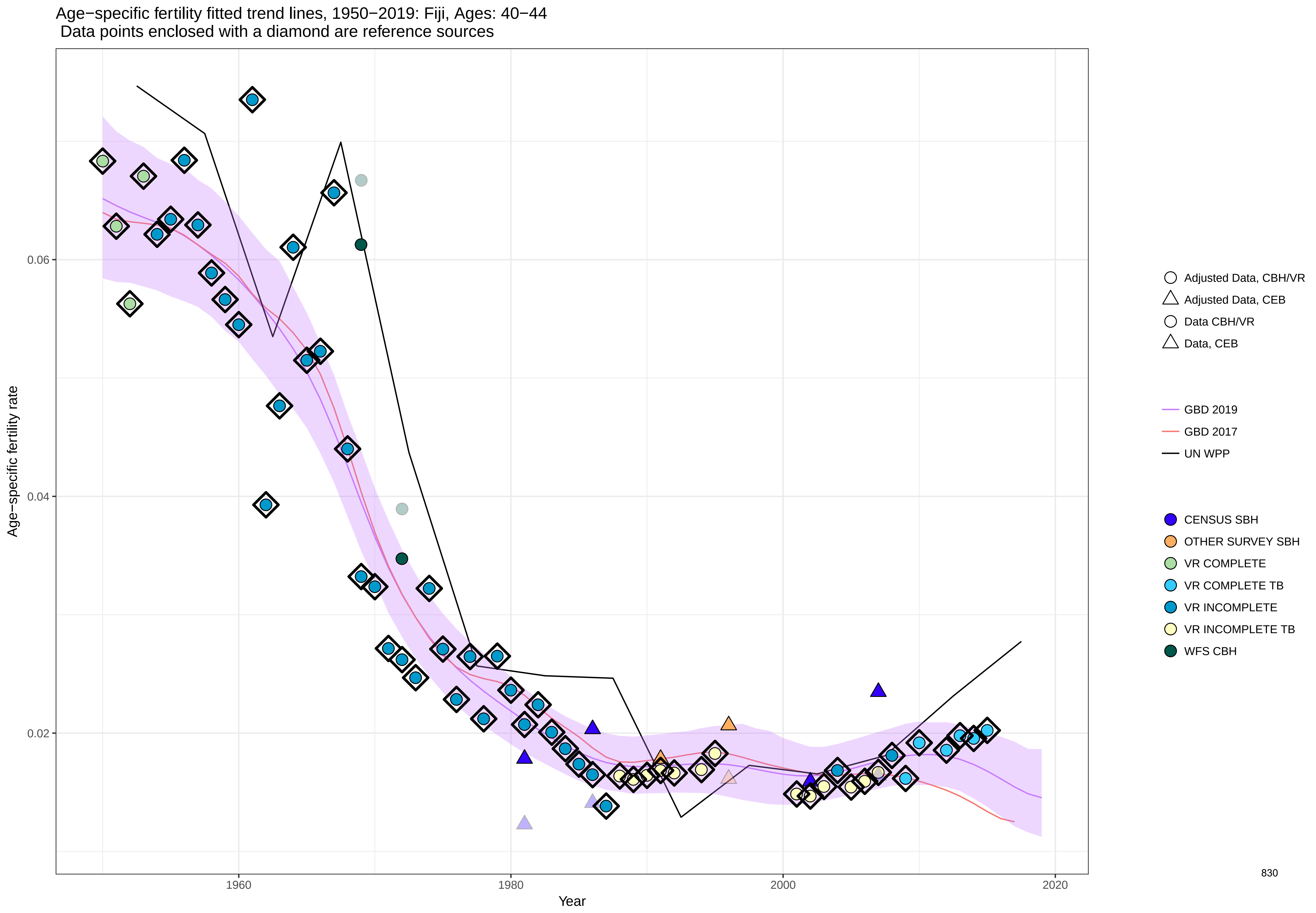

Age−specific fertility fitted trend lines, 1950−2019: Fiji, Ages: 40−44
Data points enclosed with a diamond are reference sources
Age−specific fertility rate
0.06
0.04
0.02
1960
1980
2000
2020
Year
Adjusted Data, CBH/VR
Adjusted Data, CEB
Data CBH/VR
Data, CEB
GBD 2019
GBD 2017
UN WPP
CENSUS SBH
OTHER SURVEY SBH
VR COMPLETE
VR COMPLETE TB
VR INCOMPLETE
VR INCOMPLETE TB
WFS CBH

Age−specific fertility fitted trend lines, 1950−2019: Fiji, Ages: 45−49
Data points enclosed with a diamond are reference sources

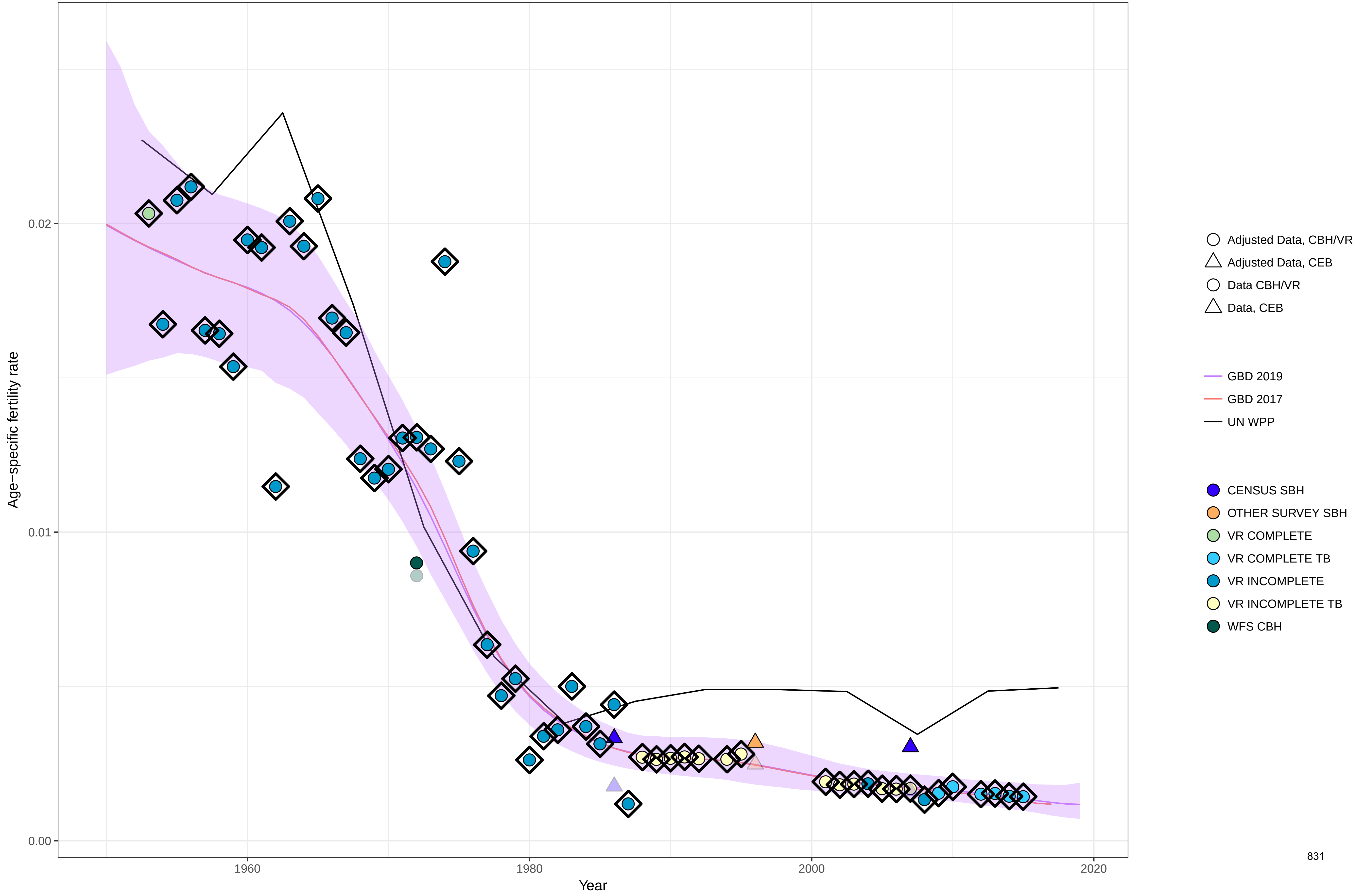

Age−specific fertility fitted trend lines, 1950−2019: Fiji, Ages: 50−54
Data points enclosed with a diamond are reference sources

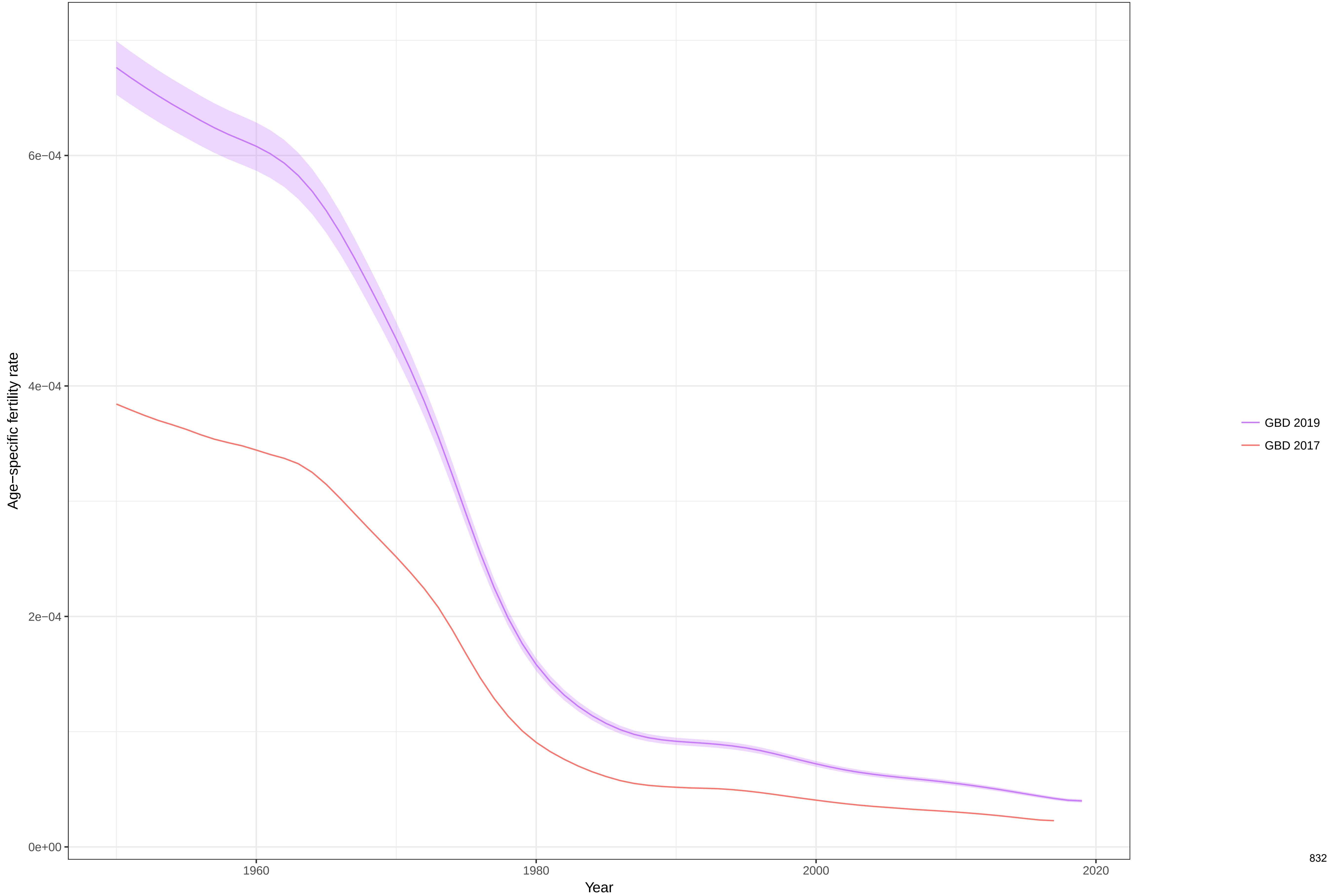

Total fertility rate fitted trend lines, 1950−2019: Fiji TFR
Data points enclosed with a diamond are reference sources

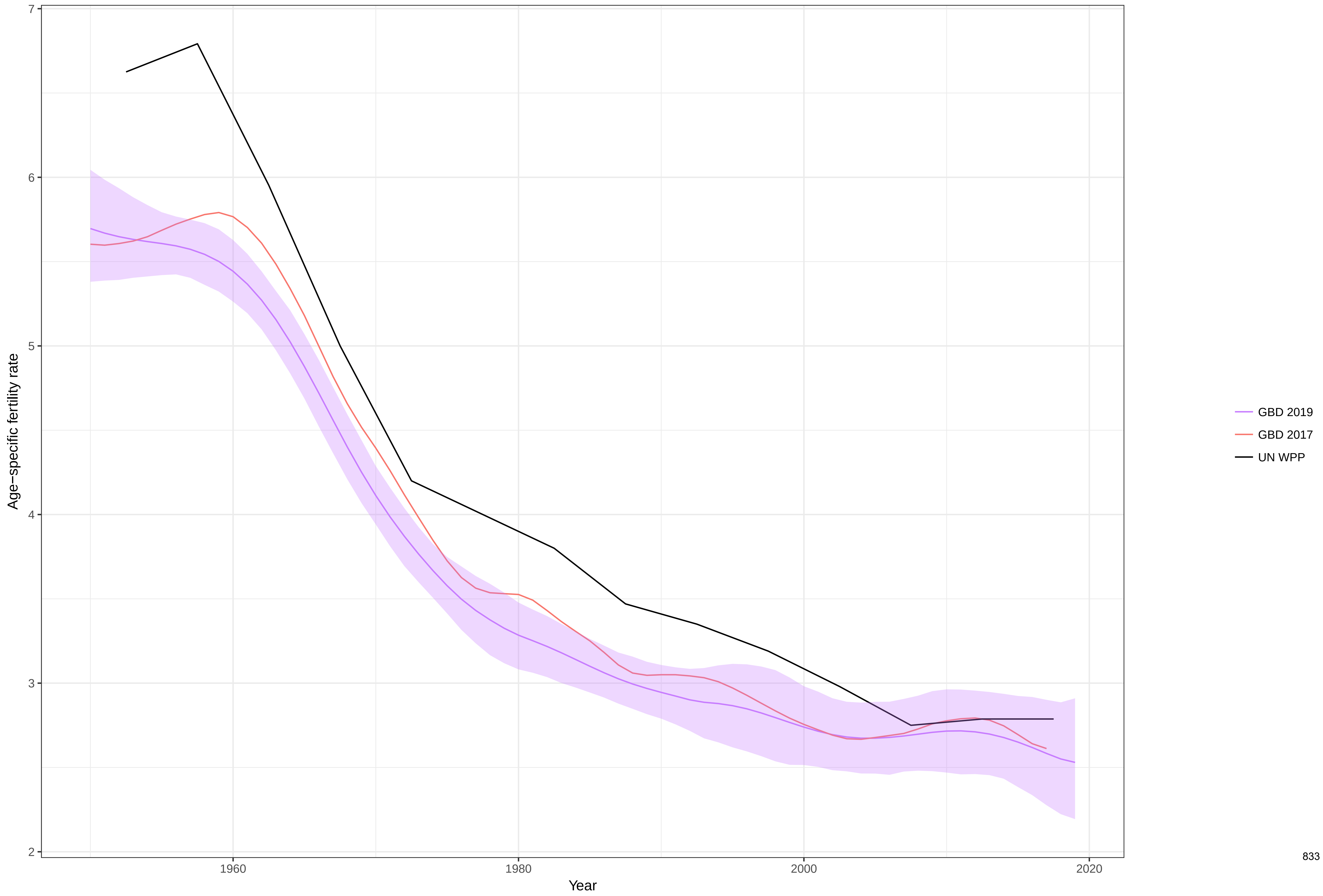

Age−specific fertility fitted trend lines, 1950−2019: France, Ages: 10−14
Data points enclosed with a diamond are reference sources

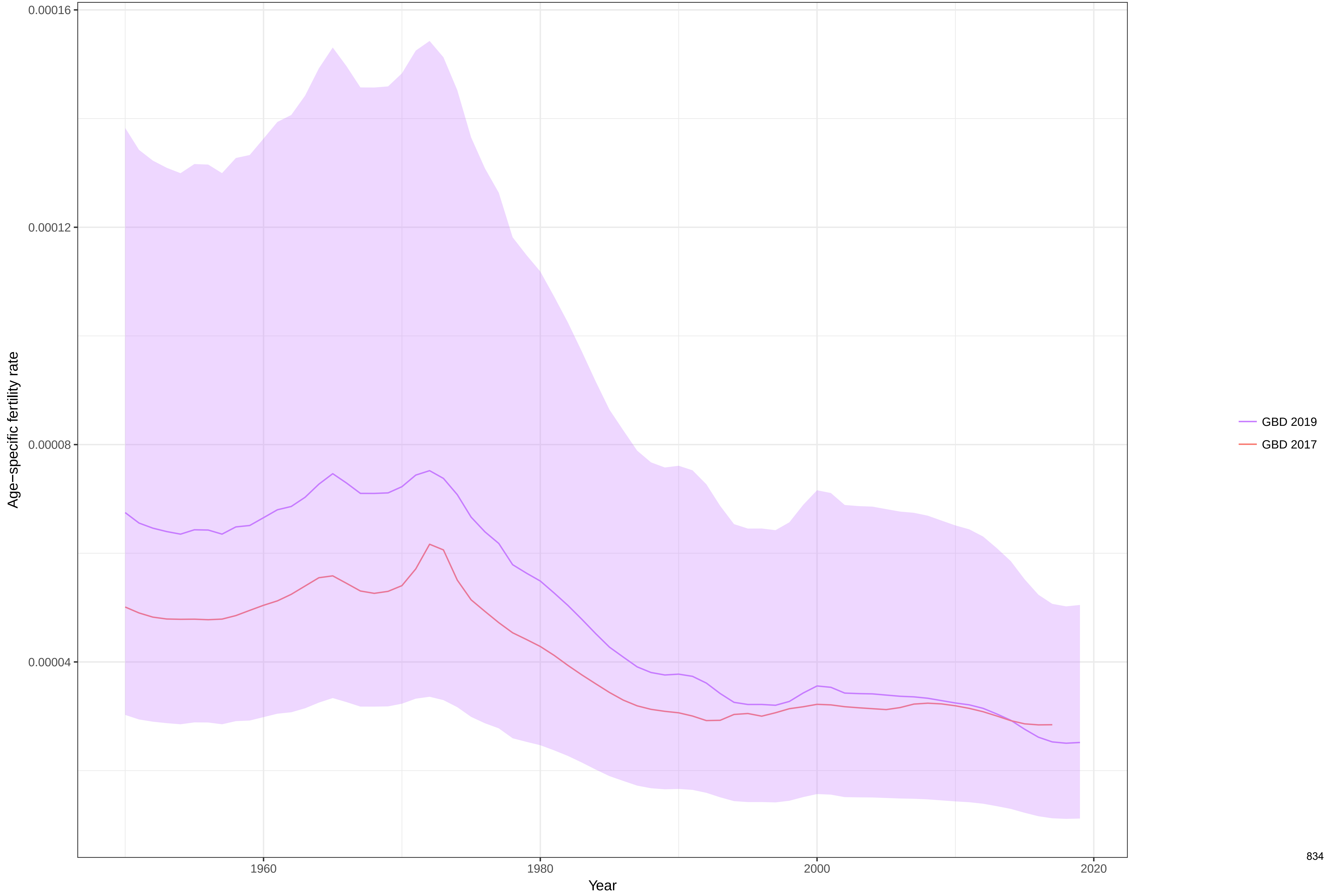

Age−specific fertility fitted trend lines, 1950−2019: France, Ages: 15−19
Data points enclosed with a diamond are reference sources

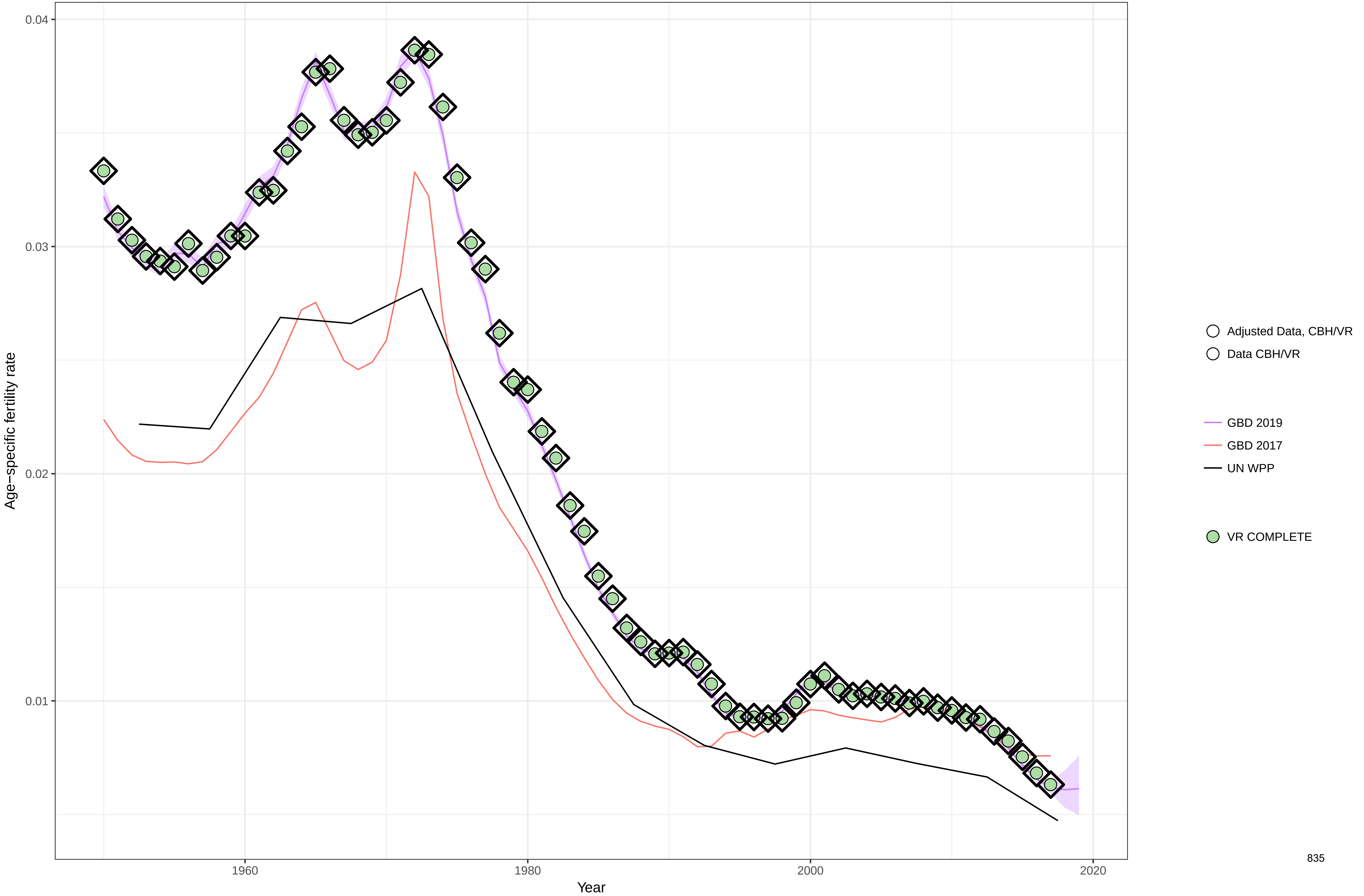

Age−specific fertility fitted trend lines, 1950−2019: France, Ages: 20−24
Data points enclosed with a diamond are reference sources

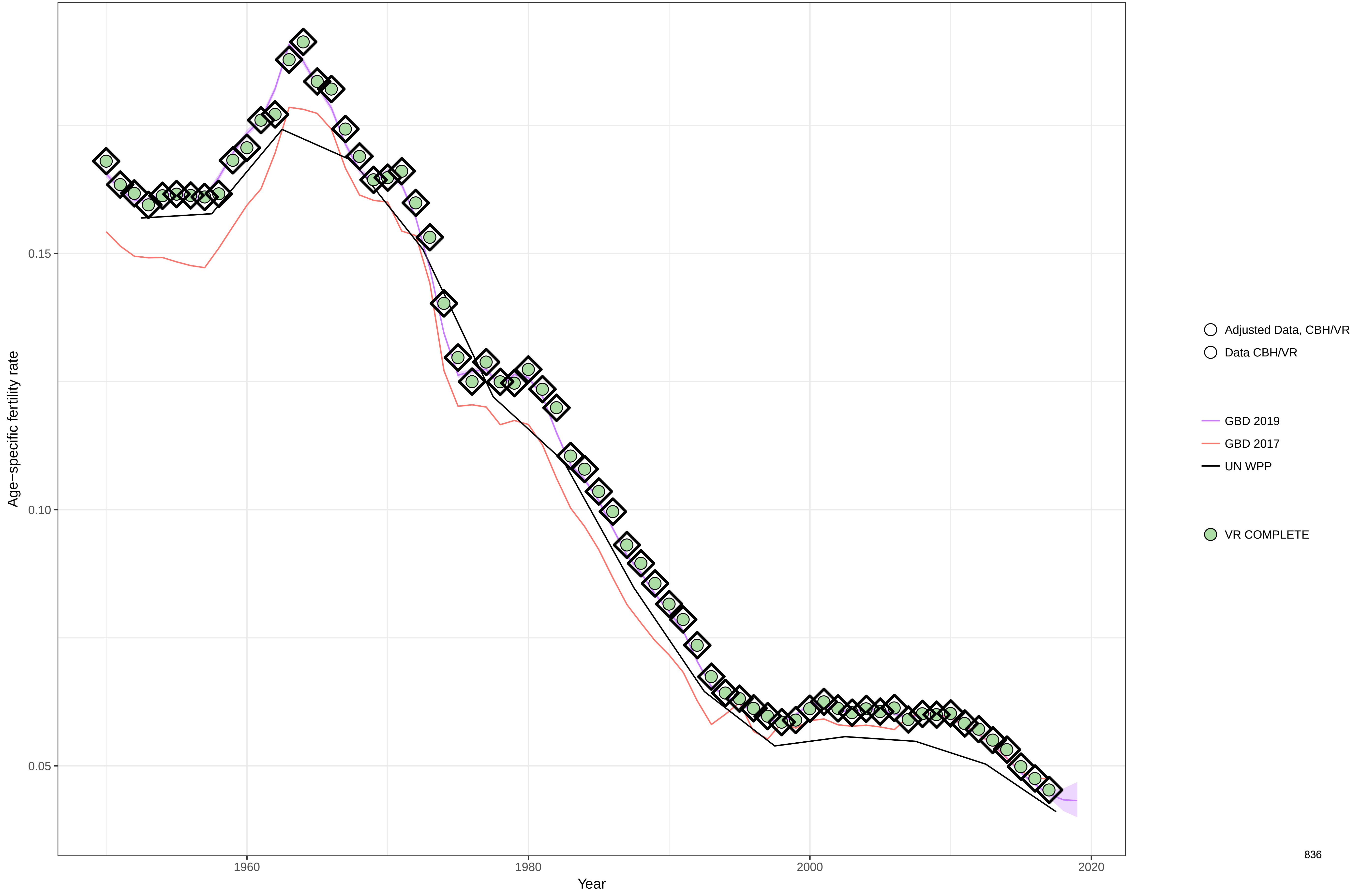

Age−specific fertility fitted trend lines, 1950−2019: France, Ages: 25−29
Data points enclosed with a diamond are reference sources

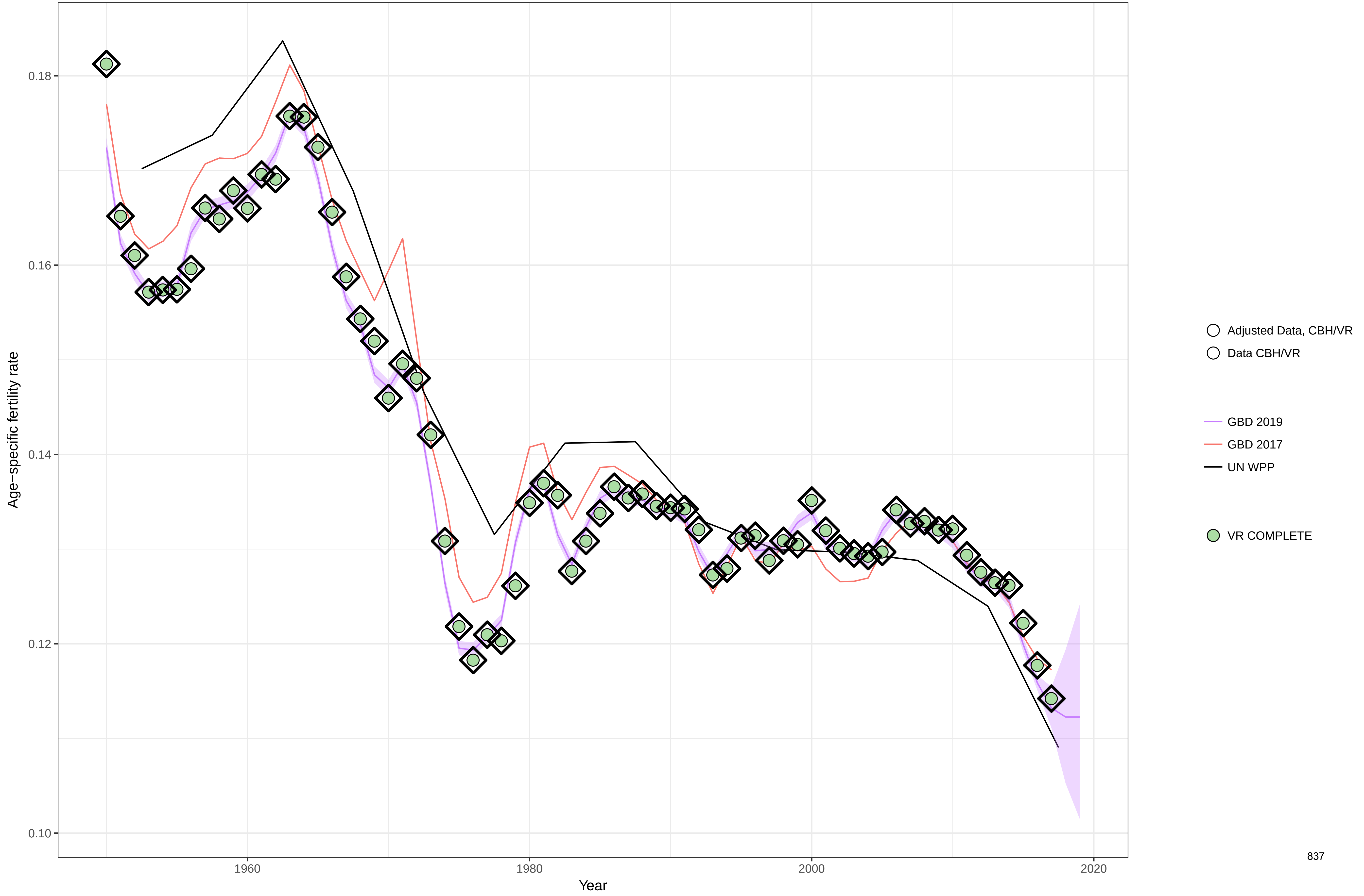

Age−specific fertility fitted trend lines, 1950−2019: France, Ages: 30−34
Data points enclosed with a diamond are reference sources

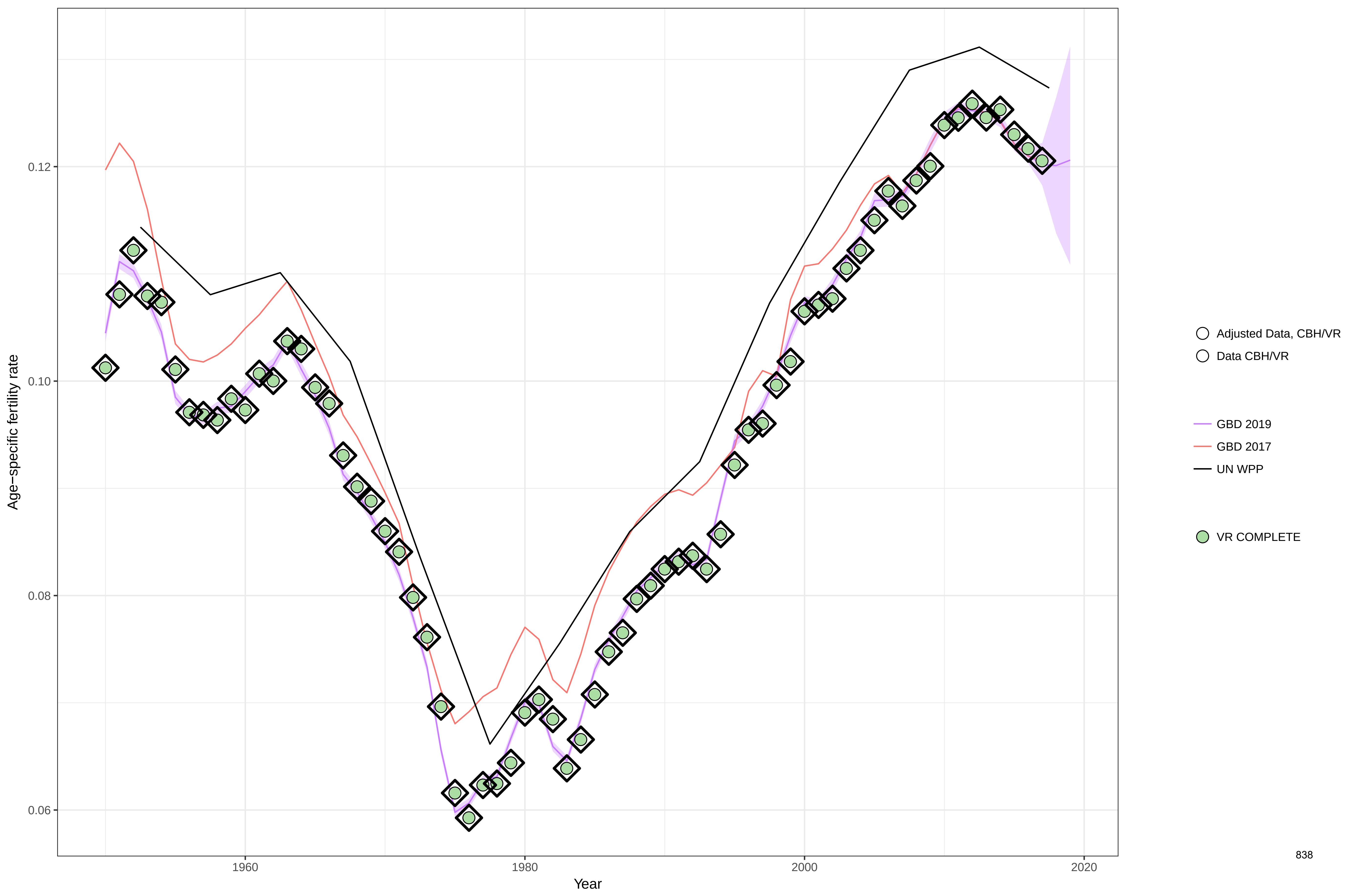

Age−specific fertility fitted trend lines, 1950−2019: France, Ages: 35−39
Data points enclosed with a diamond are reference sources

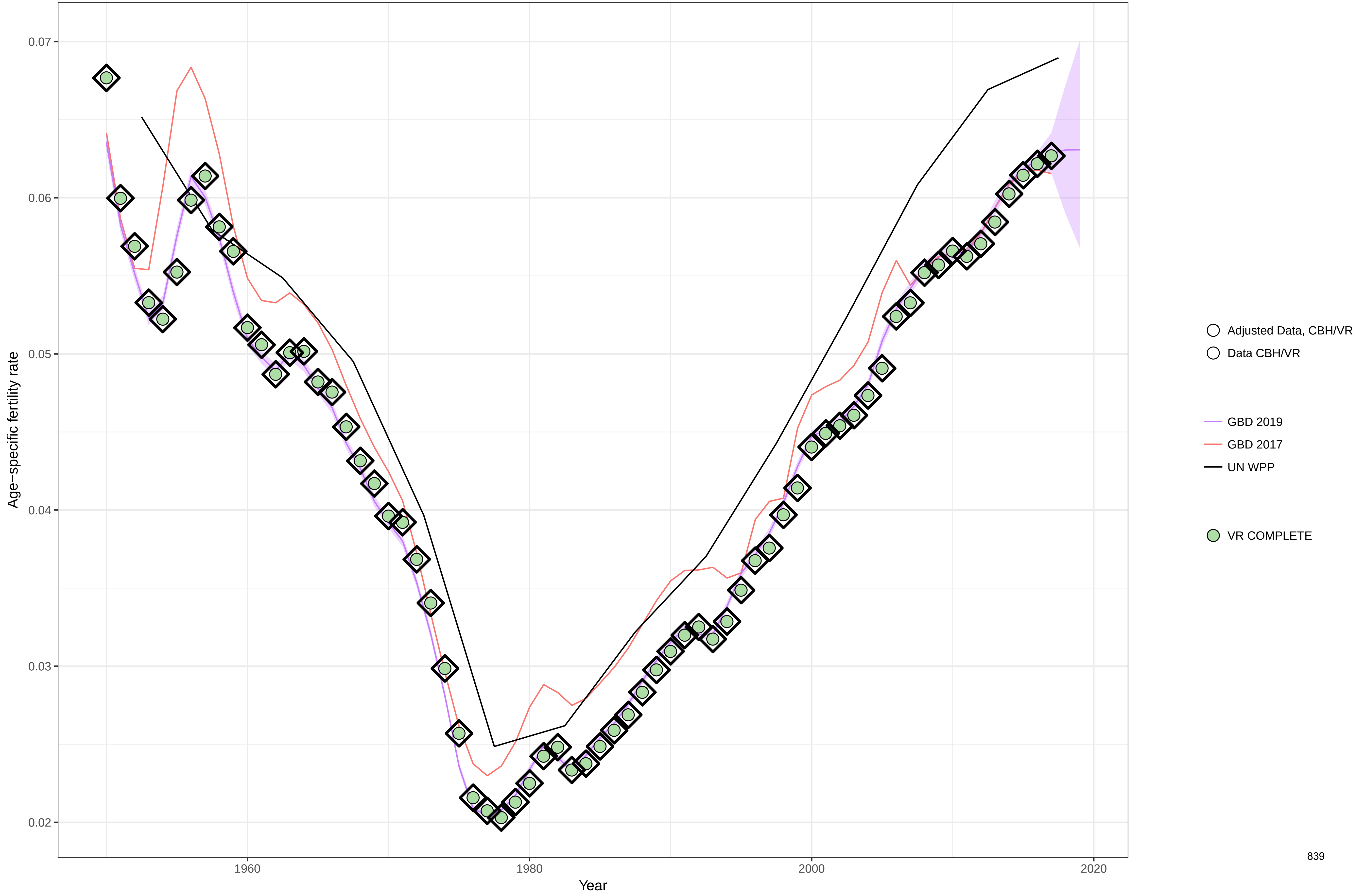

Age−specific fertility fitted trend lines, 1950−2019: France, Ages: 40−44
Data points enclosed with a diamond are reference sources

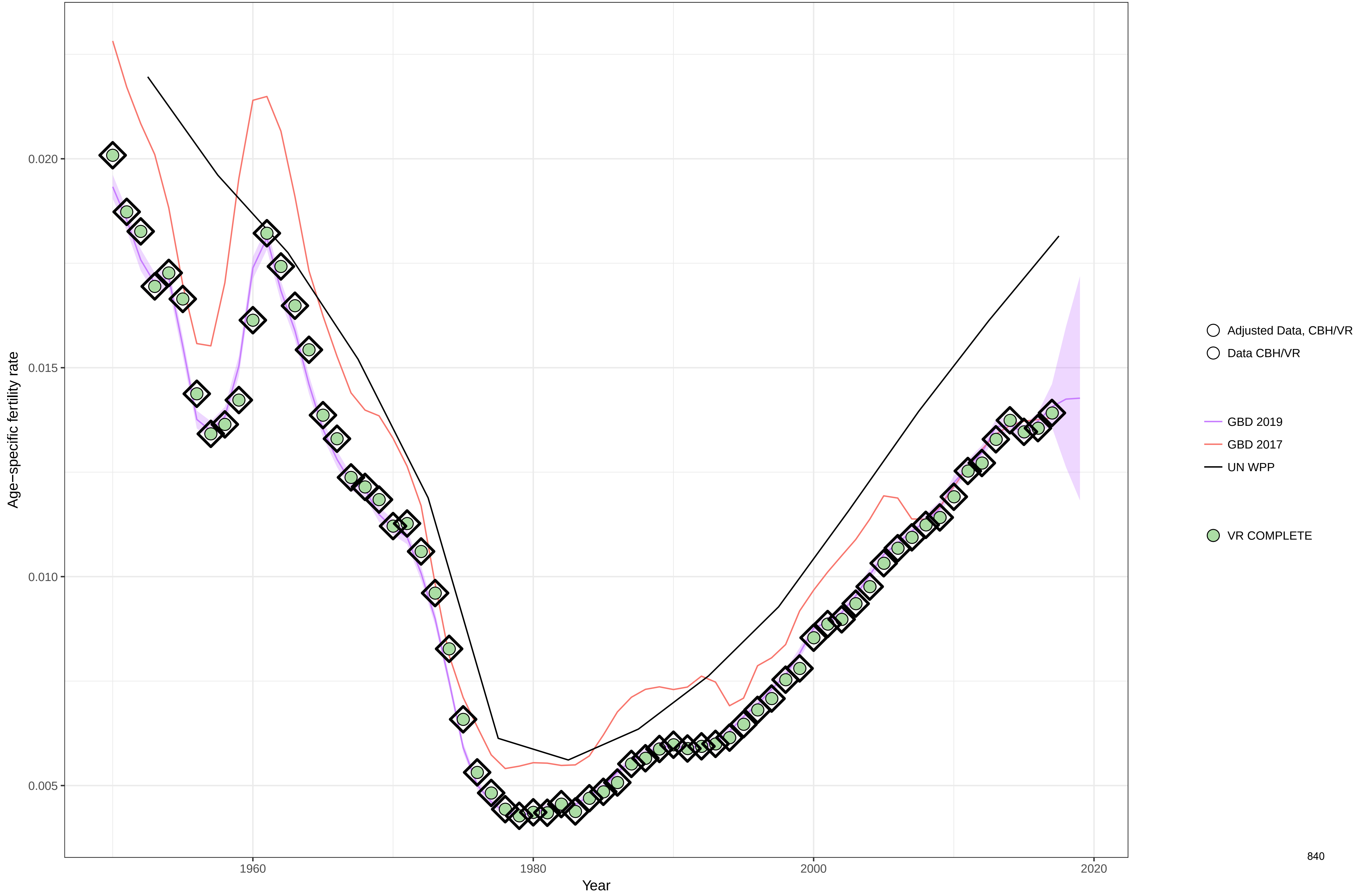

Age−specific fertility fitted trend lines, 1950−2019: France, Ages: 45−49
Data points enclosed with a diamond are reference sources

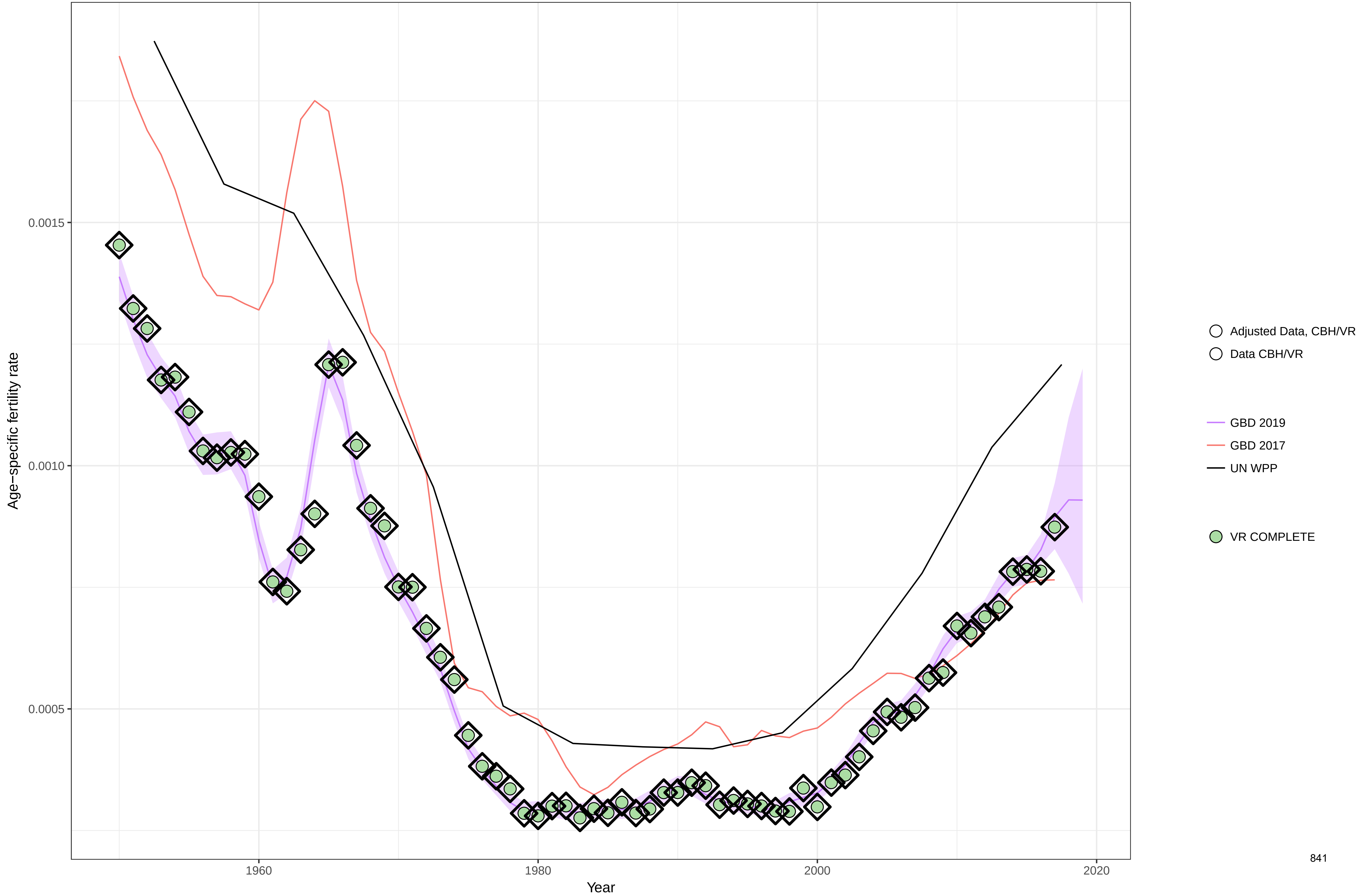

Age−specific fertility fitted trend lines, 1950−2019: France, Ages: 50−54
Data points enclosed with a diamond are reference sources

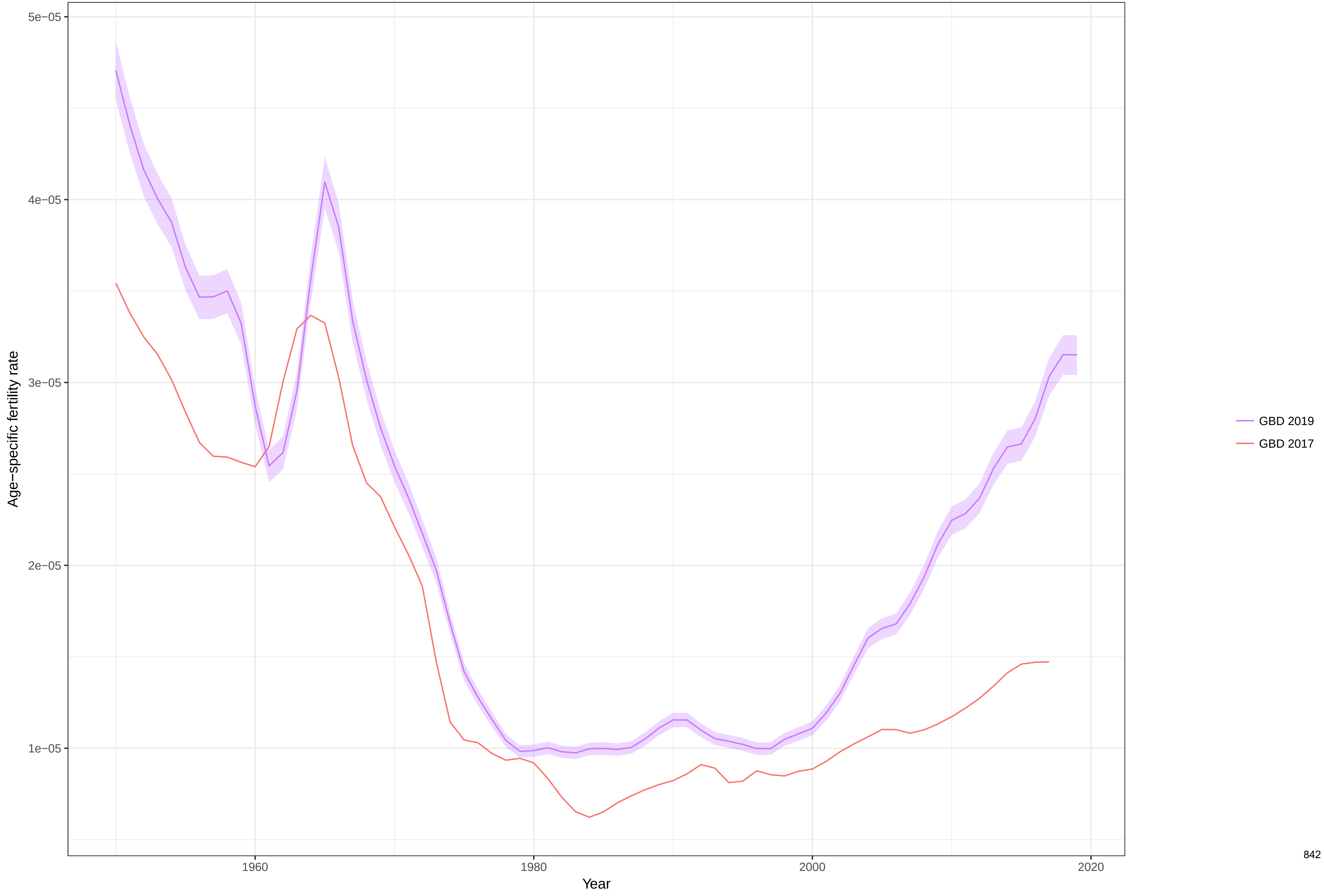

Total fertility rate fitted trend lines, 1950−2019: France TFR
Data points enclosed with a diamond are reference sources

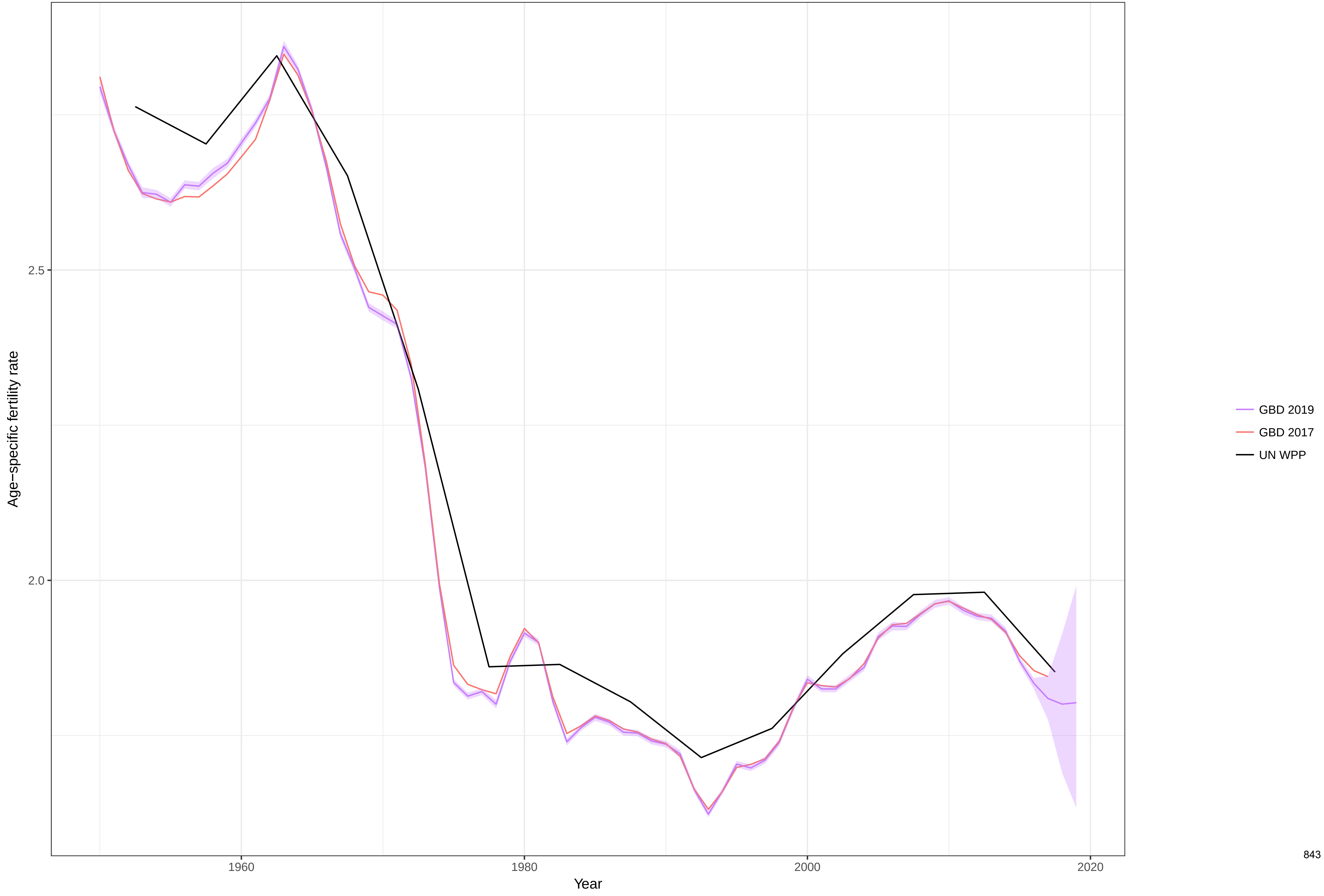

Age−specific fertility fitted trend lines, 1950−2019: Micronesia (Federated States of), Ages: 10−14
Data points enclosed with a diamond are reference sources

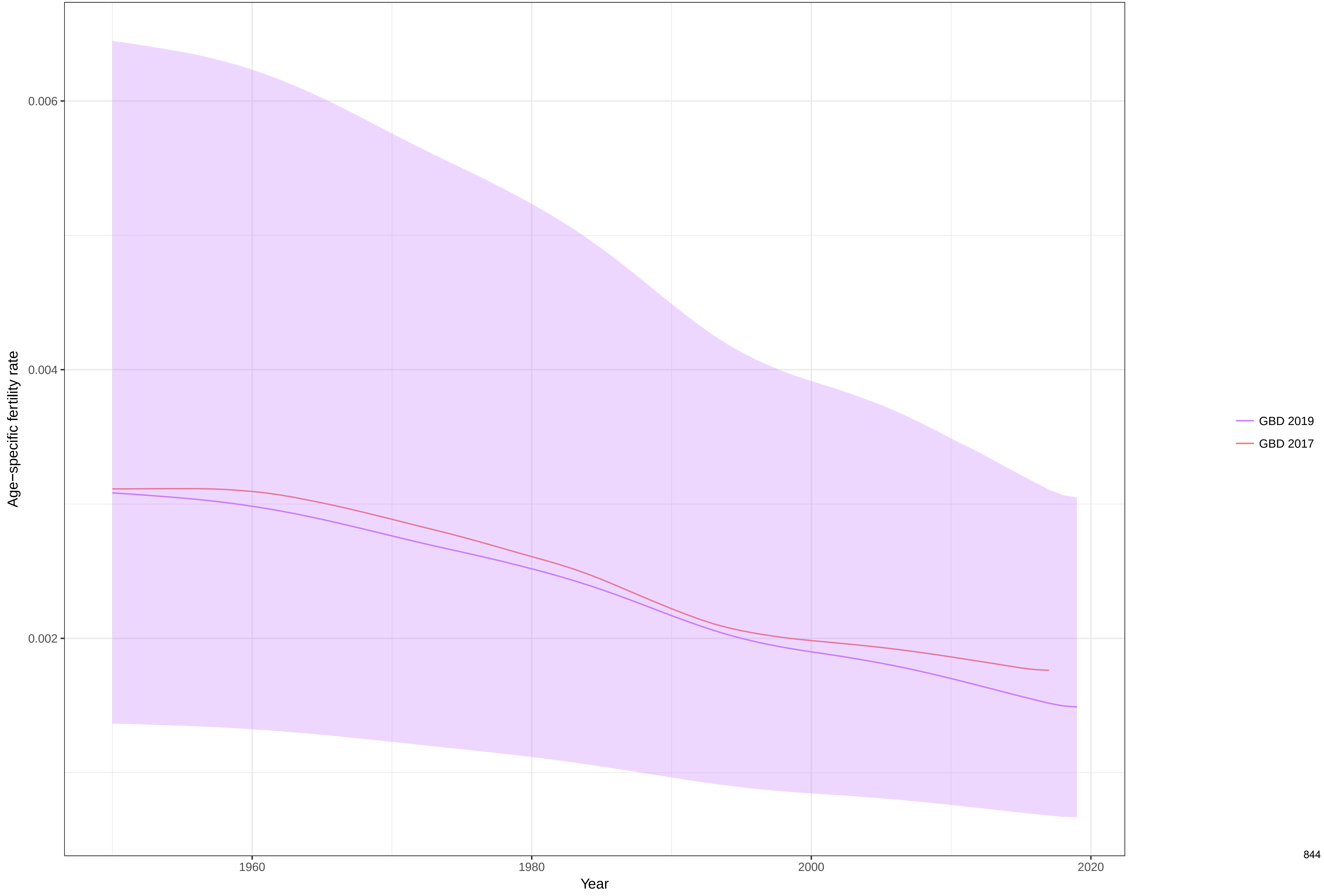

Age−specific fertility fitted trend lines, 1950−2019: Micronesia (Federated States of), Ages: 15−19
Data points enclosed with a diamond are reference sources

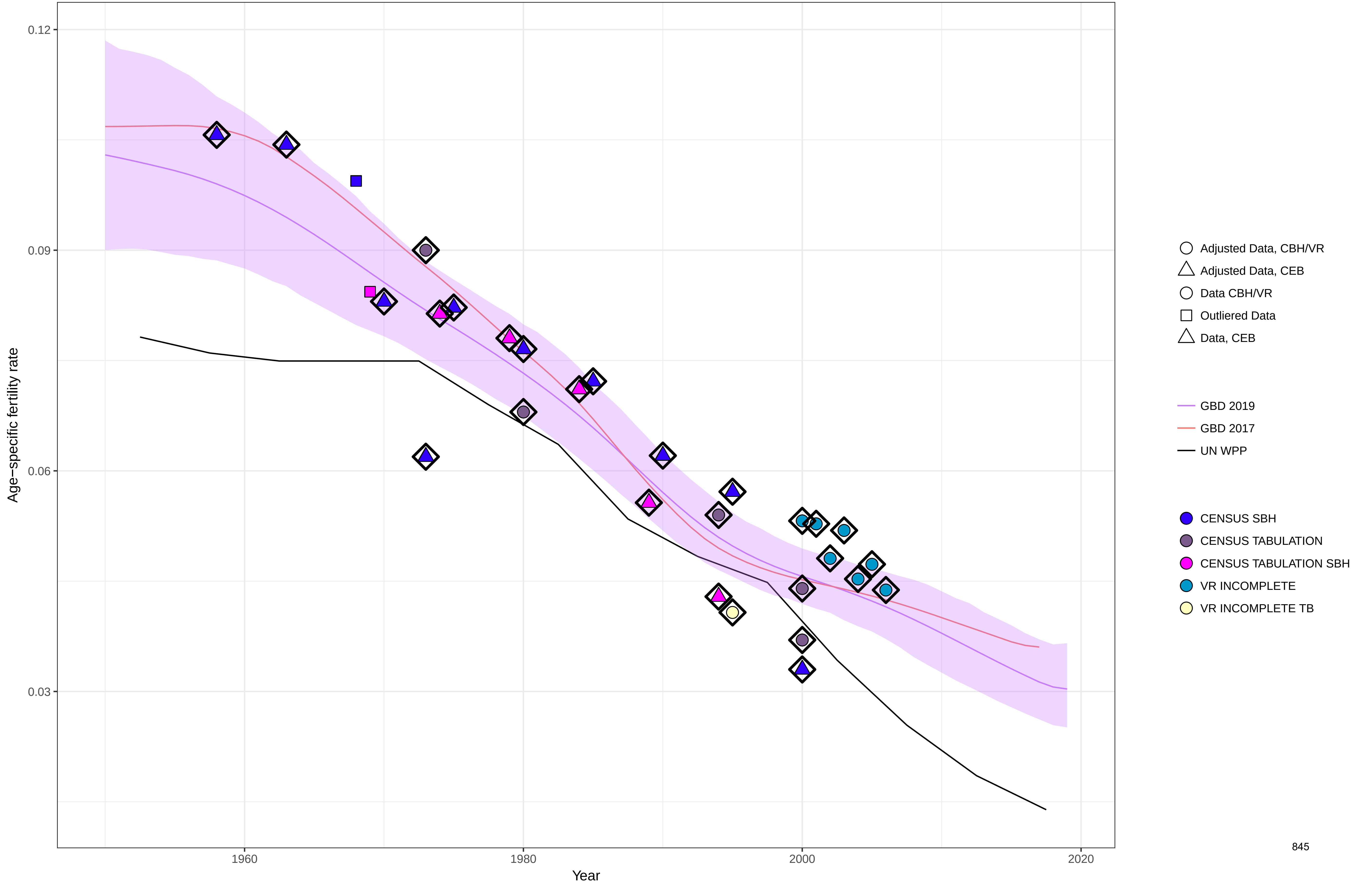

Age−specific fertility fitted trend lines, 1950−2019: Micronesia (Federated States of), Ages: 20−24
Data points enclosed with a diamond are reference sources

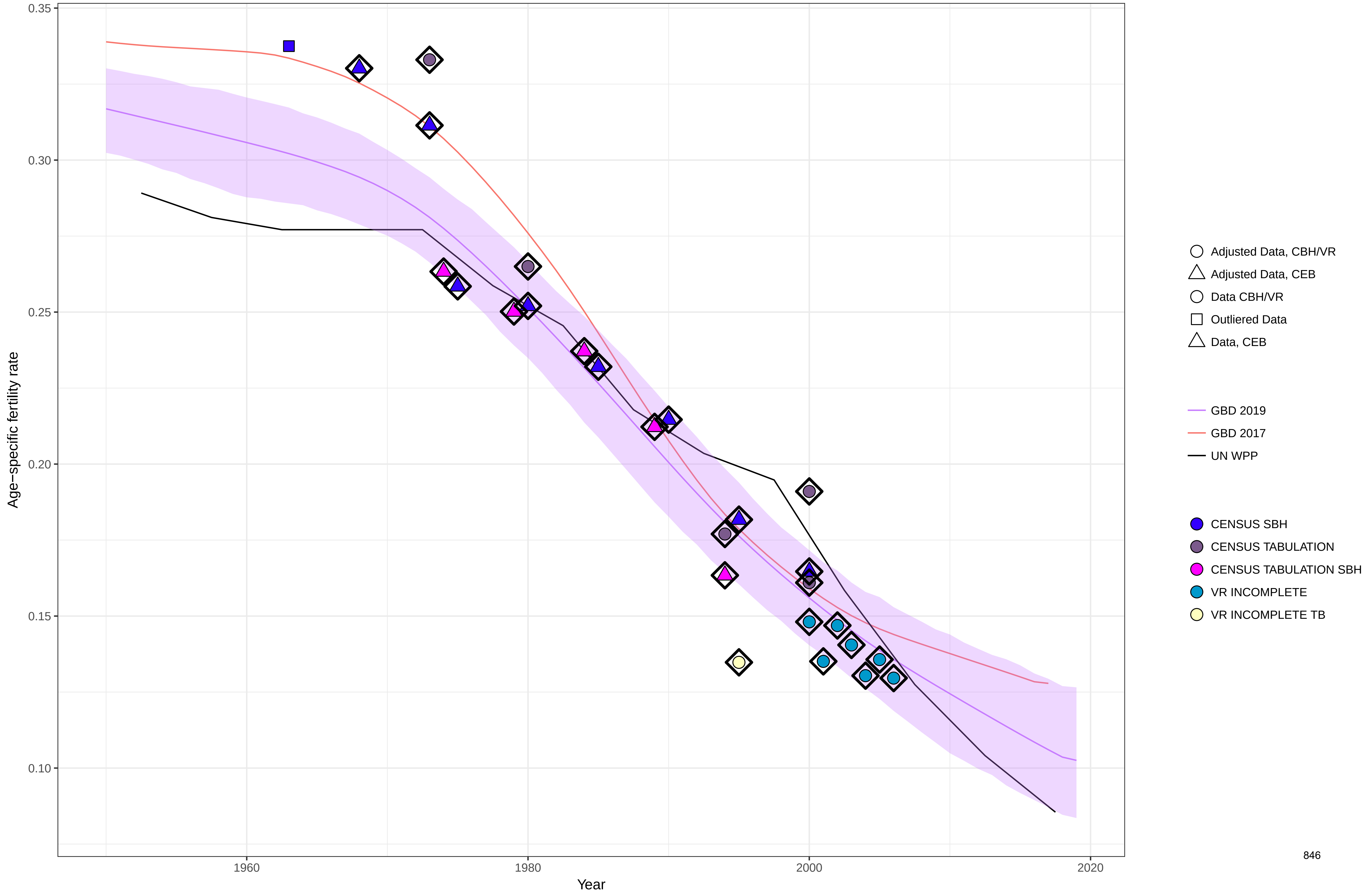

Age−specific fertility fitted trend lines, 1950−2019: Micronesia (Federated States of), Ages: 25−29
Data points enclosed with a diamond are reference sources

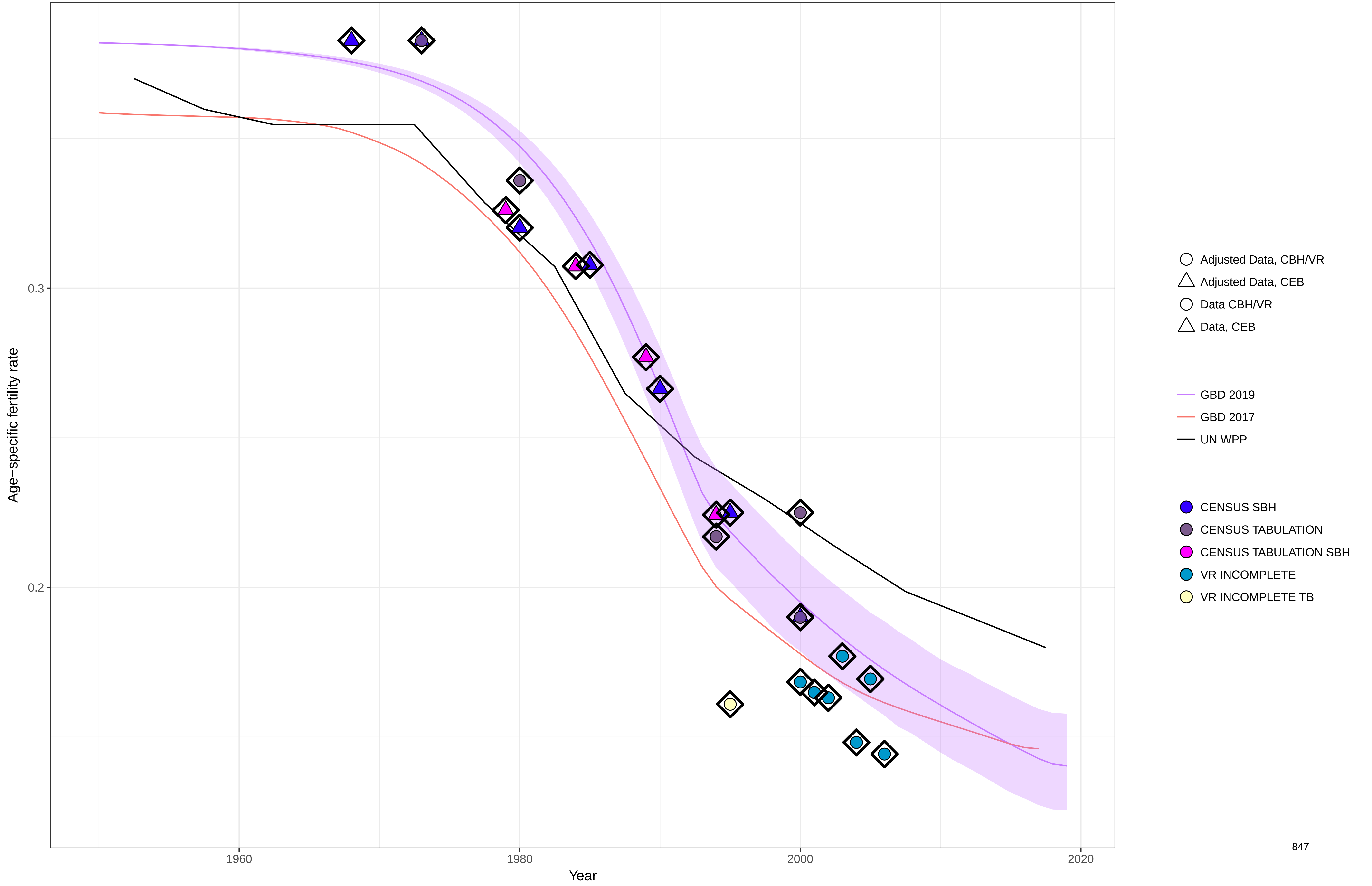

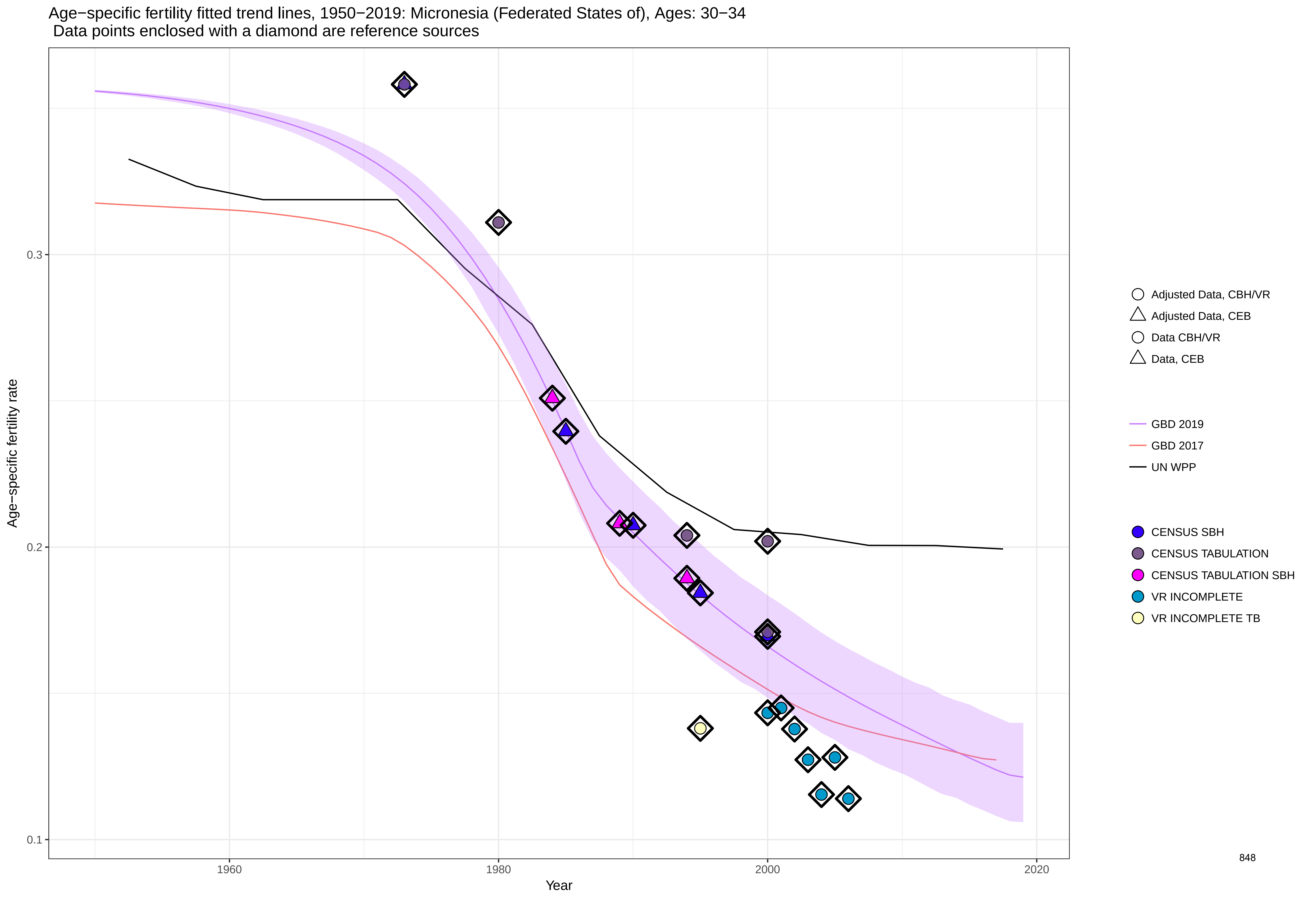
Age−specific fertility fitted trend lines, 1950−2019: Micronesia (Federated States of), Ages: 30−34
Data points enclosed with a diamond are reference sources
Age−specific fertility rate
0.1
0.2
0.3
1960
1980
2000
2020
Year
Adjusted Data, CBH/VR
Adjusted Data, CEB
Data CBH/VR
Data, CEB
GBD 2019
GBD 2017
UN WPP
CENSUS SBH
CENSUS TABULATION
CENSUS TABULATION SBH
VR INCOMPLETE
VR INCOMPLETE TB

Age−specific fertility fitted trend lines, 1950−2019: Micronesia (Federated States of), Ages: 35−39
Data points enclosed with a diamond are reference sources

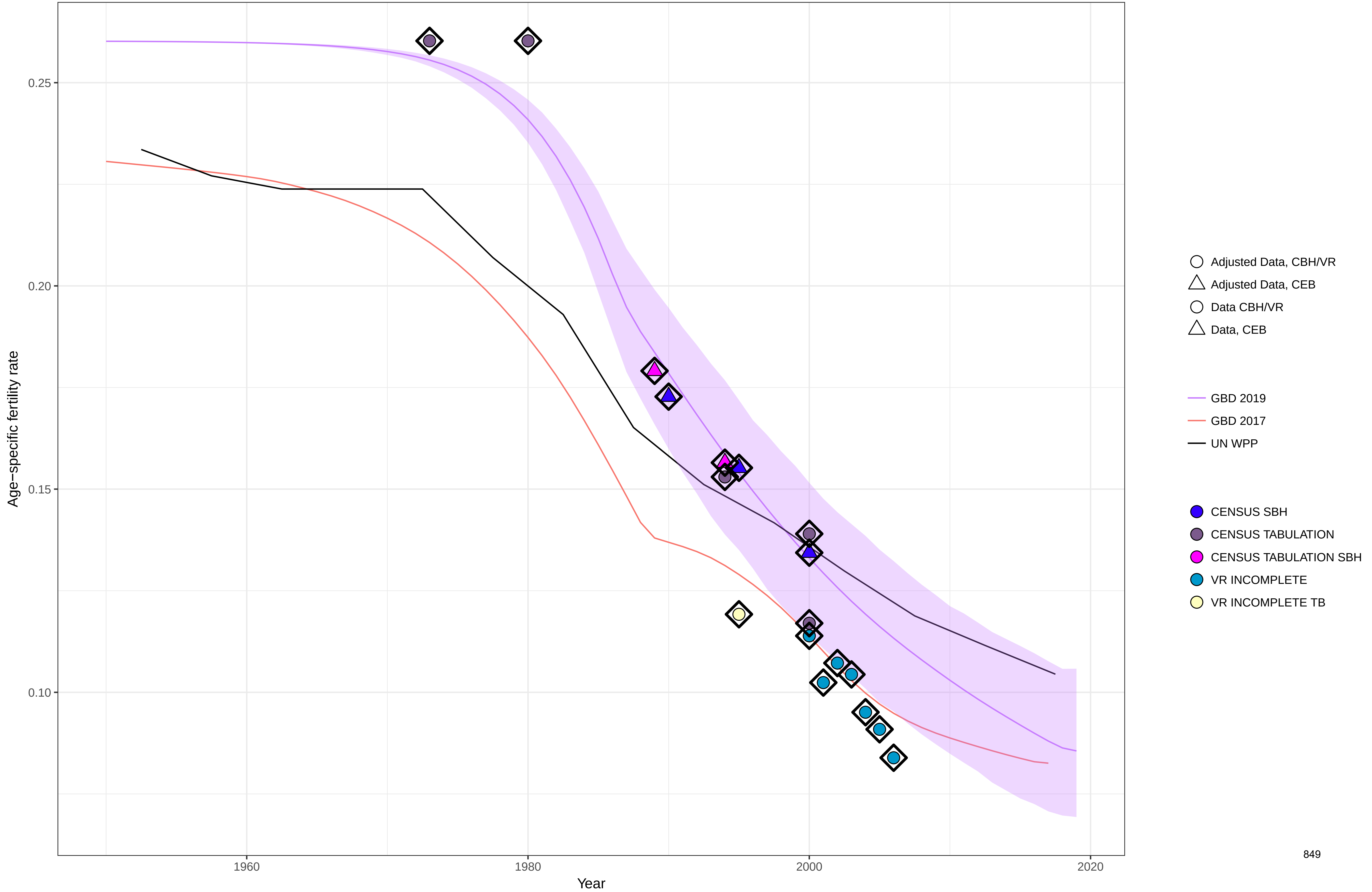

Age−specific fertility fitted trend lines, 1950−2019: Micronesia (Federated States of), Ages: 40−44
Data points enclosed with a diamond are reference sources

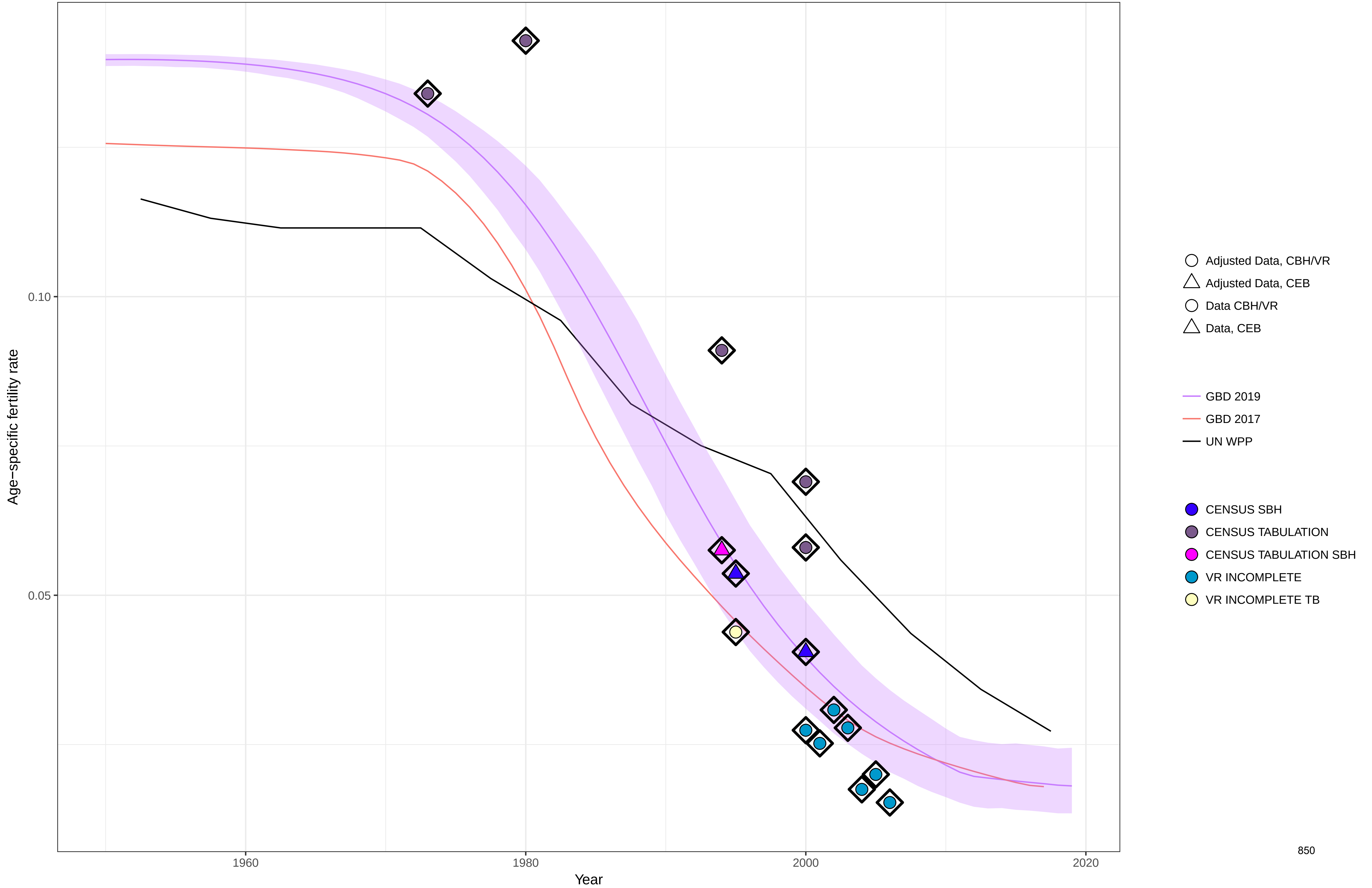

Age−specific fertility fitted trend lines, 1950−2019: Micronesia (Federated States of), Ages: 45−49
Data points enclosed with a diamond are reference sources

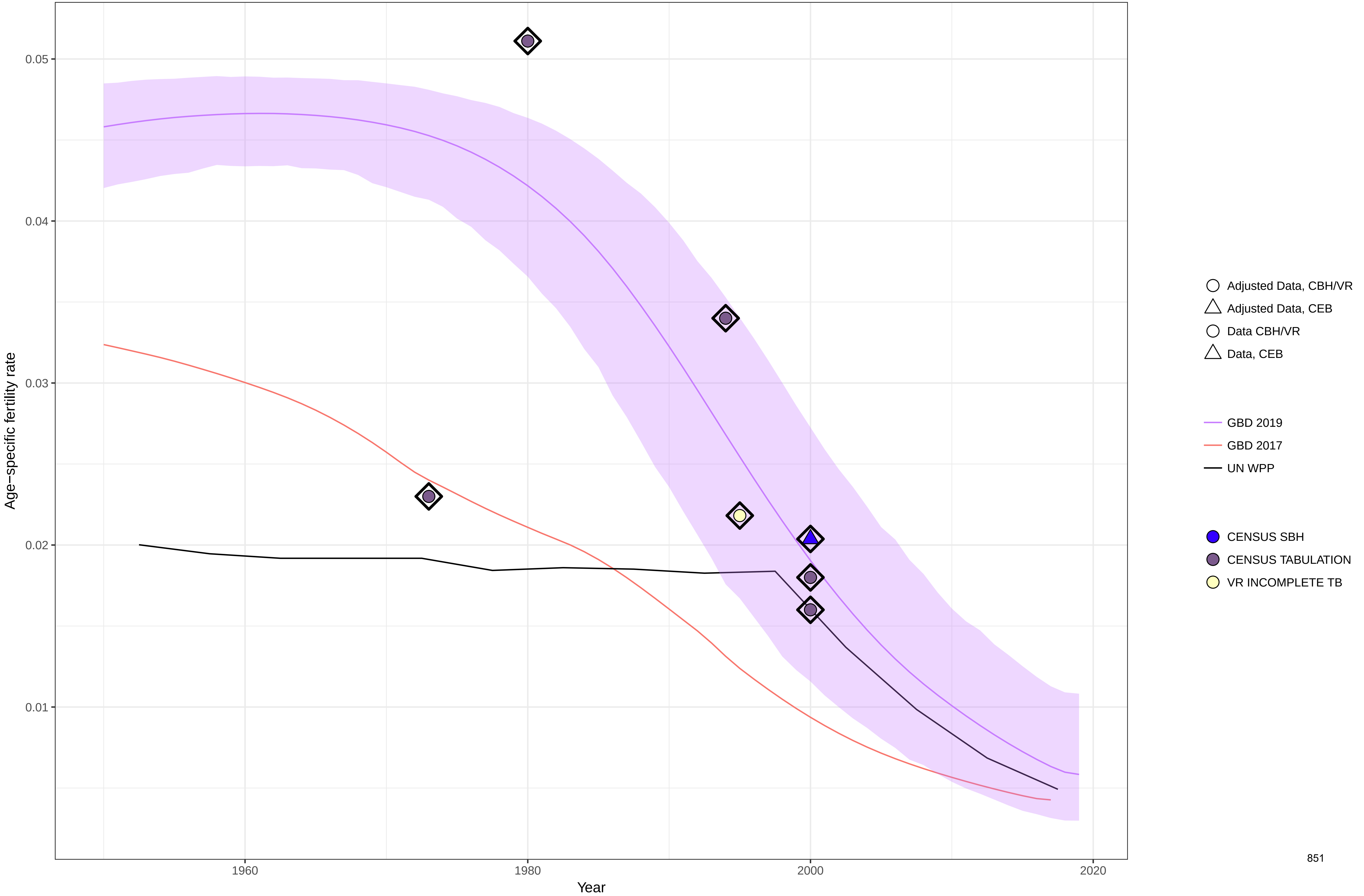

Age−specific fertility fitted trend lines, 1950−2019: Micronesia (Federated States of), Ages: 50−54
Data points enclosed with a diamond are reference sources

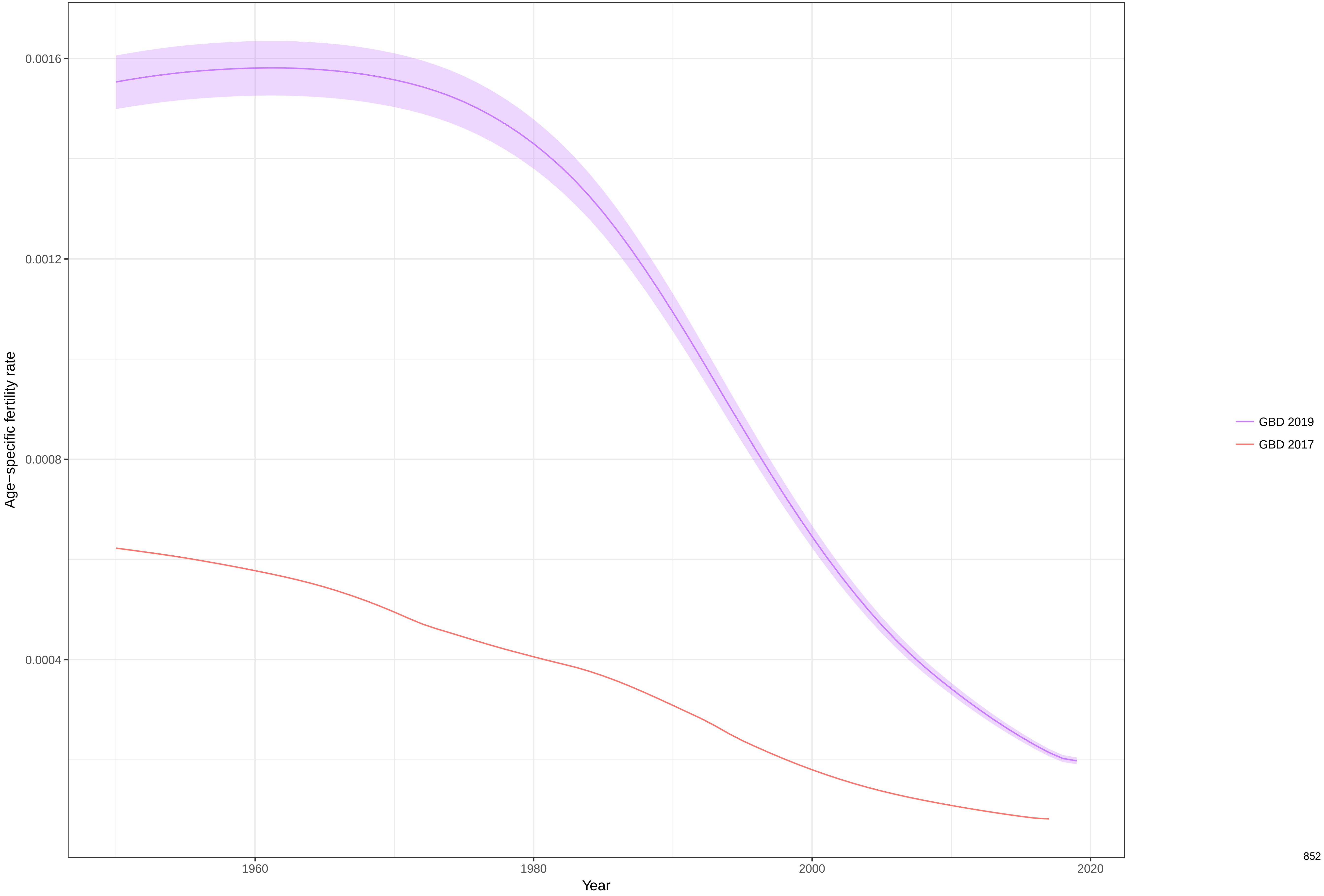

Total fertility rate fitted trend lines, 1950−2019: Micronesia (Federated States of) TFR
Data points enclosed with a diamond are reference sources

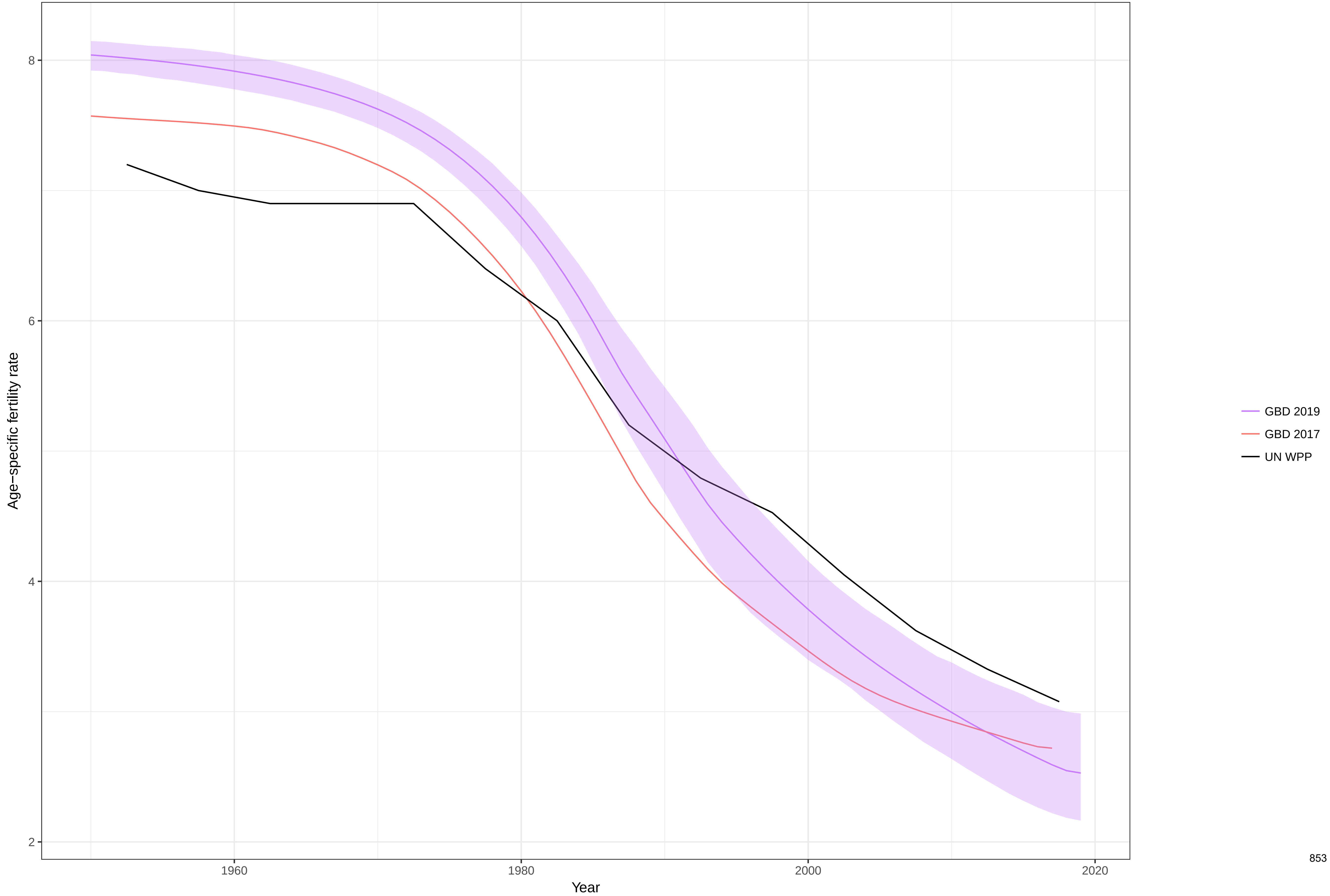

Age−specific fertility fitted trend lines, 1950−2019: Gabon, Ages: 10−14
Data points enclosed with a diamond are reference sources

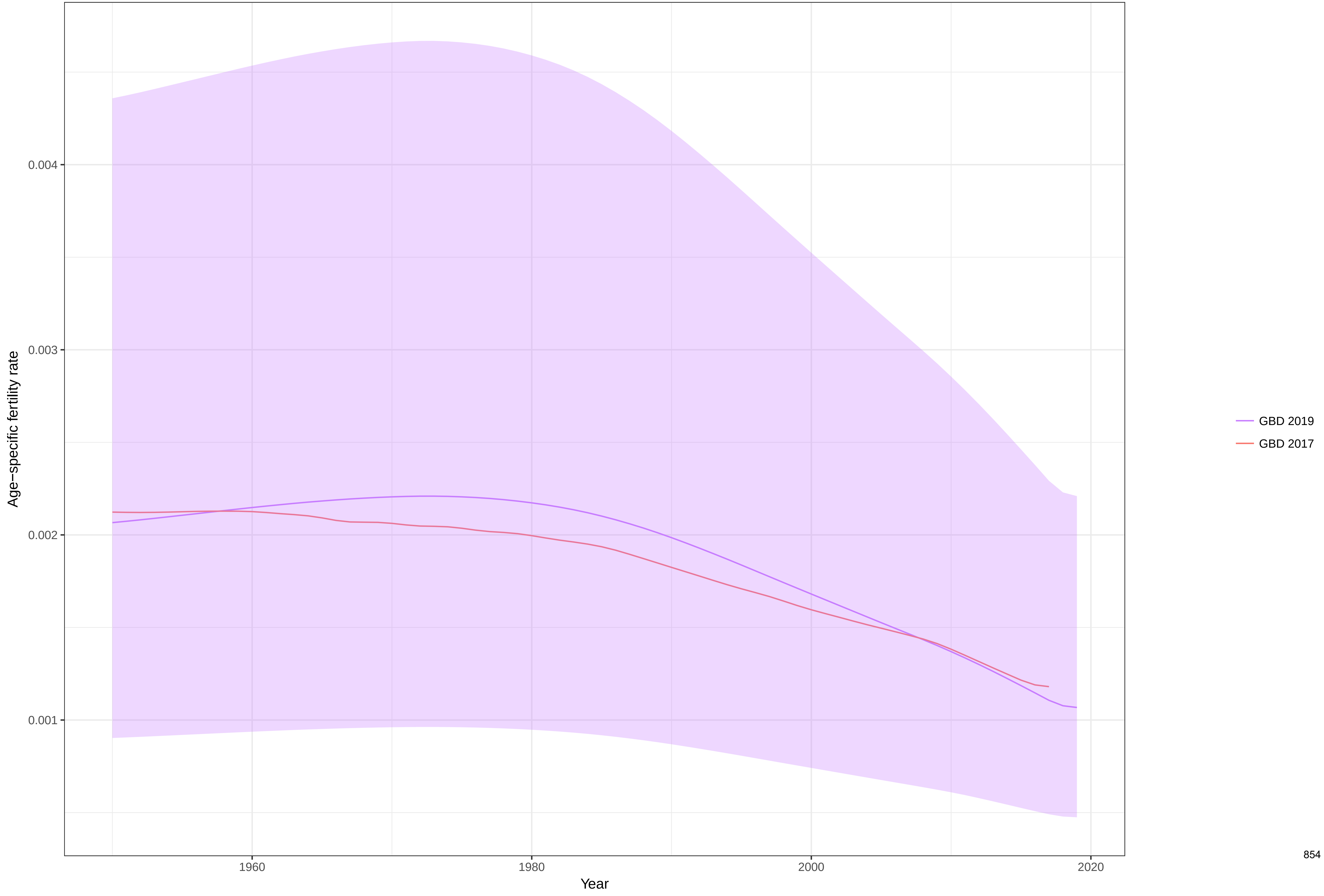

Age−specific fertility fitted trend lines, 1950−2019: Gabon, Ages: 15−19
Data points enclosed with a diamond are reference sources

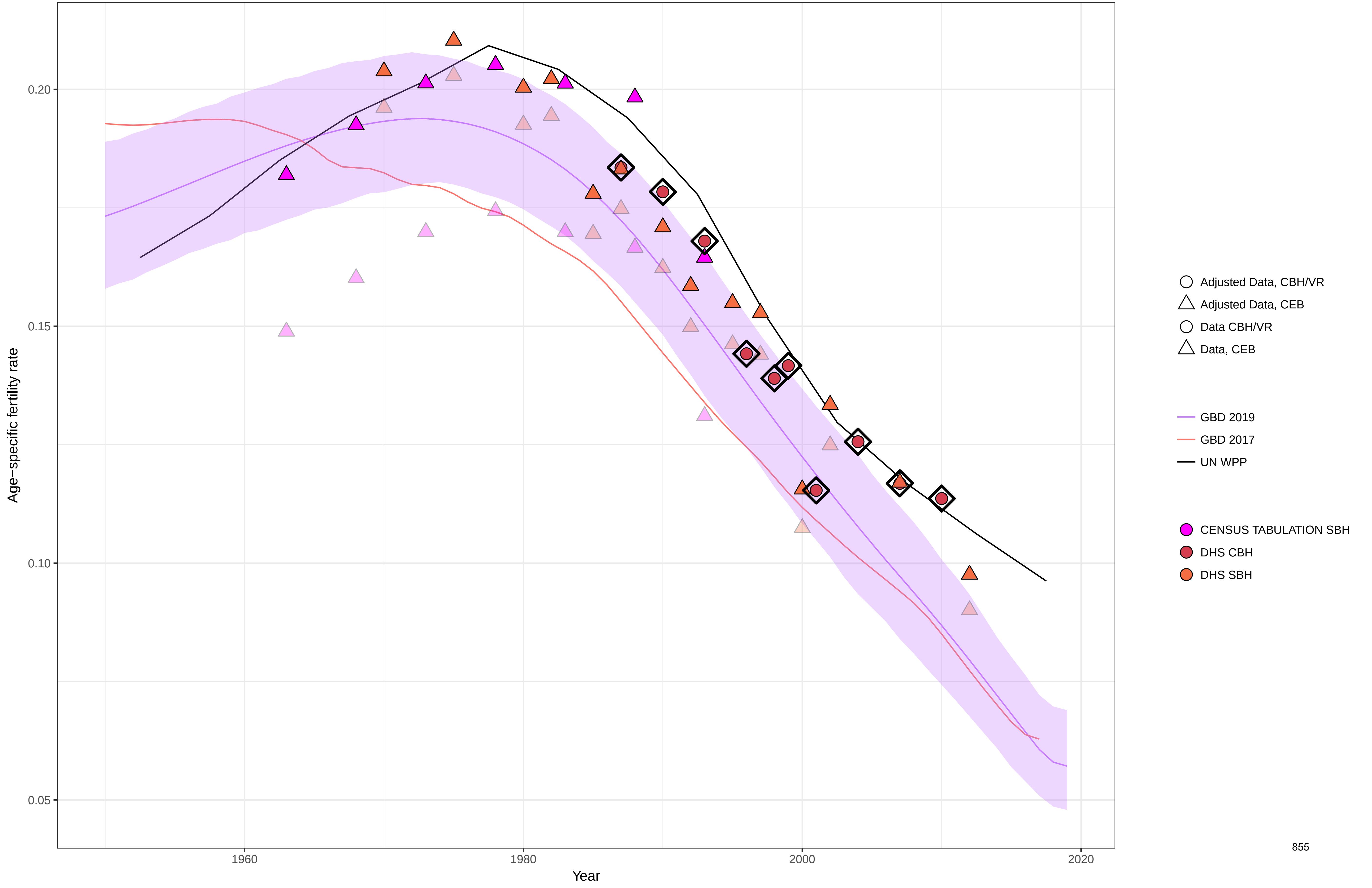

Age−specific fertility fitted trend lines, 1950−2019: Gabon, Ages: 20−24
Data points enclosed with a diamond are reference sources

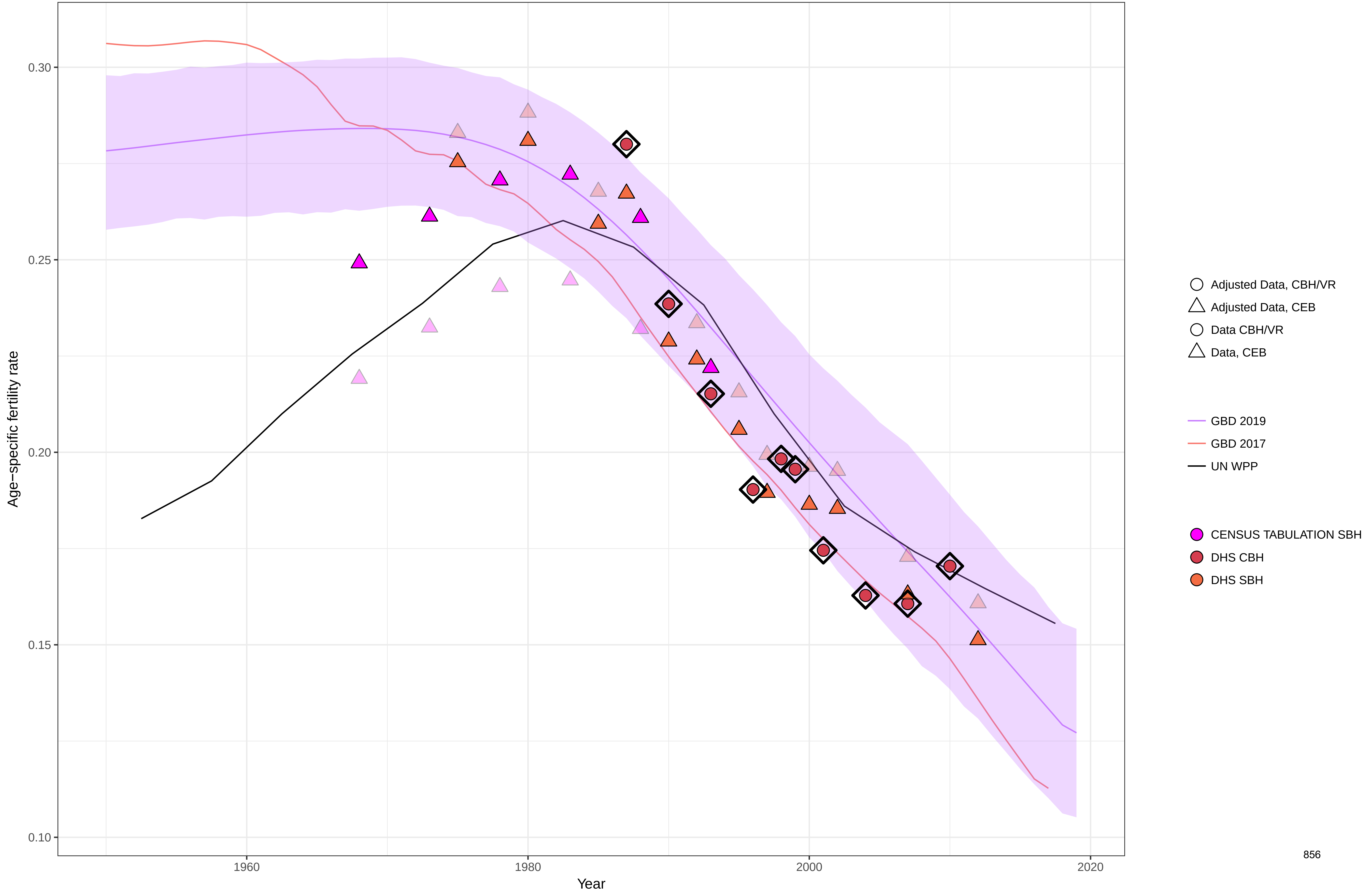

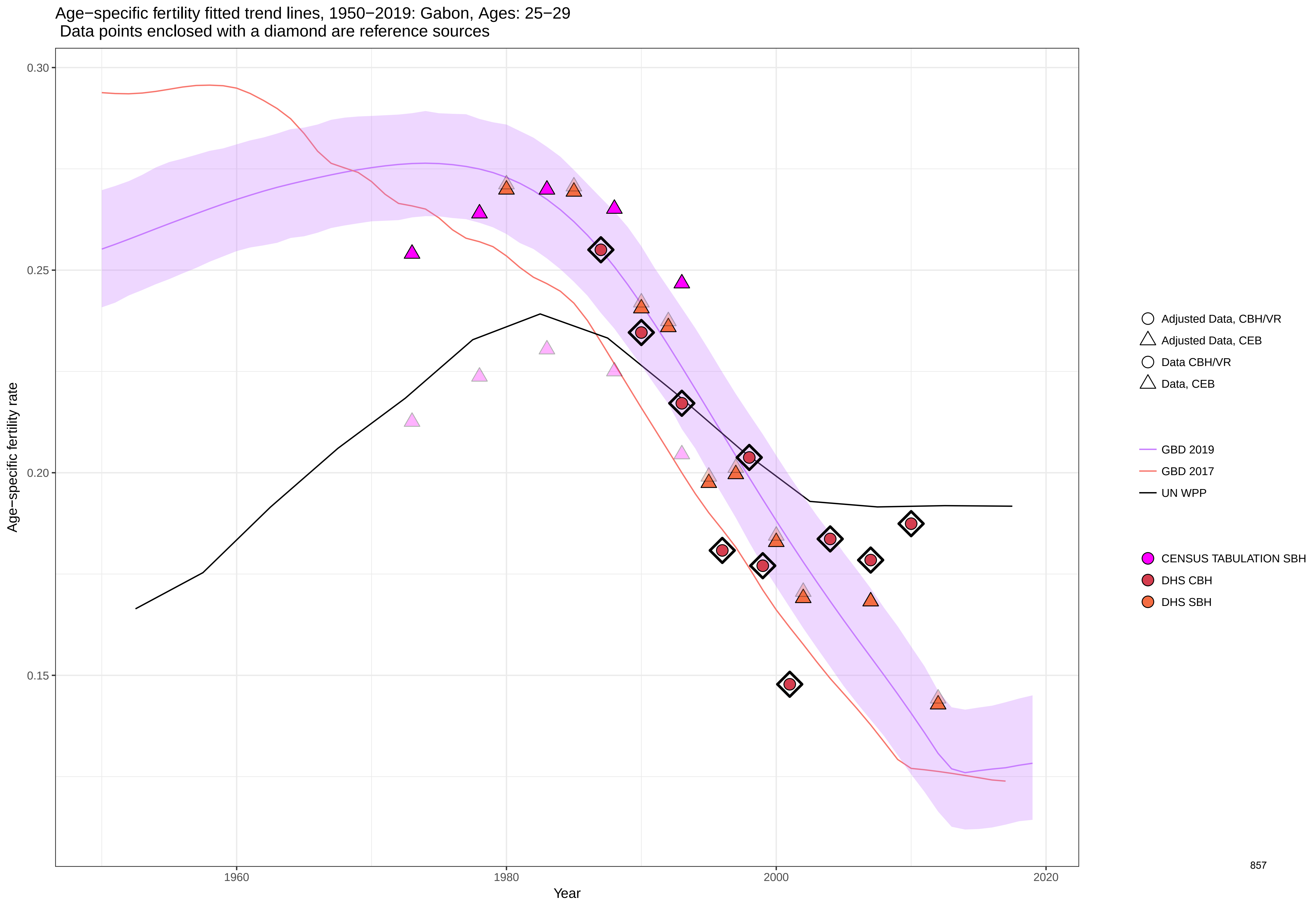
Age−specific fertility fitted trend lines, 1950−2019: Gabon, Ages: 25−29
Data points enclosed with a diamond are reference sources
Age−specific fertility rate
Year
0.30
0.25
0.20
0.15
1960
1980
2000
2020
Adjusted Data, CBH/VR
Adjusted Data, CEB
Data CBH/VR
Data, CEB
GBD 2019
GBD 2017
UN WPP
CENSUS TABULATION SBH
DHS CBH
DHS SBH

Age−specific fertility fitted trend lines, 1950−2019: Gabon, Ages: 30−34
Data points enclosed with a diamond are reference sources

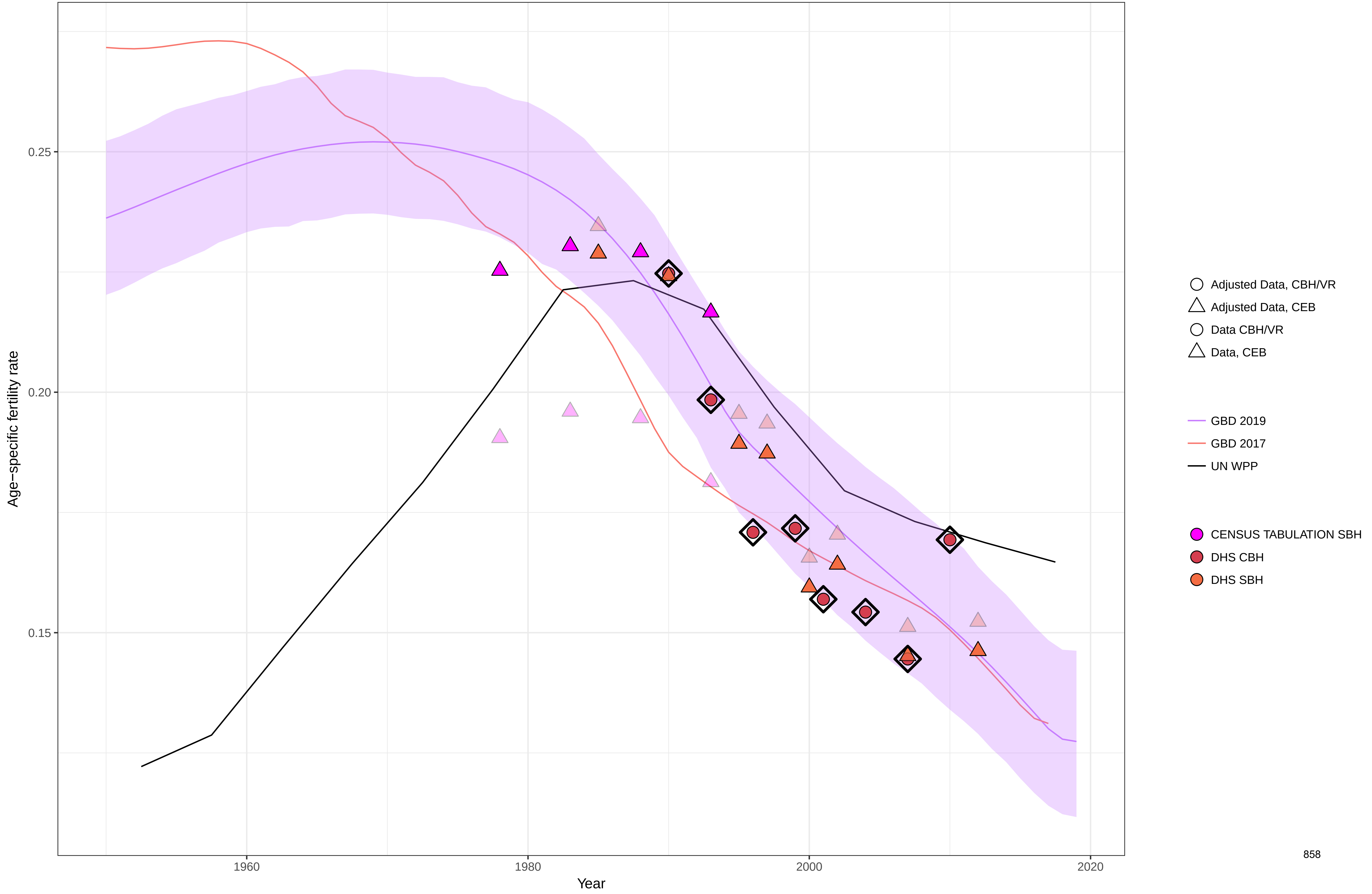

Age−specific fertility fitted trend lines, 1950−2019: Gabon, Ages: 35−39
Data points enclosed with a diamond are reference sources

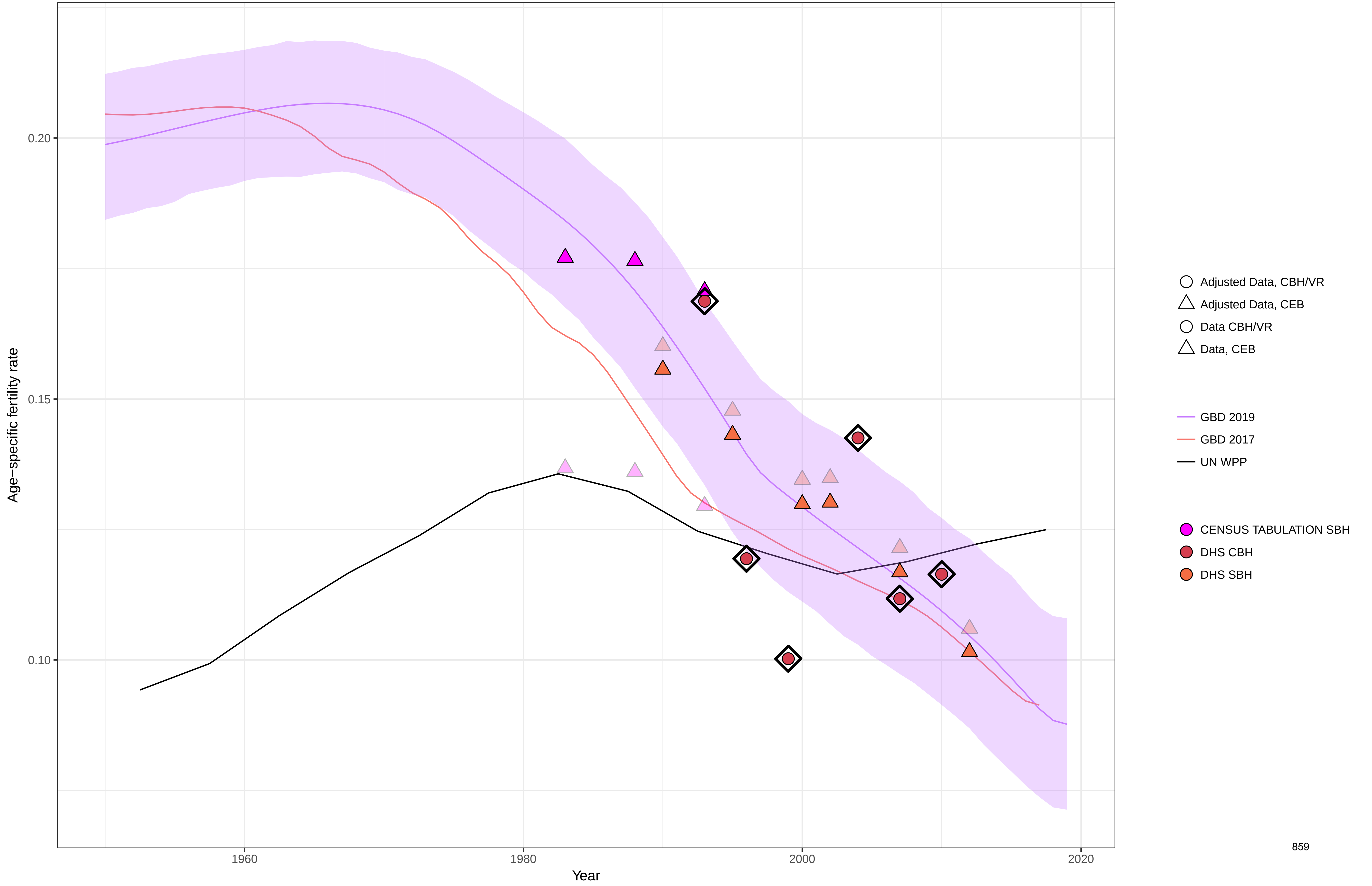

Age−specific fertility fitted trend lines, 1950−2019: Gabon, Ages: 40−44
Data points enclosed with a diamond are reference sources

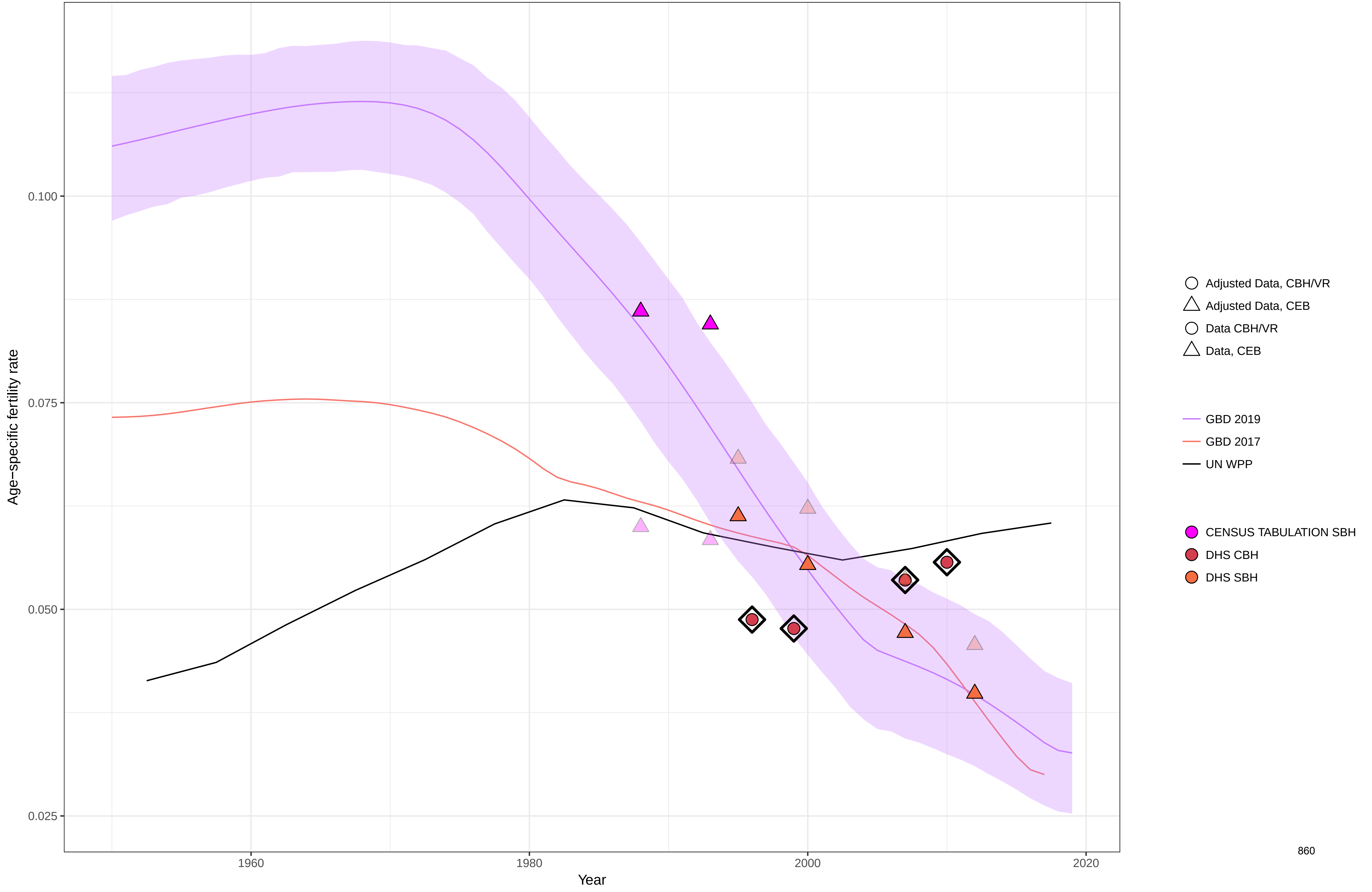

Age−specific fertility fitted trend lines, 1950−2019: Gabon, Ages: 45−49
Data points enclosed with a diamond are reference sources

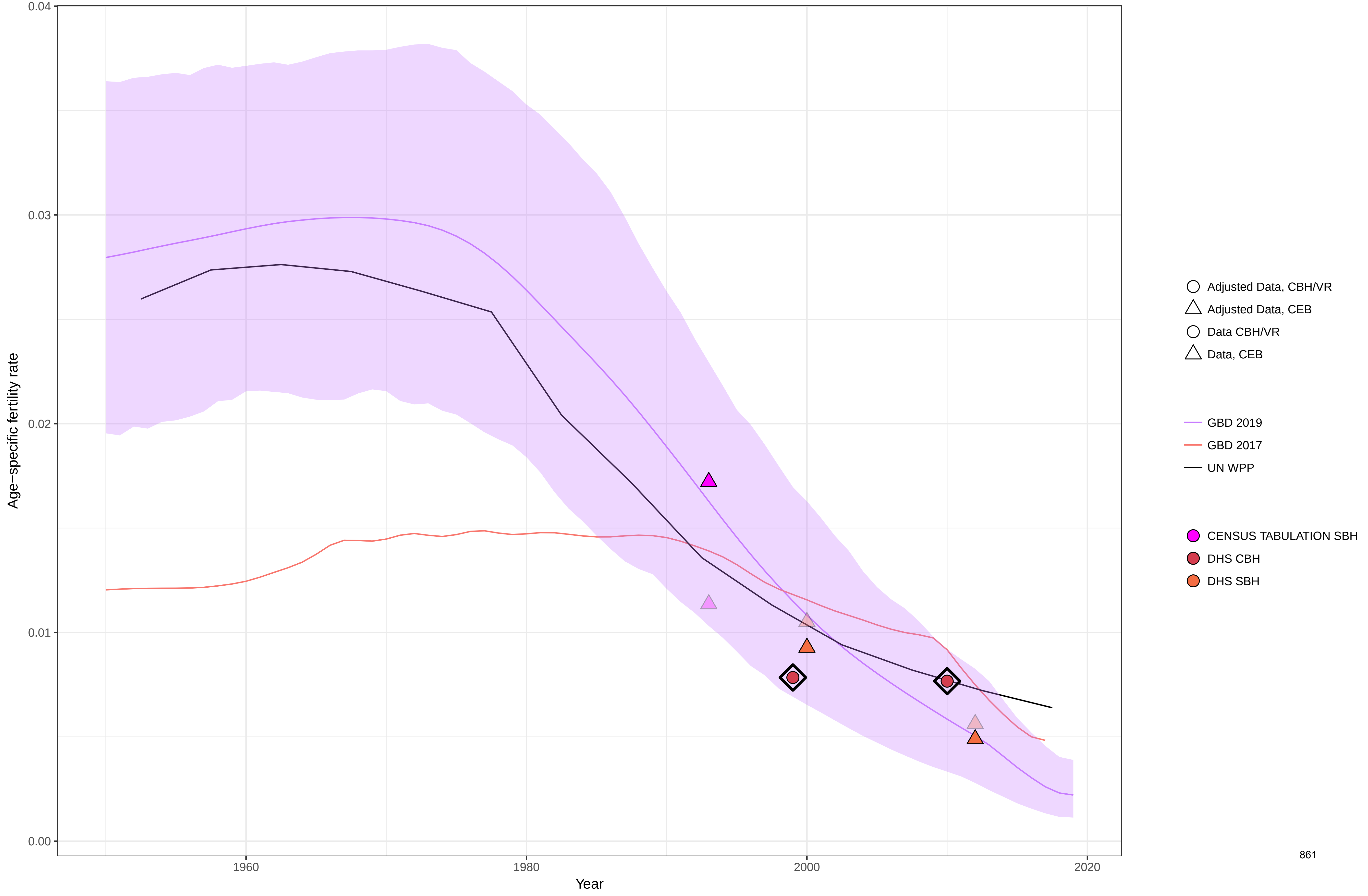

Age−specific fertility fitted trend lines, 1950−2019: Gabon, Ages: 50−54
Data points enclosed with a diamond are reference sources

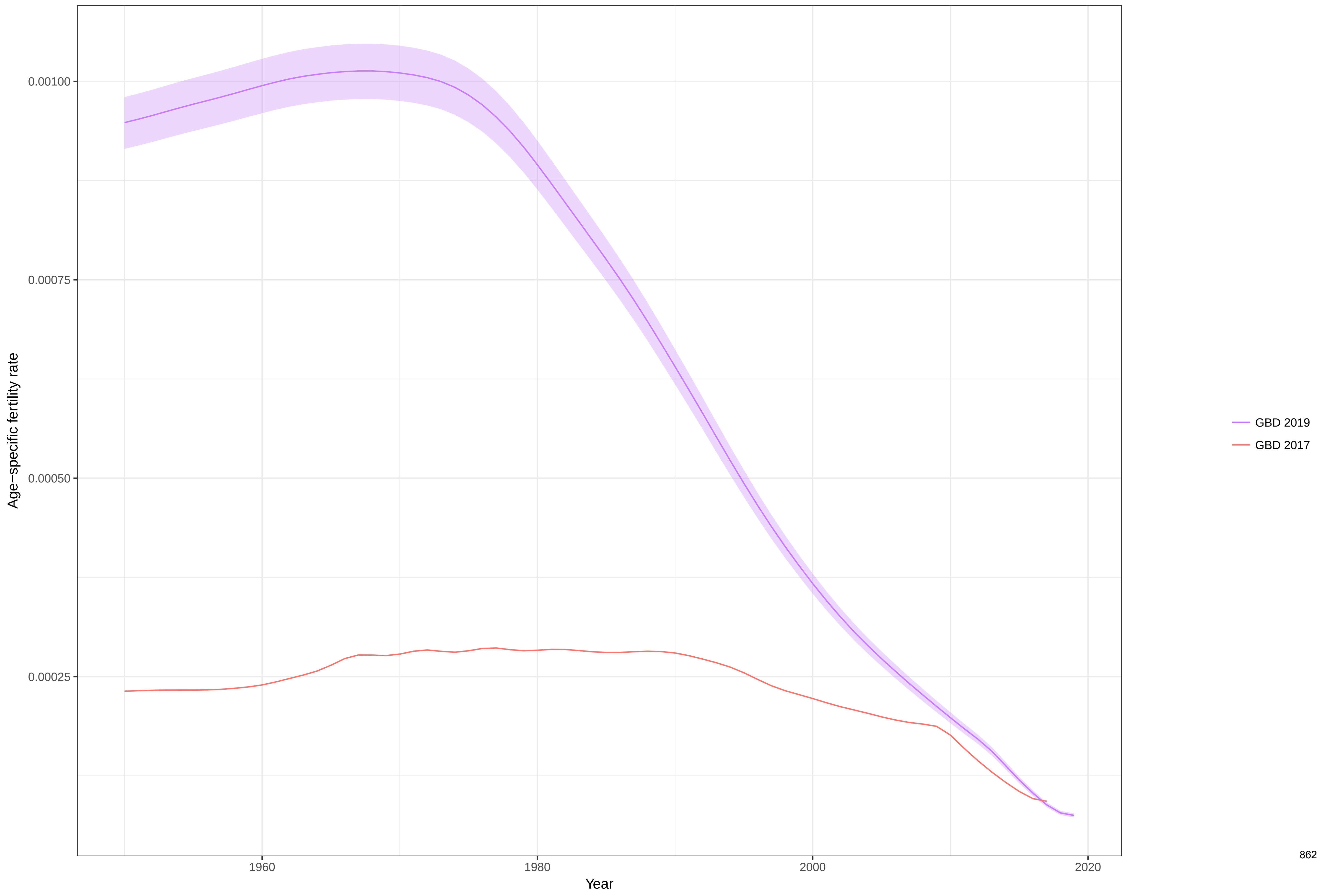

Total fertility rate fitted trend lines, 1950−2019: Gabon TFR
Data points enclosed with a diamond are reference sources

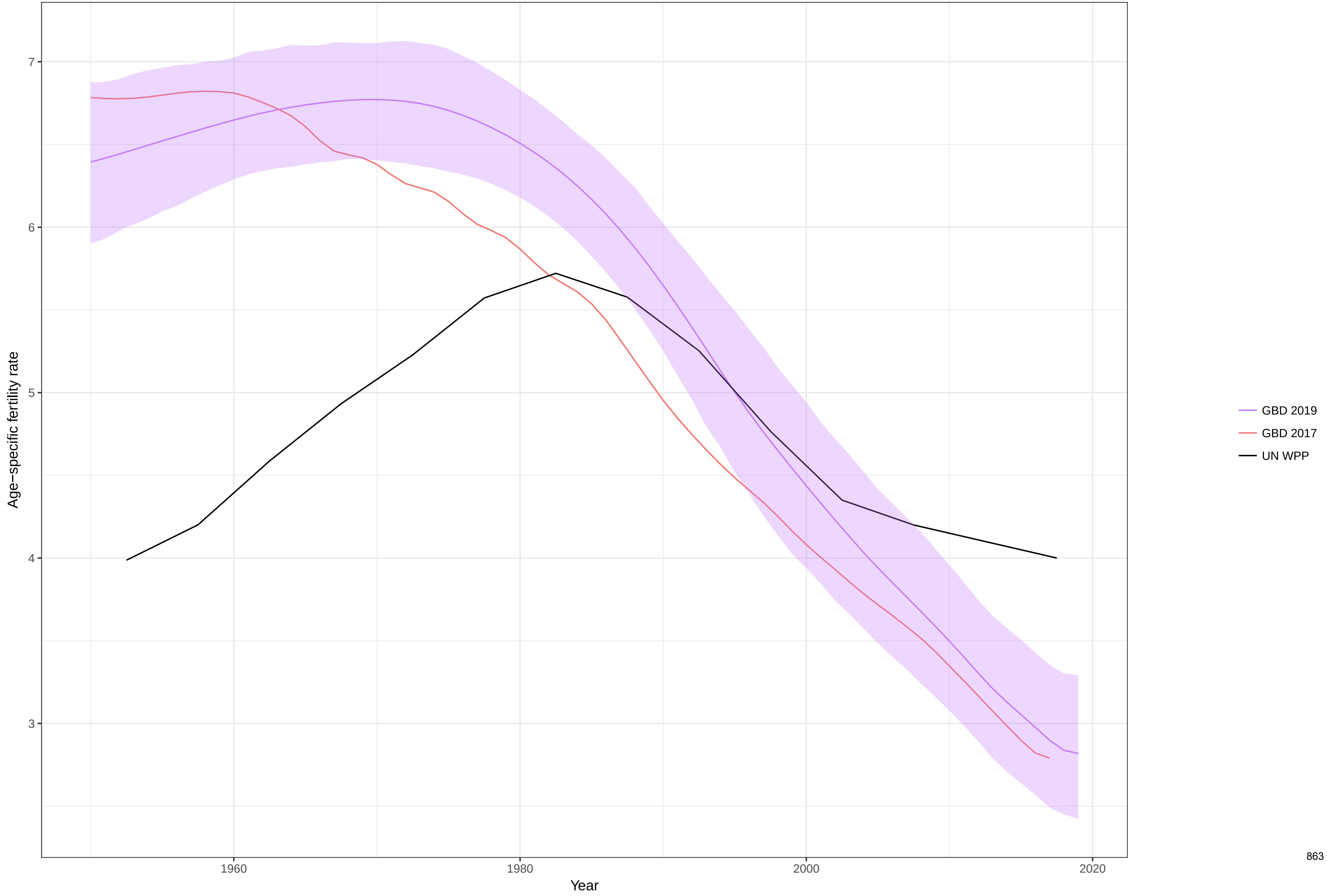

Age−specific fertility fitted trend lines, 1950−2019: United Kingdom, Ages: 10−14
Data points enclosed with a diamond are reference sources

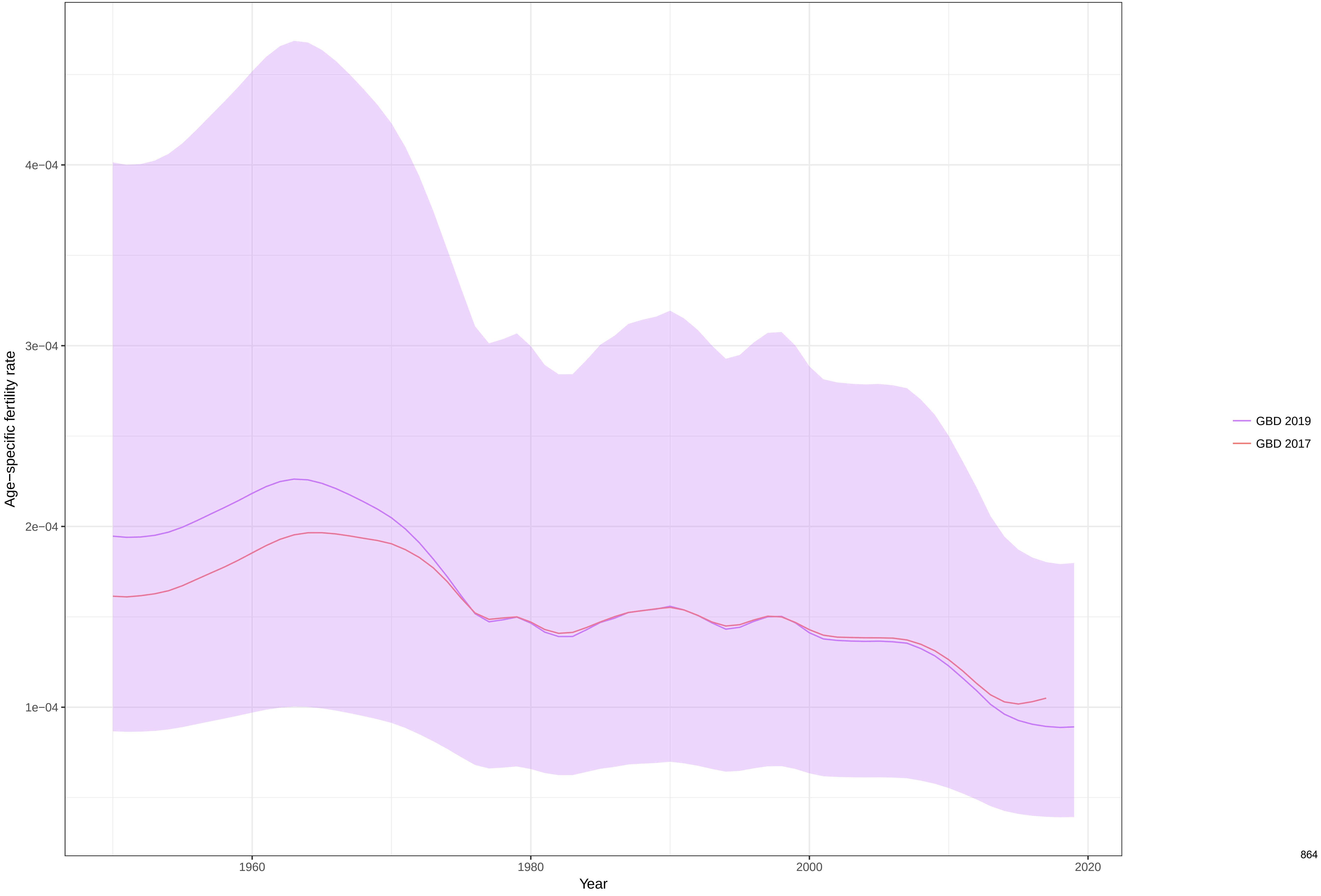

Age−specific fertility fitted trend lines, 1950−2019: United Kingdom, Ages: 15−19
Data points enclosed with a diamond are reference sources

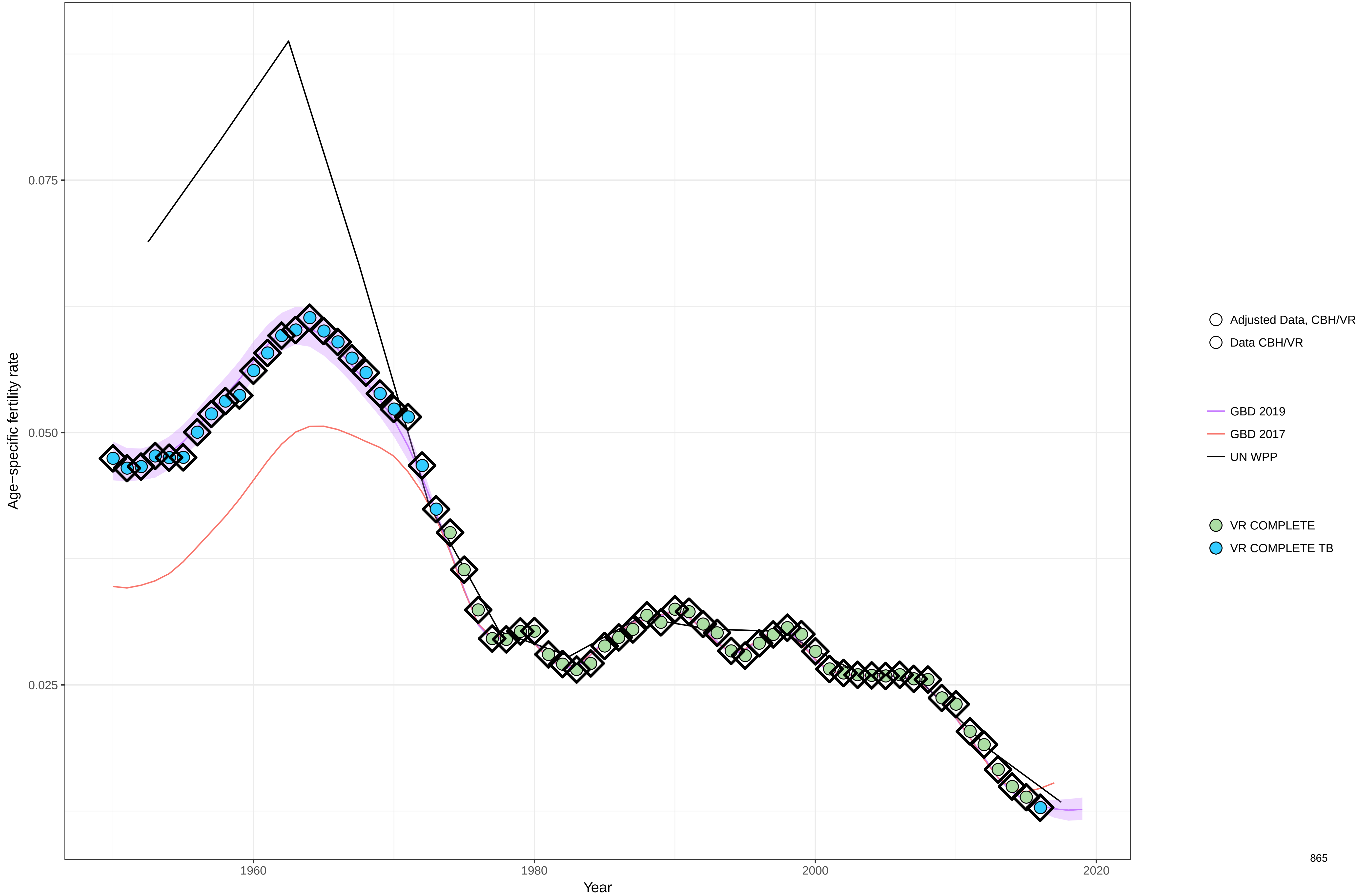

Age−specific fertility fitted trend lines, 1950−2019: United Kingdom, Ages: 20−24
Data points enclosed with a diamond are reference sources

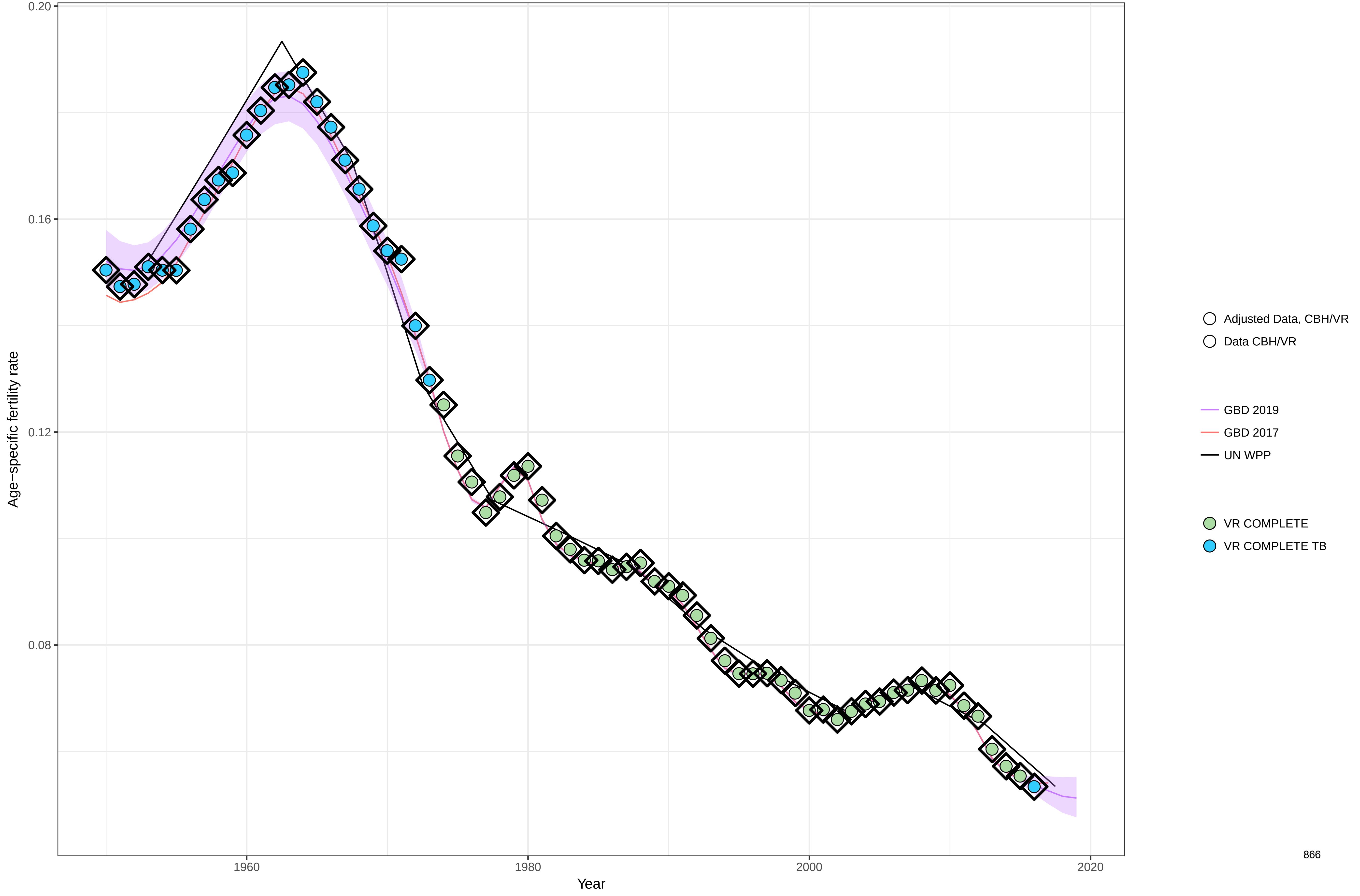

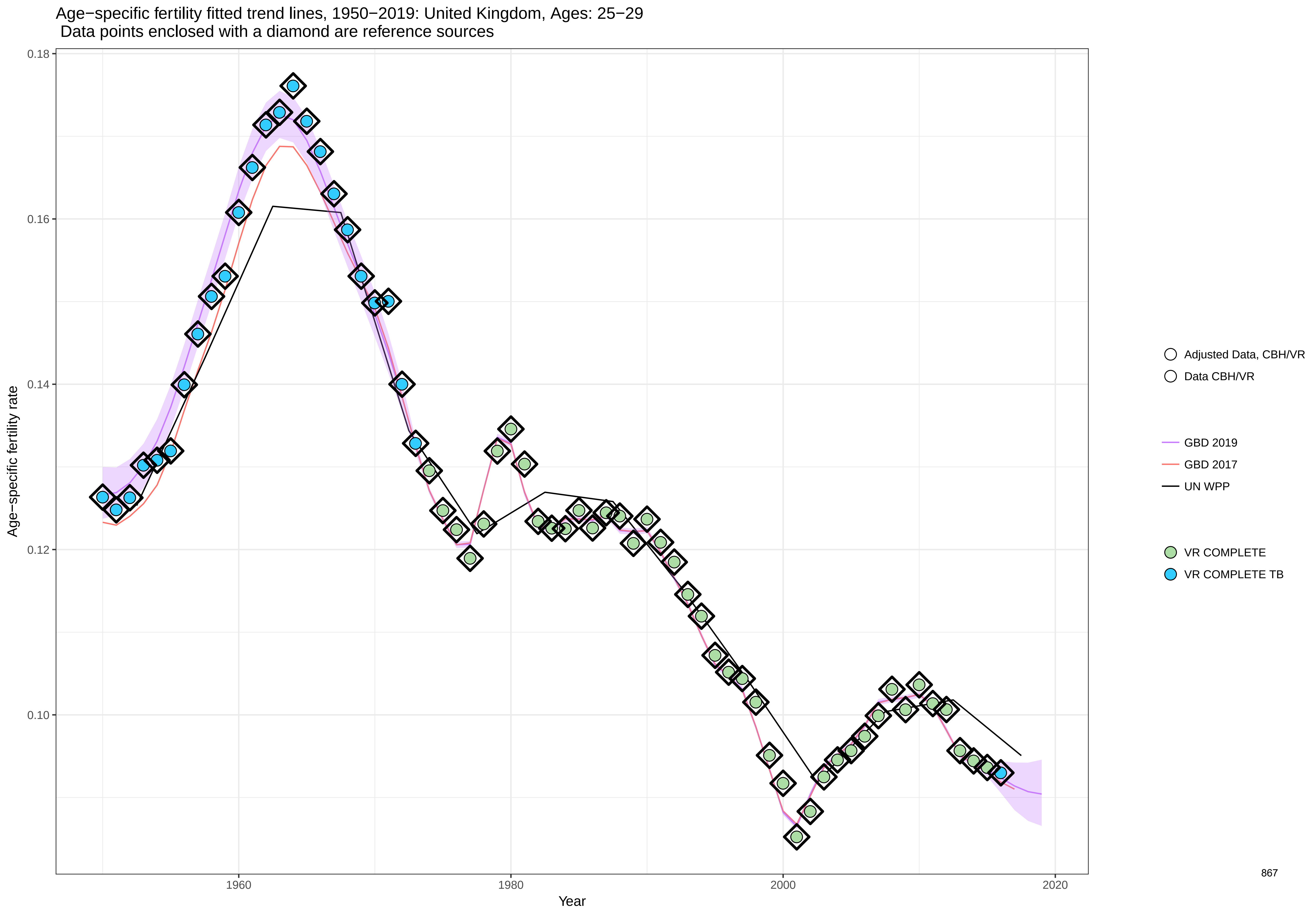

Age−specific fertility fitted trend lines, 1950−2019: United Kingdom, Ages: 25−29
Data points enclosed with a diamond are reference sources
0.18
0.16
0.14
0.12
0.10
Age−specific fertility rate
1960
1980
2000
2020
Year
Adjusted Data, CBH/VR
Data CBH/VR
GBD 2019
GBD 2017
UN WPP
VR COMPLETE
VR COMPLETE TB

Age−specific fertility fitted trend lines, 1950−2019: United Kingdom, Ages: 30−34
Data points enclosed with a diamond are reference sources

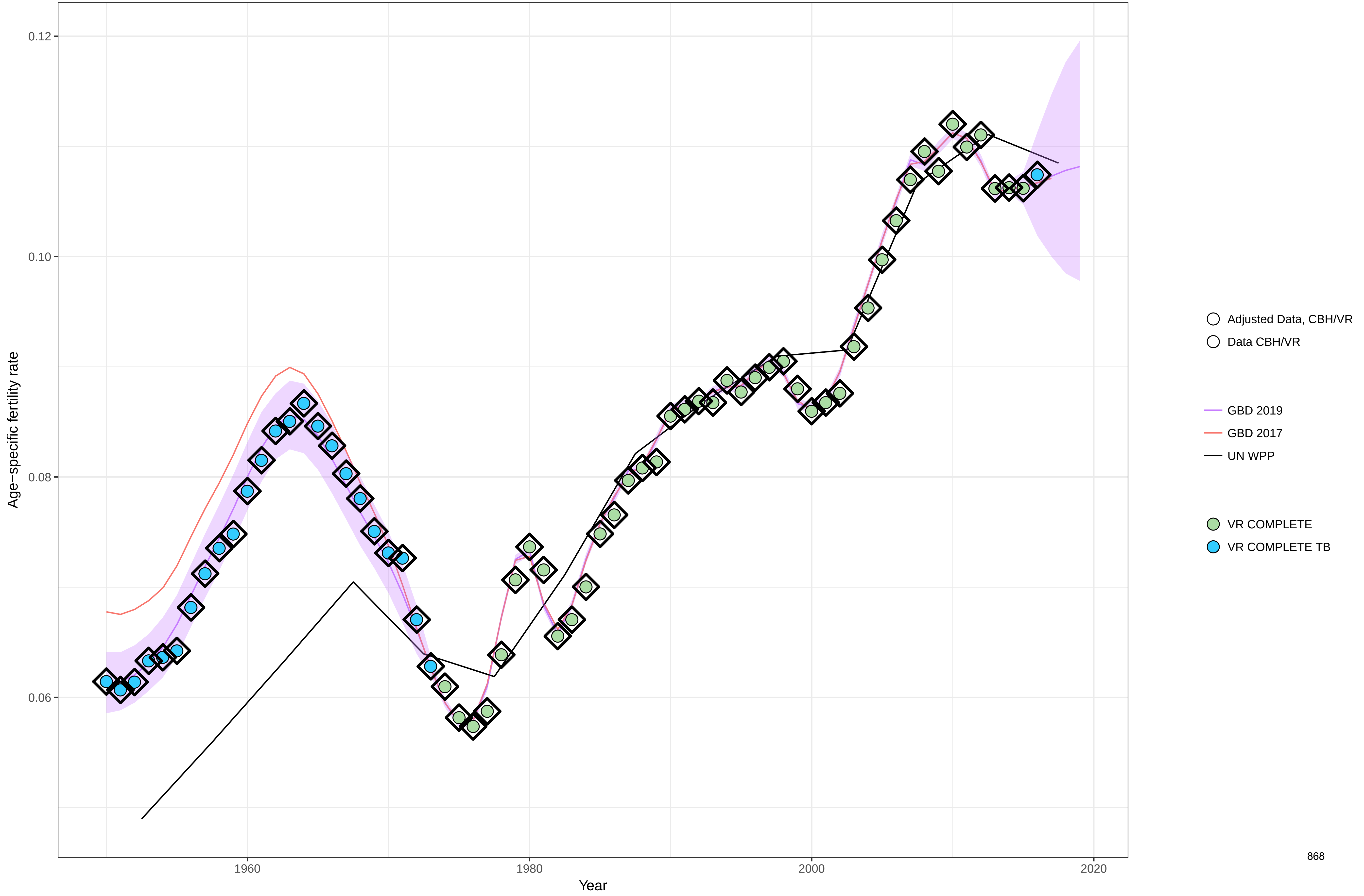

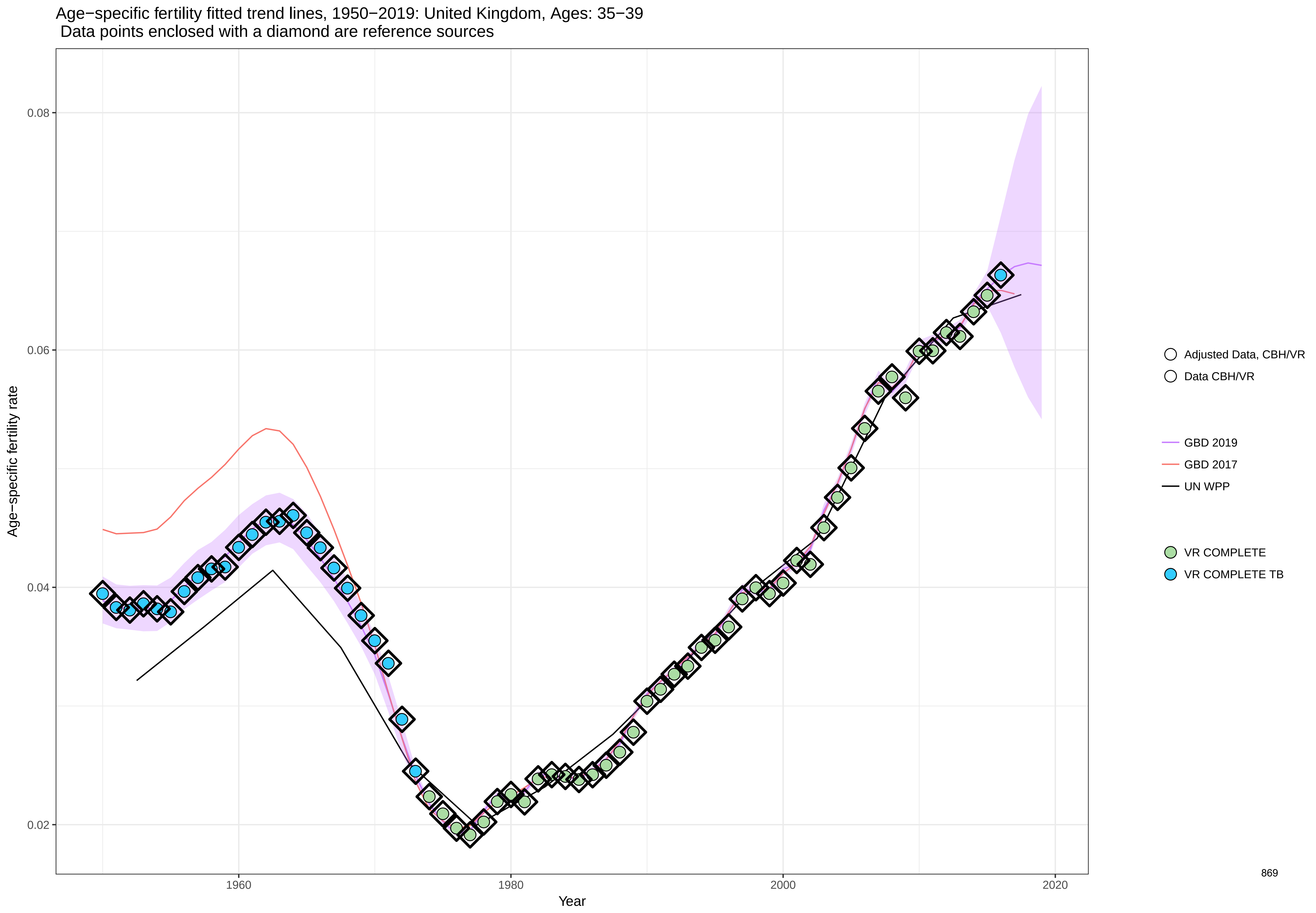

Age−specific fertility fitted trend lines, 1950−2019: United Kingdom, Ages: 35−39
Data points enclosed with a diamond are reference sources
Age−specific fertility rate
0.08
0.06
0.04
0.02
1960
1980
2000
2020
Year
Adjusted Data, CBH/VR
Data CBH/VR
GBD 2019
GBD 2017
UN WPP
VR COMPLETE
VR COMPLETE TB

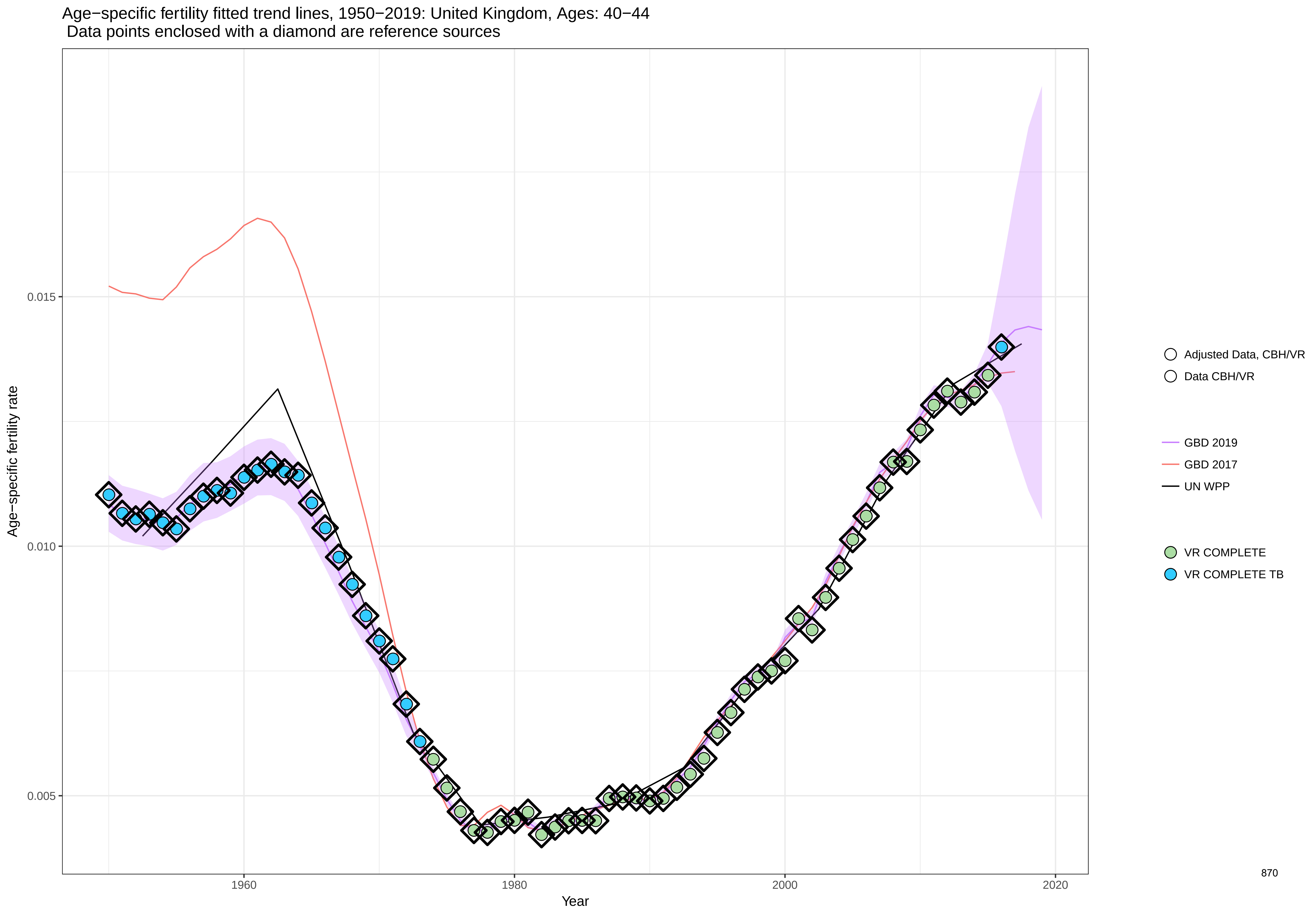
Age−specific fertility fitted trend lines, 1950−2019: United Kingdom, Ages: 40−44
Data points enclosed with a diamond are reference sources
Age−specific fertility rate
0.015
0.010
0.005
Year
1960
1980
2000
2020
Adjusted Data, CBH/VR
Data CBH/VR
GBD 2019
GBD 2017
UN WPP
VR COMPLETE
VR COMPLETE TB

Age−specific fertility fitted trend lines, 1950−2019: United Kingdom, Ages: 45−49
Data points enclosed with a diamond are reference sources

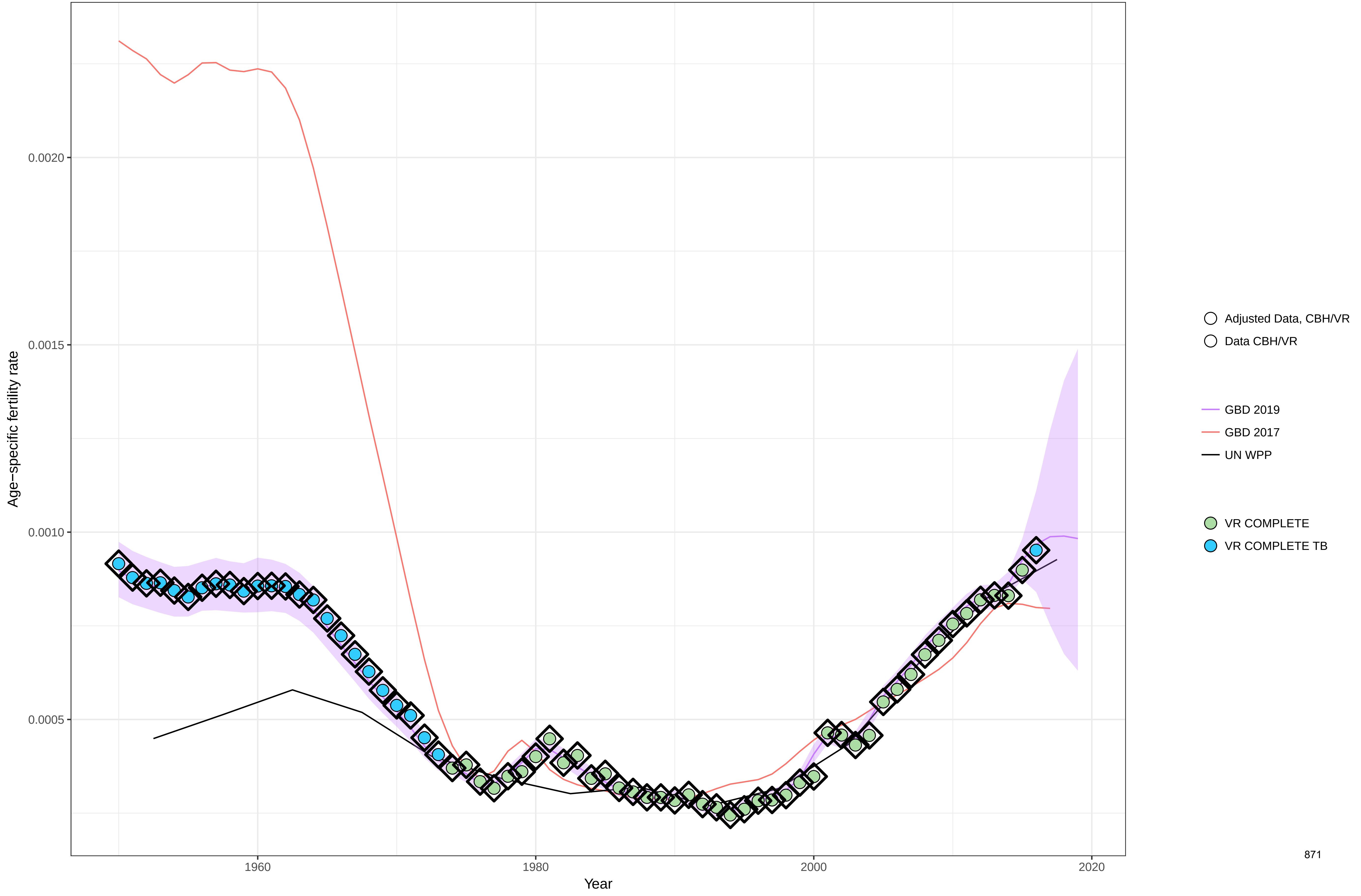

Age−specific fertility fitted trend lines, 1950−2019: United Kingdom, Ages: 50−54
Data points enclosed with a diamond are reference sources

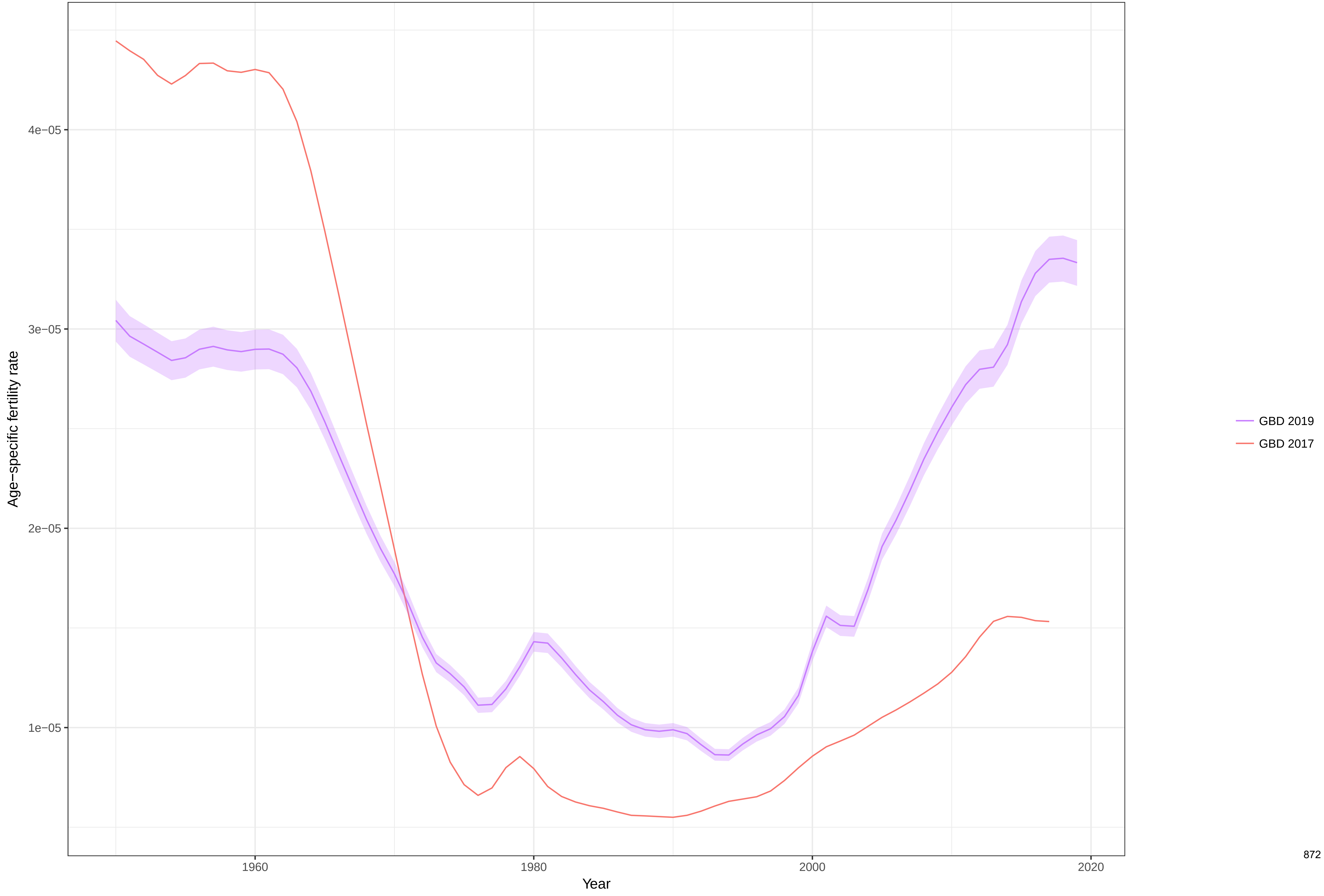

Total fertility rate fitted trend lines, 1950−2019: United Kingdom TFR
Data points enclosed with a diamond are reference sources

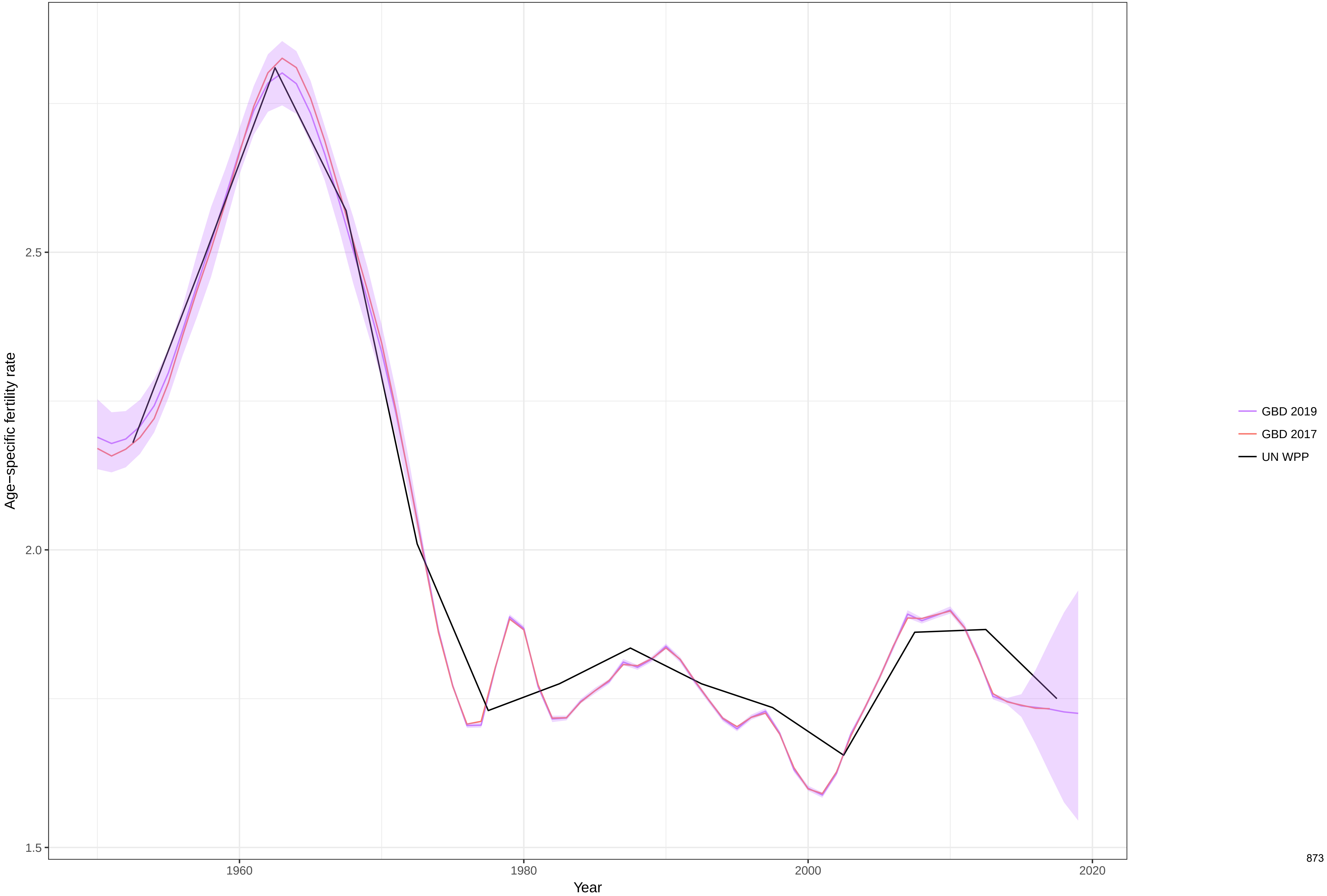

Age−specific fertility fitted trend lines, 1950−2019: Georgia, Ages: 10−14
Data points enclosed with a diamond are reference sources

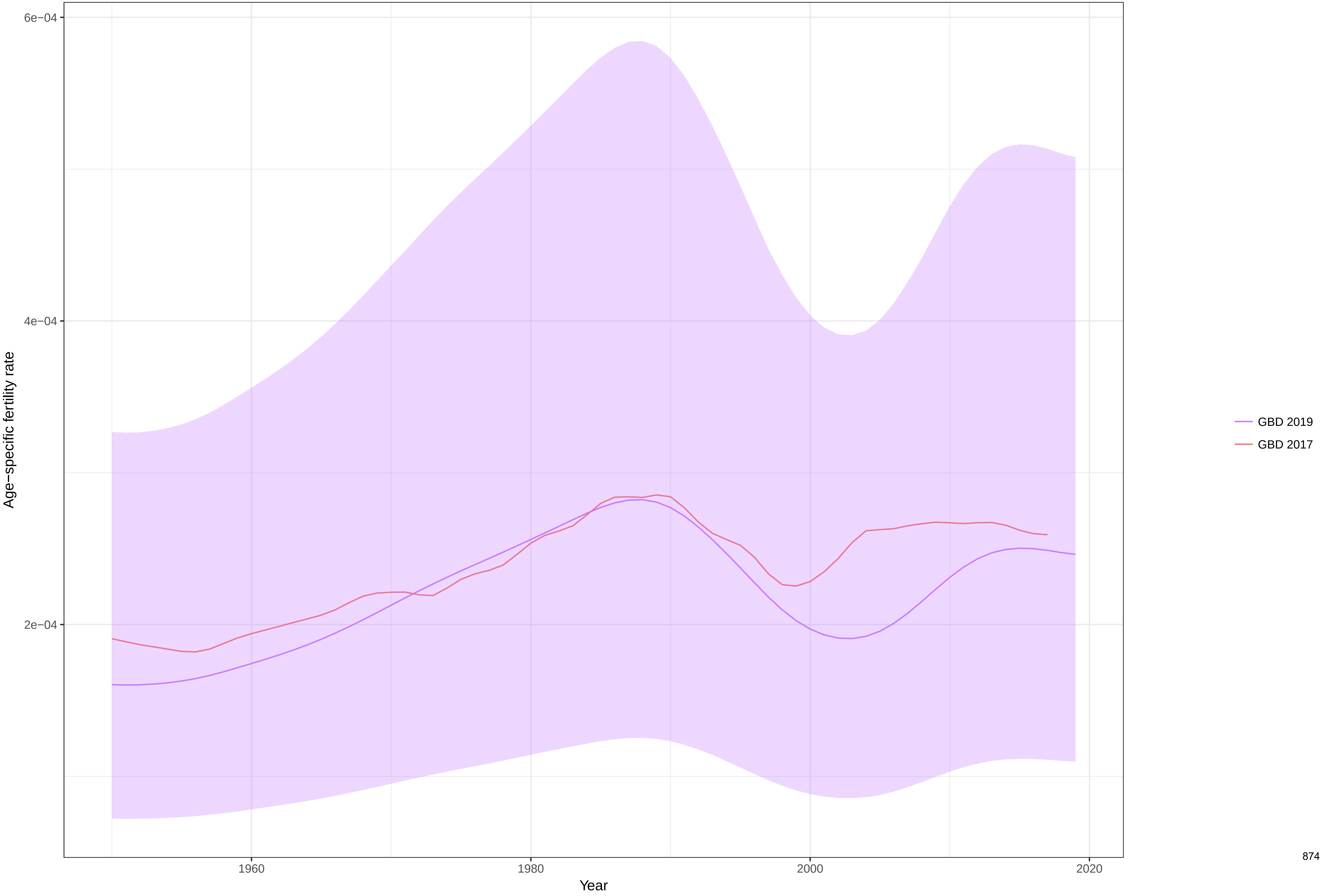

Age−specific fertility fitted trend lines, 1950−2019: Georgia, Ages: 15−19
Data points enclosed with a diamond are reference sources

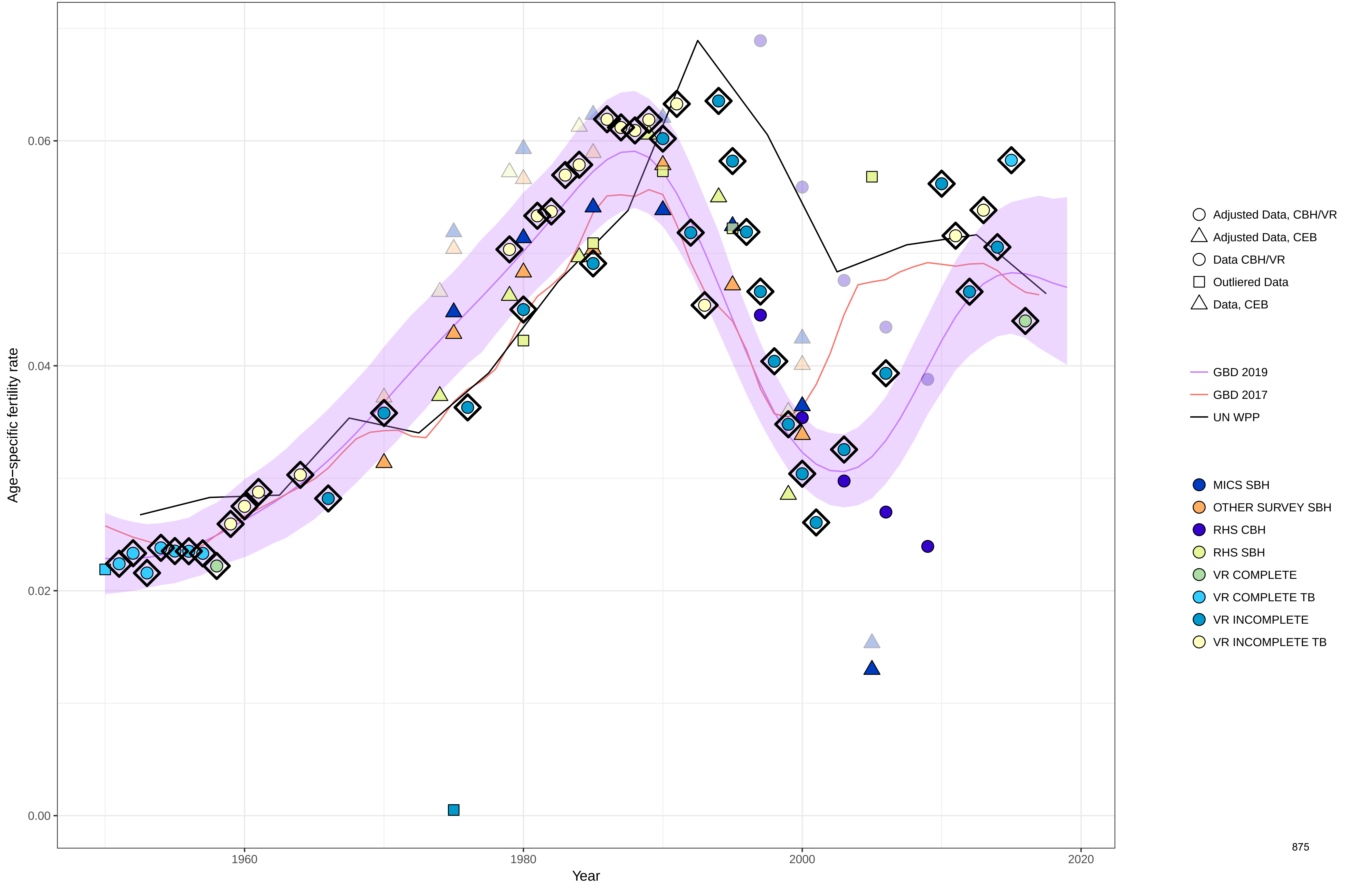

Age−specific fertility fitted trend lines, 1950−2019: Georgia, Ages: 20−24
Data points enclosed with a diamond are reference sources

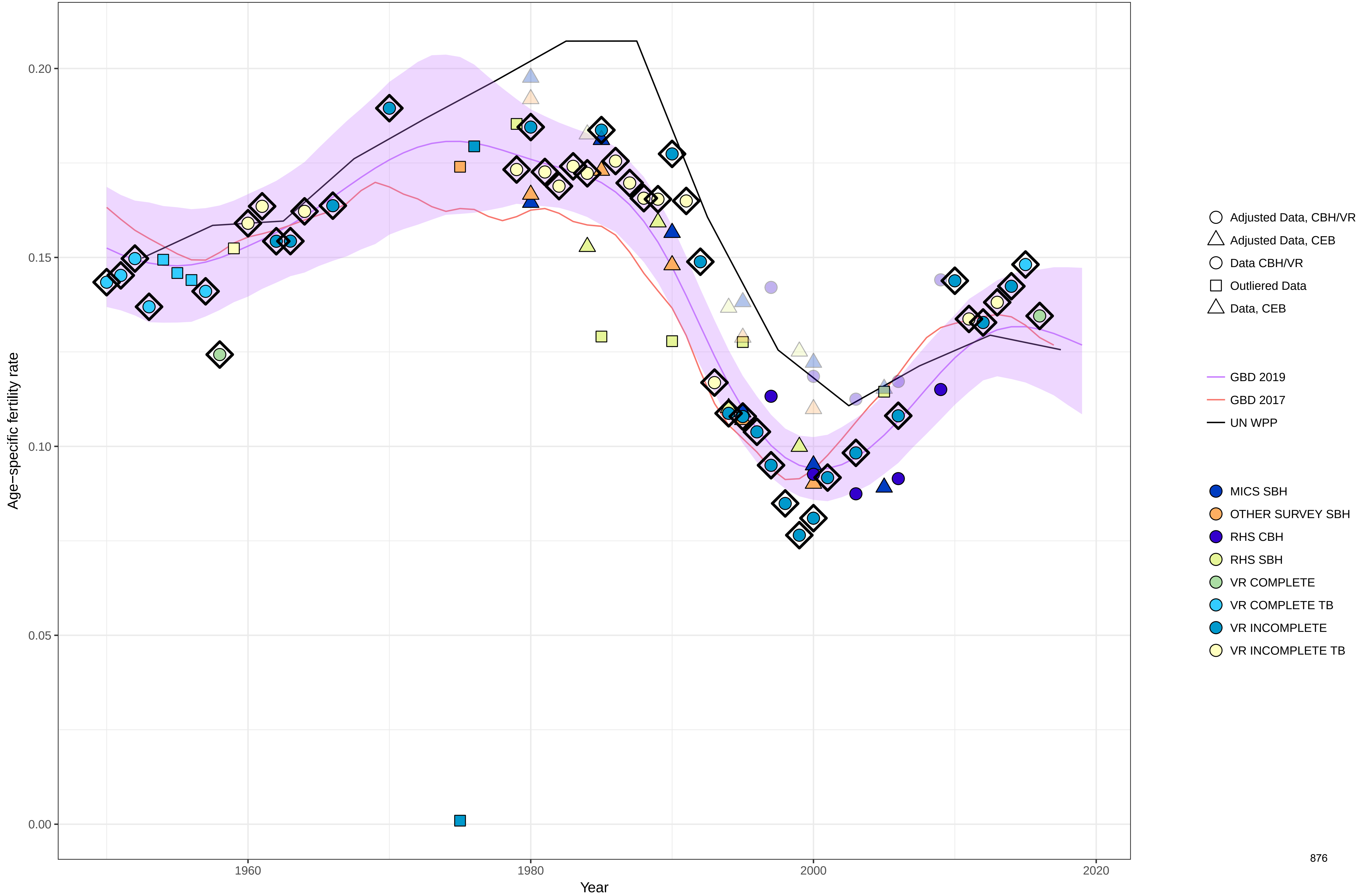

Age−specific fertility fitted trend lines, 1950−2019: Georgia, Ages: 25−29
Data points enclosed with a diamond are reference sources

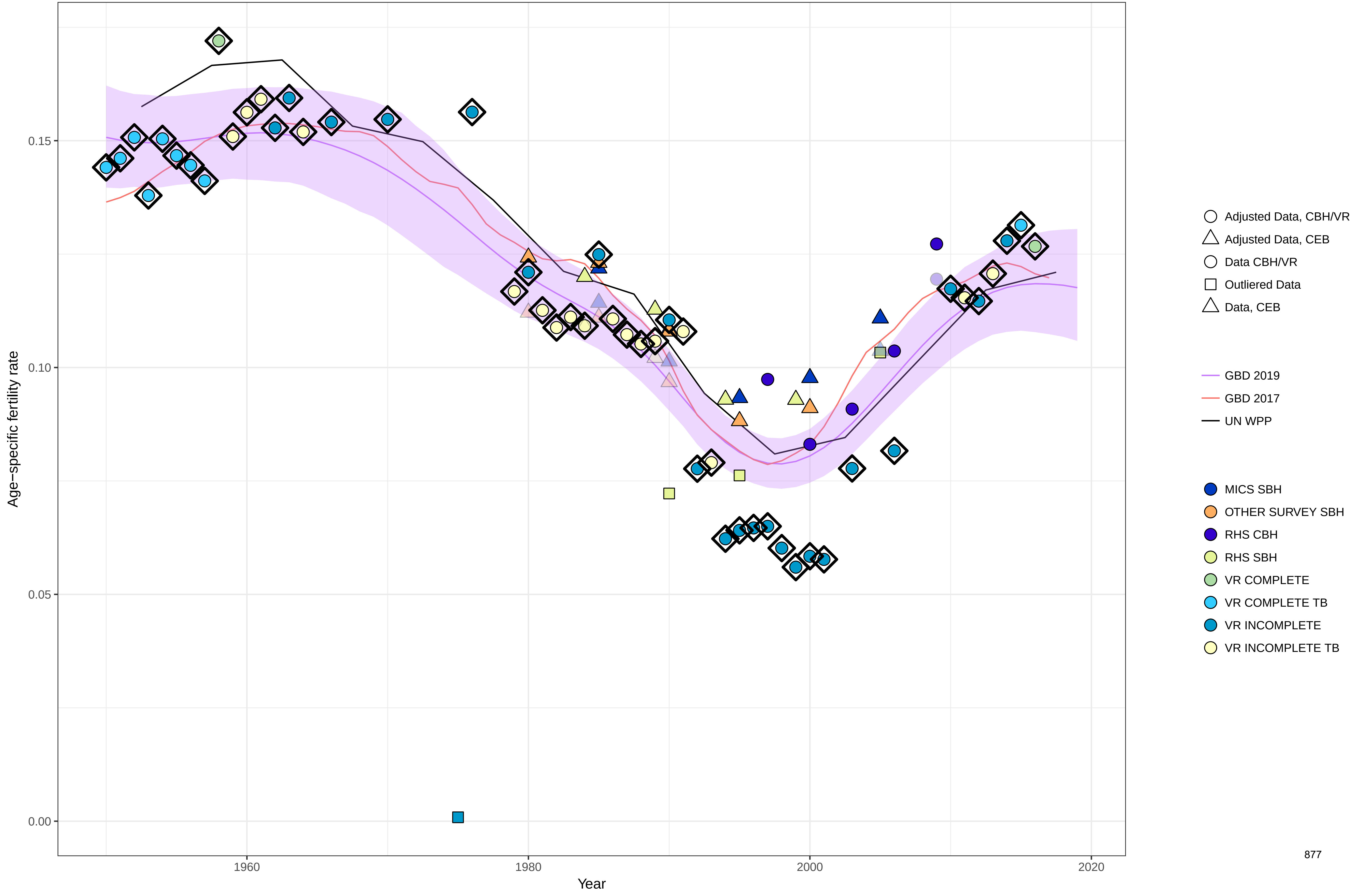

Age−specific fertility fitted trend lines, 1950−2019: Georgia, Ages: 30−34
Data points enclosed with a diamond are reference sources

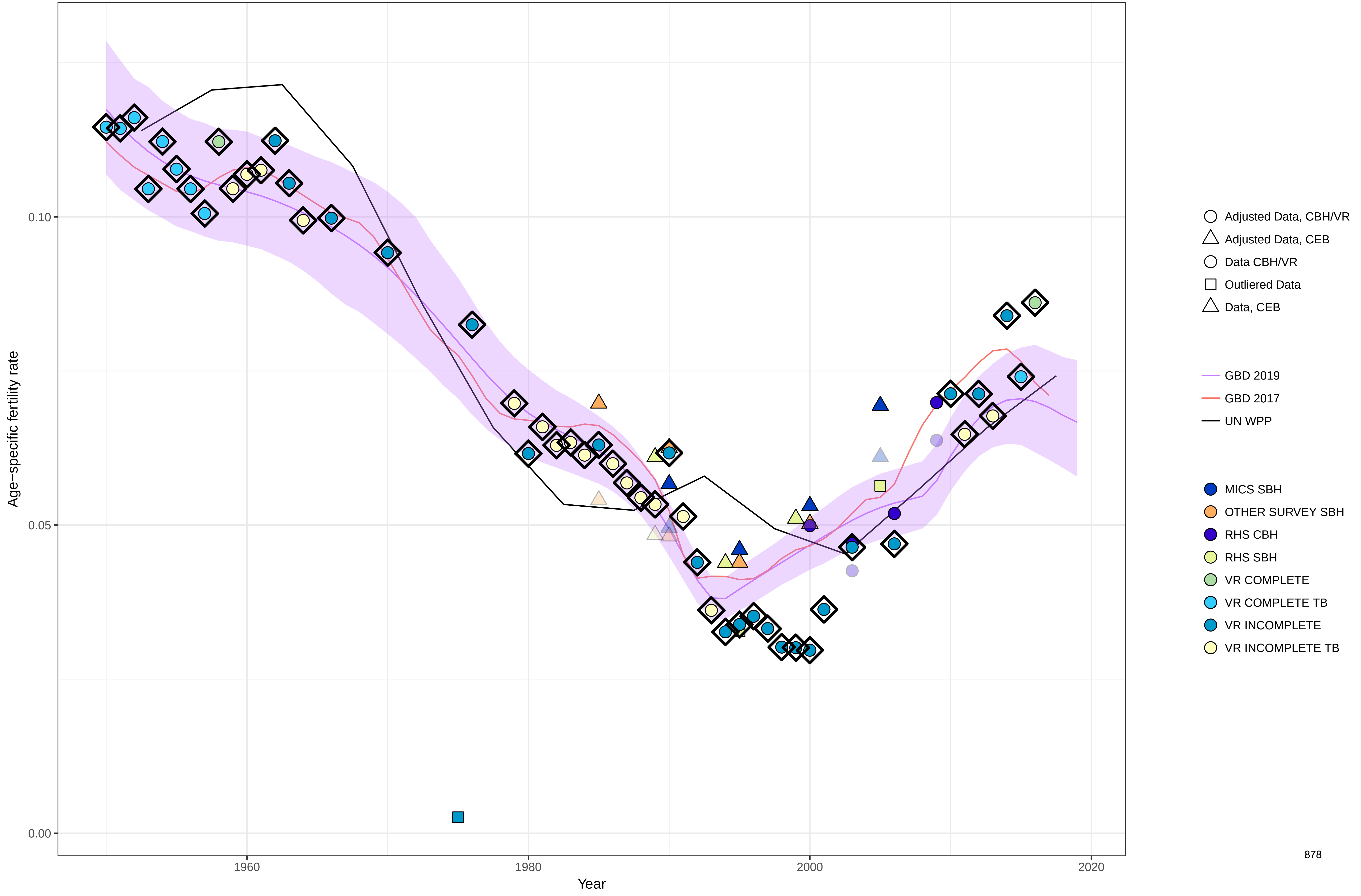

Age−specific fertility fitted trend lines, 1950−2019: Georgia, Ages: 35−39
Data points enclosed with a diamond are reference sources

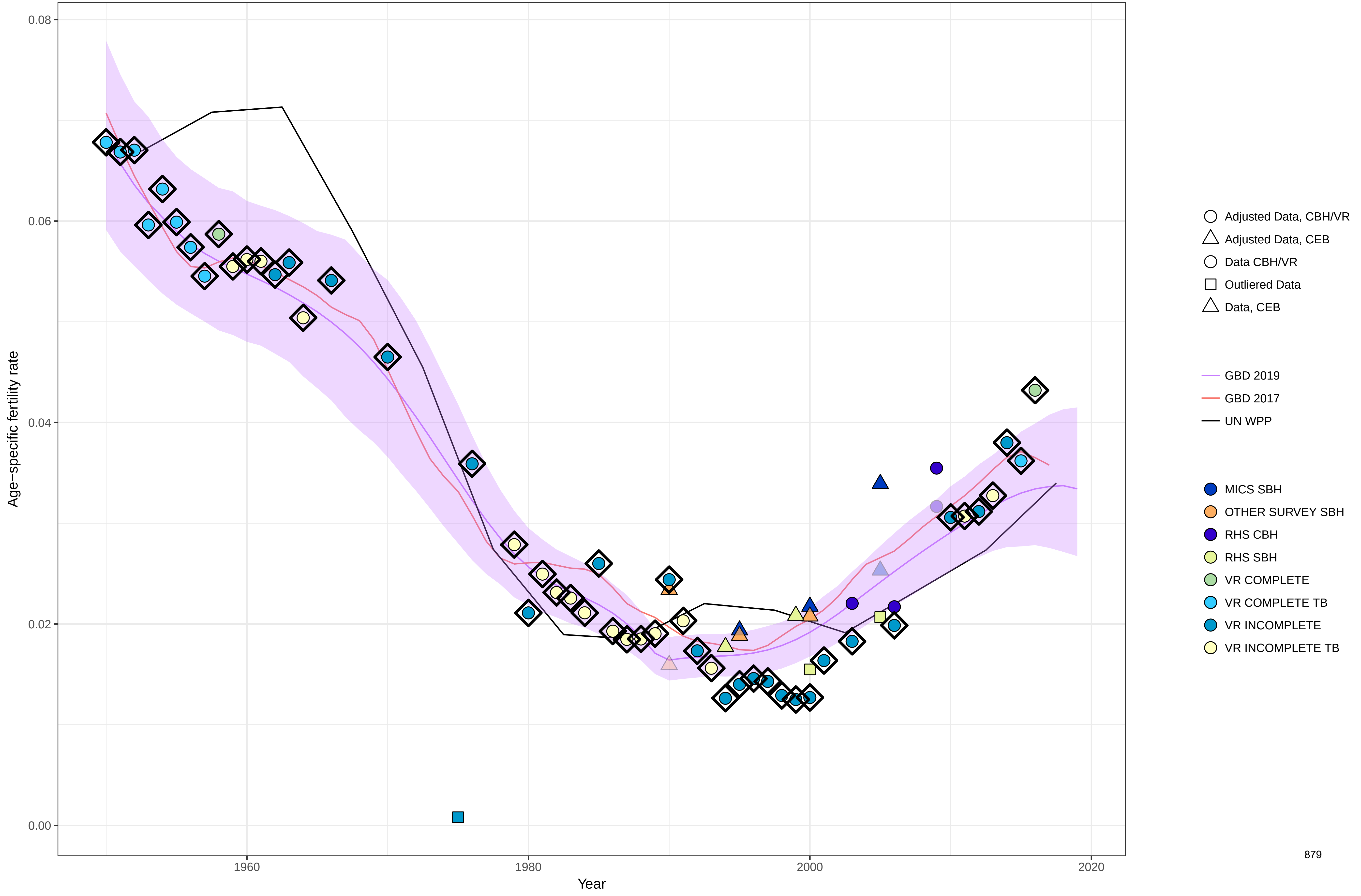

Age−specific fertility fitted trend lines, 1950−2019: Georgia, Ages: 40−44
Data points enclosed with a diamond are reference sources

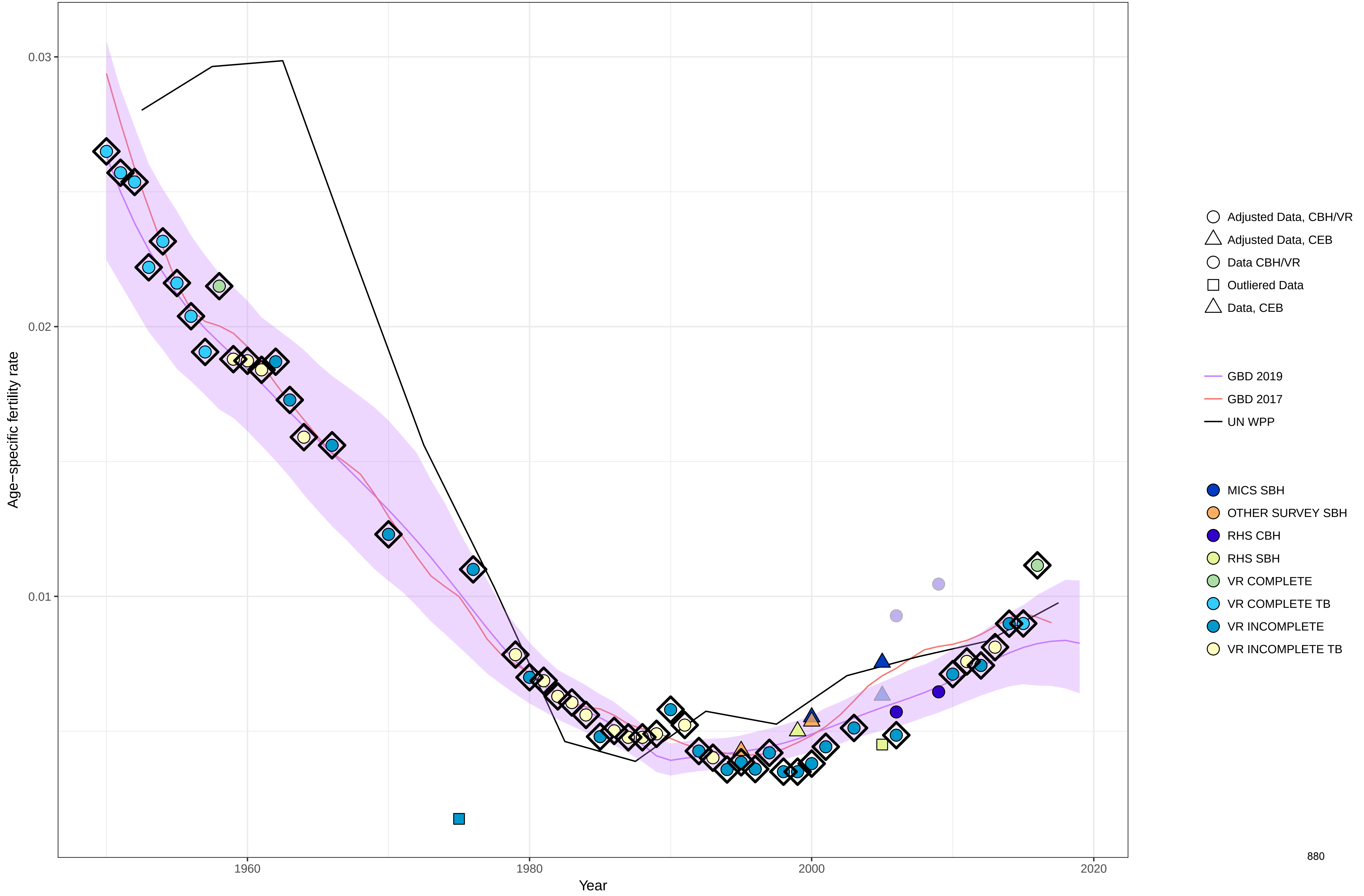

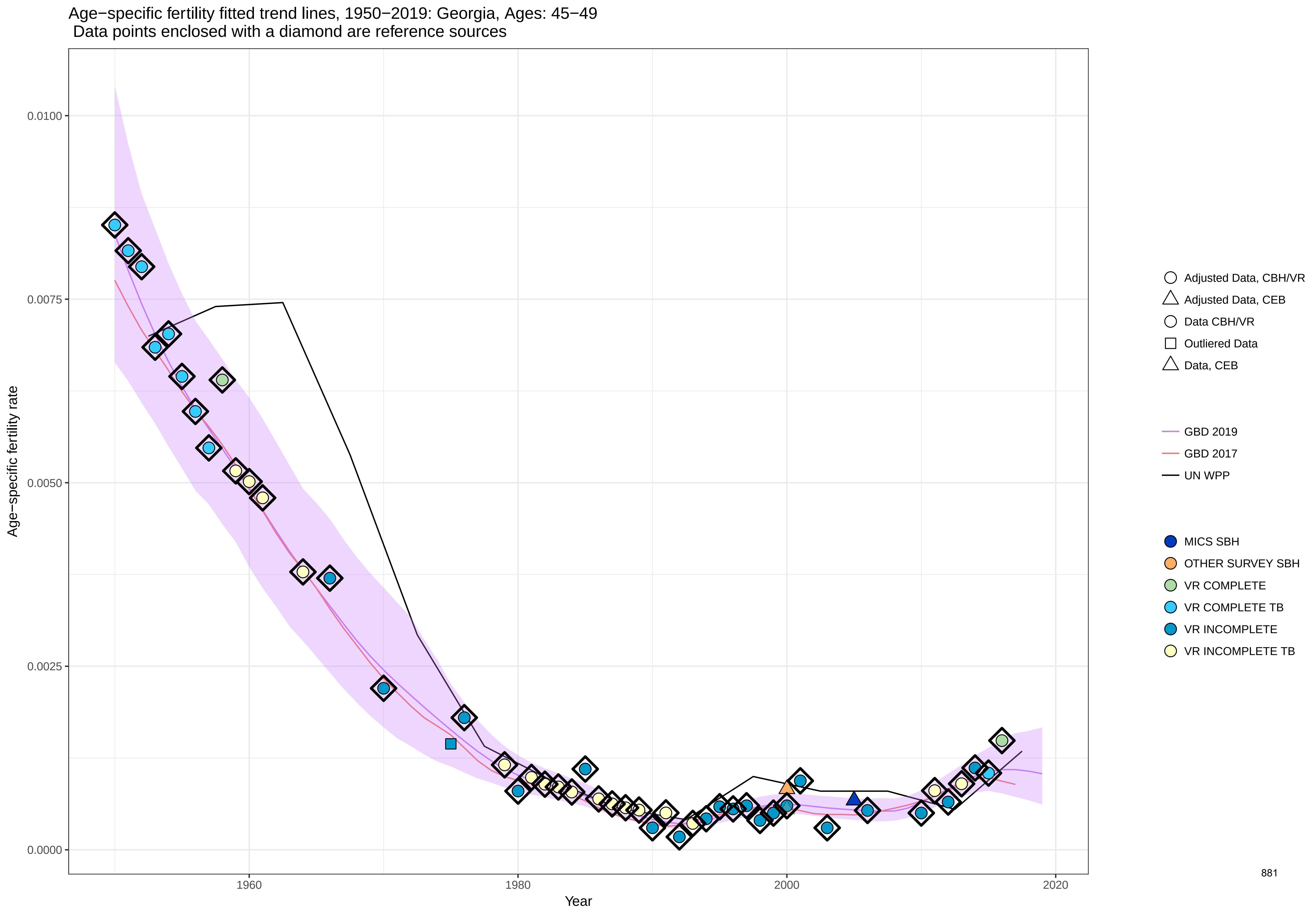

Age−specific fertility fitted trend lines, 1950−2019: Georgia, Ages: 45−49
Data points enclosed with a diamond are reference sources
Age−specific fertility rate
0.0000
0.0025
0.0050
0.0075
0.0100
1960
1980
2000
2020
Year
Adjusted Data, CBH/VR
Adjusted Data, CEB
Data CBH/VR
Outliered Data
Data, CEB
GBD 2019
GBD 2017
UN WPP
MICS SBH
OTHER SURVEY SBH
VR COMPLETE
VR COMPLETE TB
VR INCOMPLETE
VR INCOMPLETE TB

Age−specific fertility fitted trend lines, 1950−2019: Georgia, Ages: 50−54
Data points enclosed with a diamond are reference sources

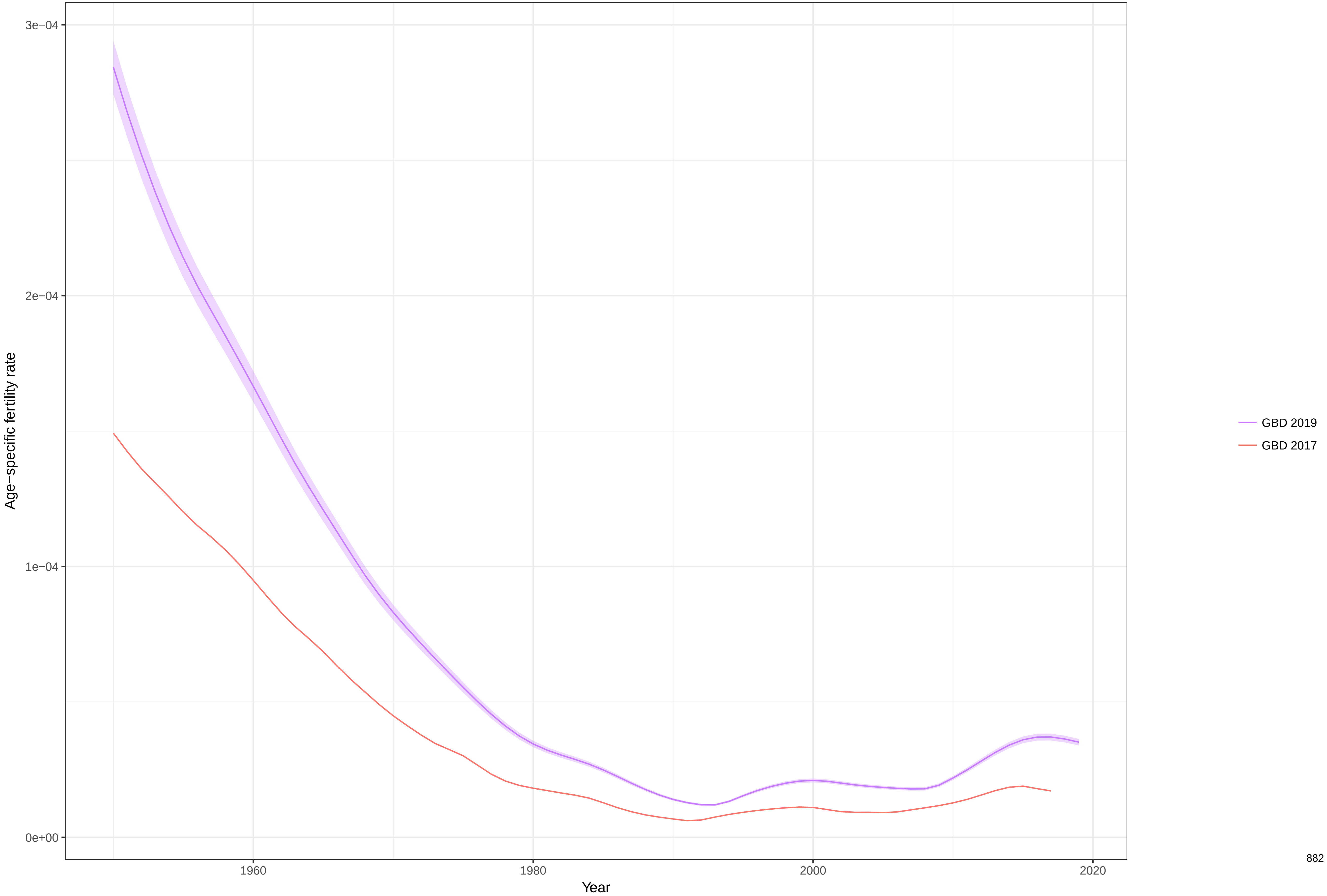

Total fertility rate fitted trend lines, 1950−2019: Georgia TFR
Data points enclosed with a diamond are reference sources

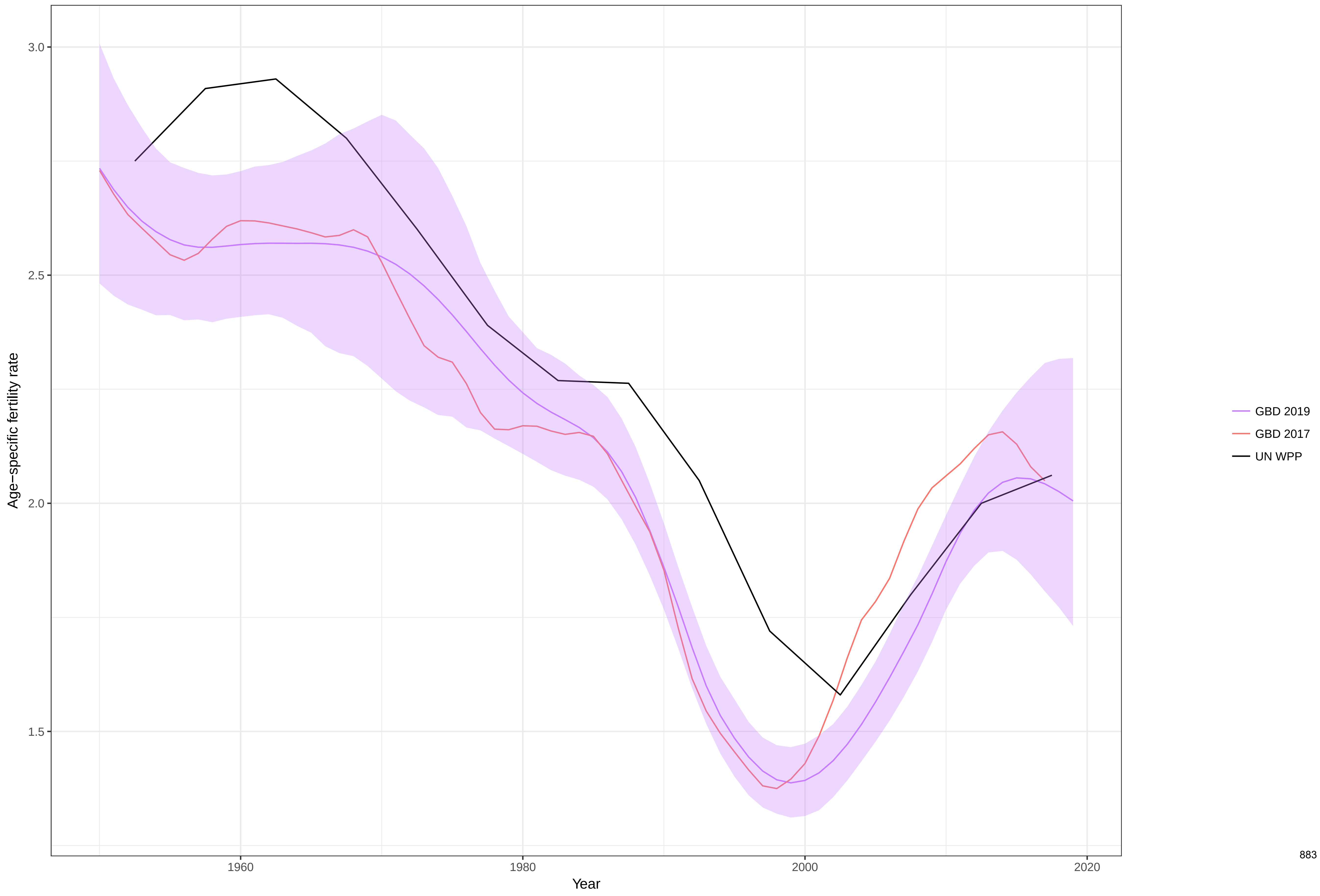

Age−specific fertility fitted trend lines, 1950−2019: Ghana, Ages: 10−14
Data points enclosed with a diamond are reference sources

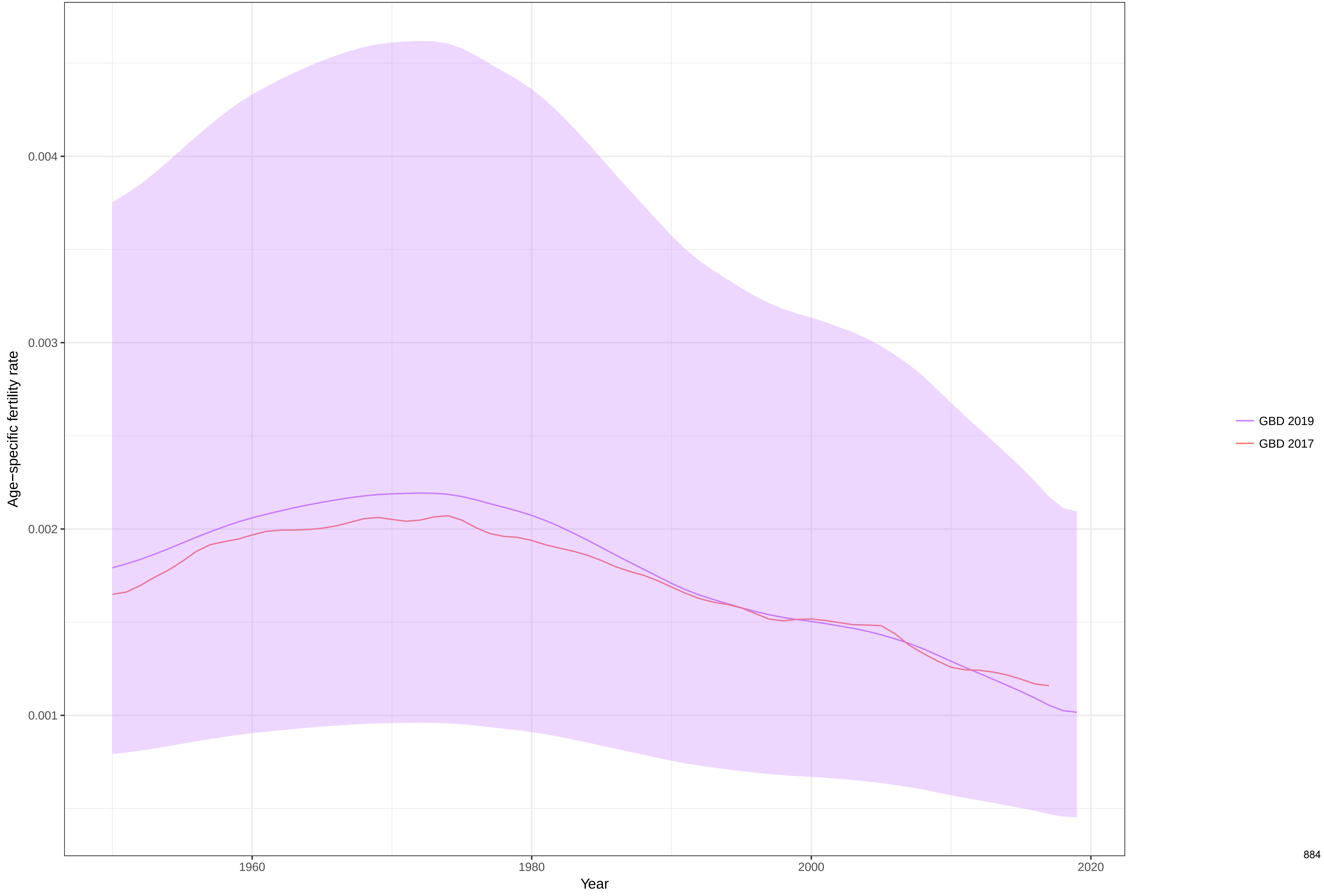

Age−specific fertility fitted trend lines, 1950−2019: Ghana, Ages: 15−19
Data points enclosed with a diamond are reference sources

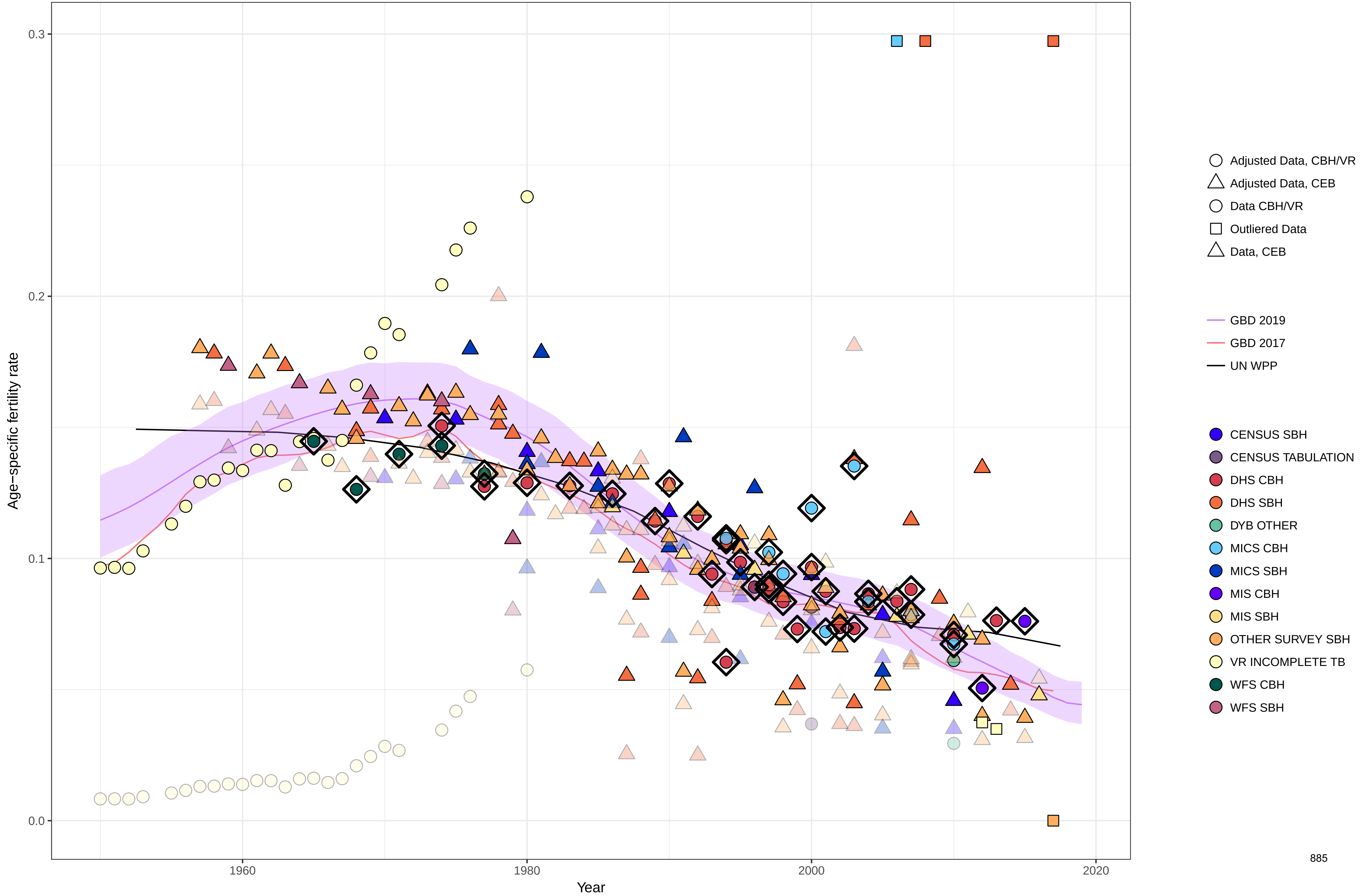

Age−specific fertility fitted trend lines, 1950−2019: Ghana, Ages: 20−24
Data points enclosed with a diamond are reference sources

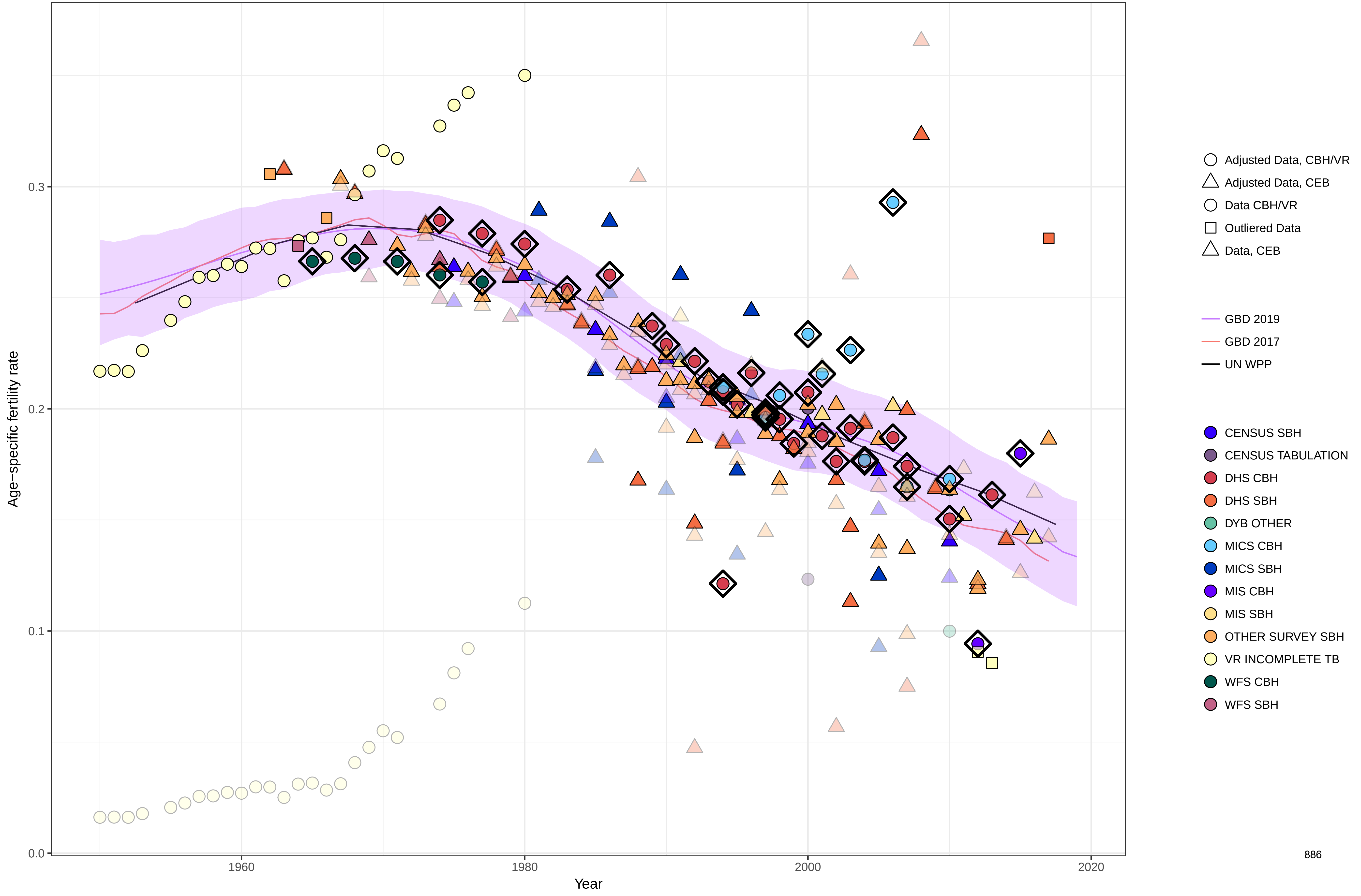

Age−specific fertility fitted trend lines, 1950−2019: Ghana, Ages: 25−29
Data points enclosed with a diamond are reference sources

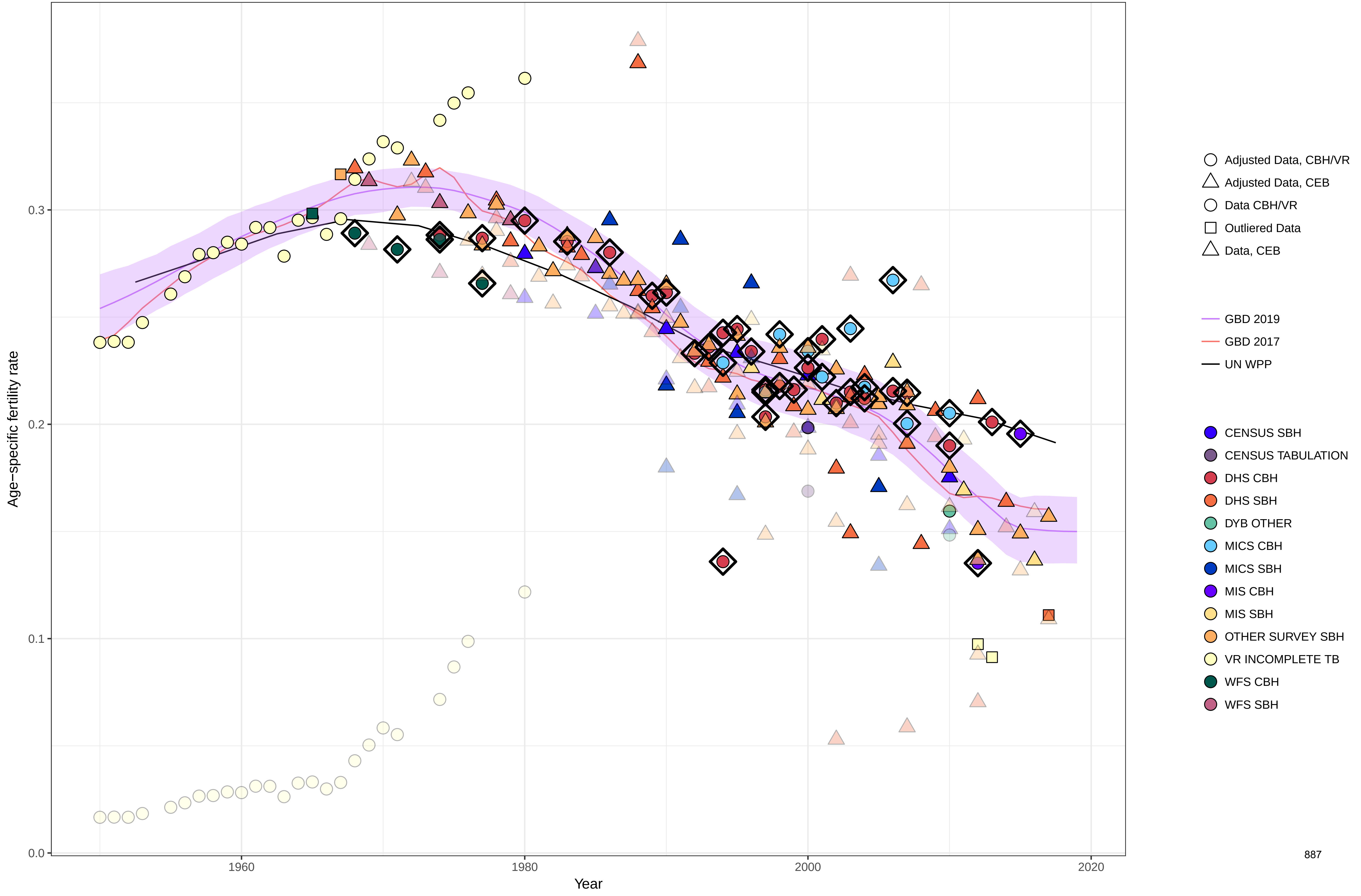

Age−specific fertility fitted trend lines, 1950−2019: Ghana, Ages: 30−34
Data points enclosed with a diamond are reference sources

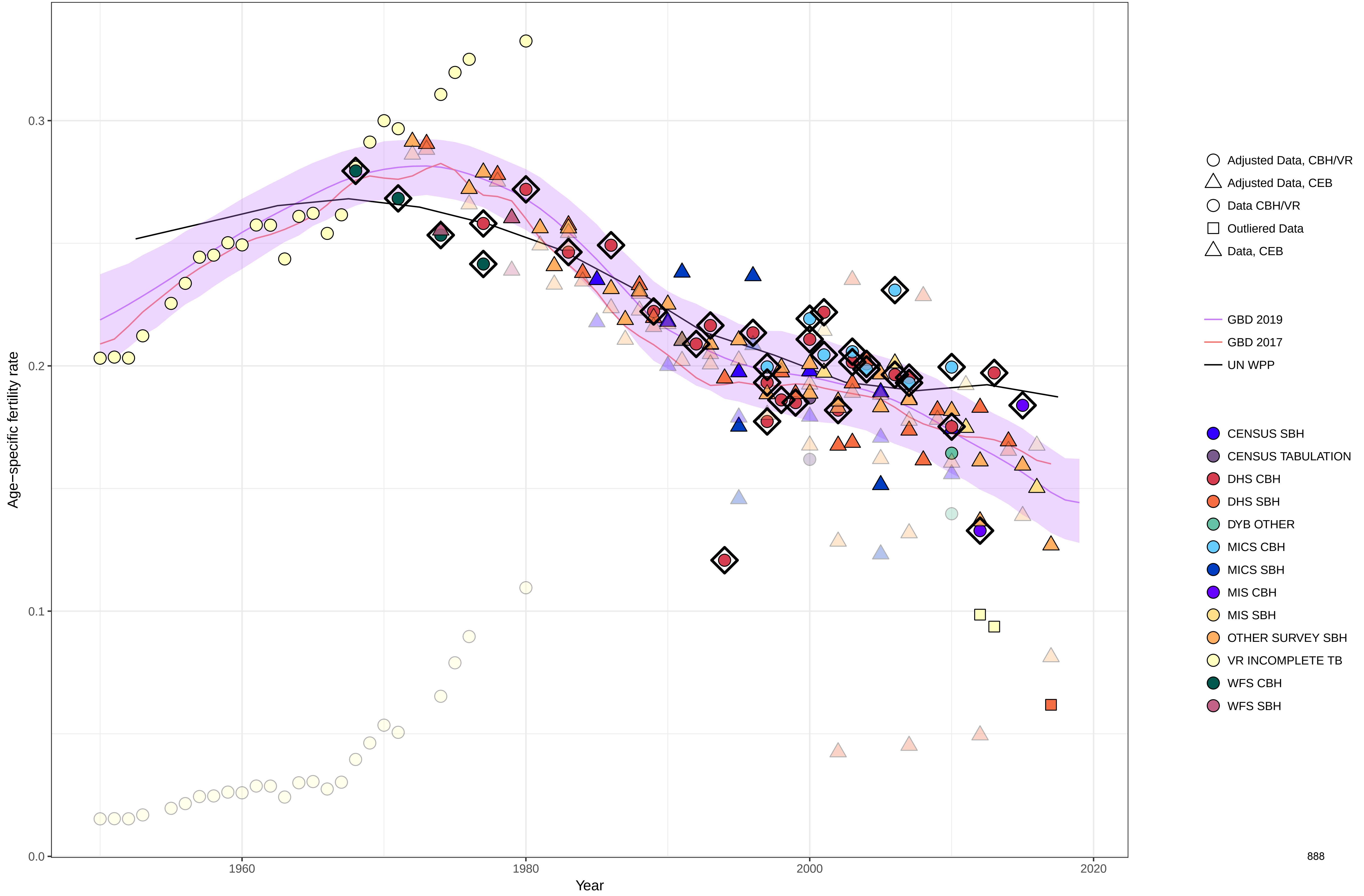

Age−specific fertility fitted trend lines, 1950−2019: Ghana, Ages: 35−39
Data points enclosed with a diamond are reference sources

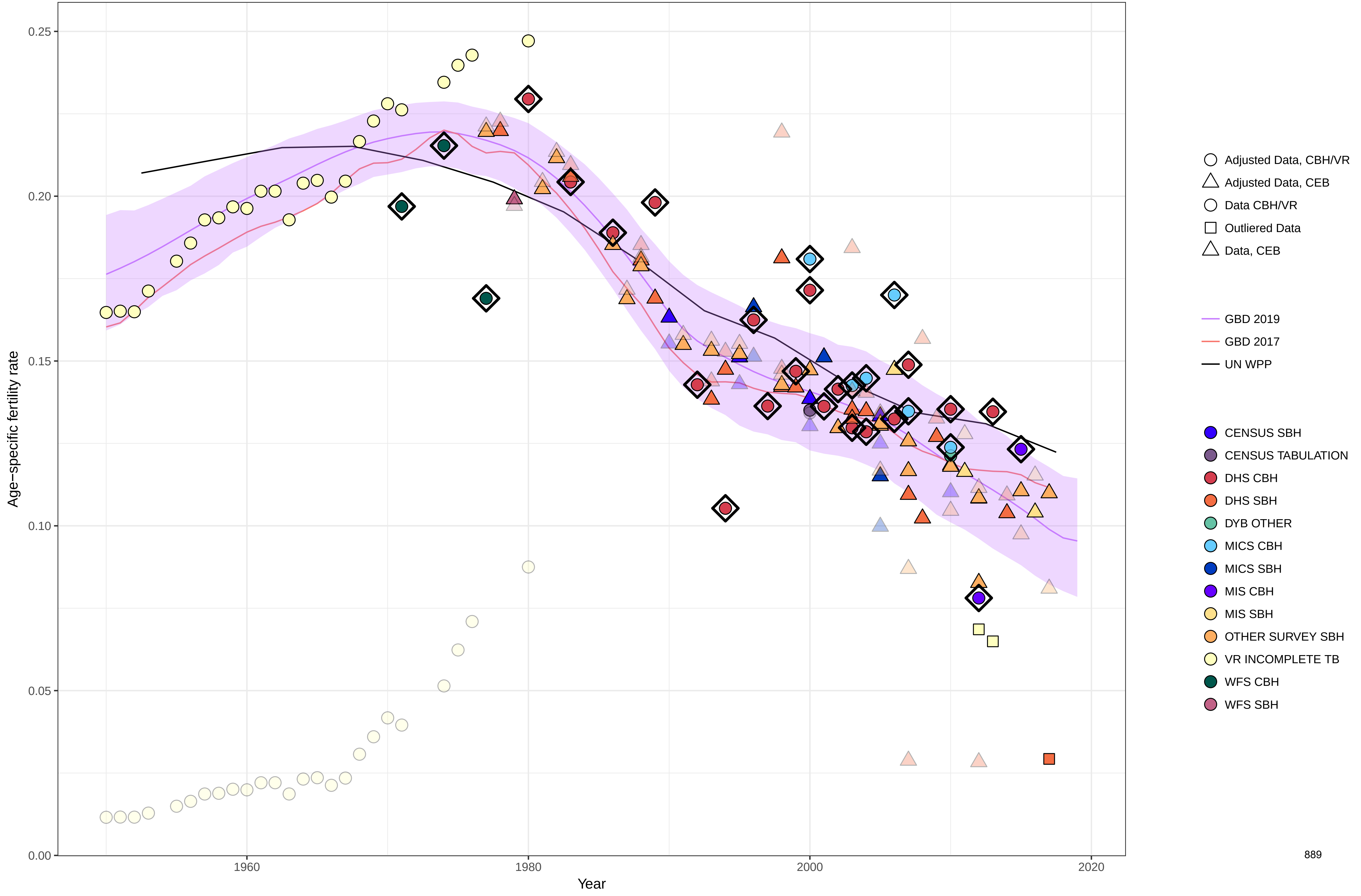

Age−specific fertility fitted trend lines, 1950−2019: Ghana, Ages: 40−44
Data points enclosed with a diamond are reference sources

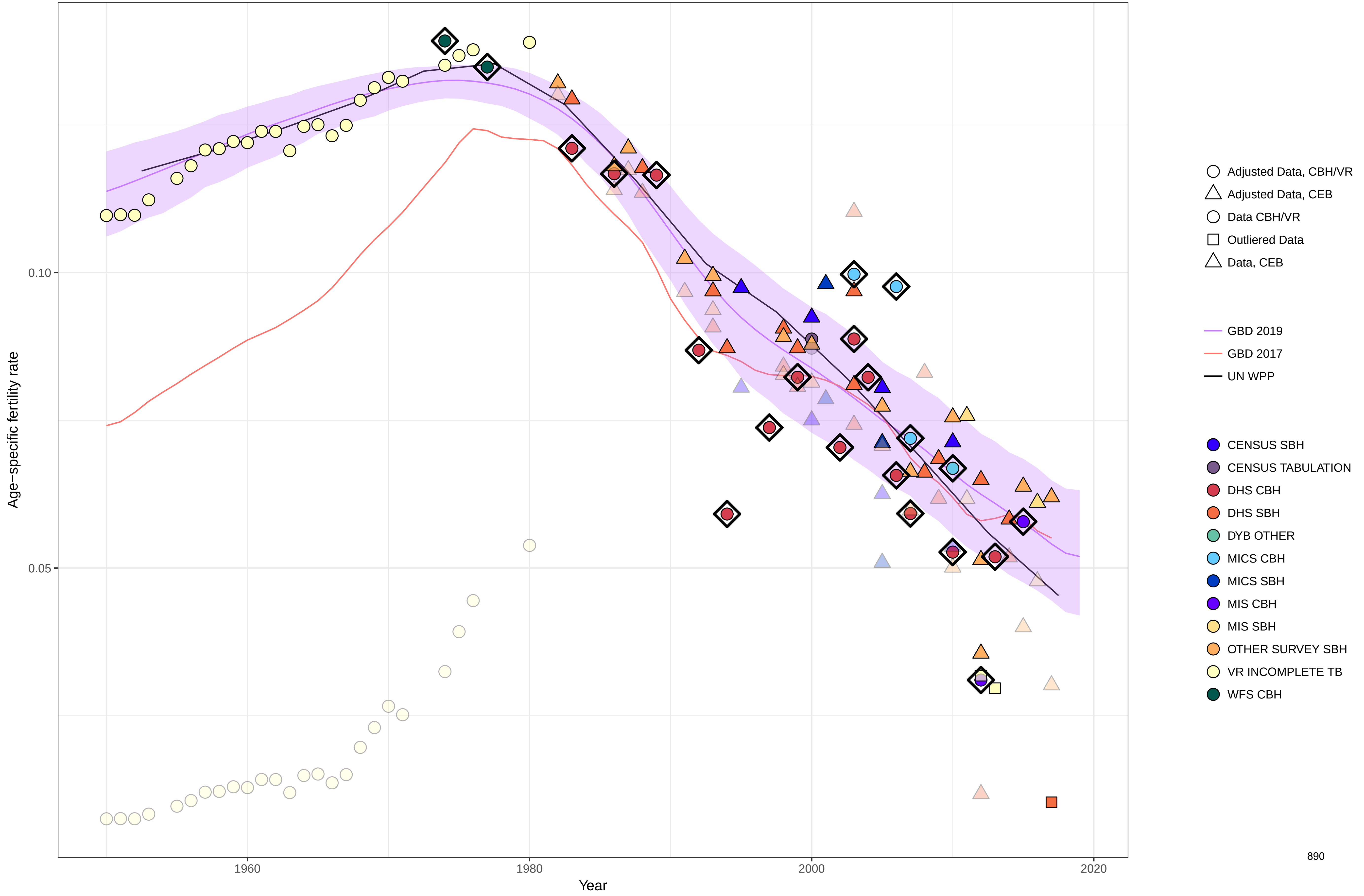

Age−specific fertility fitted trend lines, 1950−2019: Ghana, Ages: 45−49
Data points enclosed with a diamond are reference sources

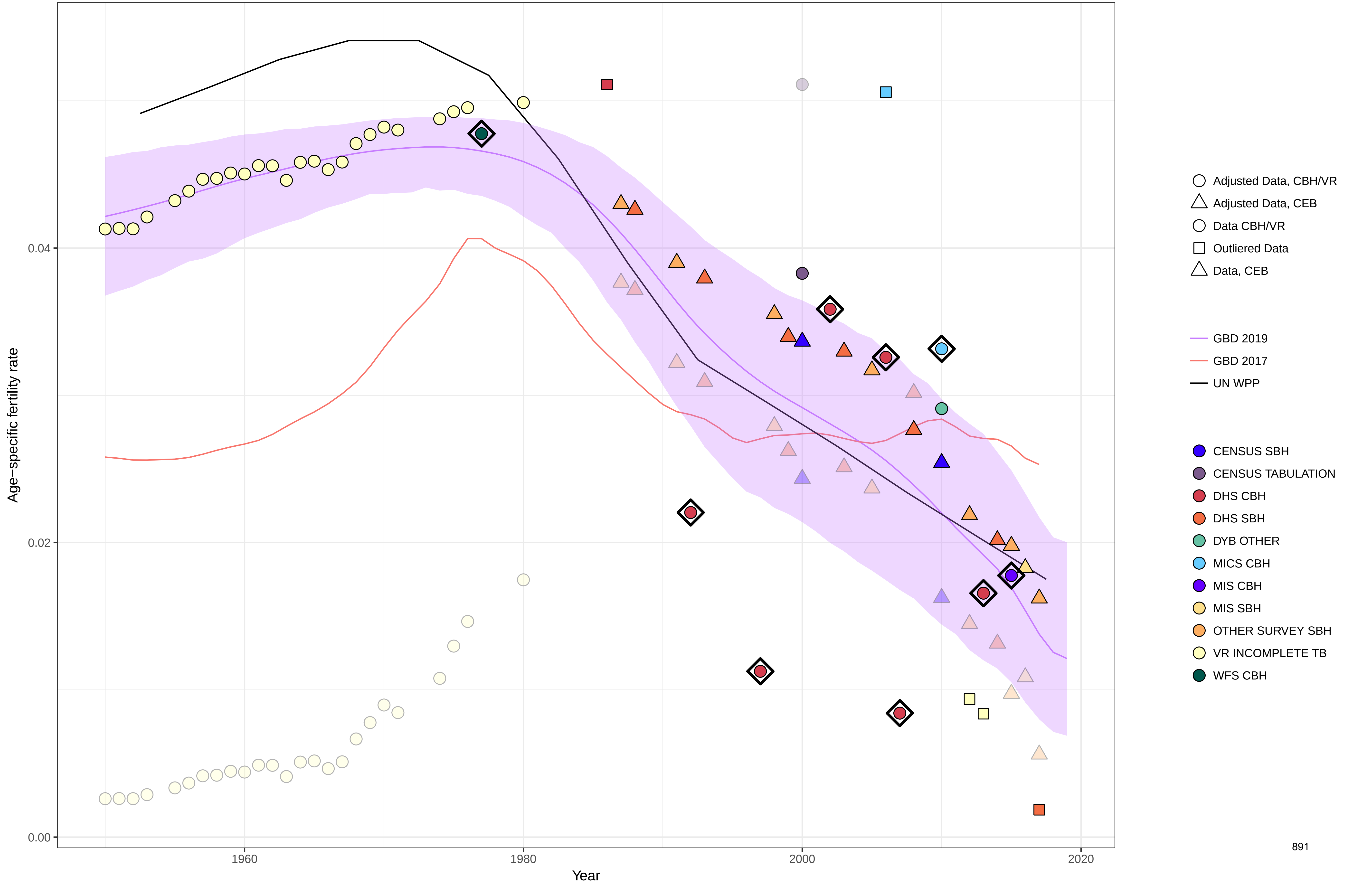

Age−specific fertility fitted trend lines, 1950−2019: Ghana, Ages: 50−54
Data points enclosed with a diamond are reference sources

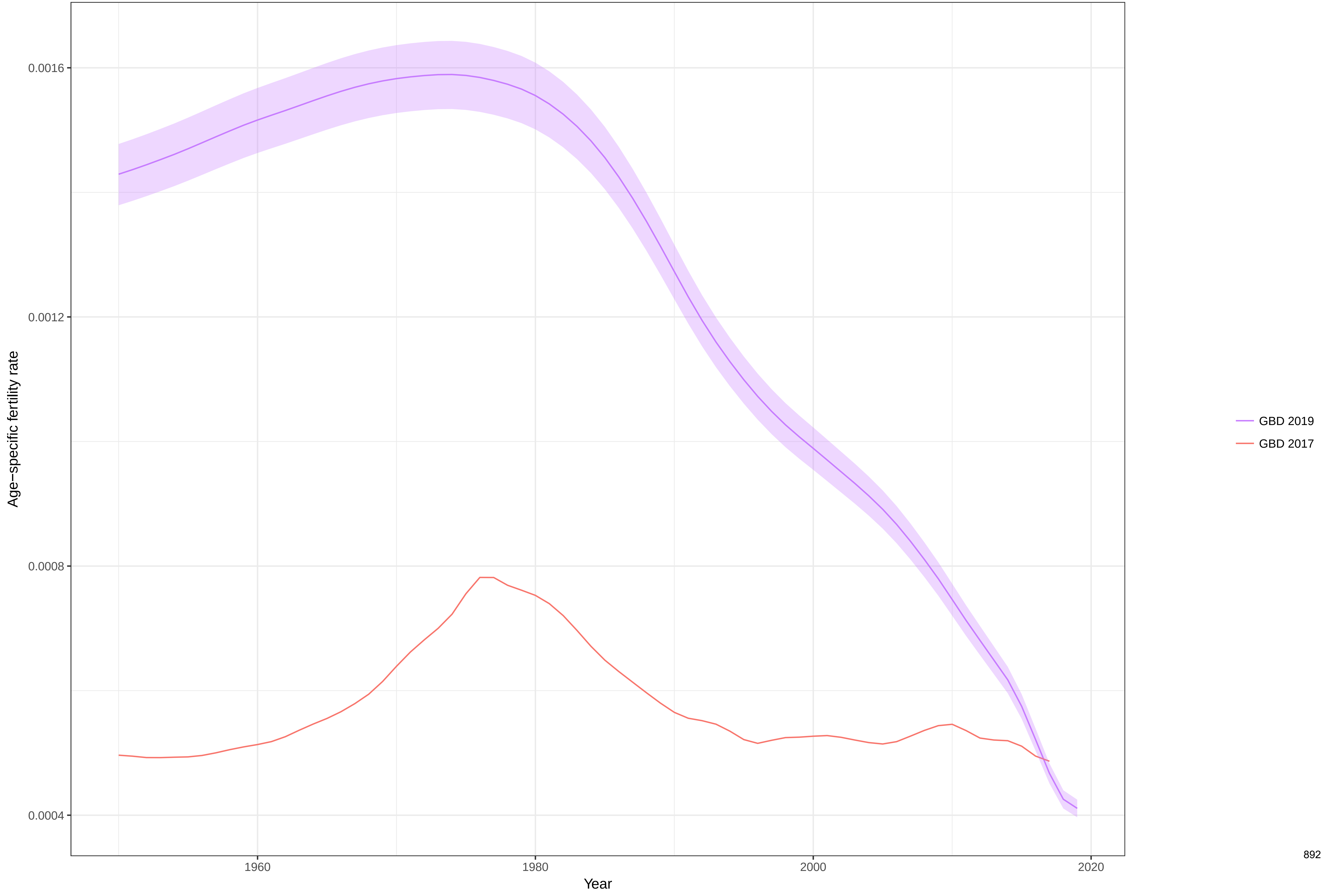

Total fertility rate fitted trend lines, 1950−2019: Ghana TFR
Data points enclosed with a diamond are reference sources

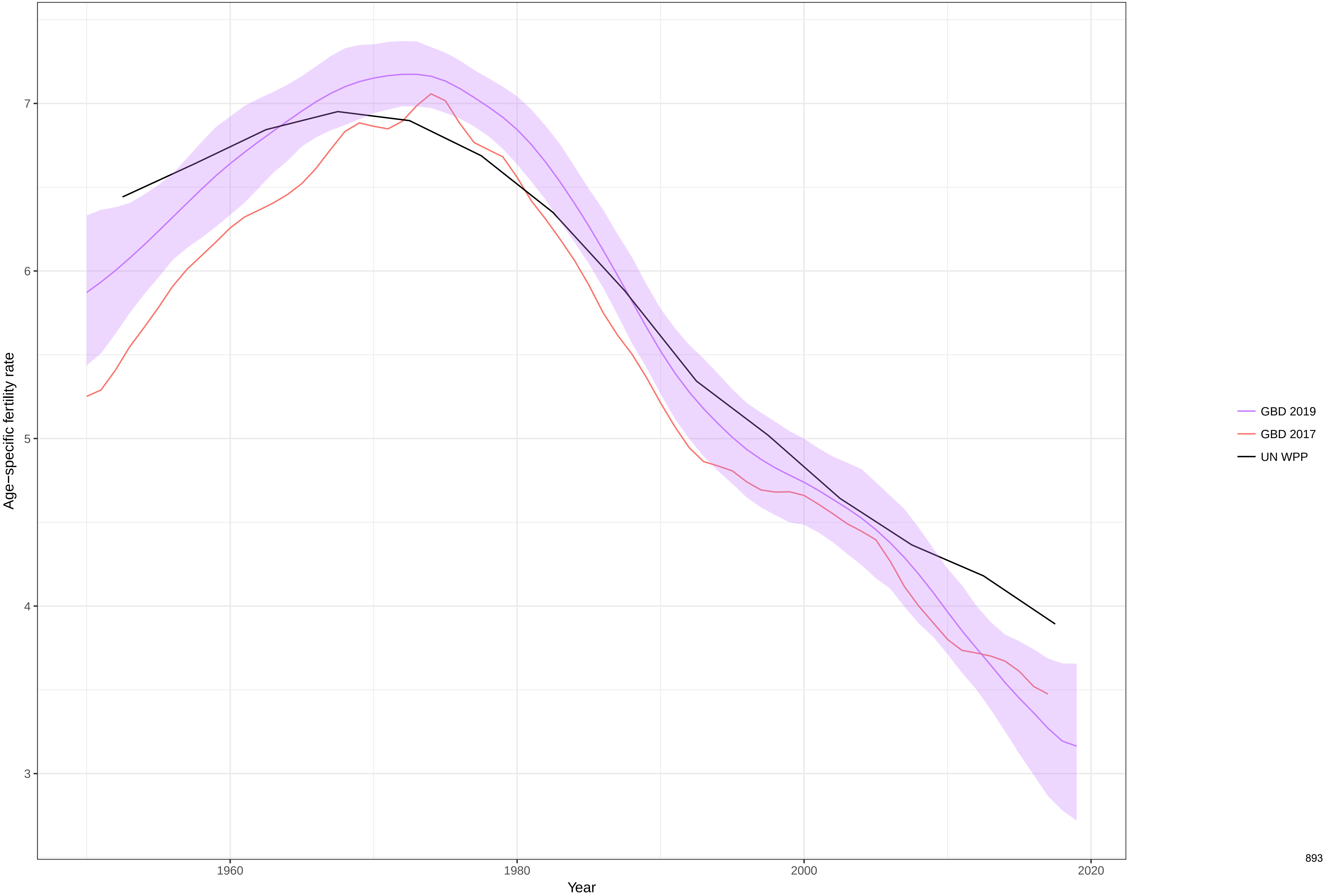

Age−specific fertility fitted trend lines, 1950−2019: Guinea, Ages: 10−14
Data points enclosed with a diamond are reference sources

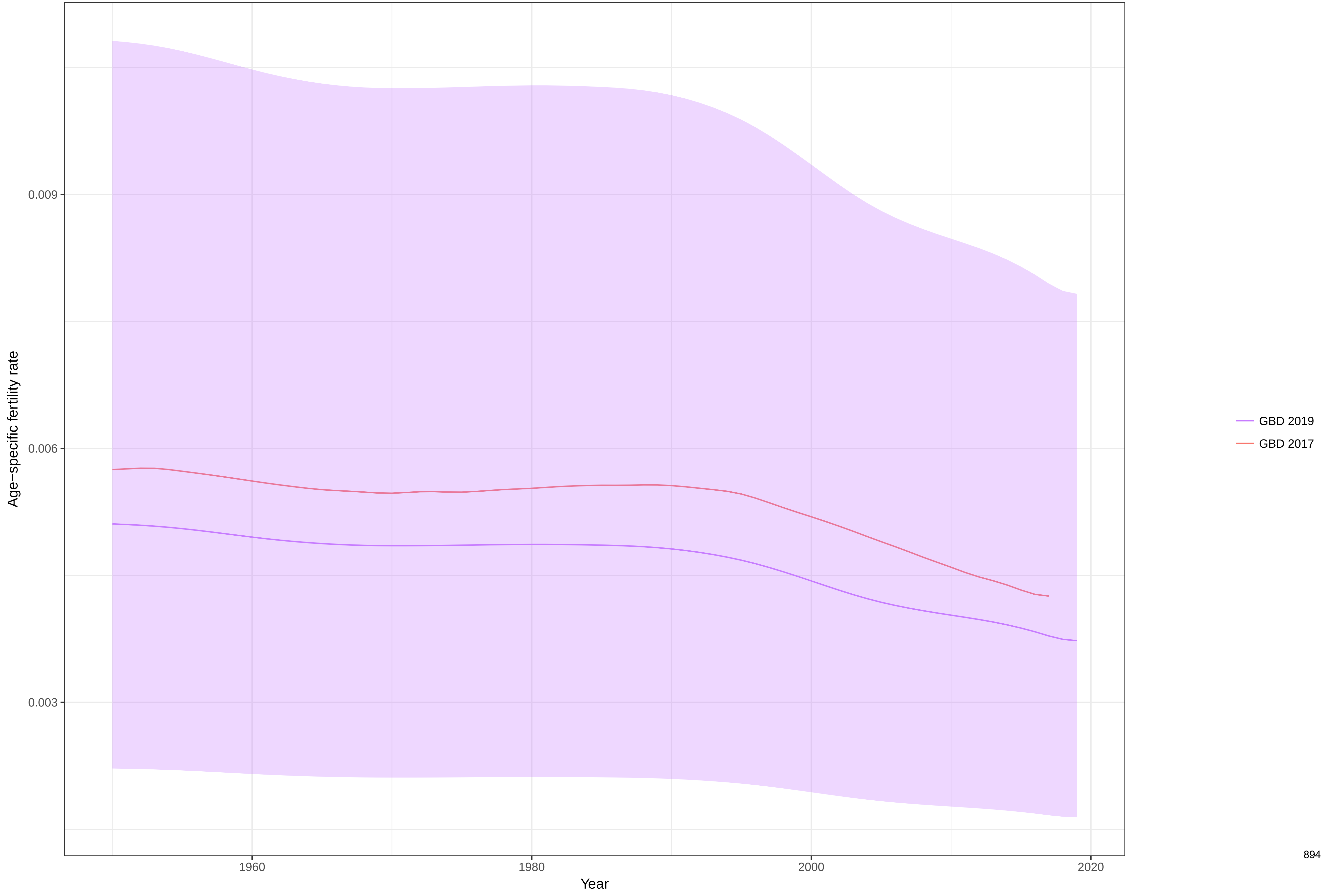

Age−specific fertility fitted trend lines, 1950−2019: Guinea, Ages: 15−19
Data points enclosed with a diamond are reference sources

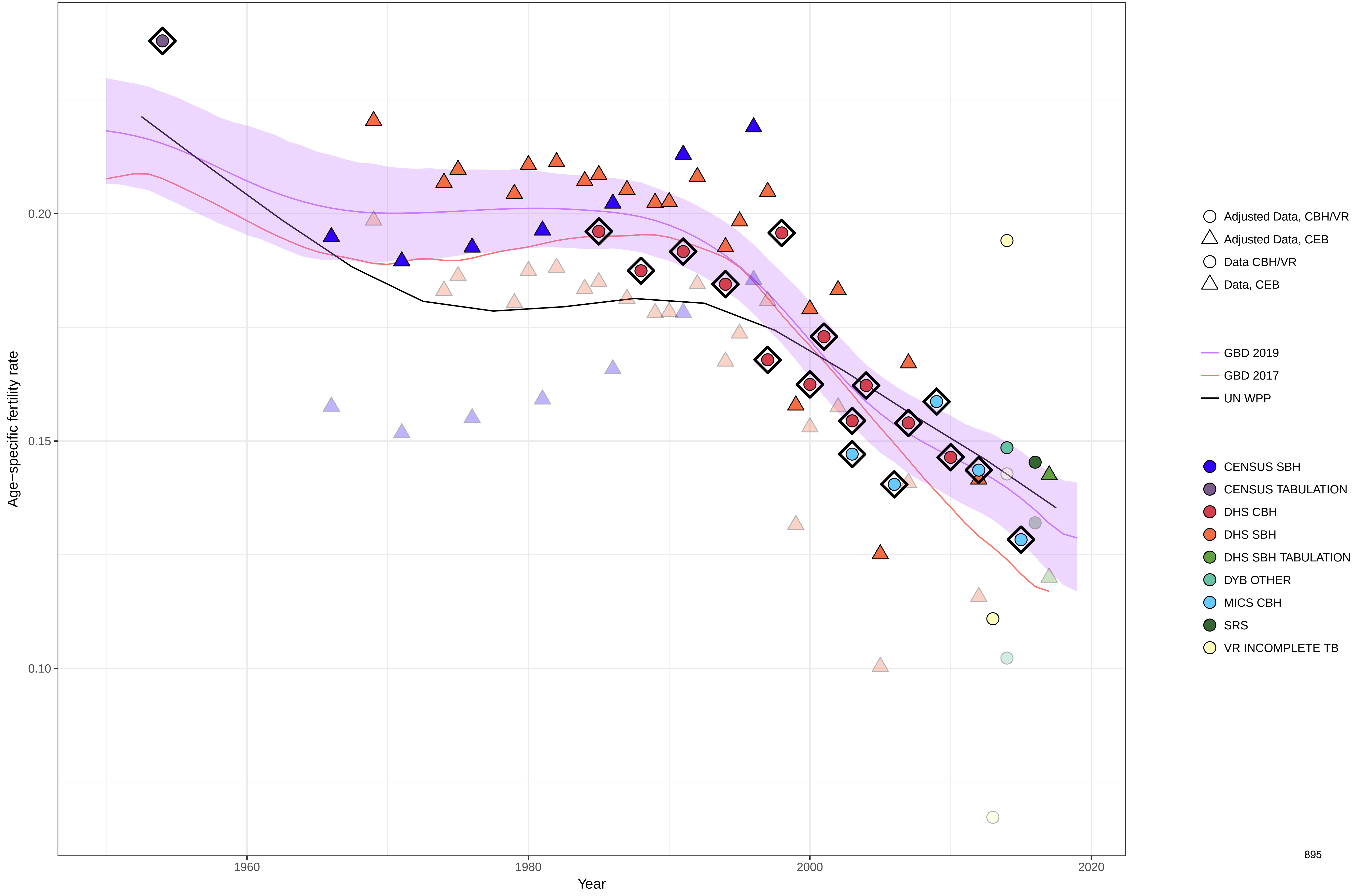

Age−specific fertility fitted trend lines, 1950−2019: Guinea, Ages: 20−24
Data points enclosed with a diamond are reference sources

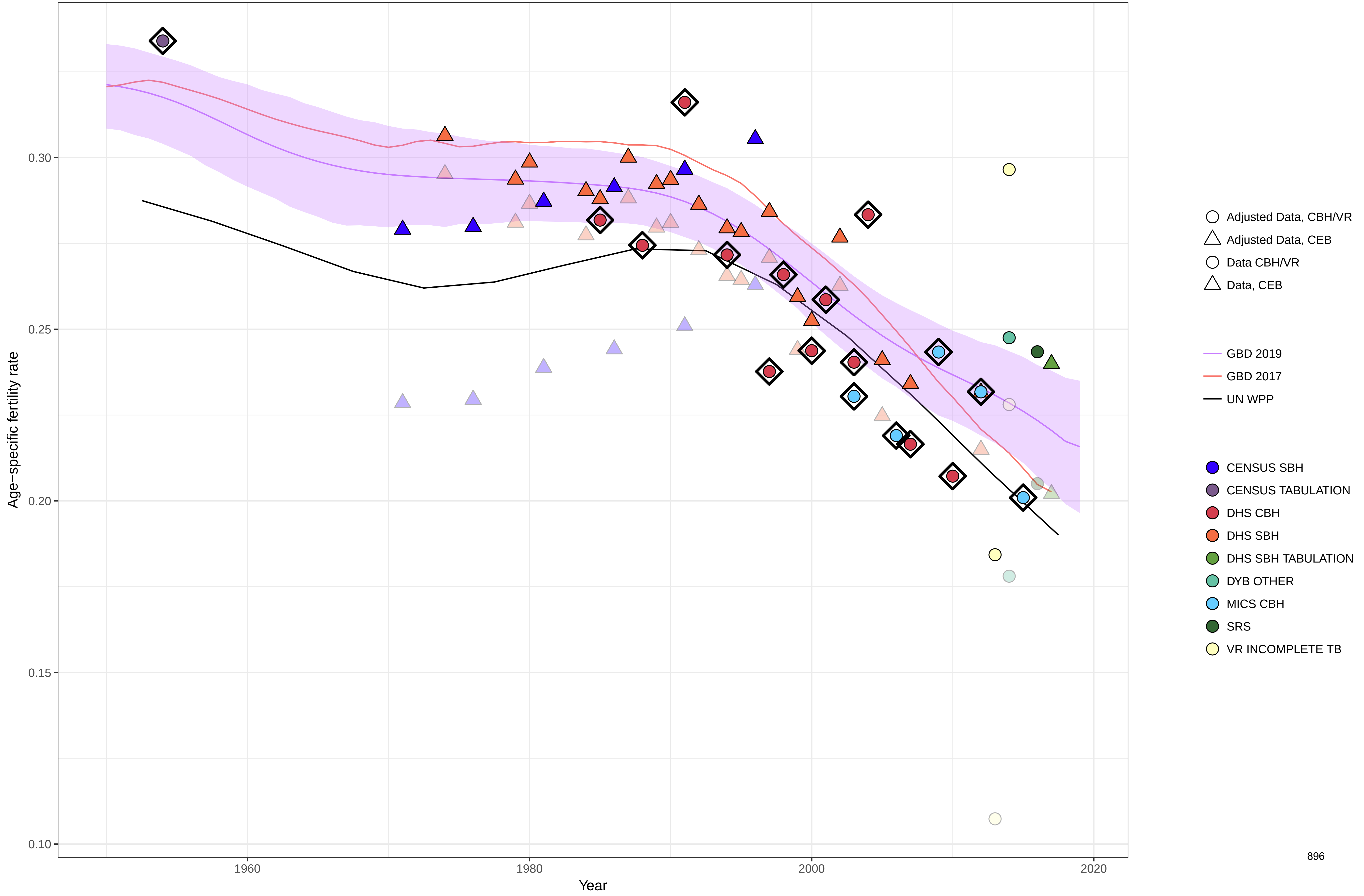

Age−specific fertility fitted trend lines, 1950−2019: Guinea, Ages: 25−29
Data points enclosed with a diamond are reference sources

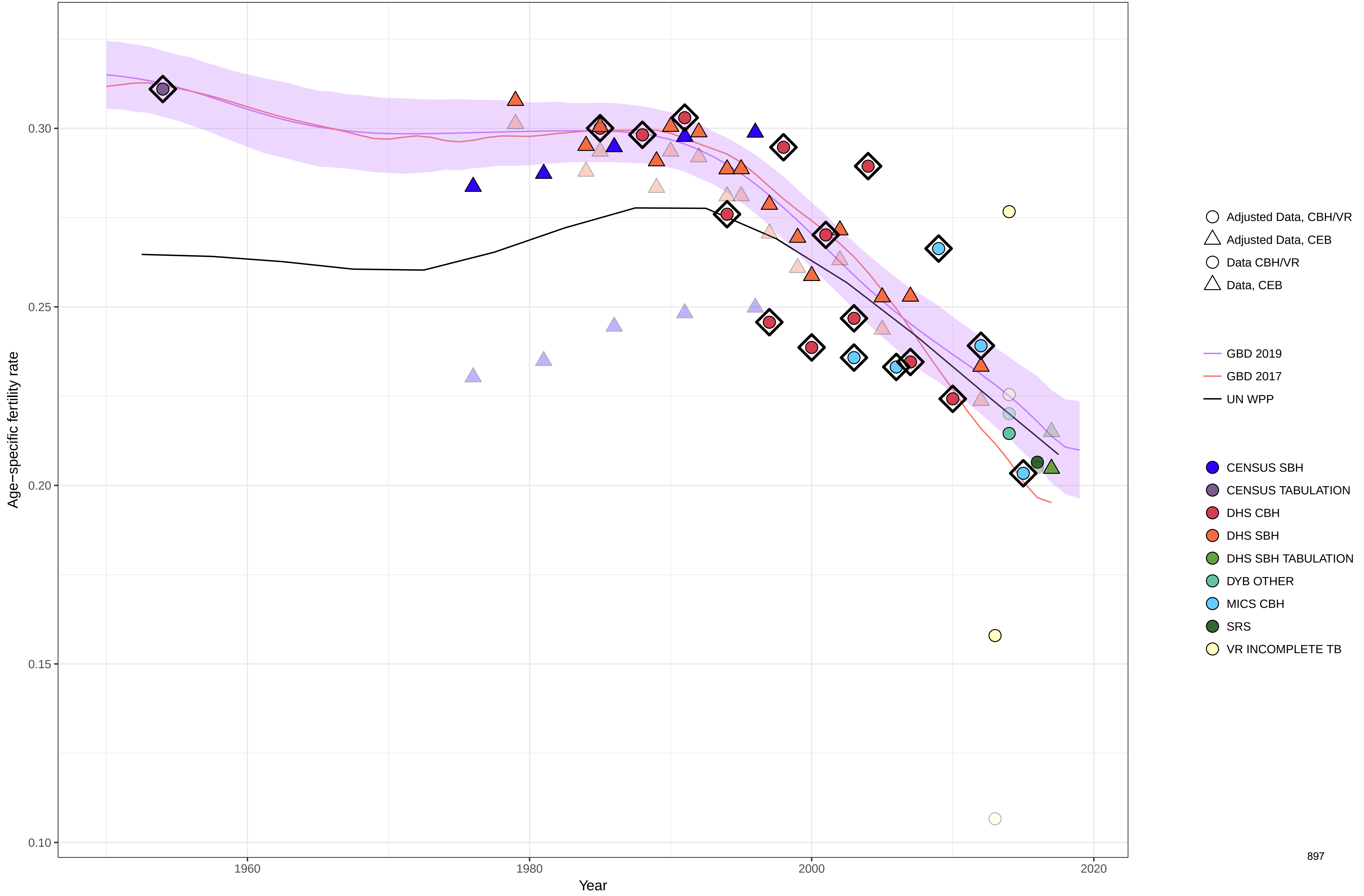

Age−specific fertility fitted trend lines, 1950−2019: Guinea, Ages: 30−34
Data points enclosed with a diamond are reference sources

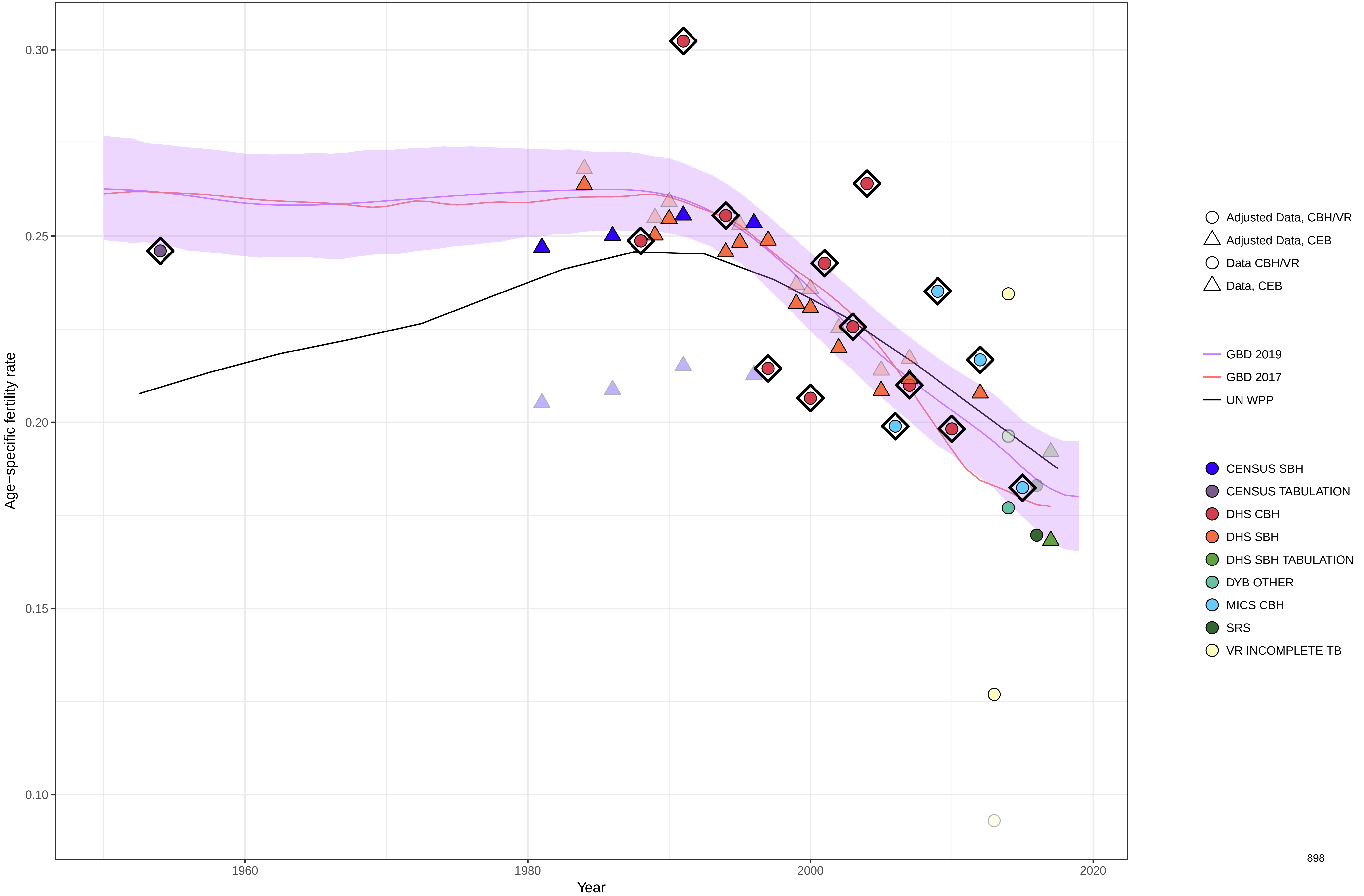

Age−specific fertility fitted trend lines, 1950−2019: Guinea, Ages: 35−39
Data points enclosed with a diamond are reference sources

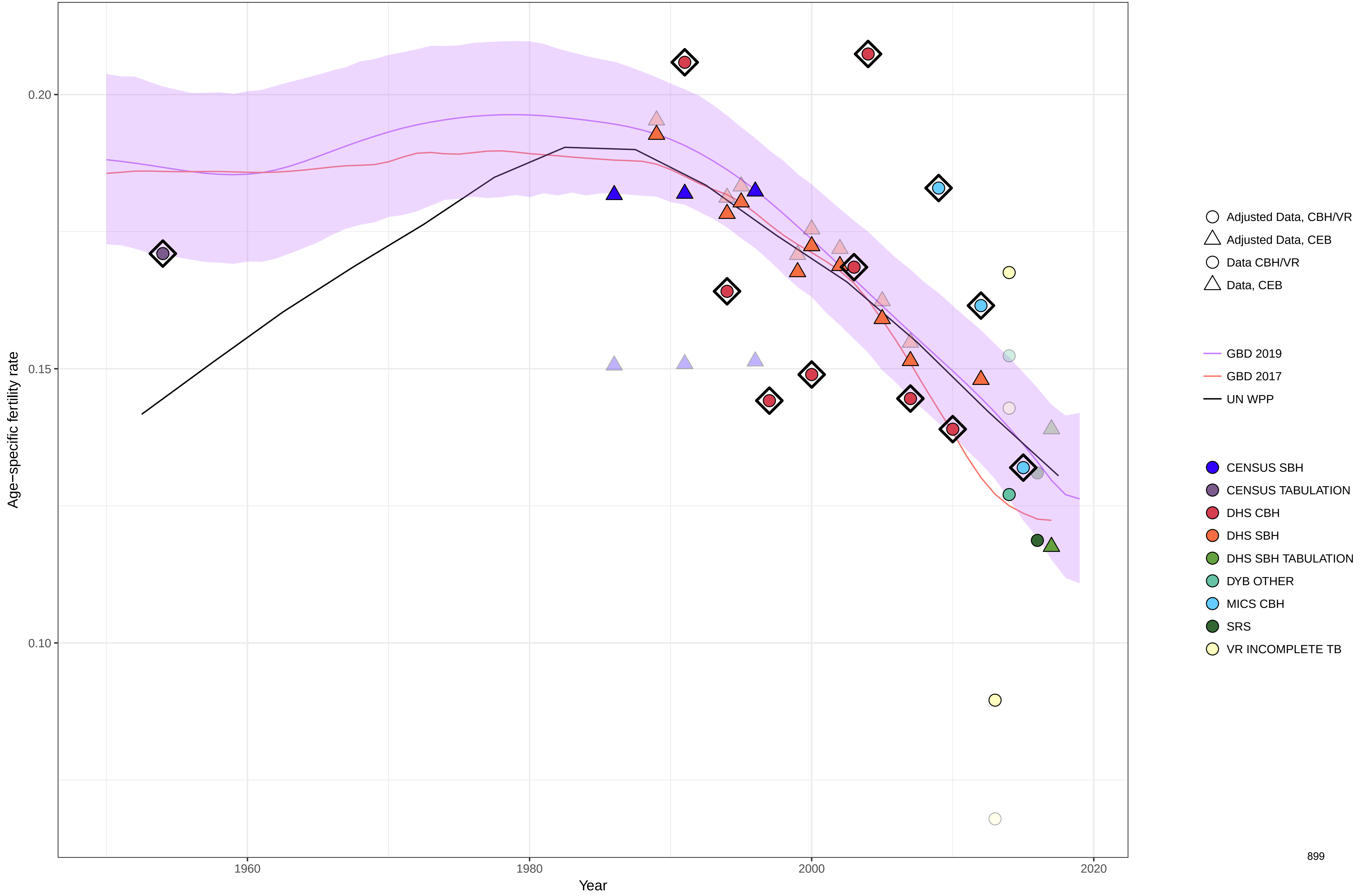

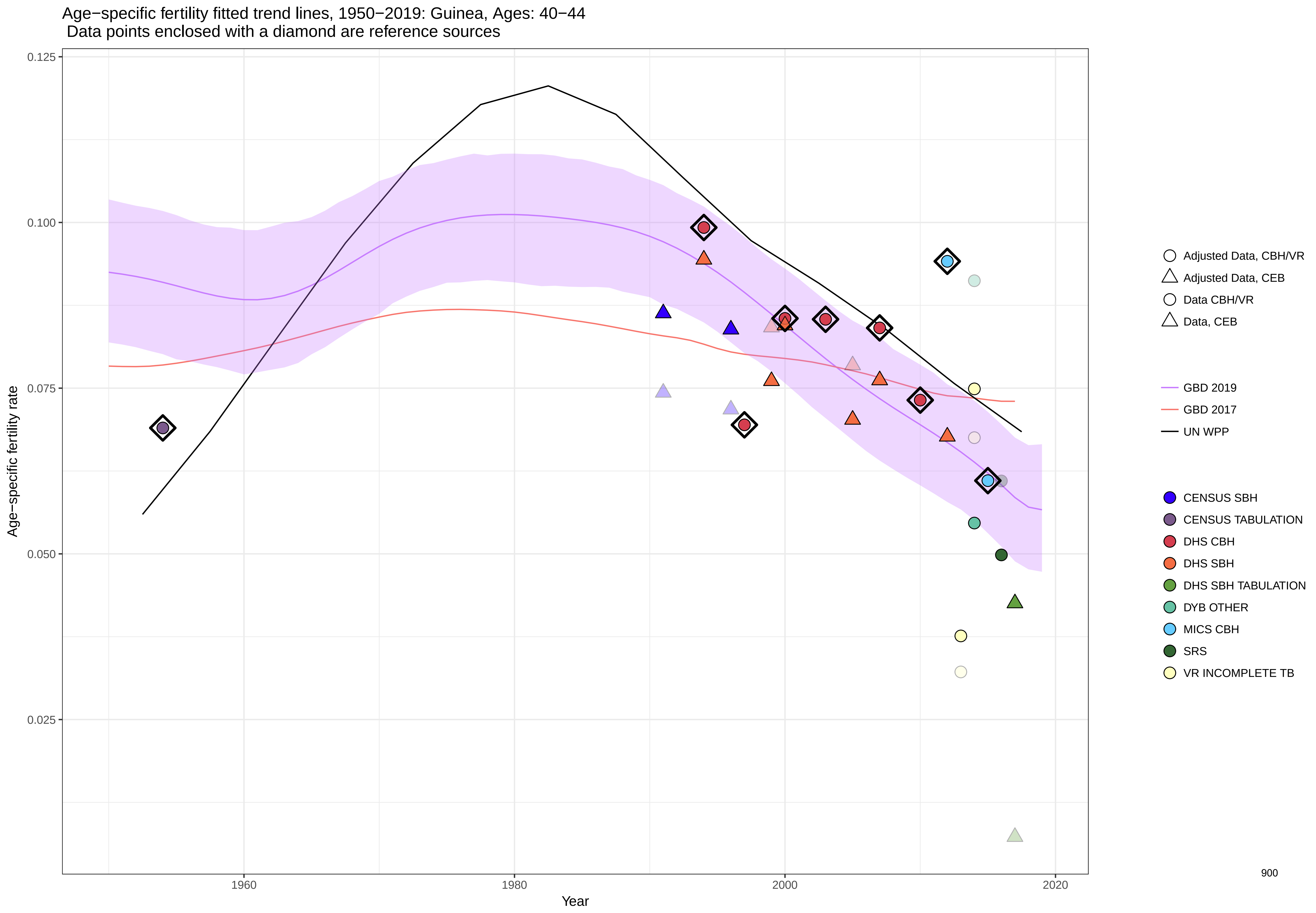
Age−specific fertility fitted trend lines, 1950−2019: Guinea, Ages: 40−44
Data points enclosed with a diamond are reference sources
0.125
0.100
0.075
0.050
0.025
Age−specific fertility rate
1960
1980
2000
2020
Year
Adjusted Data, CBH/VR
Adjusted Data, CEB
Data CBH/VR
Data, CEB
GBD 2019
GBD 2017
UN WPP
CENSUS SBH
CENSUS TABULATION
DHS CBH
DHS SBH
DHS SBH TABULATION
DYB OTHER
MICS CBH
SRS
VR INCOMPLETE TB

Age−specific fertility fitted trend lines, 1950−2019: Guinea, Ages: 45−49
Data points enclosed with a diamond are reference sources

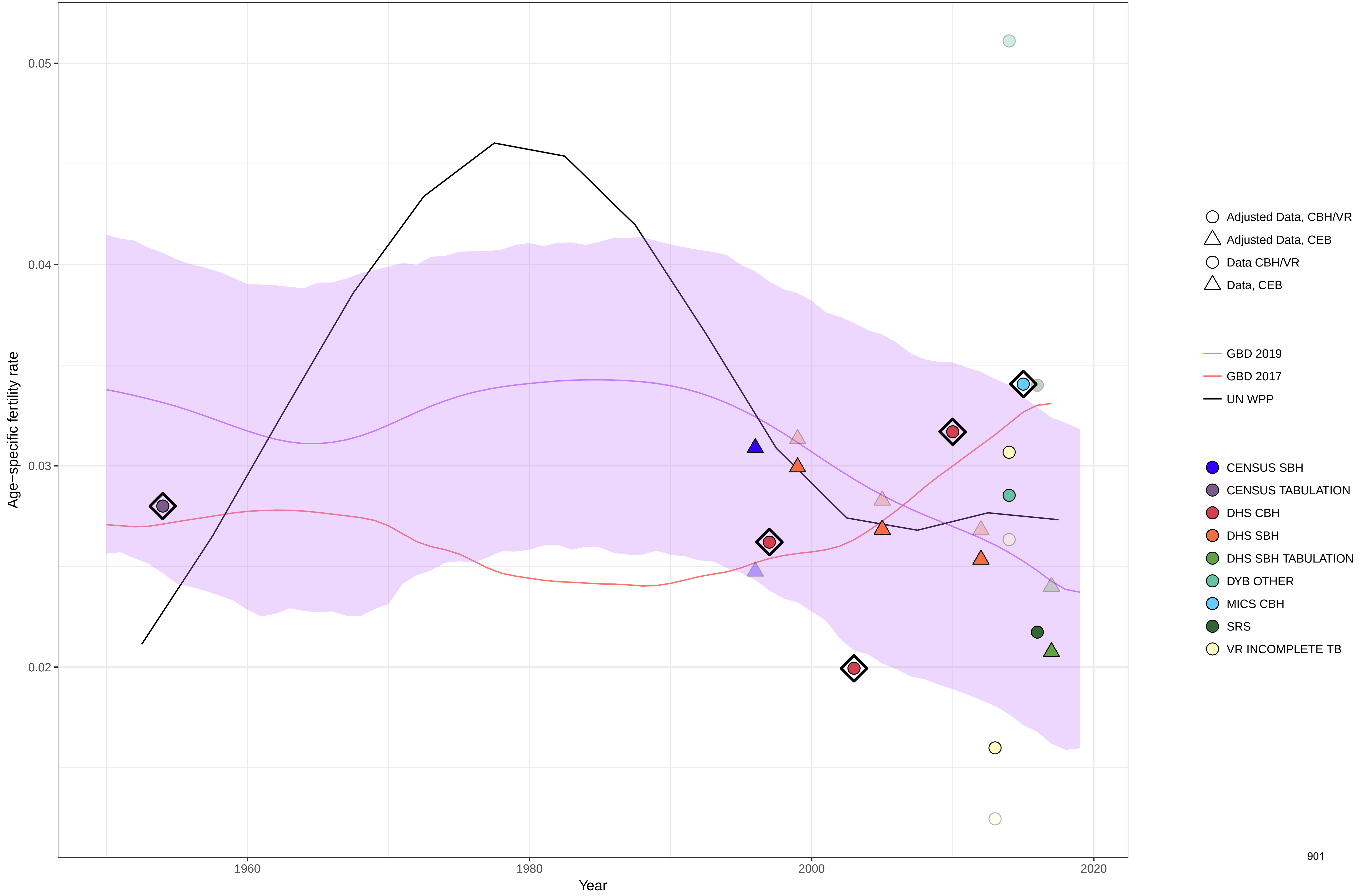

Age−specific fertility fitted trend lines, 1950−2019: Guinea, Ages: 50−54
Data points enclosed with a diamond are reference sources

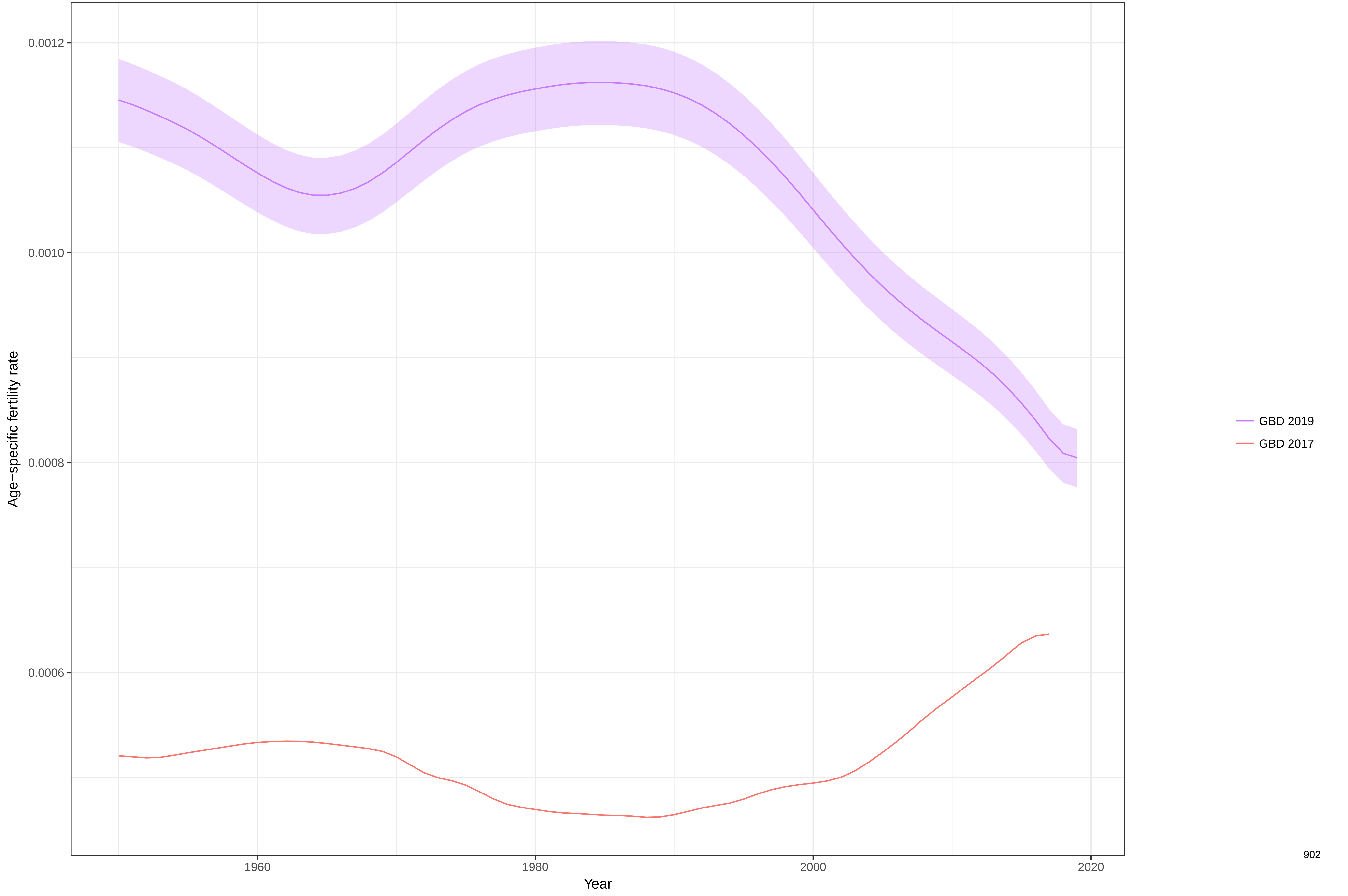

Total fertility rate fitted trend lines, 1950−2019: Guinea TFR
Data points enclosed with a diamond are reference sources

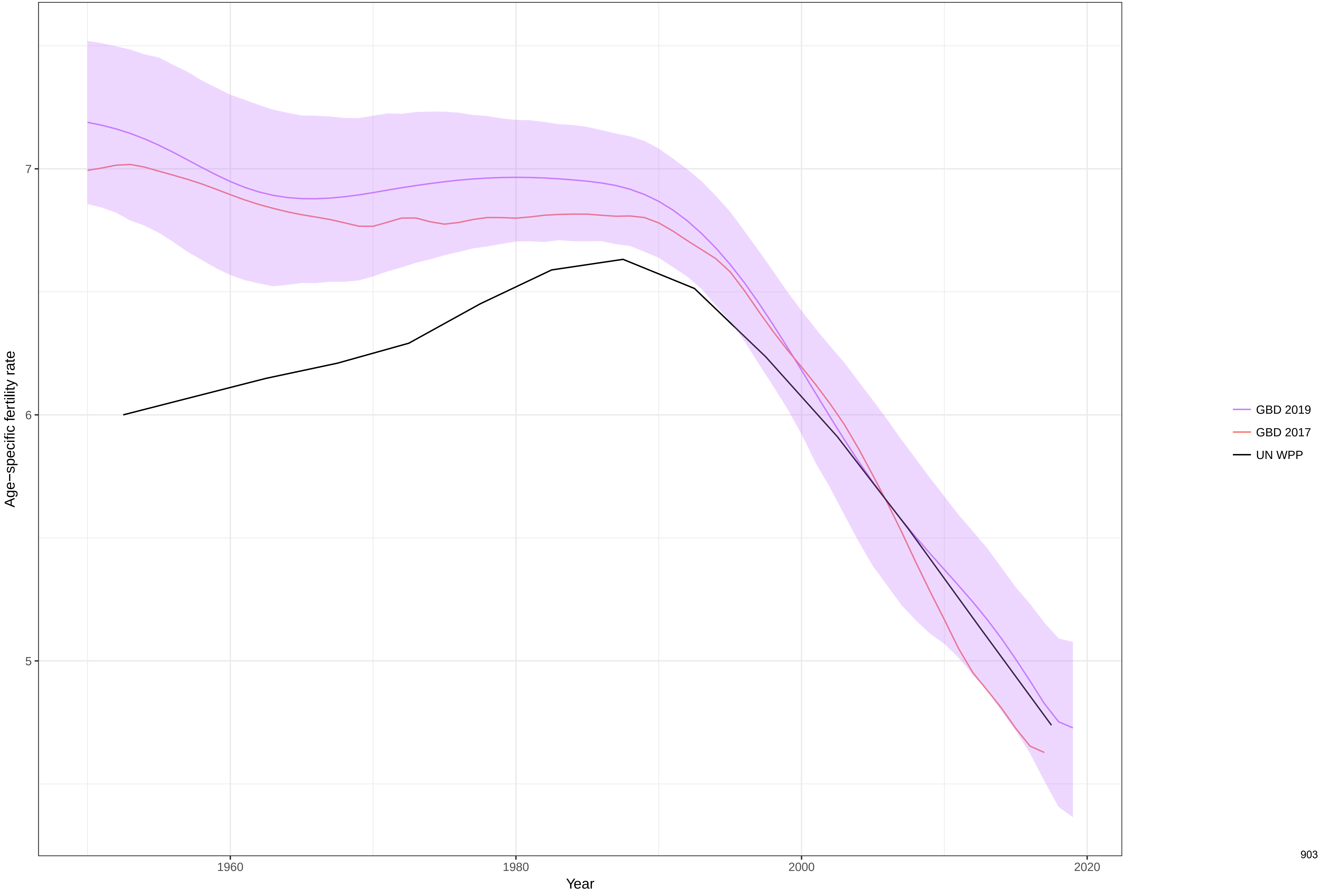

Age−specific fertility fitted trend lines, 1950−2019: Gambia, Ages: 10−14
Data points enclosed with a diamond are reference sources

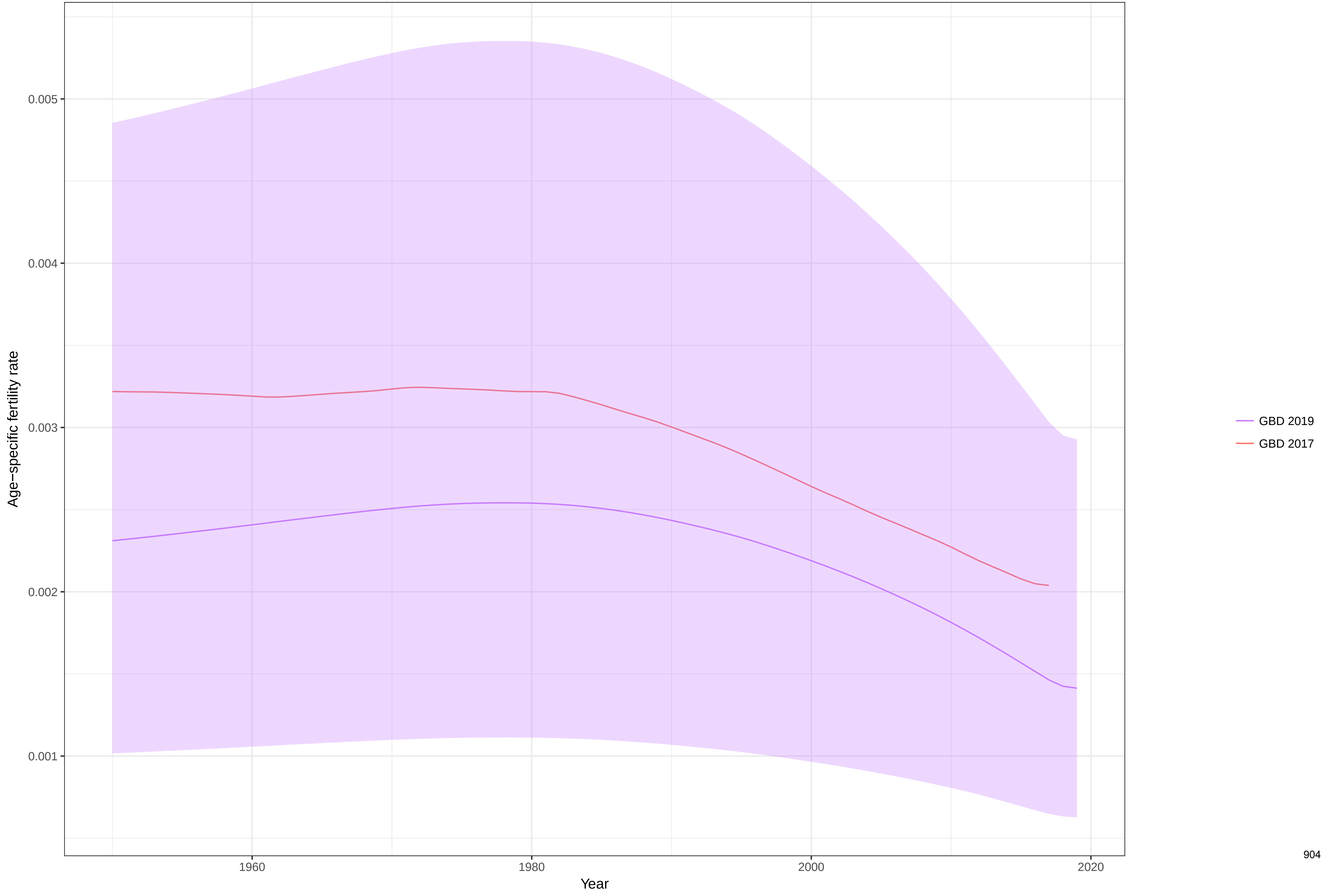

Age−specific fertility fitted trend lines, 1950−2019: Gambia, Ages: 15−19
Data points enclosed with a diamond are reference sources

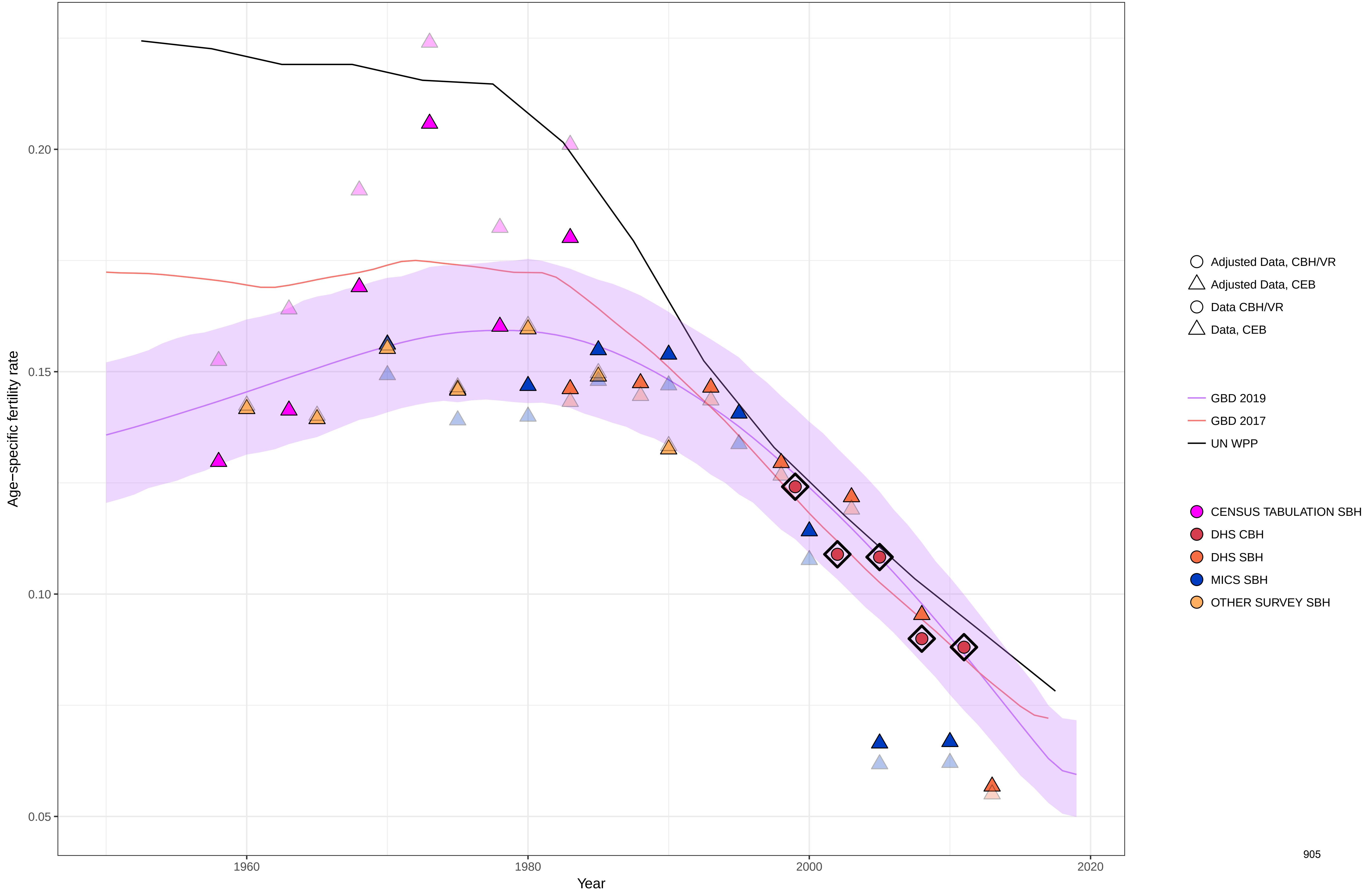

Age−specific fertility fitted trend lines, 1950−2019: Gambia, Ages: 20−24
Data points enclosed with a diamond are reference sources

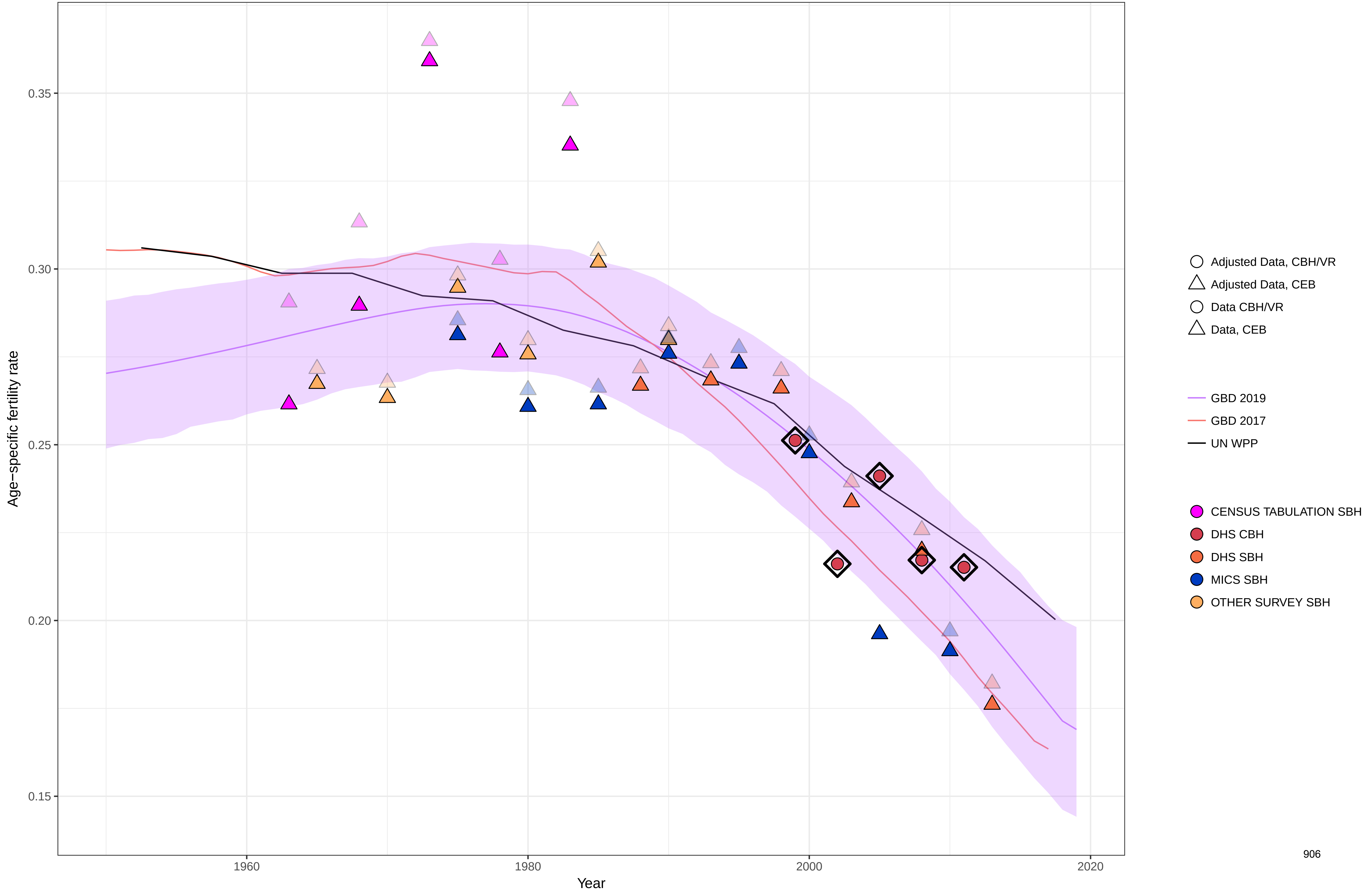

Age−specific fertility fitted trend lines, 1950−2019: Gambia, Ages: 25−29
Data points enclosed with a diamond are reference sources

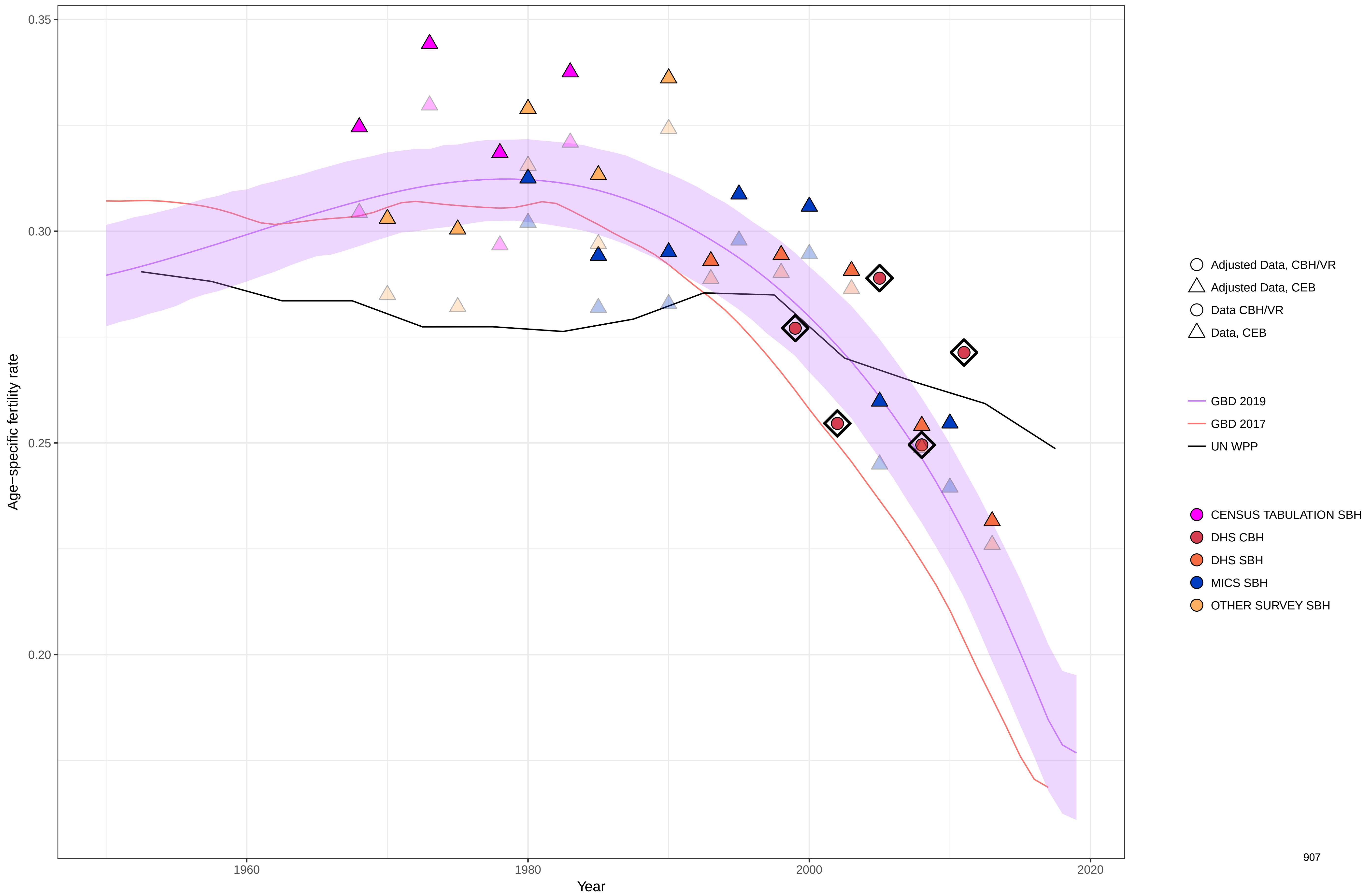

Age−specific fertility fitted trend lines, 1950−2019: Gambia, Ages: 30−34
Data points enclosed with a diamond are reference sources

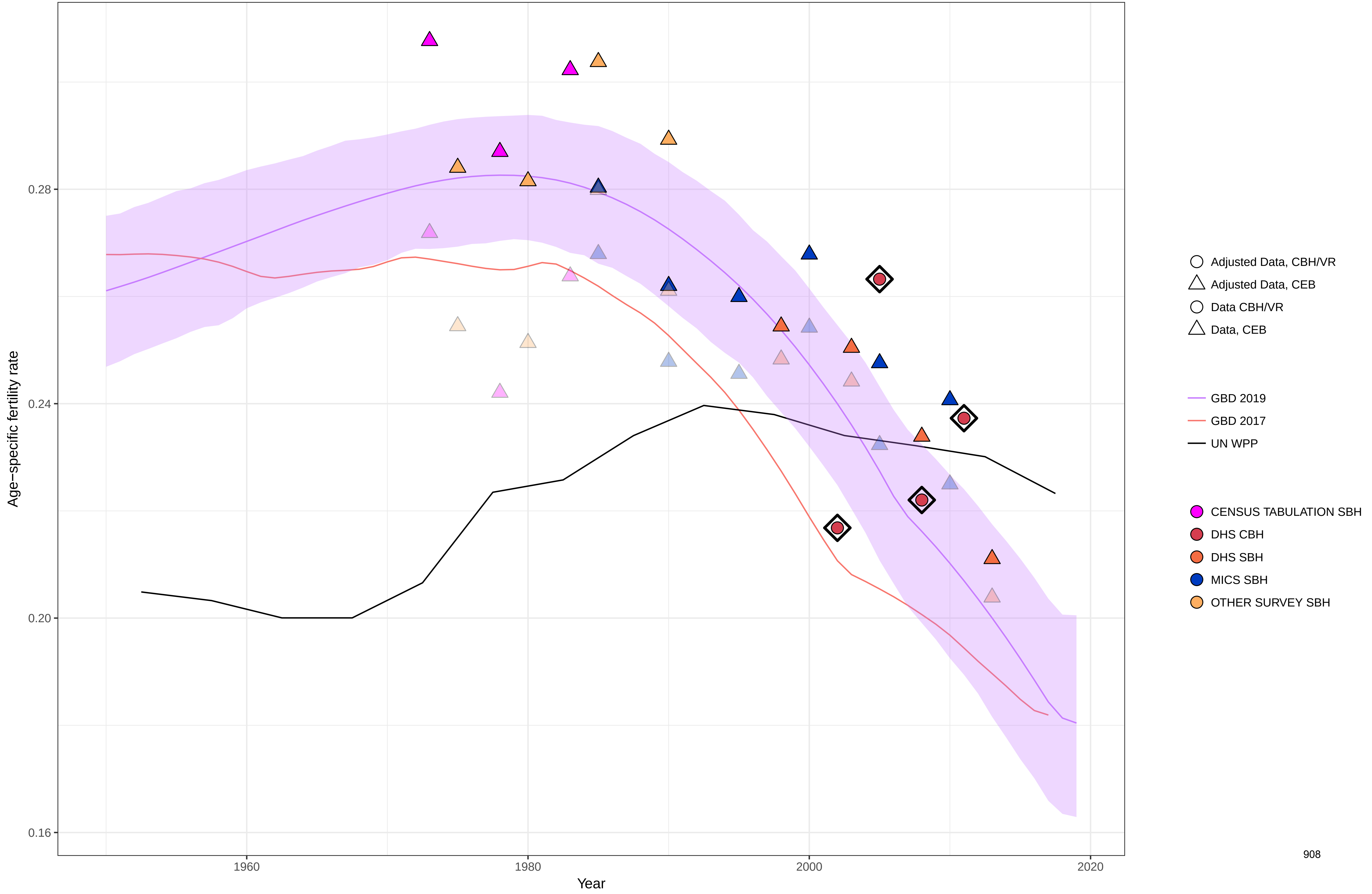

Age−specific fertility fitted trend lines, 1950−2019: Gambia, Ages: 35−39
Data points enclosed with a diamond are reference sources

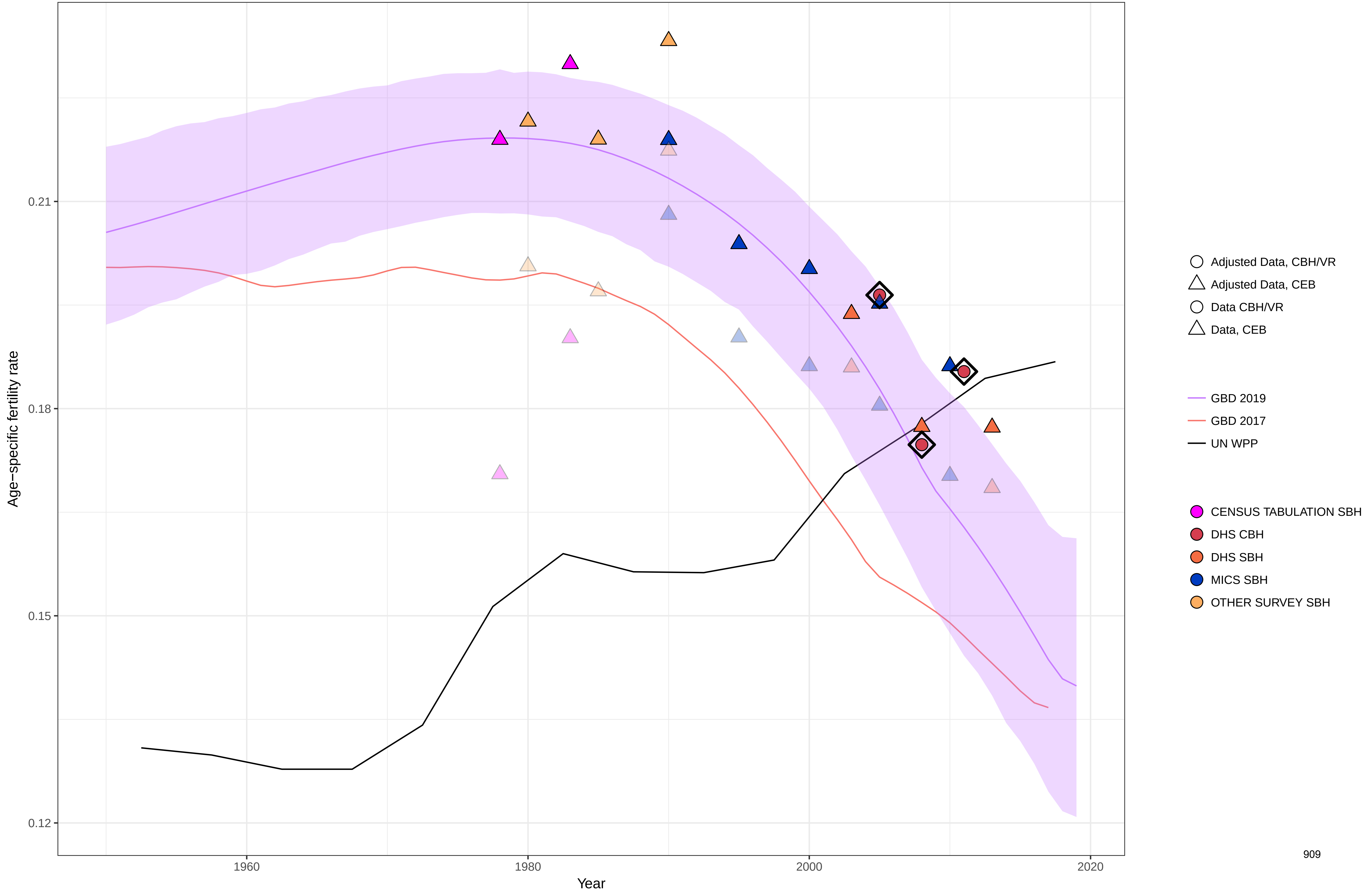

Age−specific fertility fitted trend lines, 1950−2019: Gambia, Ages: 40−44
Data points enclosed with a diamond are reference sources

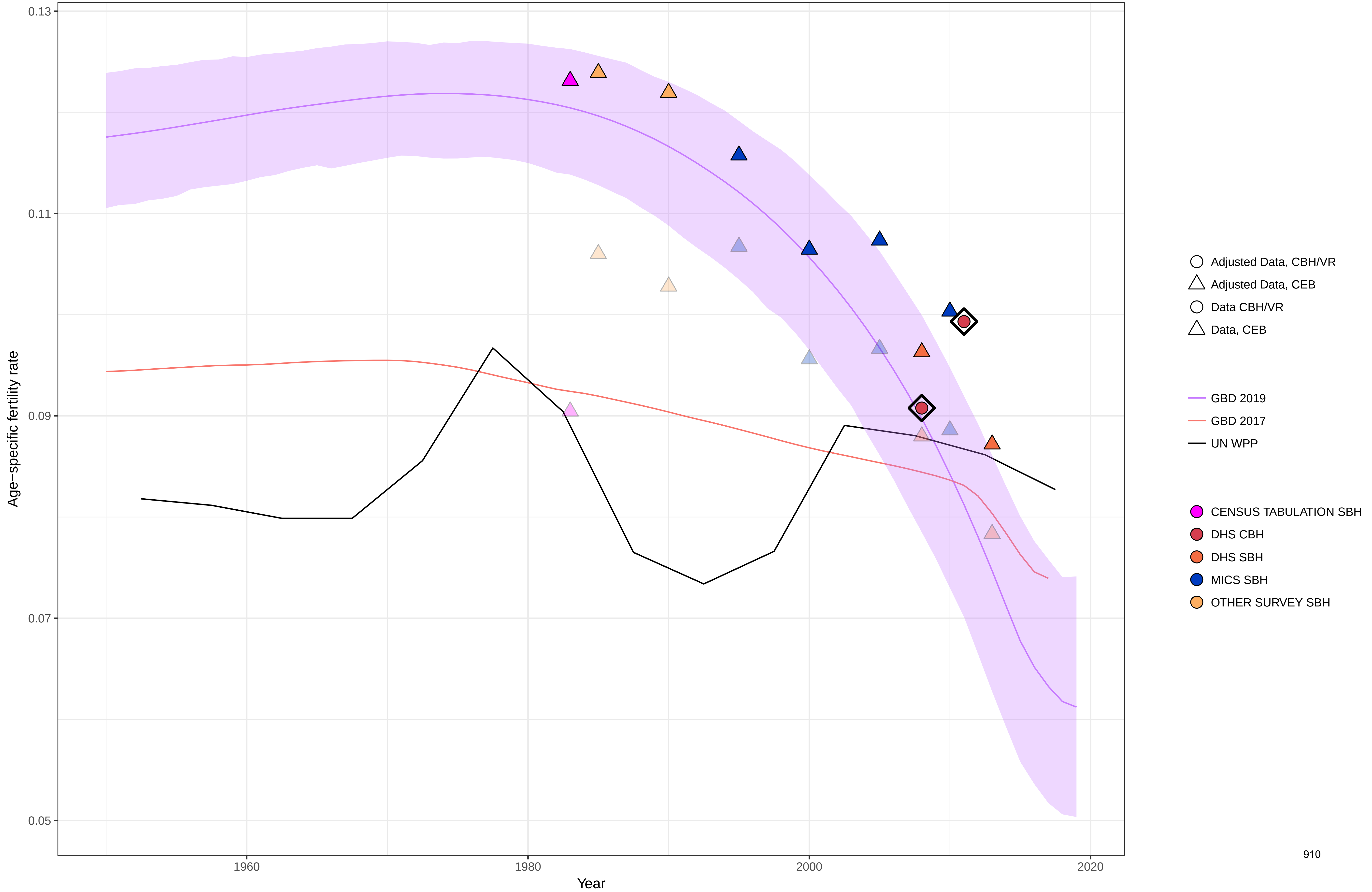

Age−specific fertility fitted trend lines, 1950−2019: Gambia, Ages: 45−49
Data points enclosed with a diamond are reference sources

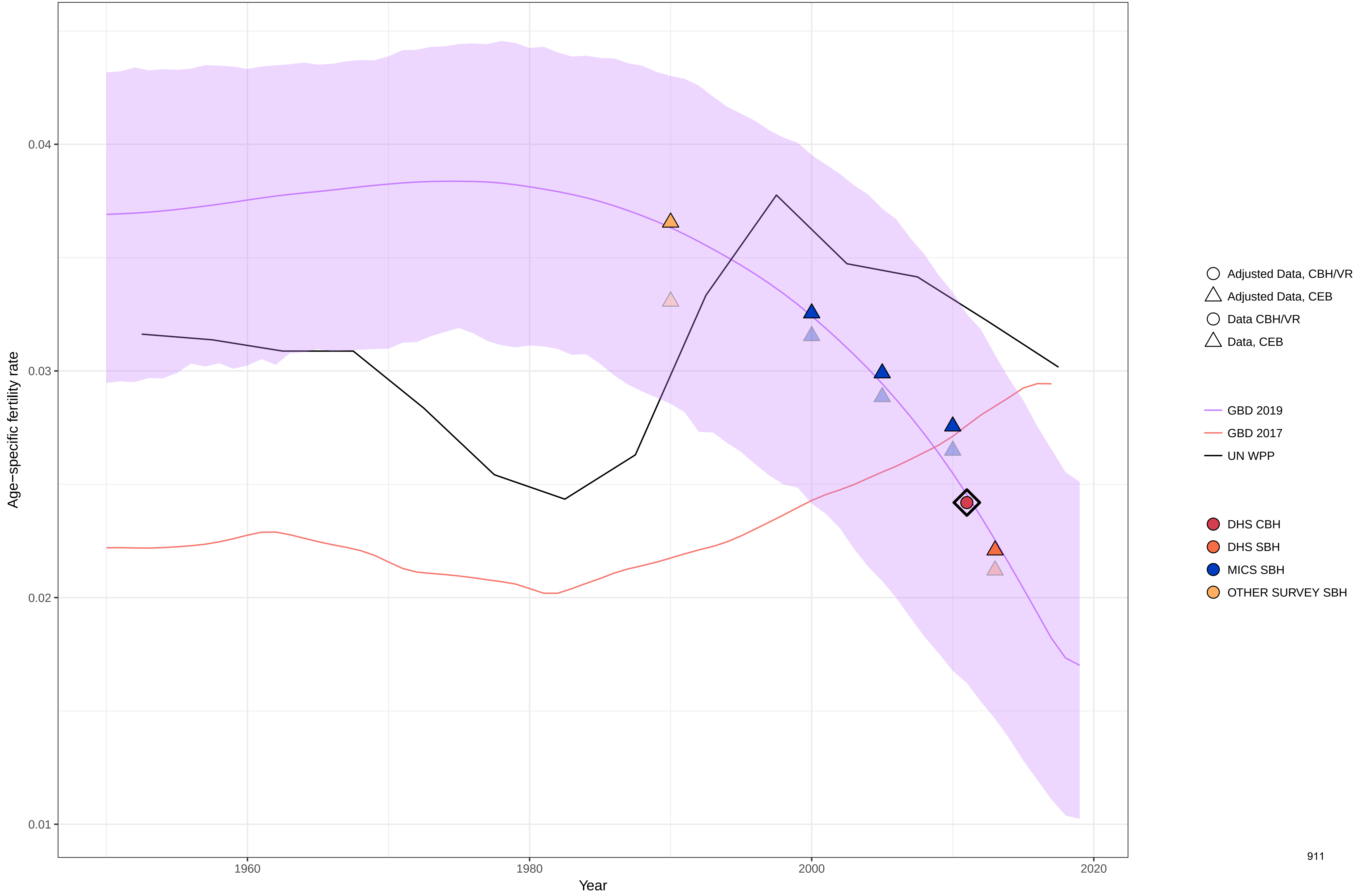

Age−specific fertility fitted trend lines, 1950−2019: Gambia, Ages: 50−54
Data points enclosed with a diamond are reference sources

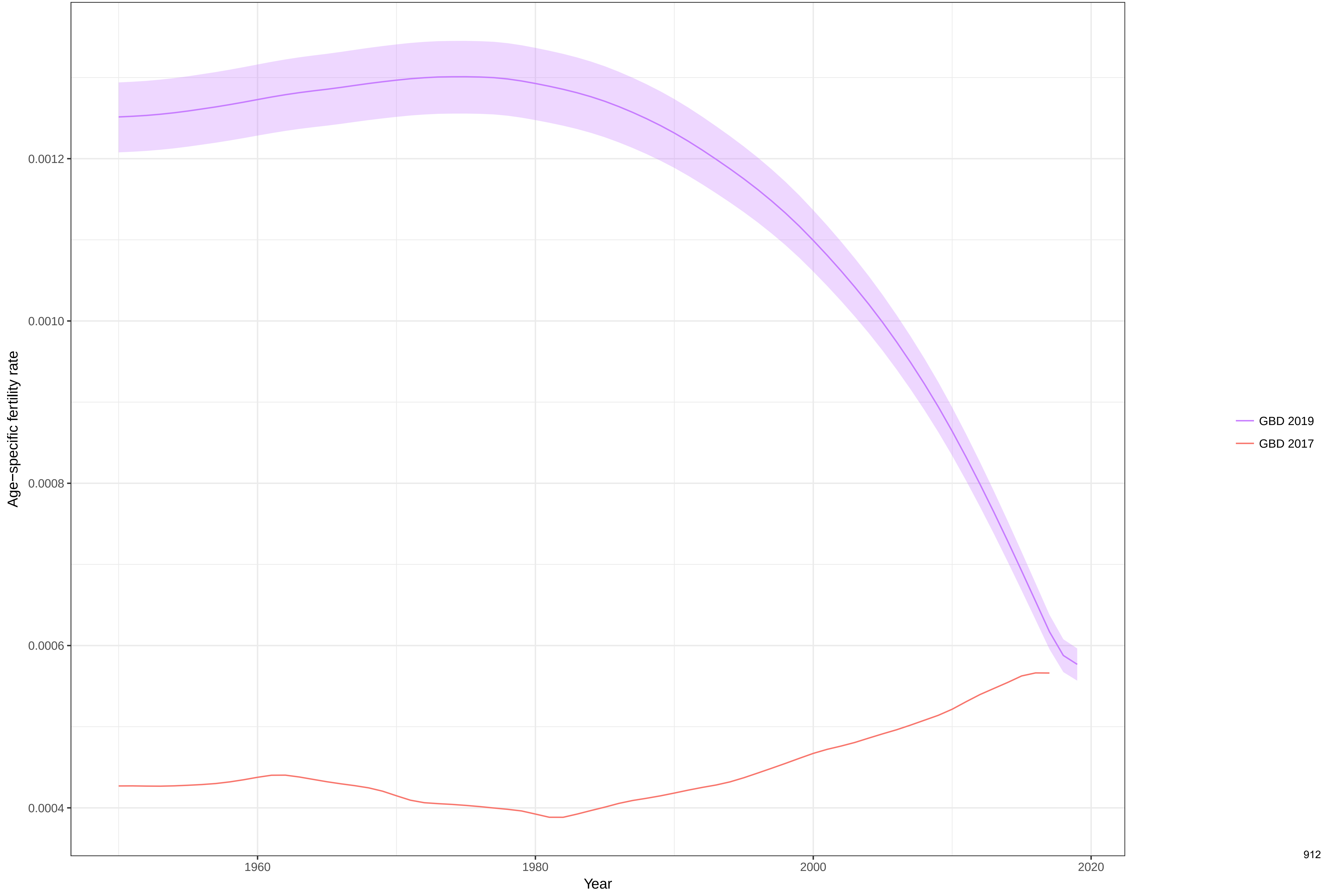

Total fertility rate fitted trend lines, 1950−2019: Gambia TFR
Data points enclosed with a diamond are reference sources

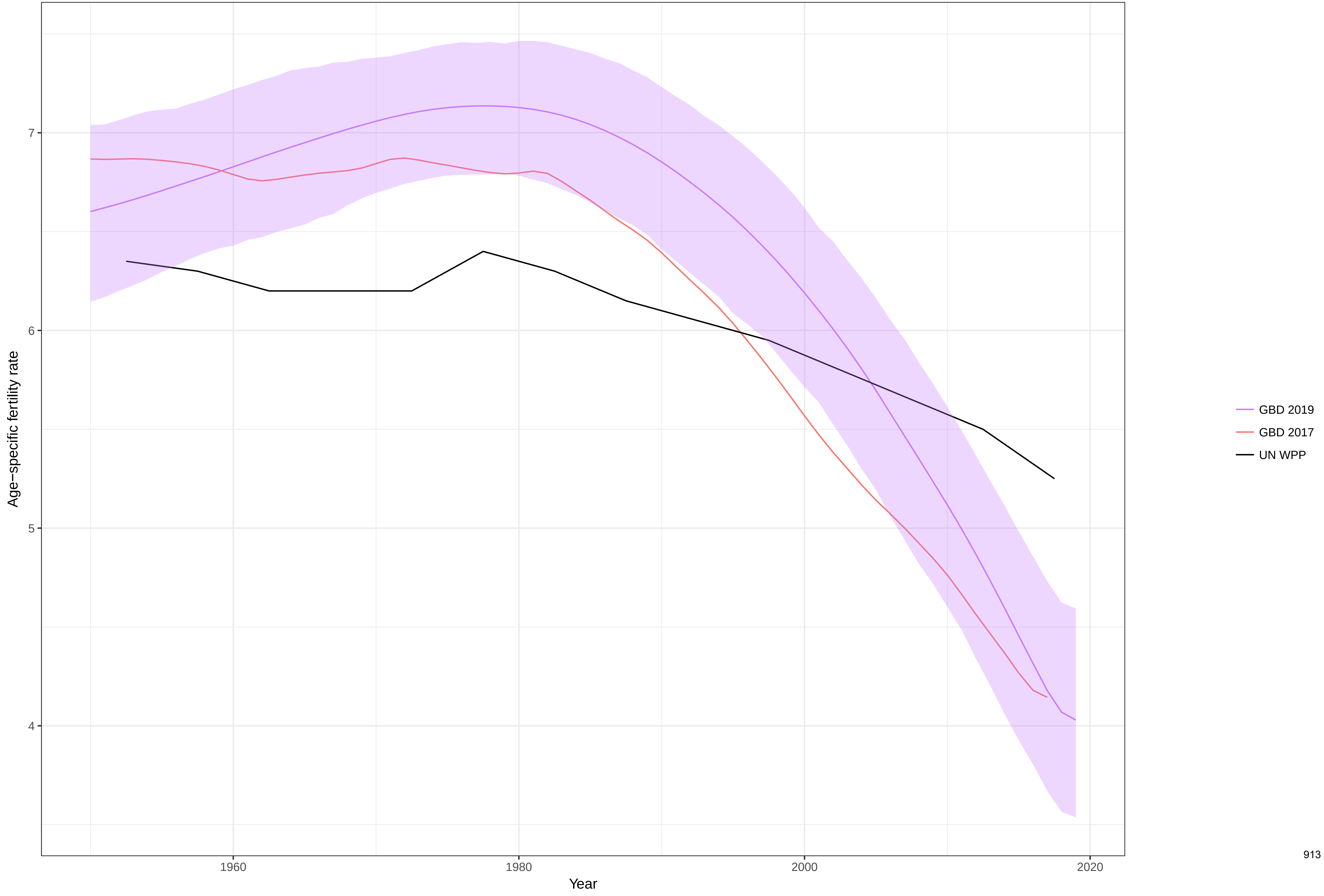

Age−specific fertility fitted trend lines, 1950−2019: Guinea−Bissau, Ages: 10−14
Data points enclosed with a diamond are reference sources

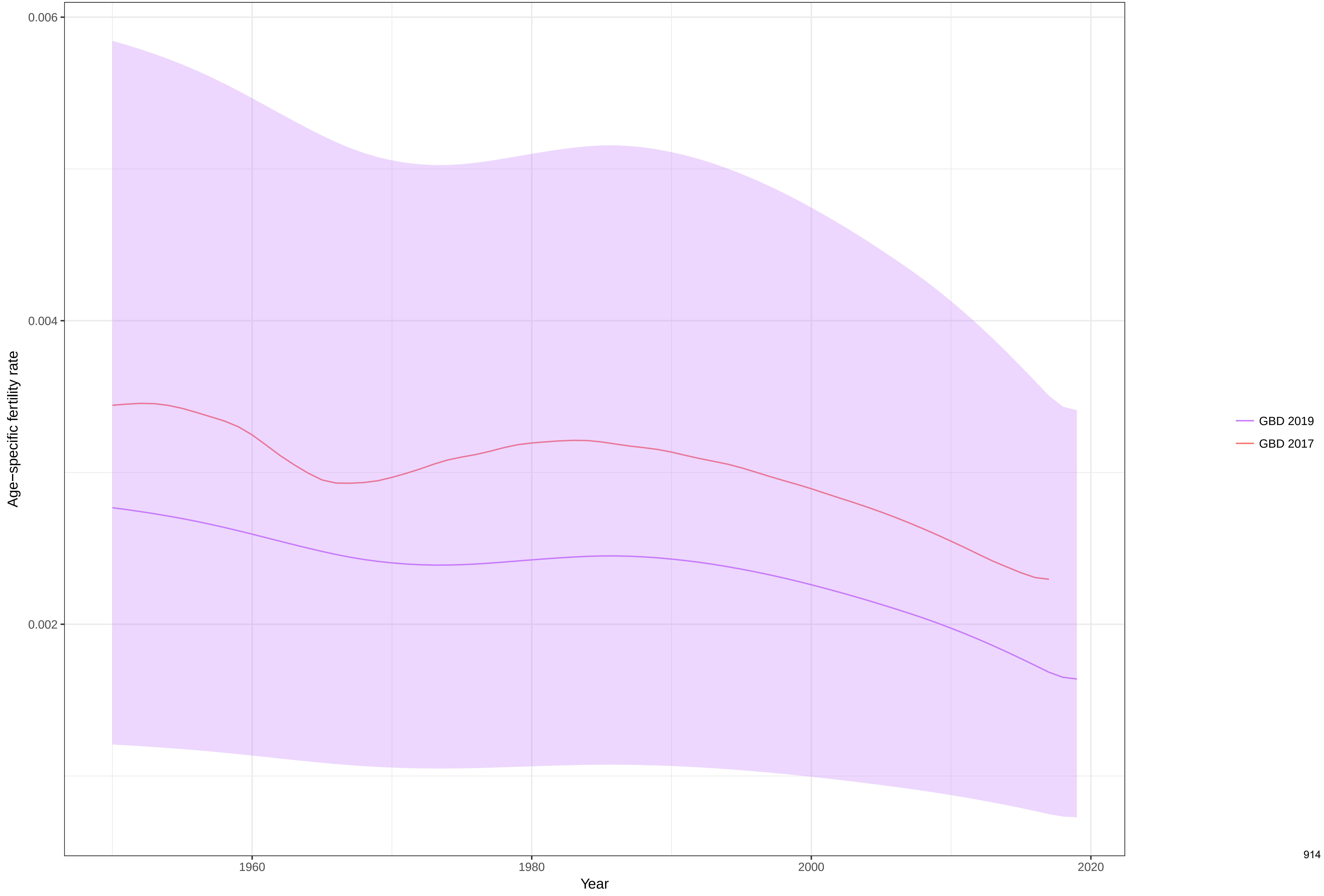

Age−specific fertility fitted trend lines, 1950−2019: Guinea−Bissau, Ages: 15−19
Data points enclosed with a diamond are reference sources

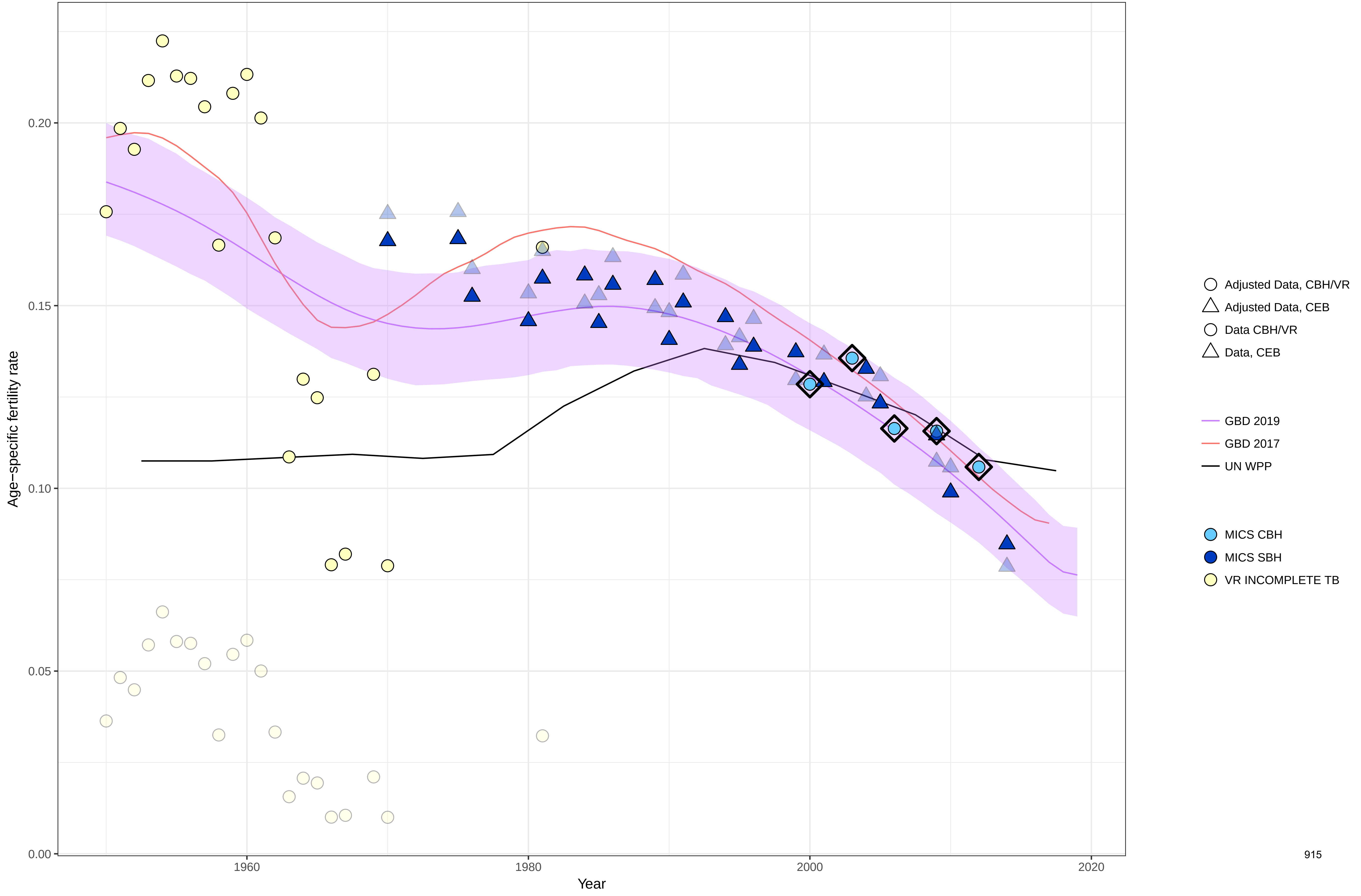

Age−specific fertility fitted trend lines, 1950−2019: Guinea−Bissau, Ages: 20−24
Data points enclosed with a diamond are reference sources

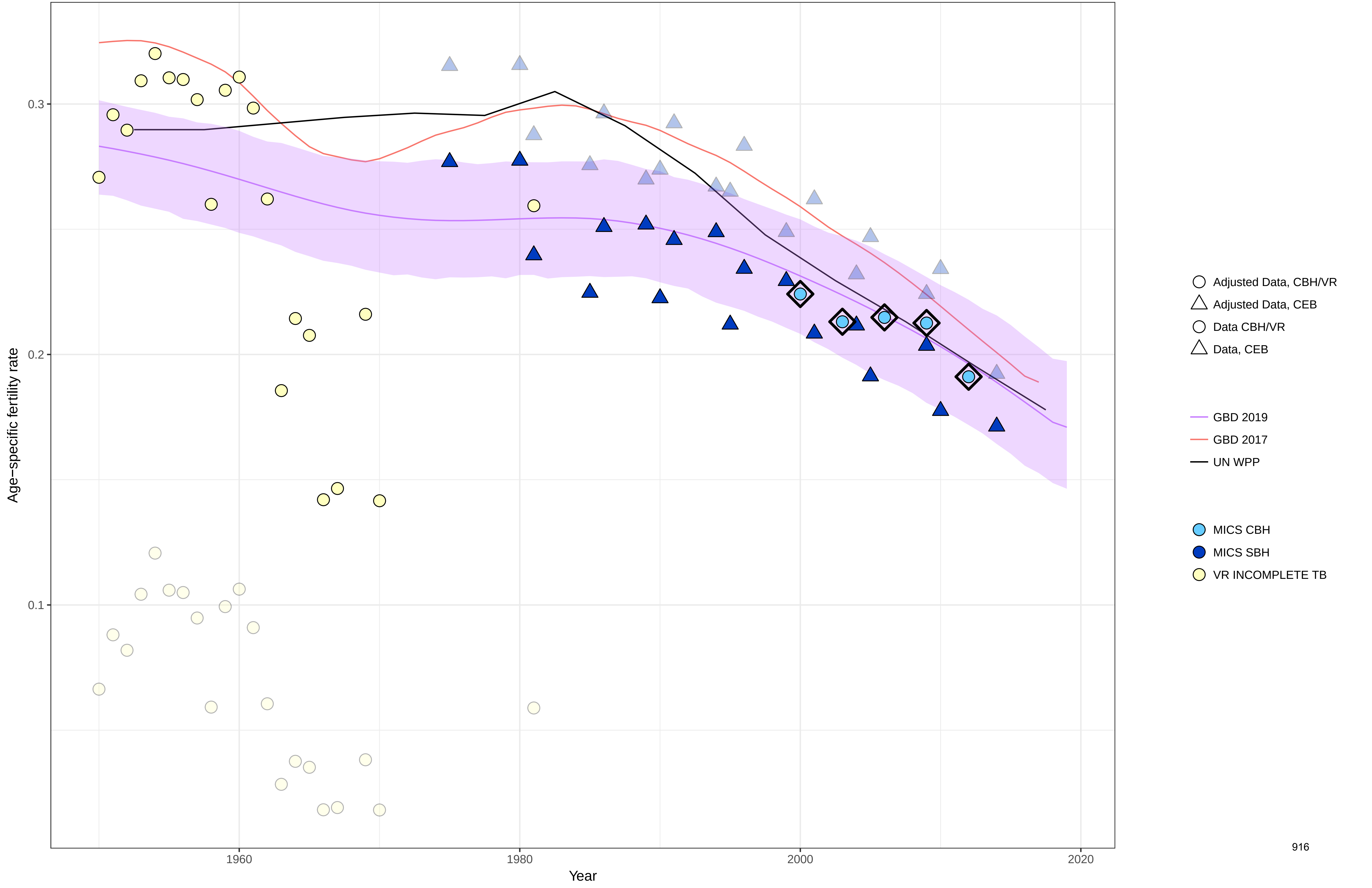

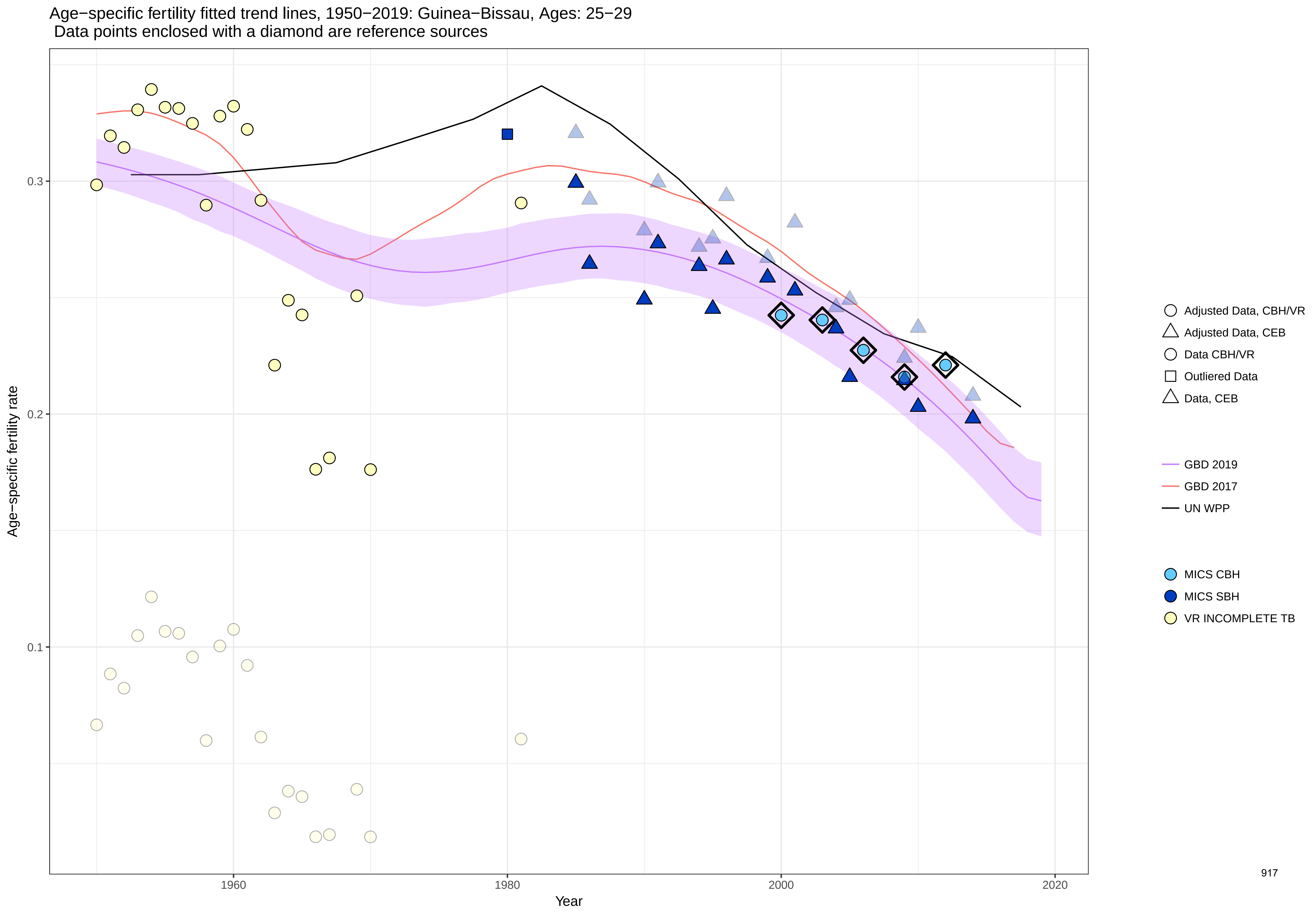

Age−specific fertility fitted trend lines, 1950−2019: Guinea−Bissau, Ages: 25−29
Data points enclosed with a diamond are reference sources
Age−specific fertility rate
0.3
0.2
0.1
1960
1980
2000
2020
Year
Adjusted Data, CBH/VR
Adjusted Data, CEB
Data CBH/VR
Outliered Data
Data, CEB
GBD 2019
GBD 2017
UN WPP
MICS CBH
MICS SBH
VR INCOMPLETE TB

Age−specific fertility fitted trend lines, 1950−2019: Guinea−Bissau, Ages: 30−34
Data points enclosed with a diamond are reference sources

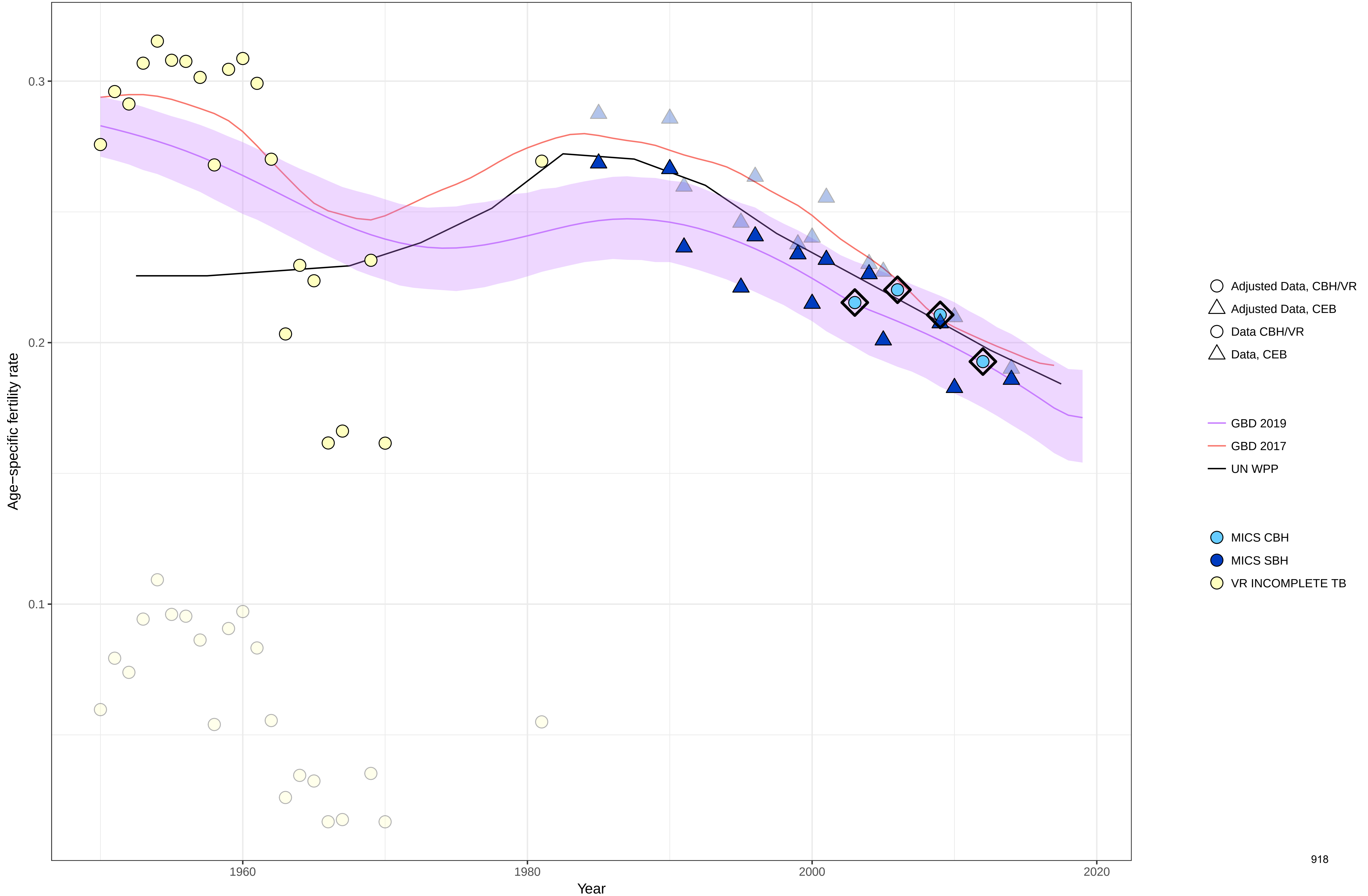

Age−specific fertility fitted trend lines, 1950−2019: Guinea−Bissau, Ages: 35−39
Data points enclosed with a diamond are reference sources

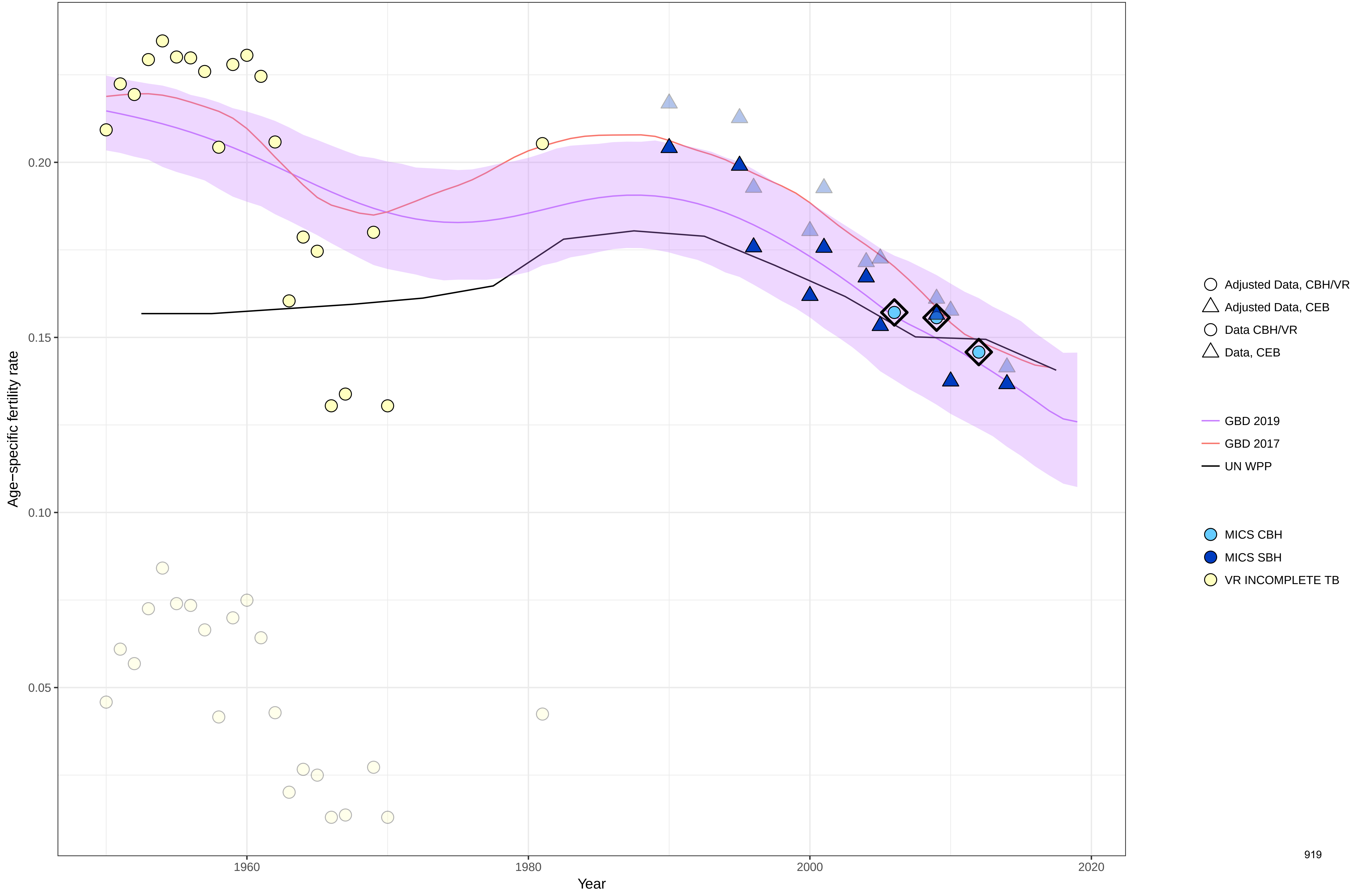

Age−specific fertility fitted trend lines, 1950−2019: Guinea−Bissau, Ages: 40−44
Data points enclosed with a diamond are reference sources

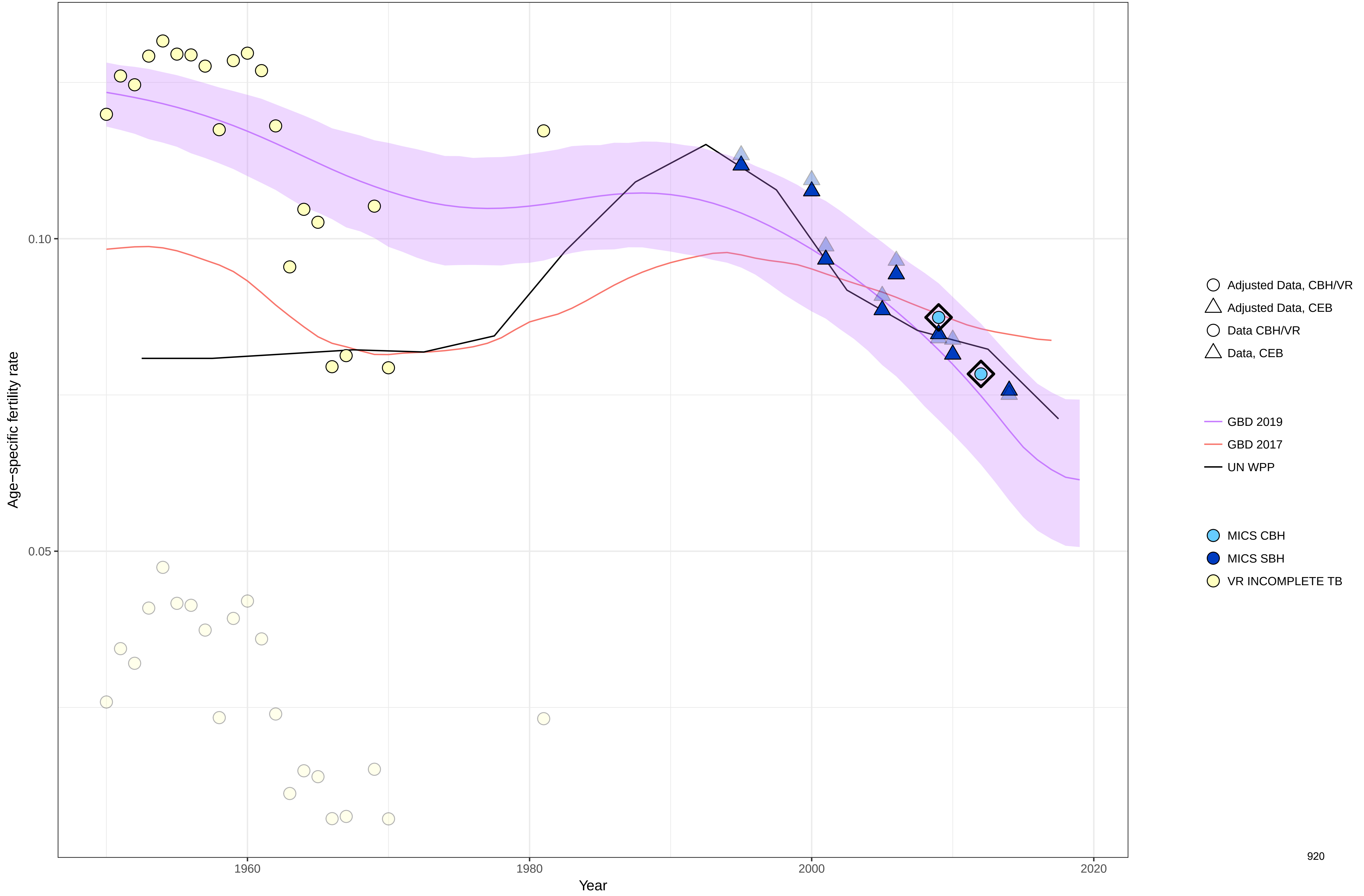

Age−specific fertility fitted trend lines, 1950−2019: Guinea−Bissau, Ages: 45−49
Data points enclosed with a diamond are reference sources

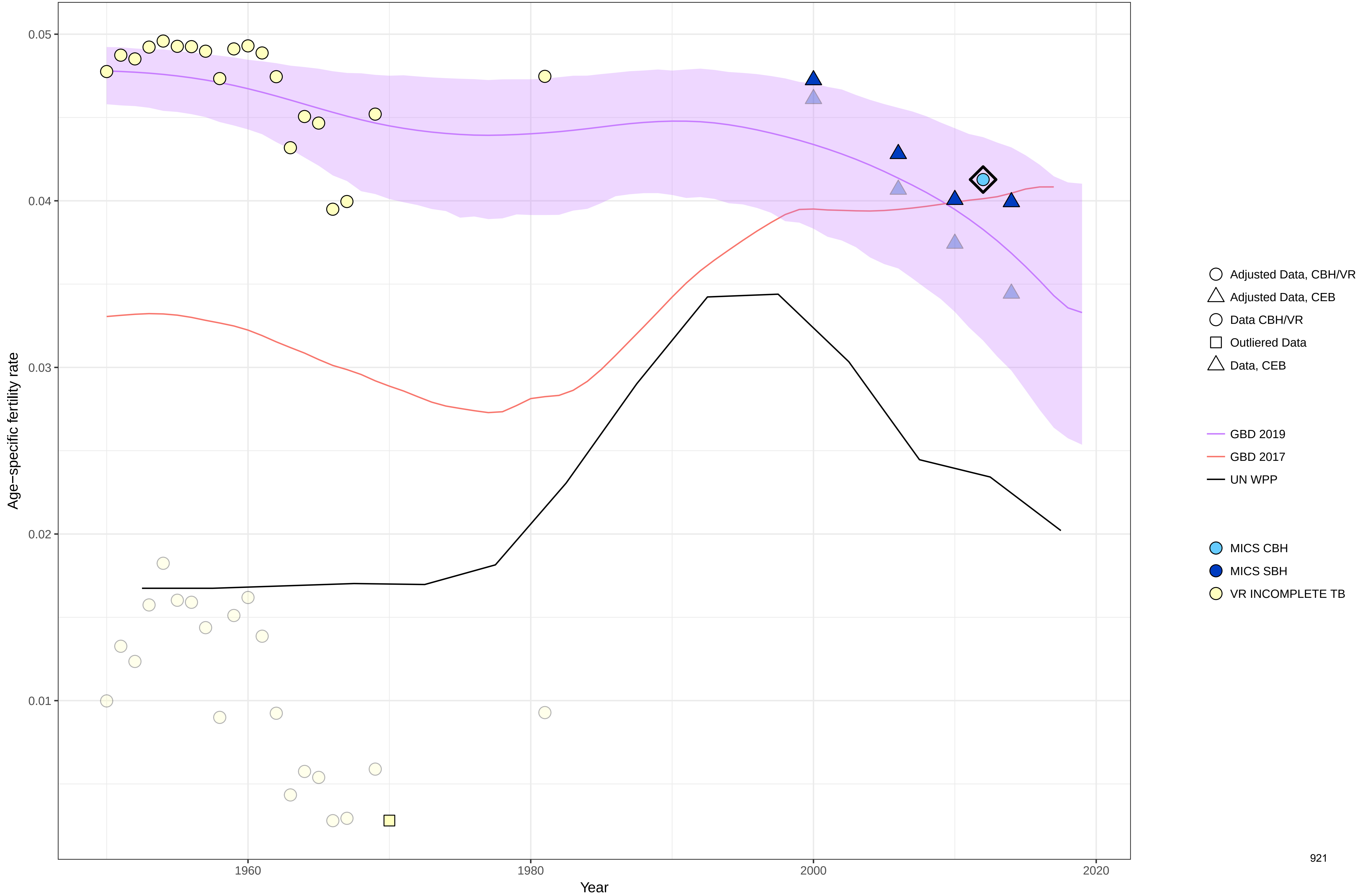

Age−specific fertility fitted trend lines, 1950−2019: Guinea−Bissau, Ages: 50−54
Data points enclosed with a diamond are reference sources

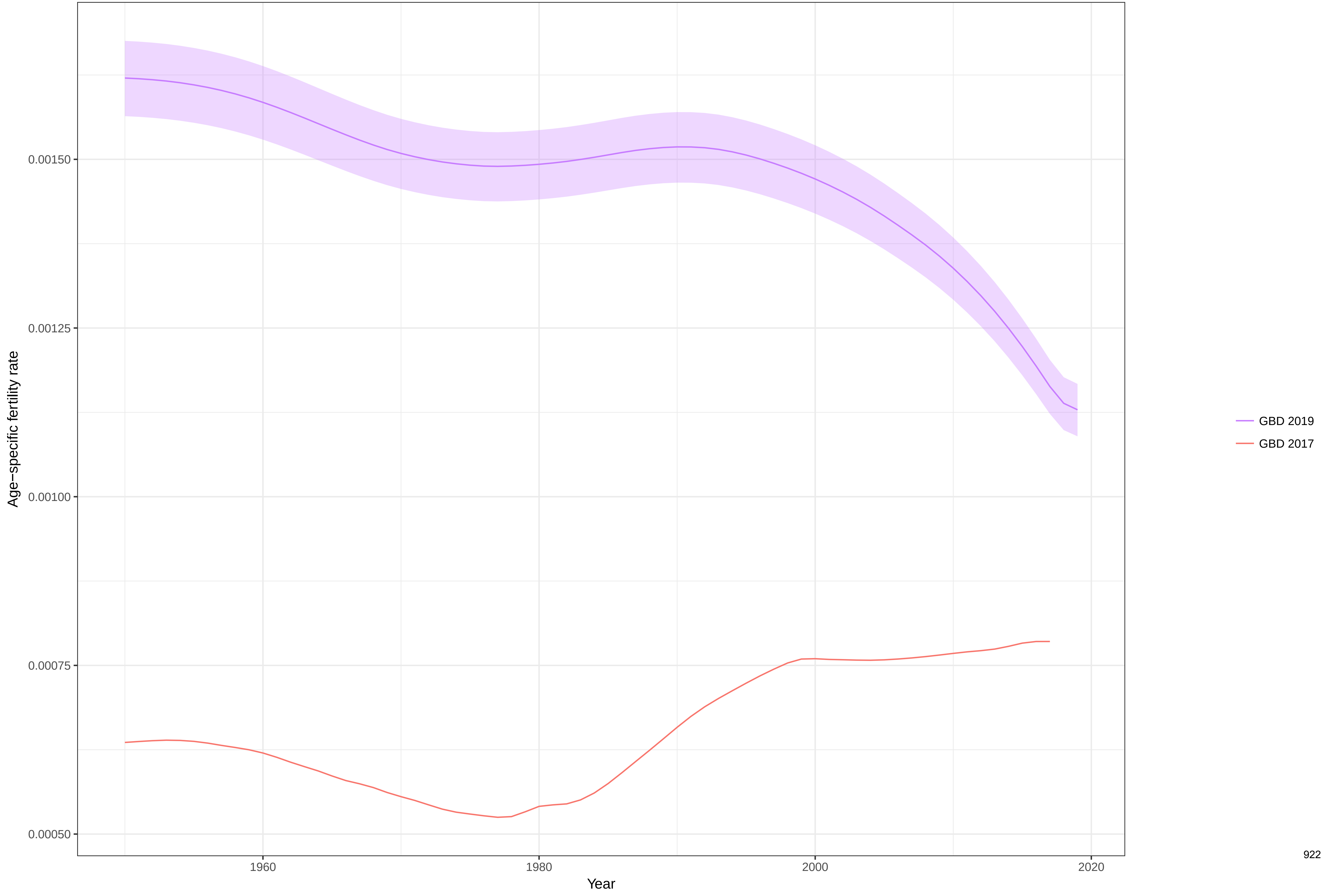

Total fertility rate fitted trend lines, 1950−2019: Guinea−Bissau TFR
Data points enclosed with a diamond are reference sources

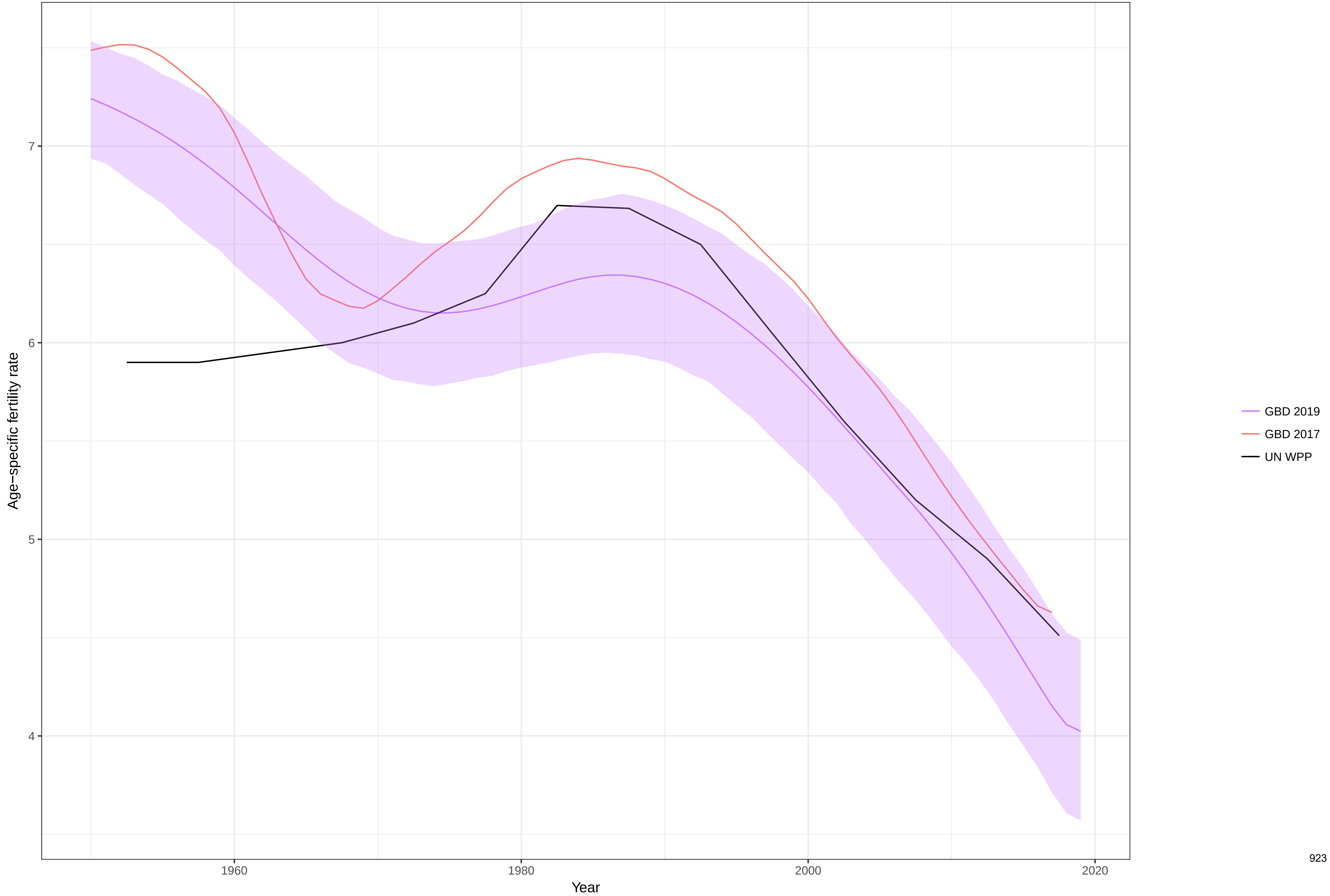

Age−specific fertility fitted trend lines, 1950−2019: Equatorial Guinea, Ages: 10−14
Data points enclosed with a diamond are reference sources

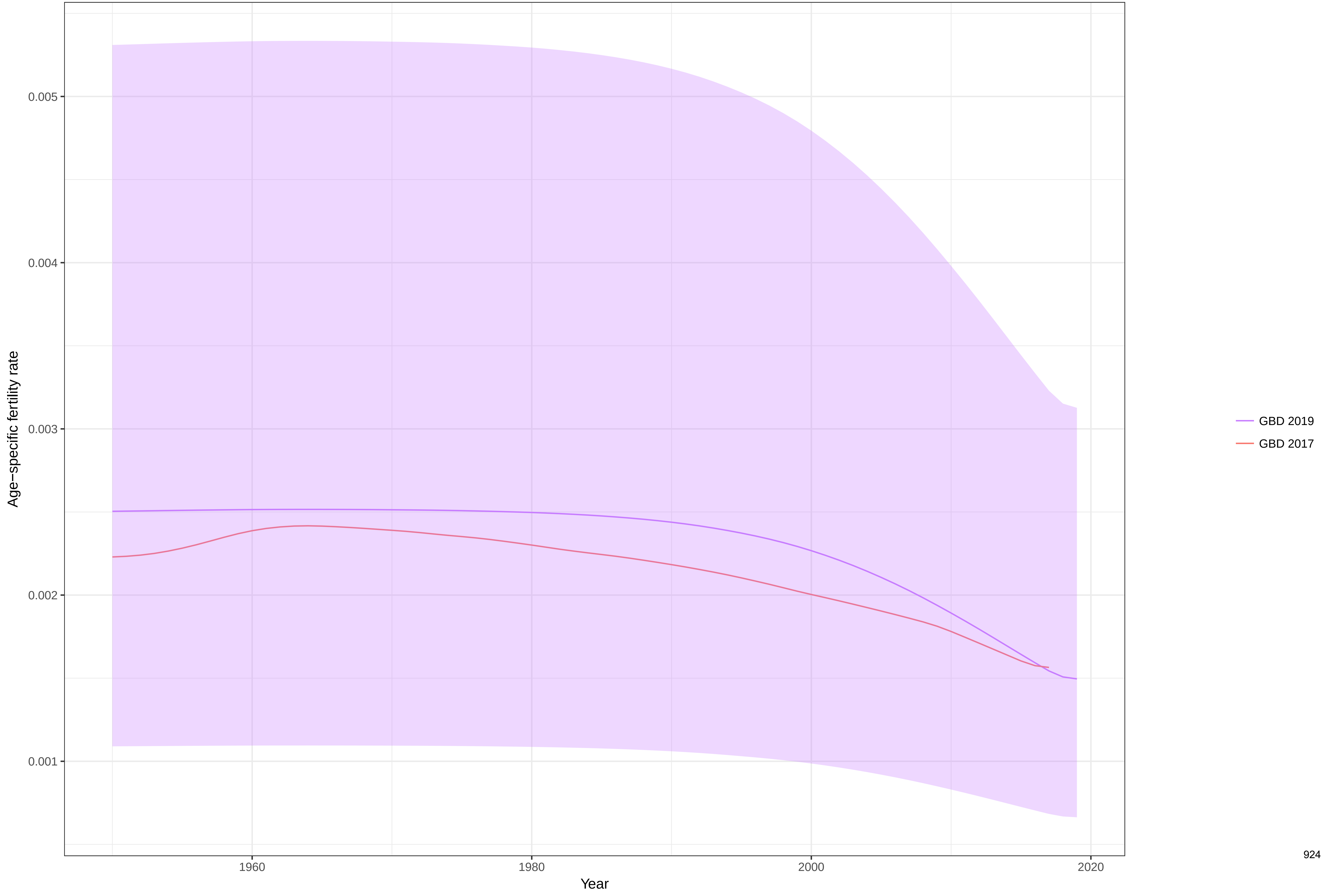

Age−specific fertility fitted trend lines, 1950−2019: Equatorial Guinea, Ages: 15−19
Data points enclosed with a diamond are reference sources

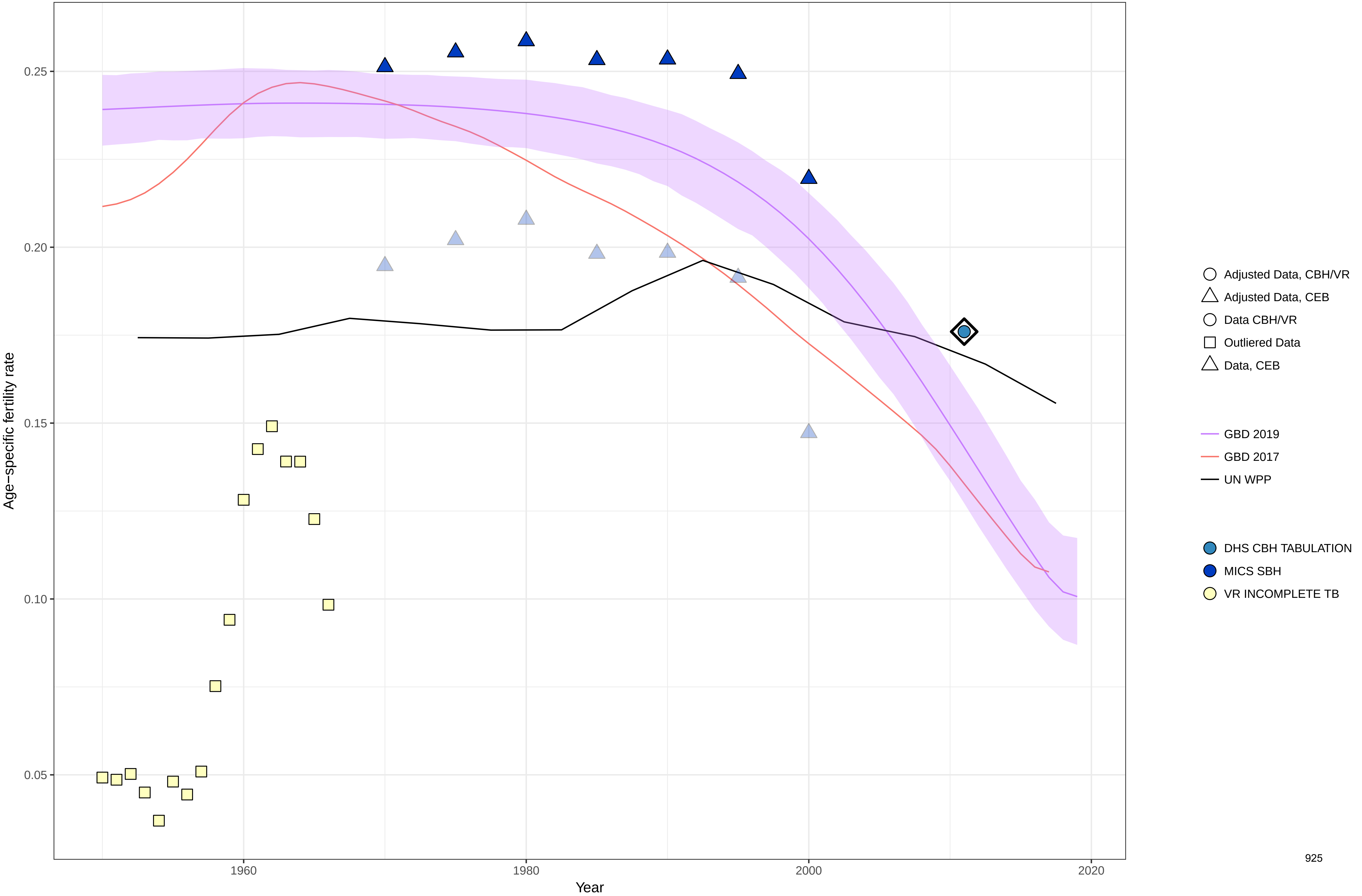

Age−specific fertility fitted trend lines, 1950−2019: Equatorial Guinea, Ages: 20−24
Data points enclosed with a diamond are reference sources

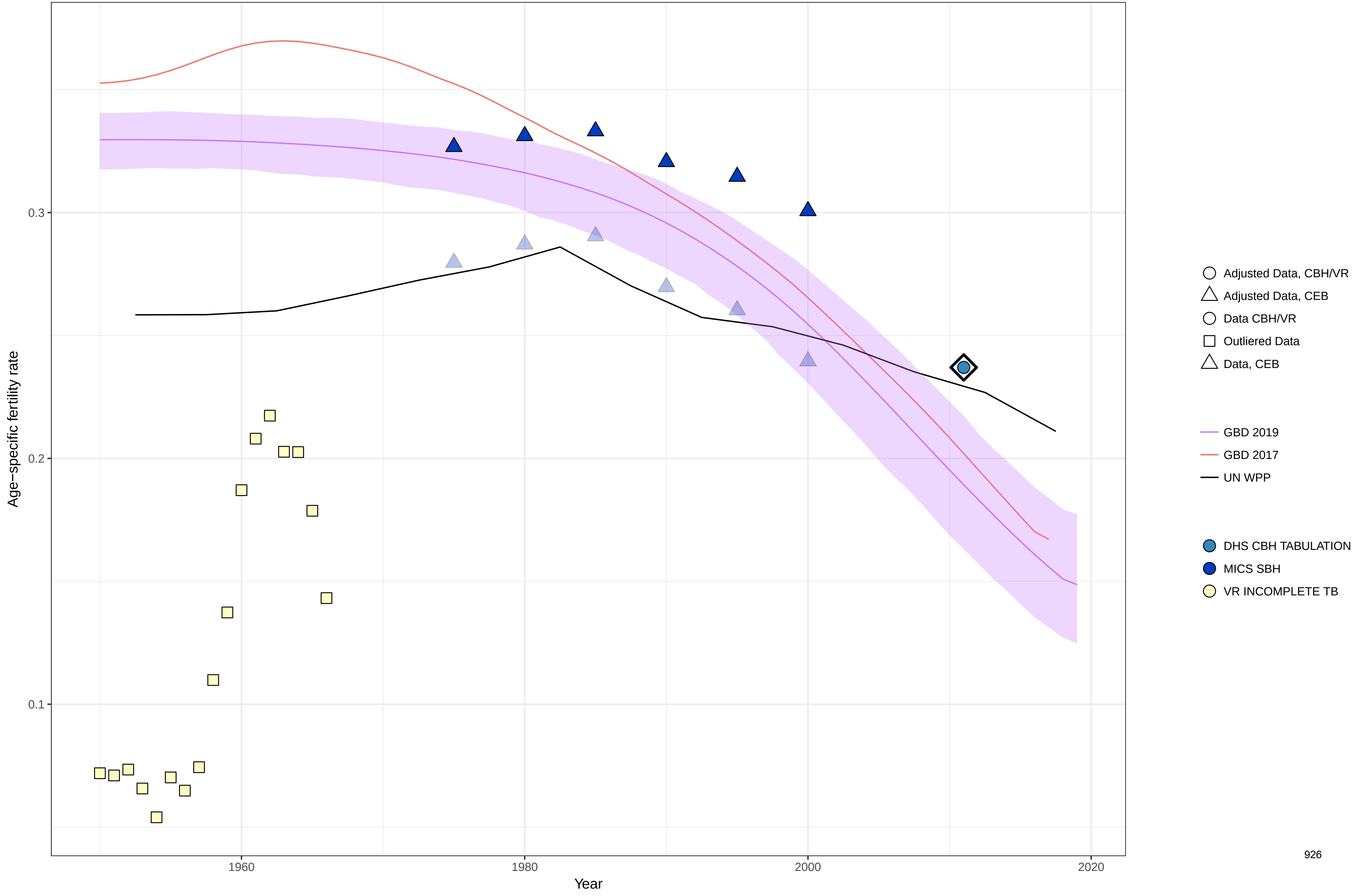

Age−specific fertility fitted trend lines, 1950−2019: Equatorial Guinea, Ages: 25−29
Data points enclosed with a diamond are reference sources

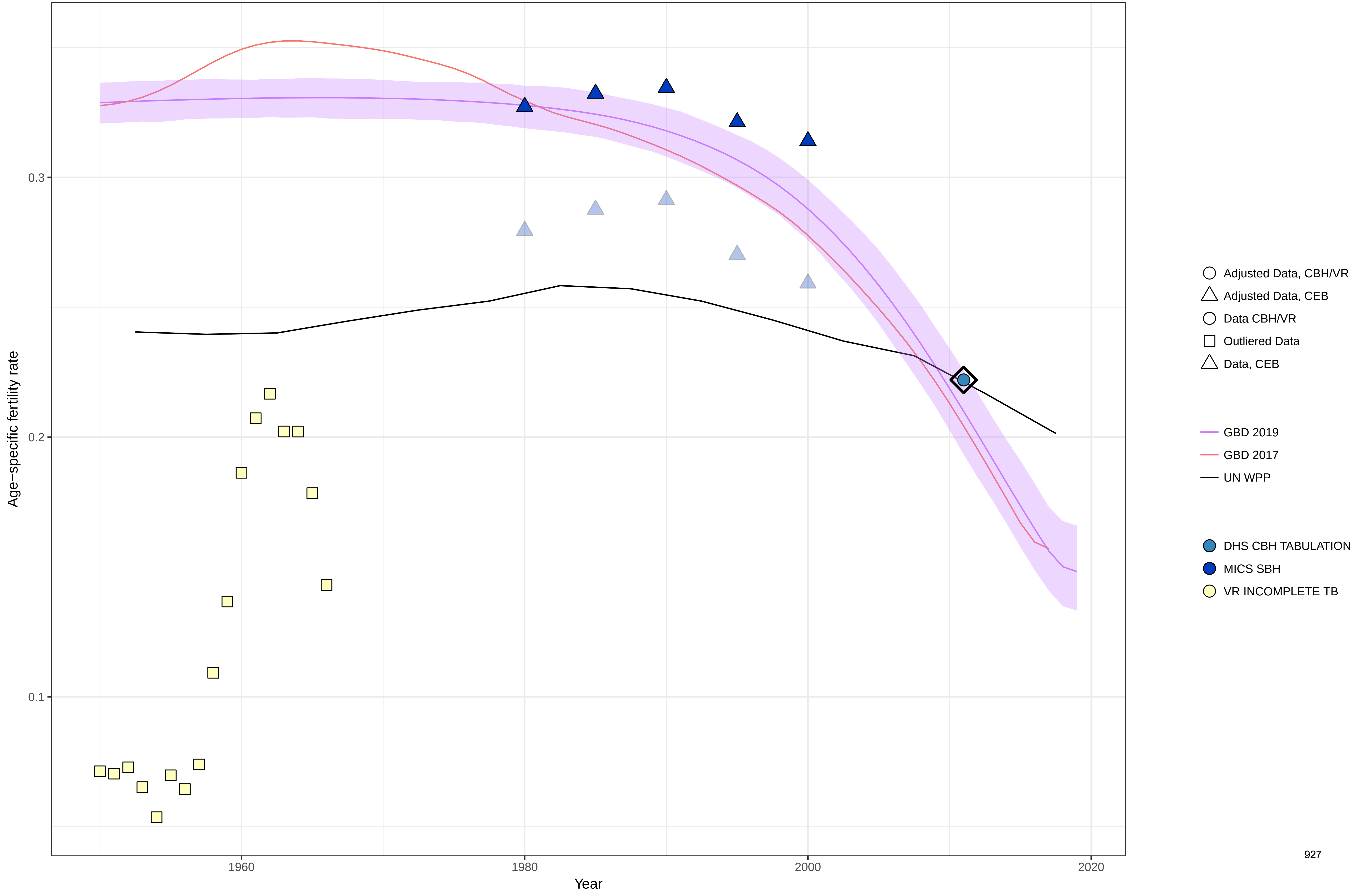

Age−specific fertility fitted trend lines, 1950−2019: Equatorial Guinea, Ages: 30−34
Data points enclosed with a diamond are reference sources

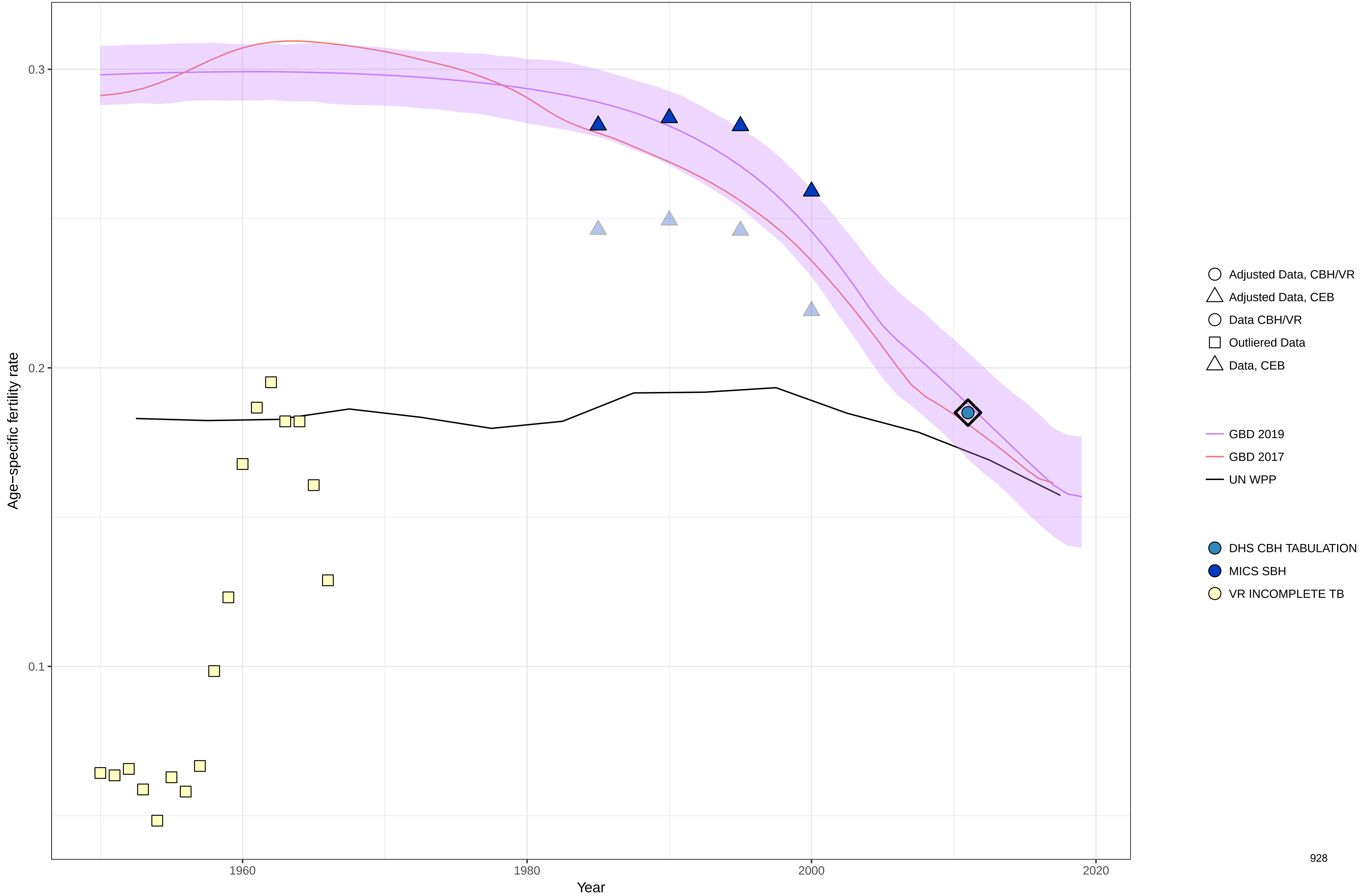

Age−specific fertility fitted trend lines, 1950−2019: Equatorial Guinea, Ages: 35−39
Data points enclosed with a diamond are reference sources

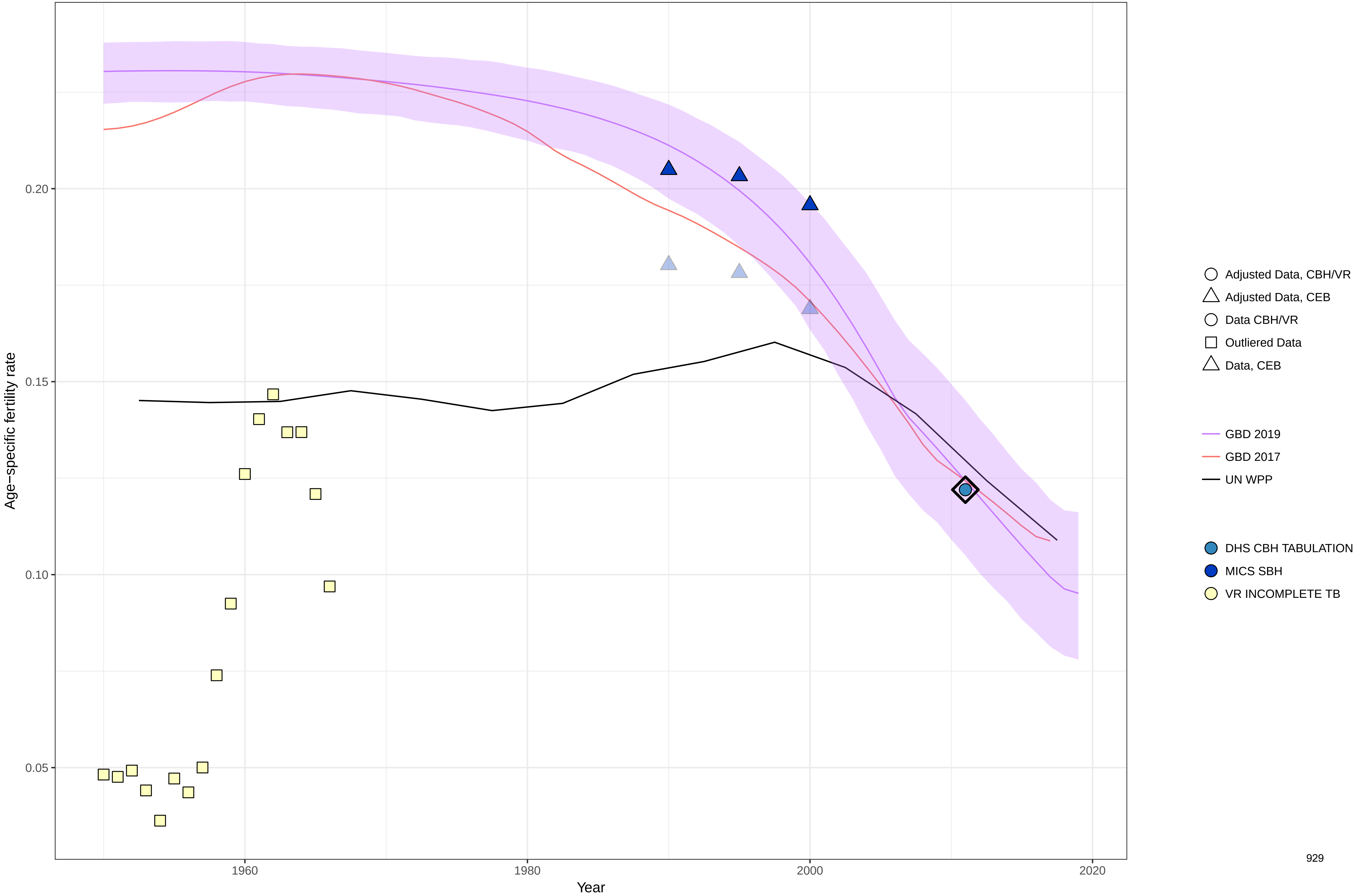

Age−specific fertility fitted trend lines, 1950−2019: Equatorial Guinea, Ages: 40−44
Data points enclosed with a diamond are reference sources

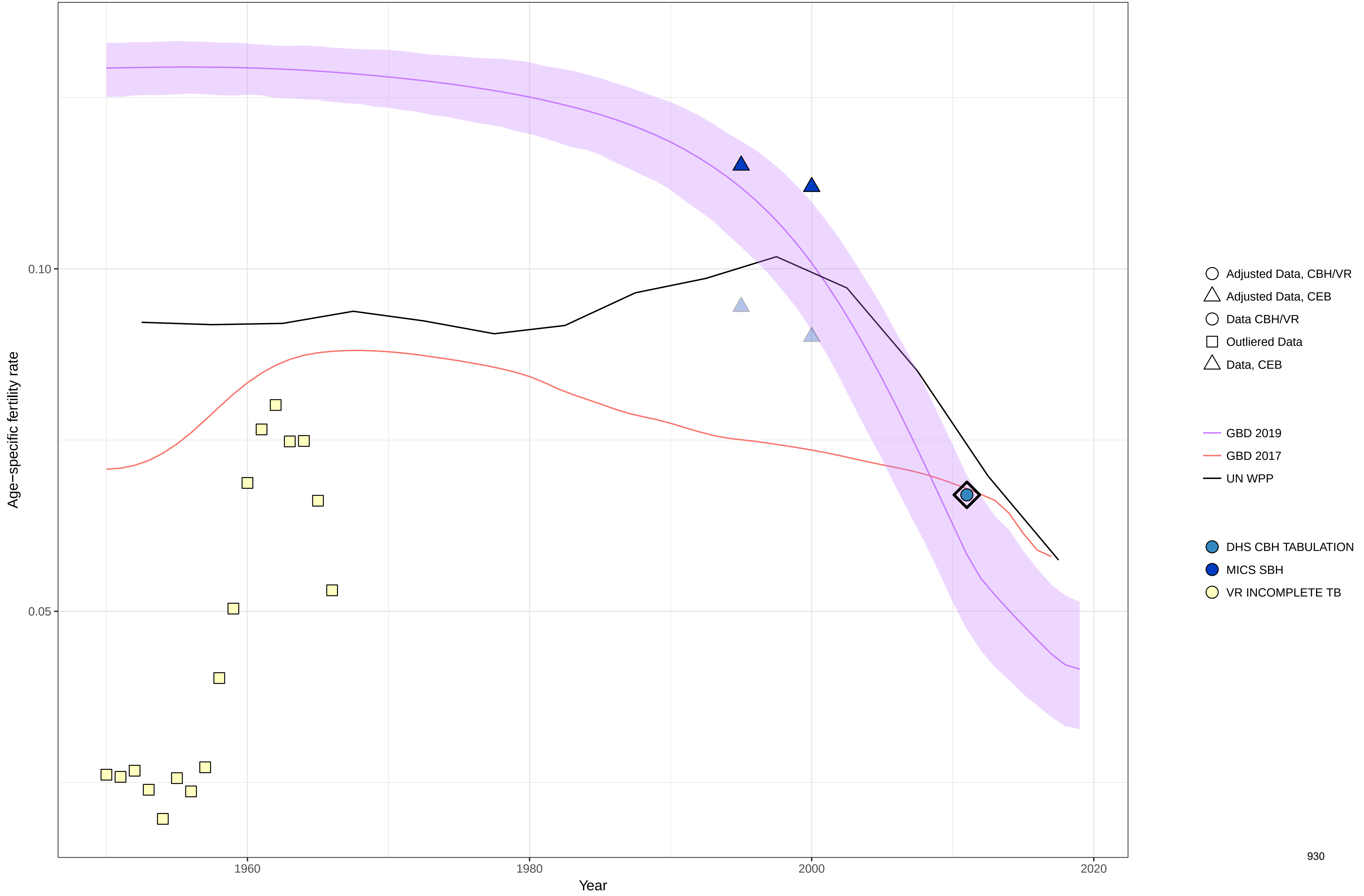

Age−specific fertility fitted trend lines, 1950−2019: Equatorial Guinea, Ages: 45−49
Data points enclosed with a diamond are reference sources

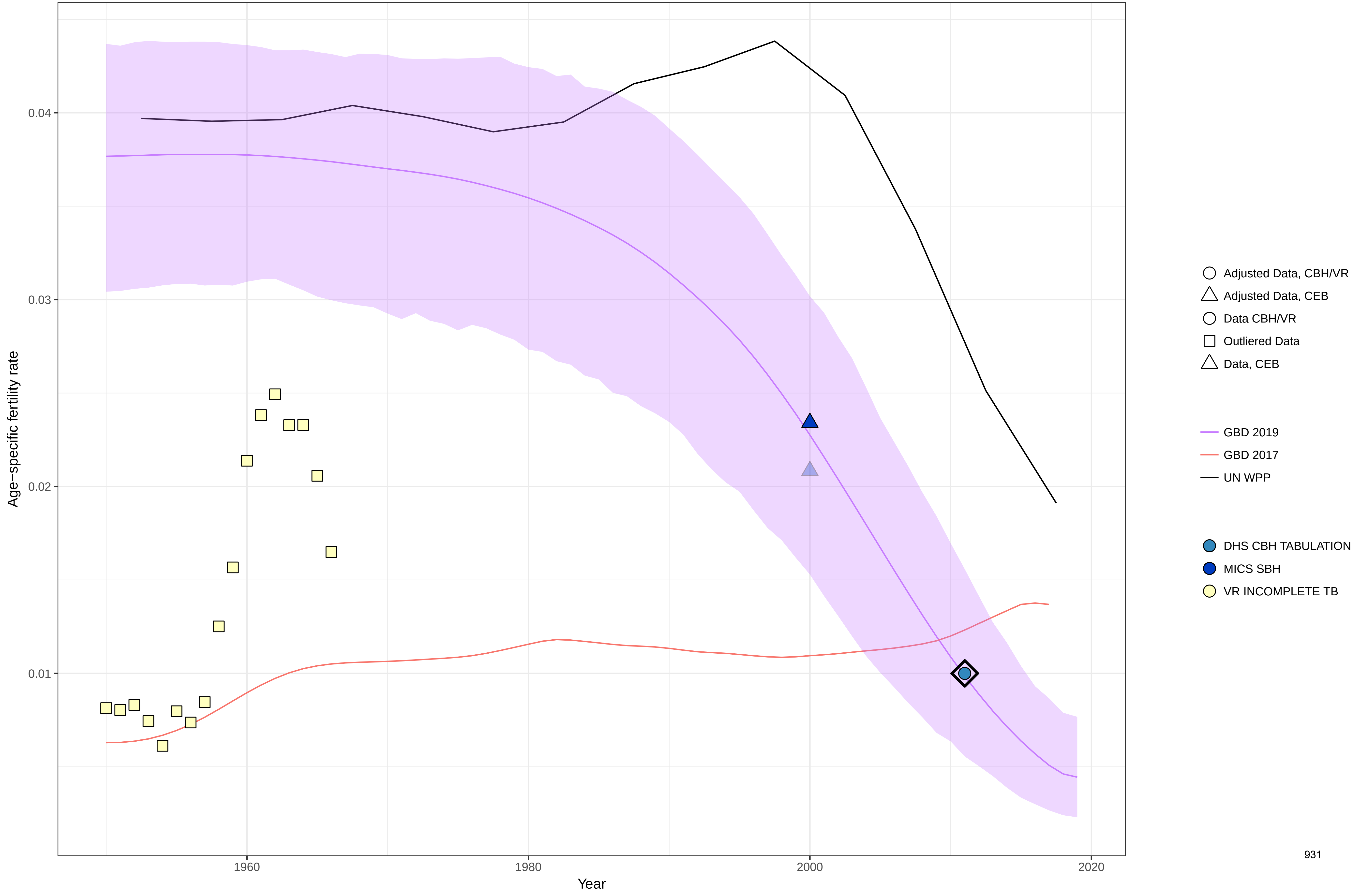

Age−specific fertility fitted trend lines, 1950−2019: Equatorial Guinea, Ages: 50−54
Data points enclosed with a diamond are reference sources

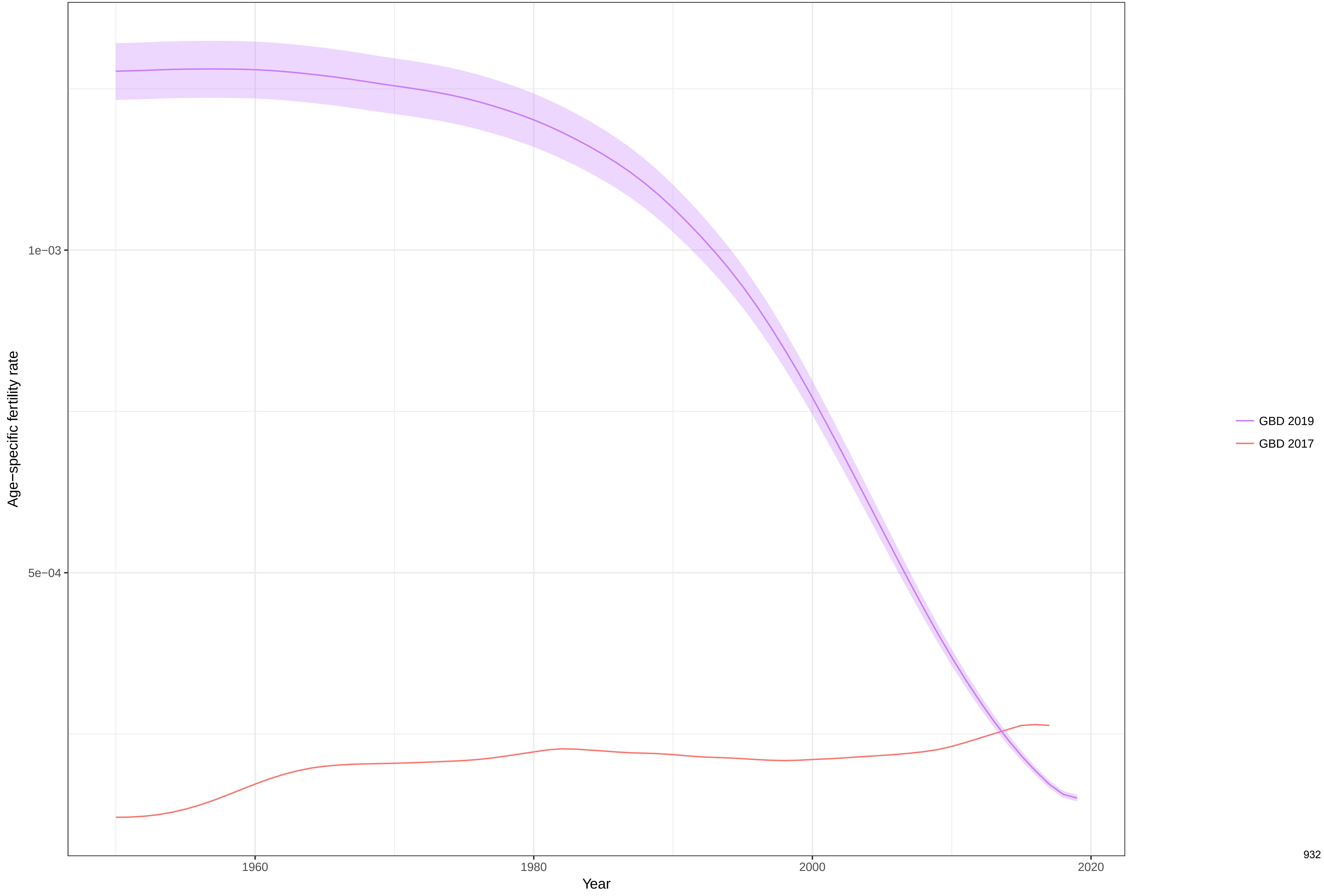

Total fertility rate fitted trend lines, 1950−2019: Equatorial Guinea TFR
Data points enclosed with a diamond are reference sources

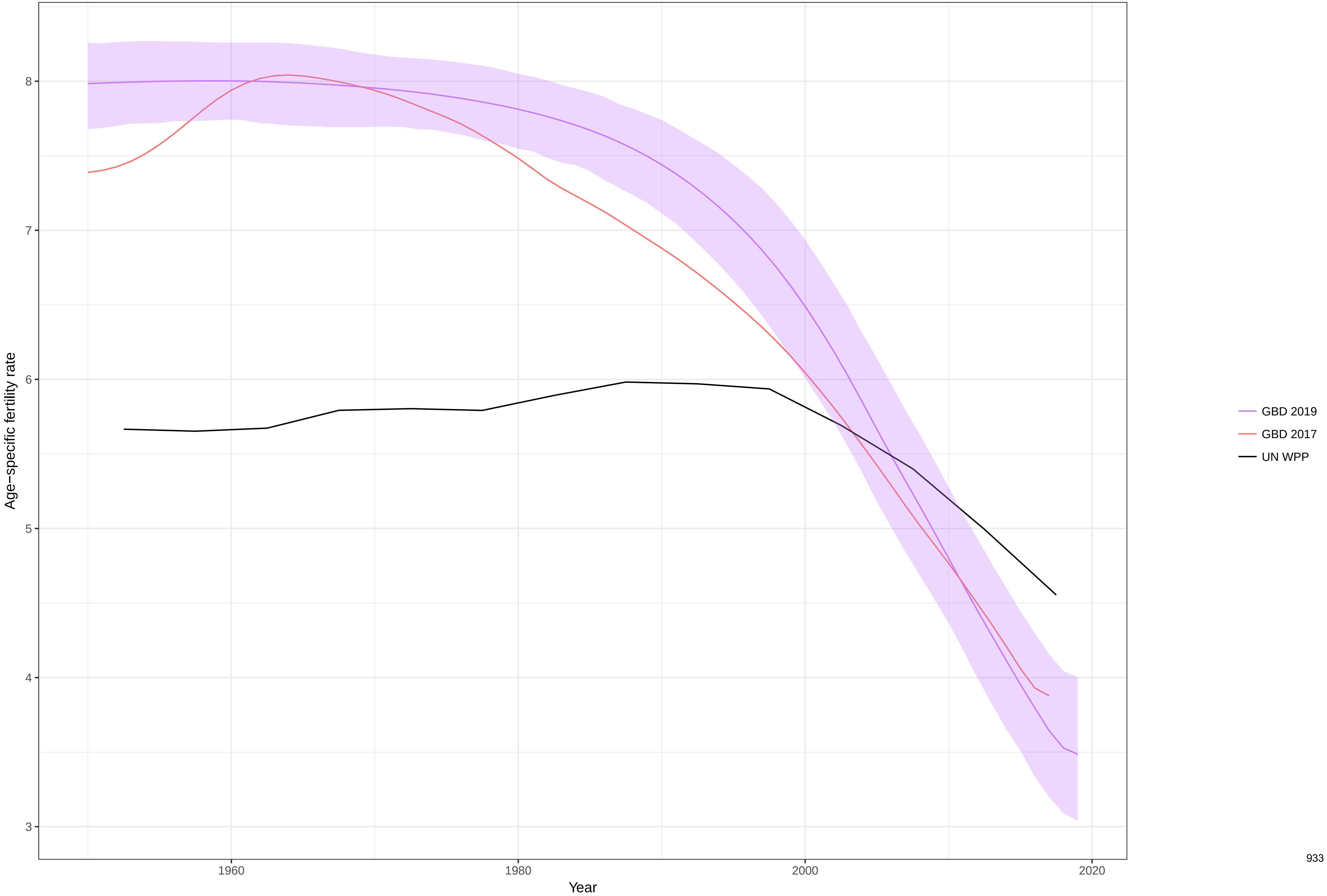

Age−specific fertility fitted trend lines, 1950−2019: Greece, Ages: 10−14
Data points enclosed with a diamond are reference sources

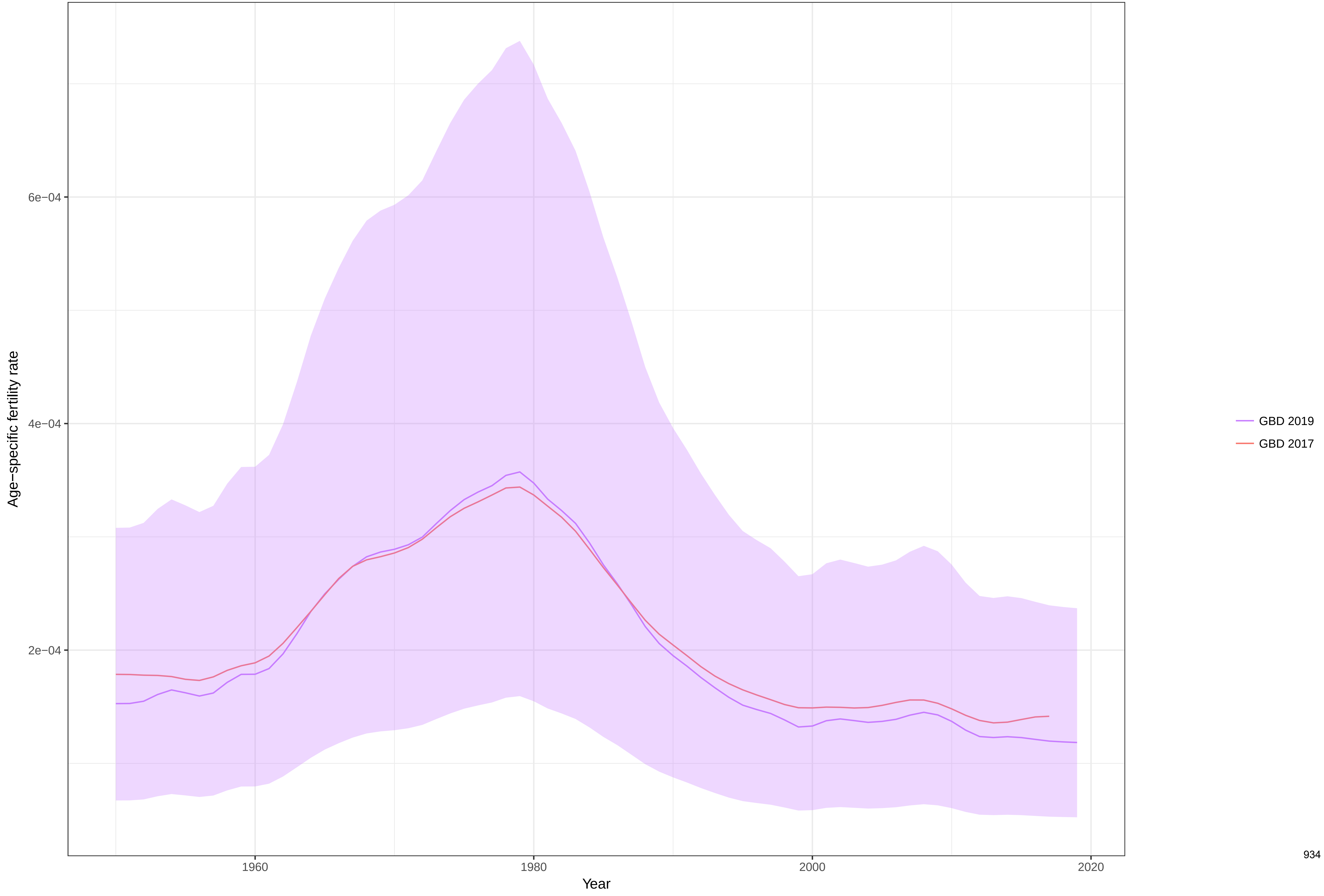

Age−specific fertility fitted trend lines, 1950−2019: Greece, Ages: 15−19
Data points enclosed with a diamond are reference sources

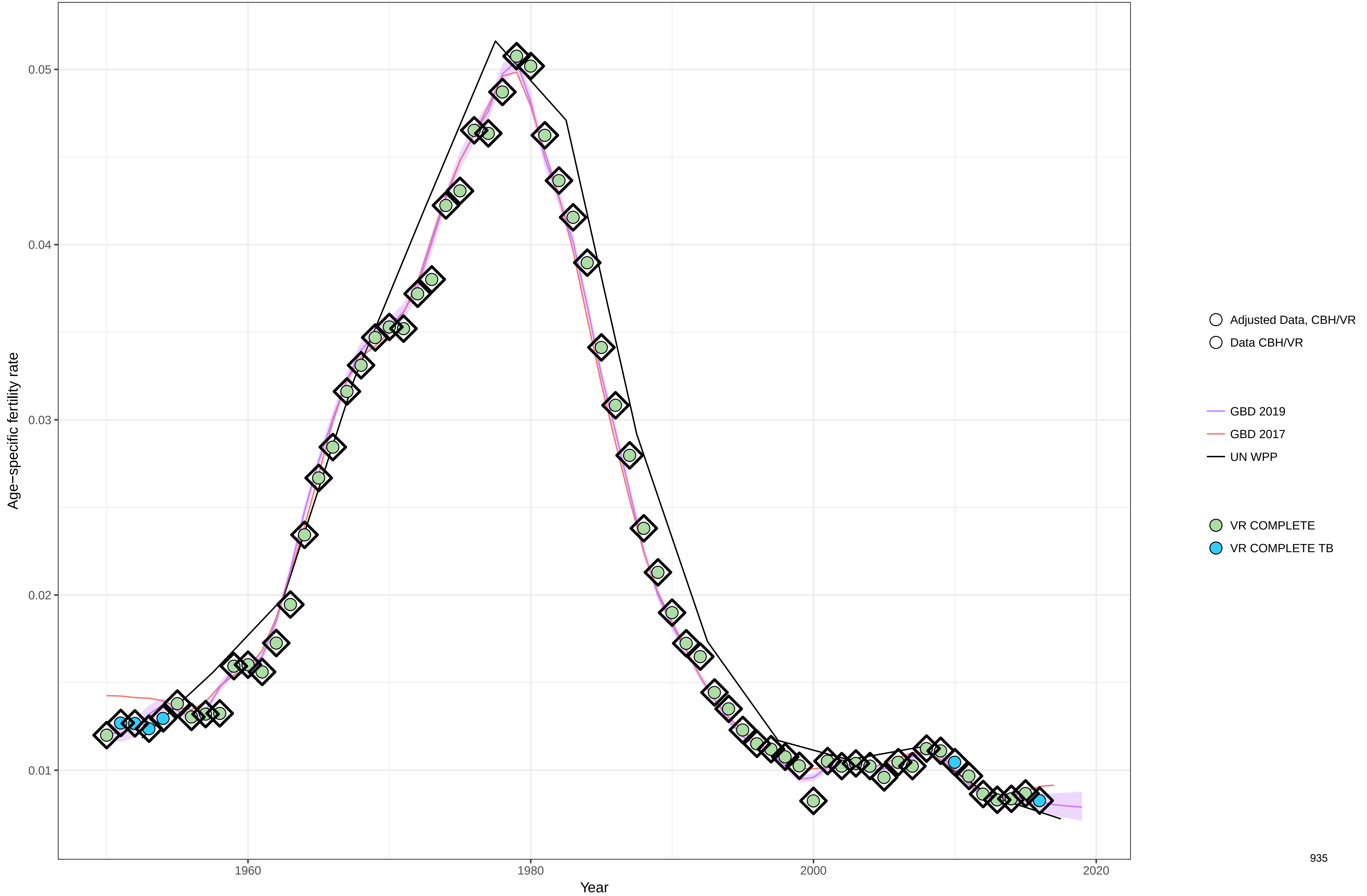

Age−specific fertility fitted trend lines, 1950−2019: Greece, Ages: 20−24
Data points enclosed with a diamond are reference sources

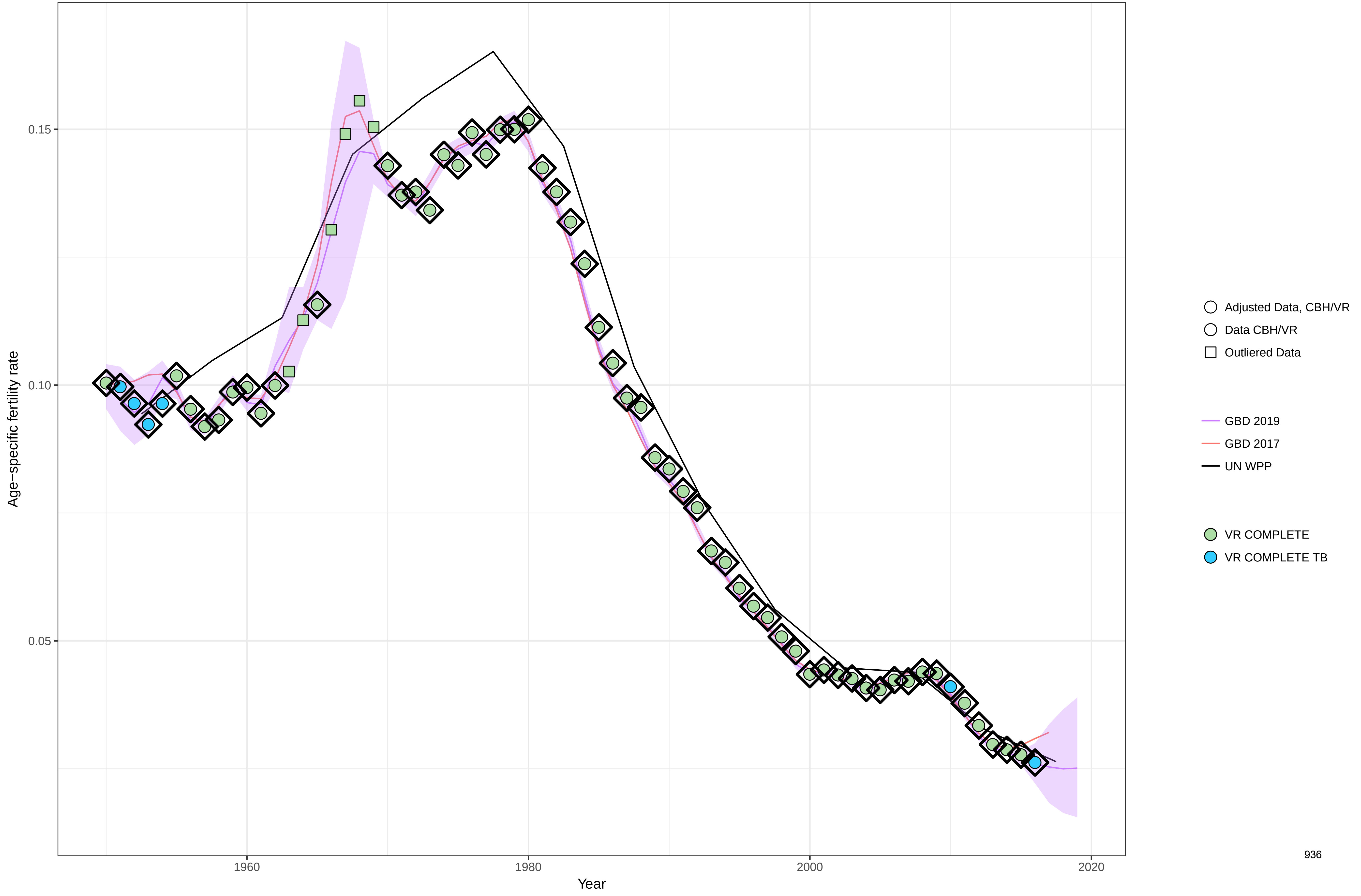

Age−specific fertility fitted trend lines, 1950−2019: Greece, Ages: 25−29
Data points enclosed with a diamond are reference sources

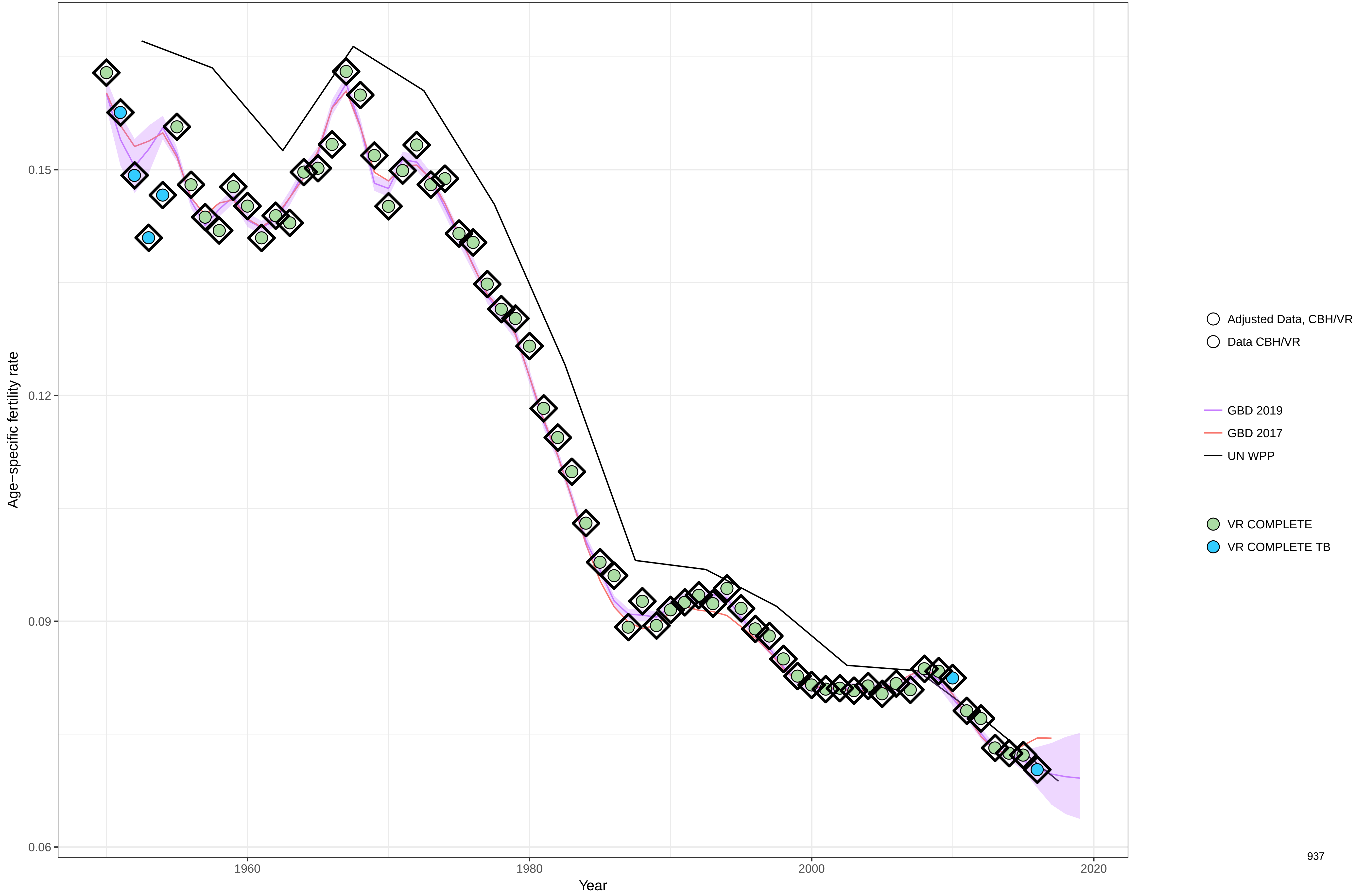

Age−specific fertility fitted trend lines, 1950−2019: Greece, Ages: 30−34
Data points enclosed with a diamond are reference sources

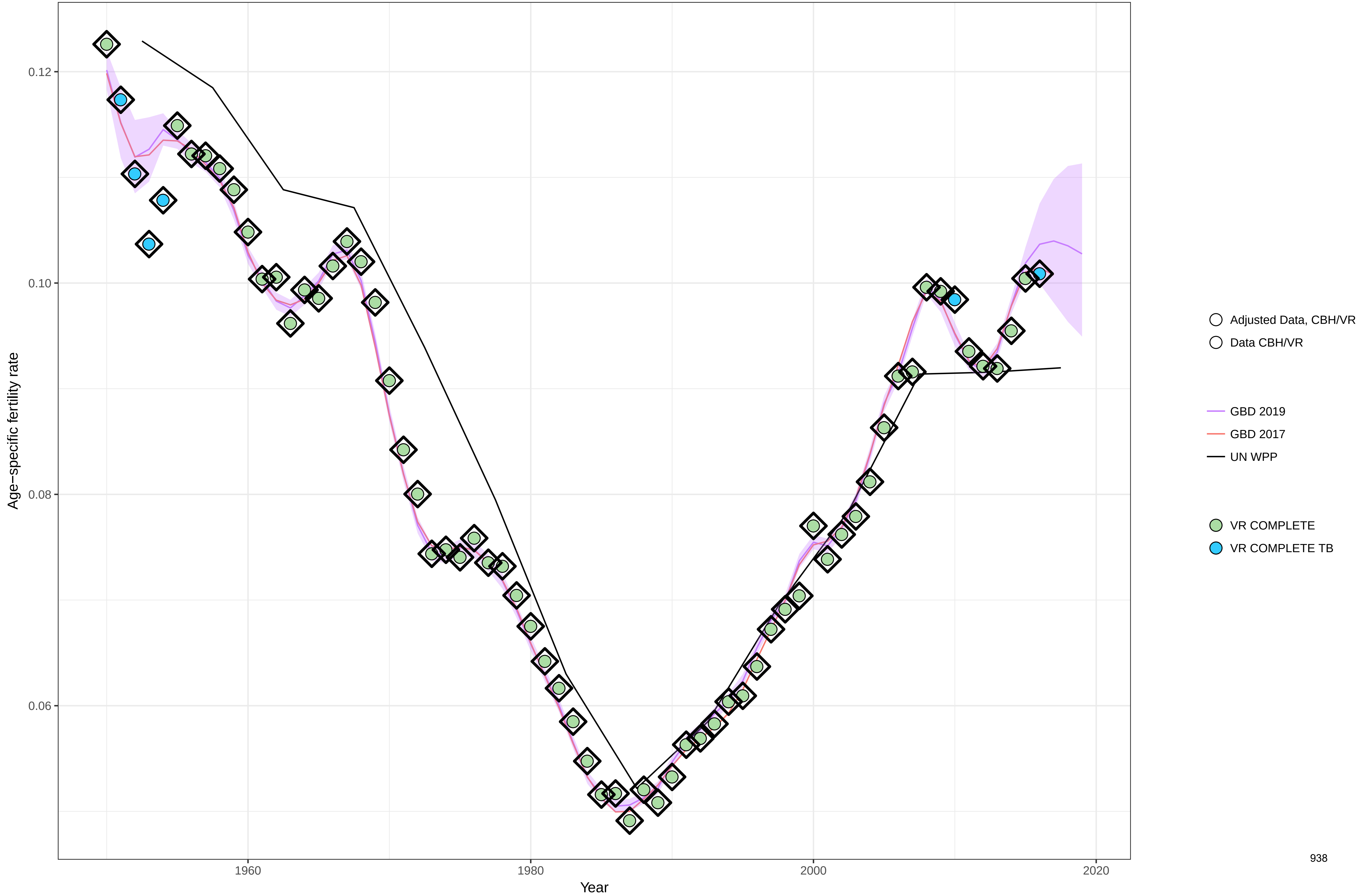

Age−specific fertility fitted trend lines, 1950−2019: Greece, Ages: 35−39
Data points enclosed with a diamond are reference sources

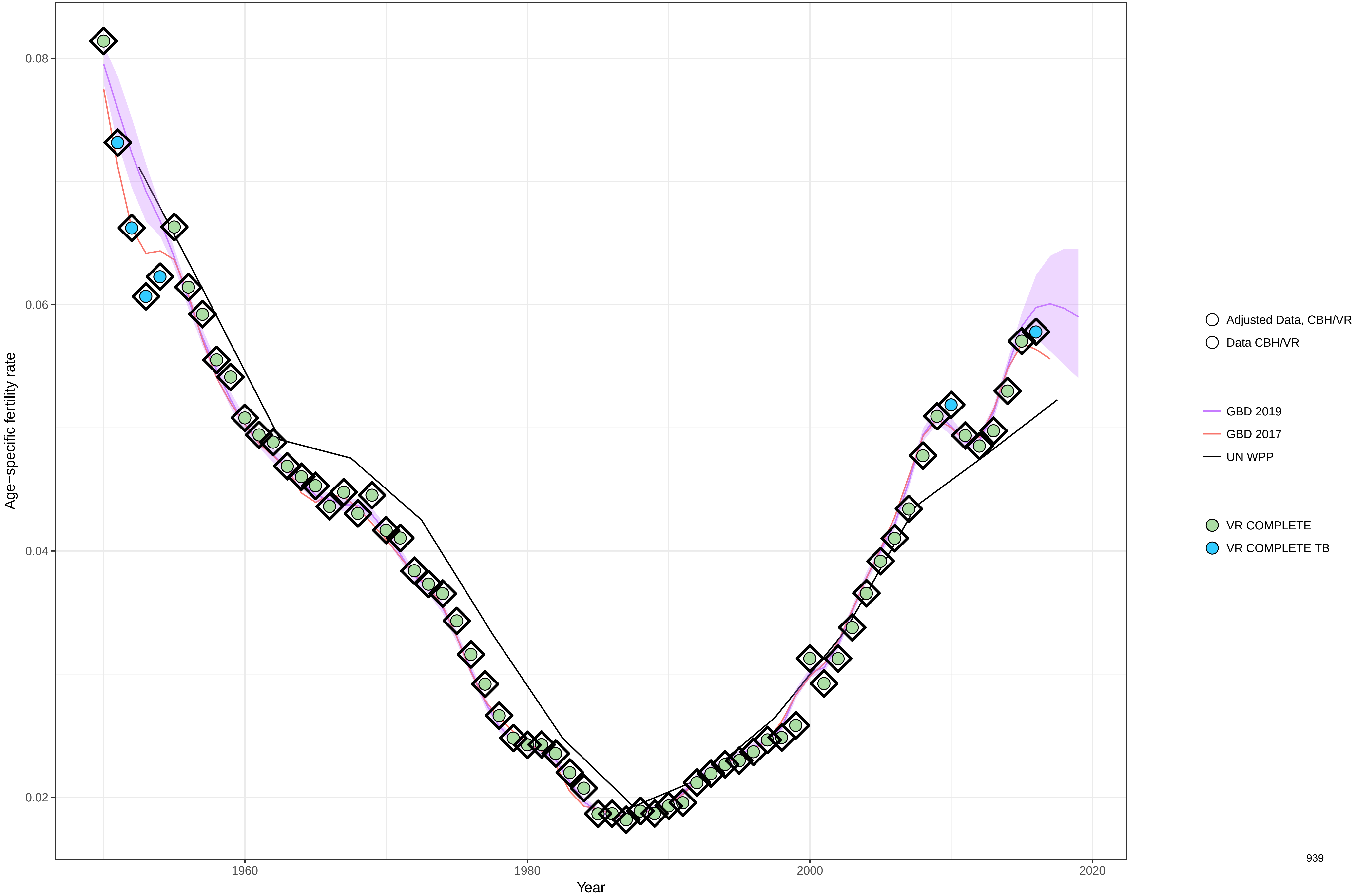

Age−specific fertility fitted trend lines, 1950−2019: Greece, Ages: 40−44
Data points enclosed with a diamond are reference sources

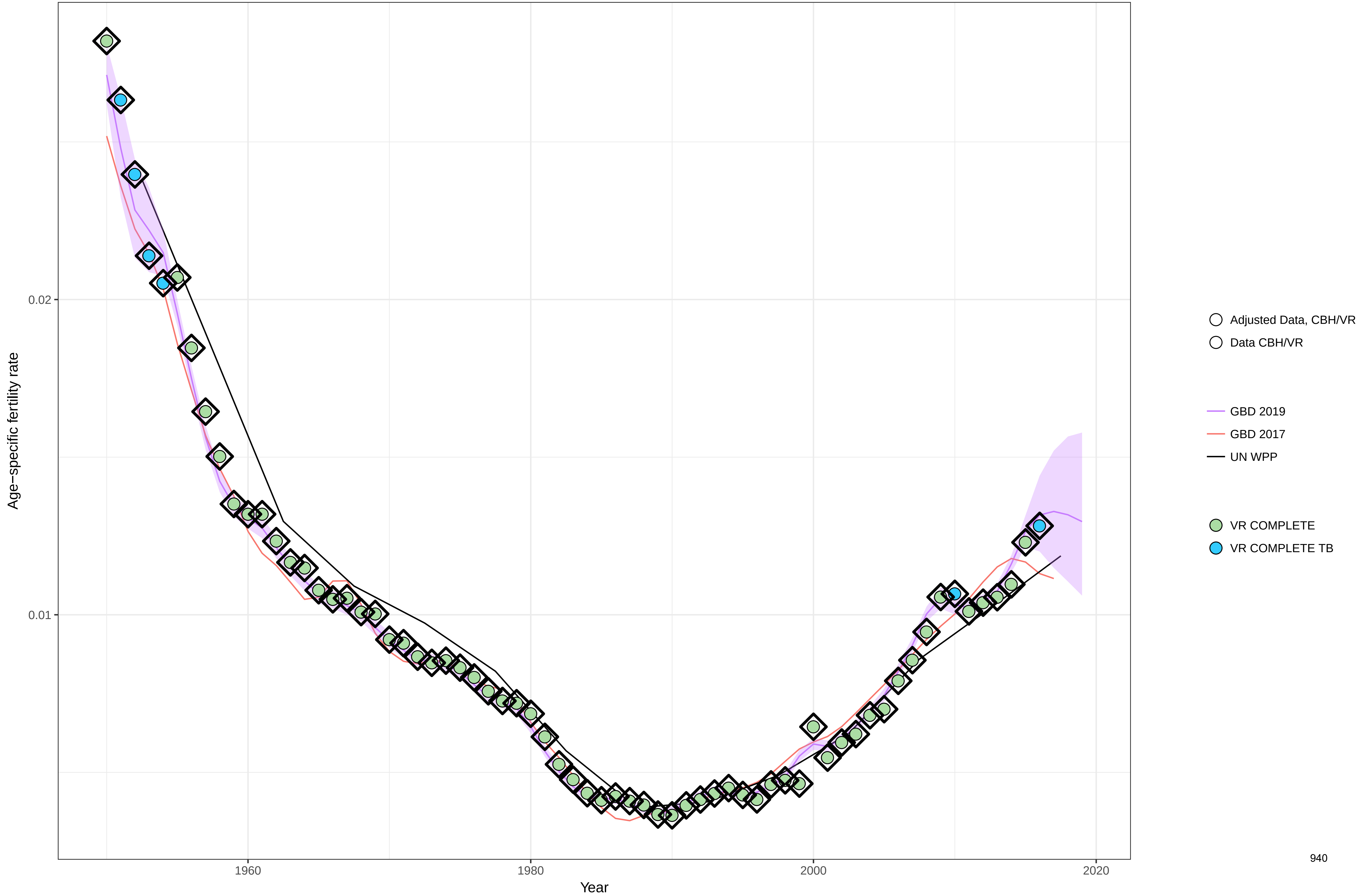

Age−specific fertility fitted trend lines, 1950−2019: Greece, Ages: 45−49
Data points enclosed with a diamond are reference sources

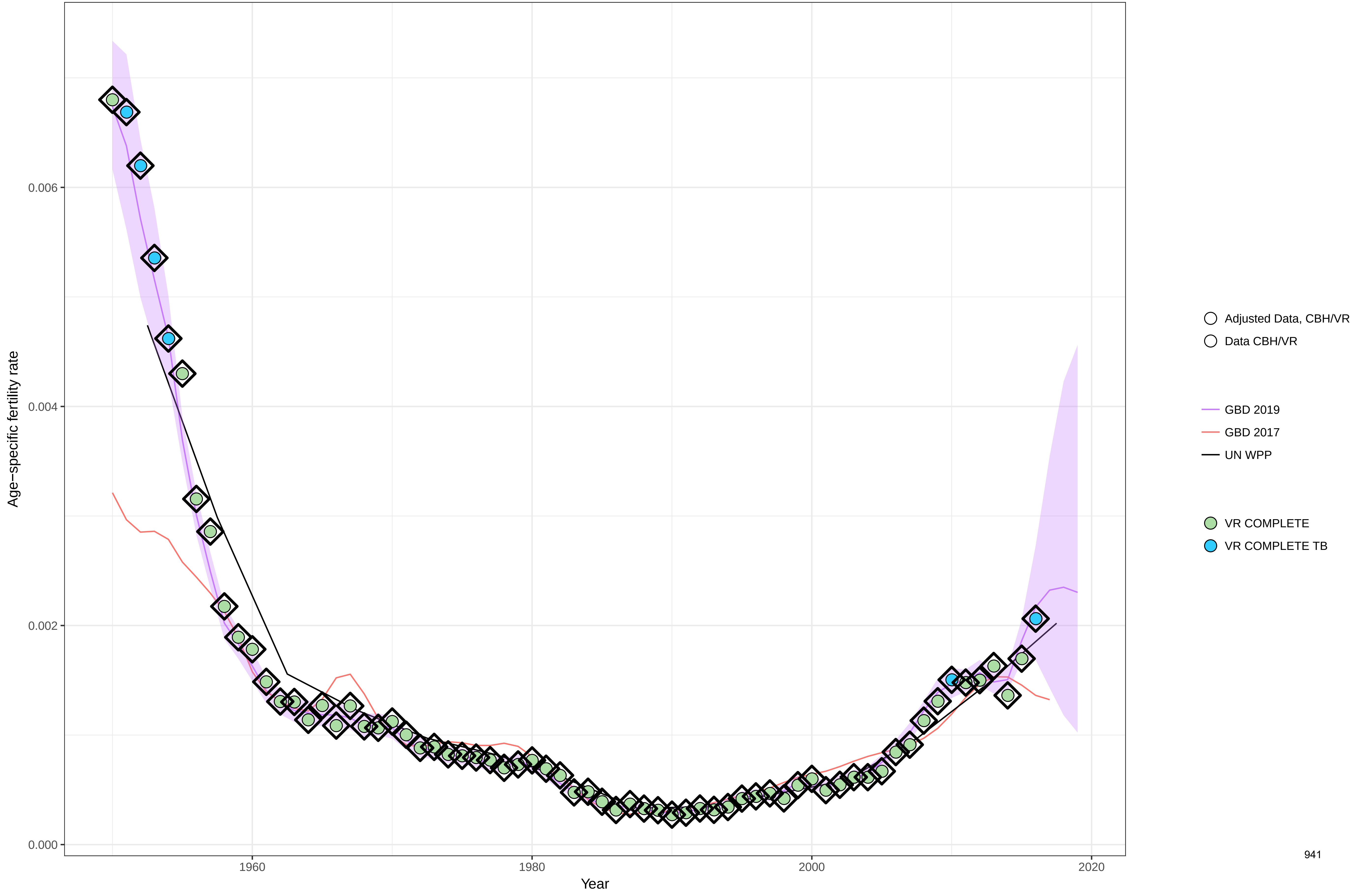

Age−specific fertility fitted trend lines, 1950−2019: Greece, Ages: 50−54
Data points enclosed with a diamond are reference sources

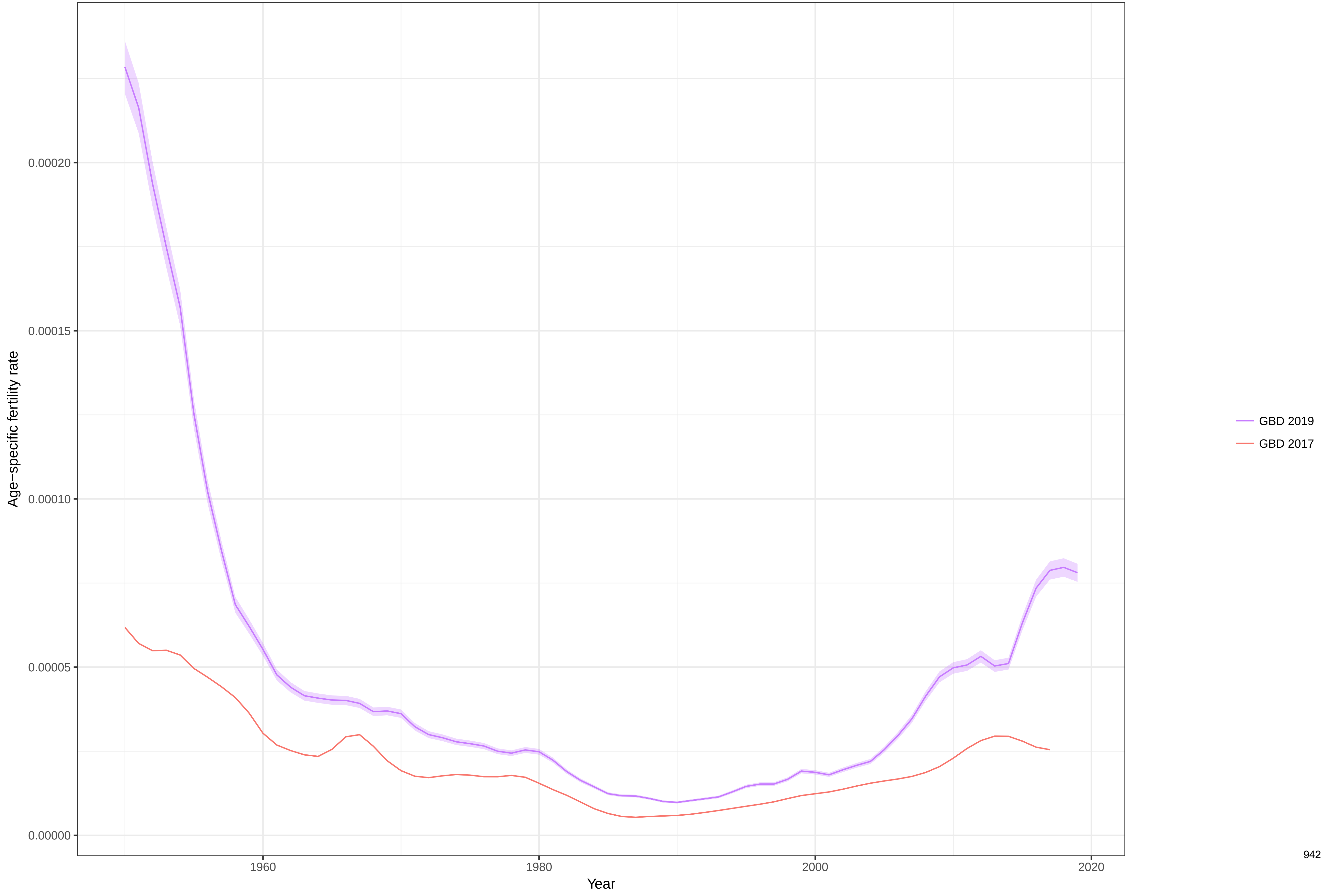

Total fertility rate fitted trend lines, 1950−2019: Greece TFR
Data points enclosed with a diamond are reference sources

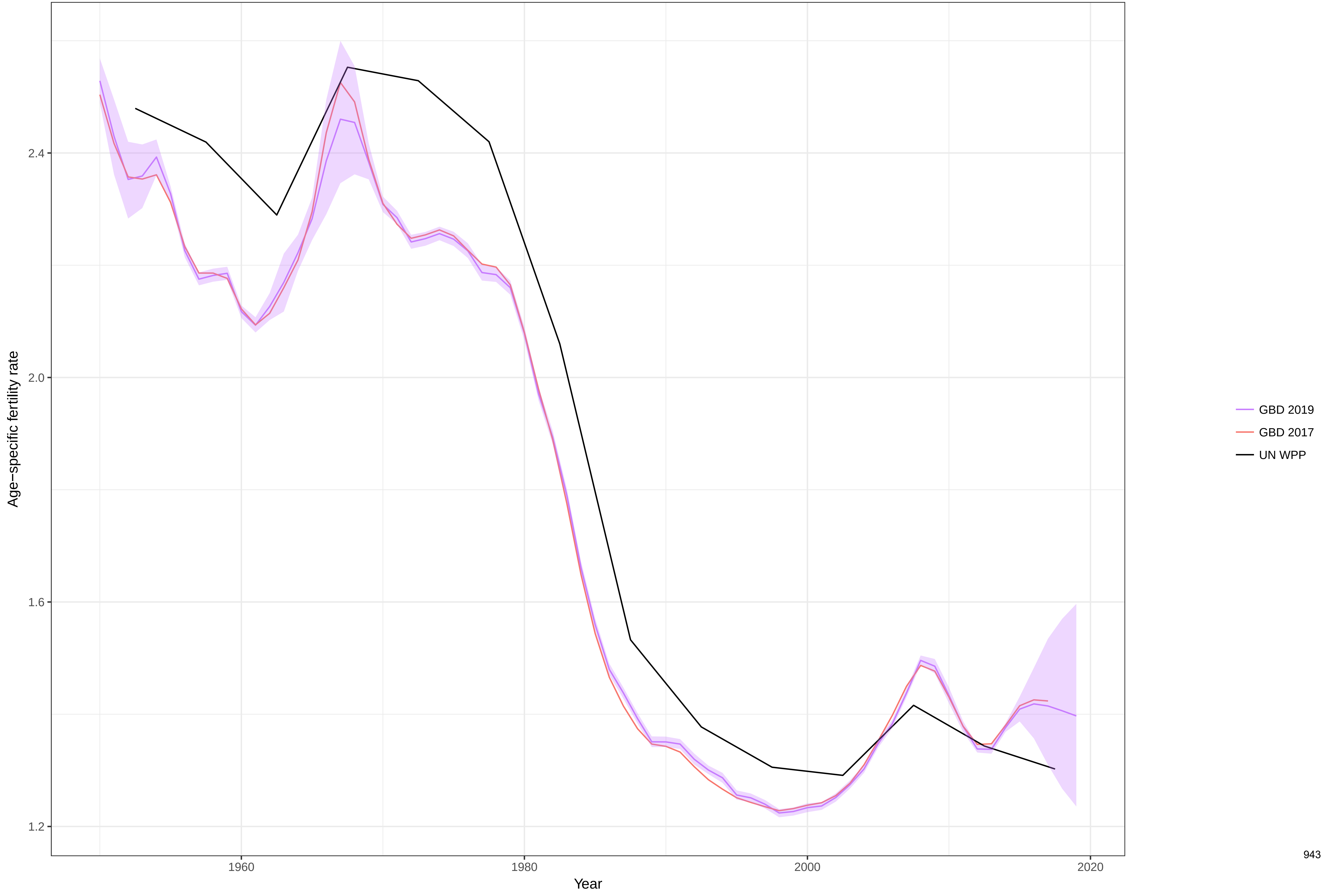

Age−specific fertility fitted trend lines, 1950−2019: Grenada, Ages: 10−14
Data points enclosed with a diamond are reference sources

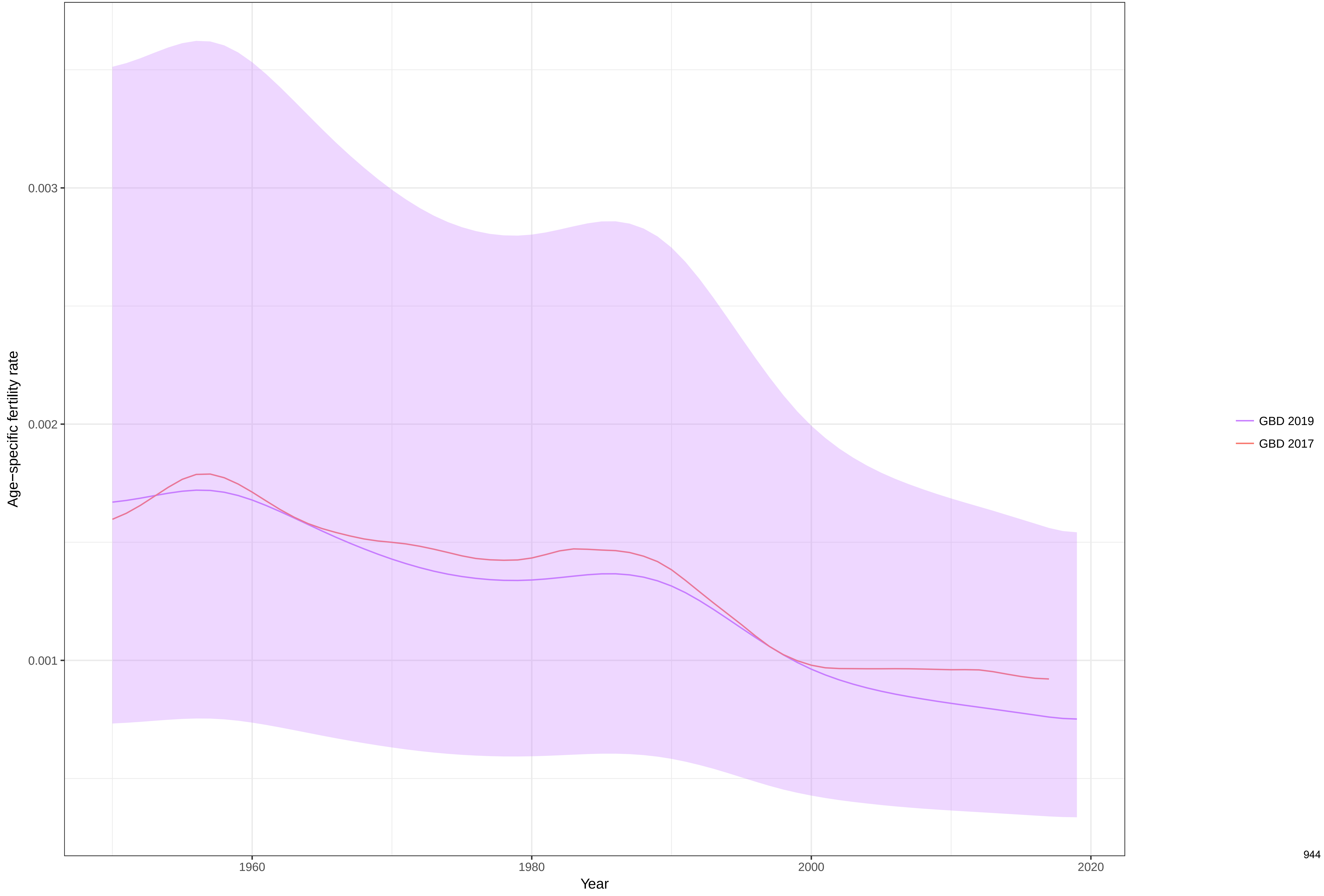

Age−specific fertility fitted trend lines, 1950−2019: Grenada, Ages: 15−19
Data points enclosed with a diamond are reference sources

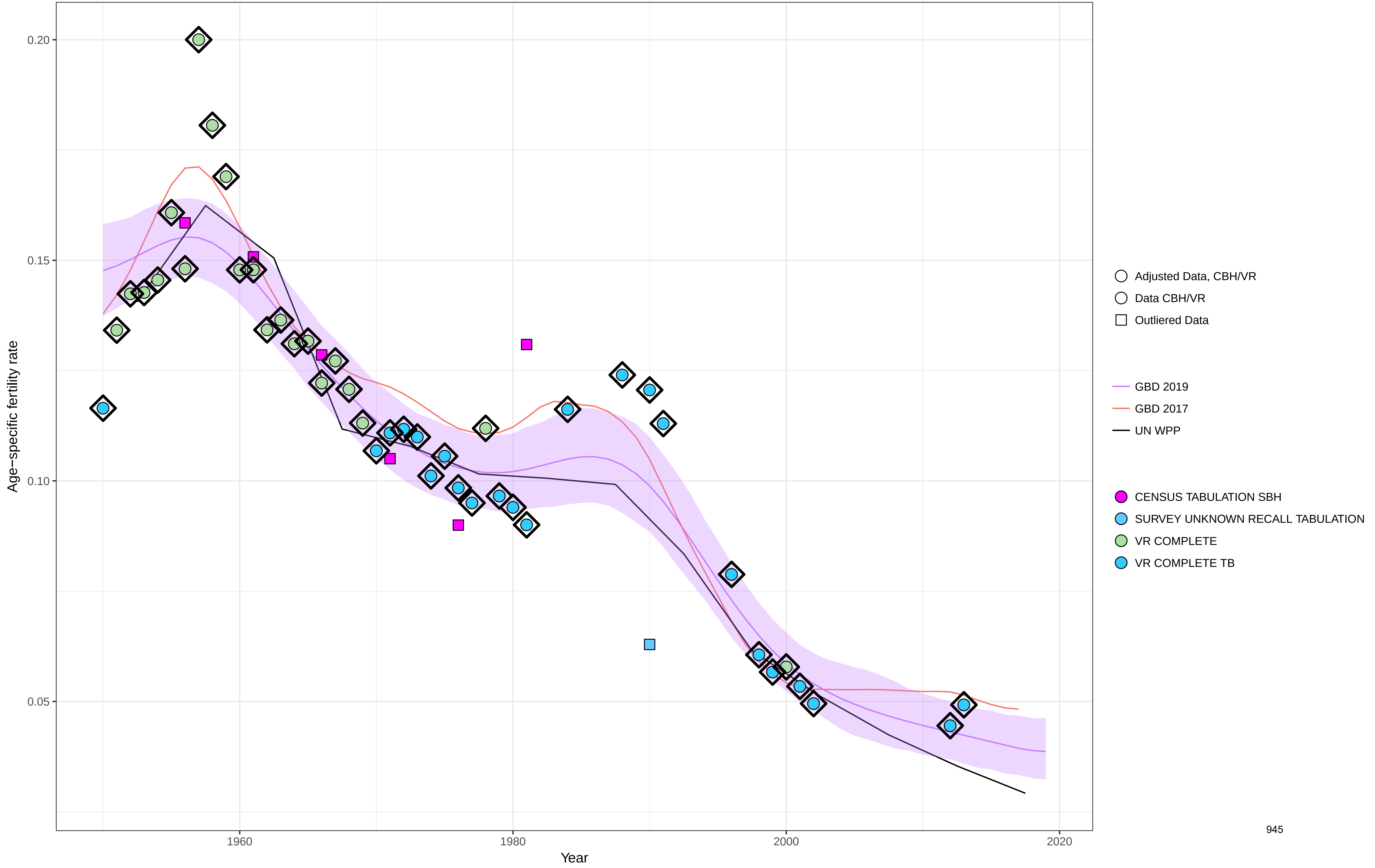

Age−specific fertility fitted trend lines, 1950−2019: Grenada, Ages: 20−24
Data points enclosed with a diamond are reference sources

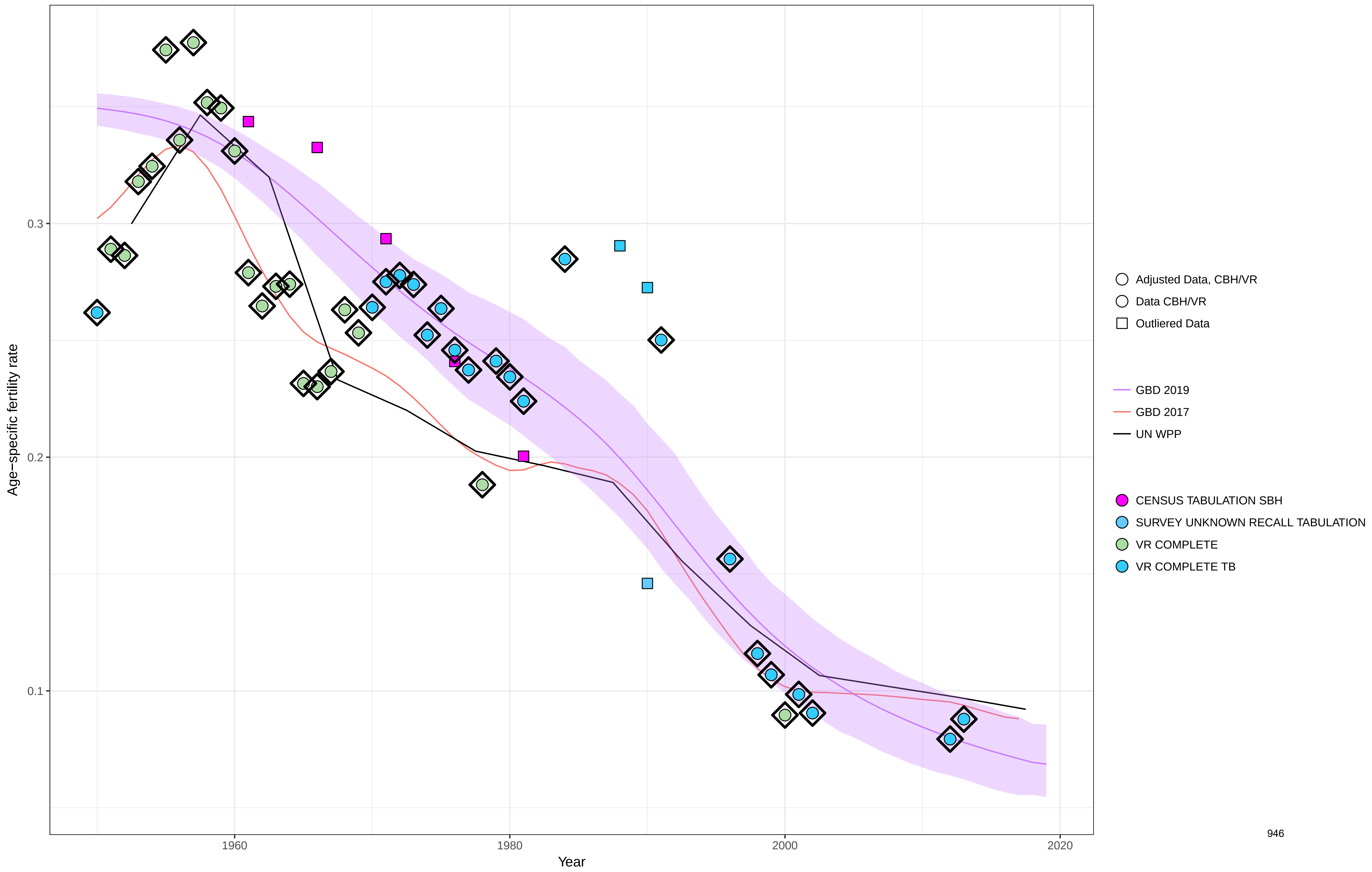

Age−specific fertility fitted trend lines, 1950−2019: Grenada, Ages: 25−29
Data points enclosed with a diamond are reference sources

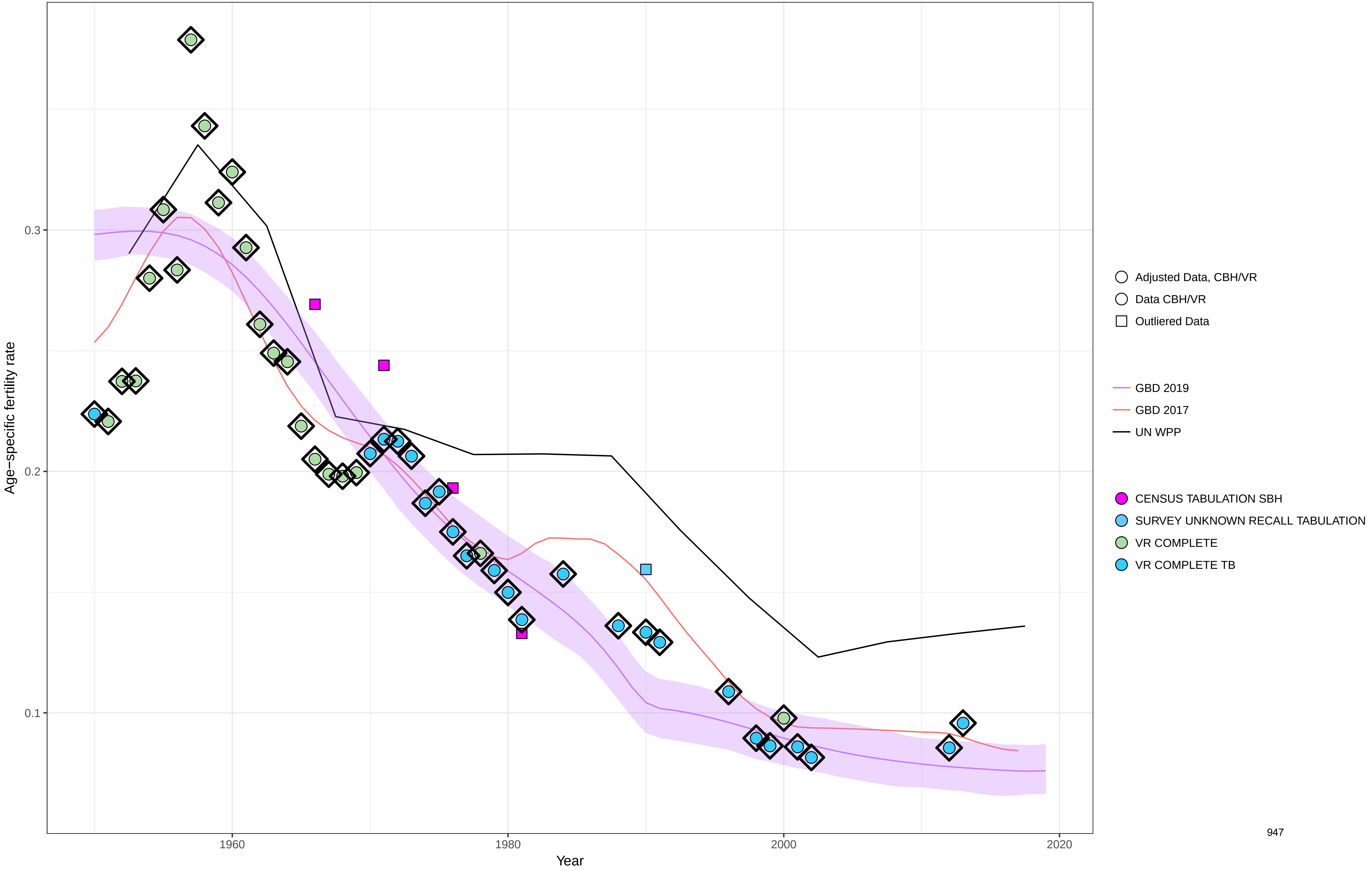

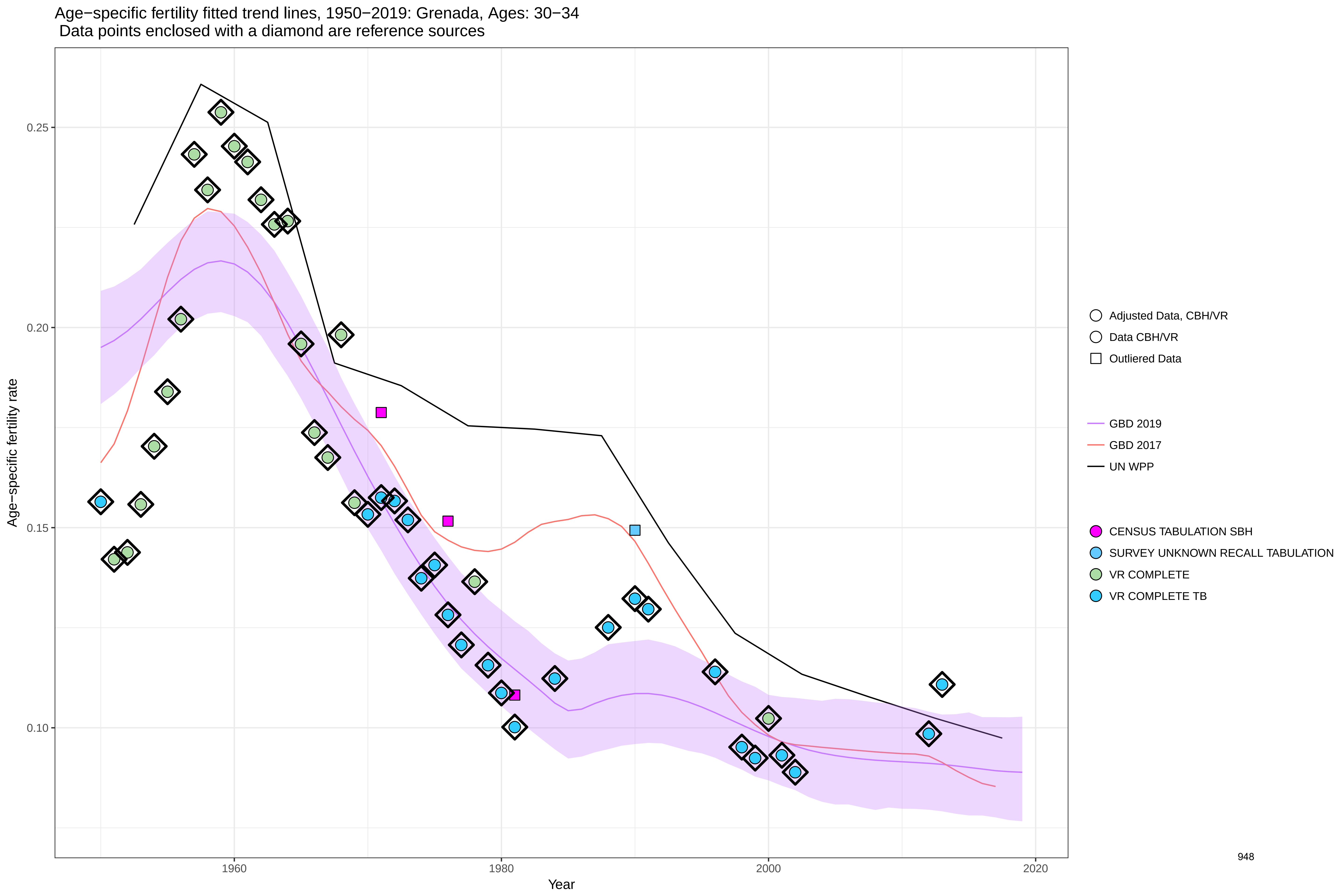

Age−specific fertility fitted trend lines, 1950−2019: Grenada, Ages: 30−34
Data points enclosed with a diamond are reference sources
0.25
0.20
0.15
0.10
Age−specific fertility rate
1960
1980
2000
2020
Year
Adjusted Data, CBH/VR
Data CBH/VR
Outliered Data
GBD 2019
GBD 2017
UN WPP
CENSUS TABULATION SBH
SURVEY UNKNOWN RECALL TABULATION
VR COMPLETE
VR COMPLETE TB

Age−specific fertility fitted trend lines, 1950−2019: Grenada, Ages: 35−39
Data points enclosed with a diamond are reference sources

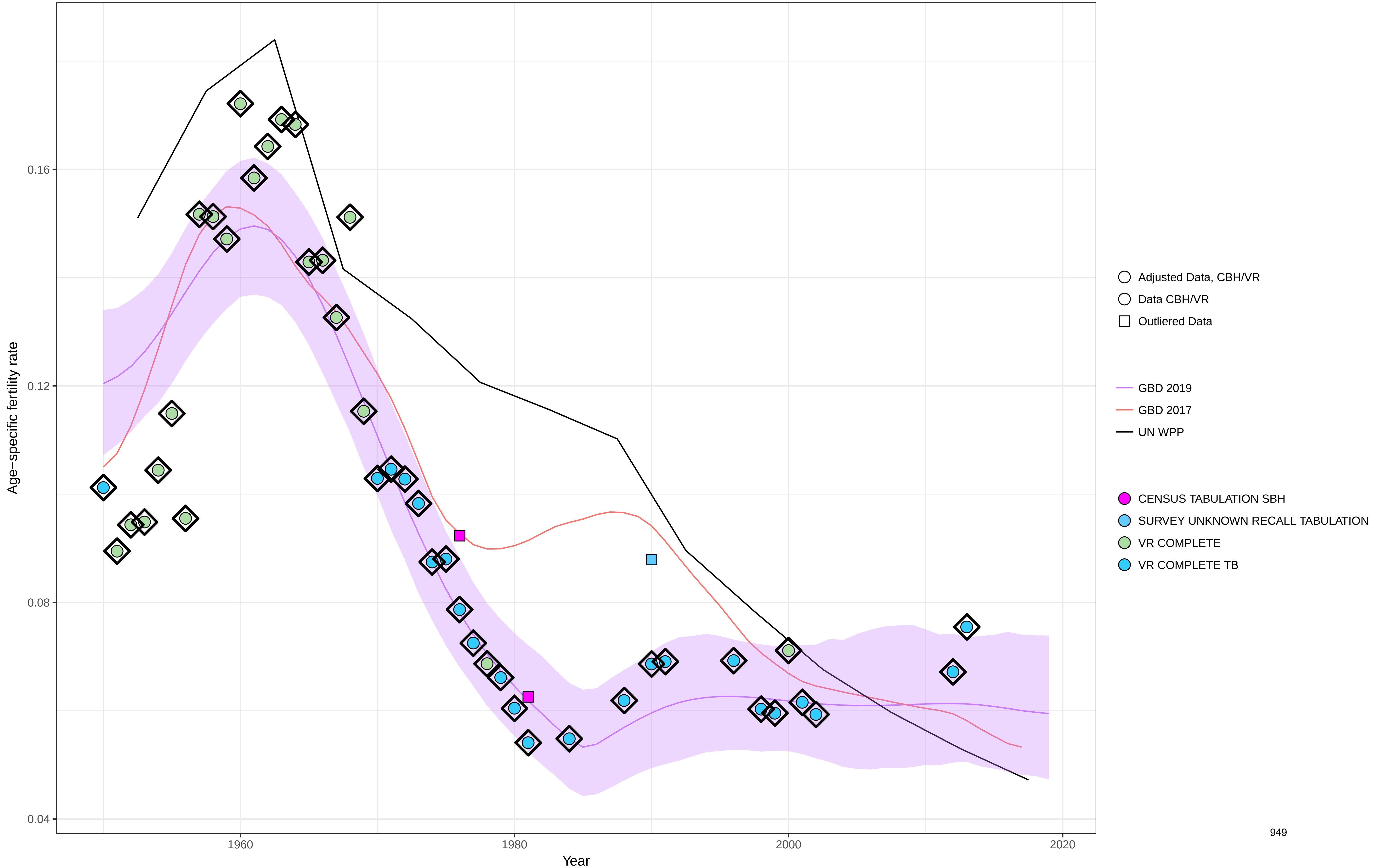

Age−specific fertility fitted trend lines, 1950−2019: Grenada, Ages: 40−44
Data points enclosed with a diamond are reference sources

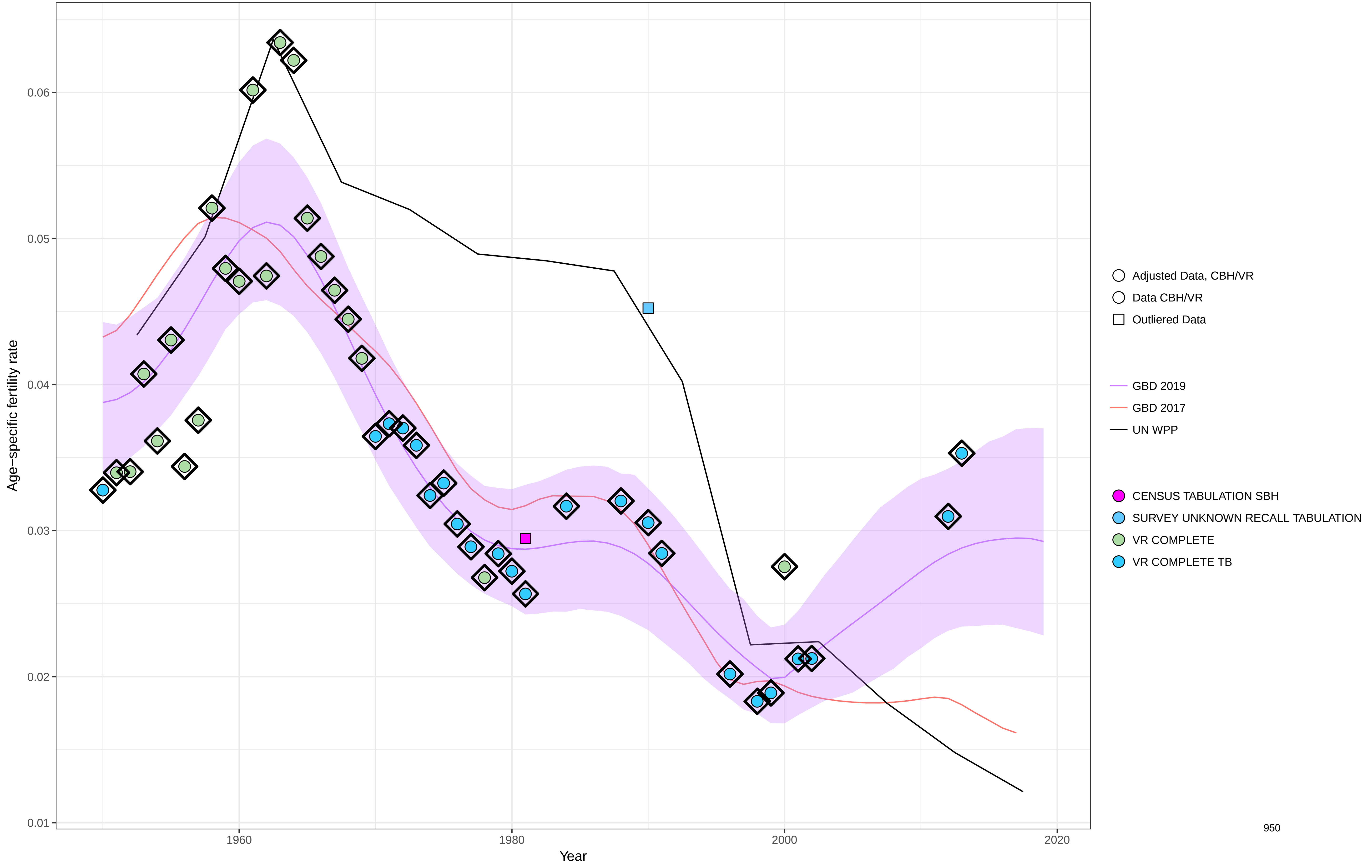

Age−specific fertility fitted trend lines, 1950−2019: Grenada, Ages: 45−49
Data points enclosed with a diamond are reference sources

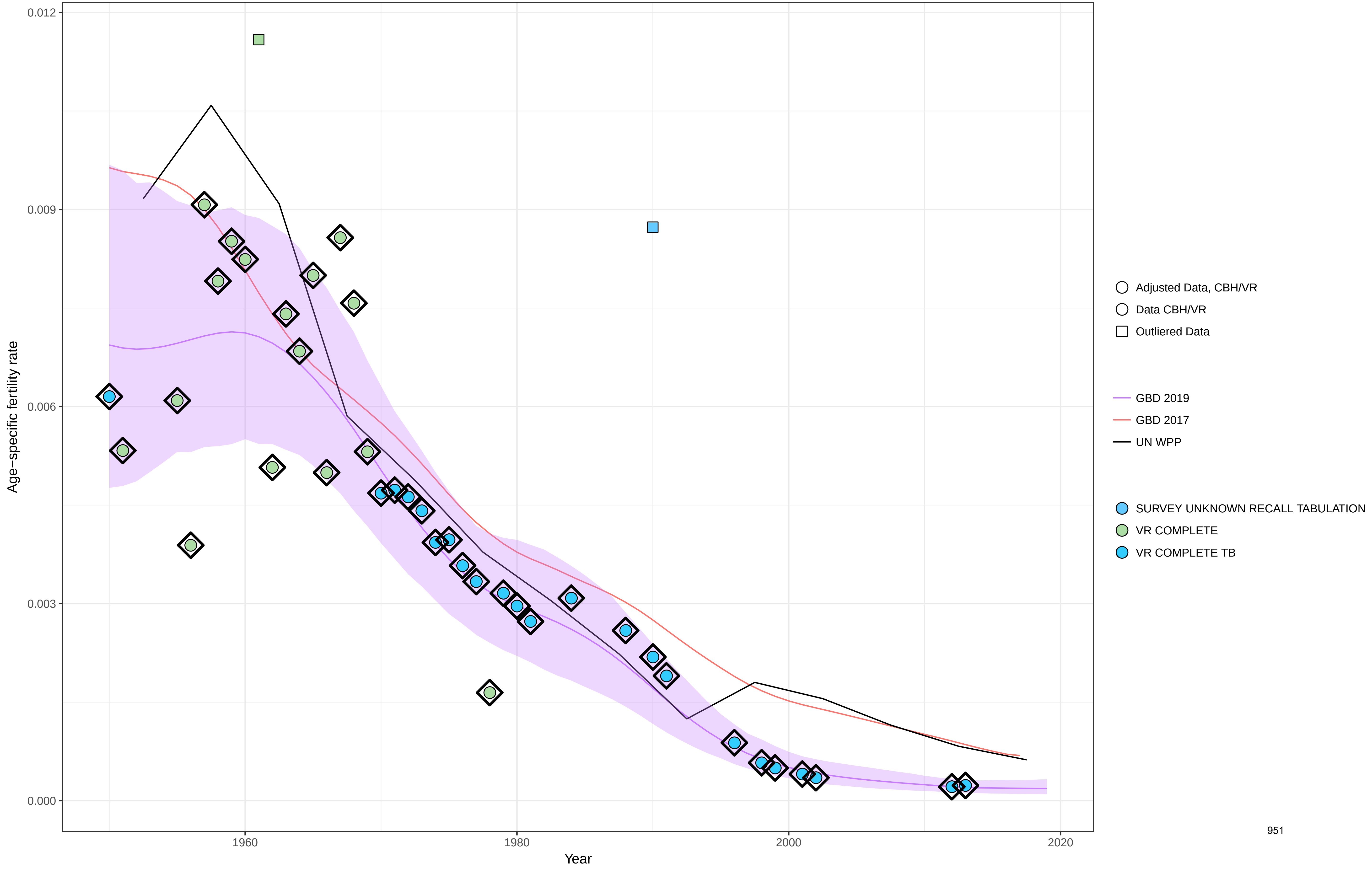

Age−specific fertility fitted trend lines, 1950−2019: Grenada, Ages: 50−54
Data points enclosed with a diamond are reference sources

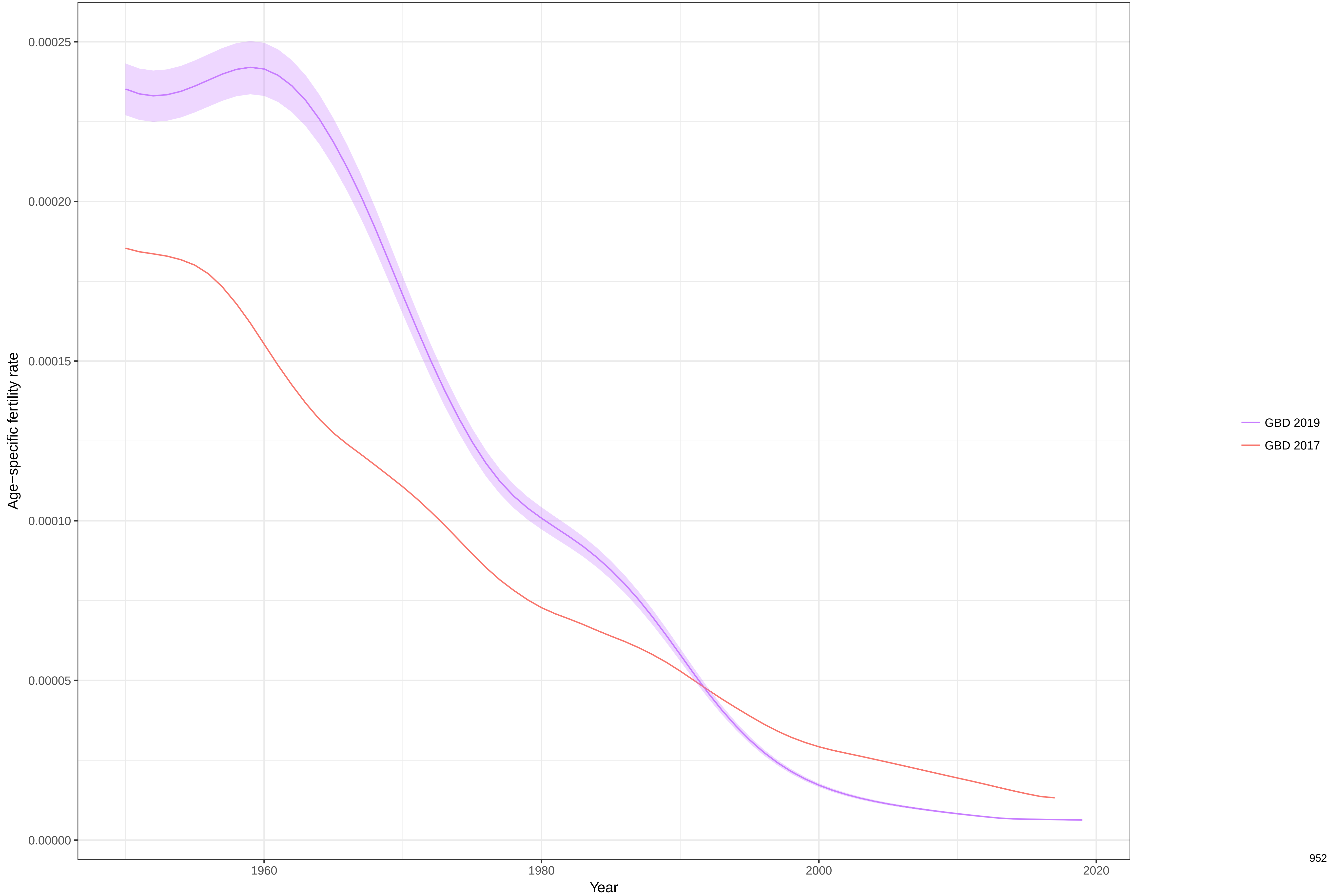

Total fertility rate fitted trend lines, 1950−2019: Grenada TFR
Data points enclosed with a diamond are reference sources

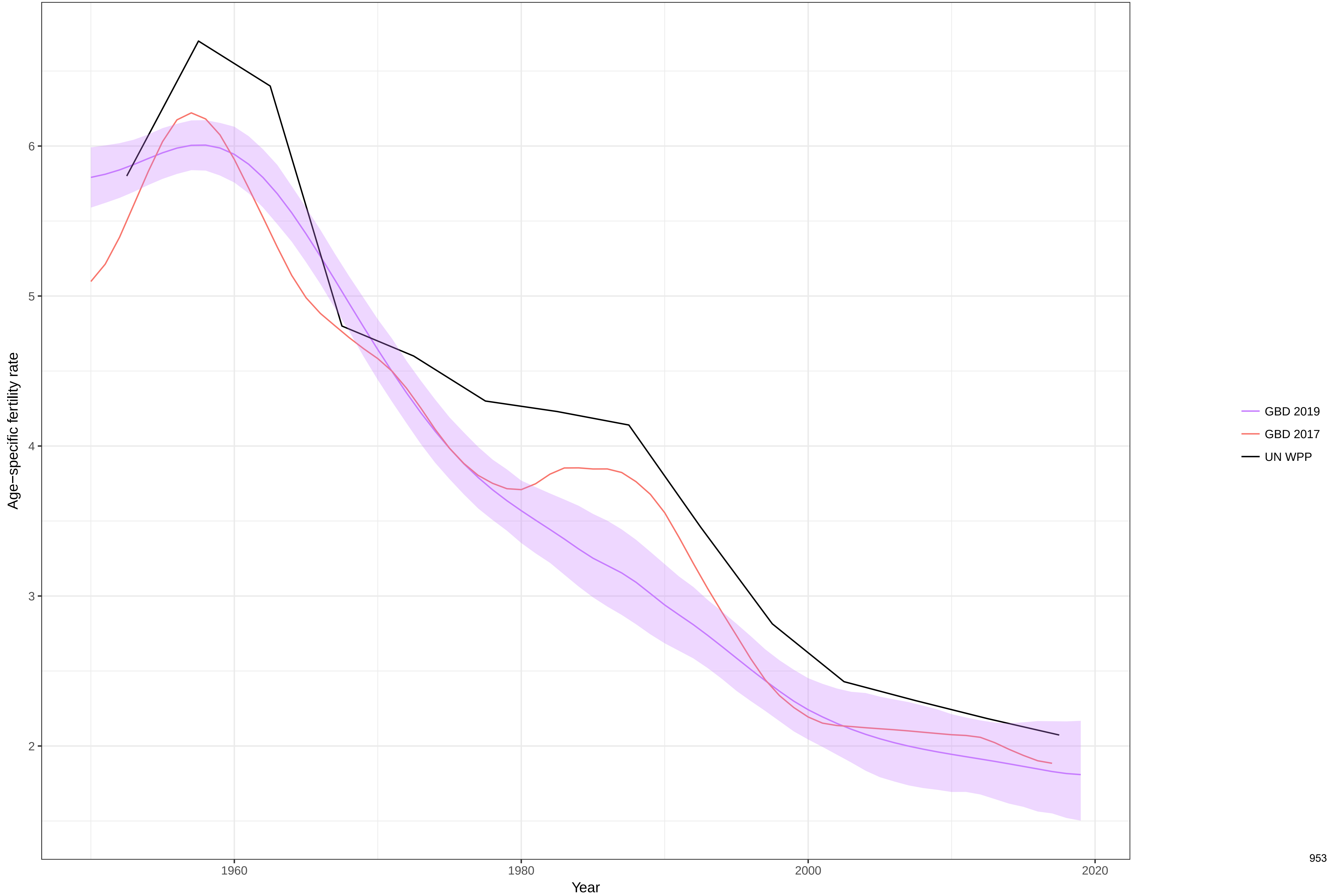

Age−specific fertility fitted trend lines, 1950−2019: Greenland, Ages: 10−14
Data points enclosed with a diamond are reference sources

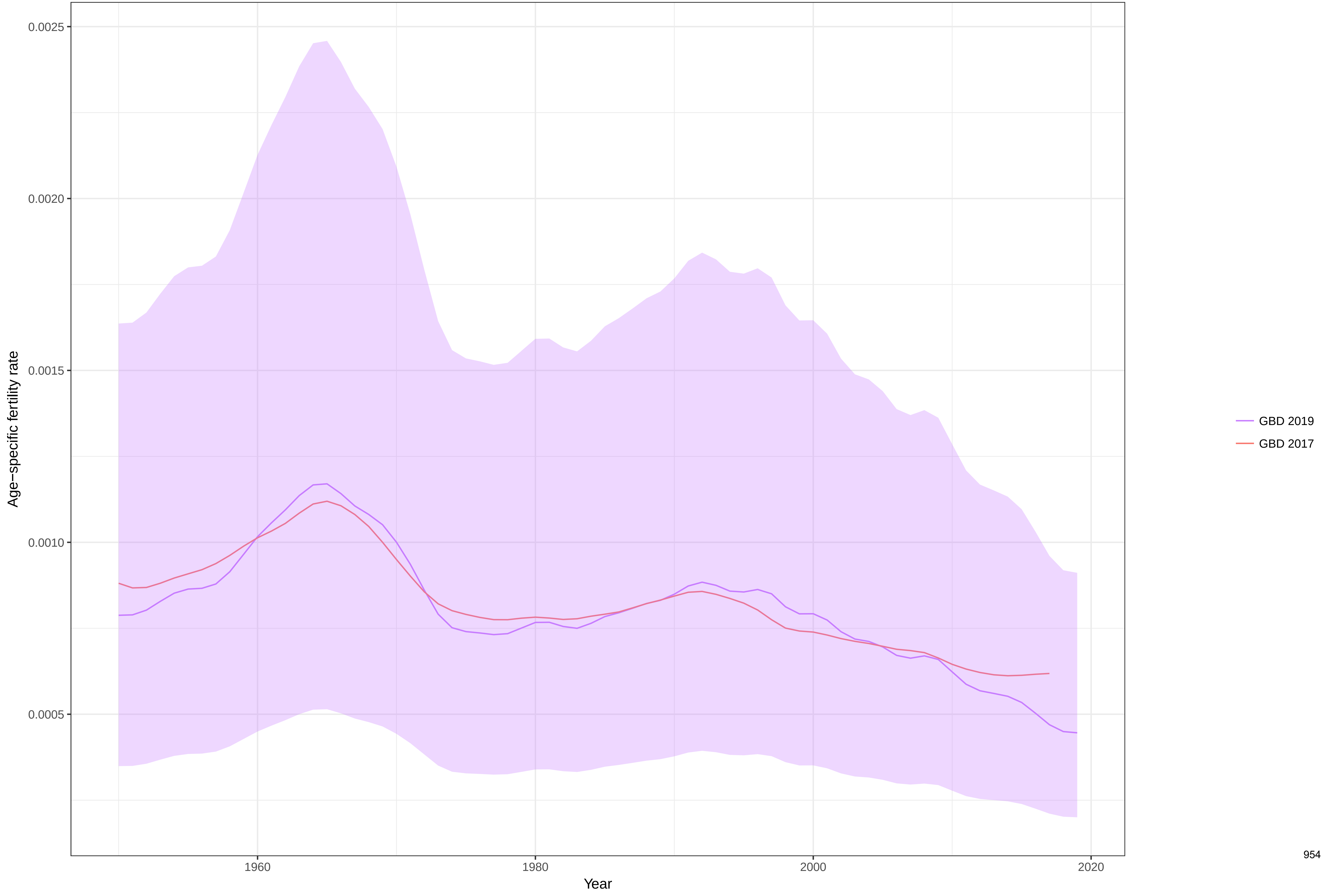

Age−specific fertility fitted trend lines, 1950−2019: Greenland, Ages: 15−19
Data points enclosed with a diamond are reference sources

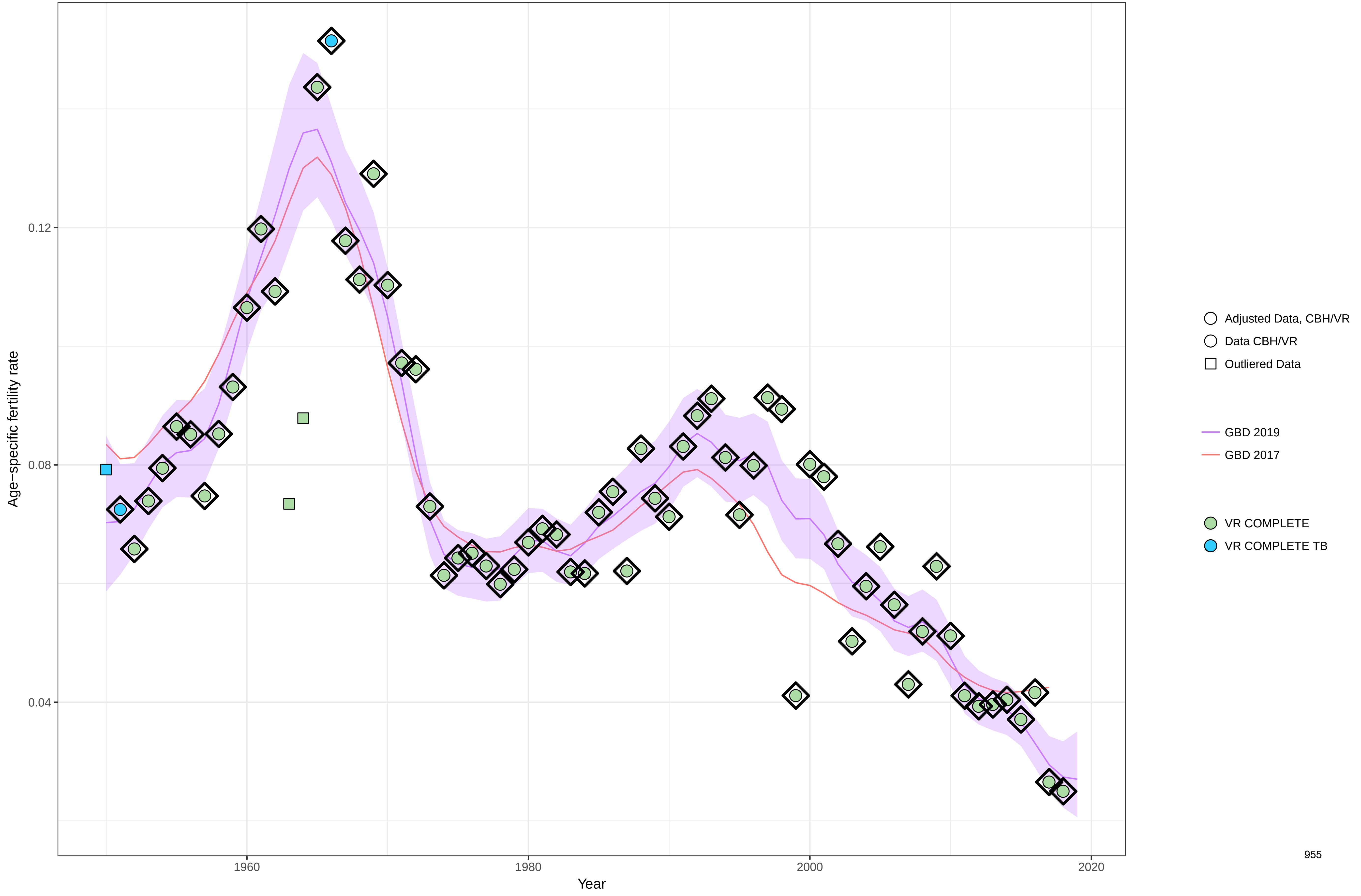

Age−specific fertility fitted trend lines, 1950−2019: Greenland, Ages: 20−24
Data points enclosed with a diamond are reference sources

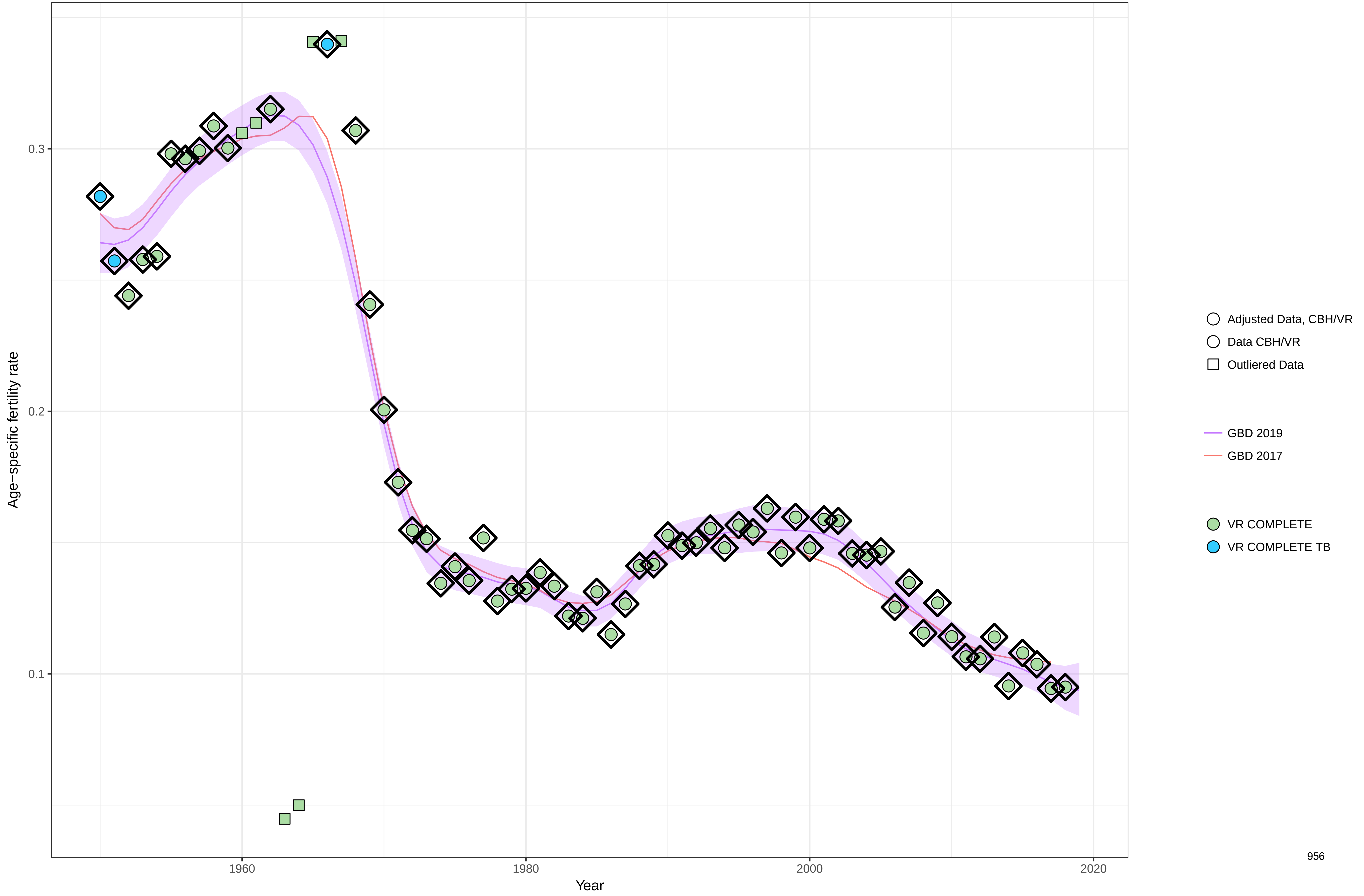

Age−specific fertility fitted trend lines, 1950−2019: Greenland, Ages: 25−29
Data points enclosed with a diamond are reference sources

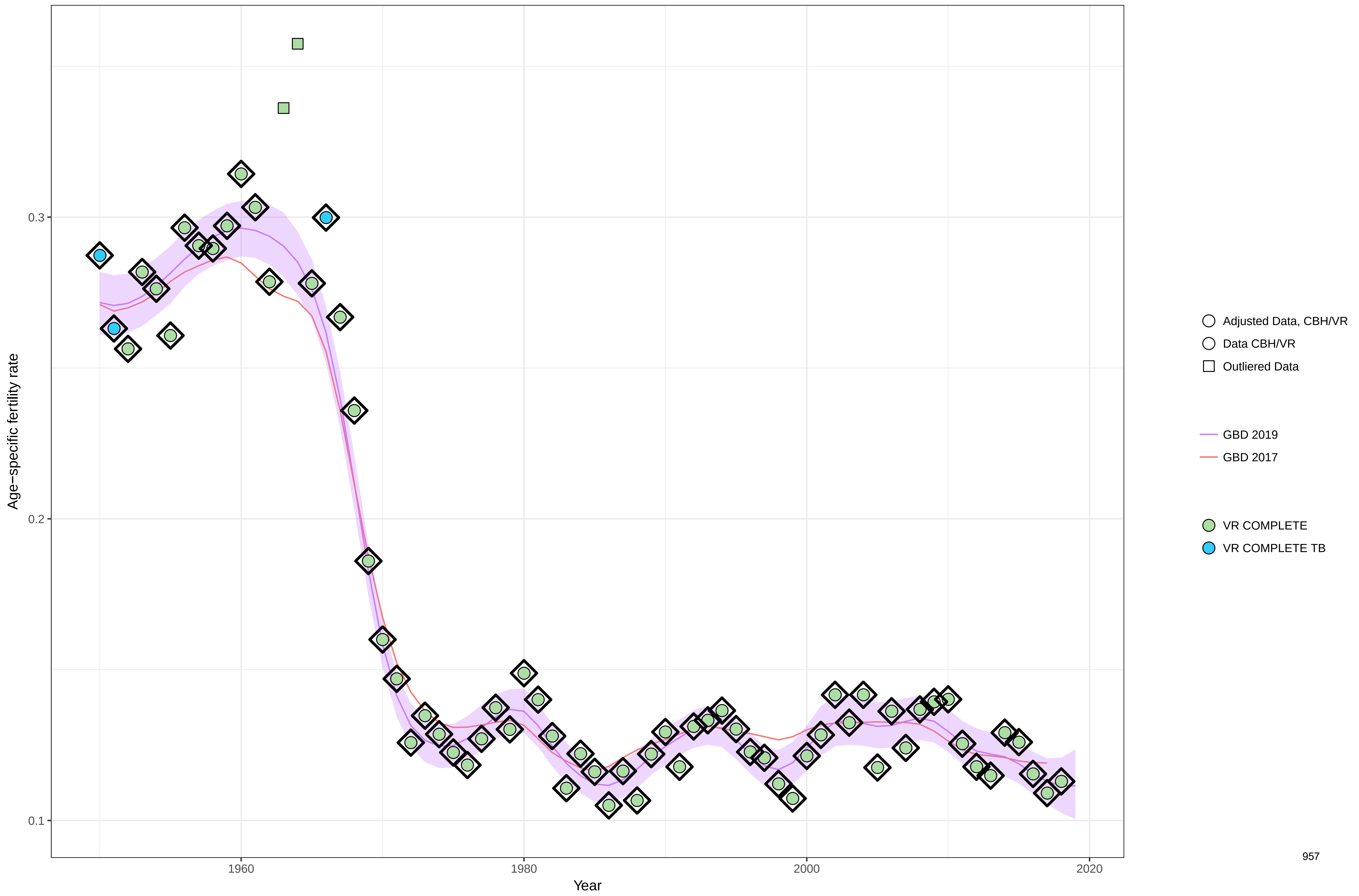

Age−specific fertility fitted trend lines, 1950−2019: Greenland, Ages: 30−34
Data points enclosed with a diamond are reference sources

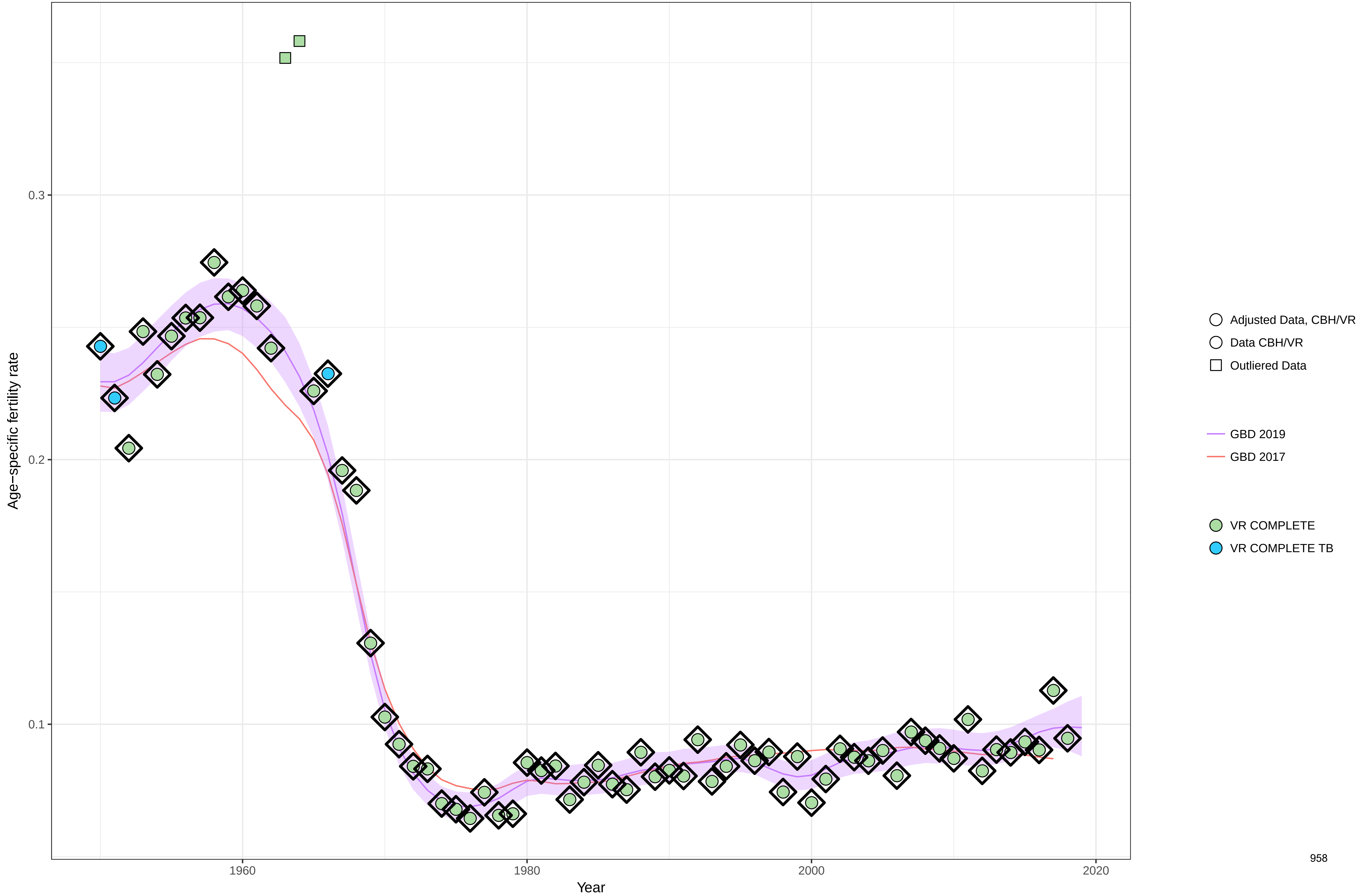

Age−specific fertility fitted trend lines, 1950−2019: Greenland, Ages: 35−39
Data points enclosed with a diamond are reference sources

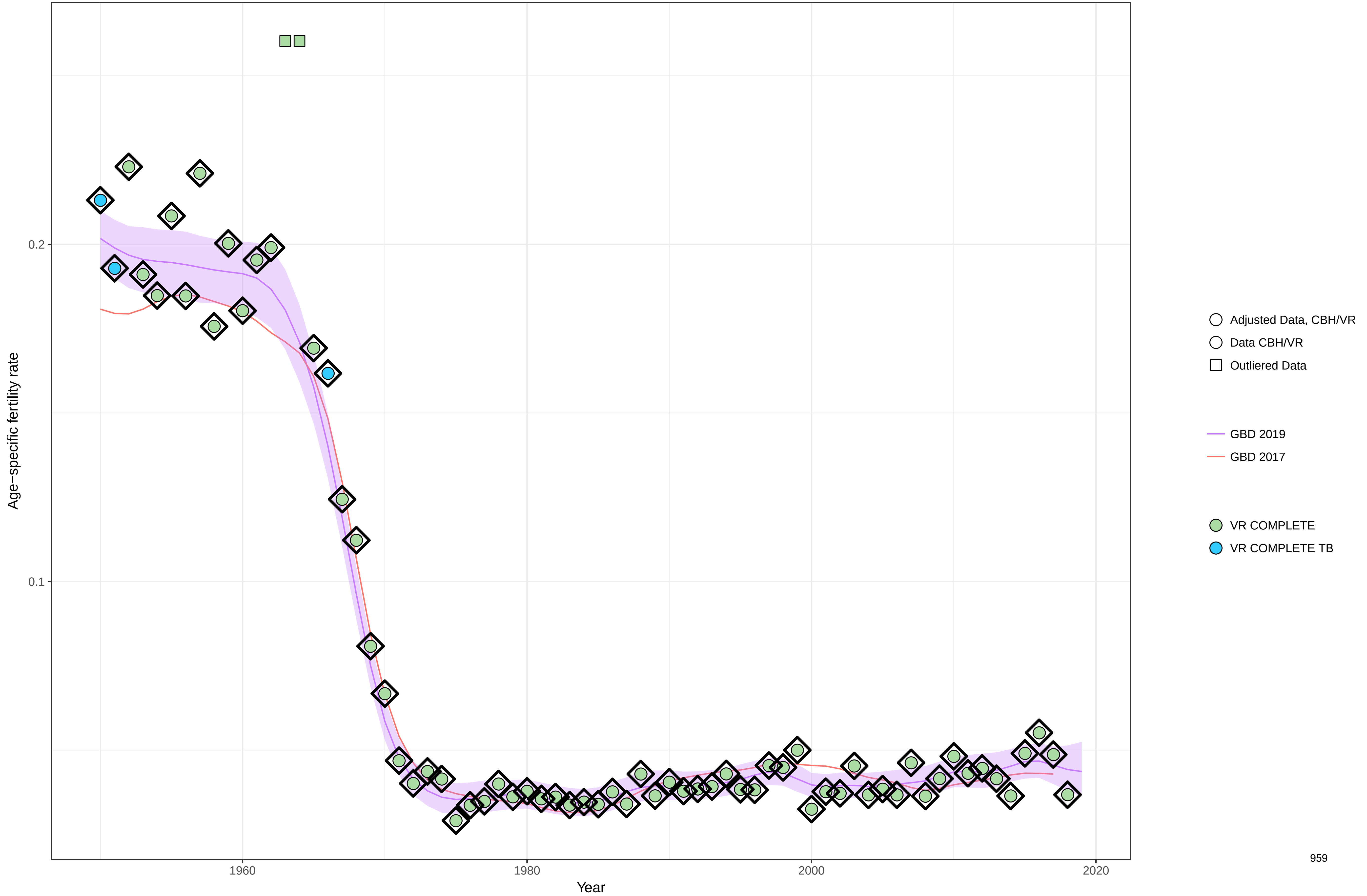

Age−specific fertility fitted trend lines, 1950−2019: Greenland, Ages: 40−44
Data points enclosed with a diamond are reference sources

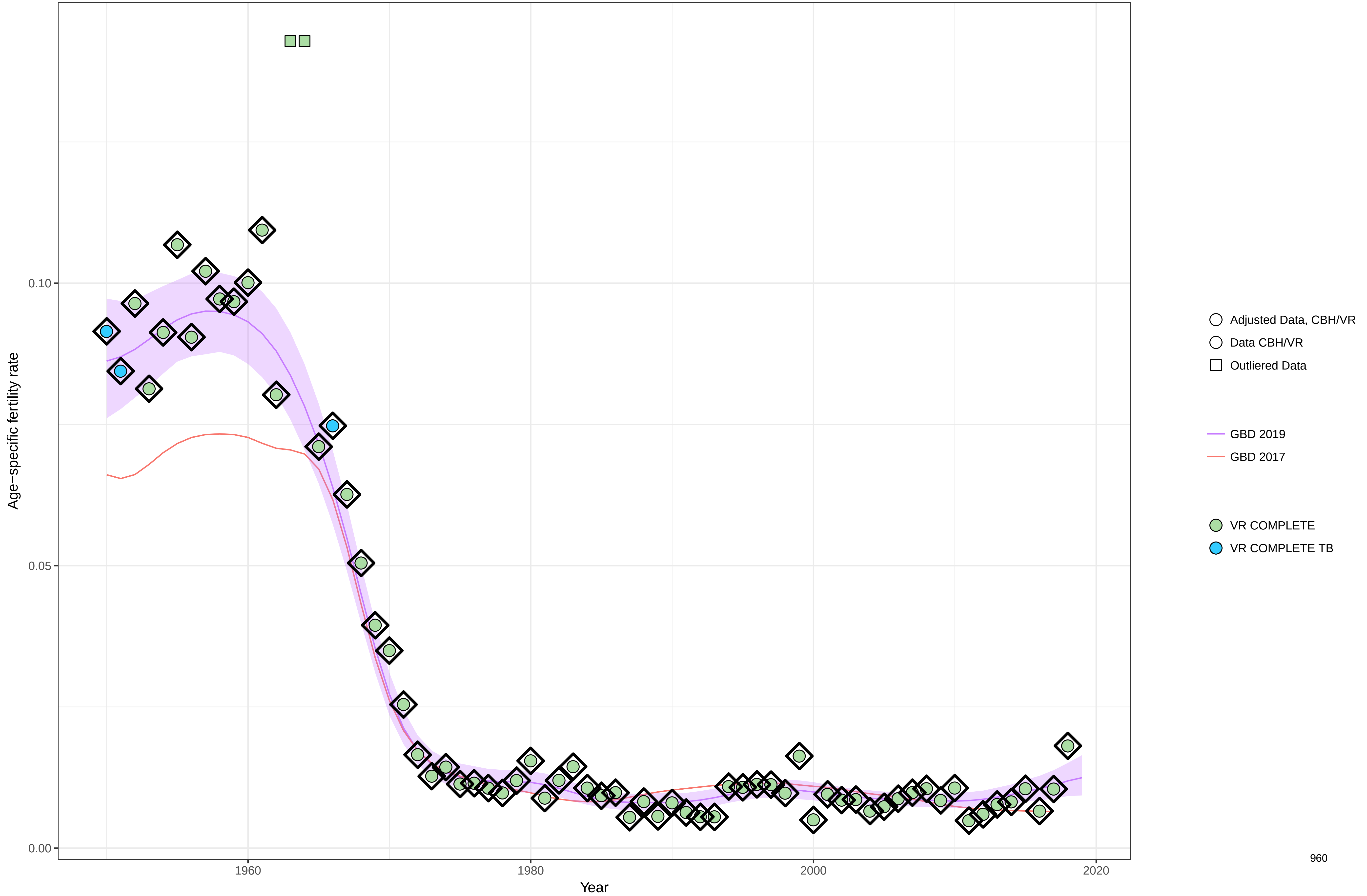

Age−specific fertility fitted trend lines, 1950−2019: Greenland, Ages: 45−49
Data points enclosed with a diamond are reference sources

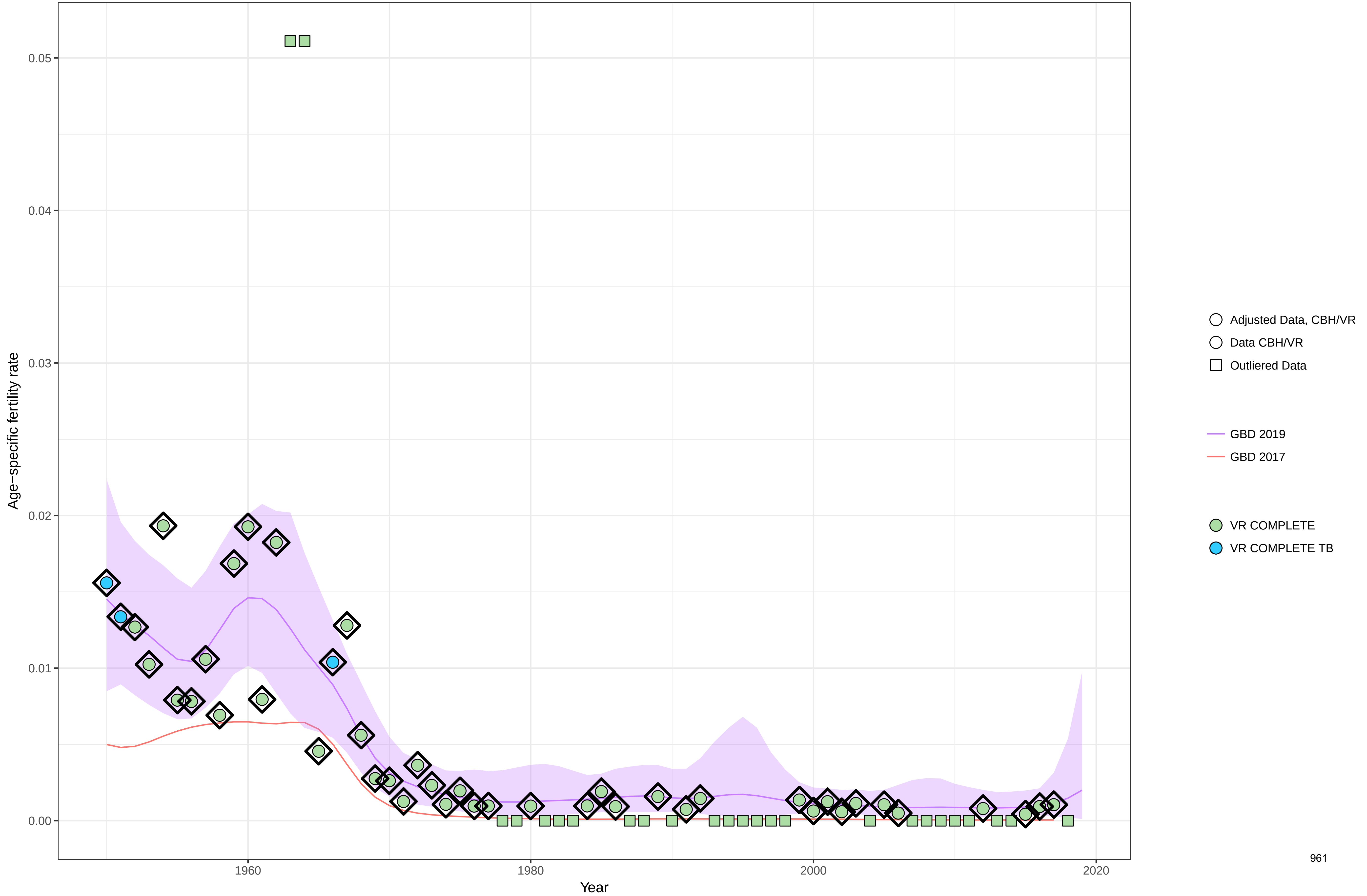

Age−specific fertility fitted trend lines, 1950−2019: Greenland, Ages: 50−54
Data points enclosed with a diamond are reference sources

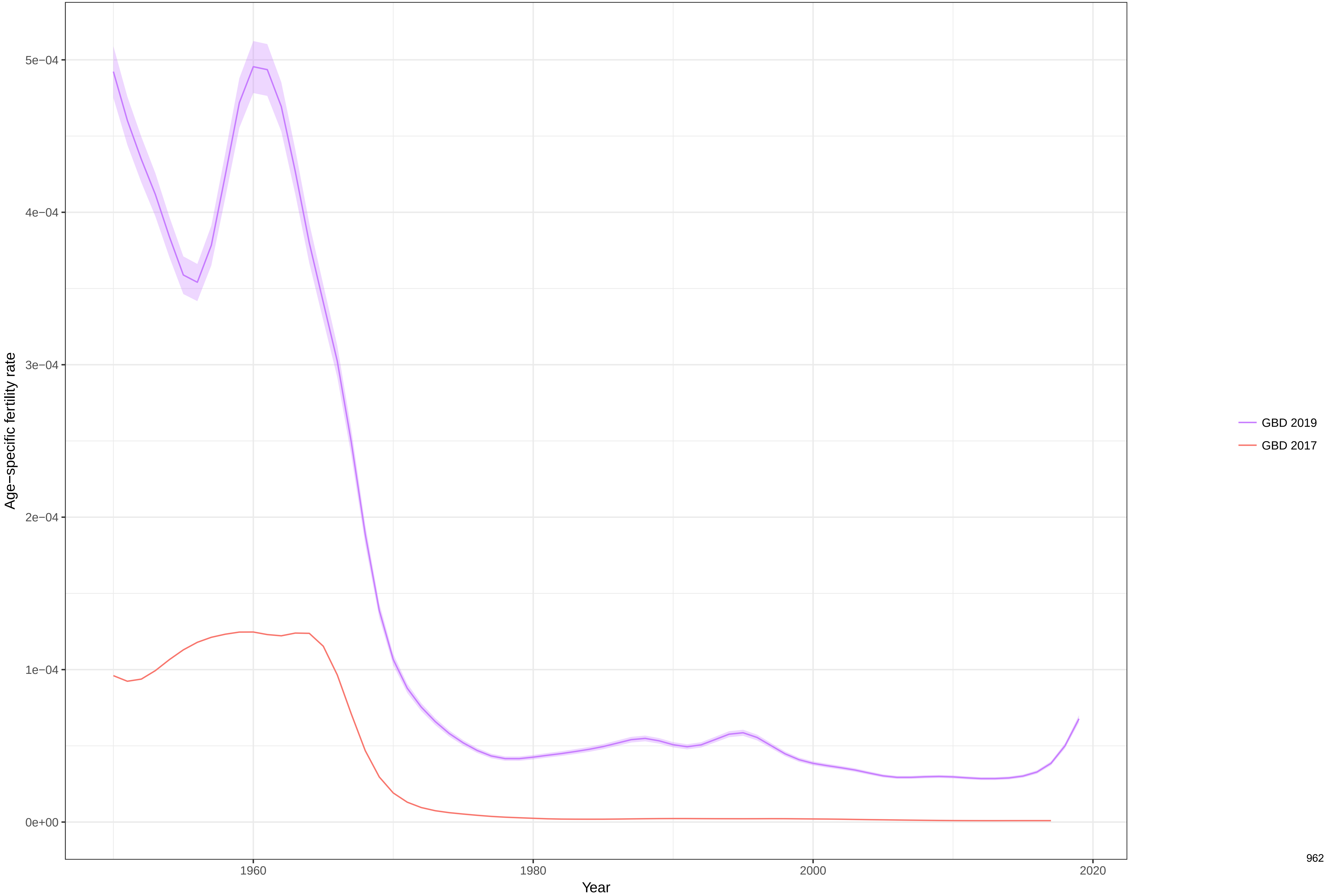

Total fertility rate fitted trend lines, 1950−2019: Greenland TFR
Data points enclosed with a diamond are reference sources

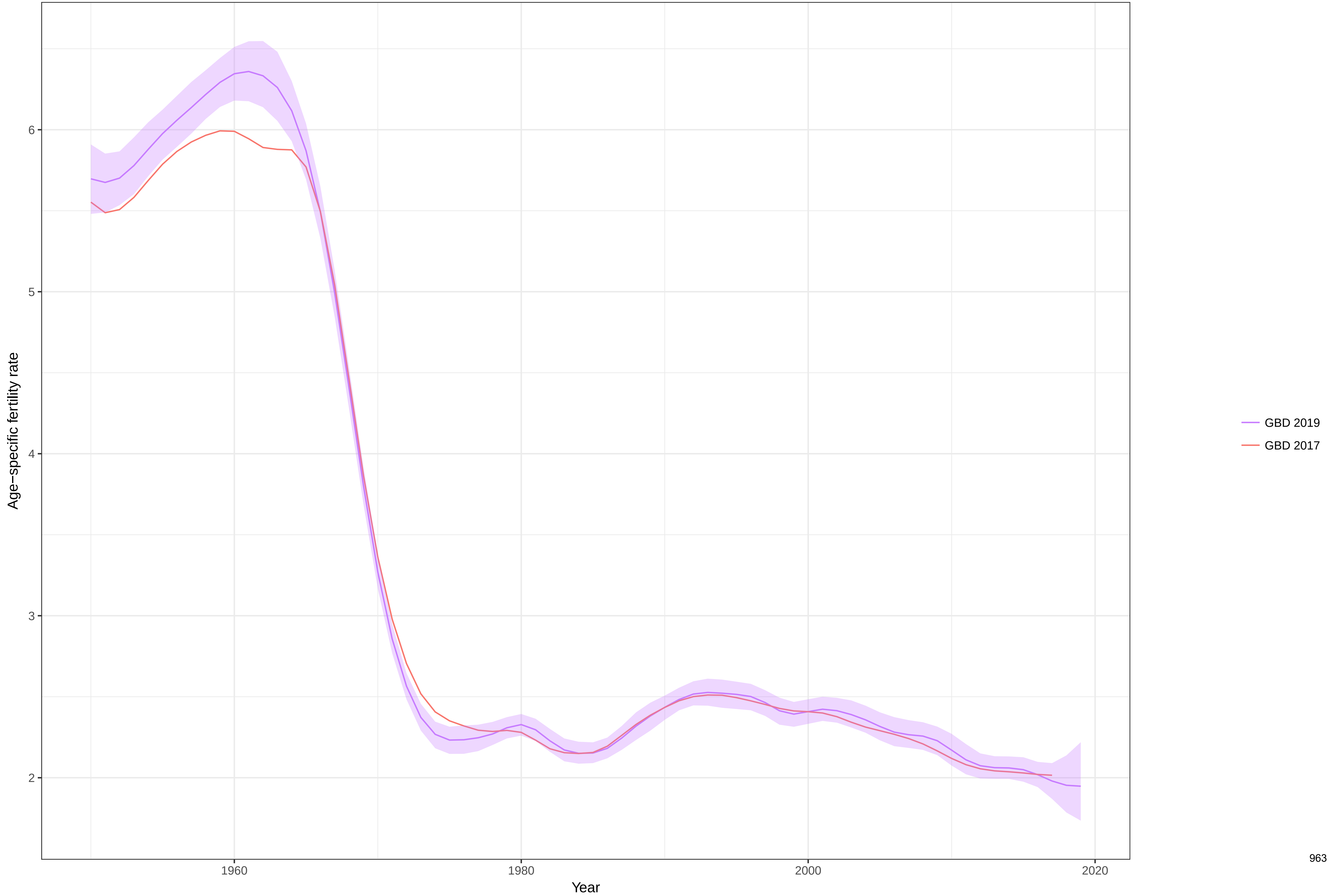

Age−specific fertility fitted trend lines, 1950−2019: Guatemala, Ages: 10−14
Data points enclosed with a diamond are reference sources

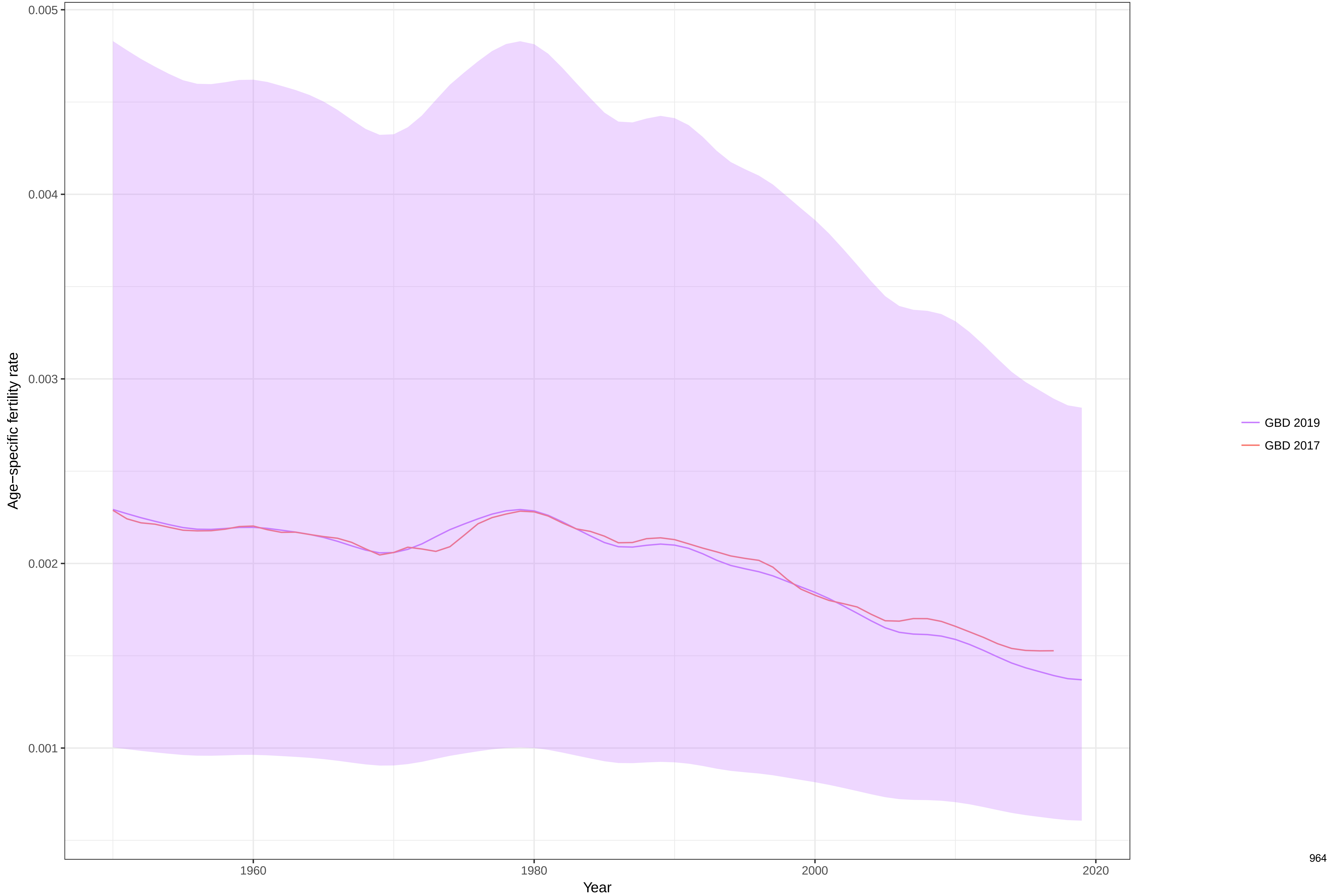

Age−specific fertility fitted trend lines, 1950−2019: Guatemala, Ages: 15−19
Data points enclosed with a diamond are reference sources

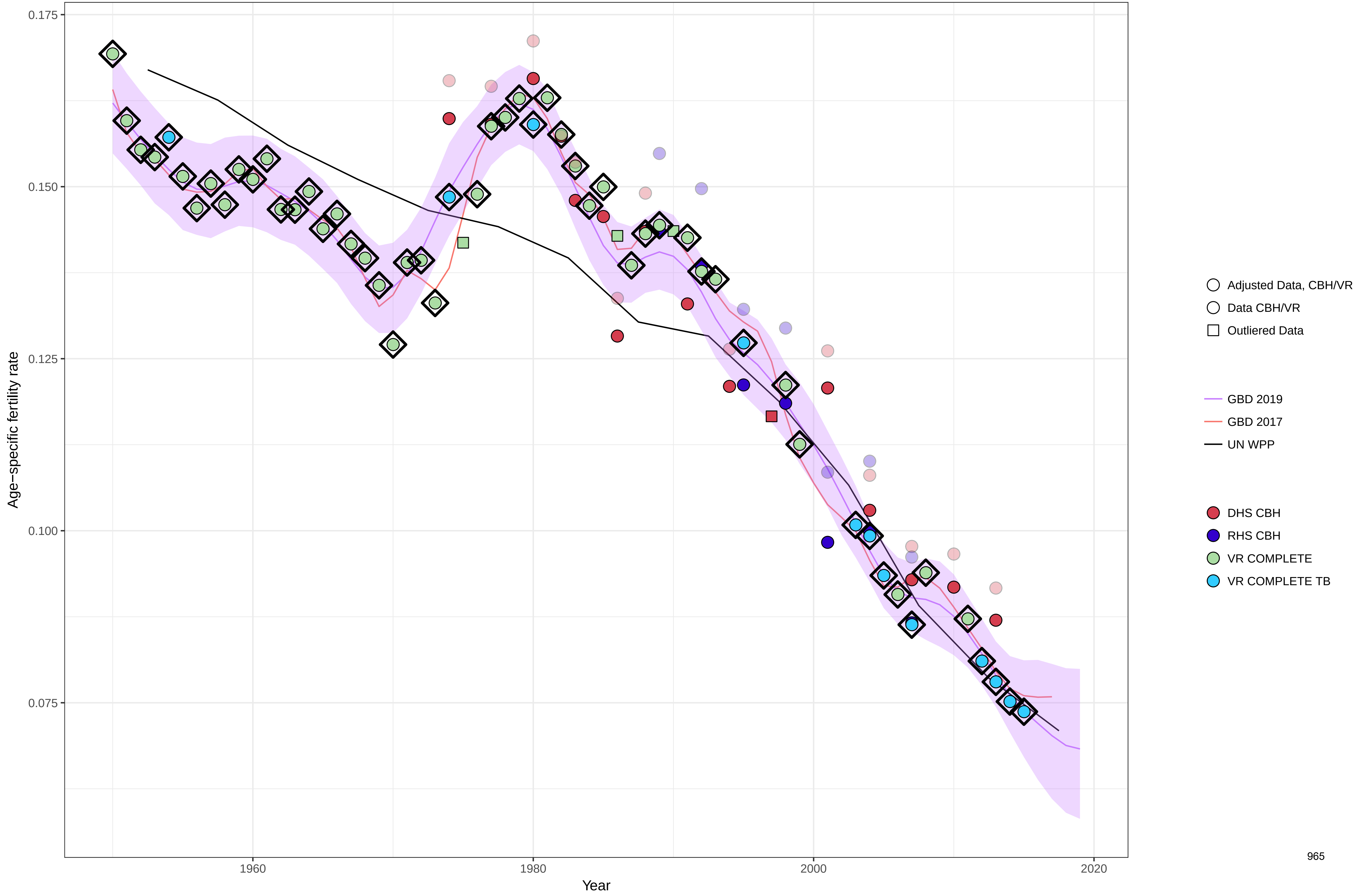

Age−specific fertility fitted trend lines, 1950−2019: Guatemala, Ages: 20−24
Data points enclosed with a diamond are reference sources

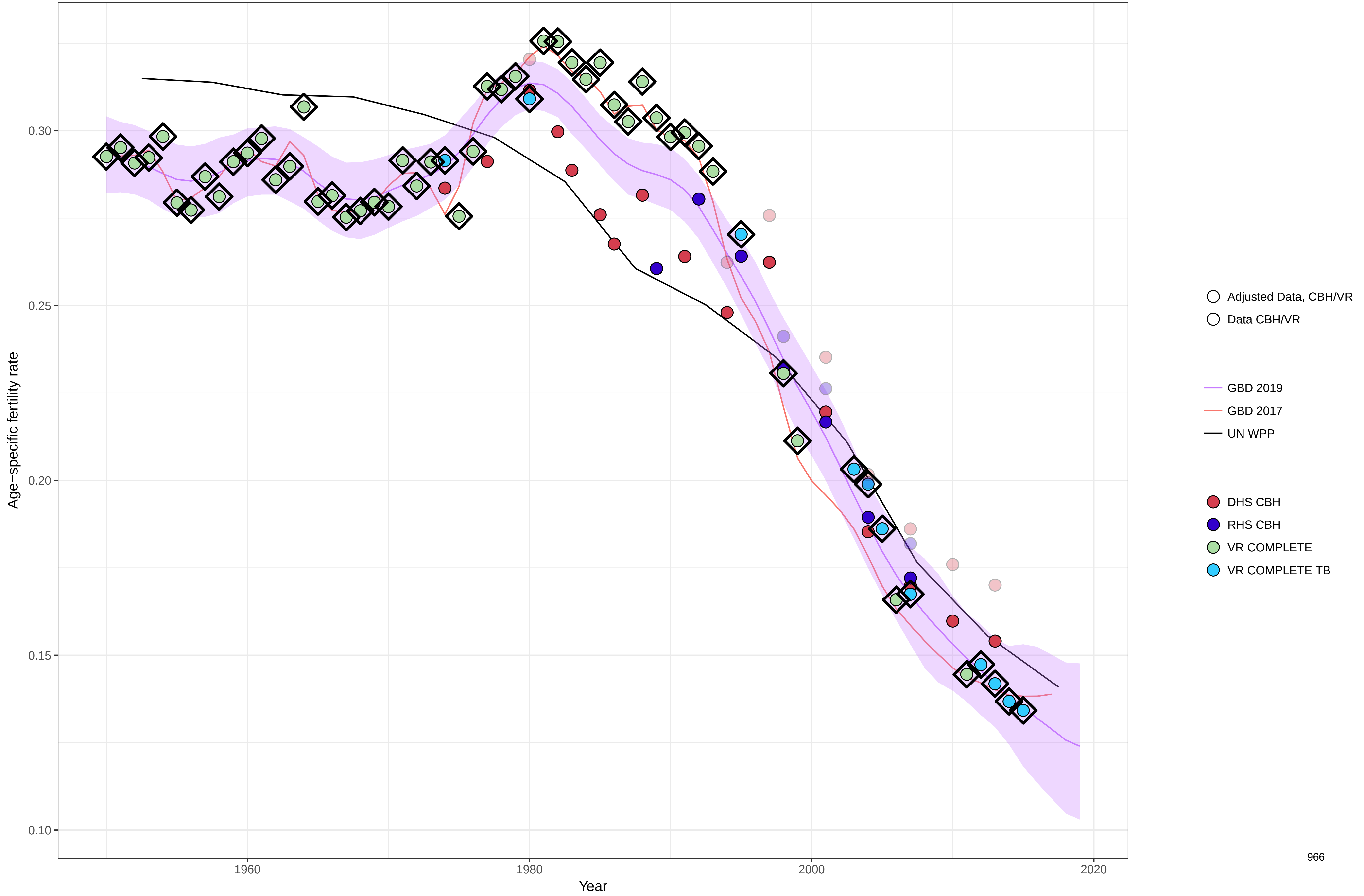

Age−specific fertility fitted trend lines, 1950−2019: Guatemala, Ages: 25−29
Data points enclosed with a diamond are reference sources

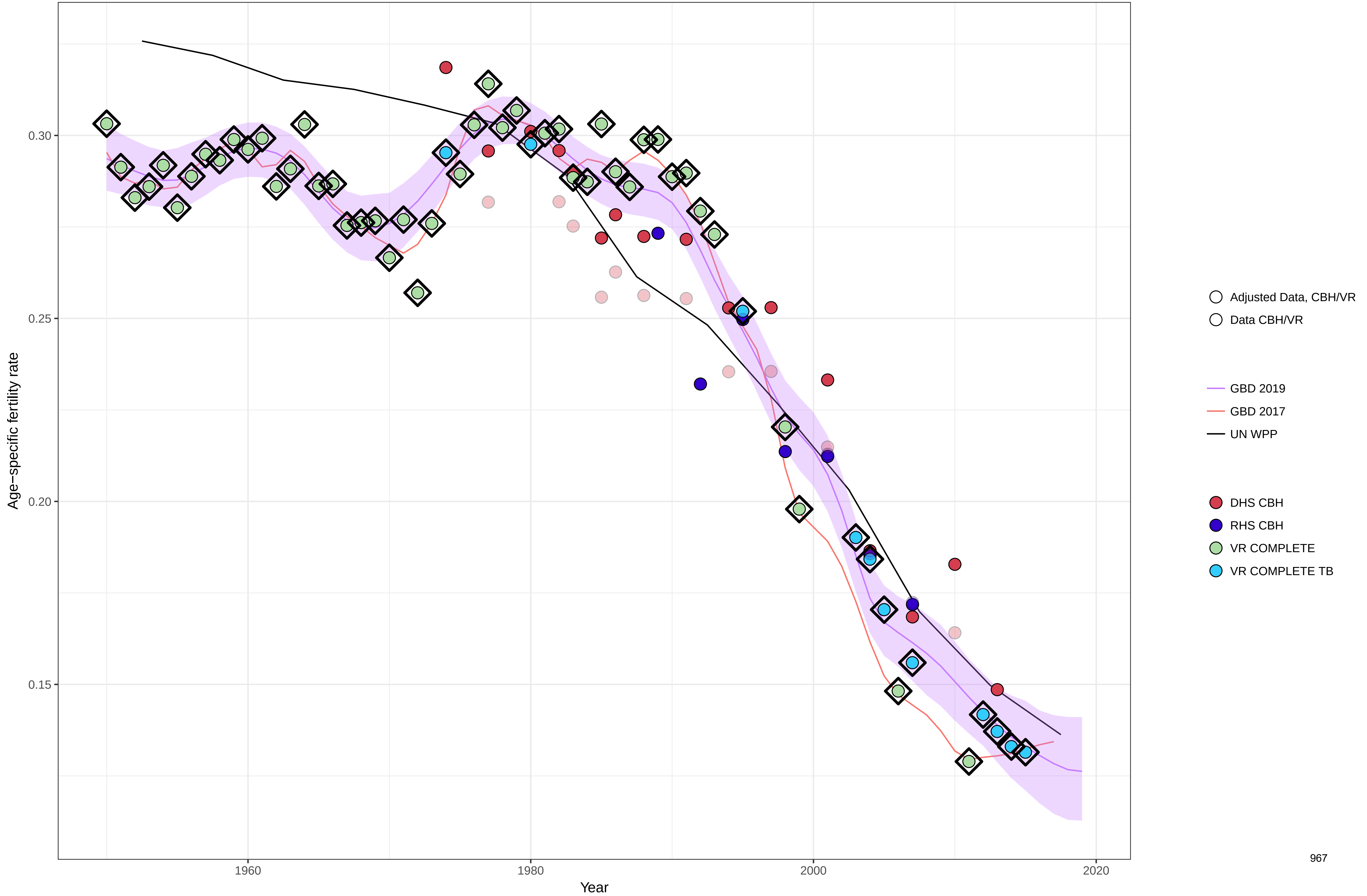

Age−specific fertility fitted trend lines, 1950−2019: Guatemala, Ages: 30−34
Data points enclosed with a diamond are reference sources

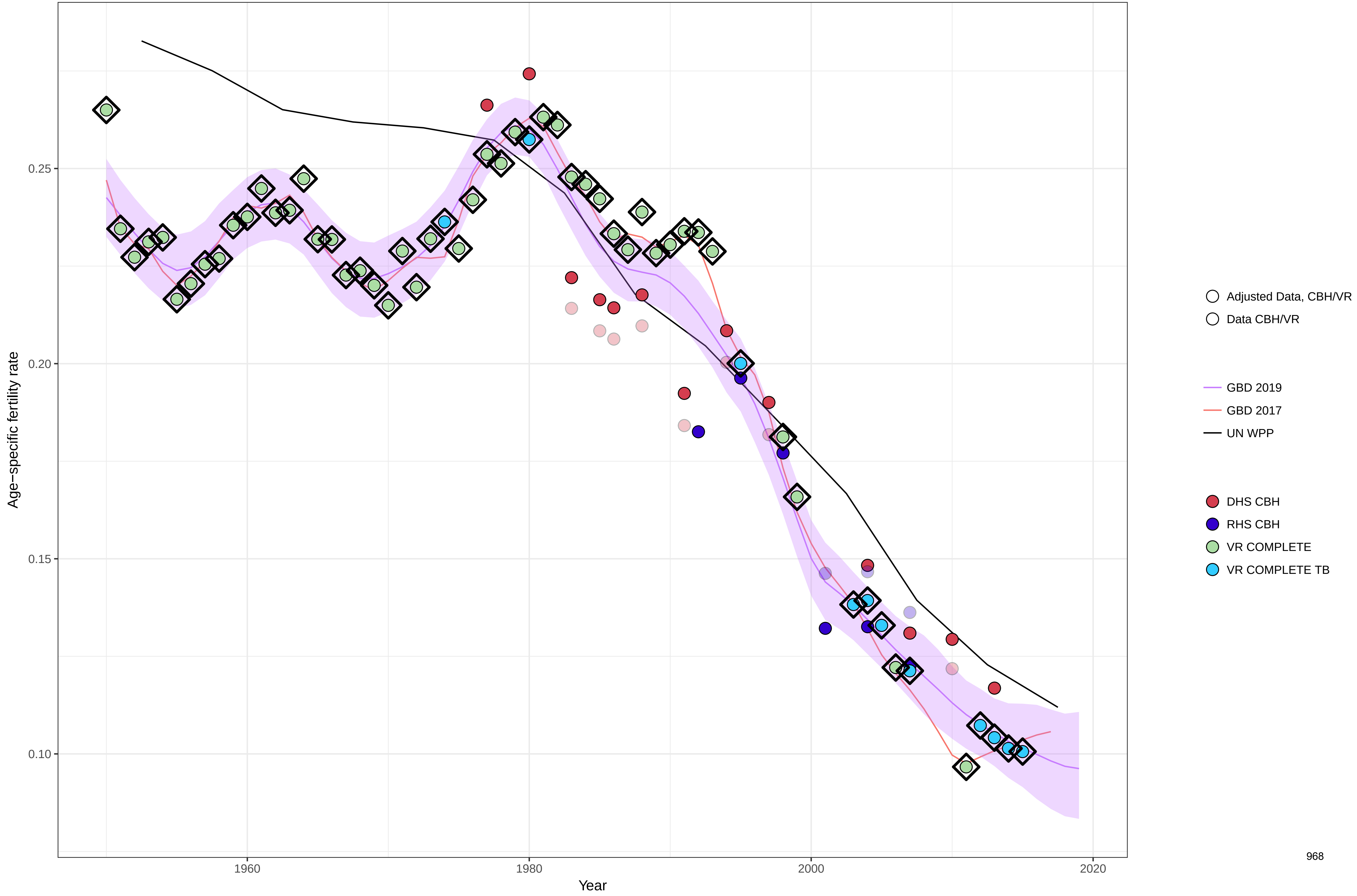

Age−specific fertility fitted trend lines, 1950−2019: Guatemala, Ages: 35−39
Data points enclosed with a diamond are reference sources

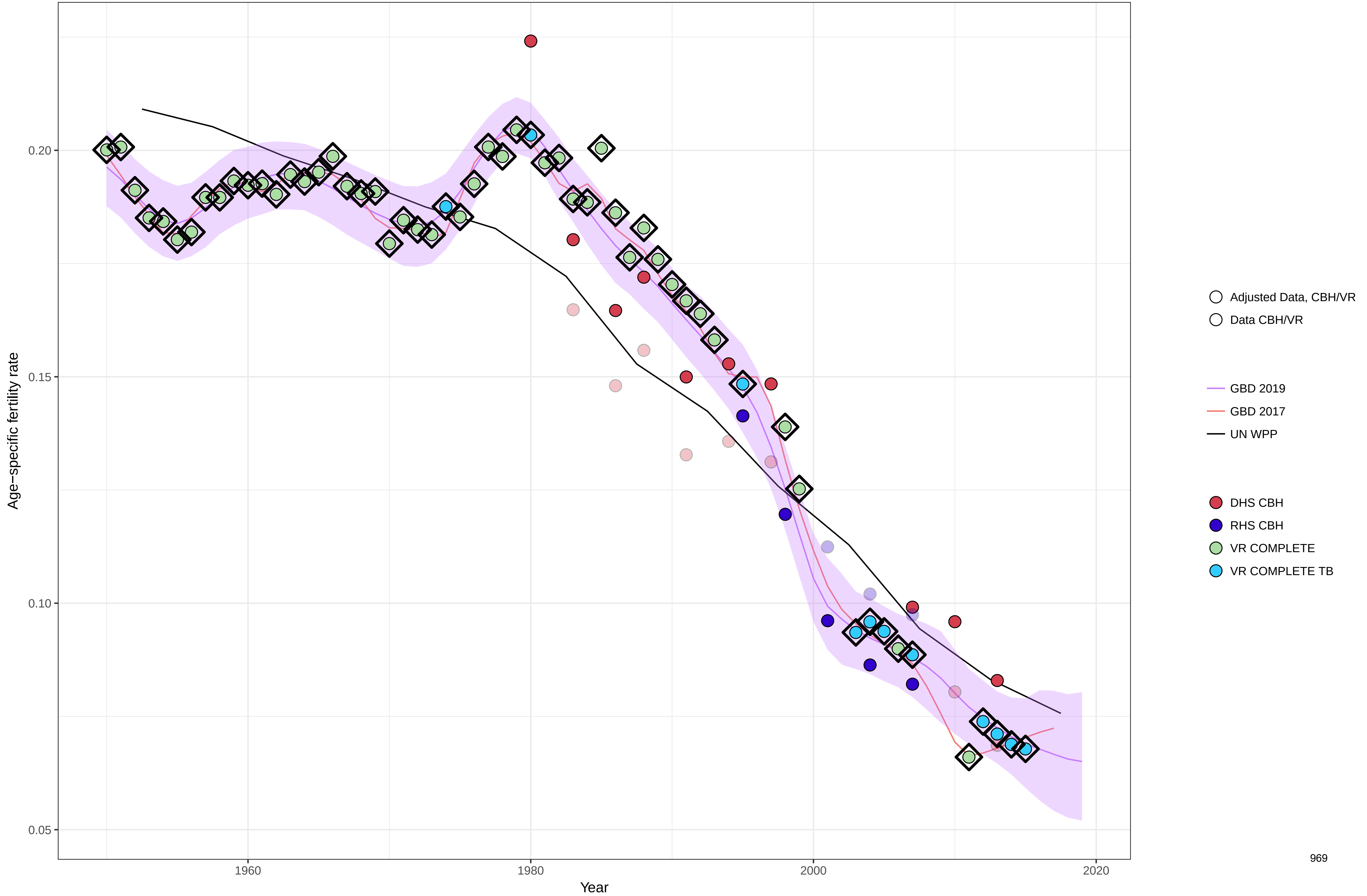

Age−specific fertility fitted trend lines, 1950−2019: Guatemala, Ages: 40−44
Data points enclosed with a diamond are reference sources

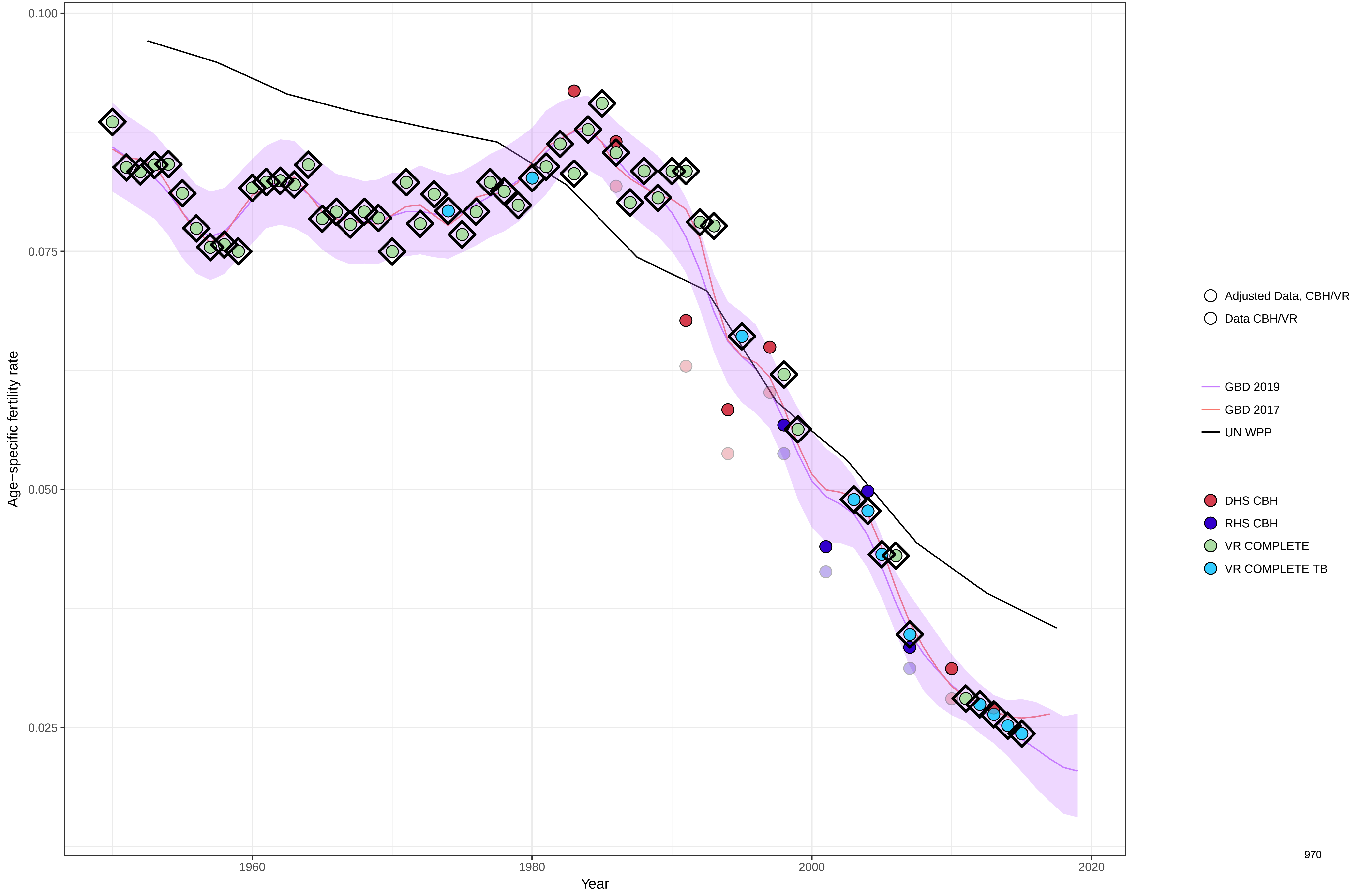

Age−specific fertility fitted trend lines, 1950−2019: Guatemala, Ages: 45−49
Data points enclosed with a diamond are reference sources

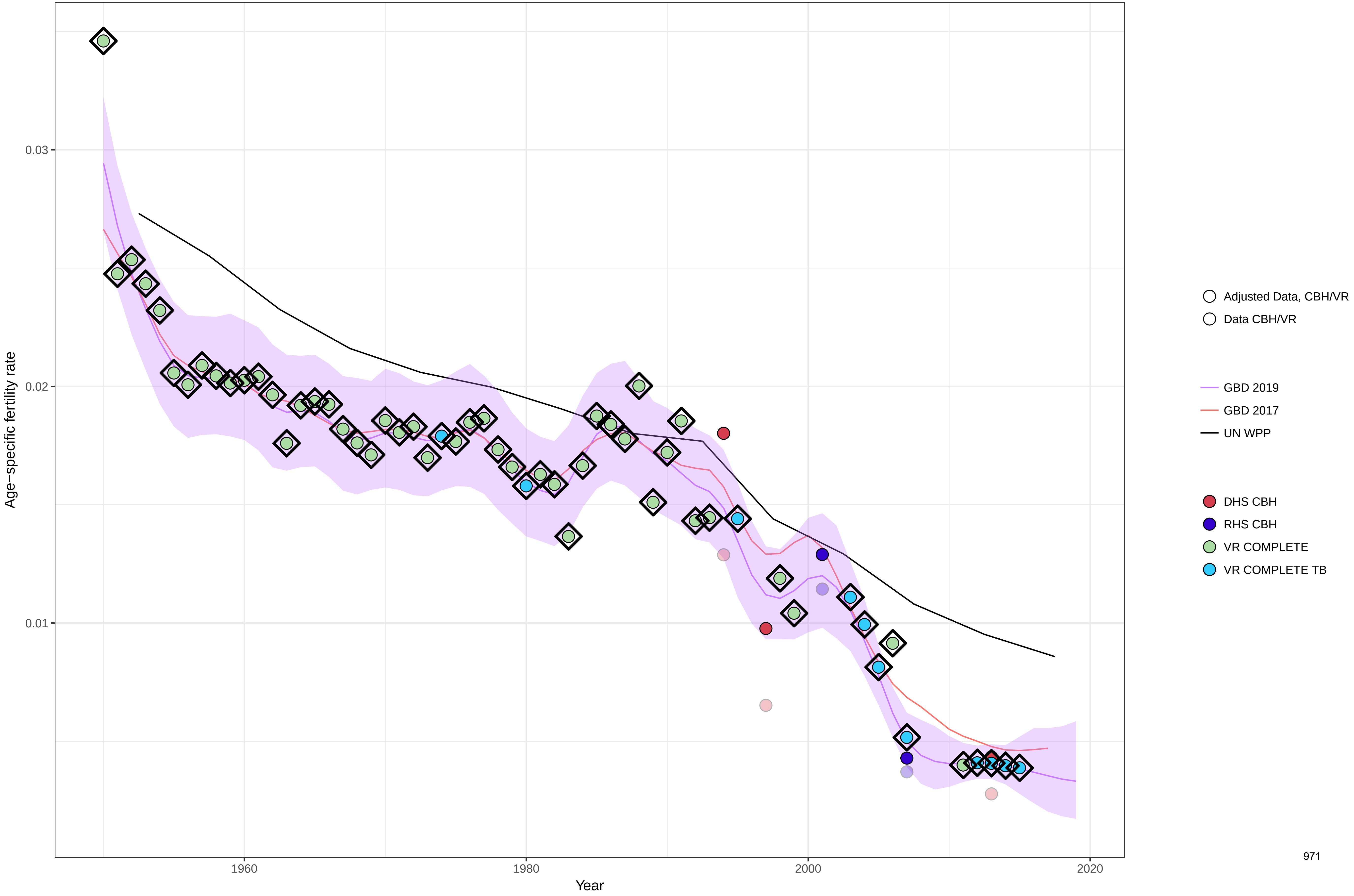

Age−specific fertility fitted trend lines, 1950−2019: Guatemala, Ages: 50−54
Data points enclosed with a diamond are reference sources

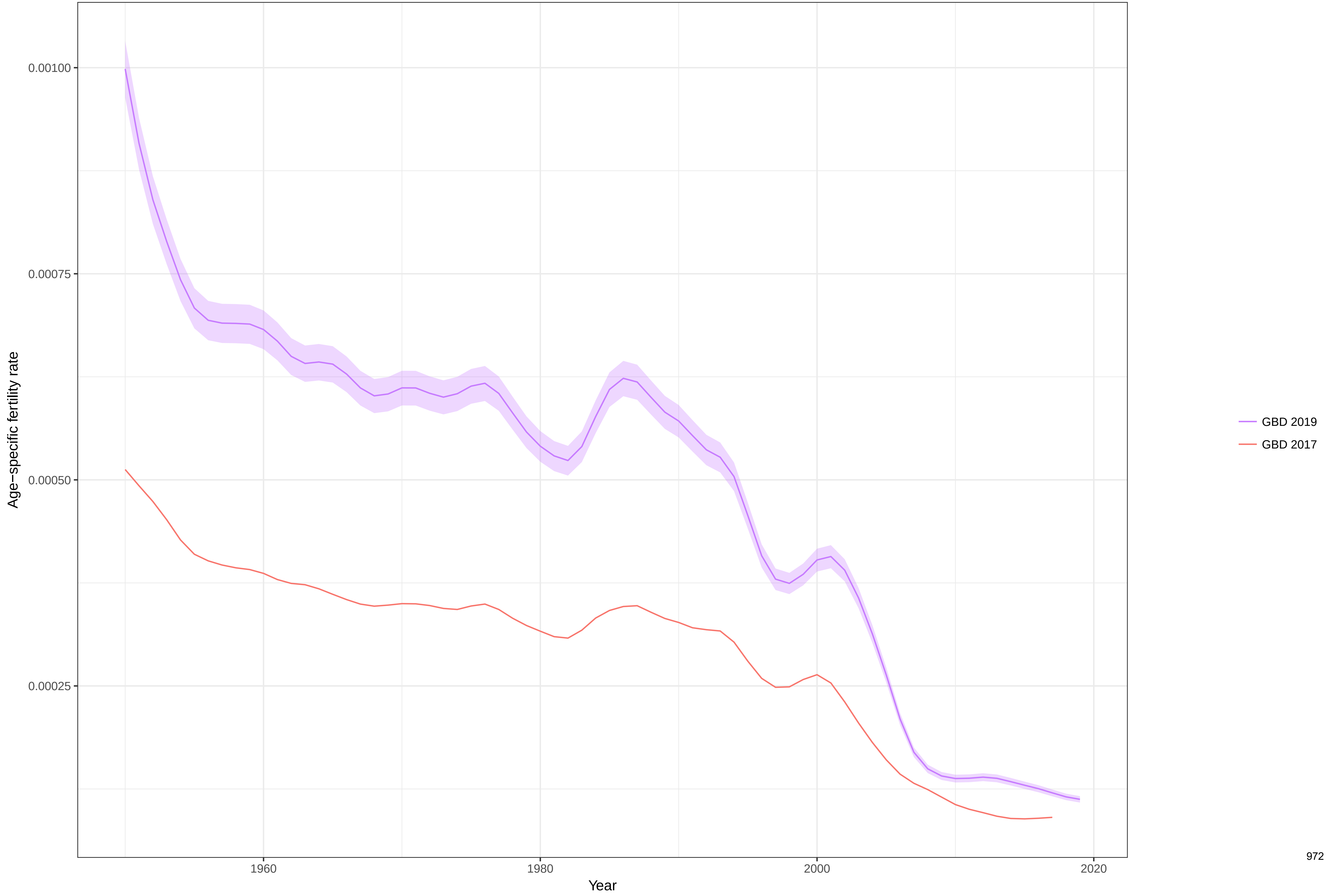

Total fertility rate fitted trend lines, 1950−2019: Guatemala TFR
Data points enclosed with a diamond are reference sources

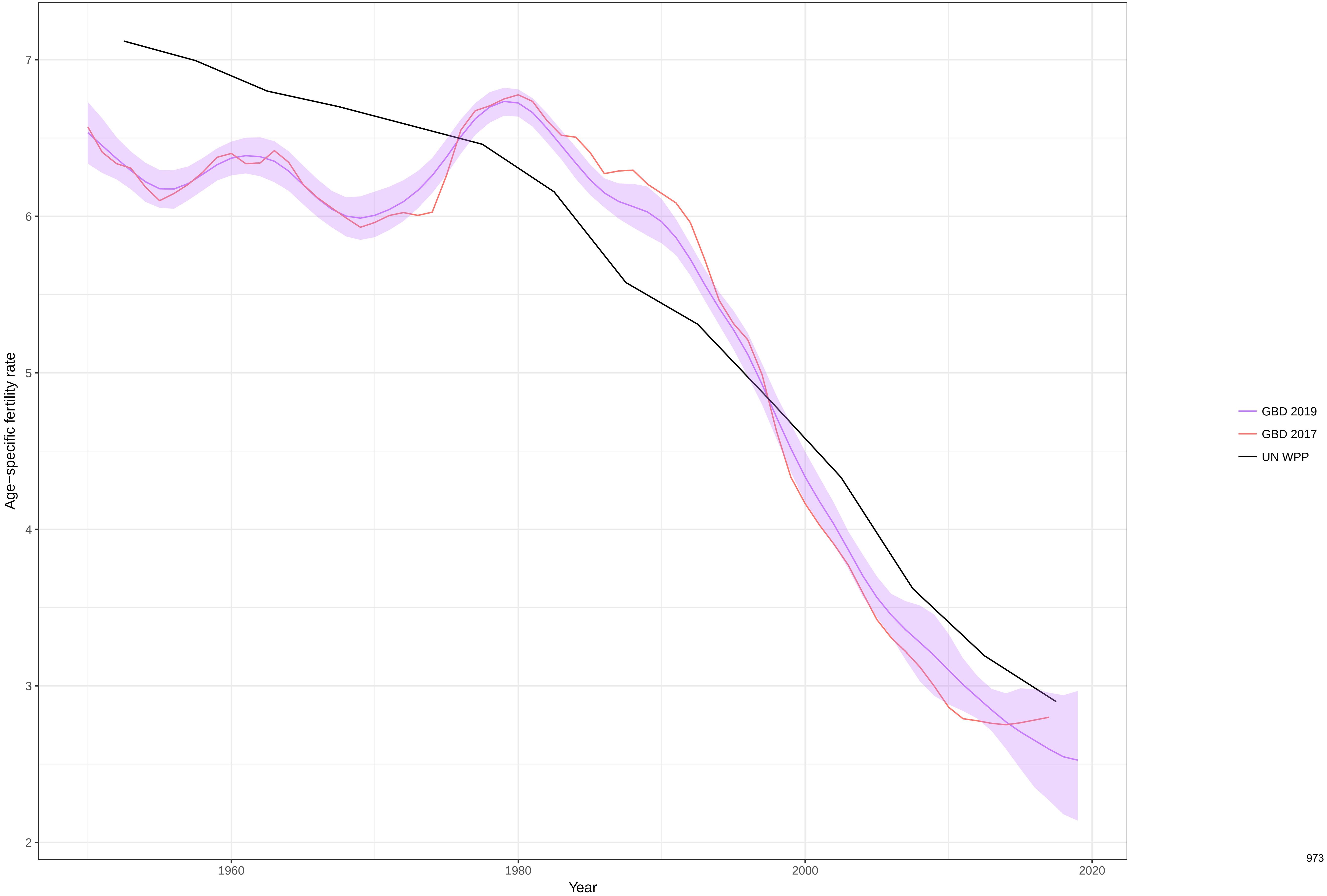

Age−specific fertility fitted trend lines, 1950−2019: Guam, Ages: 10−14
Data points enclosed with a diamond are reference sources

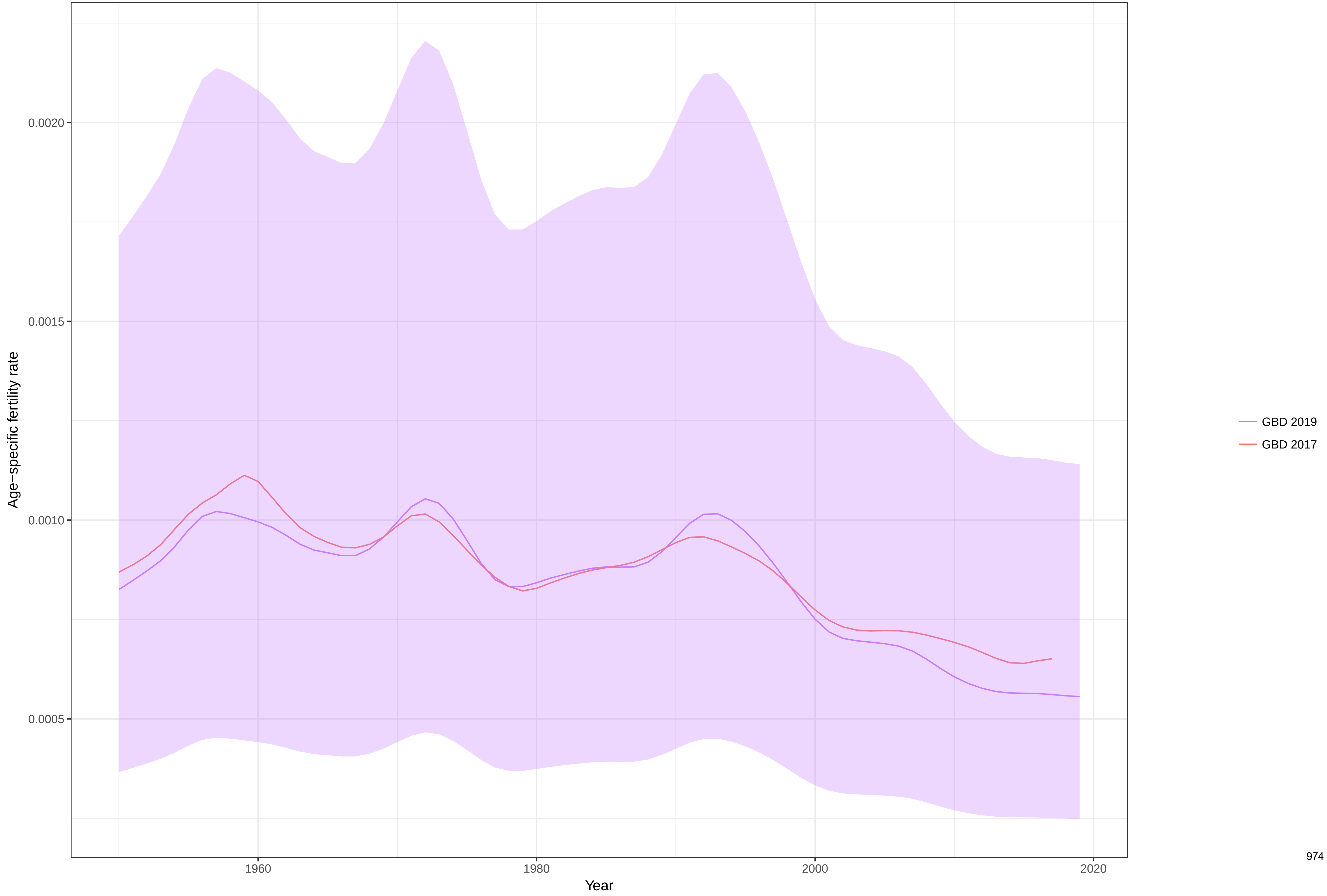

Age−specific fertility fitted trend lines, 1950−2019: Guam, Ages: 15−19
Data points enclosed with a diamond are reference sources

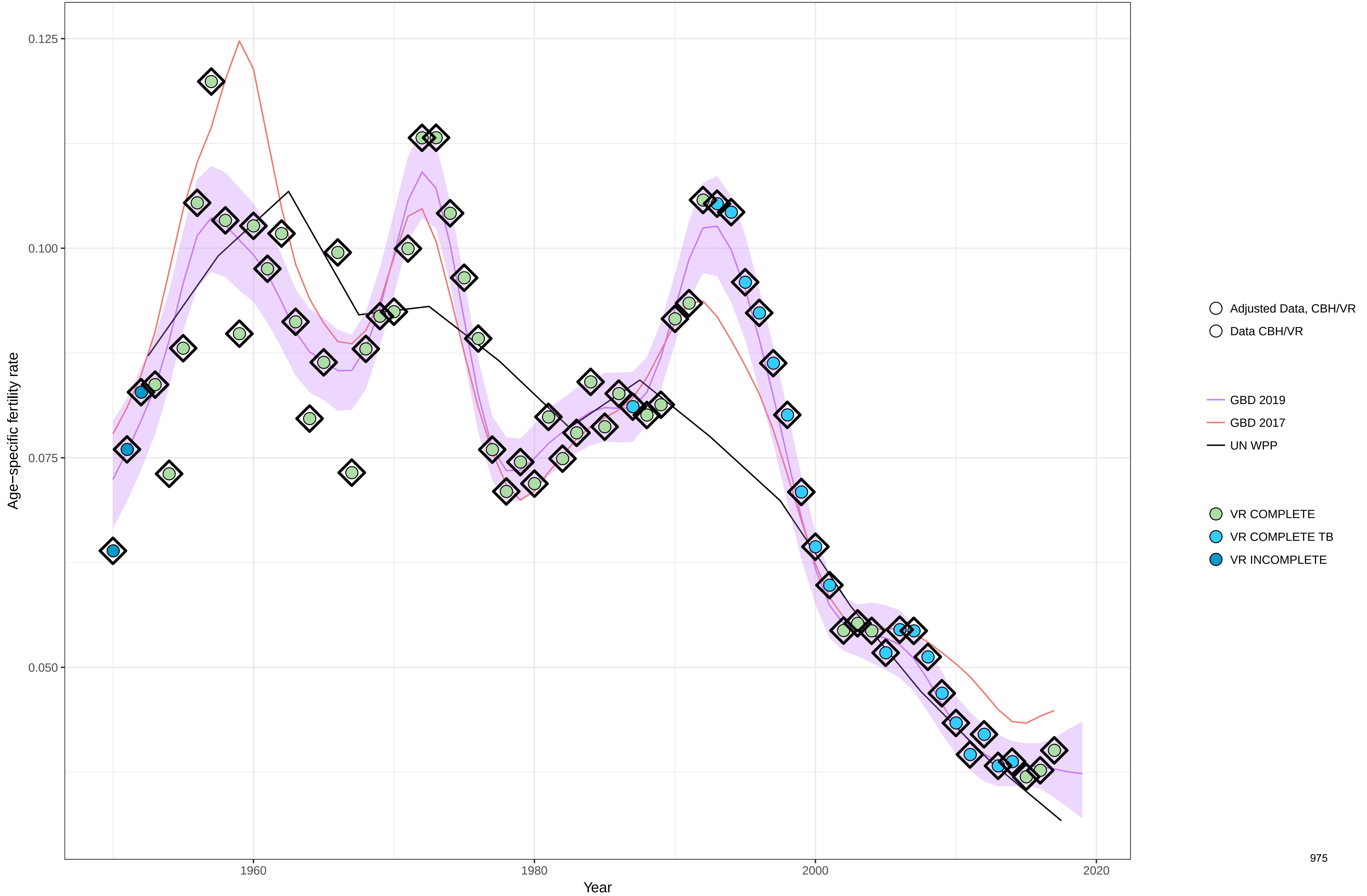

Age−specific fertility fitted trend lines, 1950−2019: Guam, Ages: 20−24
Data points enclosed with a diamond are reference sources

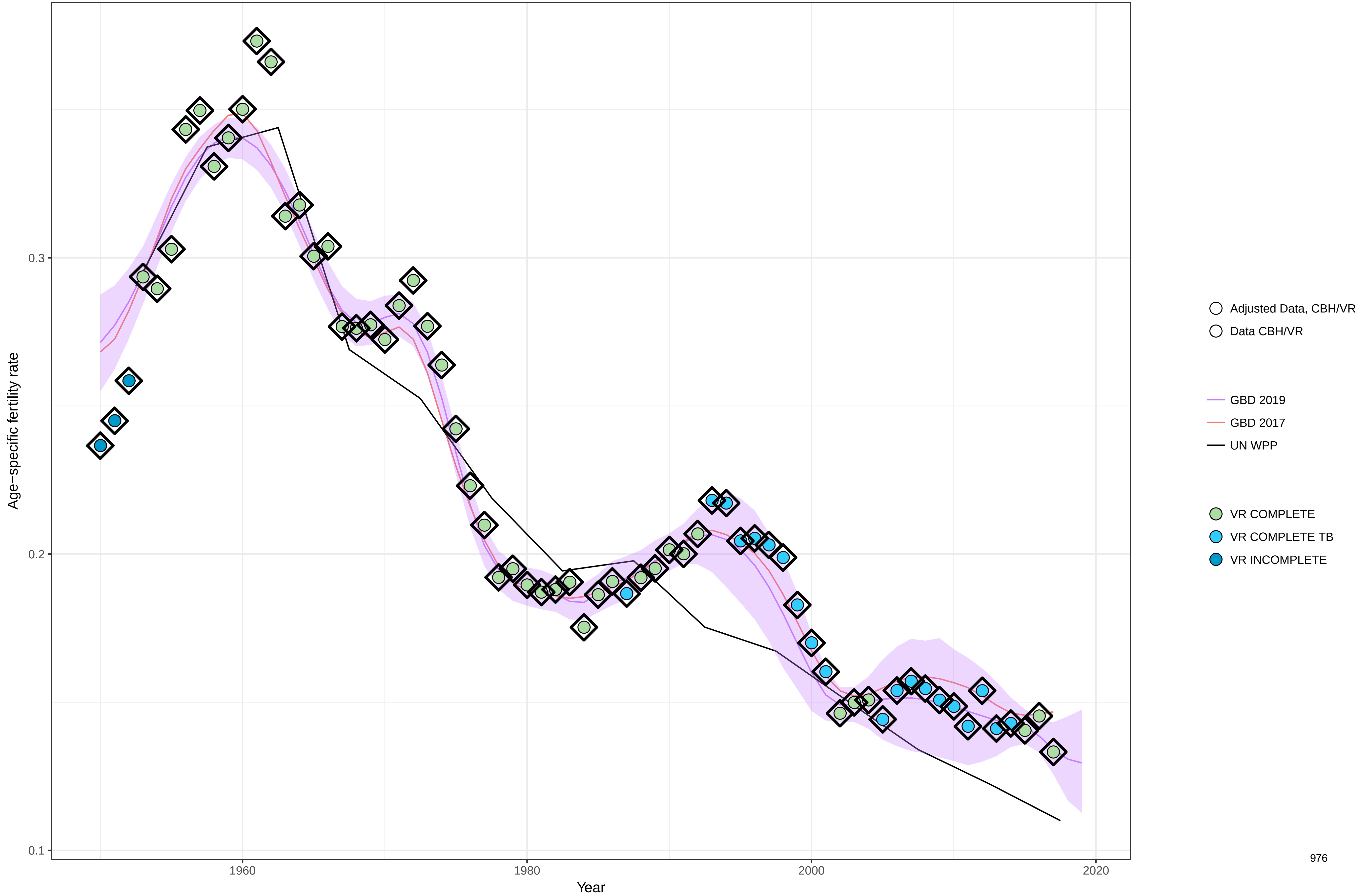

Age−specific fertility fitted trend lines, 1950−2019: Guam, Ages: 25−29
Data points enclosed with a diamond are reference sources

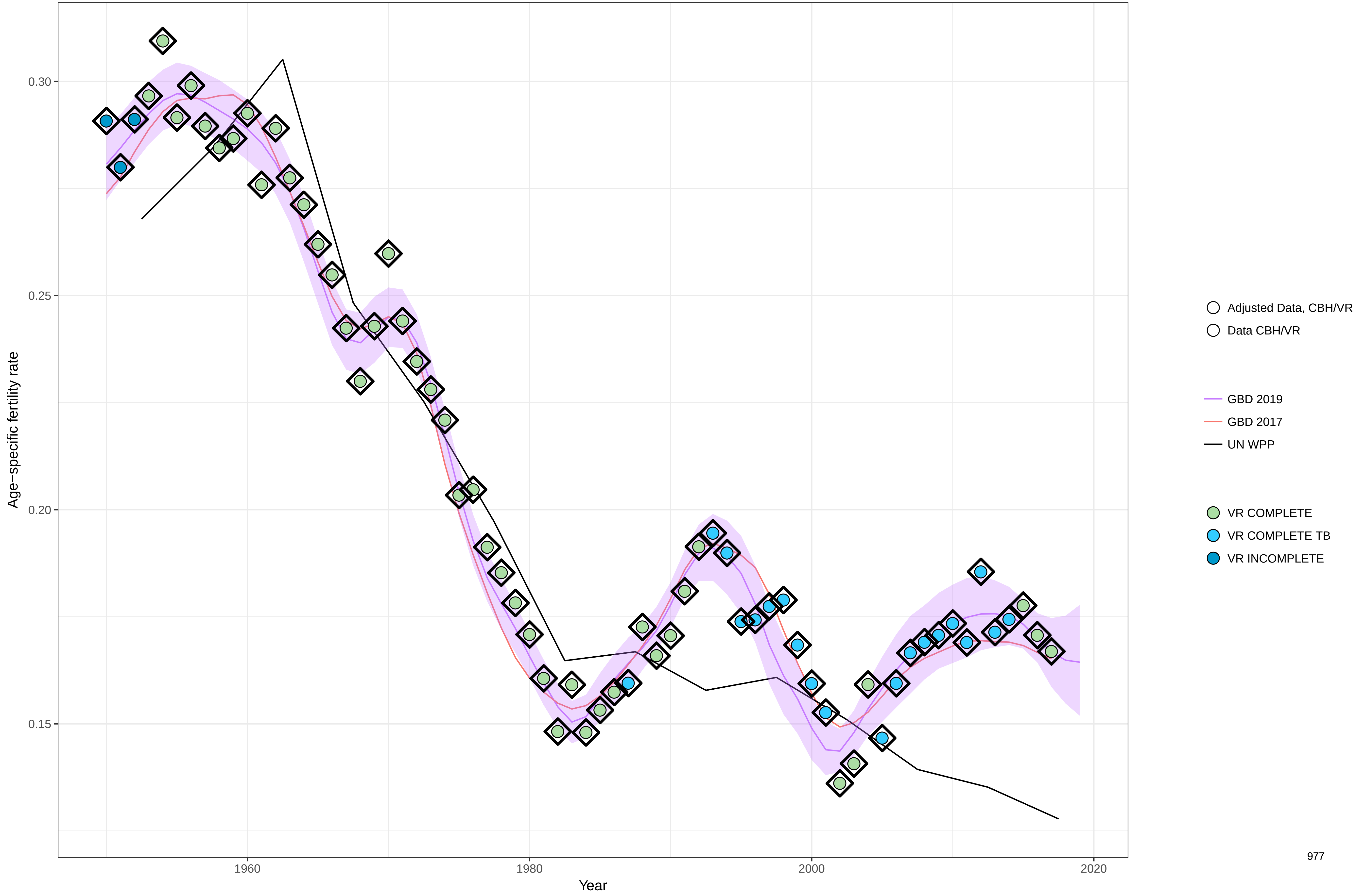

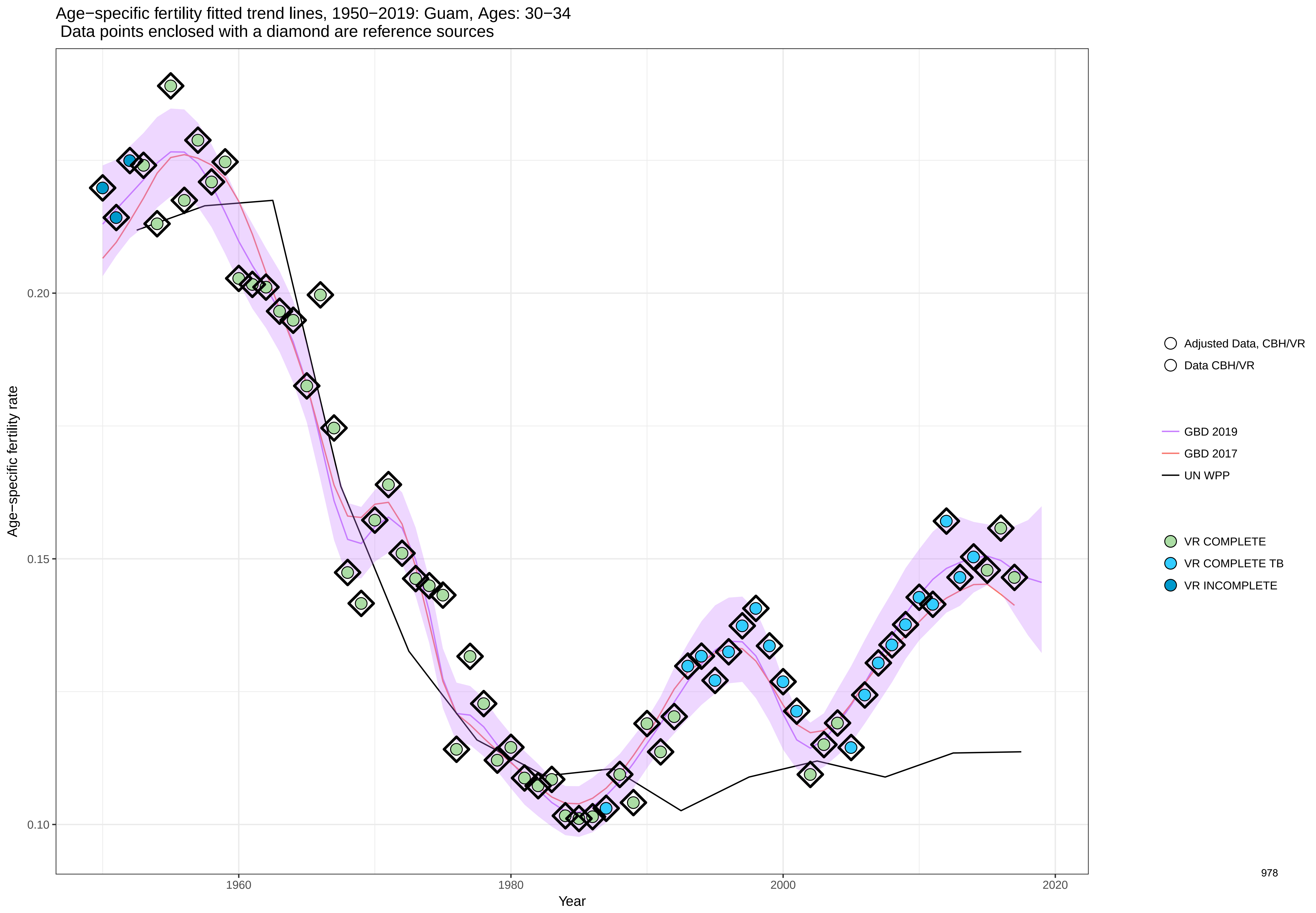

Age−specific fertility fitted trend lines, 1950−2019: Guam, Ages: 30−34
Data points enclosed with a diamond are reference sources
Age−specific fertility rate
0.20
0.15
0.10
1960
1980
2000
2020
Year
Adjusted Data, CBH/VR
Data CBH/VR
GBD 2019
GBD 2017
UN WPP
VR COMPLETE
VR COMPLETE TB
VR INCOMPLETE

Age−specific fertility fitted trend lines, 1950−2019: Guam, Ages: 35−39
Data points enclosed with a diamond are reference sources

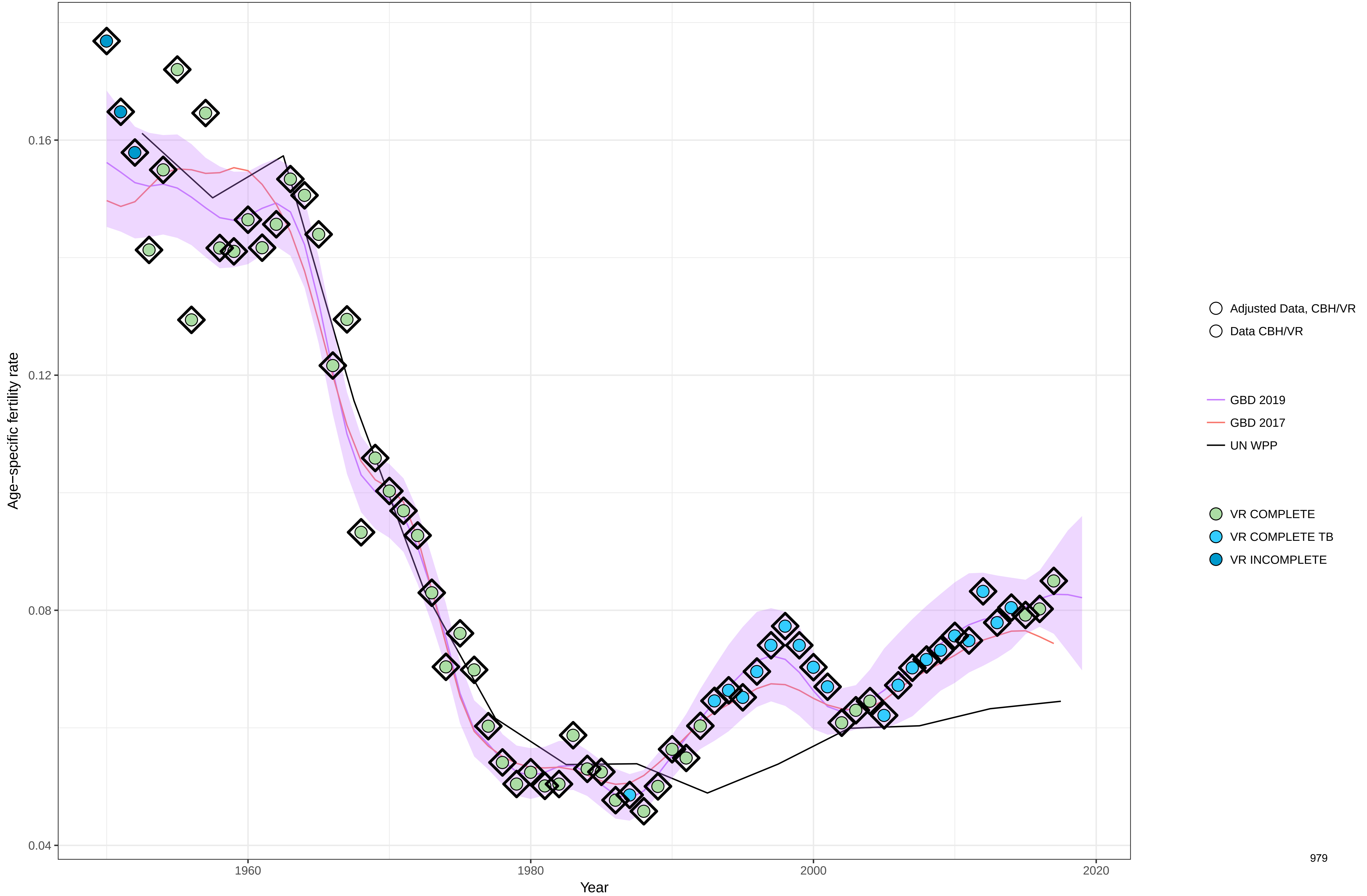

Age−specific fertility fitted trend lines, 1950−2019: Guam, Ages: 40−44
Data points enclosed with a diamond are reference sources

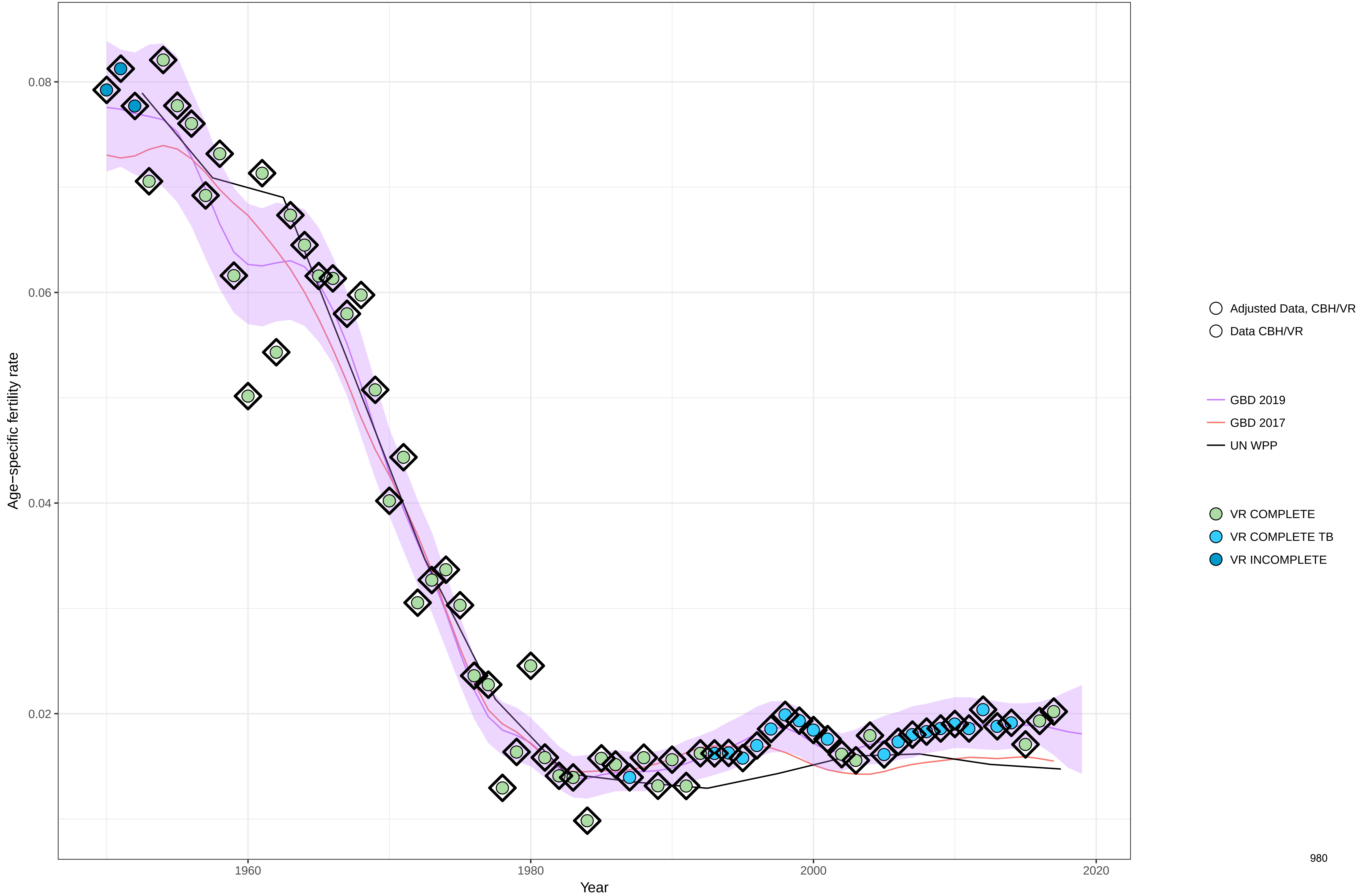

Age−specific fertility fitted trend lines, 1950−2019: Guam, Ages: 45−49
Data points enclosed with a diamond are reference sources

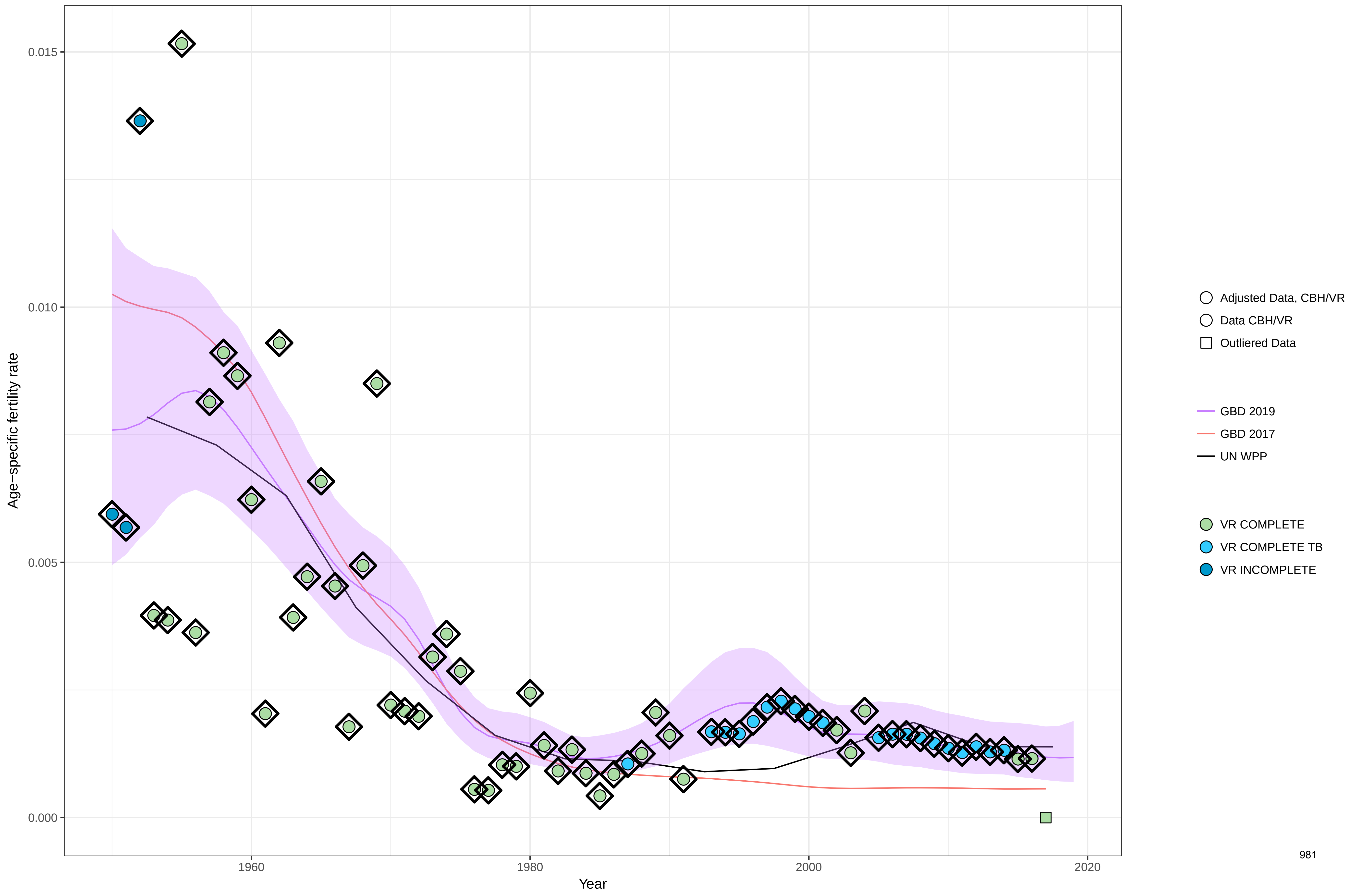

Age−specific fertility fitted trend lines, 1950−2019: Guam, Ages: 50−54
Data points enclosed with a diamond are reference sources

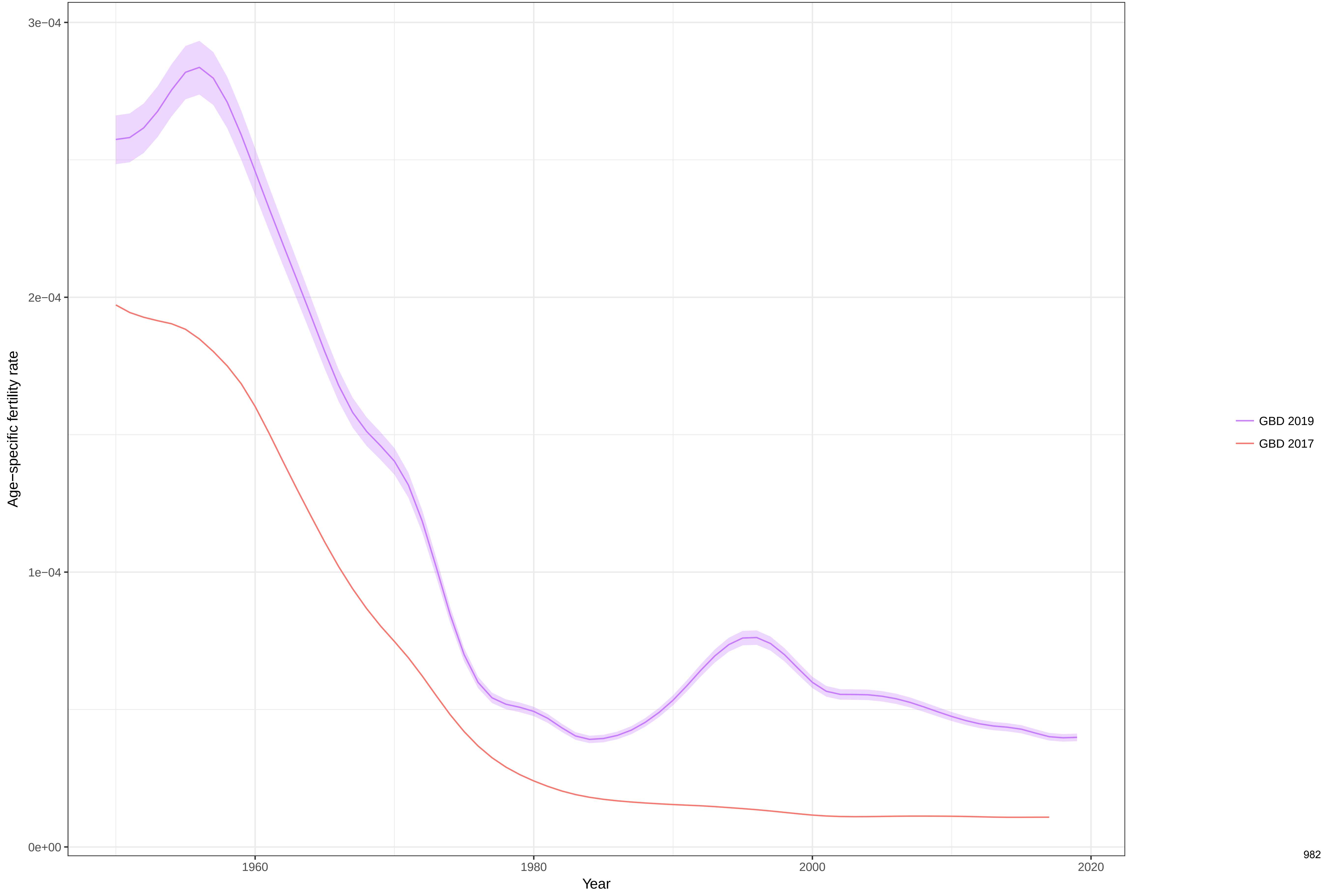

Total fertility rate fitted trend lines, 1950−2019: Guam TFR
Data points enclosed with a diamond are reference sources

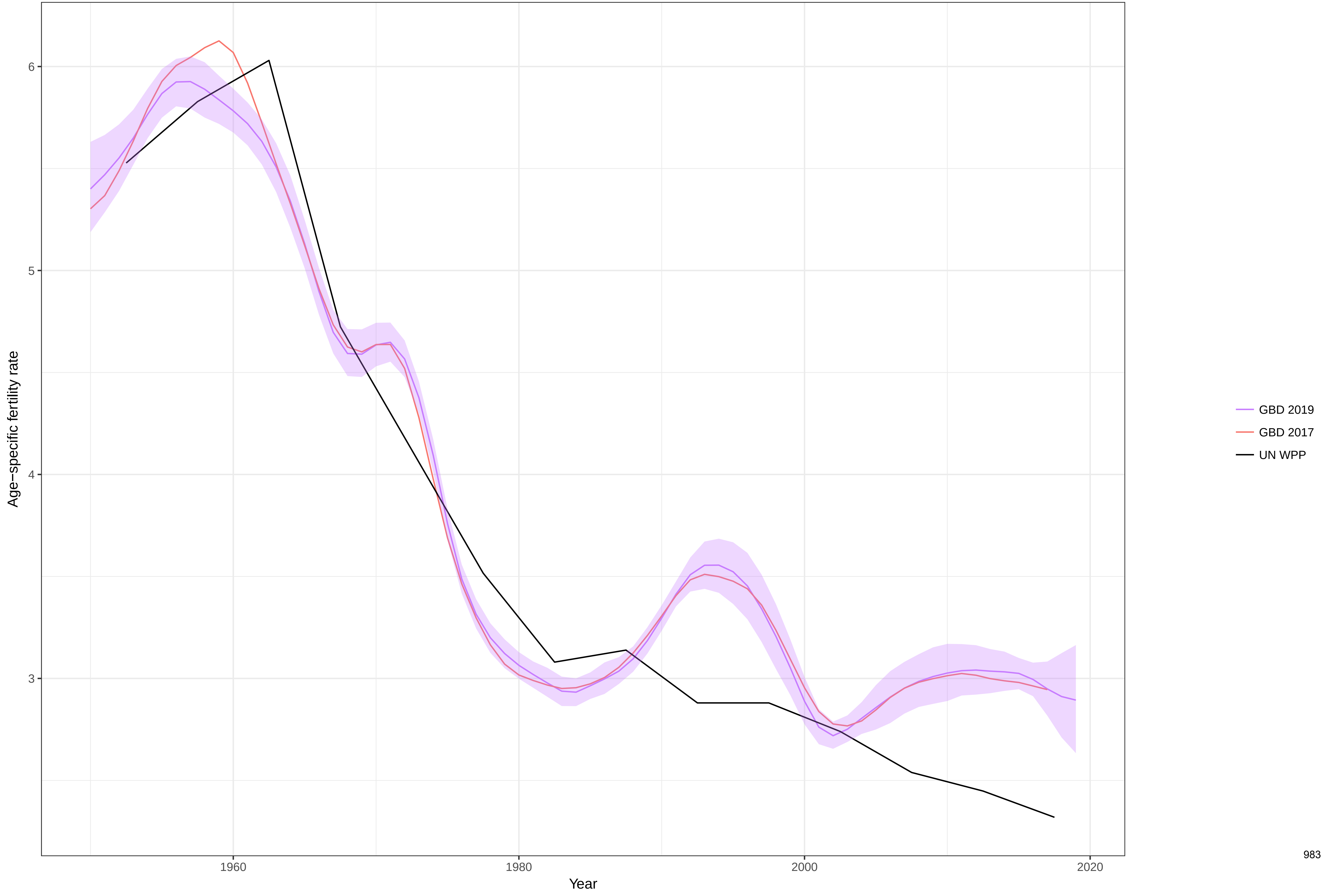

983

Age−specific fertility fitted trend lines, 1950−2019: Guyana, Ages: 10−14
Data points enclosed with a diamond are reference sources

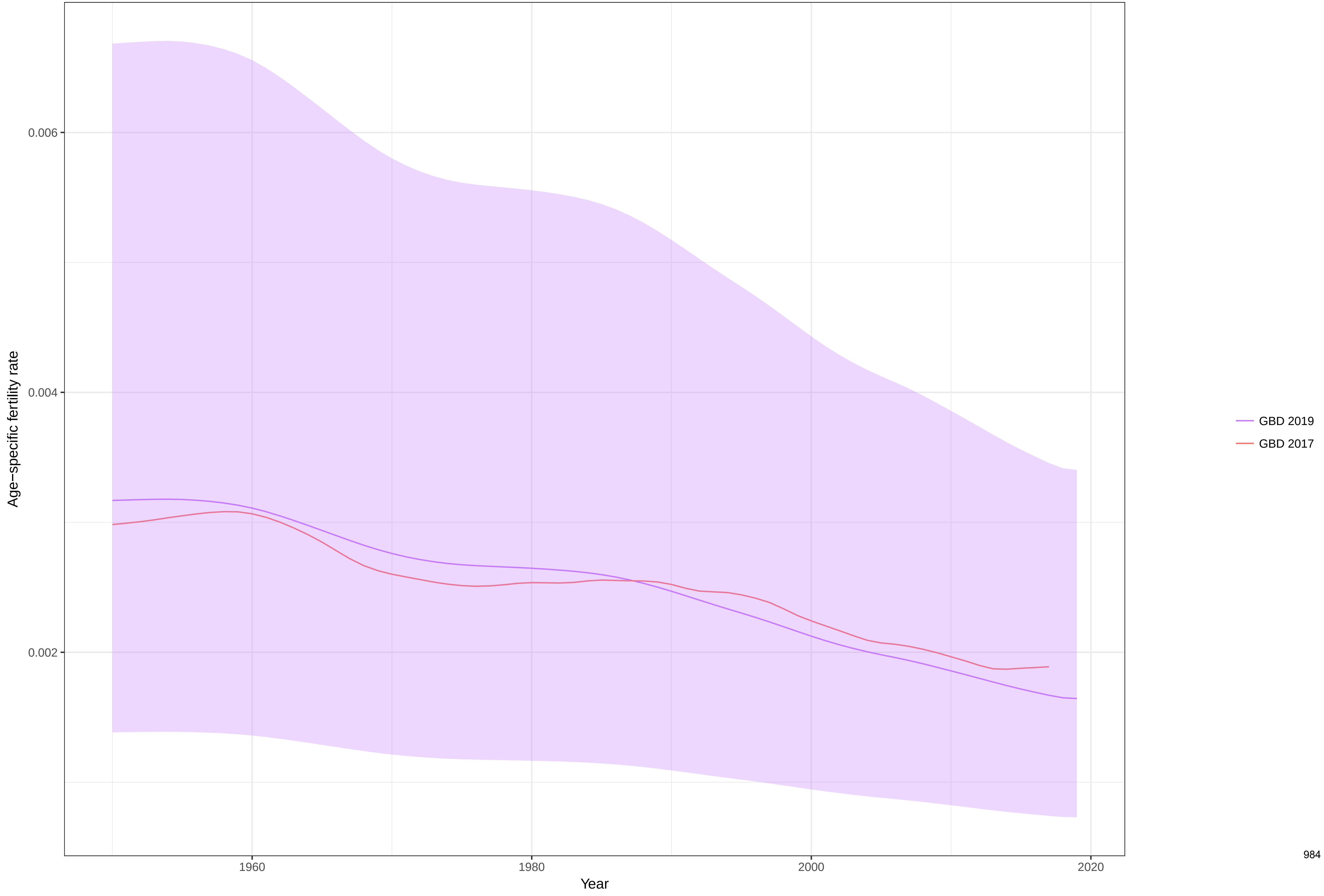

Age−specific fertility fitted trend lines, 1950−2019: Guyana, Ages: 15−19
Data points enclosed with a diamond are reference sources

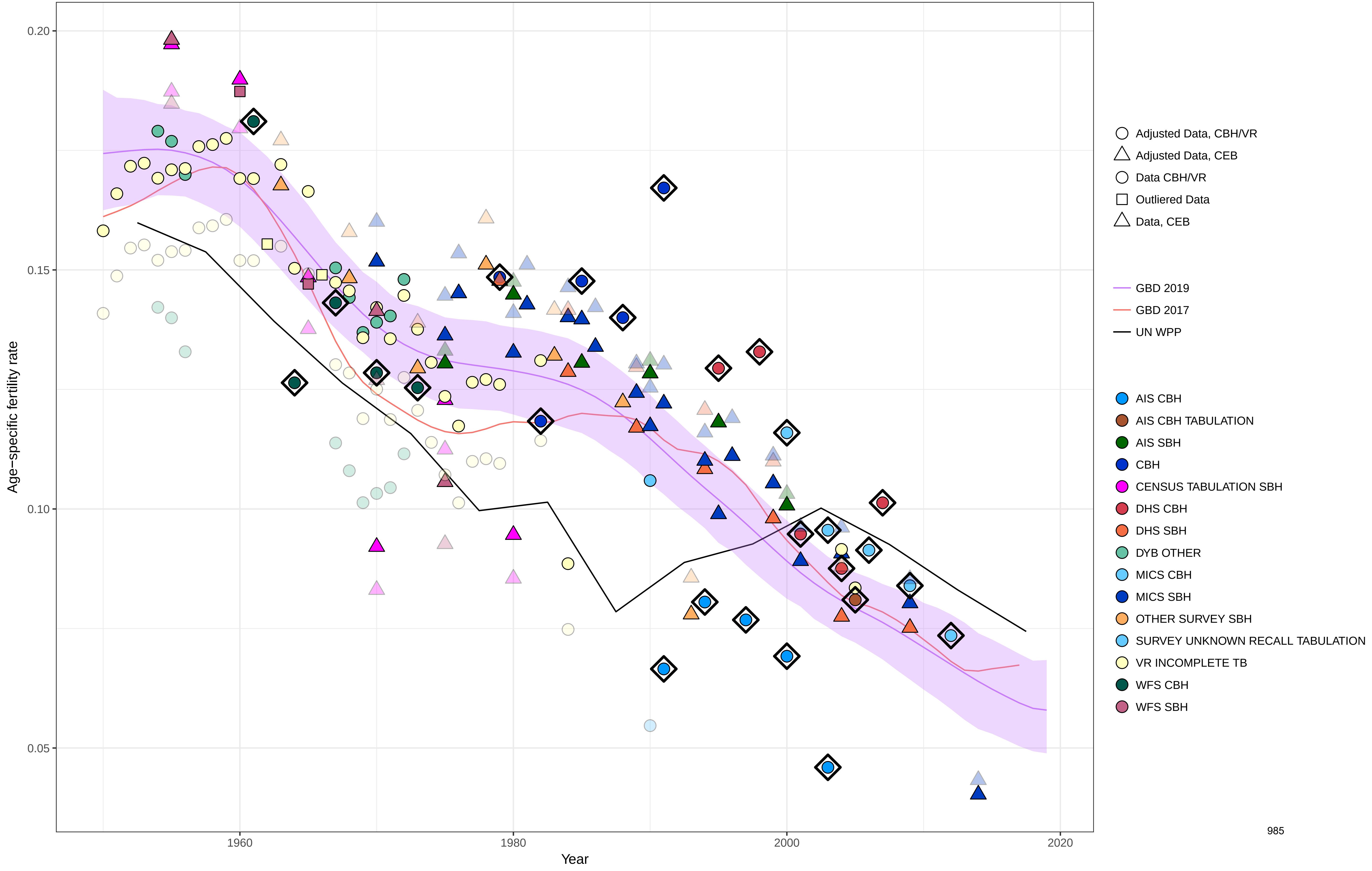

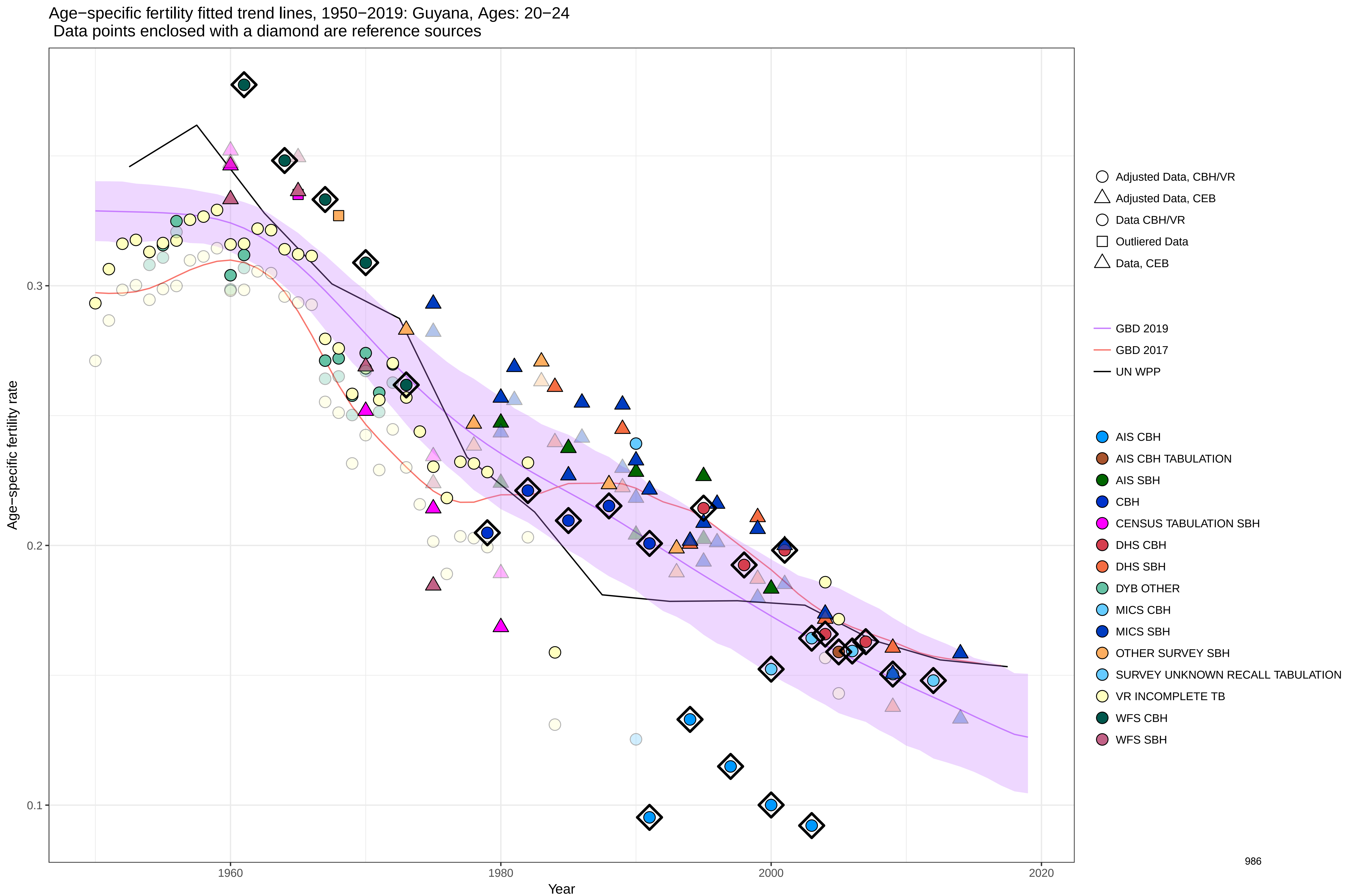
Age−specific fertility fitted trend lines, 1950−2019: Guyana, Ages: 20−24
Data points enclosed with a diamond are reference sources
Age−specific fertility rate
0.1
0.2
0.3
Year
1960
1980
2000
2020
Adjusted Data, CBH/VR
Adjusted Data, CEB
Data CBH/VR
Outliered Data
Data, CEB
GBD 2019
GBD 2017
UN WPP
AIS CBH
AIS CBH TABULATION
AIS SBH
CBH
CENSUS TABULATION SBH
DHS CBH
DHS SBH
DYB OTHER
MICS CBH
MICS SBH
OTHER SURVEY SBH
SURVEY UNKNOWN RECALL TABULATION
VR INCOMPLETE TB
WFS CBH
WFS SBH

Age−specific fertility fitted trend lines, 1950−2019: Guyana, Ages: 25−29
Data points enclosed with a diamond are reference sources

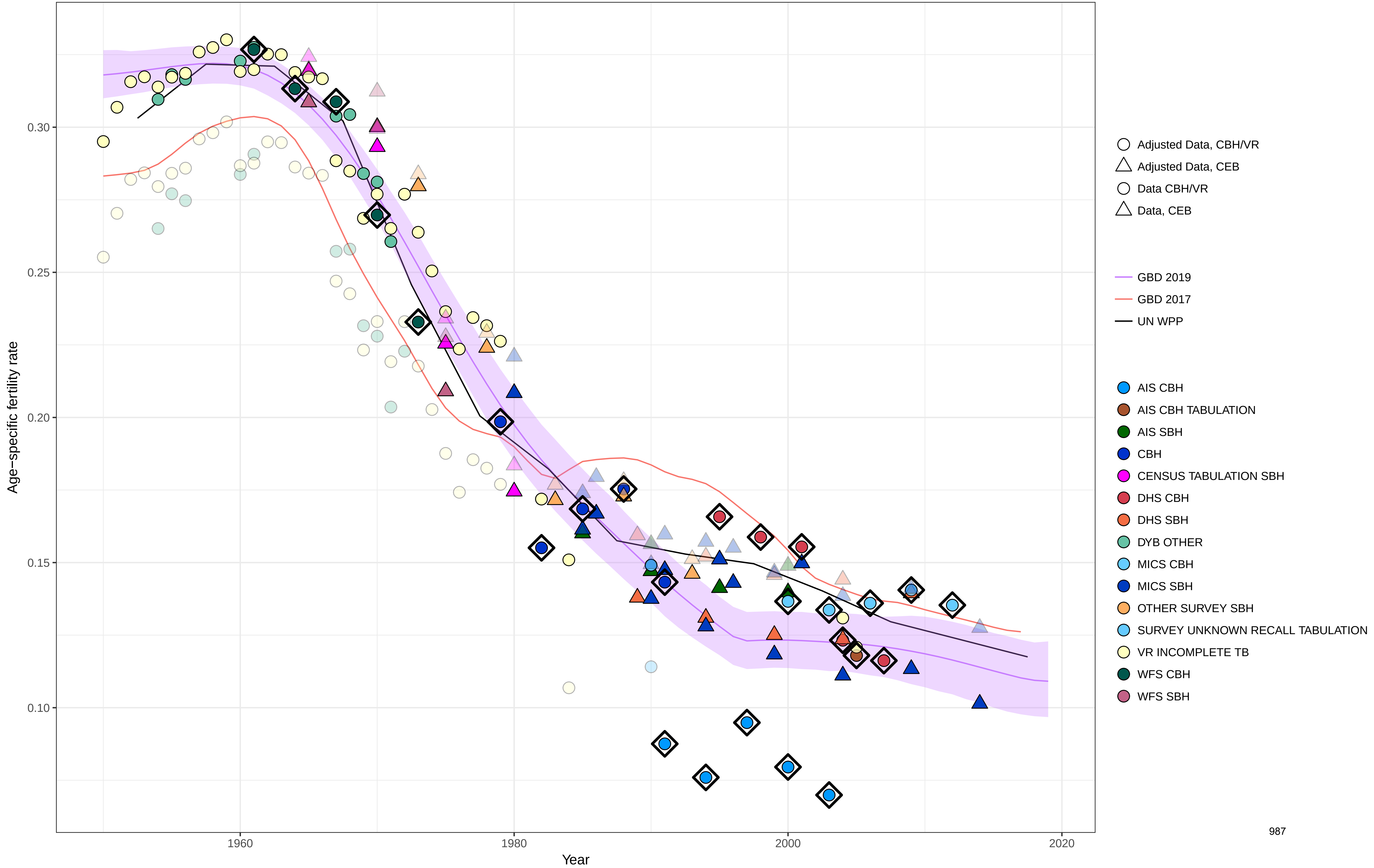

Age−specific fertility fitted trend lines, 1950−2019: Guyana, Ages: 30−34
Data points enclosed with a diamond are reference sources

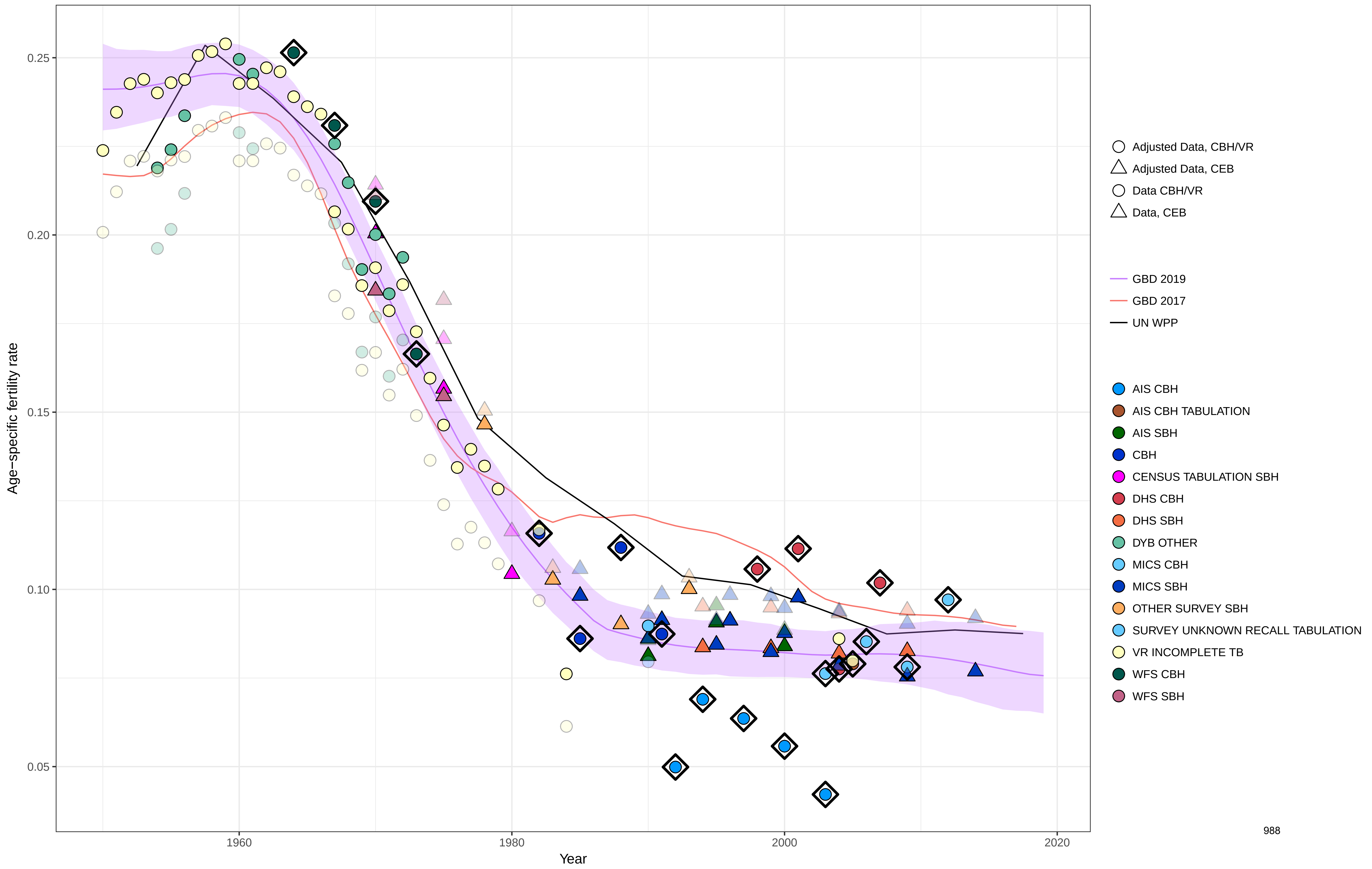

Age−specific fertility fitted trend lines, 1950−2019: Guyana, Ages: 35−39
Data points enclosed with a diamond are reference sources

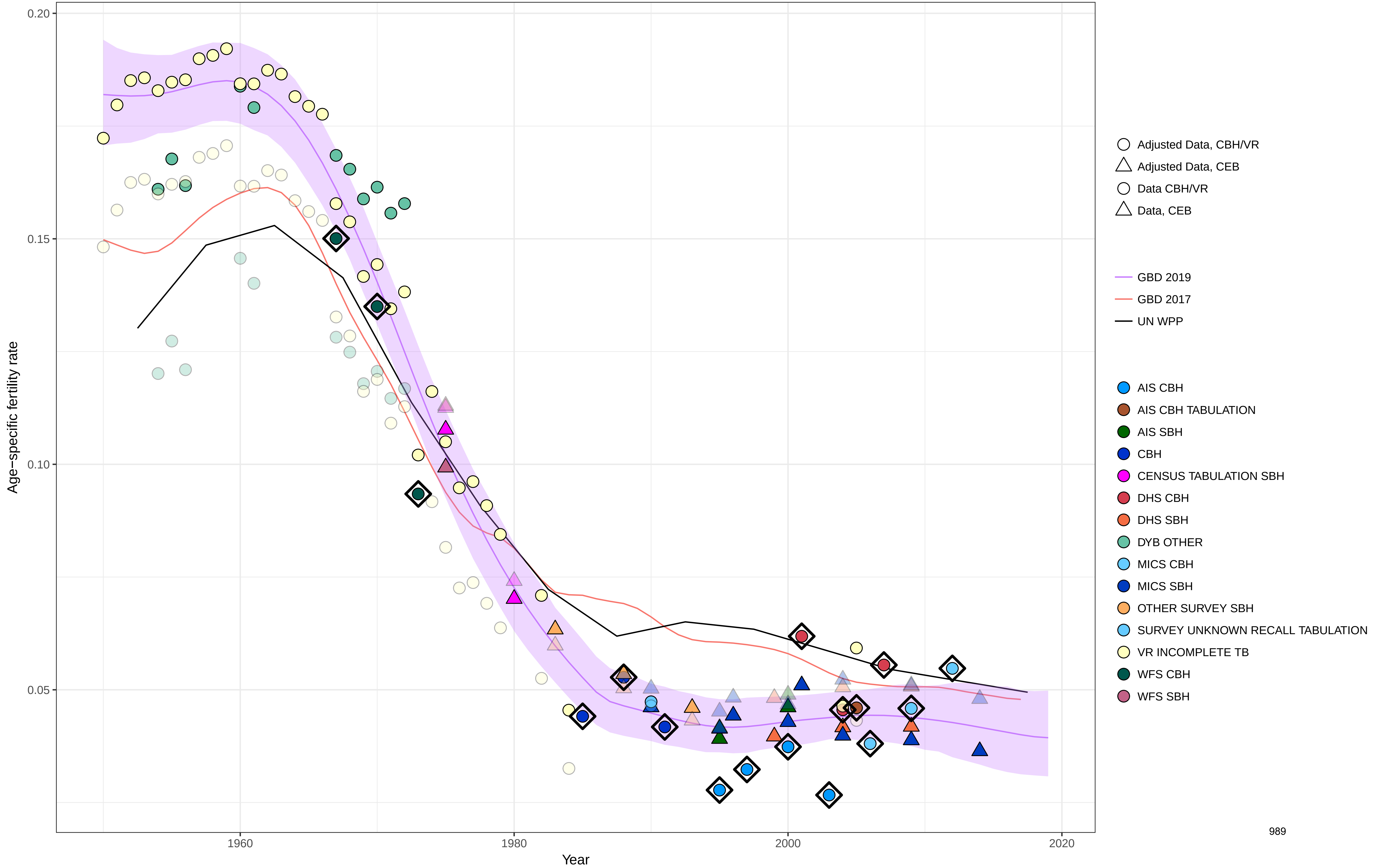

Age−specific fertility fitted trend lines, 1950−2019: Guyana, Ages: 40−44
Data points enclosed with a diamond are reference sources

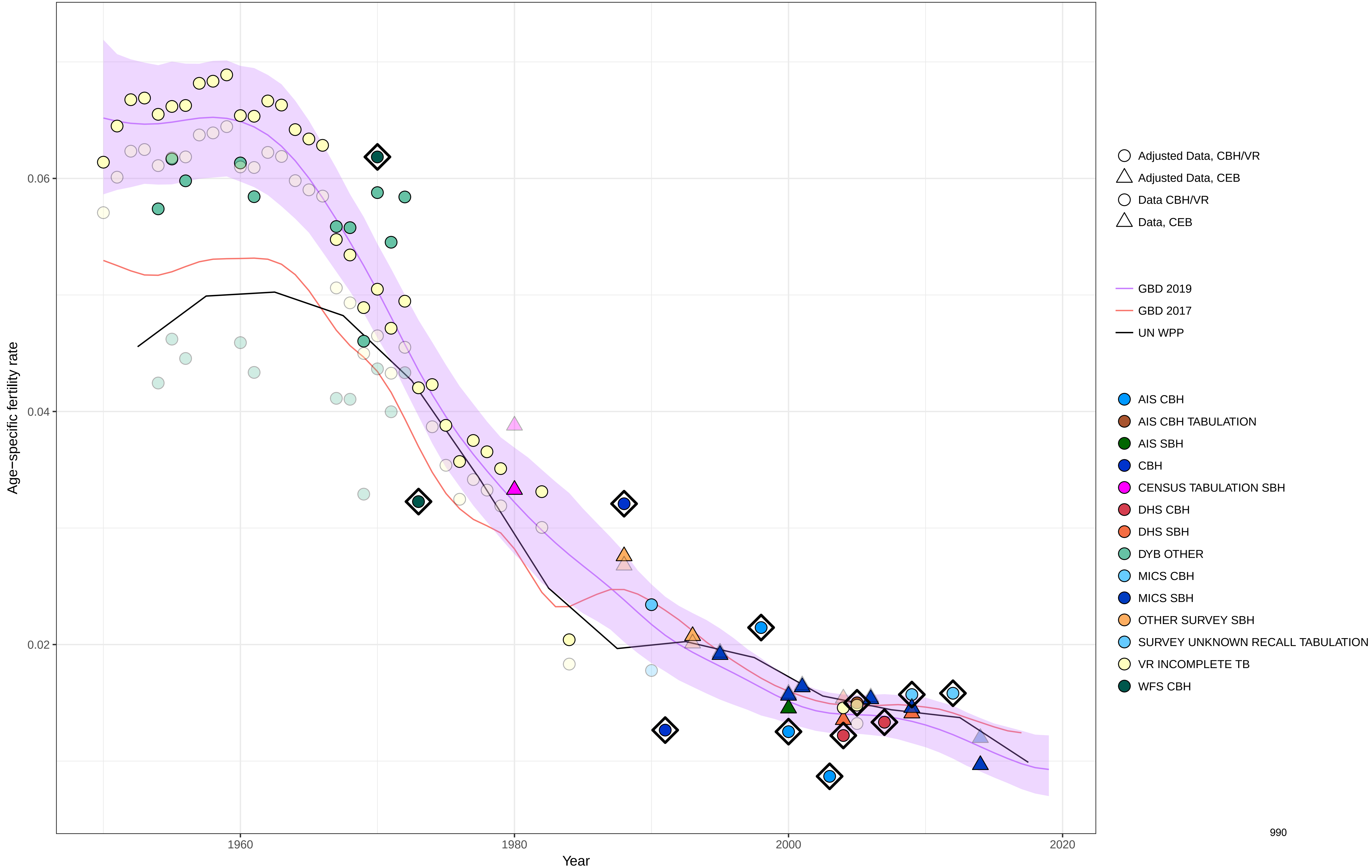

Age−specific fertility fitted trend lines, 1950−2019: Guyana, Ages: 45−49
Data points enclosed with a diamond are reference sources

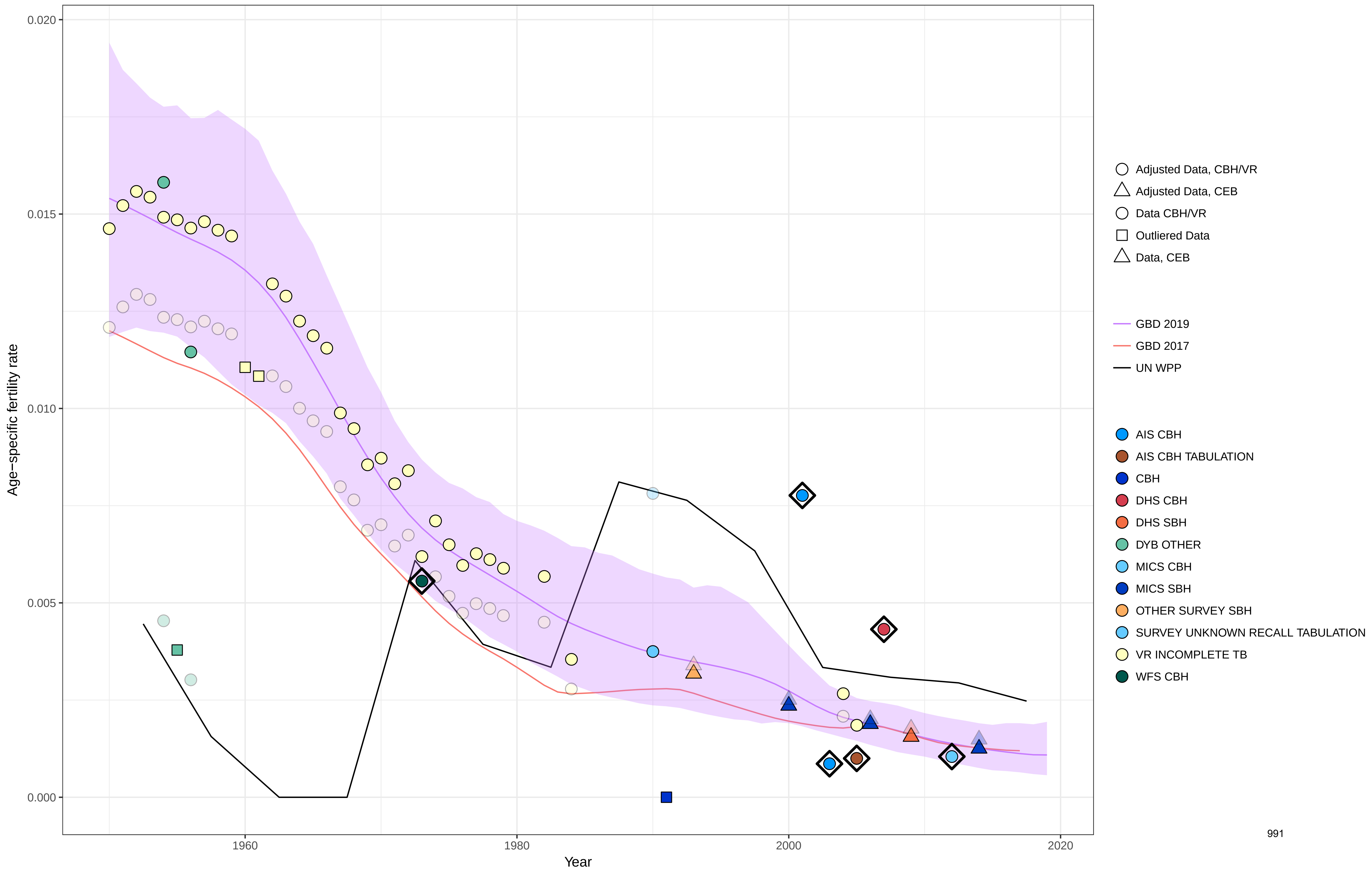

Age−specific fertility fitted trend lines, 1950−2019: Guyana, Ages: 50−54
Data points enclosed with a diamond are reference sources

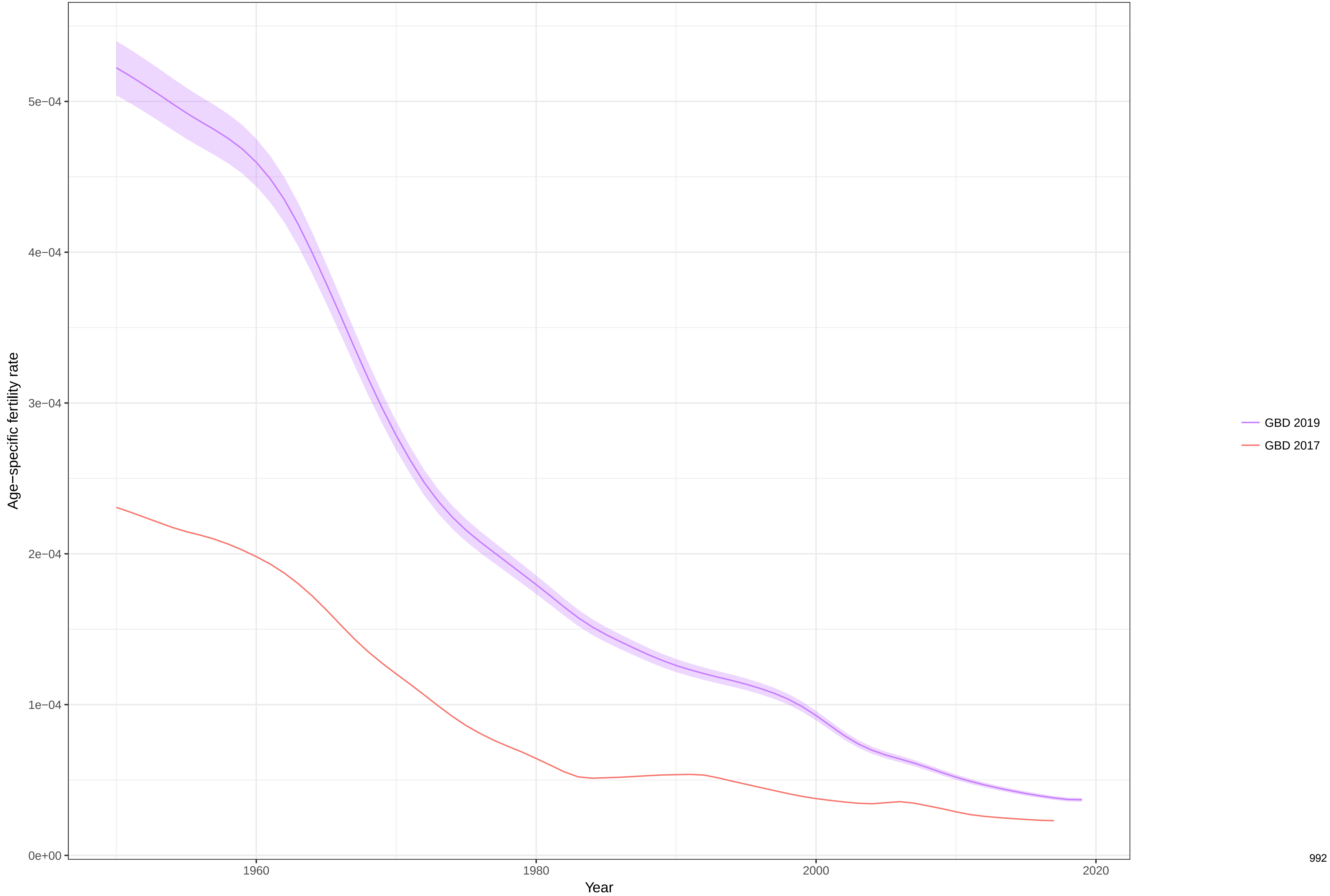

Total fertility rate fitted trend lines, 1950−2019: Guyana TFR
Data points enclosed with a diamond are reference sources

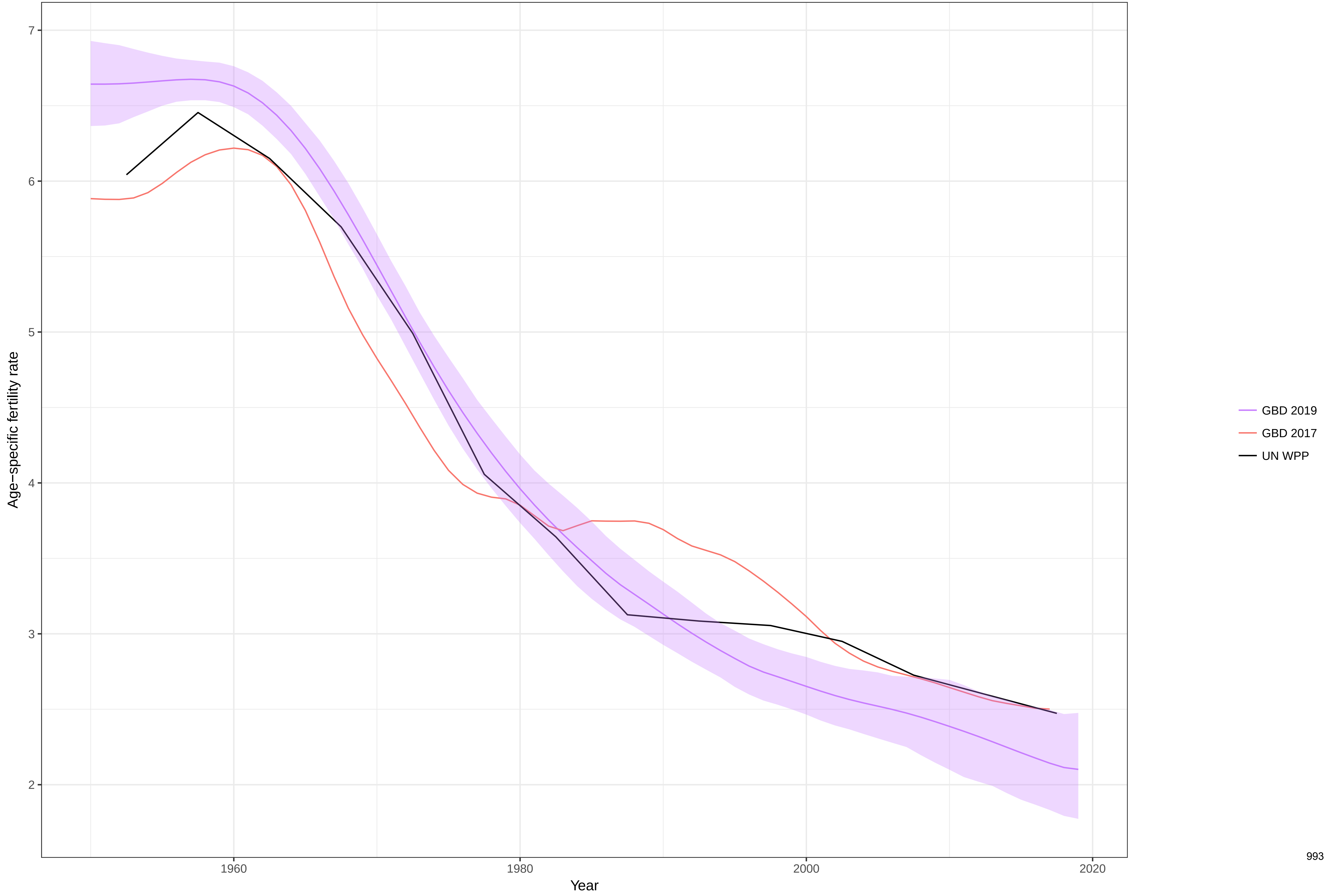

Age−specific fertility fitted trend lines, 1950−2019: Honduras, Ages: 10−14
Data points enclosed with a diamond are reference sources

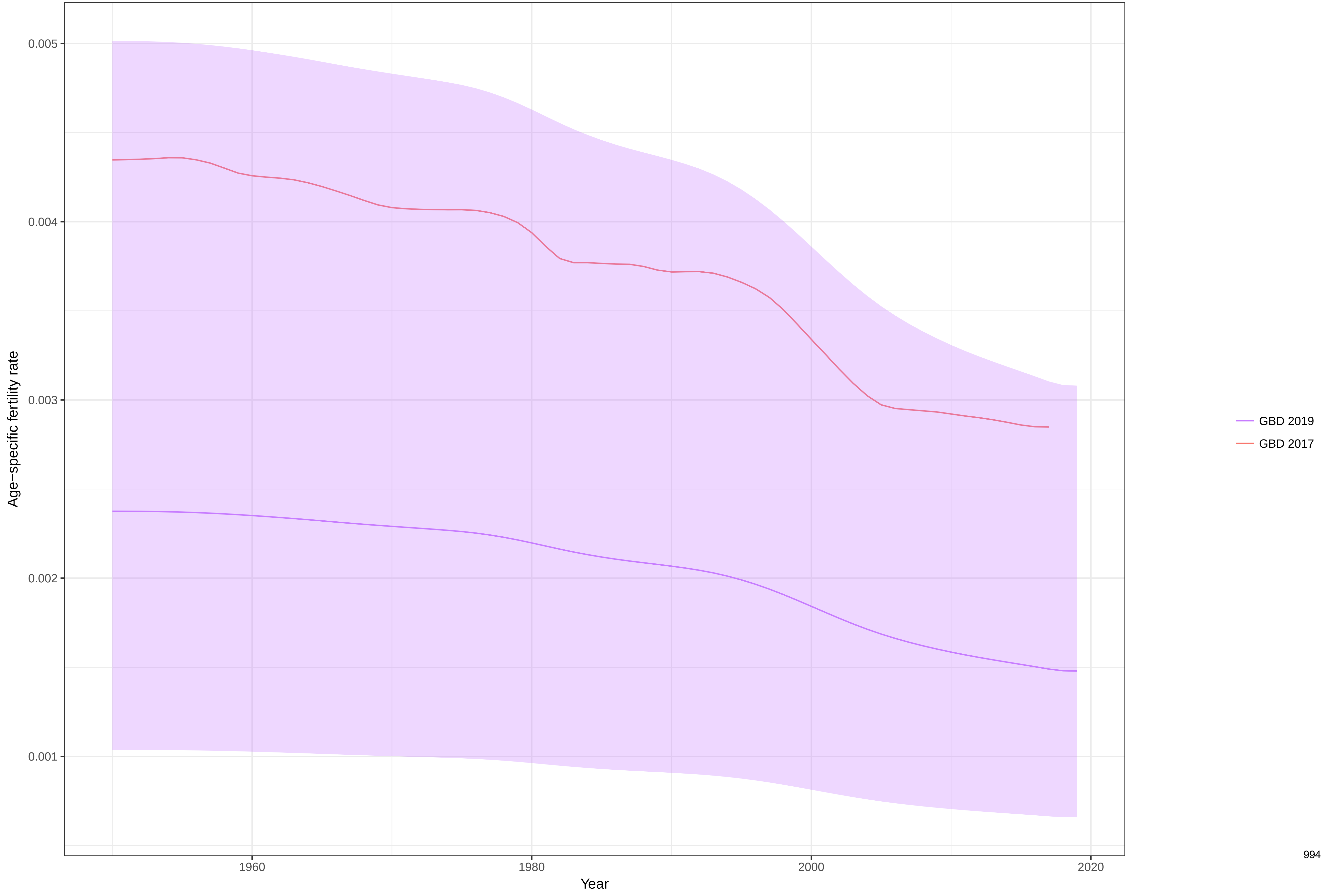

Age−specific fertility fitted trend lines, 1950−2019: Honduras, Ages: 15−19
Data points enclosed with a diamond are reference sources

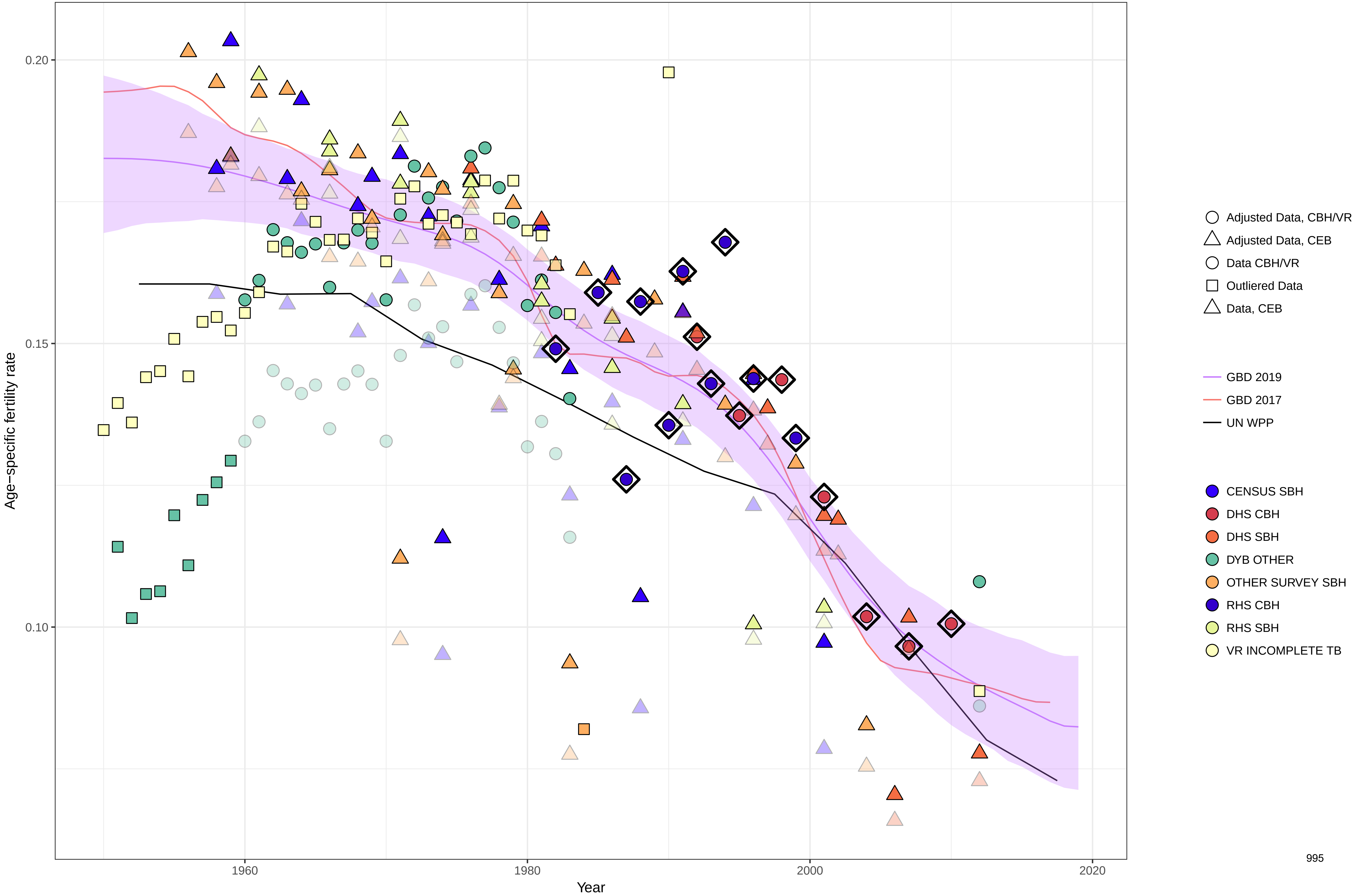

Age−specific fertility fitted trend lines, 1950−2019: Honduras, Ages: 20−24
Data points enclosed with a diamond are reference sources

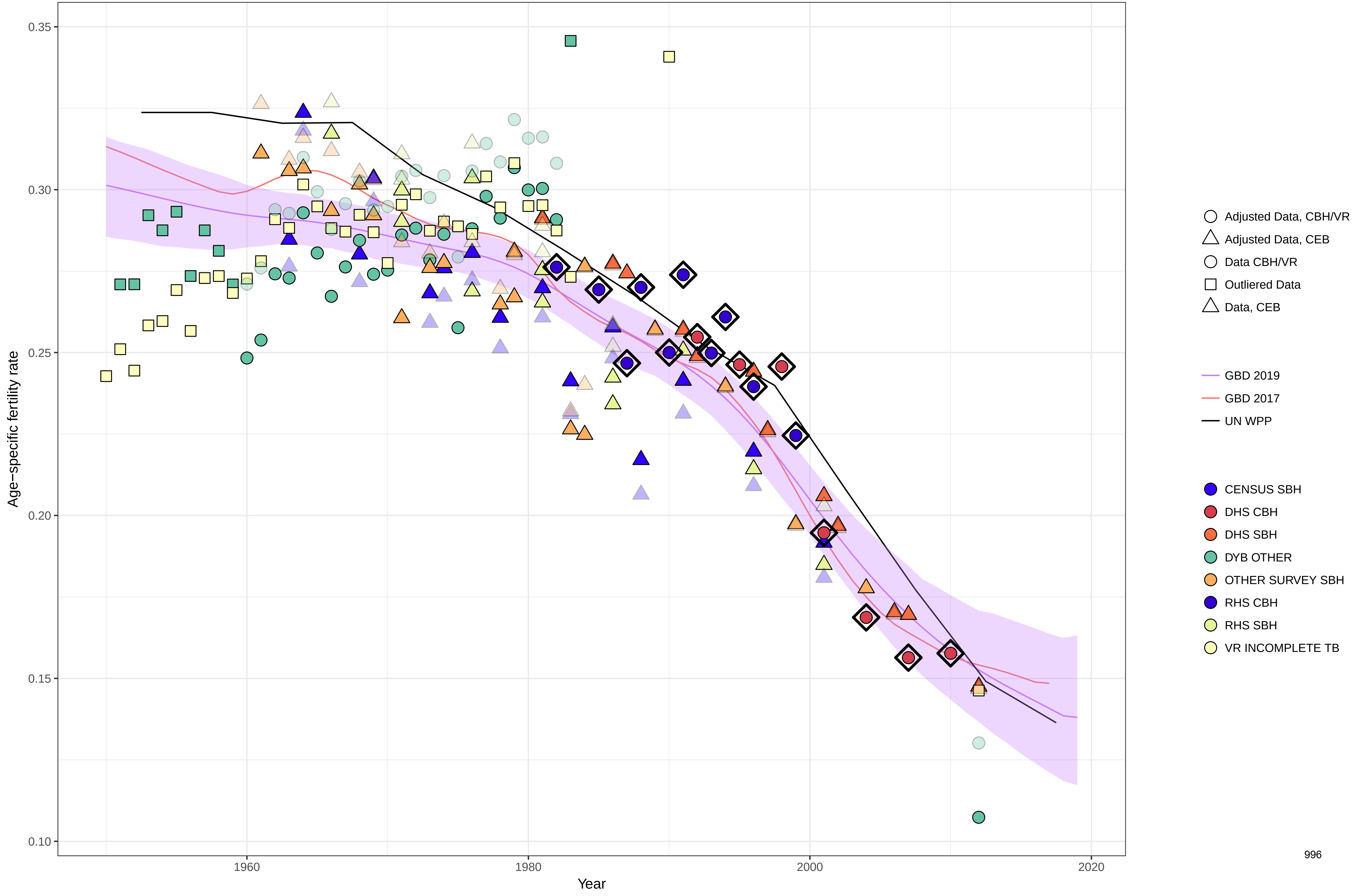

Age−specific fertility fitted trend lines, 1950−2019: Honduras, Ages: 25−29
Data points enclosed with a diamond are reference sources

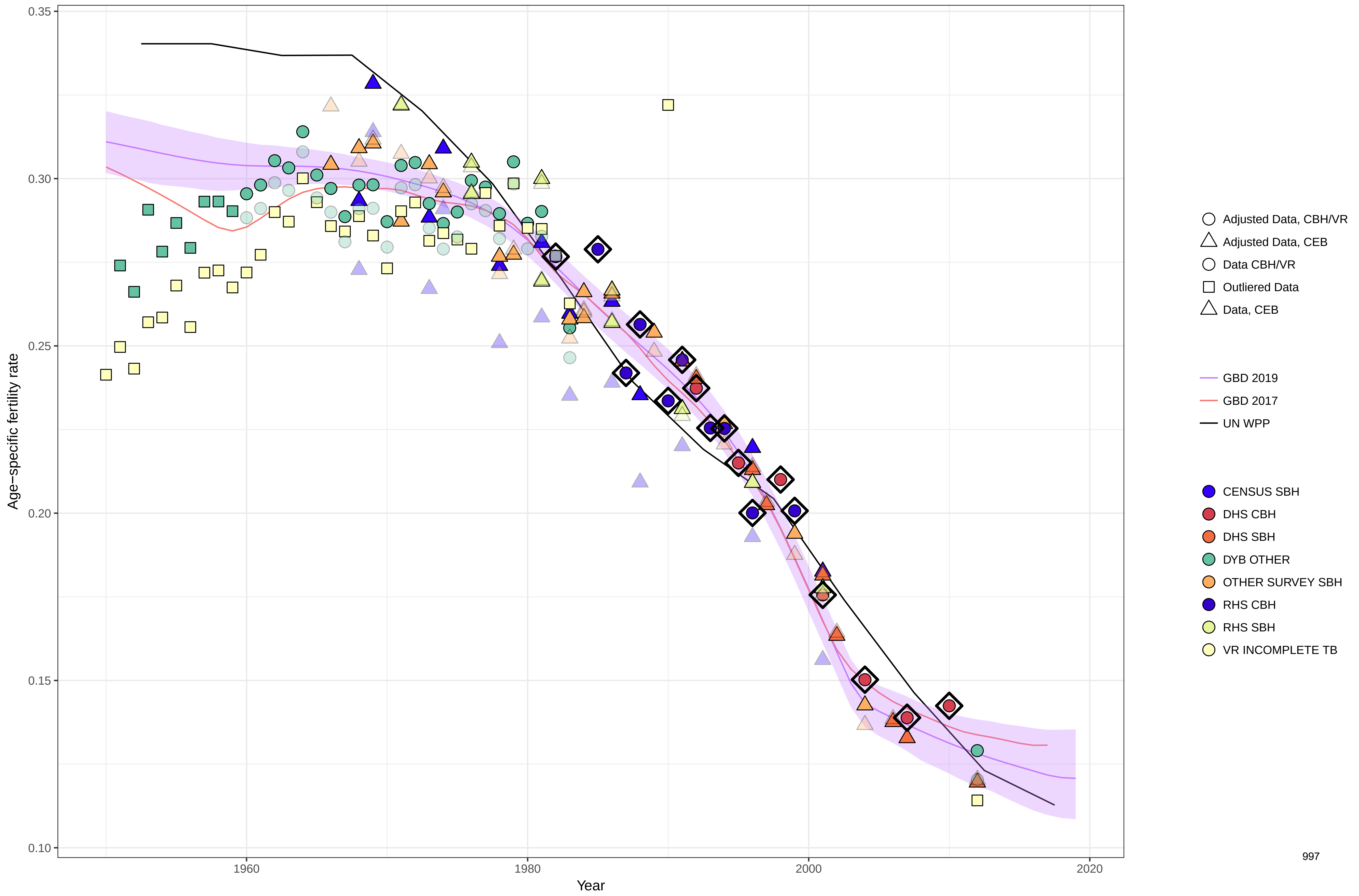

Age−specific fertility fitted trend lines, 1950−2019: Honduras, Ages: 30−34
Data points enclosed with a diamond are reference sources

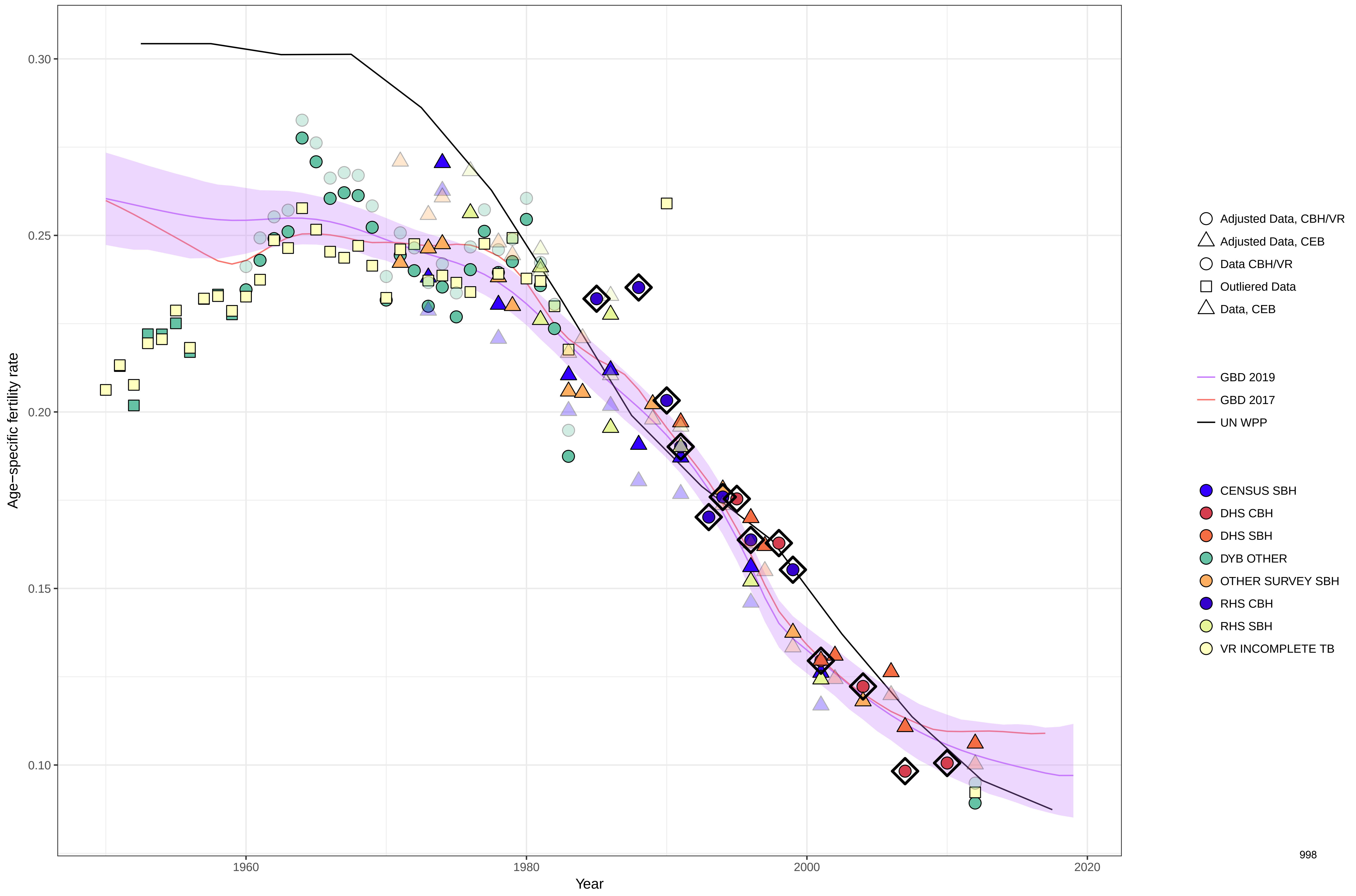

Age−specific fertility fitted trend lines, 1950−2019: Honduras, Ages: 35−39
Data points enclosed with a diamond are reference sources

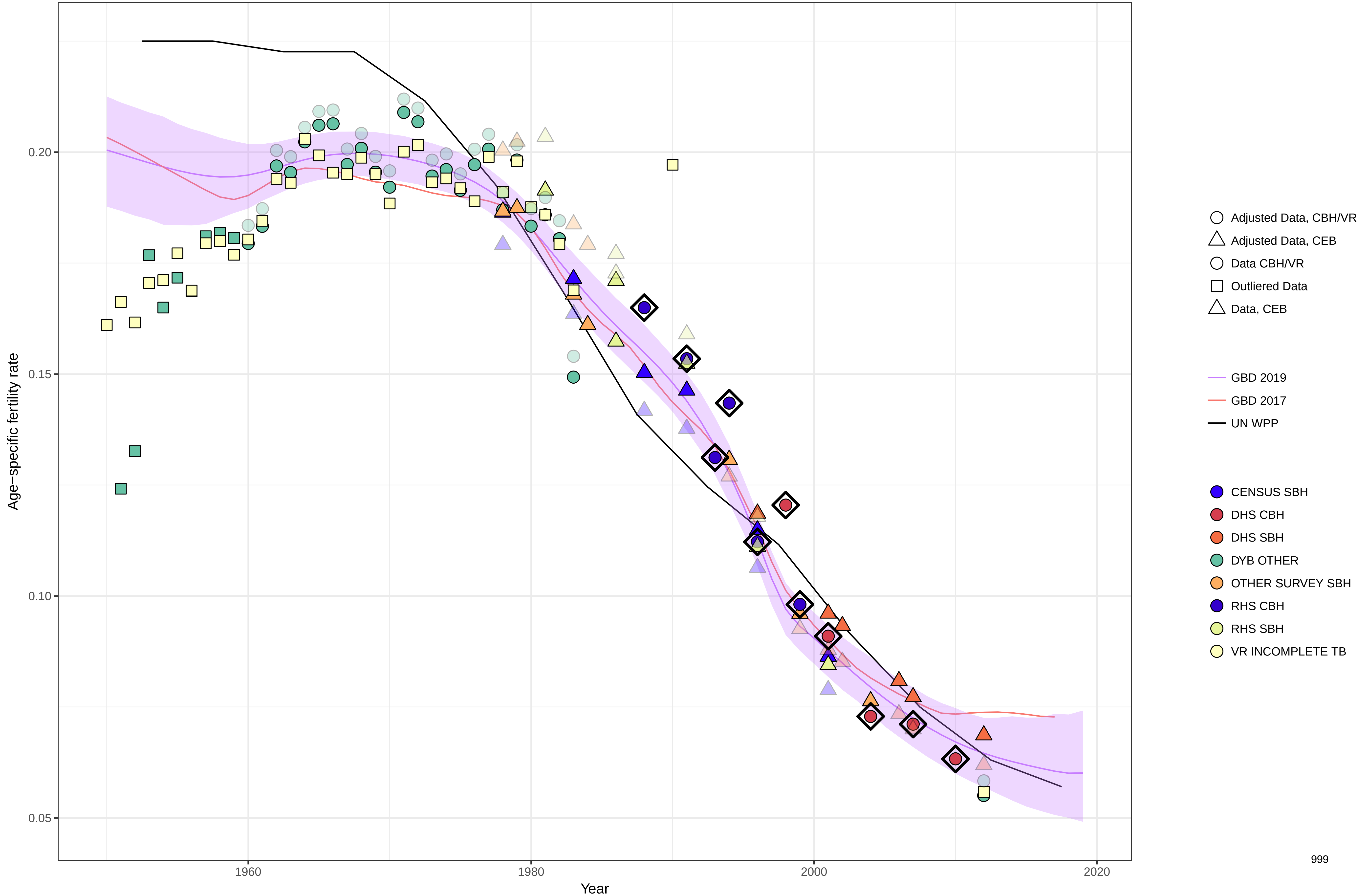

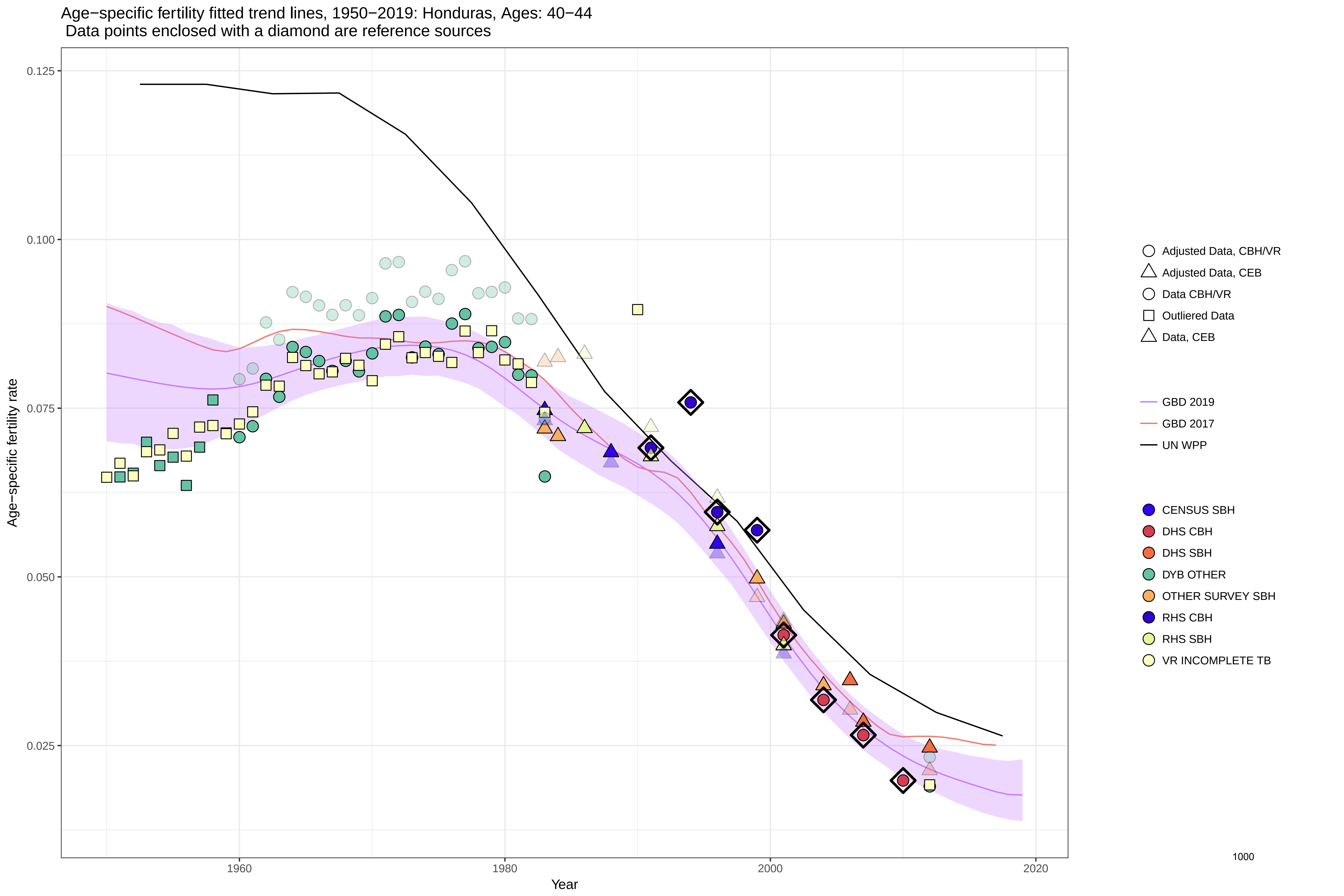

Age−specific fertility fitted trend lines, 1950−2019: Honduras, Ages: 40−44
Data points enclosed with a diamond are reference sources
Age−specific fertility rate
0.125
0.100
0.075
0.050
0.025
1960
1980
2000
2020
Year
Adjusted Data, CBH/VR
Adjusted Data, CEB
Data CBH/VR
Outliered Data
Data, CEB
GBD 2019
GBD 2017
UN WPP
CENSUS SBH
DHS CBH
DHS SBH
DYB OTHER
OTHER SURVEY SBH
RHS CBH
RHS SBH
VR INCOMPLETE TB
1000

Age−specific fertility fitted trend lines, 1950−2019: Honduras, Ages: 45−49
Data points enclosed with a diamond are reference sources

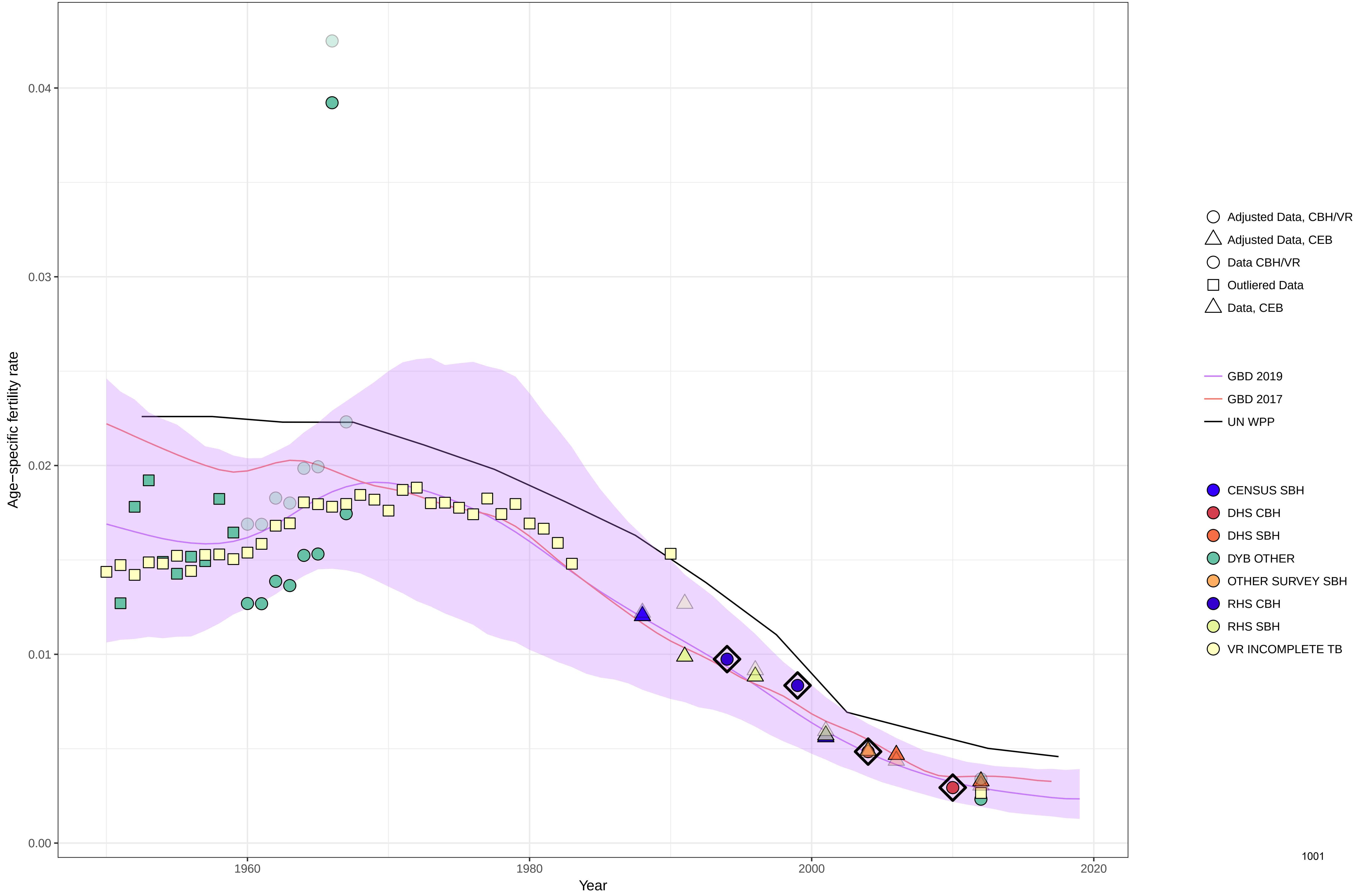

Age−specific fertility fitted trend lines, 1950−2019: Honduras, Ages: 50−54
Data points enclosed with a diamond are reference sources

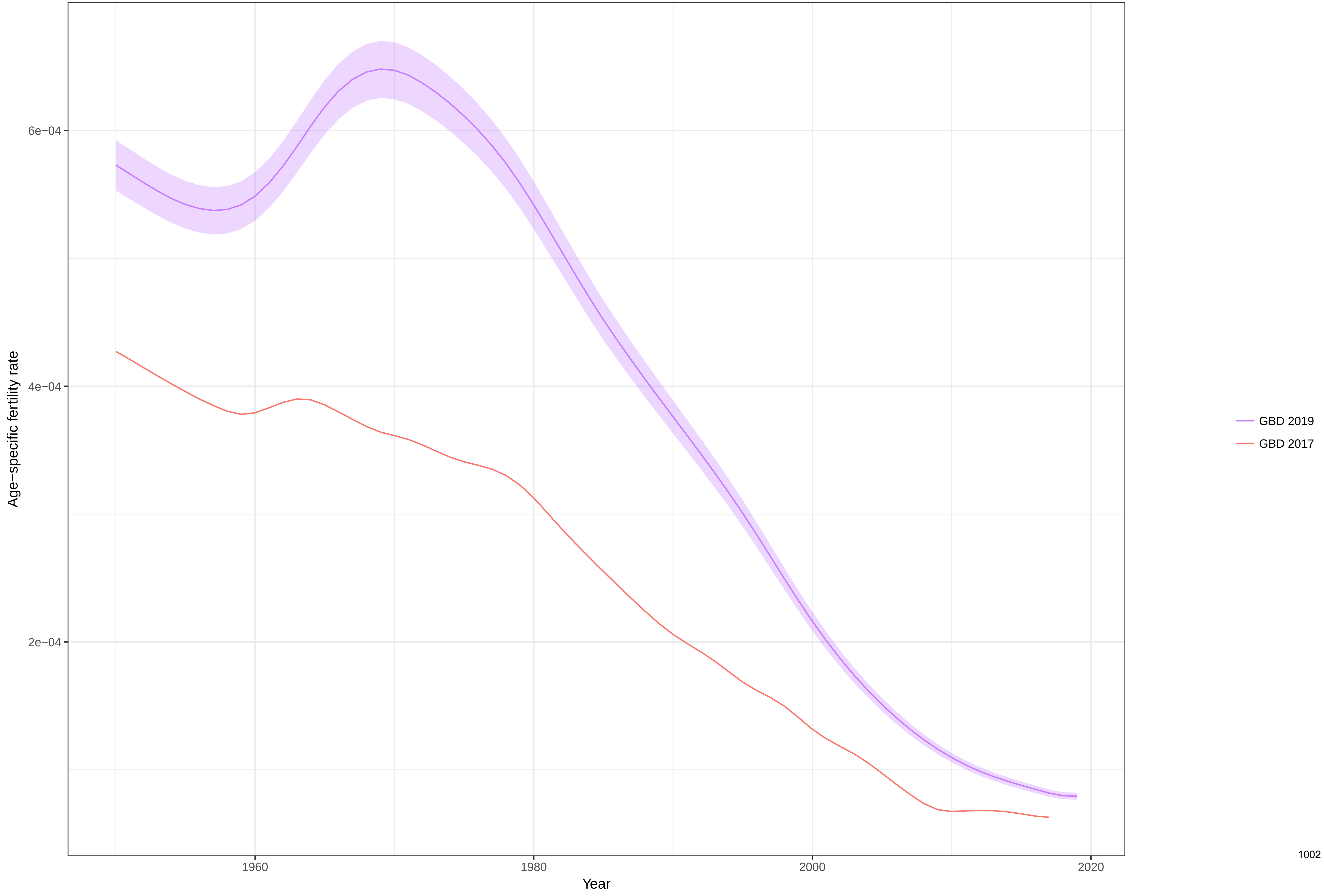

Total fertility rate fitted trend lines, 1950−2019: Honduras TFR
Data points enclosed with a diamond are reference sources

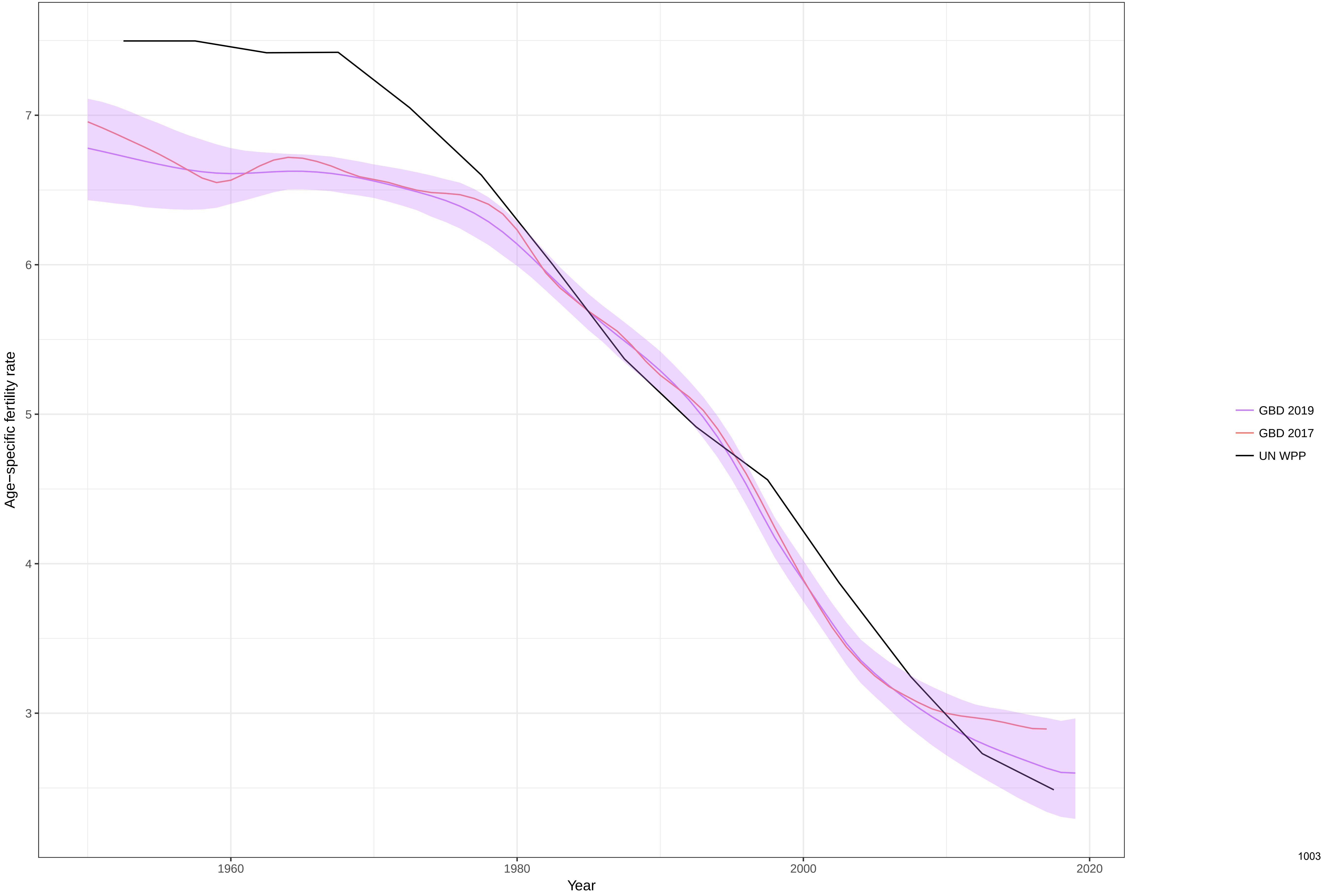

Age−specific fertility fitted trend lines, 1950−2019: Croatia, Ages: 10−14
Data points enclosed with a diamond are reference sources

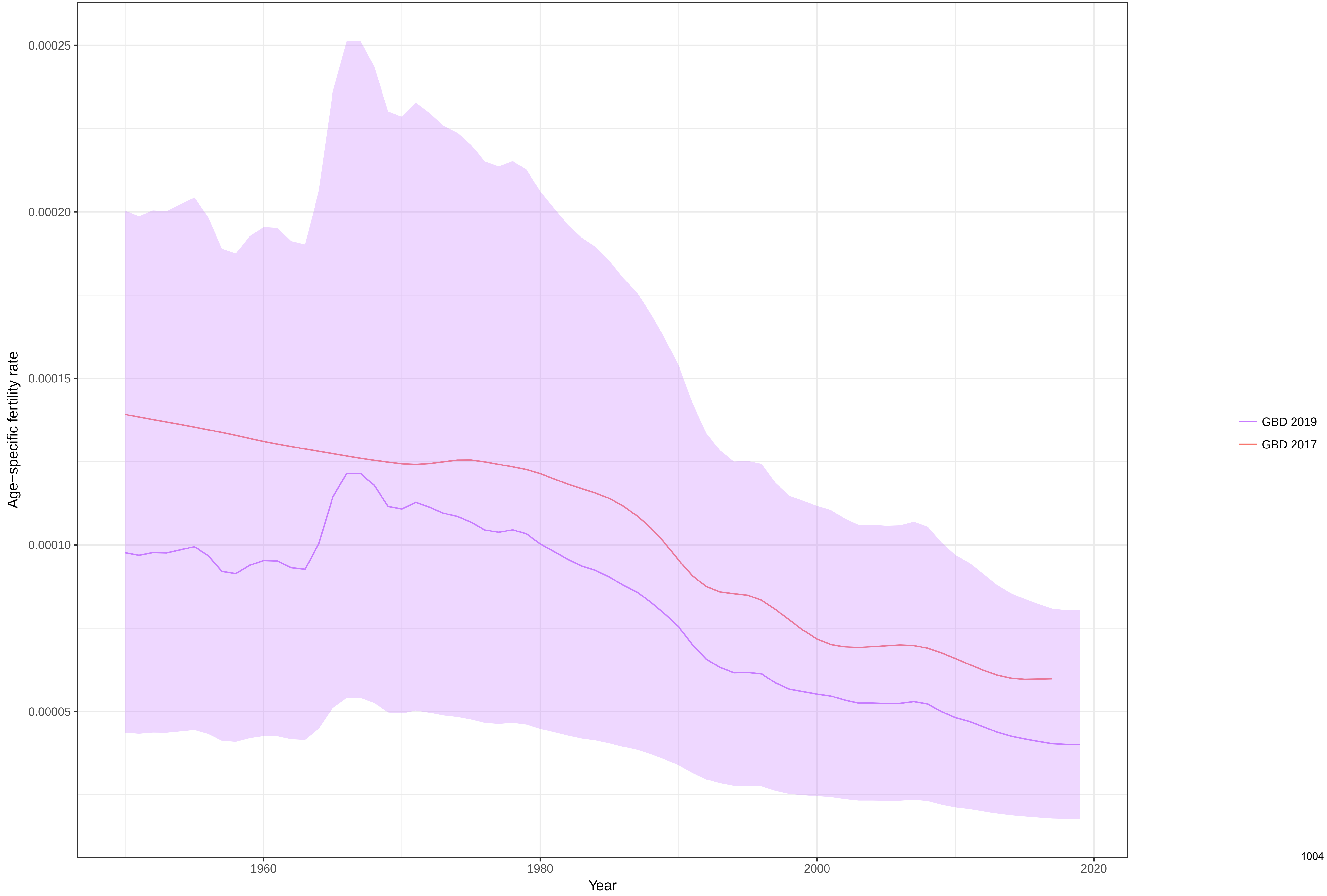

Age−specific fertility fitted trend lines, 1950−2019: Croatia, Ages: 15−19
Data points enclosed with a diamond are reference sources

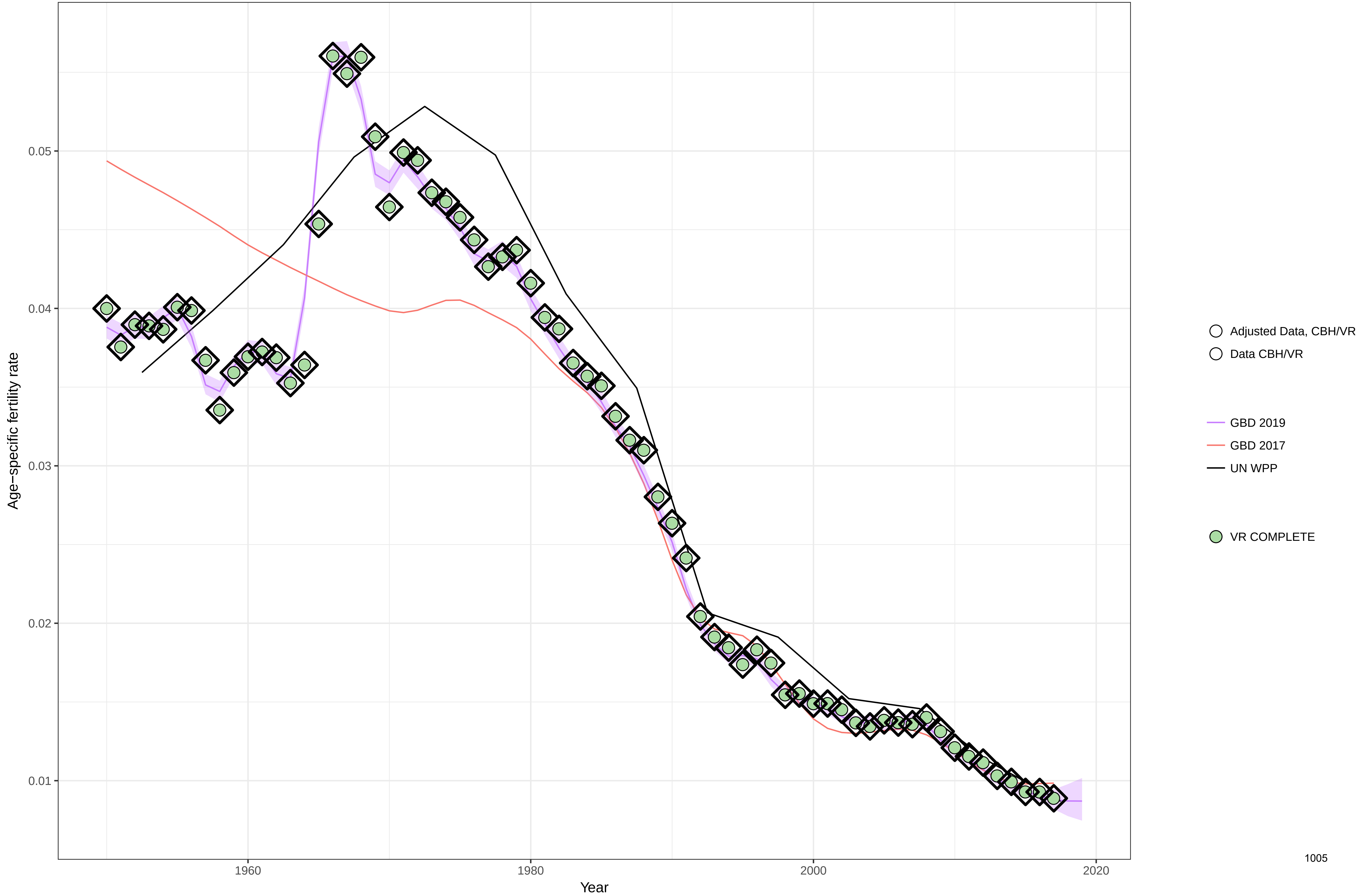

Age−specific fertility fitted trend lines, 1950−2019: Croatia, Ages: 20−24
Data points enclosed with a diamond are reference sources

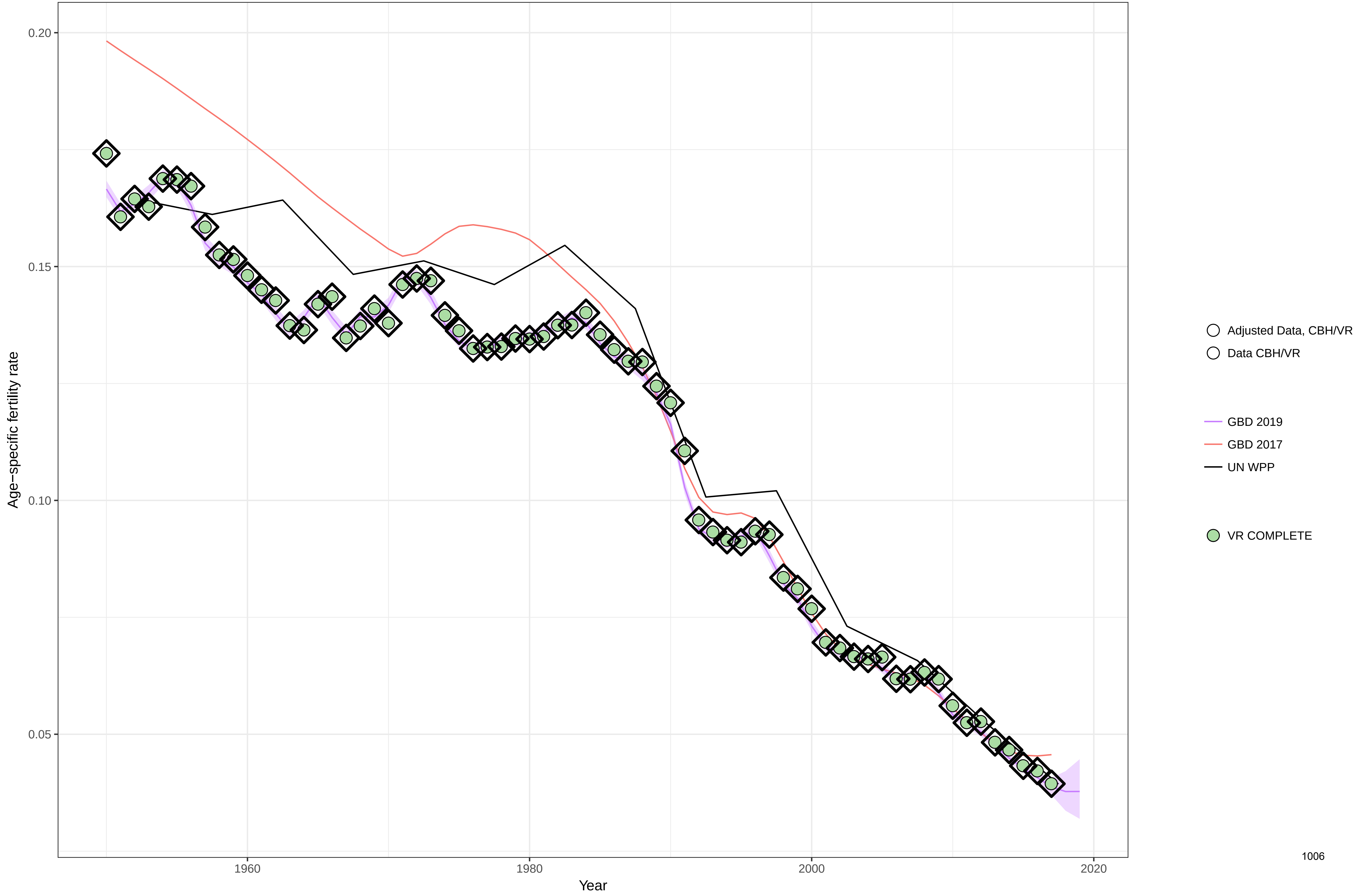

Age−specific fertility fitted trend lines, 1950−2019: Croatia, Ages: 25−29
Data points enclosed with a diamond are reference sources

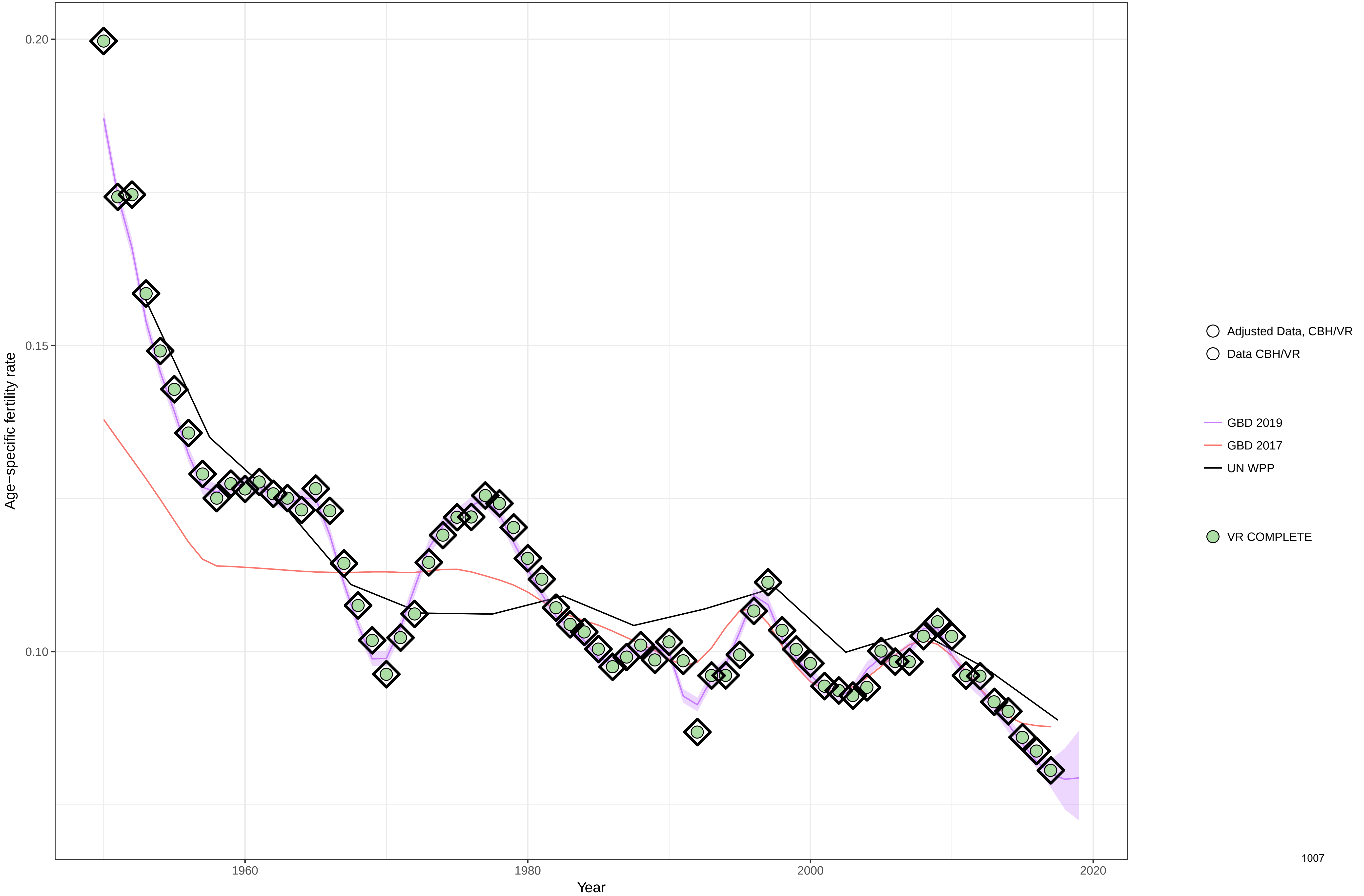

Age−specific fertility fitted trend lines, 1950−2019: Croatia, Ages: 30−34
Data points enclosed with a diamond are reference sources

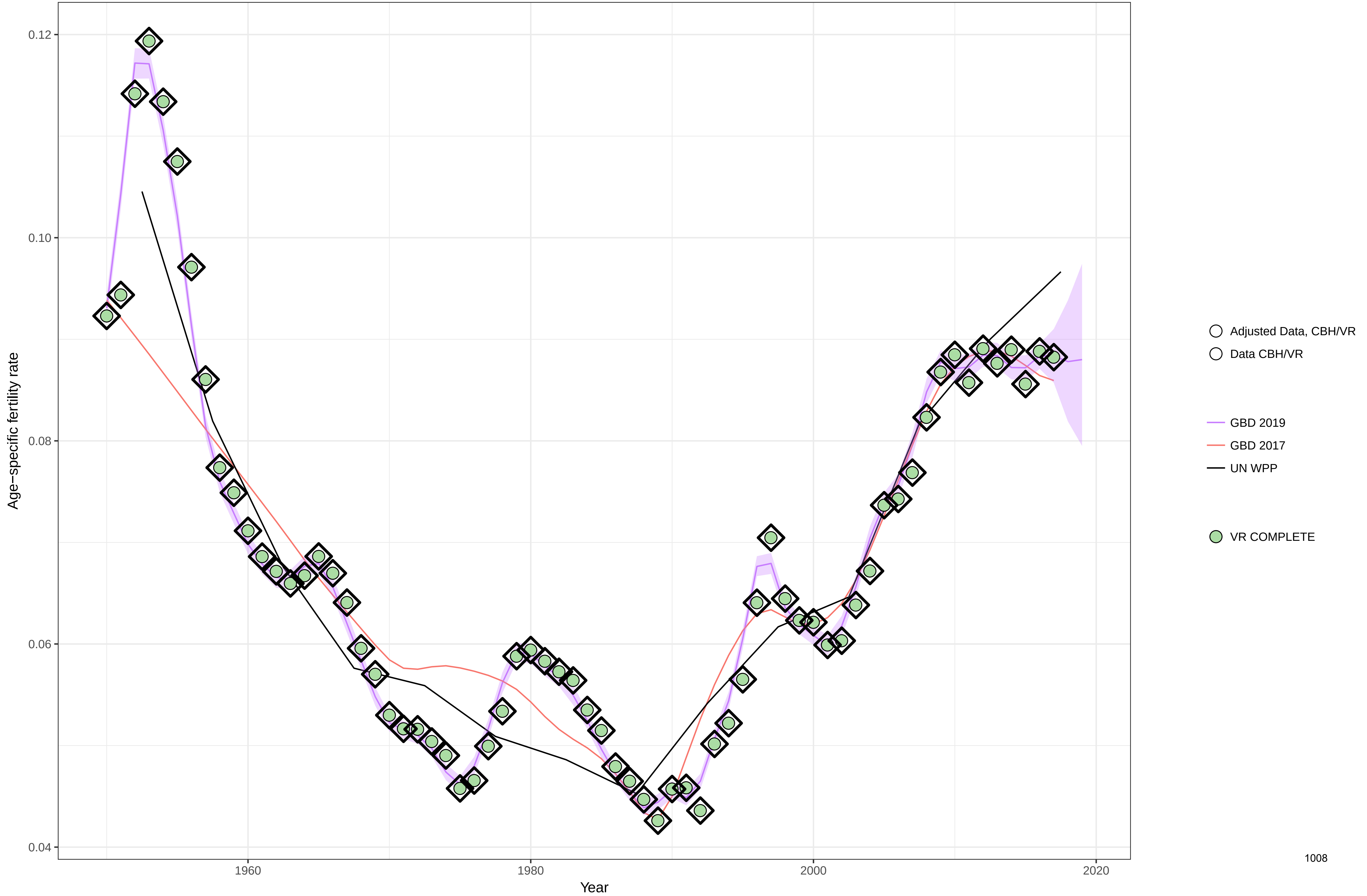

Age−specific fertility fitted trend lines, 1950−2019: Croatia, Ages: 35−39
Data points enclosed with a diamond are reference sources

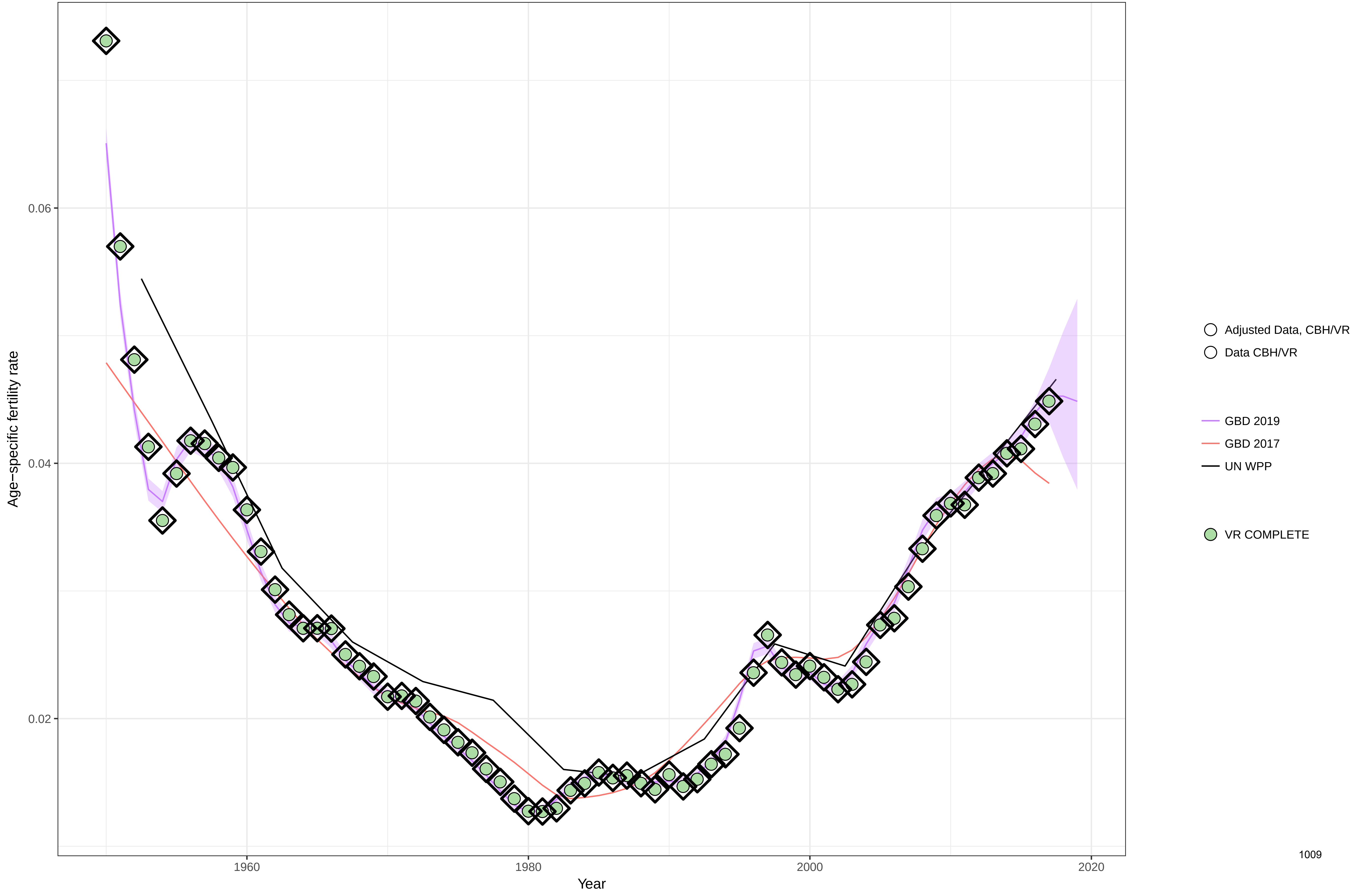

Age−specific fertility fitted trend lines, 1950−2019: Croatia, Ages: 40−44
Data points enclosed with a diamond are reference sources

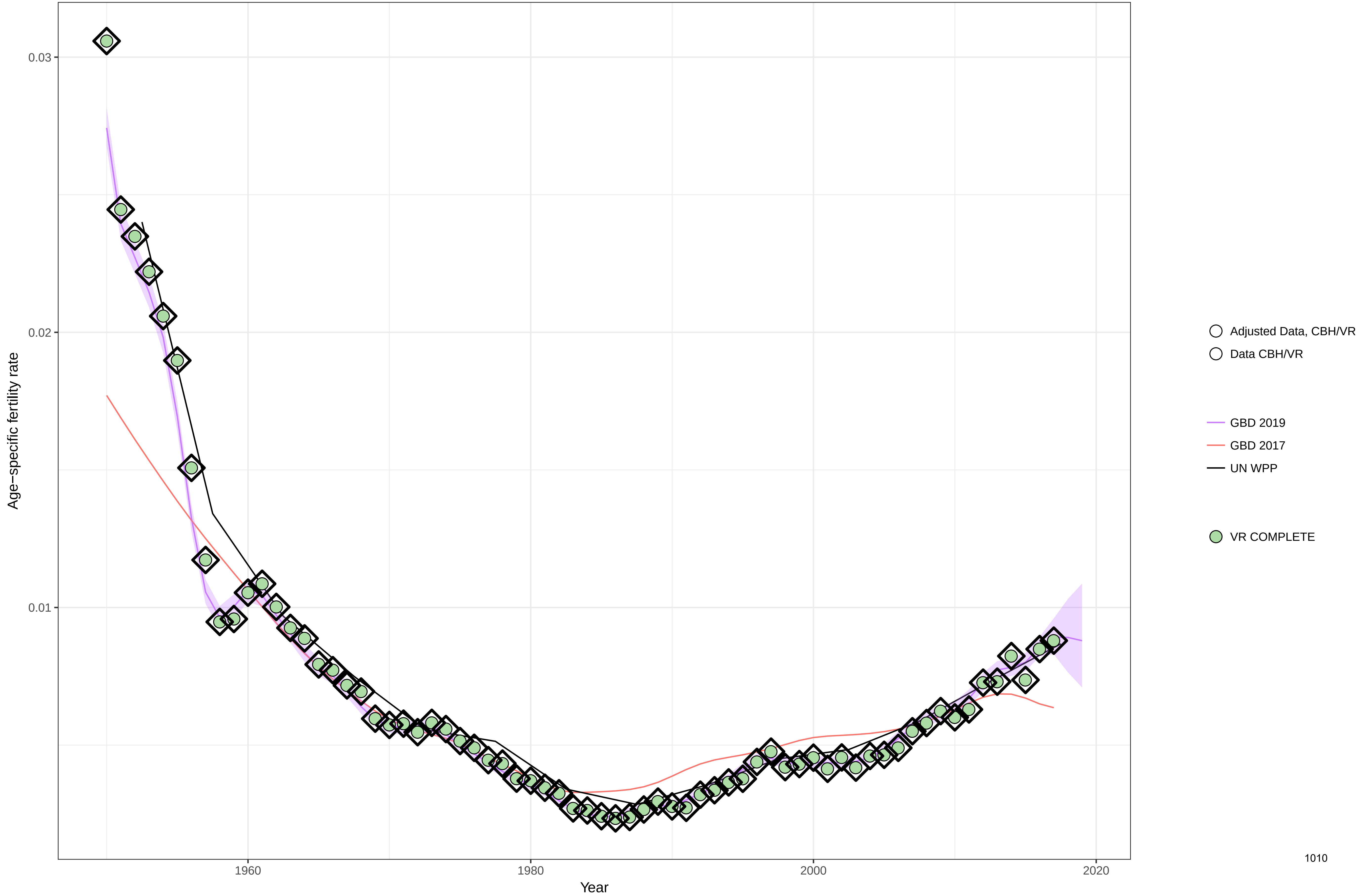

Age−specific fertility fitted trend lines, 1950−2019: Croatia, Ages: 45−49
Data points enclosed with a diamond are reference sources

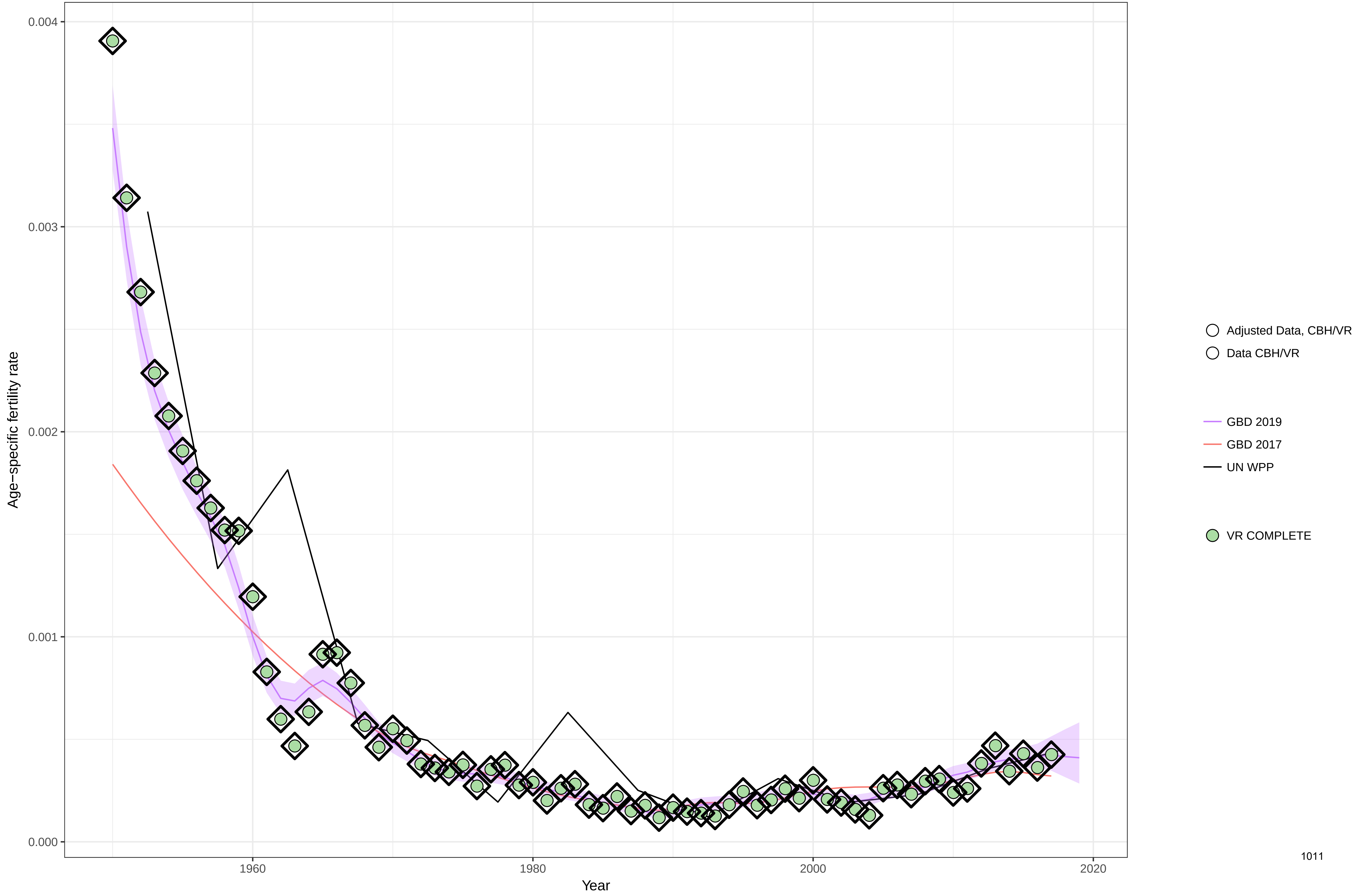

Age−specific fertility fitted trend lines, 1950−2019: Croatia, Ages: 50−54
Data points enclosed with a diamond are reference sources

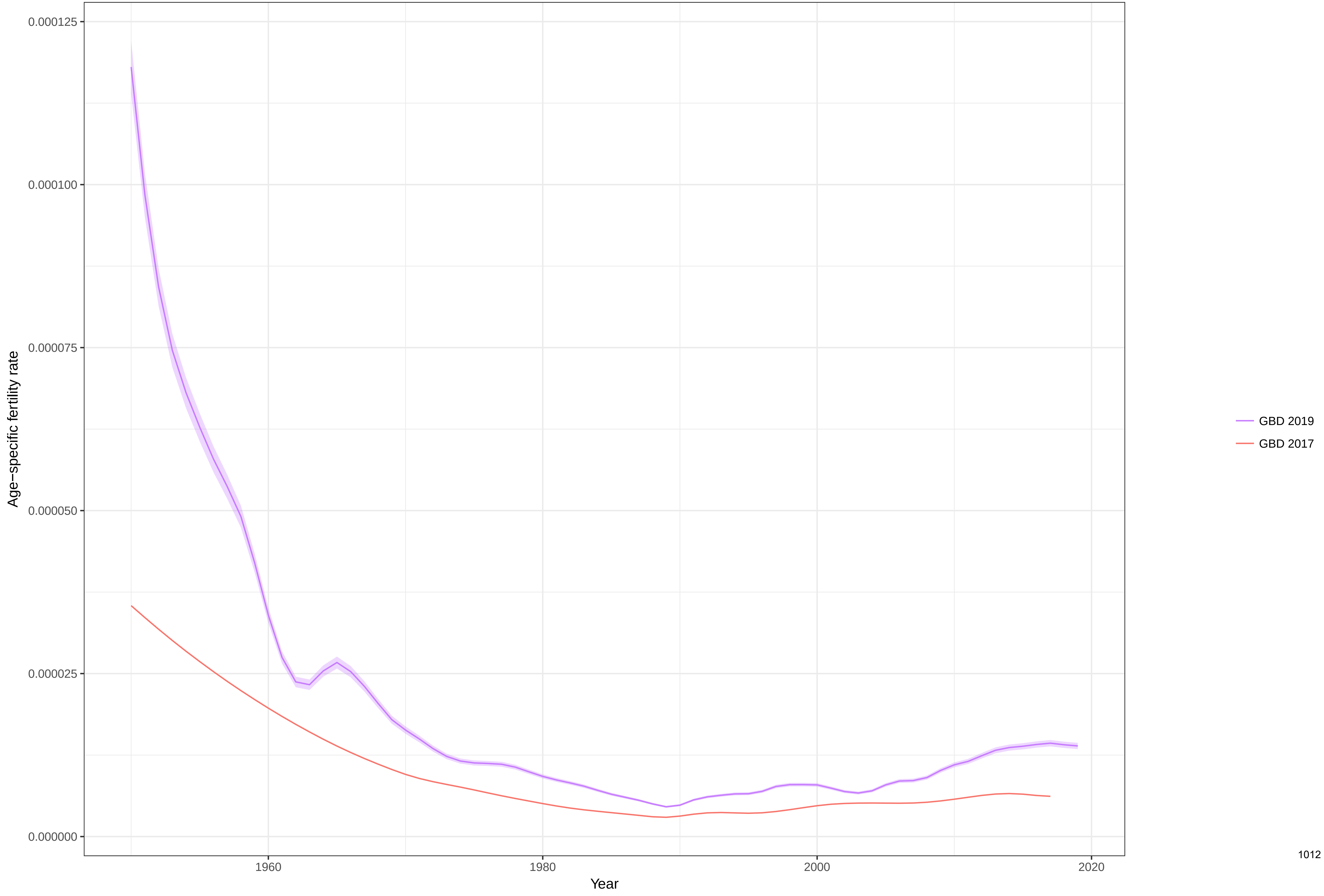

Total fertility rate fitted trend lines, 1950−2019: Croatia TFR
Data points enclosed with a diamond are reference sources

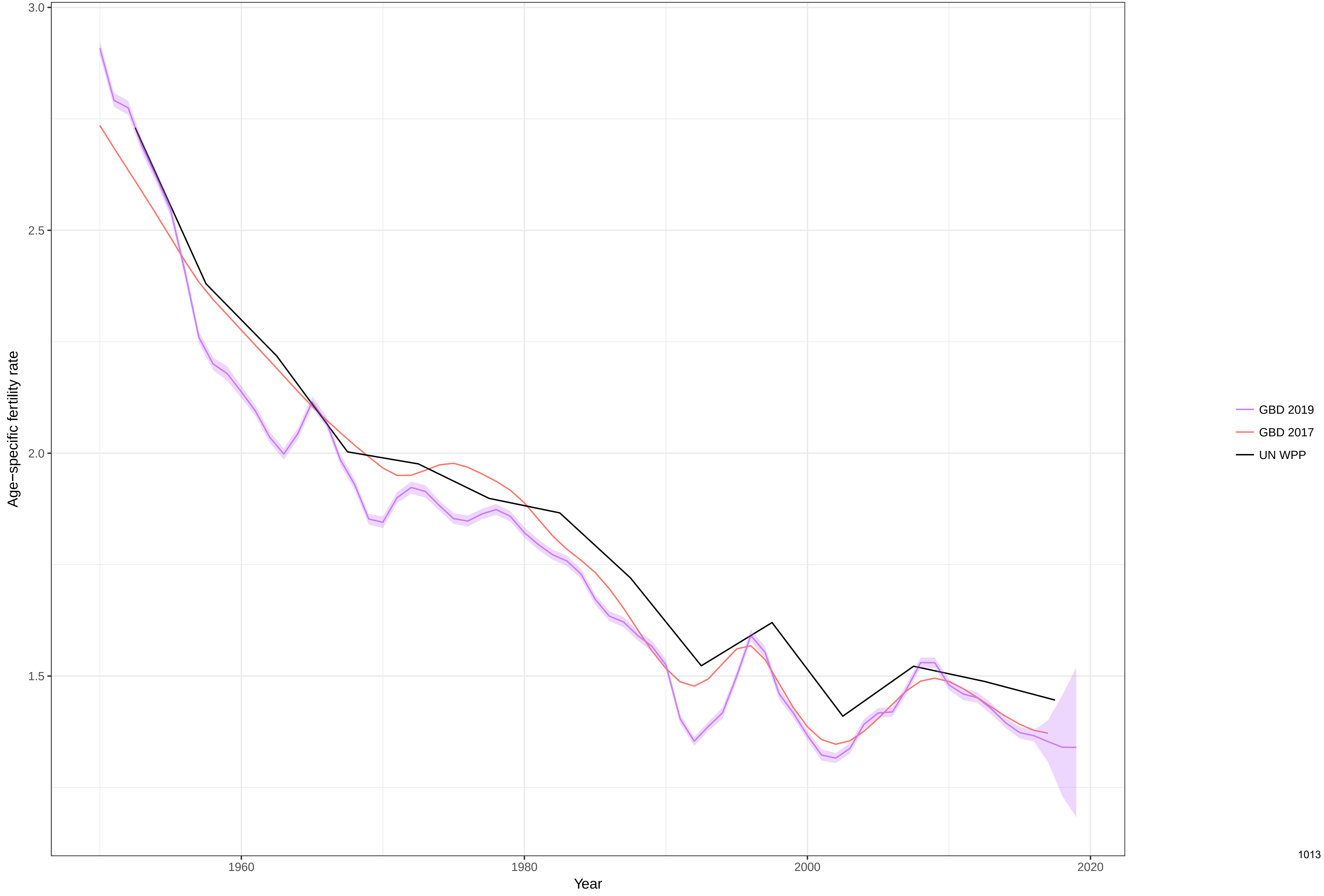

Age−specific fertility fitted trend lines, 1950−2019: Haiti, Ages: 10−14
Data points enclosed with a diamond are reference sources

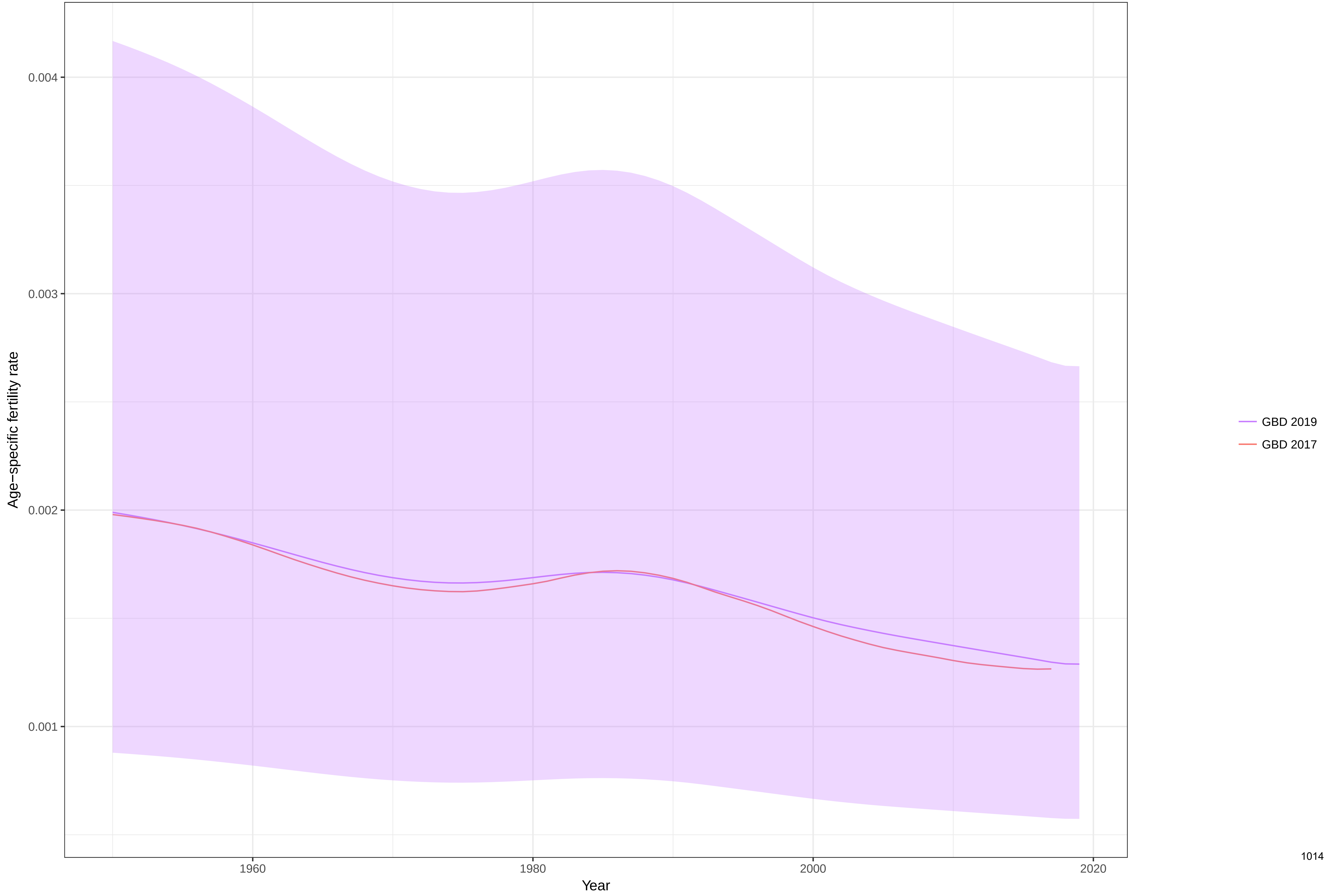

Age−specific fertility fitted trend lines, 1950−2019: Haiti, Ages: 15−19
Data points enclosed with a diamond are reference sources

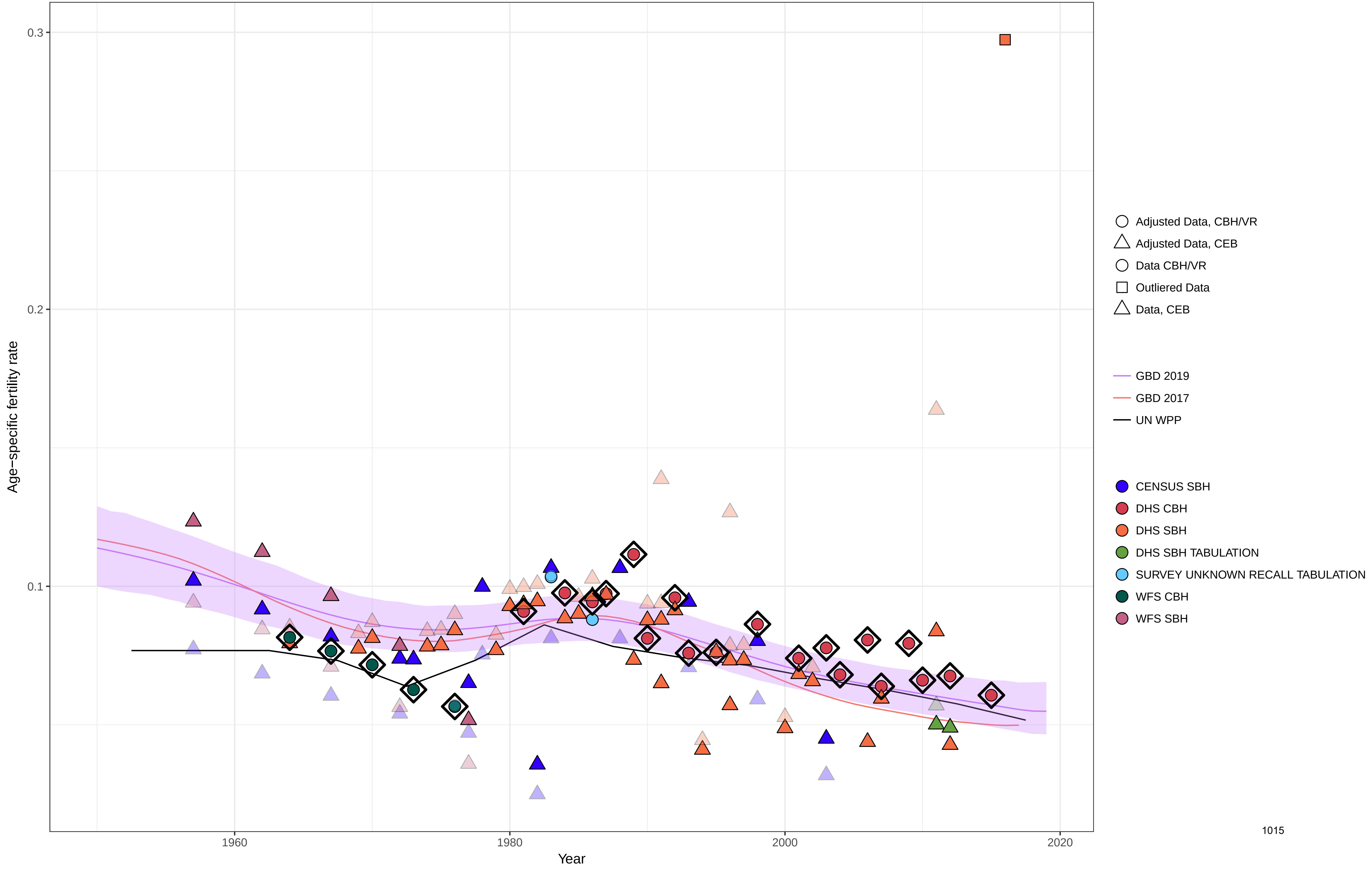

Age−specific fertility fitted trend lines, 1950−2019: Haiti, Ages: 20−24
Data points enclosed with a diamond are reference sources

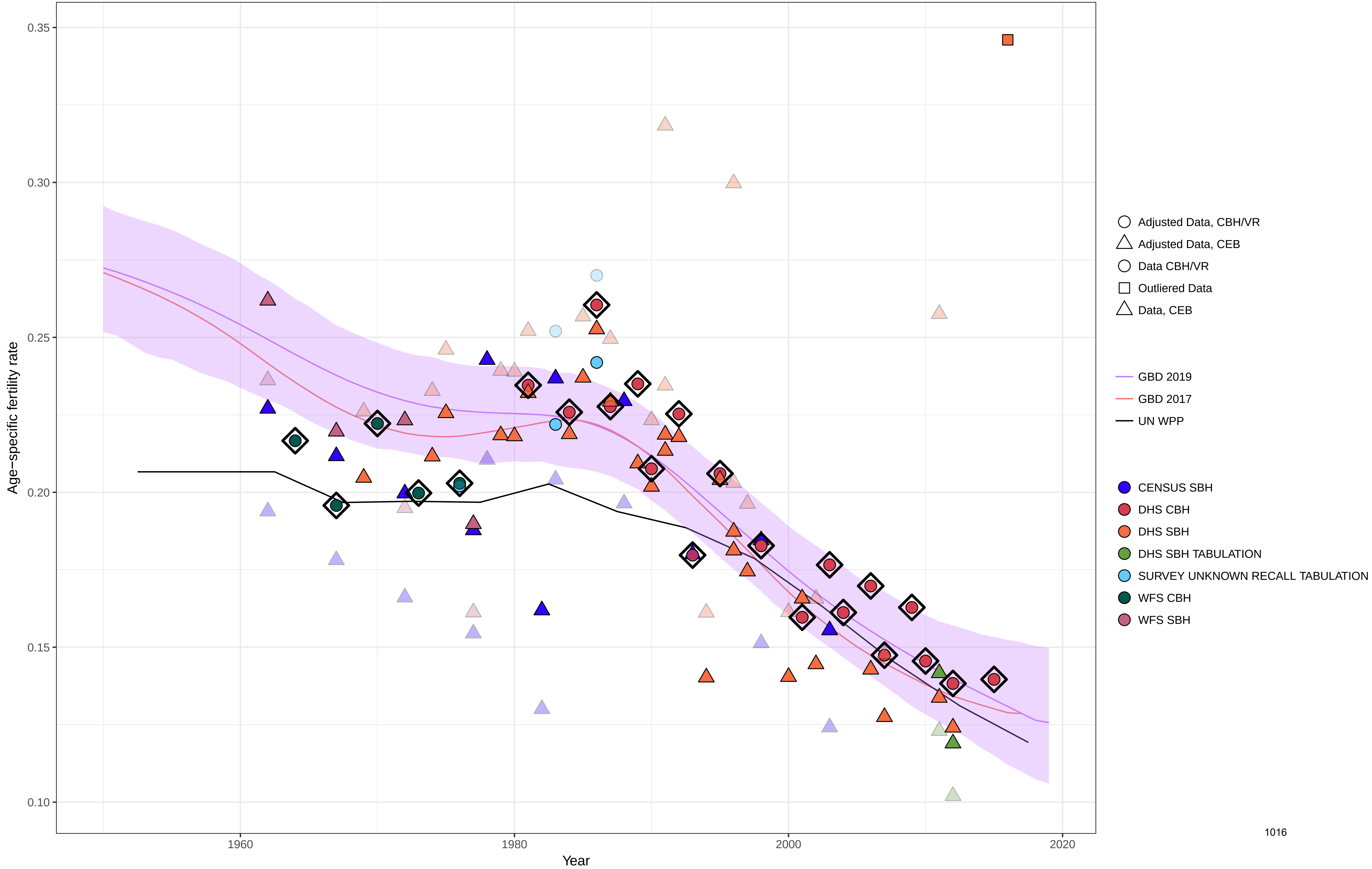

1016

Age−specific fertility fitted trend lines, 1950−2019: Haiti, Ages: 25−29
Data points enclosed with a diamond are reference sources

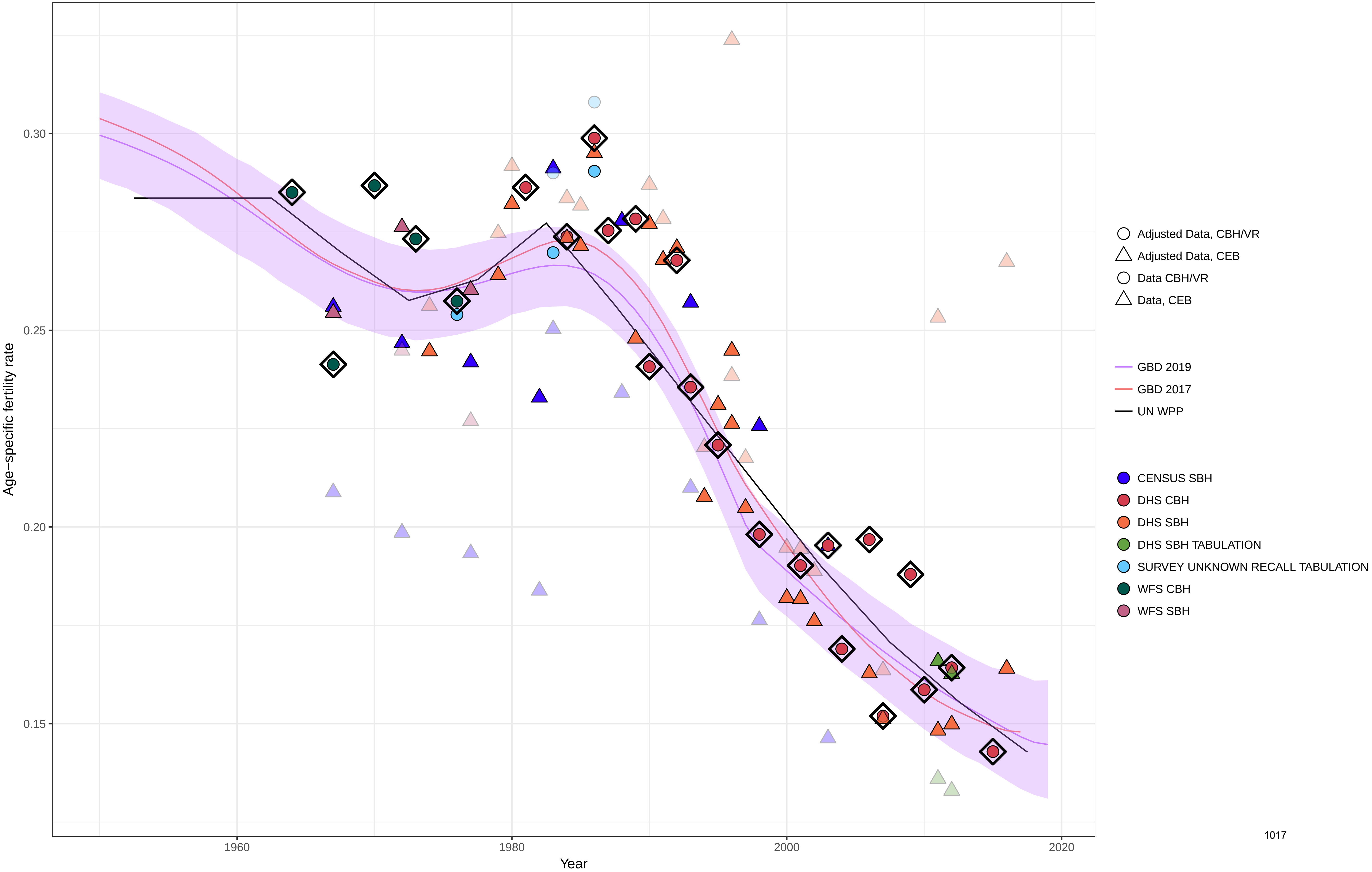

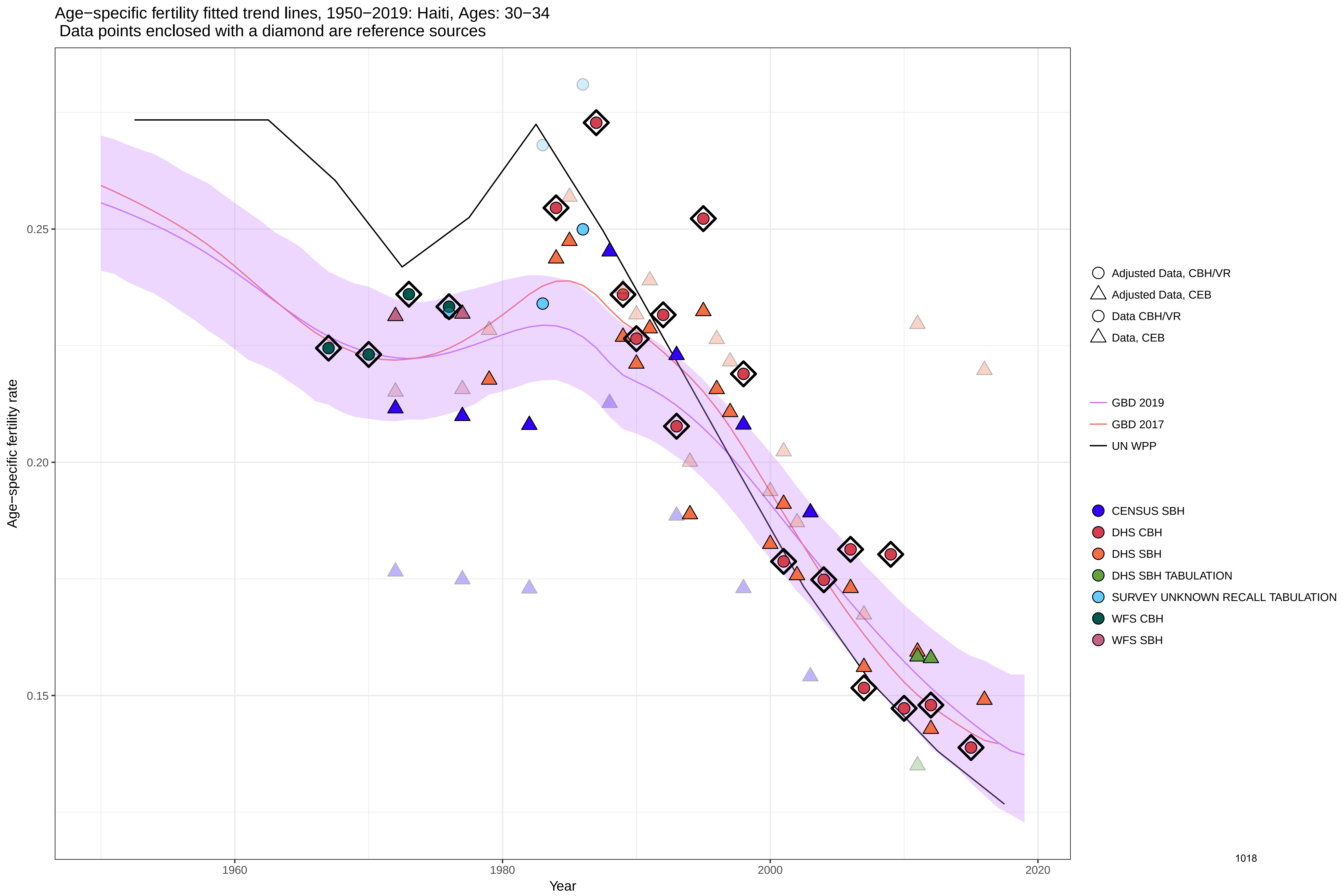

Age−specific fertility fitted trend lines, 1950−2019: Haiti, Ages: 30−34
Data points enclosed with a diamond are reference sources
Age−specific fertility rate
0.25
0.20
0.15
1960
1980
2000
2020
Year
Adjusted Data, CBH/VR
Adjusted Data, CEB
Data CBH/VR
Data, CEB
GBD 2019
GBD 2017
UN WPP
CENSUS SBH
DHS CBH
DHS SBH
DHS SBH TABULATION
SURVEY UNKNOWN RECALL TABULATION
WFS CBH
WFS SBH

Age−specific fertility fitted trend lines, 1950−2019: Haiti, Ages: 35−39
Data points enclosed with a diamond are reference sources

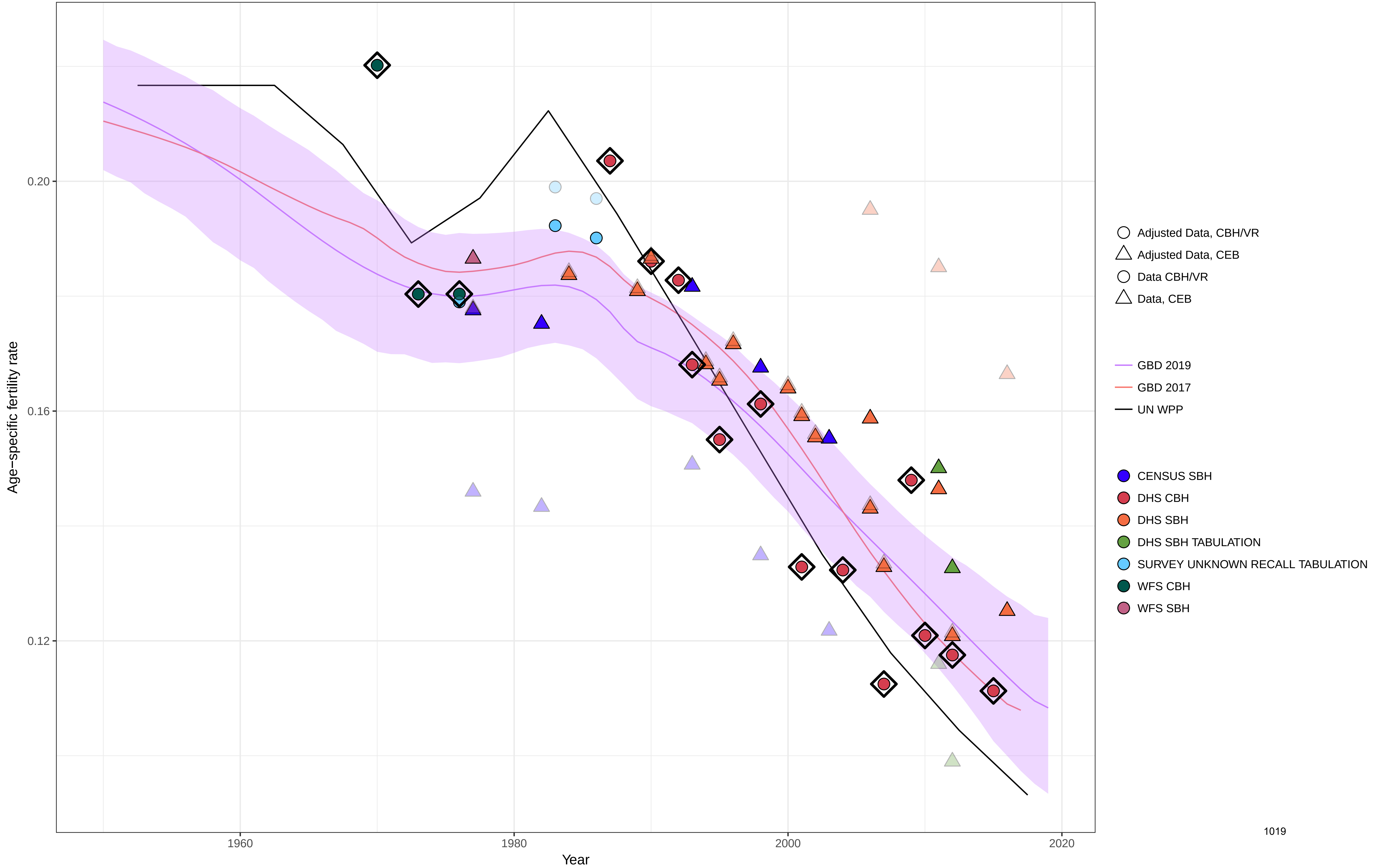

Age−specific fertility fitted trend lines, 1950−2019: Haiti, Ages: 40−44
Data points enclosed with a diamond are reference sources

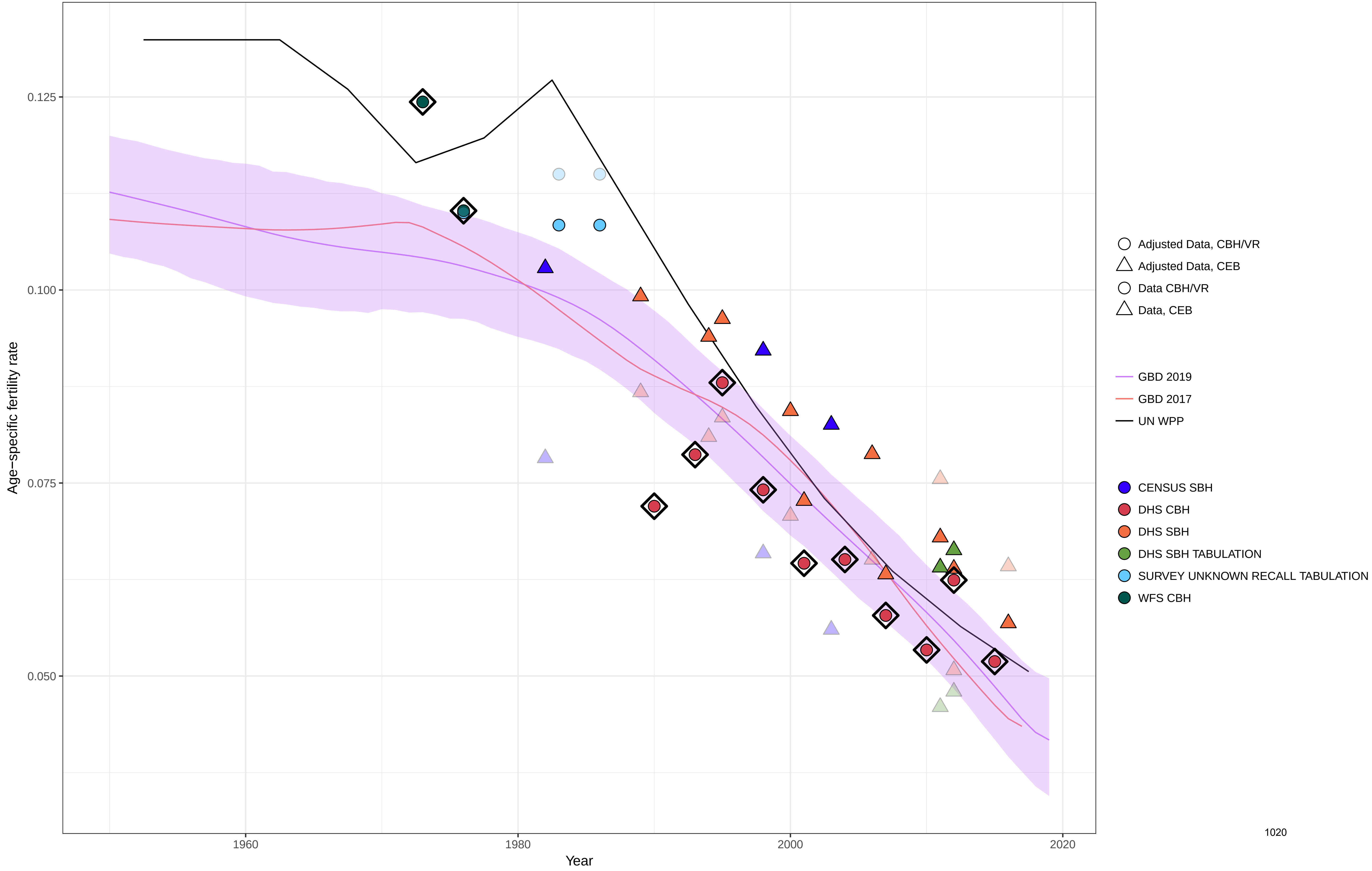

Age−specific fertility fitted trend lines, 1950−2019: Haiti, Ages: 45−49
Data points enclosed with a diamond are reference sources

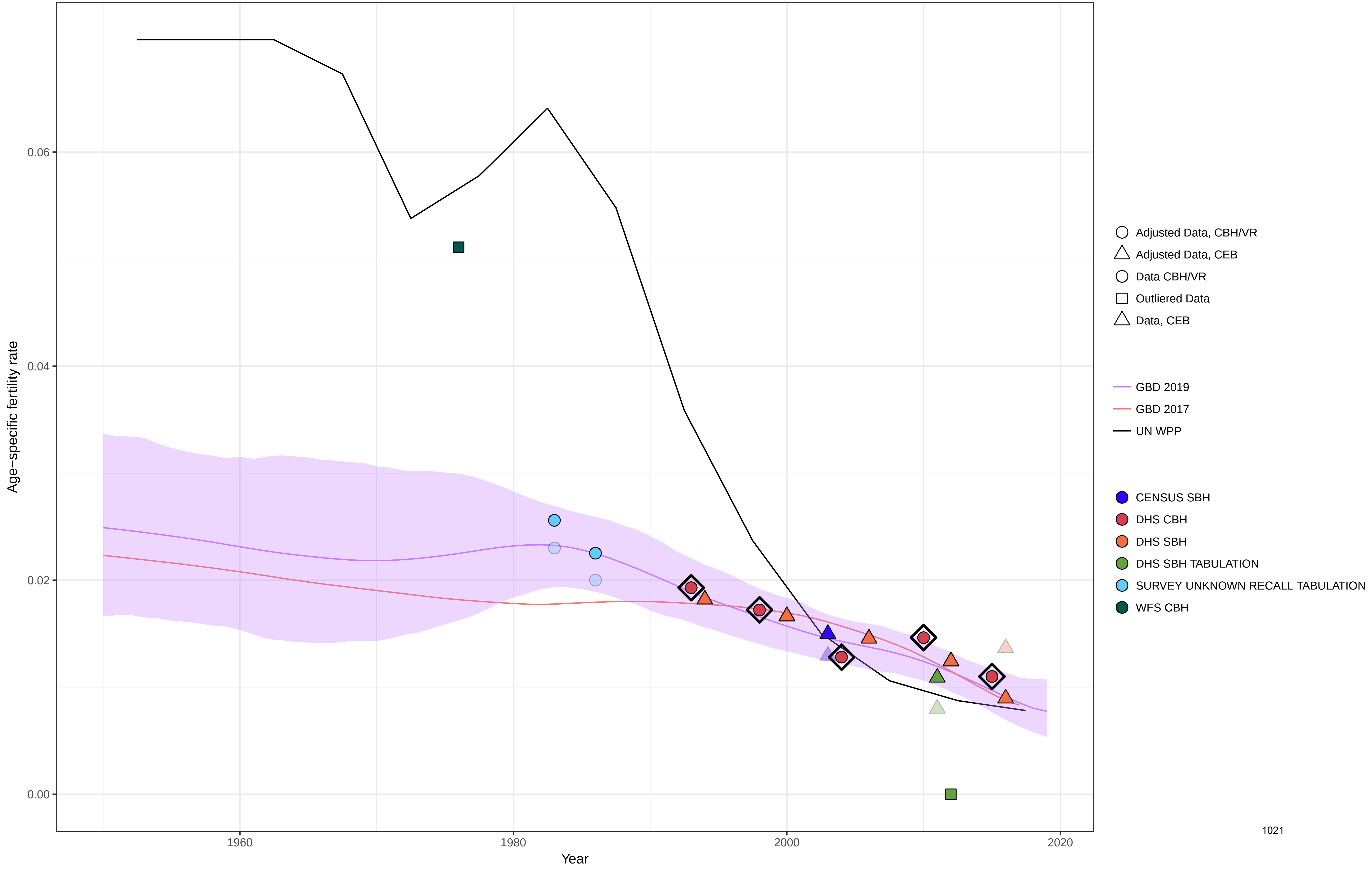

Age−specific fertility fitted trend lines, 1950−2019: Haiti, Ages: 50−54
Data points enclosed with a diamond are reference sources

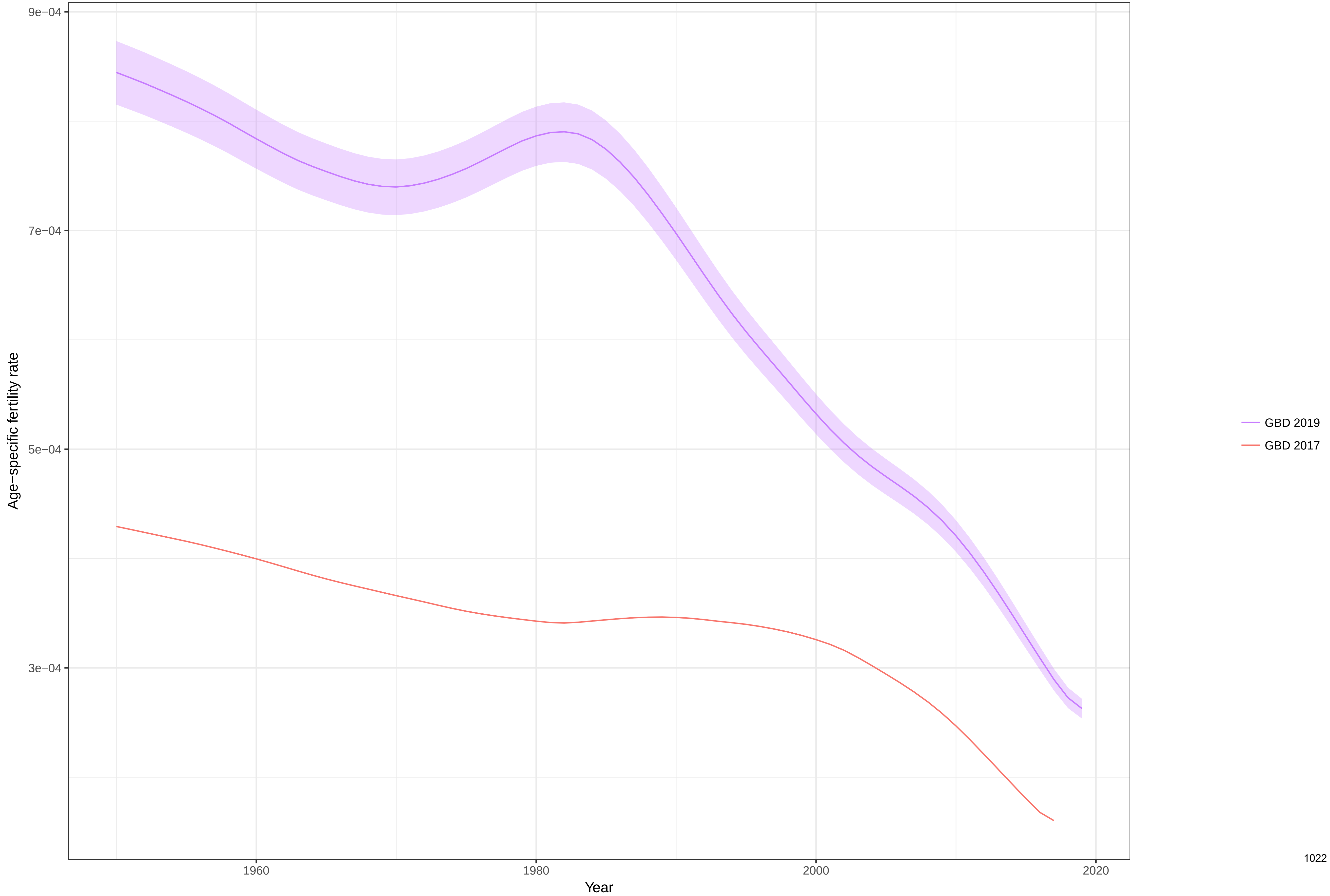

Total fertility rate fitted trend lines, 1950−2019: Haiti TFR
Data points enclosed with a diamond are reference sources

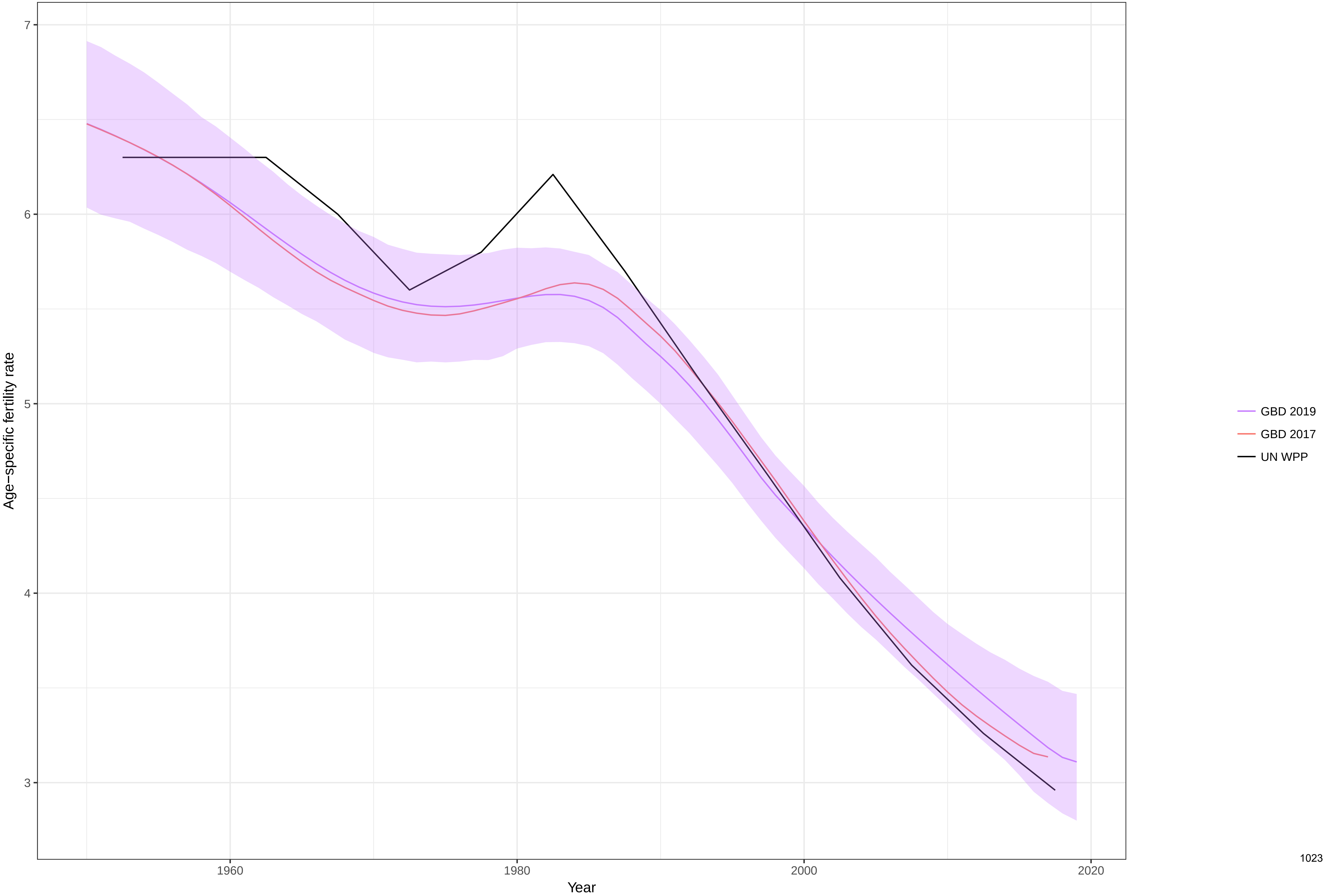

Age−specific fertility fitted trend lines, 1950−2019: Hungary, Ages: 10−14
Data points enclosed with a diamond are reference sources

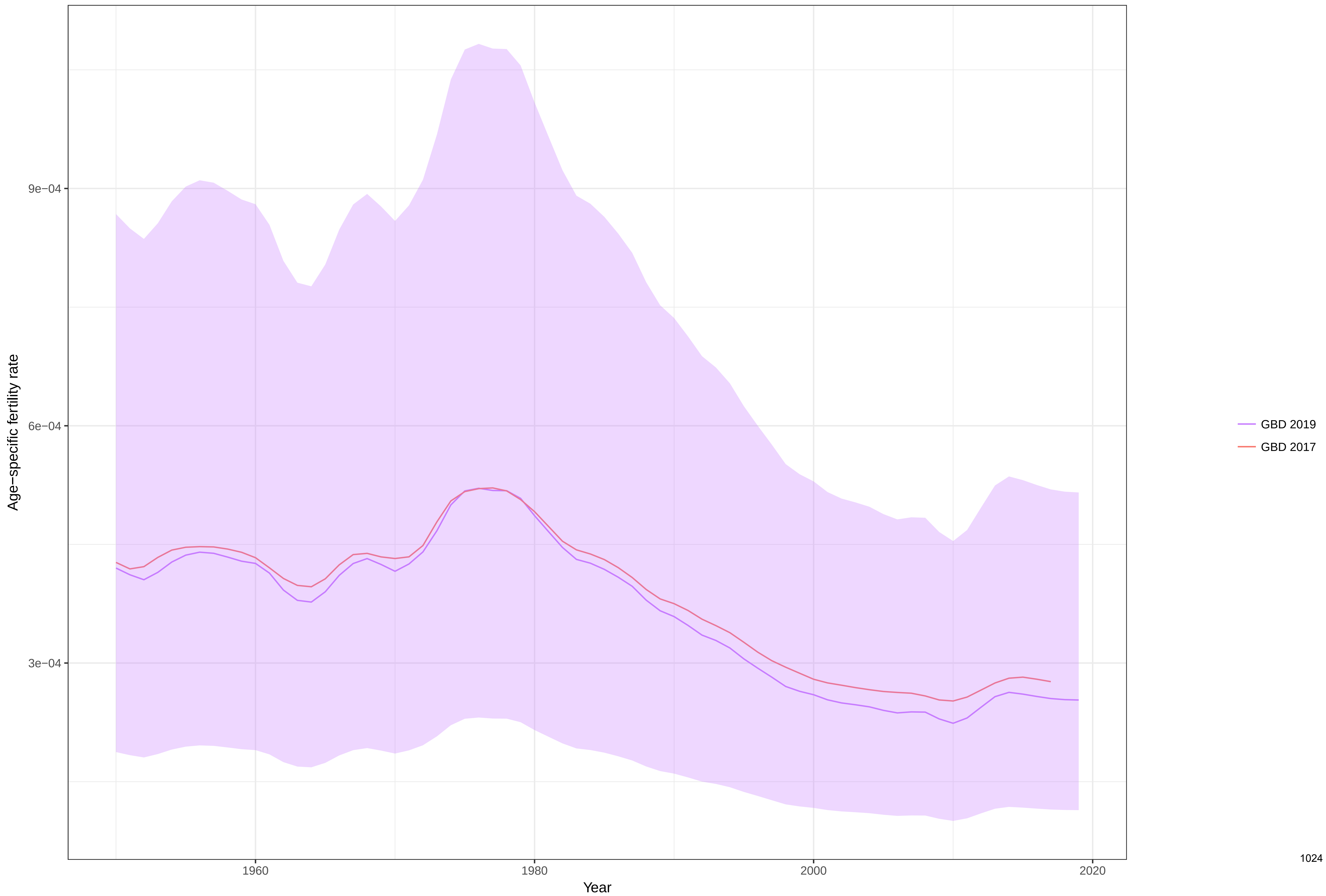

Age−specific fertility fitted trend lines, 1950−2019: Hungary, Ages: 15−19
Data points enclosed with a diamond are reference sources

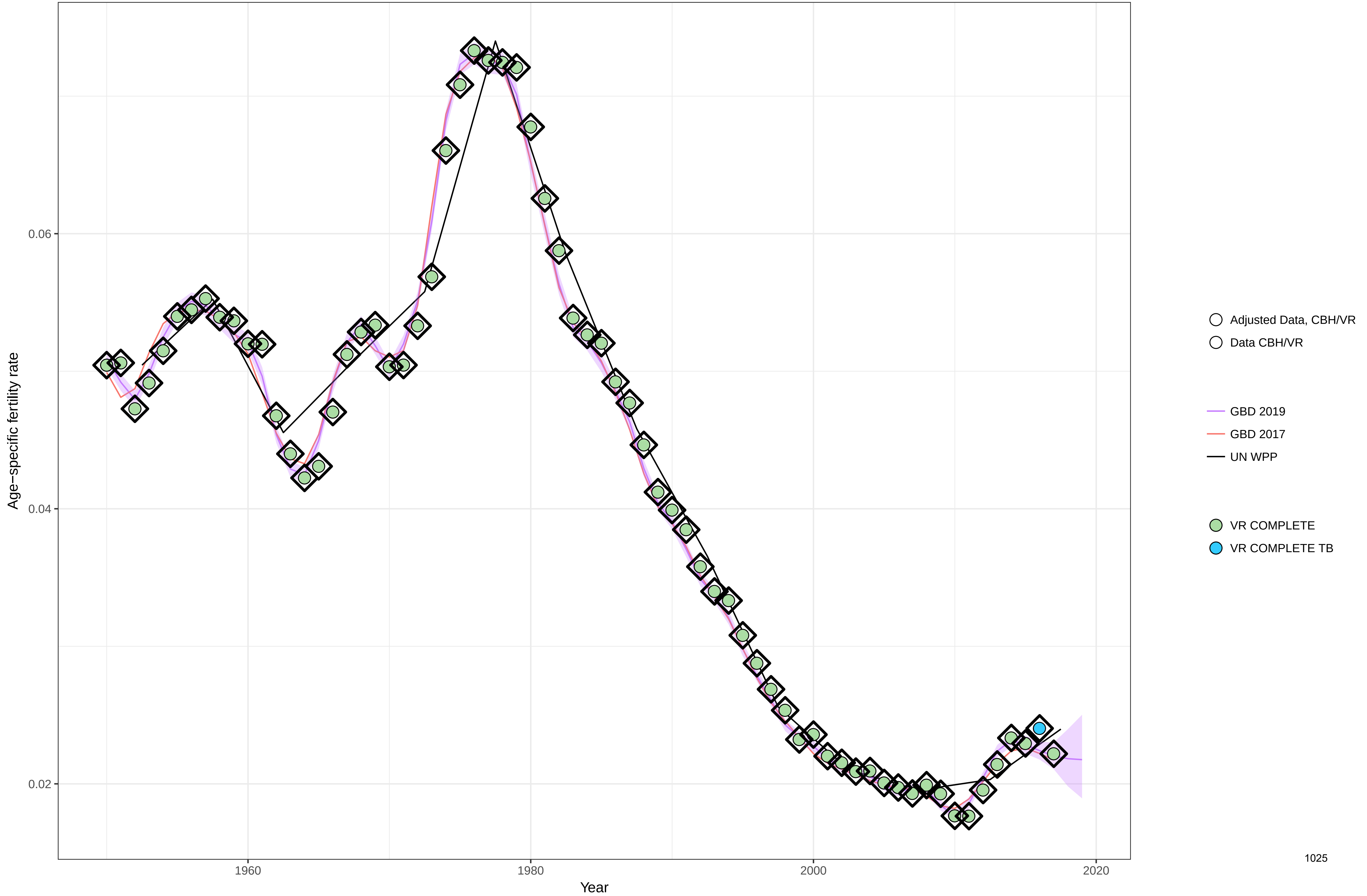

Age−specific fertility fitted trend lines, 1950−2019: Hungary, Ages: 20−24
Data points enclosed with a diamond are reference sources

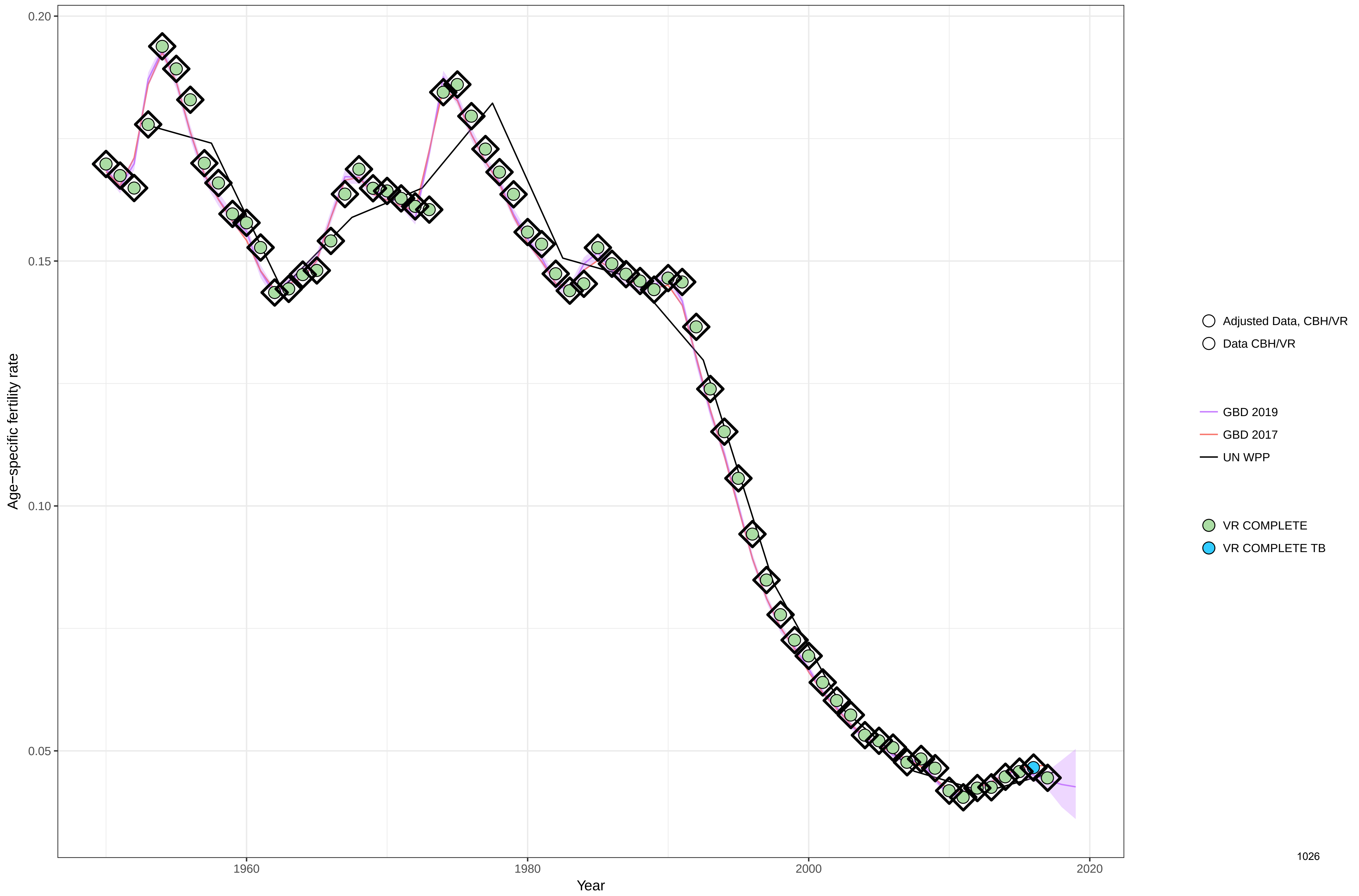

Age−specific fertility fitted trend lines, 1950−2019: Hungary, Ages: 25−29
Data points enclosed with a diamond are reference sources

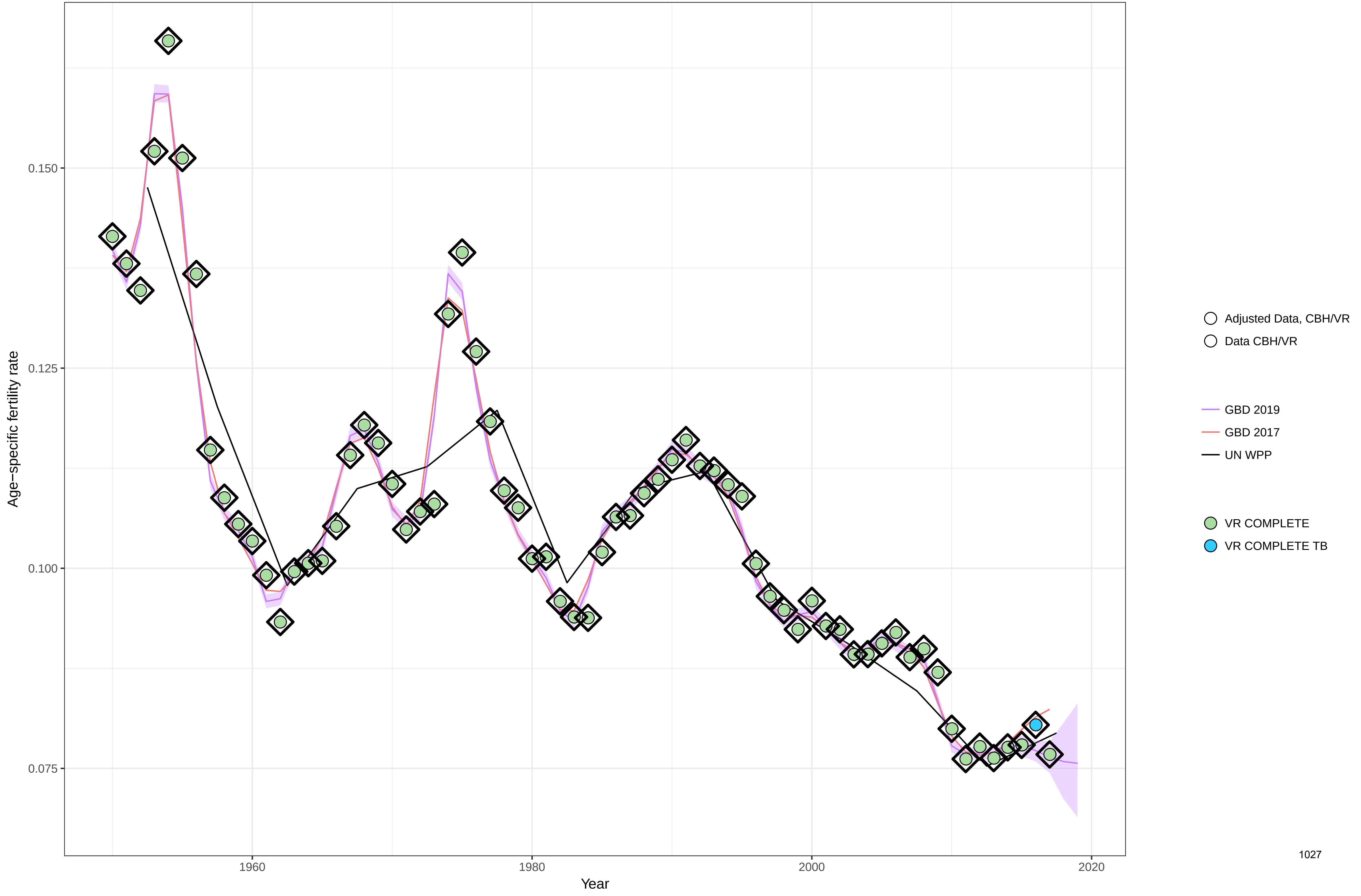

Age−specific fertility fitted trend lines, 1950−2019: Hungary, Ages: 30−34
Data points enclosed with a diamond are reference sources

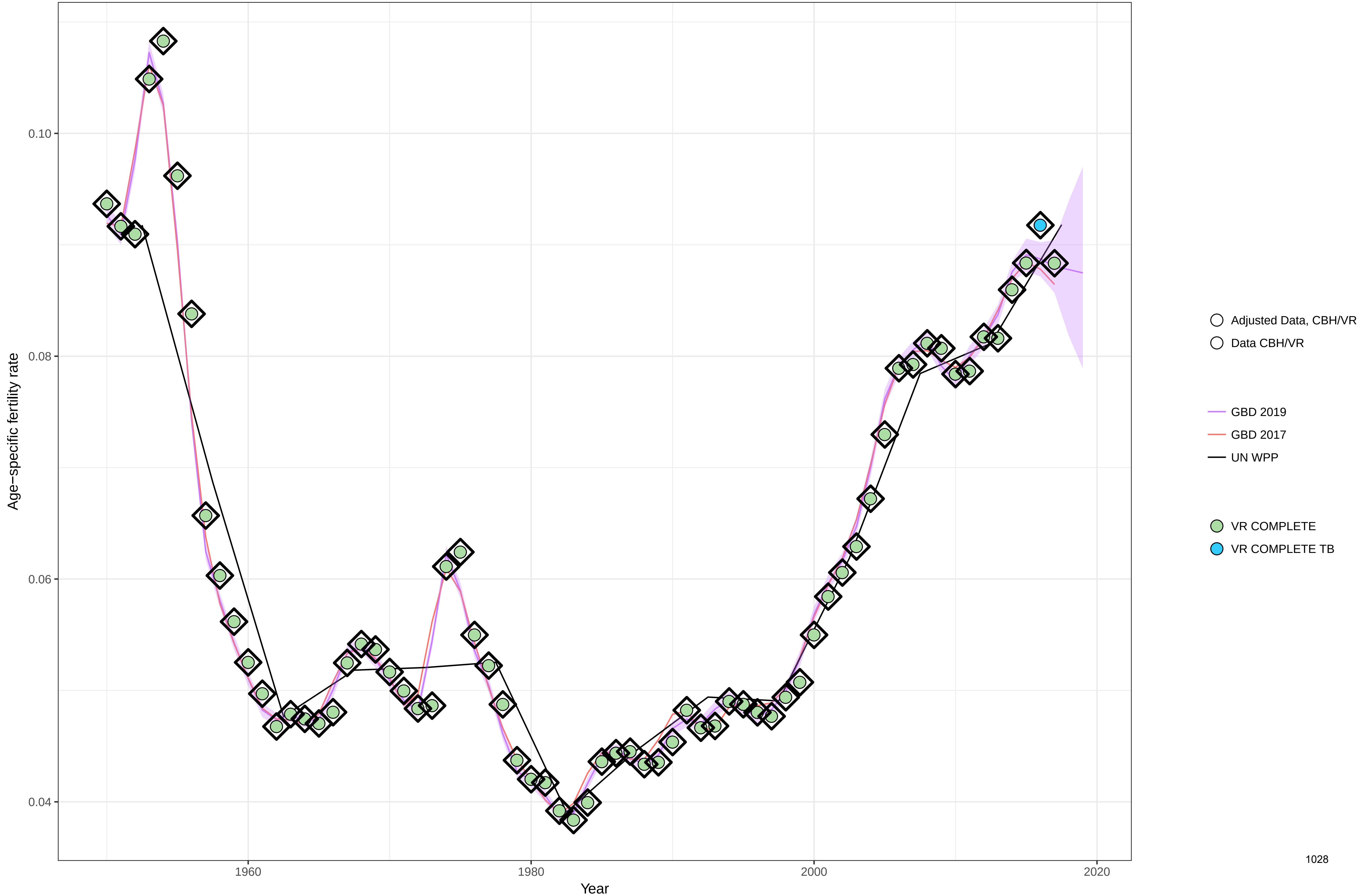

Age−specific fertility fitted trend lines, 1950−2019: Hungary, Ages: 35−39
Data points enclosed with a diamond are reference sources

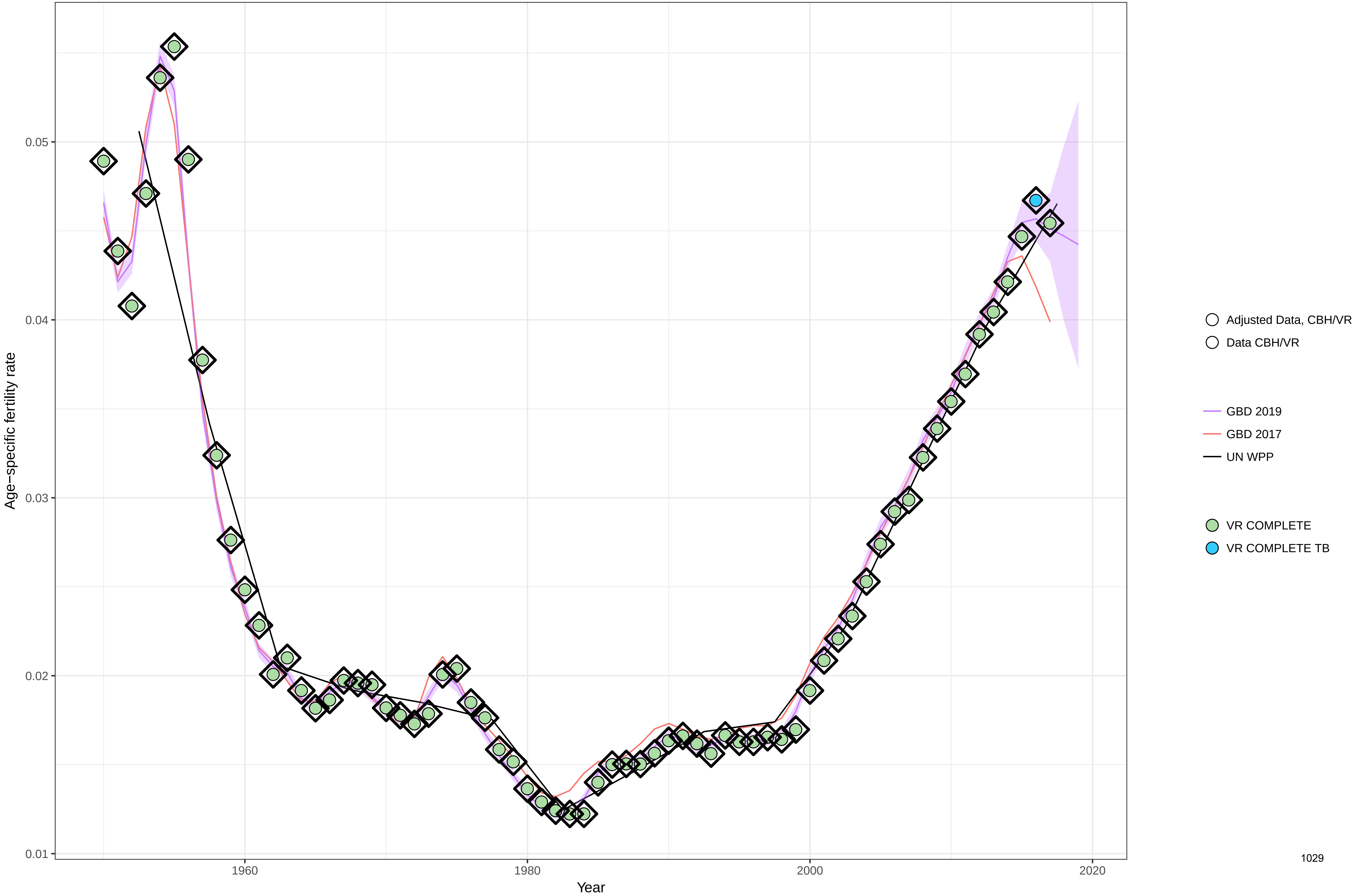

Age−specific fertility fitted trend lines, 1950−2019: Hungary, Ages: 40−44
Data points enclosed with a diamond are reference sources

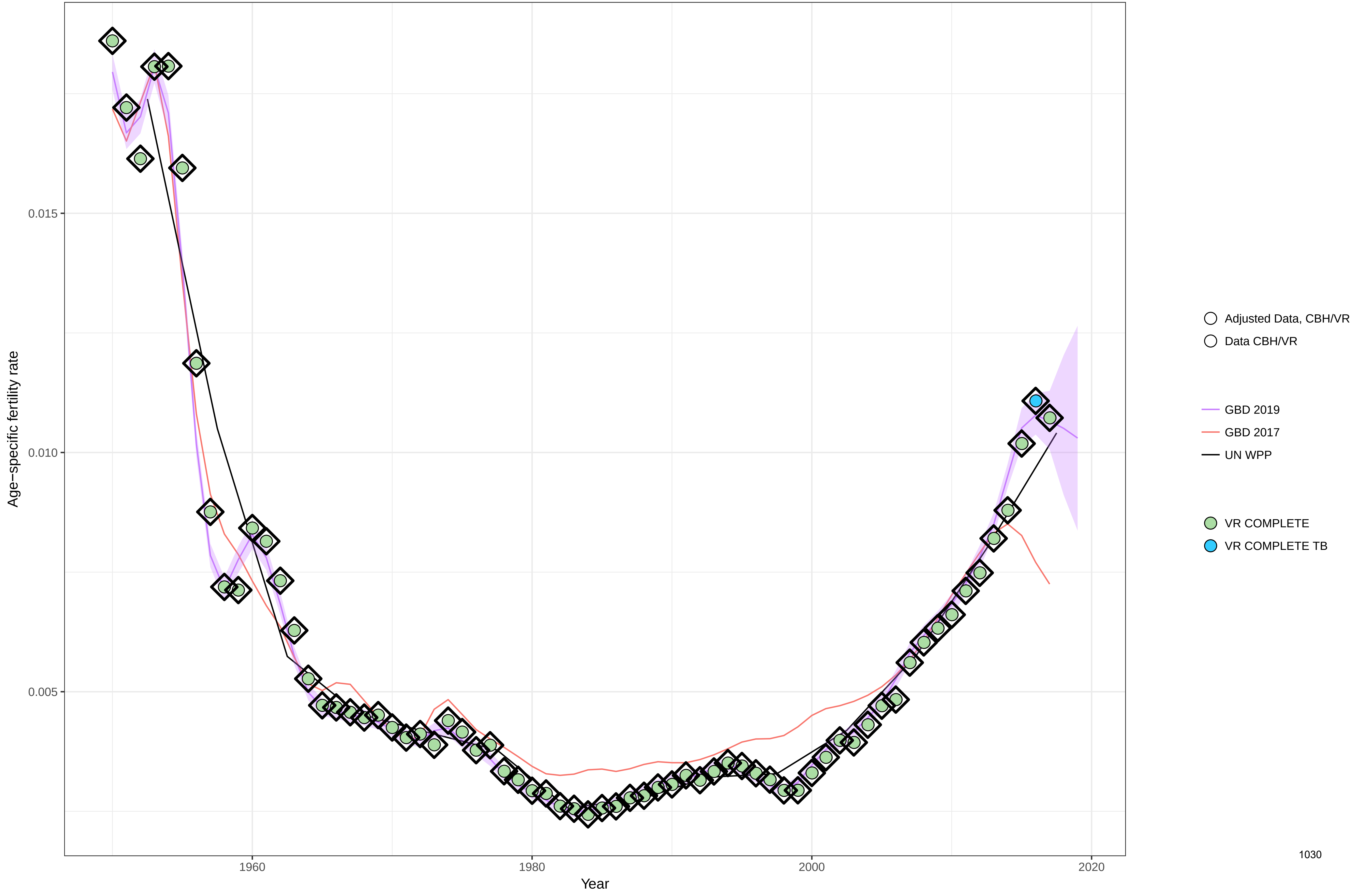

Age−specific fertility fitted trend lines, 1950−2019: Hungary, Ages: 45−49
Data points enclosed with a diamond are reference sources

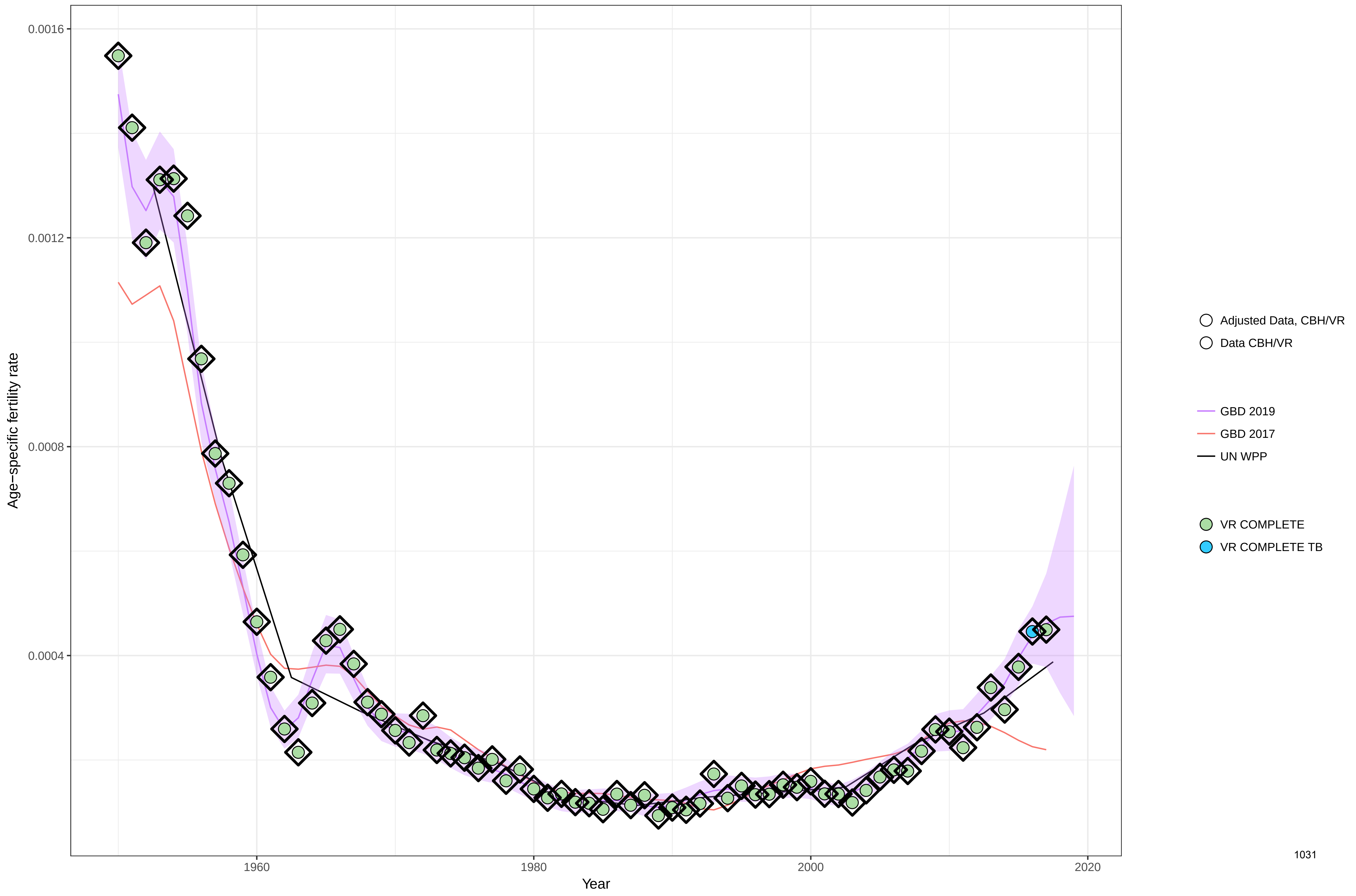

Age−specific fertility fitted trend lines, 1950−2019: Hungary, Ages: 50−54
Data points enclosed with a diamond are reference sources

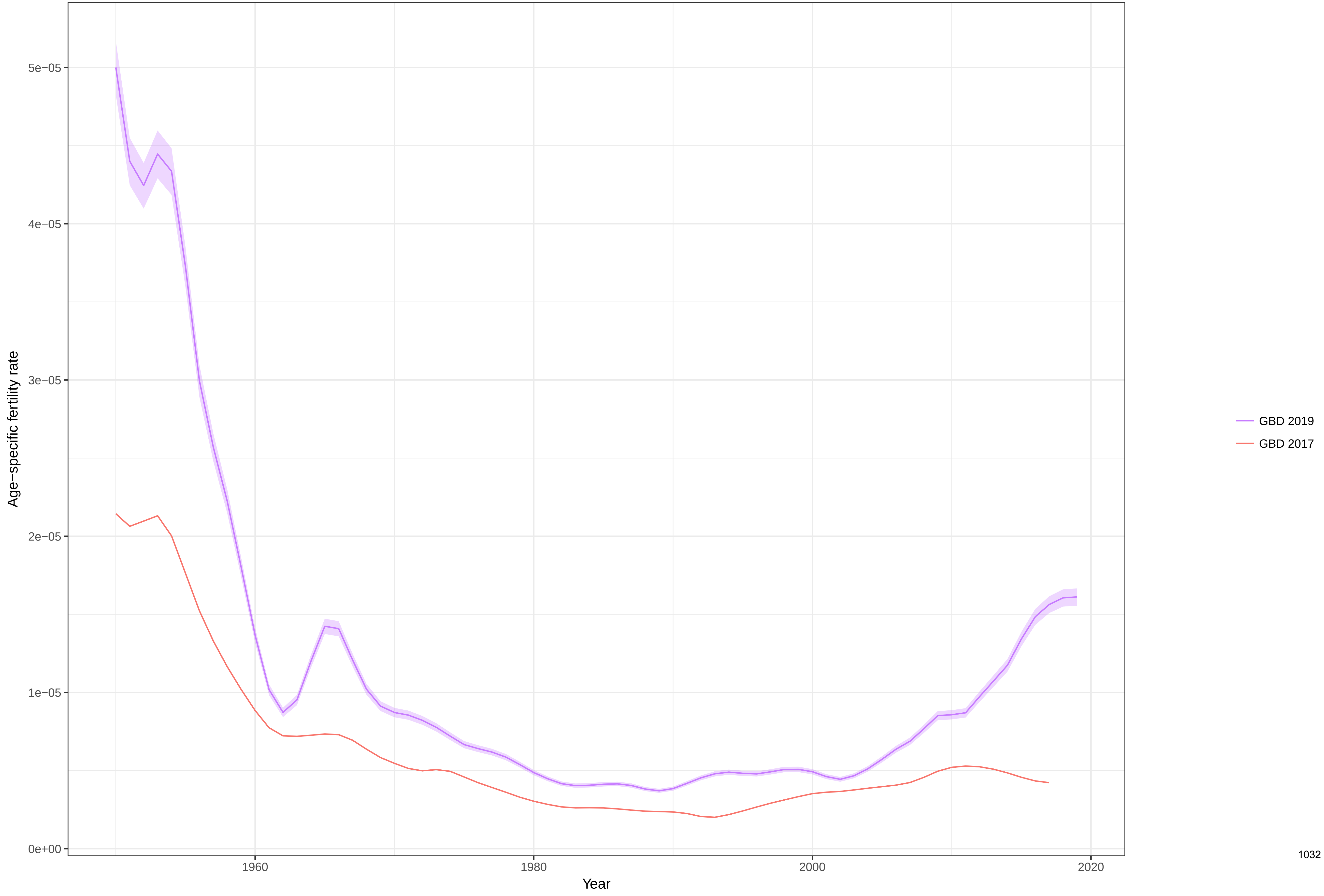

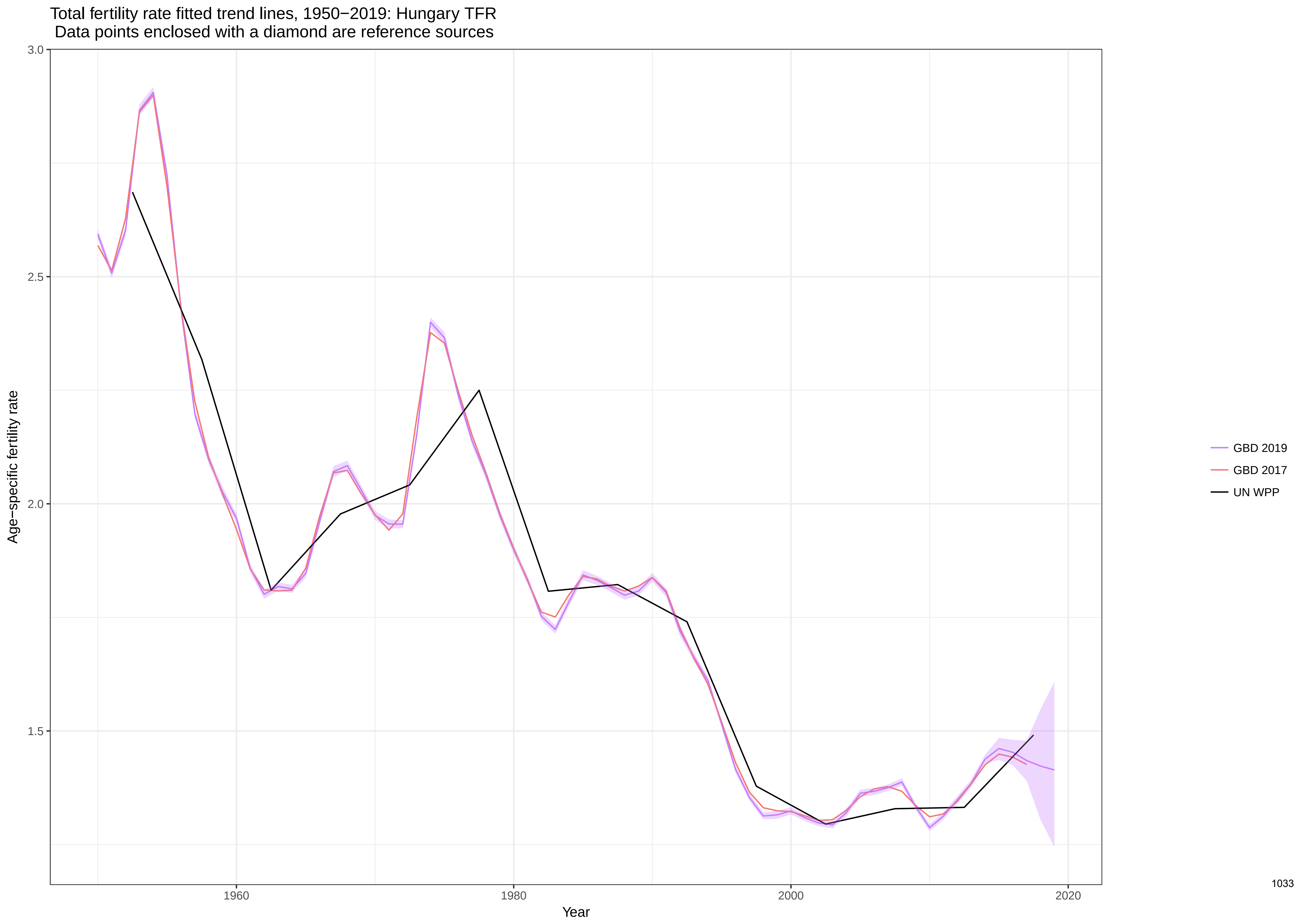
Total fertility rate fitted trend lines, 1950−2019: Hungary TFR
Data points enclosed with a diamond are reference sources
3.0
2.5
2.0
1.5
Age−specific fertility rate
1960
1980
2000
2020
Year
GBD 2019
GBD 2017
UN WPP

Age−specific fertility fitted trend lines, 1950−2019: Indonesia, Ages: 10−14
Data points enclosed with a diamond are reference sources

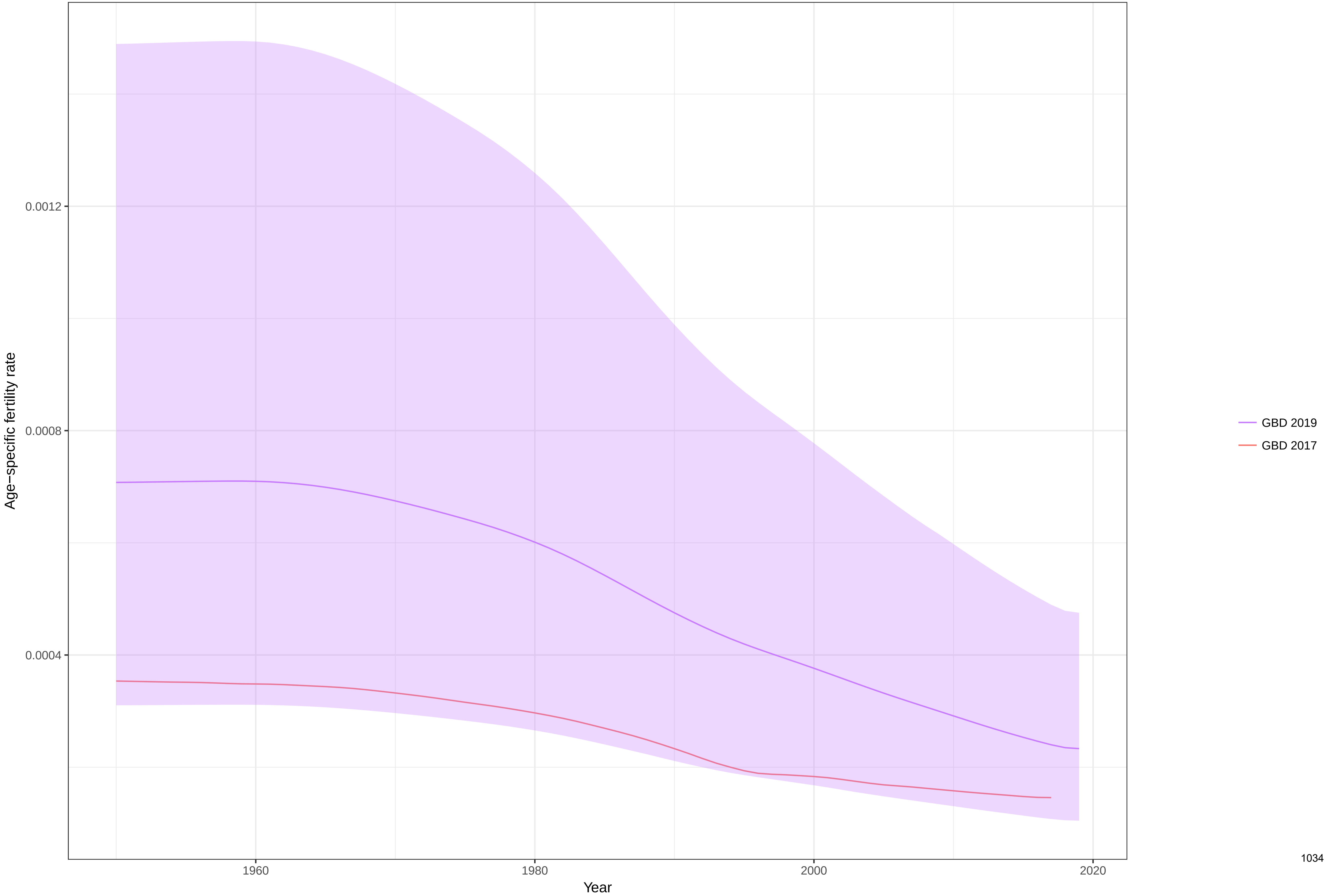

Age−specific fertility fitted trend lines, 1950−2019: Indonesia, Ages: 15−19
Data points enclosed with a diamond are reference sources

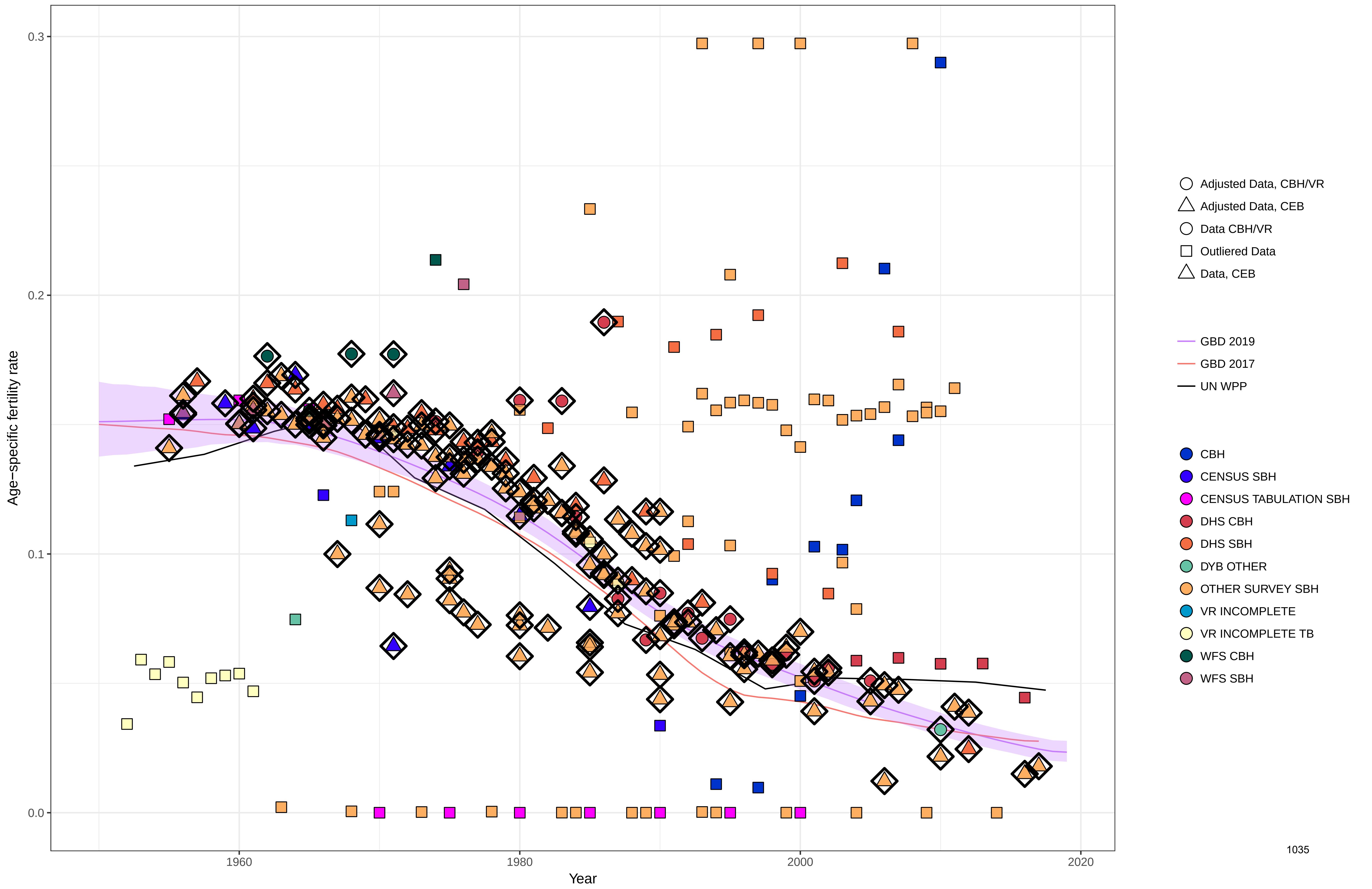

Age−specific fertility fitted trend lines, 1950−2019: Indonesia, Ages: 20−24
Data points enclosed with a diamond are reference sources

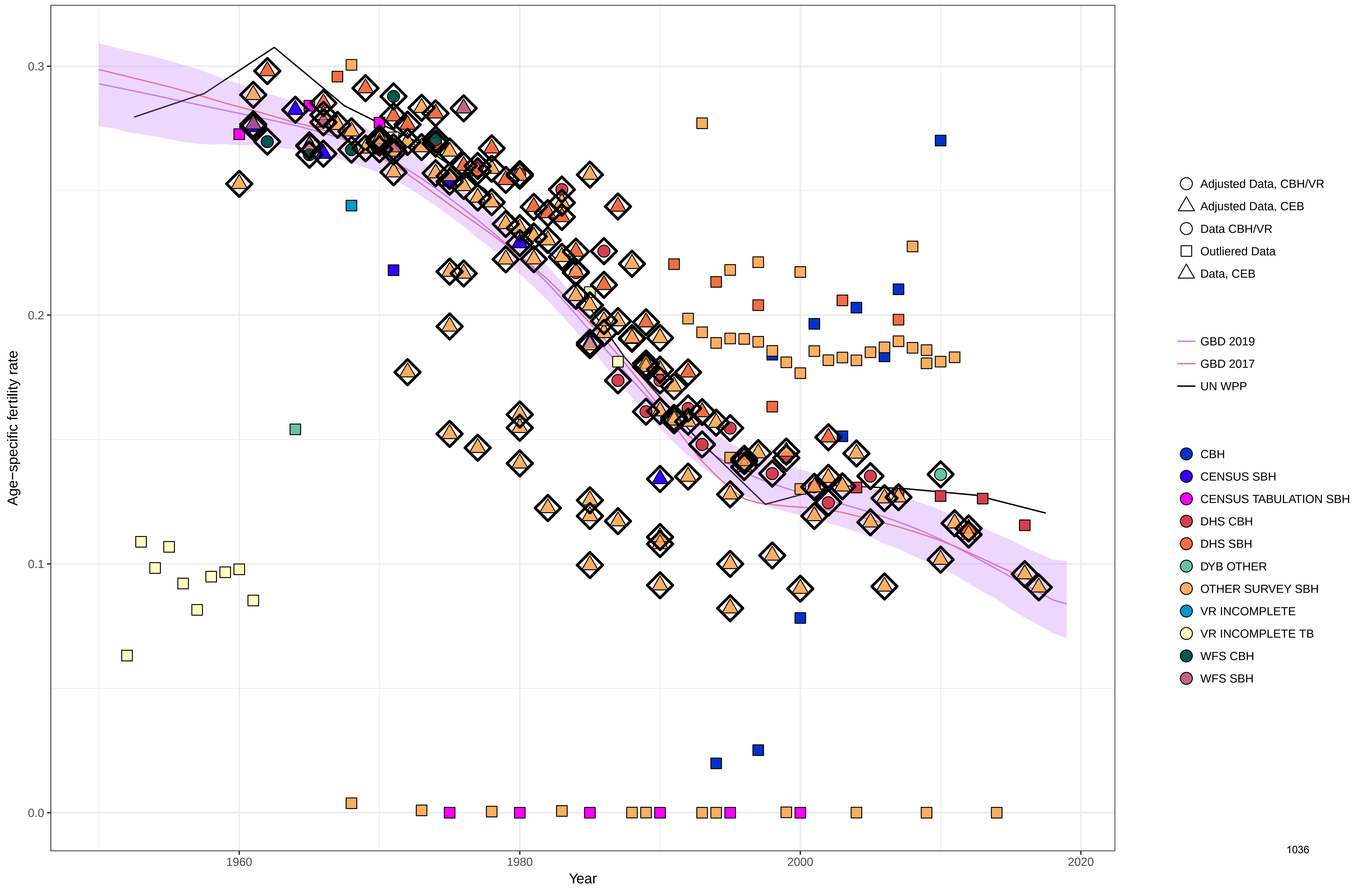

Age−specific fertility fitted trend lines, 1950−2019: Indonesia, Ages: 25−29
Data points enclosed with a diamond are reference sources

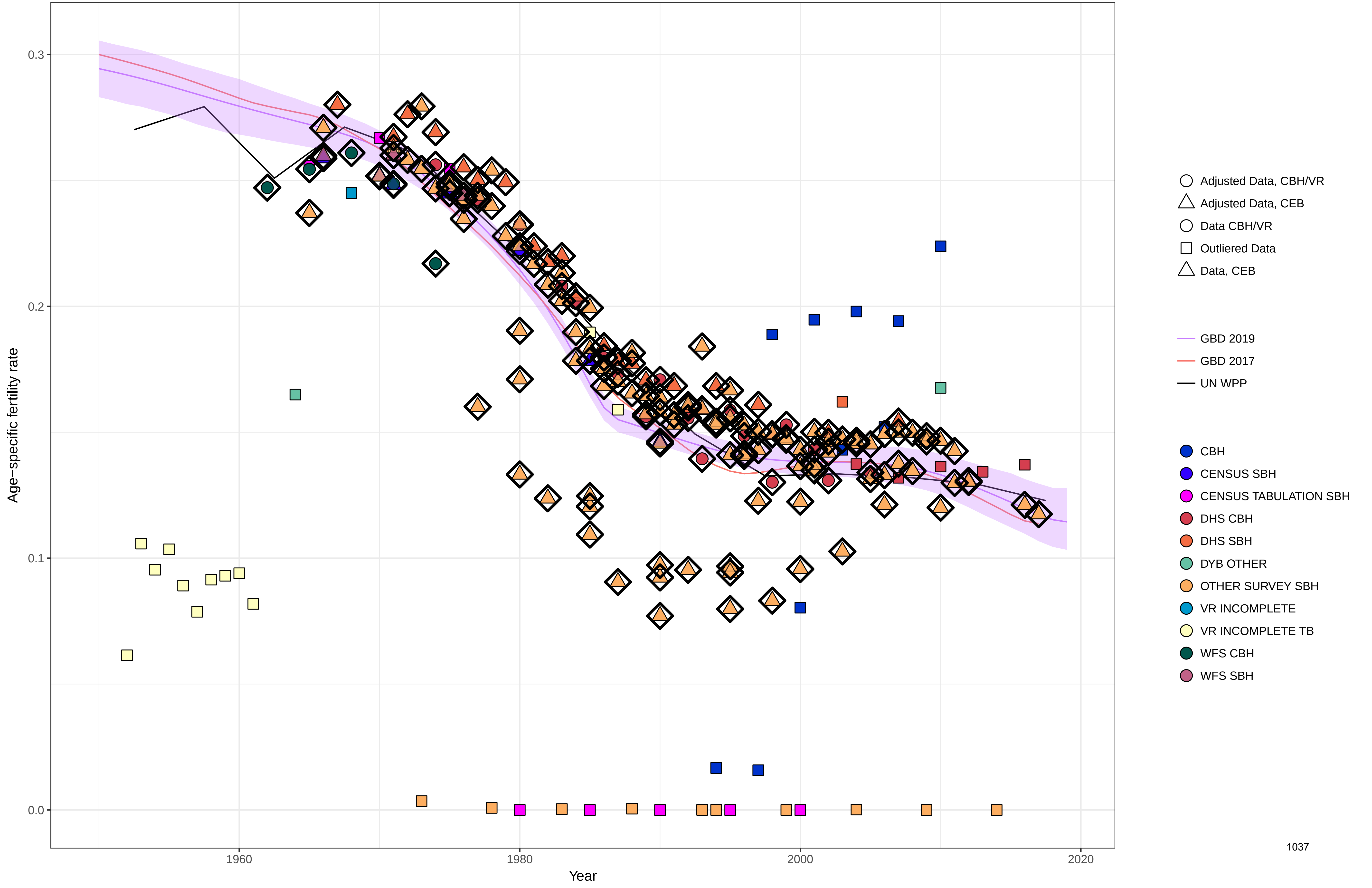

Age−specific fertility fitted trend lines, 1950−2019: Indonesia, Ages: 30−34
Data points enclosed with a diamond are reference sources

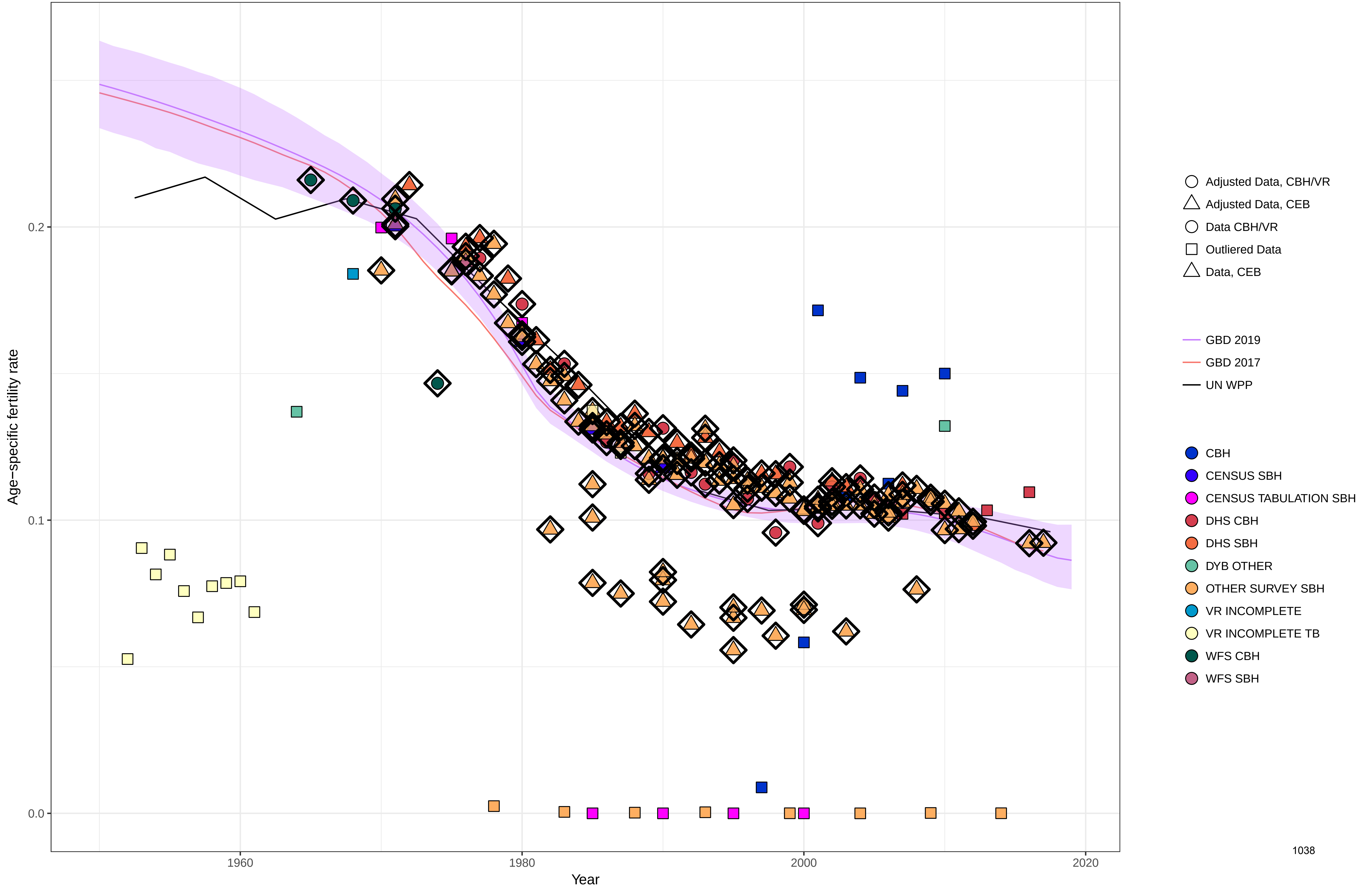

Age−specific fertility fitted trend lines, 1950−2019: Indonesia, Ages: 35−39
Data points enclosed with a diamond are reference sources

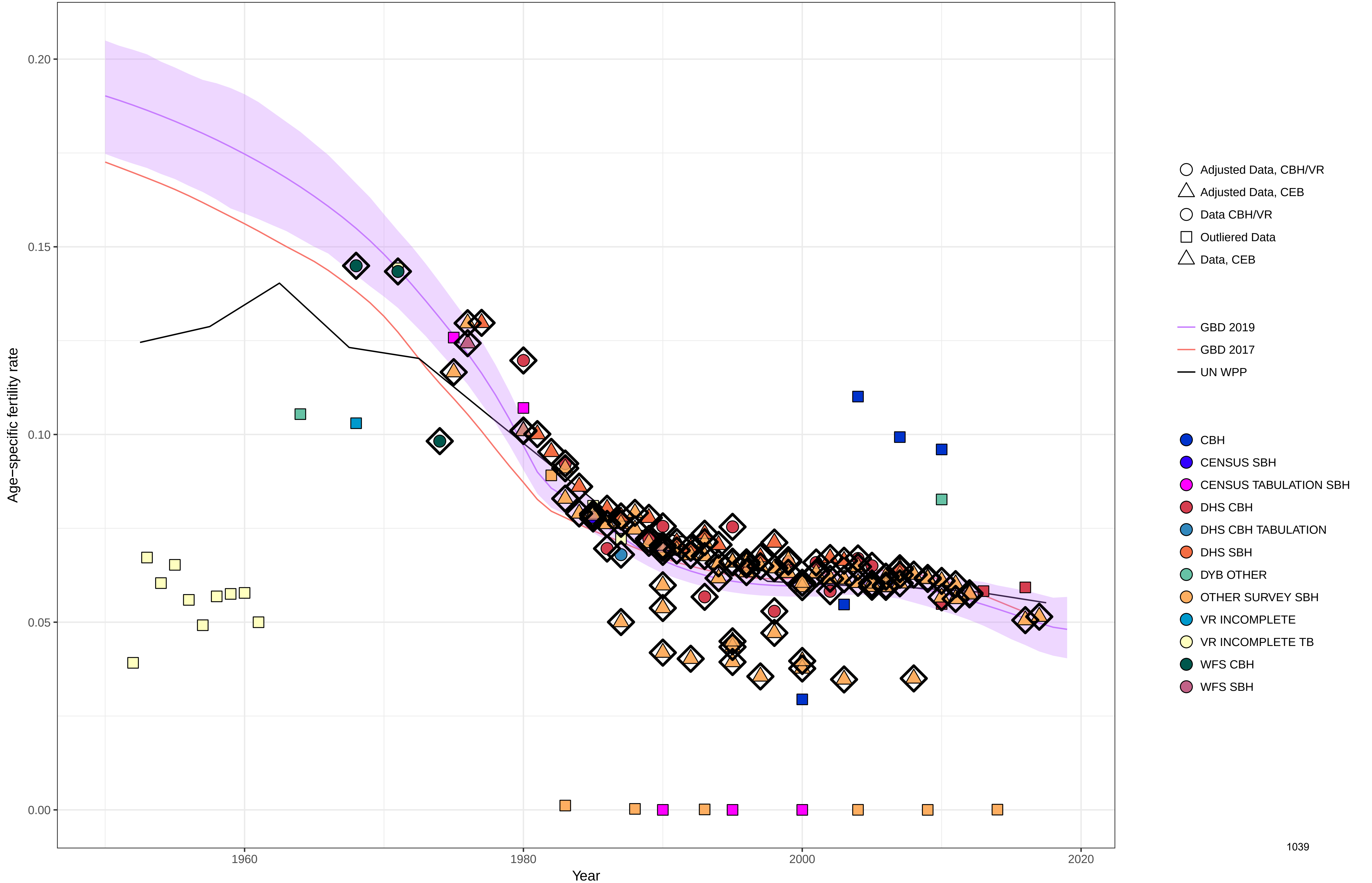

Age−specific fertility fitted trend lines, 1950−2019: Indonesia, Ages: 40−44
Data points enclosed with a diamond are reference sources

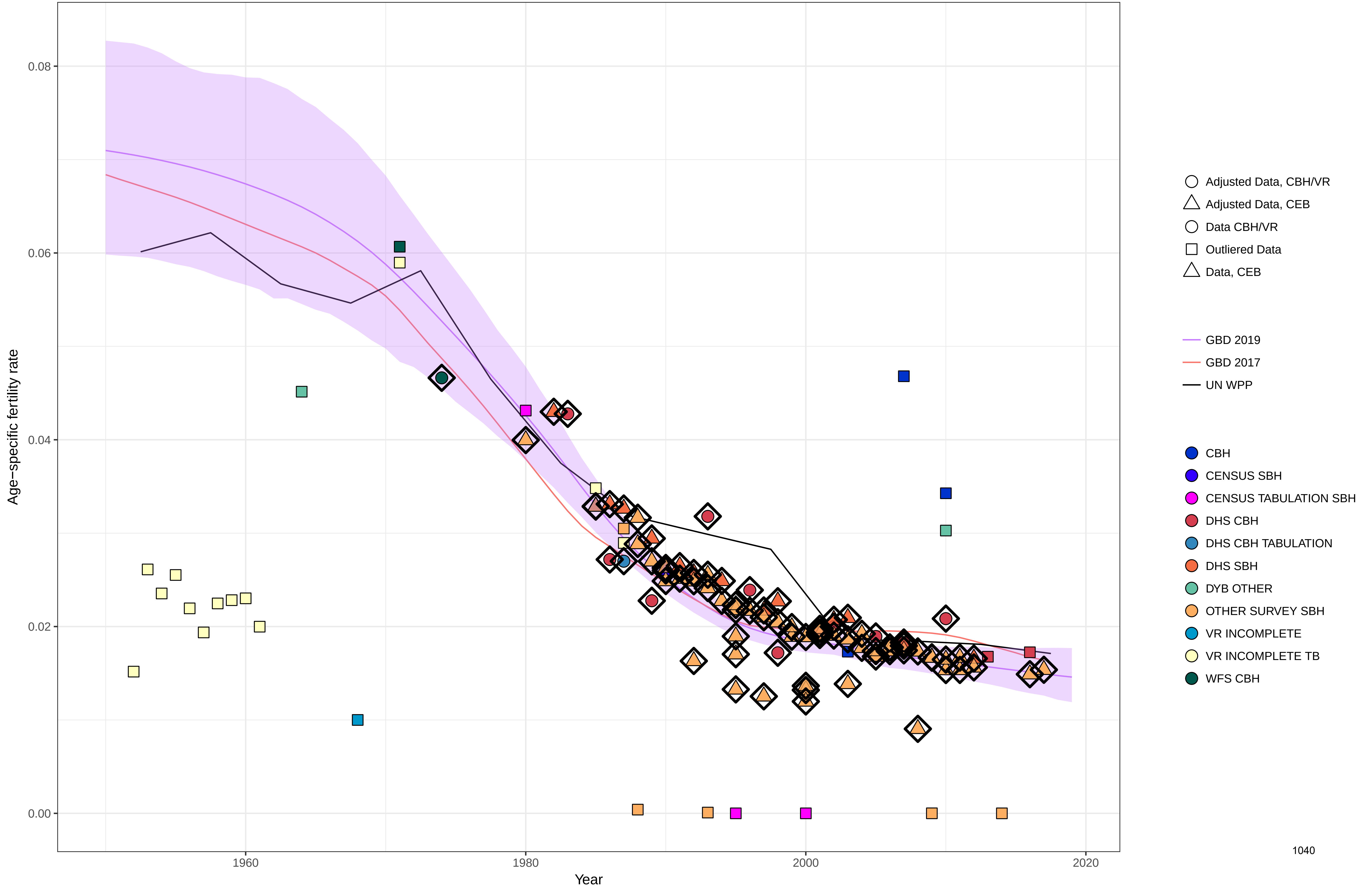

Age−specific fertility fitted trend lines, 1950−2019: Indonesia, Ages: 45−49
Data points enclosed with a diamond are reference sources

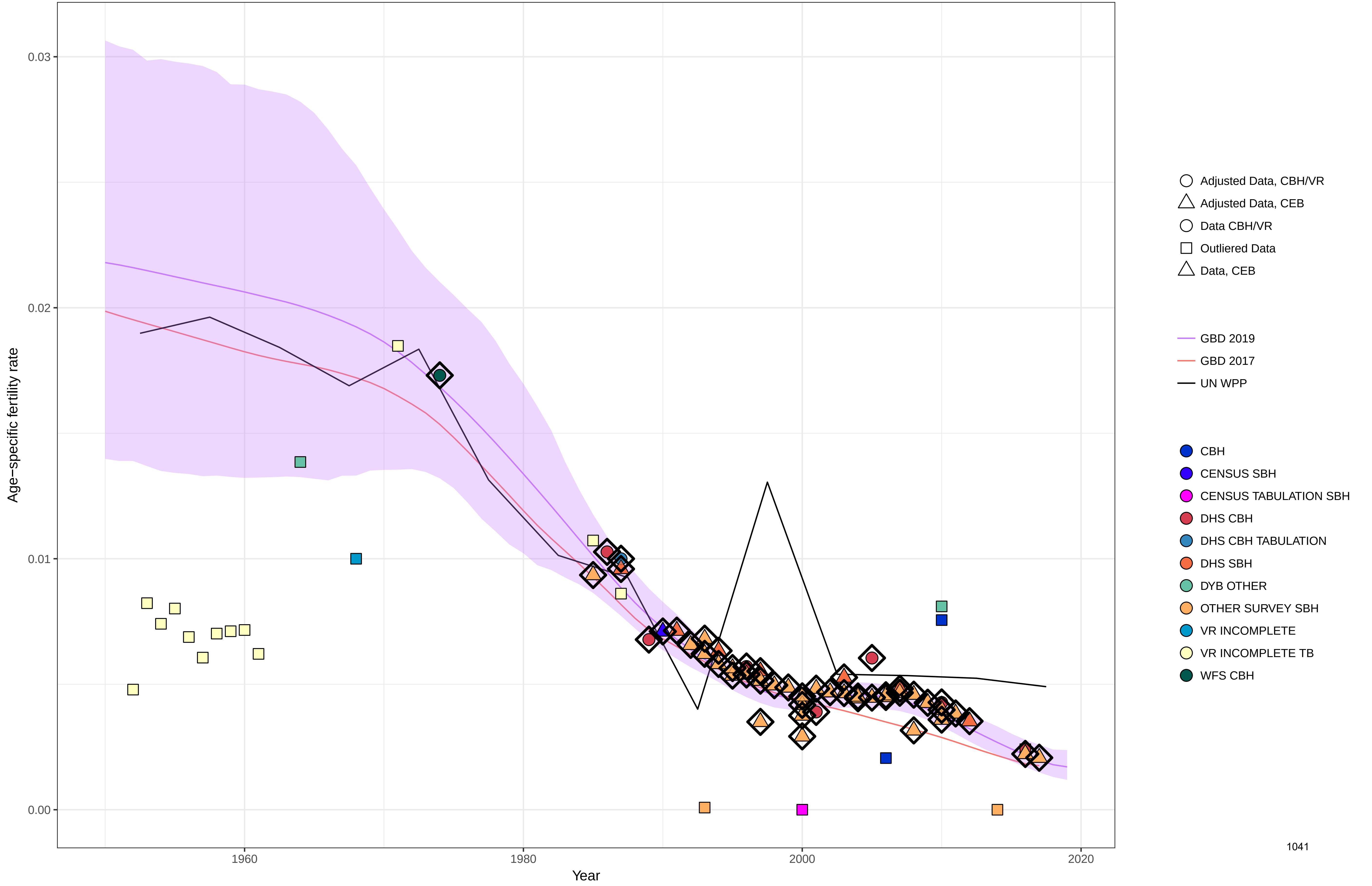

Age−specific fertility fitted trend lines, 1950−2019: Indonesia, Ages: 50−54
Data points enclosed with a diamond are reference sources

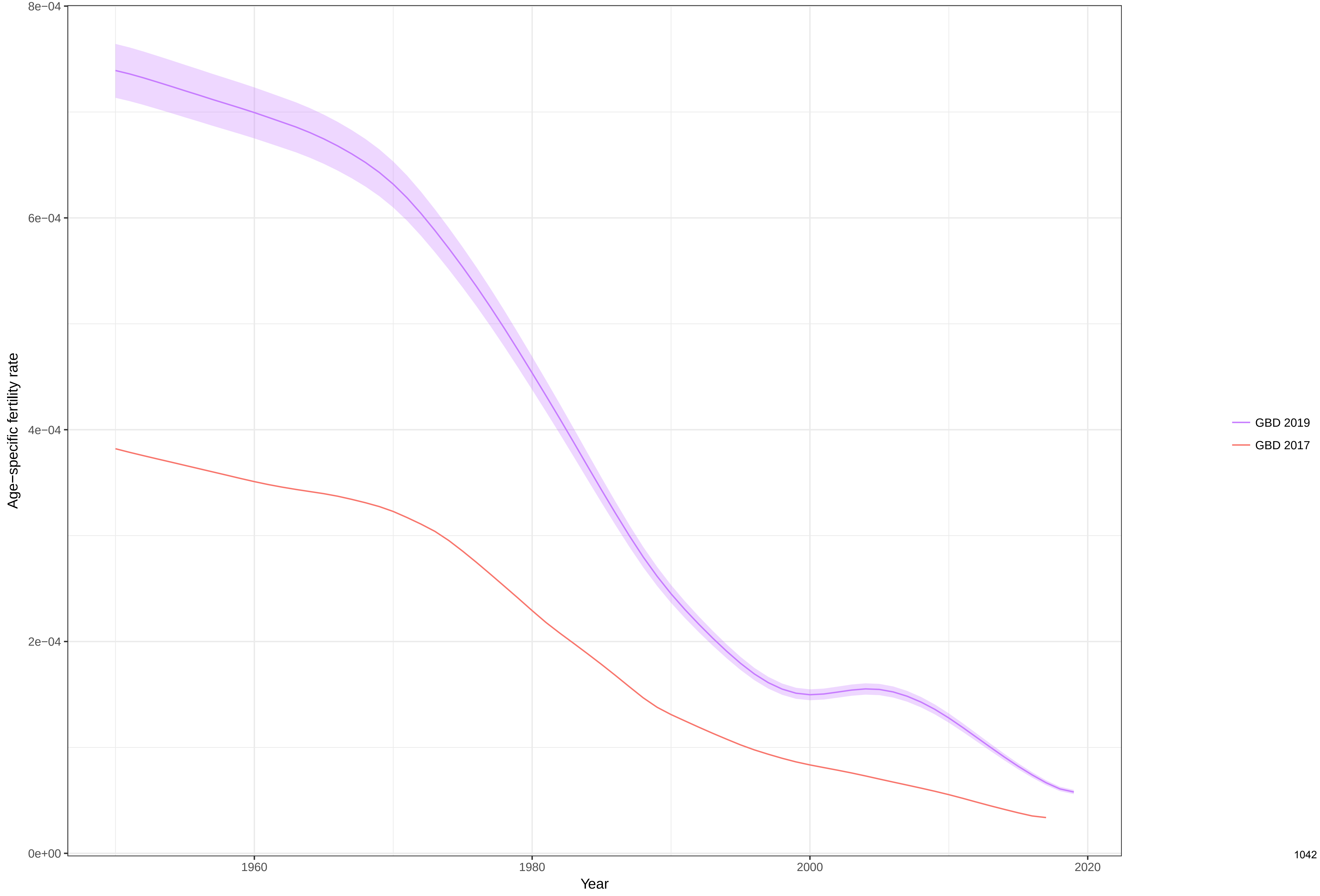

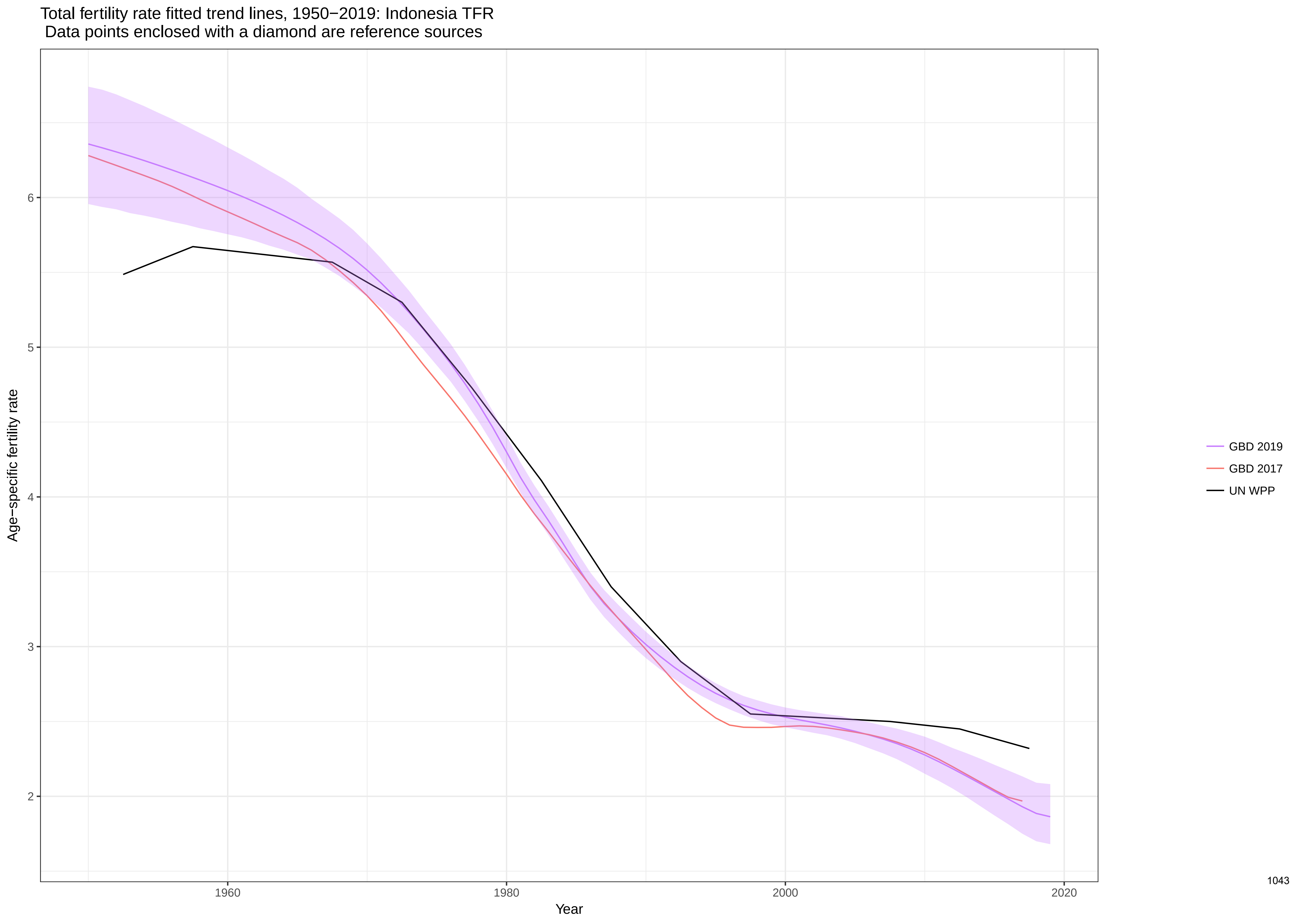

Total fertility rate fitted trend lines, 1950−2019: Indonesia TFR
Data points enclosed with a diamond are reference sources
Age−specific fertility rate
6
5
4
3
2
1960
1980
2000
2020
Year
GBD 2019
GBD 2017
UN WPP

Age−specific fertility fitted trend lines, 1950−2019: India, Ages: 10−14
Data points enclosed with a diamond are reference sources

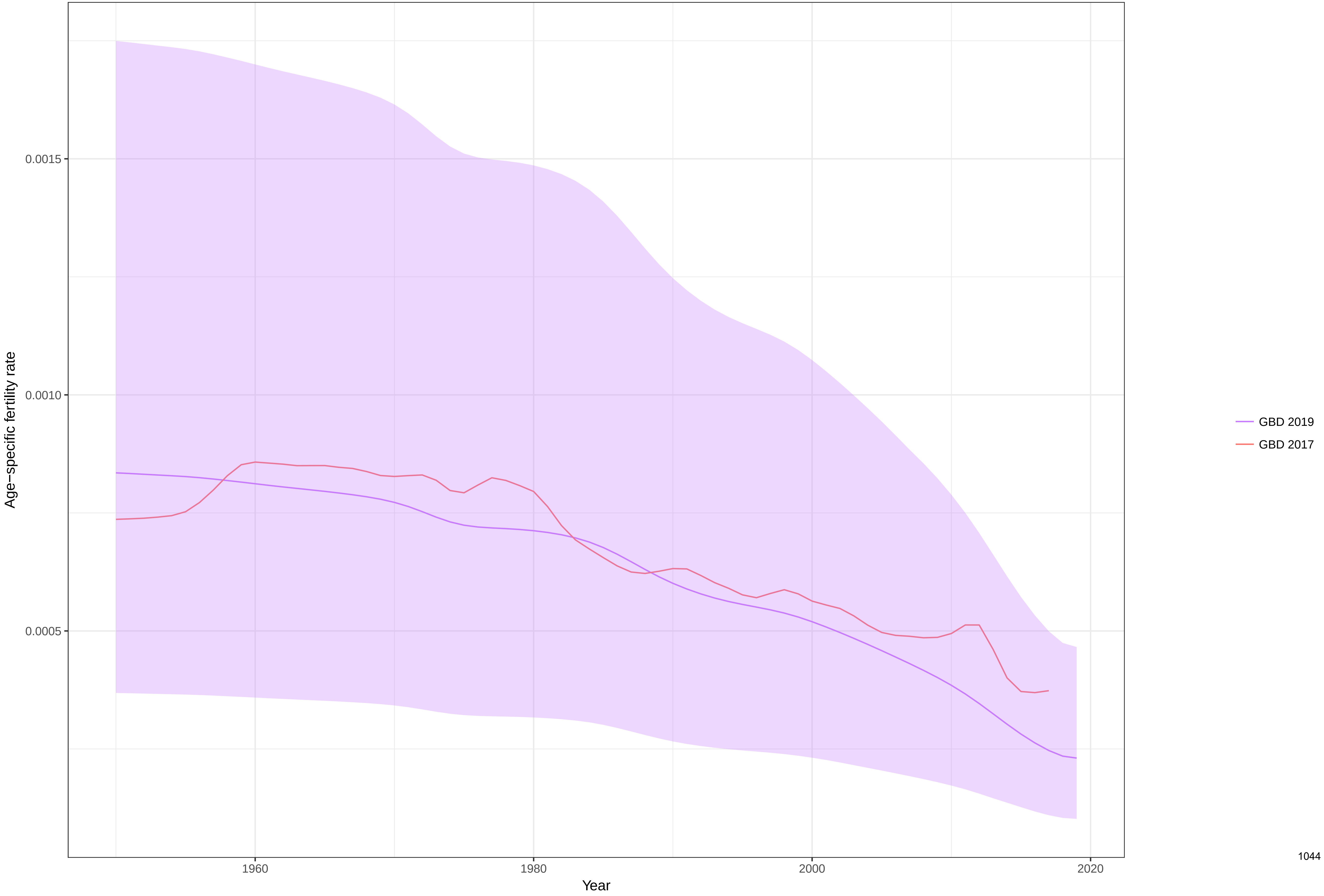

Age−specific fertility fitted trend lines, 1950−2019: India, Ages: 15−19
Data points enclosed with a diamond are reference sources

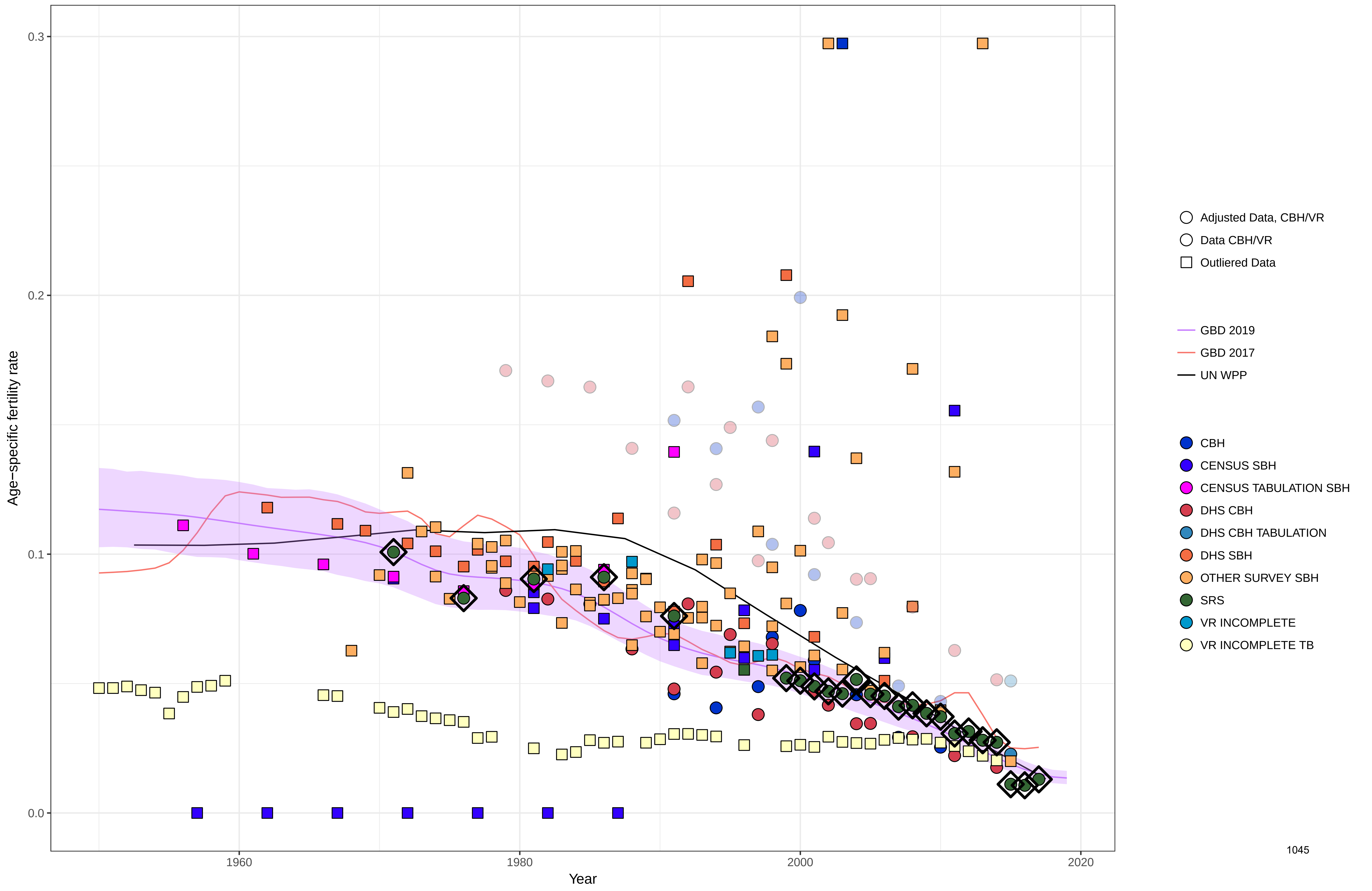

Age−specific fertility fitted trend lines, 1950−2019: India, Ages: 20−24
Data points enclosed with a diamond are reference sources

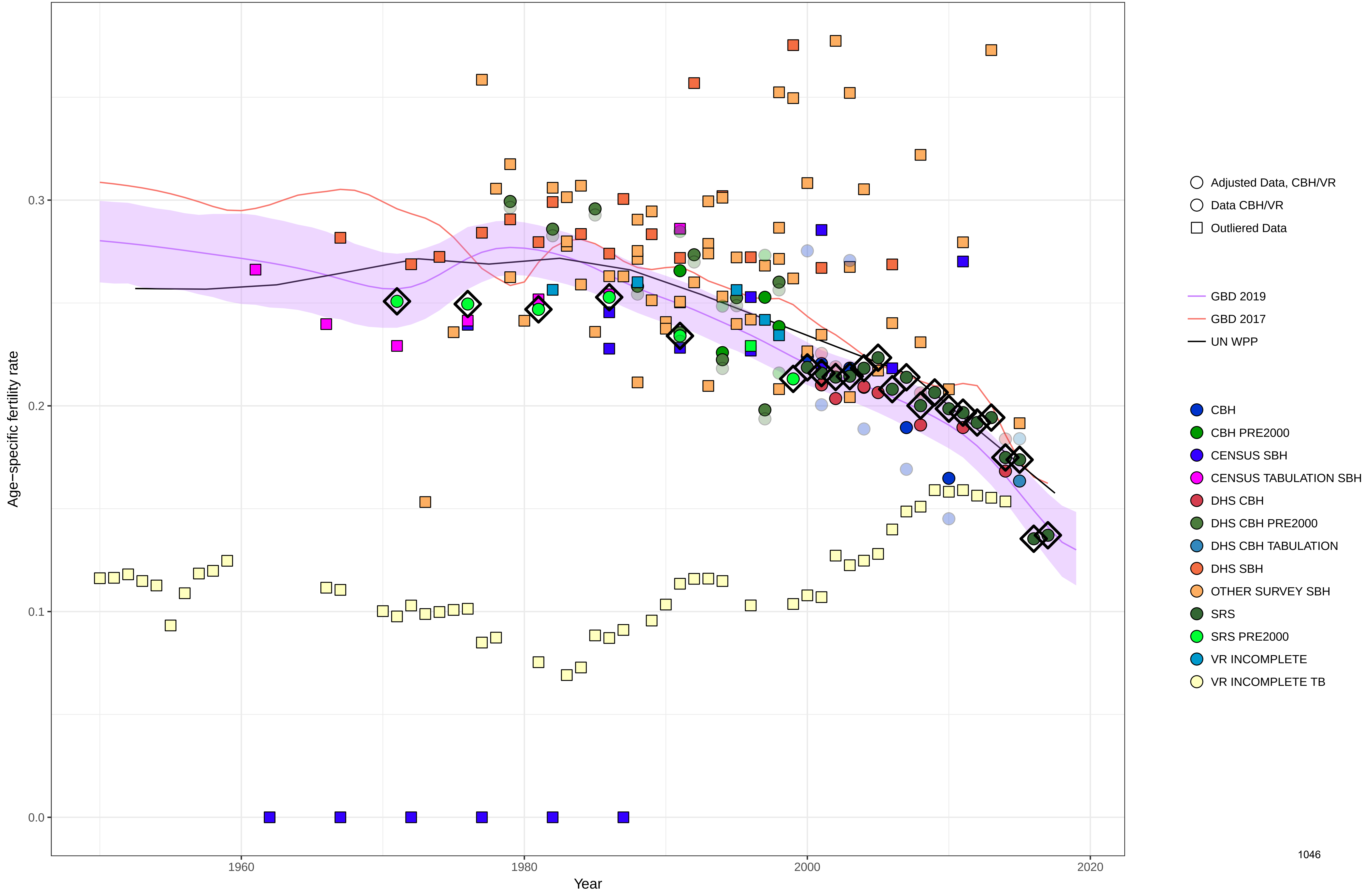

Age−specific fertility fitted trend lines, 1950−2019: India, Ages: 25−29
Data points enclosed with a diamond are reference sources

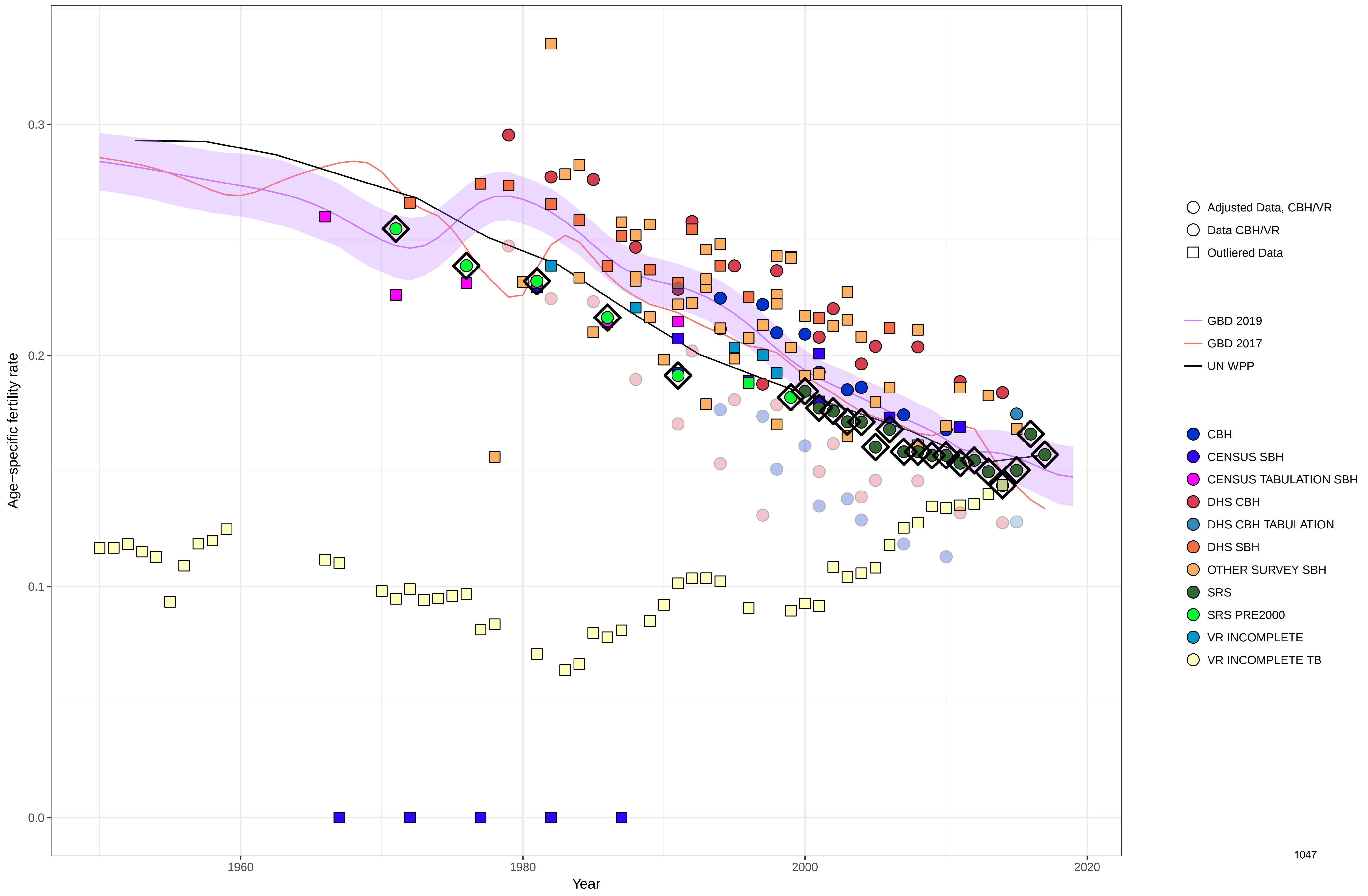

Age−specific fertility fitted trend lines, 1950−2019: India, Ages: 30−34
Data points enclosed with a diamond are reference sources

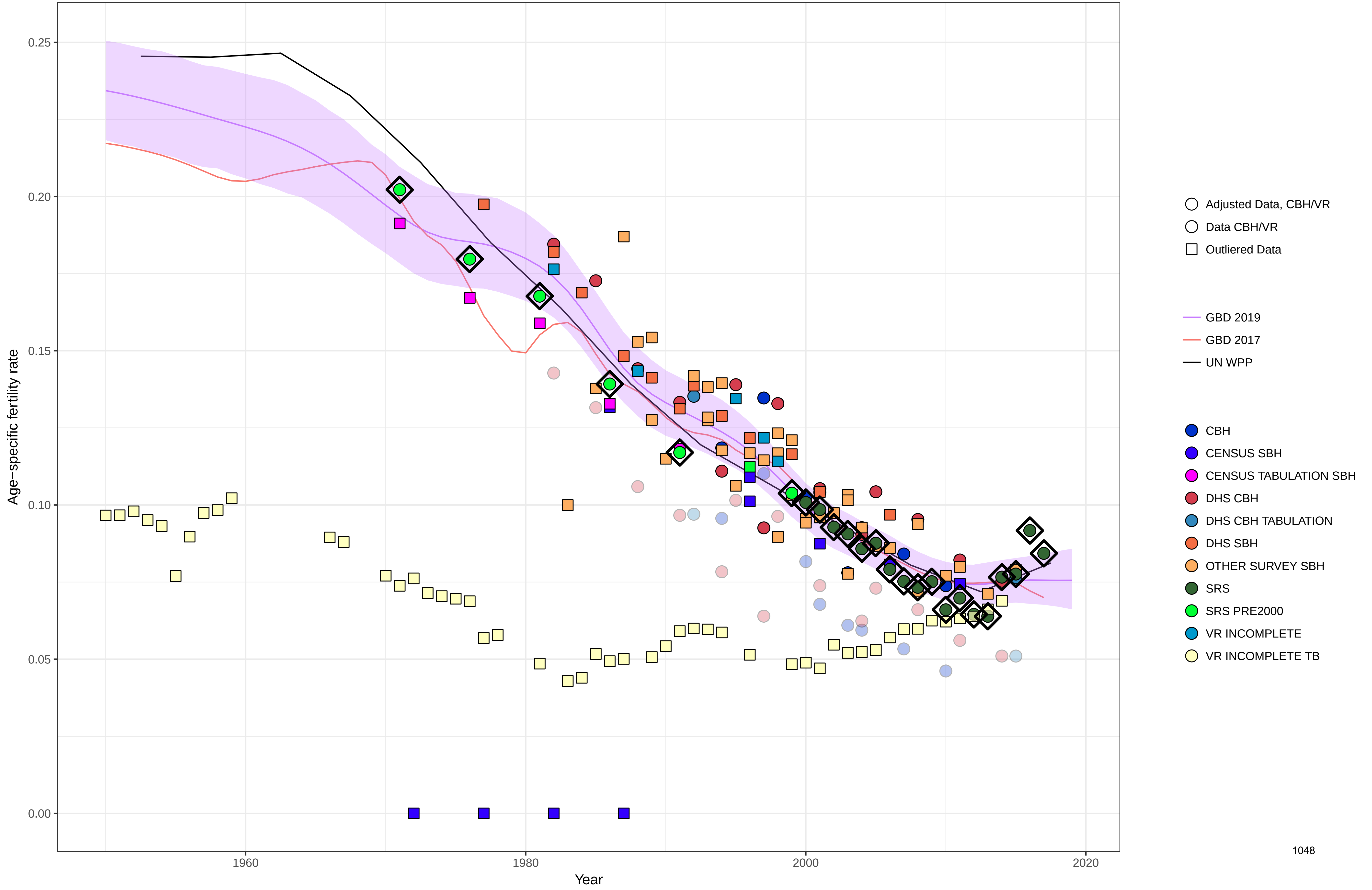

Age−specific fertility fitted trend lines, 1950−2019: India, Ages: 35−39
Data points enclosed with a diamond are reference sources

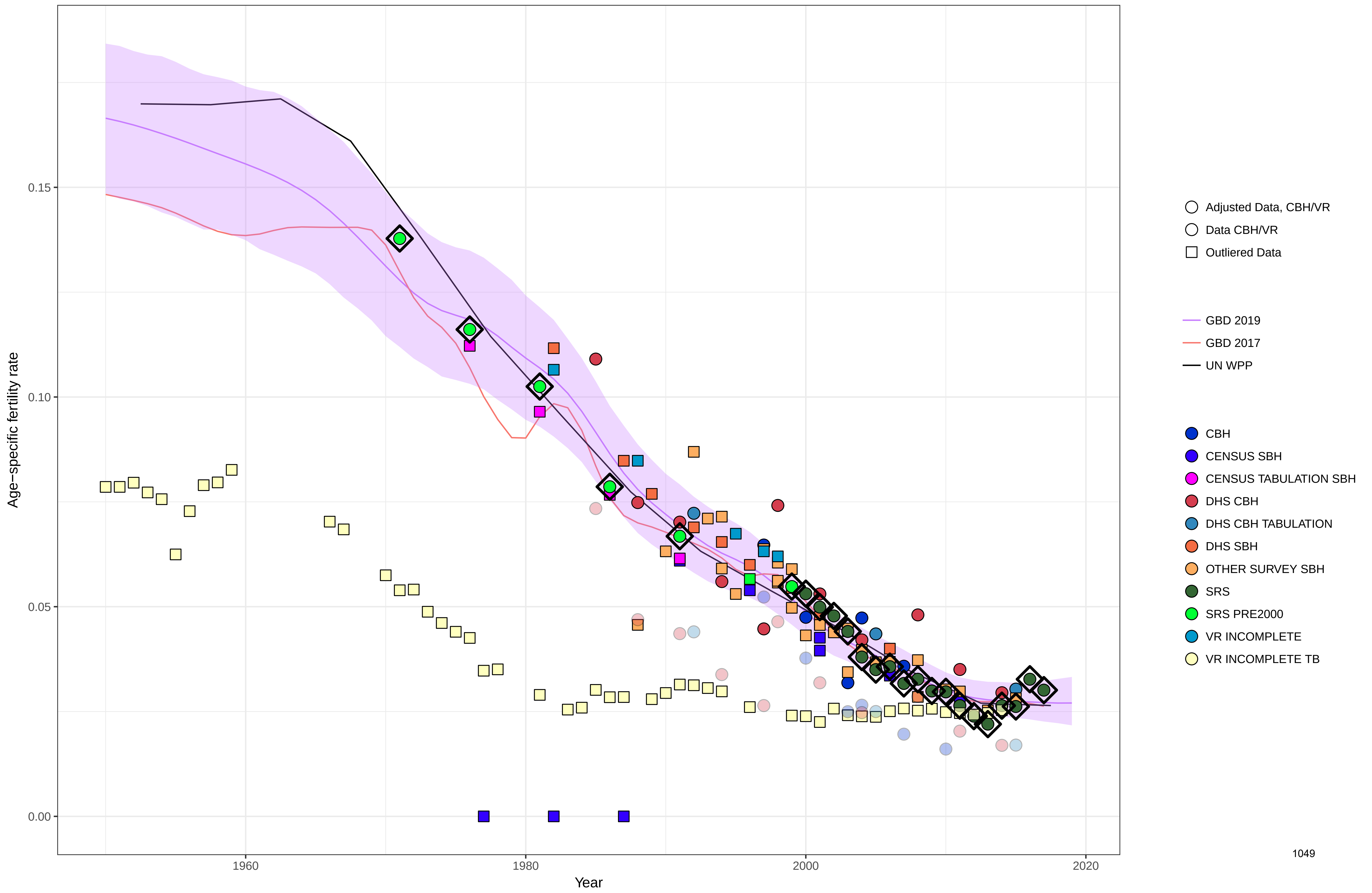

Age−specific fertility fitted trend lines, 1950−2019: India, Ages: 40−44
Data points enclosed with a diamond are reference sources

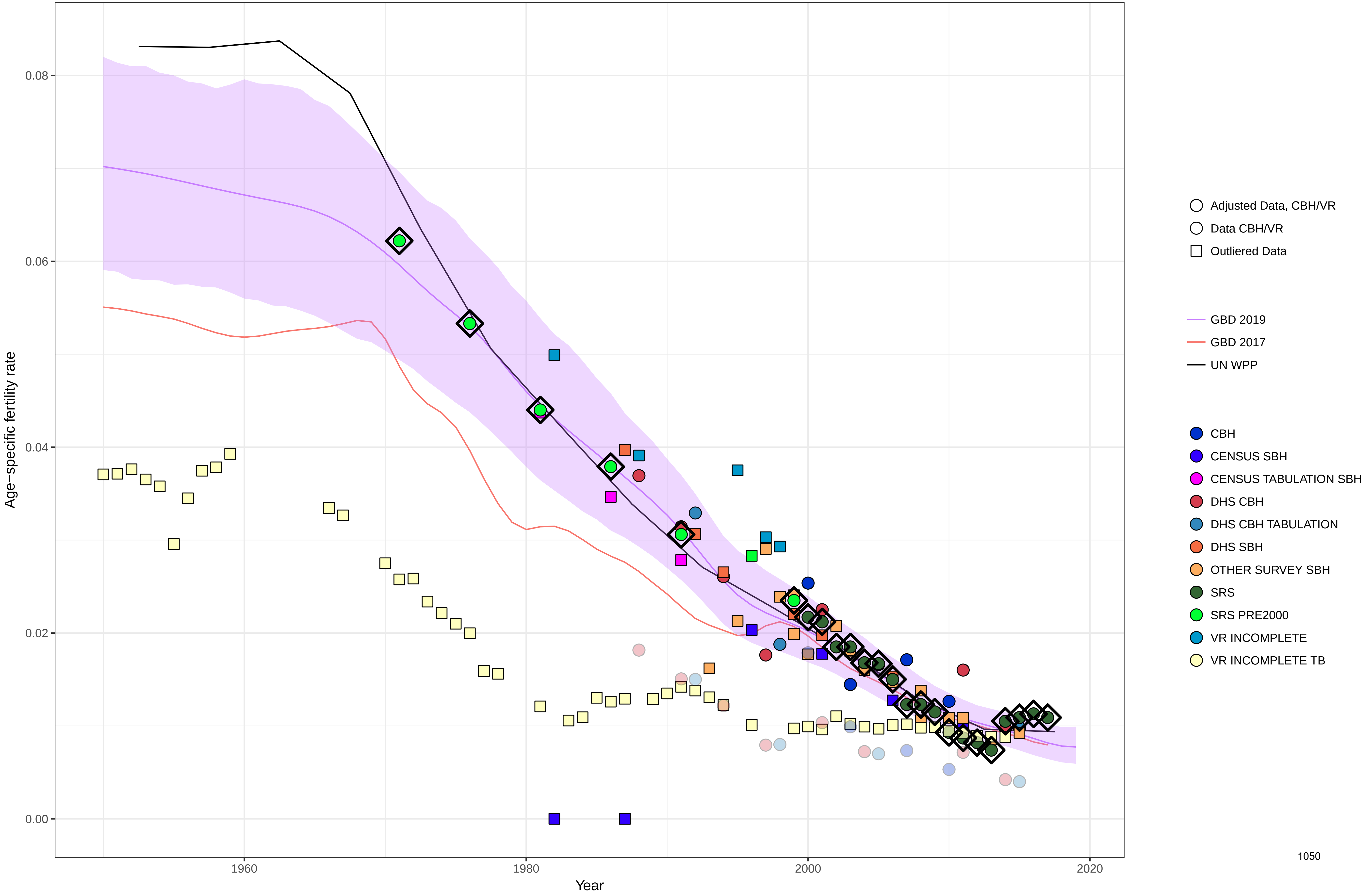

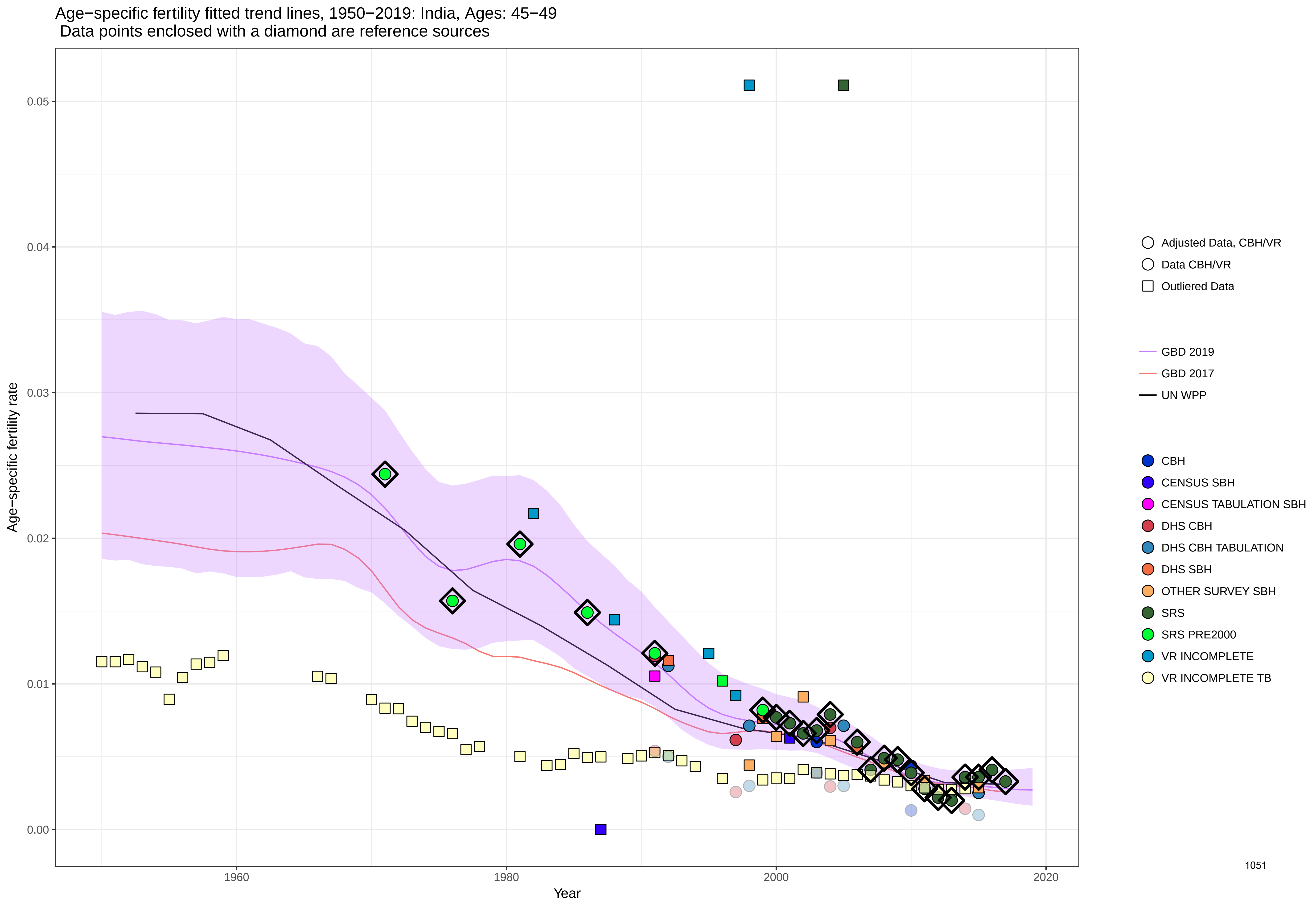

Age−specific fertility fitted trend lines, 1950−2019: India, Ages: 45−49
Data points enclosed with a diamond are reference sources
Age−specific fertility rate
0.00
0.01
0.02
0.03
0.04
0.05
Year
1960
1980
2000
2020
Adjusted Data, CBH/VR
Data CBH/VR
Outliered Data
GBD 2019
GBD 2017
UN WPP
CBH
CENSUS SBH
CENSUS TABULATION SBH
DHS CBH
DHS CBH TABULATION
DHS SBH
OTHER SURVEY SBH
SRS
SRS PRE2000
VR INCOMPLETE
VR INCOMPLETE TB

Age−specific fertility fitted trend lines, 1950−2019: India, Ages: 50−54
Data points enclosed with a diamond are reference sources

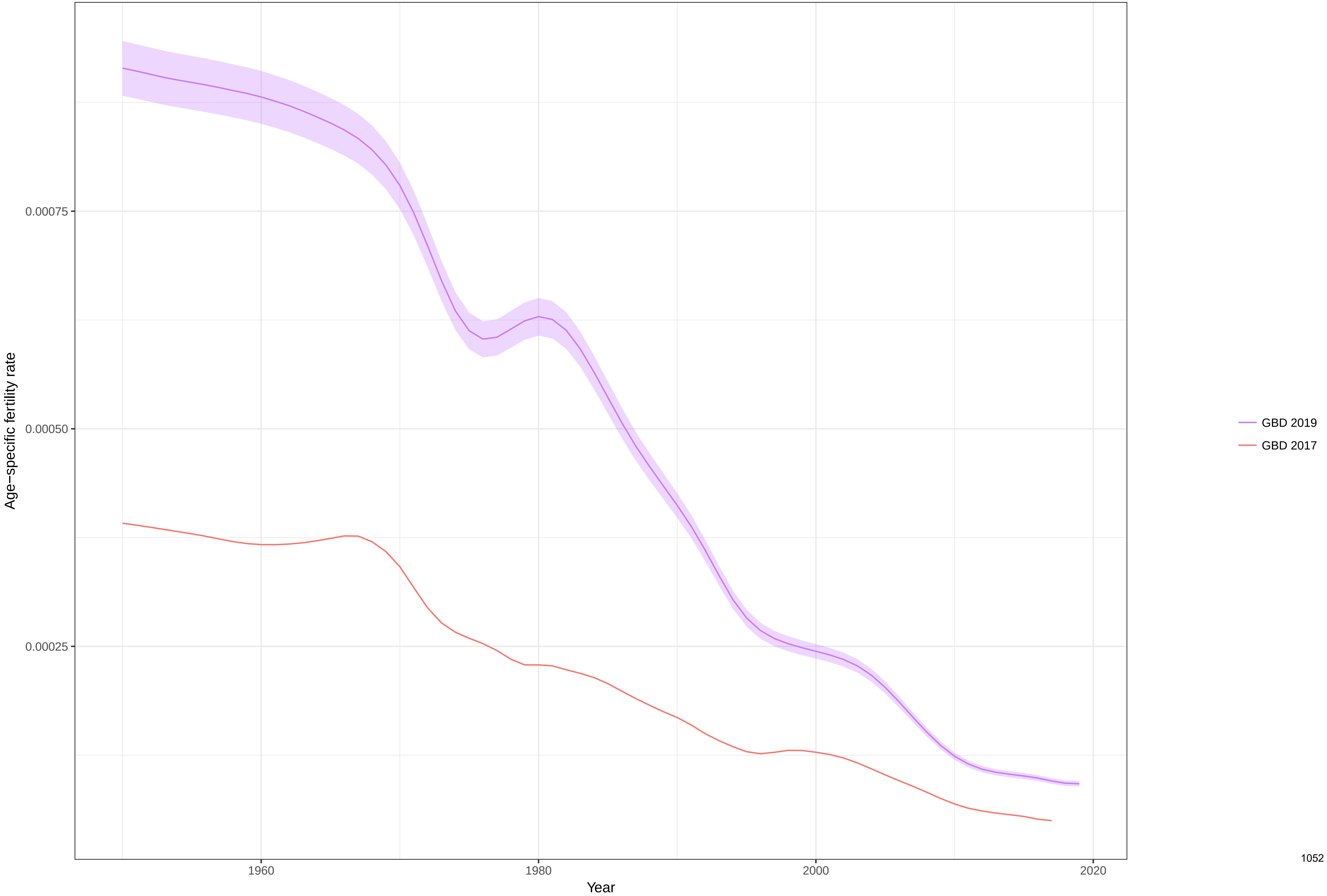

Total fertility rate fitted trend lines, 1950−2019: India TFR
Data points enclosed with a diamond are reference sources

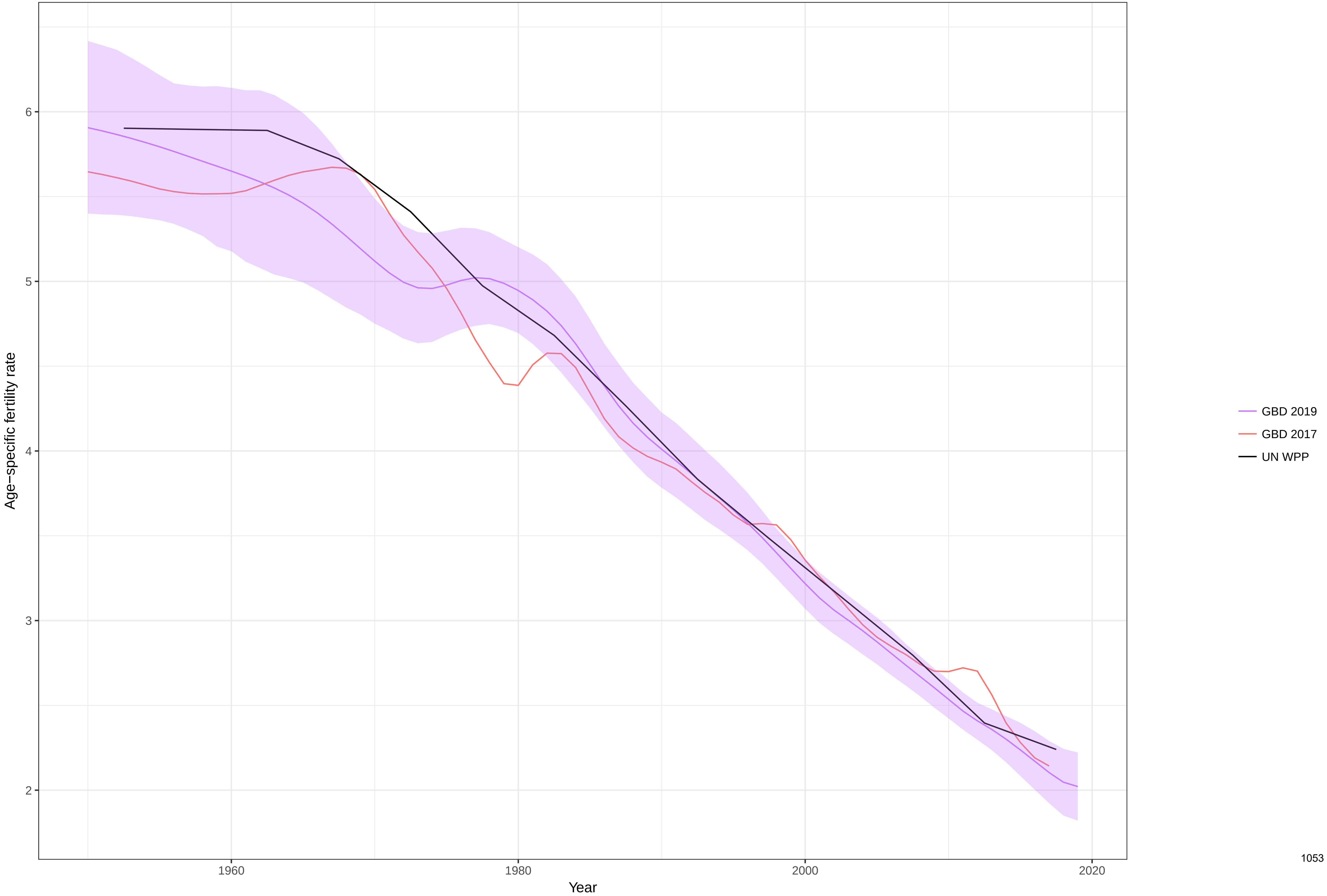

Age−specific fertility fitted trend lines, 1950−2019: Ireland, Ages: 10−14
Data points enclosed with a diamond are reference sources

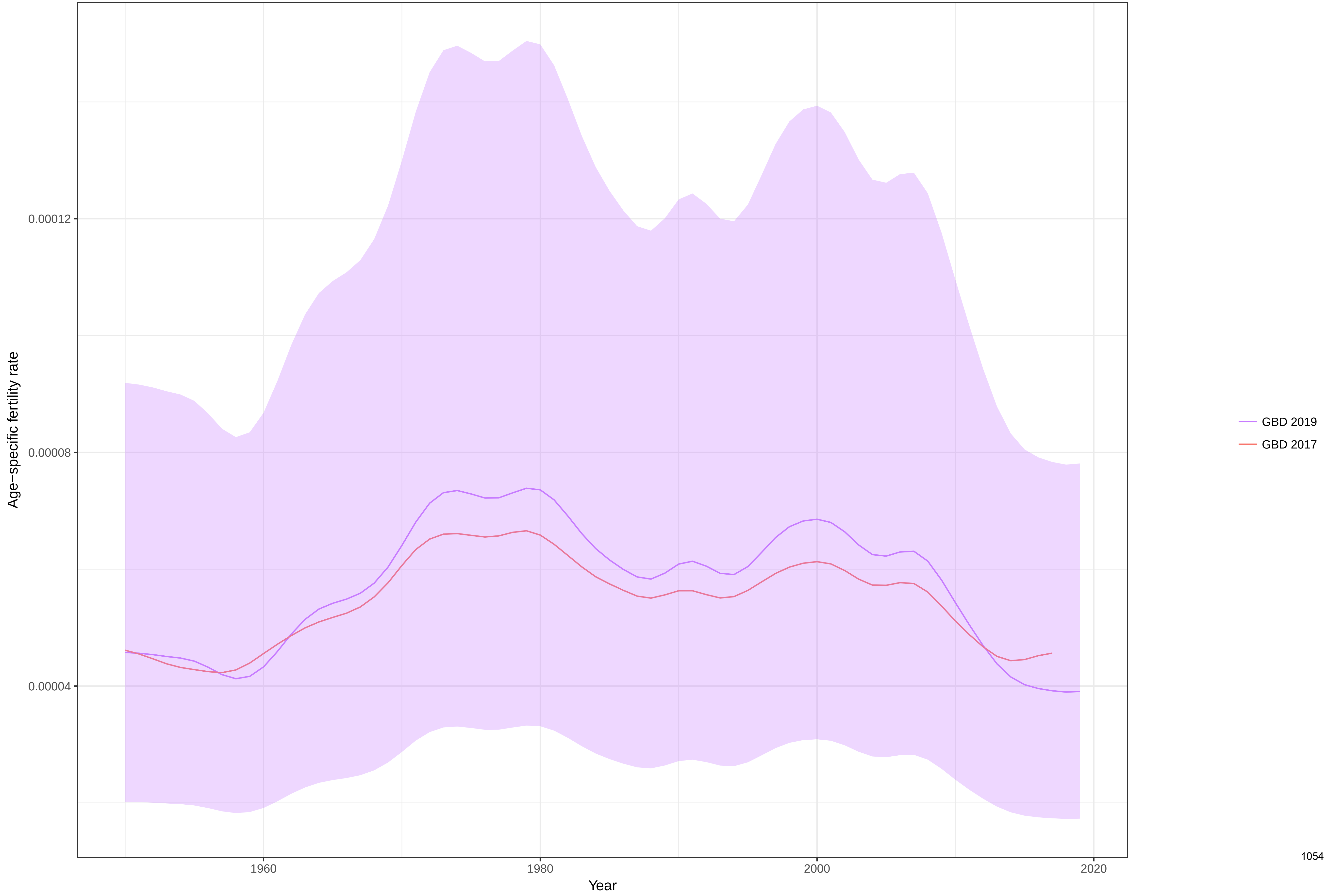

Age−specific fertility fitted trend lines, 1950−2019: Ireland, Ages: 15−19
Data points enclosed with a diamond are reference sources

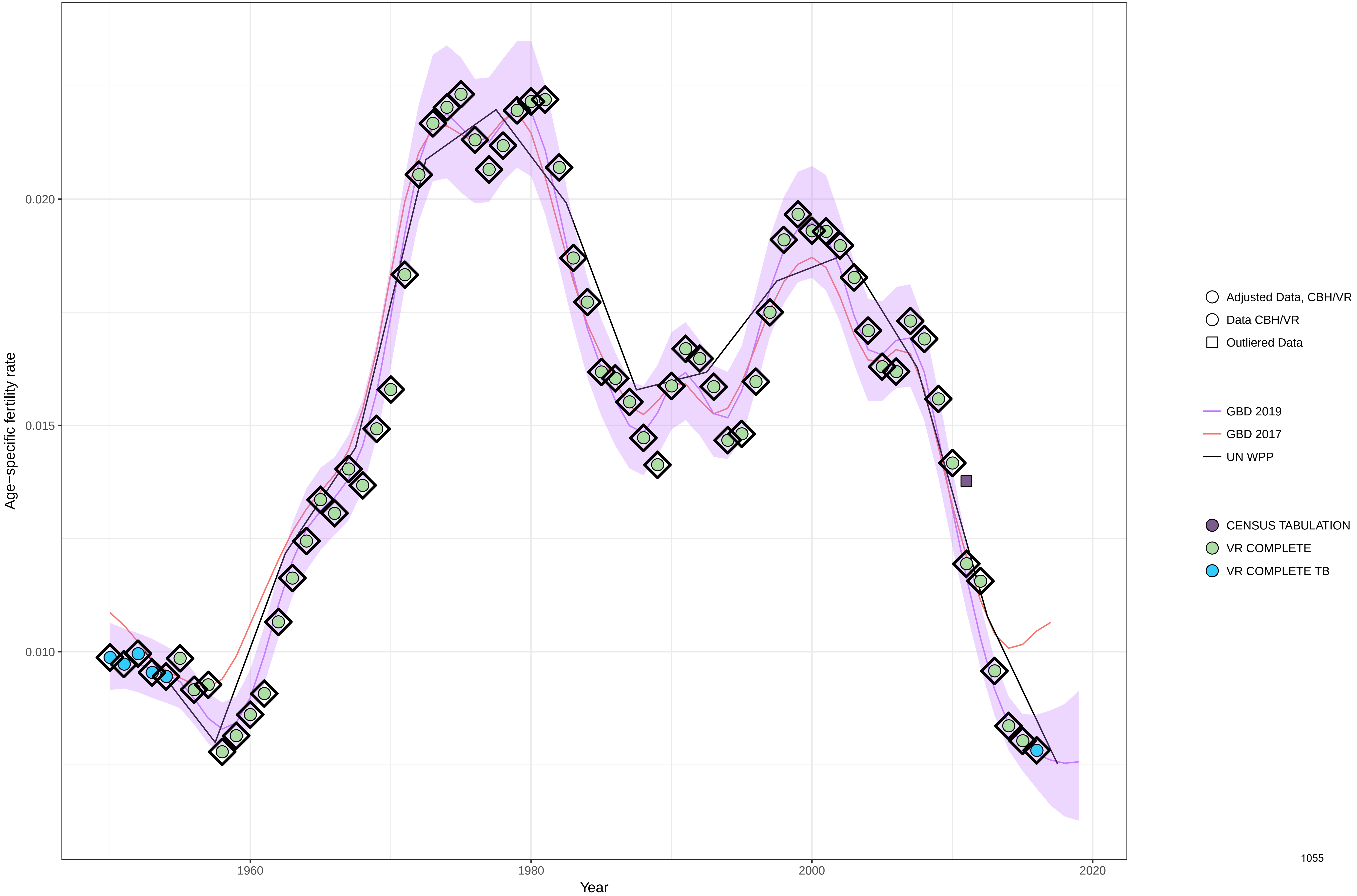

Age−specific fertility fitted trend lines, 1950−2019: Ireland, Ages: 20−24
Data points enclosed with a diamond are reference sources

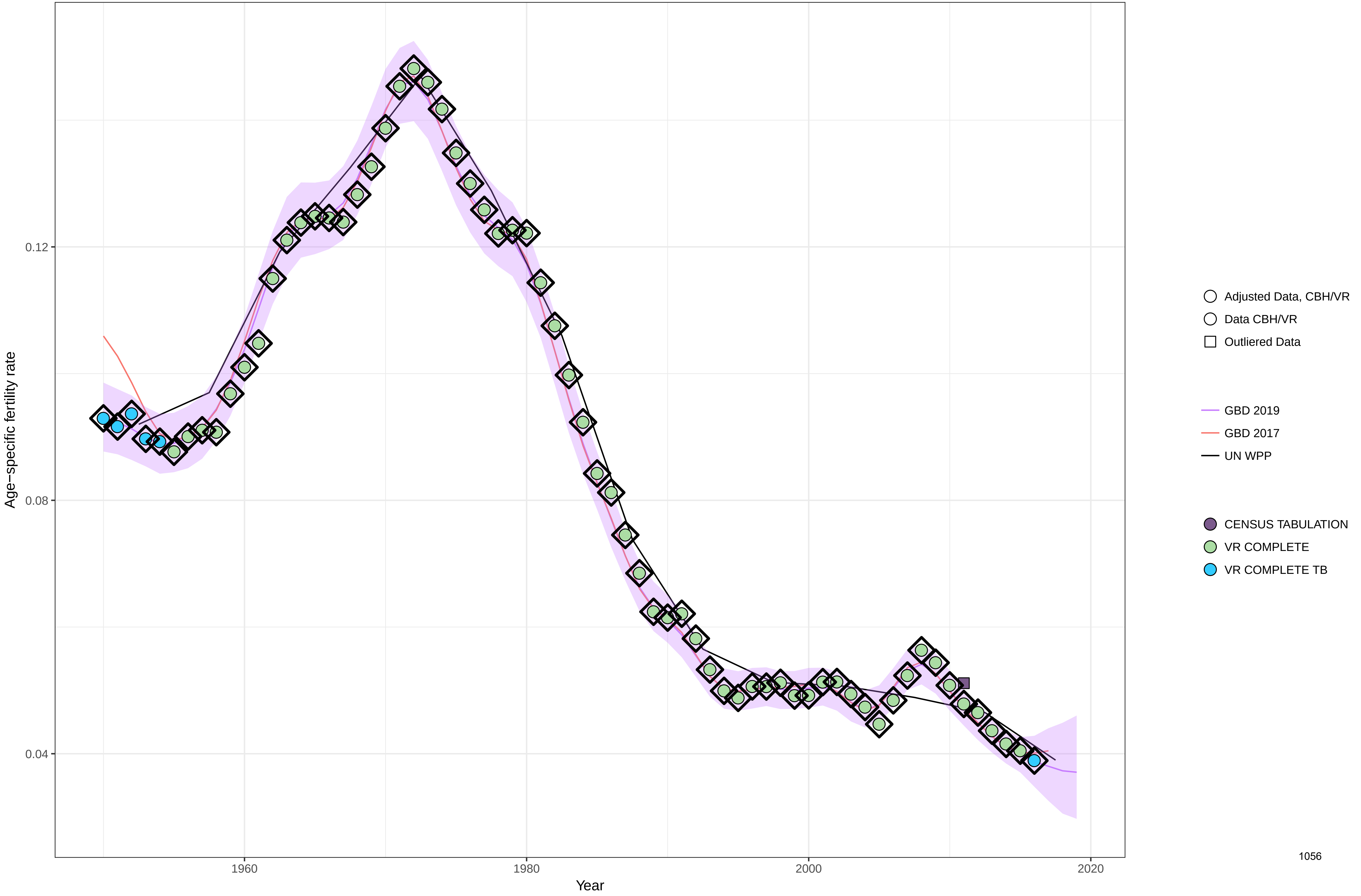

Age−specific fertility fitted trend lines, 1950−2019: Ireland, Ages: 25−29
Data points enclosed with a diamond are reference sources

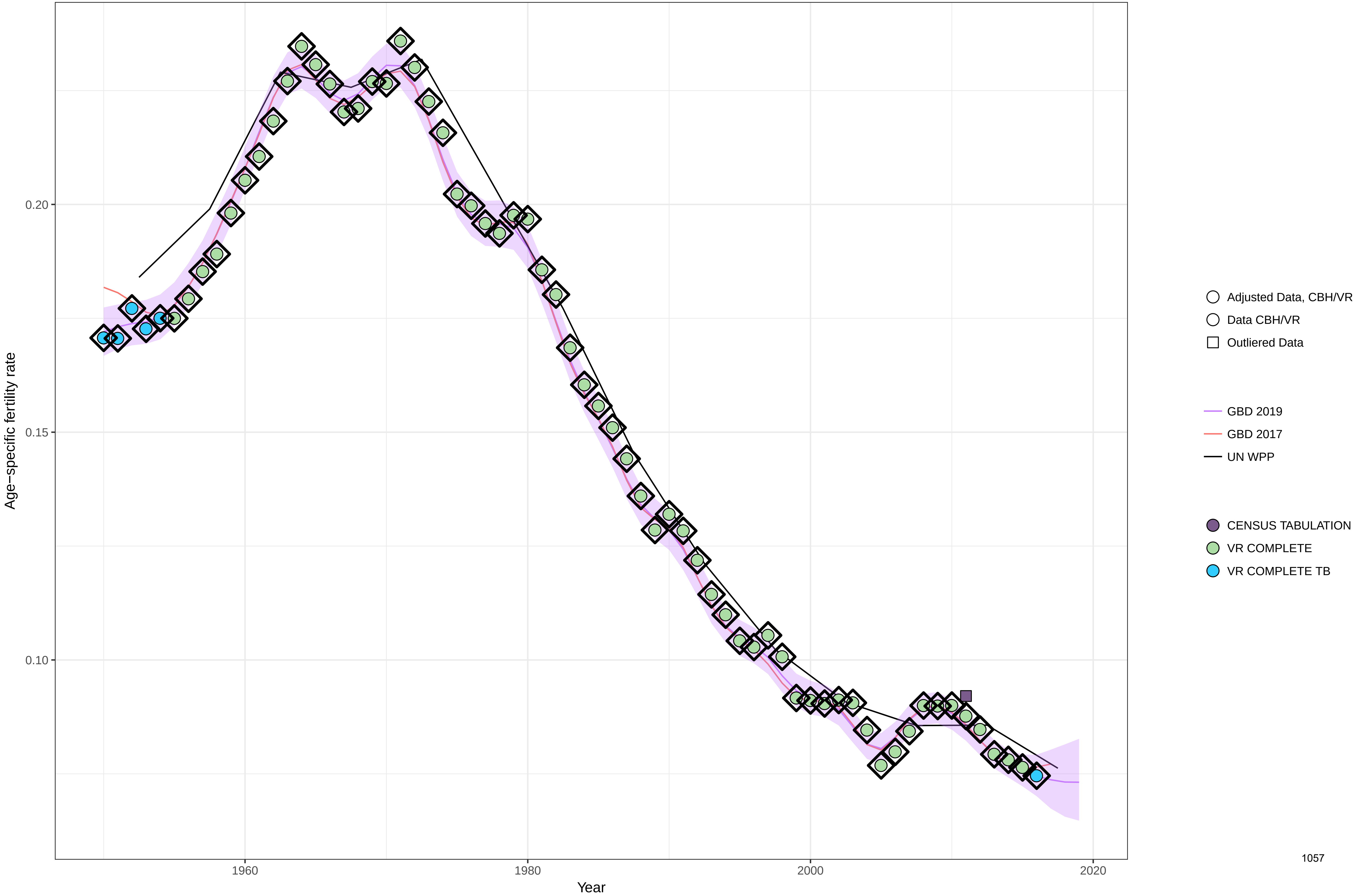

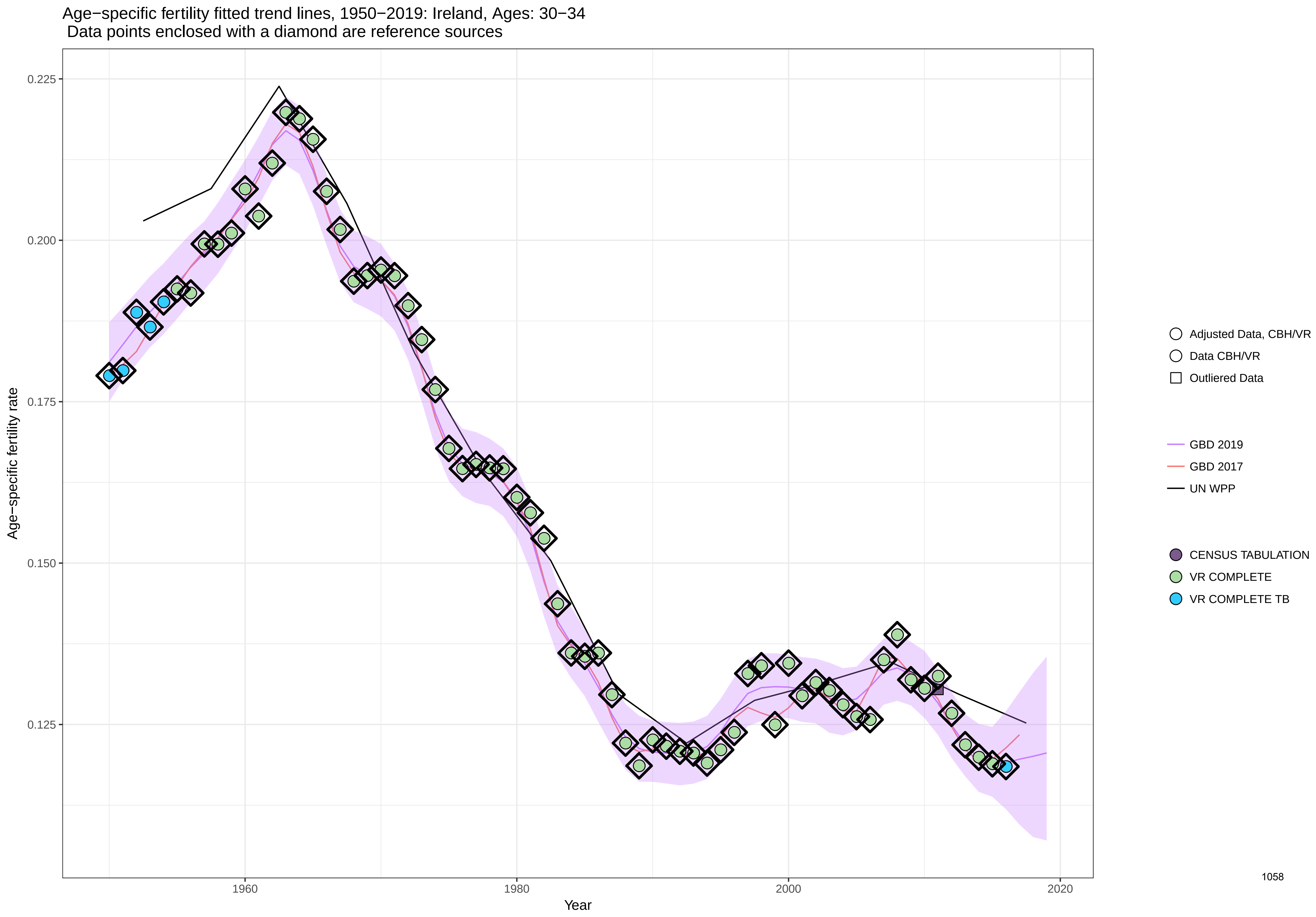

Age−specific fertility fitted trend lines, 1950−2019: Ireland, Ages: 30−34
Data points enclosed with a diamond are reference sources
0.225
0.200
0.175
0.150
0.125
Age−specific fertility rate
1960
1980
2000
2020
Year
Adjusted Data, CBH/VR
Data CBH/VR
Outliered Data
GBD 2019
GBD 2017
UN WPP
CENSUS TABULATION
VR COMPLETE
VR COMPLETE TB

Age−specific fertility fitted trend lines, 1950−2019: Ireland, Ages: 35−39
Data points enclosed with a diamond are reference sources

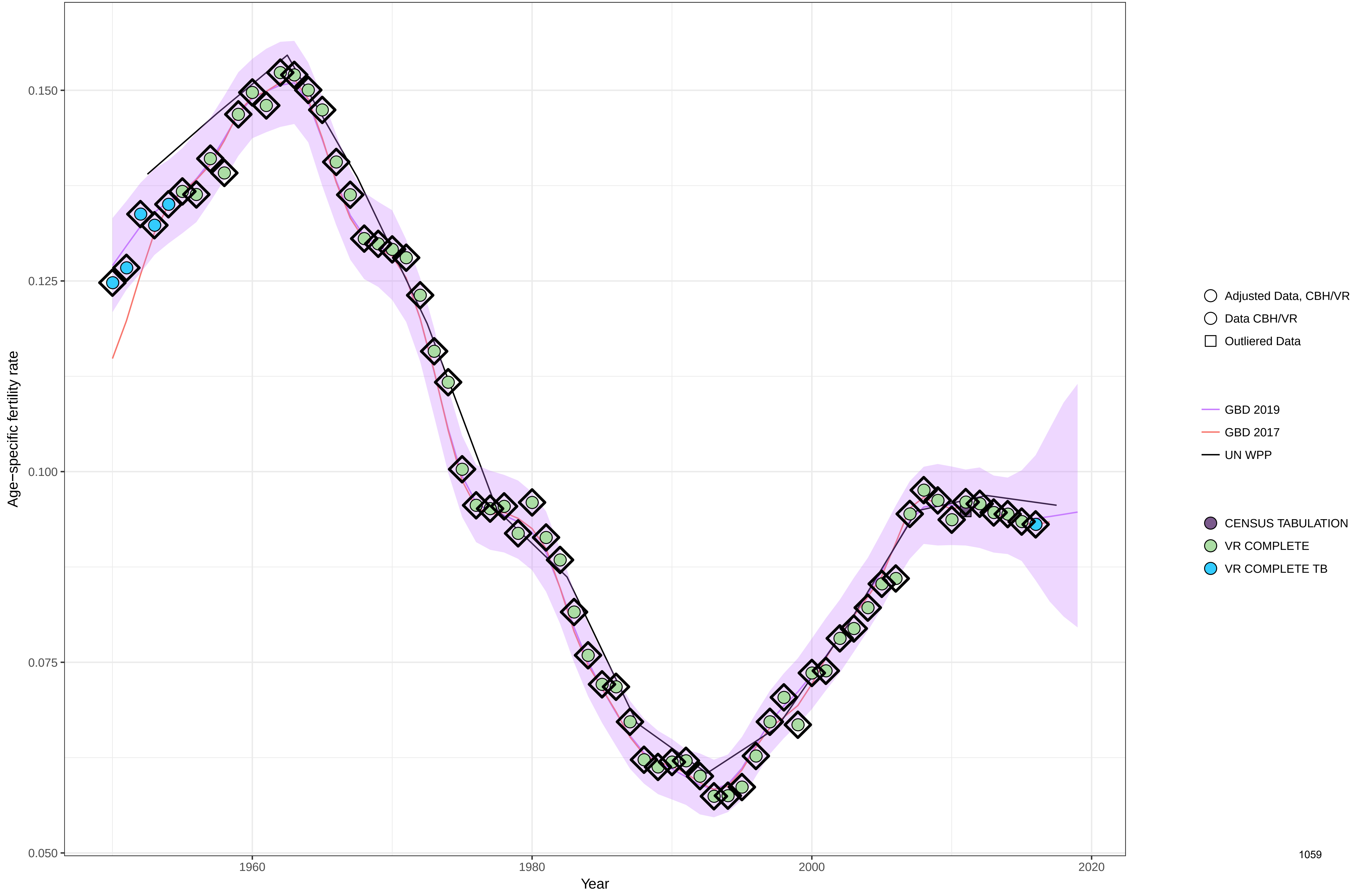

Age−specific fertility fitted trend lines, 1950−2019: Ireland, Ages: 40−44
Data points enclosed with a diamond are reference sources

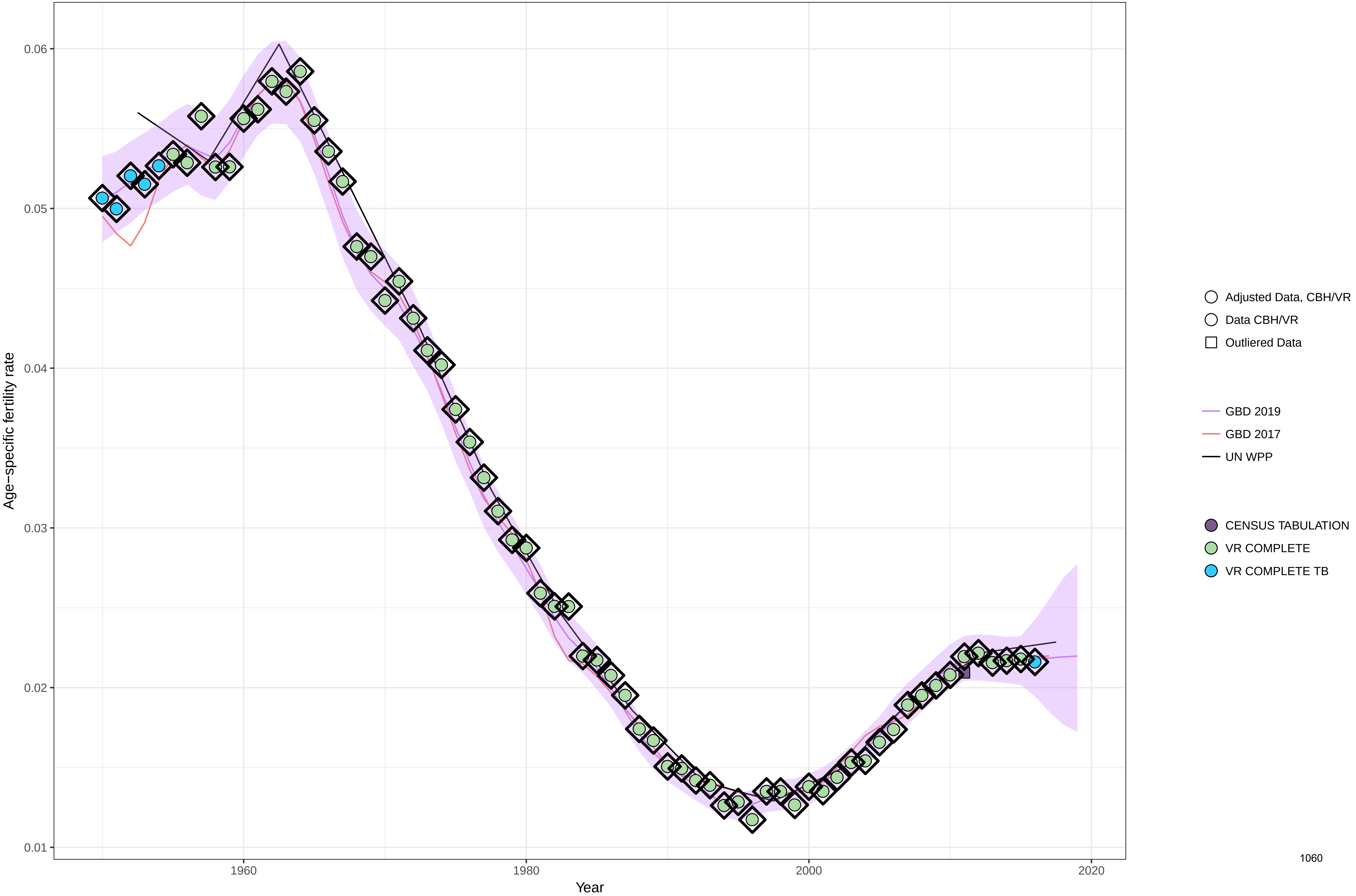

Age−specific fertility fitted trend lines, 1950−2019: Ireland, Ages: 45−49
Data points enclosed with a diamond are reference sources

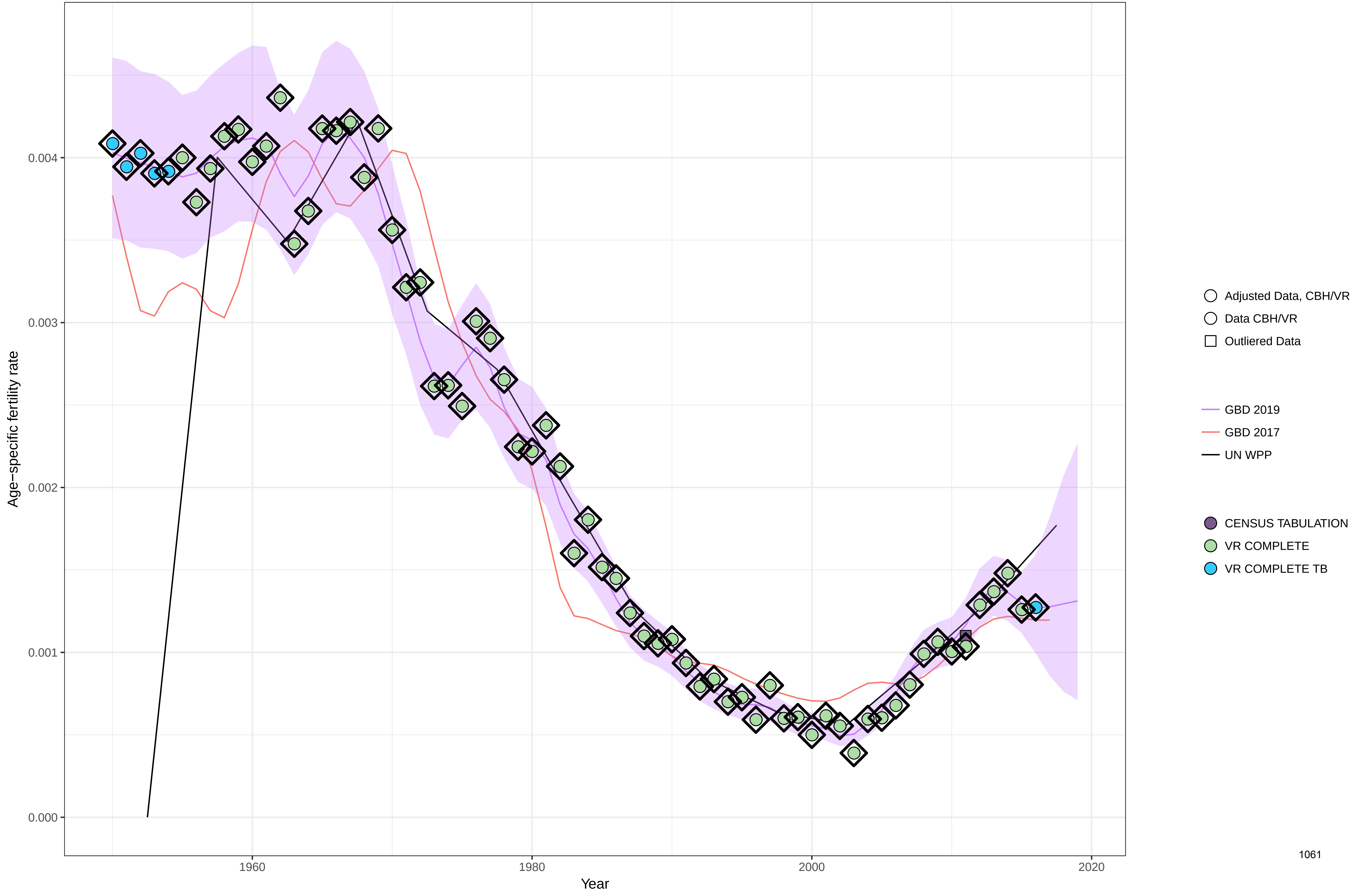

Age−specific fertility fitted trend lines, 1950−2019: Ireland, Ages: 50−54
Data points enclosed with a diamond are reference sources

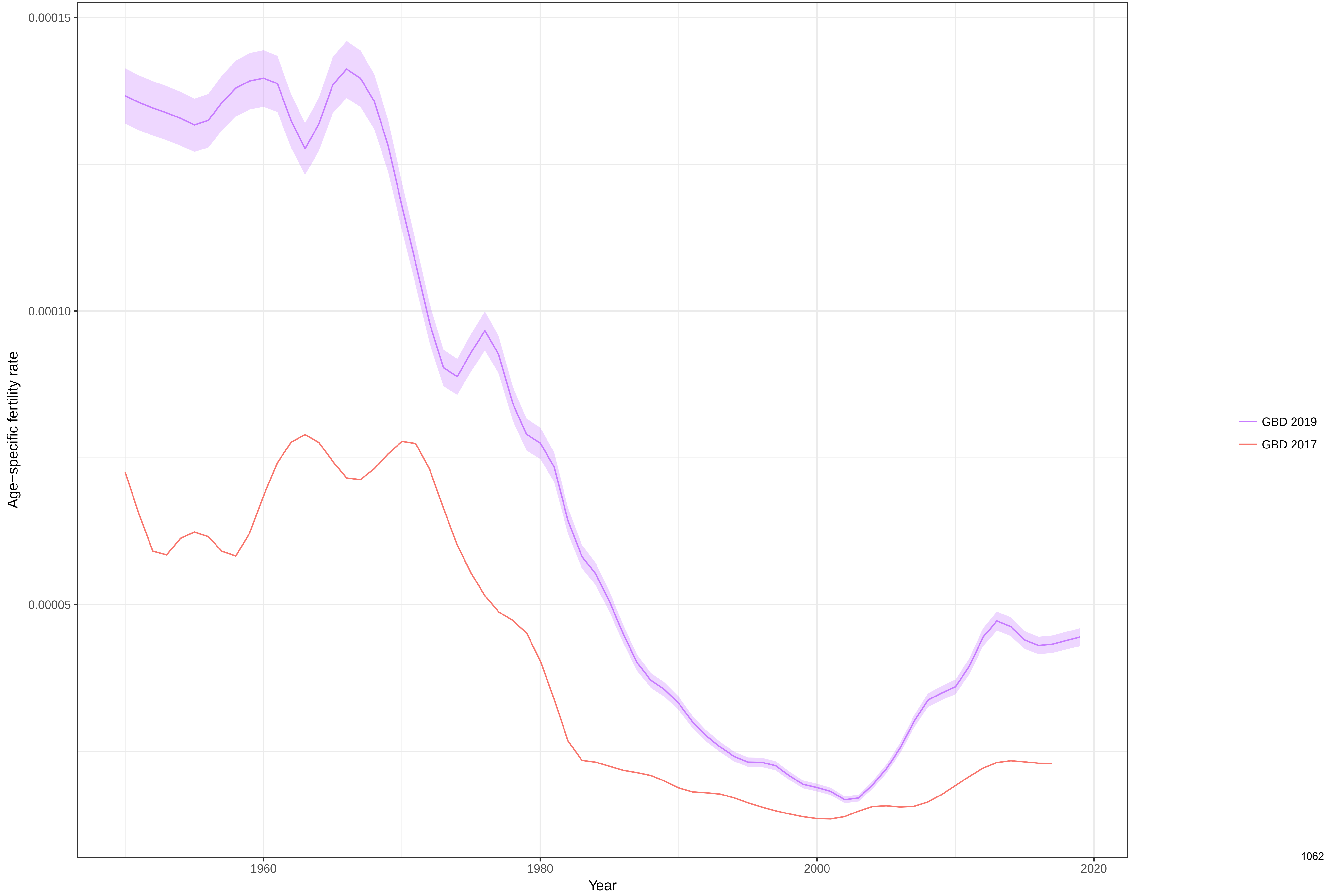

Total fertility rate fitted trend lines, 1950−2019: Ireland TFR
Data points enclosed with a diamond are reference sources

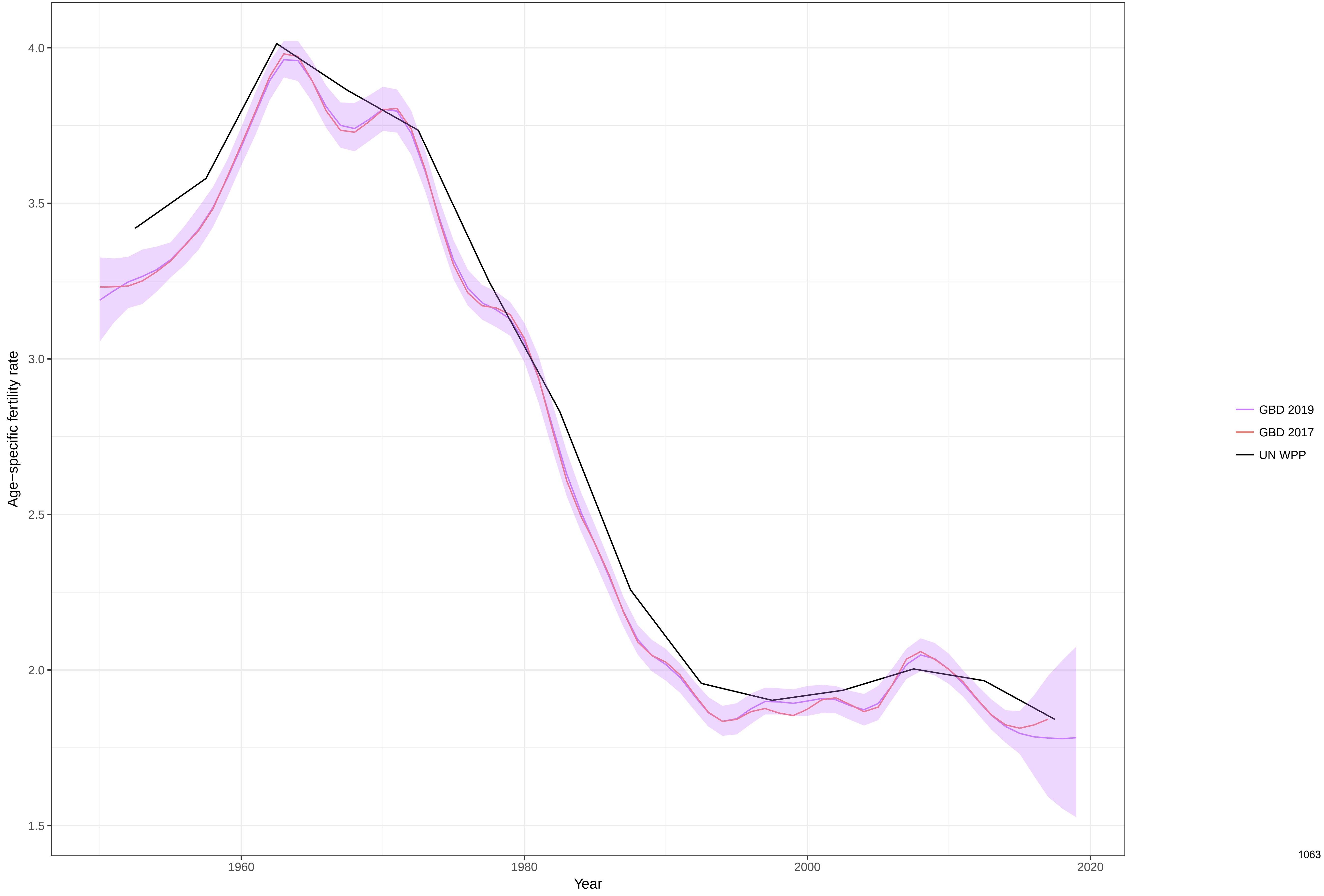

Age−specific fertility fitted trend lines, 1950−2019: Iran (Islamic Republic of), Ages: 10−14
Data points enclosed with a diamond are reference sources

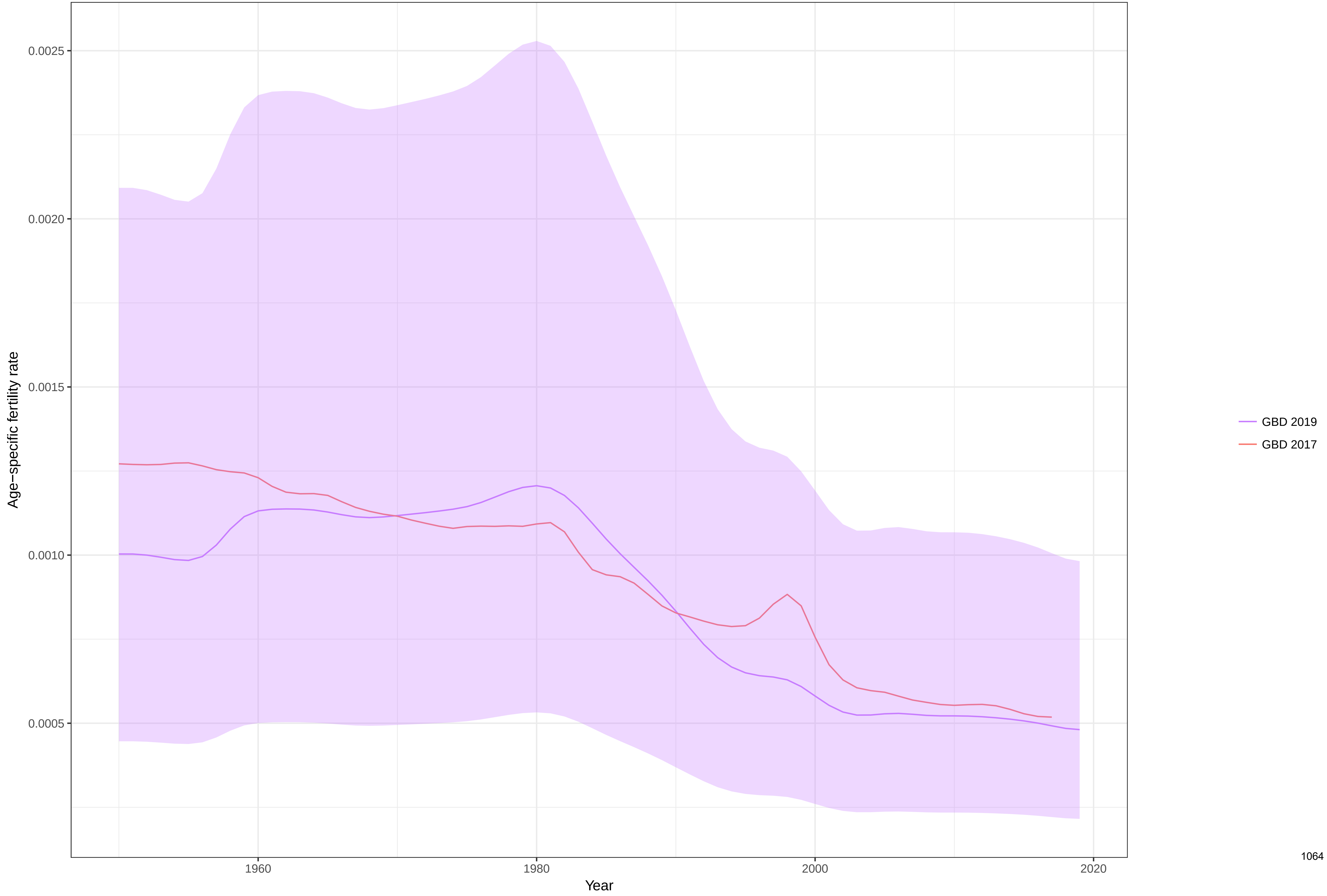

Age−specific fertility fitted trend lines, 1950−2019: Iran (Islamic Republic of), Ages: 15−19
Data points enclosed with a diamond are reference sources

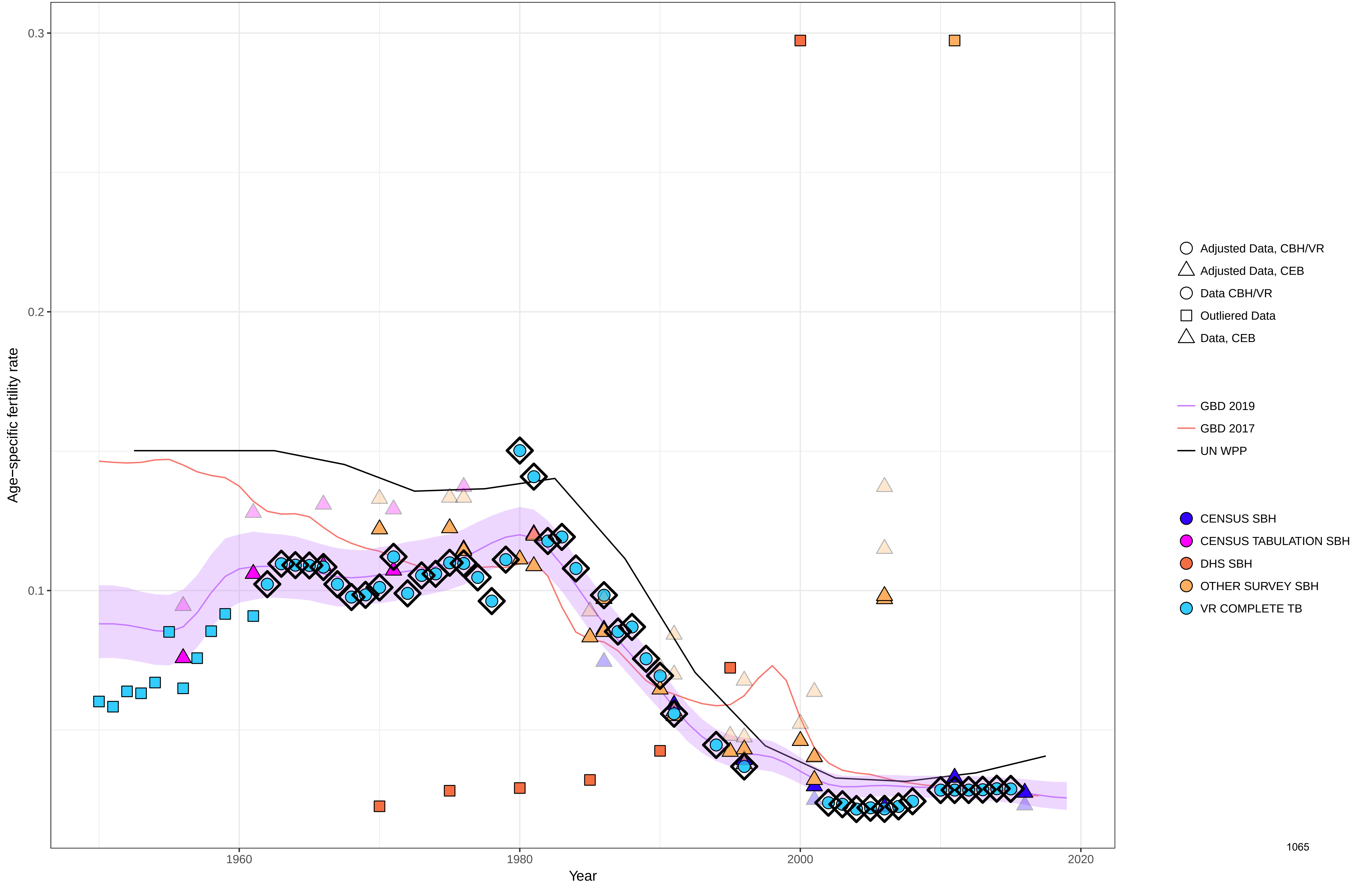

Age−specific fertility fitted trend lines, 1950−2019: Iran (Islamic Republic of), Ages: 20−24
Data points enclosed with a diamond are reference sources

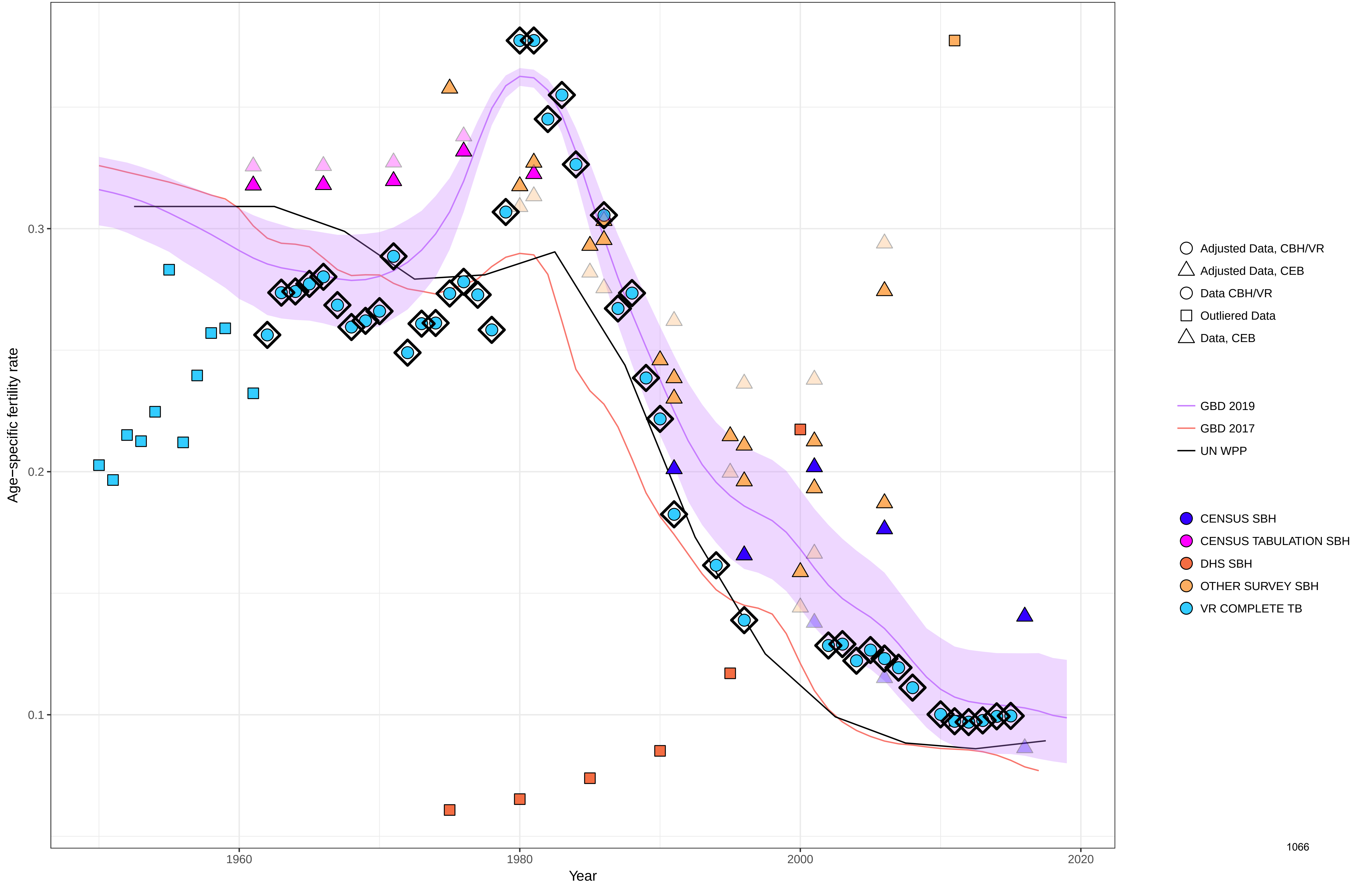

Age−specific fertility fitted trend lines, 1950−2019: Iran (Islamic Republic of), Ages: 25−29
Data points enclosed with a diamond are reference sources

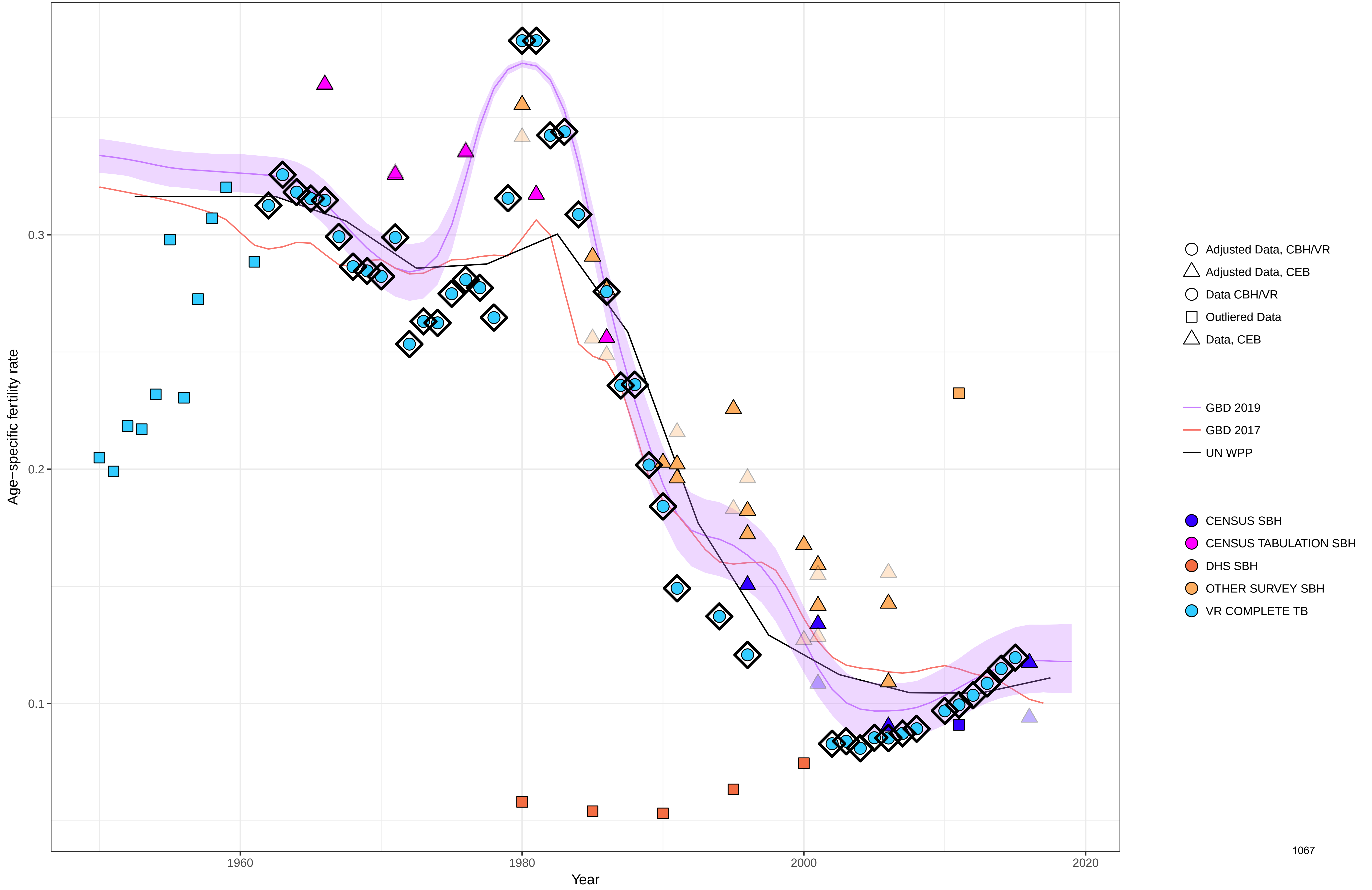

Age−specific fertility fitted trend lines, 1950−2019: Iran (Islamic Republic of), Ages: 30−34
Data points enclosed with a diamond are reference sources

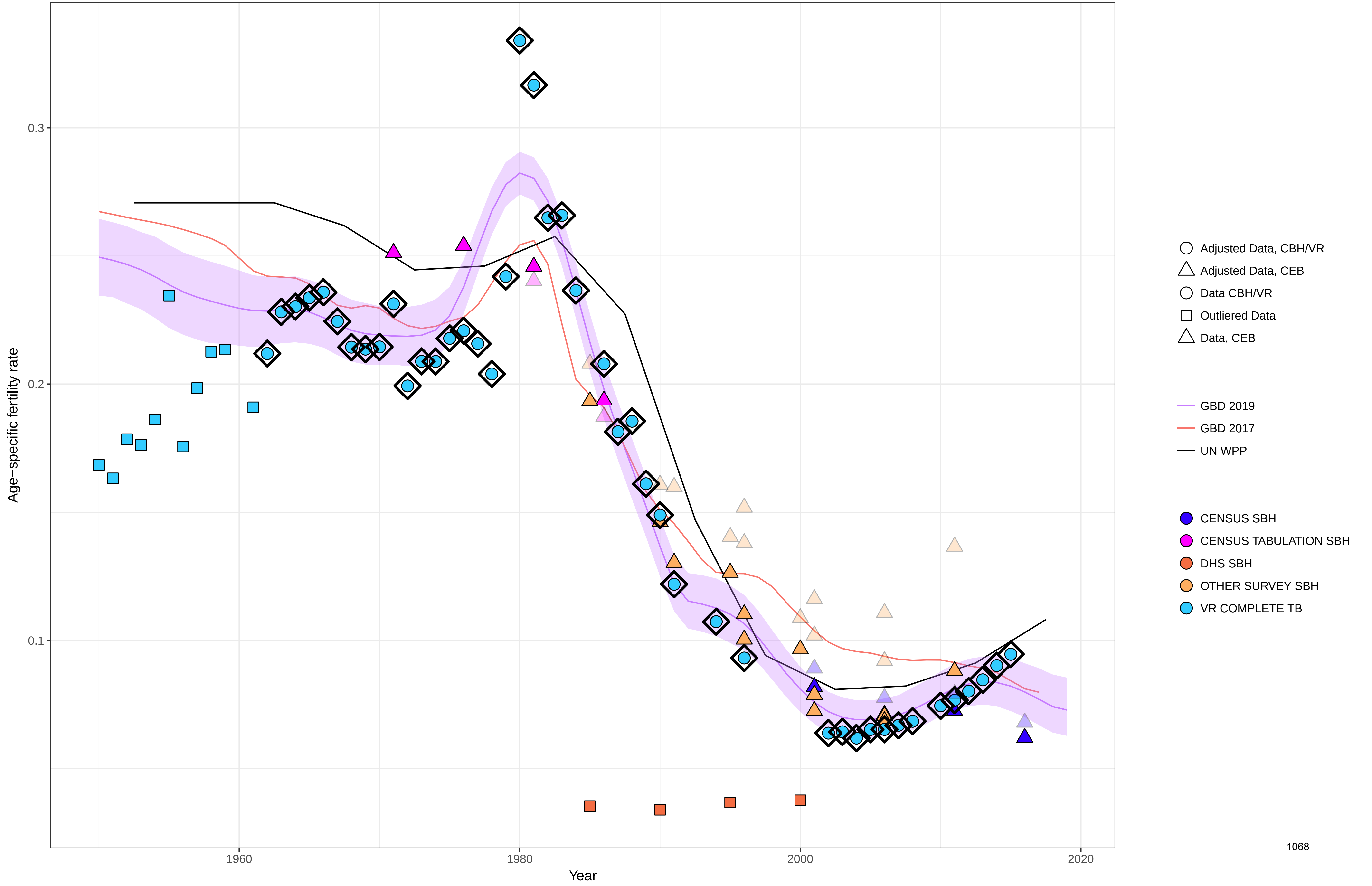

Age−specific fertility fitted trend lines, 1950−2019: Iran (Islamic Republic of), Ages: 35−39
Data points enclosed with a diamond are reference sources

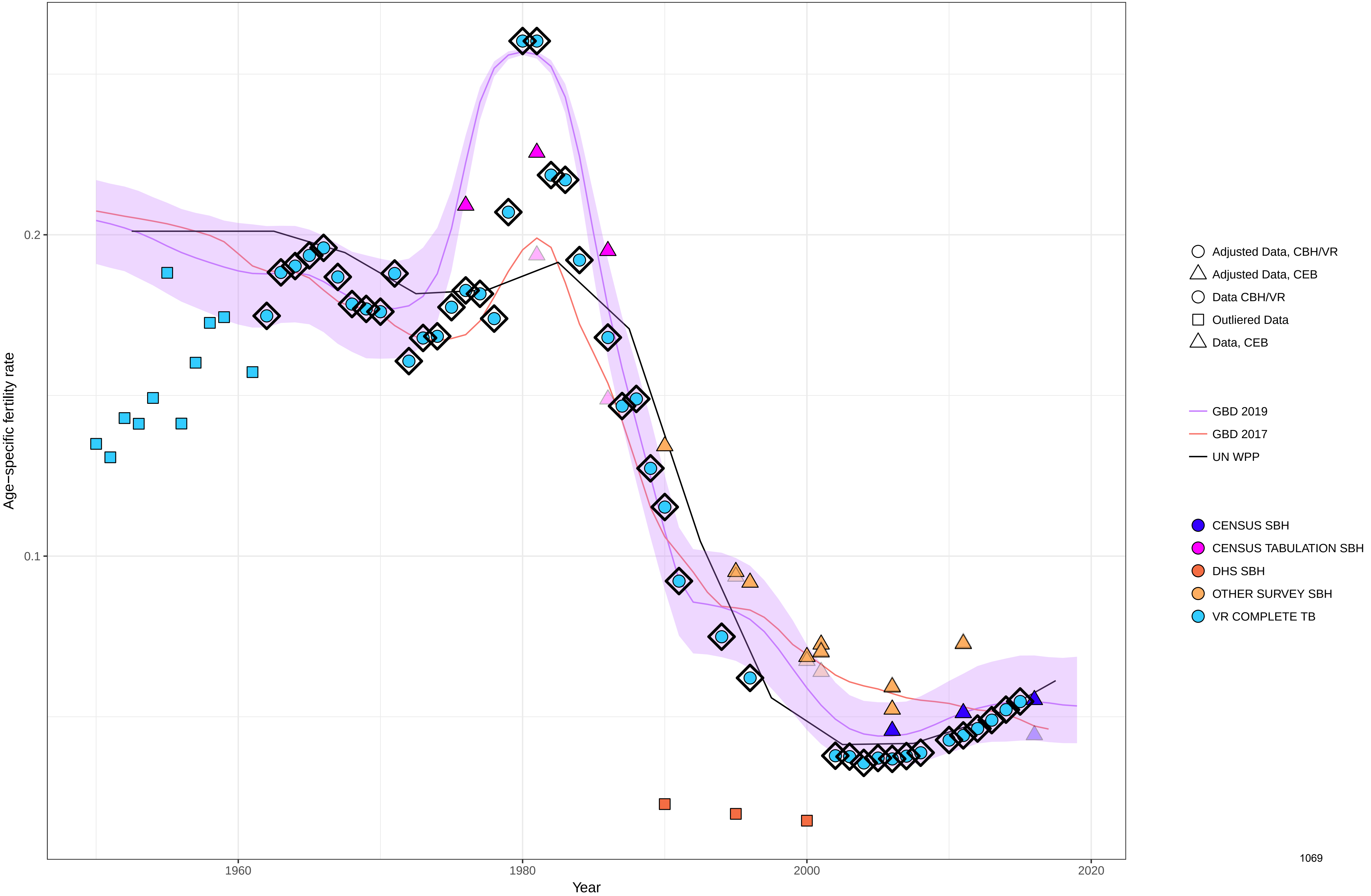

Age−specific fertility fitted trend lines, 1950−2019: Iran (Islamic Republic of), Ages: 40−44
Data points enclosed with a diamond are reference sources

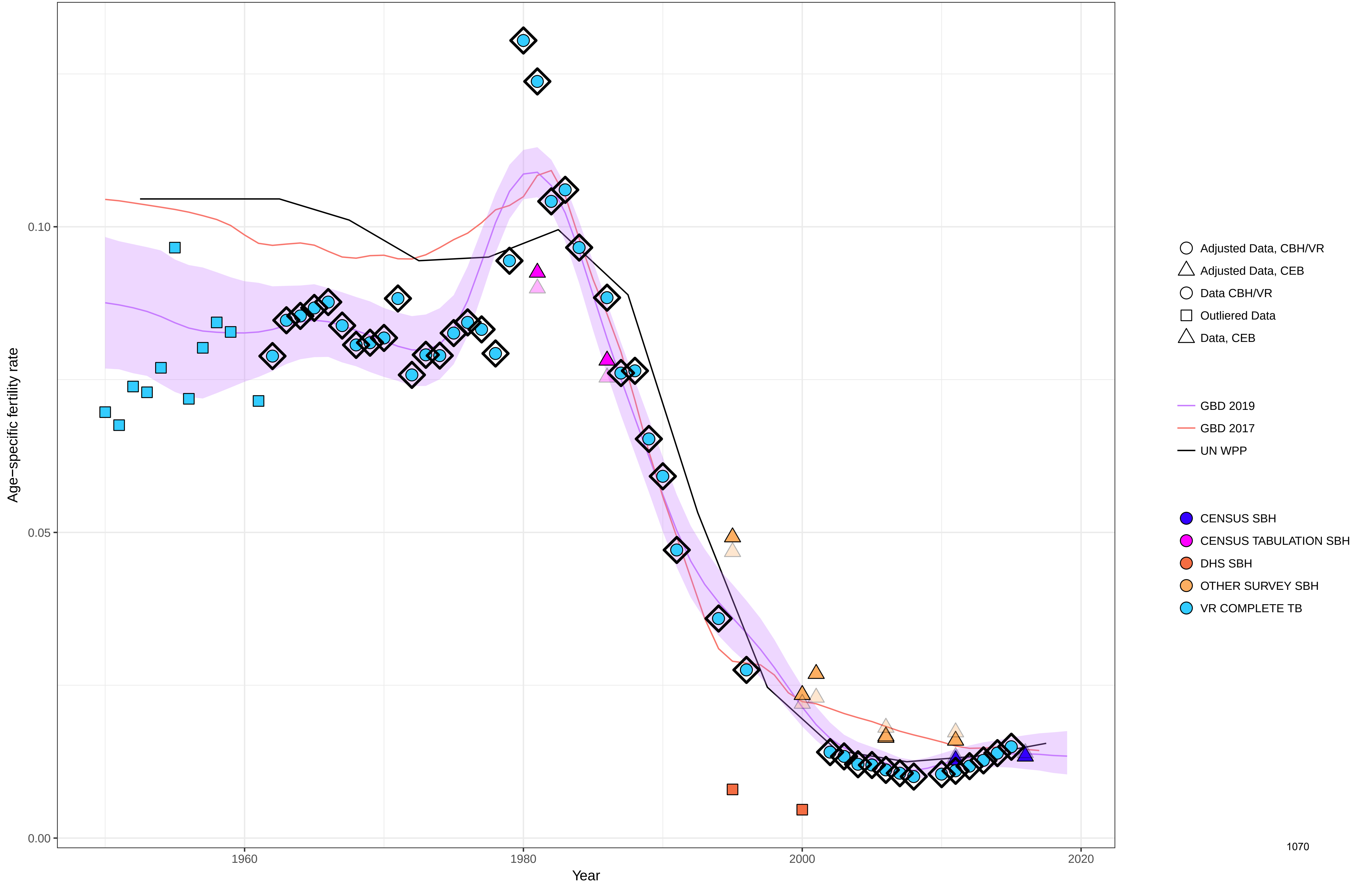

Age−specific fertility fitted trend lines, 1950−2019: Iran (Islamic Republic of), Ages: 45−49
Data points enclosed with a diamond are reference sources

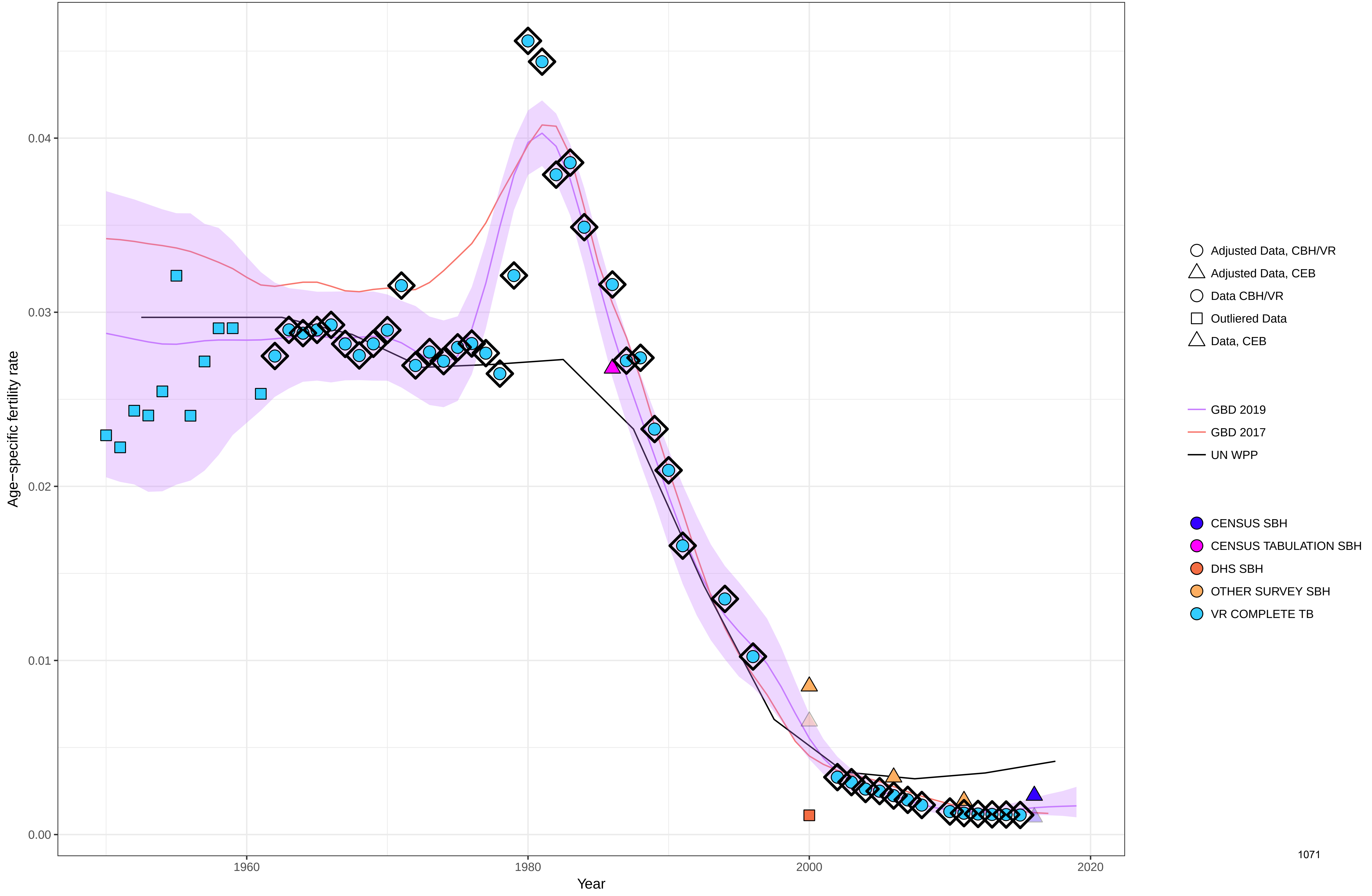

Age−specific fertility fitted trend lines, 1950−2019: Iran (Islamic Republic of), Ages: 50−54
Data points enclosed with a diamond are reference sources

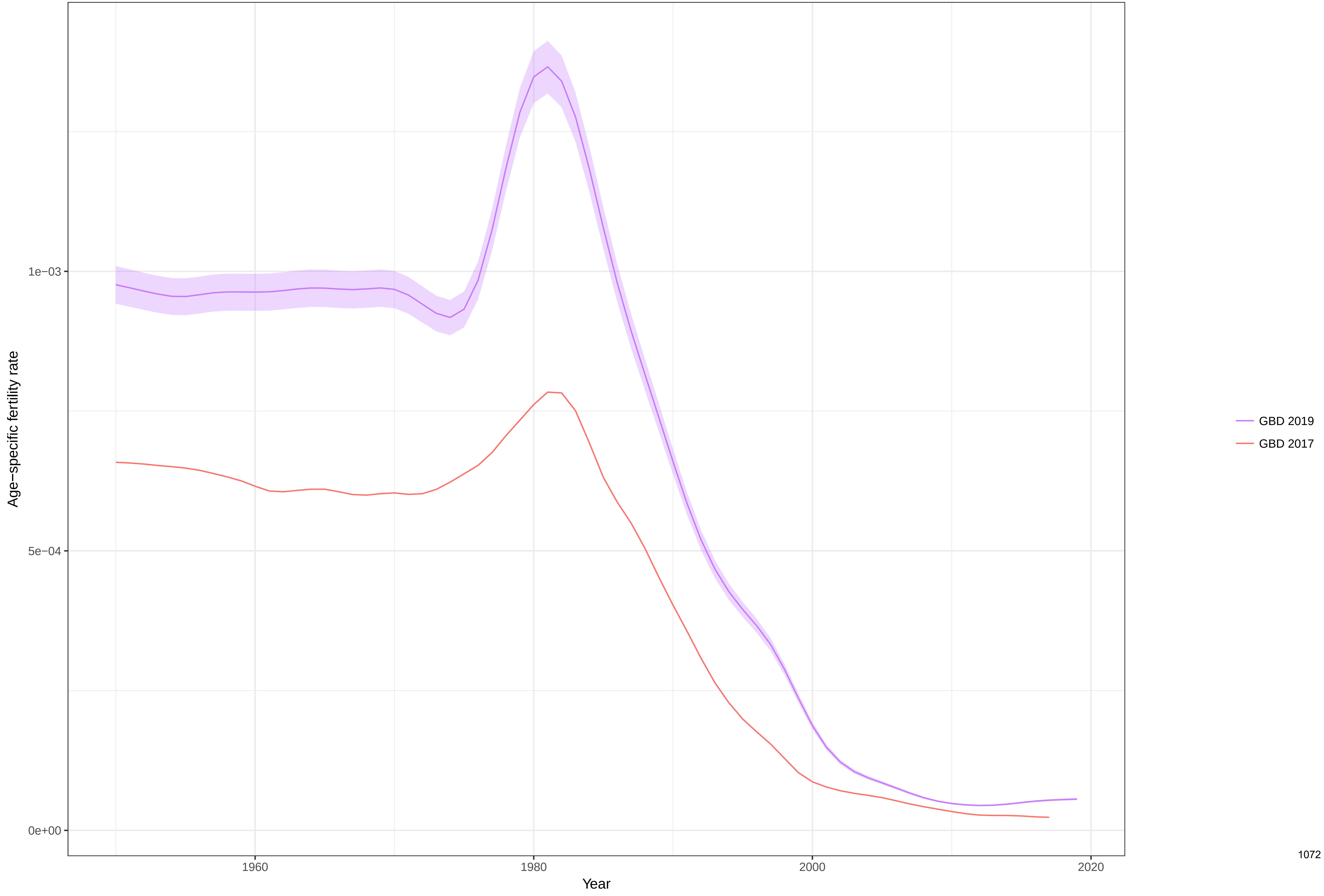

Total fertility rate fitted trend lines, 1950−2019: Iran (Islamic Republic of) TFR
Data points enclosed with a diamond are reference sources

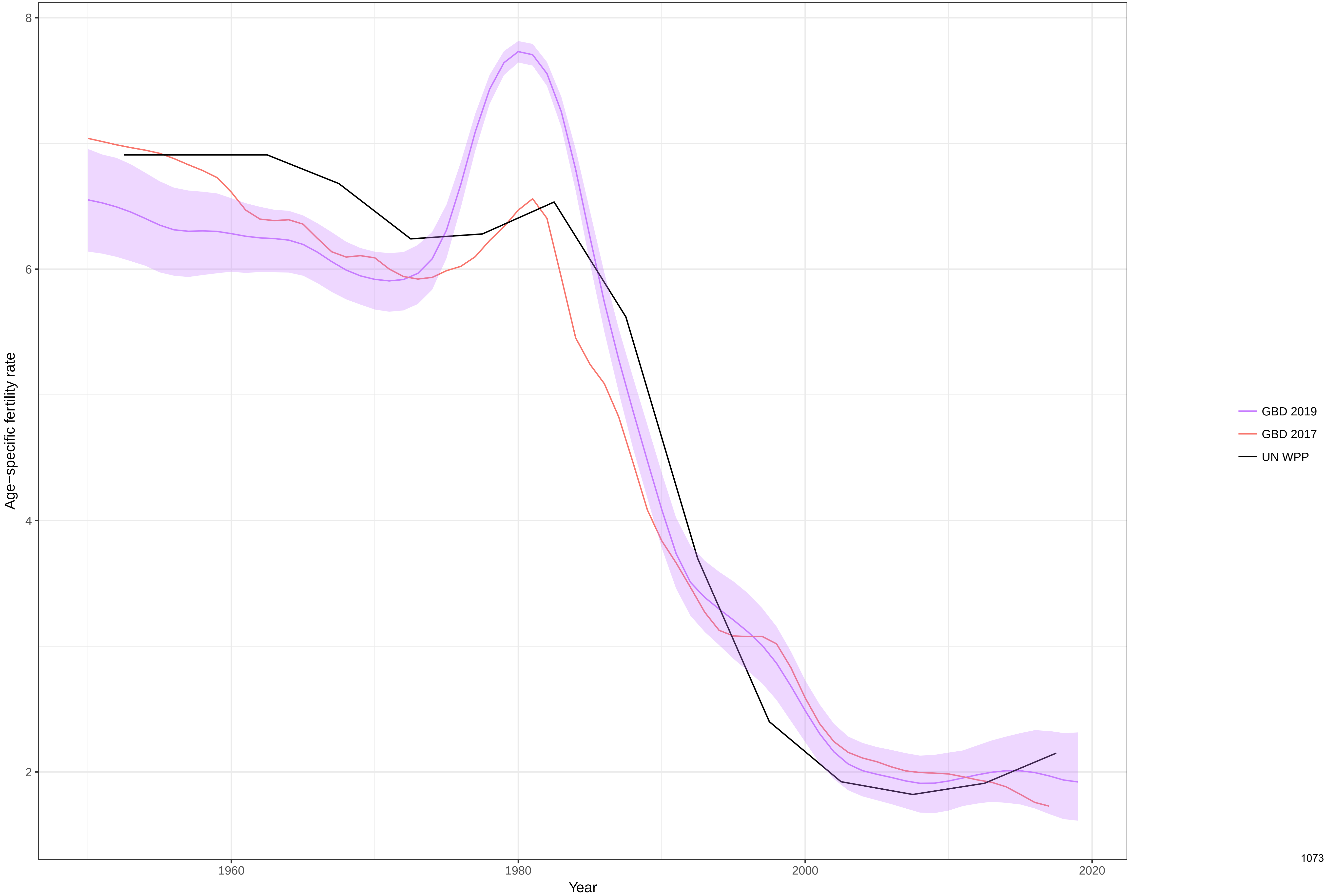

Age−specific fertility fitted trend lines, 1950−2019: Iraq, Ages: 10−14
Data points enclosed with a diamond are reference sources

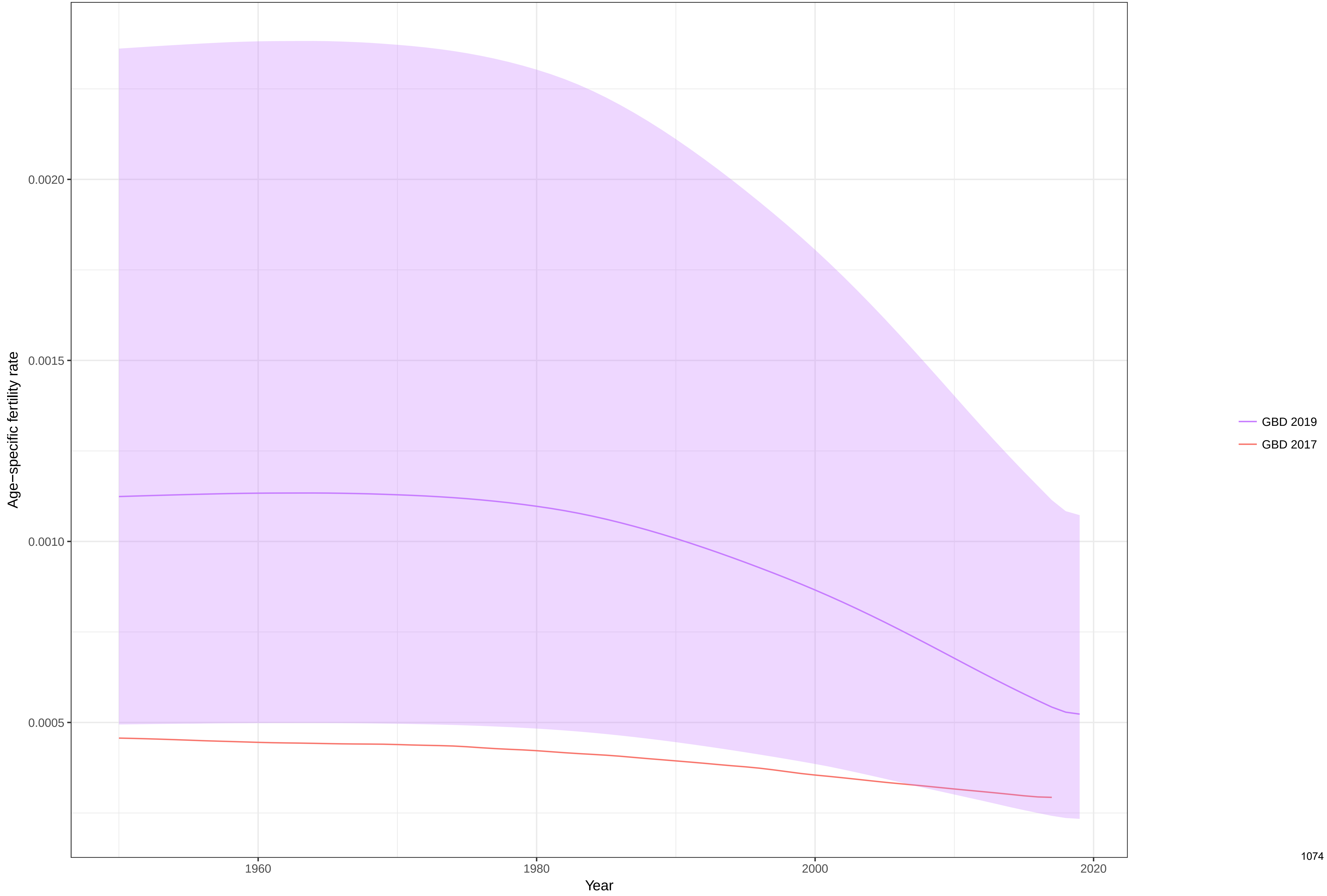

Age−specific fertility fitted trend lines, 1950−2019: Iraq, Ages: 15−19
Data points enclosed with a diamond are reference sources

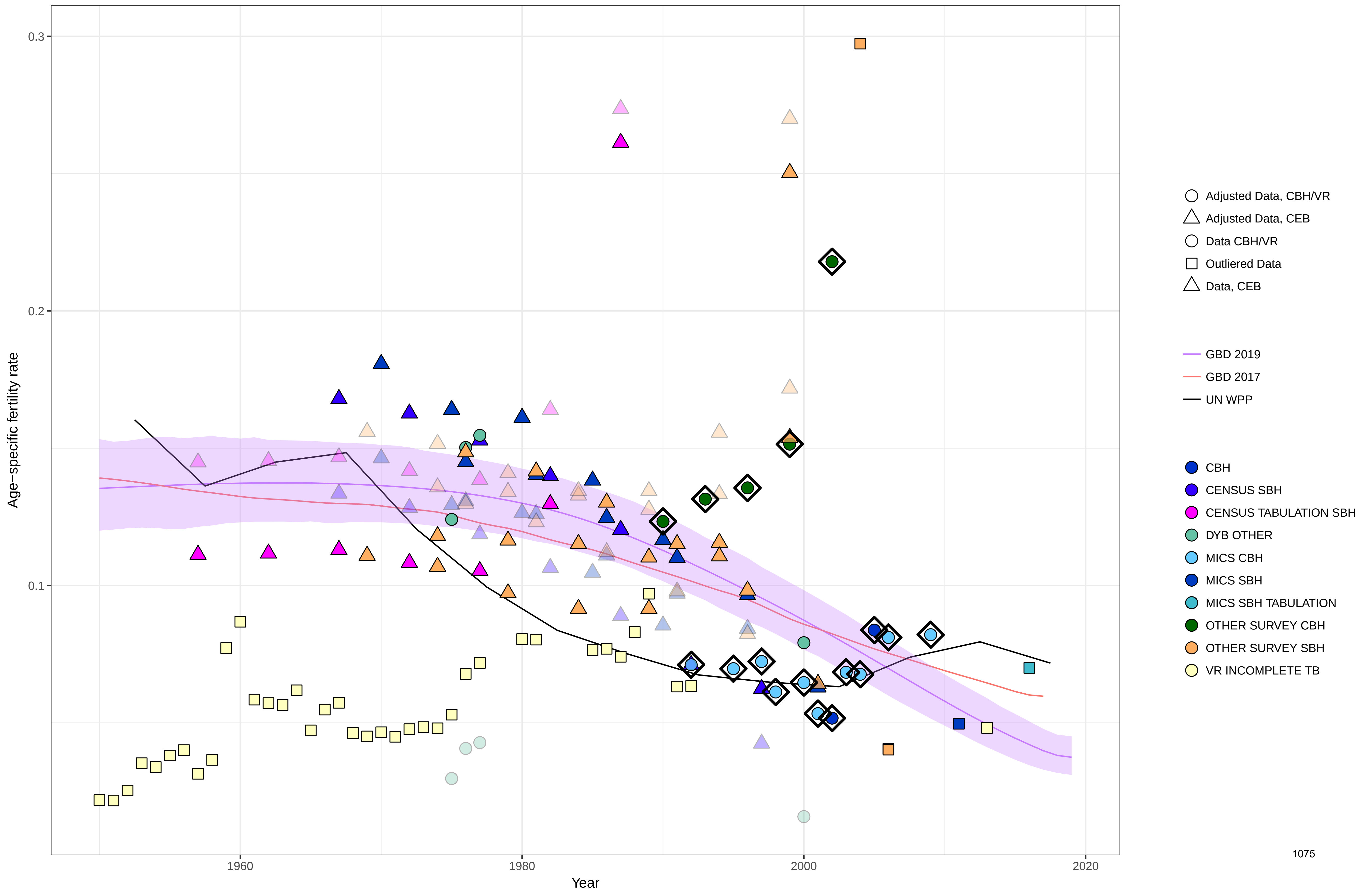

Age−specific fertility fitted trend lines, 1950−2019: Iraq, Ages: 20−24
Data points enclosed with a diamond are reference sources

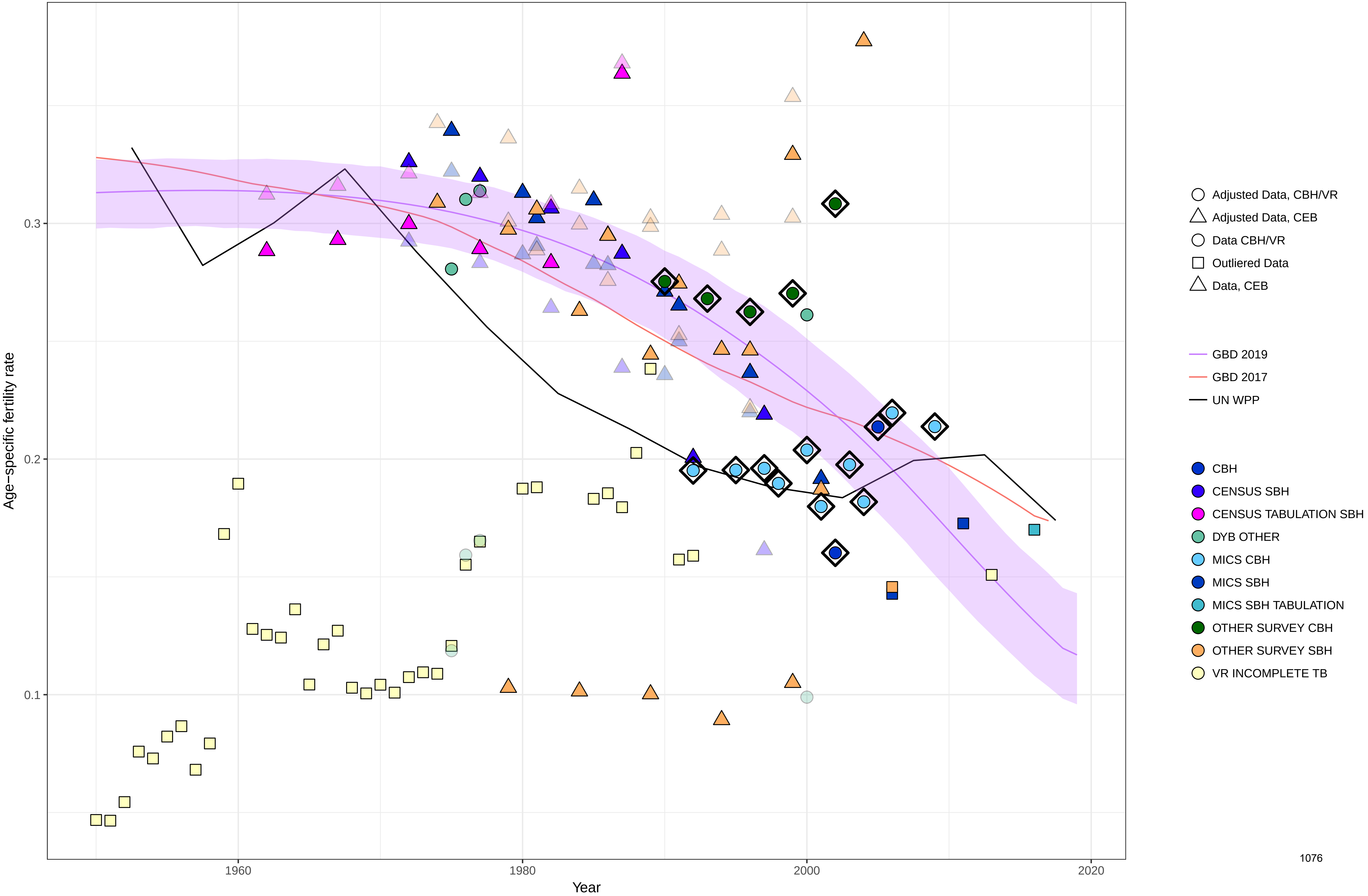

Age−specific fertility fitted trend lines, 1950−2019: Iraq, Ages: 25−29
Data points enclosed with a diamond are reference sources

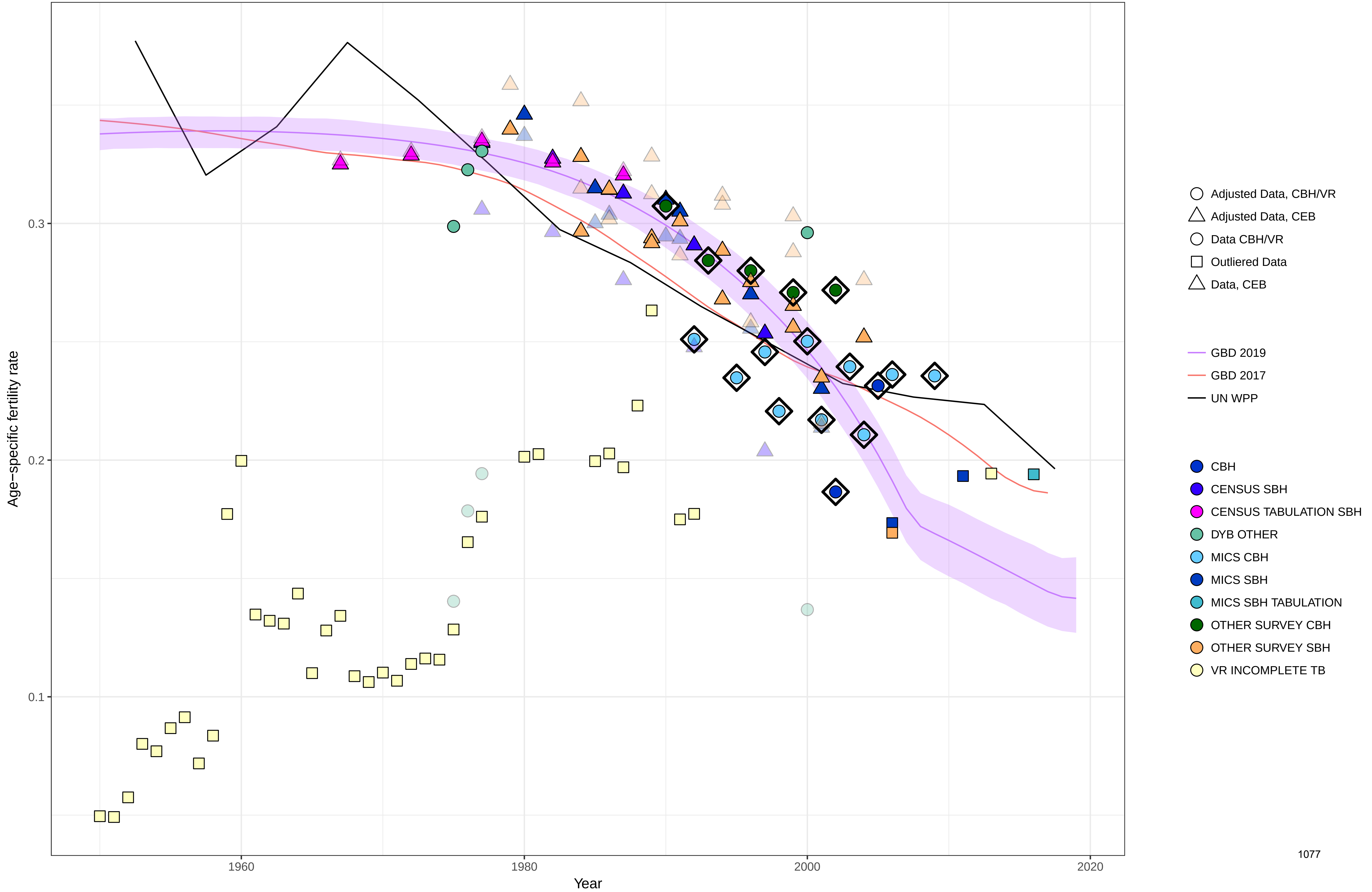

Age−specific fertility fitted trend lines, 1950−2019: Iraq, Ages: 30−34
Data points enclosed with a diamond are reference sources

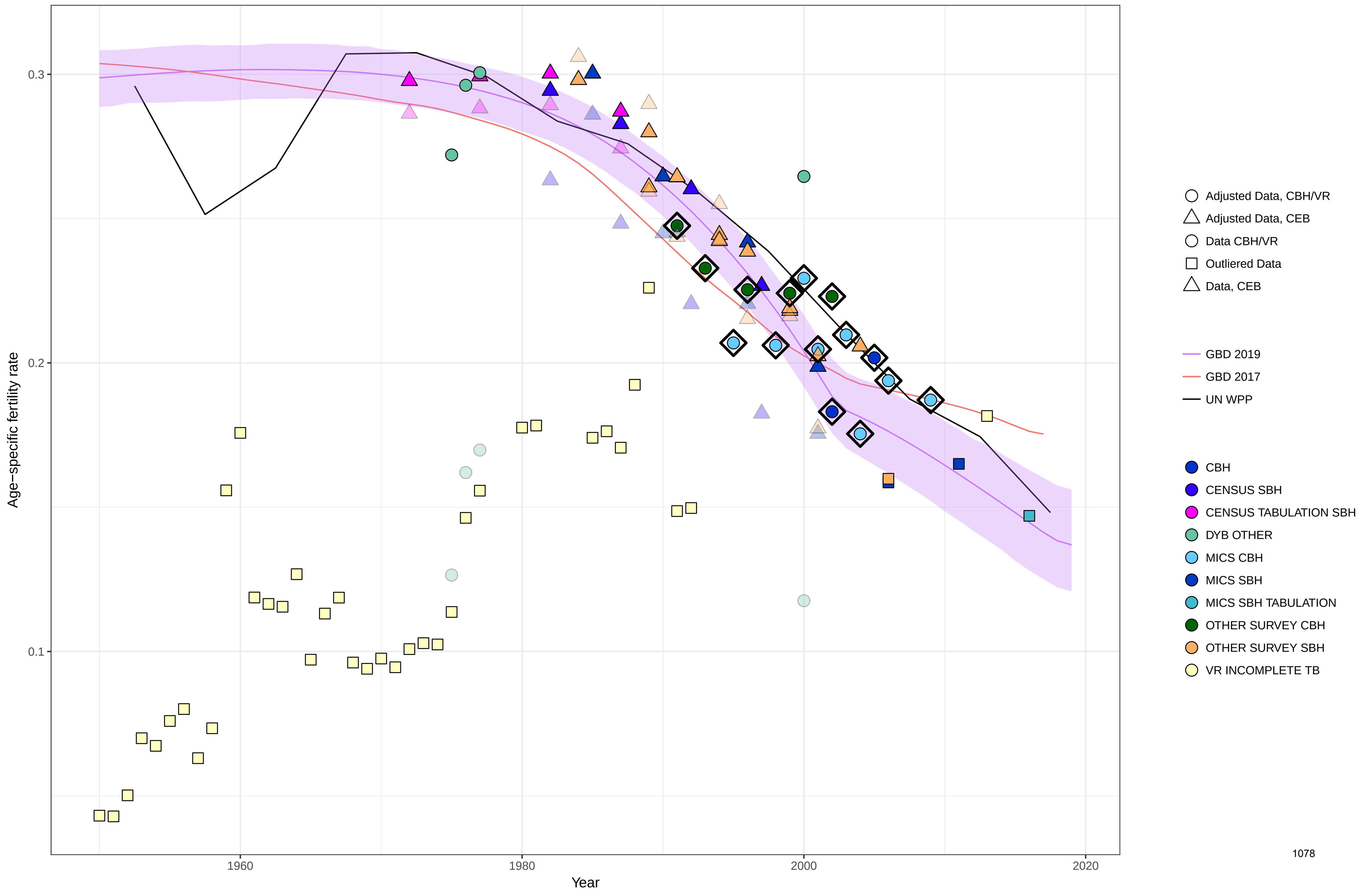

Age−specific fertility fitted trend lines, 1950−2019: Iraq, Ages: 35−39
Data points enclosed with a diamond are reference sources

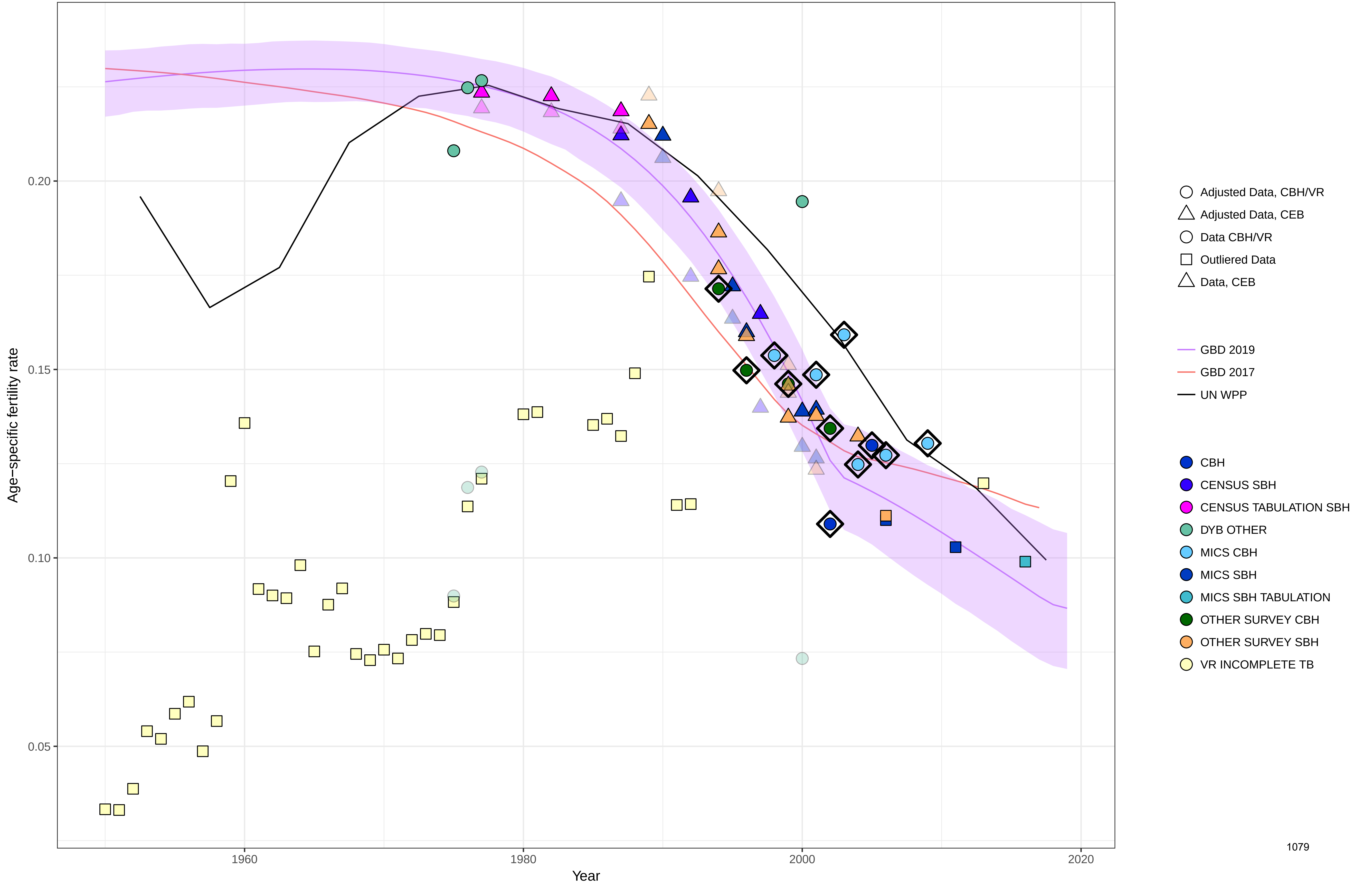

Age−specific fertility fitted trend lines, 1950−2019: Iraq, Ages: 40−44
Data points enclosed with a diamond are reference sources

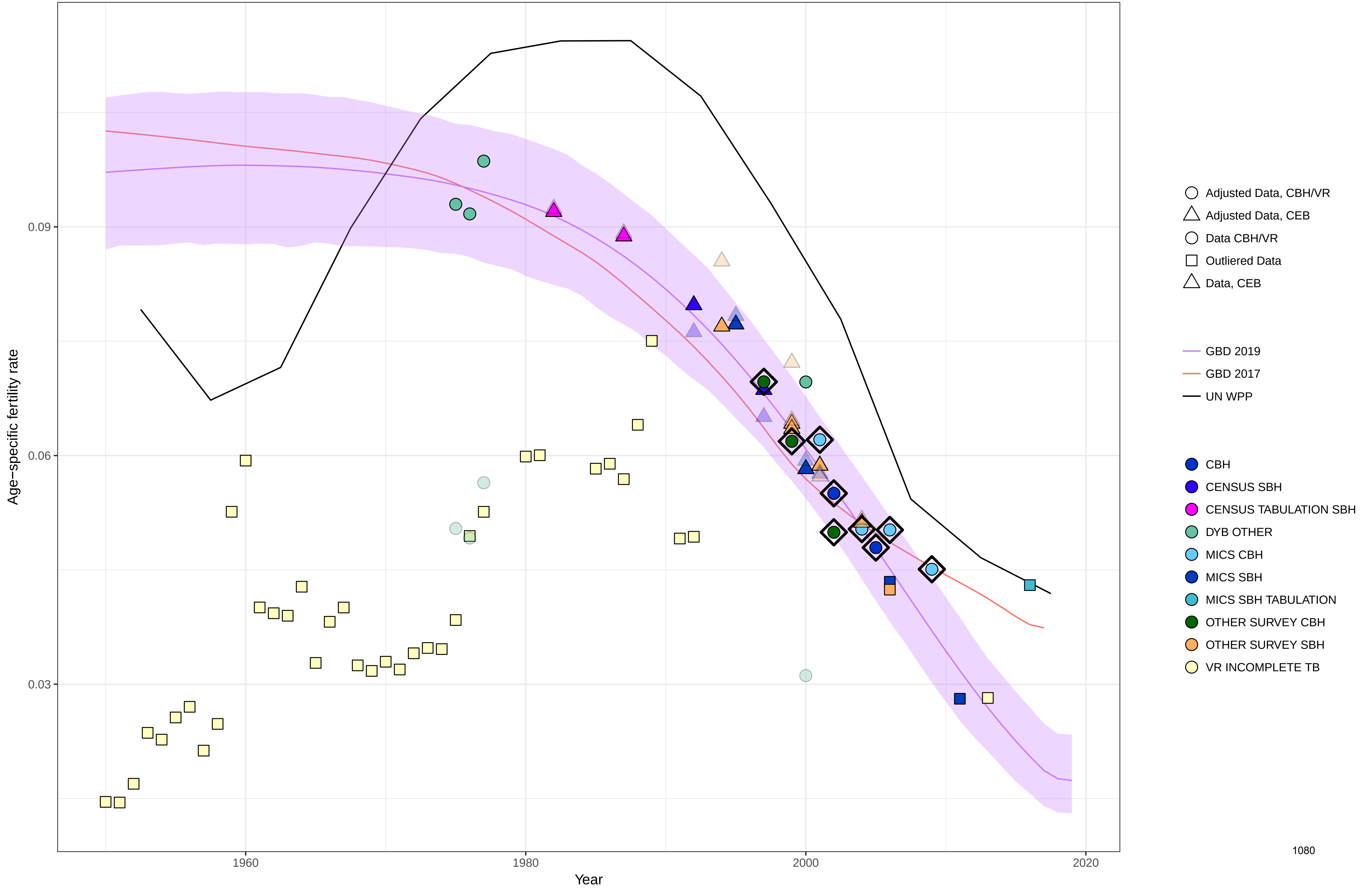

Age−specific fertility fitted trend lines, 1950−2019: Iraq, Ages: 45−49
Data points enclosed with a diamond are reference sources

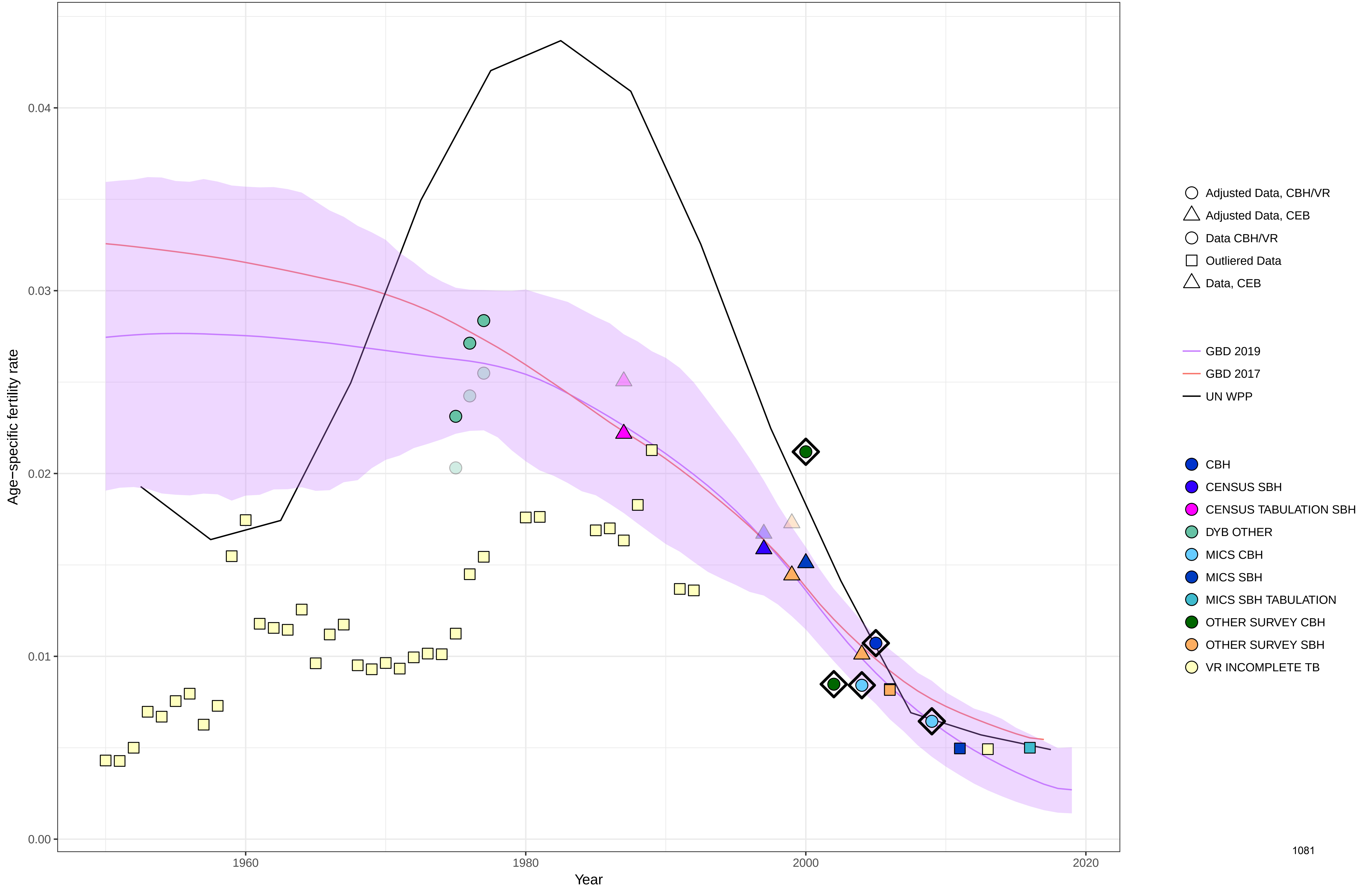

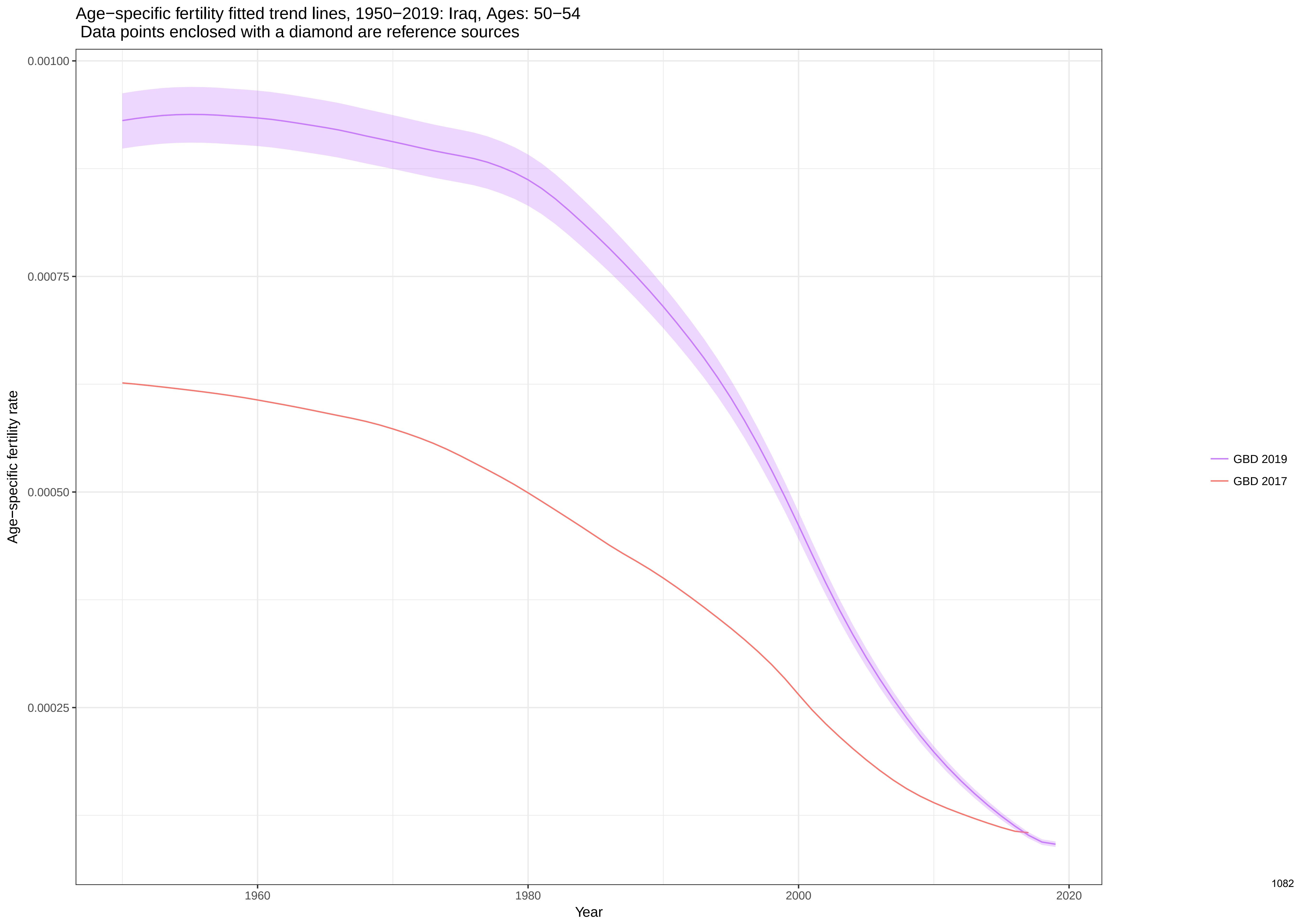
Age−specific fertility fitted trend lines, 1950−2019: Iraq, Ages: 50−54
Data points enclosed with a diamond are reference sources
0.00100
0.00075
0.00050
0.00025
Age−specific fertility rate
1960
1980
2000
2020
Year
GBD 2019
GBD 2017

Total fertility rate fitted trend lines, 1950−2019: Iraq TFR
Data points enclosed with a diamond are reference sources

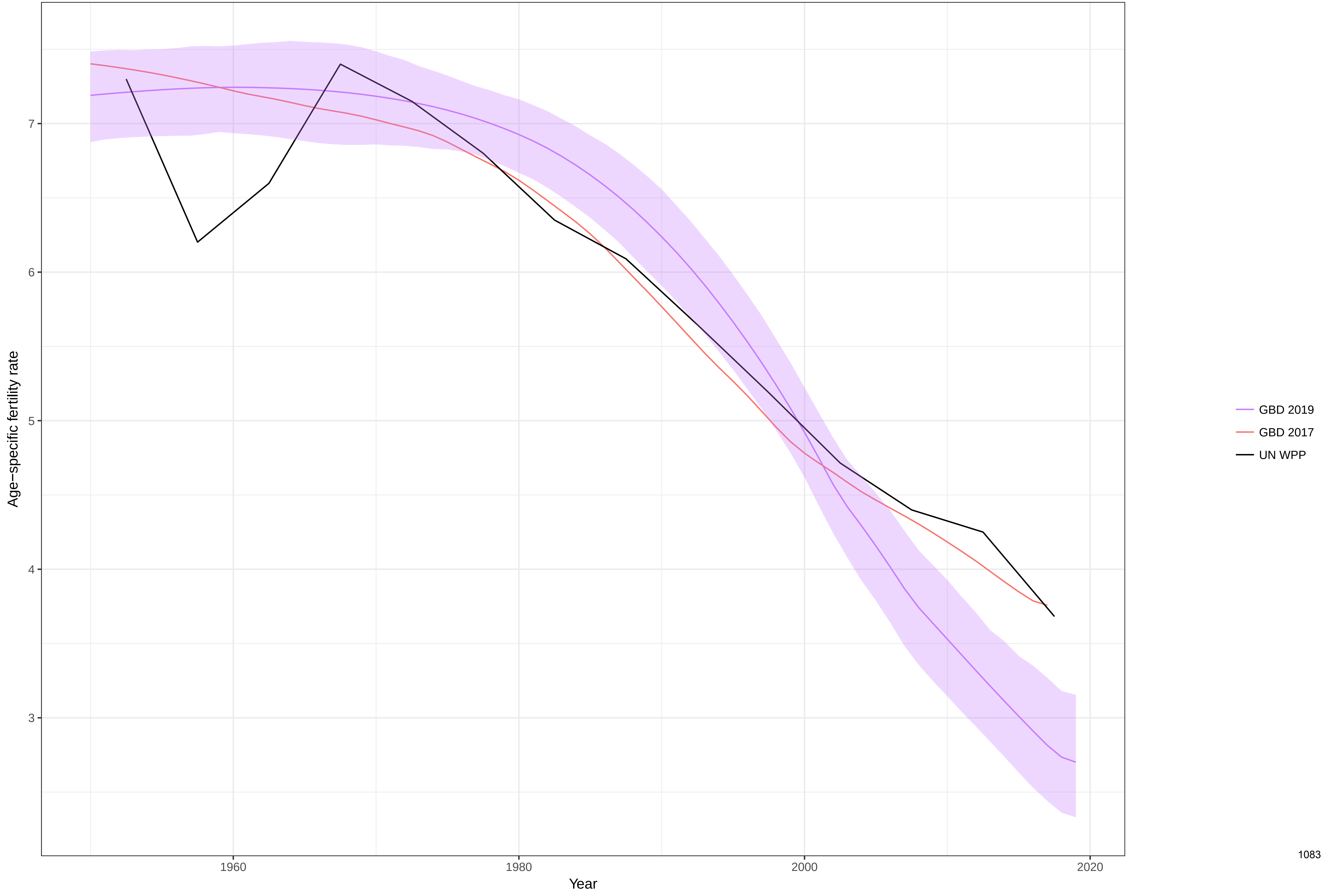

Age−specific fertility fitted trend lines, 1950−2019: Iceland, Ages: 10−14
Data points enclosed with a diamond are reference sources

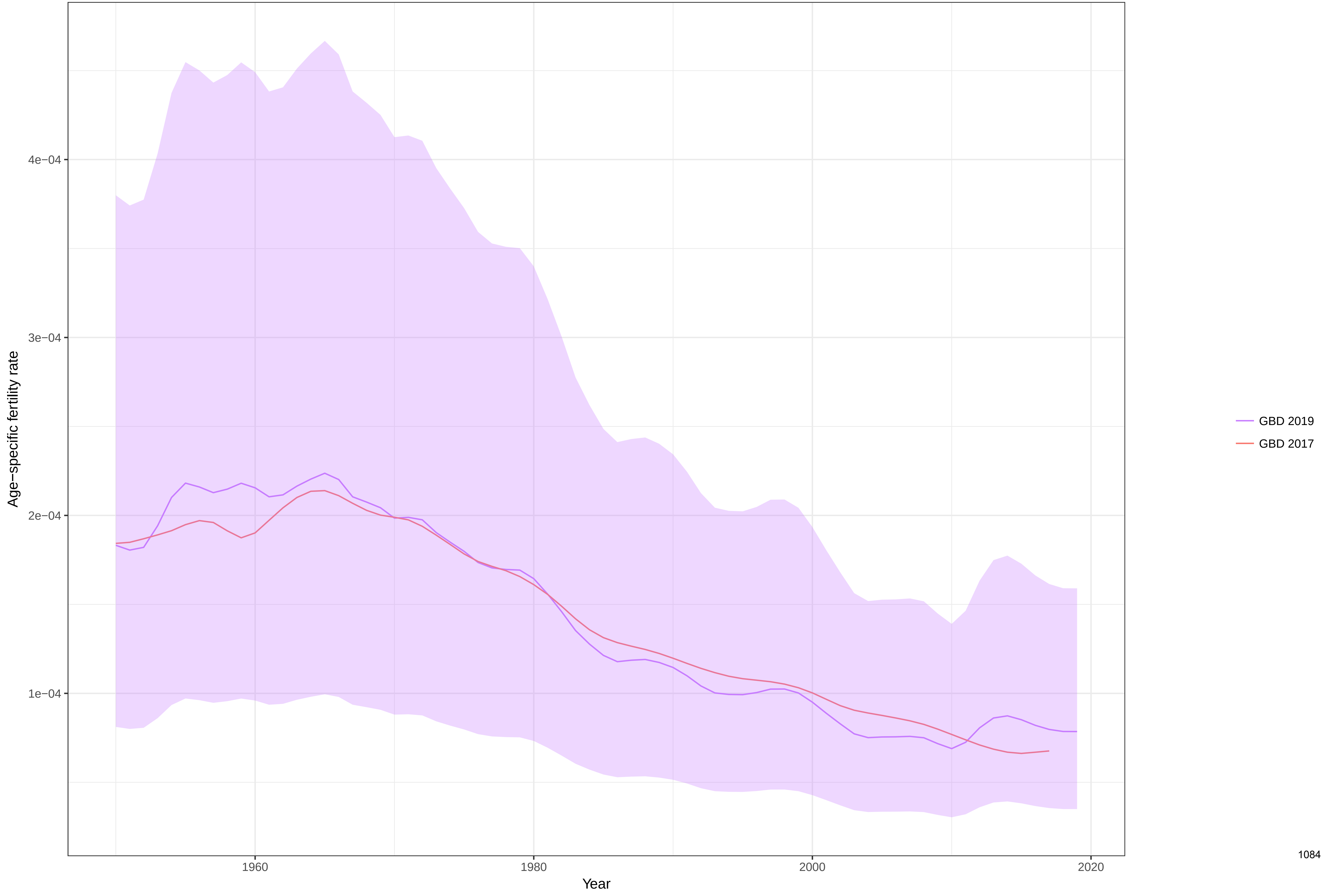

Age−specific fertility fitted trend lines, 1950−2019: Iceland, Ages: 15−19
Data points enclosed with a diamond are reference sources

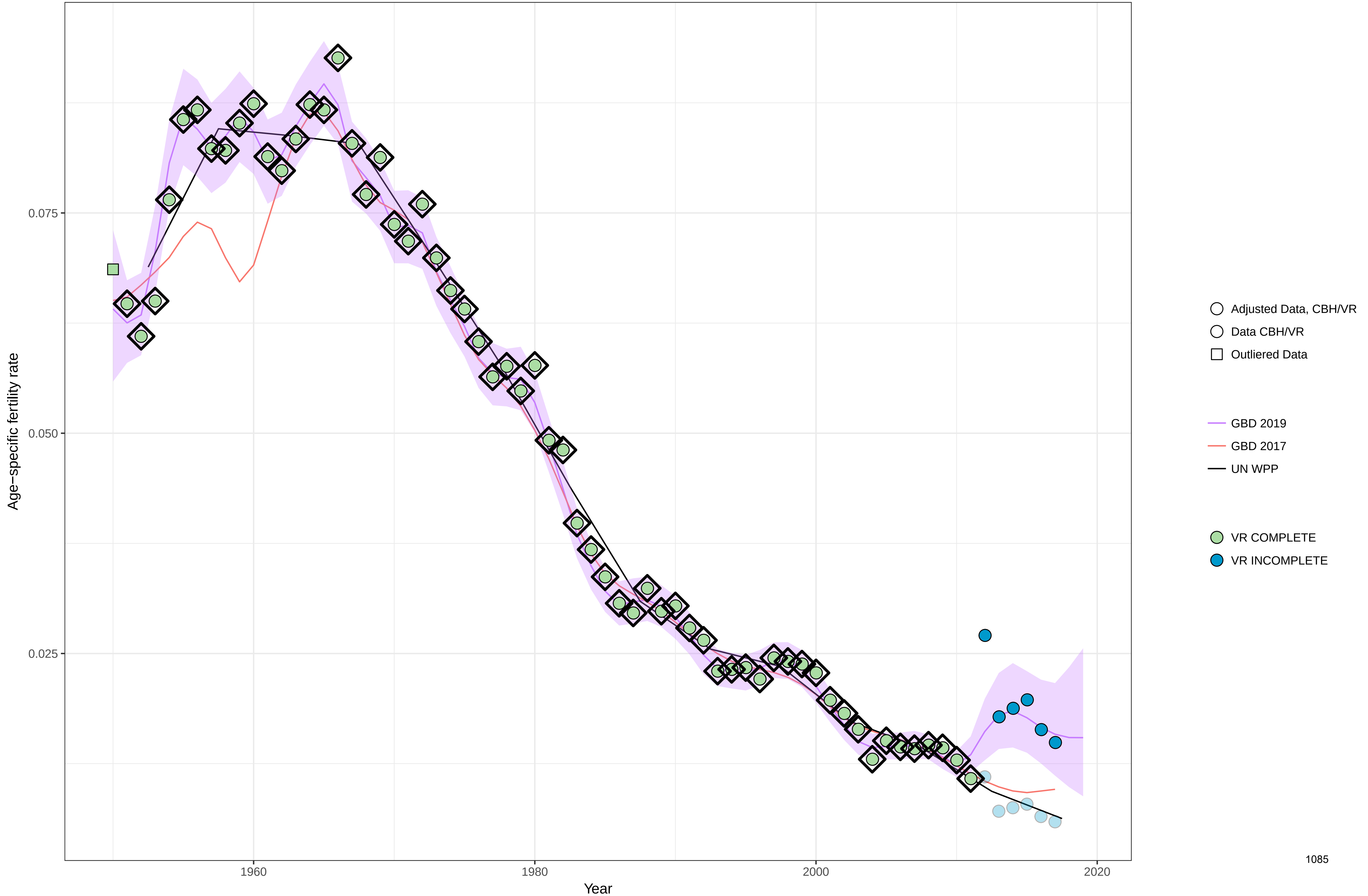

Age−specific fertility fitted trend lines, 1950−2019: Iceland, Ages: 20−24
Data points enclosed with a diamond are reference sources

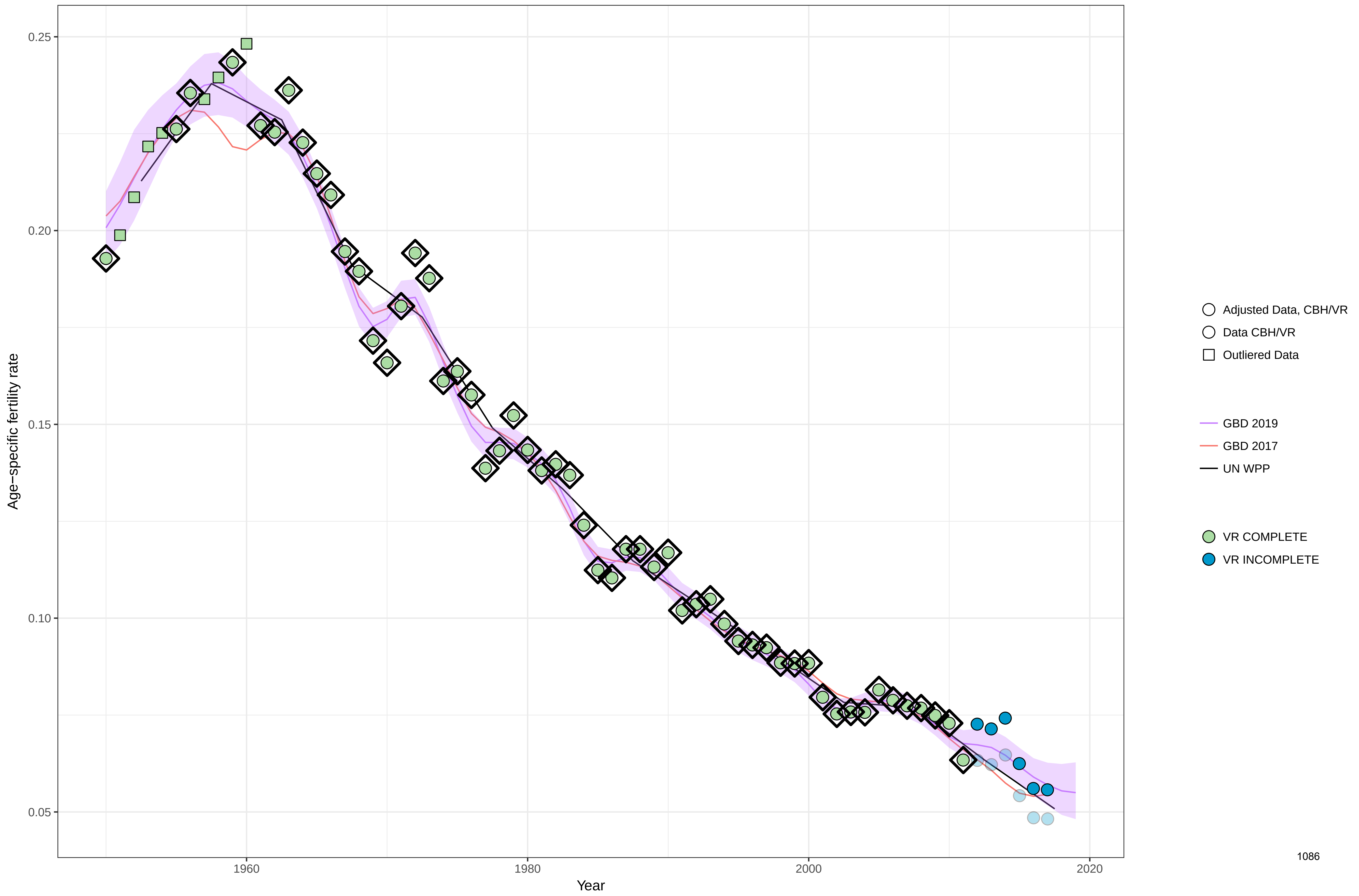

Age−specific fertility fitted trend lines, 1950−2019: Iceland, Ages: 25−29
Data points enclosed with a diamond are reference sources

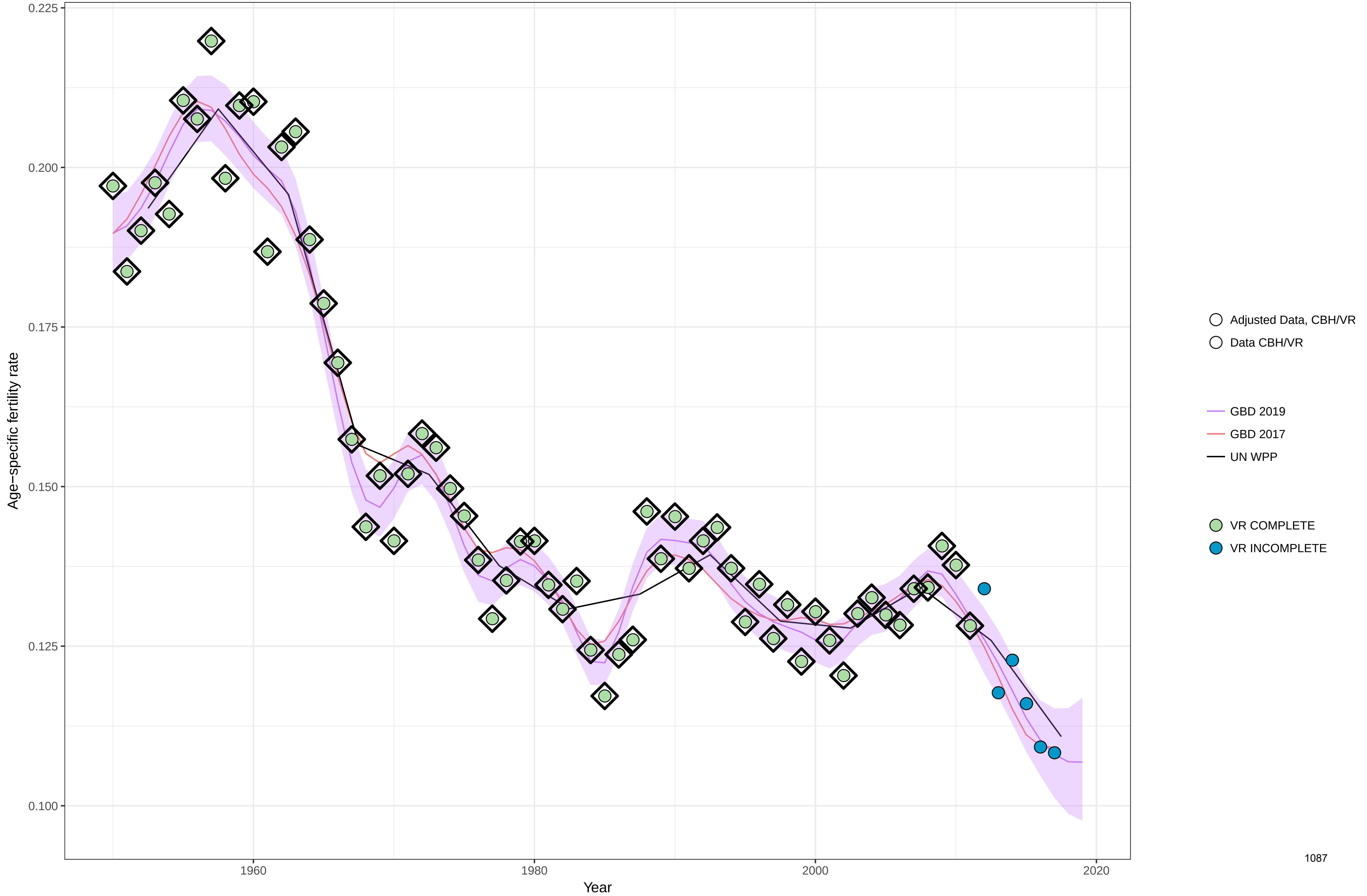

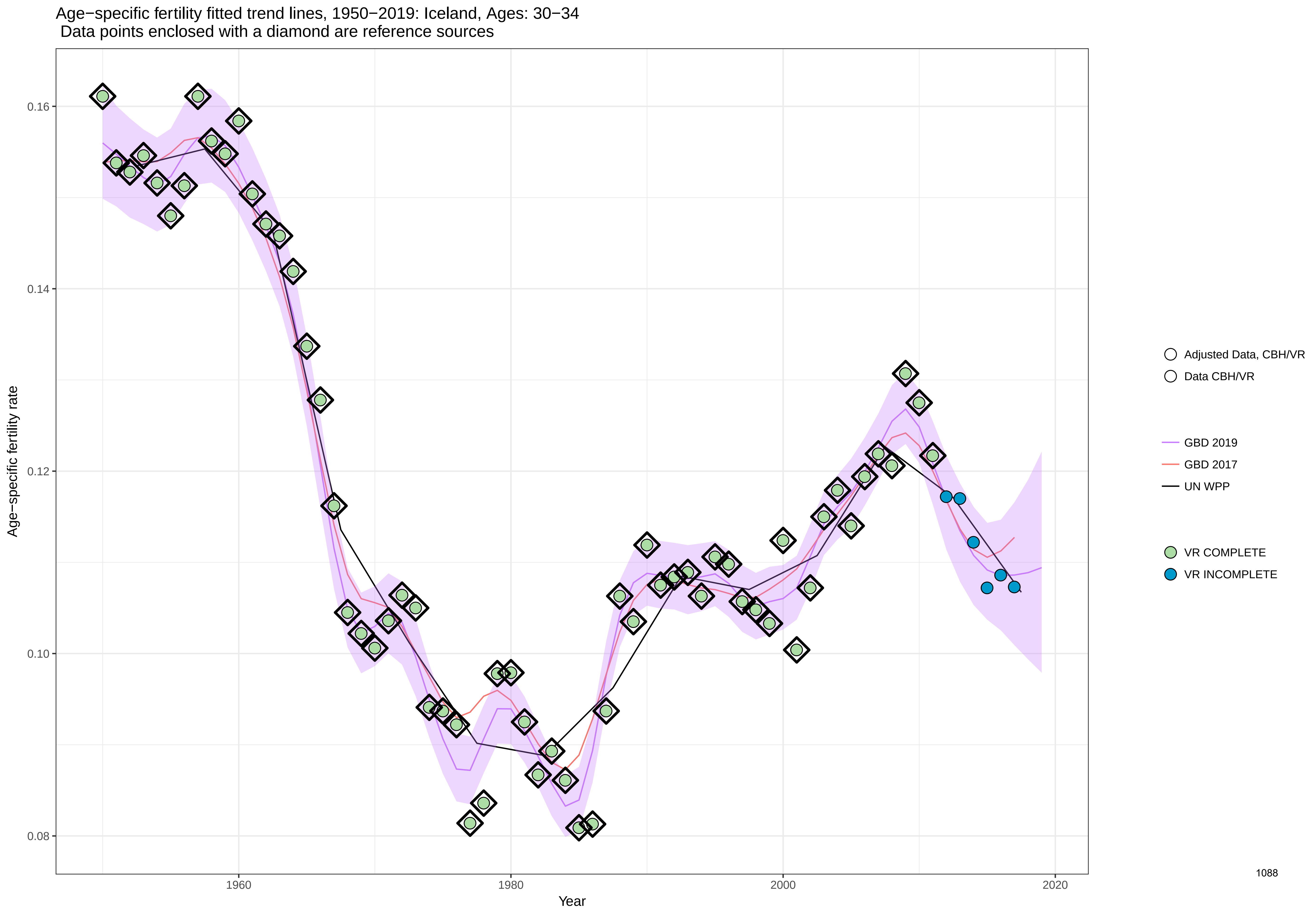

Age−specific fertility fitted trend lines, 1950−2019: Iceland, Ages: 30−34
Data points enclosed with a diamond are reference sources
Age−specific fertility rate
Year
0.16
0.14
0.12
0.10
0.08
1960
1980
2000
2020
Adjusted Data, CBH/VR
Data CBH/VR
GBD 2019
GBD 2017
UN WPP
VR COMPLETE
VR INCOMPLETE

Age−specific fertility fitted trend lines, 1950−2019: Iceland, Ages: 35−39
Data points enclosed with a diamond are reference sources

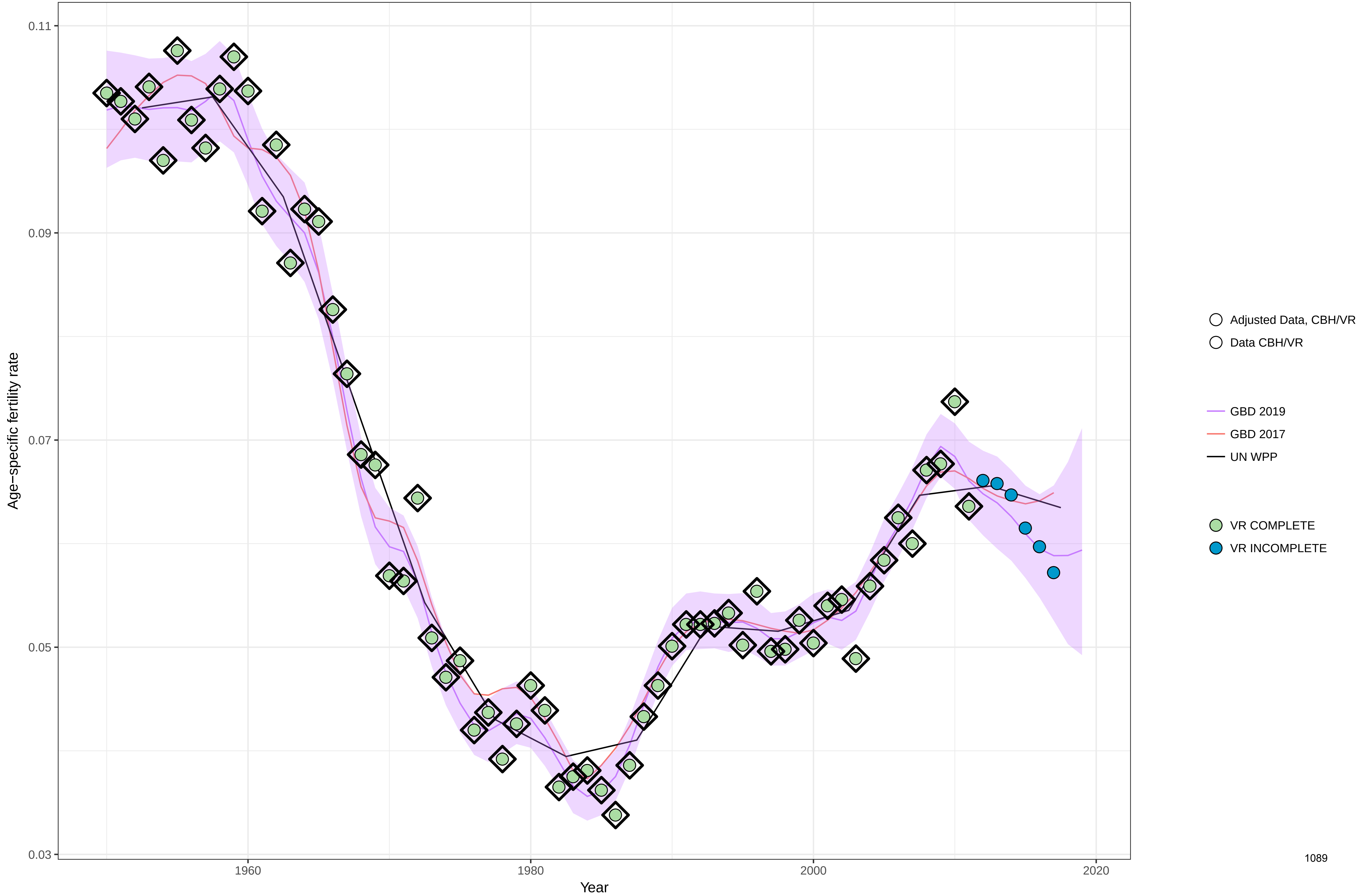

Age−specific fertility fitted trend lines, 1950−2019: Iceland, Ages: 40−44
Data points enclosed with a diamond are reference sources

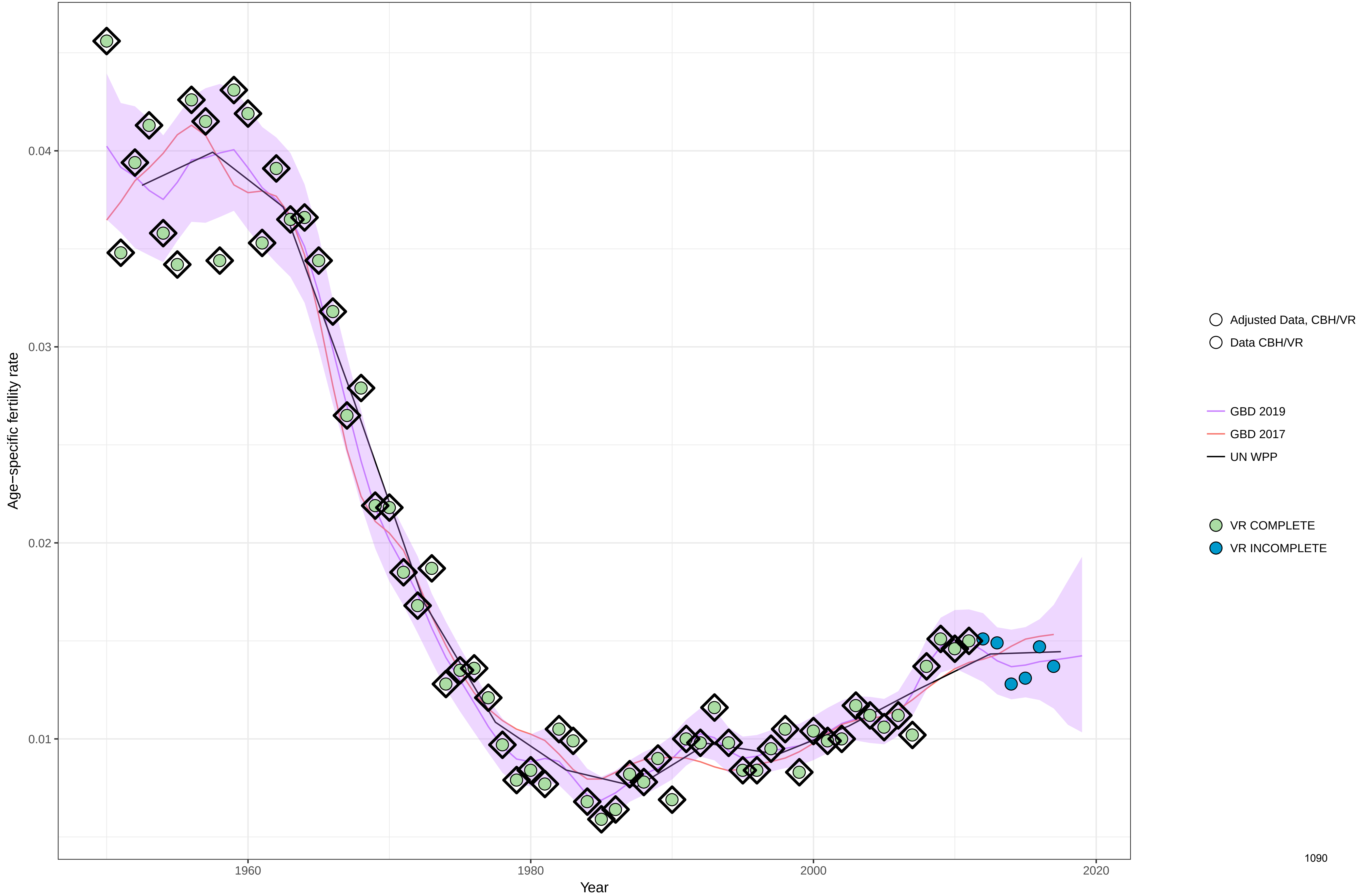

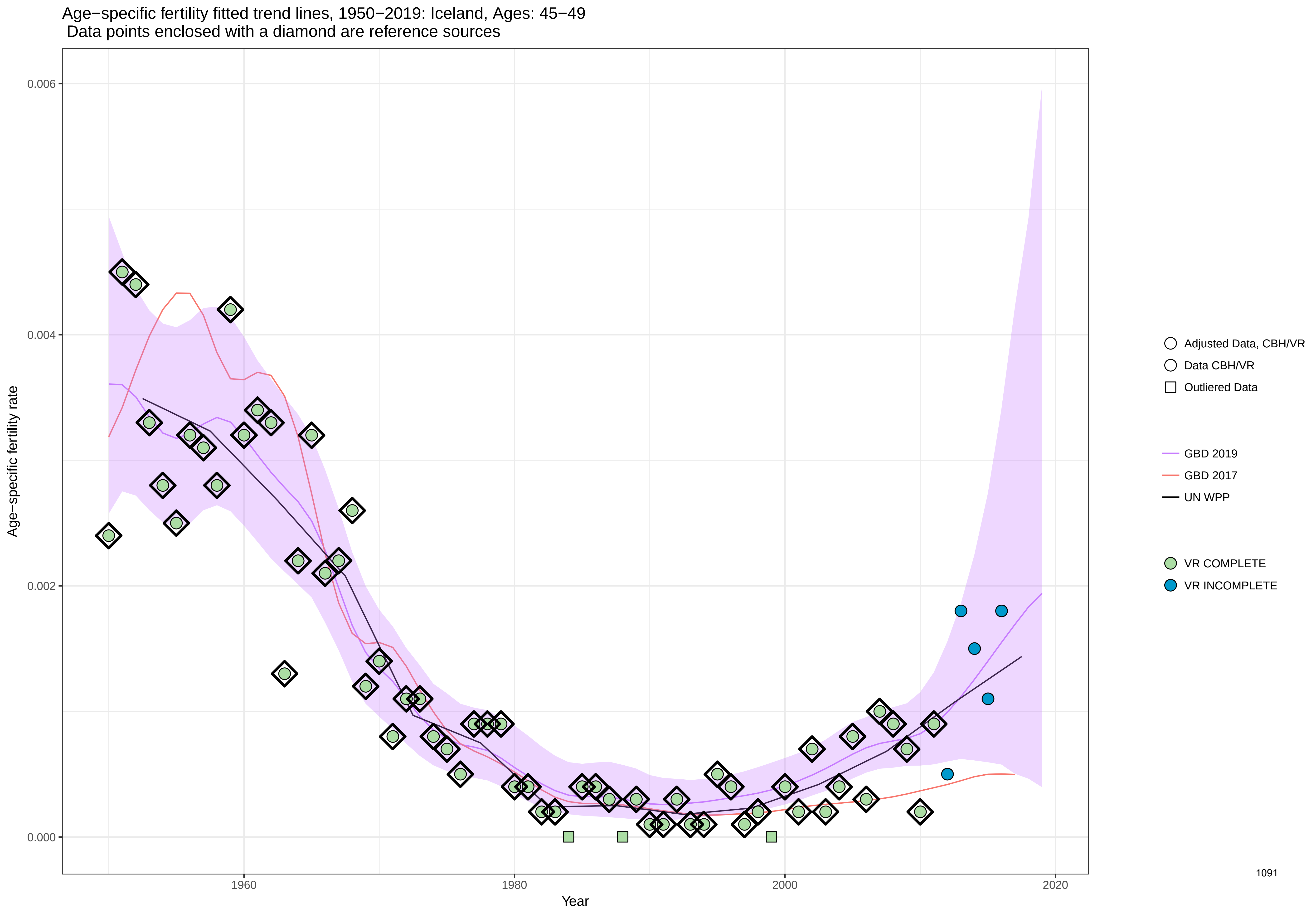
Age−specific fertility fitted trend lines, 1950−2019: Iceland, Ages: 45−49
Data points enclosed with a diamond are reference sources
Age−specific fertility rate
0.006
0.004
0.002
0.000
1960
1980
2000
2020
Year
Adjusted Data, CBH/VR
Data CBH/VR
Outliered Data
GBD 2019
GBD 2017
UN WPP
VR COMPLETE
VR INCOMPLETE

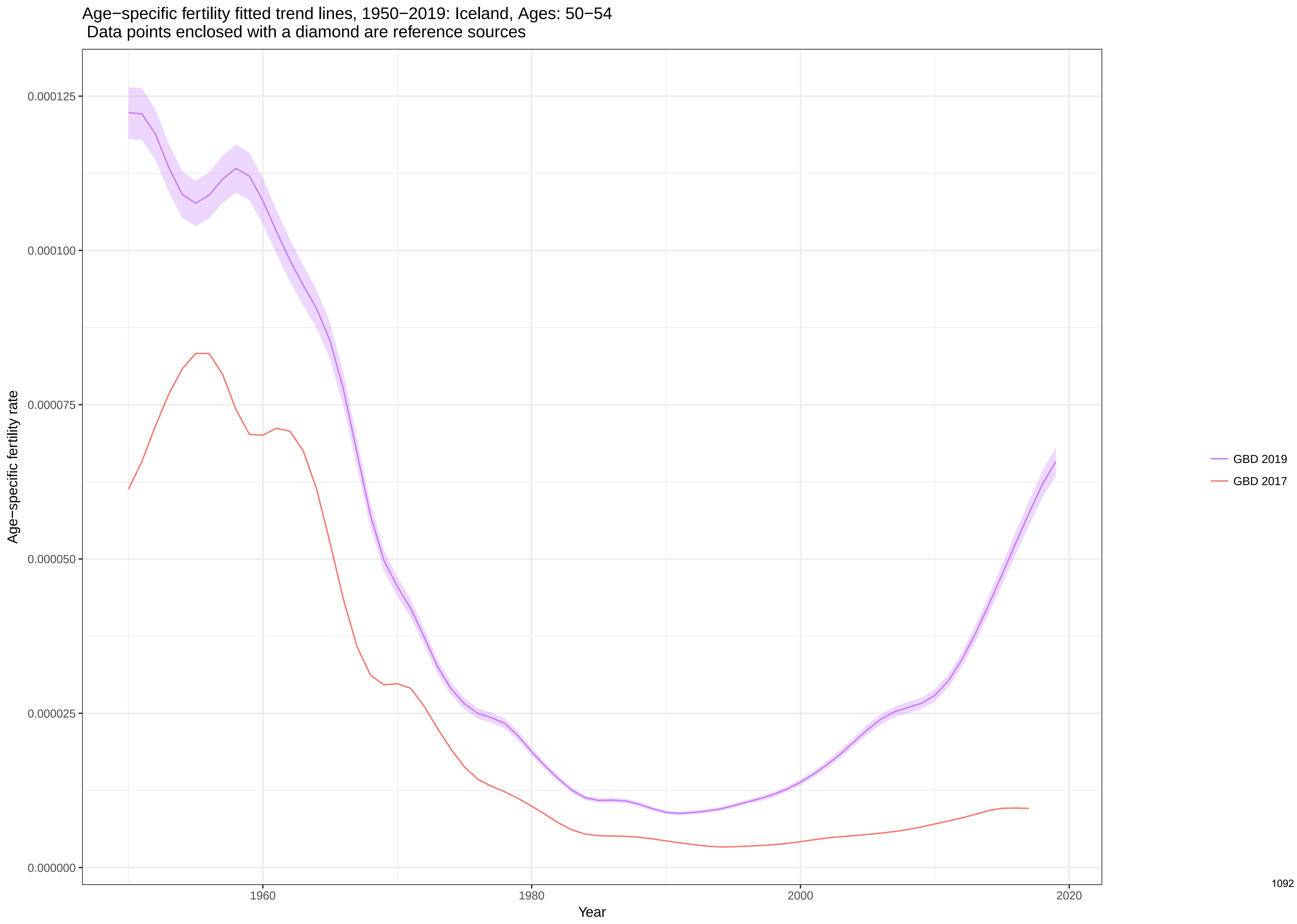
Age−specific fertility fitted trend lines, 1950−2019: Iceland, Ages: 50−54
Data points enclosed with a diamond are reference sources
0.000125
0.000100
0.000075
0.000050
0.000025
0.000000
Age−specific fertility rate
1960
1980
2000
2020
Year
GBD 2019
GBD 2017

Total fertility rate fitted trend lines, 1950−2019: Iceland TFR
Data points enclosed with a diamond are reference sources

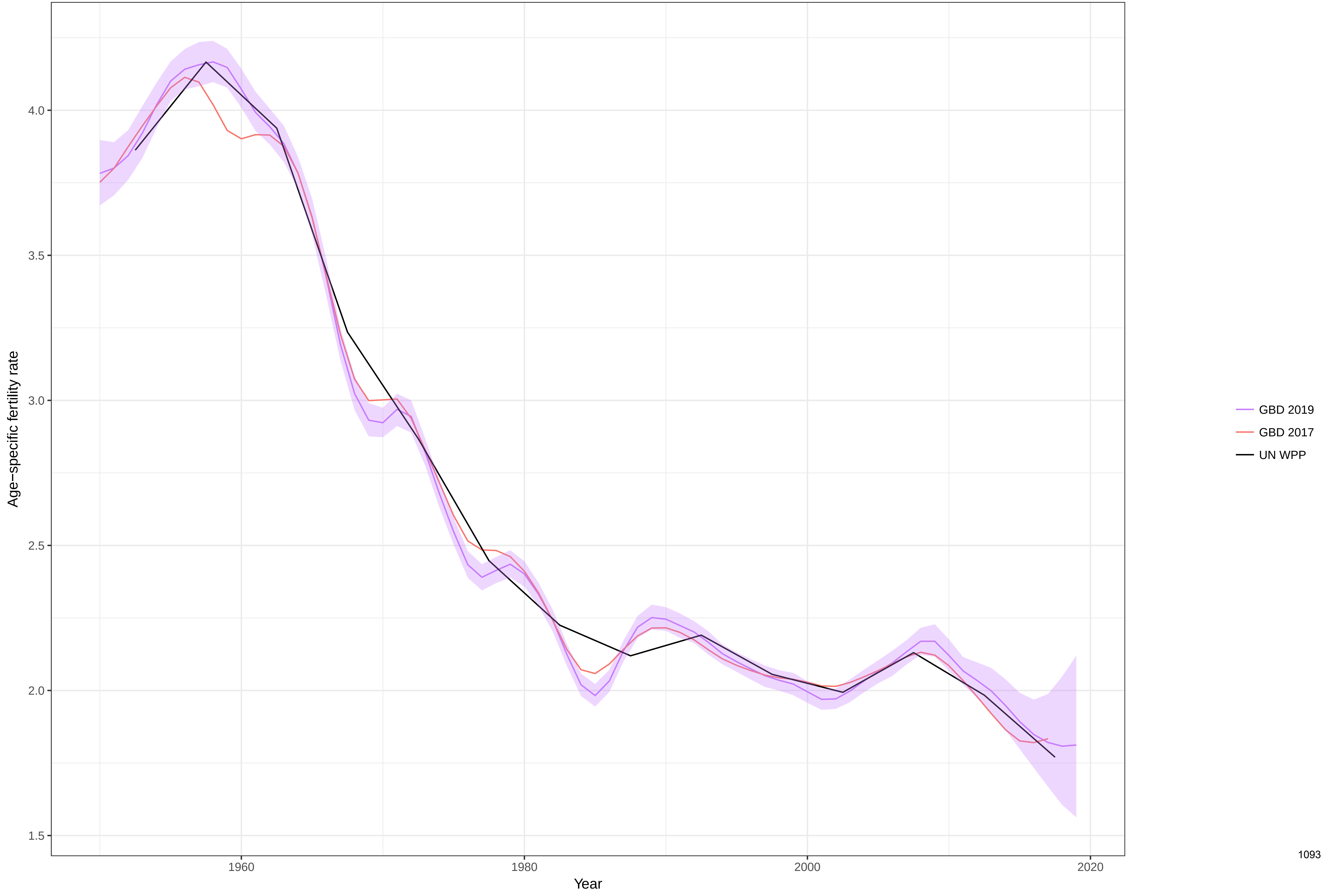

Age−specific fertility fitted trend lines, 1950−2019: Israel, Ages: 10−14
Data points enclosed with a diamond are reference sources

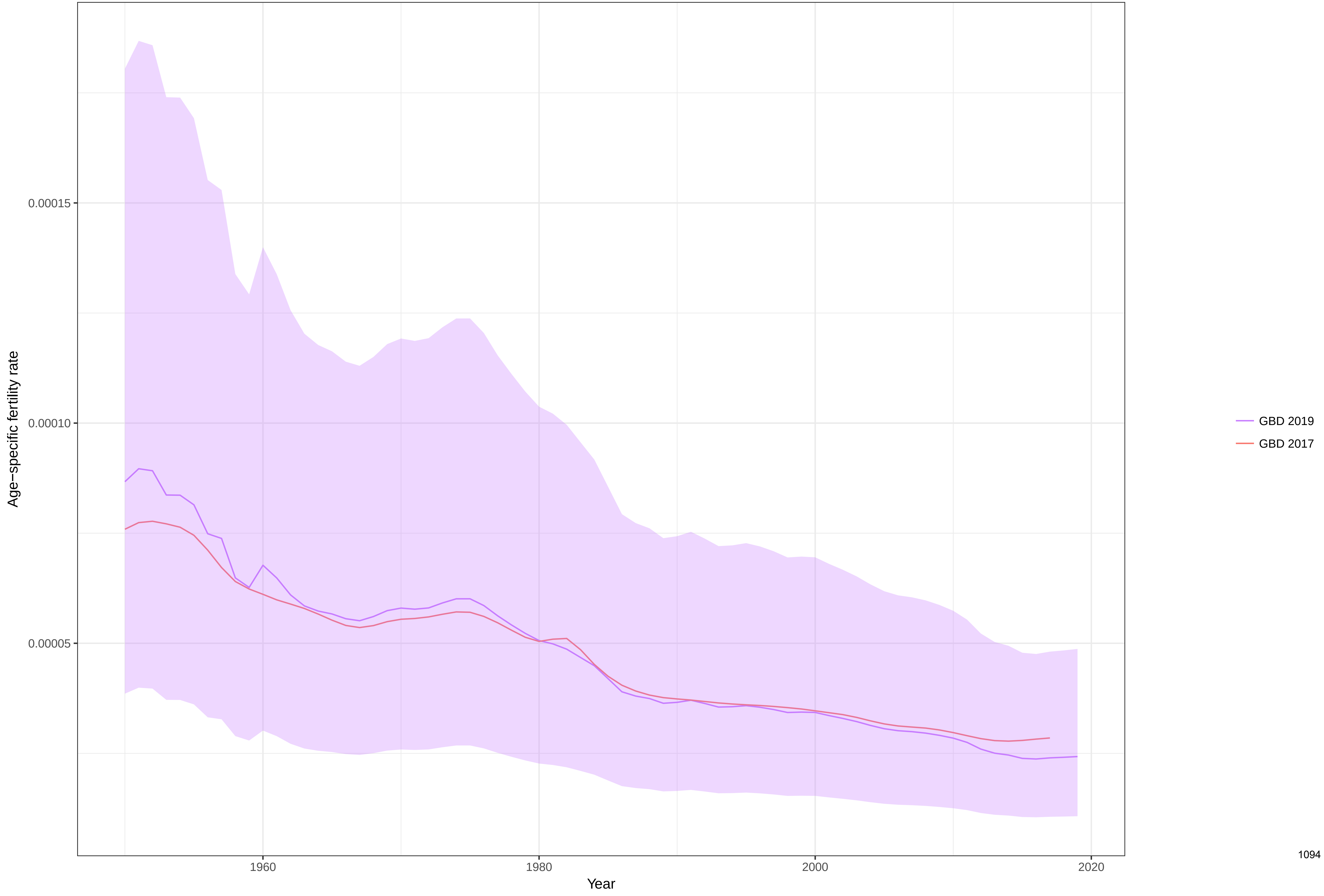

Age−specific fertility fitted trend lines, 1950−2019: Israel, Ages: 15−19
Data points enclosed with a diamond are reference sources

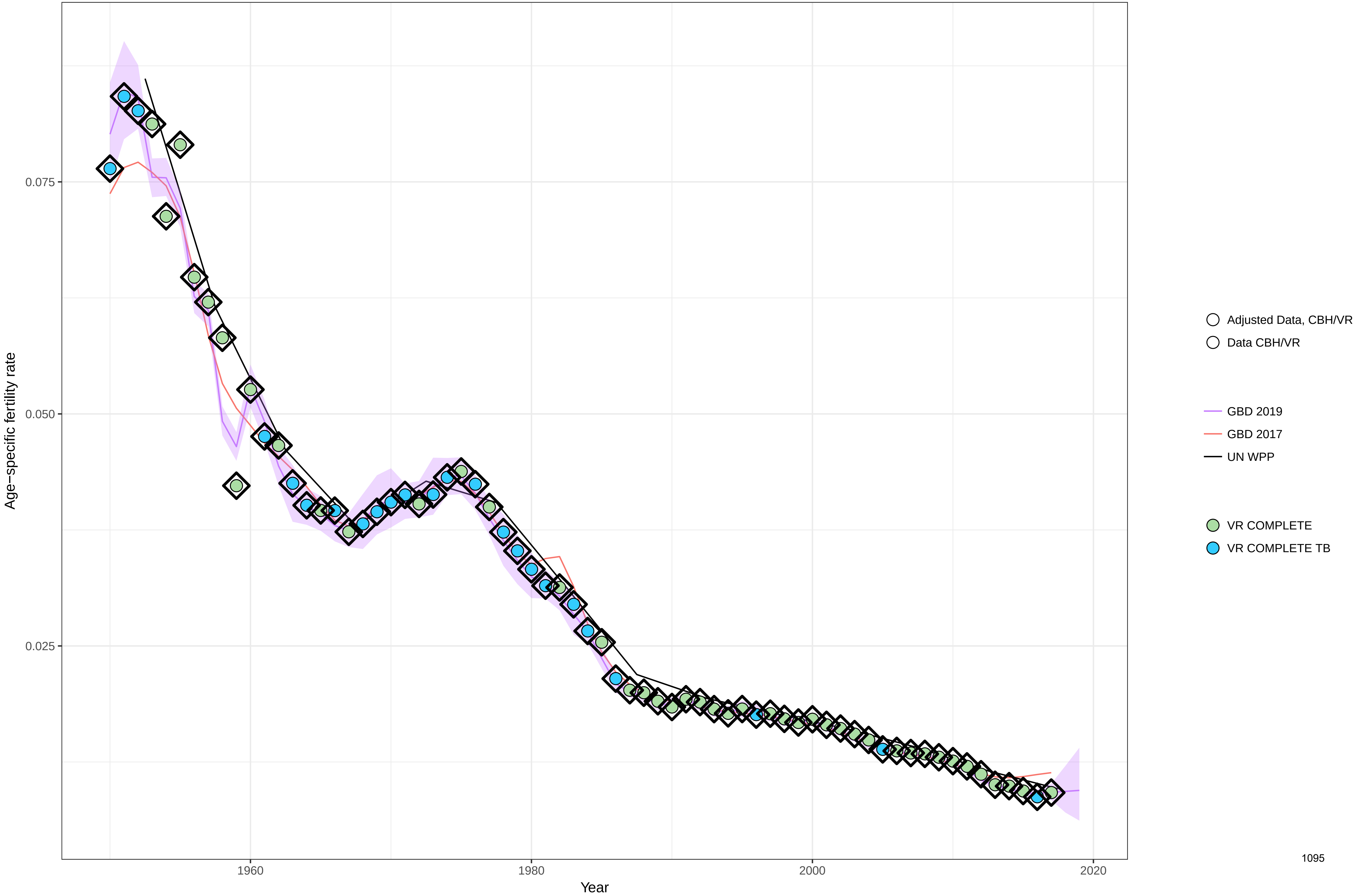

Age−specific fertility fitted trend lines, 1950−2019: Israel, Ages: 20−24
Data points enclosed with a diamond are reference sources

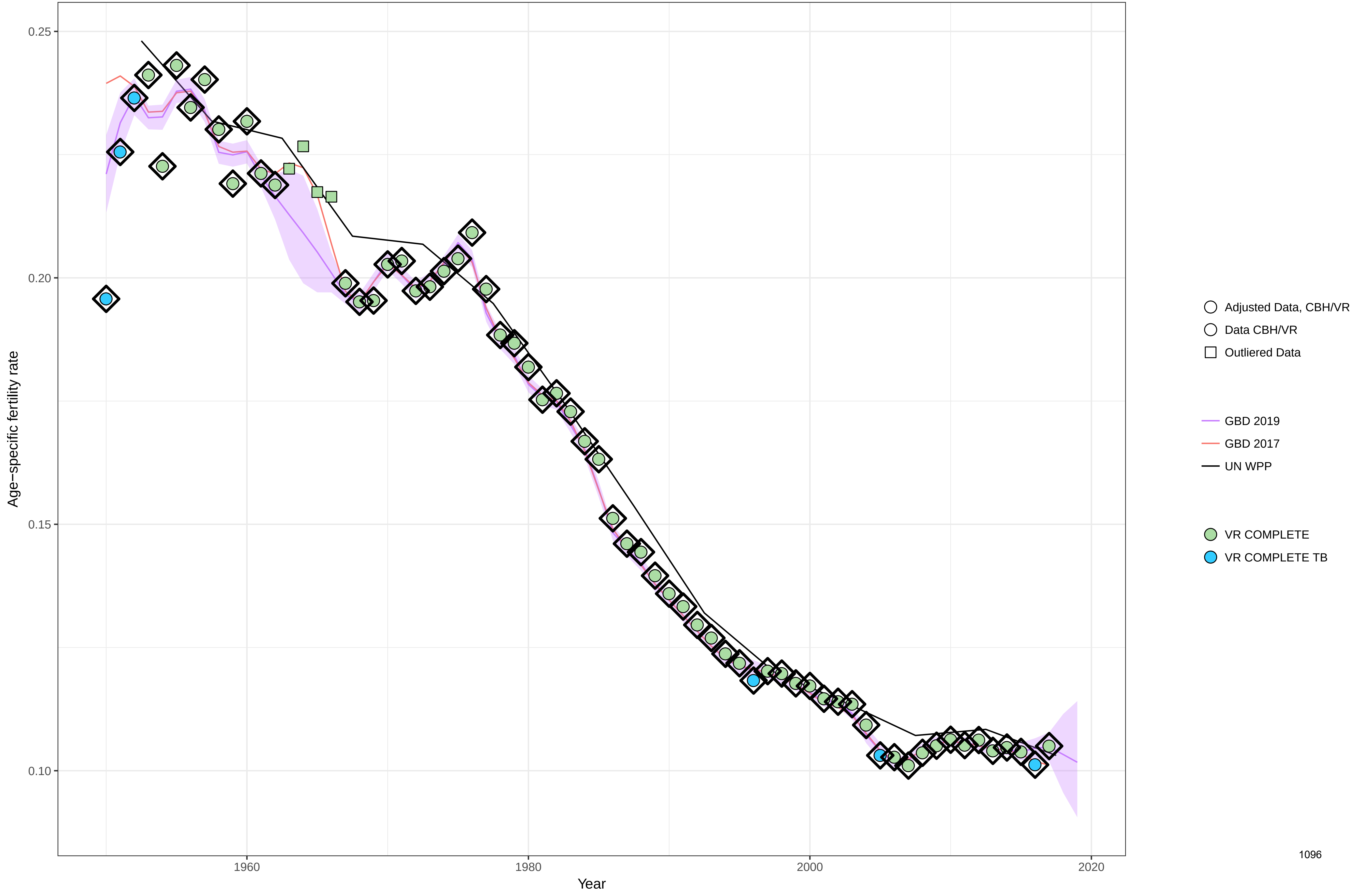

Age−specific fertility fitted trend lines, 1950−2019: Israel, Ages: 25−29
Data points enclosed with a diamond are reference sources

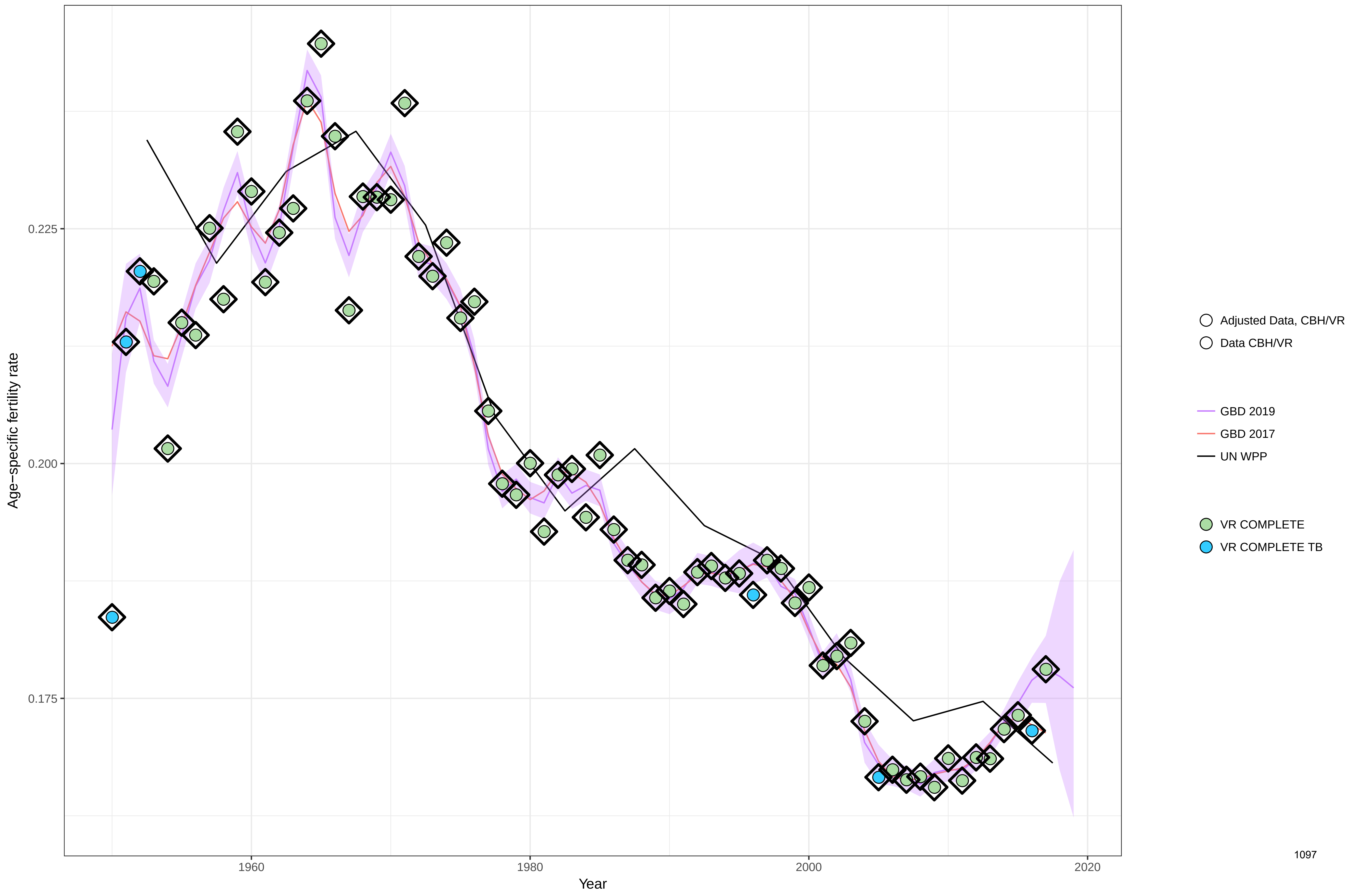

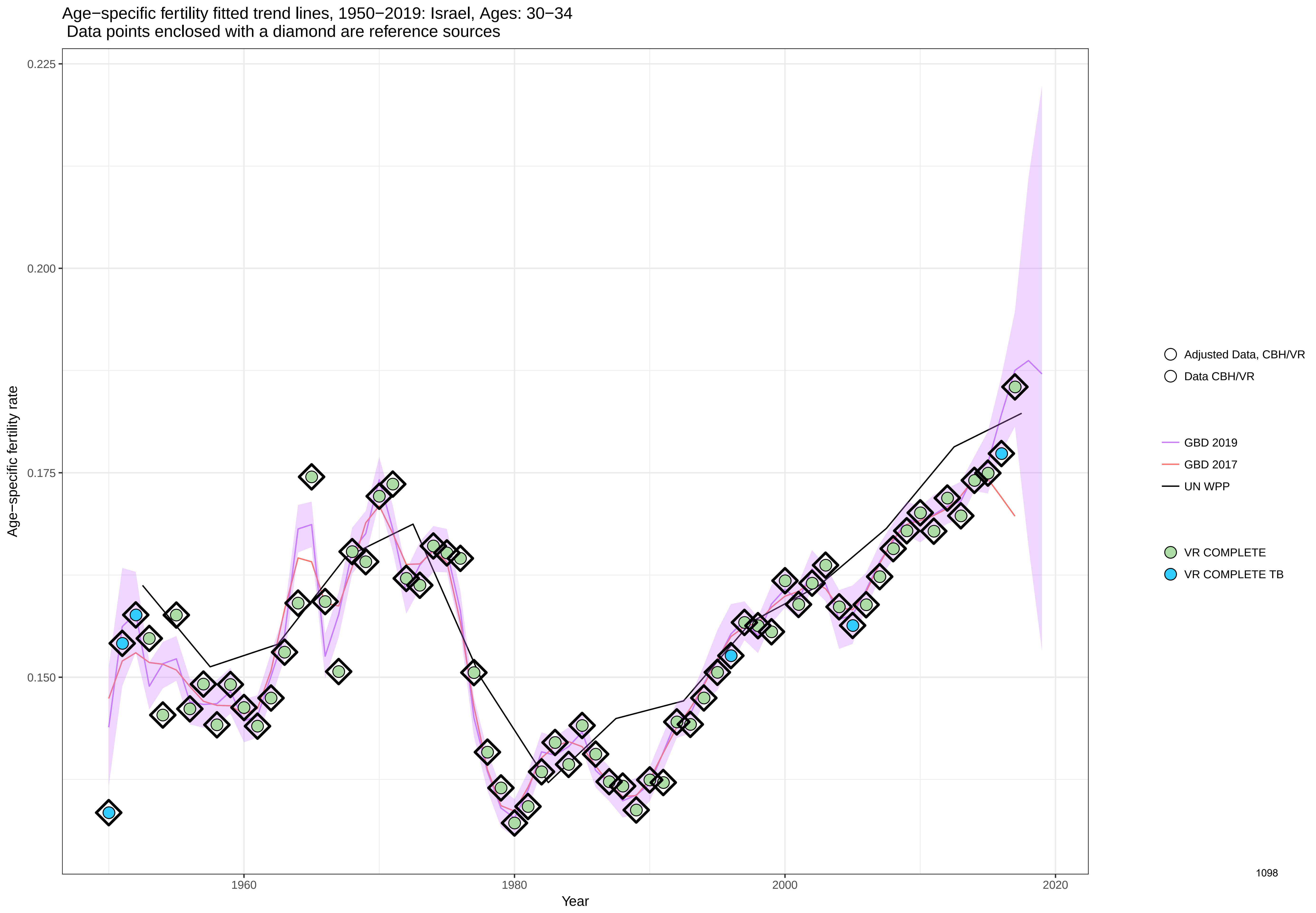
Age−specific fertility fitted trend lines, 1950−2019: Israel, Ages: 30−34
Data points enclosed with a diamond are reference sources
Age−specific fertility rate
0.225
0.200
0.175
0.150
1960
1980
2000
2020
Year
Adjusted Data, CBH/VR
Data CBH/VR
GBD 2019
GBD 2017
UN WPP
VR COMPLETE
VR COMPLETE TB

Age−specific fertility fitted trend lines, 1950−2019: Israel, Ages: 35−39
Data points enclosed with a diamond are reference sources

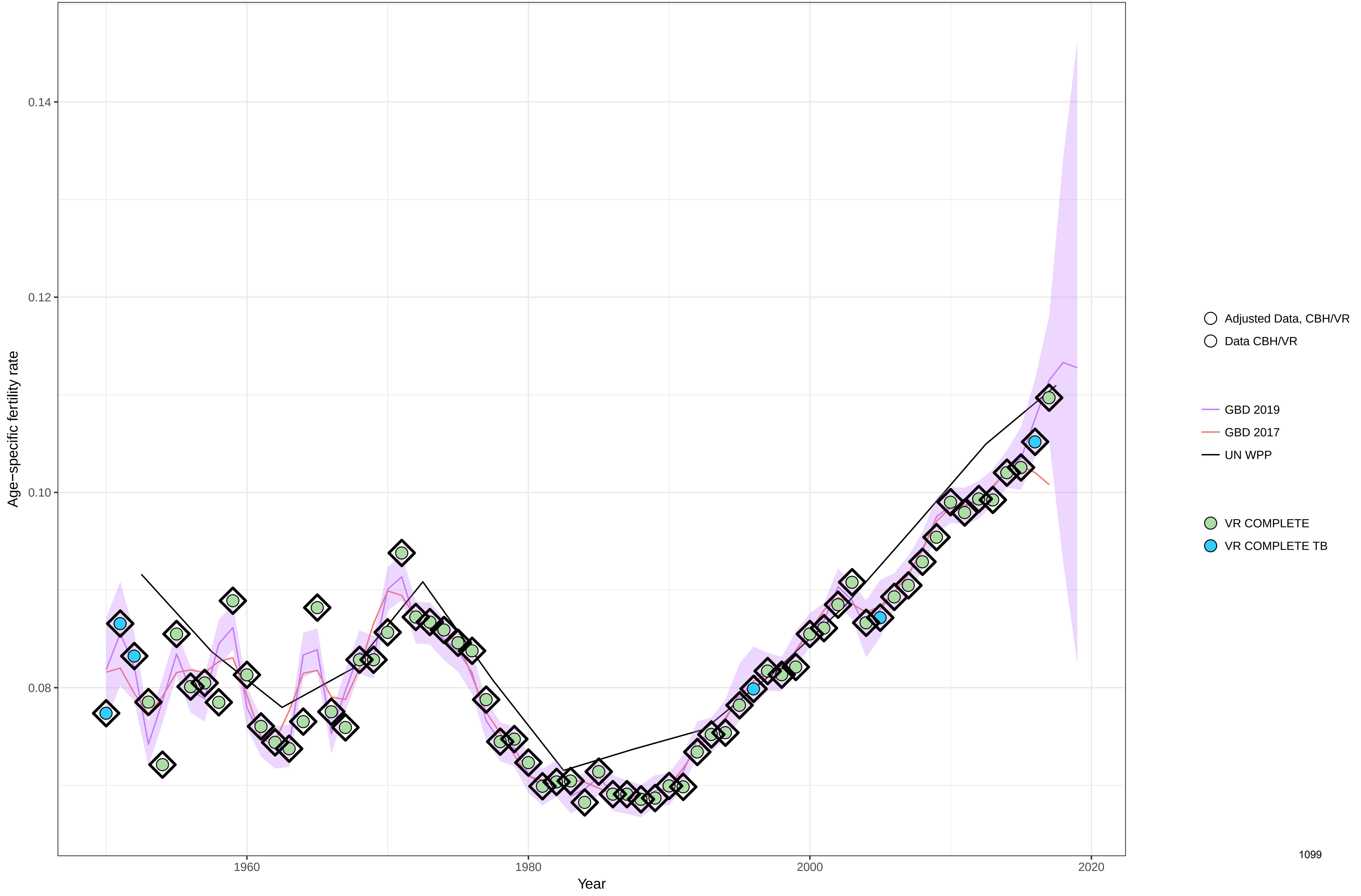

Age−specific fertility fitted trend lines, 1950−2019: Israel, Ages: 40−44
Data points enclosed with a diamond are reference sources

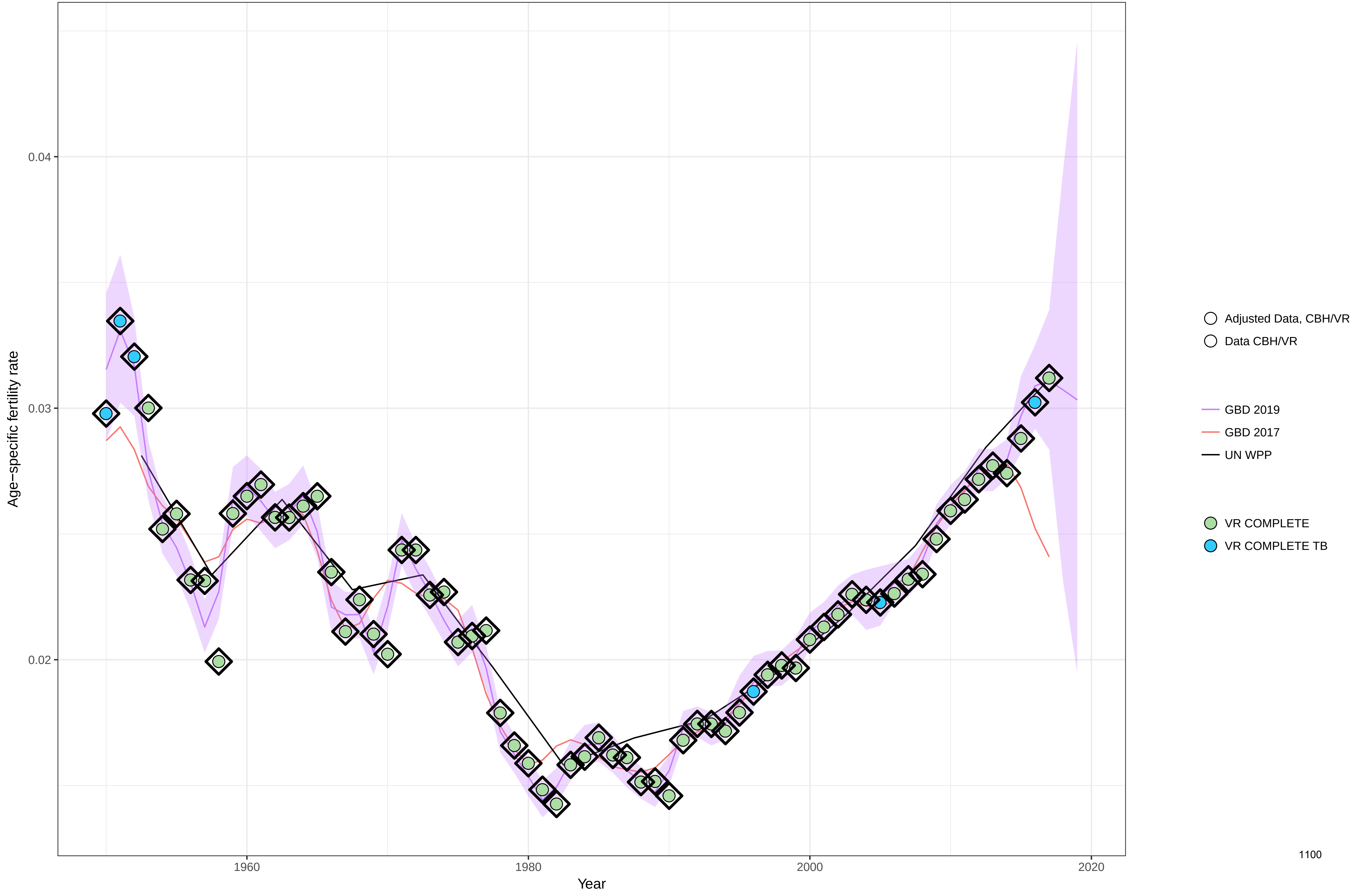

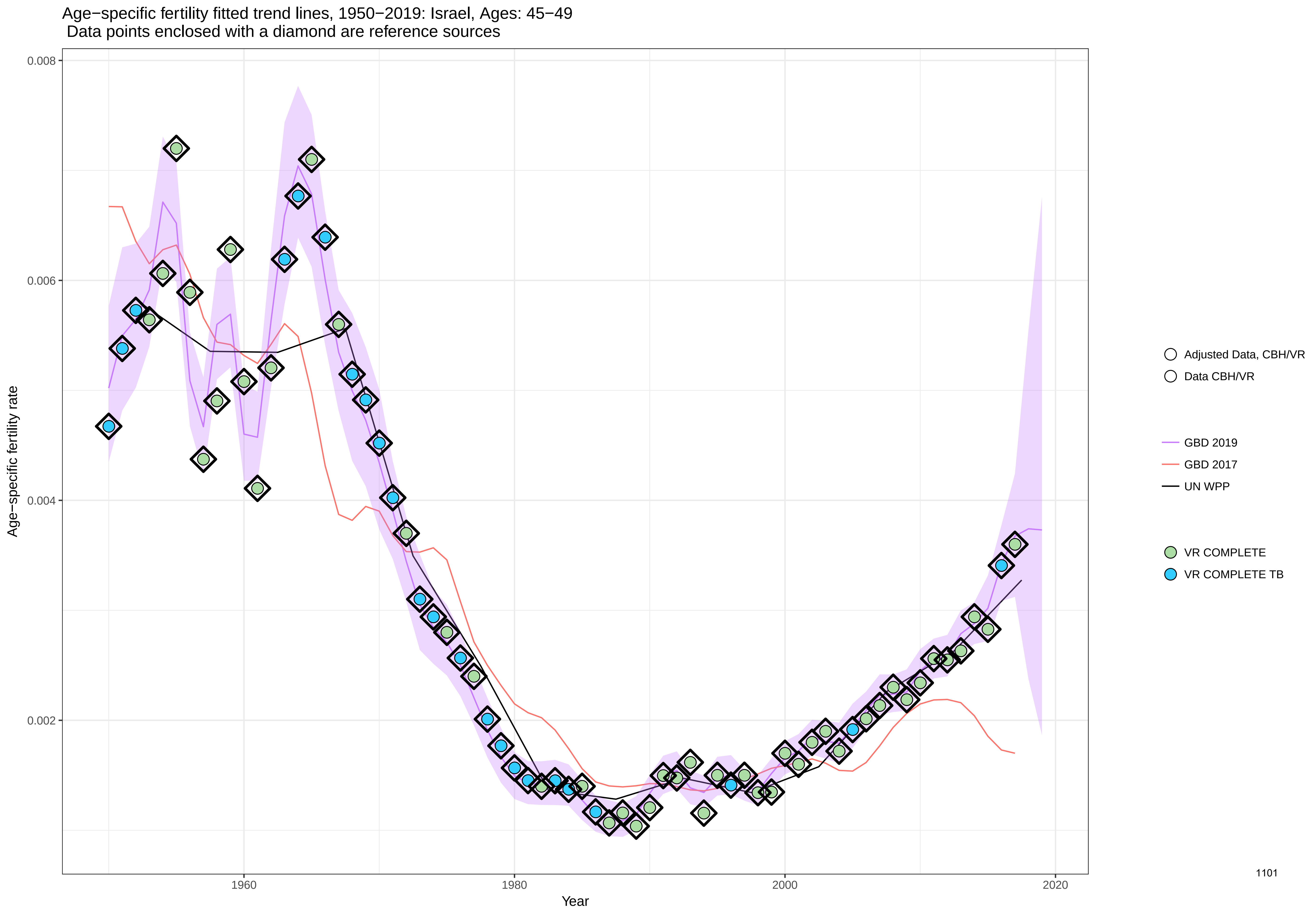

Age−specific fertility fitted trend lines, 1950−2019: Israel, Ages: 45−49
Data points enclosed with a diamond are reference sources
0.008
0.006
0.004
0.002
Age−specific fertility rate
1960
1980
2000
2020
Year
Adjusted Data, CBH/VR
Data CBH/VR
GBD 2019
GBD 2017
UN WPP
VR COMPLETE
VR COMPLETE TB

Age−specific fertility fitted trend lines, 1950−2019: Israel, Ages: 50−54
Data points enclosed with a diamond are reference sources

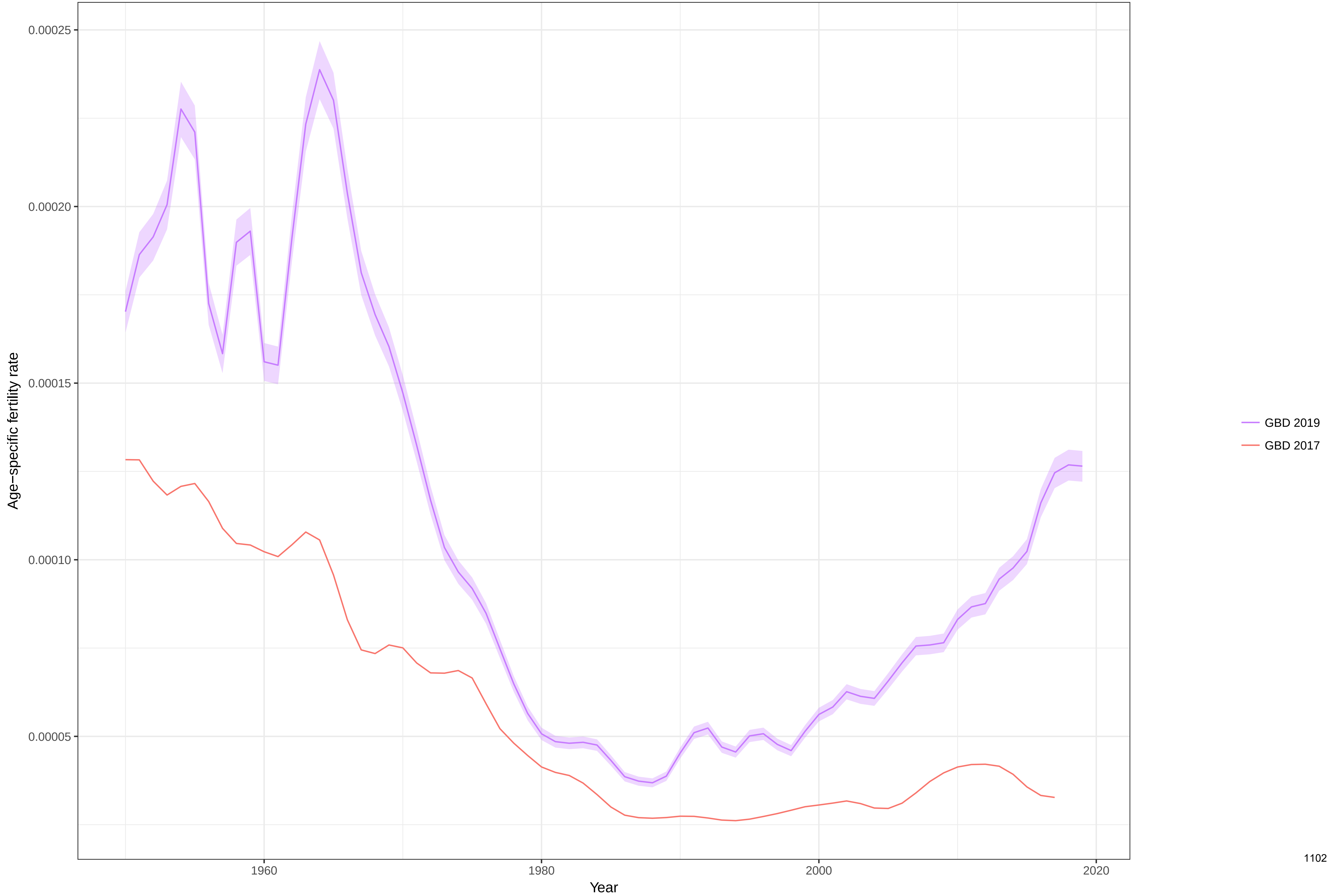

Total fertility rate fitted trend lines, 1950−2019: Israel TFR
Data points enclosed with a diamond are reference sources

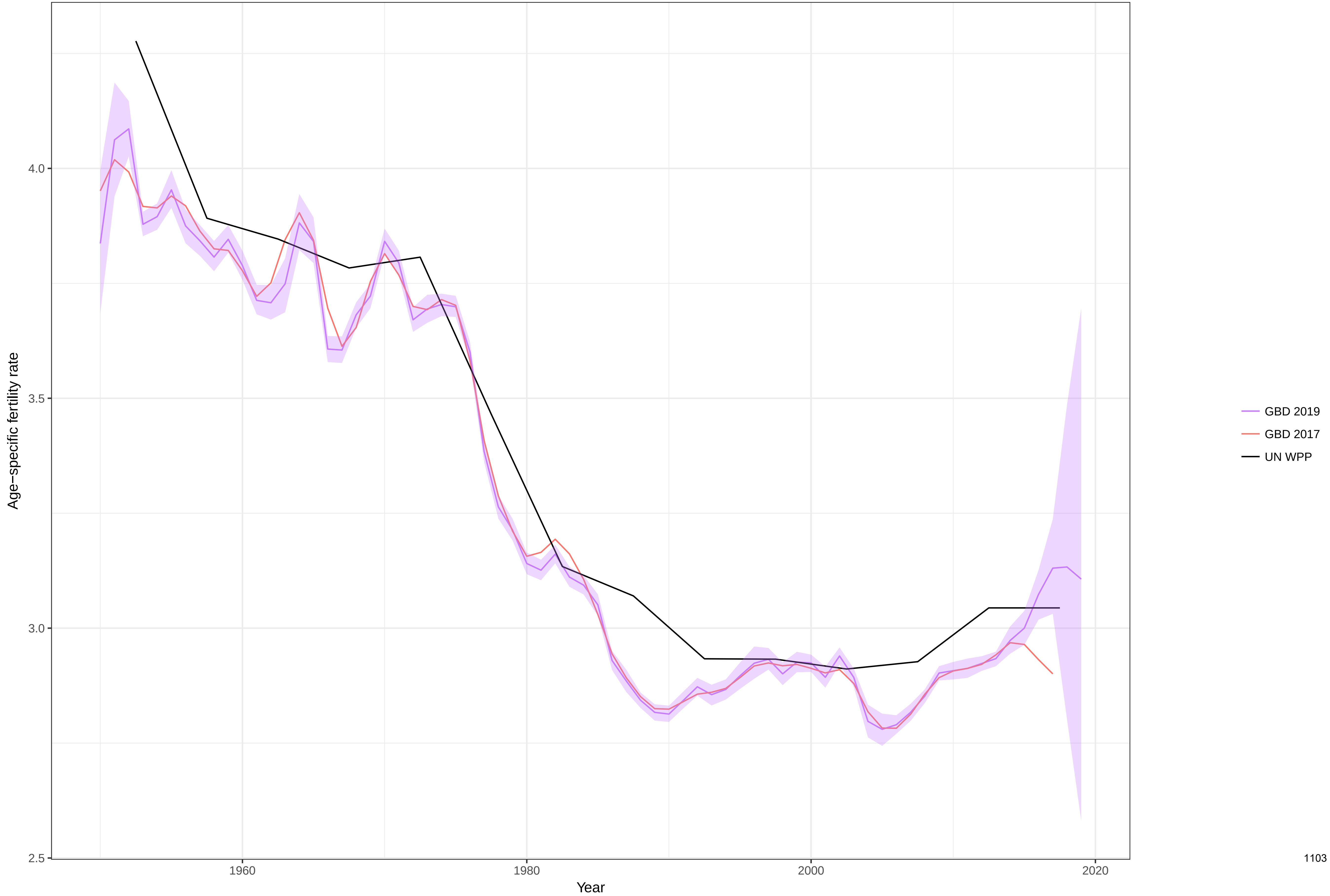

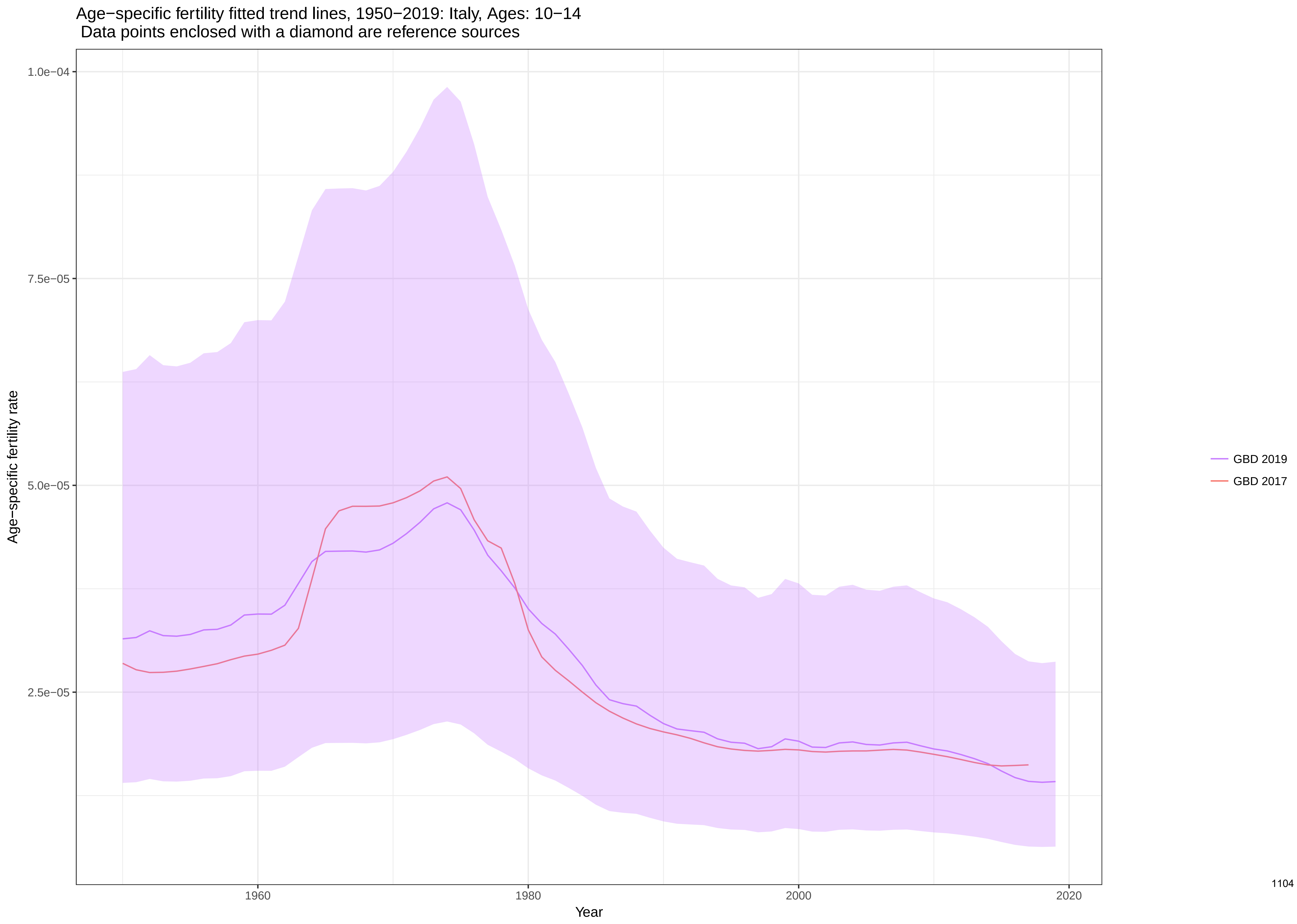
Age−specific fertility fitted trend lines, 1950−2019: Italy, Ages: 10−14
Data points enclosed with a diamond are reference sources
1.0e−04
7.5e−05
5.0e−05
2.5e−05
Age−specific fertility rate
1960
1980
2000
2020
Year
GBD 2019
GBD 2017

Age−specific fertility fitted trend lines, 1950−2019: Italy, Ages: 15−19
Data points enclosed with a diamond are reference sources

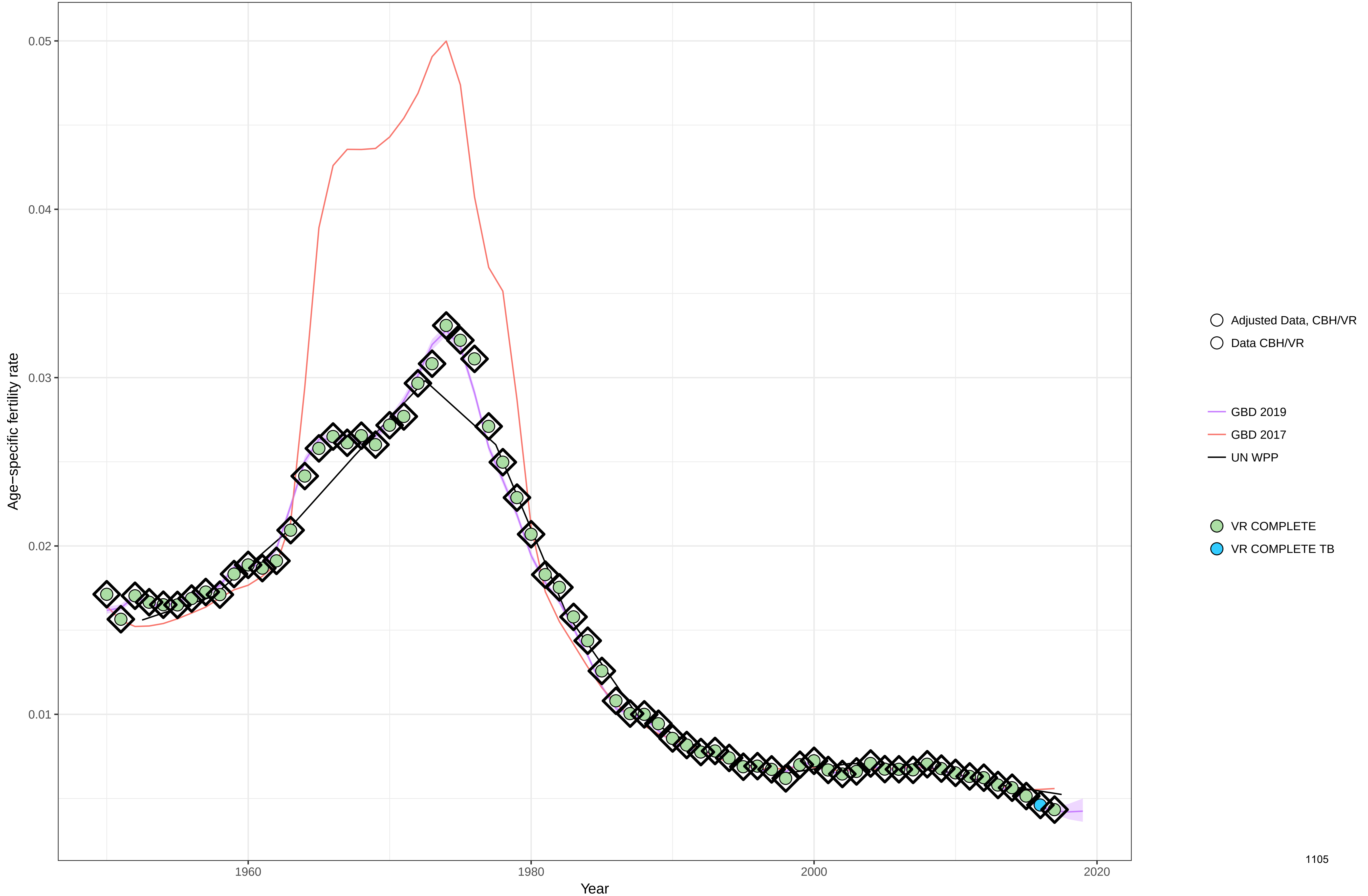

Age−specific fertility fitted trend lines, 1950−2019: Italy, Ages: 20−24
Data points enclosed with a diamond are reference sources

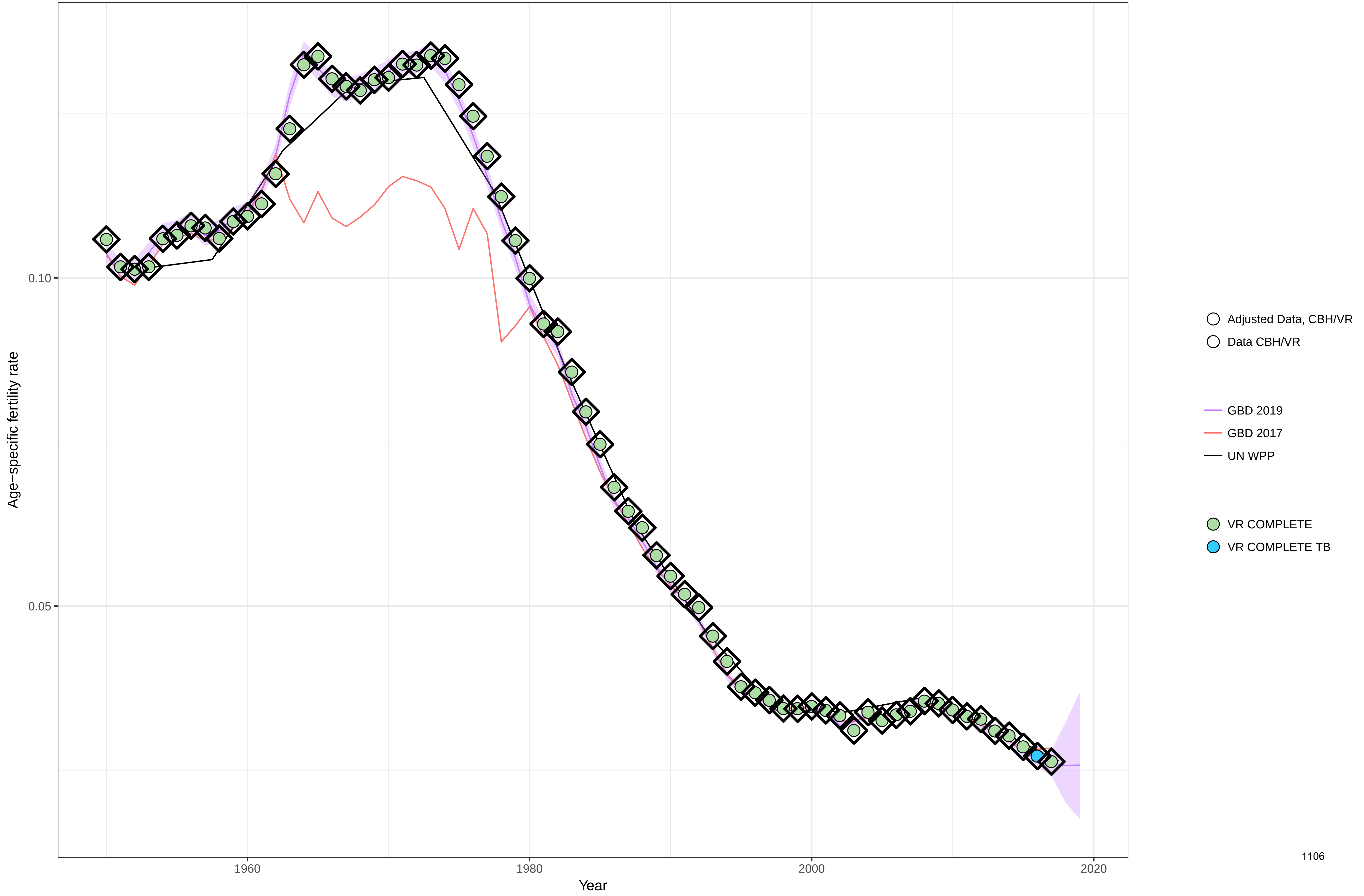

Age−specific fertility fitted trend lines, 1950−2019: Italy, Ages: 25−29
Data points enclosed with a diamond are reference sources

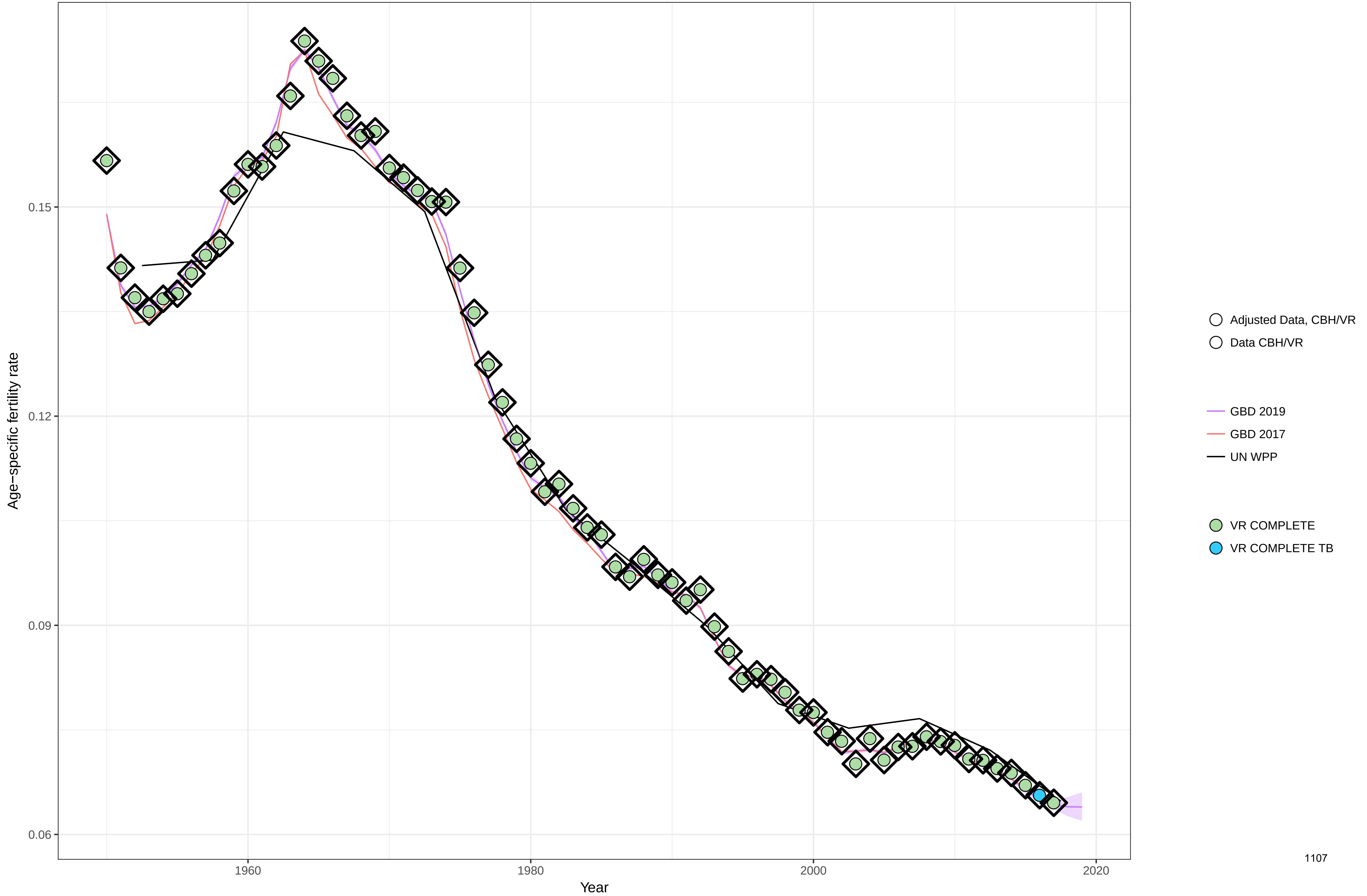

Age−specific fertility fitted trend lines, 1950−2019: Italy, Ages: 30−34
Data points enclosed with a diamond are reference sources

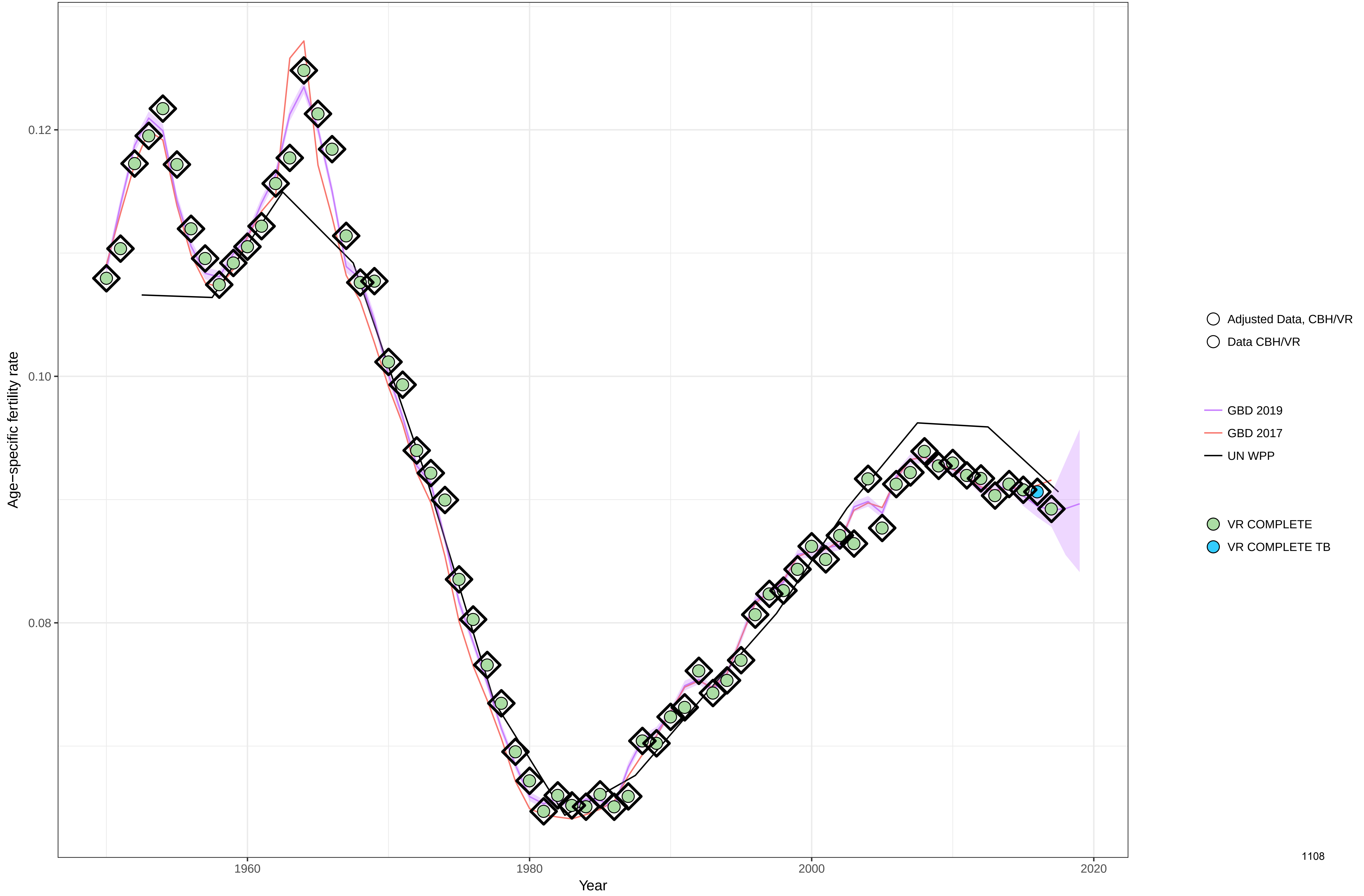

Age−specific fertility fitted trend lines, 1950−2019: Italy, Ages: 35−39
Data points enclosed with a diamond are reference sources

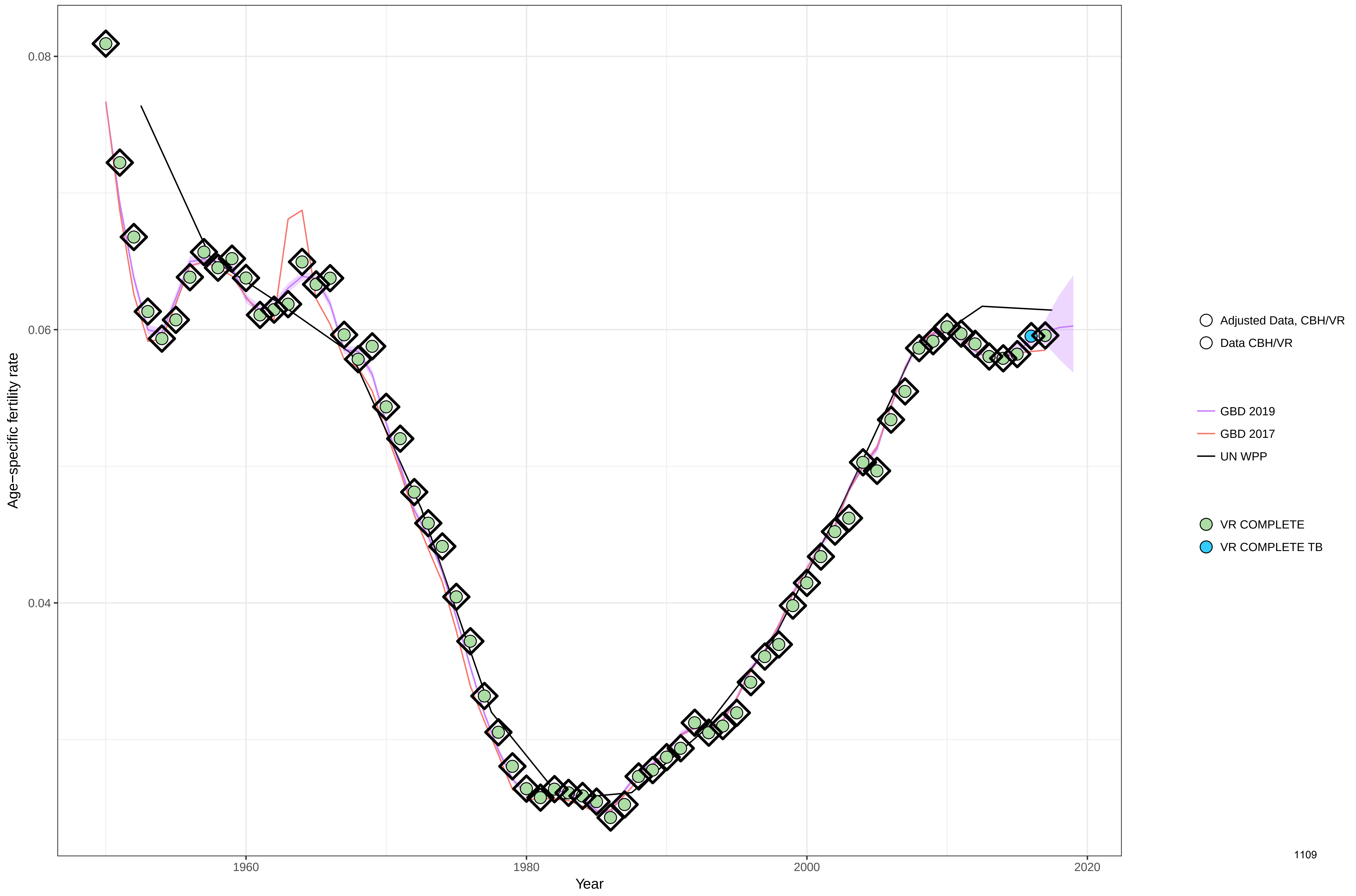

Age−specific fertility fitted trend lines, 1950−2019: Italy, Ages: 40−44
Data points enclosed with a diamond are reference sources

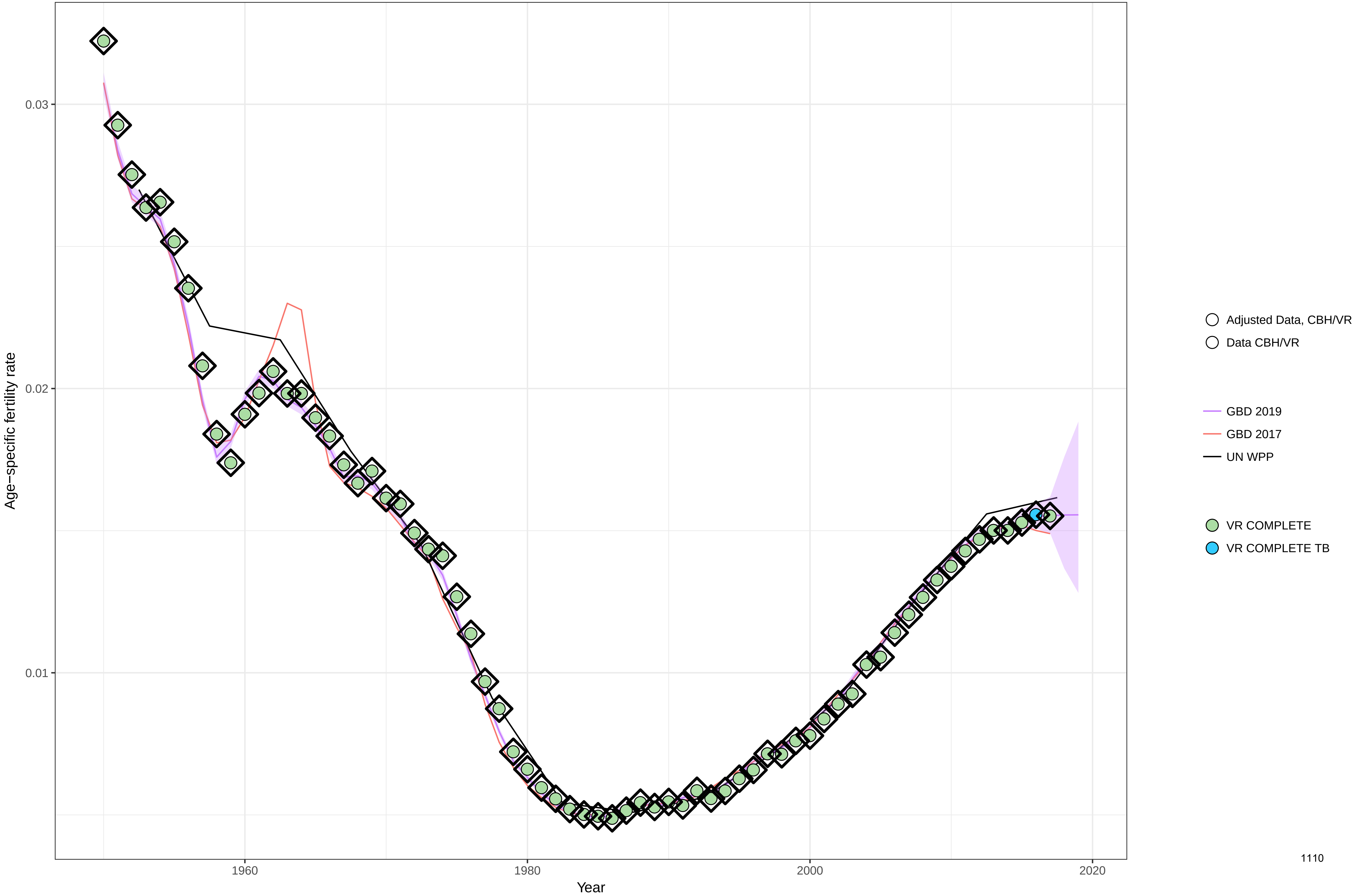

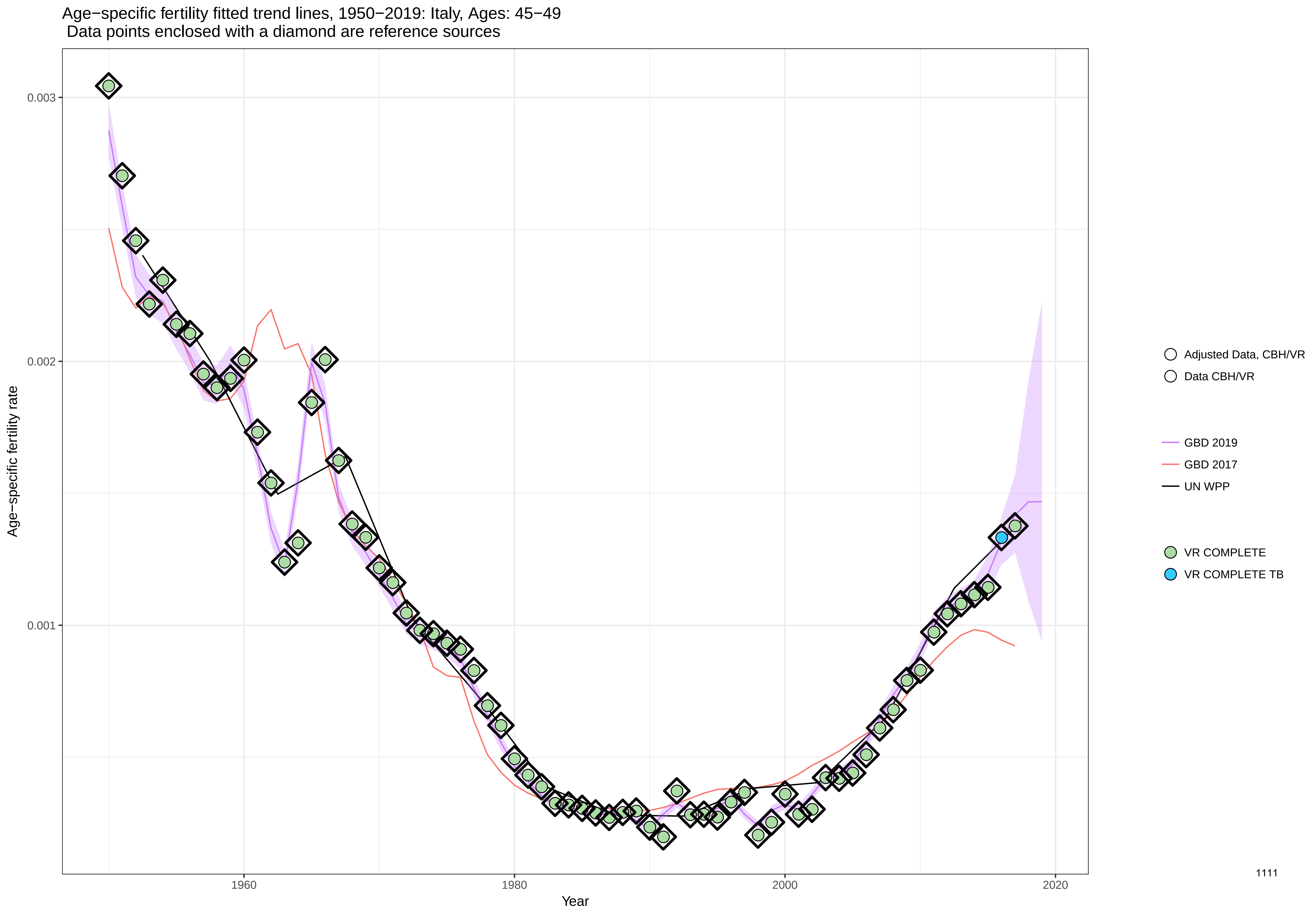

Age−specific fertility fitted trend lines, 1950−2019: Italy, Ages: 45−49
Data points enclosed with a diamond are reference sources
0.003
0.002
0.001
Age−specific fertility rate
1960
1980
2000
2020
Year
Adjusted Data, CBH/VR
Data CBH/VR
GBD 2019
GBD 2017
UN WPP
VR COMPLETE
VR COMPLETE TB

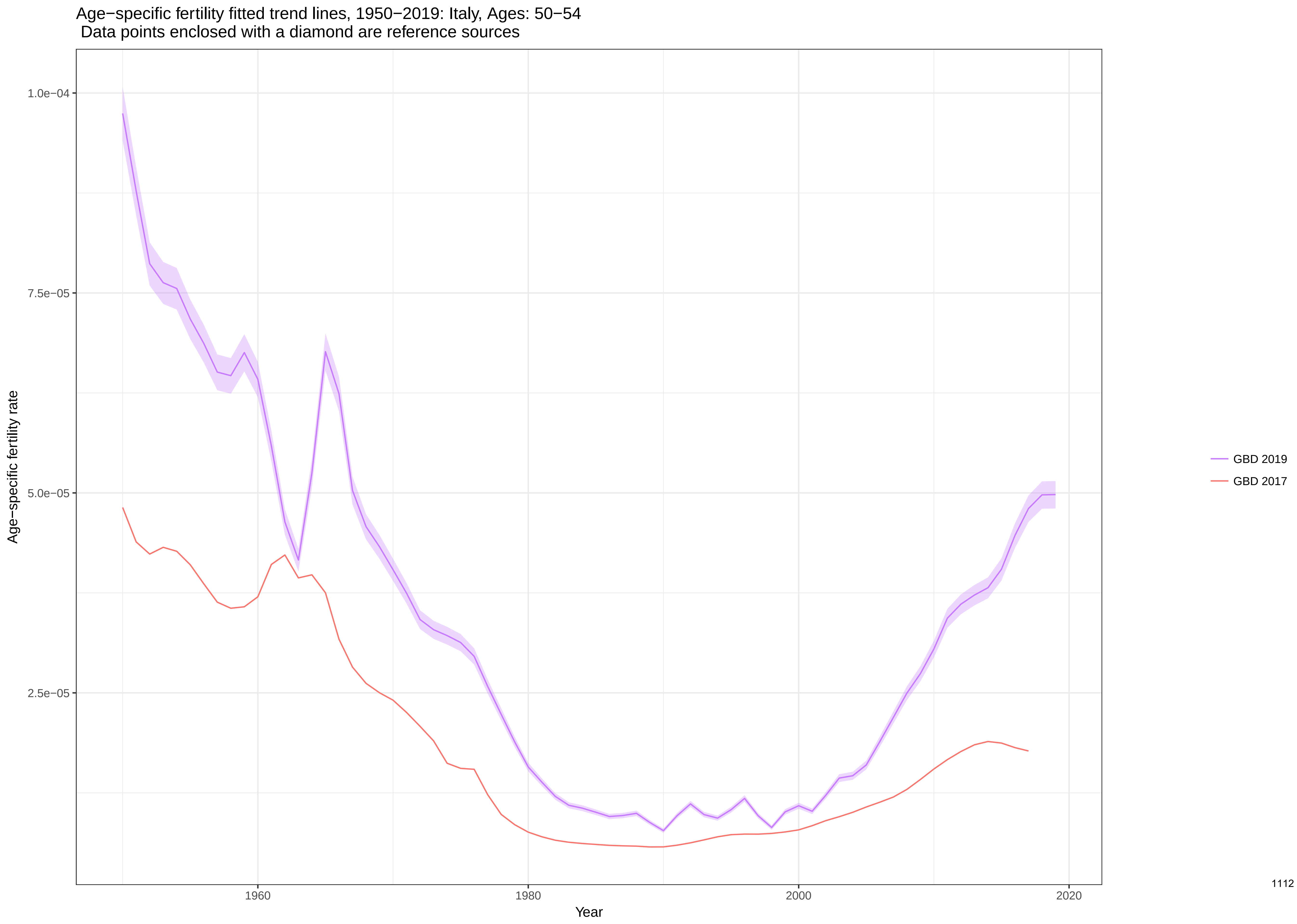

Age−specific fertility fitted trend lines, 1950−2019: Italy, Ages: 50−54
Data points enclosed with a diamond are reference sources
1.0e−04
7.5e−05
5.0e−05
2.5e−05
Age−specific fertility rate
1960
1980
2000
2020
Year
GBD 2019
GBD 2017

1112

Total fertility rate fitted trend lines, 1950−2019: Italy TFR
Data points enclosed with a diamond are reference sources

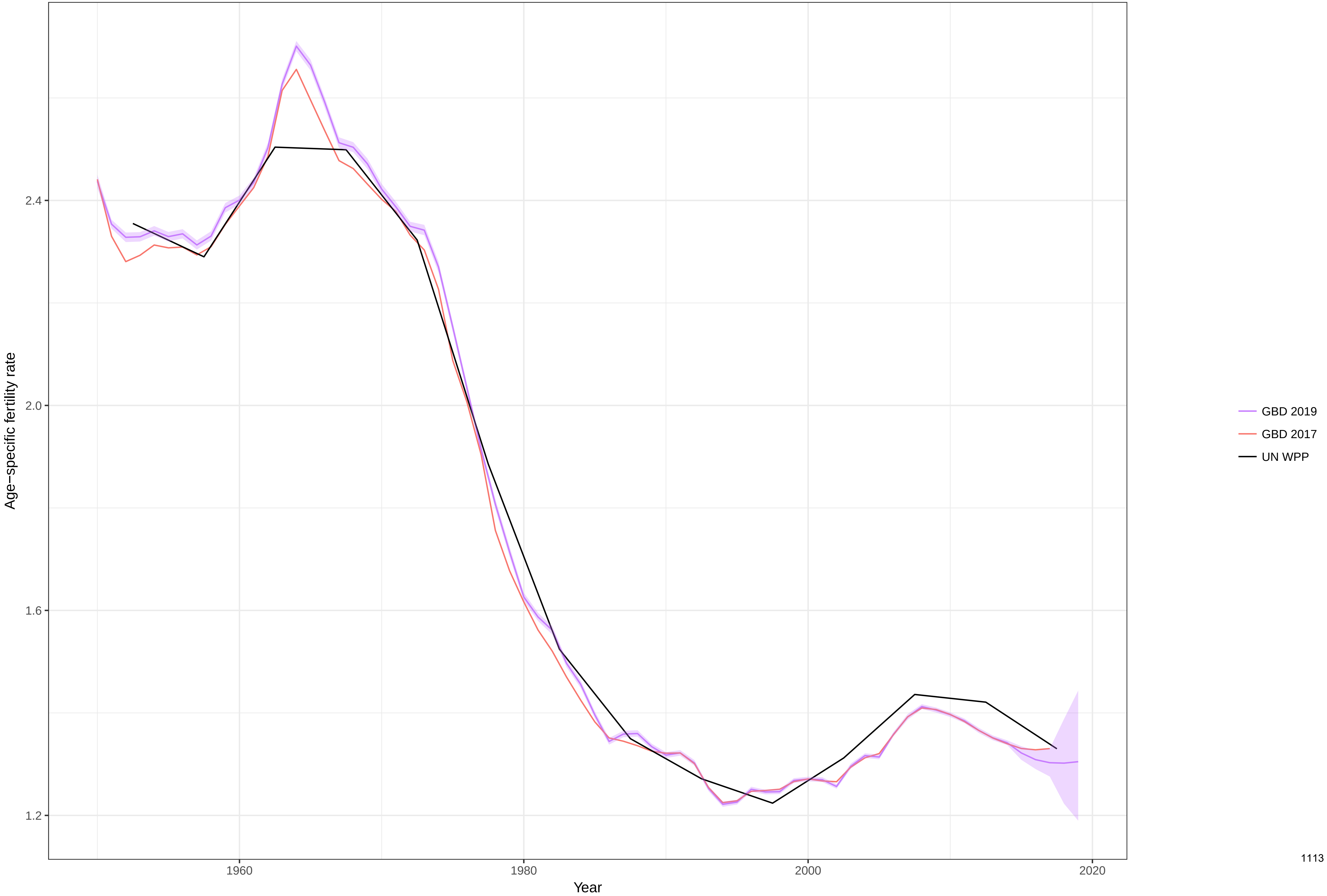

Age−specific fertility fitted trend lines, 1950−2019: Jamaica, Ages: 10−14
Data points enclosed with a diamond are reference sources

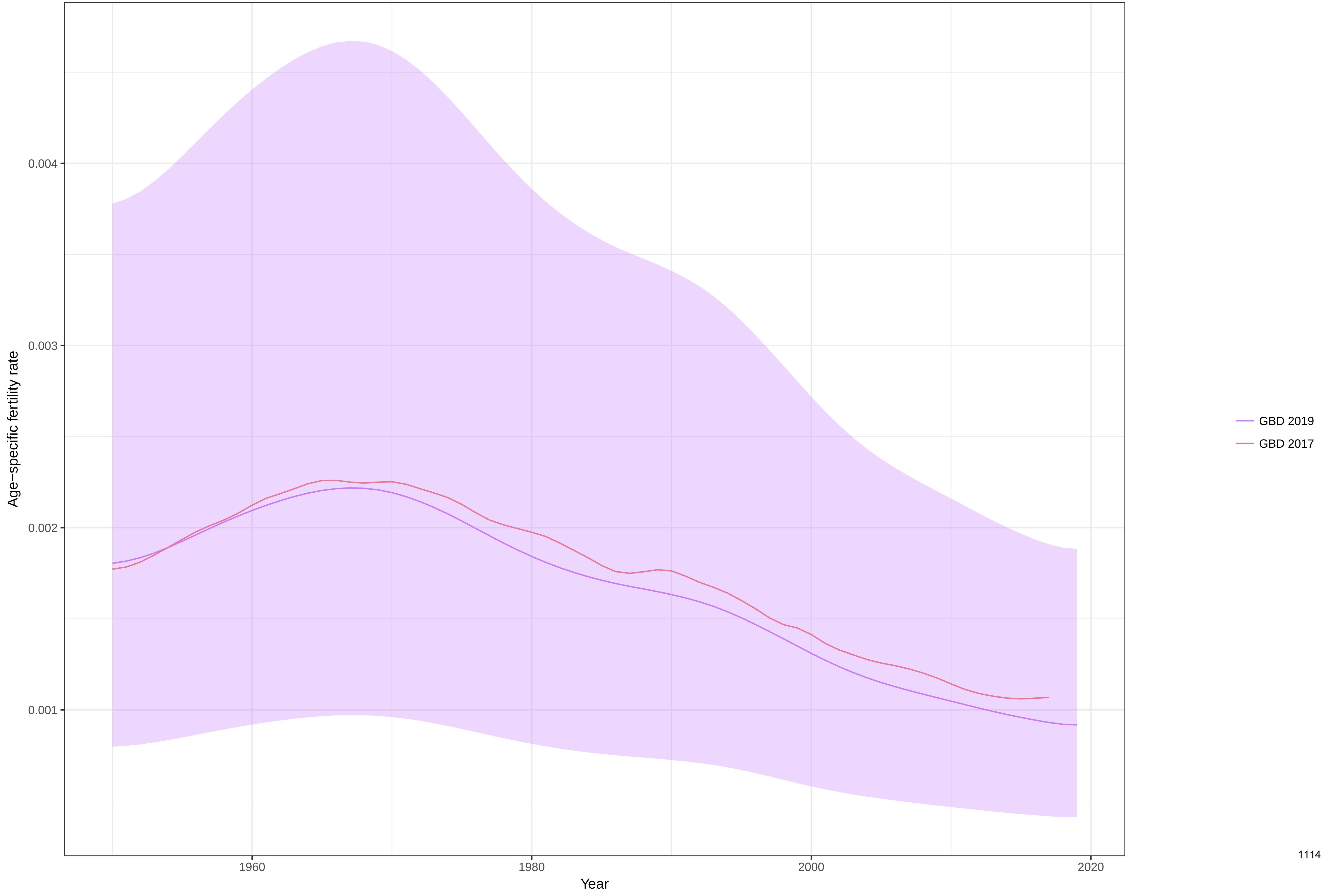

Age−specific fertility fitted trend lines, 1950−2019: Jamaica, Ages: 15−19
Data points enclosed with a diamond are reference sources

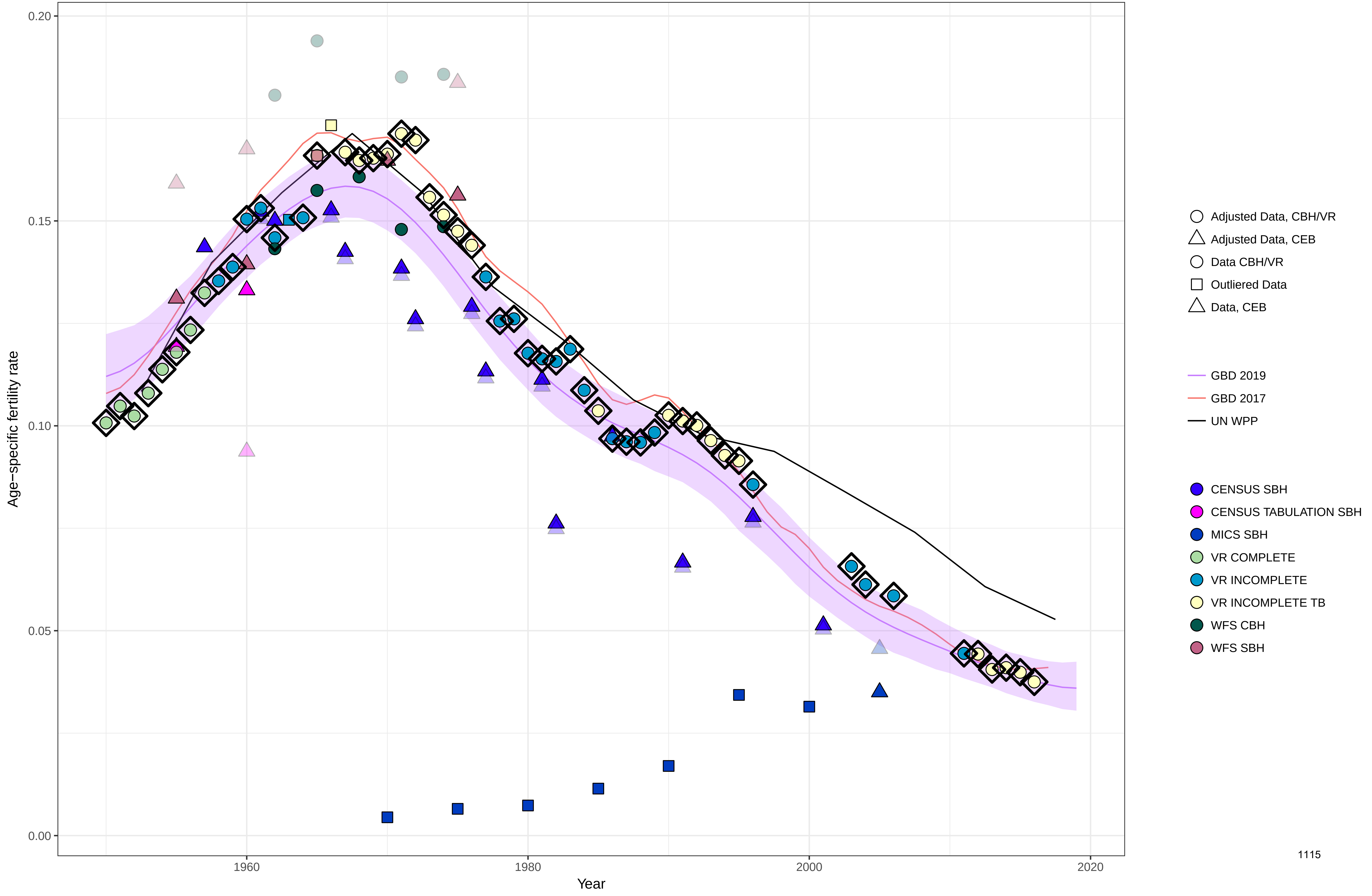

Age−specific fertility fitted trend lines, 1950−2019: Jamaica, Ages: 20−24
Data points enclosed with a diamond are reference sources

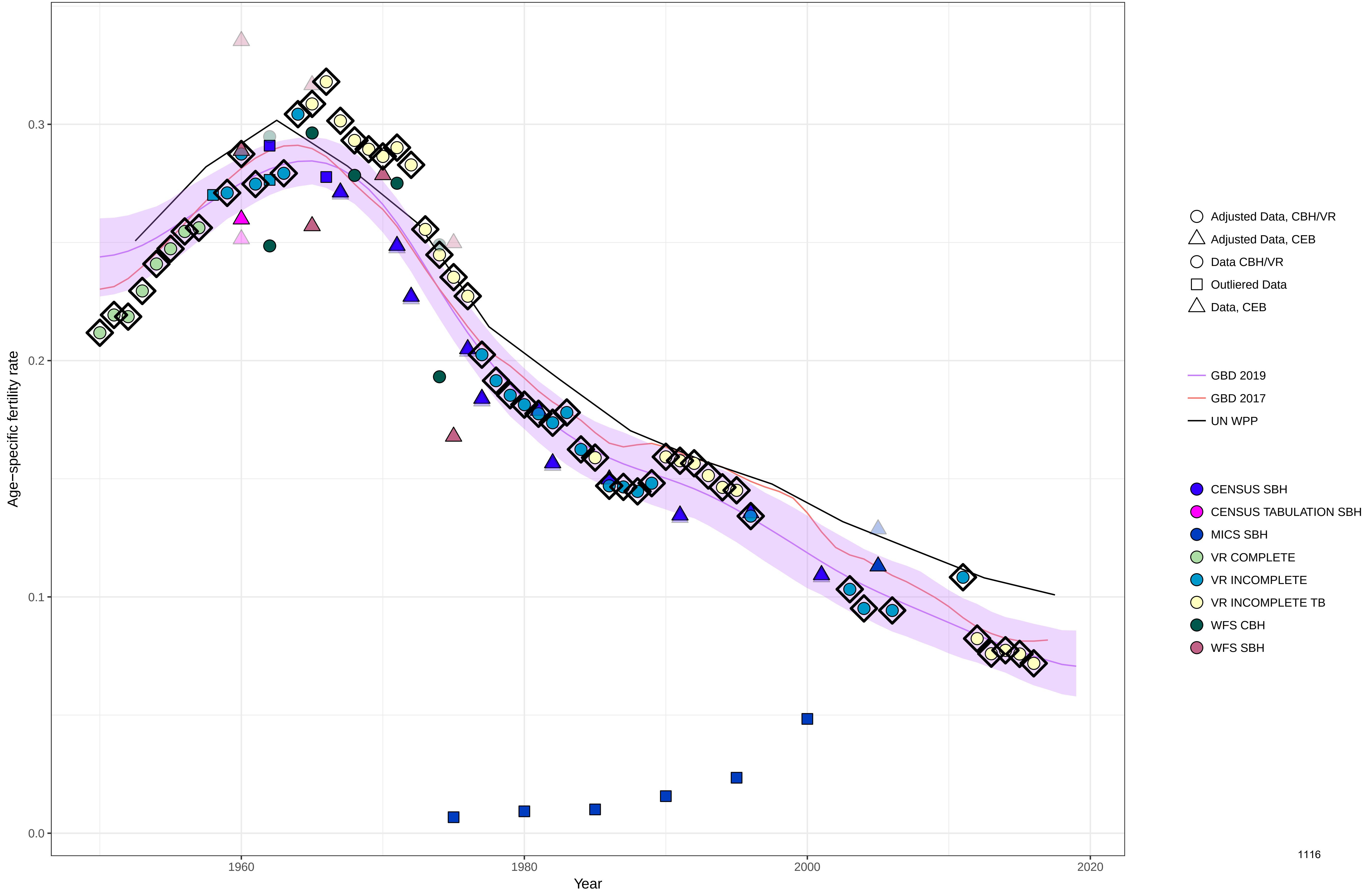

Age−specific fertility fitted trend lines, 1950−2019: Jamaica, Ages: 25−29
Data points enclosed with a diamond are reference sources

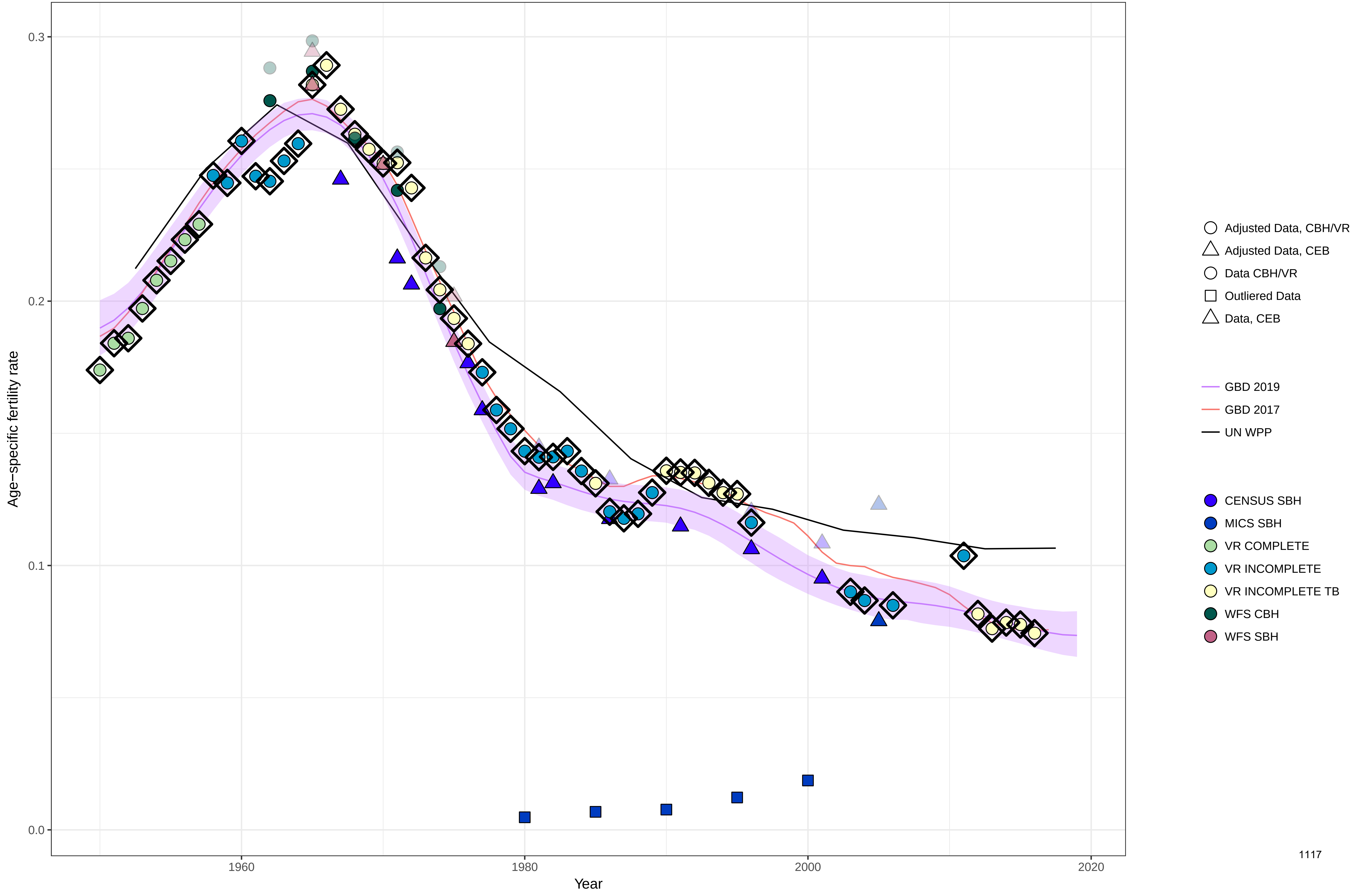

Age−specific fertility fitted trend lines, 1950−2019: Jamaica, Ages: 30−34
Data points enclosed with a diamond are reference sources

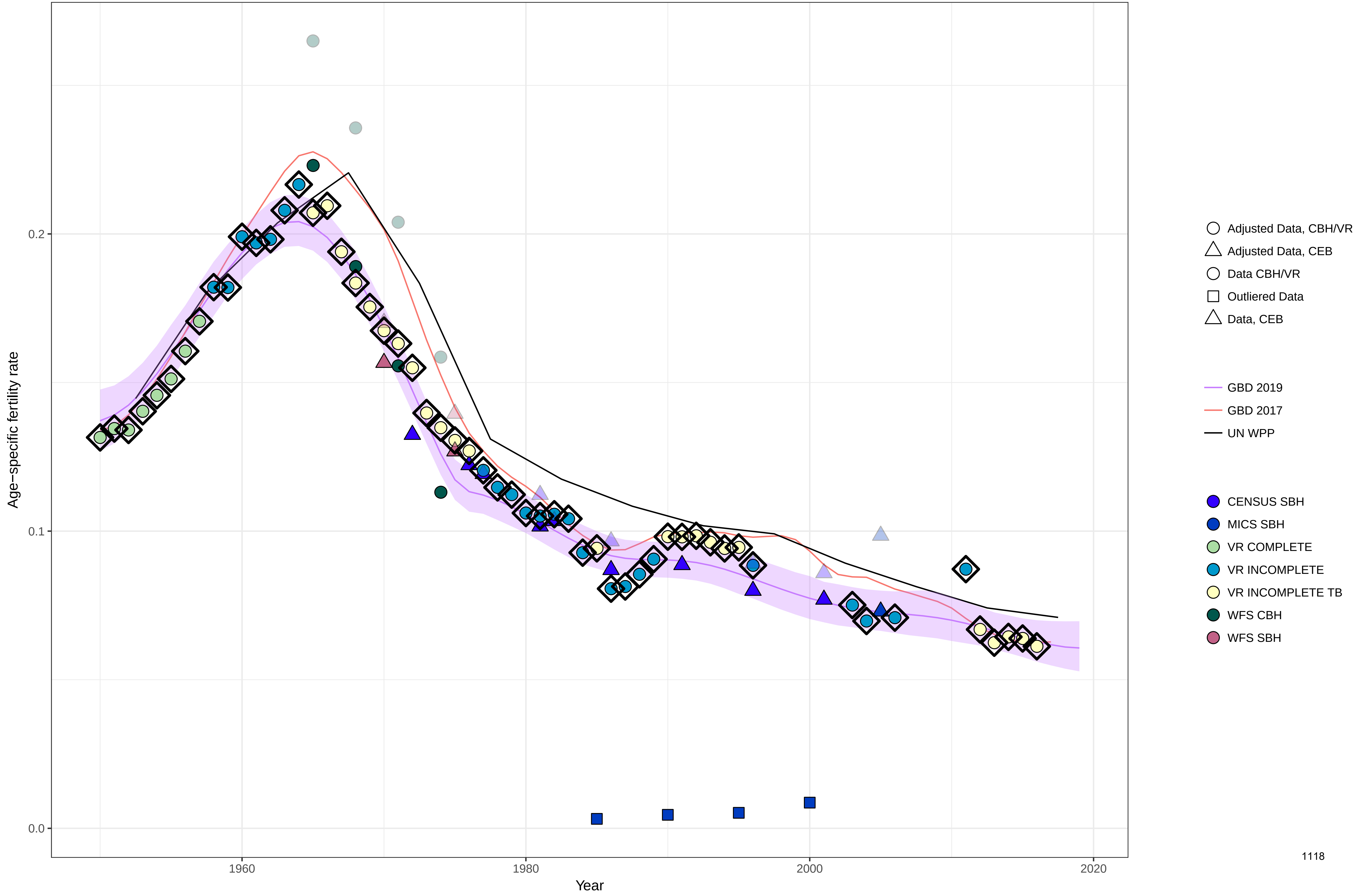

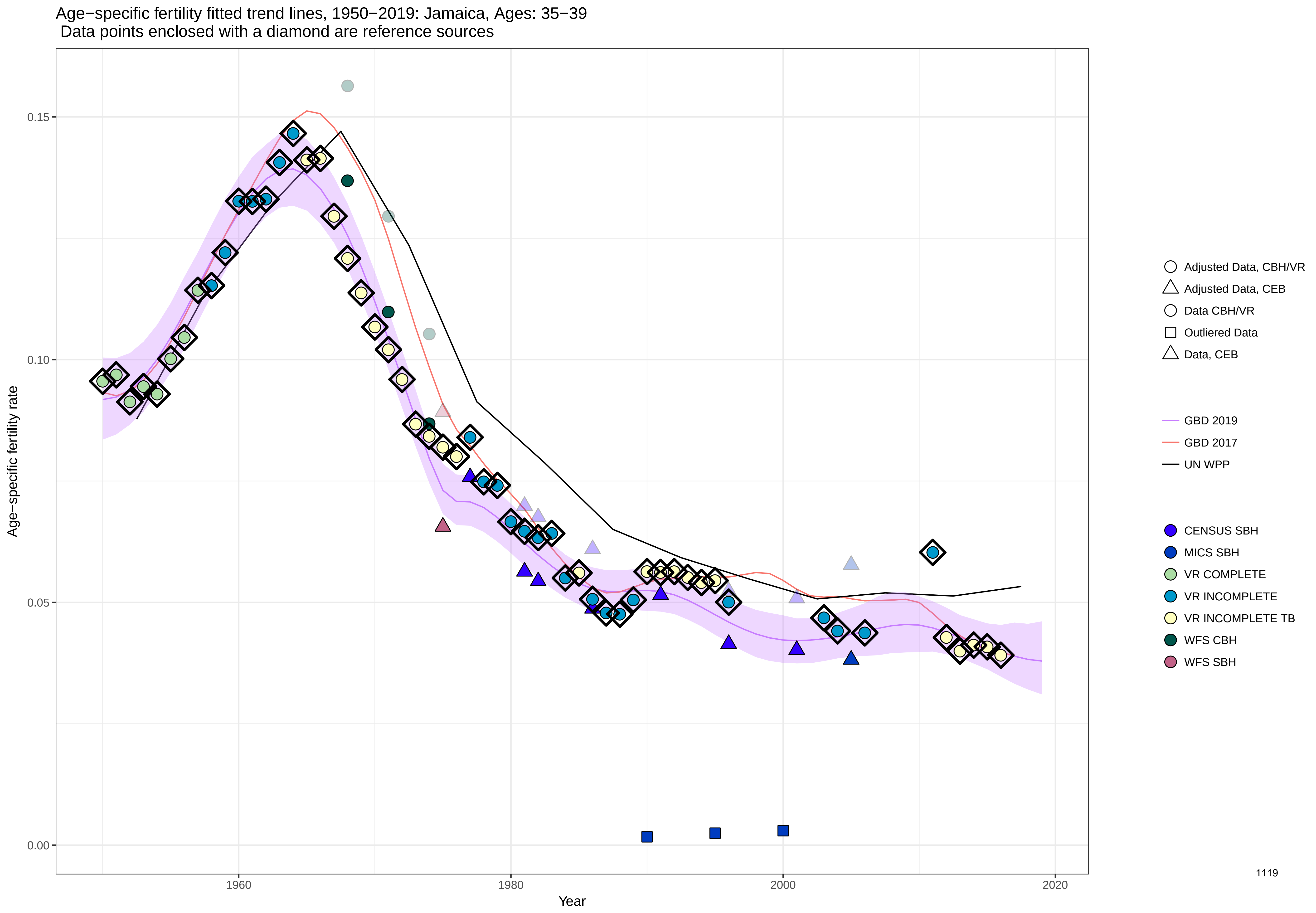

Age−specific fertility fitted trend lines, 1950−2019: Jamaica, Ages: 35−39
Data points enclosed with a diamond are reference sources
Age−specific fertility rate
0.15
0.10
0.05
0.00
1960
1980
2000
2020
Year
Adjusted Data, CBH/VR
Adjusted Data, CEB
Data CBH/VR
Outliered Data
Data, CEB
GBD 2019
GBD 2017
UN WPP
CENSUS SBH
MICS SBH
VR COMPLETE
VR INCOMPLETE
VR INCOMPLETE TB
WFS CBH
WFS SBH

Age−specific fertility fitted trend lines, 1950−2019: Jamaica, Ages: 40−44
Data points enclosed with a diamond are reference sources

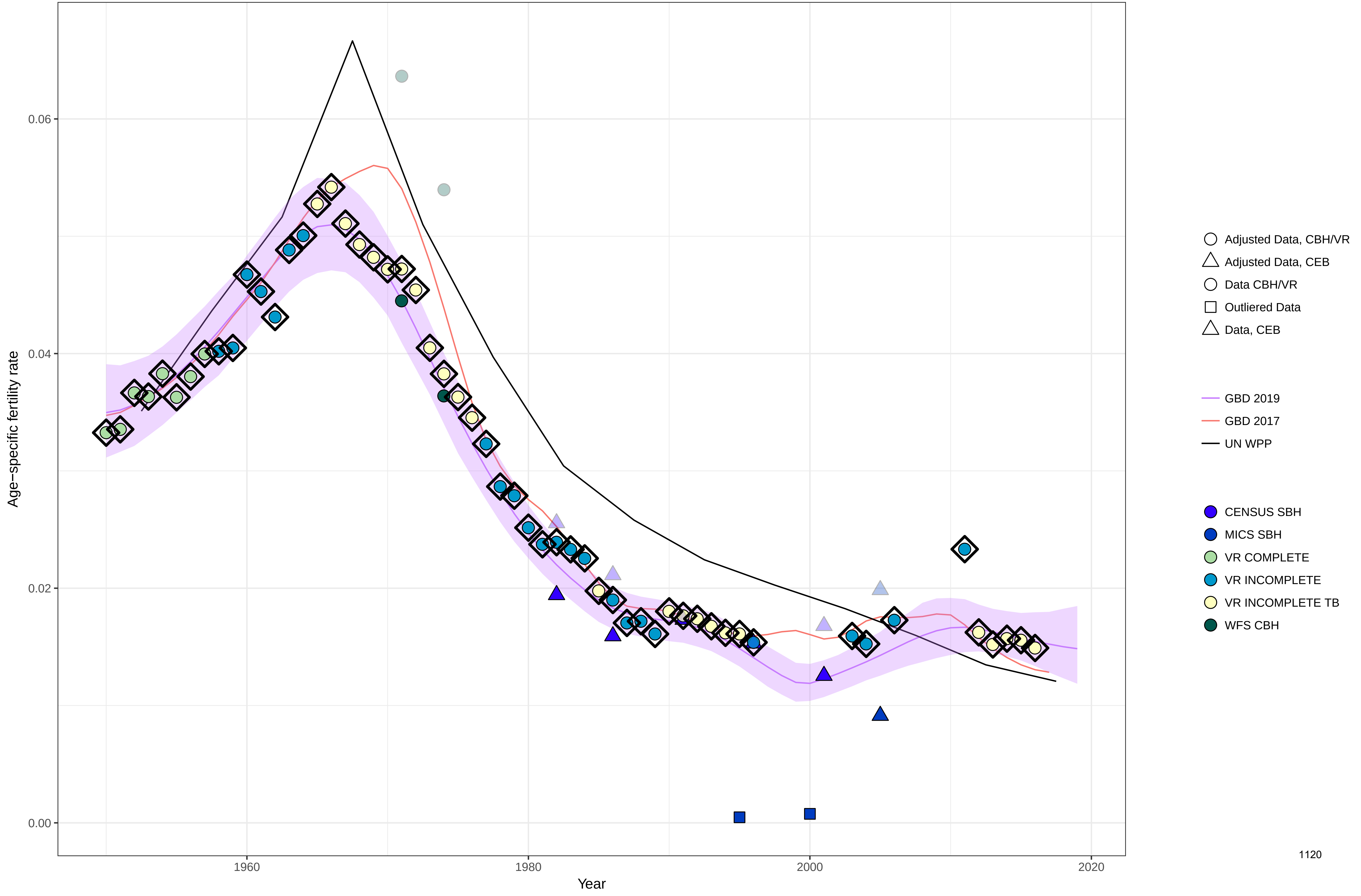

Age−specific fertility fitted trend lines, 1950−2019: Jamaica, Ages: 45−49
Data points enclosed with a diamond are reference sources

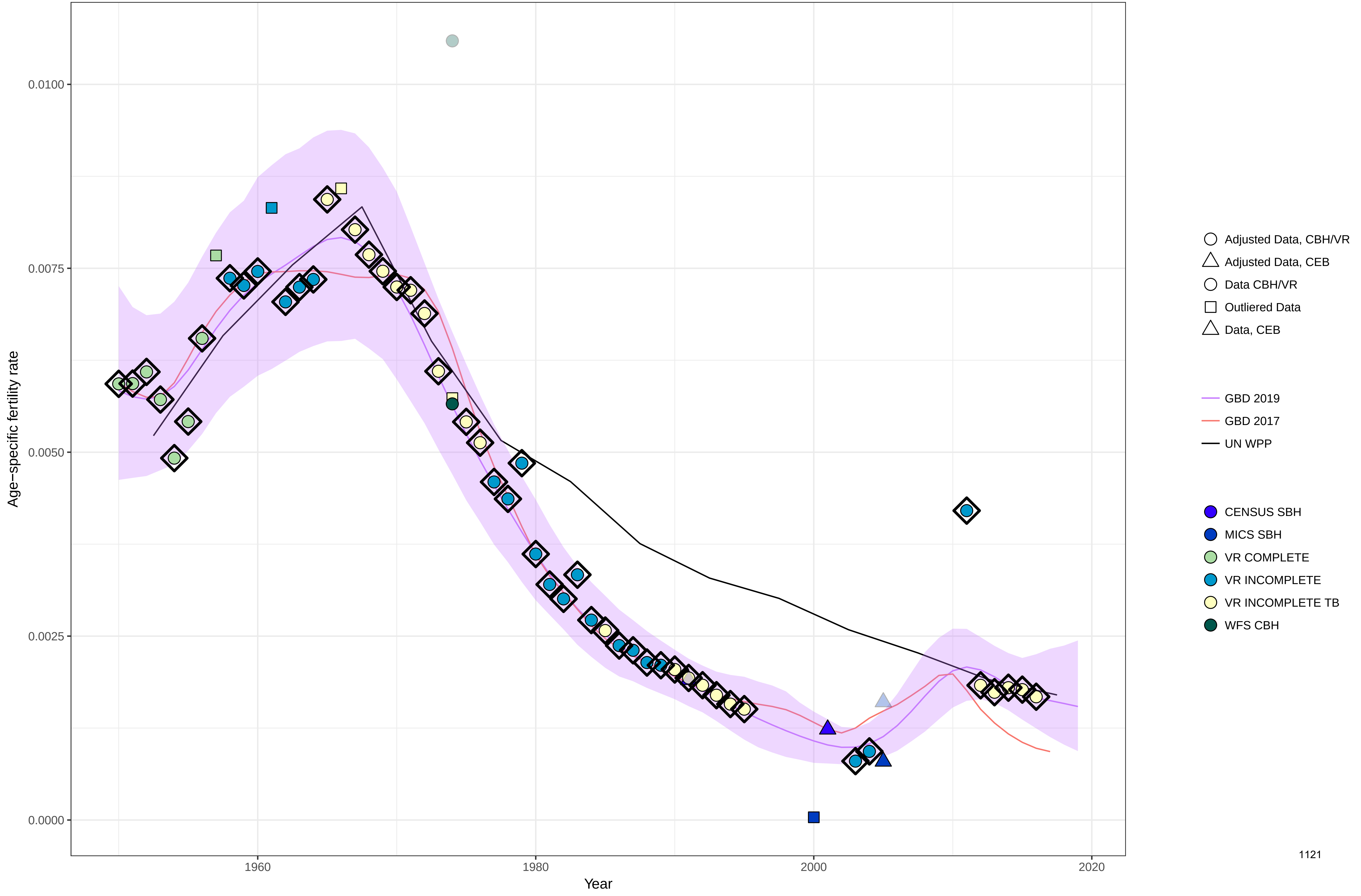

Age−specific fertility fitted trend lines, 1950−2019: Jamaica, Ages: 50−54
Data points enclosed with a diamond are reference sources

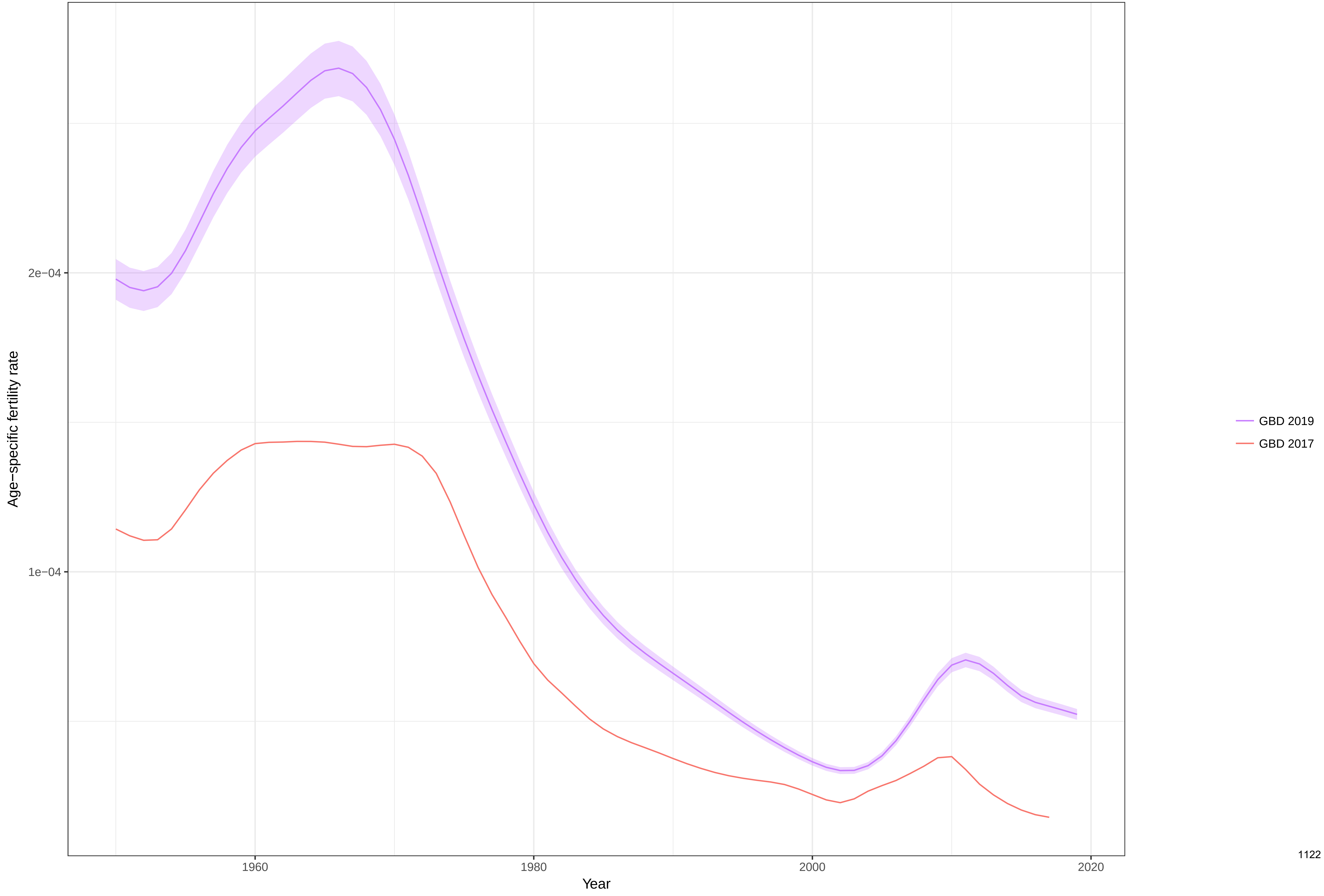

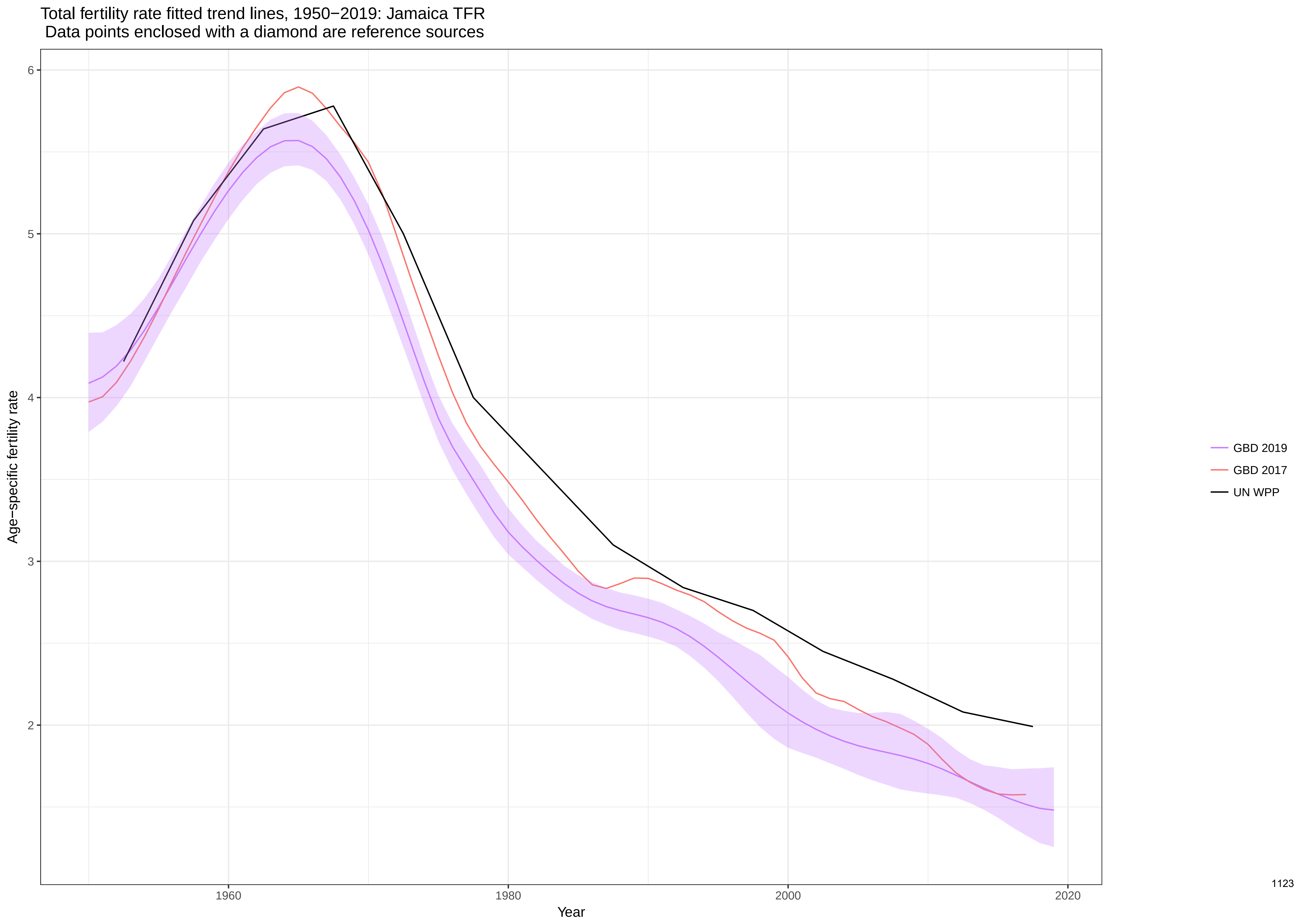
Total fertility rate fitted trend lines, 1950−2019: Jamaica TFR
Data points enclosed with a diamond are reference sources
Age−specific fertility rate
Year
6
5
4
3
2
1960
1980
2000
2020
GBD 2019
GBD 2017
UN WPP

Age−specific fertility fitted trend lines, 1950−2019: Jordan, Ages: 10−14
Data points enclosed with a diamond are reference sources

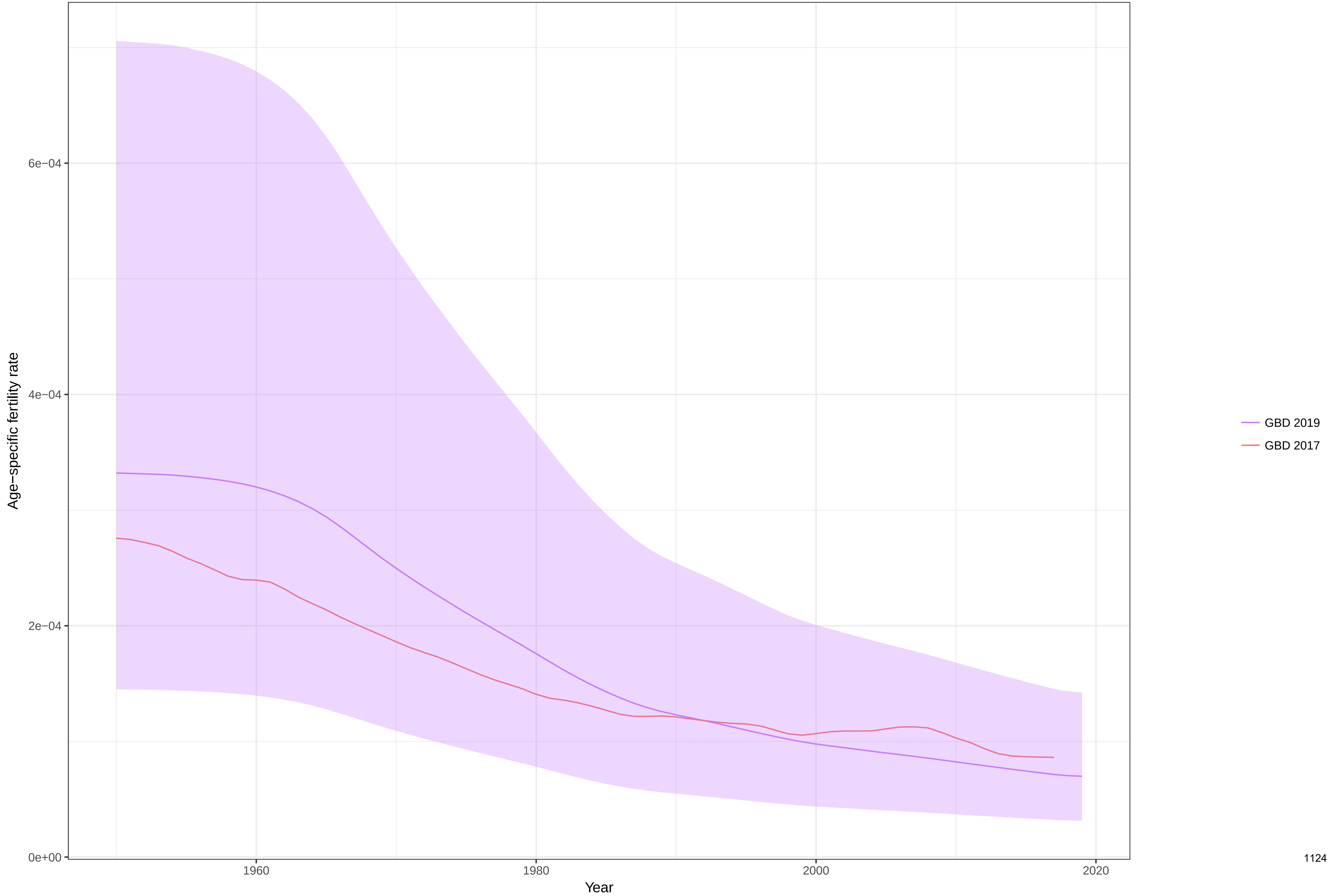

Age−specific fertility fitted trend lines, 1950−2019: Jordan, Ages: 15−19
Data points enclosed with a diamond are reference sources

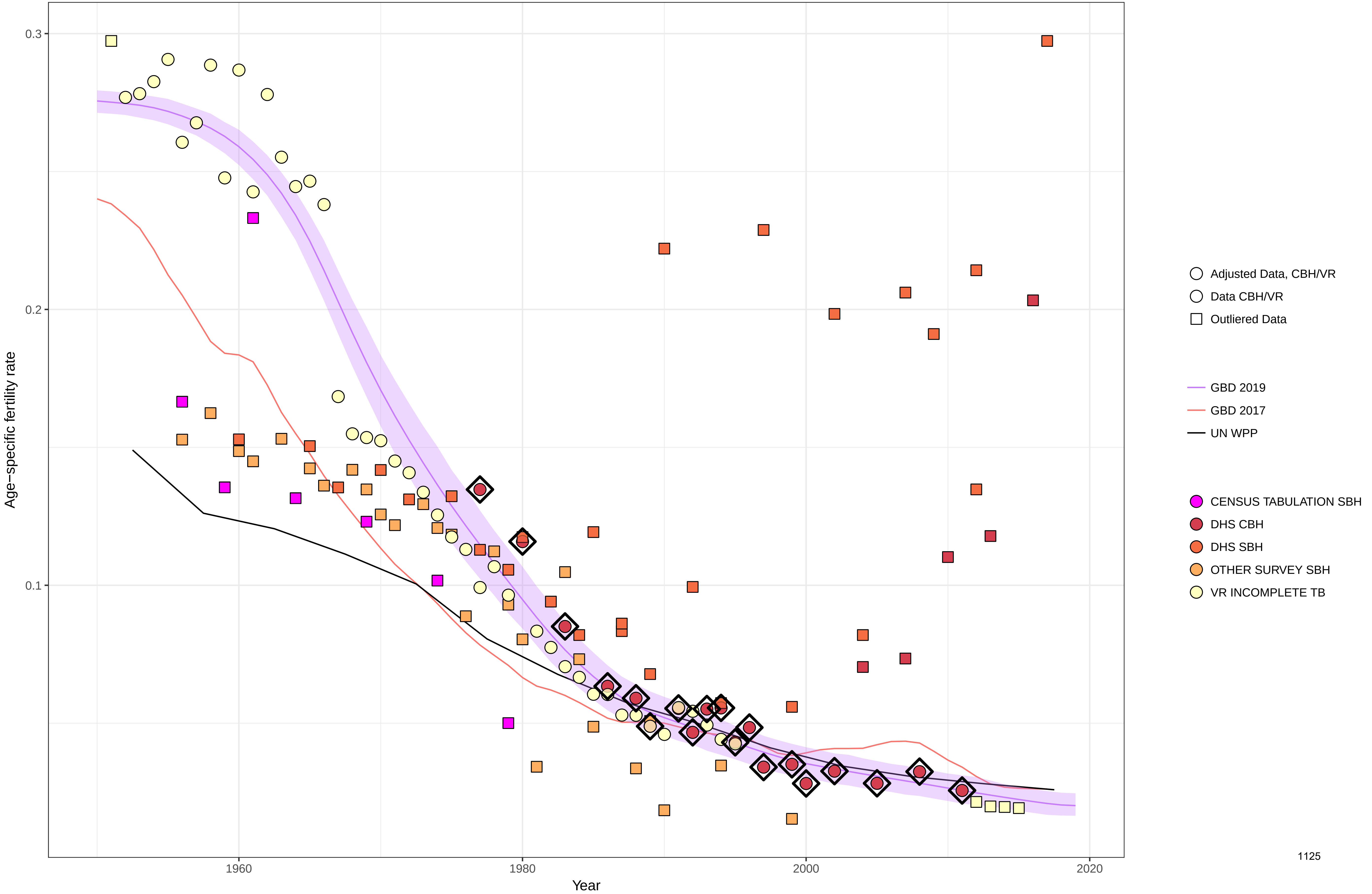

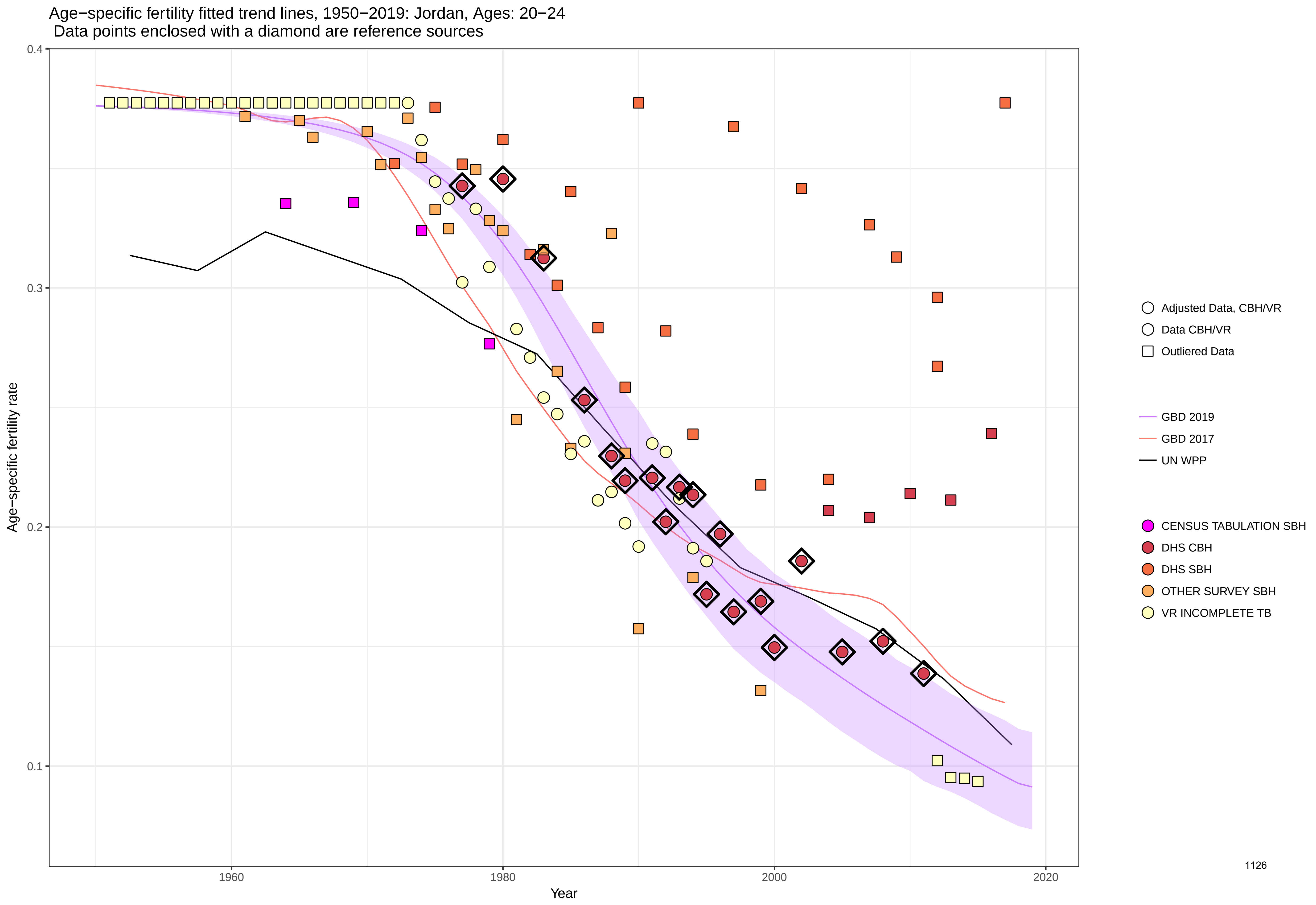

Age−specific fertility fitted trend lines, 1950−2019: Jordan, Ages: 20−24
Data points enclosed with a diamond are reference sources
0.4
0.3
0.2
0.1
Age−specific fertility rate
1960
1980
2000
2020
Year
Adjusted Data, CBH/VR
Data CBH/VR
Outliered Data
GBD 2019
GBD 2017
UN WPP
CENSUS TABULATION SBH
DHS CBH
DHS SBH
OTHER SURVEY SBH
VR INCOMPLETE TB

Age−specific fertility fitted trend lines, 1950−2019: Jordan, Ages: 25−29
Data points enclosed with a diamond are reference sources

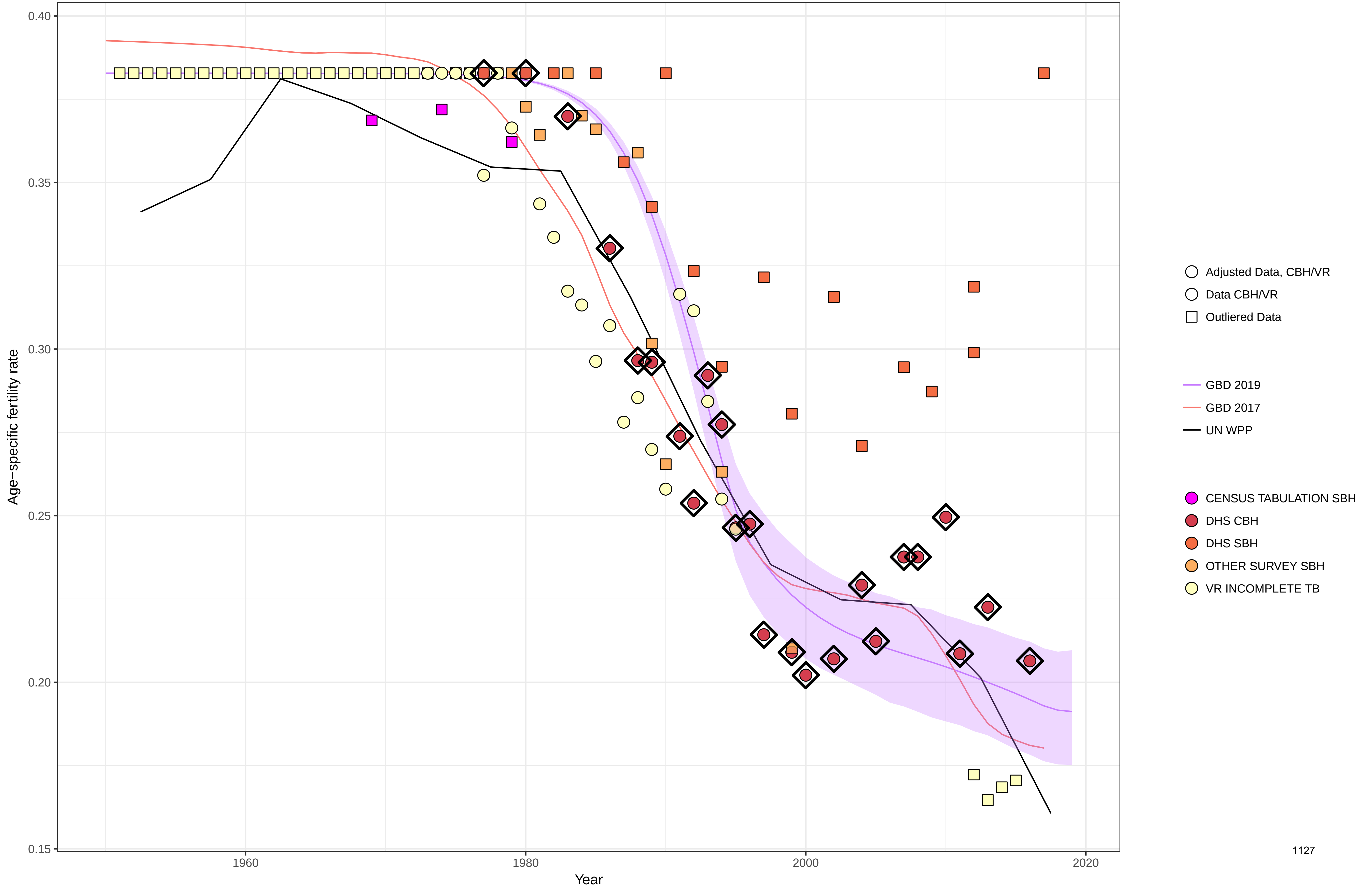

Age−specific fertility fitted trend lines, 1950−2019: Jordan, Ages: 30−34
Data points enclosed with a diamond are reference sources

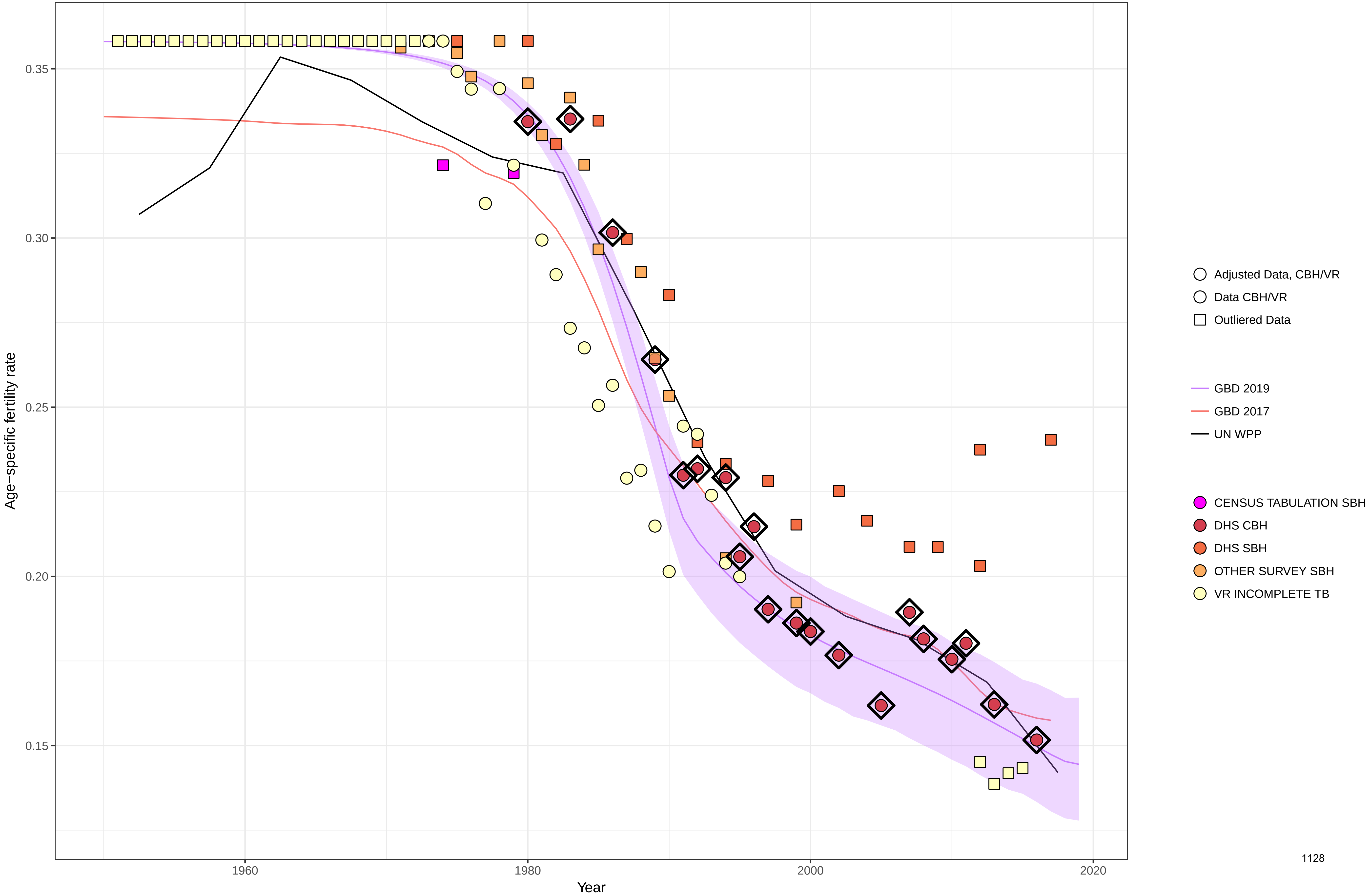

Age−specific fertility fitted trend lines, 1950−2019: Jordan, Ages: 35−39
Data points enclosed with a diamond are reference sources

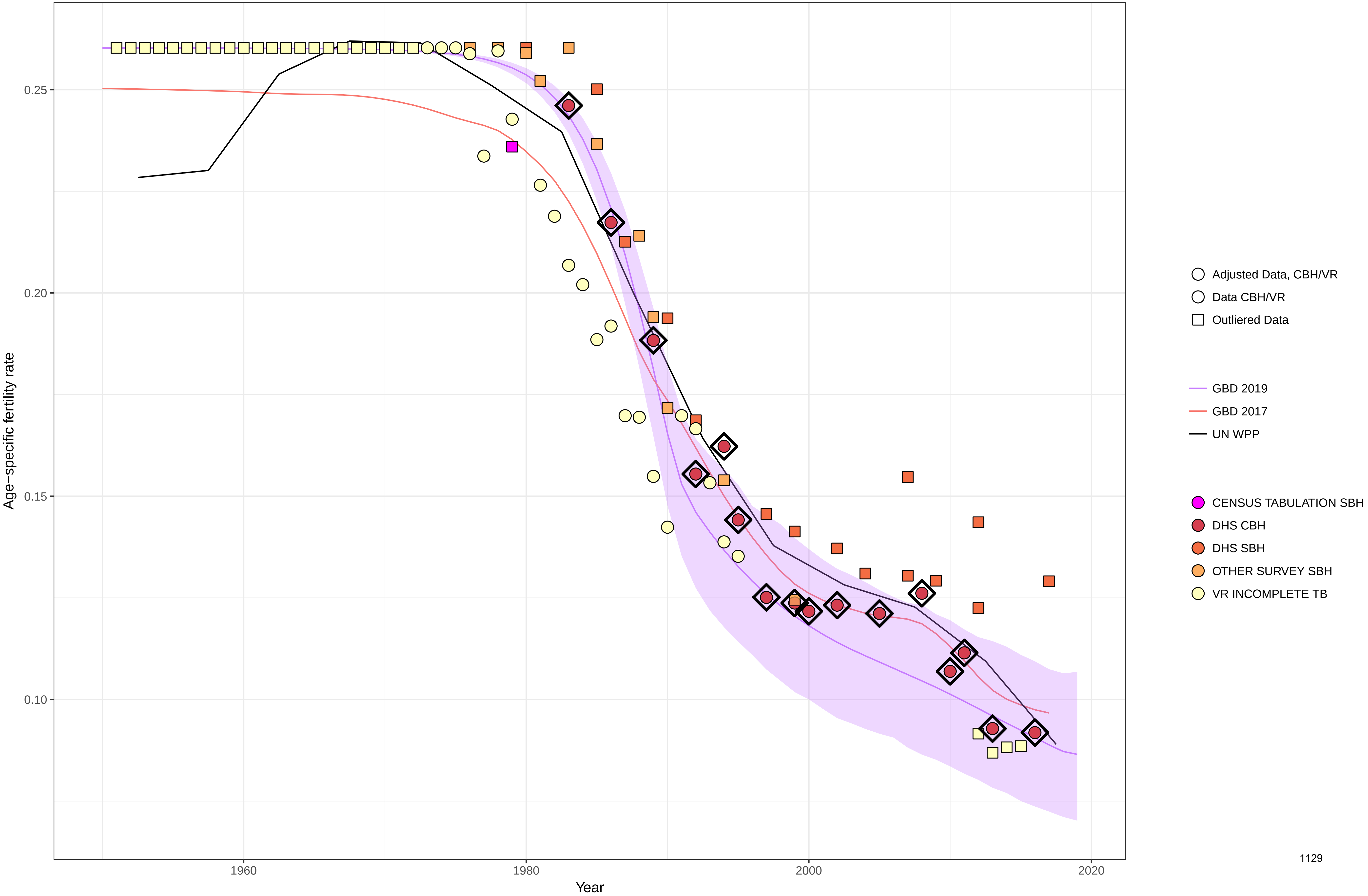

Age−specific fertility fitted trend lines, 1950−2019: Jordan, Ages: 40−44
Data points enclosed with a diamond are reference sources

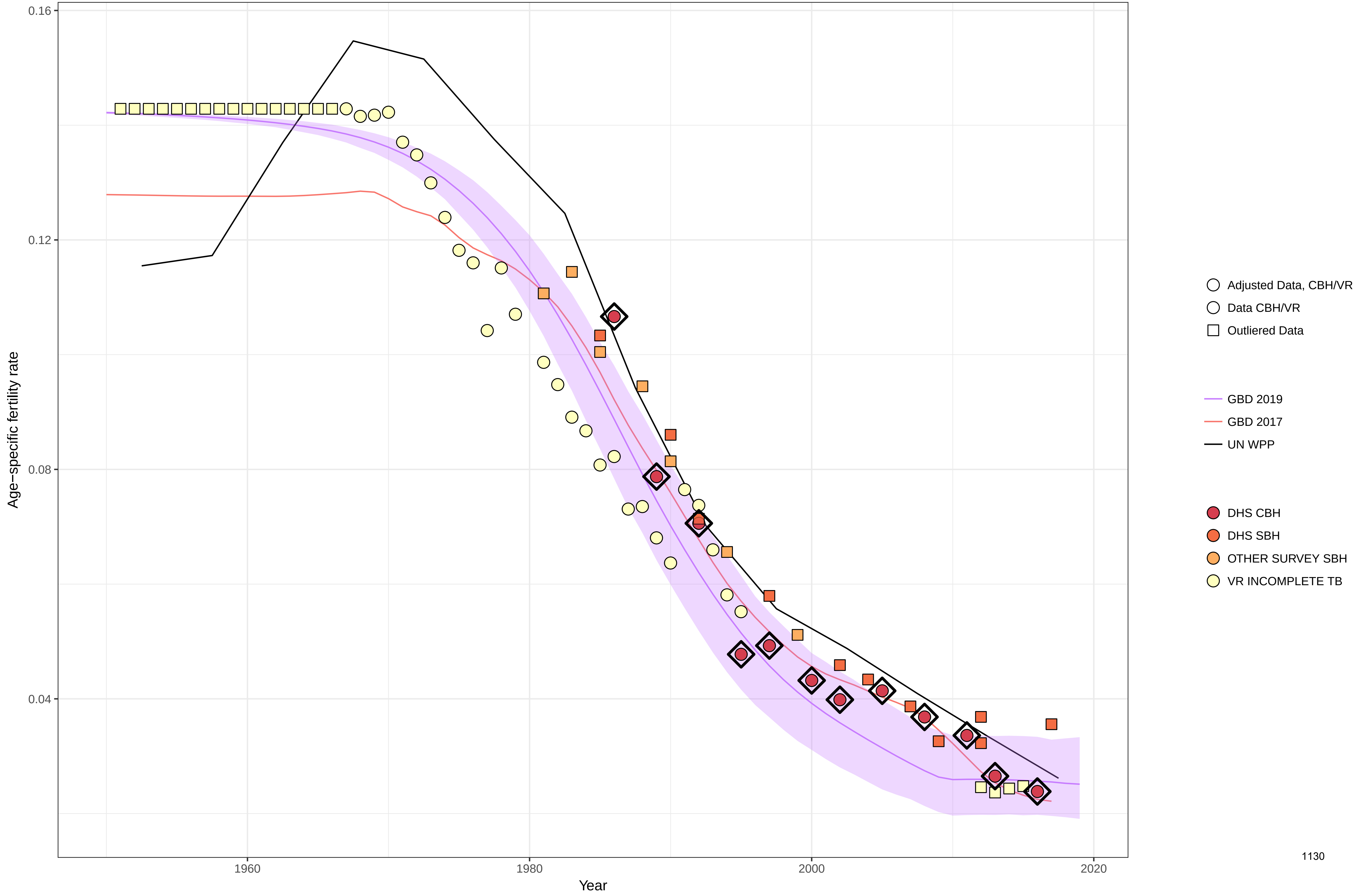

Age−specific fertility fitted trend lines, 1950−2019: Jordan, Ages: 45−49
Data points enclosed with a diamond are reference sources

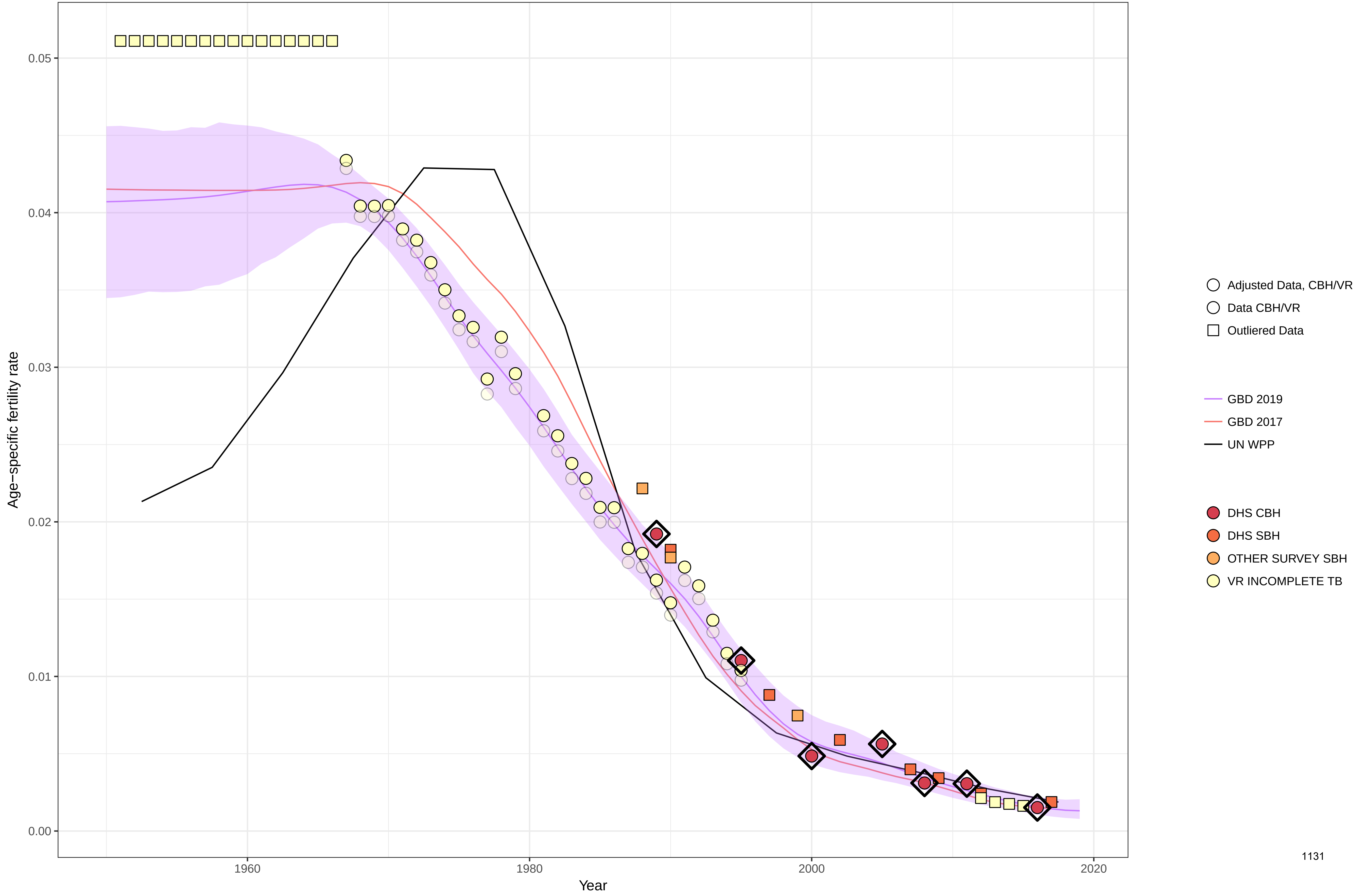

Age−specific fertility fitted trend lines, 1950−2019: Jordan, Ages: 50−54
Data points enclosed with a diamond are reference sources

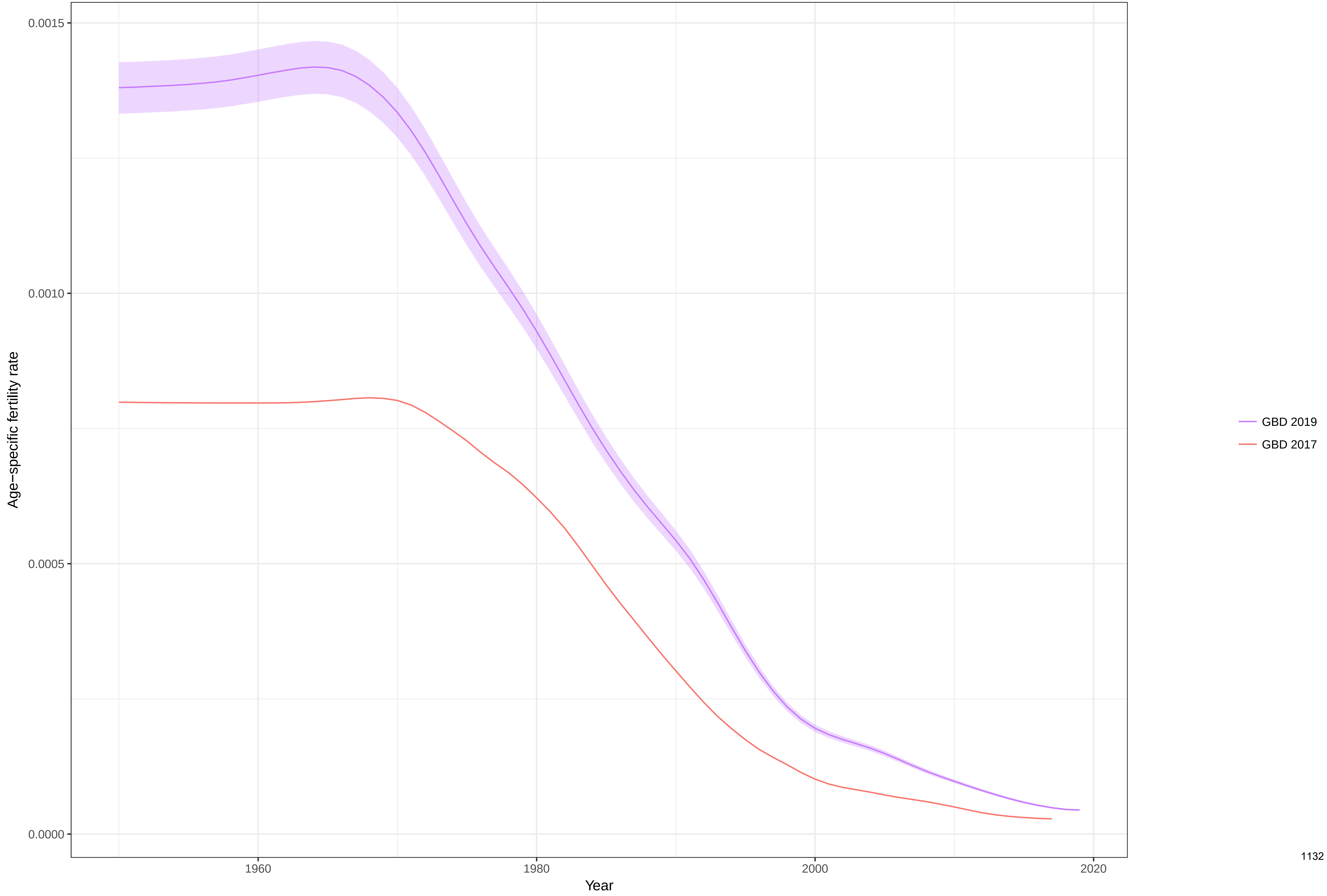

Total fertility rate fitted trend lines, 1950−2019: Jordan TFR
Data points enclosed with a diamond are reference sources

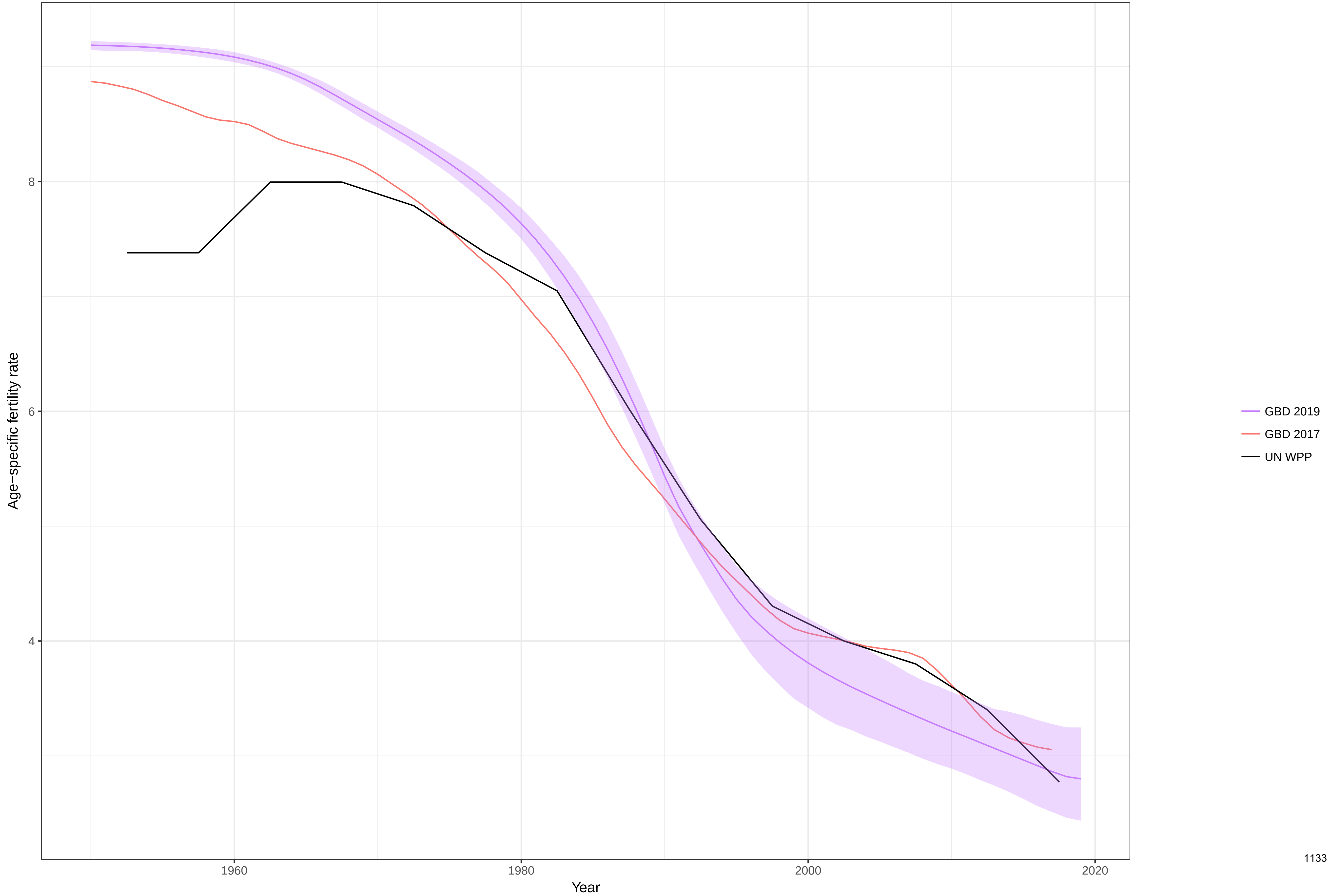

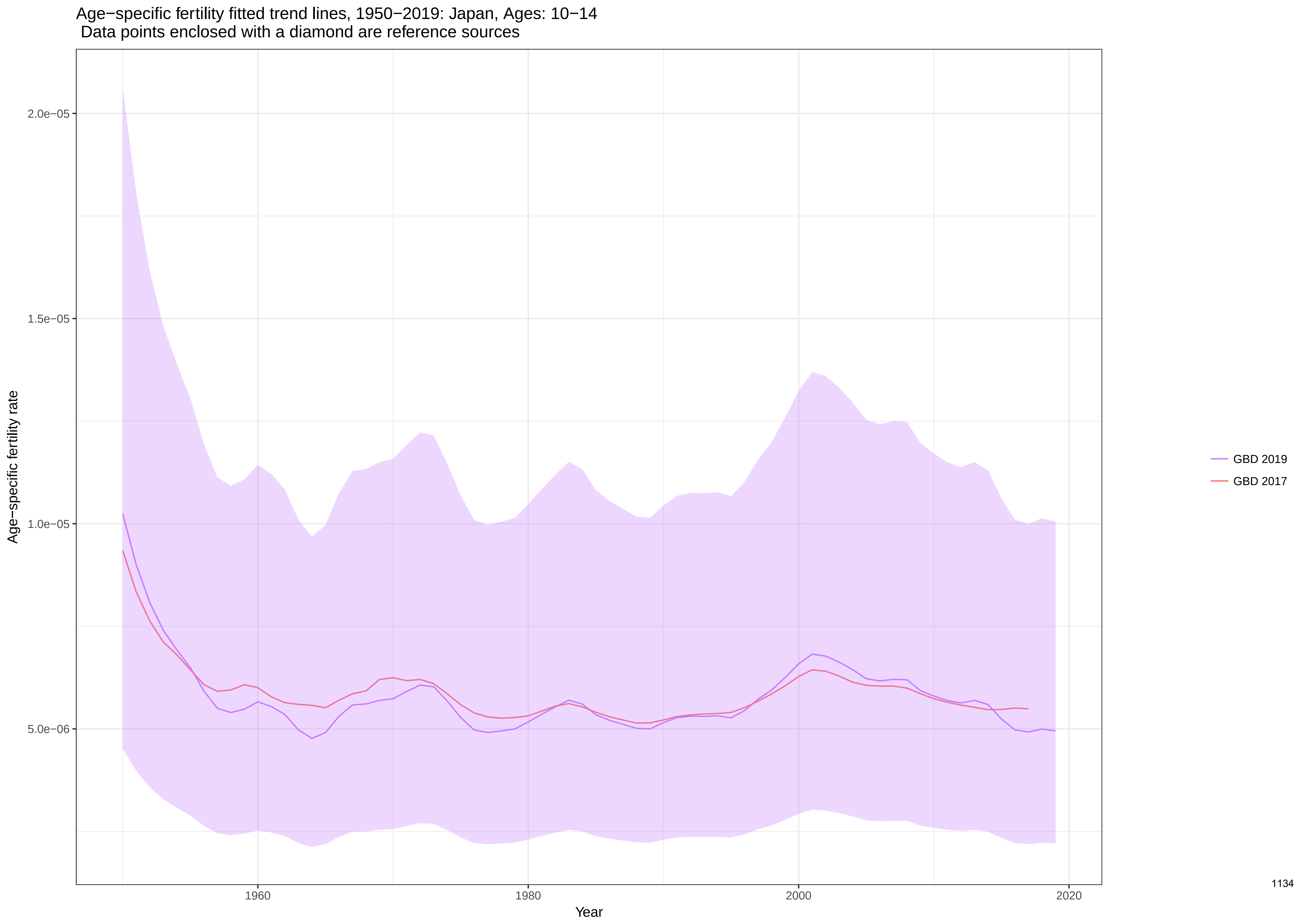

Age−specific fertility fitted trend lines, 1950−2019: Japan, Ages: 10−14
Data points enclosed with a diamond are reference sources
Age−specific fertility rate
2.0e−05
1.5e−05
1.0e−05
5.0e−06
1960
1980
2000
2020
Year
GBD 2019
GBD 2017

Age−specific fertility fitted trend lines, 1950−2019: Japan, Ages: 15−19
Data points enclosed with a diamond are reference sources

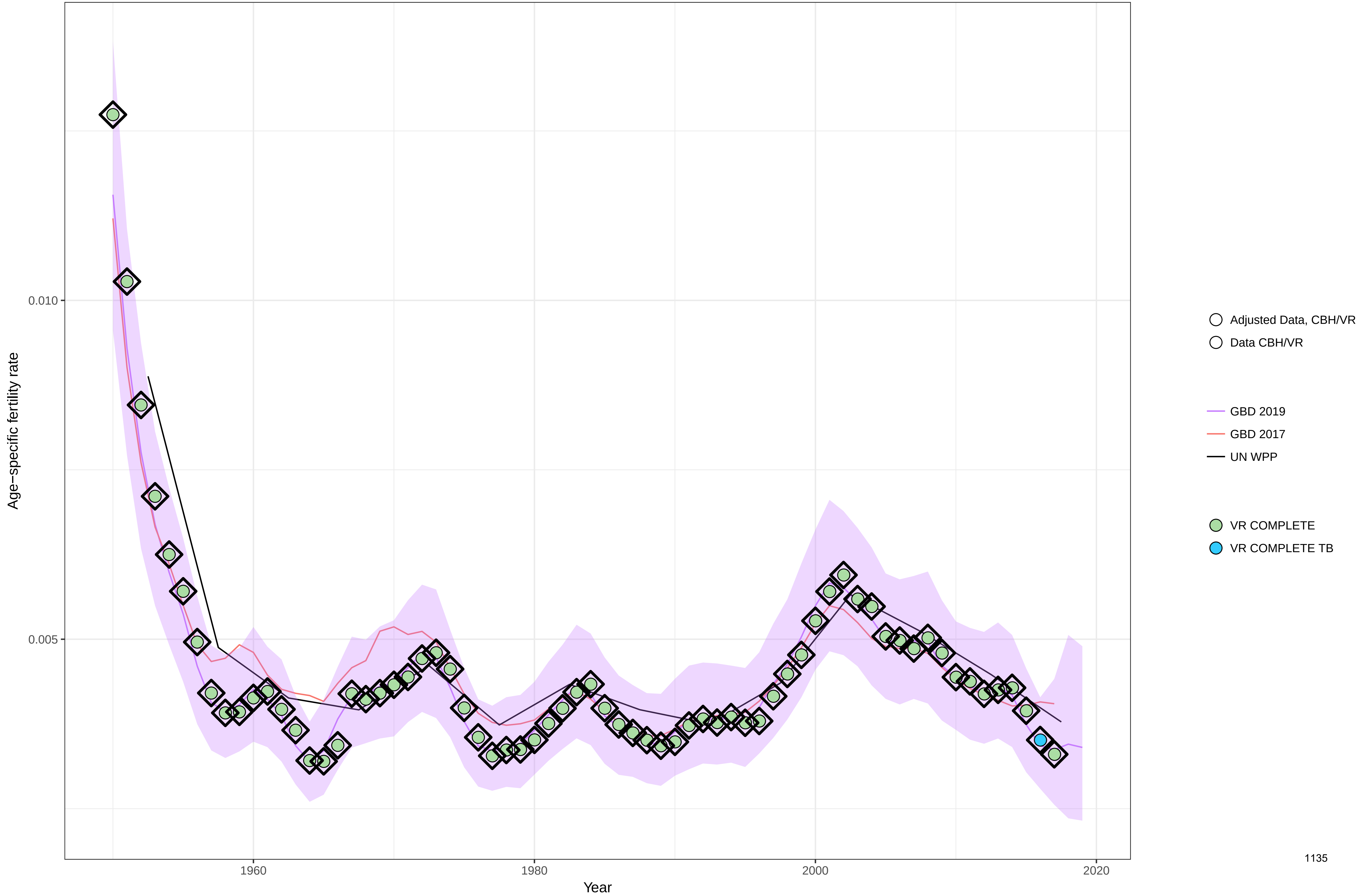

Age−specific fertility fitted trend lines, 1950−2019: Japan, Ages: 20−24
Data points enclosed with a diamond are reference sources

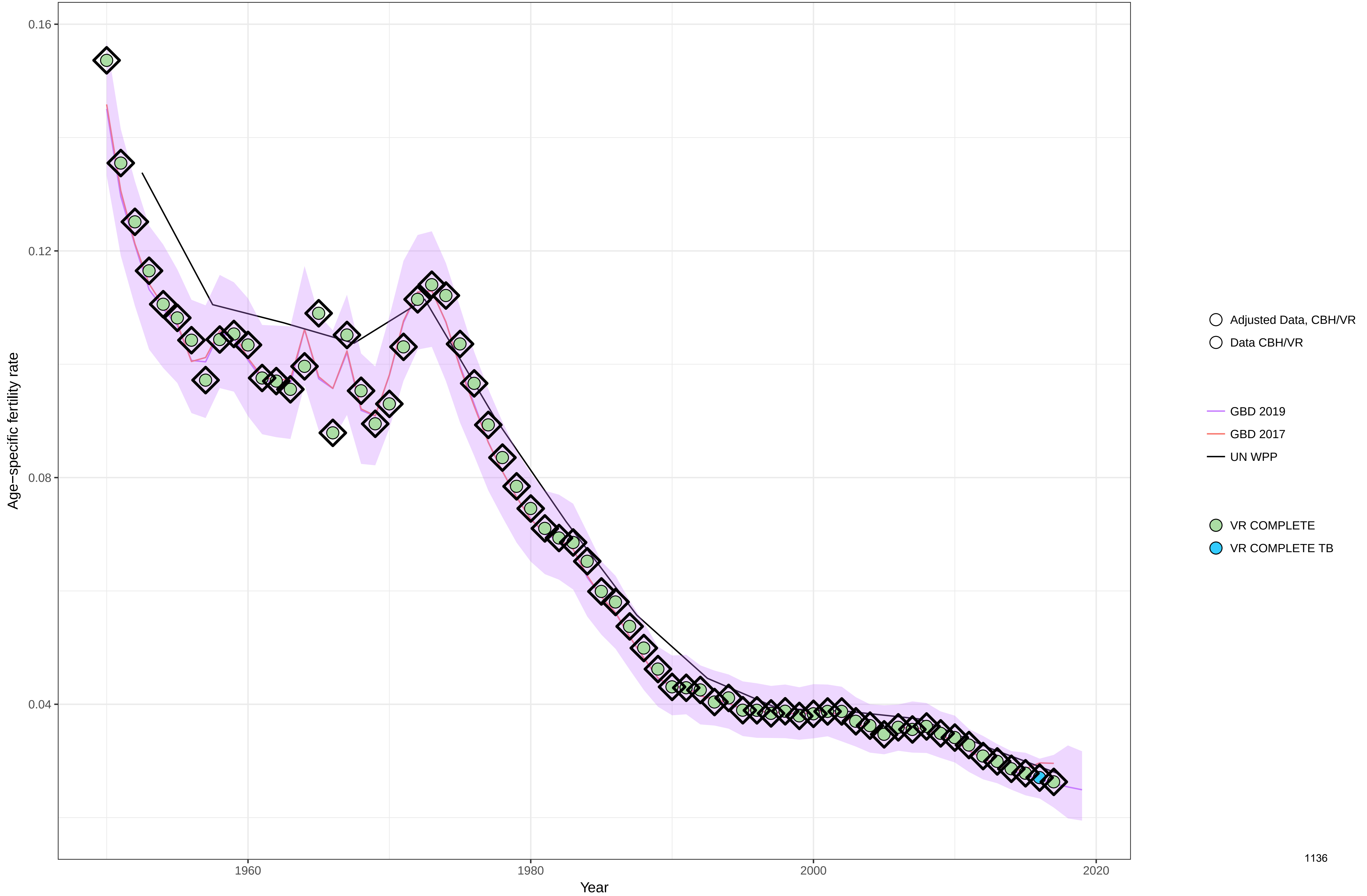

Age−specific fertility fitted trend lines, 1950−2019: Japan, Ages: 25−29
Data points enclosed with a diamond are reference sources

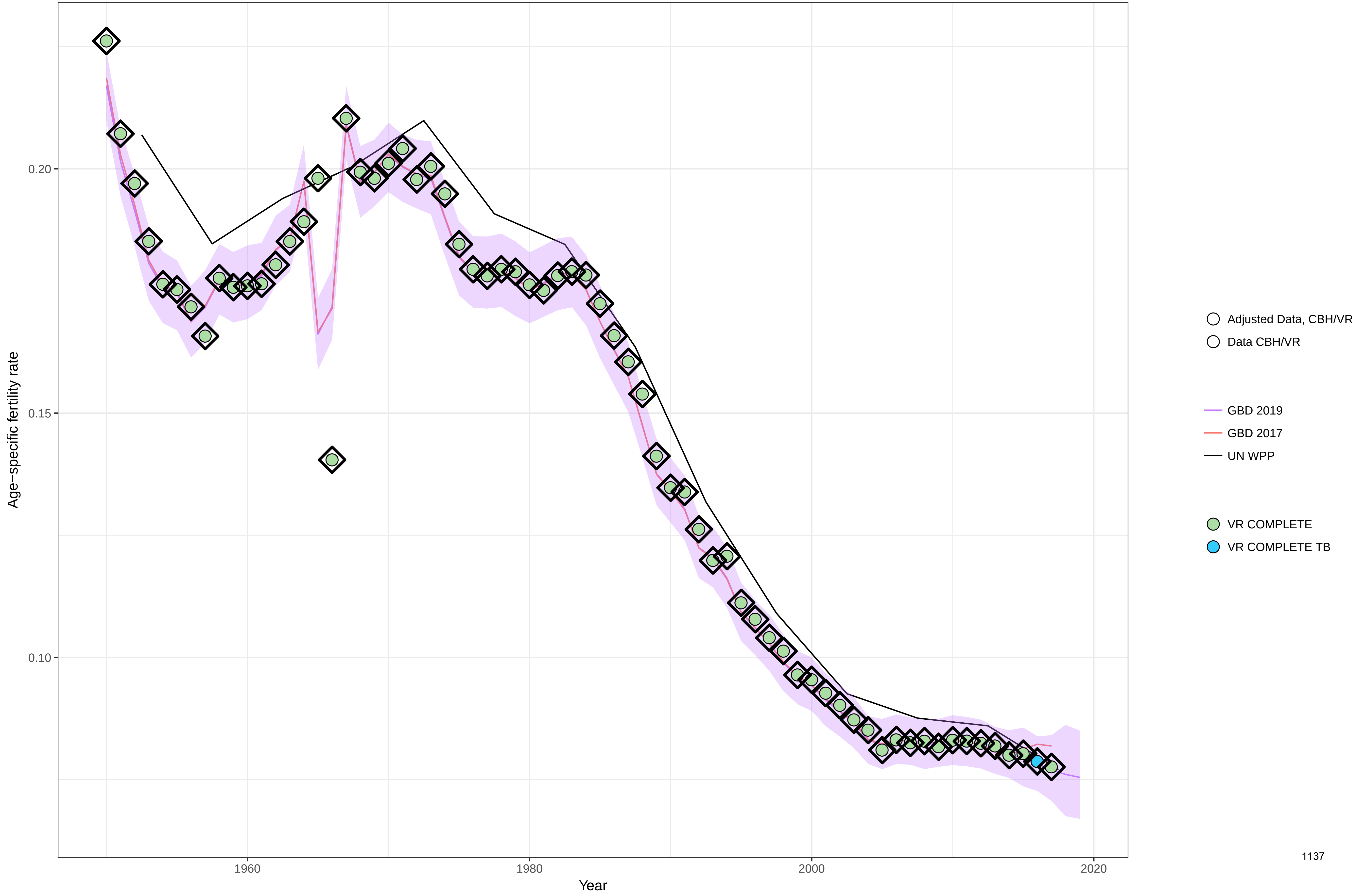

Age−specific fertility fitted trend lines, 1950−2019: Japan, Ages: 30−34
Data points enclosed with a diamond are reference sources

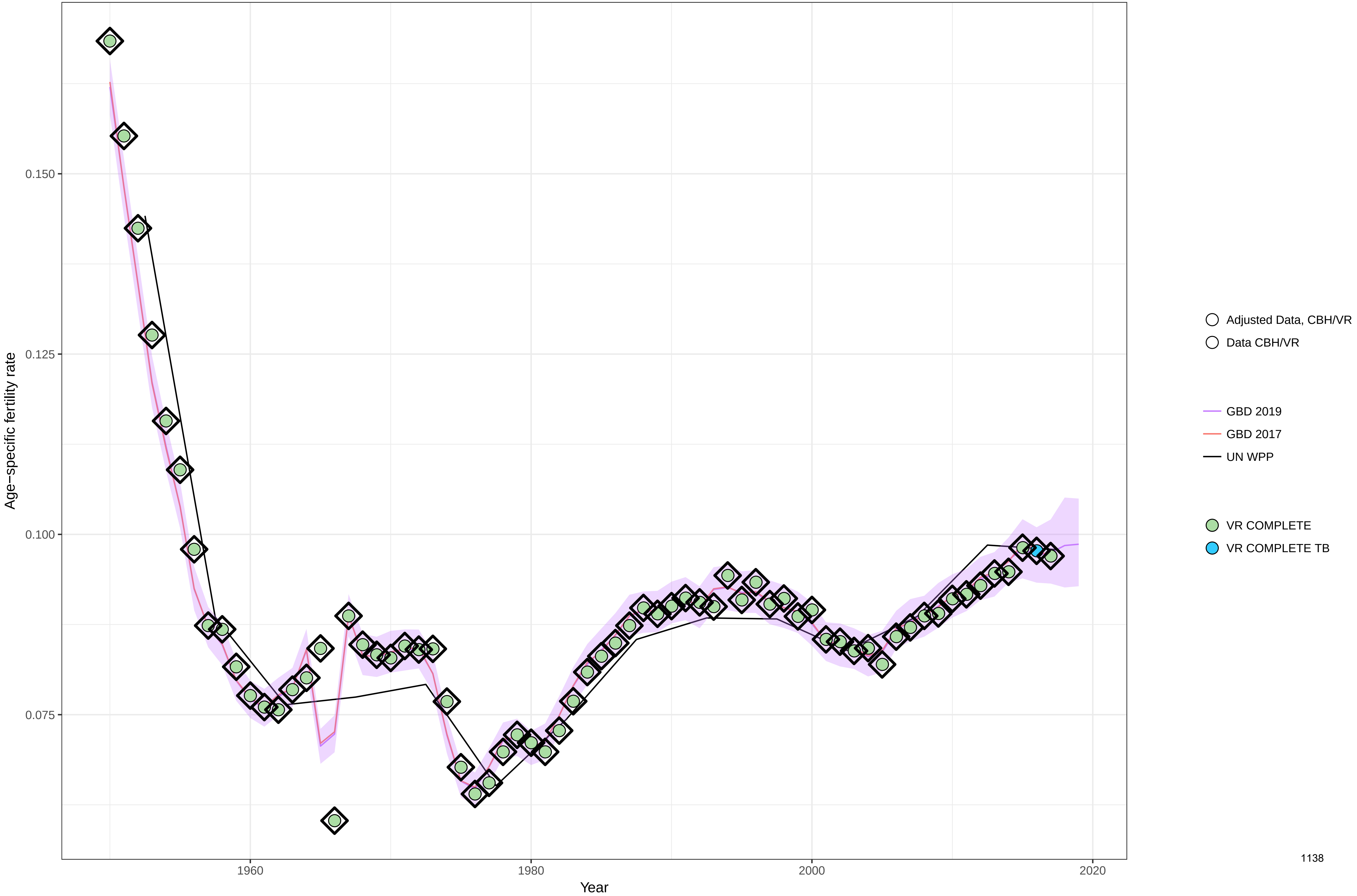

Age−specific fertility fitted trend lines, 1950−2019: Japan, Ages: 35−39
Data points enclosed with a diamond are reference sources

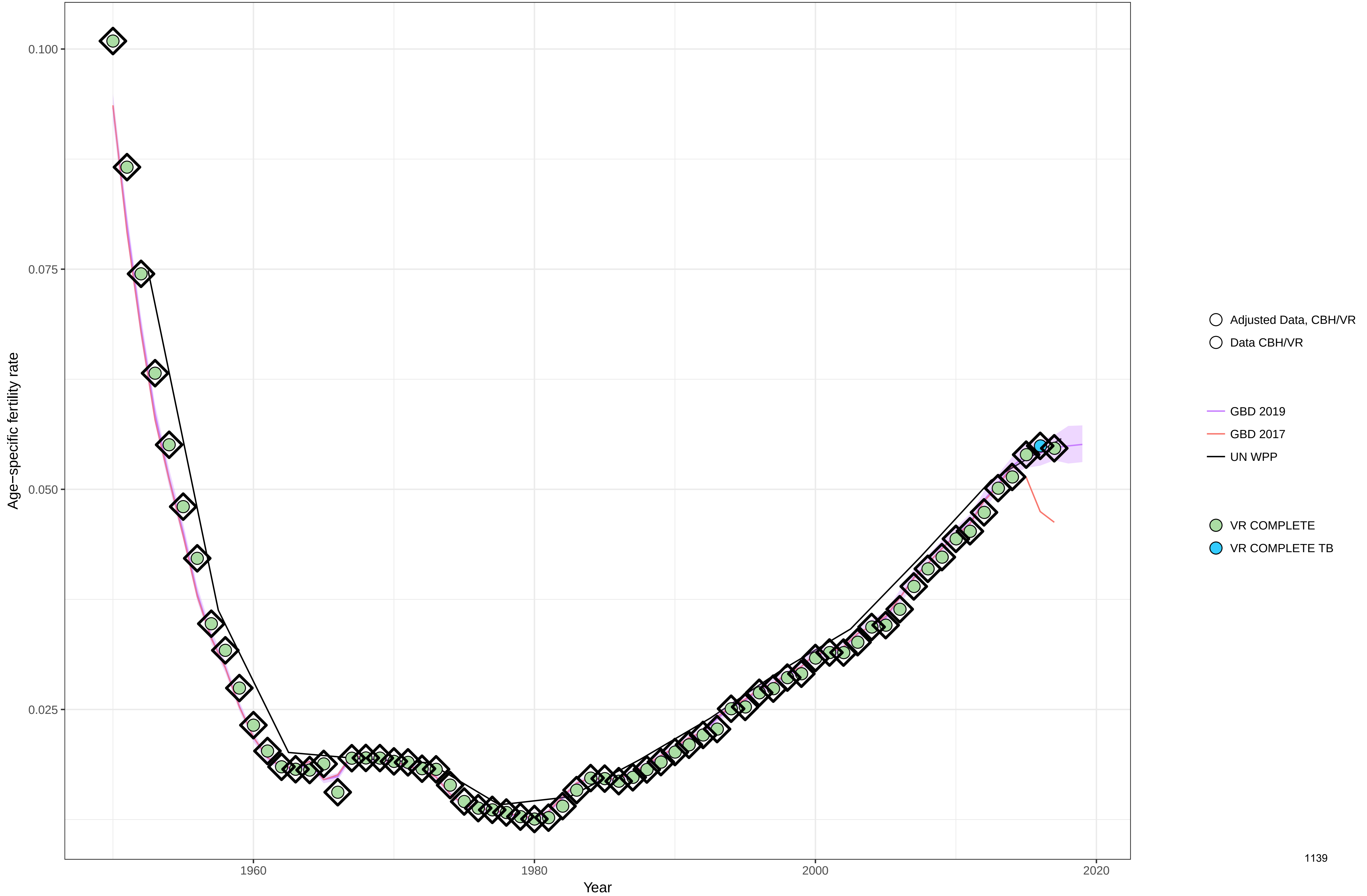

Age−specific fertility fitted trend lines, 1950−2019: Japan, Ages: 40−44
Data points enclosed with a diamond are reference sources

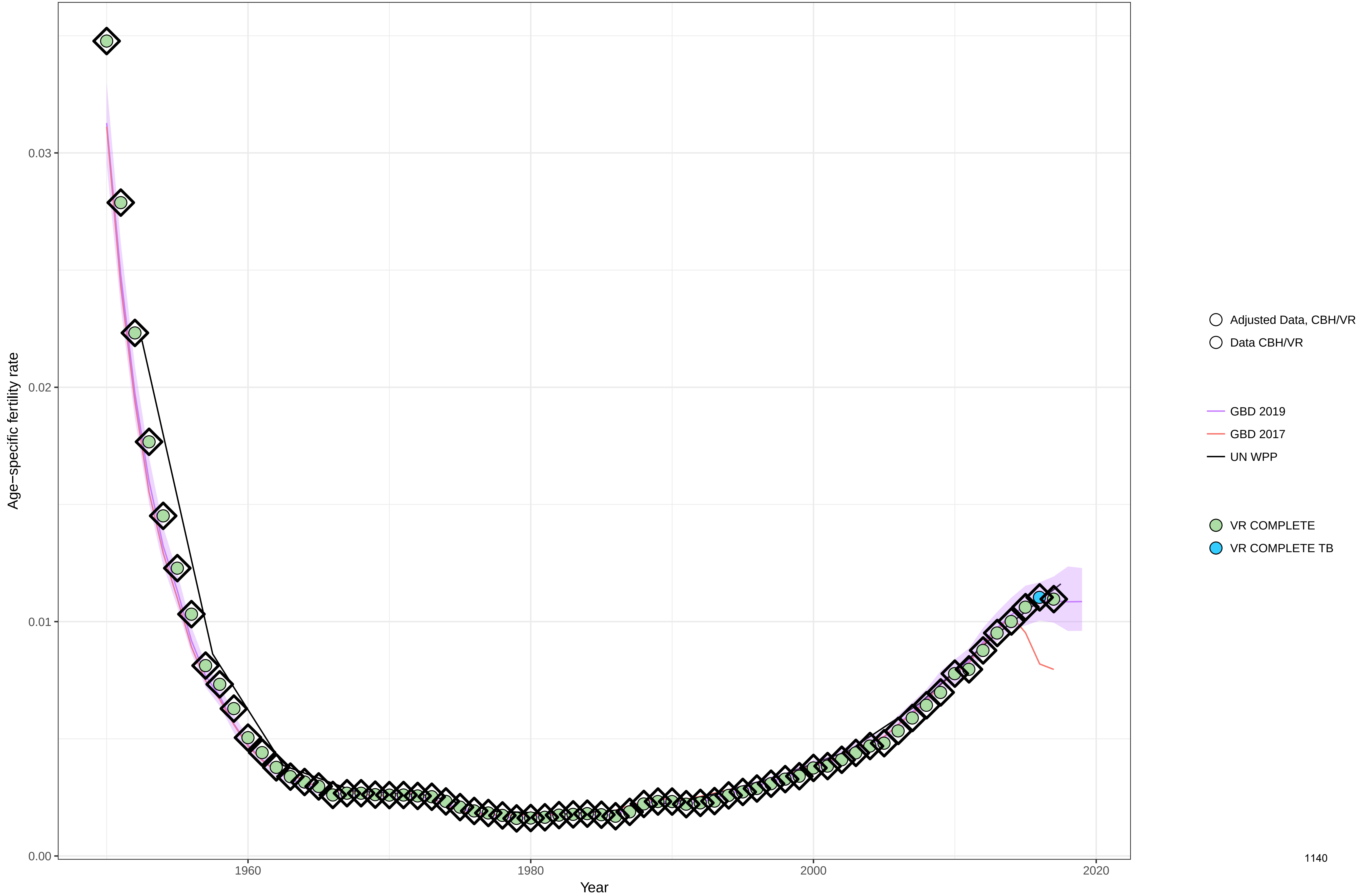

Age−specific fertility fitted trend lines, 1950−2019: Japan, Ages: 45−49
Data points enclosed with a diamond are reference sources

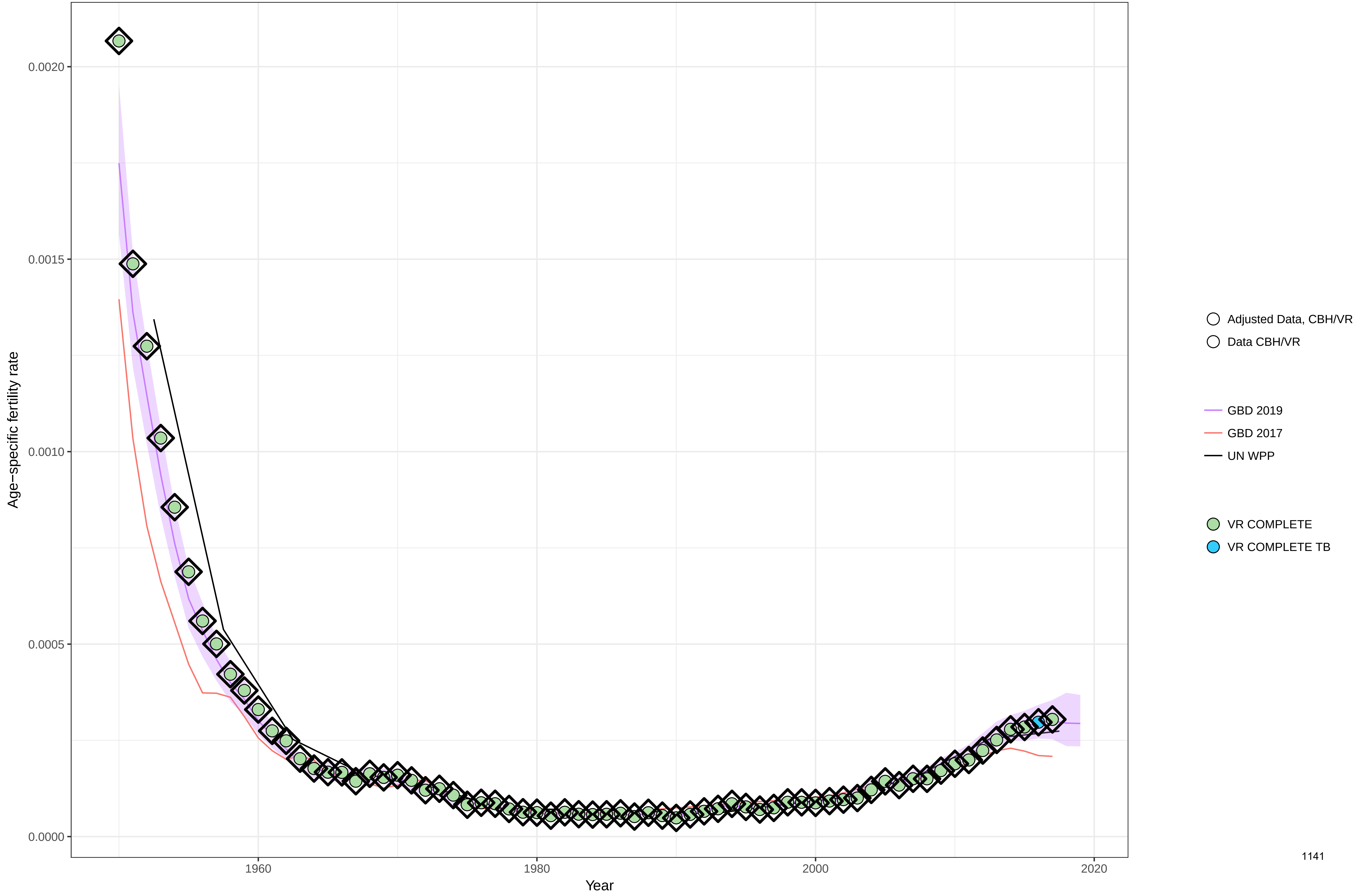

Age−specific fertility fitted trend lines, 1950−2019: Japan, Ages: 50−54
Data points enclosed with a diamond are reference sources

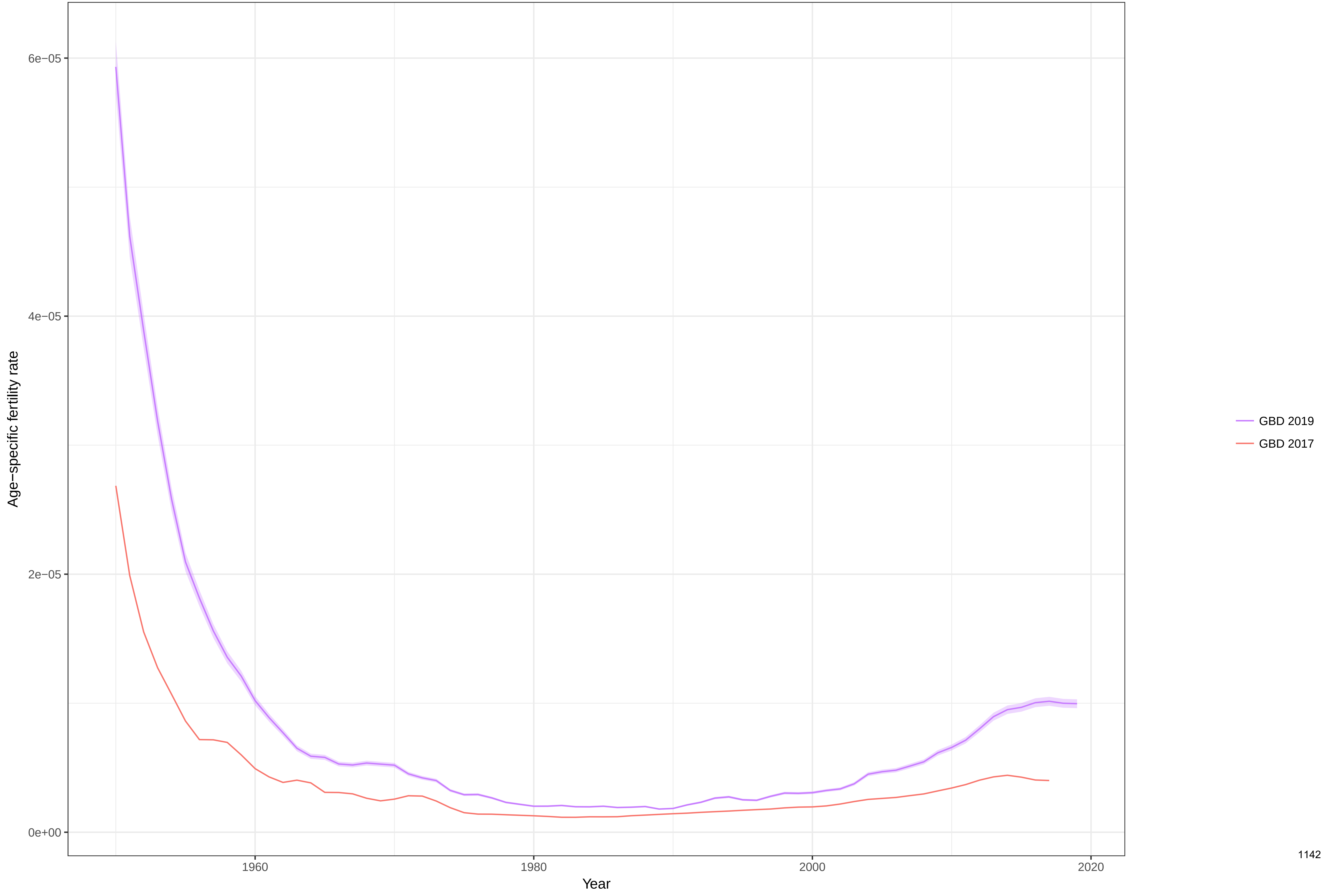

Total fertility rate fitted trend lines, 1950−2019: Japan TFR
Data points enclosed with a diamond are reference sources

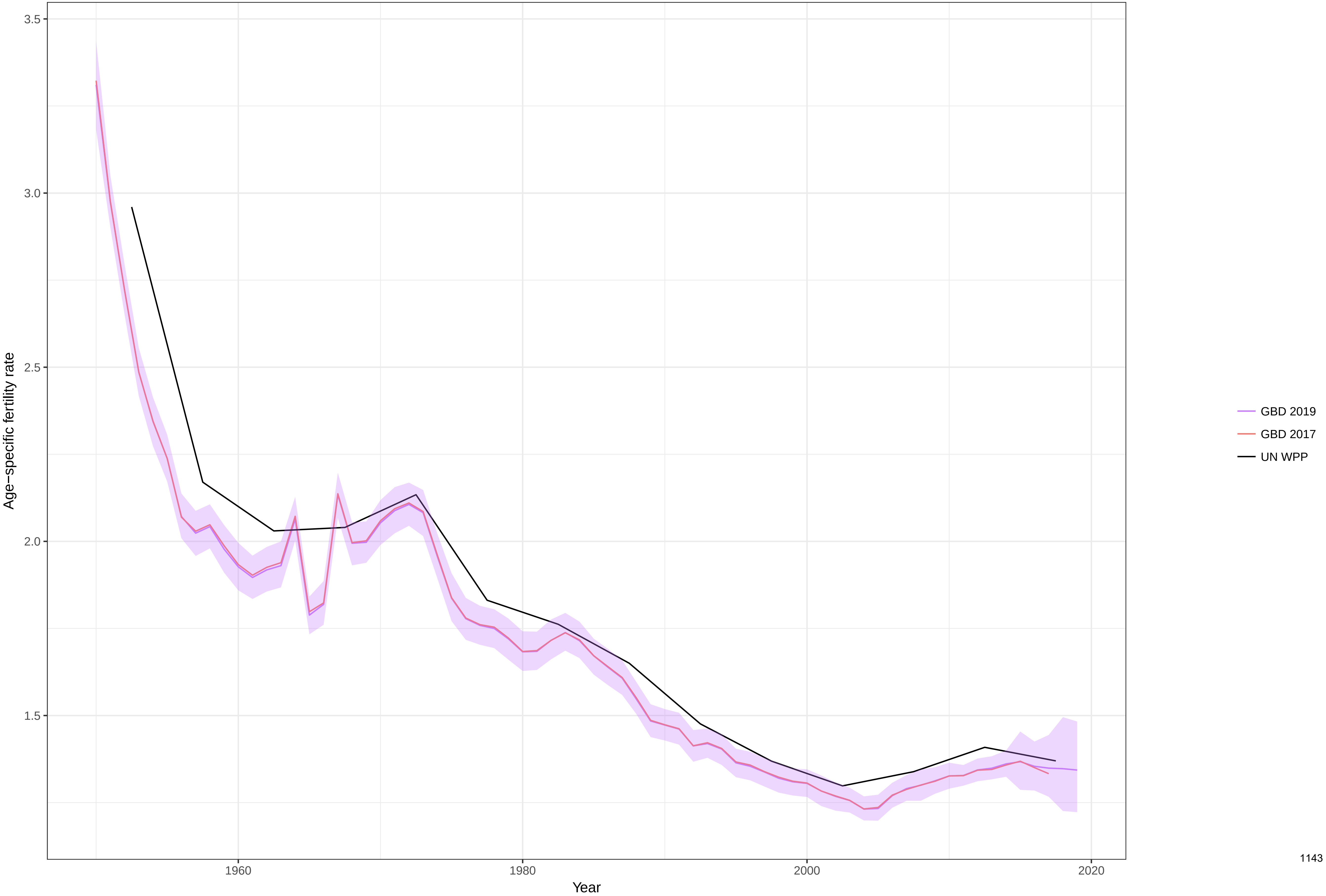

Age−specific fertility fitted trend lines, 1950−2019: Kazakhstan, Ages: 10−14
Data points enclosed with a diamond are reference sources

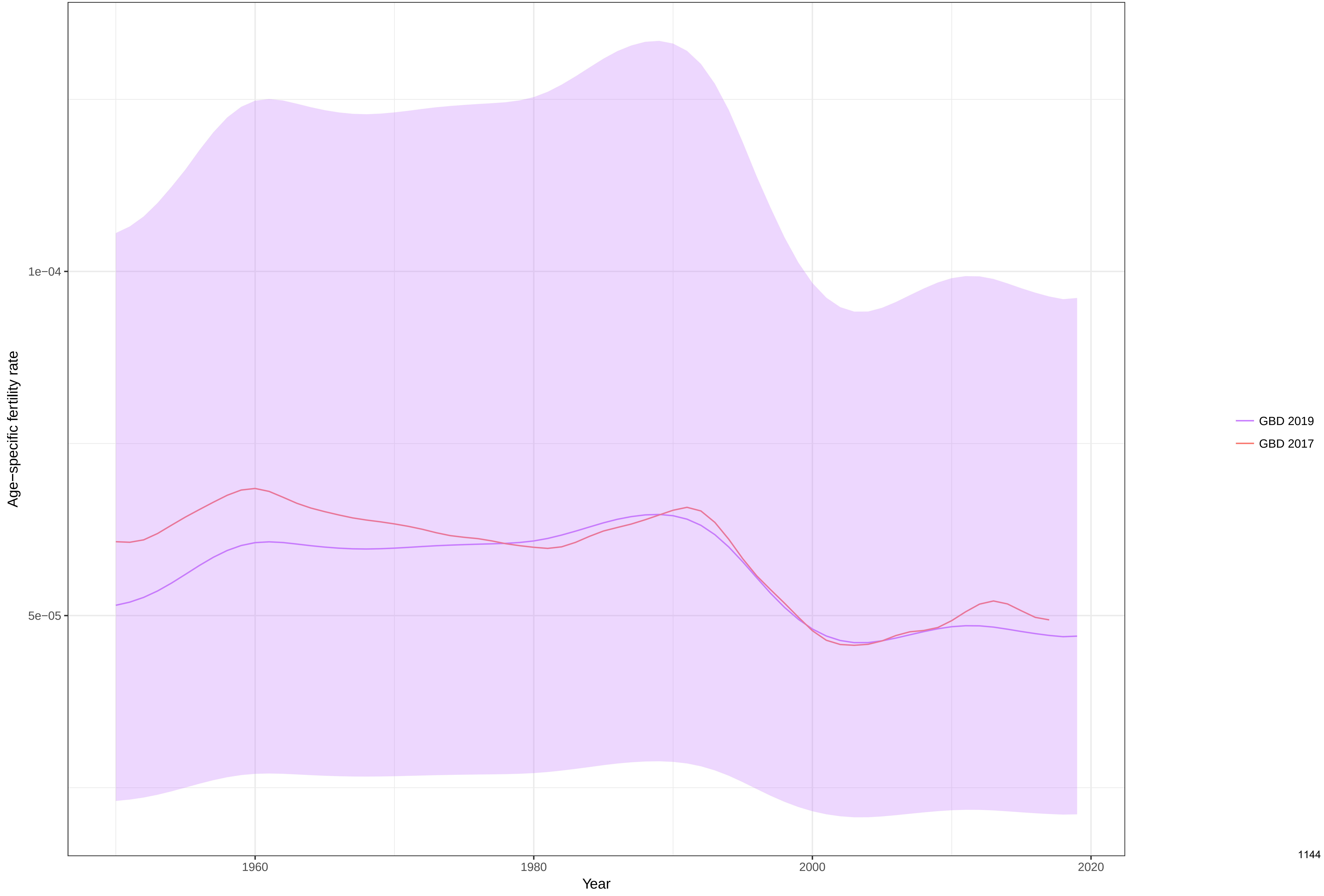

Age−specific fertility fitted trend lines, 1950−2019: Kazakhstan, Ages: 15−19
Data points enclosed with a diamond are reference sources

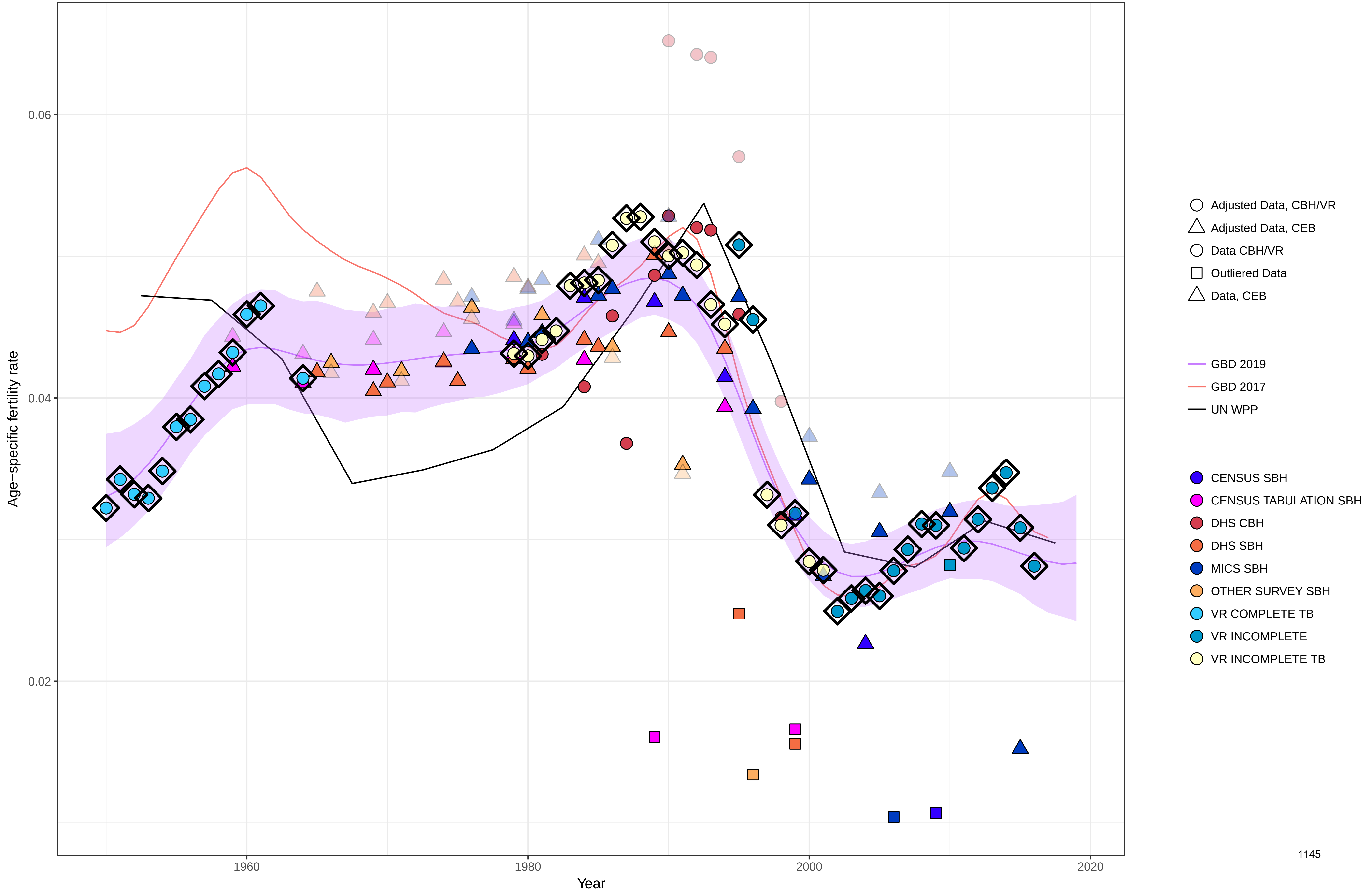

Age−specific fertility fitted trend lines, 1950−2019: Kazakhstan, Ages: 20−24
Data points enclosed with a diamond are reference sources

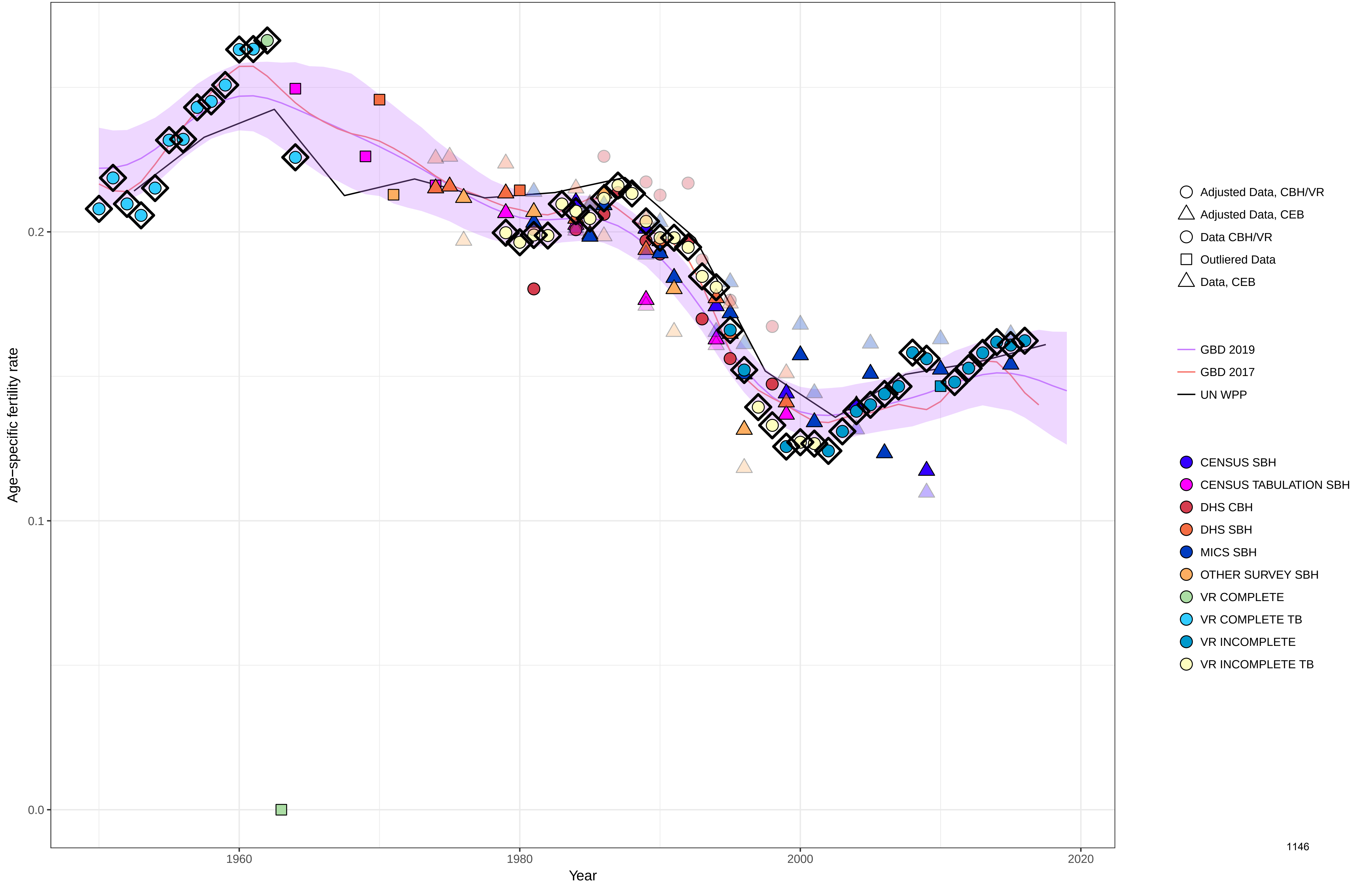

Age−specific fertility fitted trend lines, 1950−2019: Kazakhstan, Ages: 25−29
Data points enclosed with a diamond are reference sources

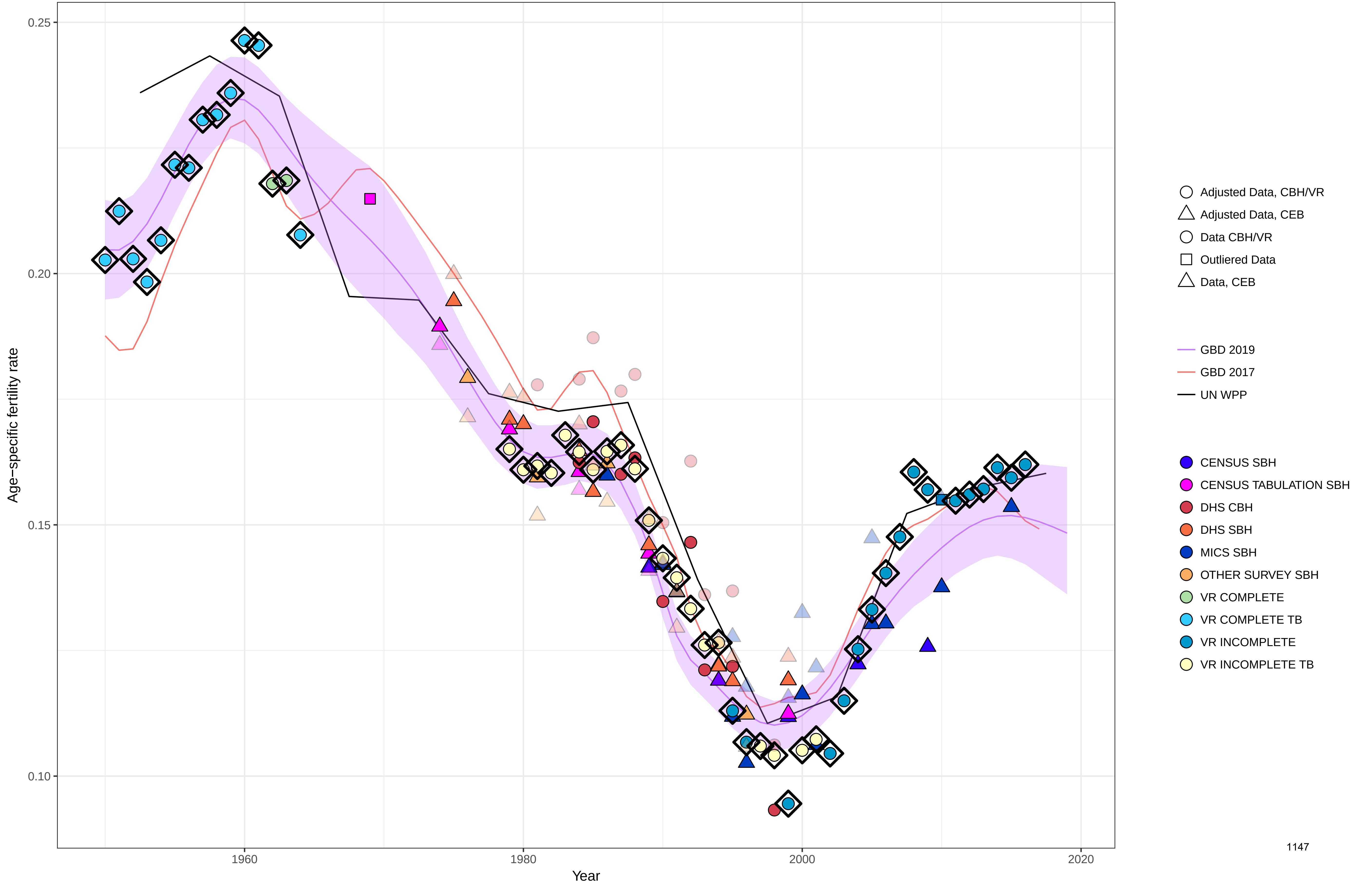

Age−specific fertility fitted trend lines, 1950−2019: Kazakhstan, Ages: 30−34
Data points enclosed with a diamond are reference sources

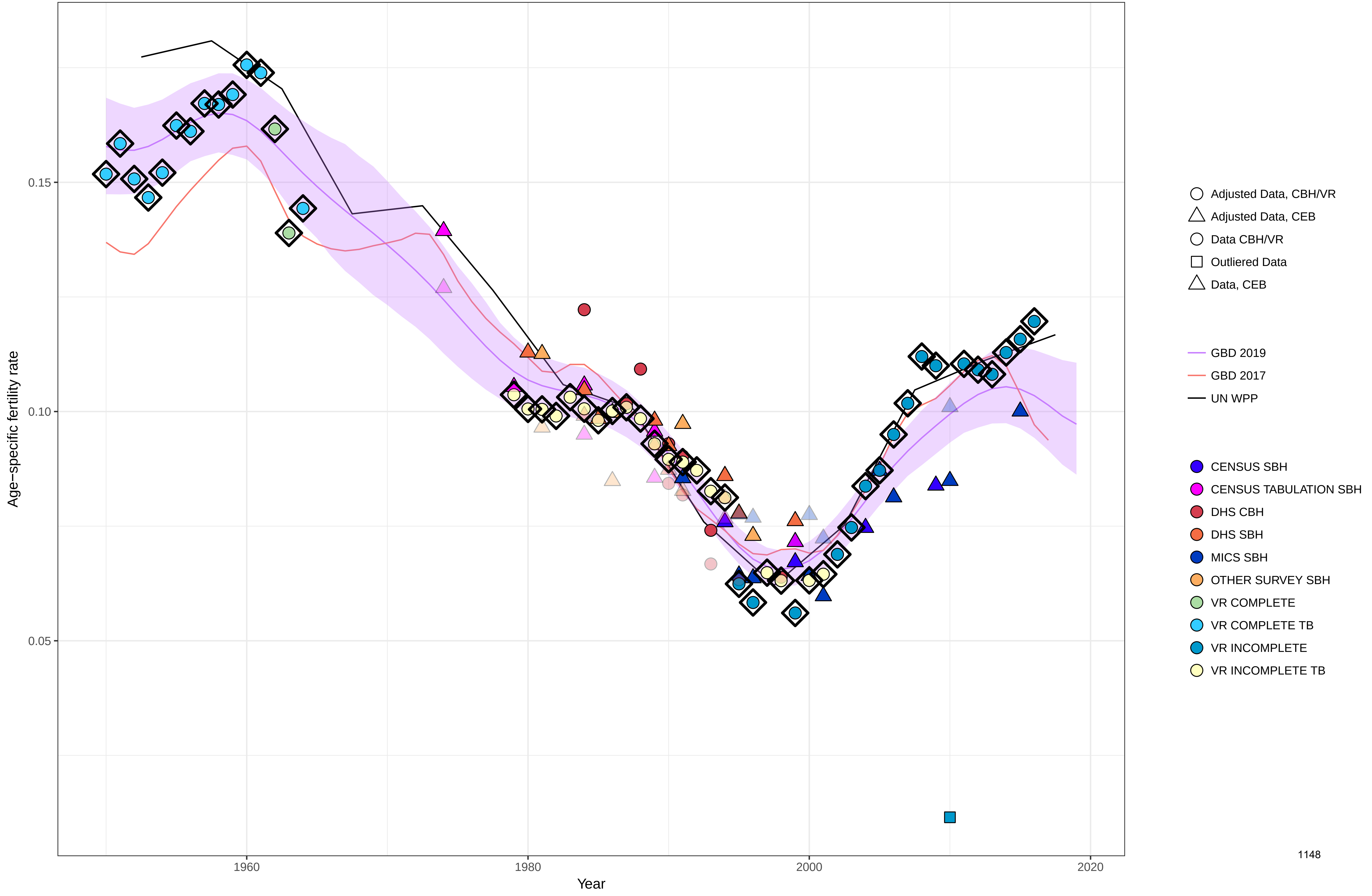

Age−specific fertility fitted trend lines, 1950−2019: Kazakhstan, Ages: 35−39
Data points enclosed with a diamond are reference sources

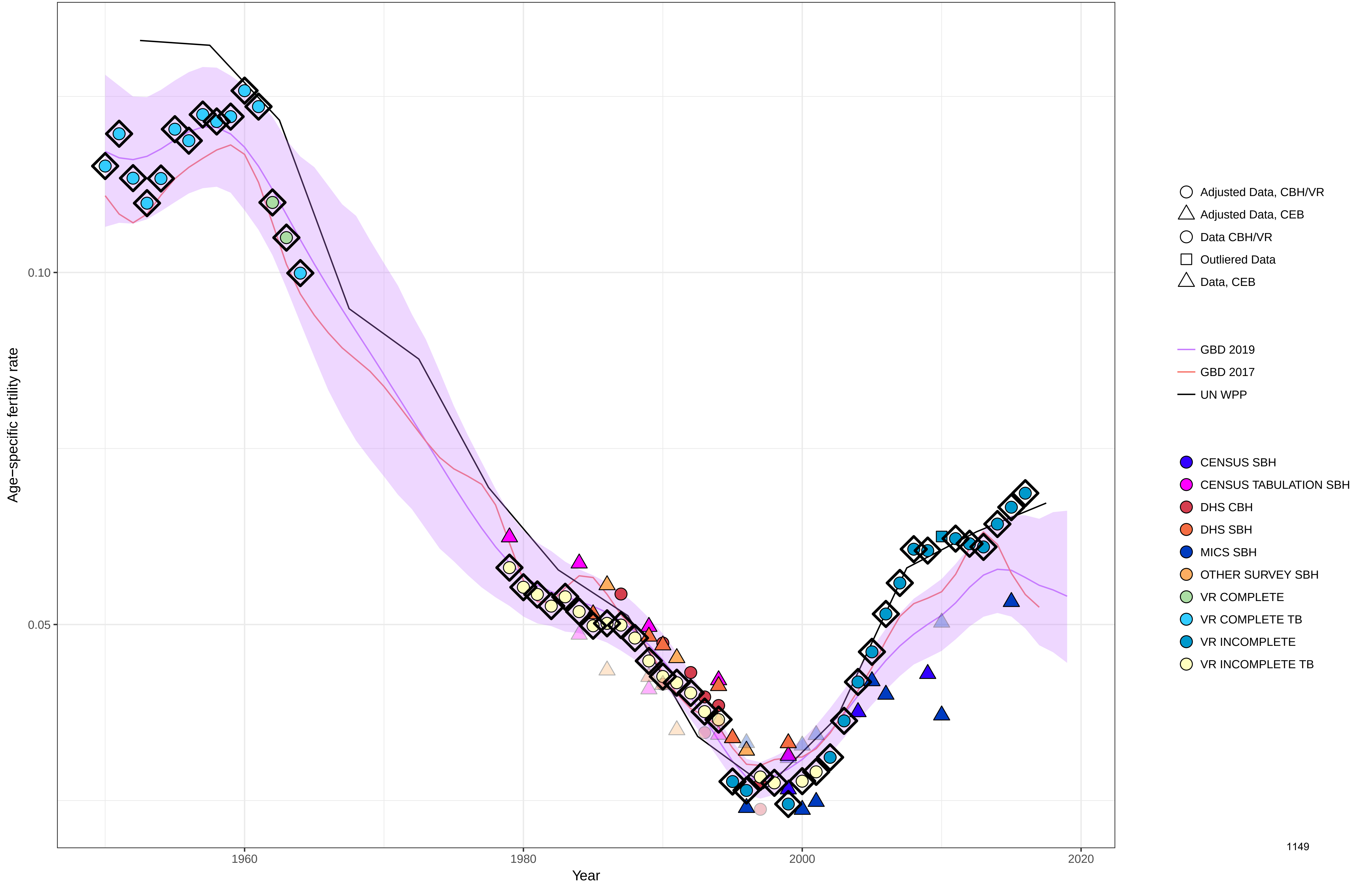

Age−specific fertility fitted trend lines, 1950−2019: Kazakhstan, Ages: 40−44
Data points enclosed with a diamond are reference sources

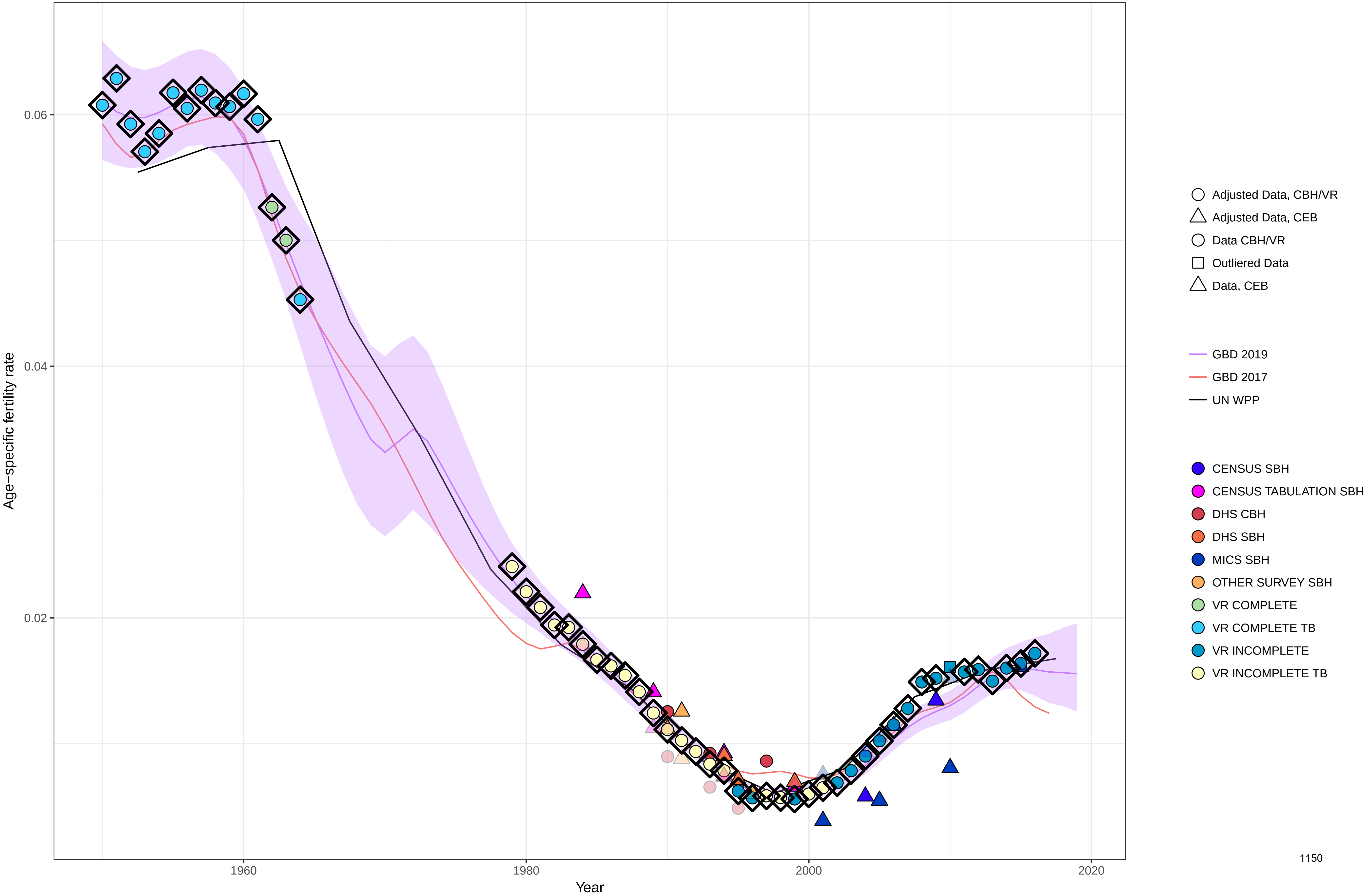

Age−specific fertility fitted trend lines, 1950−2019: Kazakhstan, Ages: 45−49
Data points enclosed with a diamond are reference sources

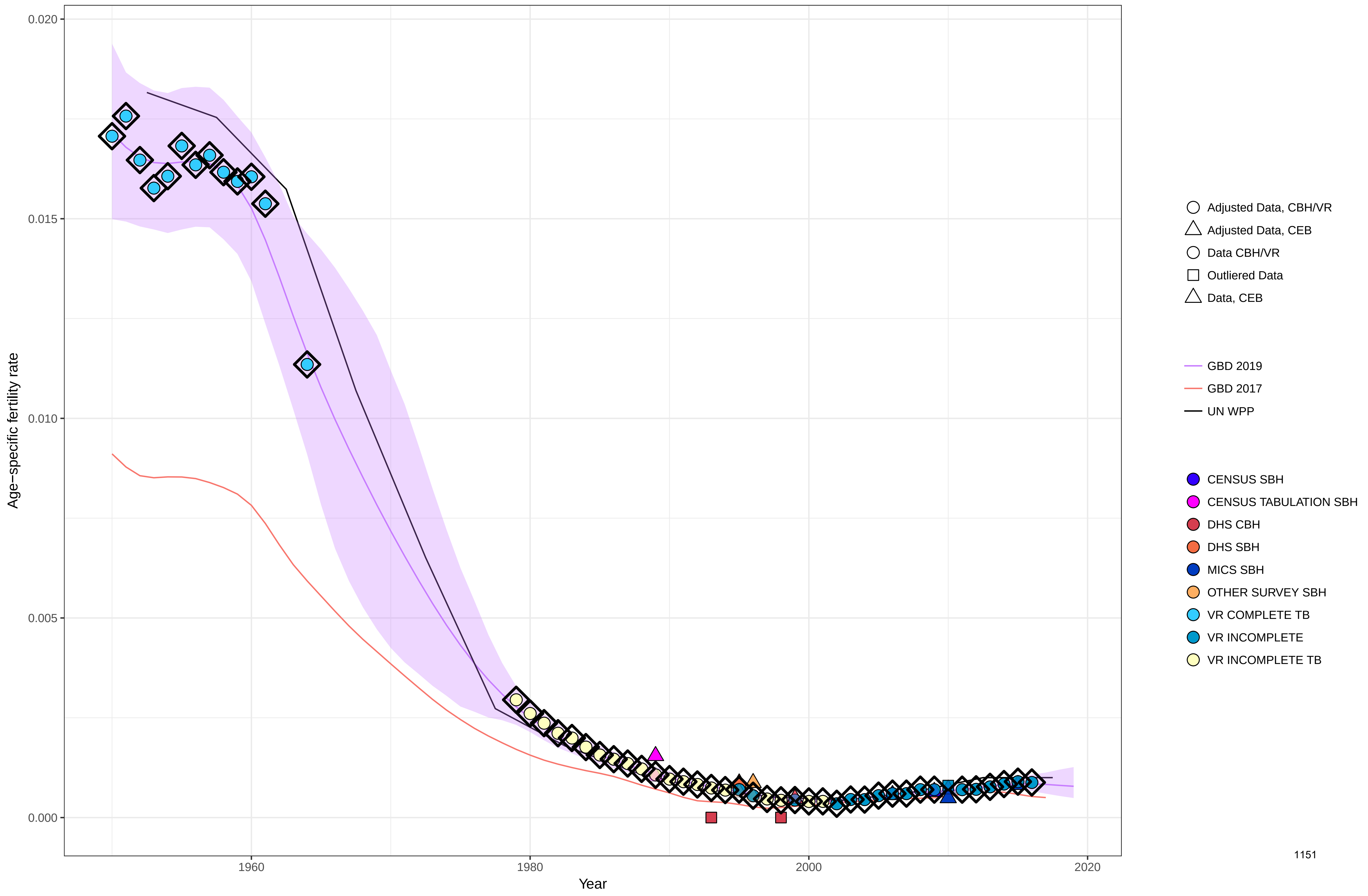

Age−specific fertility fitted trend lines, 1950−2019: Kazakhstan, Ages: 50−54
Data points enclosed with a diamond are reference sources

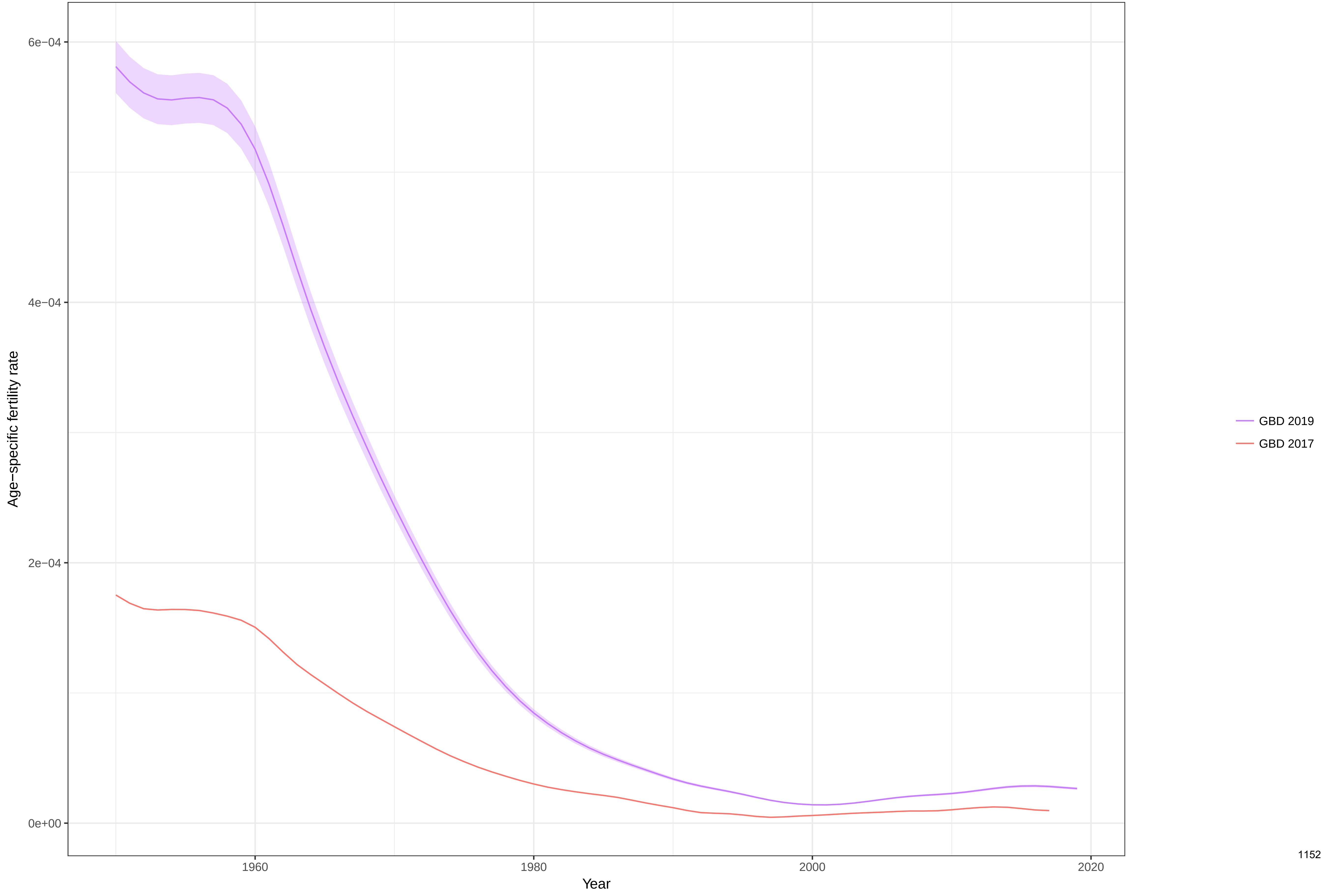

Total fertility rate fitted trend lines, 1950−2019: Kazakhstan TFR
Data points enclosed with a diamond are reference sources

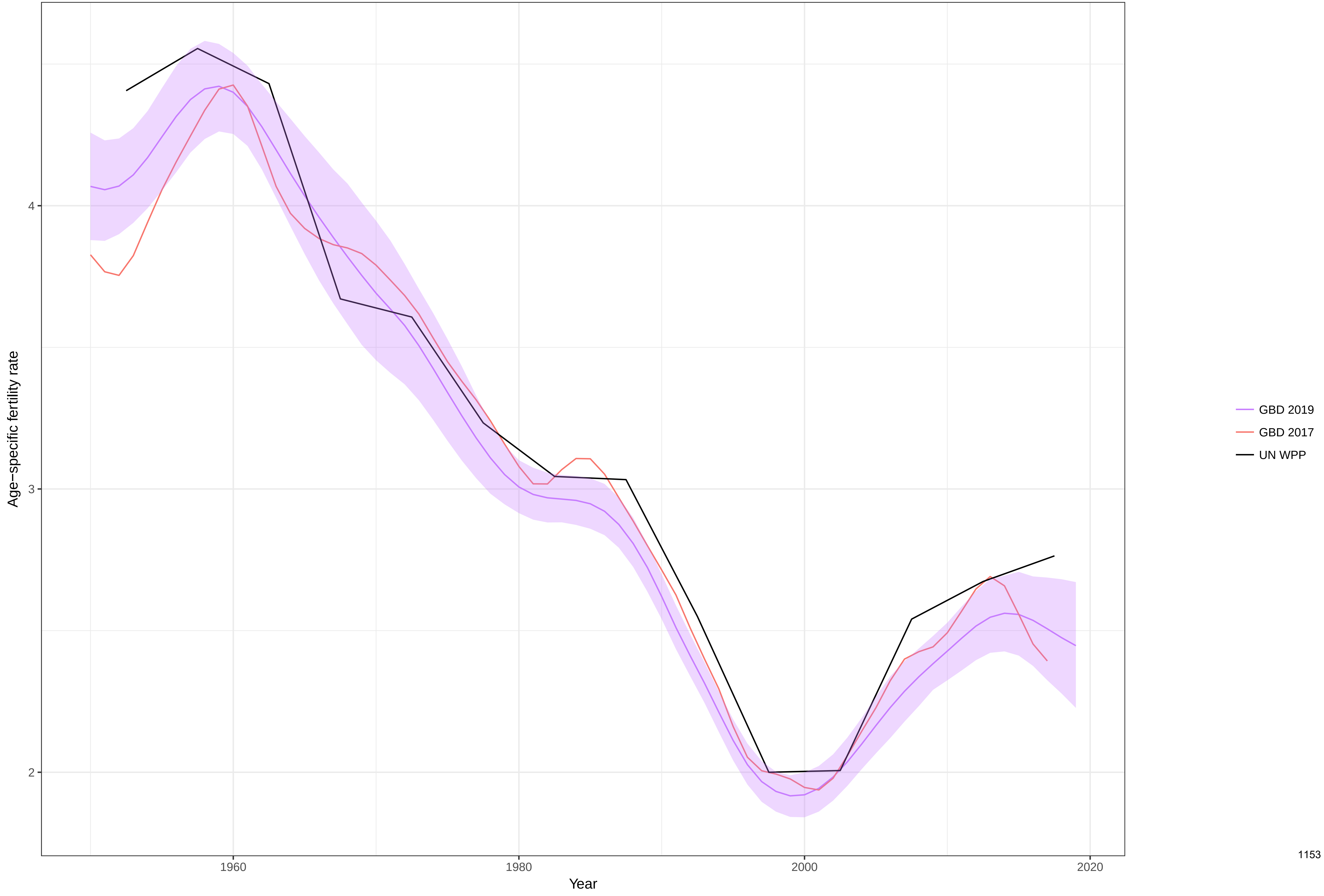

Age−specific fertility fitted trend lines, 1950−2019: Kenya, Ages: 10−14
Data points enclosed with a diamond are reference sources

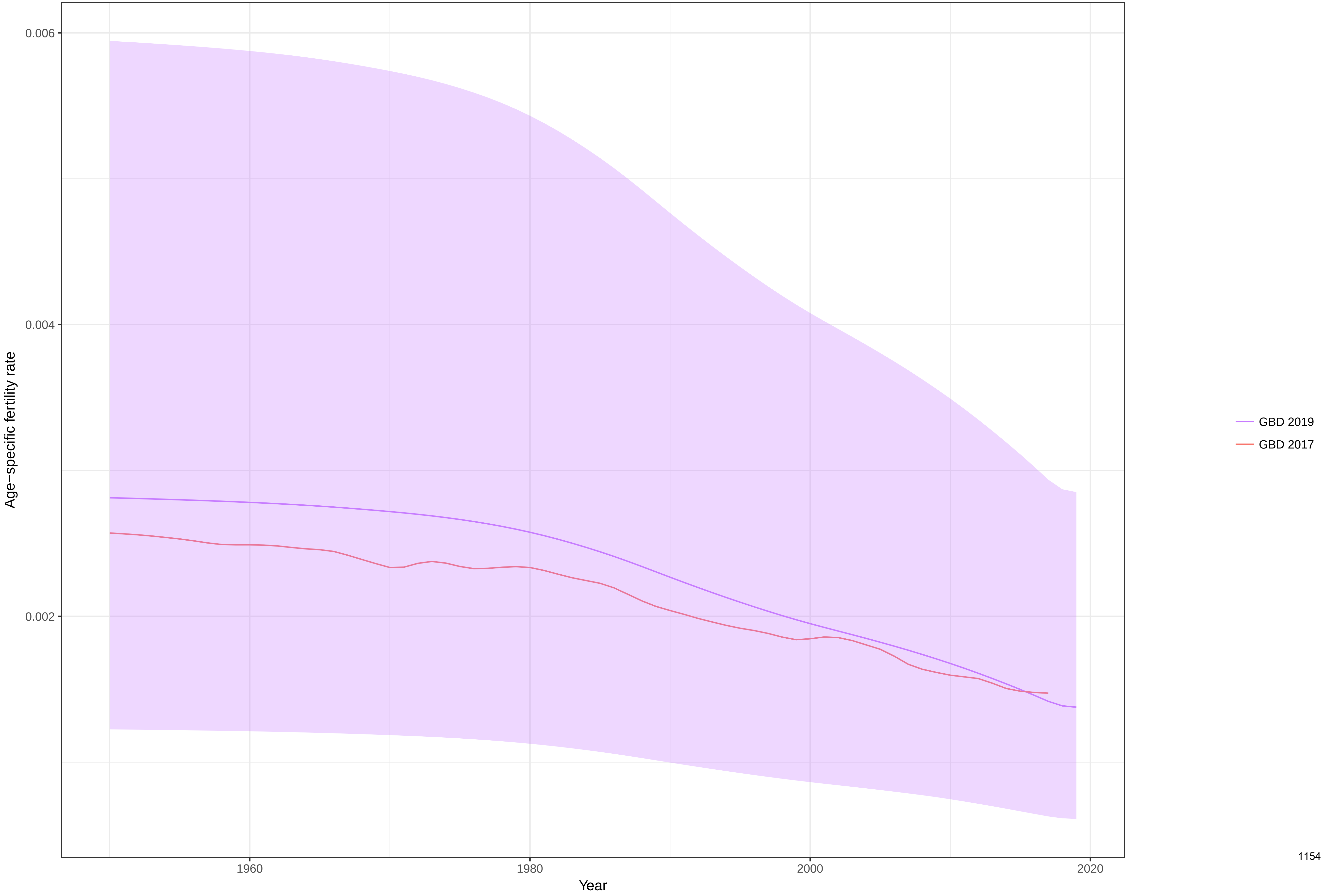

Age−specific fertility fitted trend lines, 1950−2019: Kenya, Ages: 15−19
Data points enclosed with a diamond are reference sources

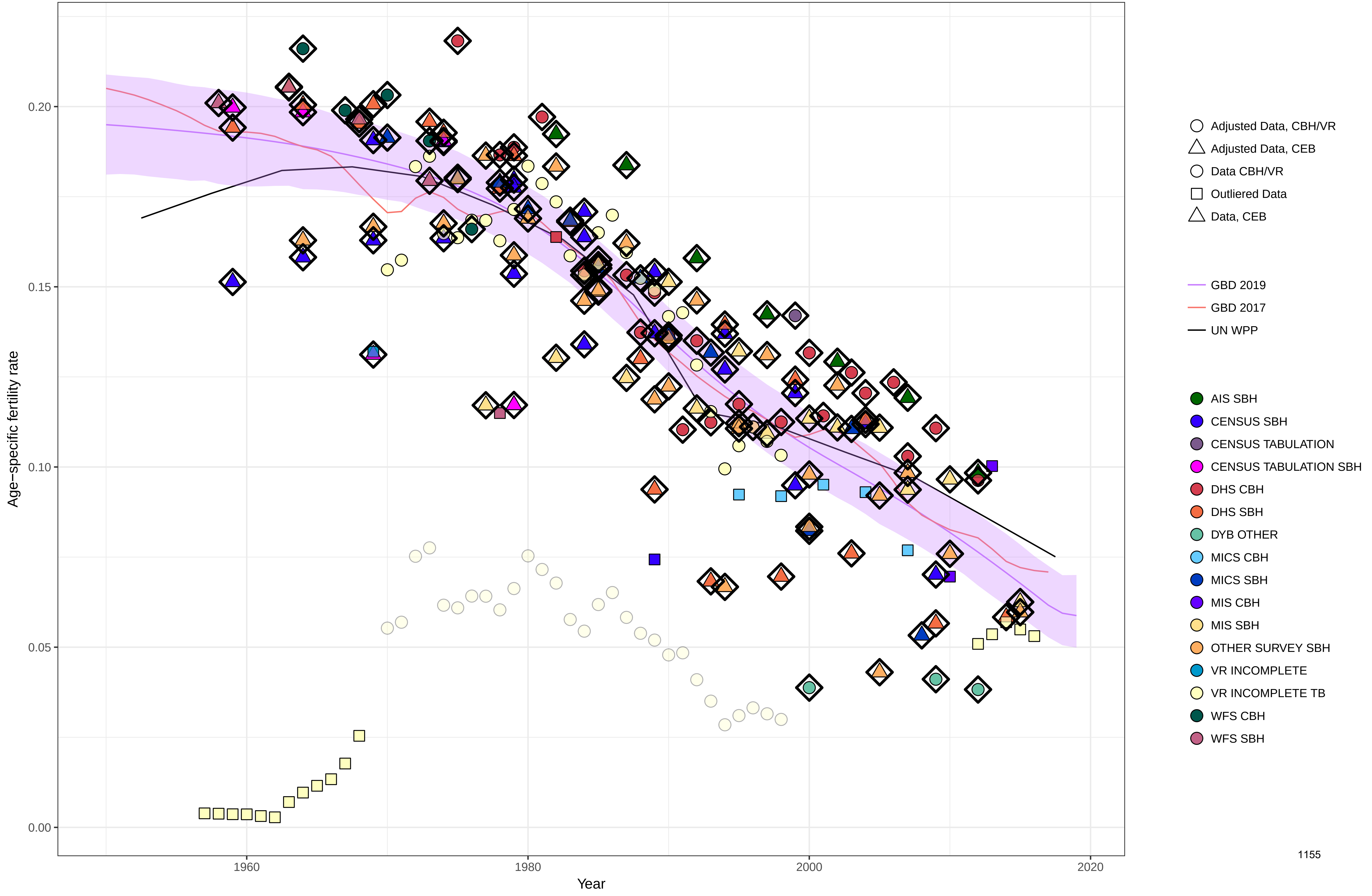

Age−specific fertility fitted trend lines, 1950−2019: Kenya, Ages: 20−24
Data points enclosed with a diamond are reference sources

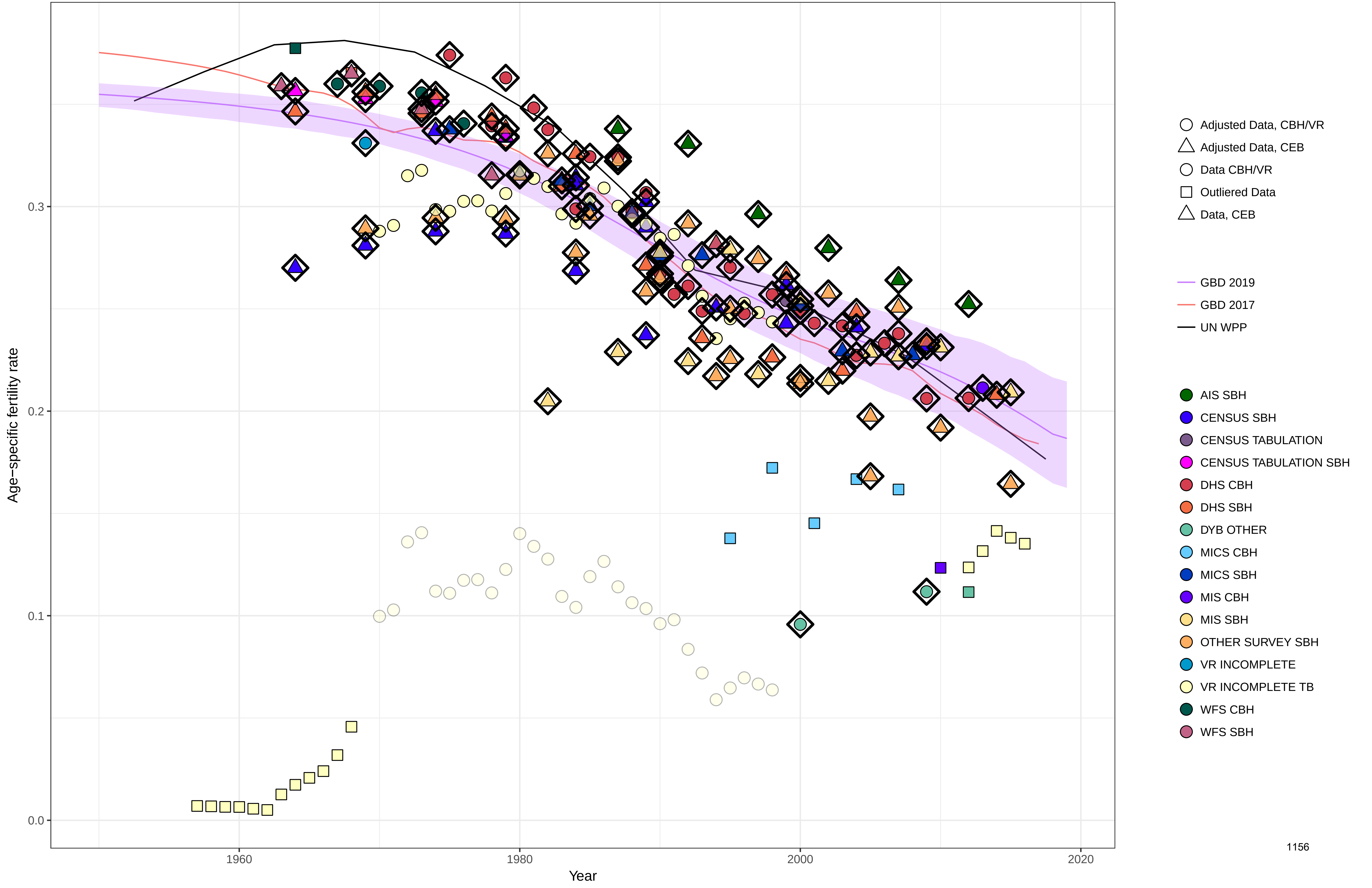

Age−specific fertility fitted trend lines, 1950−2019: Kenya, Ages: 25−29
Data points enclosed with a diamond are reference sources

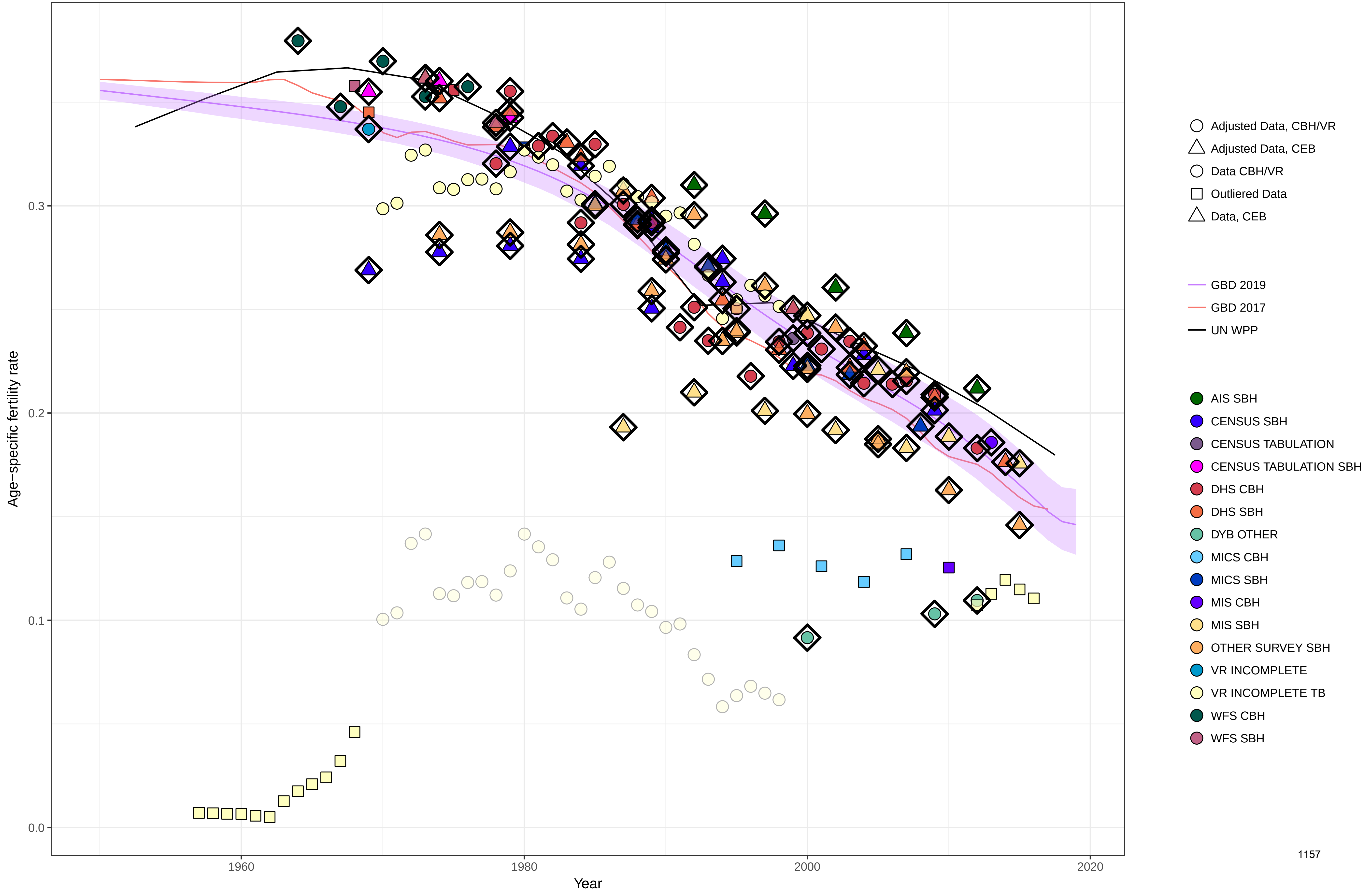

Age−specific fertility fitted trend lines, 1950−2019: Kenya, Ages: 30−34
Data points enclosed with a diamond are reference sources

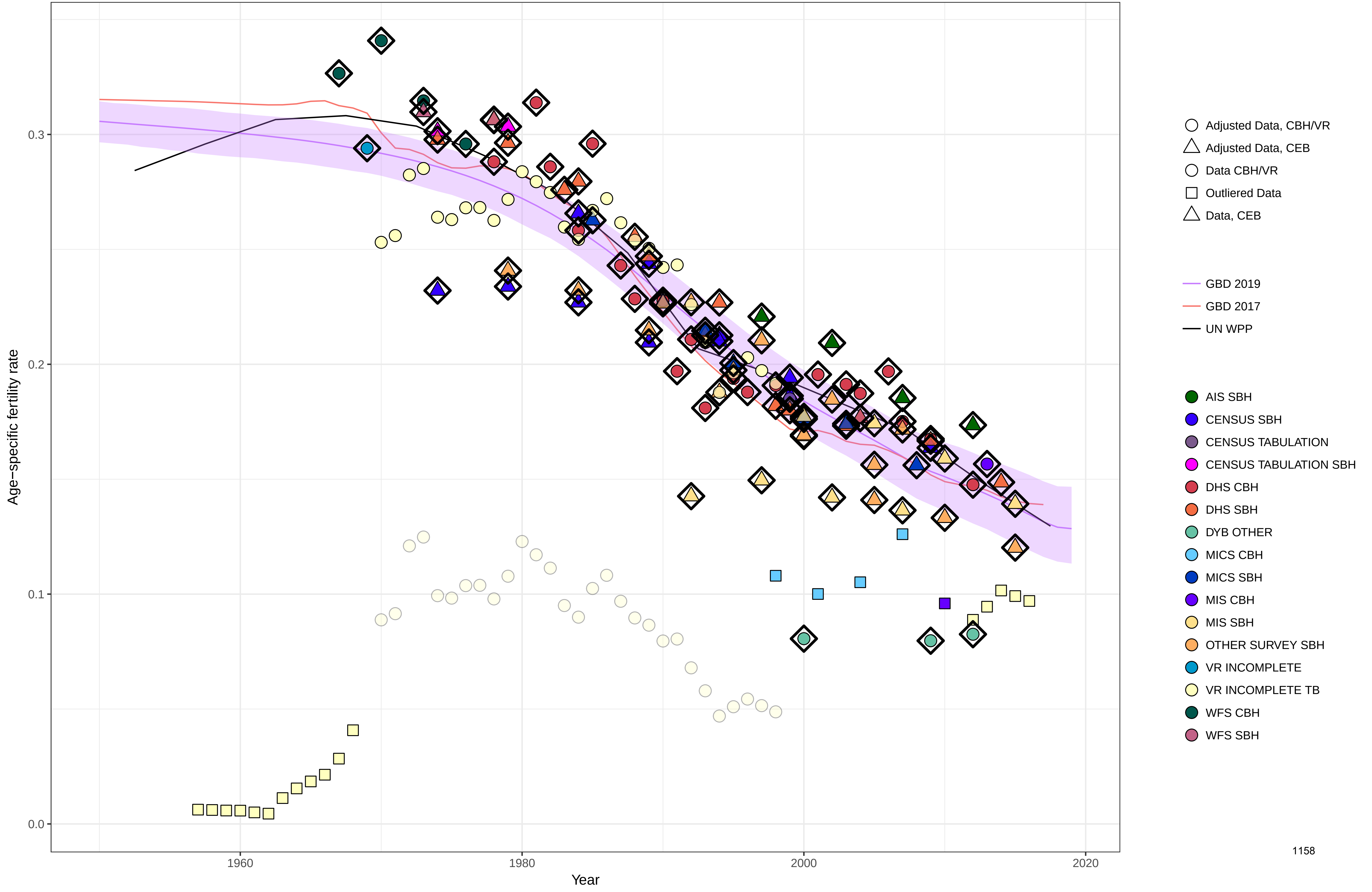

Age−specific fertility fitted trend lines, 1950−2019: Kenya, Ages: 35−39
Data points enclosed with a diamond are reference sources

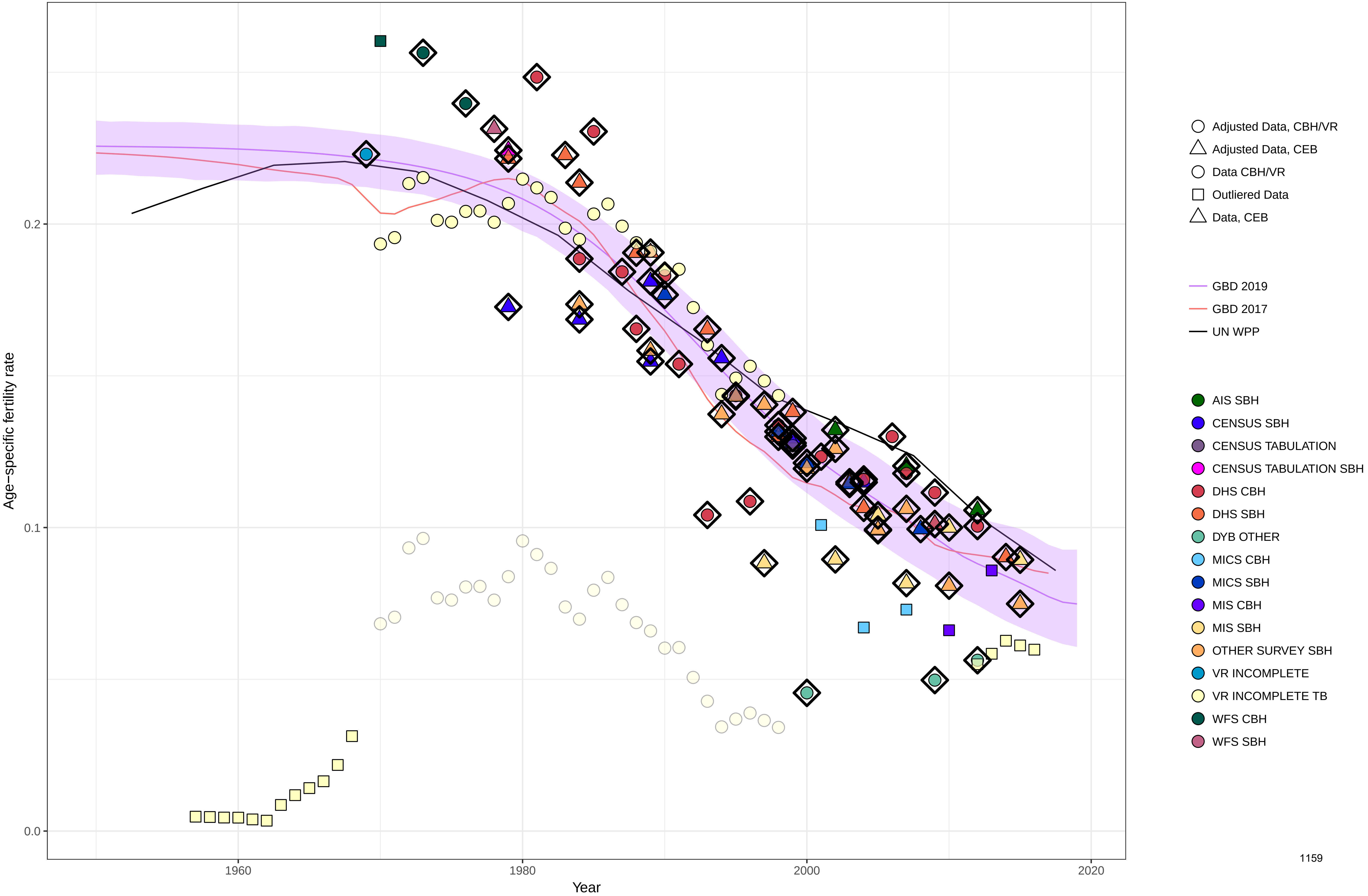

Age−specific fertility fitted trend lines, 1950−2019: Kenya, Ages: 40−44
Data points enclosed with a diamond are reference sources

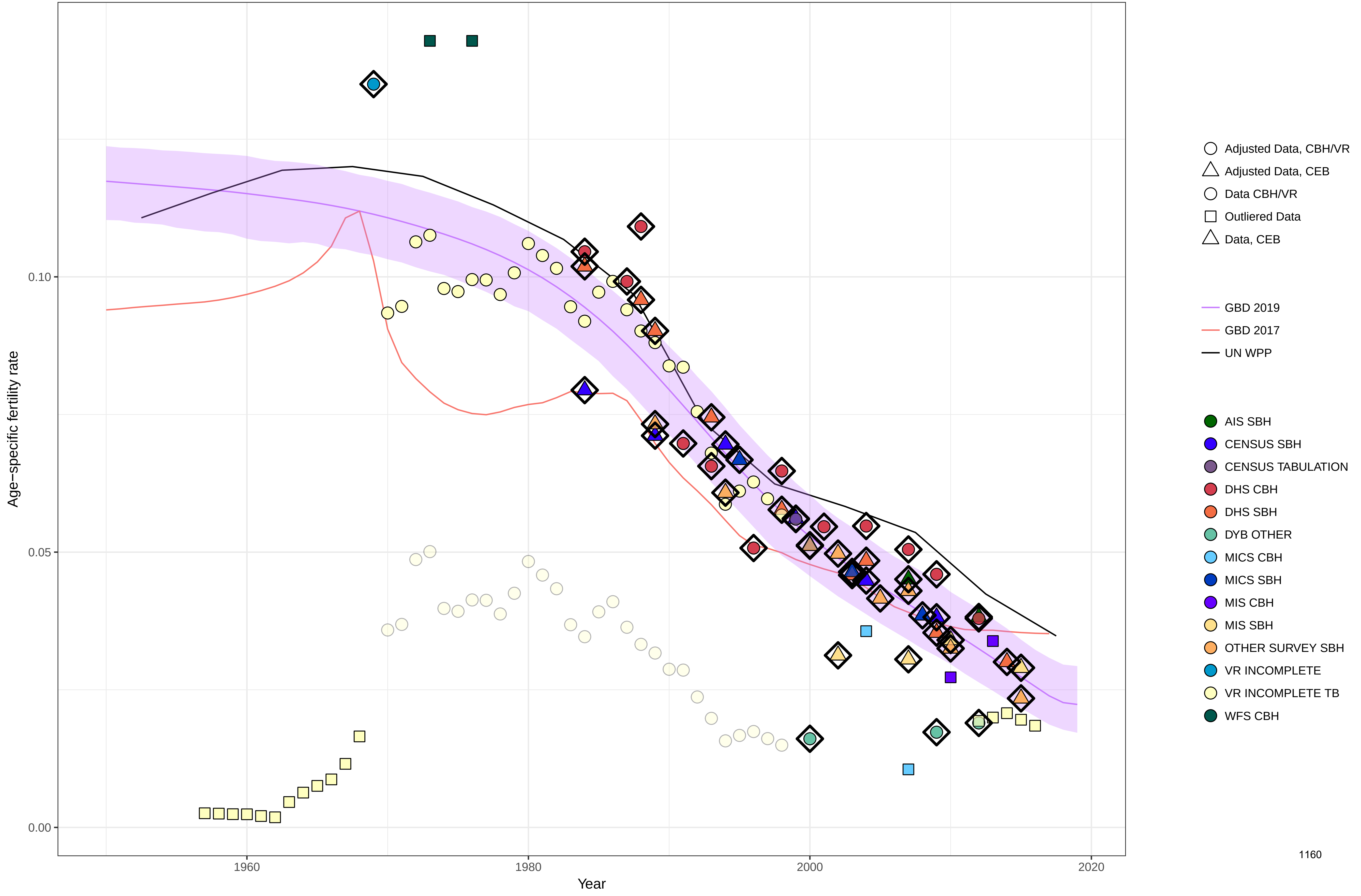

Age−specific fertility fitted trend lines, 1950−2019: Kenya, Ages: 45−49
Data points enclosed with a diamond are reference sources

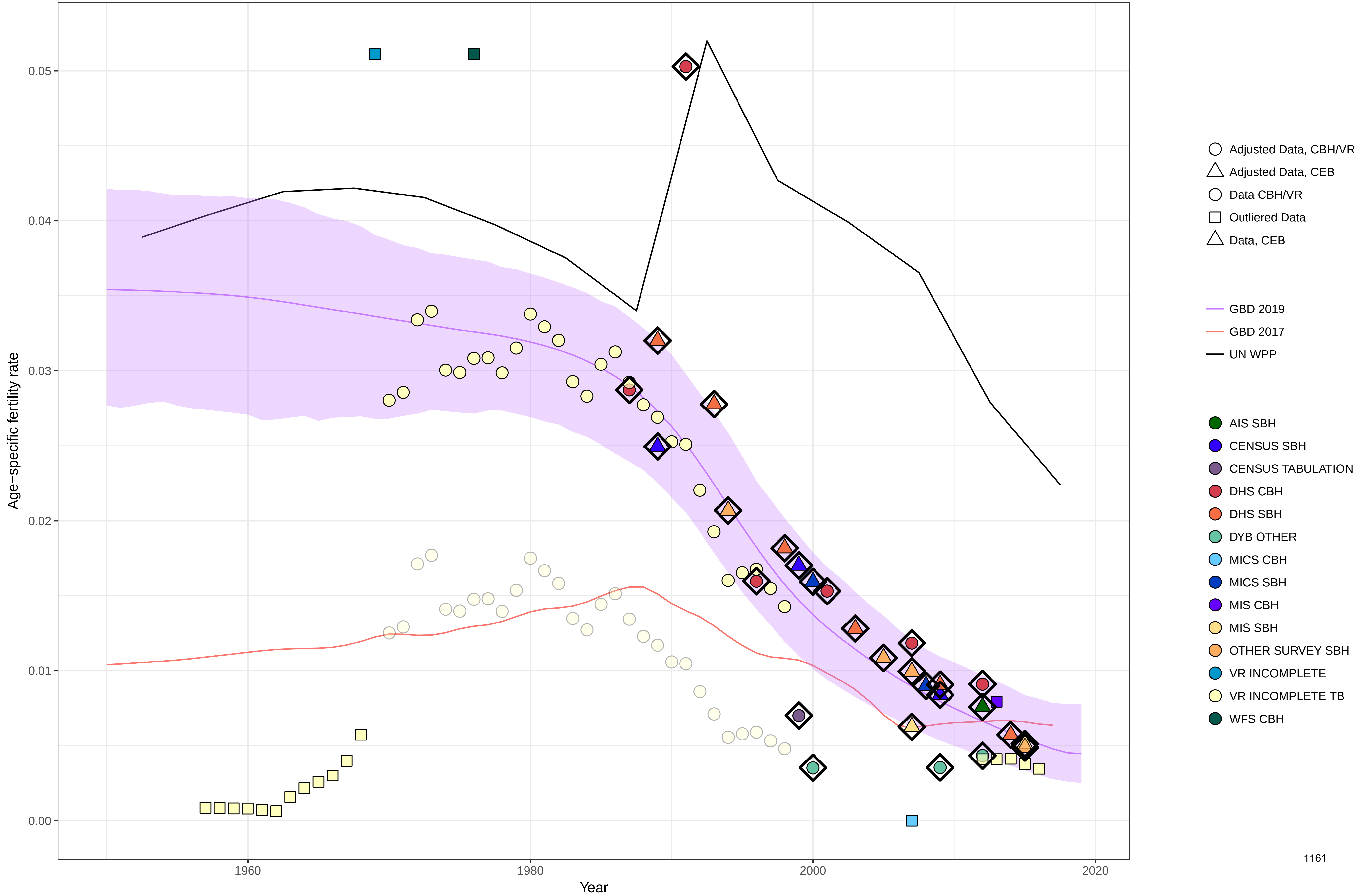

Age−specific fertility fitted trend lines, 1950−2019: Kenya, Ages: 50−54
Data points enclosed with a diamond are reference sources

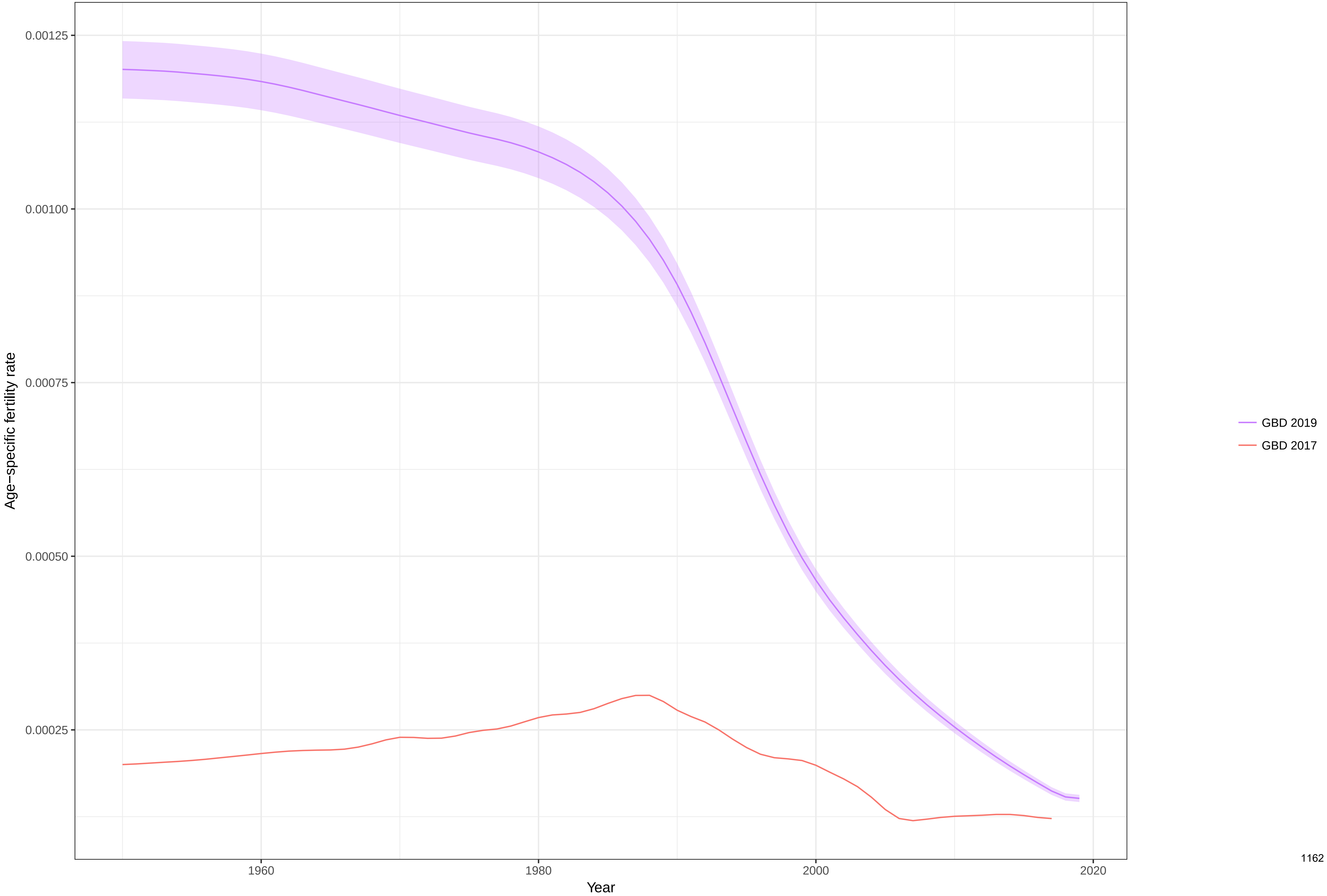

1162

Total fertility rate fitted trend lines, 1950−2019: Kenya TFR
Data points enclosed with a diamond are reference sources

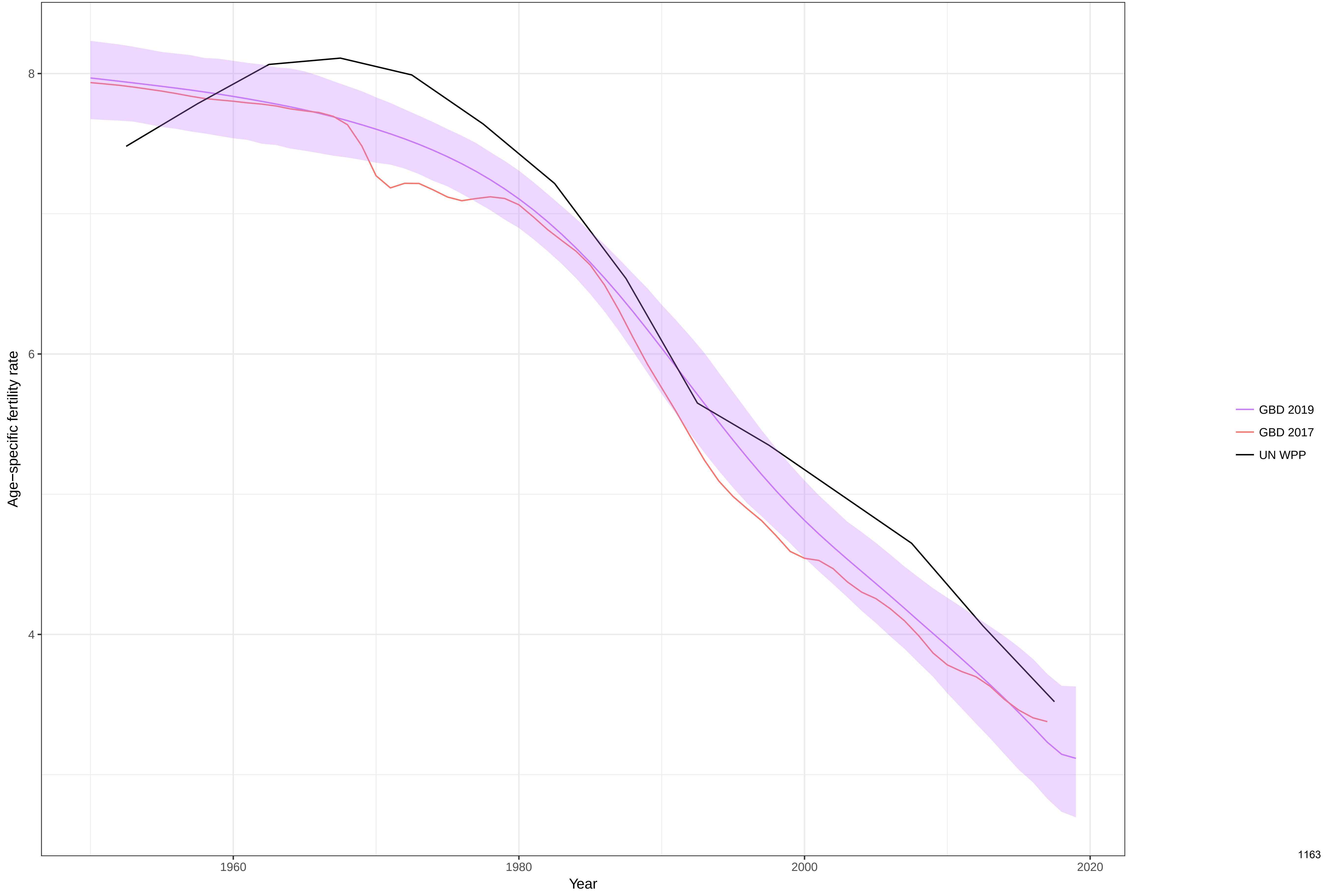

Age−specific fertility fitted trend lines, 1950−2019: Kyrgyzstan, Ages: 10−14
Data points enclosed with a diamond are reference sources

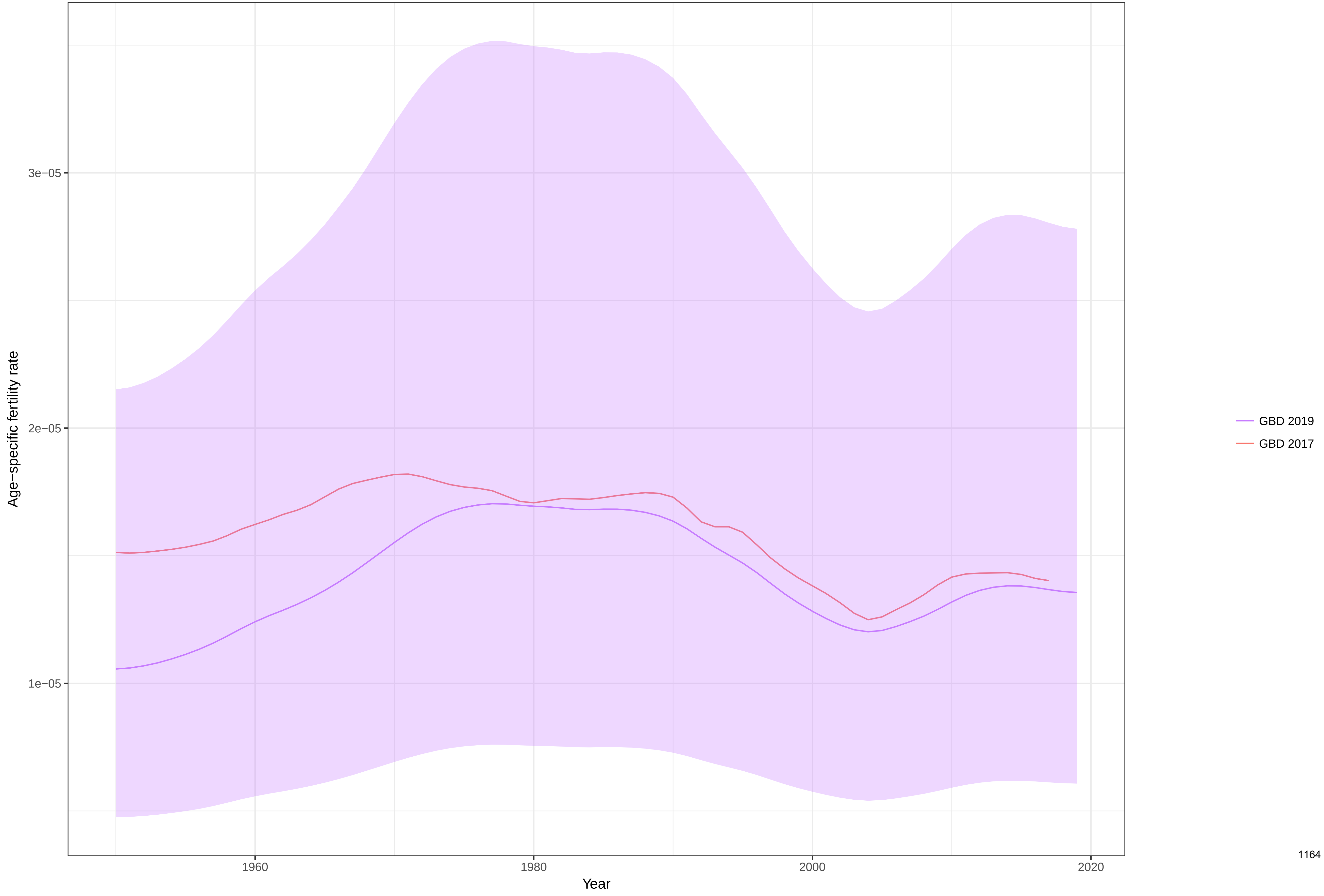

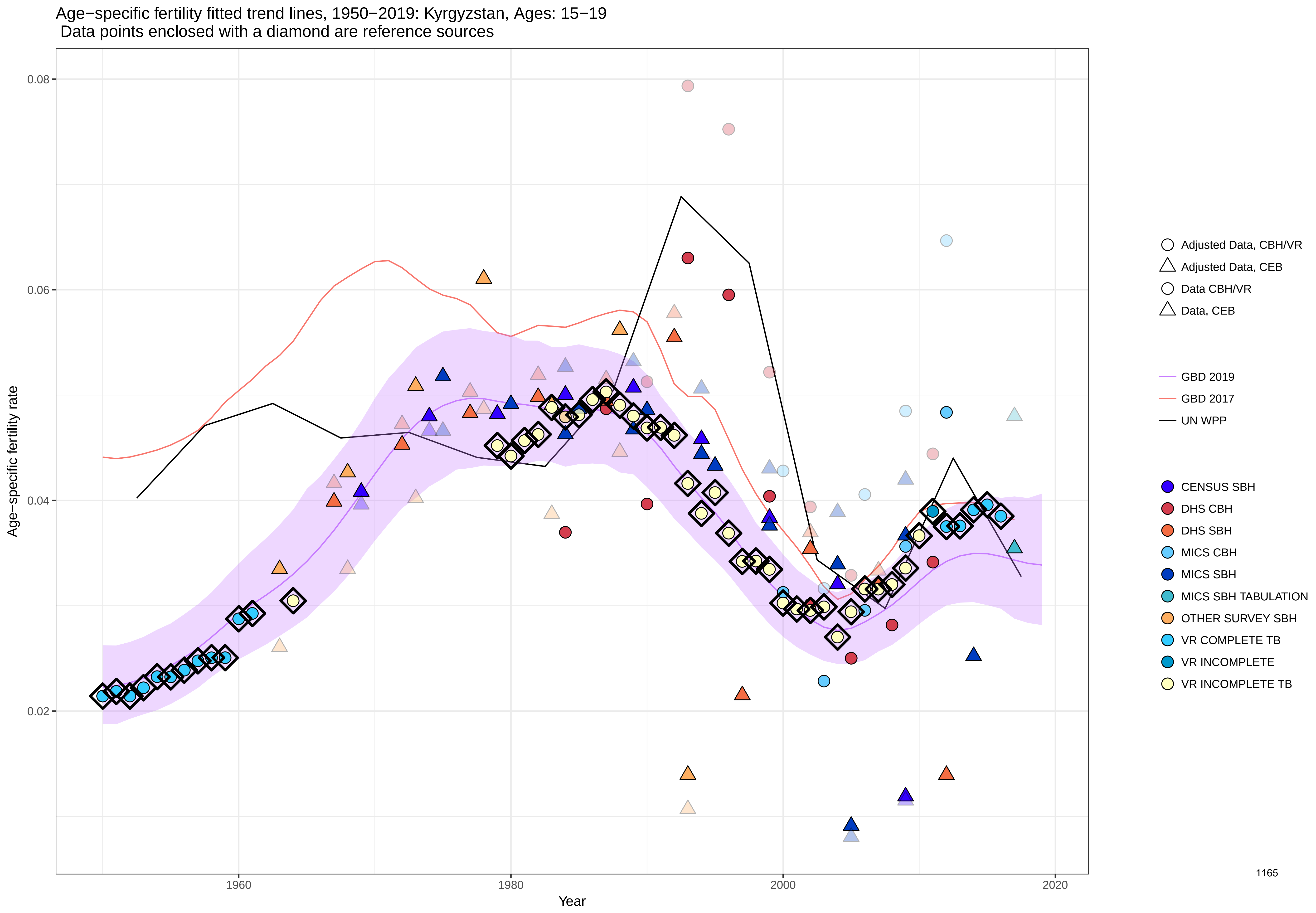

Age−specific fertility fitted trend lines, 1950−2019: Kyrgyzstan, Ages: 15−19
Data points enclosed with a diamond are reference sources
Age−specific fertility rate
Year
0.08
0.06
0.04
0.02
1960
1980
2000
2020
Adjusted Data, CBH/VR
Adjusted Data, CEB
Data CBH/VR
Data, CEB
GBD 2019
GBD 2017
UN WPP
CENSUS SBH
DHS CBH
DHS SBH
MICS CBH
MICS SBH
MICS SBH TABULATION
OTHER SURVEY SBH
VR COMPLETE TB
VR INCOMPLETE
VR INCOMPLETE TB

Age−specific fertility fitted trend lines, 1950−2019: Kyrgyzstan, Ages: 20−24
Data points enclosed with a diamond are reference sources

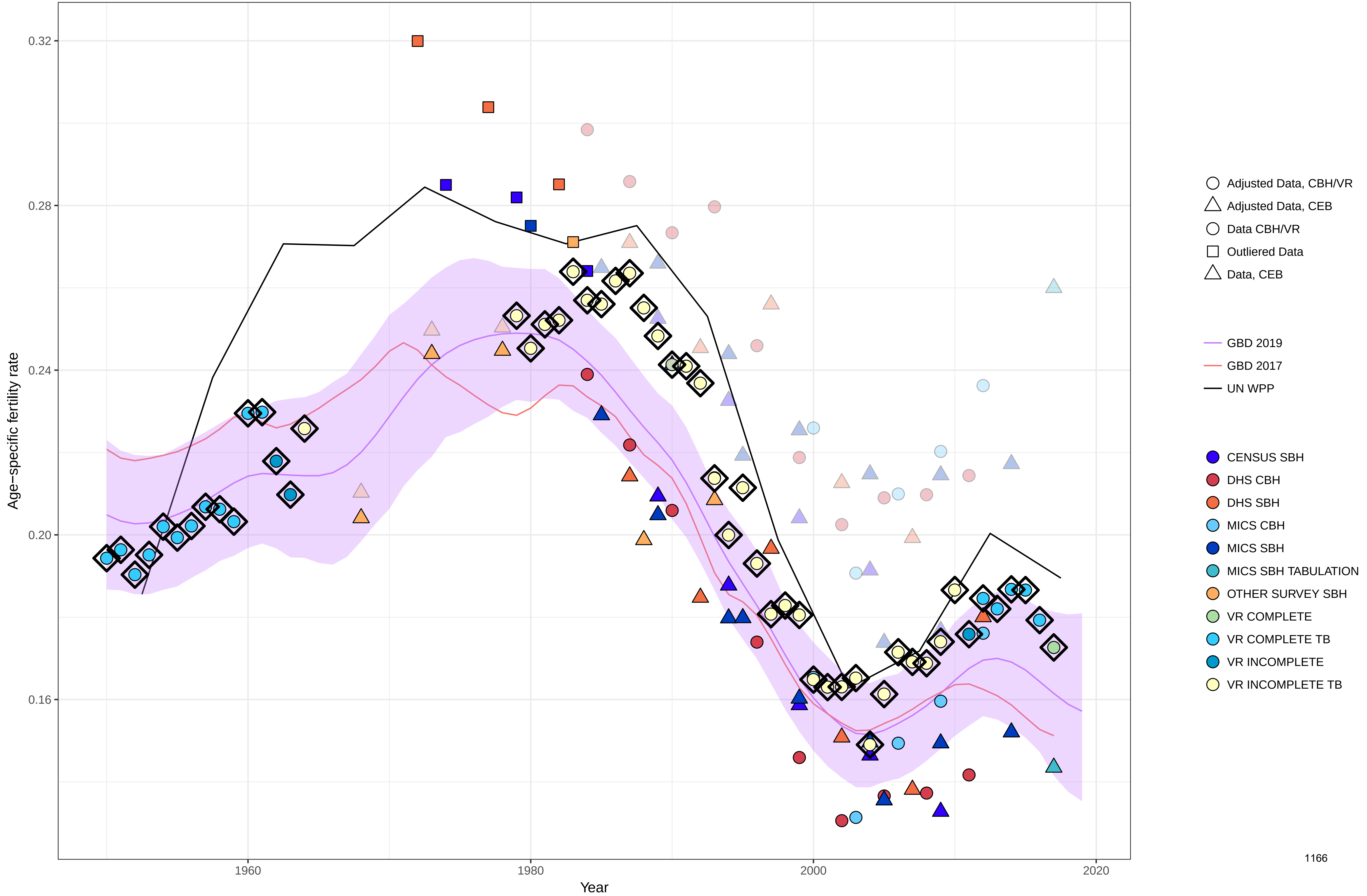

Age−specific fertility fitted trend lines, 1950−2019: Kyrgyzstan, Ages: 25−29
Data points enclosed with a diamond are reference sources

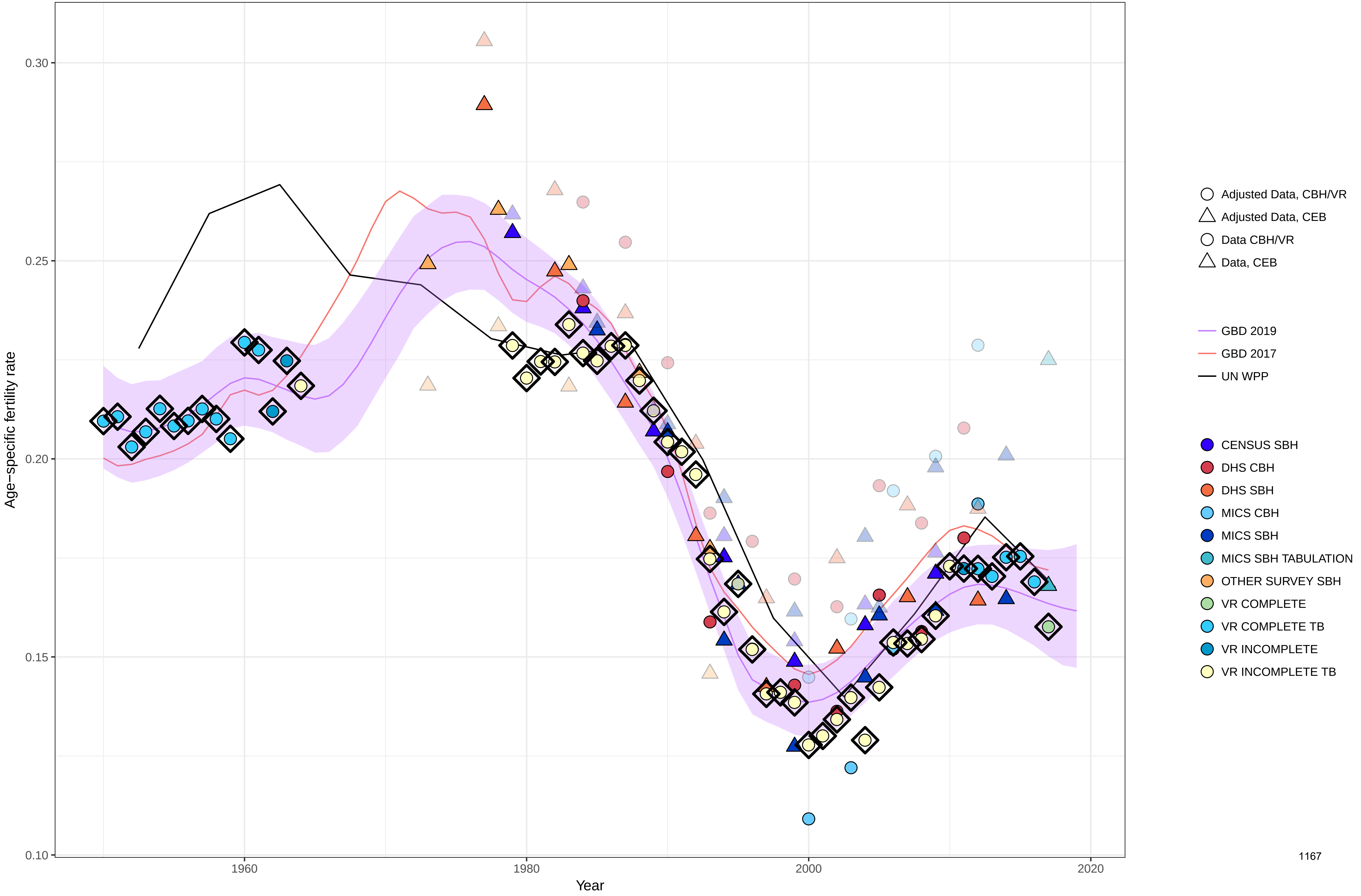

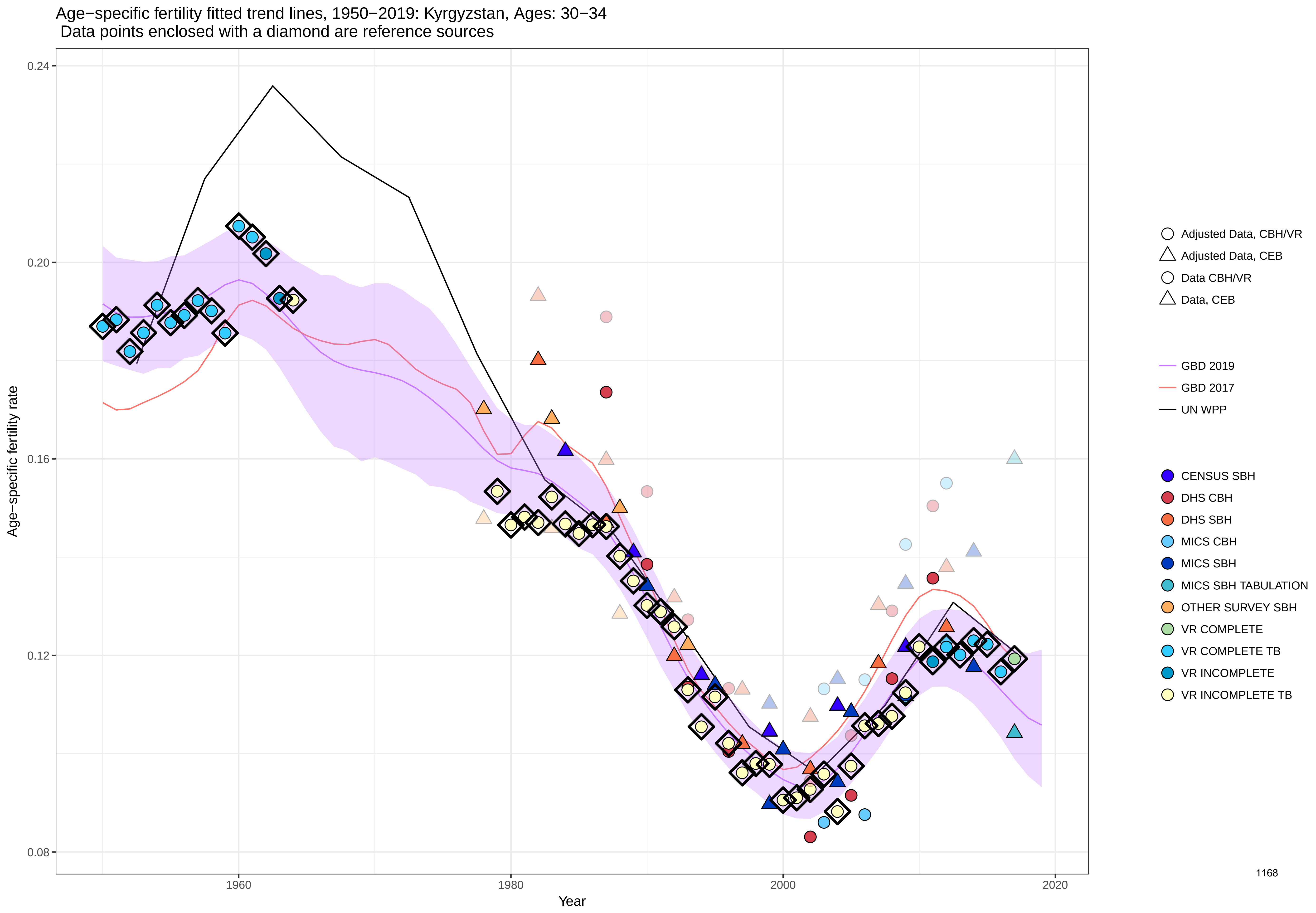
Age−specific fertility fitted trend lines, 1950−2019: Kyrgyzstan, Ages: 30−34
Data points enclosed with a diamond are reference sources
Age−specific fertility rate
Year
0.24
0.20
0.16
0.12
0.08
1960
1980
2000
2020
Adjusted Data, CBH/VR
Adjusted Data, CEB
Data CBH/VR
Data, CEB
GBD 2019
GBD 2017
UN WPP
CENSUS SBH
DHS CBH
DHS SBH
MICS CBH
MICS SBH
MICS SBH TABULATION
OTHER SURVEY SBH
VR COMPLETE
VR COMPLETE TB
VR INCOMPLETE
VR INCOMPLETE TB

Age−specific fertility fitted trend lines, 1950−2019: Kyrgyzstan, Ages: 35−39
Data points enclosed with a diamond are reference sources

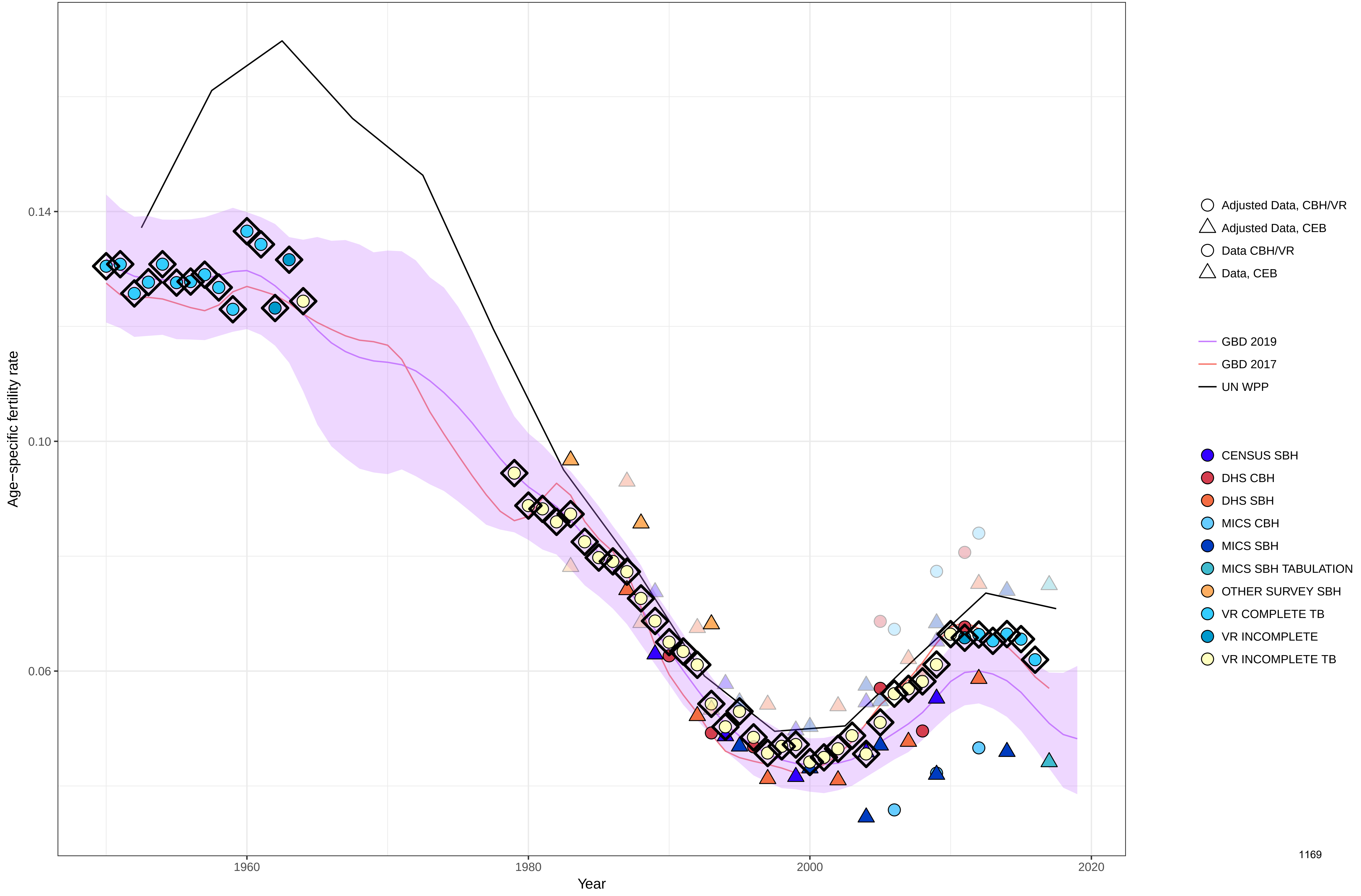

Age−specific fertility fitted trend lines, 1950−2019: Kyrgyzstan, Ages: 40−44
Data points enclosed with a diamond are reference sources

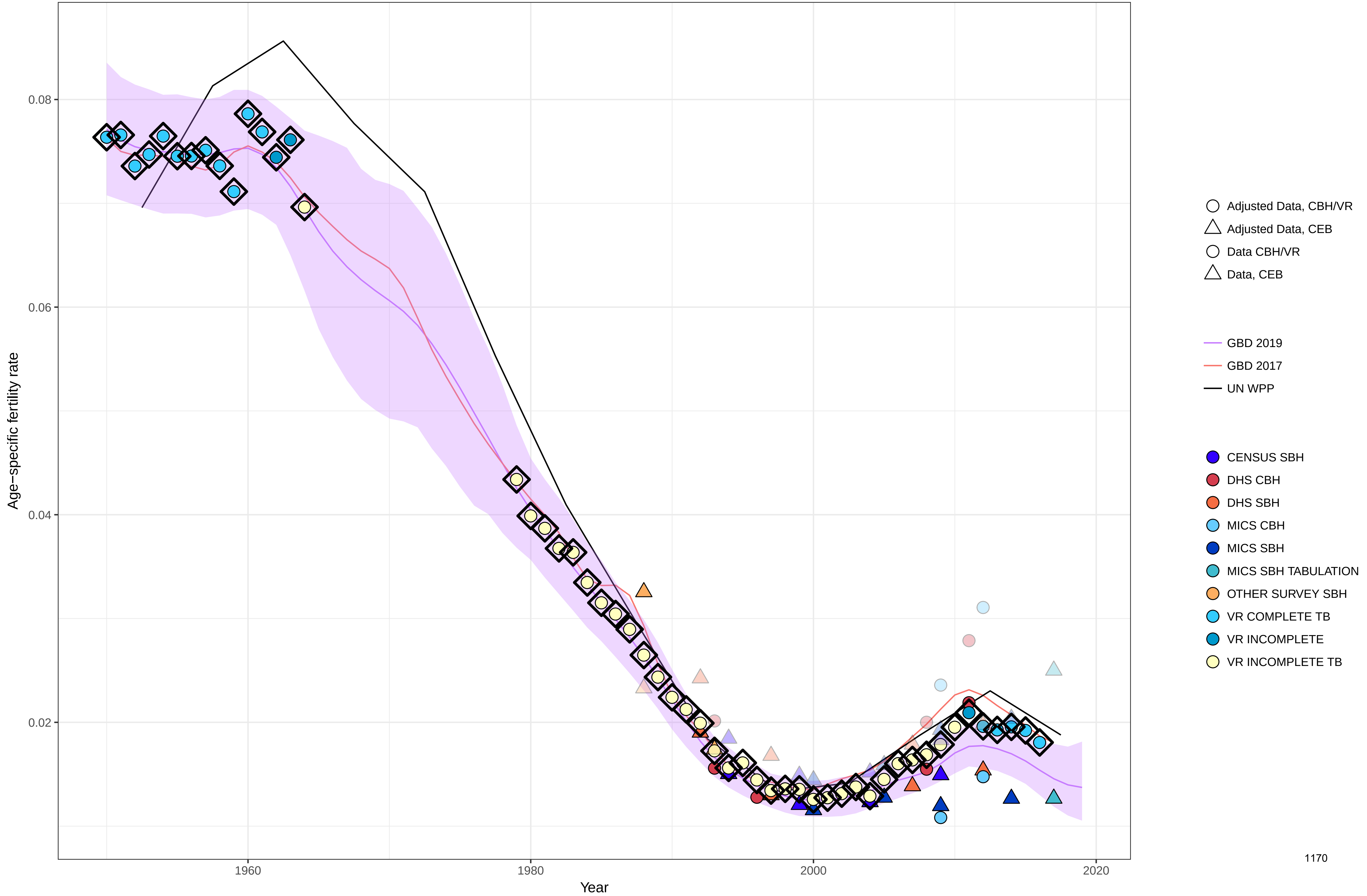

Age−specific fertility fitted trend lines, 1950−2019: Kyrgyzstan, Ages: 45−49
Data points enclosed with a diamond are reference sources

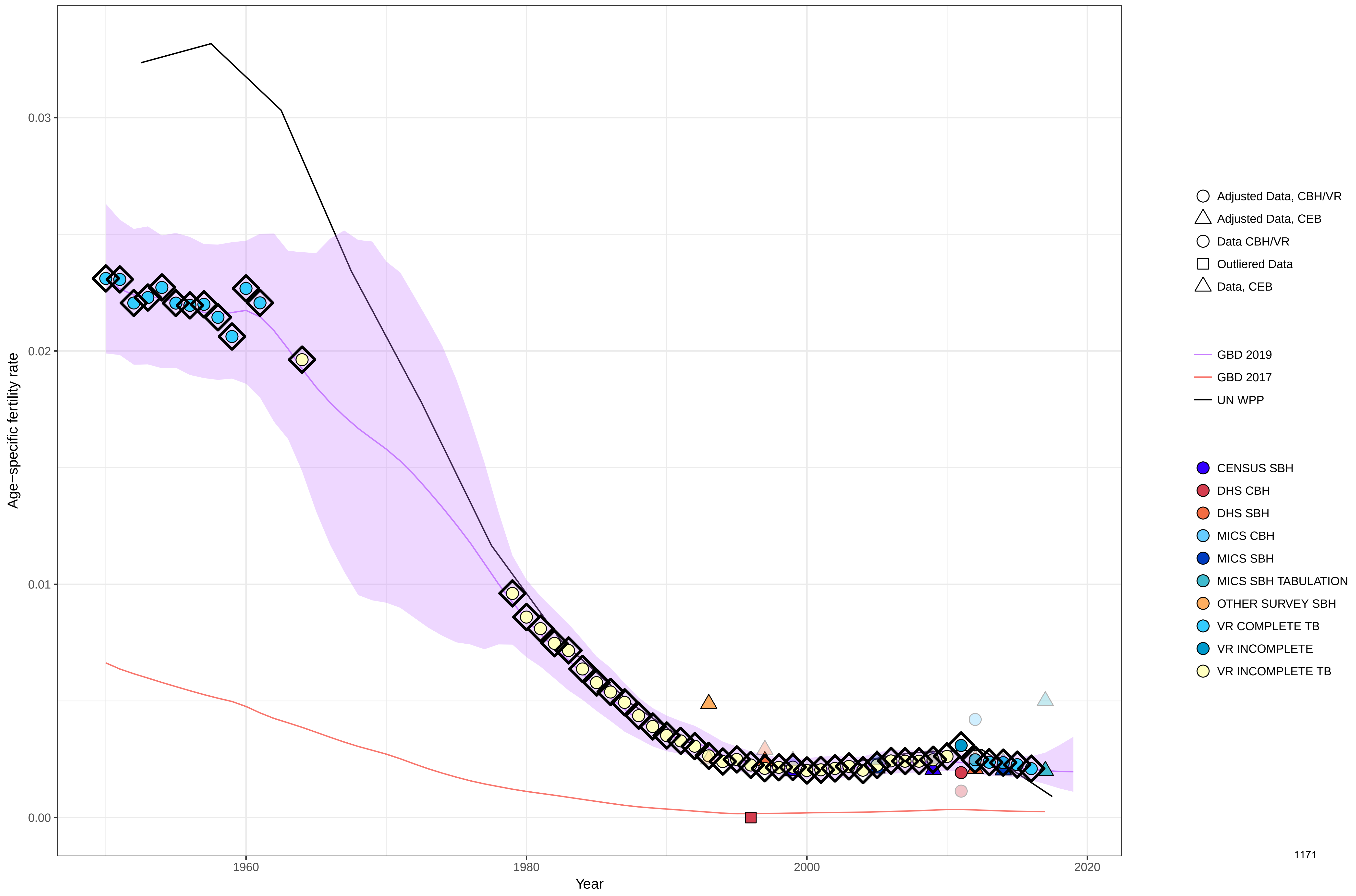

Age−specific fertility fitted trend lines, 1950−2019: Kyrgyzstan, Ages: 50−54
Data points enclosed with a diamond are reference sources

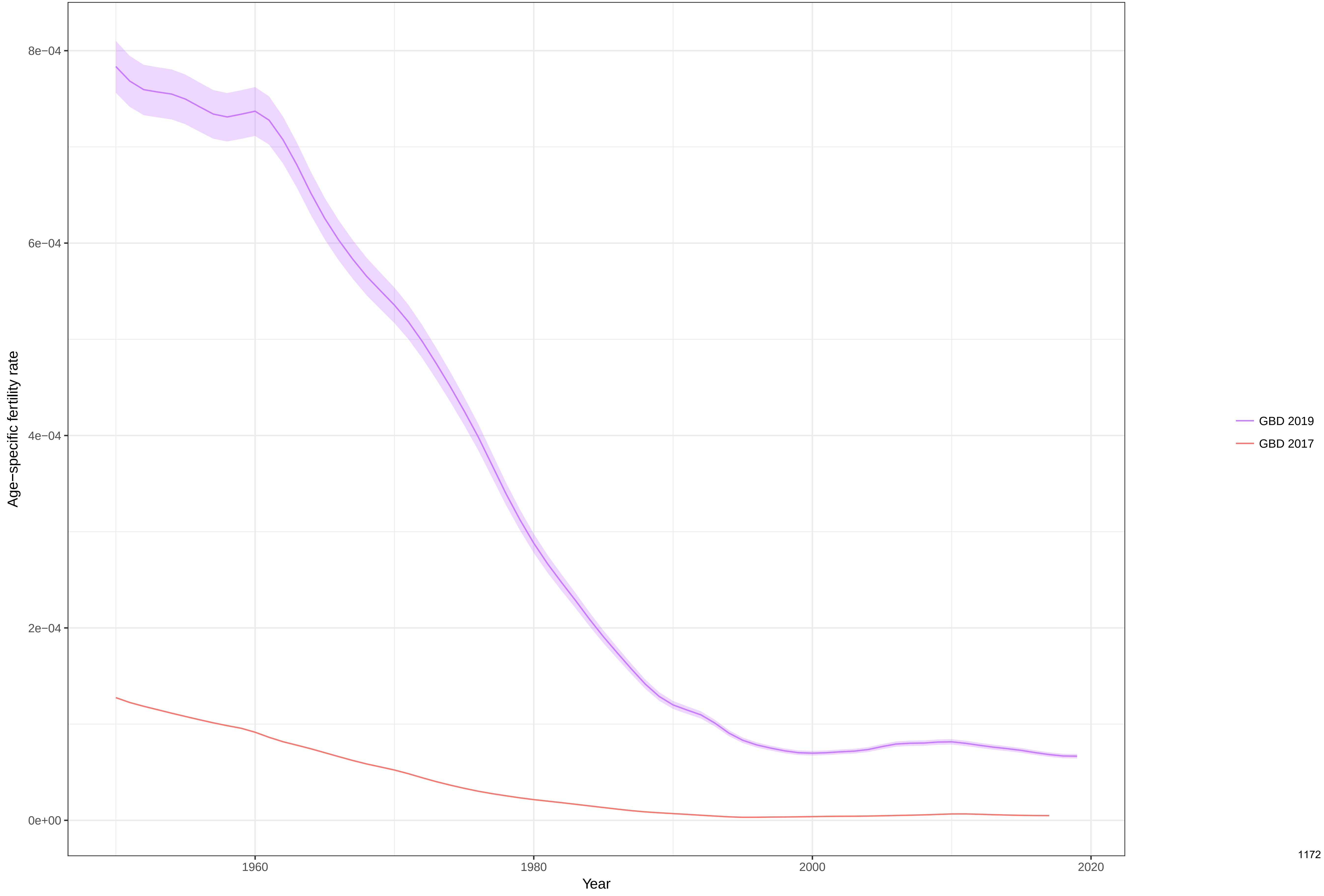

Total fertility rate fitted trend lines, 1950−2019: Kyrgyzstan TFR
Data points enclosed with a diamond are reference sources

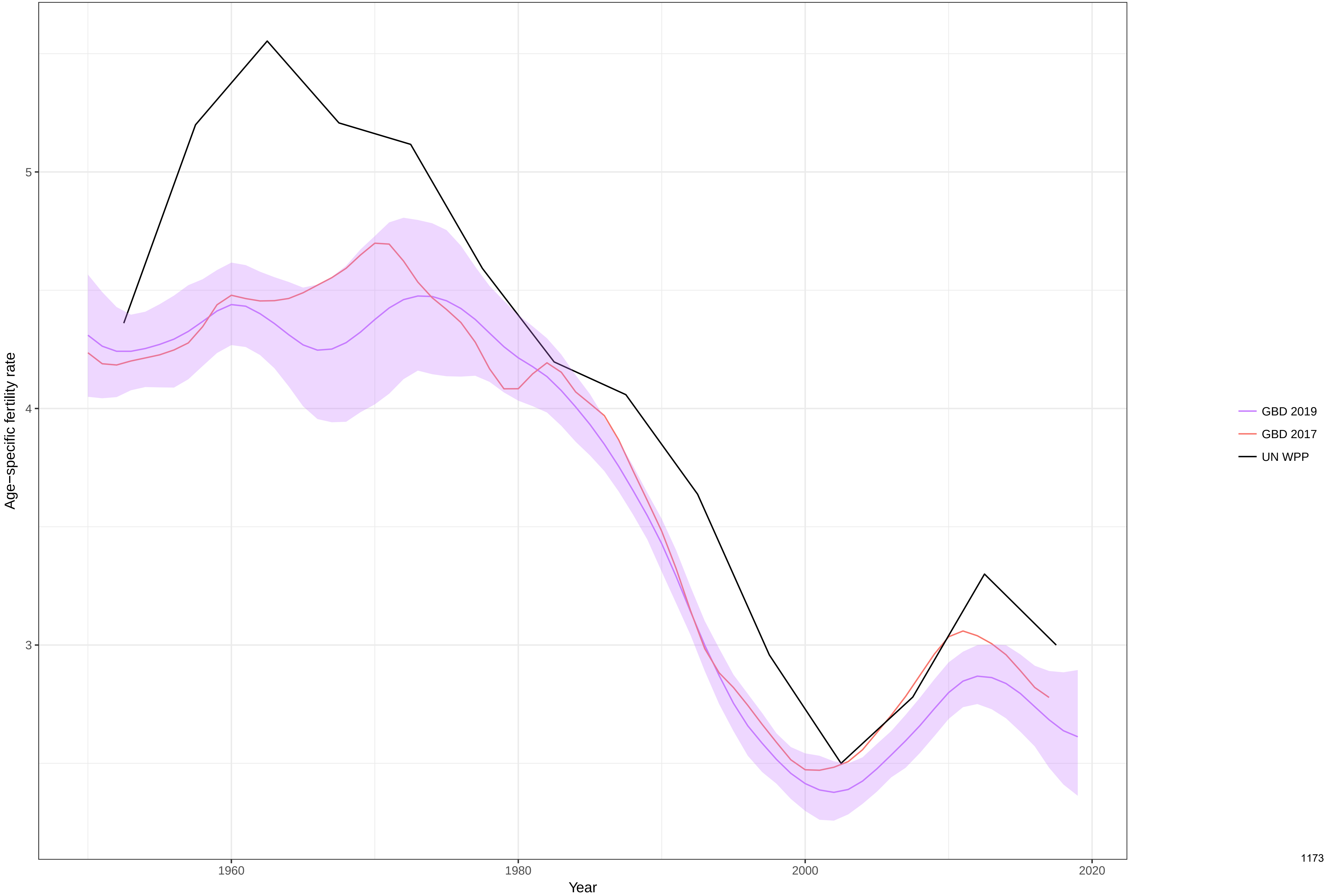

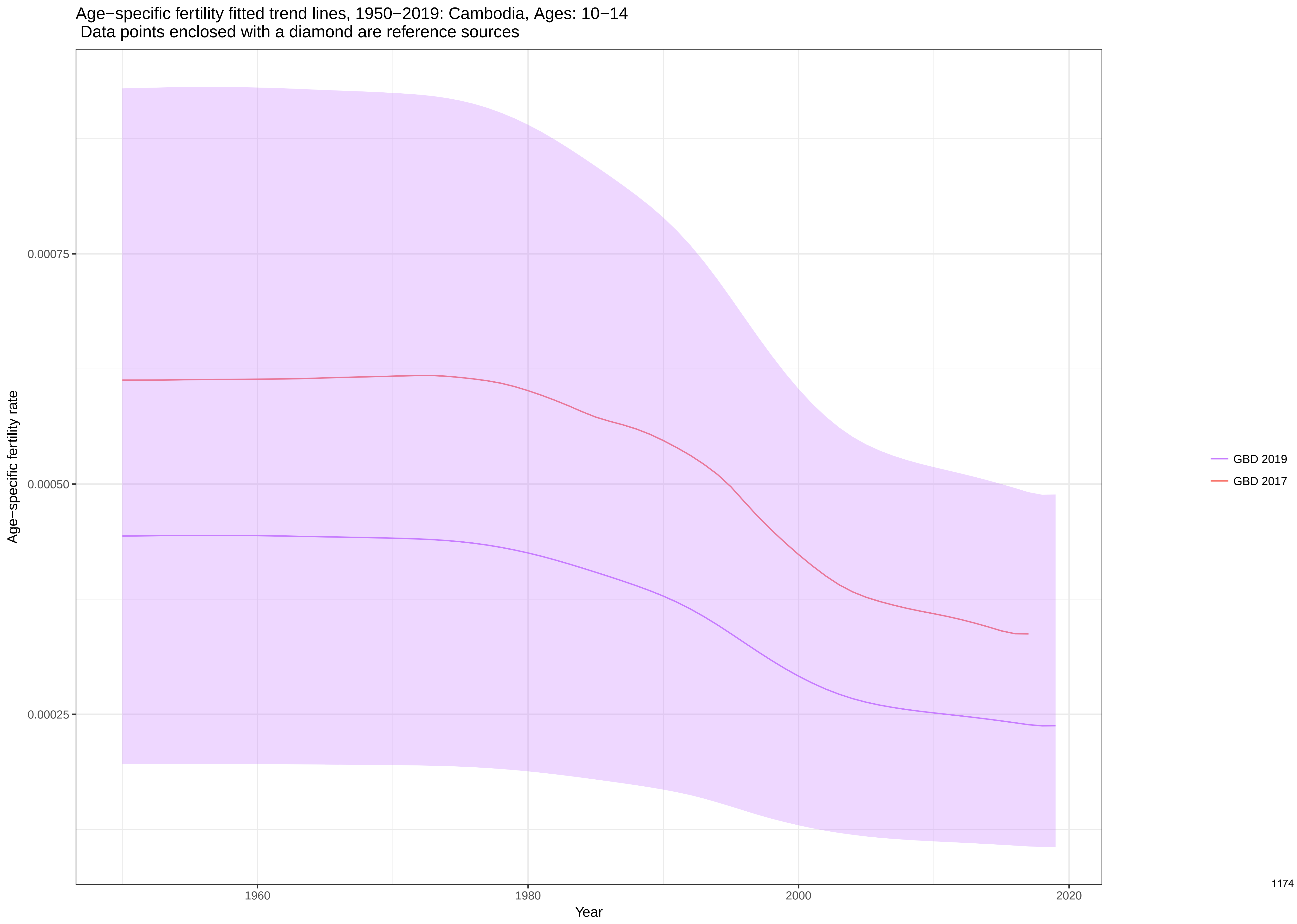
Age−specific fertility fitted trend lines, 1950−2019: Cambodia, Ages: 10−14
Data points enclosed with a diamond are reference sources
0.00075
0.00050
0.00025
Age−specific fertility rate
1960
1980
2000
2020
Year
GBD 2019
GBD 2017

Age−specific fertility fitted trend lines, 1950−2019: Cambodia, Ages: 15−19
 Data points enclosed with a diamond are reference sources

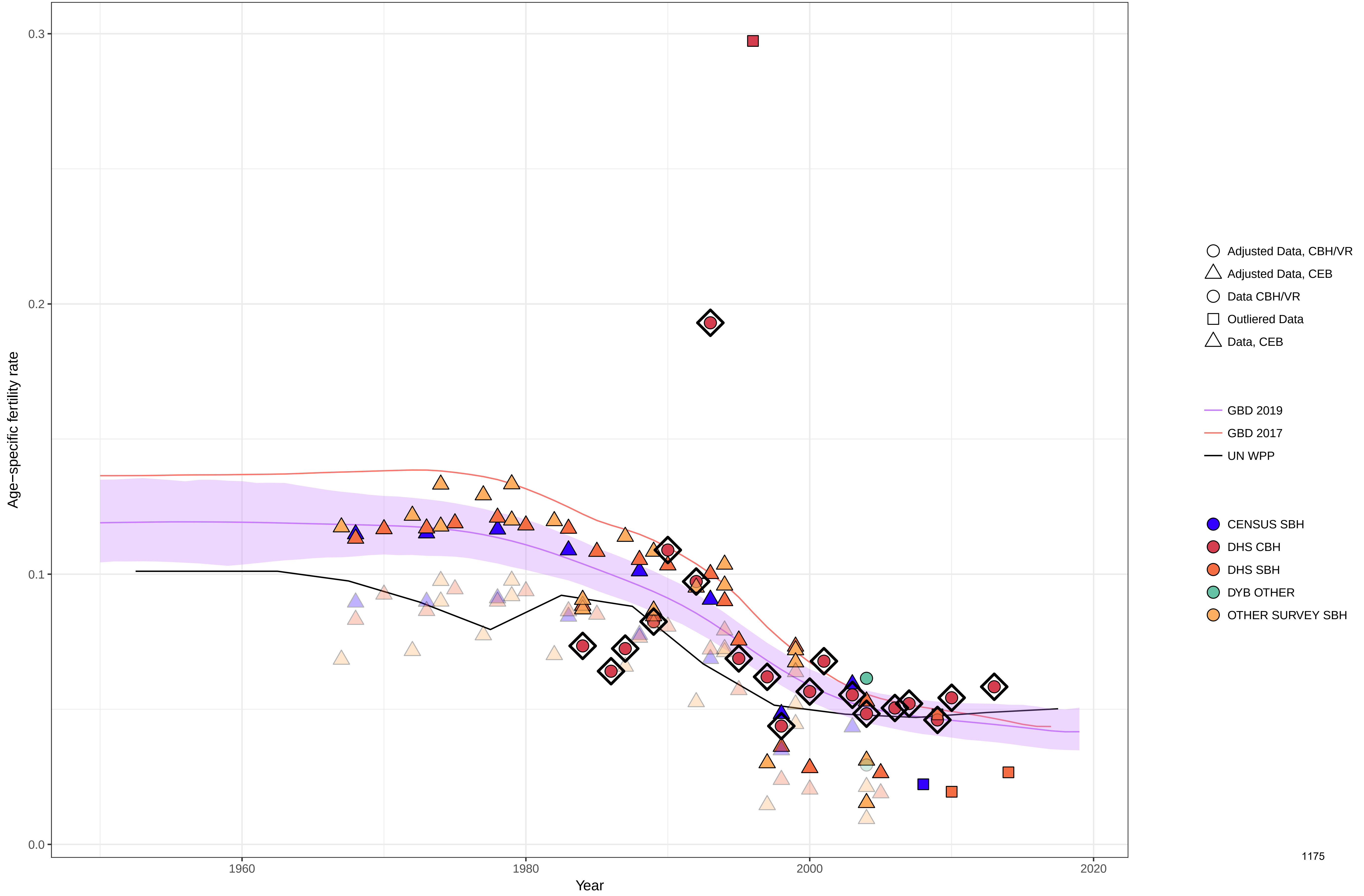

Age−specific fertility fitted trend lines, 1950−2019: Cambodia, Ages: 20−24
Data points enclosed with a diamond are reference sources

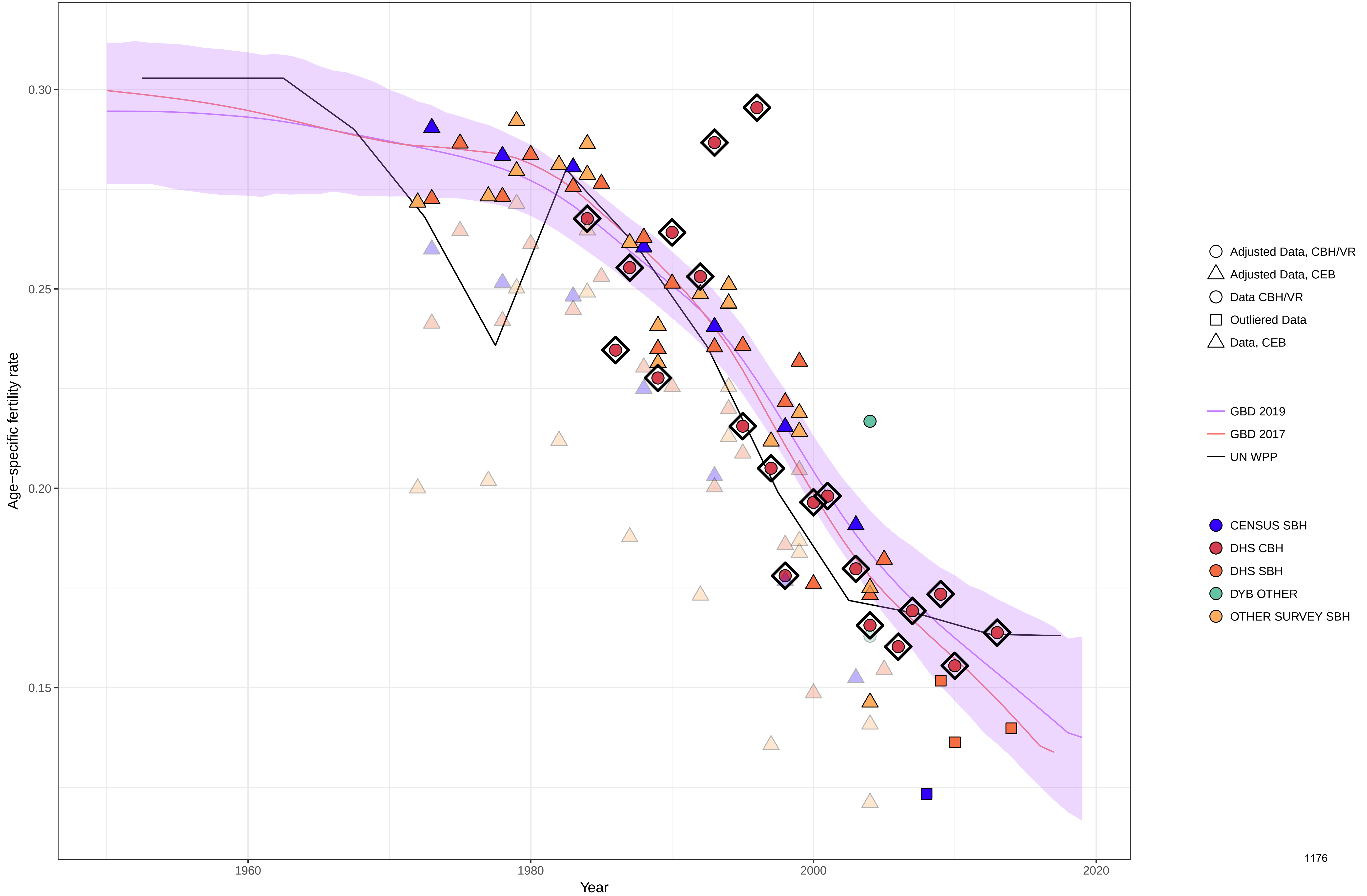

Age−specific fertility fitted trend lines, 1950−2019: Cambodia, Ages: 25−29
Data points enclosed with a diamond are reference sources

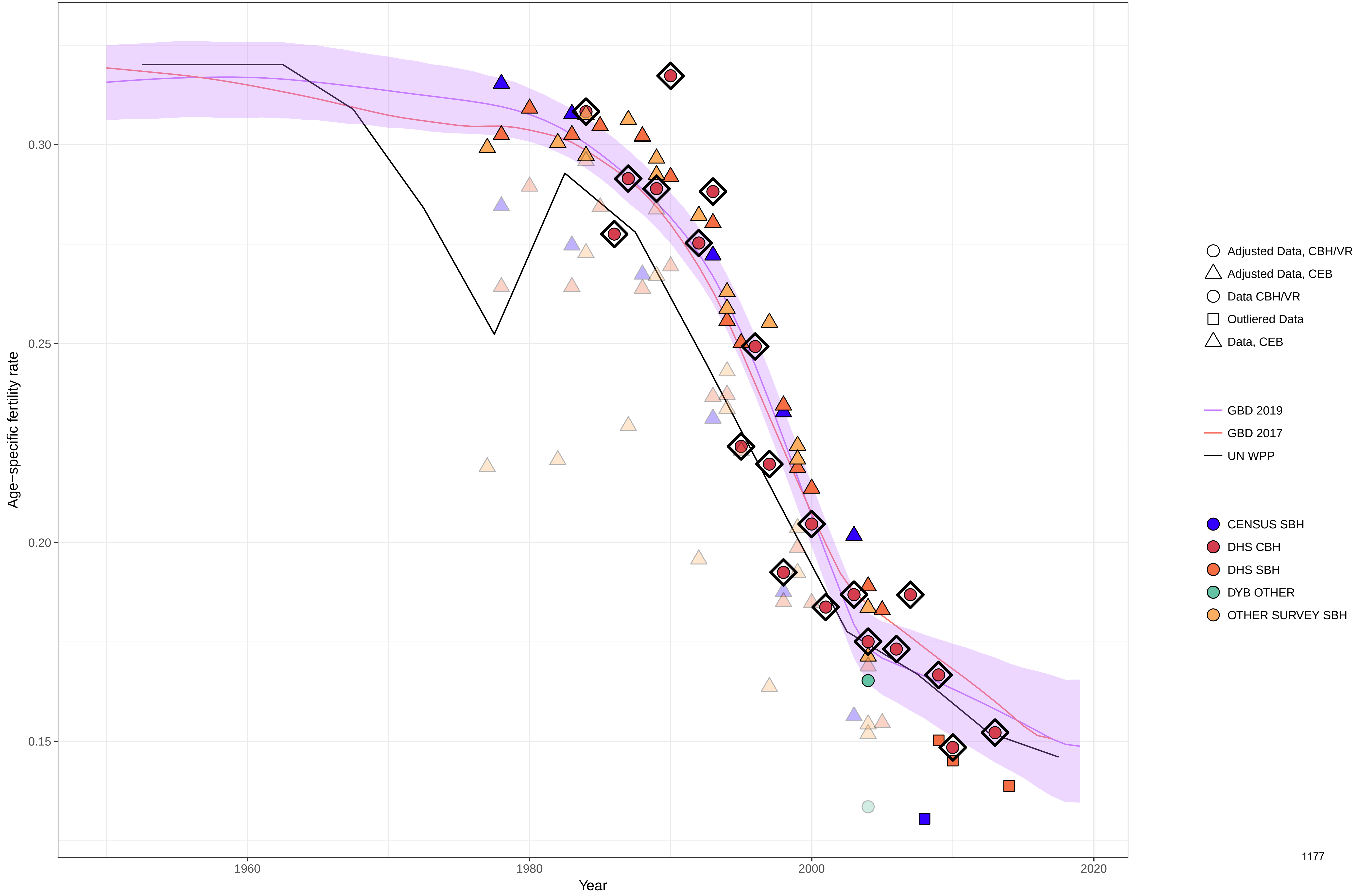

Age−specific fertility fitted trend lines, 1950−2019: Cambodia, Ages: 30−34
Data points enclosed with a diamond are reference sources

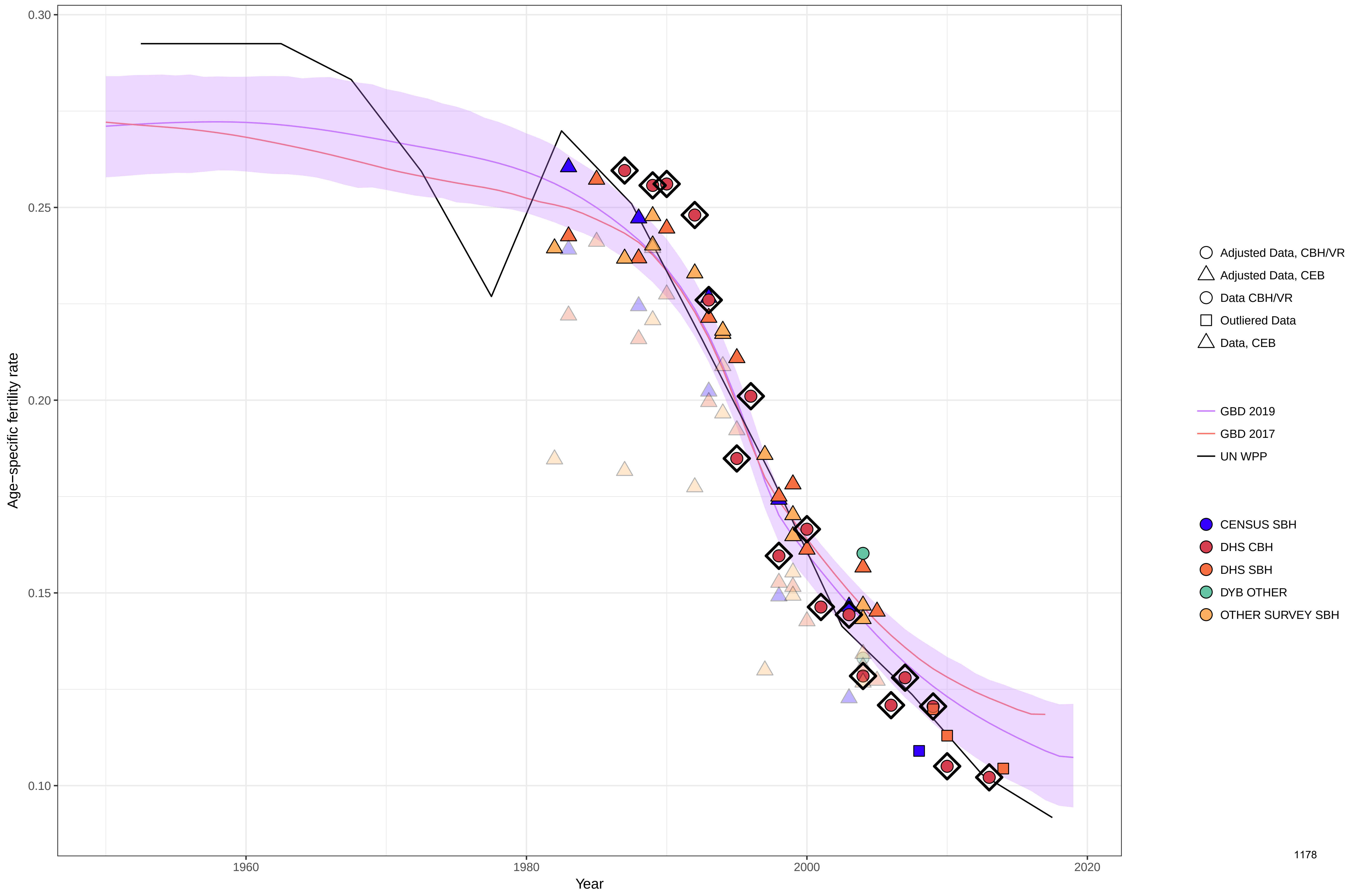

Age−specific fertility fitted trend lines, 1950−2019: Cambodia, Ages: 35−39
Data points enclosed with a diamond are reference sources

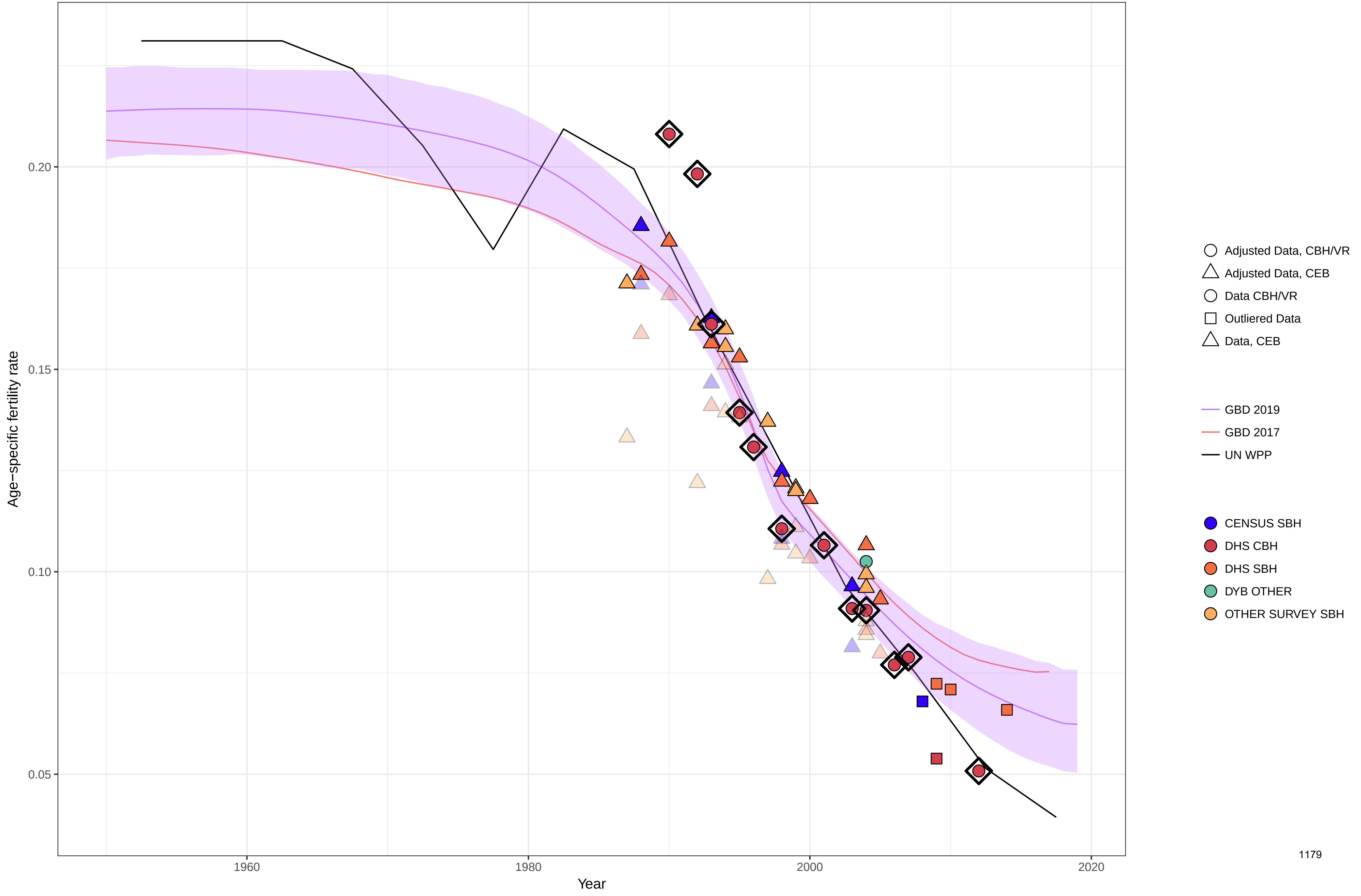

Age−specific fertility fitted trend lines, 1950−2019: Cambodia, Ages: 40−44
Data points enclosed with a diamond are reference sources

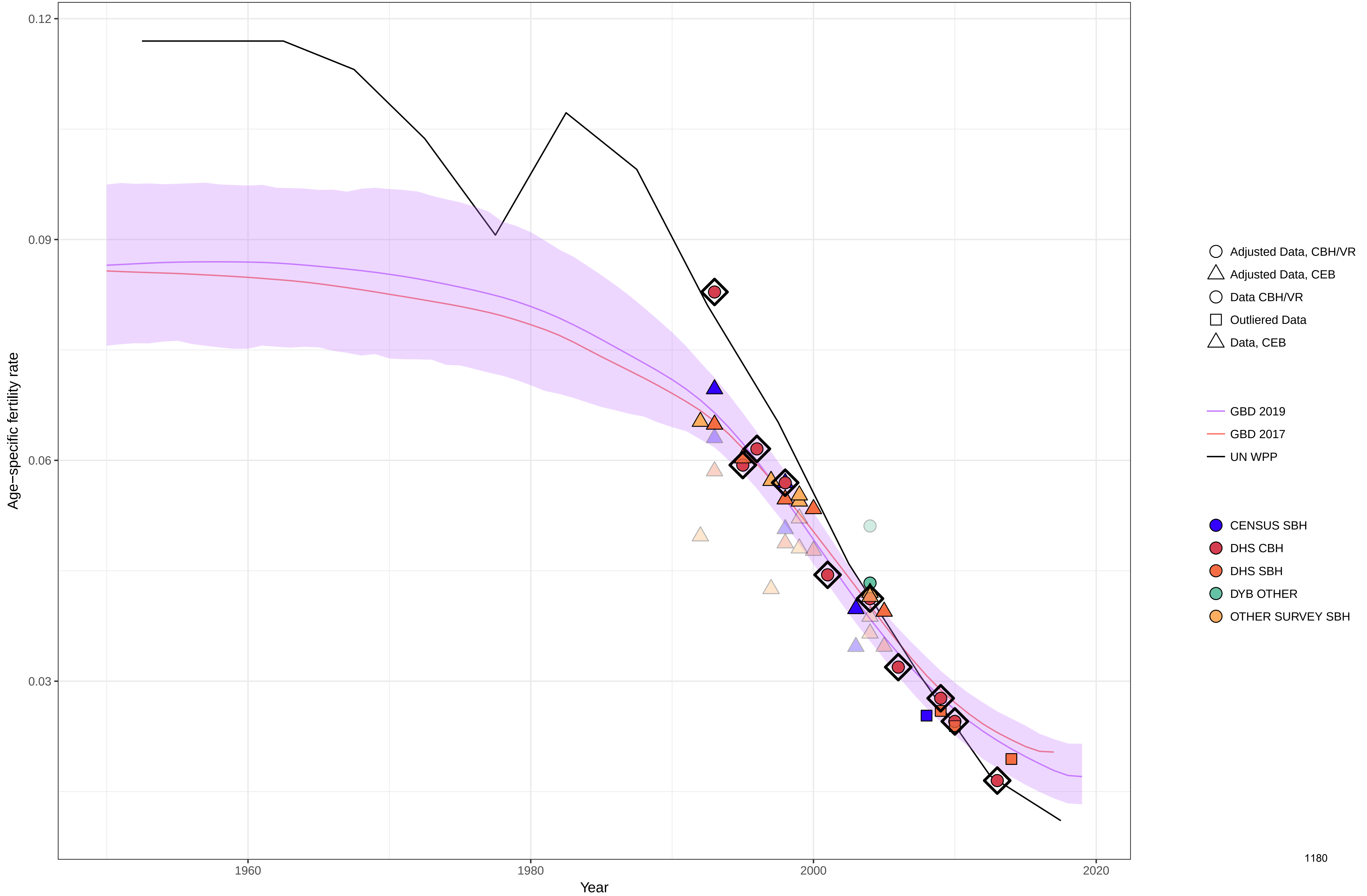

Age−specific fertility fitted trend lines, 1950−2019: Cambodia, Ages: 45−49
Data points enclosed with a diamond are reference sources

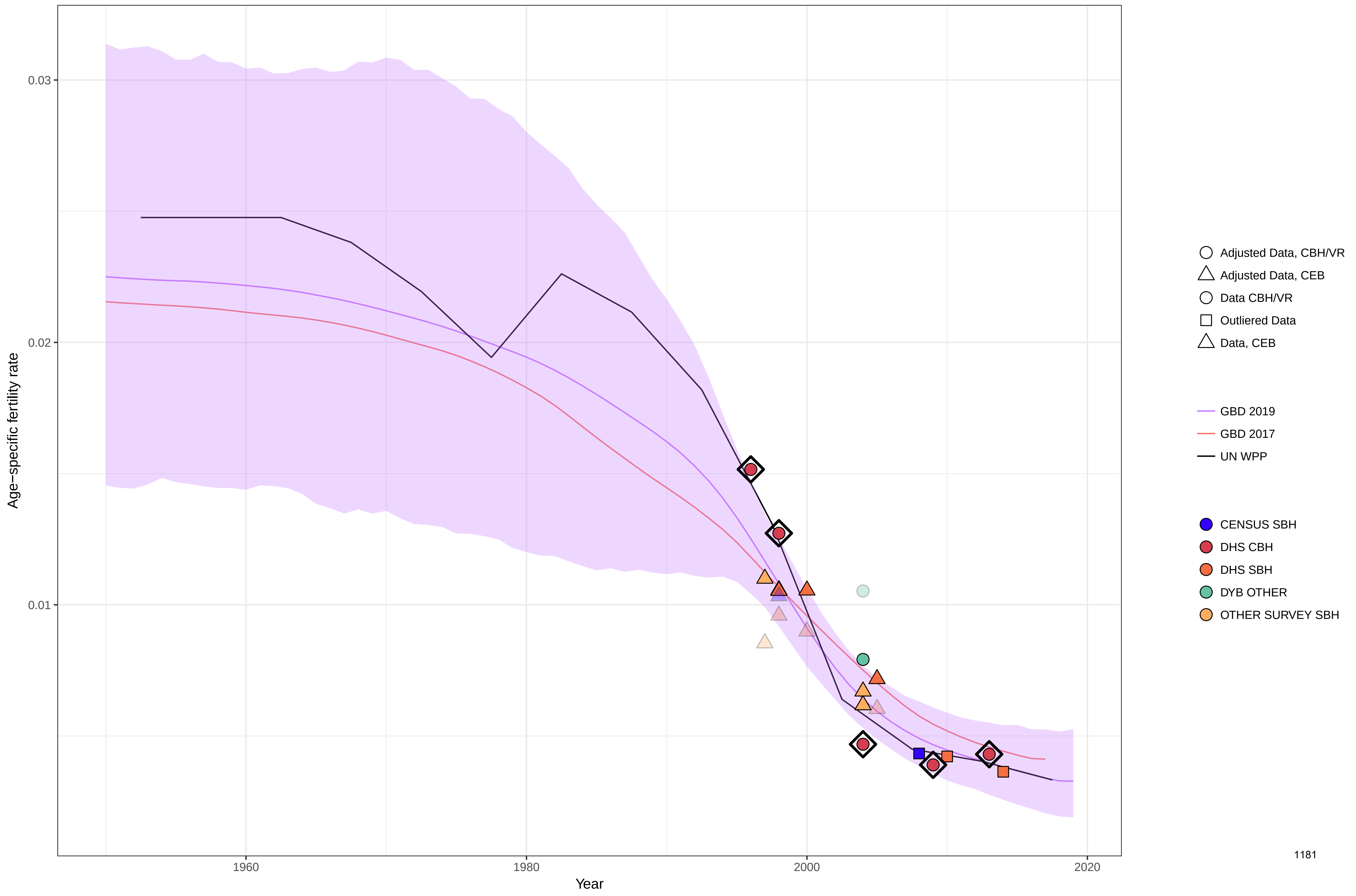

Age−specific fertility fitted trend lines, 1950−2019: Cambodia, Ages: 50−54
Data points enclosed with a diamond are reference sources

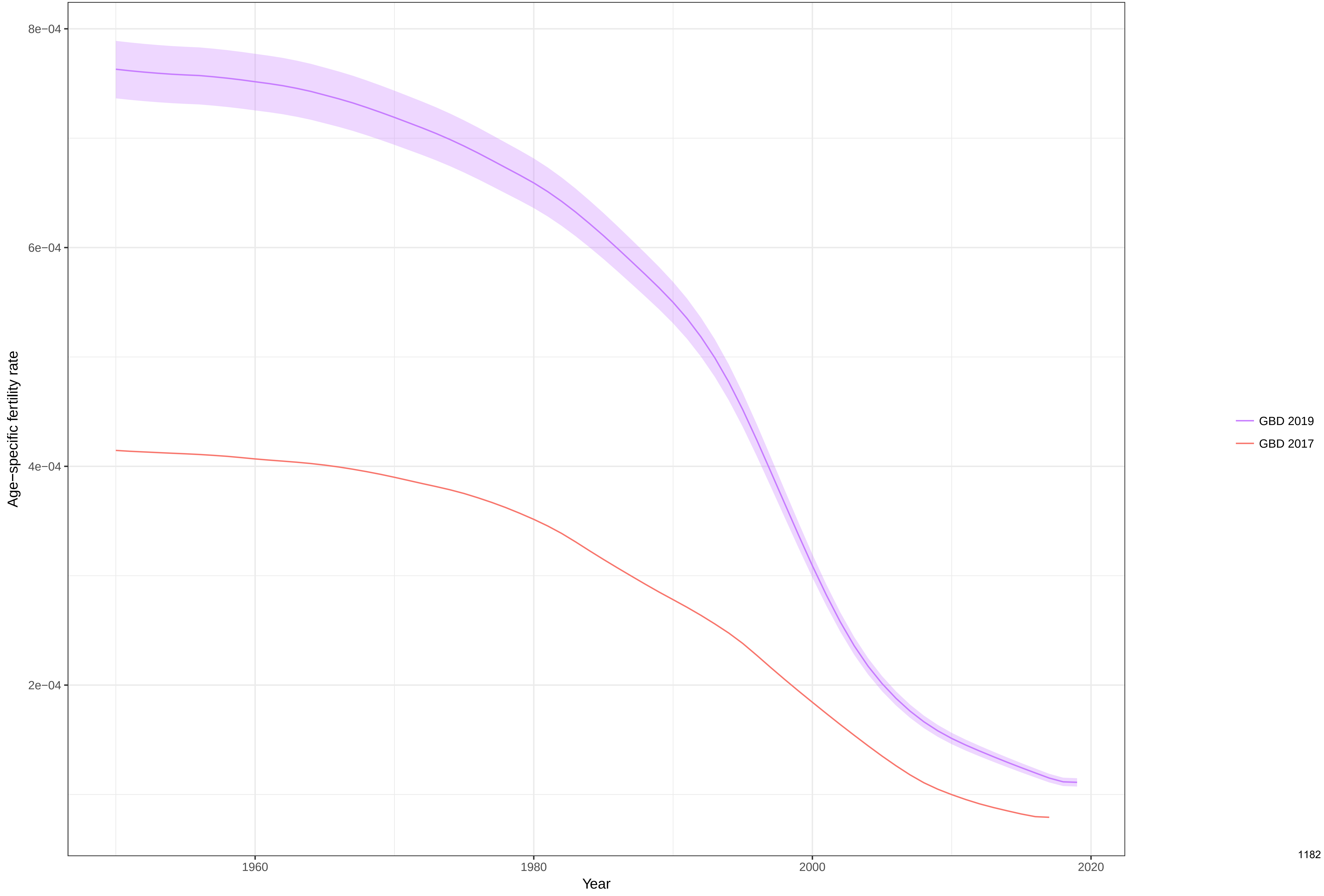

Total fertility rate fitted trend lines, 1950−2019: Cambodia TFR
Data points enclosed with a diamond are reference sources

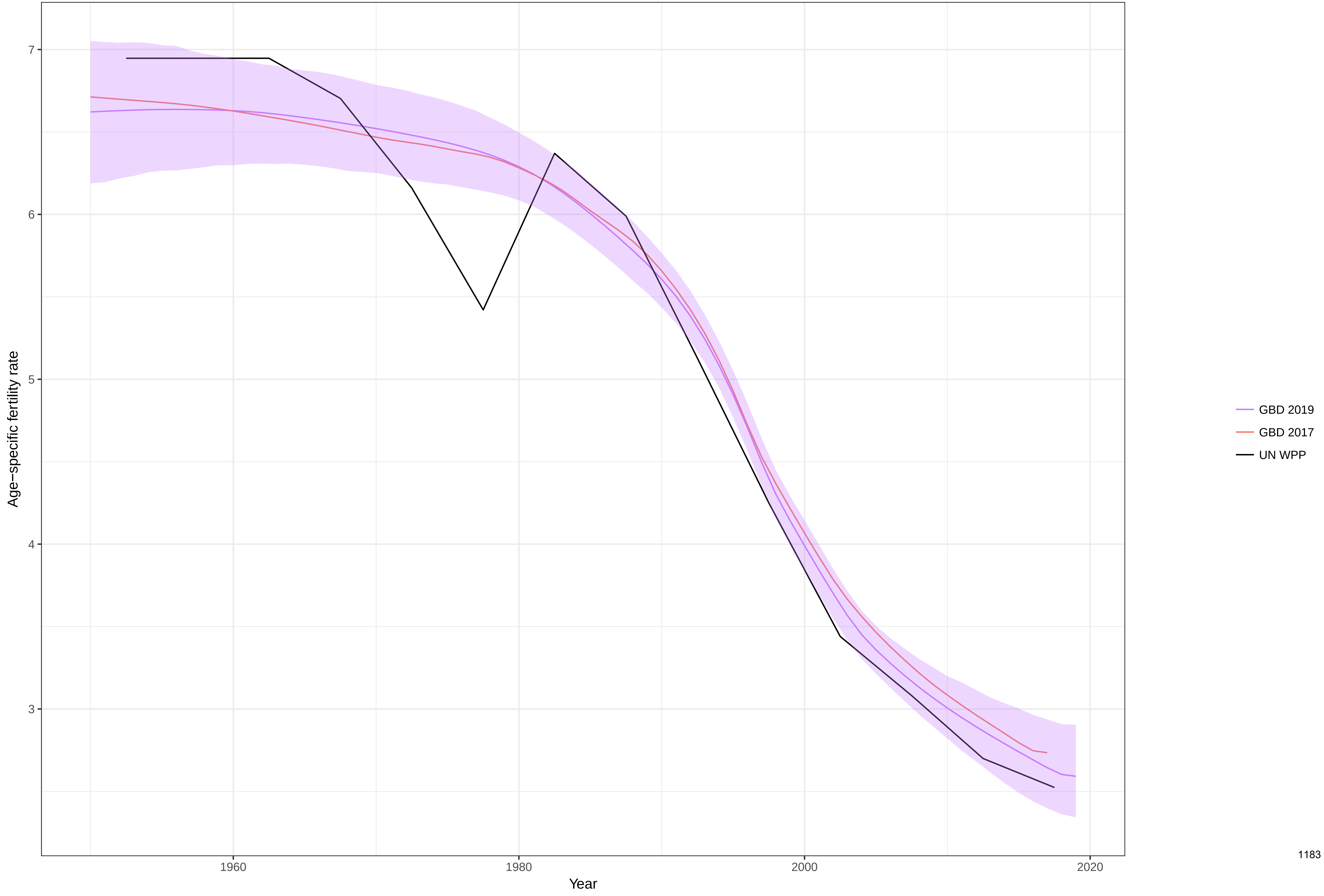

Age−specific fertility fitted trend lines, 1950−2019: Kiribati, Ages: 10−14
Data points enclosed with a diamond are reference sources

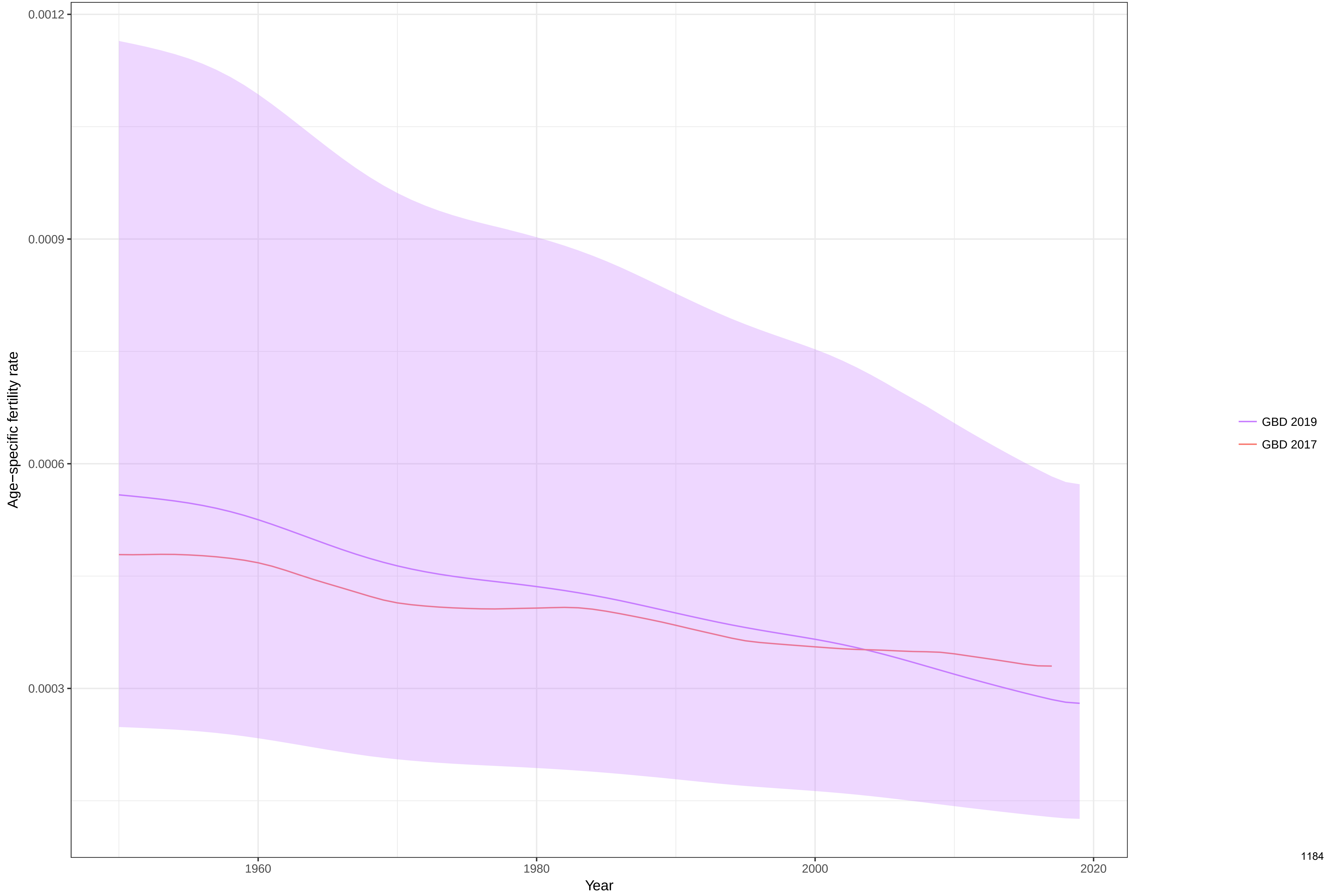

Age−specific fertility fitted trend lines, 1950−2019: Kiribati, Ages: 15−19
Data points enclosed with a diamond are reference sources

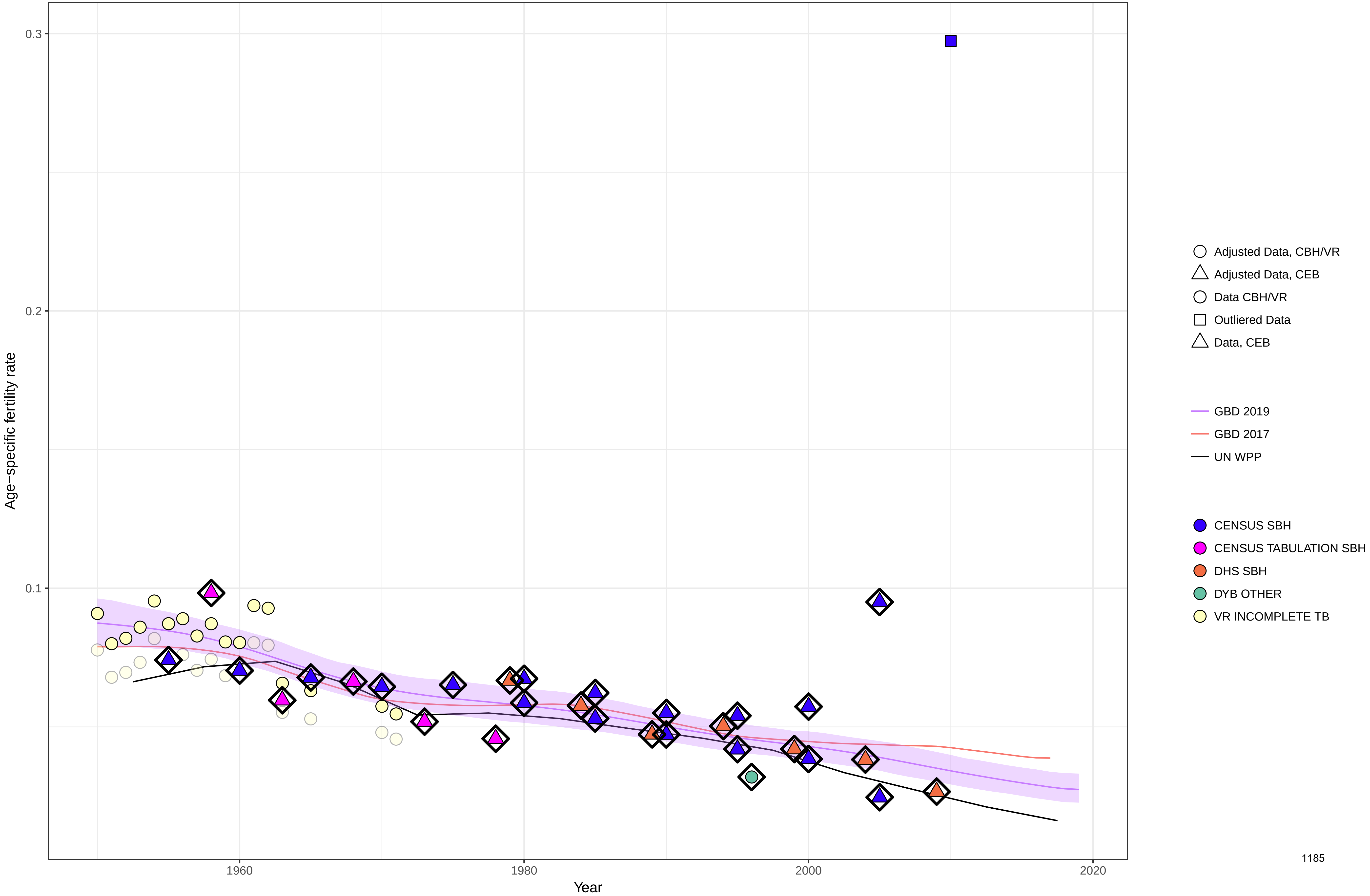

Age−specific fertility fitted trend lines, 1950−2019: Kiribati, Ages: 20−24
Data points enclosed with a diamond are reference sources

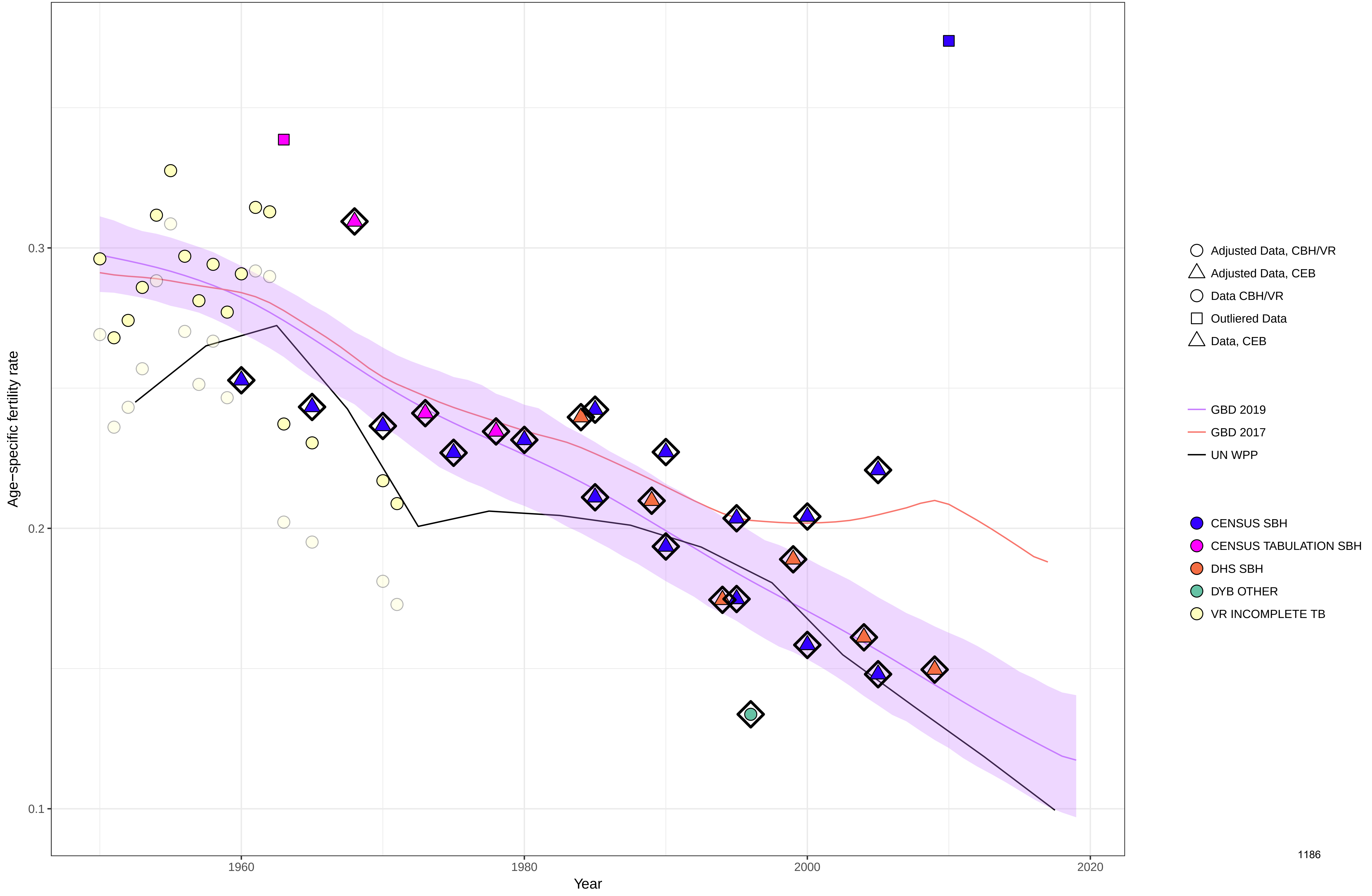

Age−specific fertility fitted trend lines, 1950−2019: Kiribati, Ages: 25−29
Data points enclosed with a diamond are reference sources

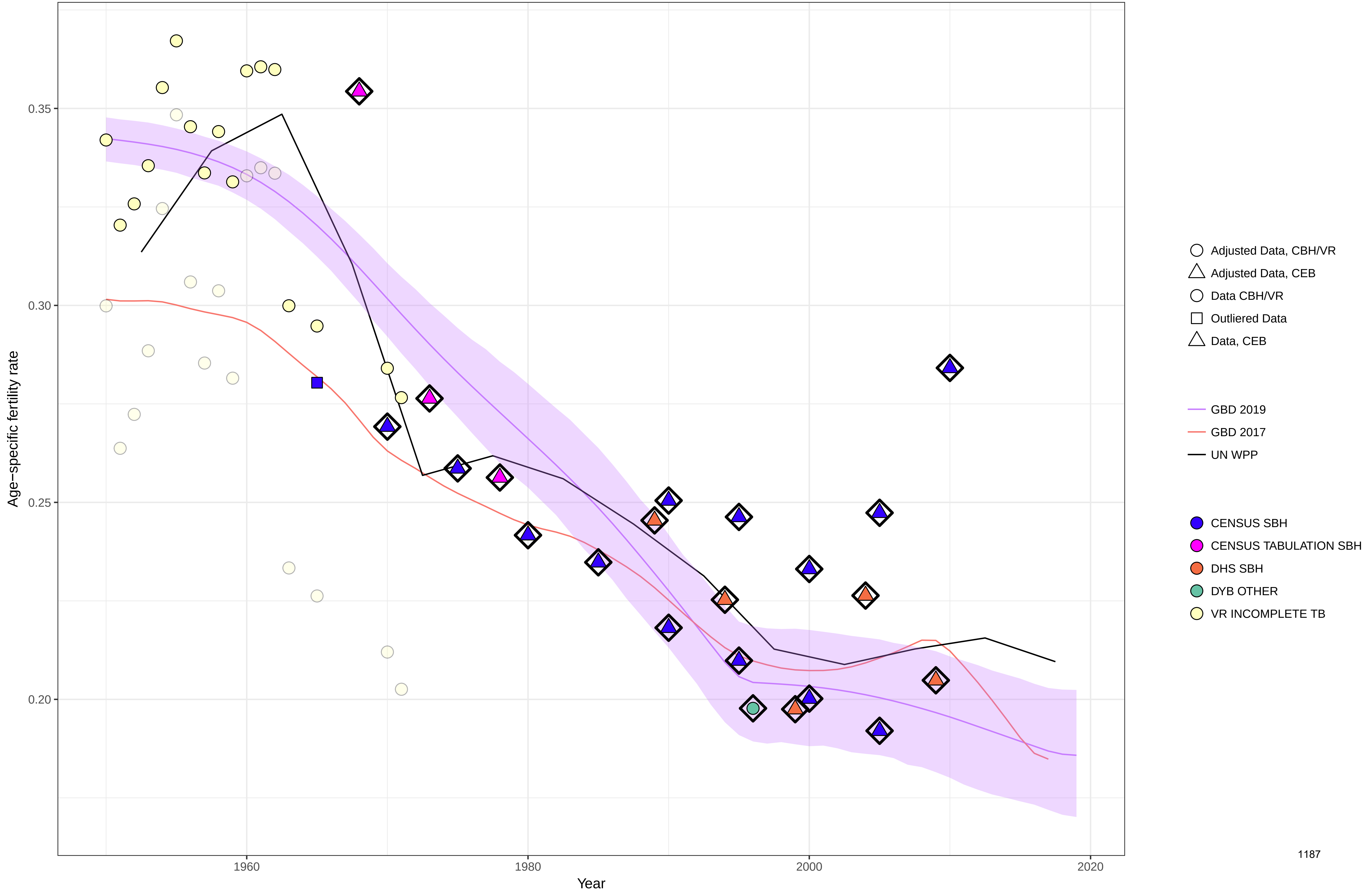

Age−specific fertility fitted trend lines, 1950−2019: Kiribati, Ages: 30−34
Data points enclosed with a diamond are reference sources

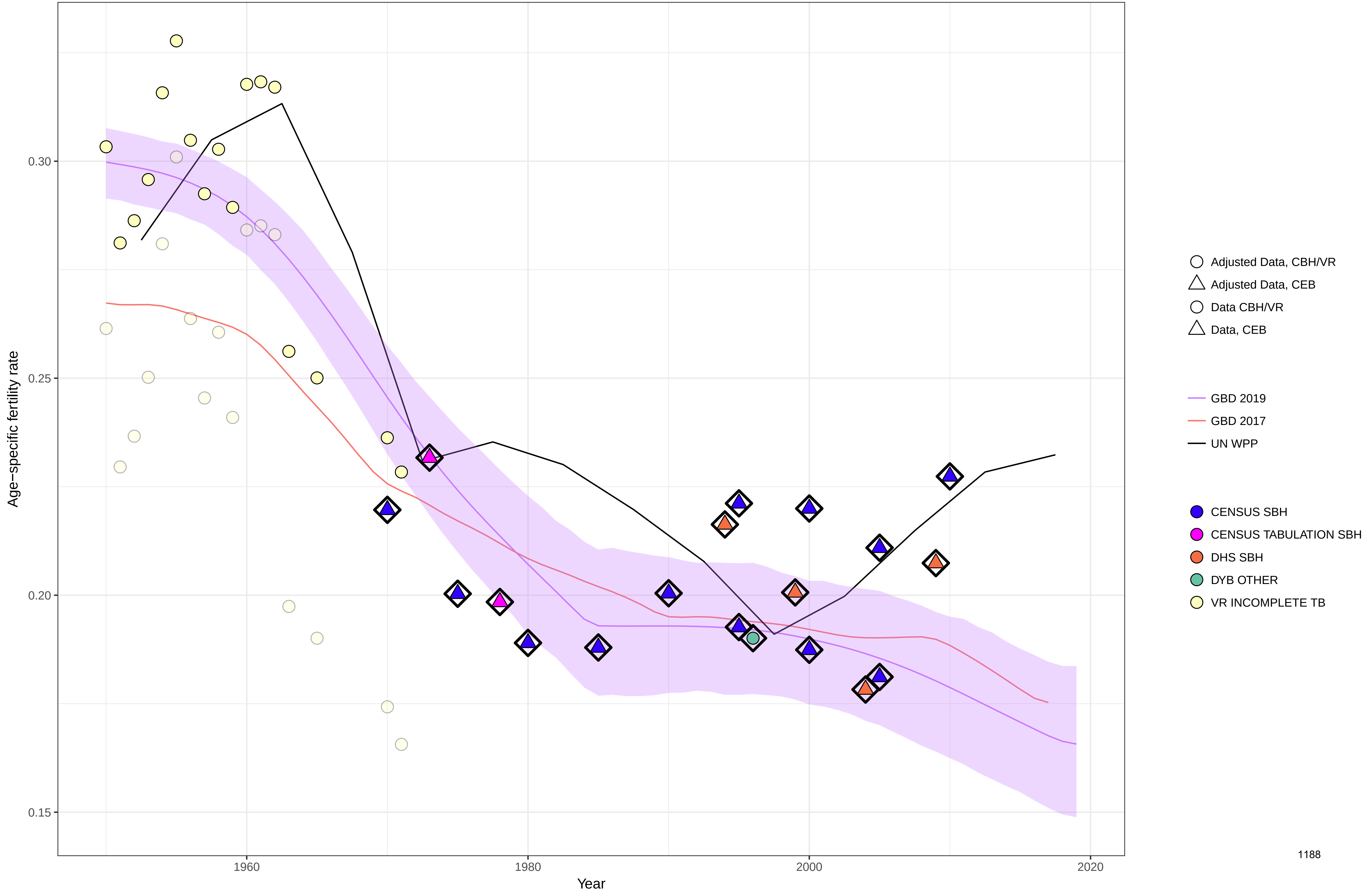

Age−specific fertility fitted trend lines, 1950−2019: Kiribati, Ages: 35−39
Data points enclosed with a diamond are reference sources

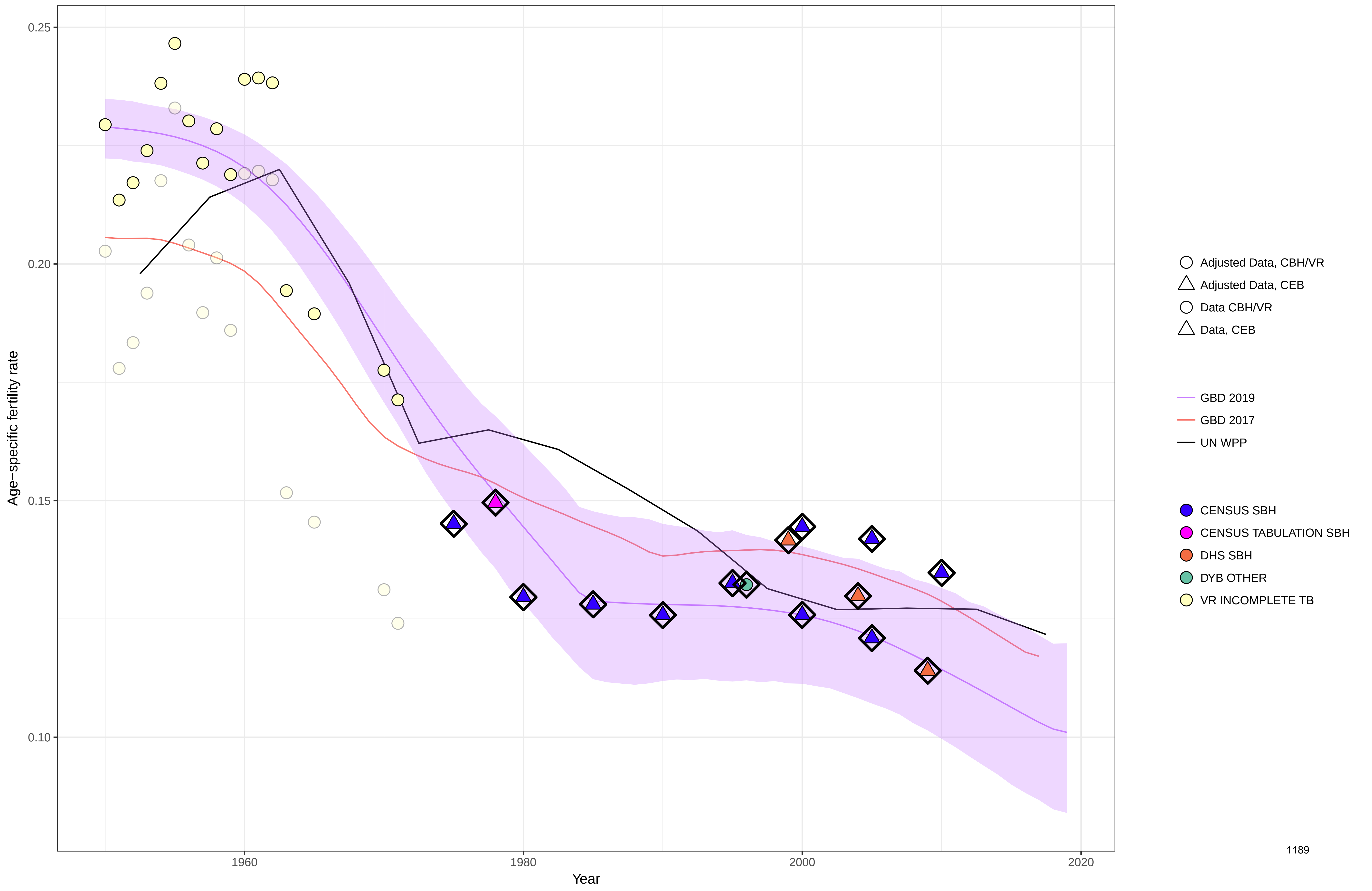

Age−specific fertility fitted trend lines, 1950−2019: Kiribati, Ages: 40−44
Data points enclosed with a diamond are reference sources

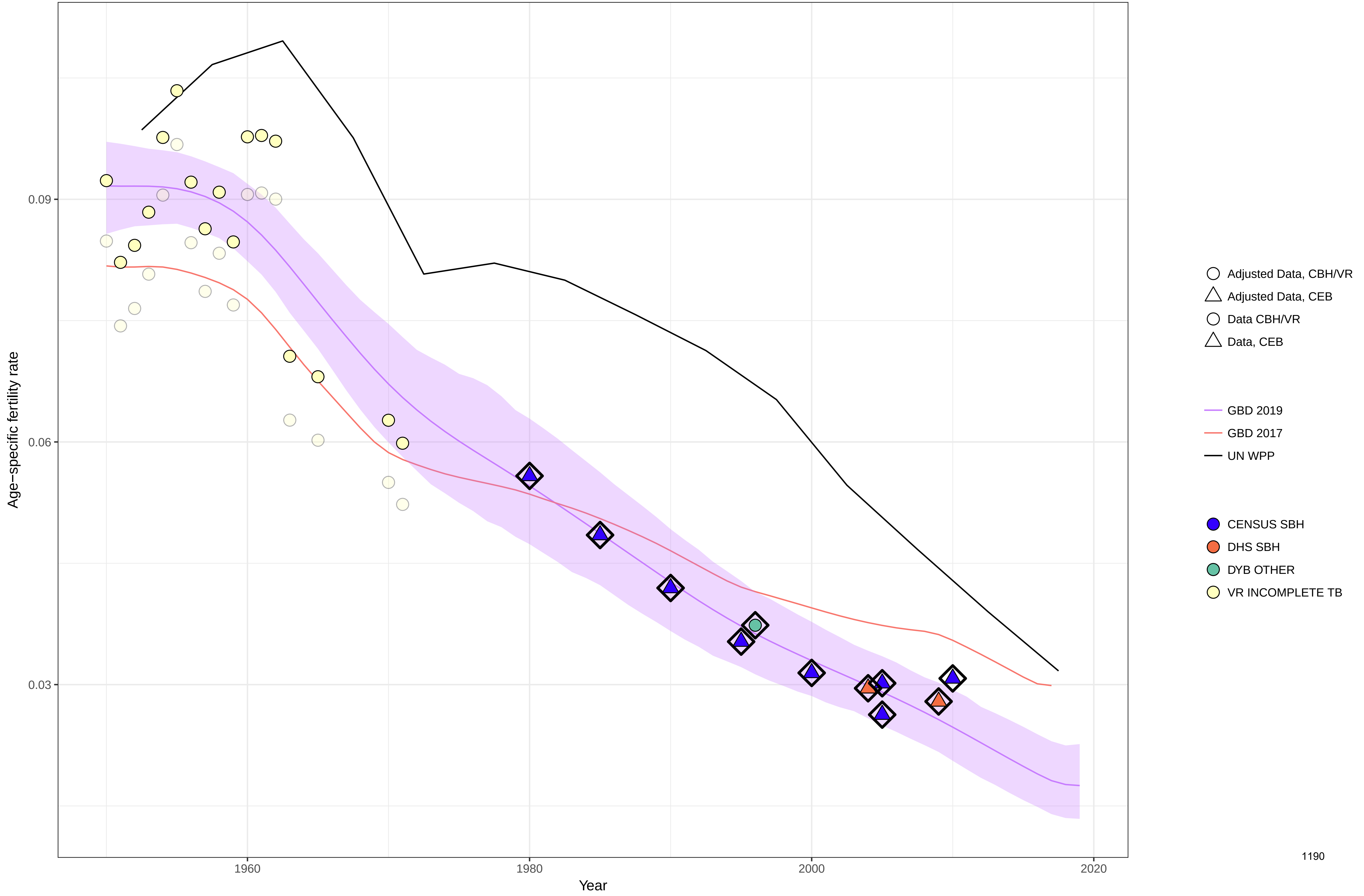

Age−specific fertility fitted trend lines, 1950−2019: Kiribati, Ages: 45−49
Data points enclosed with a diamond are reference sources

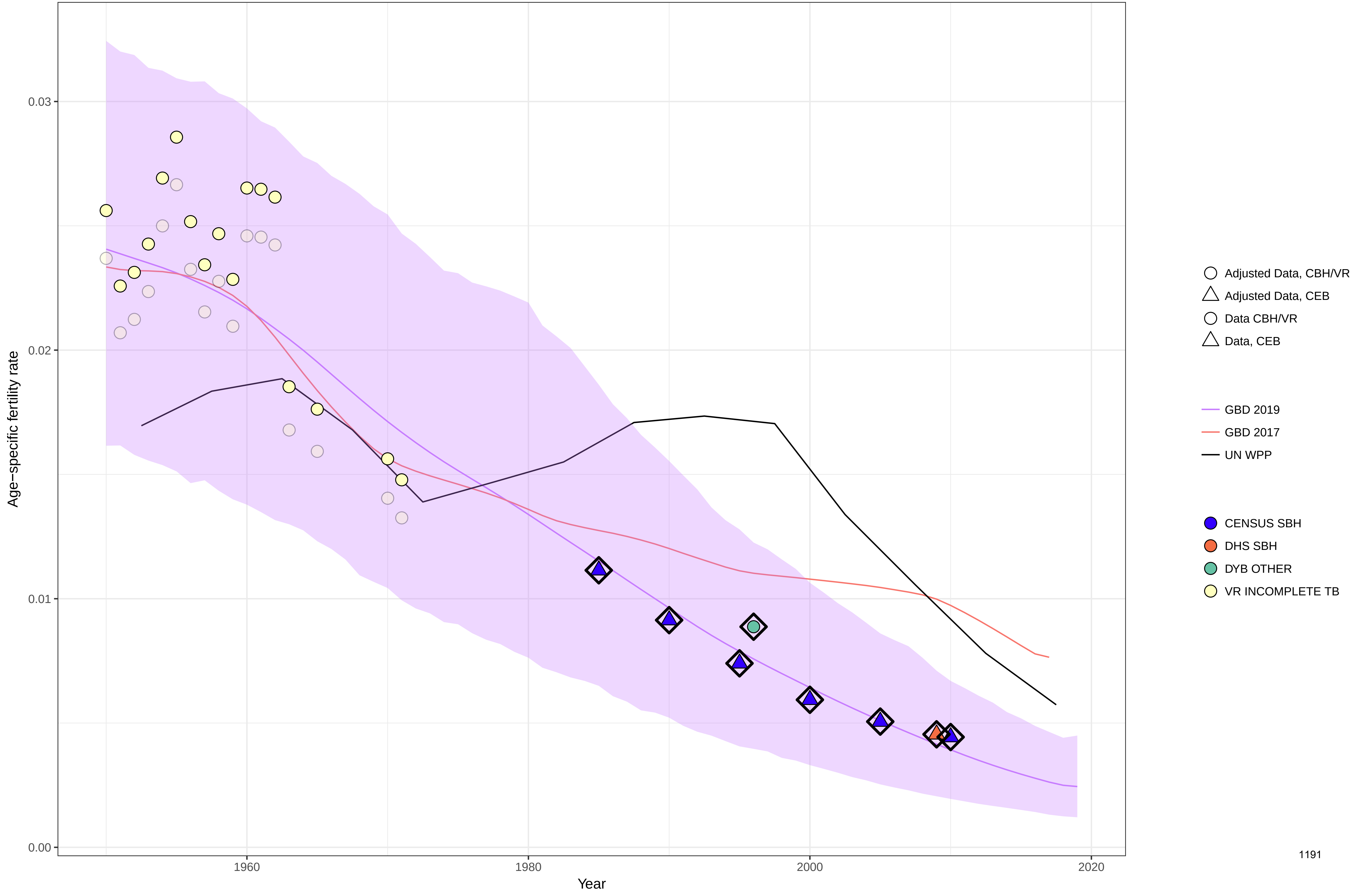

Age−specific fertility fitted trend lines, 1950−2019: Kiribati, Ages: 50−54
Data points enclosed with a diamond are reference sources

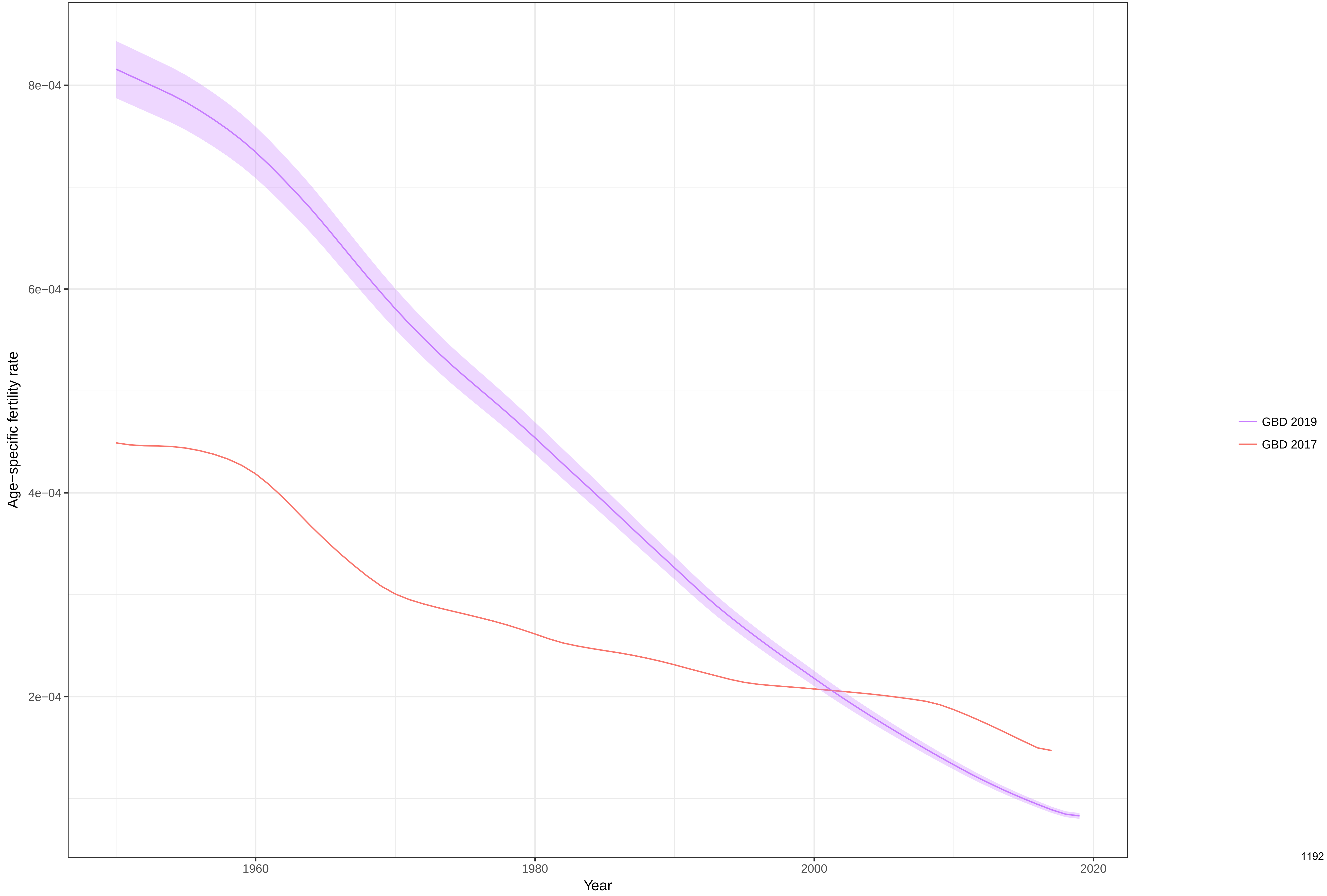

Total fertility rate fitted trend lines, 1950−2019: Kiribati TFR
Data points enclosed with a diamond are reference sources

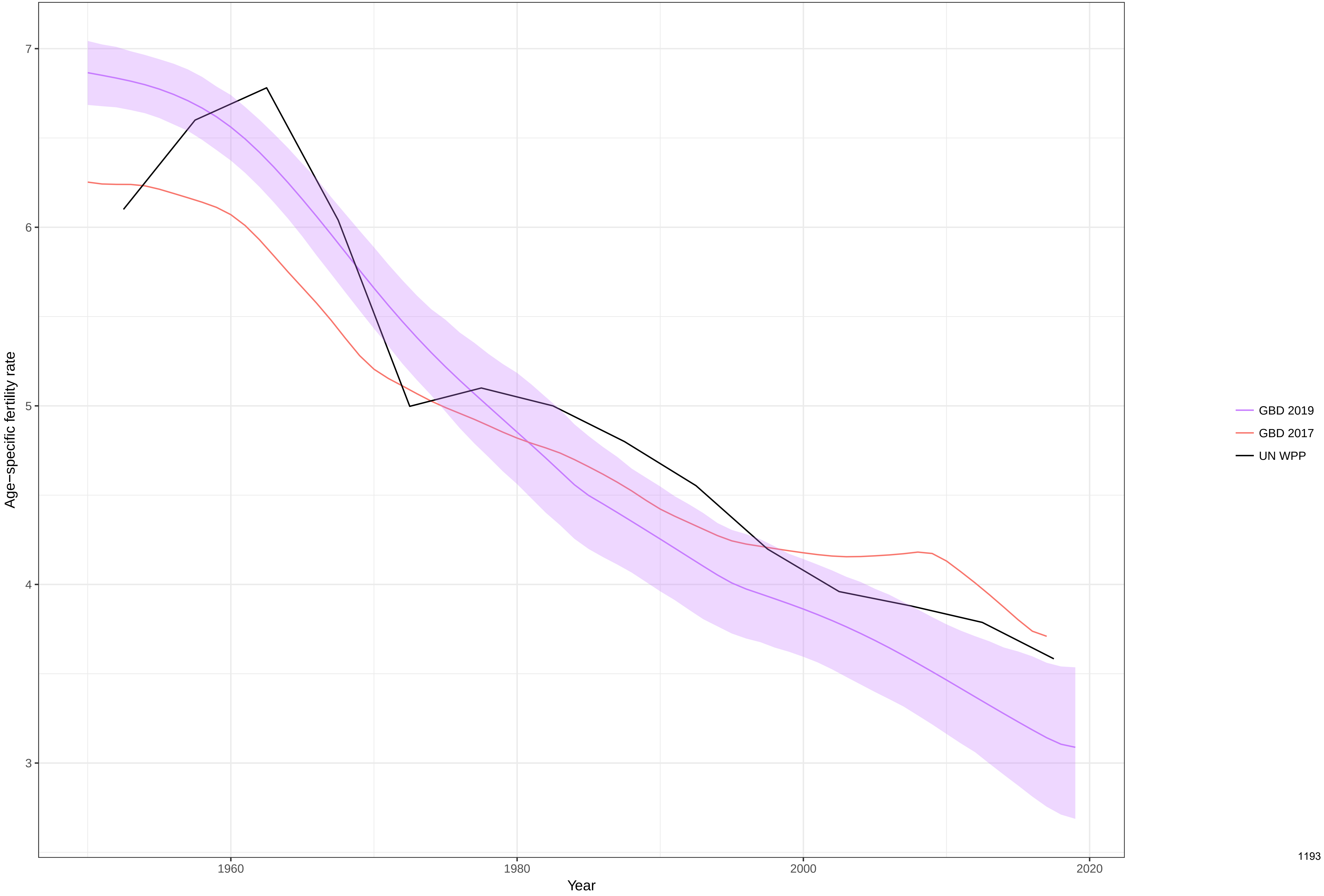

Age−specific fertility fitted trend lines, 1950−2019: Saint Kitts and Nevis, Ages: 10−14
Data points enclosed with a diamond are reference sources

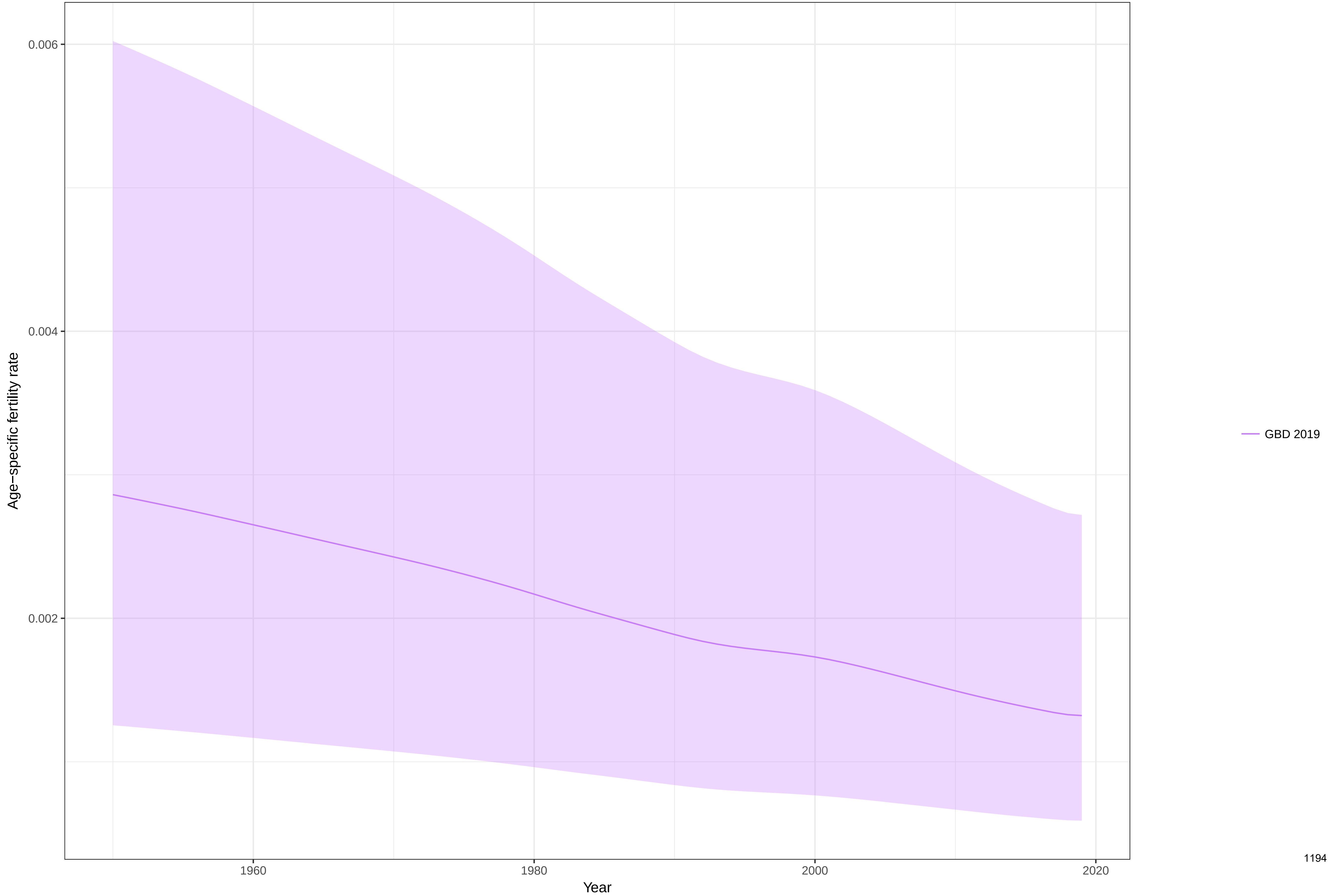

Age−specific fertility fitted trend lines, 1950−2019: Saint Kitts and Nevis, Ages: 15−19
Data points enclosed with a diamond are reference sources

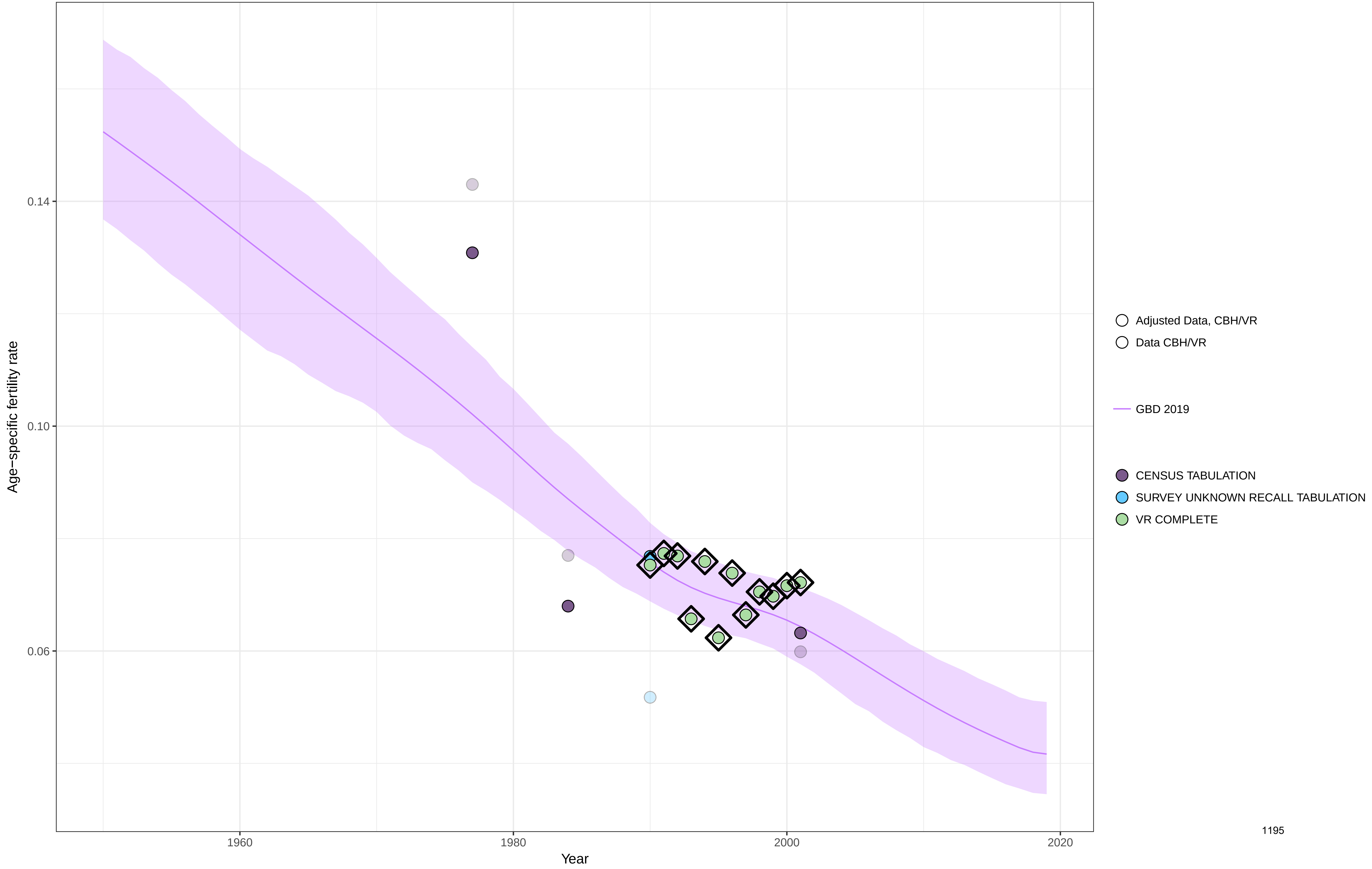

Age−specific fertility fitted trend lines, 1950−2019: Saint Kitts and Nevis, Ages: 20−24
Data points enclosed with a diamond are reference sources

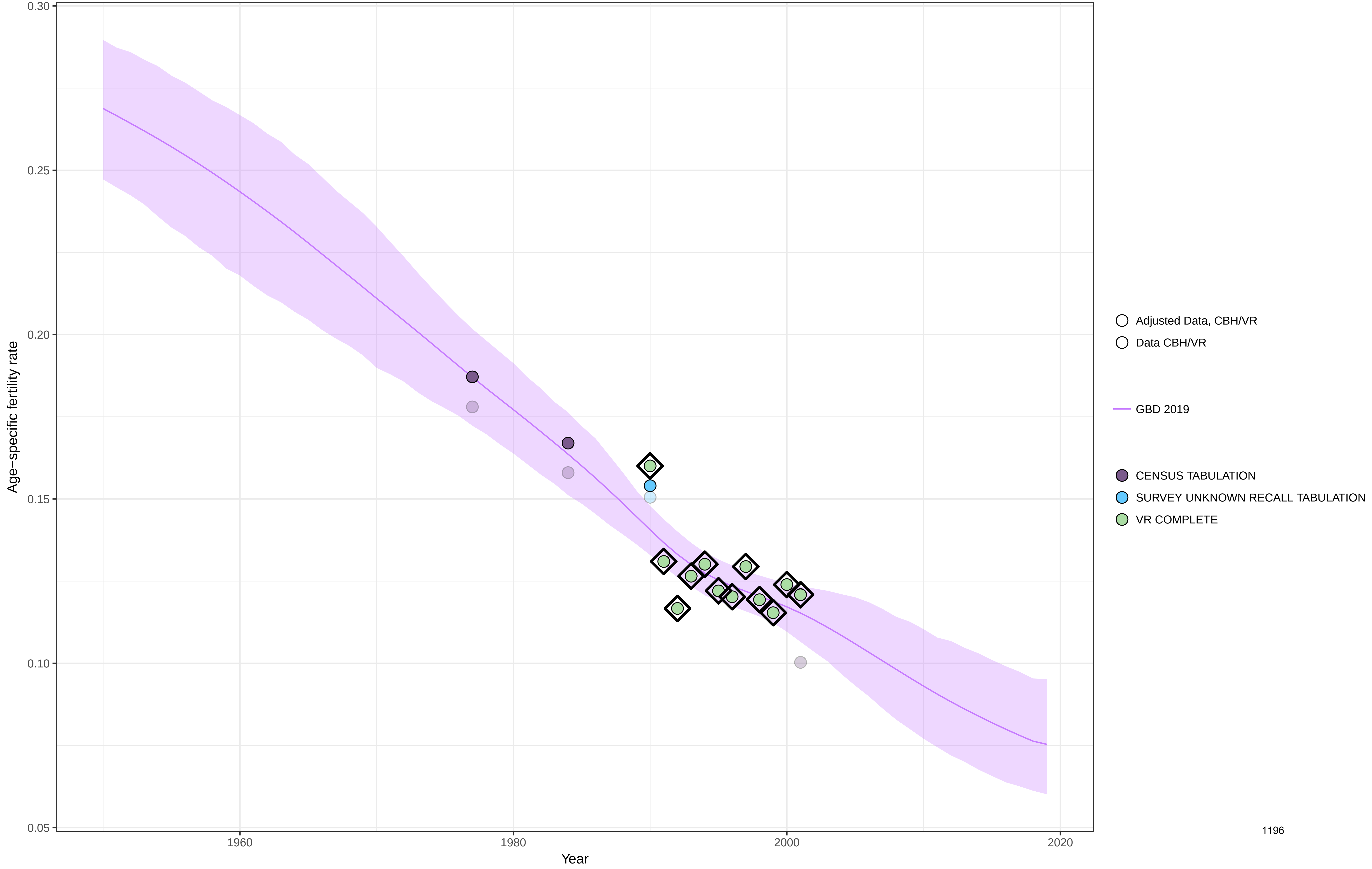

Age−specific fertility fitted trend lines, 1950−2019: Saint Kitts and Nevis, Ages: 25−29
Data points enclosed with a diamond are reference sources

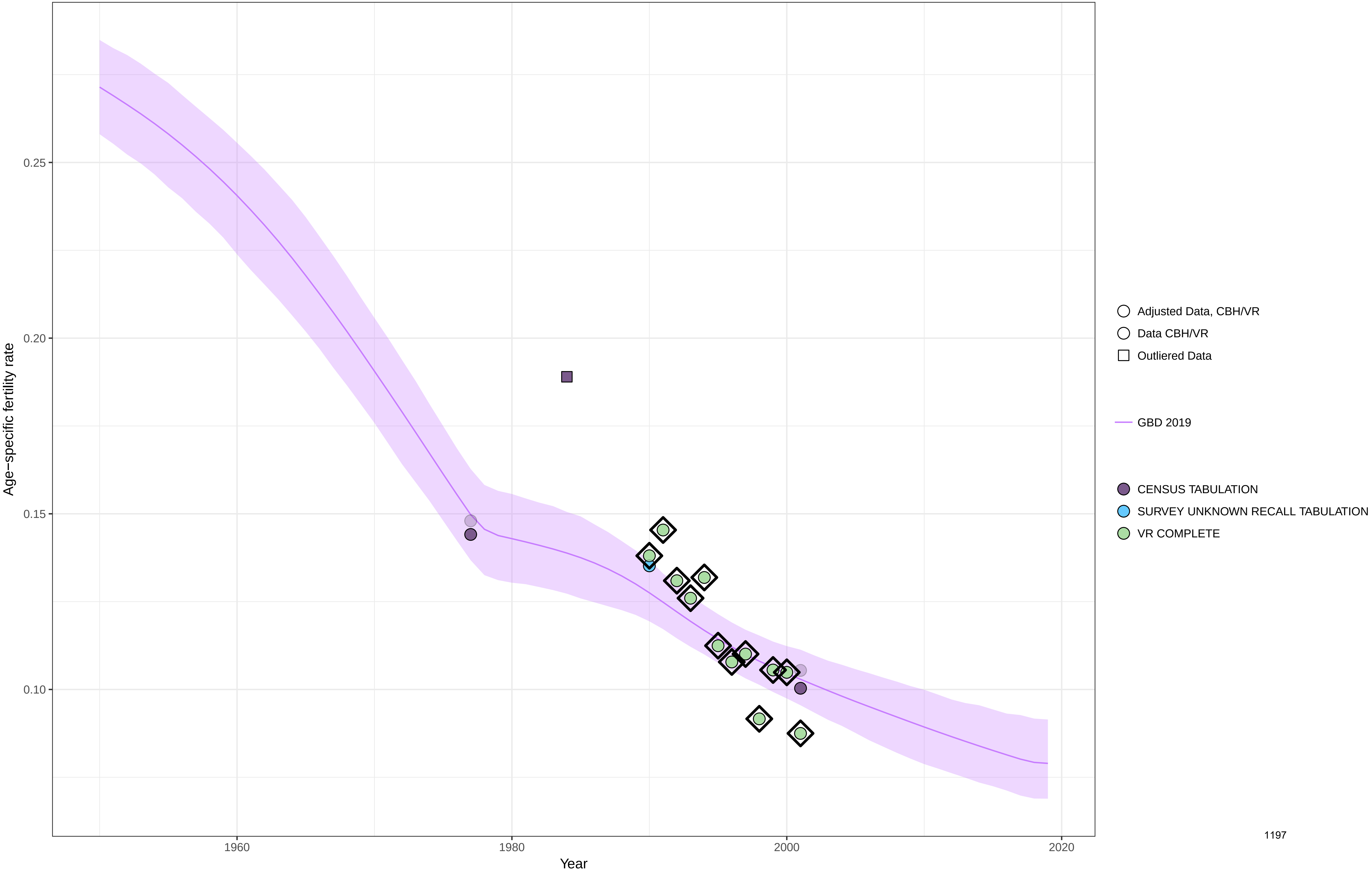

Age−specific fertility fitted trend lines, 1950−2019: Saint Kitts and Nevis, Ages: 30−34
Data points enclosed with a diamond are reference sources

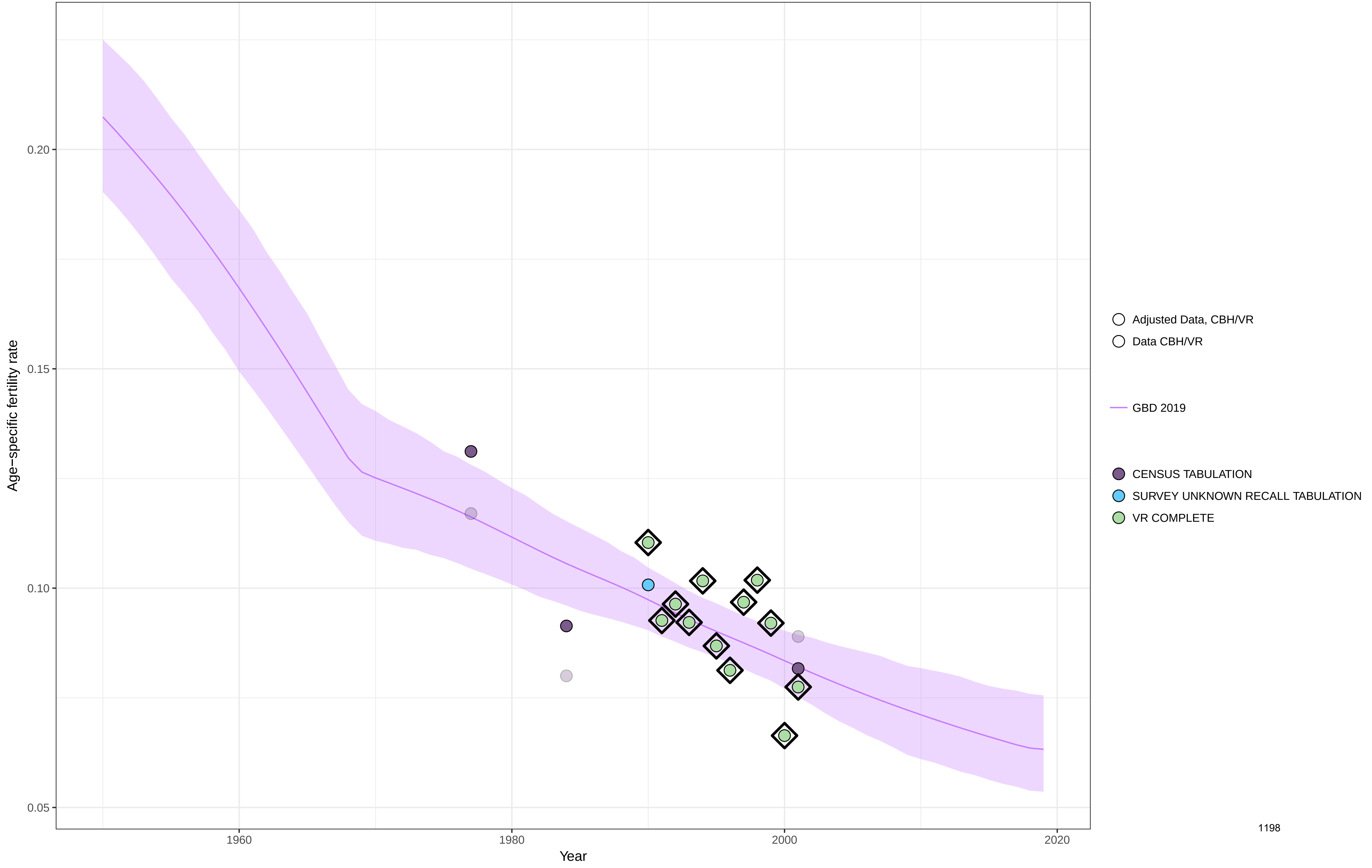

Age−specific fertility fitted trend lines, 1950−2019: Saint Kitts and Nevis, Ages: 35−39
Data points enclosed with a diamond are reference sources

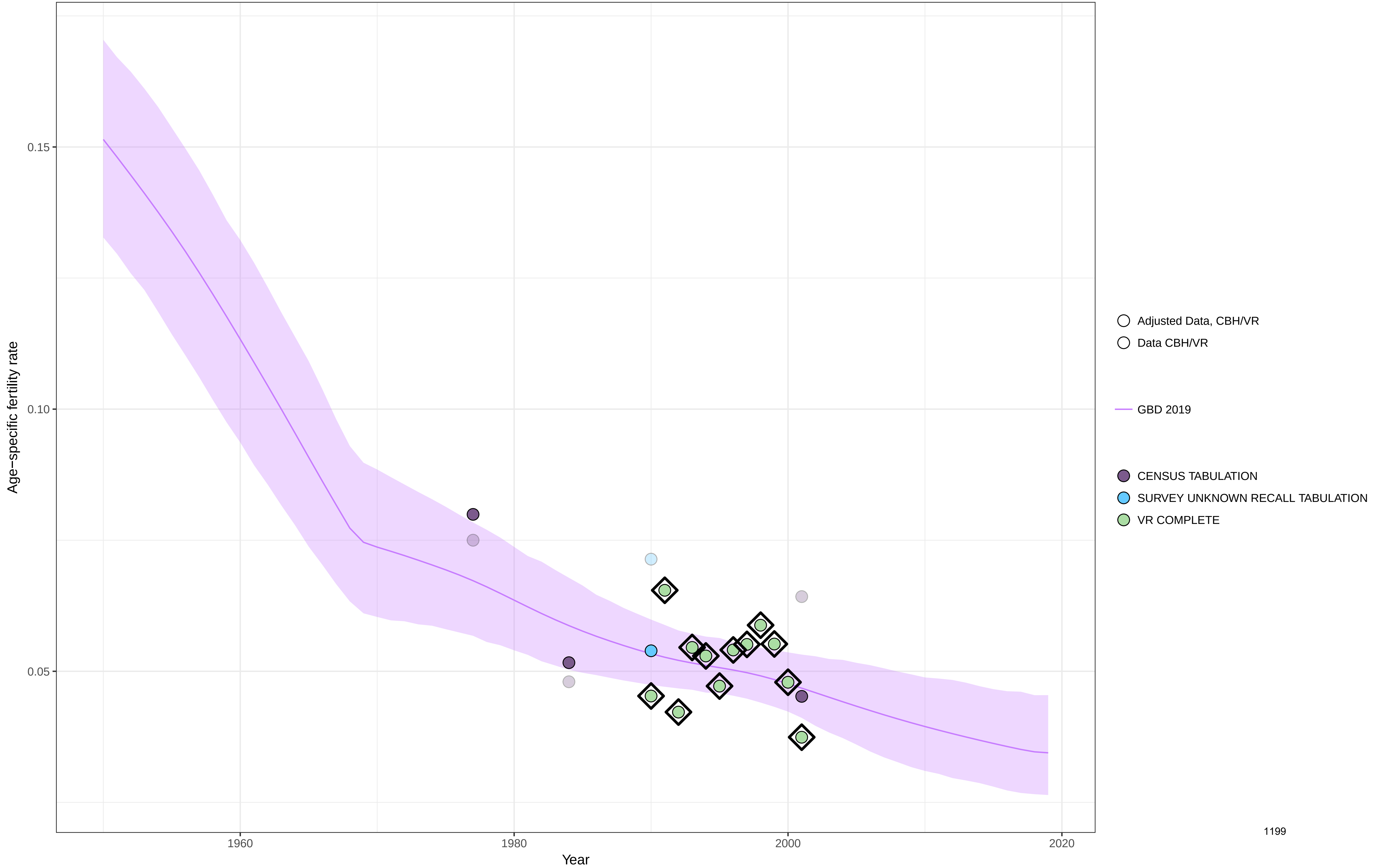

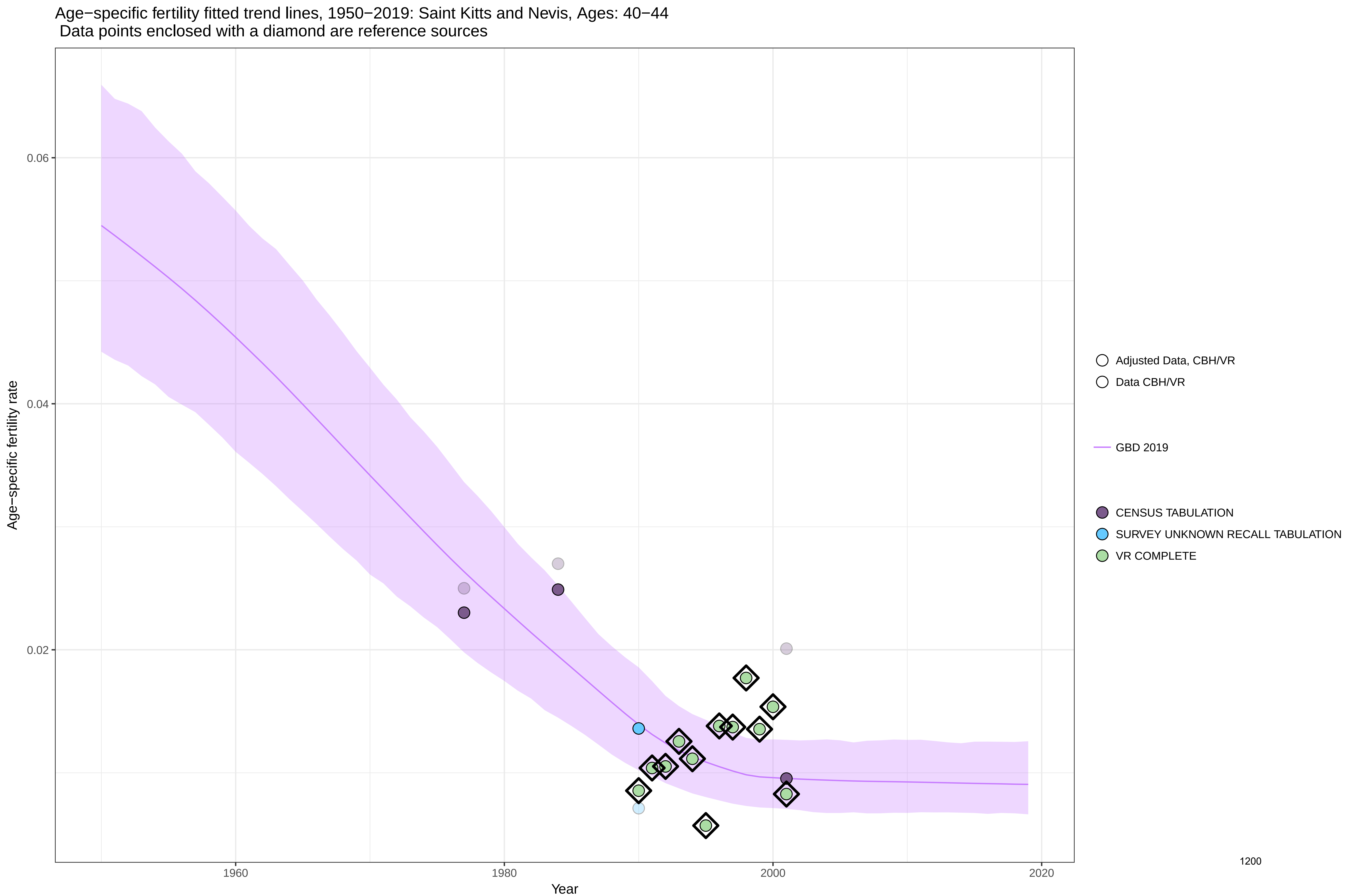
Age−specific fertility fitted trend lines, 1950−2019: Saint Kitts and Nevis, Ages: 40−44
Data points enclosed with a diamond are reference sources
0.06
0.04
0.02
1960
1980
2000
2020
Year
Age−specific fertility rate
Adjusted Data, CBH/VR
Data CBH/VR
GBD 2019
CENSUS TABULATION
SURVEY UNKNOWN RECALL TABULATION
VR COMPLETE

Age−specific fertility fitted trend lines, 1950−2019: Saint Kitts and Nevis, Ages: 45−49
Data points enclosed with a diamond are reference sources

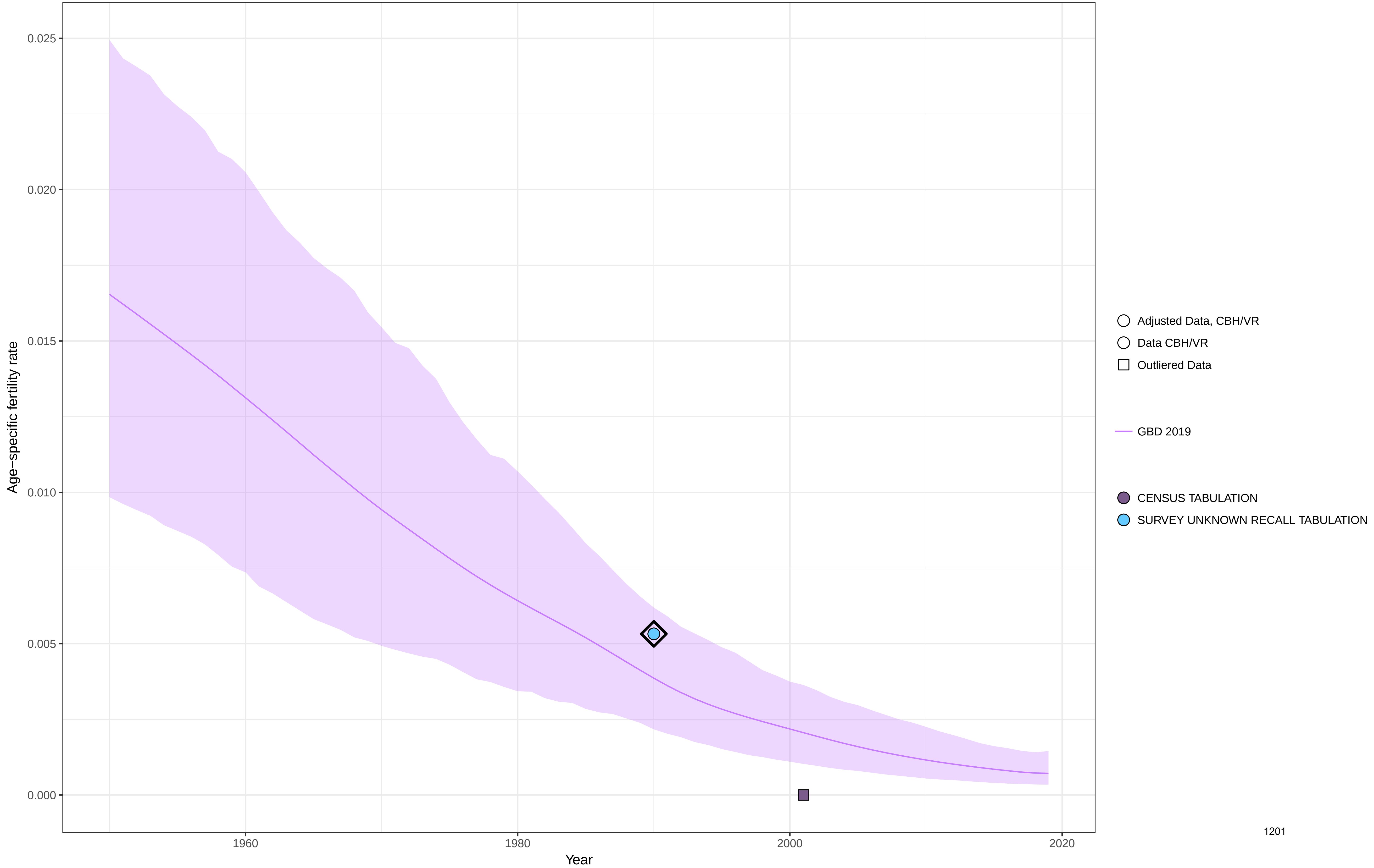

Age−specific fertility fitted trend lines, 1950−2019: Saint Kitts and Nevis, Ages: 50−54
Data points enclosed with a diamond are reference sources

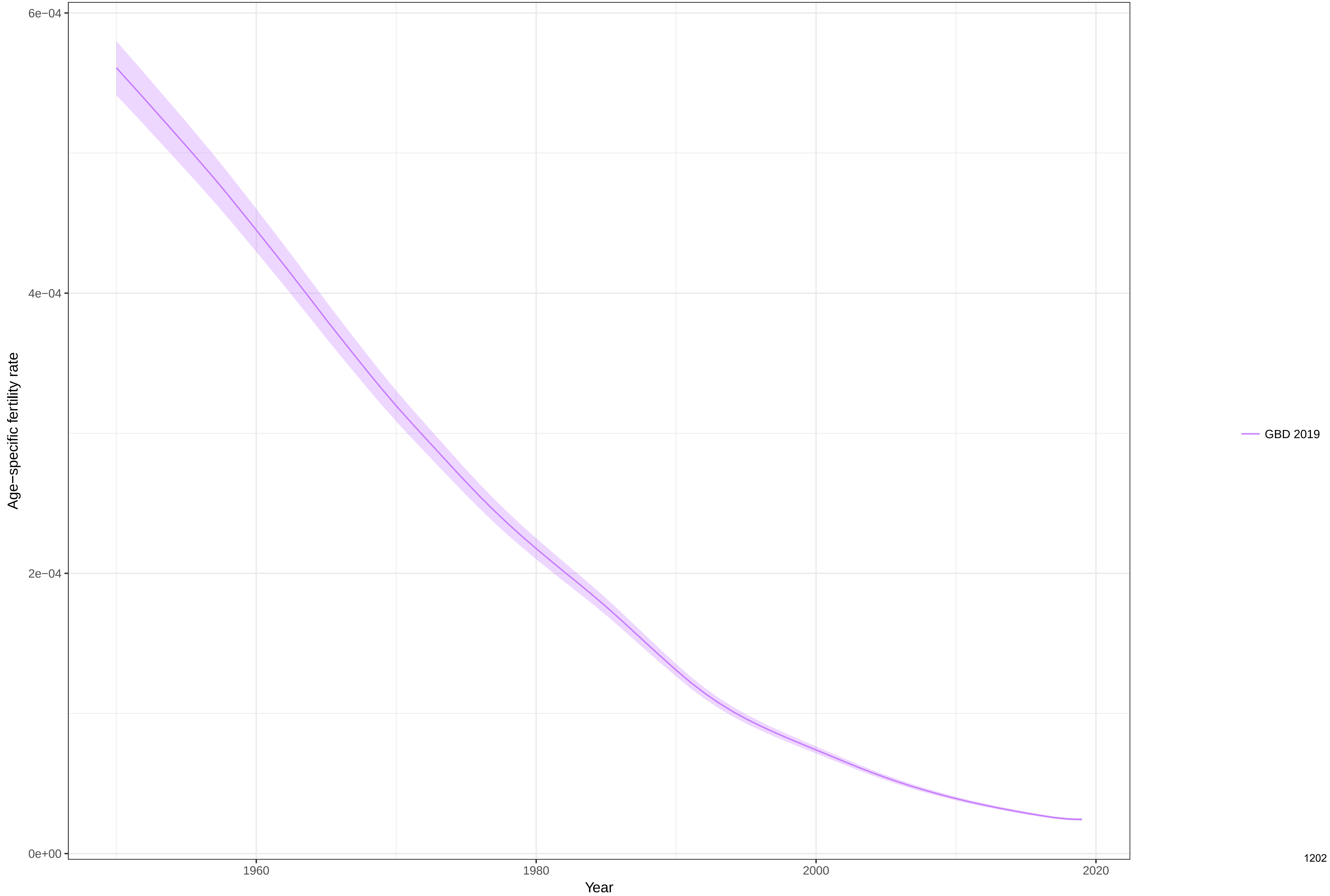

Total fertility rate fitted trend lines, 1950−2019: Saint Kitts and Nevis TFR
Data points enclosed with a diamond are reference sources

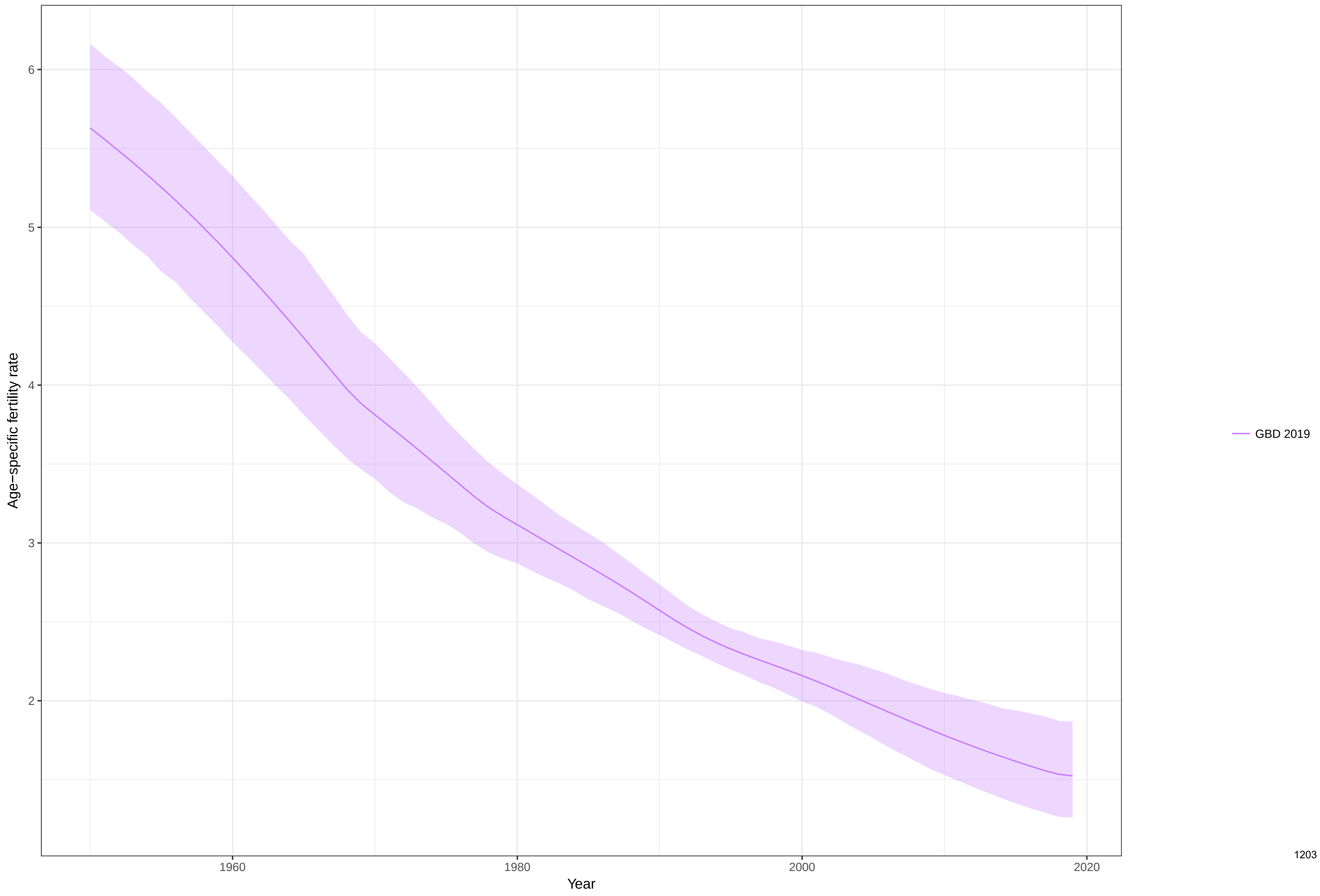

Age−specific fertility fitted trend lines, 1950−2019: Republic of Korea, Ages: 10−14
Data points enclosed with a diamond are reference sources

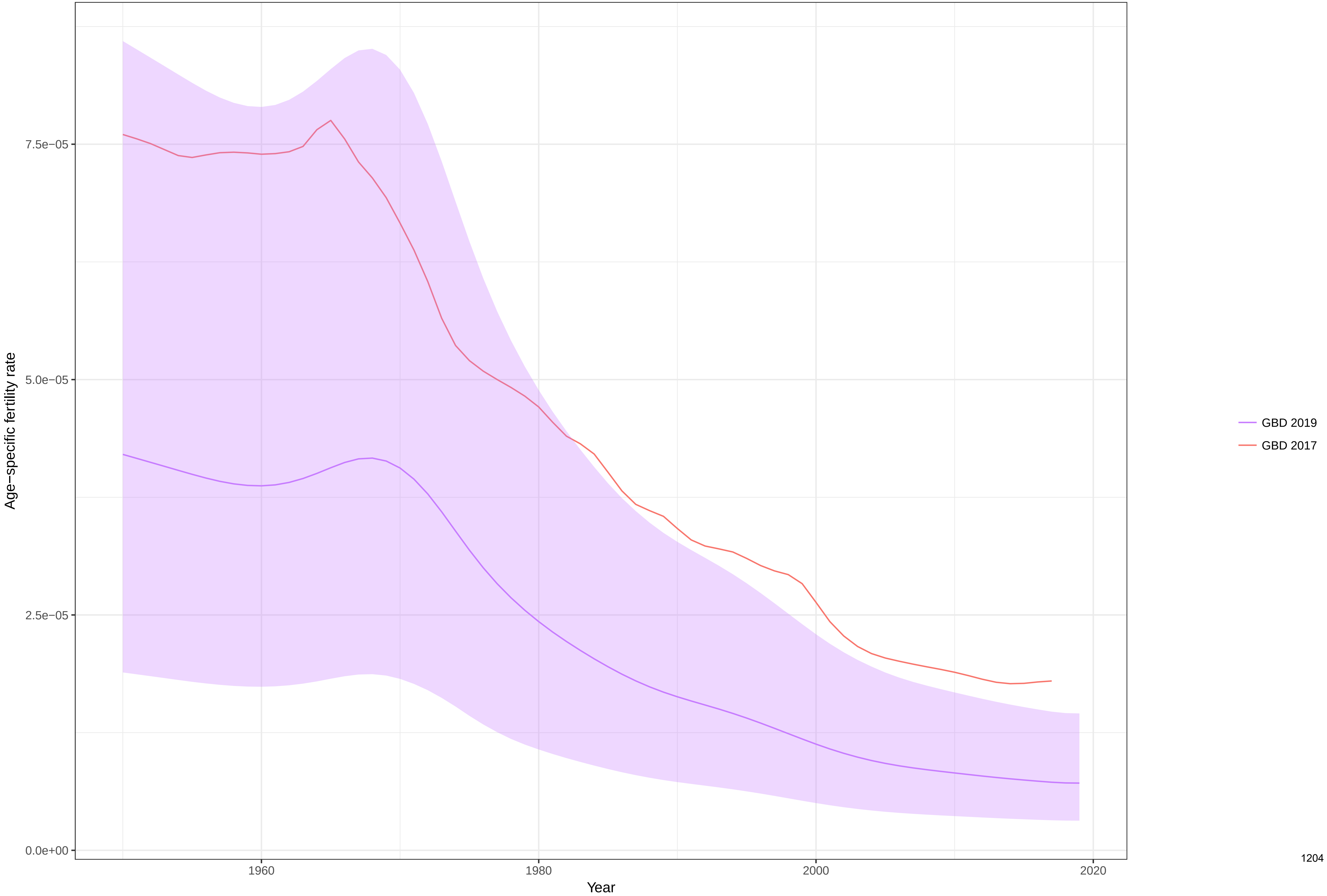

Age−specific fertility fitted trend lines, 1950−2019: Republic of Korea, Ages: 15−19
Data points enclosed with a diamond are reference sources

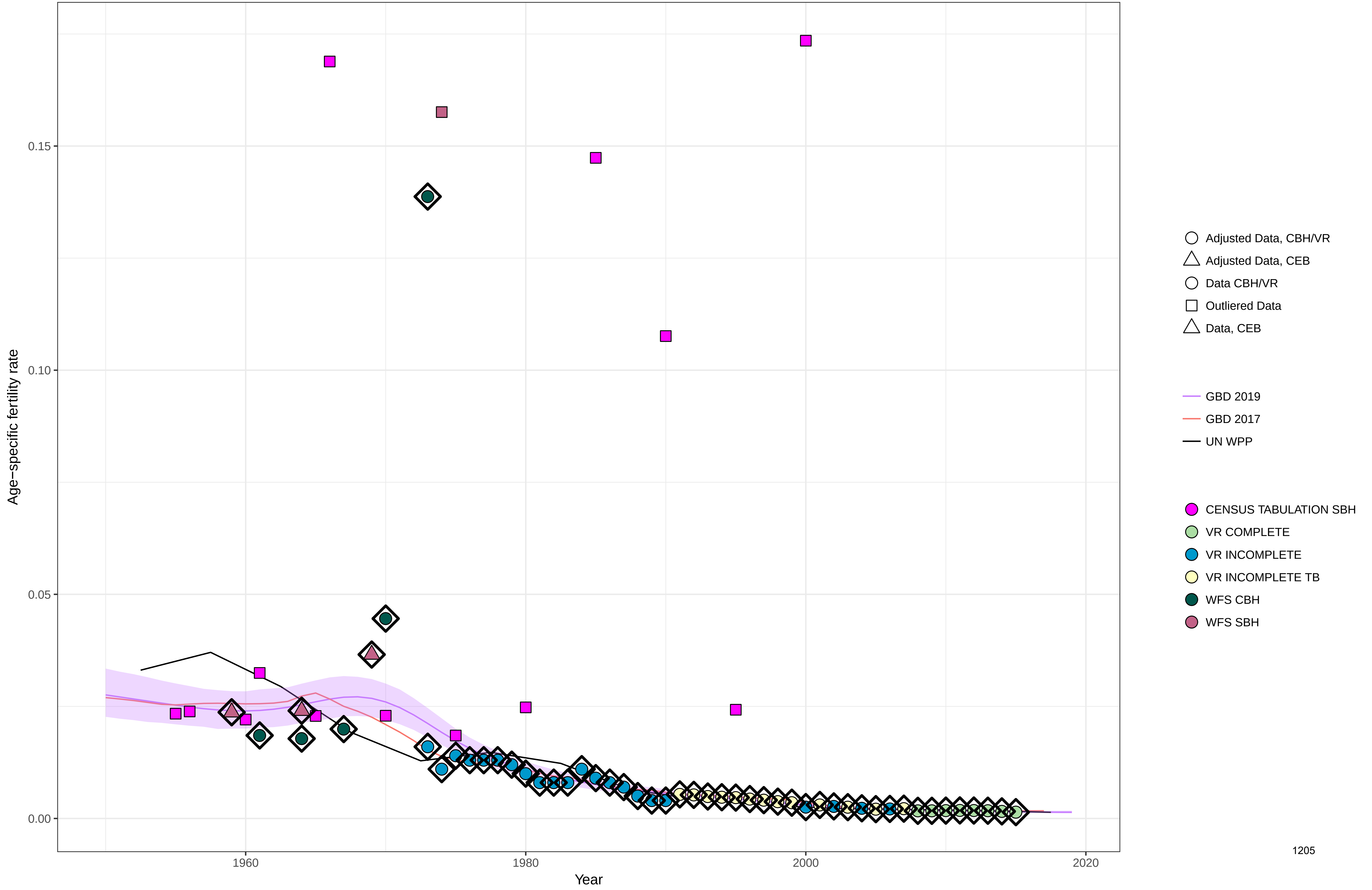

Age−specific fertility fitted trend lines, 1950−2019: Republic of Korea, Ages: 20−24
Data points enclosed with a diamond are reference sources

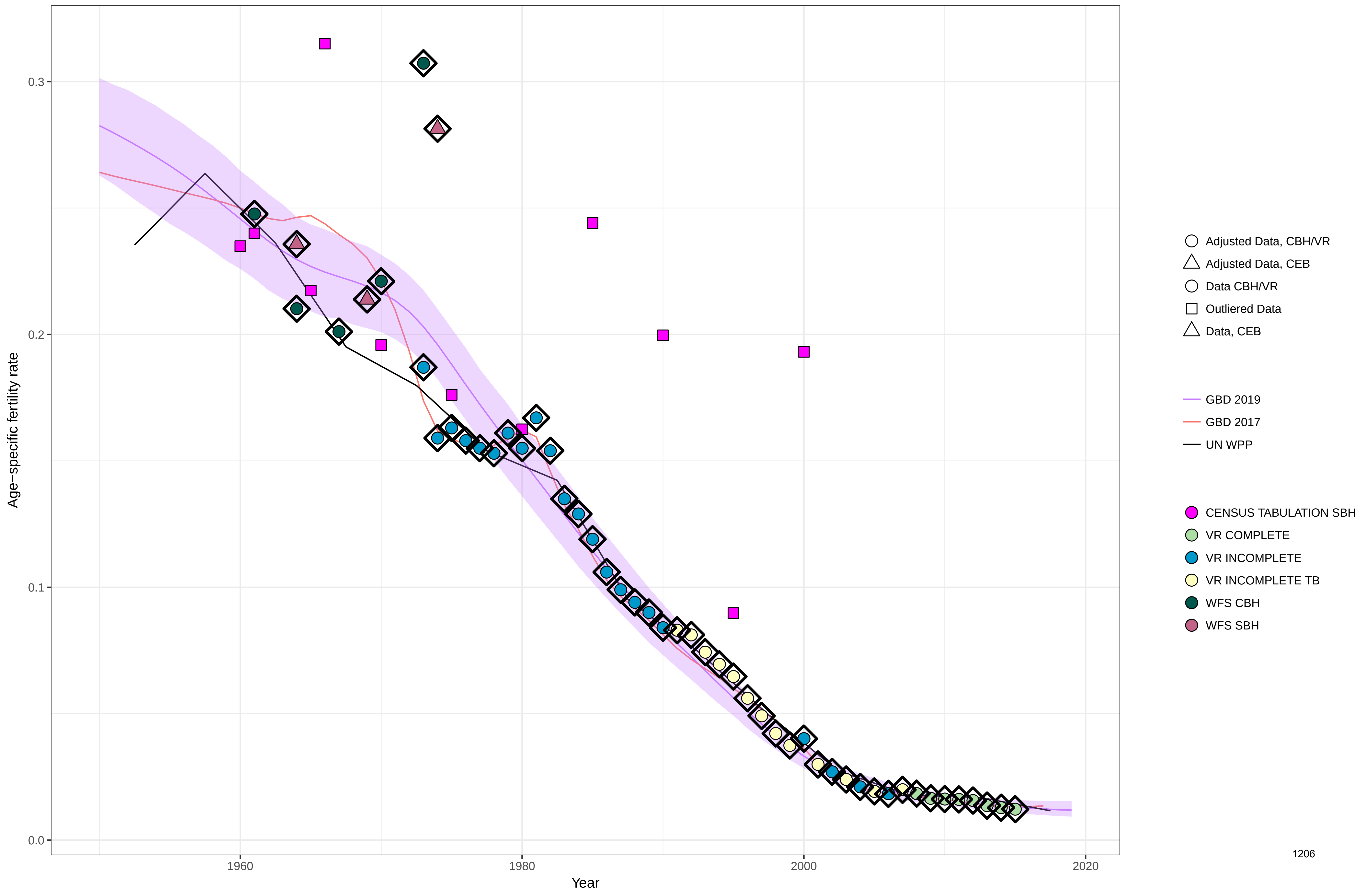

Age−specific fertility fitted trend lines, 1950−2019: Republic of Korea, Ages: 25−29
Data points enclosed with a diamond are reference sources

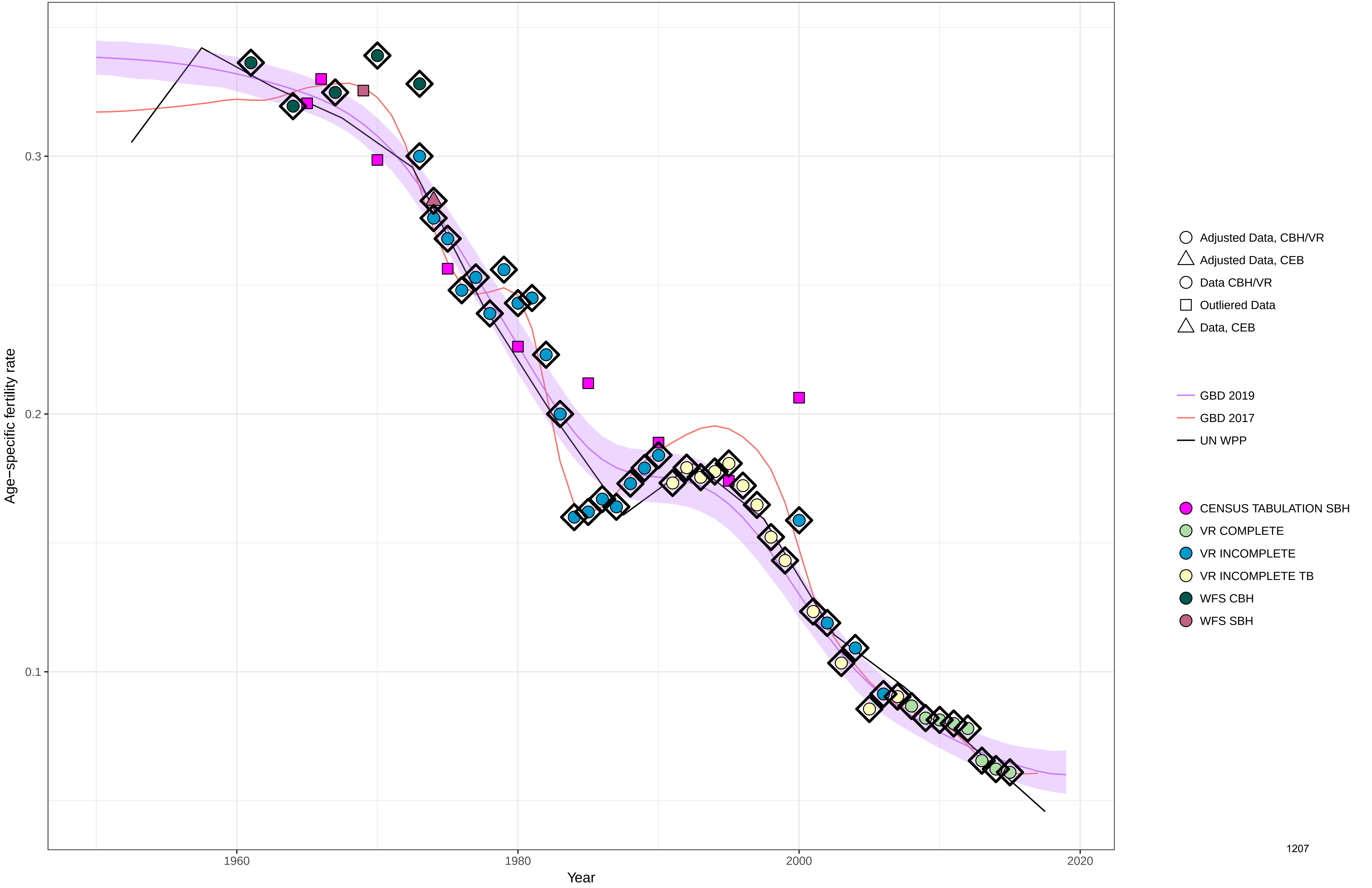

Age−specific fertility fitted trend lines, 1950−2019: Republic of Korea, Ages: 30−34
Data points enclosed with a diamond are reference sources

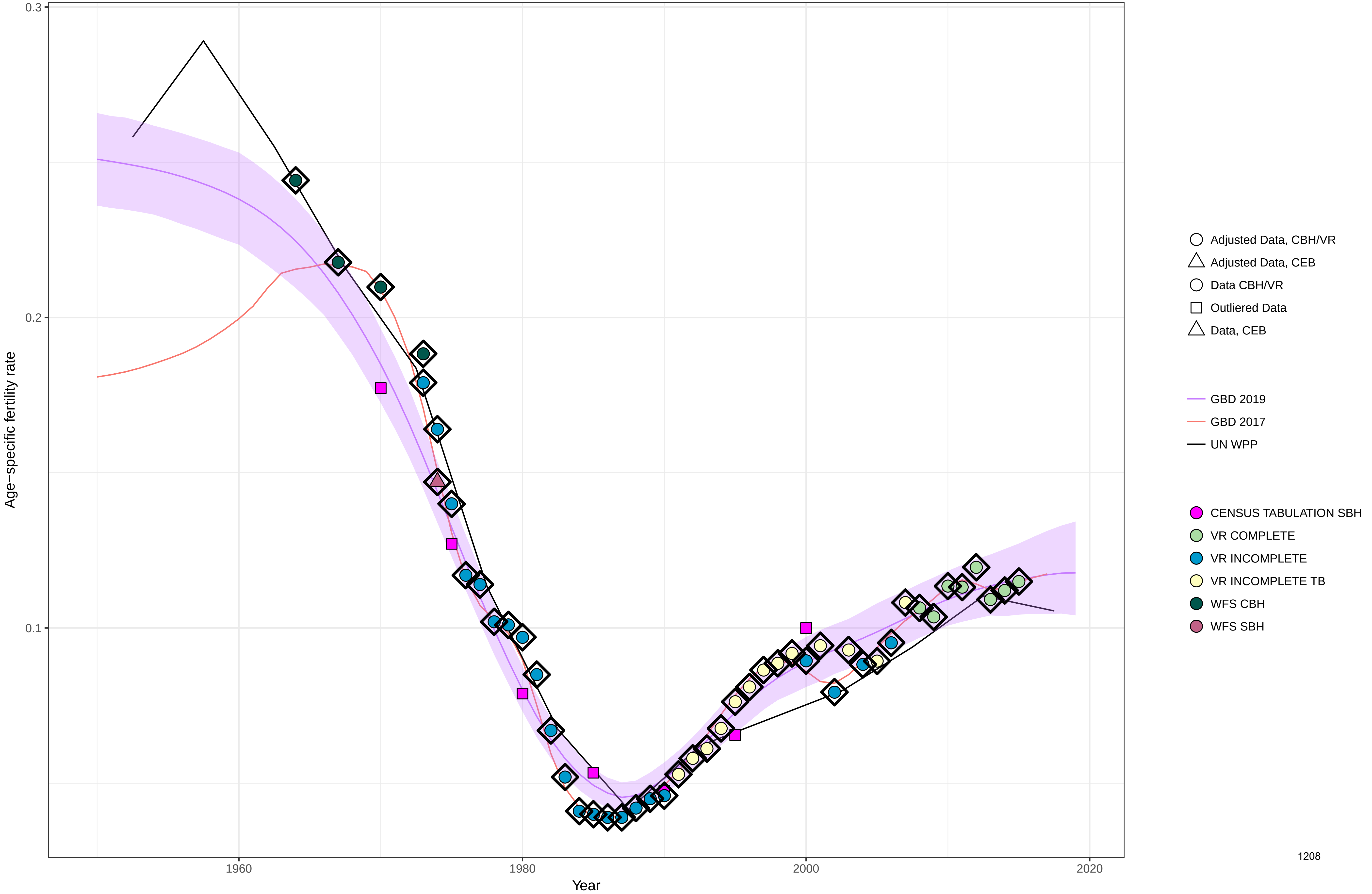

Age−specific fertility fitted trend lines, 1950−2019: Republic of Korea, Ages: 35−39
Data points enclosed with a diamond are reference sources

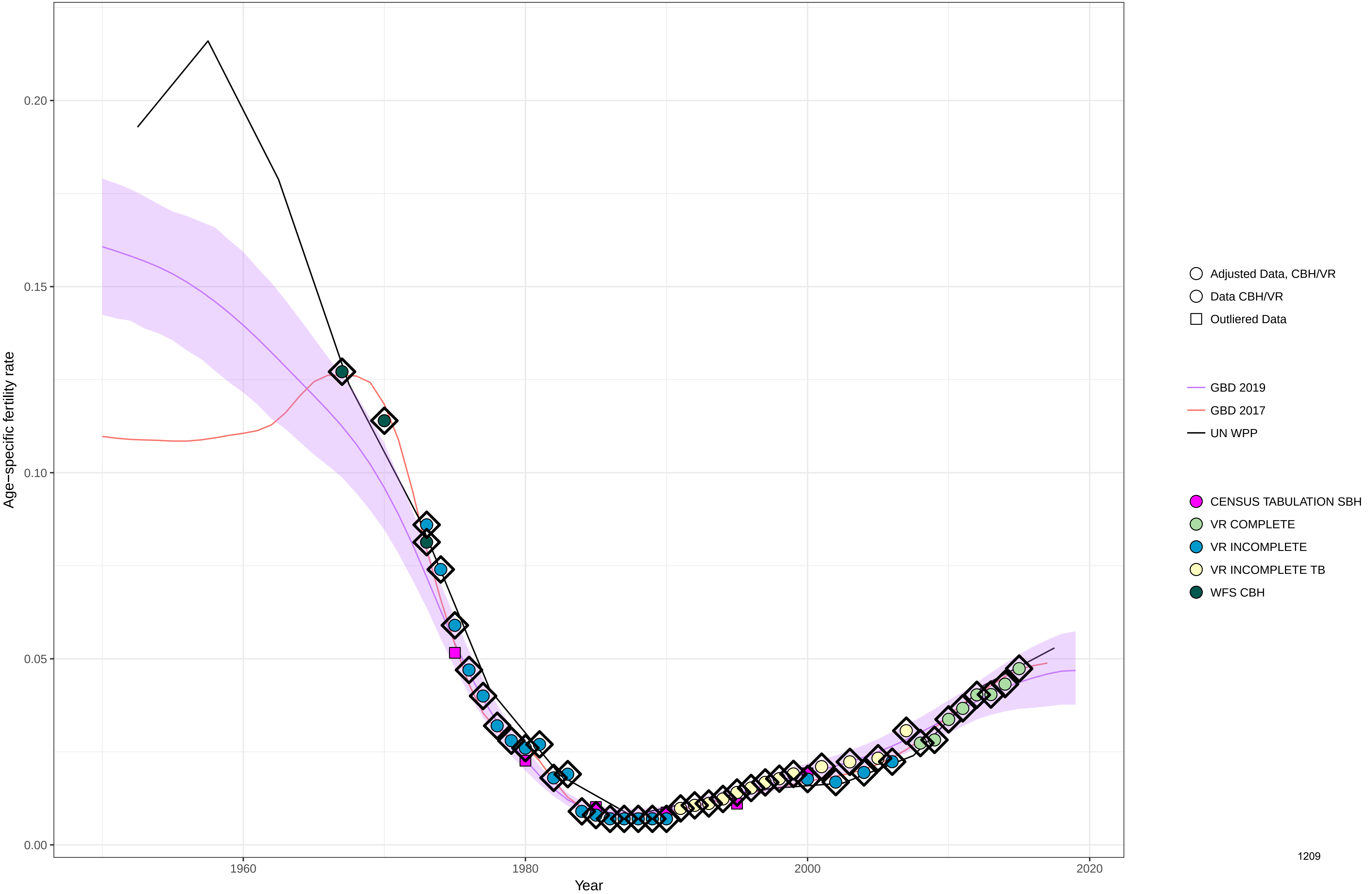

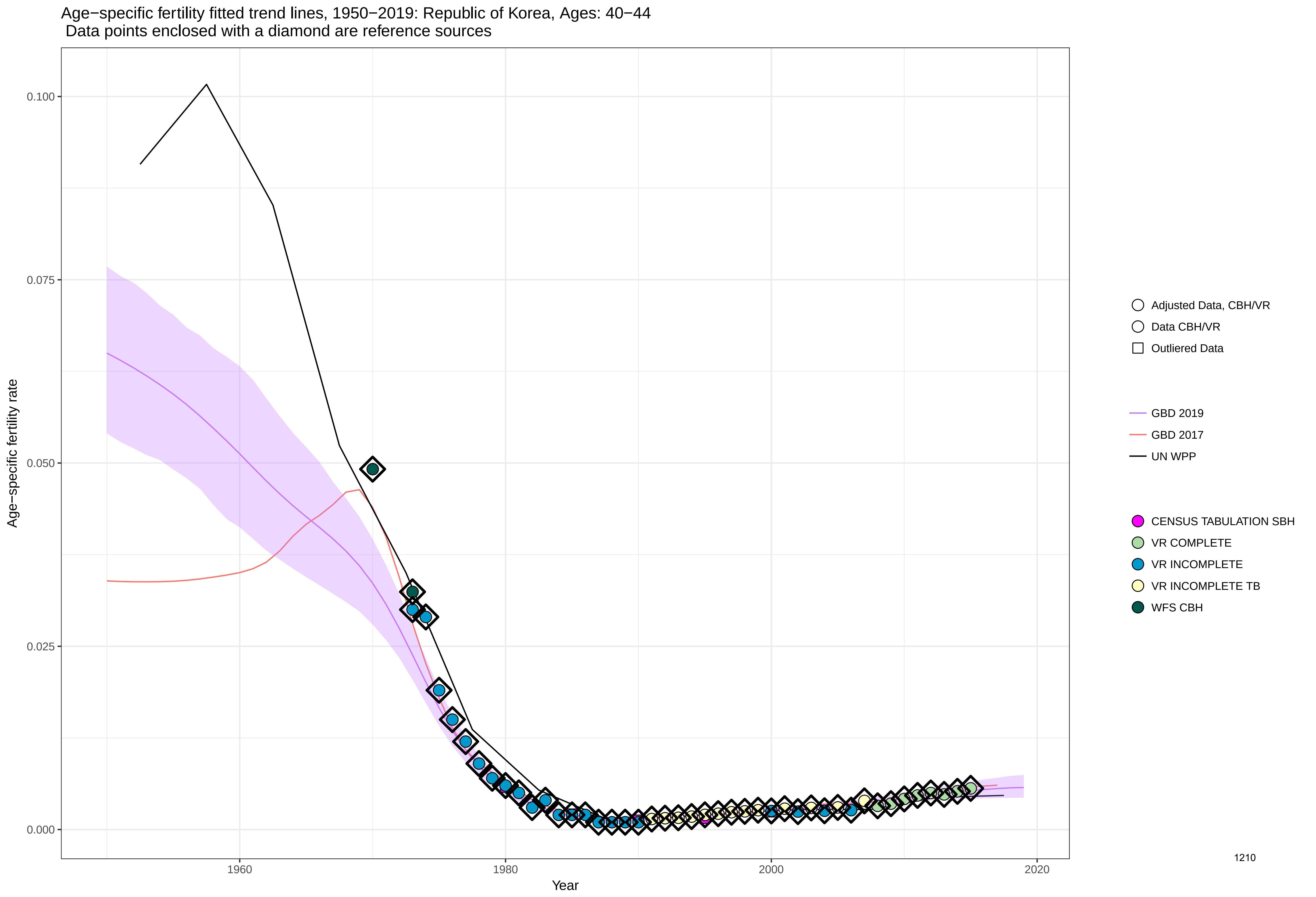
Age−specific fertility fitted trend lines, 1950−2019: Republic of Korea, Ages: 40−44
Data points enclosed with a diamond are reference sources
Age−specific fertility rate
Year
0.000
0.025
0.050
0.075
0.100
1960
1980
2000
2020
Adjusted Data, CBH/VR
Data CBH/VR
Outliered Data
GBD 2019
GBD 2017
UN WPP
CENSUS TABULATION SBH
VR COMPLETE
VR INCOMPLETE
VR INCOMPLETE TB
WFS CBH

Age−specific fertility fitted trend lines, 1950−2019: Republic of Korea, Ages: 45−49
Data points enclosed with a diamond are reference sources

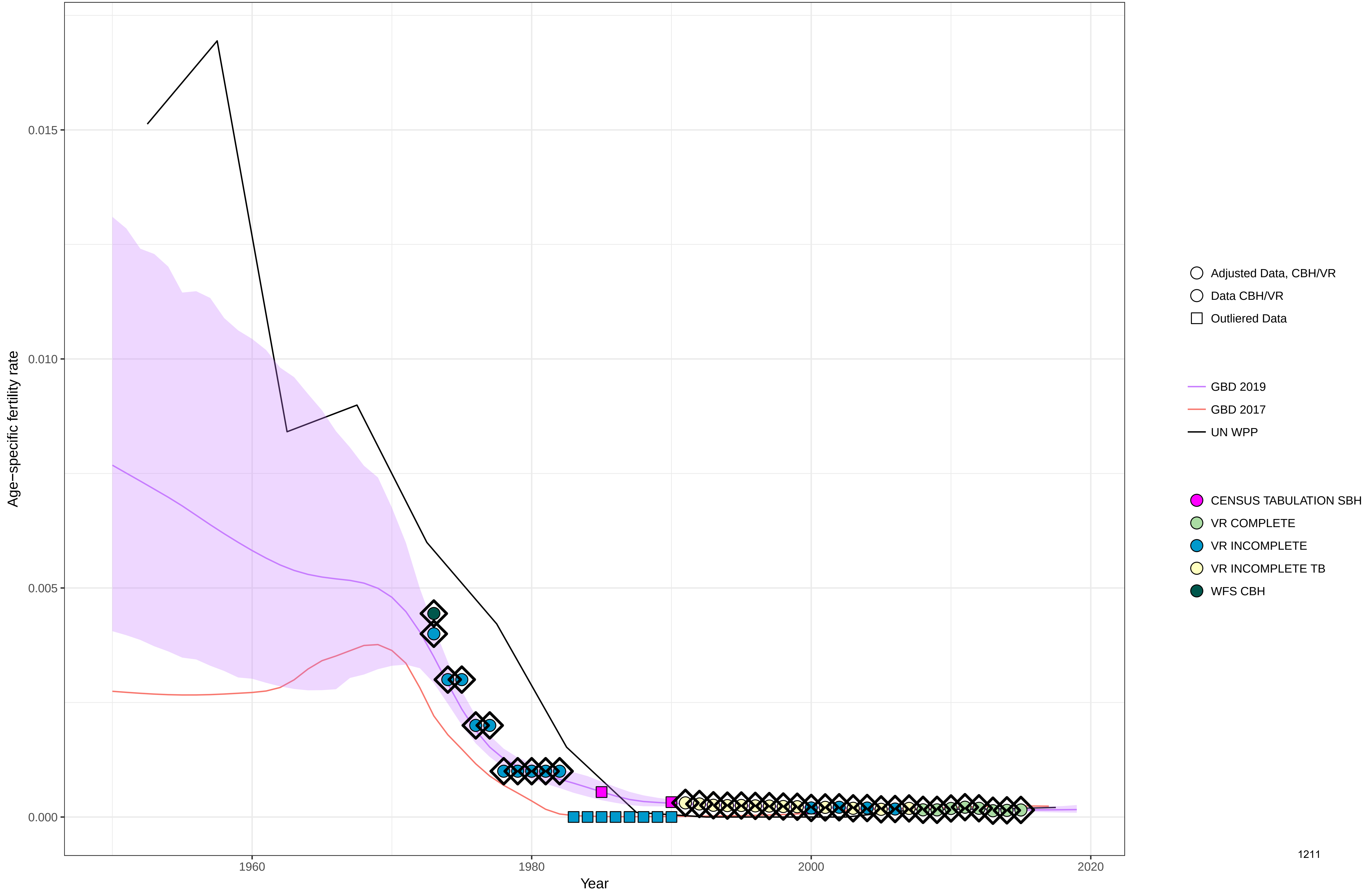

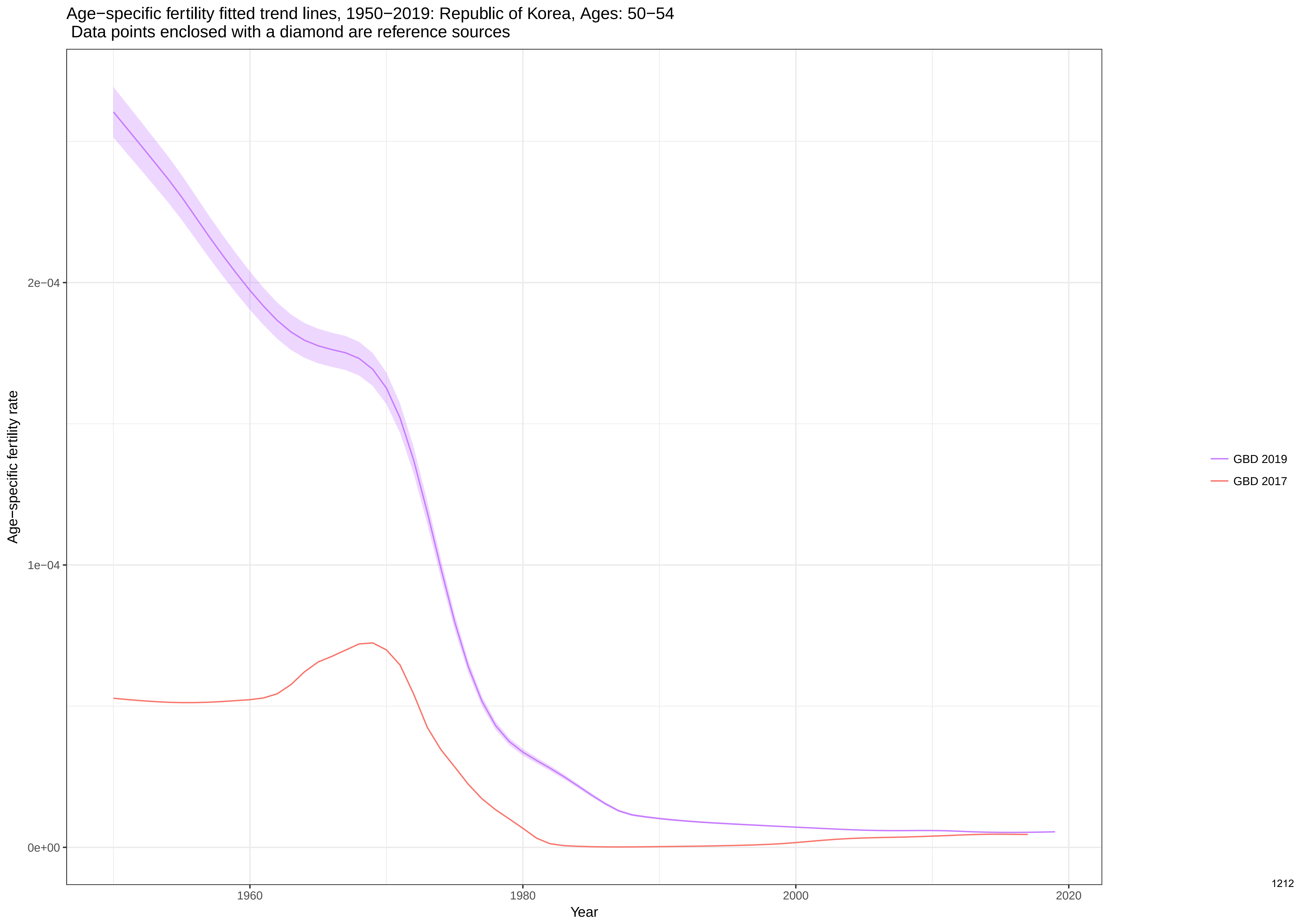

Age−specific fertility fitted trend lines, 1950−2019: Republic of Korea, Ages: 50−54
Data points enclosed with a diamond are reference sources
2e−04
1e−04
0e+00
Age−specific fertility rate
1960
1980
2000
2020
Year
GBD 2019
GBD 2017

1212

Total fertility rate fitted trend lines, 1950−2019: Republic of Korea TFR
Data points enclosed with a diamond are reference sources

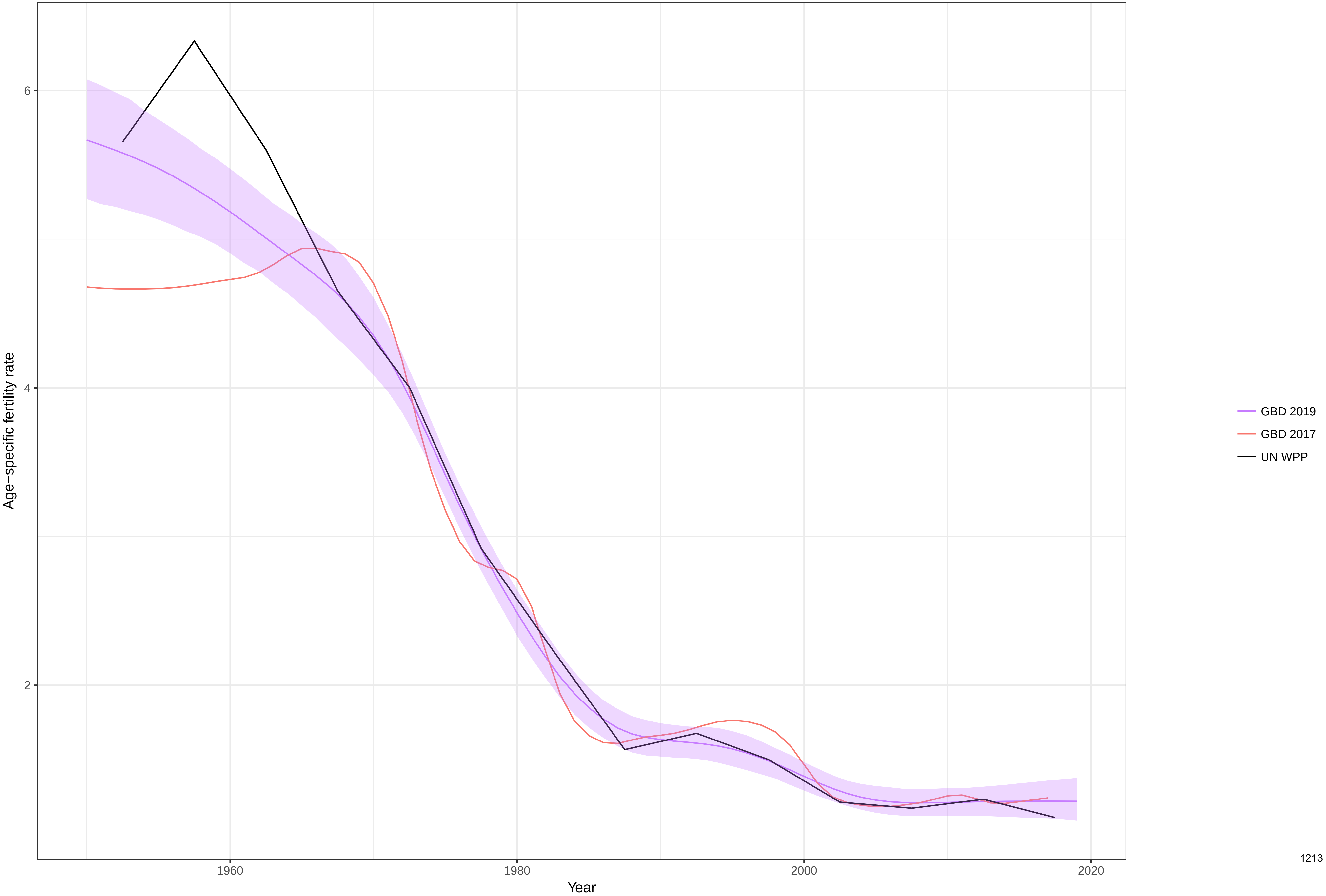

Age−specific fertility fitted trend lines, 1950−2019: Kuwait, Ages: 10−14
Data points enclosed with a diamond are reference sources

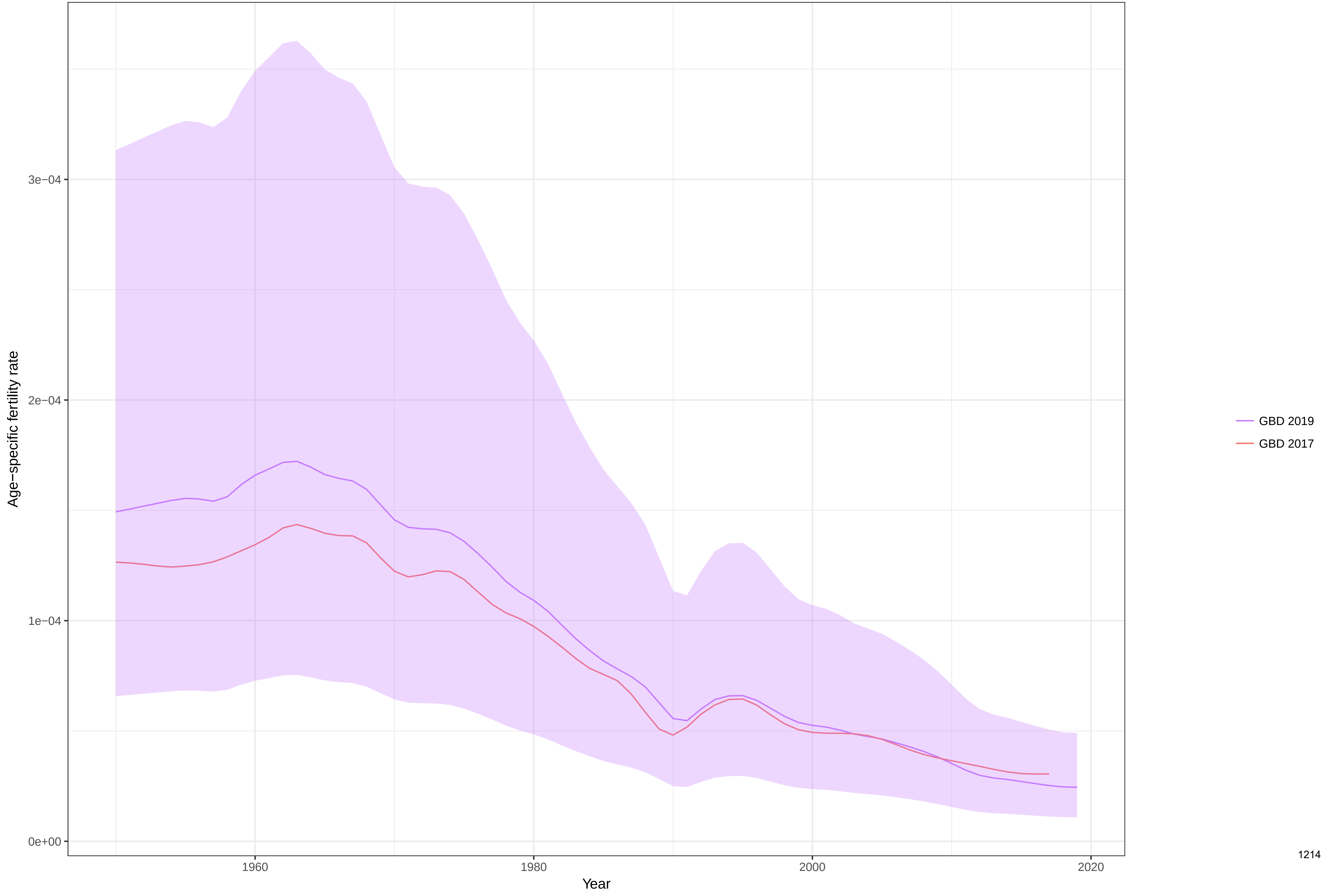

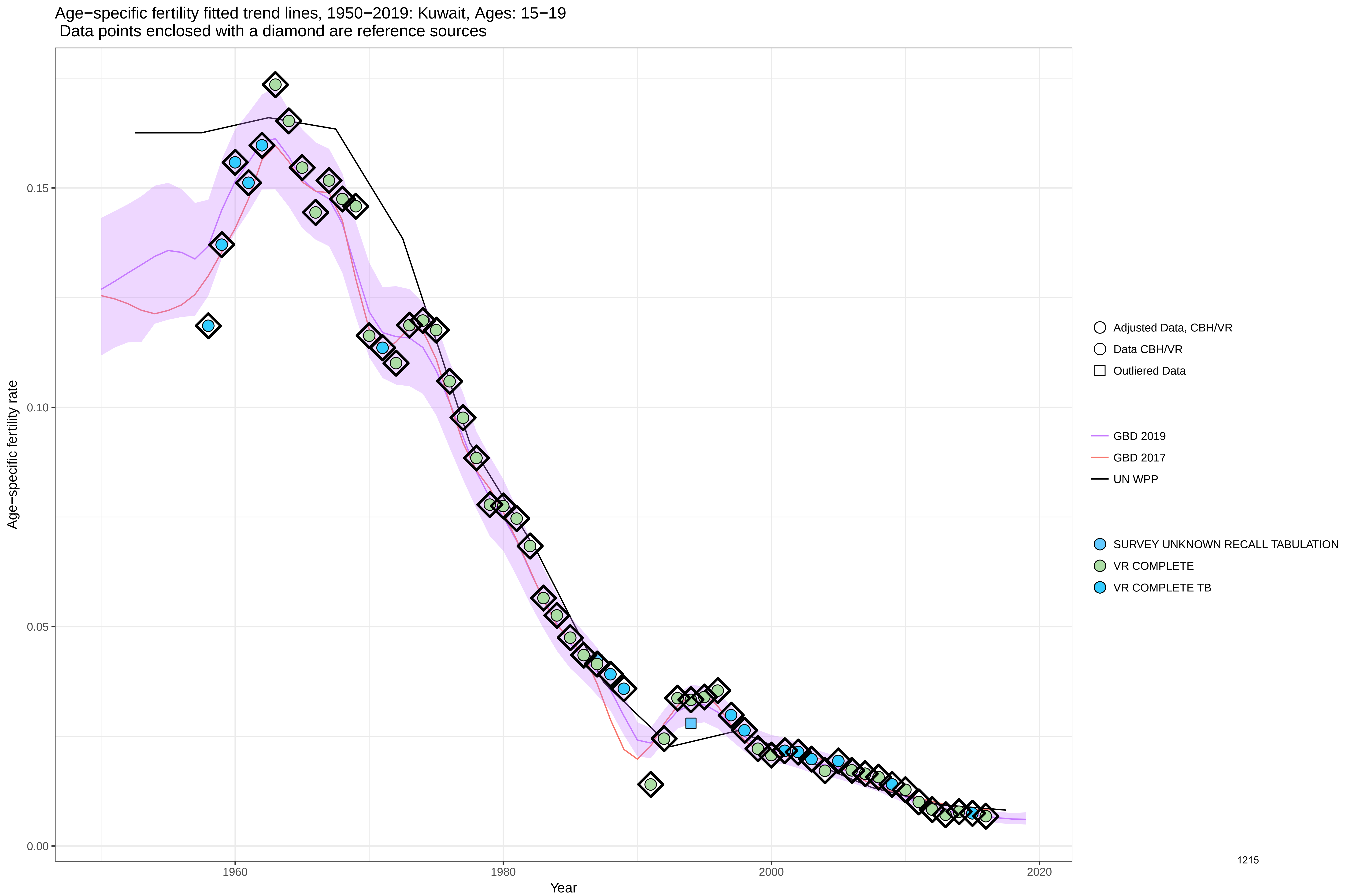
Age−specific fertility fitted trend lines, 1950−2019: Kuwait, Ages: 15−19
Data points enclosed with a diamond are reference sources
Age−specific fertility rate
Year
0.00
0.05
0.10
0.15
1960
1980
2000
2020
Adjusted Data, CBH/VR
Data CBH/VR
Outliered Data
GBD 2019
GBD 2017
UN WPP
SURVEY UNKNOWN RECALL TABULATION
VR COMPLETE
VR COMPLETE TB

Age−specific fertility fitted trend lines, 1950−2019: Kuwait, Ages: 20−24
Data points enclosed with a diamond are reference sources

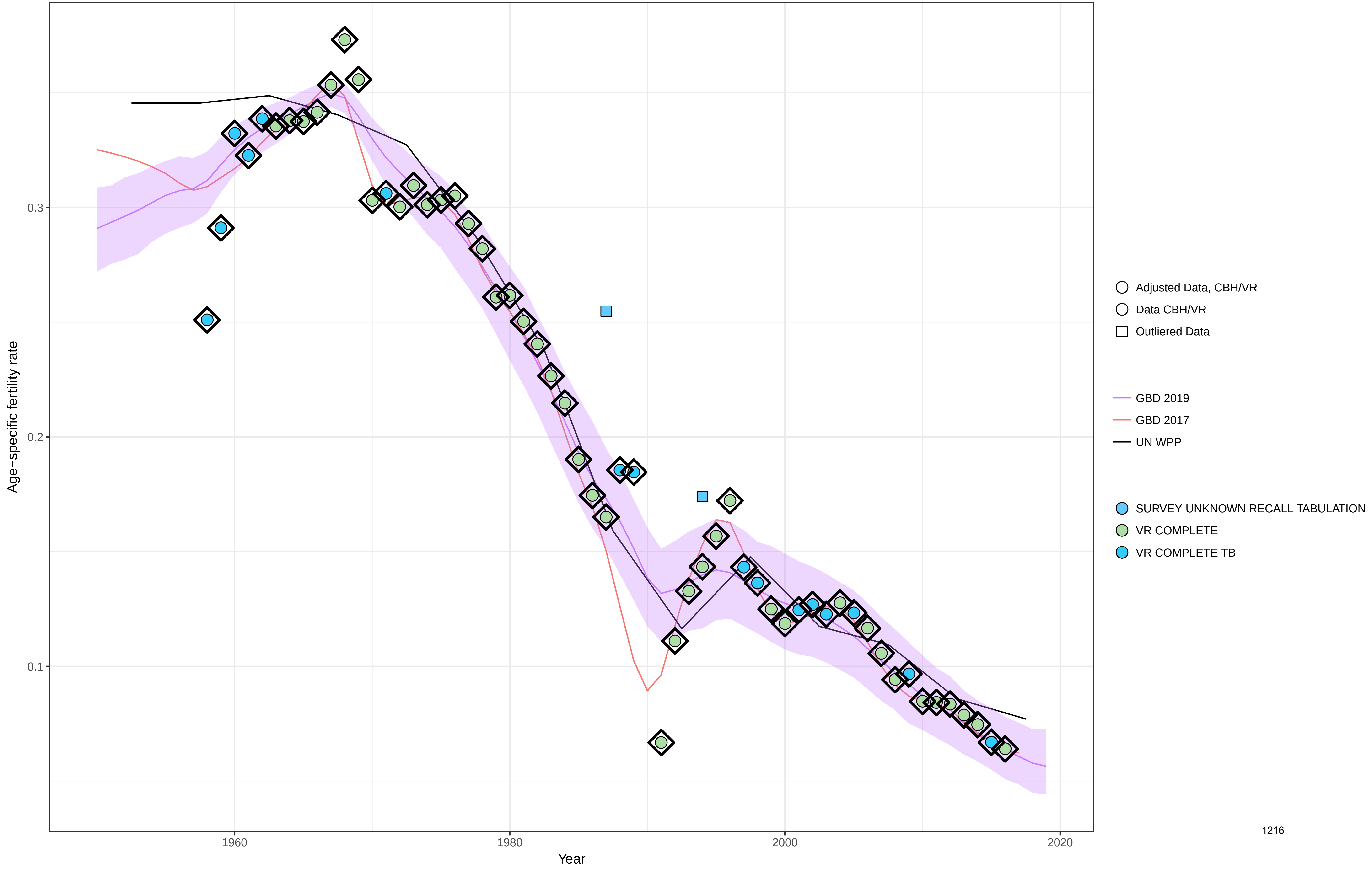

Age−specific fertility fitted trend lines, 1950−2019: Kuwait, Ages: 25−29
Data points enclosed with a diamond are reference sources

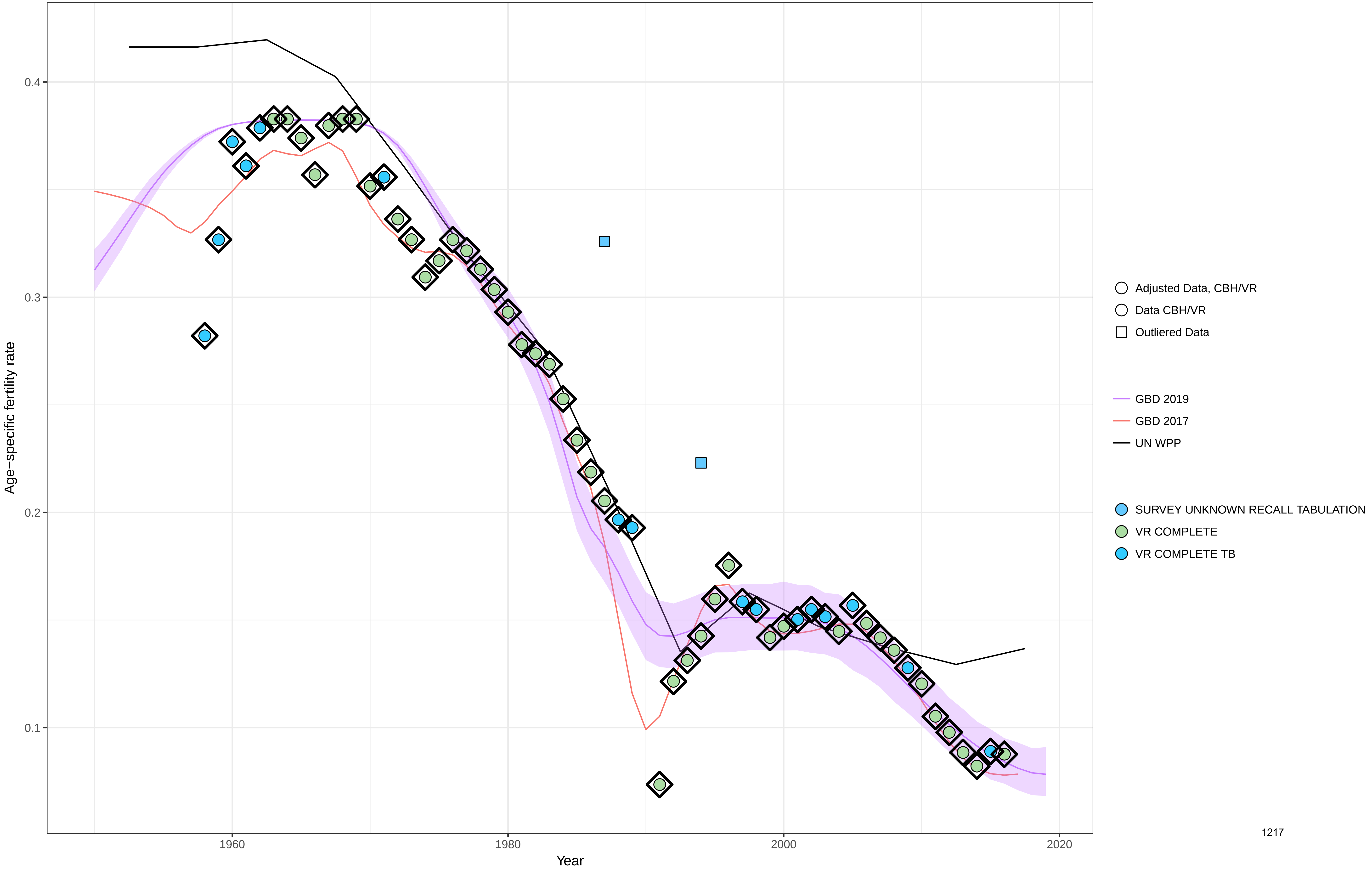

Age−specific fertility fitted trend lines, 1950−2019: Kuwait, Ages: 30−34
Data points enclosed with a diamond are reference sources

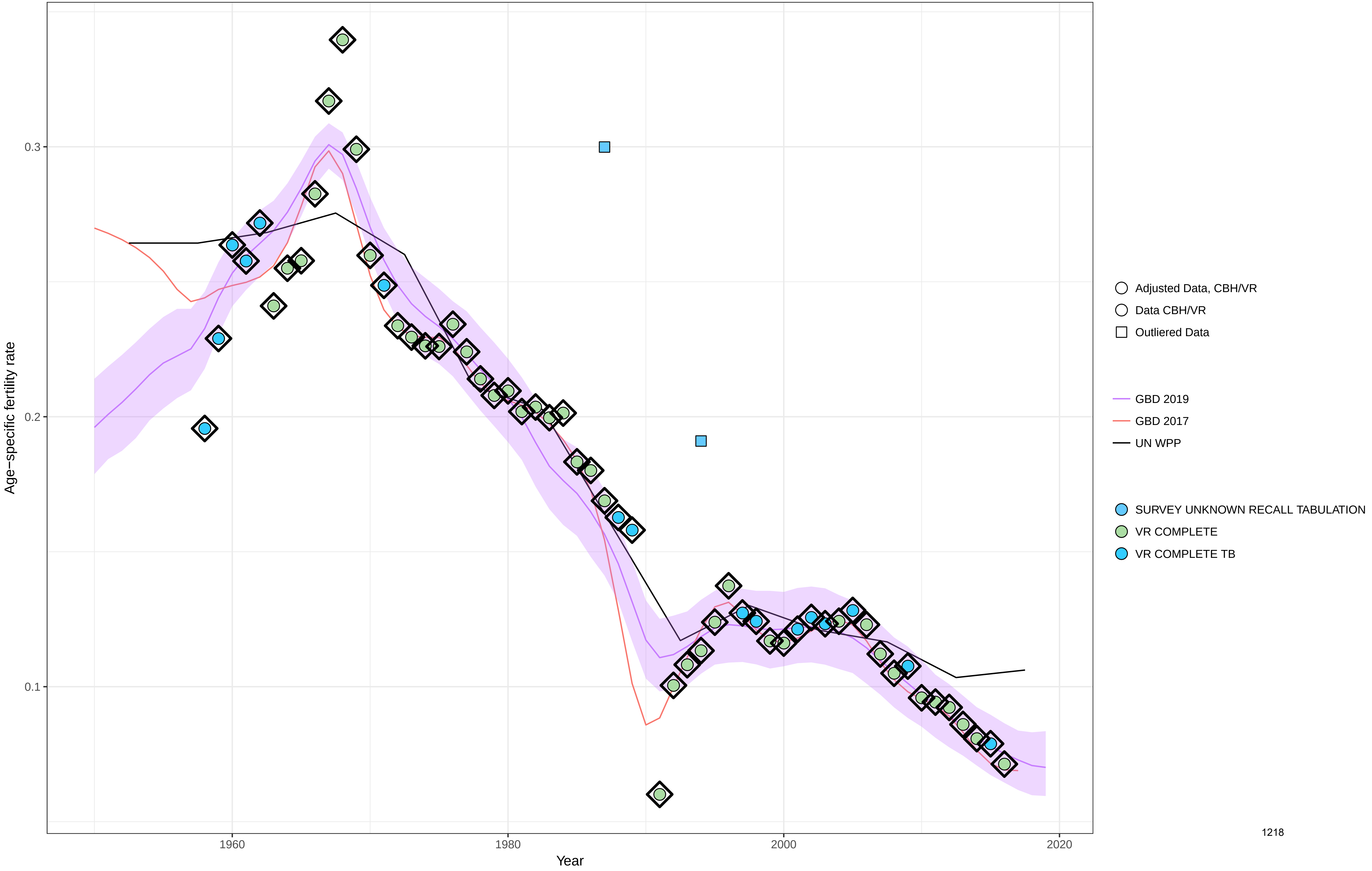

Age−specific fertility fitted trend lines, 1950−2019: Kuwait, Ages: 35−39
Data points enclosed with a diamond are reference sources

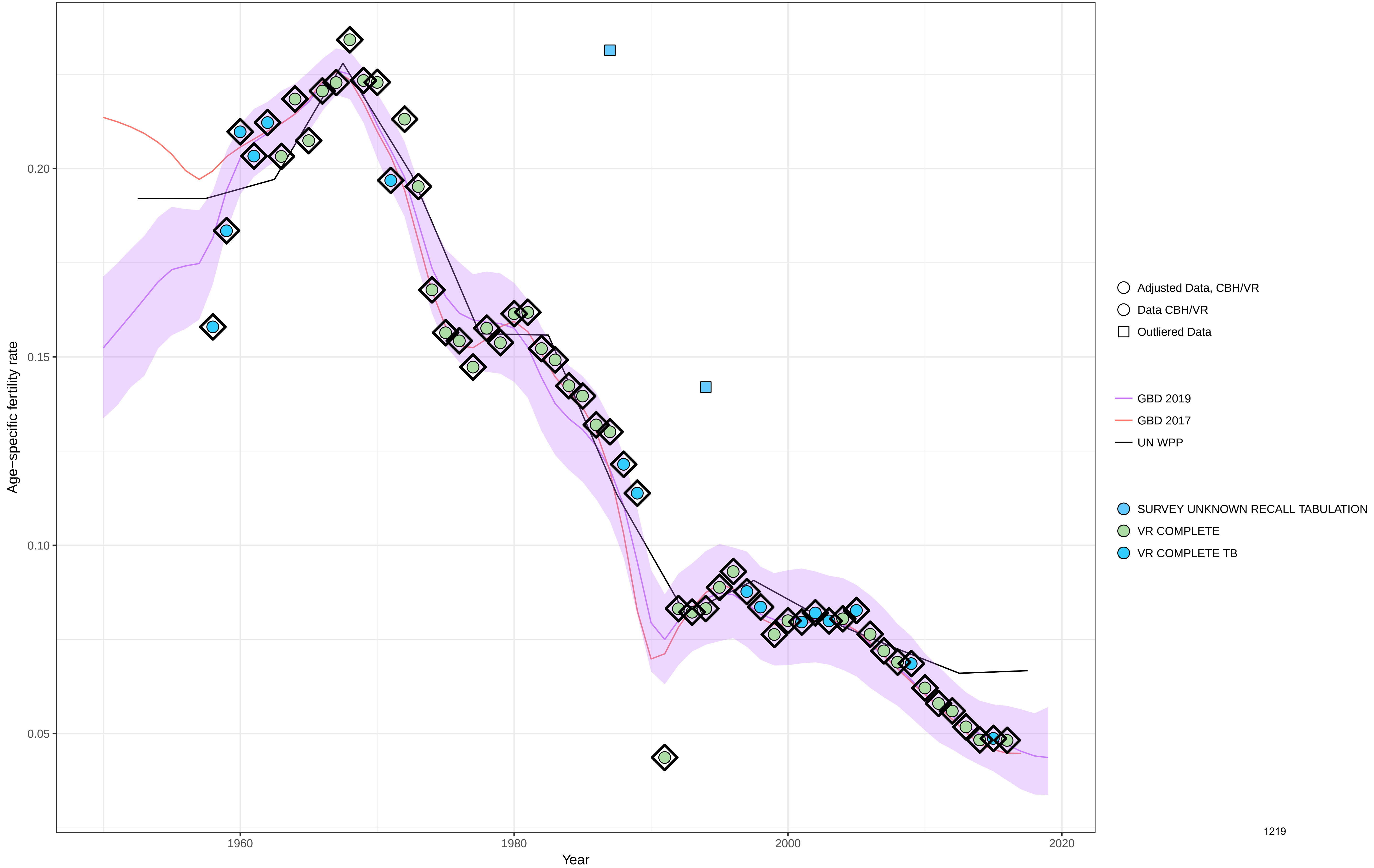

Age−specific fertility fitted trend lines, 1950−2019: Kuwait, Ages: 40−44
Data points enclosed with a diamond are reference sources

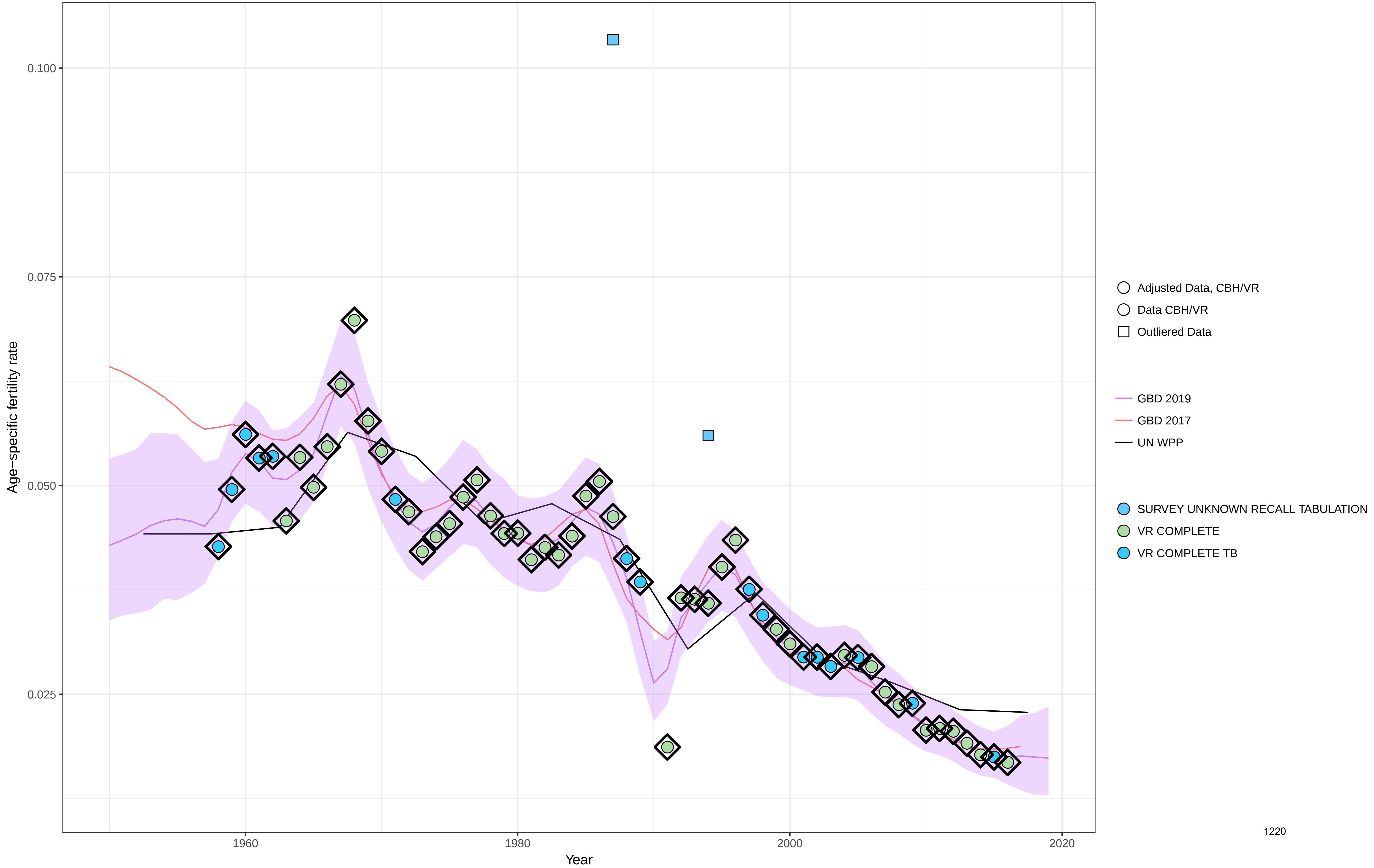

Age−specific fertility fitted trend lines, 1950−2019: Kuwait, Ages: 45−49
Data points enclosed with a diamond are reference sources

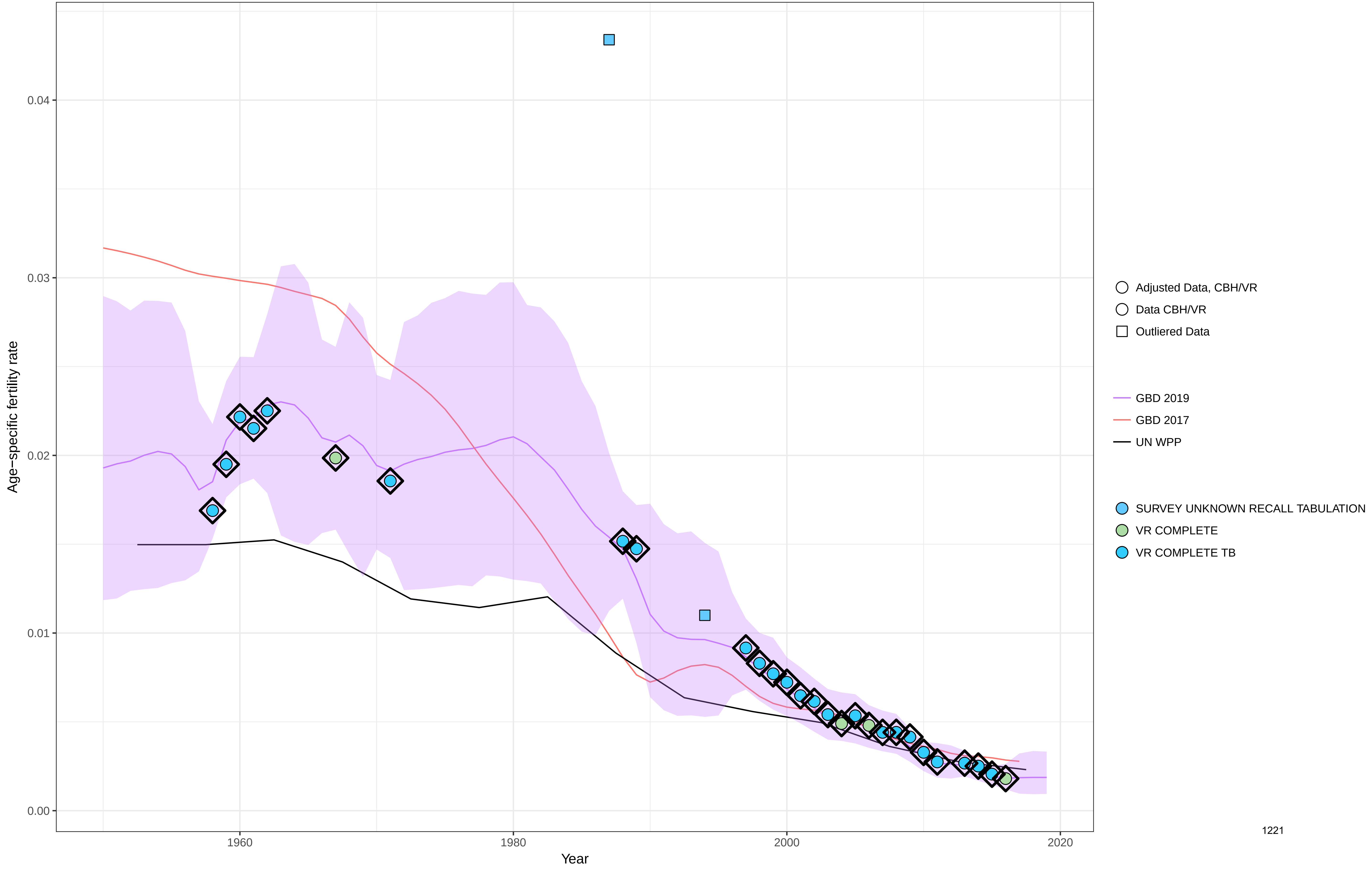

Age−specific fertility fitted trend lines, 1950−2019: Kuwait, Ages: 50−54
Data points enclosed with a diamond are reference sources

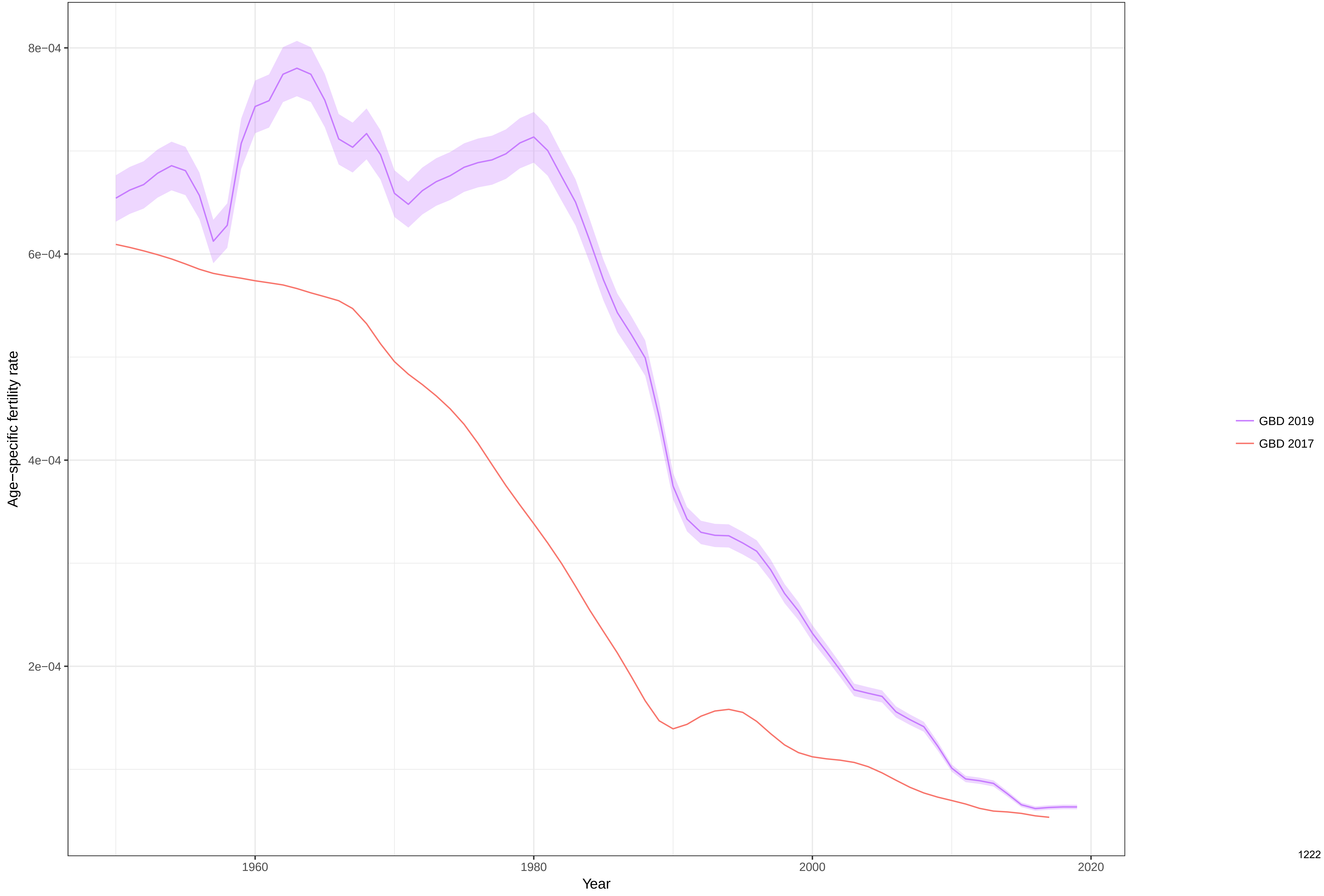

Total fertility rate fitted trend lines, 1950−2019: Kuwait TFR
Data points enclosed with a diamond are reference sources

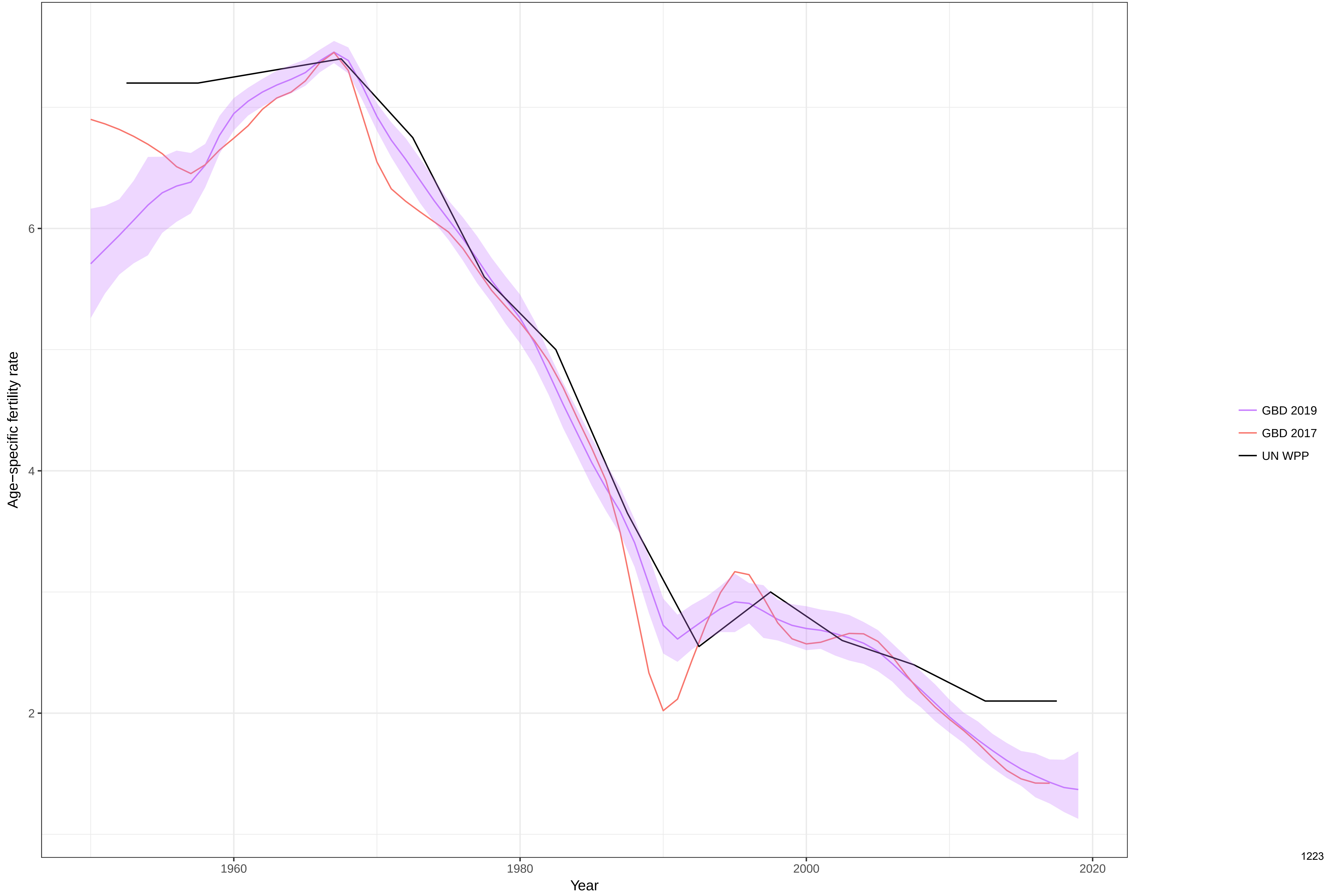

Age−specific fertility fitted trend lines, 1950−2019: Lao People's Democratic Republic, Ages: 10−14
Data points enclosed with a diamond are reference sources

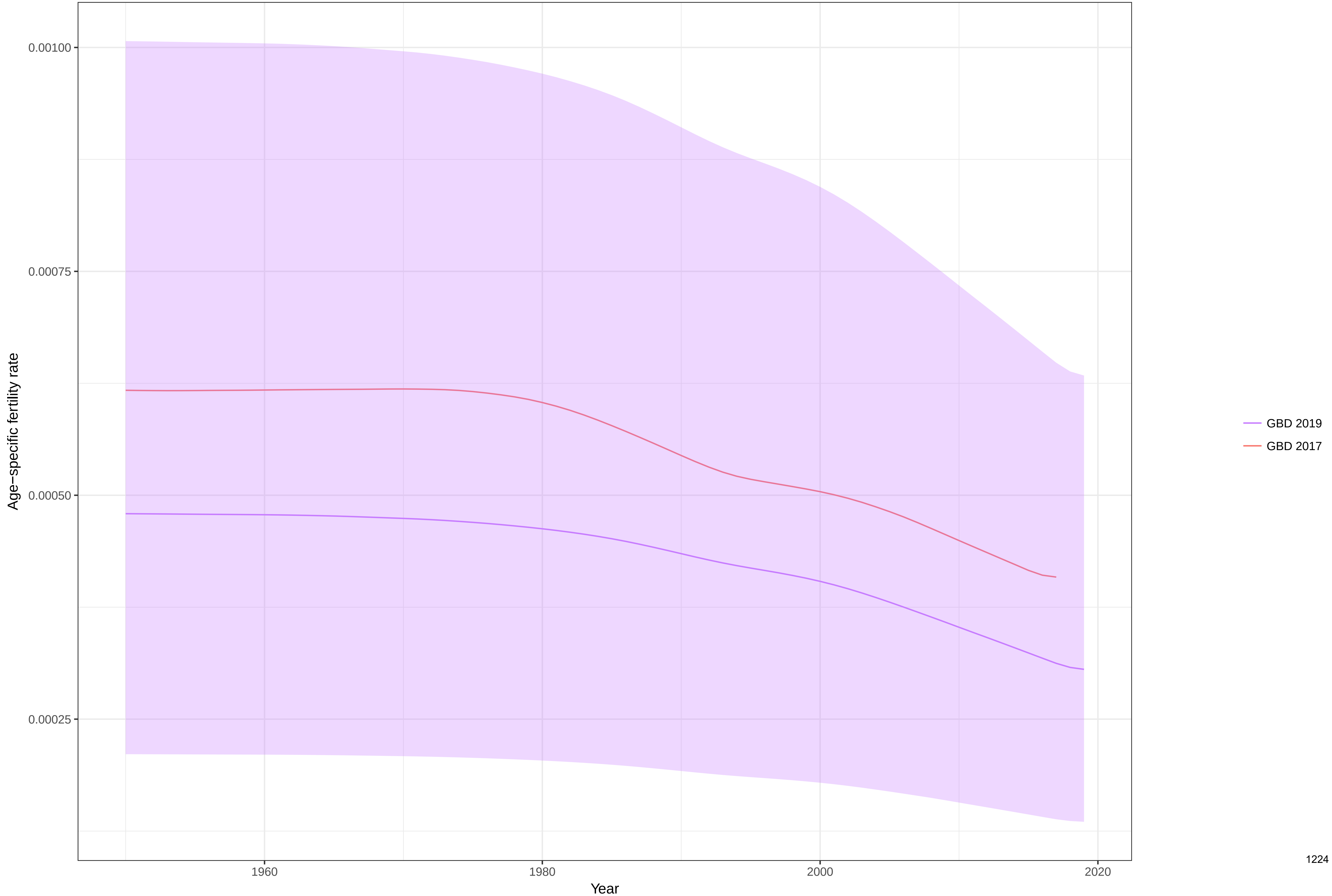

Age−specific fertility fitted trend lines, 1950−2019: Lao People's Democratic Republic, Ages: 15−19
Data points enclosed with a diamond are reference sources

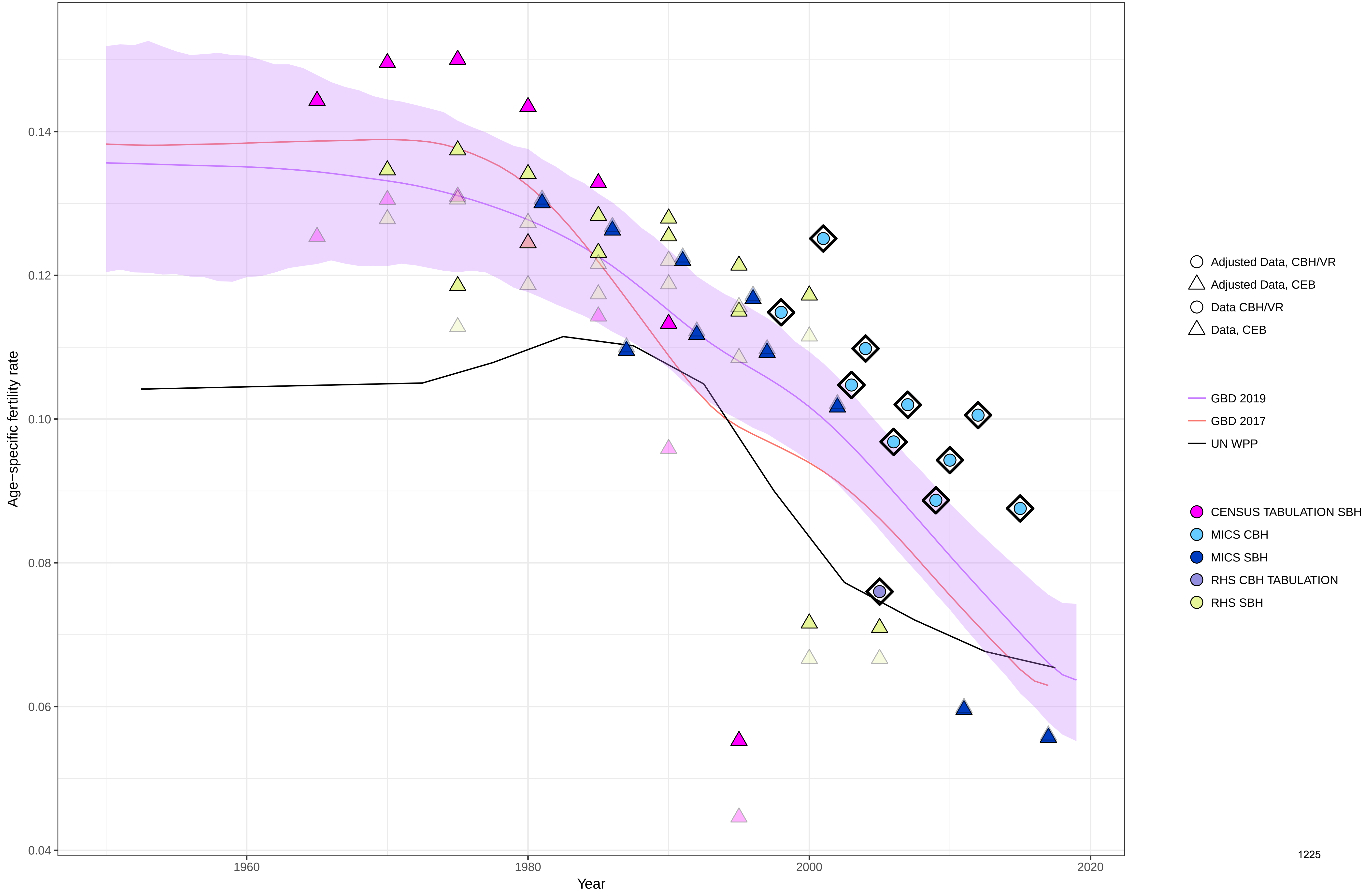

Age−specific fertility fitted trend lines, 1950−2019: Lao People's Democratic Republic, Ages: 20−24
Data points enclosed with a diamond are reference sources

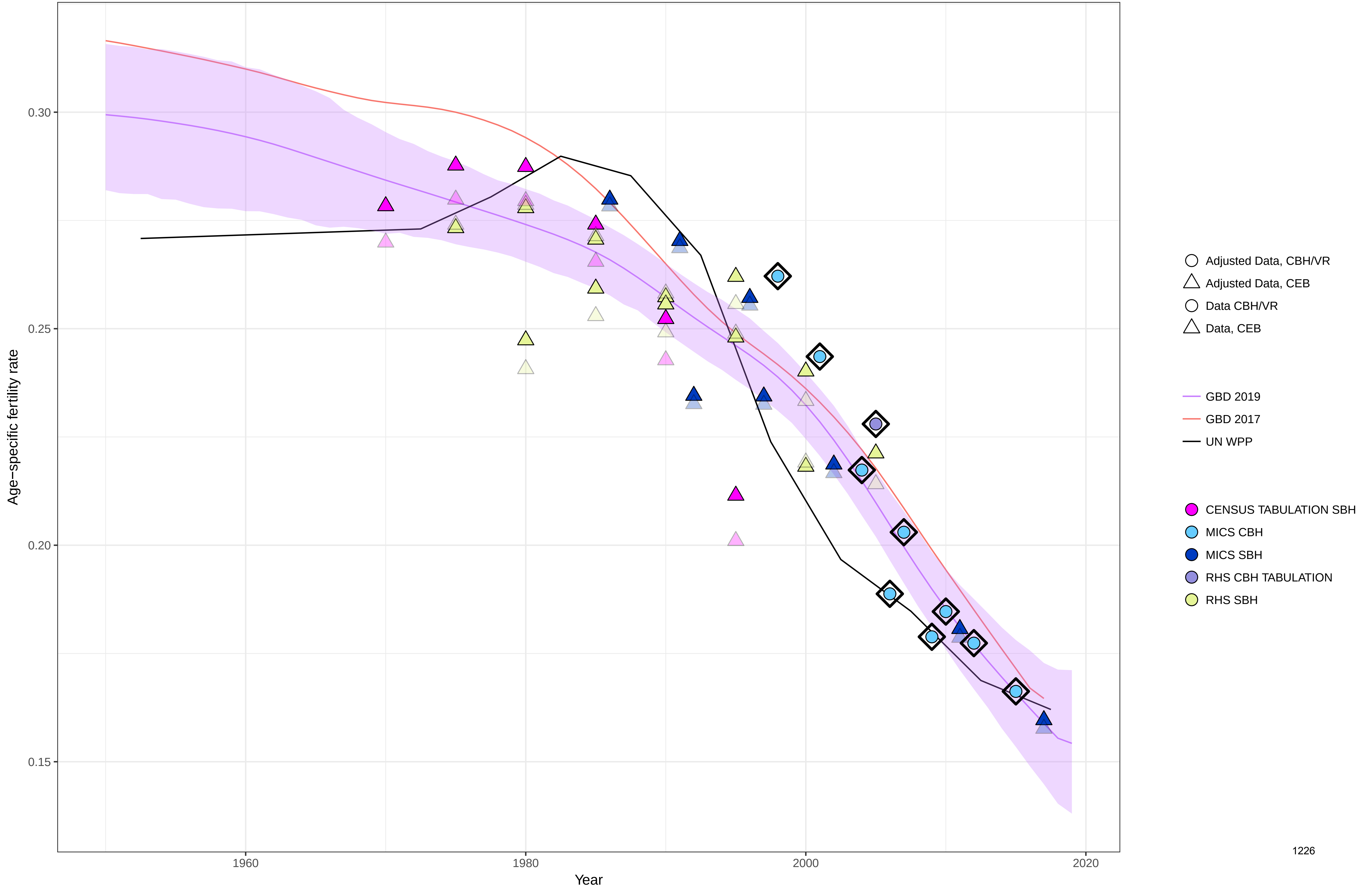

Age−specific fertility fitted trend lines, 1950−2019: Lao People's Democratic Republic, Ages: 25−29
Data points enclosed with a diamond are reference sources

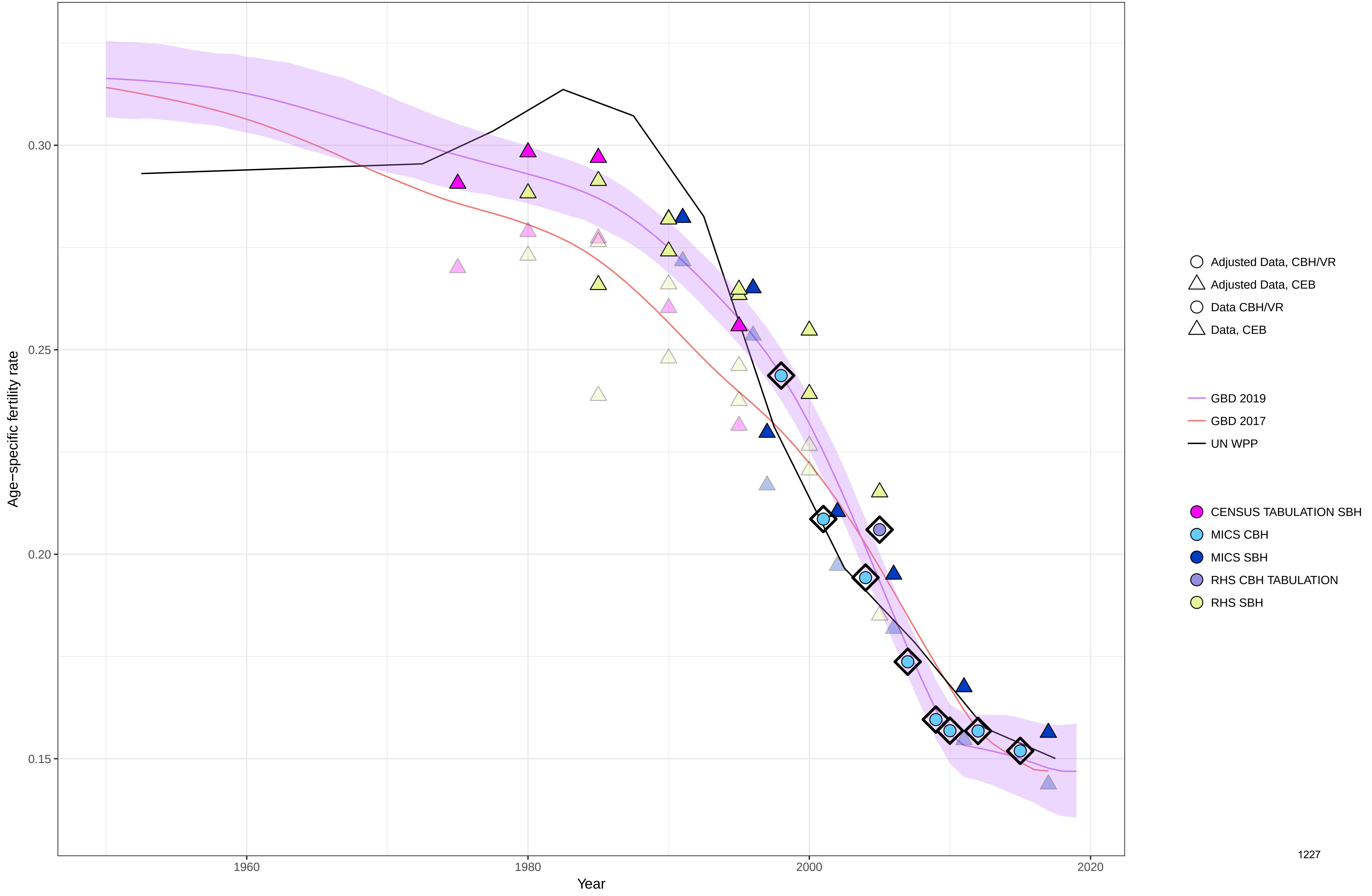

Age−specific fertility fitted trend lines, 1950−2019: Lao People's Democratic Republic, Ages: 30−34
Data points enclosed with a diamond are reference sources

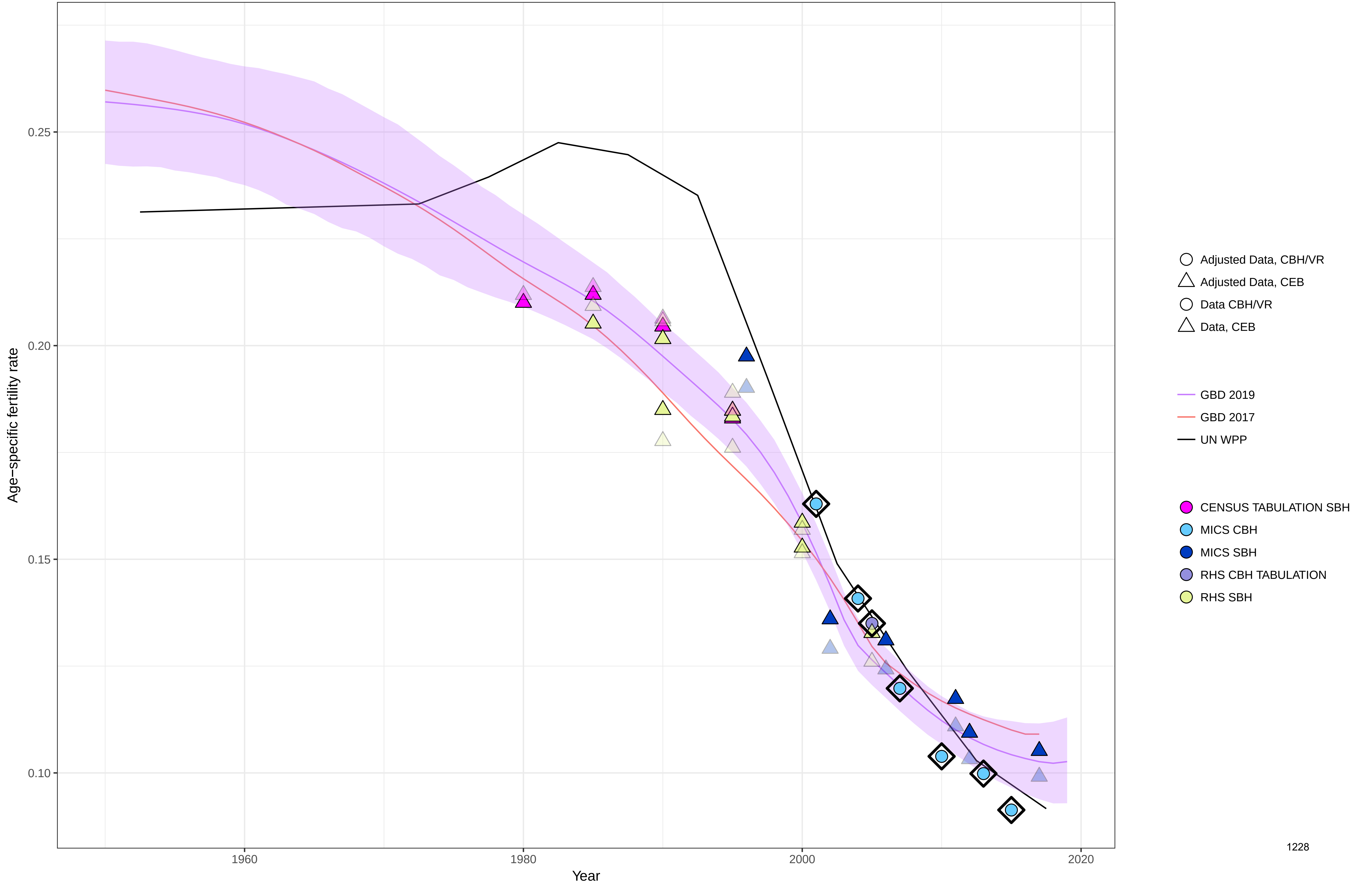

Age−specific fertility fitted trend lines, 1950−2019: Lao People's Democratic Republic, Ages: 35−39
Data points enclosed with a diamond are reference sources

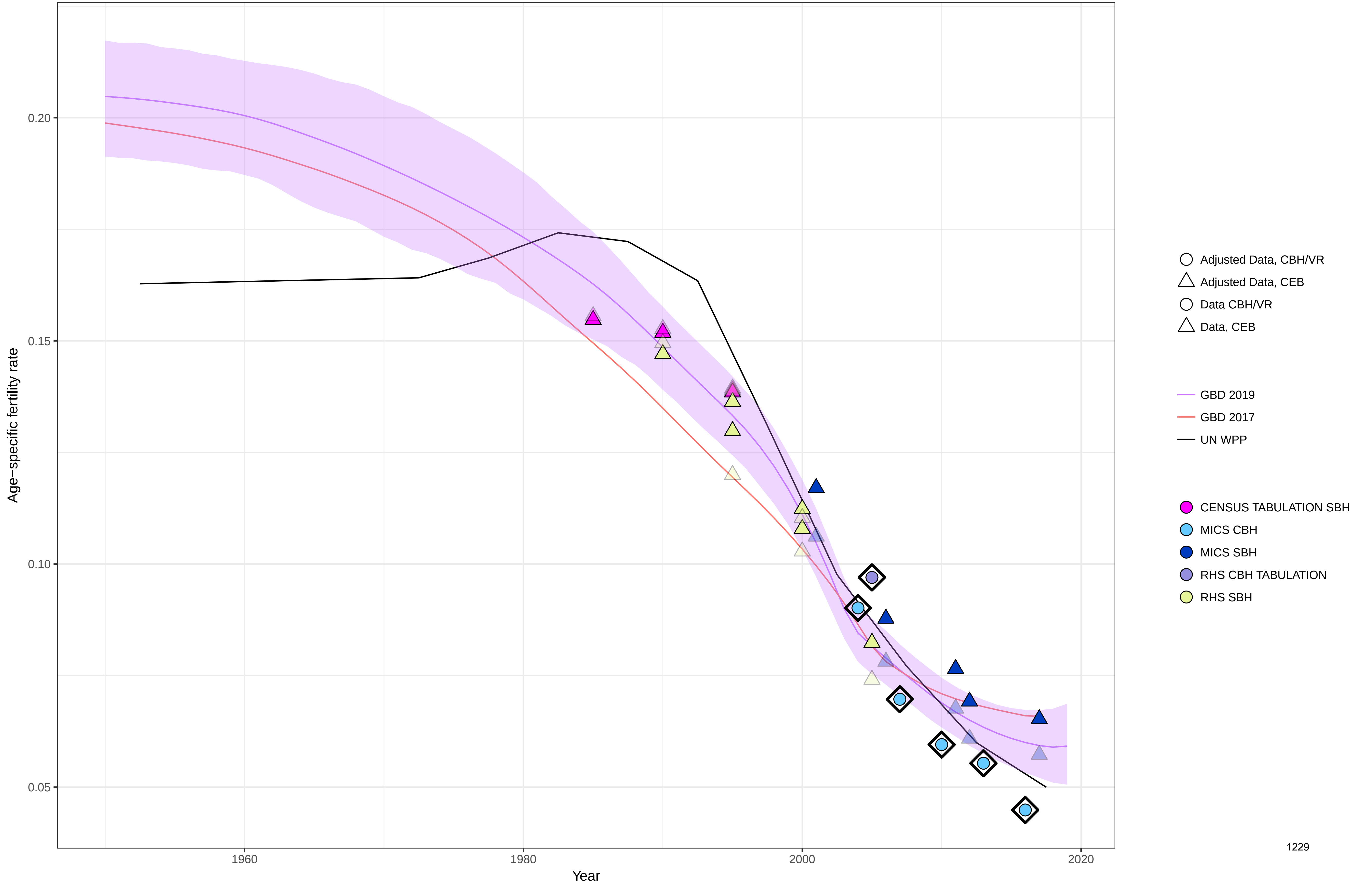

Age−specific fertility fitted trend lines, 1950−2019: Lao People's Democratic Republic, Ages: 40−44
Data points enclosed with a diamond are reference sources

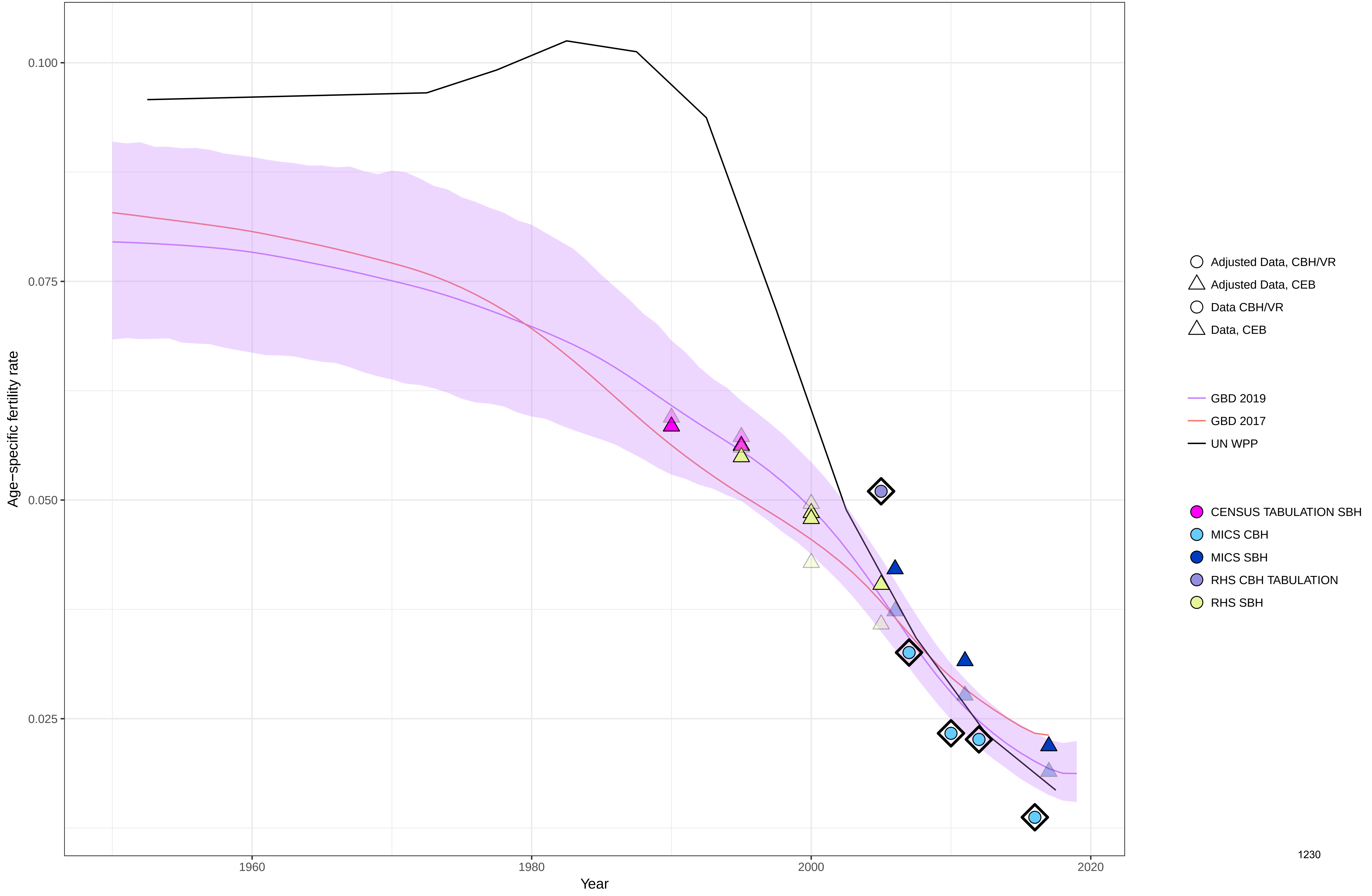

Age−specific fertility fitted trend lines, 1950−2019: Lao People's Democratic Republic, Ages: 45−49
Data points enclosed with a diamond are reference sources

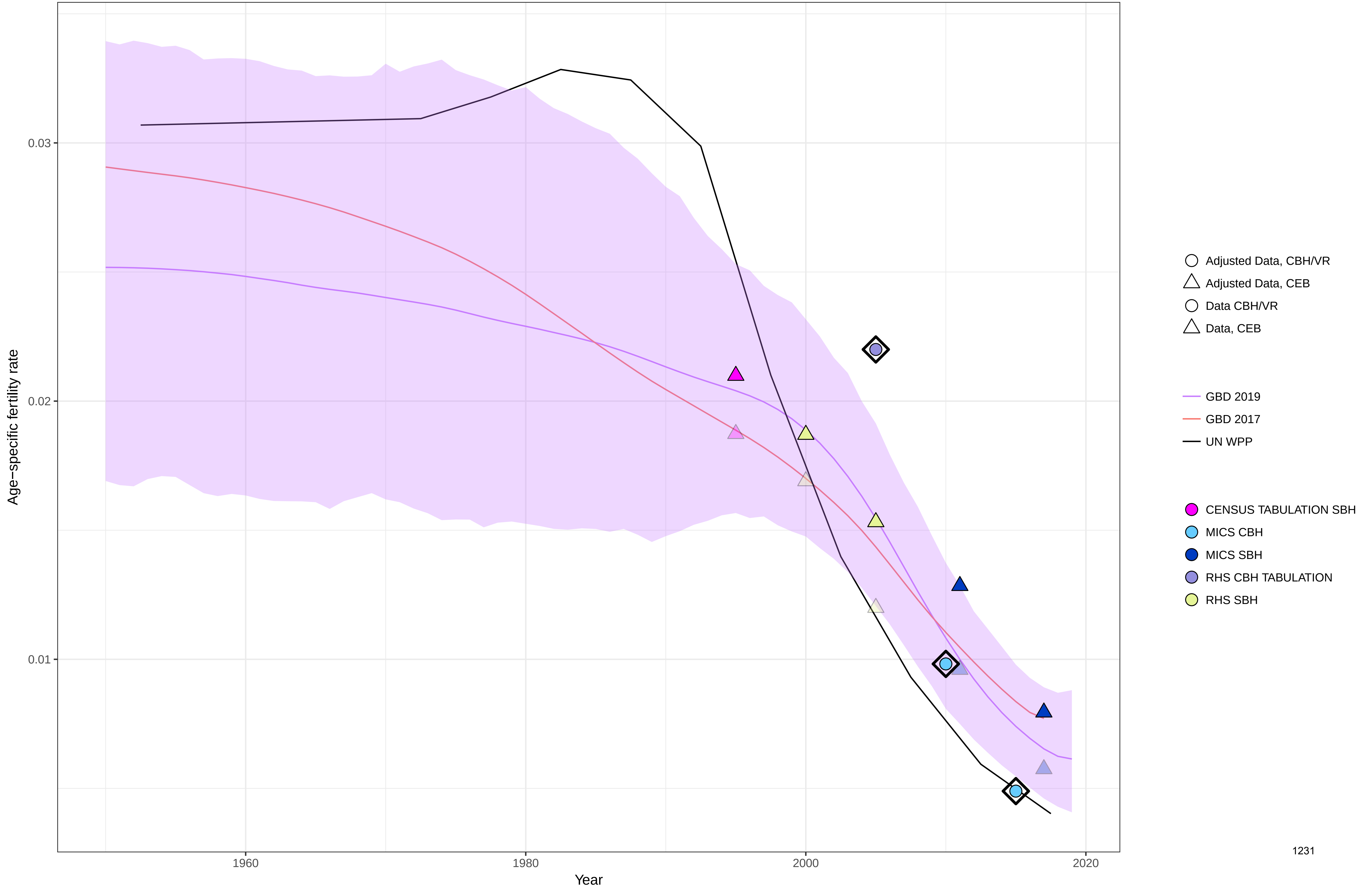

Age−specific fertility fitted trend lines, 1950−2019: Lao People's Democratic Republic, Ages: 50−54
Data points enclosed with a diamond are reference sources

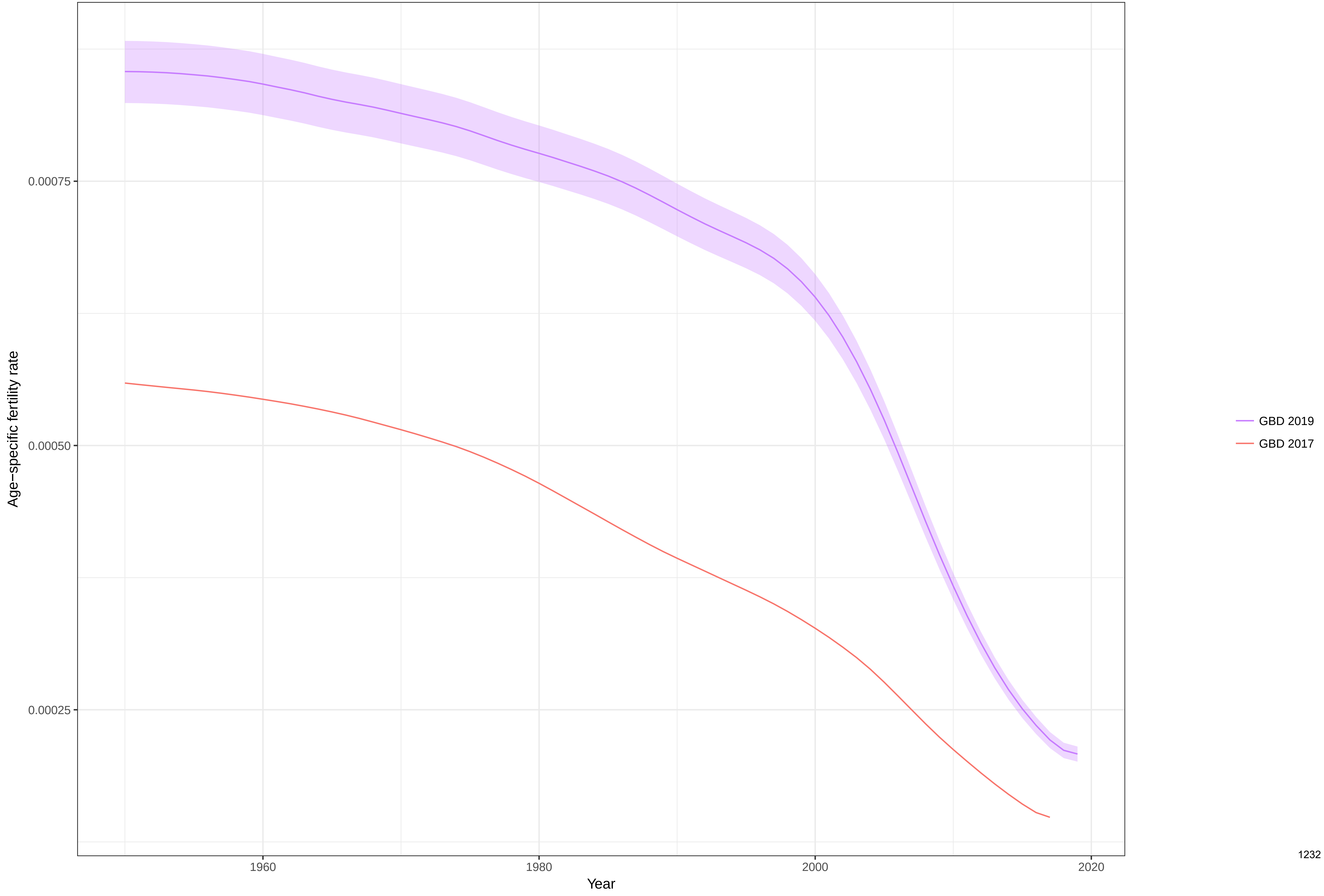

Total fertility rate fitted trend lines, 1950−2019: Lao People's Democratic Republic TFR
Data points enclosed with a diamond are reference sources

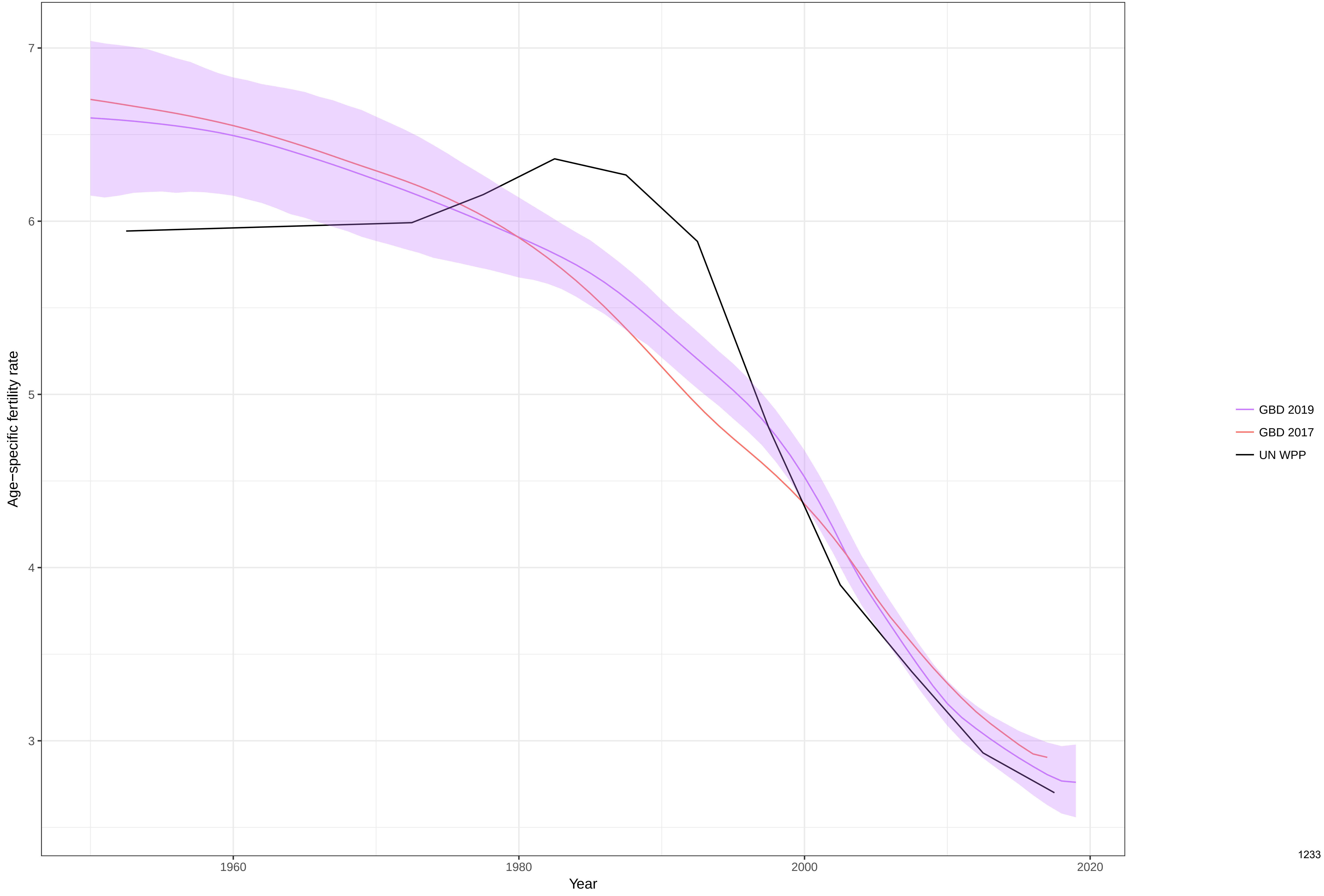

Age−specific fertility fitted trend lines, 1950−2019: Lebanon, Ages: 10−14
Data points enclosed with a diamond are reference sources

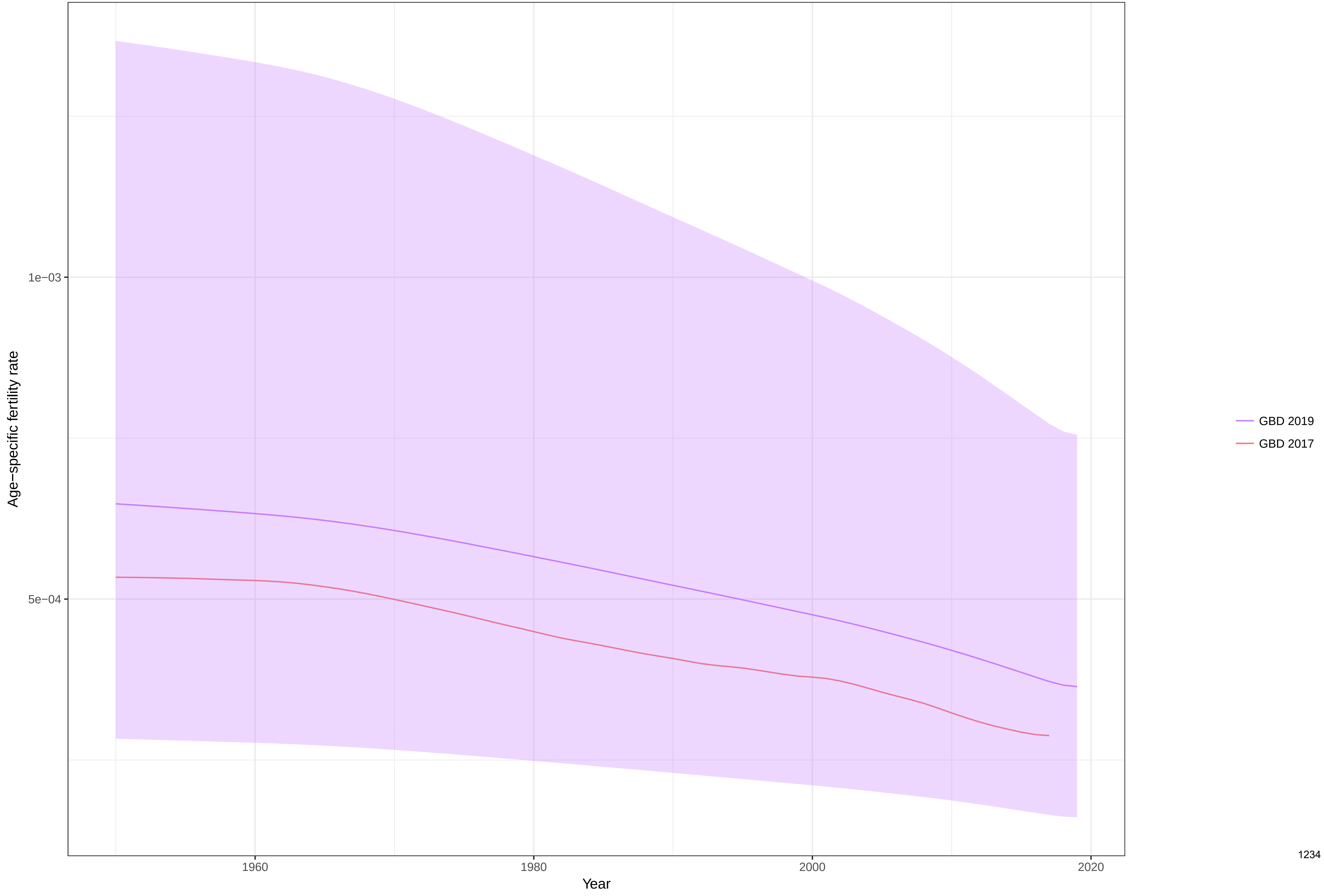

Age−specific fertility fitted trend lines, 1950−2019: Lebanon, Ages: 15−19
 Data points enclosed with a diamond are reference sources

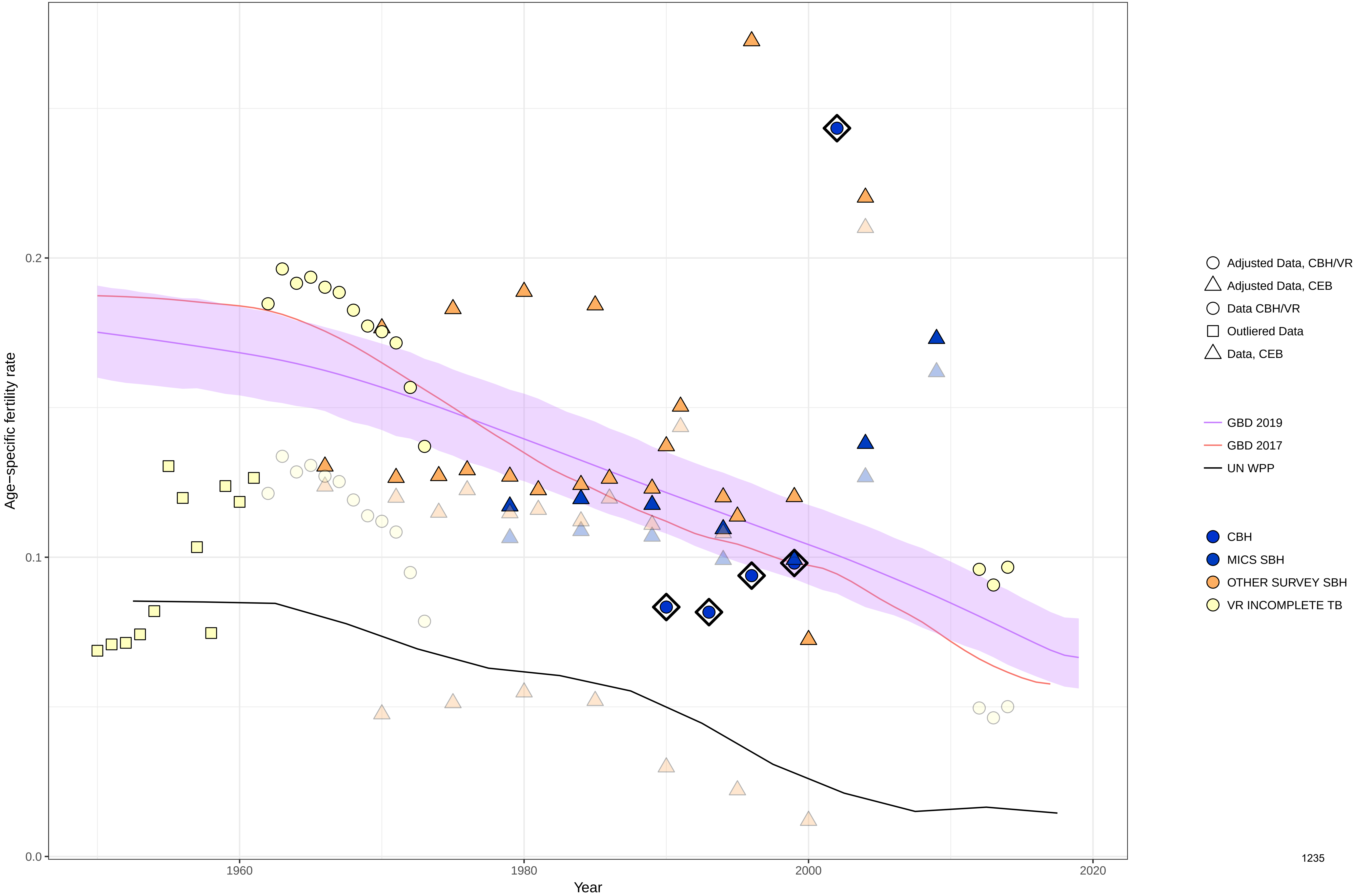

Age−specific fertility fitted trend lines, 1950−2019: Lebanon, Ages: 20−24
Data points enclosed with a diamond are reference sources

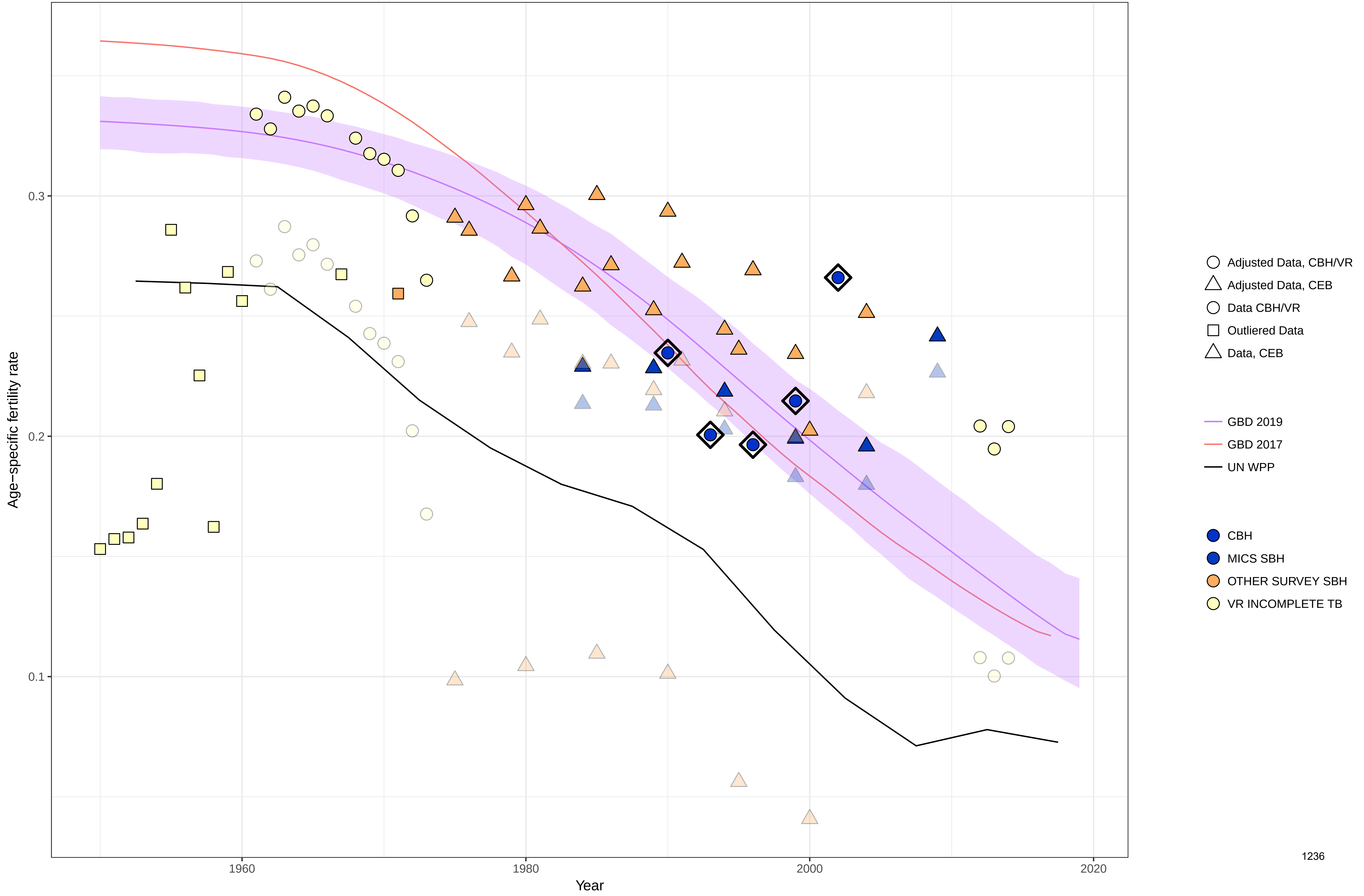

Age−specific fertility fitted trend lines, 1950−2019: Lebanon, Ages: 25−29
Data points enclosed with a diamond are reference sources

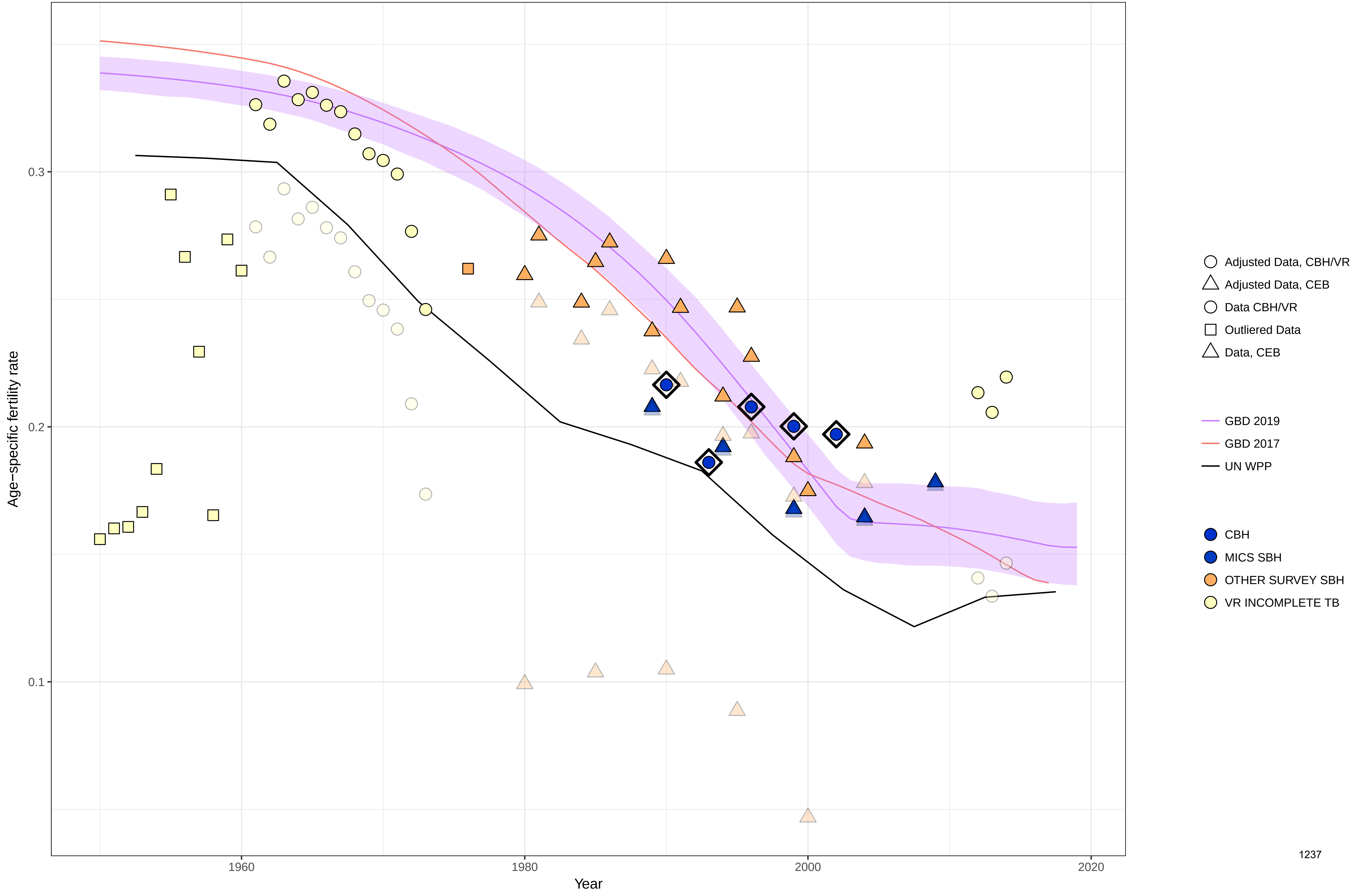

Age−specific fertility fitted trend lines, 1950−2019: Lebanon, Ages: 30−34
Data points enclosed with a diamond are reference sources

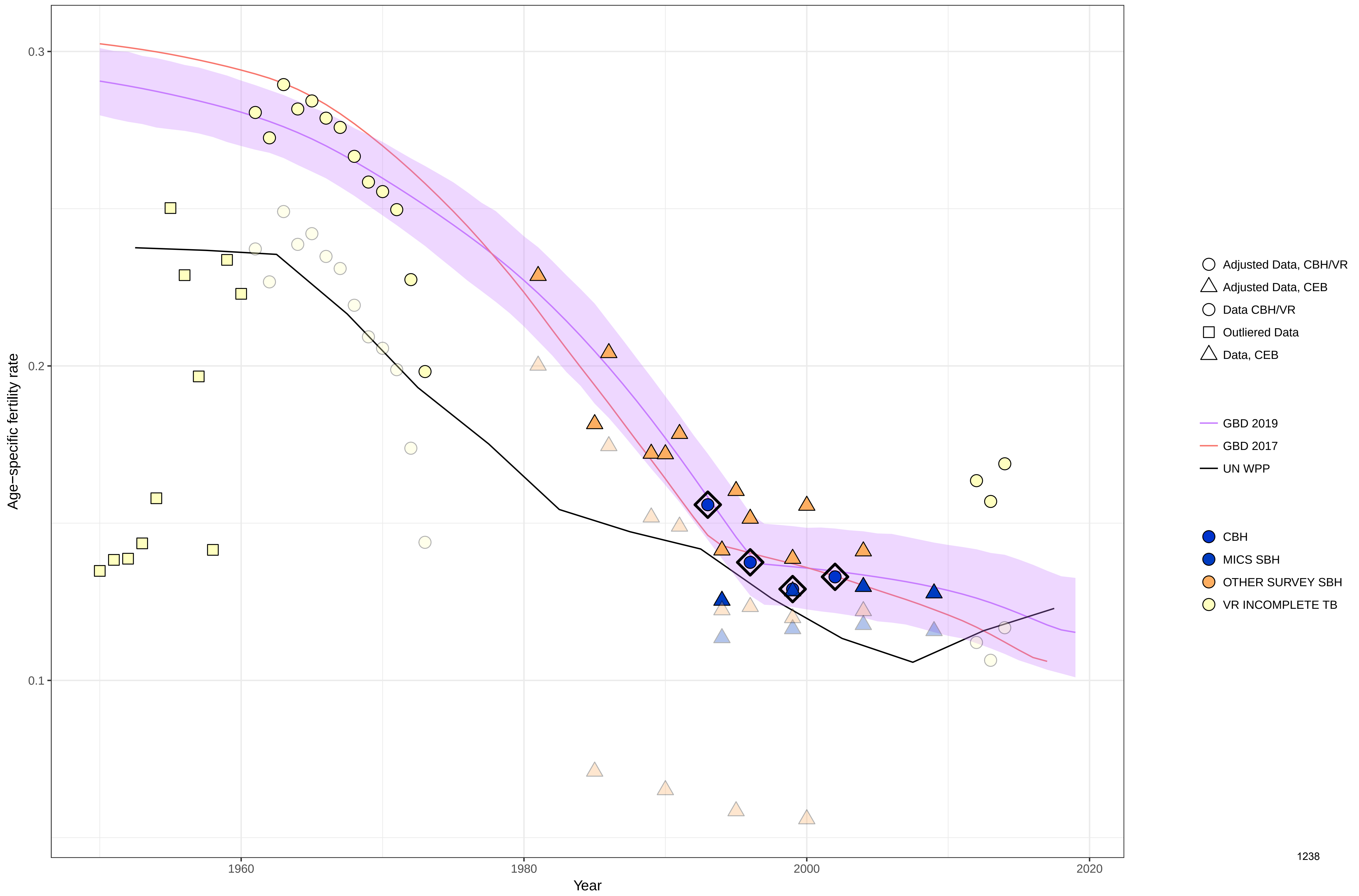

Age−specific fertility fitted trend lines, 1950−2019: Lebanon, Ages: 35−39
Data points enclosed with a diamond are reference sources

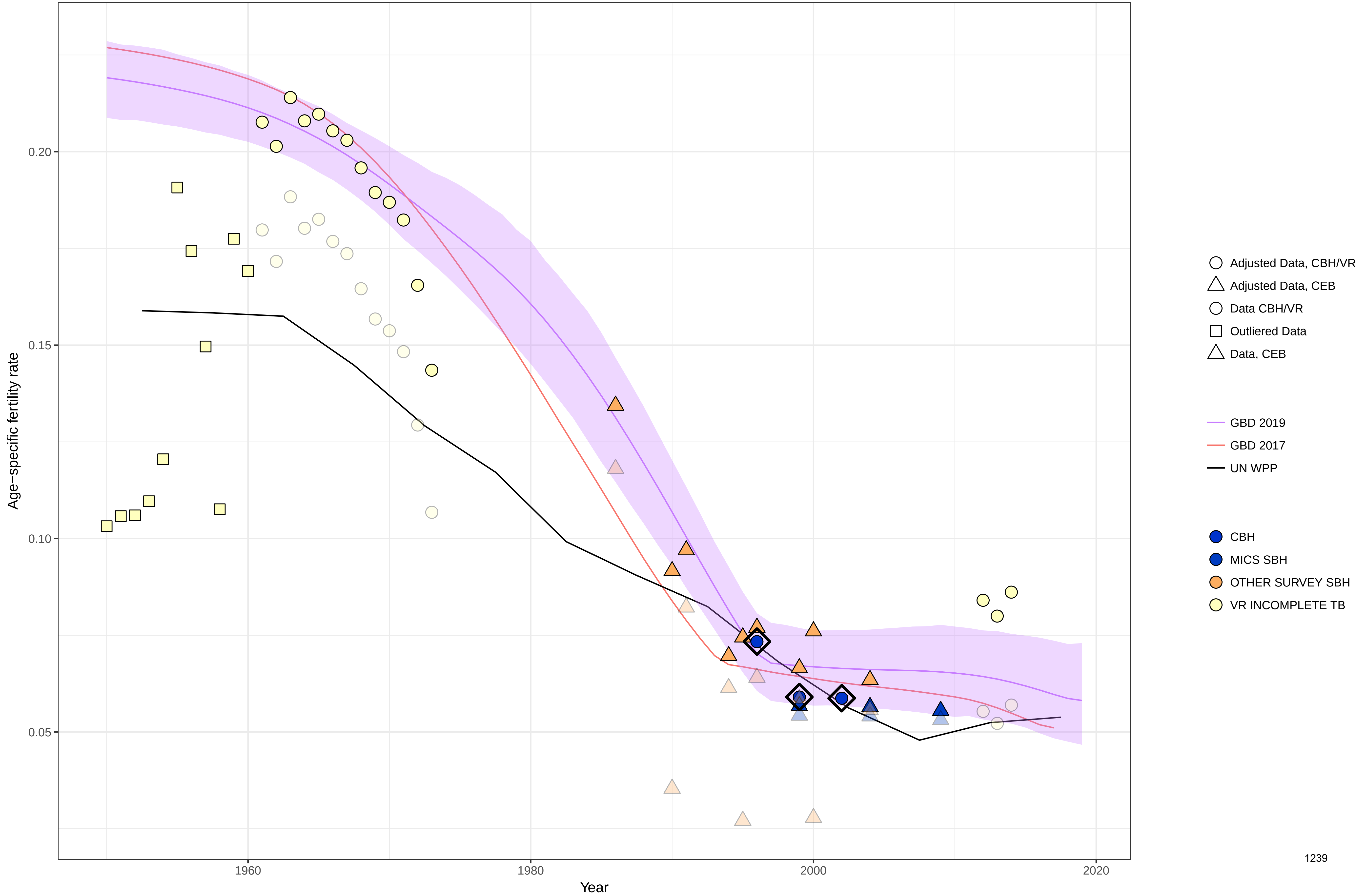

Age−specific fertility fitted trend lines, 1950−2019: Lebanon, Ages: 40−44
Data points enclosed with a diamond are reference sources

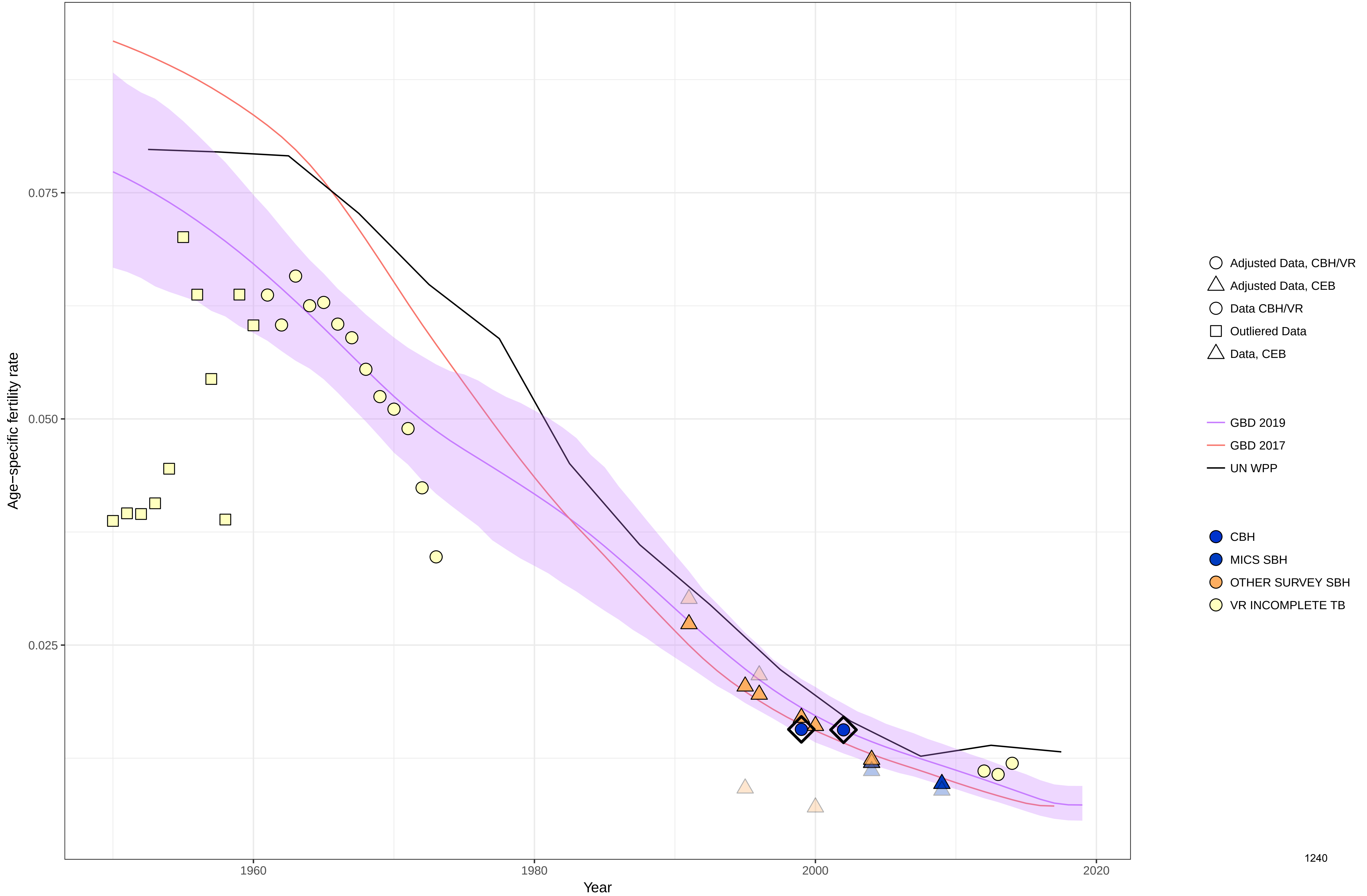

Age−specific fertility fitted trend lines, 1950−2019: Lebanon, Ages: 45−49
Data points enclosed with a diamond are reference sources

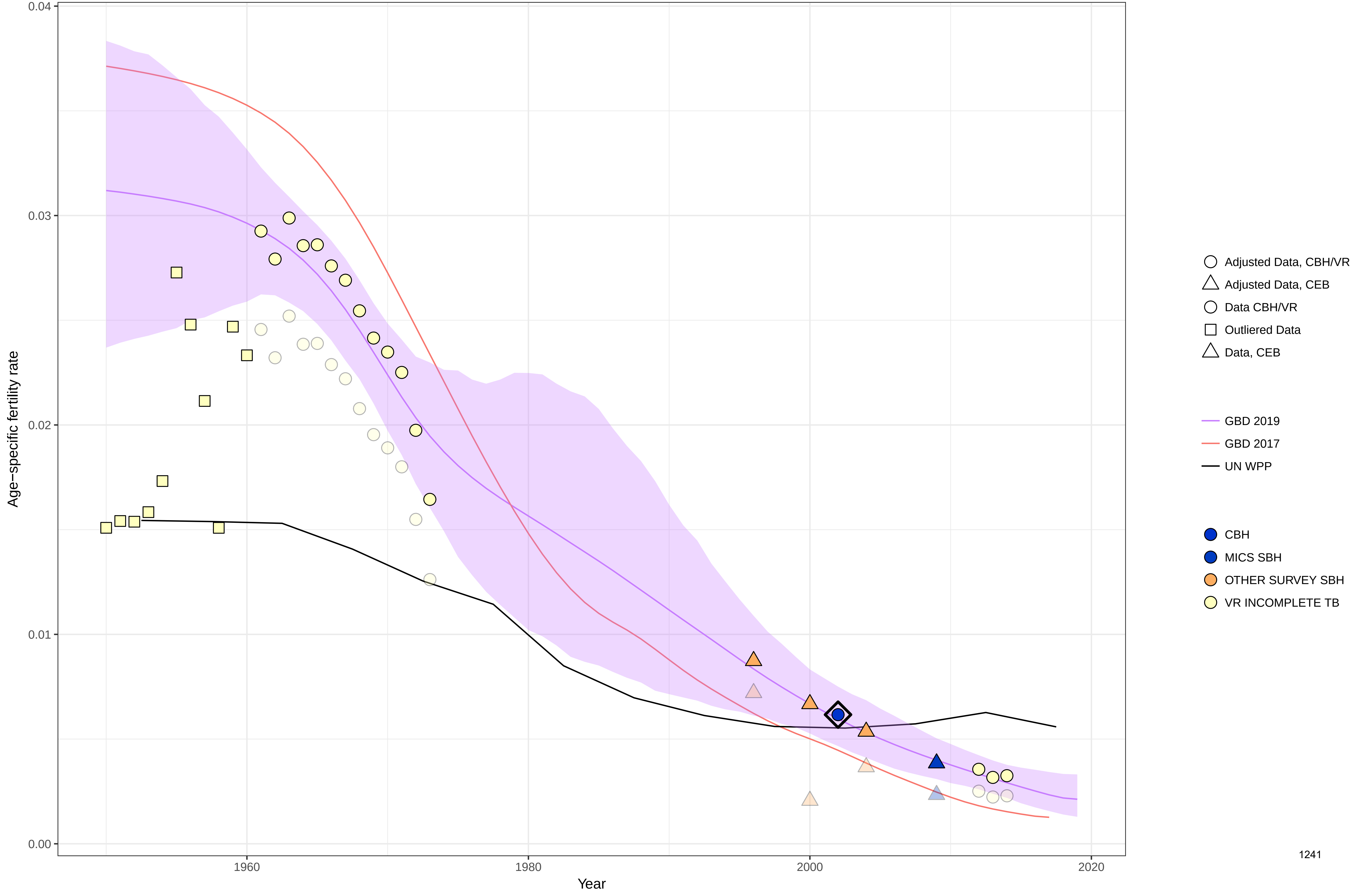

Age−specific fertility fitted trend lines, 1950−2019: Lebanon, Ages: 50−54
Data points enclosed with a diamond are reference sources

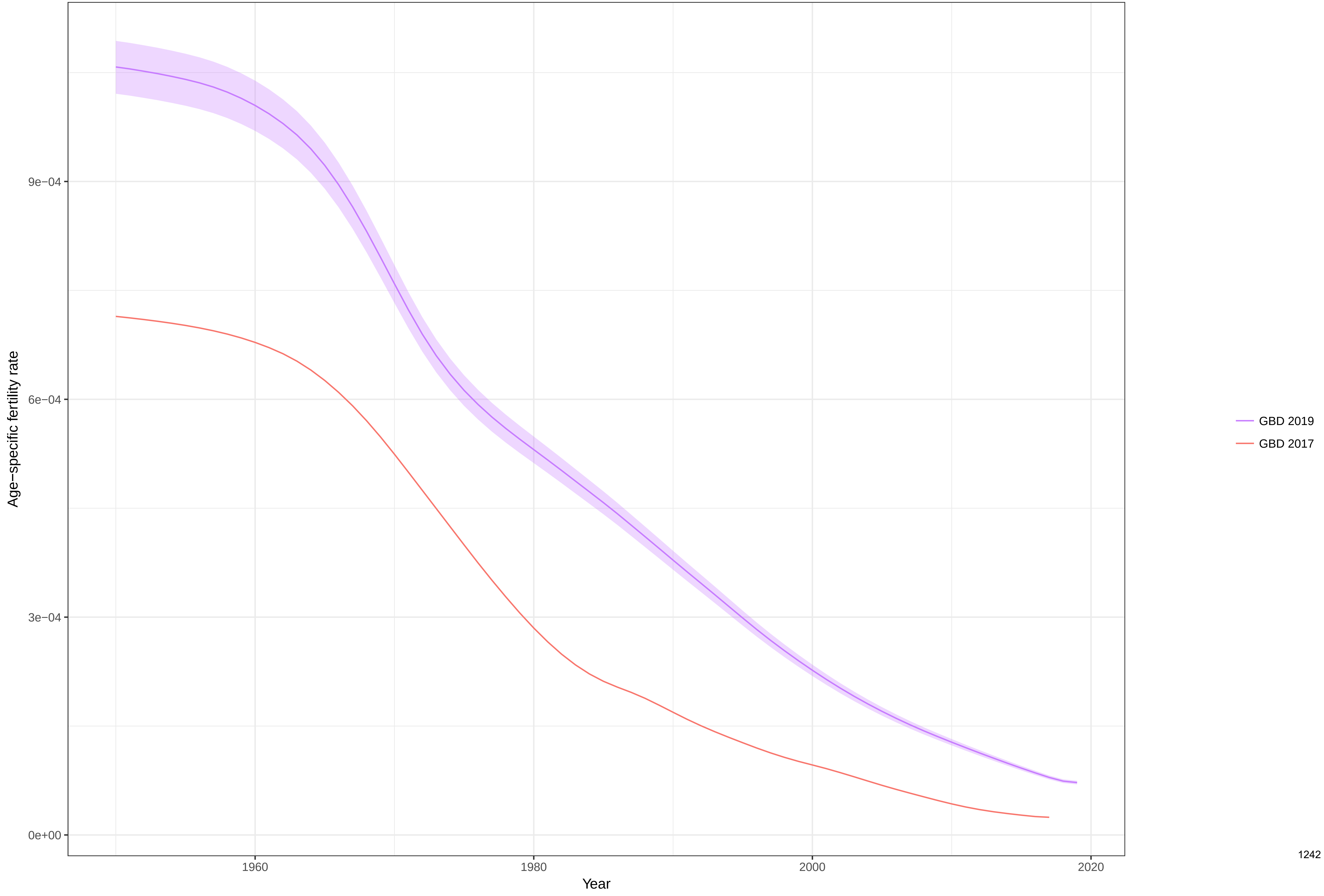

Total fertility rate fitted trend lines, 1950−2019: Lebanon TFR
Data points enclosed with a diamond are reference sources

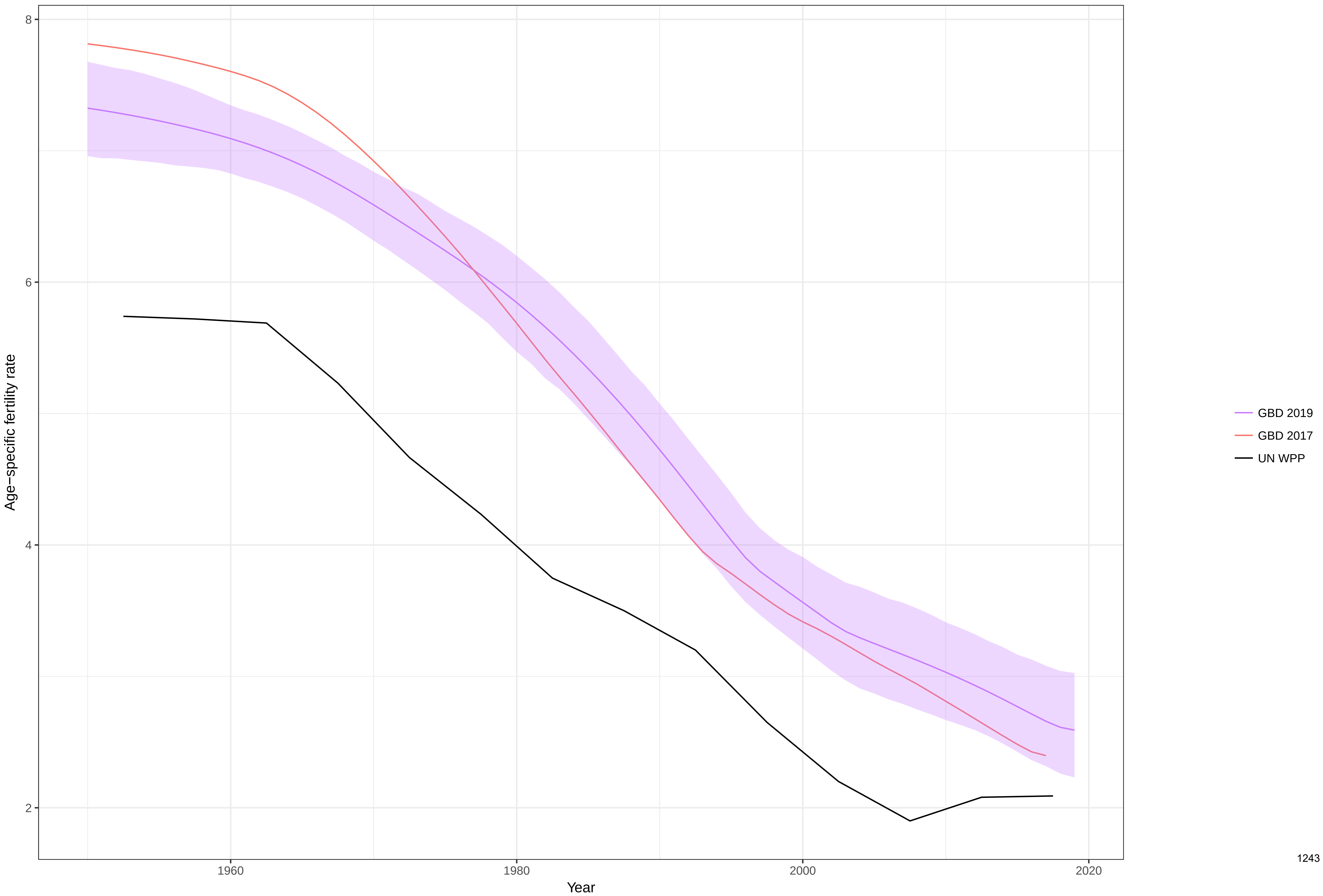

Age−specific fertility fitted trend lines, 1950−2019: Liberia, Ages: 10−14
Data points enclosed with a diamond are reference sources

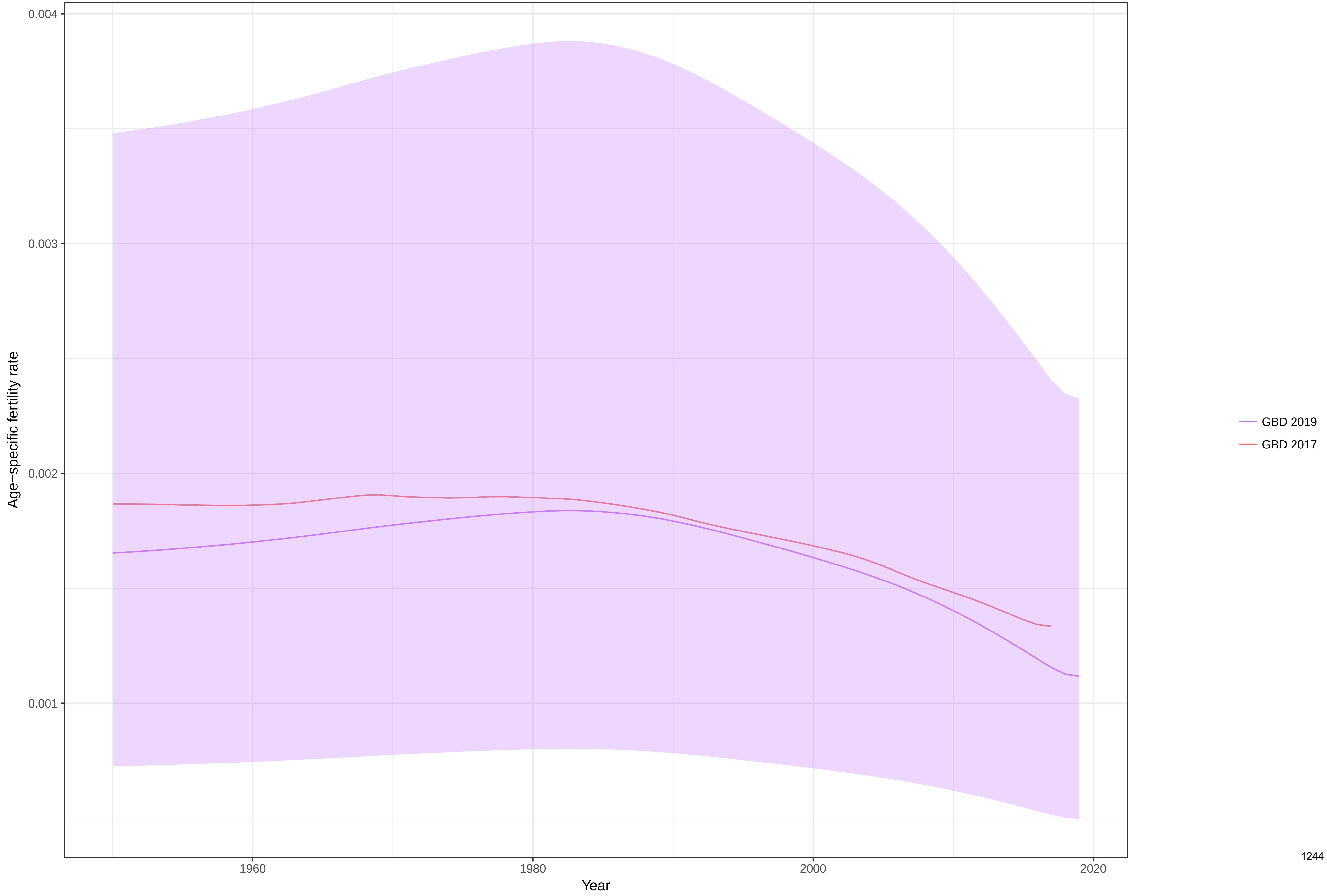

Age−specific fertility fitted trend lines, 1950−2019: Liberia, Ages: 15−19
Data points enclosed with a diamond are reference sources

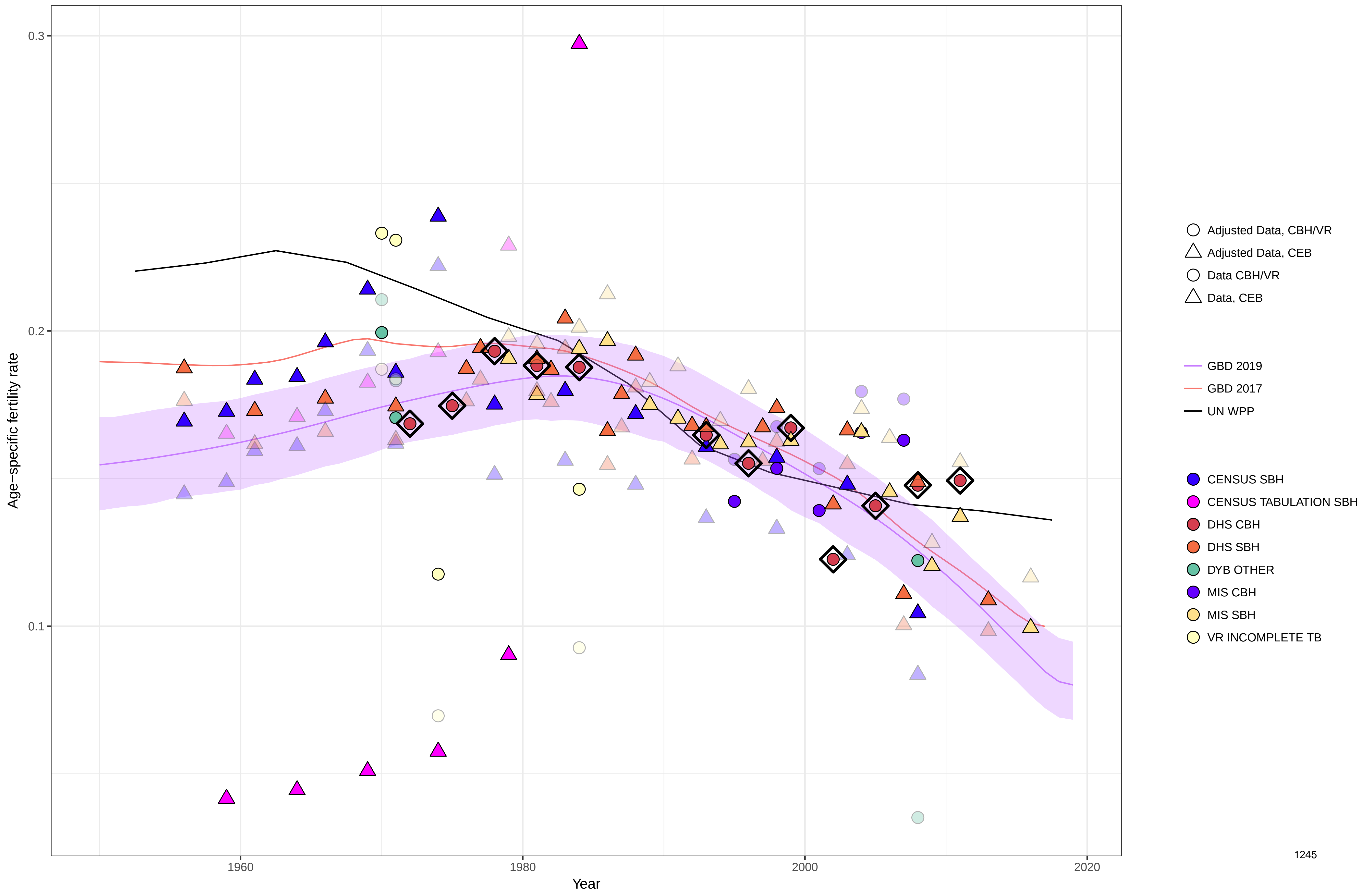

Age−specific fertility fitted trend lines, 1950−2019: Liberia, Ages: 20−24
Data points enclosed with a diamond are reference sources

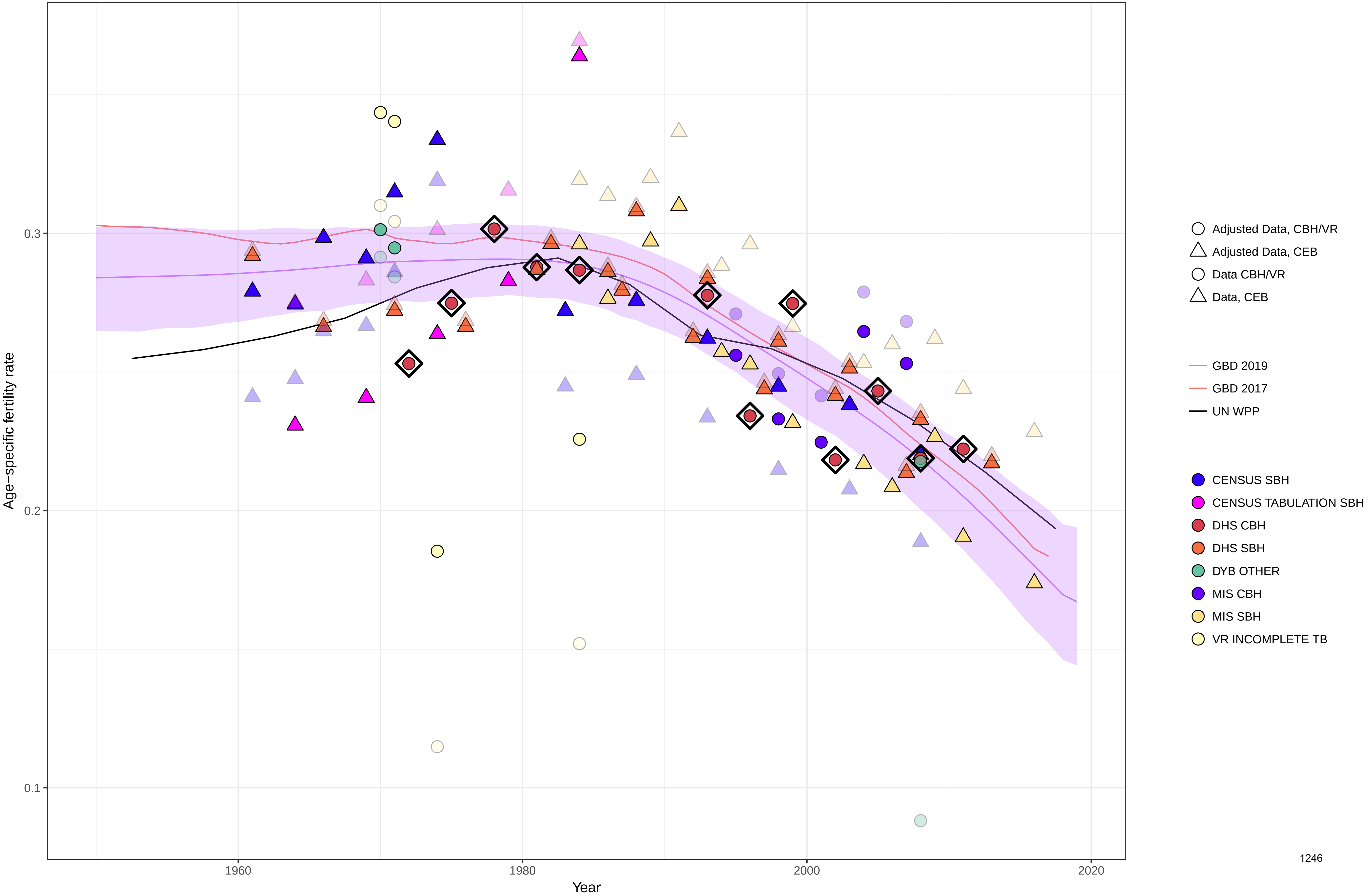

Age−specific fertility fitted trend lines, 1950−2019: Liberia, Ages: 25−29
Data points enclosed with a diamond are reference sources

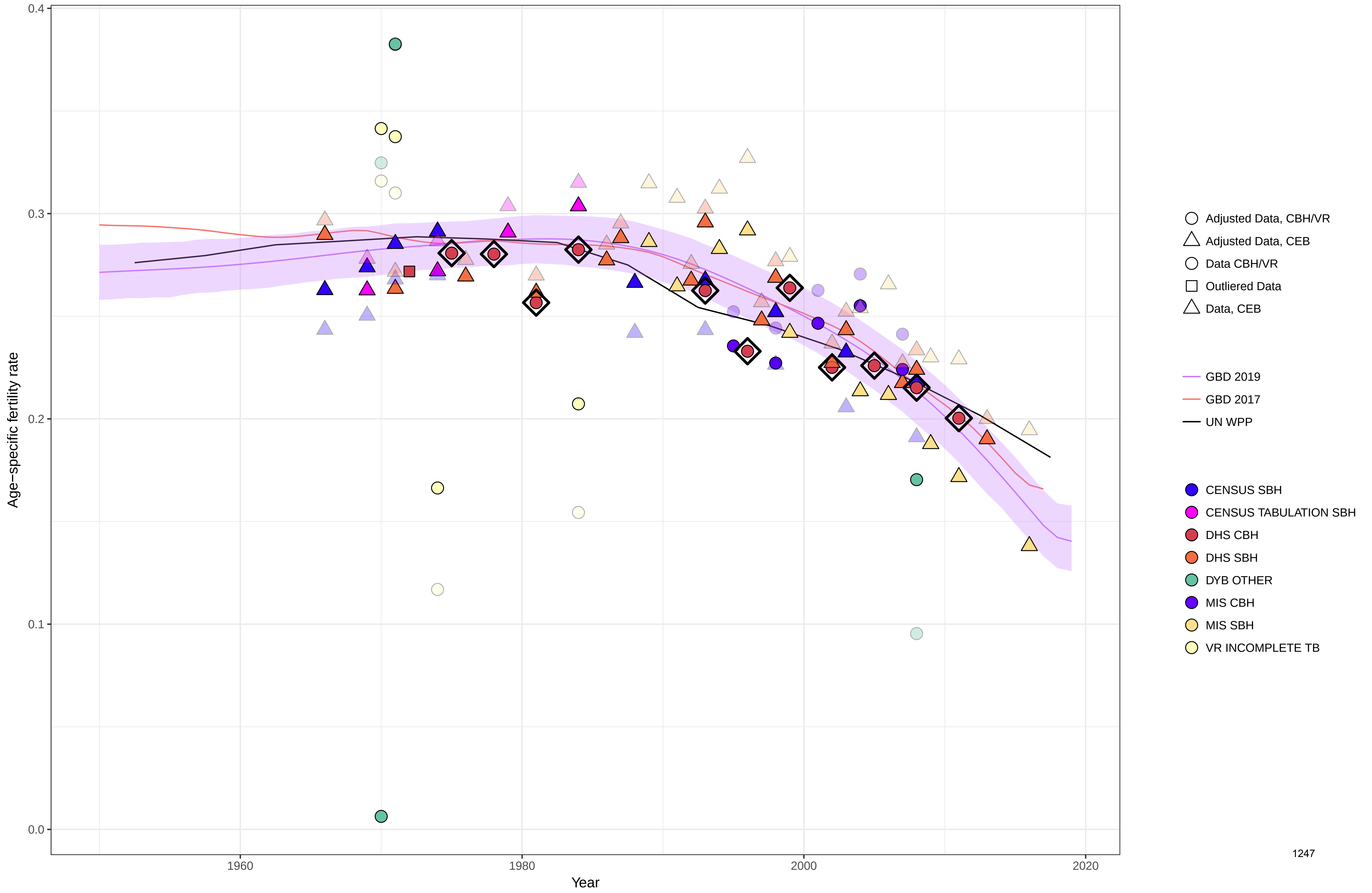

Age−specific fertility fitted trend lines, 1950−2019: Liberia, Ages: 30−34
Data points enclosed with a diamond are reference sources

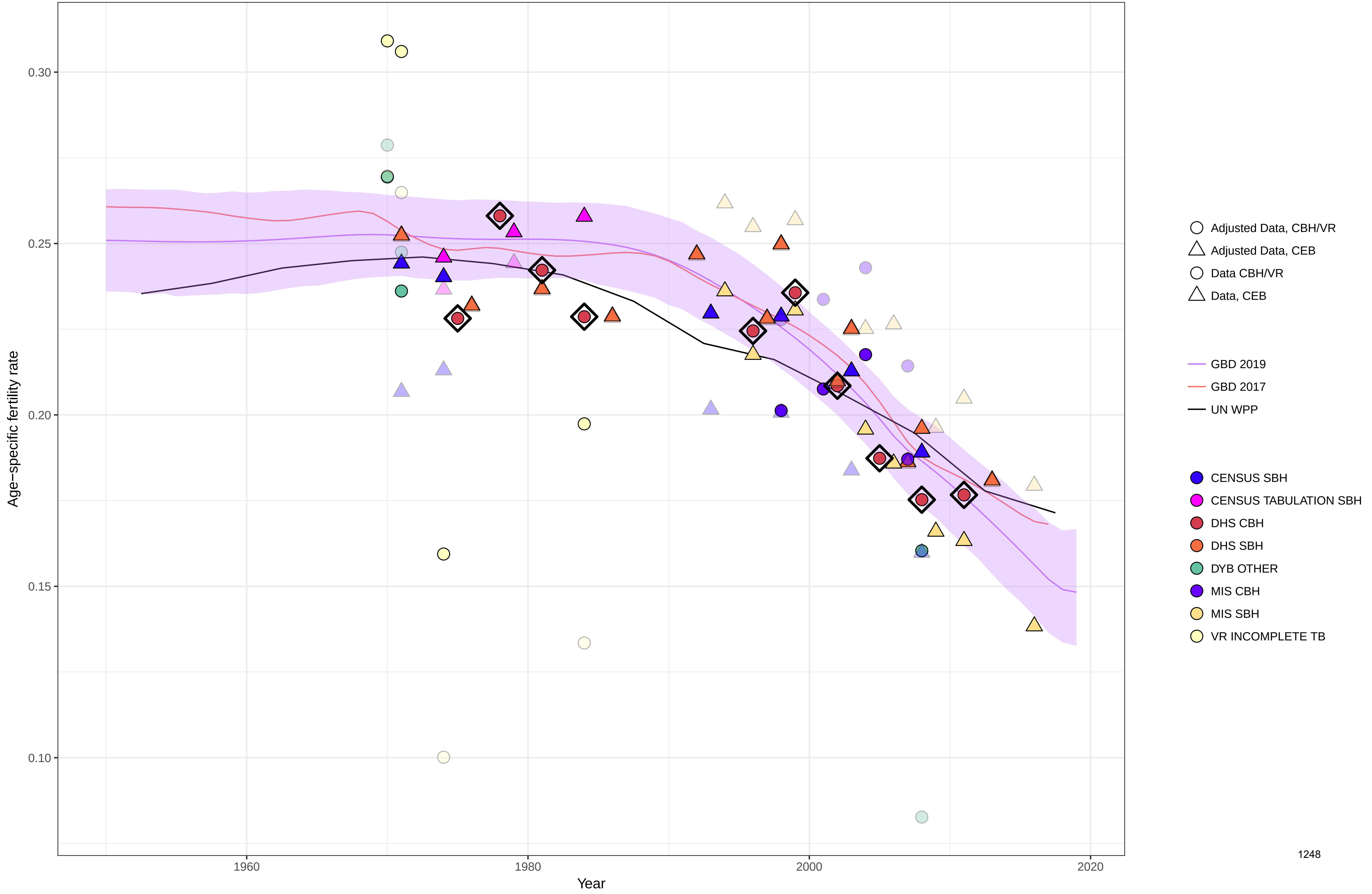

Age−specific fertility fitted trend lines, 1950−2019: Liberia, Ages: 35−39
Data points enclosed with a diamond are reference sources

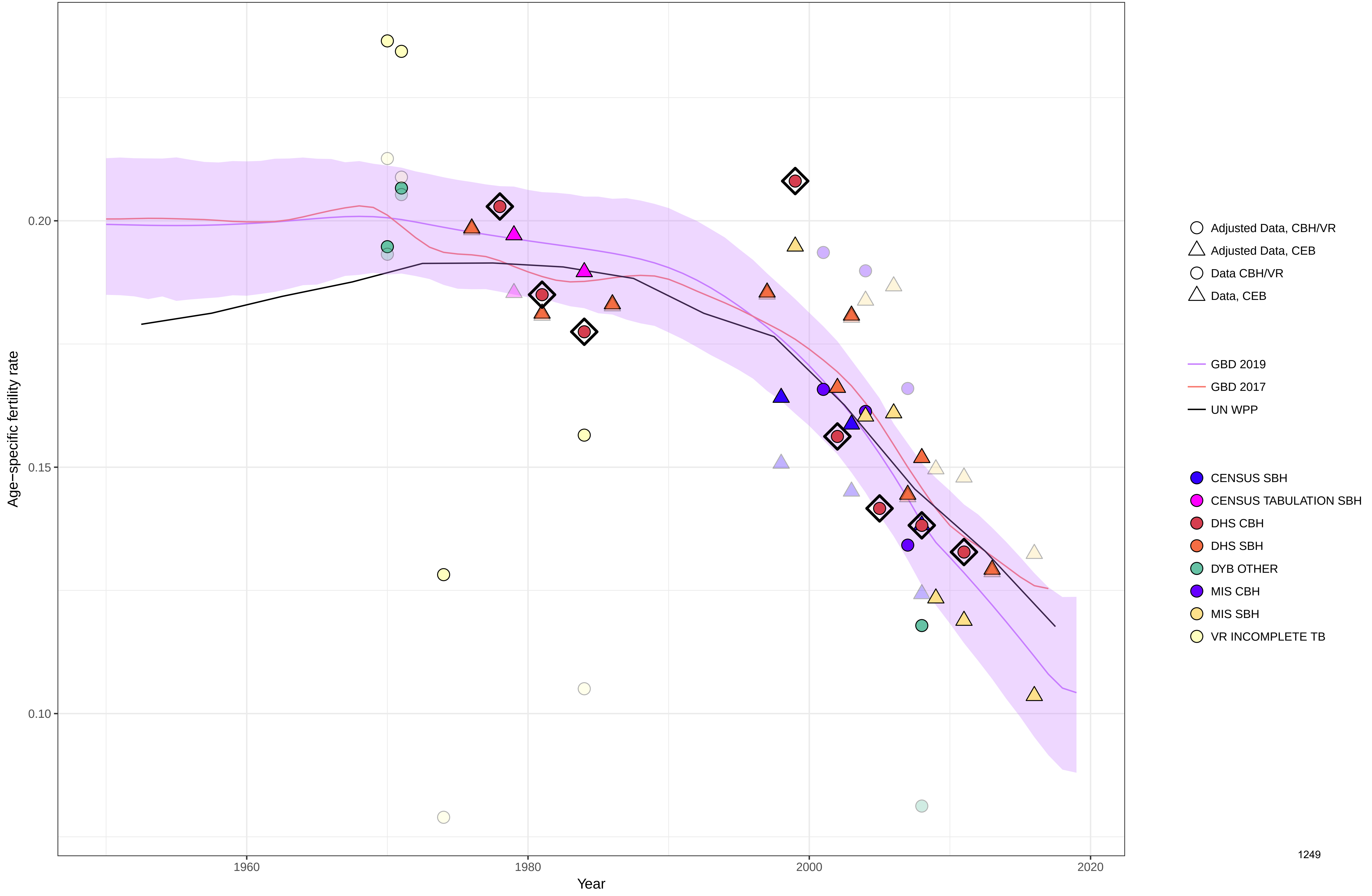

Age−specific fertility fitted trend lines, 1950−2019: Liberia, Ages: 40−44
Data points enclosed with a diamond are reference sources

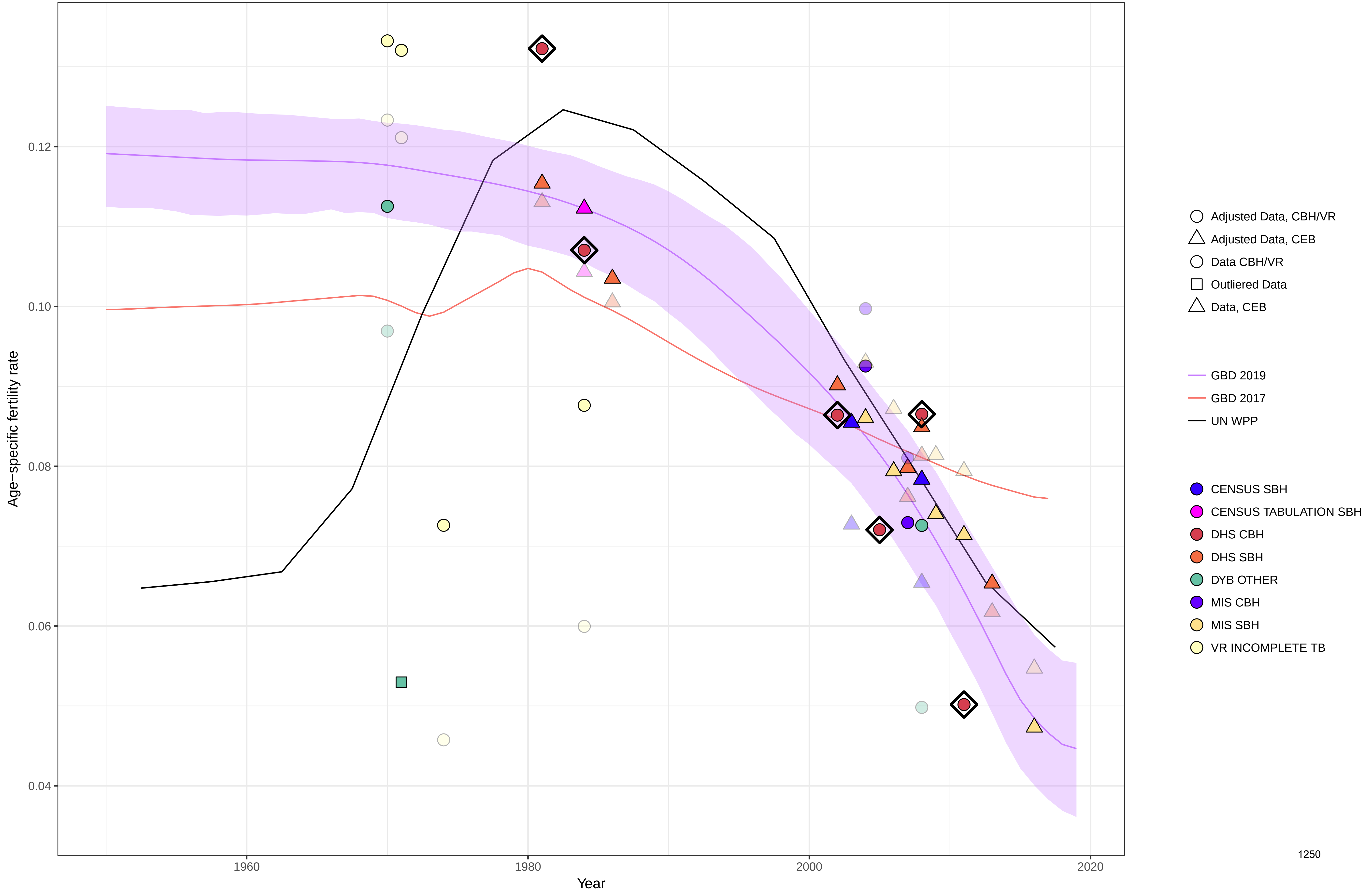

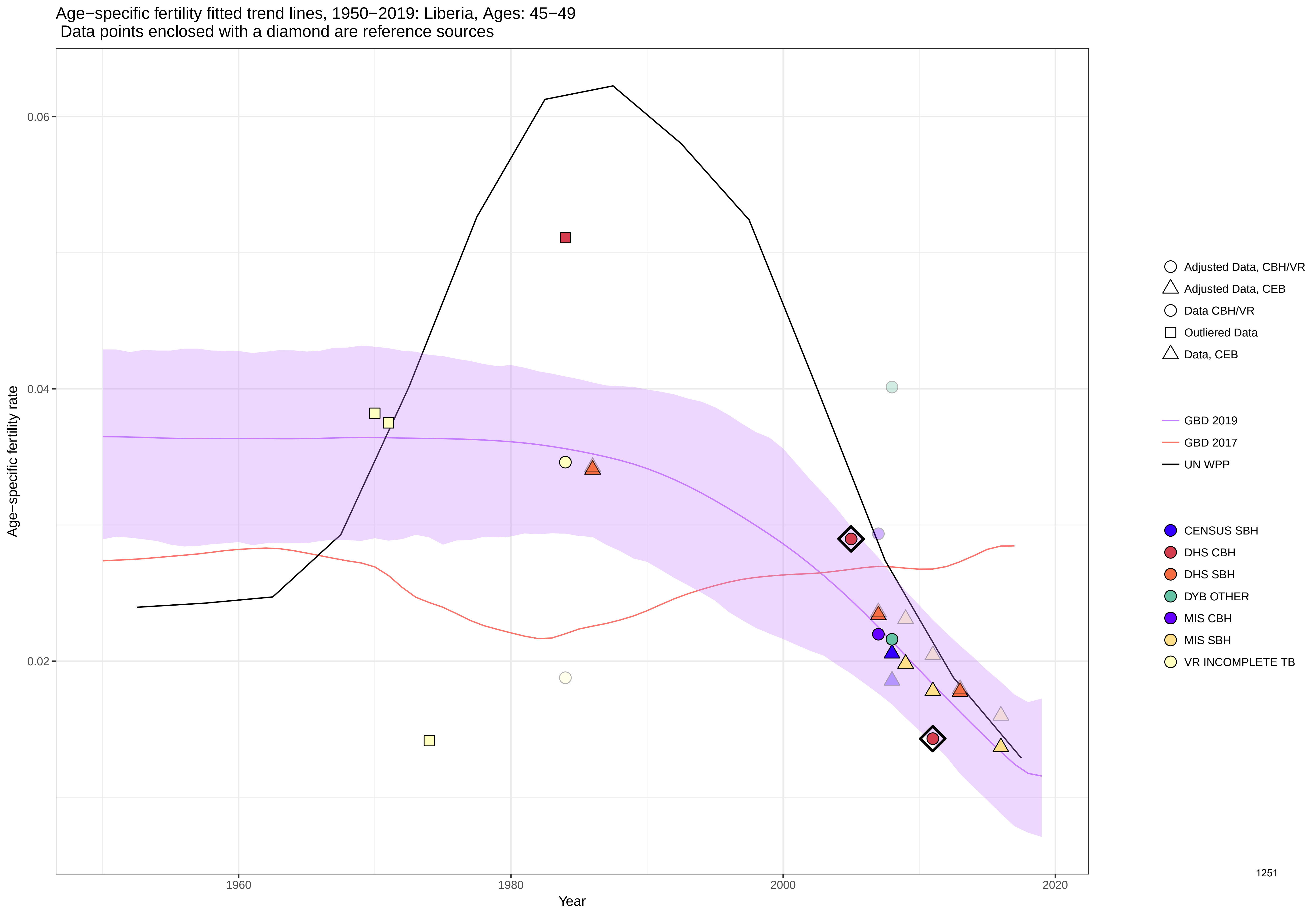

Age−specific fertility fitted trend lines, 1950−2019: Liberia, Ages: 45−49
Data points enclosed with a diamond are reference sources
Age−specific fertility rate
0.06
0.04
0.02
Year
1960
1980
2000
2020
Adjusted Data, CBH/VR
Adjusted Data, CEB
Data CBH/VR
Outliered Data
Data, CEB
GBD 2019
GBD 2017
UN WPP
CENSUS SBH
DHS CBH
DHS SBH
DYB OTHER
MIS CBH
MIS SBH
VR INCOMPLETE TB

Age−specific fertility fitted trend lines, 1950−2019: Liberia, Ages: 50−54
Data points enclosed with a diamond are reference sources

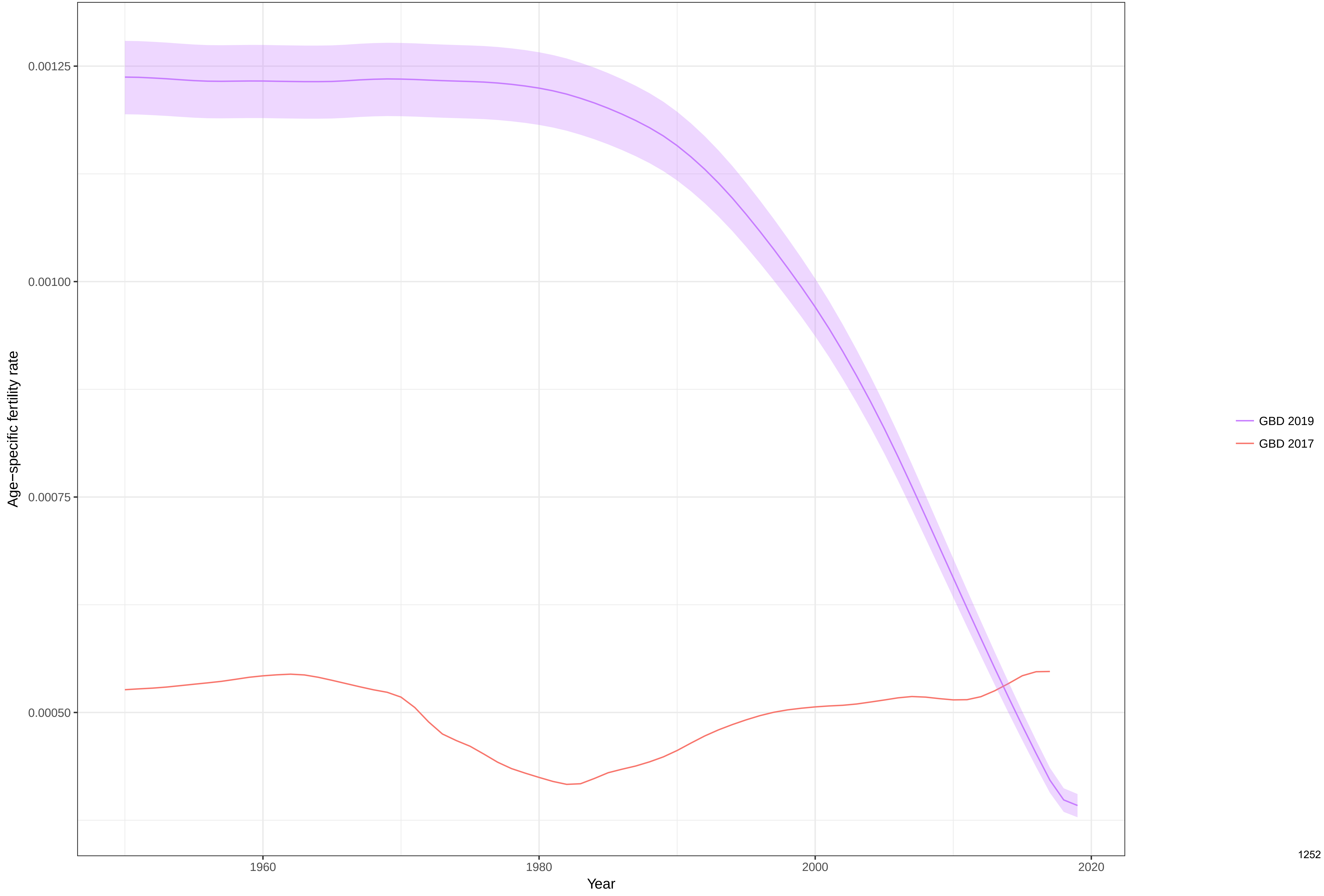

Total fertility rate fitted trend lines, 1950−2019: Liberia TFR
Data points enclosed with a diamond are reference sources

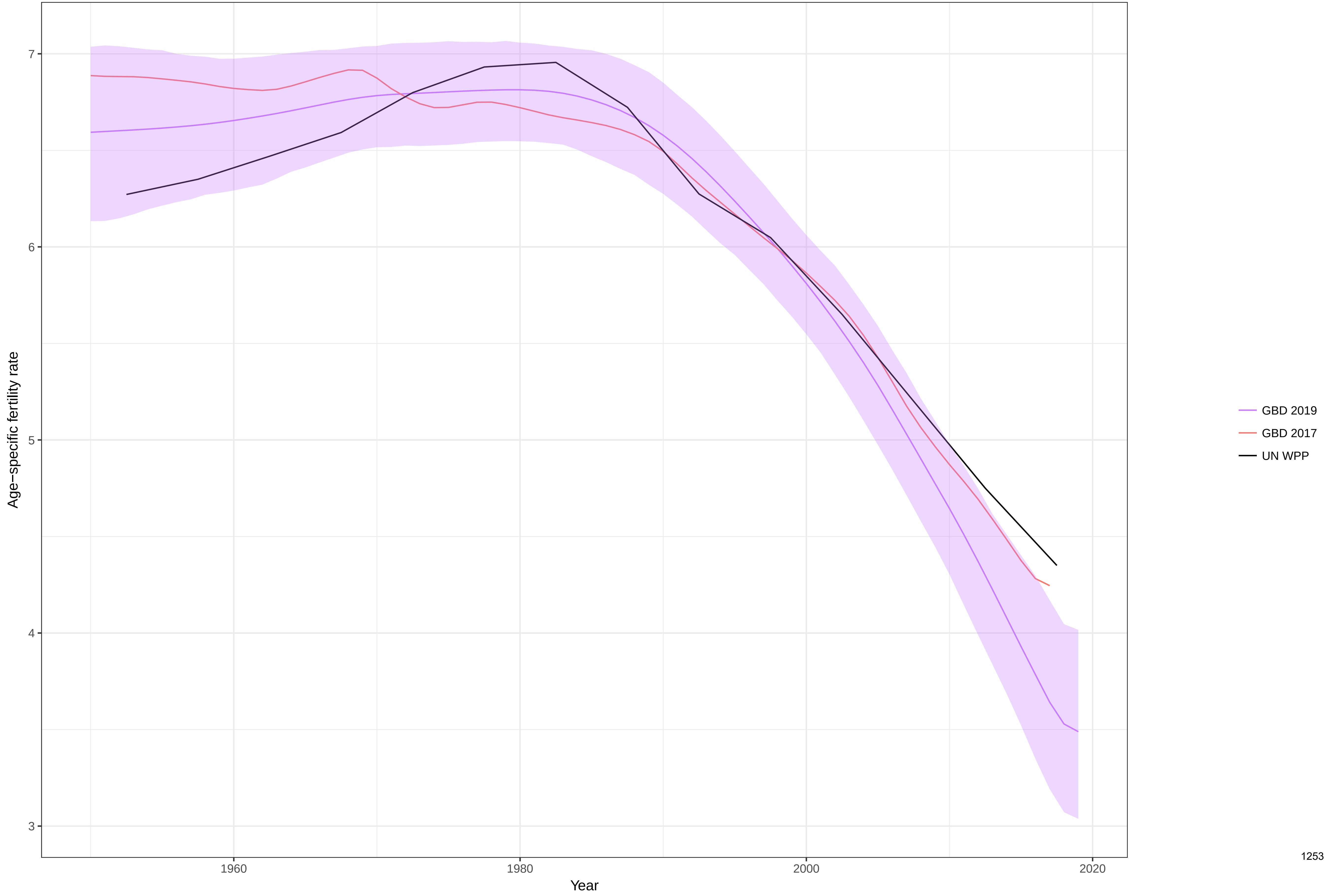

Age−specific fertility fitted trend lines, 1950−2019: Libya, Ages: 10−14
Data points enclosed with a diamond are reference sources

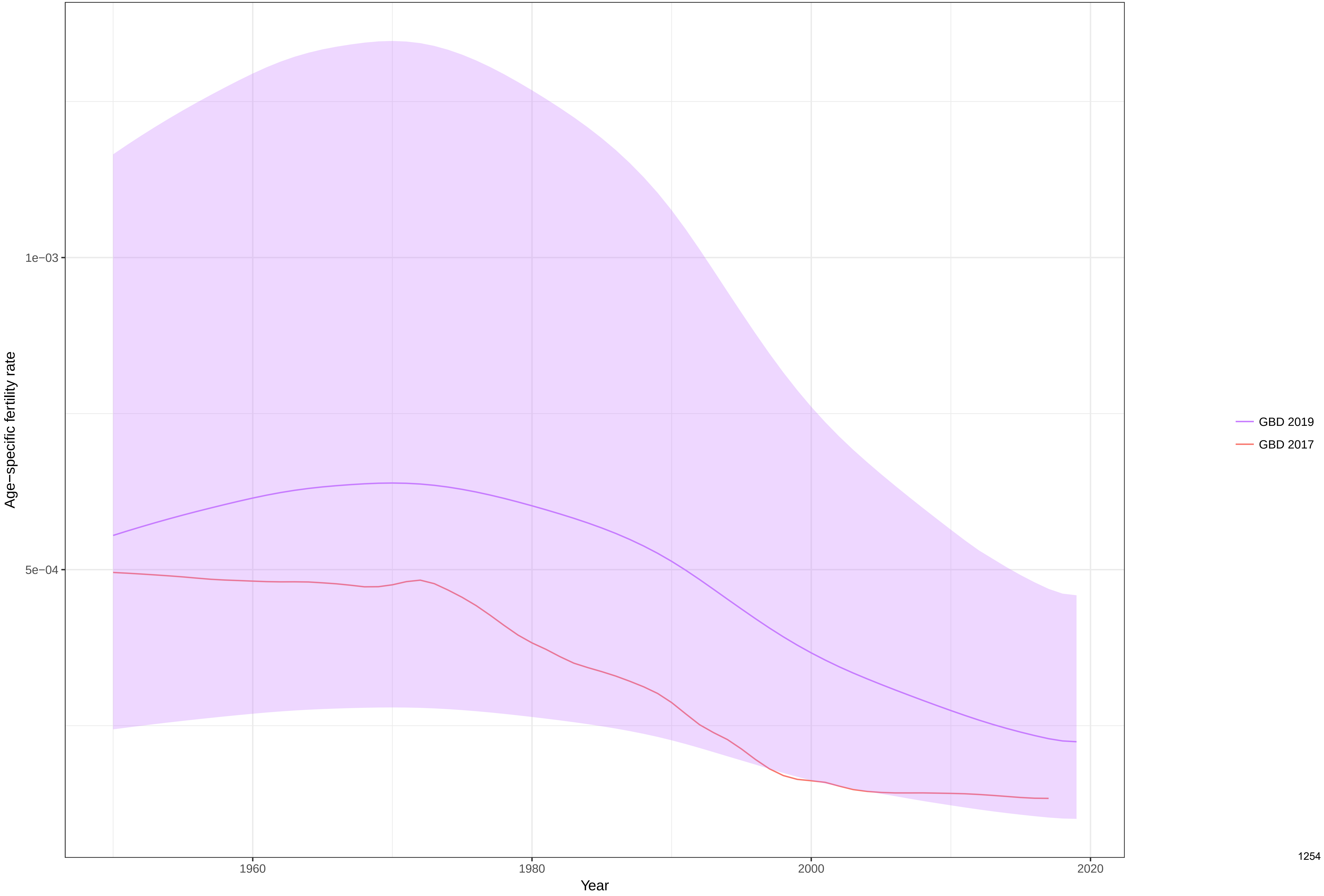

Age−specific fertility fitted trend lines, 1950−2019: Libya, Ages: 15−19
Data points enclosed with a diamond are reference sources

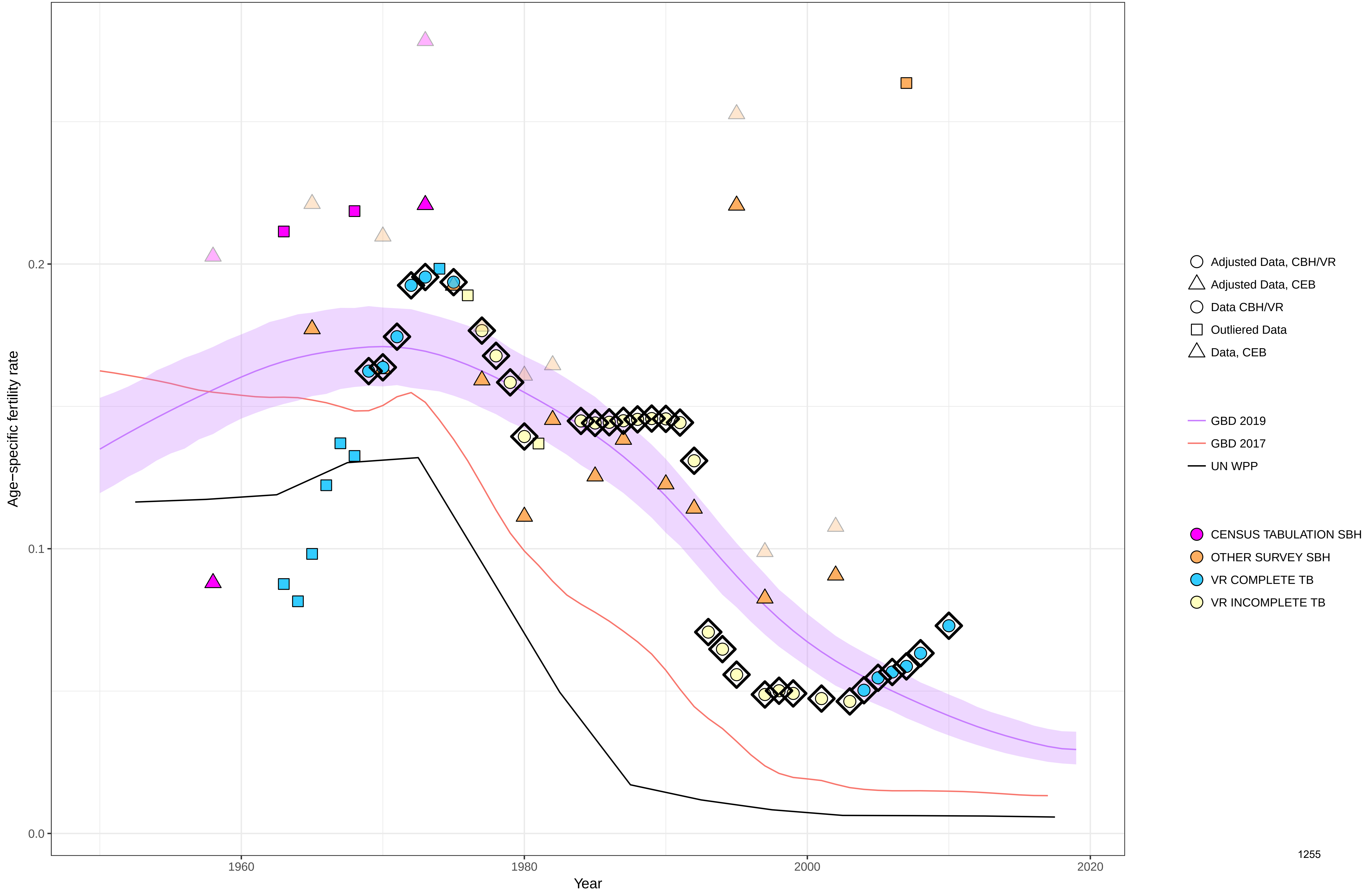

Age−specific fertility fitted trend lines, 1950−2019: Libya, Ages: 20−24
Data points enclosed with a diamond are reference sources

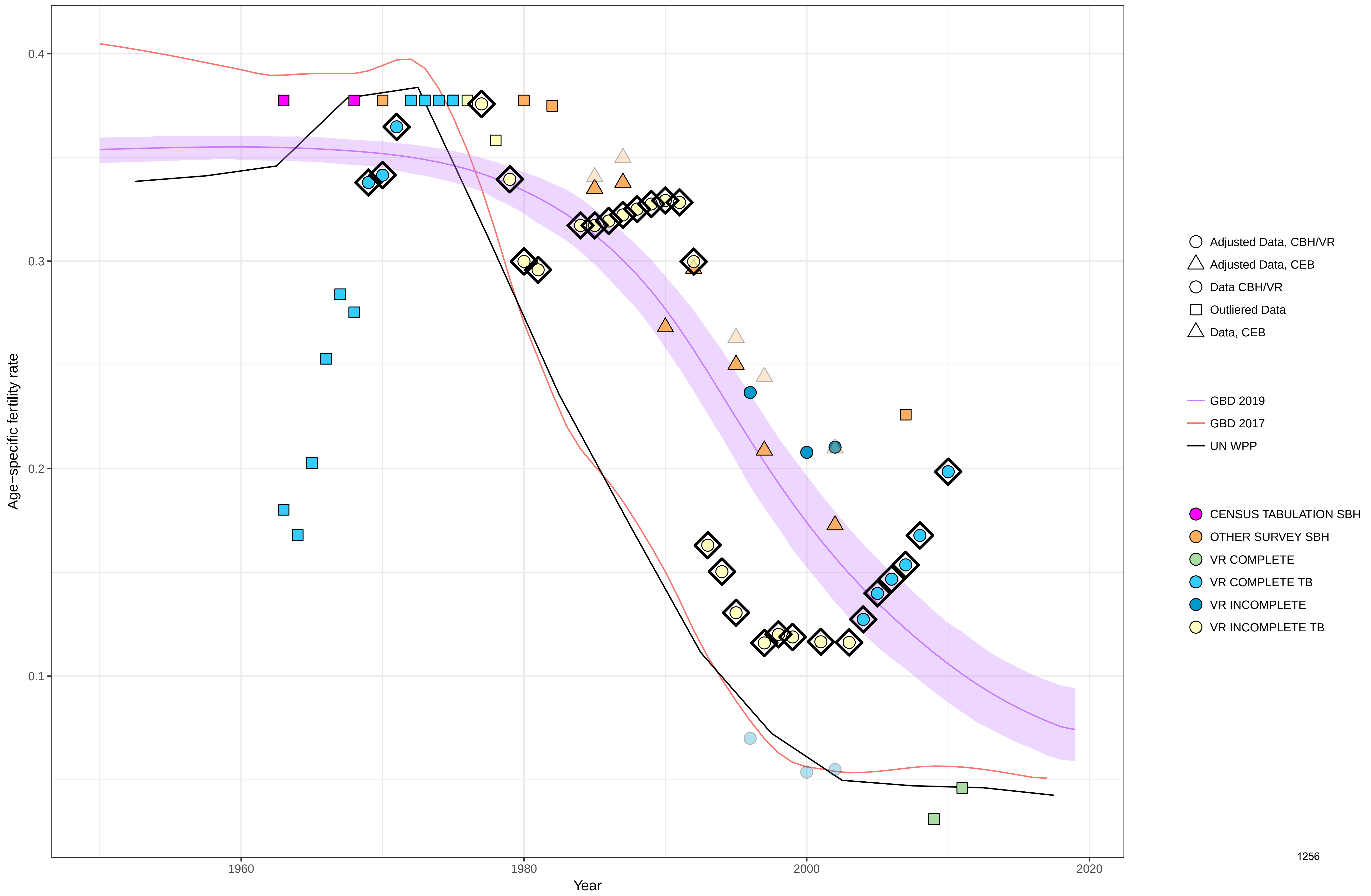

Age−specific fertility fitted trend lines, 1950−2019: Libya, Ages: 25−29
Data points enclosed with a diamond are reference sources

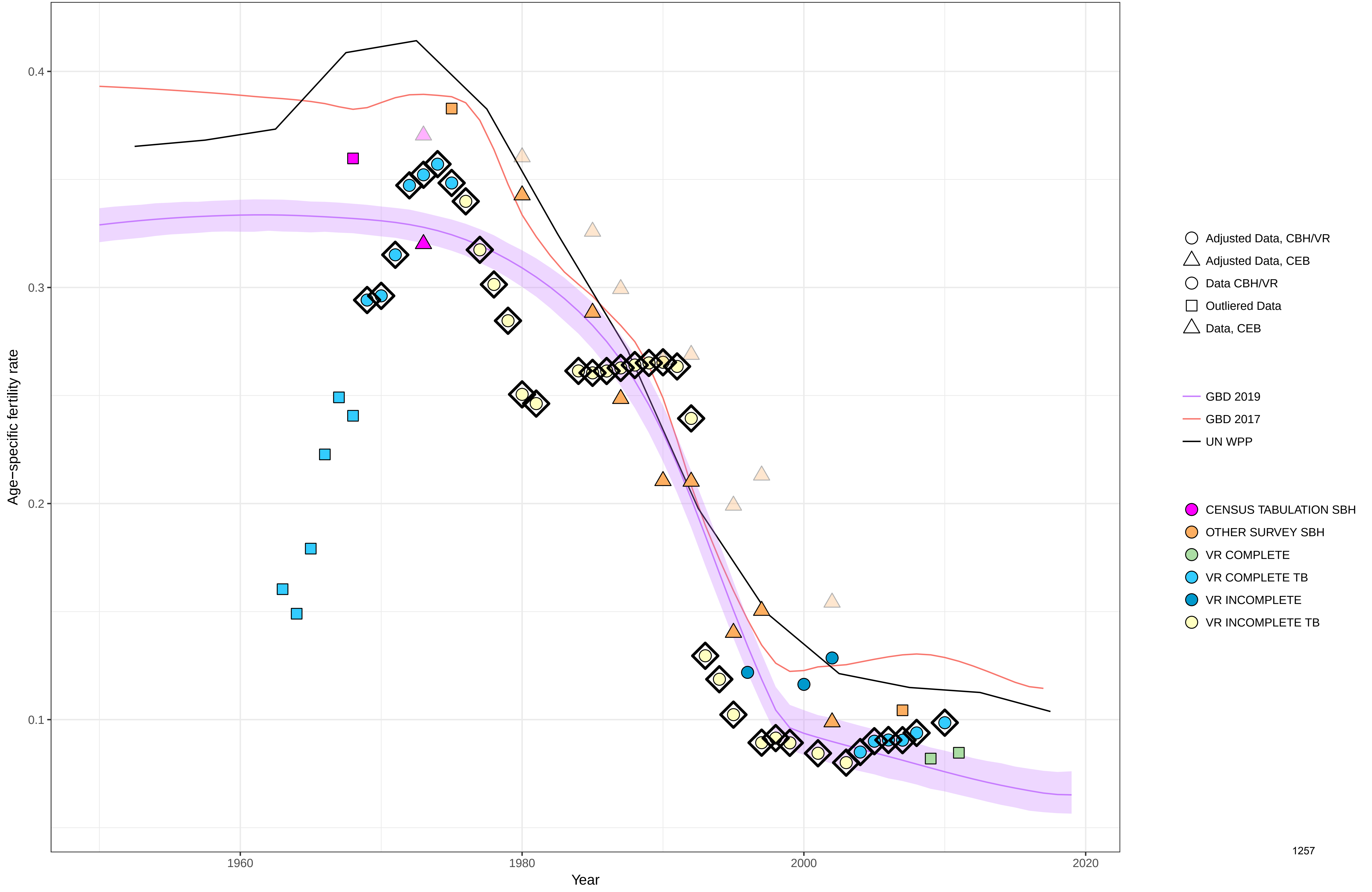

Age−specific fertility fitted trend lines, 1950−2019: Libya, Ages: 30−34
Data points enclosed with a diamond are reference sources

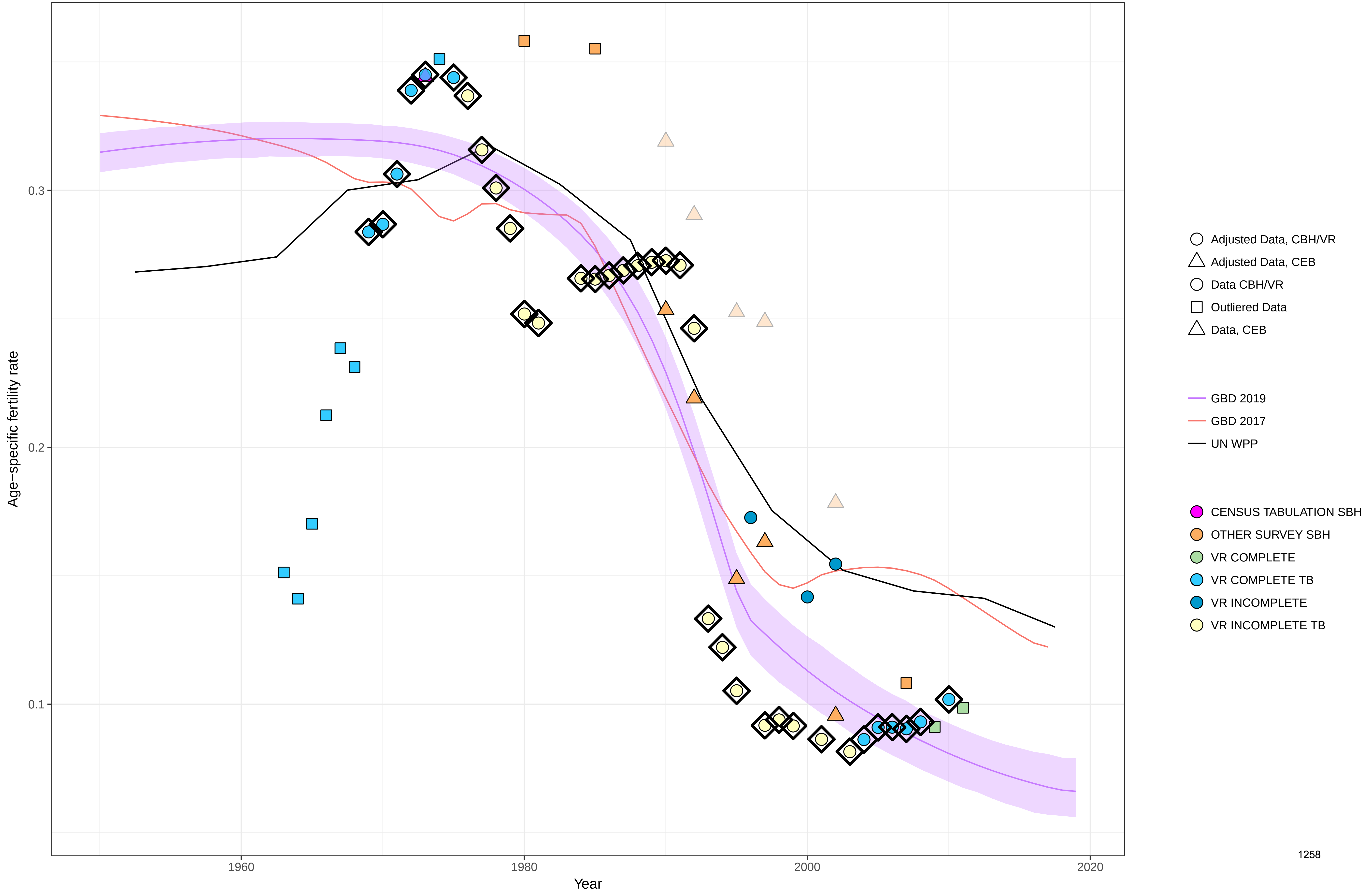

Age−specific fertility fitted trend lines, 1950−2019: Libya, Ages: 35−39
Data points enclosed with a diamond are reference sources

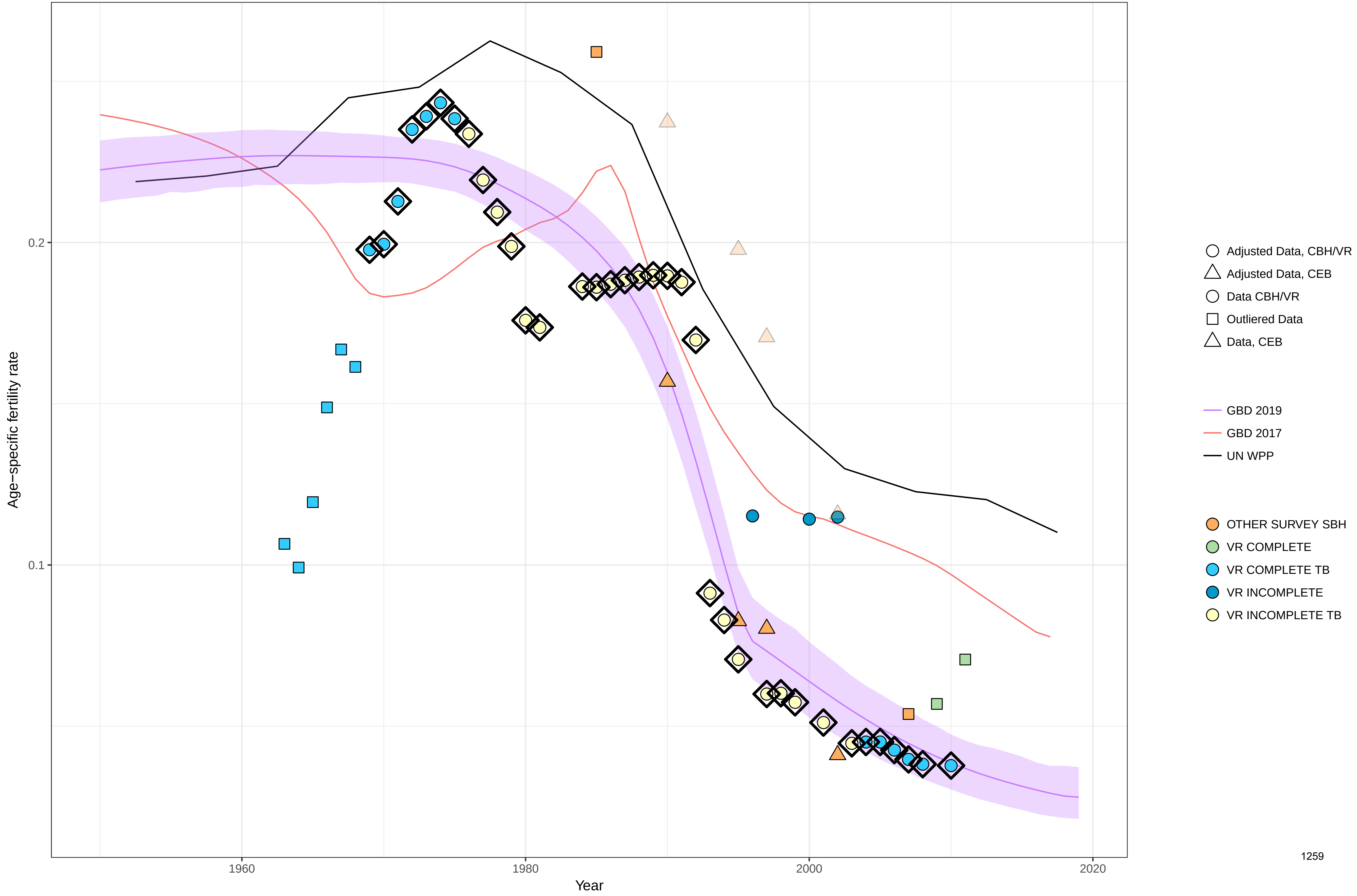

Age−specific fertility fitted trend lines, 1950−2019: Libya, Ages: 40−44
Data points enclosed with a diamond are reference sources

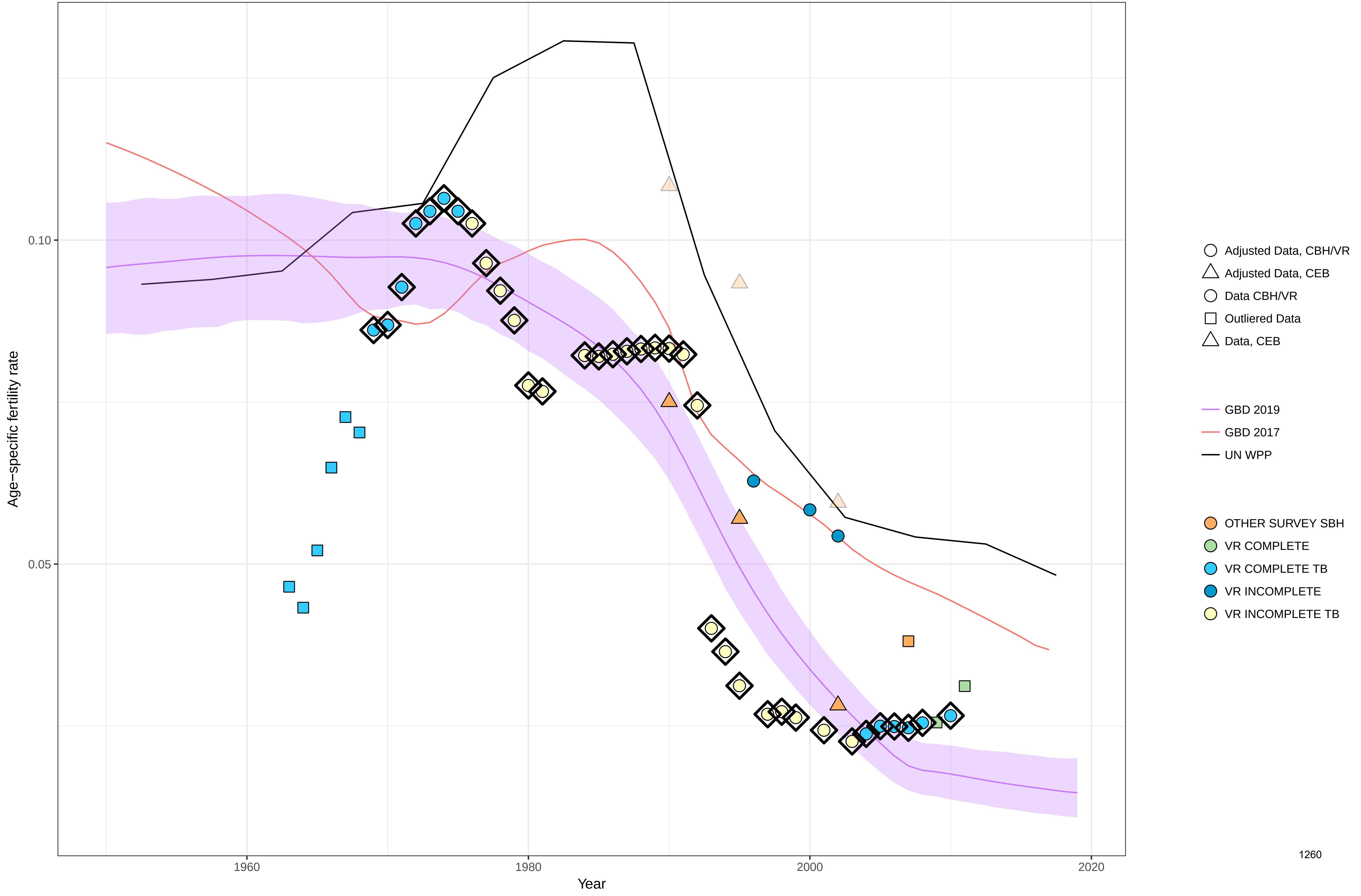

Age−specific fertility fitted trend lines, 1950−2019: Libya, Ages: 45−49
Data points enclosed with a diamond are reference sources

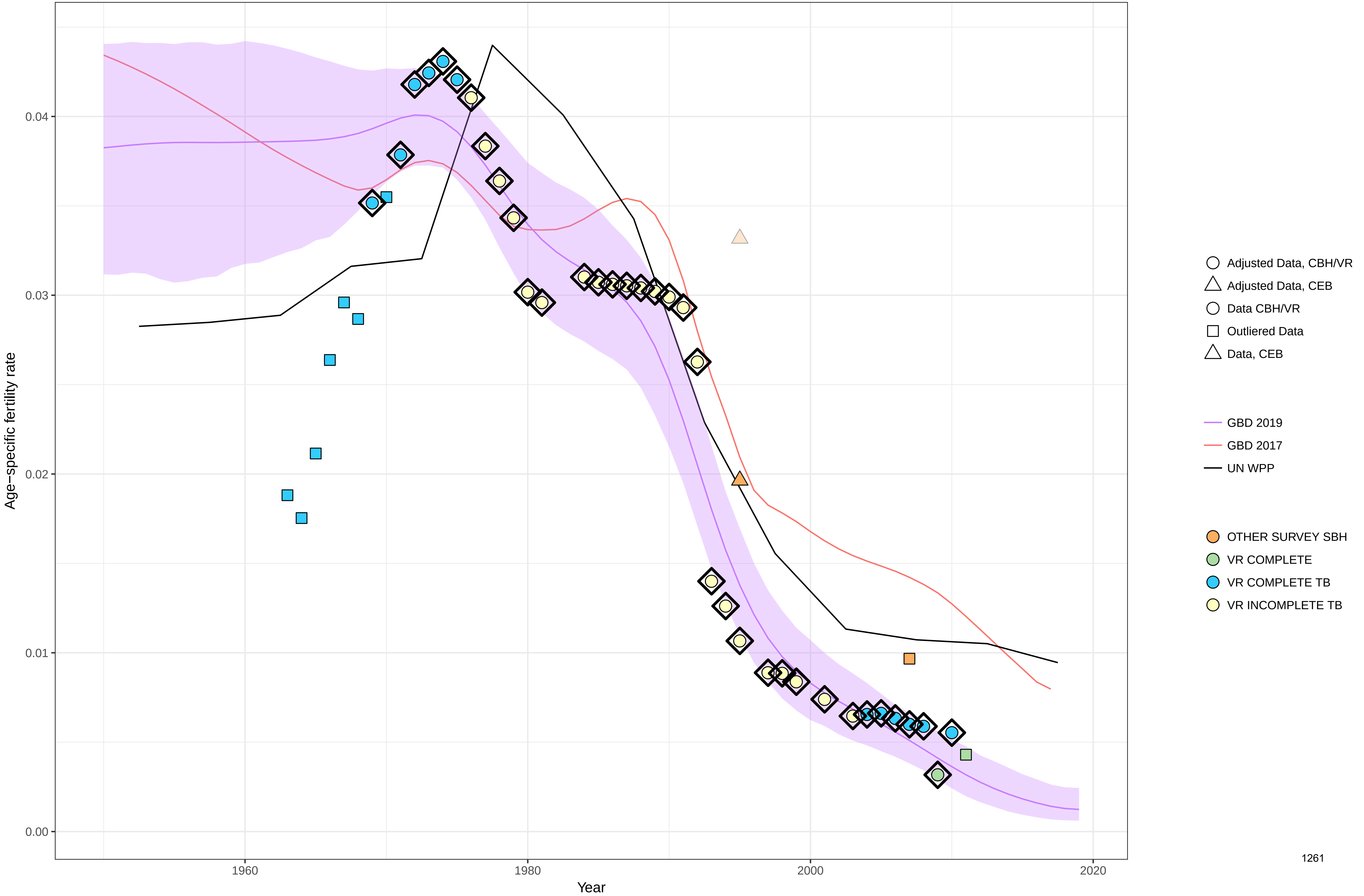

Age−specific fertility fitted trend lines, 1950−2019: Libya, Ages: 50−54
Data points enclosed with a diamond are reference sources

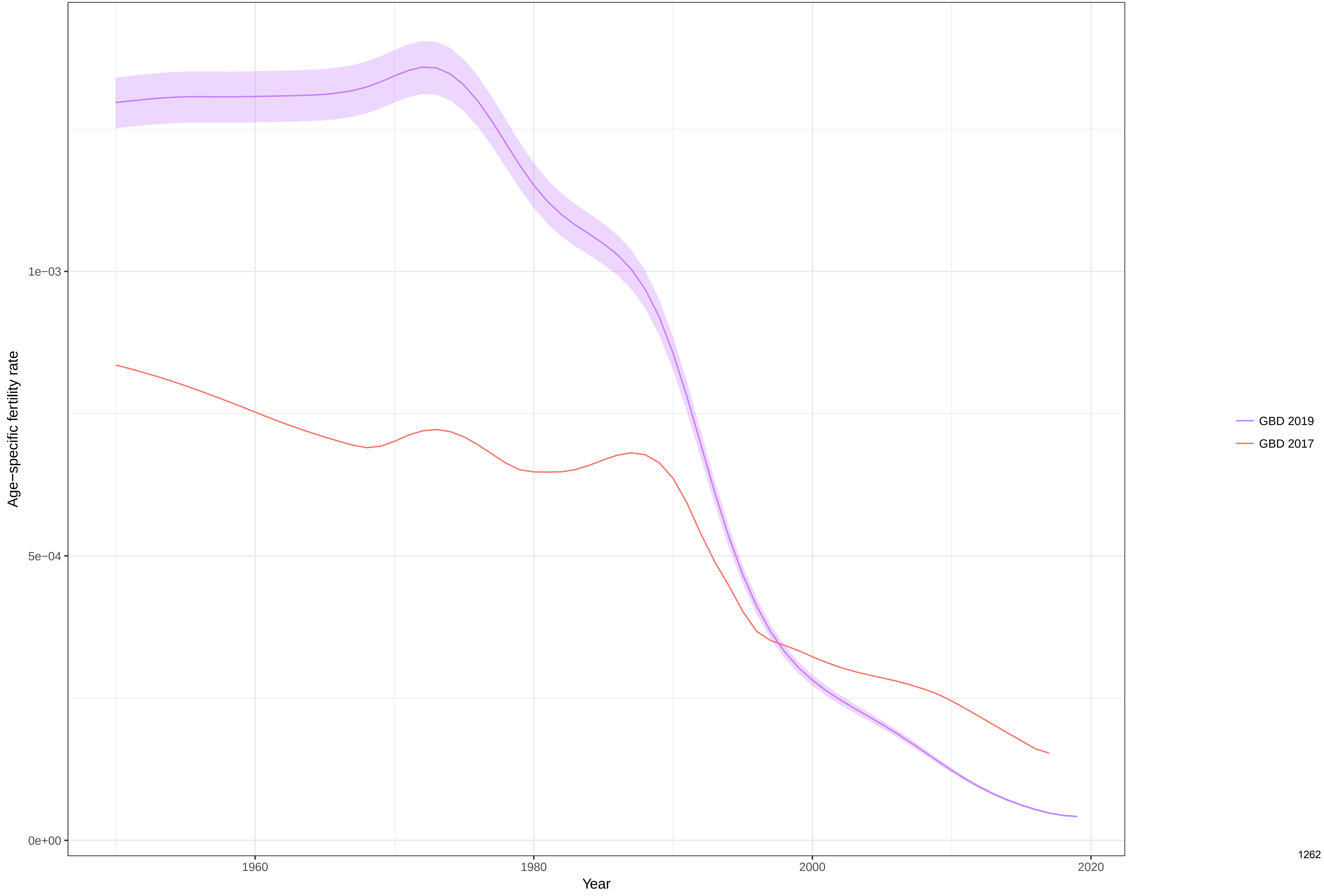

Total fertility rate fitted trend lines, 1950−2019: Libya TFR
Data points enclosed with a diamond are reference sources

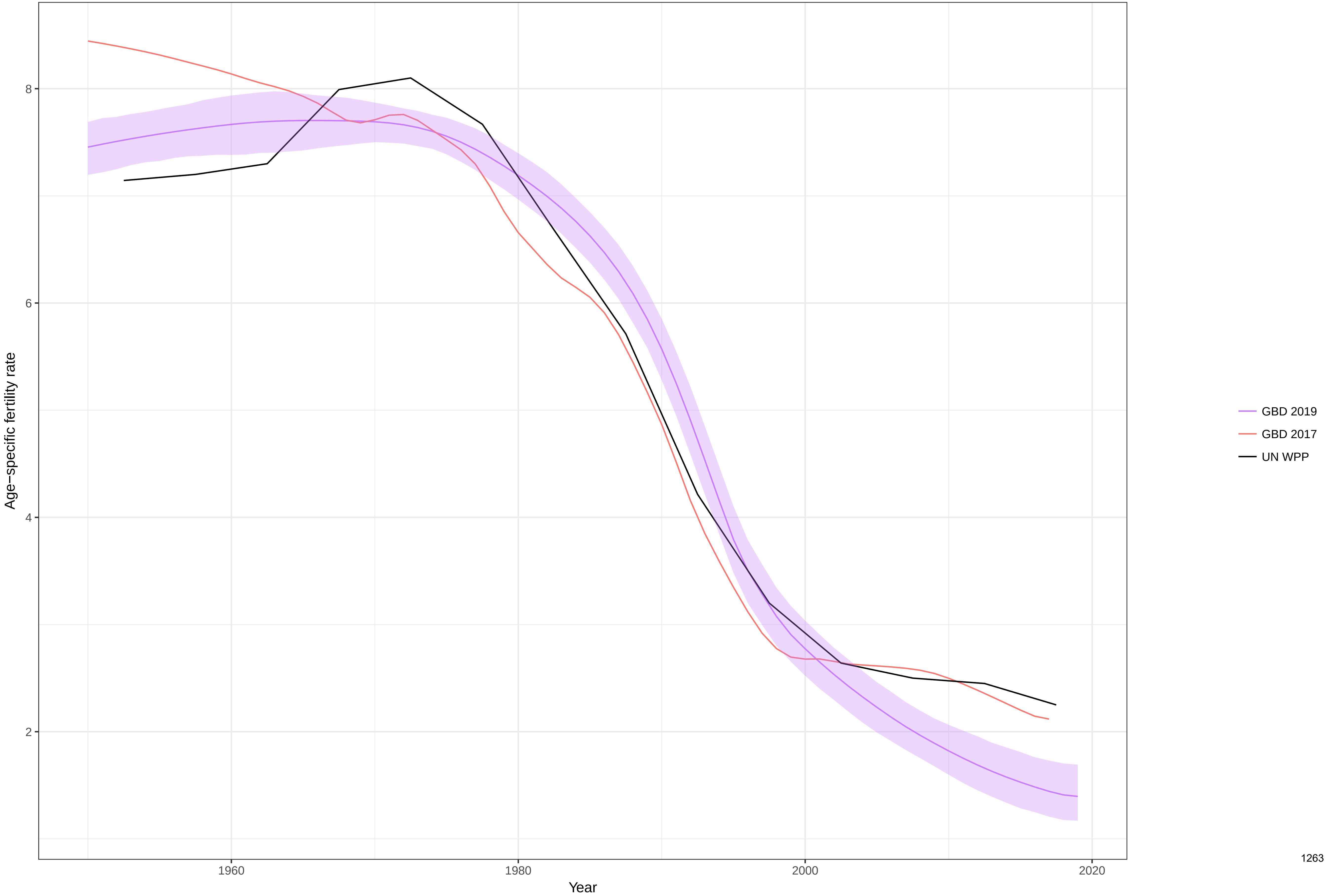

Age−specific fertility fitted trend lines, 1950−2019: Saint Lucia, Ages: 10−14
Data points enclosed with a diamond are reference sources

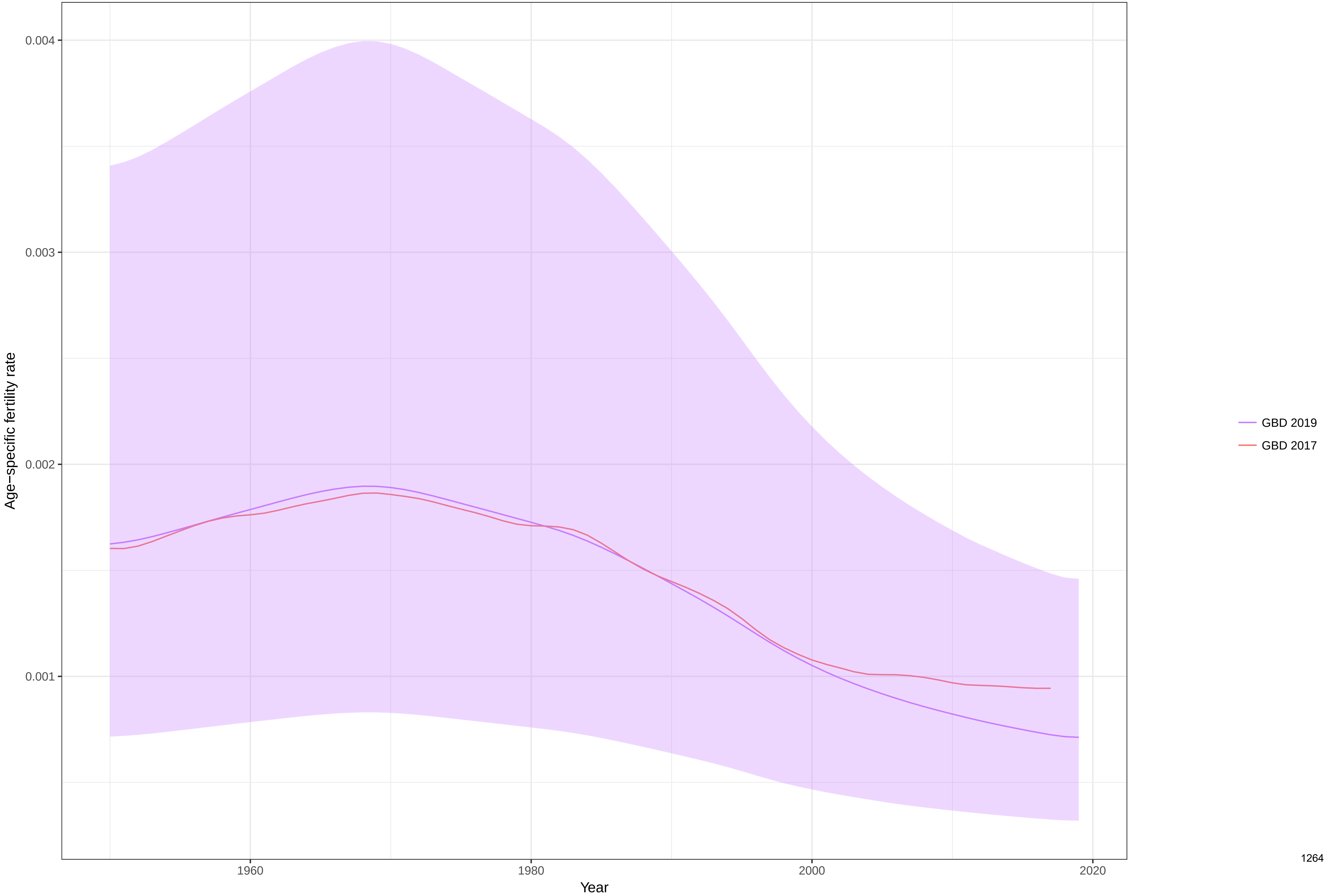

Age−specific fertility fitted trend lines, 1950−2019: Saint Lucia, Ages: 15−19
Data points enclosed with a diamond are reference sources

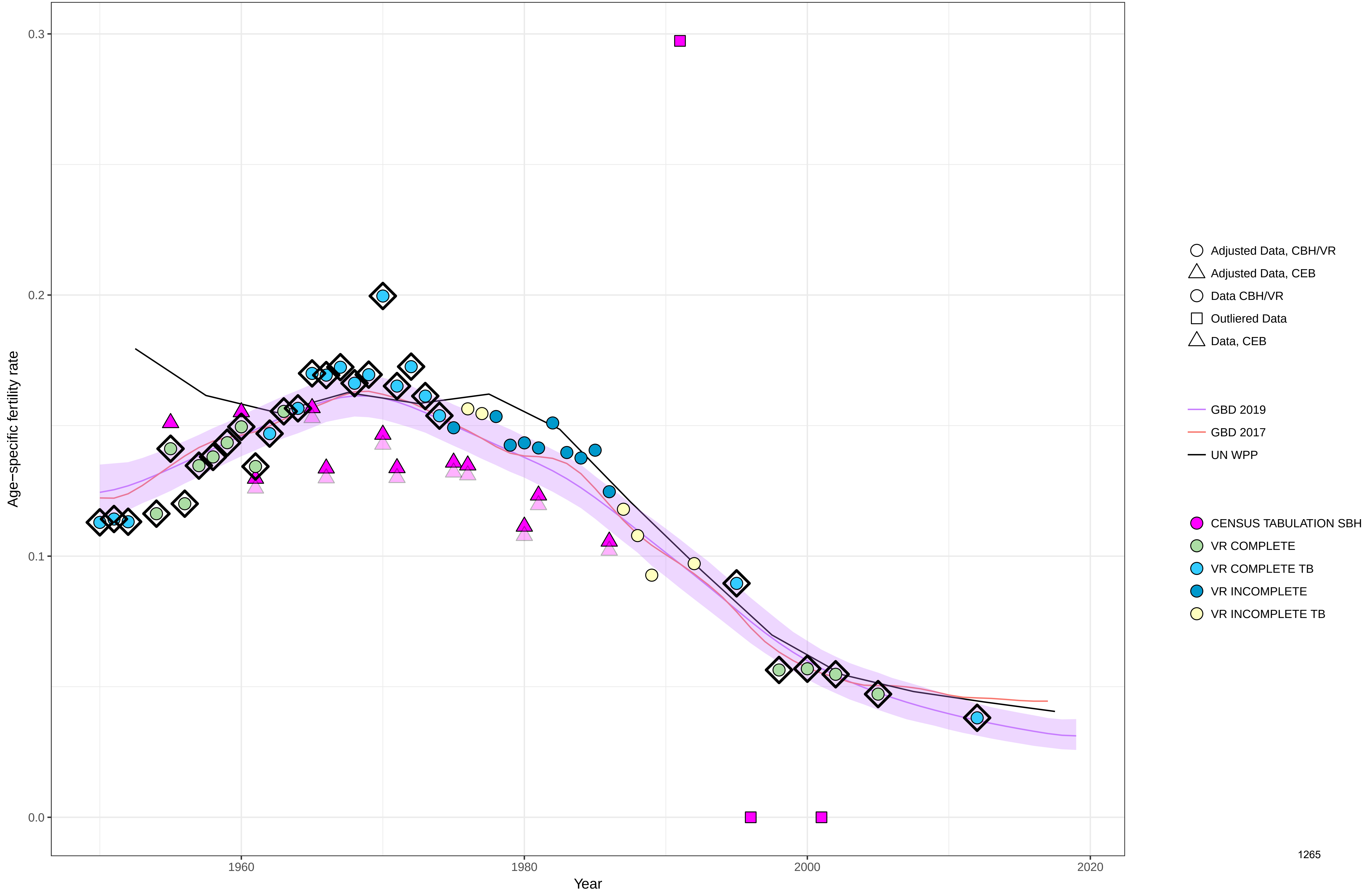

Age−specific fertility fitted trend lines, 1950−2019: Saint Lucia, Ages: 20−24
Data points enclosed with a diamond are reference sources

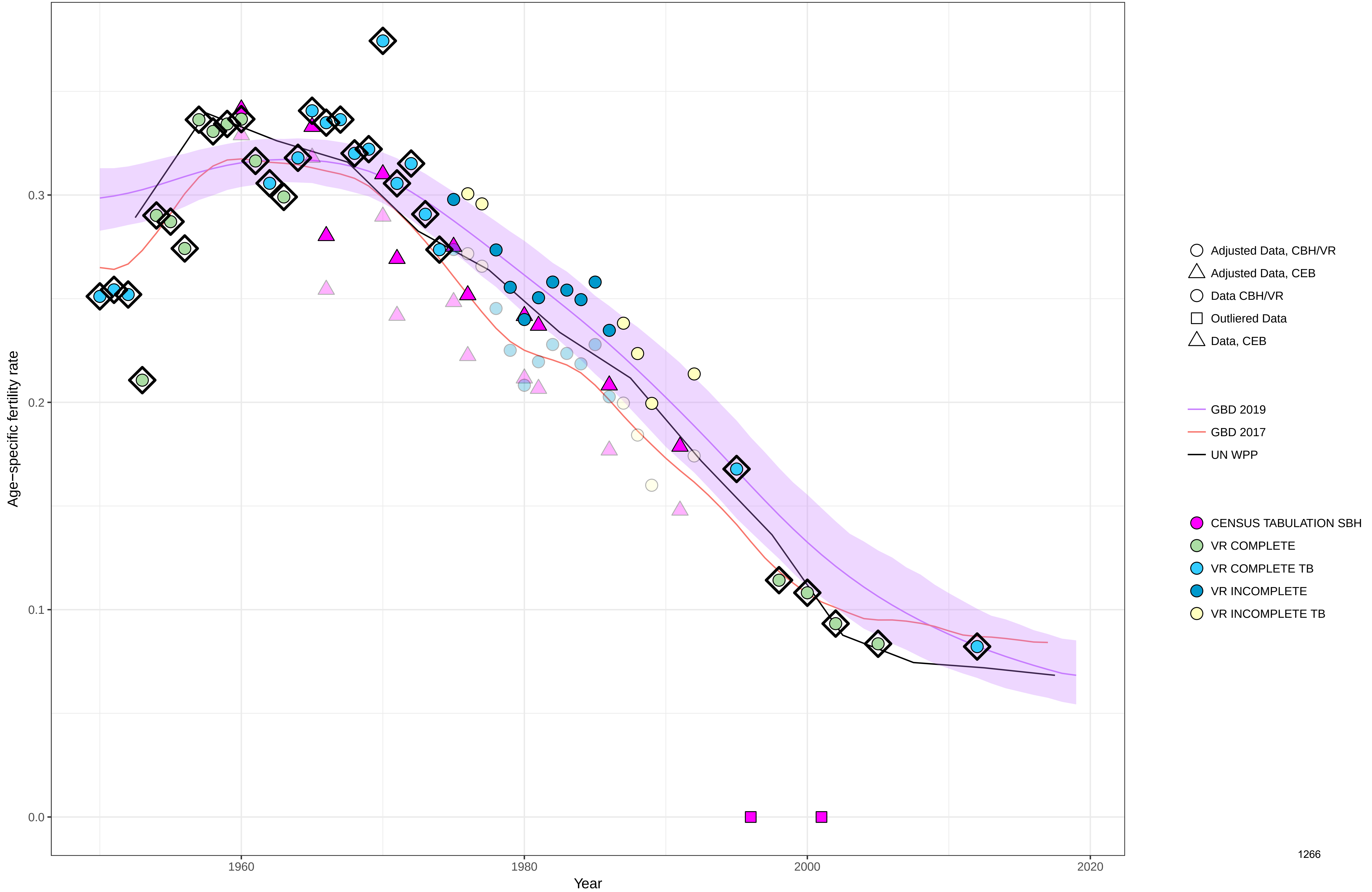

Age−specific fertility fitted trend lines, 1950−2019: Saint Lucia, Ages: 25−29
Data points enclosed with a diamond are reference sources

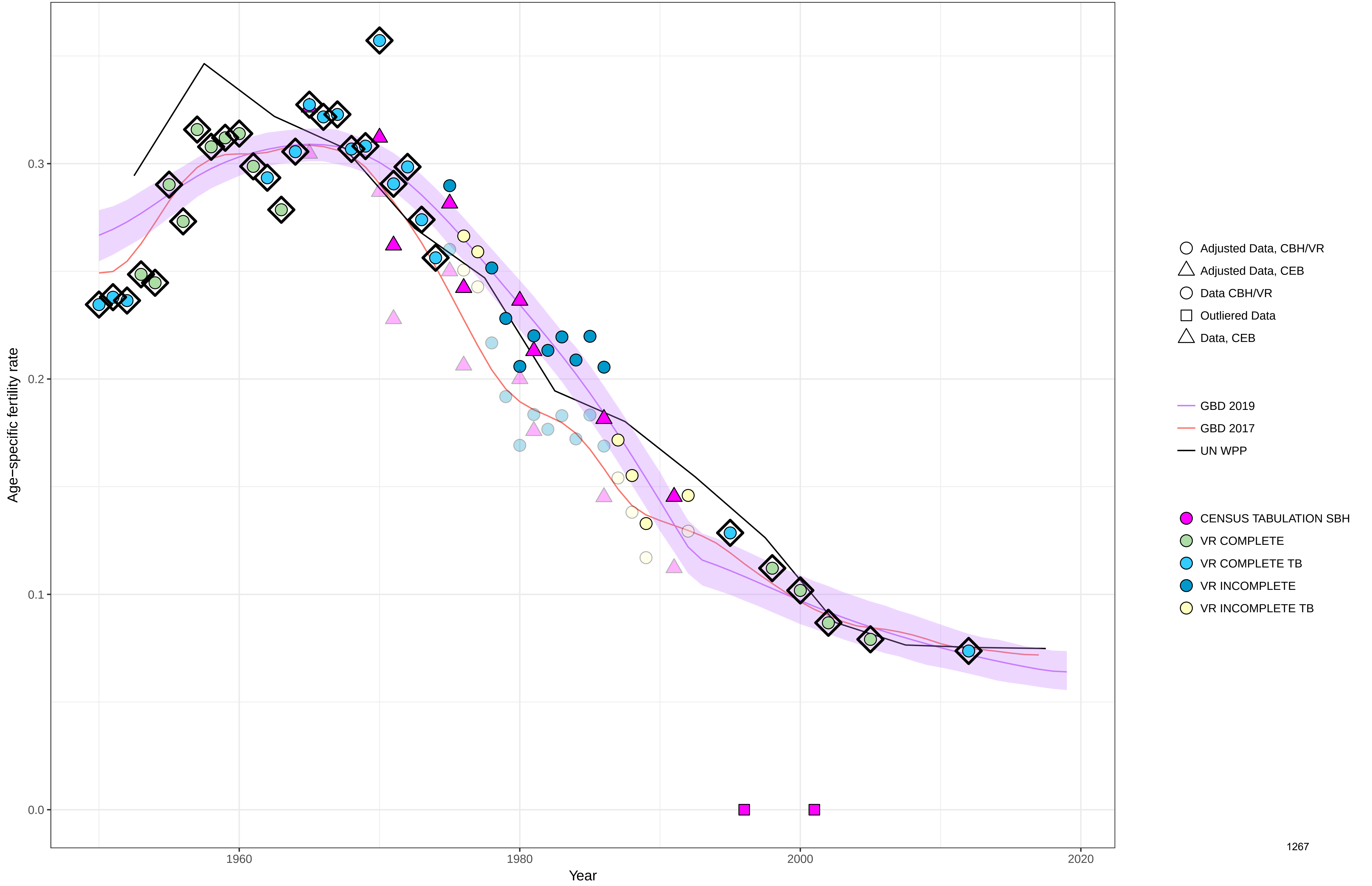

Age−specific fertility fitted trend lines, 1950−2019: Saint Lucia, Ages: 30−34
Data points enclosed with a diamond are reference sources

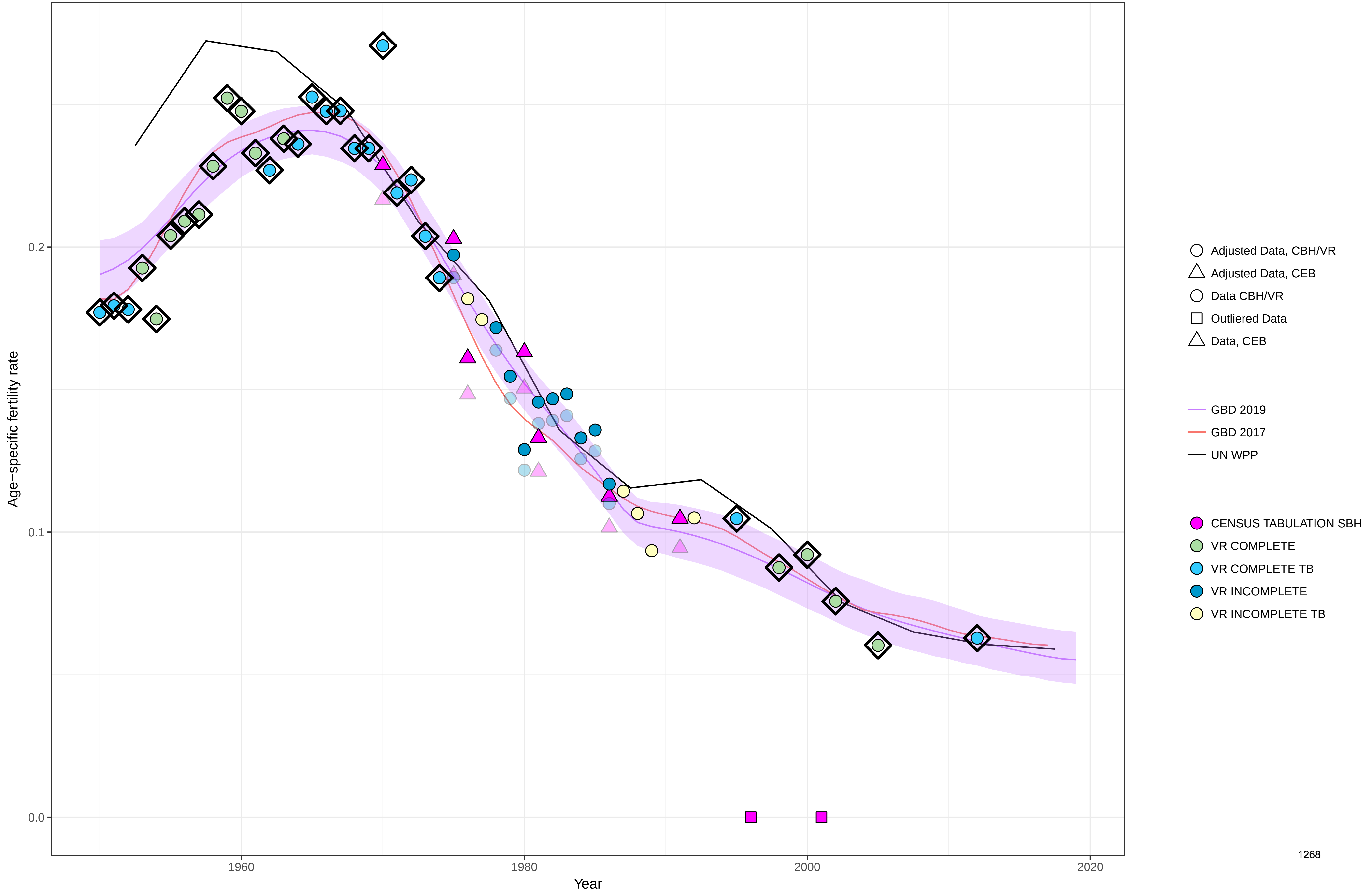

Age−specific fertility fitted trend lines, 1950−2019: Saint Lucia, Ages: 35−39
Data points enclosed with a diamond are reference sources

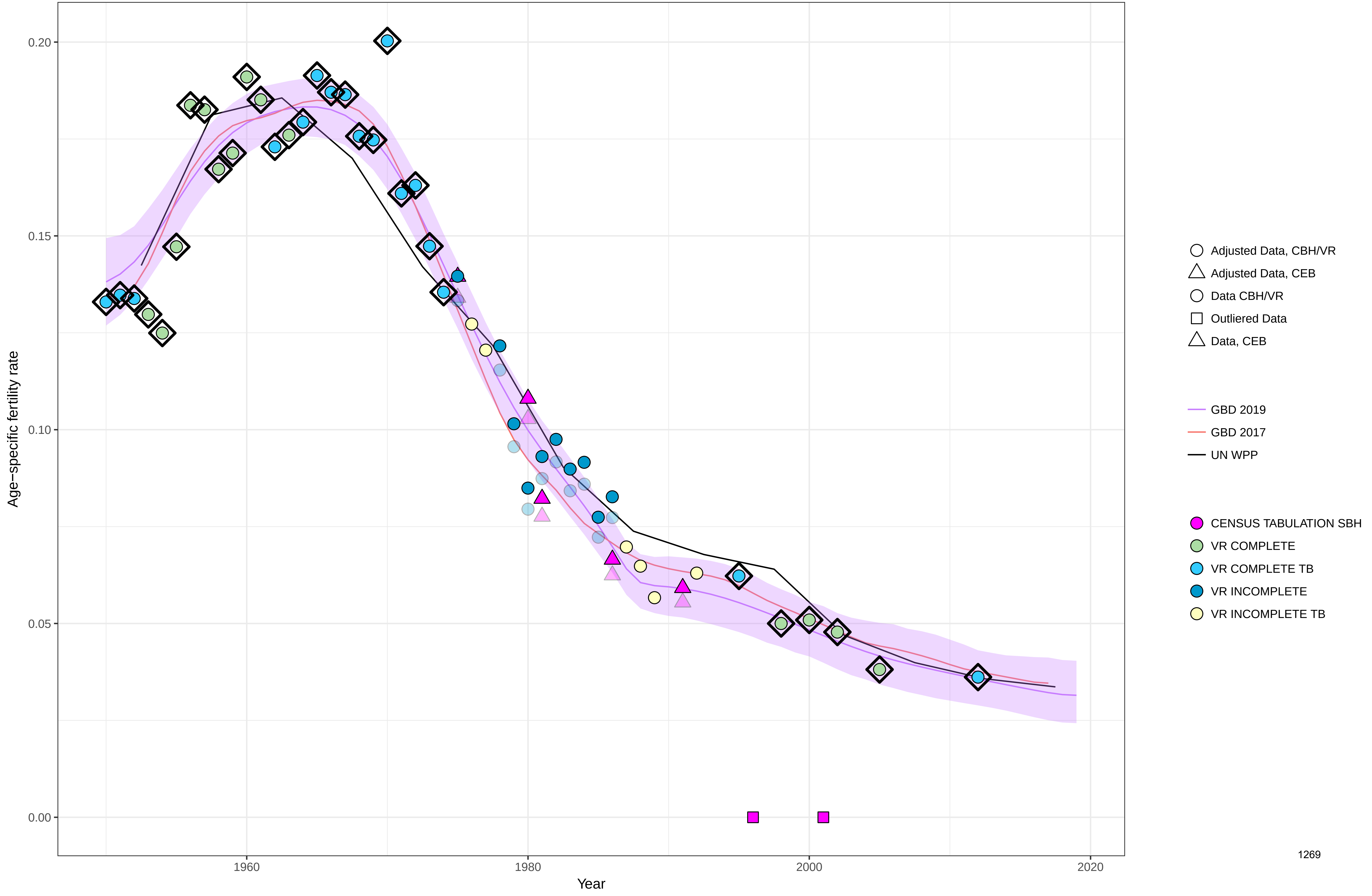

Age−specific fertility fitted trend lines, 1950−2019: Saint Lucia, Ages: 40−44
Data points enclosed with a diamond are reference sources

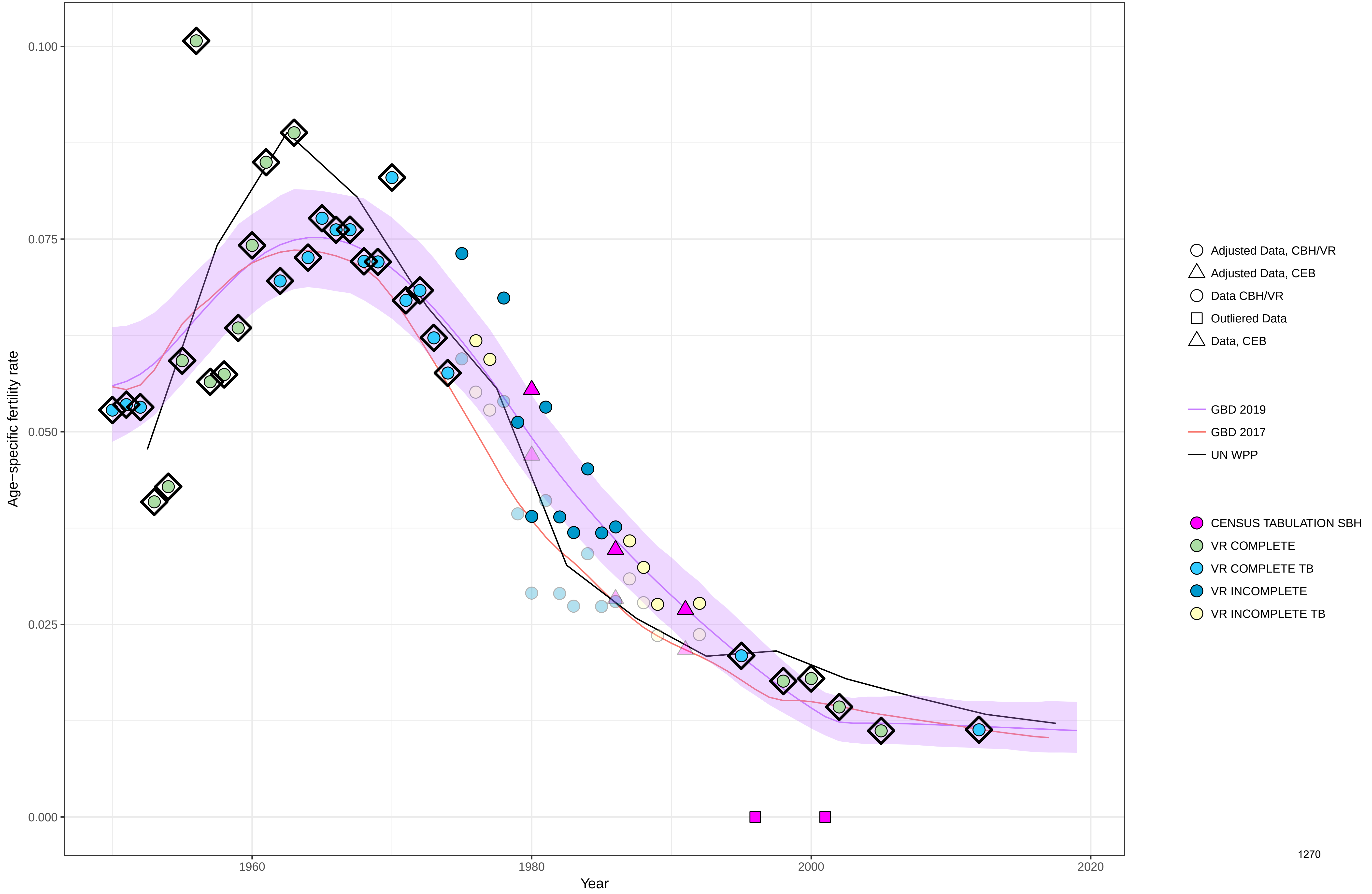

Age−specific fertility fitted trend lines, 1950−2019: Saint Lucia, Ages: 45−49
Data points enclosed with a diamond are reference sources

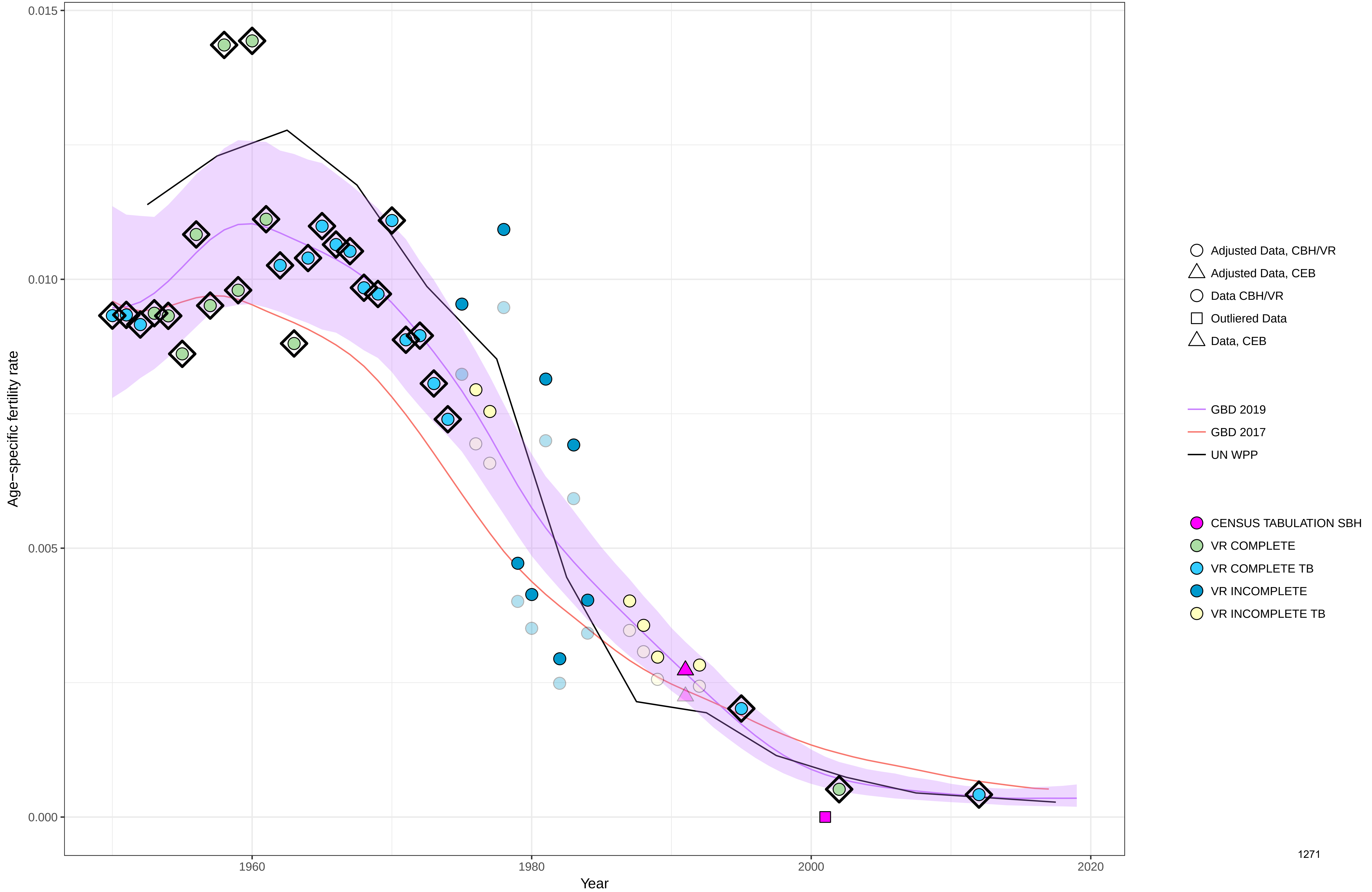

Age−specific fertility fitted trend lines, 1950−2019: Saint Lucia, Ages: 50−54
Data points enclosed with a diamond are reference sources

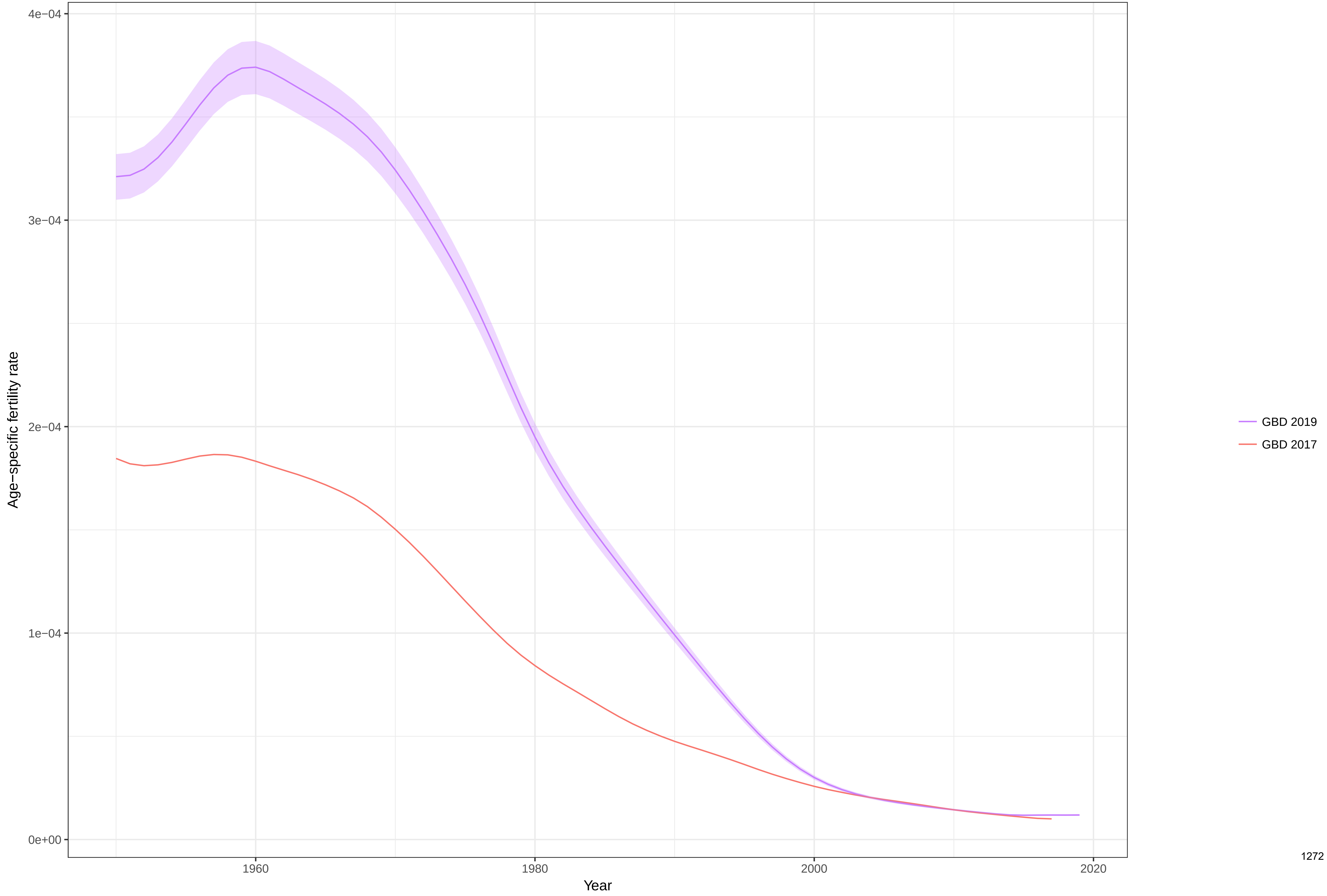

Total fertility rate fitted trend lines, 1950−2019: Saint Lucia TFR
Data points enclosed with a diamond are reference sources

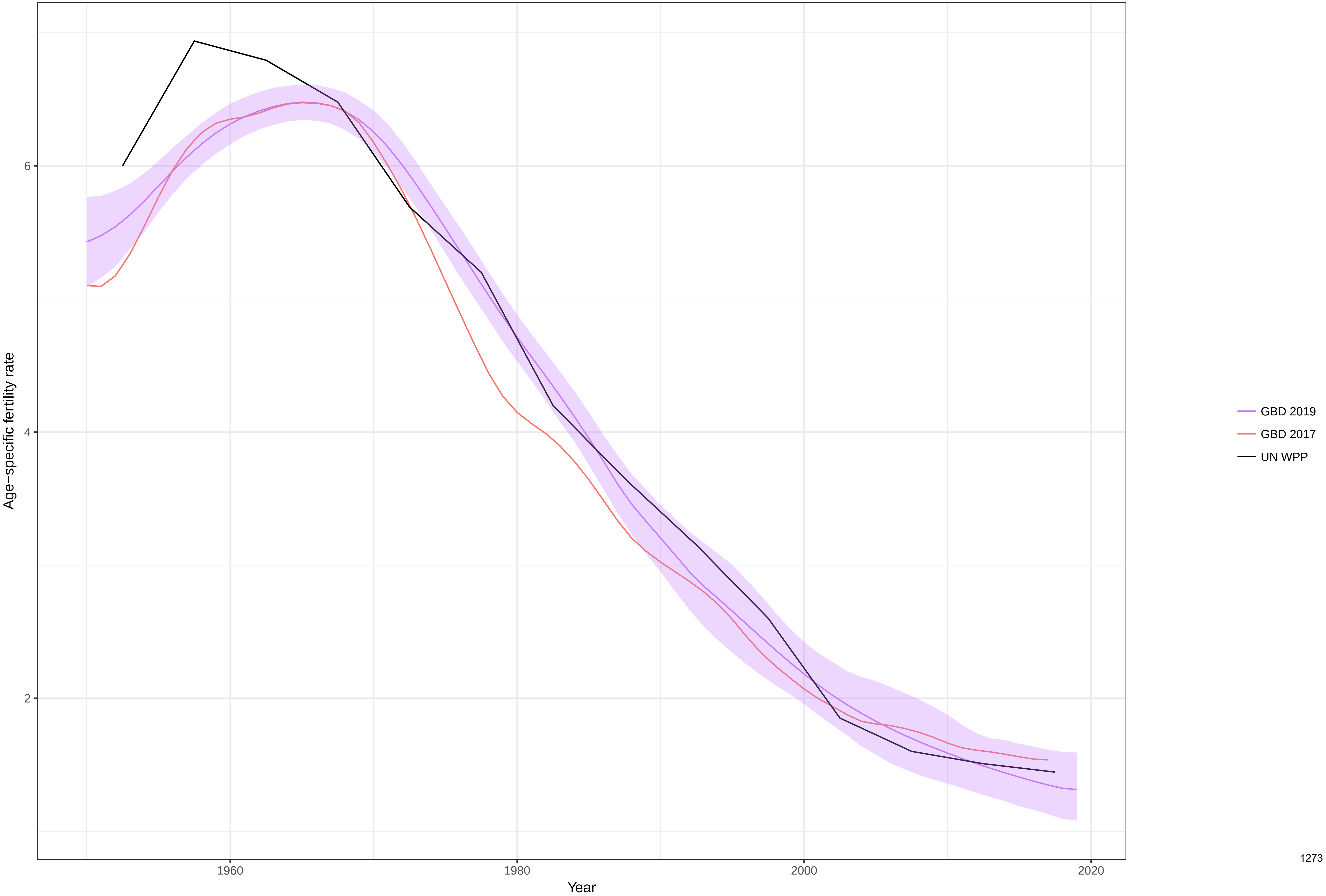

Age−specific fertility fitted trend lines, 1950−2019: Sri Lanka, Ages: 10−14
Data points enclosed with a diamond are reference sources

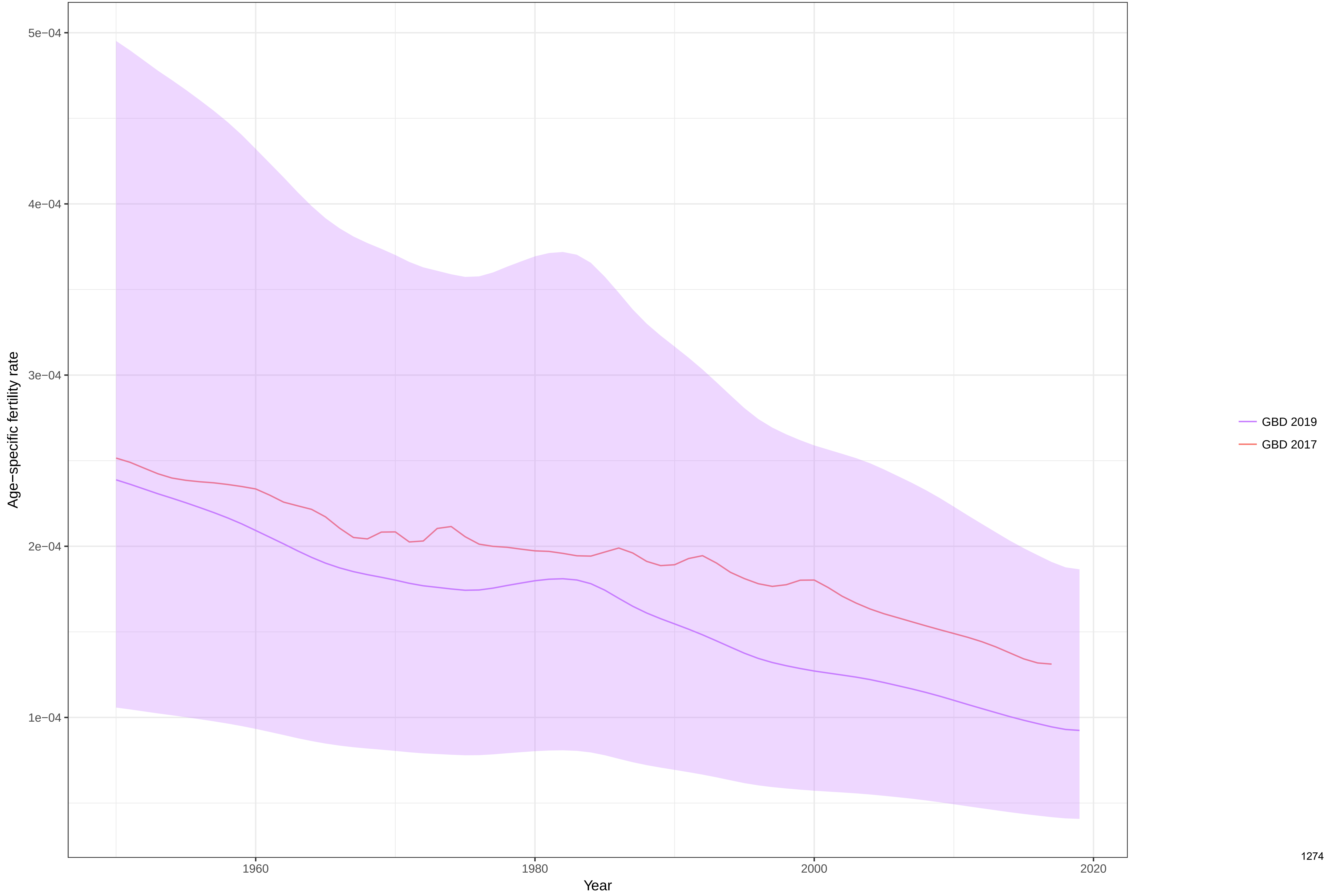

Age−specific fertility fitted trend lines, 1950−2019: Sri Lanka, Ages: 15−19
Data points enclosed with a diamond are reference sources

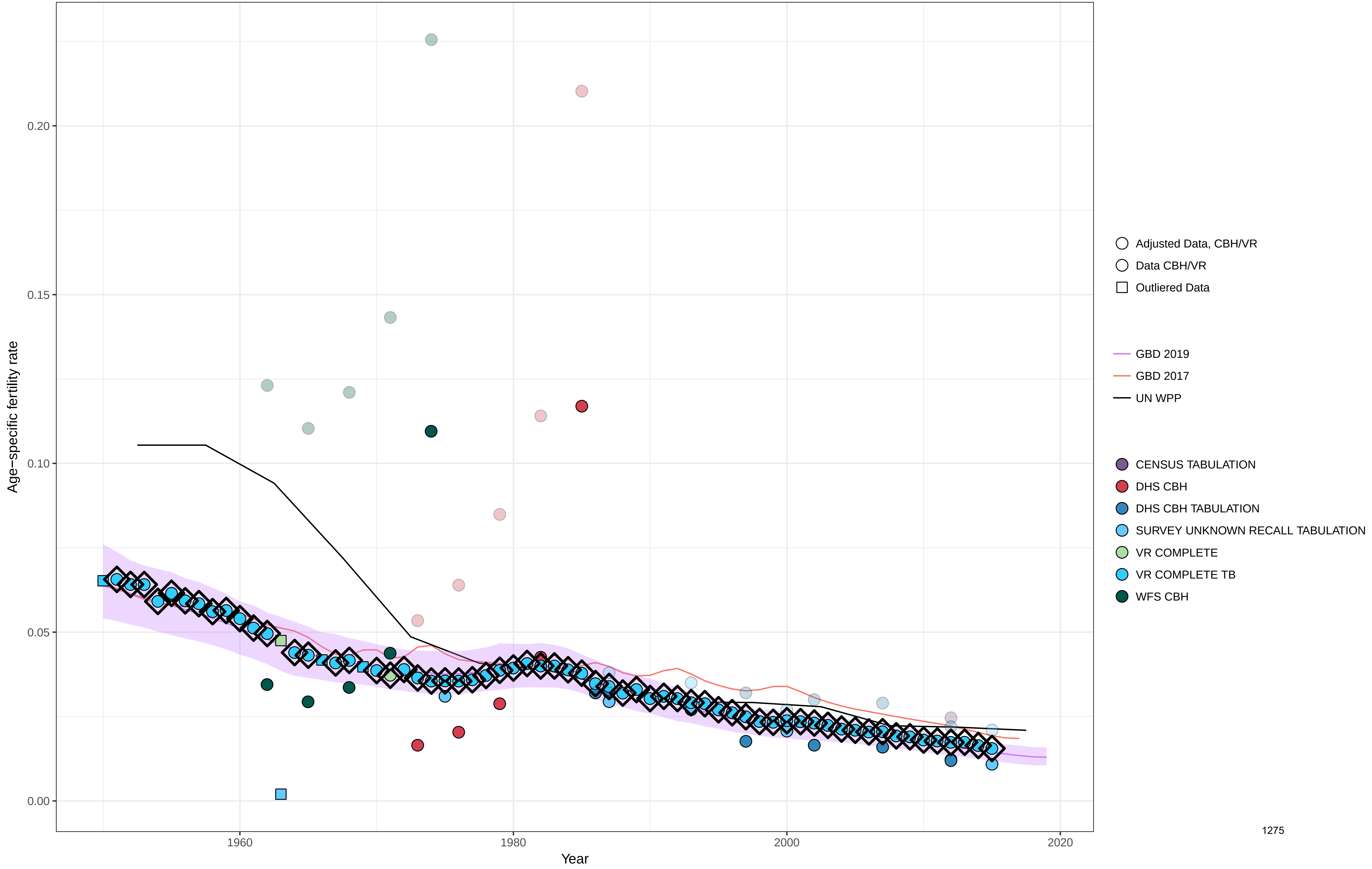

Age−specific fertility fitted trend lines, 1950−2019: Sri Lanka, Ages: 20−24
Data points enclosed with a diamond are reference sources

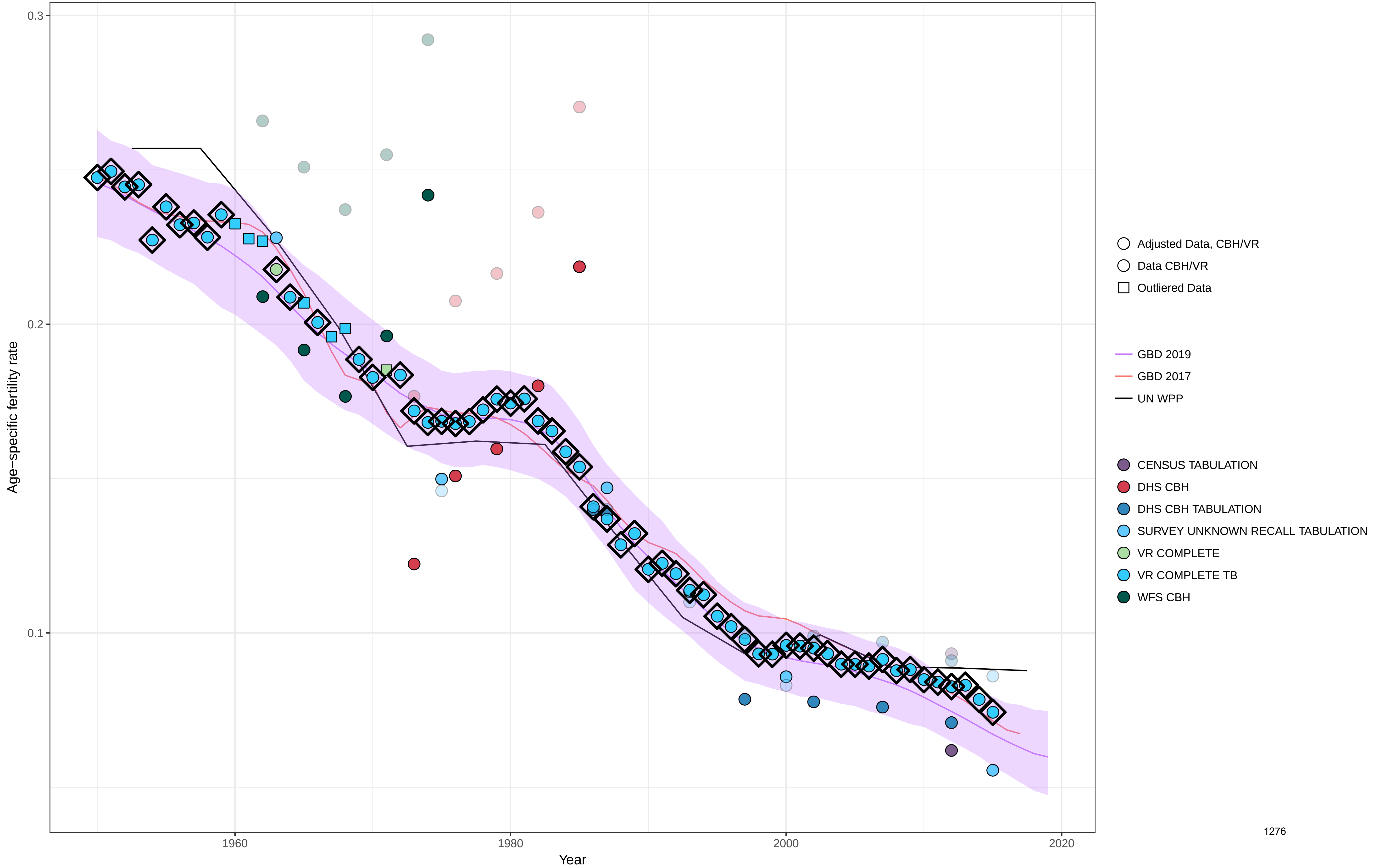

Age−specific fertility fitted trend lines, 1950−2019: Sri Lanka, Ages: 25−29
Data points enclosed with a diamond are reference sources

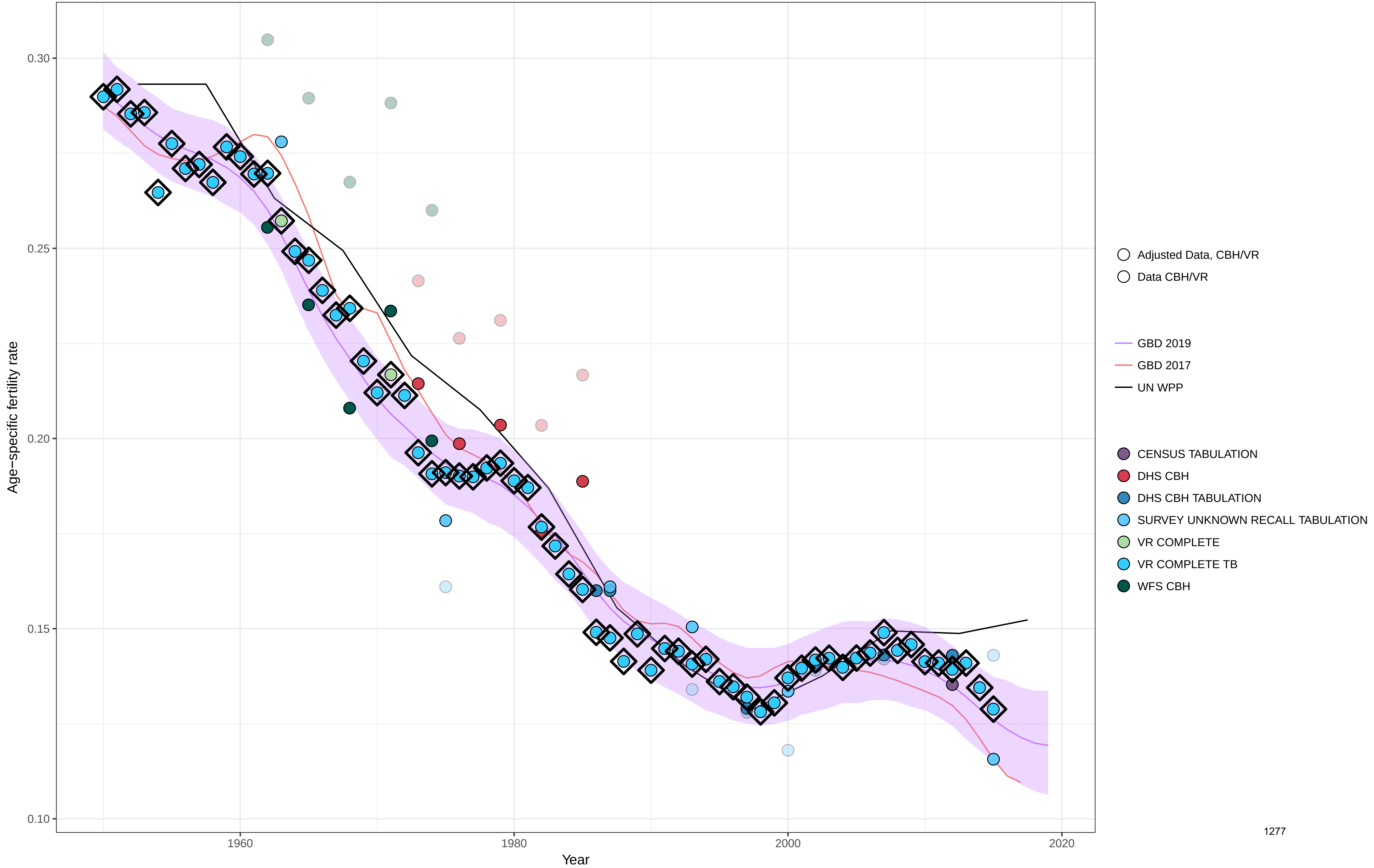

Age−specific fertility fitted trend lines, 1950−2019: Sri Lanka, Ages: 30−34
Data points enclosed with a diamond are reference sources

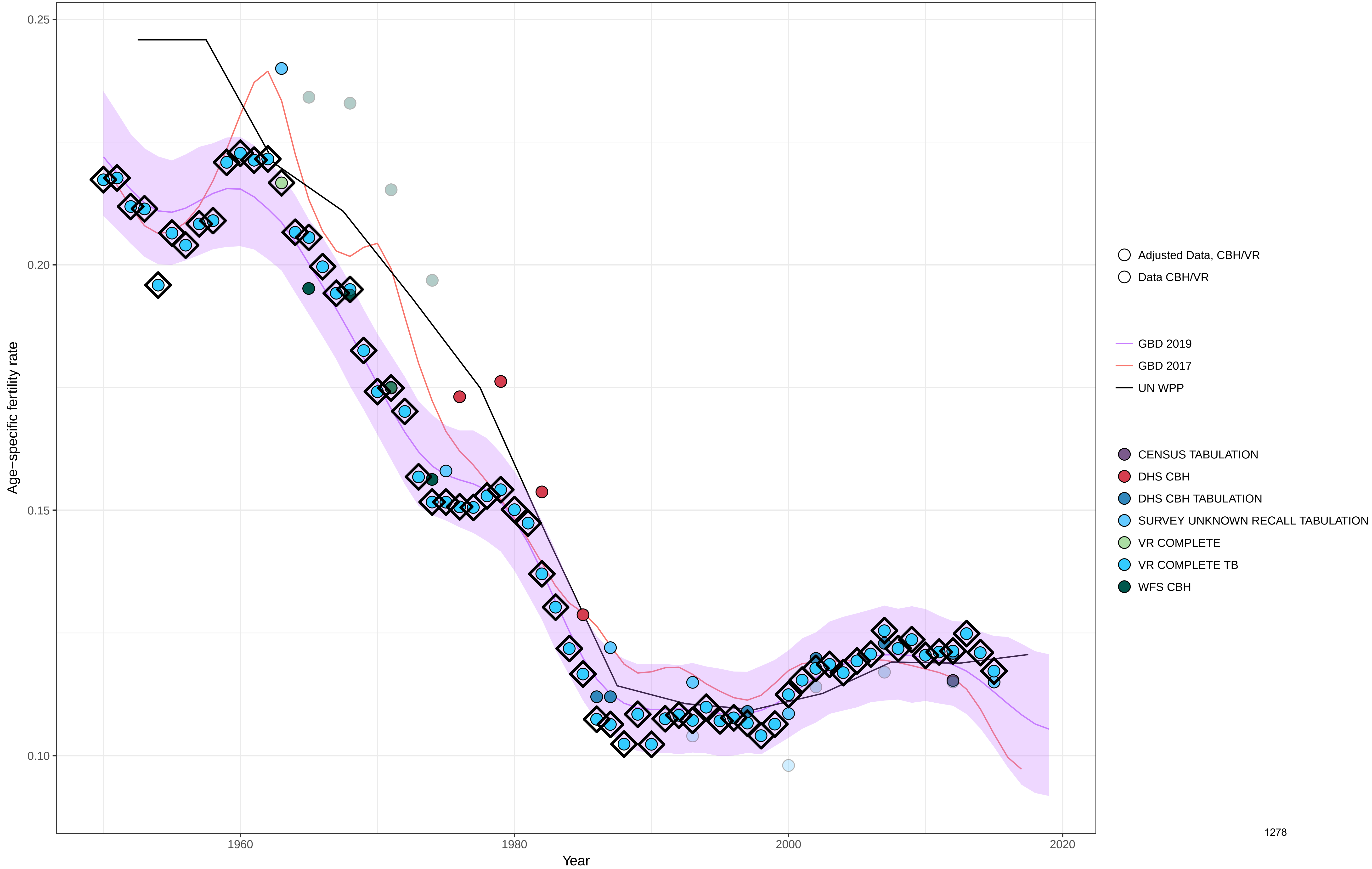

Age−specific fertility fitted trend lines, 1950−2019: Sri Lanka, Ages: 35−39
Data points enclosed with a diamond are reference sources

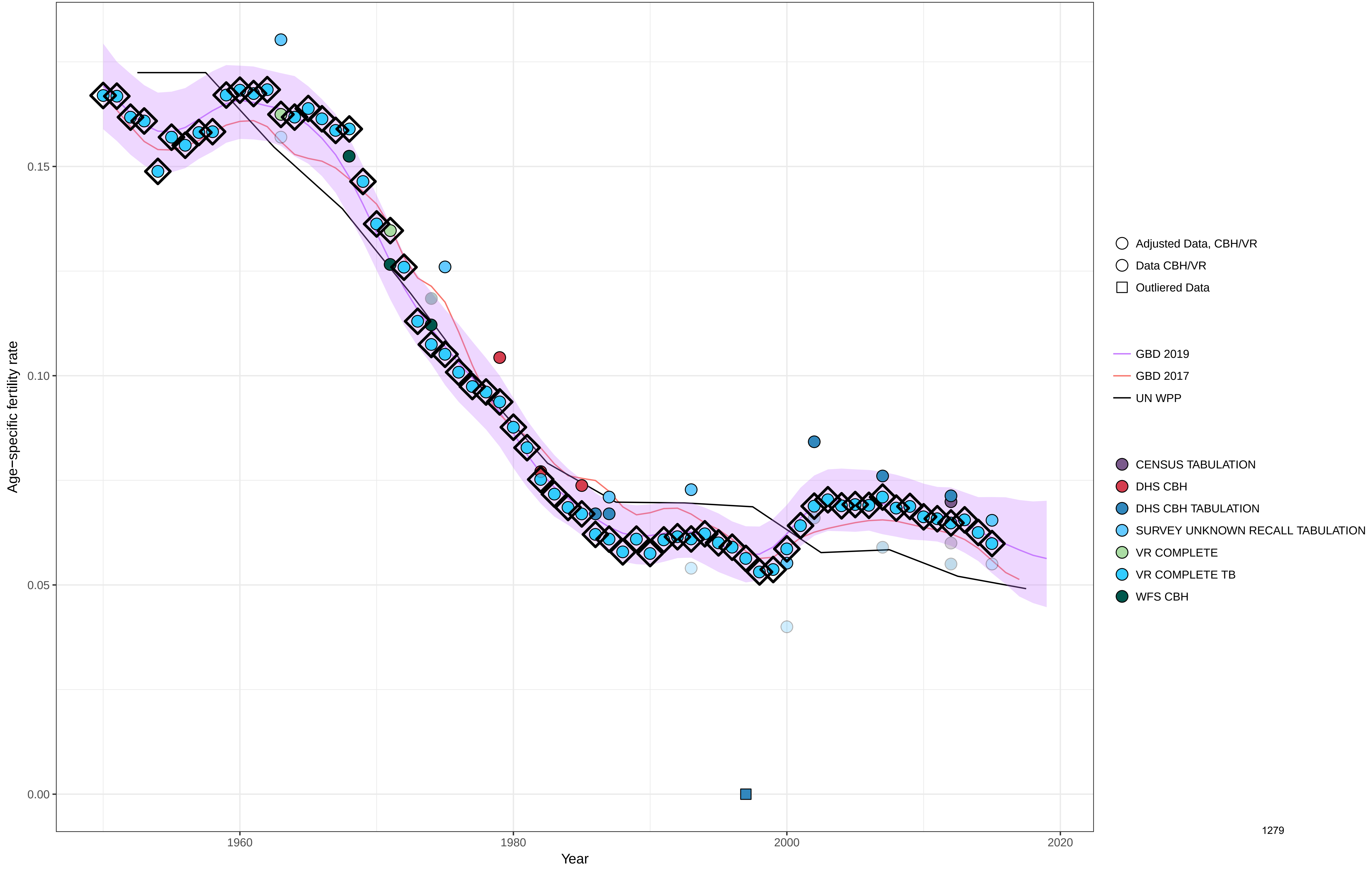

Age−specific fertility fitted trend lines, 1950−2019: Sri Lanka, Ages: 40−44
Data points enclosed with a diamond are reference sources

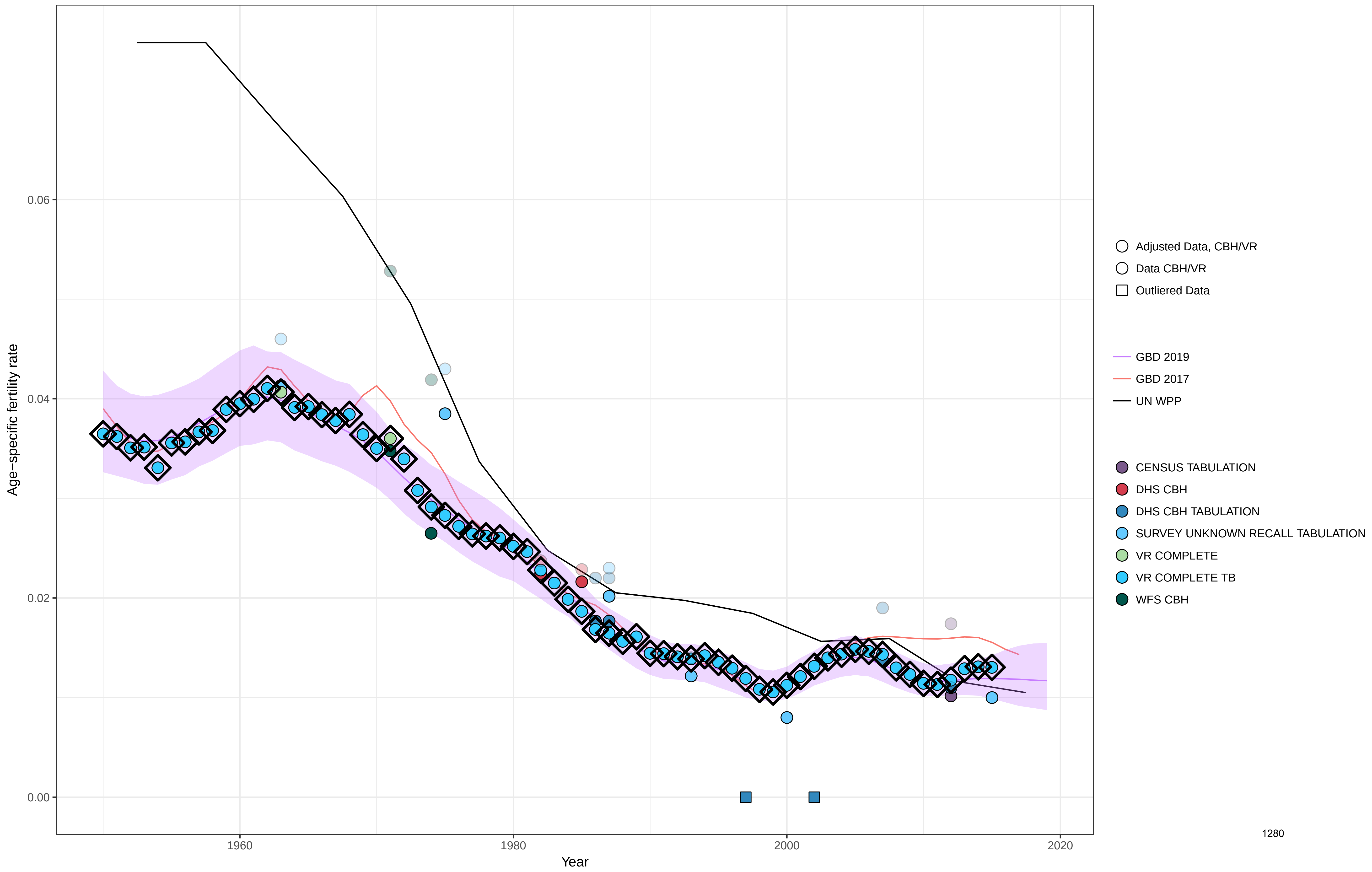

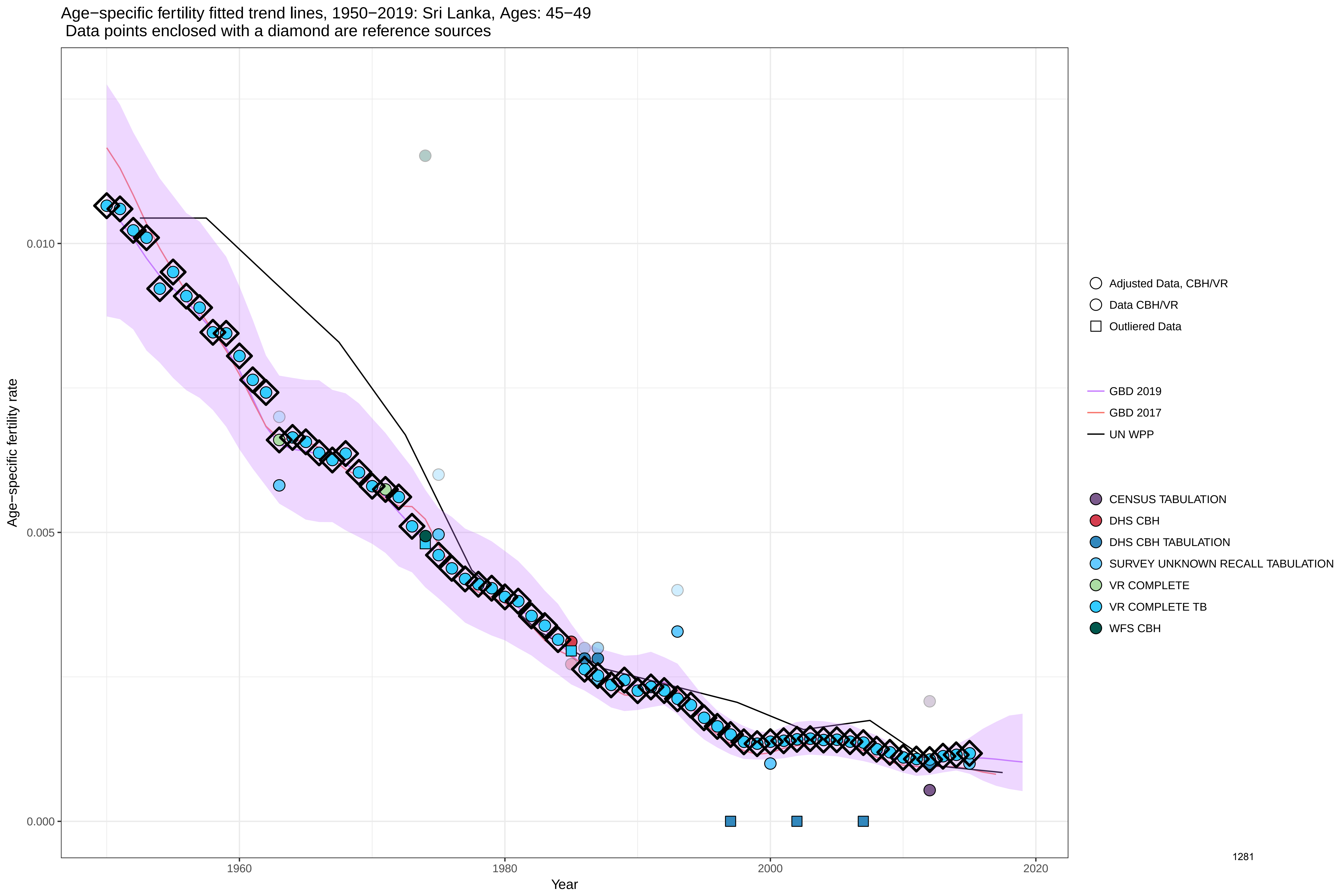
Age−specific fertility fitted trend lines, 1950−2019: Sri Lanka, Ages: 45−49
Data points enclosed with a diamond are reference sources
Age−specific fertility rate
0.000
0.005
0.010
Year
1960
1980
2000
2020
Adjusted Data, CBH/VR
Data CBH/VR
Outliered Data
GBD 2019
GBD 2017
UN WPP
CENSUS TABULATION
DHS CBH
DHS CBH TABULATION
SURVEY UNKNOWN RECALL TABULATION
VR COMPLETE
VR COMPLETE TB
WFS CBH

Age−specific fertility fitted trend lines, 1950−2019: Sri Lanka, Ages: 50−54
Data points enclosed with a diamond are reference sources

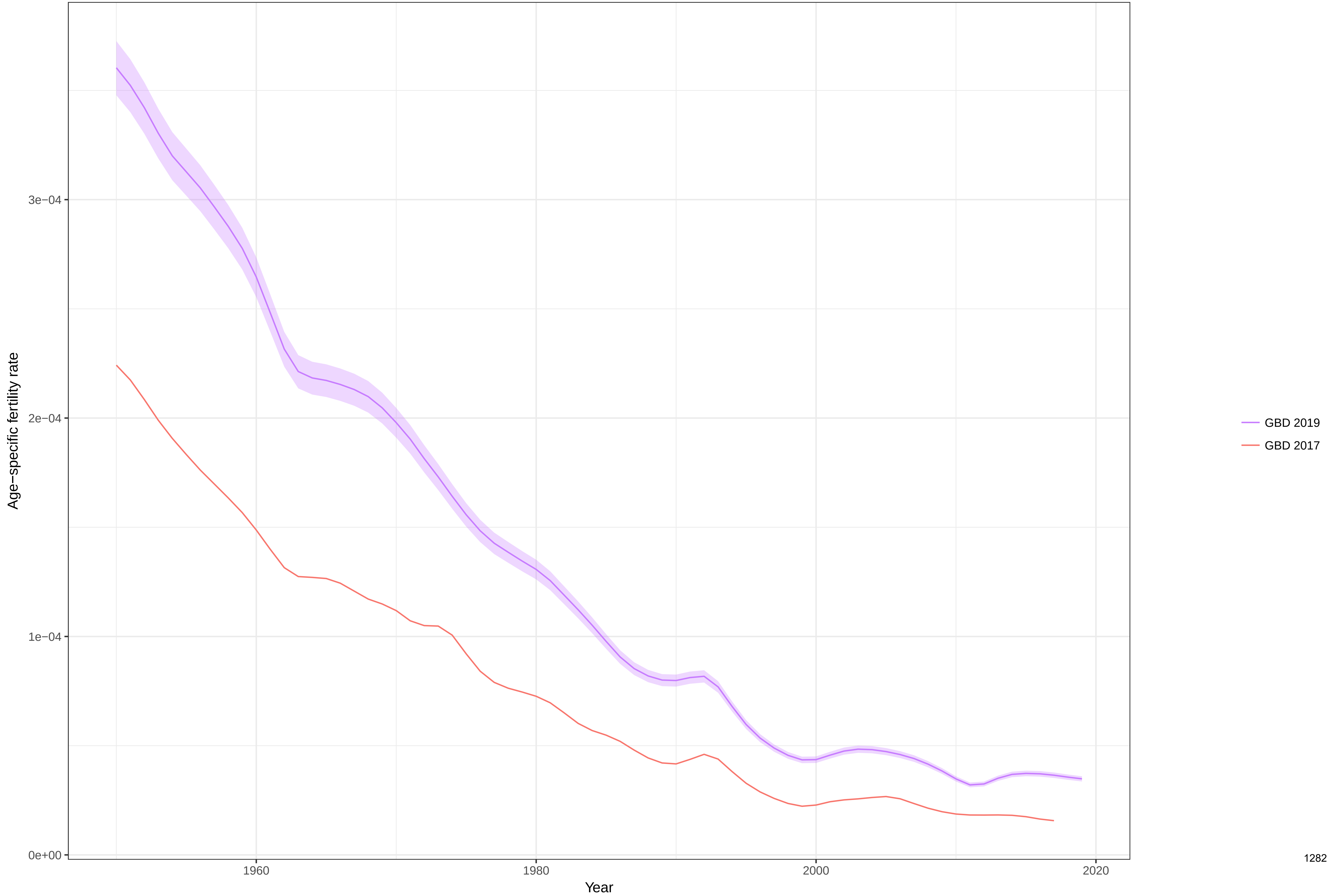

Total fertility rate fitted trend lines, 1950−2019: Sri Lanka TFR
Data points enclosed with a diamond are reference sources

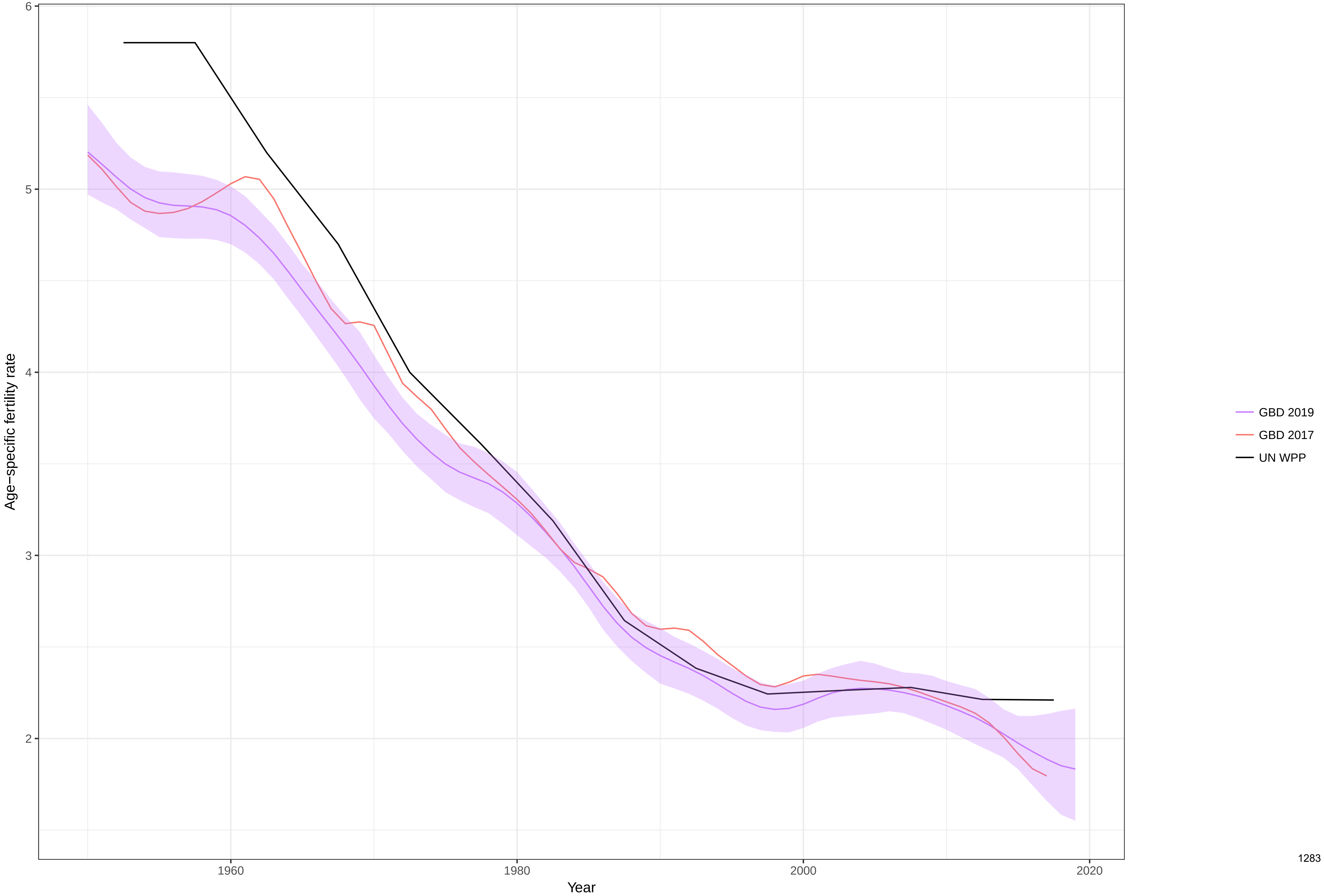

Age−specific fertility fitted trend lines, 1950−2019: Lesotho, Ages: 10−14
Data points enclosed with a diamond are reference sources

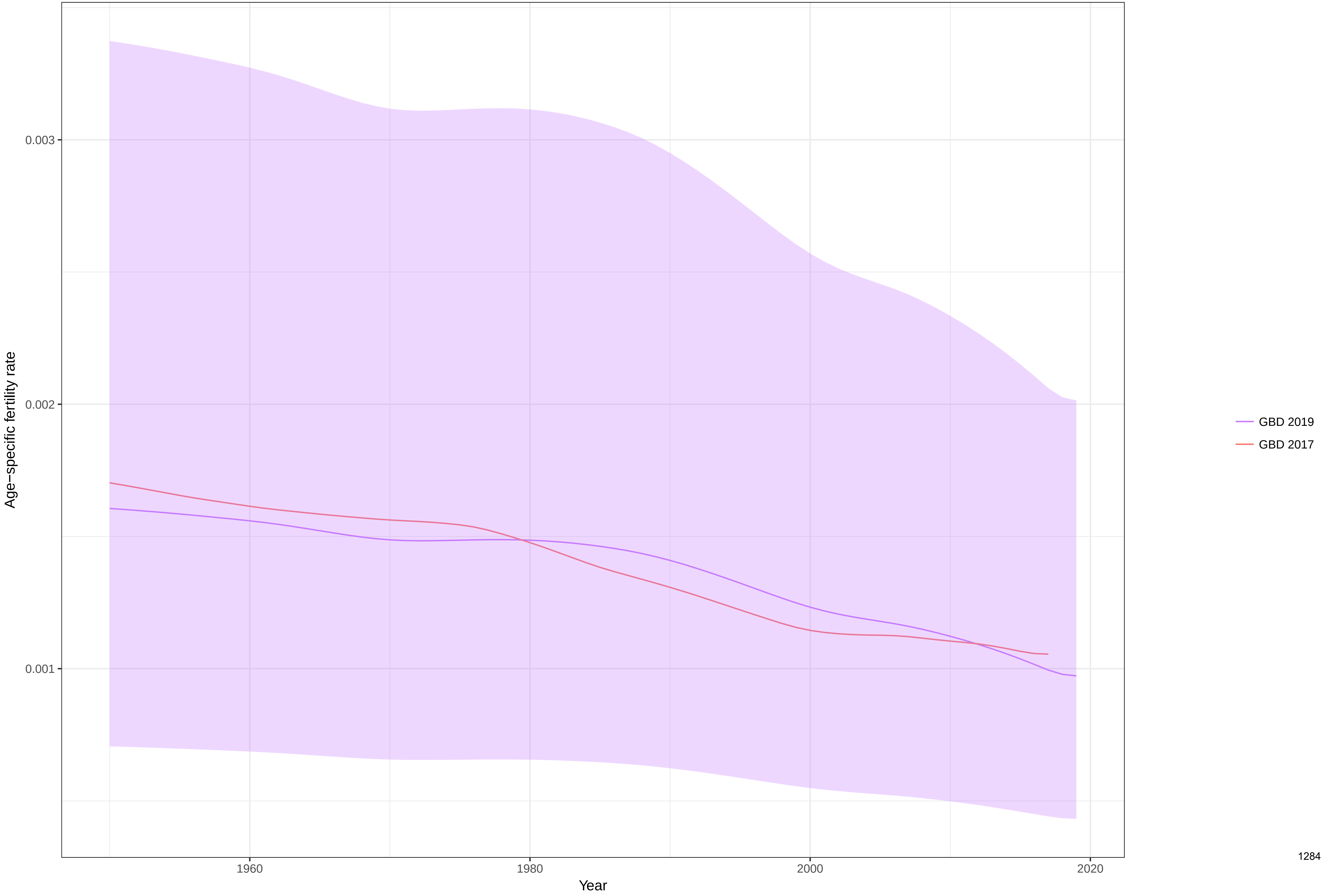

Age−specific fertility fitted trend lines, 1950−2019: Lesotho, Ages: 15−19
Data points enclosed with a diamond are reference sources

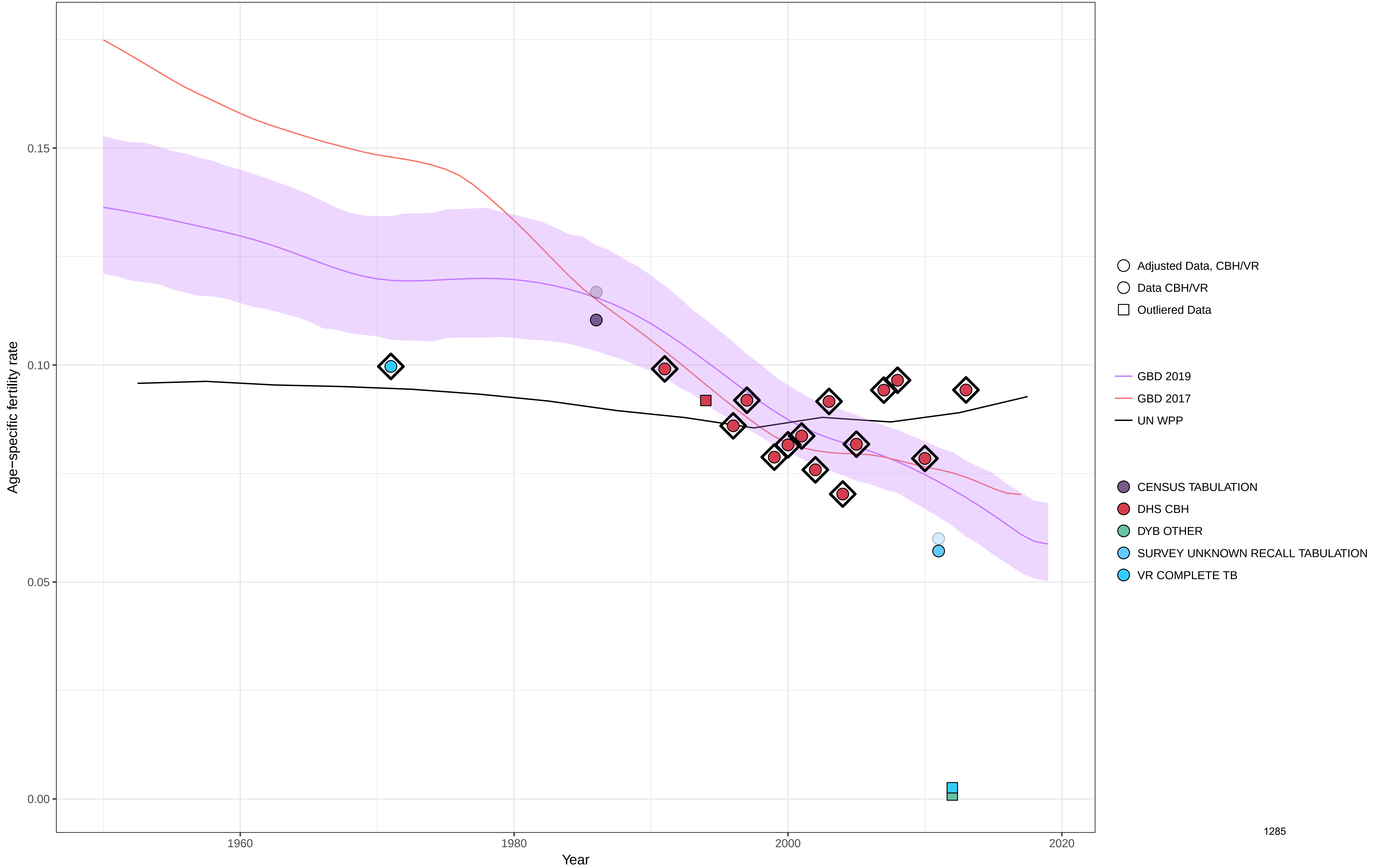

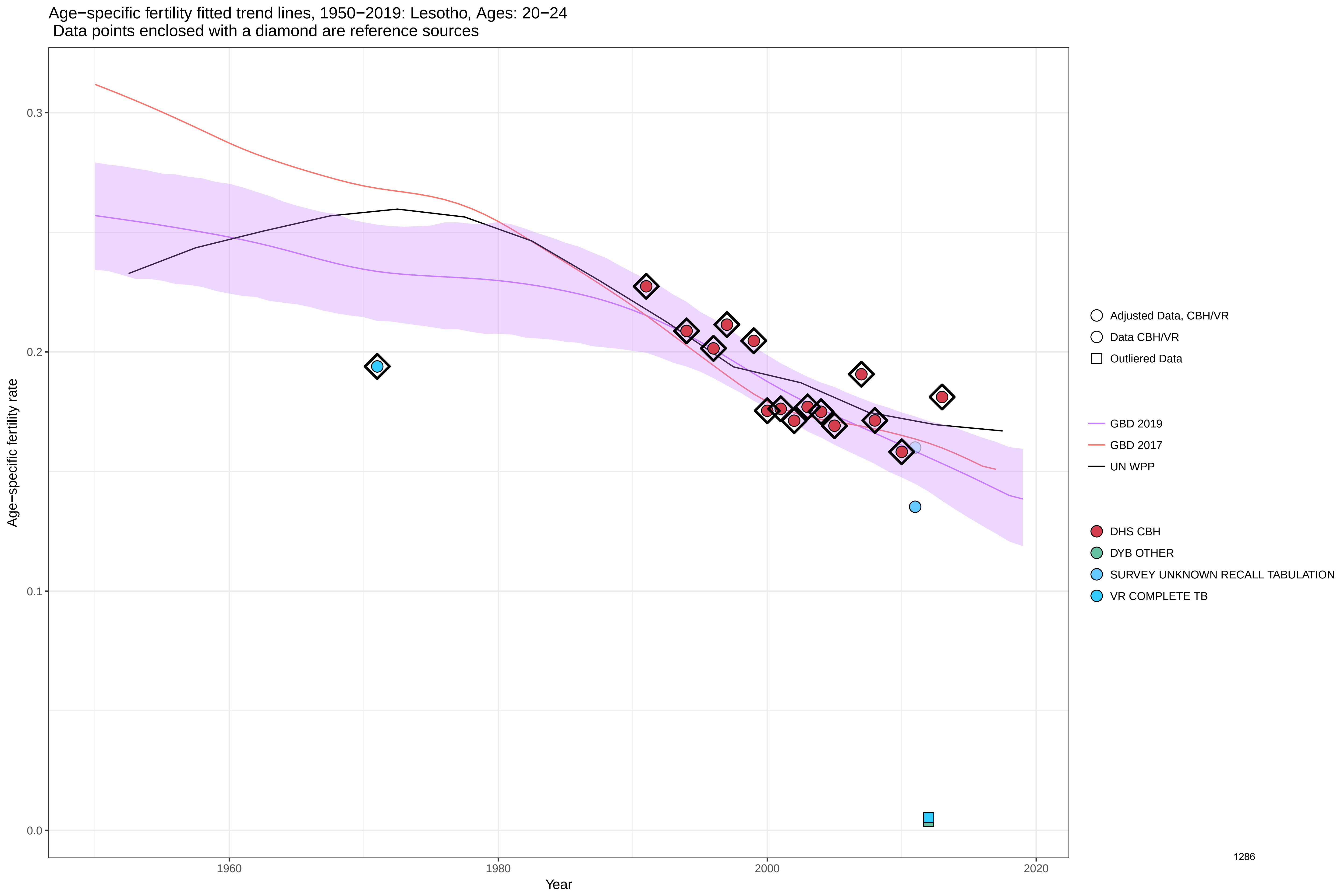

Age−specific fertility fitted trend lines, 1950−2019: Lesotho, Ages: 20−24
Data points enclosed with a diamond are reference sources
0.3
0.2
0.1
0.0
Age−specific fertility rate
1960
1980
2000
2020
Year
Adjusted Data, CBH/VR
Data CBH/VR
Outliered Data
GBD 2019
GBD 2017
UN WPP
DHS CBH
DYB OTHER
SURVEY UNKNOWN RECALL TABULATION
VR COMPLETE TB

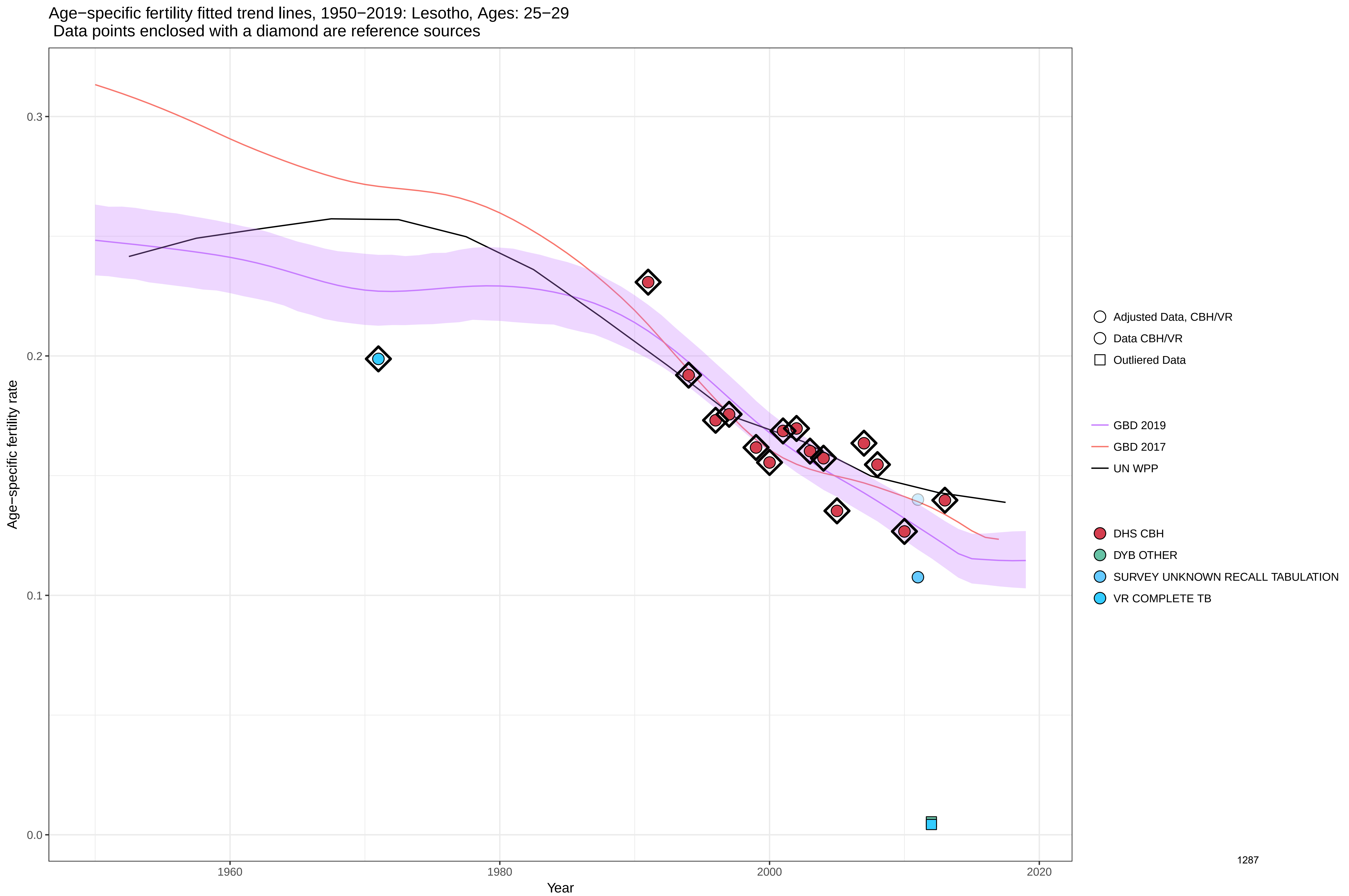

Age−specific fertility fitted trend lines, 1950−2019: Lesotho, Ages: 25−29
Data points enclosed with a diamond are reference sources
Age−specific fertility rate
0.0
0.1
0.2
0.3
Year
1960
1980
2000
2020
Adjusted Data, CBH/VR
Data CBH/VR
Outliered Data
GBD 2019
GBD 2017
UN WPP
DHS CBH
DYB OTHER
SURVEY UNKNOWN RECALL TABULATION
VR COMPLETE TB

Age−specific fertility fitted trend lines, 1950−2019: Lesotho, Ages: 30−34
Data points enclosed with a diamond are reference sources

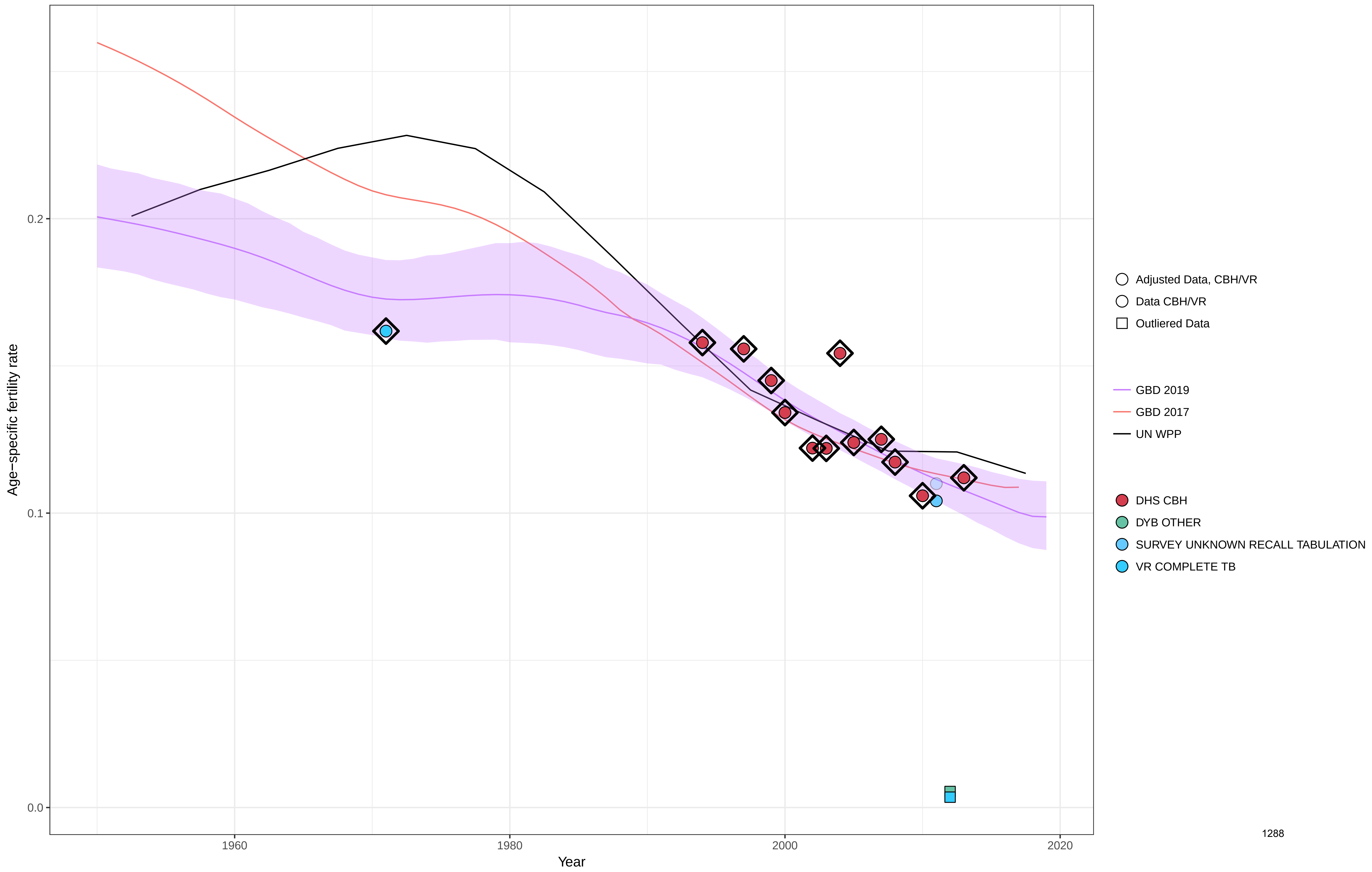

Age−specific fertility fitted trend lines, 1950−2019: Lesotho, Ages: 35−39
Data points enclosed with a diamond are reference sources

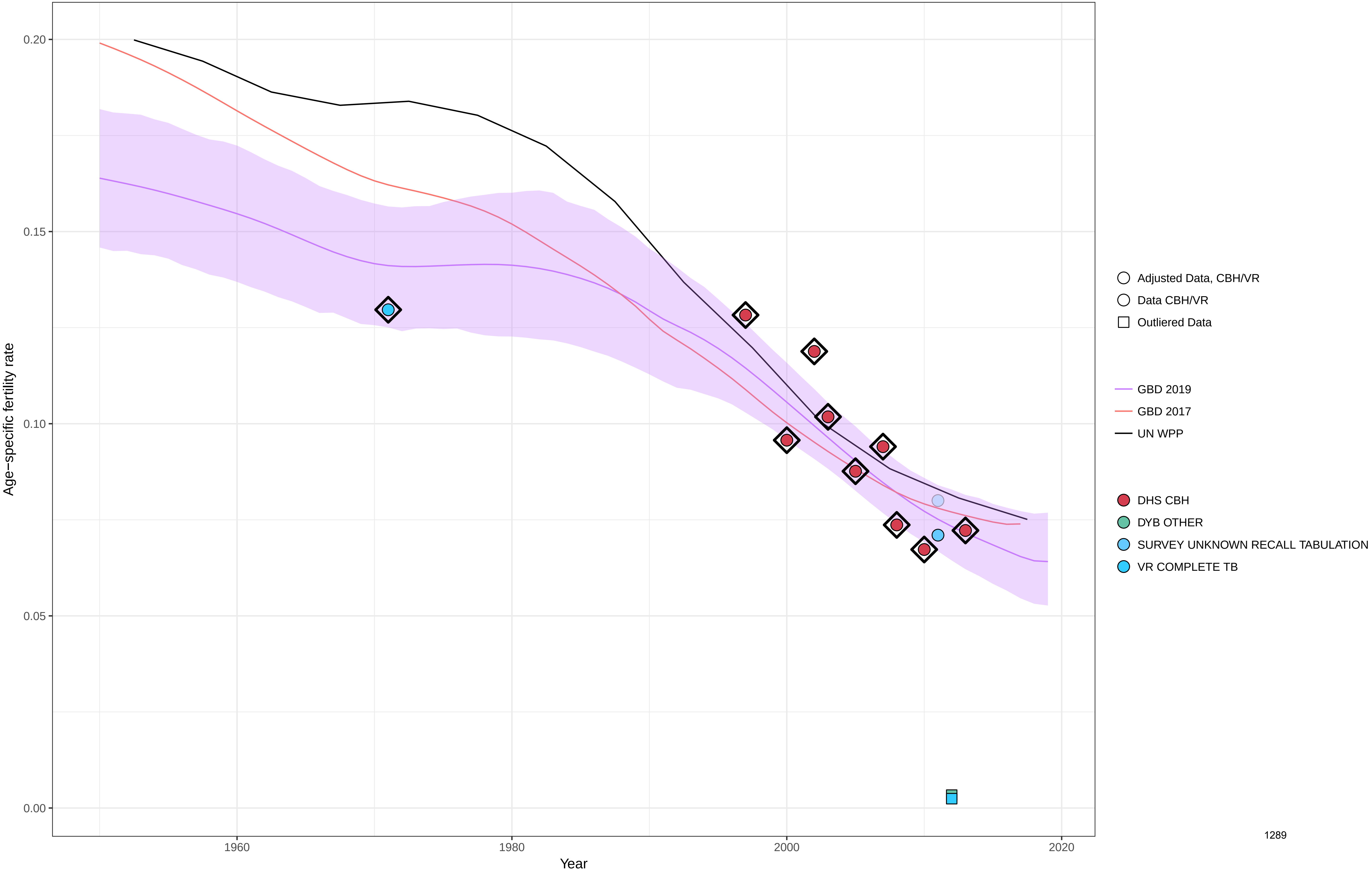

Age−specific fertility fitted trend lines, 1950−2019: Lesotho, Ages: 40−44
Data points enclosed with a diamond are reference sources

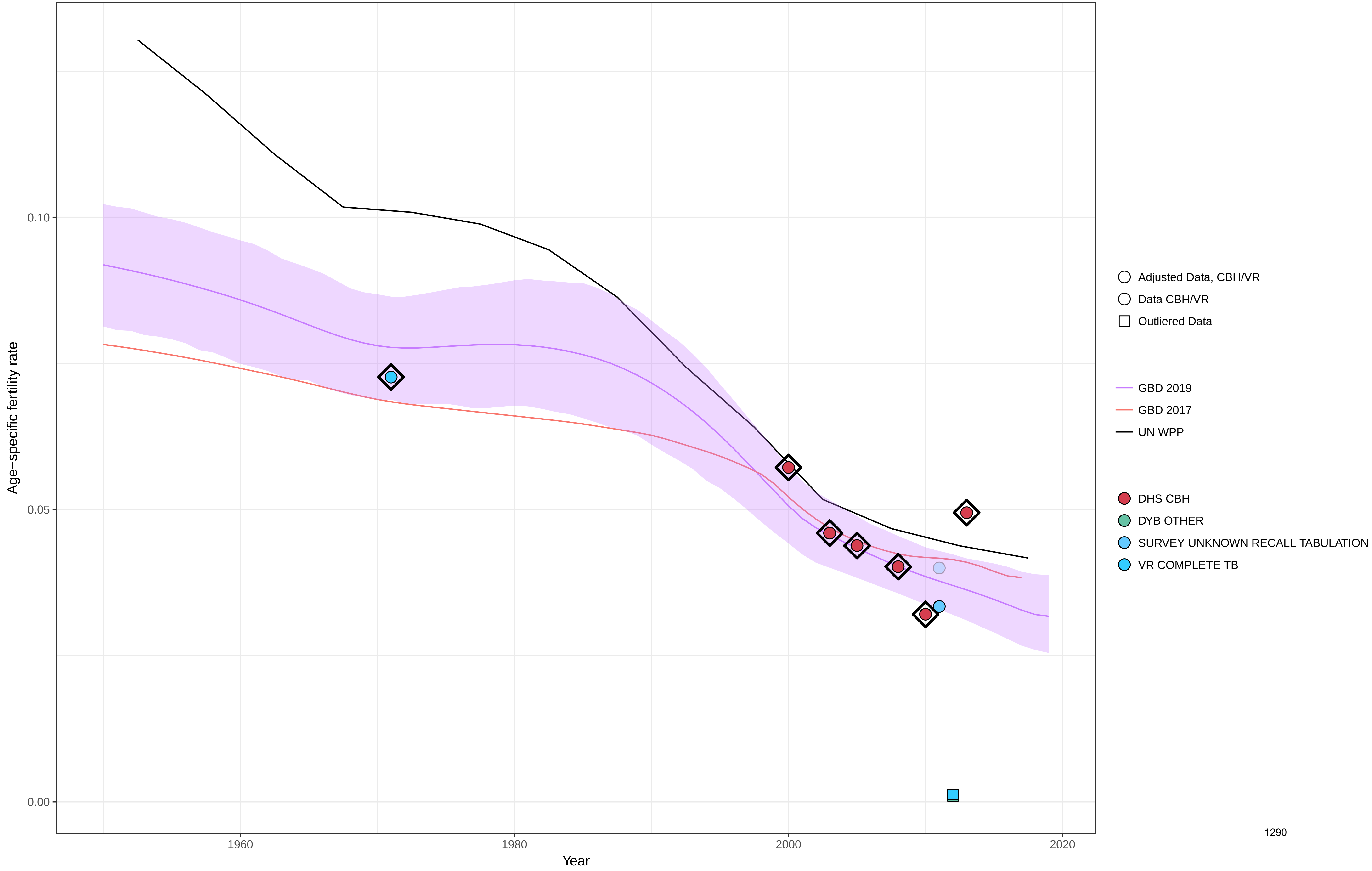

Age−specific fertility fitted trend lines, 1950−2019: Lesotho, Ages: 45−49
Data points enclosed with a diamond are reference sources

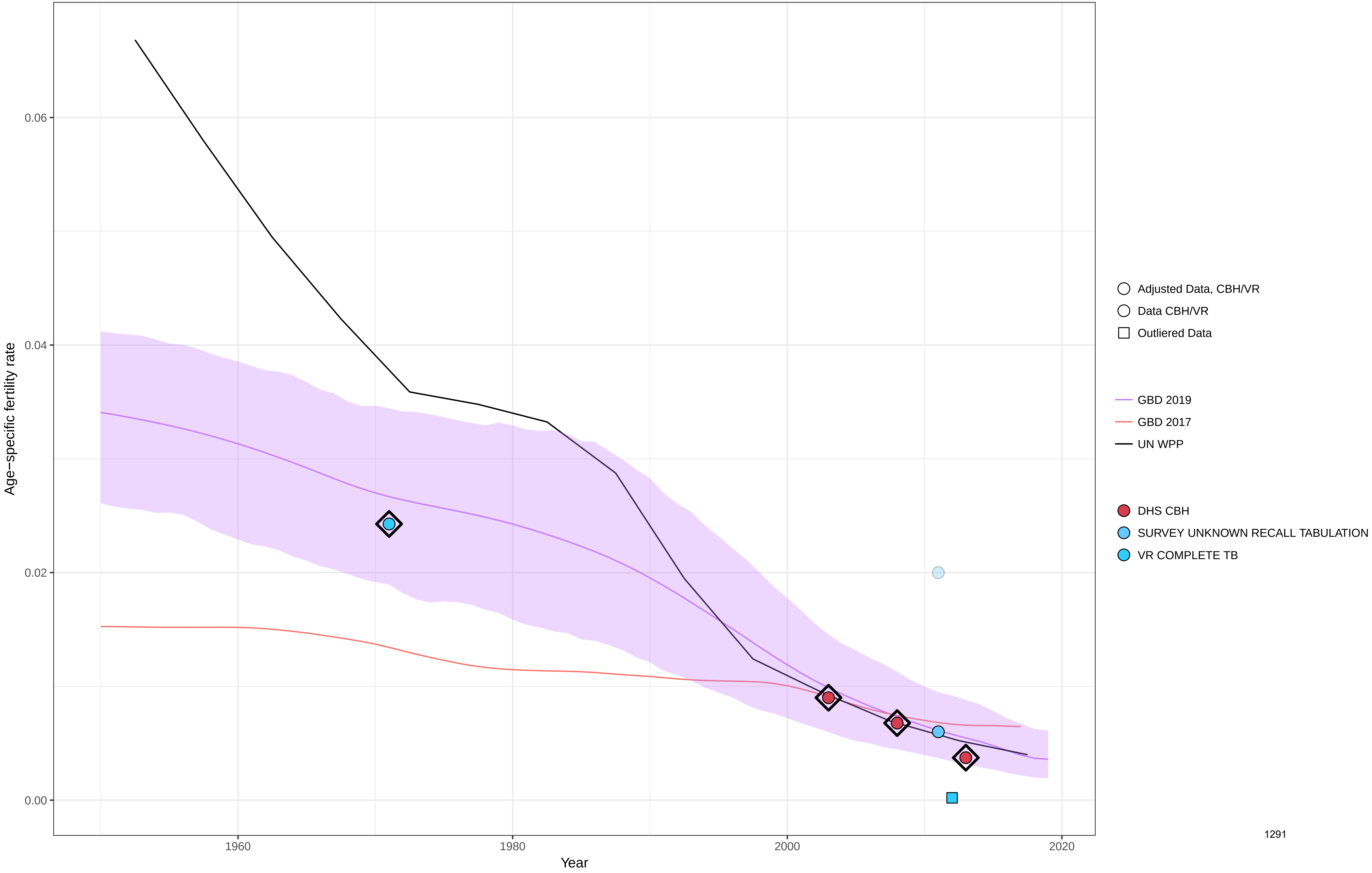

Age−specific fertility fitted trend lines, 1950−2019: Lesotho, Ages: 50−54
Data points enclosed with a diamond are reference sources

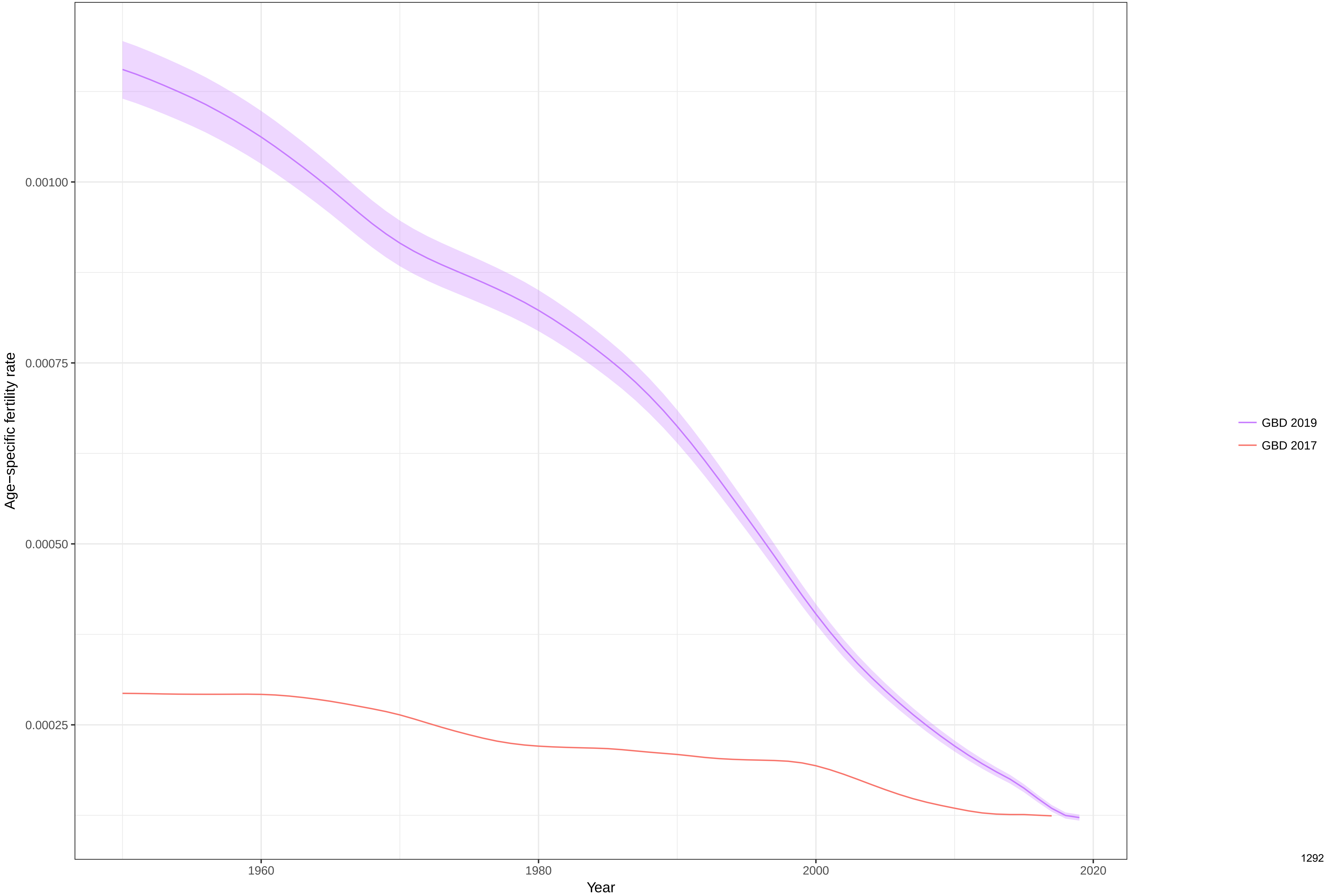

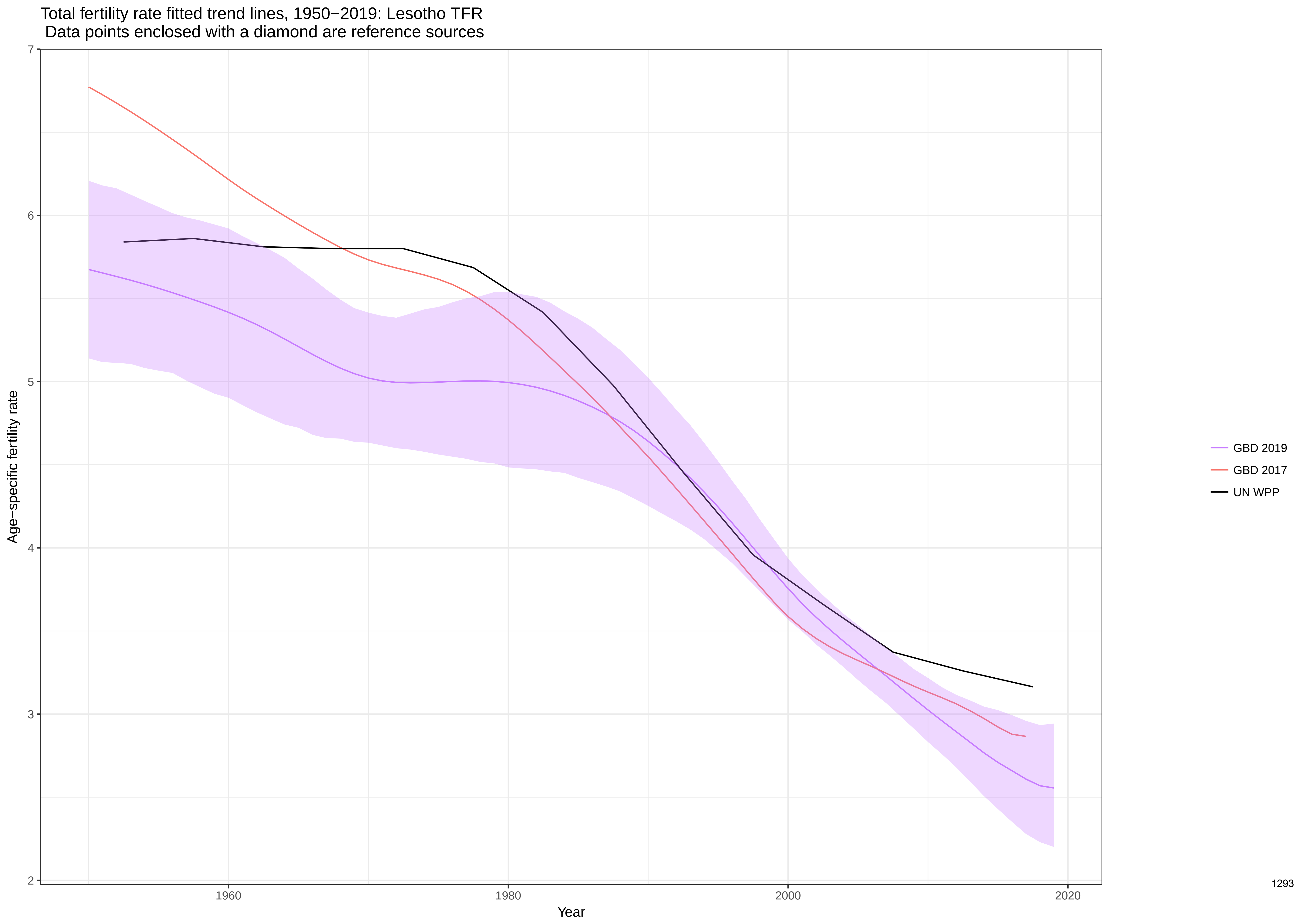
Total fertility rate fitted trend lines, 1950−2019: Lesotho TFR
Data points enclosed with a diamond are reference sources
Age−specific fertility rate
Year
7
6
5
4
3
2
1960
1980
2000
2020
GBD 2019
GBD 2017
UN WPP

Age−specific fertility fitted trend lines, 1950−2019: Lithuania, Ages: 10−14
Data points enclosed with a diamond are reference sources

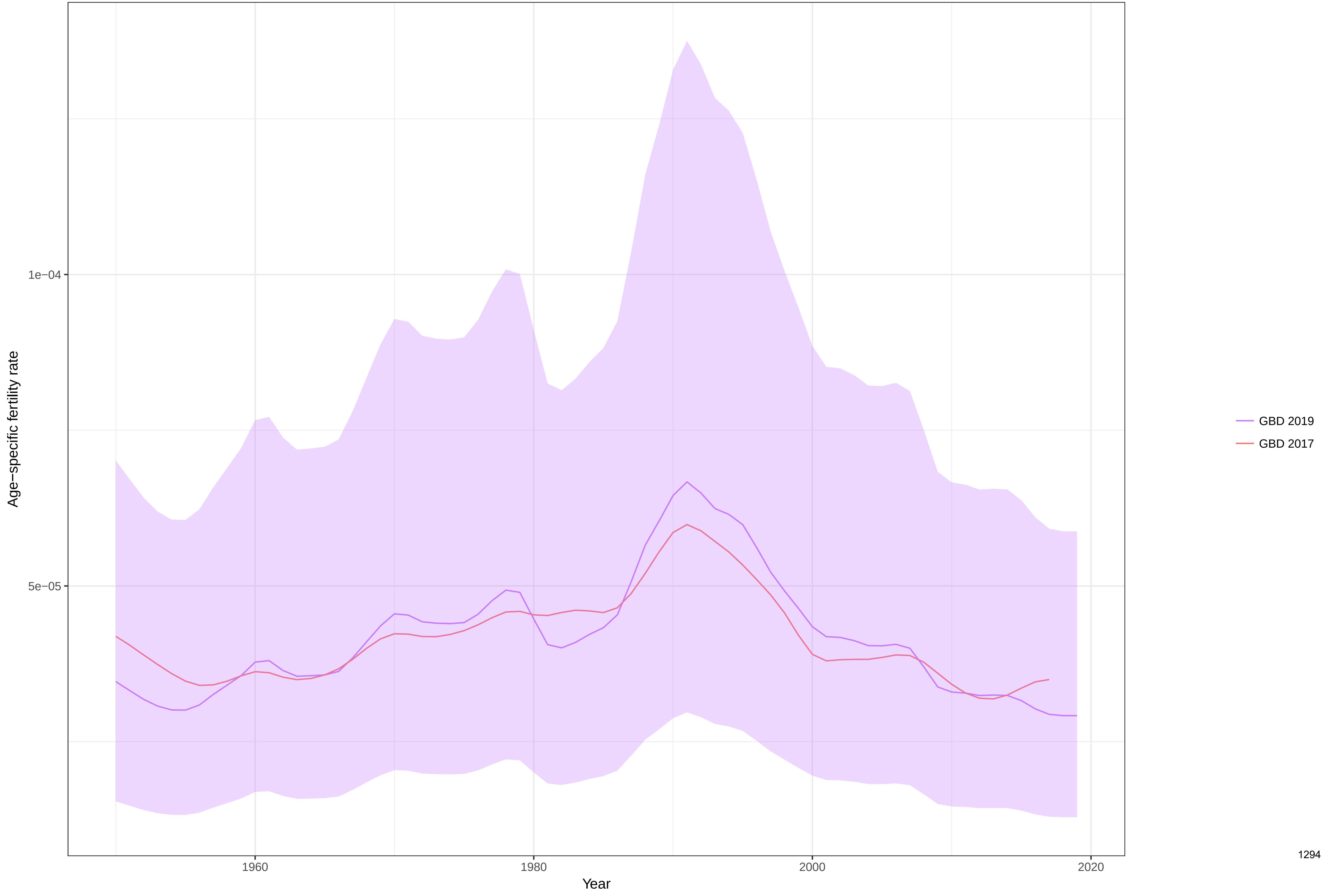

Age−specific fertility fitted trend lines, 1950−2019: Lithuania, Ages: 15−19
Data points enclosed with a diamond are reference sources

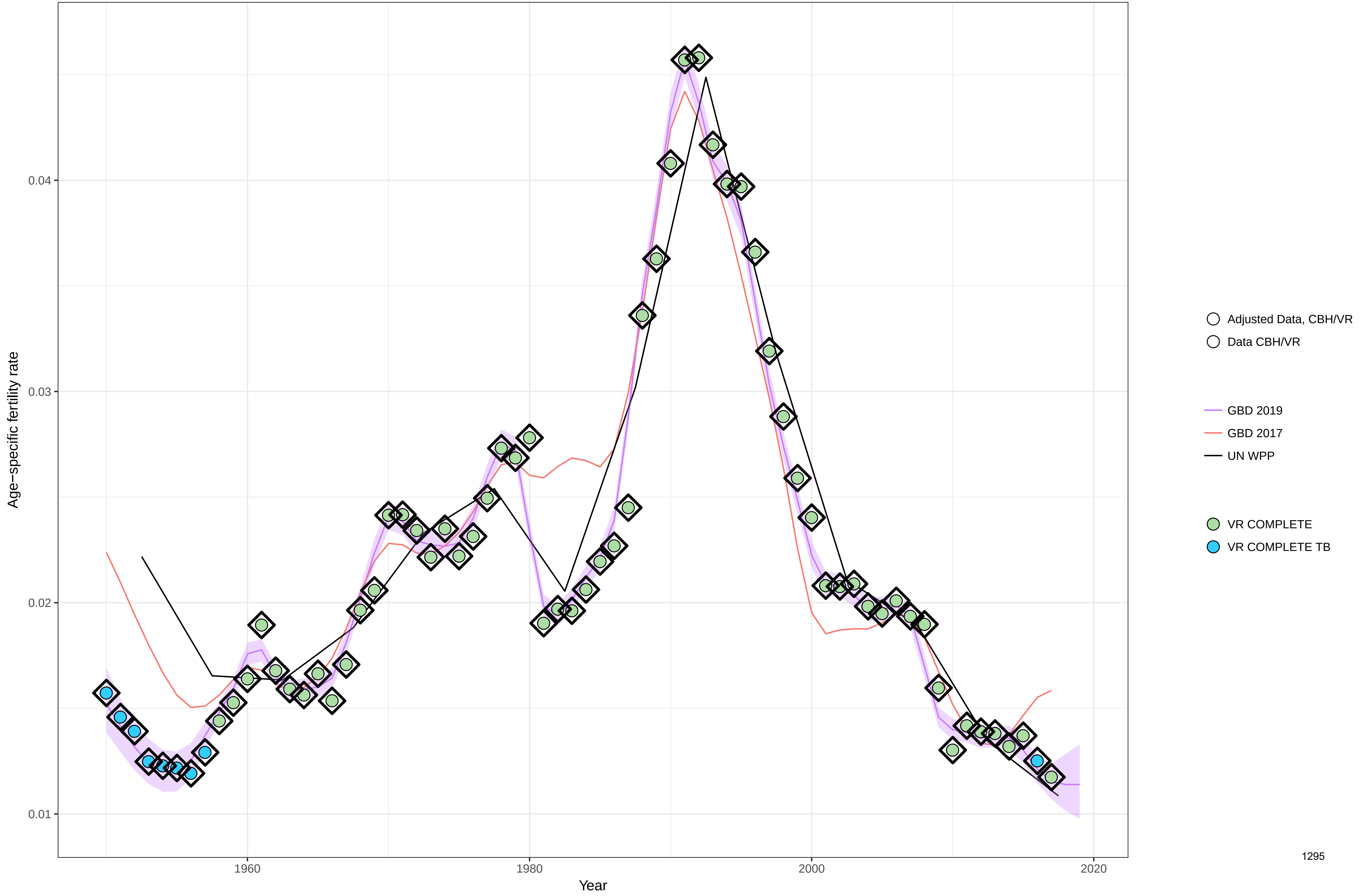

Age−specific fertility fitted trend lines, 1950−2019: Lithuania, Ages: 20−24
Data points enclosed with a diamond are reference sources

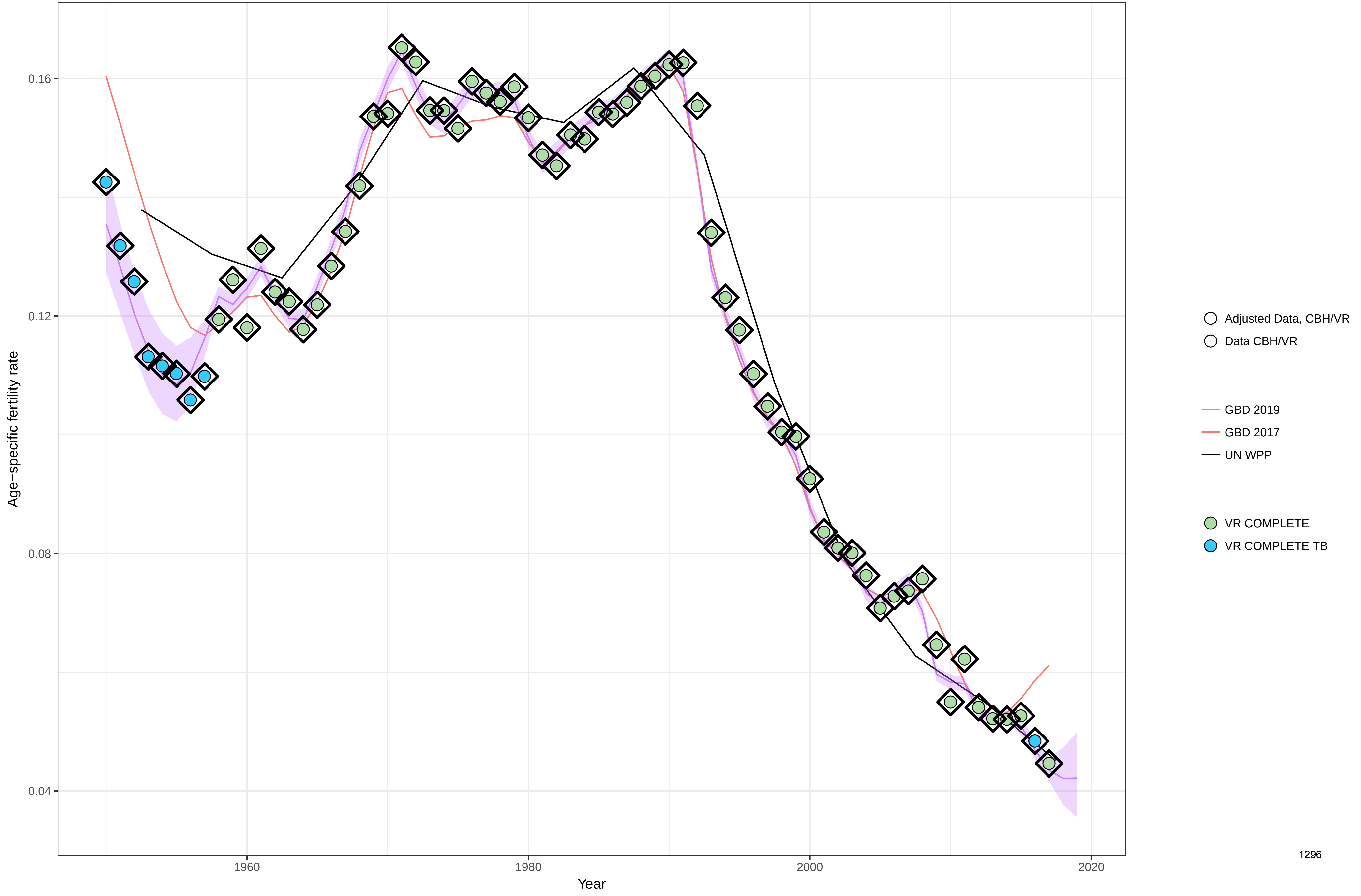

Age−specific fertility fitted trend lines, 1950−2019: Lithuania, Ages: 25−29
Data points enclosed with a diamond are reference sources

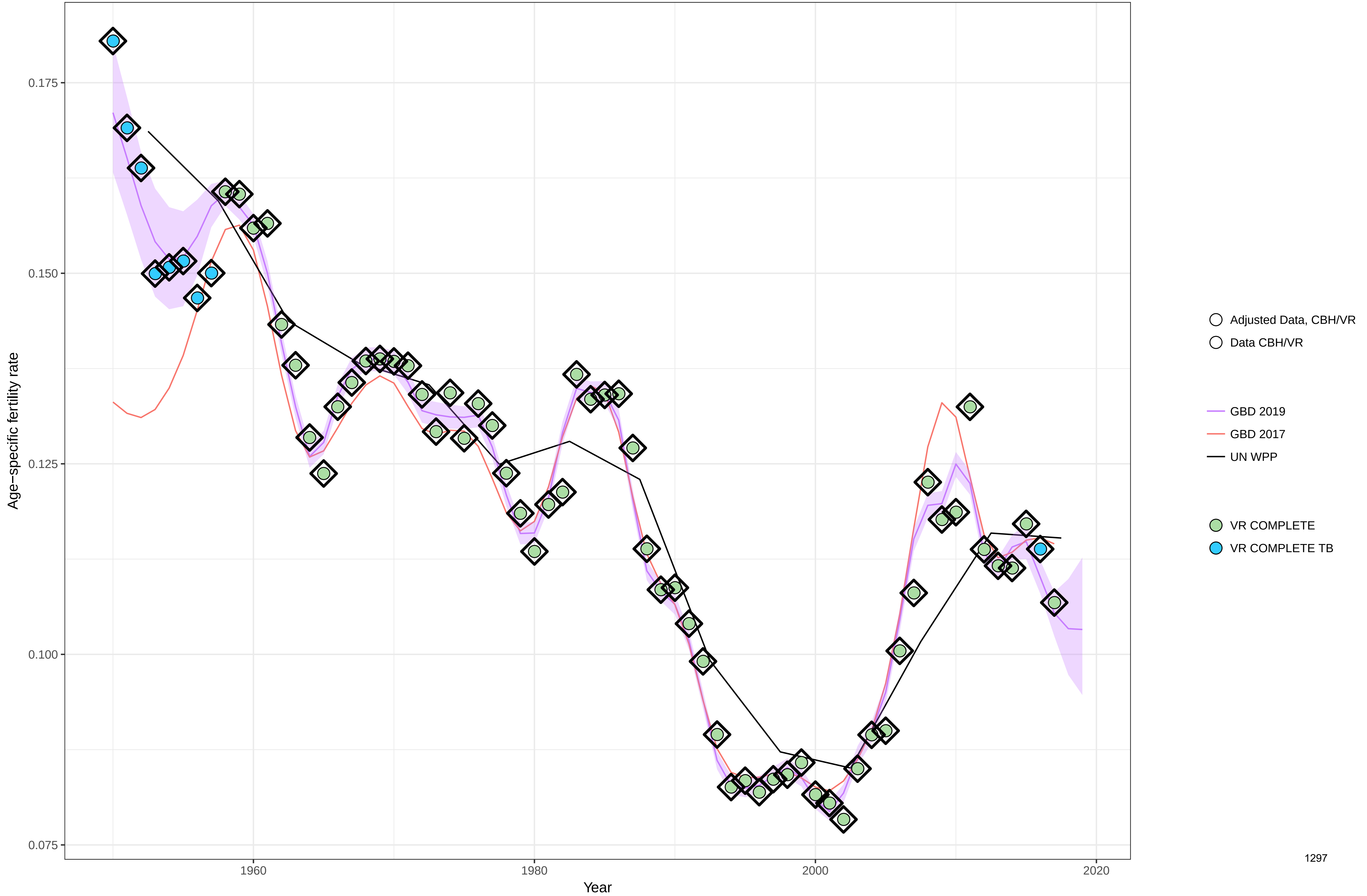

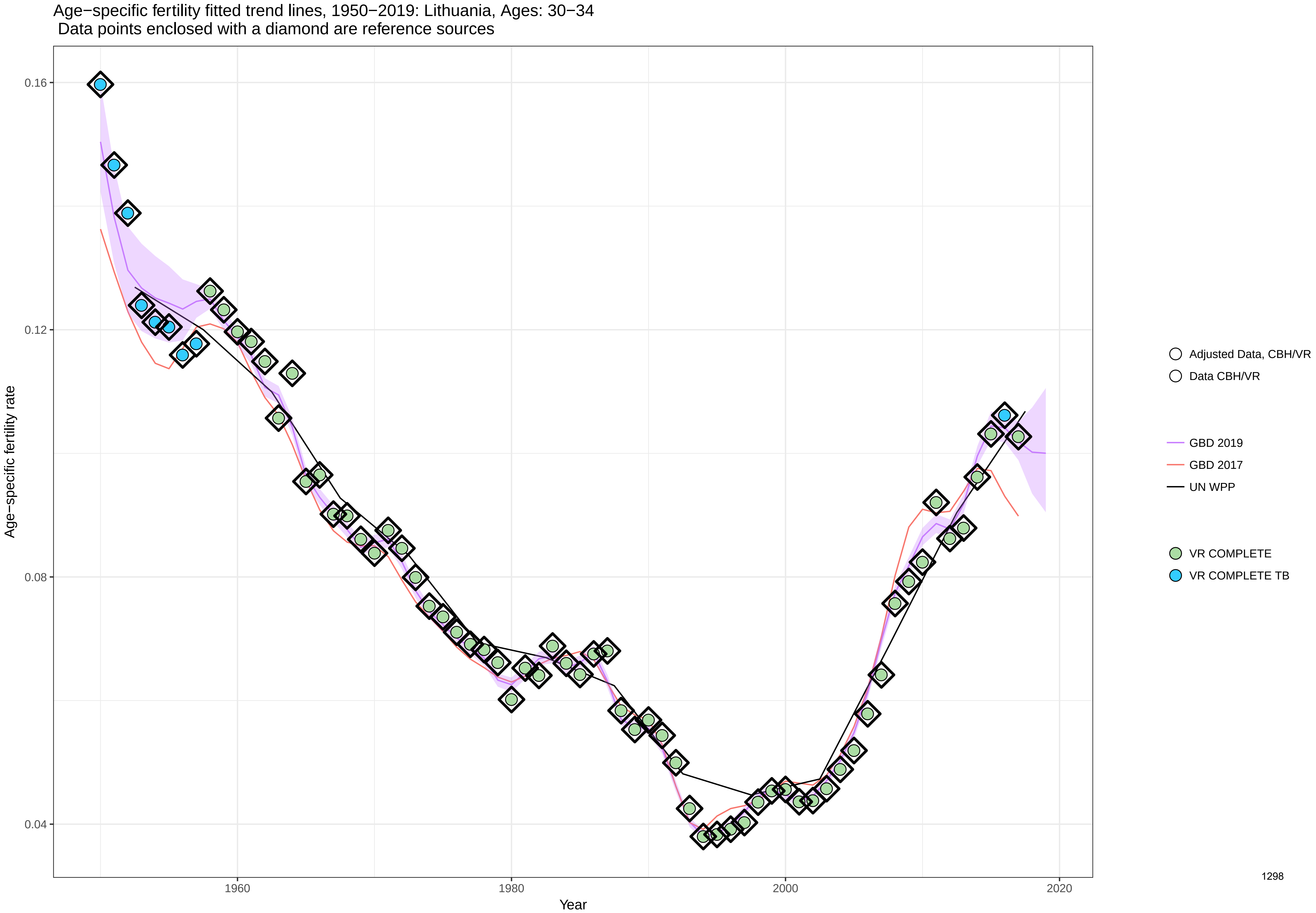

Age−specific fertility fitted trend lines, 1950−2019: Lithuania, Ages: 30−34
Data points enclosed with a diamond are reference sources
0.16
0.12
0.08
0.04
Age−specific fertility rate
1960
1980
2000
2020
Year
Adjusted Data, CBH/VR
Data CBH/VR
GBD 2019
GBD 2017
UN WPP
VR COMPLETE
VR COMPLETE TB

Age−specific fertility fitted trend lines, 1950−2019: Lithuania, Ages: 35−39
Data points enclosed with a diamond are reference sources

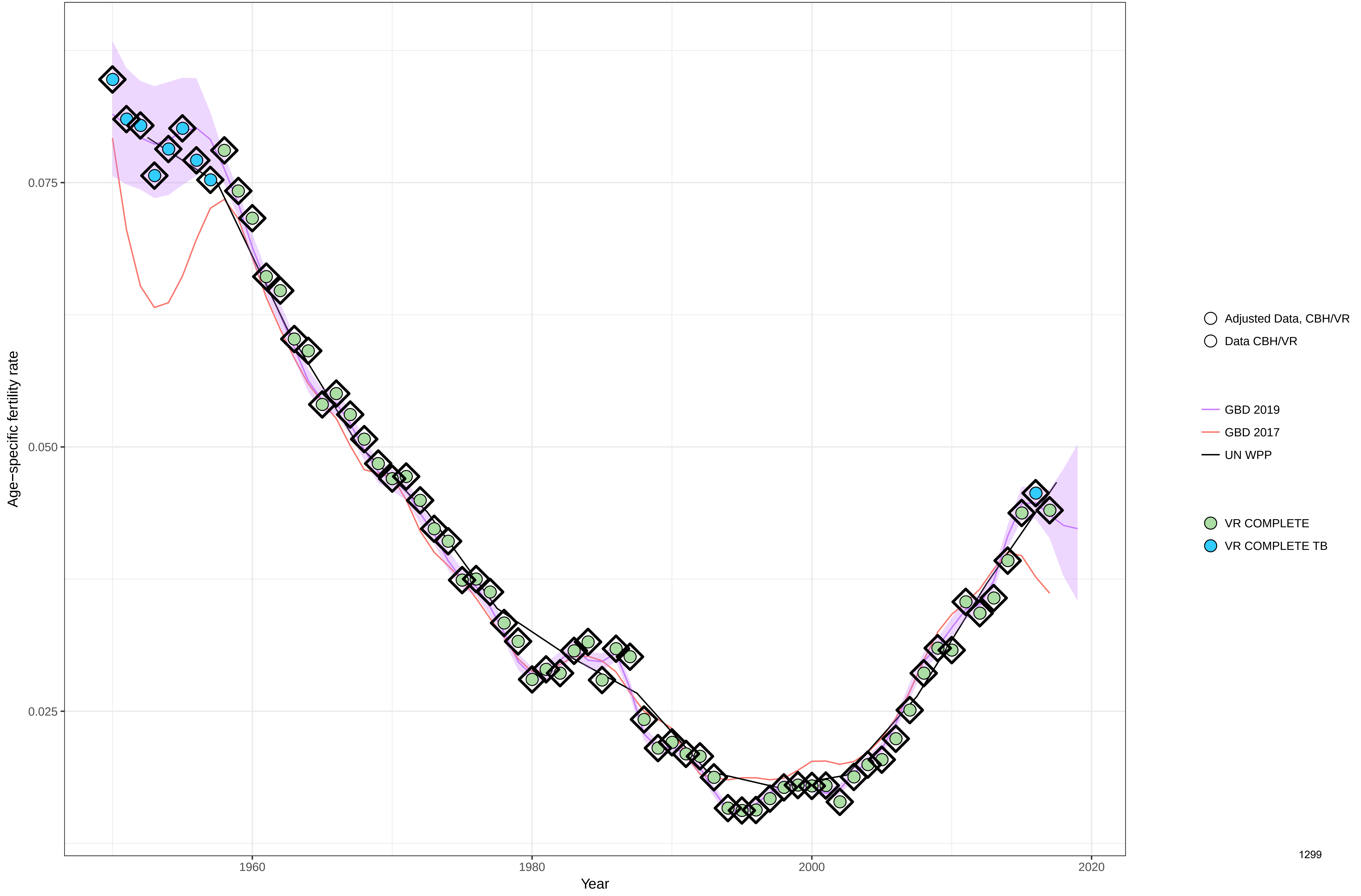

Age−specific fertility fitted trend lines, 1950−2019: Lithuania, Ages: 40−44
Data points enclosed with a diamond are reference sources

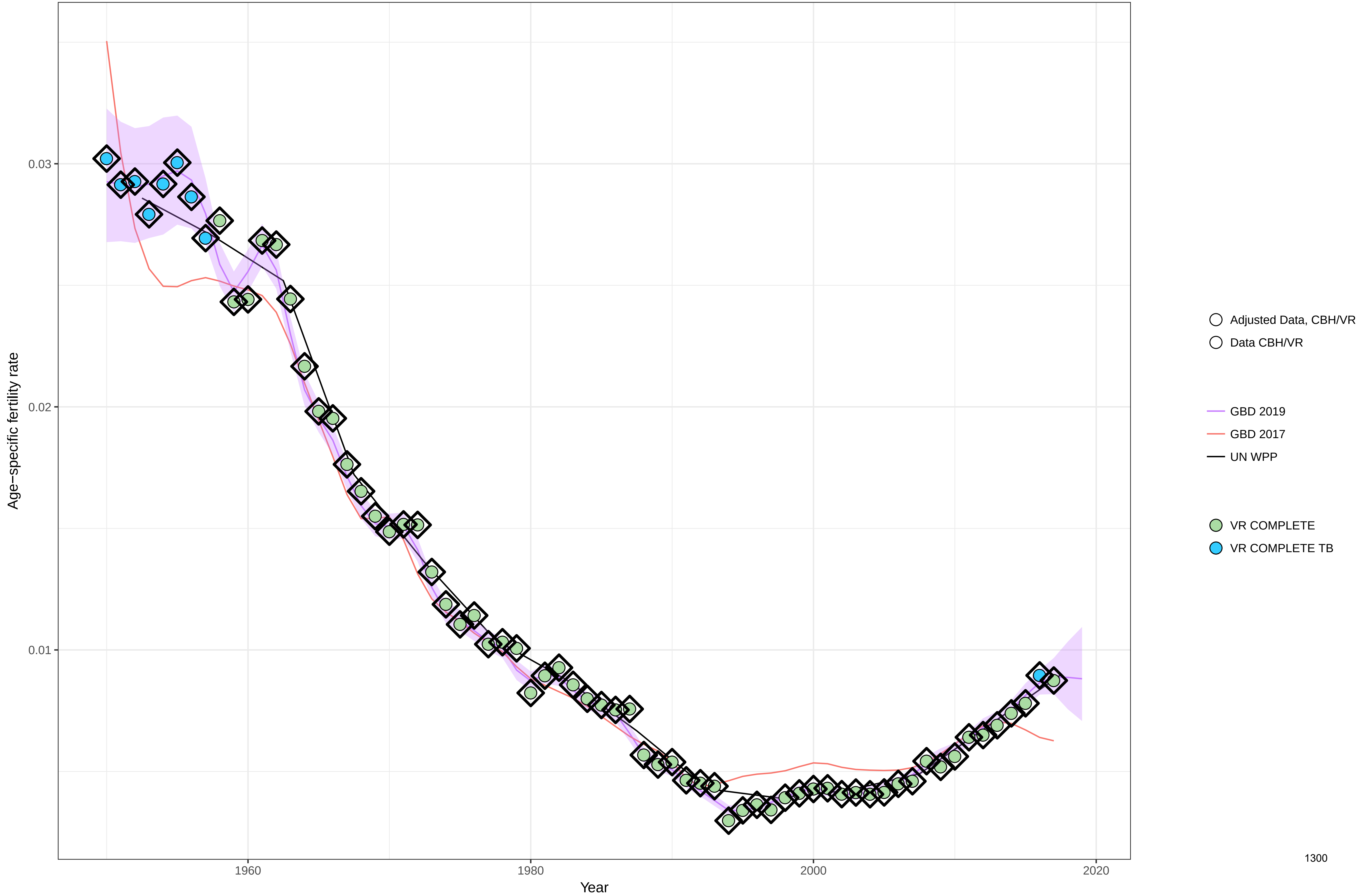

Age−specific fertility fitted trend lines, 1950−2019: Lithuania, Ages: 45−49
Data points enclosed with a diamond are reference sources

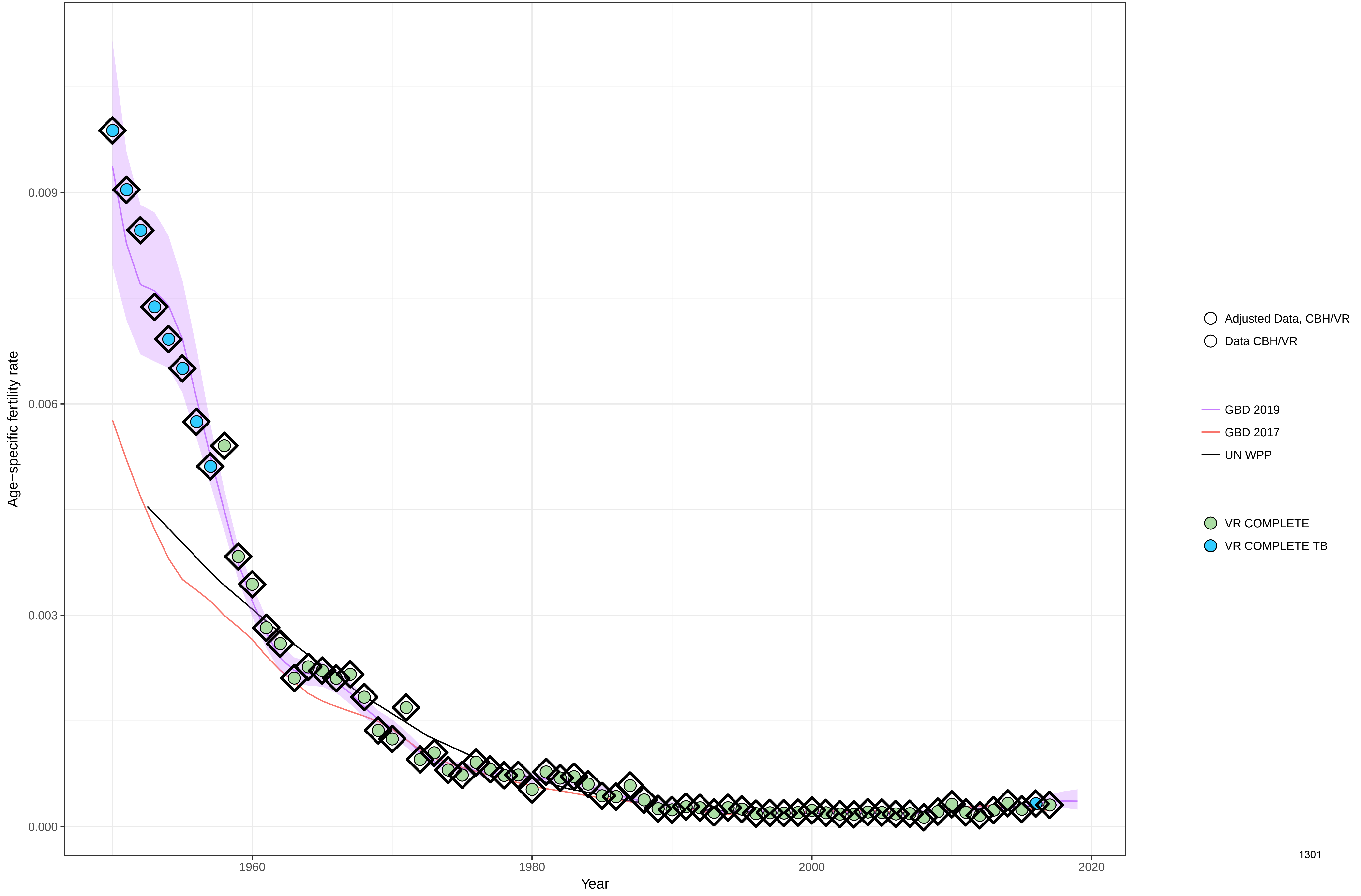

Age−specific fertility fitted trend lines, 1950−2019: Lithuania, Ages: 50−54
Data points enclosed with a diamond are reference sources

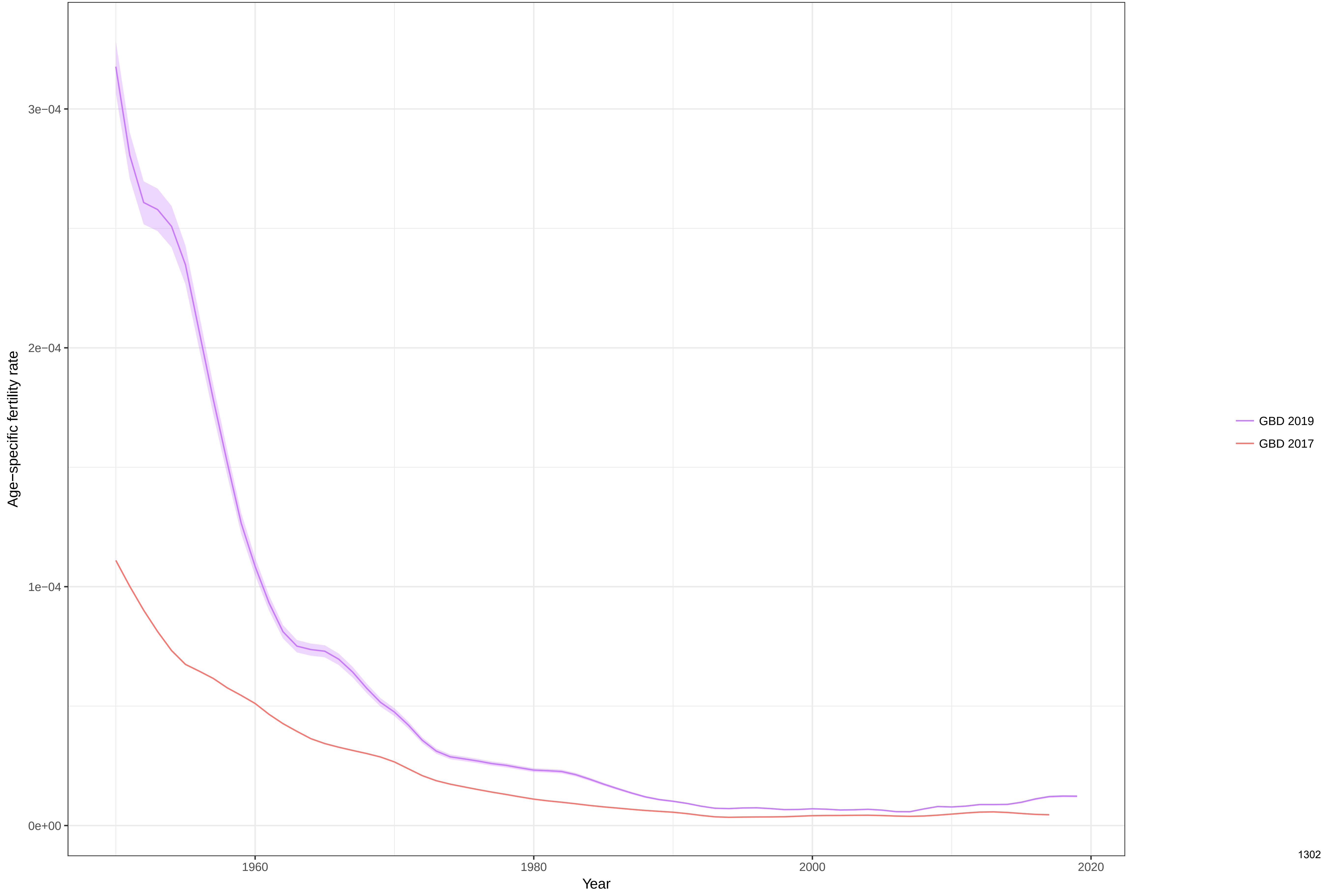

Total fertility rate fitted trend lines, 1950−2019: Lithuania TFR
Data points enclosed with a diamond are reference sources

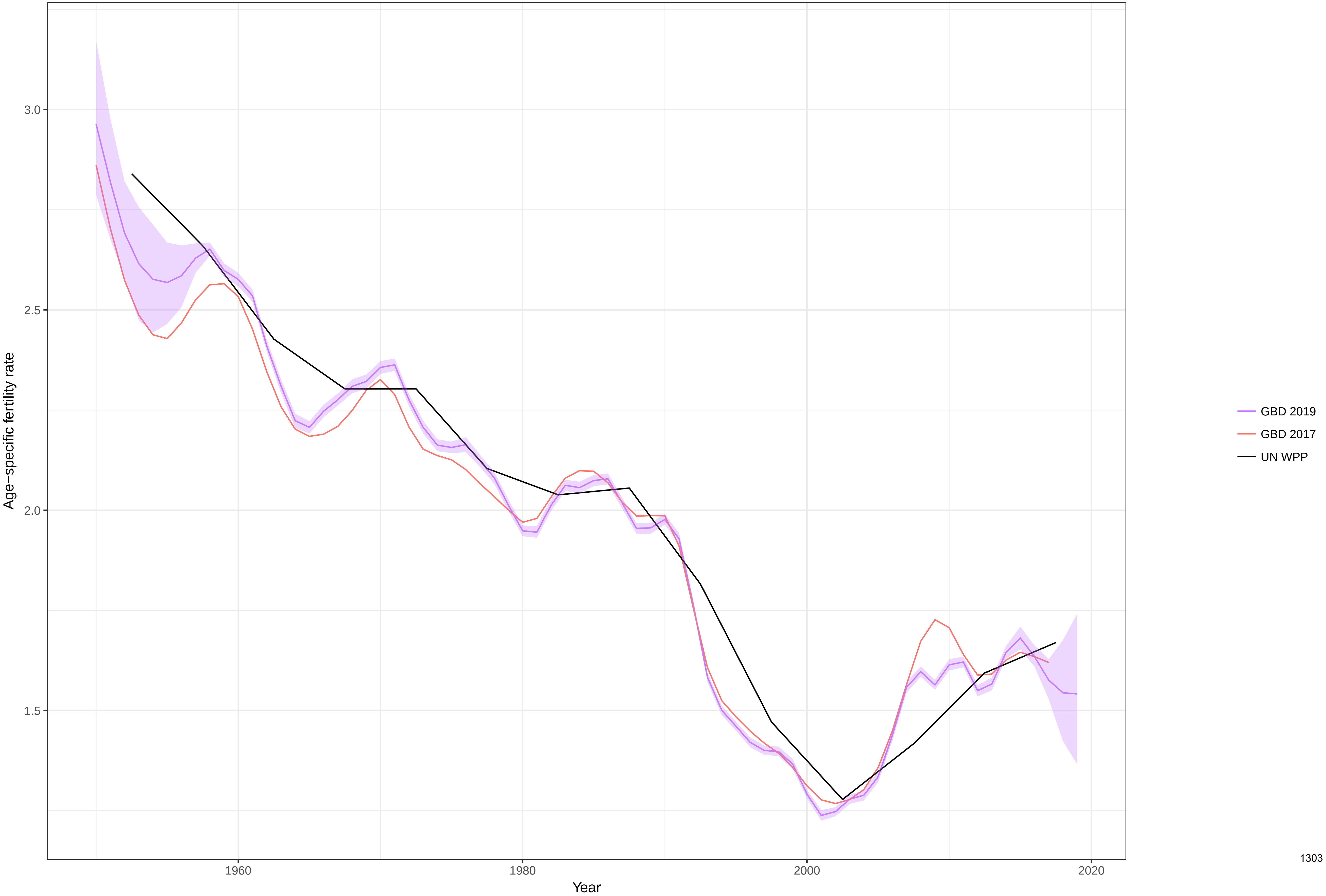

Age−specific fertility fitted trend lines, 1950−2019: Luxembourg, Ages: 10−14
Data points enclosed with a diamond are reference sources

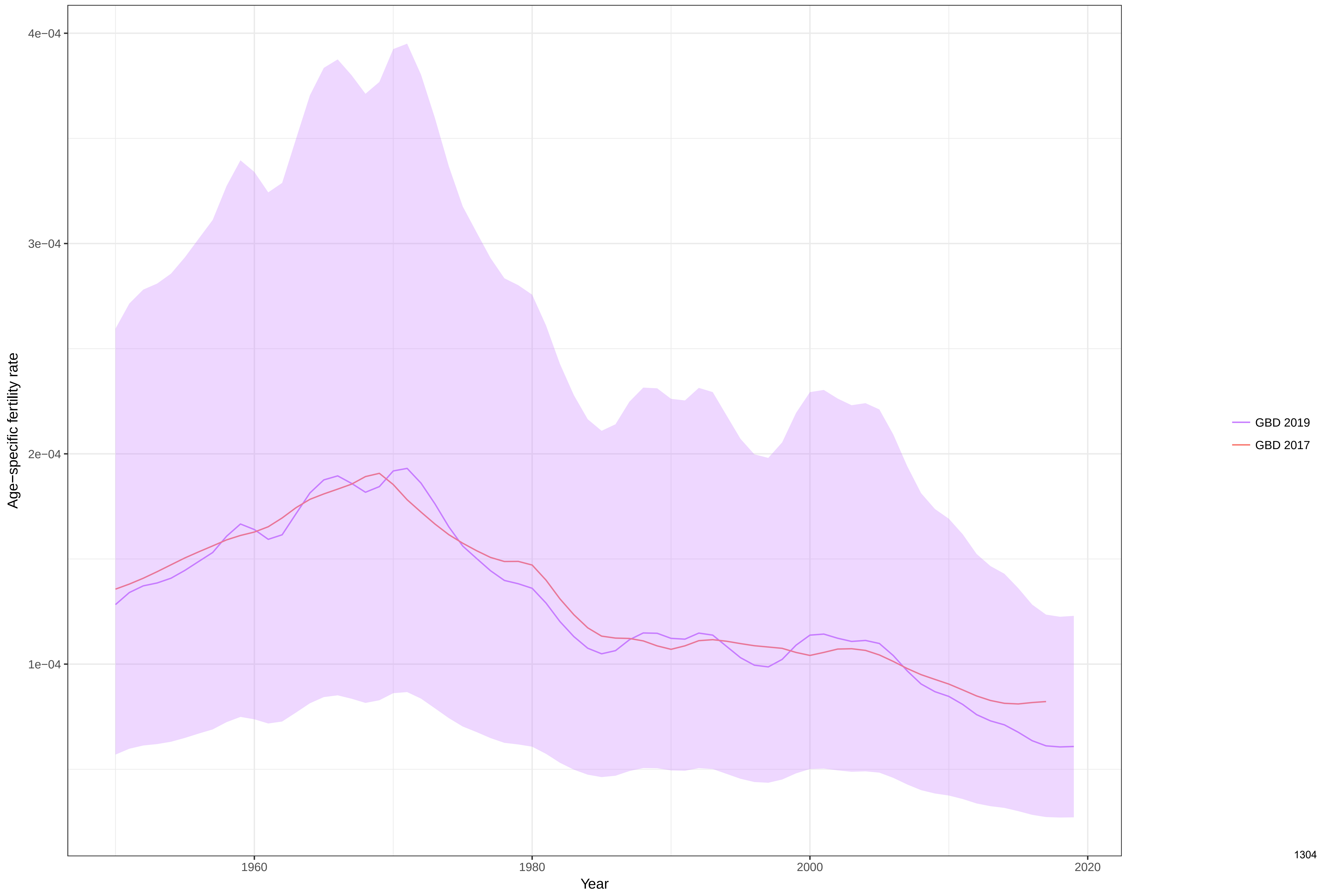

Age−specific fertility fitted trend lines, 1950−2019: Luxembourg, Ages: 15−19
Data points enclosed with a diamond are reference sources

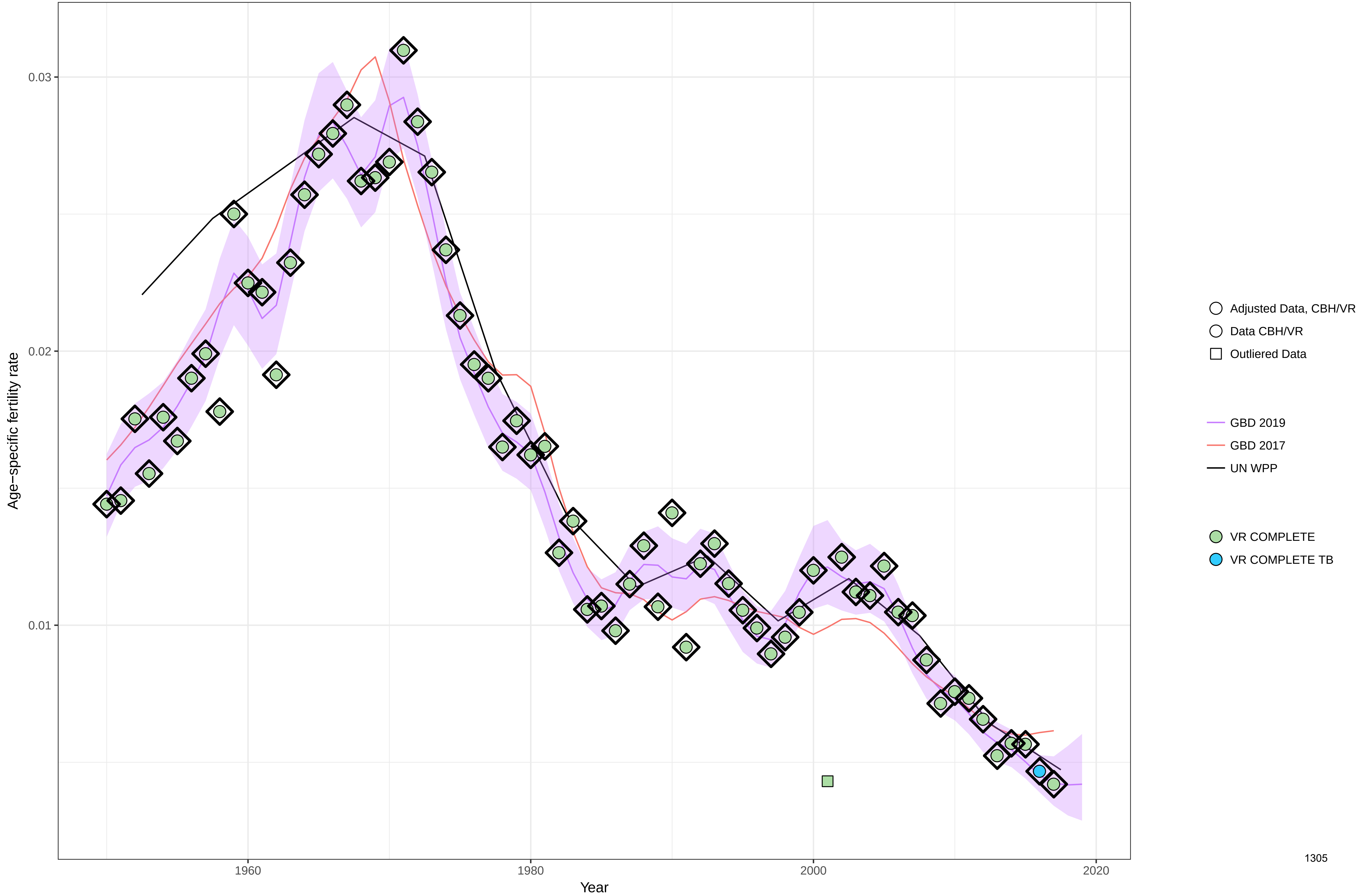

Age−specific fertility fitted trend lines, 1950−2019: Luxembourg, Ages: 20−24
Data points enclosed with a diamond are reference sources

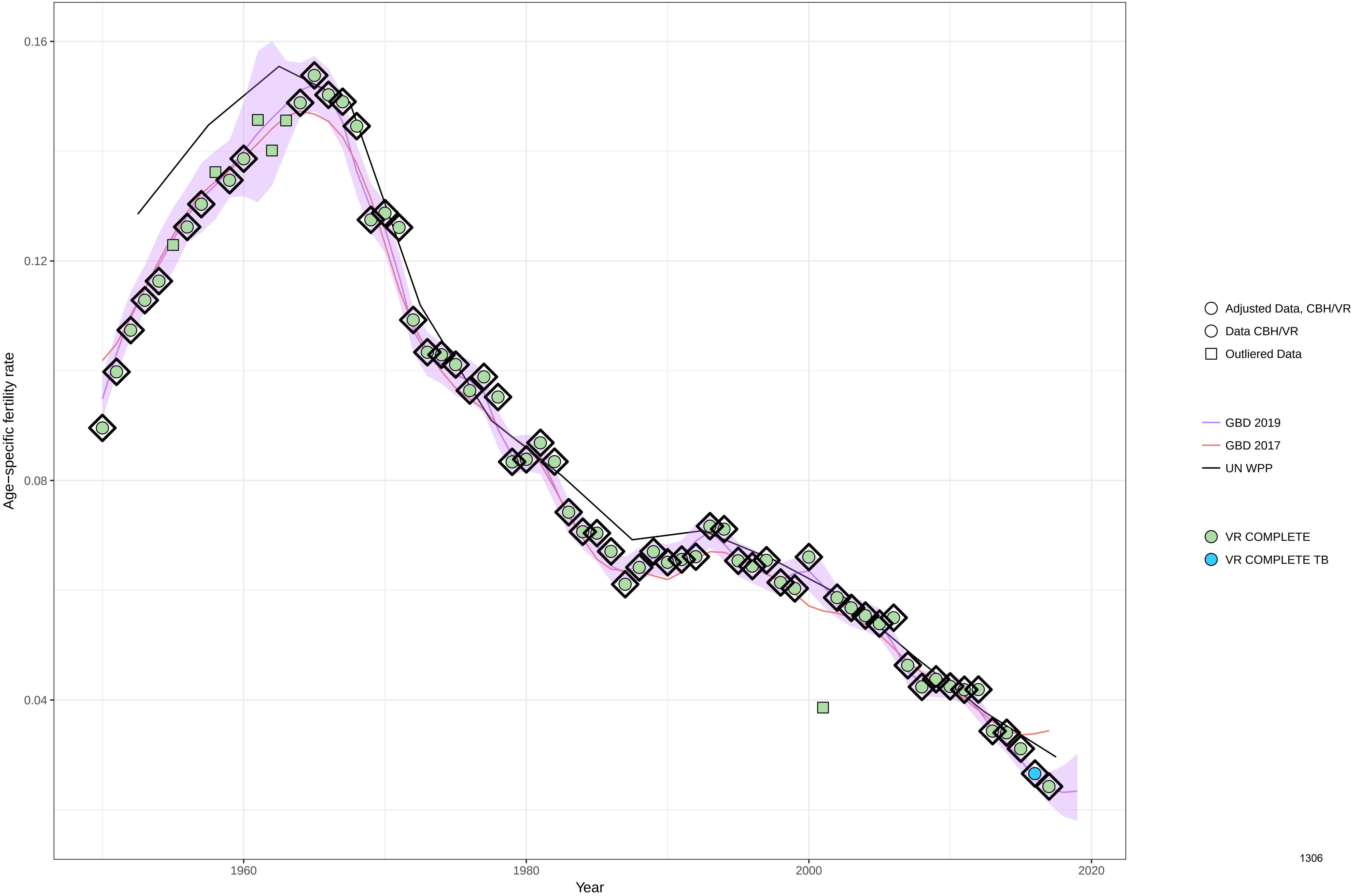

Age−specific fertility fitted trend lines, 1950−2019: Luxembourg, Ages: 25−29
Data points enclosed with a diamond are reference sources

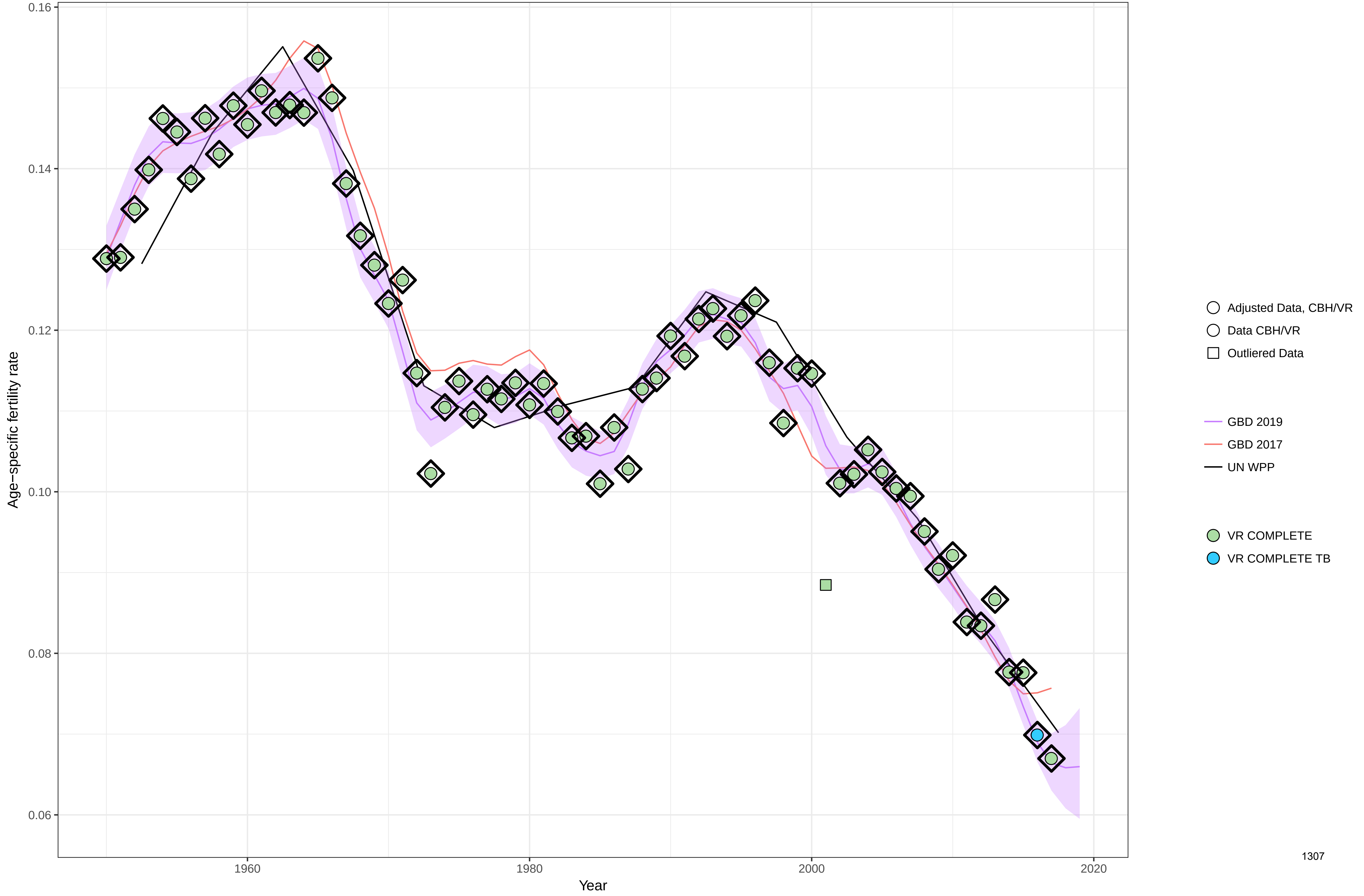

Age−specific fertility fitted trend lines, 1950−2019: Luxembourg, Ages: 30−34
Data points enclosed with a diamond are reference sources

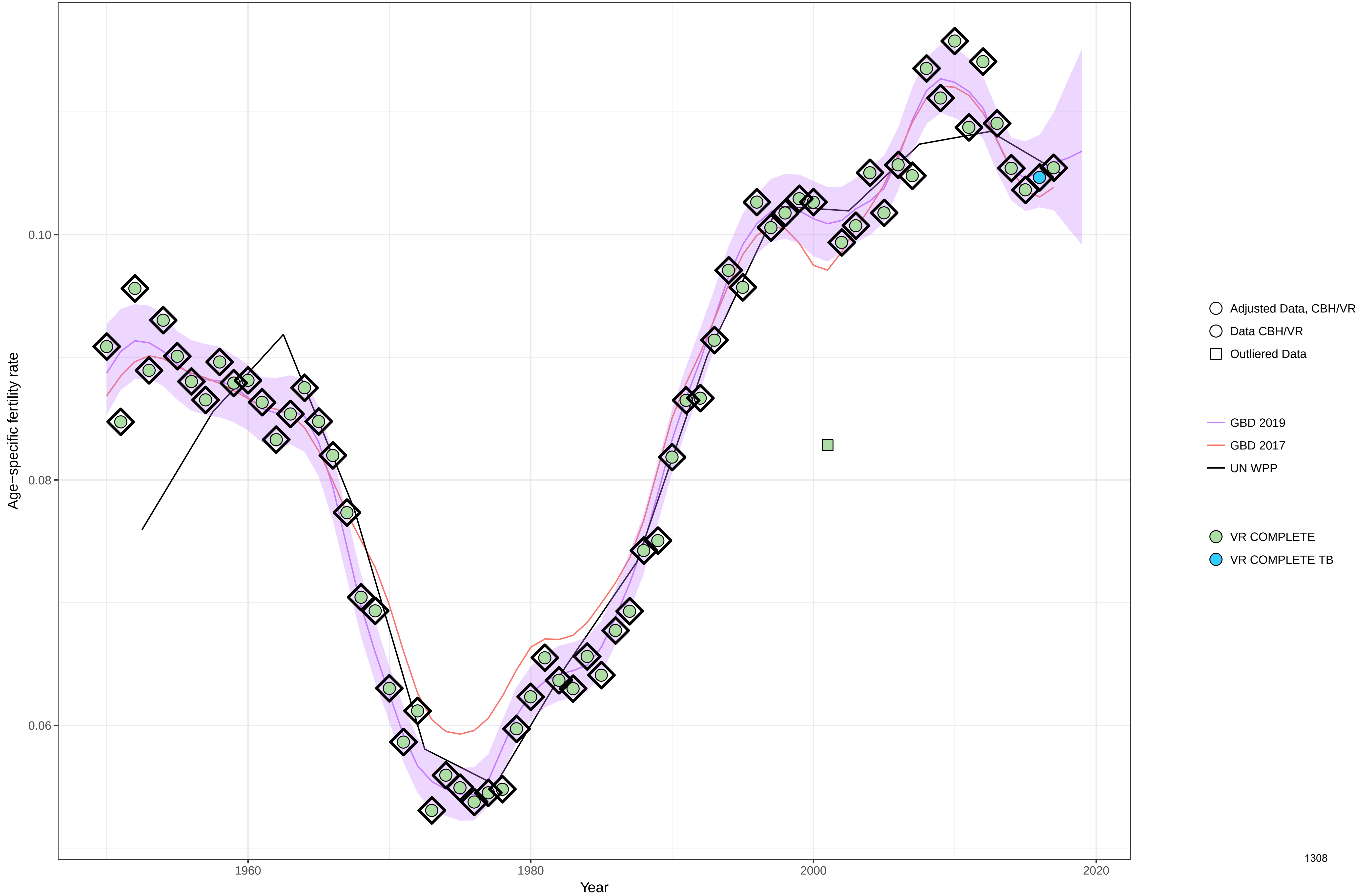

Age−specific fertility fitted trend lines, 1950−2019: Luxembourg, Ages: 35−39
Data points enclosed with a diamond are reference sources

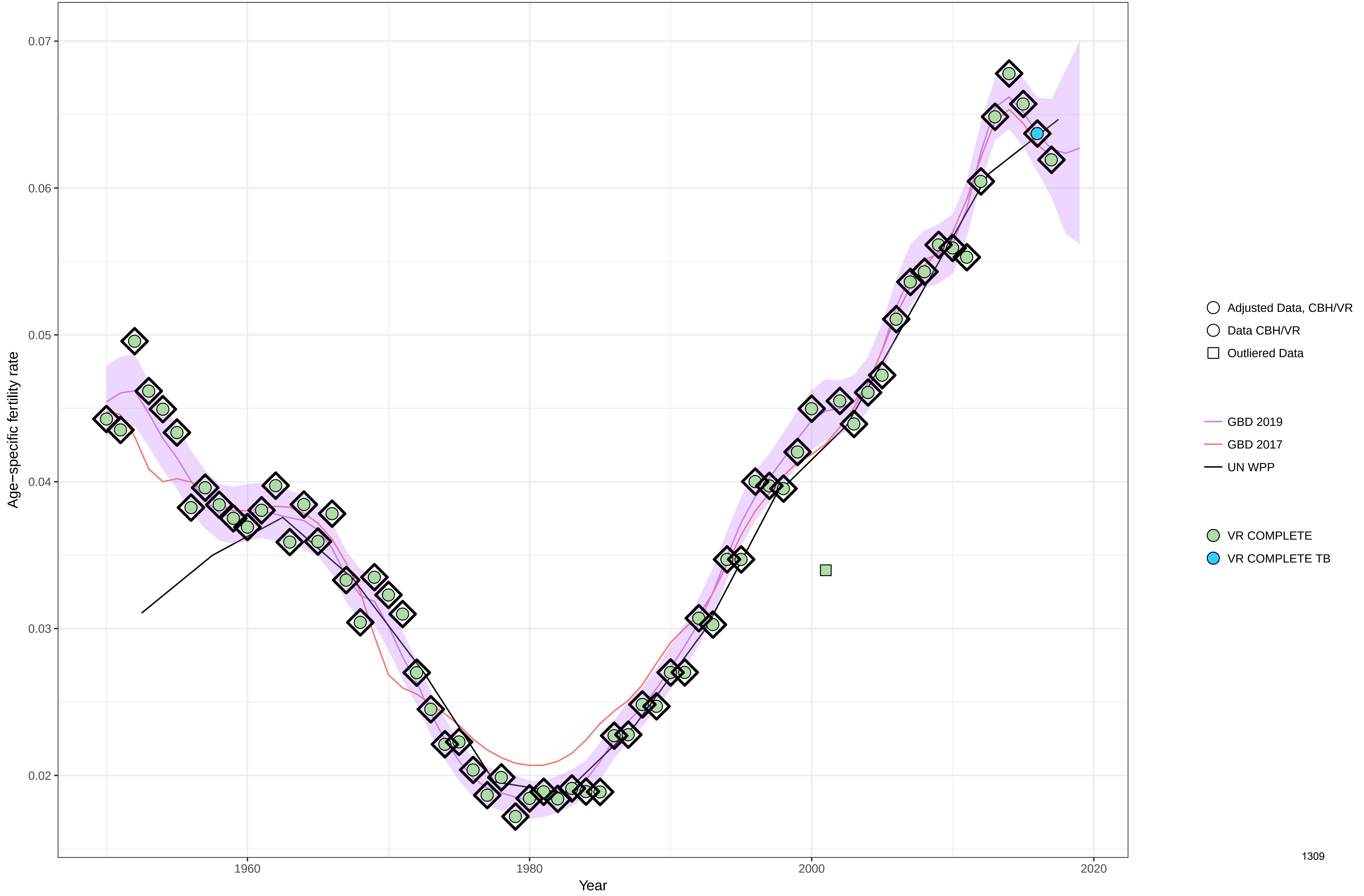

Age−specific fertility fitted trend lines, 1950−2019: Luxembourg, Ages: 40−44
Data points enclosed with a diamond are reference sources

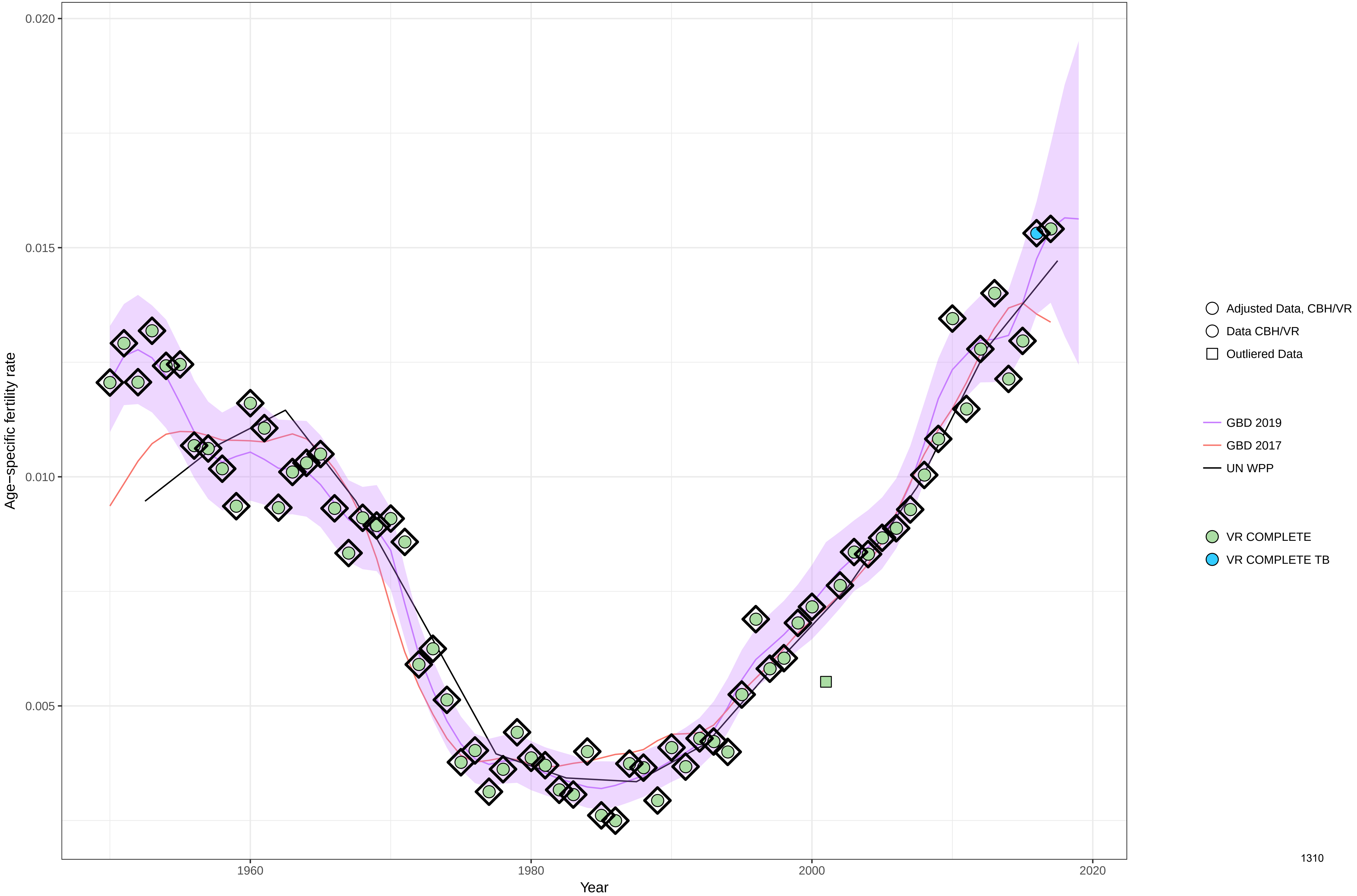

Age−specific fertility fitted trend lines, 1950−2019: Luxembourg, Ages: 45−49
Data points enclosed with a diamond are reference sources

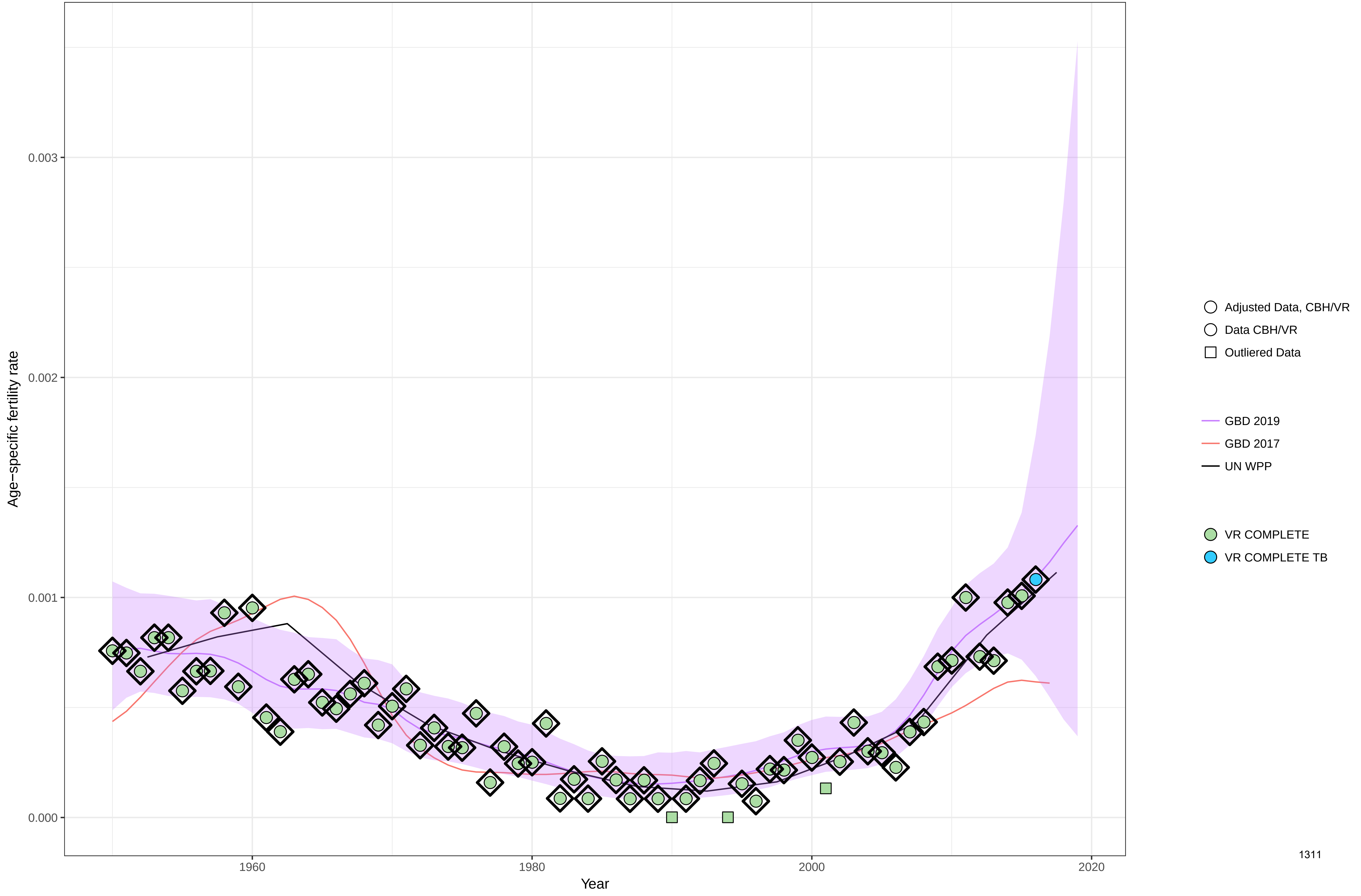

Age−specific fertility fitted trend lines, 1950−2019: Luxembourg, Ages: 50−54
Data points enclosed with a diamond are reference sources

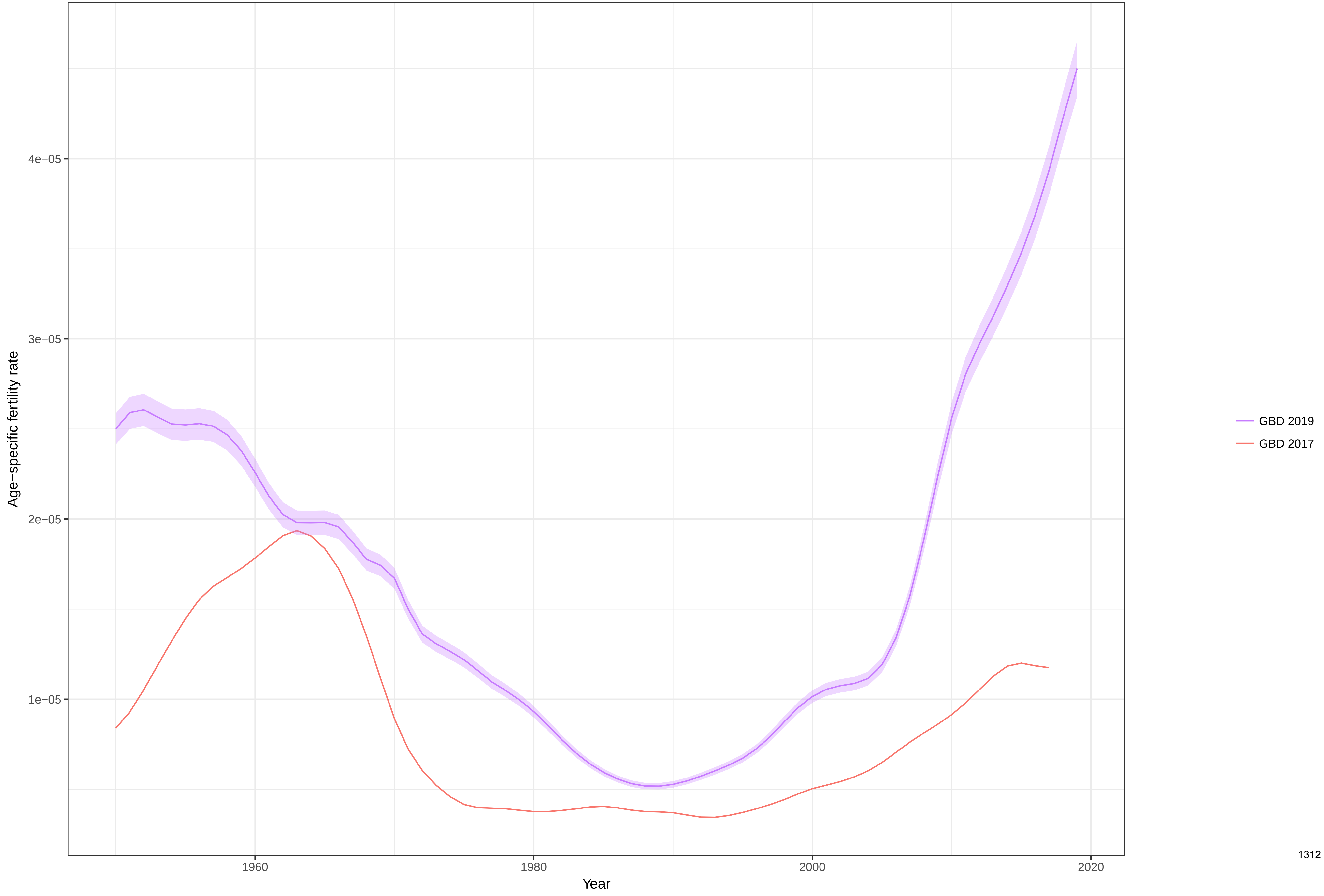

Total fertility rate fitted trend lines, 1950−2019: Luxembourg TFR
Data points enclosed with a diamond are reference sources

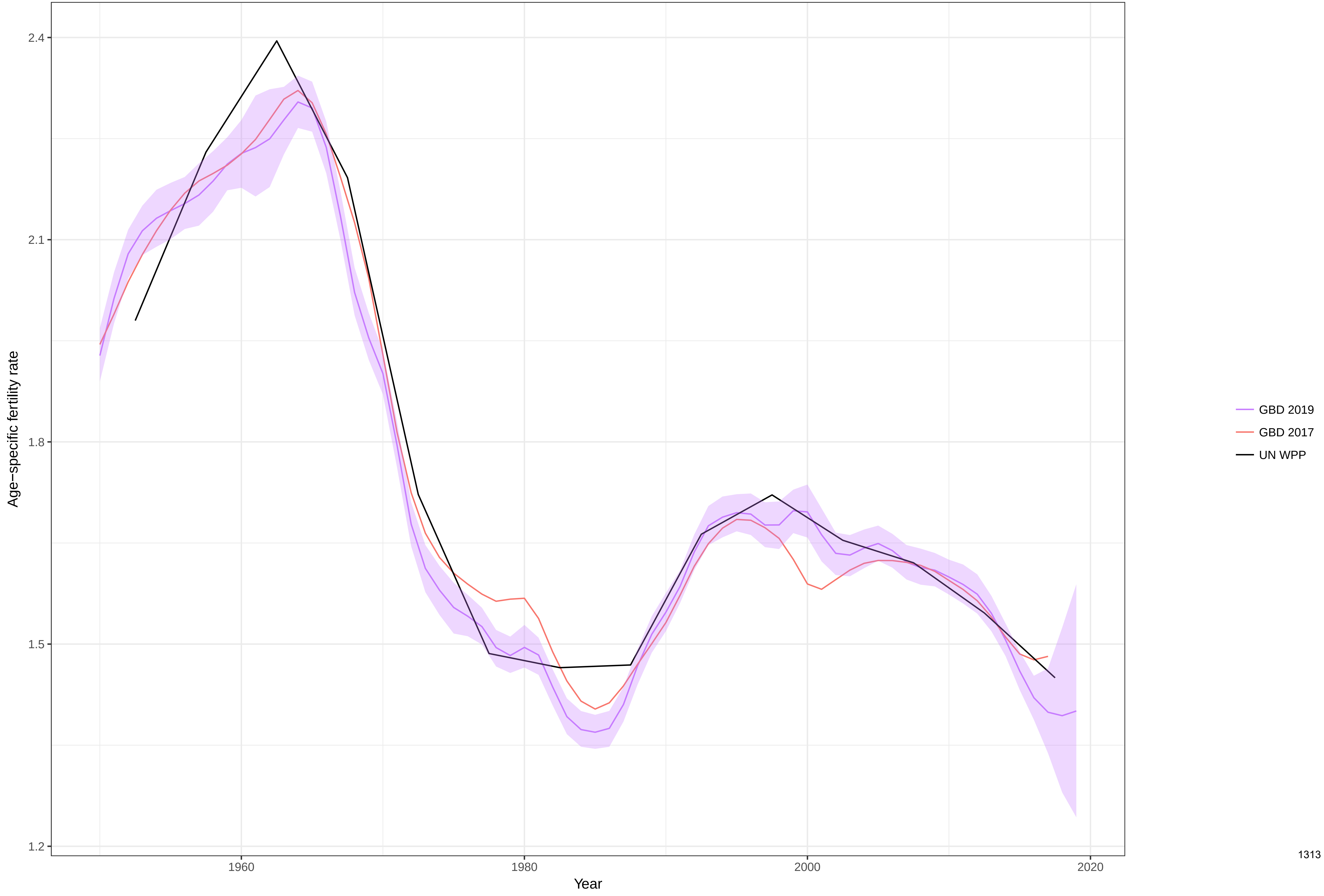

Age−specific fertility fitted trend lines, 1950−2019: Latvia, Ages: 10−14
Data points enclosed with a diamond are reference sources

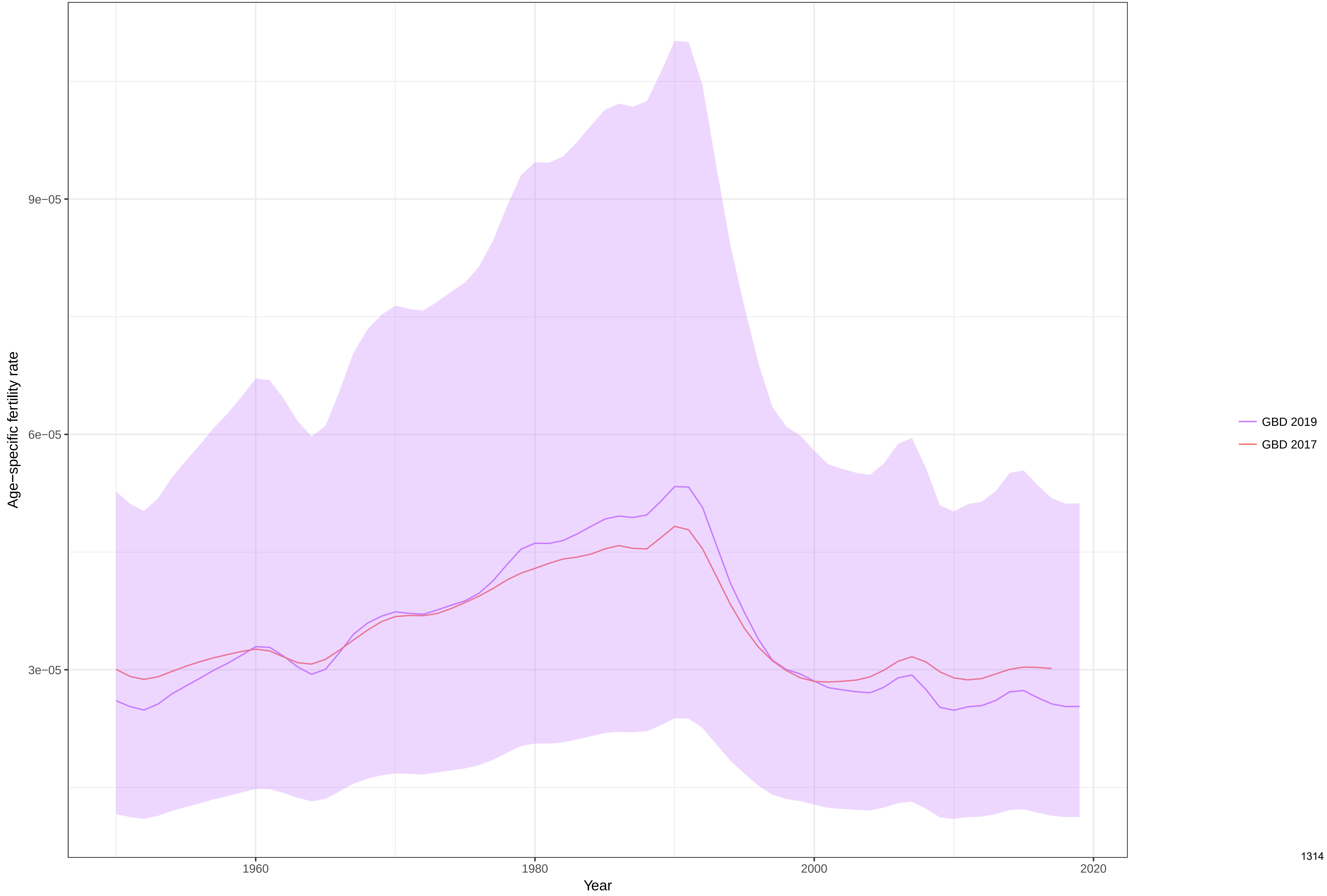

Age−specific fertility fitted trend lines, 1950−2019: Latvia, Ages: 15−19
Data points enclosed with a diamond are reference sources

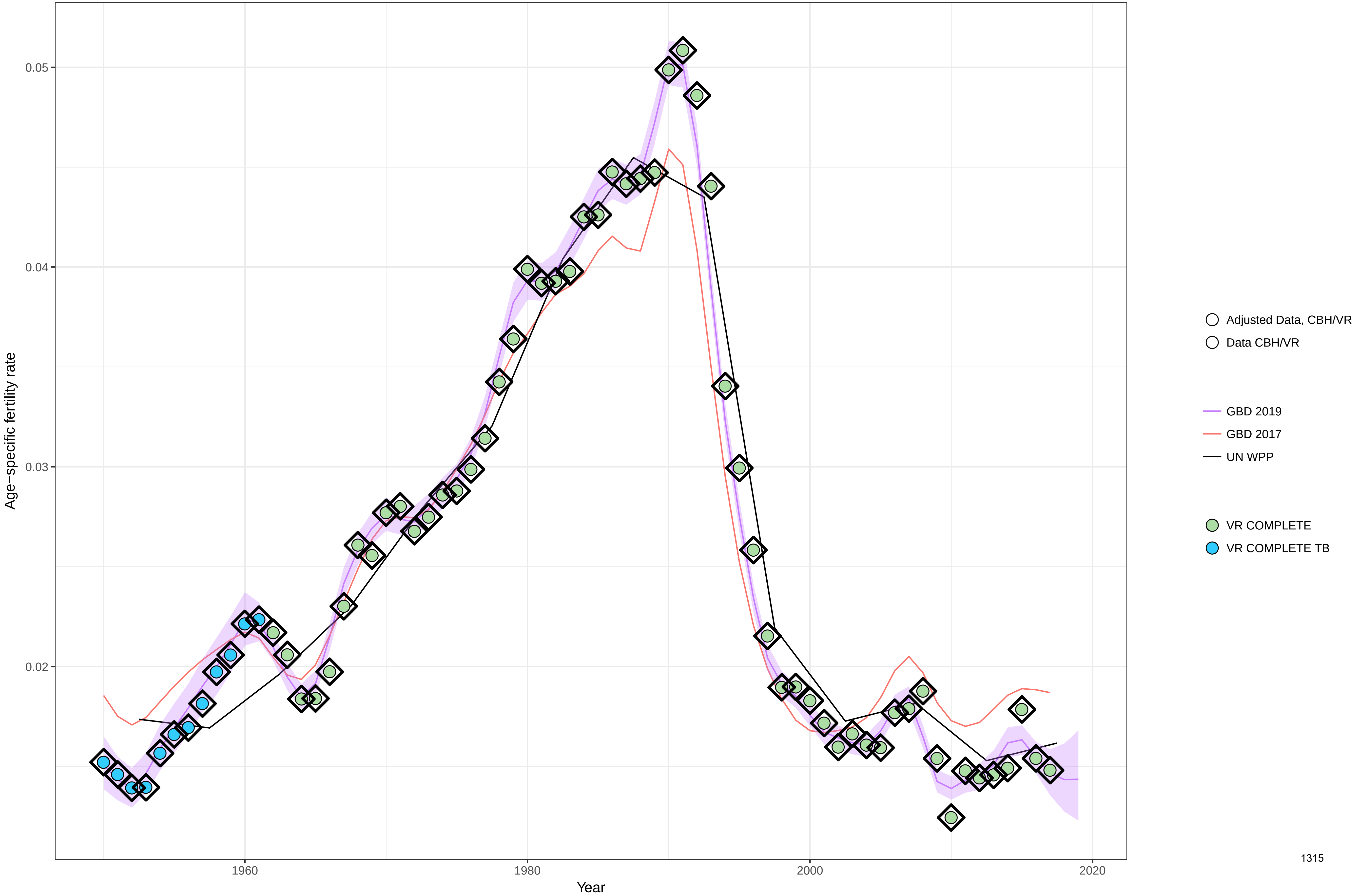

Age−specific fertility fitted trend lines, 1950−2019: Latvia, Ages: 20−24
Data points enclosed with a diamond are reference sources

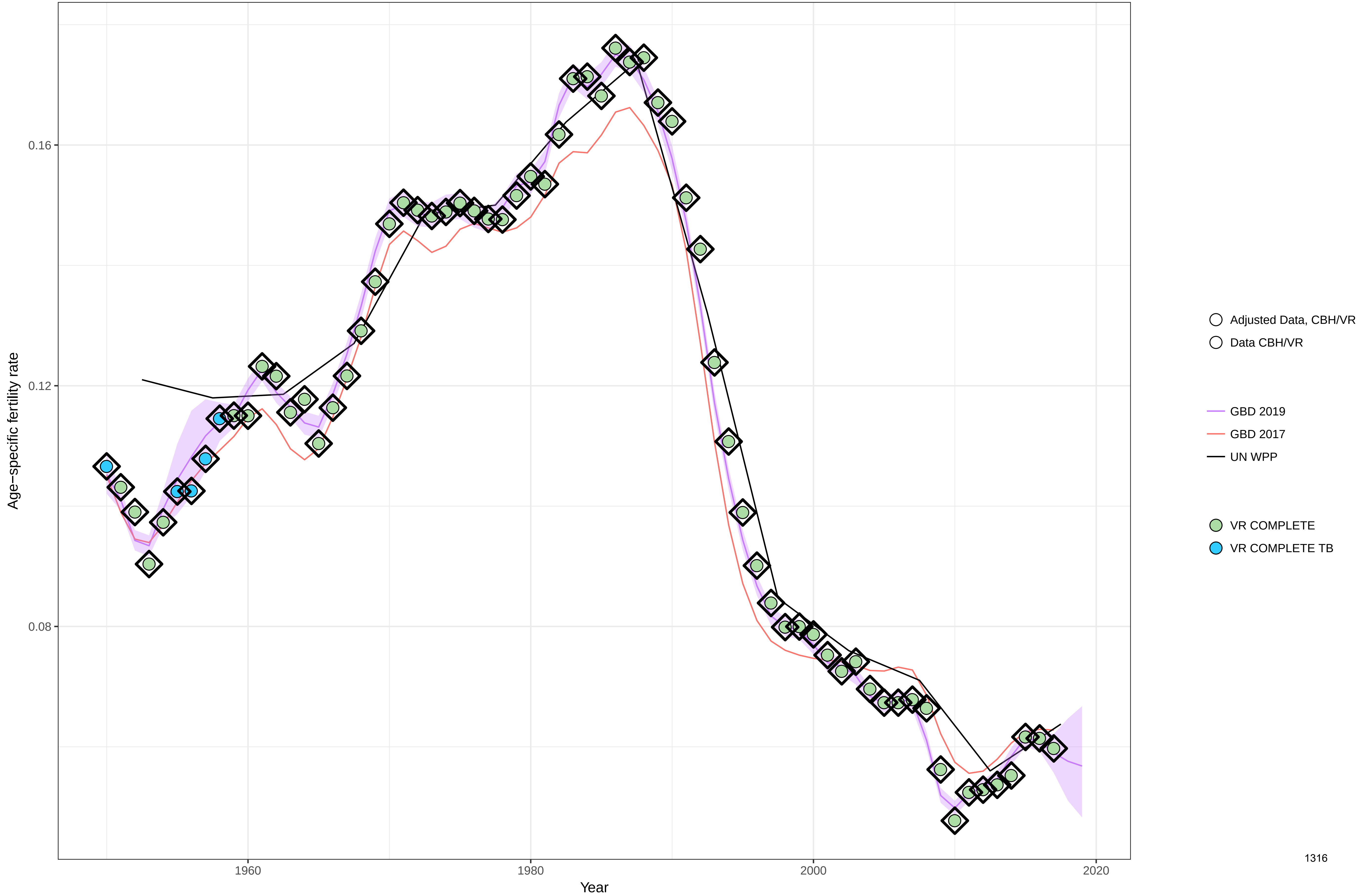

Age−specific fertility fitted trend lines, 1950−2019: Latvia, Ages: 25−29
Data points enclosed with a diamond are reference sources

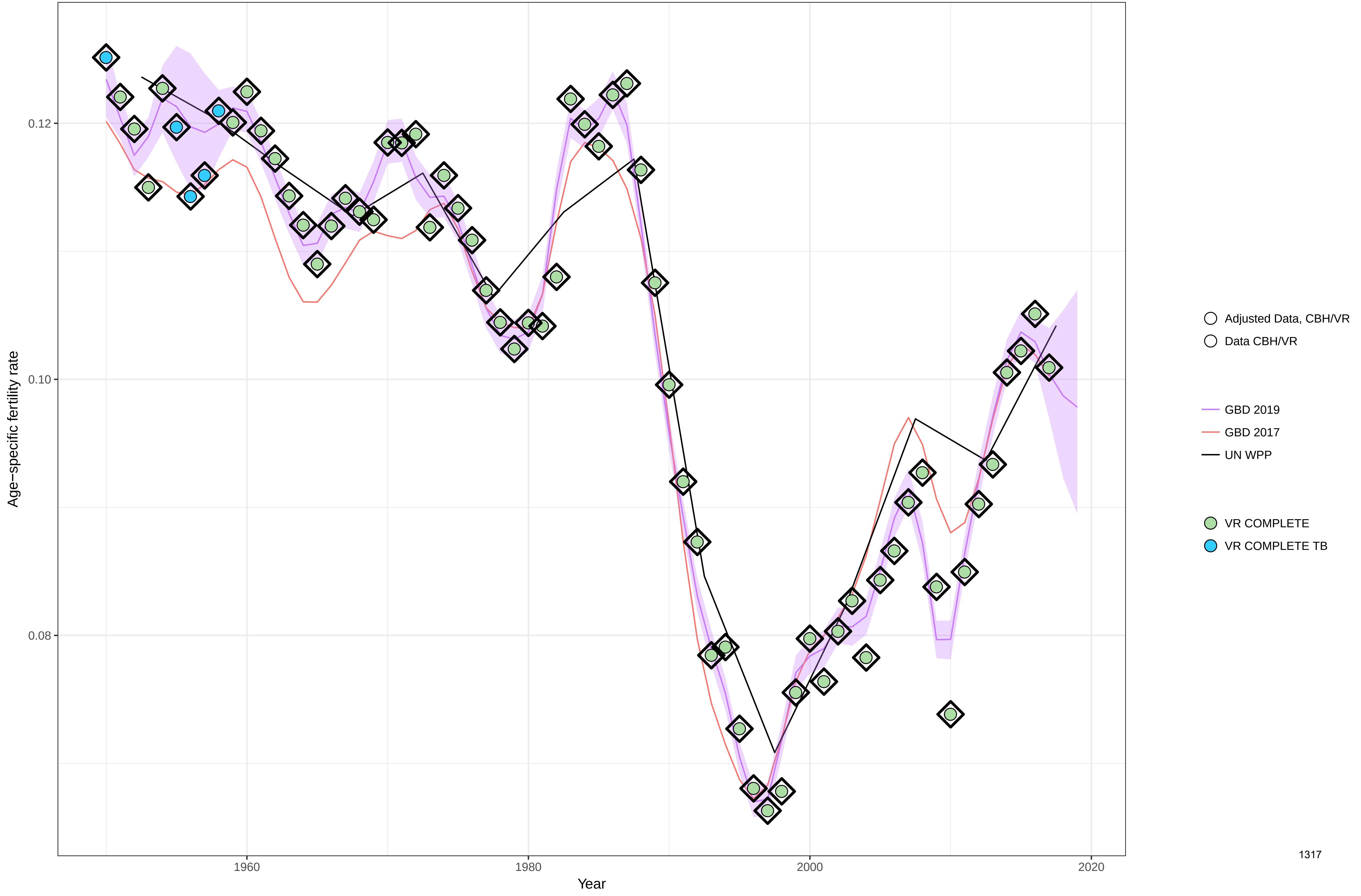

Age−specific fertility fitted trend lines, 1950−2019: Latvia, Ages: 30−34
Data points enclosed with a diamond are reference sources

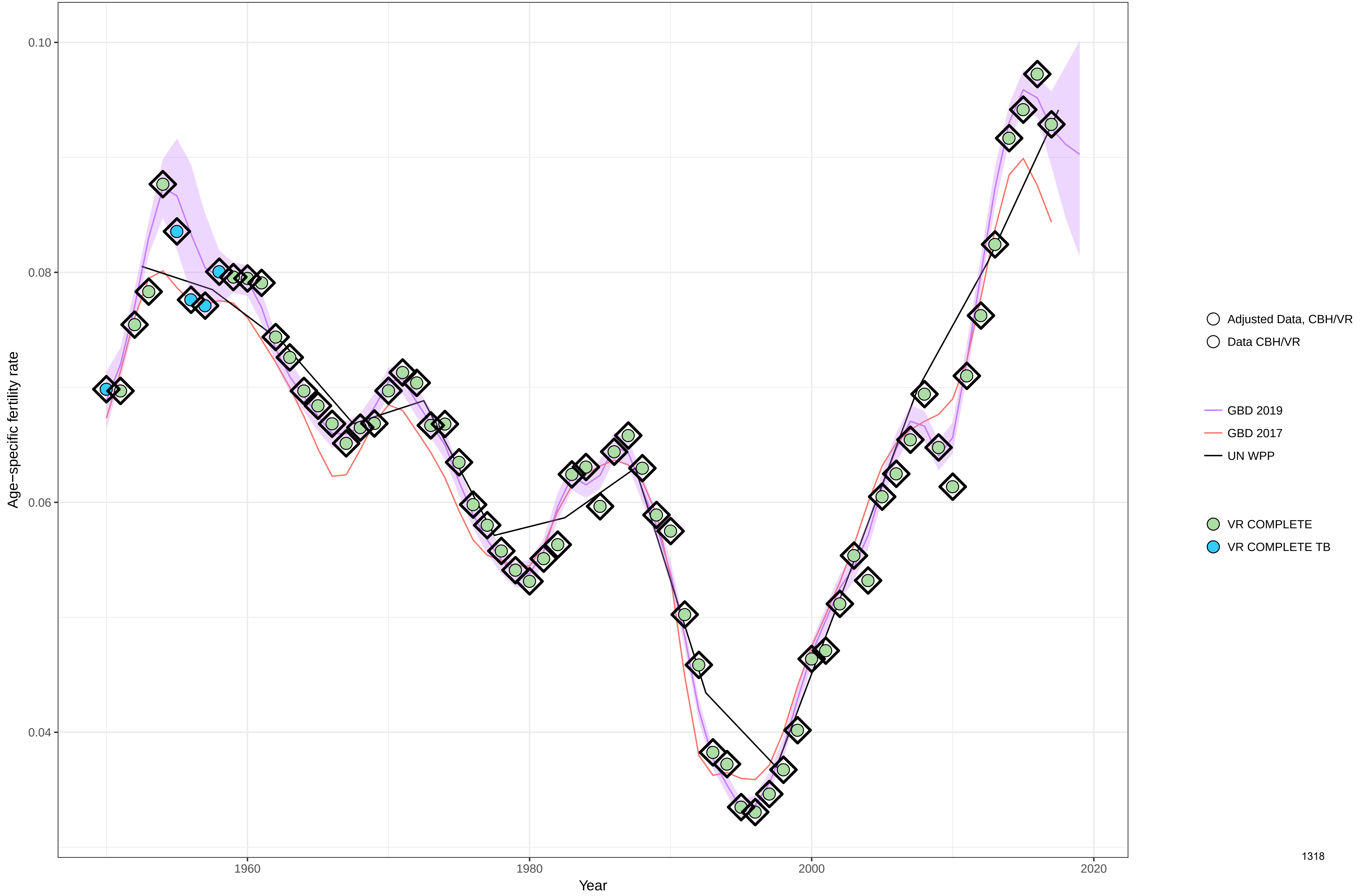

Age−specific fertility fitted trend lines, 1950−2019: Latvia, Ages: 35−39
Data points enclosed with a diamond are reference sources

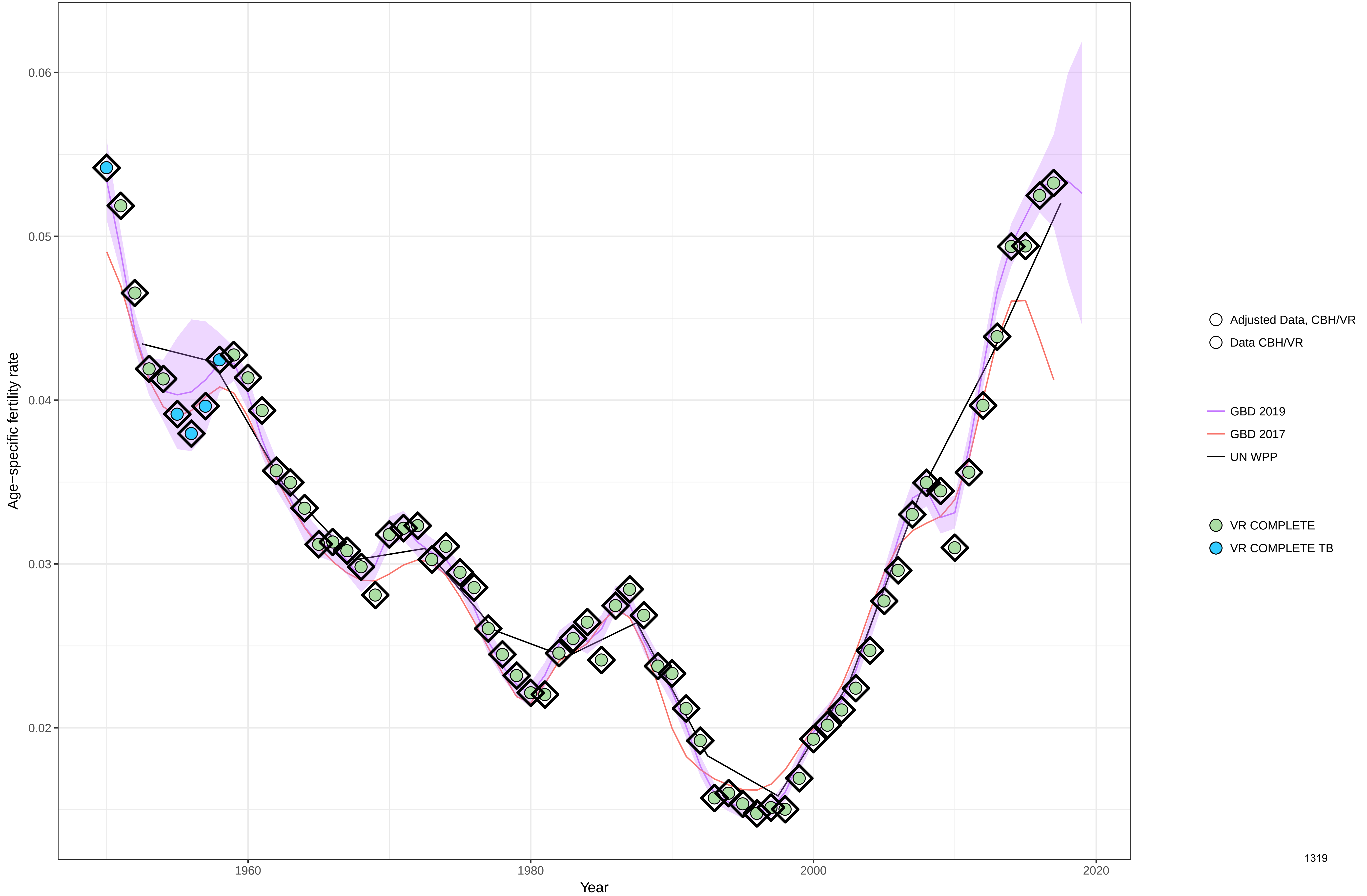

Age−specific fertility fitted trend lines, 1950−2019: Latvia, Ages: 40−44
Data points enclosed with a diamond are reference sources

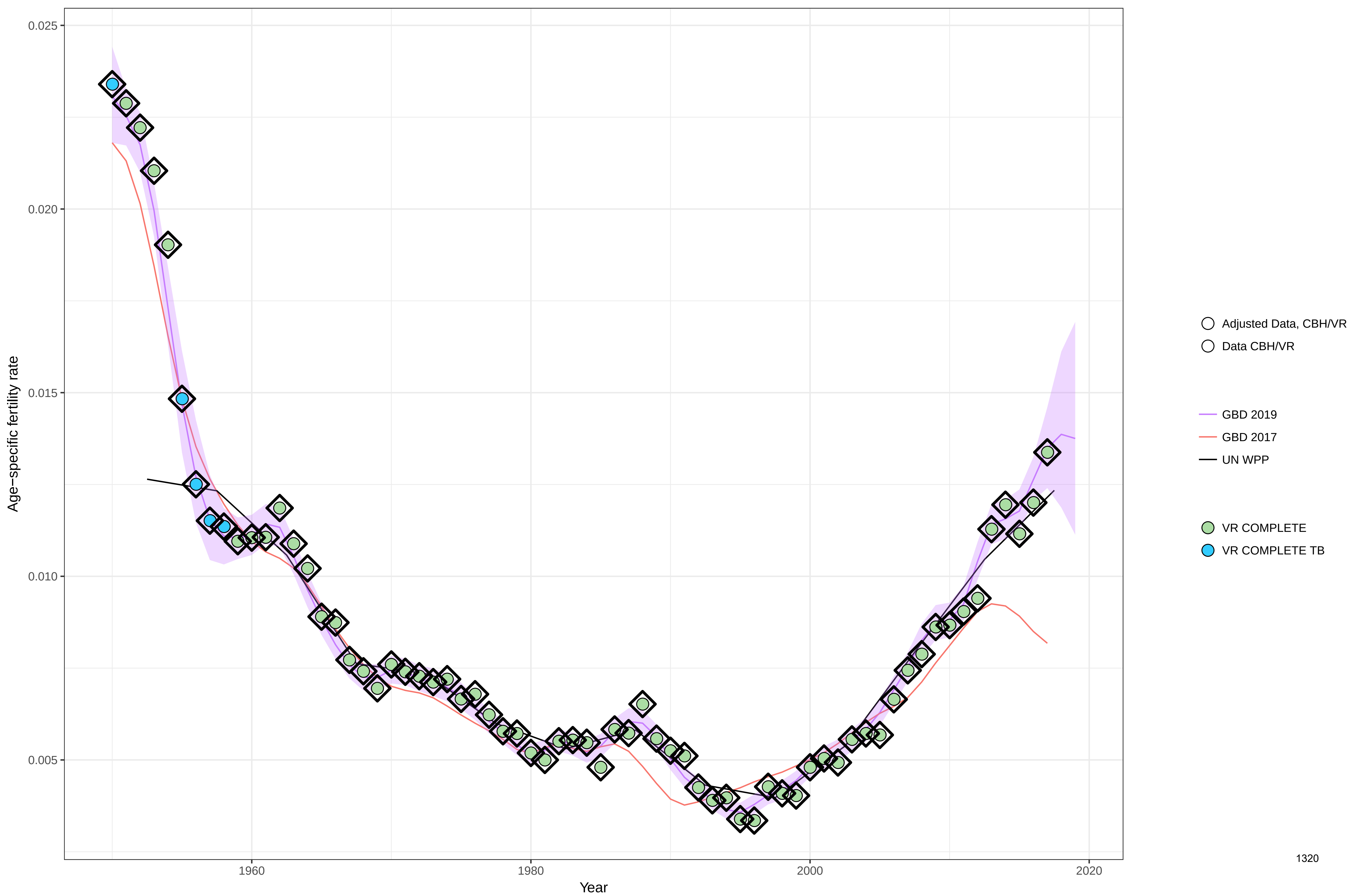

Age−specific fertility fitted trend lines, 1950−2019: Latvia, Ages: 45−49
Data points enclosed with a diamond are reference sources

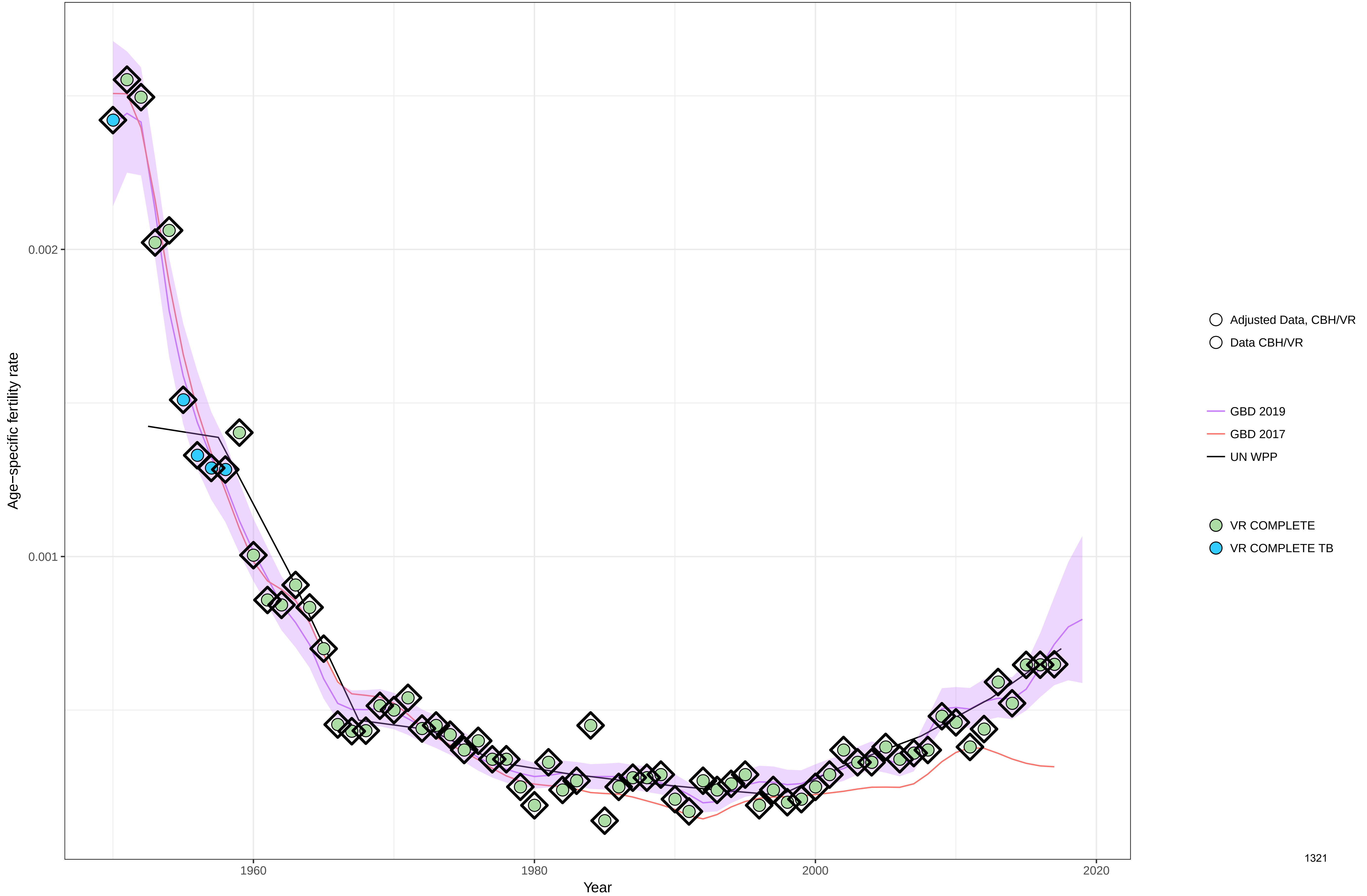

Age−specific fertility fitted trend lines, 1950−2019: Latvia, Ages: 50−54
Data points enclosed with a diamond are reference sources

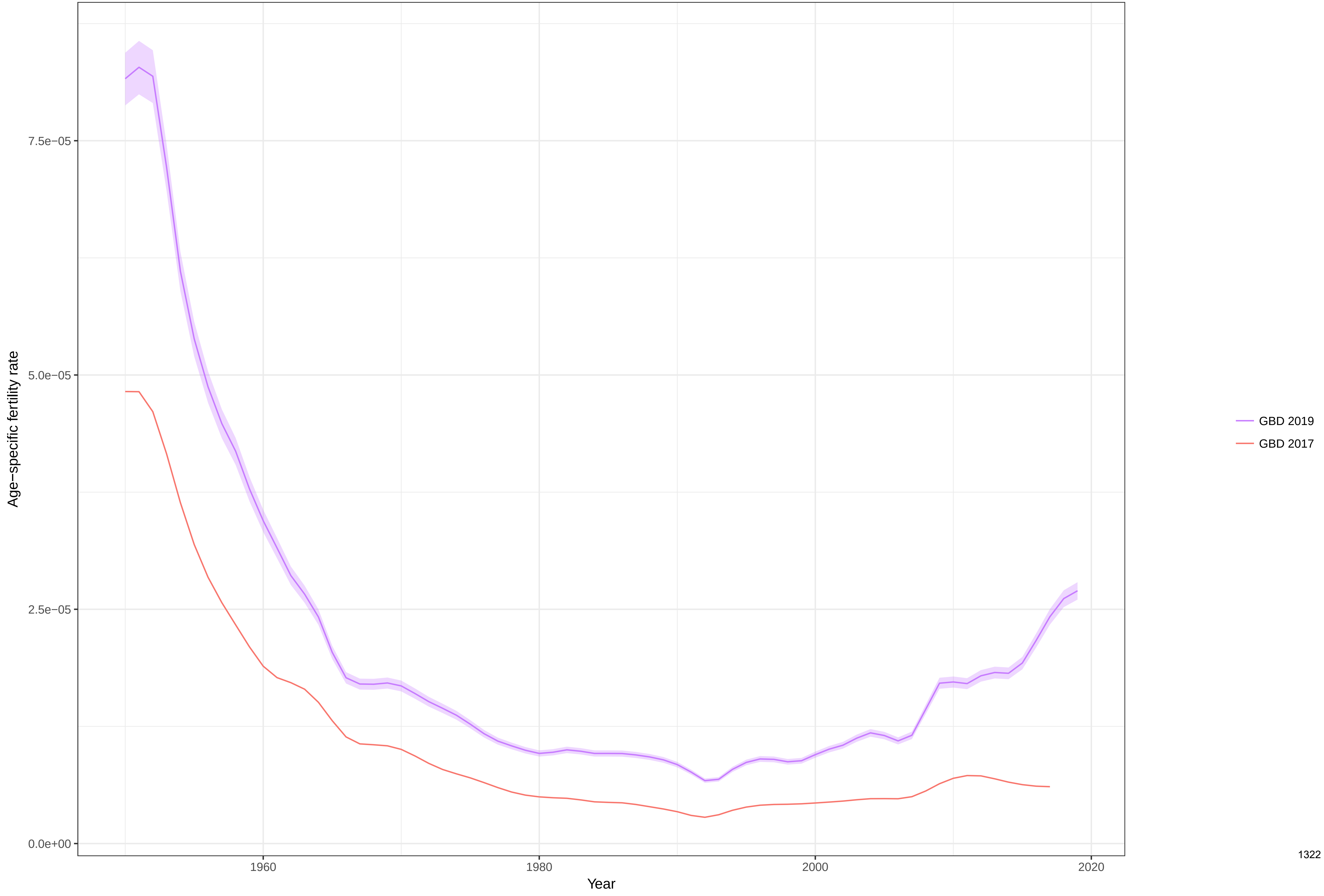

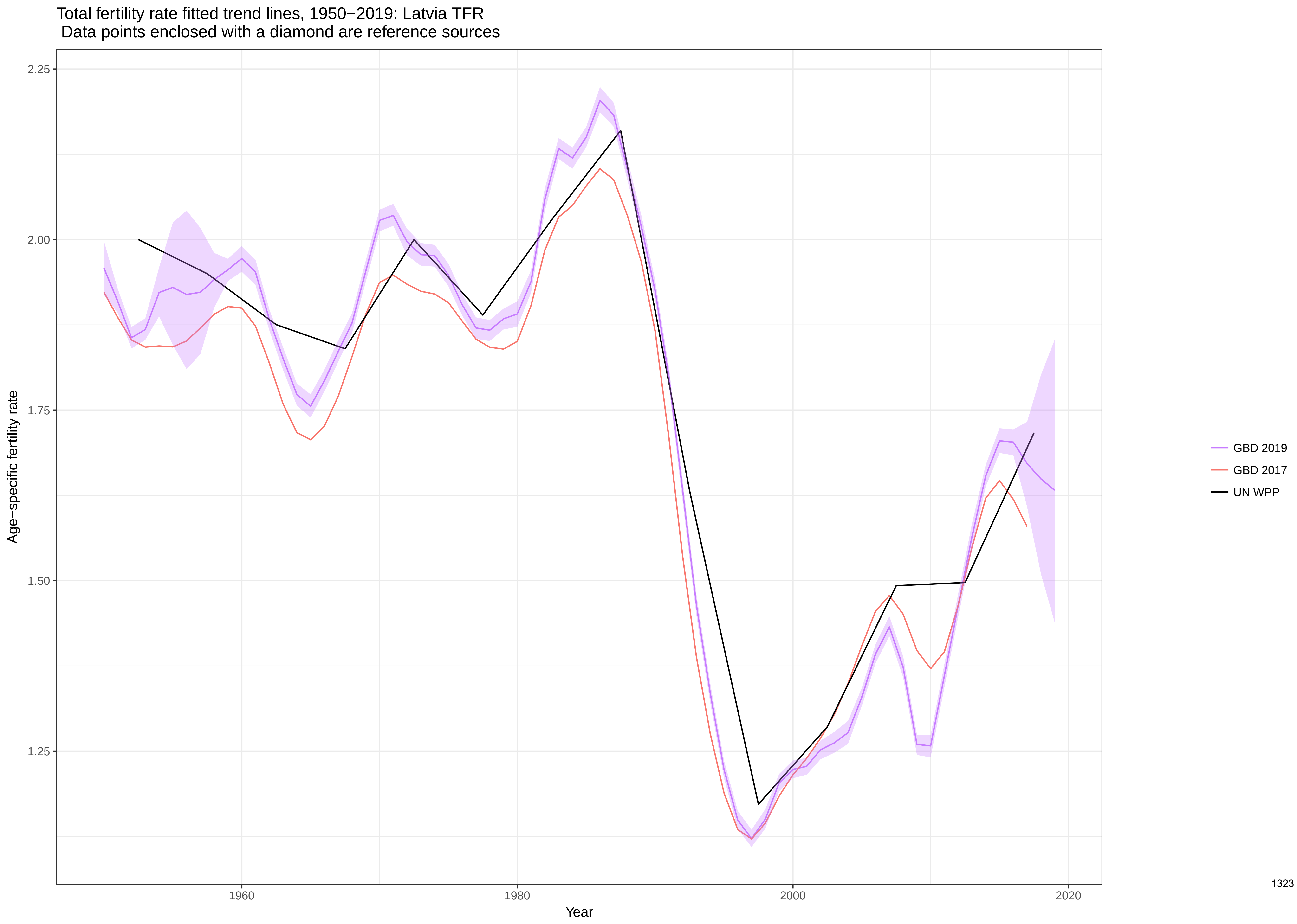
Total fertility rate fitted trend lines, 1950−2019: Latvia TFR
Data points enclosed with a diamond are reference sources
2.25
2.00
1.75
1.50
1.25
Age−specific fertility rate
1960
1980
2000
2020
Year
GBD 2019
GBD 2017
UN WPP

Age−specific fertility fitted trend lines, 1950−2019: Morocco, Ages: 10−14
Data points enclosed with a diamond are reference sources

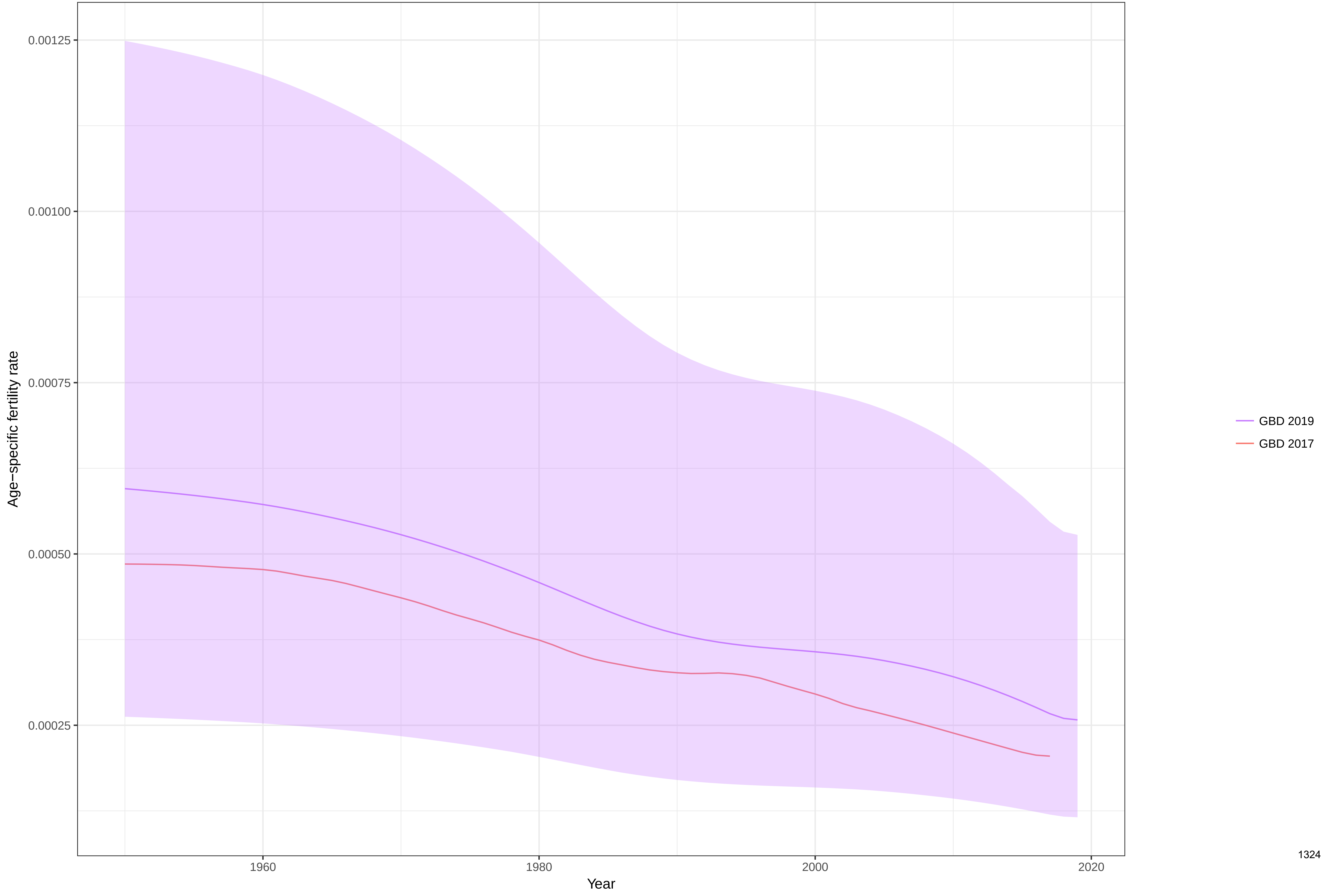

Age−specific fertility fitted trend lines, 1950−2019: Morocco, Ages: 15−19
Data points enclosed with a diamond are reference sources

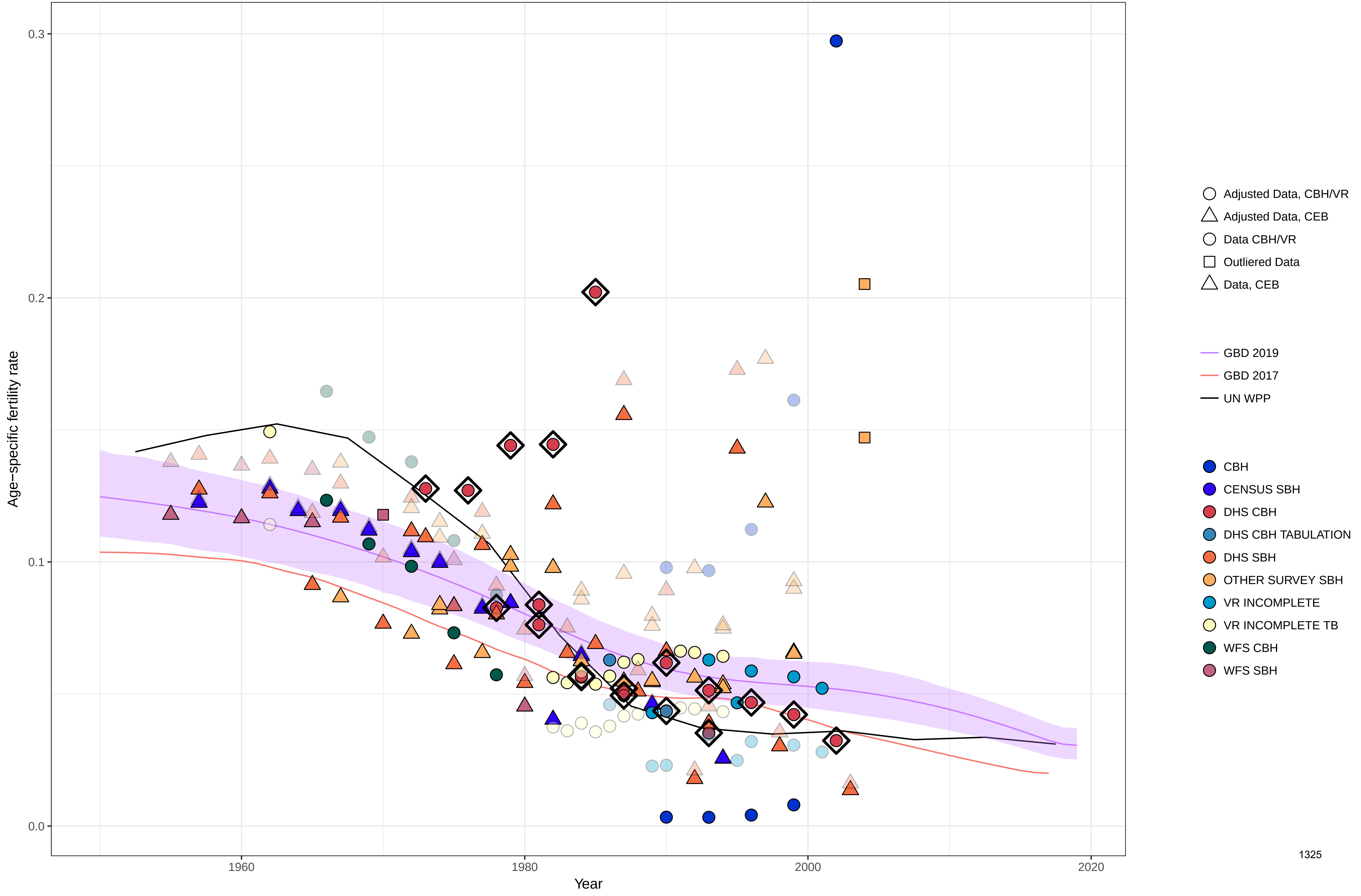

Age−specific fertility fitted trend lines, 1950−2019: Morocco, Ages: 20−24
Data points enclosed with a diamond are reference sources

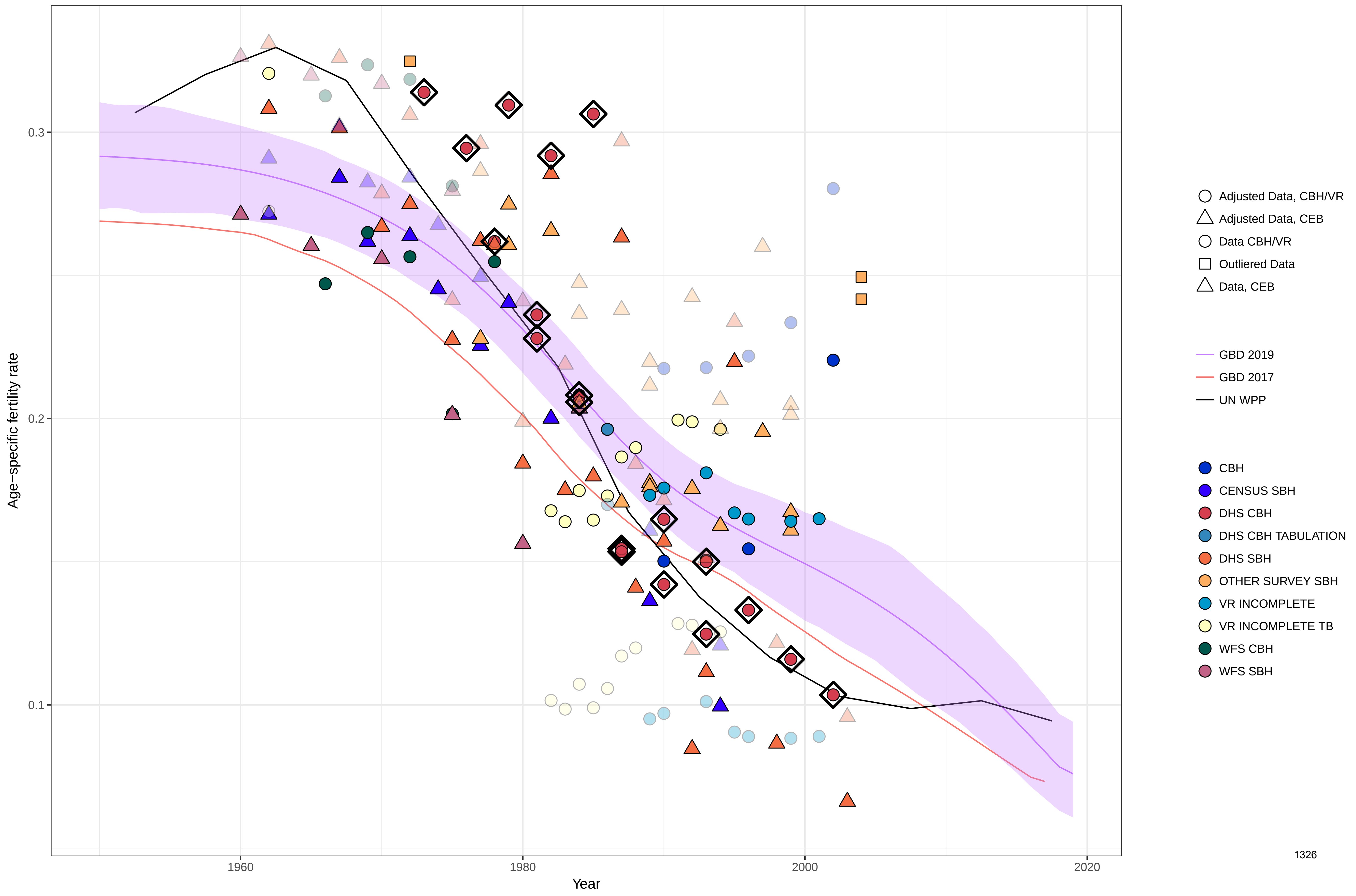

Age−specific fertility fitted trend lines, 1950−2019: Morocco, Ages: 25−29
Data points enclosed with a diamond are reference sources

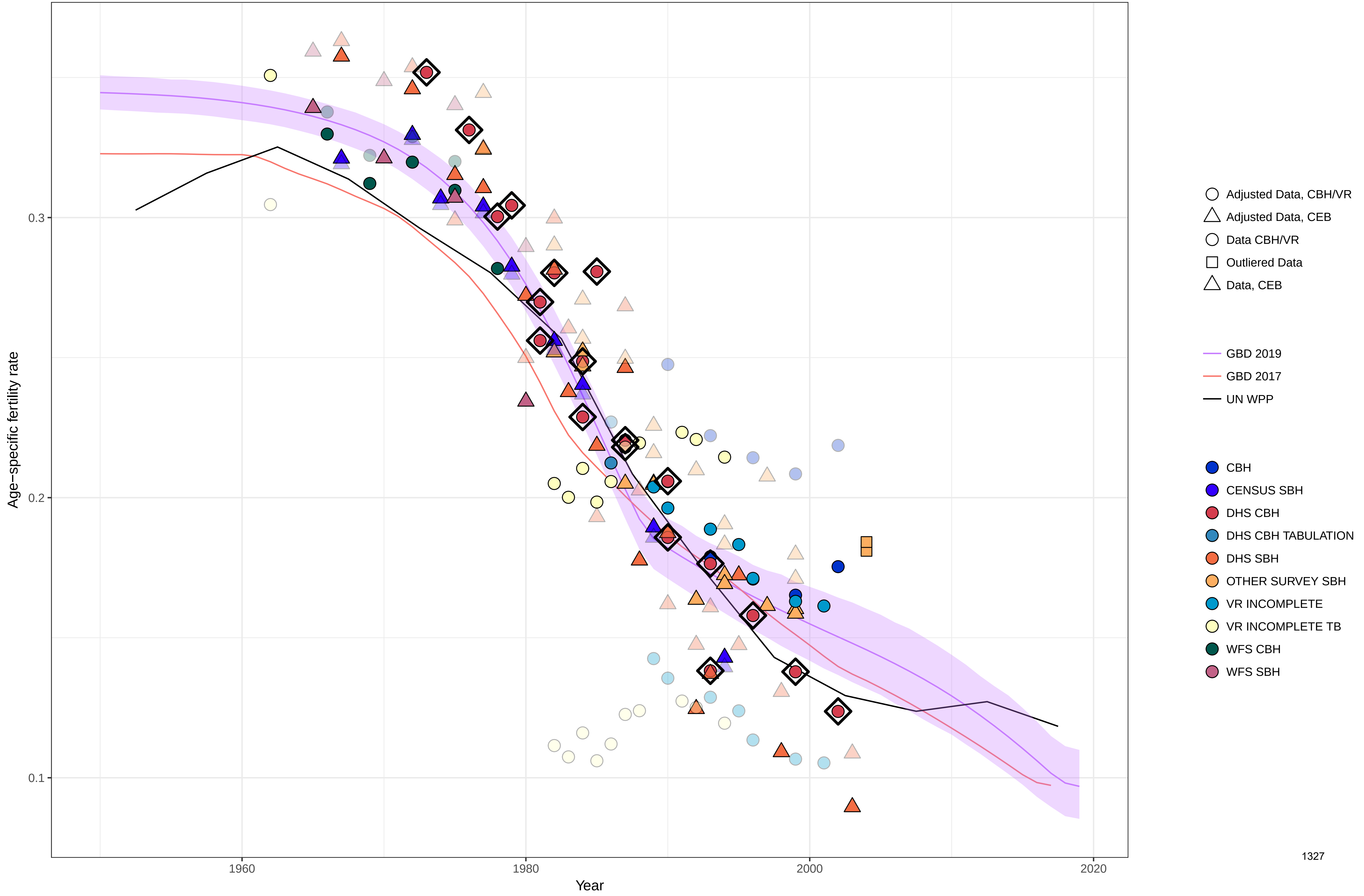

Age−specific fertility fitted trend lines, 1950−2019: Morocco, Ages: 30−34
Data points enclosed with a diamond are reference sources

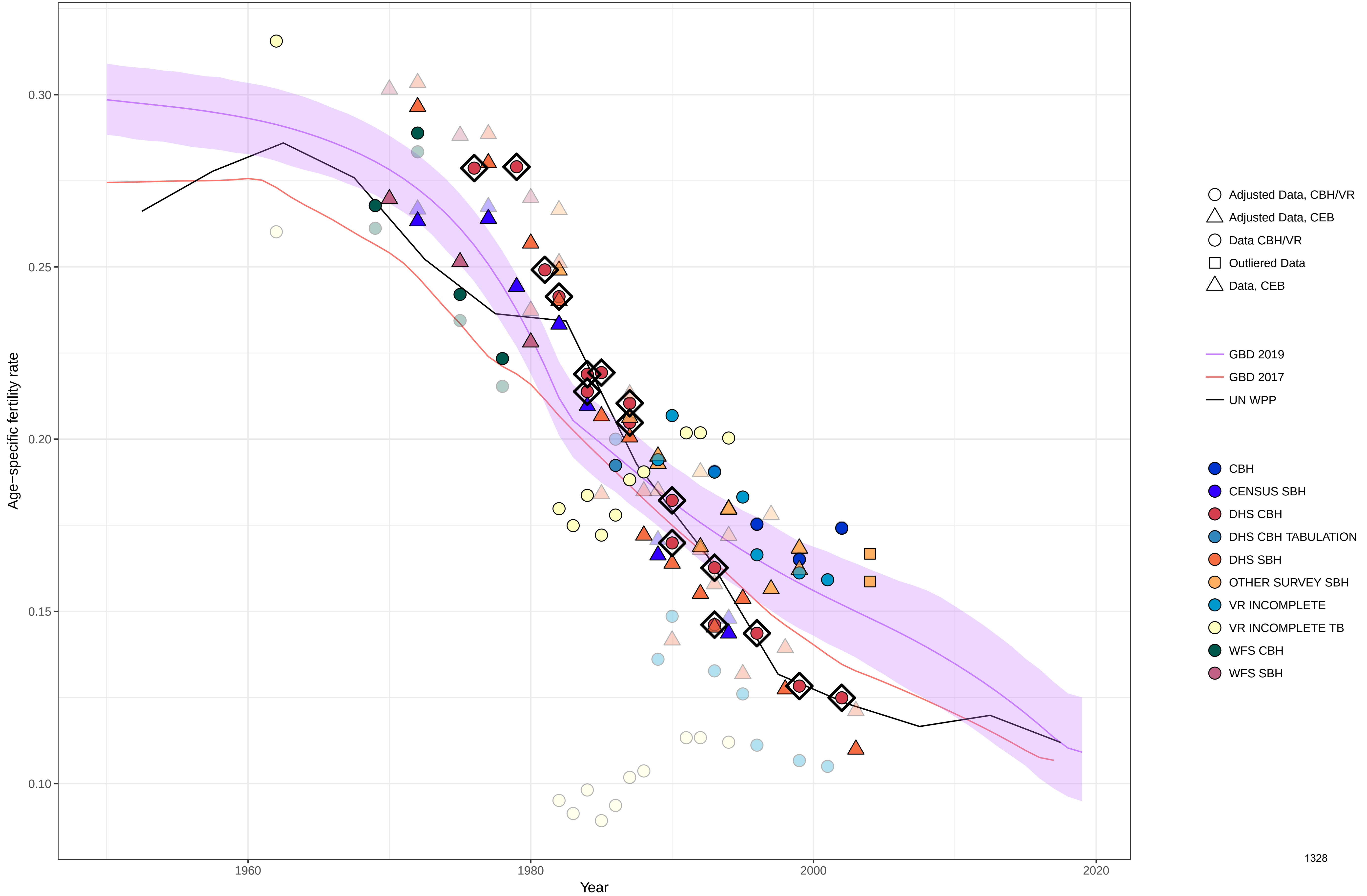

Age−specific fertility fitted trend lines, 1950−2019: Morocco, Ages: 35−39
Data points enclosed with a diamond are reference sources

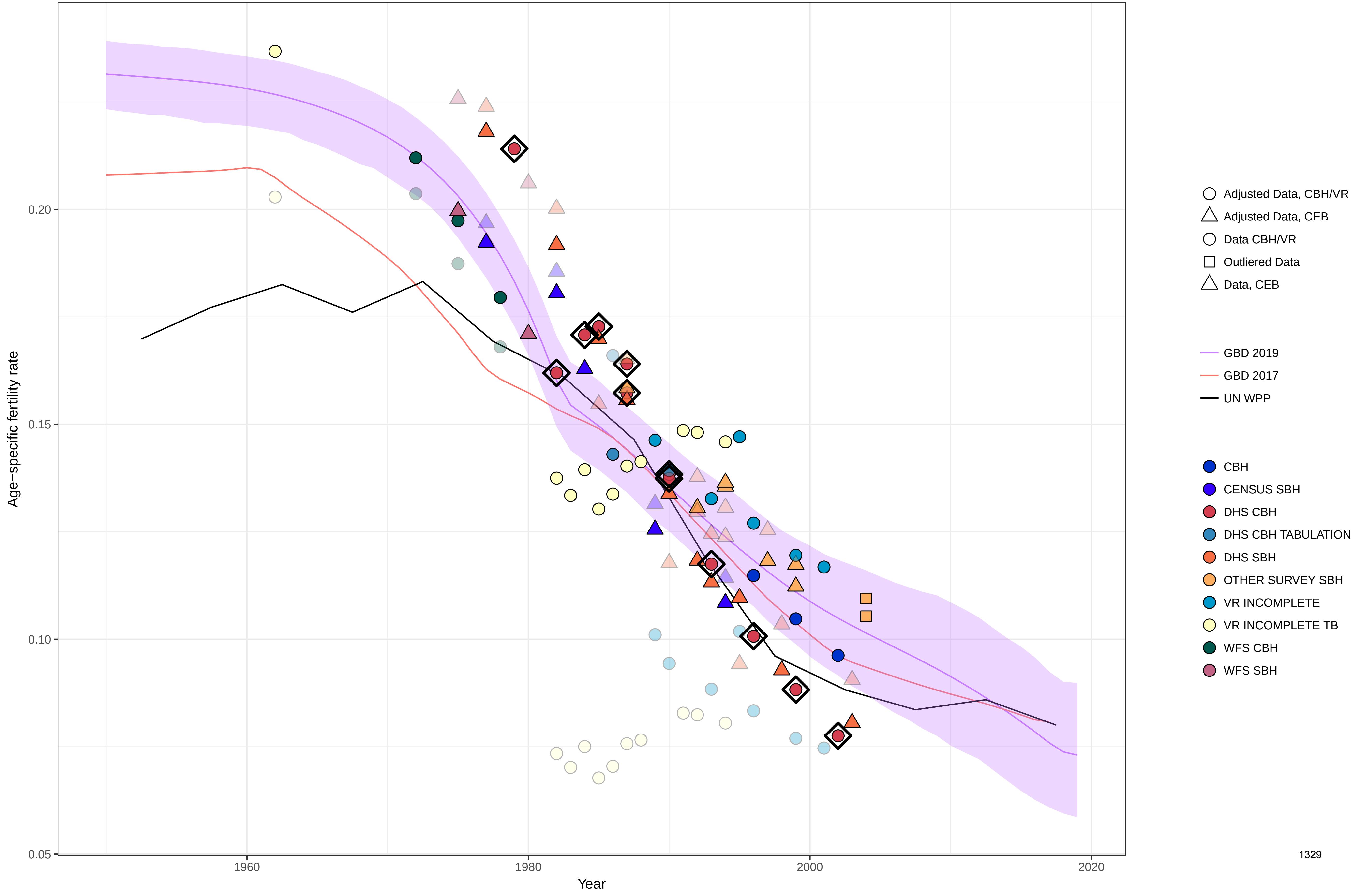

Age−specific fertility fitted trend lines, 1950−2019: Morocco, Ages: 40−44
Data points enclosed with a diamond are reference sources

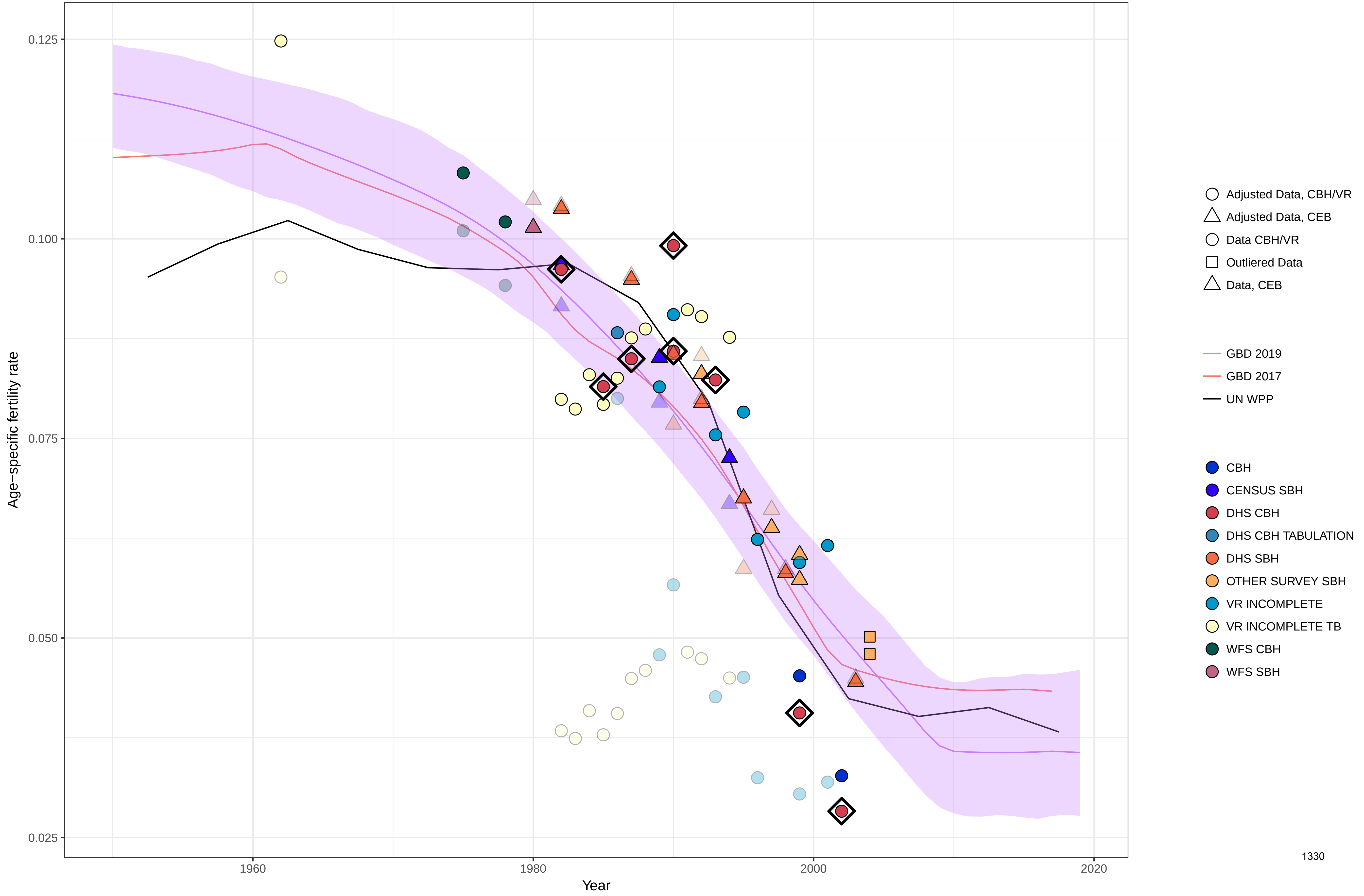

Age−specific fertility fitted trend lines, 1950−2019: Morocco, Ages: 45−49
Data points enclosed with a diamond are reference sources

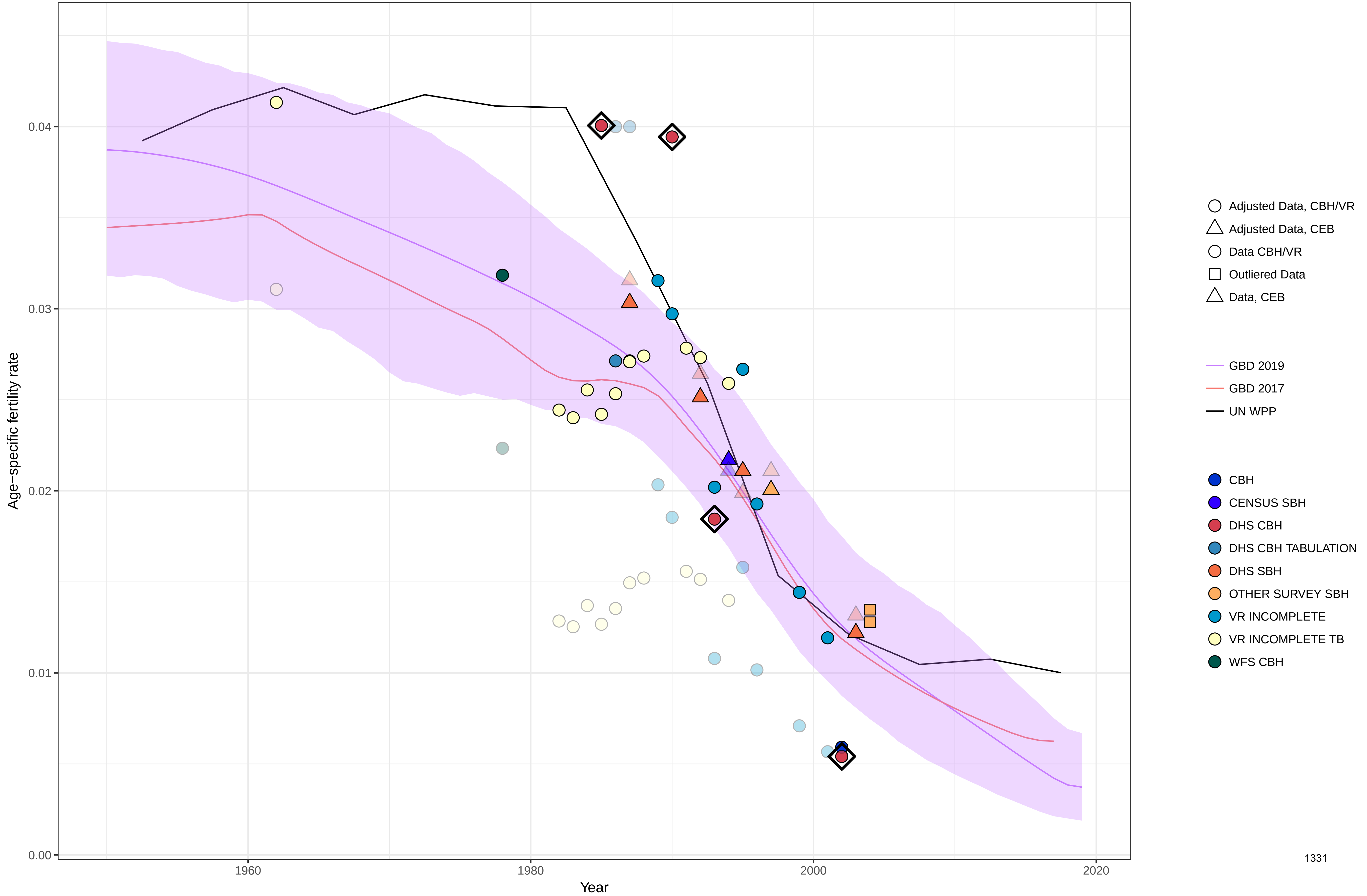

Age−specific fertility fitted trend lines, 1950−2019: Morocco, Ages: 50−54
Data points enclosed with a diamond are reference sources

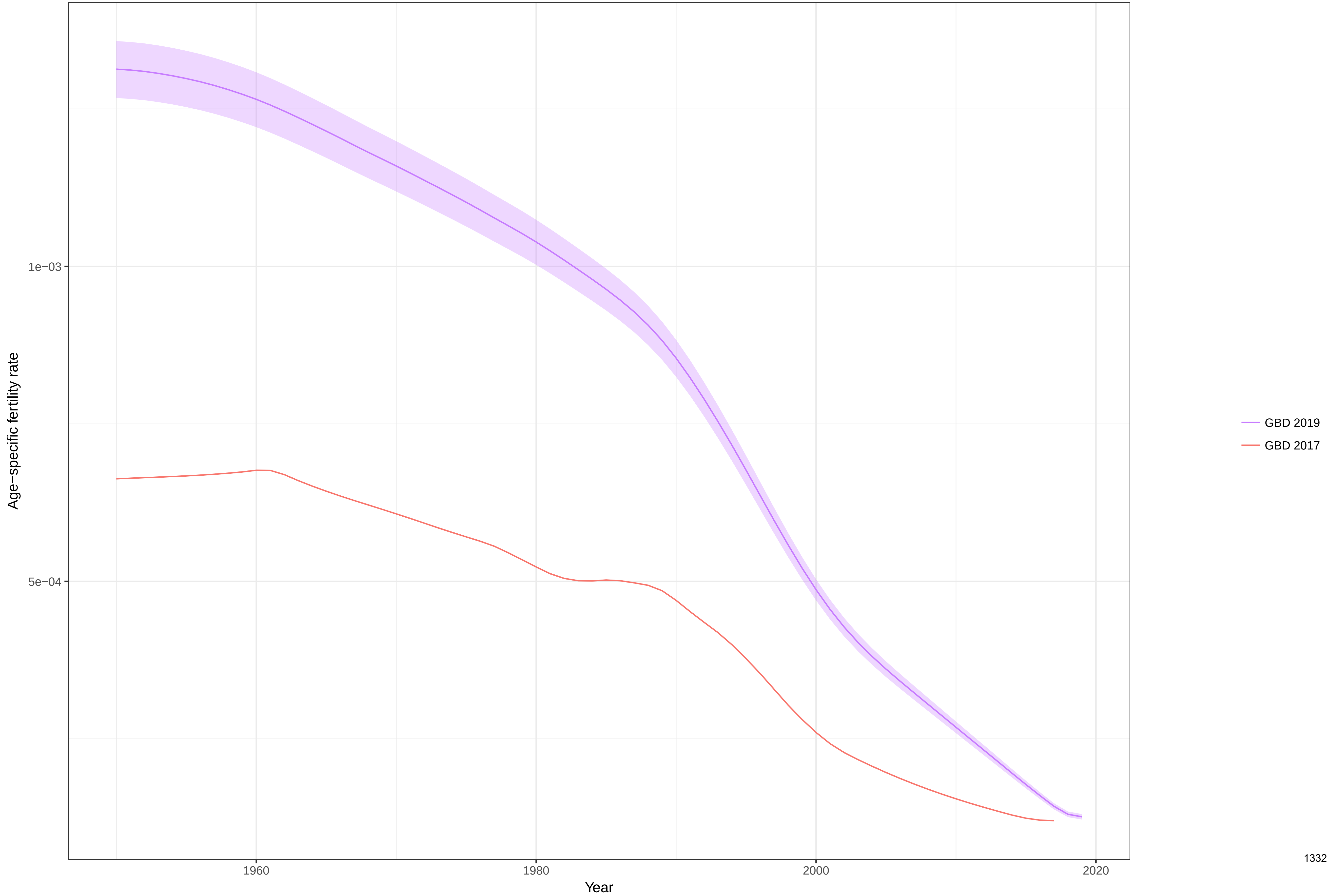

Total fertility rate fitted trend lines, 1950−2019: Morocco TFR
Data points enclosed with a diamond are reference sources

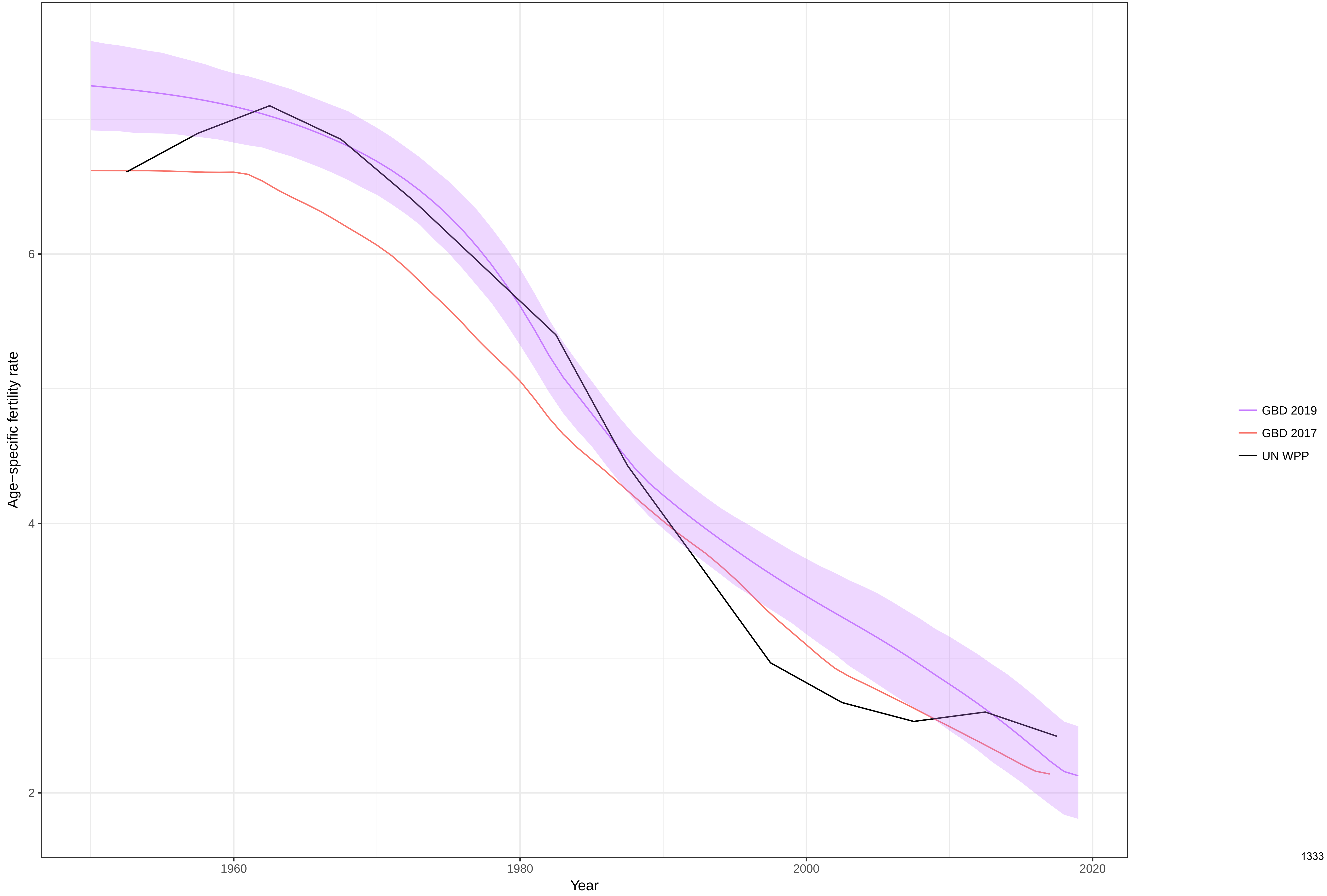

Age−specific fertility fitted trend lines, 1950−2019: Monaco, Ages: 10−14
Data points enclosed with a diamond are reference sources

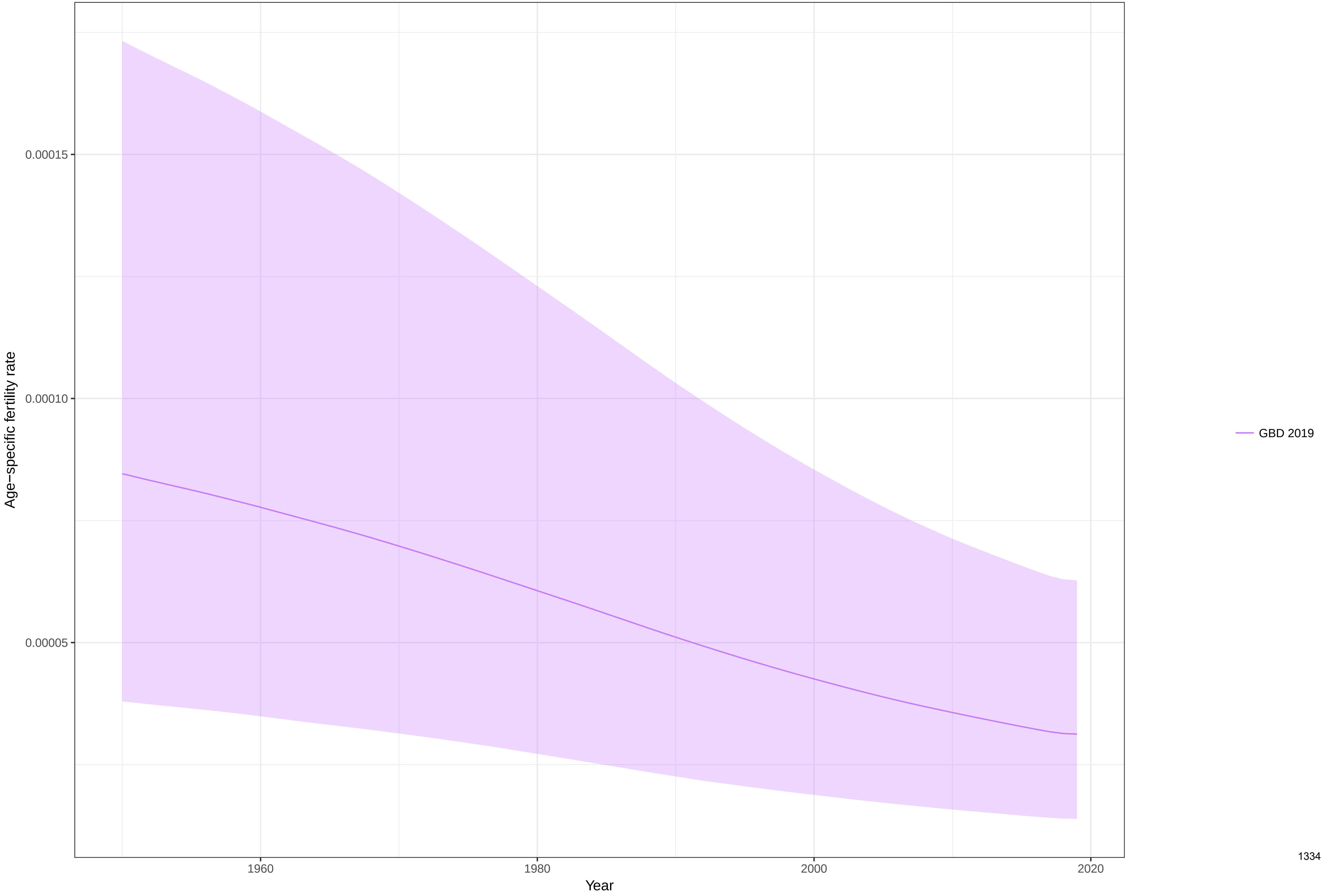

Age−specific fertility fitted trend lines, 1950−2019: Monaco, Ages: 15−19
Data points enclosed with a diamond are reference sources

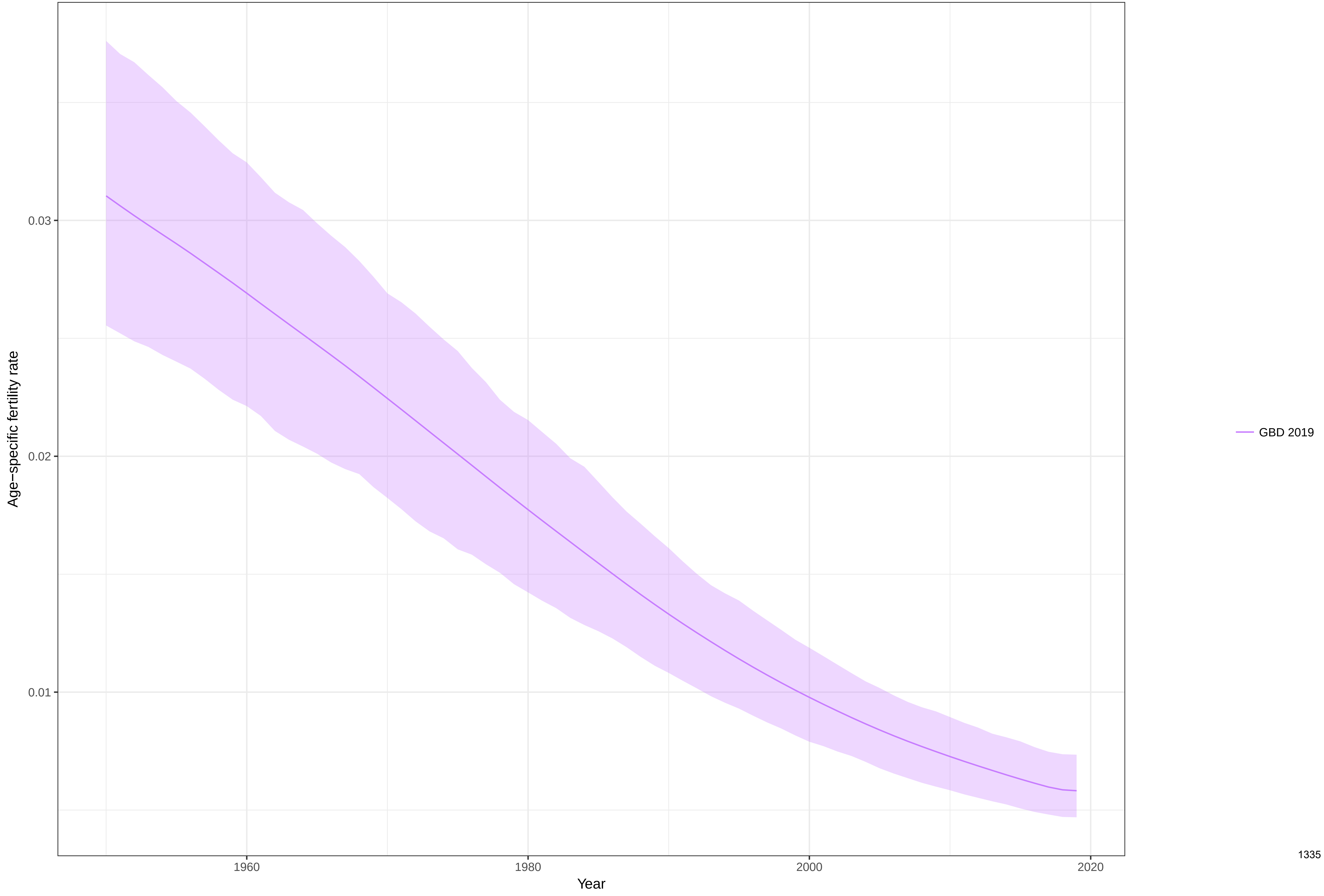

Age−specific fertility fitted trend lines, 1950−2019: Monaco, Ages: 20−24
Data points enclosed with a diamond are reference sources

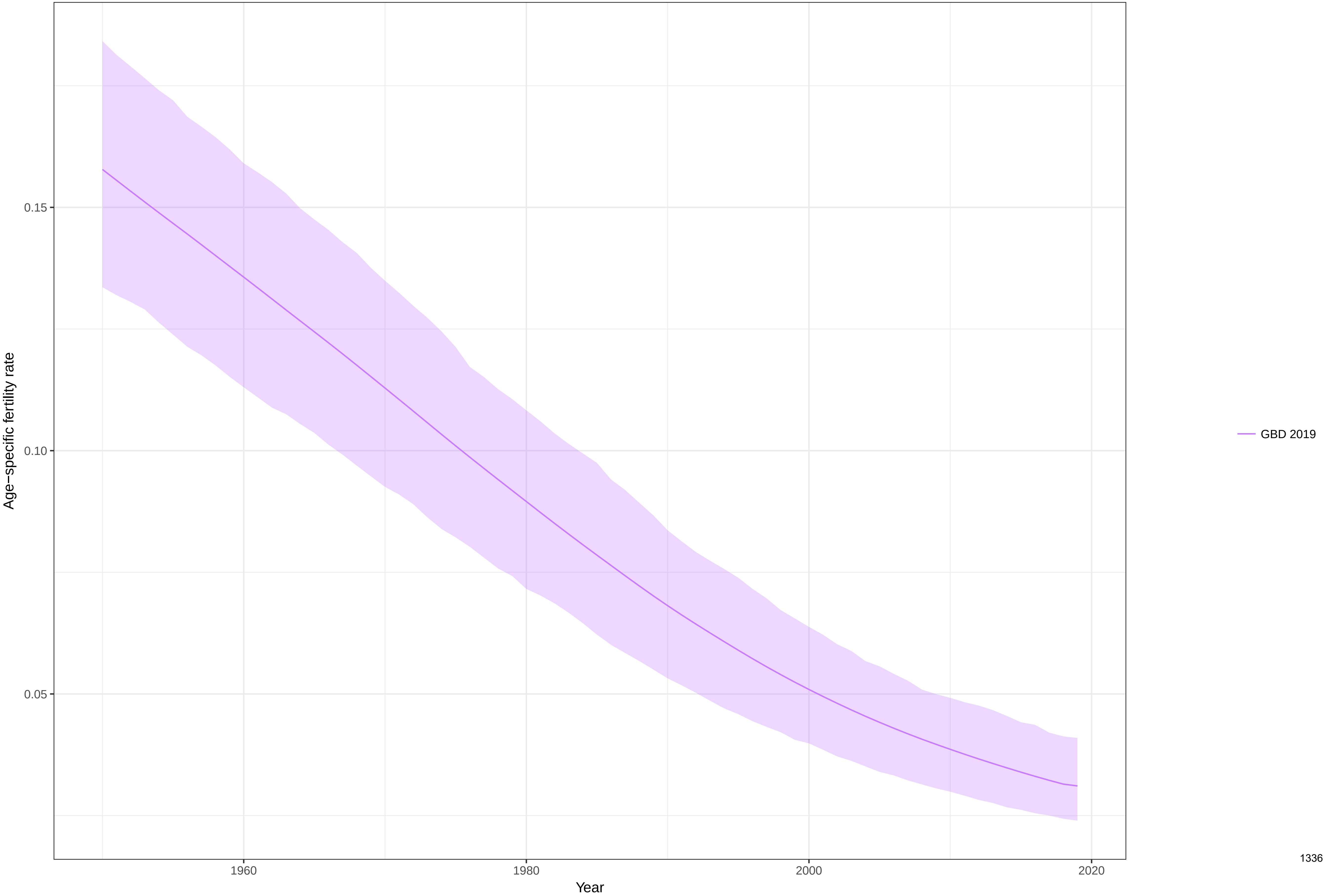

Age−specific fertility fitted trend lines, 1950−2019: Monaco, Ages: 25−29
Data points enclosed with a diamond are reference sources

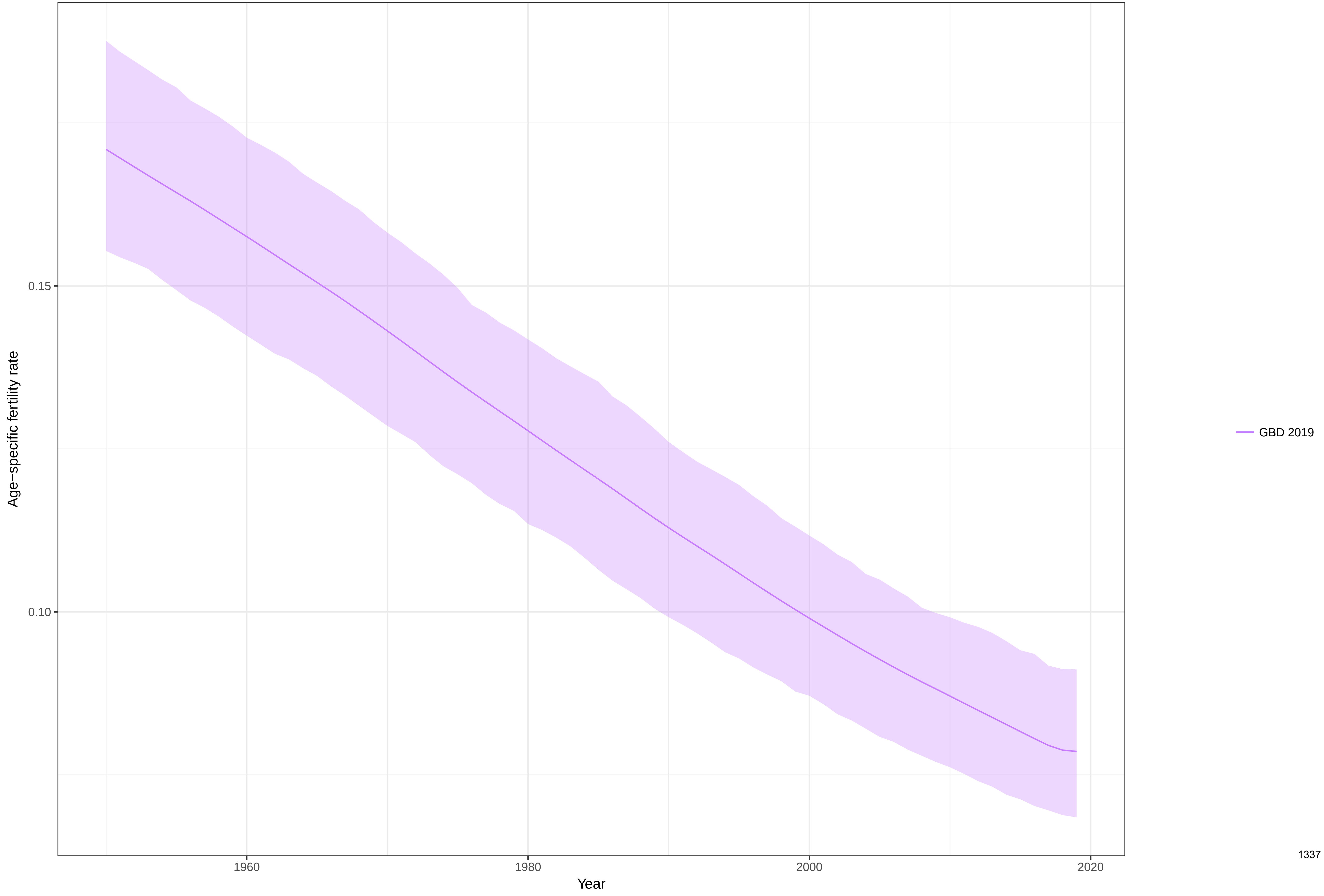

Age−specific fertility fitted trend lines, 1950−2019: Monaco, Ages: 30−34
Data points enclosed with a diamond are reference sources

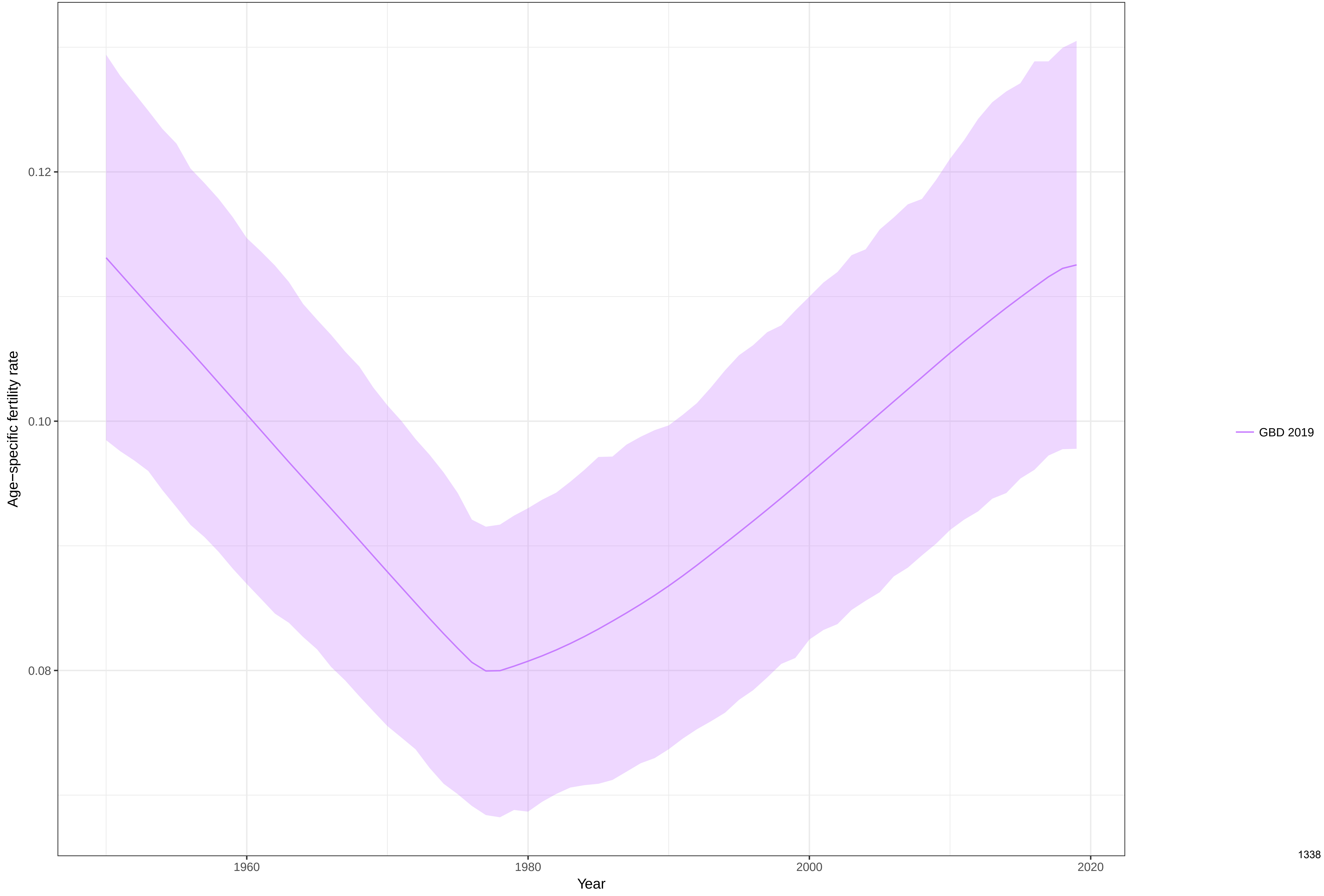

Age−specific fertility fitted trend lines, 1950−2019: Monaco, Ages: 35−39
Data points enclosed with a diamond are reference sources

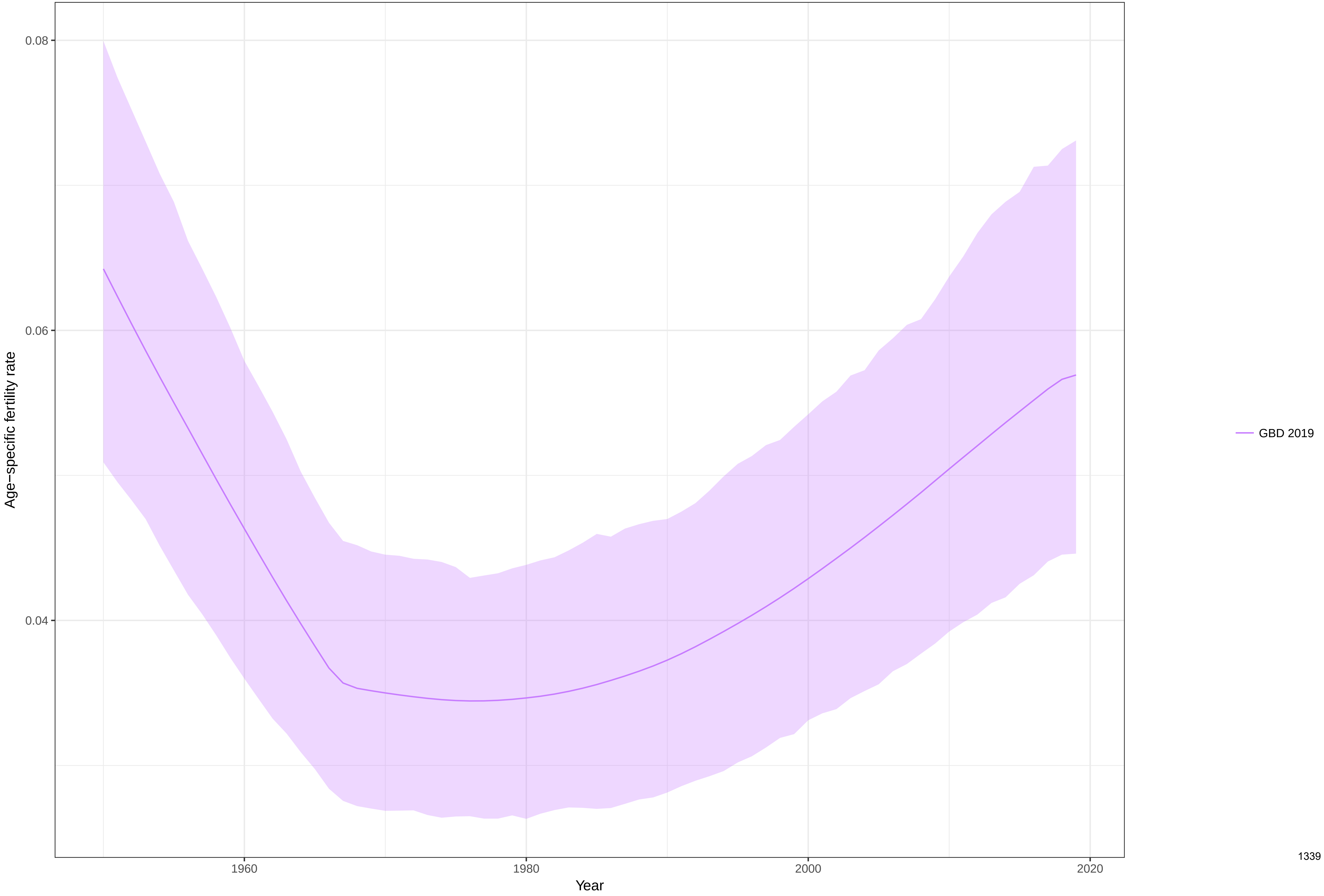

Age−specific fertility fitted trend lines, 1950−2019: Monaco, Ages: 40−44
Data points enclosed with a diamond are reference sources

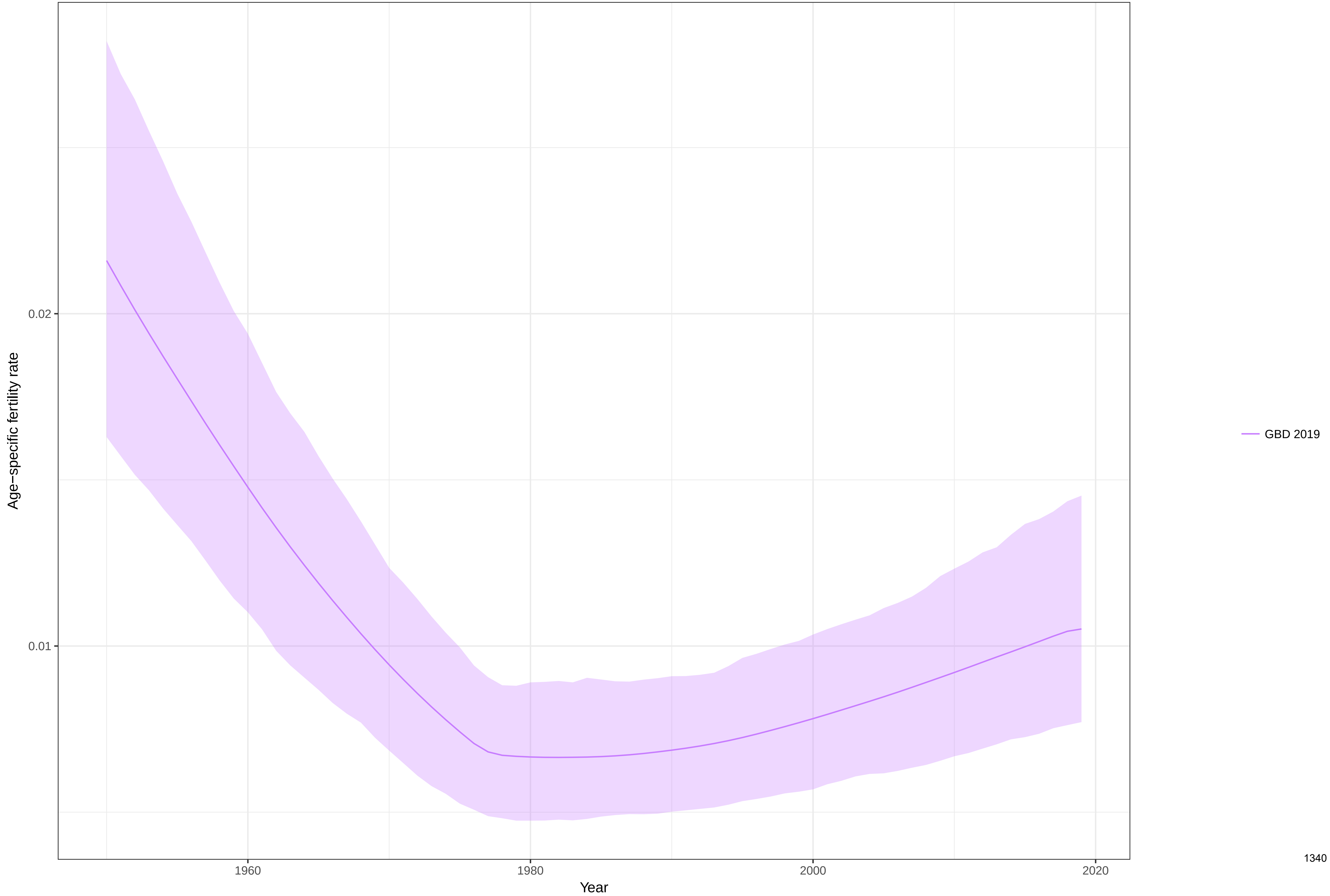

Age−specific fertility fitted trend lines, 1950−2019: Monaco, Ages: 45−49
Data points enclosed with a diamond are reference sources

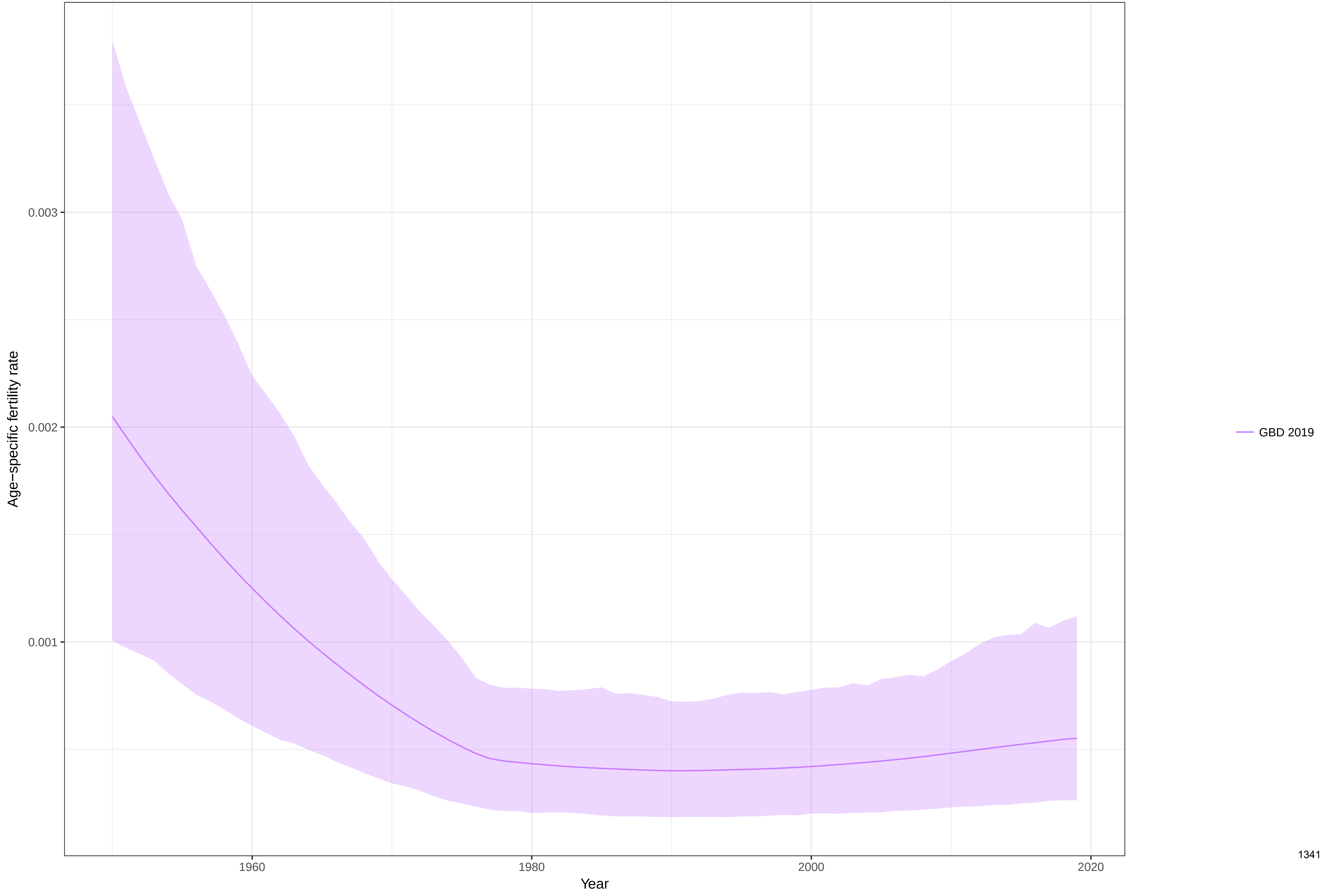

Age−specific fertility fitted trend lines, 1950−2019: Monaco, Ages: 50−54
Data points enclosed with a diamond are reference sources

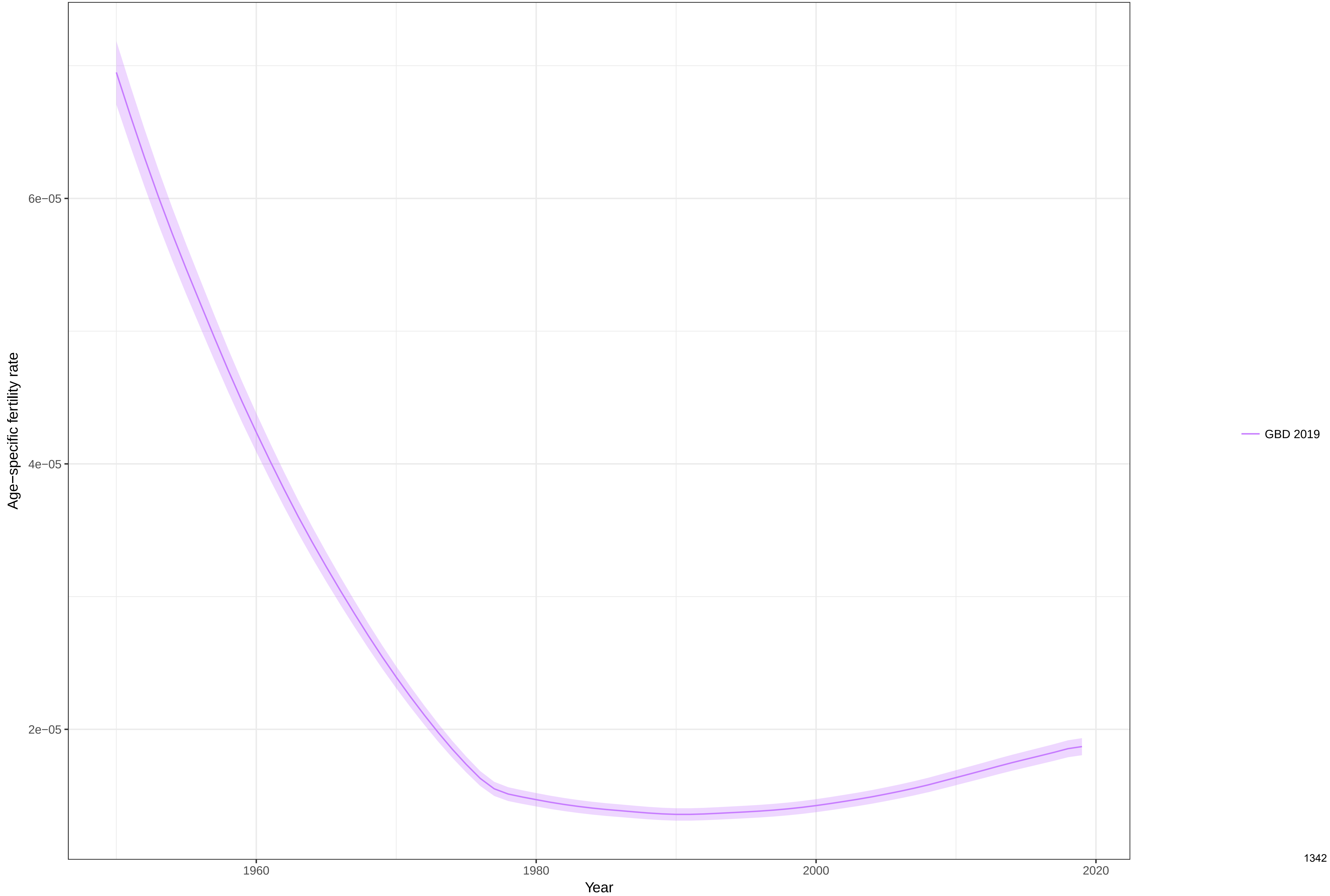

Total fertility rate fitted trend lines, 1950−2019: Monaco TFR
Data points enclosed with a diamond are reference sources

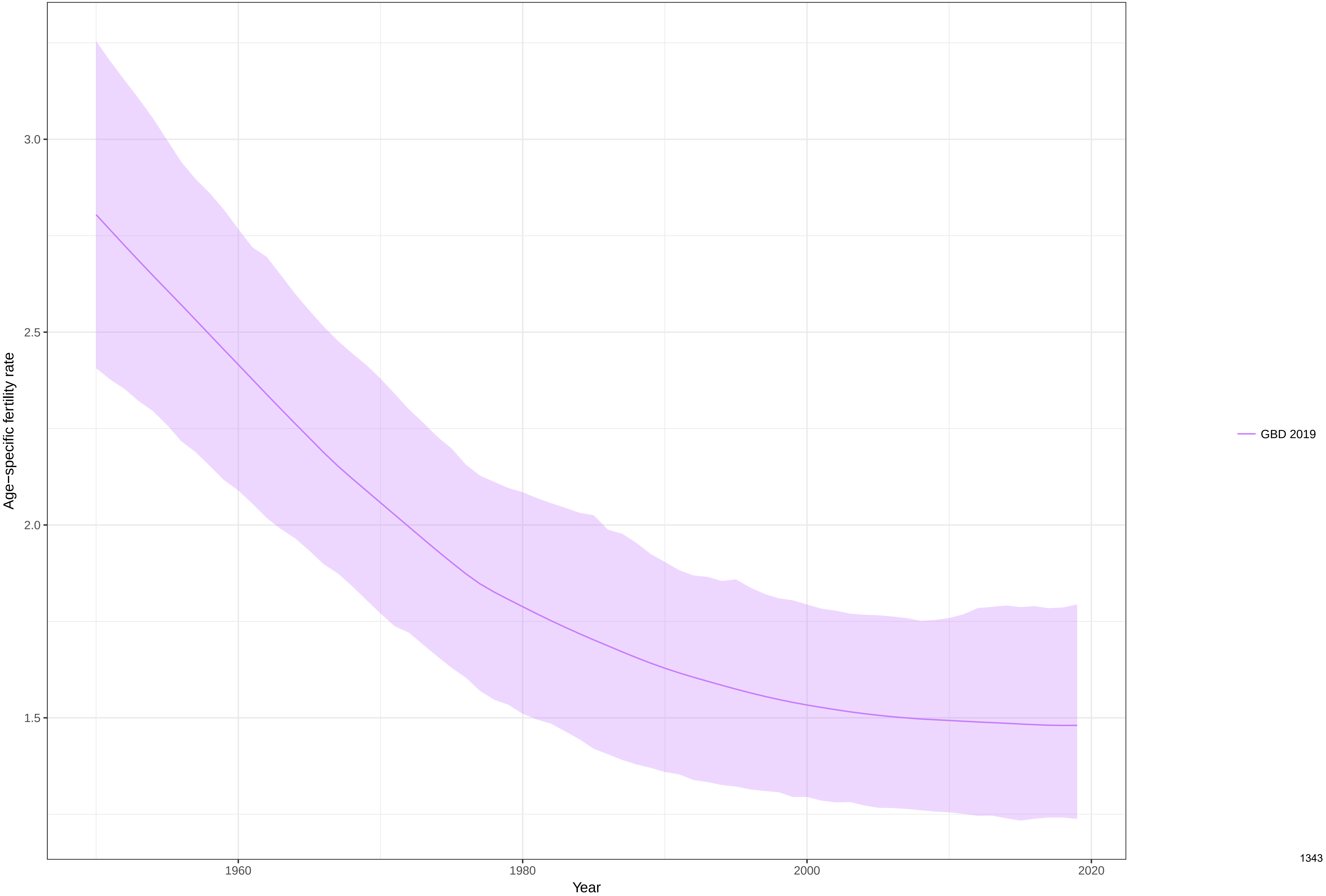

Age−specific fertility fitted trend lines, 1950−2019: Republic of Moldova, Ages: 10−14
Data points enclosed with a diamond are reference sources

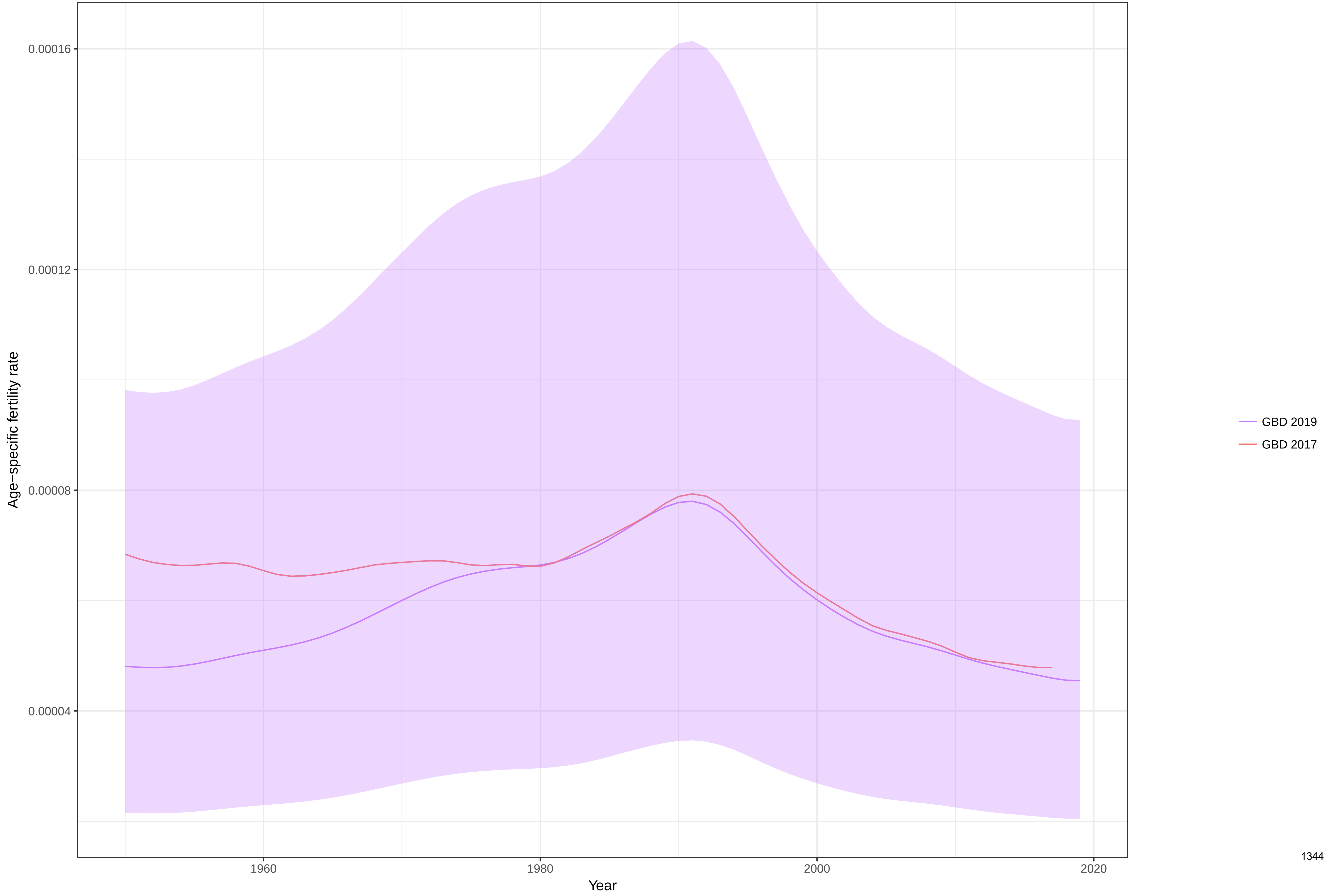

Age−specific fertility fitted trend lines, 1950−2019: Republic of Moldova, Ages: 15−19
Data points enclosed with a diamond are reference sources

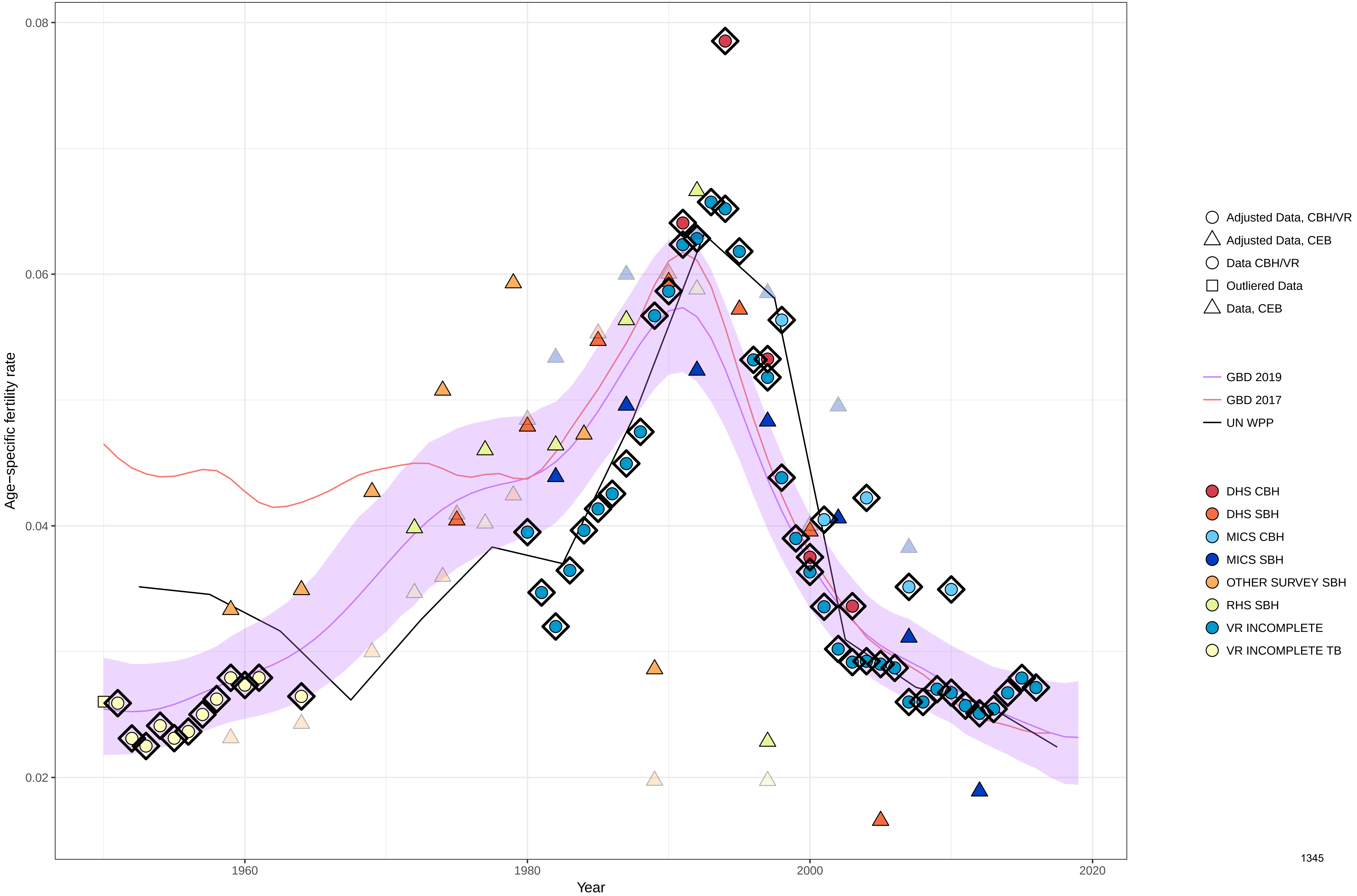

Age−specific fertility fitted trend lines, 1950−2019: Republic of Moldova, Ages: 20−24
Data points enclosed with a diamond are reference sources

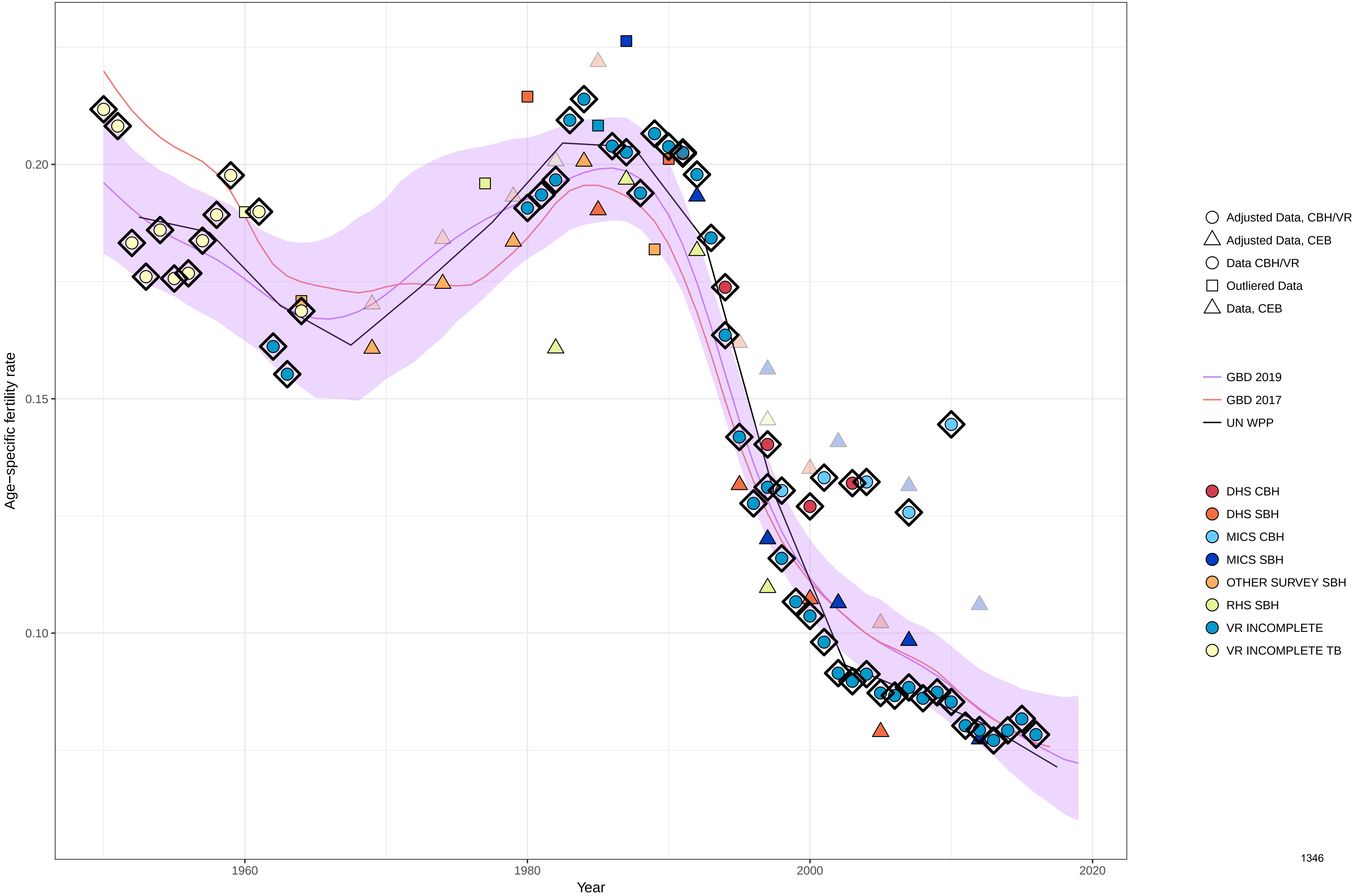

Age−specific fertility fitted trend lines, 1950−2019: Republic of Moldova, Ages: 25−29
Data points enclosed with a diamond are reference sources

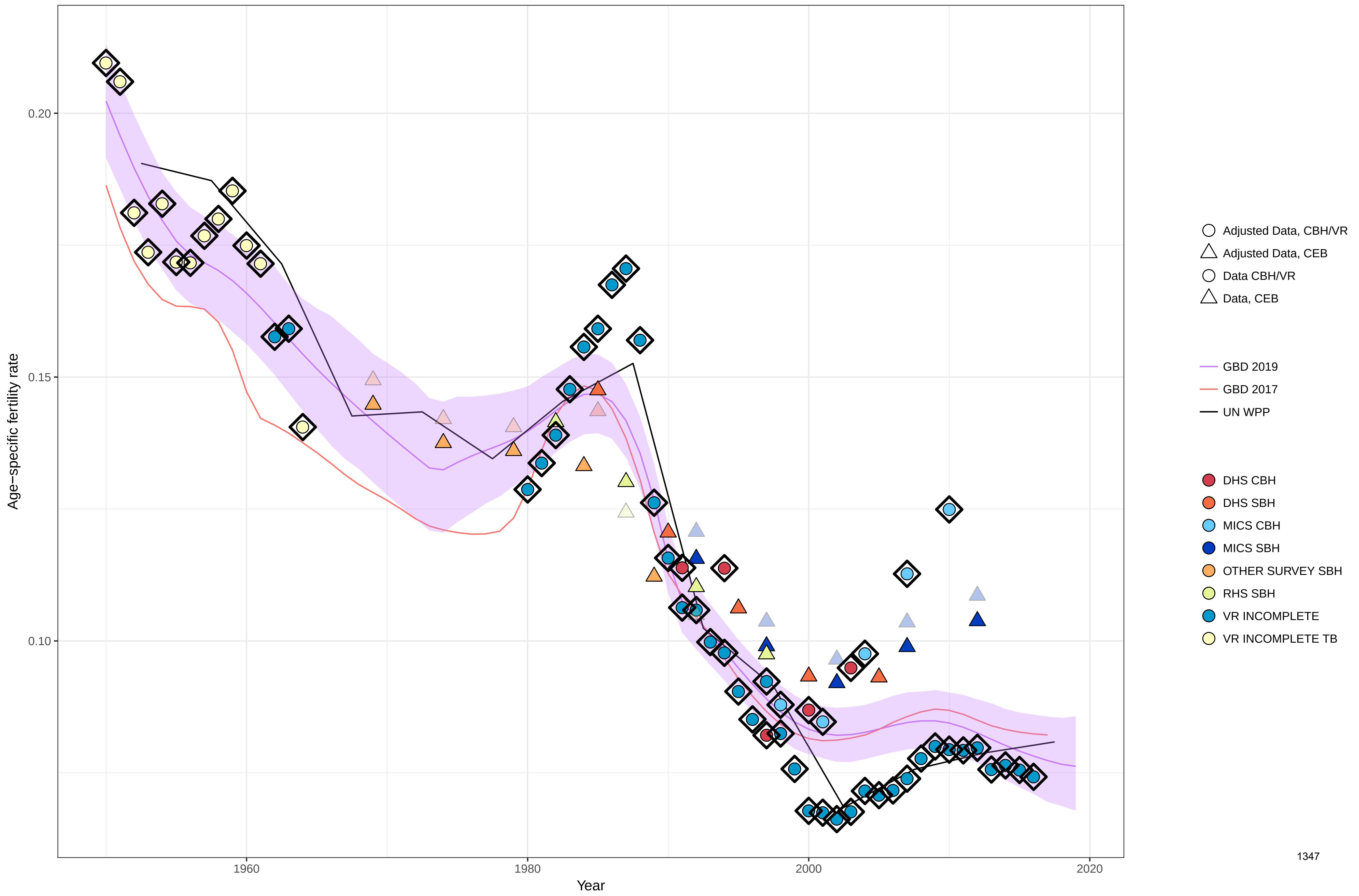

Age−specific fertility fitted trend lines, 1950−2019: Republic of Moldova, Ages: 30−34
Data points enclosed with a diamond are reference sources

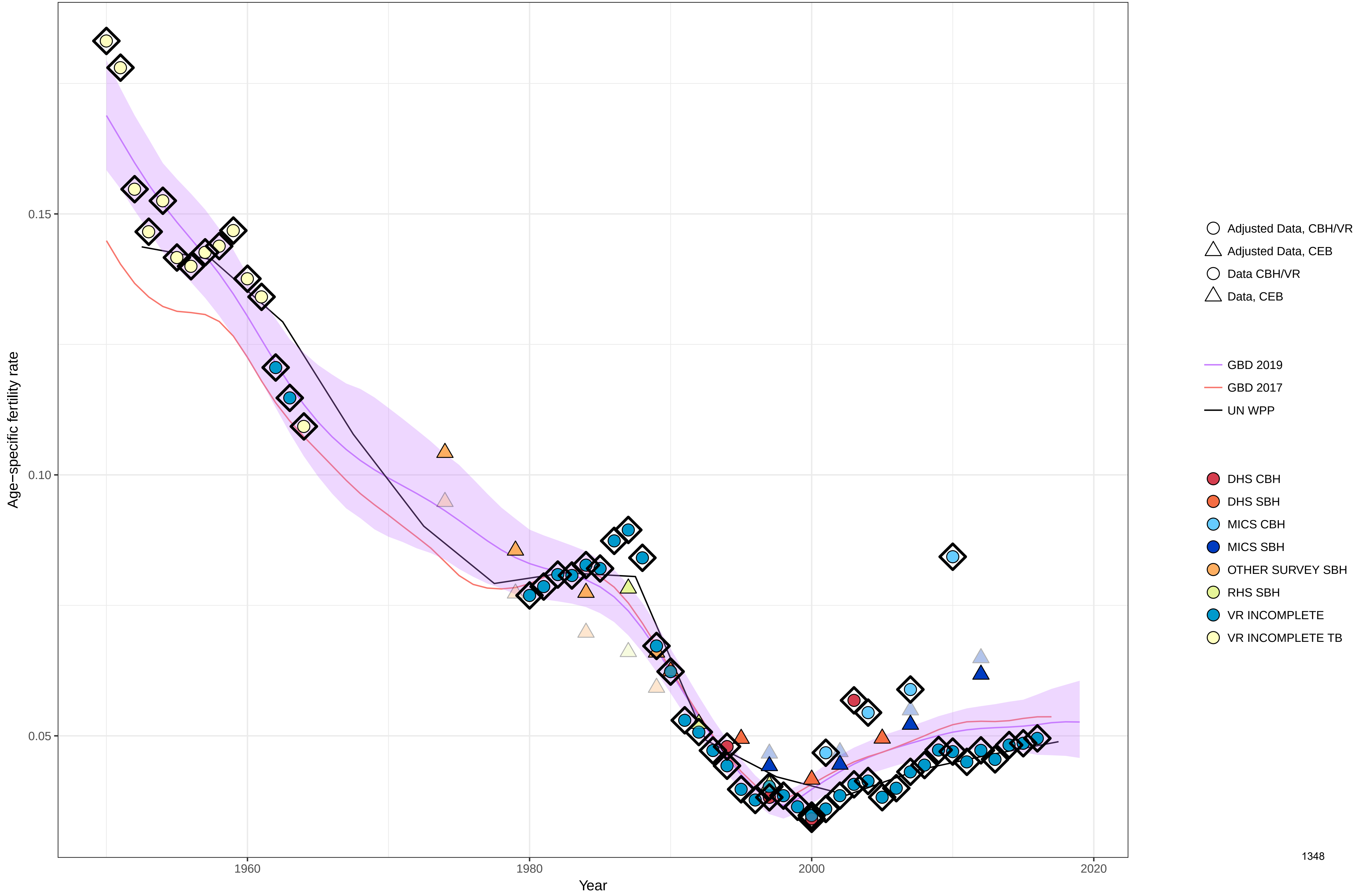

Age−specific fertility fitted trend lines, 1950−2019: Republic of Moldova, Ages: 35−39
Data points enclosed with a diamond are reference sources

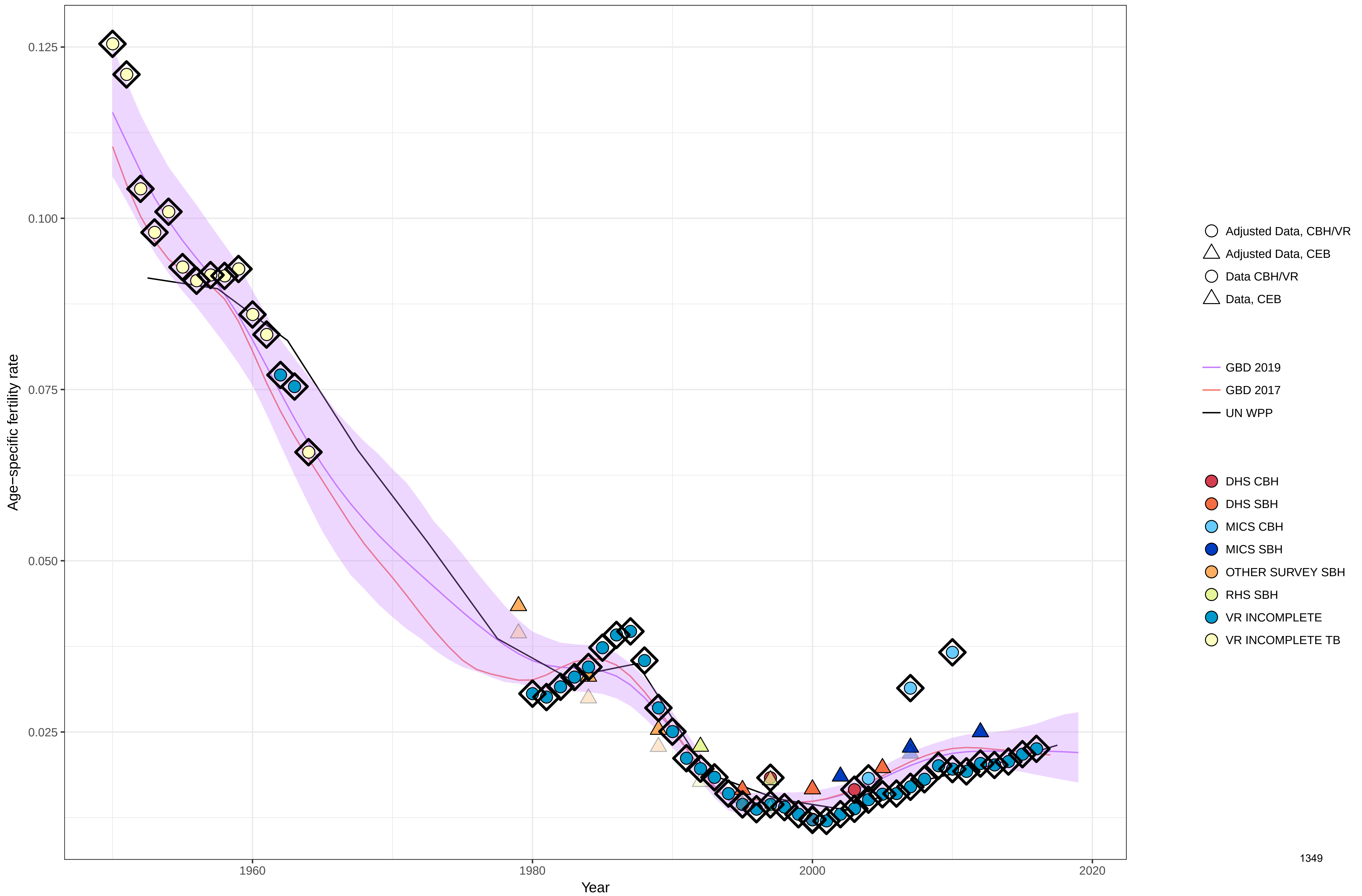

Age−specific fertility fitted trend lines, 1950−2019: Republic of Moldova, Ages: 40−44
Data points enclosed with a diamond are reference sources

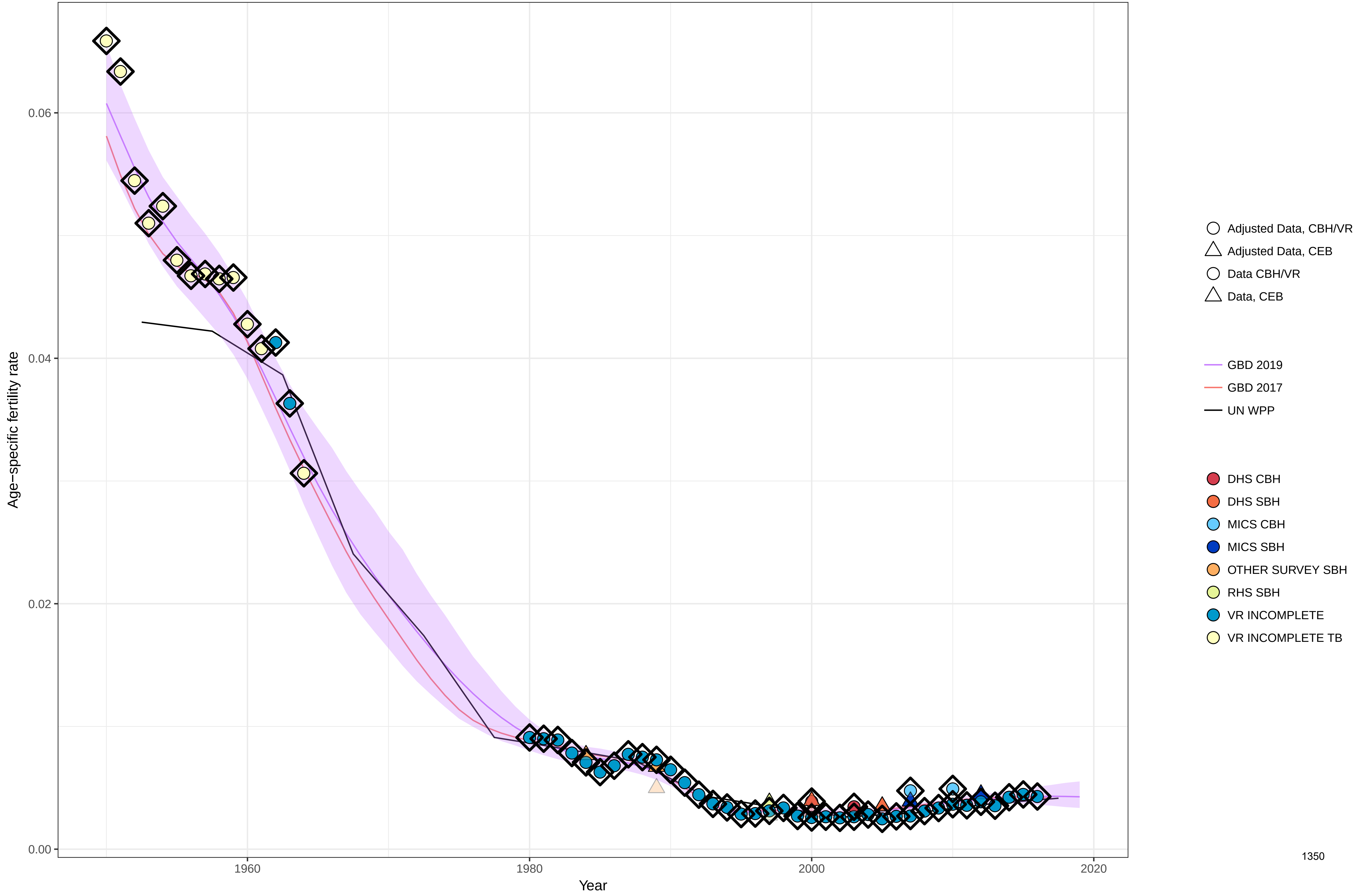

Age−specific fertility fitted trend lines, 1950−2019: Republic of Moldova, Ages: 45−49
Data points enclosed with a diamond are reference sources

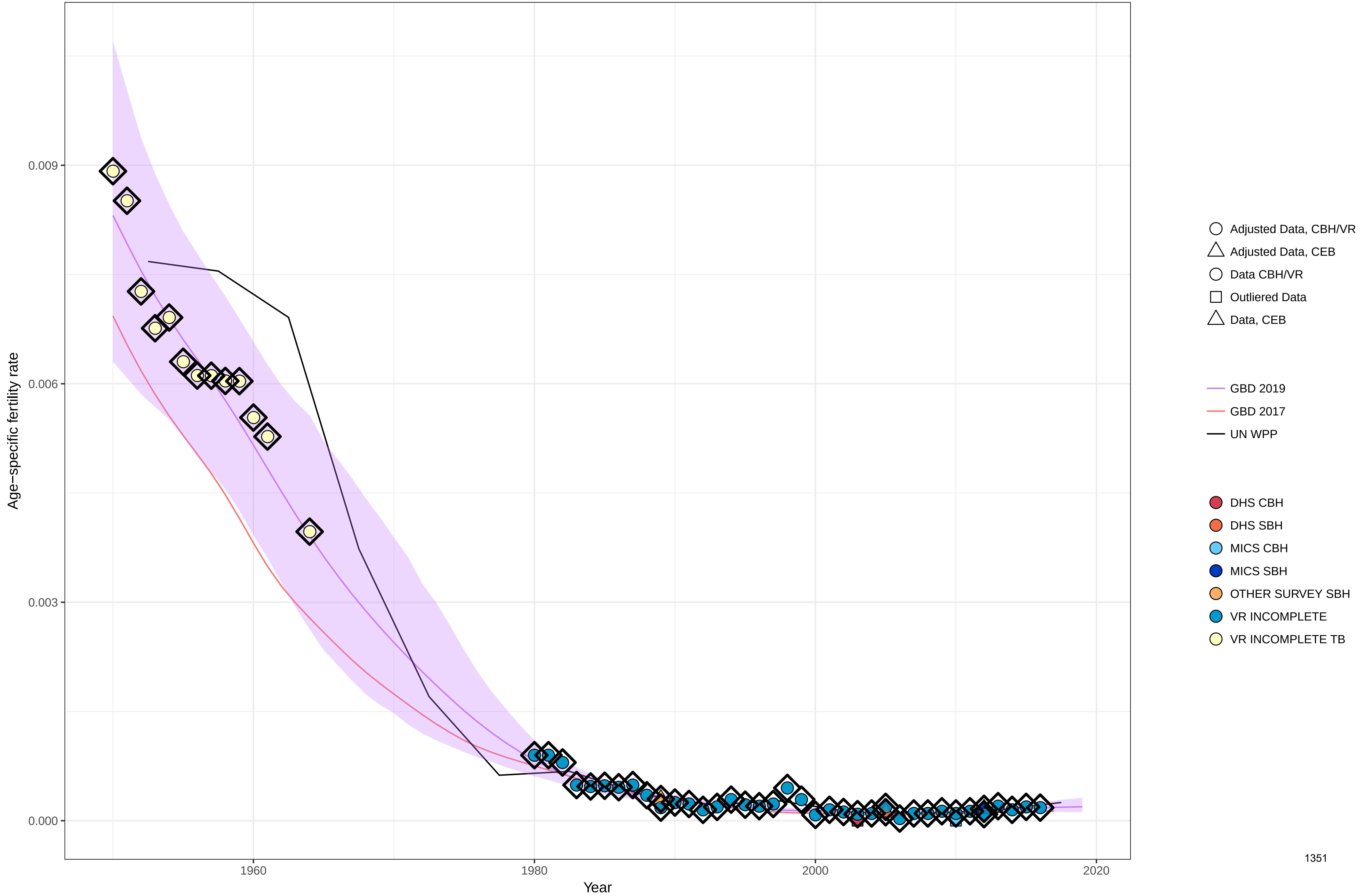

Age−specific fertility fitted trend lines, 1950−2019: Republic of Moldova, Ages: 50−54
Data points enclosed with a diamond are reference sources

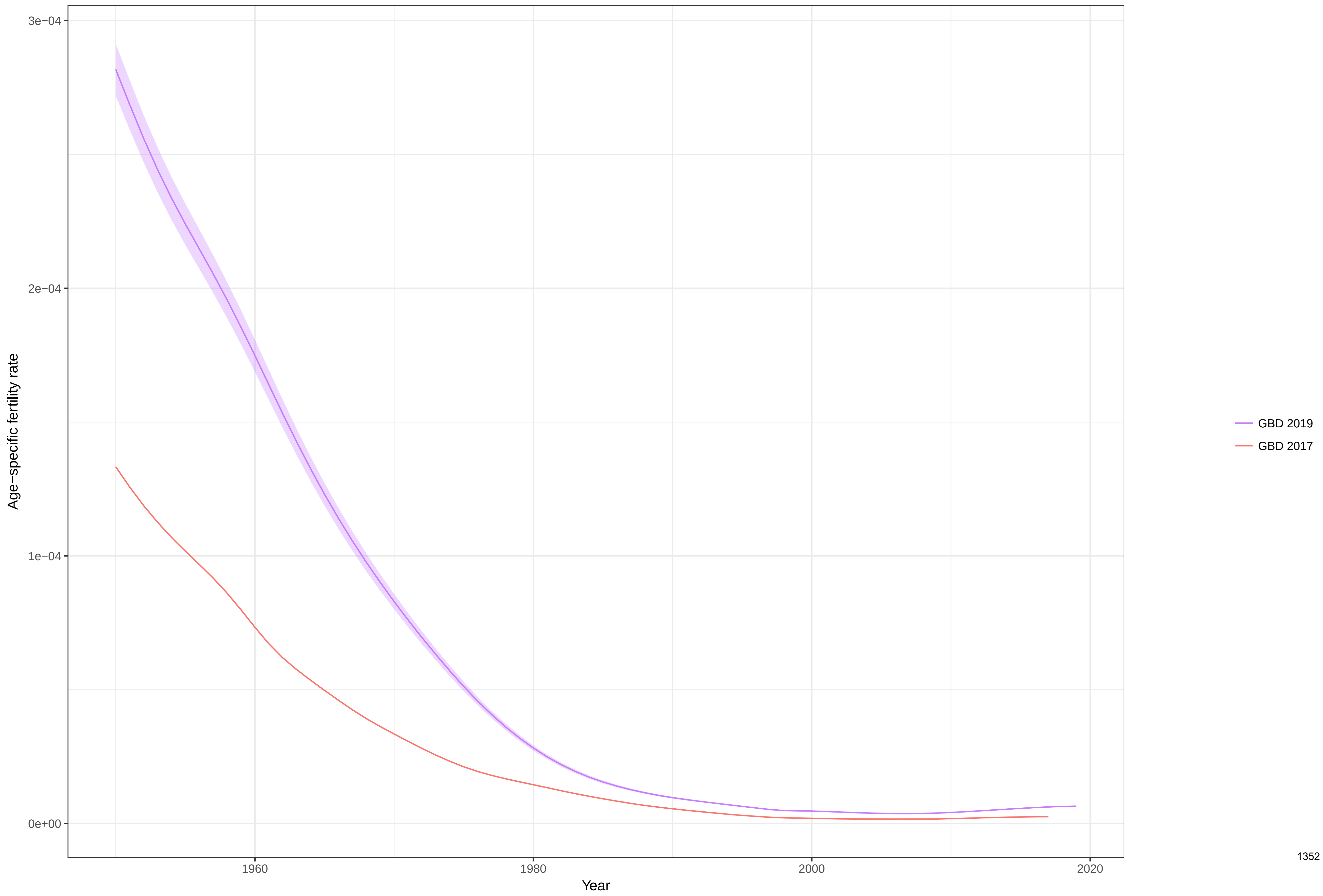

Total fertility rate fitted trend lines, 1950−2019: Republic of Moldova TFR
Data points enclosed with a diamond are reference sources

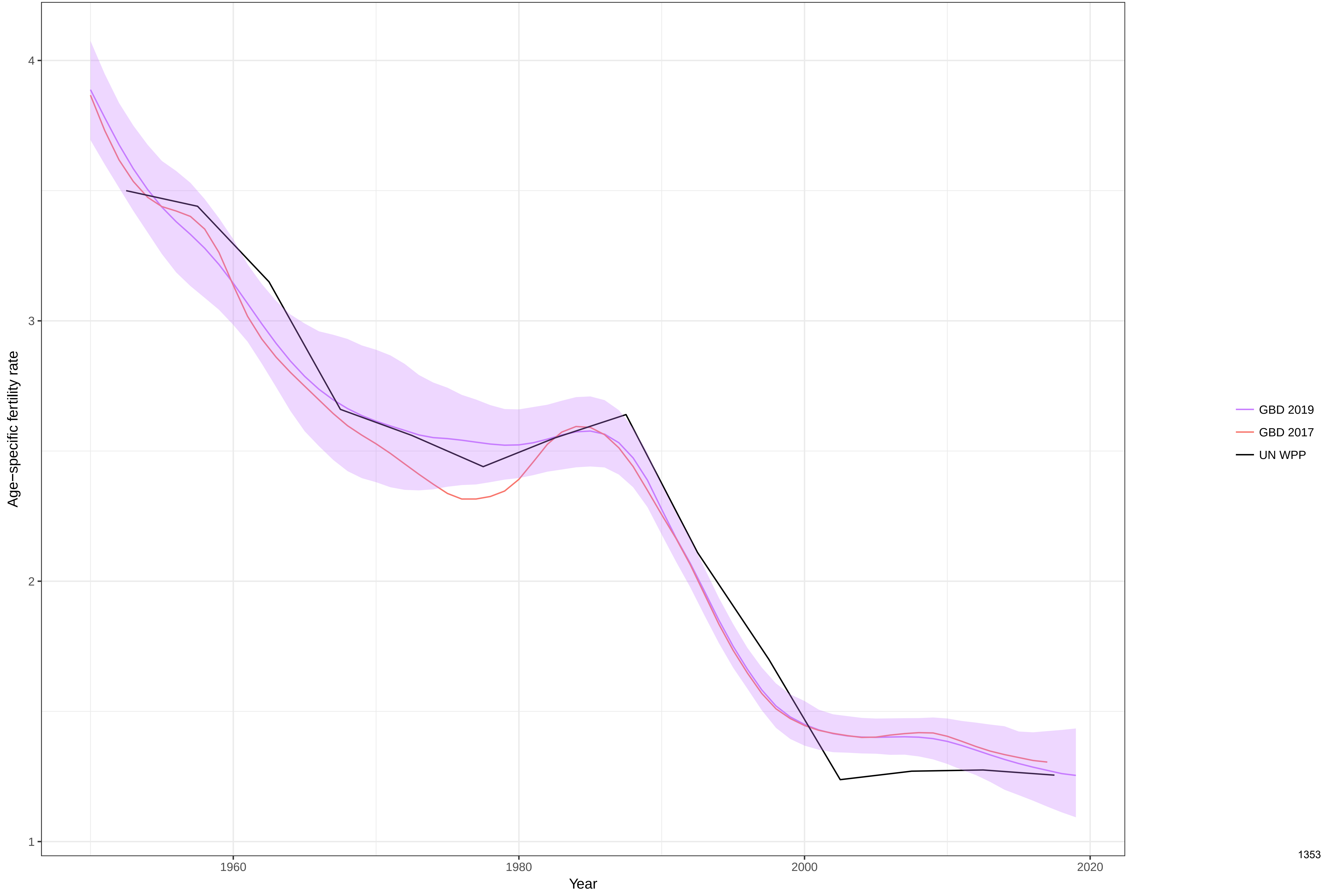

Age−specific fertility fitted trend lines, 1950−2019: Madagascar, Ages: 10−14
Data points enclosed with a diamond are reference sources

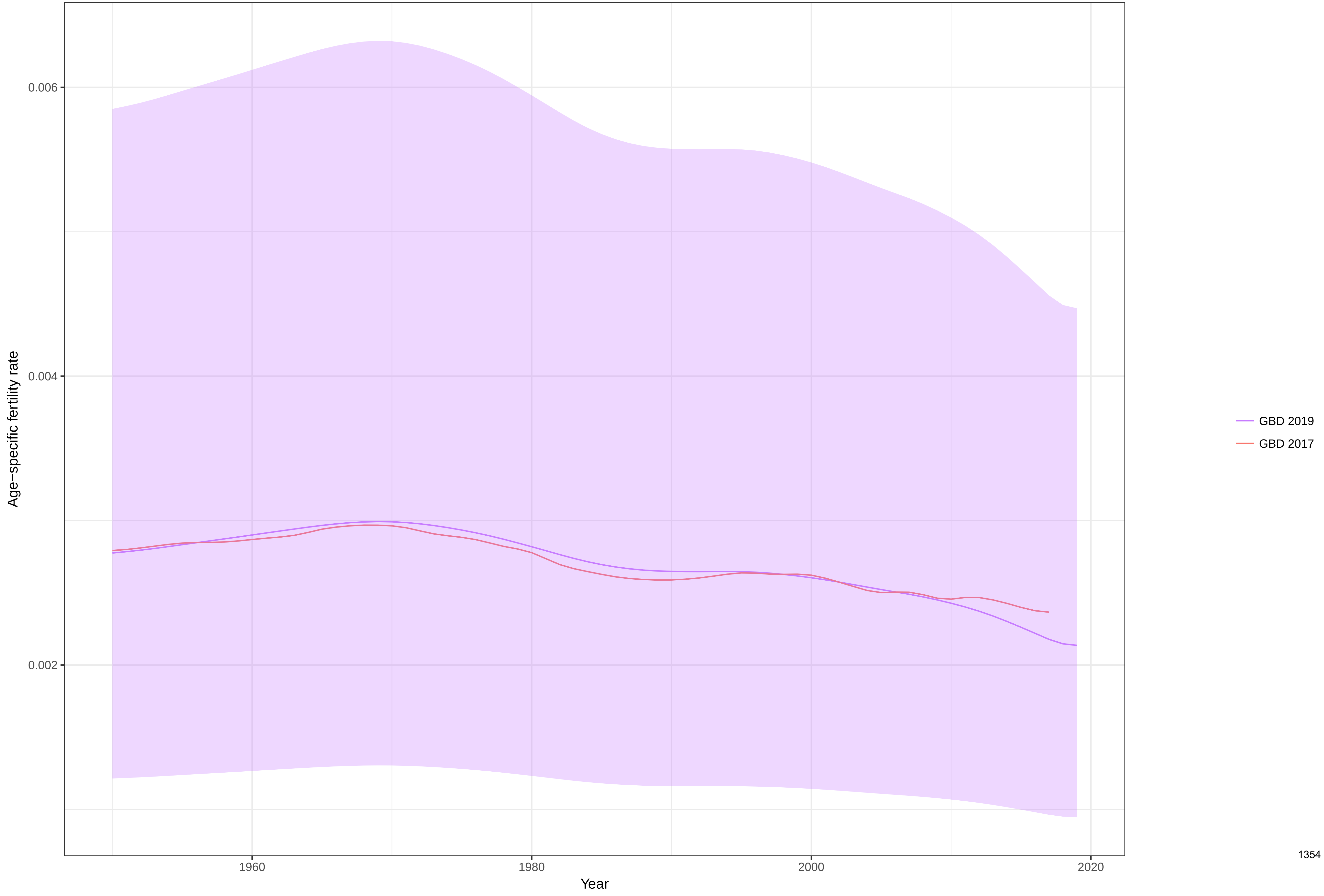

Age−specific fertility fitted trend lines, 1950−2019: Madagascar, Ages: 15−19
Data points enclosed with a diamond are reference sources

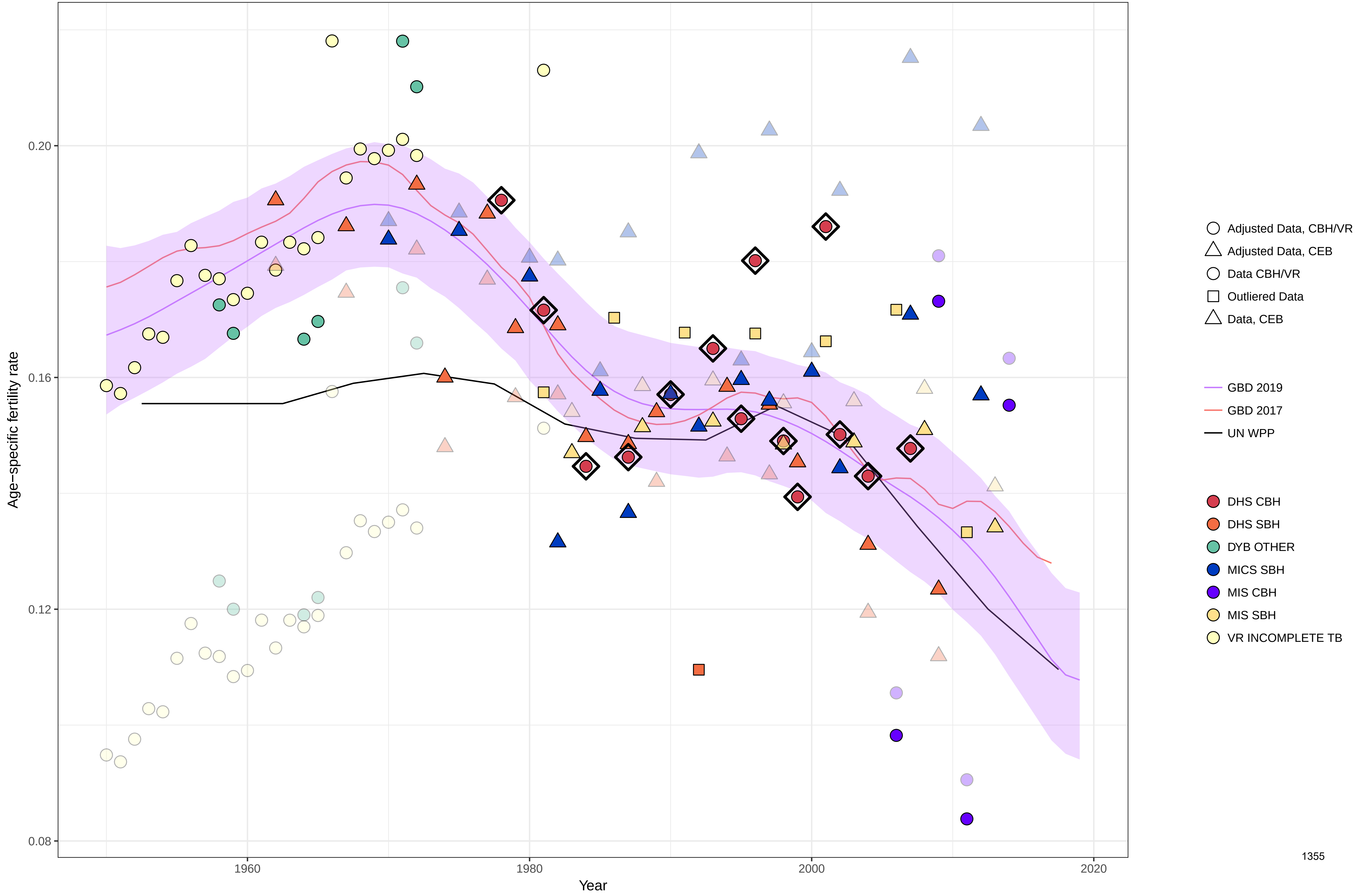

Age−specific fertility fitted trend lines, 1950−2019: Madagascar, Ages: 20−24
Data points enclosed with a diamond are reference sources

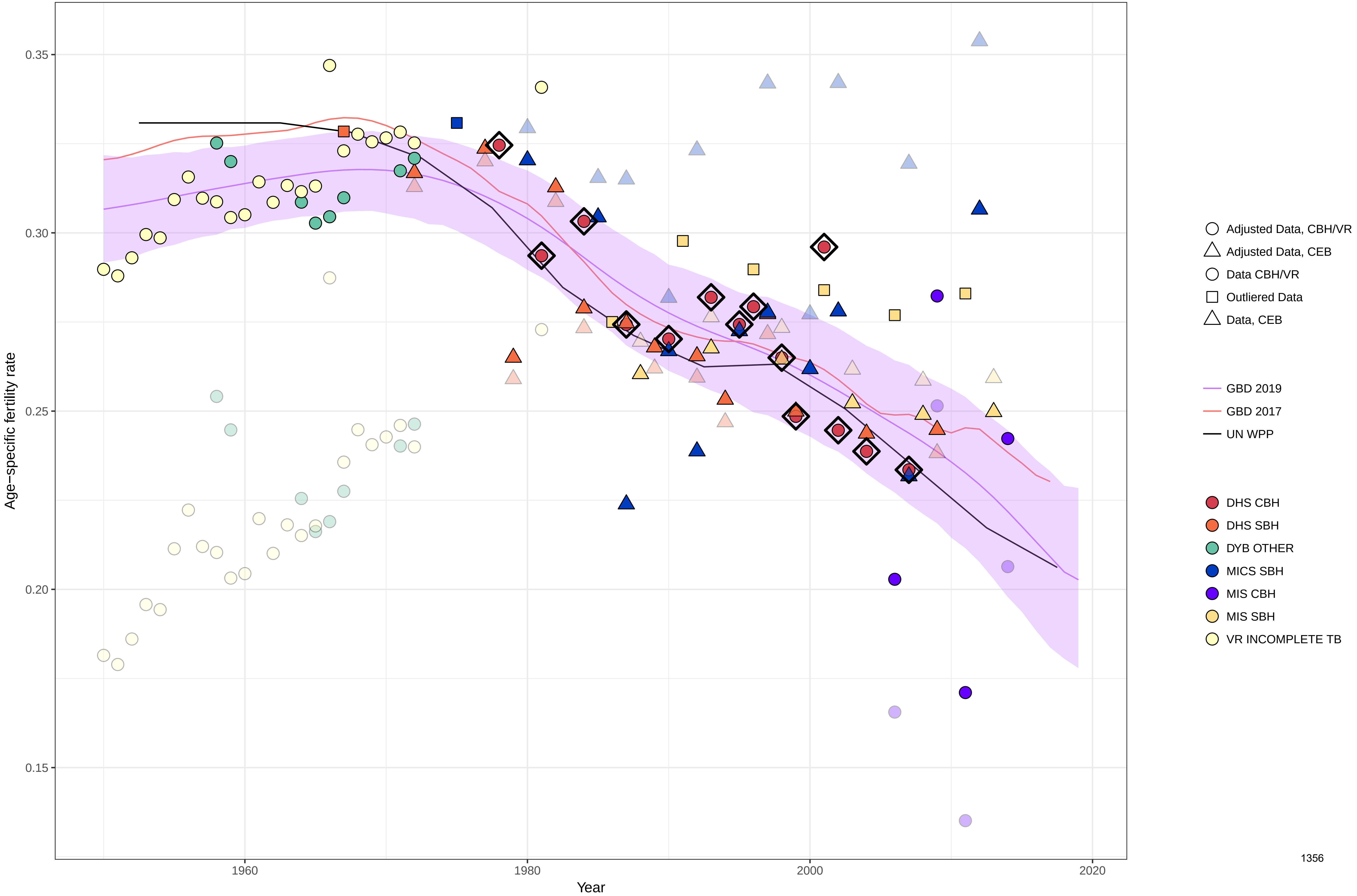

Age−specific fertility fitted trend lines, 1950−2019: Madagascar, Ages: 25−29
Data points enclosed with a diamond are reference sources

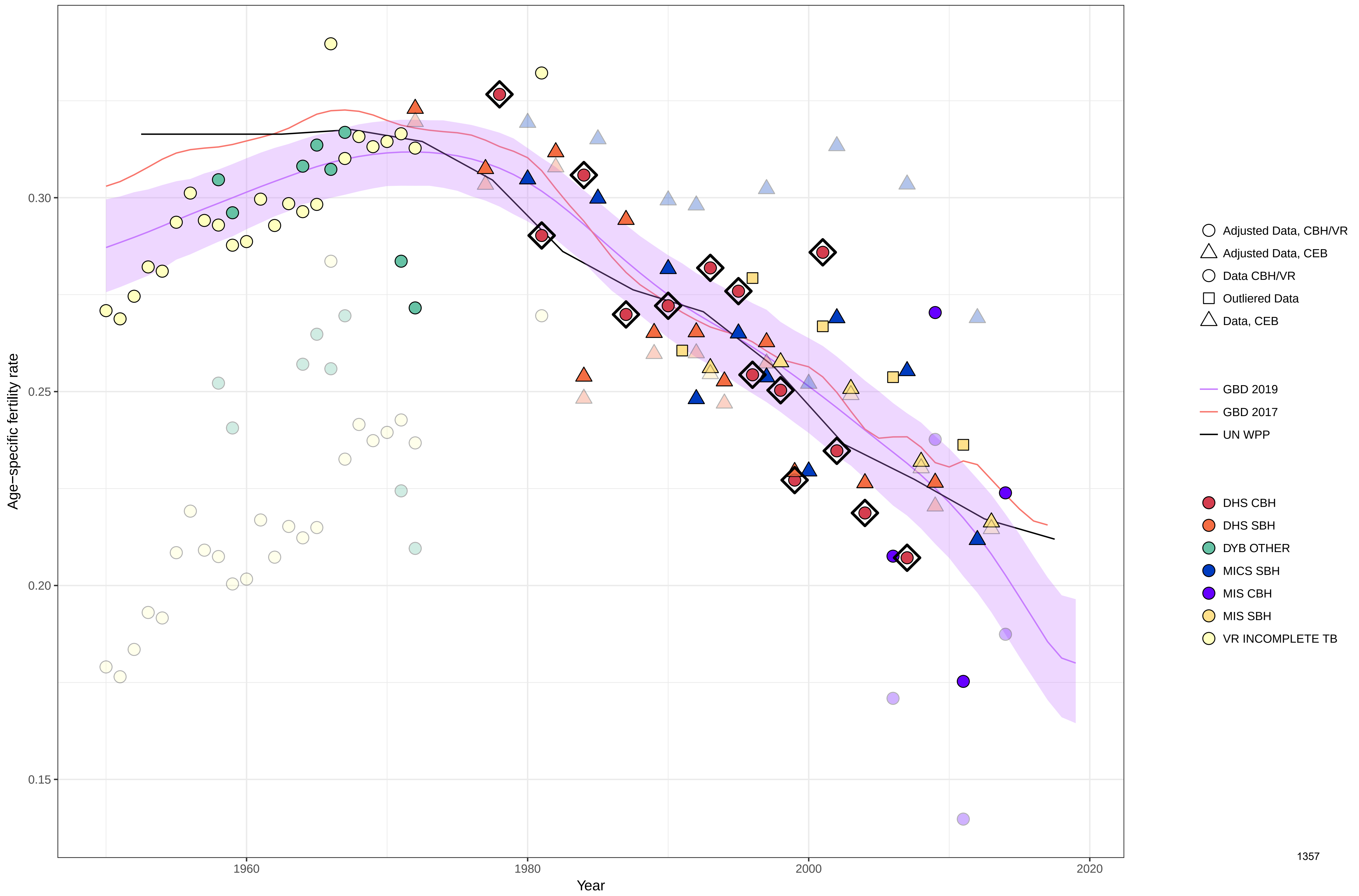

Age−specific fertility fitted trend lines, 1950−2019: Madagascar, Ages: 30−34
Data points enclosed with a diamond are reference sources

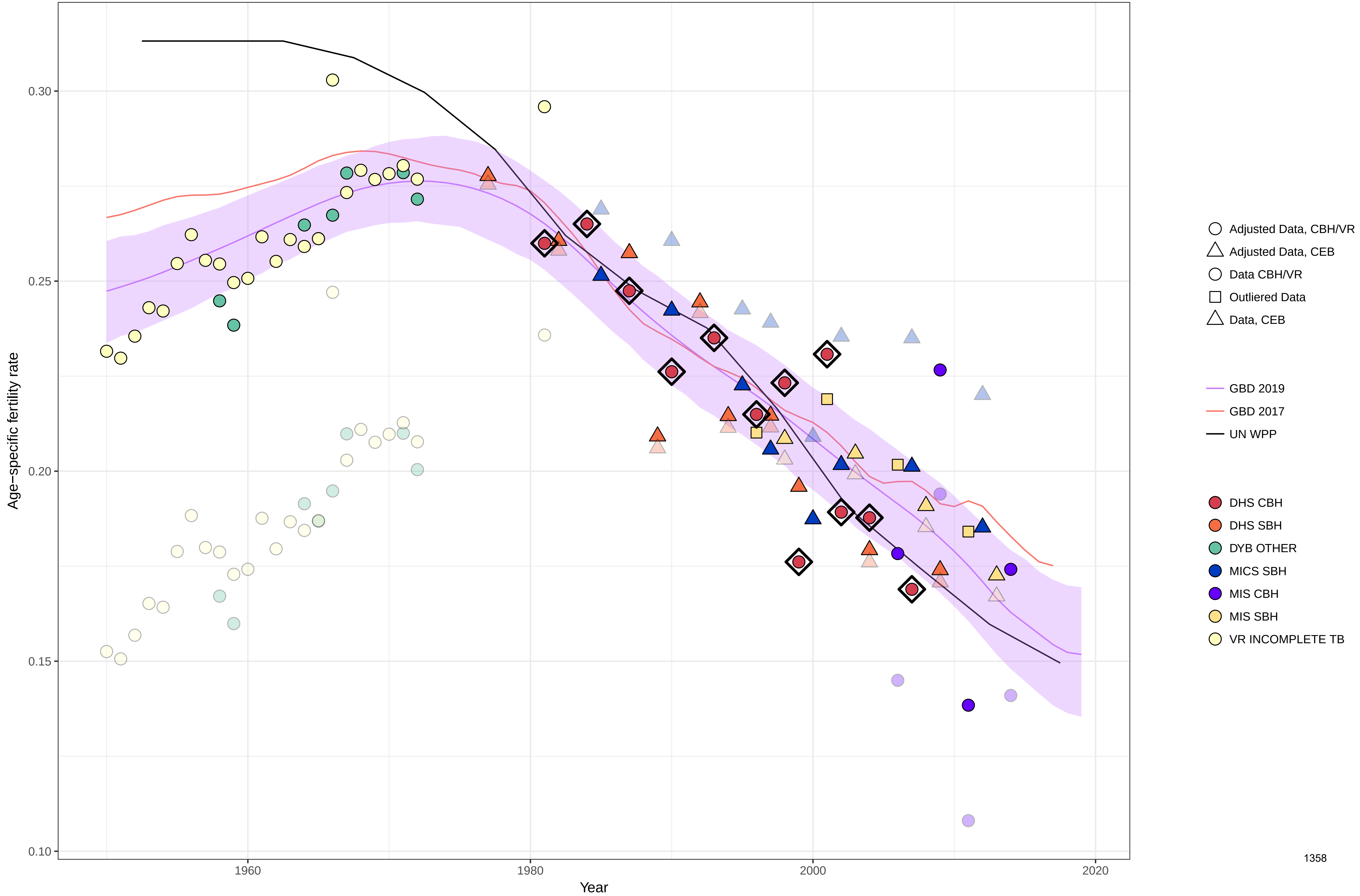

Age−specific fertility fitted trend lines, 1950−2019: Madagascar, Ages: 35−39
Data points enclosed with a diamond are reference sources

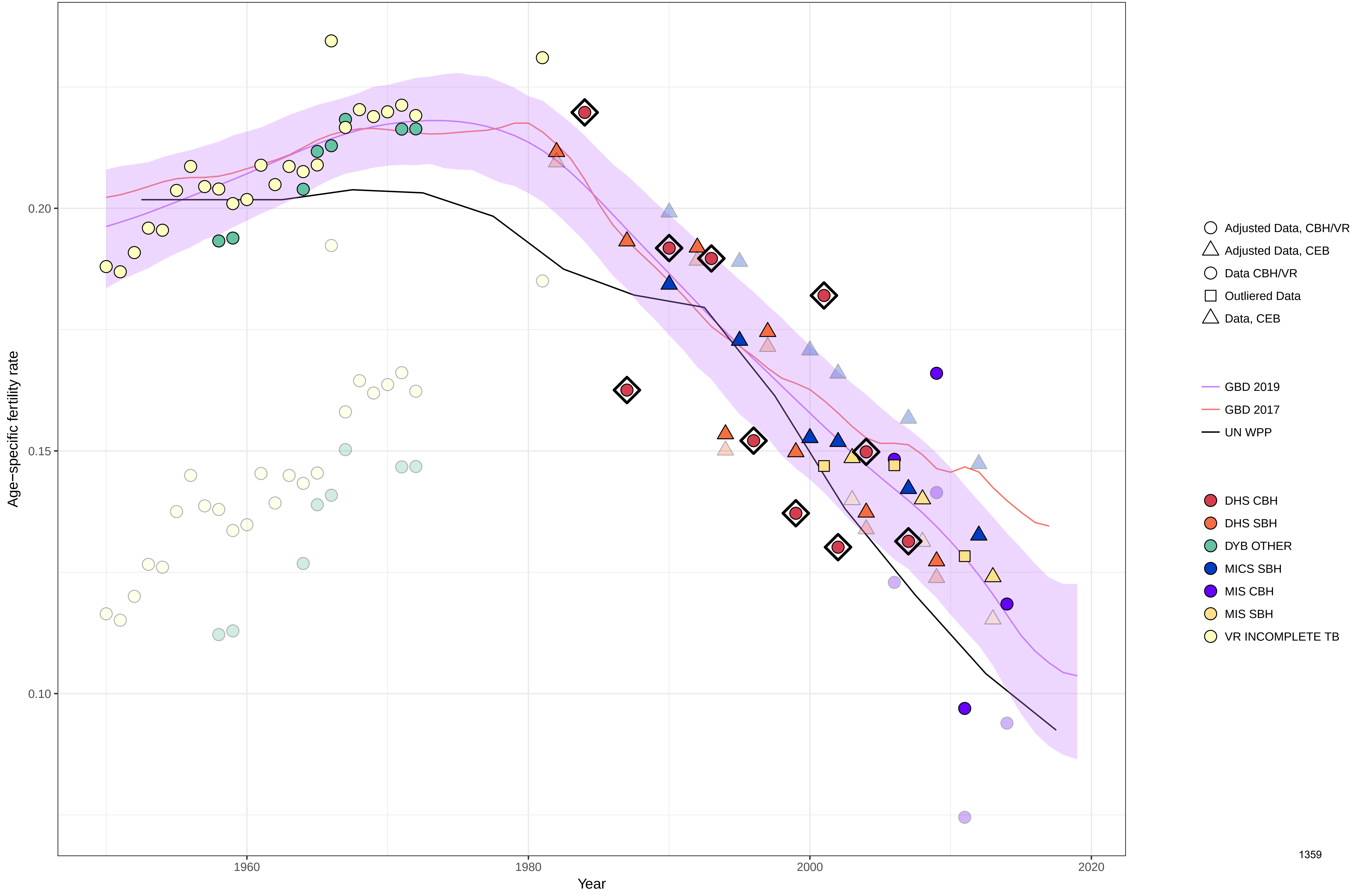

Age−specific fertility fitted trend lines, 1950−2019: Madagascar, Ages: 40−44
Data points enclosed with a diamond are reference sources

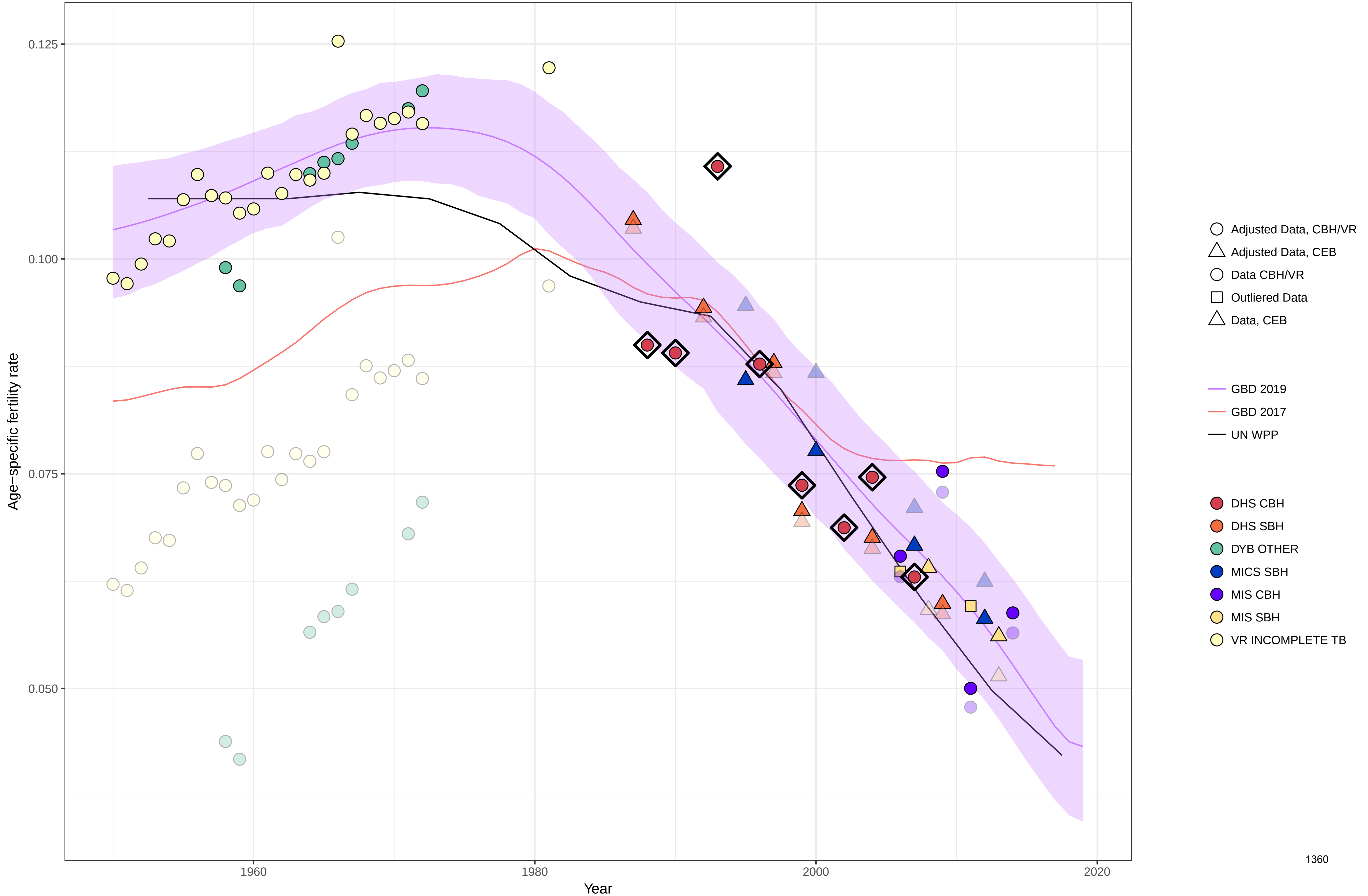

Age−specific fertility fitted trend lines, 1950−2019: Madagascar, Ages: 45−49
Data points enclosed with a diamond are reference sources

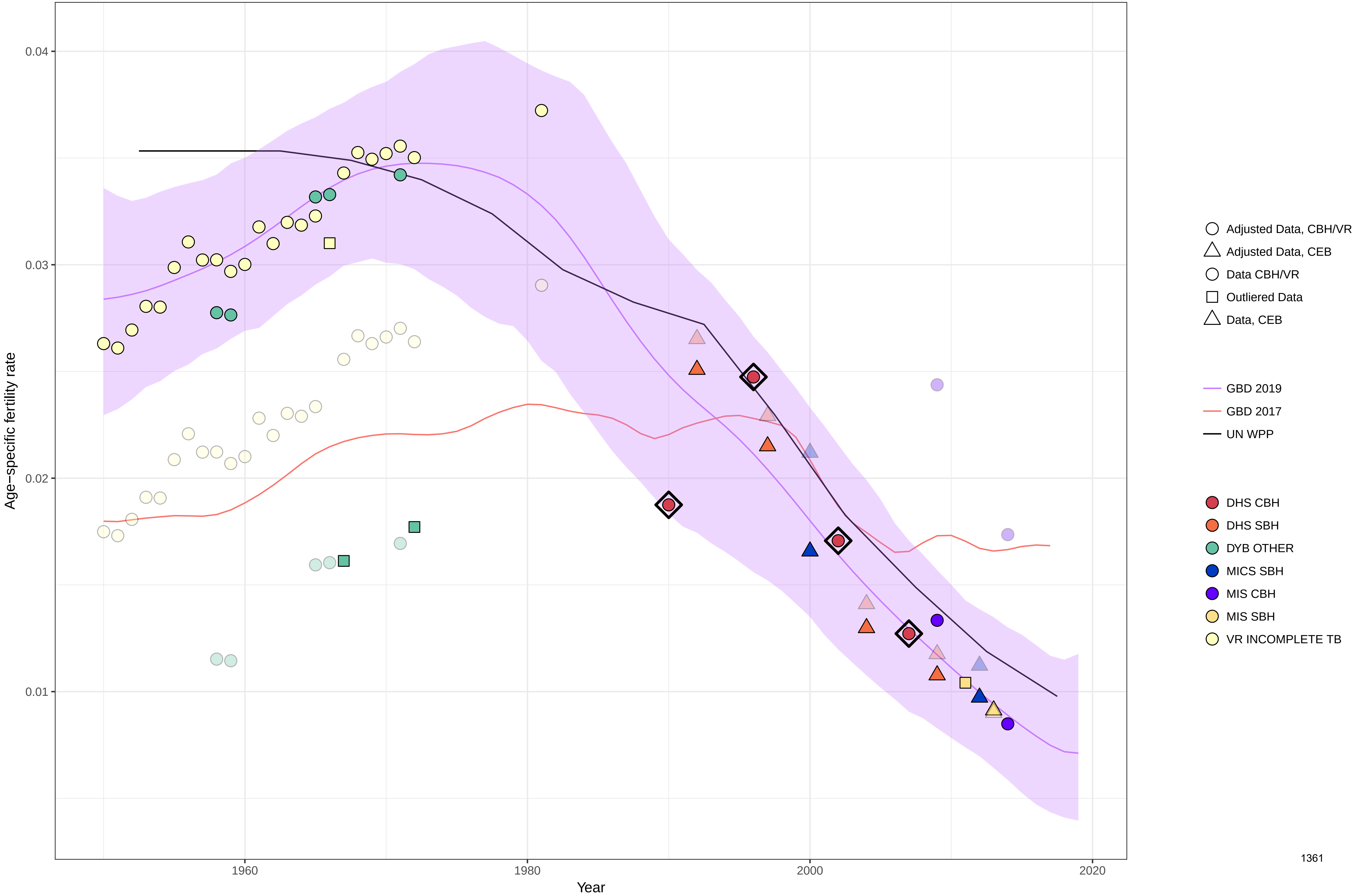

Age−specific fertility fitted trend lines, 1950−2019: Madagascar, Ages: 50−54
Data points enclosed with a diamond are reference sources

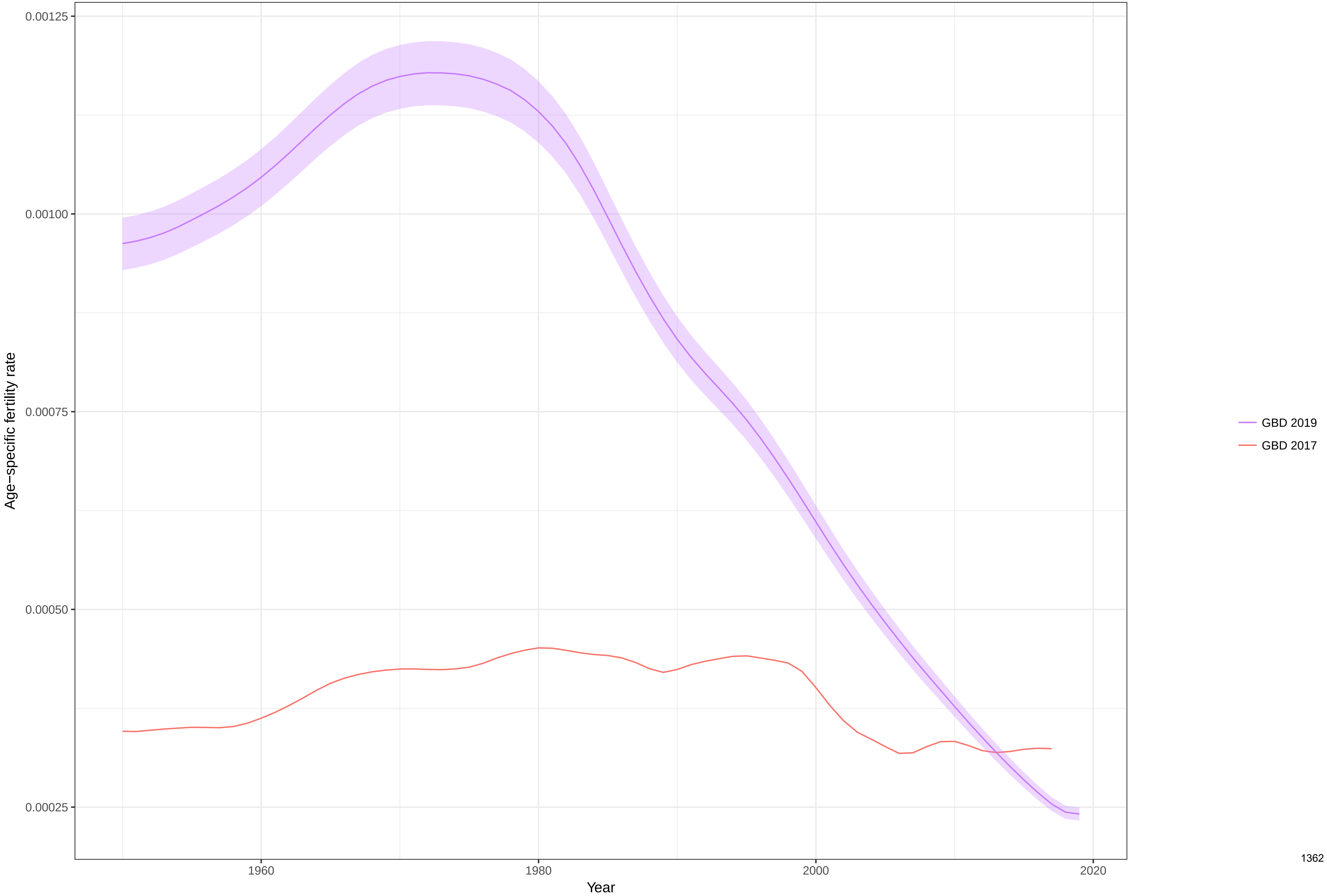

Total fertility rate fitted trend lines, 1950−2019: Madagascar TFR
Data points enclosed with a diamond are reference sources

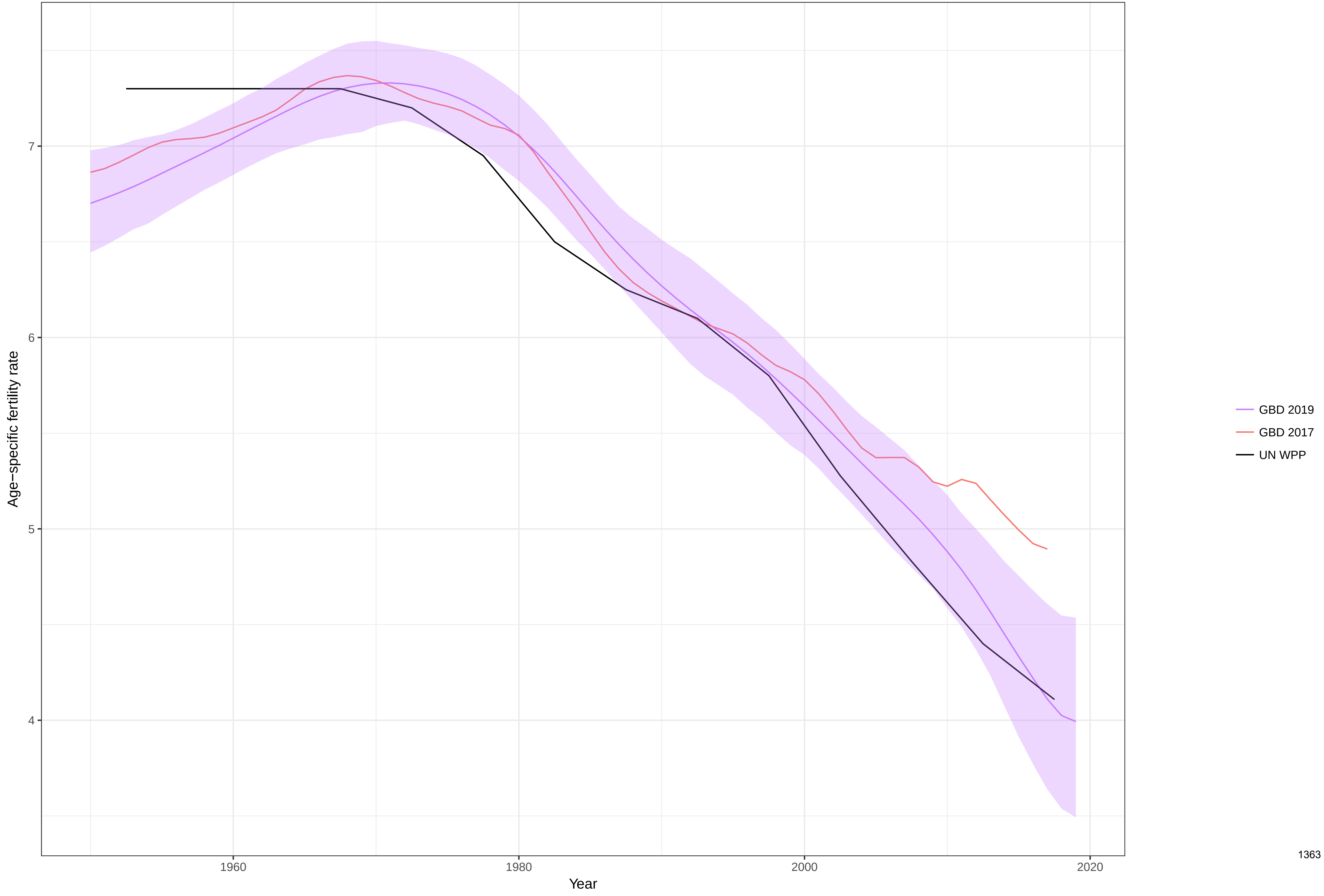

Age−specific fertility fitted trend lines, 1950−2019: Maldives, Ages: 10−14
Data points enclosed with a diamond are reference sources

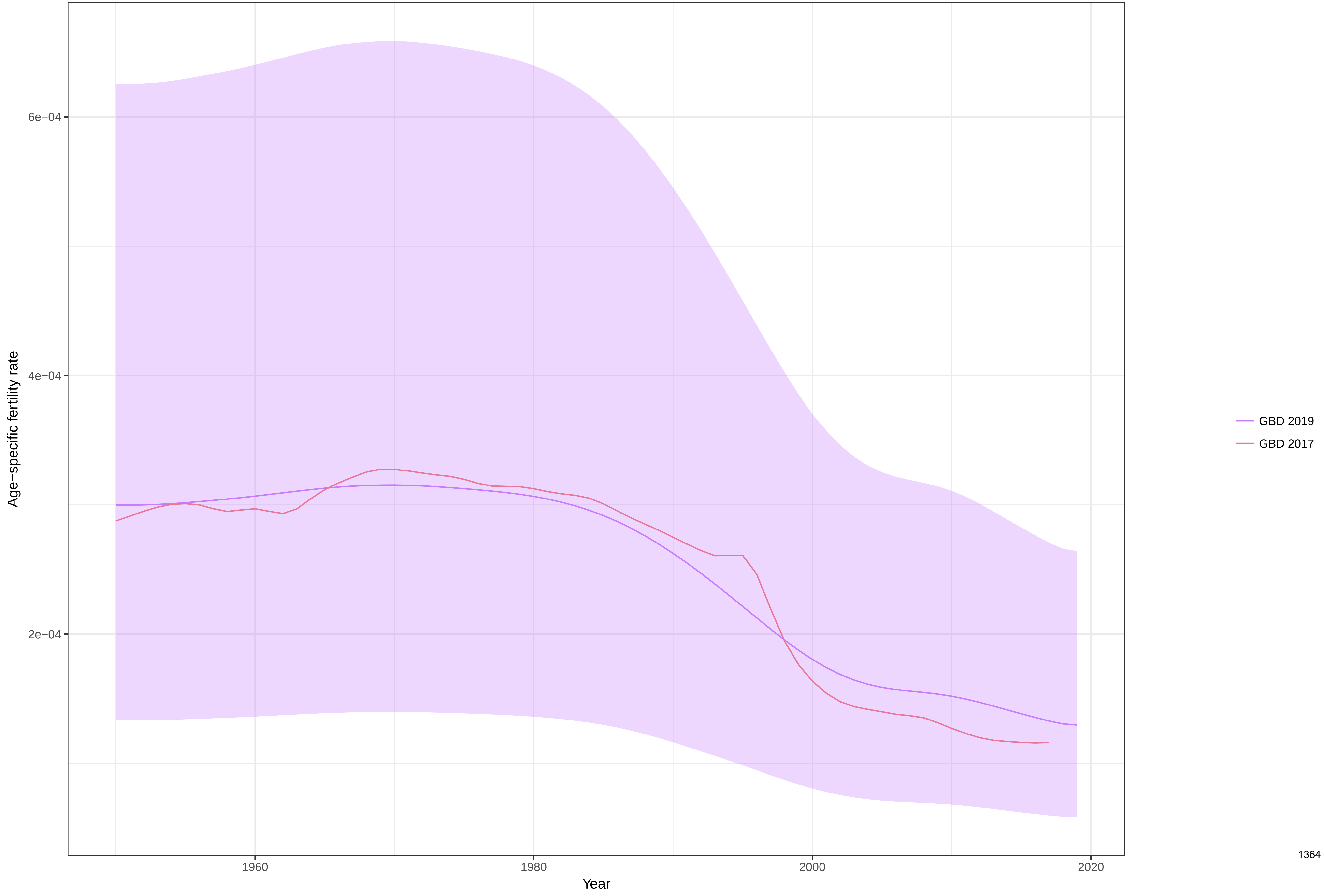

Age−specific fertility fitted trend lines, 1950−2019: Maldives, Ages: 15−19
Data points enclosed with a diamond are reference sources

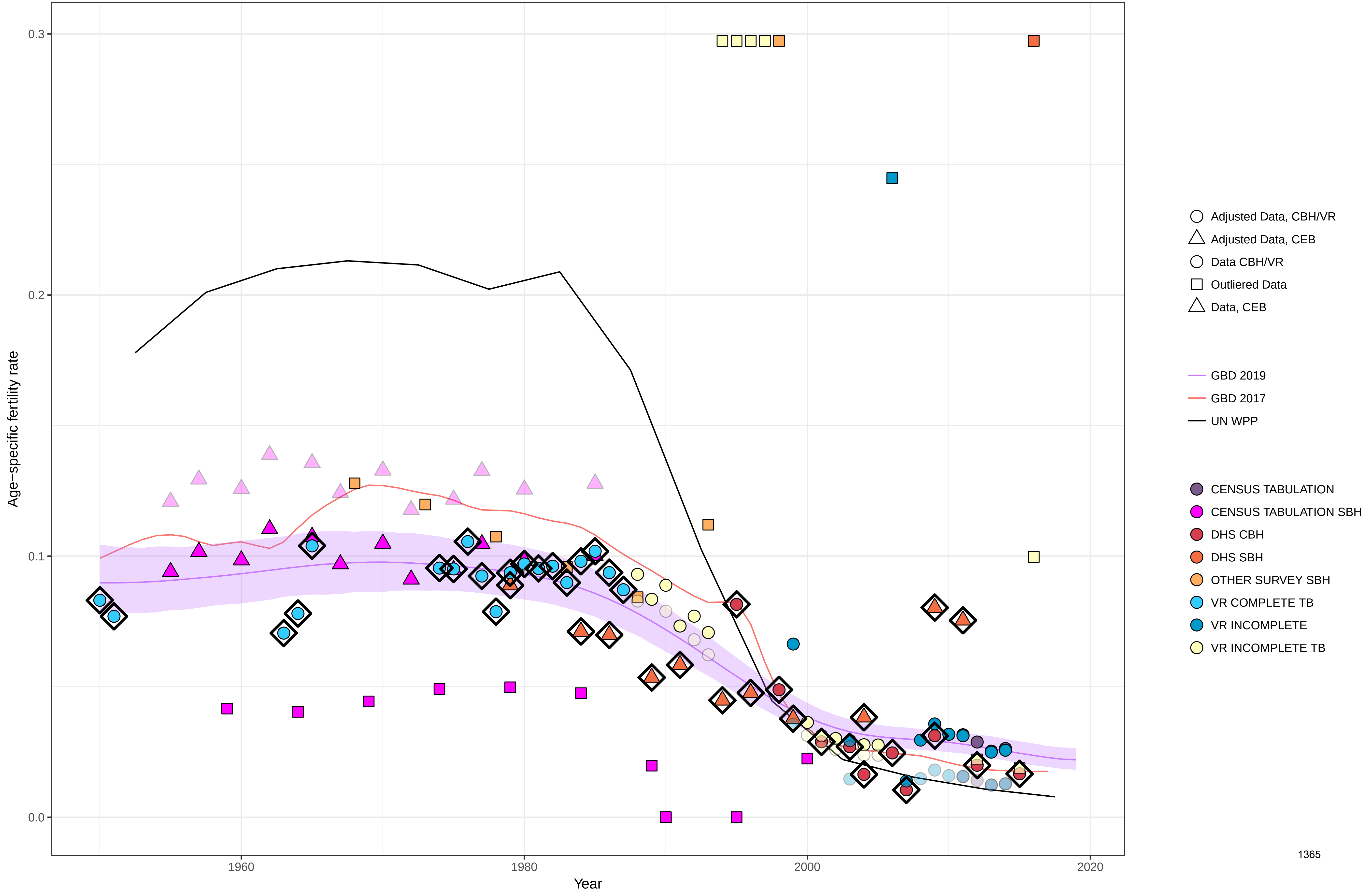

Age−specific fertility fitted trend lines, 1950−2019: Maldives, Ages: 20−24
Data points enclosed with a diamond are reference sources

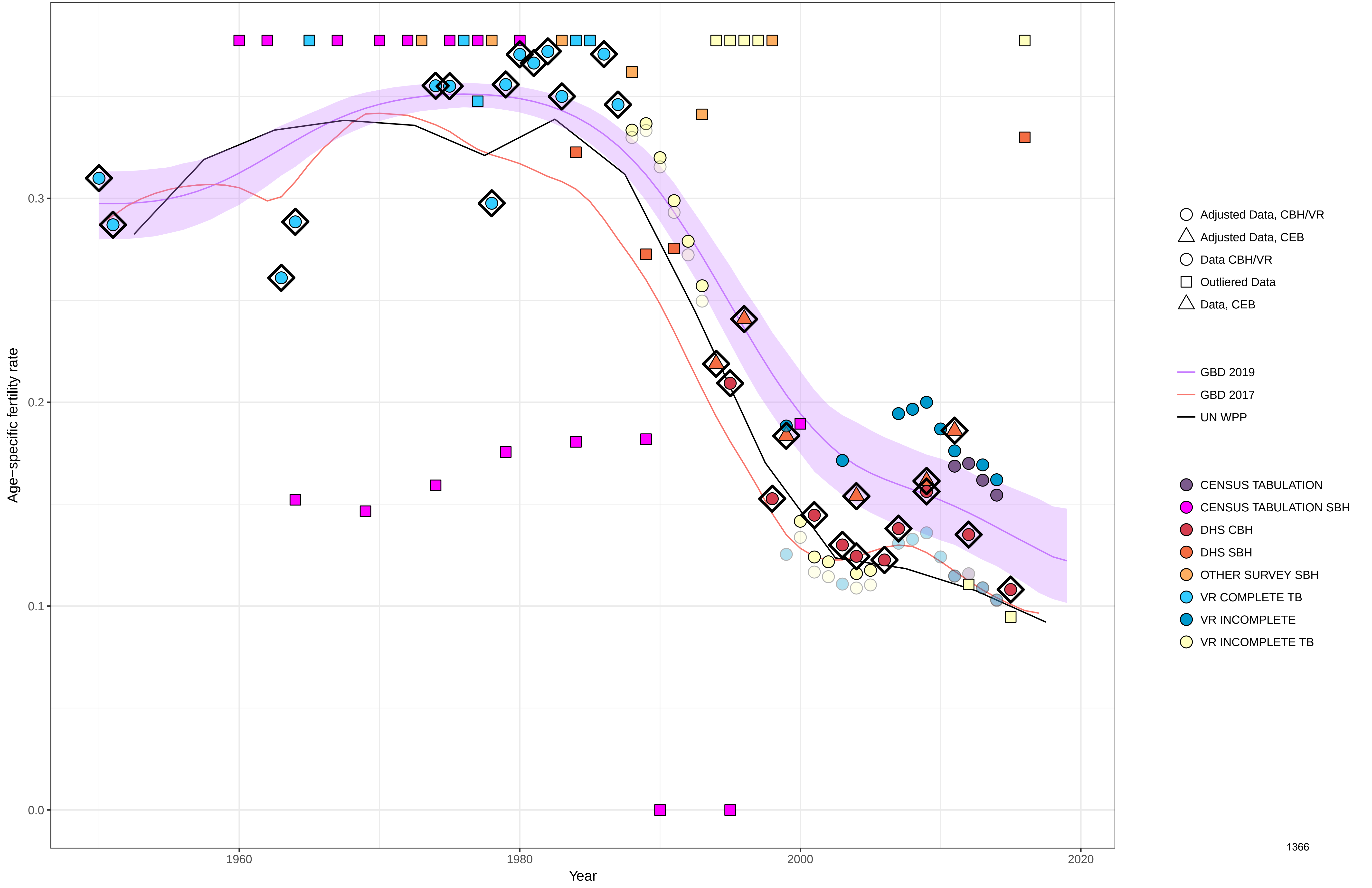

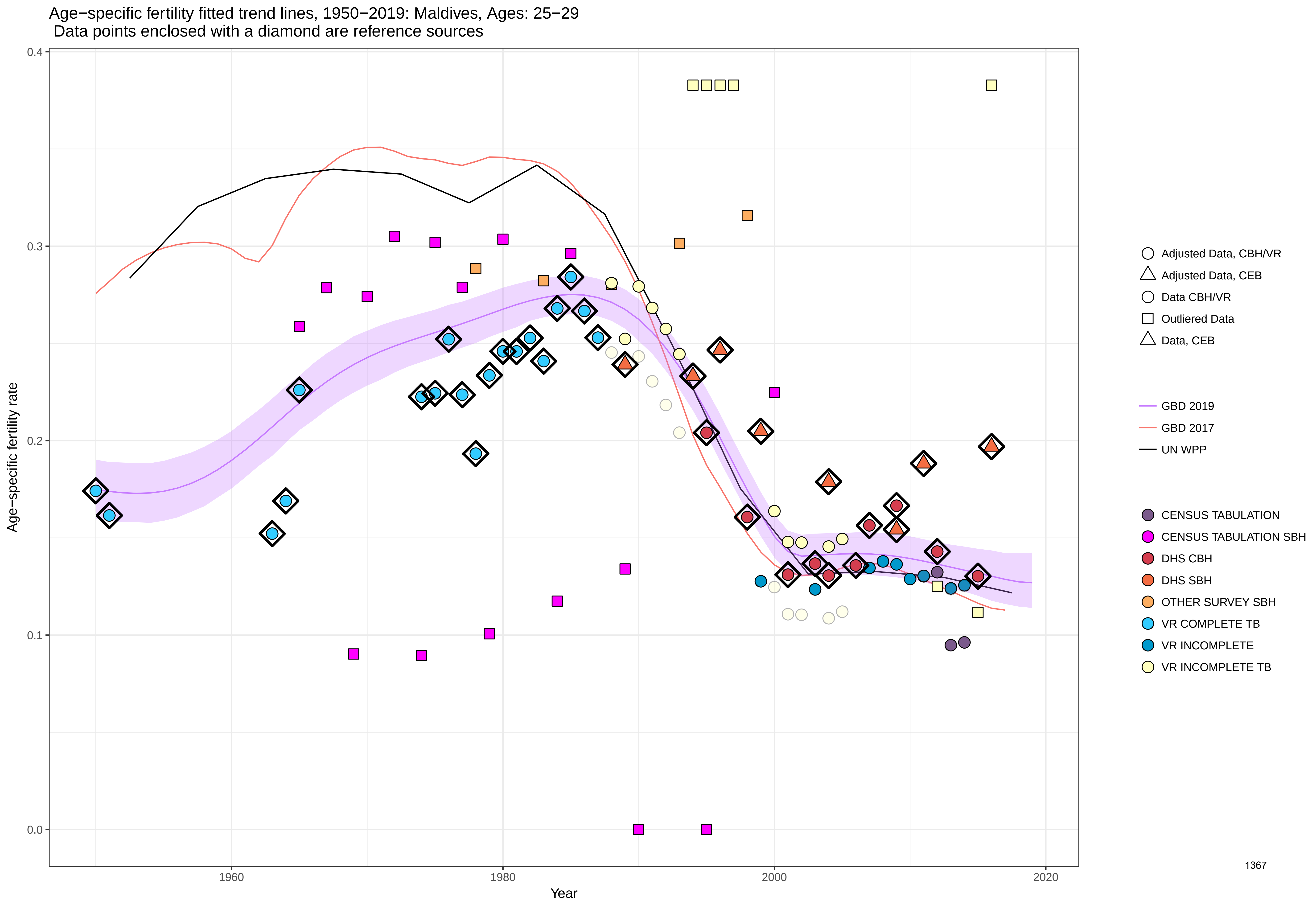

Age−specific fertility fitted trend lines, 1950−2019: Maldives, Ages: 25−29
Data points enclosed with a diamond are reference sources
Age−specific fertility rate
0.0
0.1
0.2
0.3
0.4
1960
1980
2000
2020
Year
Adjusted Data, CBH/VR
Adjusted Data, CEB
Data CBH/VR
Outliered Data
Data, CEB
GBD 2019
GBD 2017
UN WPP
CENSUS TABULATION
CENSUS TABULATION SBH
DHS CBH
DHS SBH
OTHER SURVEY SBH
VR COMPLETE TB
VR INCOMPLETE
VR INCOMPLETE TB

Age−specific fertility fitted trend lines, 1950−2019: Maldives, Ages: 30−34
Data points enclosed with a diamond are reference sources

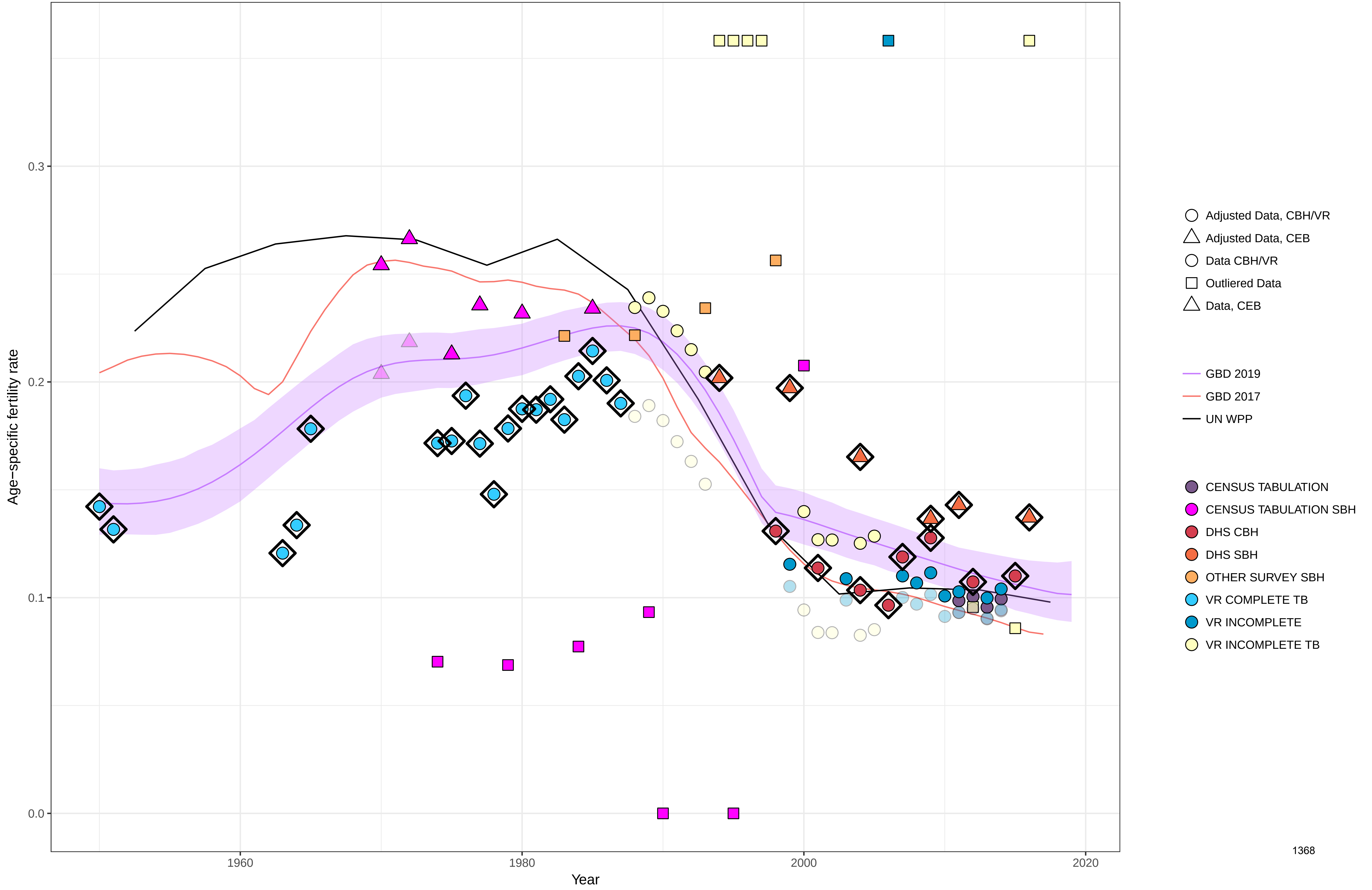

Age−specific fertility fitted trend lines, 1950−2019: Maldives, Ages: 35−39
Data points enclosed with a diamond are reference sources

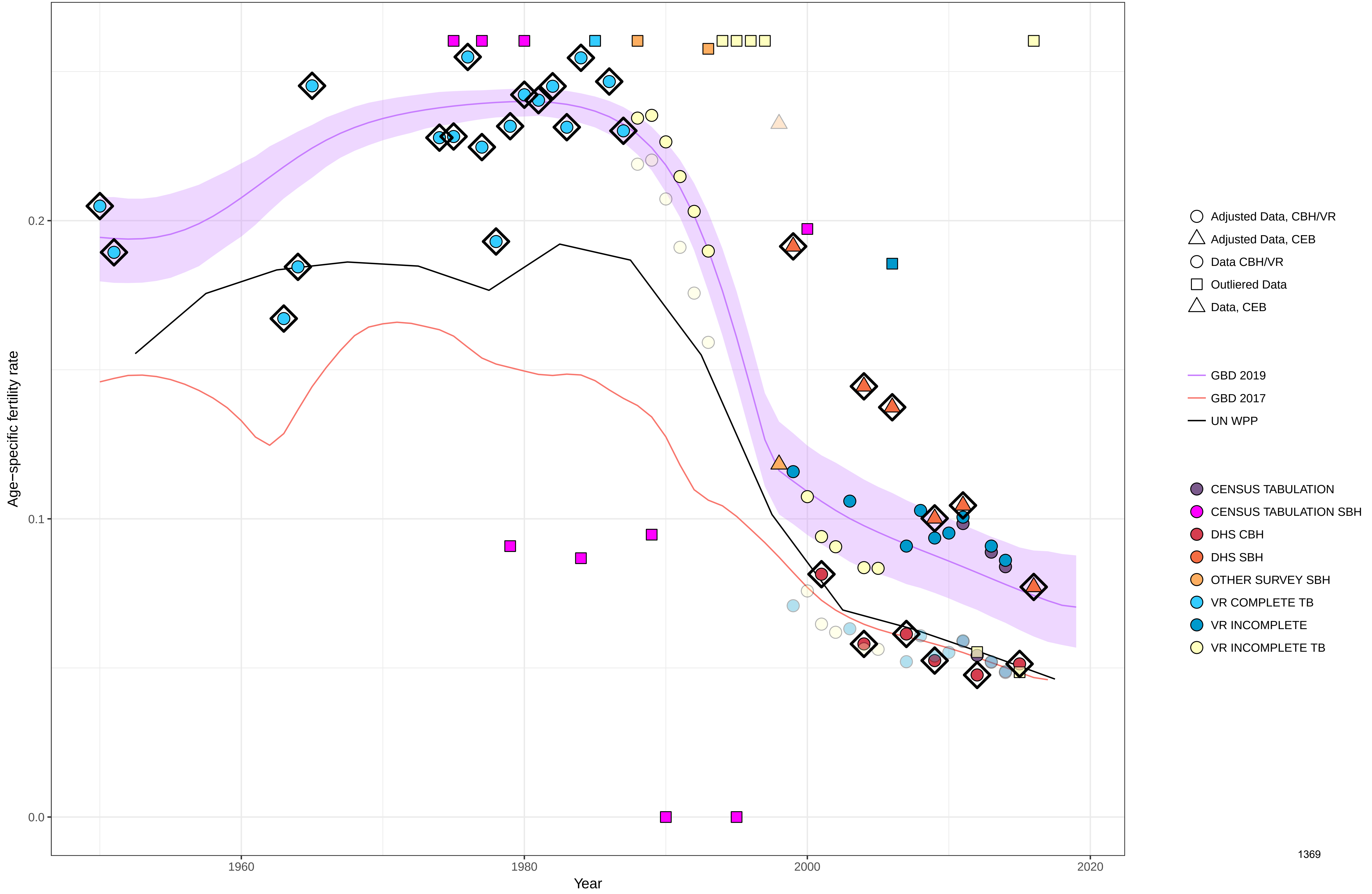

Age−specific fertility fitted trend lines, 1950−2019: Maldives, Ages: 40−44
Data points enclosed with a diamond are reference sources

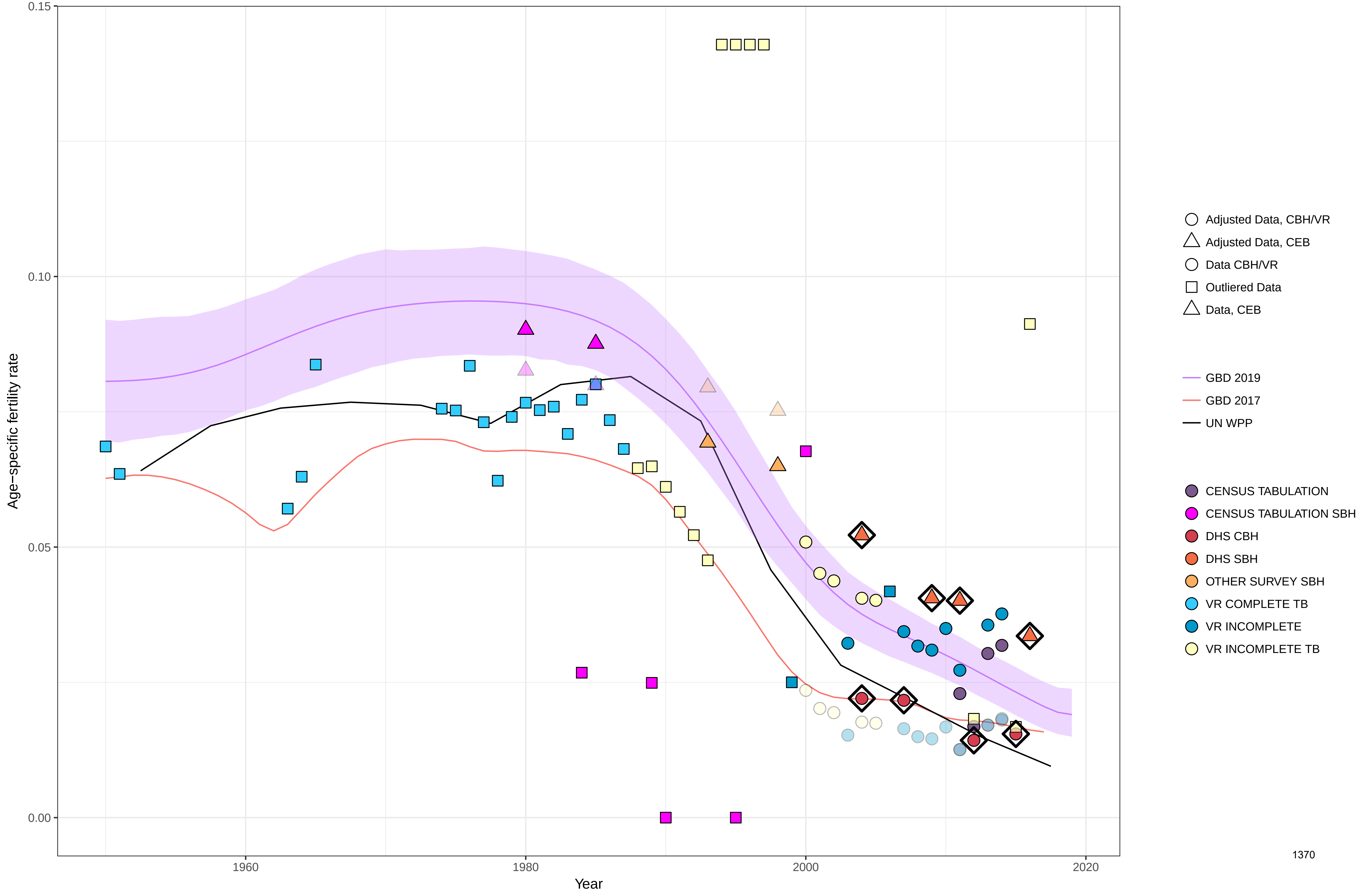

Age−specific fertility fitted trend lines, 1950−2019: Maldives, Ages: 45−49
Data points enclosed with a diamond are reference sources

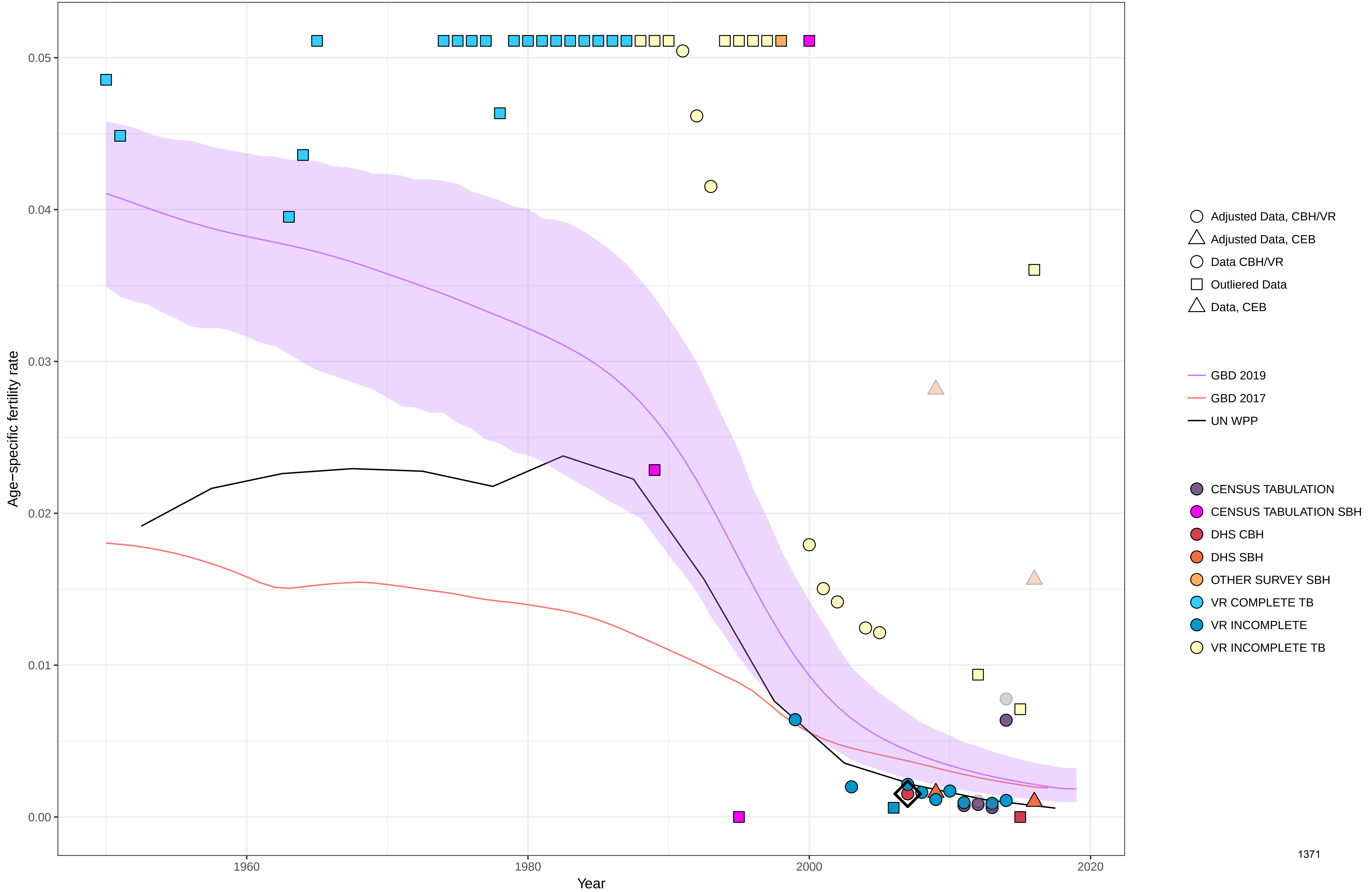

Age−specific fertility fitted trend lines, 1950−2019: Maldives, Ages: 50−54
Data points enclosed with a diamond are reference sources

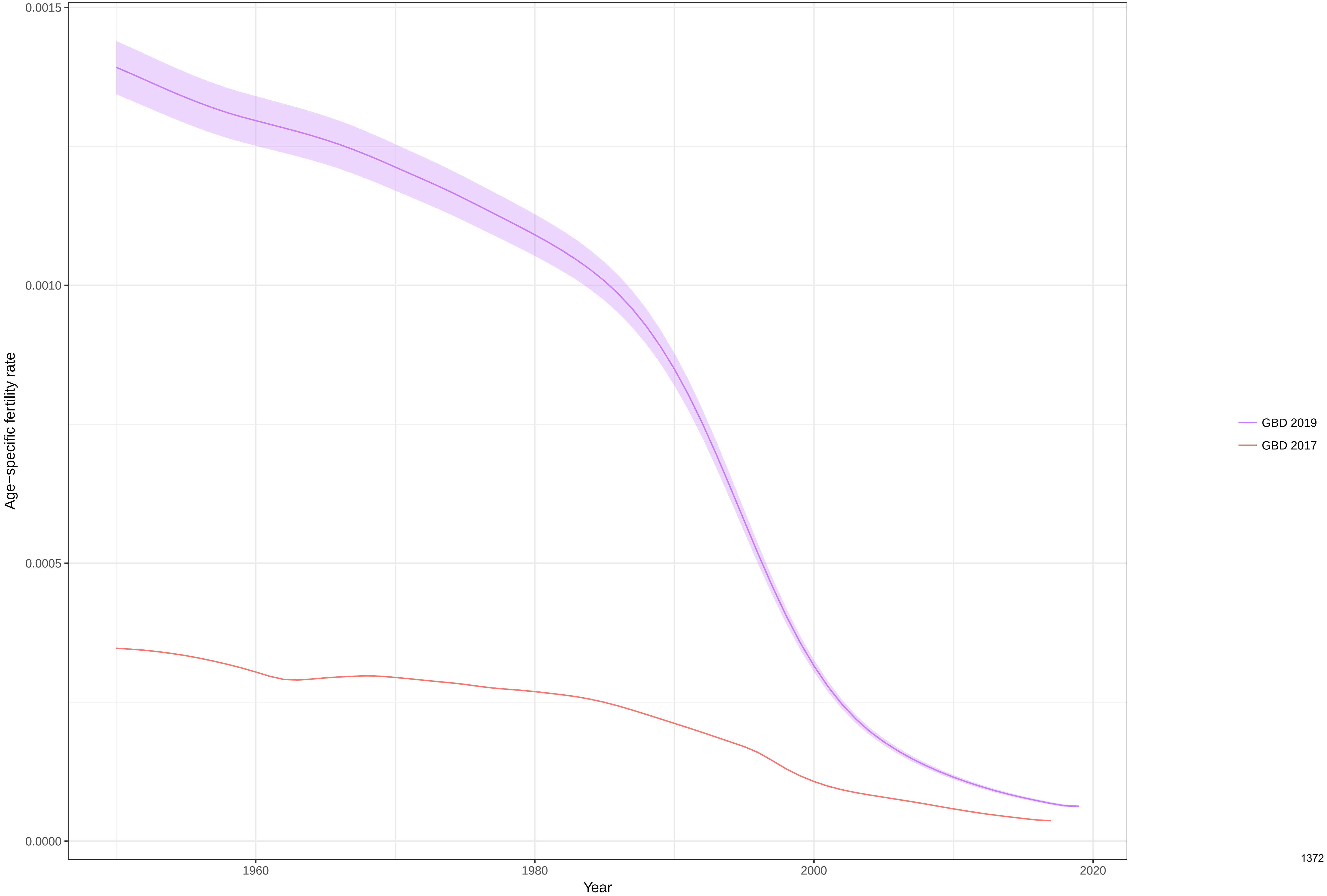

Total fertility rate fitted trend lines, 1950−2019: Maldives TFR
Data points enclosed with a diamond are reference sources

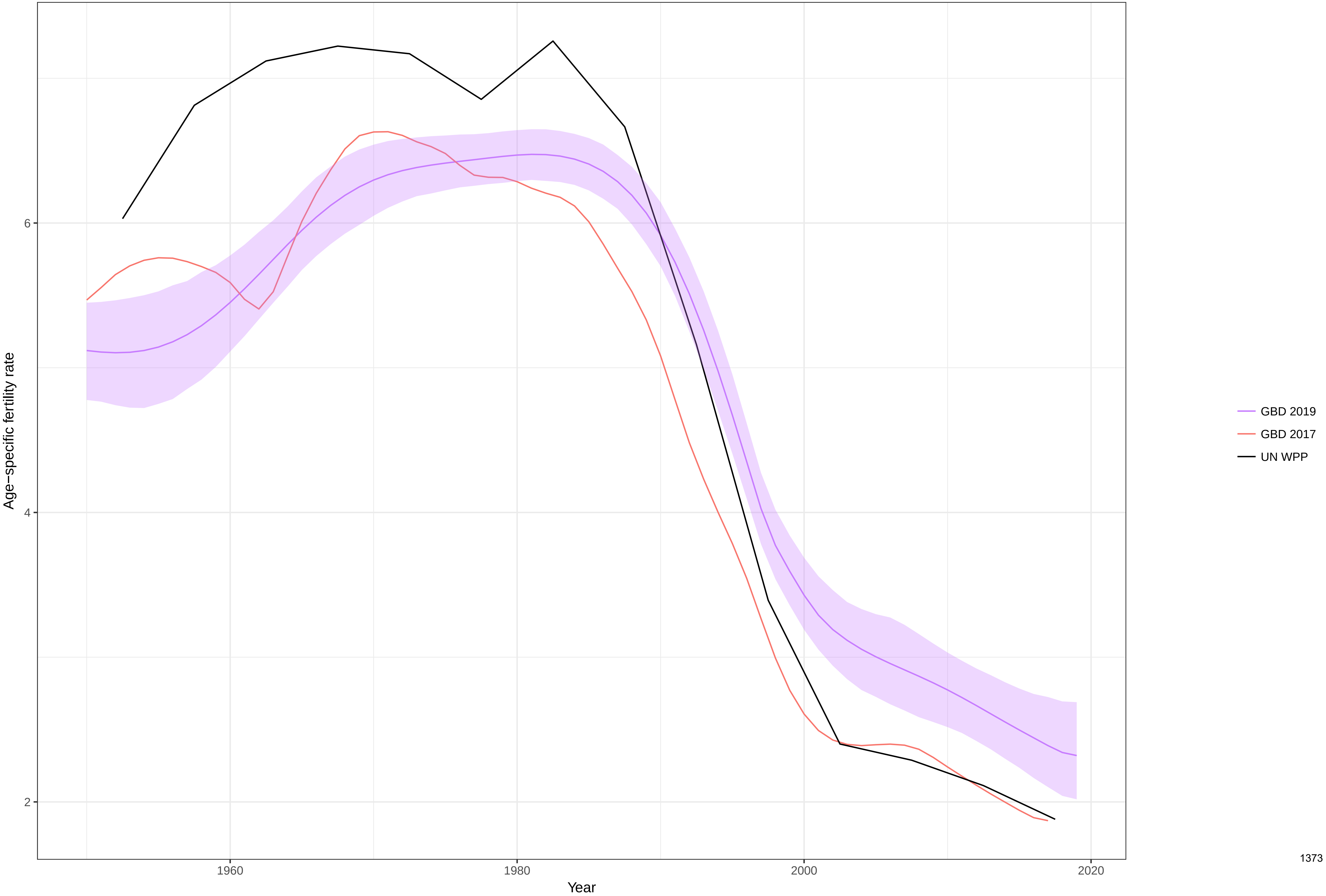

Age−specific fertility fitted trend lines, 1950−2019: Mexico, Ages: 10−14
Data points enclosed with a diamond are reference sources

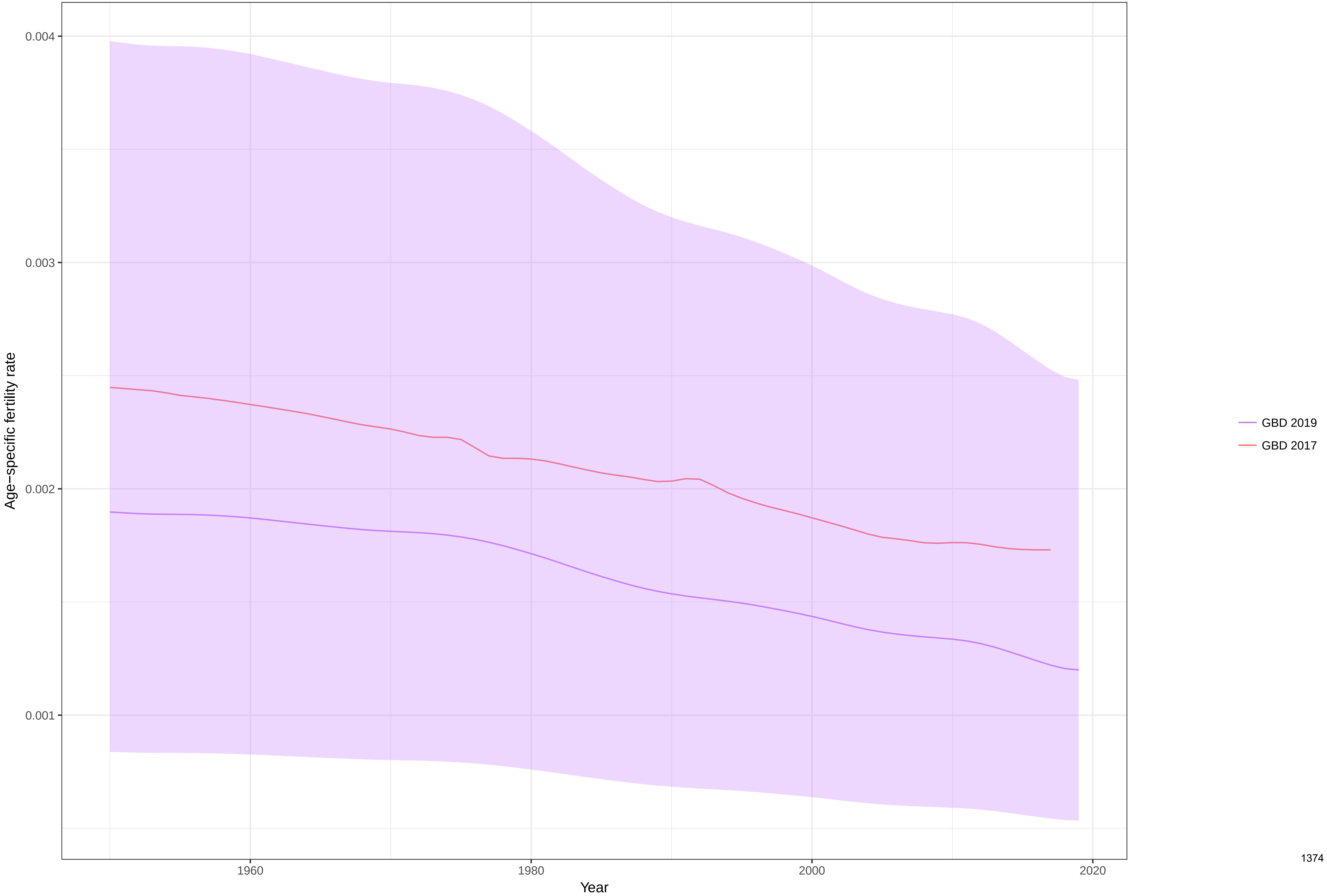

Age−specific fertility fitted trend lines, 1950−2019: Mexico, Ages: 15−19
Data points enclosed with a diamond are reference sources

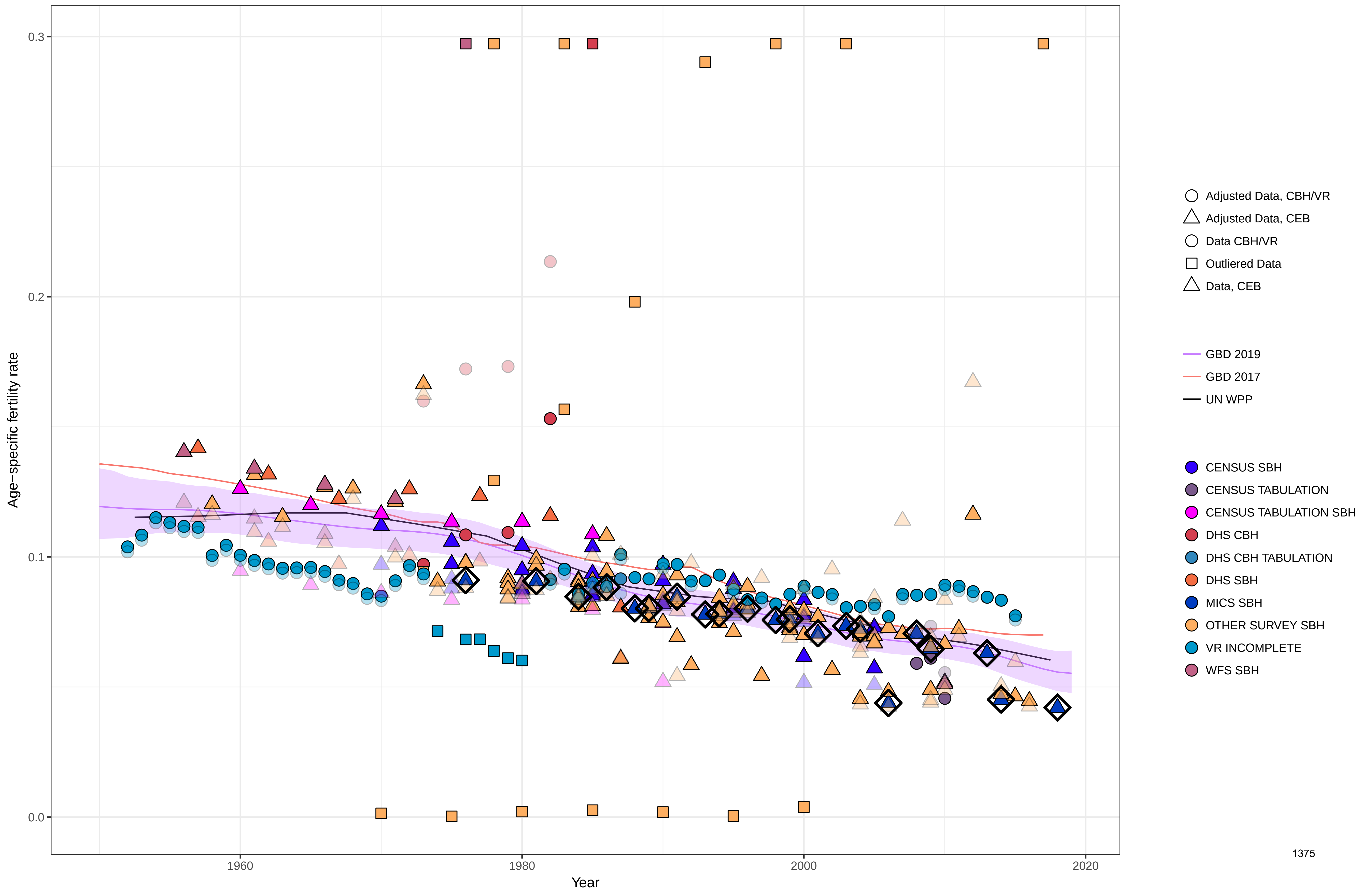

Age−specific fertility fitted trend lines, 1950−2019: Mexico, Ages: 20−24
Data points enclosed with a diamond are reference sources

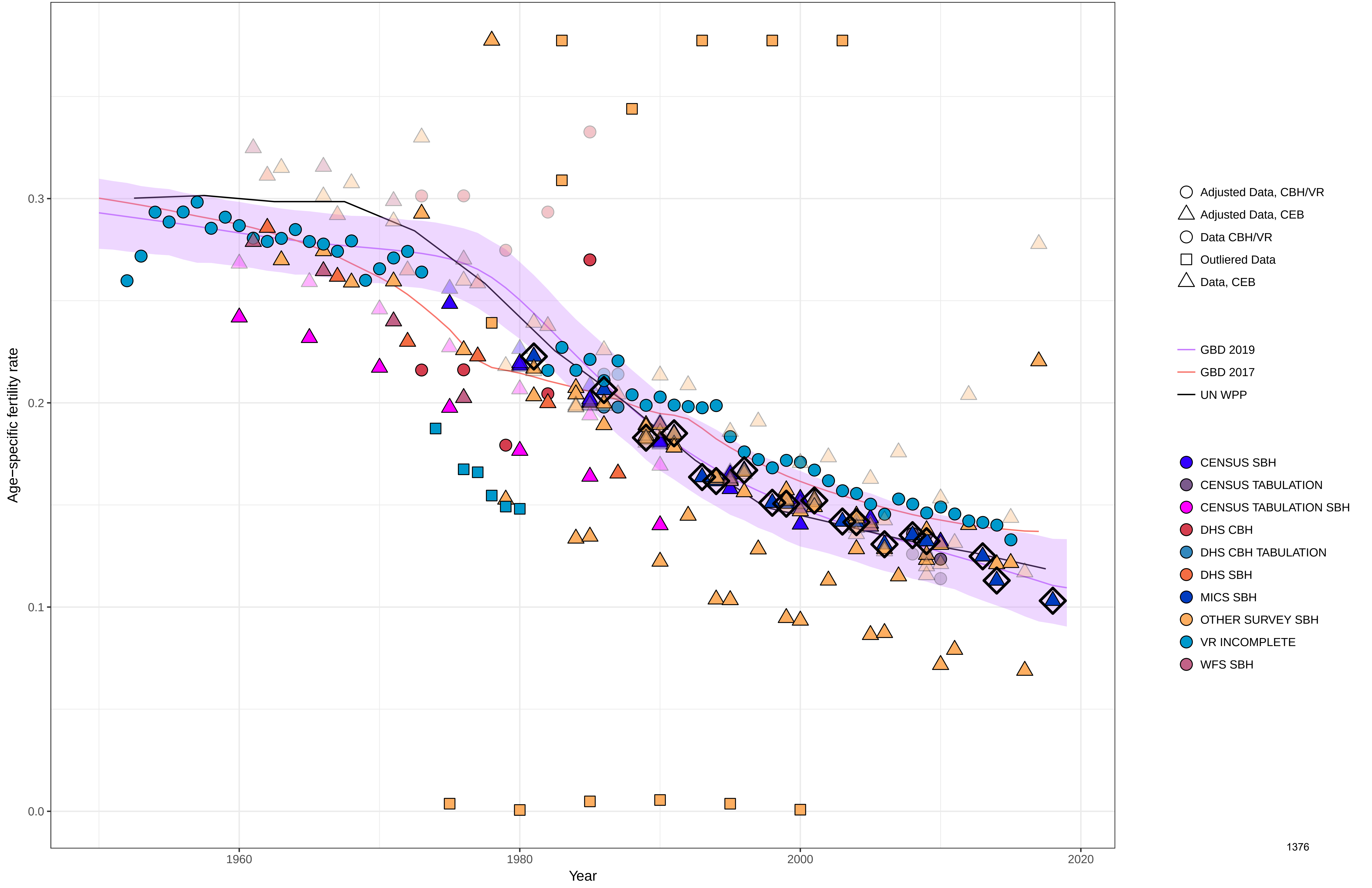

Age−specific fertility fitted trend lines, 1950−2019: Mexico, Ages: 25−29
Data points enclosed with a diamond are reference sources

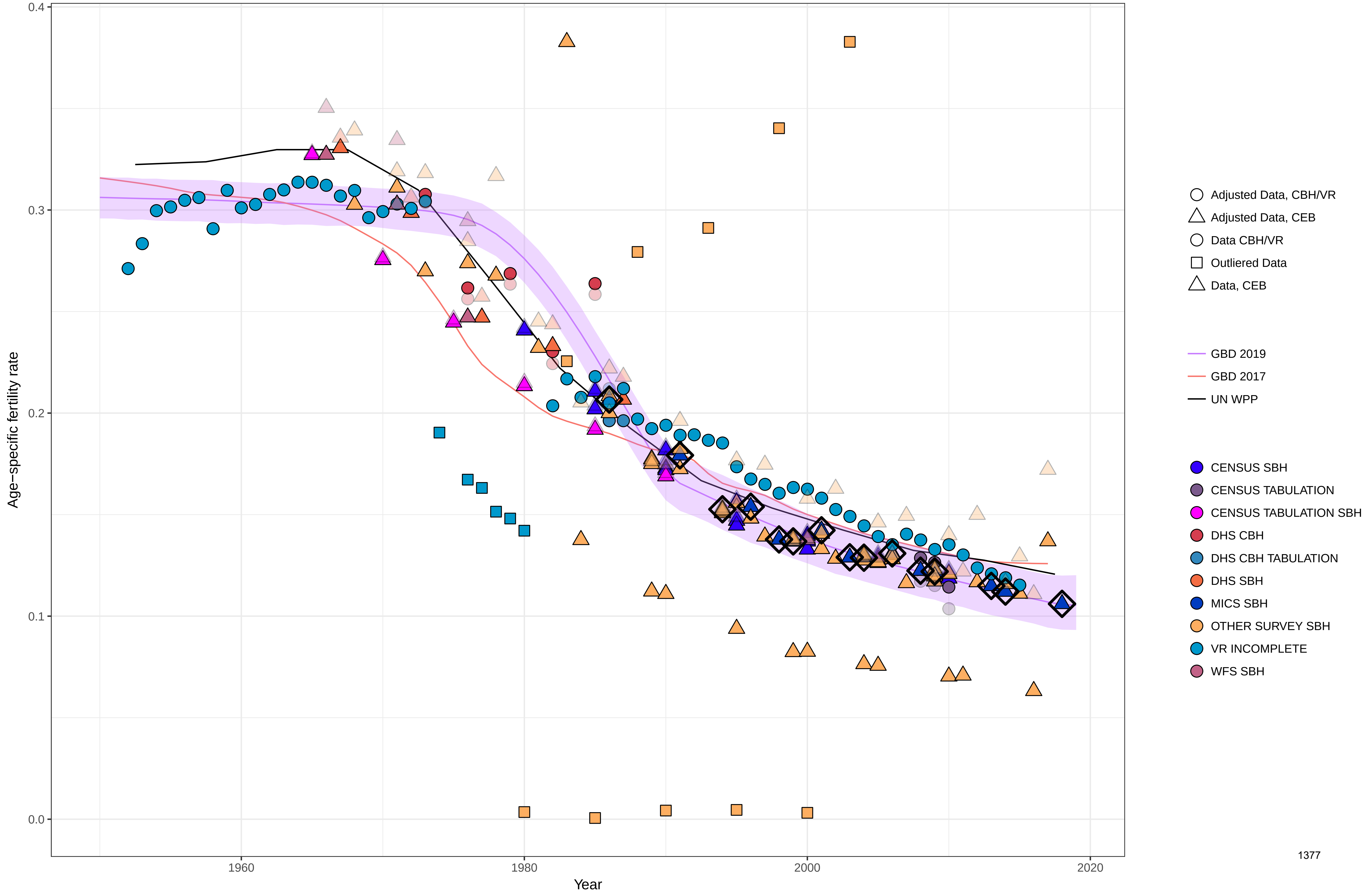

Age−specific fertility fitted trend lines, 1950−2019: Mexico, Ages: 30−34
Data points enclosed with a diamond are reference sources

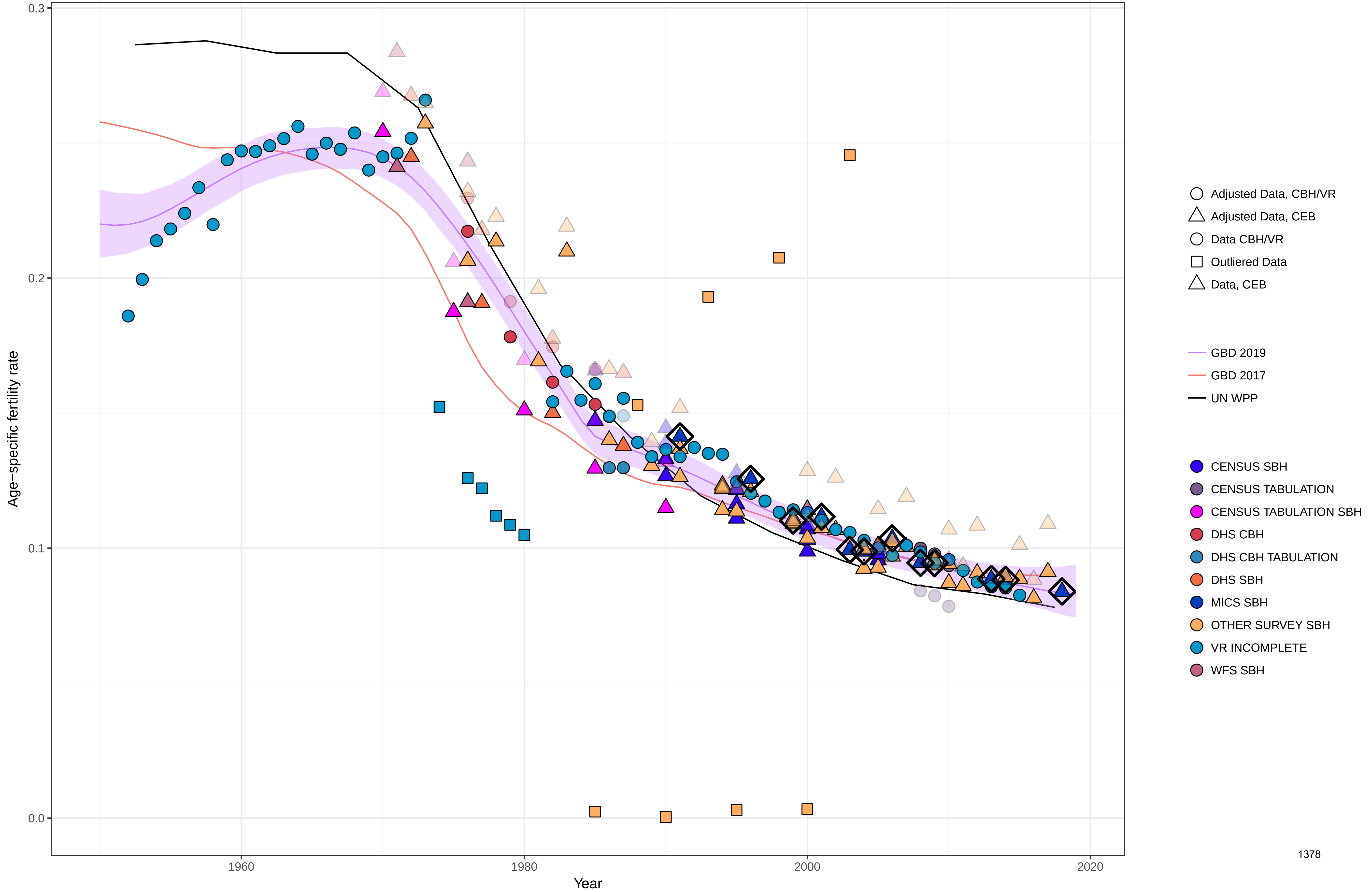

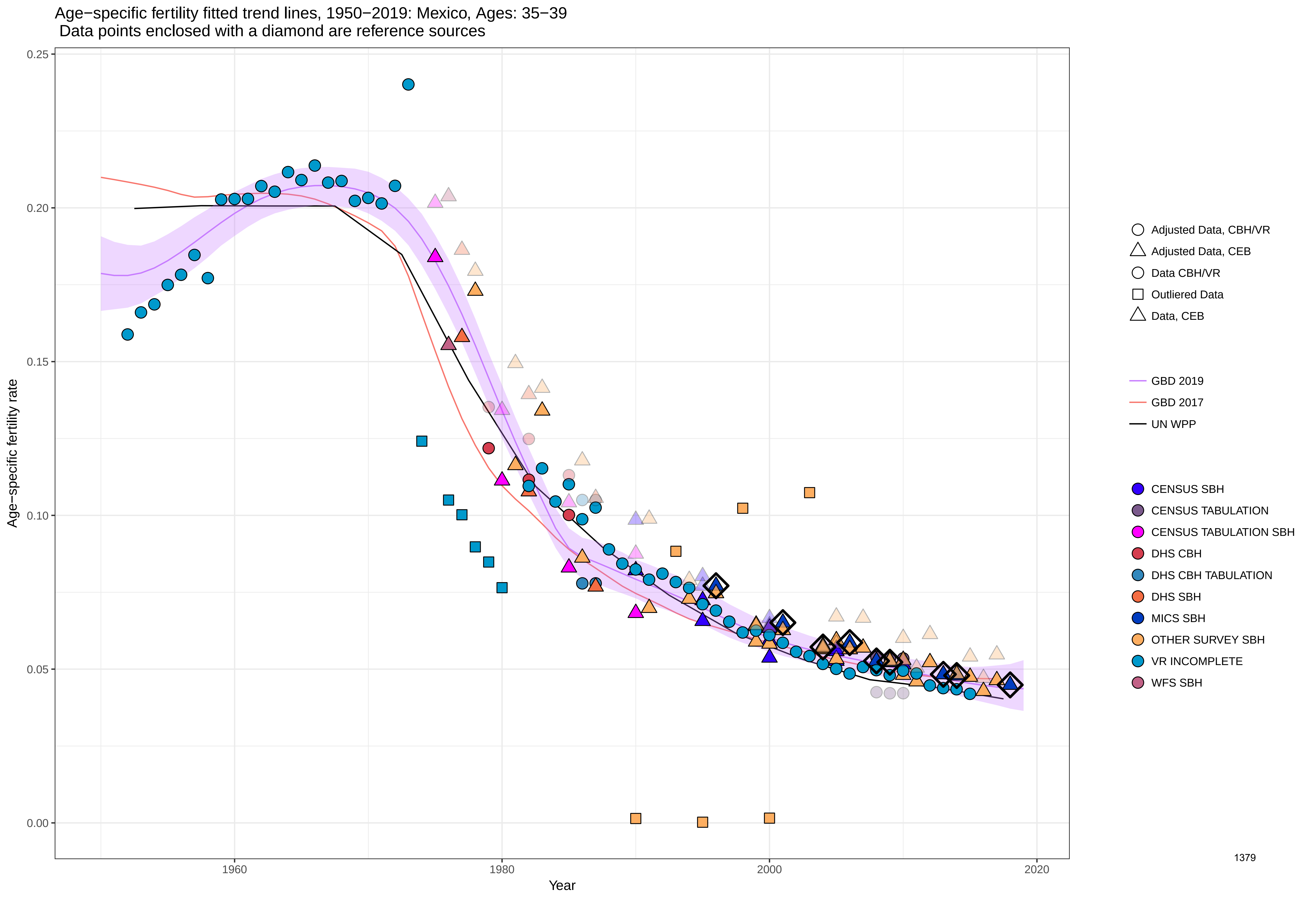

Age−specific fertility fitted trend lines, 1950−2019: Mexico, Ages: 35−39
Data points enclosed with a diamond are reference sources
Age−specific fertility rate
Year
0.00
0.05
0.10
0.15
0.20
0.25
1960
1980
2000
2020
Adjusted Data, CBH/VR
Adjusted Data, CEB
Data CBH/VR
Outliered Data
Data, CEB
GBD 2019
GBD 2017
UN WPP
CENSUS SBH
CENSUS TABULATION
CENSUS TABULATION SBH
DHS CBH
DHS CBH TABULATION
DHS SBH
MICS SBH
OTHER SURVEY SBH
VR INCOMPLETE
WFS SBH

Age−specific fertility fitted trend lines, 1950−2019: Mexico, Ages: 40−44
Data points enclosed with a diamond are reference sources

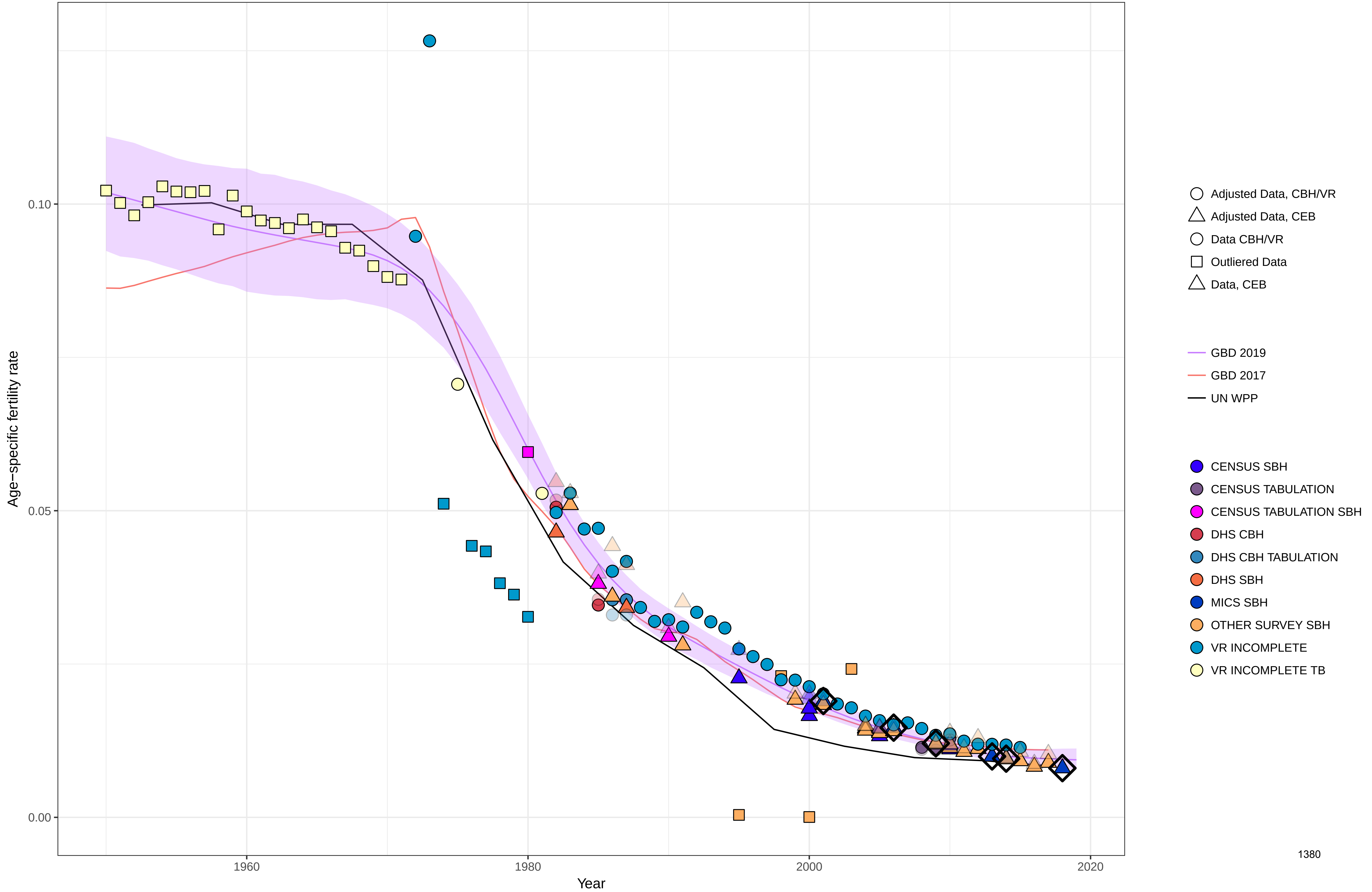

Age−specific fertility fitted trend lines, 1950−2019: Mexico, Ages: 45−49
Data points enclosed with a diamond are reference sources

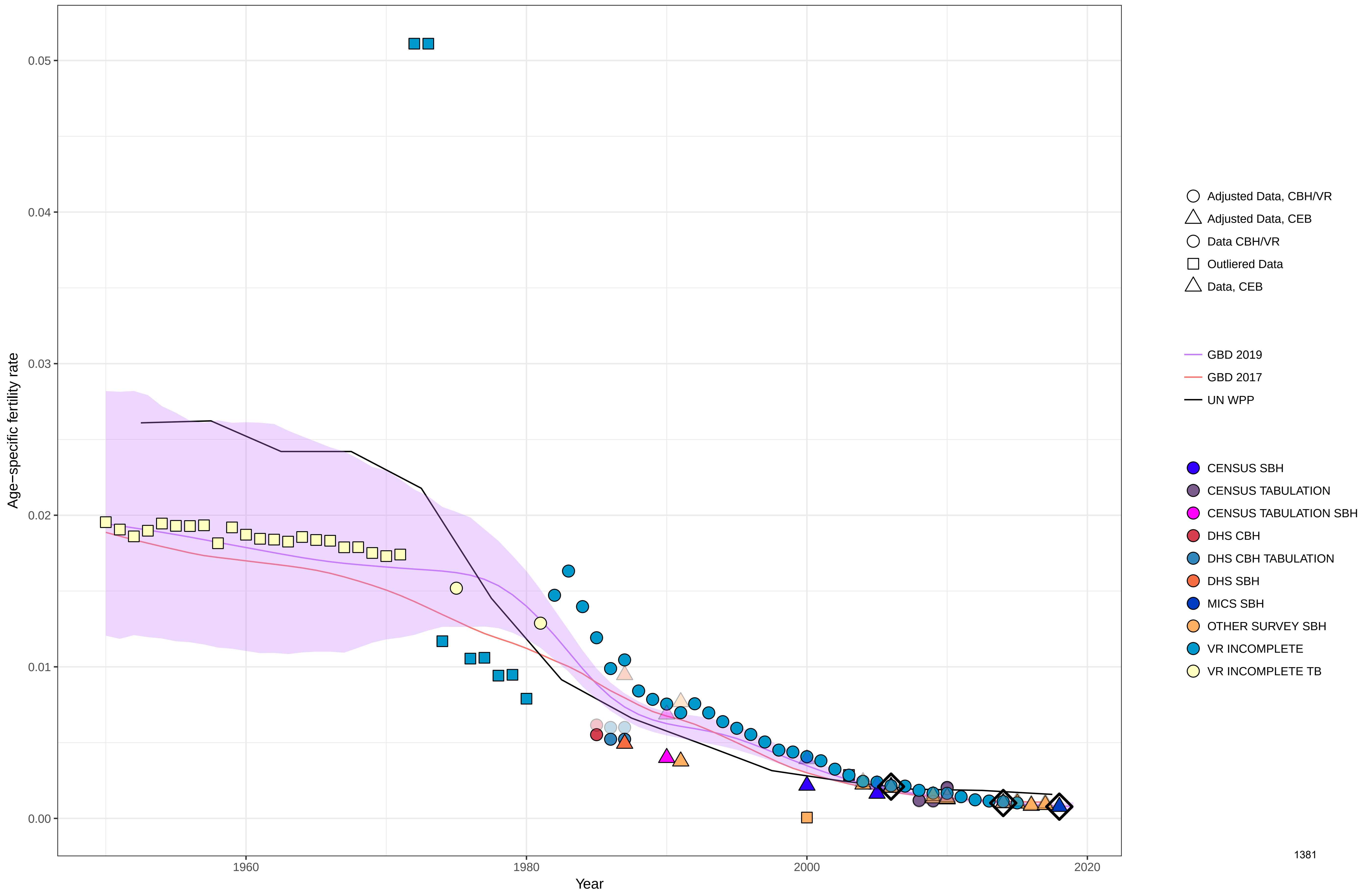

Age−specific fertility fitted trend lines, 1950−2019: Mexico, Ages: 50−54
Data points enclosed with a diamond are reference sources

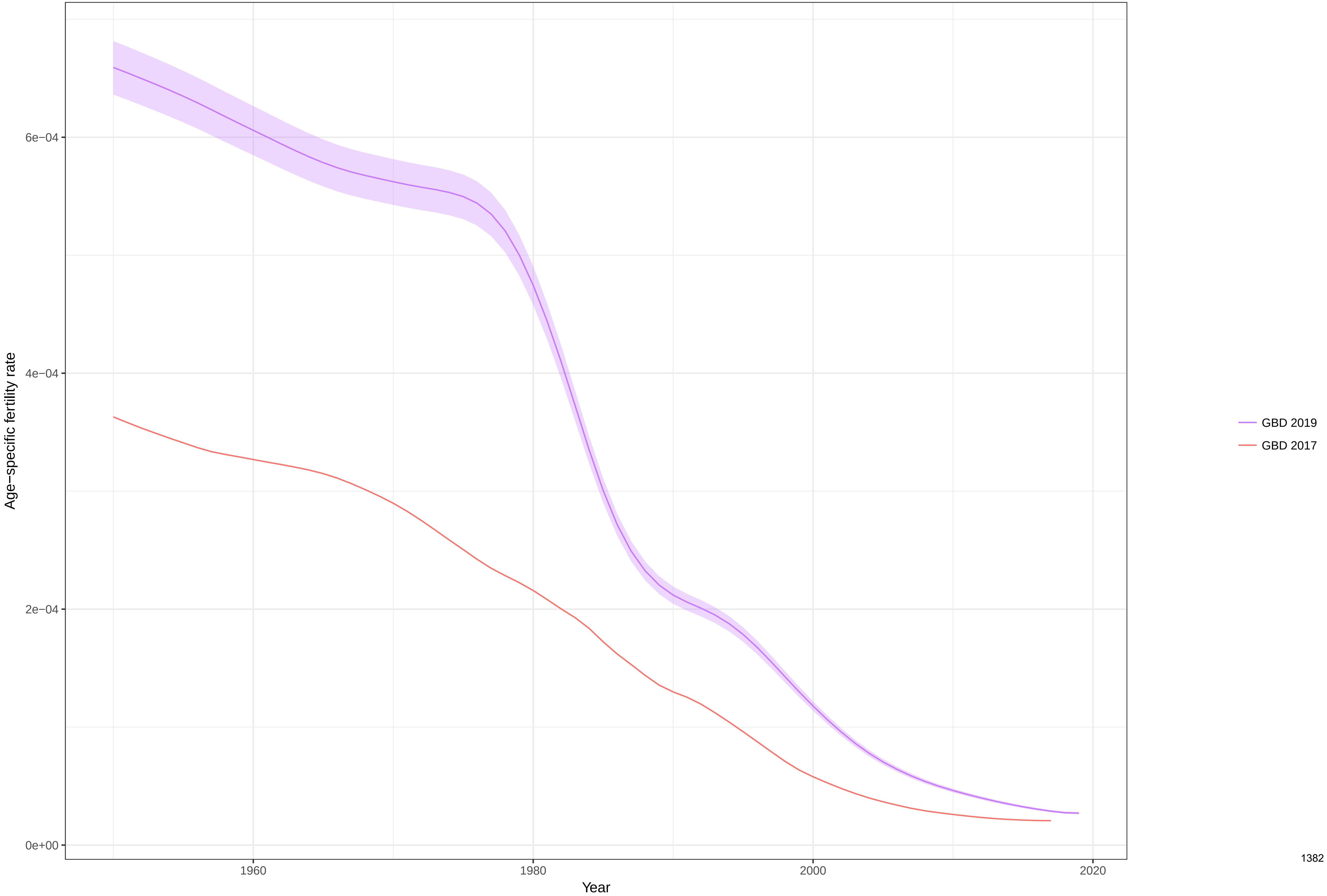

Total fertility rate fitted trend lines, 1950−2019: Mexico TFR
Data points enclosed with a diamond are reference sources

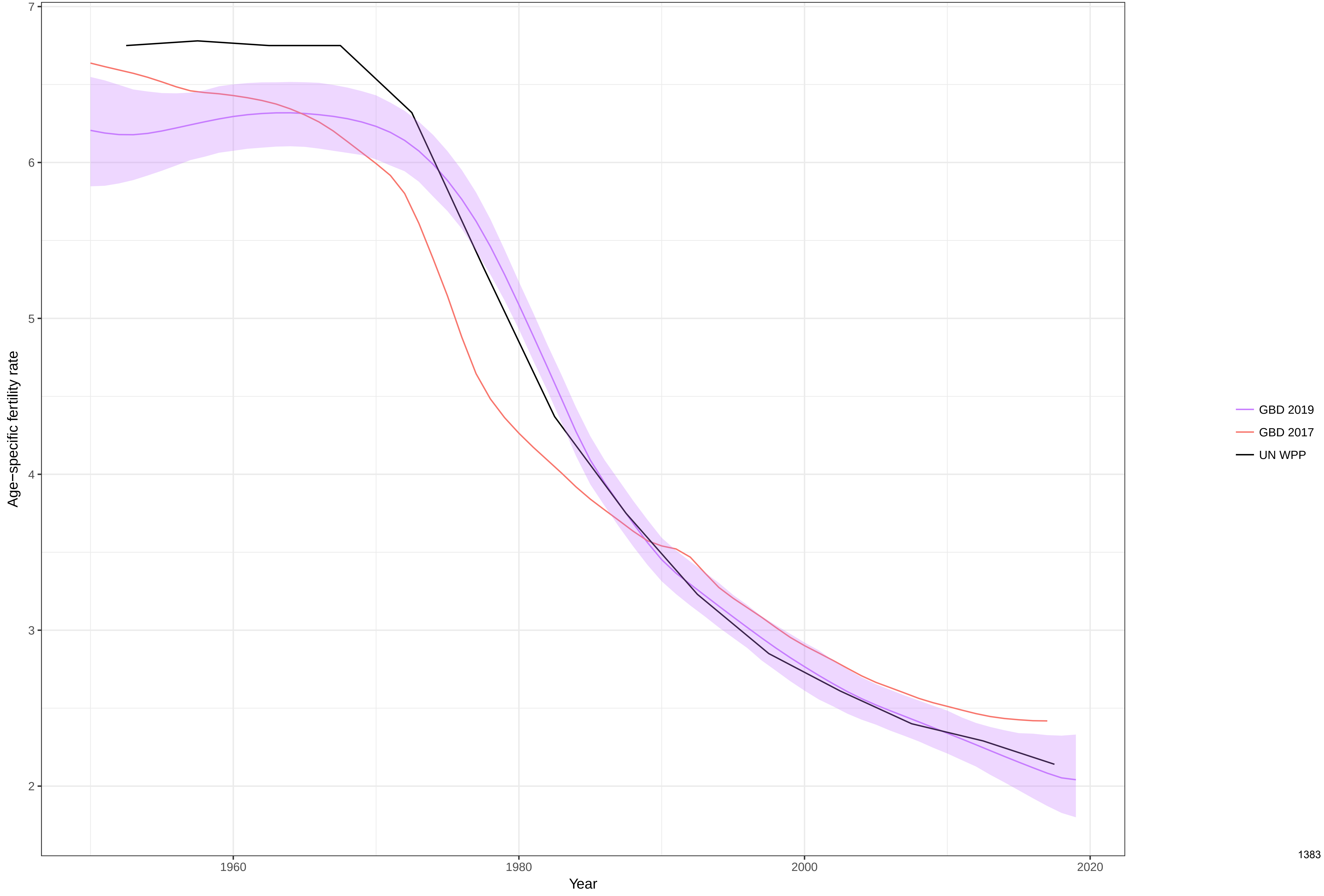

Age−specific fertility fitted trend lines, 1950−2019: Marshall Islands, Ages: 10−14
Data points enclosed with a diamond are reference sources

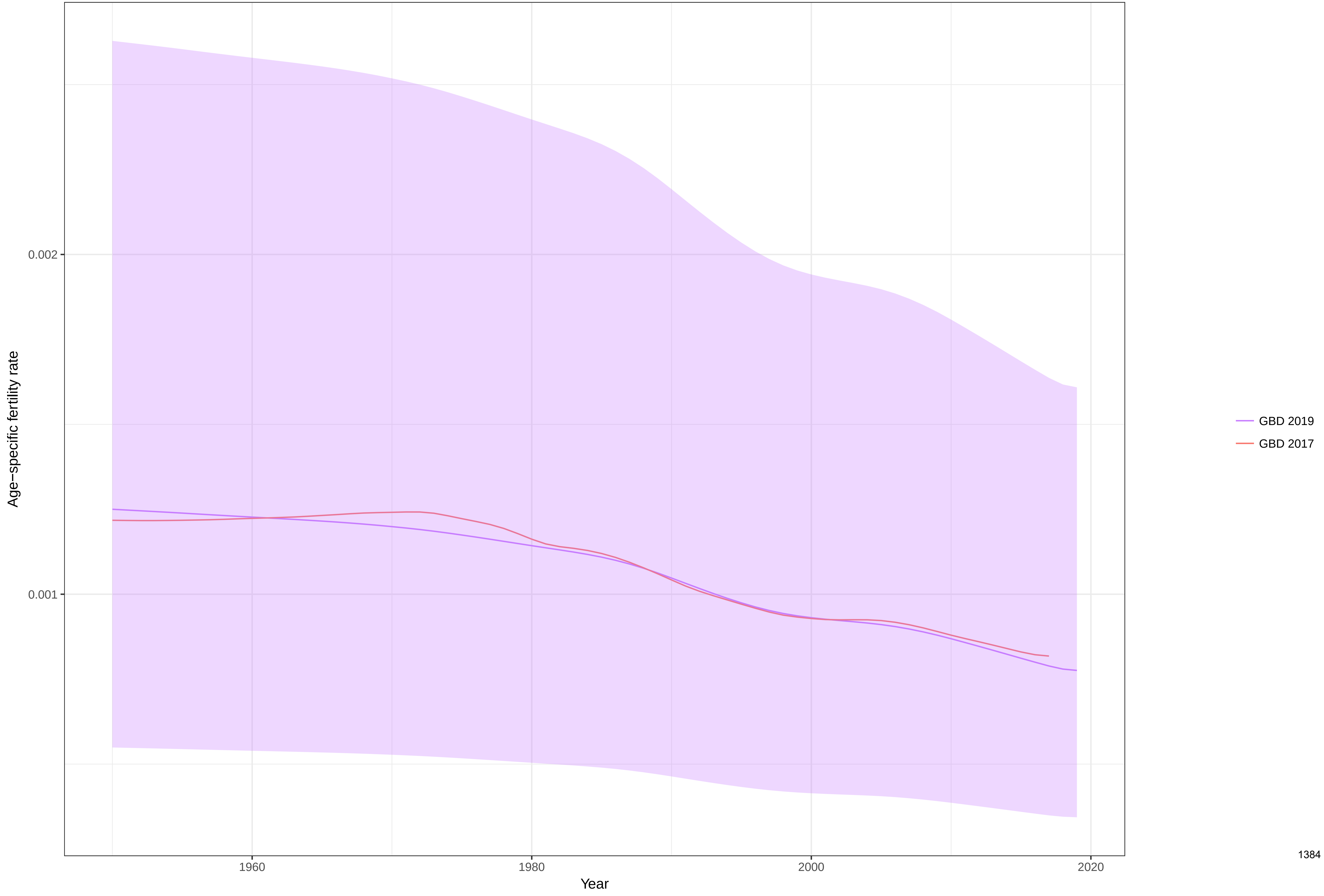

Age−specific fertility fitted trend lines, 1950−2019: Marshall Islands, Ages: 15−19
Data points enclosed with a diamond are reference sources

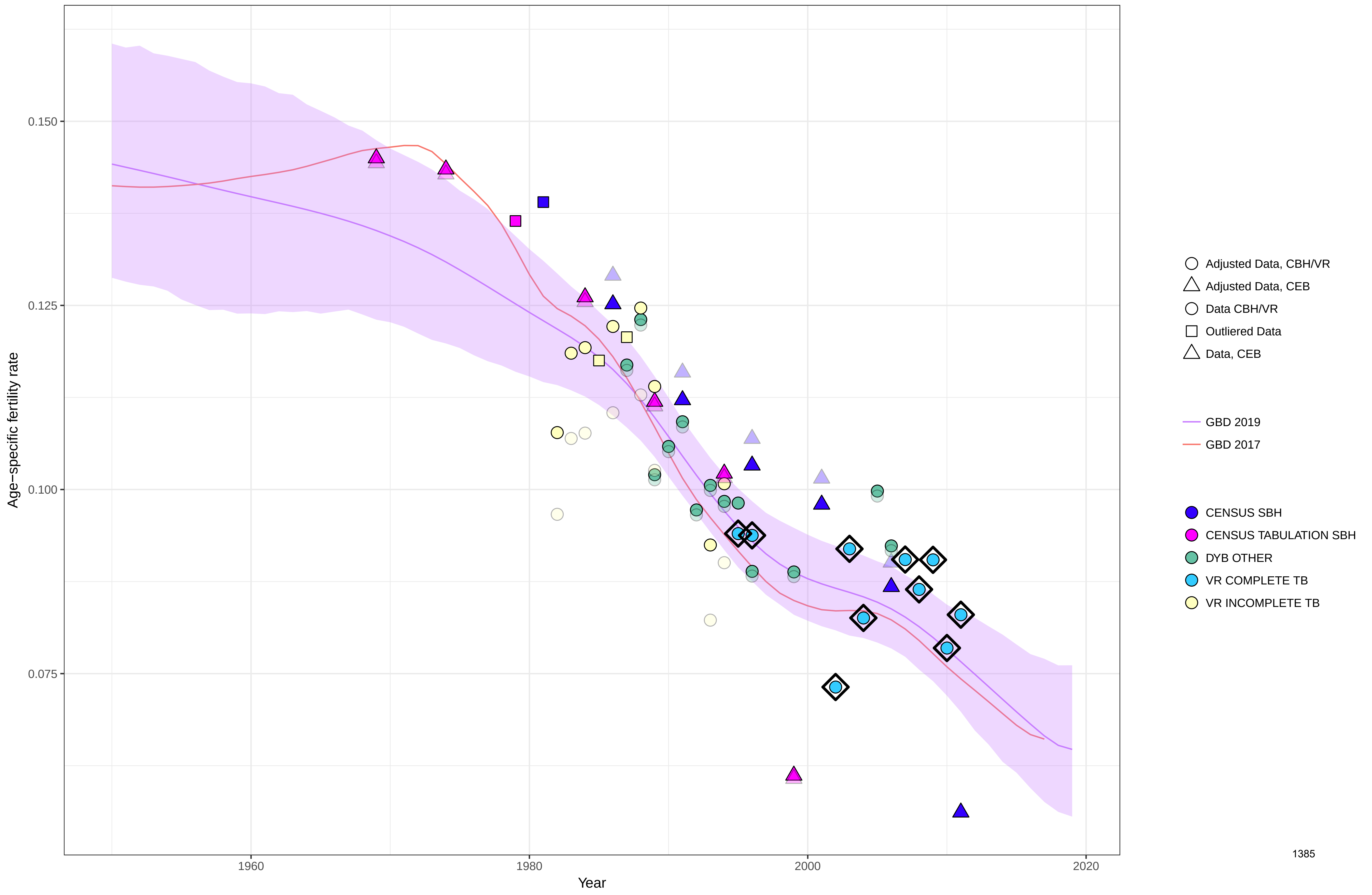

Age−specific fertility fitted trend lines, 1950−2019: Marshall Islands, Ages: 20−24
Data points enclosed with a diamond are reference sources

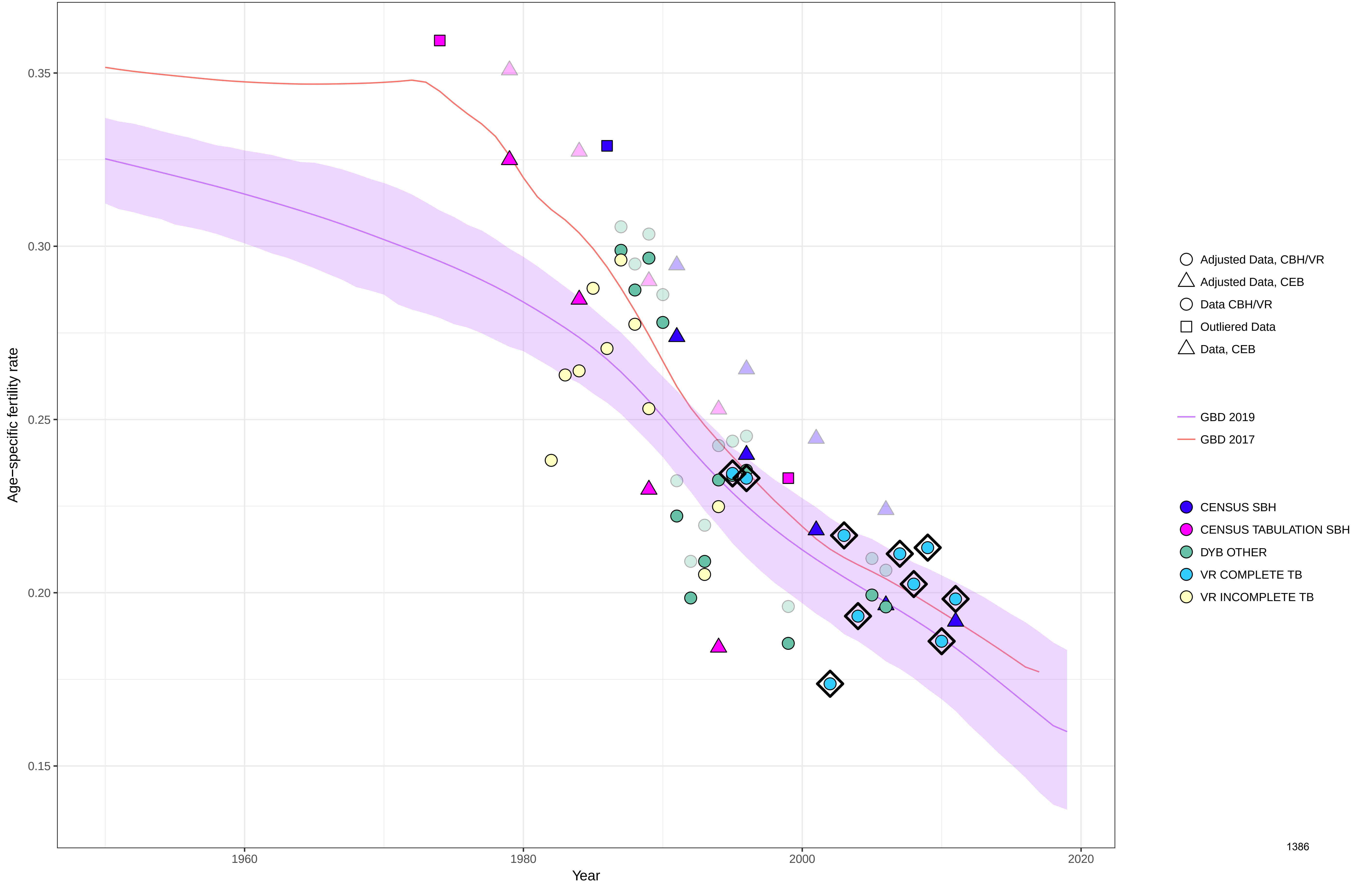

Age−specific fertility fitted trend lines, 1950−2019: Marshall Islands, Ages: 25−29
Data points enclosed with a diamond are reference sources

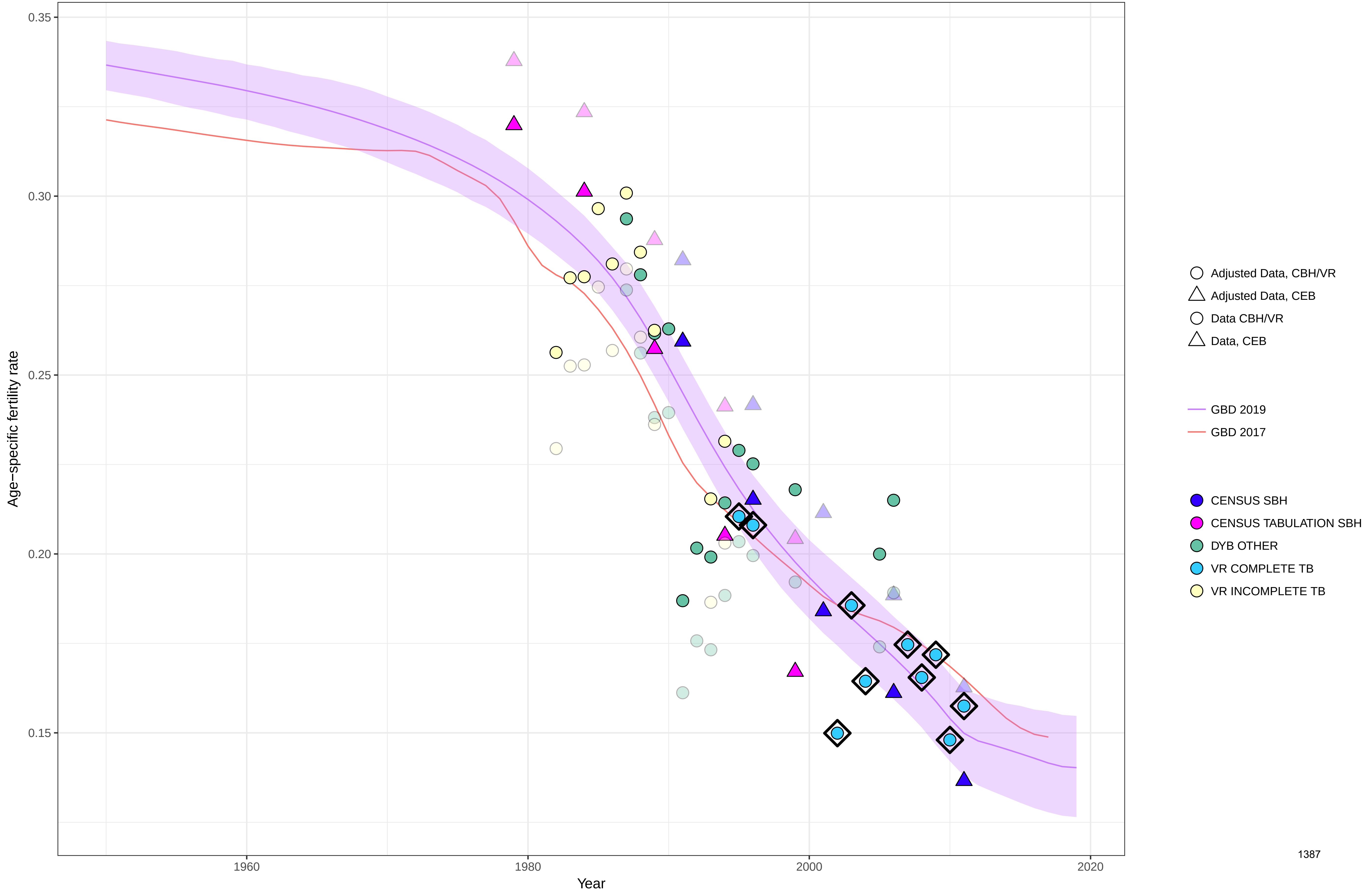

Age−specific fertility fitted trend lines, 1950−2019: Marshall Islands, Ages: 30−34
Data points enclosed with a diamond are reference sources

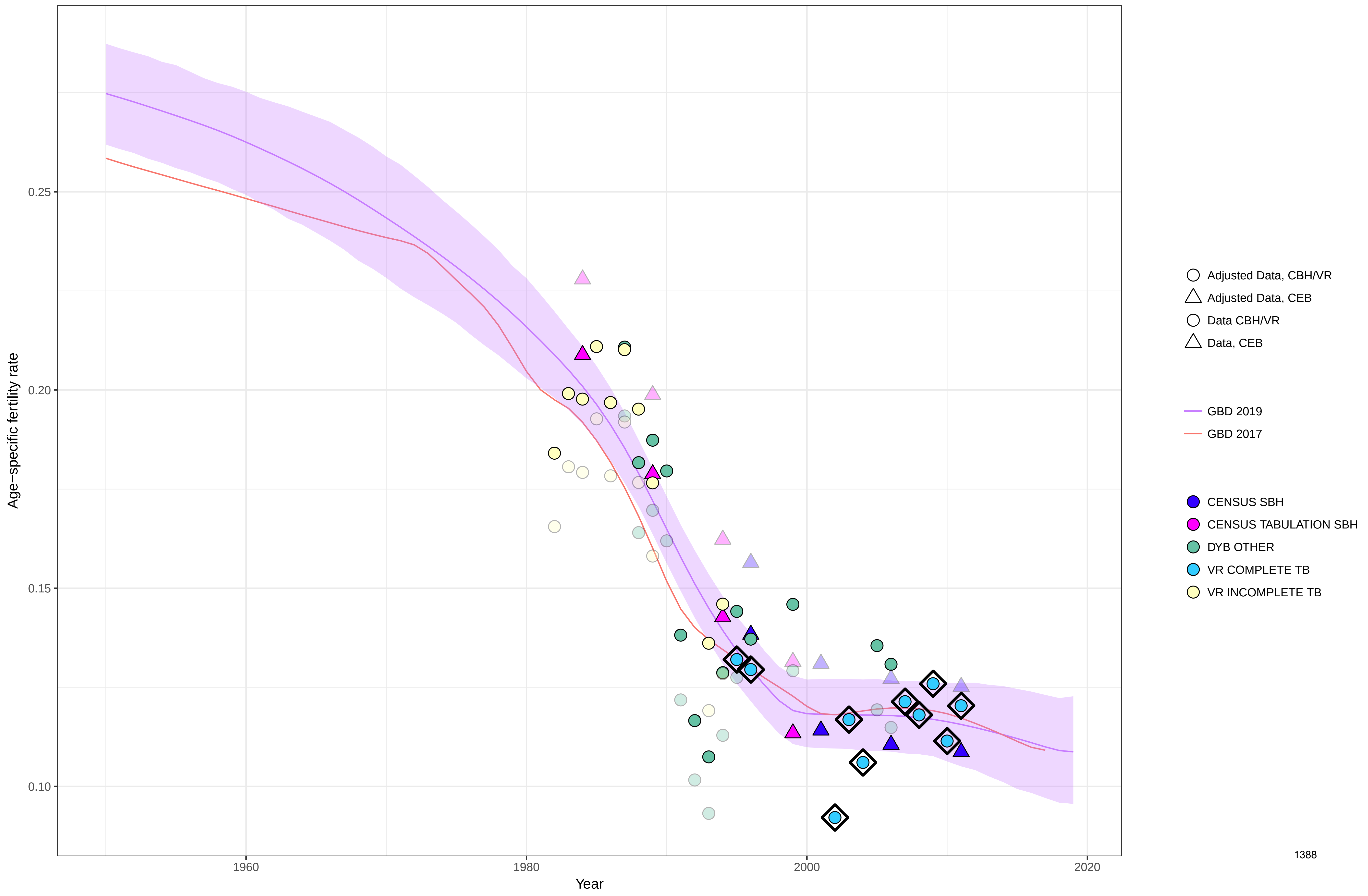

Age−specific fertility fitted trend lines, 1950−2019: Marshall Islands, Ages: 35−39
Data points enclosed with a diamond are reference sources

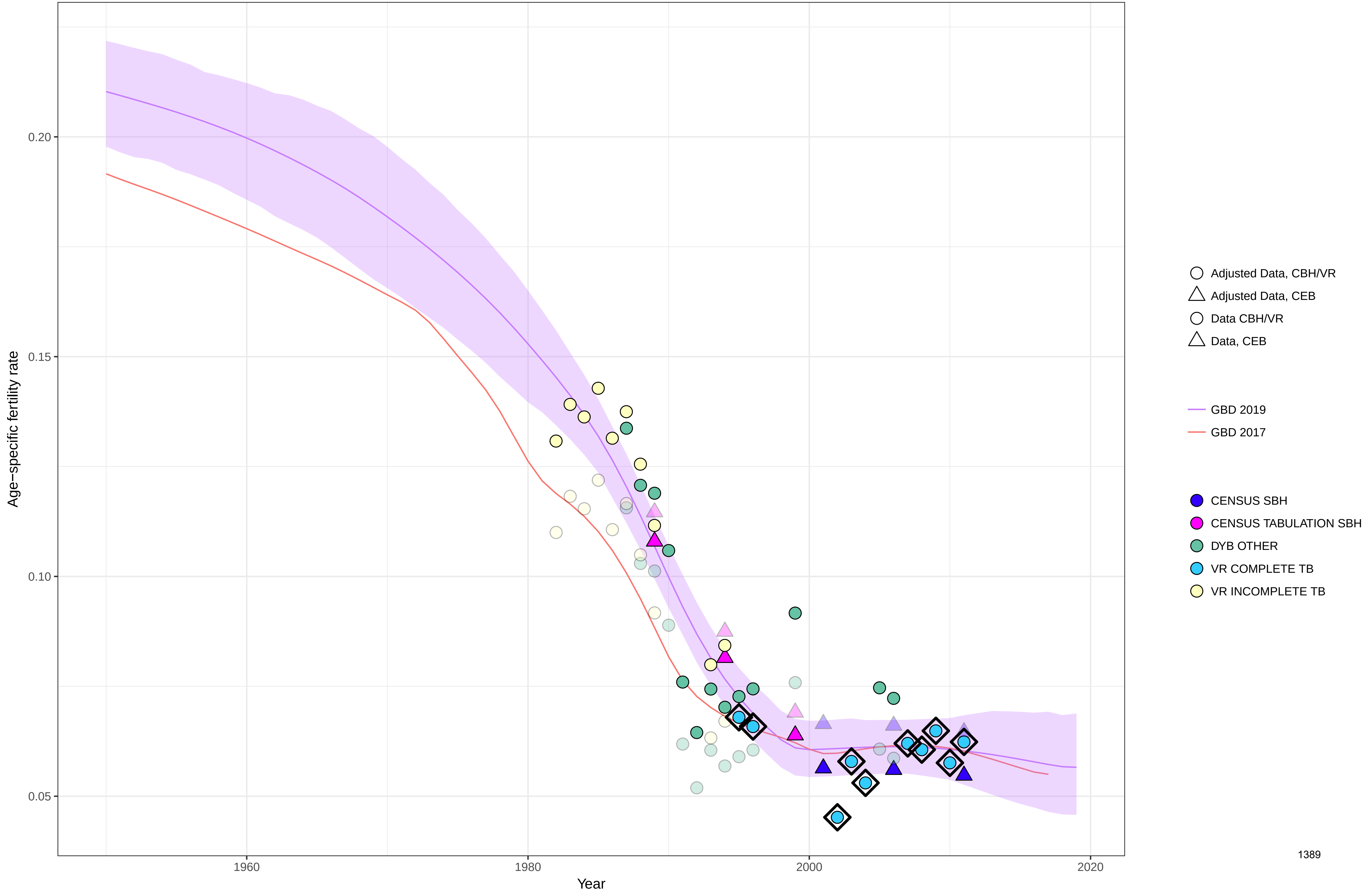

Age−specific fertility fitted trend lines, 1950−2019: Marshall Islands, Ages: 40−44
Data points enclosed with a diamond are reference sources

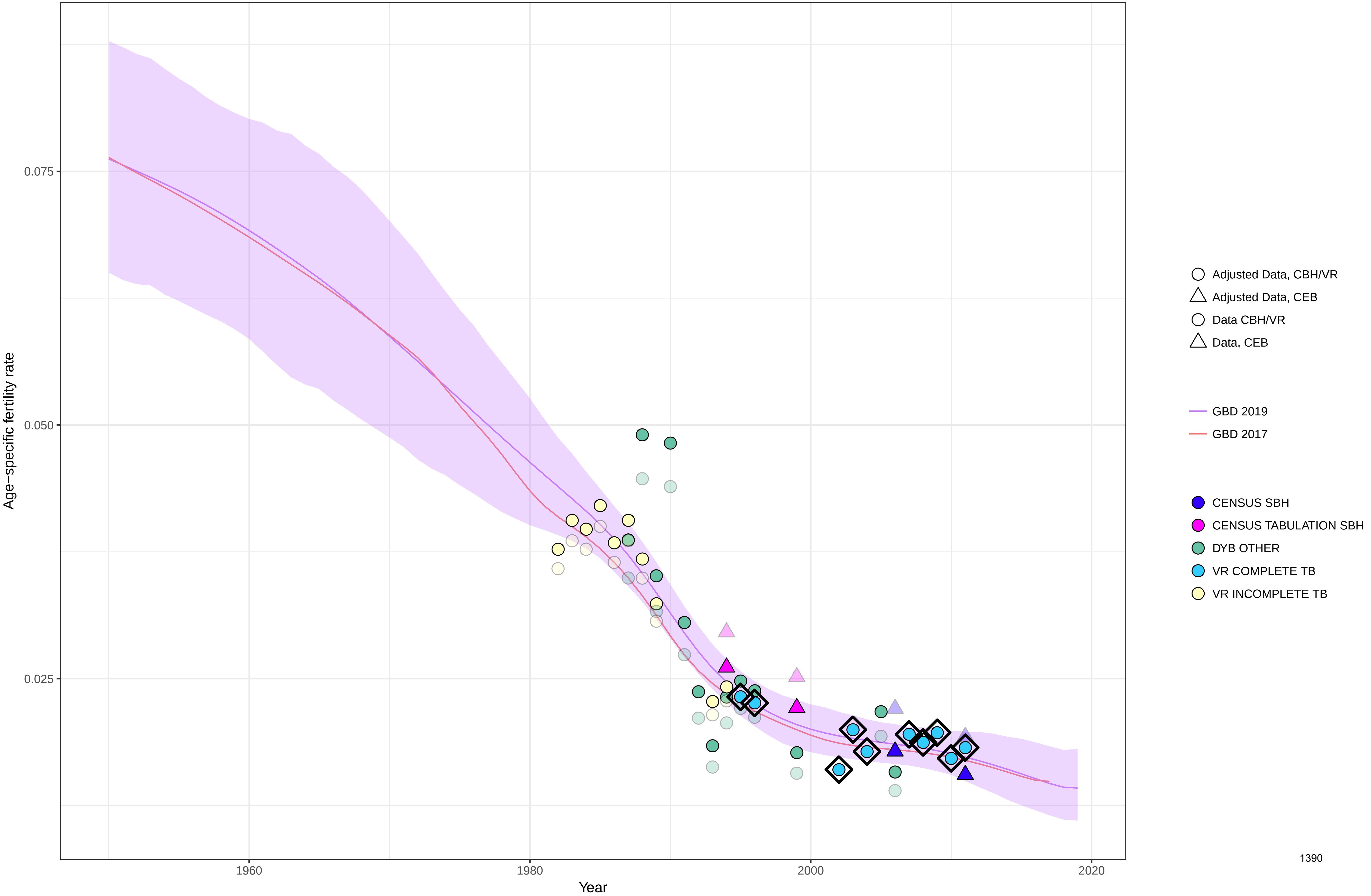

Age−specific fertility fitted trend lines, 1950−2019: Marshall Islands, Ages: 45−49
Data points enclosed with a diamond are reference sources

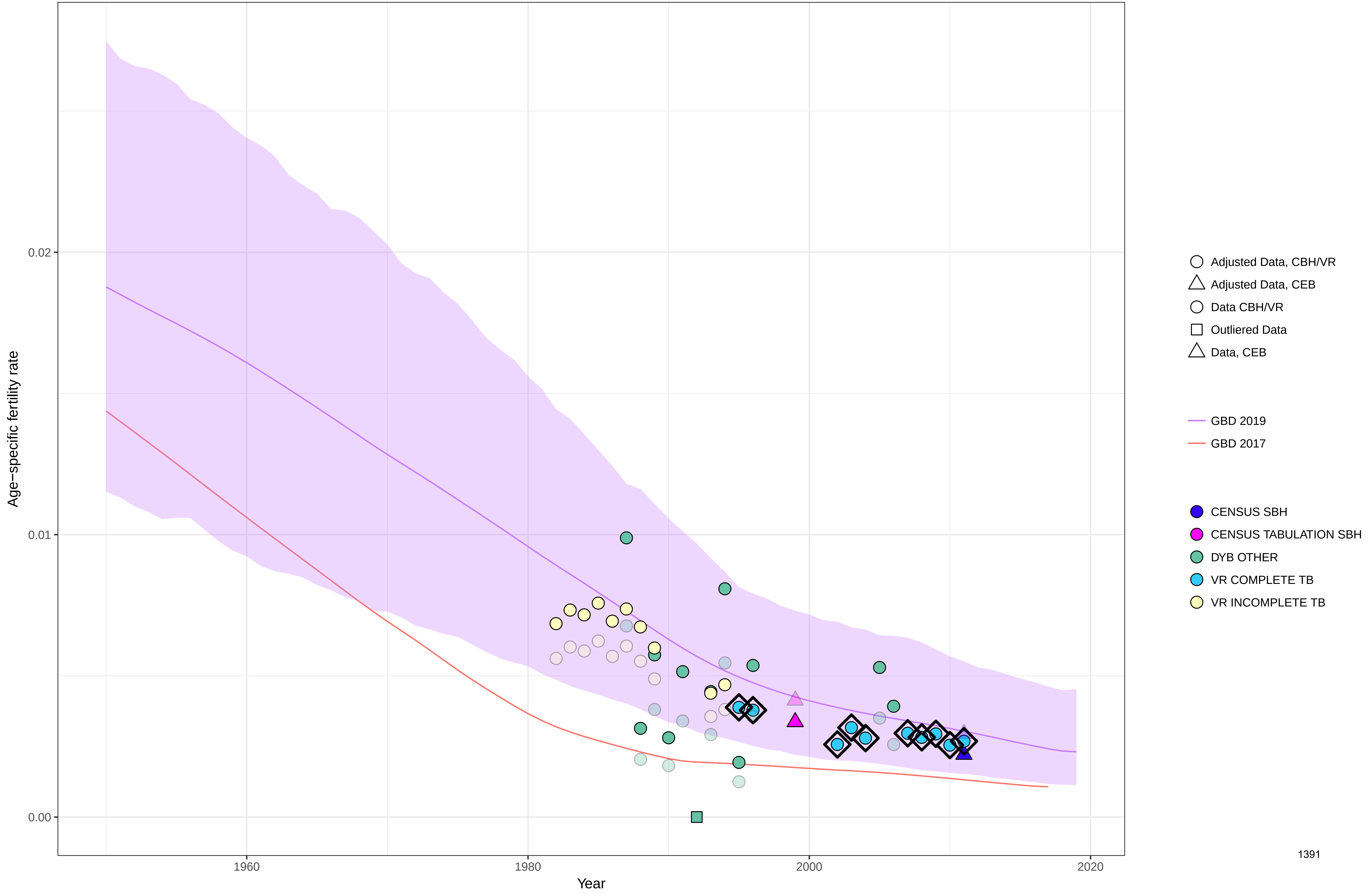

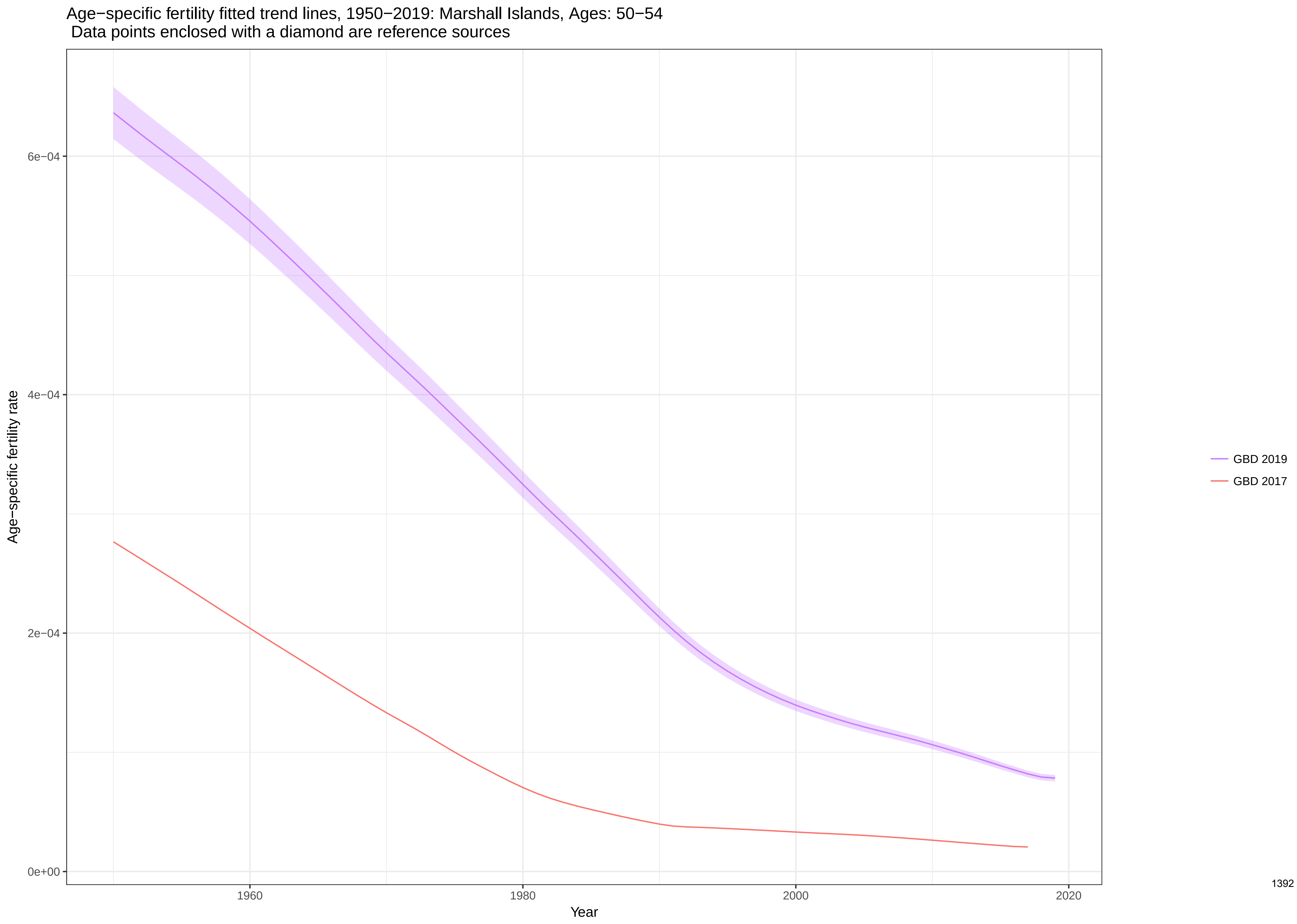

Age−specific fertility fitted trend lines, 1950−2019: Marshall Islands, Ages: 50−54
Data points enclosed with a diamond are reference sources
6e−04
4e−04
2e−04
0e+00
Age−specific fertility rate
1960
1980
2000
2020
Year
GBD 2019
GBD 2017

Total fertility rate fitted trend lines, 1950−2019: Marshall Islands TFR
Data points enclosed with a diamond are reference sources

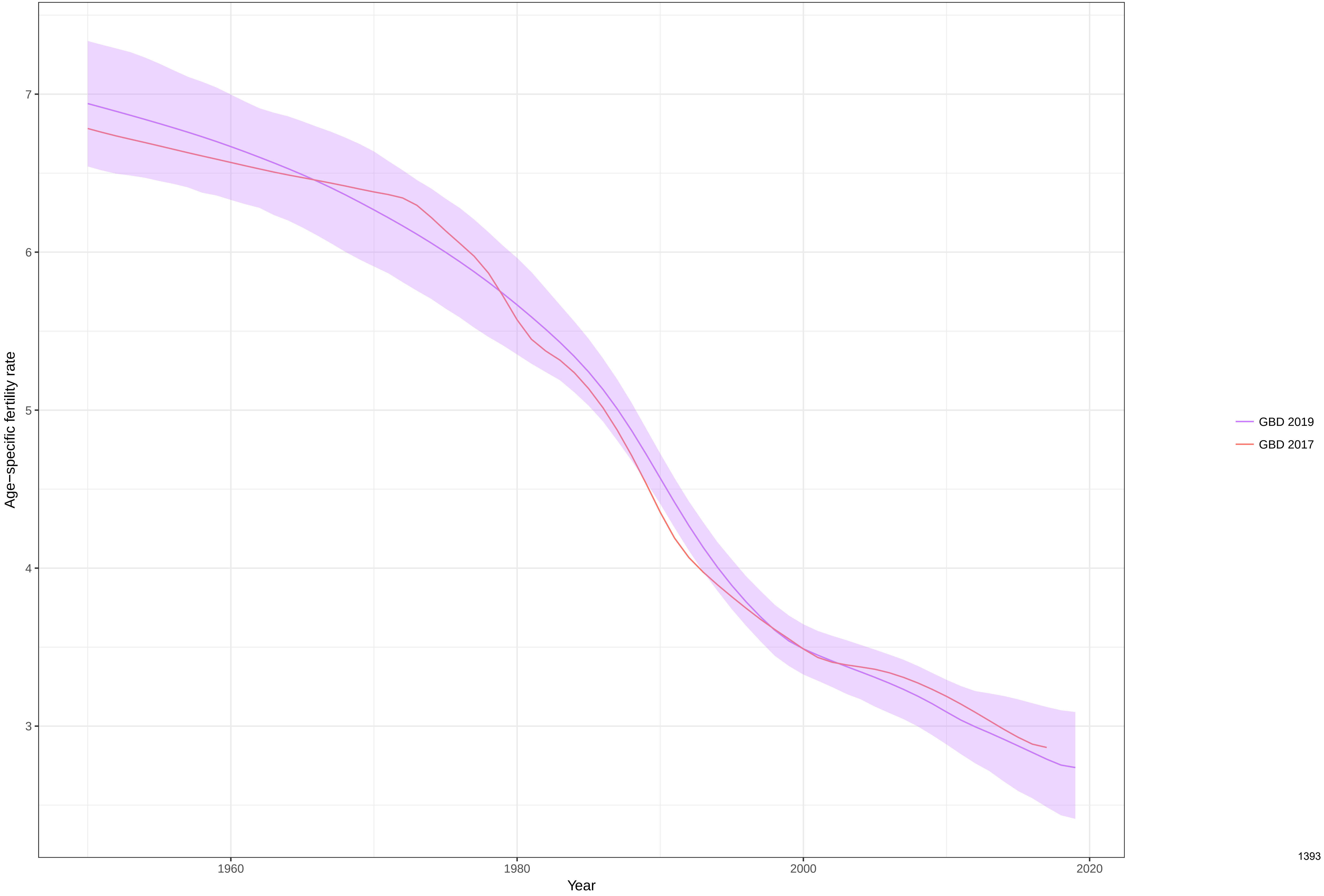

Age−specific fertility fitted trend lines, 1950−2019: North Macedonia, Ages: 10−14
Data points enclosed with a diamond are reference sources

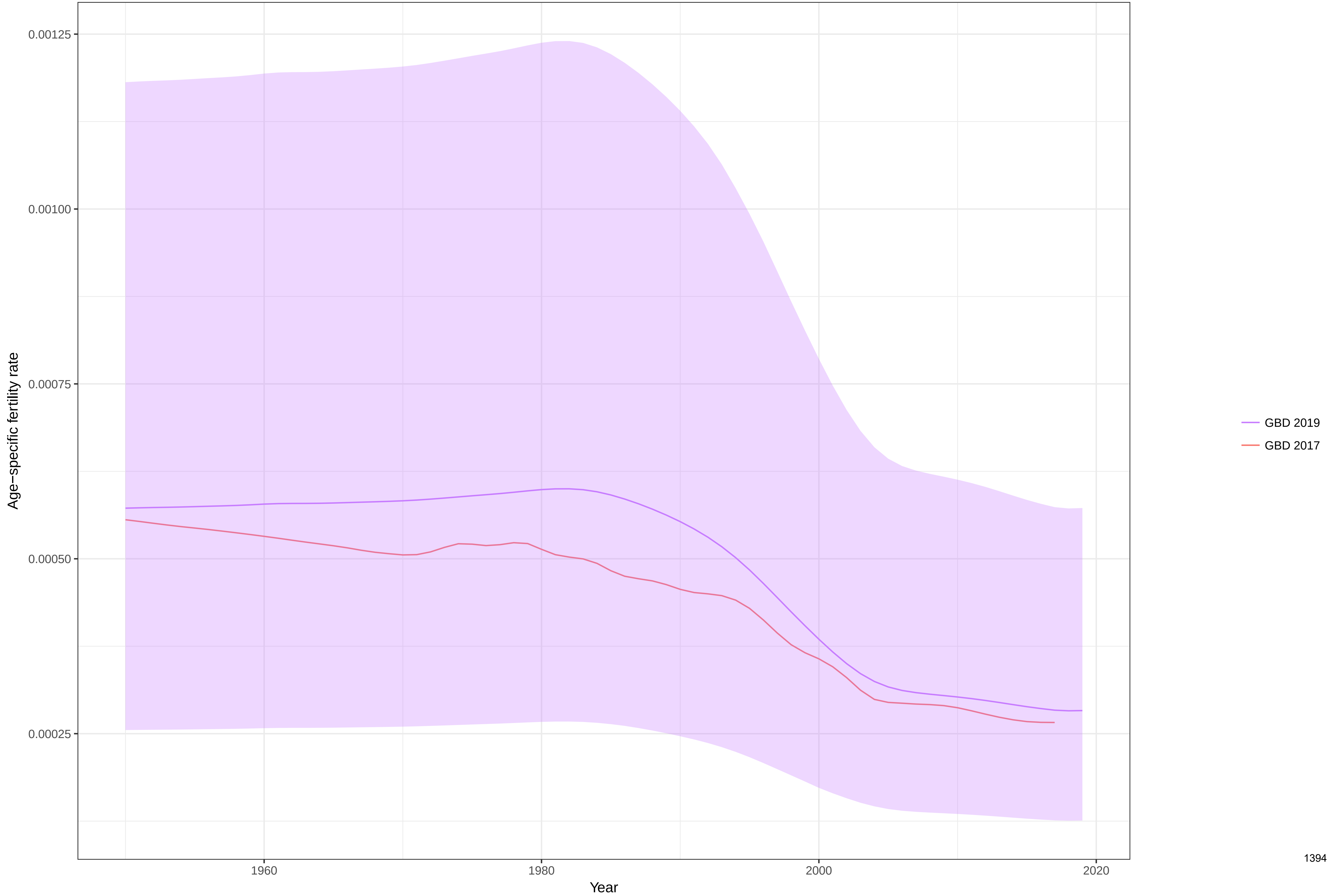

Age−specific fertility fitted trend lines, 1950−2019: North Macedonia, Ages: 15−19
Data points enclosed with a diamond are reference sources

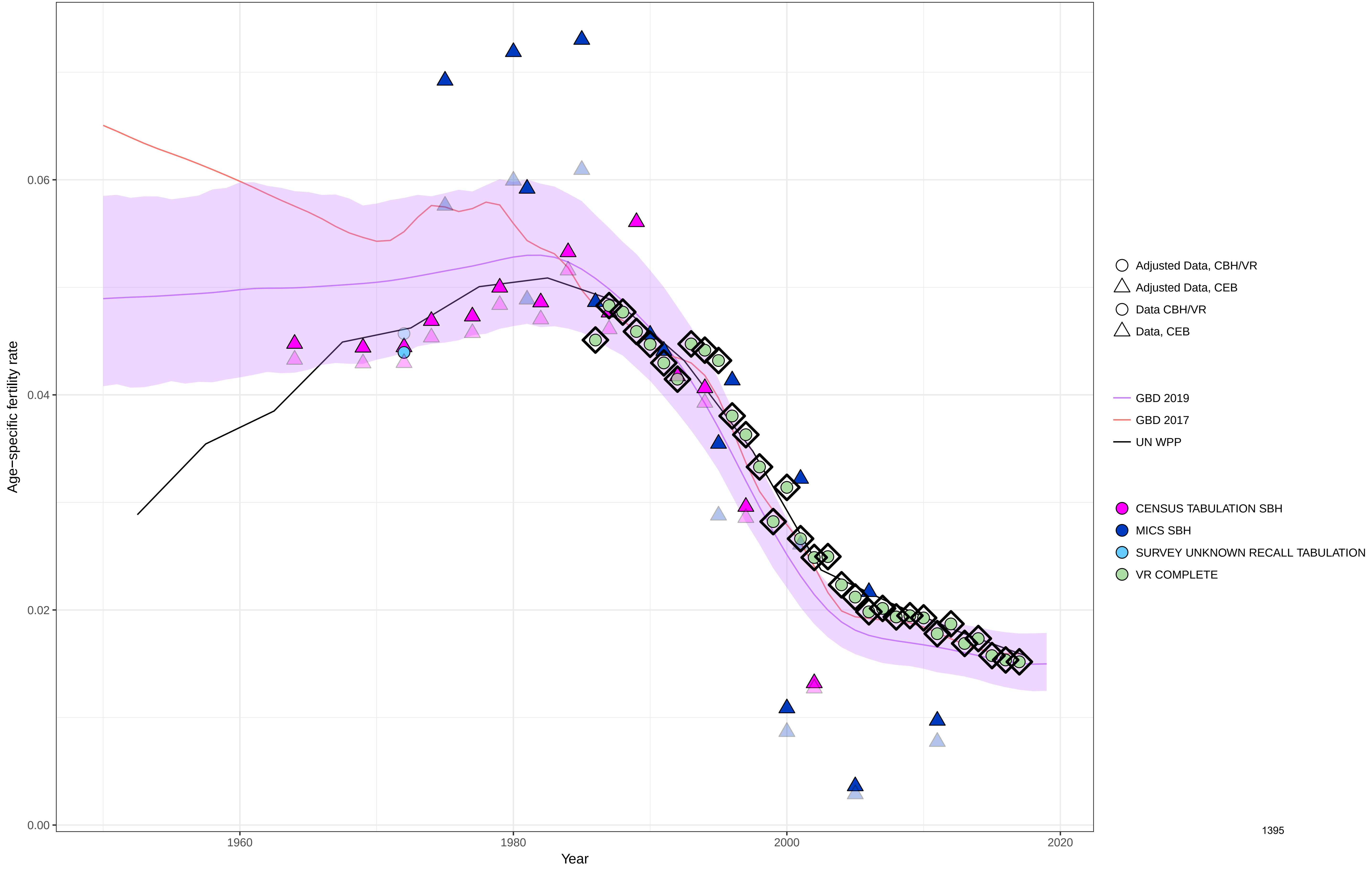

Age−specific fertility fitted trend lines, 1950−2019: North Macedonia, Ages: 20−24
Data points enclosed with a diamond are reference sources

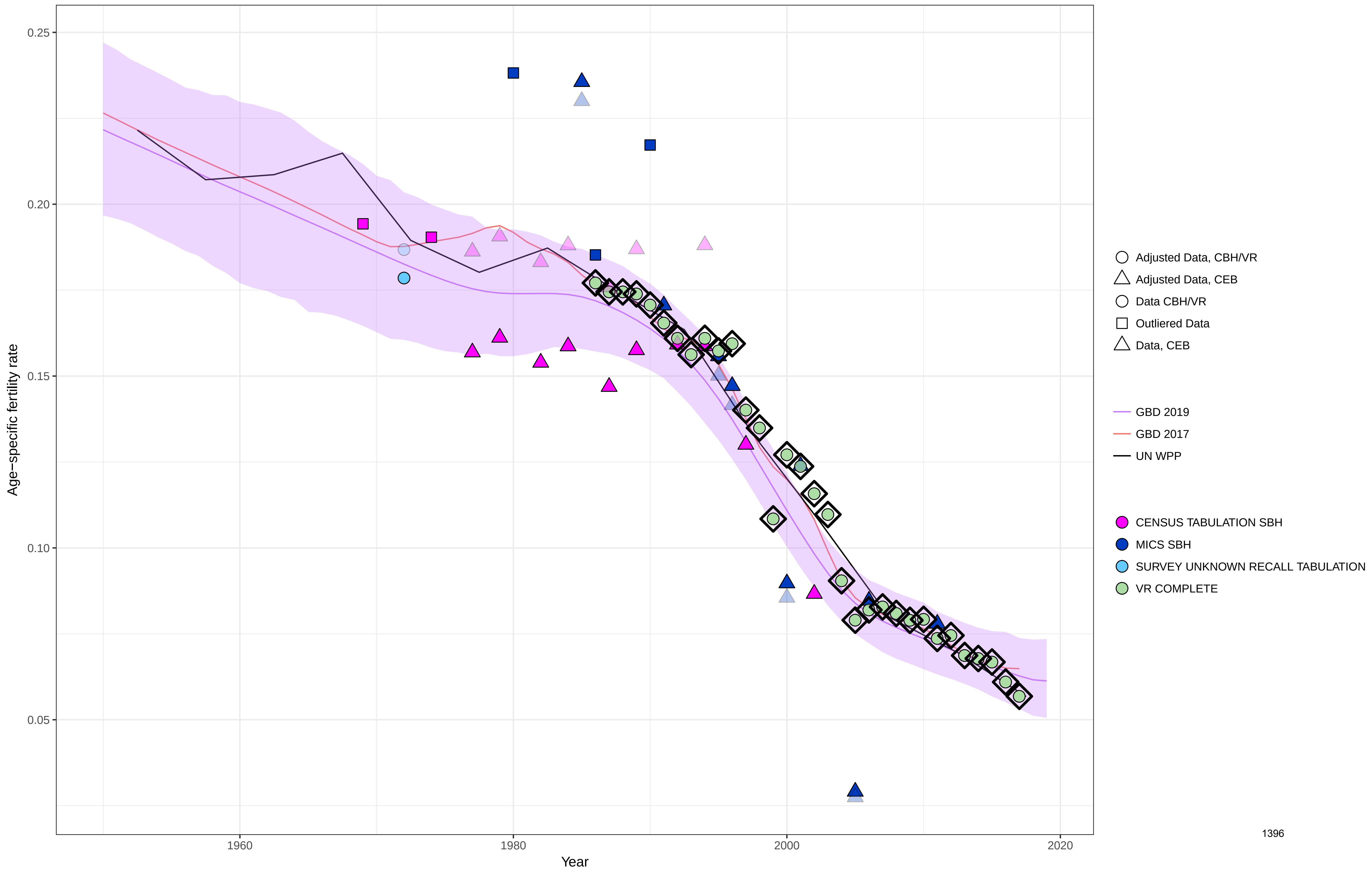

Age−specific fertility fitted trend lines, 1950−2019: North Macedonia, Ages: 25−29
Data points enclosed with a diamond are reference sources

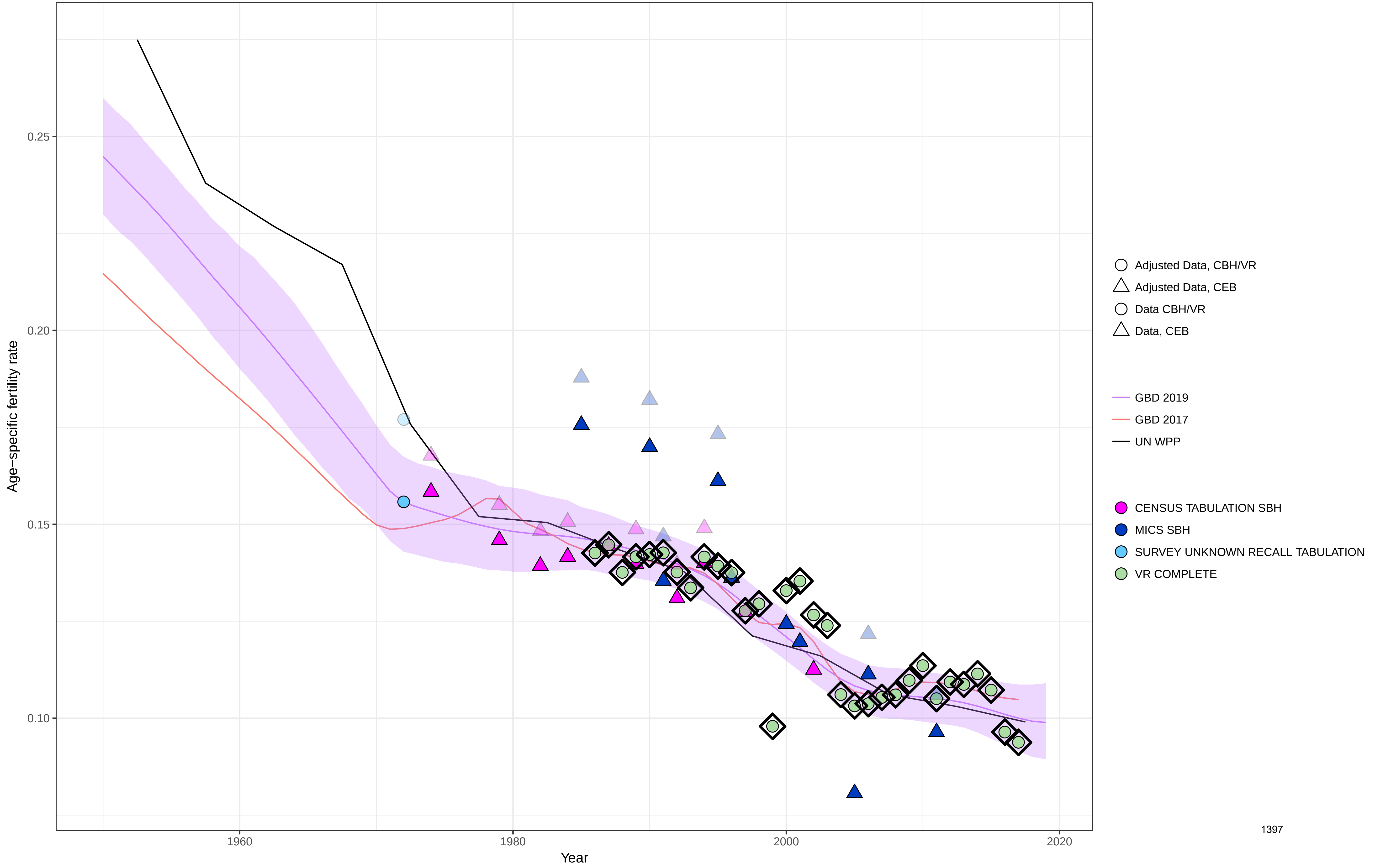

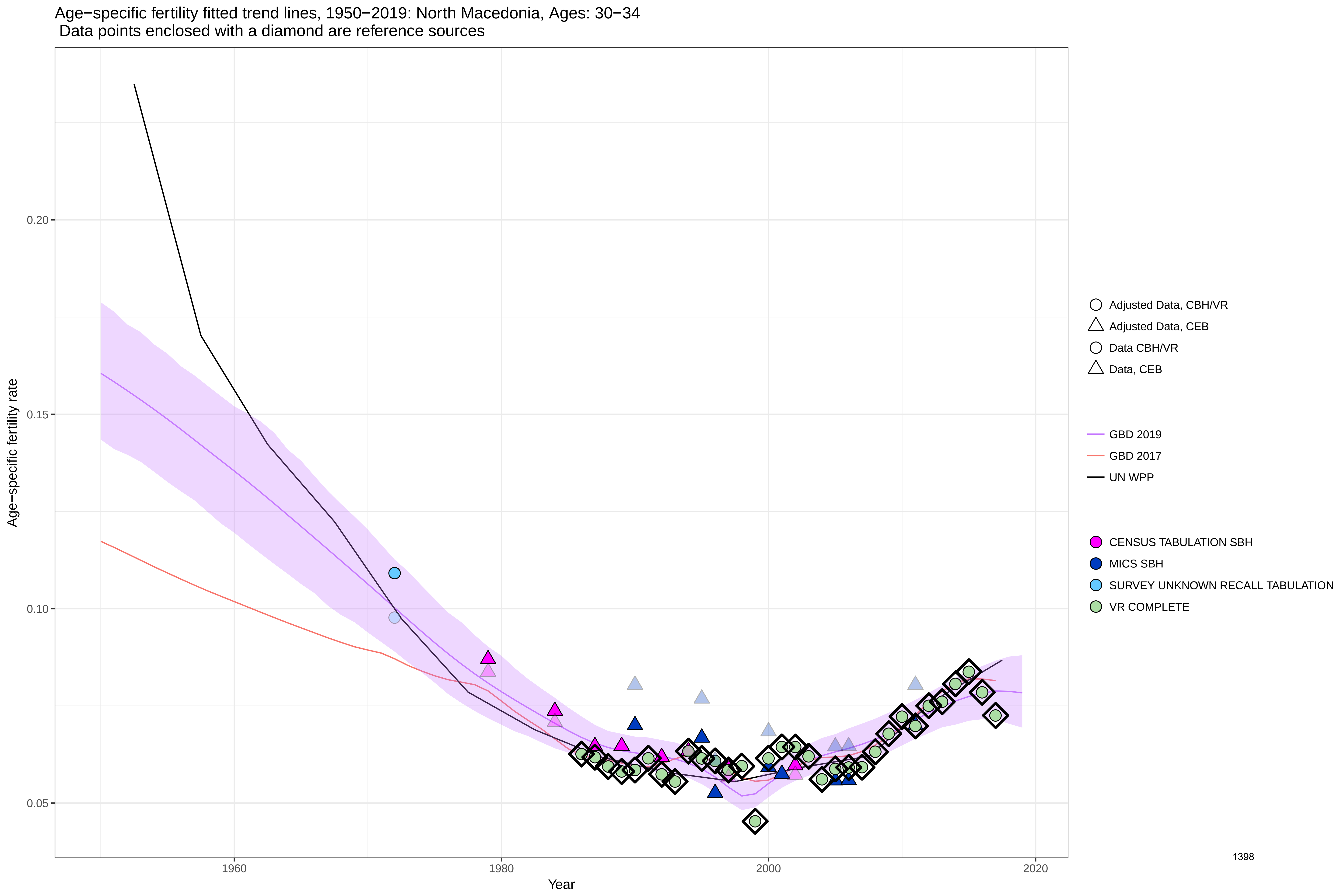

Age−specific fertility fitted trend lines, 1950−2019: North Macedonia, Ages: 30−34
Data points enclosed with a diamond are reference sources
Age−specific fertility rate
Year
0.05
0.10
0.15
0.20
1960
1980
2000
2020
Adjusted Data, CBH/VR
Adjusted Data, CEB
Data CBH/VR
Data, CEB
GBD 2019
GBD 2017
UN WPP
CENSUS TABULATION SBH
MICS SBH
SURVEY UNKNOWN RECALL TABULATION
VR COMPLETE

Age−specific fertility fitted trend lines, 1950−2019: North Macedonia, Ages: 35−39
Data points enclosed with a diamond are reference sources

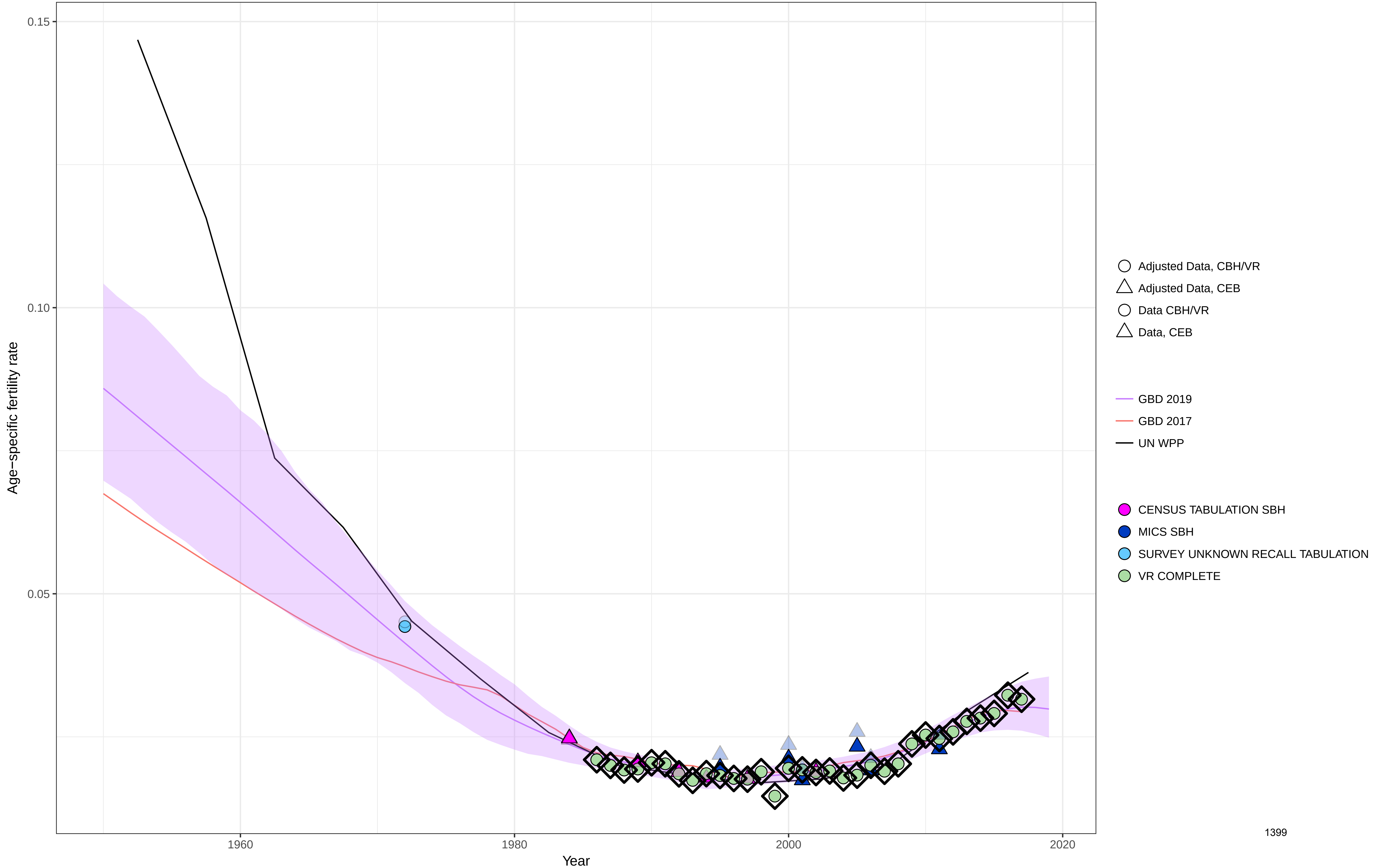

Age−specific fertility fitted trend lines, 1950−2019: North Macedonia, Ages: 40−44
Data points enclosed with a diamond are reference sources

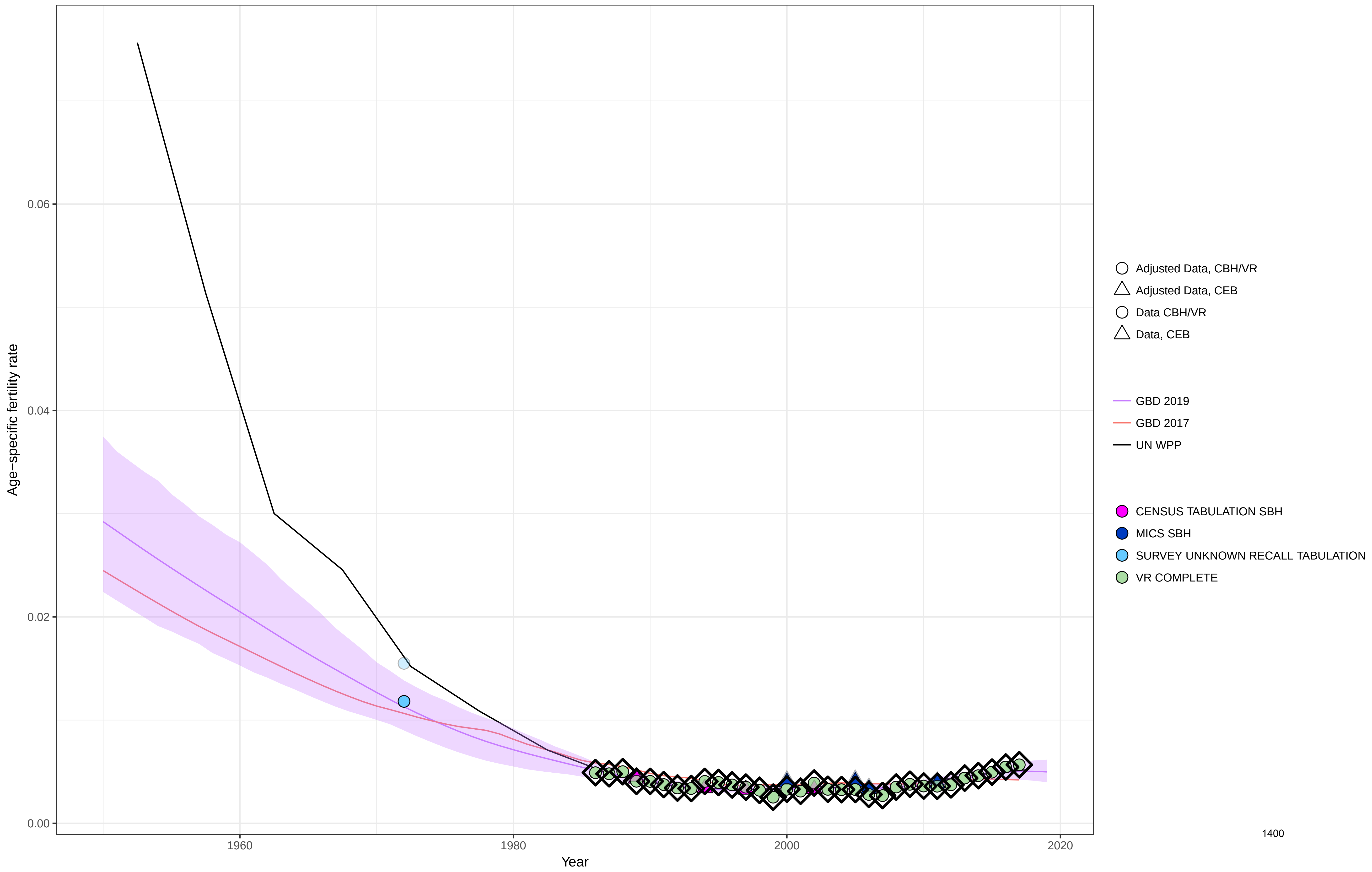

Age−specific fertility fitted trend lines, 1950−2019: North Macedonia, Ages: 45−49
Data points enclosed with a diamond are reference sources

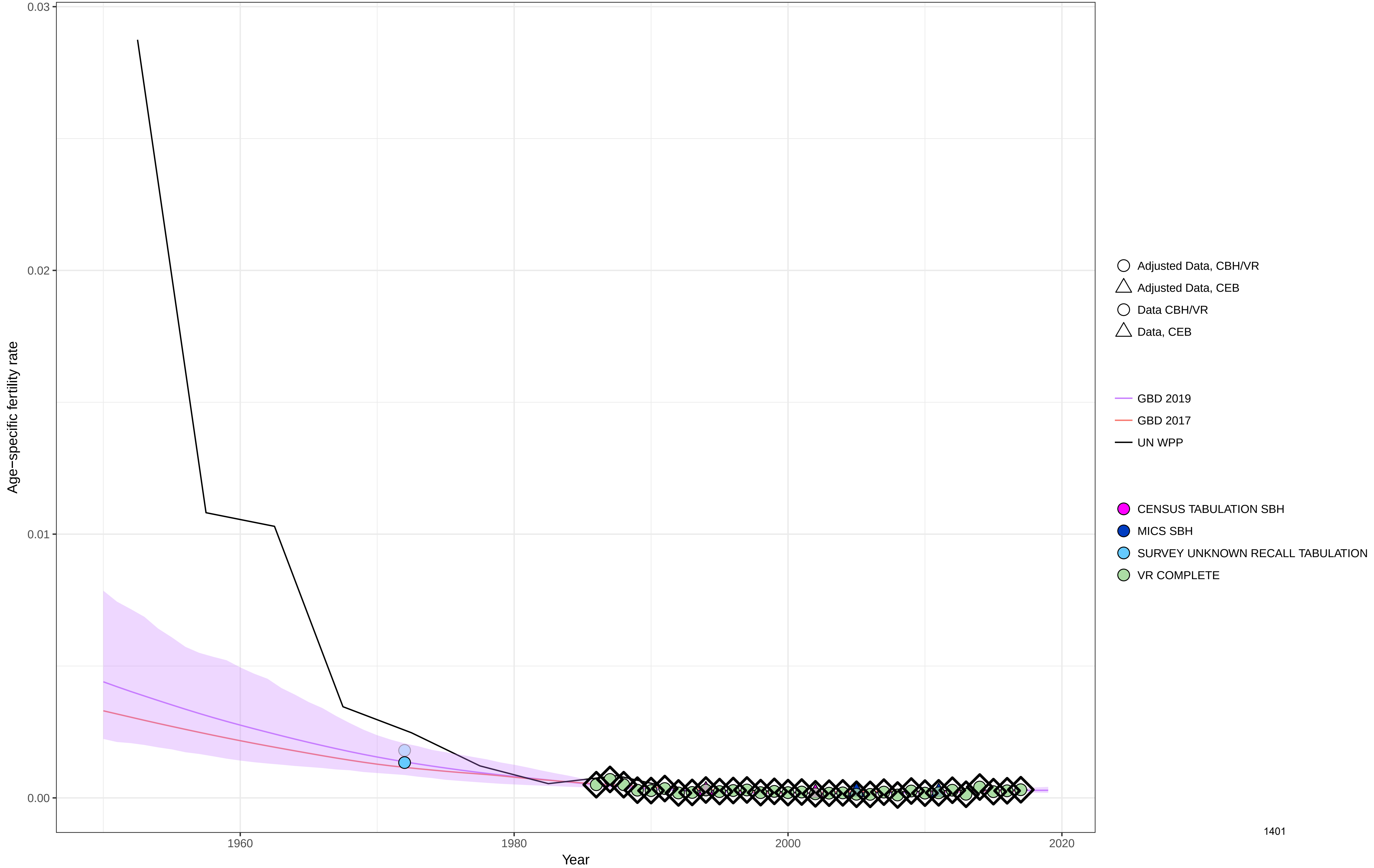

Age−specific fertility fitted trend lines, 1950−2019: North Macedonia, Ages: 50−54
Data points enclosed with a diamond are reference sources

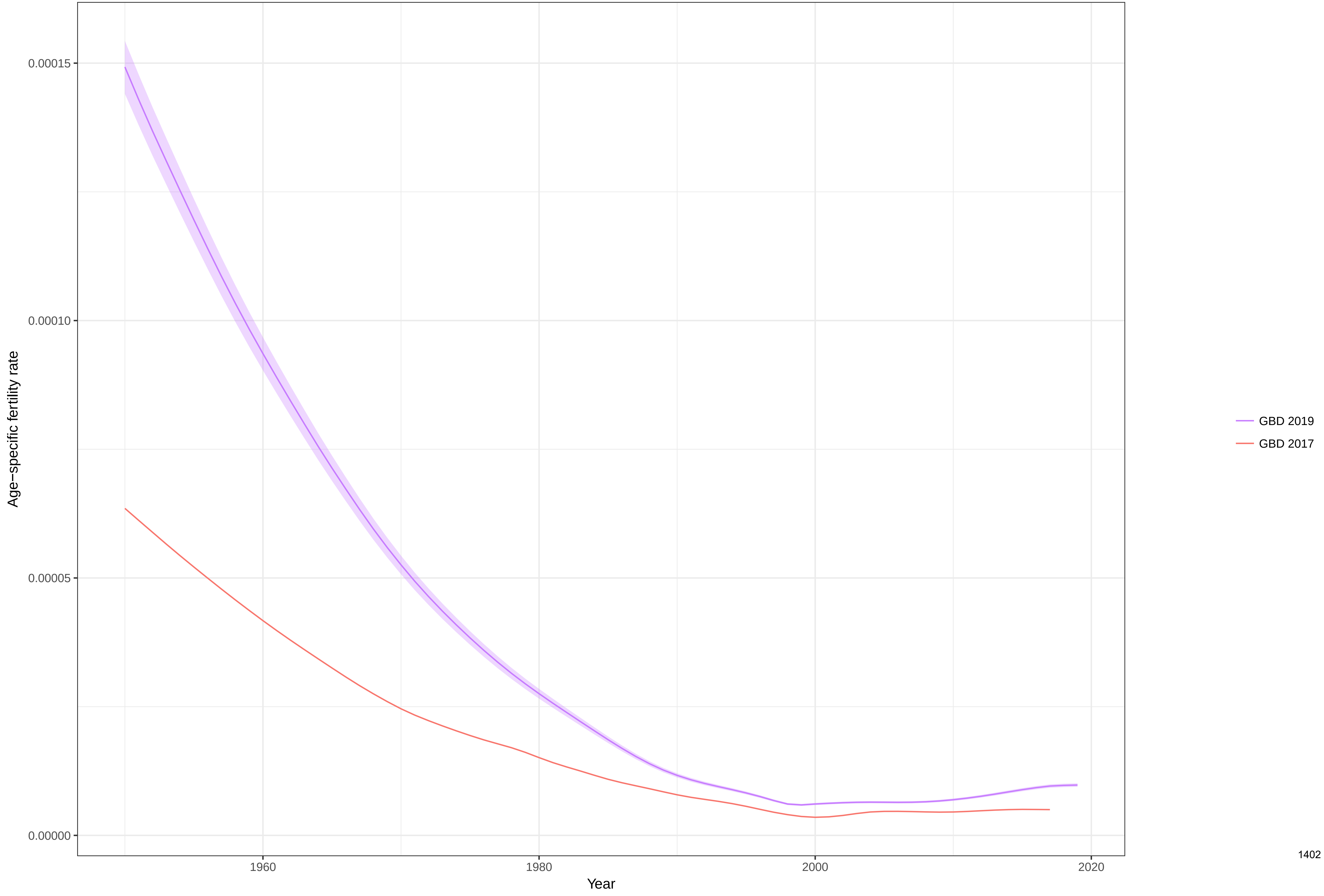

Total fertility rate fitted trend lines, 1950−2019: North Macedonia TFR
Data points enclosed with a diamond are reference sources

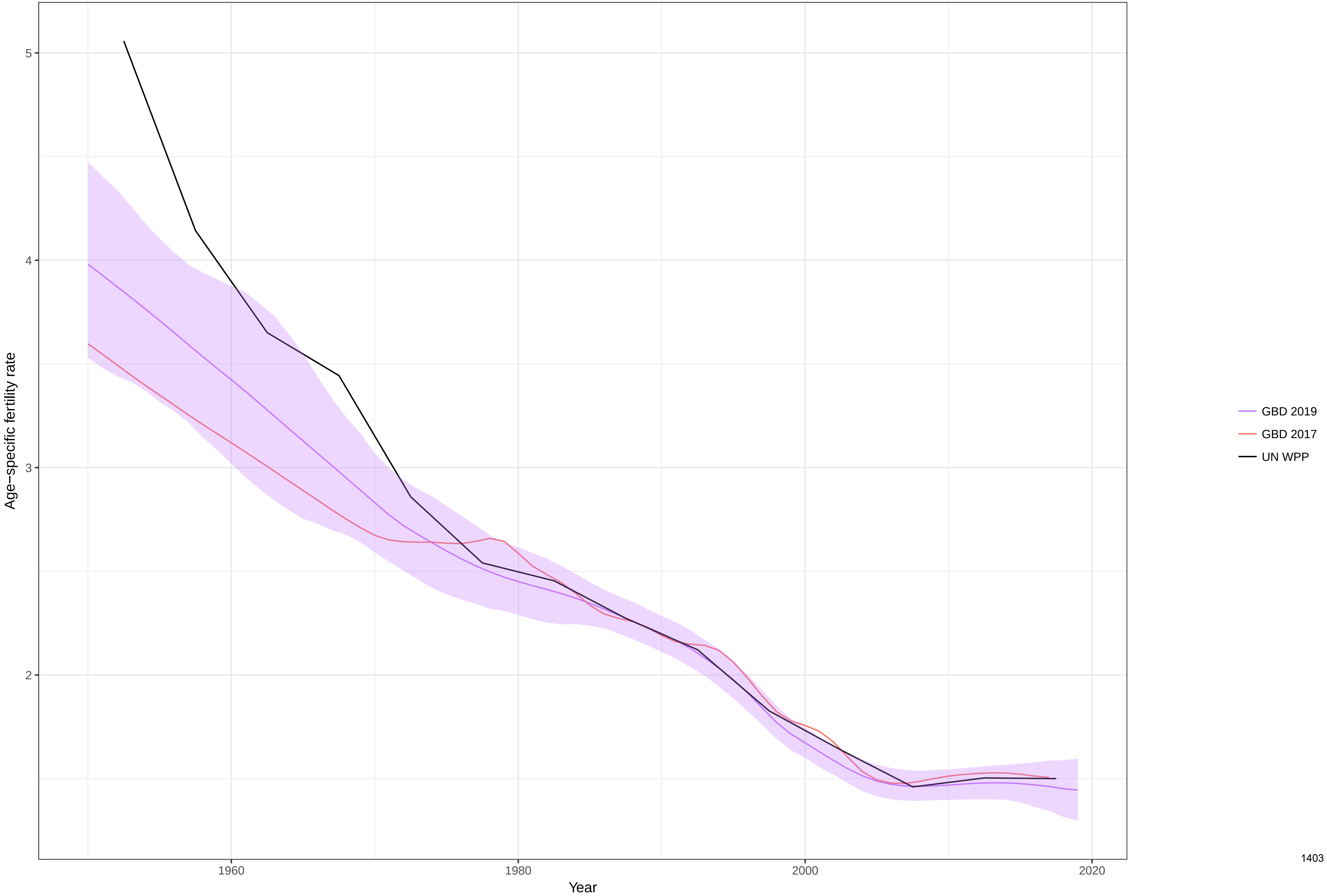

Age−specific fertility fitted trend lines, 1950−2019: Mali, Ages: 10−14
Data points enclosed with a diamond are reference sources

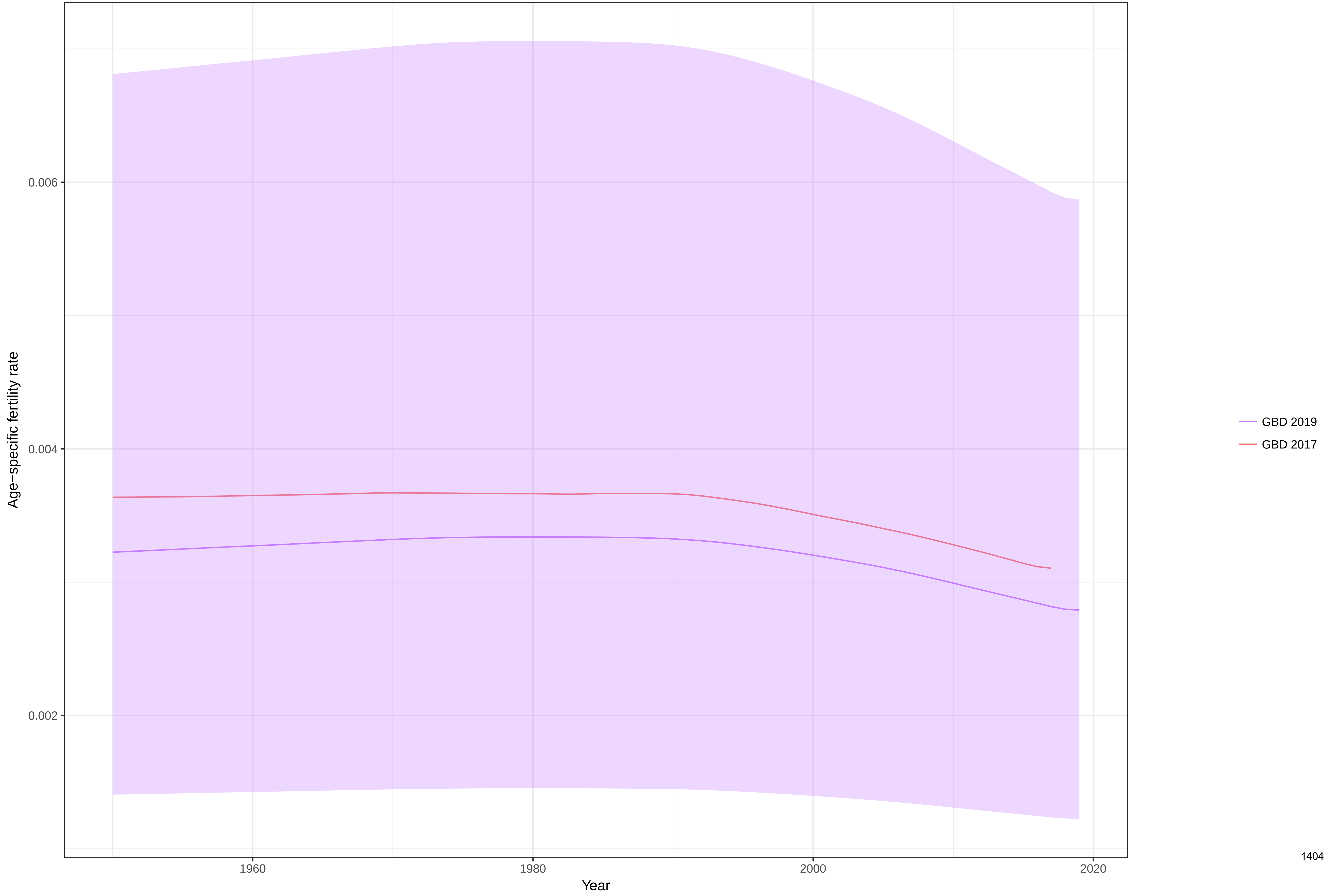

Age−specific fertility fitted trend lines, 1950−2019: Mali, Ages: 15−19
Data points enclosed with a diamond are reference sources

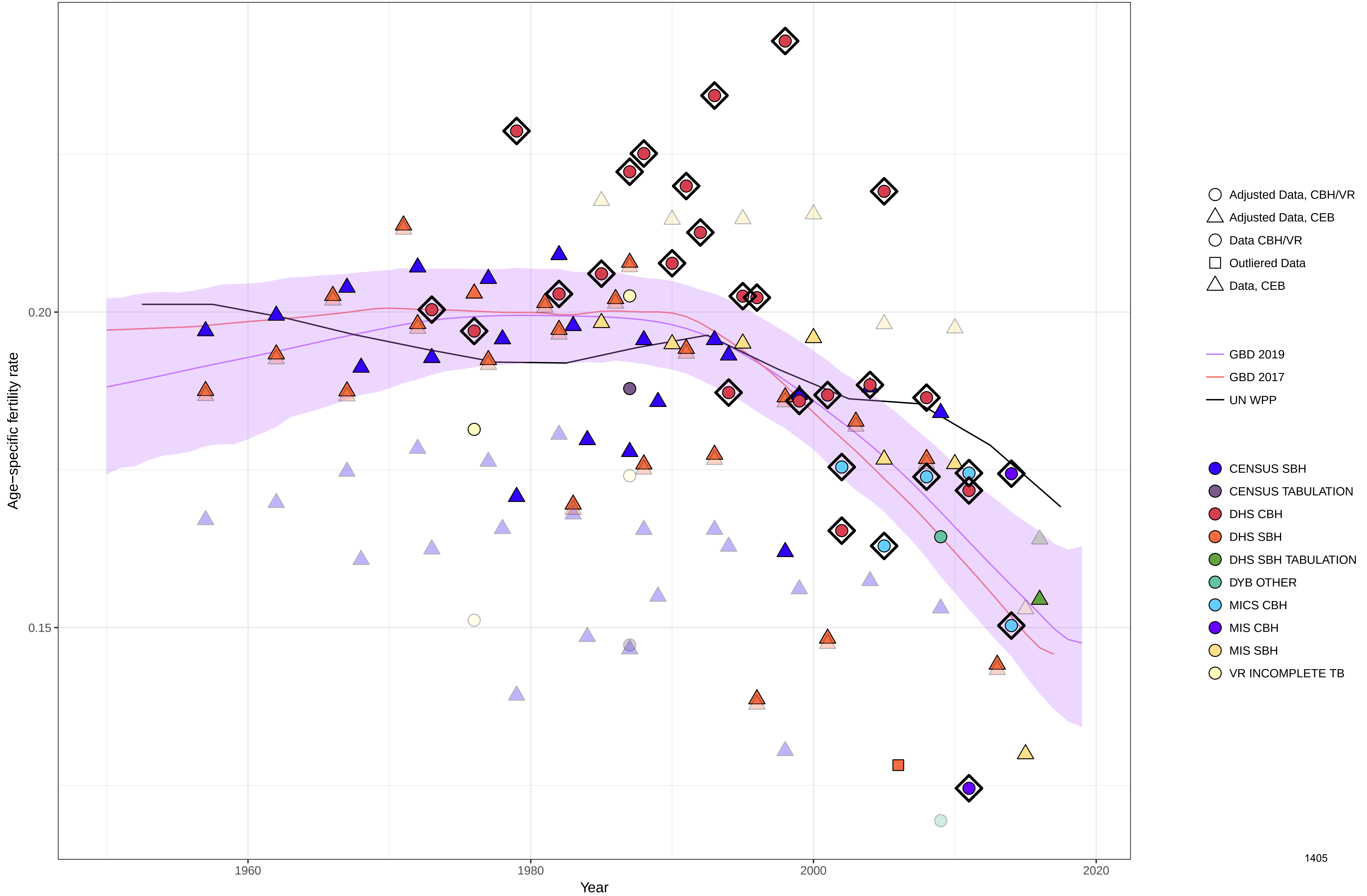

Age−specific fertility fitted trend lines, 1950−2019: Mali, Ages: 20−24
Data points enclosed with a diamond are reference sources

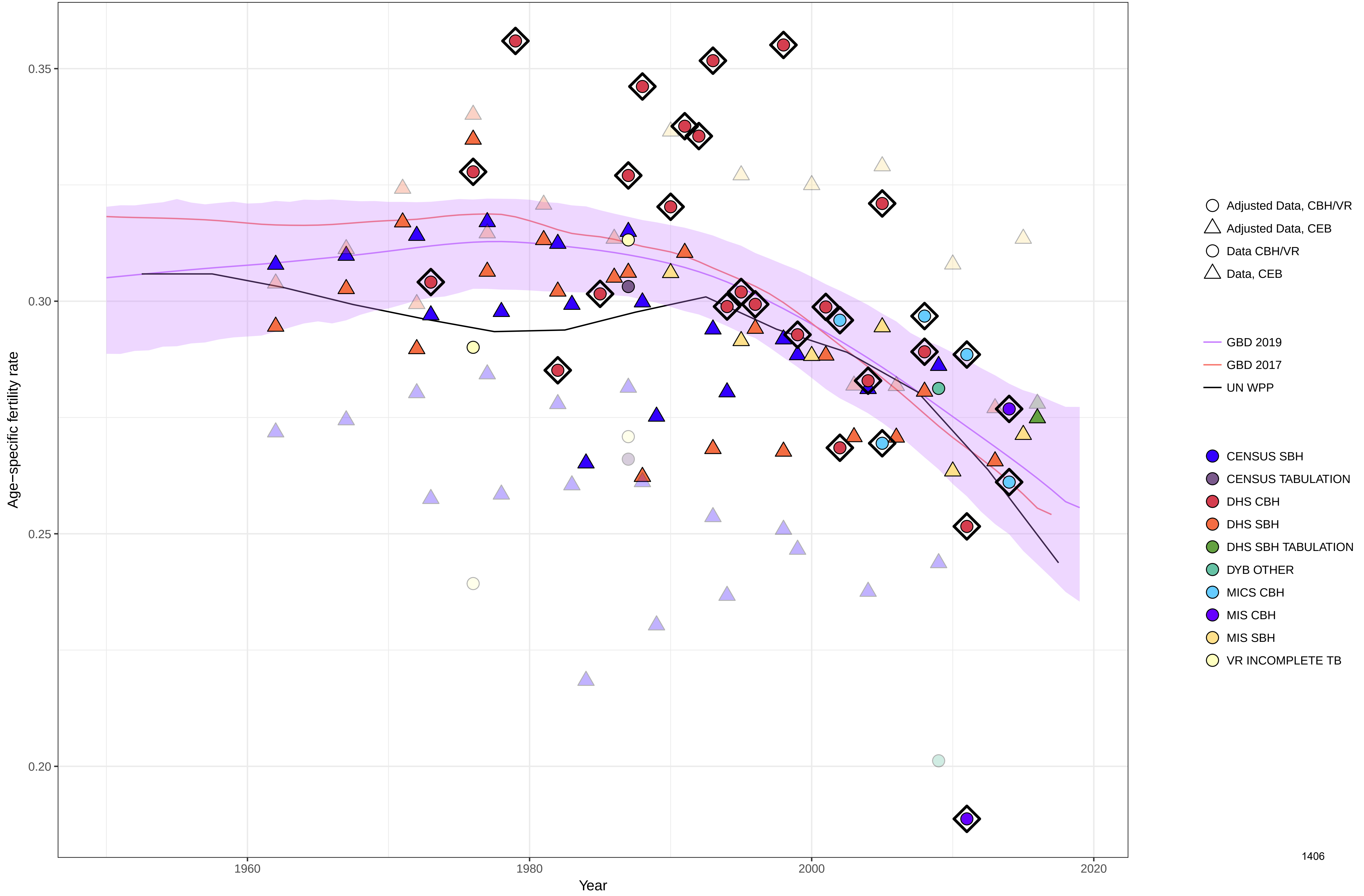

Age−specific fertility fitted trend lines, 1950−2019: Mali, Ages: 25−29
Data points enclosed with a diamond are reference sources

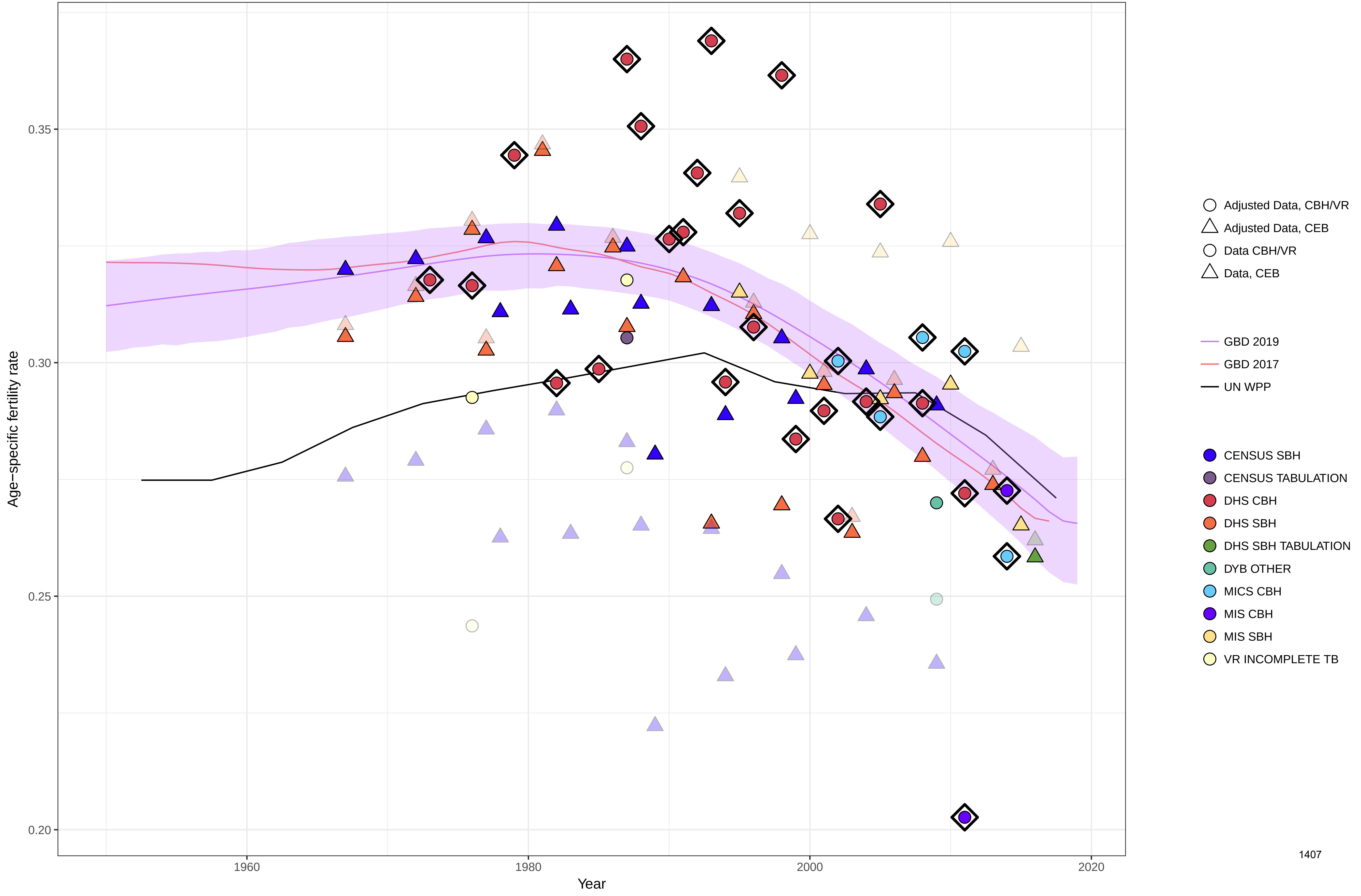

Age−specific fertility fitted trend lines, 1950−2019: Mali, Ages: 30−34
Data points enclosed with a diamond are reference sources

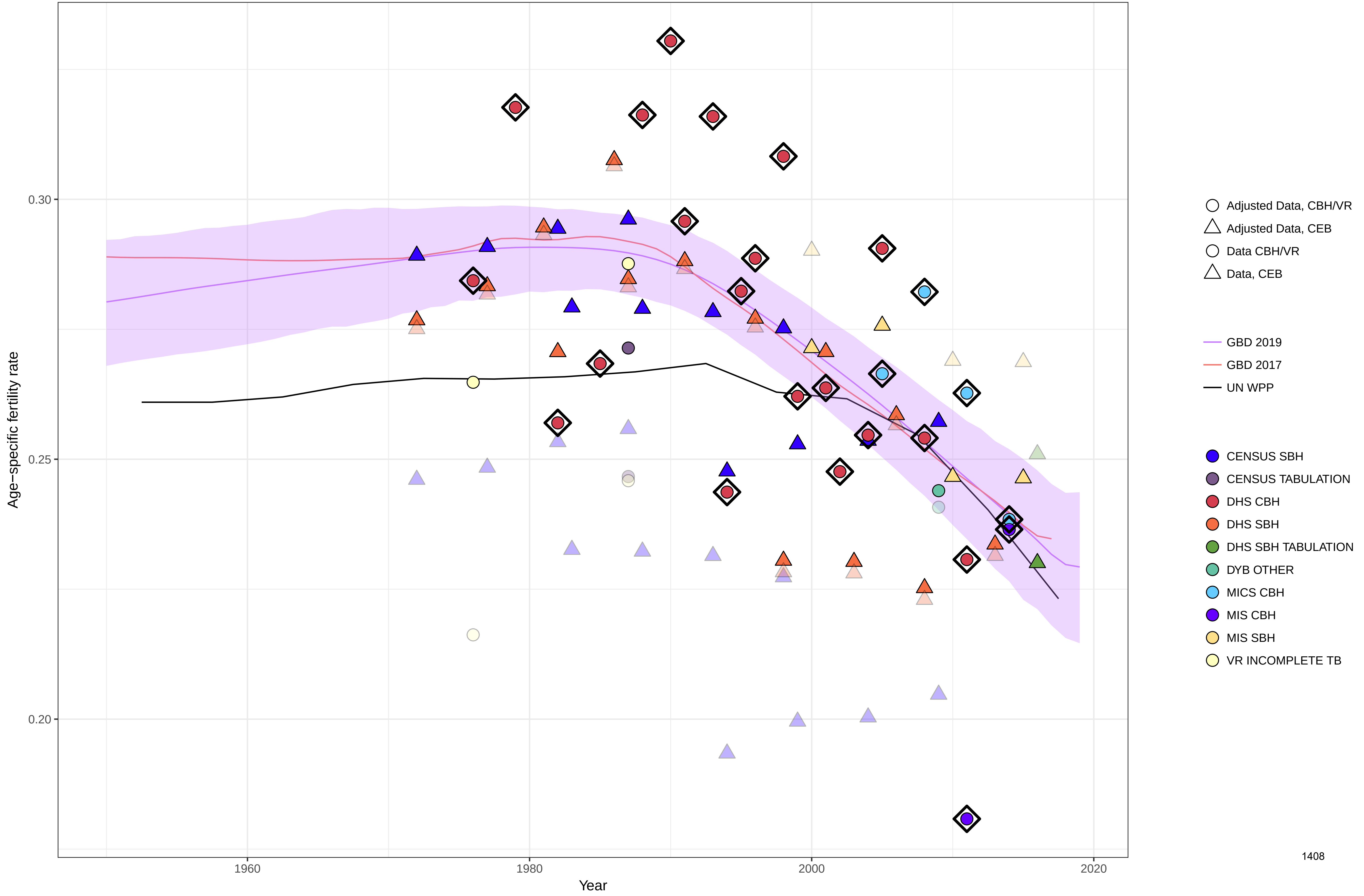

Age−specific fertility fitted trend lines, 1950−2019: Mali, Ages: 35−39
Data points enclosed with a diamond are reference sources

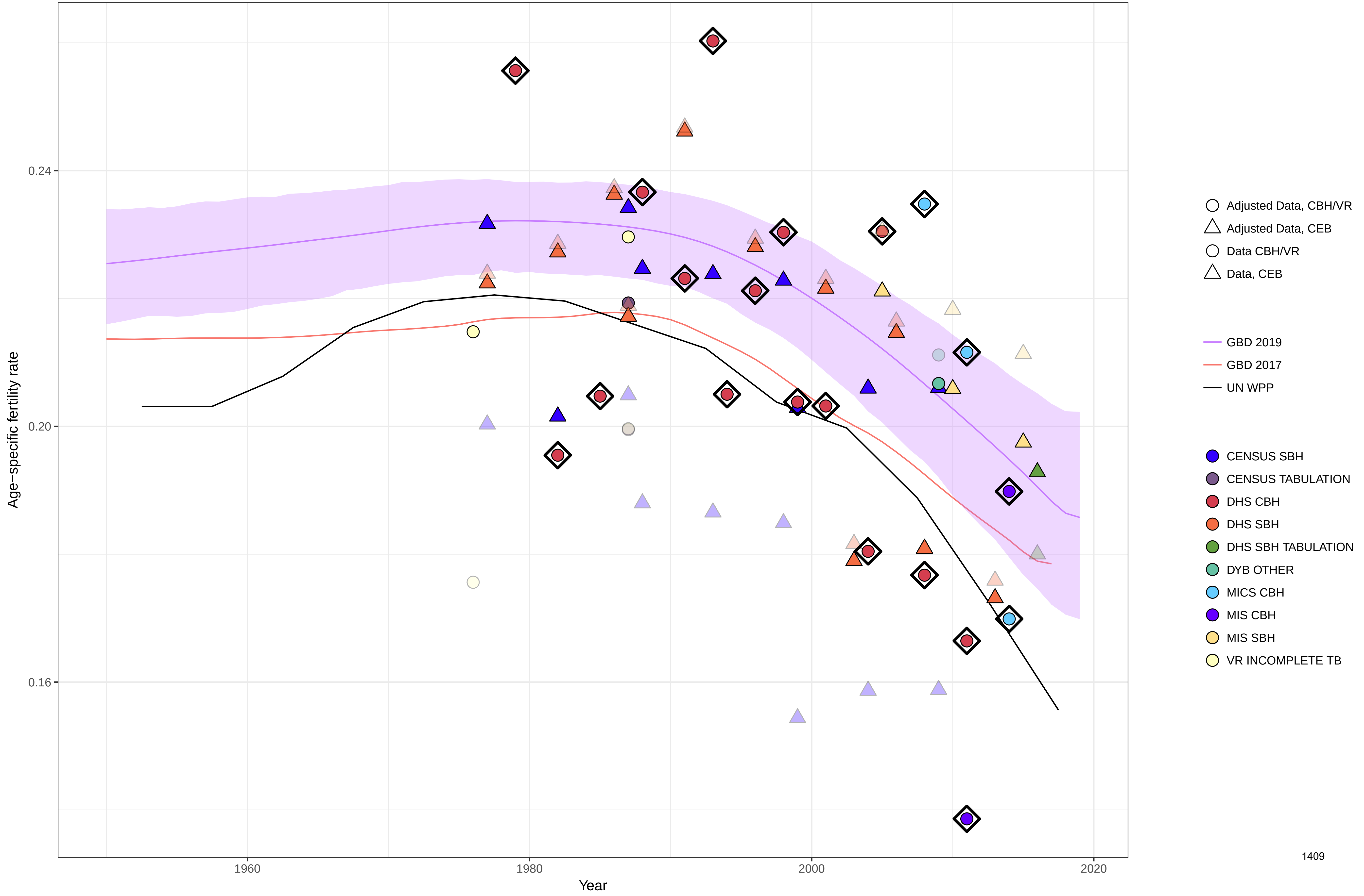

Age−specific fertility fitted trend lines, 1950−2019: Mali, Ages: 40−44
Data points enclosed with a diamond are reference sources

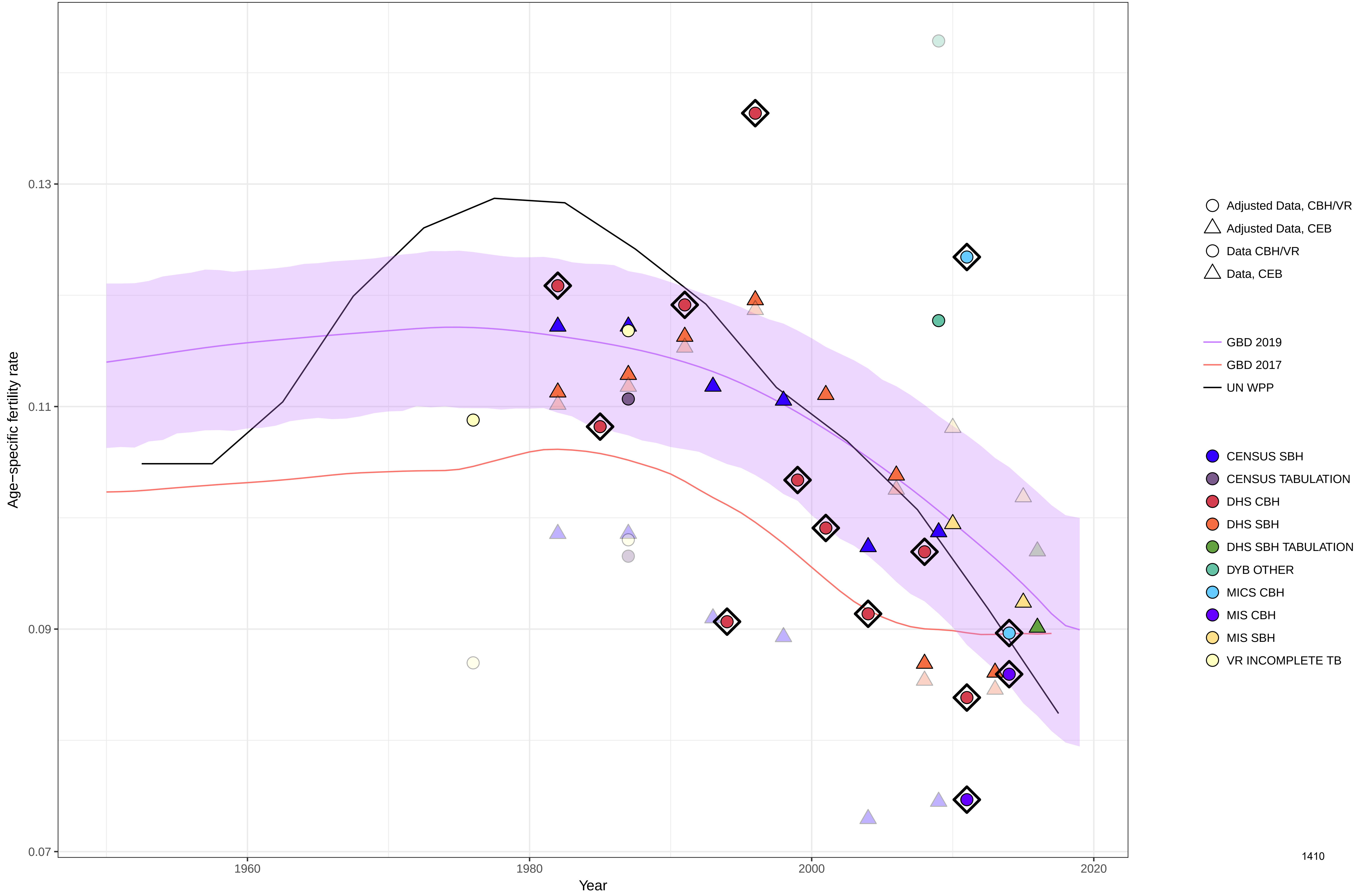

Age−specific fertility fitted trend lines, 1950−2019: Mali, Ages: 45−49
Data points enclosed with a diamond are reference sources

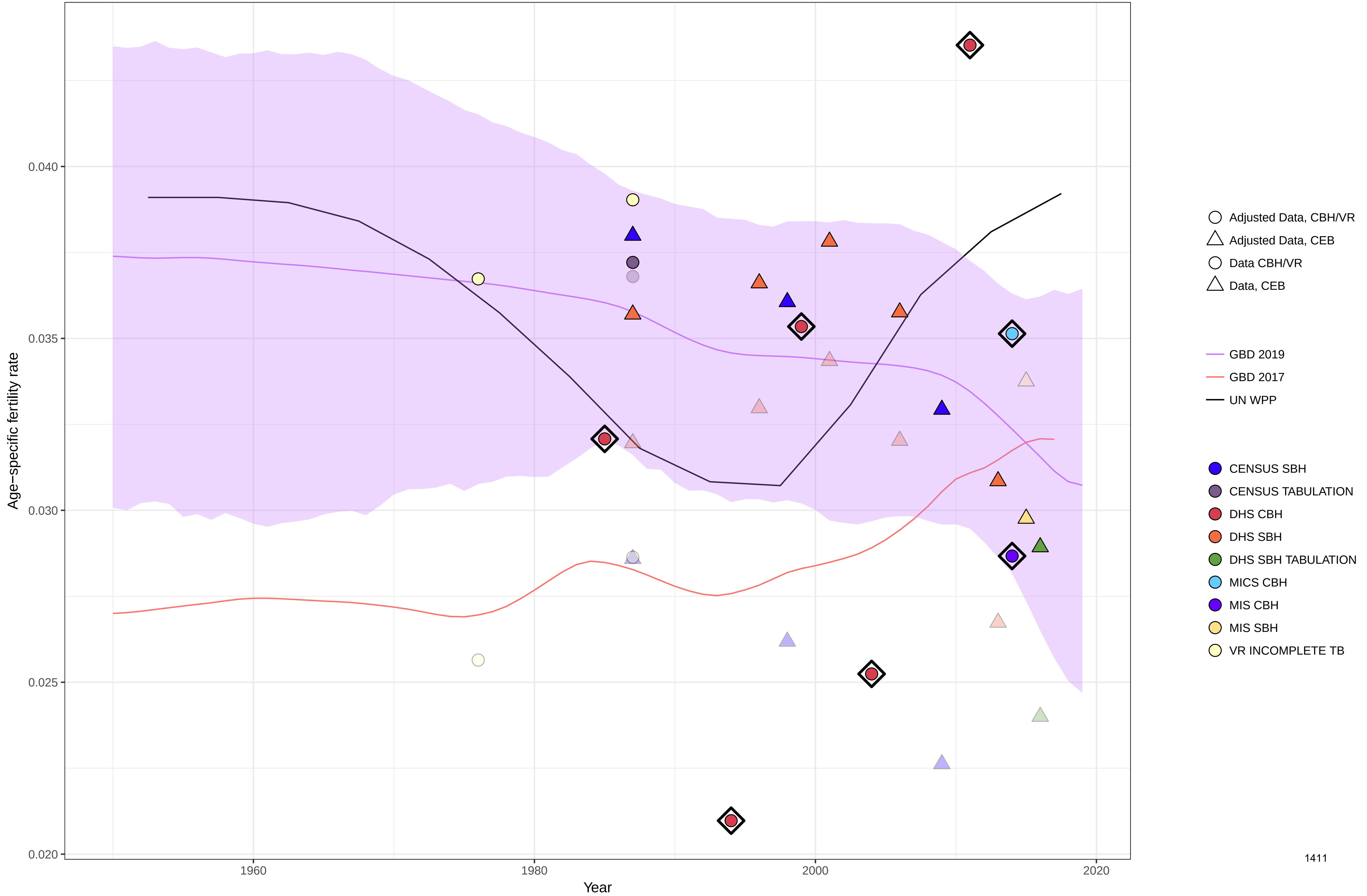

Age−specific fertility fitted trend lines, 1950−2019: Mali, Ages: 50−54
Data points enclosed with a diamond are reference sources

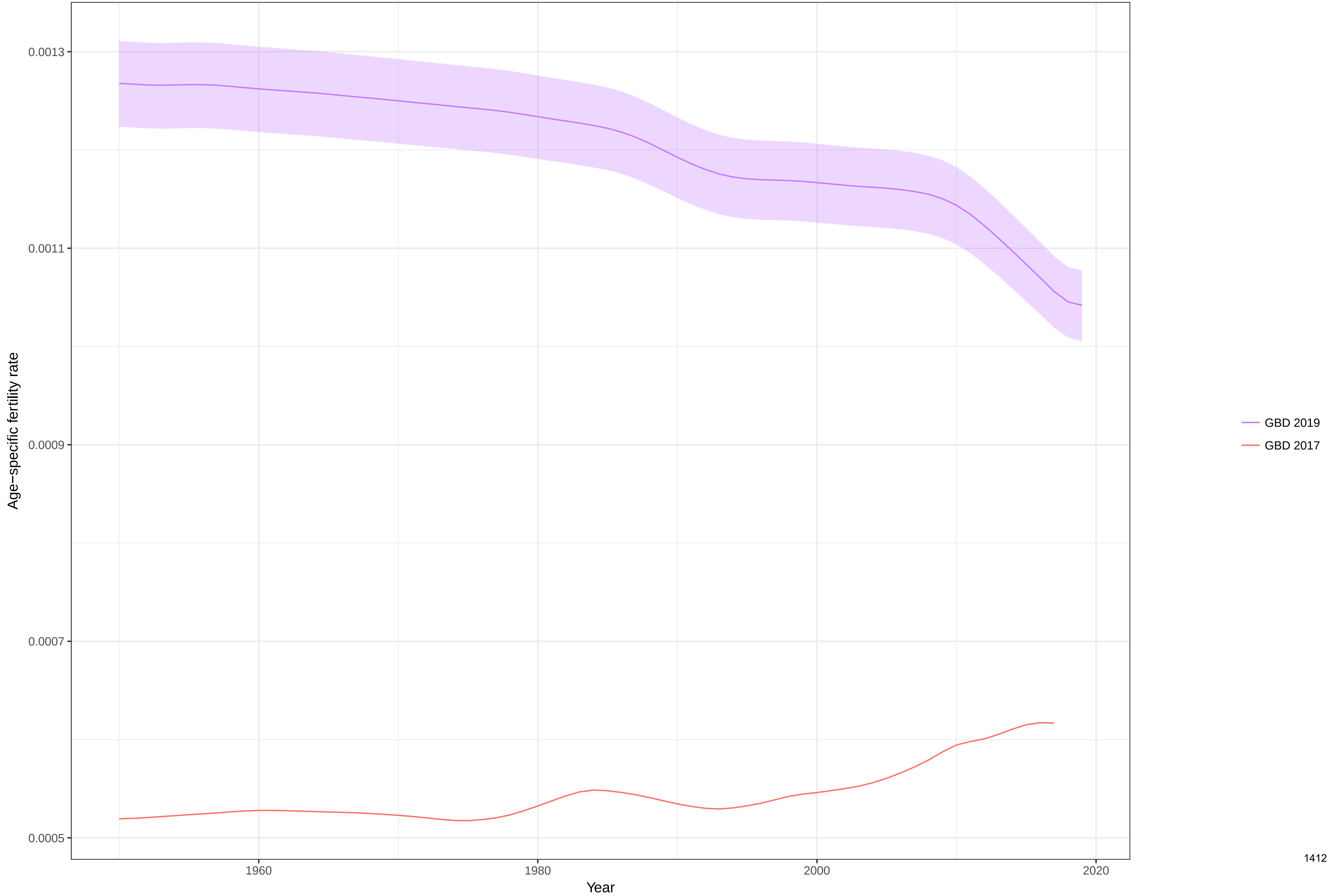

Total fertility rate fitted trend lines, 1950−2019: Mali TFR
Data points enclosed with a diamond are reference sources

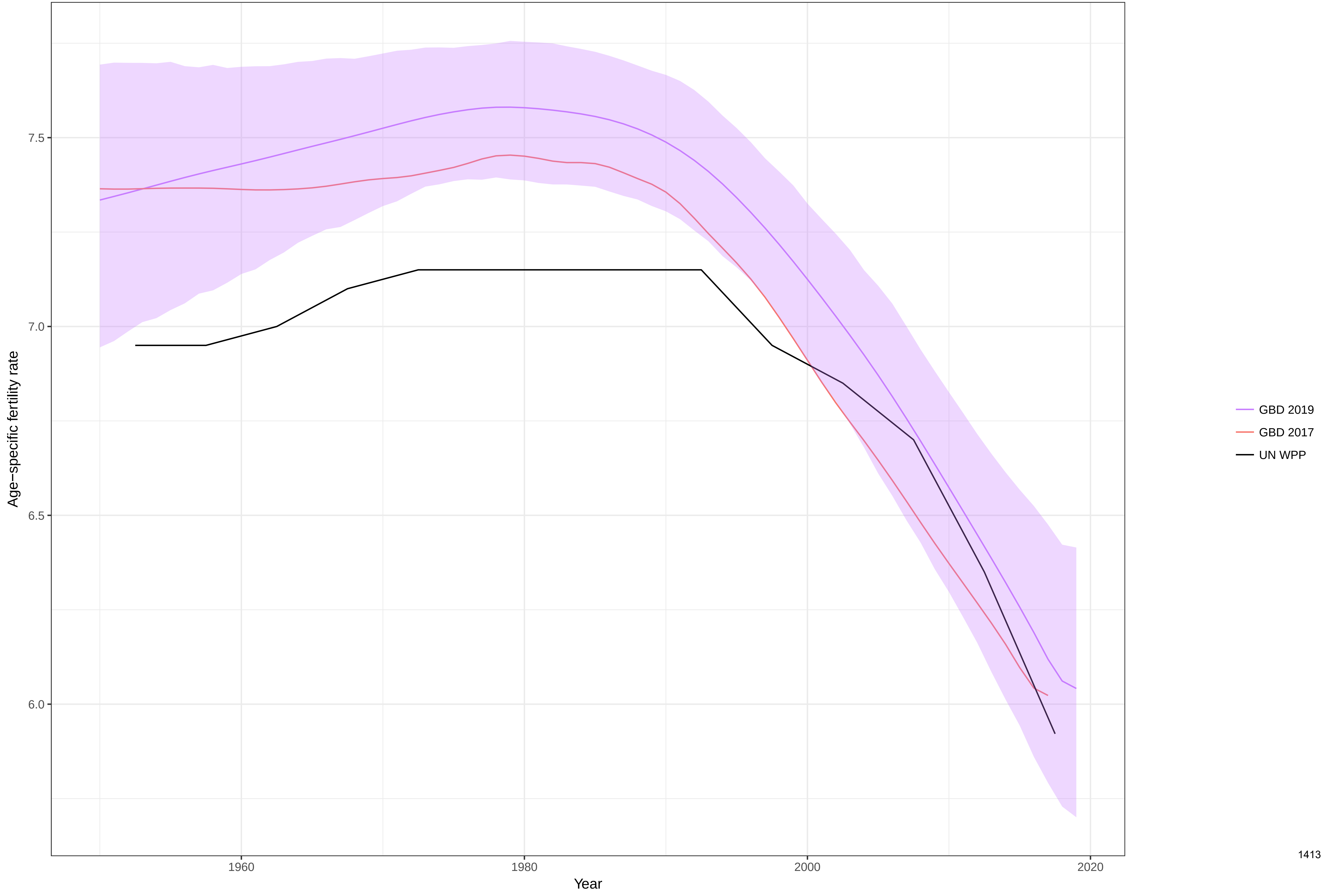

Age−specific fertility fitted trend lines, 1950−2019: Malta, Ages: 10−14
Data points enclosed with a diamond are reference sources

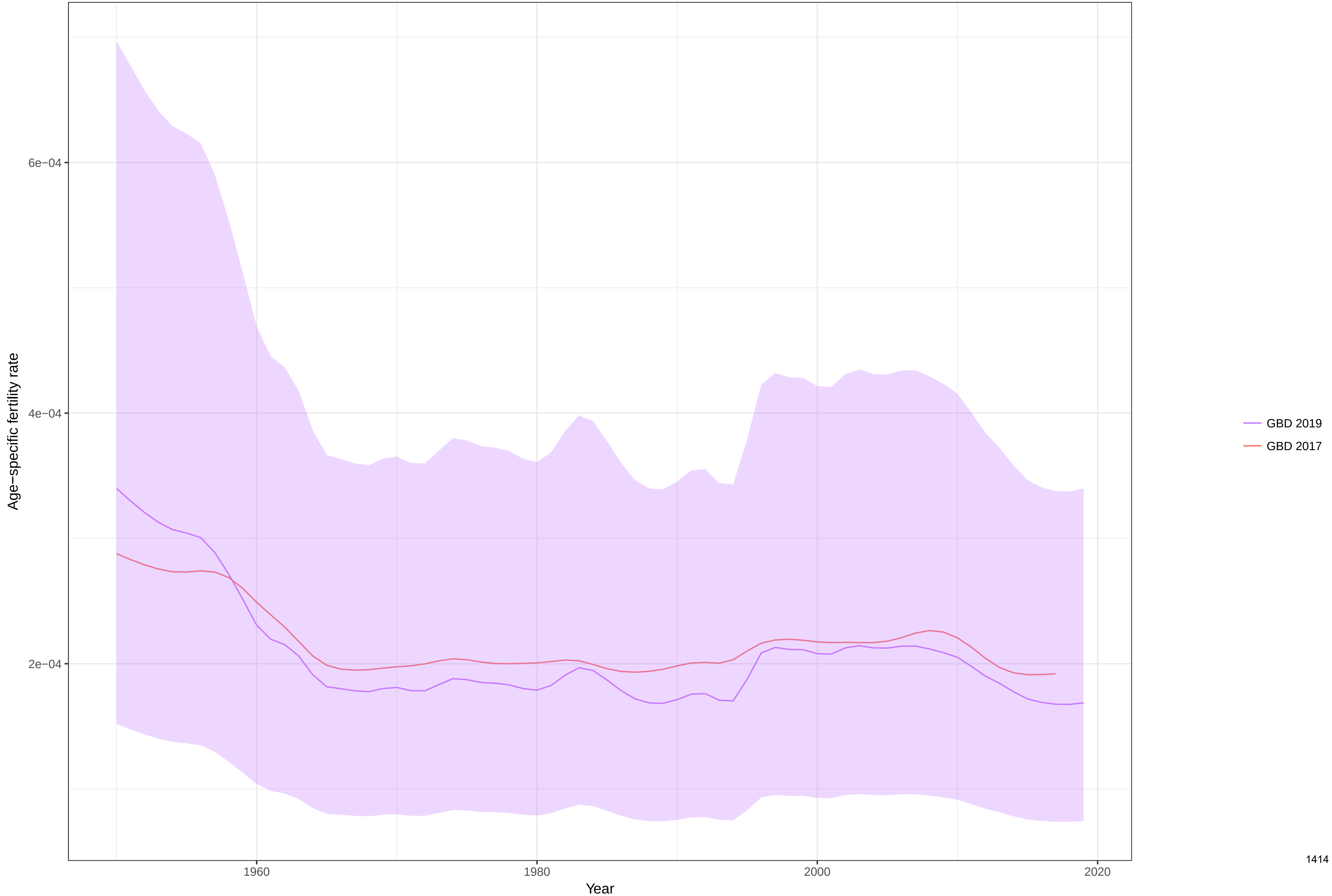

Age−specific fertility fitted trend lines, 1950−2019: Malta, Ages: 15−19
Data points enclosed with a diamond are reference sources

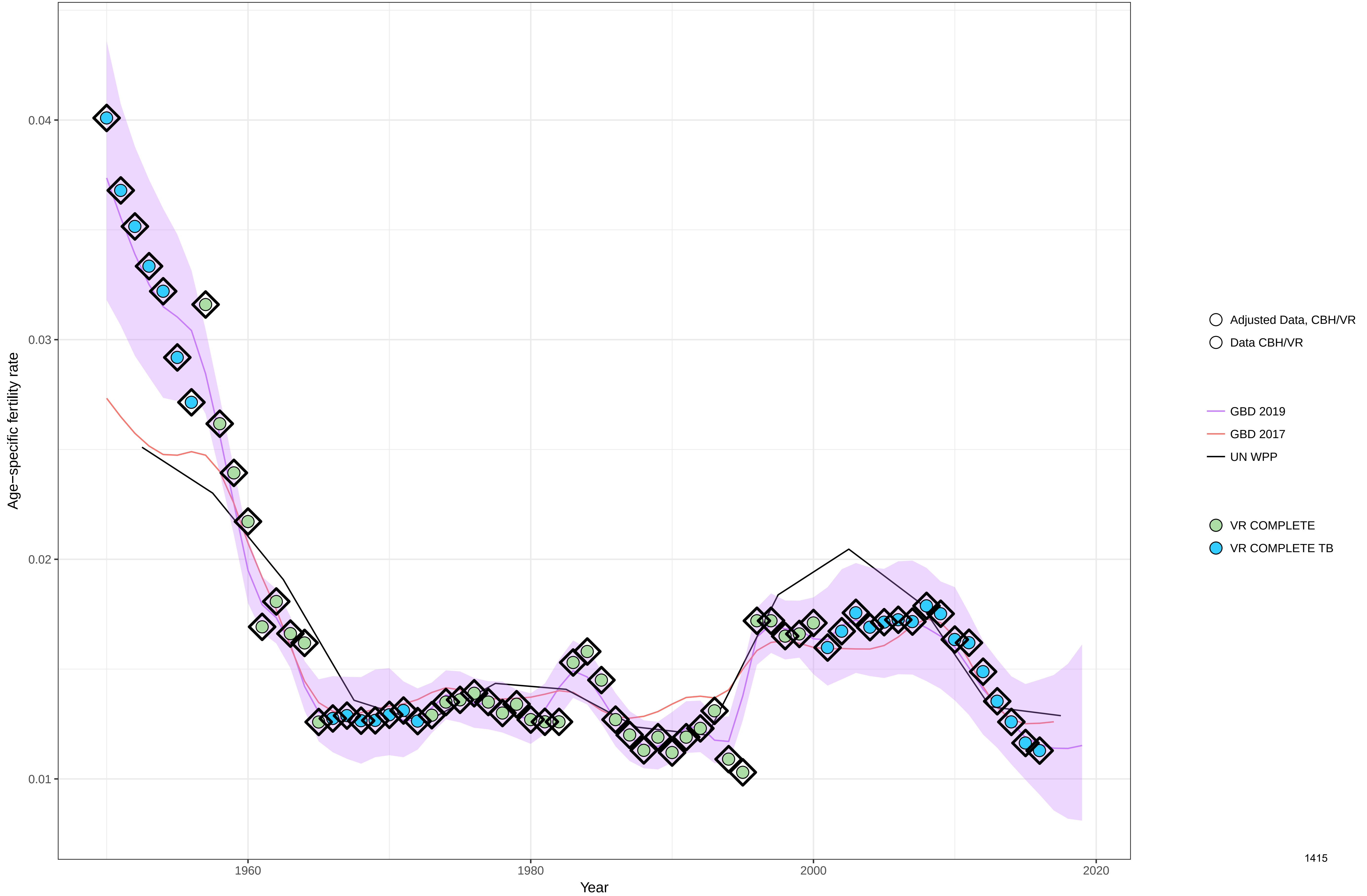

Age−specific fertility fitted trend lines, 1950−2019: Malta, Ages: 20−24
Data points enclosed with a diamond are reference sources

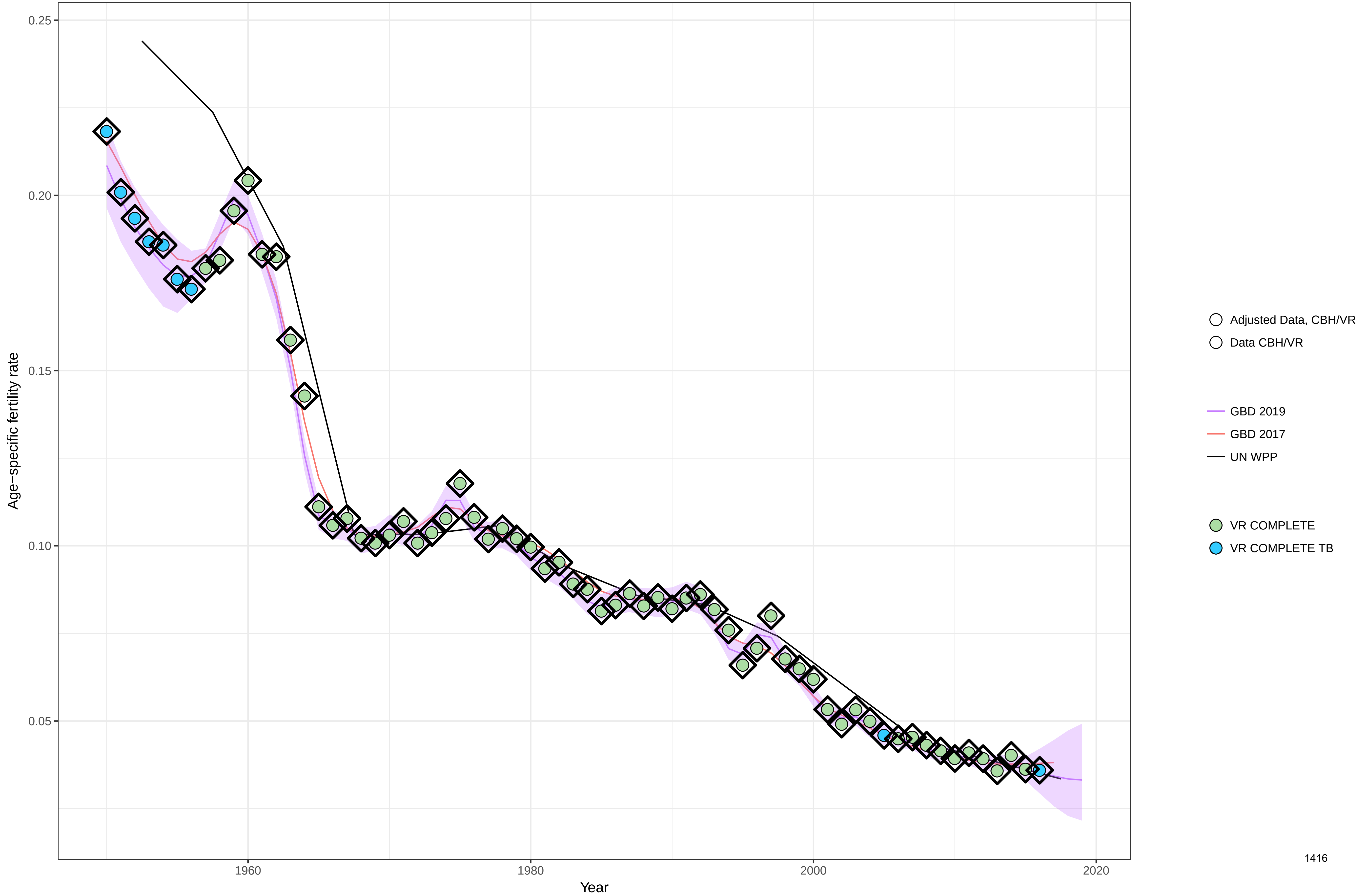

Age−specific fertility fitted trend lines, 1950−2019: Malta, Ages: 25−29
Data points enclosed with a diamond are reference sources

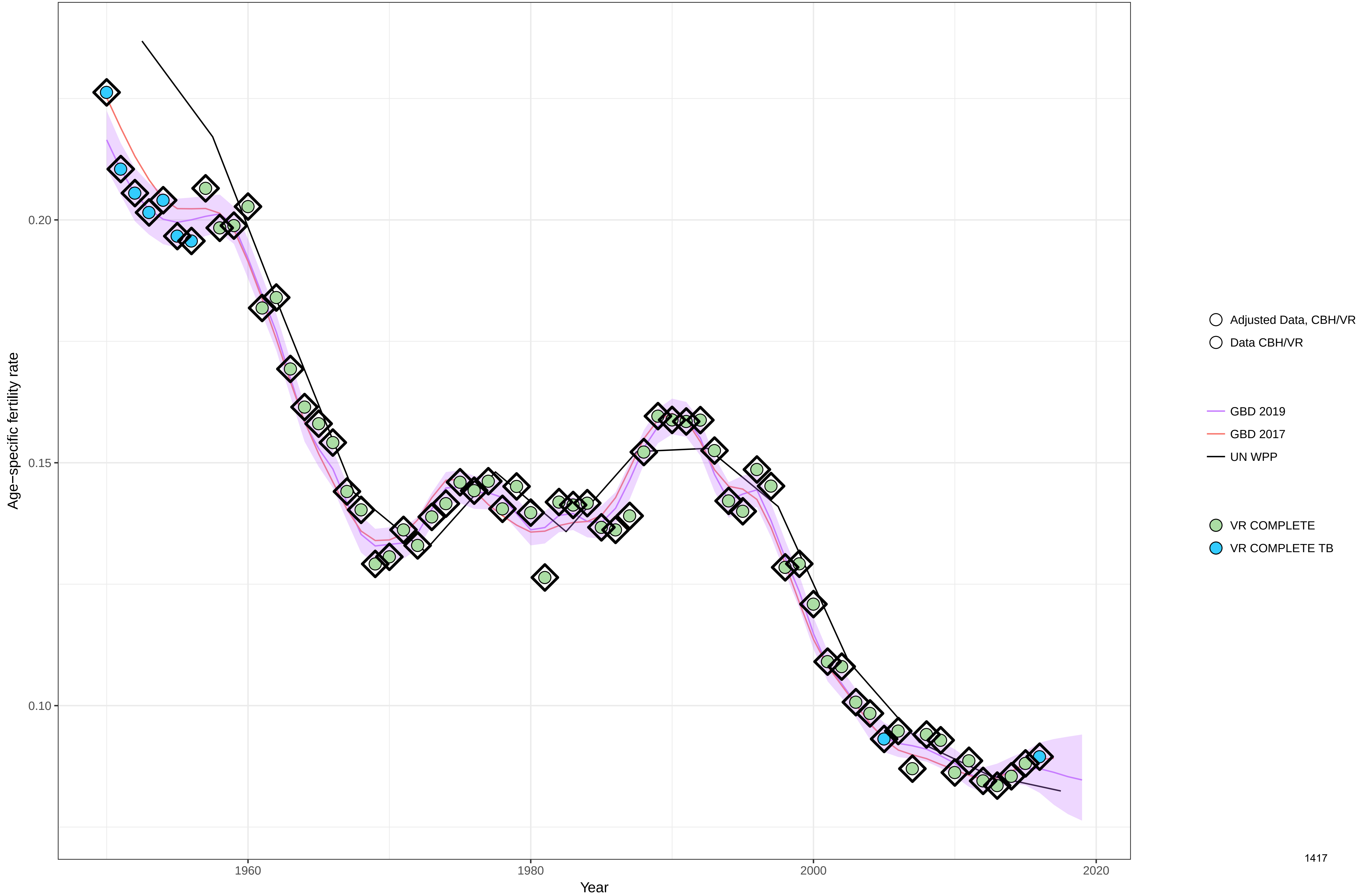

Age−specific fertility fitted trend lines, 1950−2019: Malta, Ages: 30−34
Data points enclosed with a diamond are reference sources

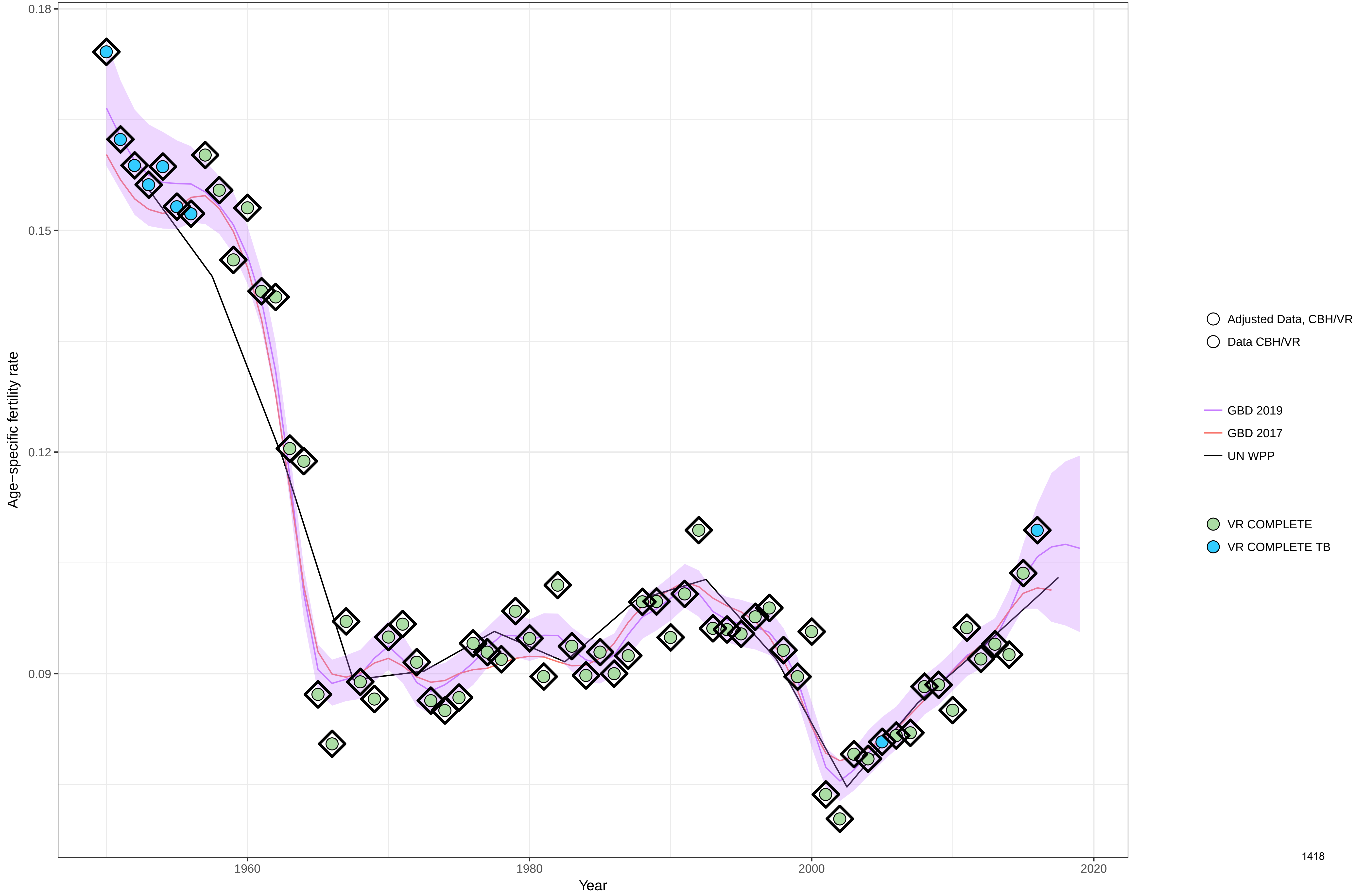

Age−specific fertility fitted trend lines, 1950−2019: Malta, Ages: 35−39
Data points enclosed with a diamond are reference sources

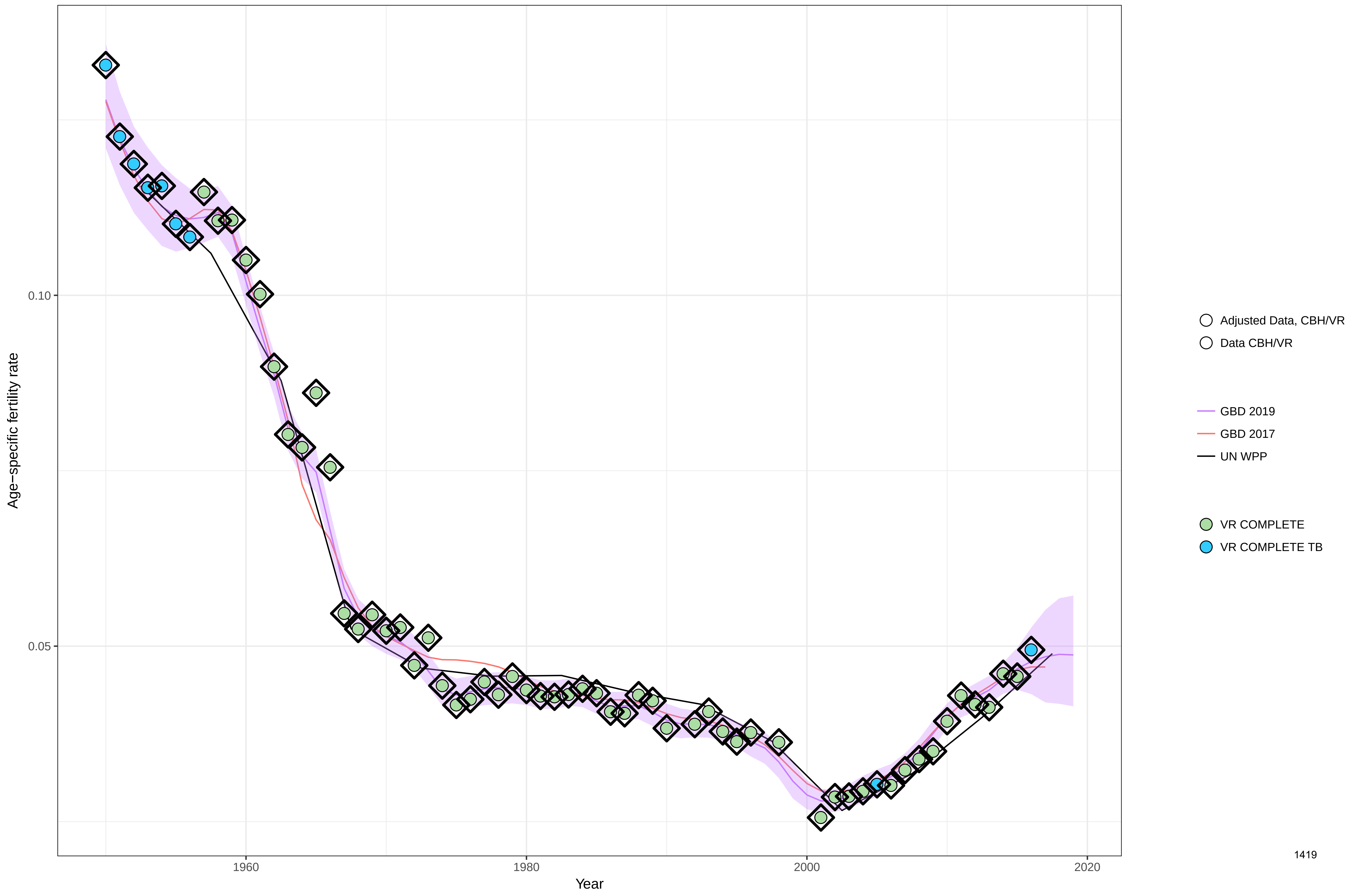

Age−specific fertility fitted trend lines, 1950−2019: Malta, Ages: 40−44
Data points enclosed with a diamond are reference sources

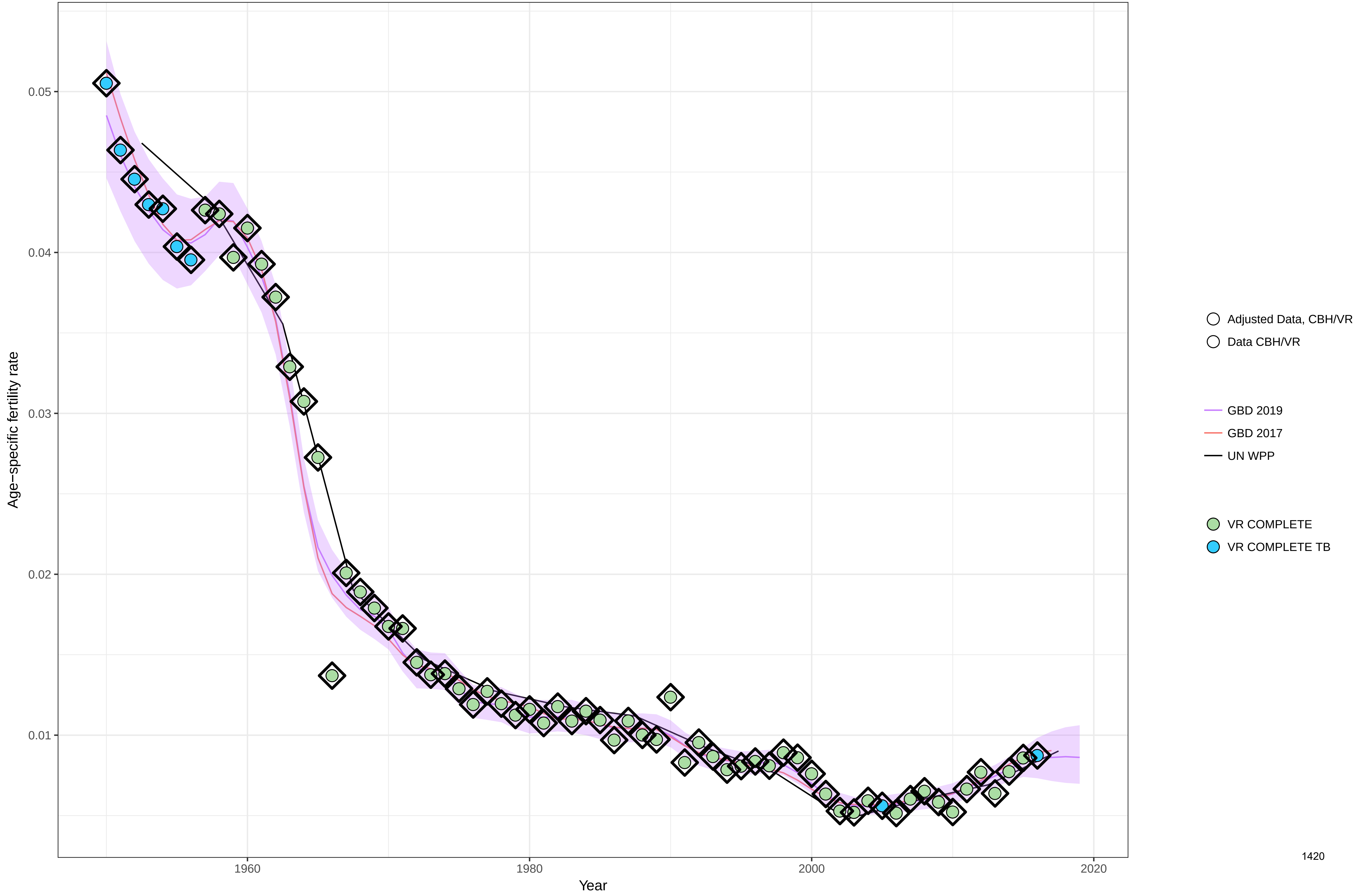

Age−specific fertility fitted trend lines, 1950−2019: Malta, Ages: 45−49
Data points enclosed with a diamond are reference sources

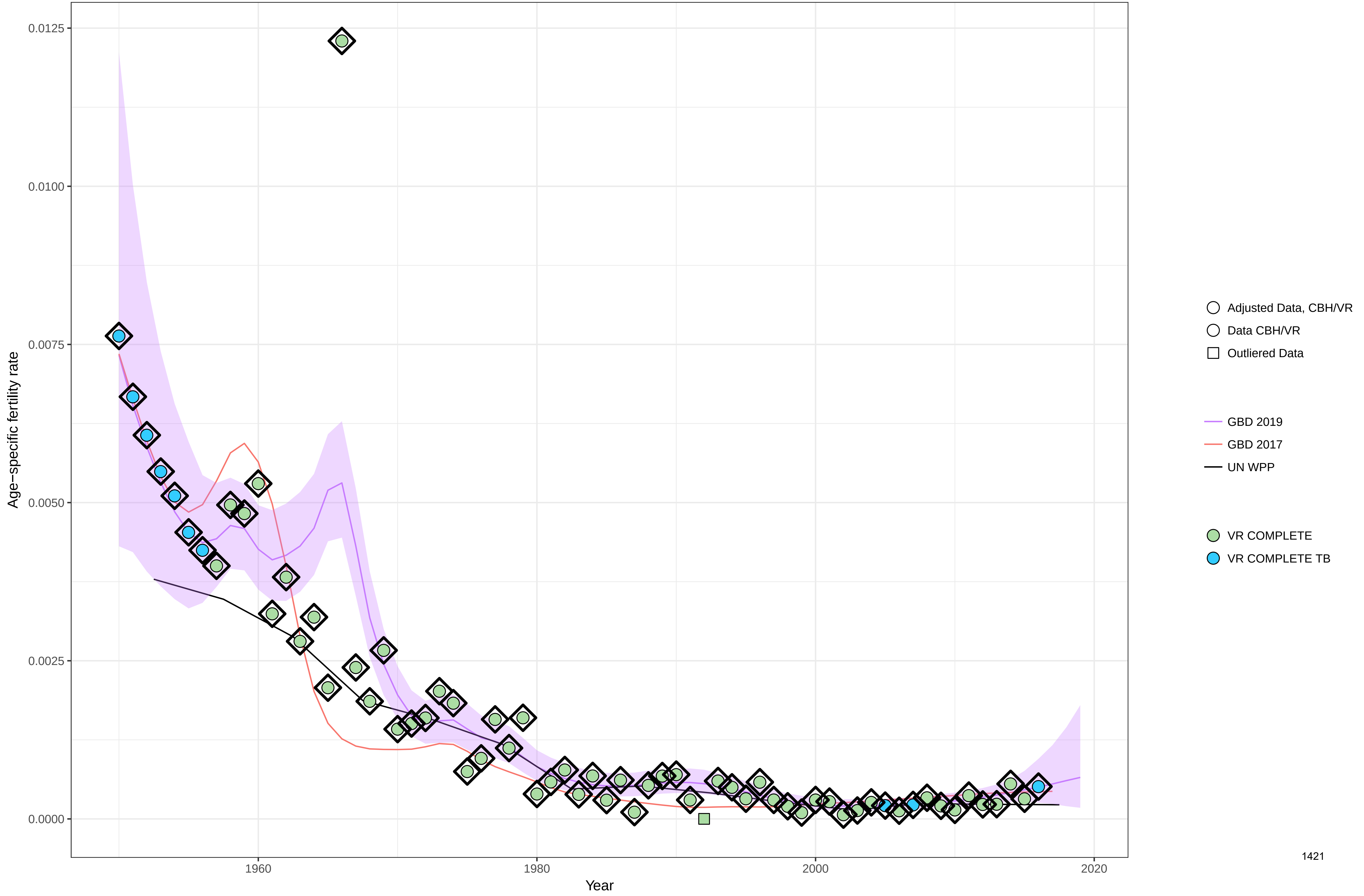

Age−specific fertility fitted trend lines, 1950−2019: Malta, Ages: 50−54
Data points enclosed with a diamond are reference sources

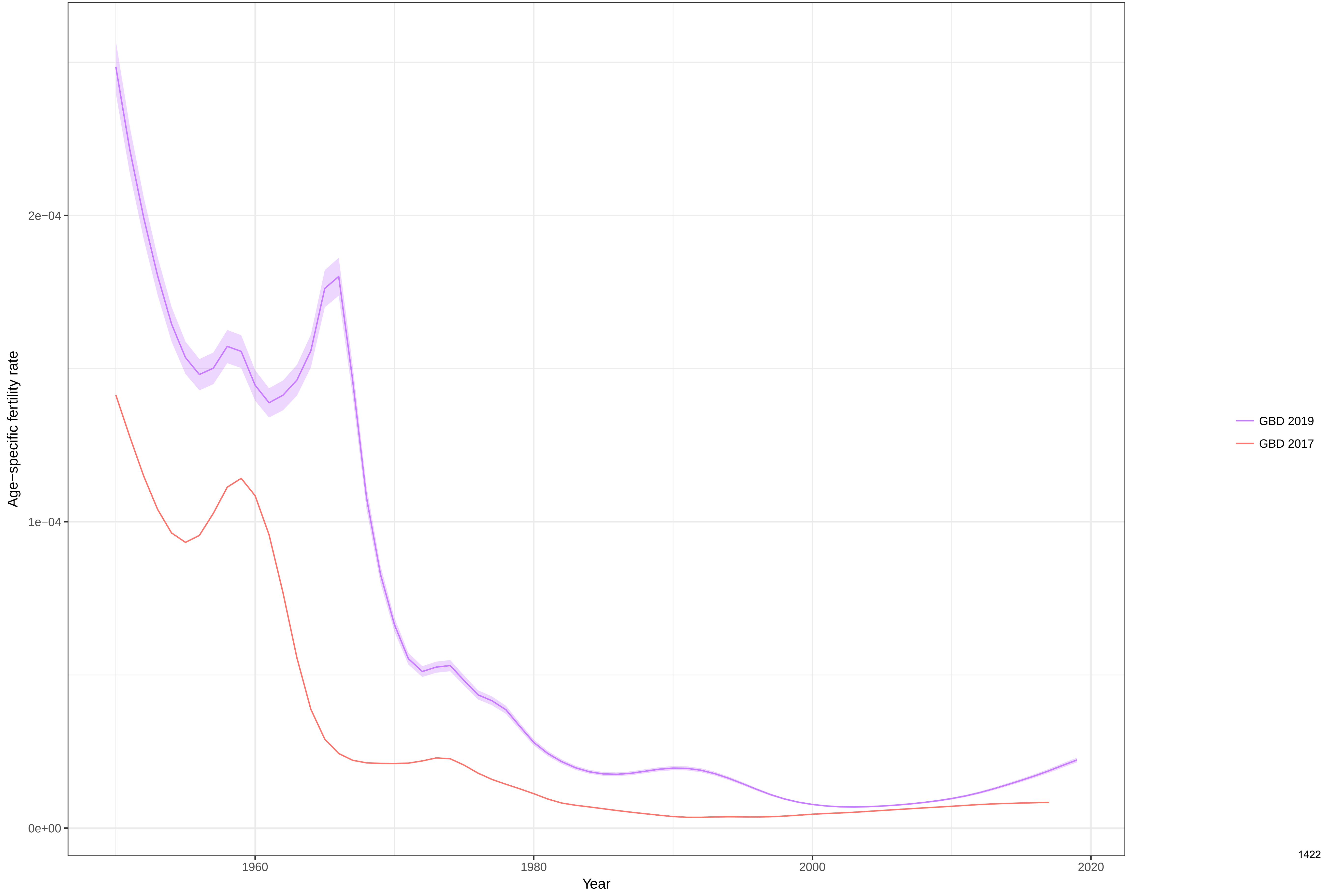

Total fertility rate fitted trend lines, 1950−2019: Malta TFR
Data points enclosed with a diamond are reference sources

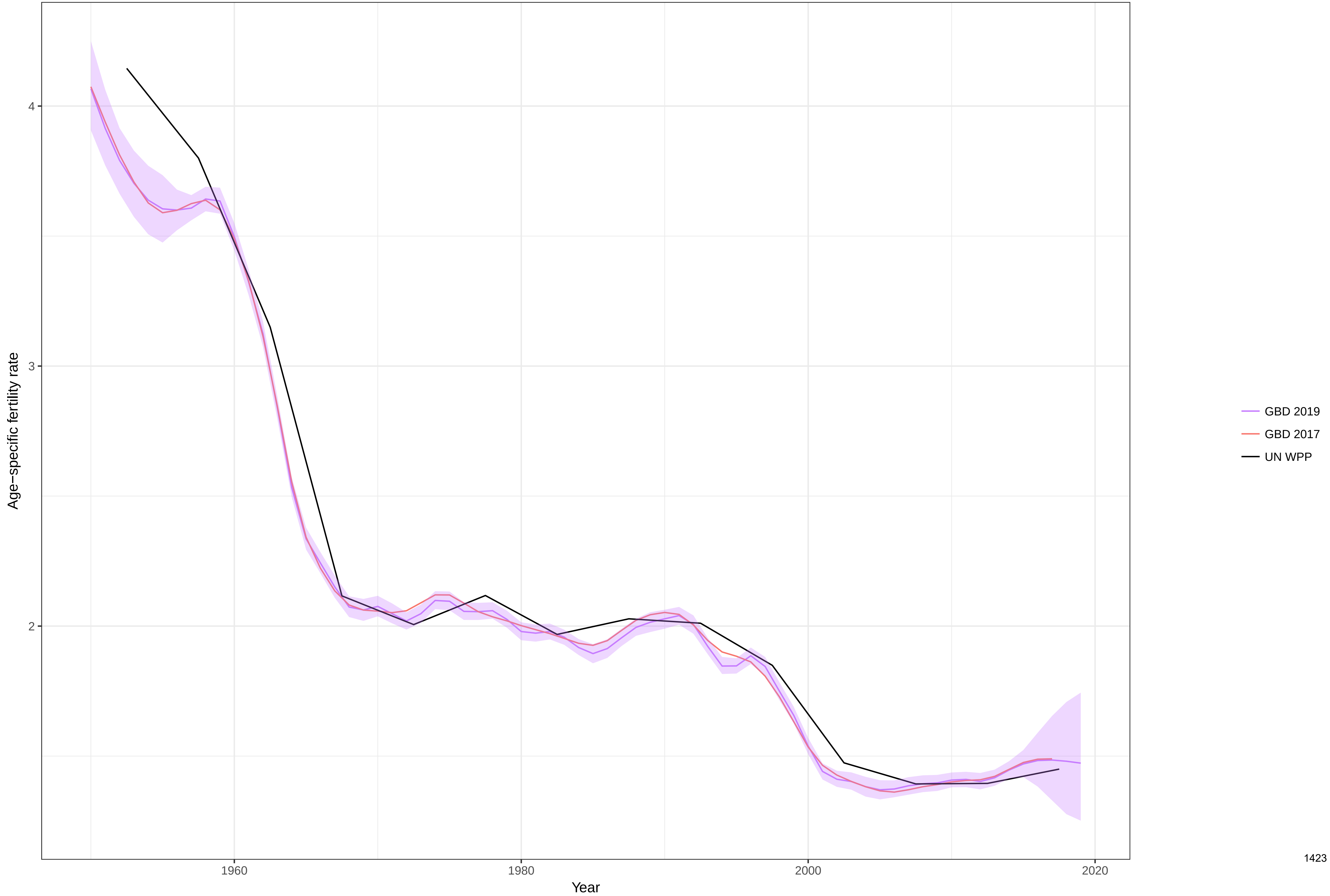

Age−specific fertility fitted trend lines, 1950−2019: Myanmar, Ages: 10−14
Data points enclosed with a diamond are reference sources

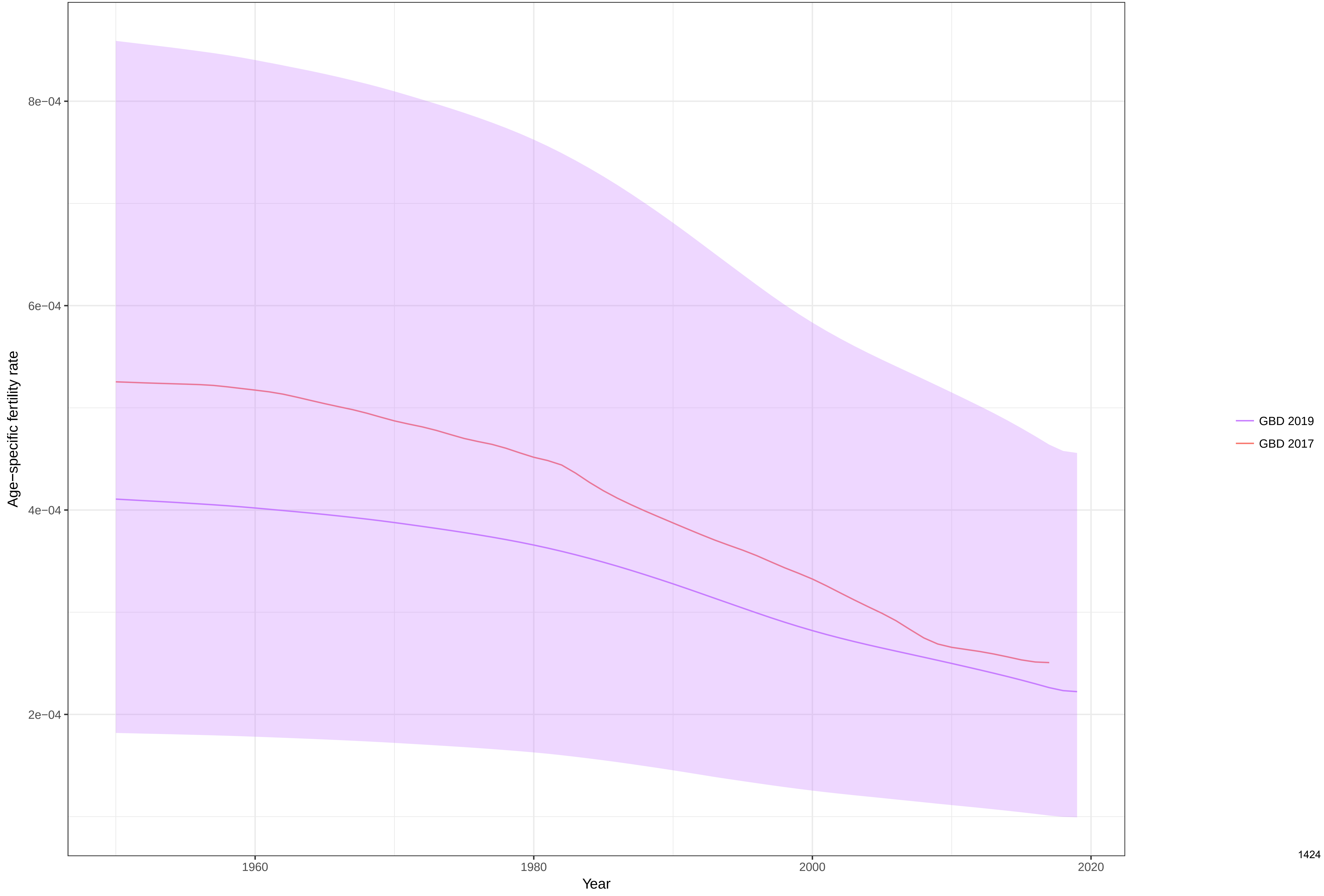

Age−specific fertility fitted trend lines, 1950−2019: Myanmar, Ages: 15−19
Data points enclosed with a diamond are reference sources

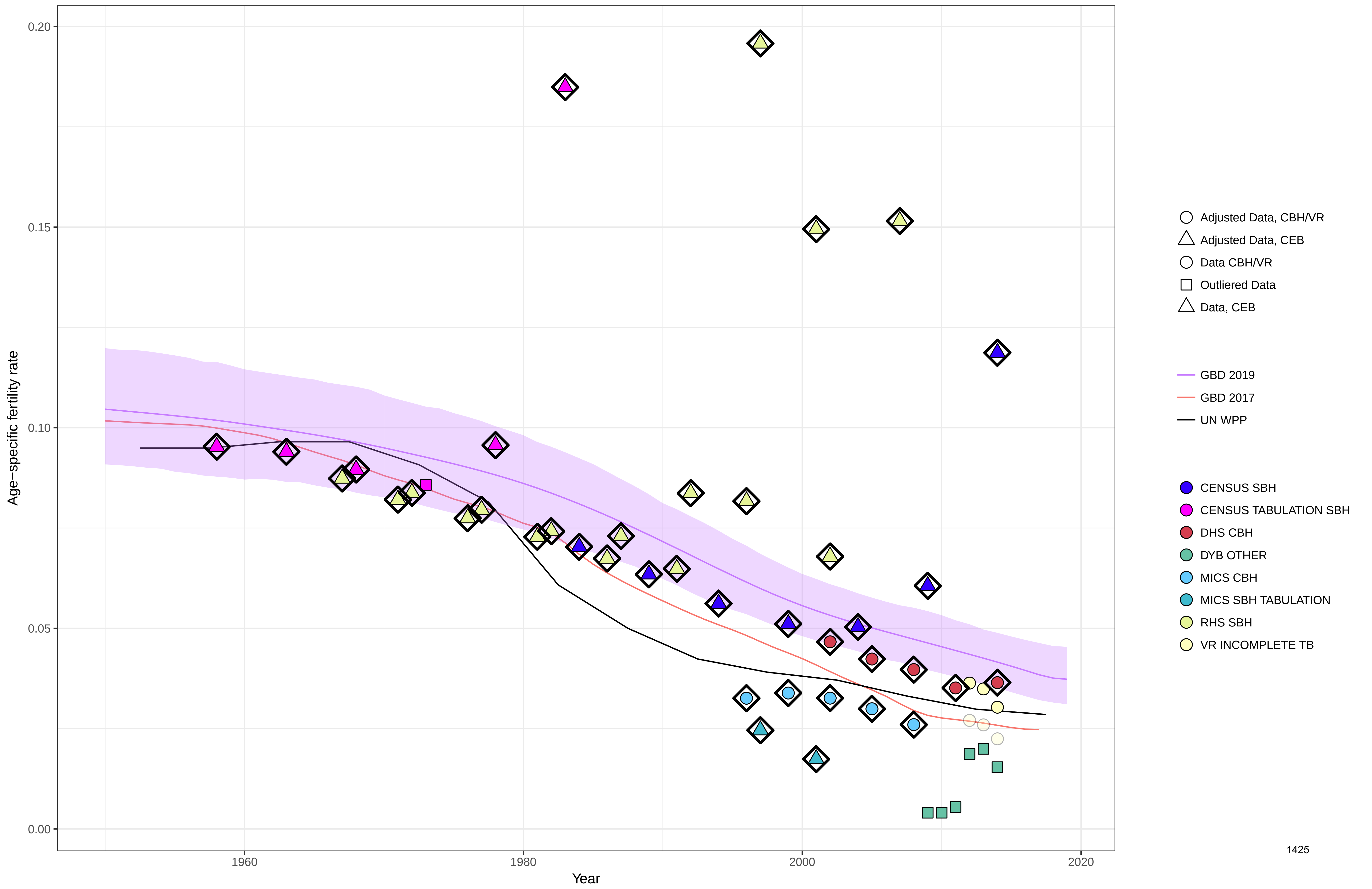

Age−specific fertility fitted trend lines, 1950−2019: Myanmar, Ages: 20−24
Data points enclosed with a diamond are reference sources

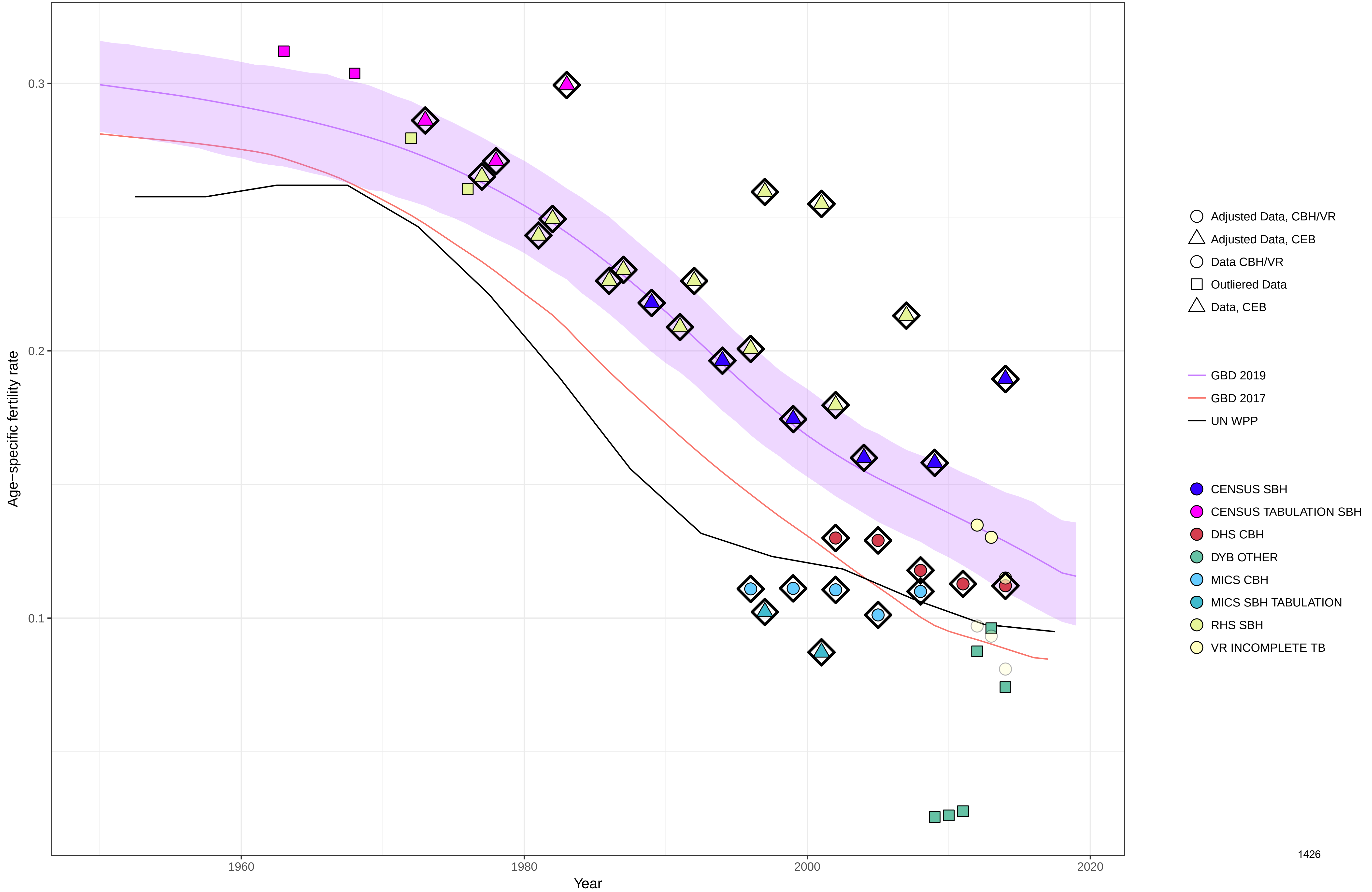

Age−specific fertility fitted trend lines, 1950−2019: Myanmar, Ages: 25−29
Data points enclosed with a diamond are reference sources

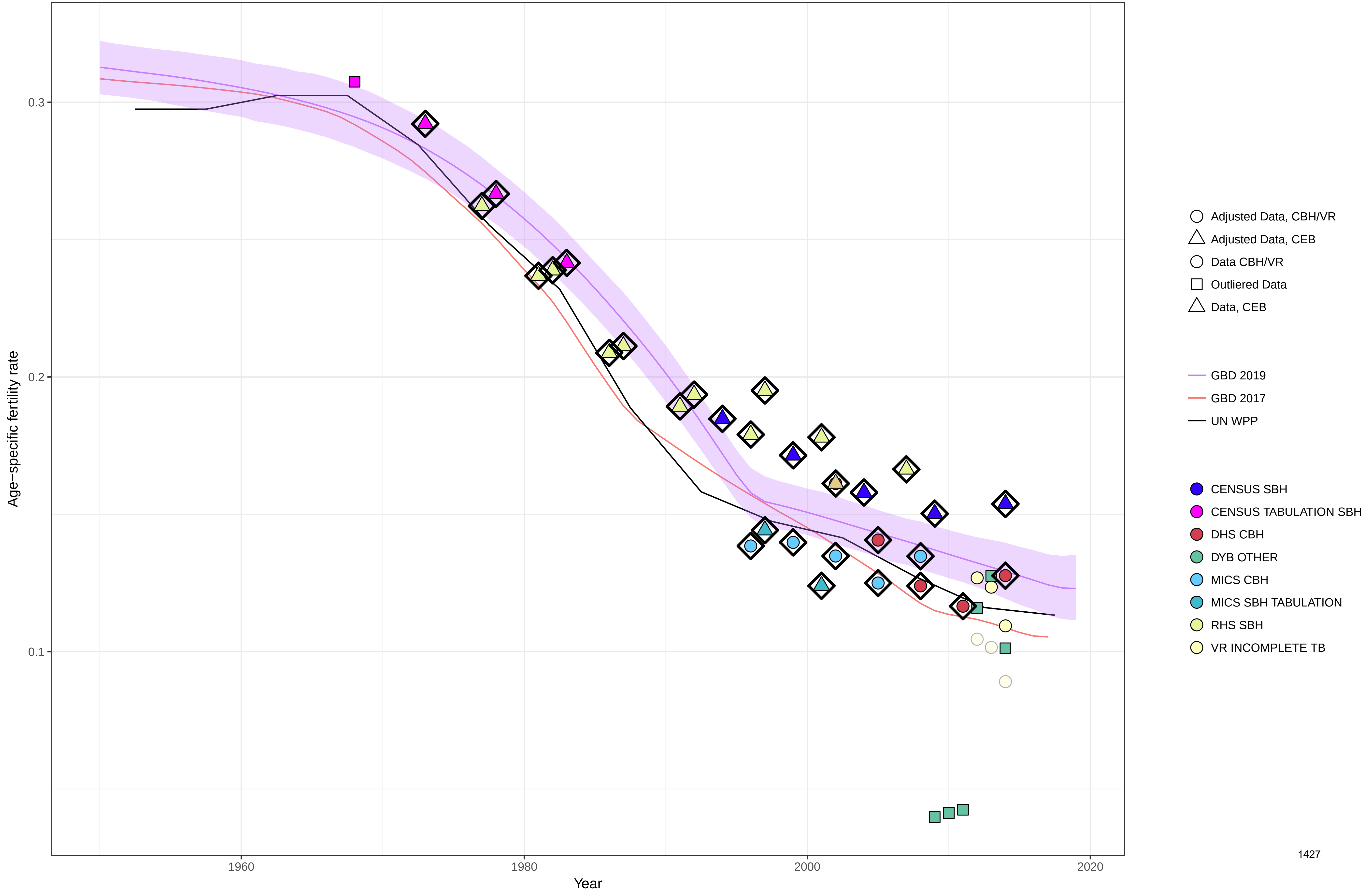

Age−specific fertility fitted trend lines, 1950−2019: Myanmar, Ages: 30−34
Data points enclosed with a diamond are reference sources

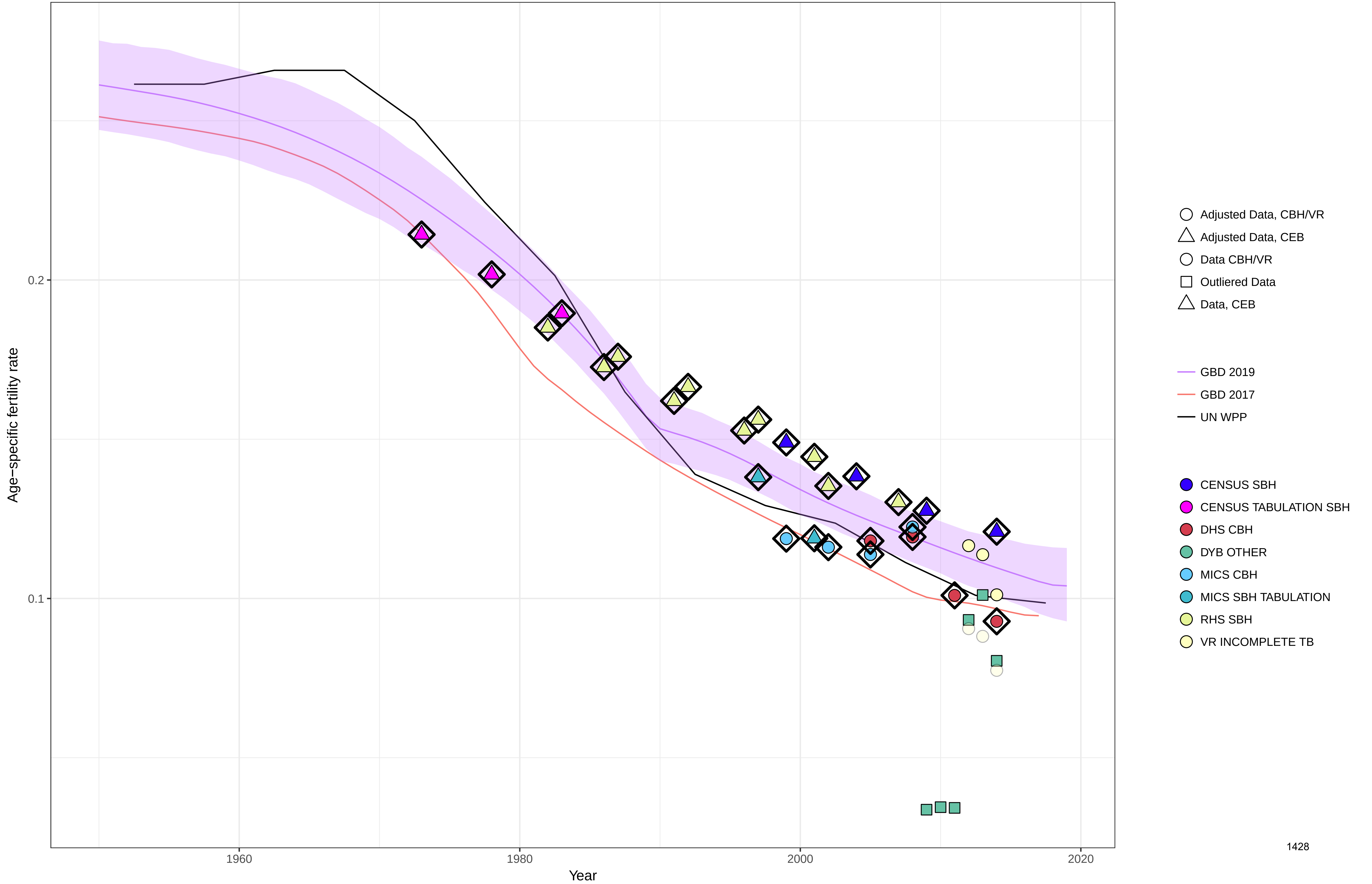

Age−specific fertility fitted trend lines, 1950−2019: Myanmar, Ages: 35−39
Data points enclosed with a diamond are reference sources

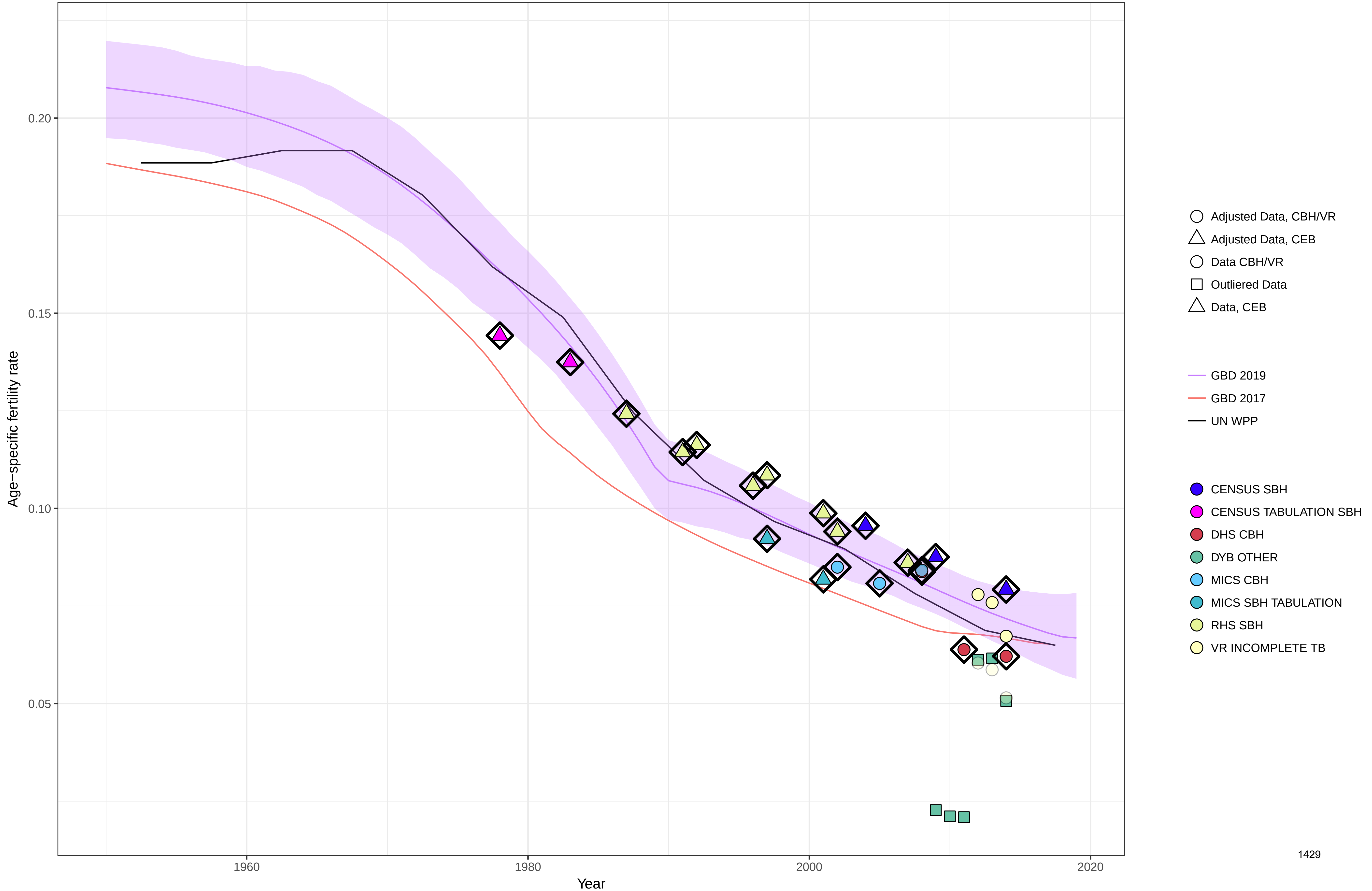

Age−specific fertility fitted trend lines, 1950−2019: Myanmar, Ages: 40−44
Data points enclosed with a diamond are reference sources

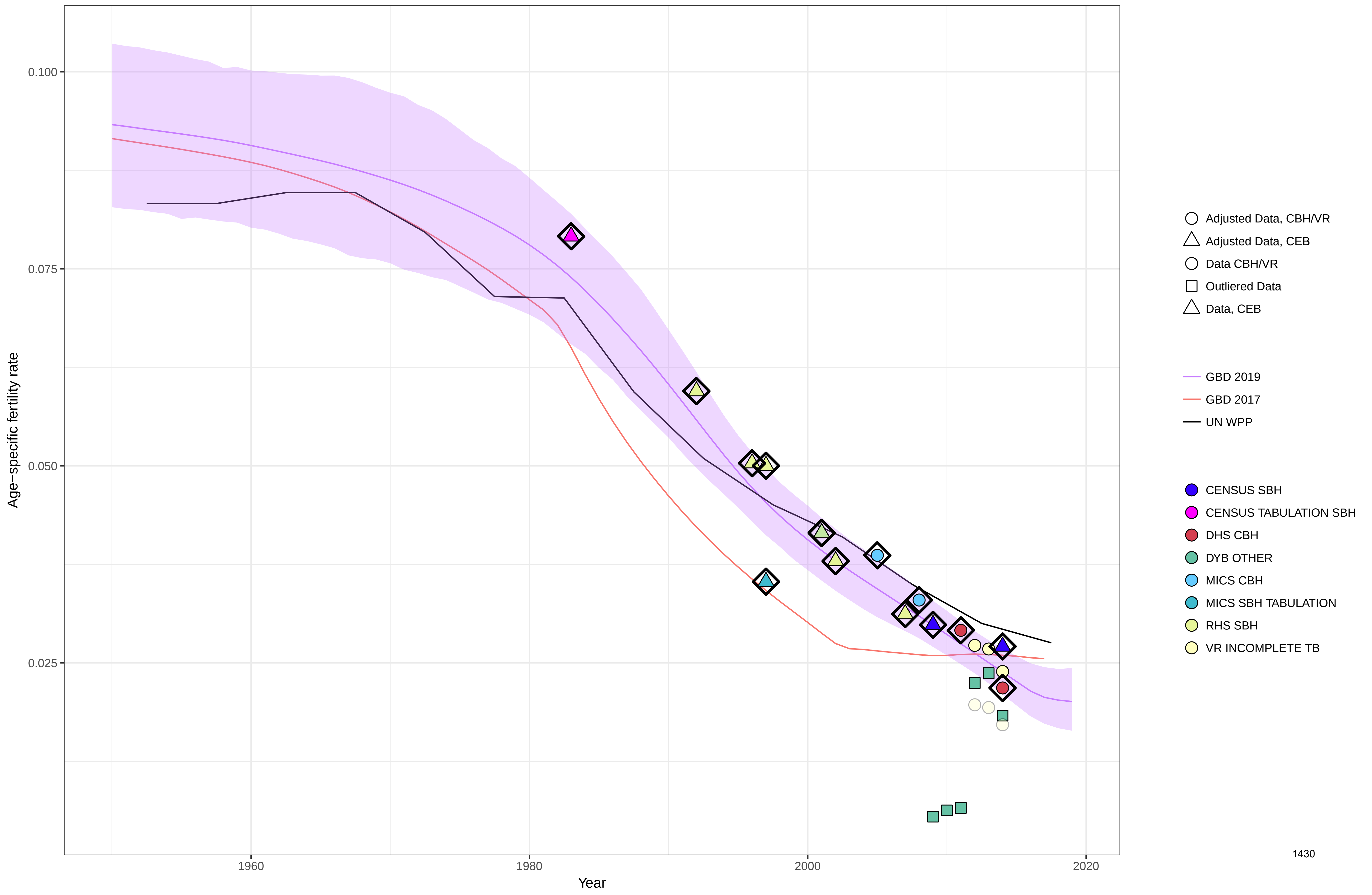

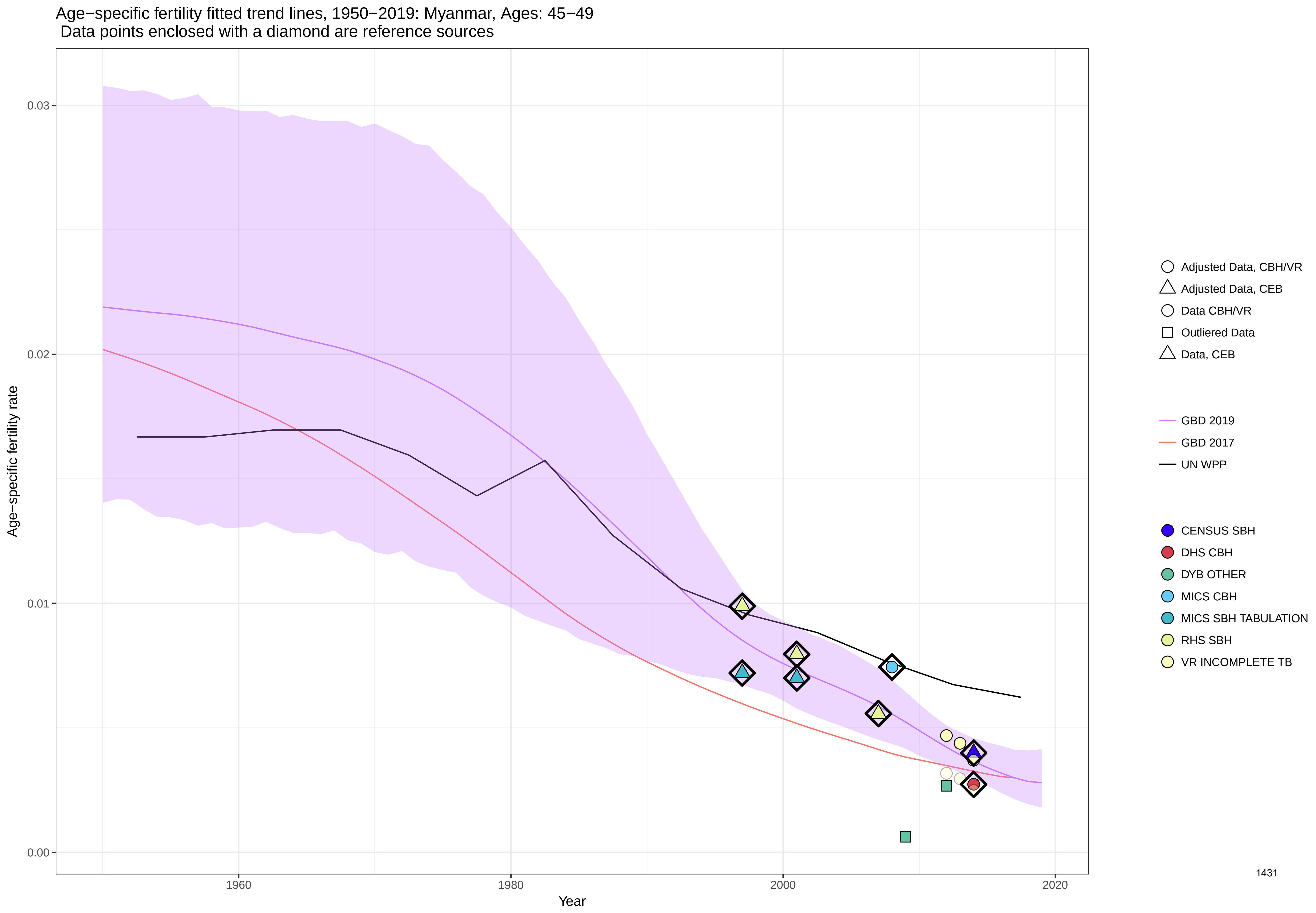

Age−specific fertility fitted trend lines, 1950−2019: Myanmar, Ages: 45−49
Data points enclosed with a diamond are reference sources
Age−specific fertility rate
0.00
0.01
0.02
0.03
Year
1960
1980
2000
2020
Adjusted Data, CBH/VR
Adjusted Data, CEB
Data CBH/VR
Outliered Data
Data, CEB
GBD 2019
GBD 2017
UN WPP
CENSUS SBH
DHS CBH
DYB OTHER
MICS CBH
MICS SBH TABULATION
RHS SBH
VR INCOMPLETE TB

Age−specific fertility fitted trend lines, 1950−2019: Myanmar, Ages: 50−54
Data points enclosed with a diamond are reference sources

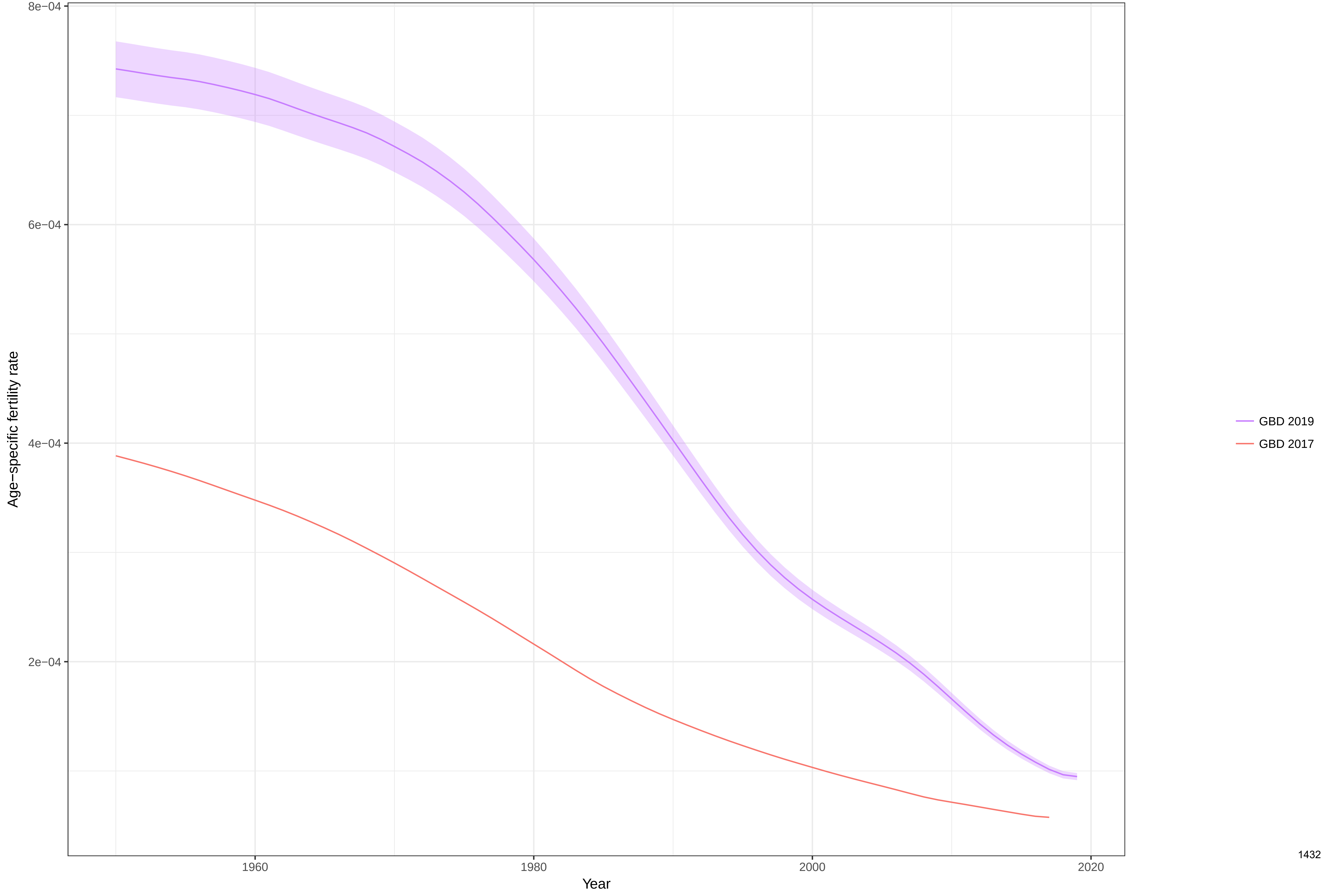

Total fertility rate fitted trend lines, 1950−2019: Myanmar TFR
Data points enclosed with a diamond are reference sources

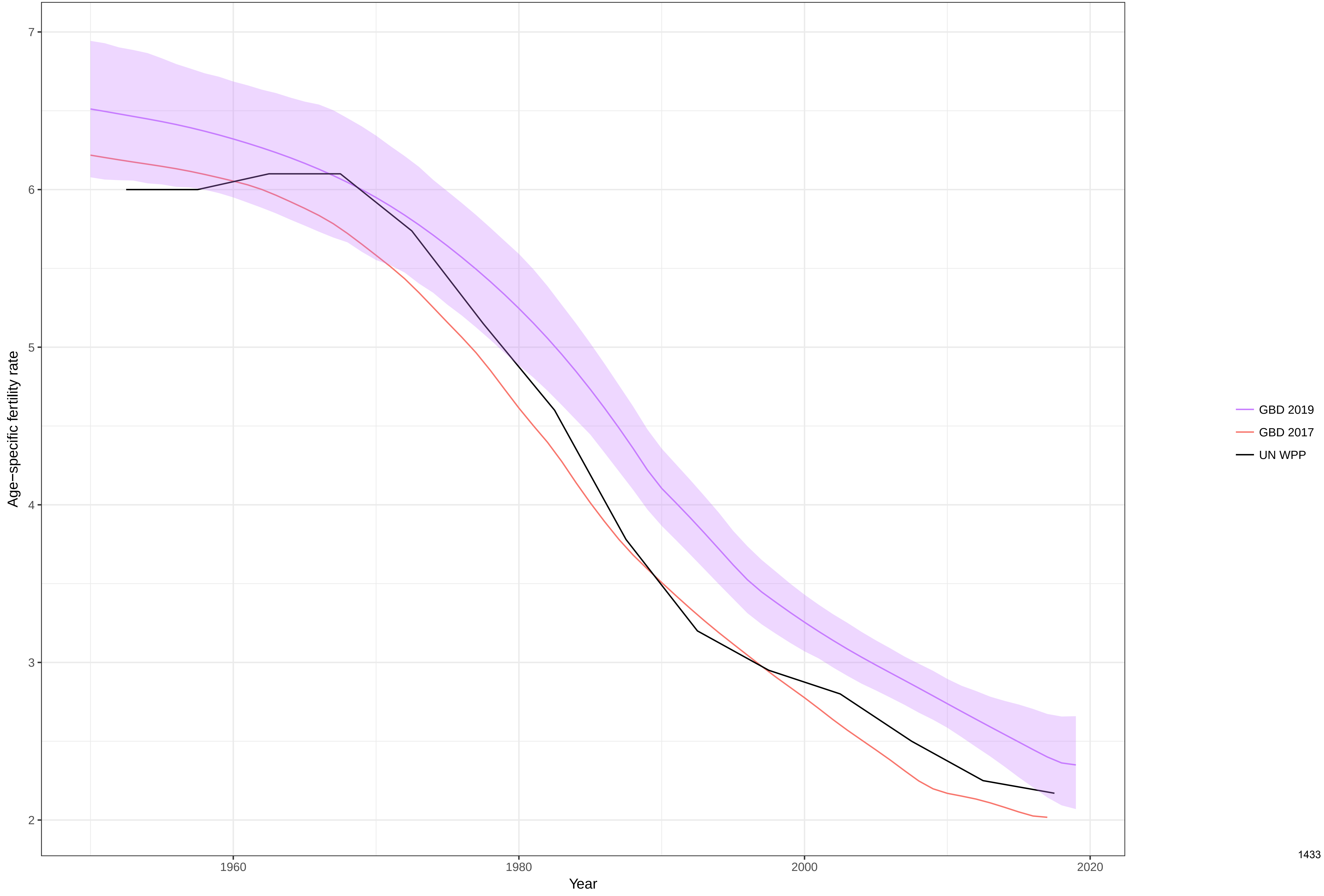

Age−specific fertility fitted trend lines, 1950−2019: Montenegro, Ages: 10−14
Data points enclosed with a diamond are reference sources

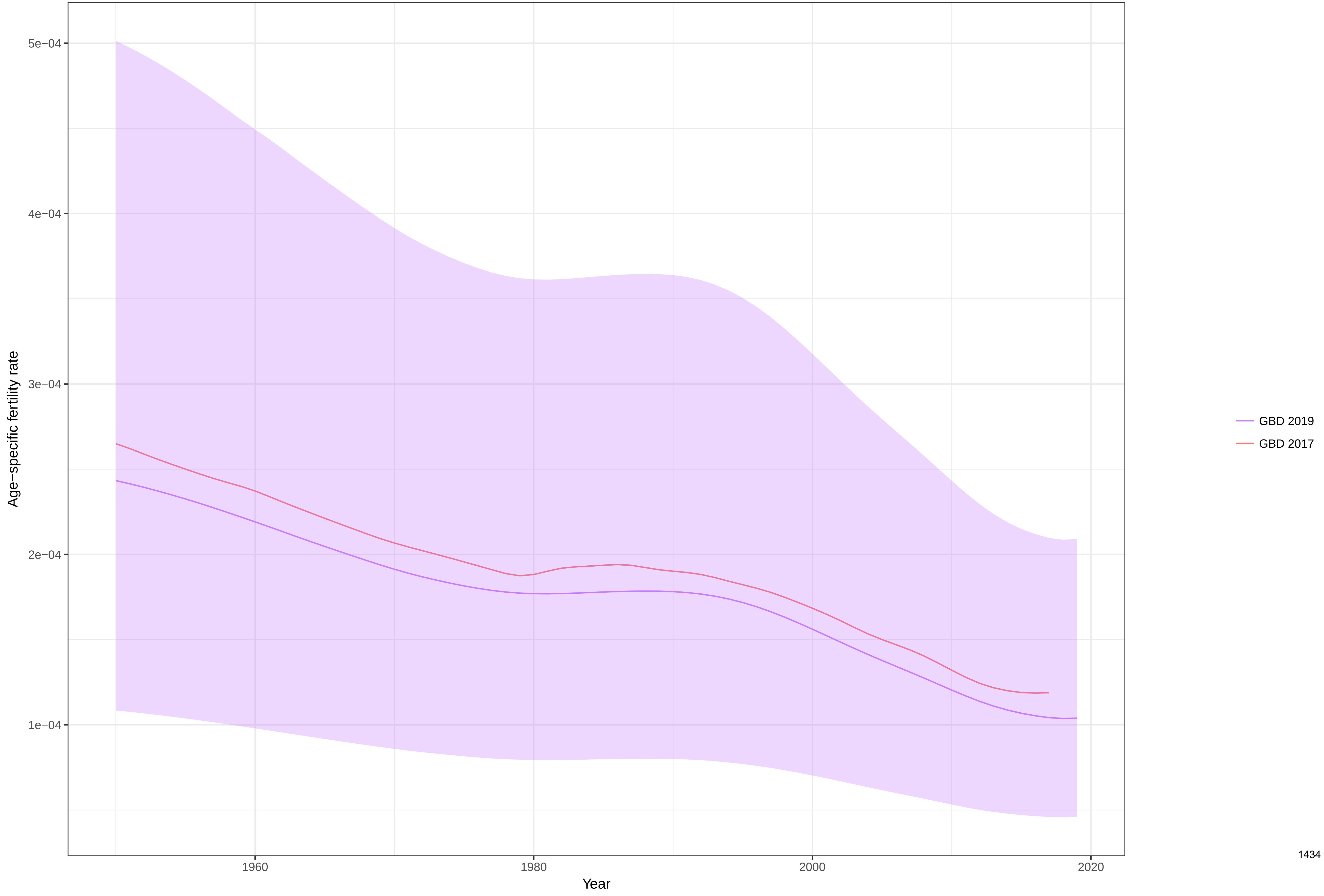

Age−specific fertility fitted trend lines, 1950−2019: Montenegro, Ages: 15−19
Data points enclosed with a diamond are reference sources

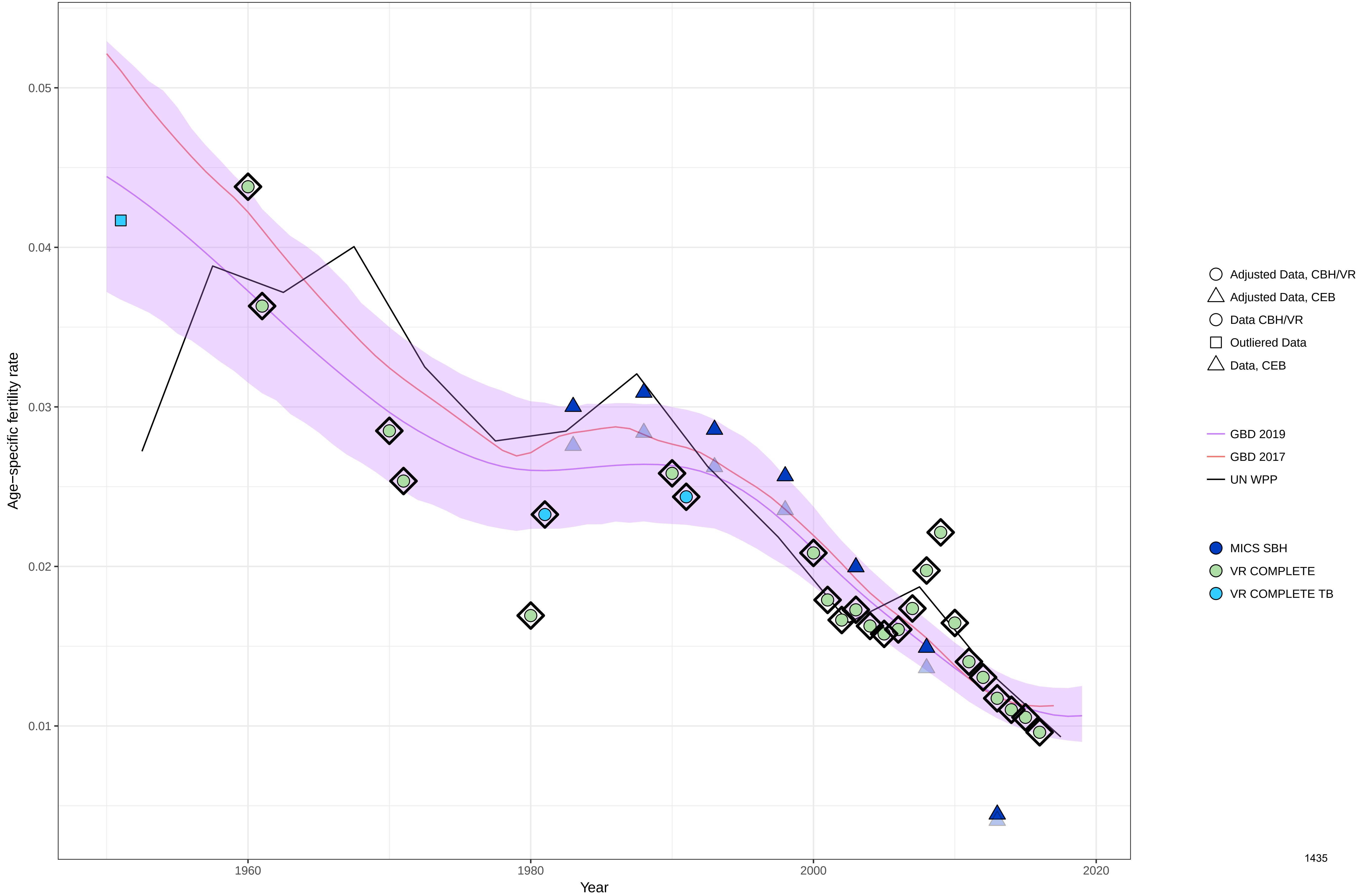

Age−specific fertility fitted trend lines, 1950−2019: Montenegro, Ages: 20−24
Data points enclosed with a diamond are reference sources

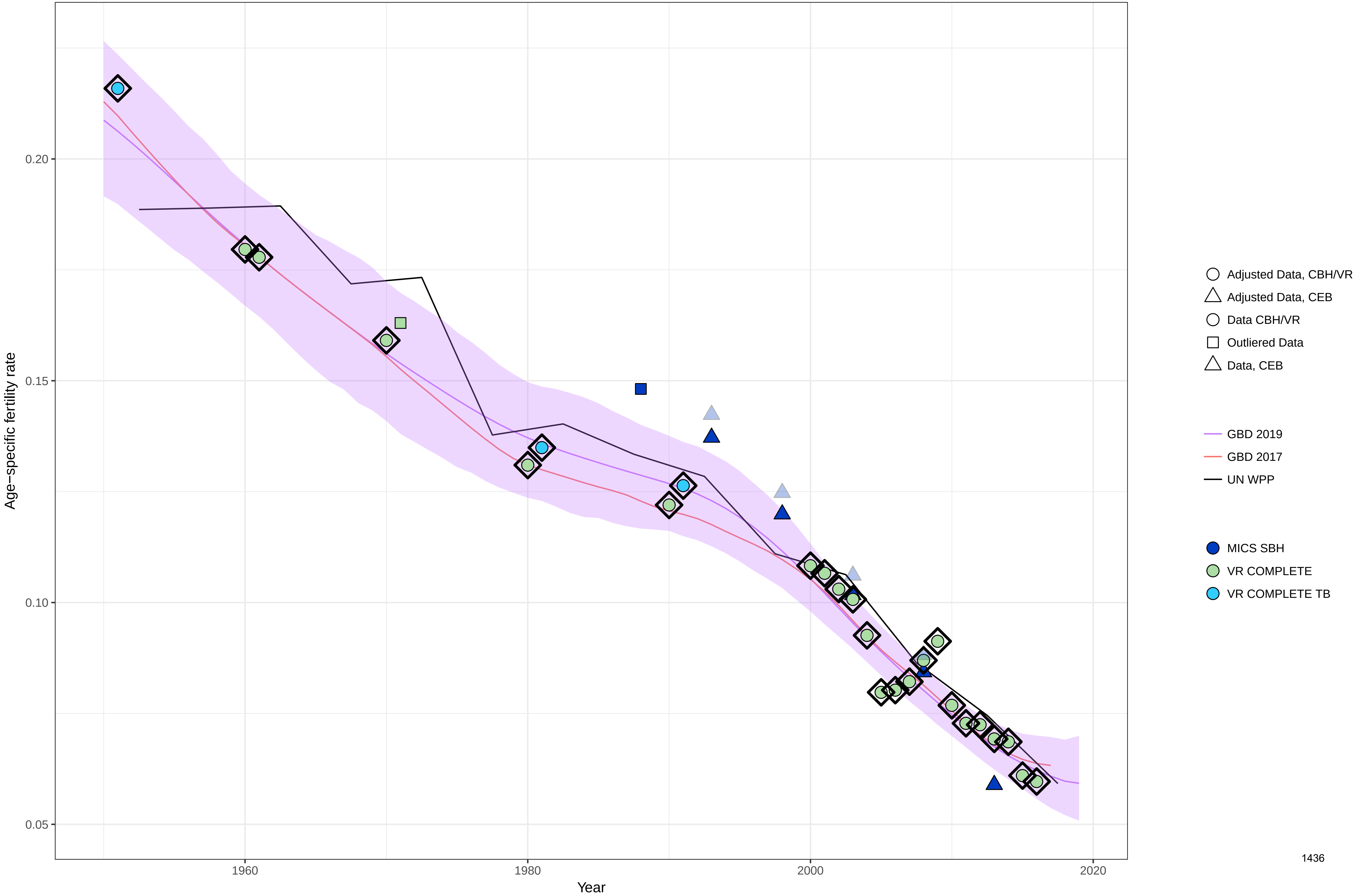

Age−specific fertility fitted trend lines, 1950−2019: Montenegro, Ages: 25−29
Data points enclosed with a diamond are reference sources

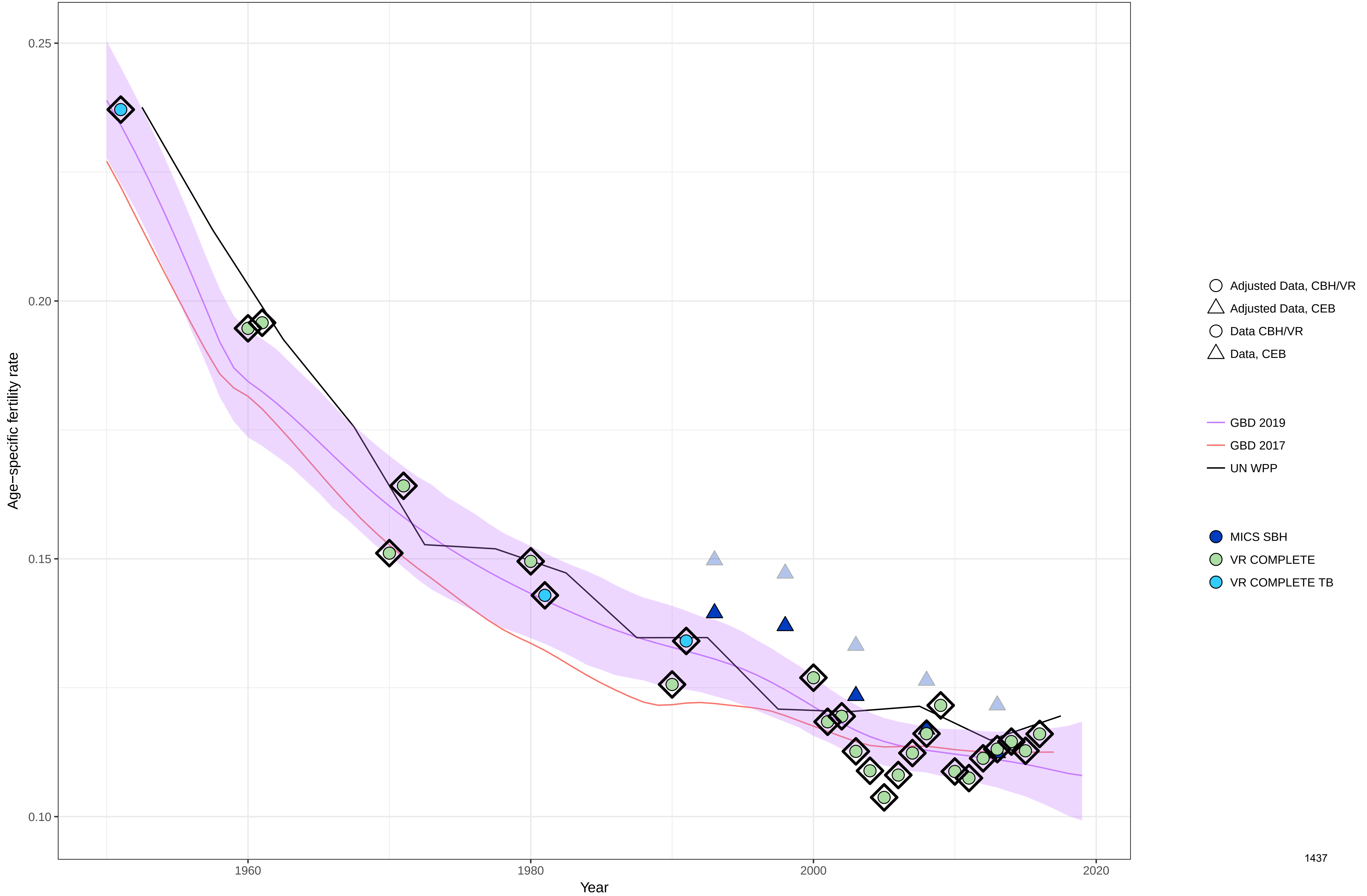

Age−specific fertility fitted trend lines, 1950−2019: Montenegro, Ages: 30−34
Data points enclosed with a diamond are reference sources

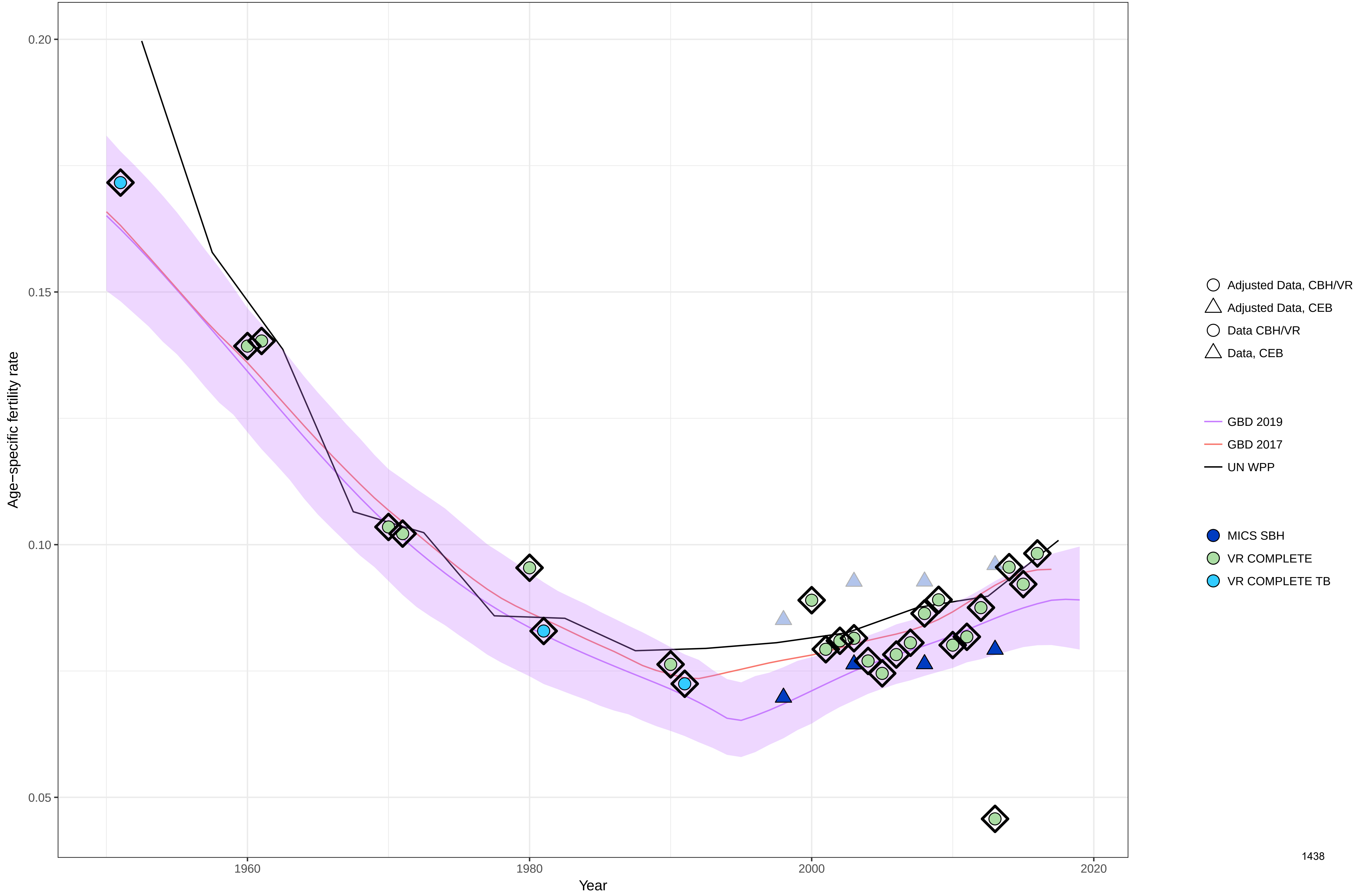

Age−specific fertility fitted trend lines, 1950−2019: Montenegro, Ages: 35−39
Data points enclosed with a diamond are reference sources

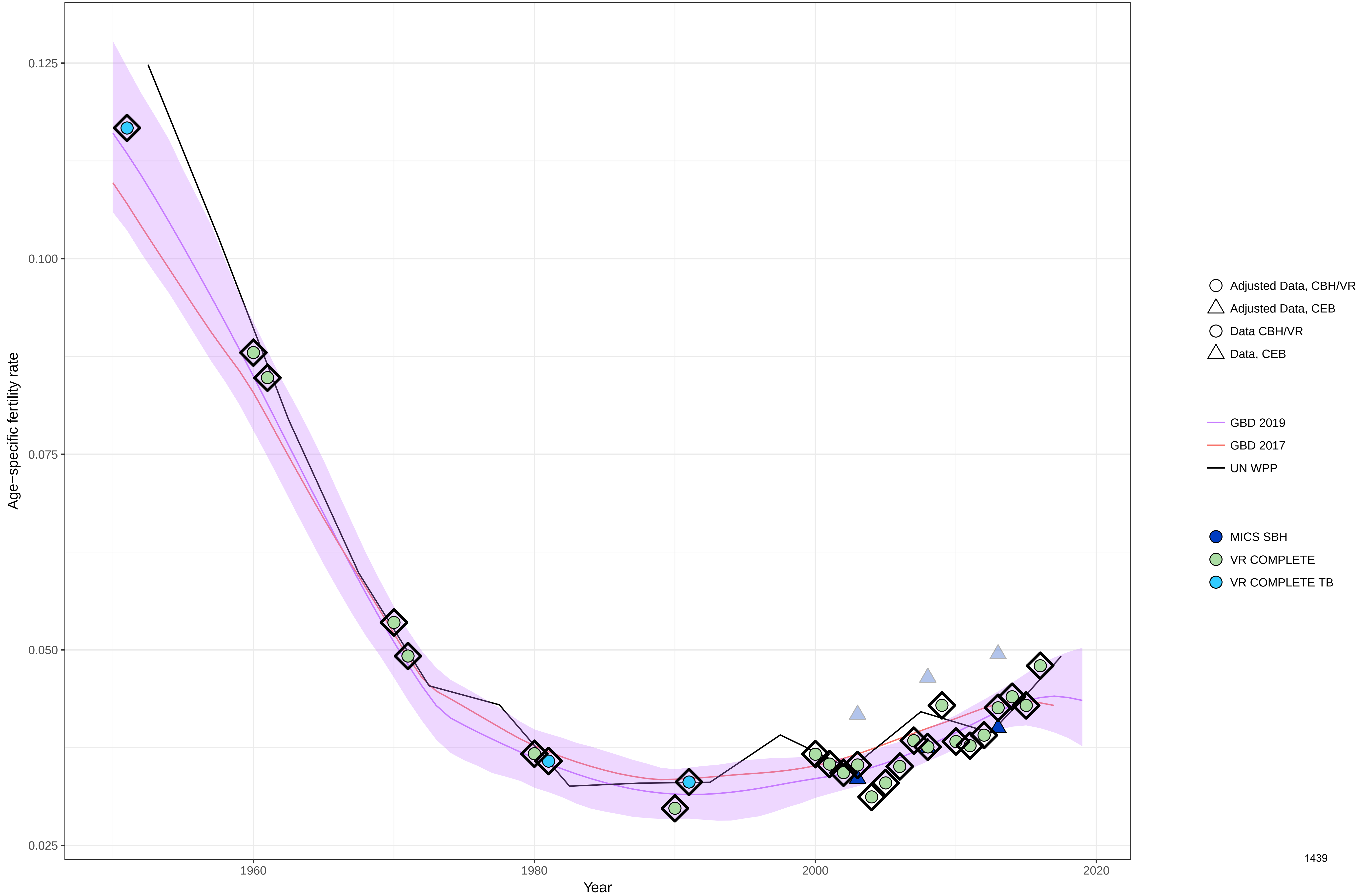

Age−specific fertility fitted trend lines, 1950−2019: Montenegro, Ages: 40−44
Data points enclosed with a diamond are reference sources

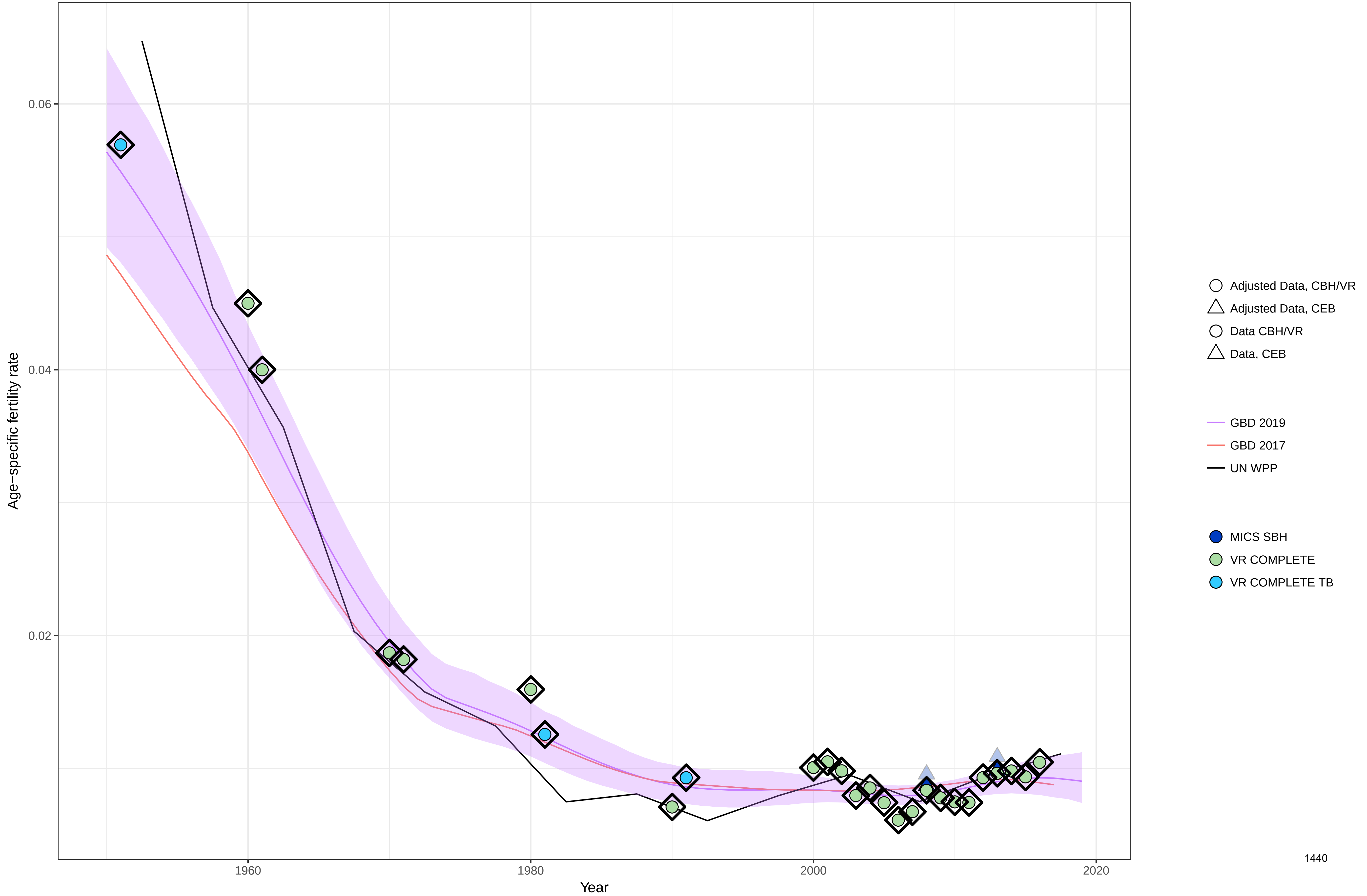

Age−specific fertility fitted trend lines, 1950−2019: Montenegro, Ages: 45−49
Data points enclosed with a diamond are reference sources

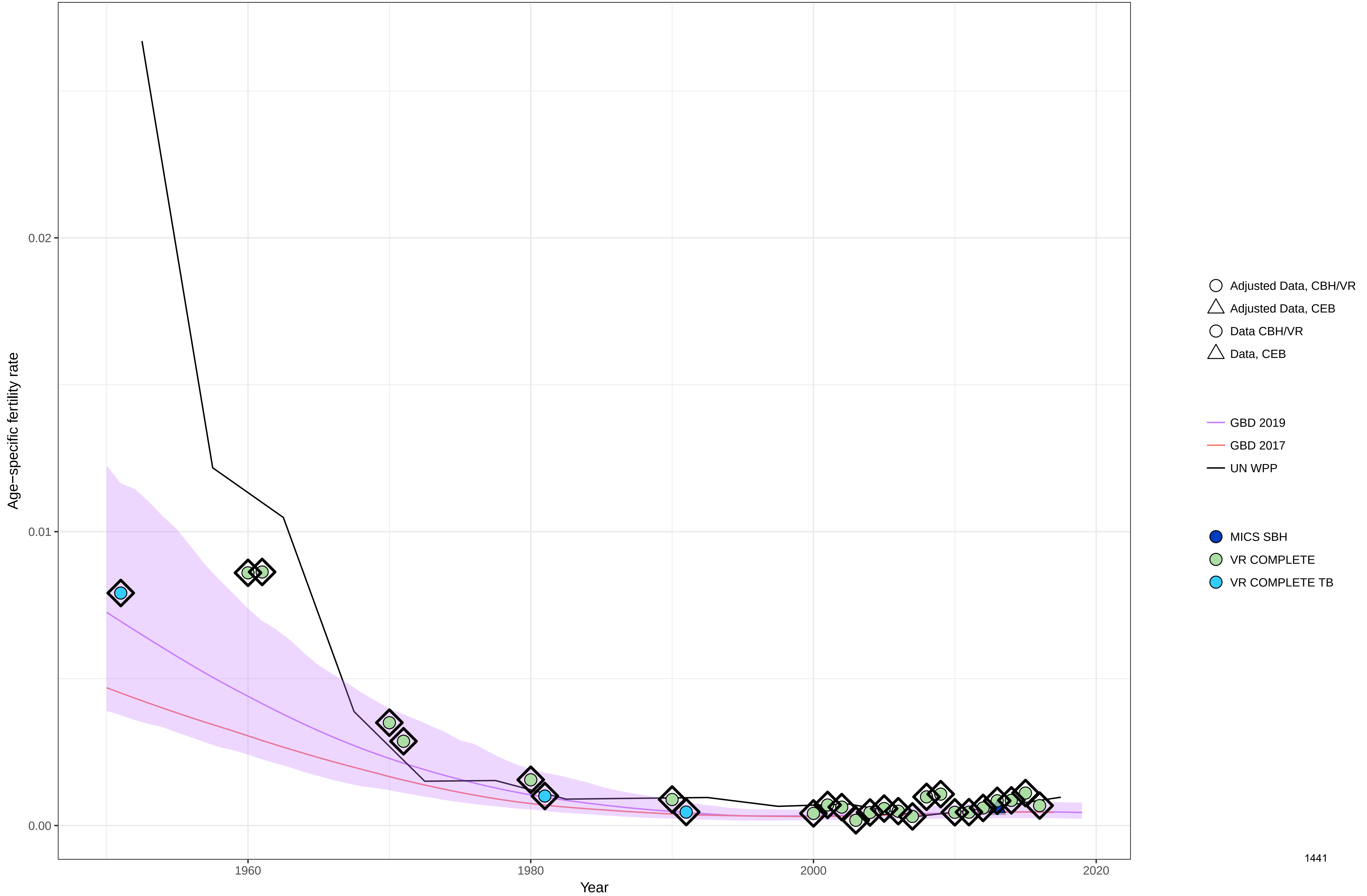

Age−specific fertility fitted trend lines, 1950−2019: Montenegro, Ages: 50−54
Data points enclosed with a diamond are reference sources

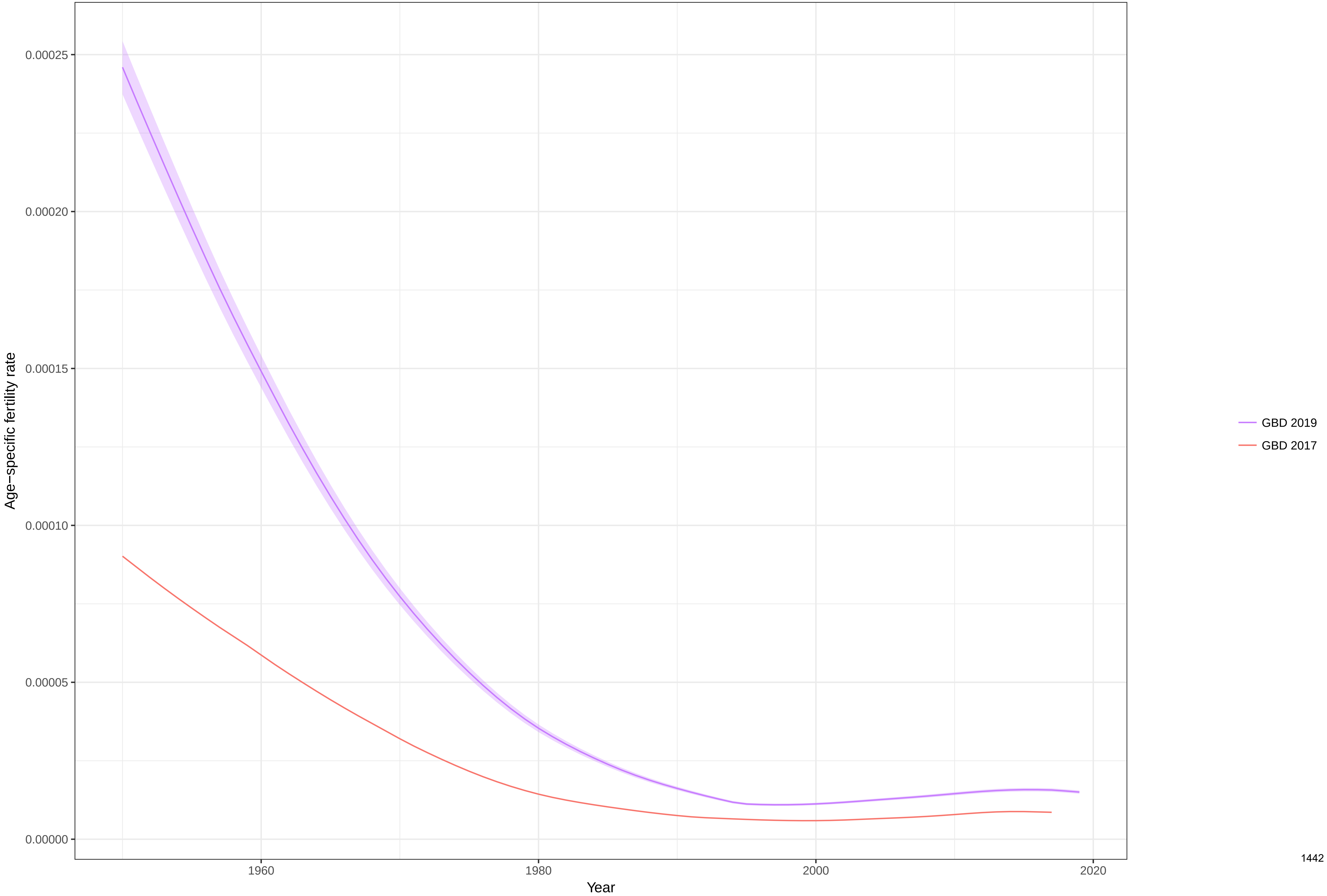

Total fertility rate fitted trend lines, 1950−2019: Montenegro TFR
Data points enclosed with a diamond are reference sources

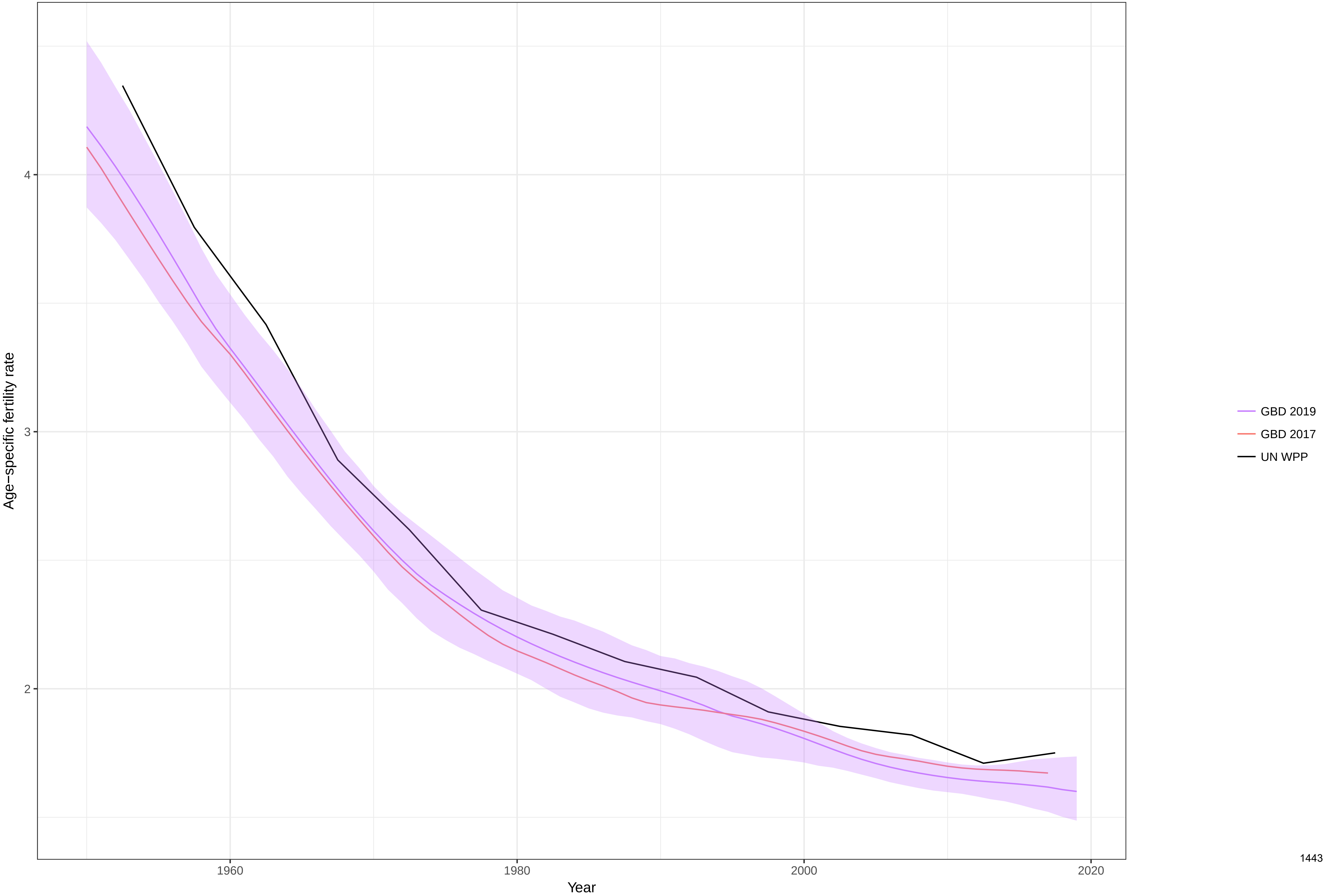

Age−specific fertility fitted trend lines, 1950−2019: Mongolia, Ages: 10−14
Data points enclosed with a diamond are reference sources

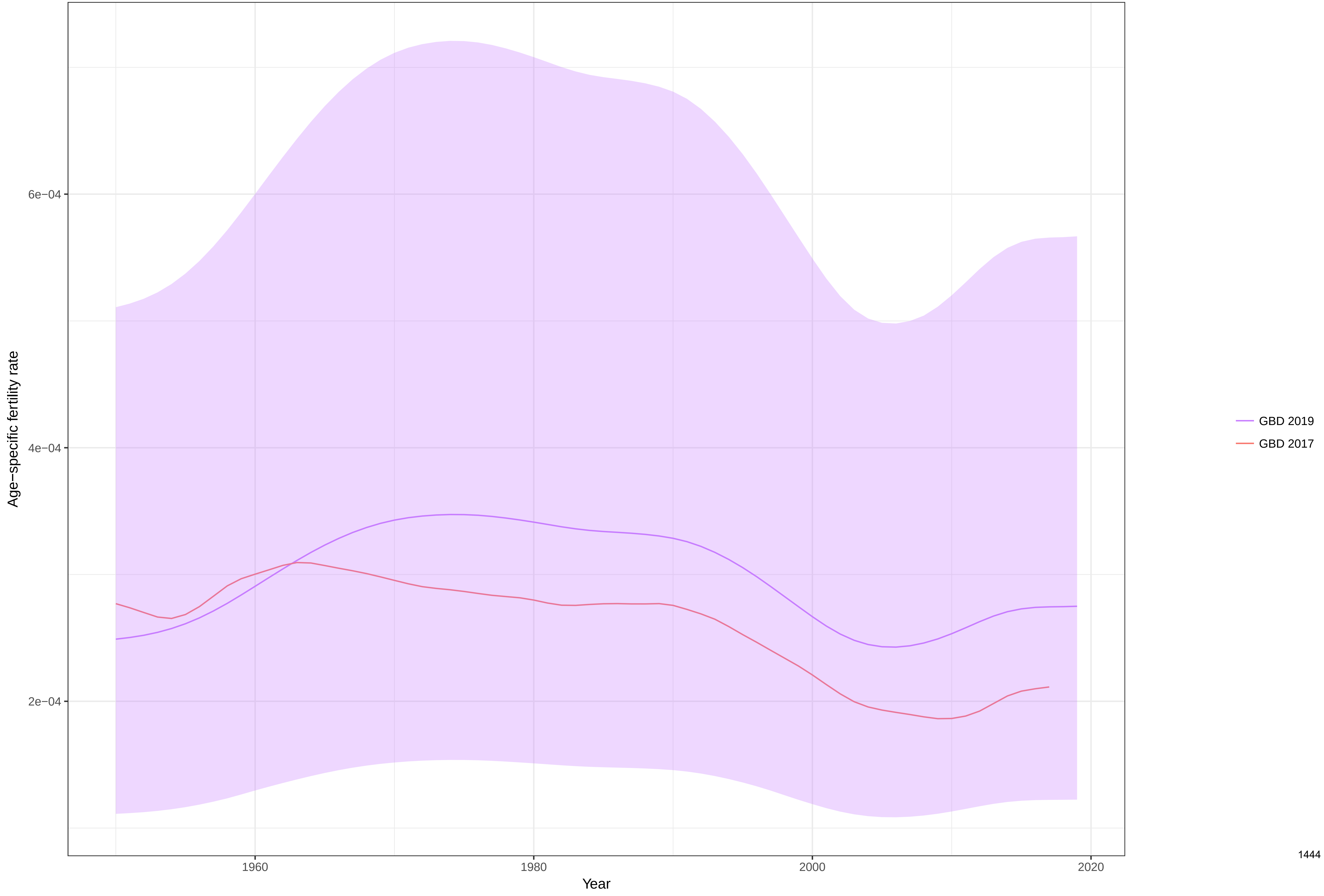

Age−specific fertility fitted trend lines, 1950−2019: Mongolia, Ages: 15−19
Data points enclosed with a diamond are reference sources

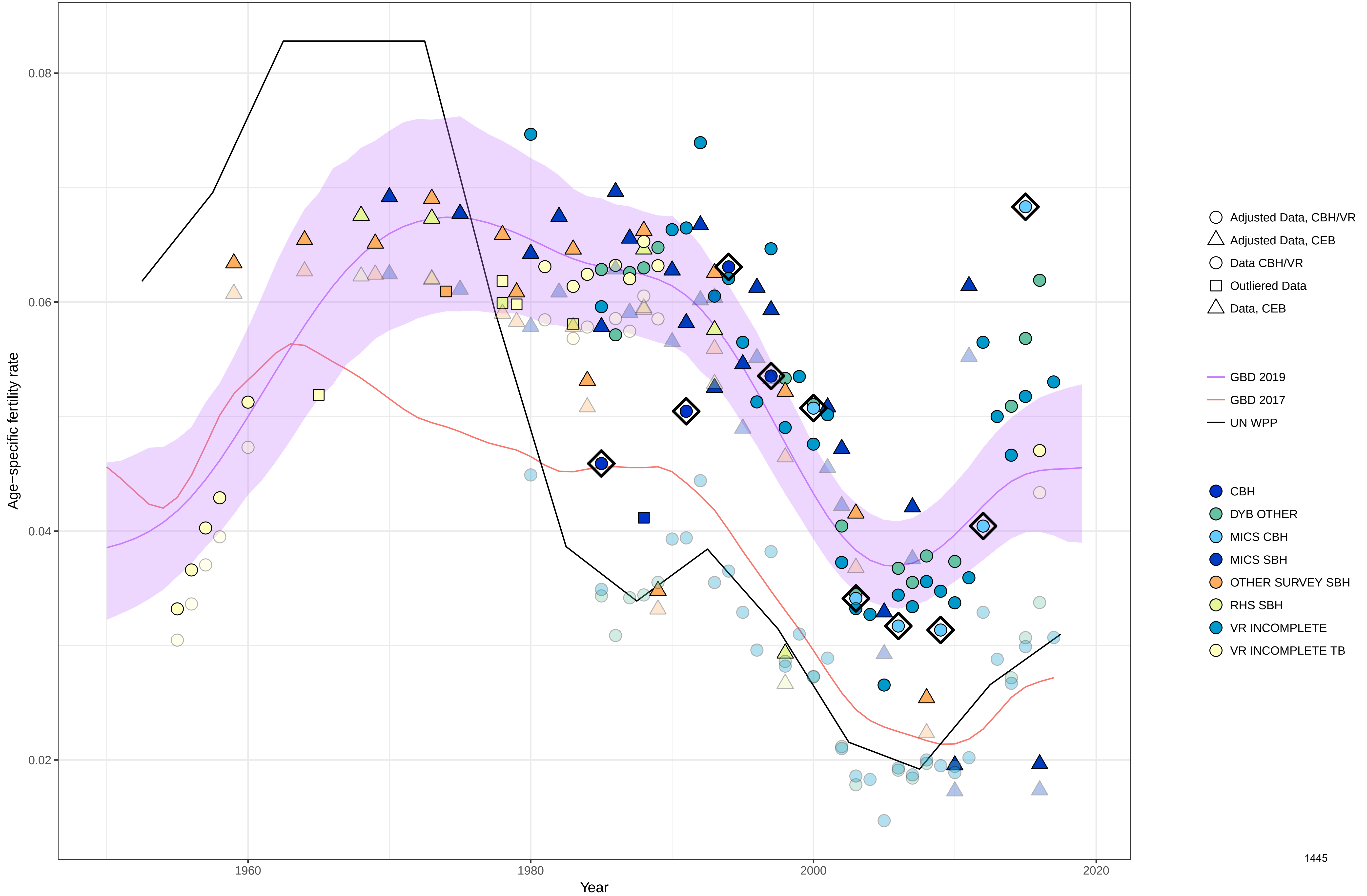

Age−specific fertility fitted trend lines, 1950−2019: Mongolia, Ages: 20−24
Data points enclosed with a diamond are reference sources

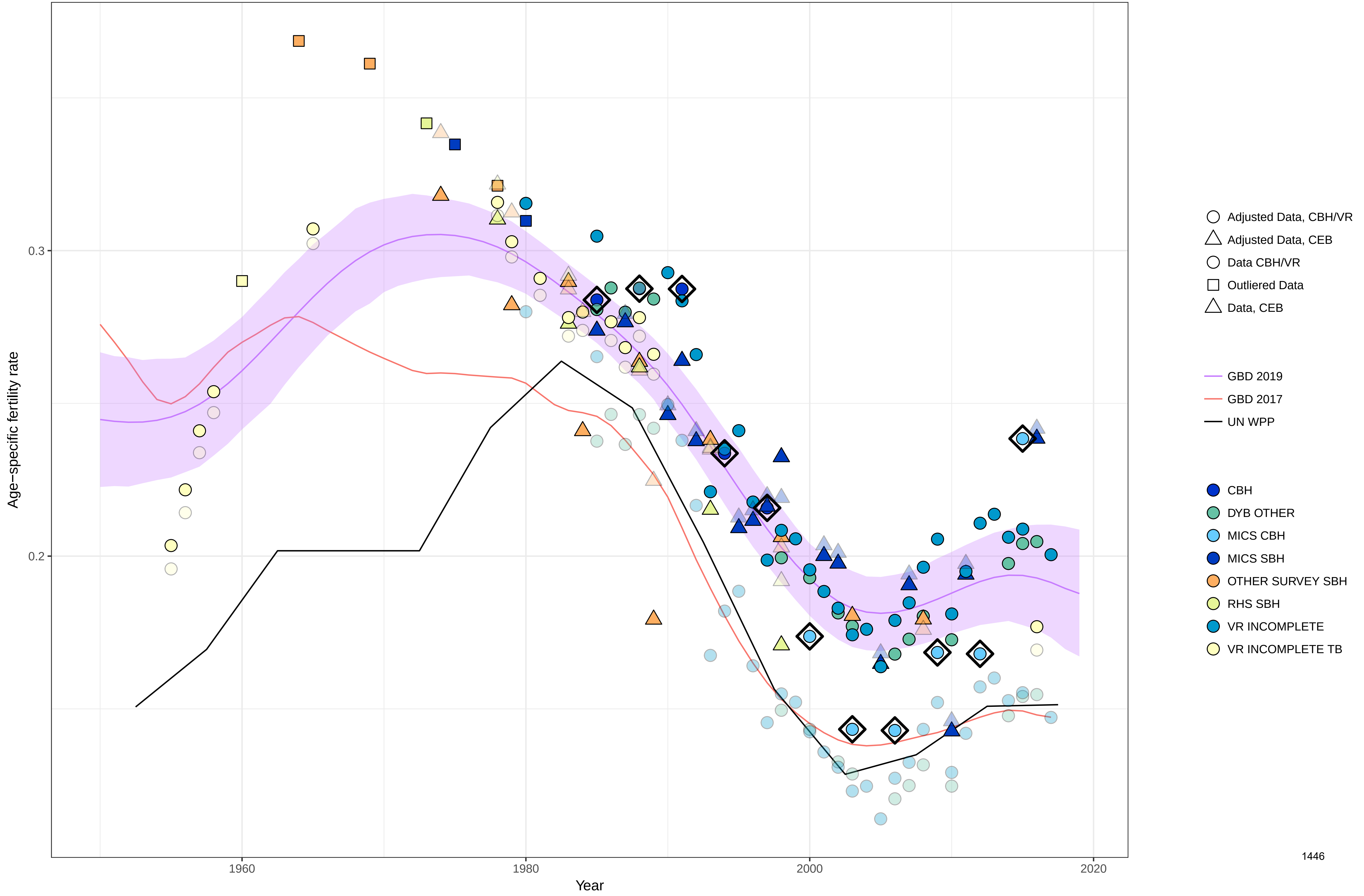

Age−specific fertility fitted trend lines, 1950−2019: Mongolia, Ages: 25−29
Data points enclosed with a diamond are reference sources

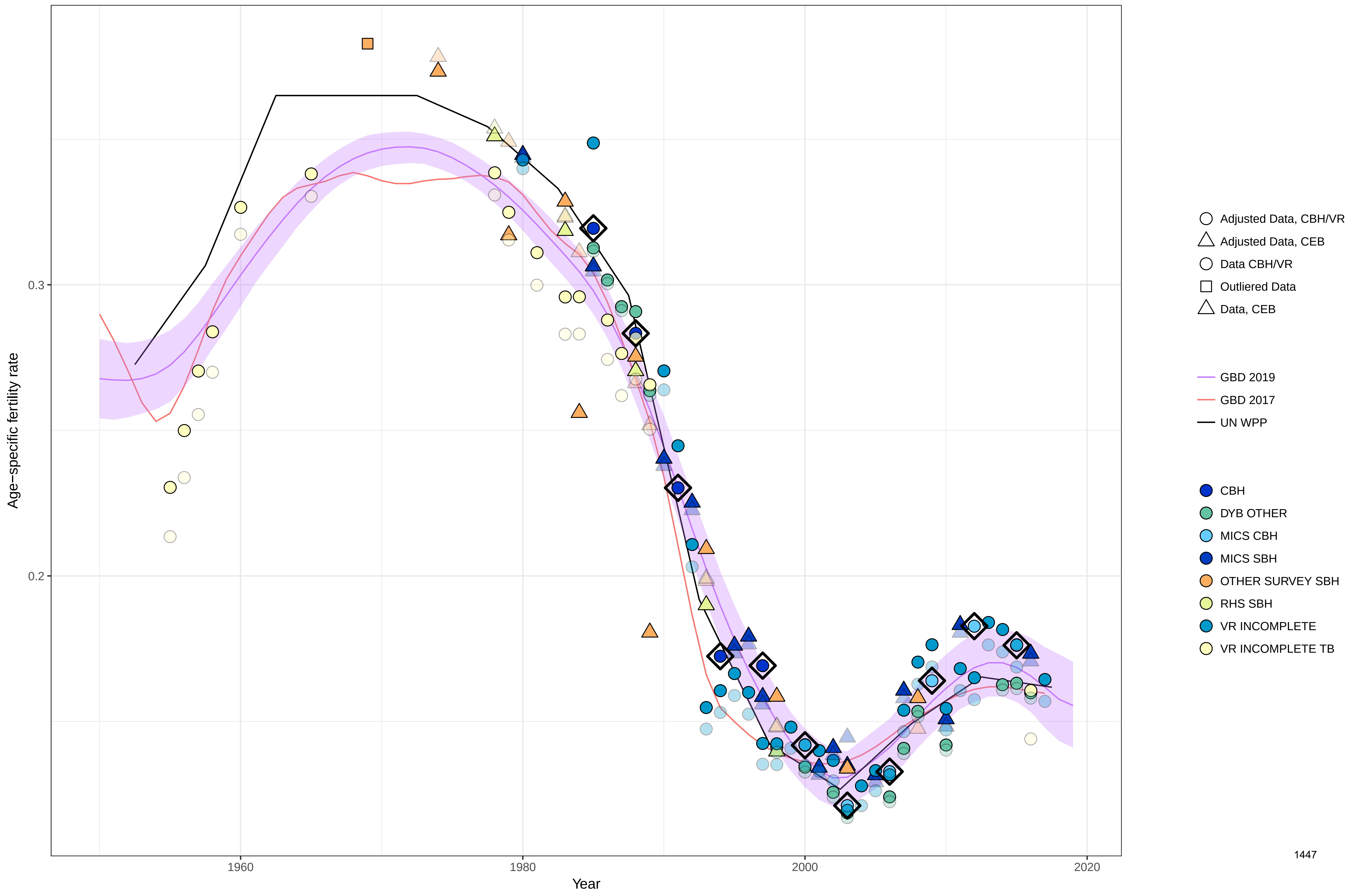

Age−specific fertility fitted trend lines, 1950−2019: Mongolia, Ages: 30−34
Data points enclosed with a diamond are reference sources

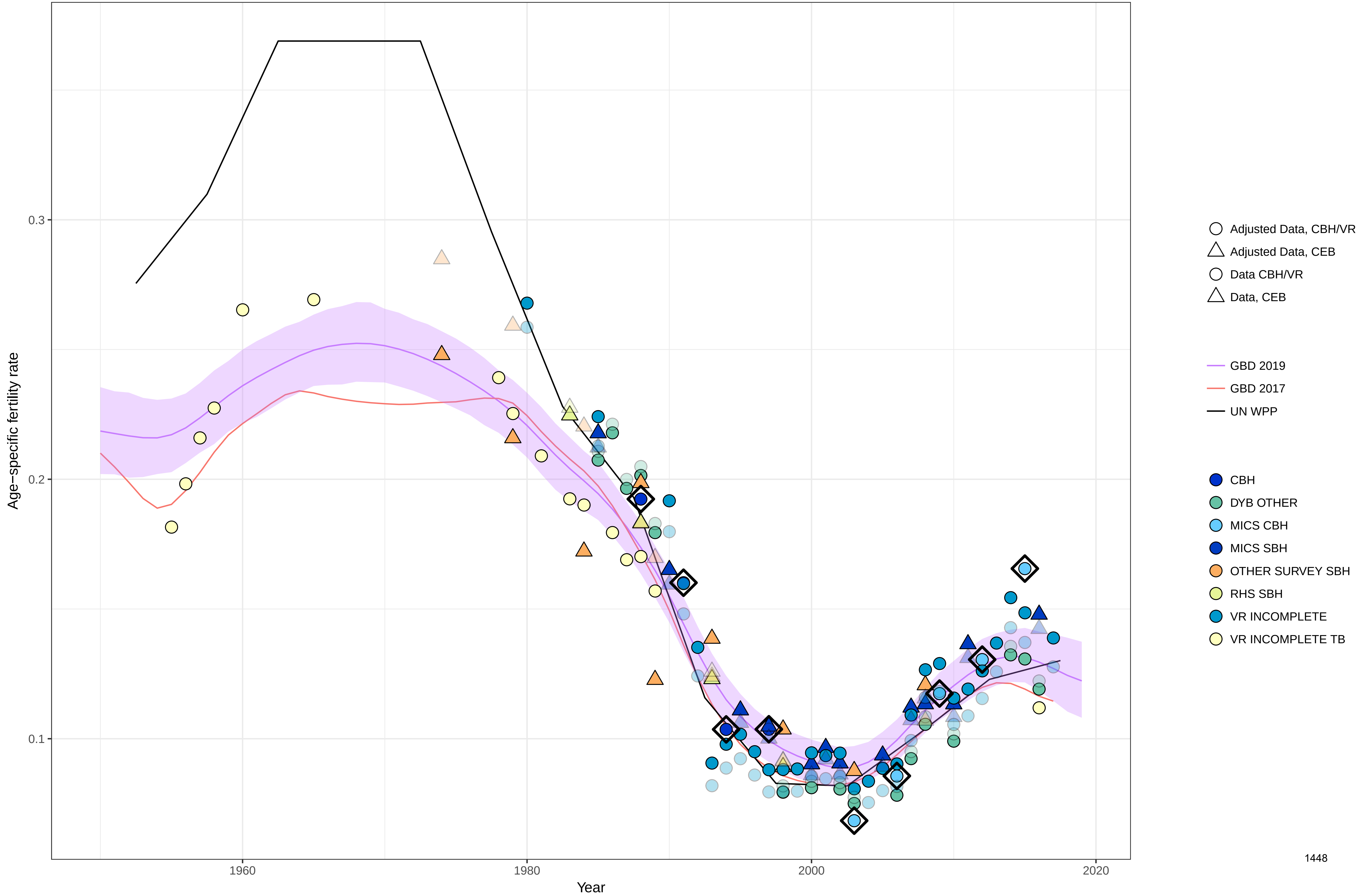

Age−specific fertility fitted trend lines, 1950−2019: Mongolia, Ages: 35−39
Data points enclosed with a diamond are reference sources

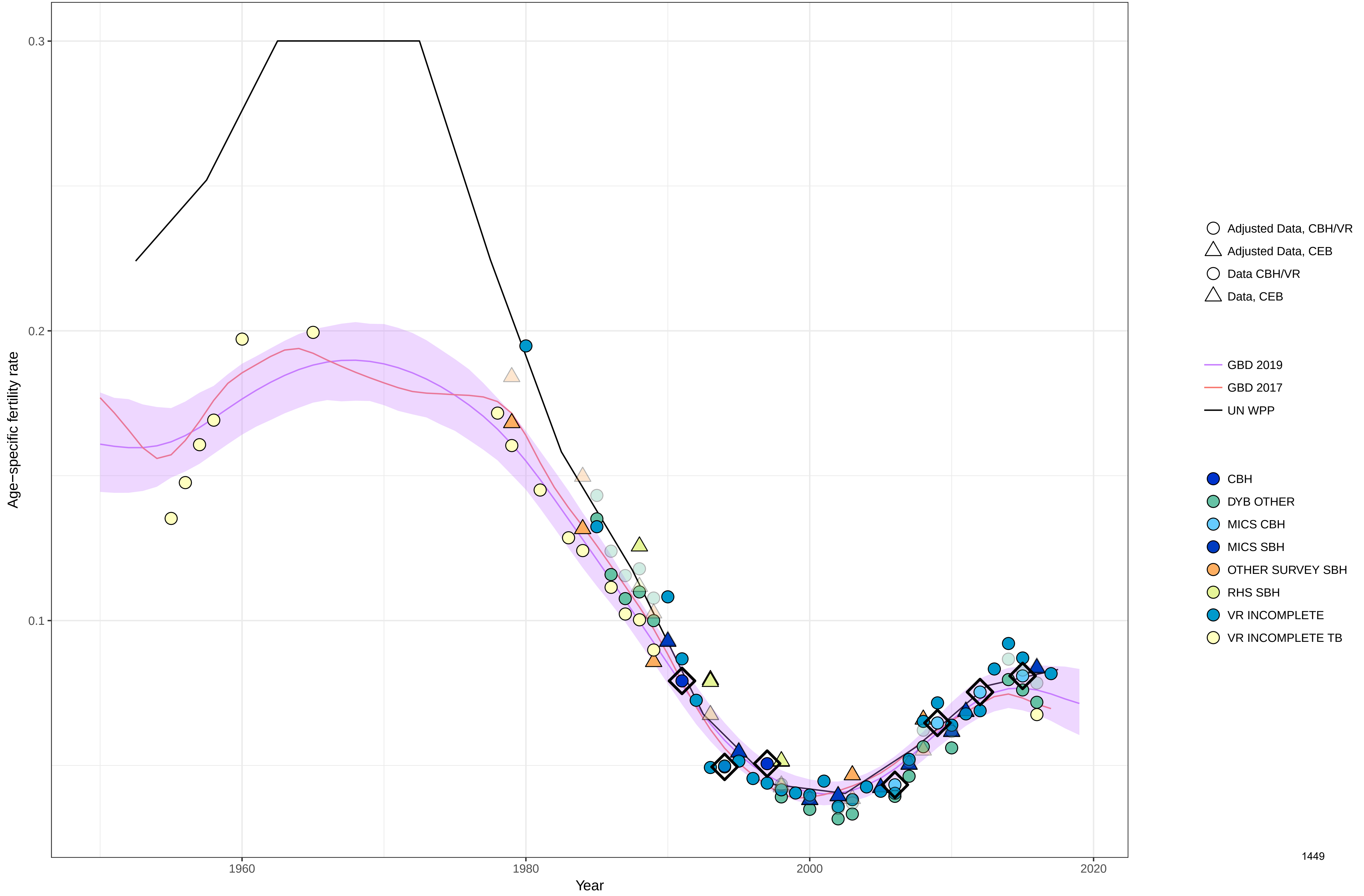

Age−specific fertility fitted trend lines, 1950−2019: Mongolia, Ages: 40−44
Data points enclosed with a diamond are reference sources

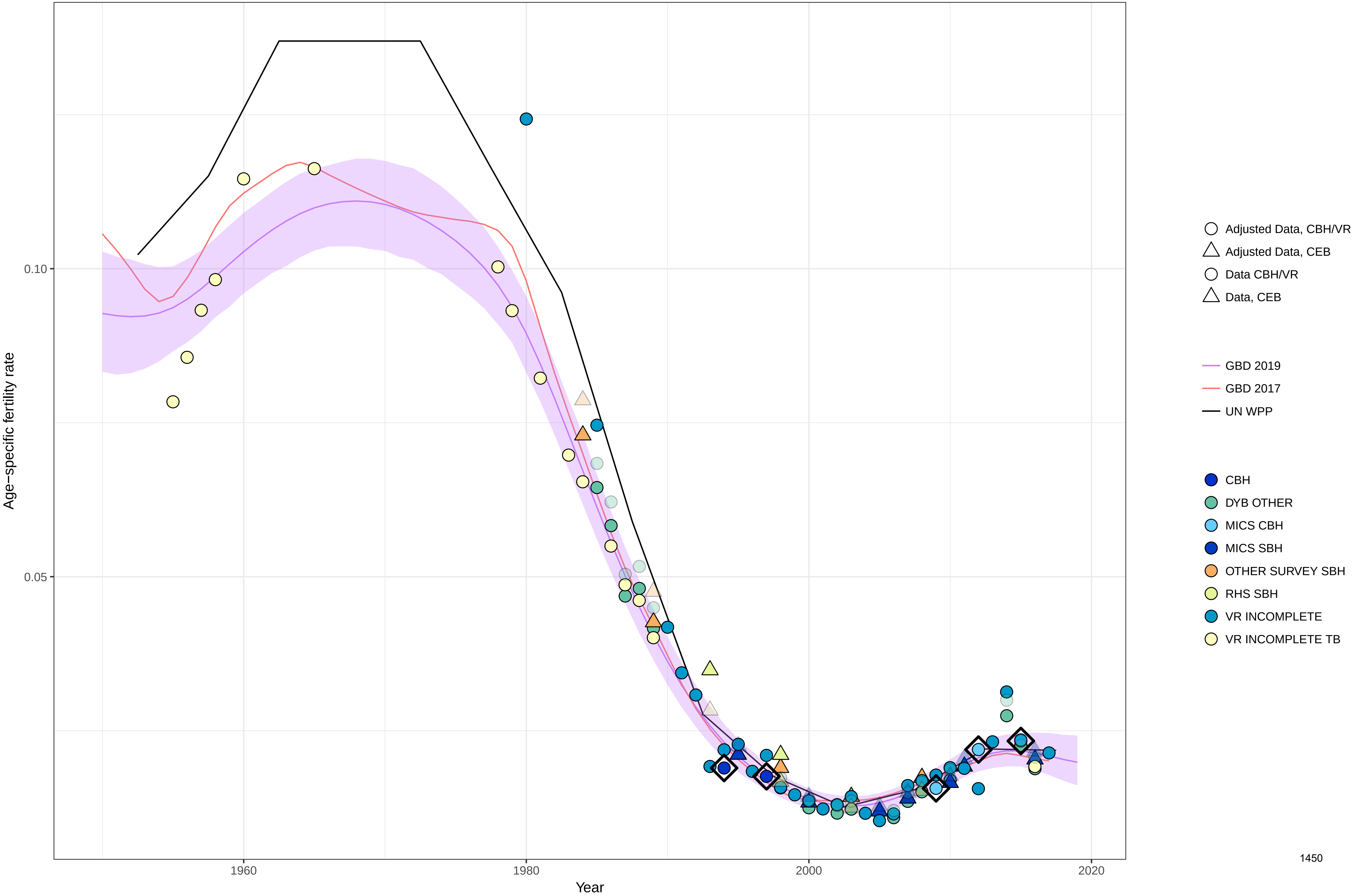

Age−specific fertility fitted trend lines, 1950−2019: Mongolia, Ages: 45−49
Data points enclosed with a diamond are reference sources

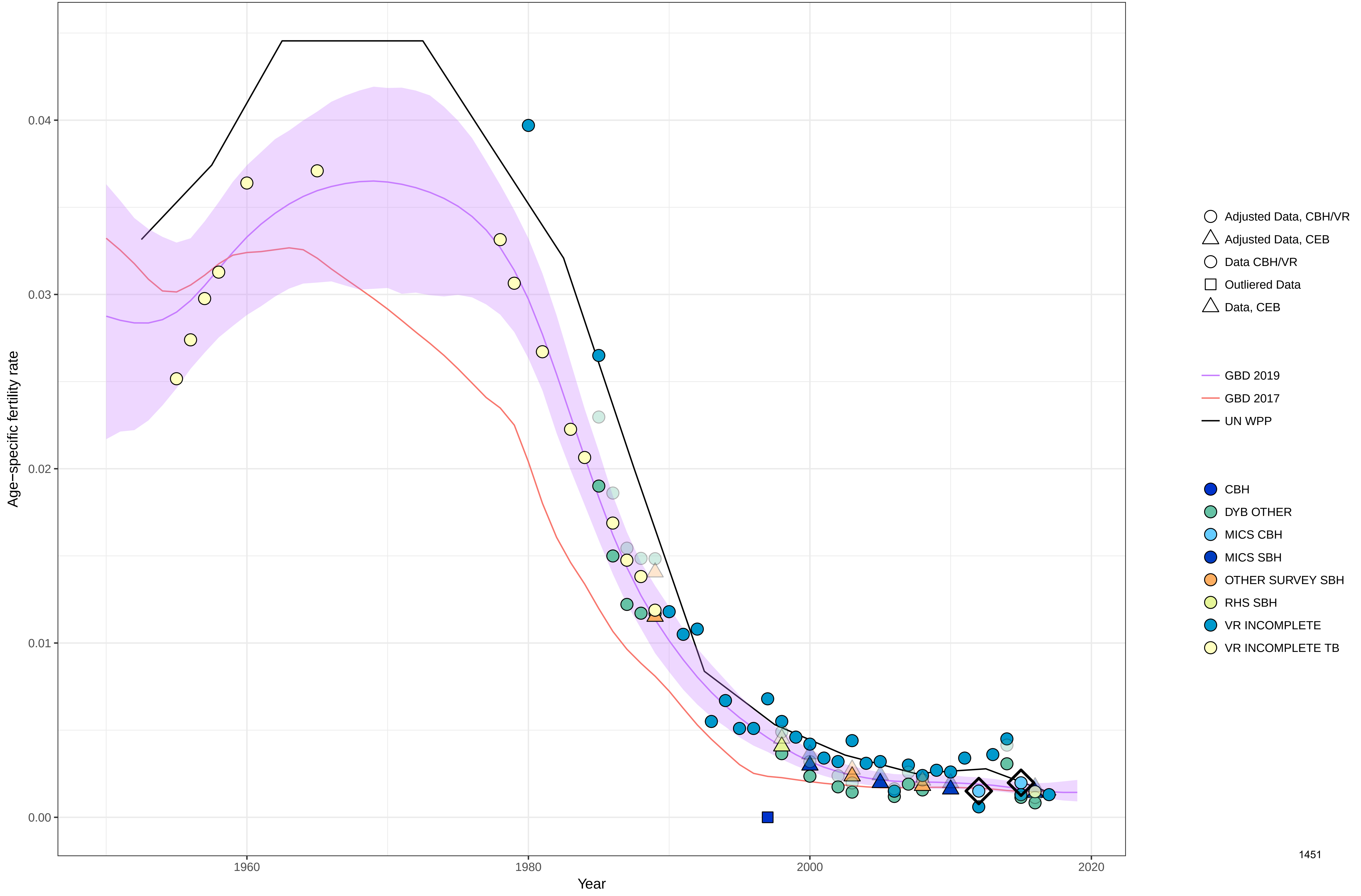

Age−specific fertility fitted trend lines, 1950−2019: Mongolia, Ages: 50−54
Data points enclosed with a diamond are reference sources

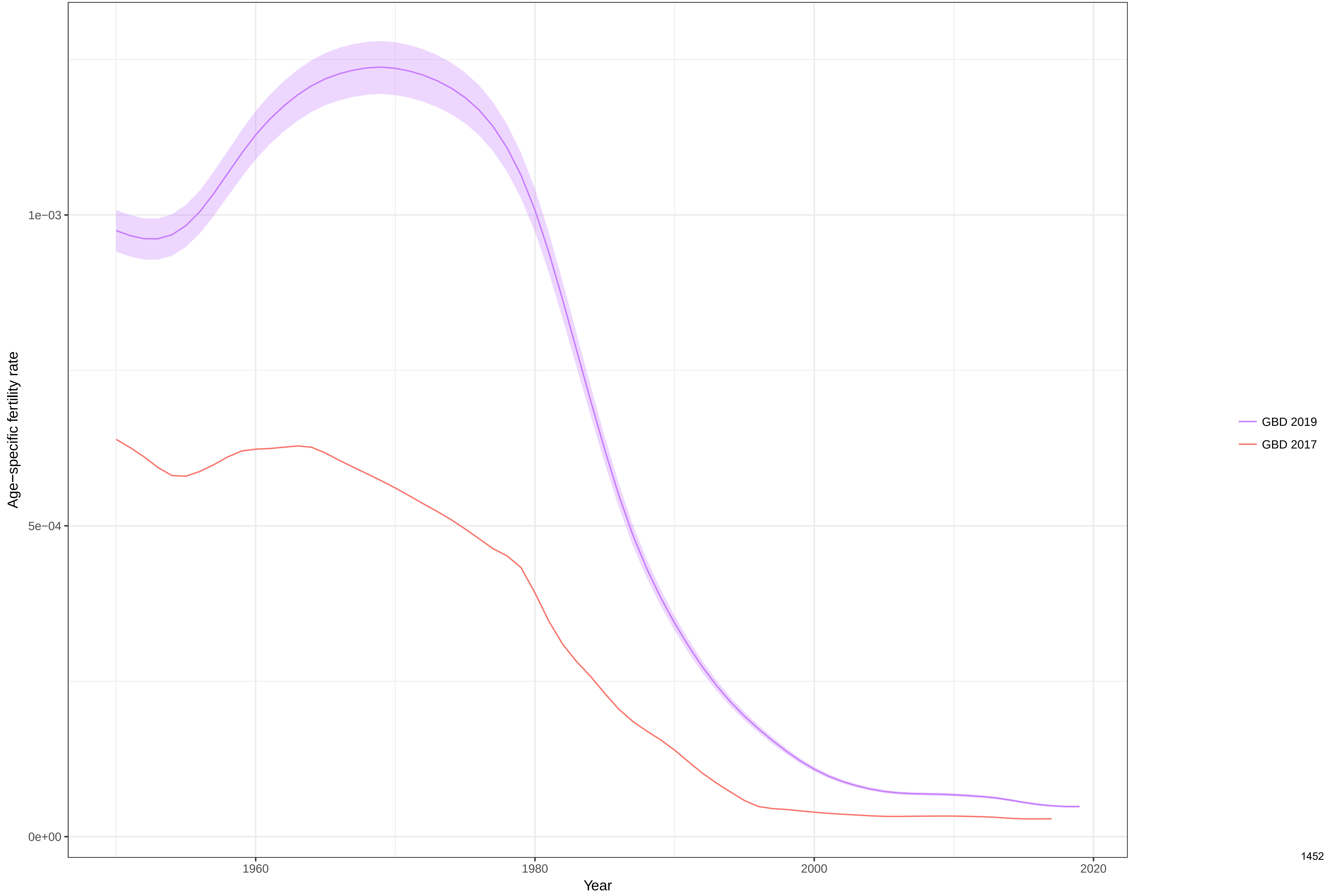

Total fertility rate fitted trend lines, 1950−2019: Mongolia TFR
Data points enclosed with a diamond are reference sources

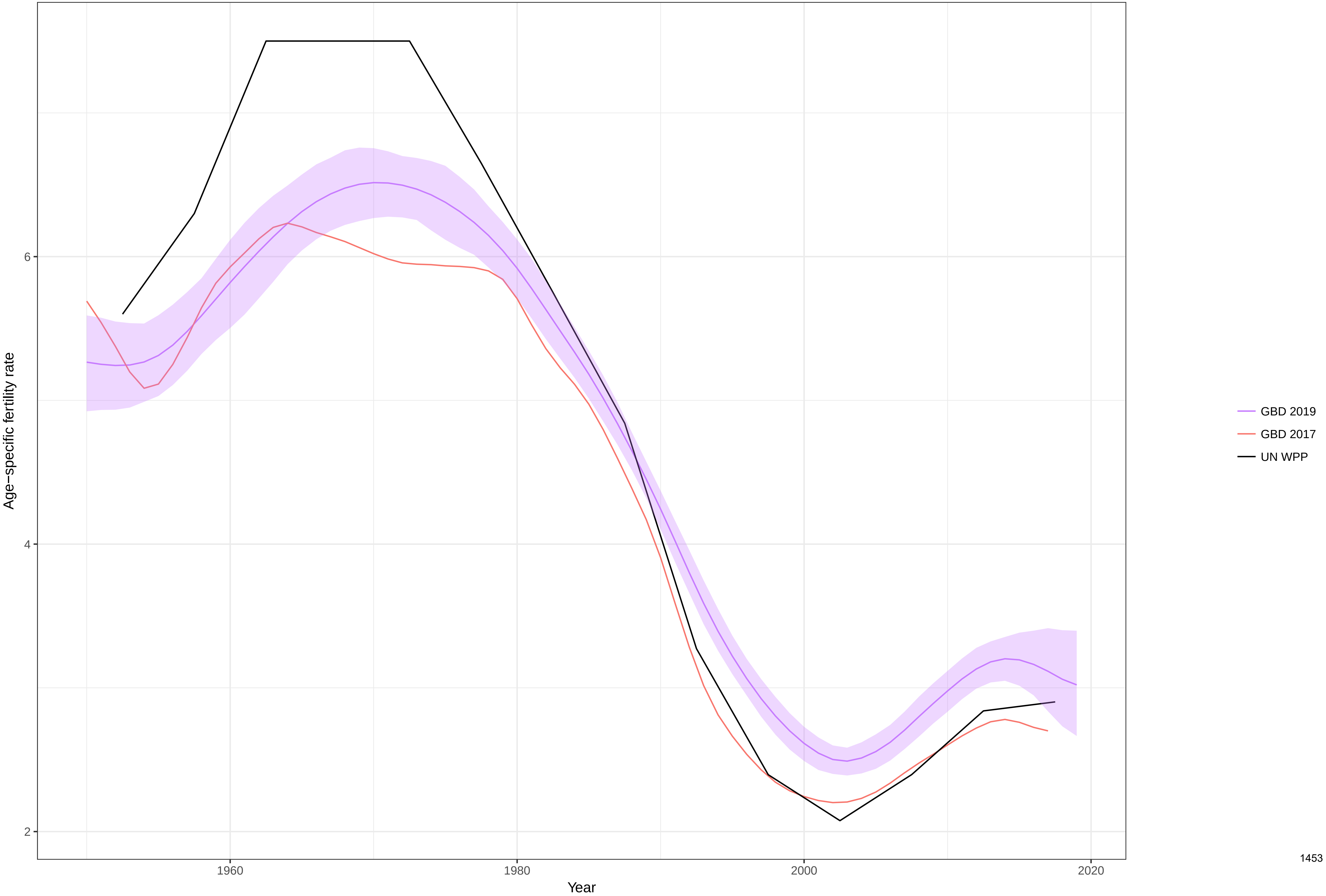

Age−specific fertility fitted trend lines, 1950−2019: Northern Mariana Islands, Ages: 10−14
Data points enclosed with a diamond are reference sources

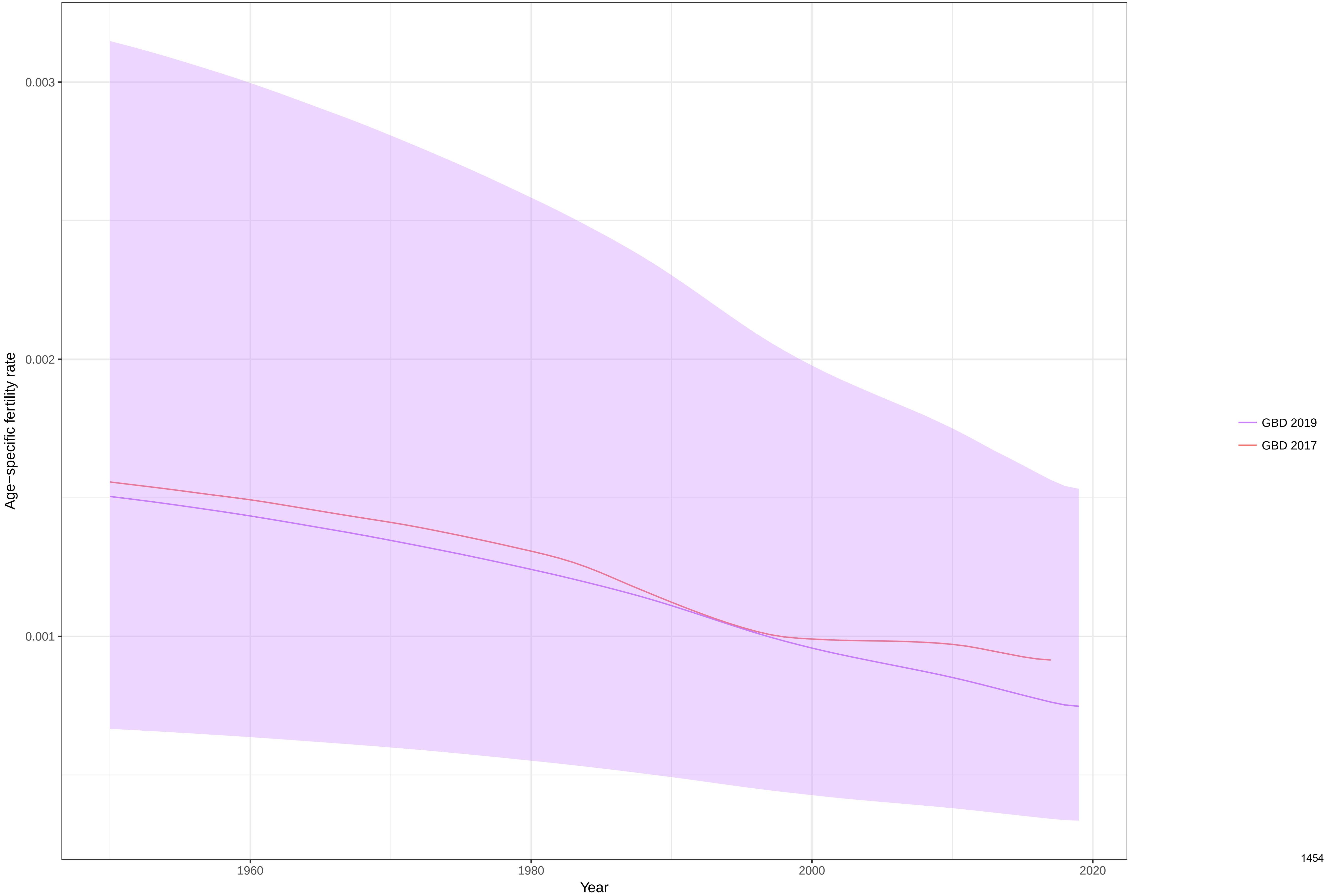

Age−specific fertility fitted trend lines, 1950−2019: Northern Mariana Islands, Ages: 15−19
Data points enclosed with a diamond are reference sources

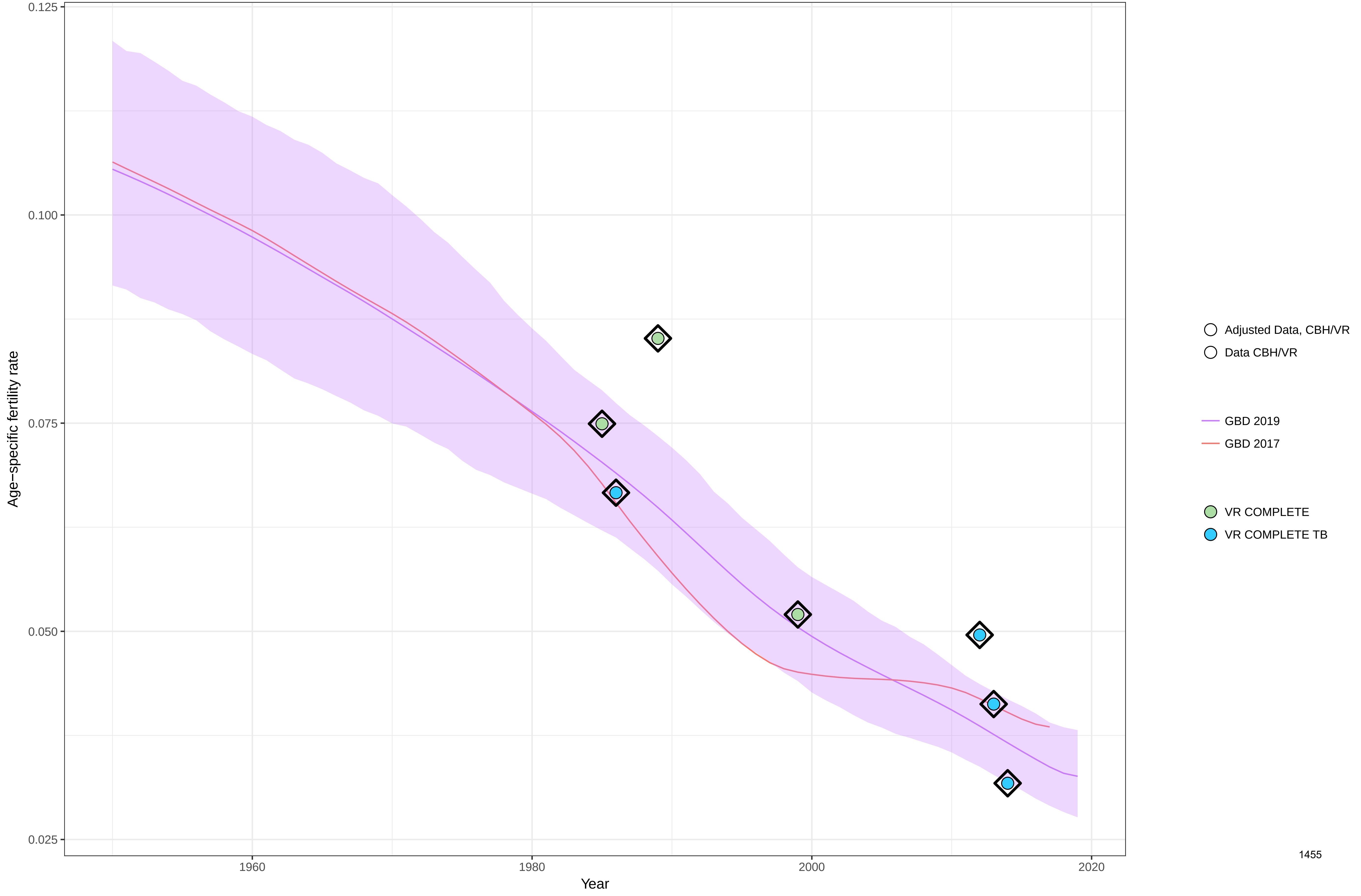

Age−specific fertility fitted trend lines, 1950−2019: Northern Mariana Islands, Ages: 20−24
Data points enclosed with a diamond are reference sources

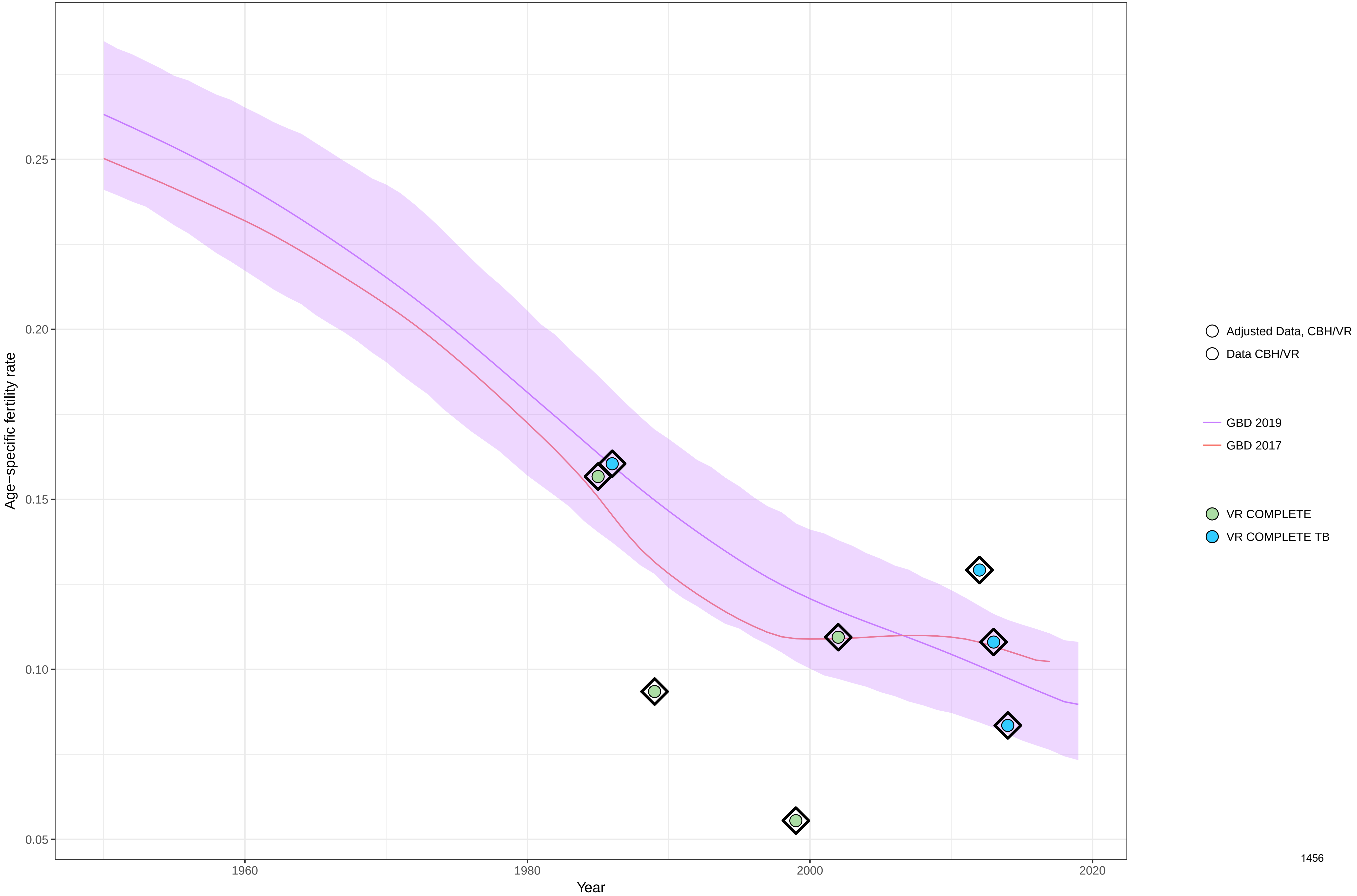

Age−specific fertility fitted trend lines, 1950−2019: Northern Mariana Islands, Ages: 25−29
Data points enclosed with a diamond are reference sources

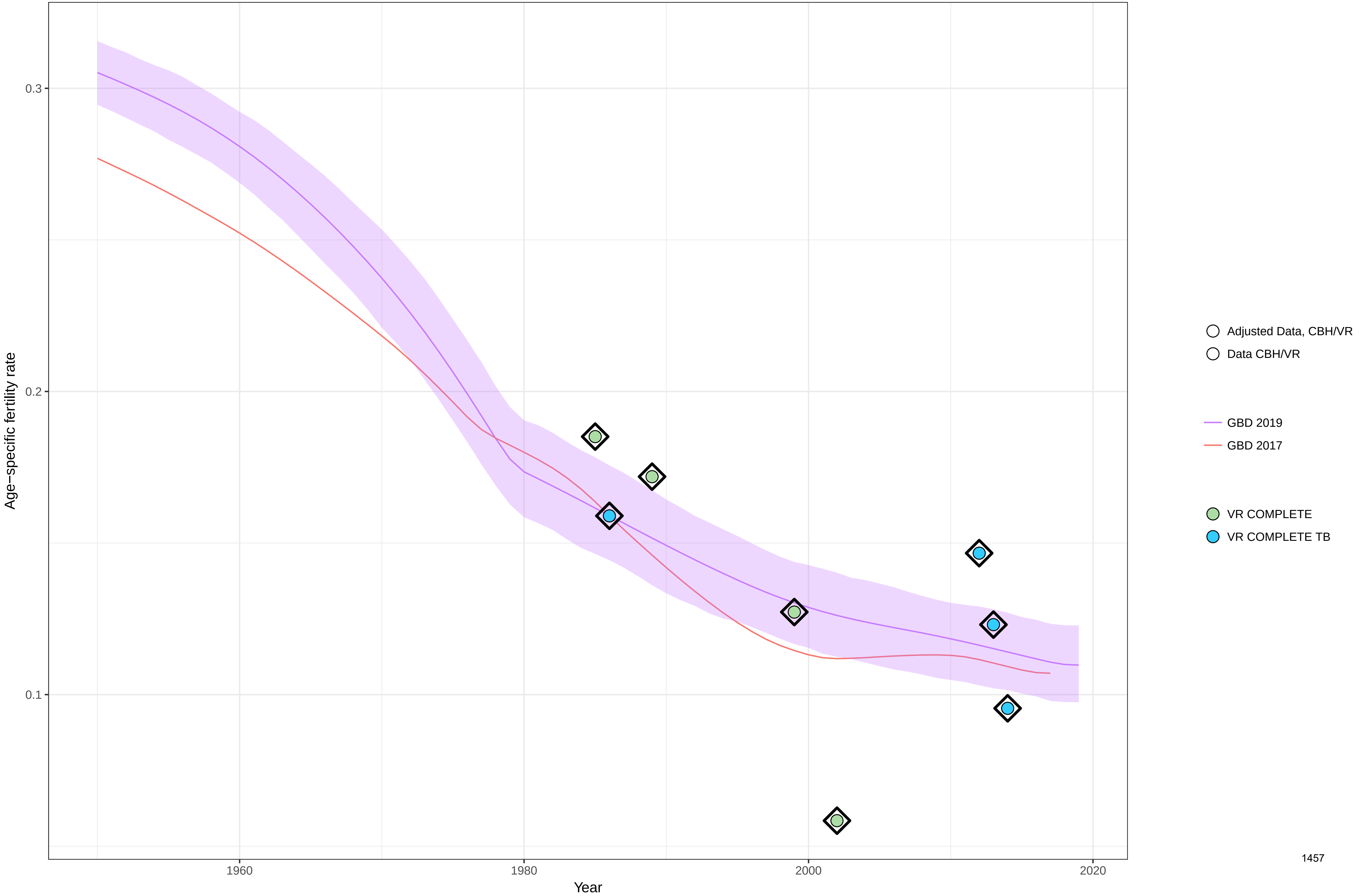

Age−specific fertility fitted trend lines, 1950−2019: Northern Mariana Islands, Ages: 30−34
Data points enclosed with a diamond are reference sources

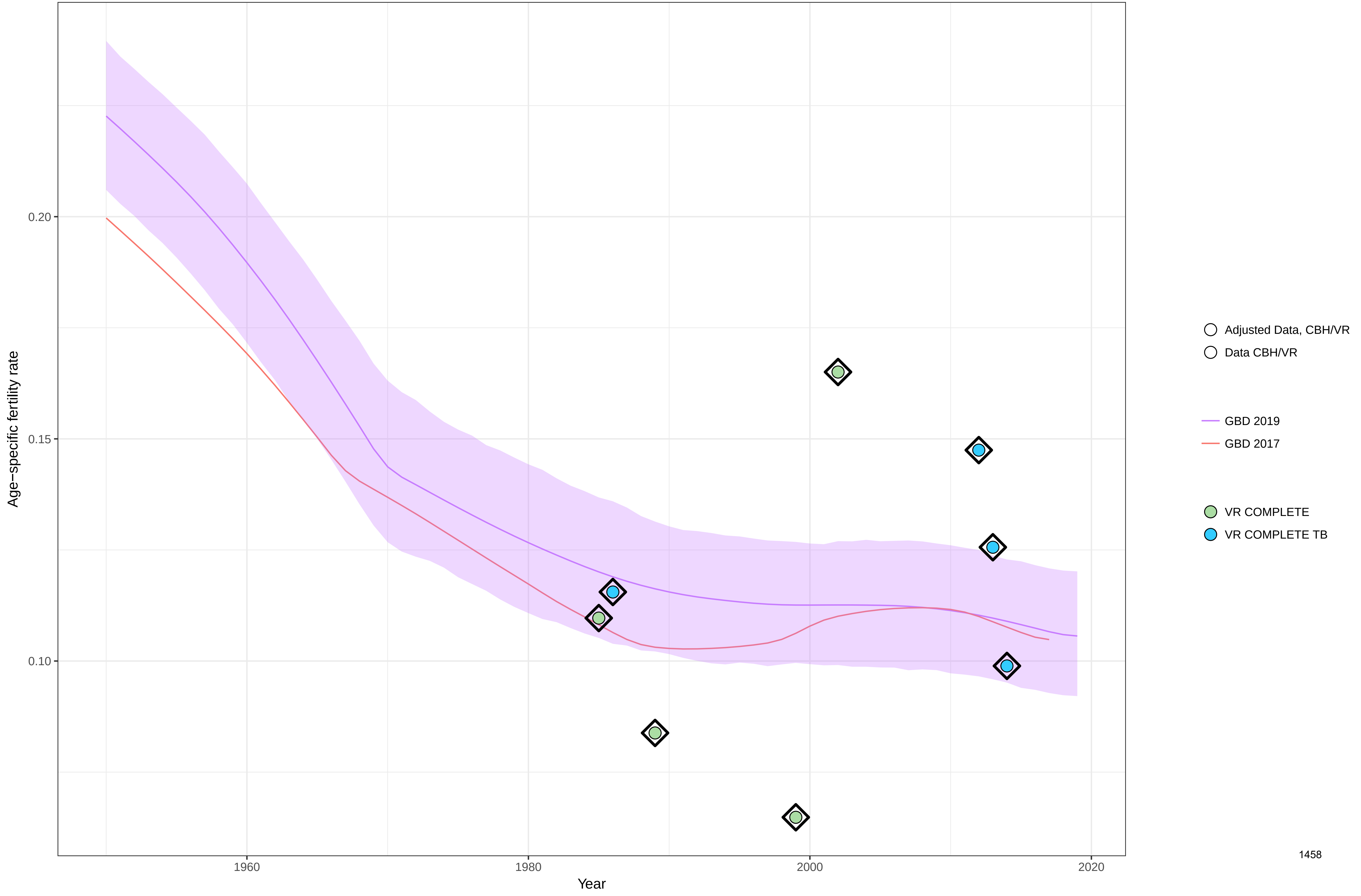

Age−specific fertility fitted trend lines, 1950−2019: Northern Mariana Islands, Ages: 35−39
Data points enclosed with a diamond are reference sources

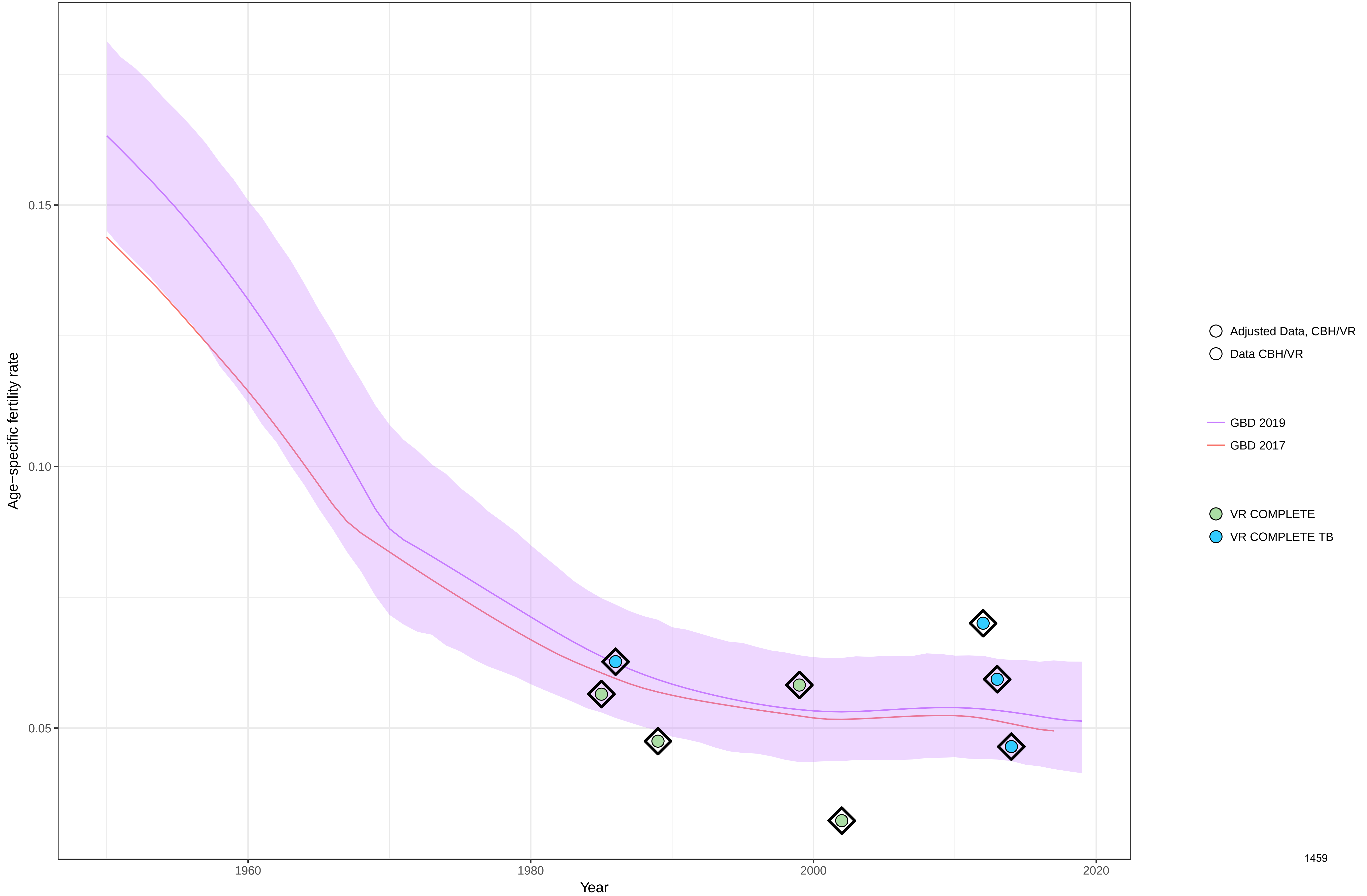

Age−specific fertility fitted trend lines, 1950−2019: Northern Mariana Islands, Ages: 40−44
Data points enclosed with a diamond are reference sources

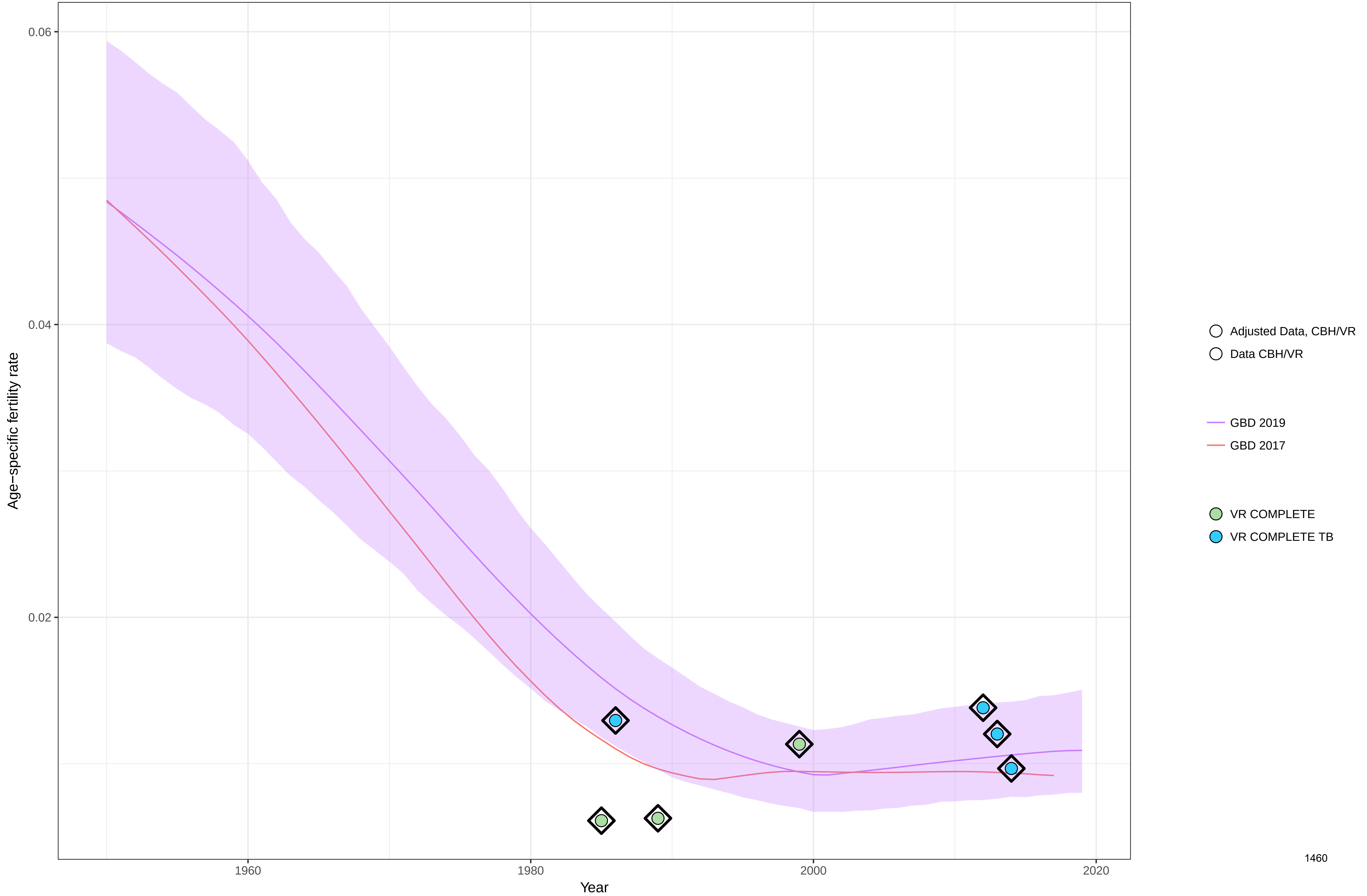

Age−specific fertility fitted trend lines, 1950−2019: Northern Mariana Islands, Ages: 45−49
Data points enclosed with a diamond are reference sources

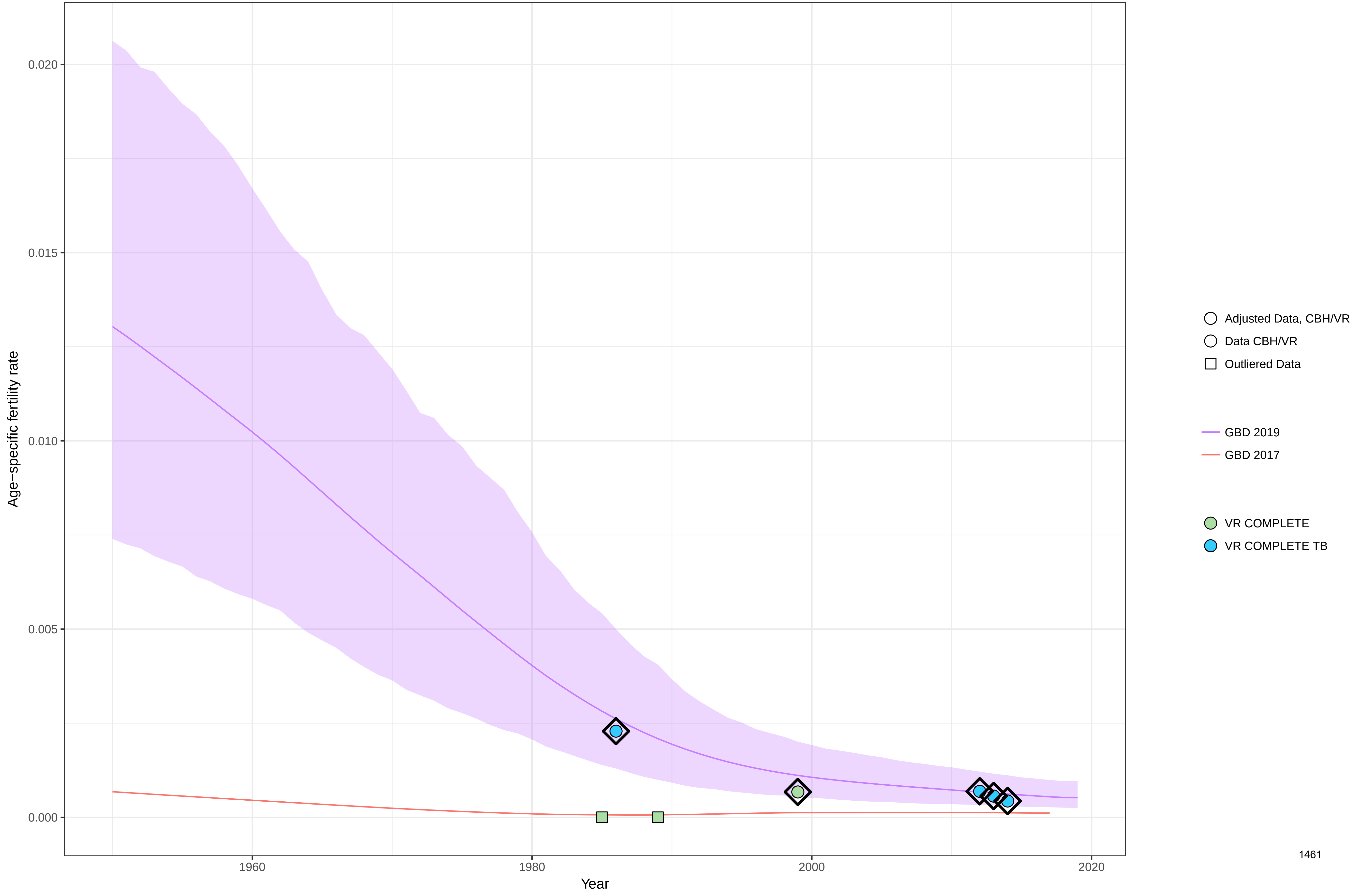

Age−specific fertility fitted trend lines, 1950−2019: Northern Mariana Islands, Ages: 50−54
Data points enclosed with a diamond are reference sources

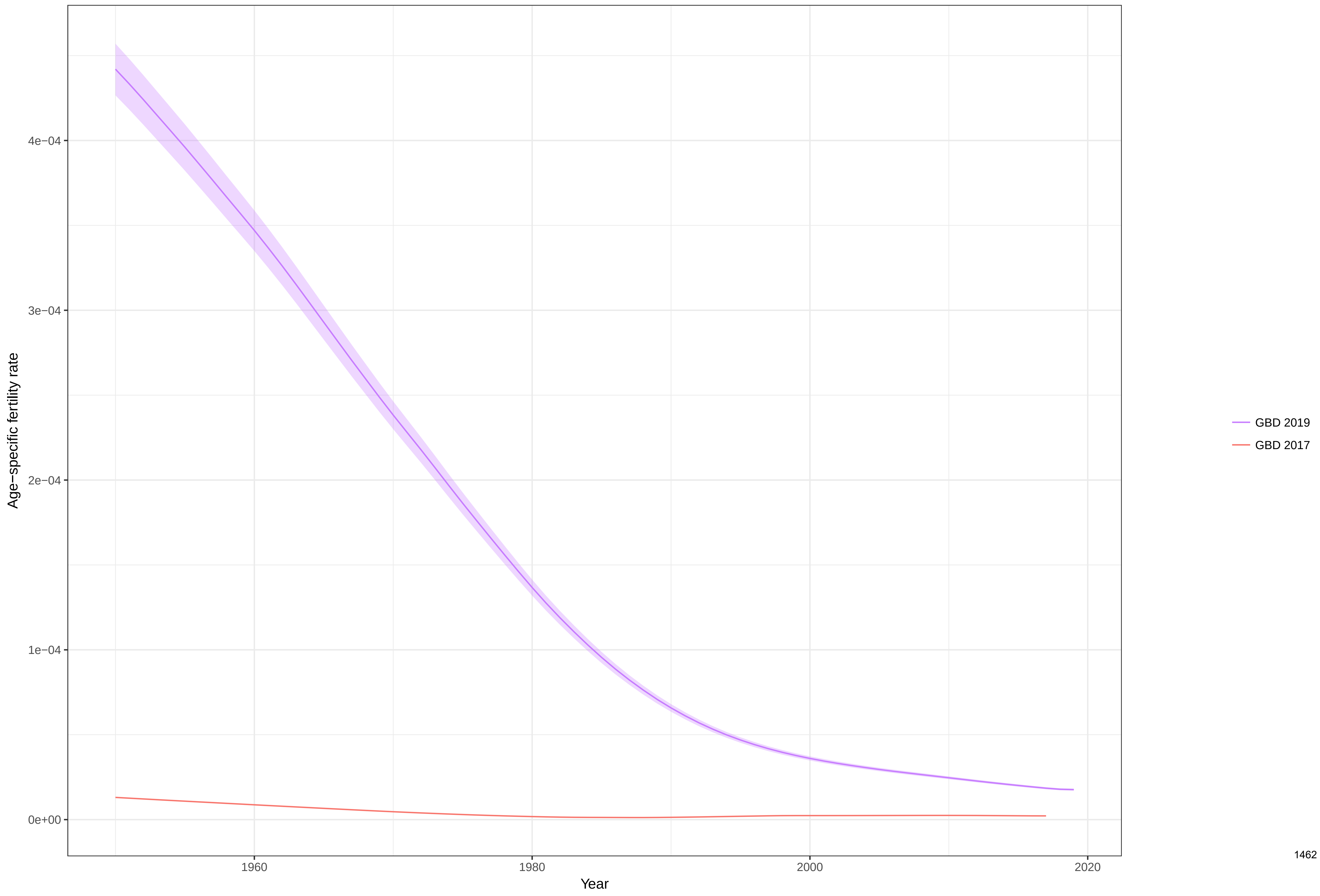

Total fertility rate fitted trend lines, 1950−2019: Northern Mariana Islands TFR
Data points enclosed with a diamond are reference sources

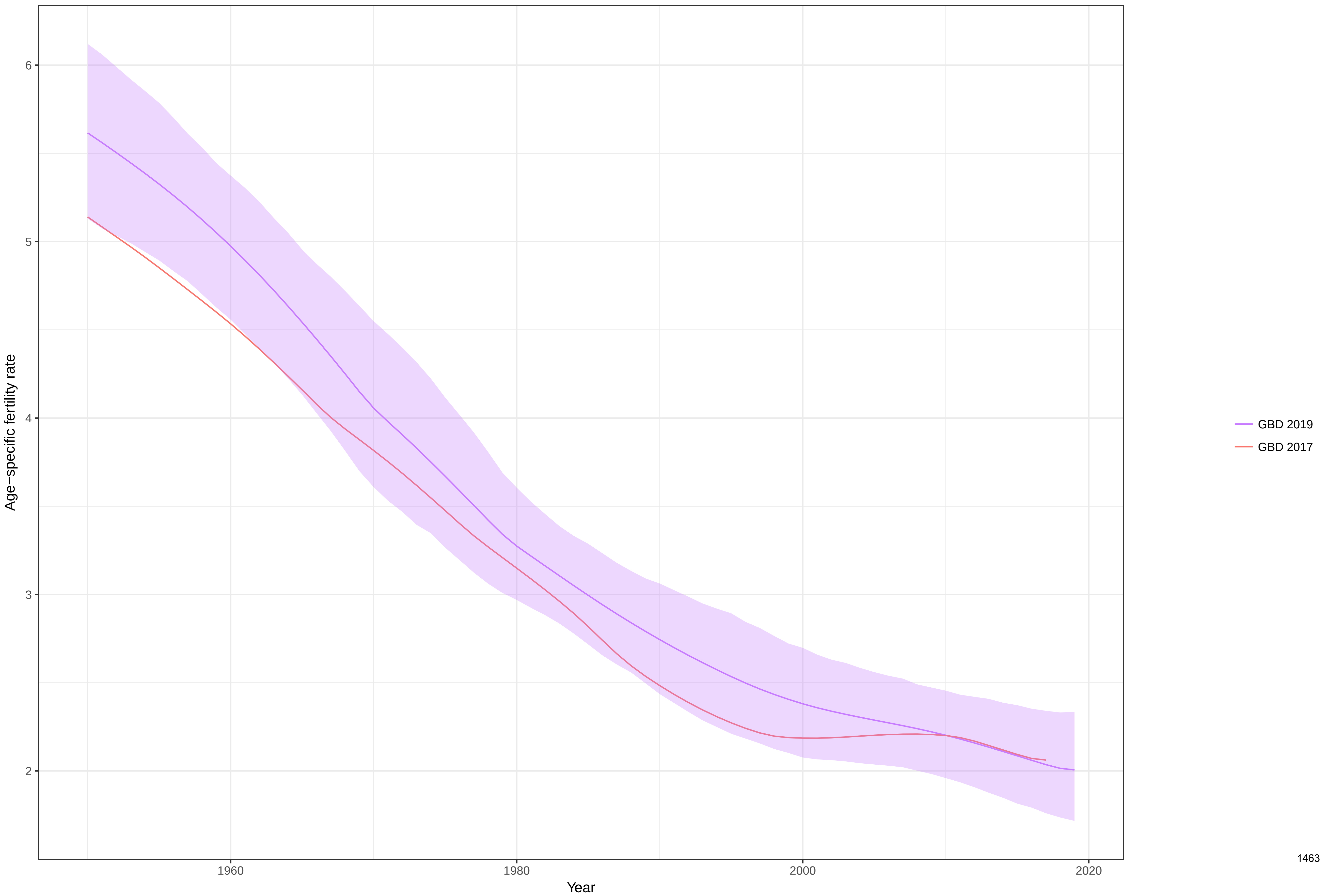

Age−specific fertility fitted trend lines, 1950−2019: Mozambique, Ages: 10−14
Data points enclosed with a diamond are reference sources

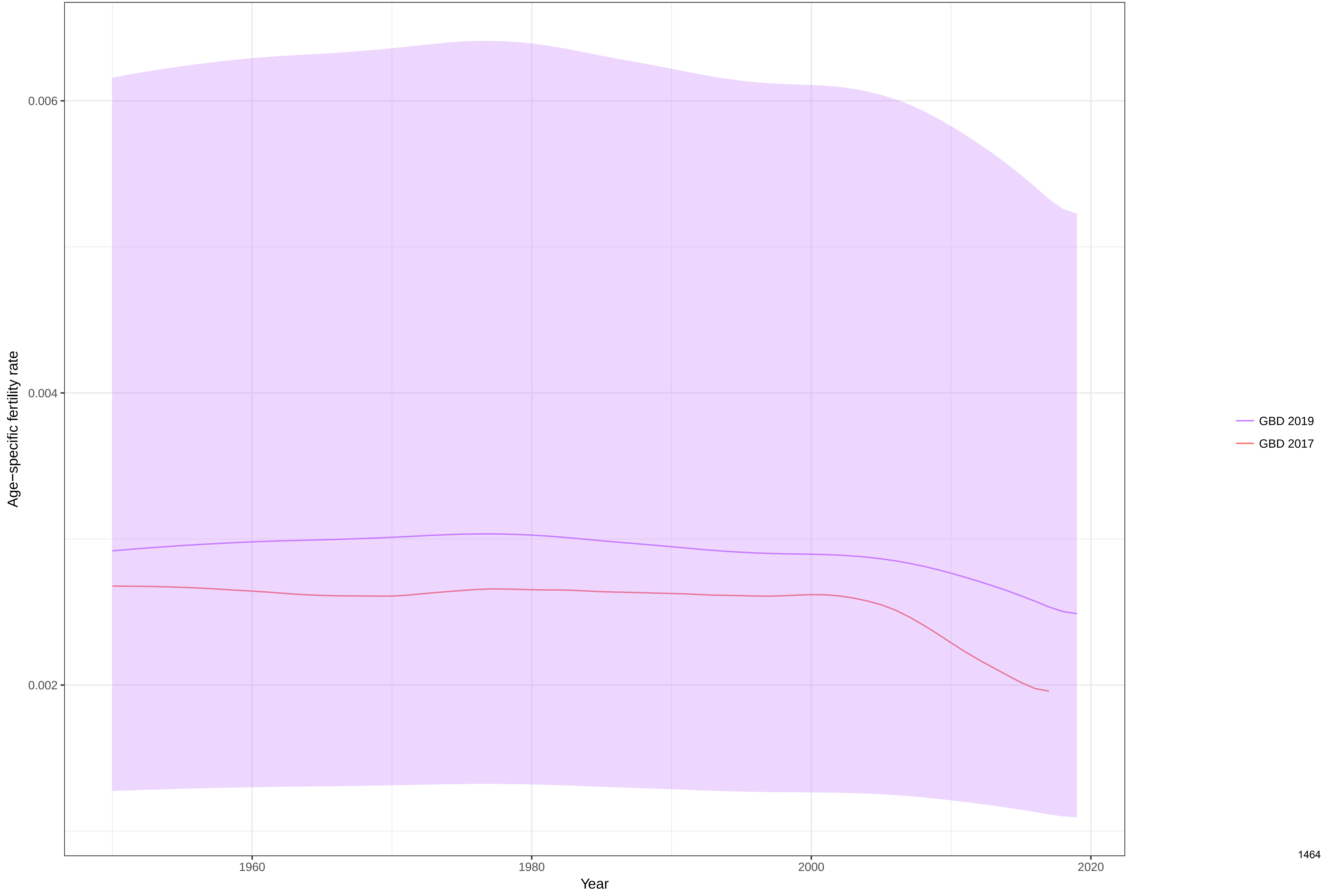

Age−specific fertility fitted trend lines, 1950−2019: Mozambique, Ages: 15−19
Data points enclosed with a diamond are reference sources

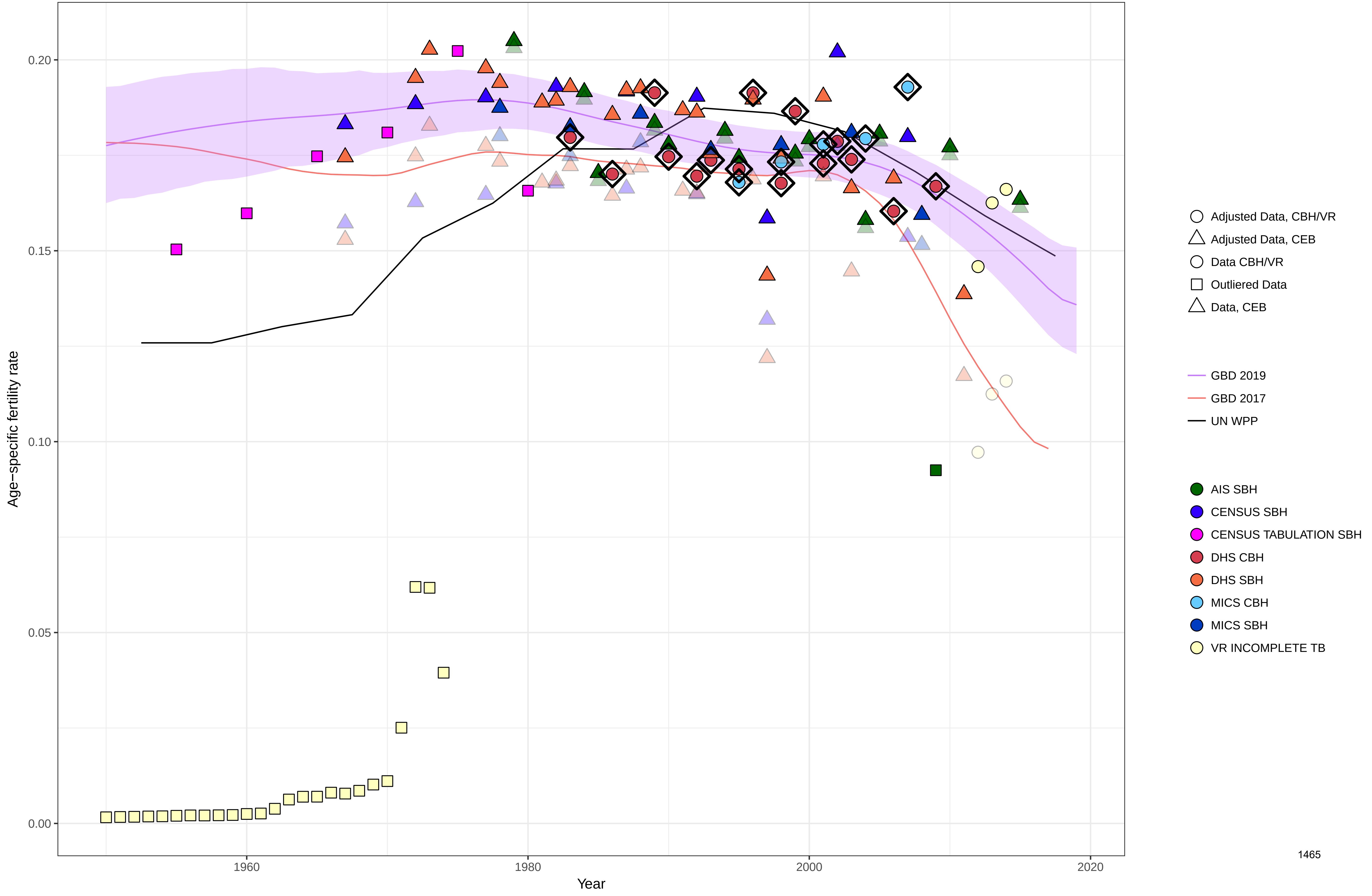

Age−specific fertility fitted trend lines, 1950−2019: Mozambique, Ages: 20−24
Data points enclosed with a diamond are reference sources

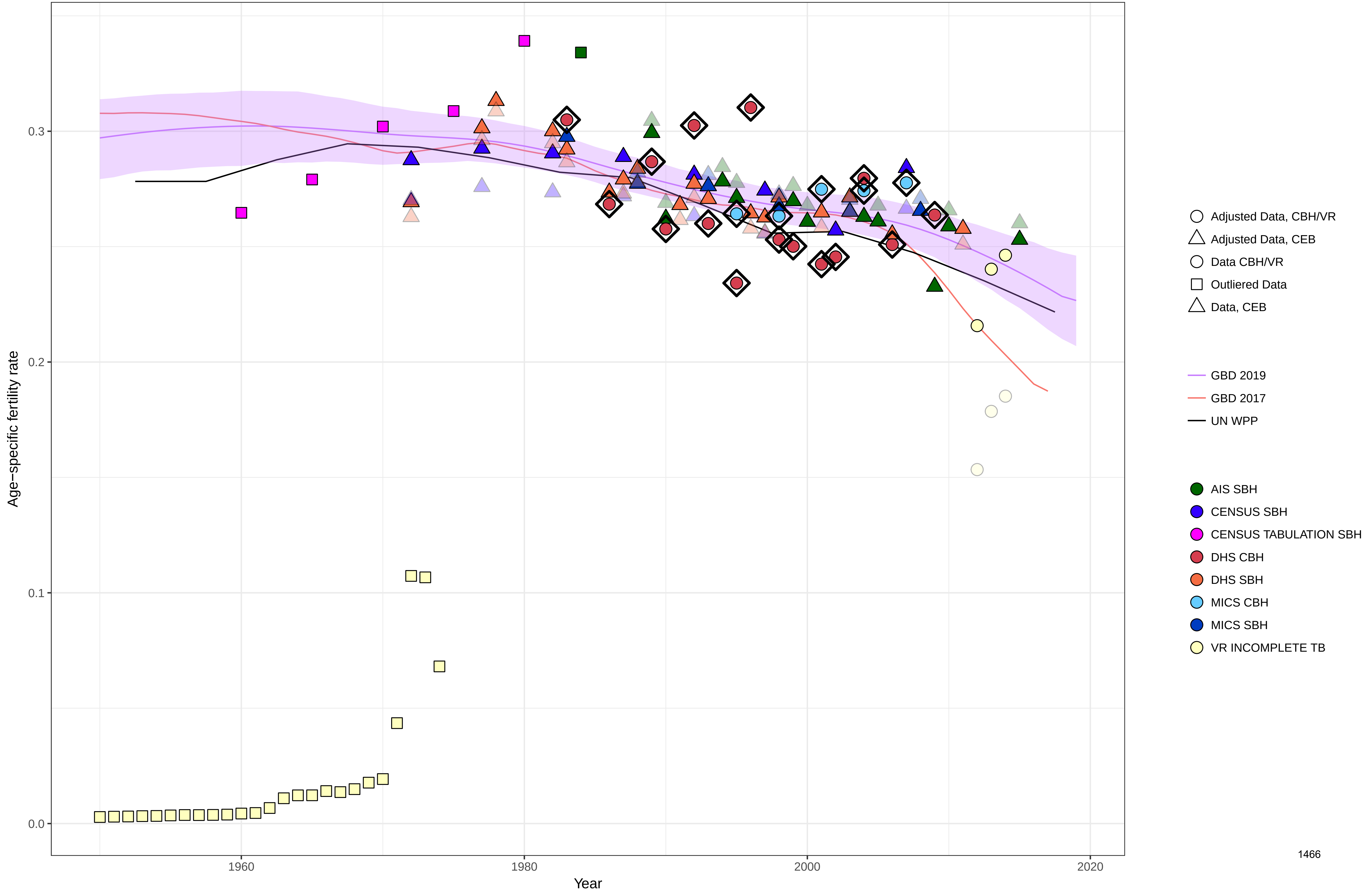

Age−specific fertility fitted trend lines, 1950−2019: Mozambique, Ages: 25−29
Data points enclosed with a diamond are reference sources

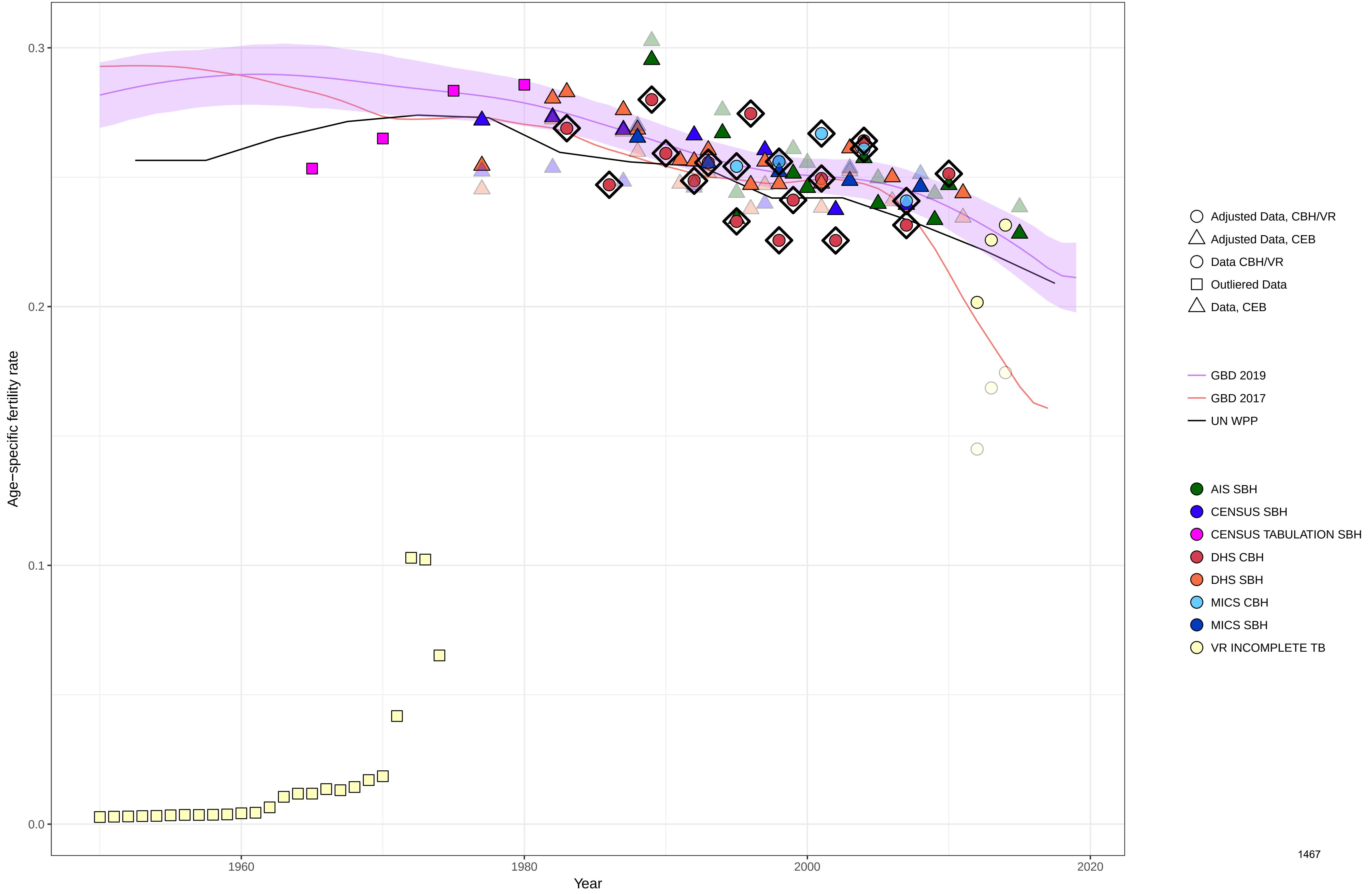

Age−specific fertility fitted trend lines, 1950−2019: Mozambique, Ages: 30−34
Data points enclosed with a diamond are reference sources

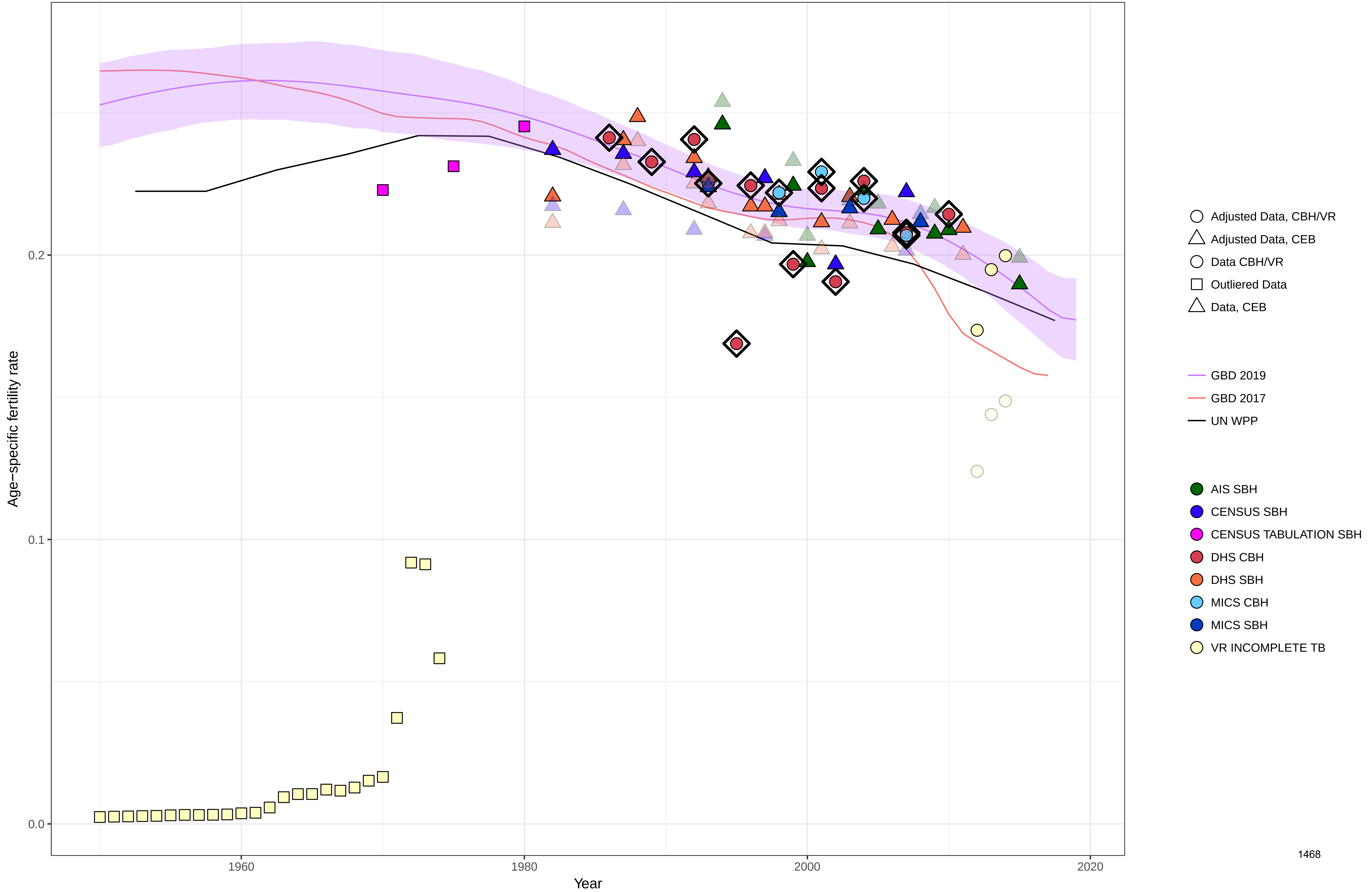

Age−specific fertility fitted trend lines, 1950−2019: Mozambique, Ages: 35−39
Data points enclosed with a diamond are reference sources

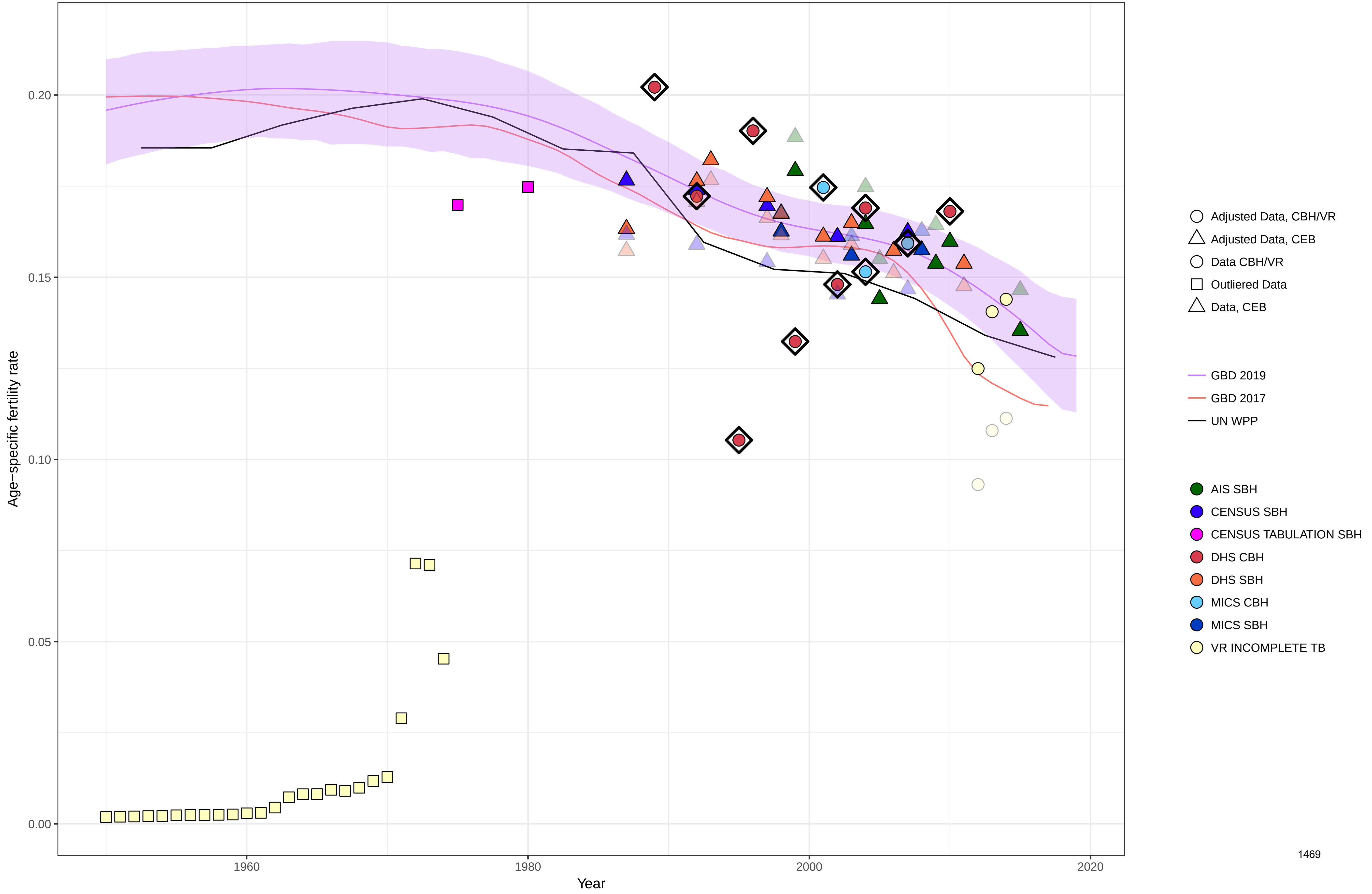

Age−specific fertility fitted trend lines, 1950−2019: Mozambique, Ages: 40−44
Data points enclosed with a diamond are reference sources

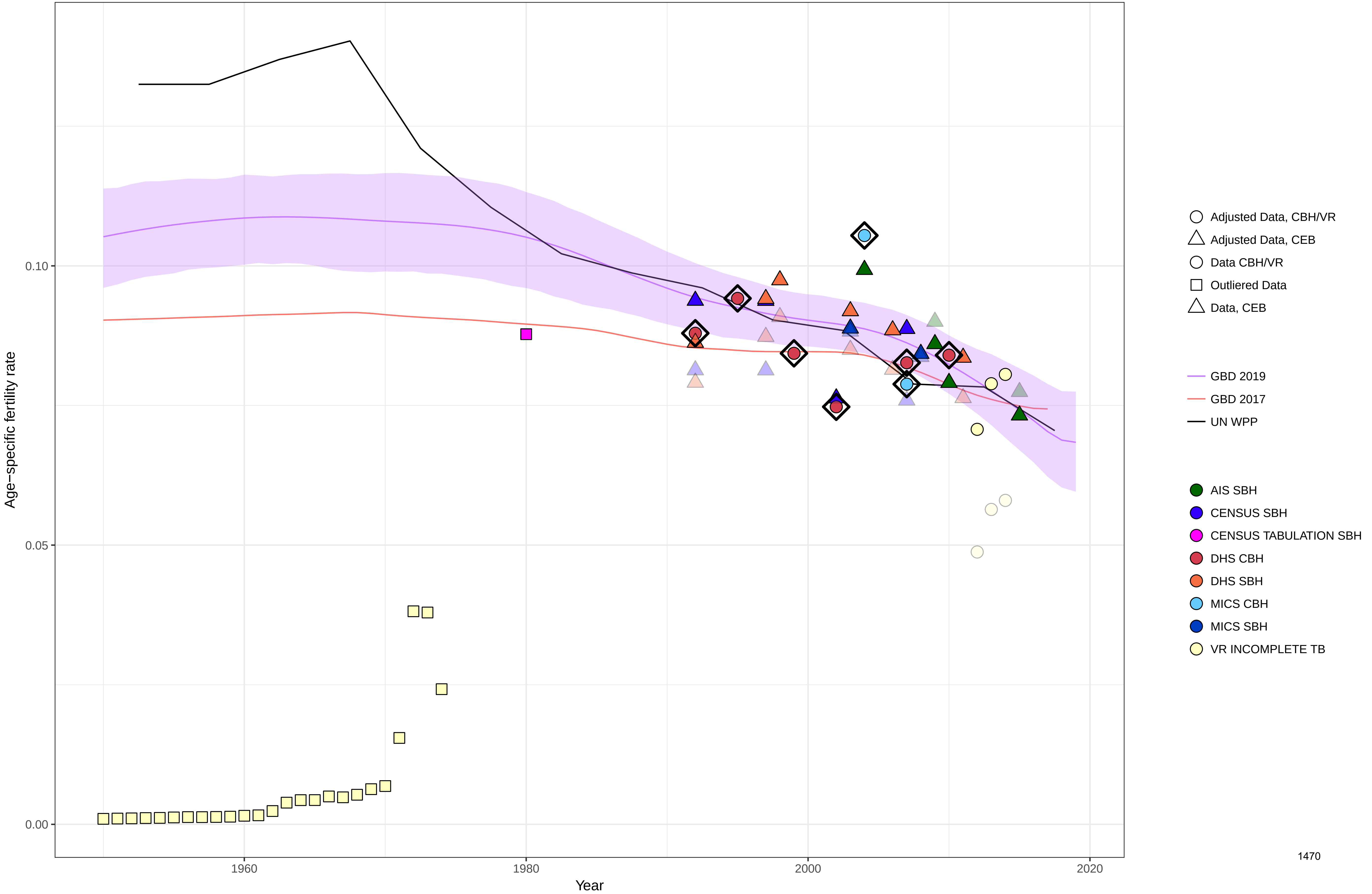

Age−specific fertility fitted trend lines, 1950−2019: Mozambique, Ages: 45−49
Data points enclosed with a diamond are reference sources

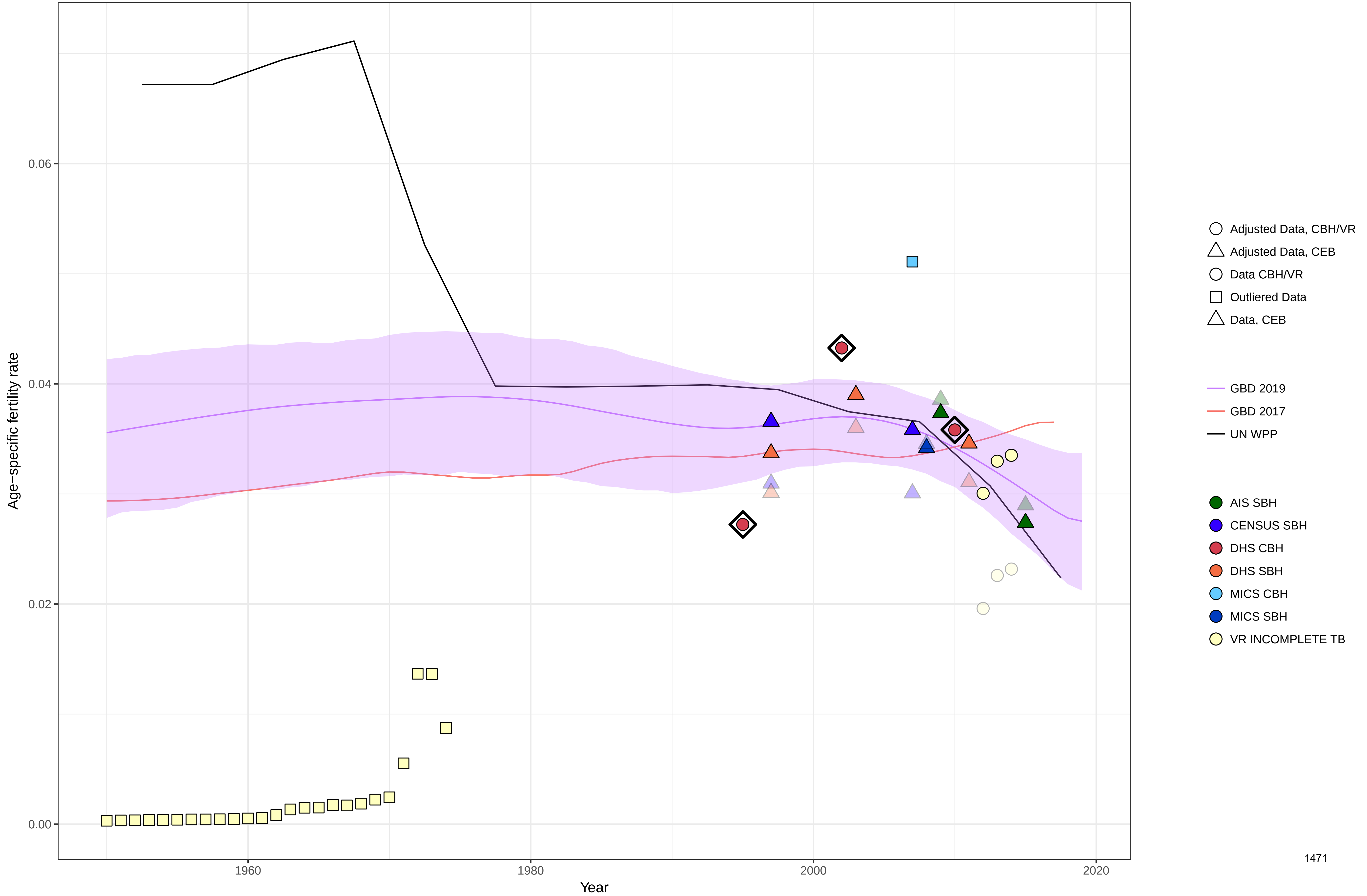

Age−specific fertility fitted trend lines, 1950−2019: Mozambique, Ages: 50−54
Data points enclosed with a diamond are reference sources

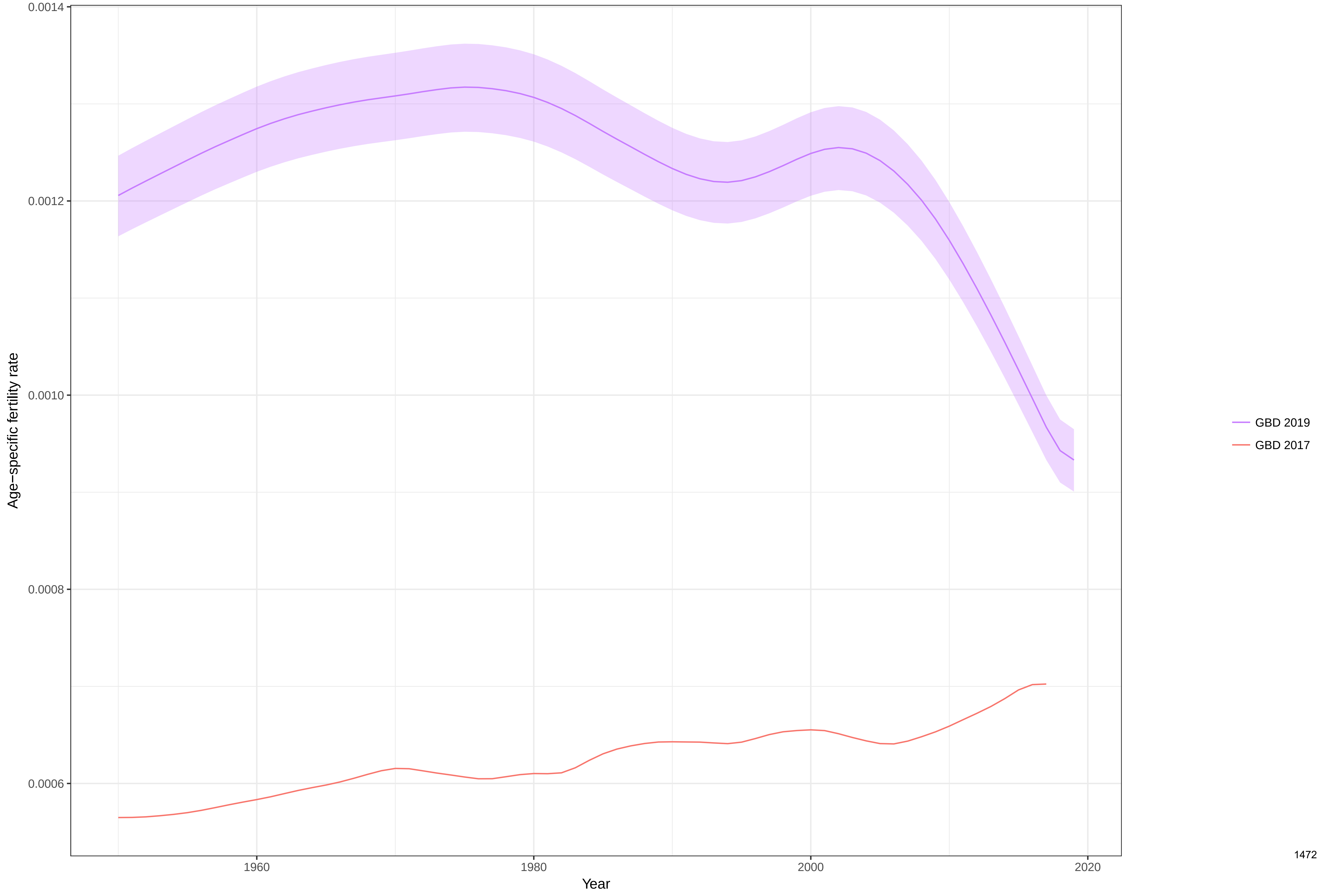

Total fertility rate fitted trend lines, 1950−2019: Mozambique TFR
Data points enclosed with a diamond are reference sources

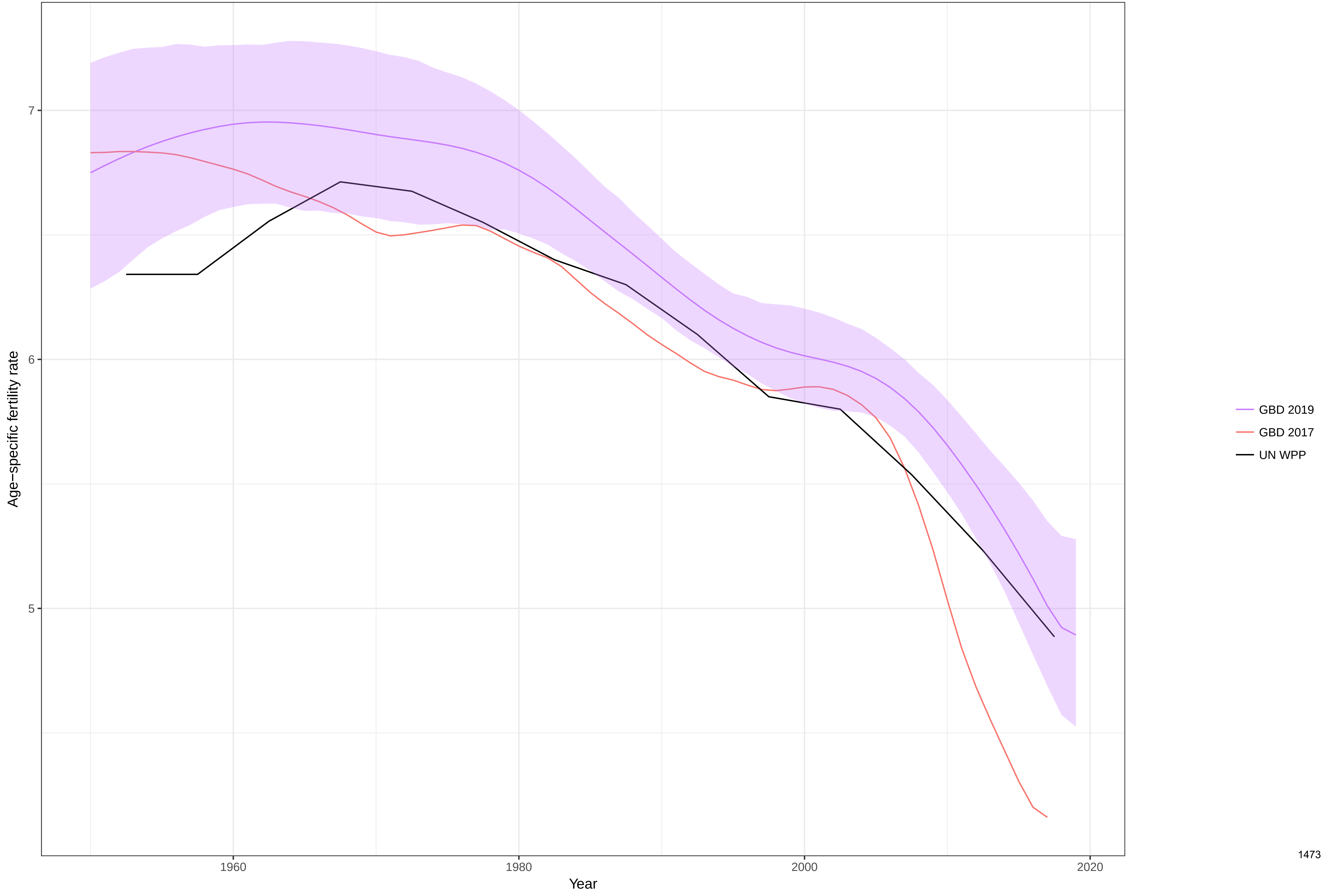

Age−specific fertility fitted trend lines, 1950−2019: Mauritania, Ages: 10−14
Data points enclosed with a diamond are reference sources

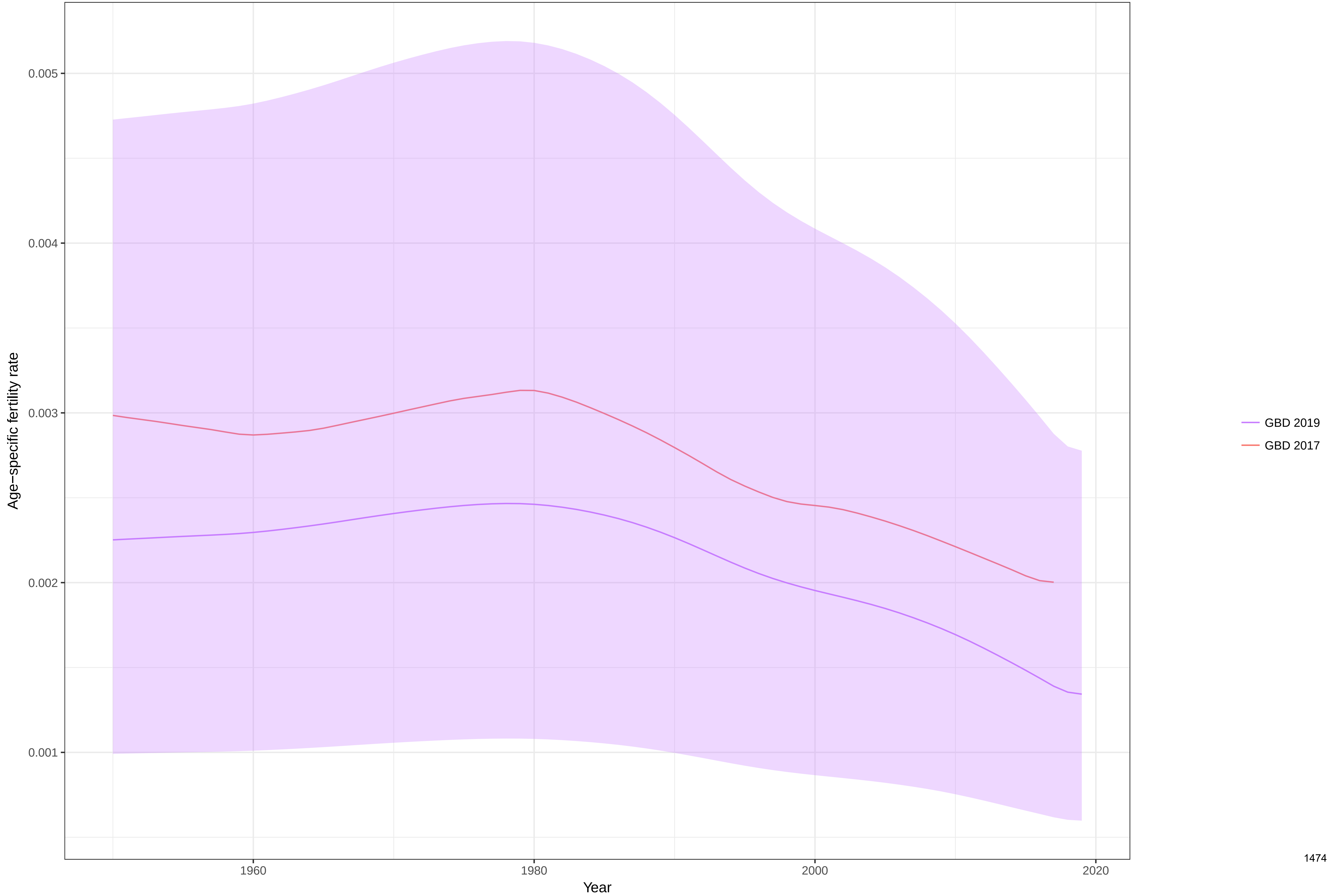

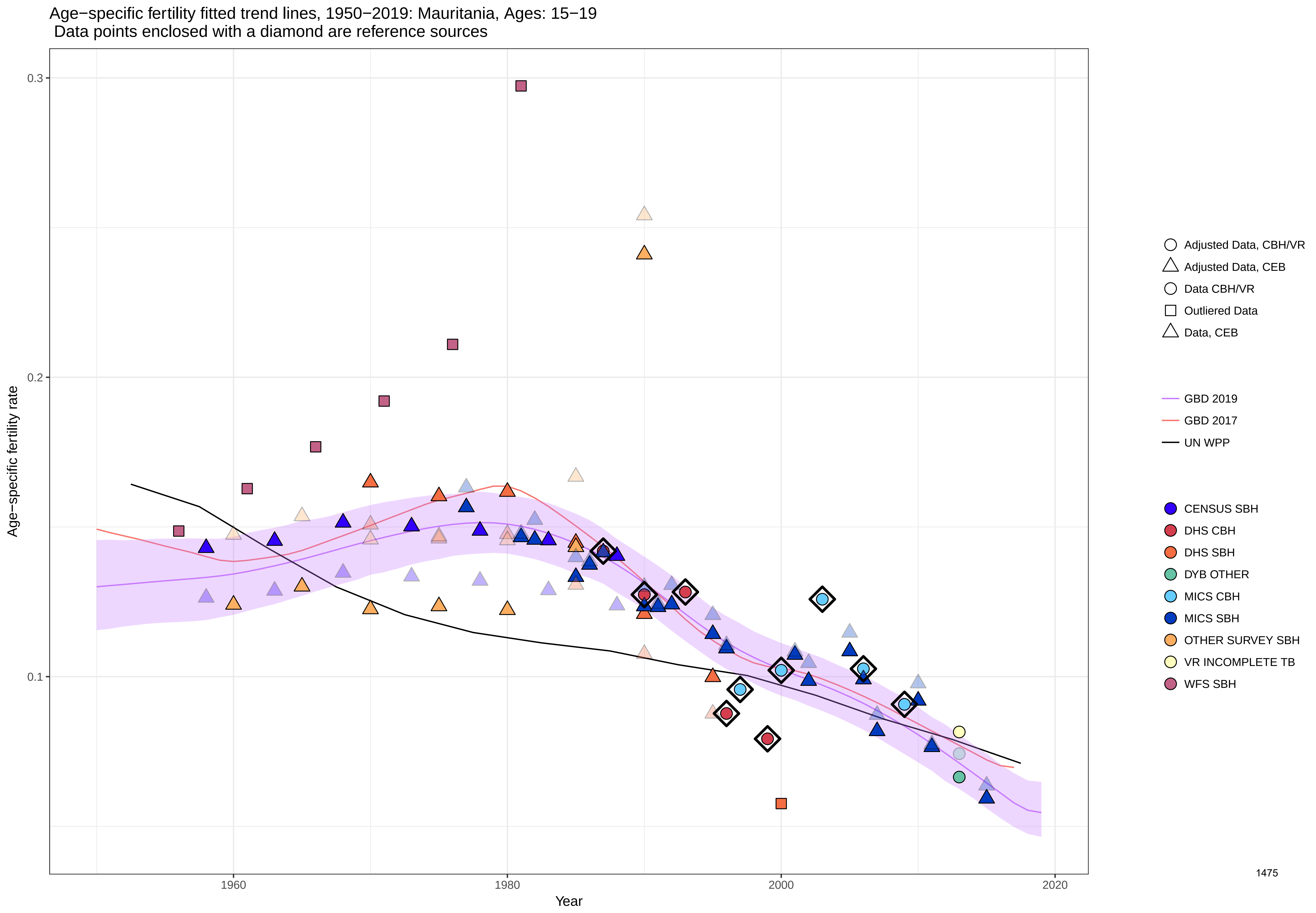

Age−specific fertility fitted trend lines, 1950−2019: Mauritania, Ages: 15−19
Data points enclosed with a diamond are reference sources
Age−specific fertility rate
0.3
0.2
0.1
1960
1980
2000
2020
Year
Adjusted Data, CBH/VR
Adjusted Data, CEB
Data CBH/VR
Outliered Data
Data, CEB
GBD 2019
GBD 2017
UN WPP
CENSUS SBH
DHS CBH
DHS SBH
DYB OTHER
MICS CBH
MICS SBH
OTHER SURVEY SBH
VR INCOMPLETE TB
WFS SBH

Age−specific fertility fitted trend lines, 1950−2019: Mauritania, Ages: 20−24
Data points enclosed with a diamond are reference sources

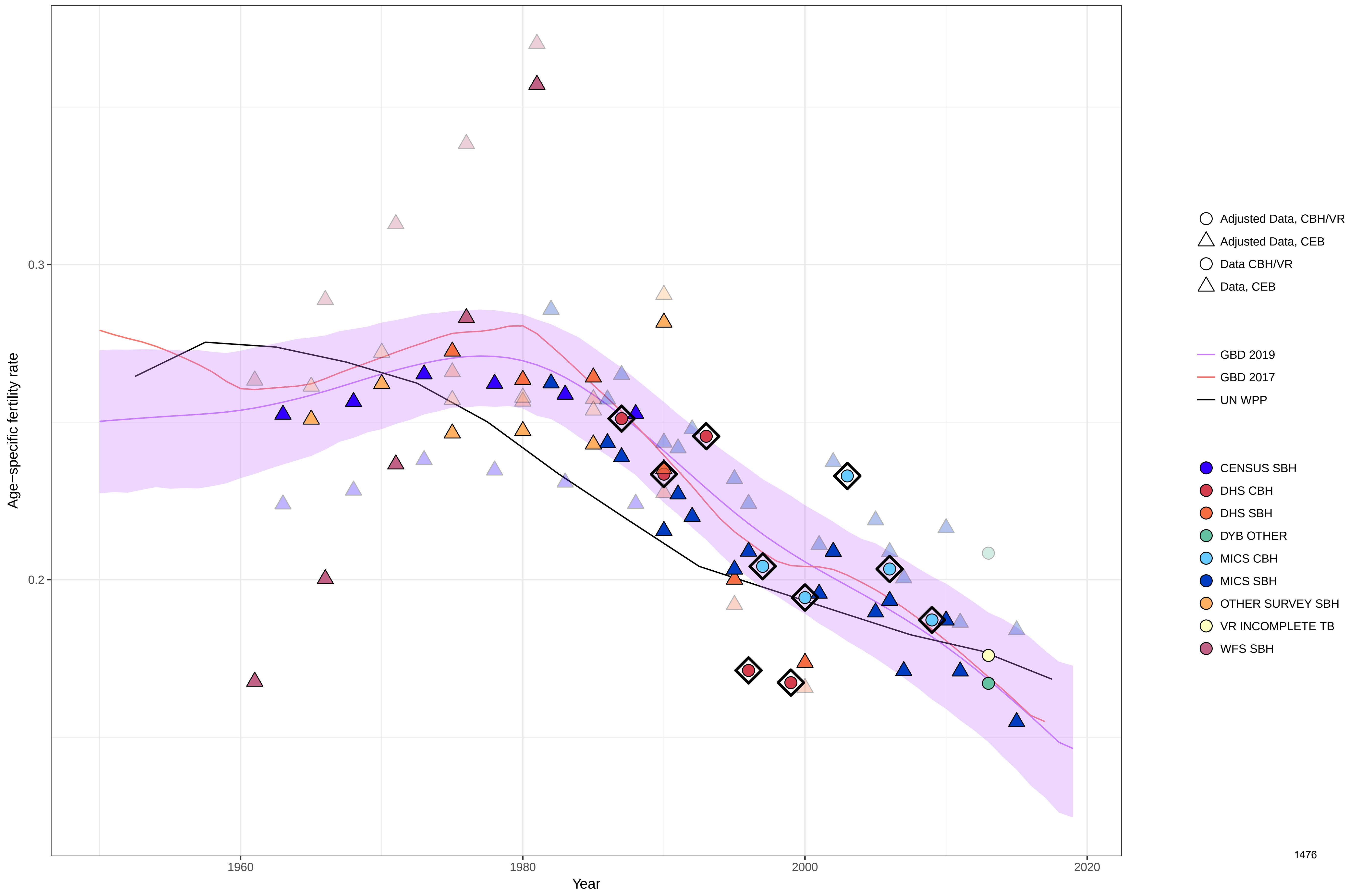

Age−specific fertility fitted trend lines, 1950−2019: Mauritania, Ages: 25−29
Data points enclosed with a diamond are reference sources

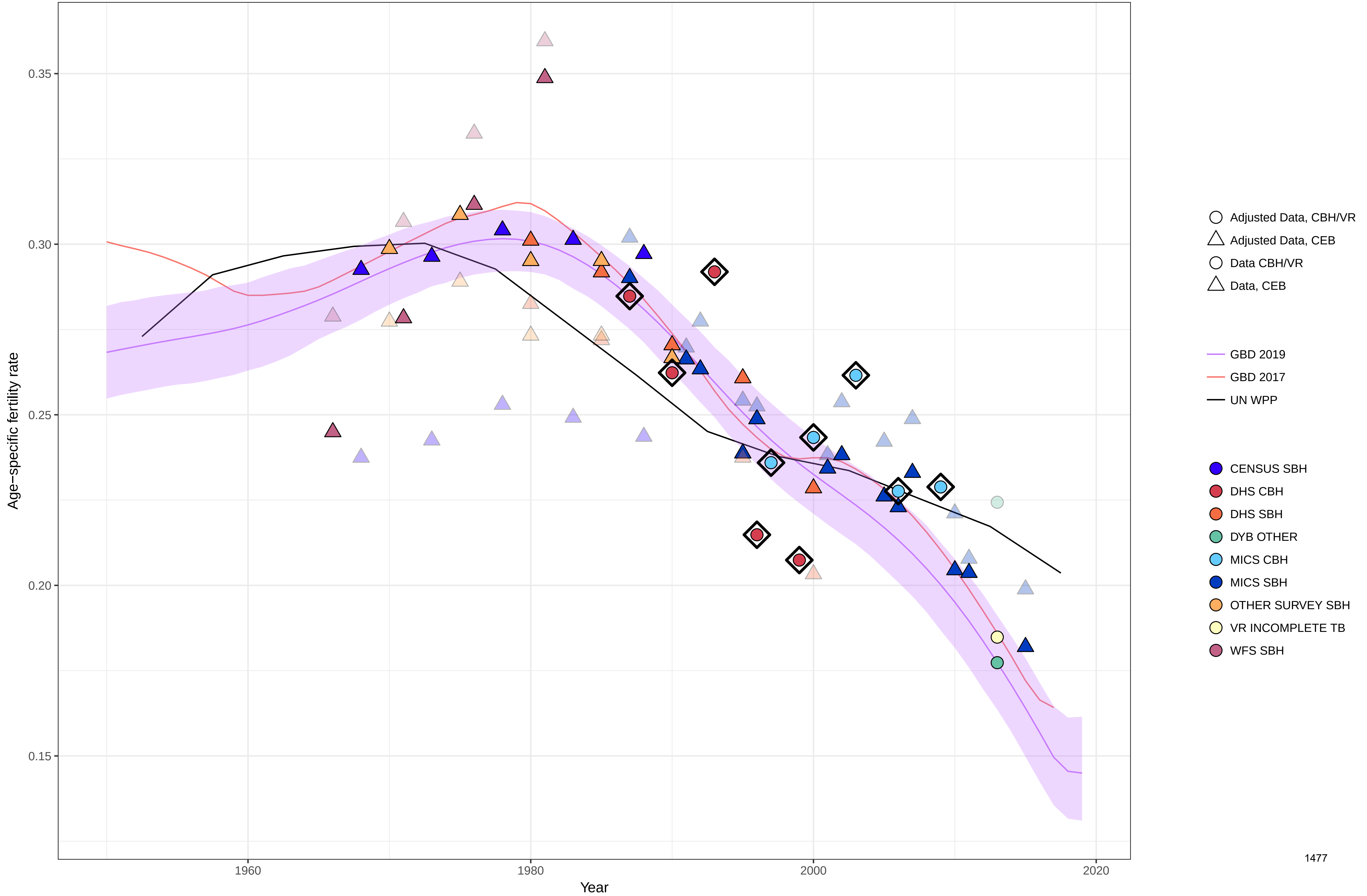

Age−specific fertility fitted trend lines, 1950−2019: Mauritania, Ages: 30−34
Data points enclosed with a diamond are reference sources

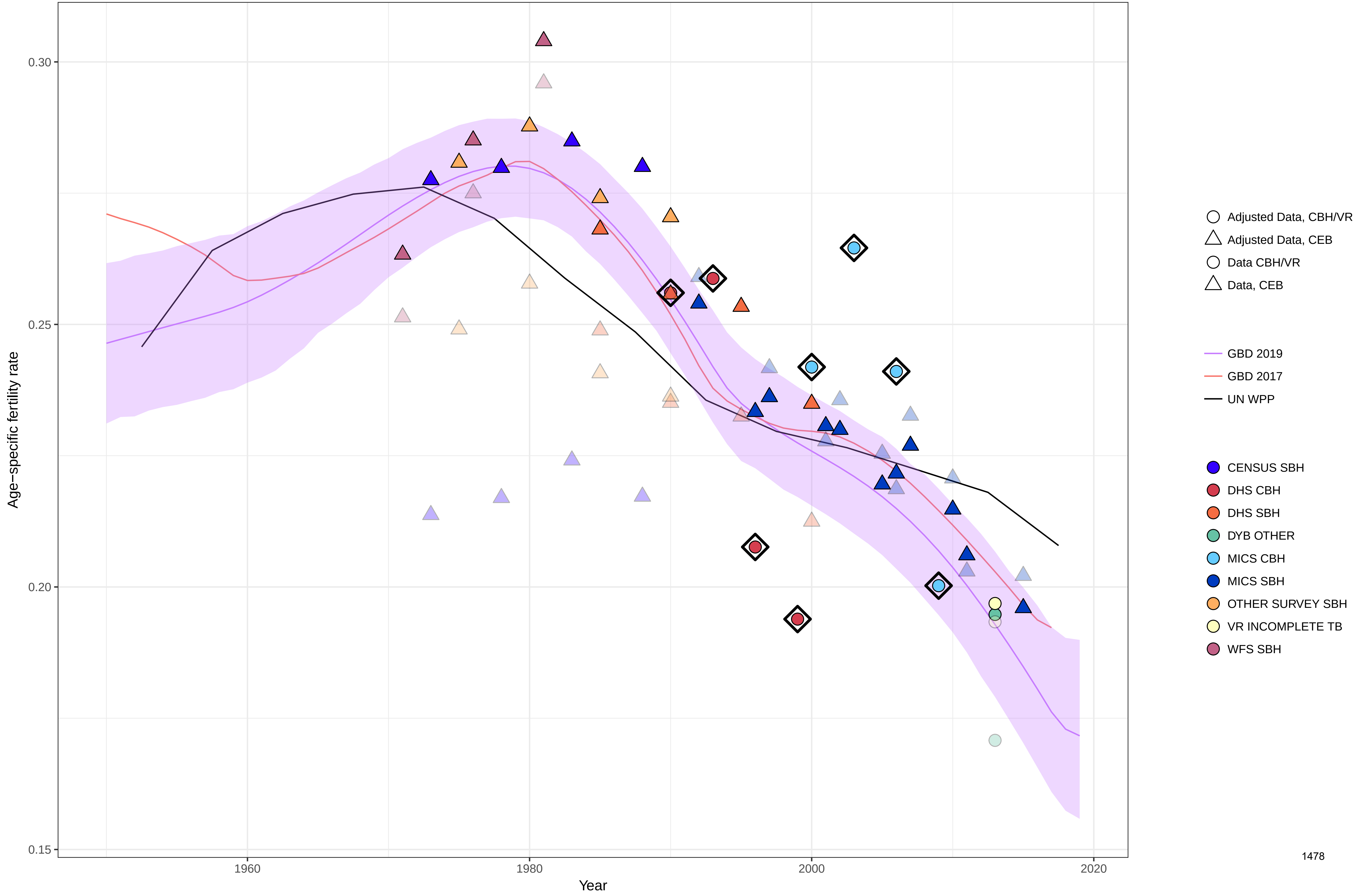

Age−specific fertility fitted trend lines, 1950−2019: Mauritania, Ages: 35−39
Data points enclosed with a diamond are reference sources

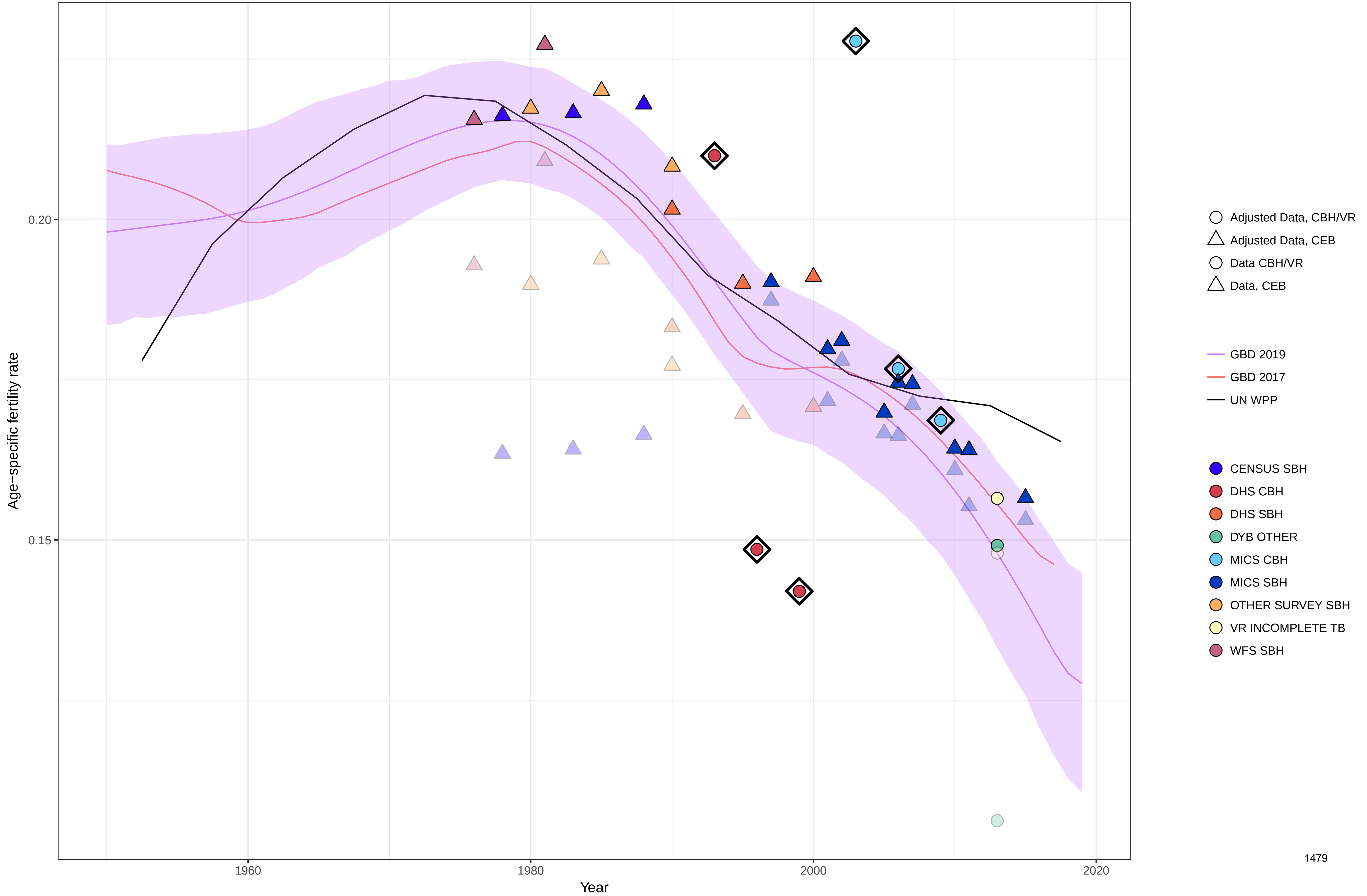

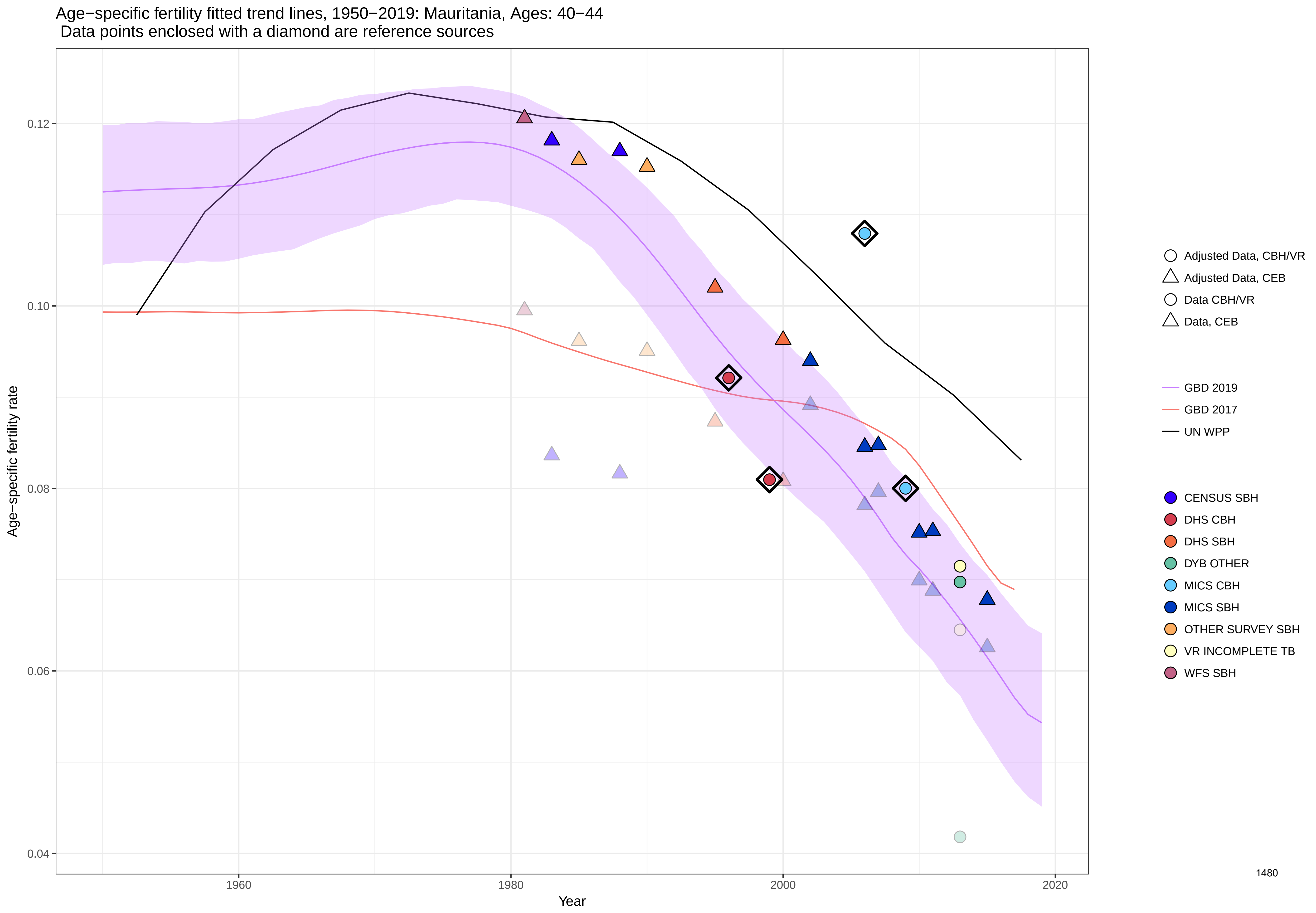
Age−specific fertility fitted trend lines, 1950−2019: Mauritania, Ages: 40−44
Data points enclosed with a diamond are reference sources
Age−specific fertility rate
Year
0.04
0.06
0.08
0.10
0.12
1960
1980
2000
2020
Adjusted Data, CBH/VR
Adjusted Data, CEB
Data CBH/VR
Data, CEB
GBD 2019
GBD 2017
UN WPP
CENSUS SBH
DHS CBH
DHS SBH
DYB OTHER
MICS CBH
MICS SBH
OTHER SURVEY SBH
VR INCOMPLETE TB
WFS SBH

Age−specific fertility fitted trend lines, 1950−2019: Mauritania, Ages: 45−49
Data points enclosed with a diamond are reference sources

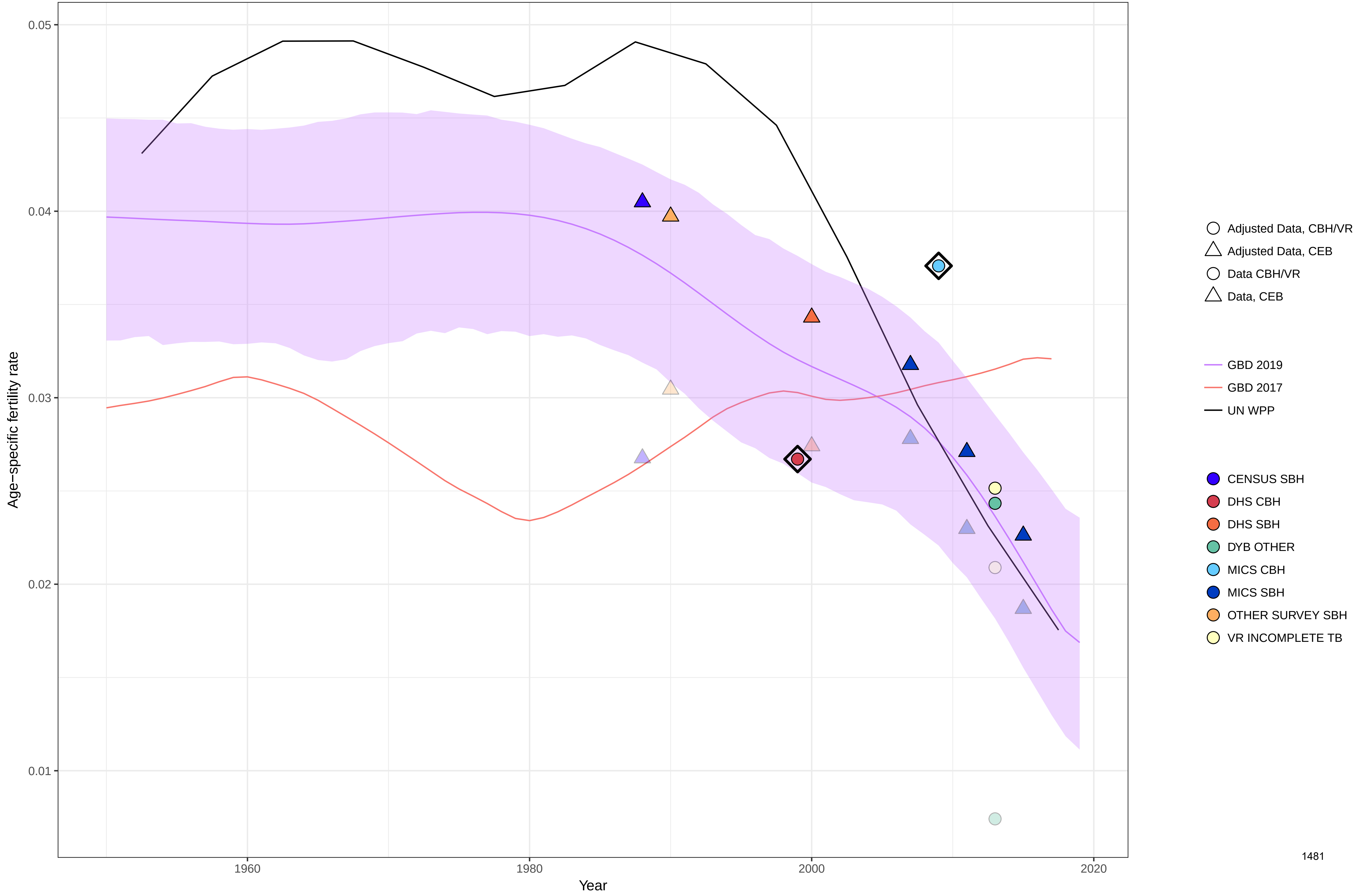

Age−specific fertility fitted trend lines, 1950−2019: Mauritania, Ages: 50−54
Data points enclosed with a diamond are reference sources

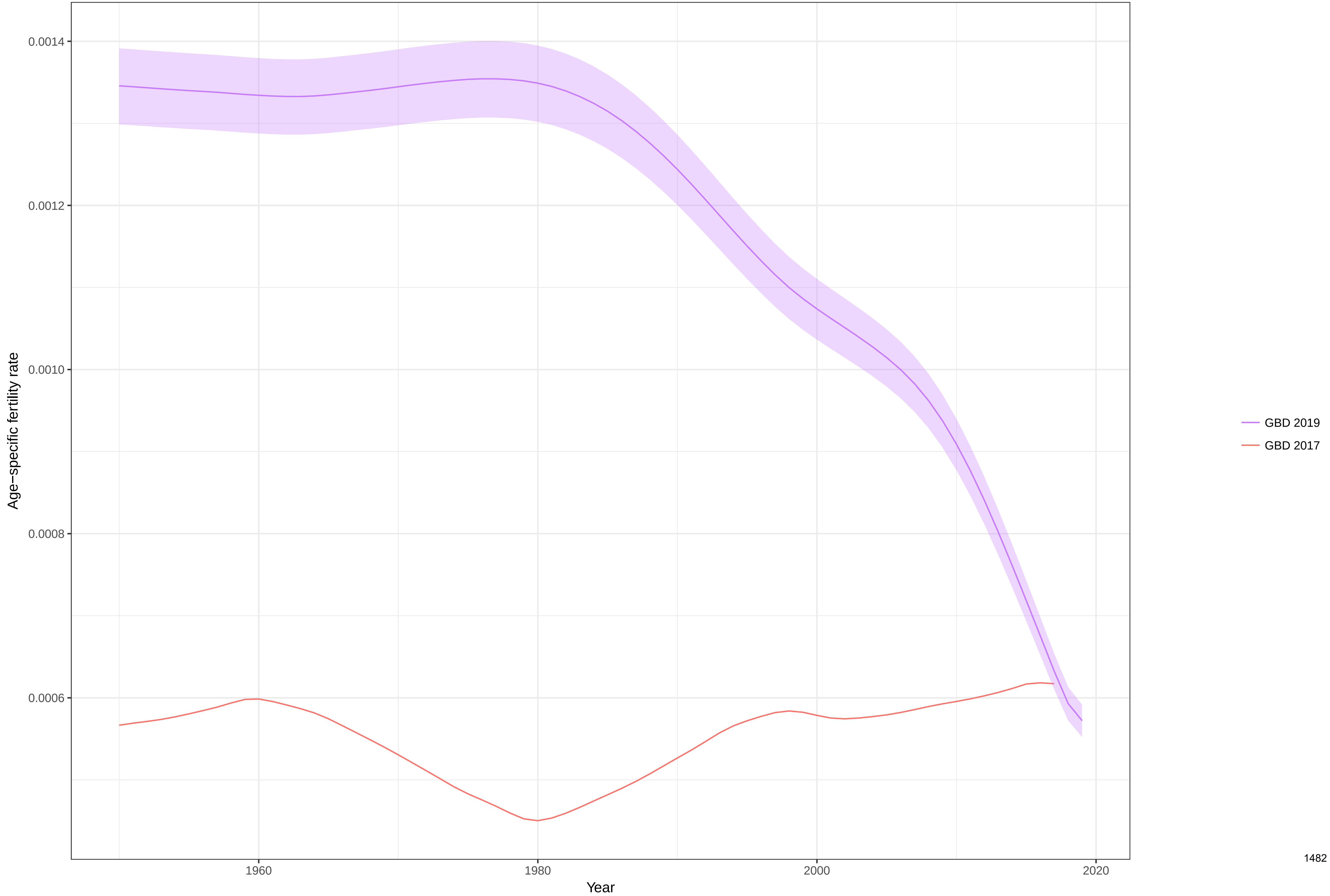

Total fertility rate fitted trend lines, 1950−2019: Mauritania TFR
Data points enclosed with a diamond are reference sources

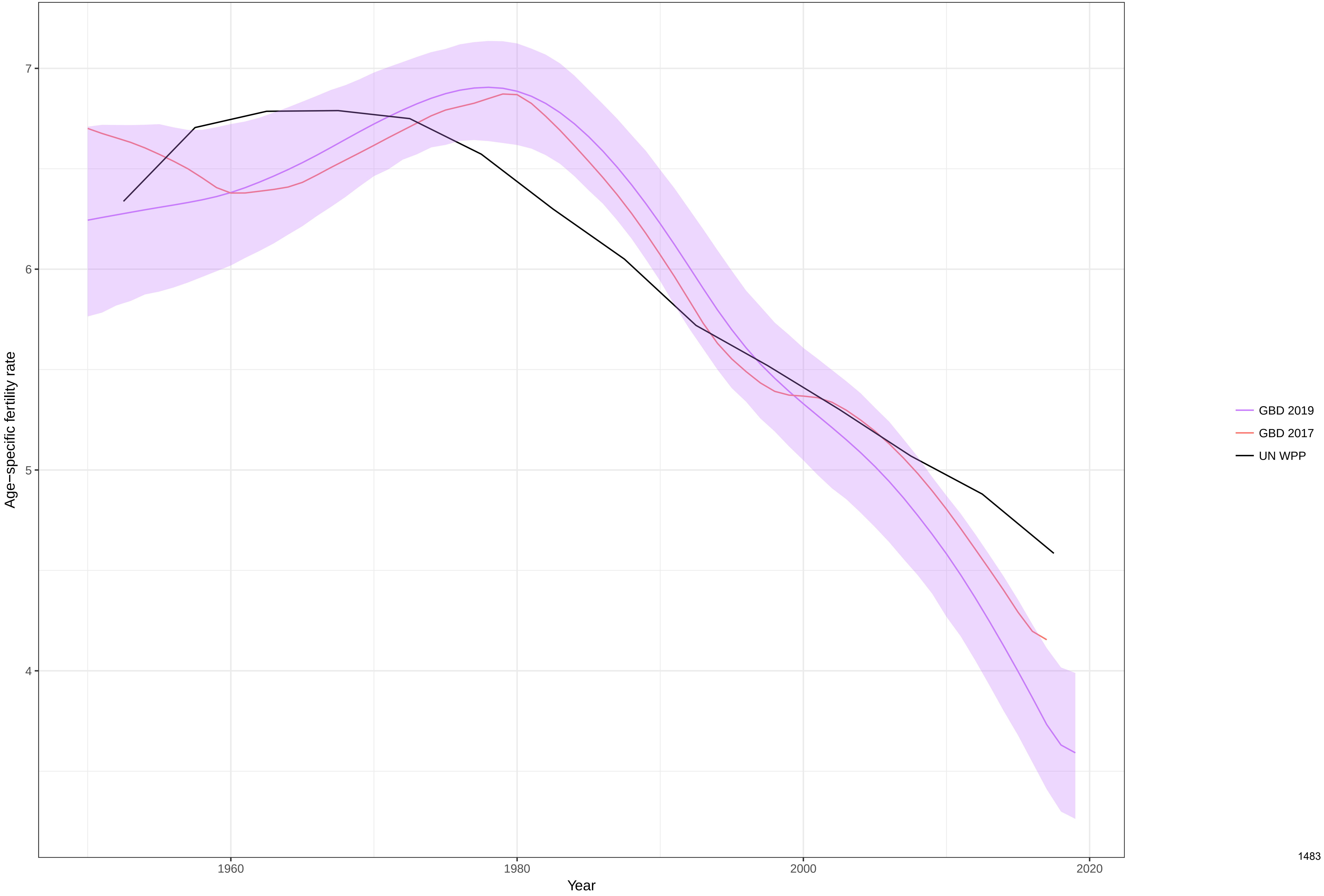

Age−specific fertility fitted trend lines, 1950−2019: Mauritius, Ages: 10−14
Data points enclosed with a diamond are reference sources

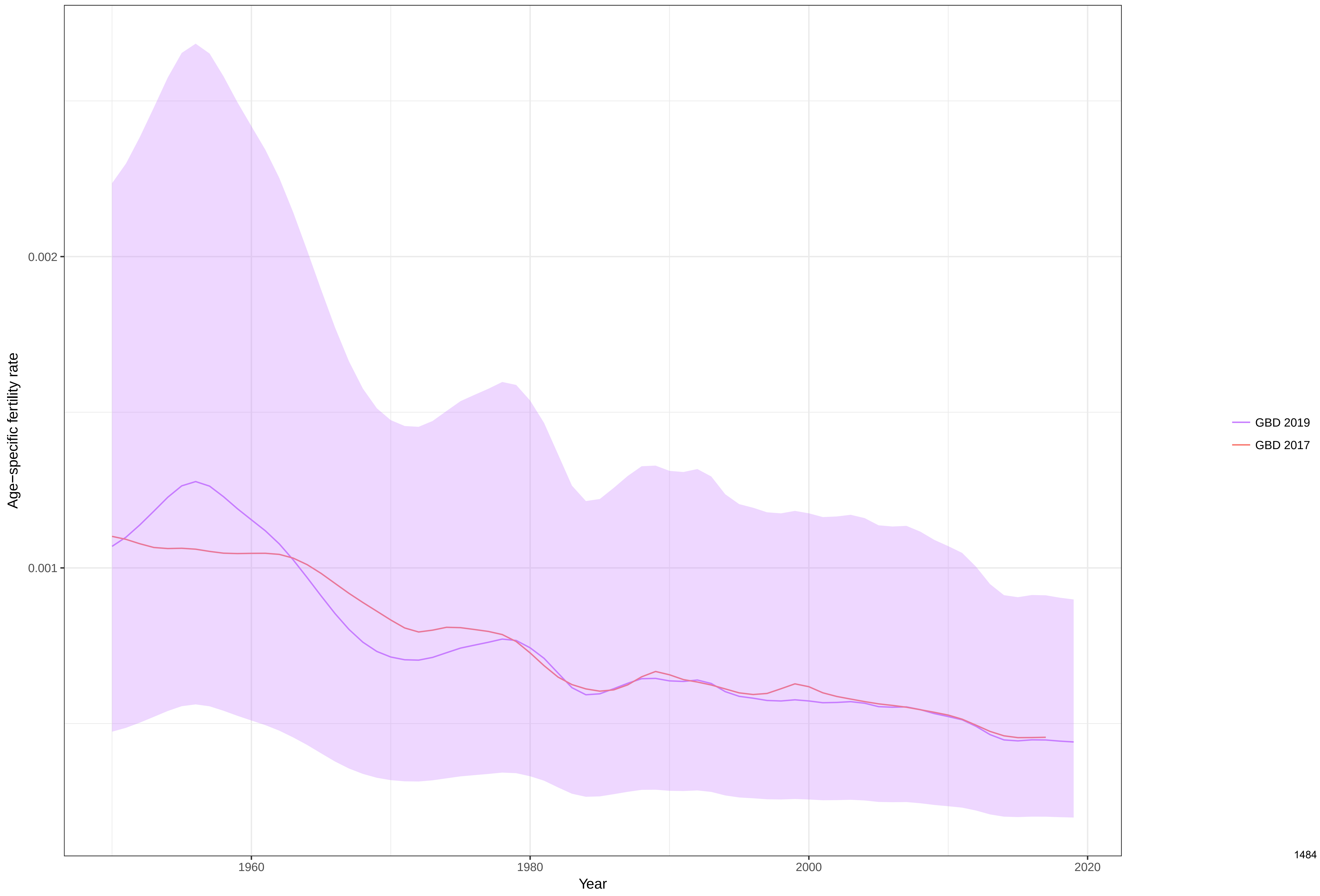

Age−specific fertility fitted trend lines, 1950−2019: Mauritius, Ages: 15−19
Data points enclosed with a diamond are reference sources

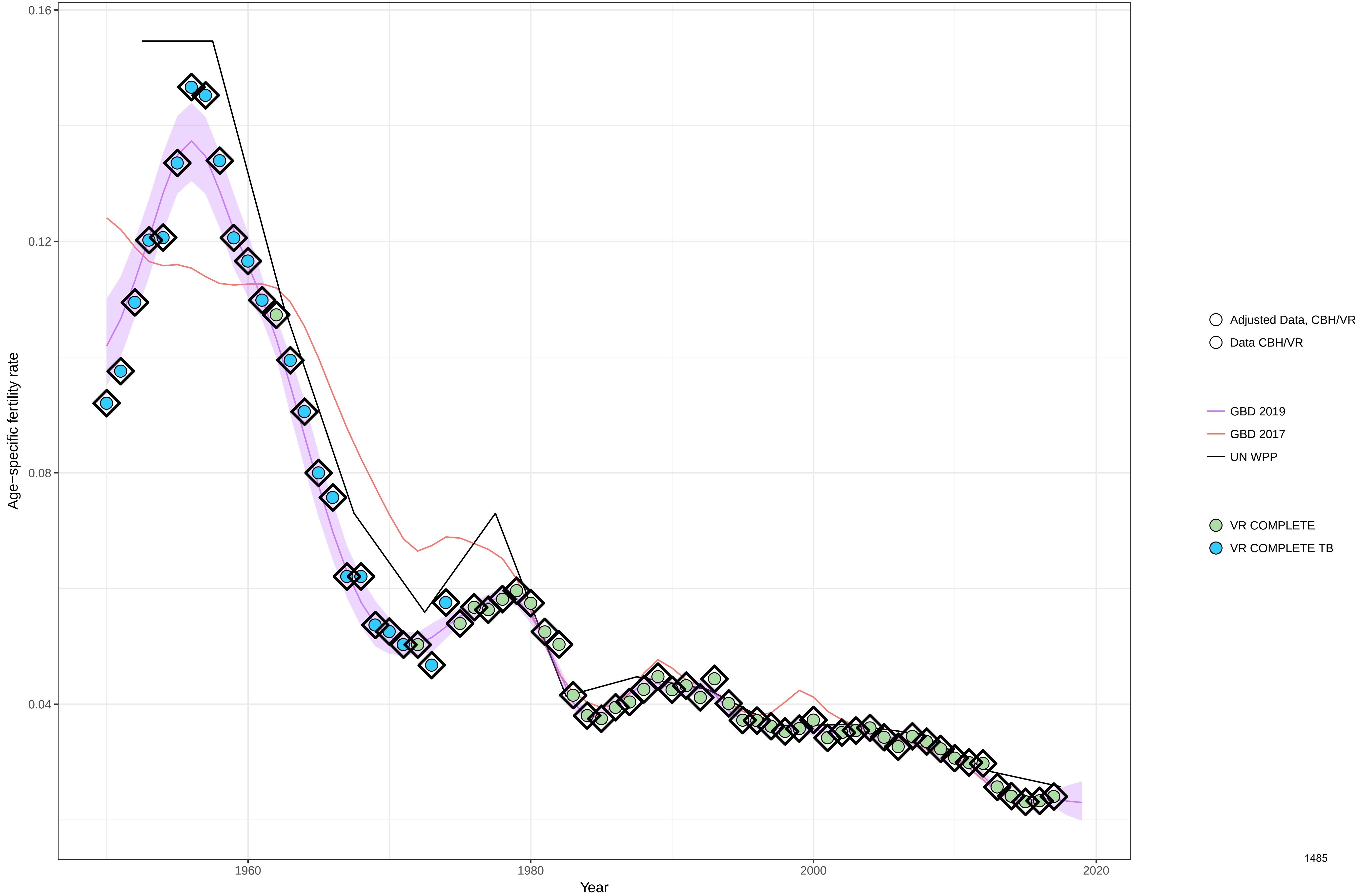

Age−specific fertility fitted trend lines, 1950−2019: Mauritius, Ages: 20−24
Data points enclosed with a diamond are reference sources

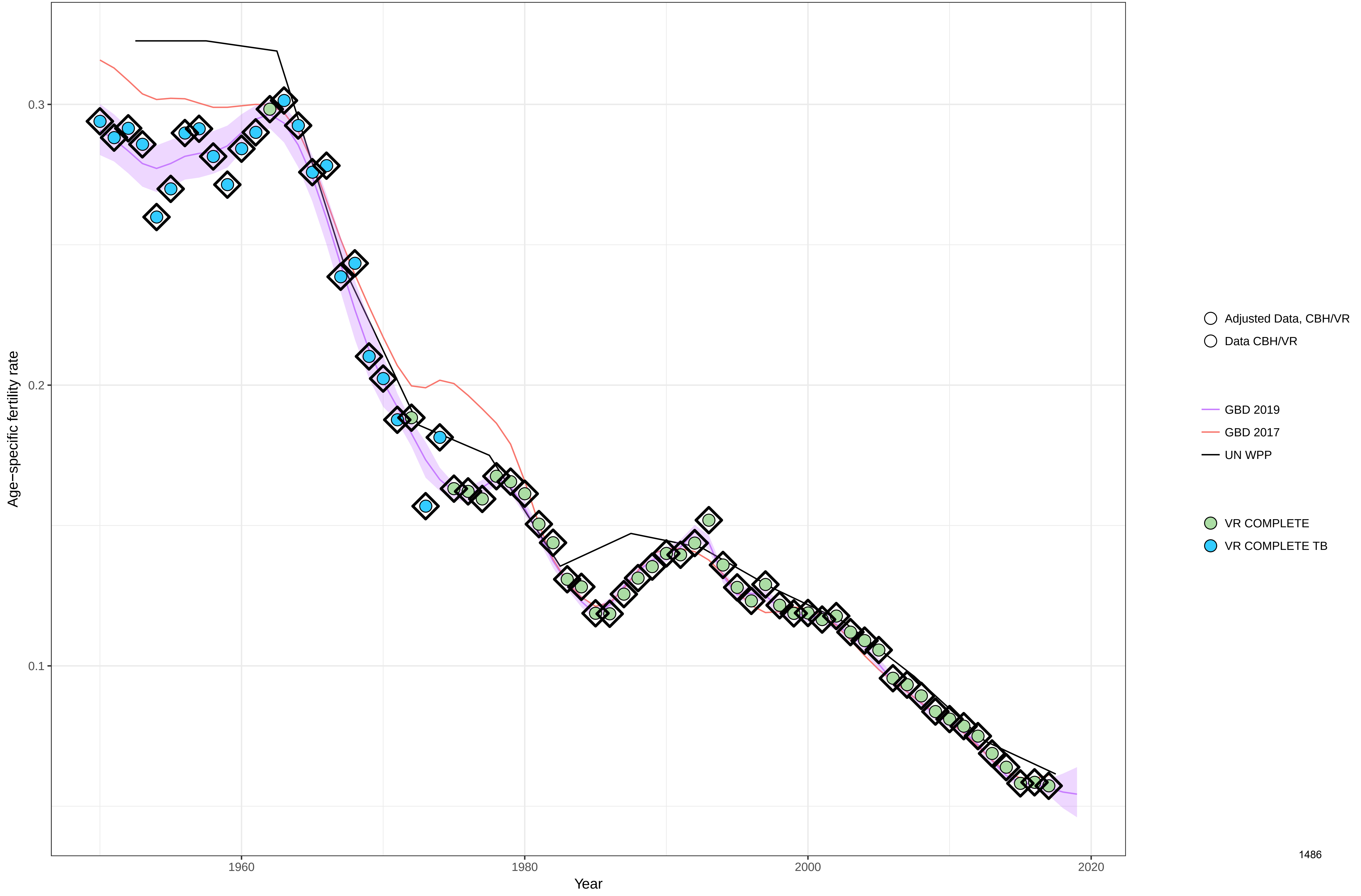

Age−specific fertility fitted trend lines, 1950−2019: Mauritius, Ages: 25−29
Data points enclosed with a diamond are reference sources

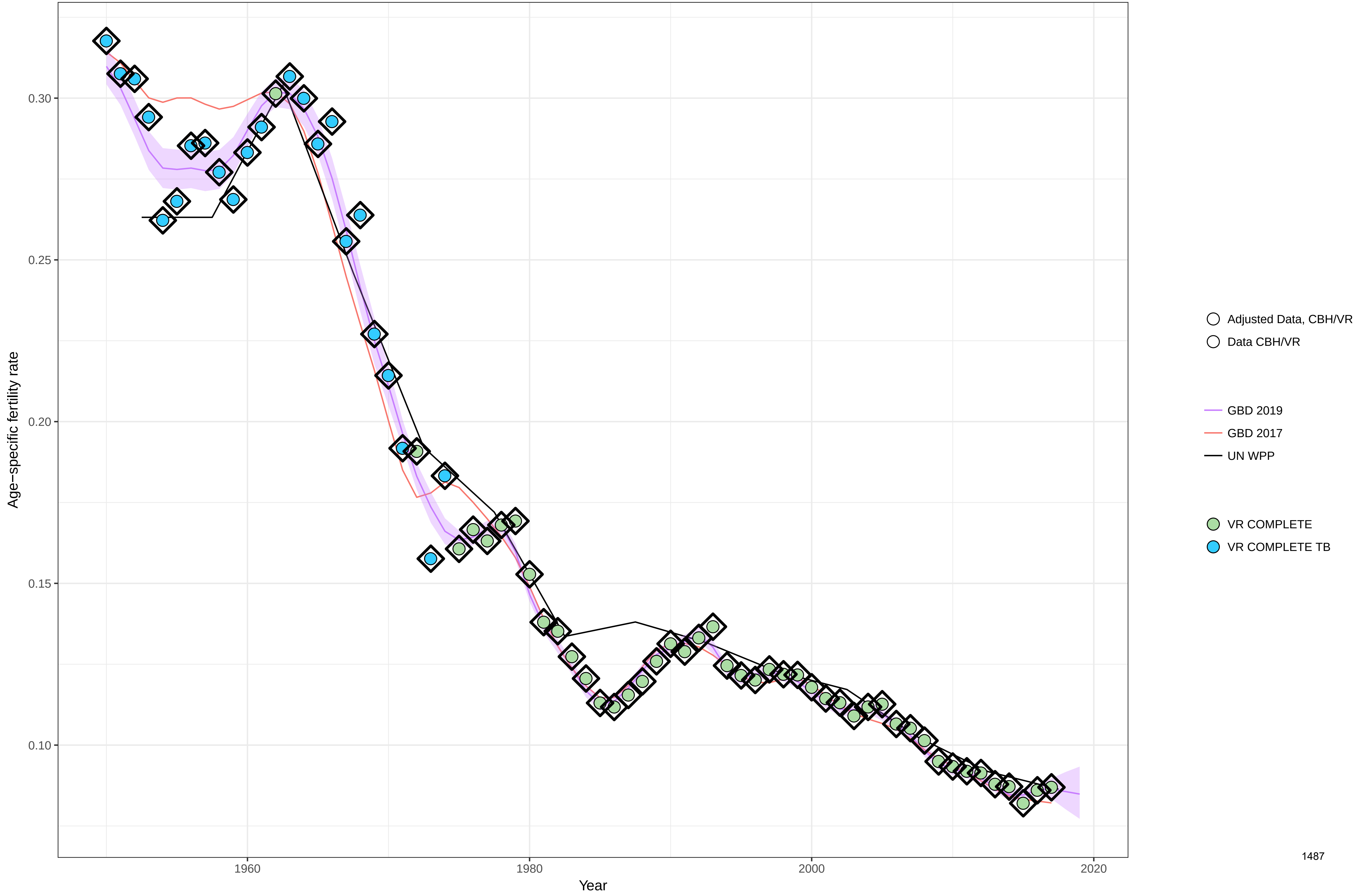

Age−specific fertility fitted trend lines, 1950−2019: Mauritius, Ages: 30−34
 Data points enclosed with a diamond are reference sources

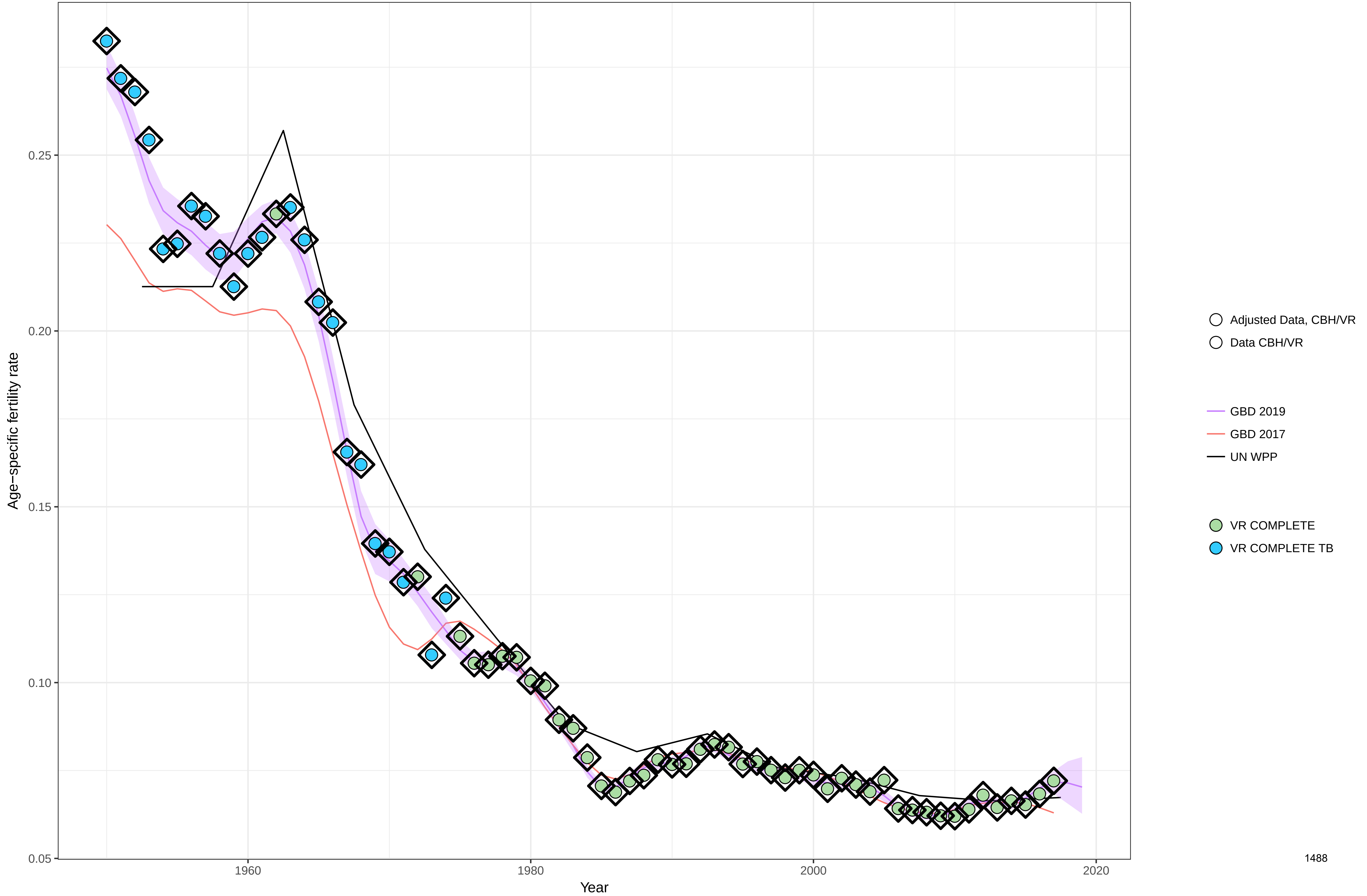

Age−specific fertility fitted trend lines, 1950−2019: Mauritius, Ages: 35−39
Data points enclosed with a diamond are reference sources

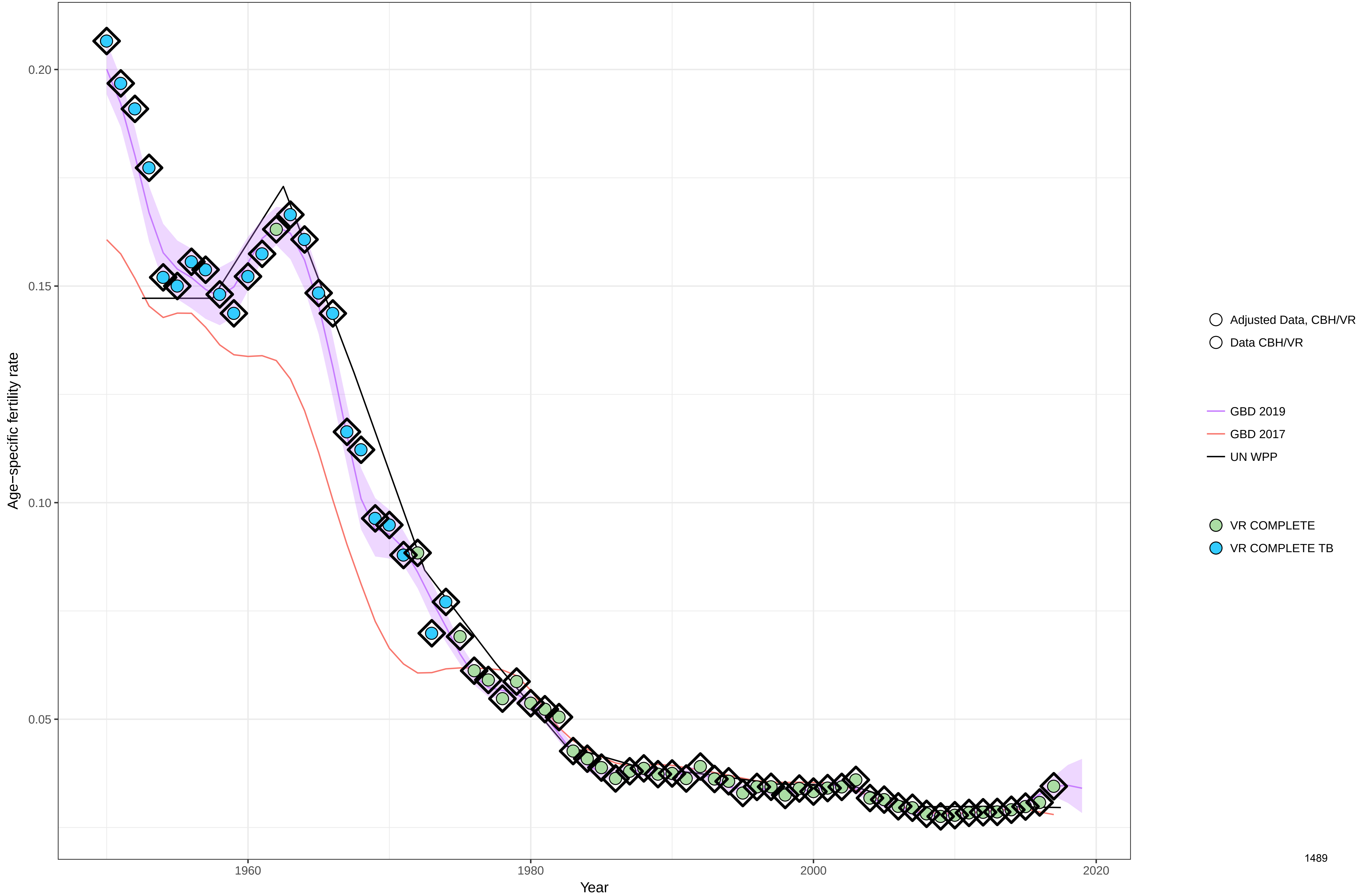

Age−specific fertility fitted trend lines, 1950−2019: Mauritius, Ages: 40−44
Data points enclosed with a diamond are reference sources

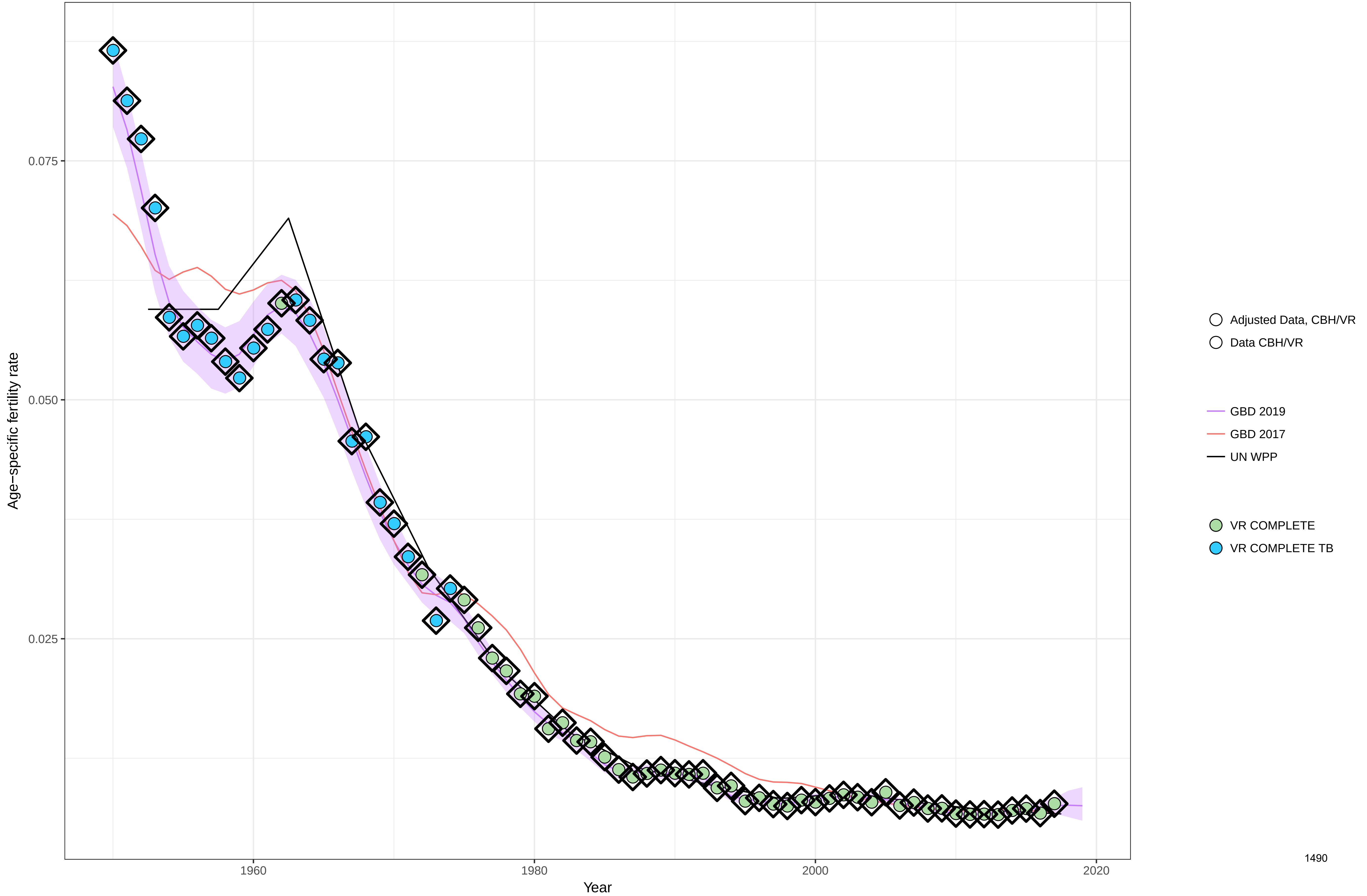

Age−specific fertility fitted trend lines, 1950−2019: Mauritius, Ages: 45−49
Data points enclosed with a diamond are reference sources

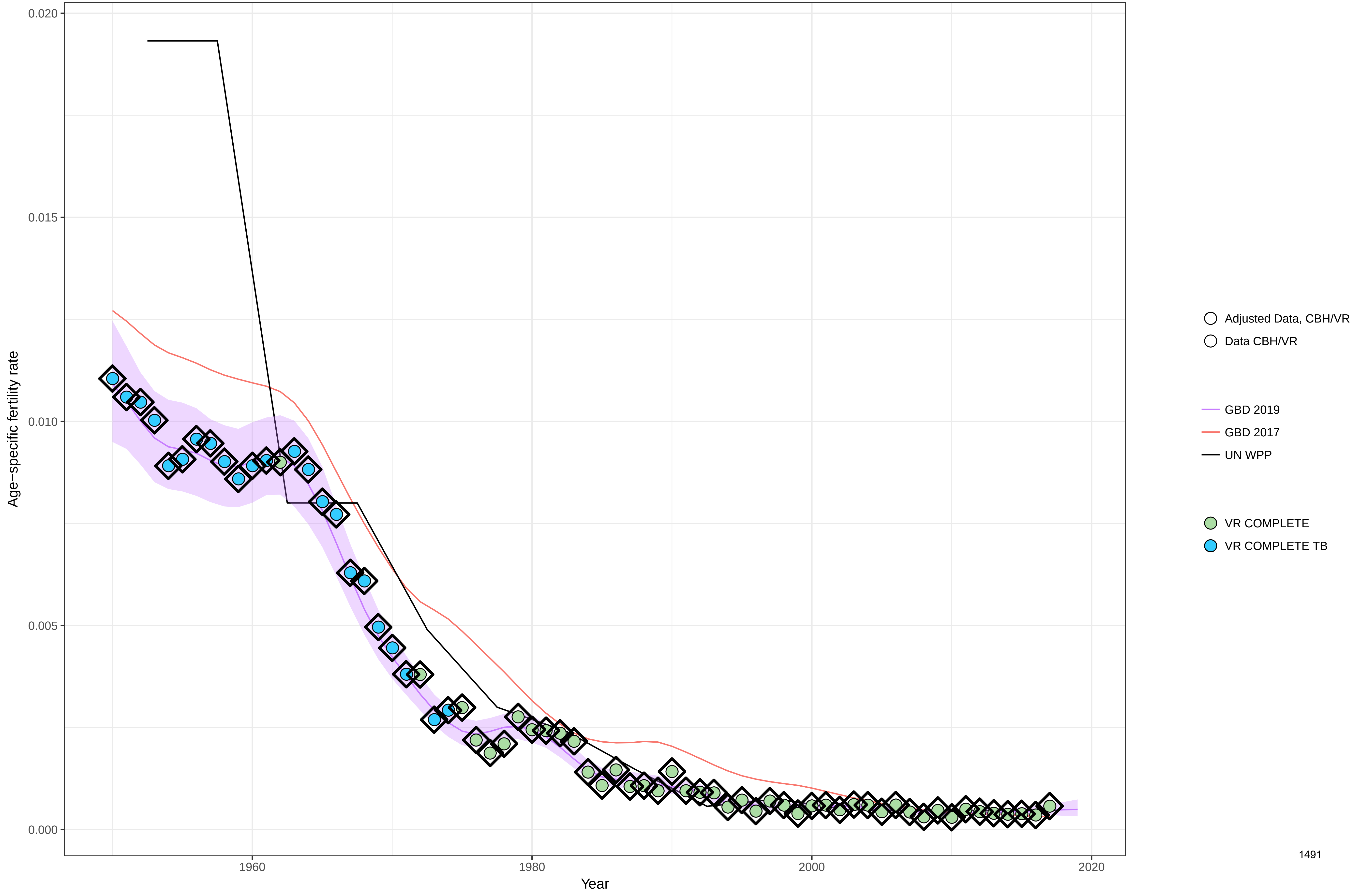

Age−specific fertility fitted trend lines, 1950−2019: Mauritius, Ages: 50−54
Data points enclosed with a diamond are reference sources

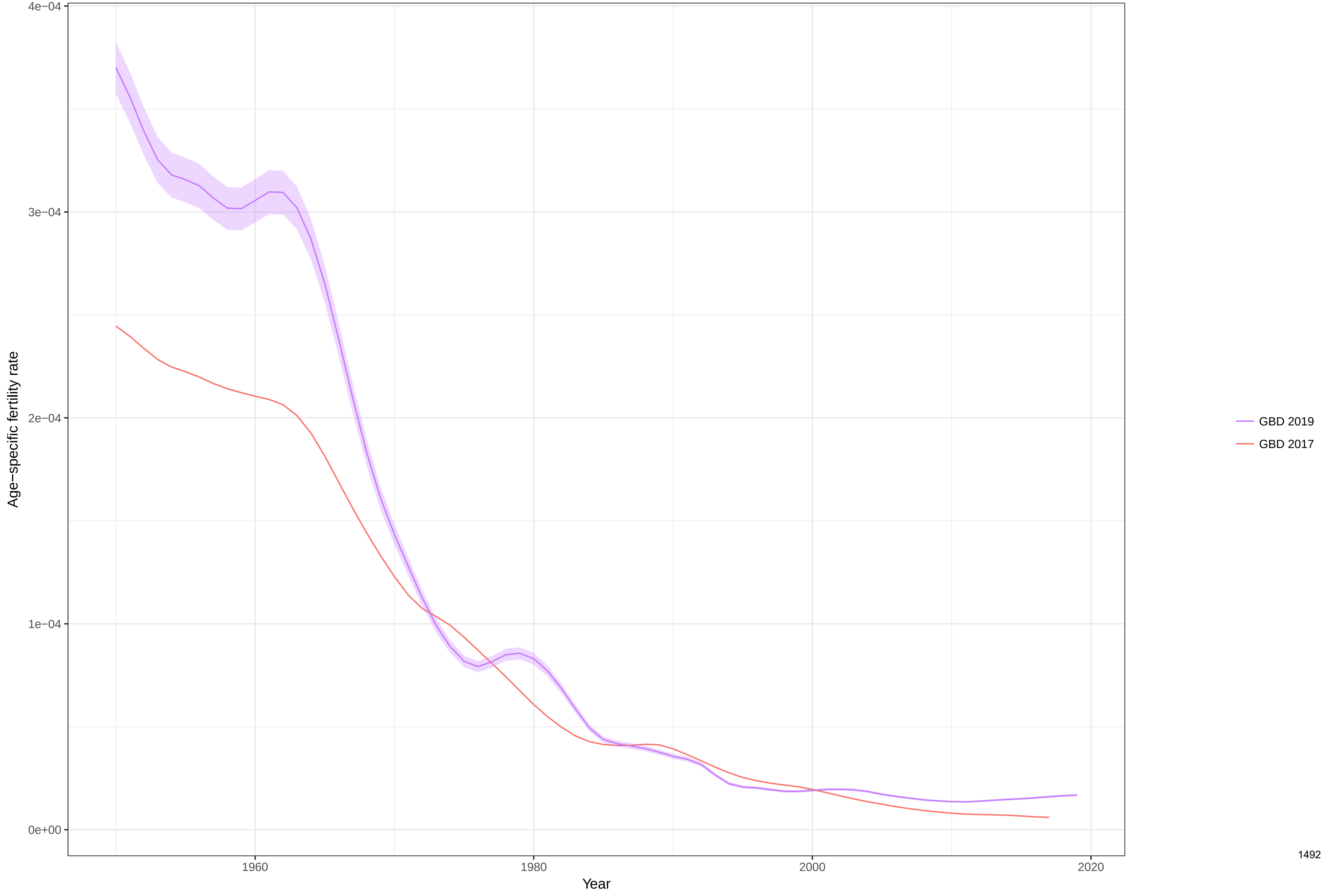

Total fertility rate fitted trend lines, 1950−2019: Mauritius TFR
Data points enclosed with a diamond are reference sources

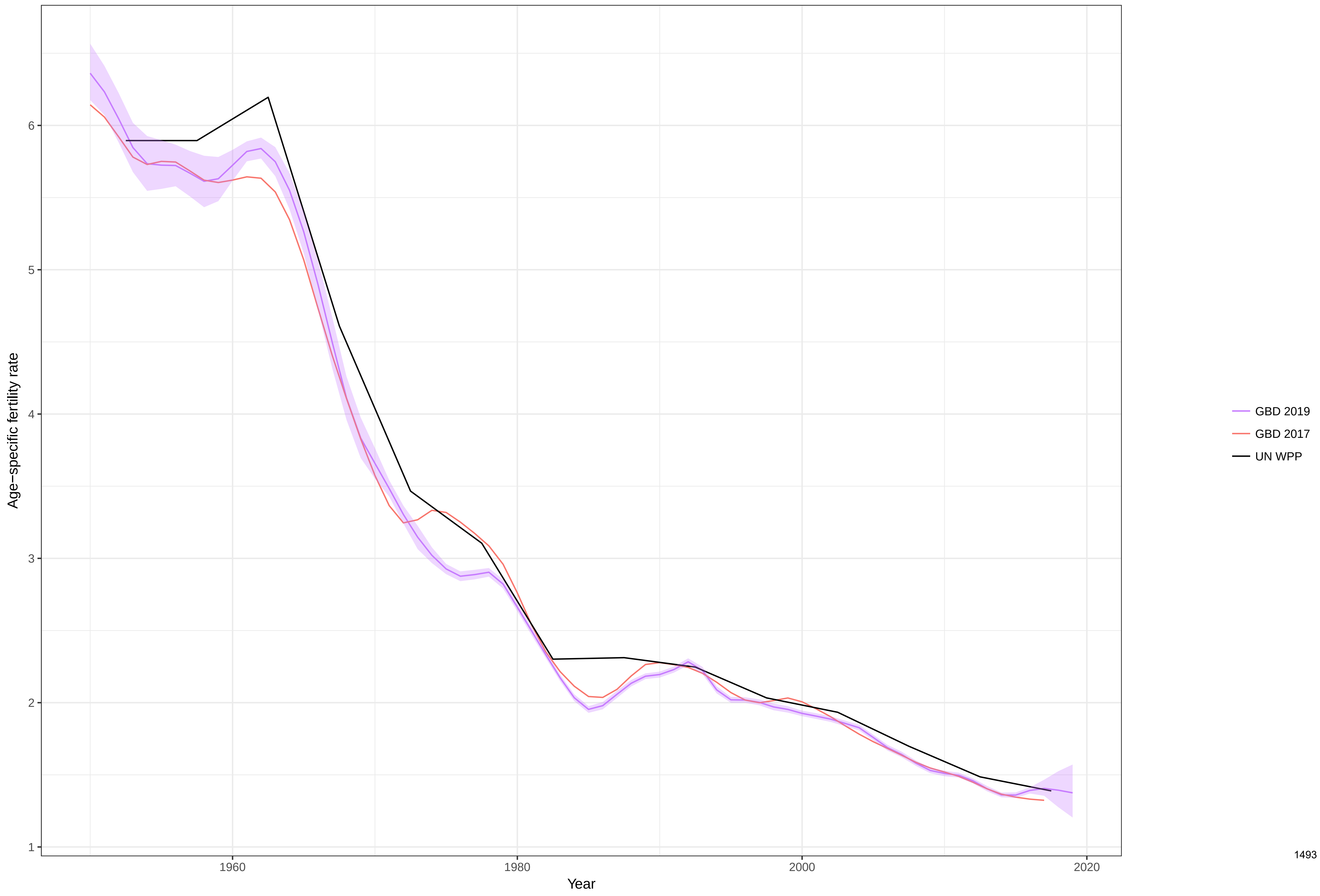

Age−specific fertility fitted trend lines, 1950−2019: Malawi, Ages: 10−14
Data points enclosed with a diamond are reference sources

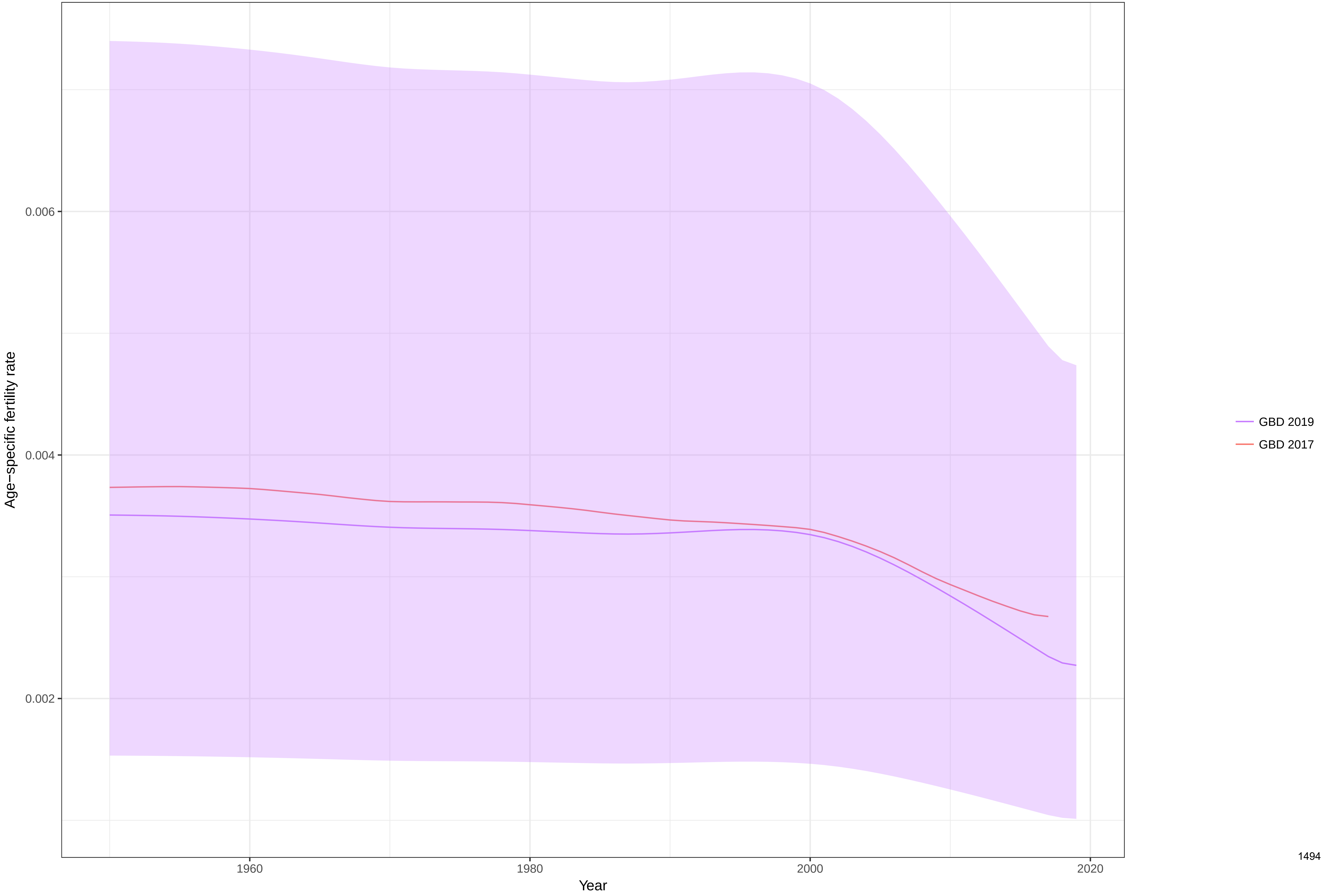

Age−specific fertility fitted trend lines, 1950−2019: Malawi, Ages: 15−19
Data points enclosed with a diamond are reference sources

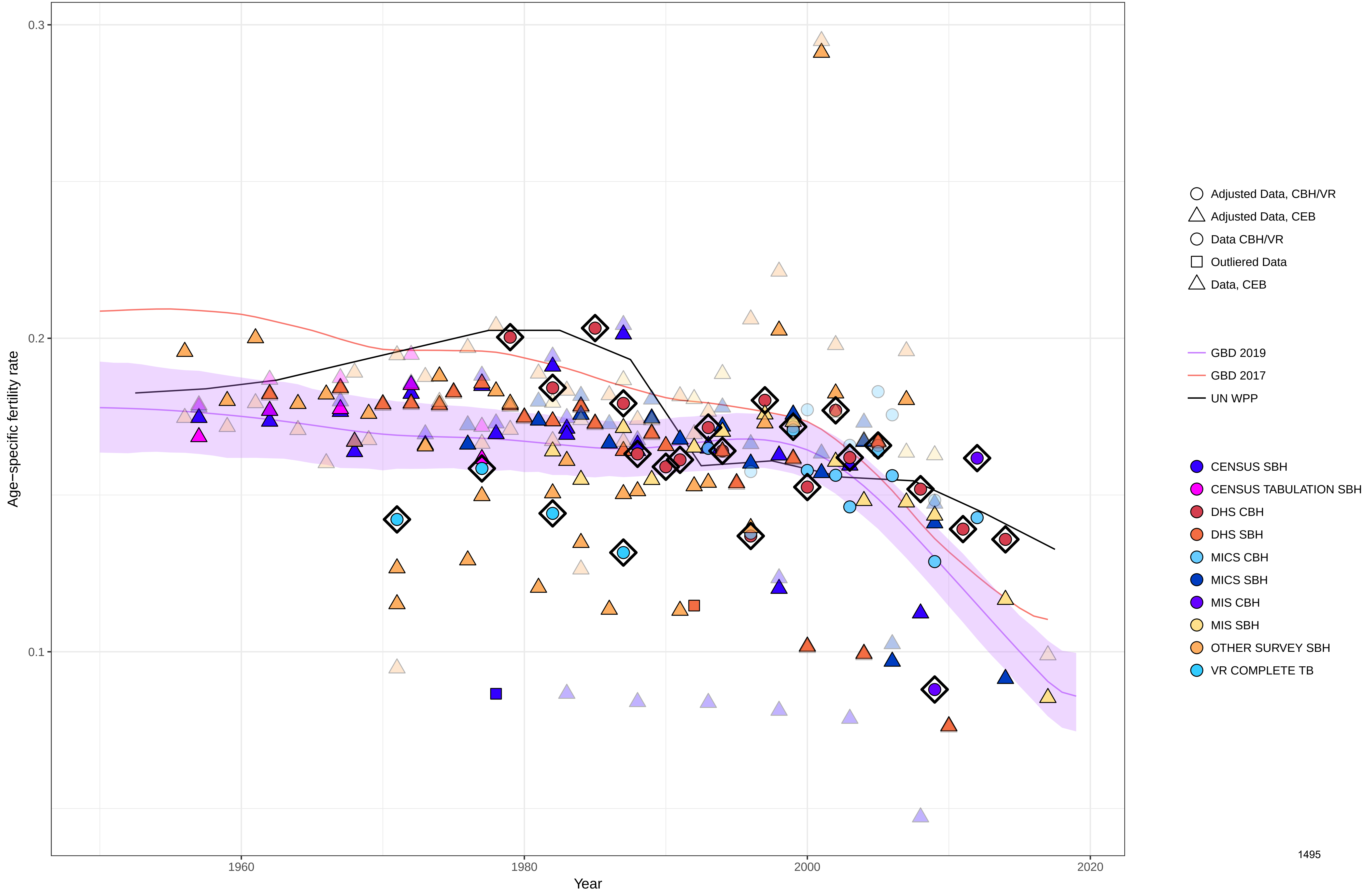

Age−specific fertility fitted trend lines, 1950−2019: Malawi, Ages: 20−24
Data points enclosed with a diamond are reference sources

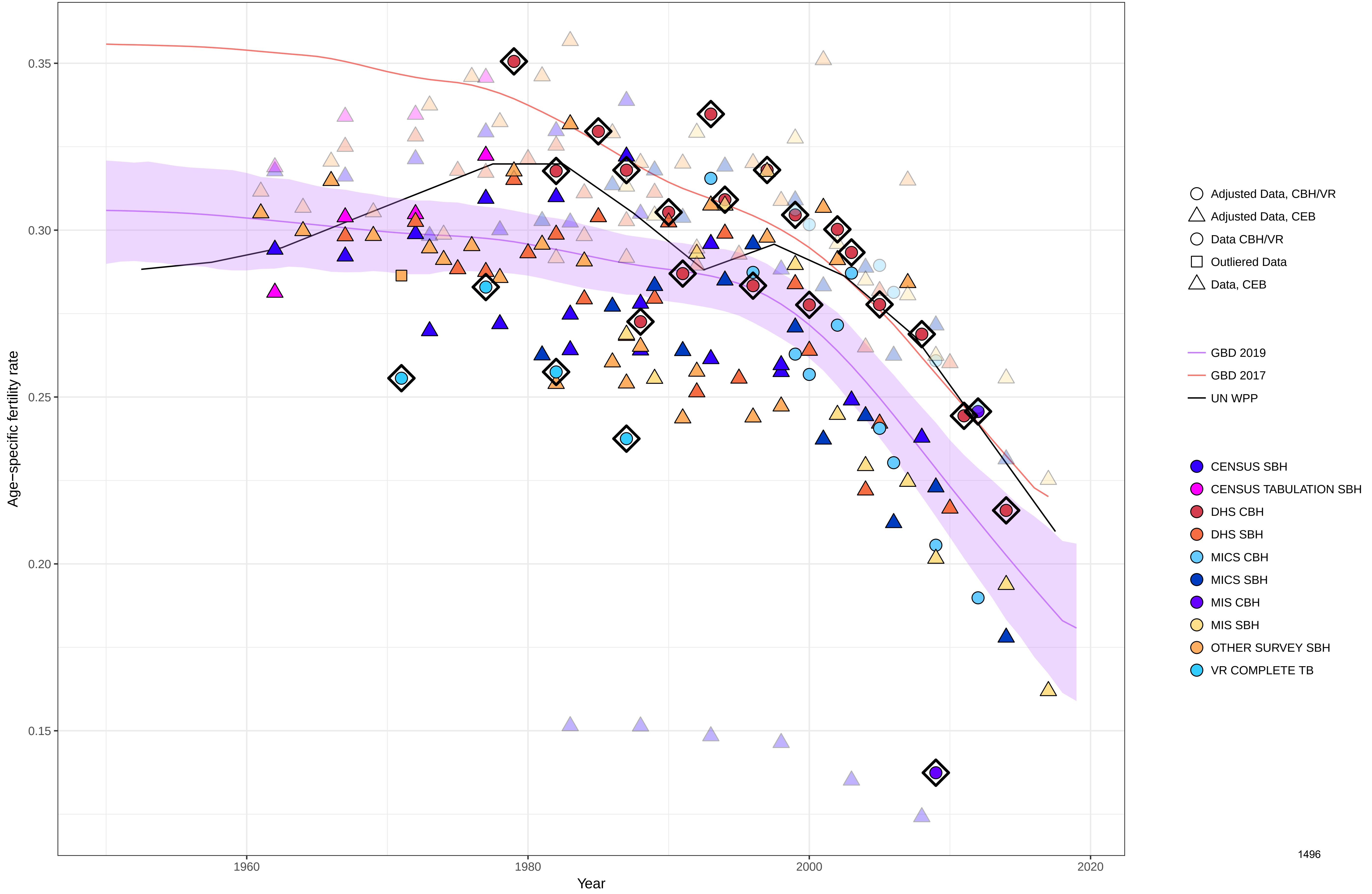

Age−specific fertility fitted trend lines, 1950−2019: Malawi, Ages: 25−29
Data points enclosed with a diamond are reference sources

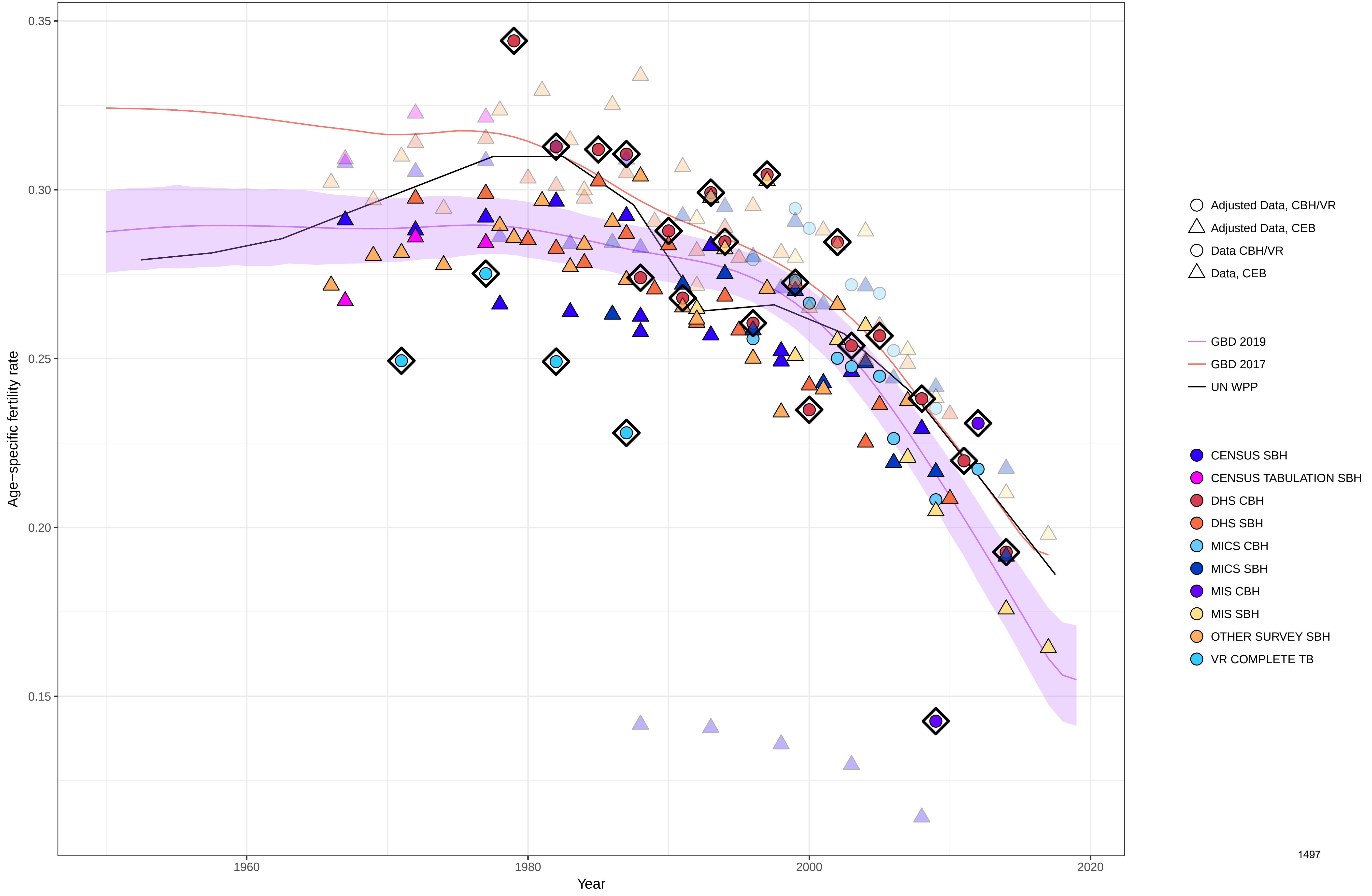

Age−specific fertility fitted trend lines, 1950−2019: Malawi, Ages: 30−34
Data points enclosed with a diamond are reference sources

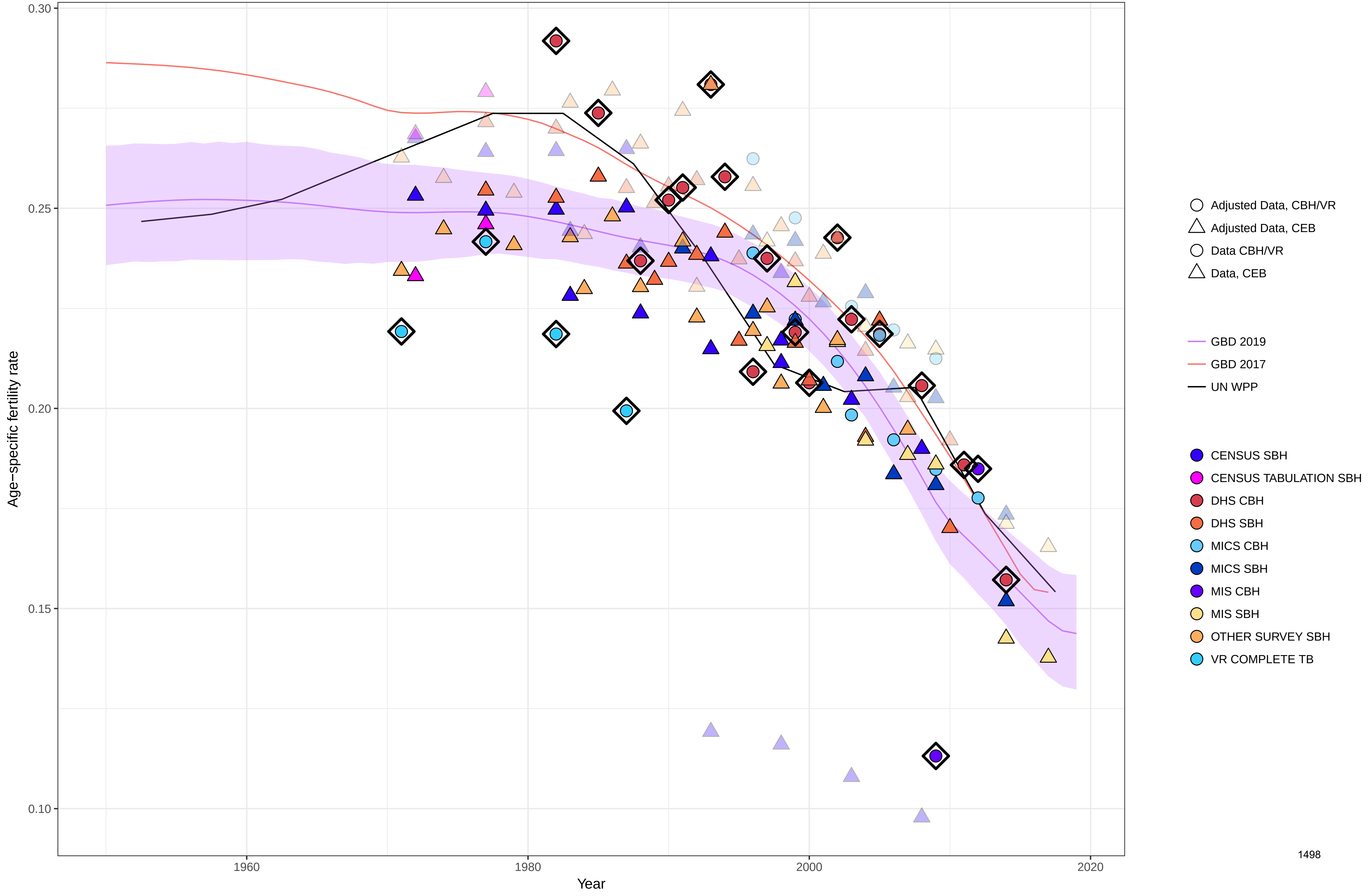

Age−specific fertility fitted trend lines, 1950−2019: Malawi, Ages: 35−39
Data points enclosed with a diamond are reference sources

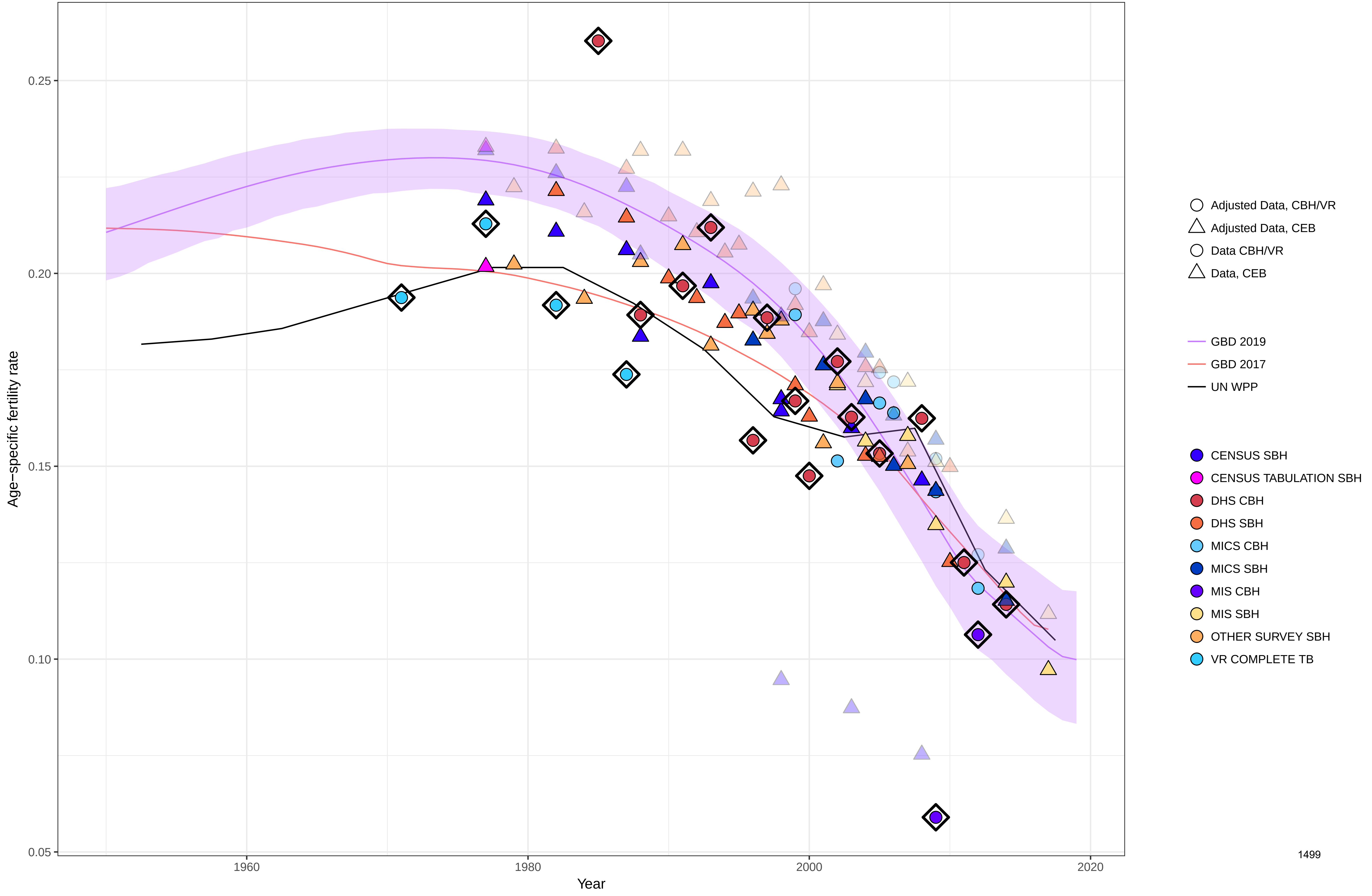

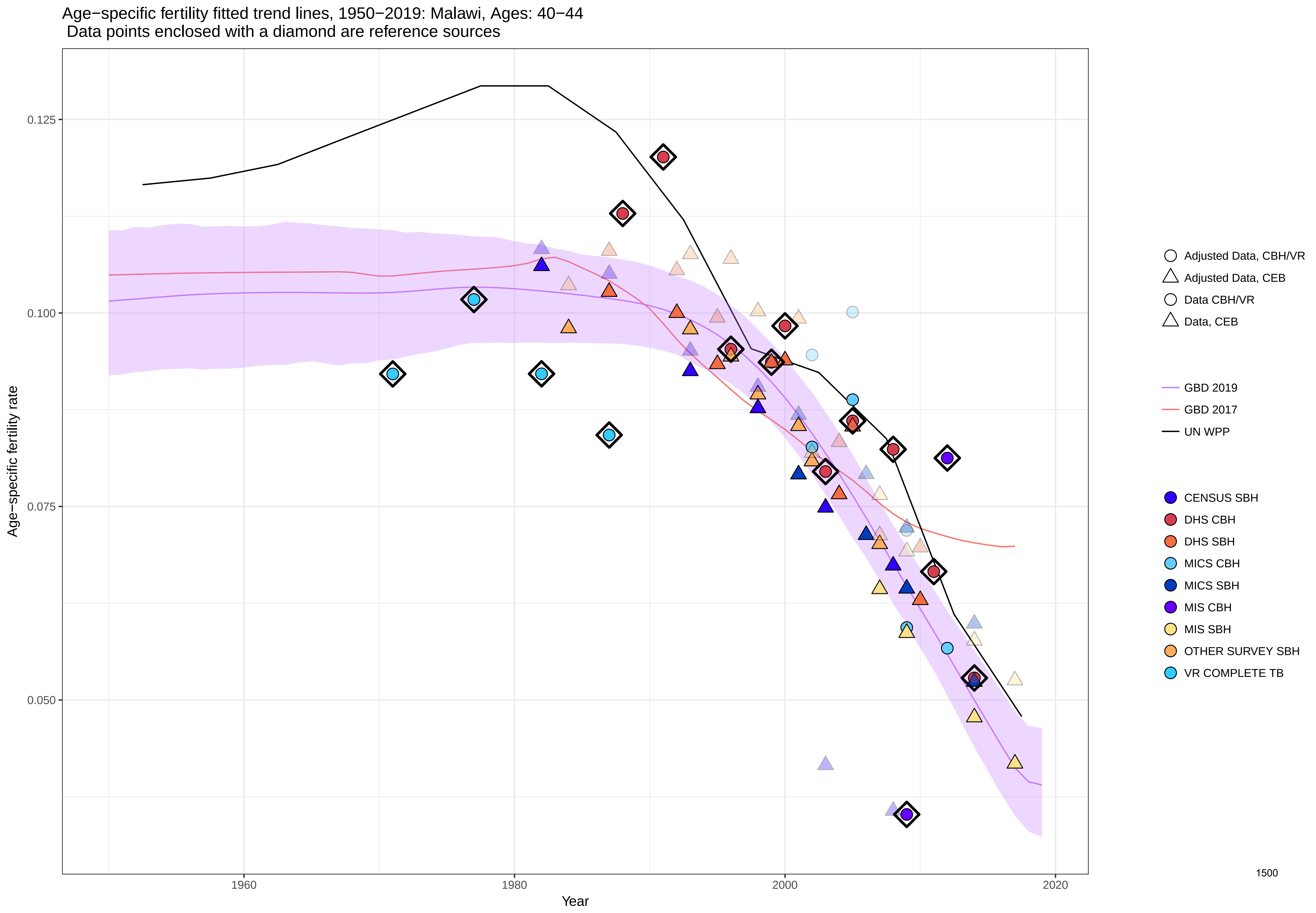
Age−specific fertility fitted trend lines, 1950−2019: Malawi, Ages: 40−44
Data points enclosed with a diamond are reference sources
Age−specific fertility rate
Year
0.125
0.100
0.075
0.050
1960
1980
2000
2020
Adjusted Data, CBH/VR
Adjusted Data, CEB
Data CBH/VR
Data, CEB
GBD 2019
GBD 2017
UN WPP
CENSUS SBH
DHS CBH
DHS SBH
MICS CBH
MICS SBH
MIS CBH
MIS SBH
OTHER SURVEY SBH
VR COMPLETE TB

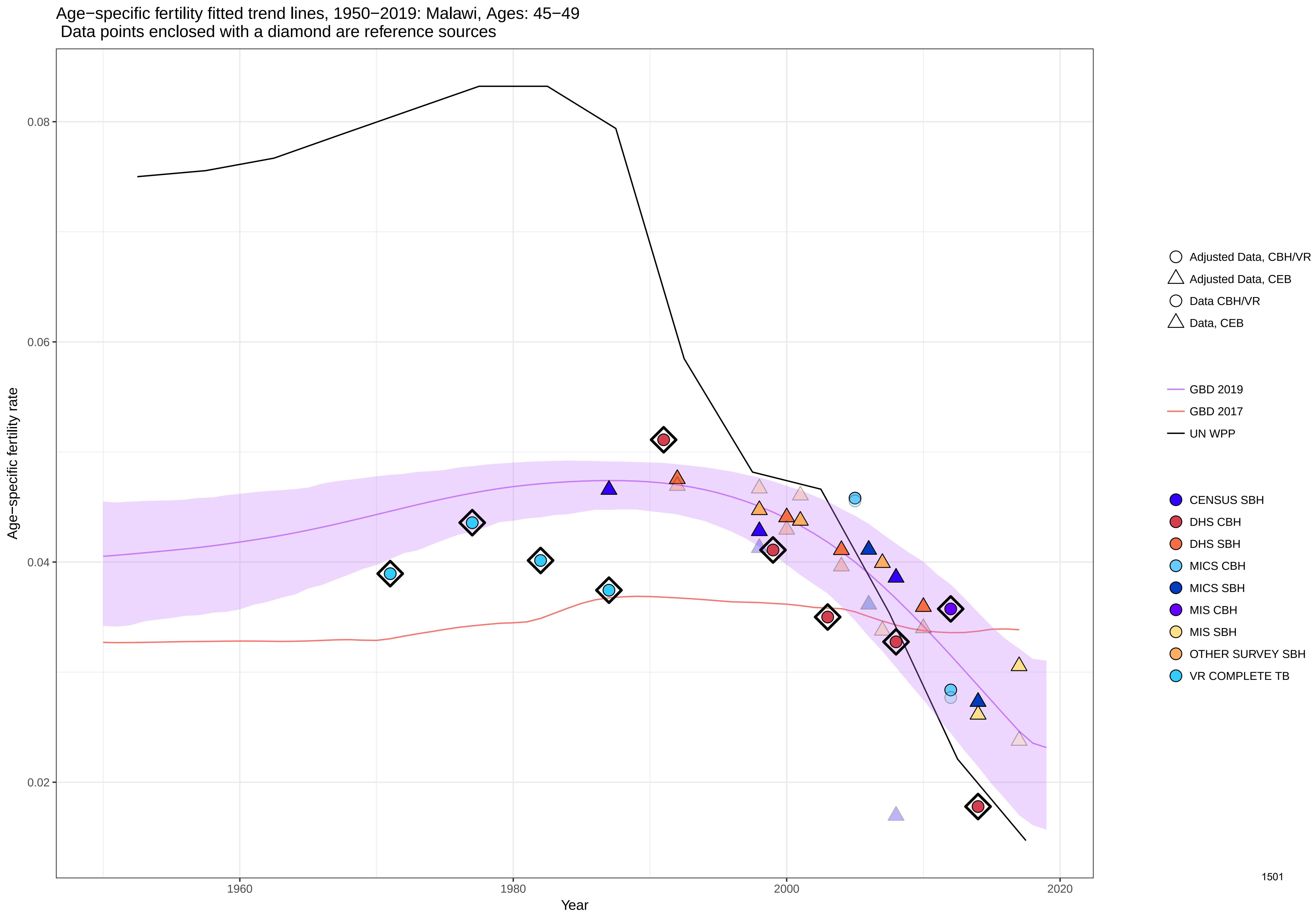

Age−specific fertility fitted trend lines, 1950−2019: Malawi, Ages: 45−49
Data points enclosed with a diamond are reference sources
Age−specific fertility rate
0.08
0.06
0.04
0.02
1960
1980
2000
2020
Year
Adjusted Data, CBH/VR
Adjusted Data, CEB
Data CBH/VR
Data, CEB
GBD 2019
GBD 2017
UN WPP
CENSUS SBH
DHS CBH
DHS SBH
MICS CBH
MICS SBH
MIS CBH
MIS SBH
OTHER SURVEY SBH
VR COMPLETE TB

Age−specific fertility fitted trend lines, 1950−2019: Malawi, Ages: 50−54
Data points enclosed with a diamond are reference sources

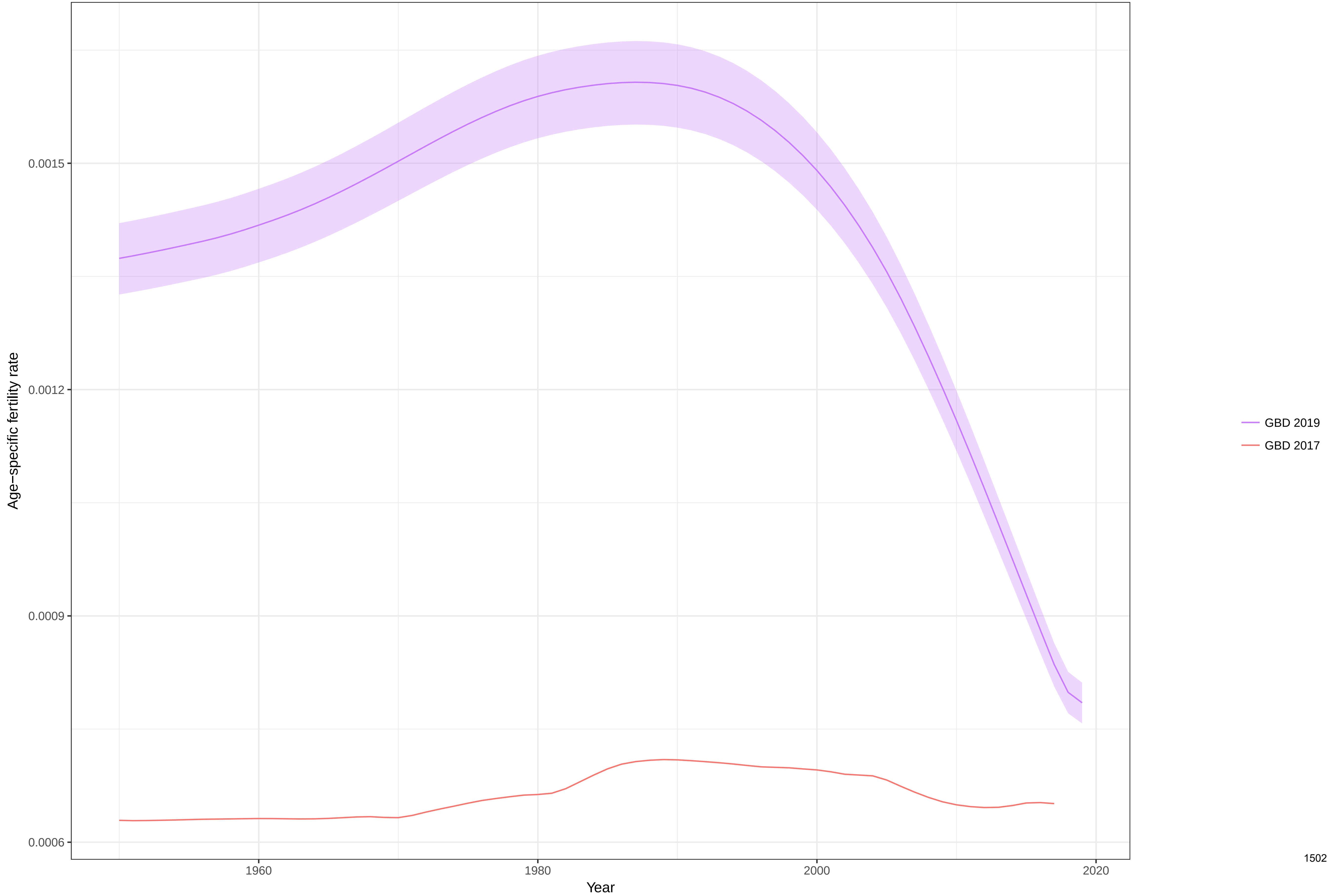

Total fertility rate fitted trend lines, 1950−2019: Malawi TFR
Data points enclosed with a diamond are reference sources

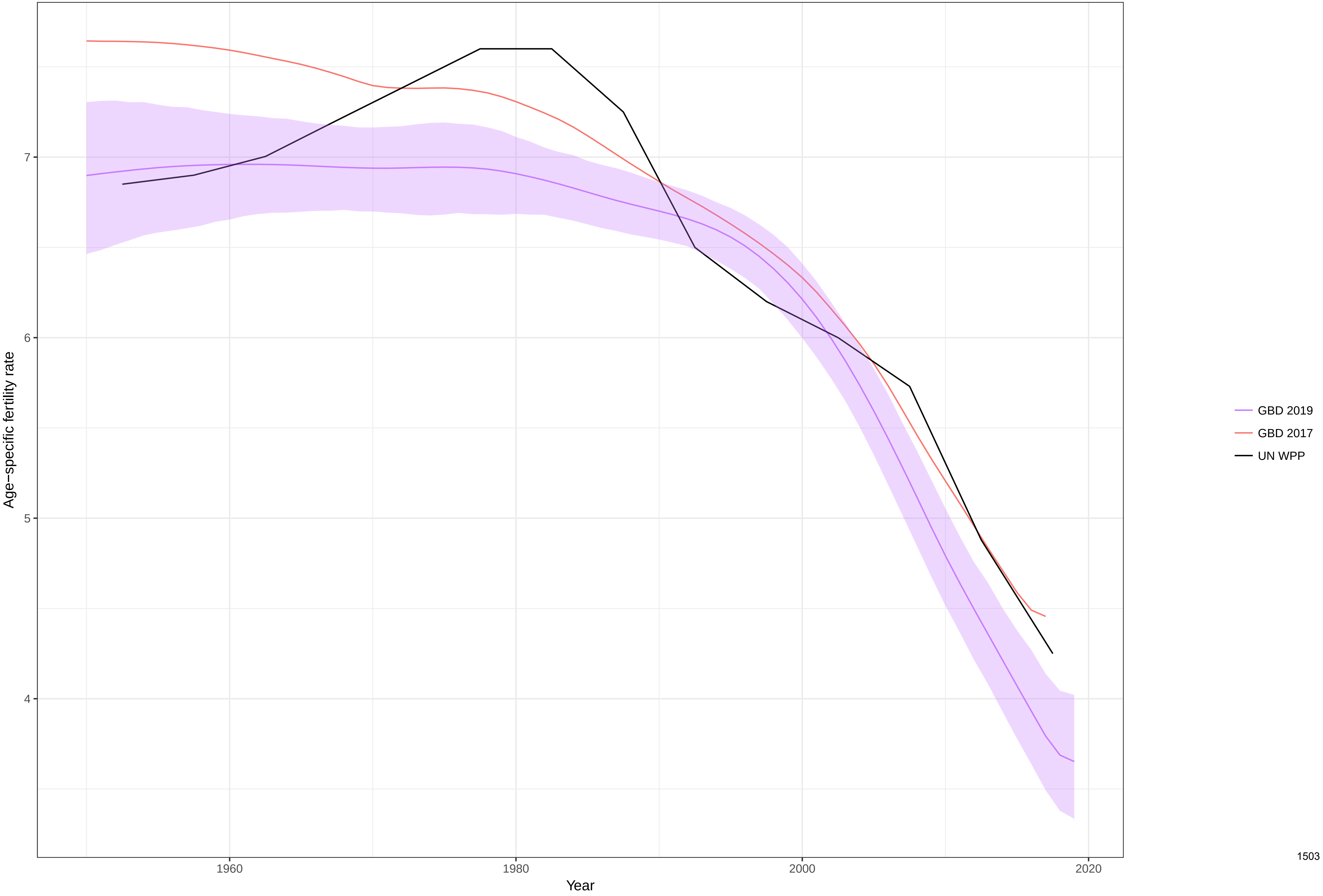

Age−specific fertility fitted trend lines, 1950−2019: Malaysia, Ages: 10−14
Data points enclosed with a diamond are reference sources

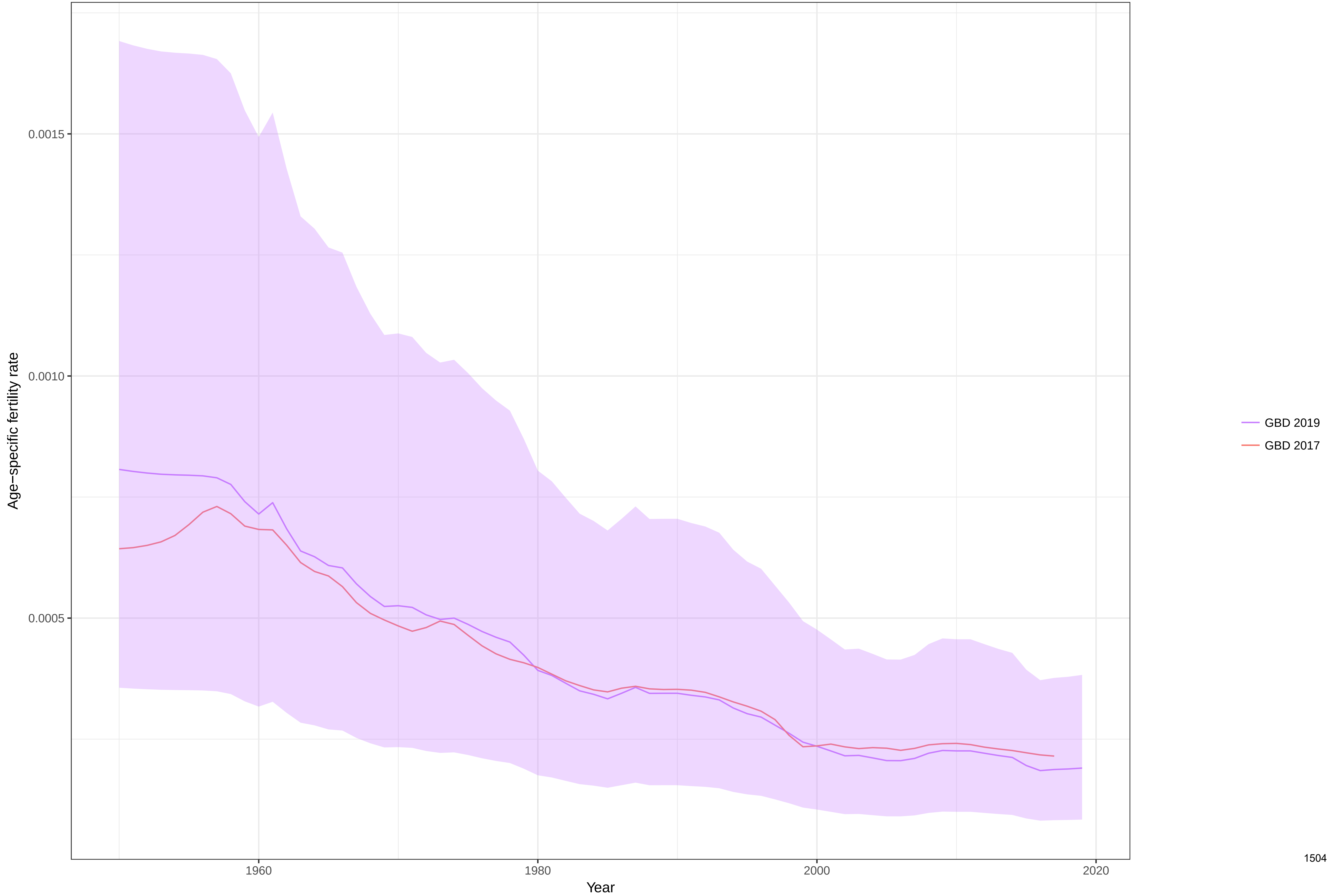

Age−specific fertility fitted trend lines, 1950−2019: Malaysia, Ages: 15−19
Data points enclosed with a diamond are reference sources

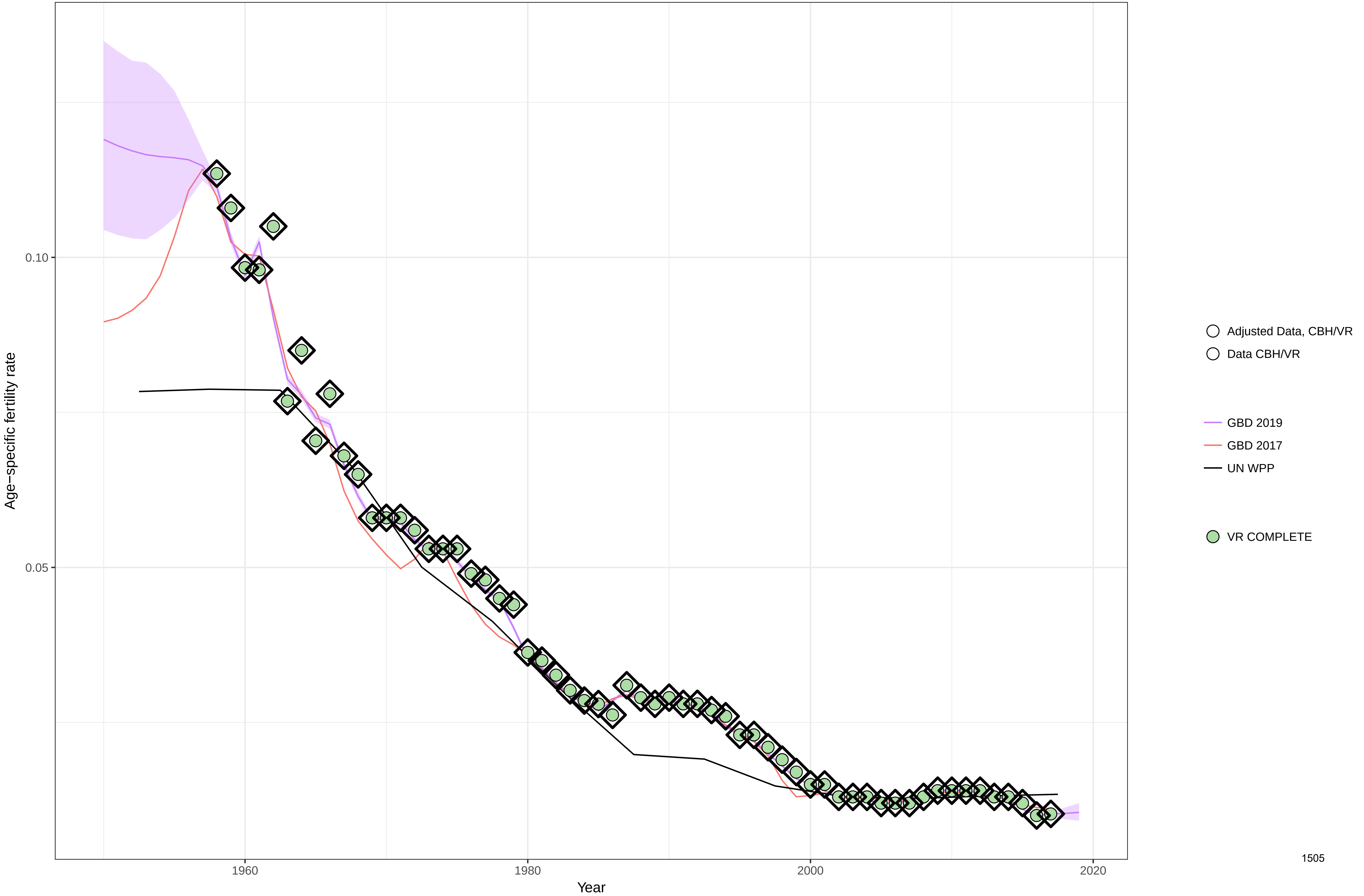

Age−specific fertility fitted trend lines, 1950−2019: Malaysia, Ages: 20−24
Data points enclosed with a diamond are reference sources

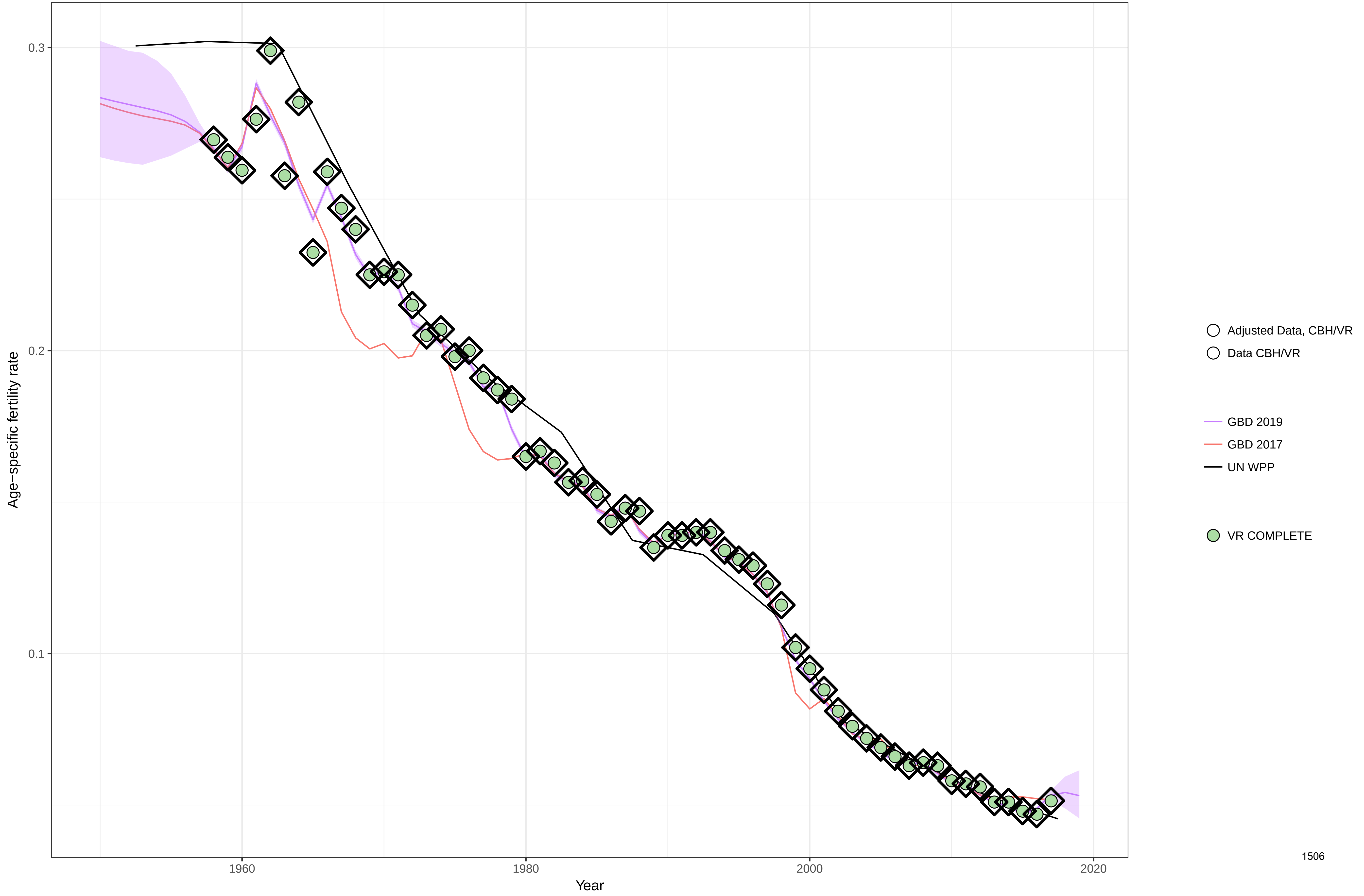

Age−specific fertility fitted trend lines, 1950−2019: Malaysia, Ages: 25−29
Data points enclosed with a diamond are reference sources

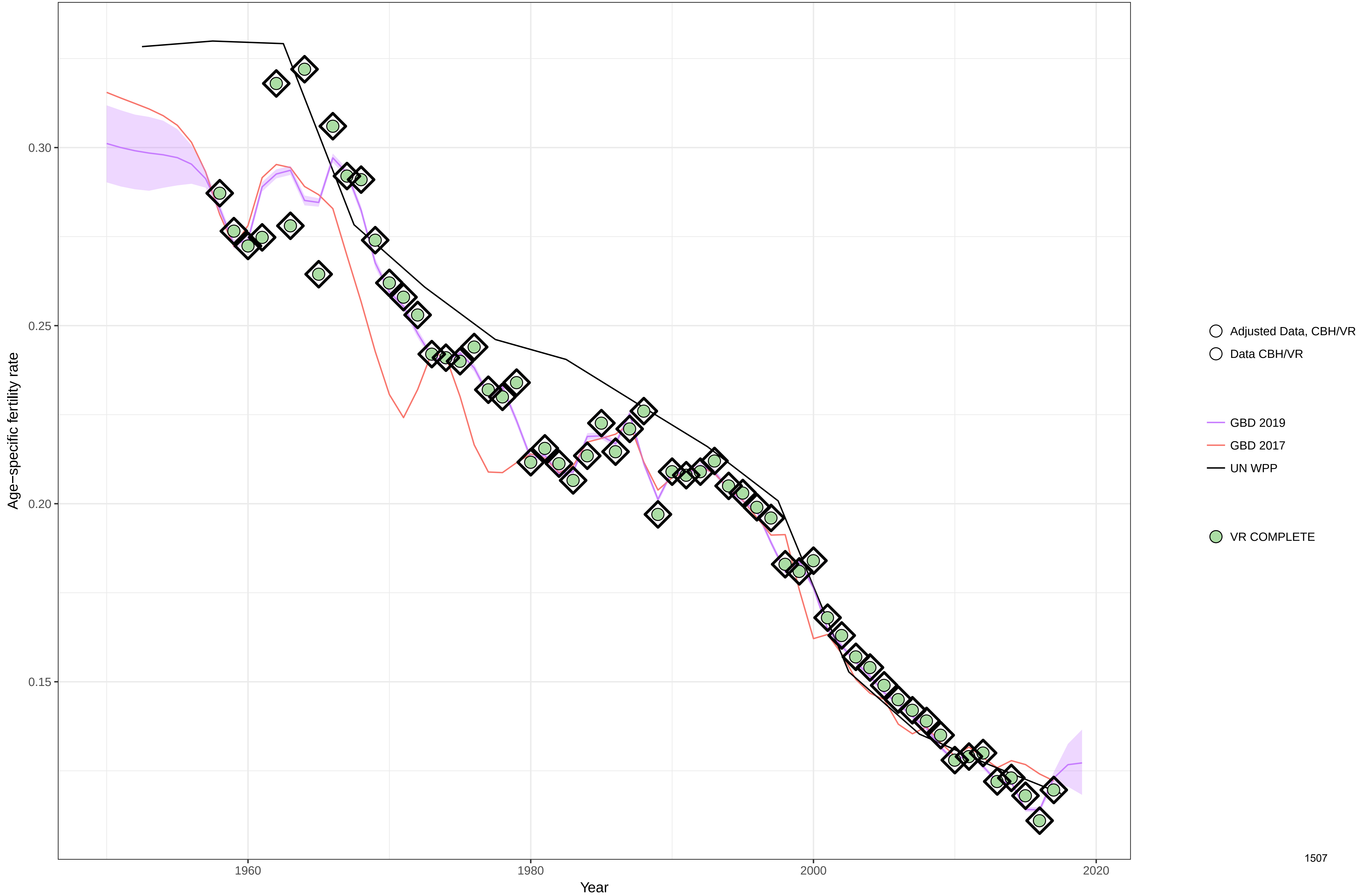

Age−specific fertility fitted trend lines, 1950−2019: Malaysia, Ages: 30−34
Data points enclosed with a diamond are reference sources

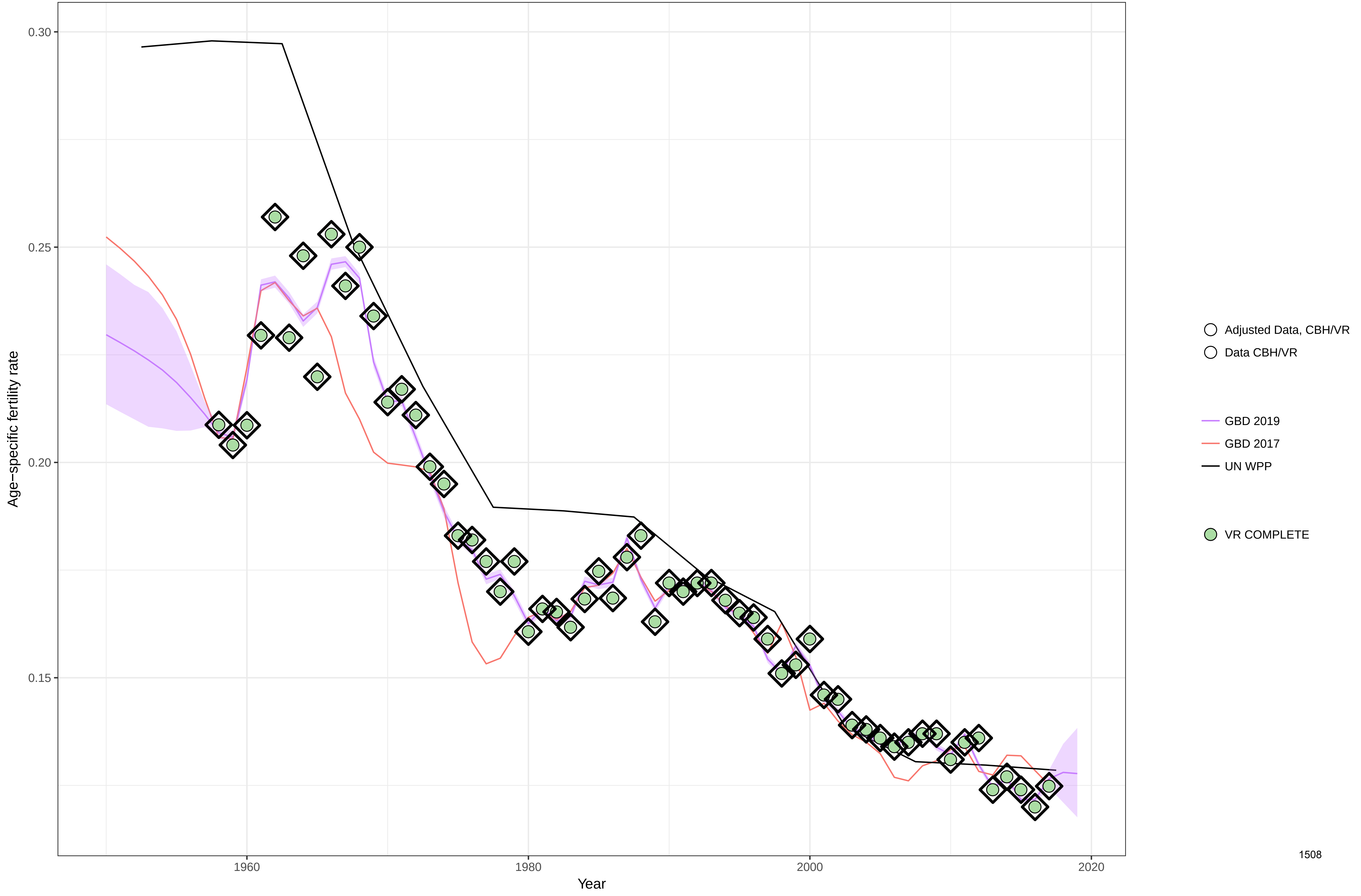

Age−specific fertility fitted trend lines, 1950−2019: Malaysia, Ages: 35−39
Data points enclosed with a diamond are reference sources

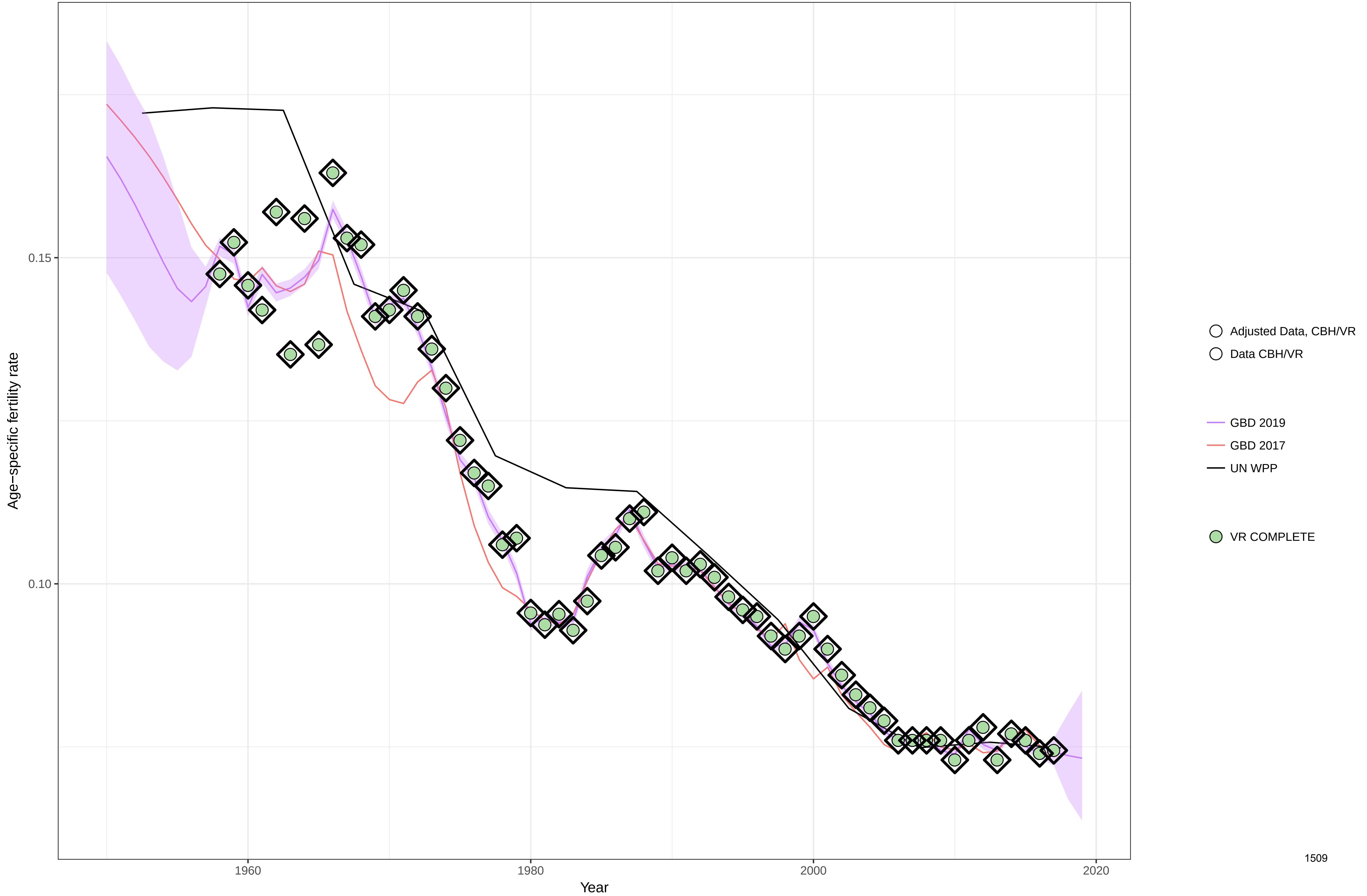

Age−specific fertility fitted trend lines, 1950−2019: Malaysia, Ages: 40−44
Data points enclosed with a diamond are reference sources

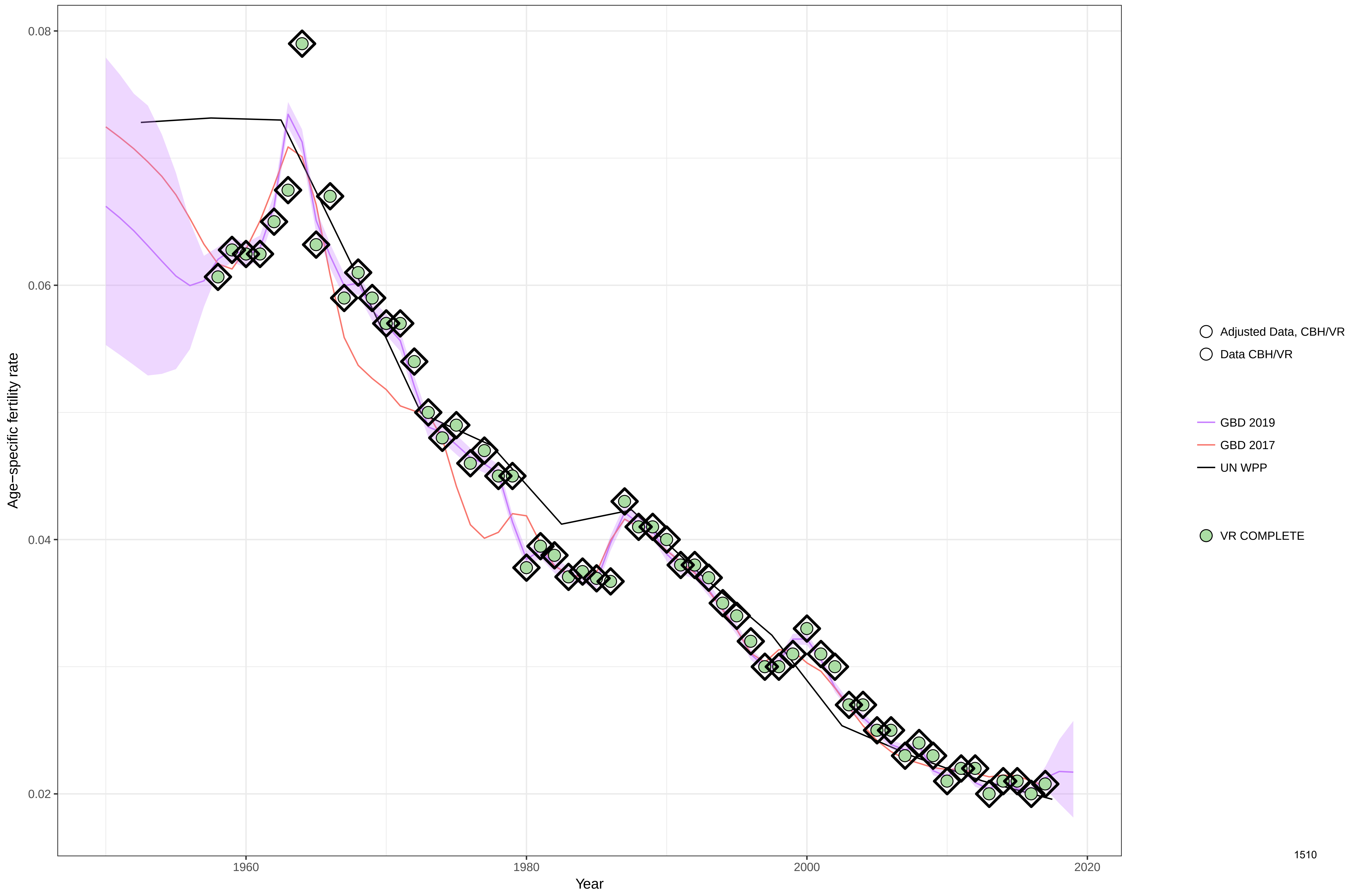

Age−specific fertility fitted trend lines, 1950−2019: Malaysia, Ages: 45−49
Data points enclosed with a diamond are reference sources

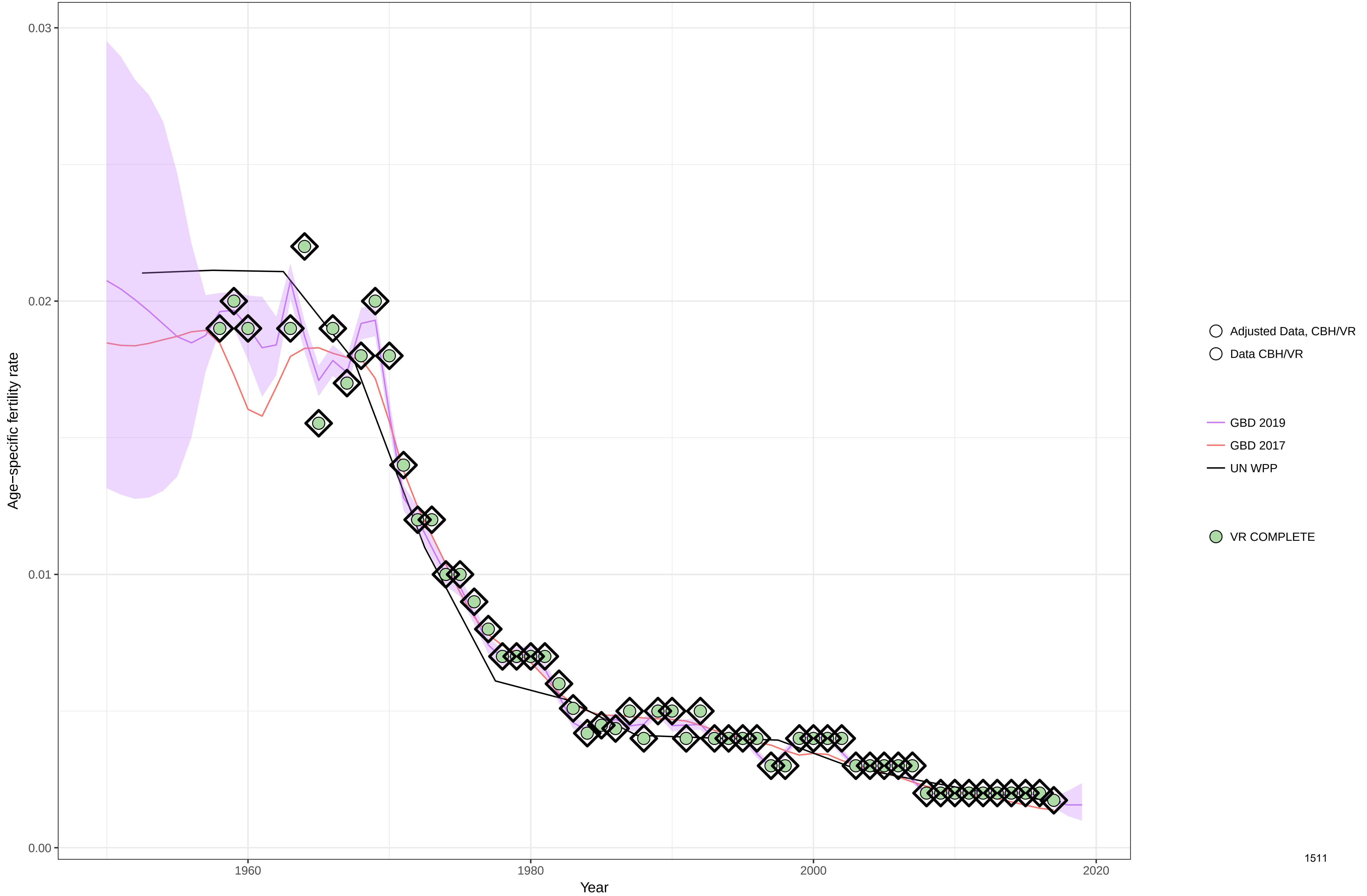

Age−specific fertility fitted trend lines, 1950−2019: Malaysia, Ages: 50−54
Data points enclosed with a diamond are reference sources

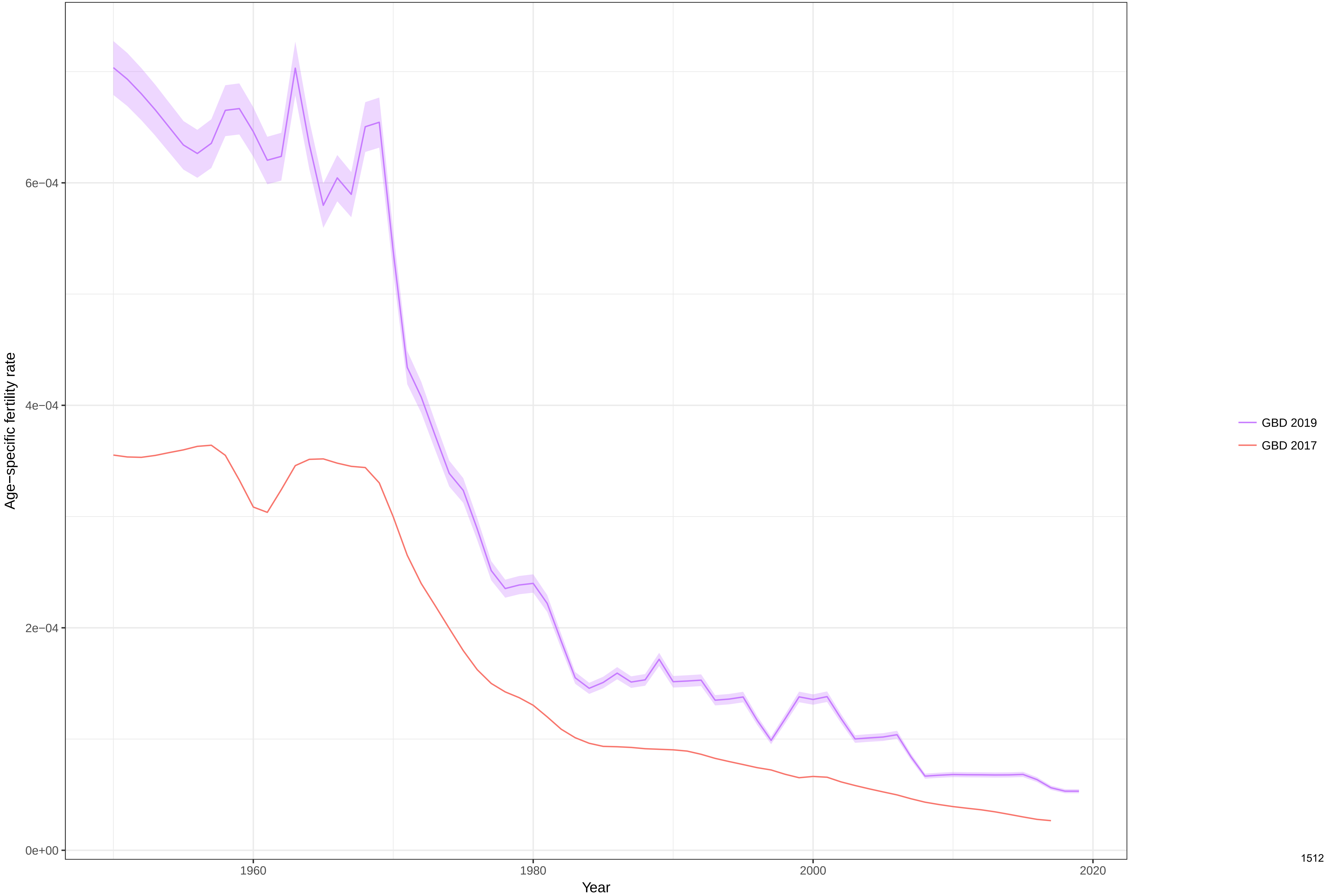

Total fertility rate fitted trend lines, 1950−2019: Malaysia TFR
Data points enclosed with a diamond are reference sources

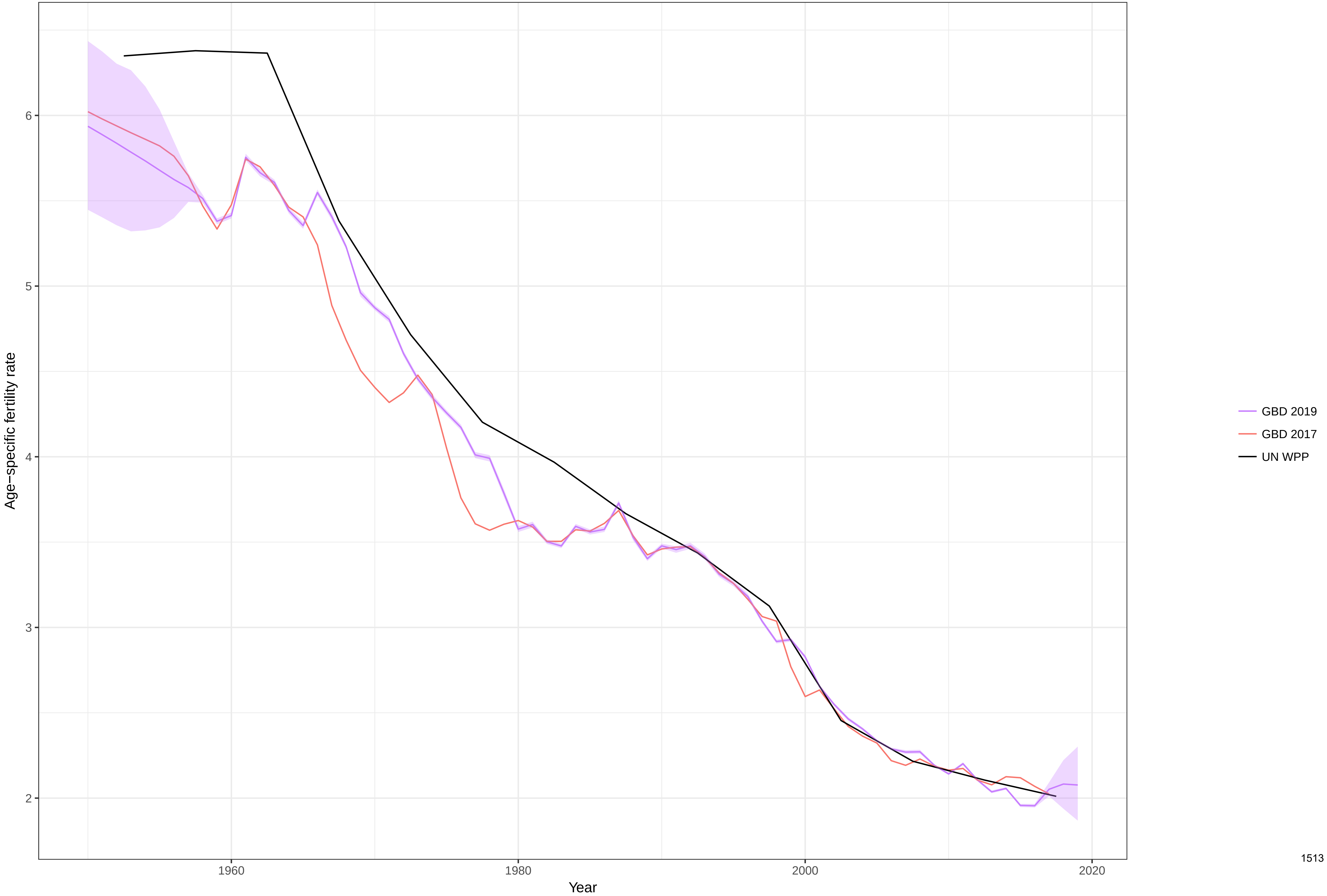

Age−specific fertility fitted trend lines, 1950−2019: Namibia, Ages: 10−14
Data points enclosed with a diamond are reference sources

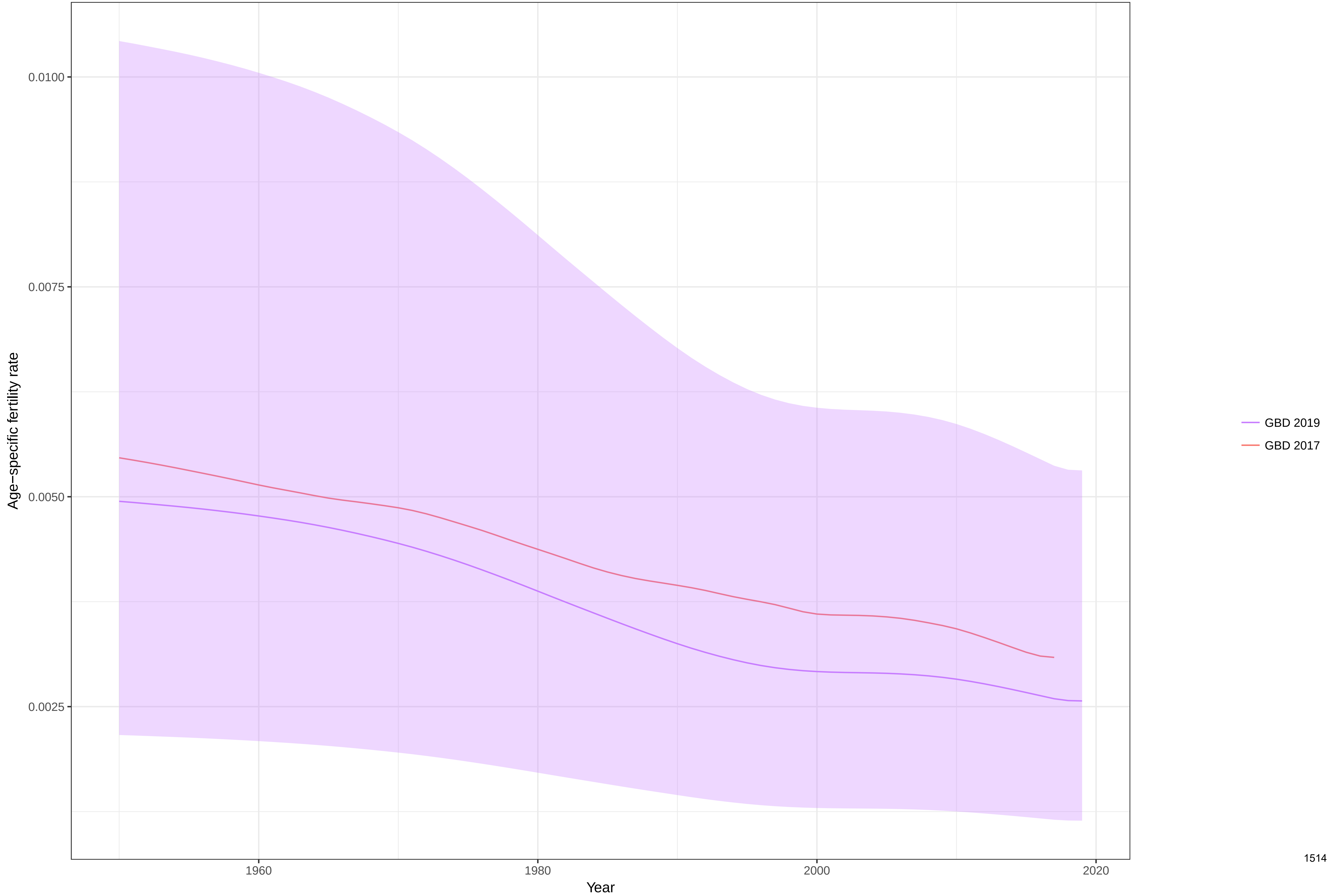

Age−specific fertility fitted trend lines, 1950−2019: Namibia, Ages: 15−19
Data points enclosed with a diamond are reference sources

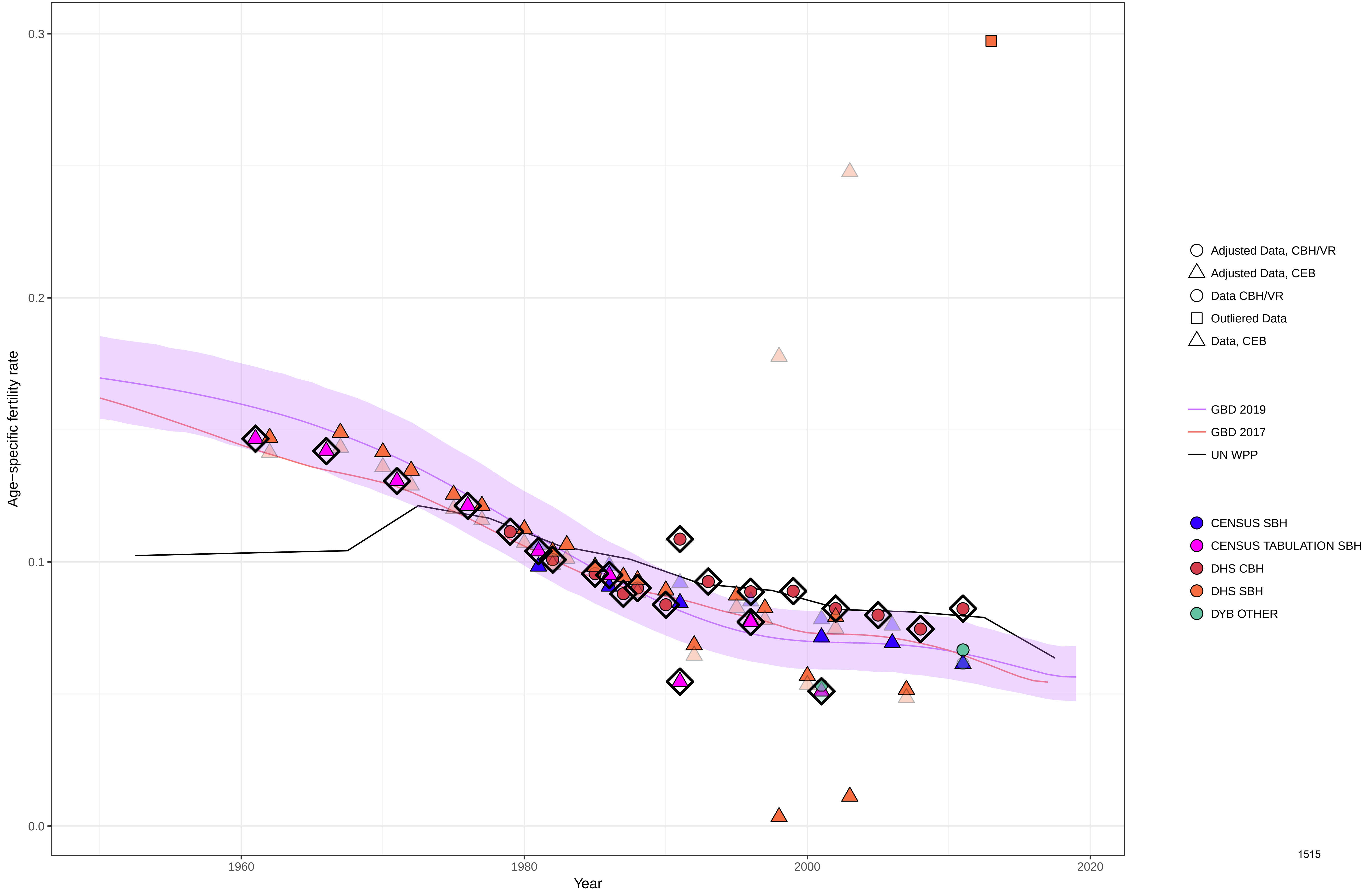

Age−specific fertility fitted trend lines, 1950−2019: Namibia, Ages: 20−24
Data points enclosed with a diamond are reference sources

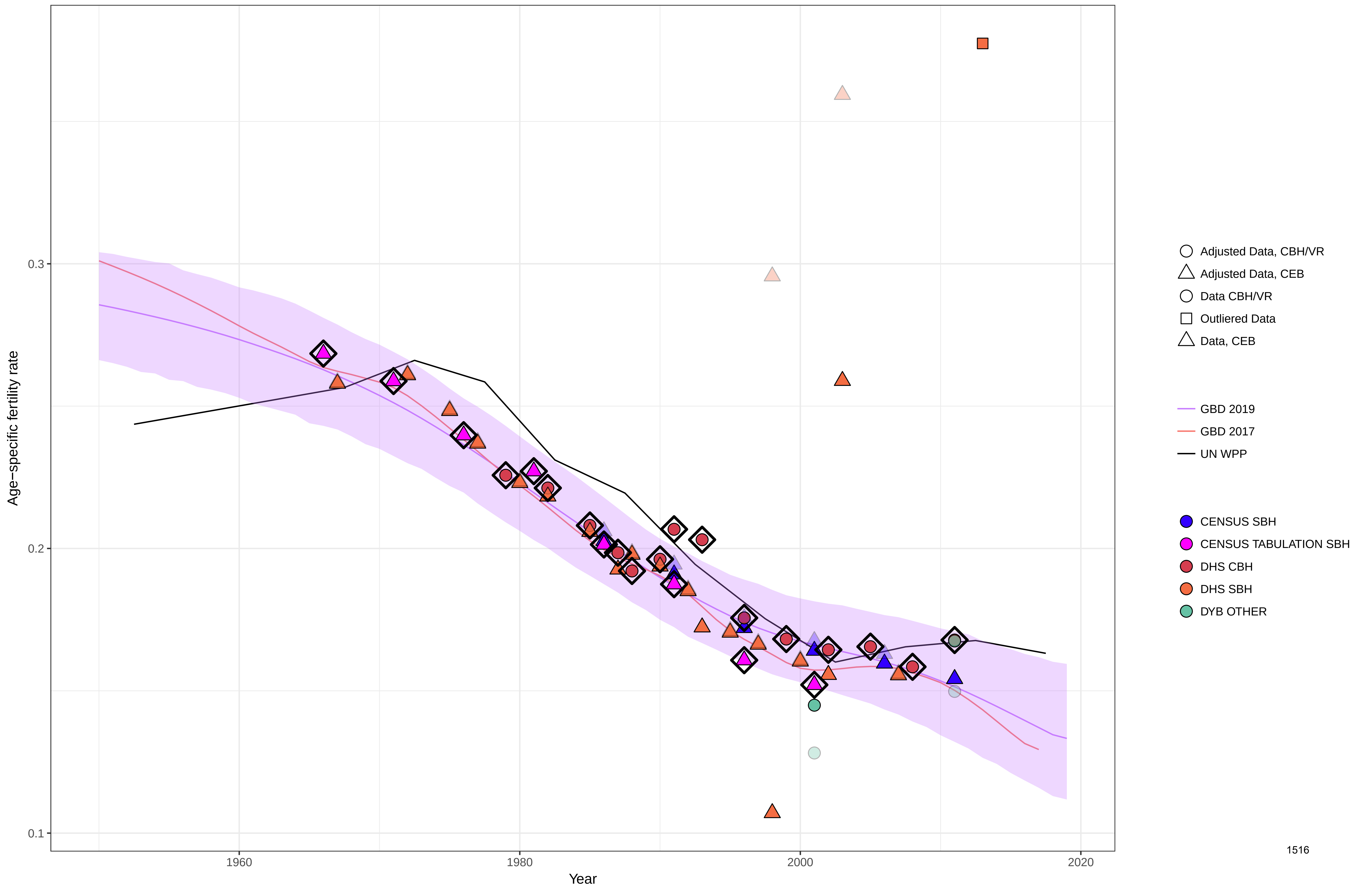

Age−specific fertility fitted trend lines, 1950−2019: Namibia, Ages: 25−29
Data points enclosed with a diamond are reference sources

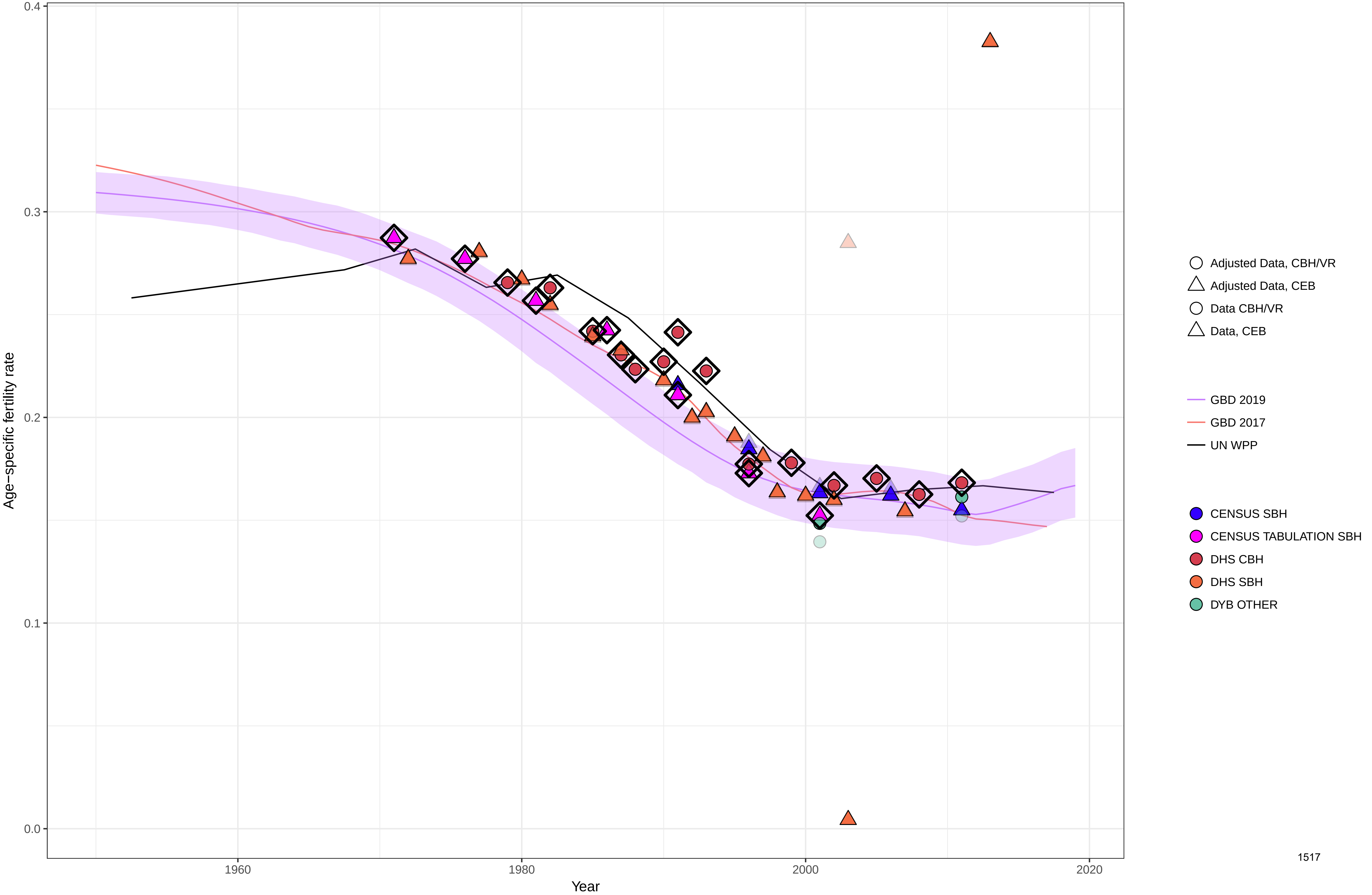

Age−specific fertility fitted trend lines, 1950−2019: Namibia, Ages: 30−34
Data points enclosed with a diamond are reference sources

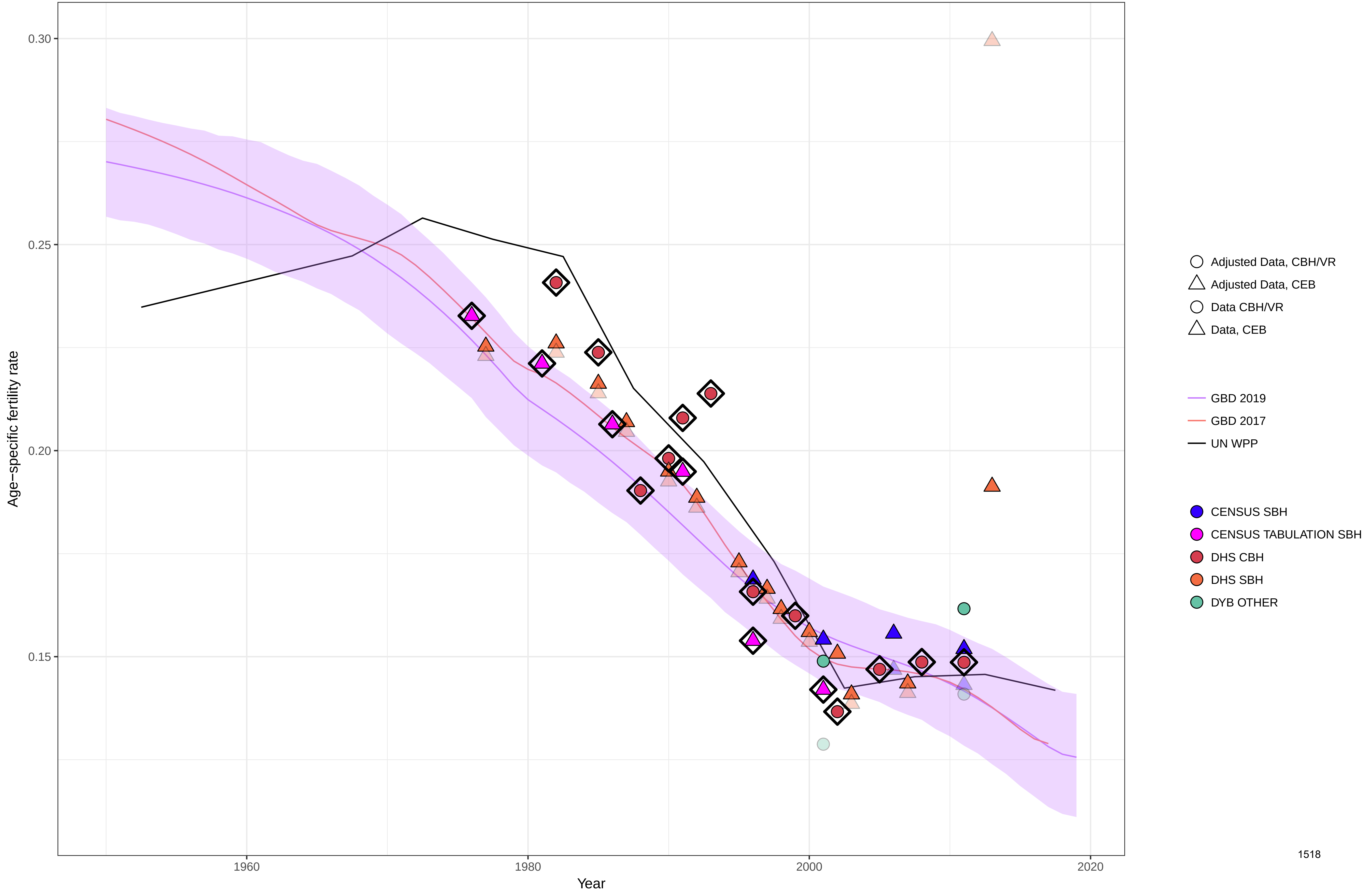

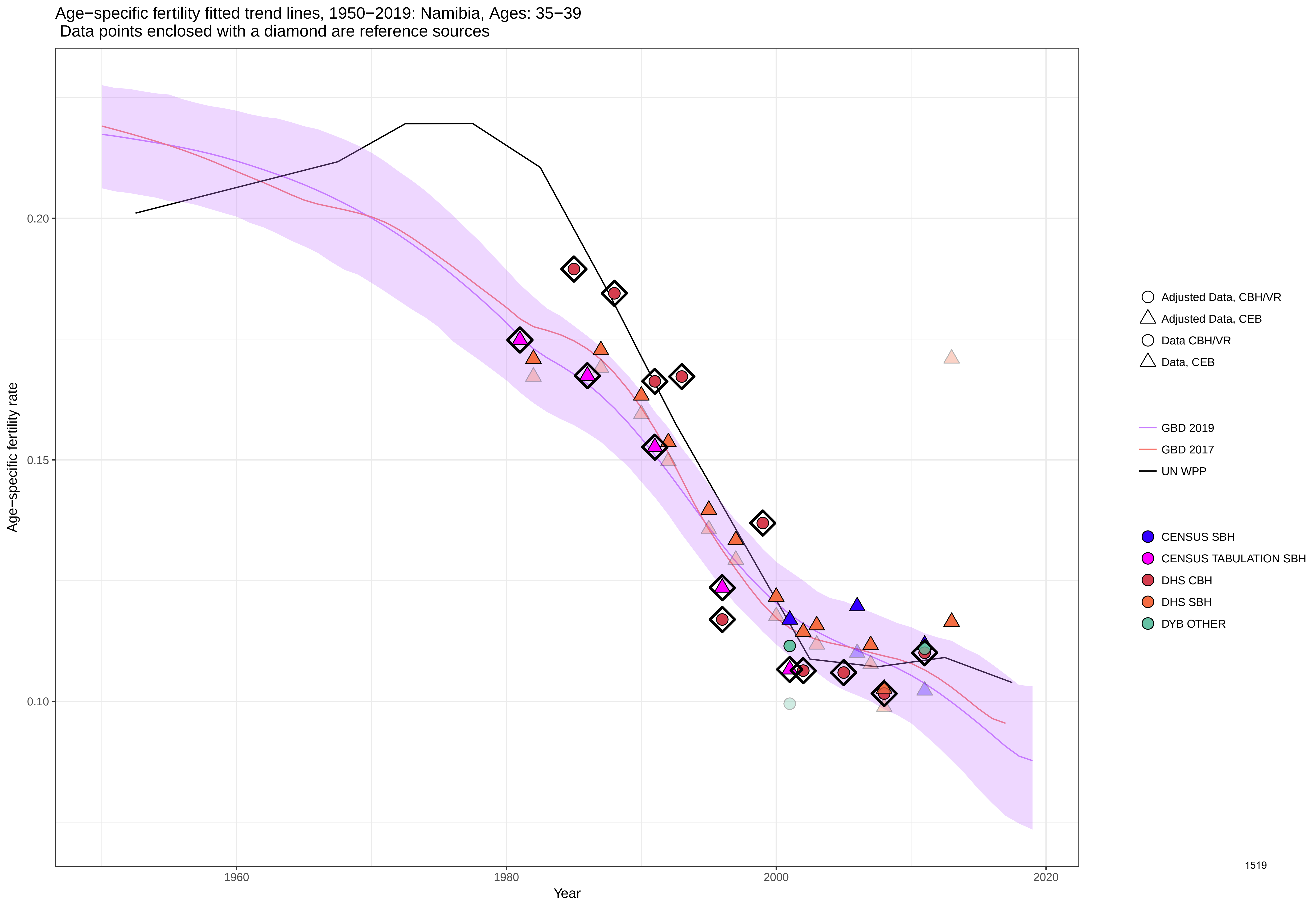

Age−specific fertility fitted trend lines, 1950−2019: Namibia, Ages: 35−39
Data points enclosed with a diamond are reference sources
Age−specific fertility rate
Year
0.20
0.15
0.10
1960
1980
2000
2020
Adjusted Data, CBH/VR
Adjusted Data, CEB
Data CBH/VR
Data, CEB
GBD 2019
GBD 2017
UN WPP
CENSUS SBH
CENSUS TABULATION SBH
DHS CBH
DHS SBH
DYB OTHER

Age−specific fertility fitted trend lines, 1950−2019: Namibia, Ages: 40−44
Data points enclosed with a diamond are reference sources

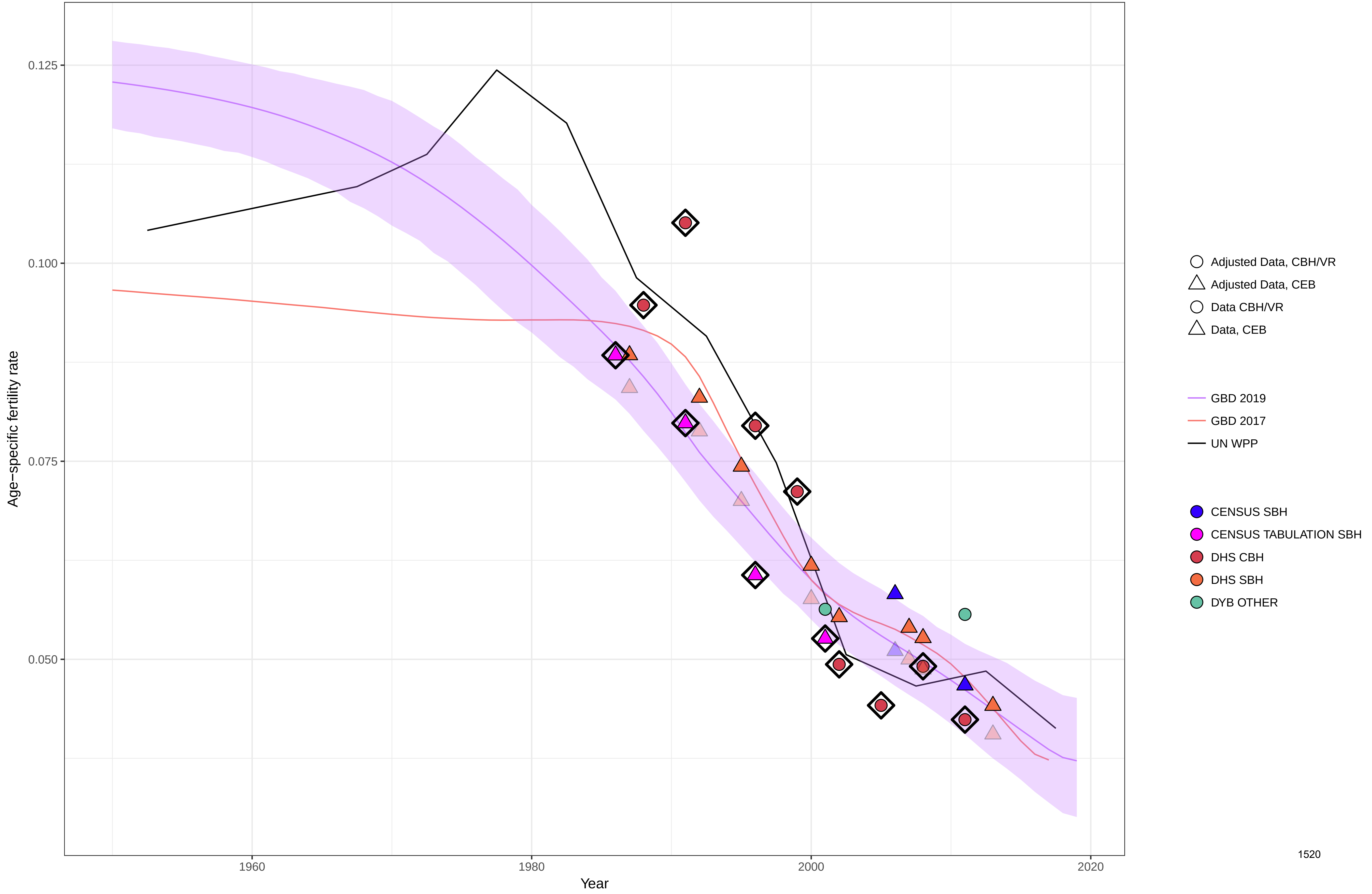

Age−specific fertility fitted trend lines, 1950−2019: Namibia, Ages: 45−49
Data points enclosed with a diamond are reference sources

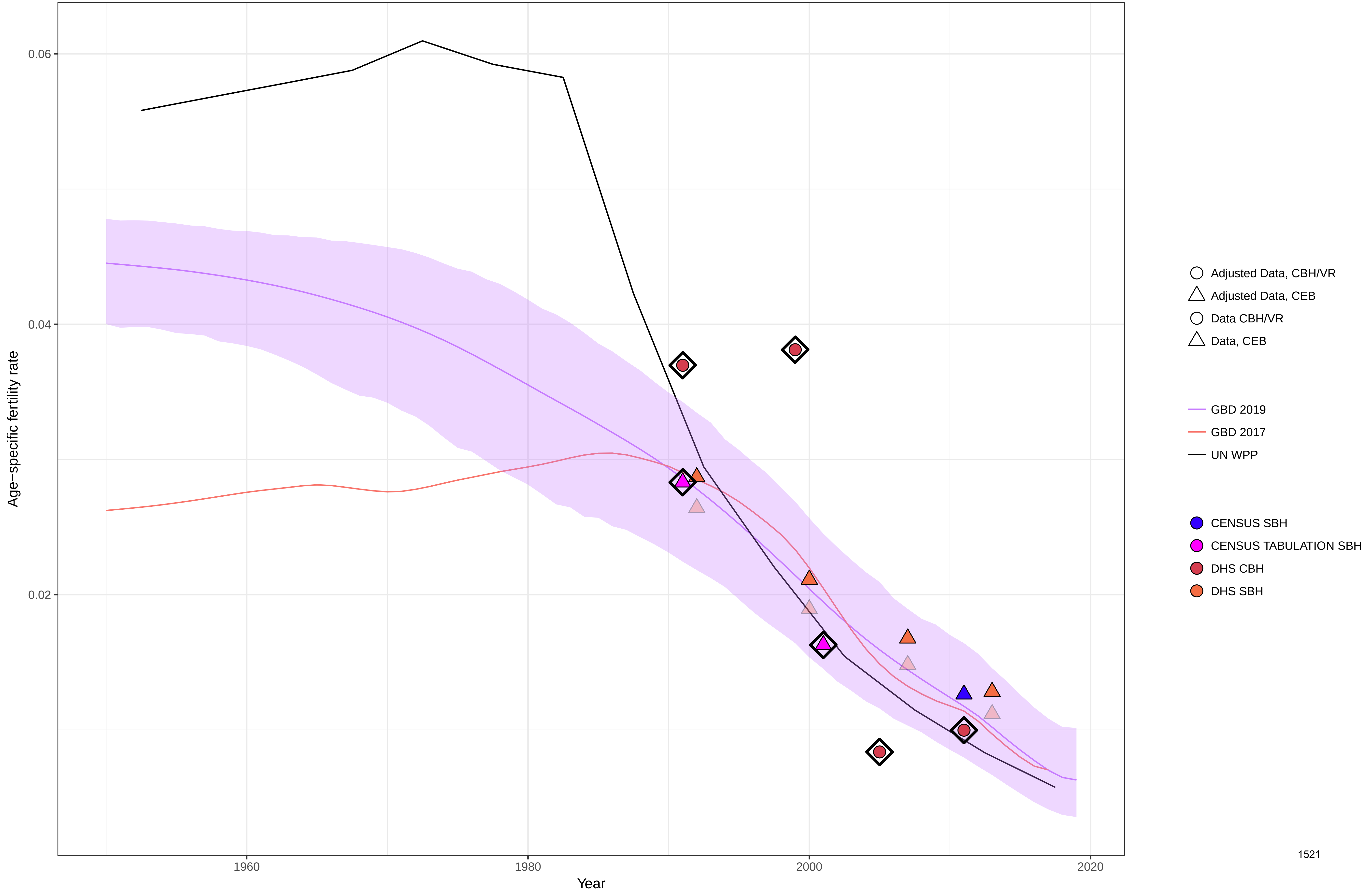

Age−specific fertility fitted trend lines, 1950−2019: Namibia, Ages: 50−54
 Data points enclosed with a diamond are reference sources

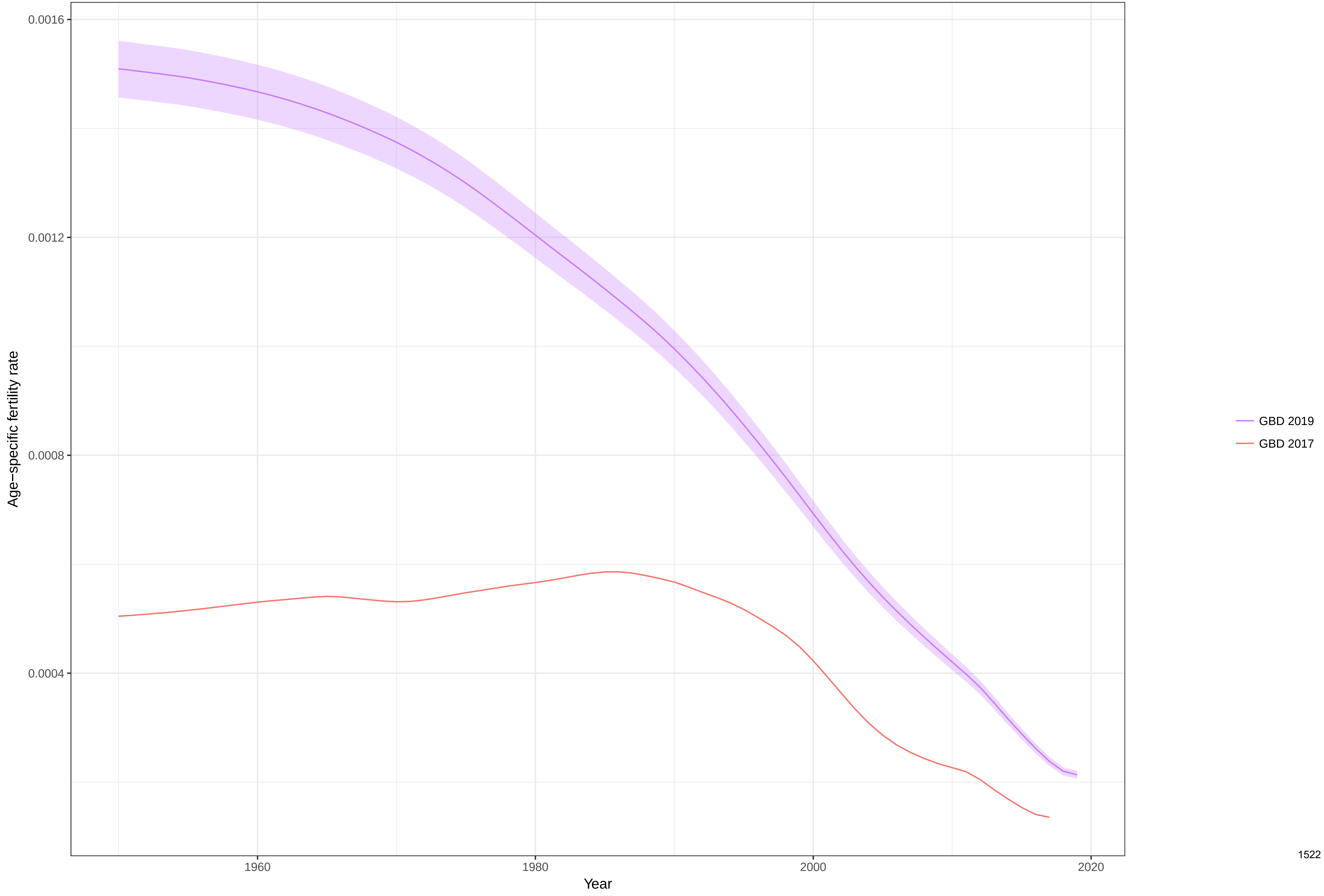

Total fertility rate fitted trend lines, 1950−2019: Namibia TFR
Data points enclosed with a diamond are reference sources

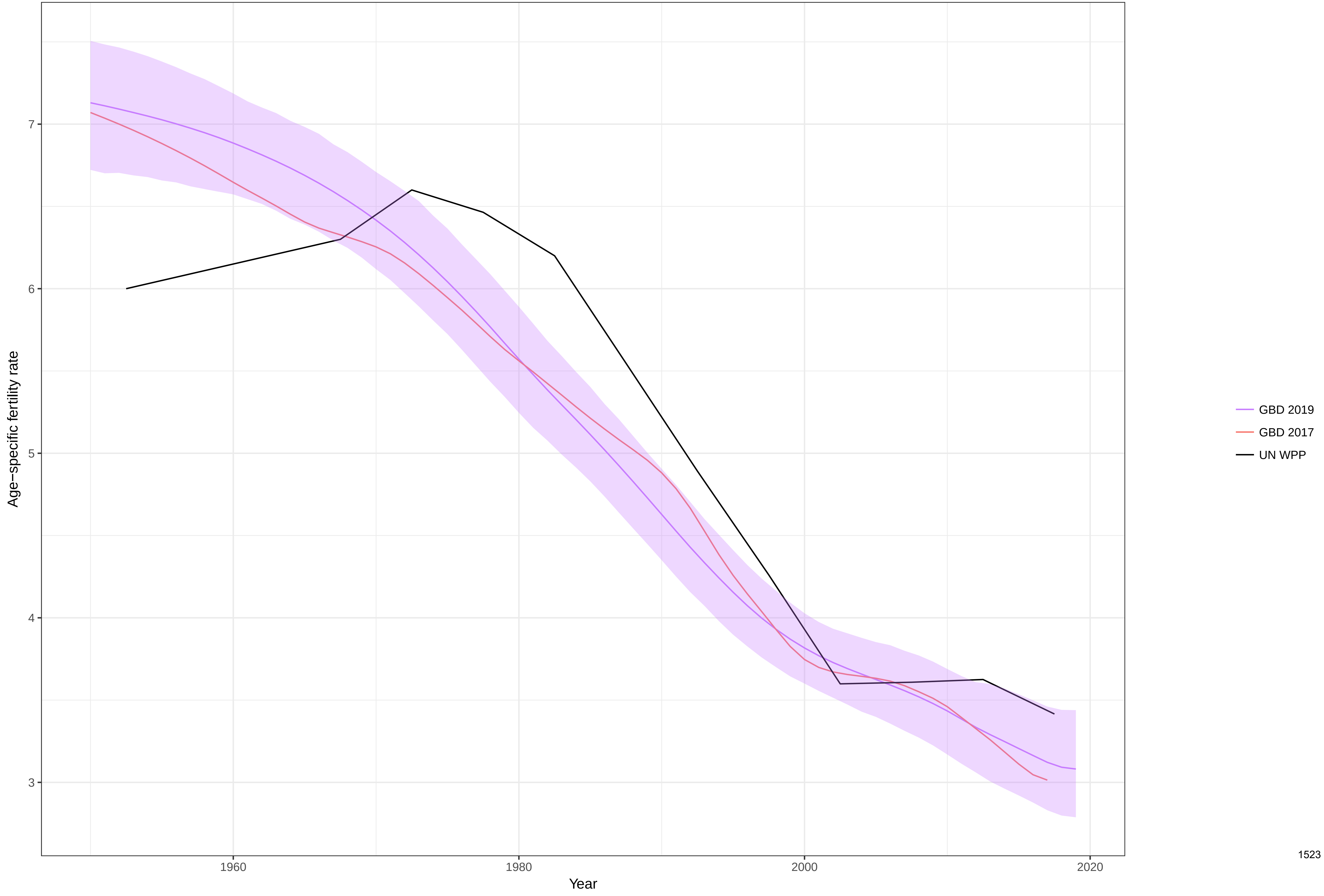

Age−specific fertility fitted trend lines, 1950−2019: Niger, Ages: 10−14
Data points enclosed with a diamond are reference sources

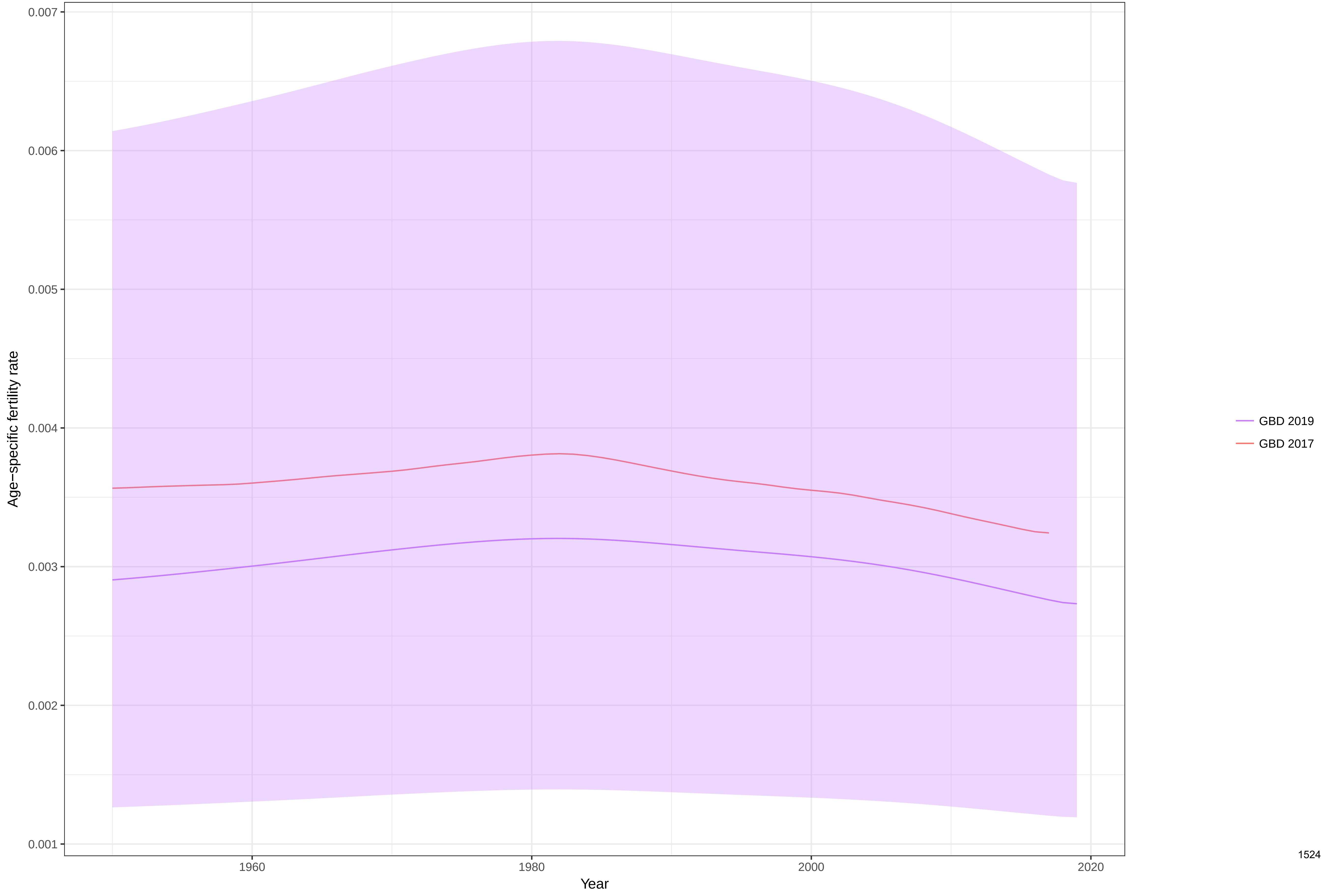

Age−specific fertility fitted trend lines, 1950−2019: Niger, Ages: 15−19
Data points enclosed with a diamond are reference sources

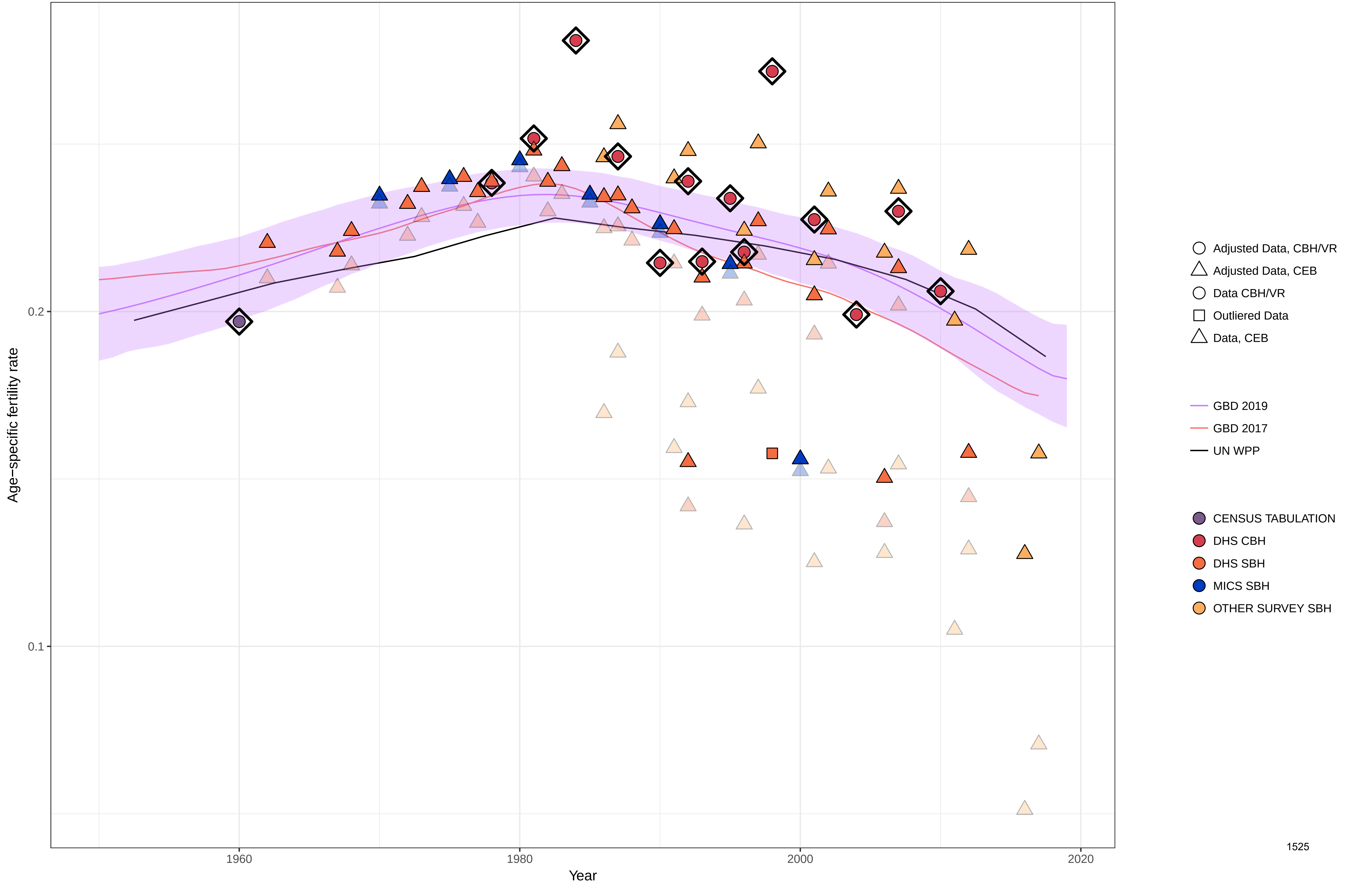

Age−specific fertility fitted trend lines, 1950−2019: Niger, Ages: 20−24
Data points enclosed with a diamond are reference sources

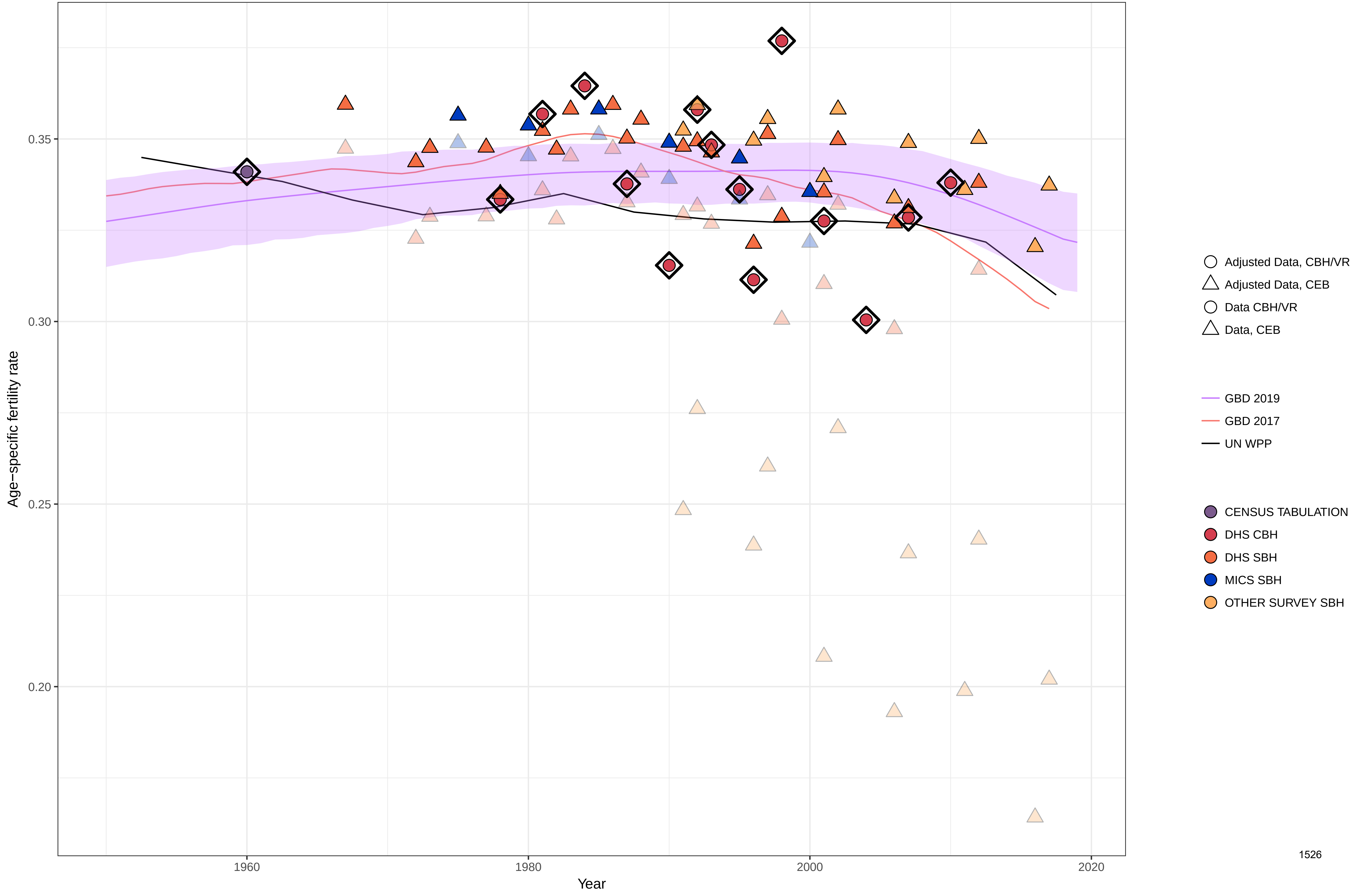

Age−specific fertility fitted trend lines, 1950−2019: Niger, Ages: 25−29
Data points enclosed with a diamond are reference sources

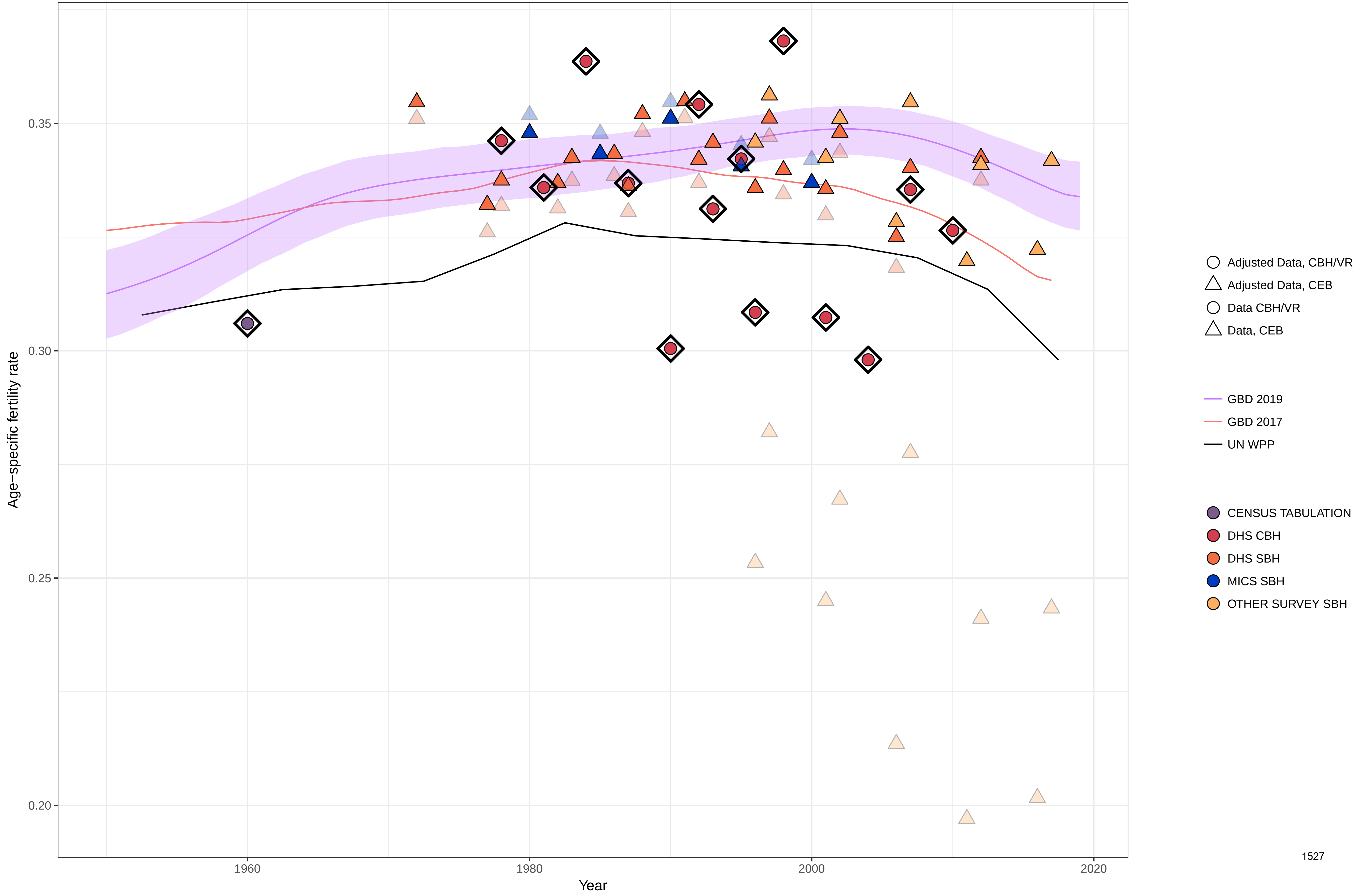

Age−specific fertility fitted trend lines, 1950−2019: Niger, Ages: 30−34
Data points enclosed with a diamond are reference sources

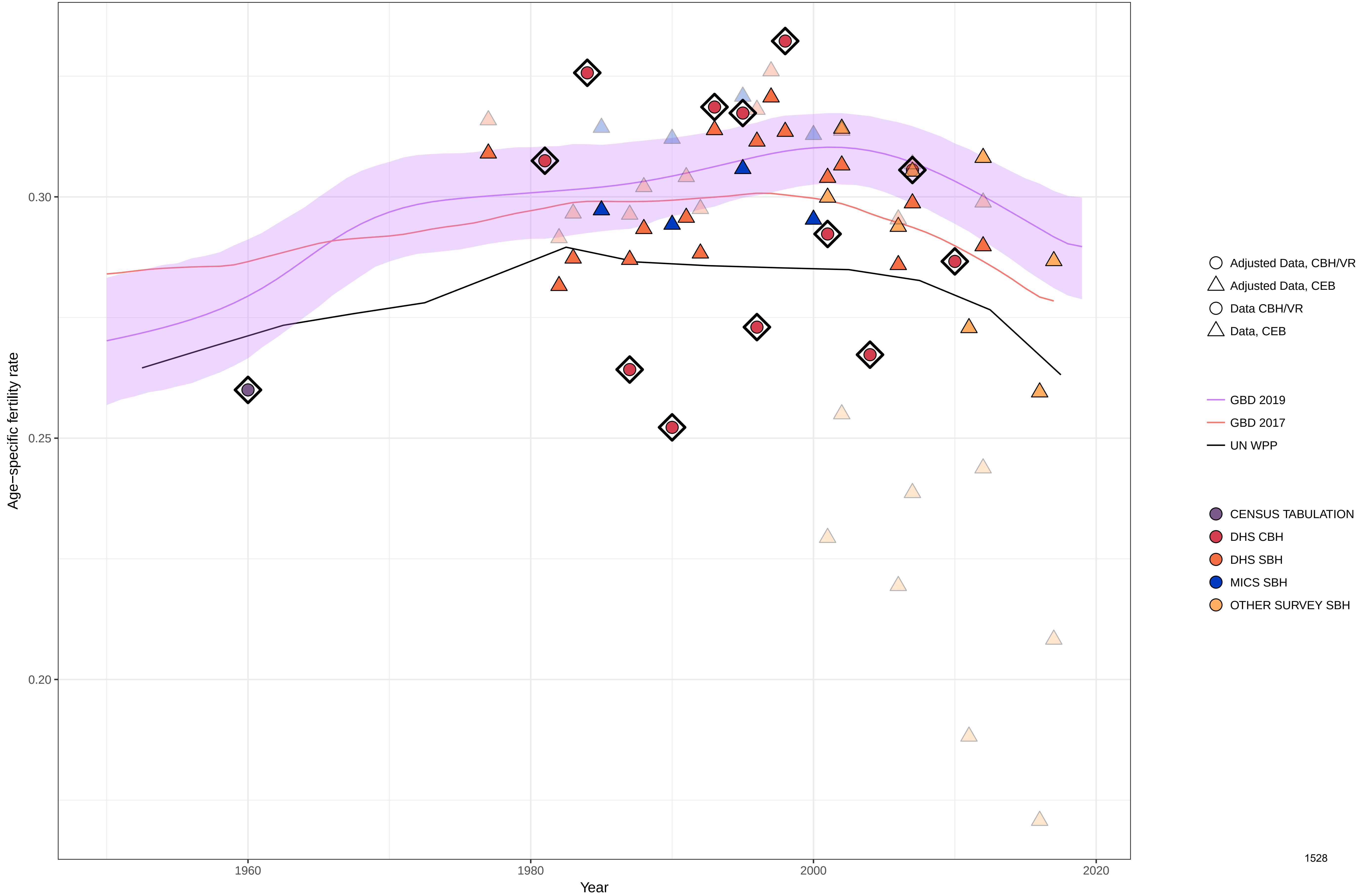

Age−specific fertility fitted trend lines, 1950−2019: Niger, Ages: 35−39
Data points enclosed with a diamond are reference sources

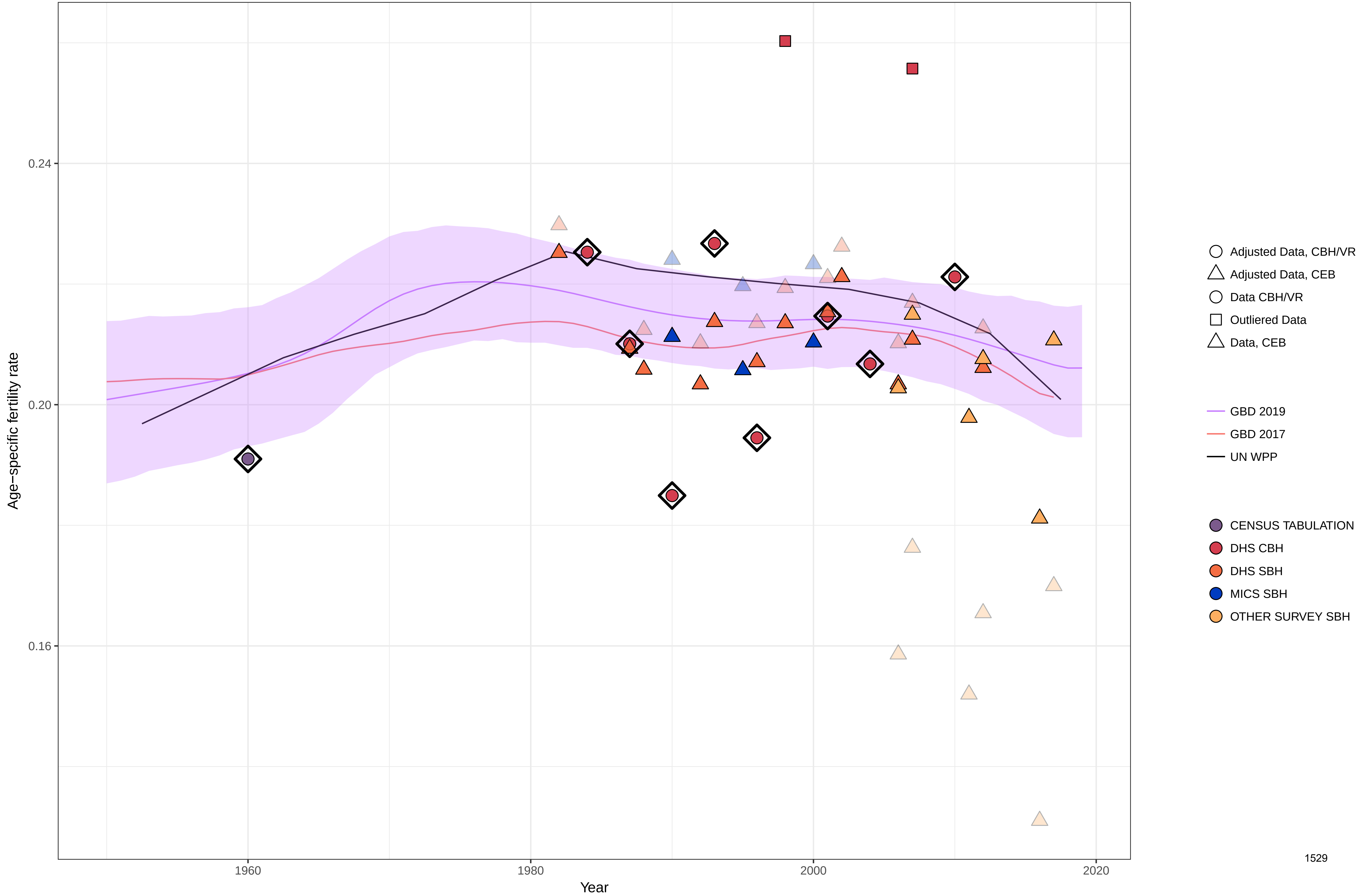

Age−specific fertility fitted trend lines, 1950−2019: Niger, Ages: 40−44
Data points enclosed with a diamond are reference sources

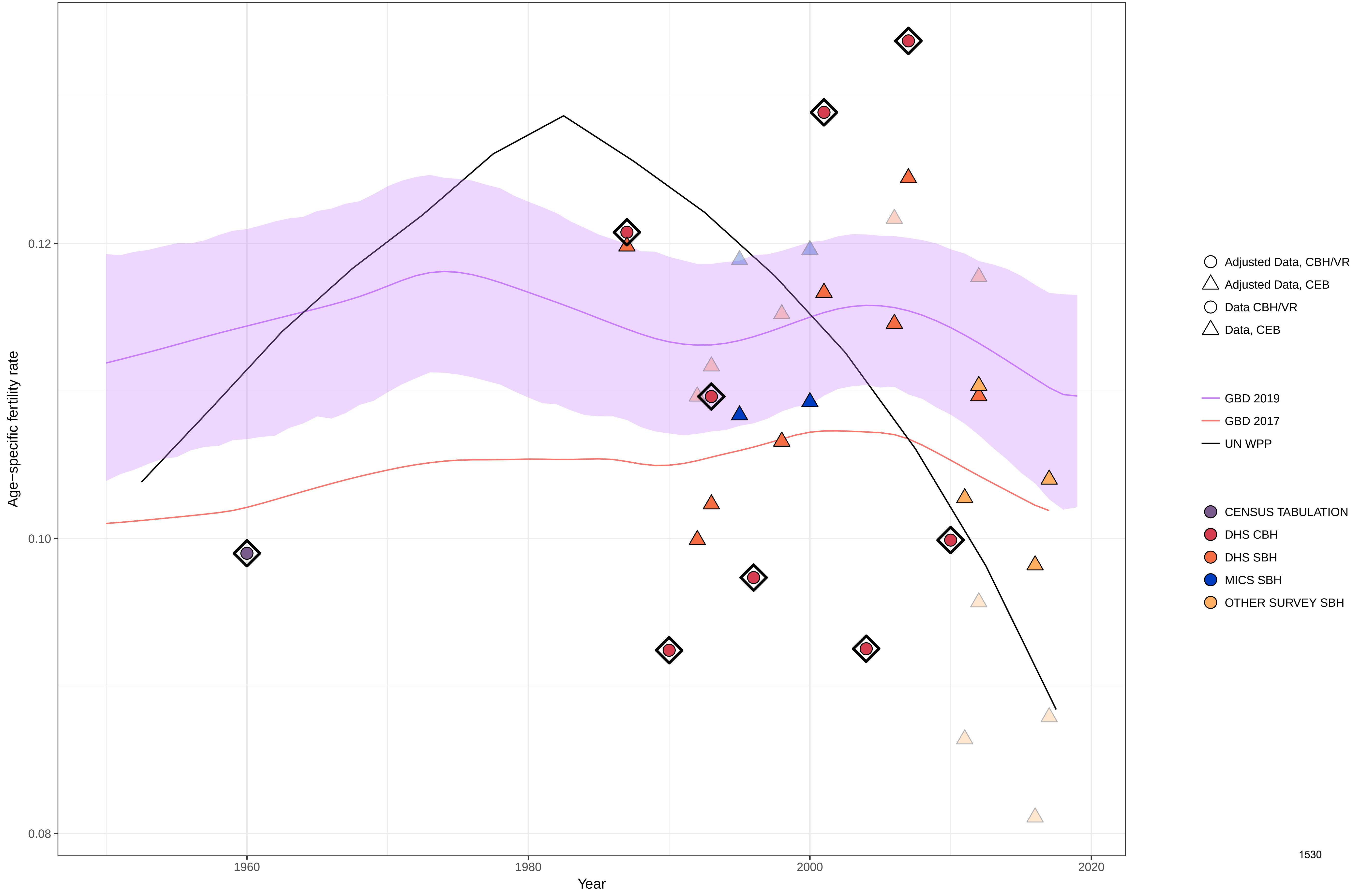

Age−specific fertility fitted trend lines, 1950−2019: Niger, Ages: 45−49
Data points enclosed with a diamond are reference sources

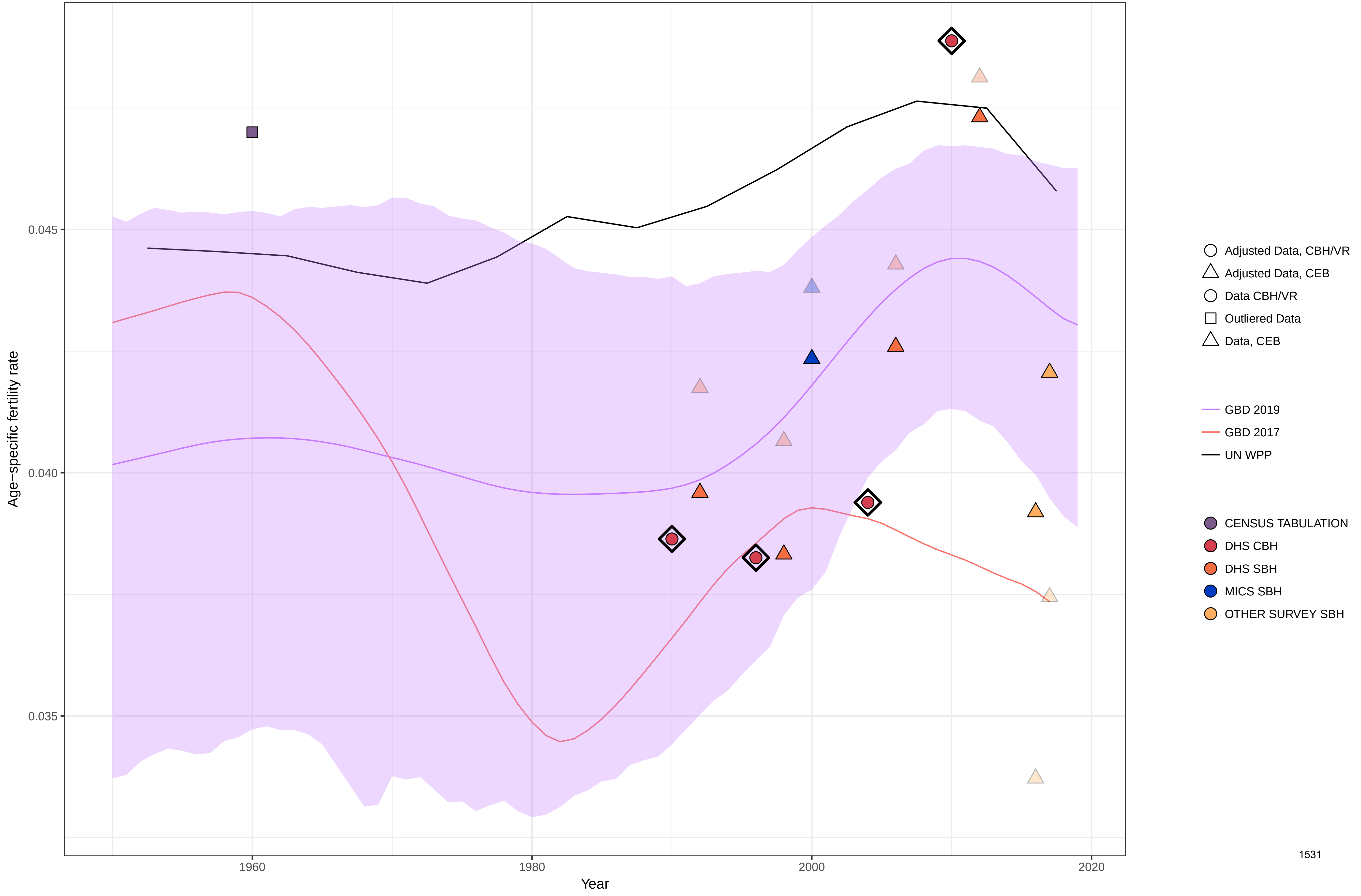

Age−specific fertility fitted trend lines, 1950−2019: Niger, Ages: 50−54
Data points enclosed with a diamond are reference sources

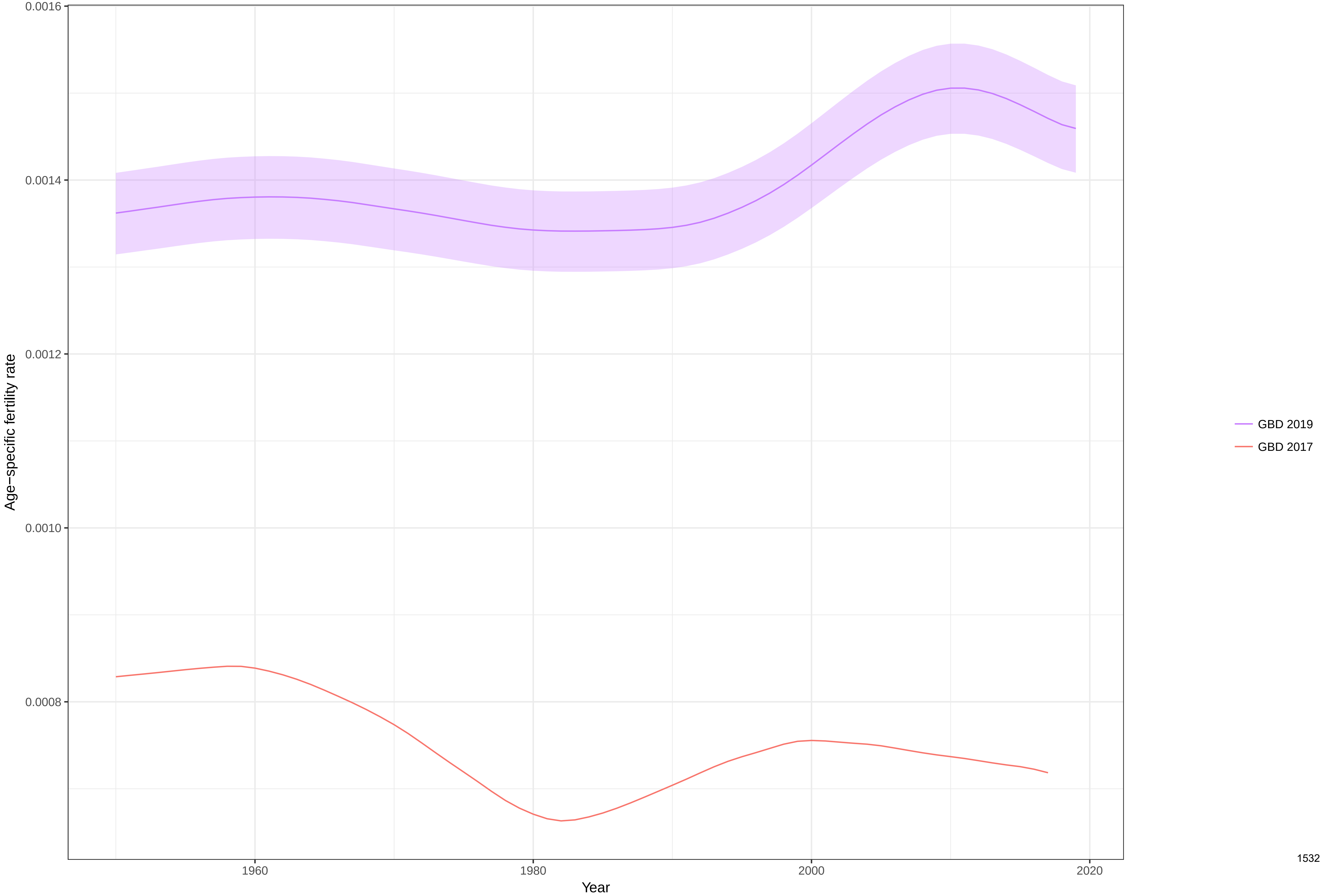

Total fertility rate fitted trend lines, 1950−2019: Niger TFR
Data points enclosed with a diamond are reference sources

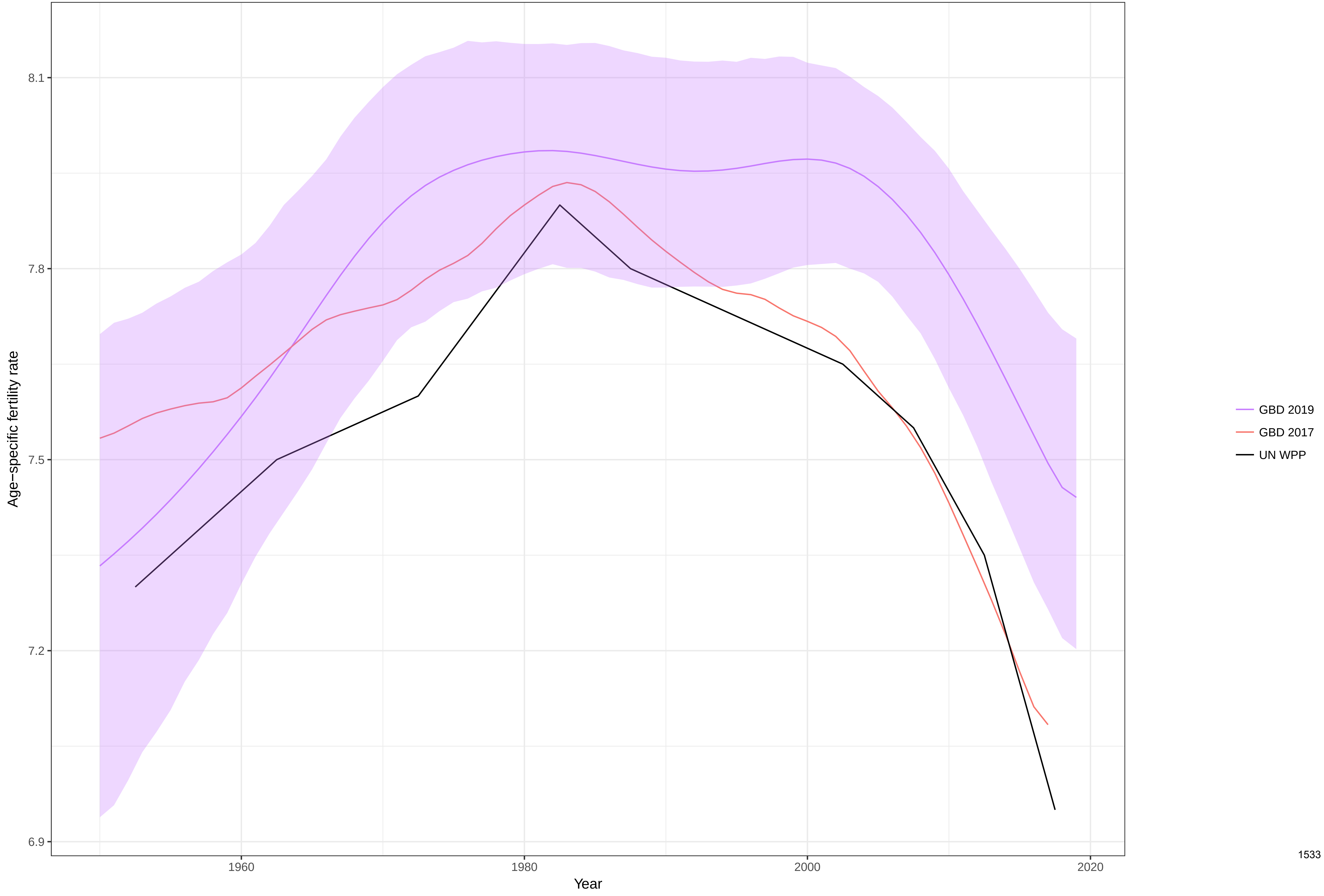

Age−specific fertility fitted trend lines, 1950−2019: Nigeria, Ages: 10−14
Data points enclosed with a diamond are reference sources

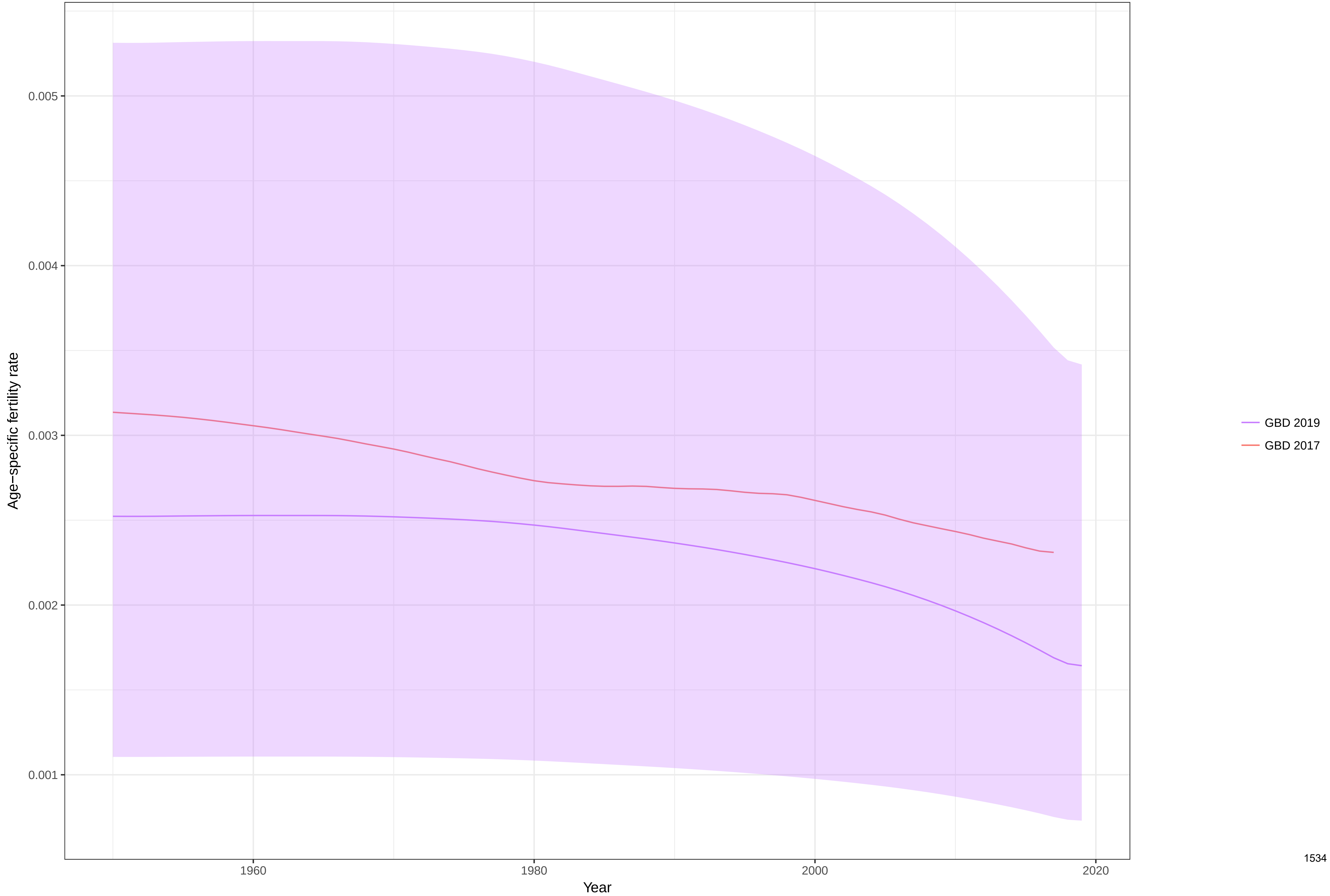

Age−specific fertility fitted trend lines, 1950−2019: Nigeria, Ages: 15−19
Data points enclosed with a diamond are reference sources

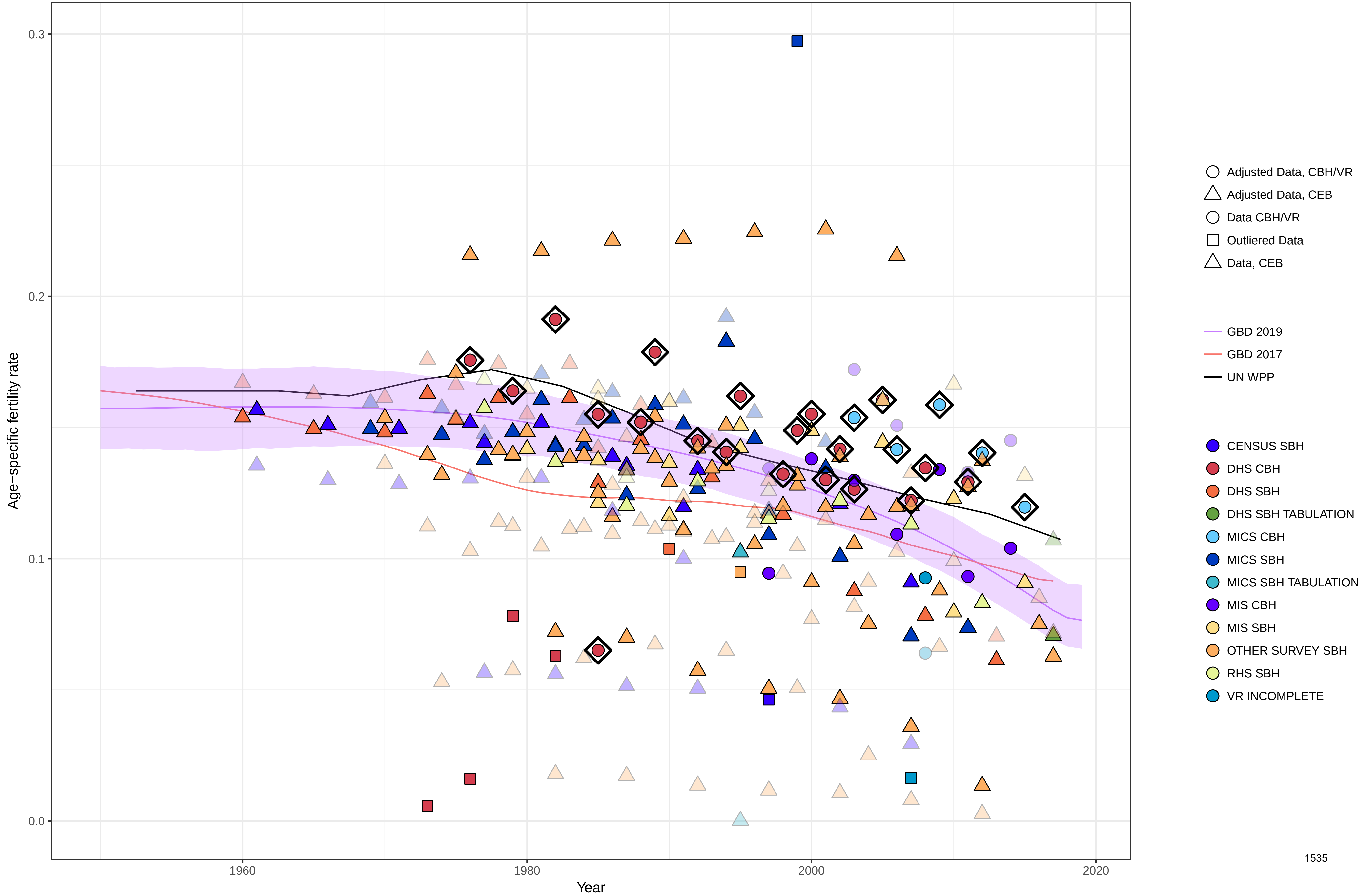

Age−specific fertility fitted trend lines, 1950−2019: Nigeria, Ages: 20−24
Data points enclosed with a diamond are reference sources

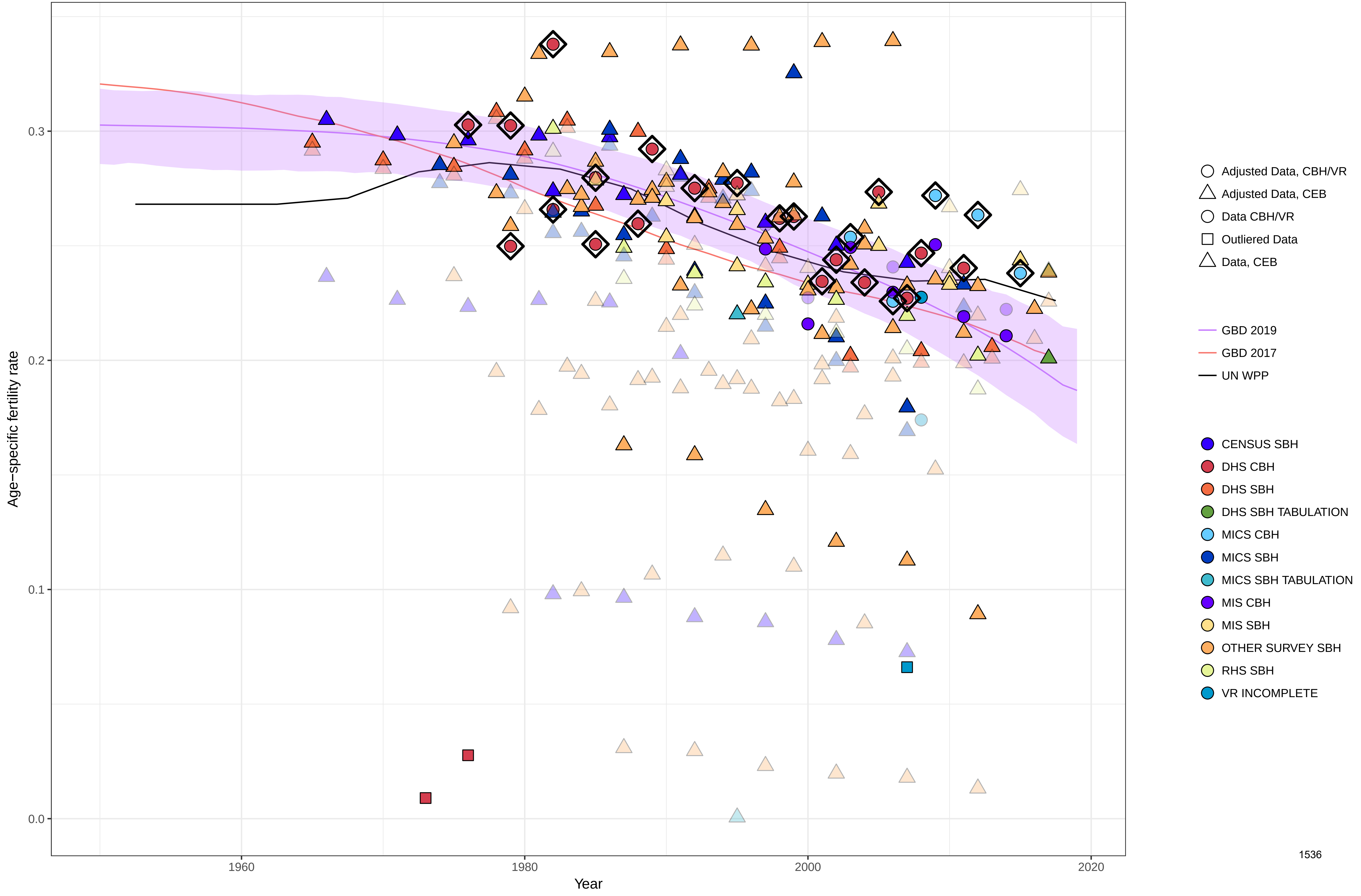

Age−specific fertility fitted trend lines, 1950−2019: Nigeria, Ages: 25−29
Data points enclosed with a diamond are reference sources

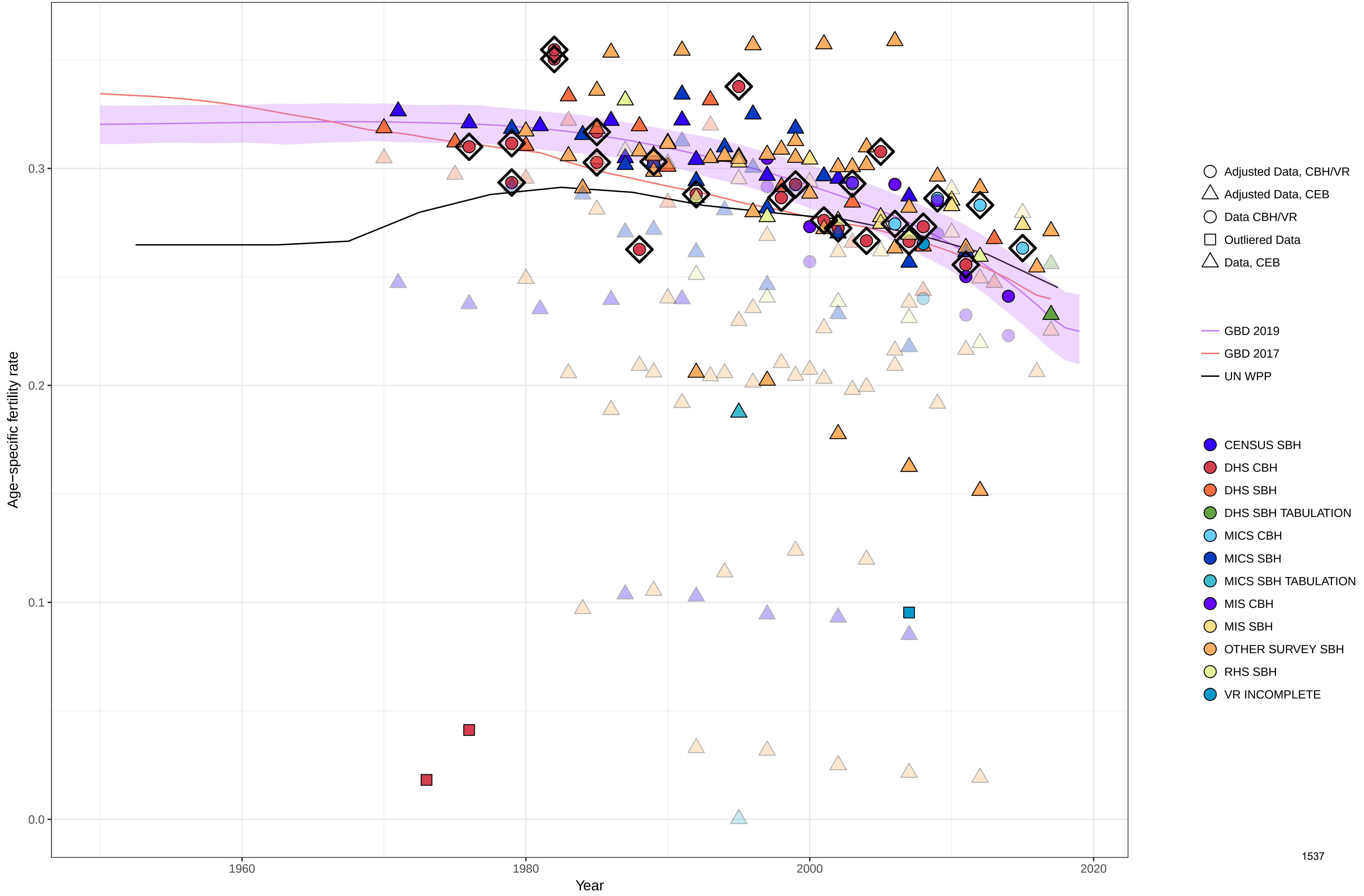

Age−specific fertility fitted trend lines, 1950−2019: Nigeria, Ages: 30−34
Data points enclosed with a diamond are reference sources

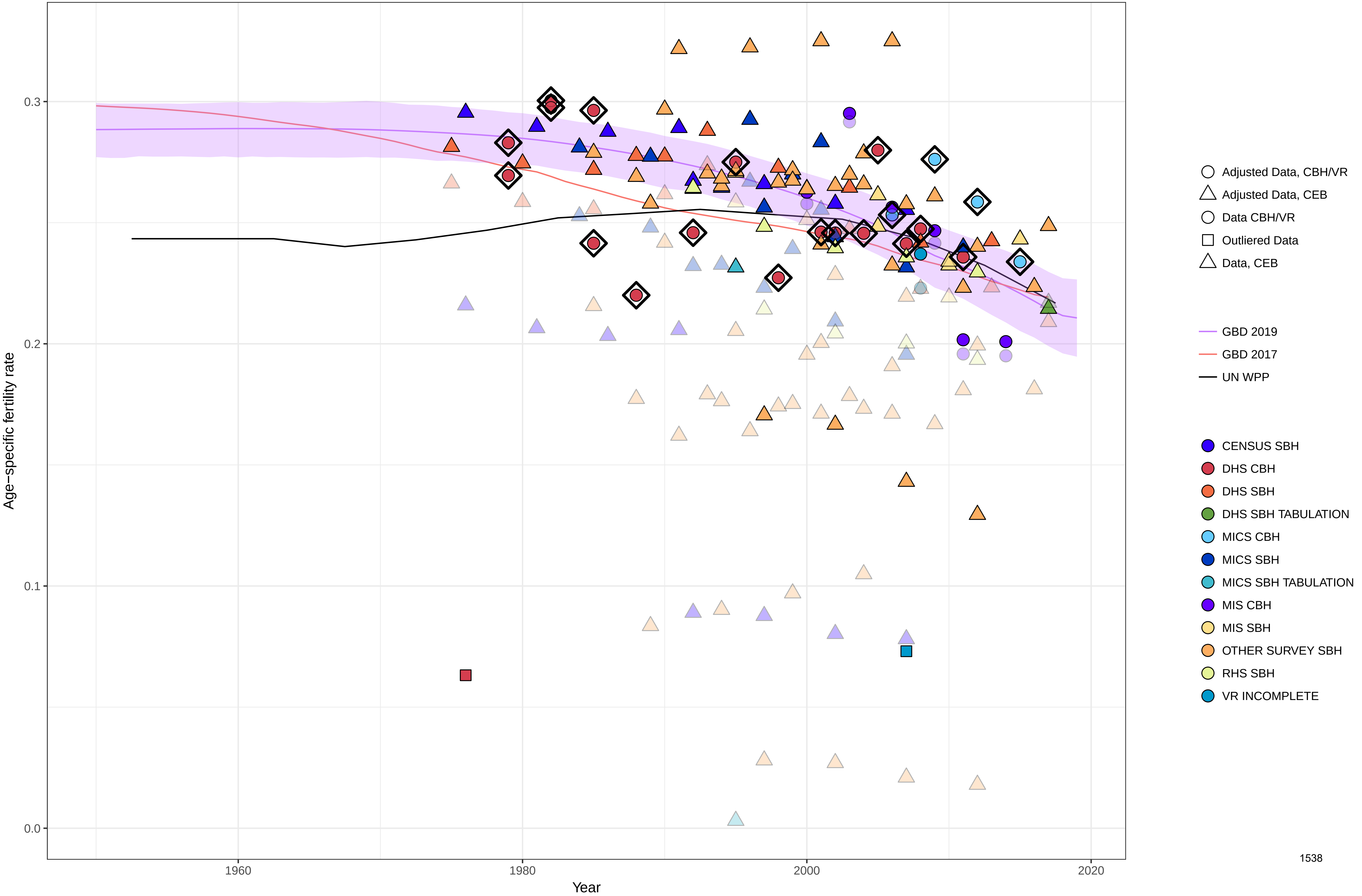

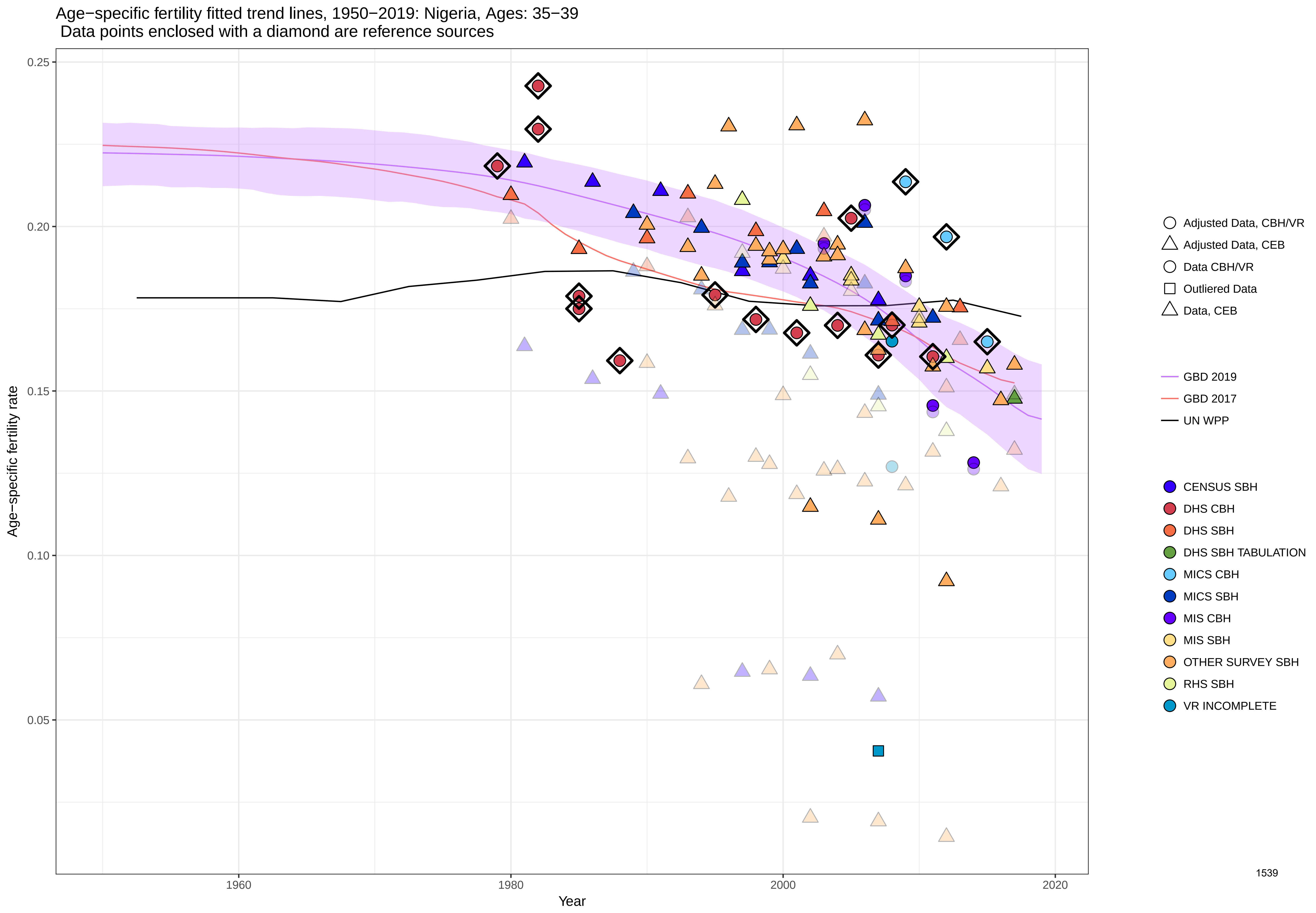

Age−specific fertility fitted trend lines, 1950−2019: Nigeria, Ages: 35−39
Data points enclosed with a diamond are reference sources
Age−specific fertility rate
0.25
0.20
0.15
0.10
0.05
Year
1960
1980
2000
2020
Adjusted Data, CBH/VR
Adjusted Data, CEB
Data CBH/VR
Outliered Data
Data, CEB
GBD 2019
GBD 2017
UN WPP
CENSUS SBH
DHS CBH
DHS SBH
DHS SBH TABULATION
MICS CBH
MICS SBH
MIS CBH
MIS SBH
OTHER SURVEY SBH
RHS SBH
VR INCOMPLETE

1539

Age−specific fertility fitted trend lines, 1950−2019: Nigeria, Ages: 40−44
Data points enclosed with a diamond are reference sources

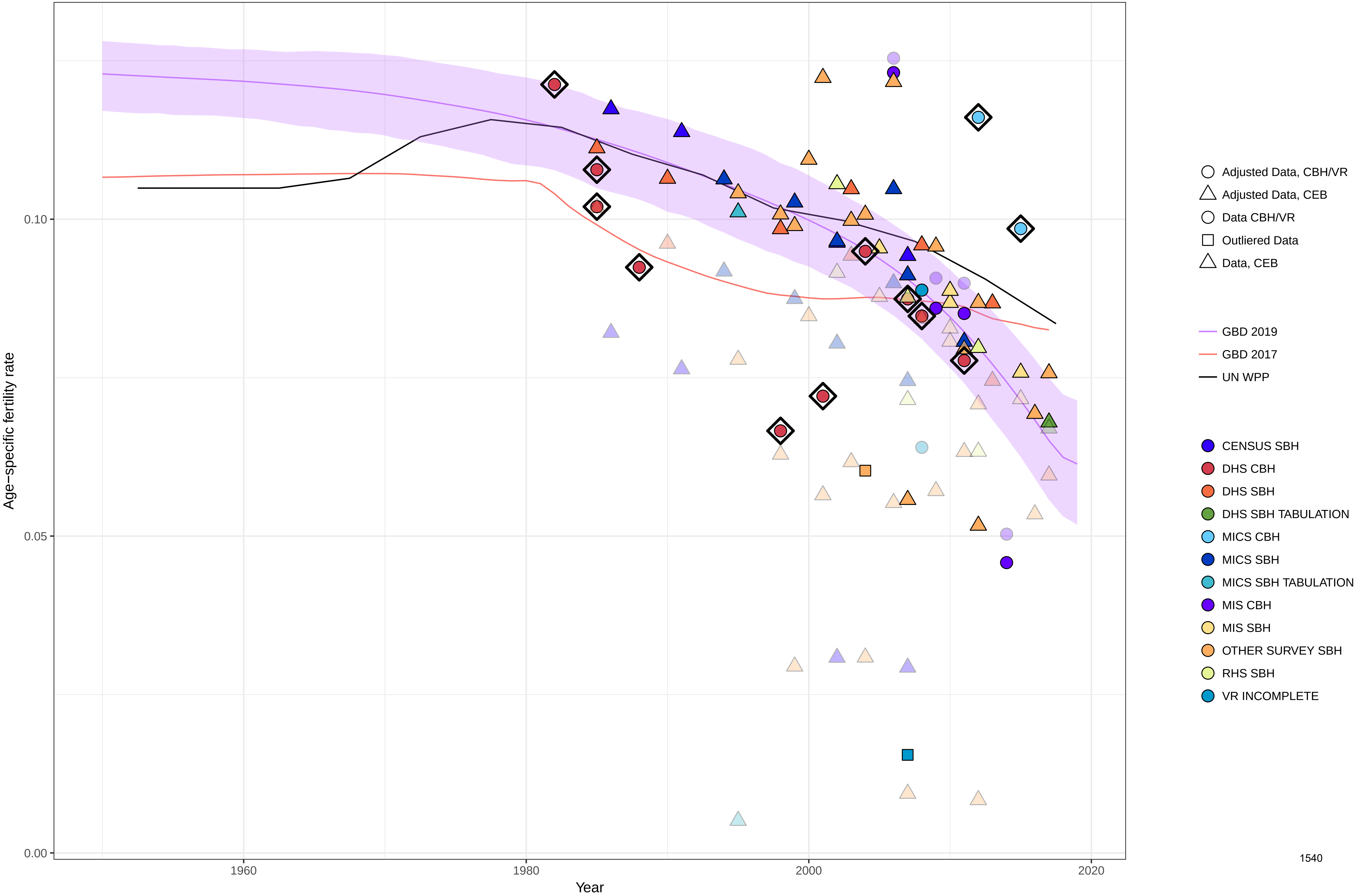

Age−specific fertility fitted trend lines, 1950−2019: Nigeria, Ages: 45−49
Data points enclosed with a diamond are reference sources

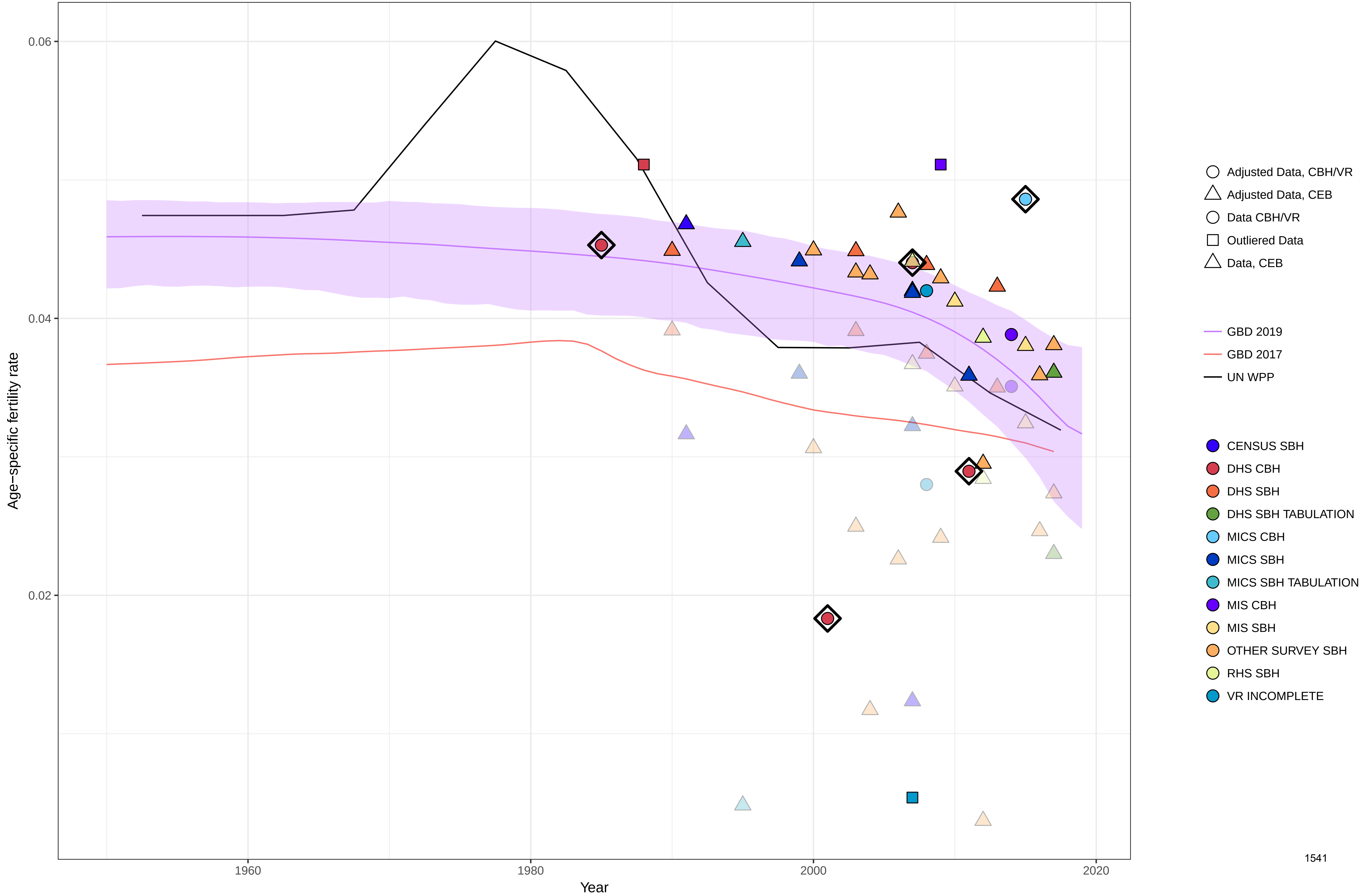

Age−specific fertility fitted trend lines, 1950−2019: Nigeria, Ages: 50−54
Data points enclosed with a diamond are reference sources

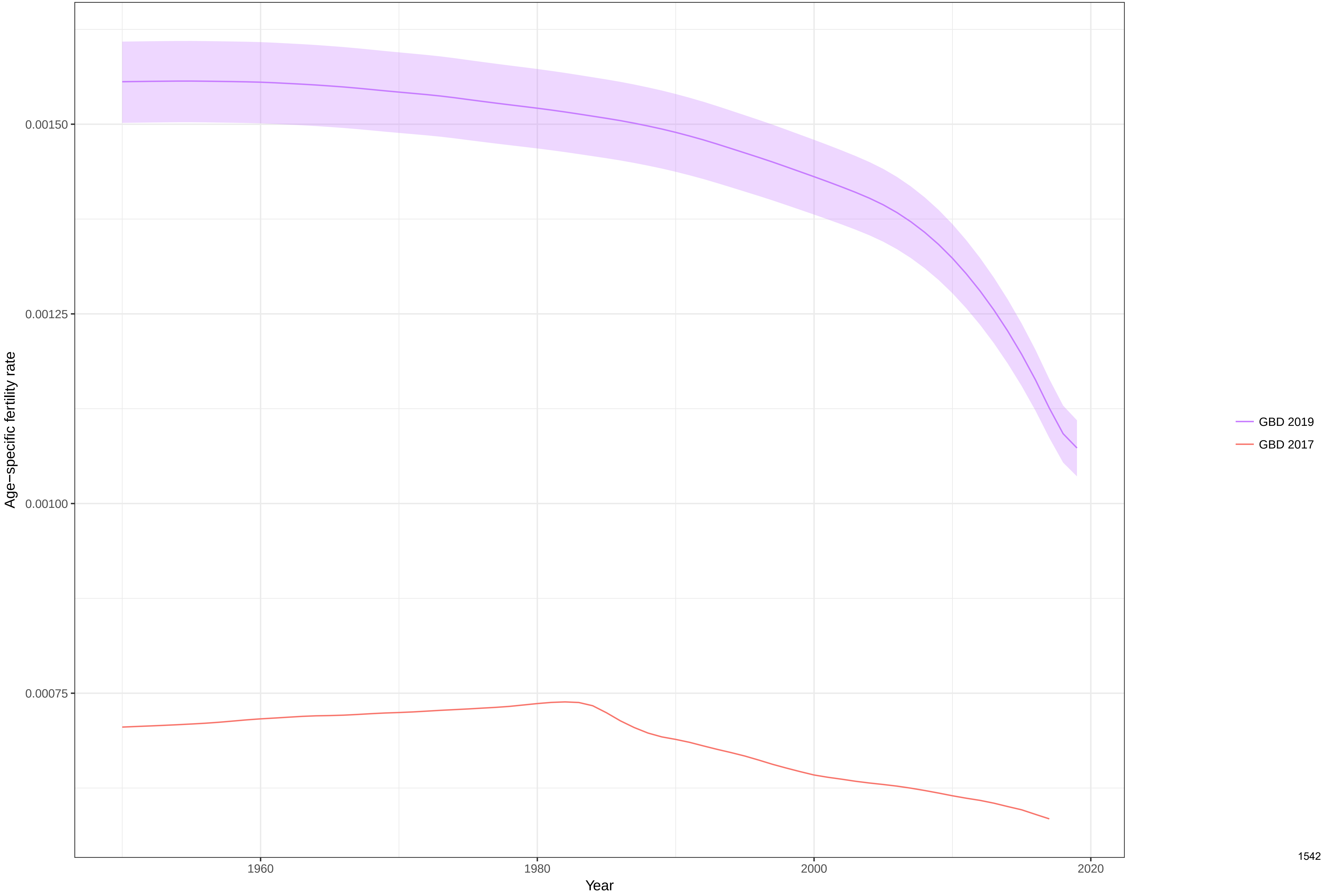

Total fertility rate fitted trend lines, 1950−2019: Nigeria TFR
Data points enclosed with a diamond are reference sources

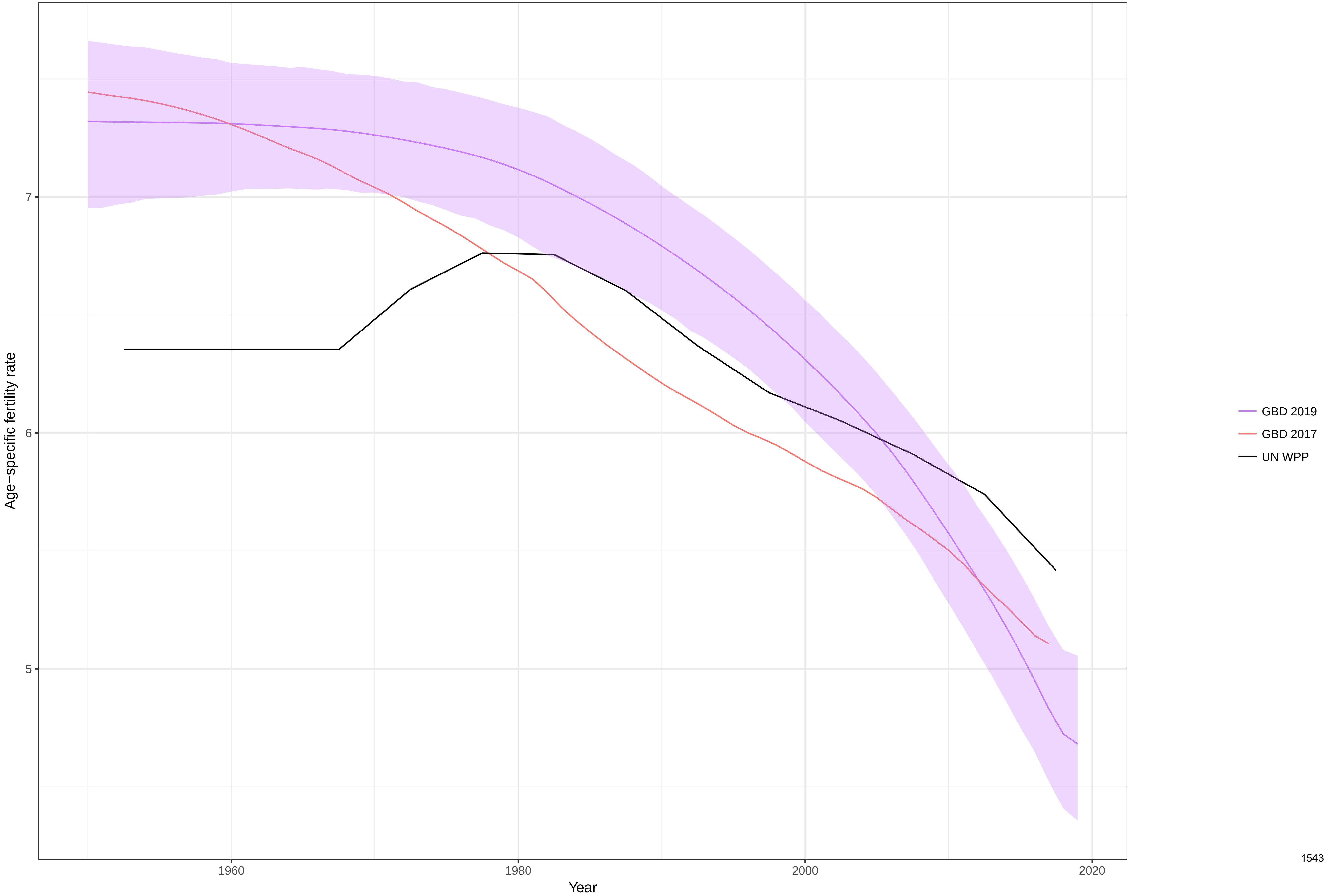

Age−specific fertility fitted trend lines, 1950−2019: Nicaragua, Ages: 10−14
Data points enclosed with a diamond are reference sources

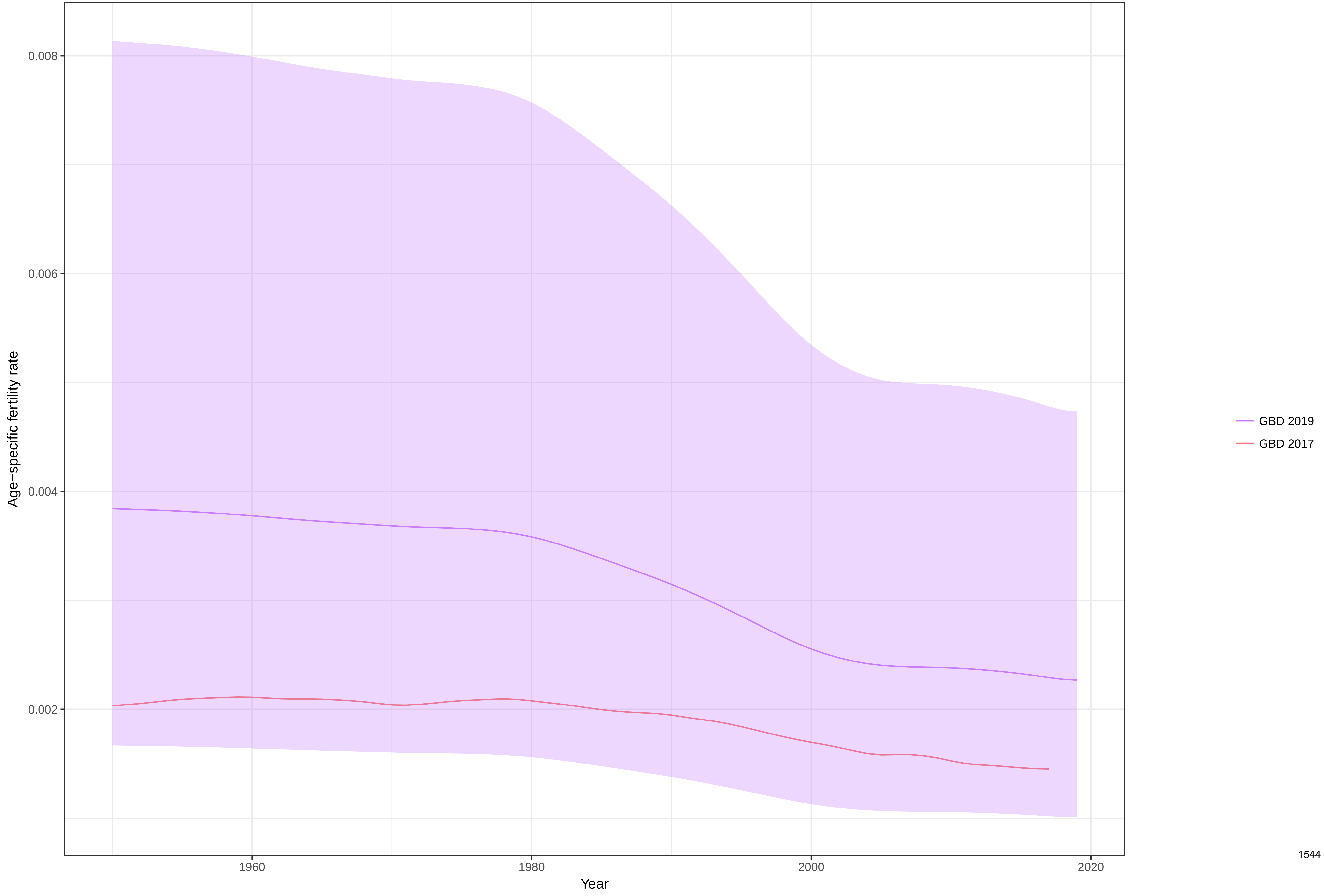

Age−specific fertility fitted trend lines, 1950−2019: Nicaragua, Ages: 15−19
Data points enclosed with a diamond are reference sources

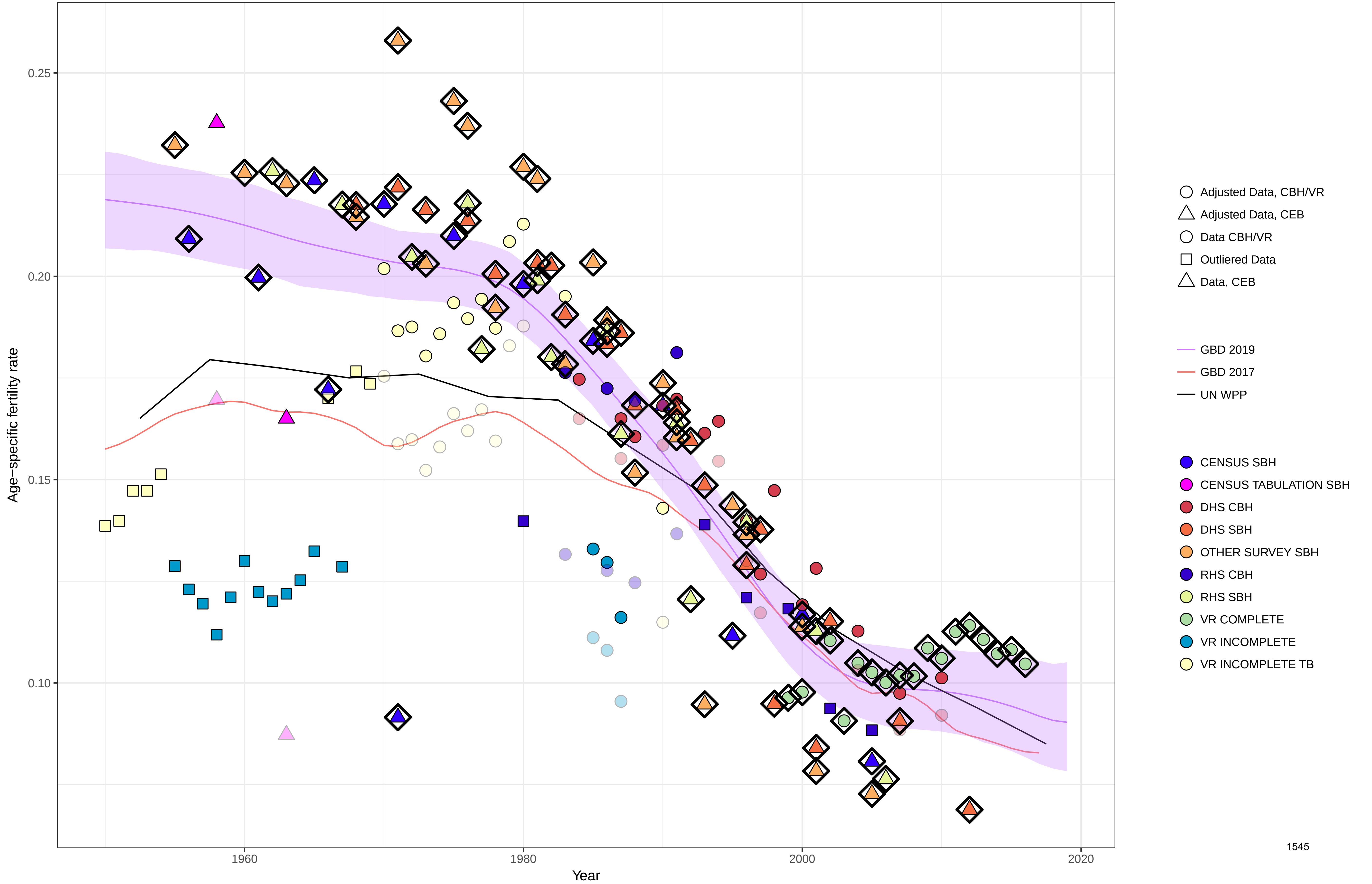

Age−specific fertility fitted trend lines, 1950−2019: Nicaragua, Ages: 20−24
Data points enclosed with a diamond are reference sources

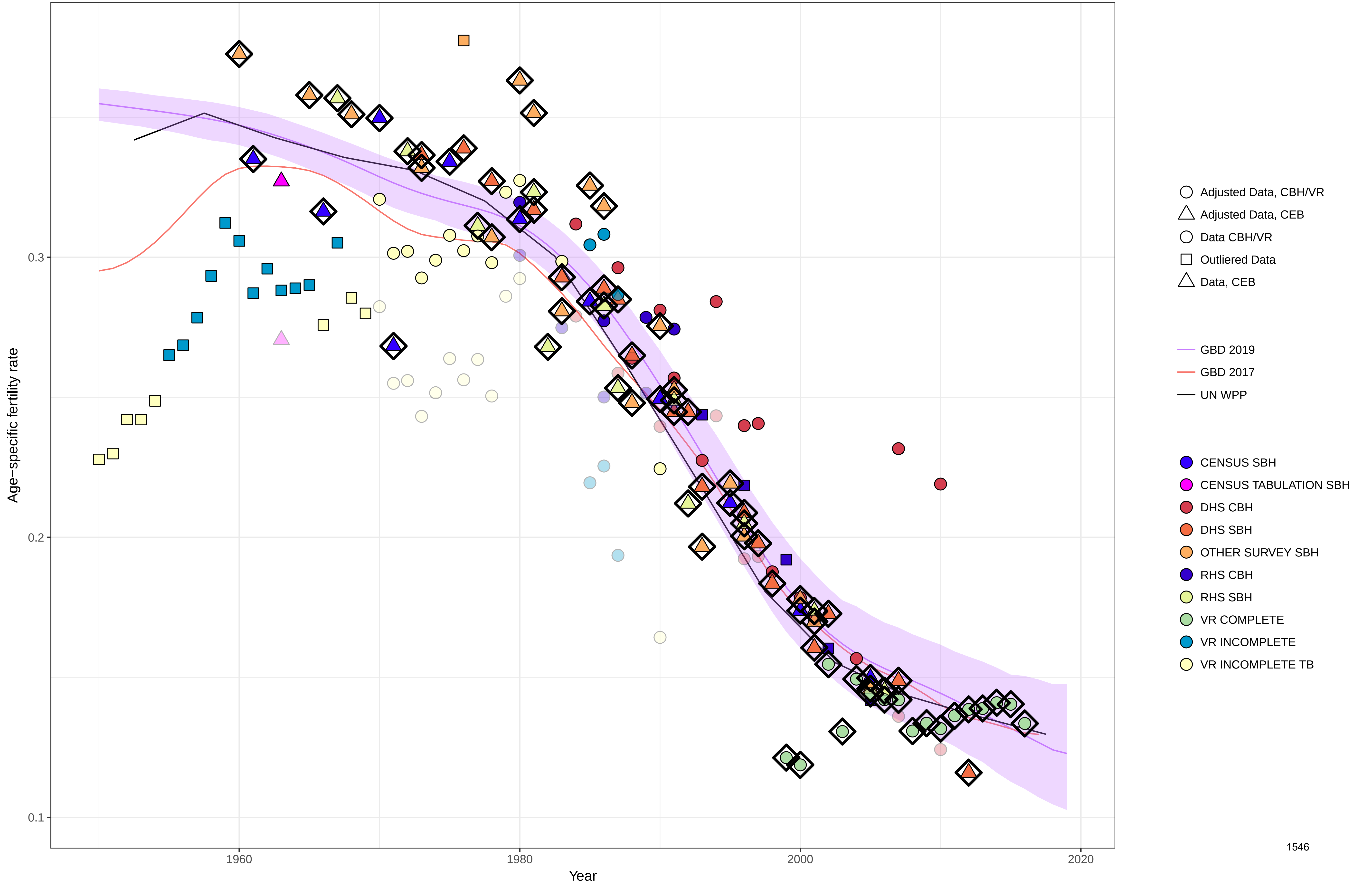

Age−specific fertility fitted trend lines, 1950−2019: Nicaragua, Ages: 25−29
Data points enclosed with a diamond are reference sources

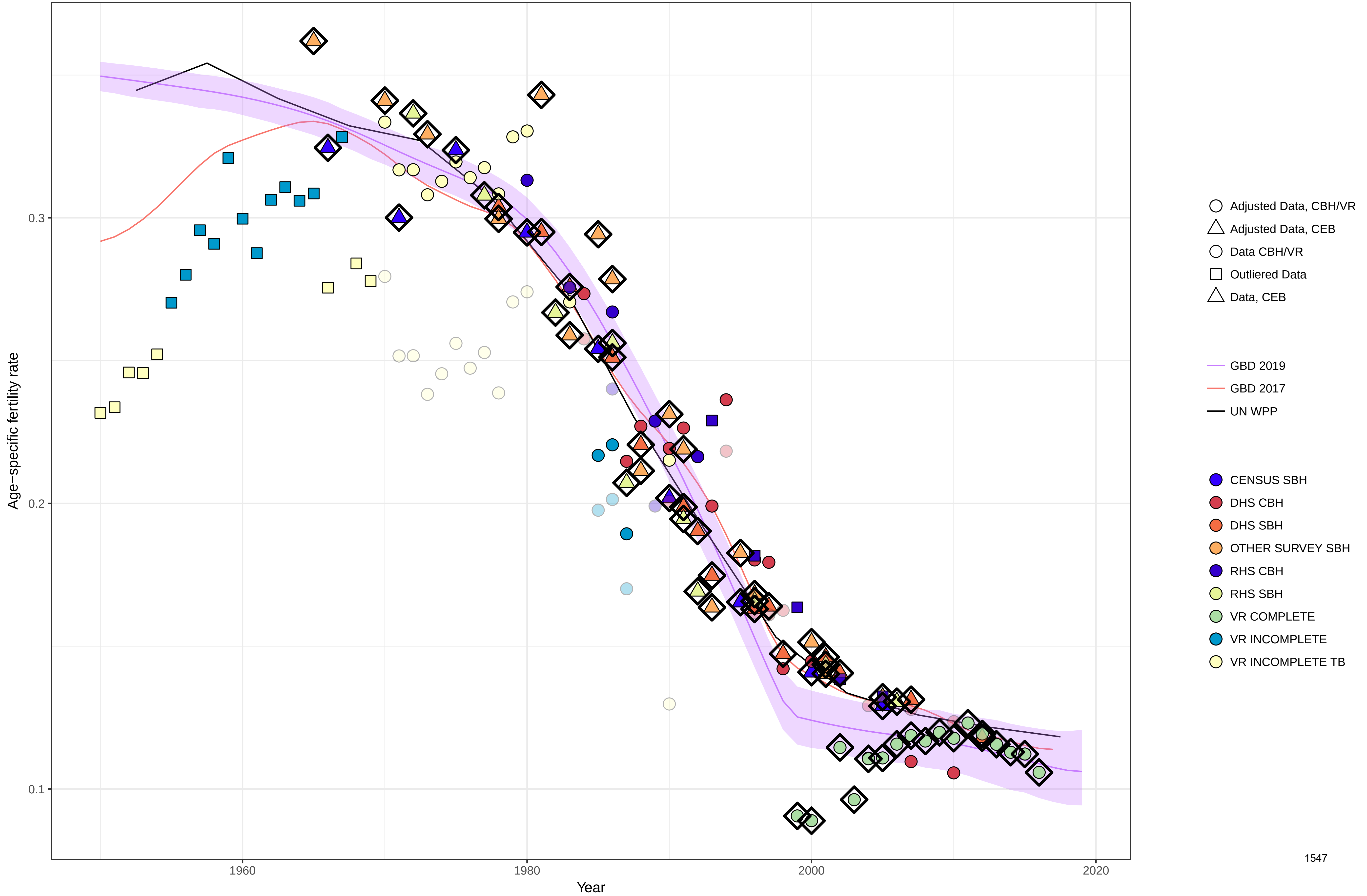

Age−specific fertility fitted trend lines, 1950−2019: Nicaragua, Ages: 30−34
Data points enclosed with a diamond are reference sources

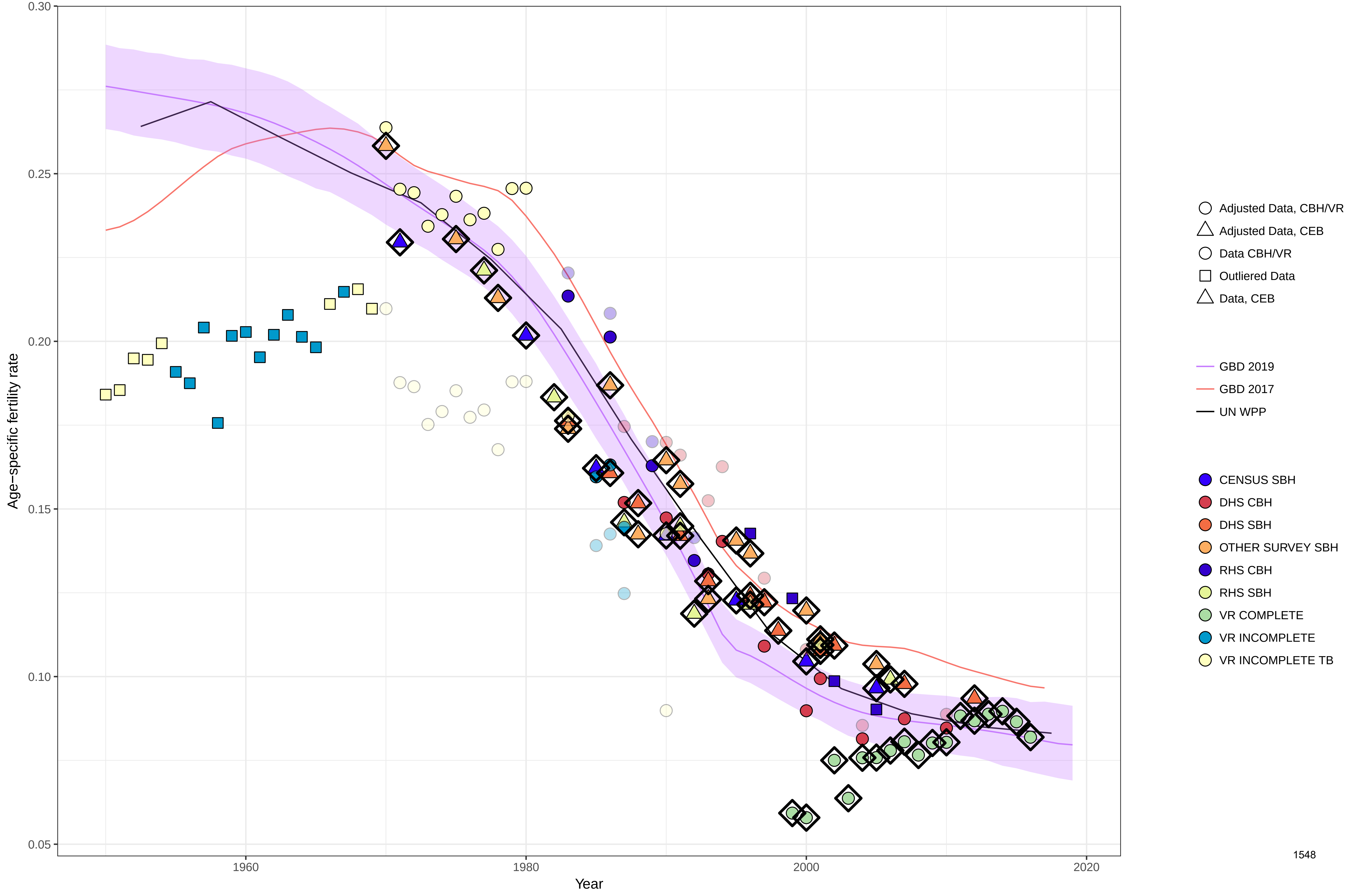

Age−specific fertility fitted trend lines, 1950−2019: Nicaragua, Ages: 35−39
Data points enclosed with a diamond are reference sources

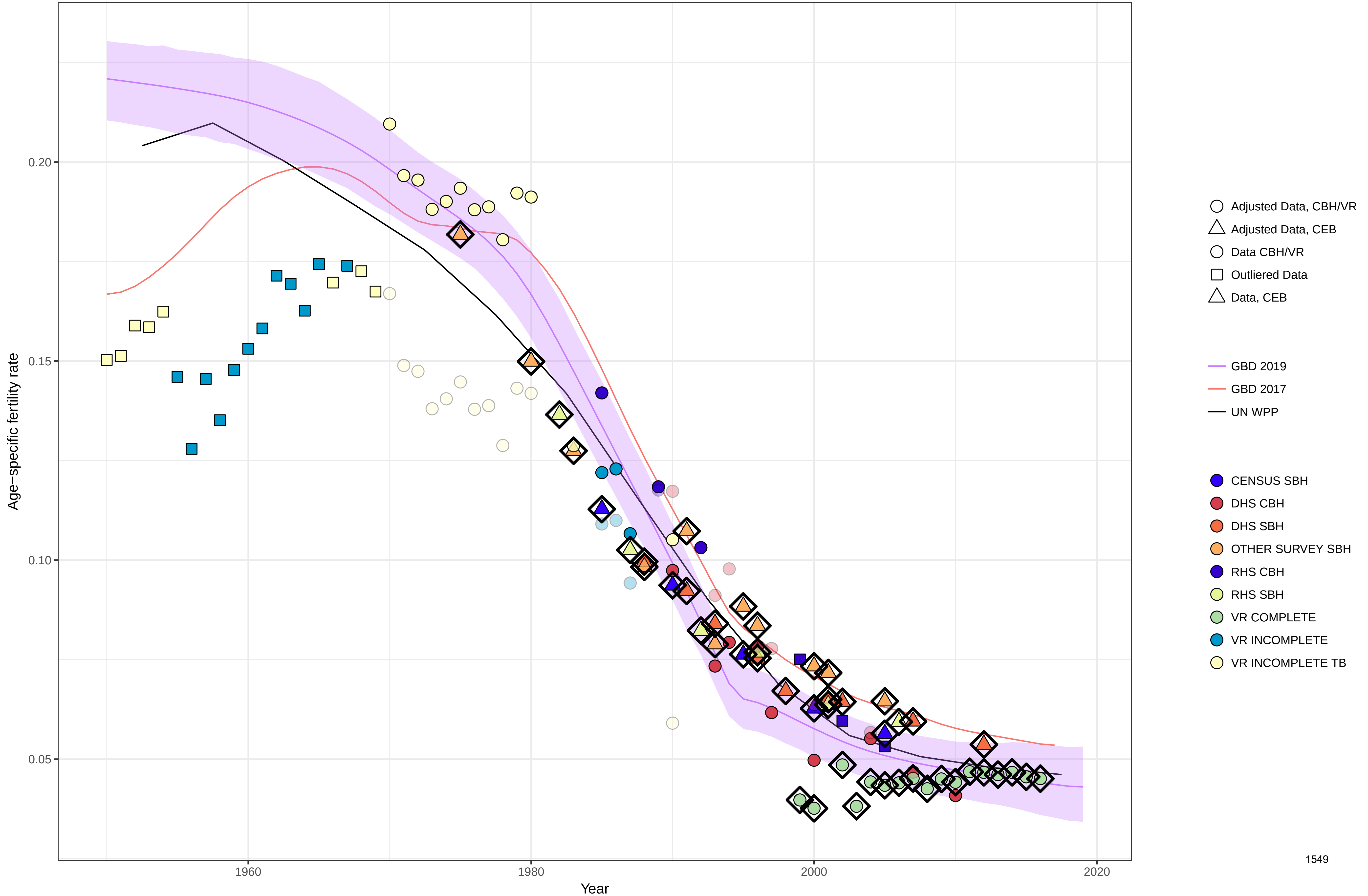

Age−specific fertility fitted trend lines, 1950−2019: Nicaragua, Ages: 40−44
Data points enclosed with a diamond are reference sources

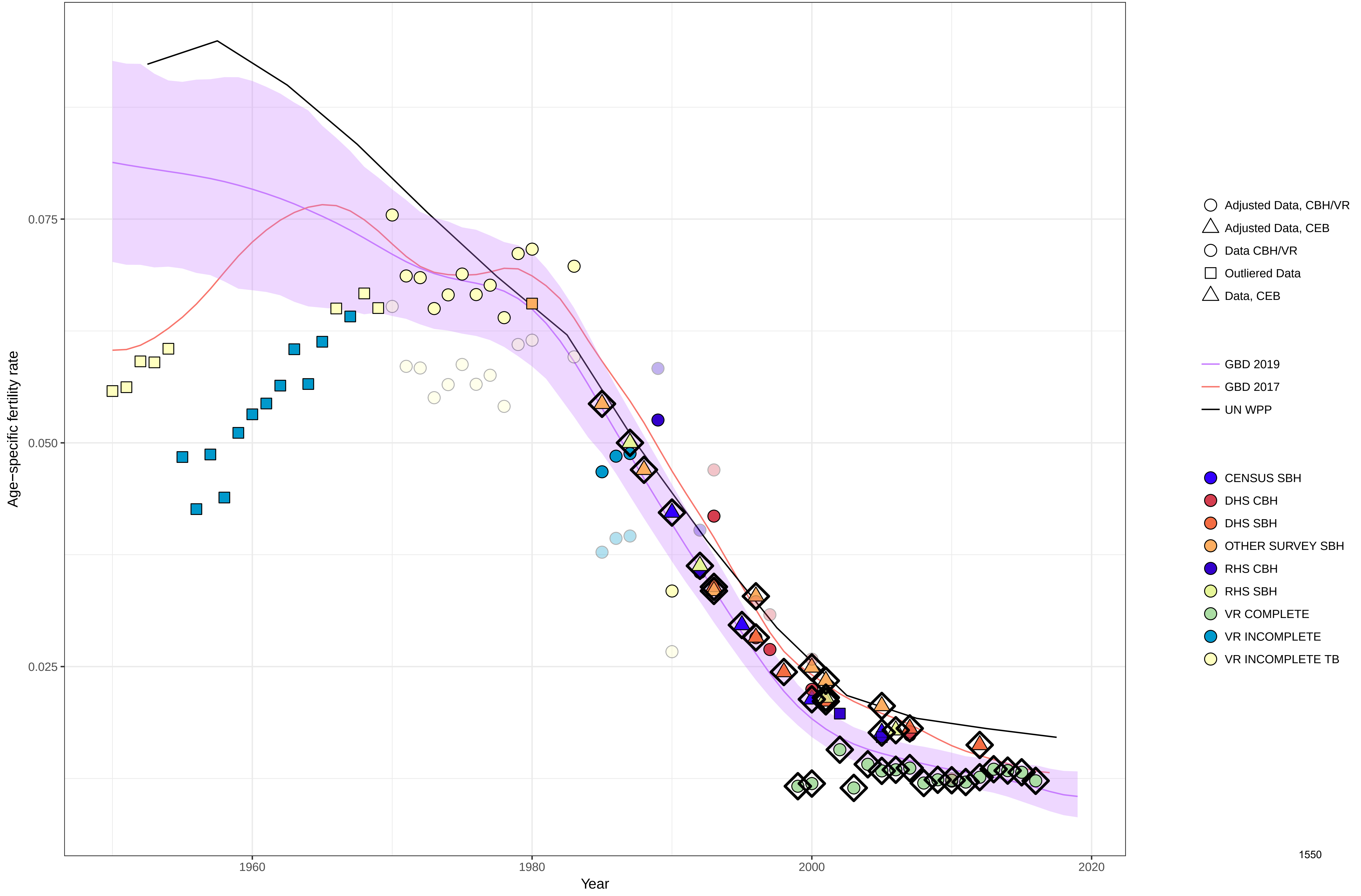

Age−specific fertility fitted trend lines, 1950−2019: Nicaragua, Ages: 45−49
Data points enclosed with a diamond are reference sources

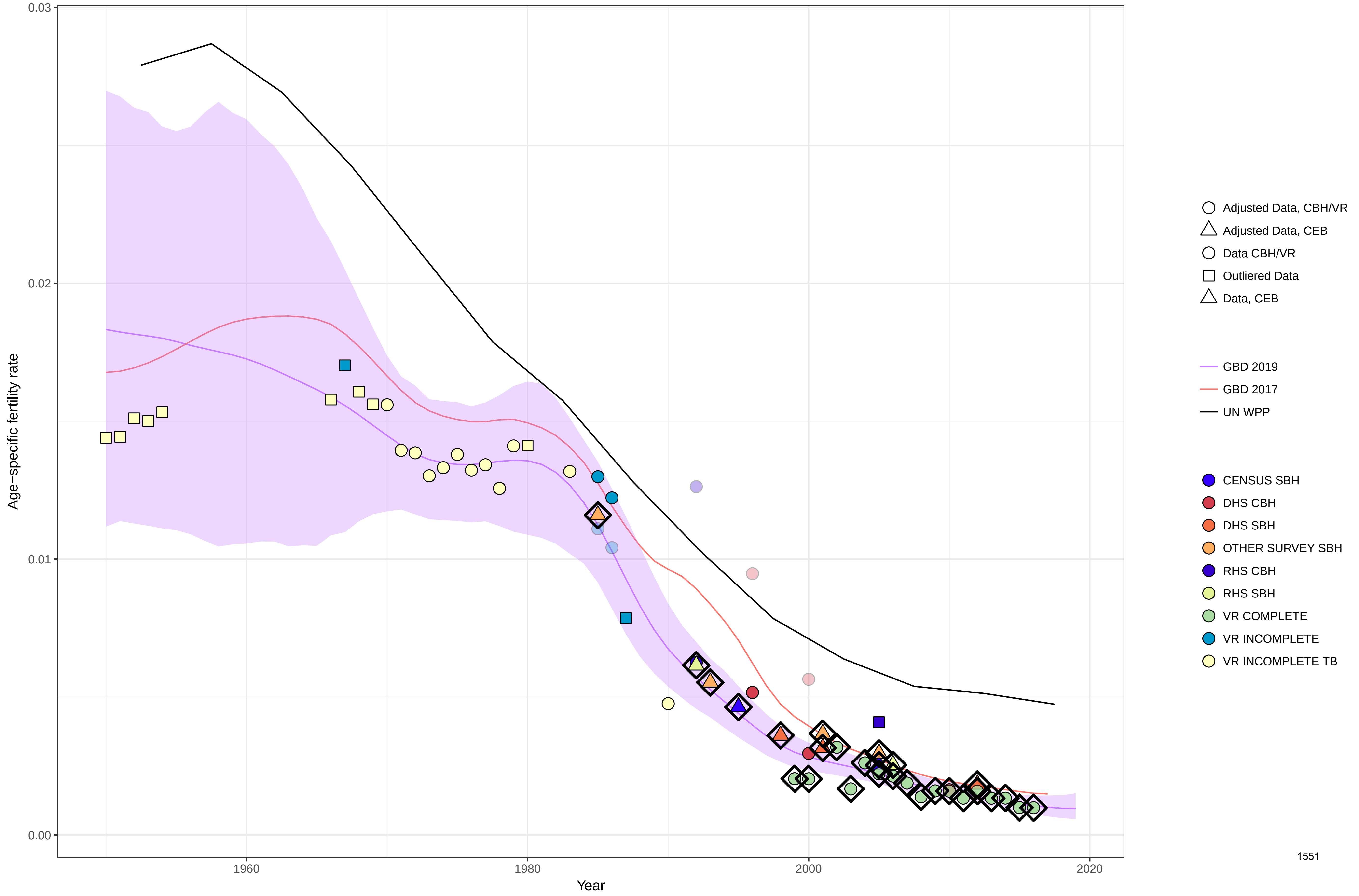

Age−specific fertility fitted trend lines, 1950−2019: Nicaragua, Ages: 50−54
Data points enclosed with a diamond are reference sources

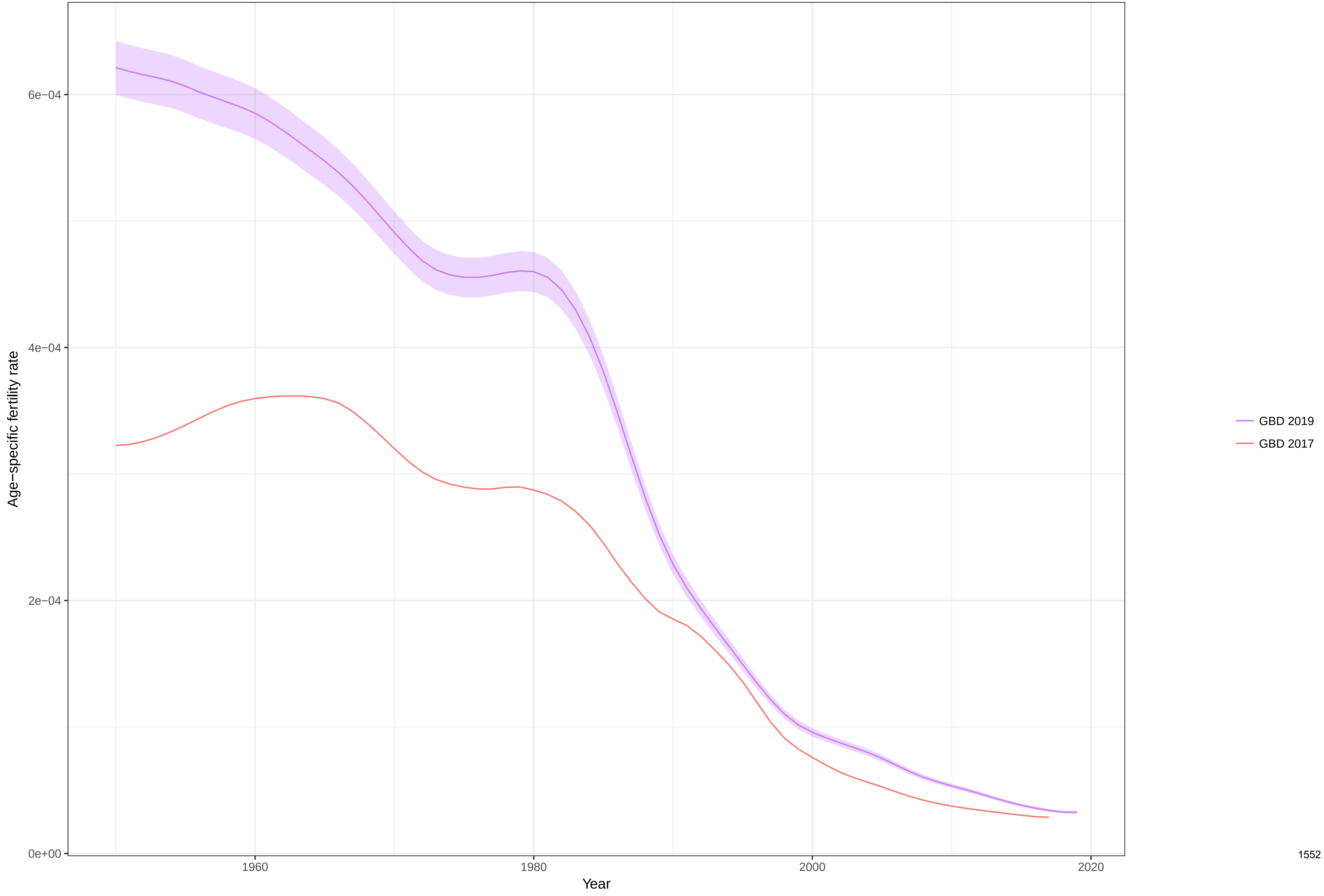

Total fertility rate fitted trend lines, 1950−2019: Nicaragua TFR
Data points enclosed with a diamond are reference sources

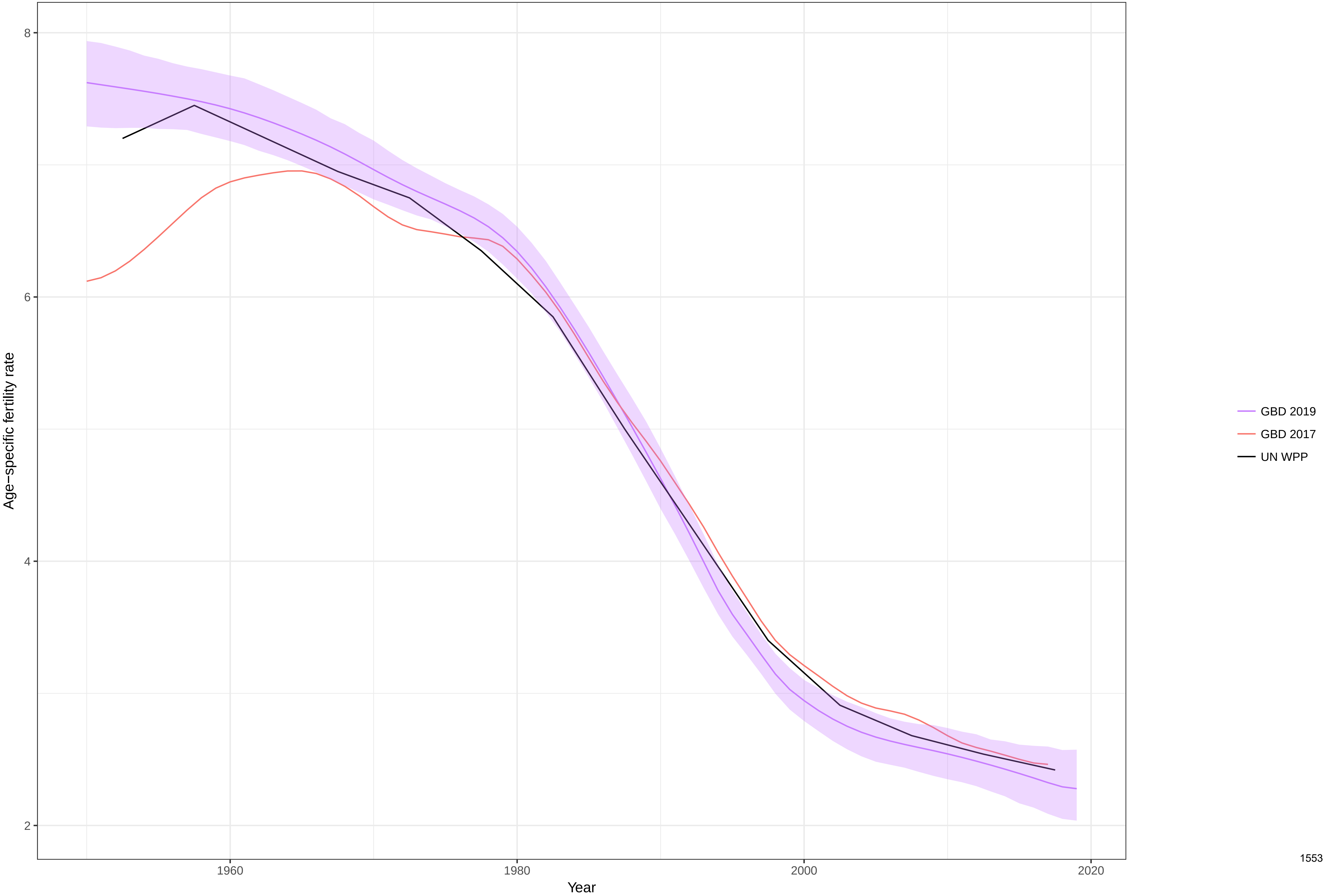

Age−specific fertility fitted trend lines, 1950−2019: Niue, Ages: 10−14
Data points enclosed with a diamond are reference sources

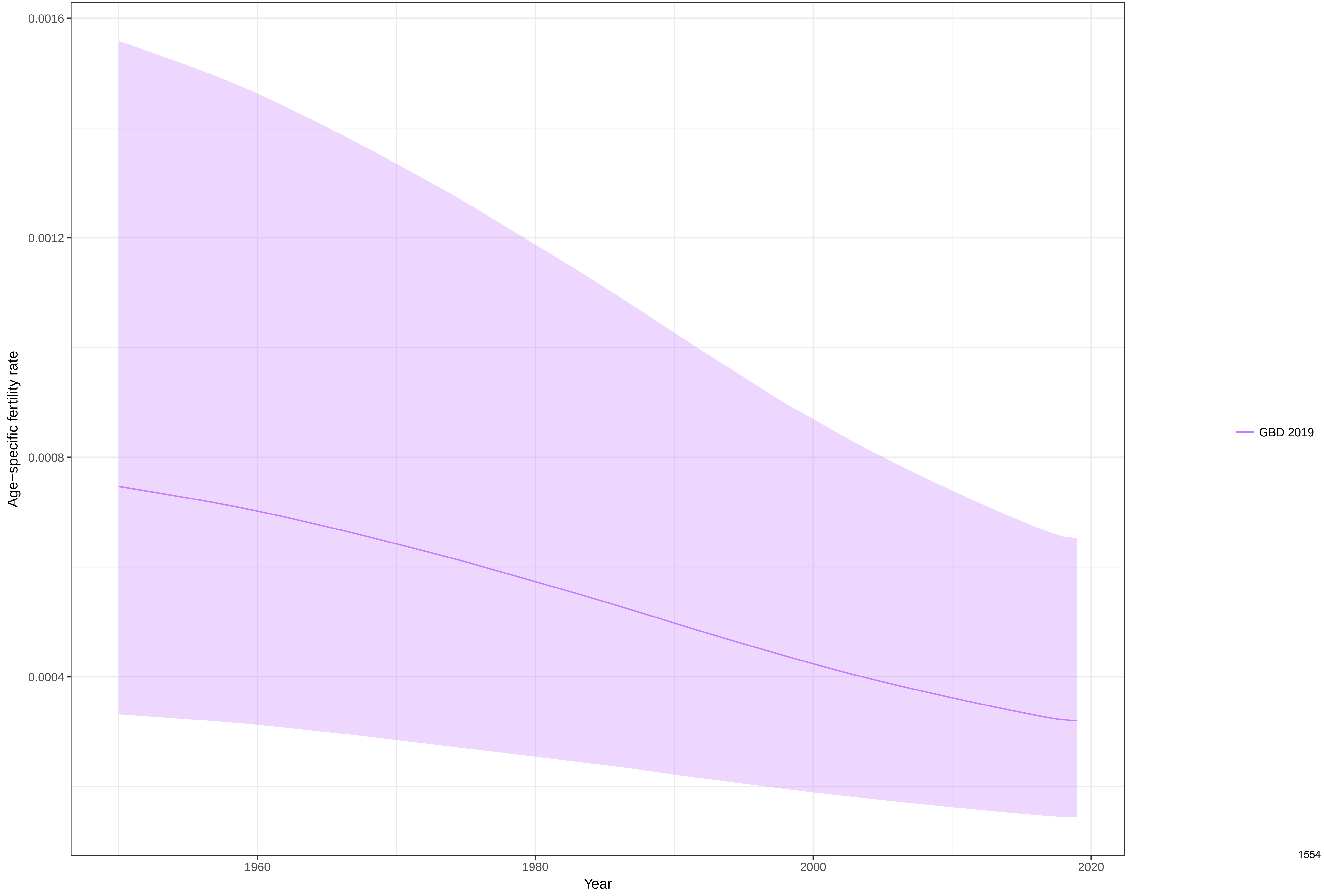

Age−specific fertility fitted trend lines, 1950−2019: Niue, Ages: 15−19
Data points enclosed with a diamond are reference sources

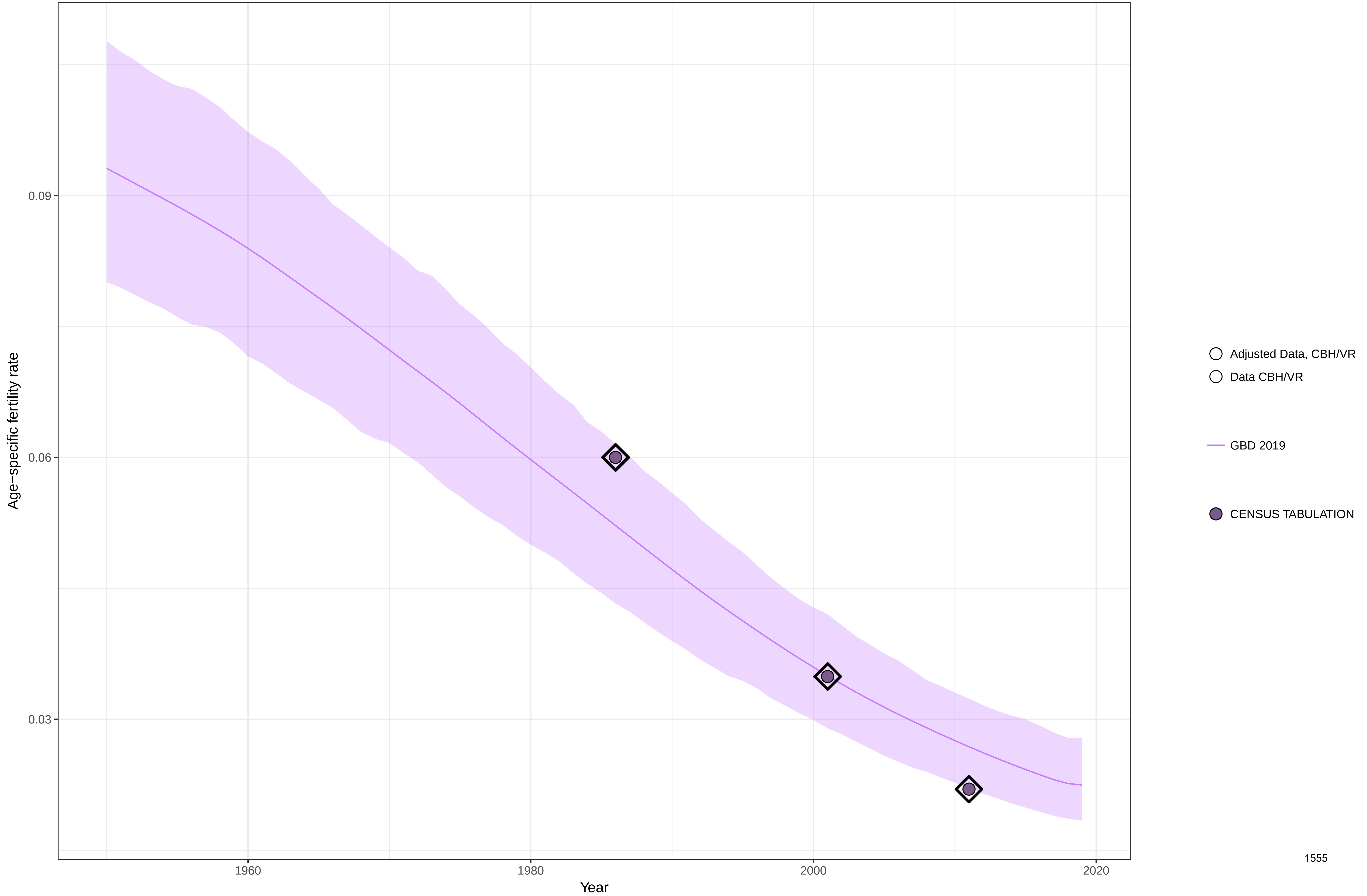

Age−specific fertility fitted trend lines, 1950−2019: Niue, Ages: 20−24
Data points enclosed with a diamond are reference sources

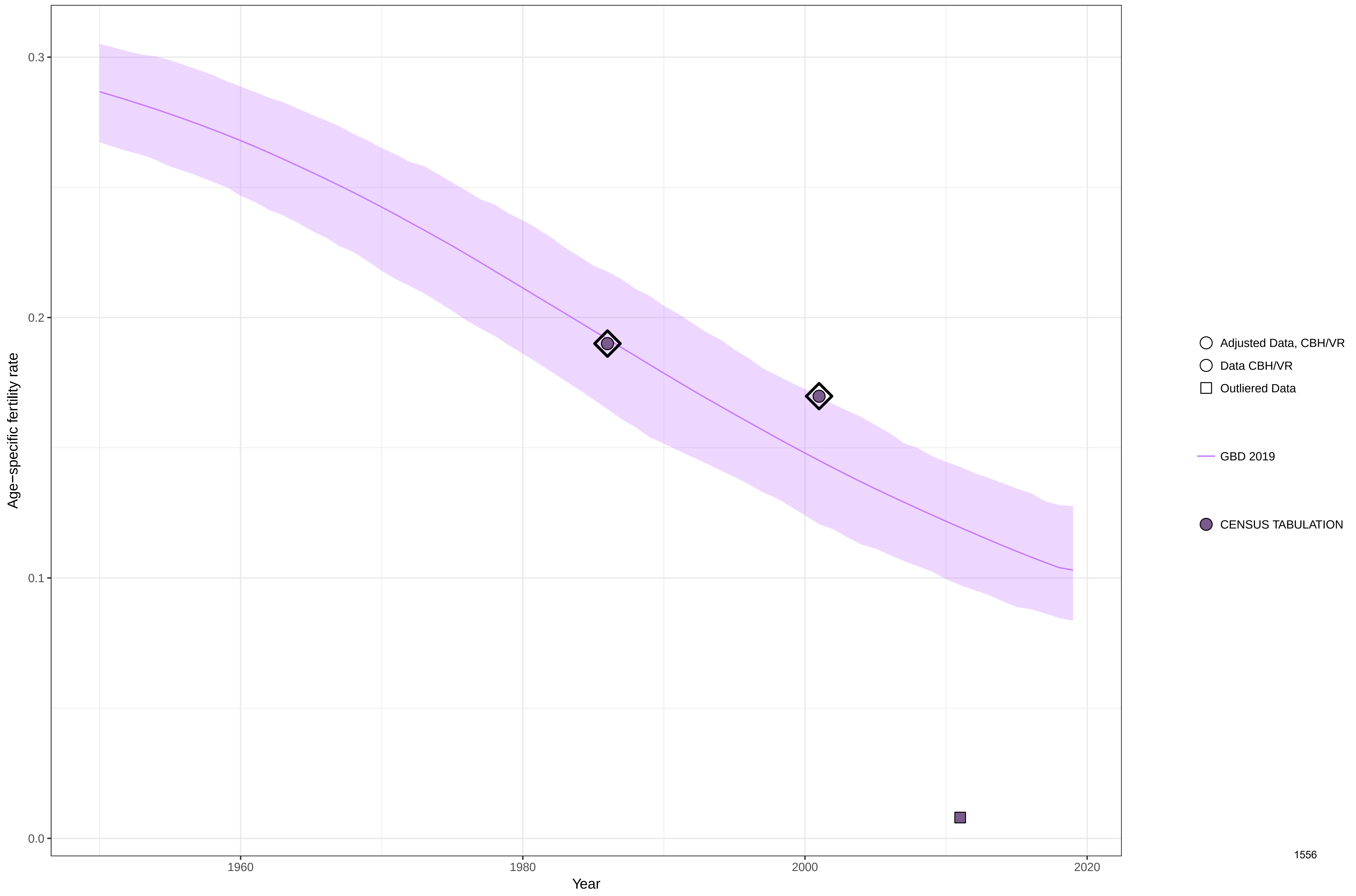

Age−specific fertility fitted trend lines, 1950−2019: Niue, Ages: 25−29
Data points enclosed with a diamond are reference sources

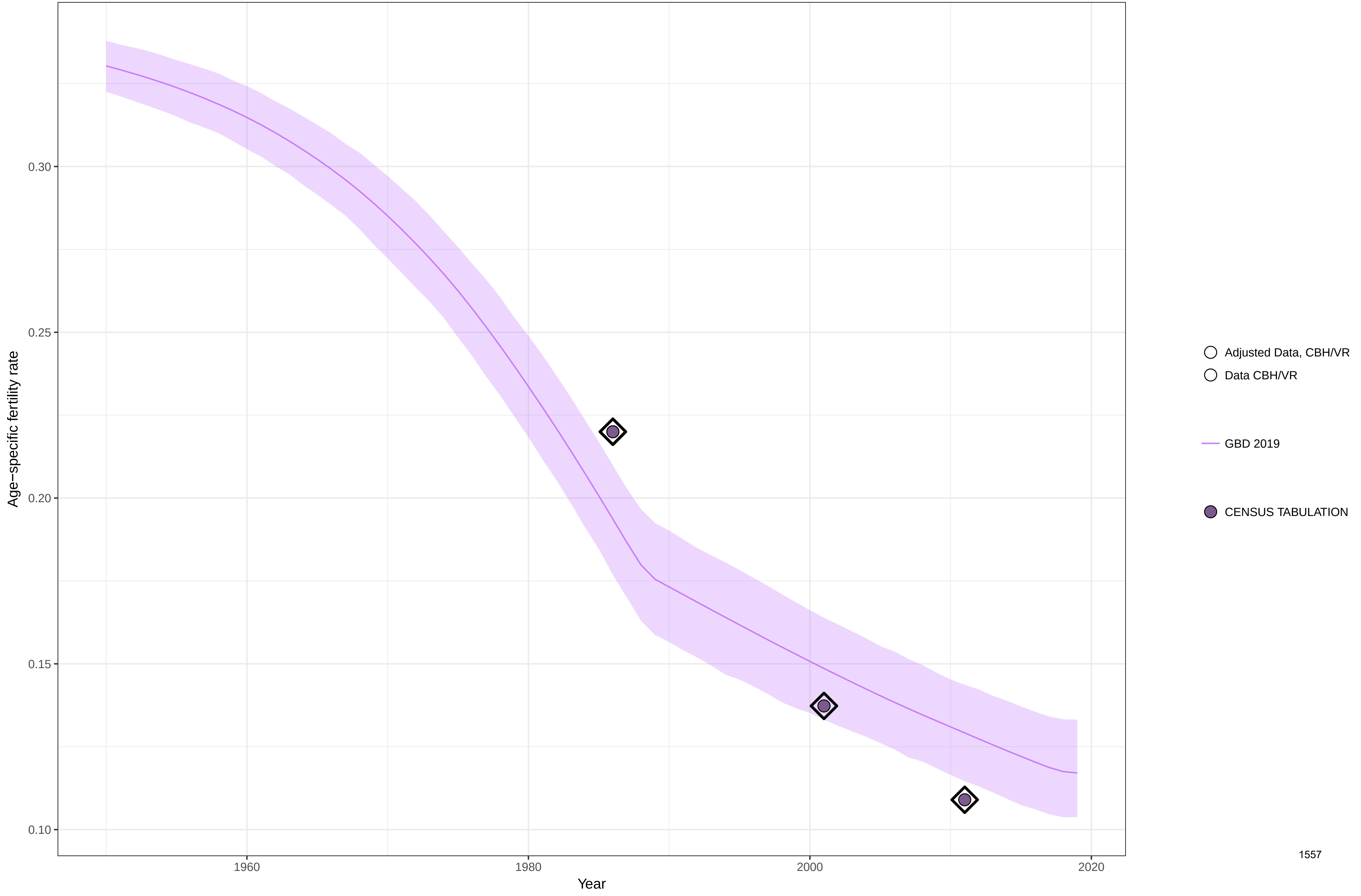

Age−specific fertility fitted trend lines, 1950−2019: Niue, Ages: 30−34
Data points enclosed with a diamond are reference sources

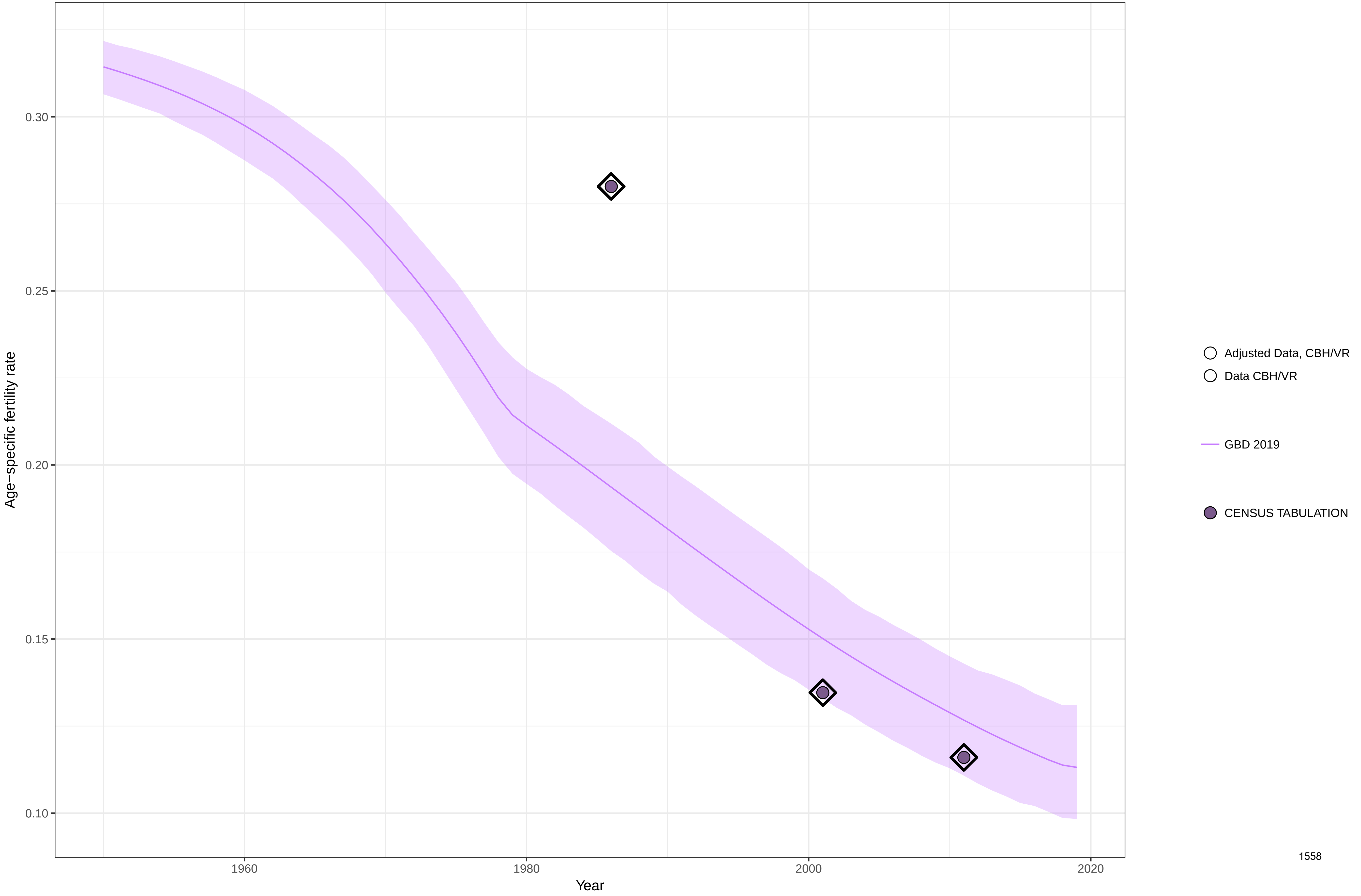

Age−specific fertility fitted trend lines, 1950−2019: Niue, Ages: 35−39
Data points enclosed with a diamond are reference sources

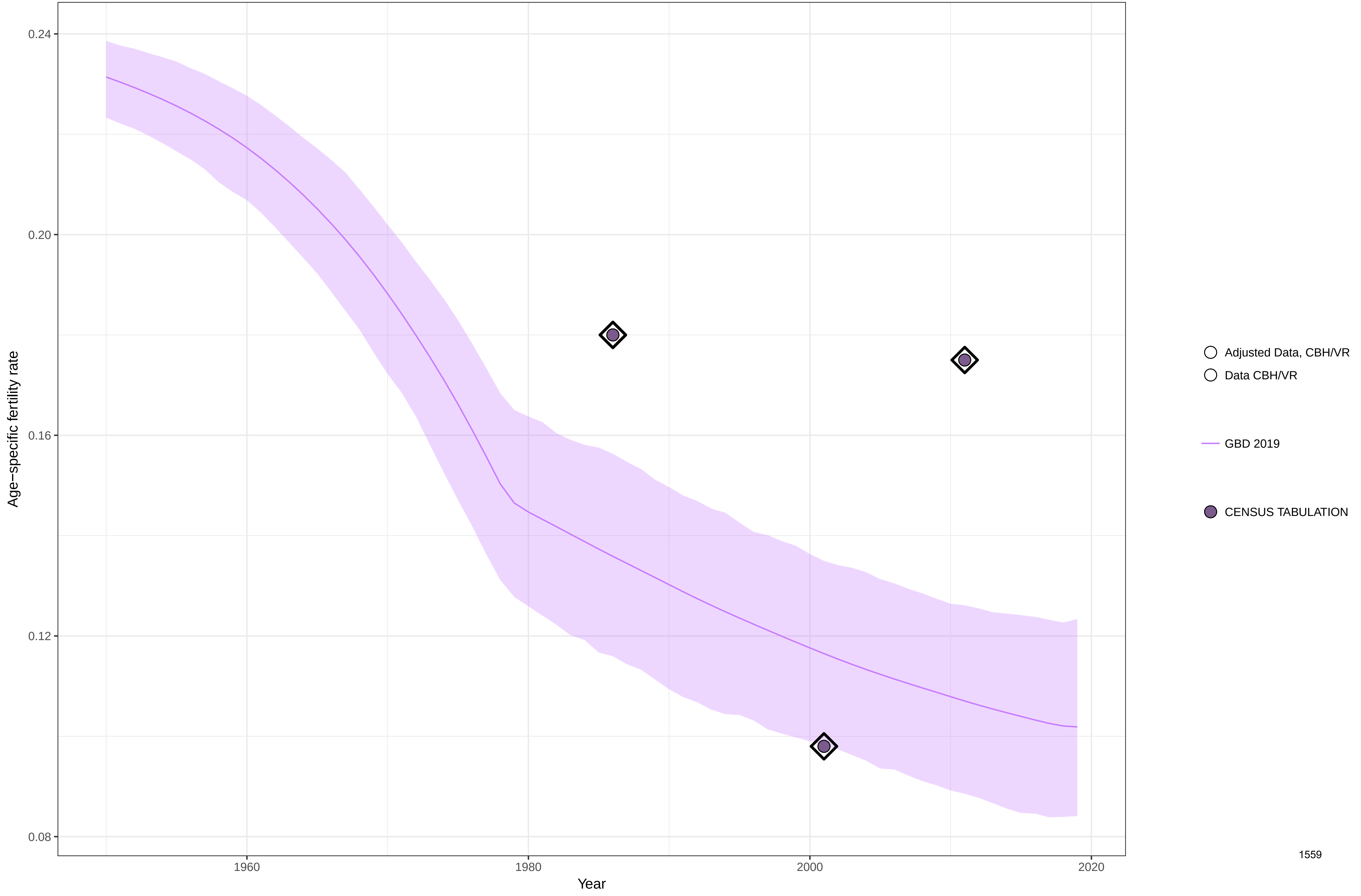

Age−specific fertility fitted trend lines, 1950−2019: Niue, Ages: 40−44
Data points enclosed with a diamond are reference sources

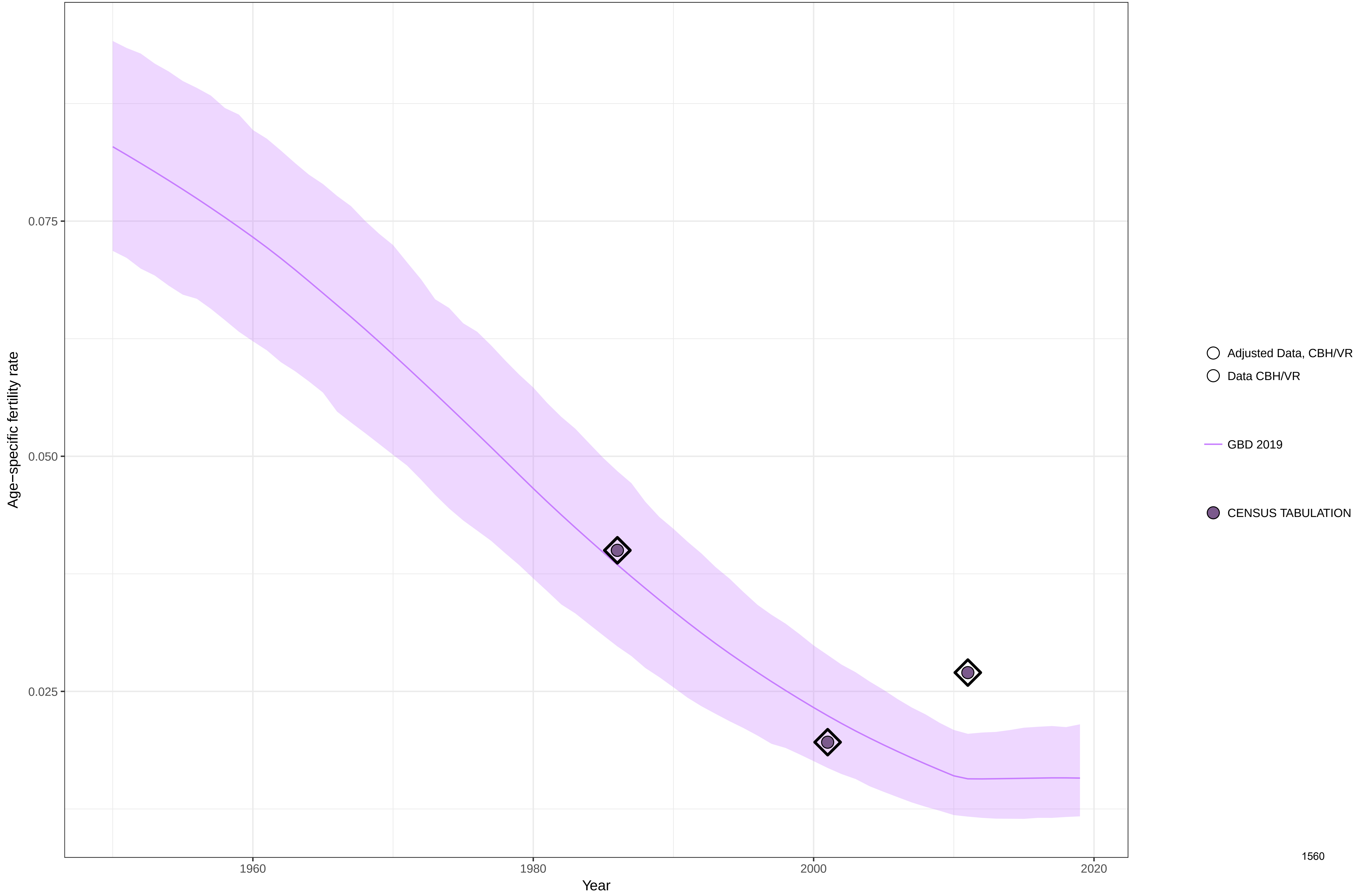

Age−specific fertility fitted trend lines, 1950−2019: Niue, Ages: 45−49
Data points enclosed with a diamond are reference sources

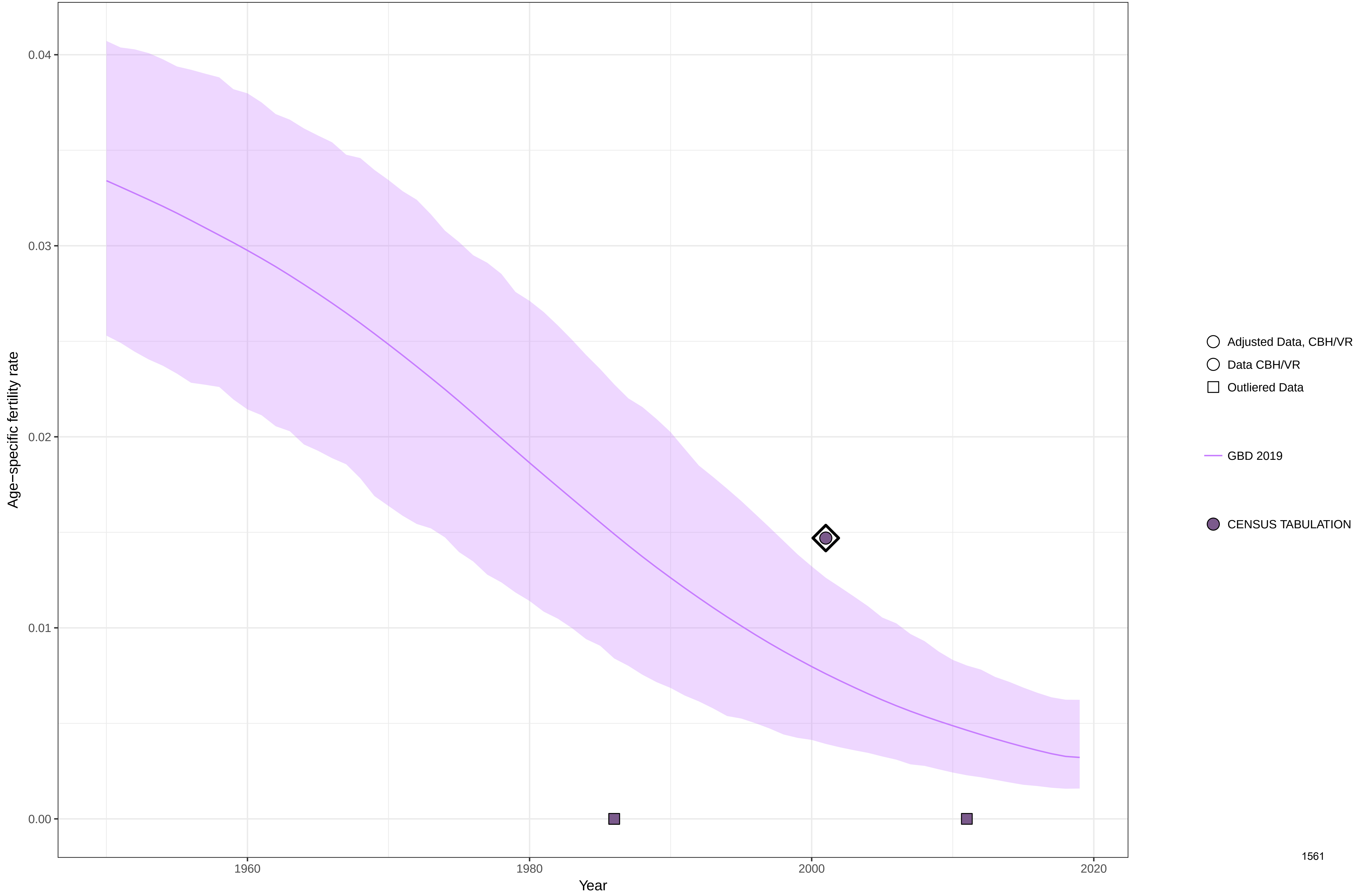

Age−specific fertility fitted trend lines, 1950−2019: Niue, Ages: 50−54
Data points enclosed with a diamond are reference sources

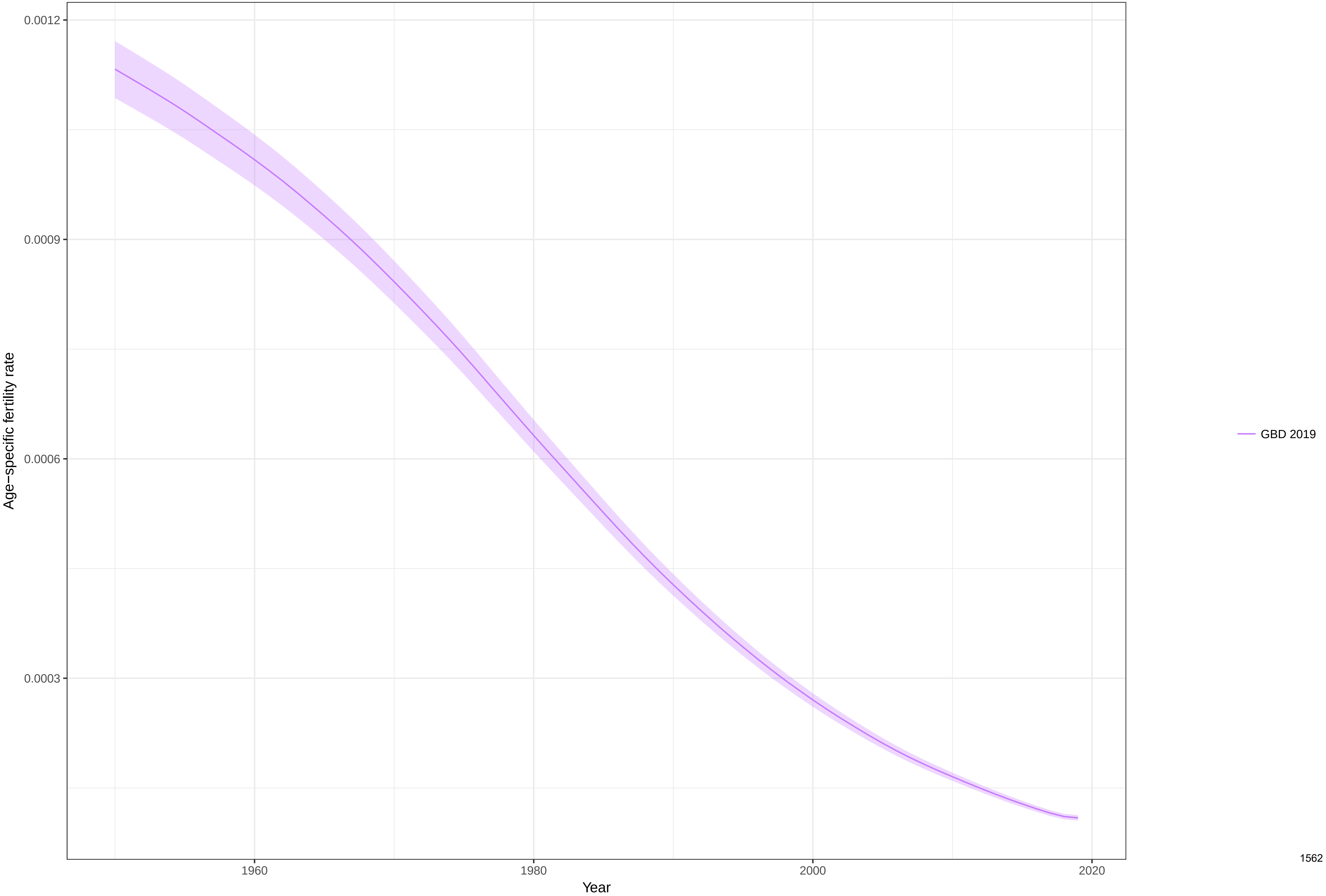

Total fertility rate fitted trend lines, 1950−2019: Niue TFR
Data points enclosed with a diamond are reference sources

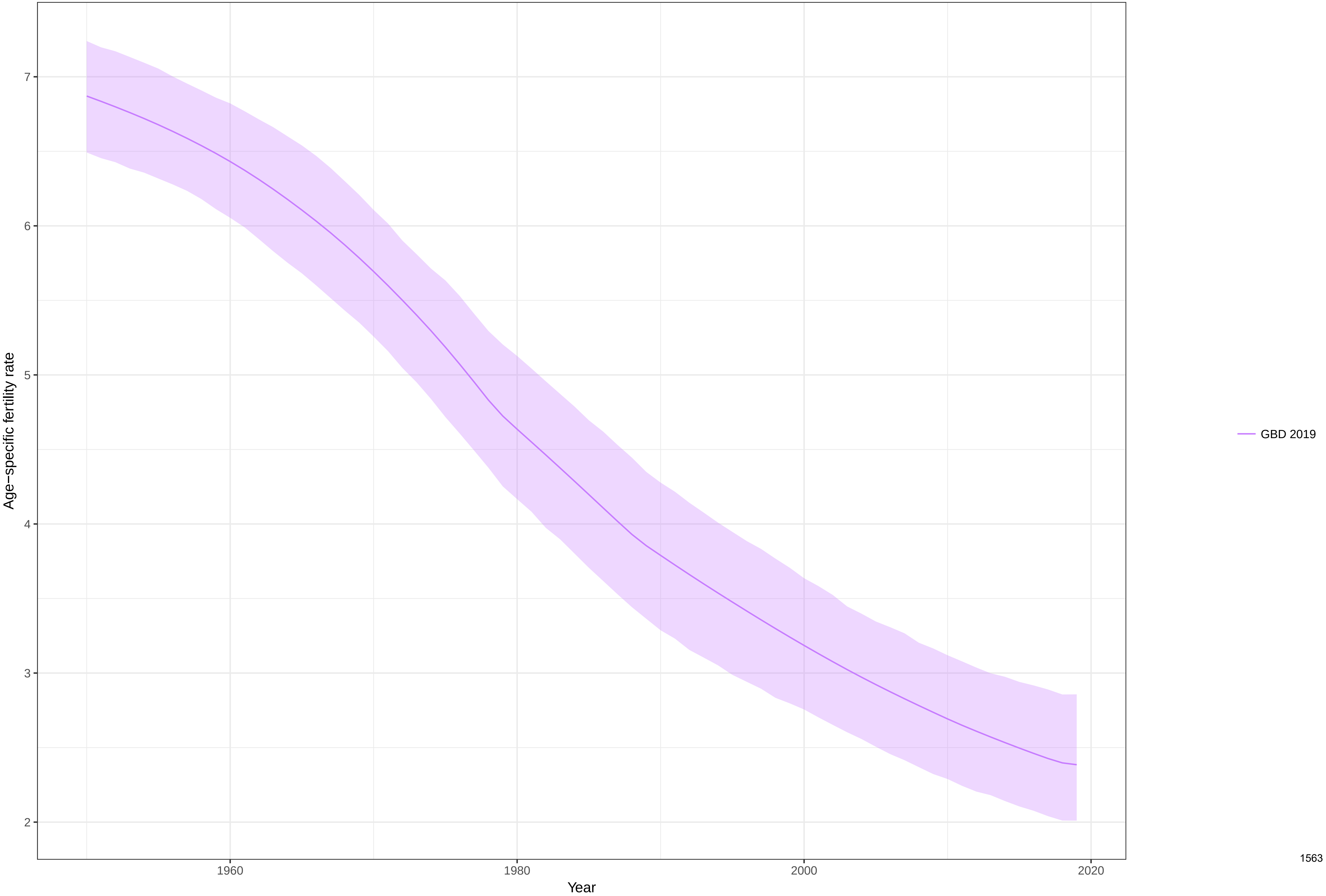

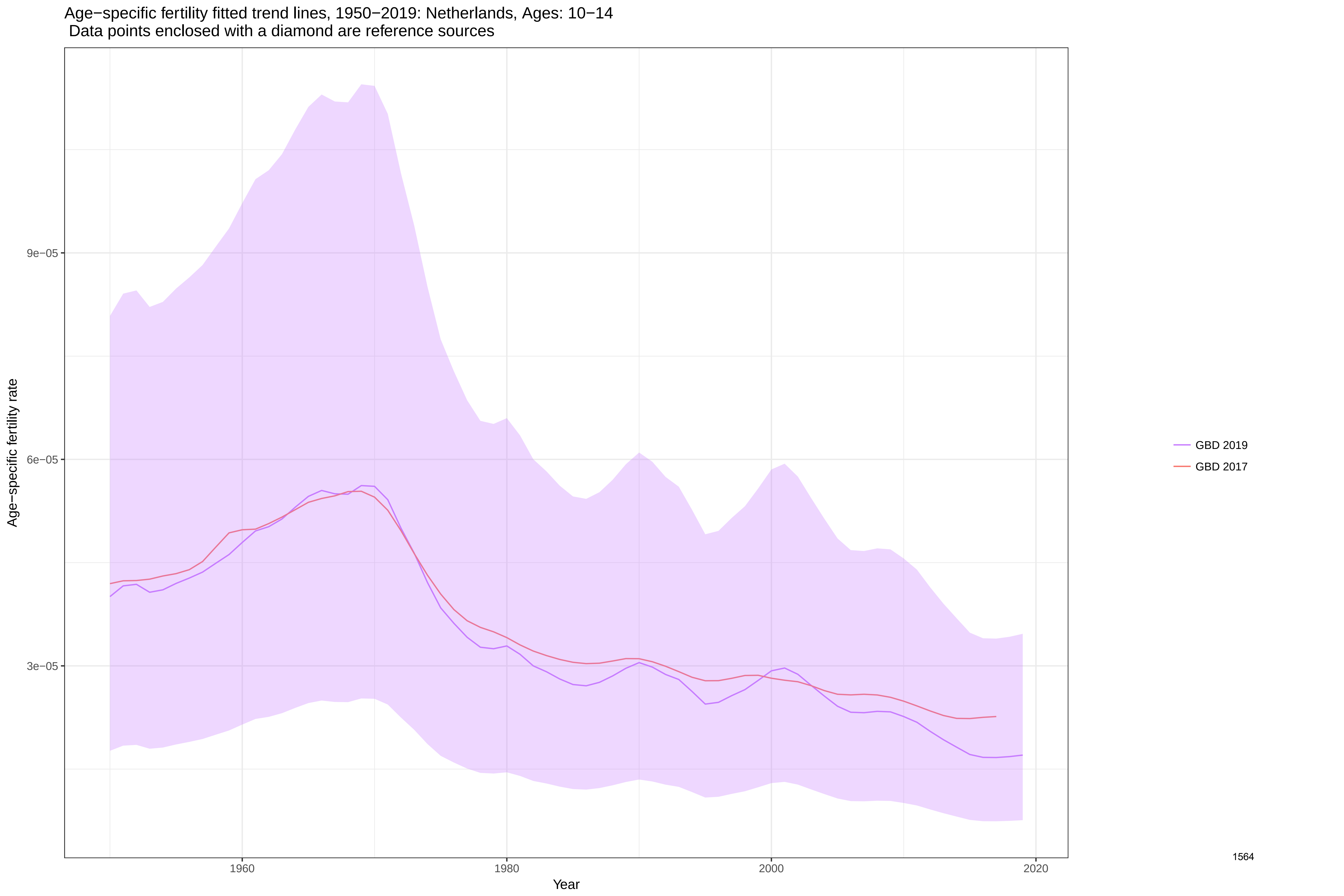
Age−specific fertility fitted trend lines, 1950−2019: Netherlands, Ages: 10−14
Data points enclosed with a diamond are reference sources
9e−05
6e−05
3e−05
Age−specific fertility rate
1960
1980
2000
2020
Year
GBD 2019
GBD 2017
1564

Age−specific fertility fitted trend lines, 1950−2019: Netherlands, Ages: 15−19
Data points enclosed with a diamond are reference sources

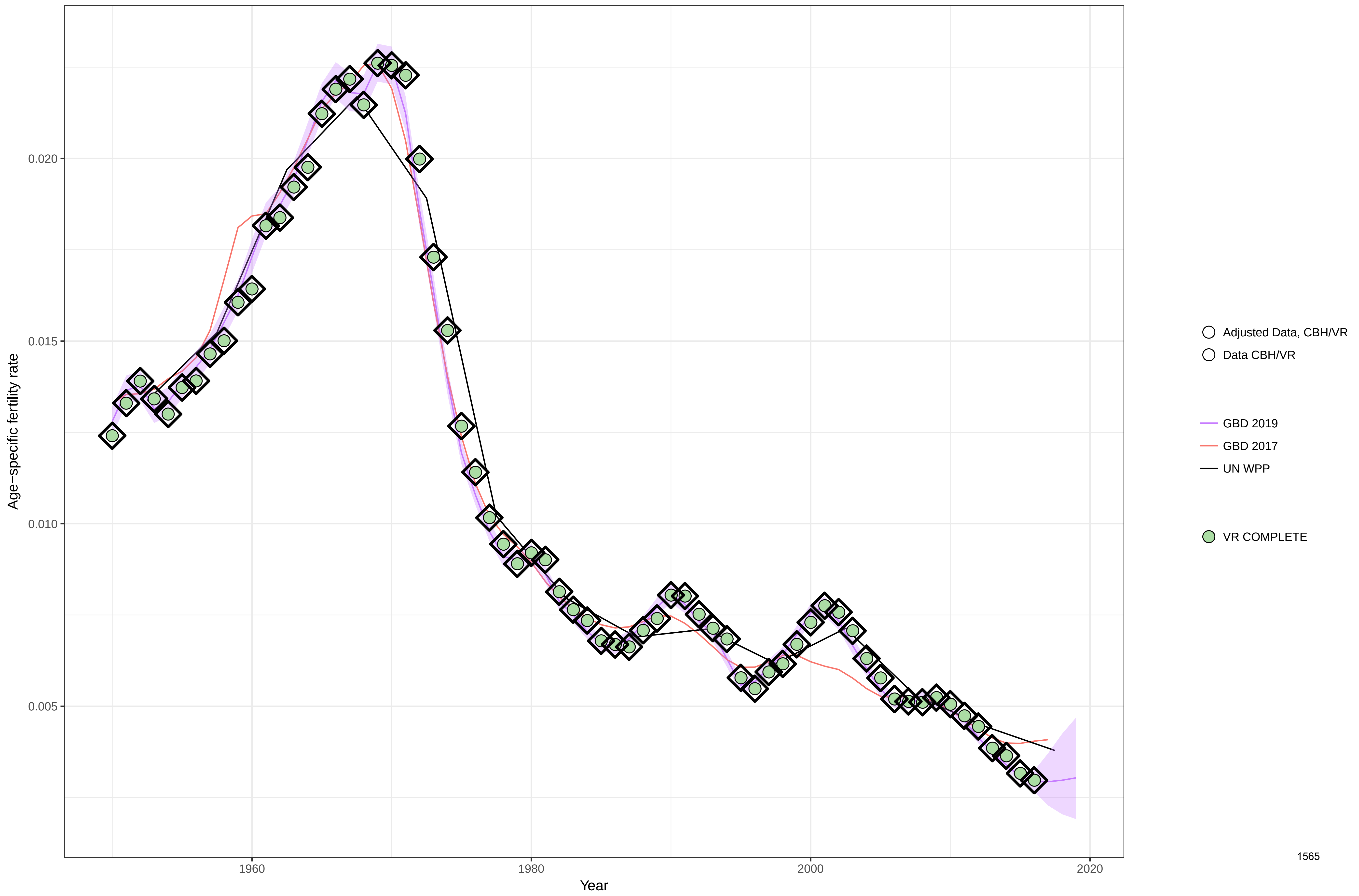

1565

Age−specific fertility fitted trend lines, 1950−2019: Netherlands, Ages: 20−24
Data points enclosed with a diamond are reference sources

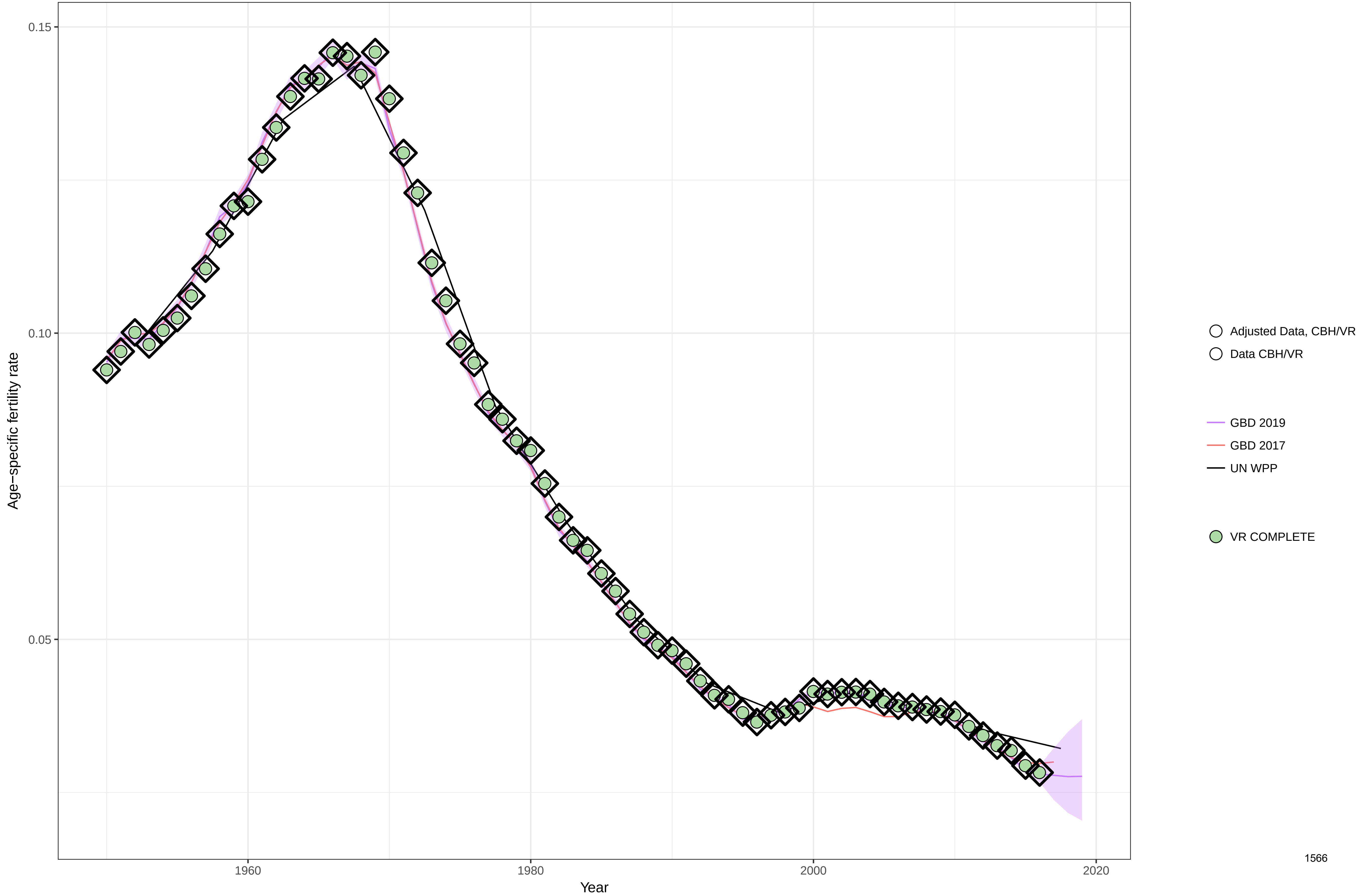

Age−specific fertility fitted trend lines, 1950−2019: Netherlands, Ages: 25−29
Data points enclosed with a diamond are reference sources

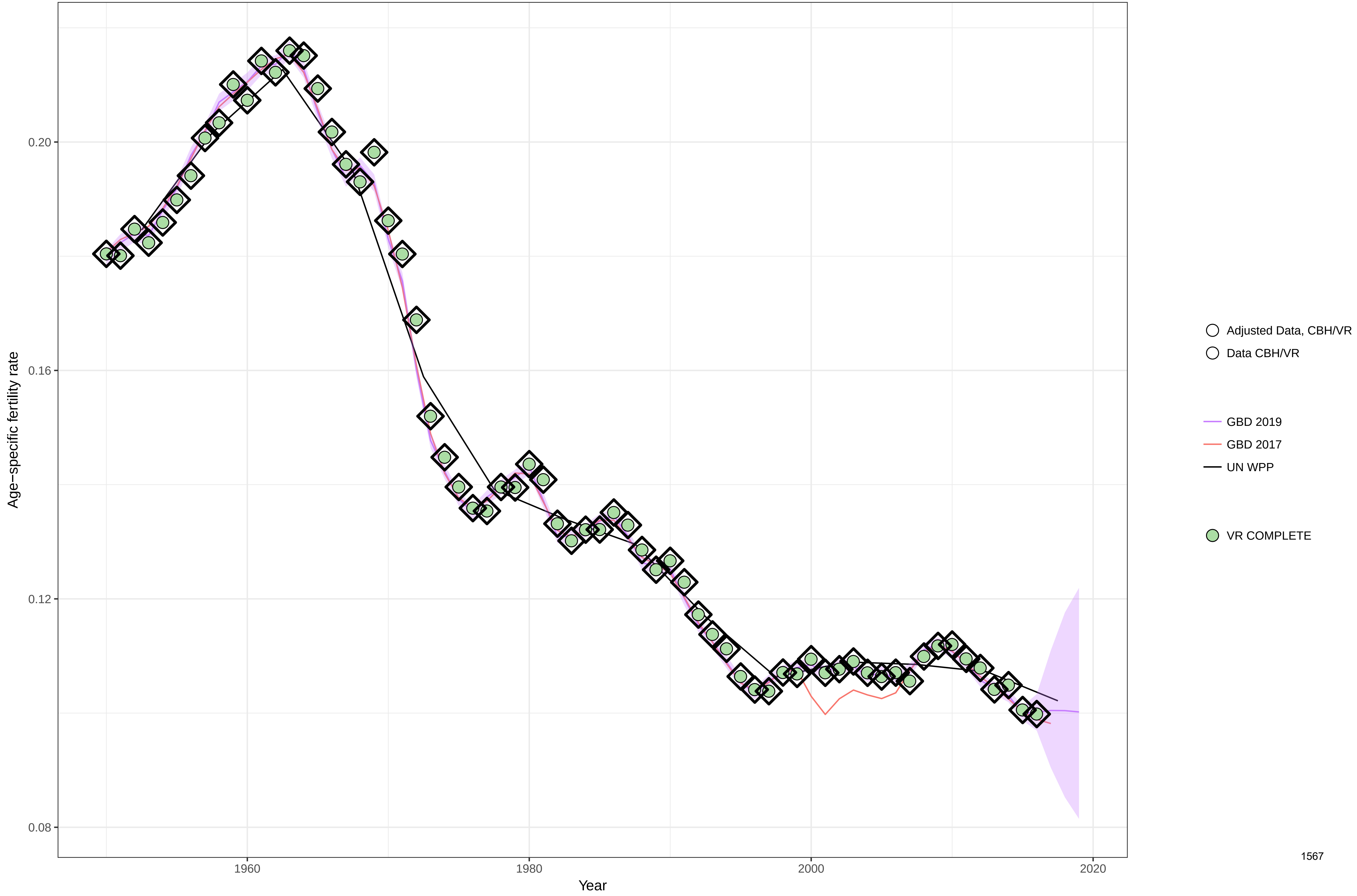

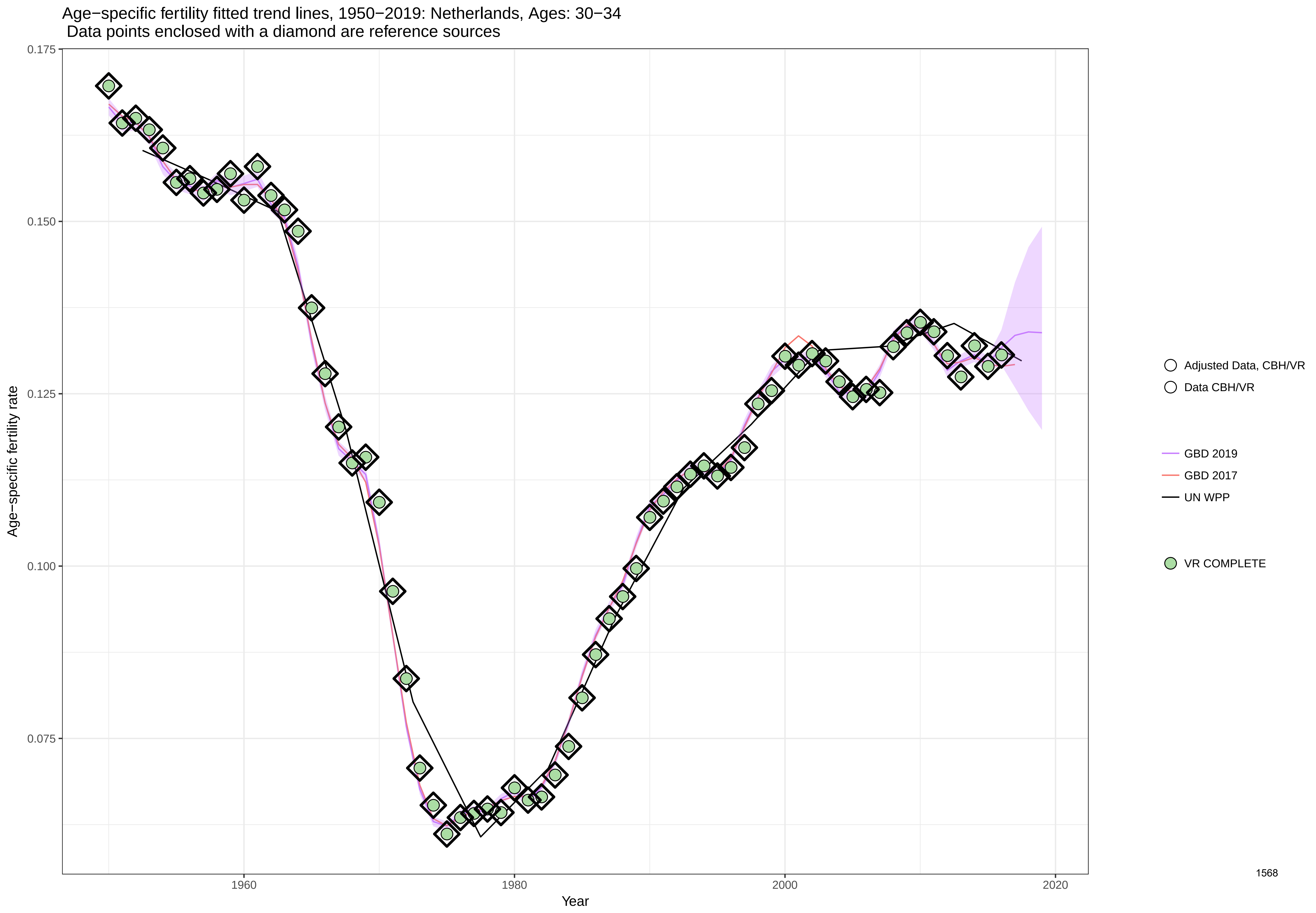

Age−specific fertility fitted trend lines, 1950−2019: Netherlands, Ages: 30−34
Data points enclosed with a diamond are reference sources
0.175
0.150
0.125
0.100
0.075
Age−specific fertility rate
1960
1980
2000
2020
Year
Adjusted Data, CBH/VR
Data CBH/VR
GBD 2019
GBD 2017
UN WPP
VR COMPLETE

Age−specific fertility fitted trend lines, 1950−2019: Netherlands, Ages: 35−39
Data points enclosed with a diamond are reference sources

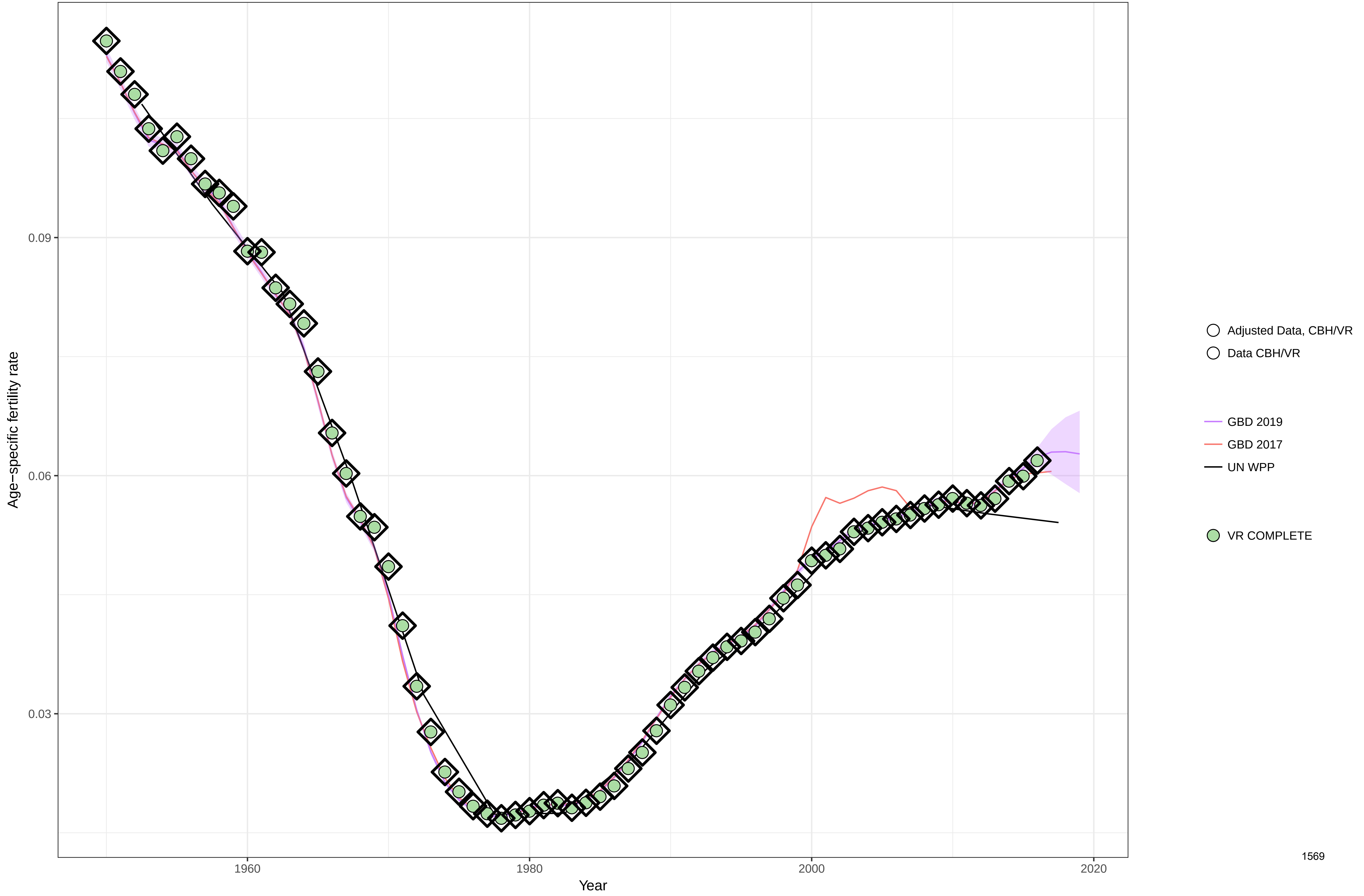

Age−specific fertility fitted trend lines, 1950−2019: Netherlands, Ages: 40−44
Data points enclosed with a diamond are reference sources

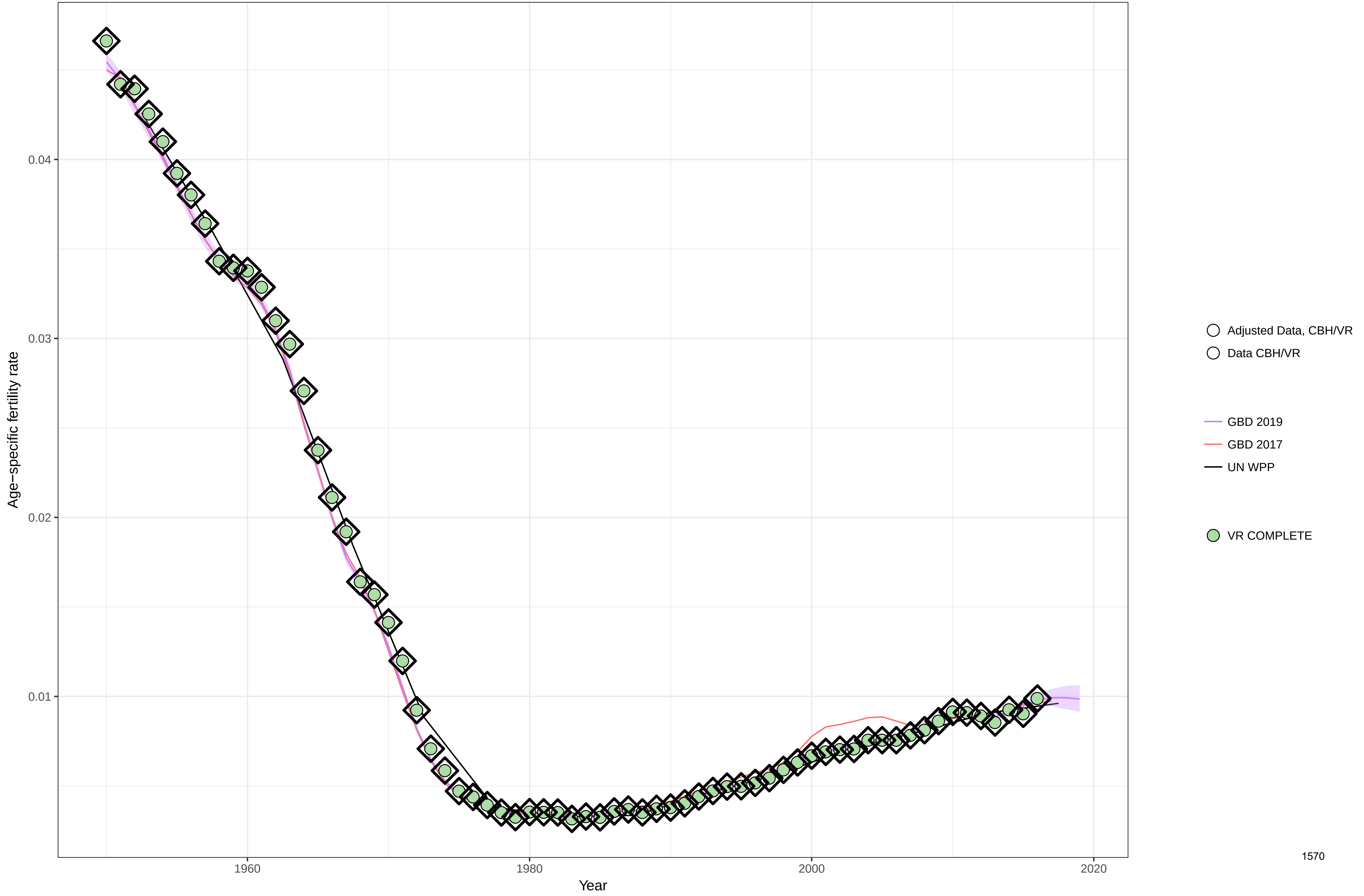

Age−specific fertility fitted trend lines, 1950−2019: Netherlands, Ages: 45−49
Data points enclosed with a diamond are reference sources

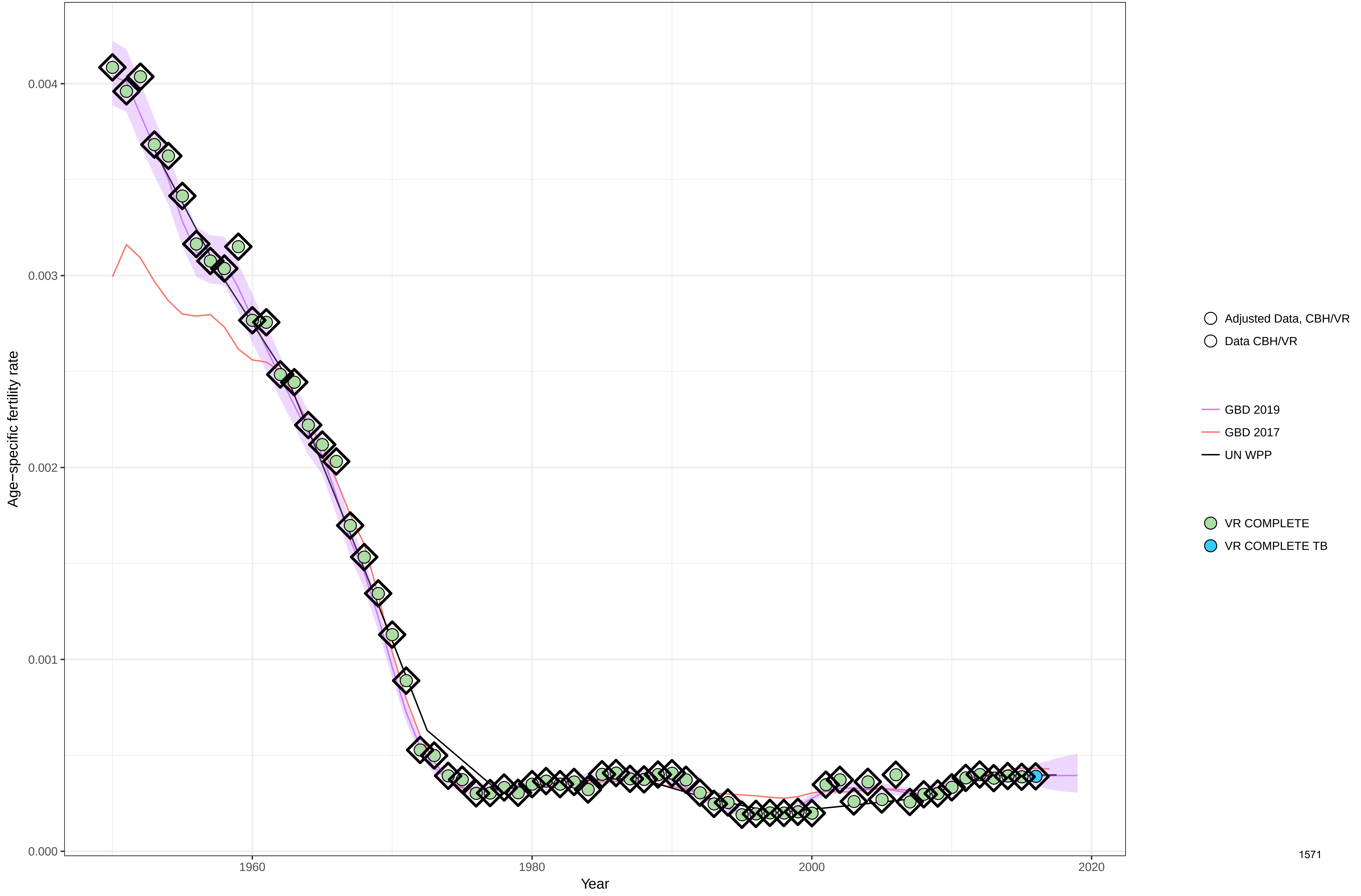

Age−specific fertility fitted trend lines, 1950−2019: Netherlands, Ages: 50−54
Data points enclosed with a diamond are reference sources

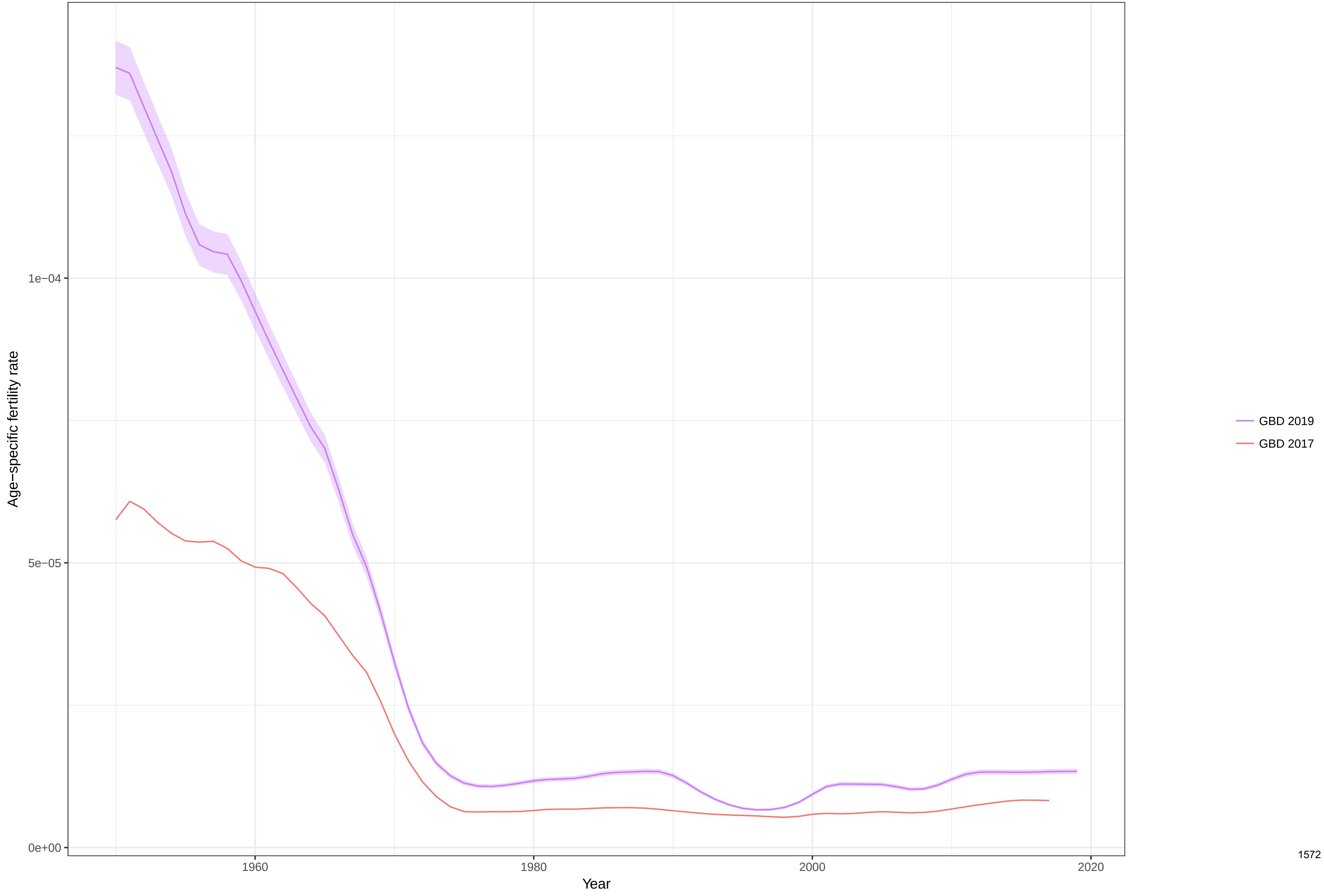

Total fertility rate fitted trend lines, 1950−2019: Netherlands TFR
Data points enclosed with a diamond are reference sources

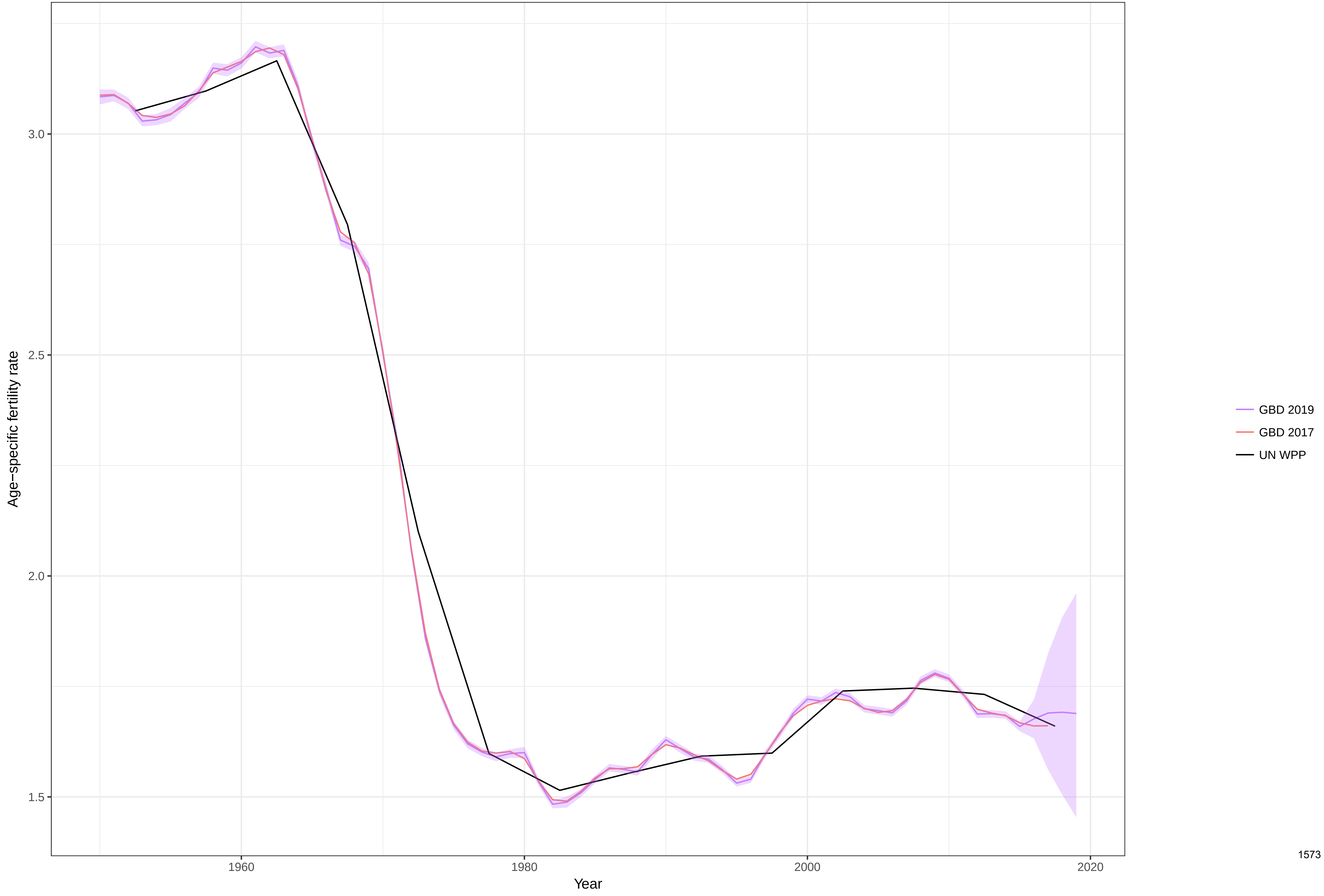

Age−specific fertility fitted trend lines, 1950−2019: Norway, Ages: 10−14
Data points enclosed with a diamond are reference sources

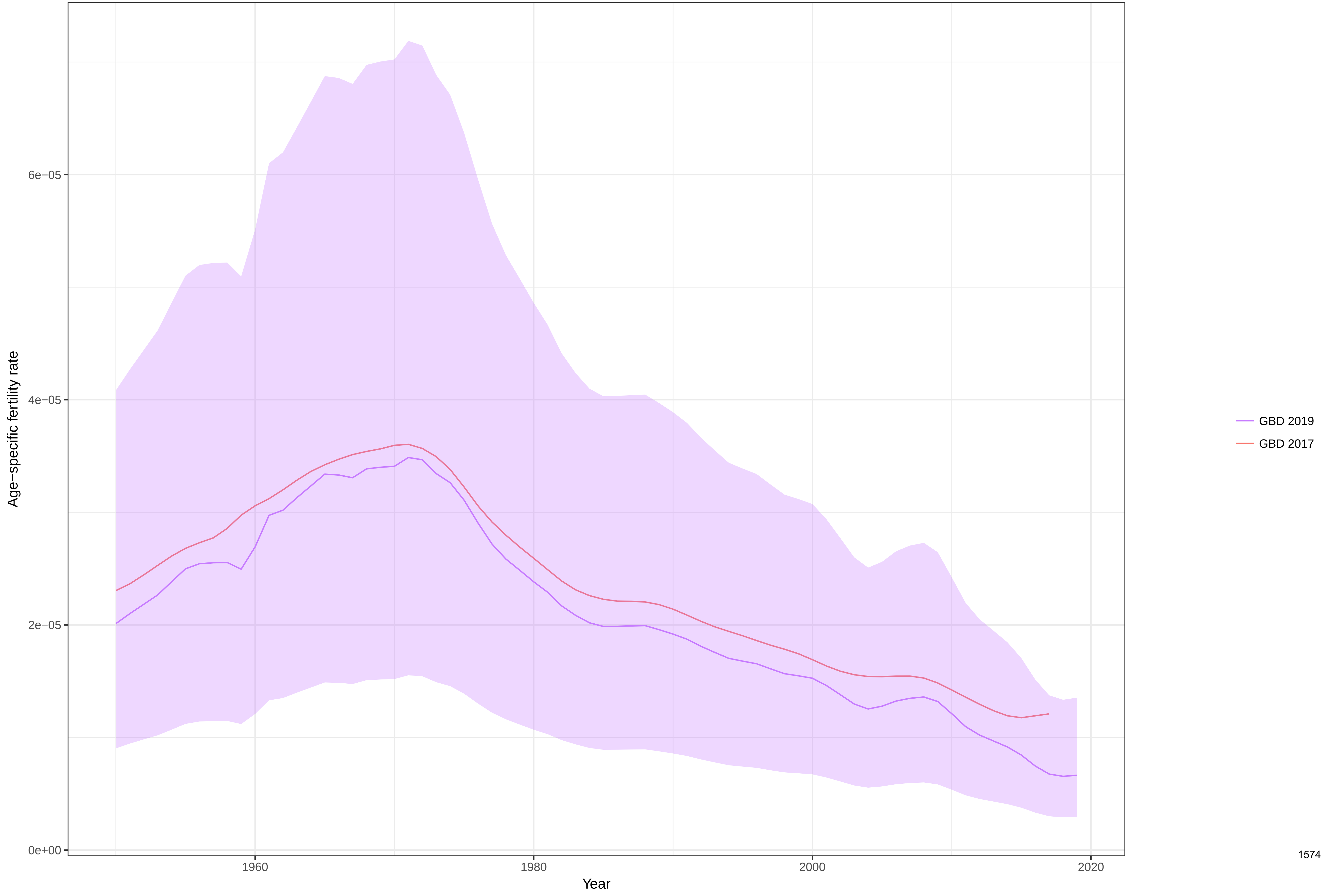

Age−specific fertility fitted trend lines, 1950−2019: Norway, Ages: 15−19
Data points enclosed with a diamond are reference sources

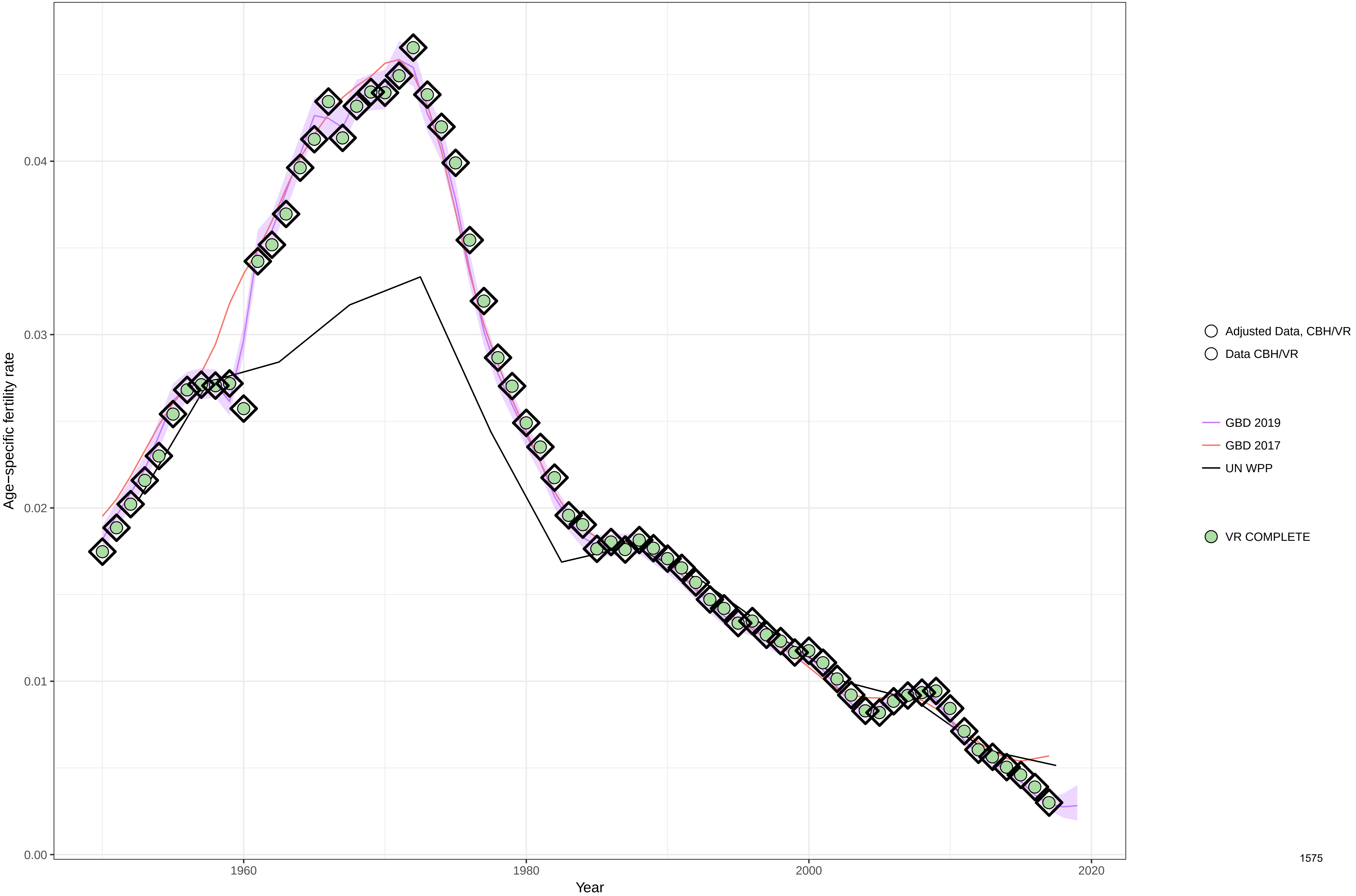

Age−specific fertility fitted trend lines, 1950−2019: Norway, Ages: 20−24
Data points enclosed with a diamond are reference sources

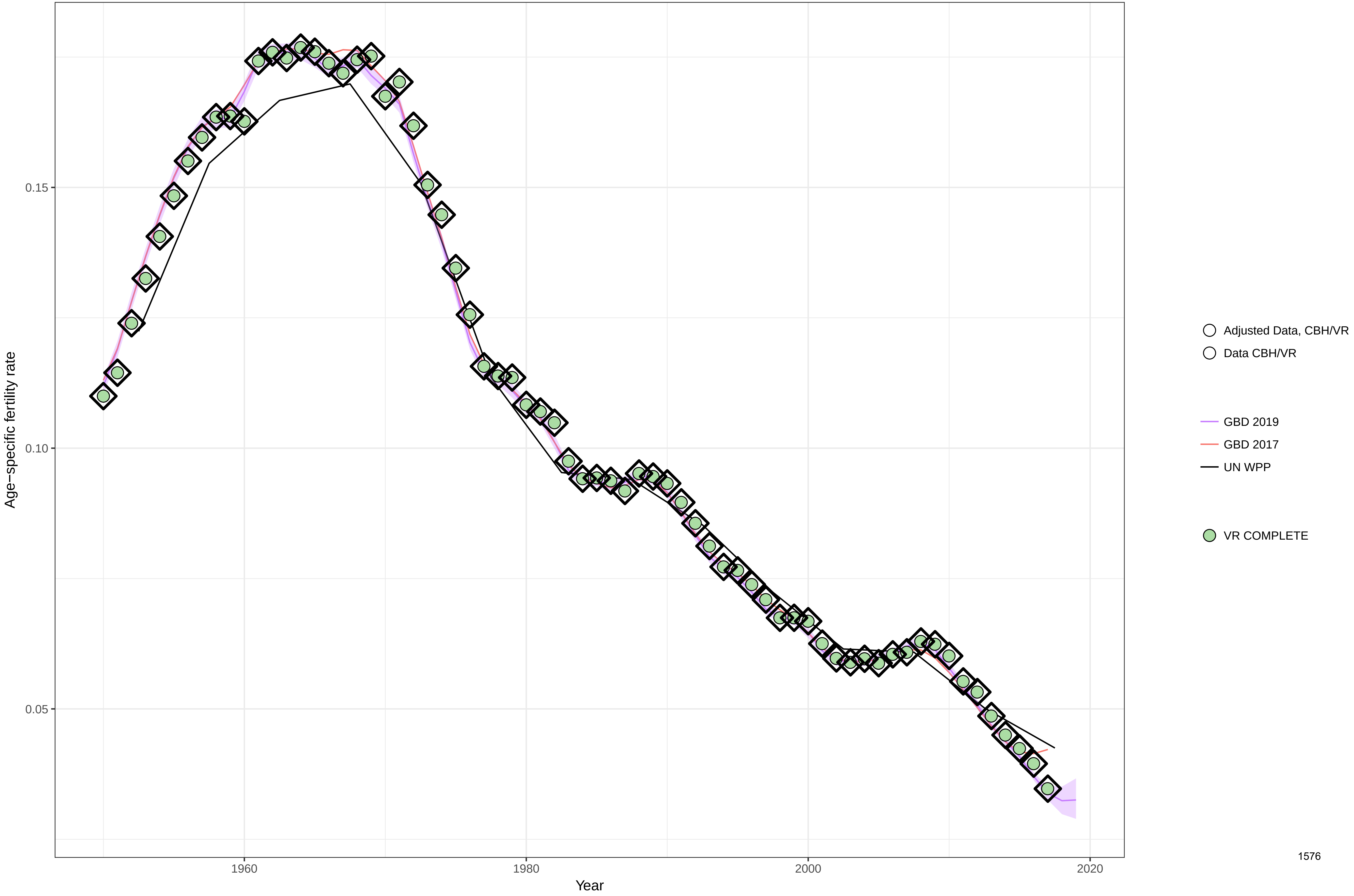

Age−specific fertility fitted trend lines, 1950−2019: Norway, Ages: 25−29
Data points enclosed with a diamond are reference sources

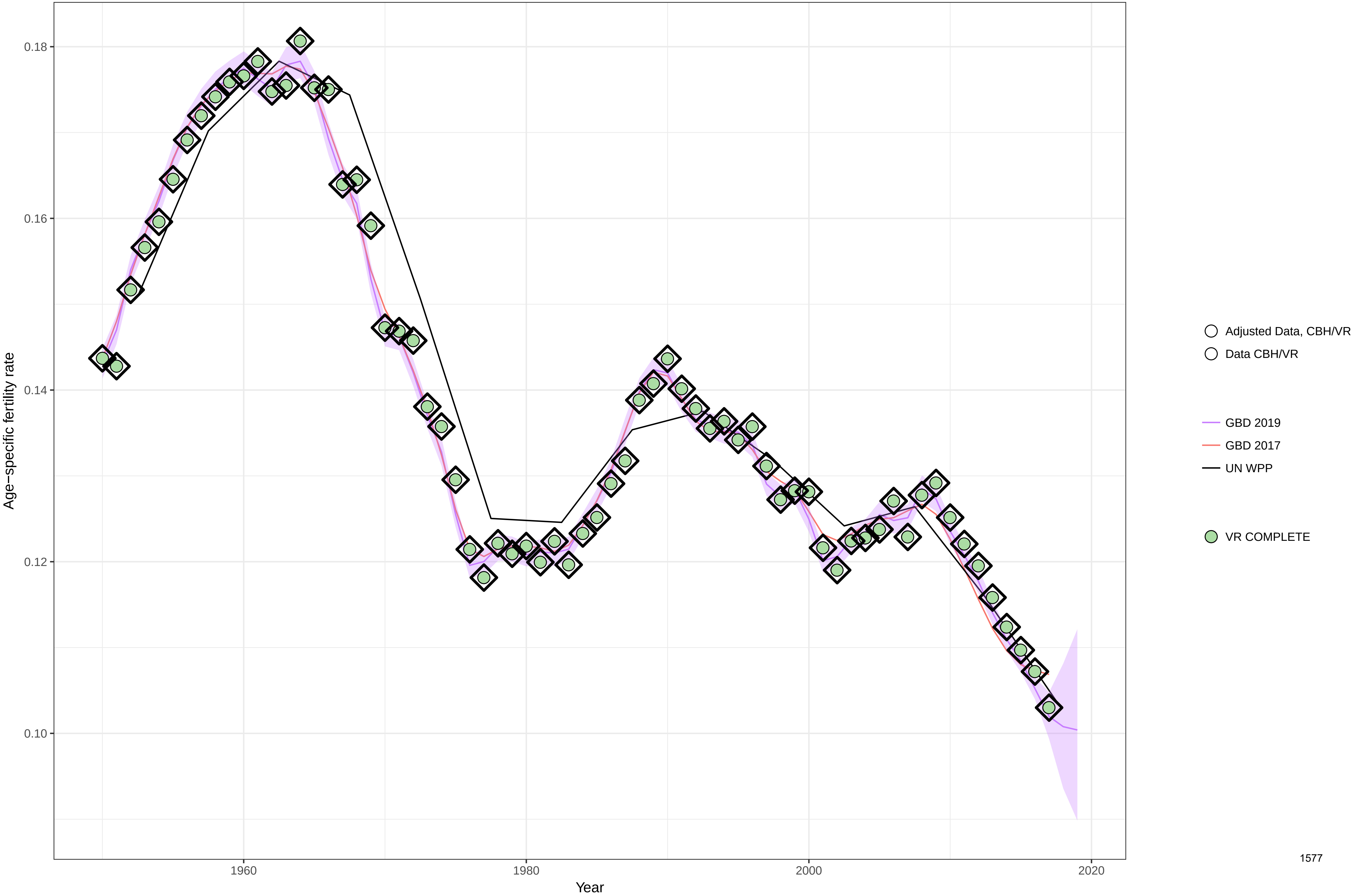

Age−specific fertility fitted trend lines, 1950−2019: Norway, Ages: 30−34
Data points enclosed with a diamond are reference sources

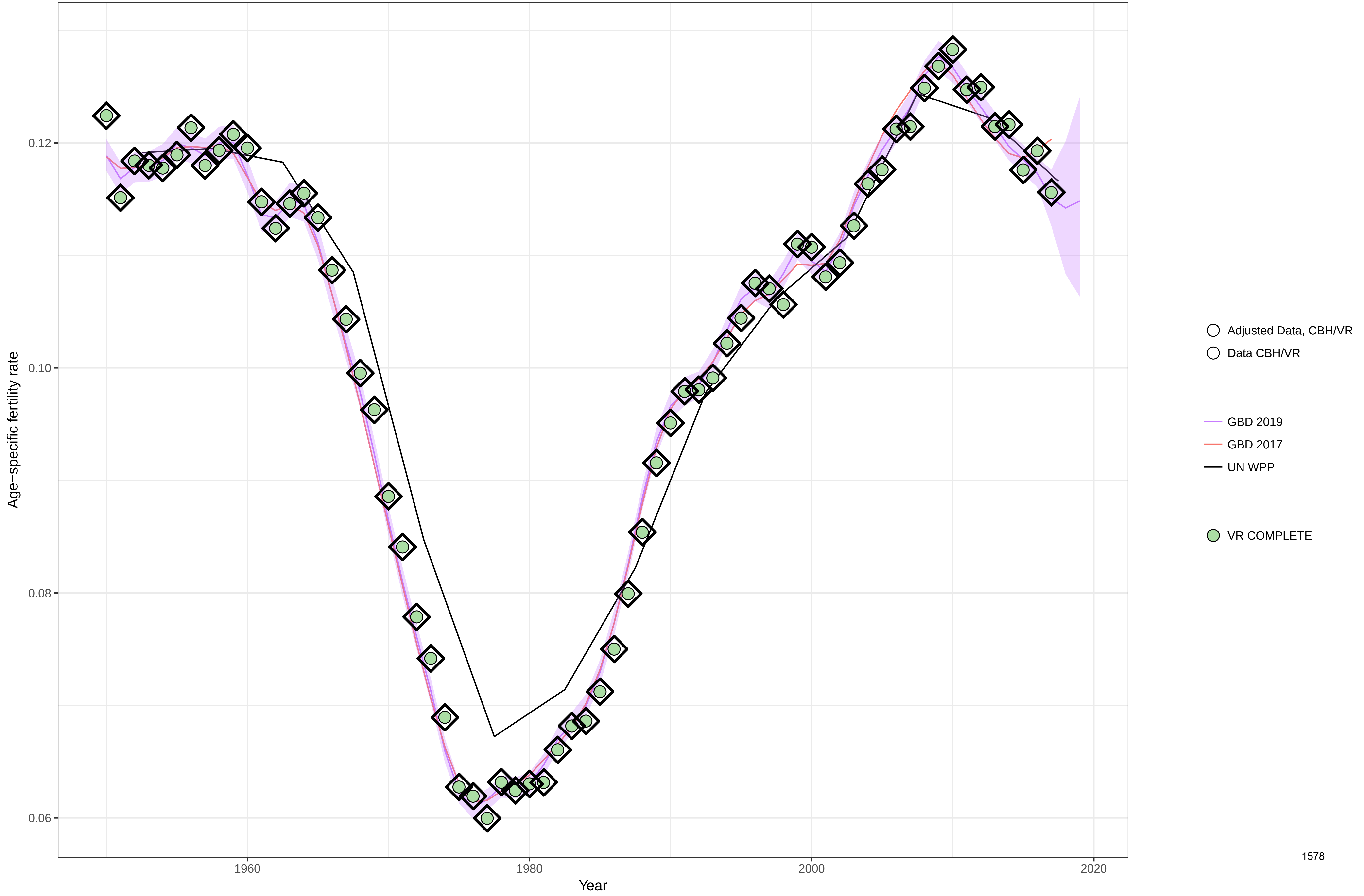

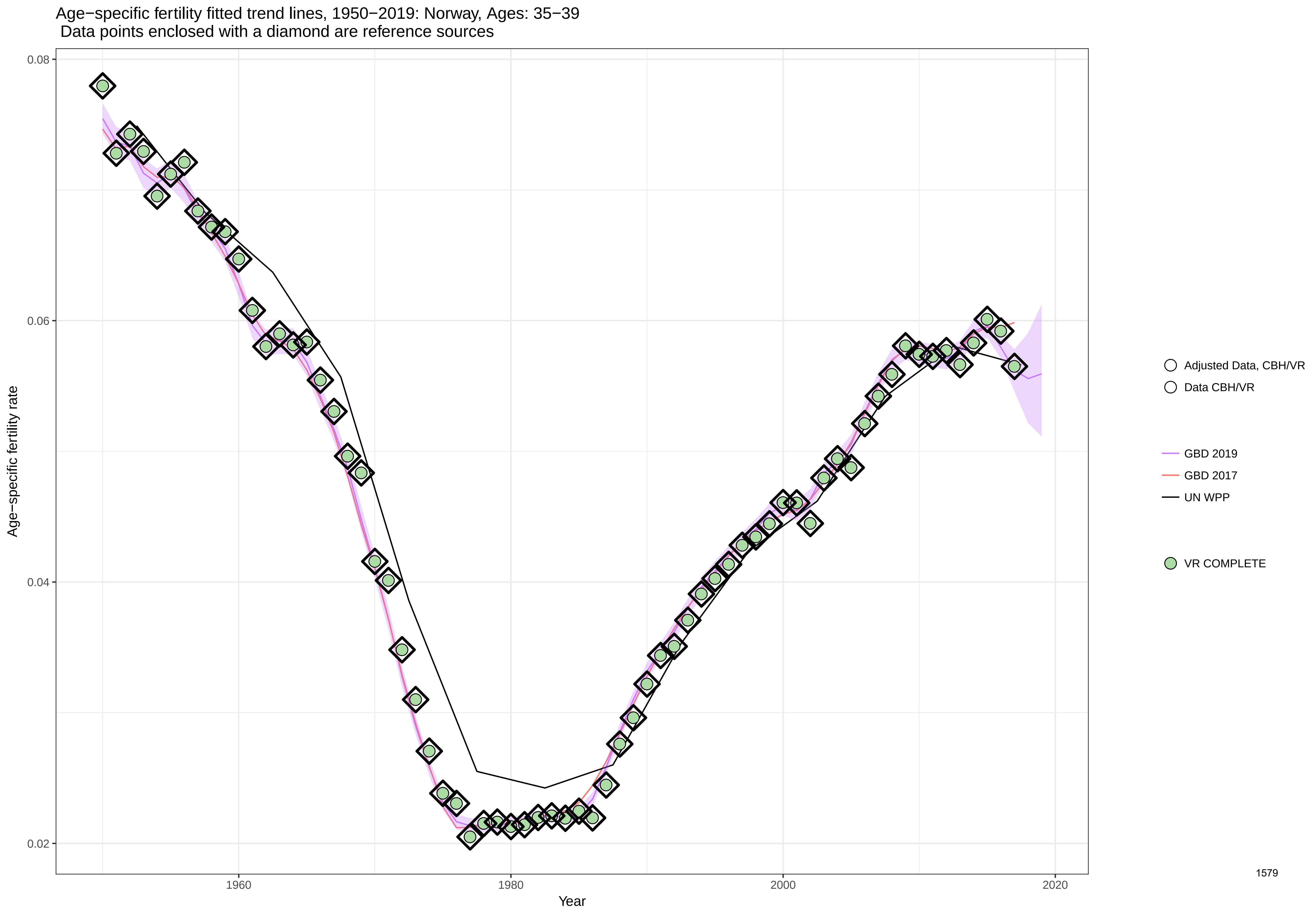

Age−specific fertility fitted trend lines, 1950−2019: Norway, Ages: 35−39
Data points enclosed with a diamond are reference sources
0.08
0.06
0.04
0.02
Age−specific fertility rate
1960
1980
2000
2020
Year
Adjusted Data, CBH/VR
Data CBH/VR
GBD 2019
GBD 2017
UN WPP
VR COMPLETE

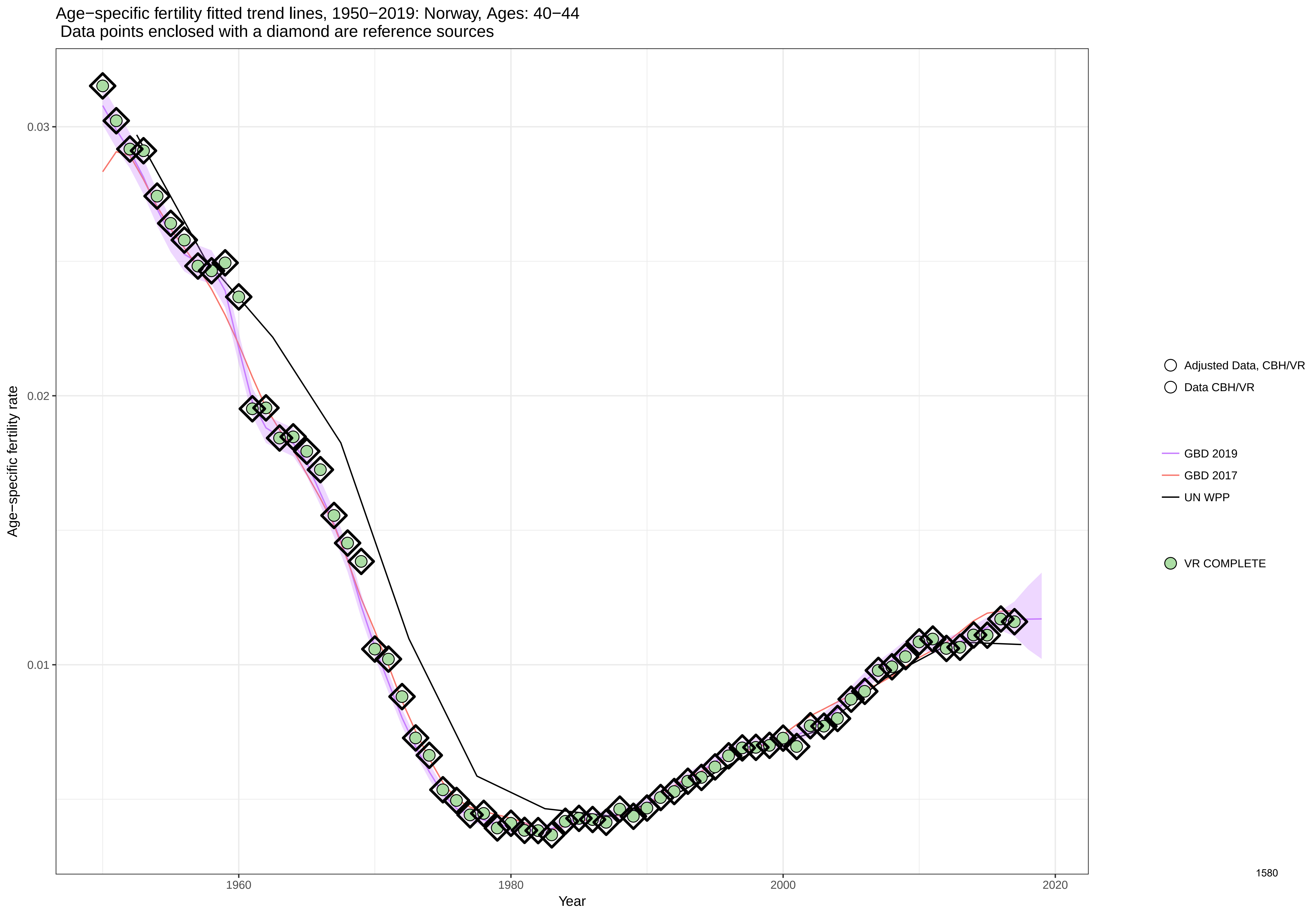
Age−specific fertility fitted trend lines, 1950−2019: Norway, Ages: 40−44
Data points enclosed with a diamond are reference sources
Age−specific fertility rate
0.03
0.02
0.01
1960
1980
2000
2020
Year
Adjusted Data, CBH/VR
Data CBH/VR
GBD 2019
GBD 2017
UN WPP
VR COMPLETE

Age−specific fertility fitted trend lines, 1950−2019: Norway, Ages: 45−49
Data points enclosed with a diamond are reference sources

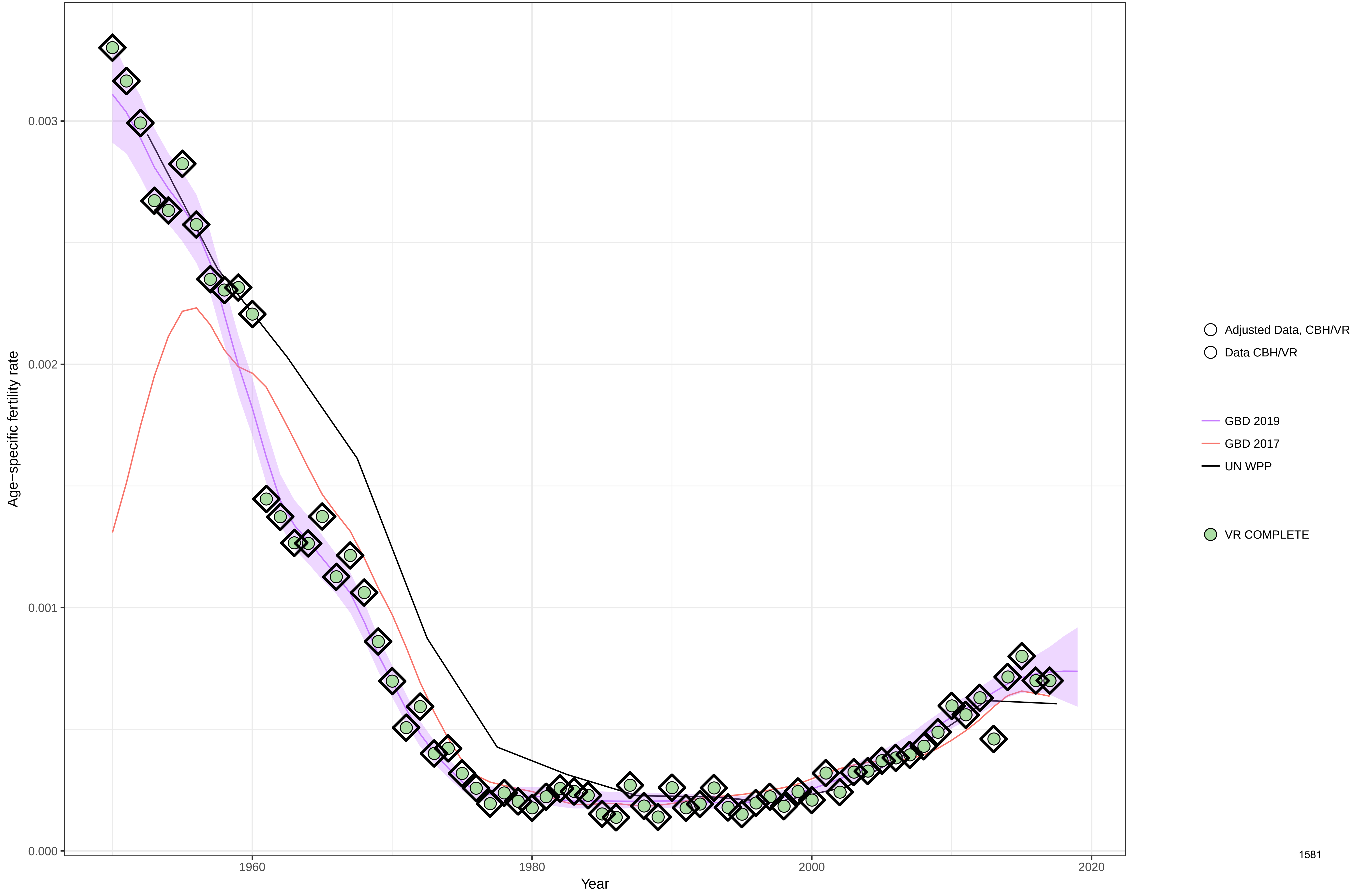

Age−specific fertility fitted trend lines, 1950−2019: Norway, Ages: 50−54
Data points enclosed with a diamond are reference sources

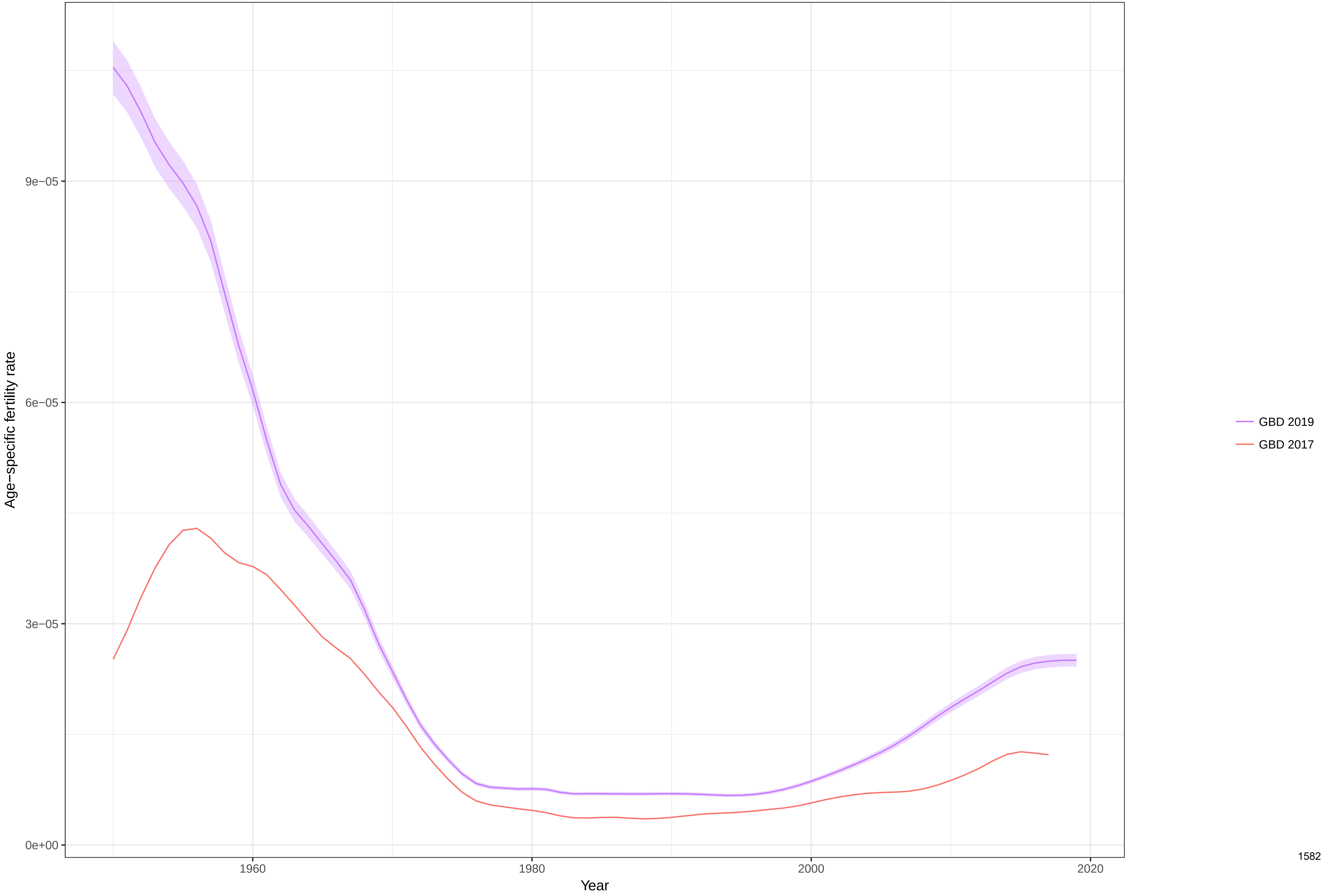

Total fertility rate fitted trend lines, 1950−2019: Norway TFR
Data points enclosed with a diamond are reference sources

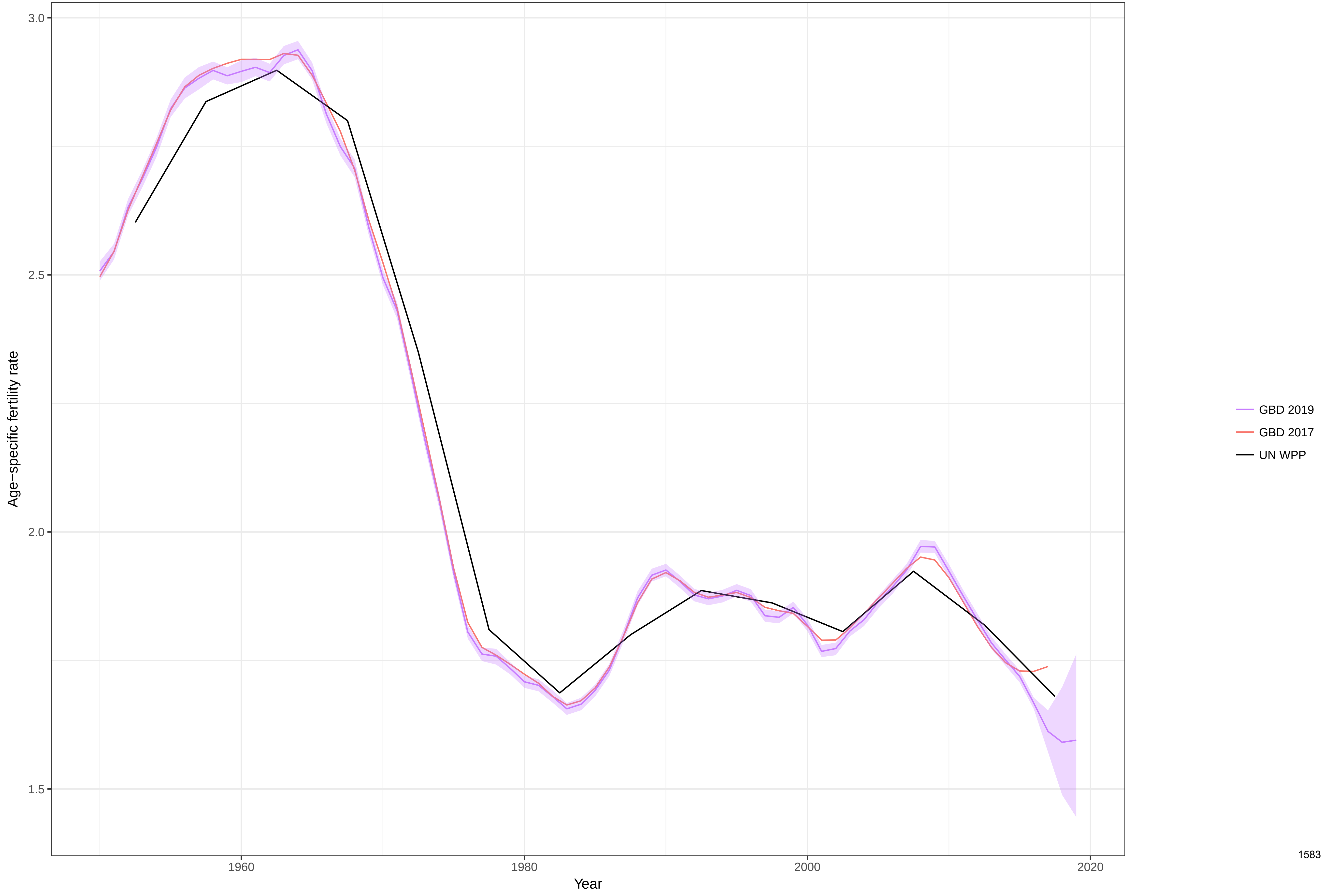

Age−specific fertility fitted trend lines, 1950−2019: Nepal, Ages: 10−14
Data points enclosed with a diamond are reference sources

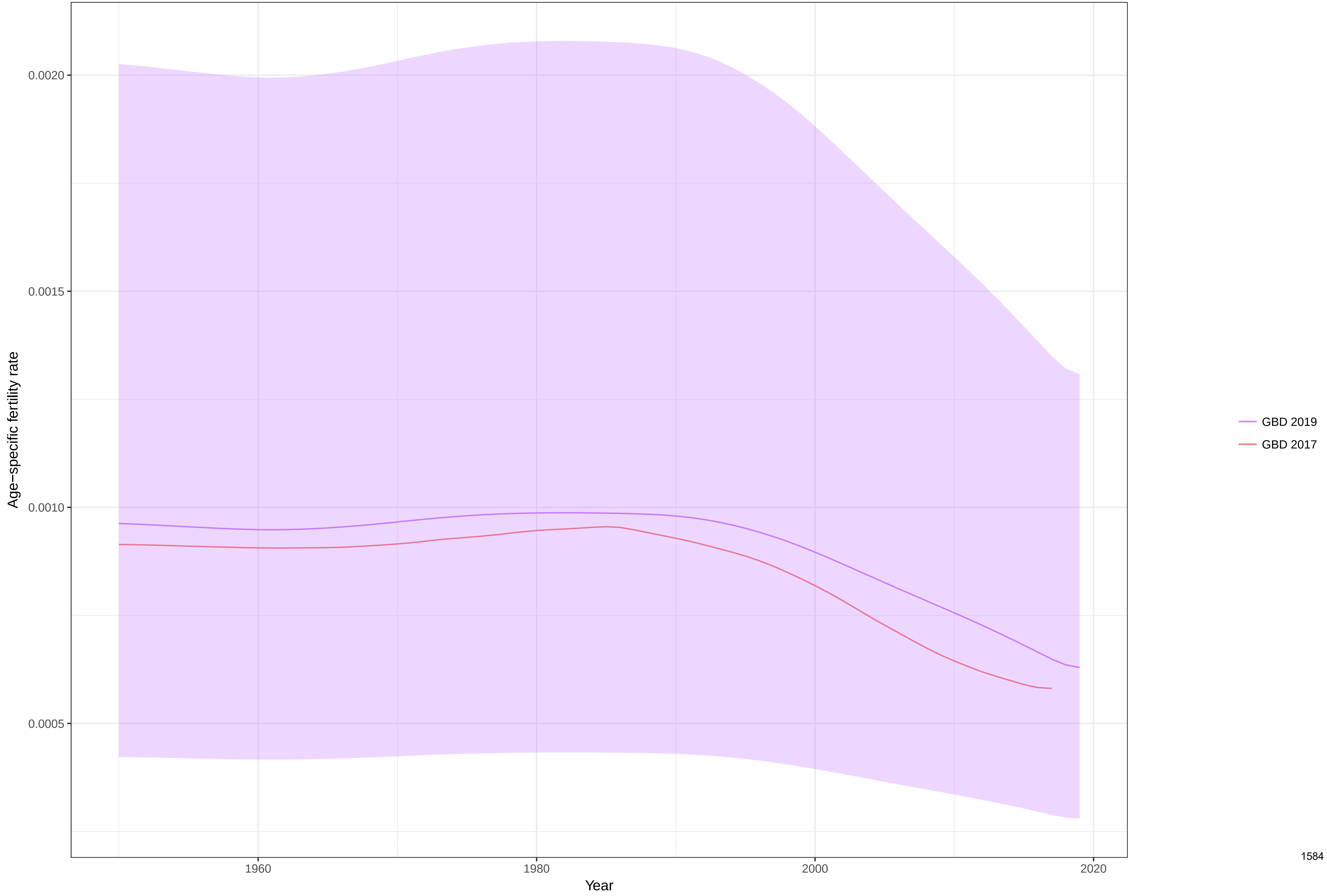

Age−specific fertility fitted trend lines, 1950−2019: Nepal, Ages: 15−19
Data points enclosed with a diamond are reference sources

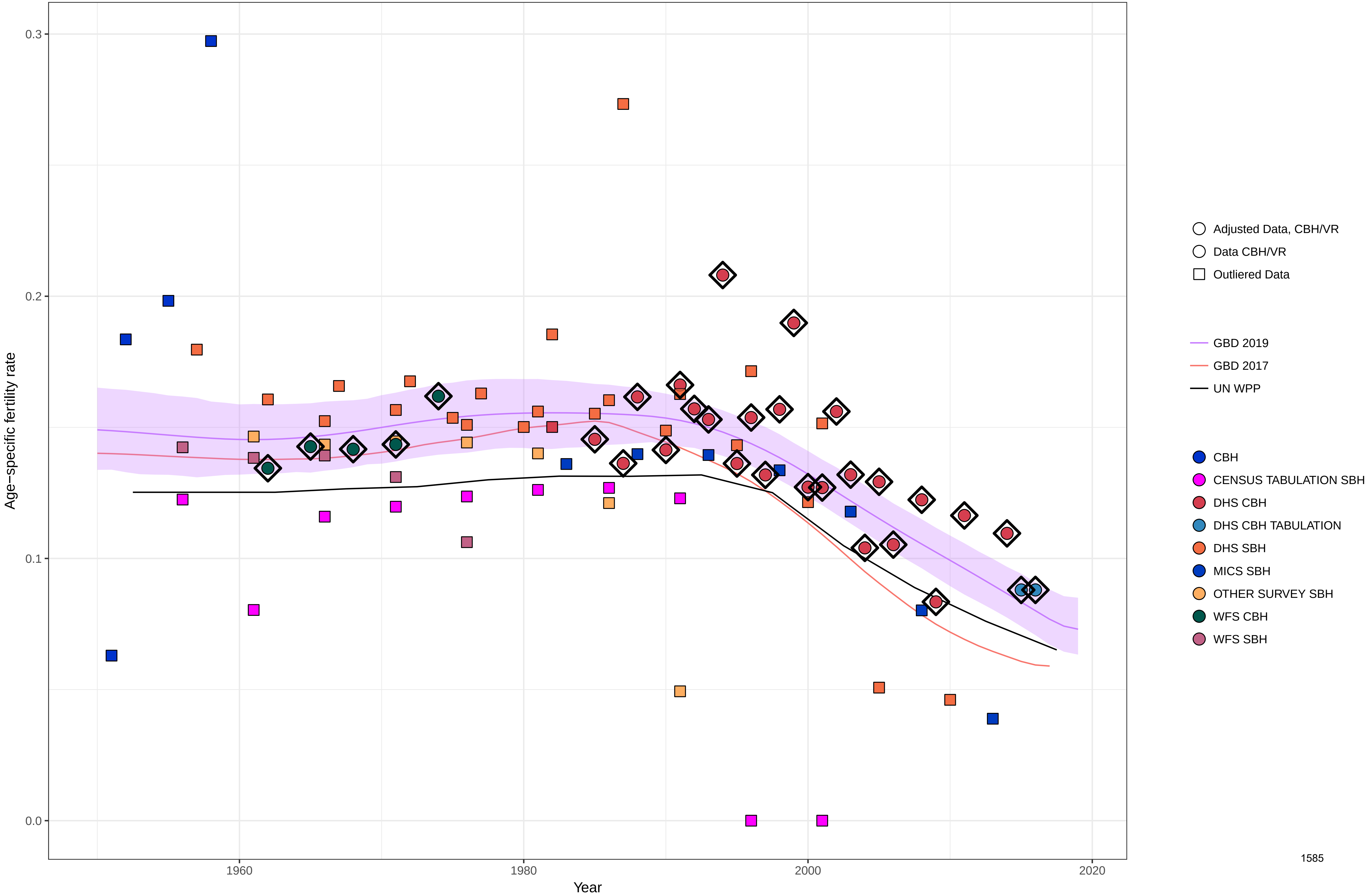

Age−specific fertility fitted trend lines, 1950−2019: Nepal, Ages: 20−24
Data points enclosed with a diamond are reference sources

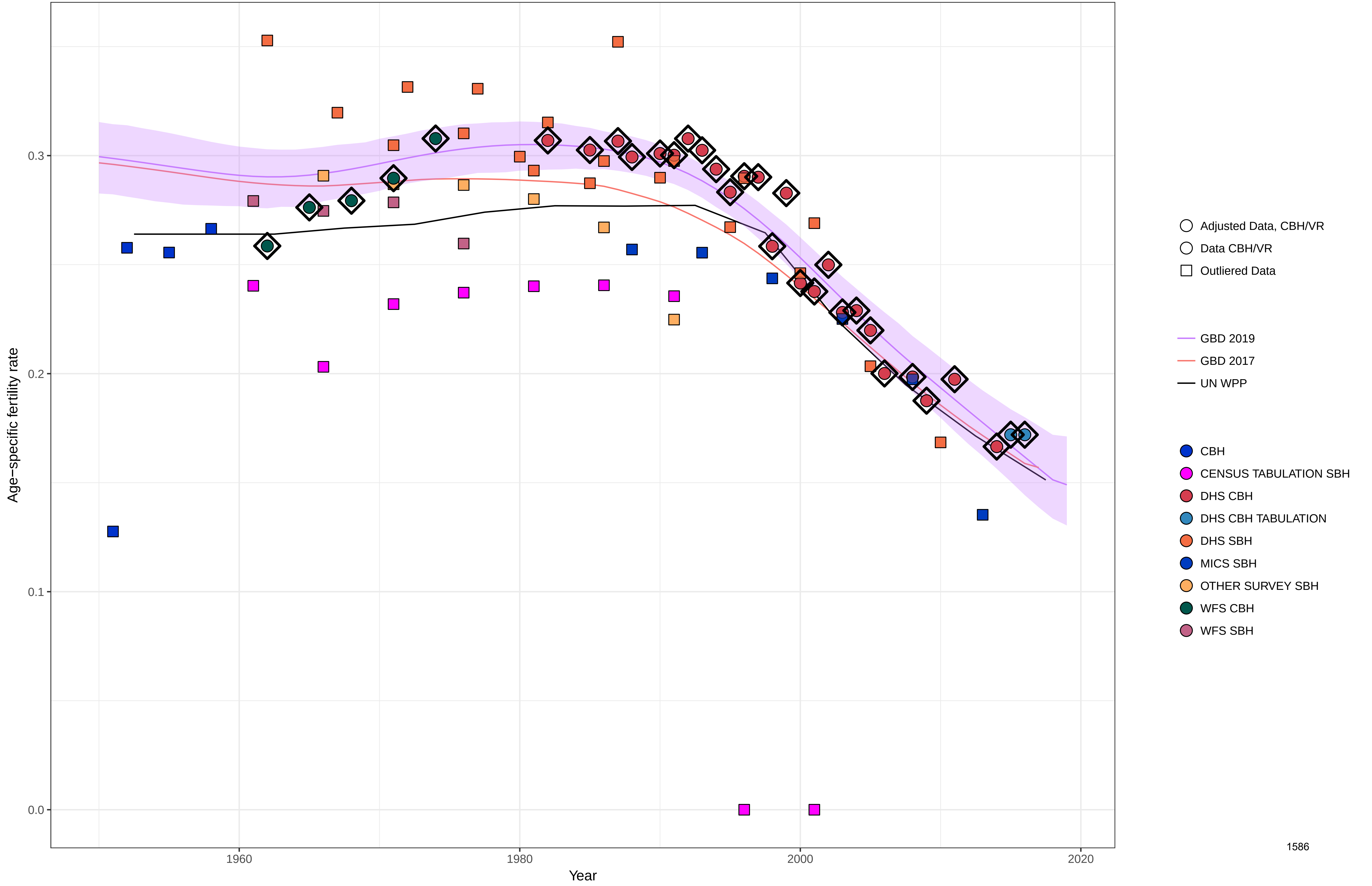

Age−specific fertility fitted trend lines, 1950−2019: Nepal, Ages: 25−29
Data points enclosed with a diamond are reference sources

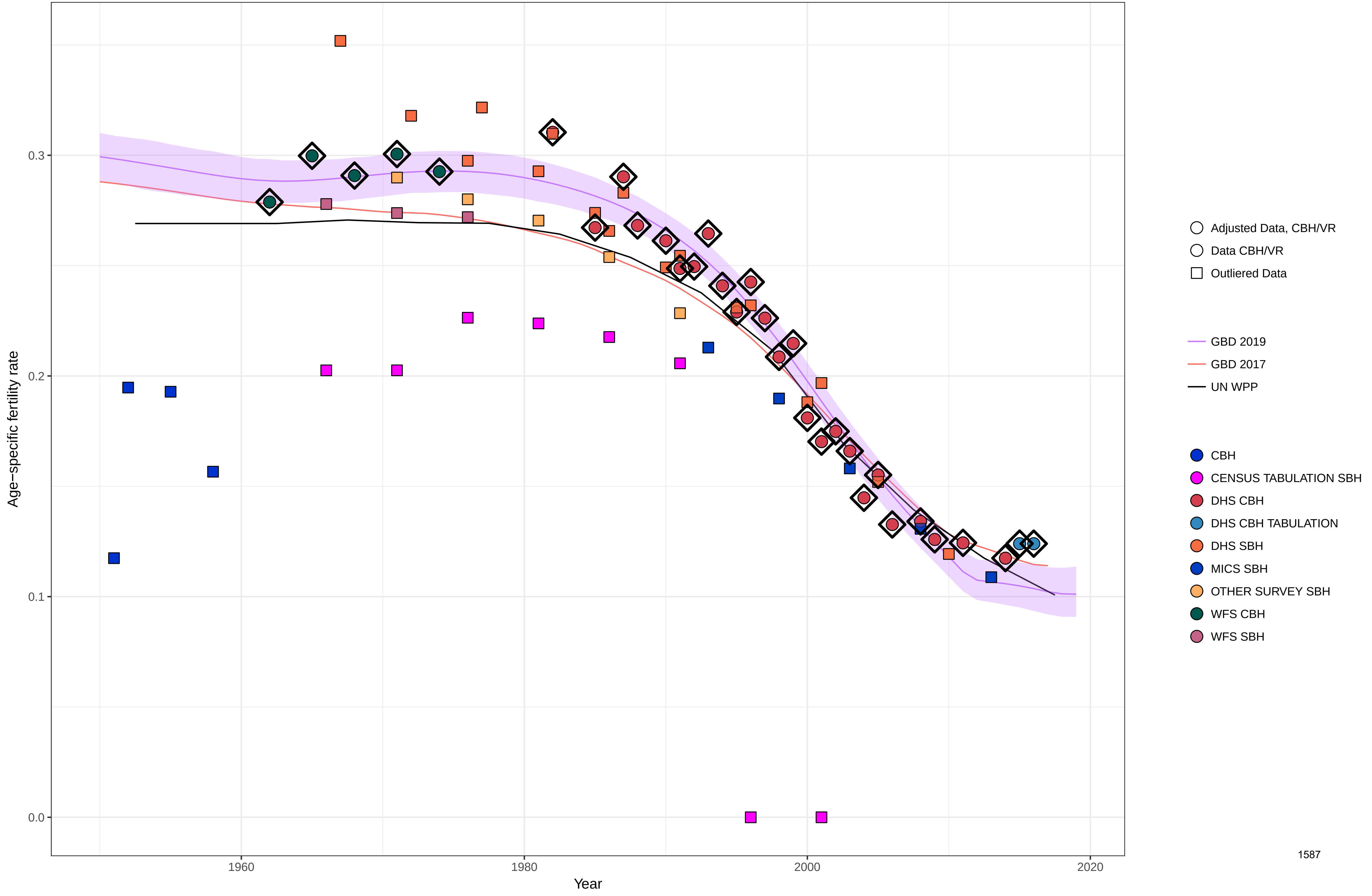

Age−specific fertility fitted trend lines, 1950−2019: Nepal, Ages: 30−34
Data points enclosed with a diamond are reference sources

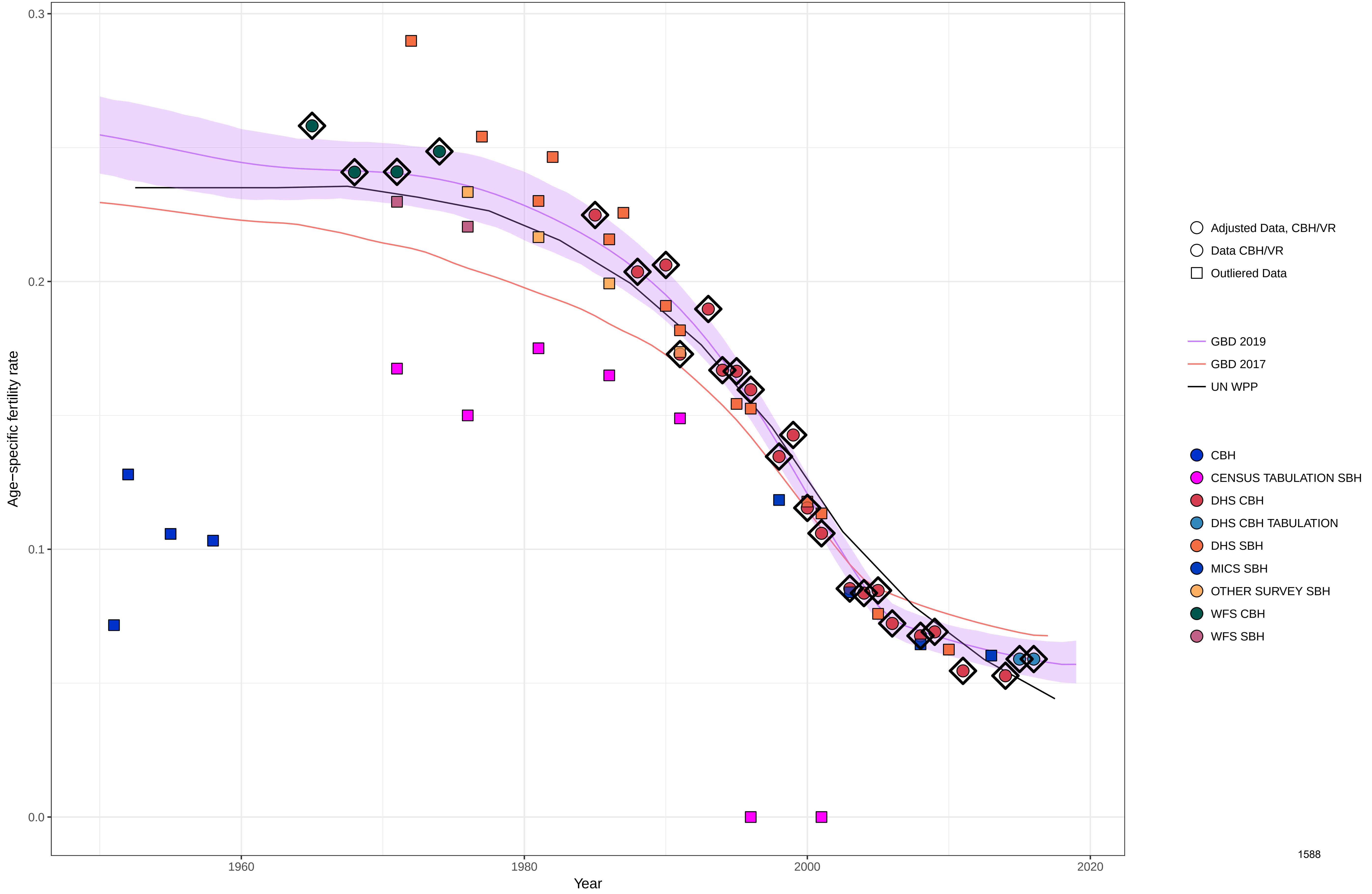

Age−specific fertility fitted trend lines, 1950−2019: Nepal, Ages: 35−39
Data points enclosed with a diamond are reference sources

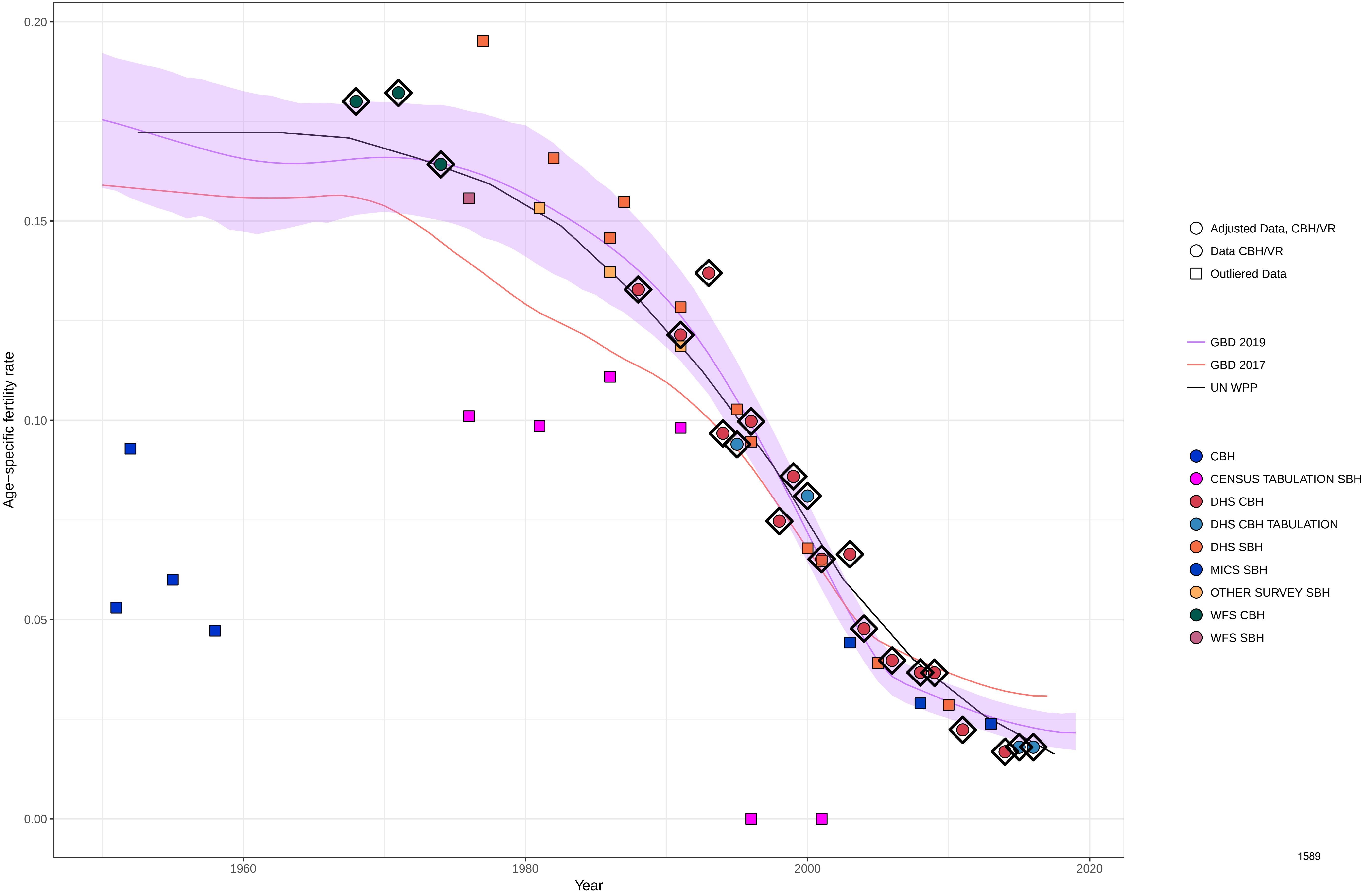

Age−specific fertility fitted trend lines, 1950−2019: Nepal, Ages: 40−44
Data points enclosed with a diamond are reference sources

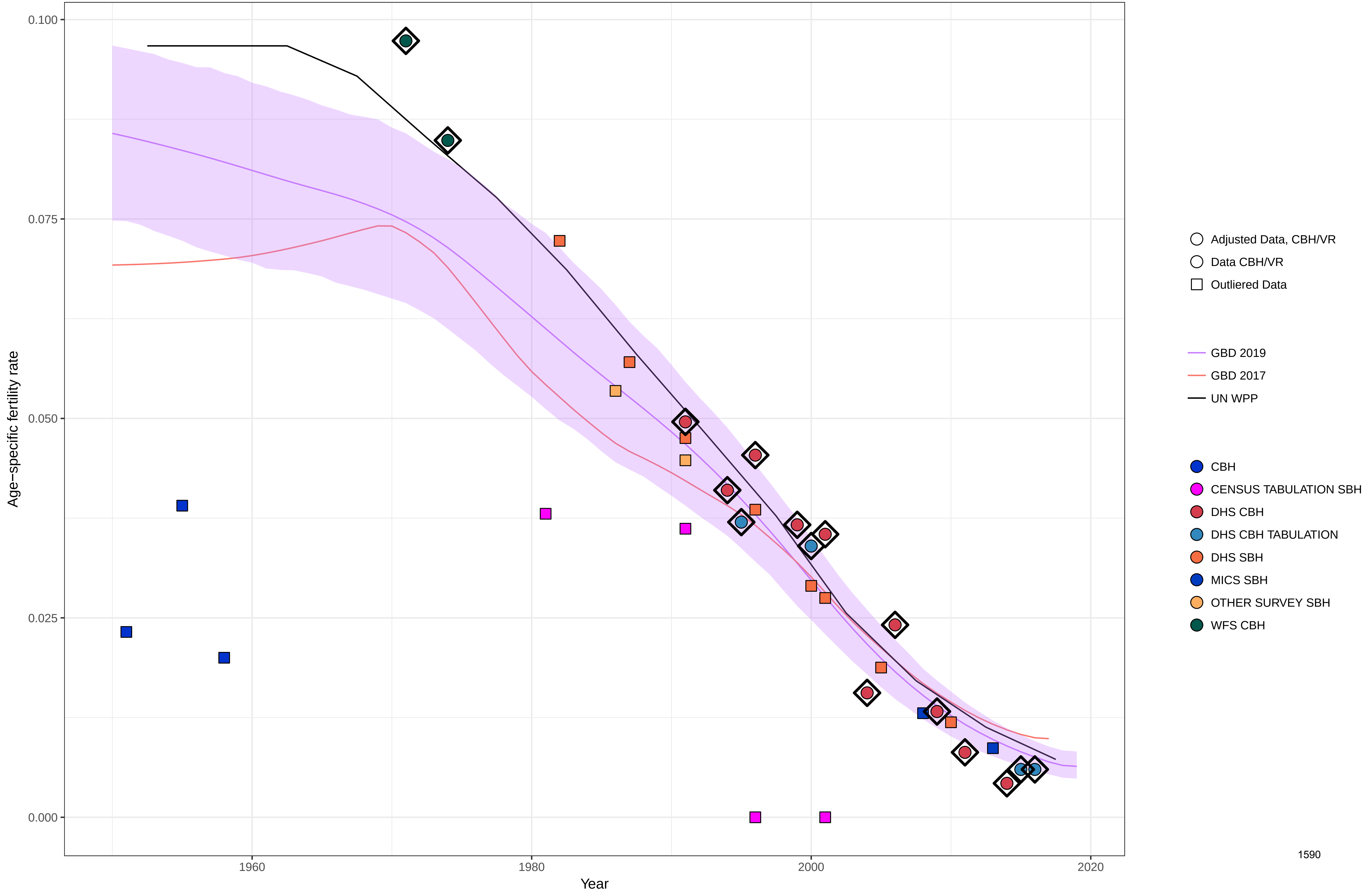

Age−specific fertility fitted trend lines, 1950−2019: Nepal, Ages: 45−49
Data points enclosed with a diamond are reference sources

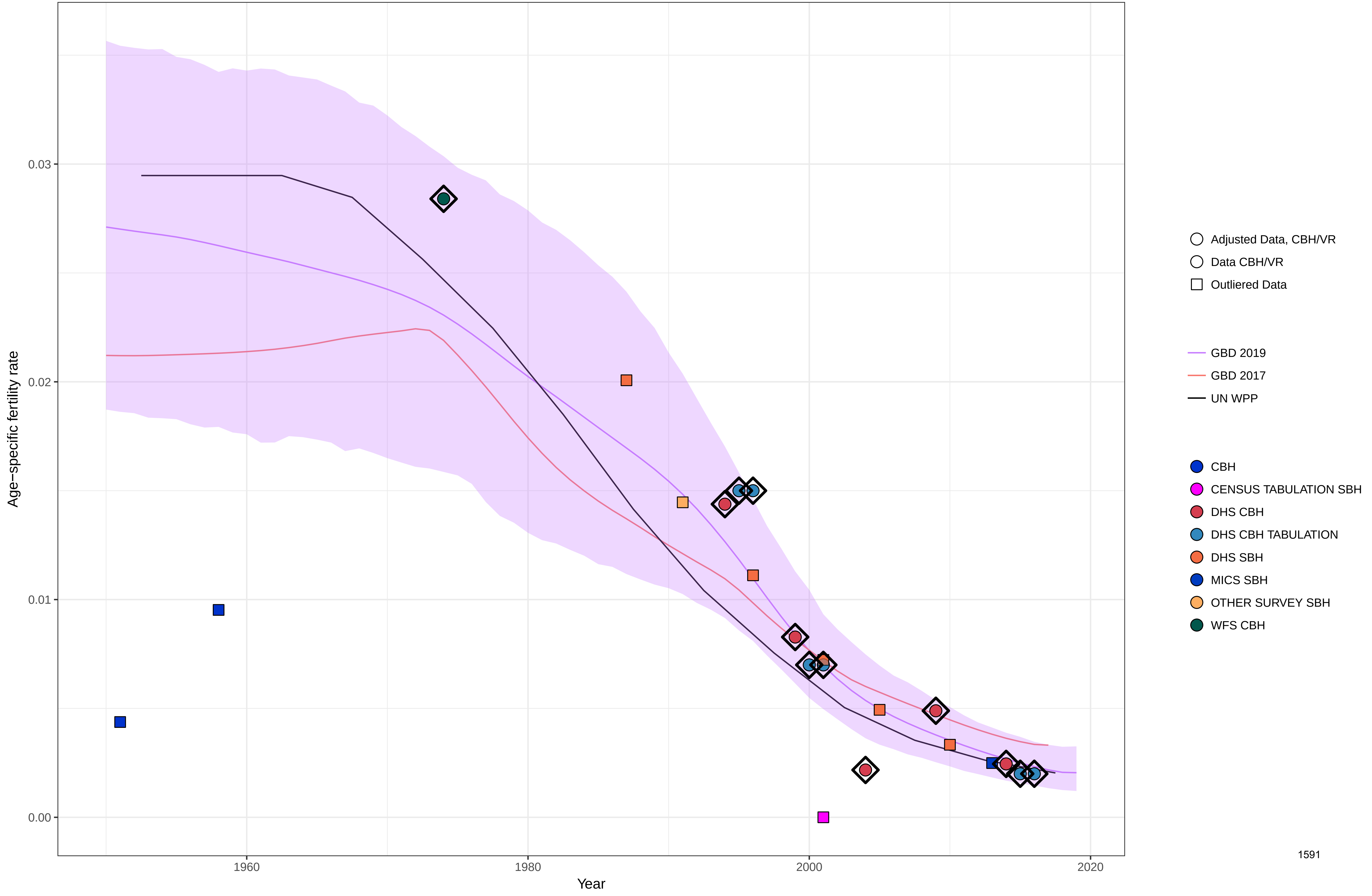

Age−specific fertility fitted trend lines, 1950−2019: Nepal, Ages: 50−54
Data points enclosed with a diamond are reference sources

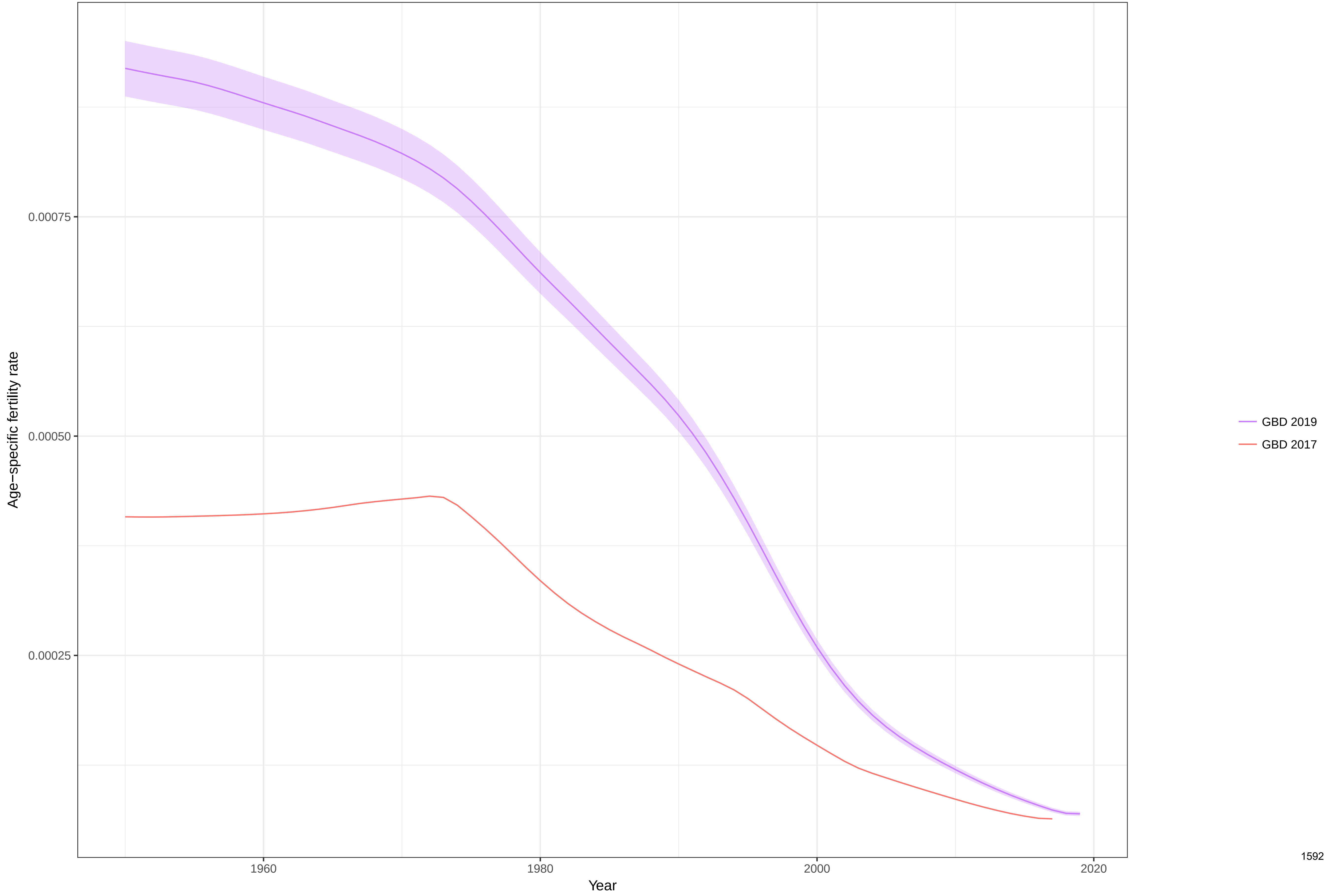

Total fertility rate fitted trend lines, 1950−2019: Nepal TFR
Data points enclosed with a diamond are reference sources

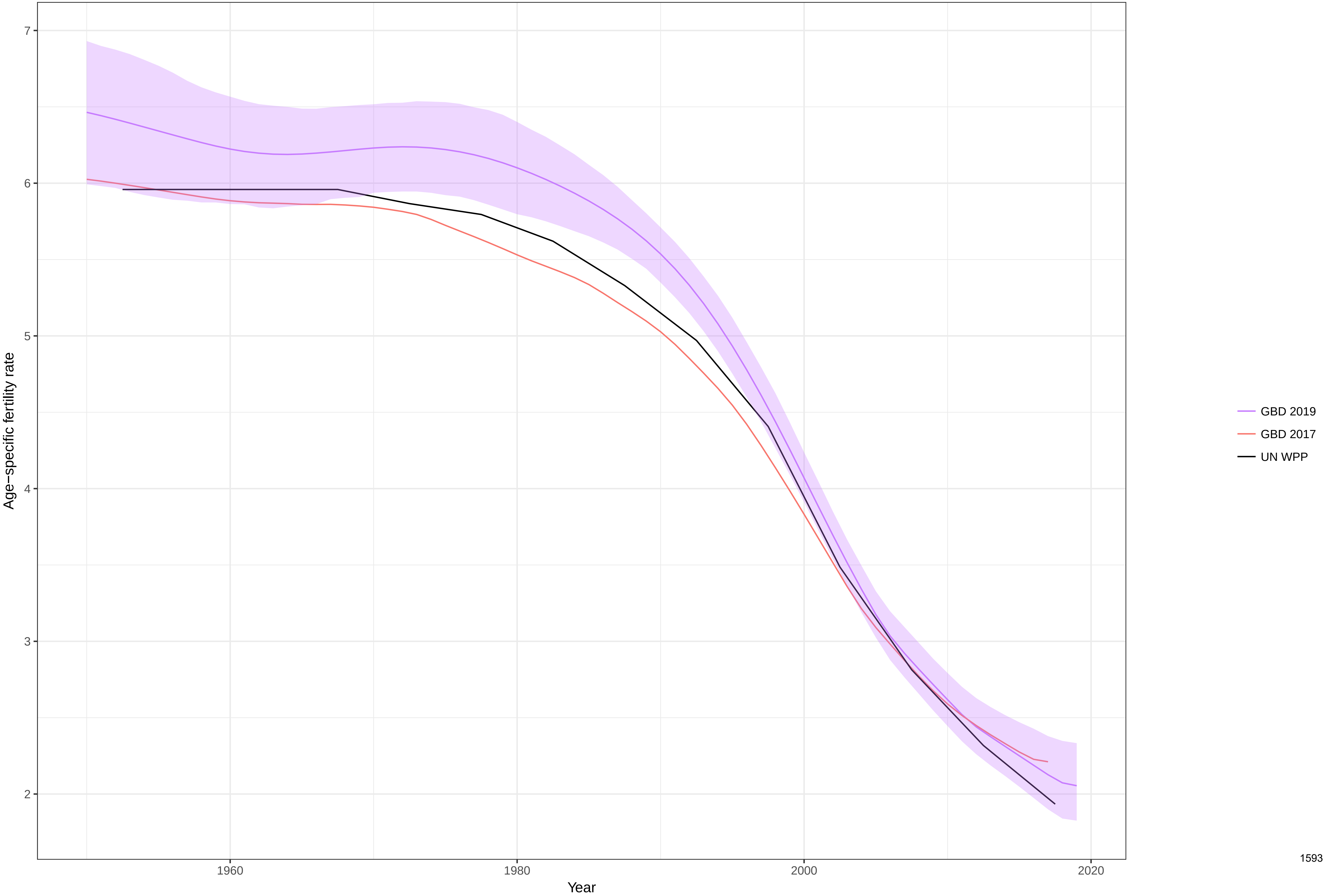

Age−specific fertility fitted trend lines, 1950−2019: Nauru, Ages: 10−14
Data points enclosed with a diamond are reference sources

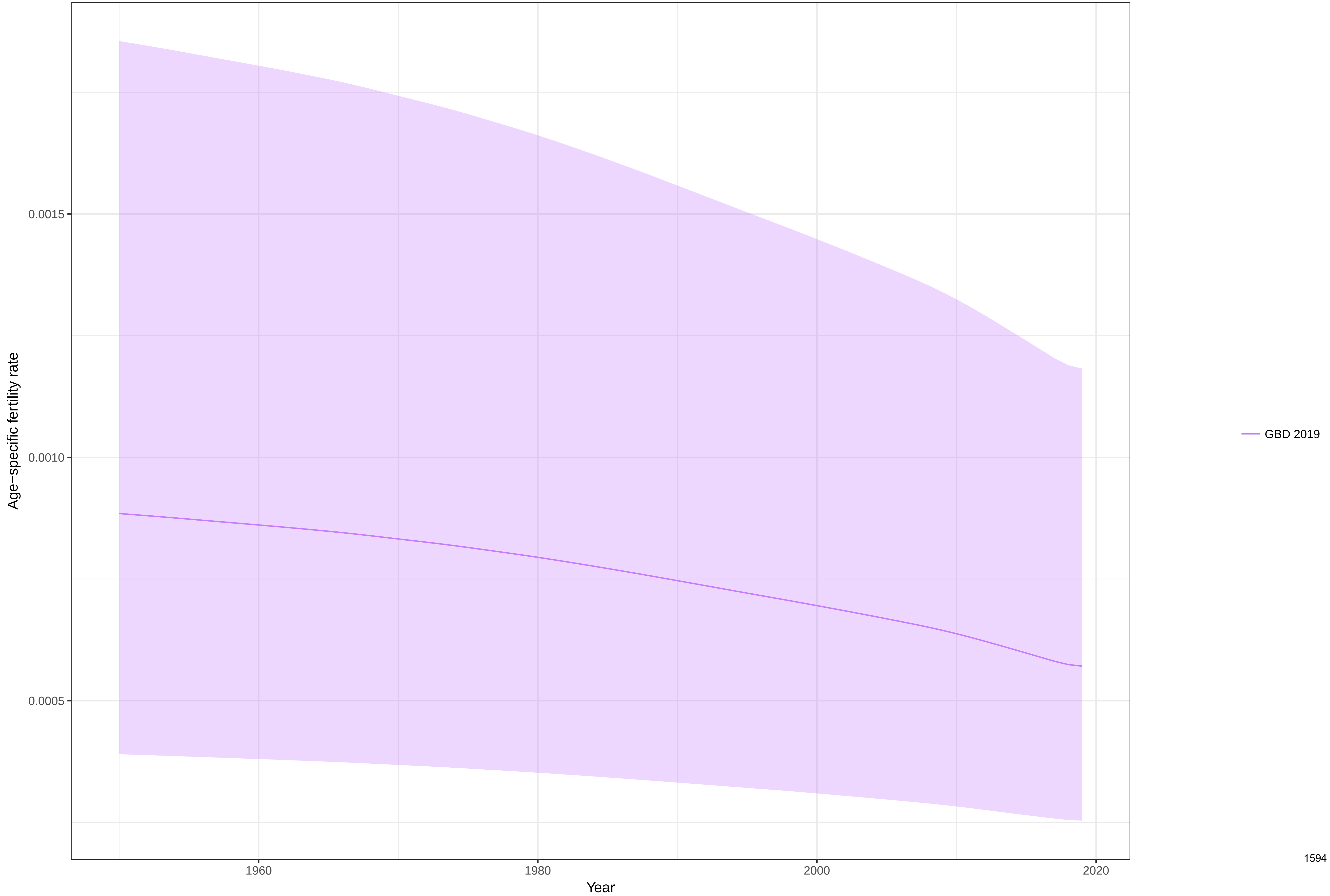

Age−specific fertility fitted trend lines, 1950−2019: Nauru, Ages: 15−19
Data points enclosed with a diamond are reference sources

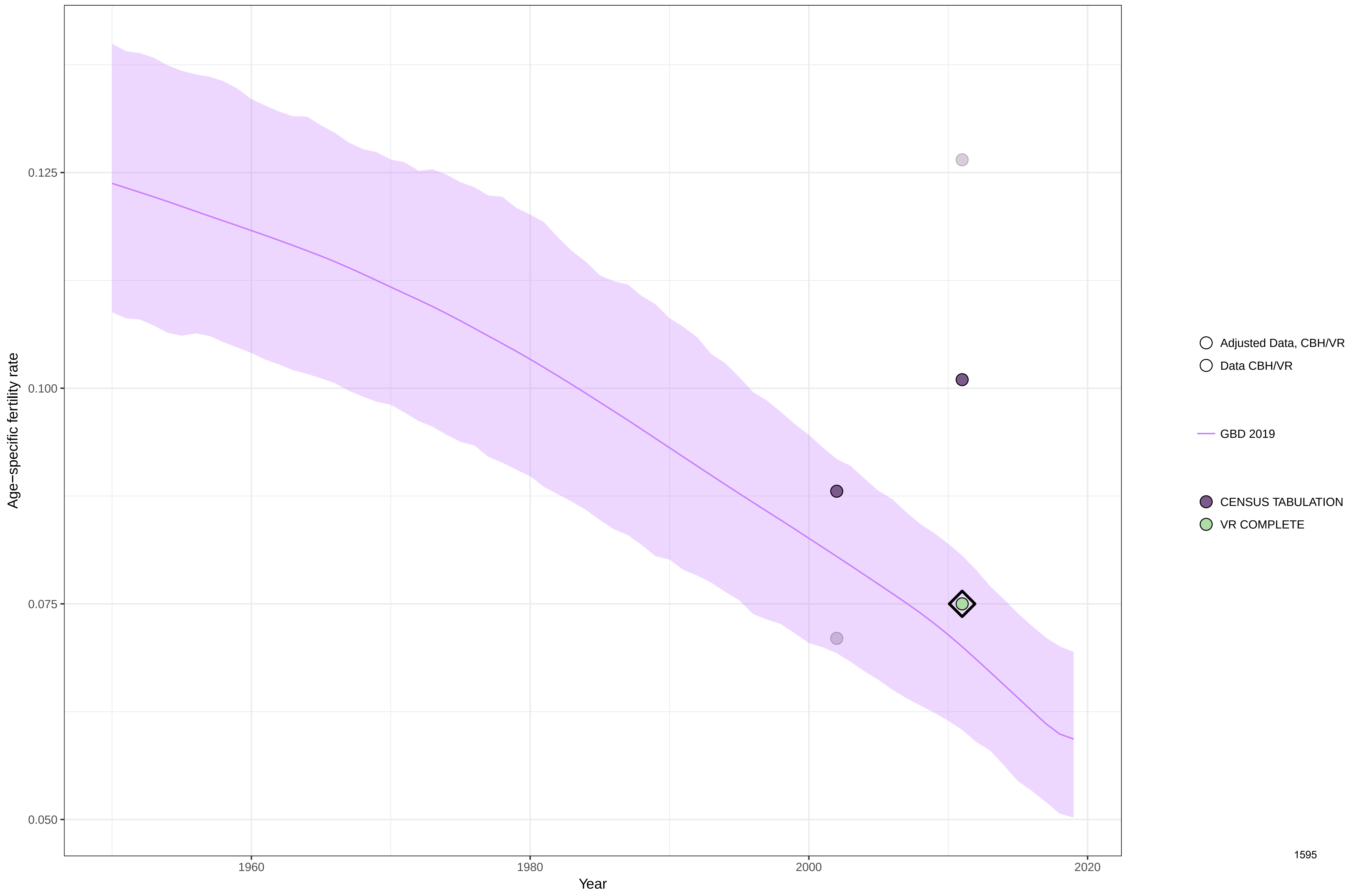

Age−specific fertility fitted trend lines, 1950−2019: Nauru, Ages: 20−24
Data points enclosed with a diamond are reference sources

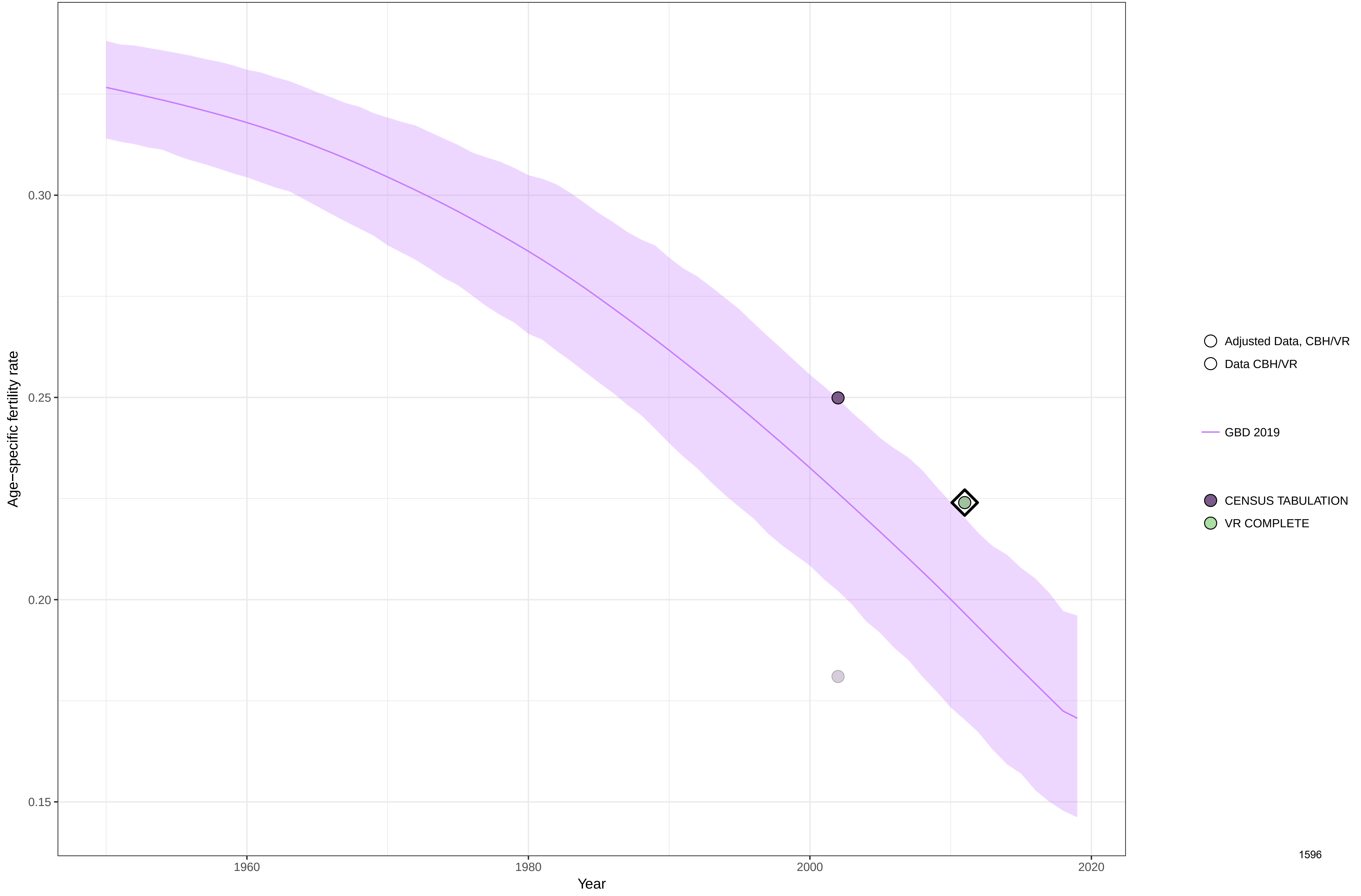

Age−specific fertility fitted trend lines, 1950−2019: Nauru, Ages: 25−29
Data points enclosed with a diamond are reference sources

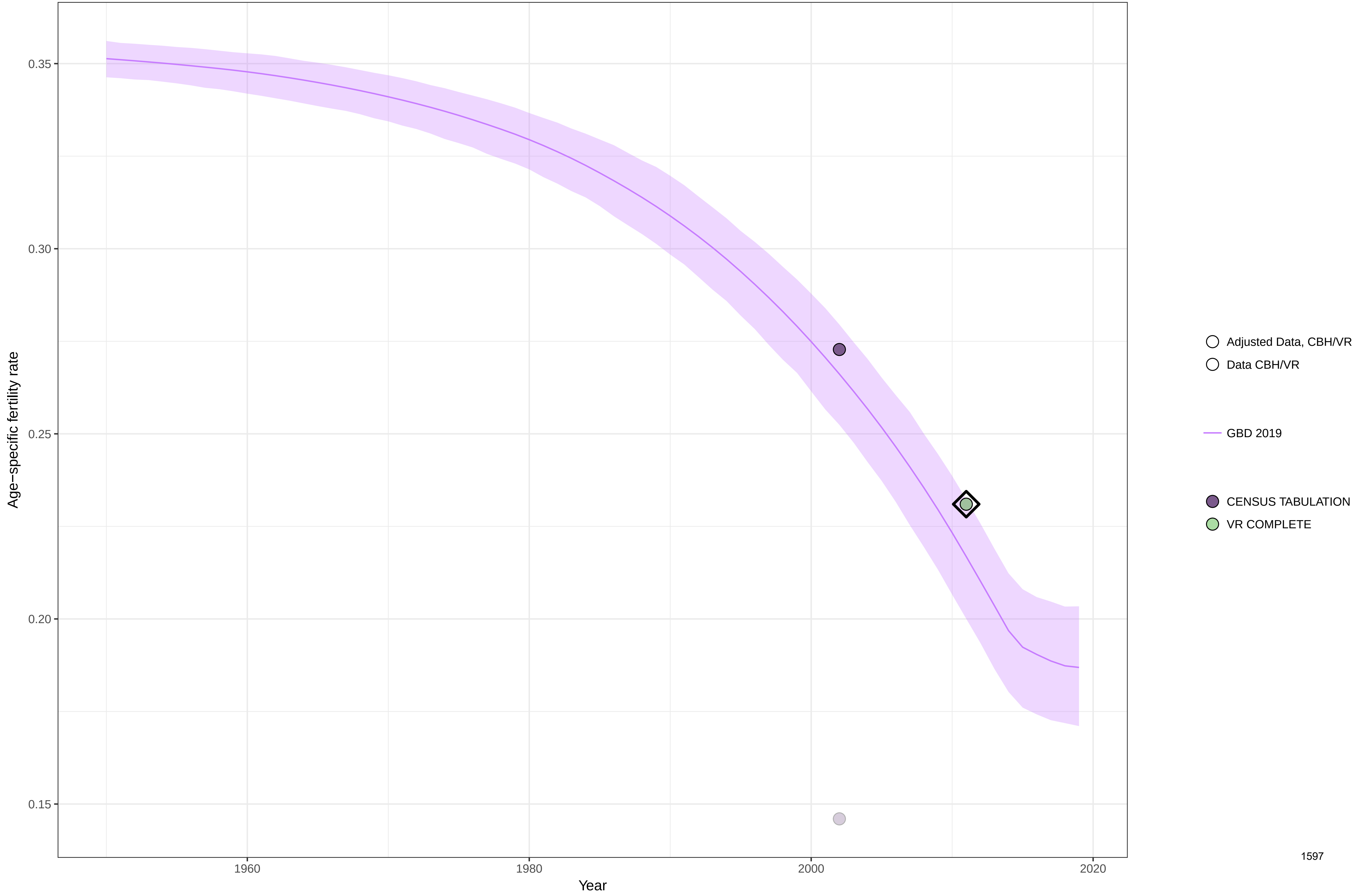

Age−specific fertility fitted trend lines, 1950−2019: Nauru, Ages: 30−34
Data points enclosed with a diamond are reference sources

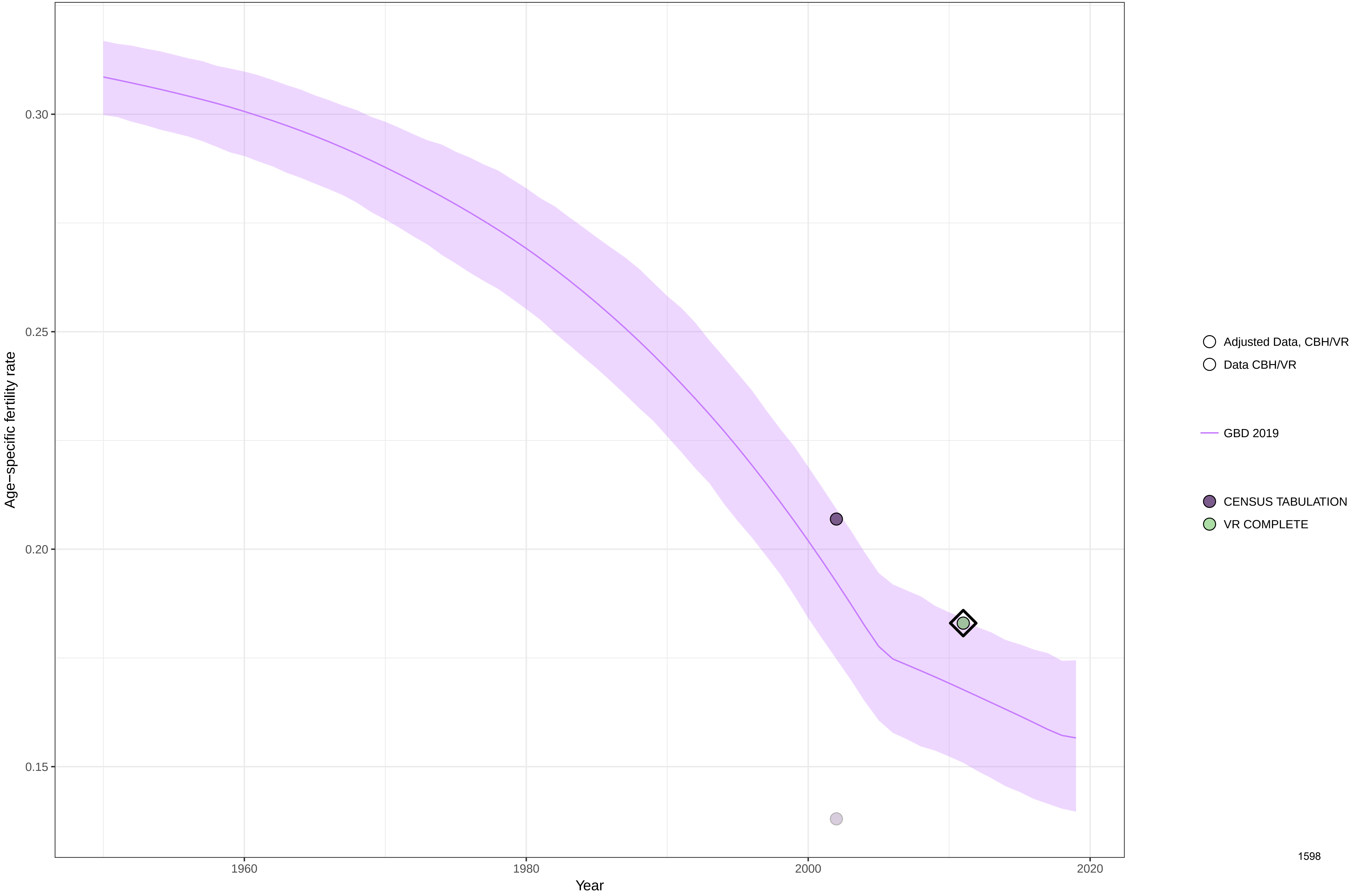

Age−specific fertility fitted trend lines, 1950−2019: Nauru, Ages: 35−39
Data points enclosed with a diamond are reference sources

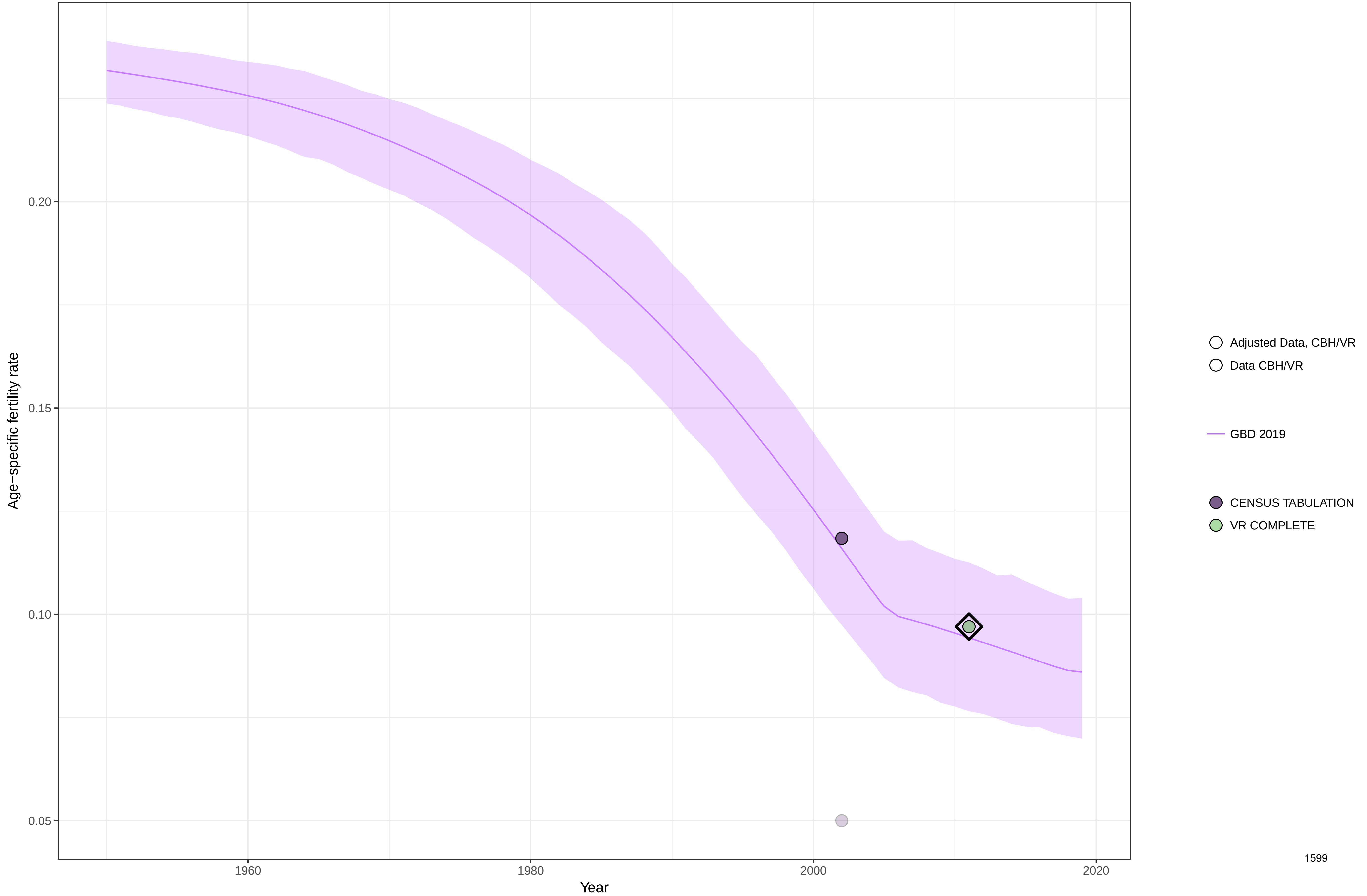

Age−specific fertility fitted trend lines, 1950−2019: Nauru, Ages: 40−44
Data points enclosed with a diamond are reference sources

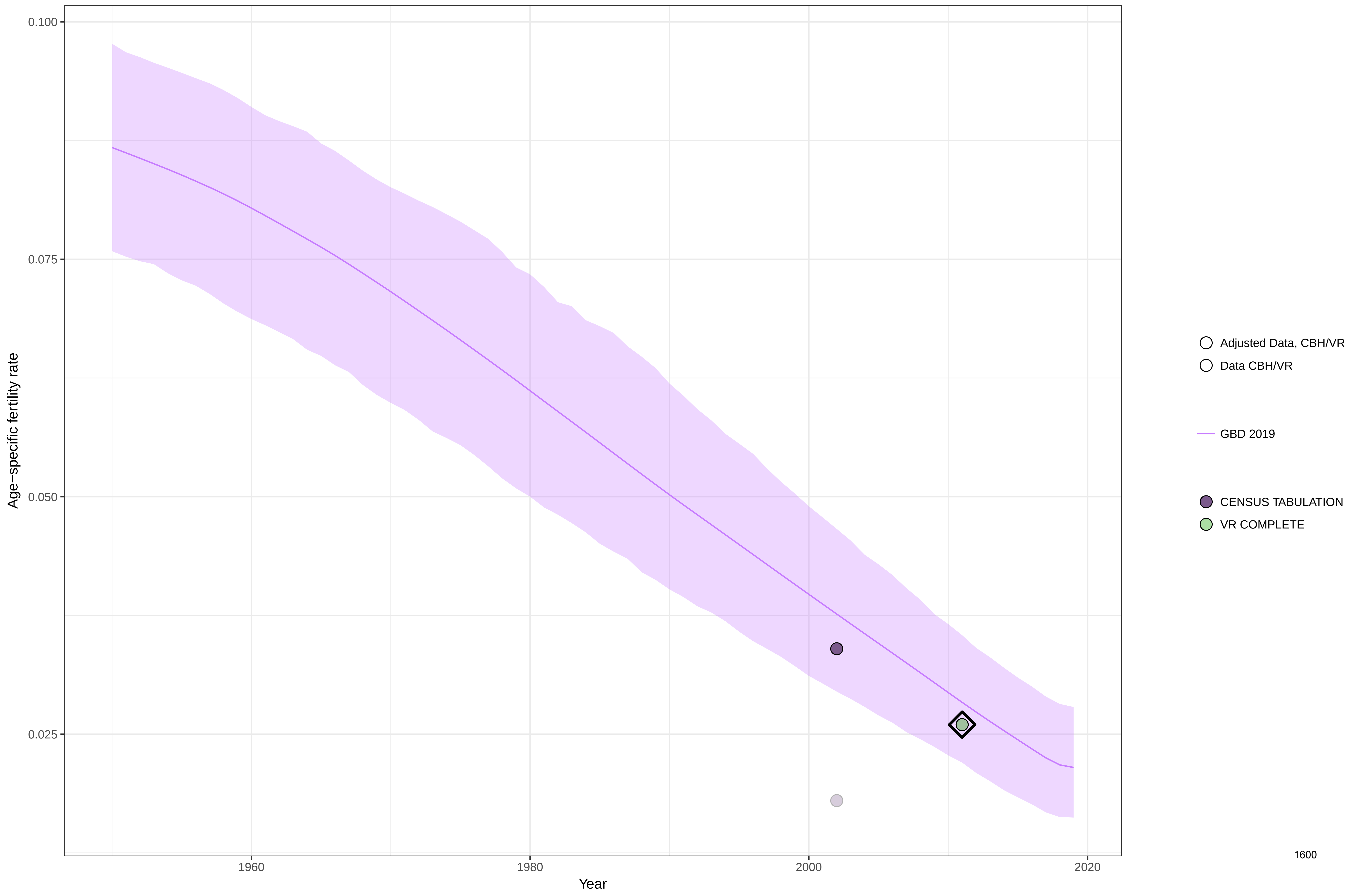

Age−specific fertility fitted trend lines, 1950−2019: Nauru, Ages: 45−49
Data points enclosed with a diamond are reference sources

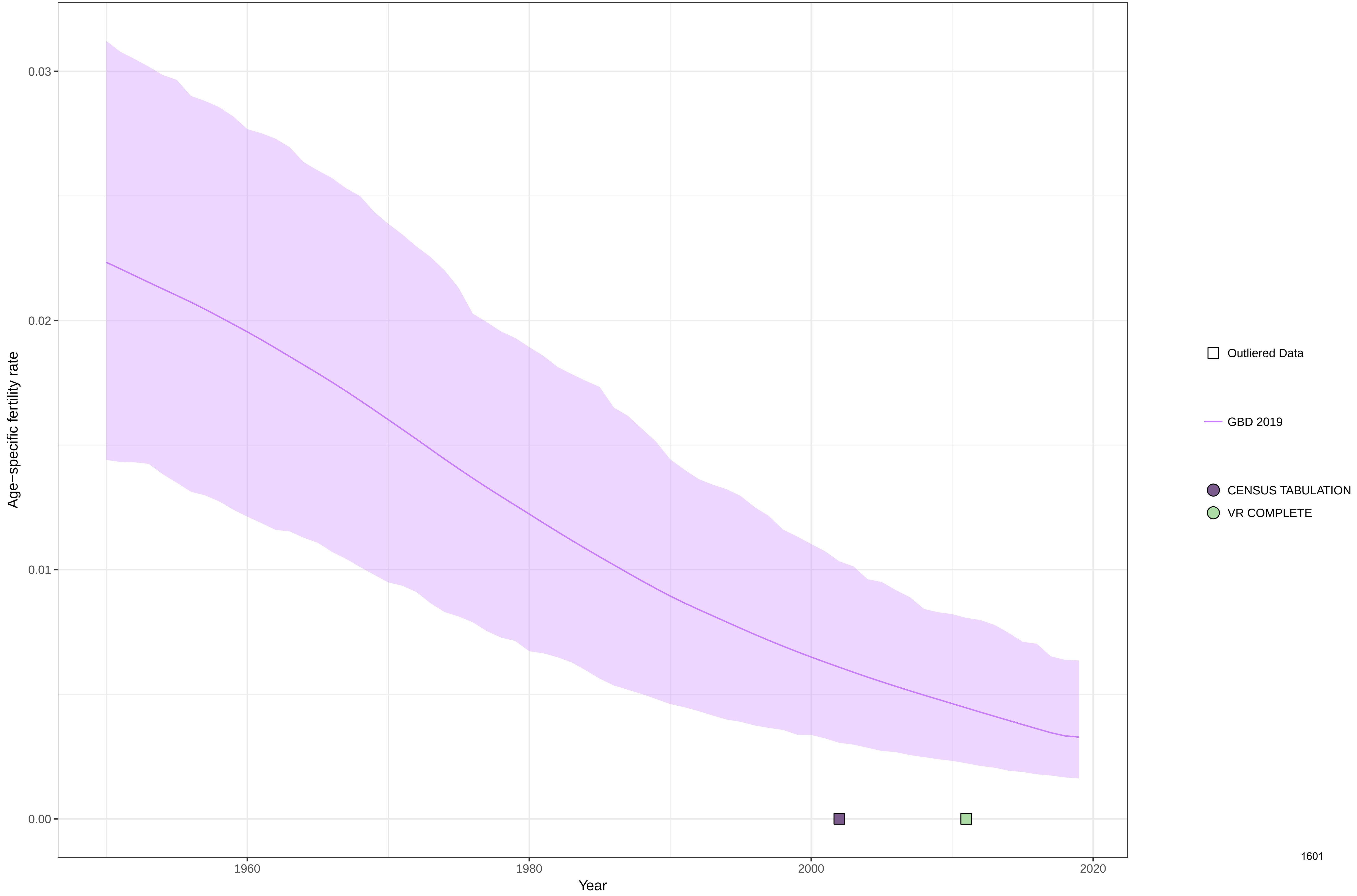

Age−specific fertility fitted trend lines, 1950−2019: Nauru, Ages: 50−54
Data points enclosed with a diamond are reference sources

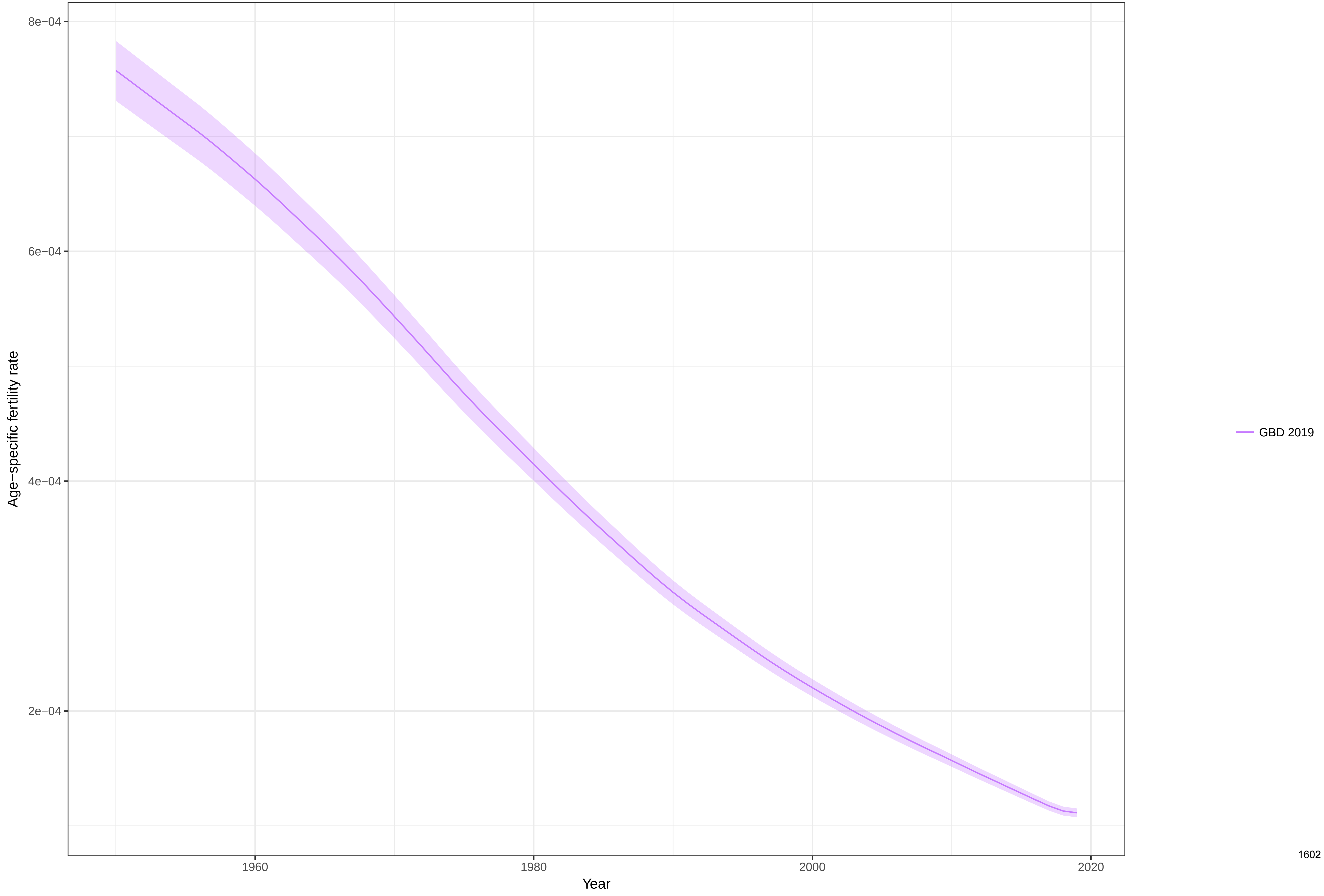

Total fertility rate fitted trend lines, 1950−2019: Nauru TFR
Data points enclosed with a diamond are reference sources

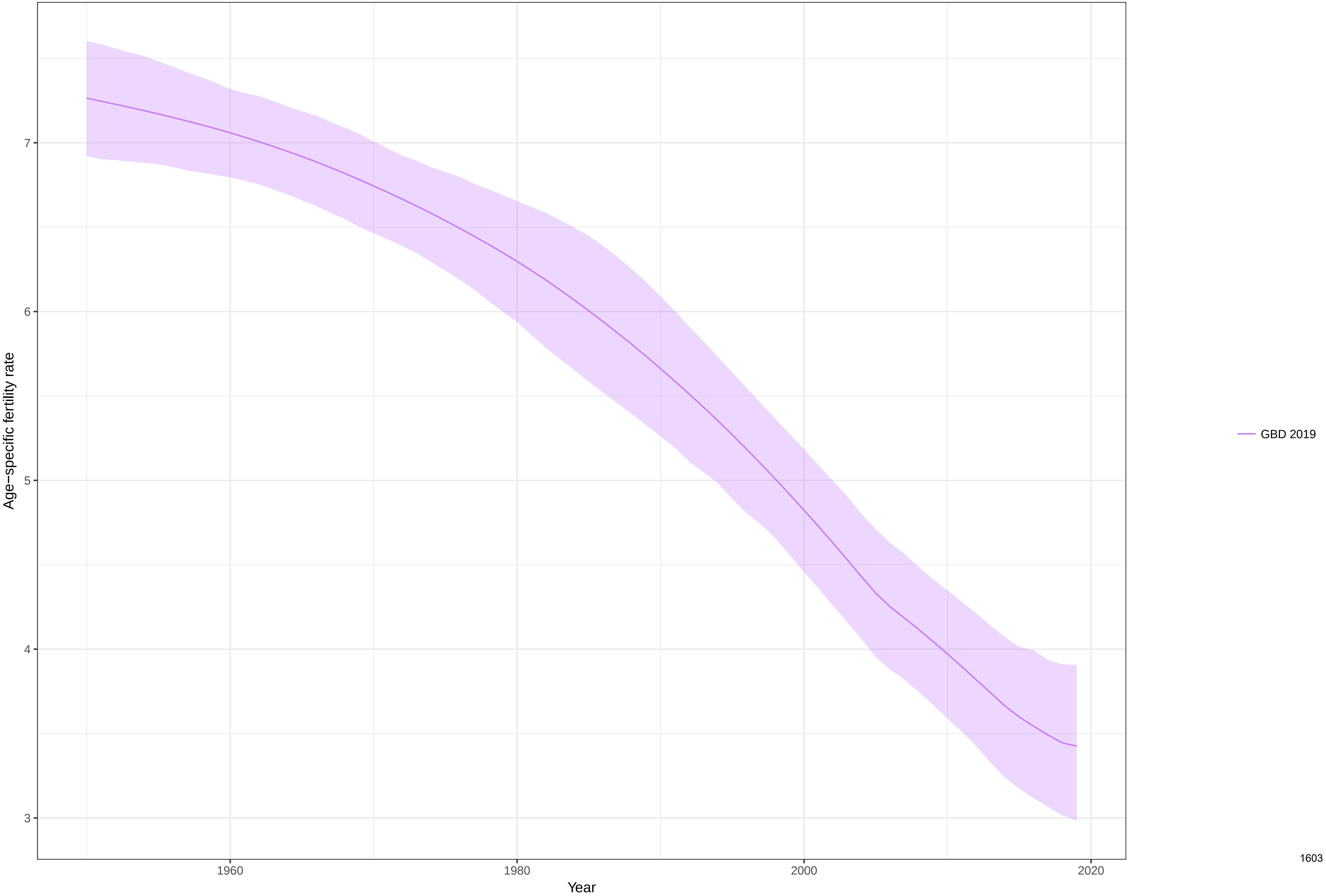

Age−specific fertility fitted trend lines, 1950−2019: New Zealand, Ages: 10−14
Data points enclosed with a diamond are reference sources

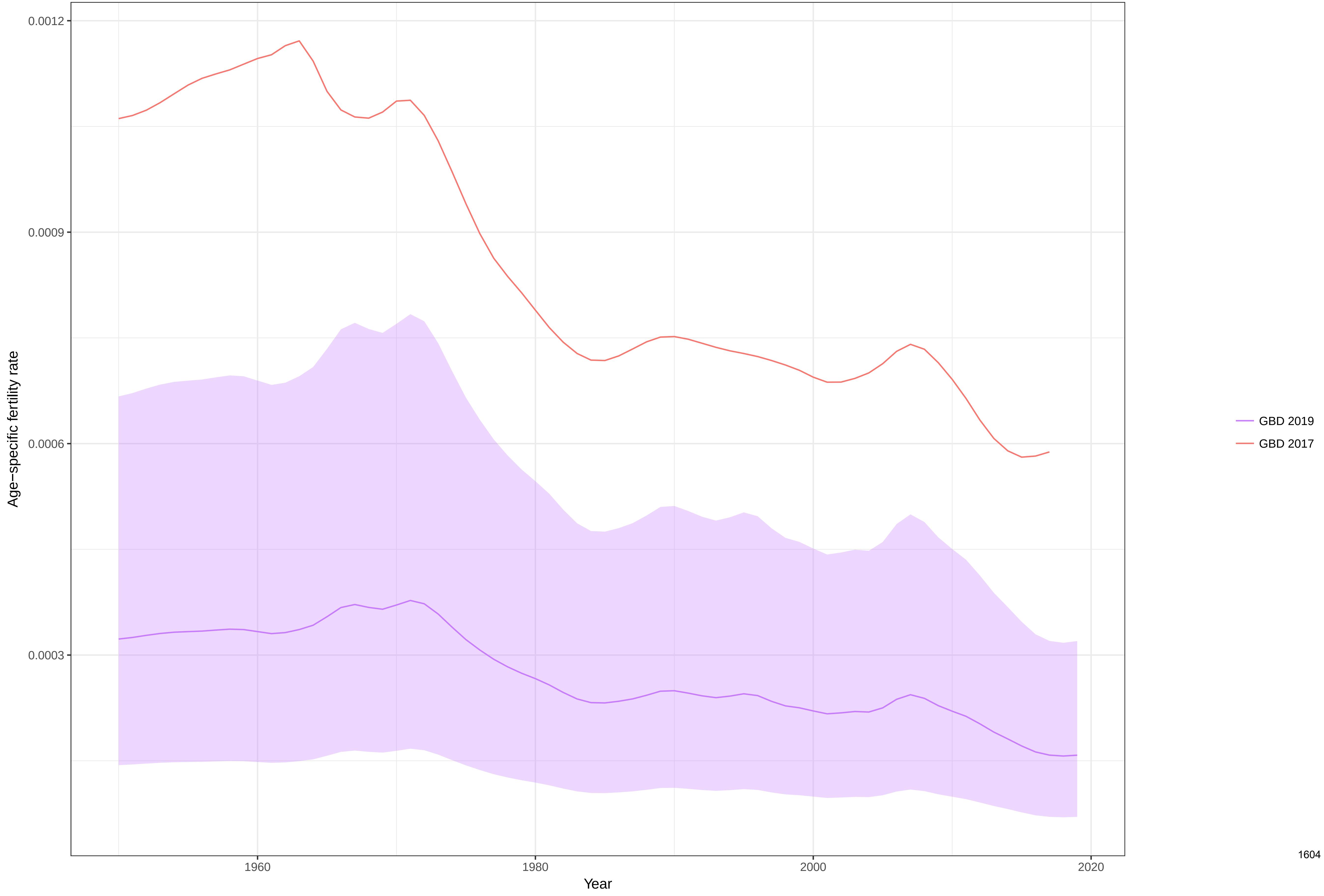

Age−specific fertility fitted trend lines, 1950−2019: New Zealand, Ages: 15−19
Data points enclosed with a diamond are reference sources

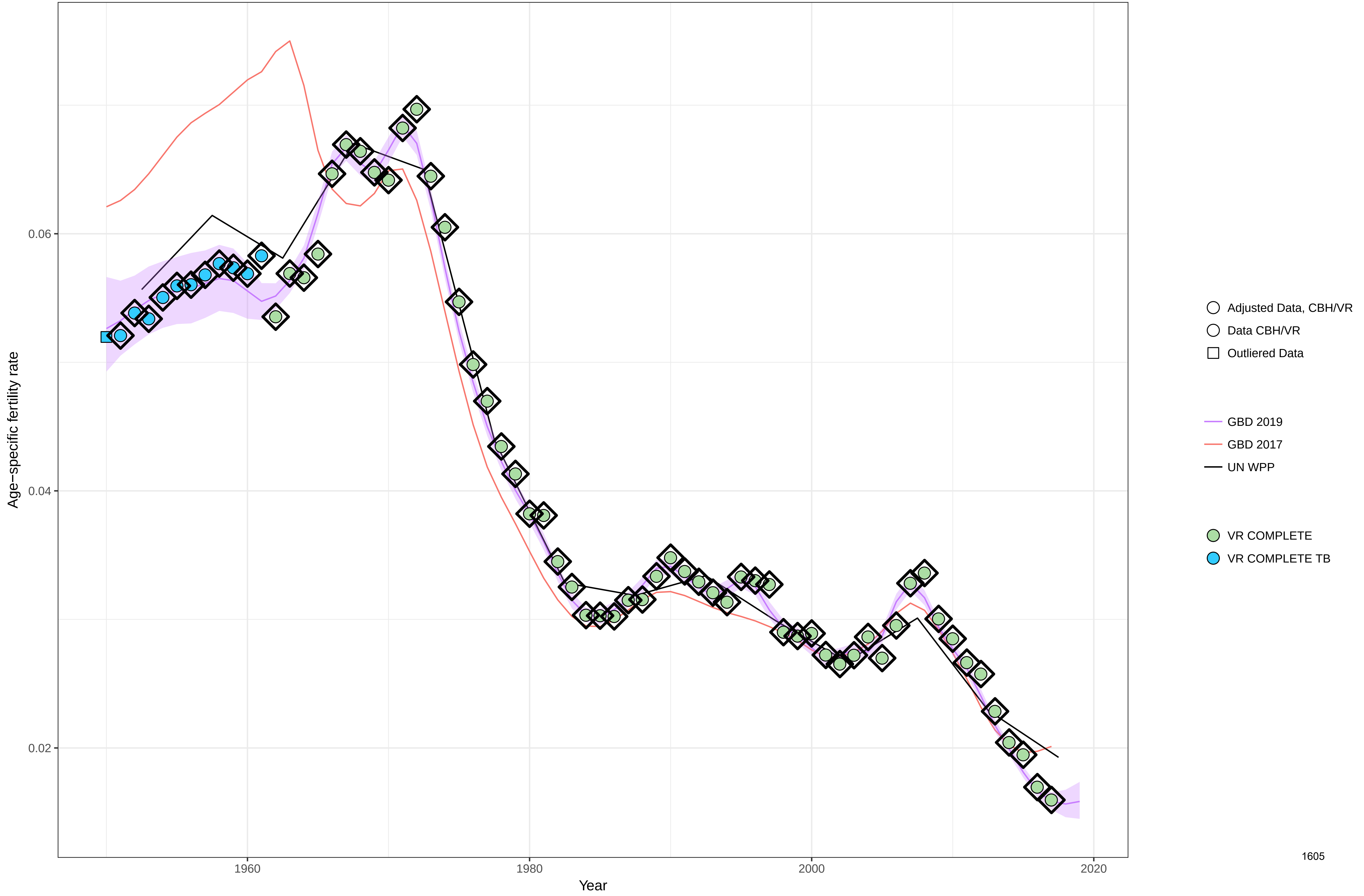

Age−specific fertility fitted trend lines, 1950−2019: New Zealand, Ages: 20−24
Data points enclosed with a diamond are reference sources

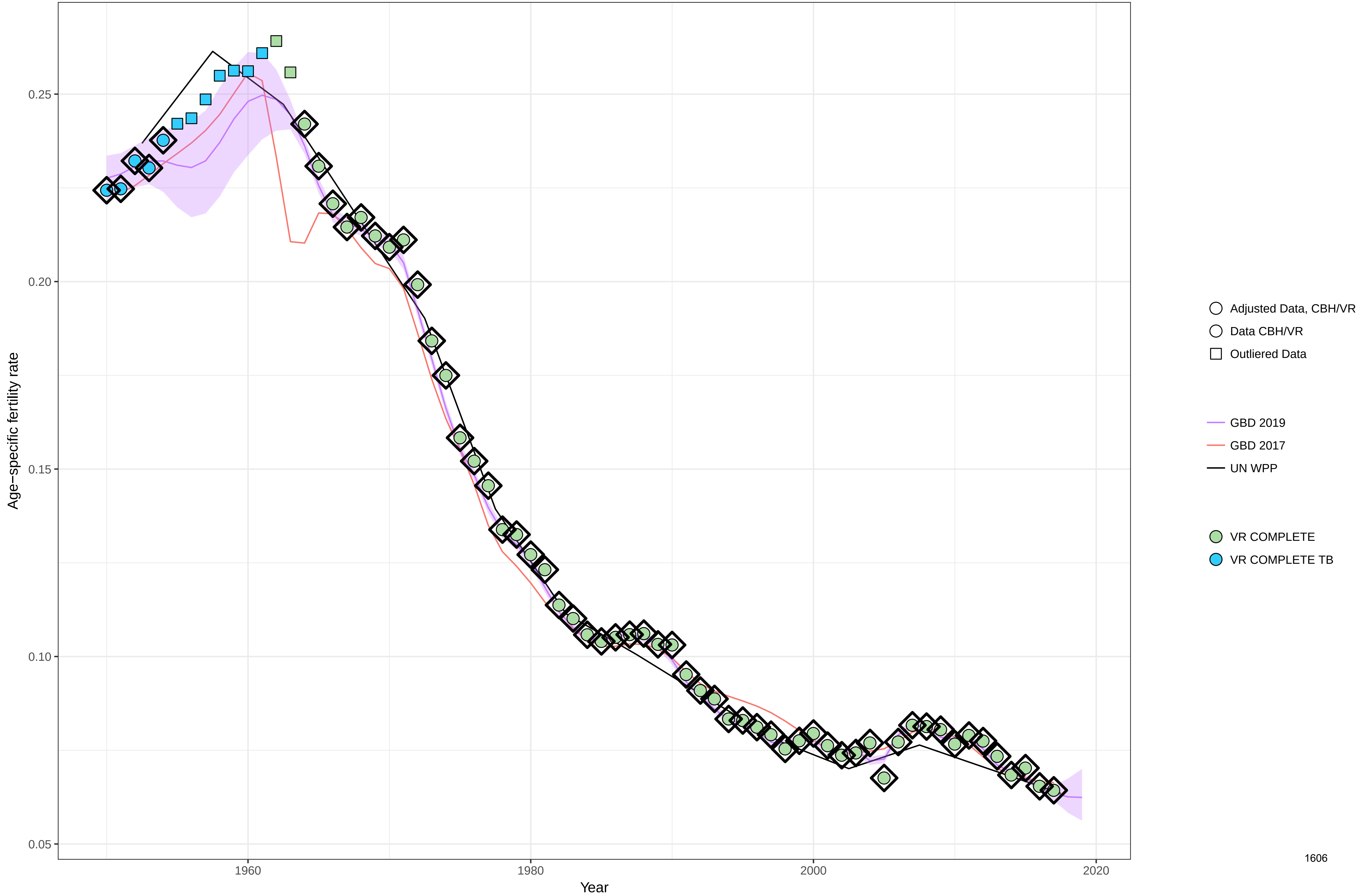

Age−specific fertility fitted trend lines, 1950−2019: New Zealand, Ages: 25−29
Data points enclosed with a diamond are reference sources

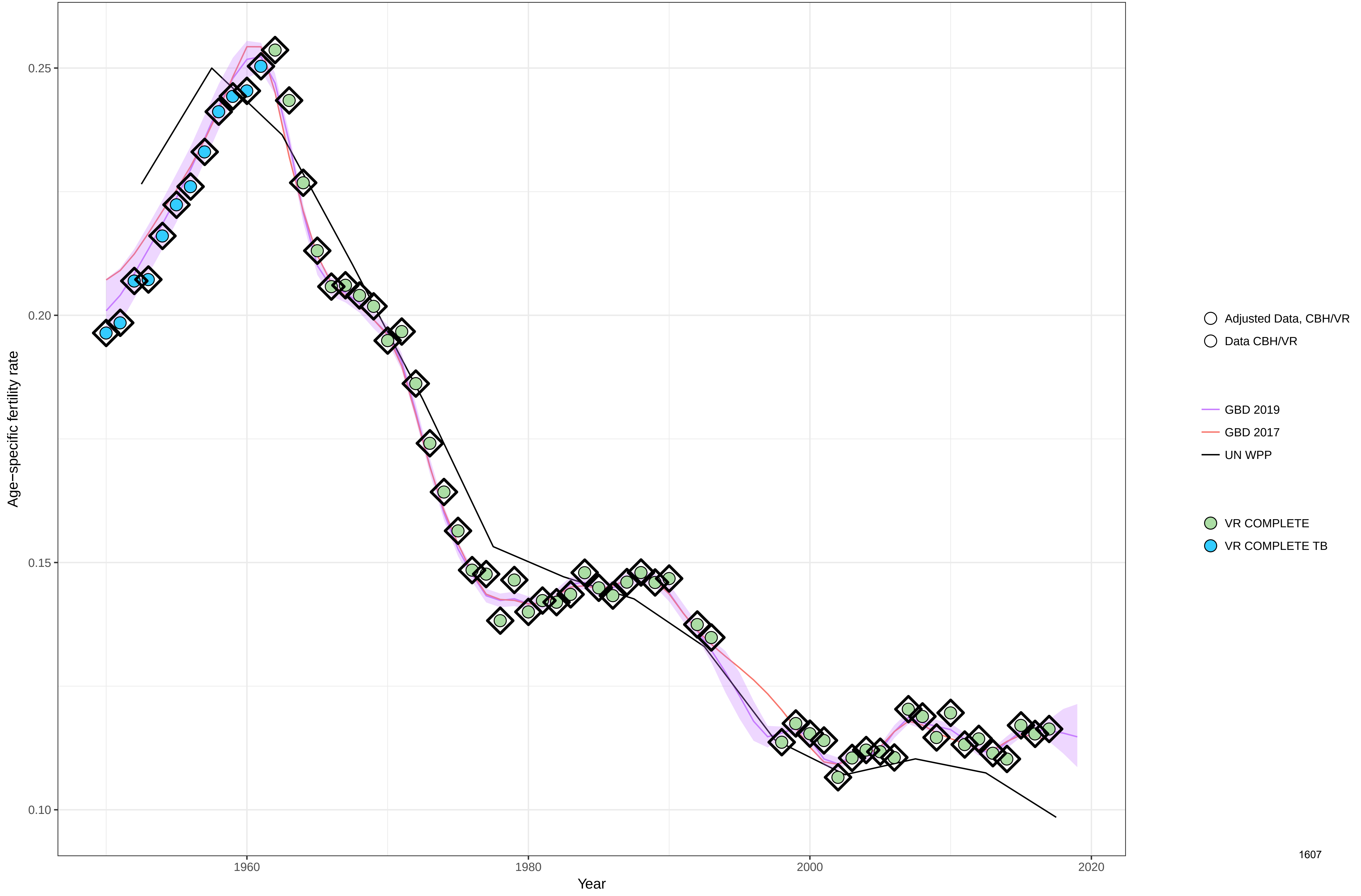

Age−specific fertility fitted trend lines, 1950−2019: New Zealand, Ages: 30−34
Data points enclosed with a diamond are reference sources

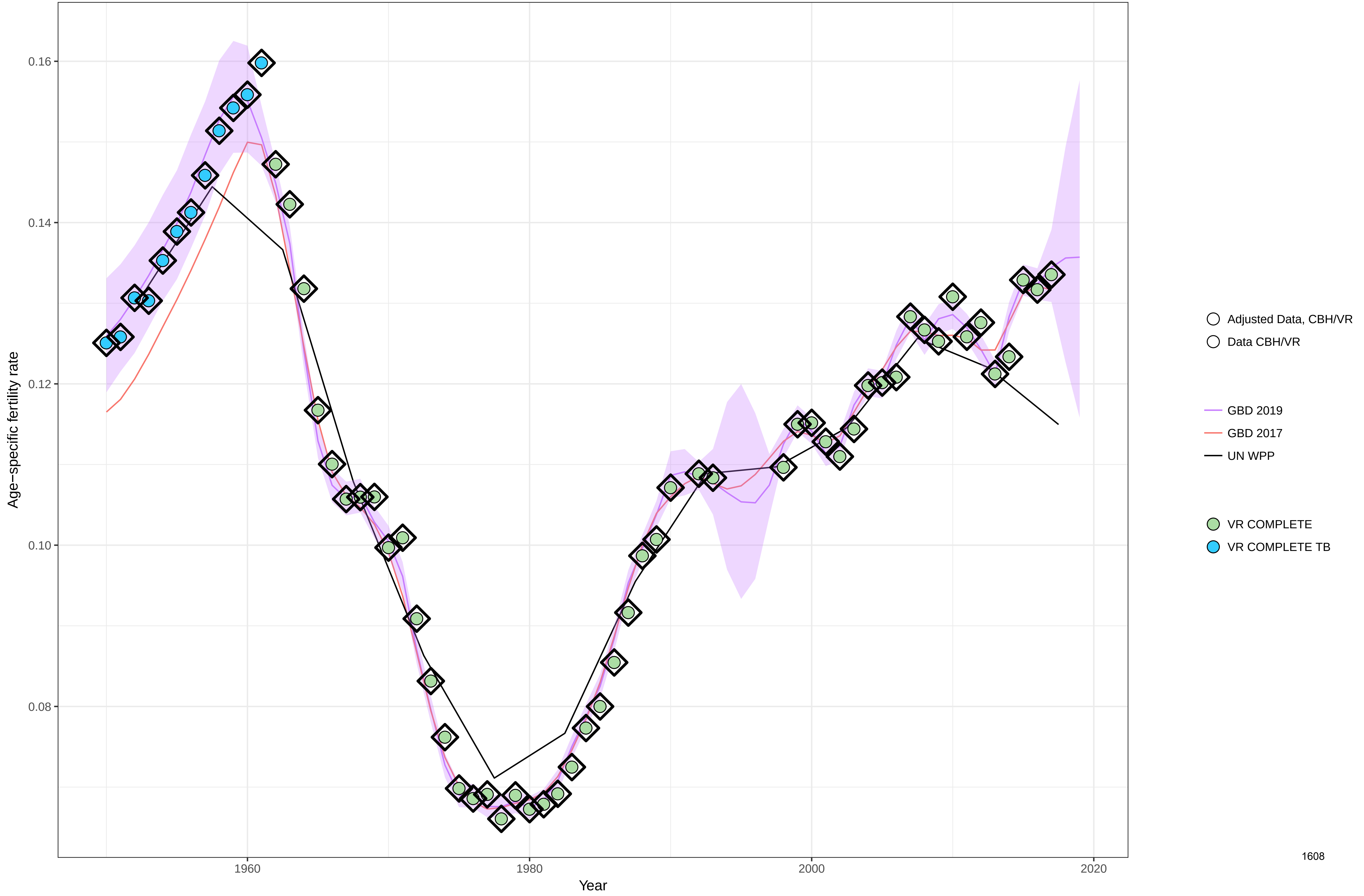

Age−specific fertility fitted trend lines, 1950−2019: New Zealand, Ages: 35−39
Data points enclosed with a diamond are reference sources

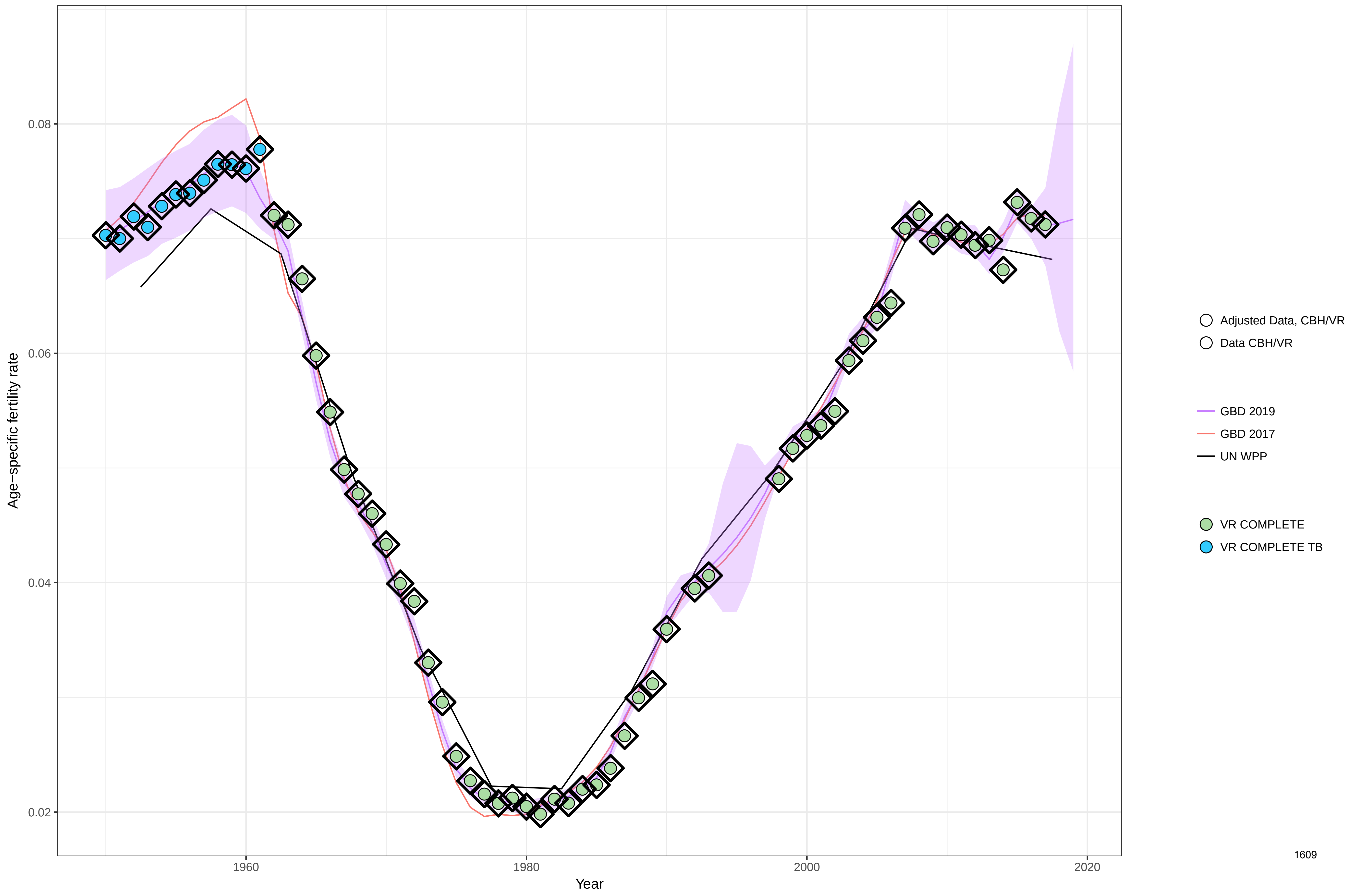

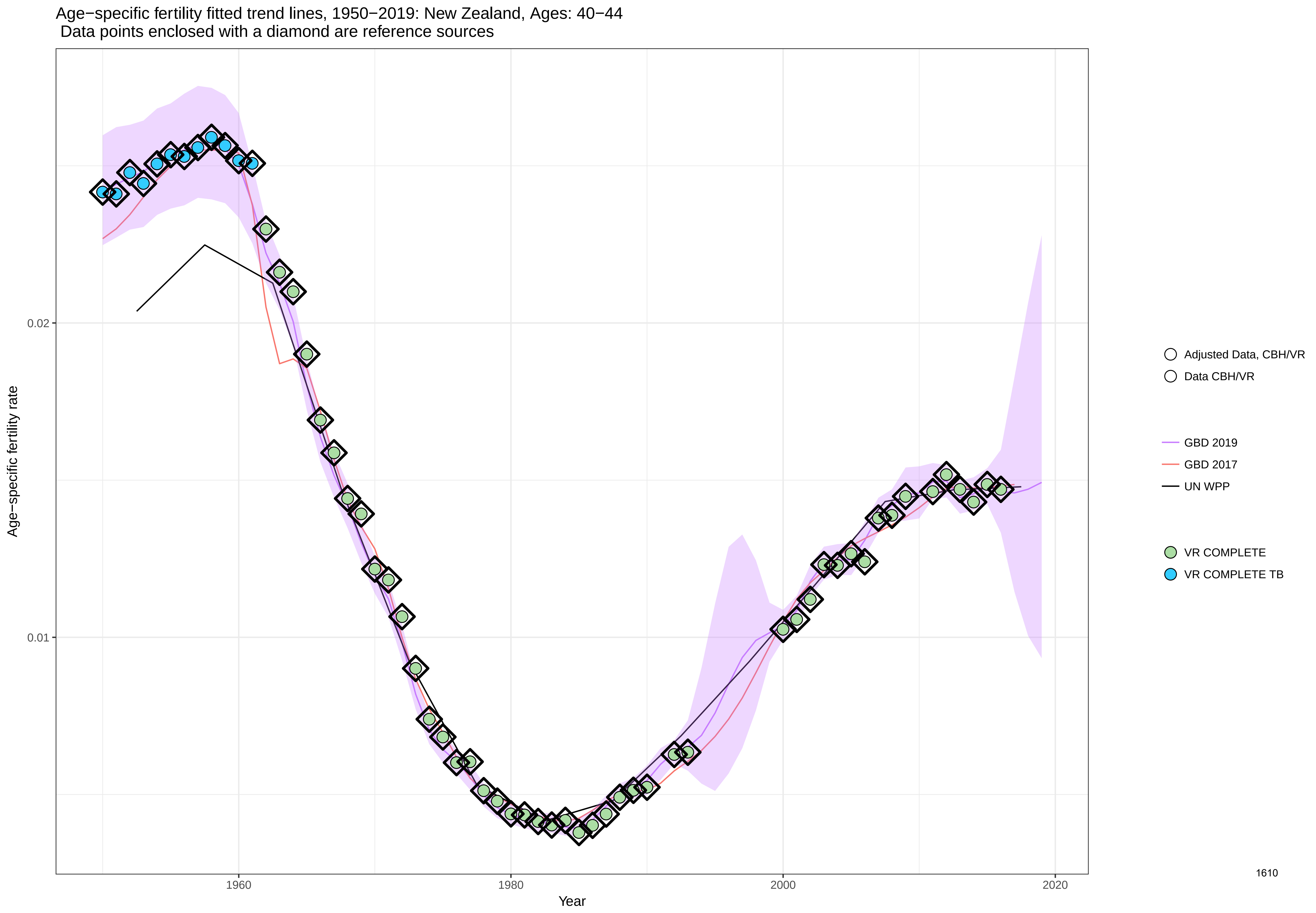
Age−specific fertility fitted trend lines, 1950−2019: New Zealand, Ages: 40−44
Data points enclosed with a diamond are reference sources
Age−specific fertility rate
0.02
0.01
1960
1980
2000
2020
Year
Adjusted Data, CBH/VR
Data CBH/VR
GBD 2019
GBD 2017
UN WPP
VR COMPLETE
VR COMPLETE TB

Age−specific fertility fitted trend lines, 1950−2019: New Zealand, Ages: 45−49
Data points enclosed with a diamond are reference sources

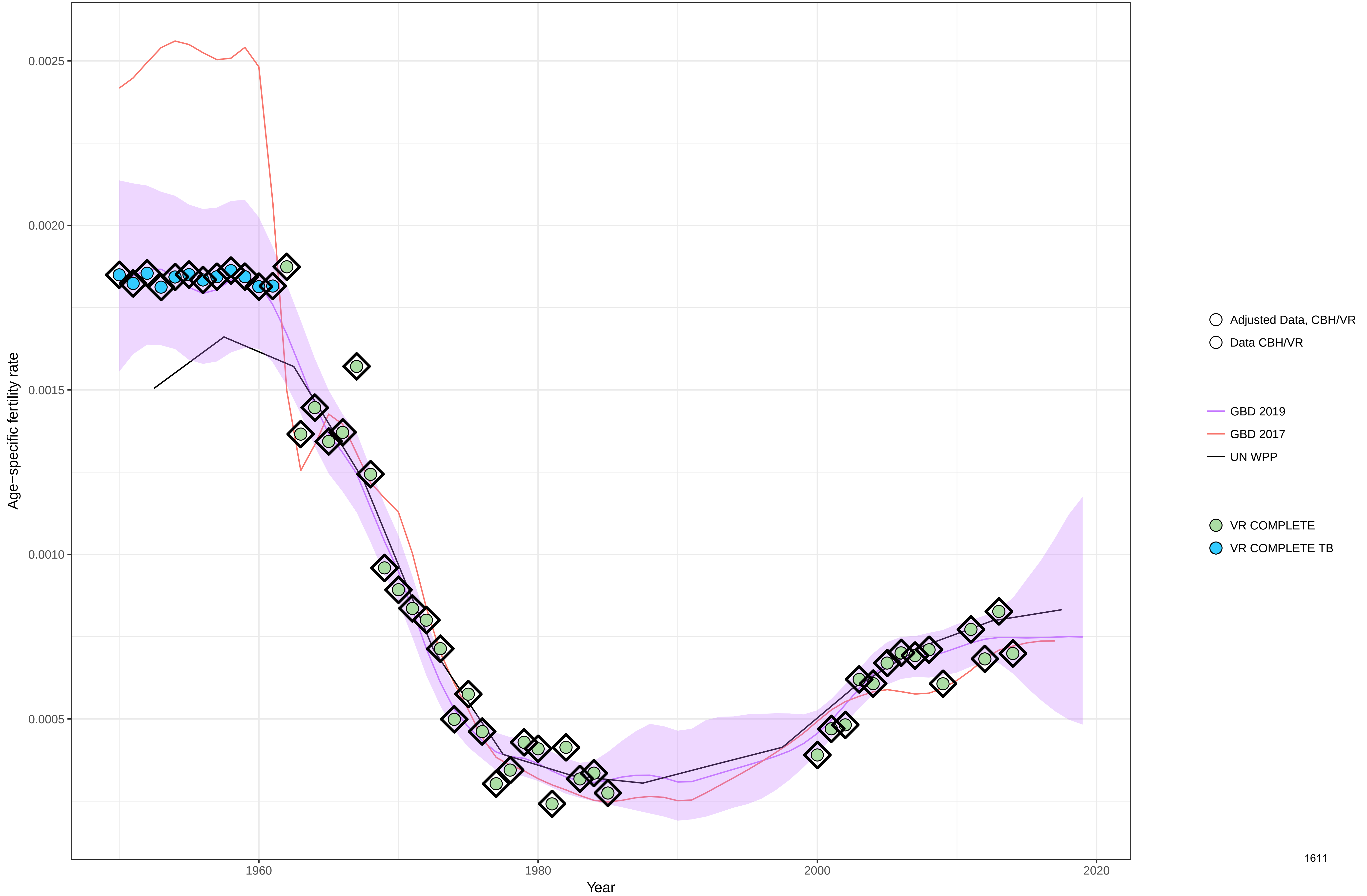

Age−specific fertility fitted trend lines, 1950−2019: New Zealand, Ages: 50−54
Data points enclosed with a diamond are reference sources

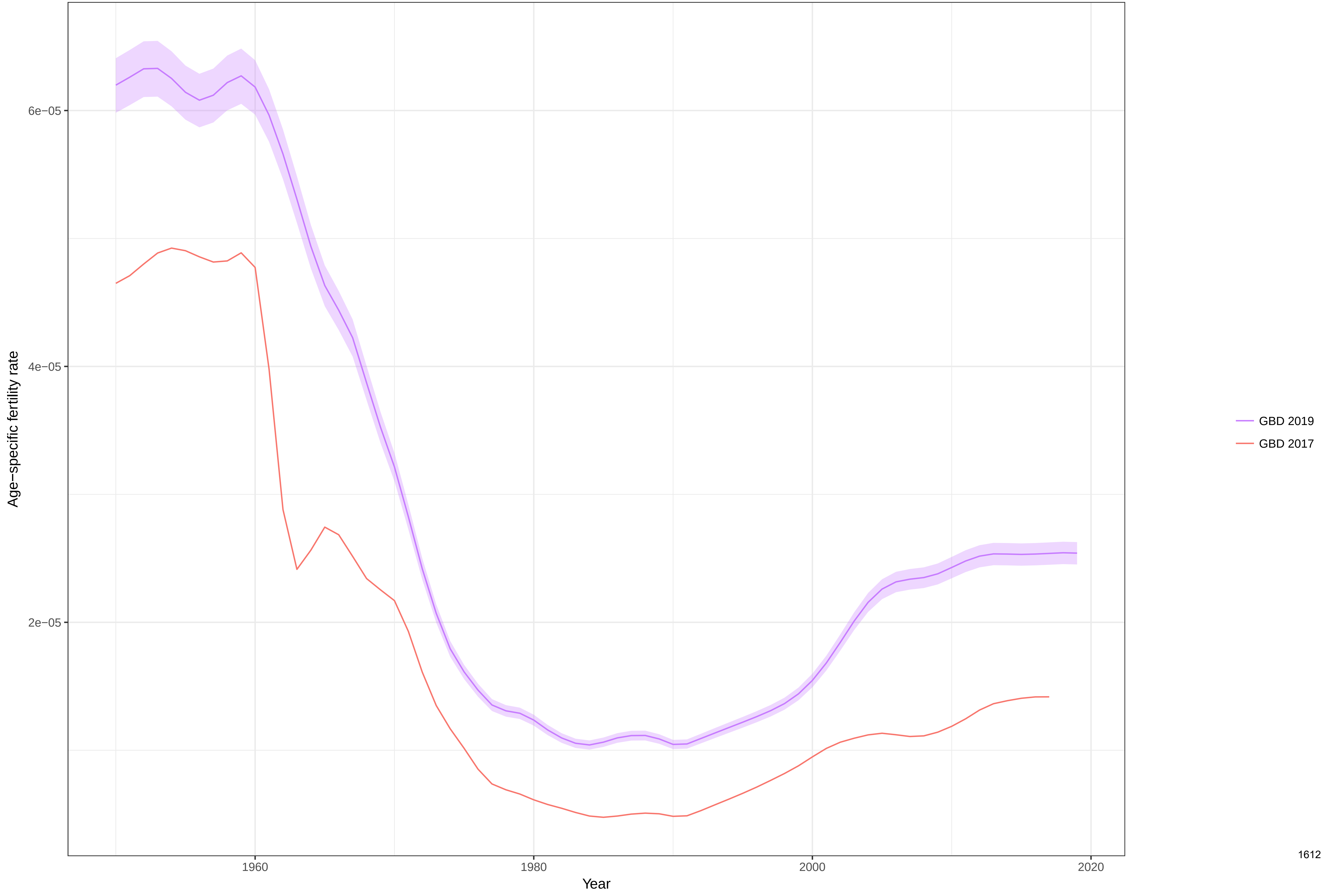

Total fertility rate fitted trend lines, 1950−2019: New Zealand TFR
Data points enclosed with a diamond are reference sources

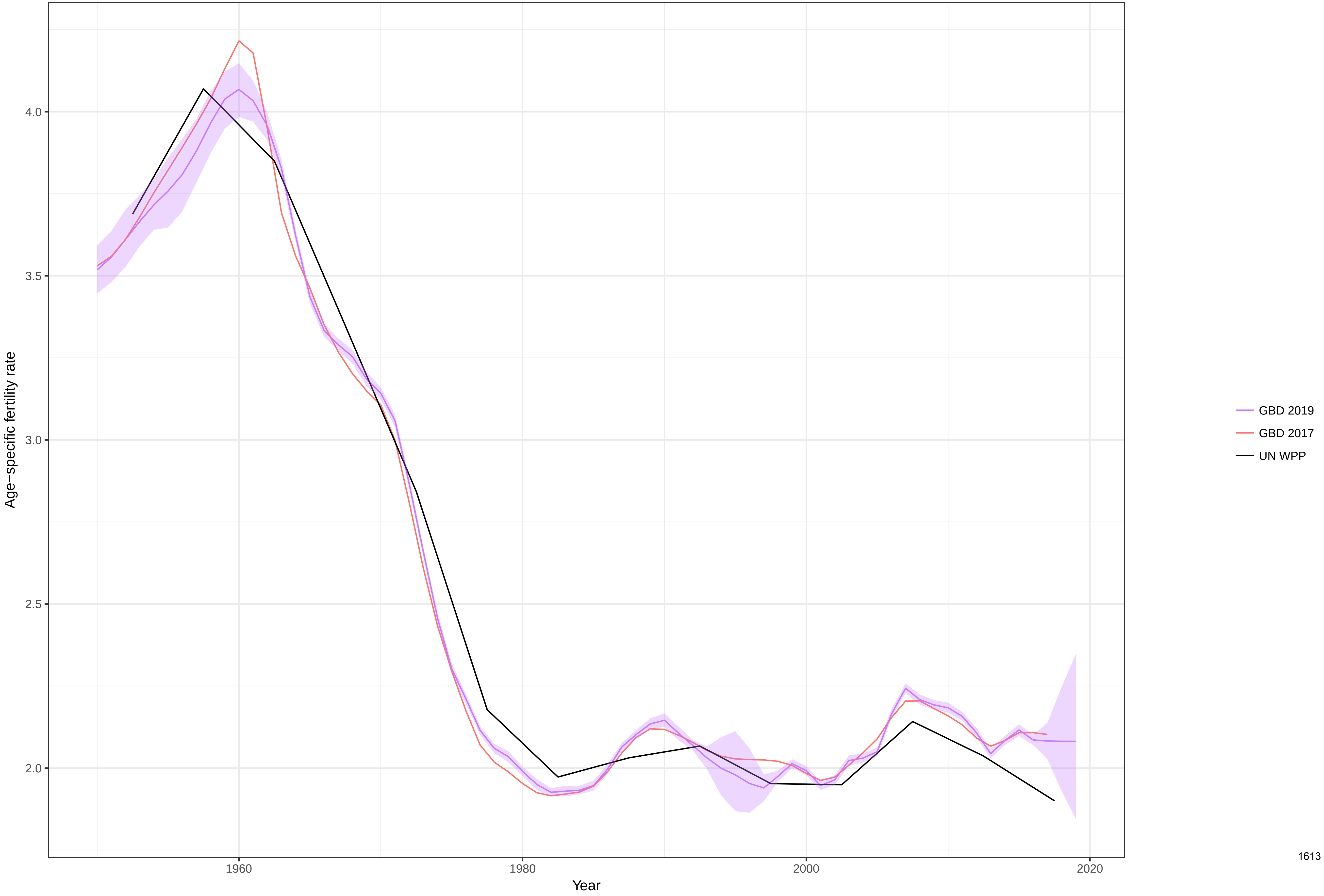

Age−specific fertility fitted trend lines, 1950−2019: Oman, Ages: 10−14
Data points enclosed with a diamond are reference sources

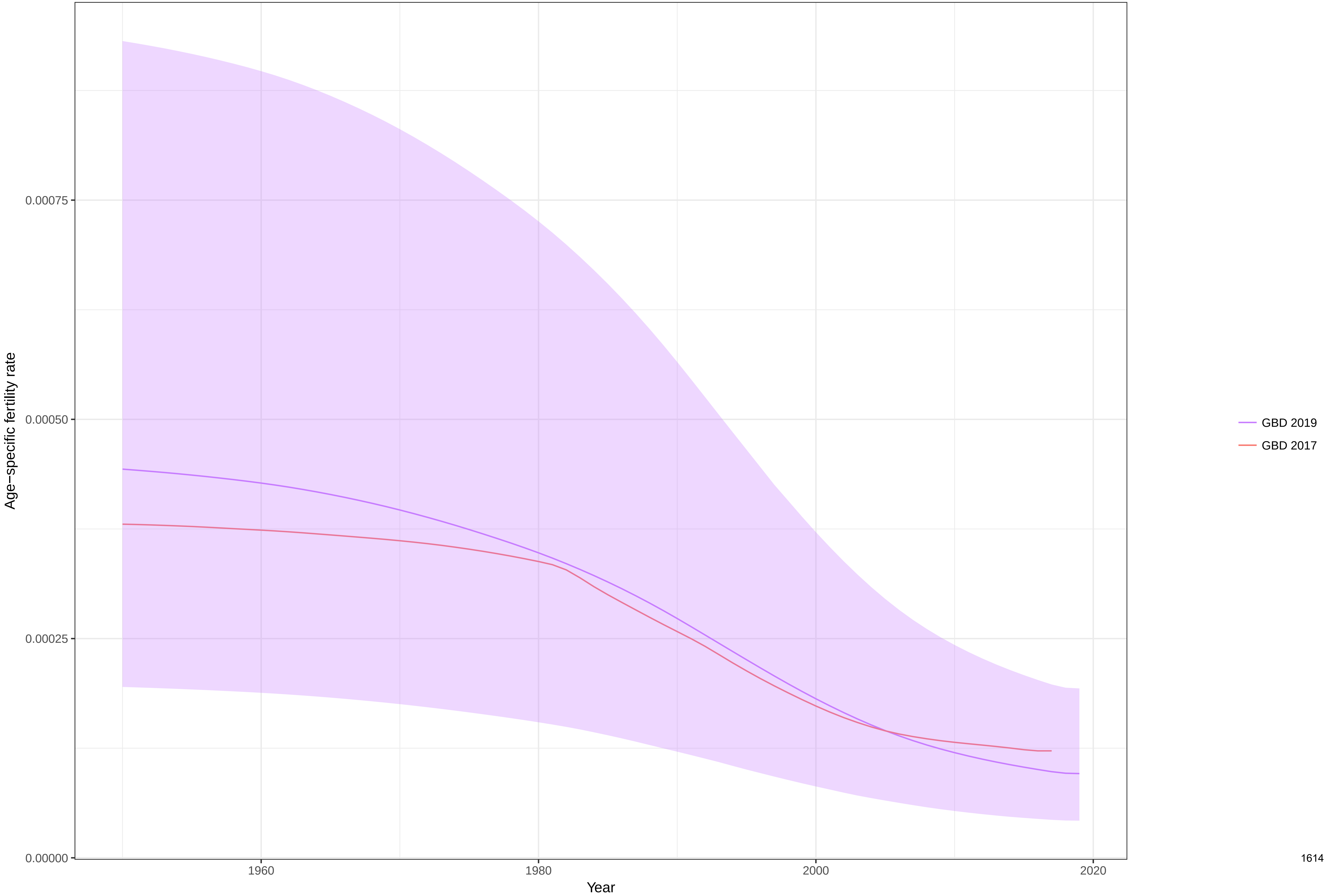

Age−specific fertility fitted trend lines, 1950−2019: Oman, Ages: 15−19
Data points enclosed with a diamond are reference sources

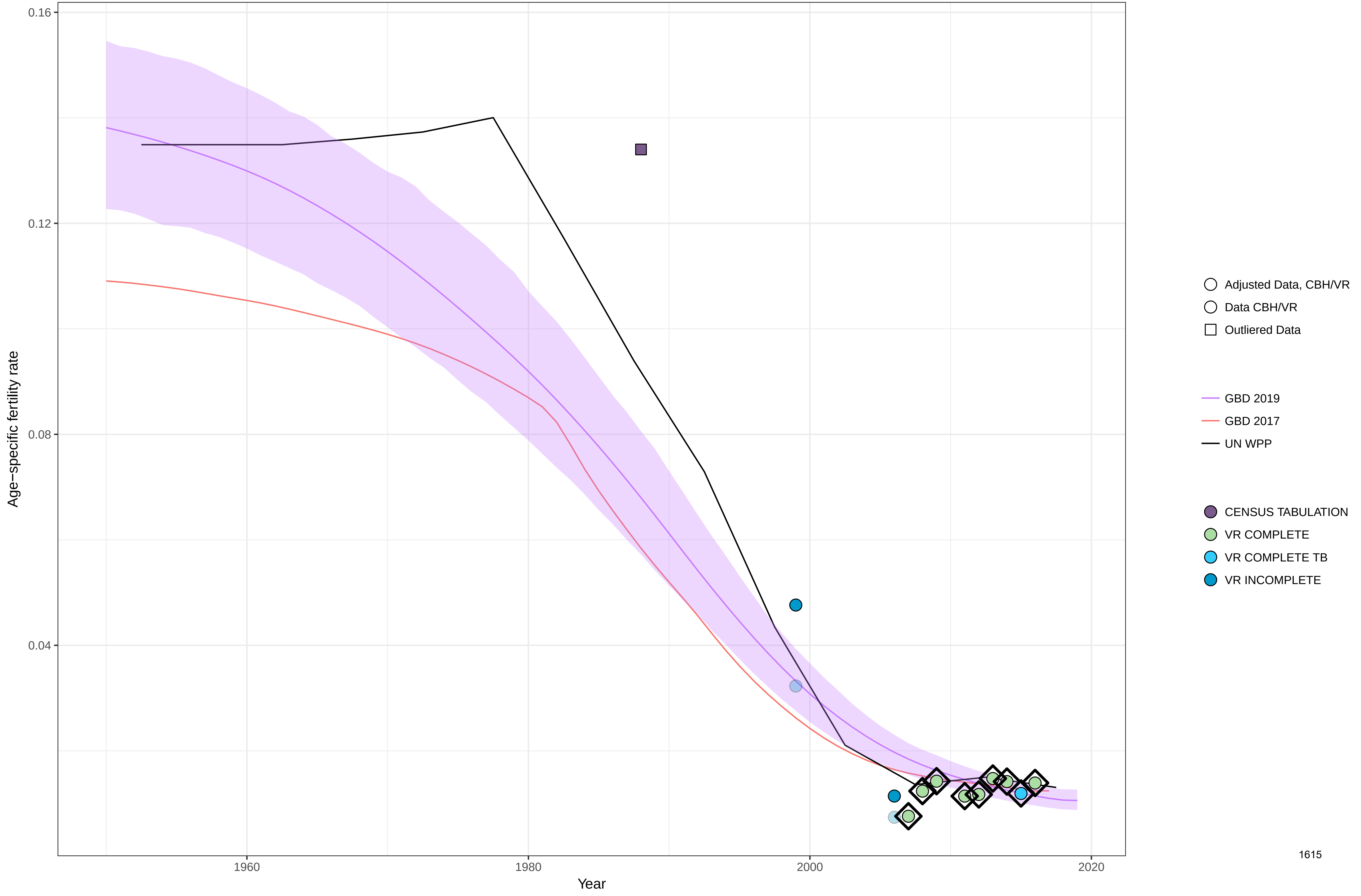

Age−specific fertility fitted trend lines, 1950−2019: Oman, Ages: 20−24
Data points enclosed with a diamond are reference sources

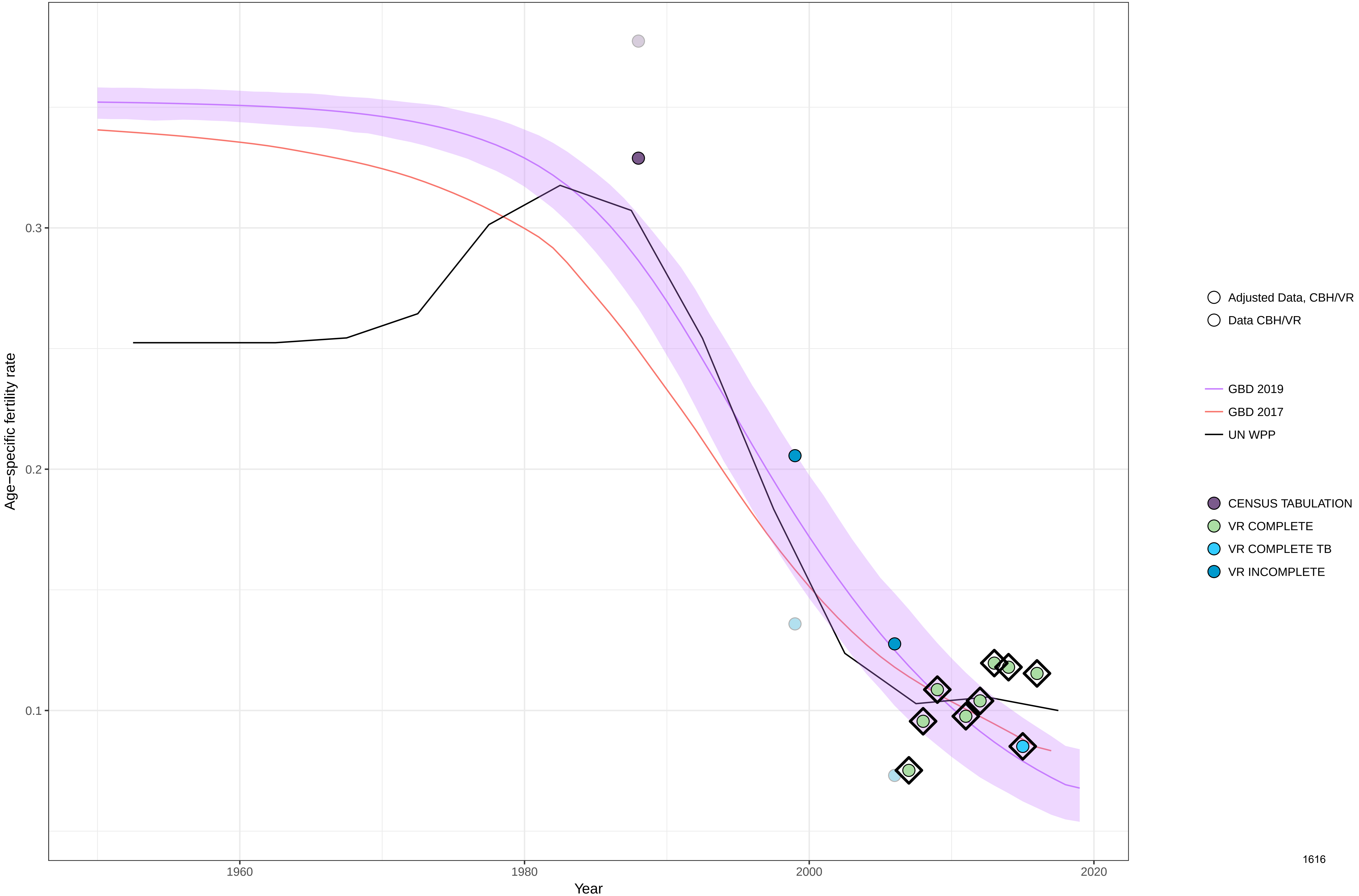

Age−specific fertility fitted trend lines, 1950−2019: Oman, Ages: 25−29
Data points enclosed with a diamond are reference sources

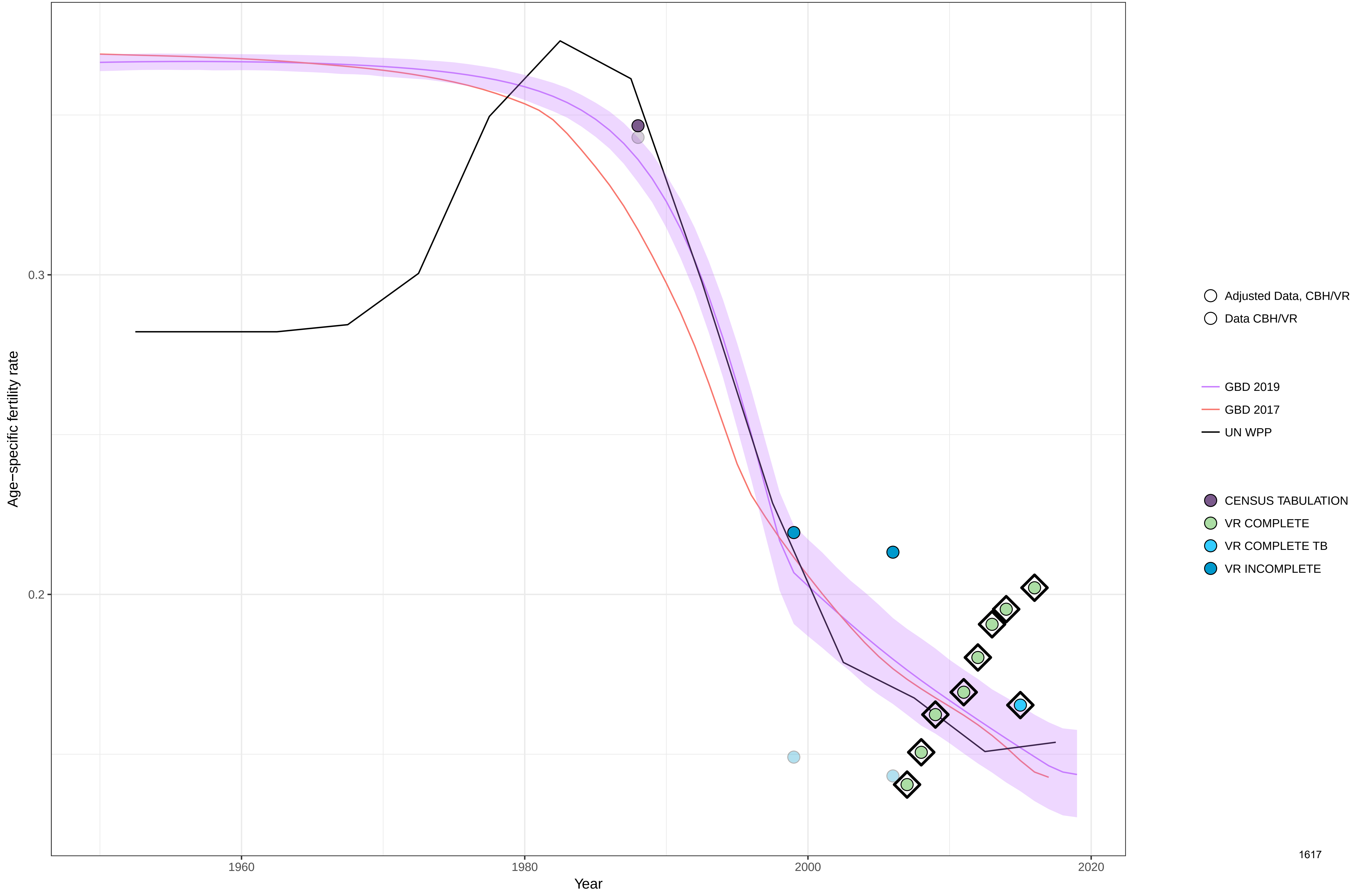

Age−specific fertility fitted trend lines, 1950−2019: Oman, Ages: 30−34
Data points enclosed with a diamond are reference sources

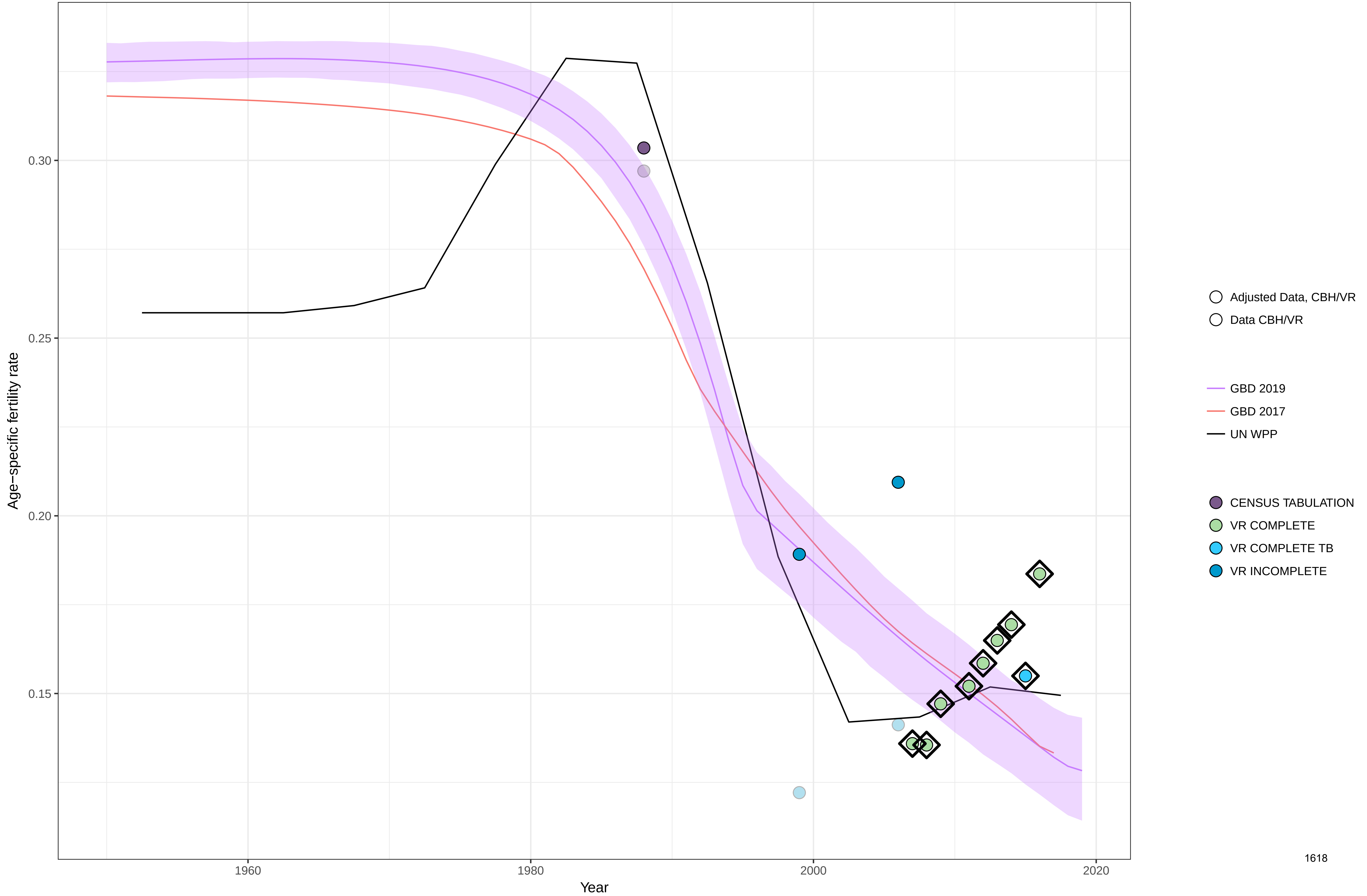

Age−specific fertility fitted trend lines, 1950−2019: Oman, Ages: 35−39
Data points enclosed with a diamond are reference sources

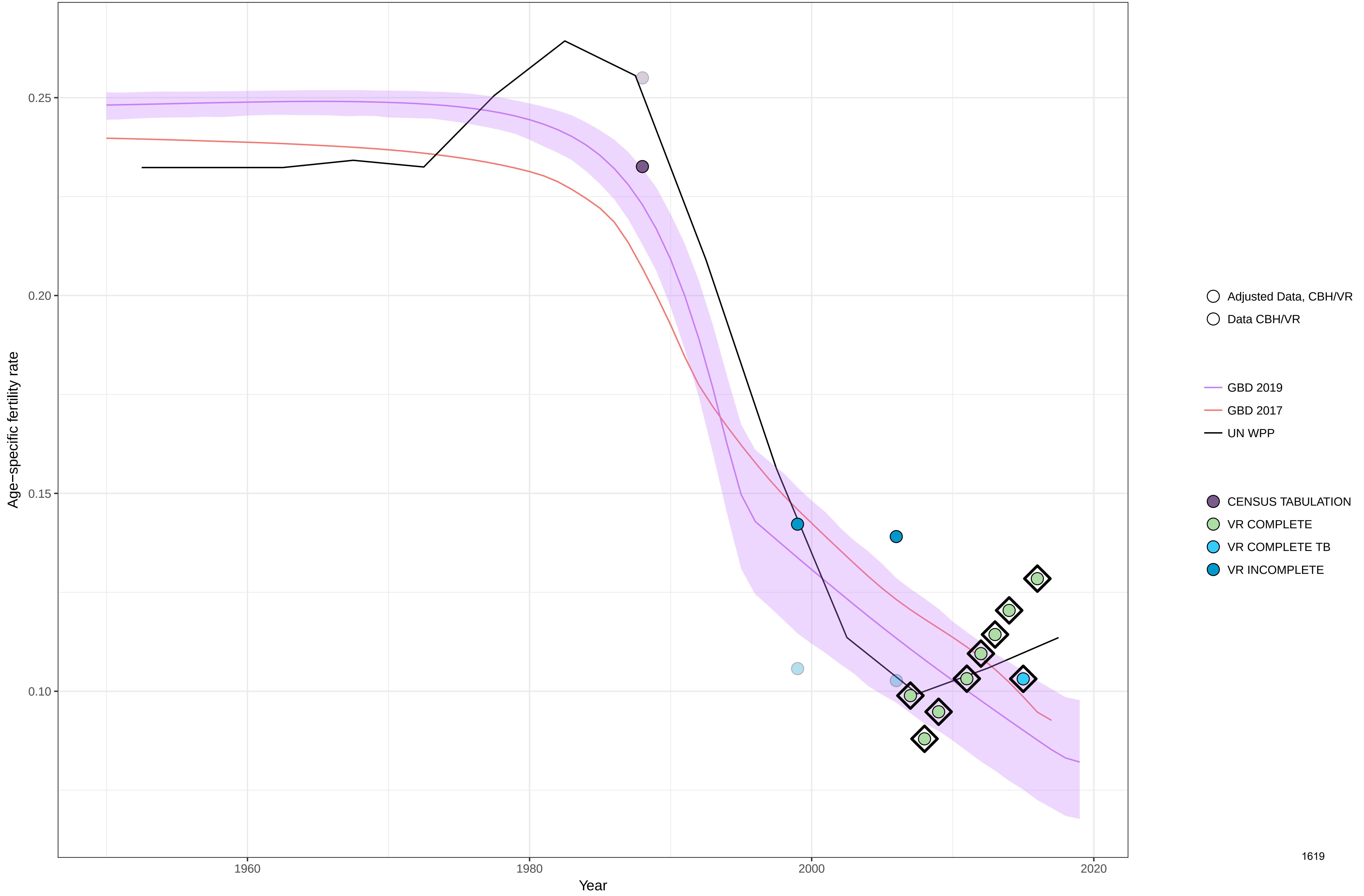

Age−specific fertility fitted trend lines, 1950−2019: Oman, Ages: 40−44
Data points enclosed with a diamond are reference sources

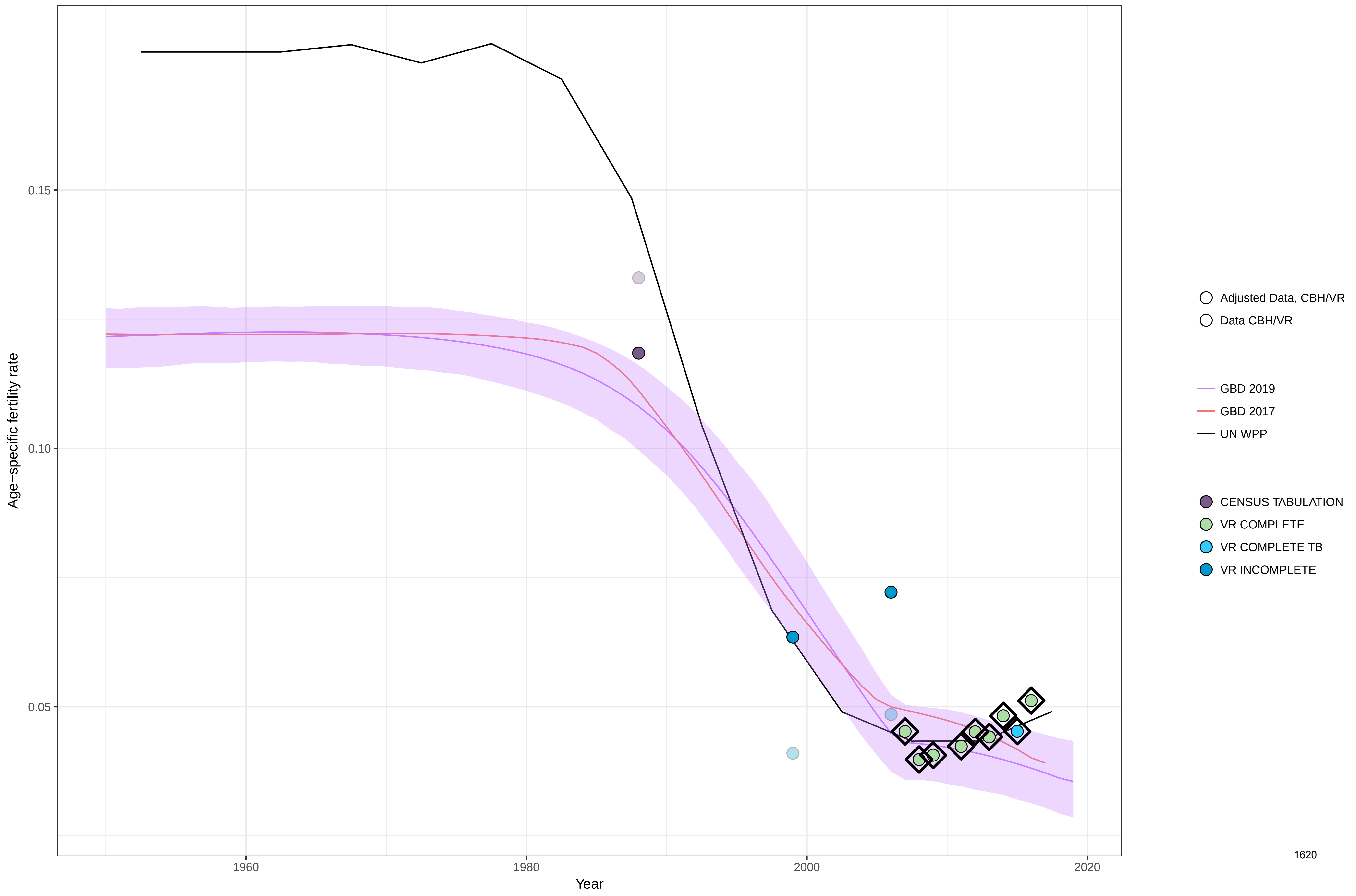

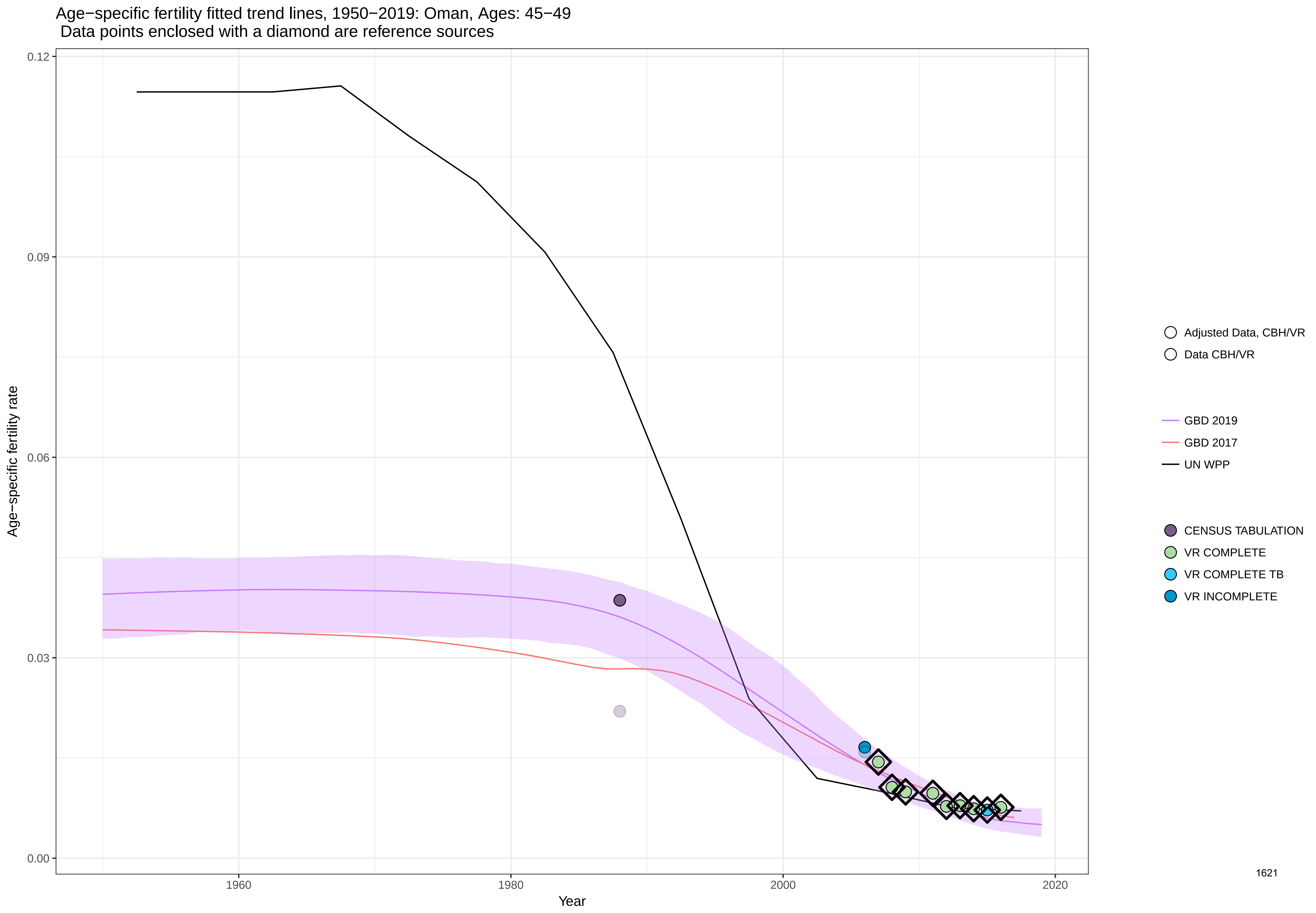

Age−specific fertility fitted trend lines, 1950−2019: Oman, Ages: 45−49
Data points enclosed with a diamond are reference sources
0.12
0.09
0.06
0.03
0.00
Age−specific fertility rate
1960
1980
2000
2020
Year
Adjusted Data, CBH/VR
Data CBH/VR
GBD 2019
GBD 2017
UN WPP
CENSUS TABULATION
VR COMPLETE
VR COMPLETE TB
VR INCOMPLETE

Age−specific fertility fitted trend lines, 1950−2019: Oman, Ages: 50−54
Data points enclosed with a diamond are reference sources

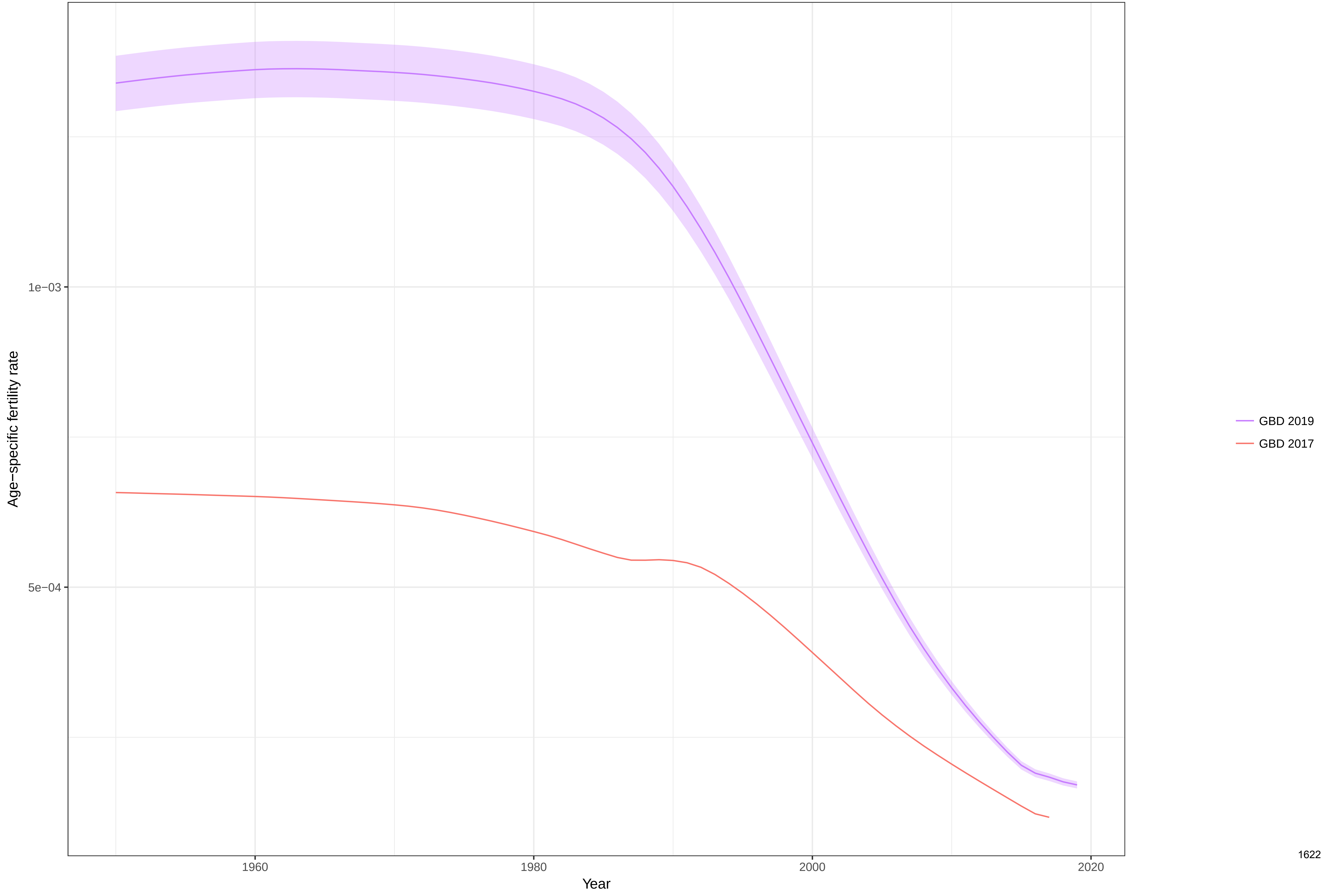

Total fertility rate fitted trend lines, 1950−2019: Oman TFR
Data points enclosed with a diamond are reference sources

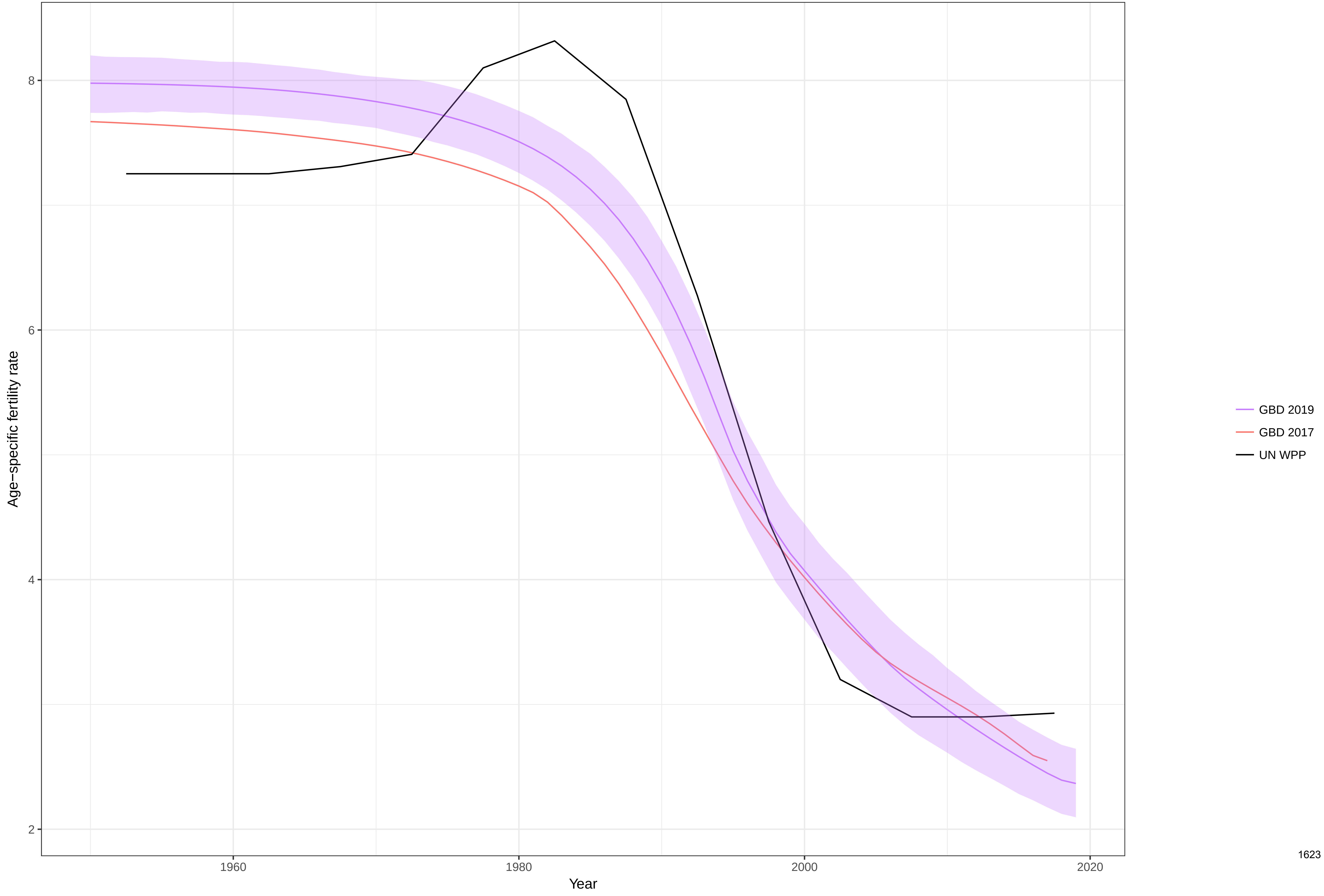

Age−specific fertility fitted trend lines, 1950−2019: Pakistan, Ages: 10−14
Data points enclosed with a diamond are reference sources

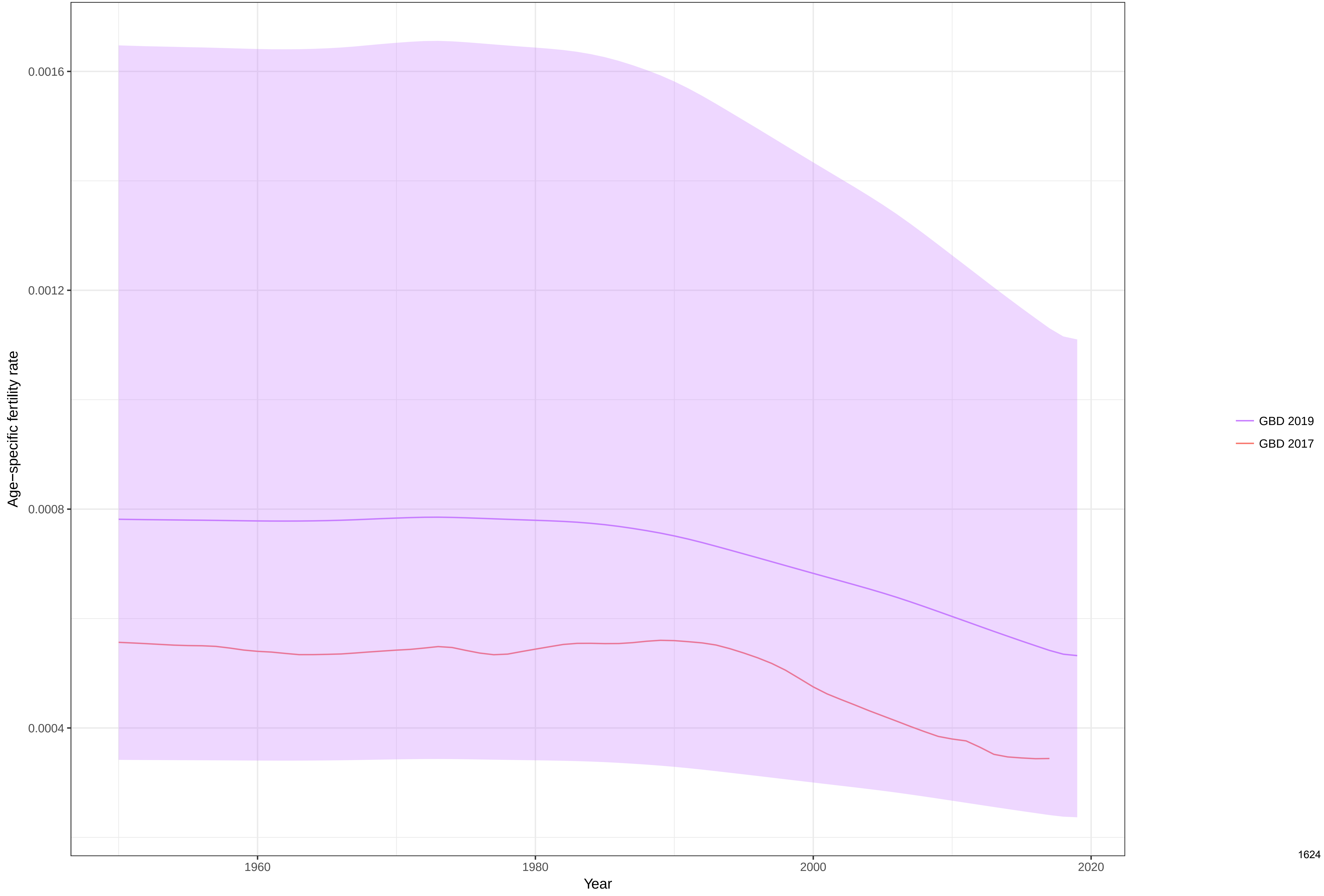

Age−specific fertility fitted trend lines, 1950−2019: Pakistan, Ages: 15−19
Data points enclosed with a diamond are reference sources

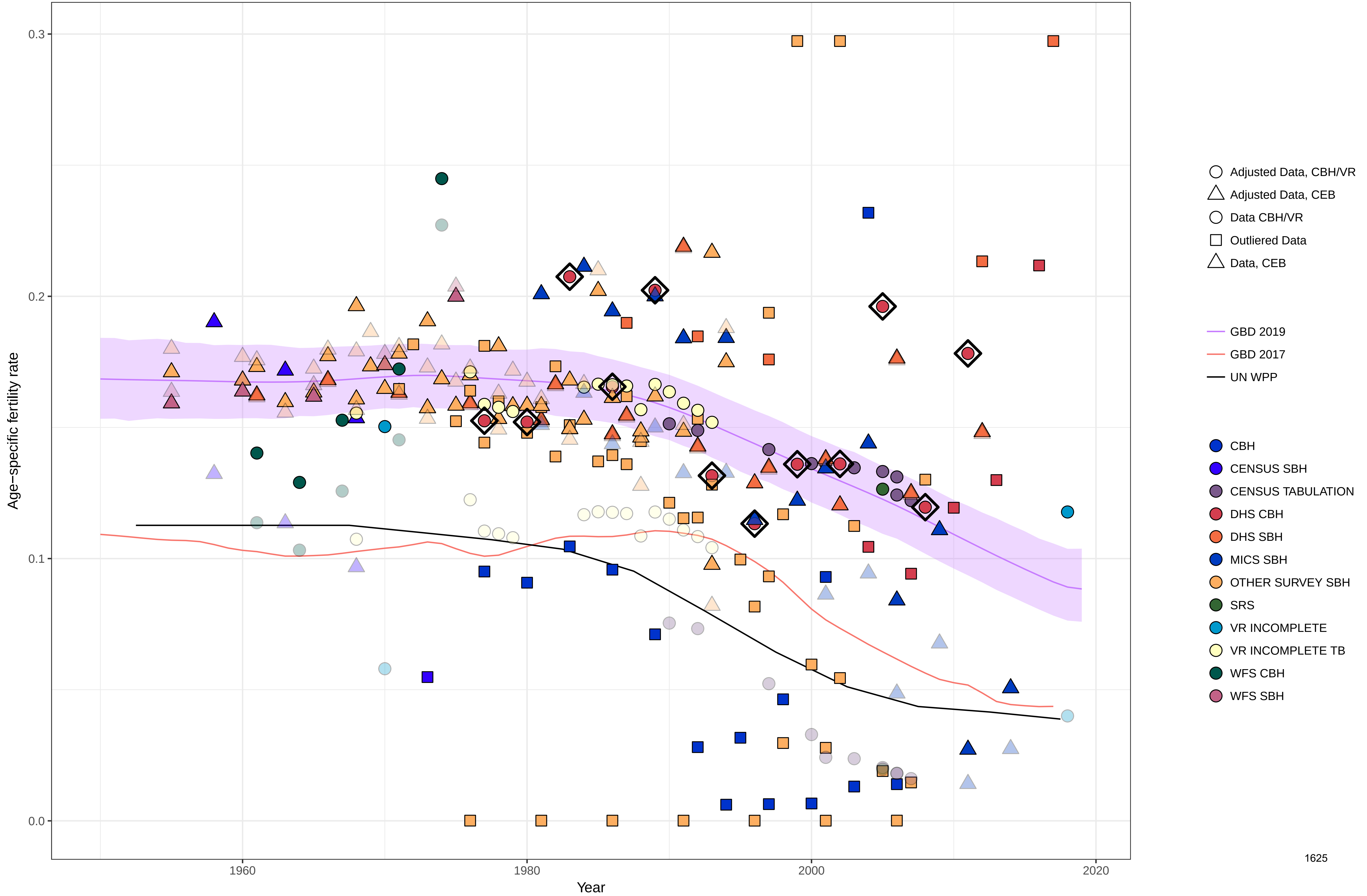

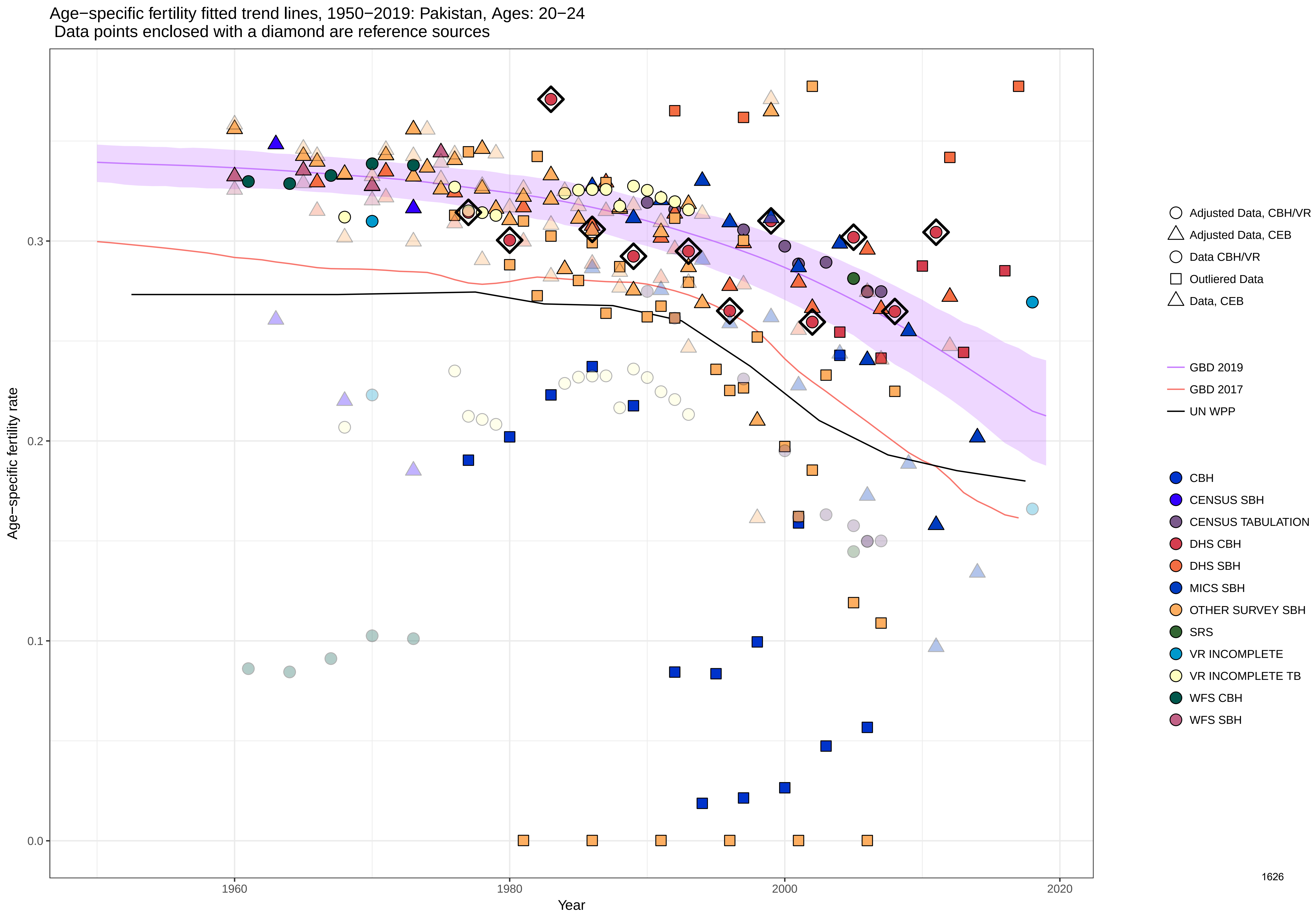

Age−specific fertility fitted trend lines, 1950−2019: Pakistan, Ages: 20−24
Data points enclosed with a diamond are reference sources
Age−specific fertility rate
Year
0.0
0.1
0.2
0.3
1960
1980
2000
2020
Adjusted Data, CBH/VR
Adjusted Data, CEB
Data CBH/VR
Outliered Data
Data, CEB
GBD 2019
GBD 2017
UN WPP
CBH
CENSUS SBH
CENSUS TABULATION
DHS CBH
DHS SBH
MICS SBH
OTHER SURVEY SBH
SRS
VR INCOMPLETE
VR INCOMPLETE TB
WFS CBH
WFS SBH

Age−specific fertility fitted trend lines, 1950−2019: Pakistan, Ages: 25−29
Data points enclosed with a diamond are reference sources

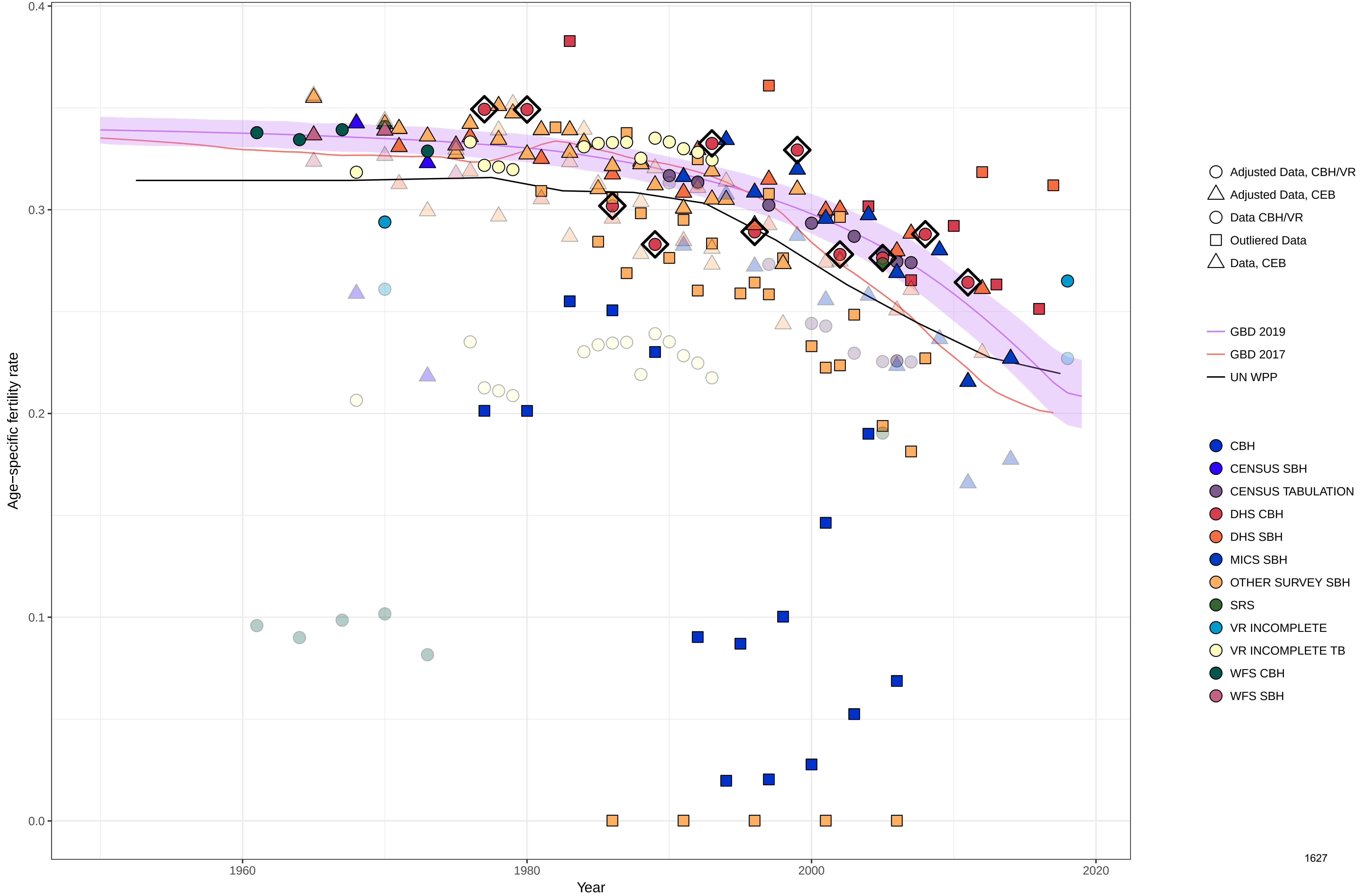

Age−specific fertility fitted trend lines, 1950−2019: Pakistan, Ages: 30−34
Data points enclosed with a diamond are reference sources

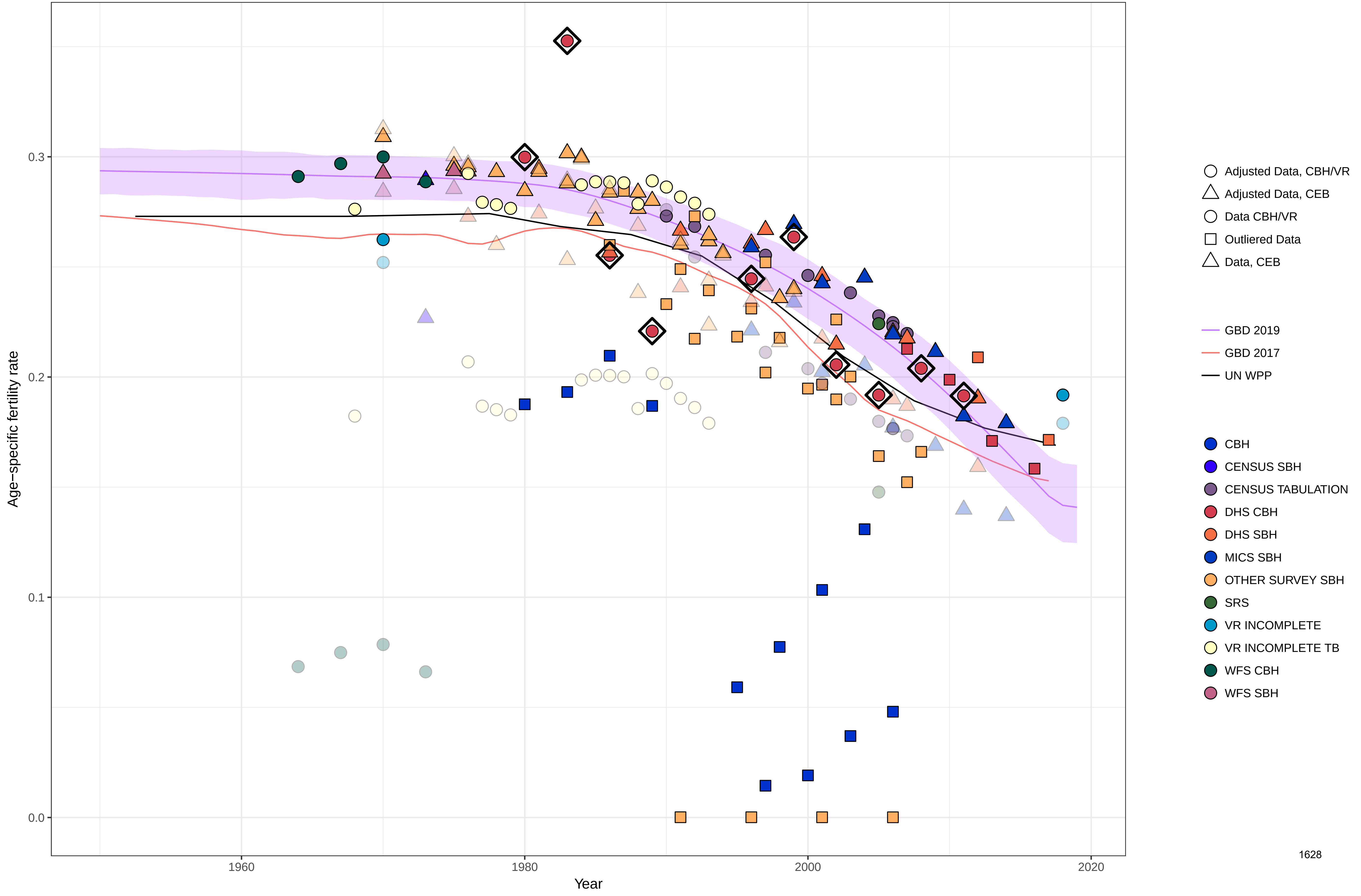

Age−specific fertility fitted trend lines, 1950−2019: Pakistan, Ages: 35−39
Data points enclosed with a diamond are reference sources

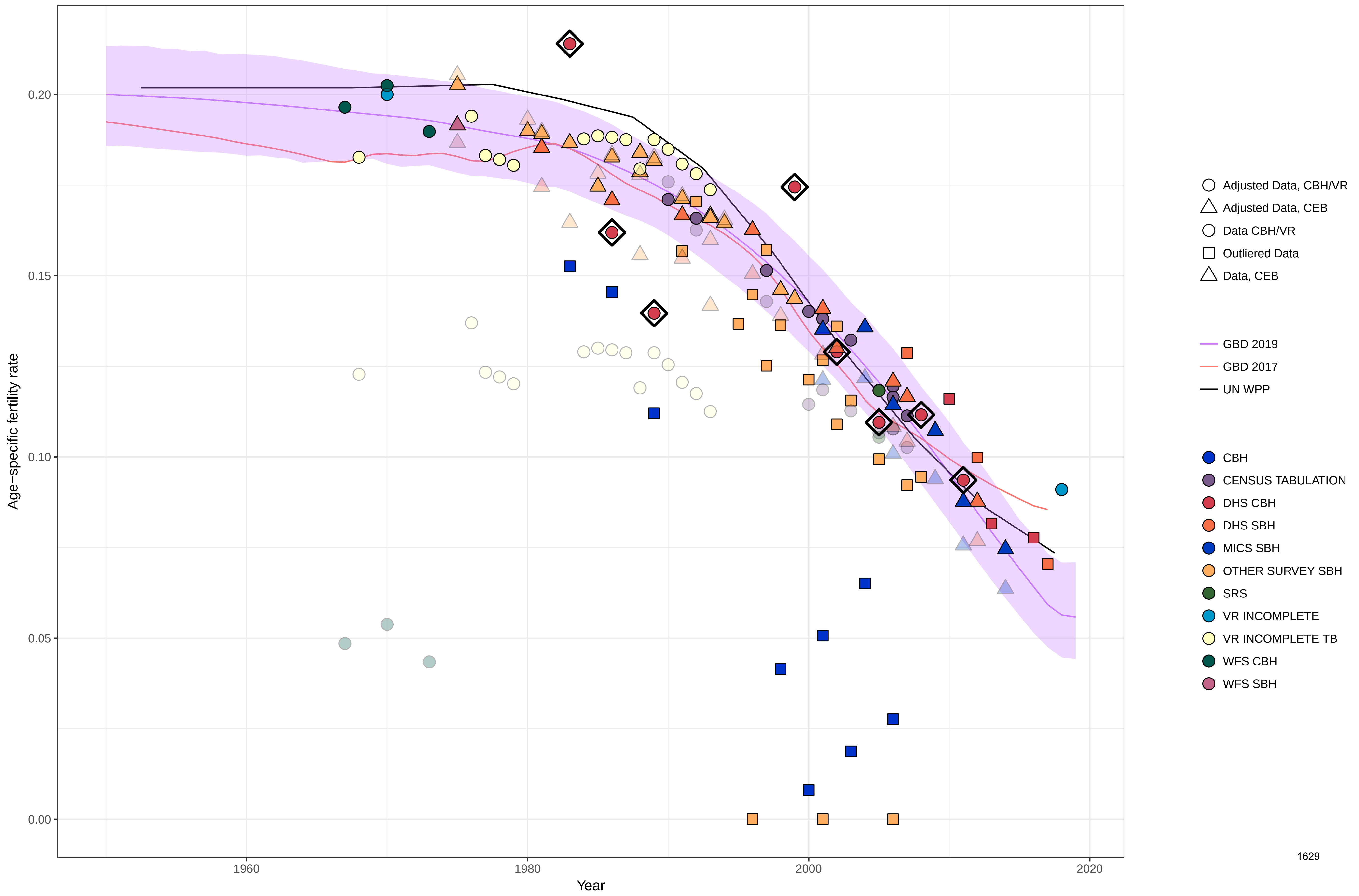

Age−specific fertility fitted trend lines, 1950−2019: Pakistan, Ages: 40−44
Data points enclosed with a diamond are reference sources

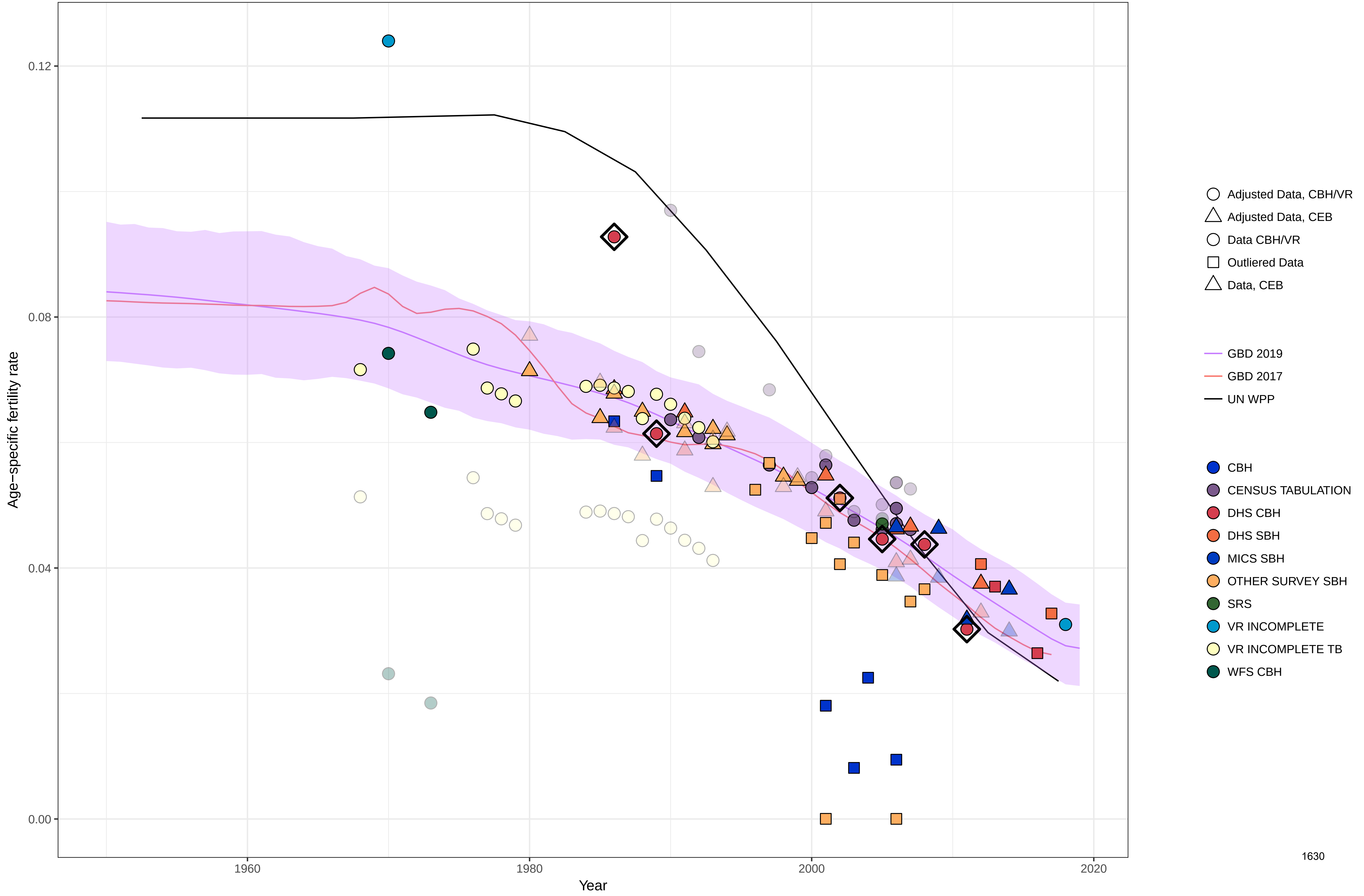

Age−specific fertility fitted trend lines, 1950−2019: Pakistan, Ages: 45−49
Data points enclosed with a diamond are reference sources

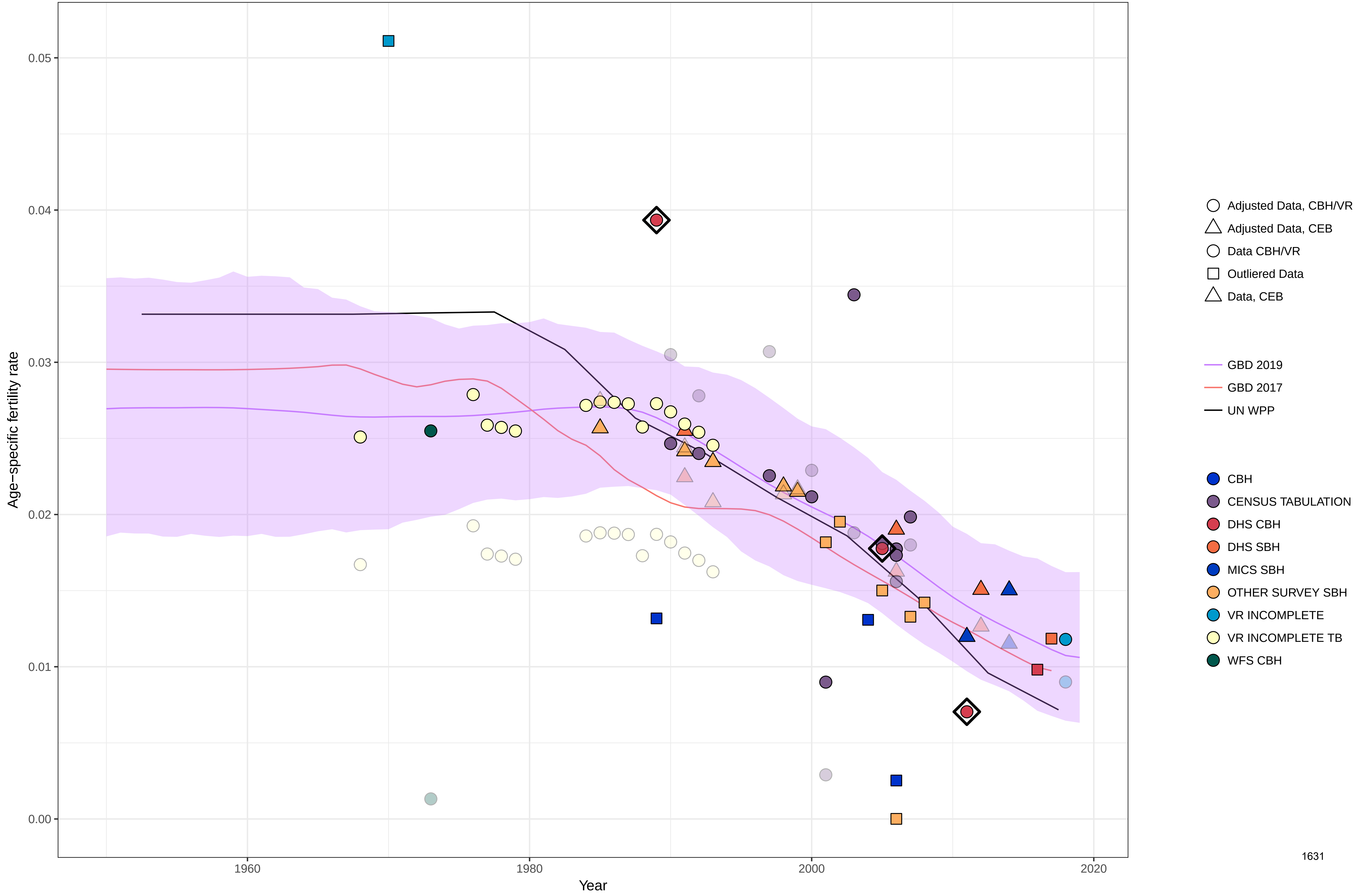

Age−specific fertility fitted trend lines, 1950−2019: Pakistan, Ages: 50−54
Data points enclosed with a diamond are reference sources

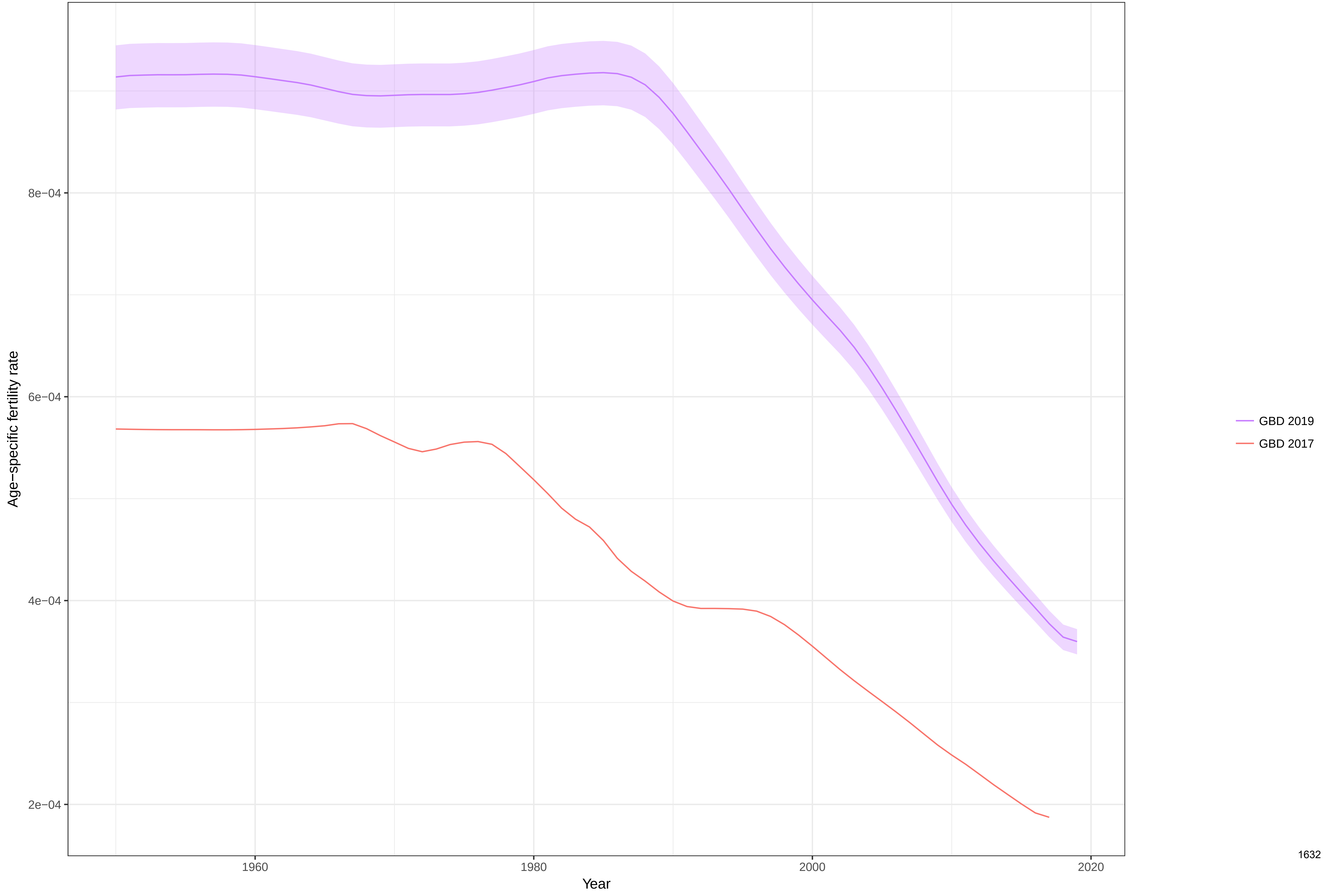

Total fertility rate fitted trend lines, 1950−2019: Pakistan TFR
Data points enclosed with a diamond are reference sources

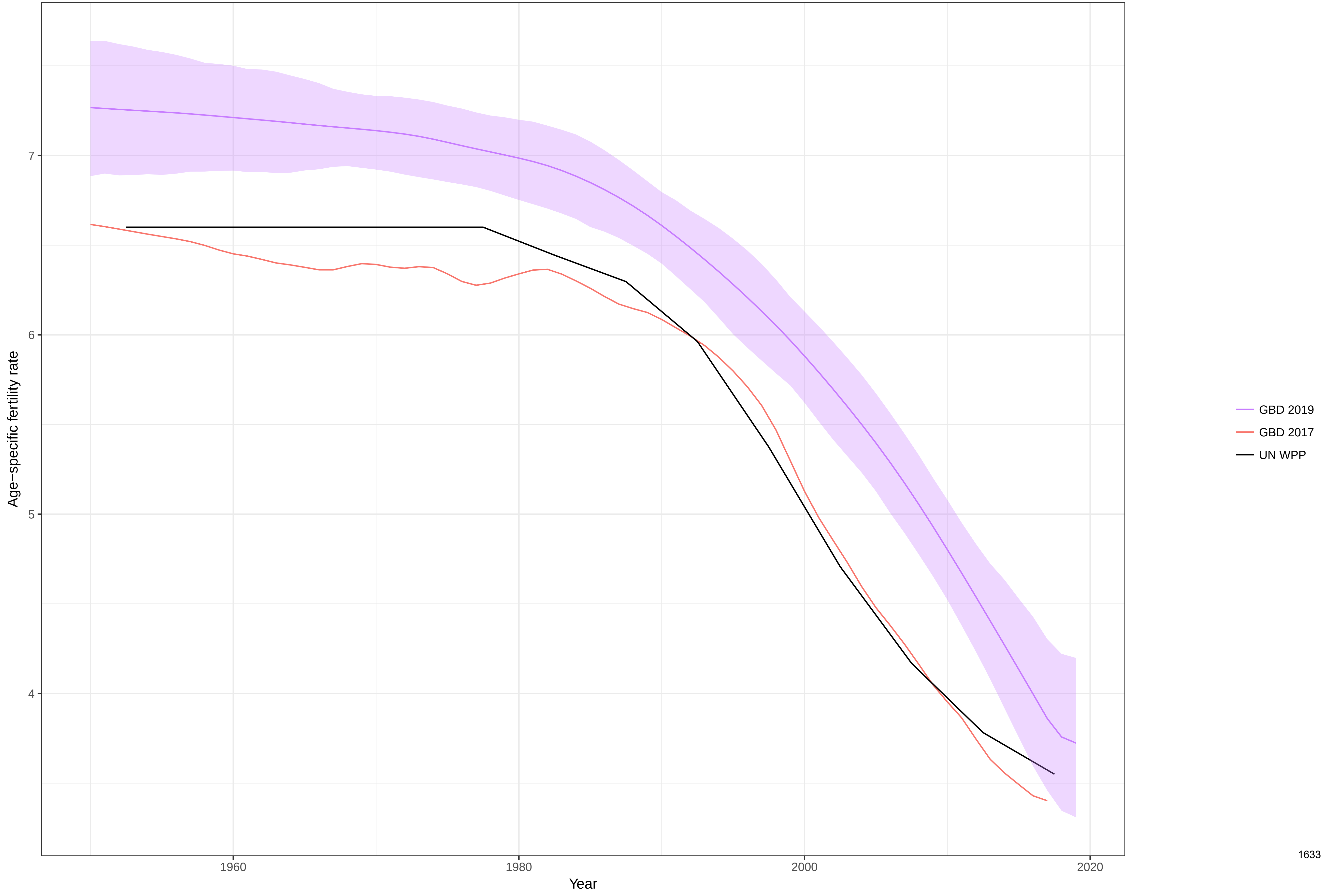

Age−specific fertility fitted trend lines, 1950−2019: Panama, Ages: 10−14
Data points enclosed with a diamond are reference sources

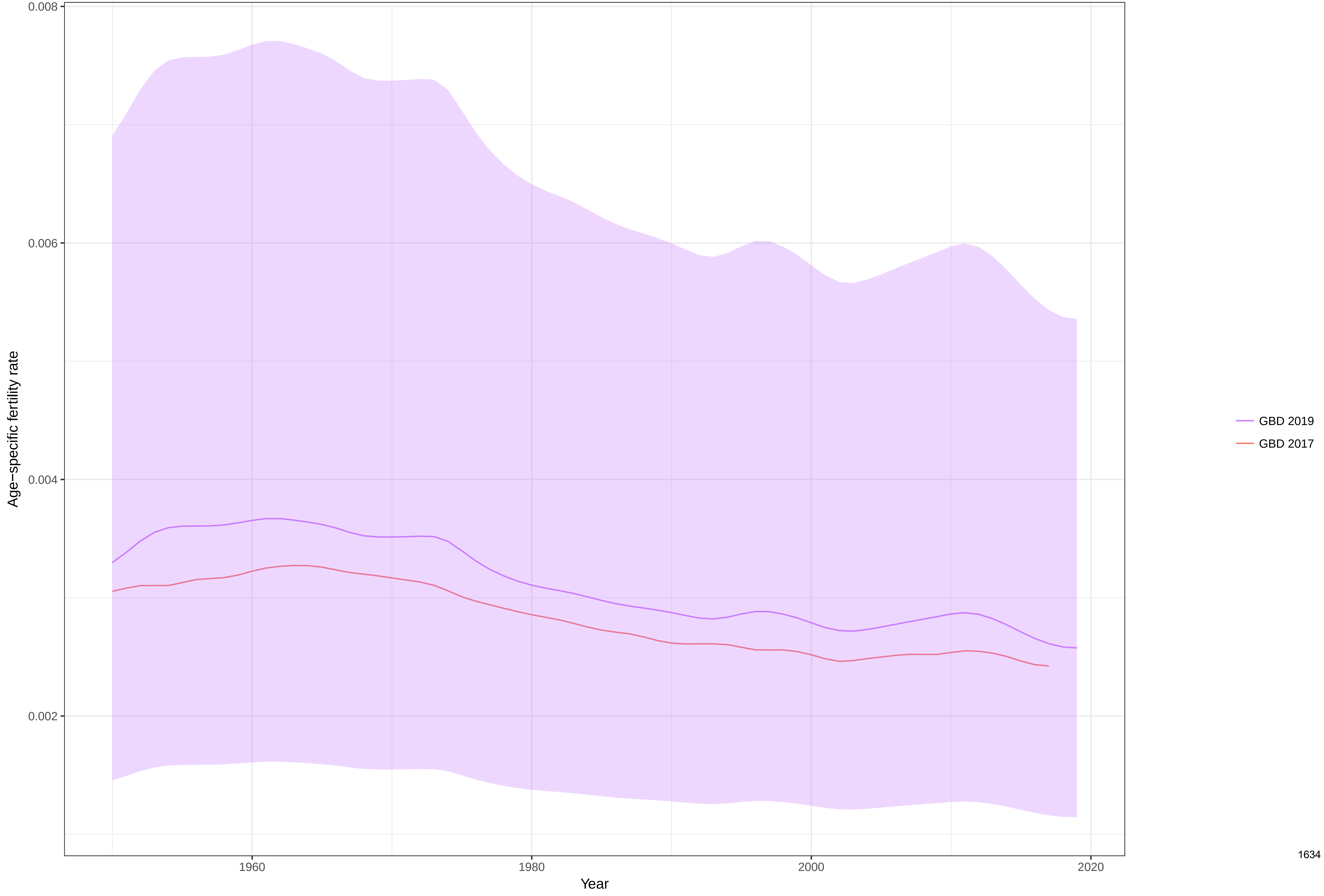

Age−specific fertility fitted trend lines, 1950−2019: Panama, Ages: 15−19
Data points enclosed with a diamond are reference sources

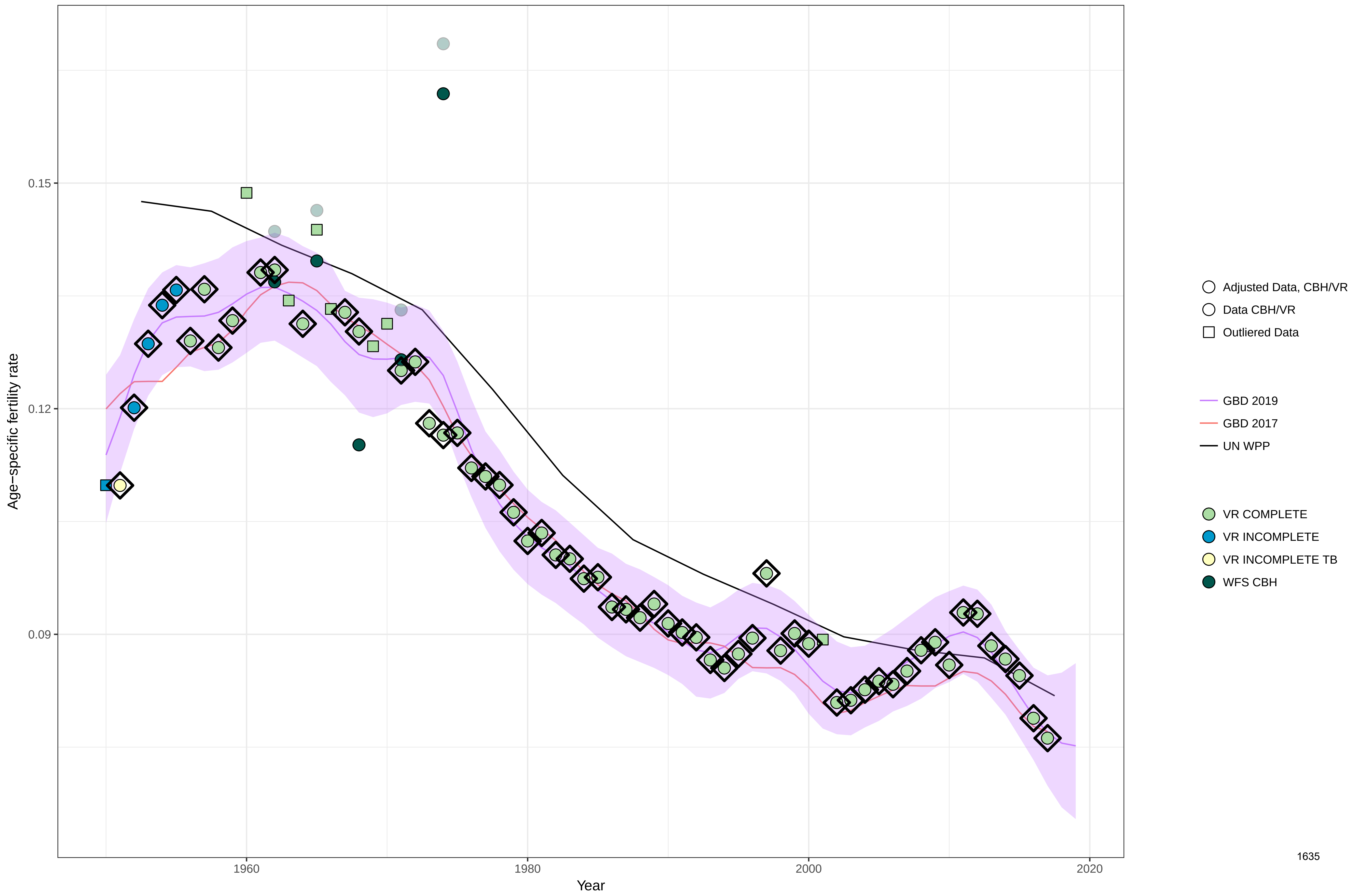

Age−specific fertility fitted trend lines, 1950−2019: Panama, Ages: 20−24
Data points enclosed with a diamond are reference sources

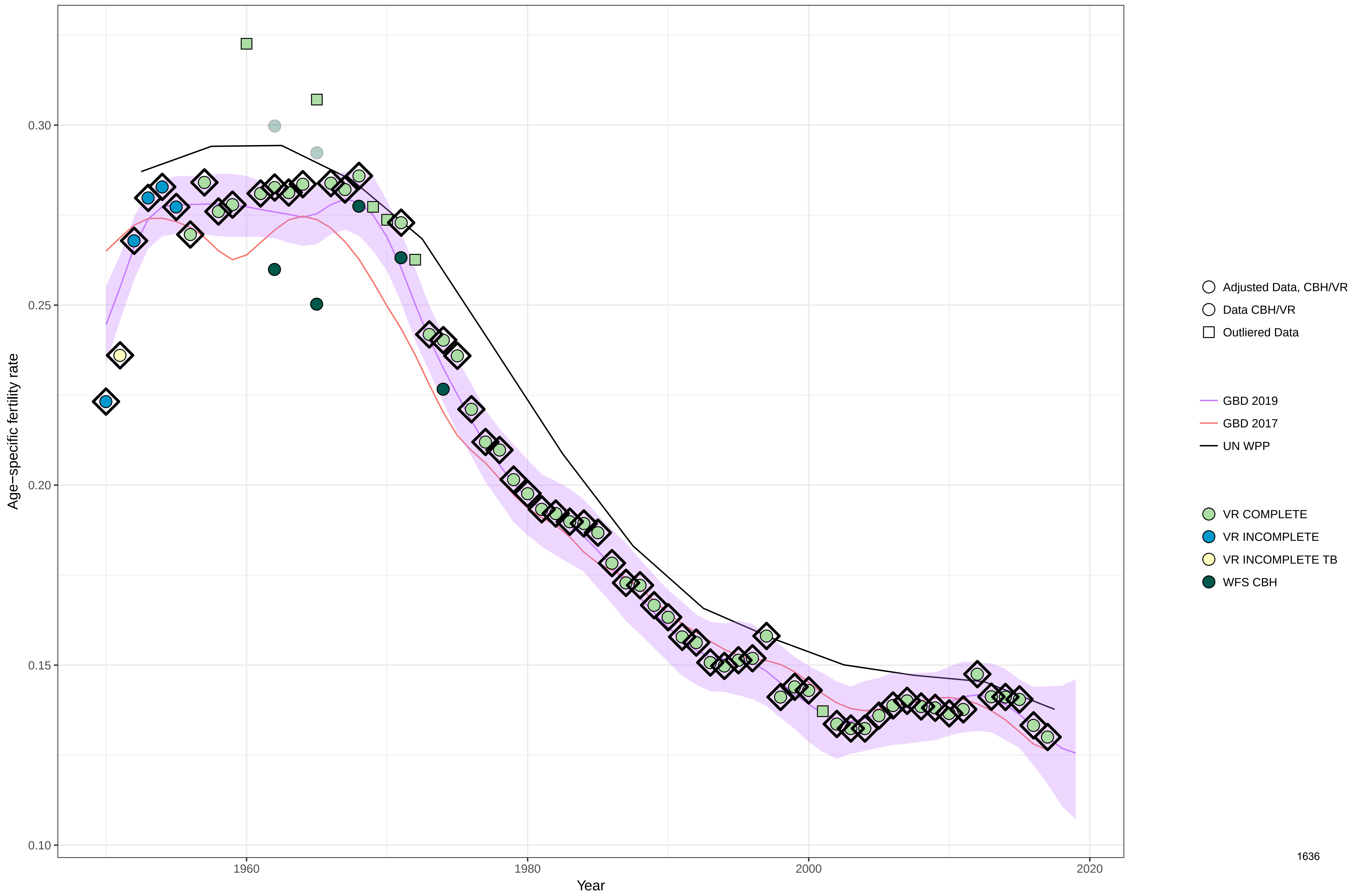

Age−specific fertility fitted trend lines, 1950−2019: Panama, Ages: 25−29
Data points enclosed with a diamond are reference sources

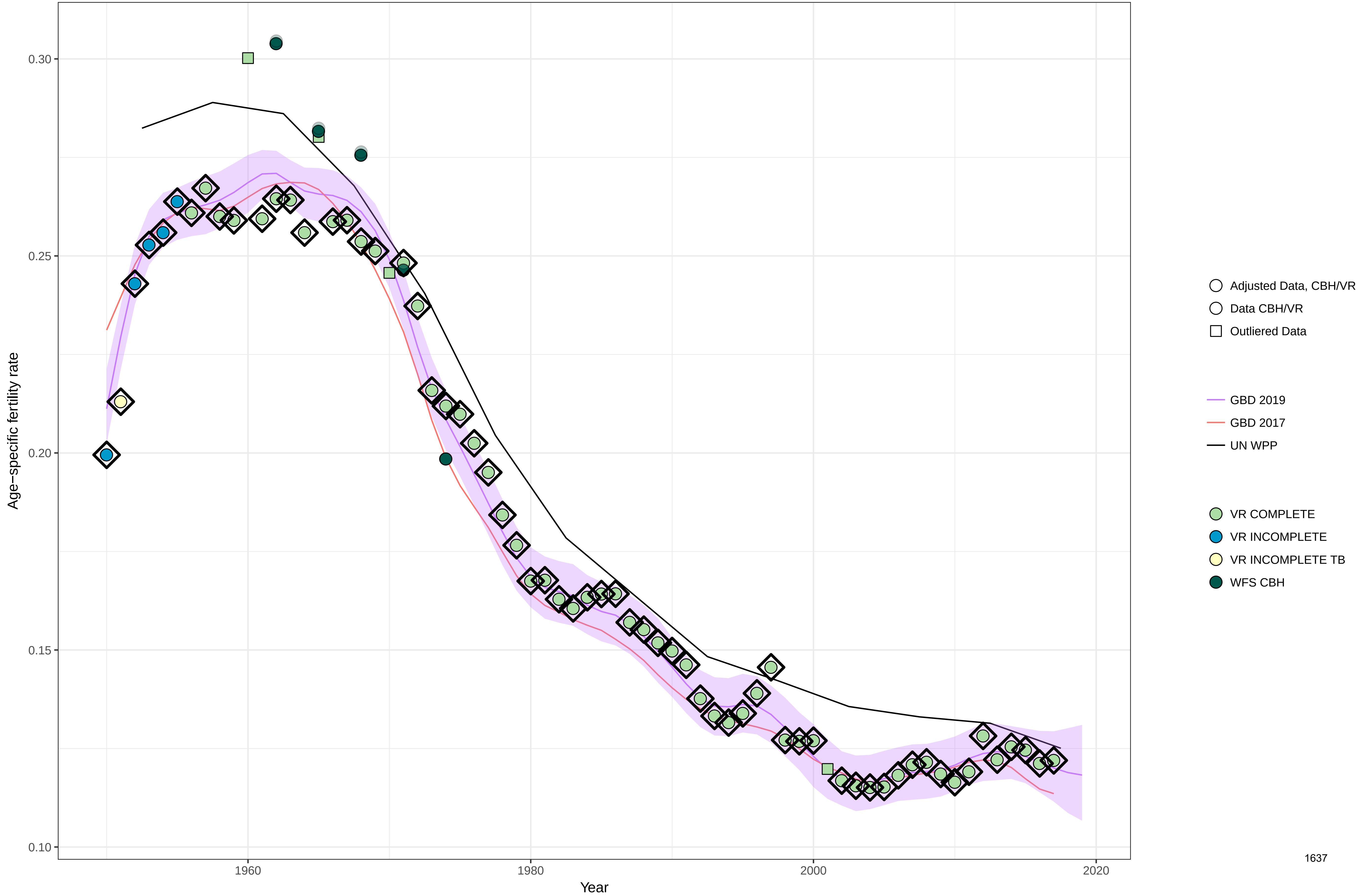

Age−specific fertility fitted trend lines, 1950−2019: Panama, Ages: 30−34
Data points enclosed with a diamond are reference sources

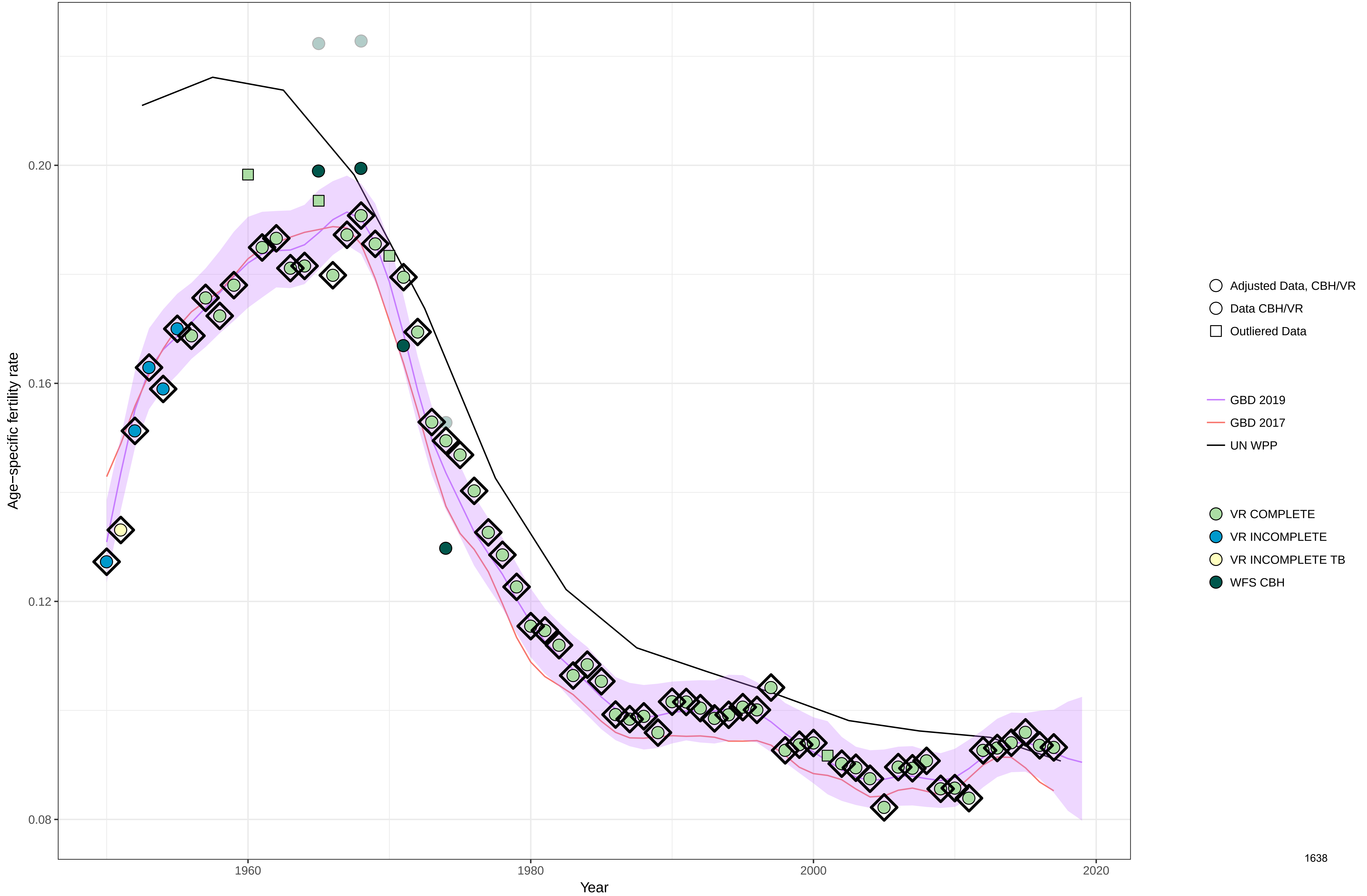

Age−specific fertility fitted trend lines, 1950−2019: Panama, Ages: 35−39
Data points enclosed with a diamond are reference sources

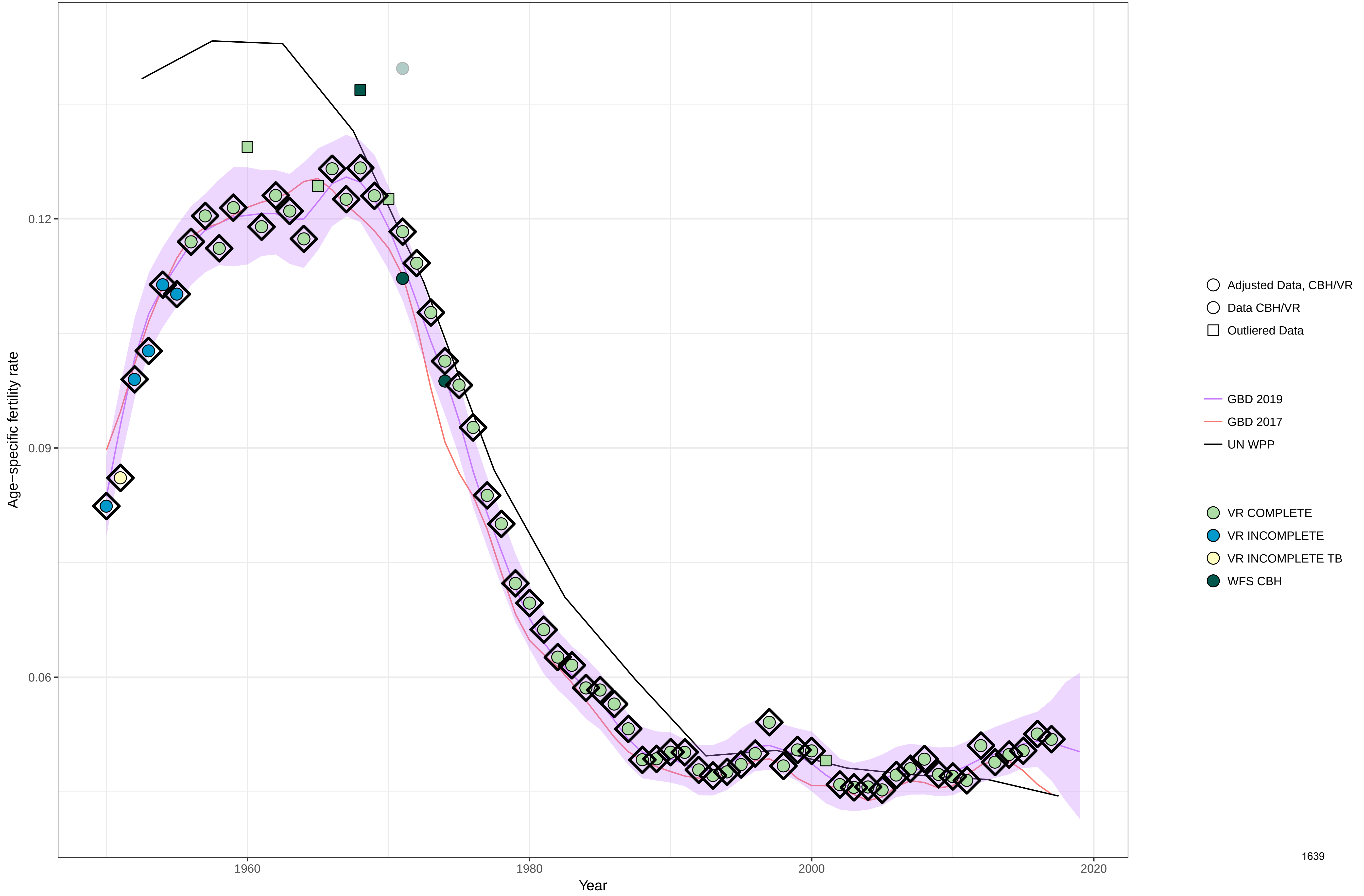

Age−specific fertility fitted trend lines, 1950−2019: Panama, Ages: 40−44
Data points enclosed with a diamond are reference sources

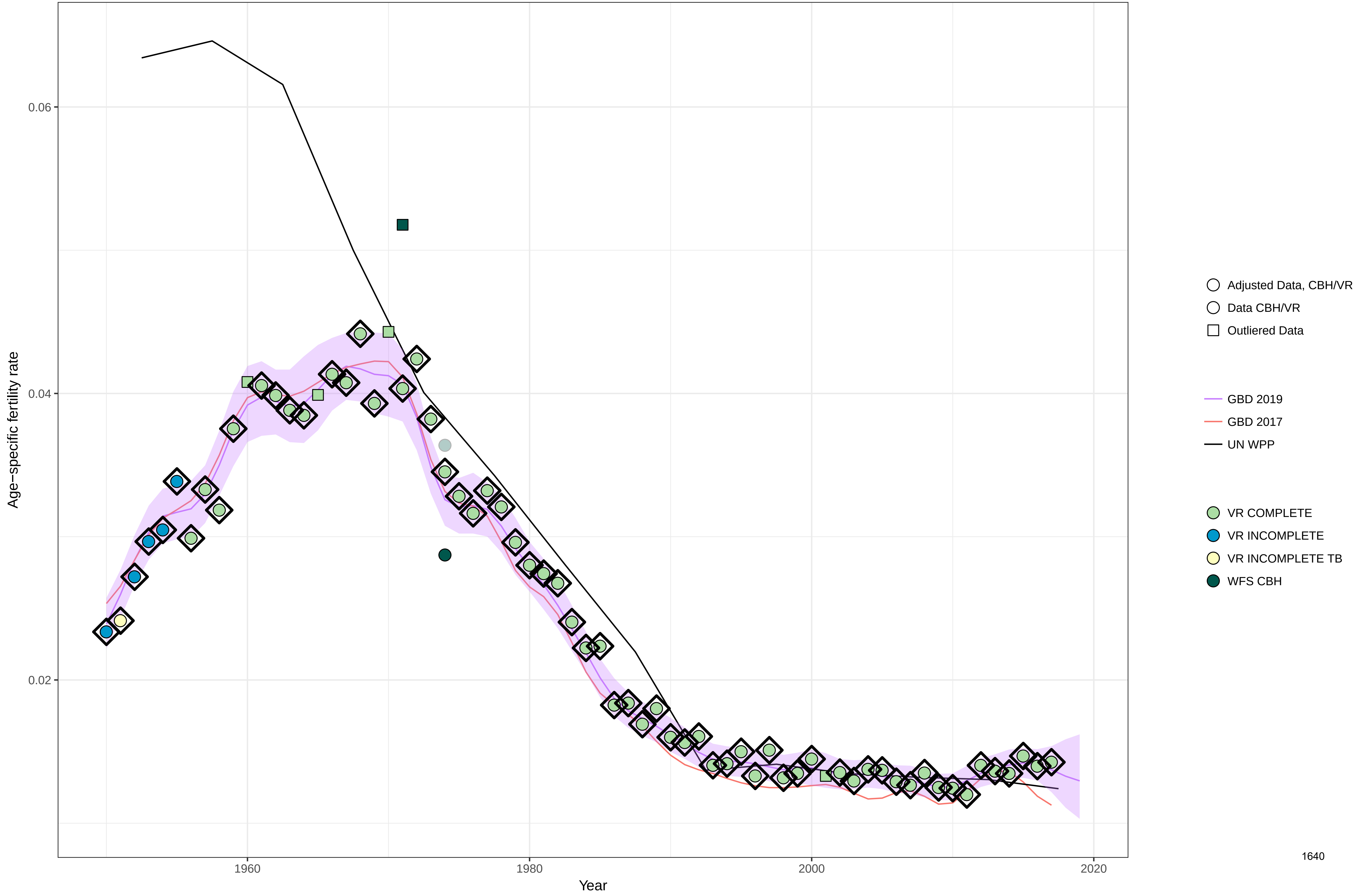

Age−specific fertility fitted trend lines, 1950−2019: Panama, Ages: 45−49
Data points enclosed with a diamond are reference sources

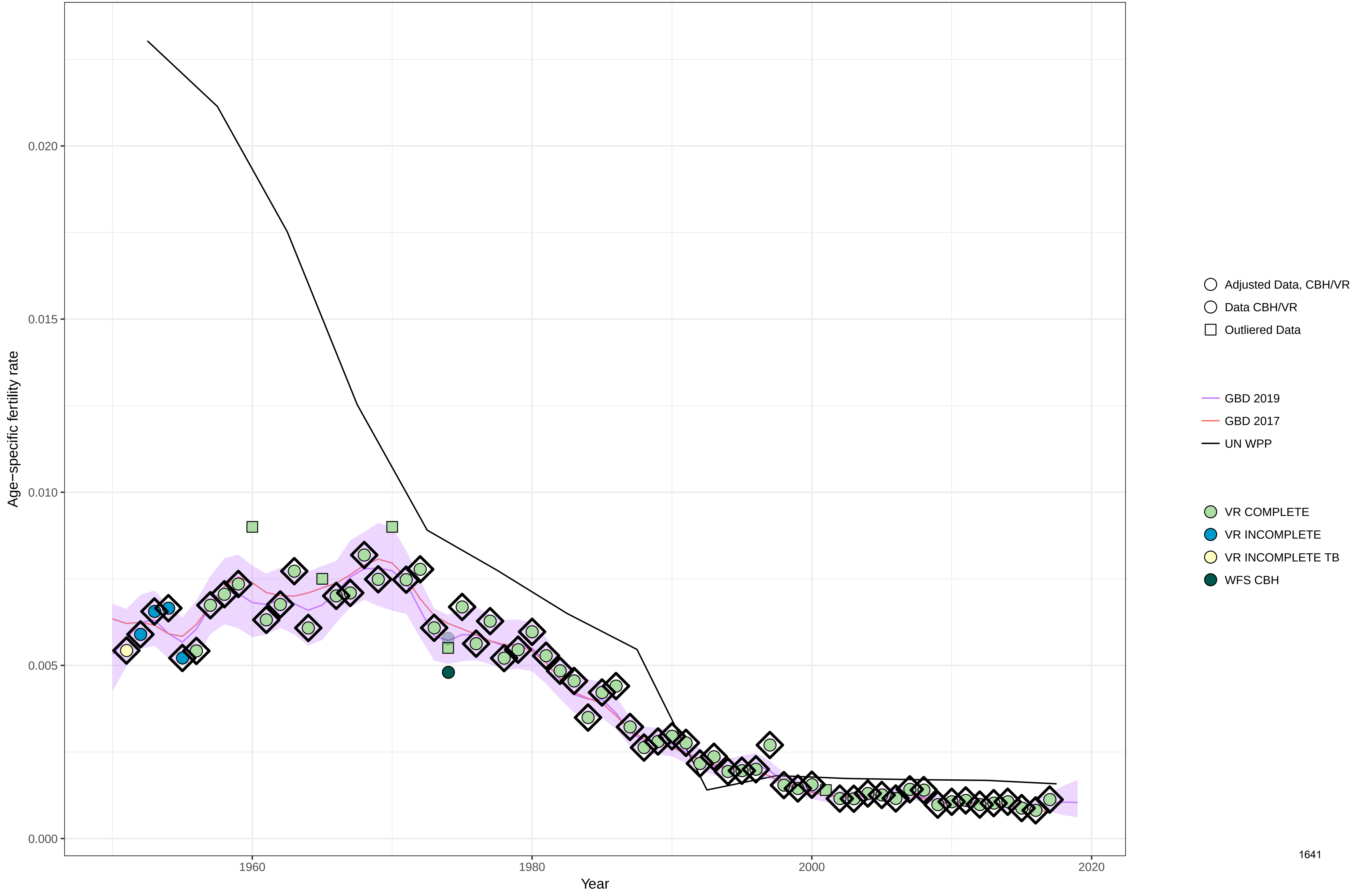

Age−specific fertility fitted trend lines, 1950−2019: Panama, Ages: 50−54
Data points enclosed with a diamond are reference sources

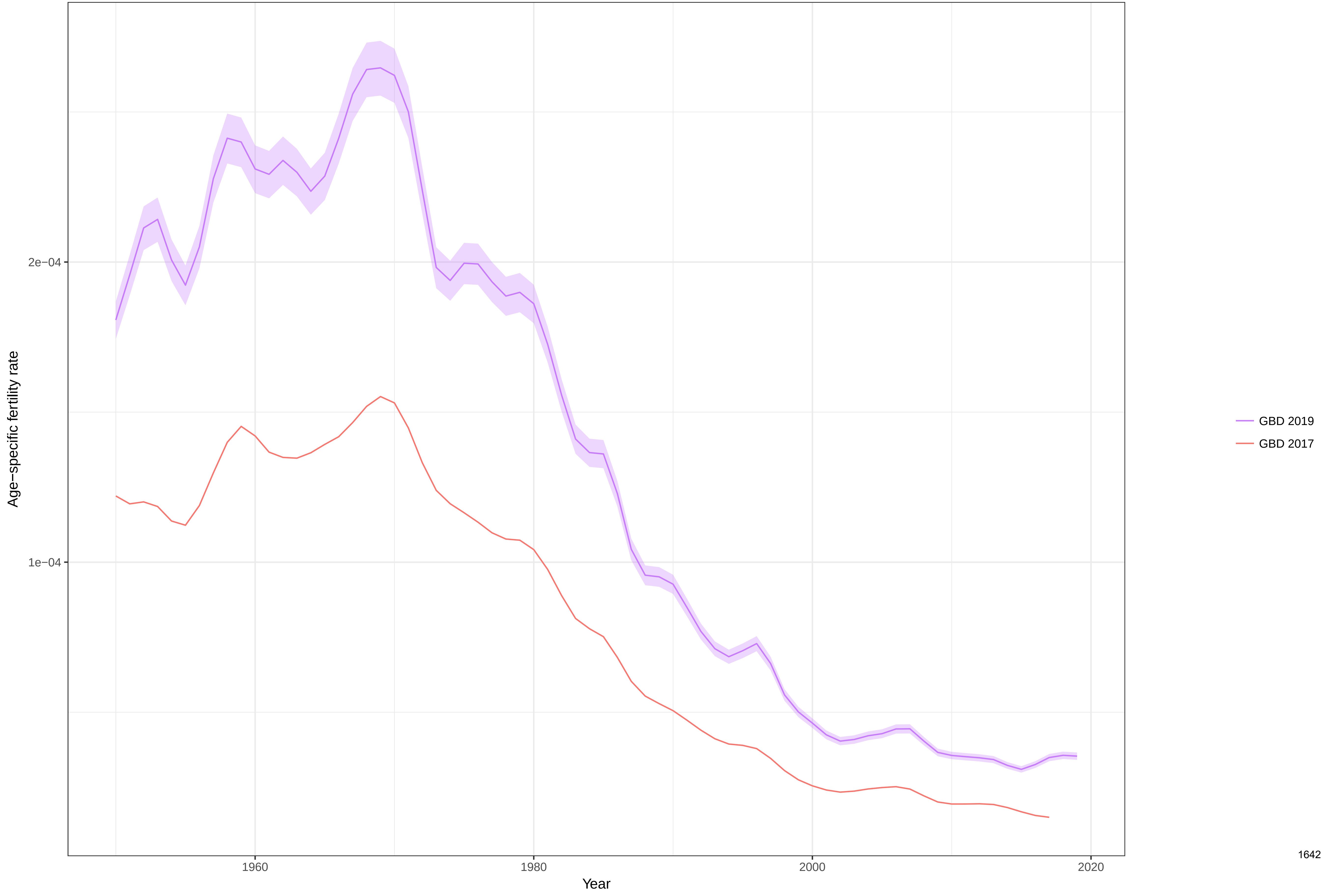

Total fertility rate fitted trend lines, 1950−2019: Panama TFR
Data points enclosed with a diamond are reference sources

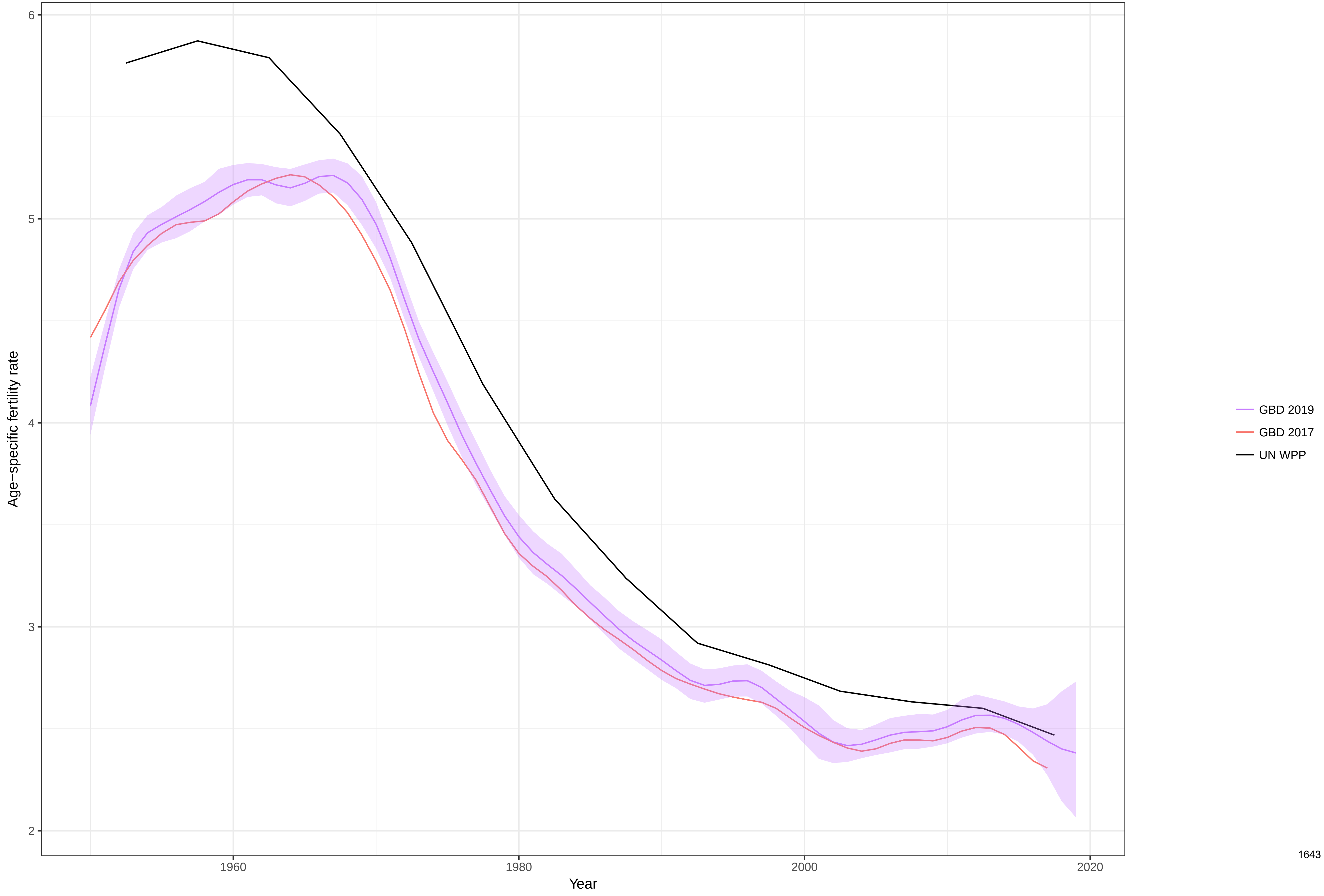

Age−specific fertility fitted trend lines, 1950−2019: Peru, Ages: 10−14
 Data points enclosed with a diamond are reference sources

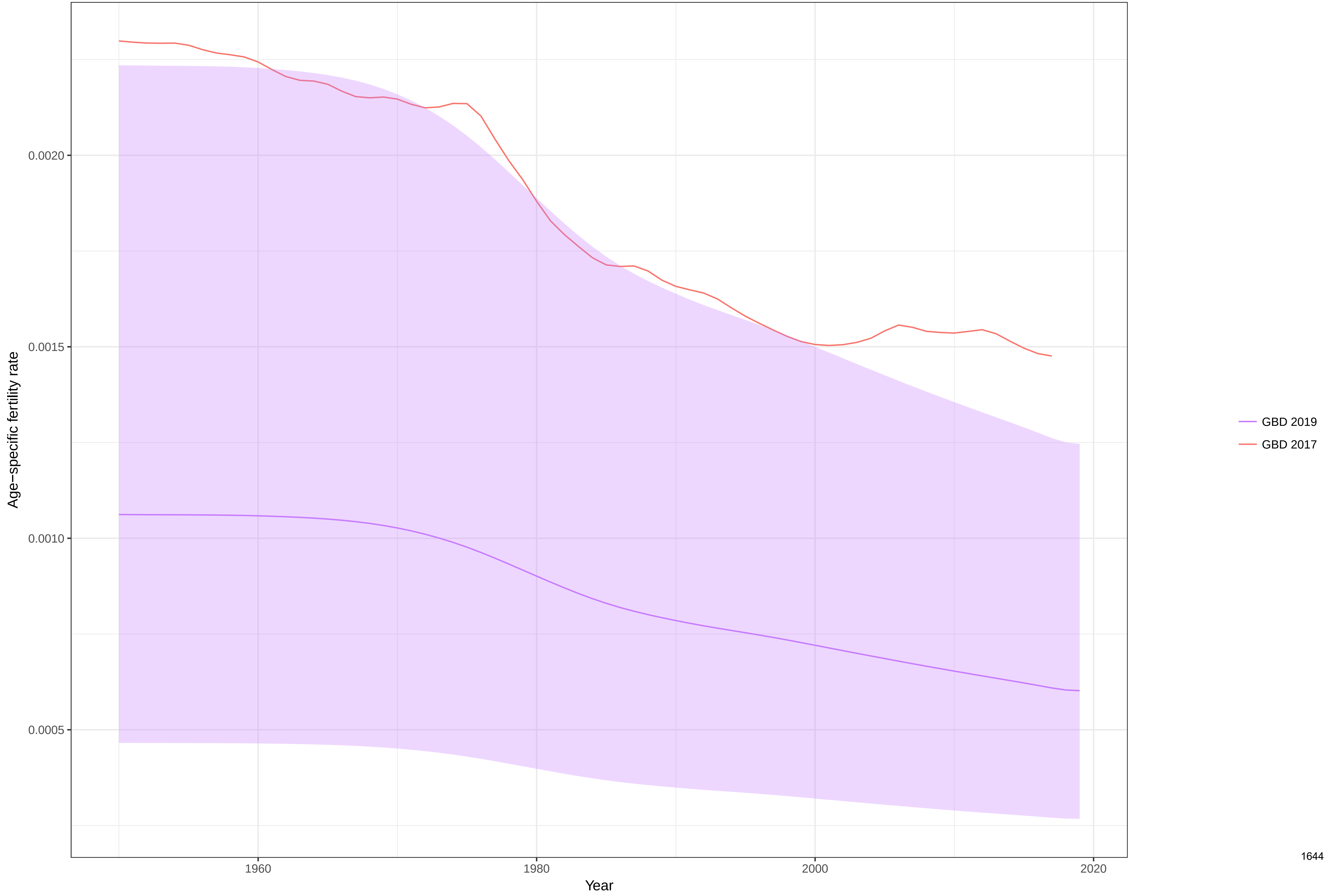

Age−specific fertility fitted trend lines, 1950−2019: Peru, Ages: 15−19
Data points enclosed with a diamond are reference sources

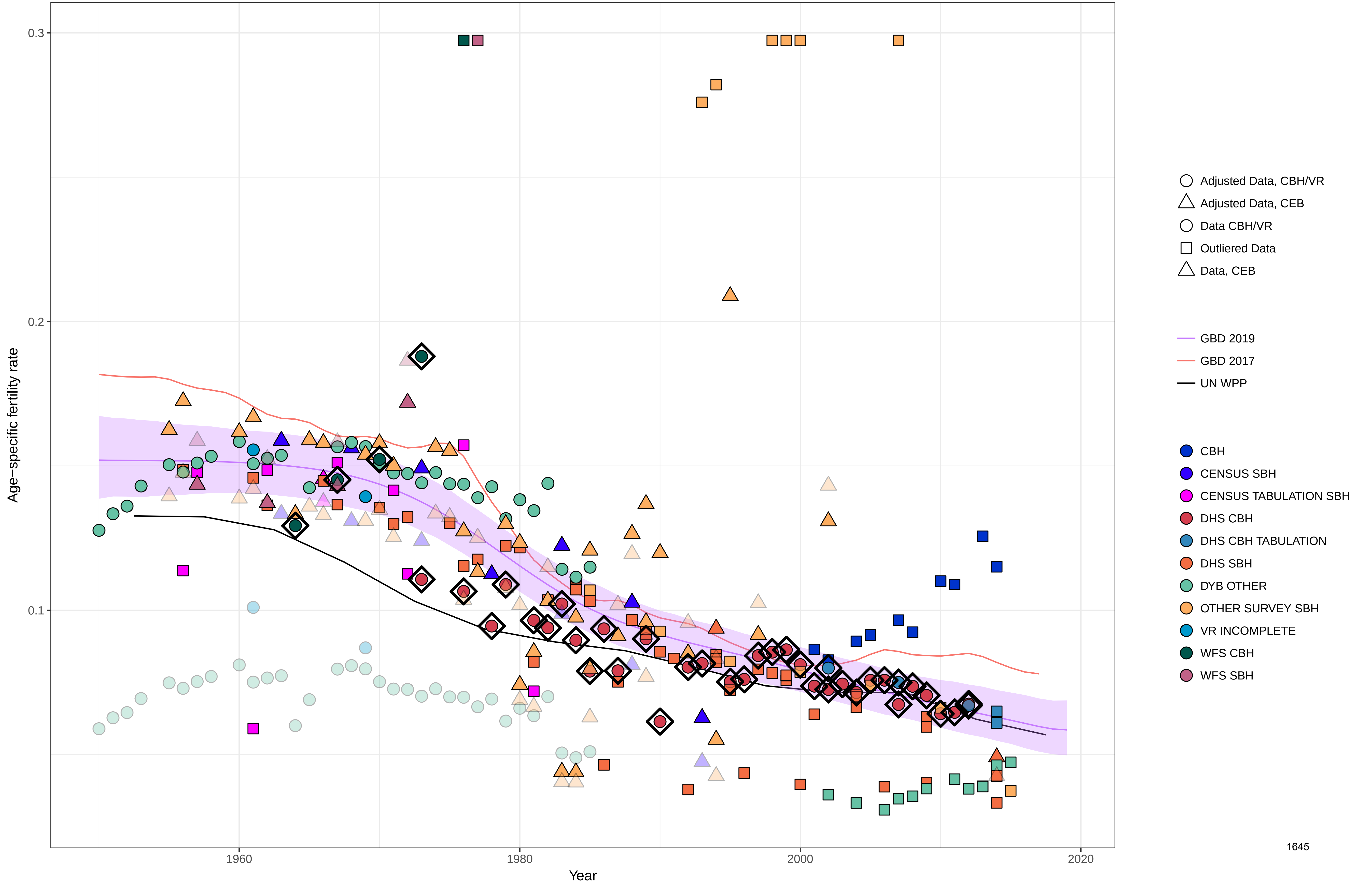

Age−specific fertility fitted trend lines, 1950−2019: Peru, Ages: 20−24
Data points enclosed with a diamond are reference sources

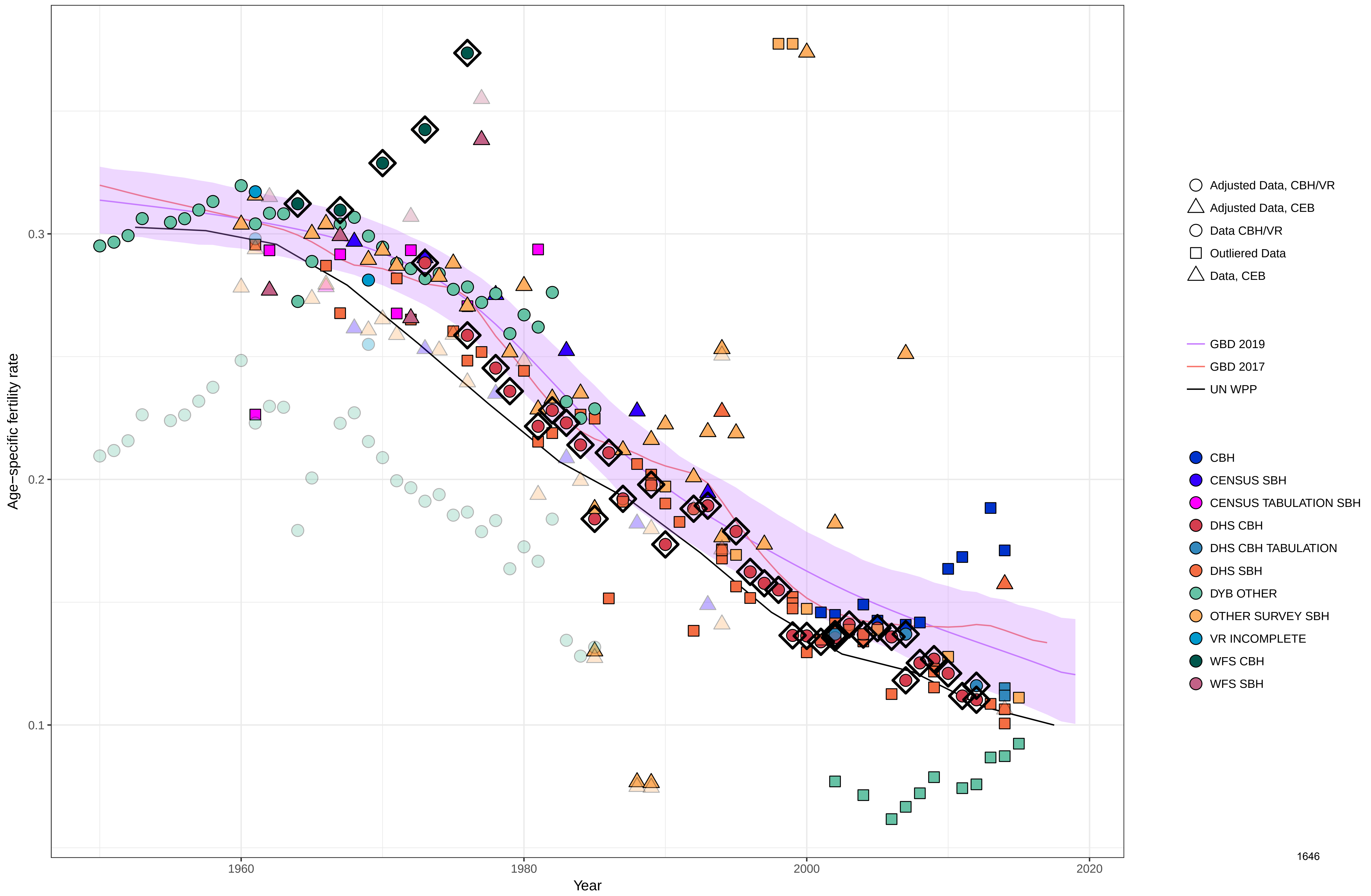

Age−specific fertility fitted trend lines, 1950−2019: Peru, Ages: 25−29
Data points enclosed with a diamond are reference sources

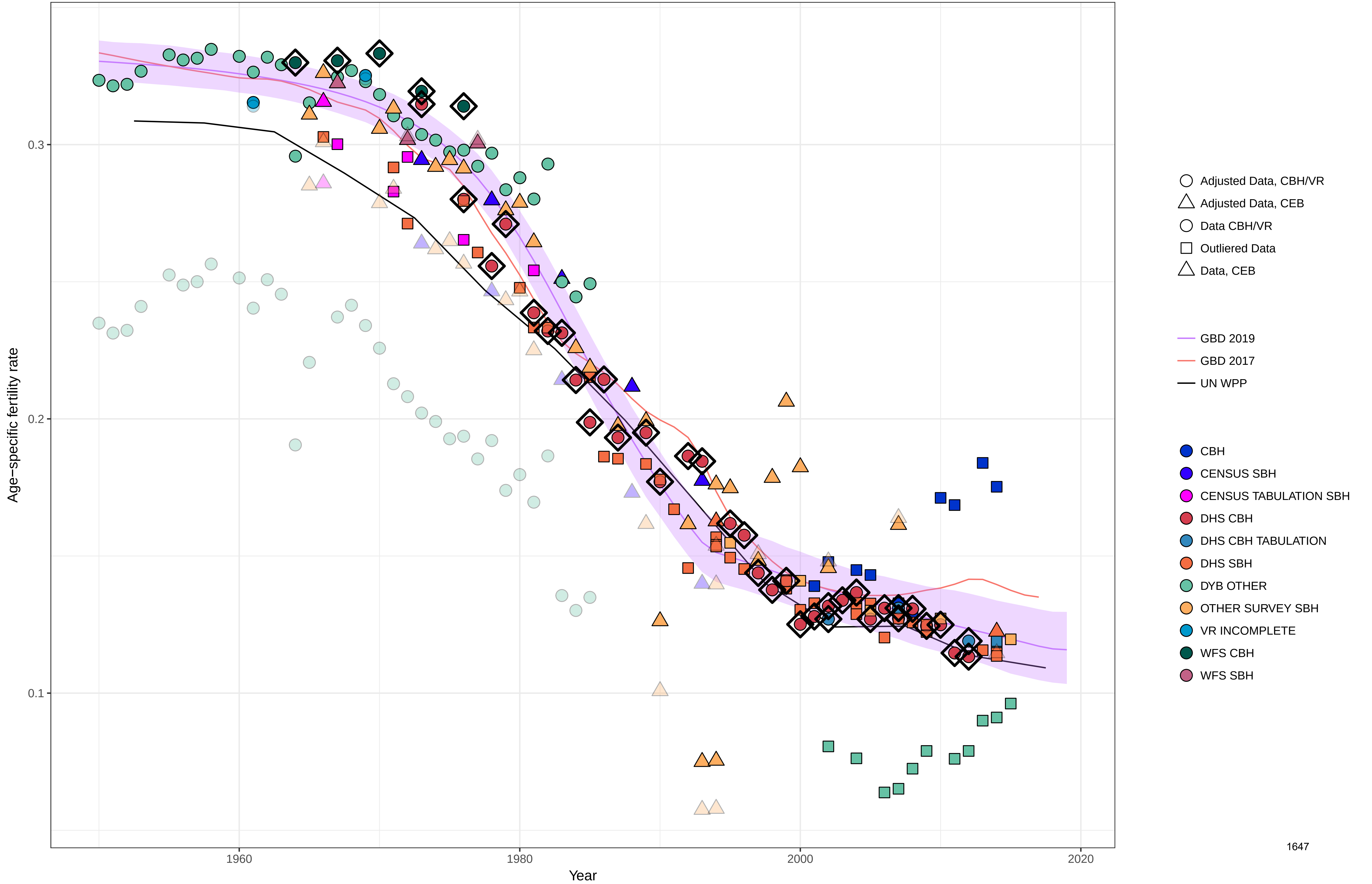

Age−specific fertility fitted trend lines, 1950−2019: Peru, Ages: 30−34
Data points enclosed with a diamond are reference sources

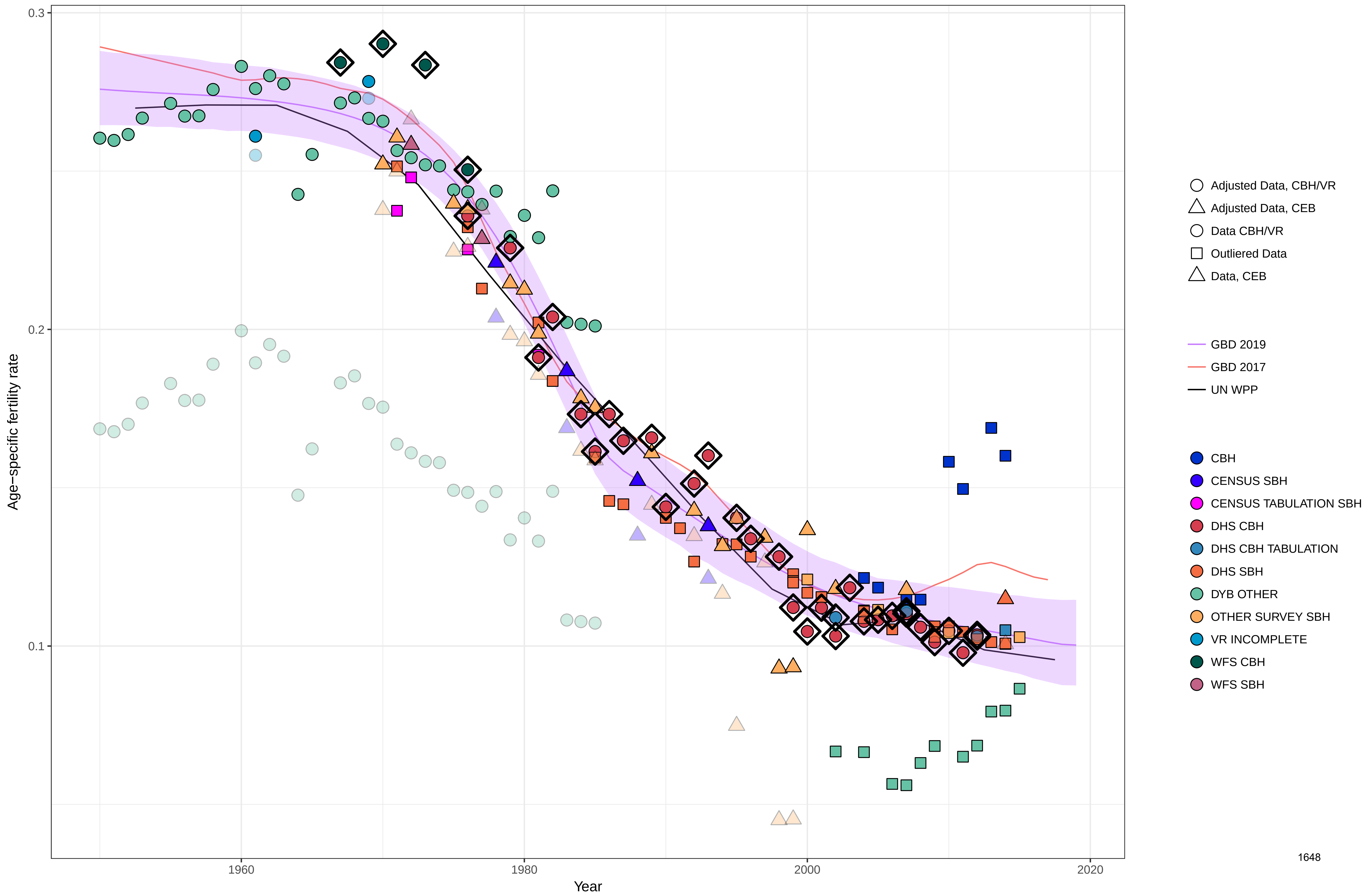

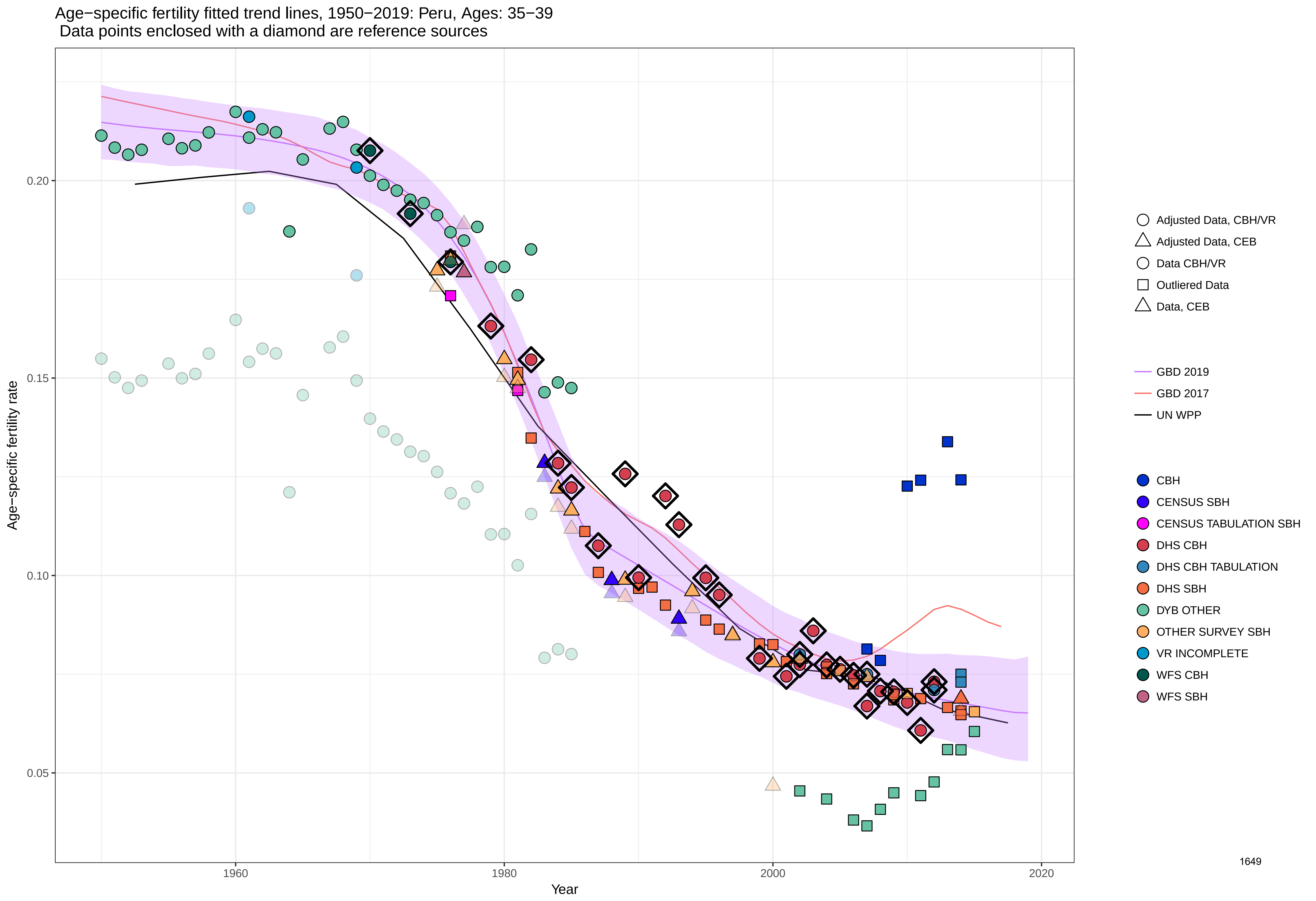
Age−specific fertility fitted trend lines, 1950−2019: Peru, Ages: 35−39
Data points enclosed with a diamond are reference sources
Age−specific fertility rate
0.20
0.15
0.10
0.05
1960
1980
2000
2020
Year
Adjusted Data, CBH/VR
Adjusted Data, CEB
Data CBH/VR
Outliered Data
Data, CEB
GBD 2019
GBD 2017
UN WPP
CBH
CENSUS SBH
CENSUS TABULATION SBH
DHS CBH
DHS CBH TABULATION
DHS SBH
DYB OTHER
OTHER SURVEY SBH
VR INCOMPLETE
WFS CBH
WFS SBH

Age−specific fertility fitted trend lines, 1950−2019: Peru, Ages: 40−44
Data points enclosed with a diamond are reference sources

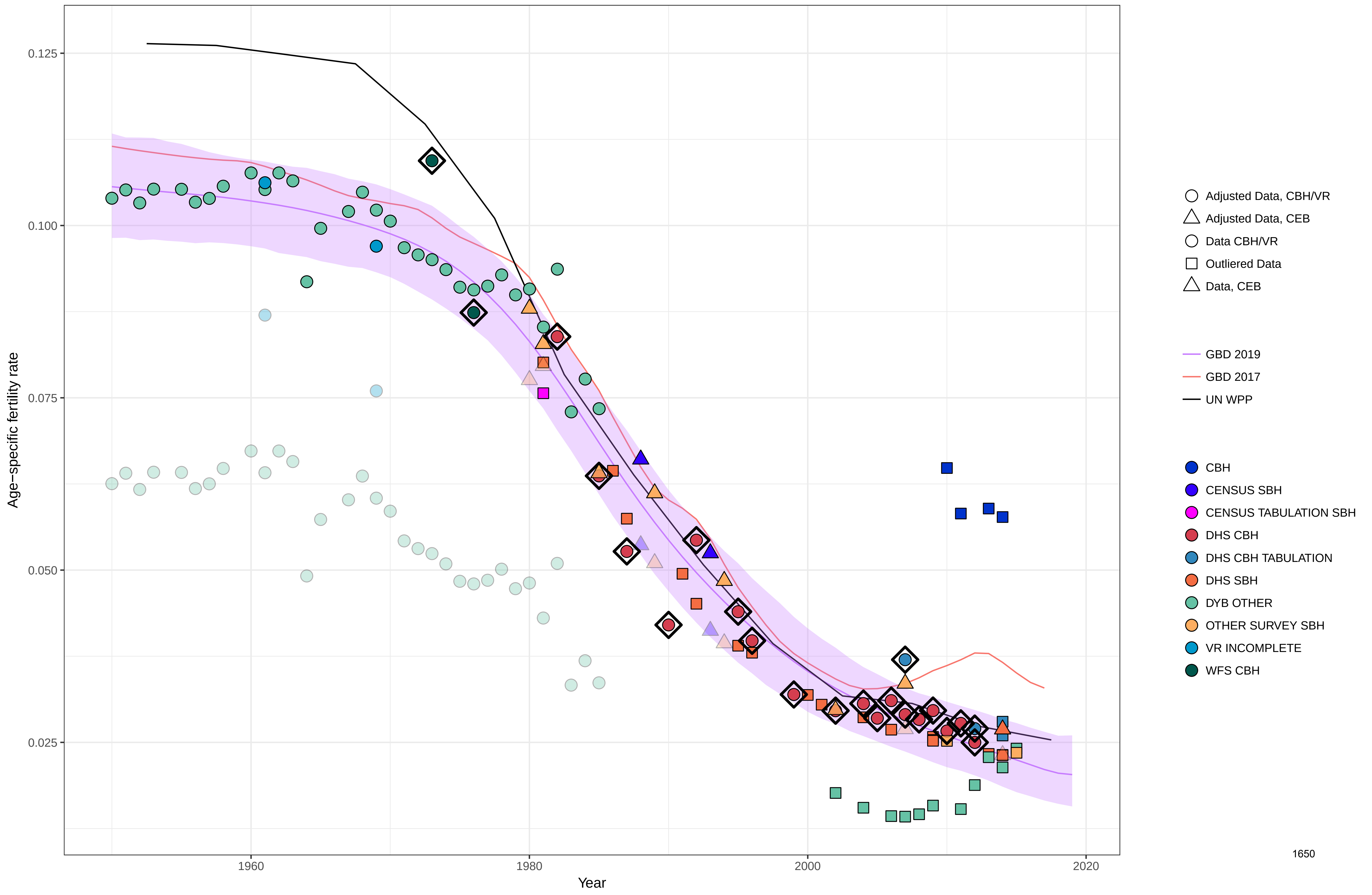

Age−specific fertility fitted trend lines, 1950−2019: Peru, Ages: 45−49
Data points enclosed with a diamond are reference sources

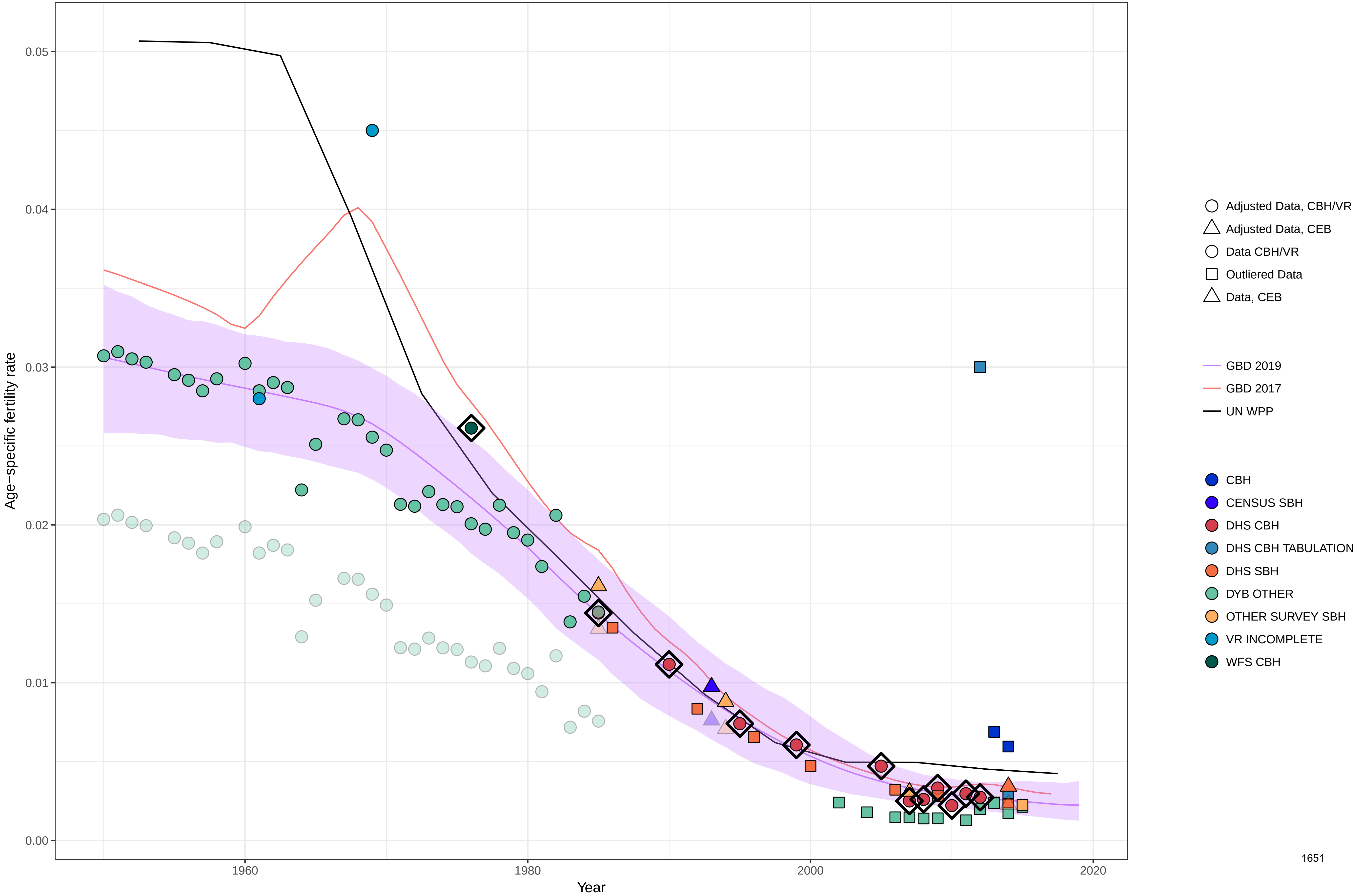

Age−specific fertility fitted trend lines, 1950−2019: Peru, Ages: 50−54
Data points enclosed with a diamond are reference sources

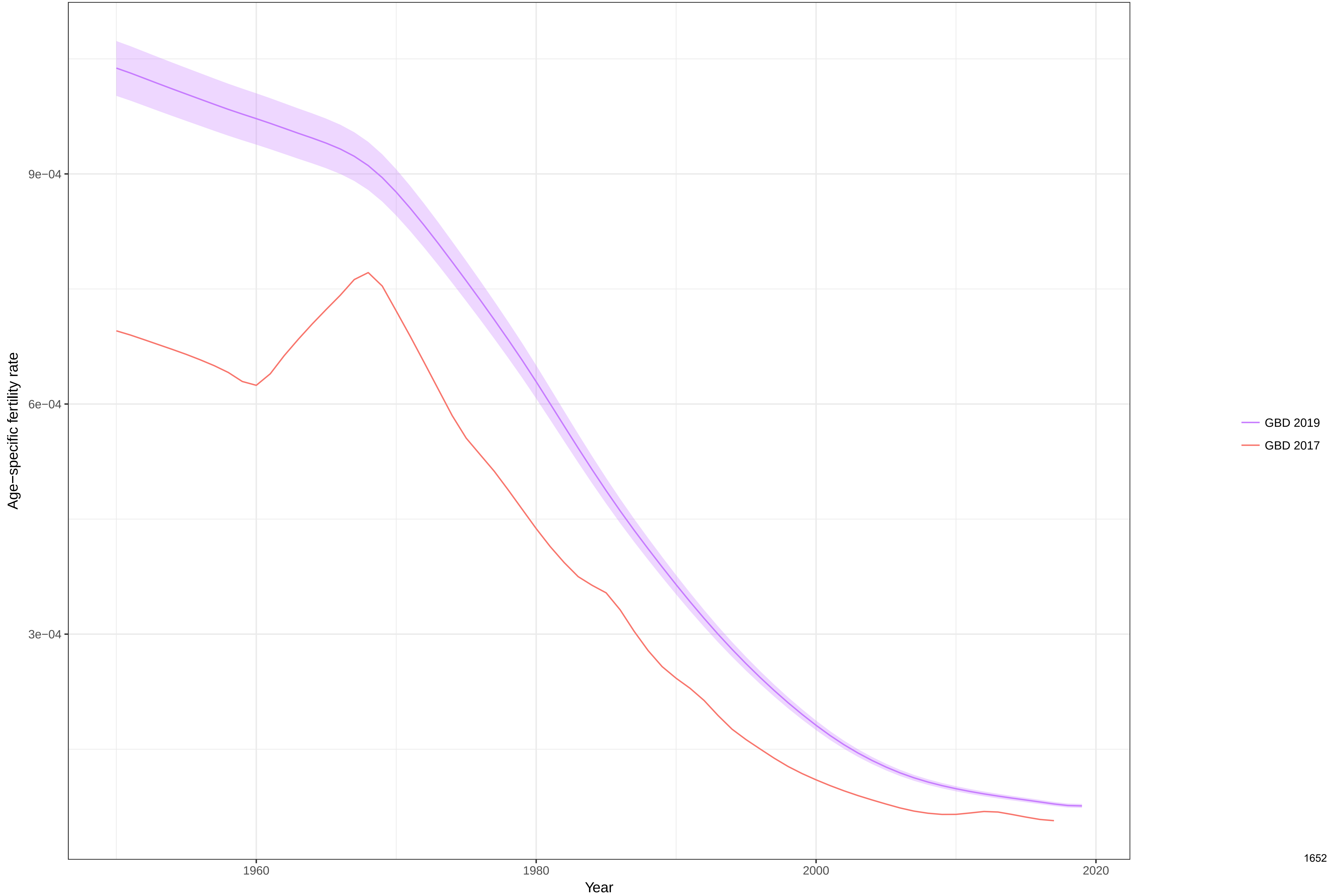

Total fertility rate fitted trend lines, 1950−2019: Peru TFR
Data points enclosed with a diamond are reference sources

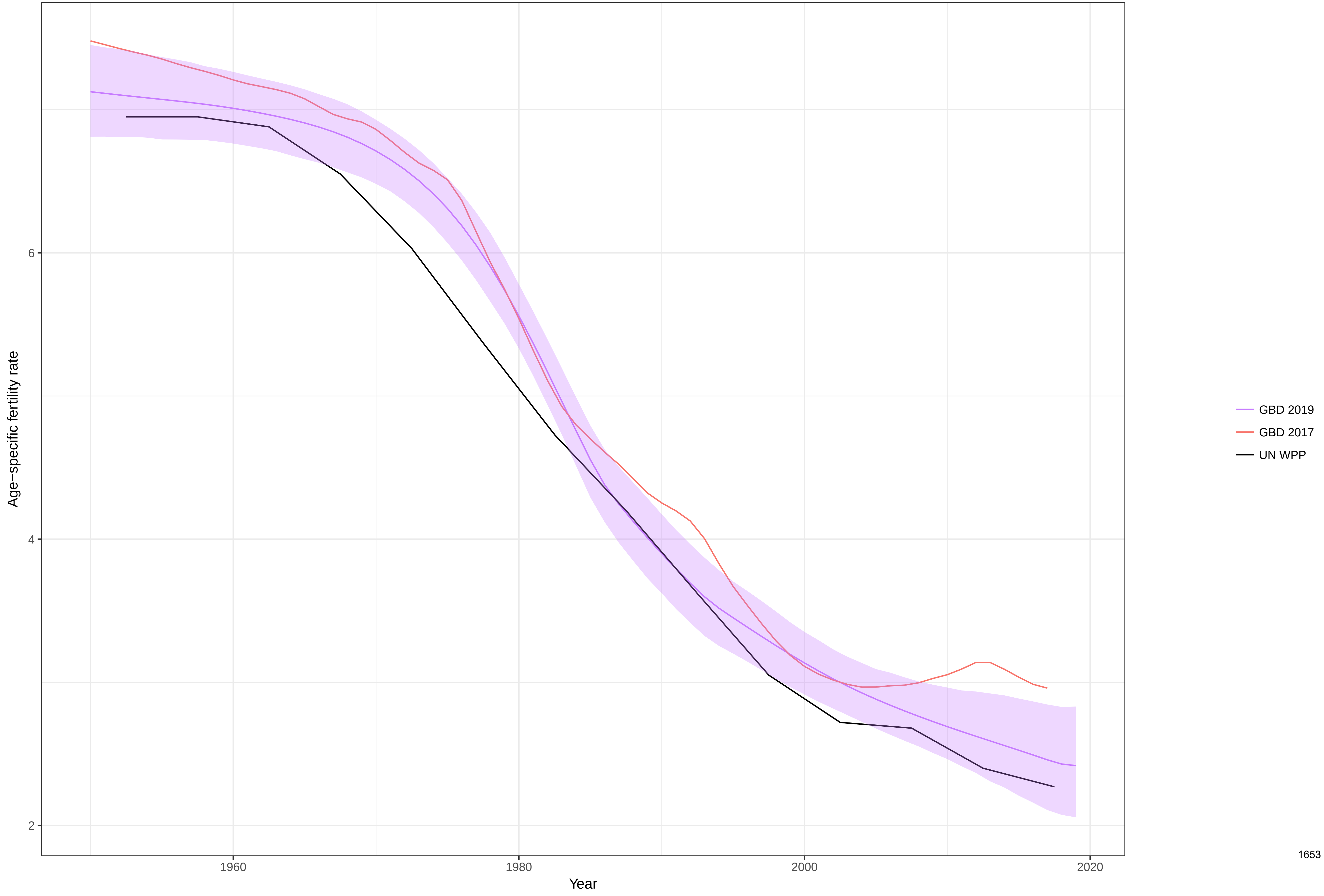

Age−specific fertility fitted trend lines, 1950−2019: Philippines, Ages: 10−14
Data points enclosed with a diamond are reference sources

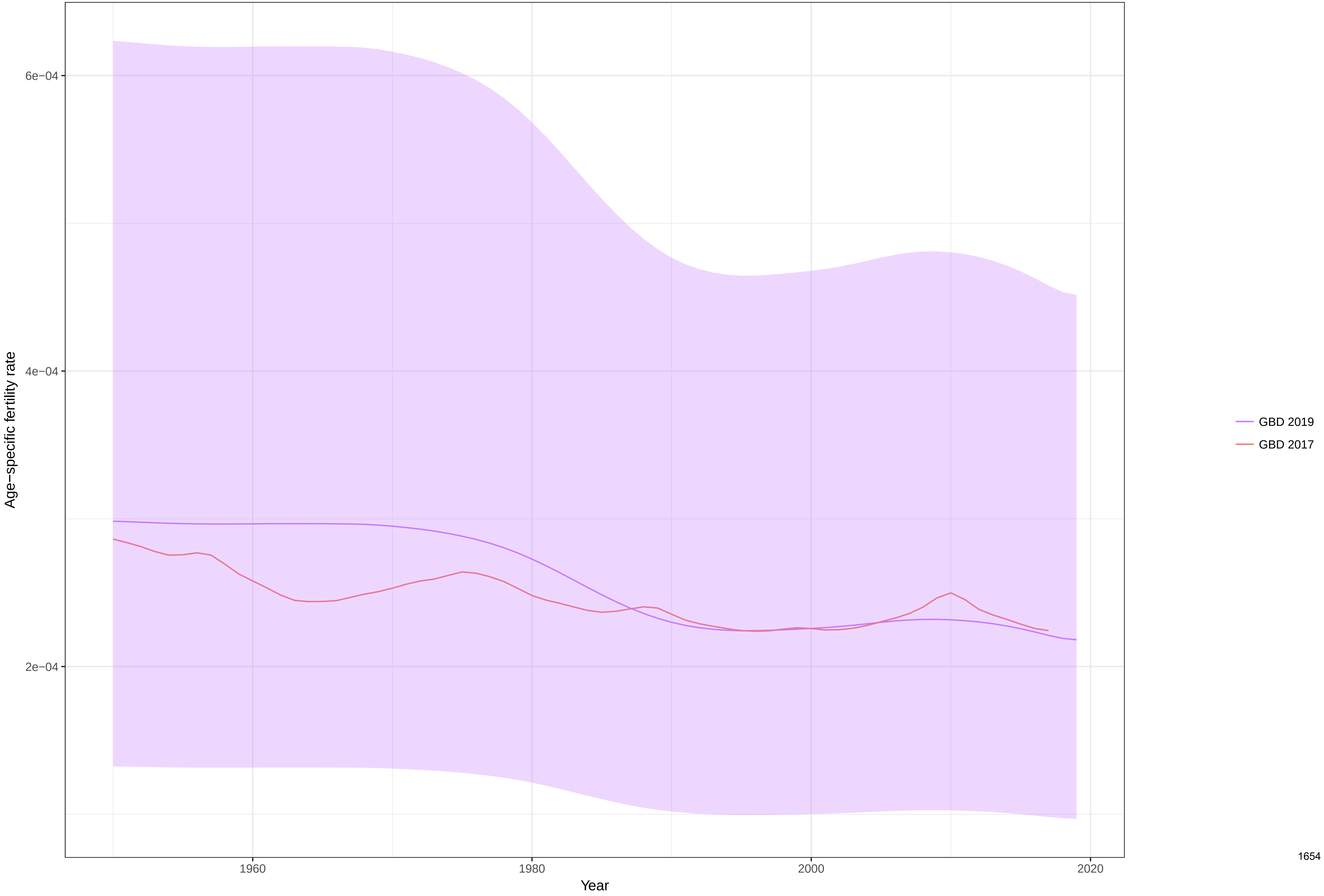

Age−specific fertility fitted trend lines, 1950−2019: Philippines, Ages: 15−19
Data points enclosed with a diamond are reference sources

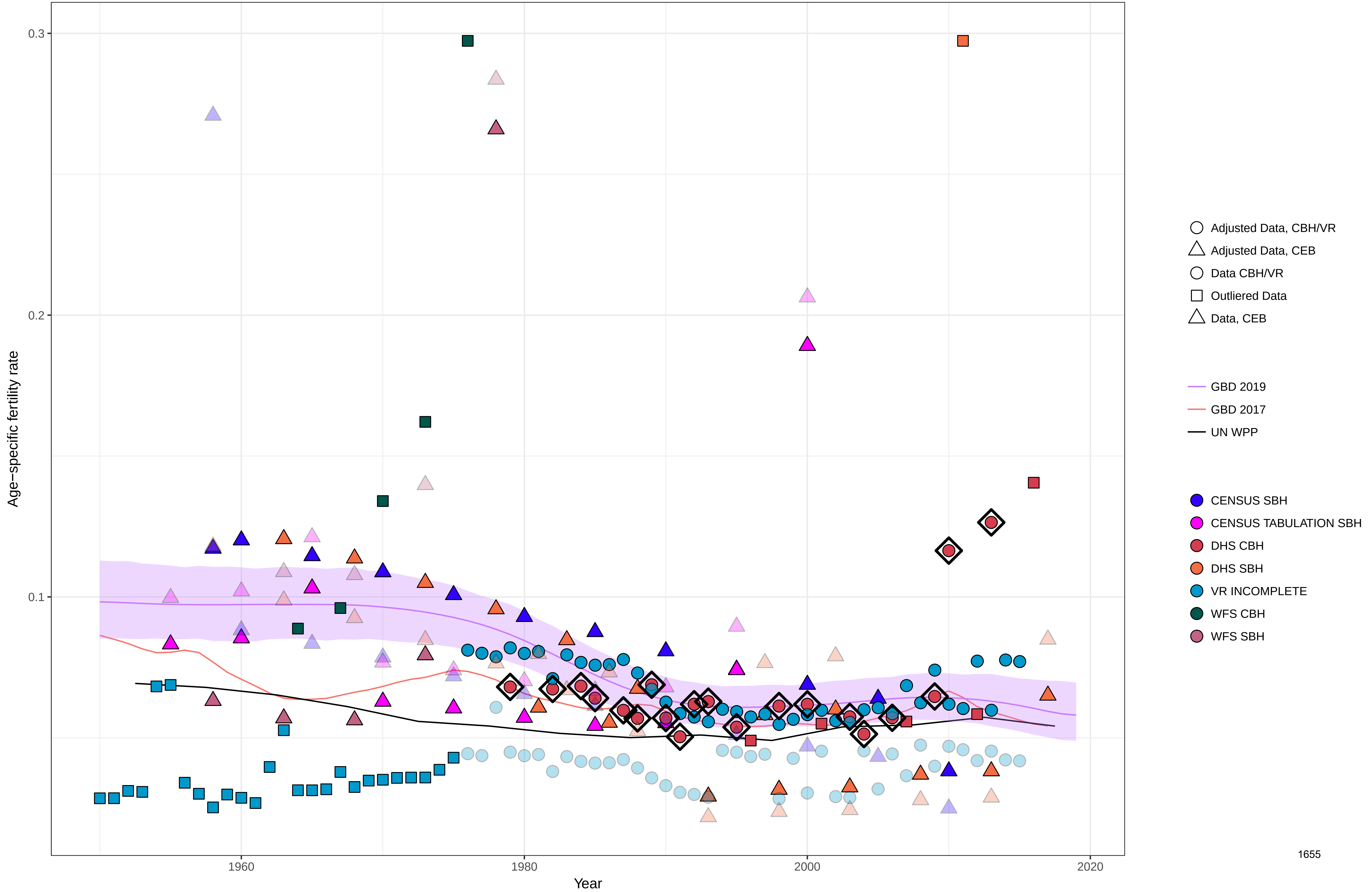

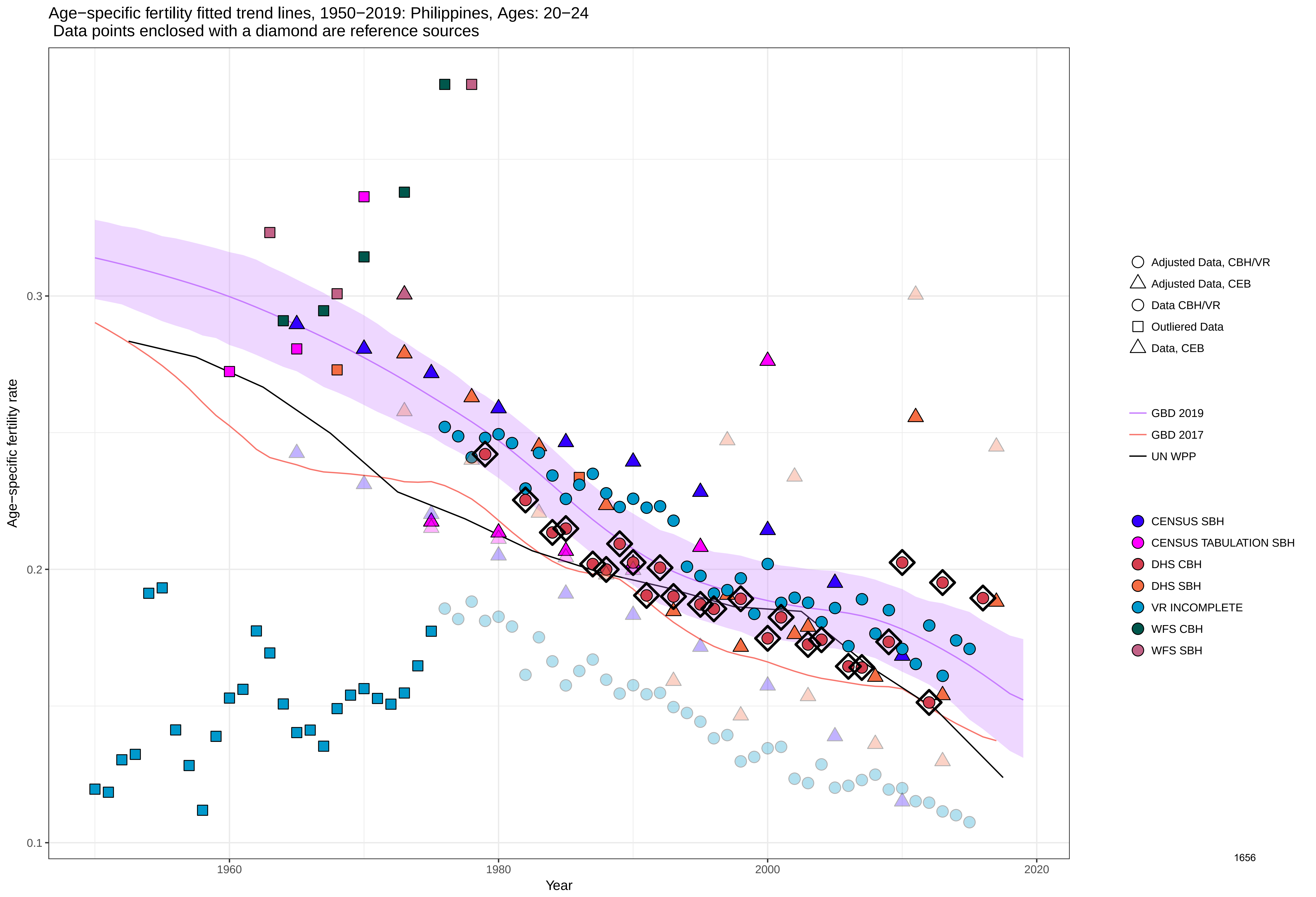

Age−specific fertility fitted trend lines, 1950−2019: Philippines, Ages: 20−24
Data points enclosed with a diamond are reference sources
Age−specific fertility rate
0.1
0.2
0.3
Year
1960
1980
2000
2020
Adjusted Data, CBH/VR
Adjusted Data, CEB
Data CBH/VR
Outliered Data
Data, CEB
GBD 2019
GBD 2017
UN WPP
CENSUS SBH
CENSUS TABULATION SBH
DHS CBH
DHS SBH
VR INCOMPLETE
WFS CBH
WFS SBH

Age−specific fertility fitted trend lines, 1950−2019: Philippines, Ages: 25−29
Data points enclosed with a diamond are reference sources

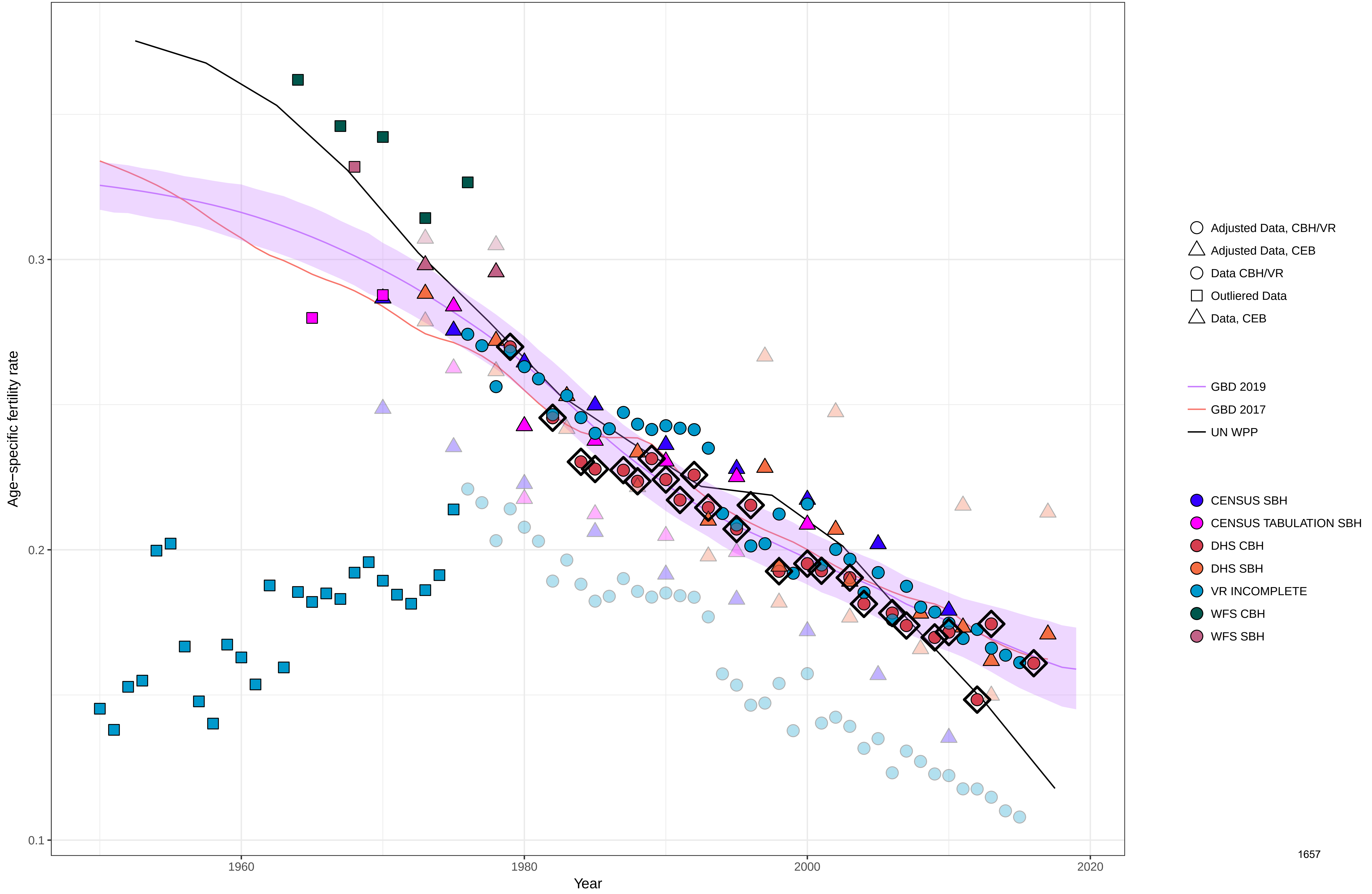

Age−specific fertility fitted trend lines, 1950−2019: Philippines, Ages: 30−34
Data points enclosed with a diamond are reference sources

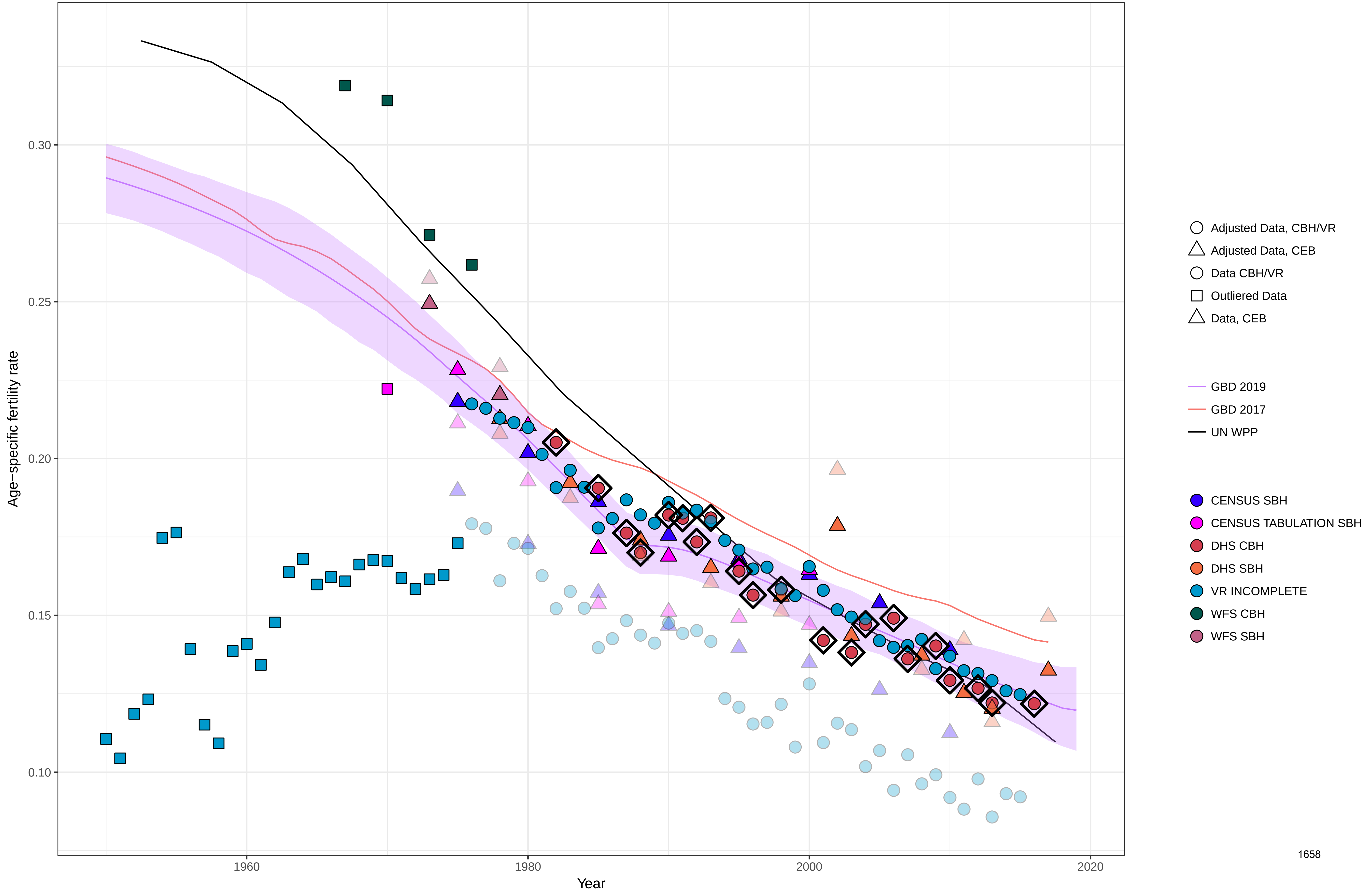

Age−specific fertility fitted trend lines, 1950−2019: Philippines, Ages: 35−39
Data points enclosed with a diamond are reference sources

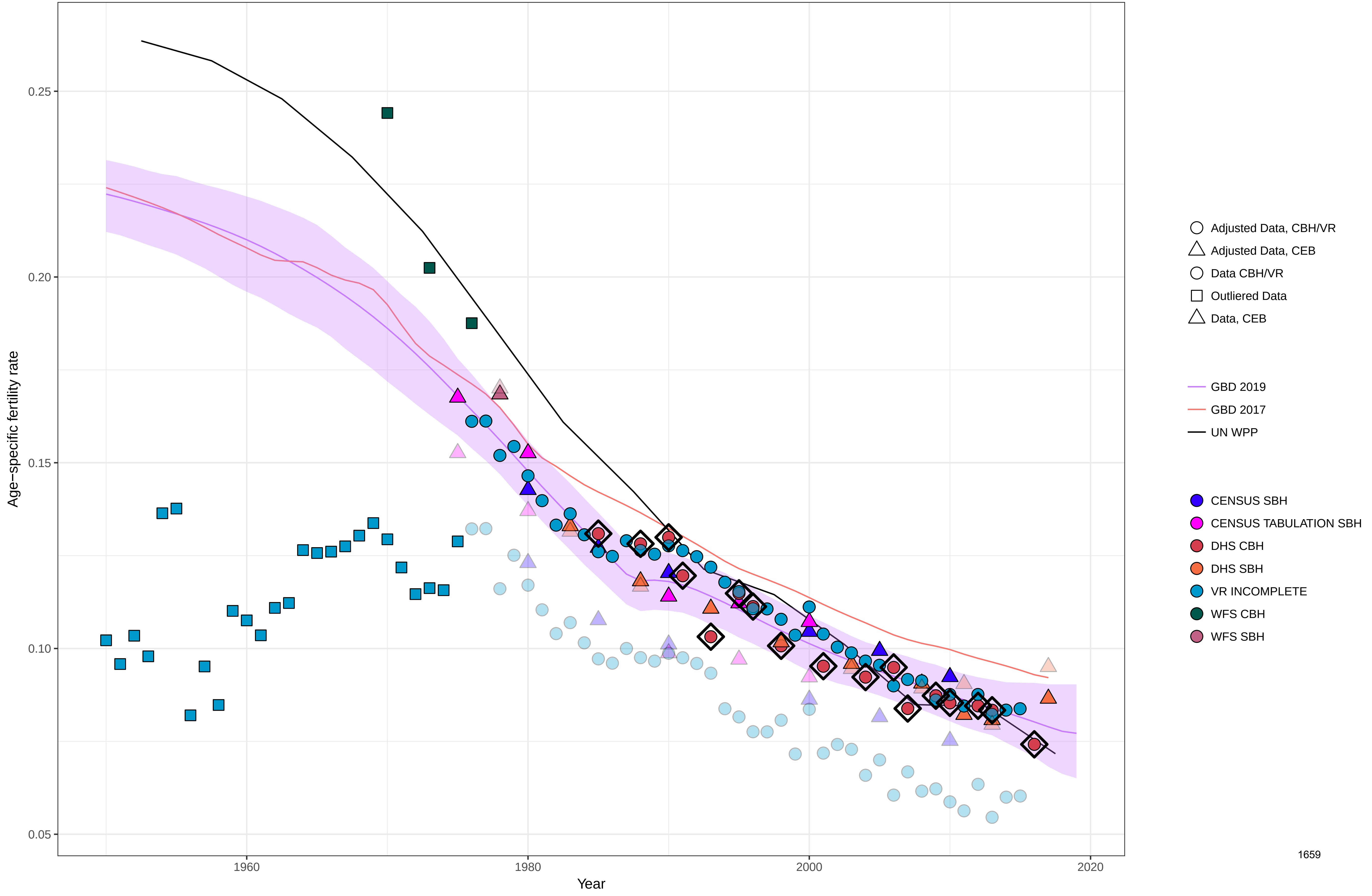

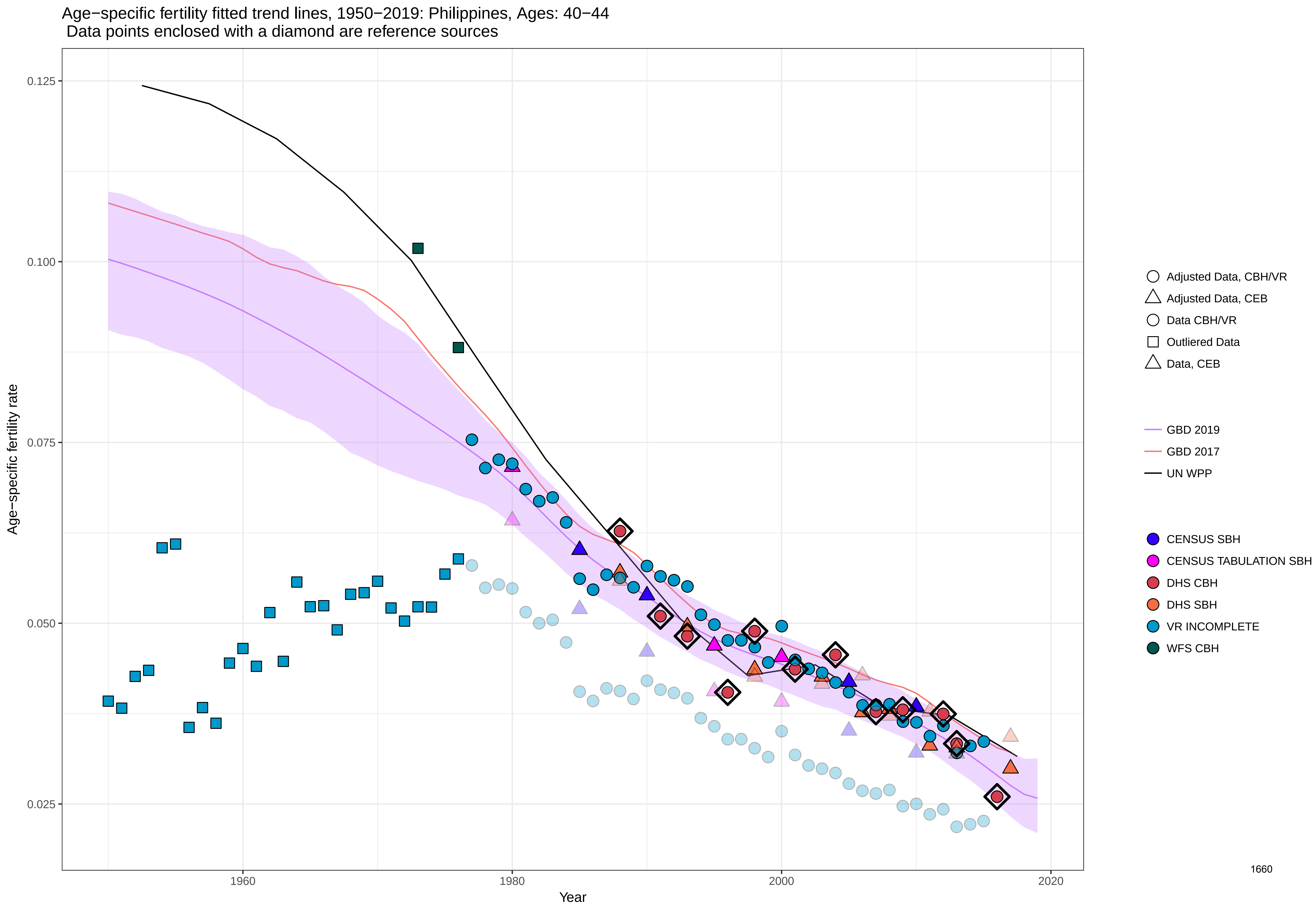

Age−specific fertility fitted trend lines, 1950−2019: Philippines, Ages: 40−44
Data points enclosed with a diamond are reference sources
Age−specific fertility rate
0.125
0.100
0.075
0.050
0.025
Year
1960
1980
2000
2020
Adjusted Data, CBH/VR
Adjusted Data, CEB
Data CBH/VR
Outliered Data
Data, CEB
GBD 2019
GBD 2017
UN WPP
CENSUS SBH
CENSUS TABULATION SBH
DHS CBH
DHS SBH
VR INCOMPLETE
WFS CBH

Age−specific fertility fitted trend lines, 1950−2019: Philippines, Ages: 45−49
Data points enclosed with a diamond are reference sources

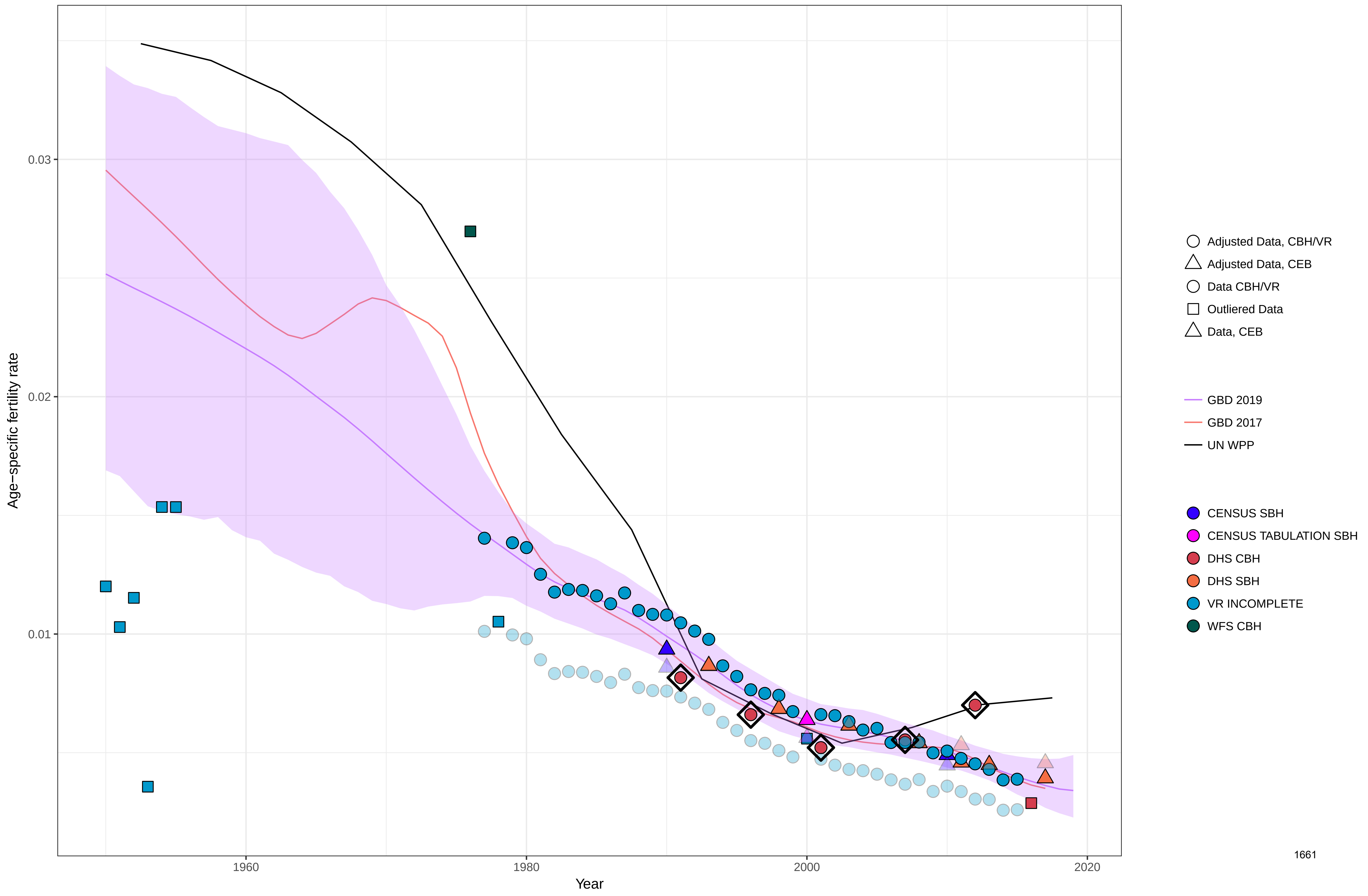

Age−specific fertility fitted trend lines, 1950−2019: Philippines, Ages: 50−54
Data points enclosed with a diamond are reference sources

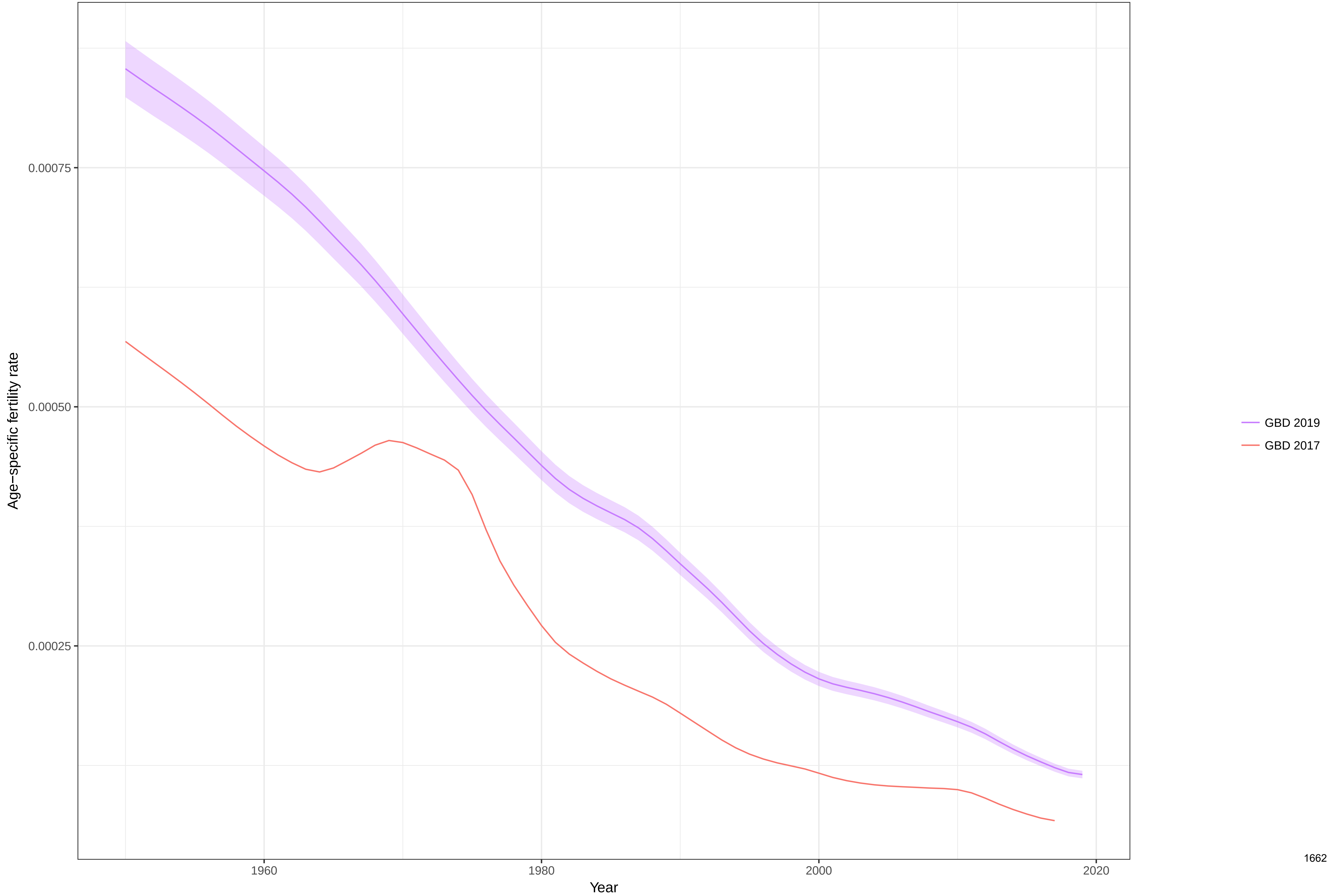

Total fertility rate fitted trend lines, 1950−2019: Philippines TFR
Data points enclosed with a diamond are reference sources

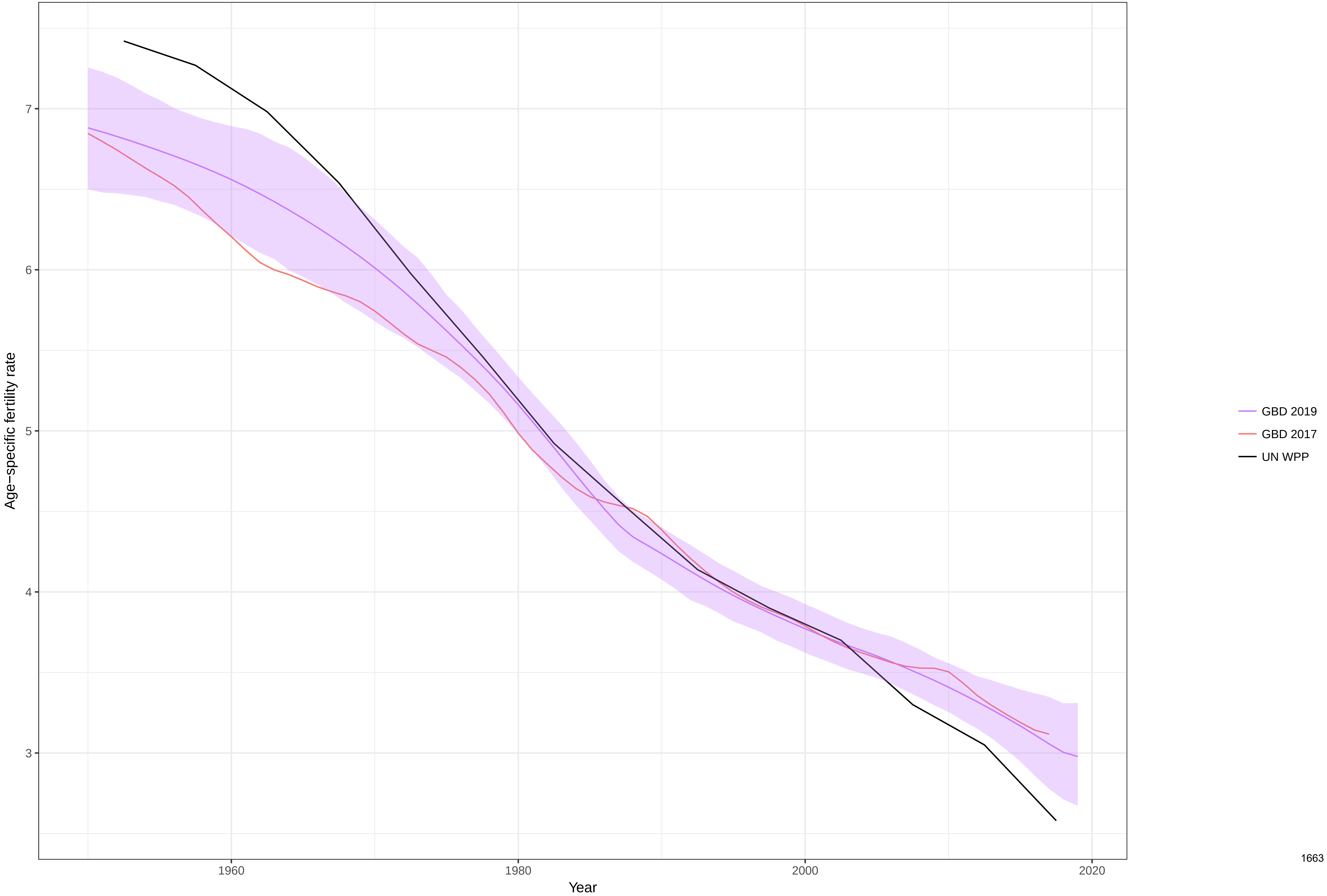

Age−specific fertility fitted trend lines, 1950−2019: Palau, Ages: 10−14
Data points enclosed with a diamond are reference sources

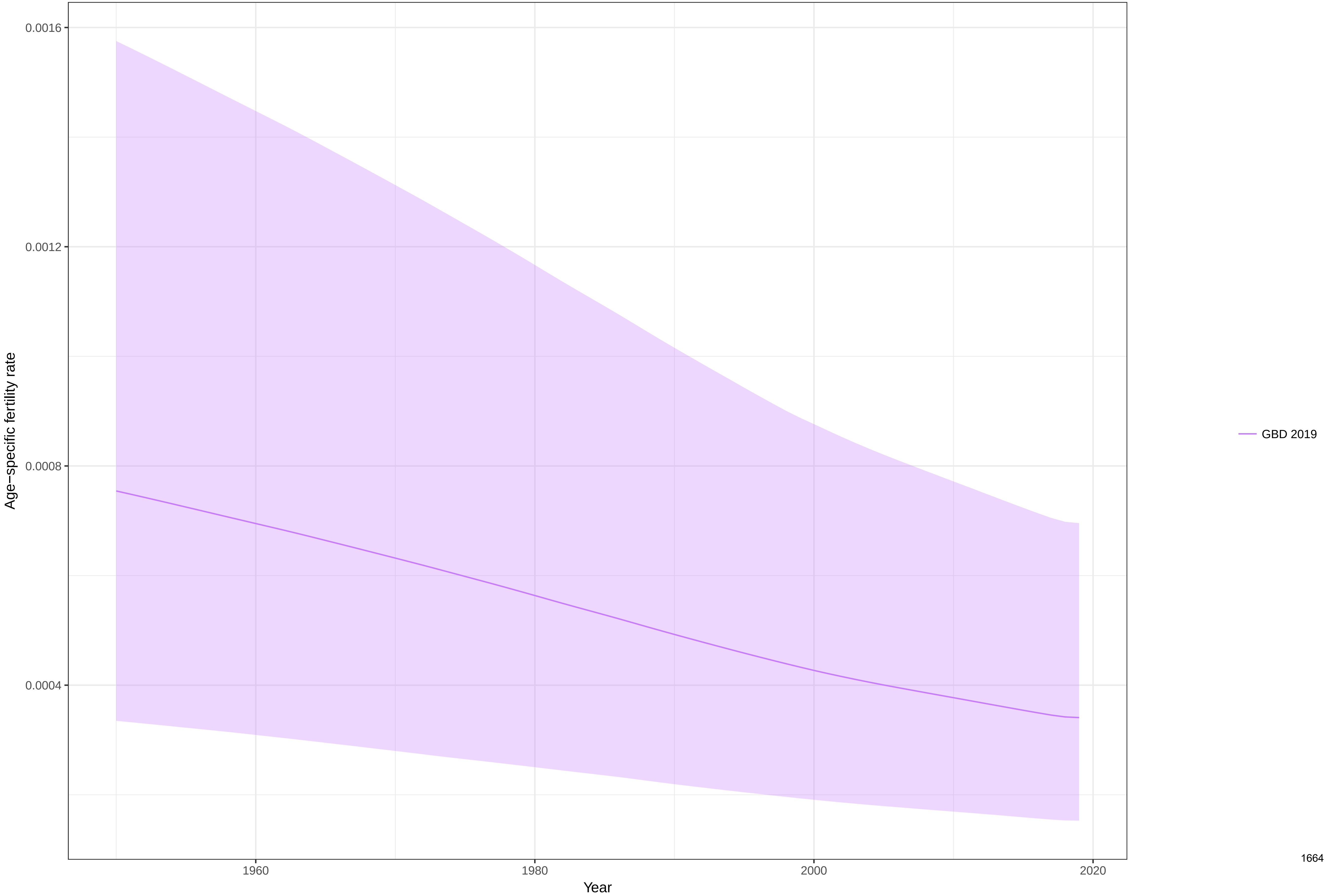

Age−specific fertility fitted trend lines, 1950−2019: Palau, Ages: 15−19
Data points enclosed with a diamond are reference sources

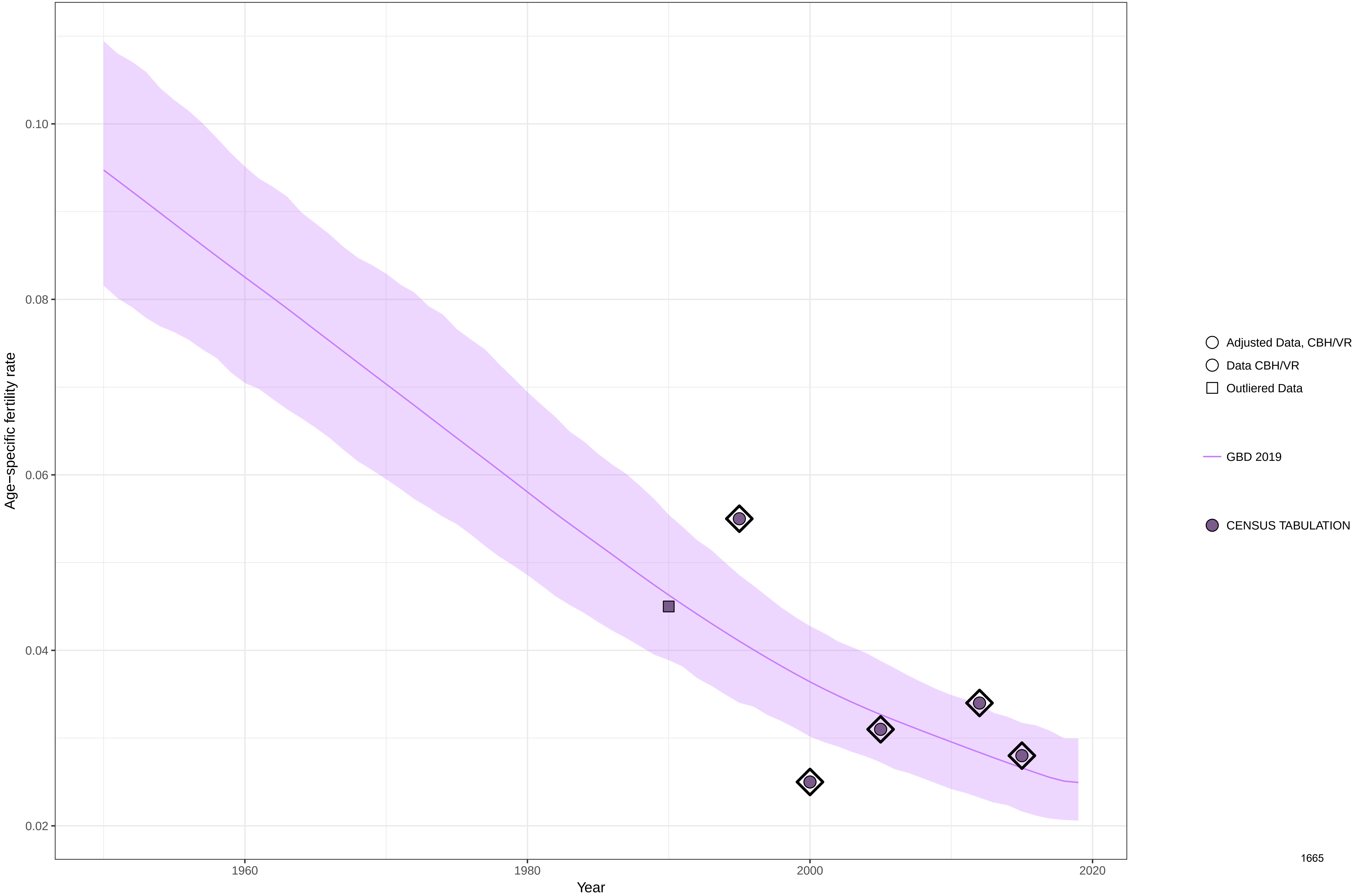

Age−specific fertility fitted trend lines, 1950−2019: Palau, Ages: 20−24
Data points enclosed with a diamond are reference sources

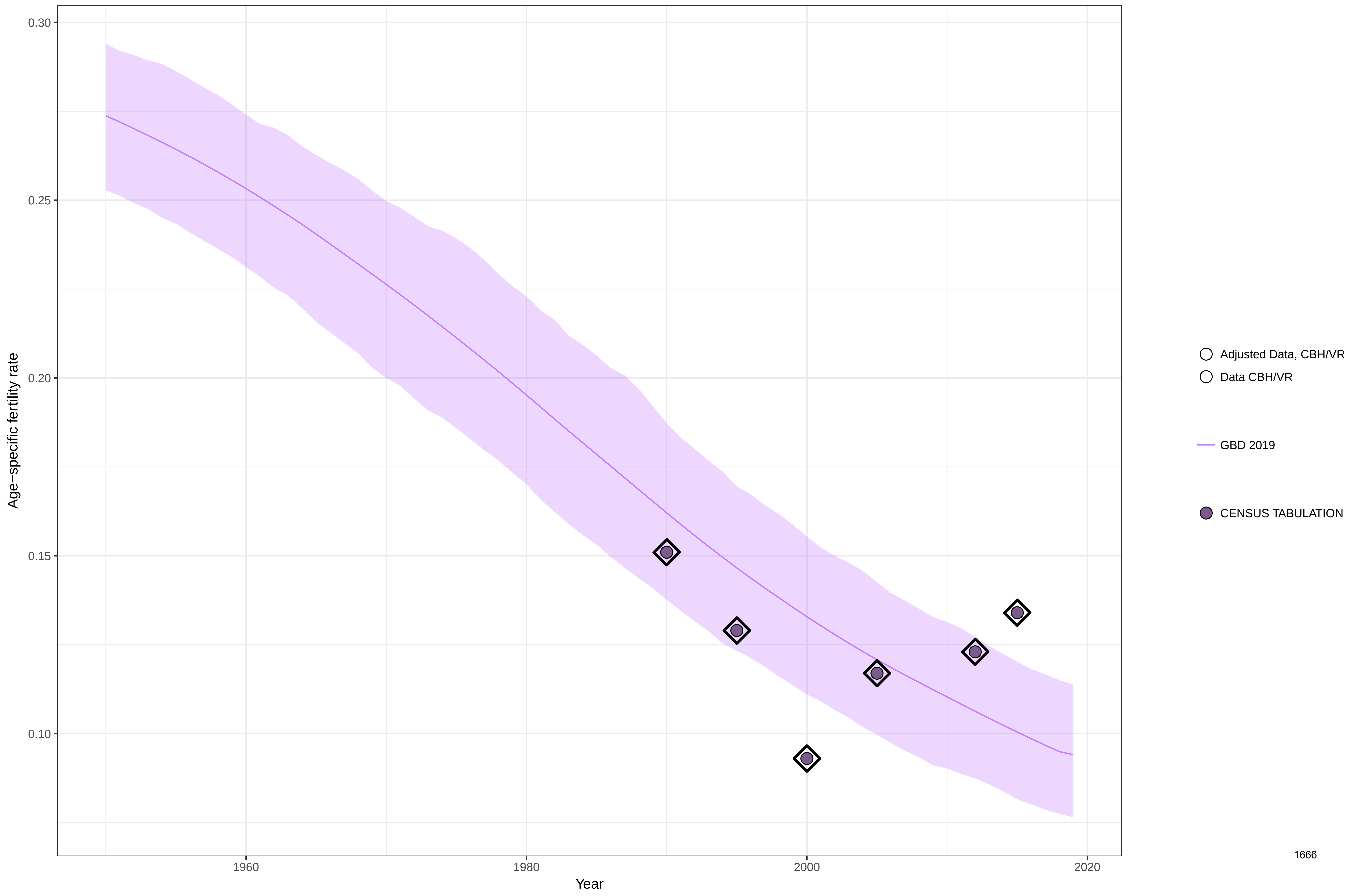

Age−specific fertility fitted trend lines, 1950−2019: Palau, Ages: 25−29
Data points enclosed with a diamond are reference sources

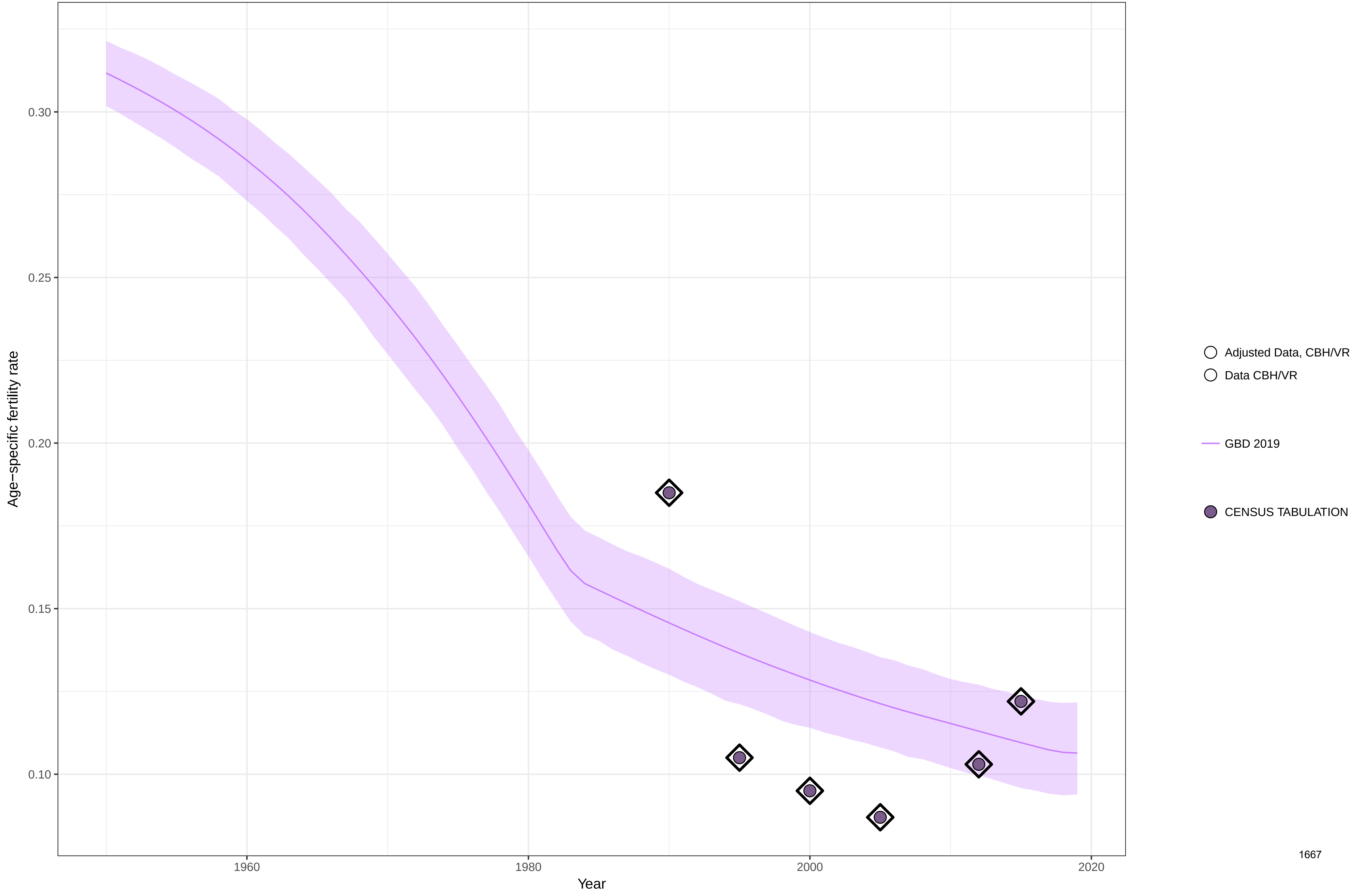

Age−specific fertility fitted trend lines, 1950−2019: Palau, Ages: 30−34
Data points enclosed with a diamond are reference sources

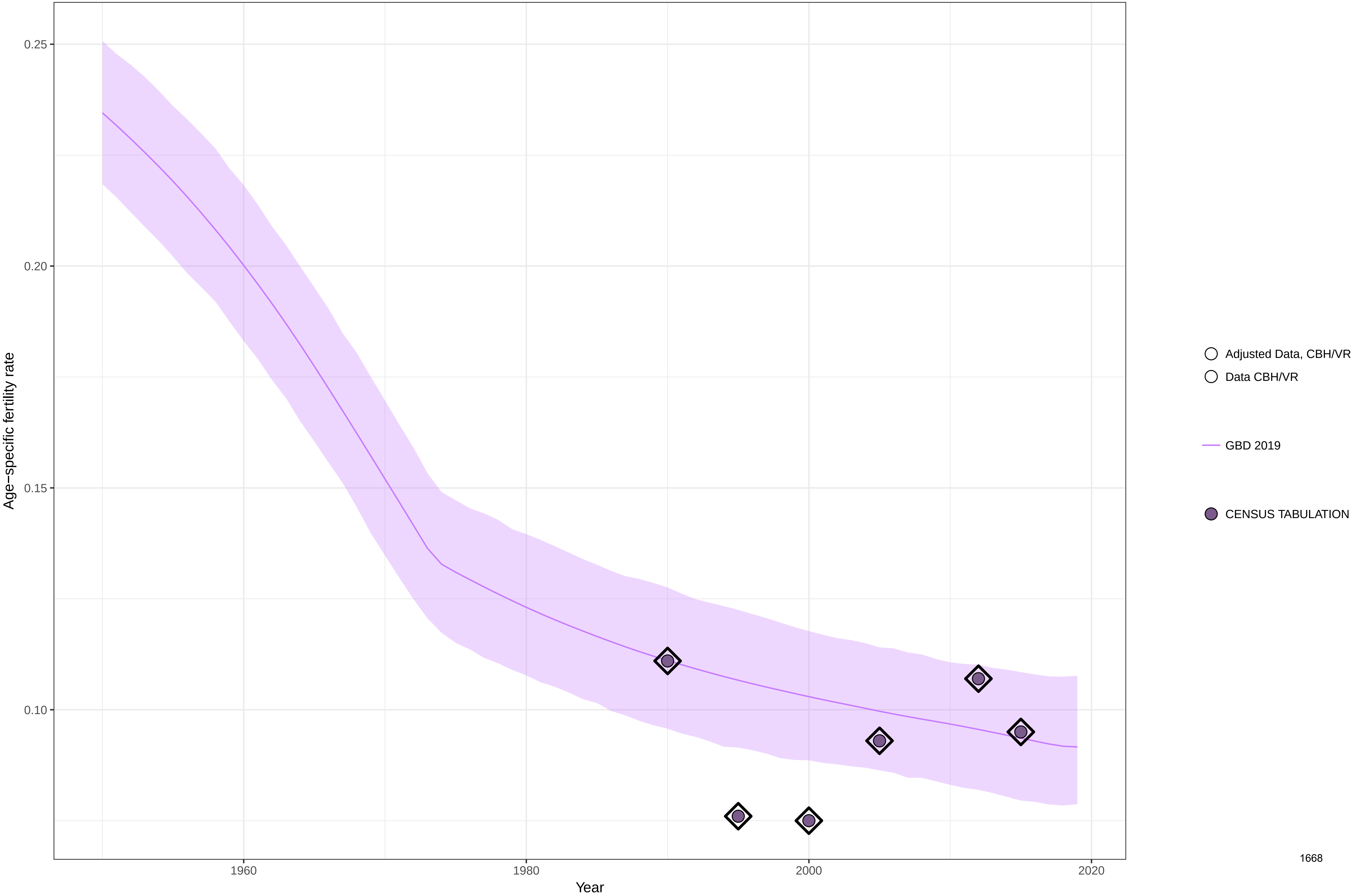

Age−specific fertility fitted trend lines, 1950−2019: Palau, Ages: 35−39
Data points enclosed with a diamond are reference sources

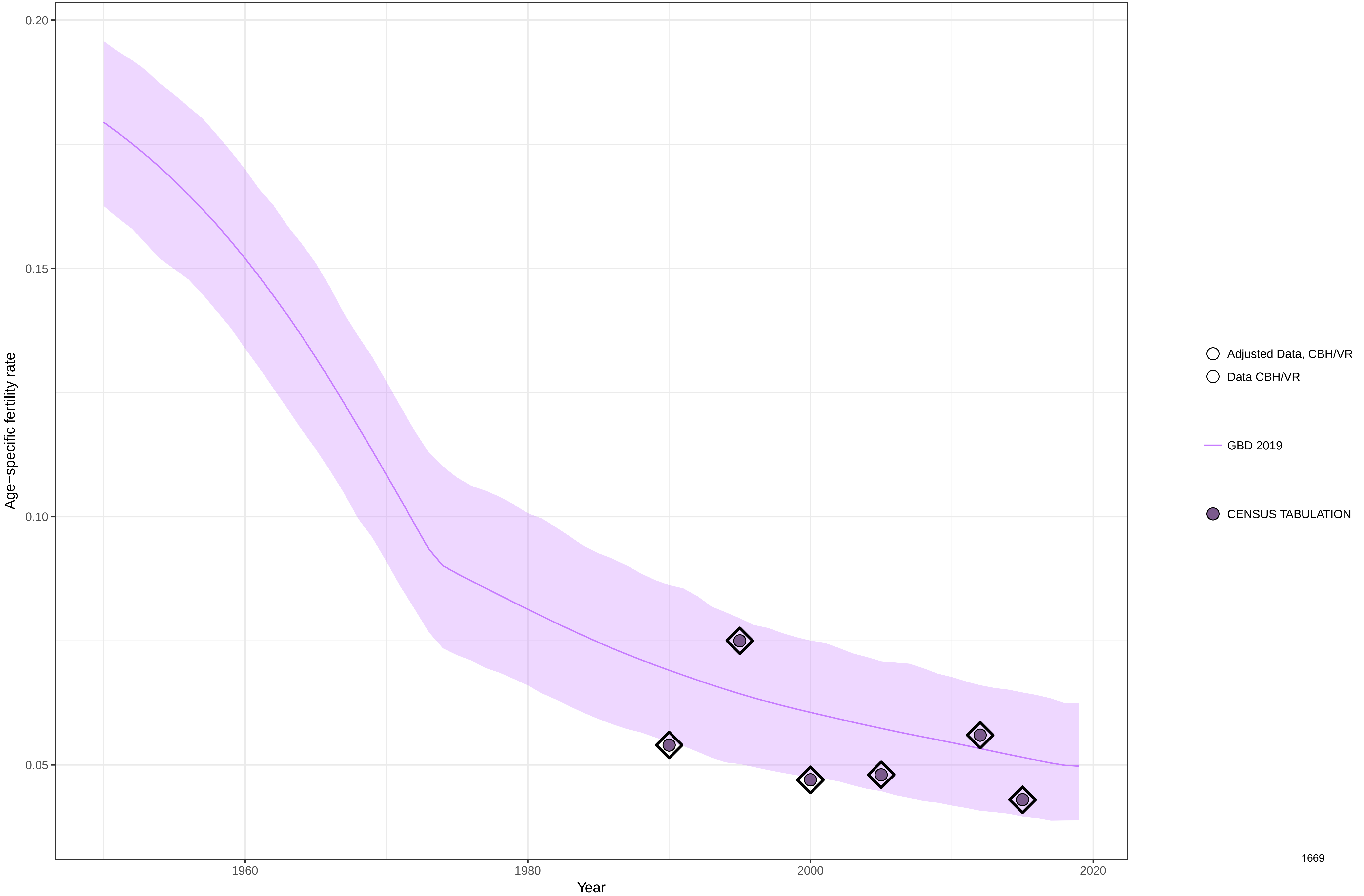

Age−specific fertility fitted trend lines, 1950−2019: Palau, Ages: 40−44
Data points enclosed with a diamond are reference sources

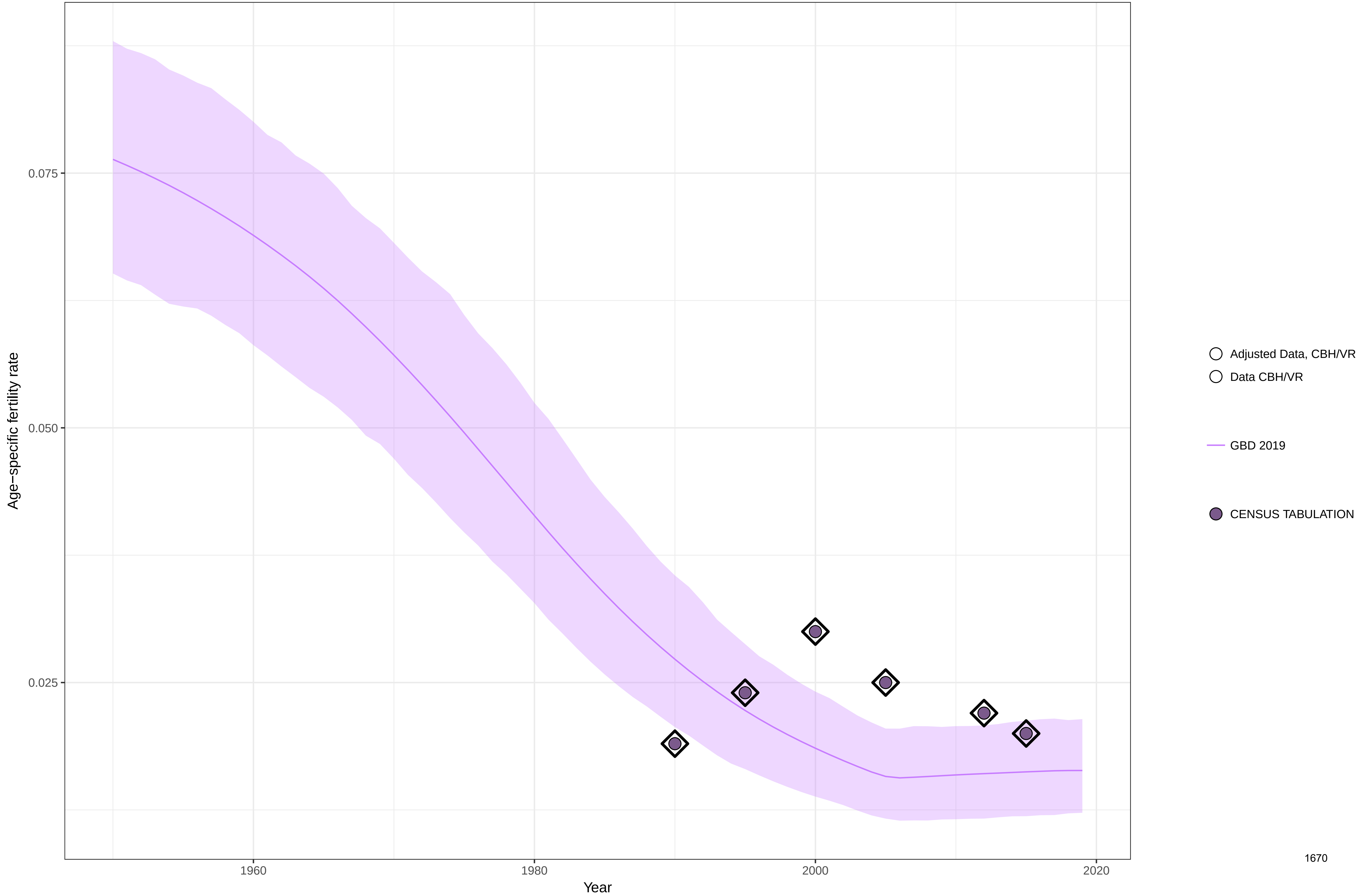

Age−specific fertility fitted trend lines, 1950−2019: Palau, Ages: 45−49
Data points enclosed with a diamond are reference sources

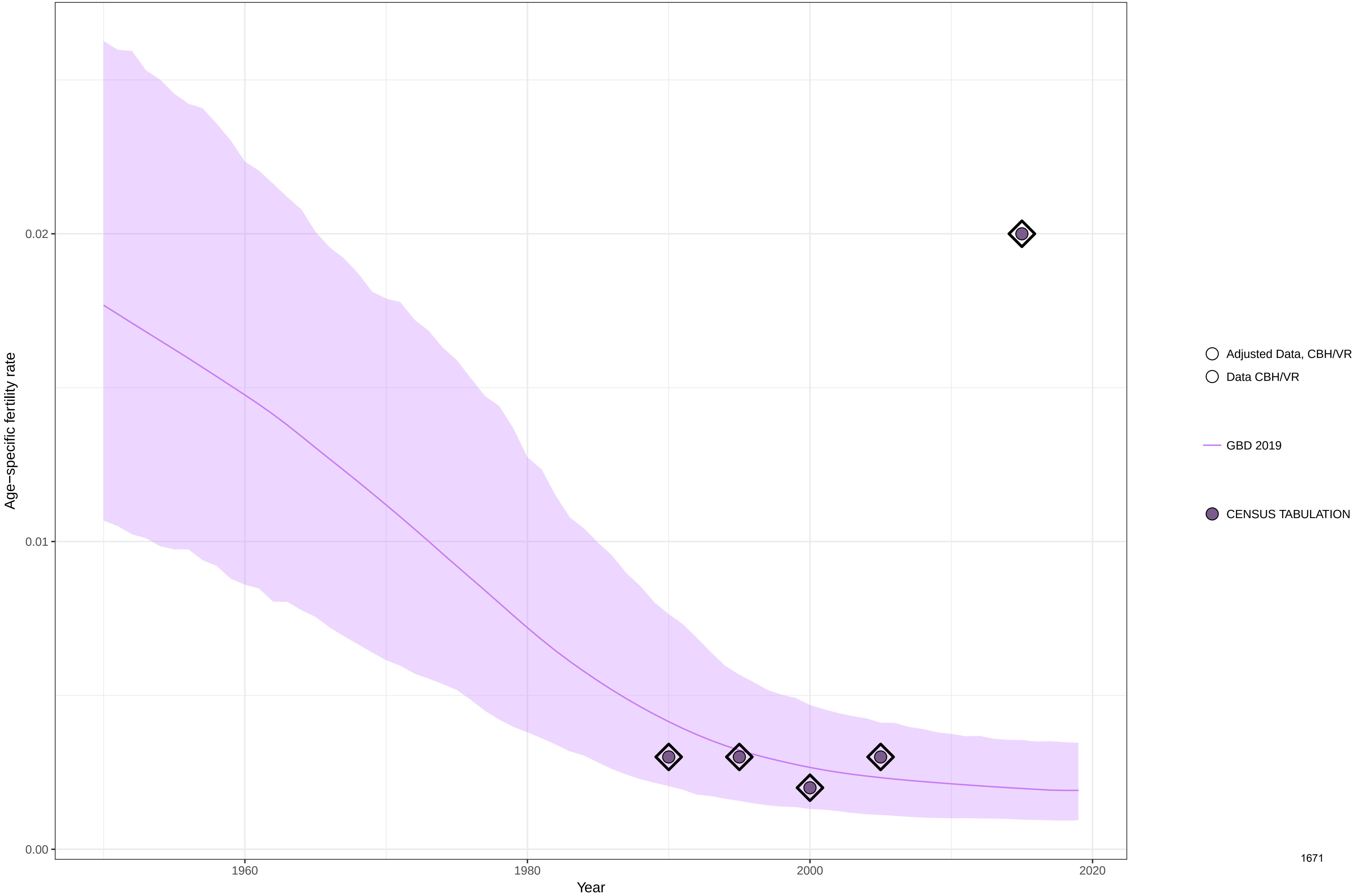

Age−specific fertility fitted trend lines, 1950−2019: Palau, Ages: 50−54
Data points enclosed with a diamond are reference sources

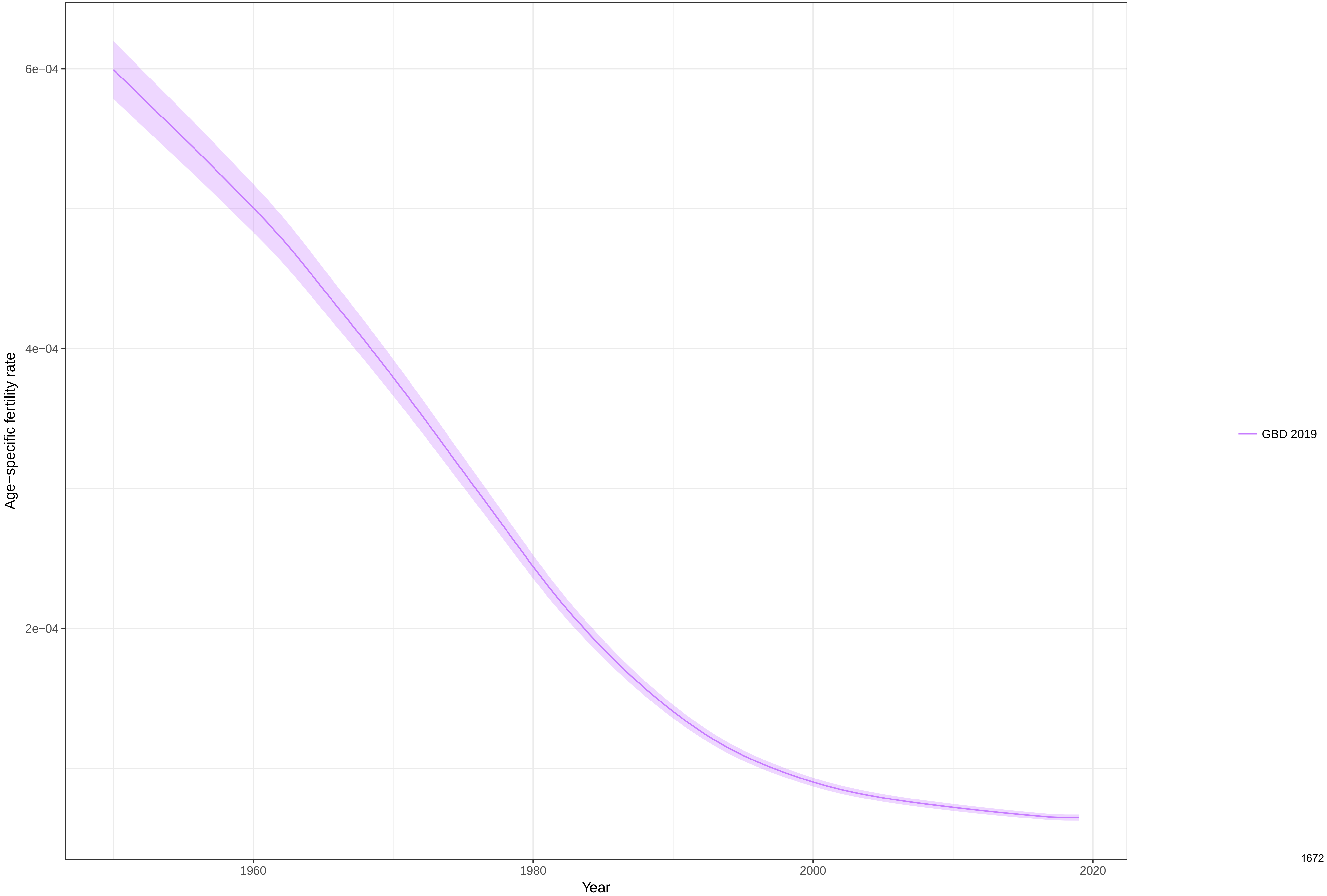

Total fertility rate fitted trend lines, 1950−2019: Palau TFR
Data points enclosed with a diamond are reference sources

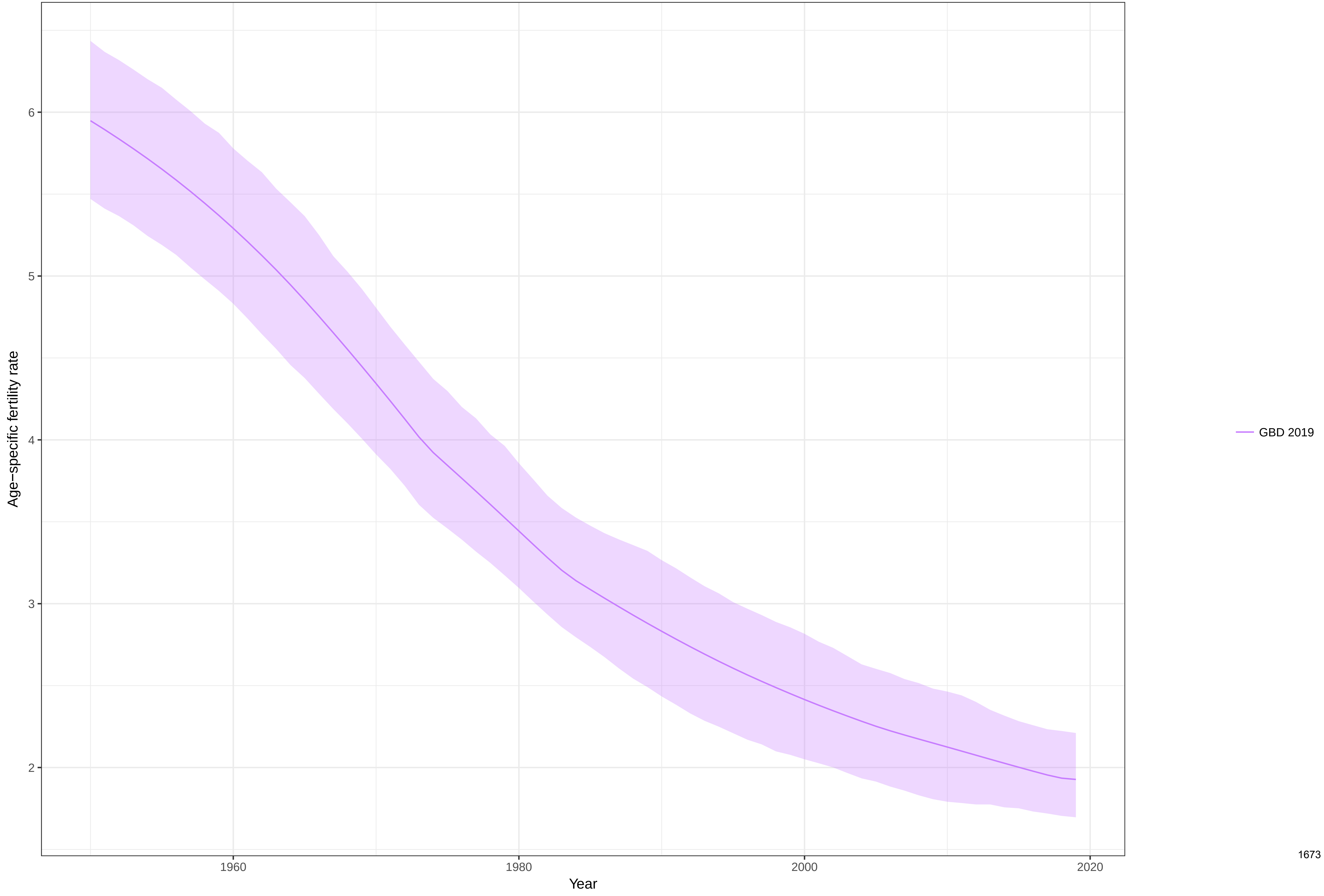

Age−specific fertility fitted trend lines, 1950−2019: Papua New Guinea, Ages: 10−14
Data points enclosed with a diamond are reference sources

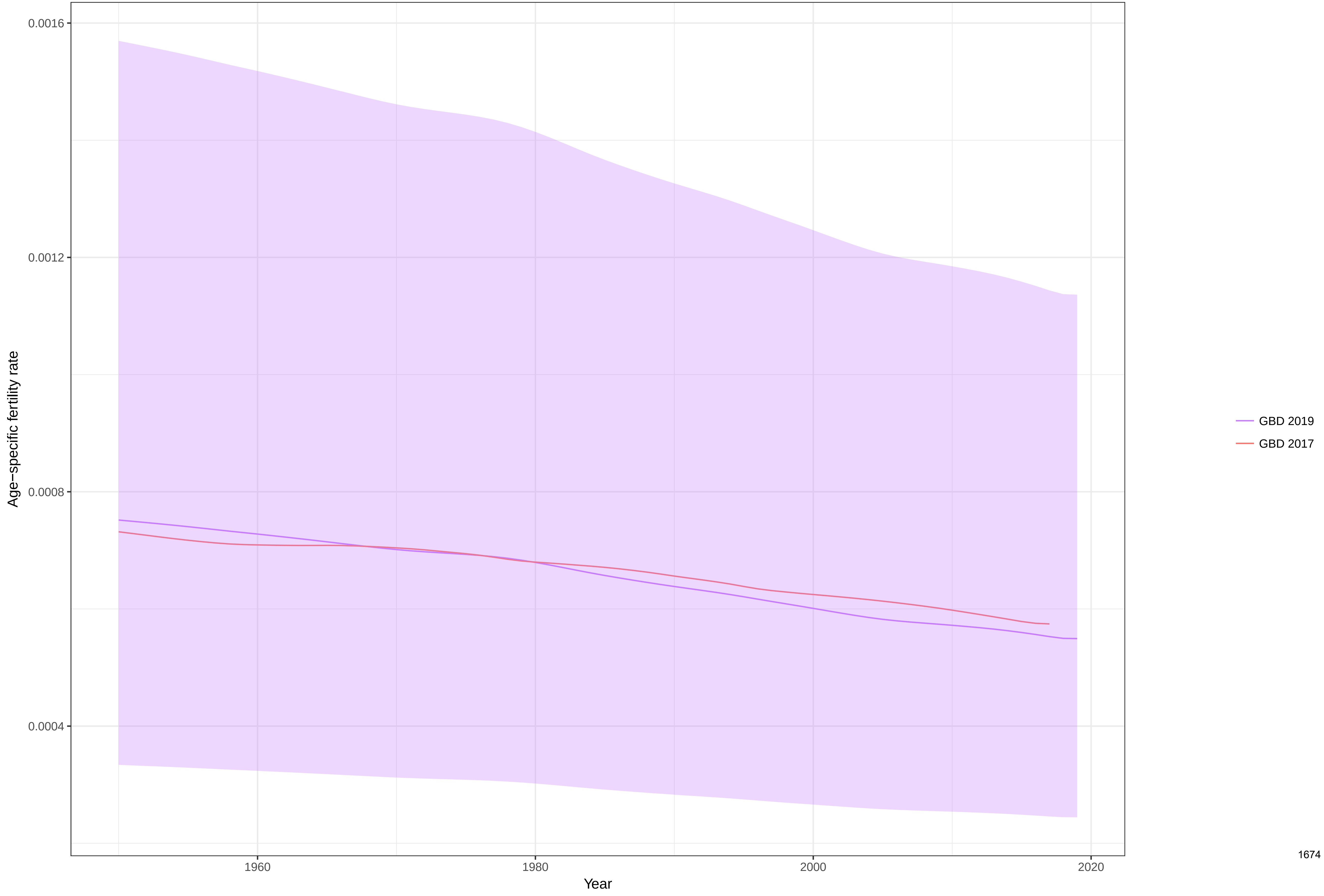

Age−specific fertility fitted trend lines, 1950−2019: Papua New Guinea, Ages: 15−19
Data points enclosed with a diamond are reference sources

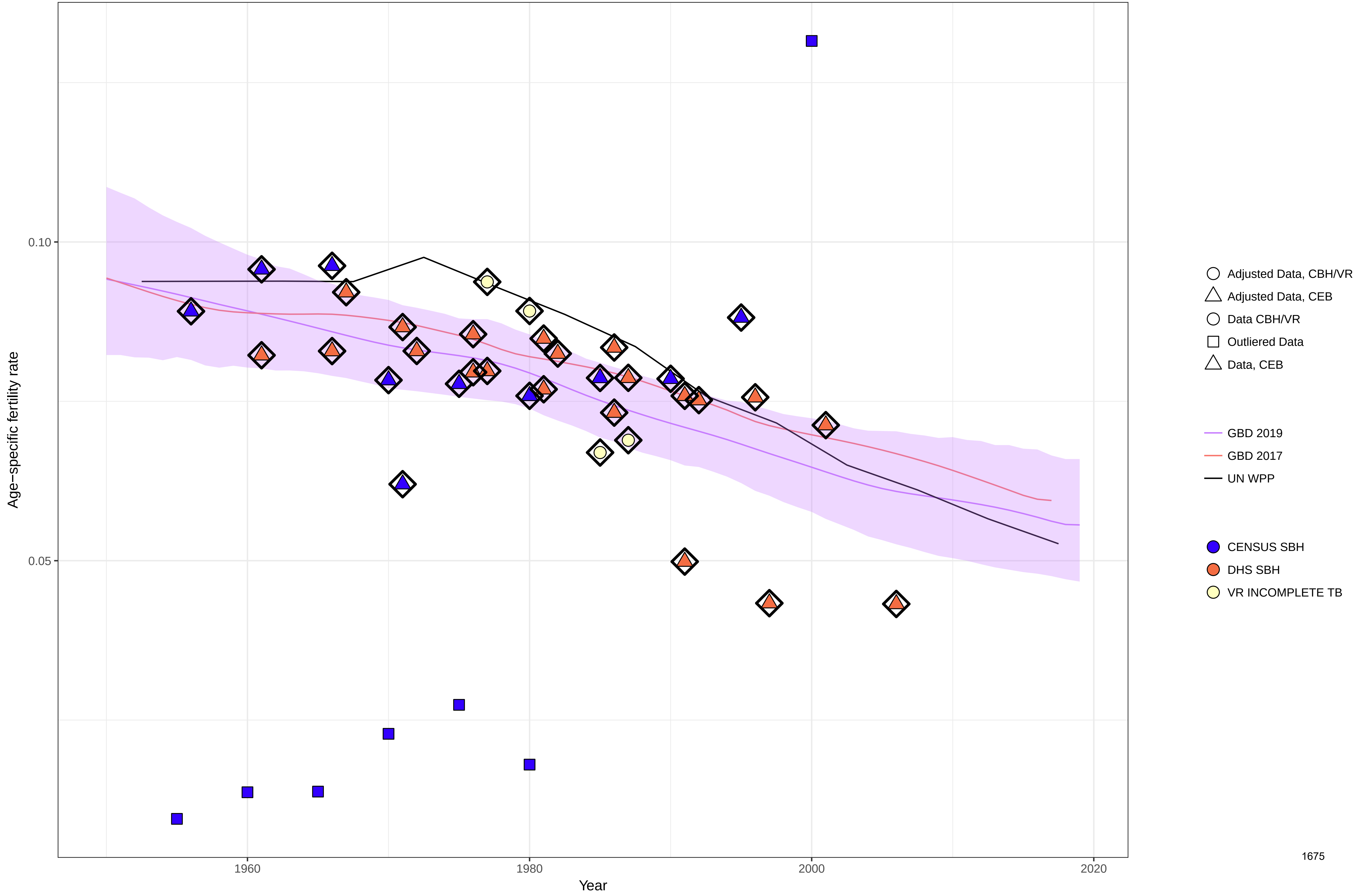

Age−specific fertility fitted trend lines, 1950−2019: Papua New Guinea, Ages: 20−24
Data points enclosed with a diamond are reference sources

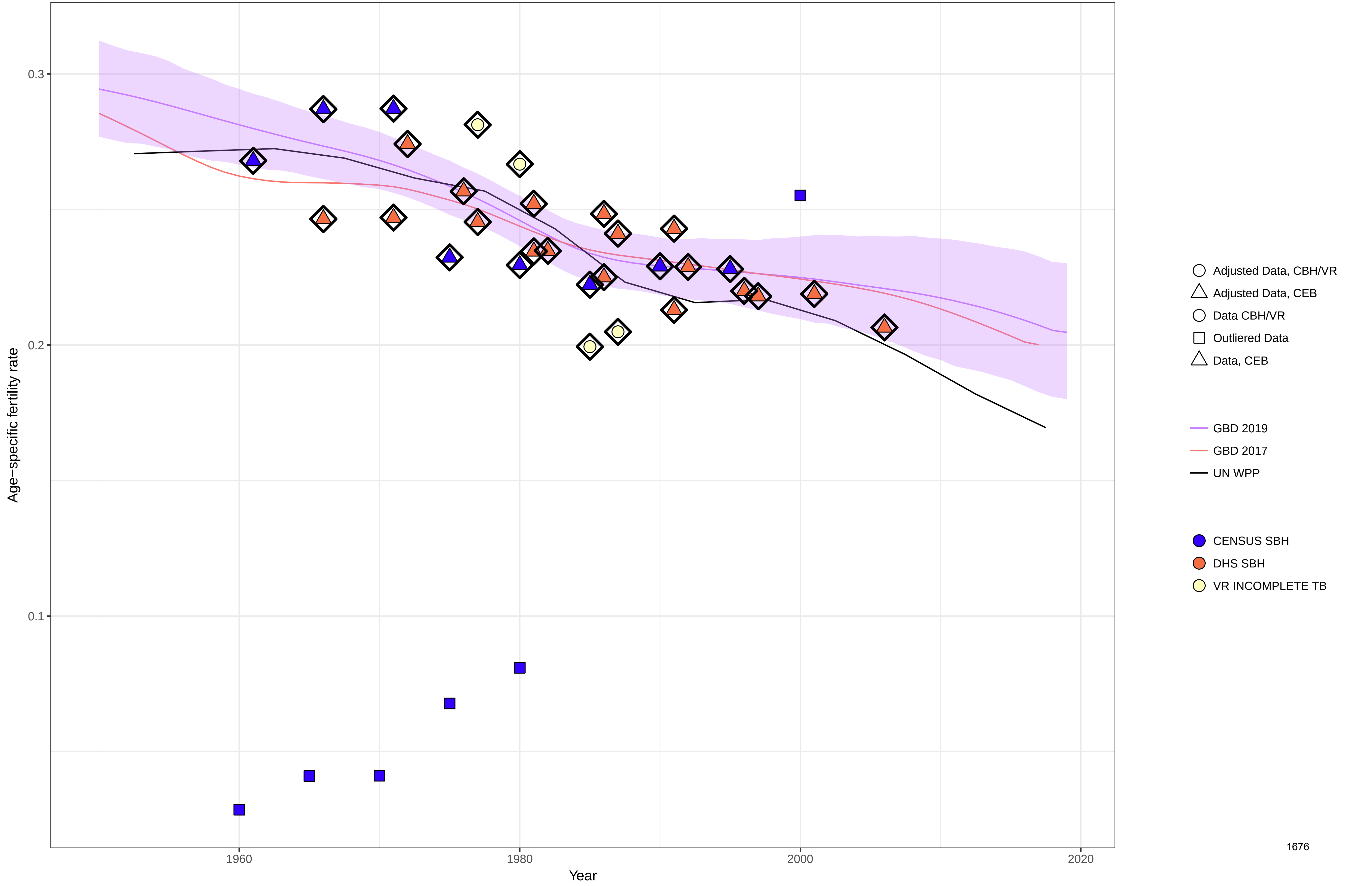

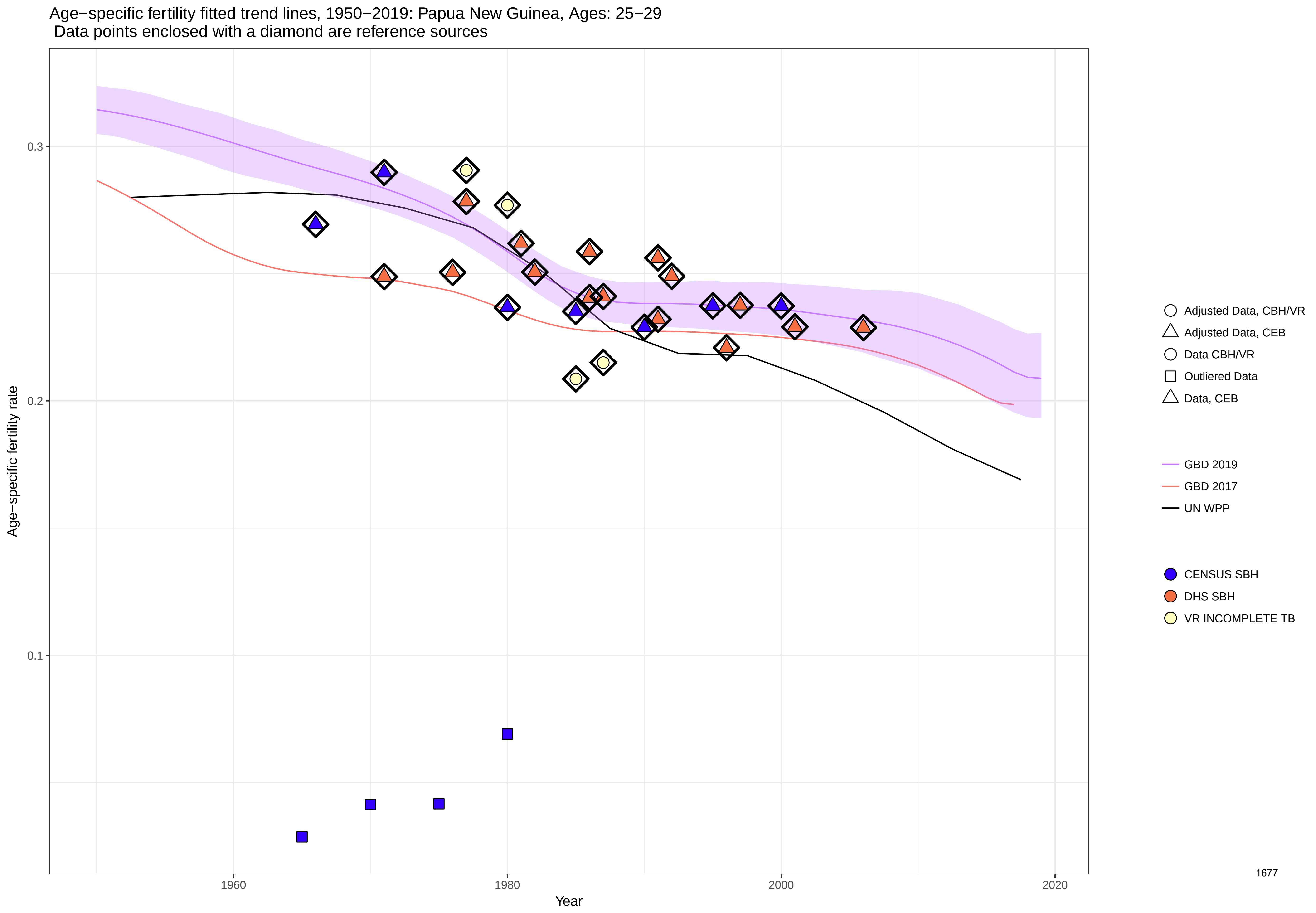

Age−specific fertility fitted trend lines, 1950−2019: Papua New Guinea, Ages: 25−29
Data points enclosed with a diamond are reference sources
Age−specific fertility rate
0.3
0.2
0.1
1960
1980
2000
2020
Year
Adjusted Data, CBH/VR
Adjusted Data, CEB
Data CBH/VR
Outliered Data
Data, CEB
GBD 2019
GBD 2017
UN WPP
CENSUS SBH
DHS SBH
VR INCOMPLETE TB

Age−specific fertility fitted trend lines, 1950−2019: Papua New Guinea, Ages: 30−34
Data points enclosed with a diamond are reference sources

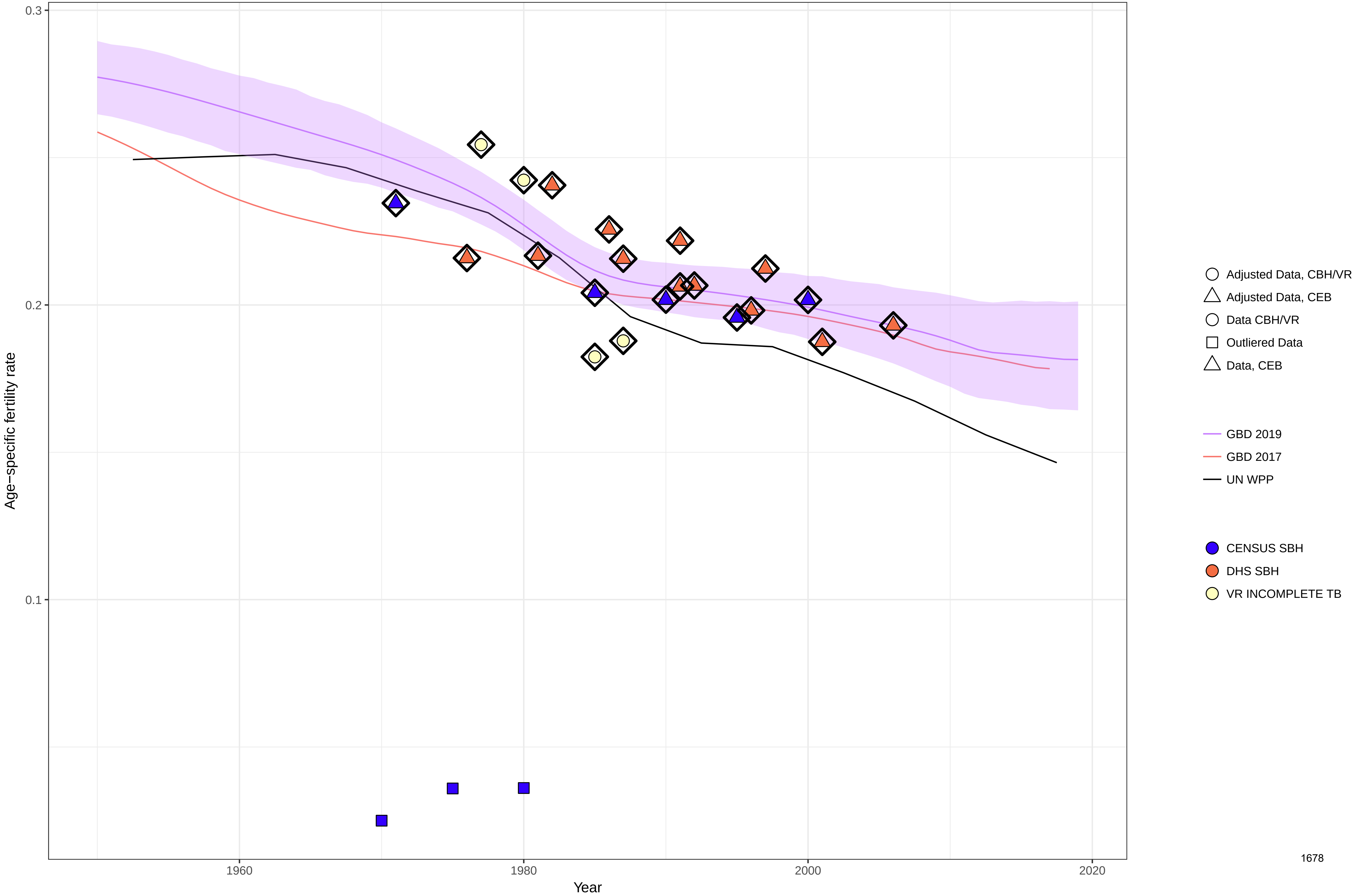

Age−specific fertility fitted trend lines, 1950−2019: Papua New Guinea, Ages: 35−39
Data points enclosed with a diamond are reference sources

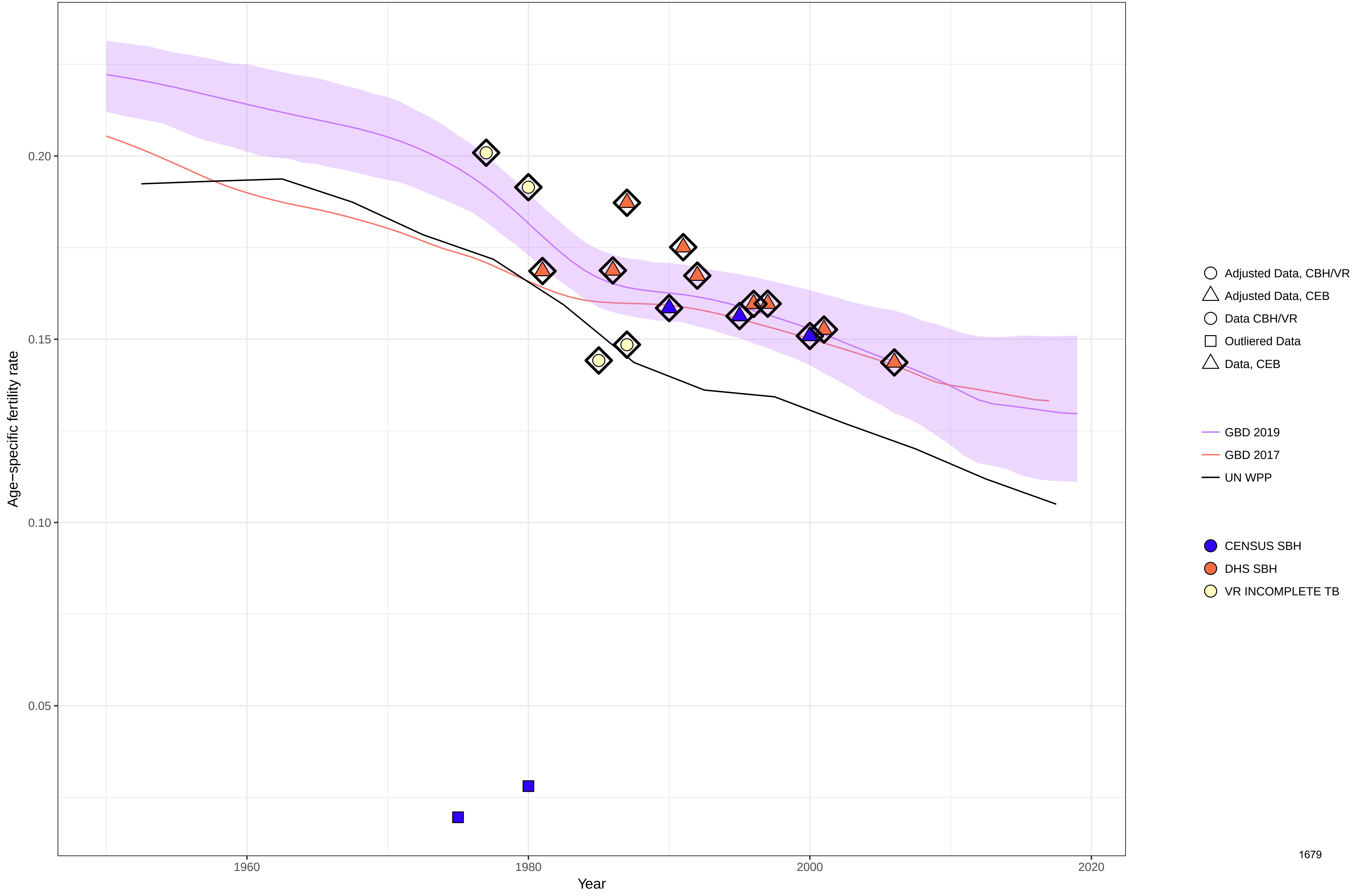

Age−specific fertility fitted trend lines, 1950−2019: Papua New Guinea, Ages: 40−44
Data points enclosed with a diamond are reference sources

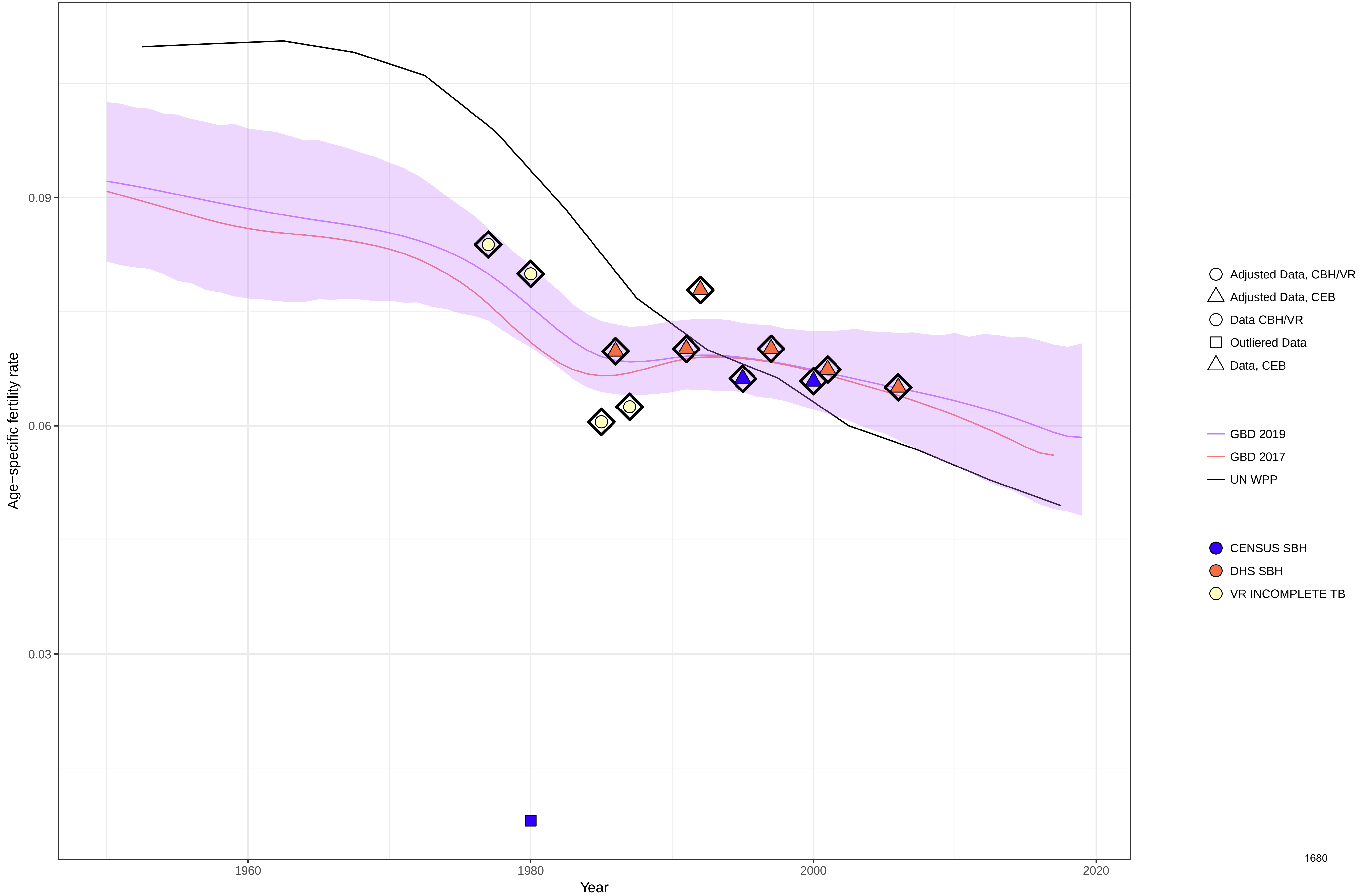

Age−specific fertility fitted trend lines, 1950−2019: Papua New Guinea, Ages: 45−49
Data points enclosed with a diamond are reference sources

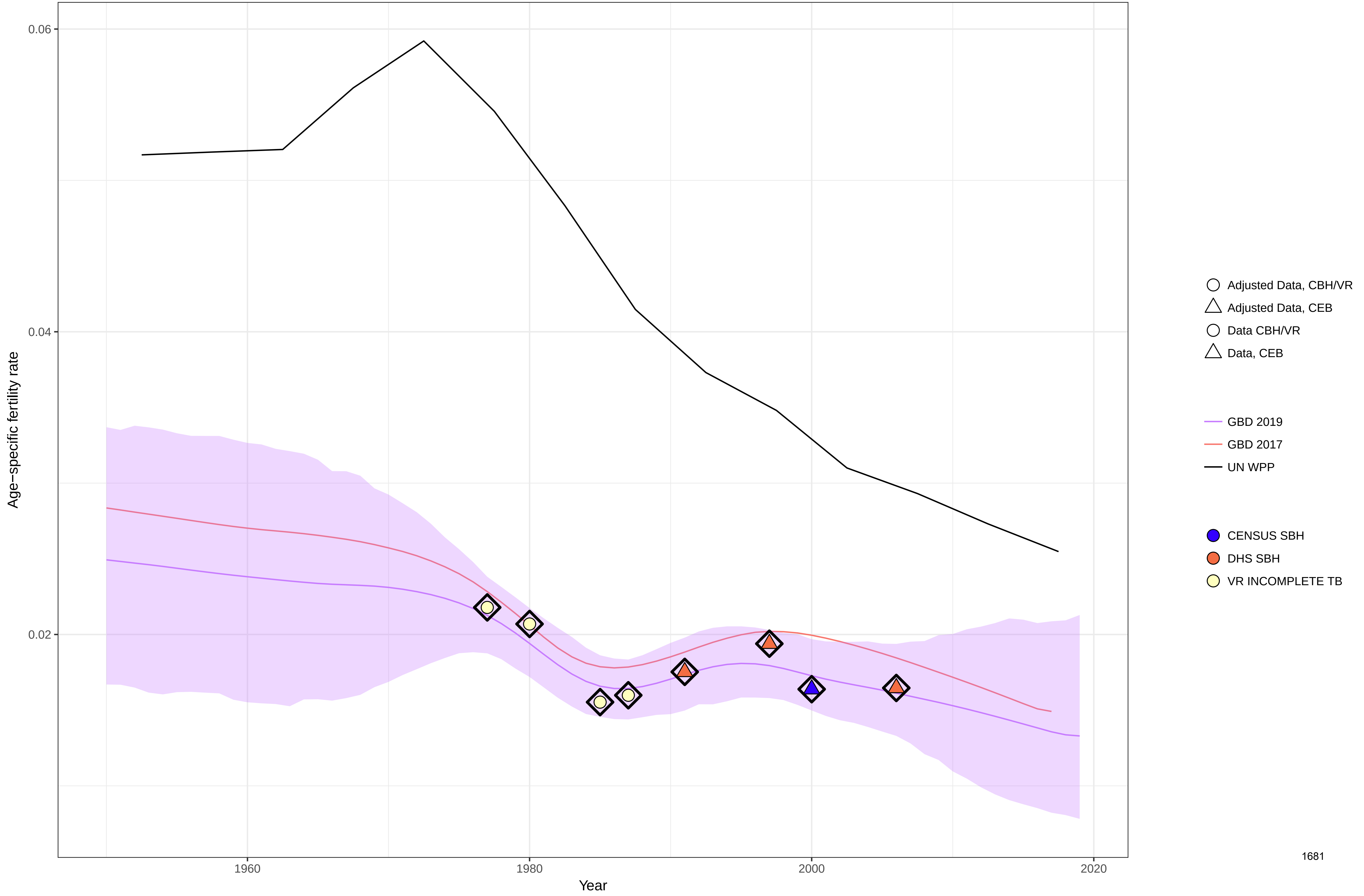

Age−specific fertility fitted trend lines, 1950−2019: Papua New Guinea, Ages: 50−54
Data points enclosed with a diamond are reference sources

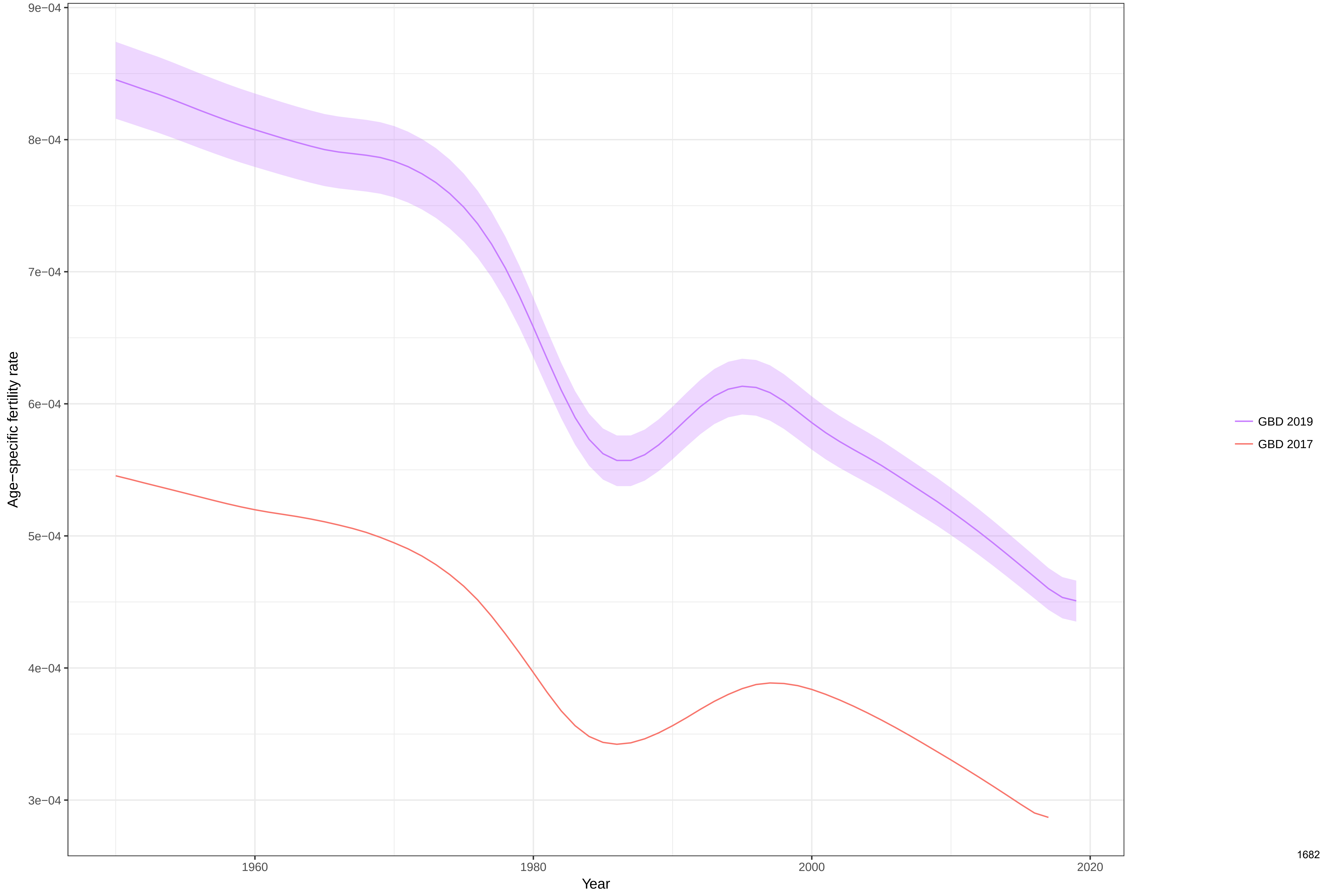

Total fertility rate fitted trend lines, 1950−2019: Papua New Guinea TFR
Data points enclosed with a diamond are reference sources

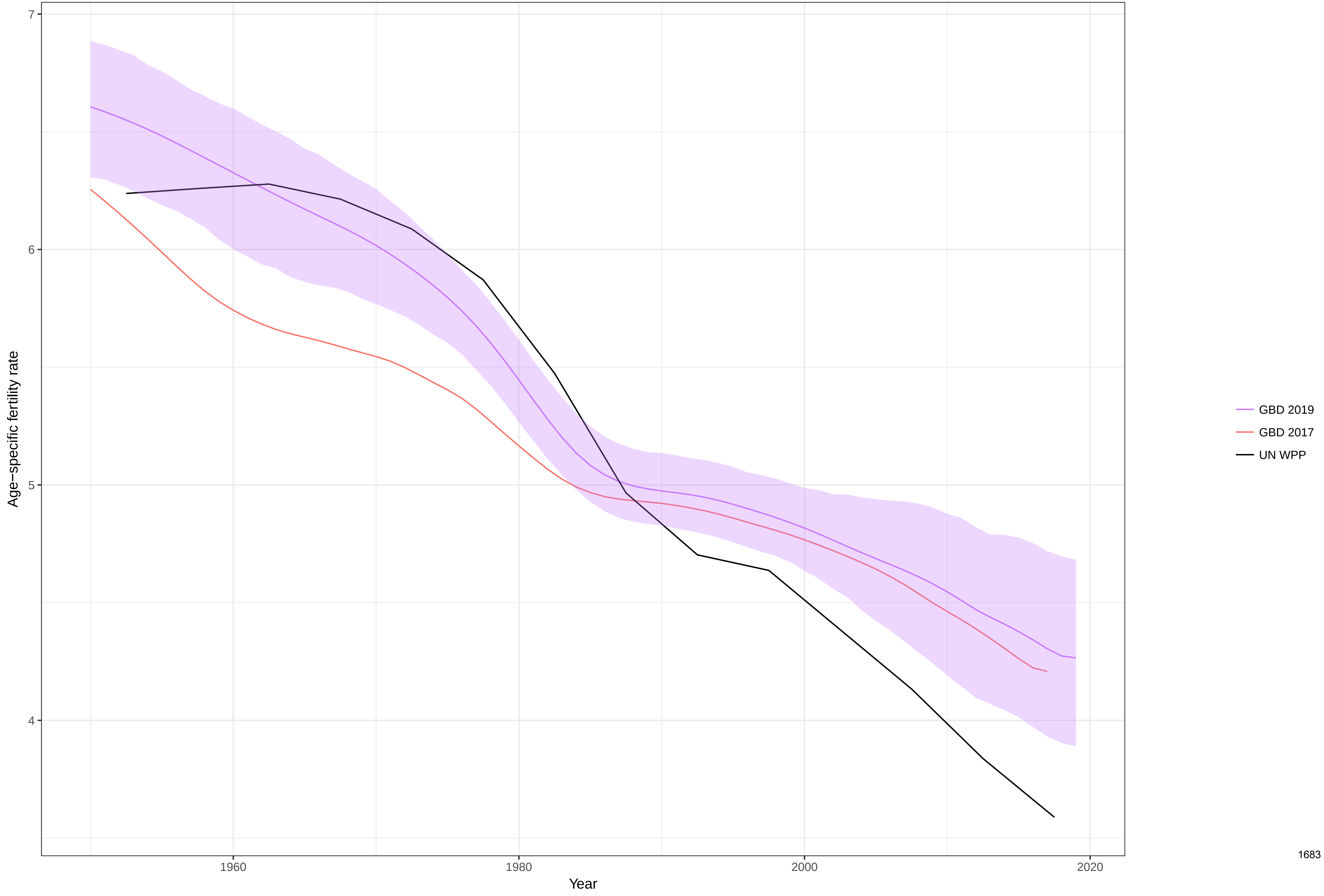

Age−specific fertility fitted trend lines, 1950−2019: Poland, Ages: 10−14
Data points enclosed with a diamond are reference sources

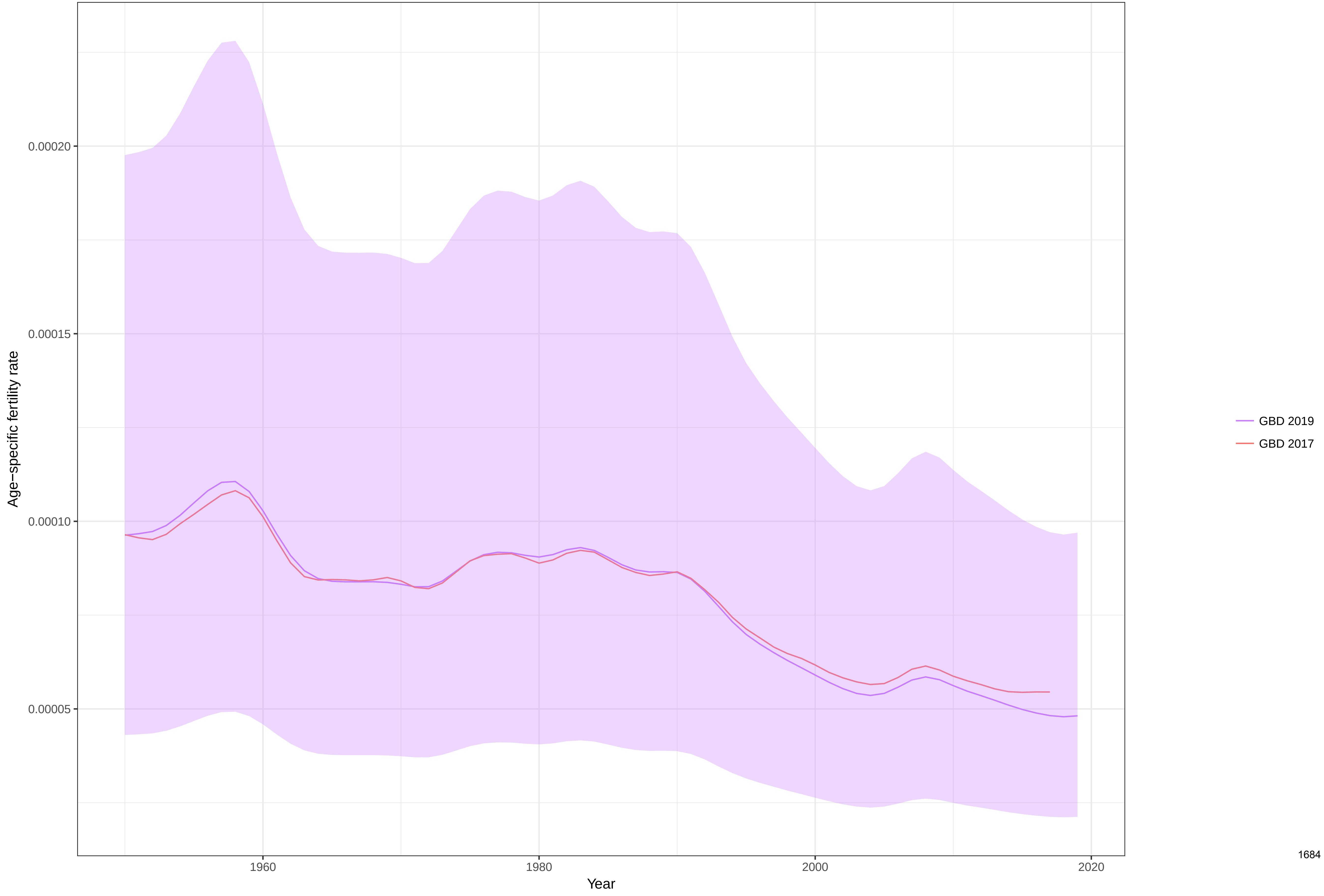

Age−specific fertility fitted trend lines, 1950−2019: Poland, Ages: 15−19
Data points enclosed with a diamond are reference sources

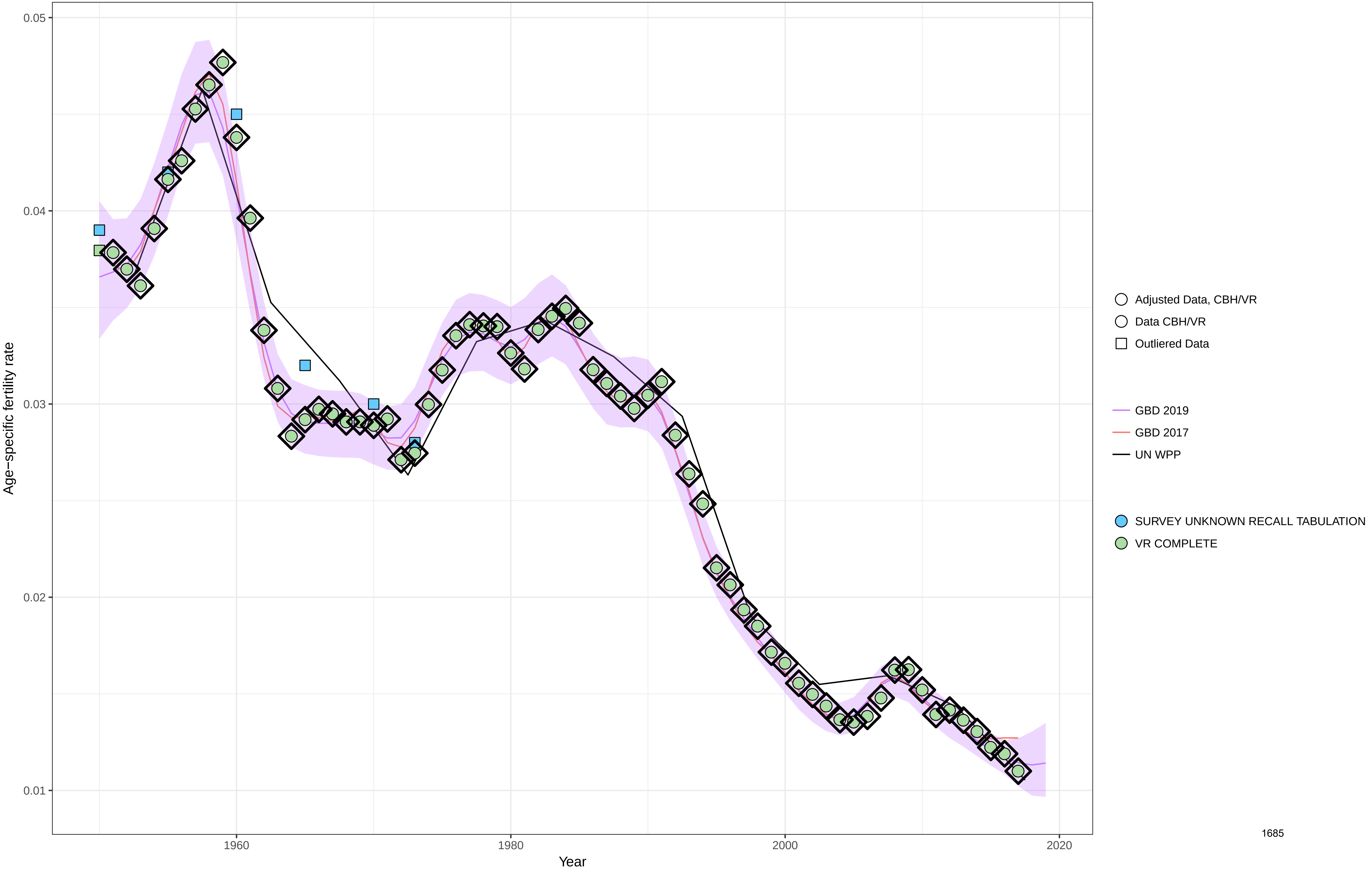

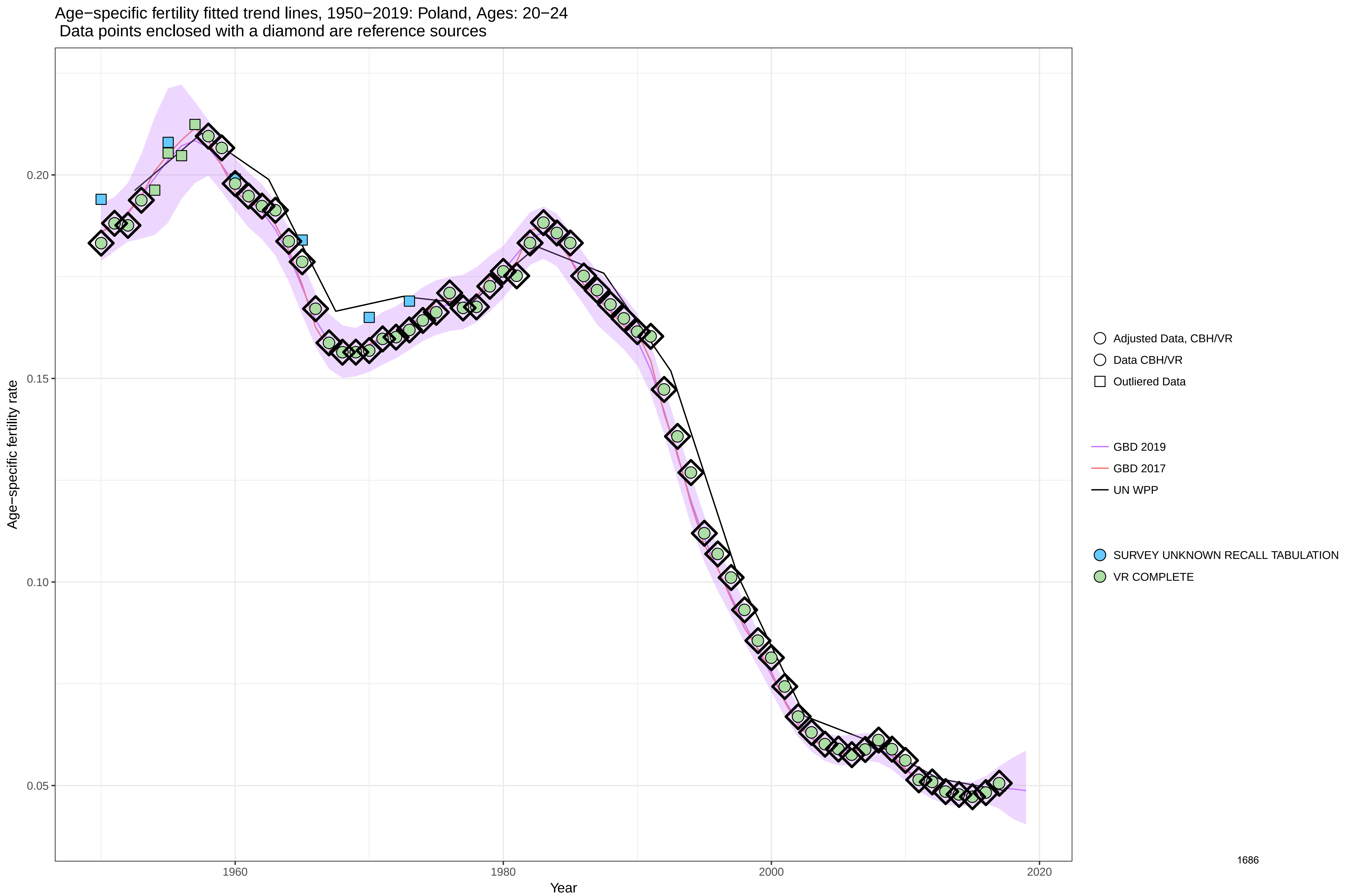
Age−specific fertility fitted trend lines, 1950−2019: Poland, Ages: 20−24
Data points enclosed with a diamond are reference sources
Age−specific fertility rate
0.20
0.15
0.10
0.05
1960
1980
2000
2020
Year
Adjusted Data, CBH/VR
Data CBH/VR
Outliered Data
GBD 2019
GBD 2017
UN WPP
SURVEY UNKNOWN RECALL TABULATION
VR COMPLETE

Age−specific fertility fitted trend lines, 1950−2019: Poland, Ages: 25−29
Data points enclosed with a diamond are reference sources

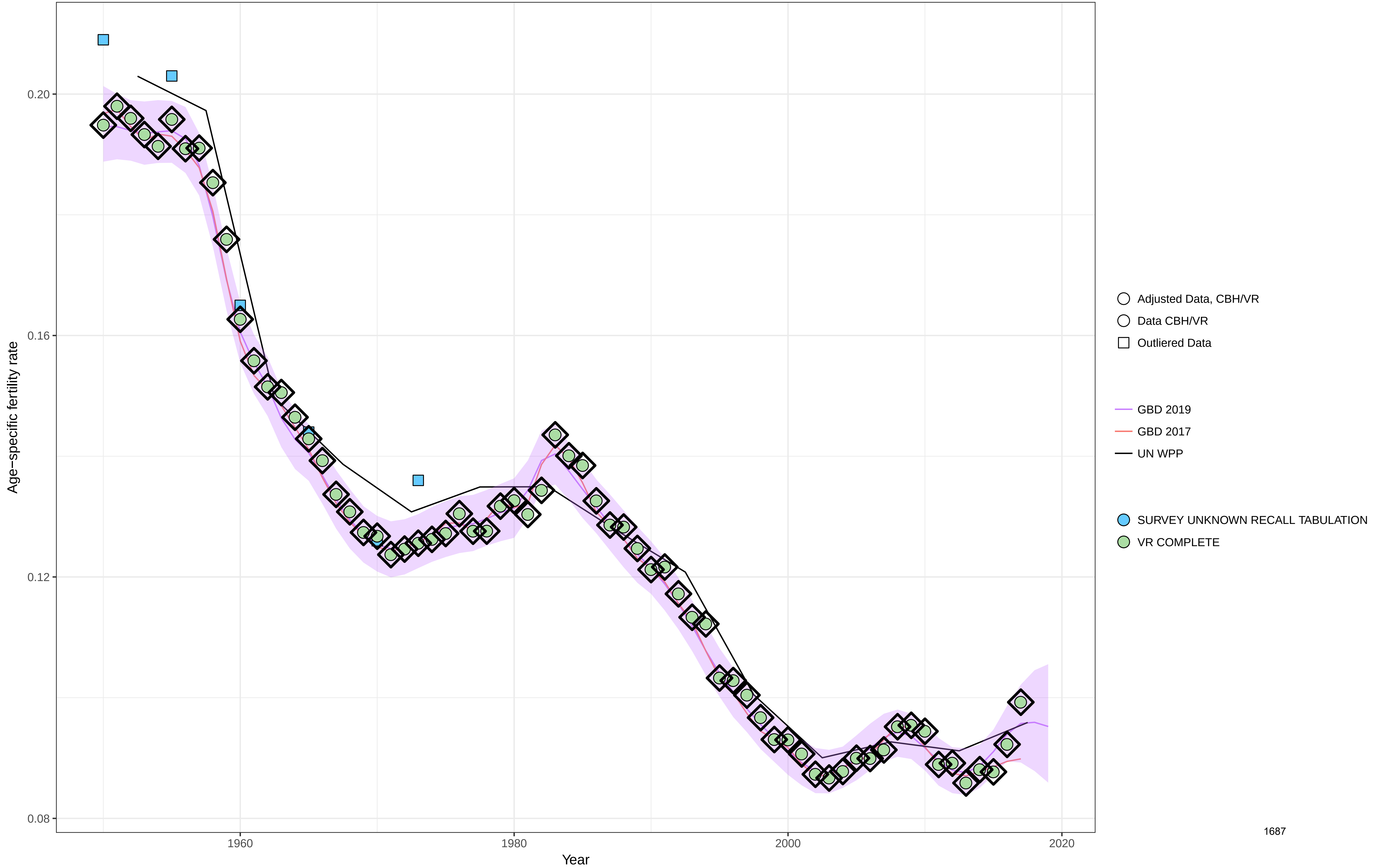

Age−specific fertility fitted trend lines, 1950−2019: Poland, Ages: 30−34
Data points enclosed with a diamond are reference sources

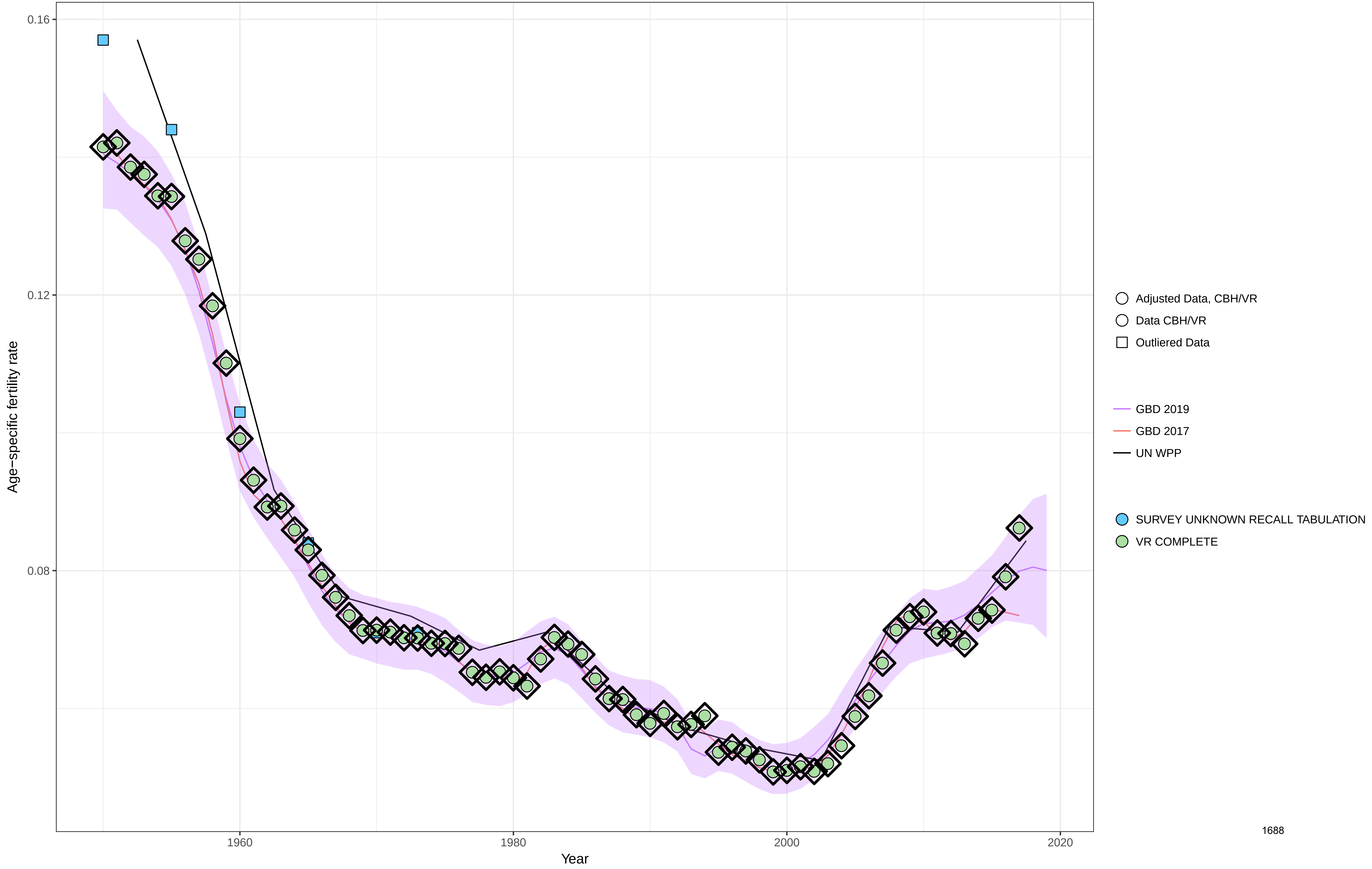

Age−specific fertility fitted trend lines, 1950−2019: Poland, Ages: 35−39
Data points enclosed with a diamond are reference sources

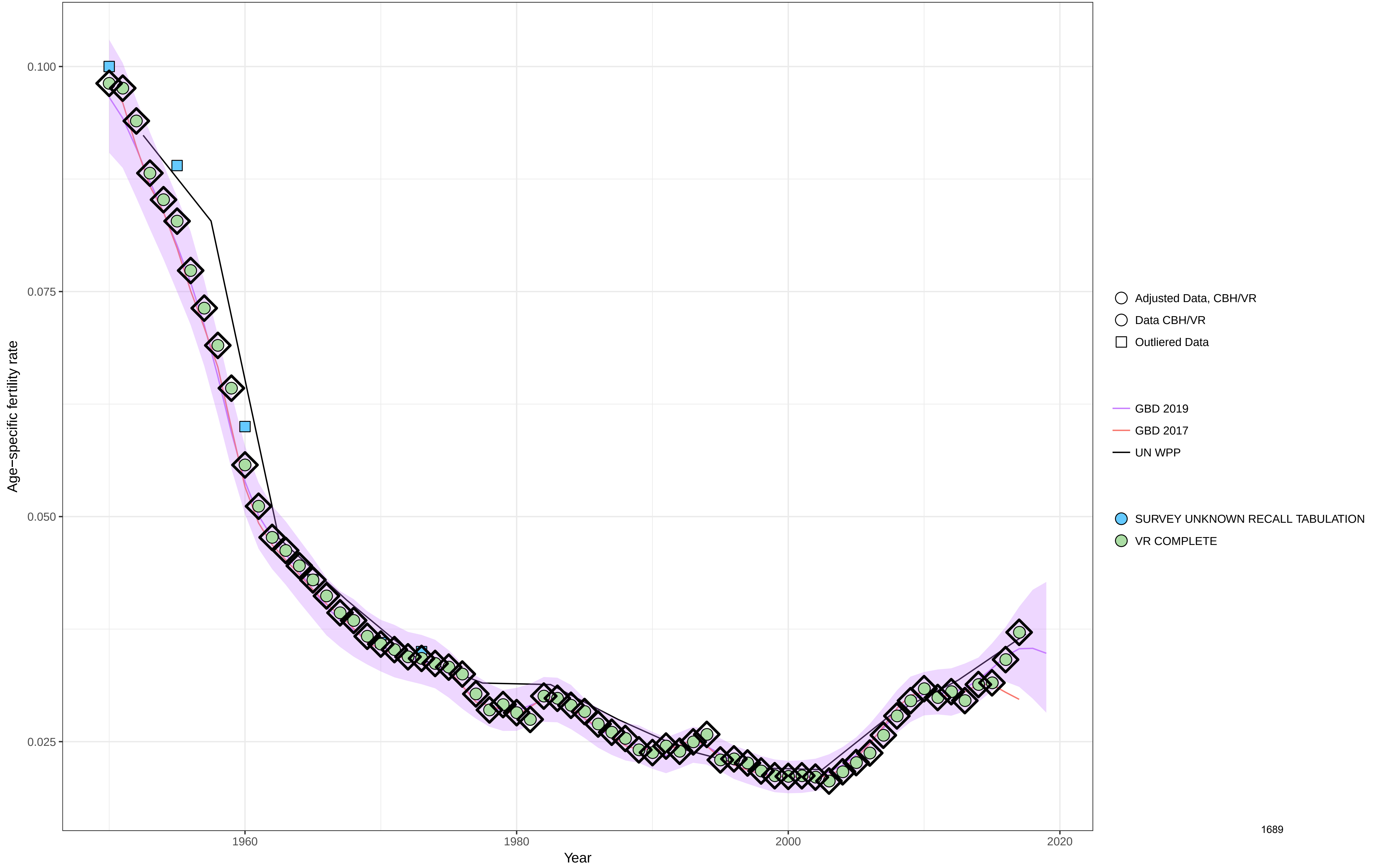

Age−specific fertility fitted trend lines, 1950−2019: Poland, Ages: 40−44
Data points enclosed with a diamond are reference sources

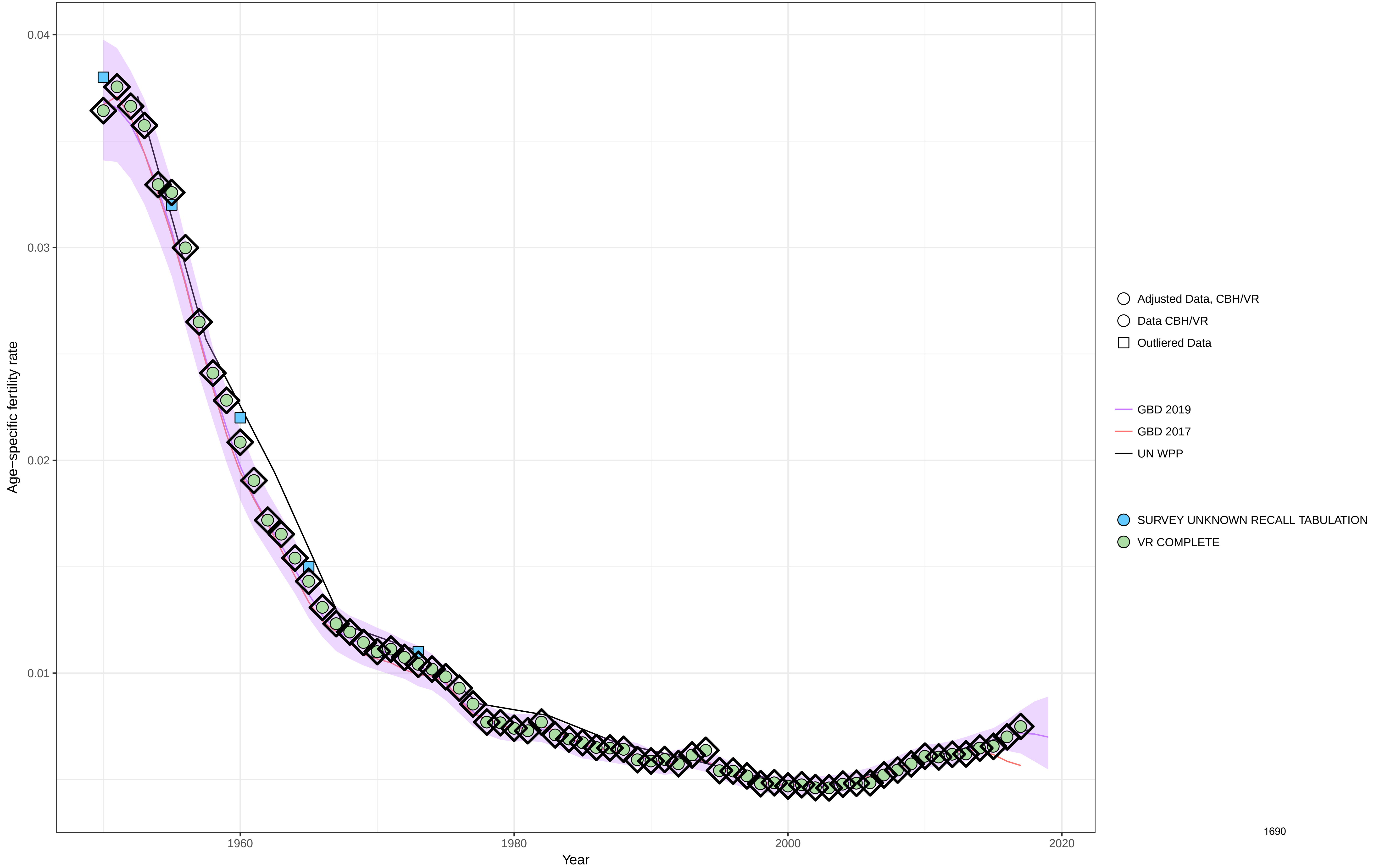

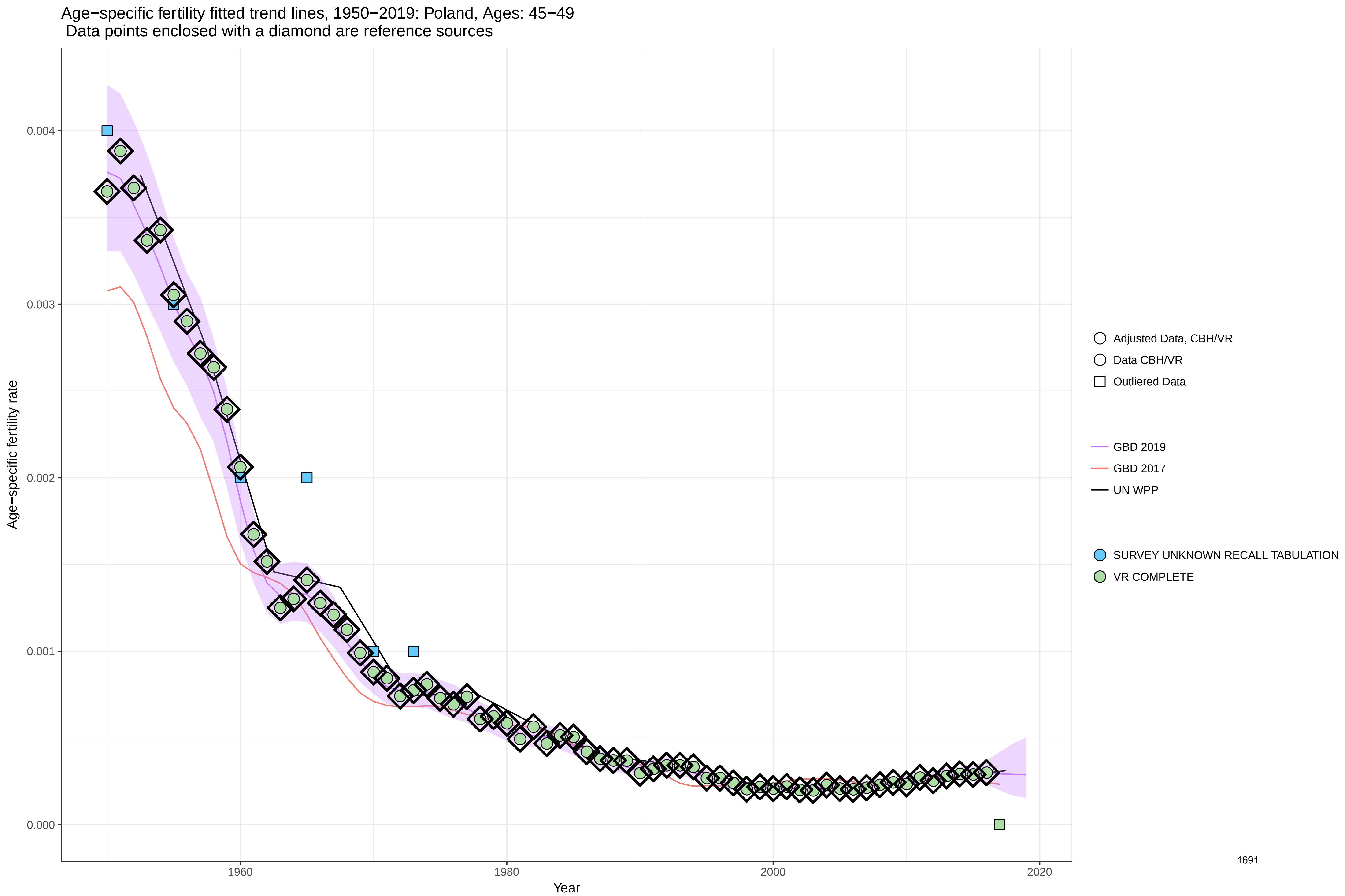
Age−specific fertility fitted trend lines, 1950−2019: Poland, Ages: 45−49
Data points enclosed with a diamond are reference sources
Age−specific fertility rate
0.000
0.001
0.002
0.003
0.004
Year
1960
1980
2000
2020
Adjusted Data, CBH/VR
Data CBH/VR
Outliered Data
GBD 2019
GBD 2017
UN WPP
SURVEY UNKNOWN RECALL TABULATION
VR COMPLETE

Age−specific fertility fitted trend lines, 1950−2019: Poland, Ages: 50−54
Data points enclosed with a diamond are reference sources

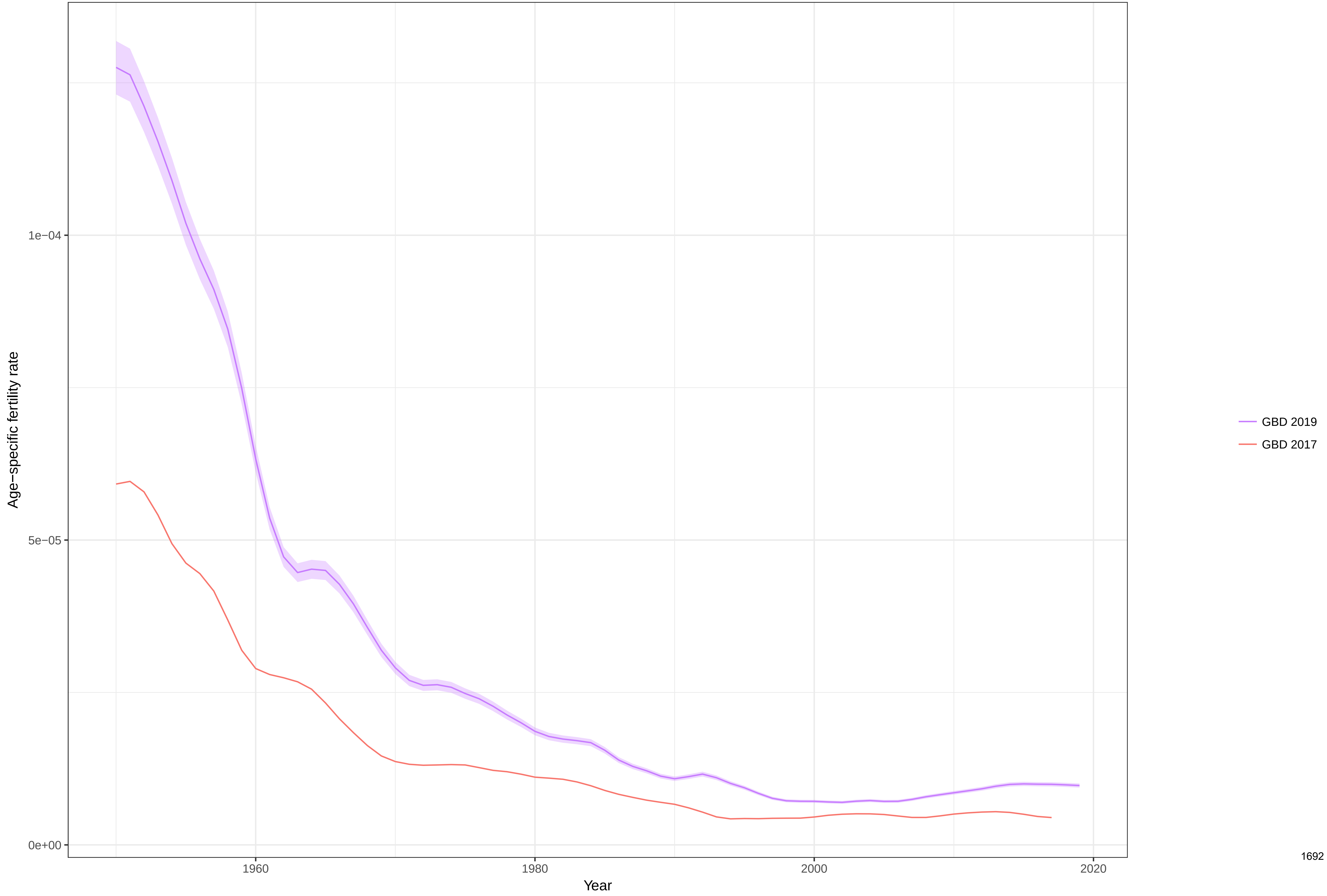

Total fertility rate fitted trend lines, 1950−2019: Poland TFR
Data points enclosed with a diamond are reference sources

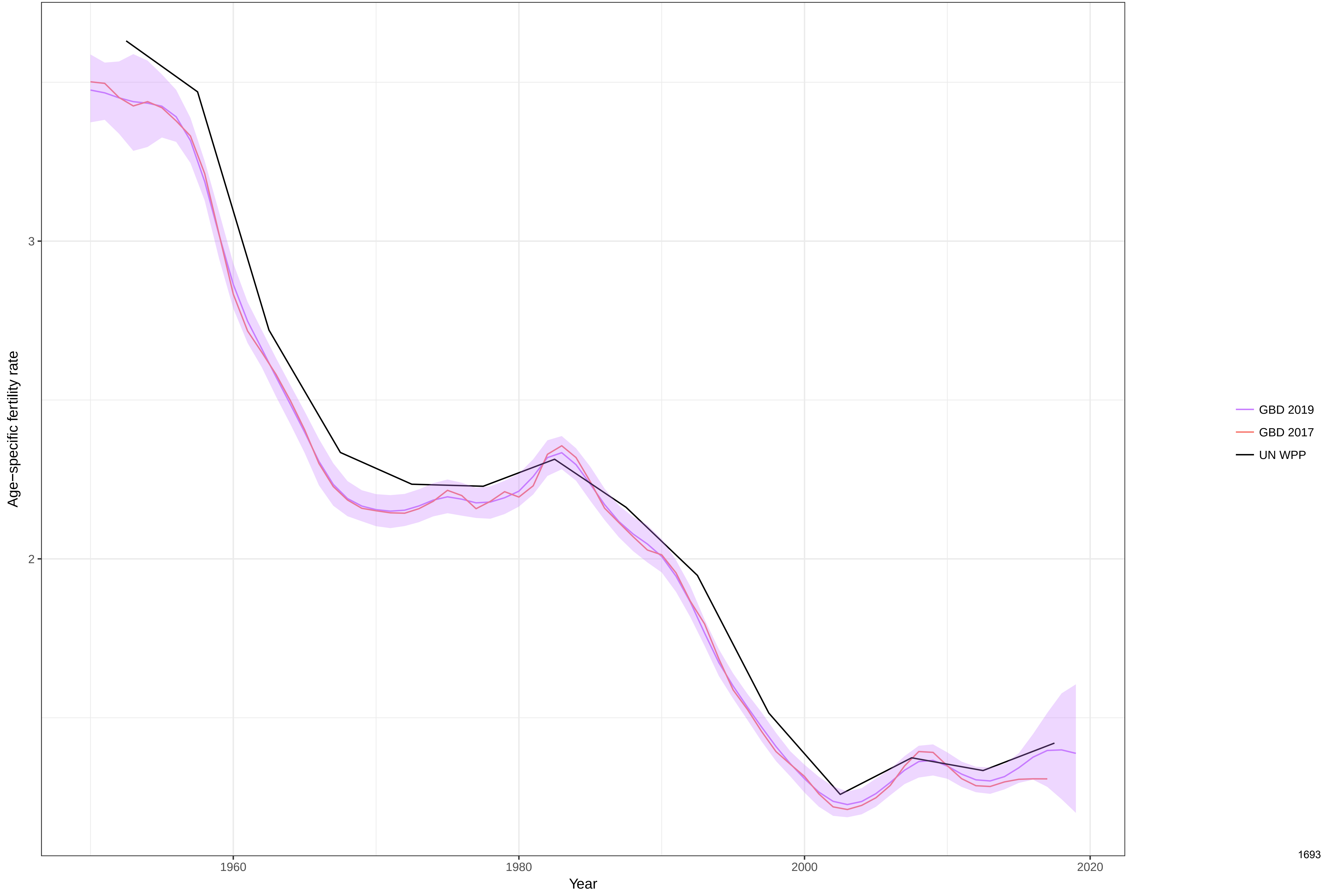

Age−specific fertility fitted trend lines, 1950−2019: Puerto Rico, Ages: 10−14
Data points enclosed with a diamond are reference sources

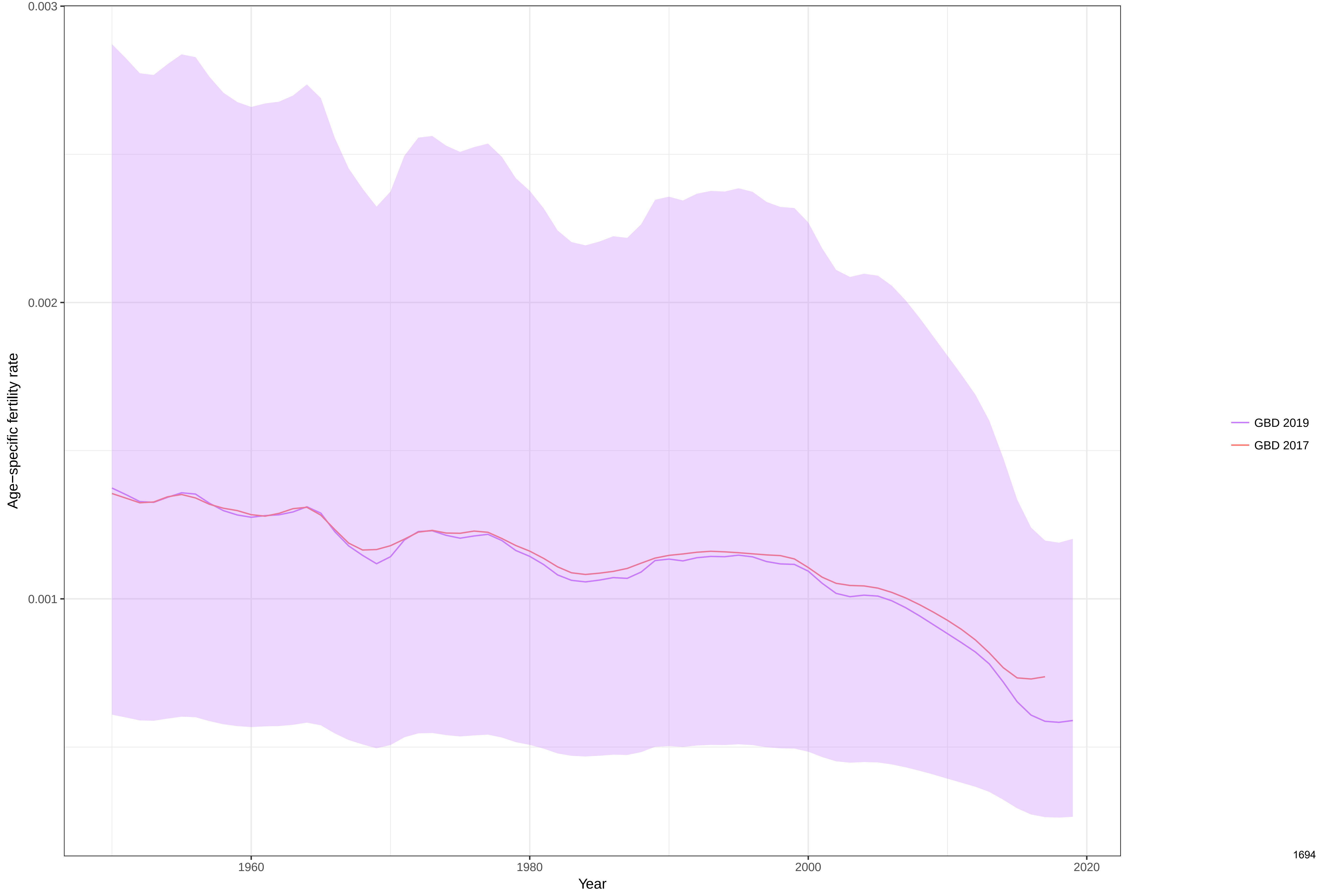

Age−specific fertility fitted trend lines, 1950−2019: Puerto Rico, Ages: 15−19
Data points enclosed with a diamond are reference sources

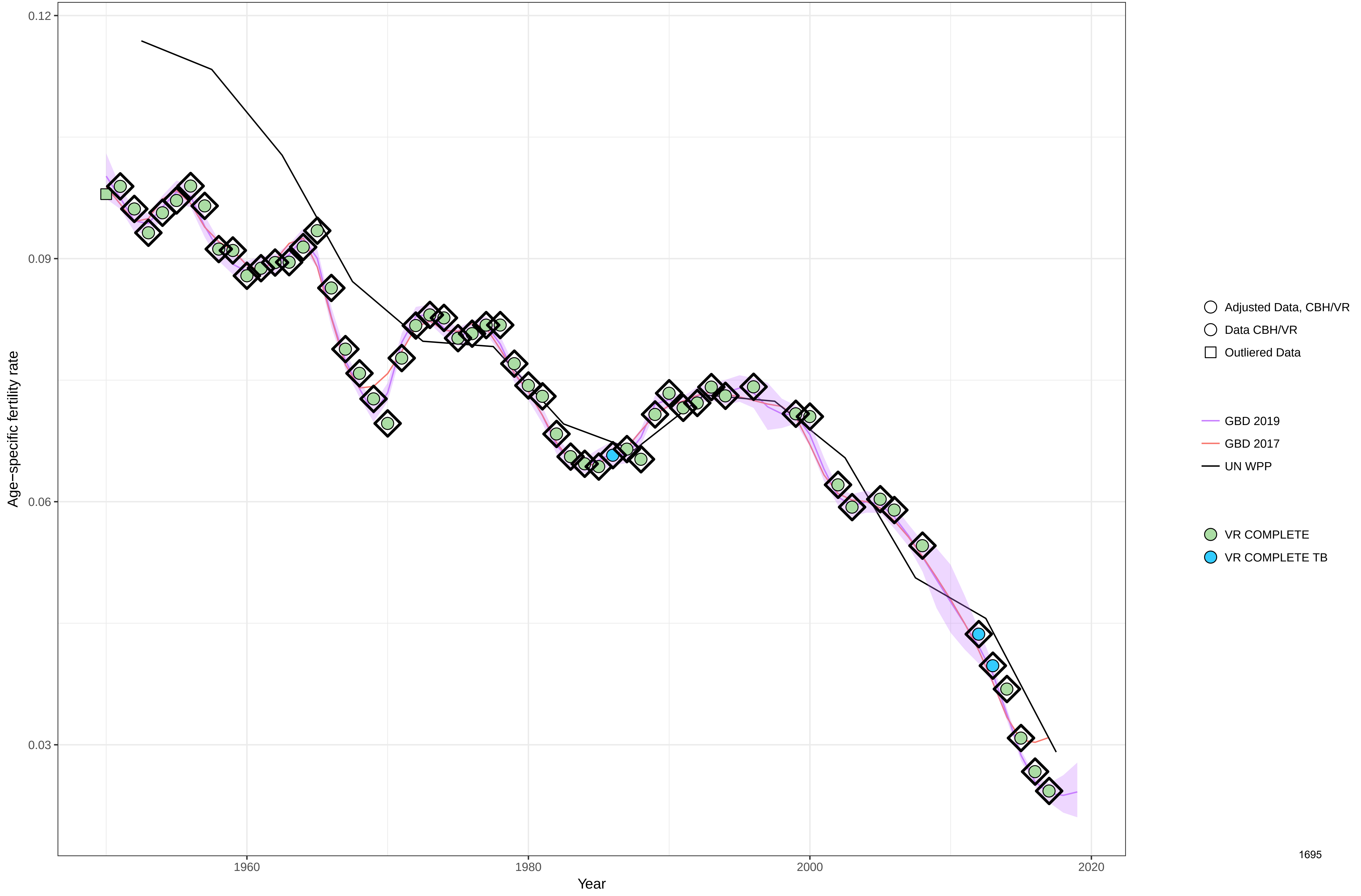

Age−specific fertility fitted trend lines, 1950−2019: Puerto Rico, Ages: 20−24
Data points enclosed with a diamond are reference sources

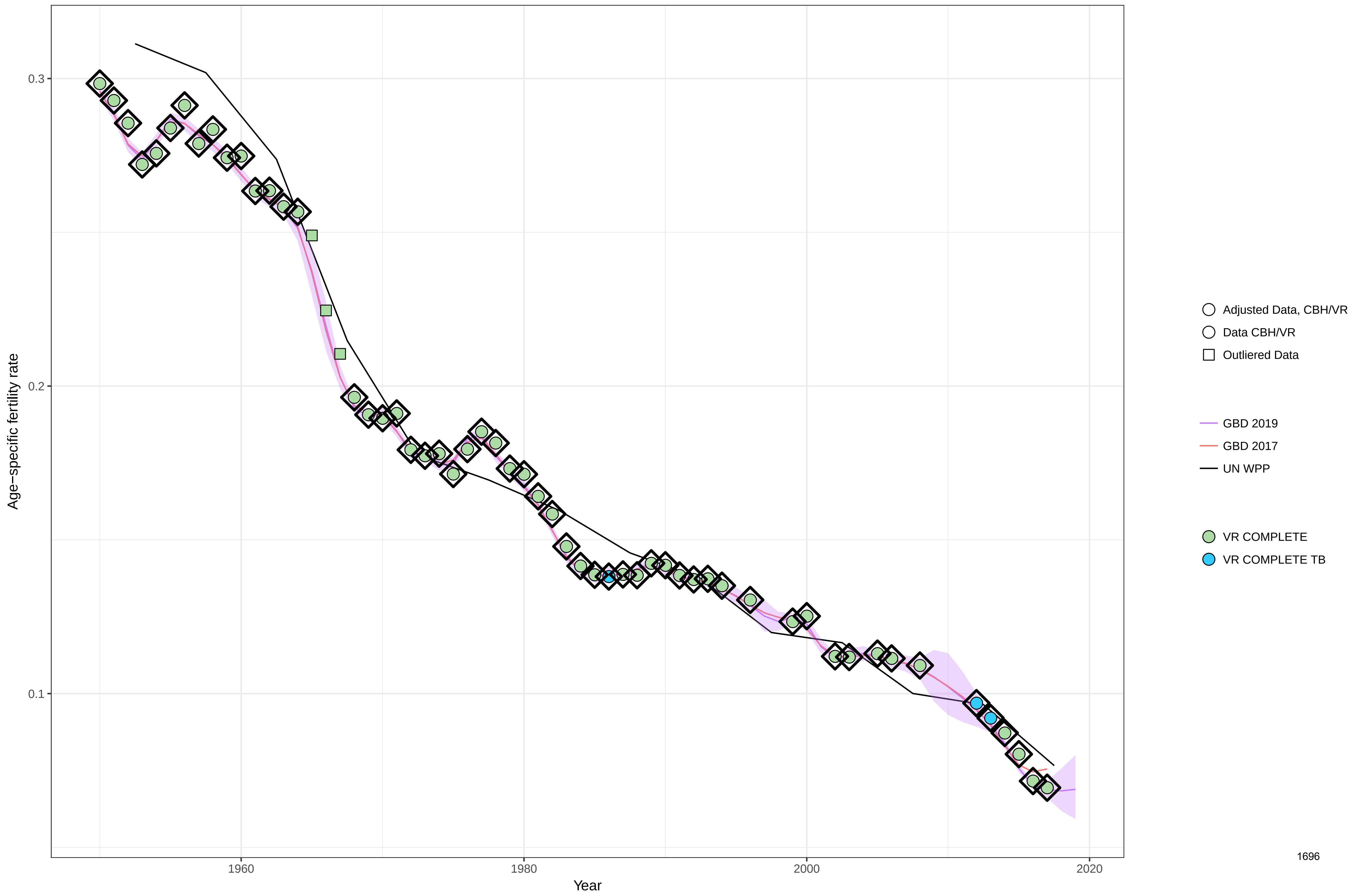

Age−specific fertility fitted trend lines, 1950−2019: Puerto Rico, Ages: 25−29
Data points enclosed with a diamond are reference sources

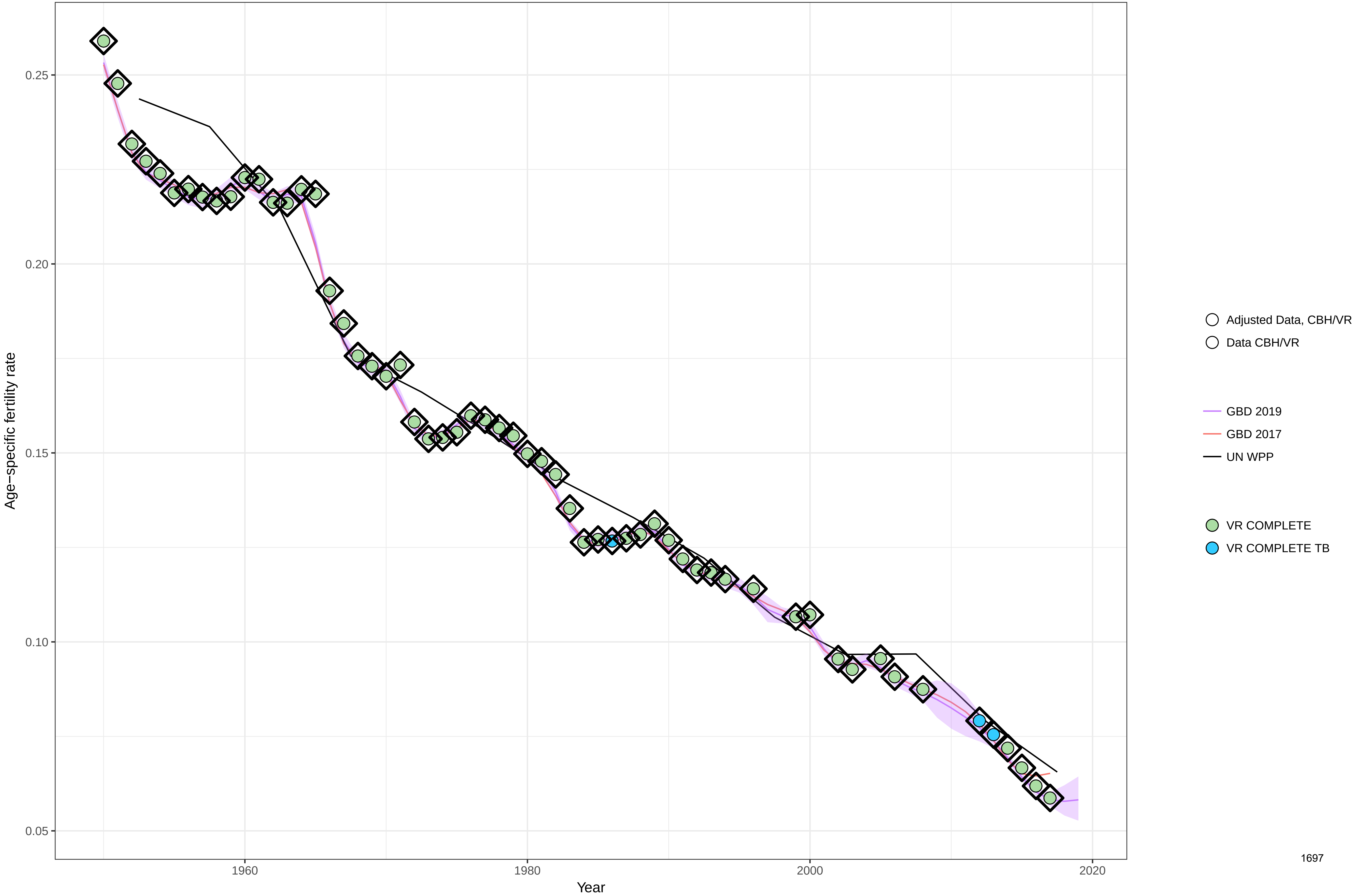

Age−specific fertility fitted trend lines, 1950−2019: Puerto Rico, Ages: 30−34
Data points enclosed with a diamond are reference sources

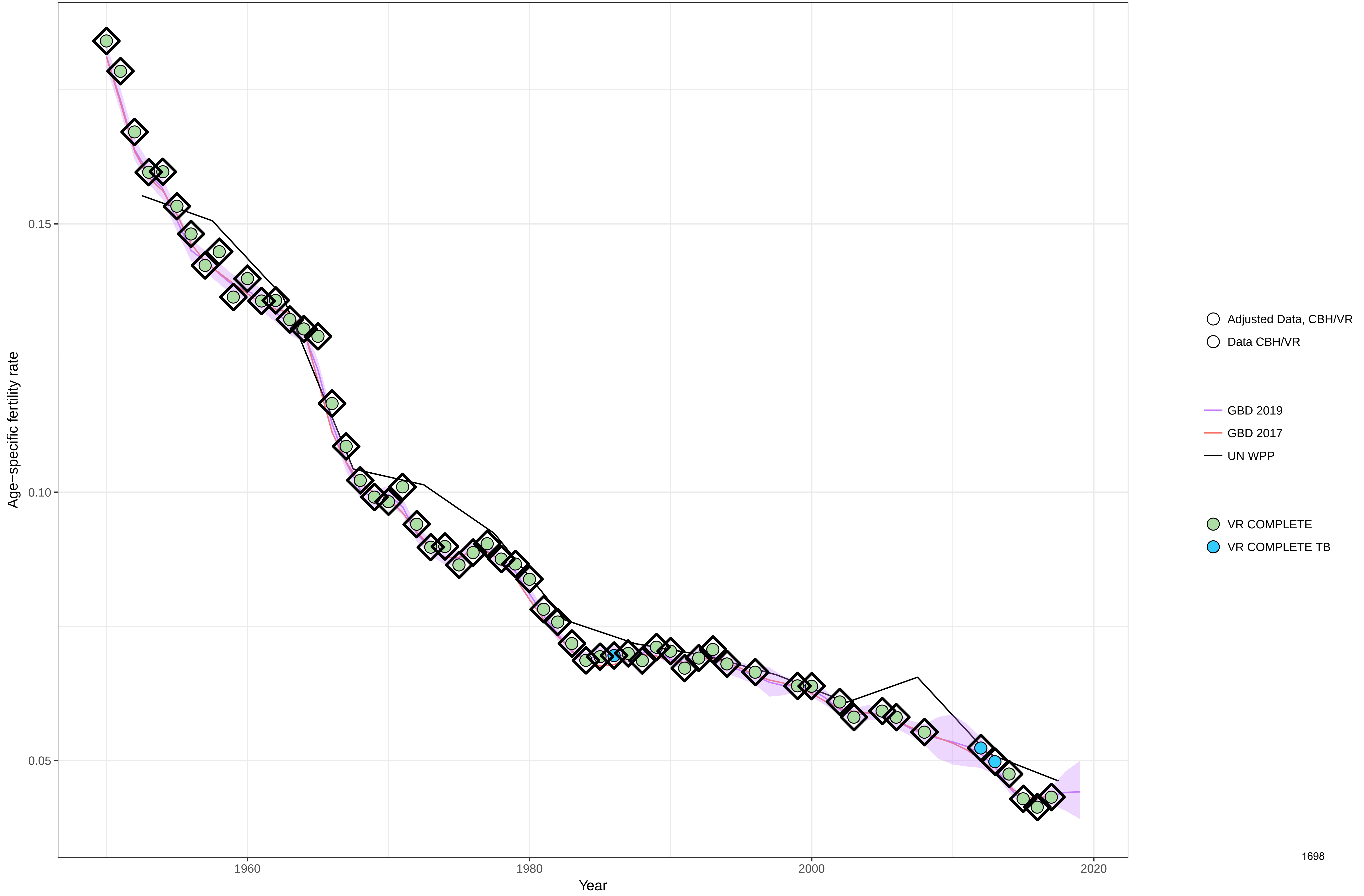

Age−specific fertility fitted trend lines, 1950−2019: Puerto Rico, Ages: 35−39
Data points enclosed with a diamond are reference sources

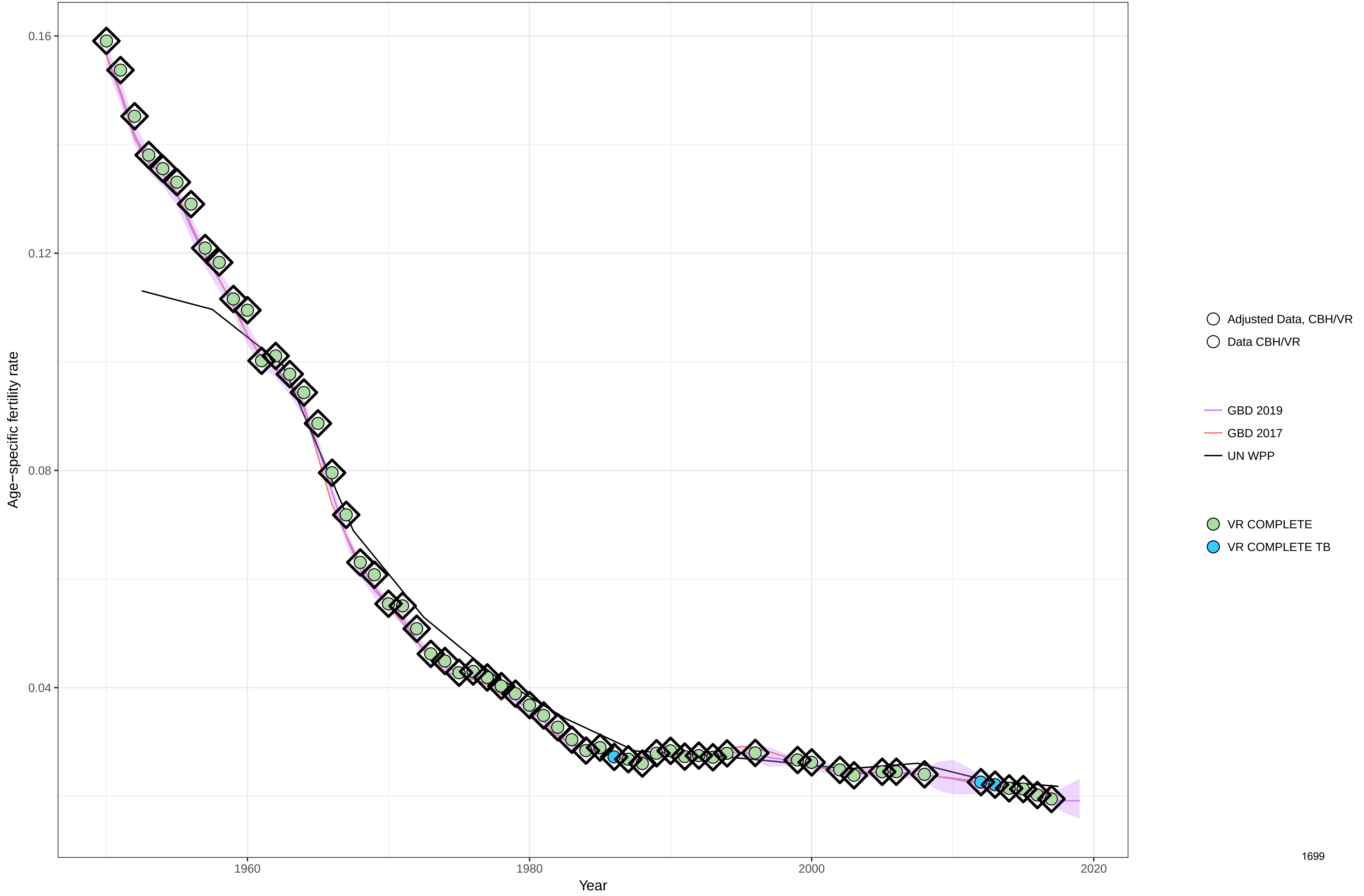

Age−specific fertility fitted trend lines, 1950−2019: Puerto Rico, Ages: 40−44
Data points enclosed with a diamond are reference sources

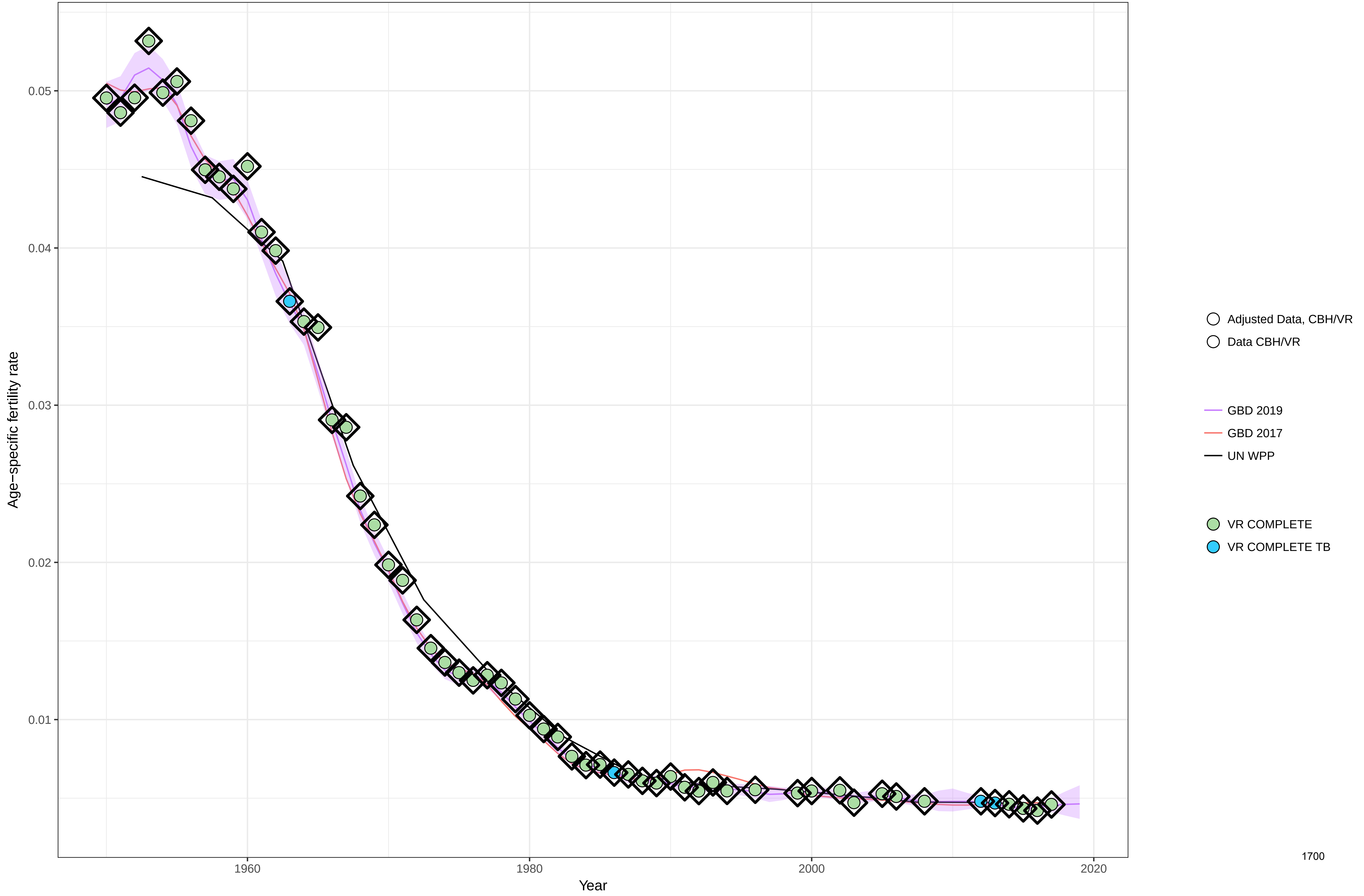

Age−specific fertility fitted trend lines, 1950−2019: Puerto Rico, Ages: 45−49
Data points enclosed with a diamond are reference sources

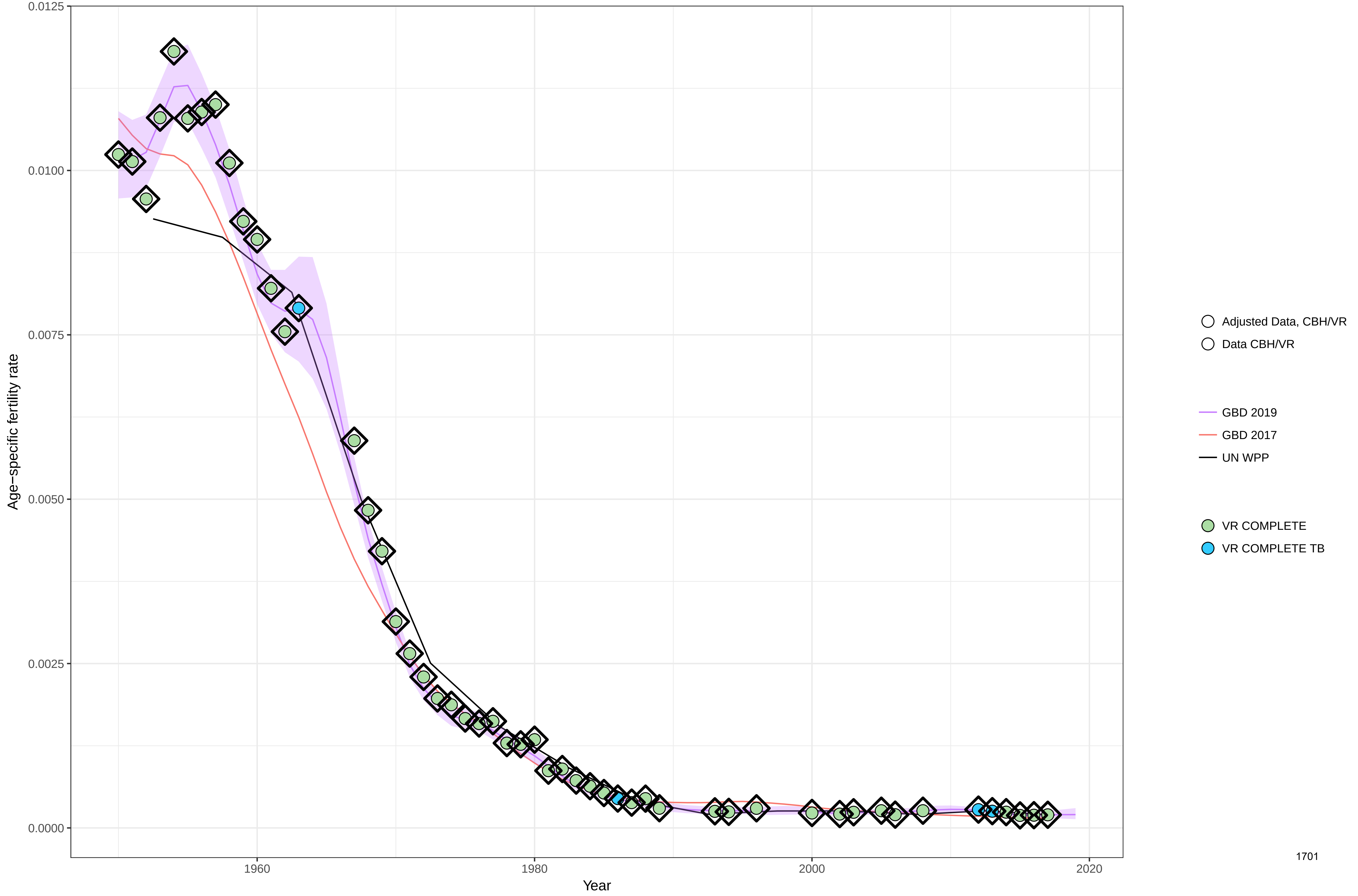

Age−specific fertility fitted trend lines, 1950−2019: Puerto Rico, Ages: 50−54
Data points enclosed with a diamond are reference sources

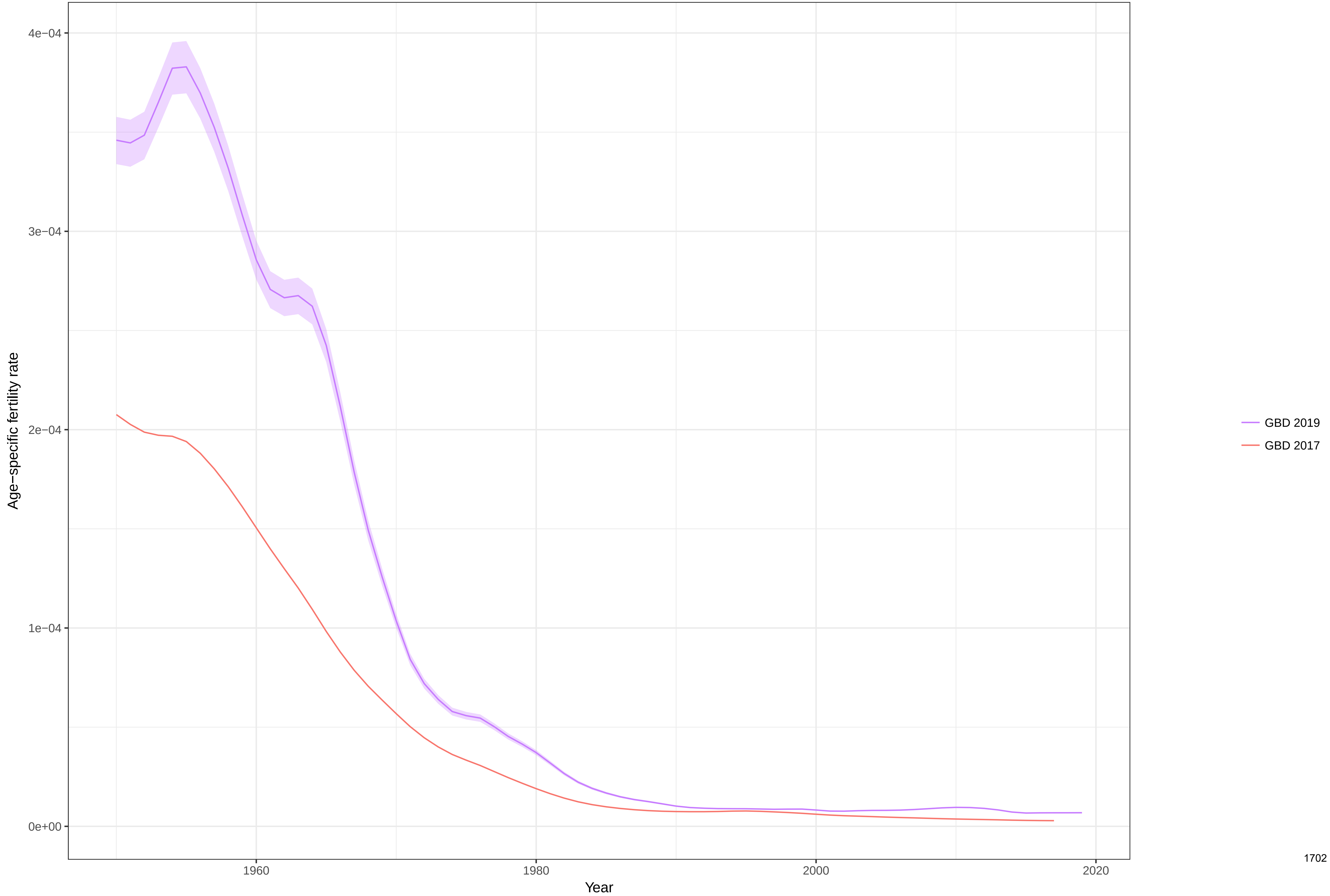

Total fertility rate fitted trend lines, 1950−2019: Puerto Rico TFR
Data points enclosed with a diamond are reference sources

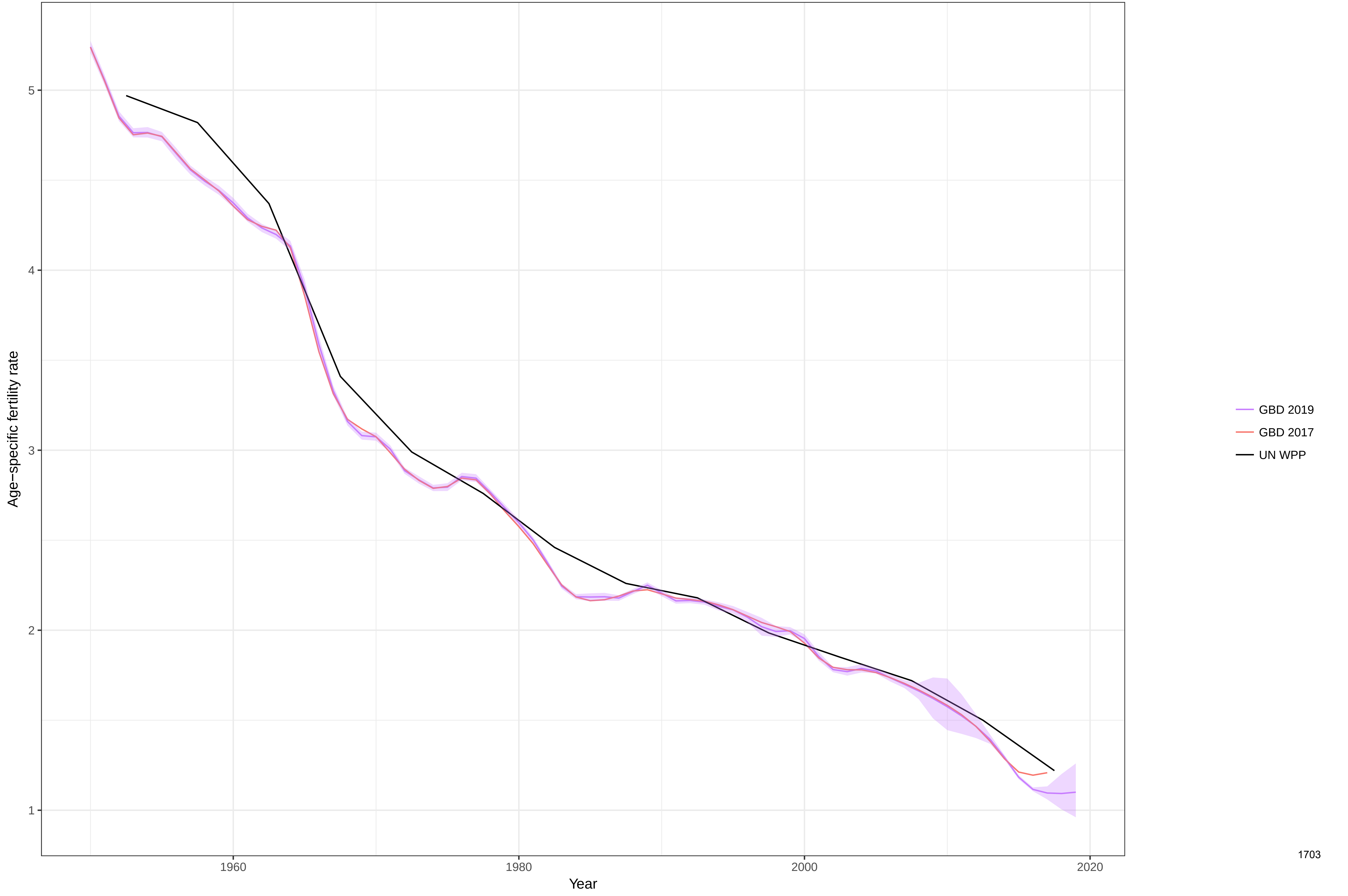

Age−specific fertility fitted trend lines, 1950−2019: Democratic People's Republic of Korea, Ages: 10−14
Data points enclosed with a diamond are reference sources

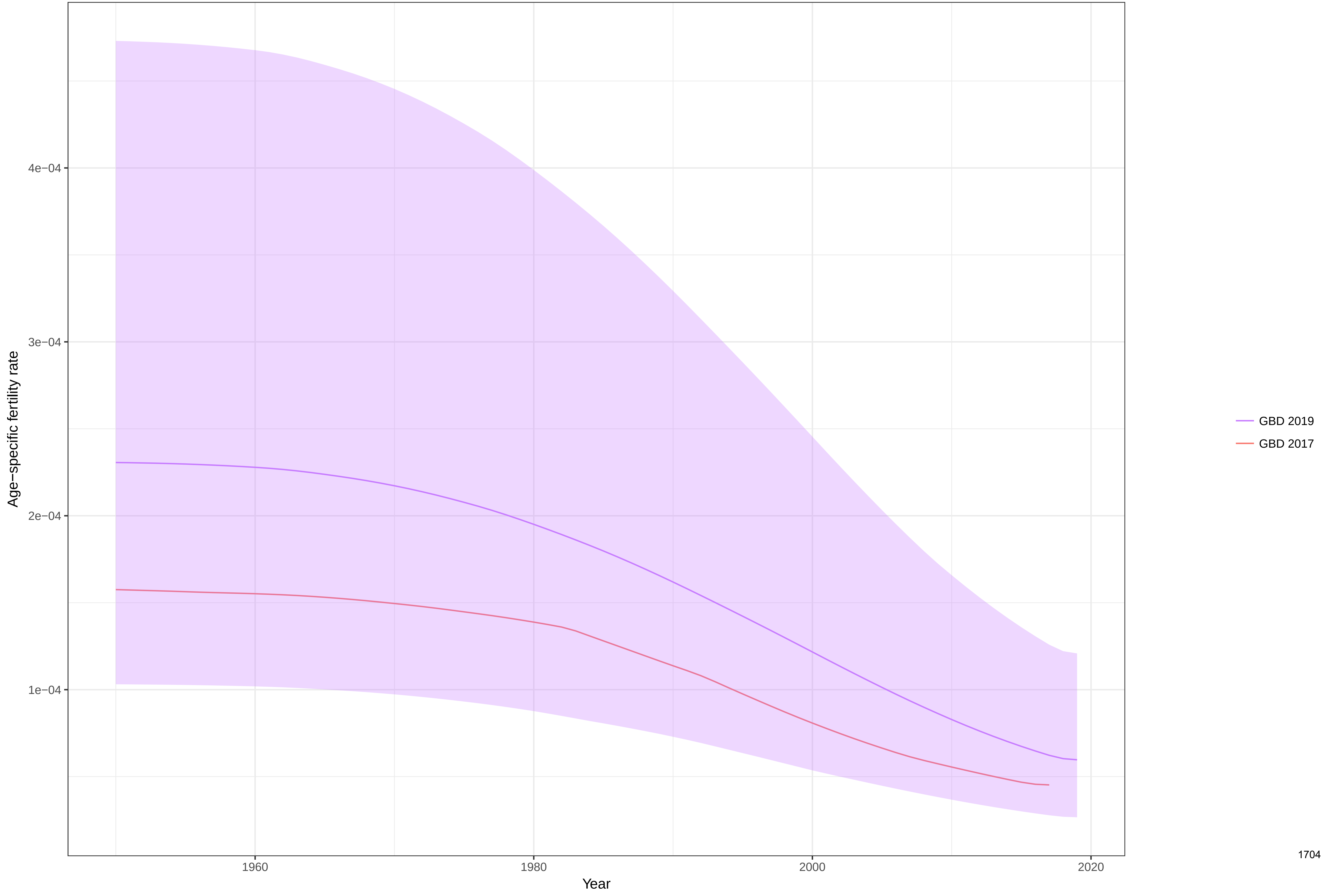

Age−specific fertility fitted trend lines, 1950−2019: Democratic People's Republic of Korea, Ages: 15−19
Data points enclosed with a diamond are reference sources

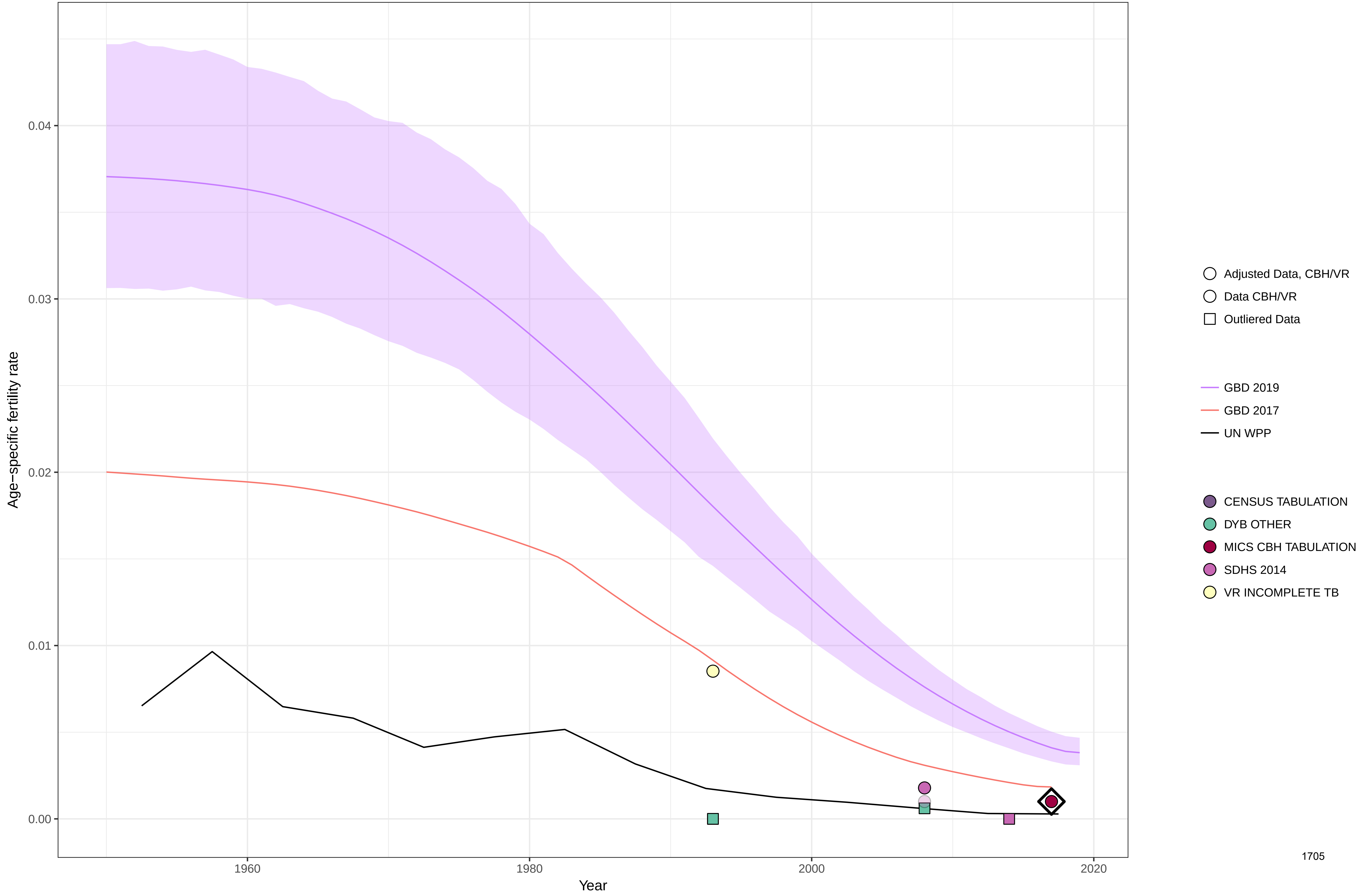

Age−specific fertility fitted trend lines, 1950−2019: Democratic People's Republic of Korea, Ages: 20−24
Data points enclosed with a diamond are reference sources

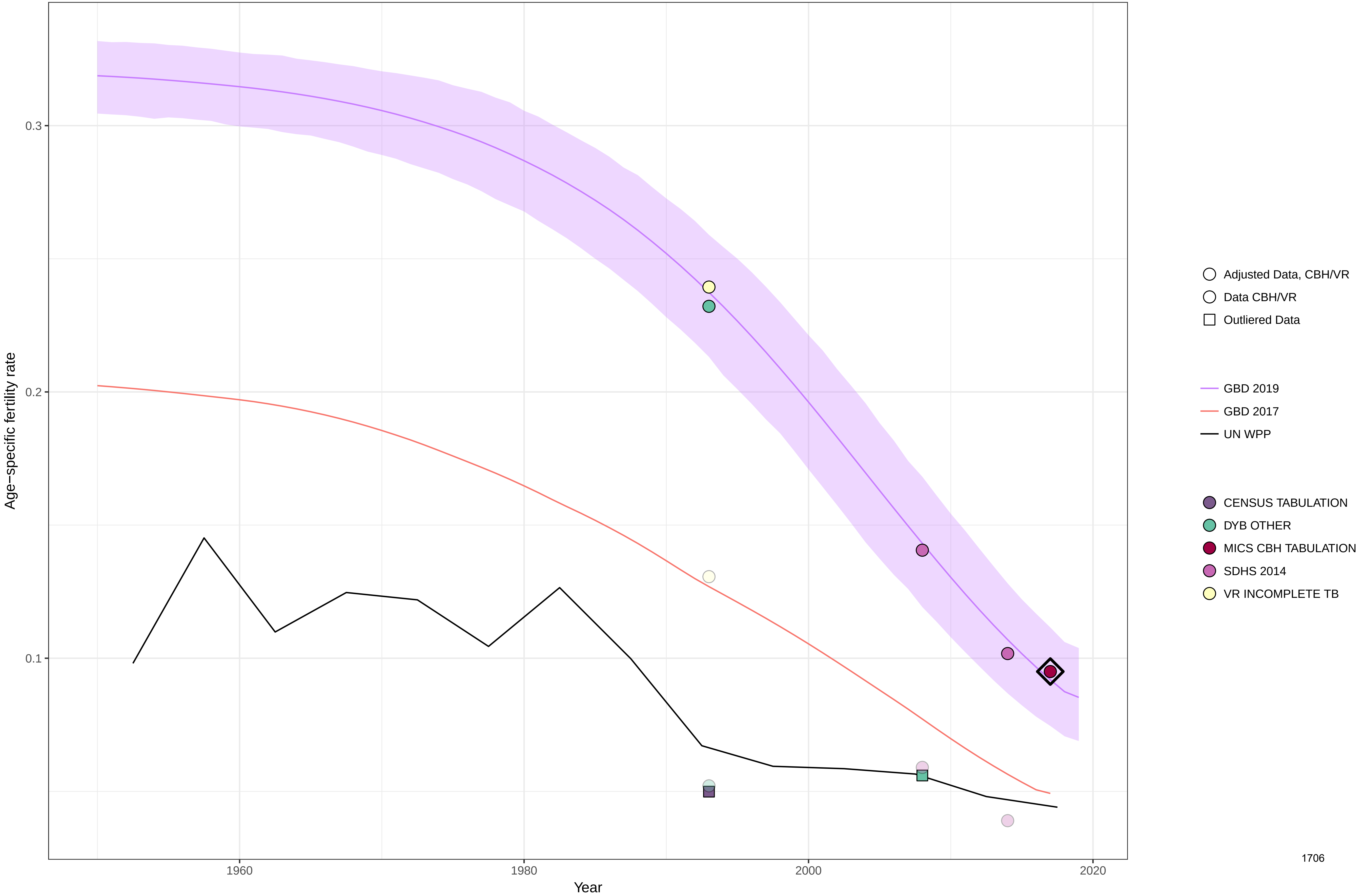

Age−specific fertility fitted trend lines, 1950−2019: Democratic People's Republic of Korea, Ages: 25−29
Data points enclosed with a diamond are reference sources

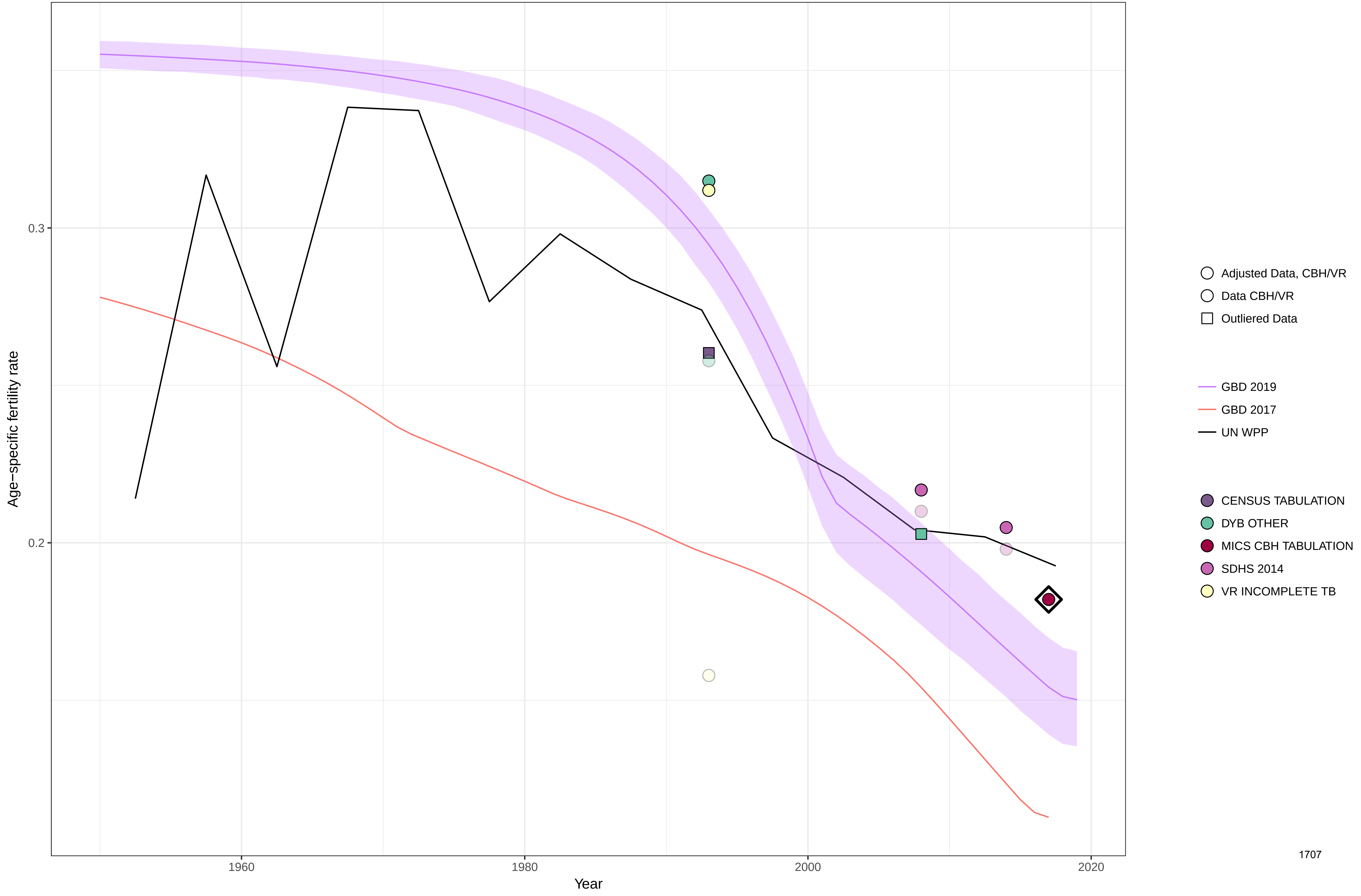

Age−specific fertility fitted trend lines, 1950−2019: Democratic People's Republic of Korea, Ages: 30−34
Data points enclosed with a diamond are reference sources

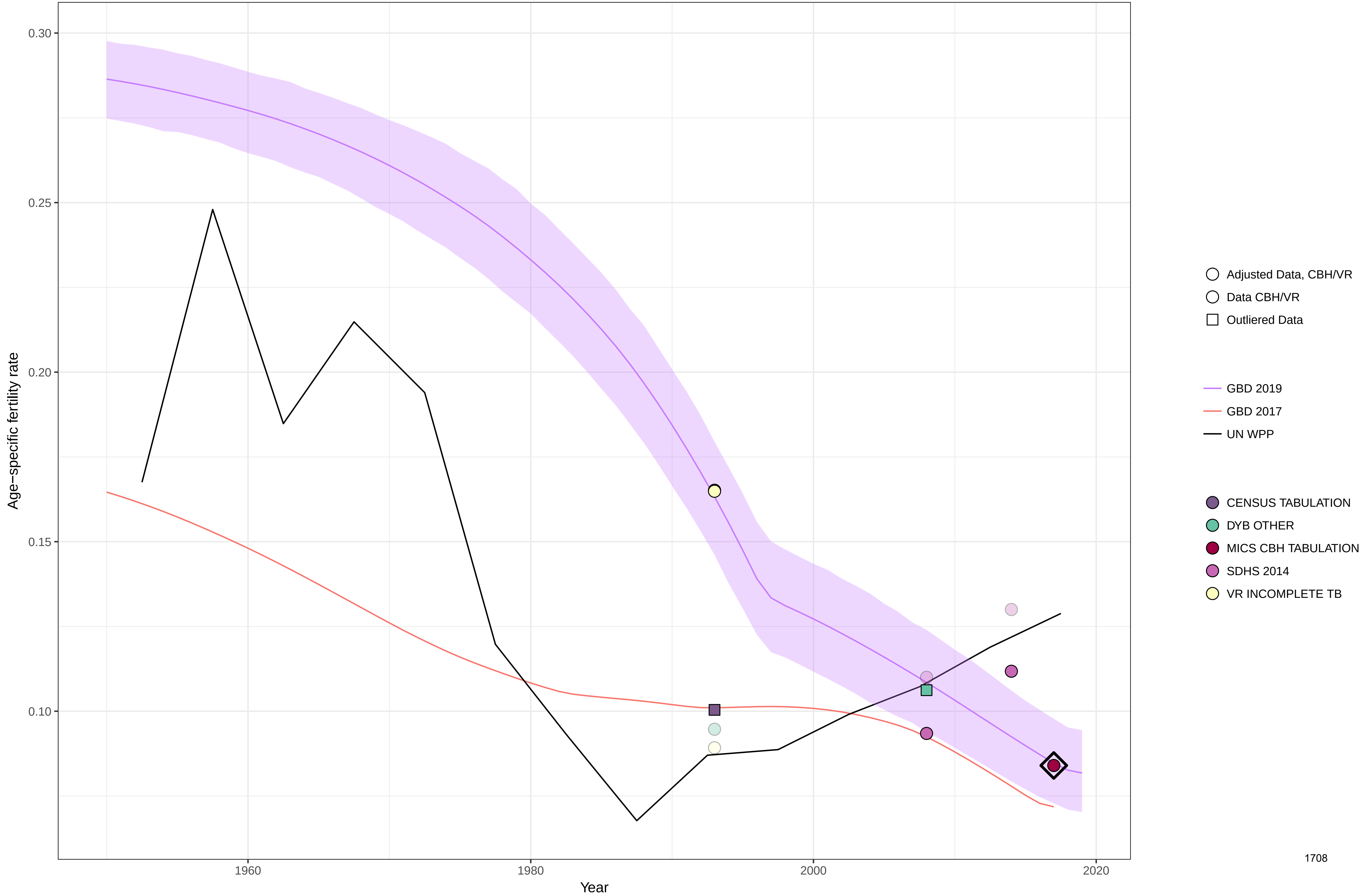

Age−specific fertility fitted trend lines, 1950−2019: Democratic People's Republic of Korea, Ages: 35−39
Data points enclosed with a diamond are reference sources

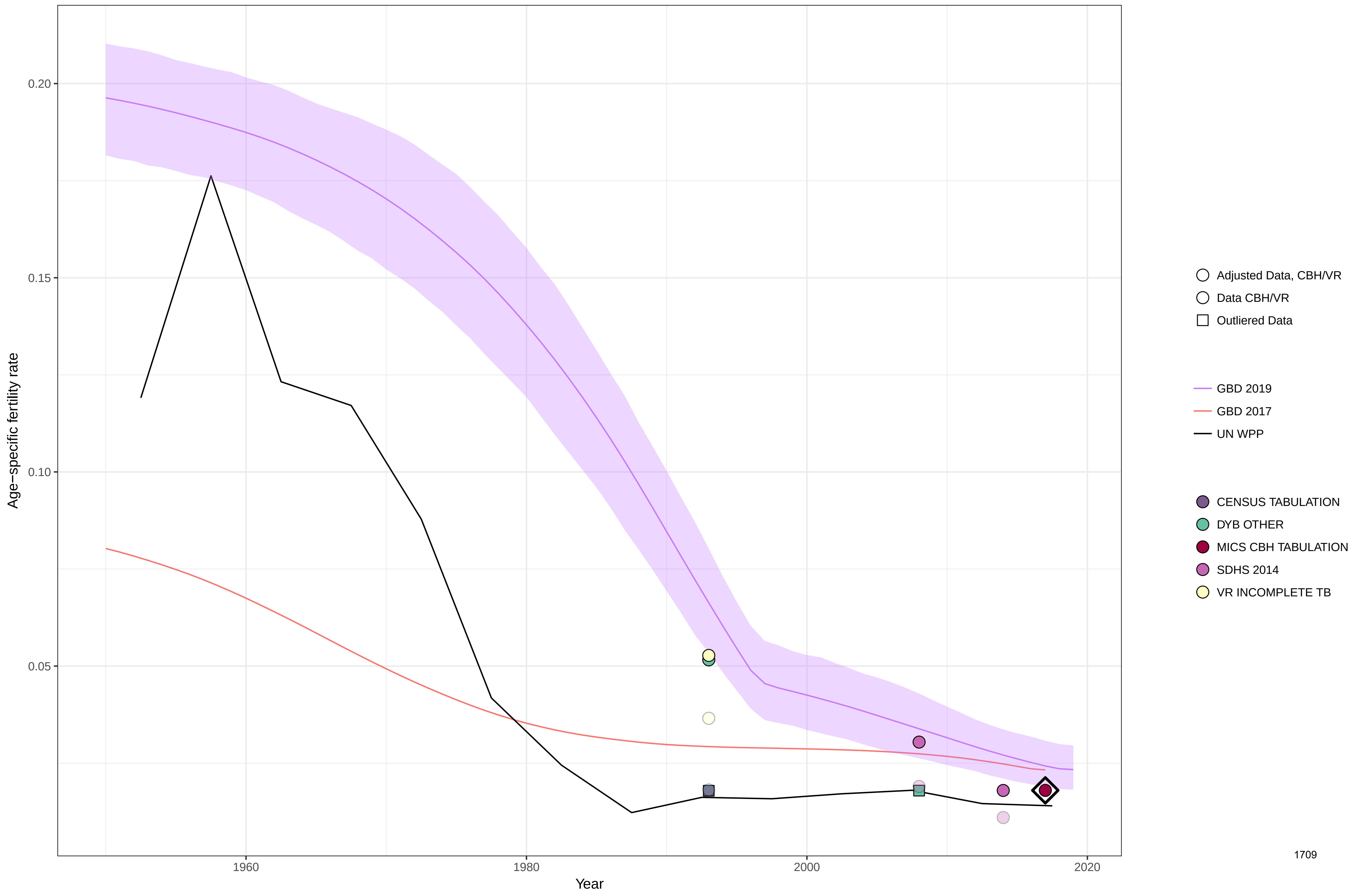

Age−specific fertility fitted trend lines, 1950−2019: Democratic People's Republic of Korea, Ages: 40−44
Data points enclosed with a diamond are reference sources

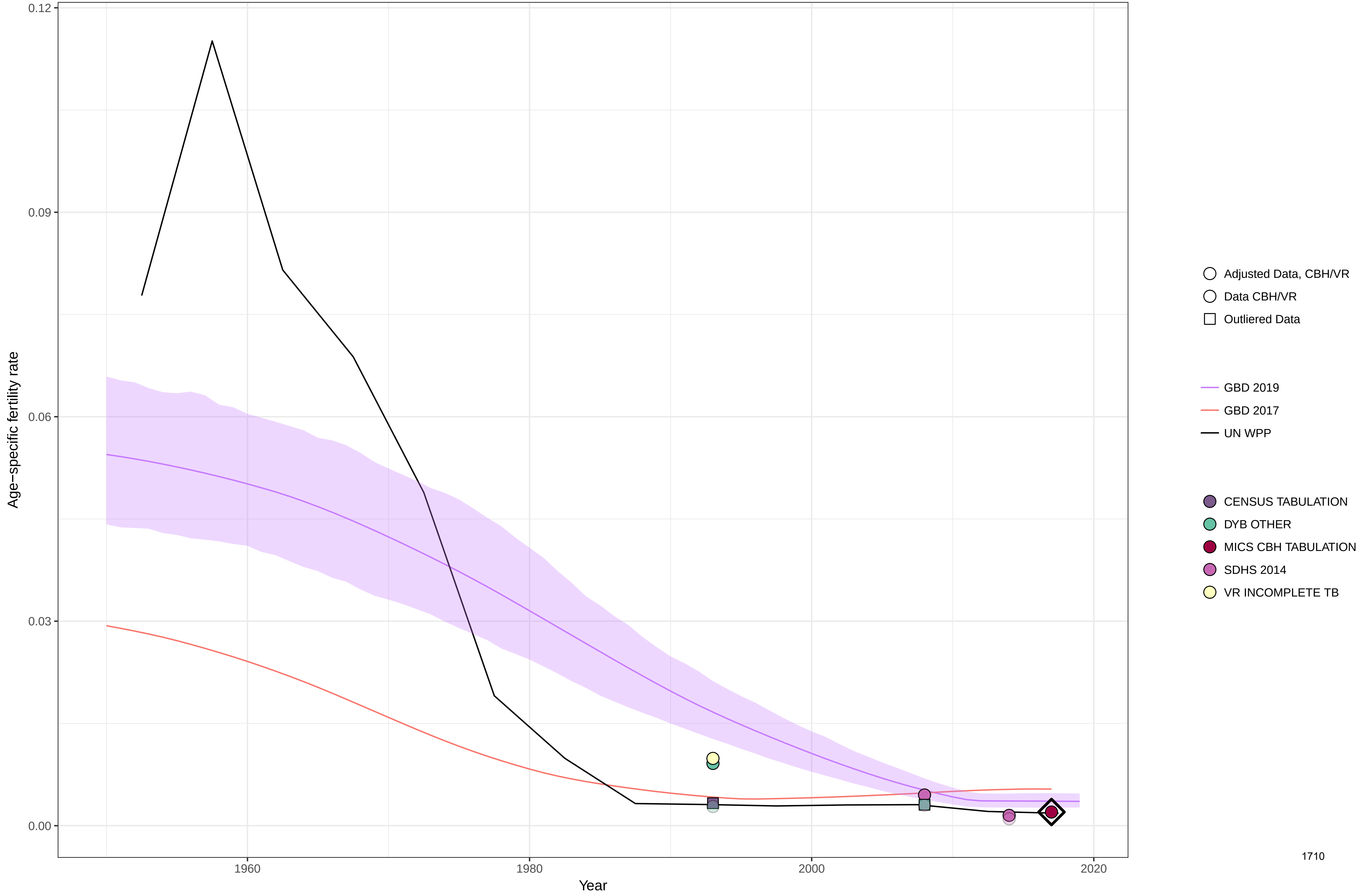

Age−specific fertility fitted trend lines, 1950−2019: Democratic People's Republic of Korea, Ages: 45−49
Data points enclosed with a diamond are reference sources

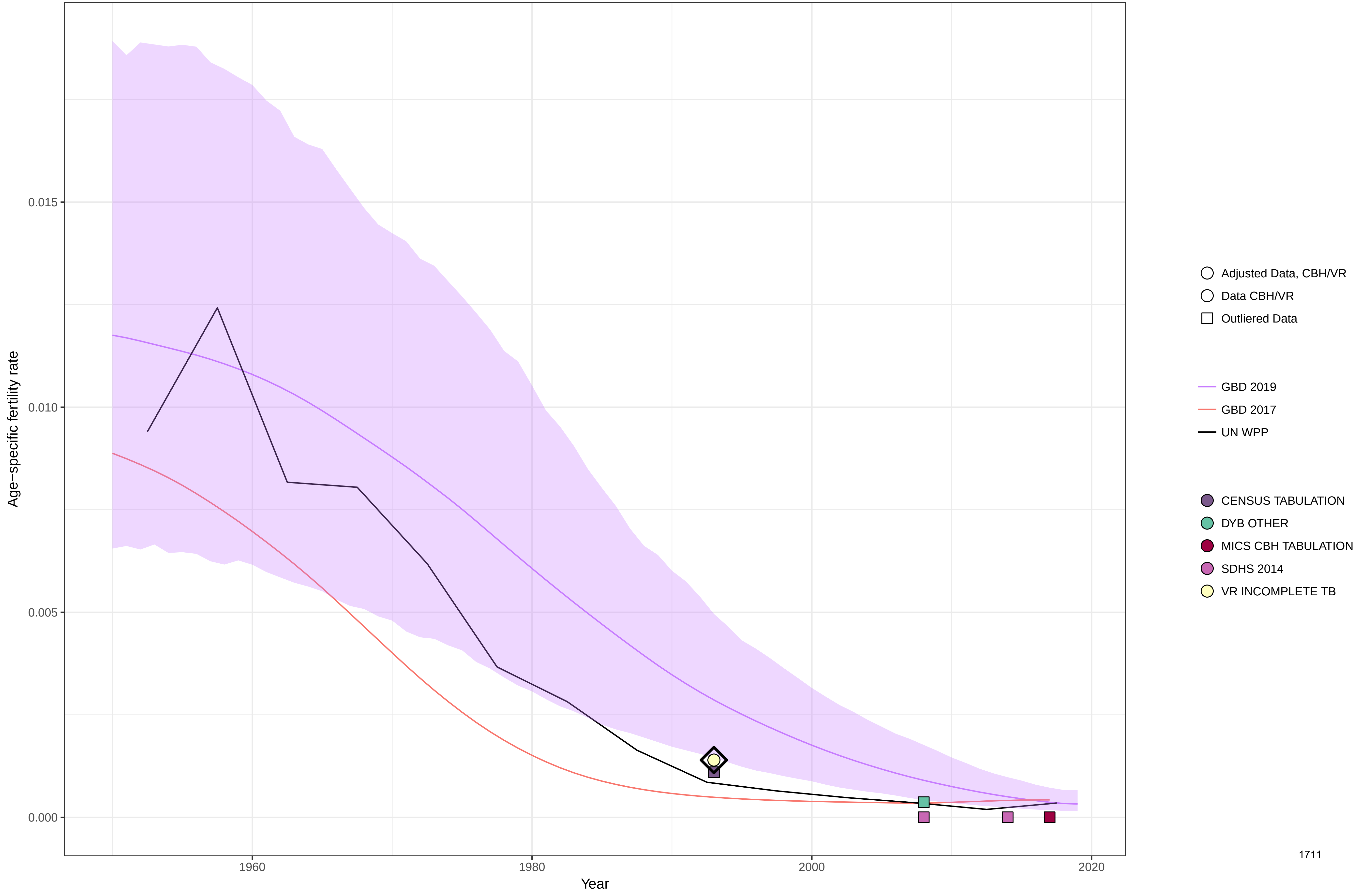

Age−specific fertility fitted trend lines, 1950−2019: Democratic People's Republic of Korea, Ages: 50−54
Data points enclosed with a diamond are reference sources

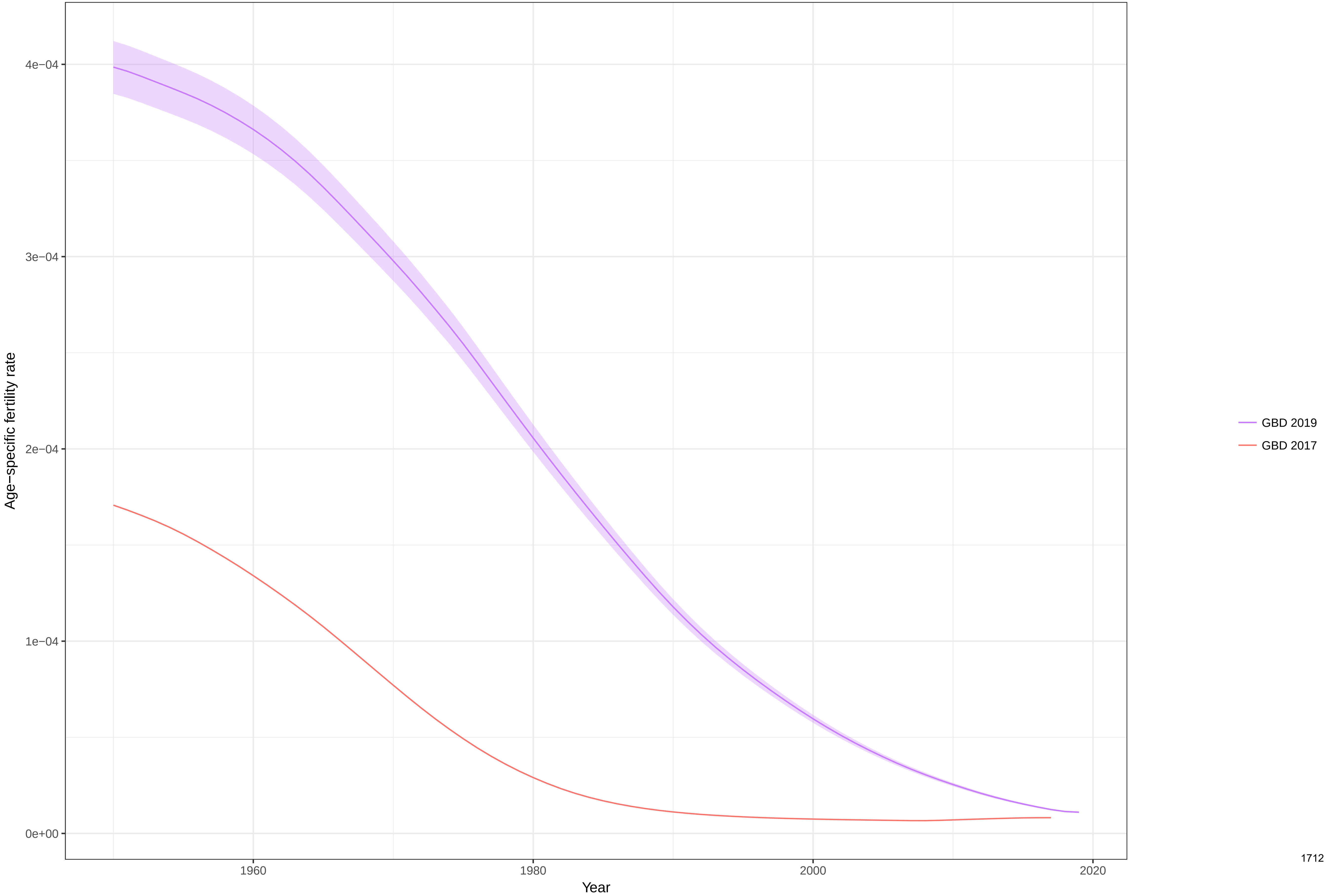

Total fertility rate fitted trend lines, 1950−2019: Democratic People's Republic of Korea TFR
Data points enclosed with a diamond are reference sources

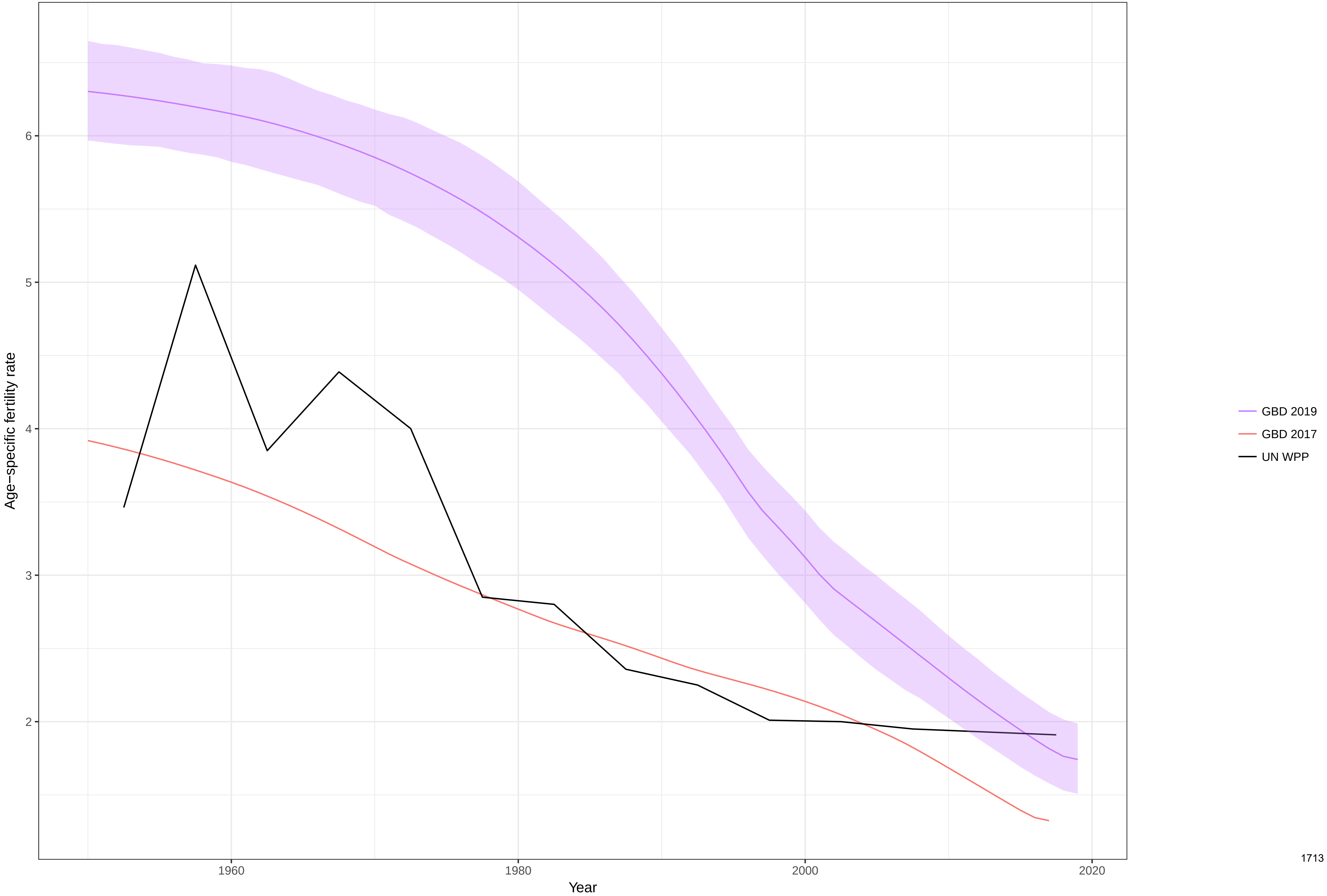

Age−specific fertility fitted trend lines, 1950−2019: Portugal, Ages: 10−14
Data points enclosed with a diamond are reference sources

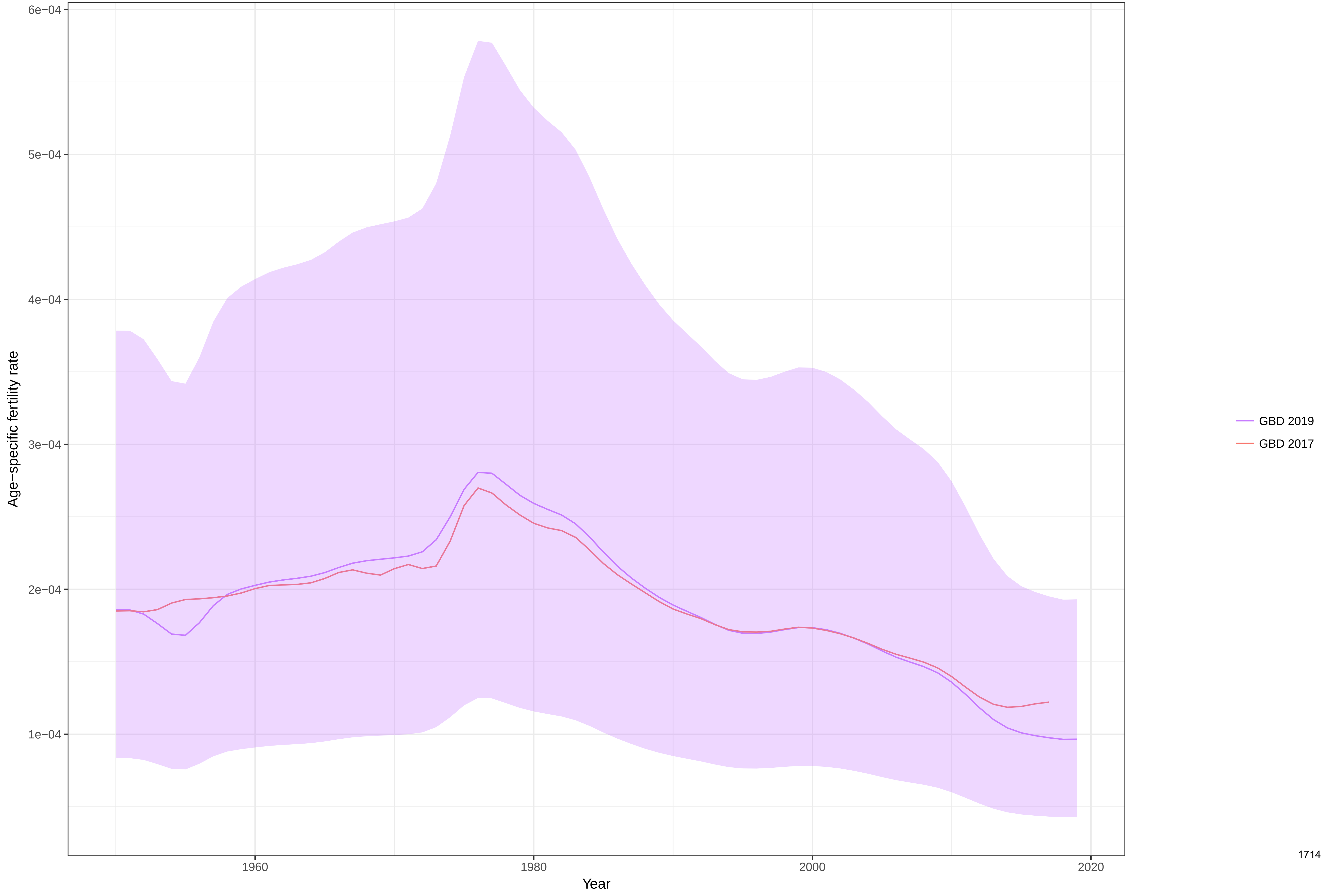

Age−specific fertility fitted trend lines, 1950−2019: Portugal, Ages: 15−19
Data points enclosed with a diamond are reference sources

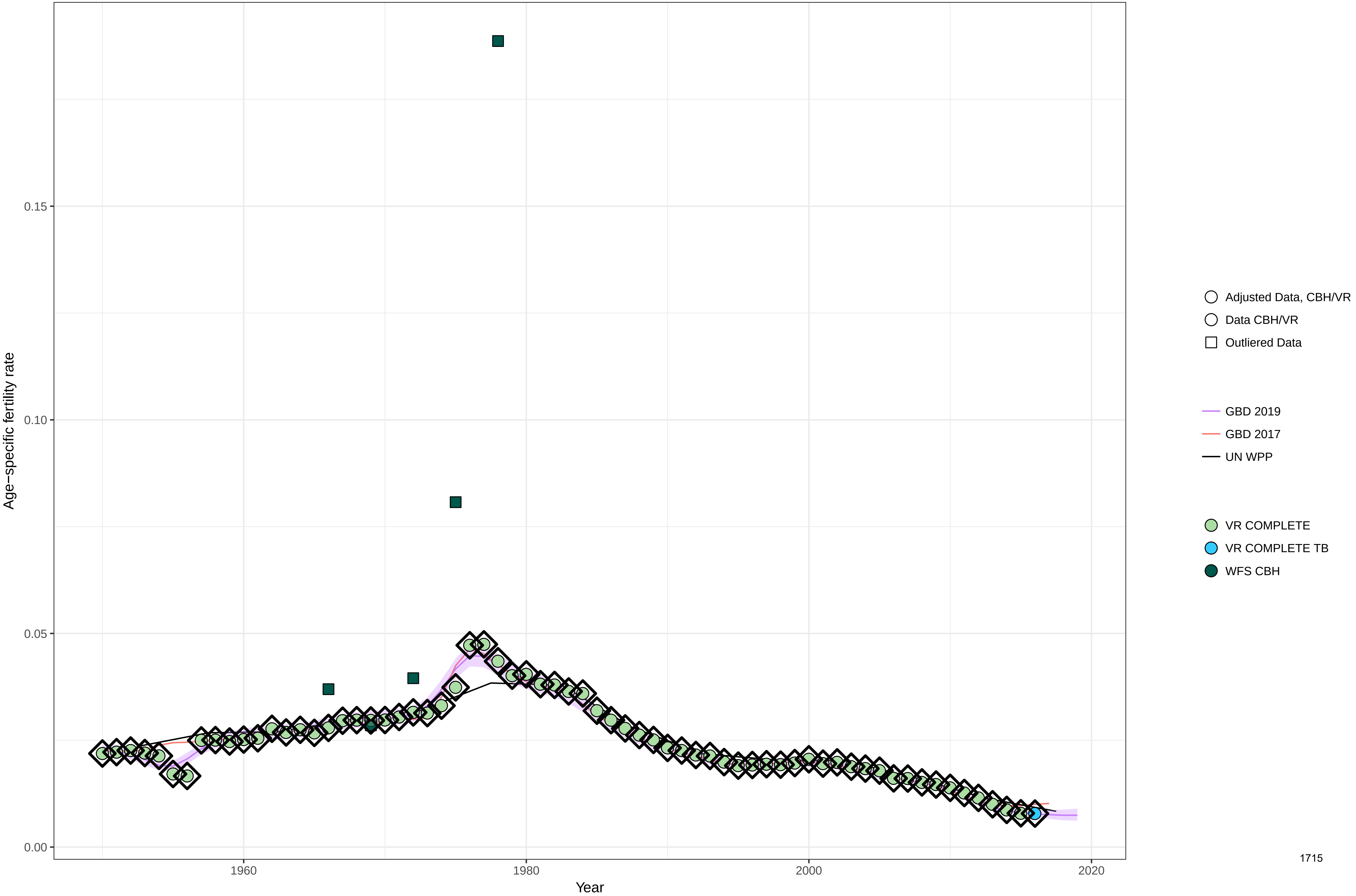

Age−specific fertility fitted trend lines, 1950−2019: Portugal, Ages: 20−24
Data points enclosed with a diamond are reference sources

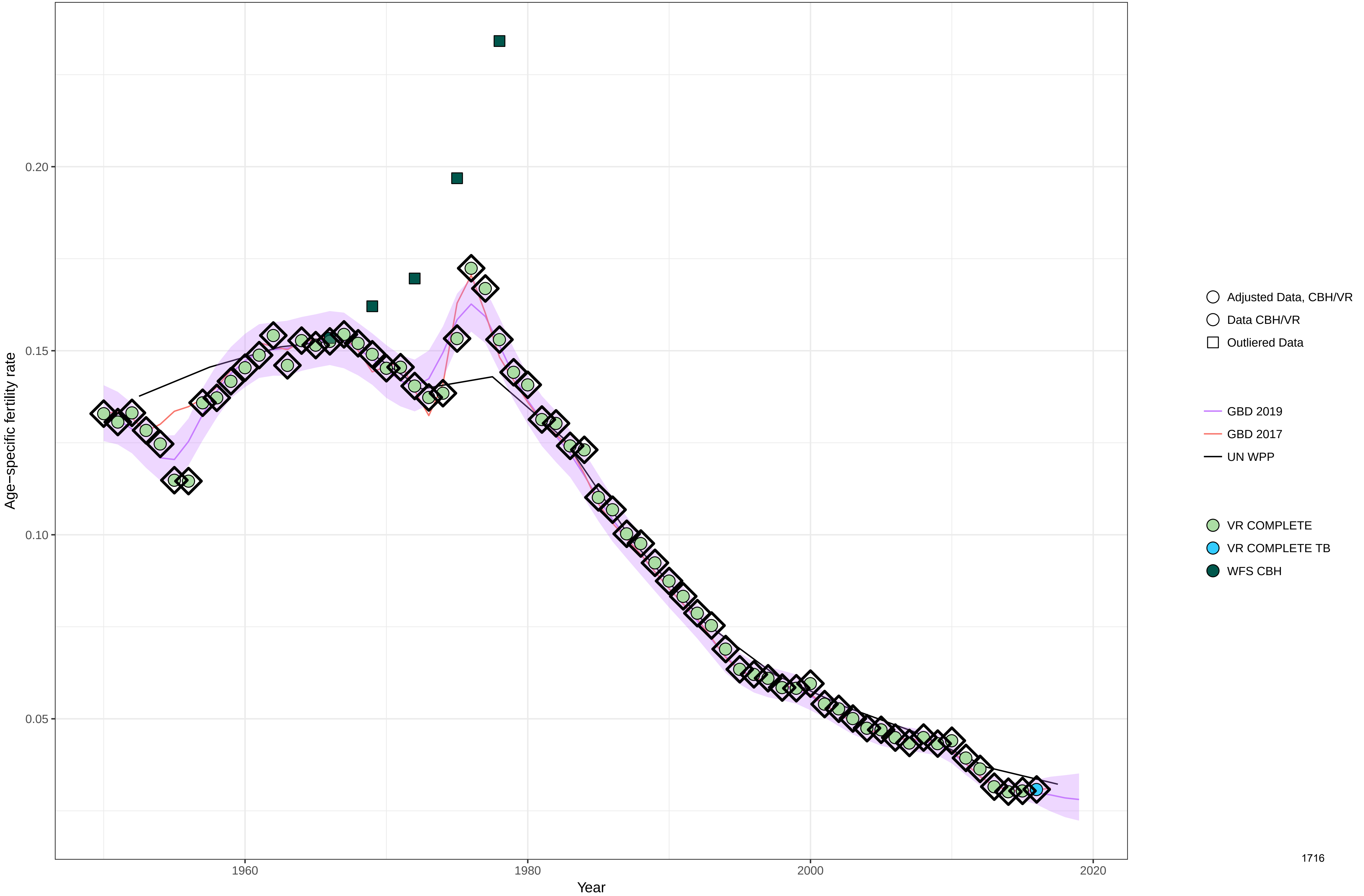

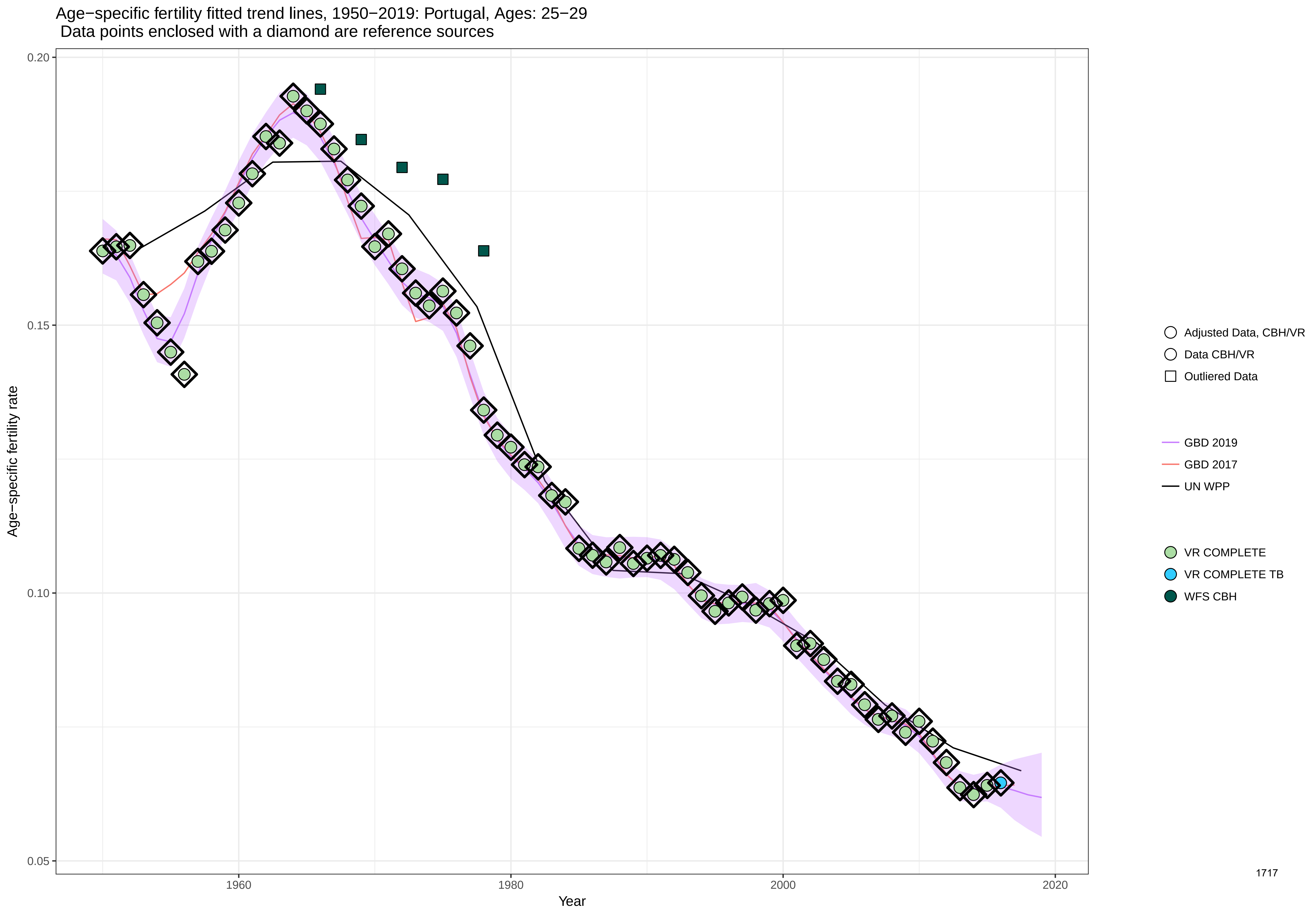

Age−specific fertility fitted trend lines, 1950−2019: Portugal, Ages: 25−29
Data points enclosed with a diamond are reference sources
Age−specific fertility rate
0.20
0.15
0.10
0.05
Year
1960
1980
2000
2020
Adjusted Data, CBH/VR
Data CBH/VR
Outliered Data
GBD 2019
GBD 2017
UN WPP
VR COMPLETE
VR COMPLETE TB
WFS CBH

Age−specific fertility fitted trend lines, 1950−2019: Portugal, Ages: 30−34
Data points enclosed with a diamond are reference sources

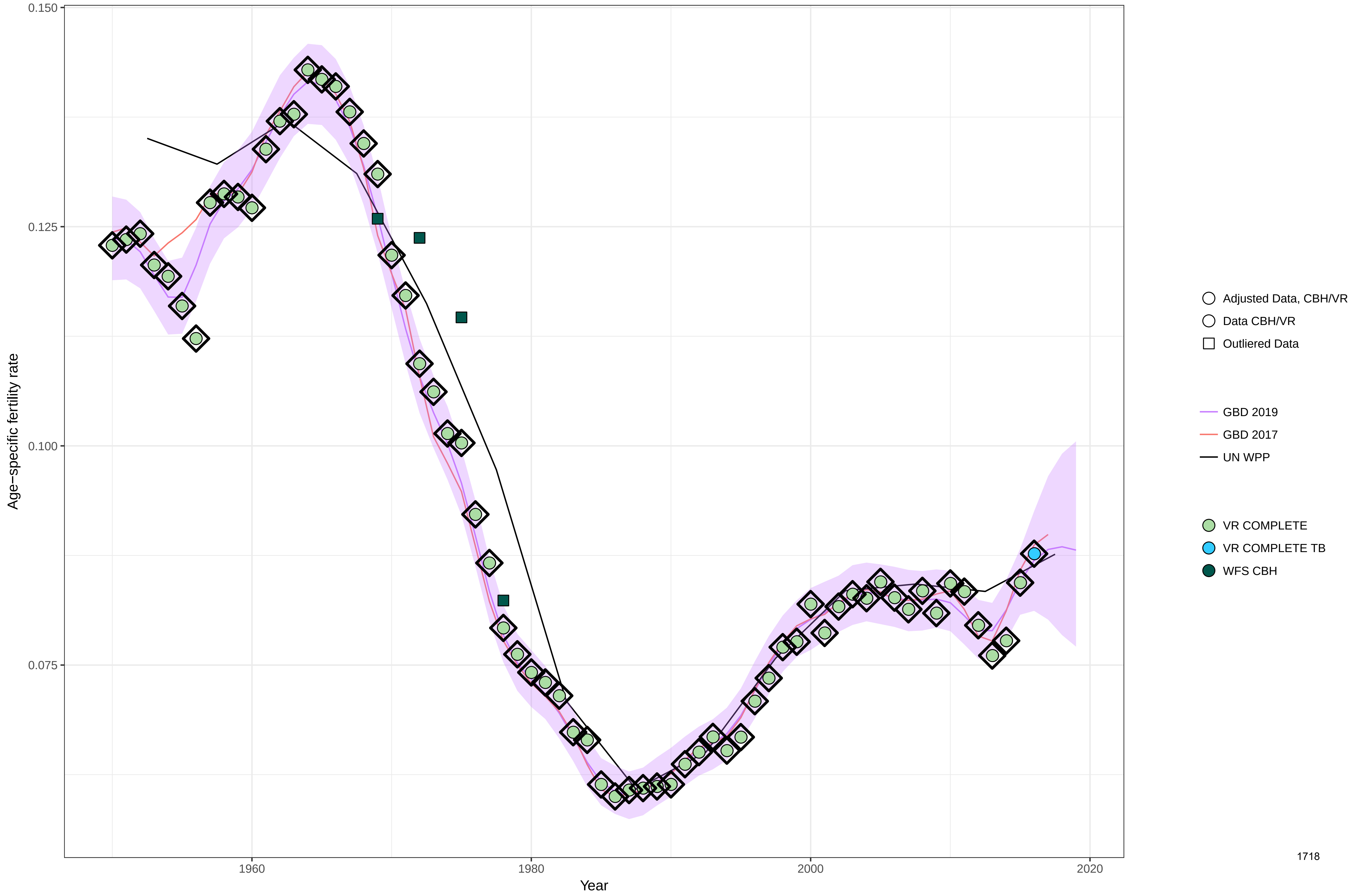

Age−specific fertility fitted trend lines, 1950−2019: Portugal, Ages: 35−39
Data points enclosed with a diamond are reference sources

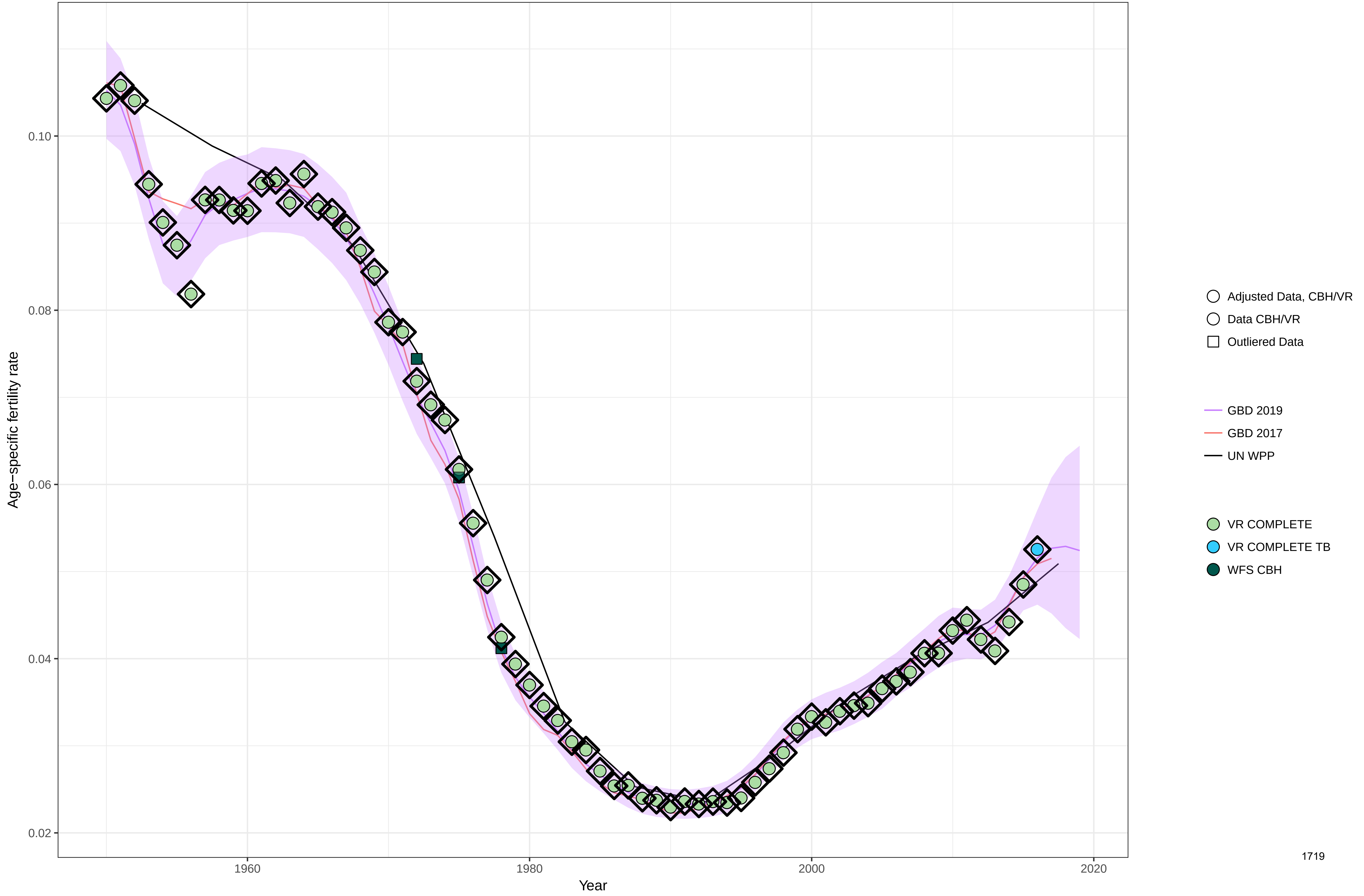

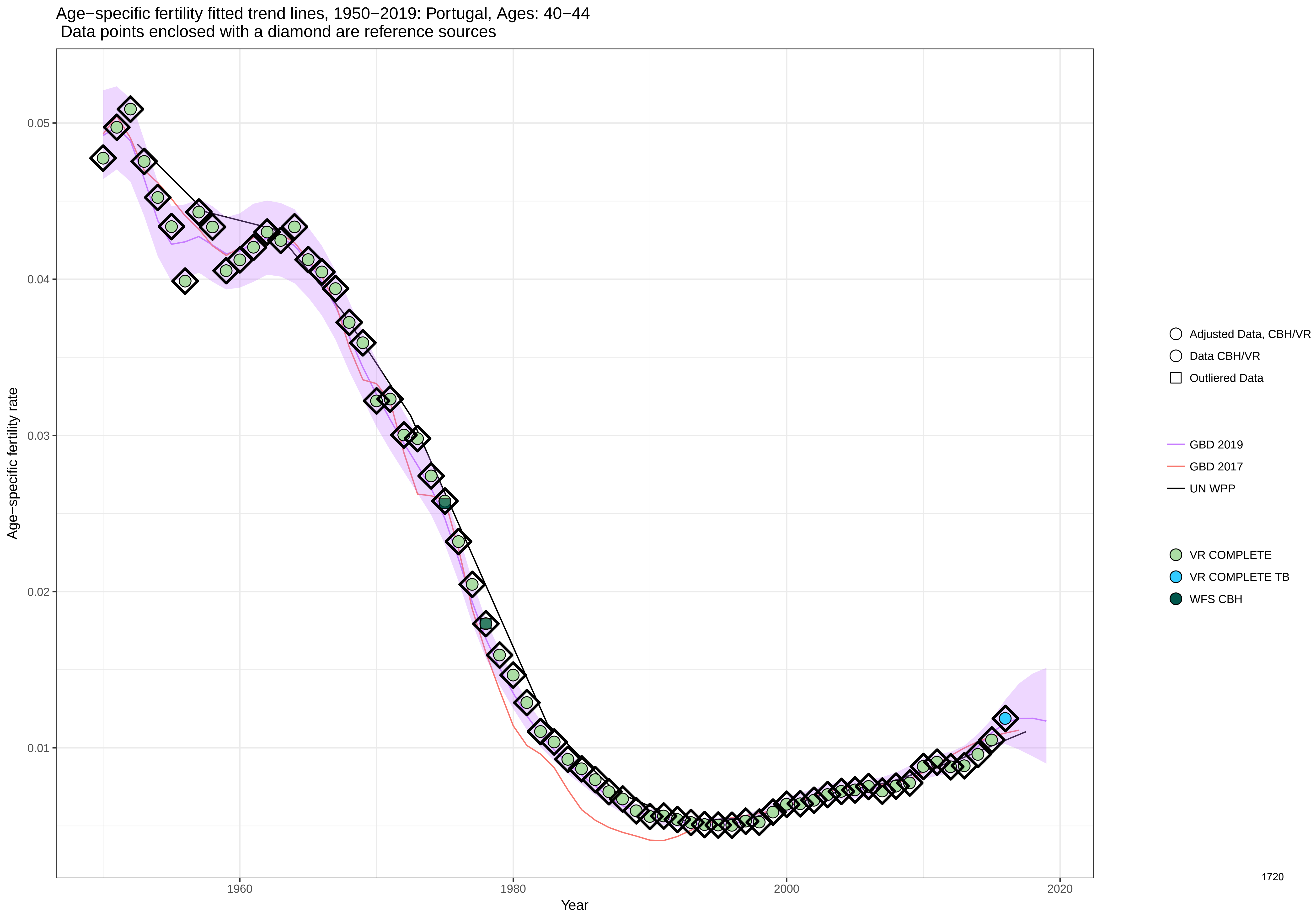

Age−specific fertility fitted trend lines, 1950−2019: Portugal, Ages: 40−44
Data points enclosed with a diamond are reference sources
0.05
0.04
0.03
0.02
0.01
Age−specific fertility rate
1960
1980
2000
2020
Year
Adjusted Data, CBH/VR
Data CBH/VR
Outliered Data
GBD 2019
GBD 2017
UN WPP
VR COMPLETE
VR COMPLETE TB
WFS CBH

Age−specific fertility fitted trend lines, 1950−2019: Portugal, Ages: 45−49
Data points enclosed with a diamond are reference sources

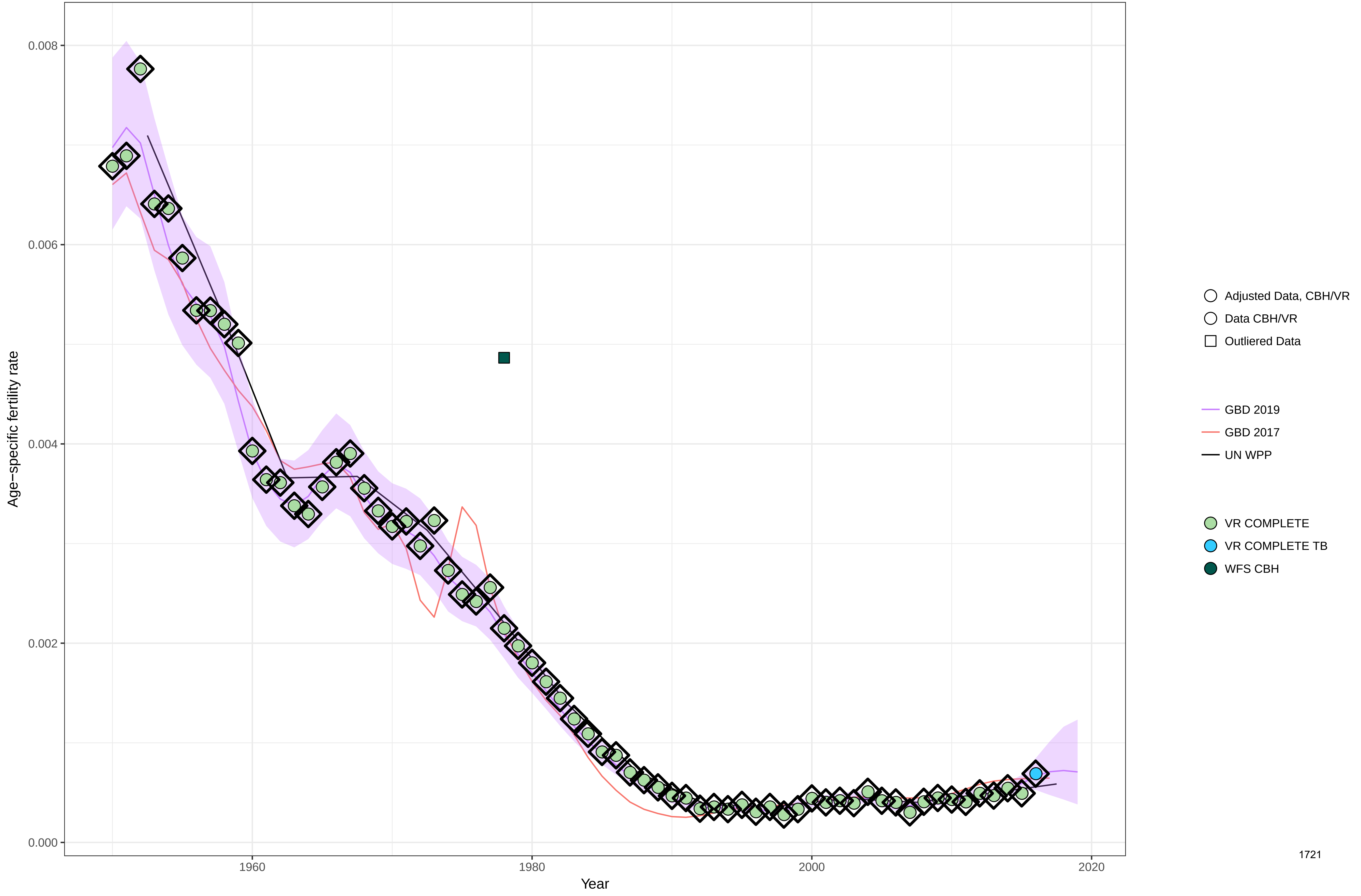

Age−specific fertility fitted trend lines, 1950−2019: Portugal, Ages: 50−54
Data points enclosed with a diamond are reference sources

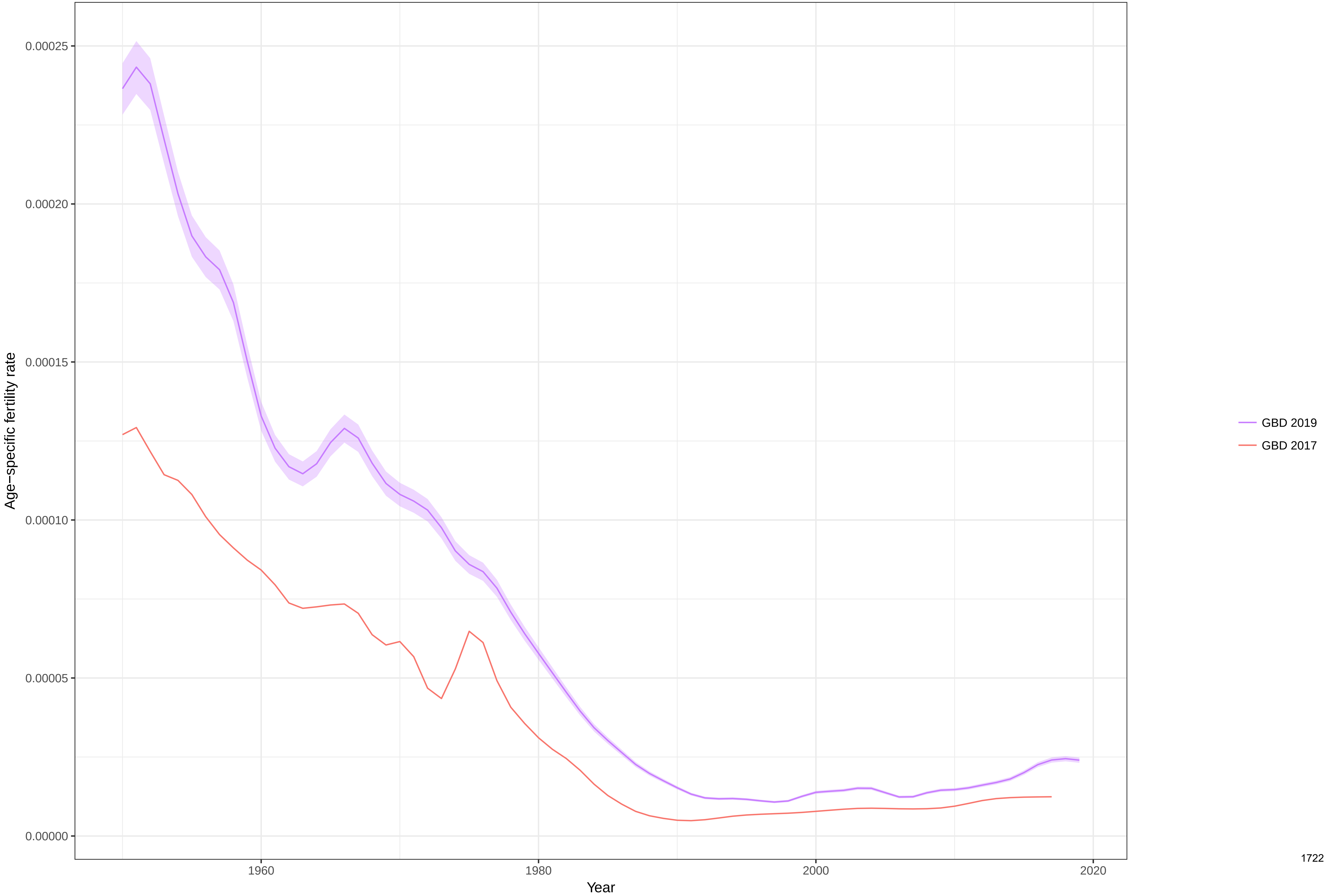

Total fertility rate fitted trend lines, 1950−2019: Portugal TFR
Data points enclosed with a diamond are reference sources

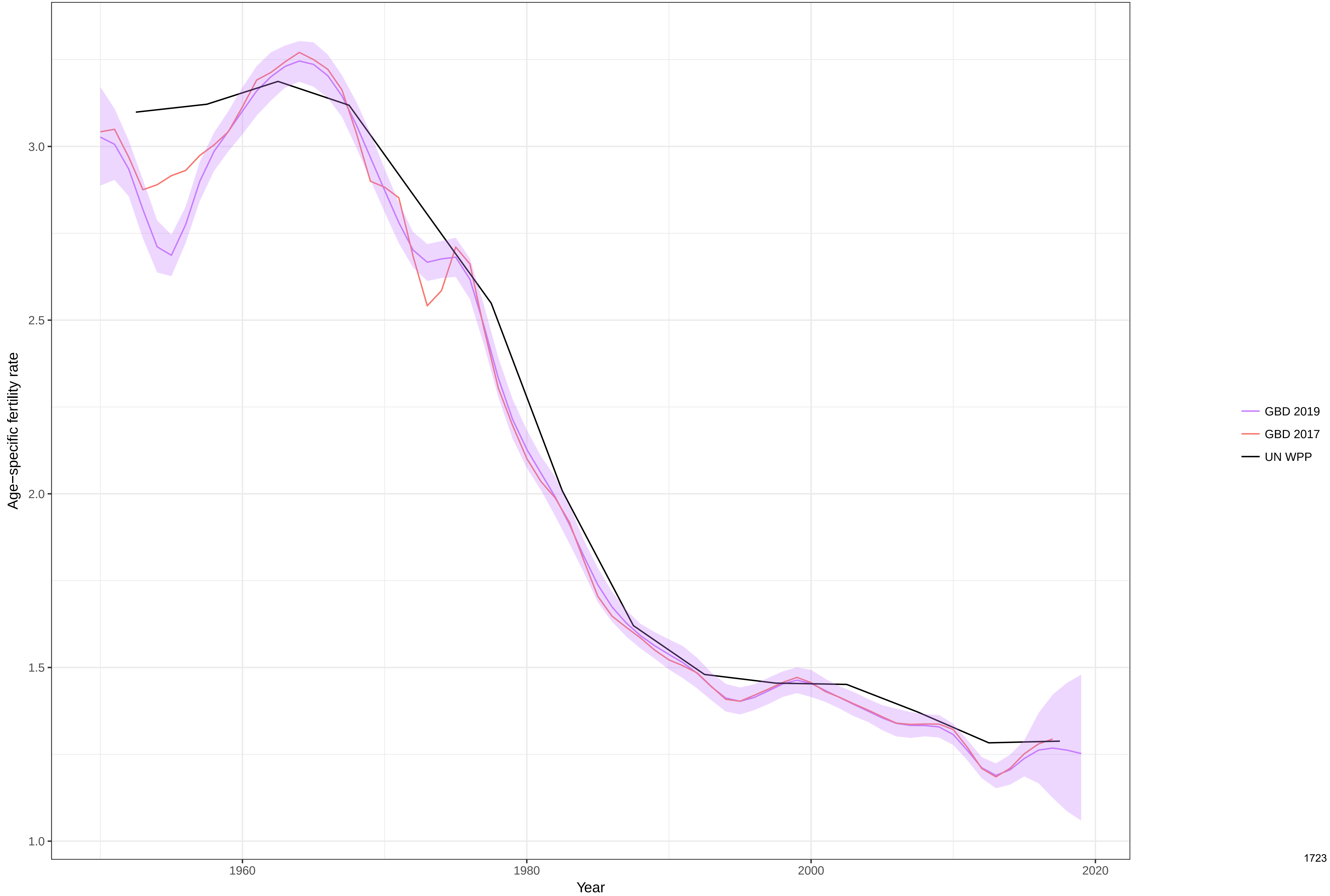

Age−specific fertility fitted trend lines, 1950−2019: Paraguay, Ages: 10−14
Data points enclosed with a diamond are reference sources

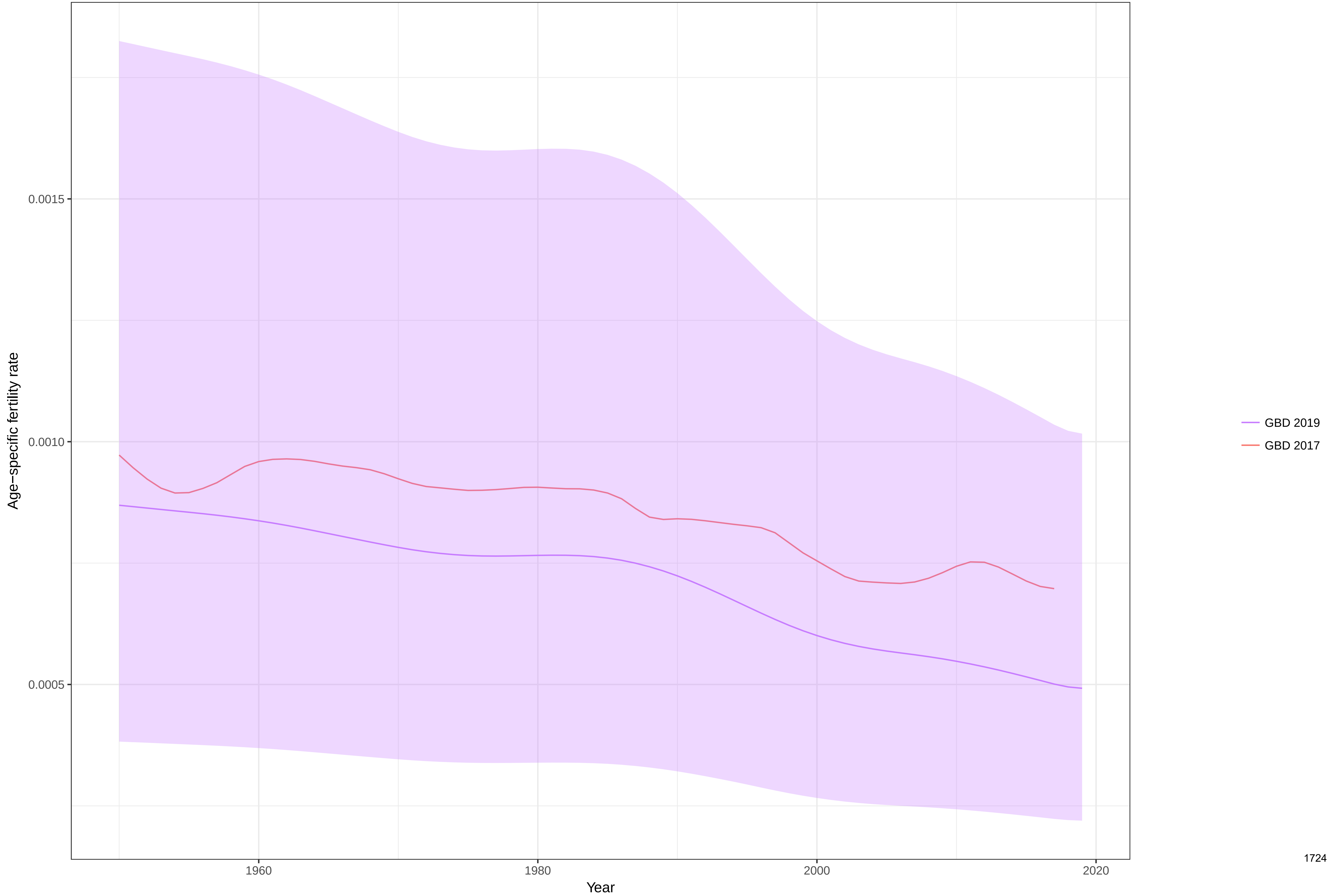

Age−specific fertility fitted trend lines, 1950−2019: Paraguay, Ages: 15−19
Data points enclosed with a diamond are reference sources

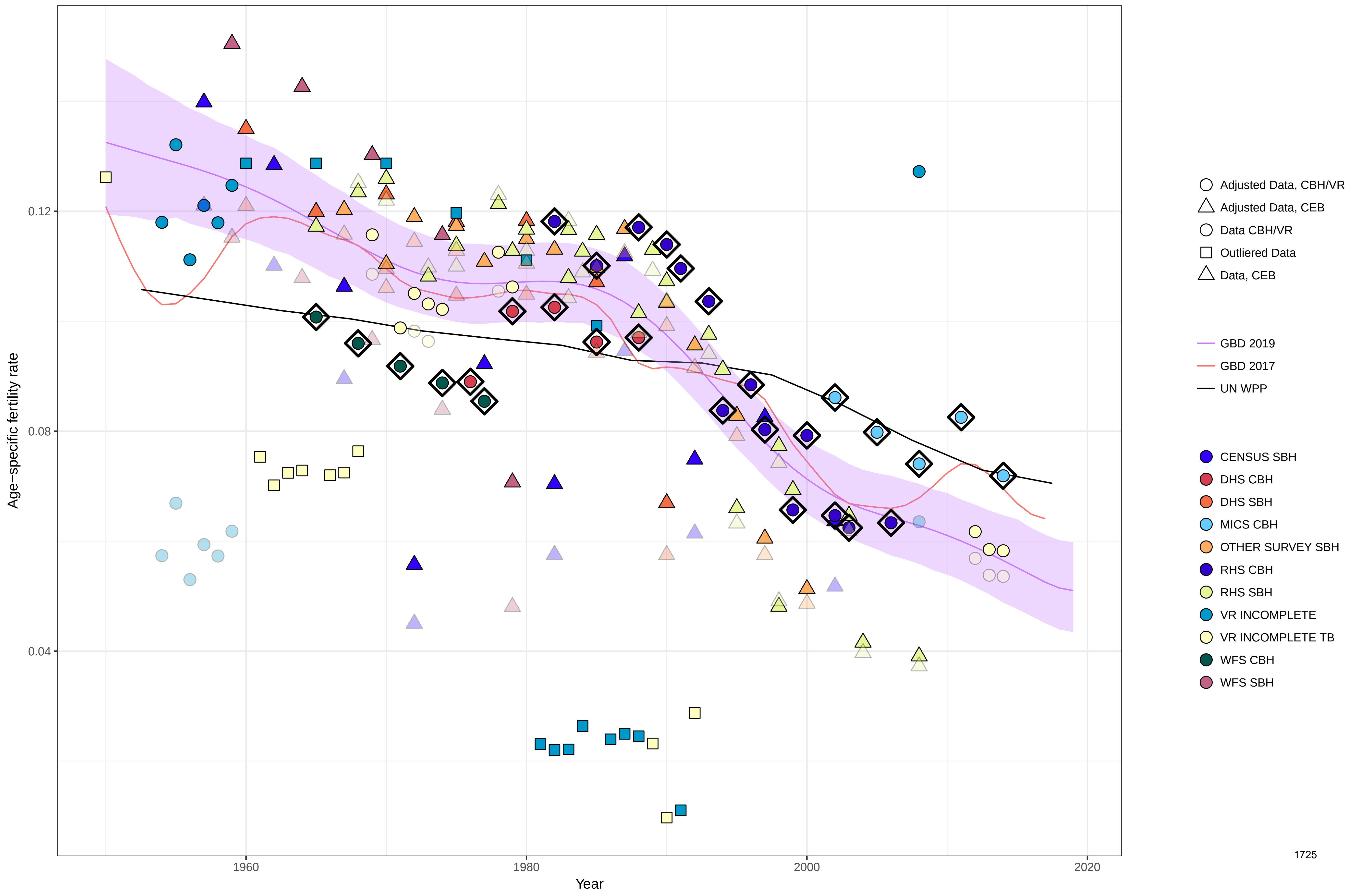

Age−specific fertility fitted trend lines, 1950−2019: Paraguay, Ages: 20−24
Data points enclosed with a diamond are reference sources

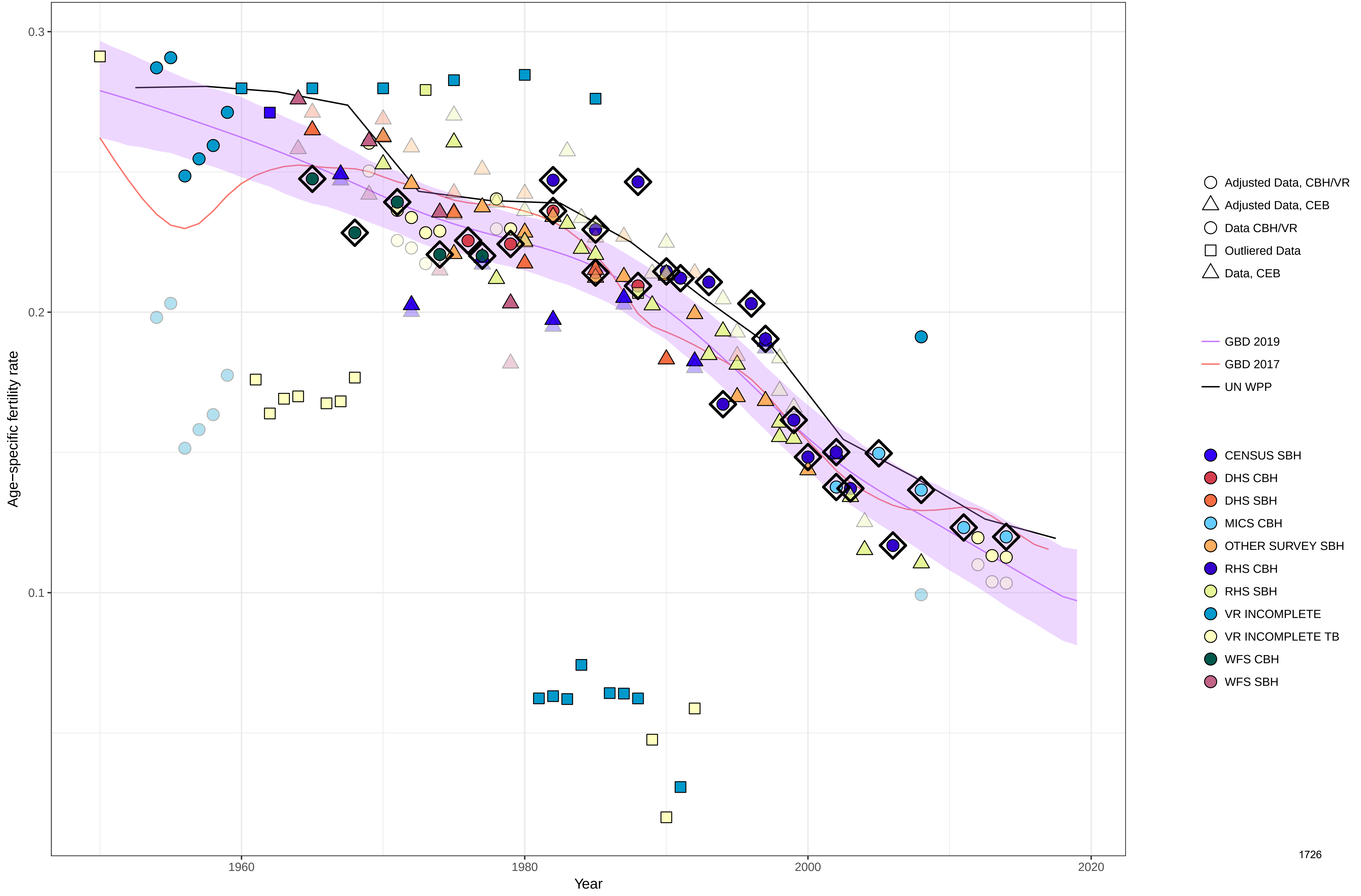

Age−specific fertility fitted trend lines, 1950−2019: Paraguay, Ages: 25−29
Data points enclosed with a diamond are reference sources

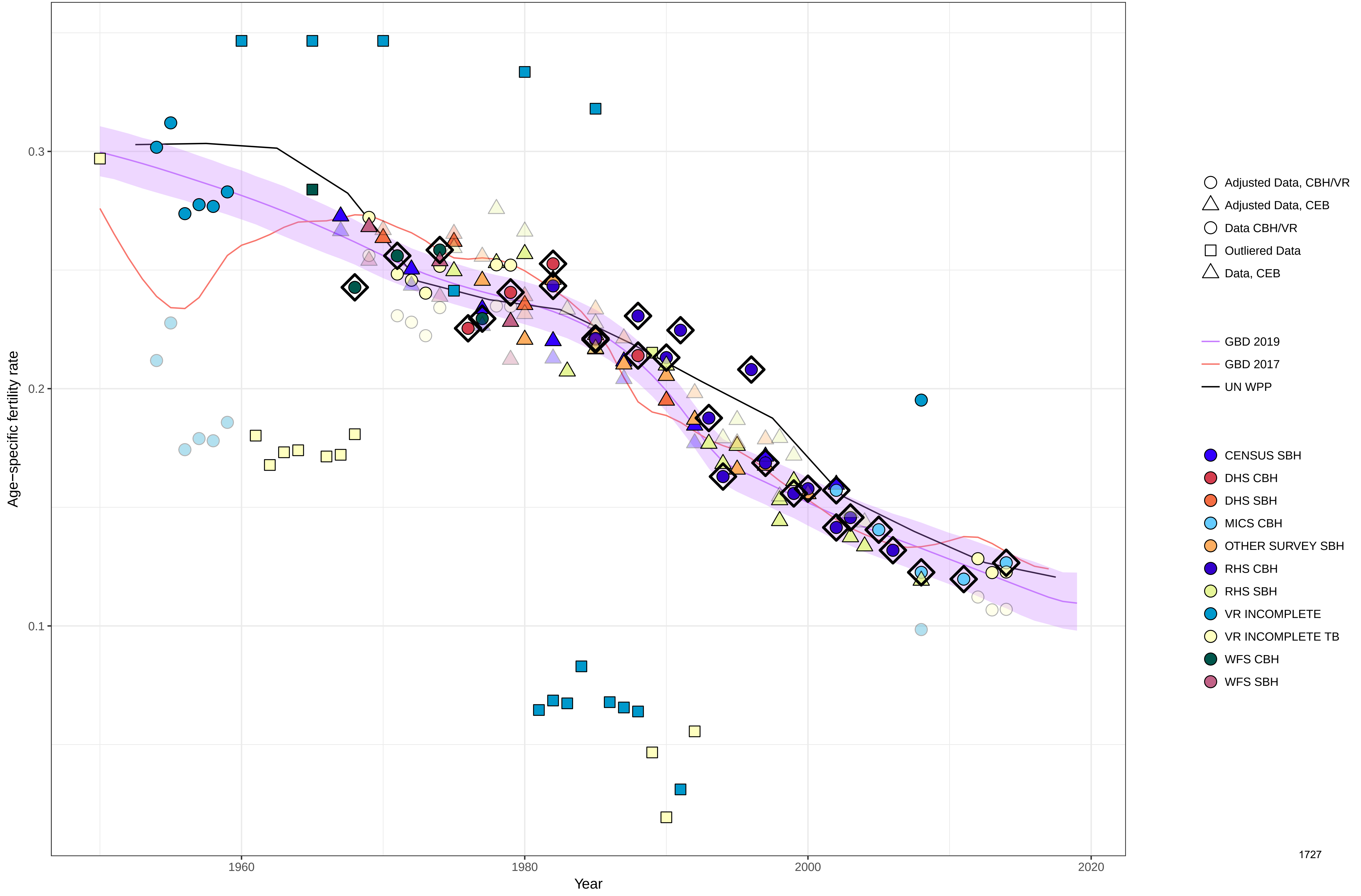

Age−specific fertility fitted trend lines, 1950−2019: Paraguay, Ages: 30−34
Data points enclosed with a diamond are reference sources

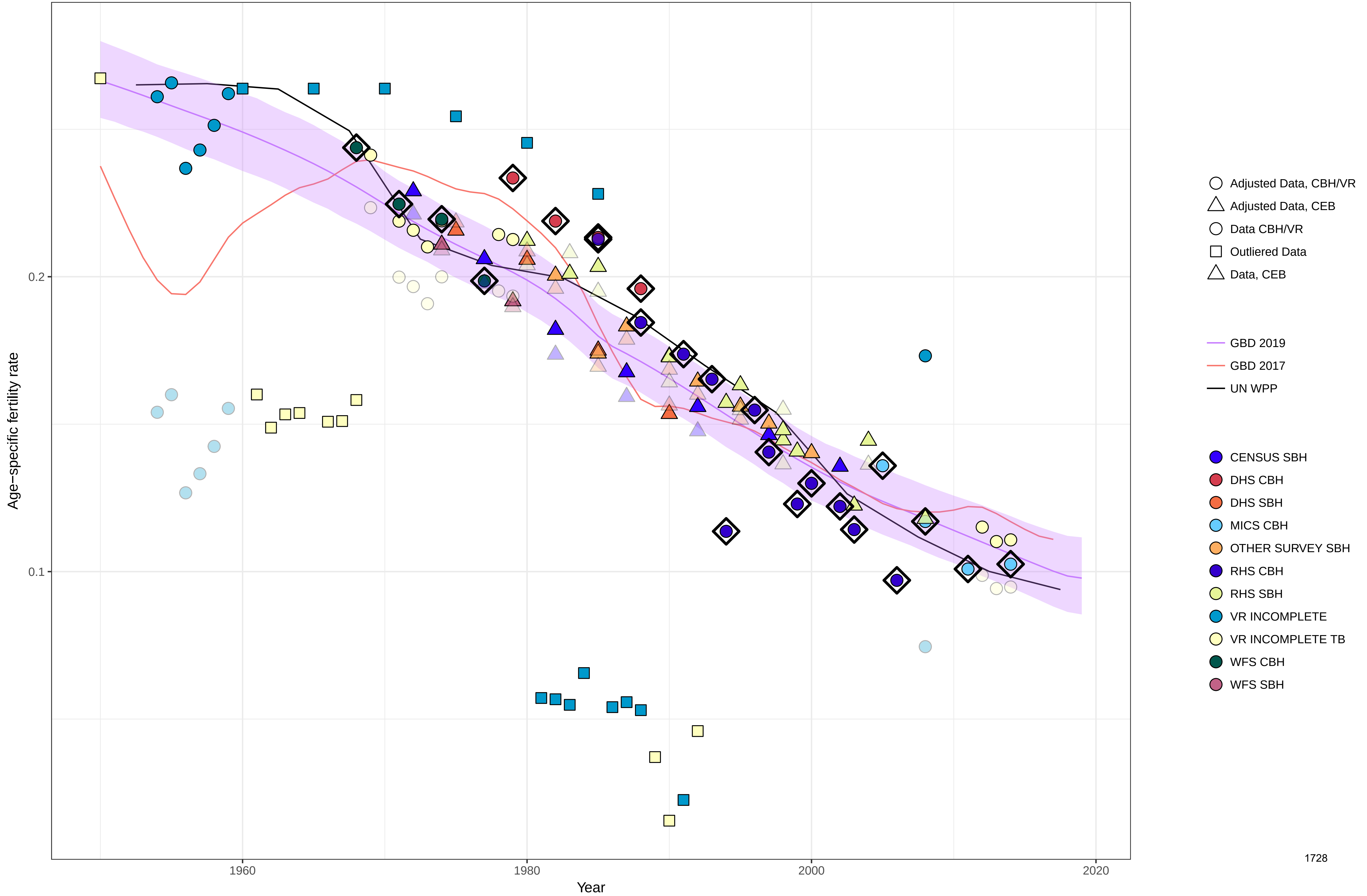

Age−specific fertility fitted trend lines, 1950−2019: Paraguay, Ages: 35−39
Data points enclosed with a diamond are reference sources

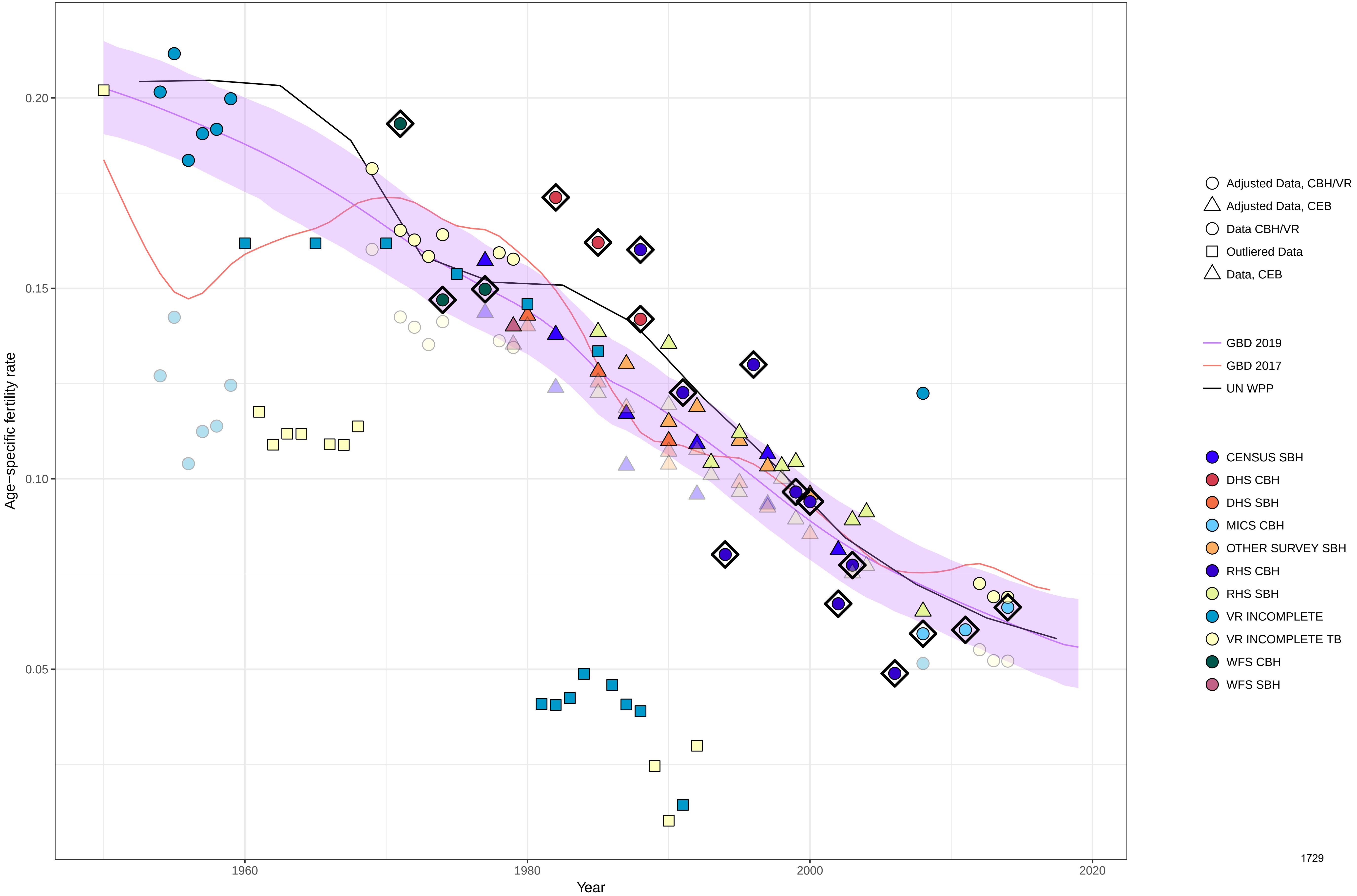

Age−specific fertility fitted trend lines, 1950−2019: Paraguay, Ages: 40−44
Data points enclosed with a diamond are reference sources

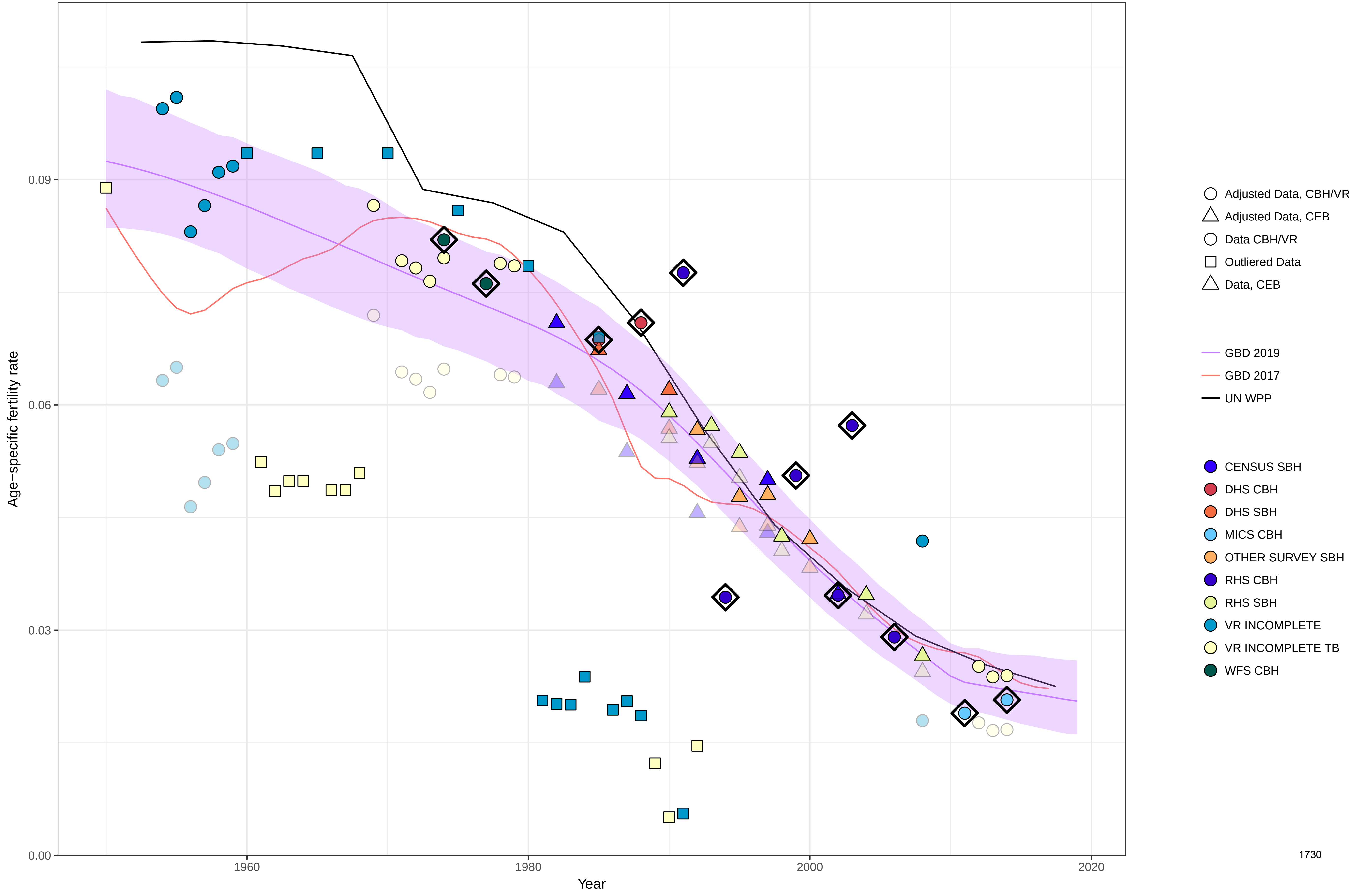

Age−specific fertility fitted trend lines, 1950−2019: Paraguay, Ages: 45−49
Data points enclosed with a diamond are reference sources

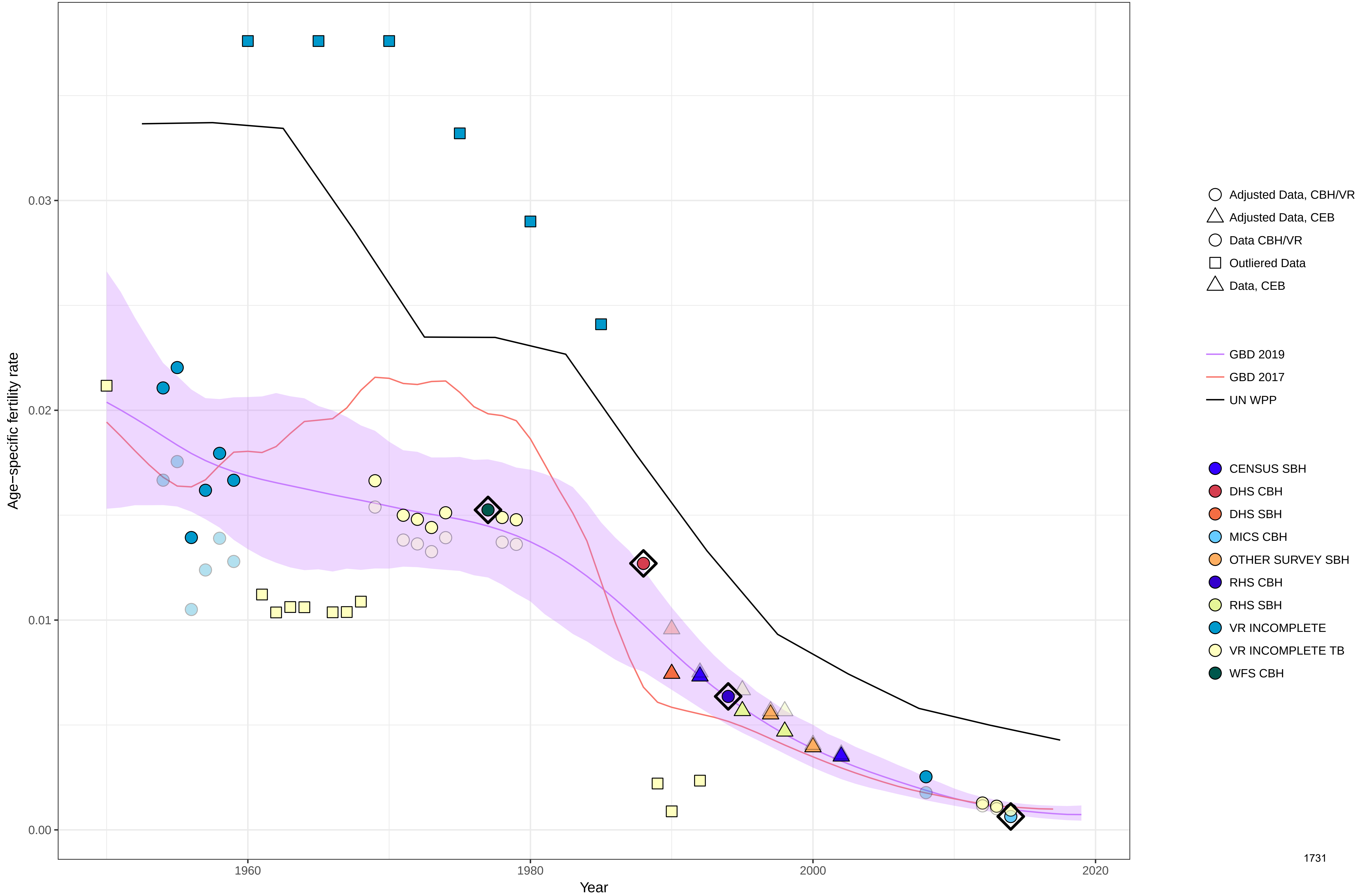

Age−specific fertility fitted trend lines, 1950−2019: Paraguay, Ages: 50−54
Data points enclosed with a diamond are reference sources

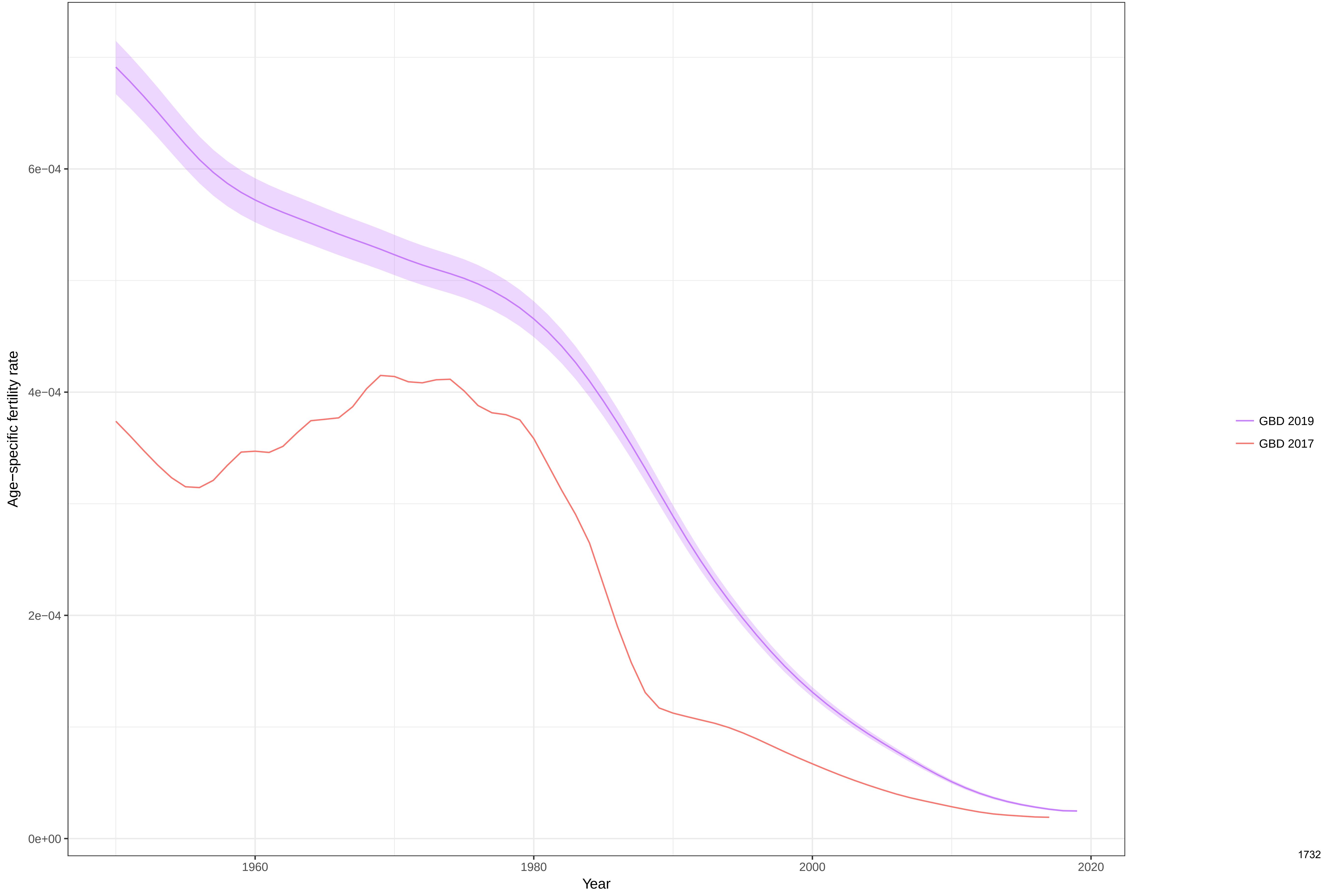

Total fertility rate fitted trend lines, 1950−2019: Paraguay TFR
Data points enclosed with a diamond are reference sources

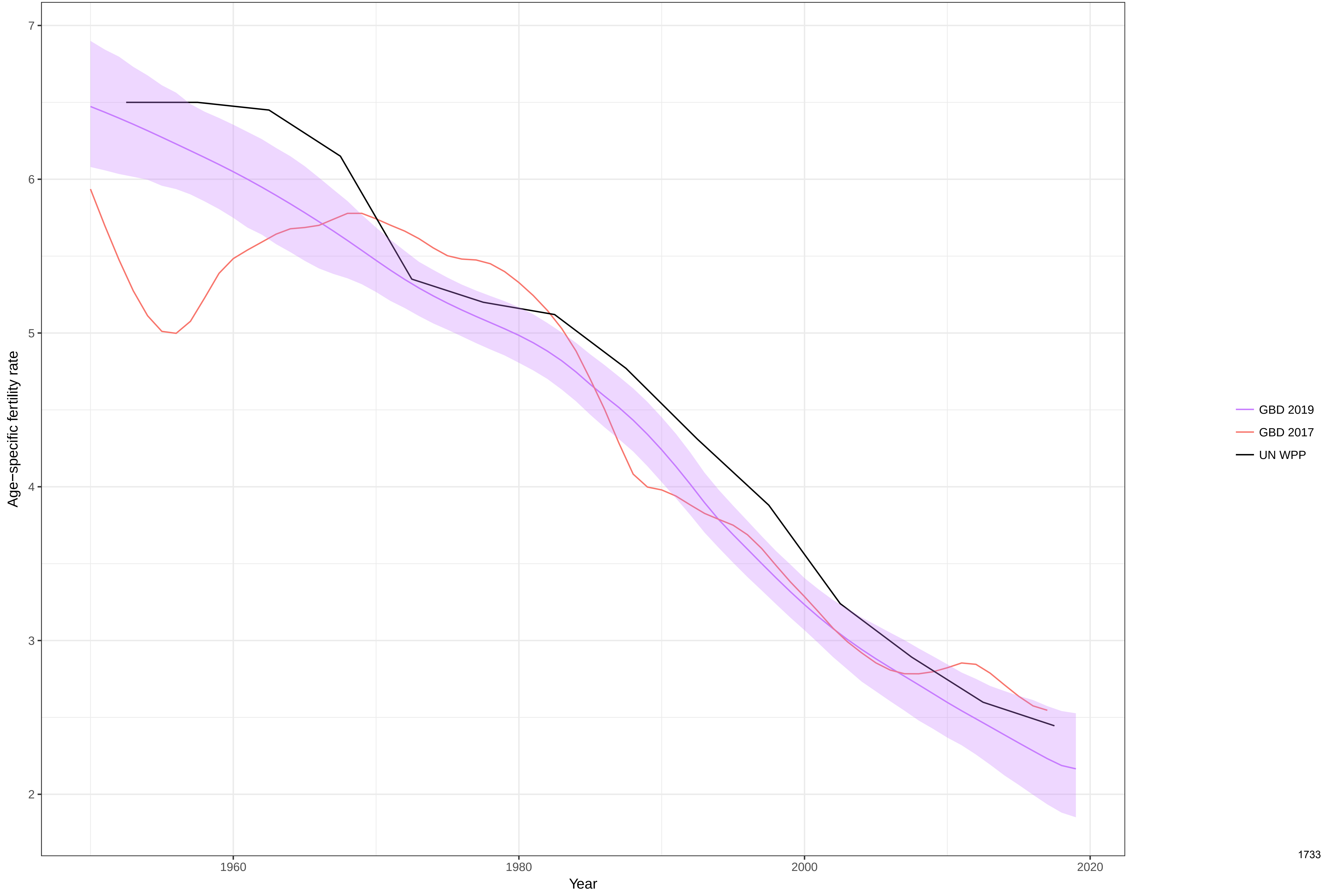

Age−specific fertility fitted trend lines, 1950−2019: Palestine, Ages: 10−14
Data points enclosed with a diamond are reference sources

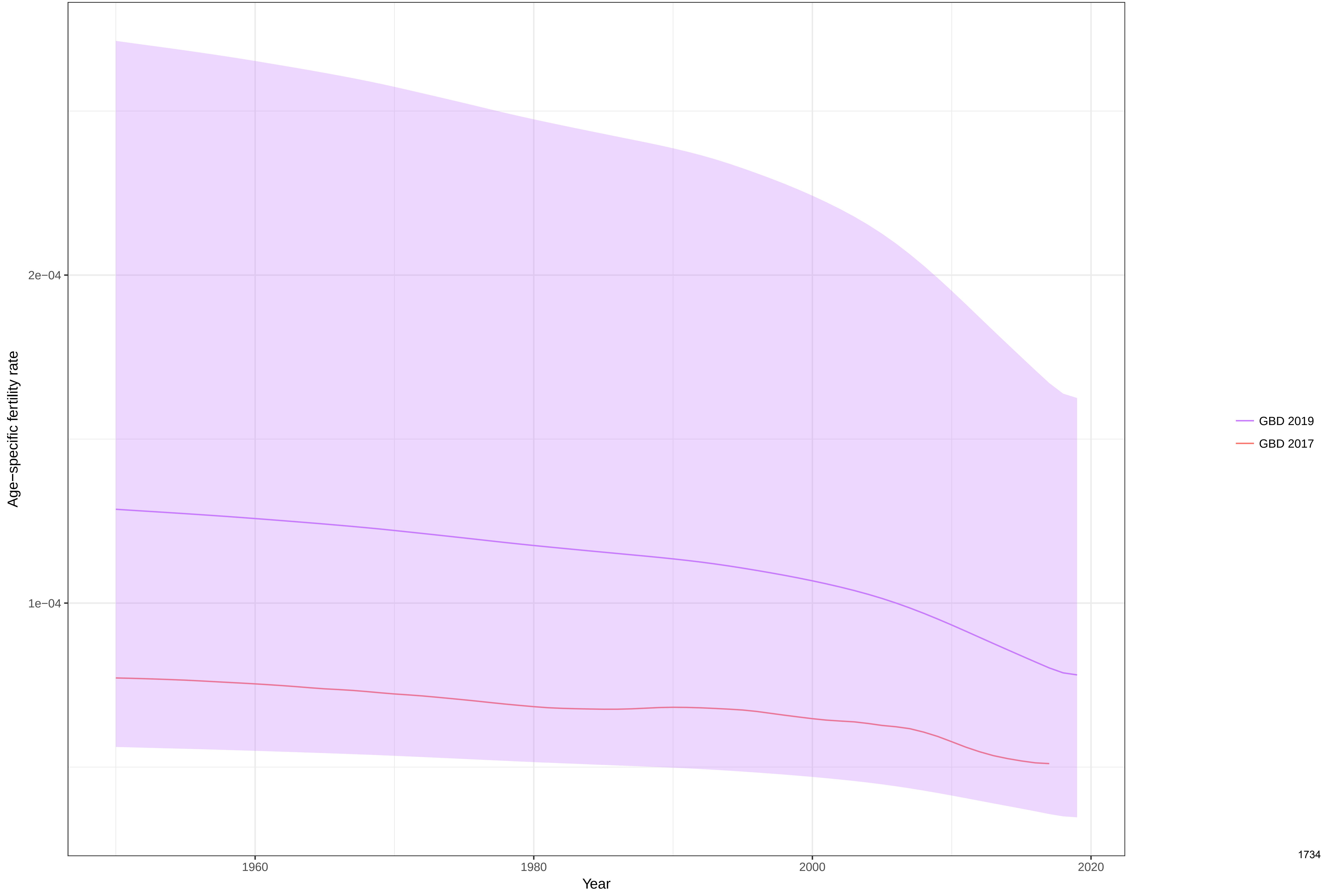

Age−specific fertility fitted trend lines, 1950−2019: Palestine, Ages: 15−19
Data points enclosed with a diamond are reference sources

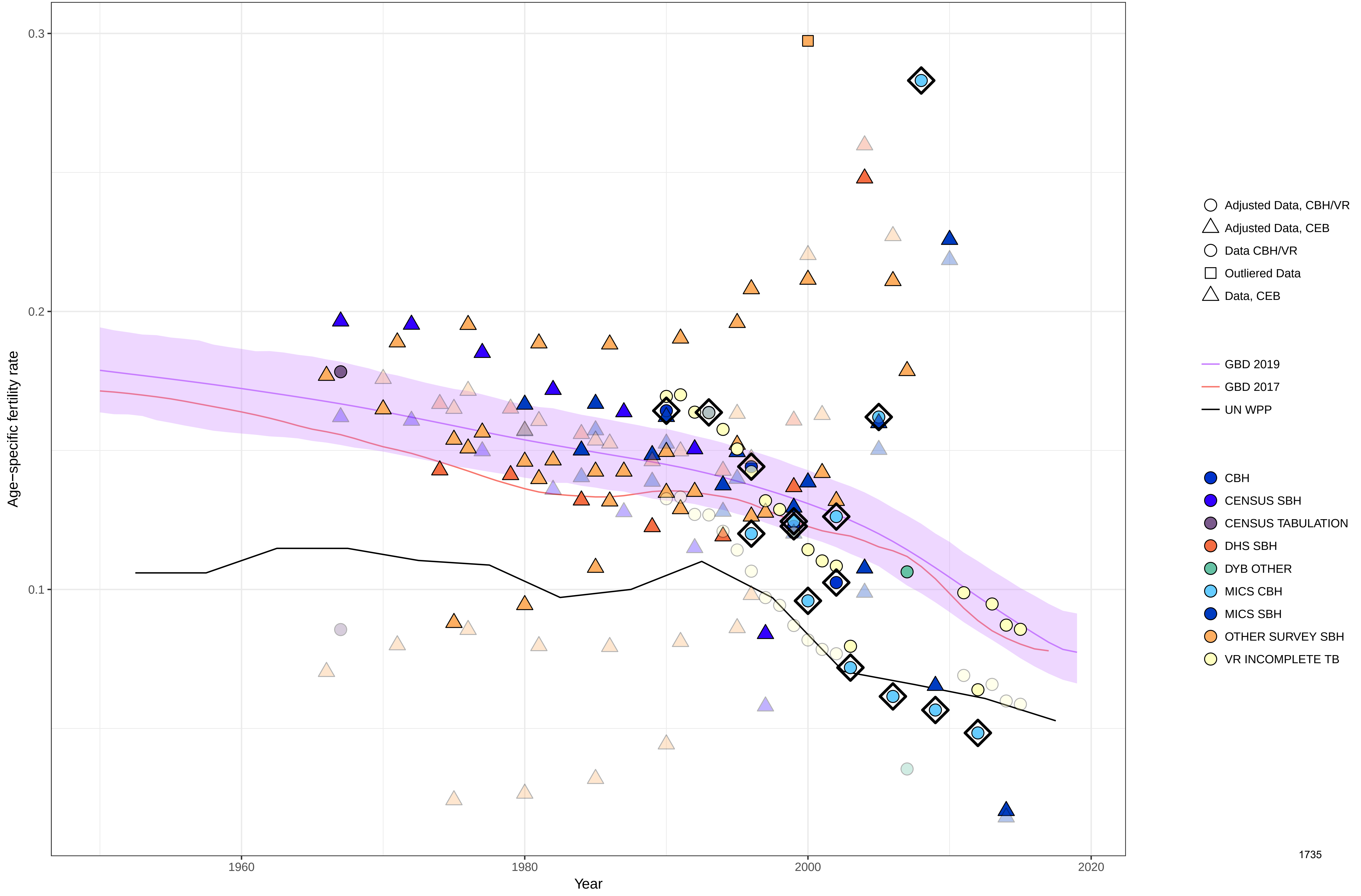

Age−specific fertility fitted trend lines, 1950−2019: Palestine, Ages: 20−24
Data points enclosed with a diamond are reference sources

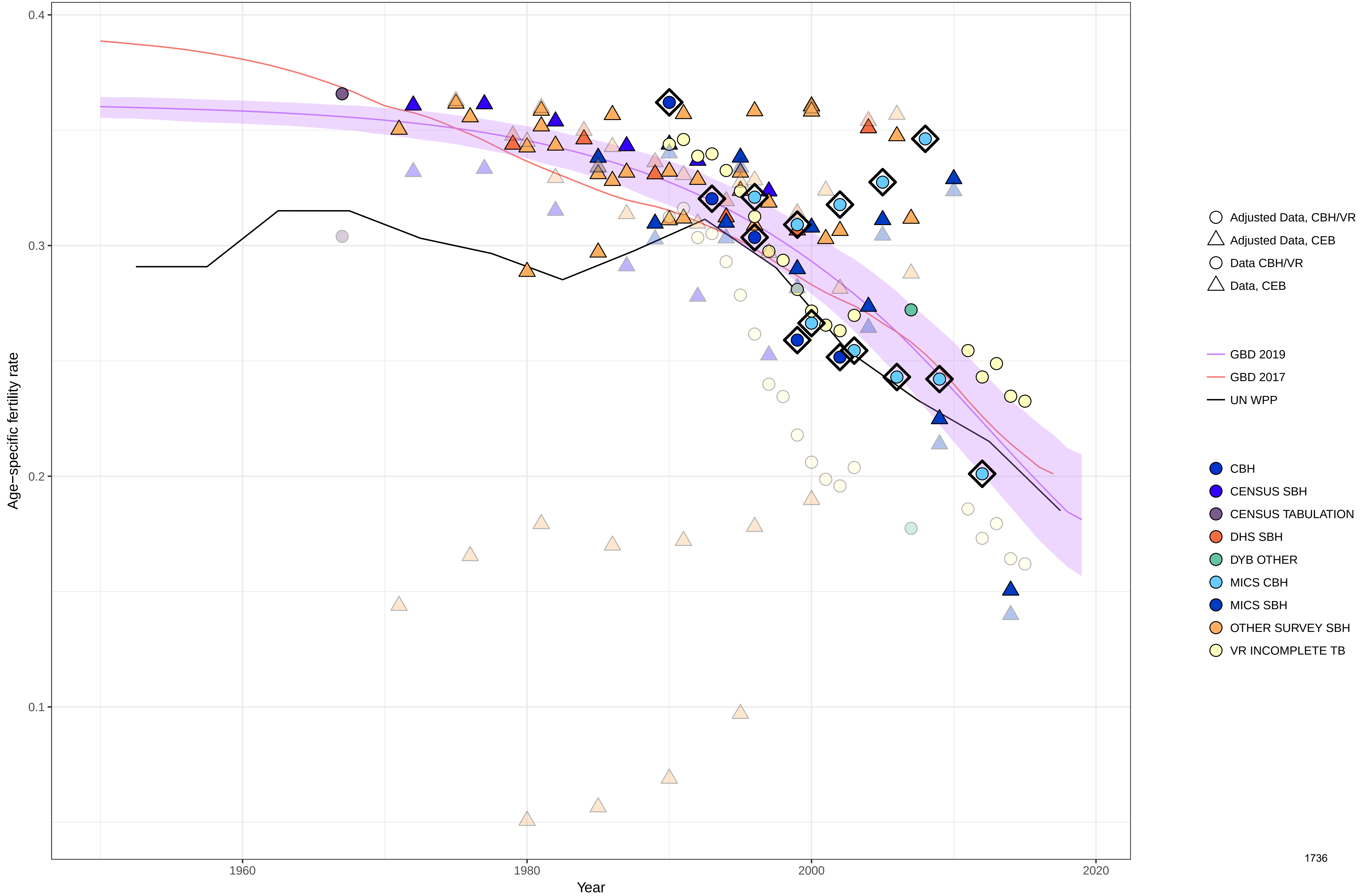

Age−specific fertility fitted trend lines, 1950−2019: Palestine, Ages: 25−29
Data points enclosed with a diamond are reference sources

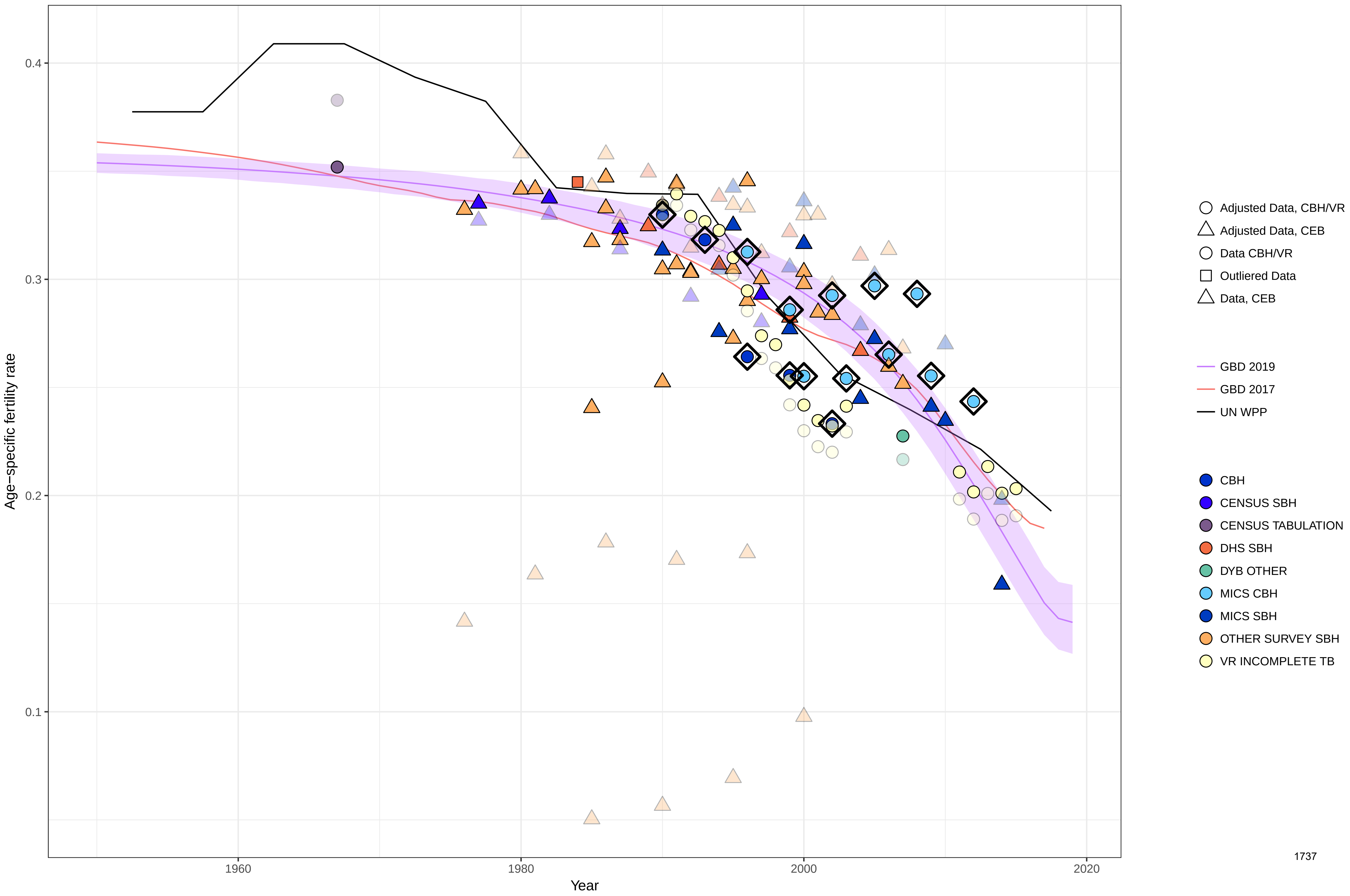

Age−specific fertility fitted trend lines, 1950−2019: Palestine, Ages: 30−34
Data points enclosed with a diamond are reference sources

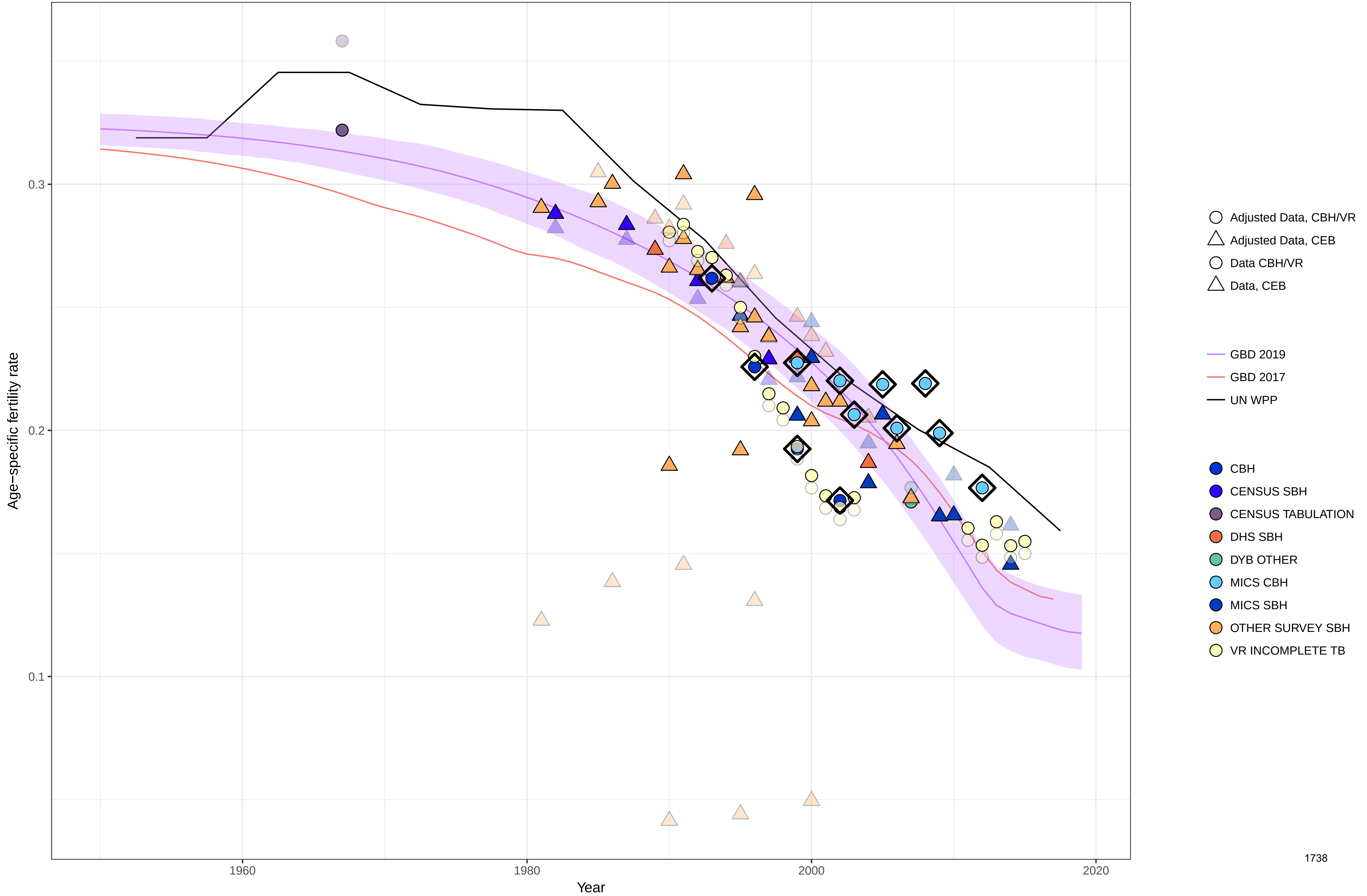

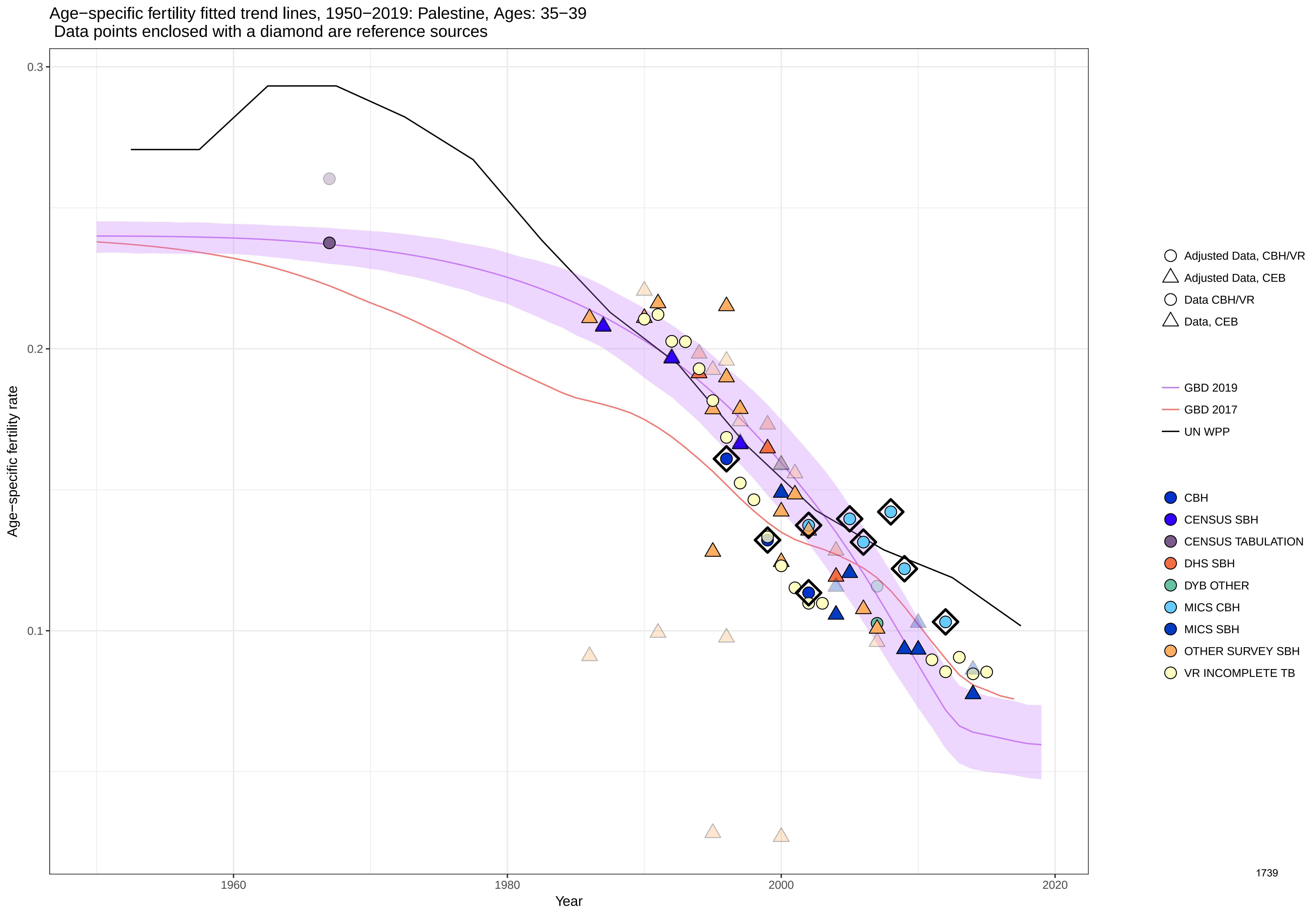

Age−specific fertility fitted trend lines, 1950−2019: Palestine, Ages: 35−39
Data points enclosed with a diamond are reference sources
Age−specific fertility rate
Year
0.1
0.2
0.3
1960
1980
2000
2020
Adjusted Data, CBH/VR
Adjusted Data, CEB
Data CBH/VR
Data, CEB
GBD 2019
GBD 2017
UN WPP
CBH
CENSUS SBH
CENSUS TABULATION
DHS SBH
DYB OTHER
MICS CBH
MICS SBH
OTHER SURVEY SBH
VR INCOMPLETE TB

Age−specific fertility fitted trend lines, 1950−2019: Palestine, Ages: 40−44
Data points enclosed with a diamond are reference sources

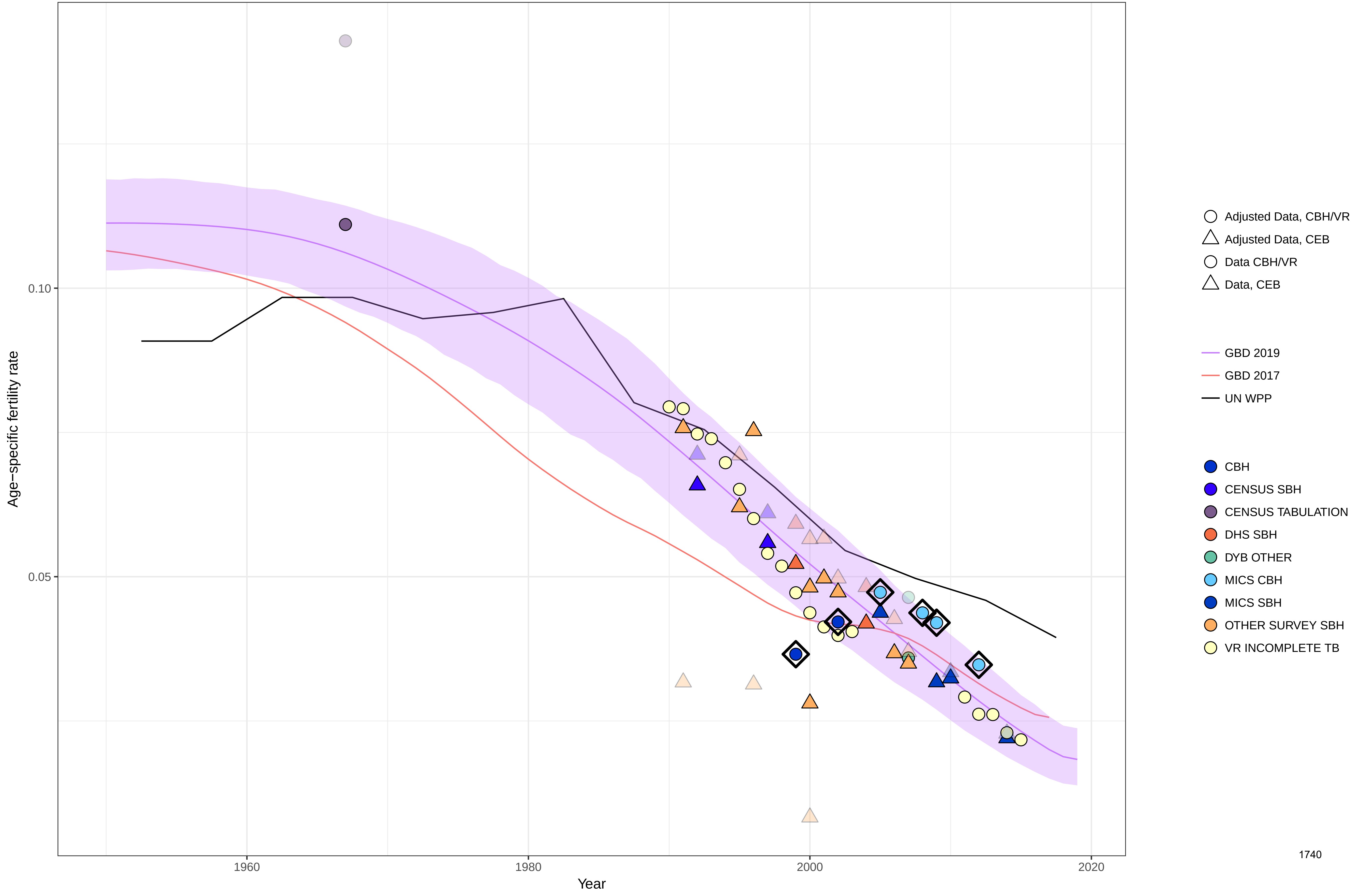

Age−specific fertility fitted trend lines, 1950−2019: Palestine, Ages: 45−49
Data points enclosed with a diamond are reference sources

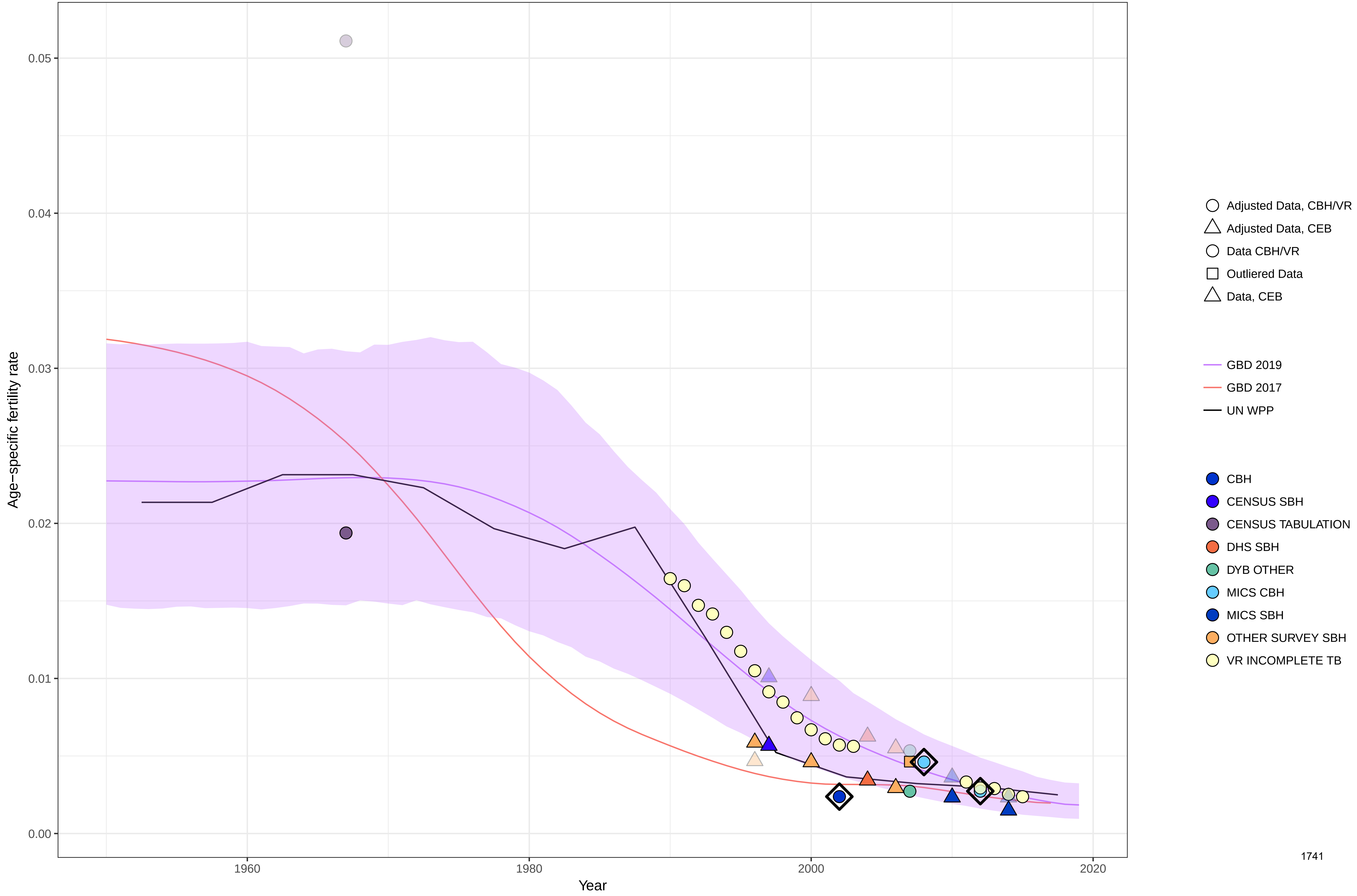

Age−specific fertility fitted trend lines, 1950−2019: Palestine, Ages: 50−54
Data points enclosed with a diamond are reference sources

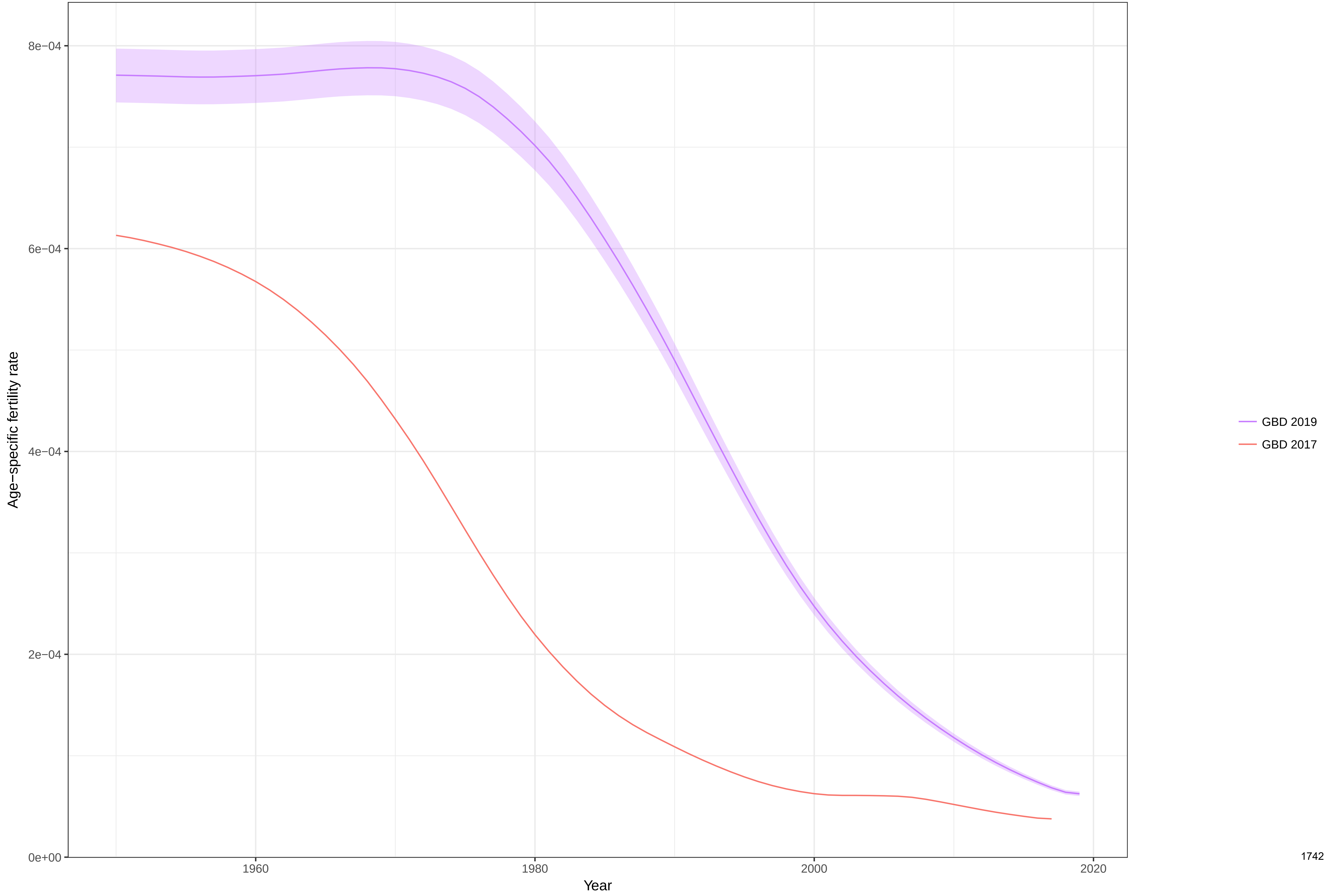

Total fertility rate fitted trend lines, 1950−2019: Palestine TFR
Data points enclosed with a diamond are reference sources

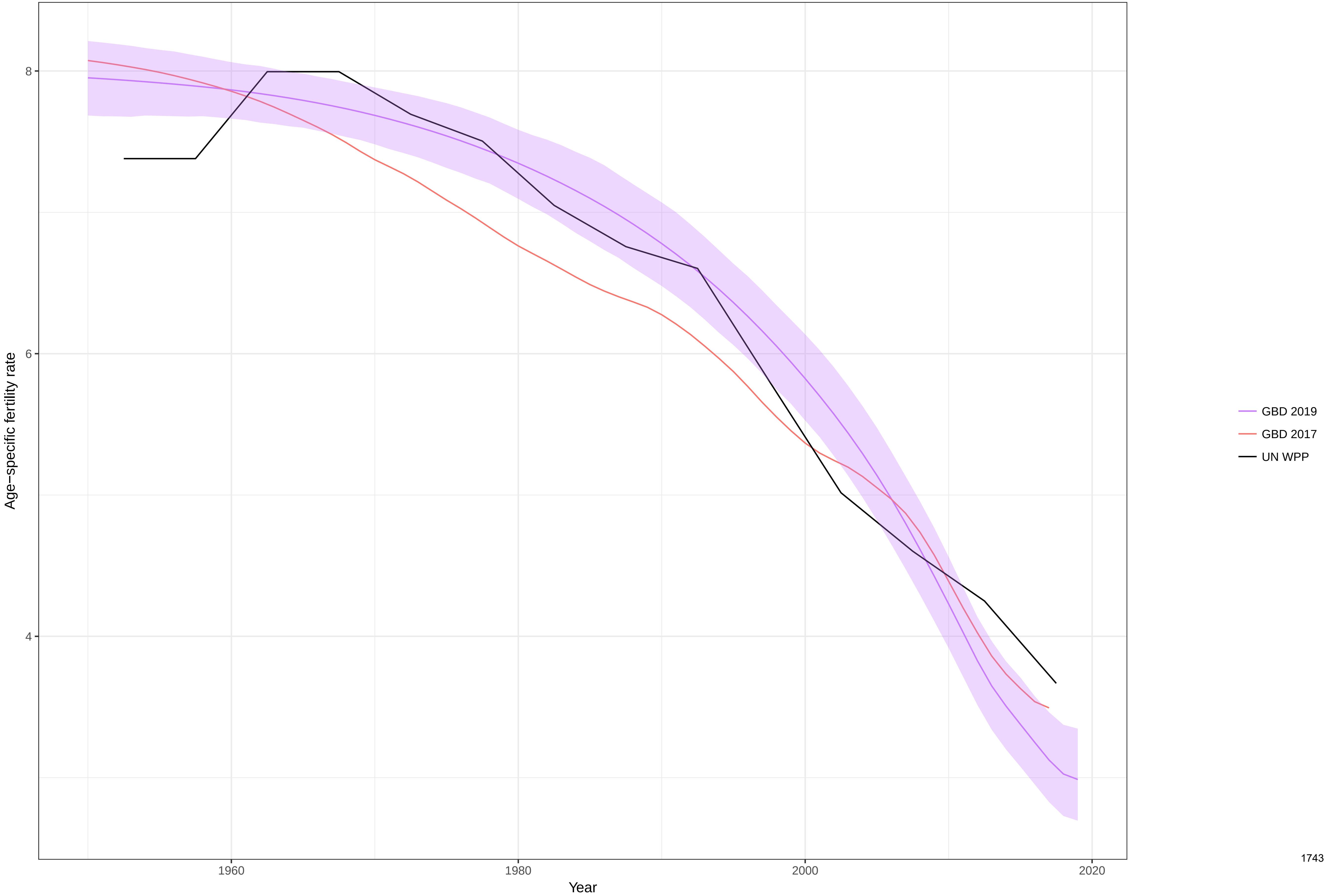

Age−specific fertility fitted trend lines, 1950−2019: Qatar, Ages: 10−14
Data points enclosed with a diamond are reference sources

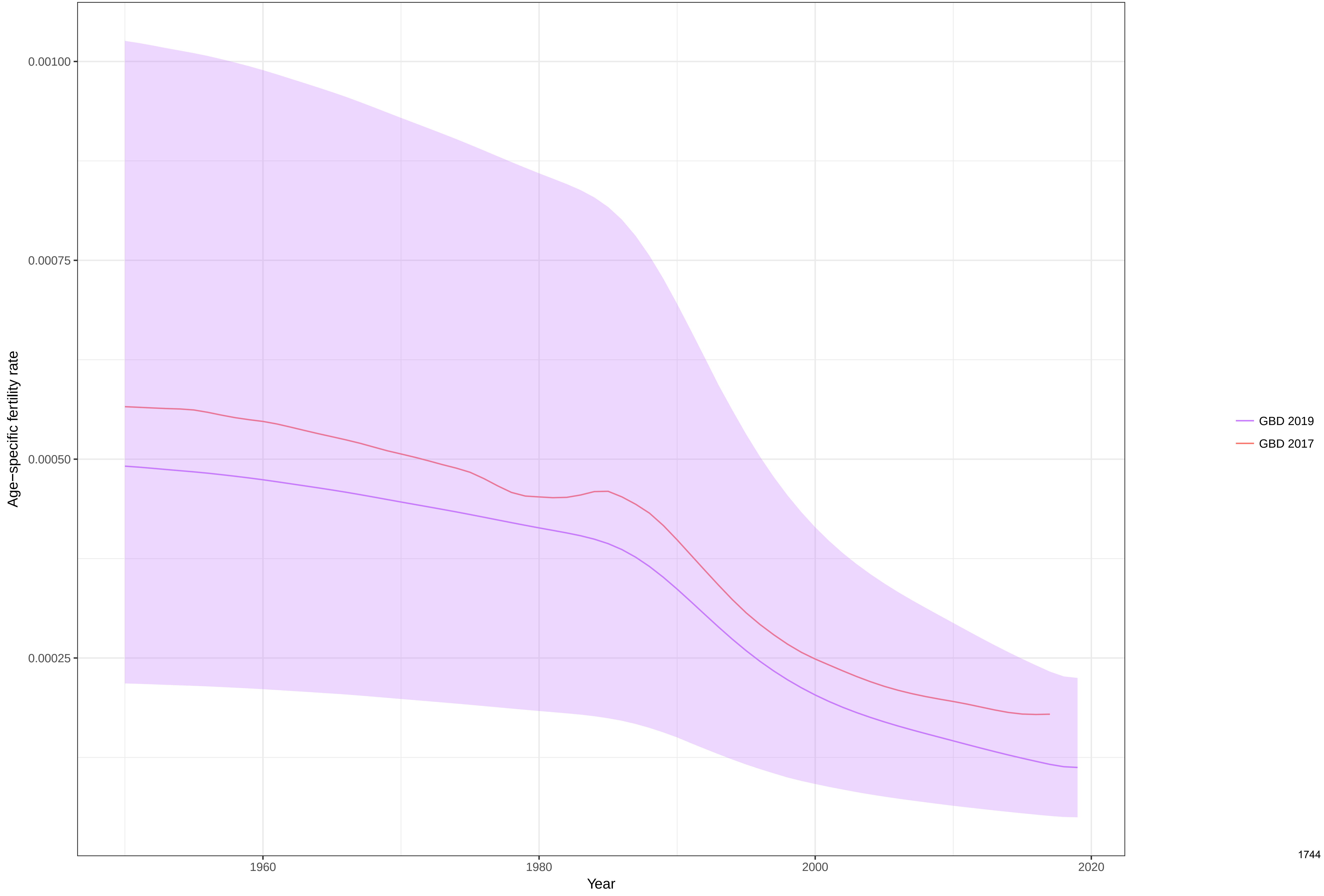

Age−specific fertility fitted trend lines, 1950−2019: Qatar, Ages: 15−19
Data points enclosed with a diamond are reference sources

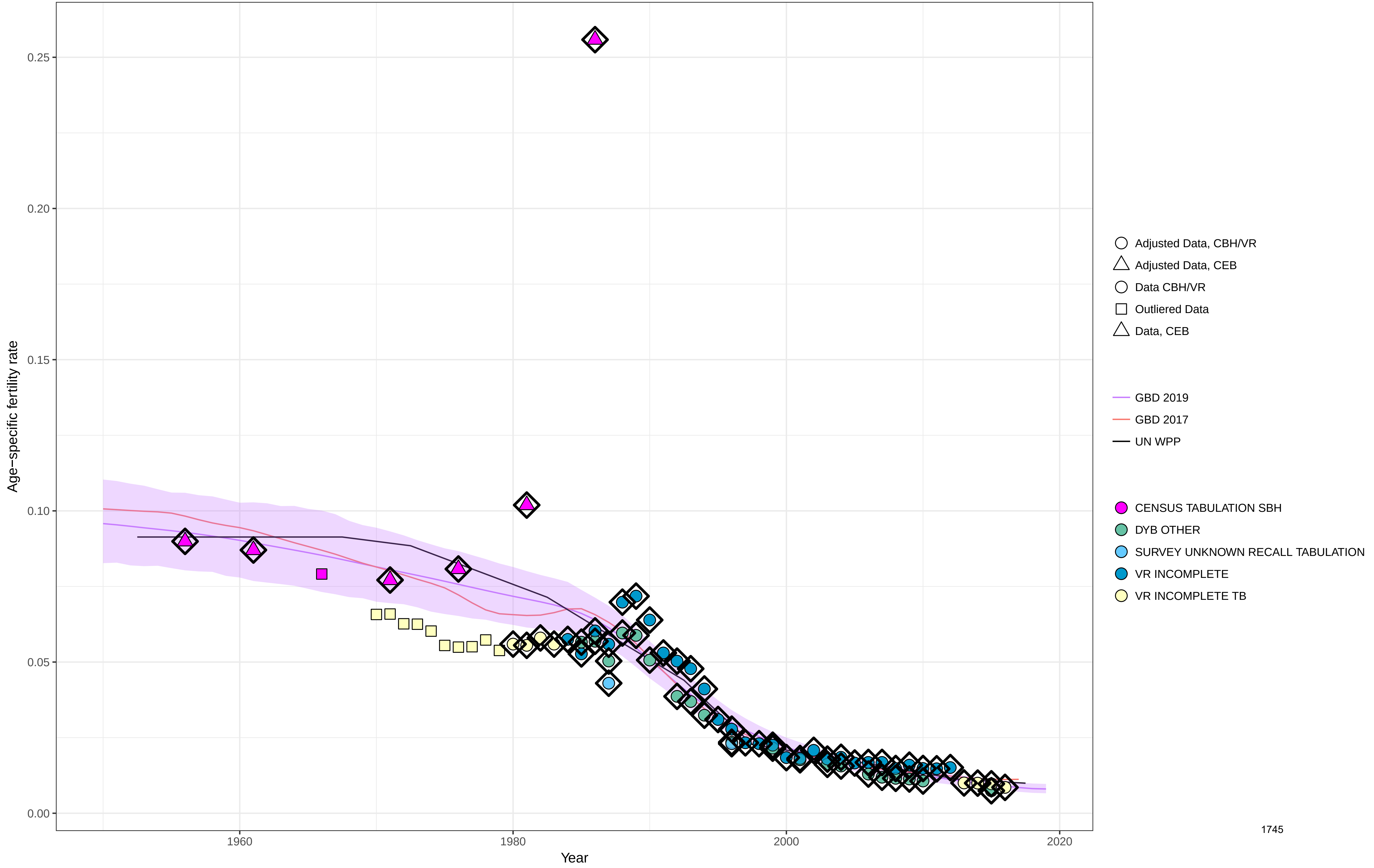

Age−specific fertility fitted trend lines, 1950−2019: Qatar, Ages: 20−24
Data points enclosed with a diamond are reference sources

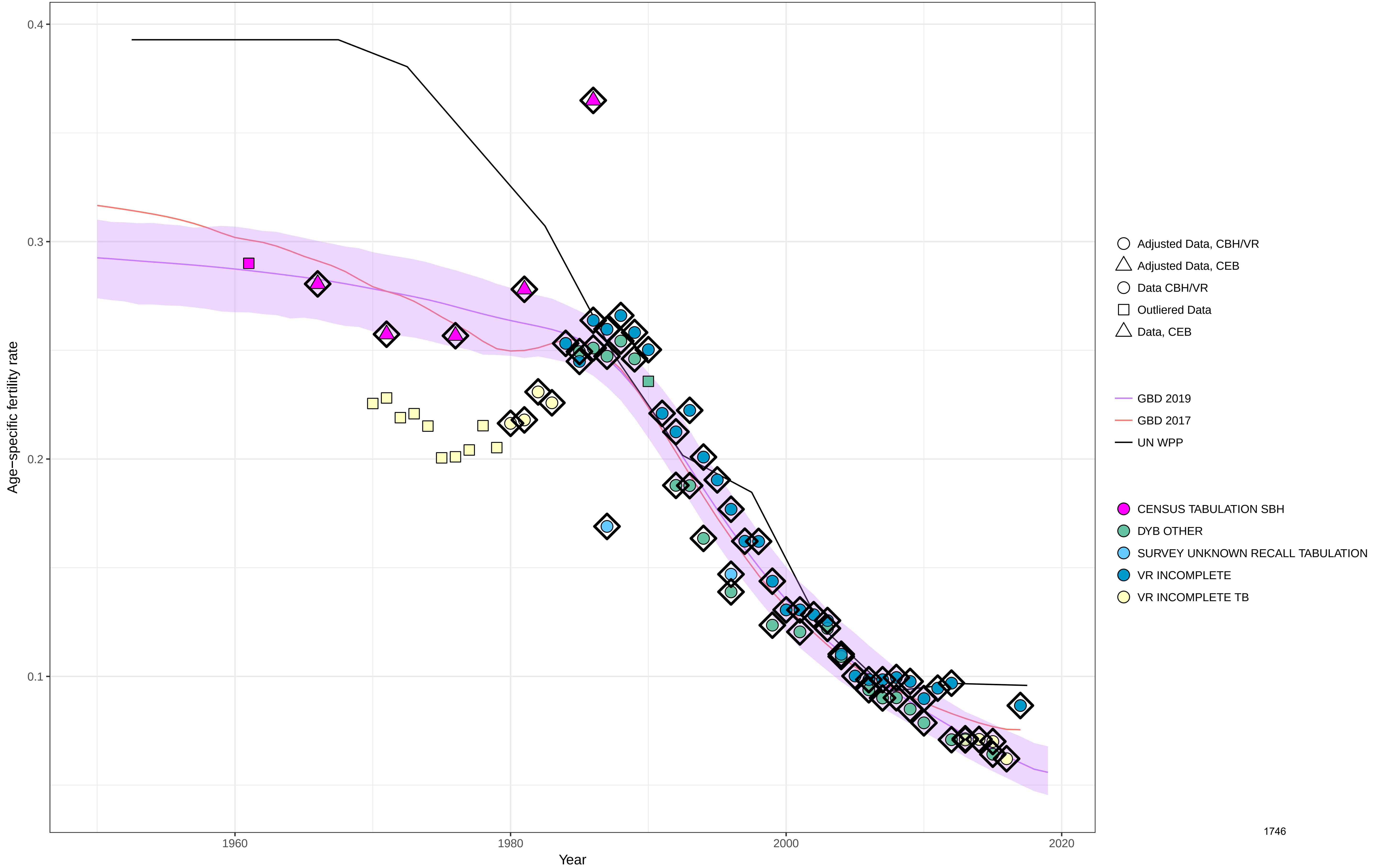

Age−specific fertility fitted trend lines, 1950−2019: Qatar, Ages: 25−29
Data points enclosed with a diamond are reference sources

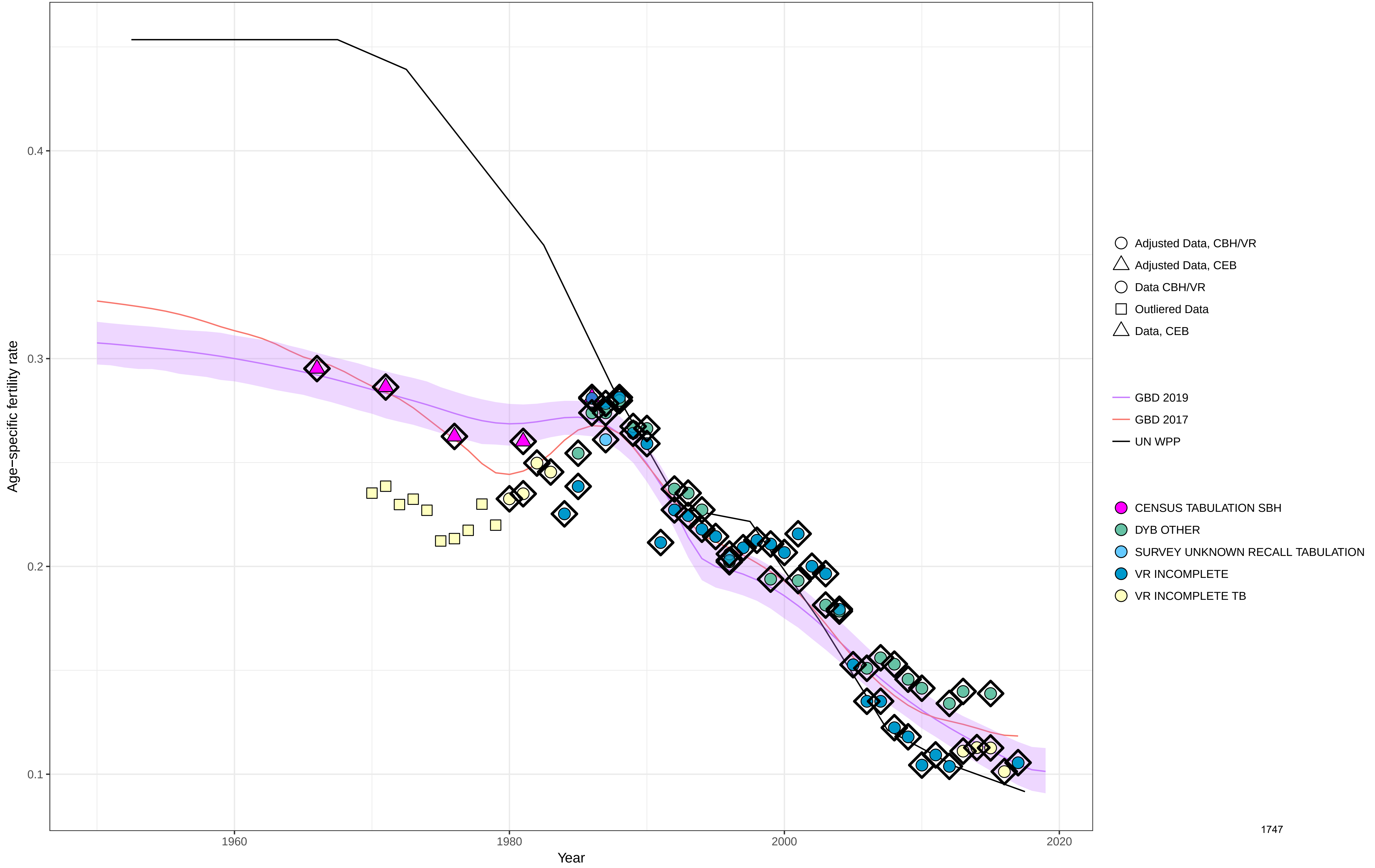

Age−specific fertility fitted trend lines, 1950−2019: Qatar, Ages: 30−34
Data points enclosed with a diamond are reference sources

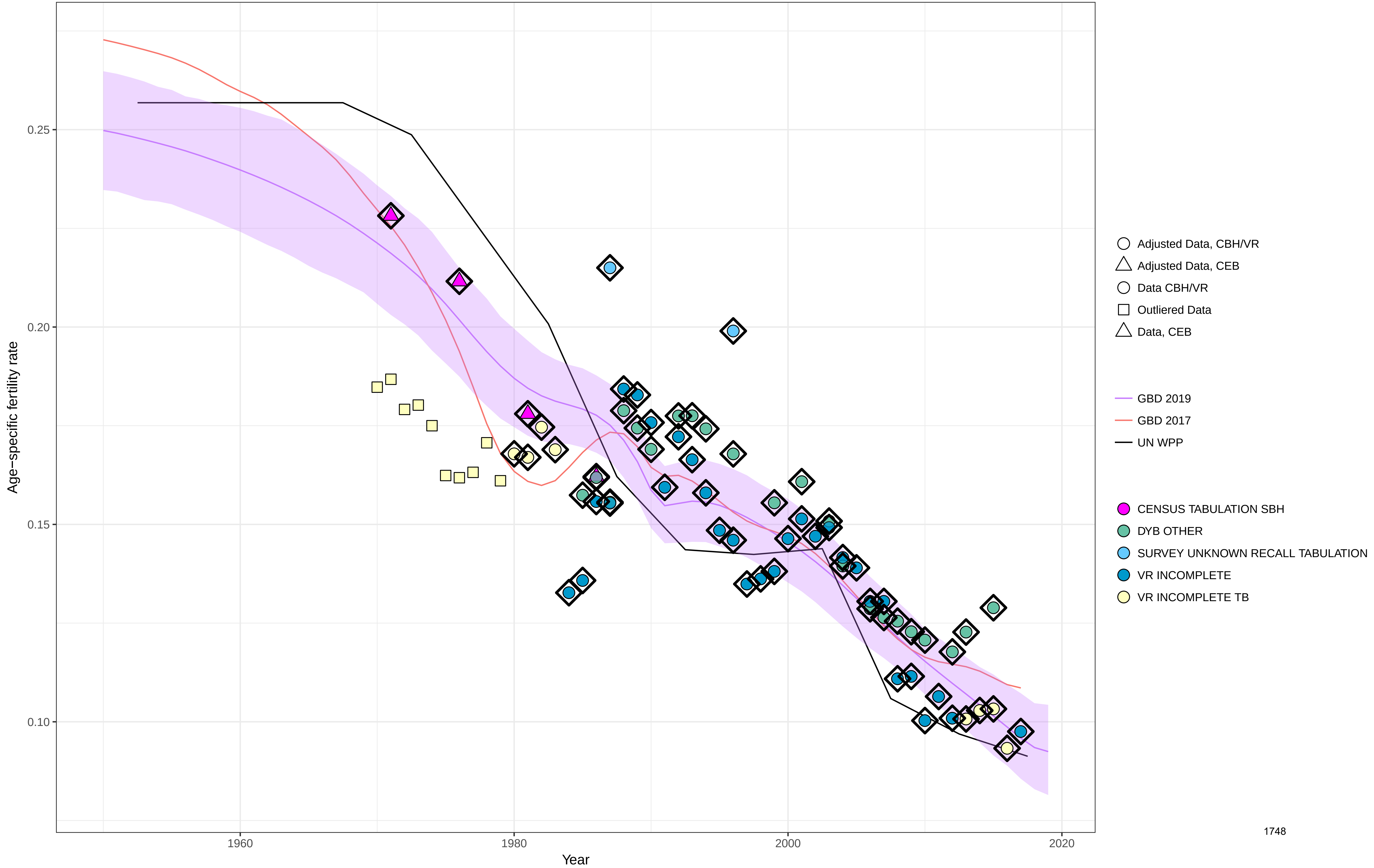

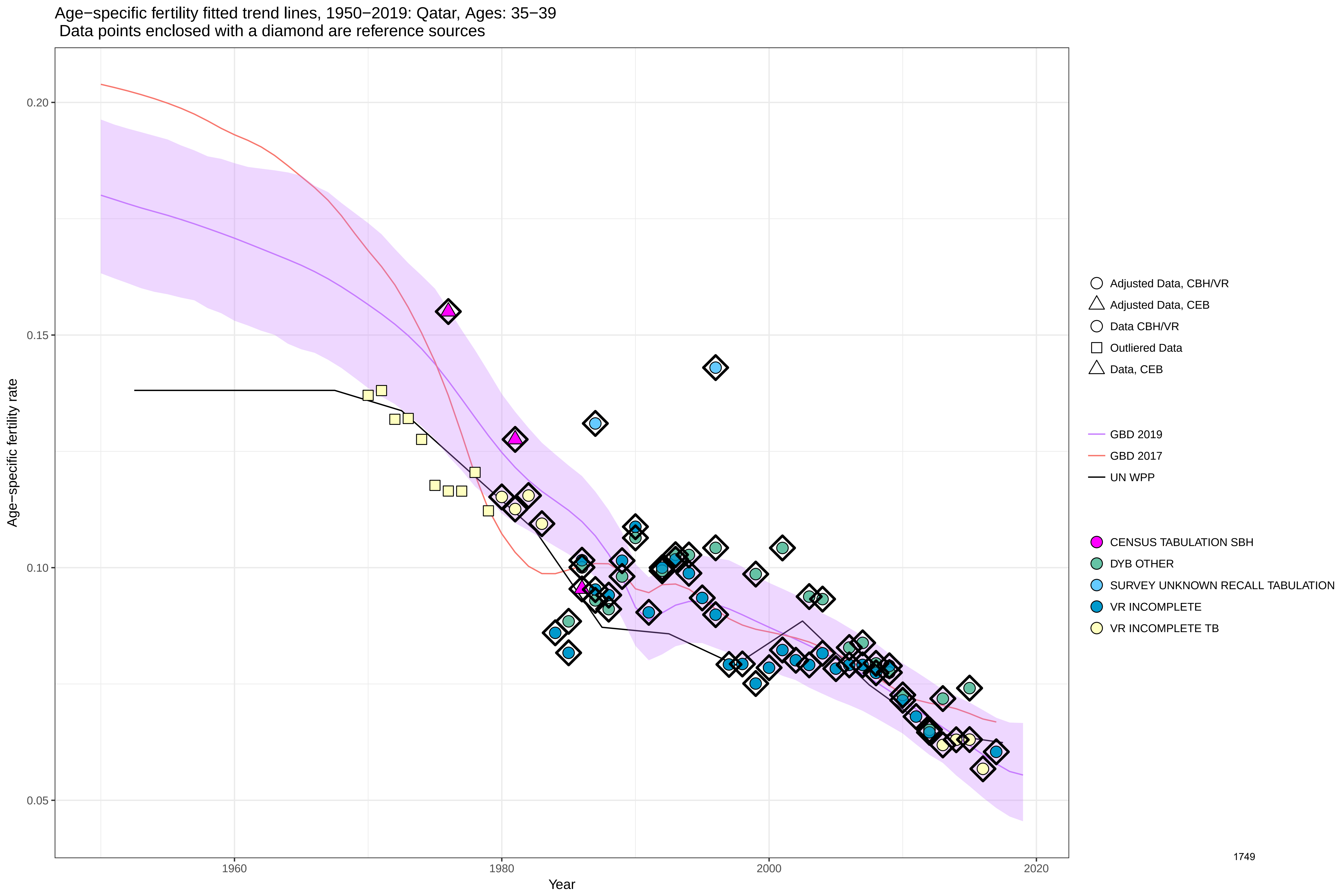
Age−specific fertility fitted trend lines, 1950−2019: Qatar, Ages: 35−39
Data points enclosed with a diamond are reference sources
Age−specific fertility rate
0.20
0.15
0.10
0.05
1960
1980
2000
2020
Year
Adjusted Data, CBH/VR
Adjusted Data, CEB
Data CBH/VR
Outliered Data
Data, CEB
GBD 2019
GBD 2017
UN WPP
CENSUS TABULATION SBH
DYB OTHER
SURVEY UNKNOWN RECALL TABULATION
VR INCOMPLETE
VR INCOMPLETE TB

Age−specific fertility fitted trend lines, 1950−2019: Qatar, Ages: 40−44
Data points enclosed with a diamond are reference sources

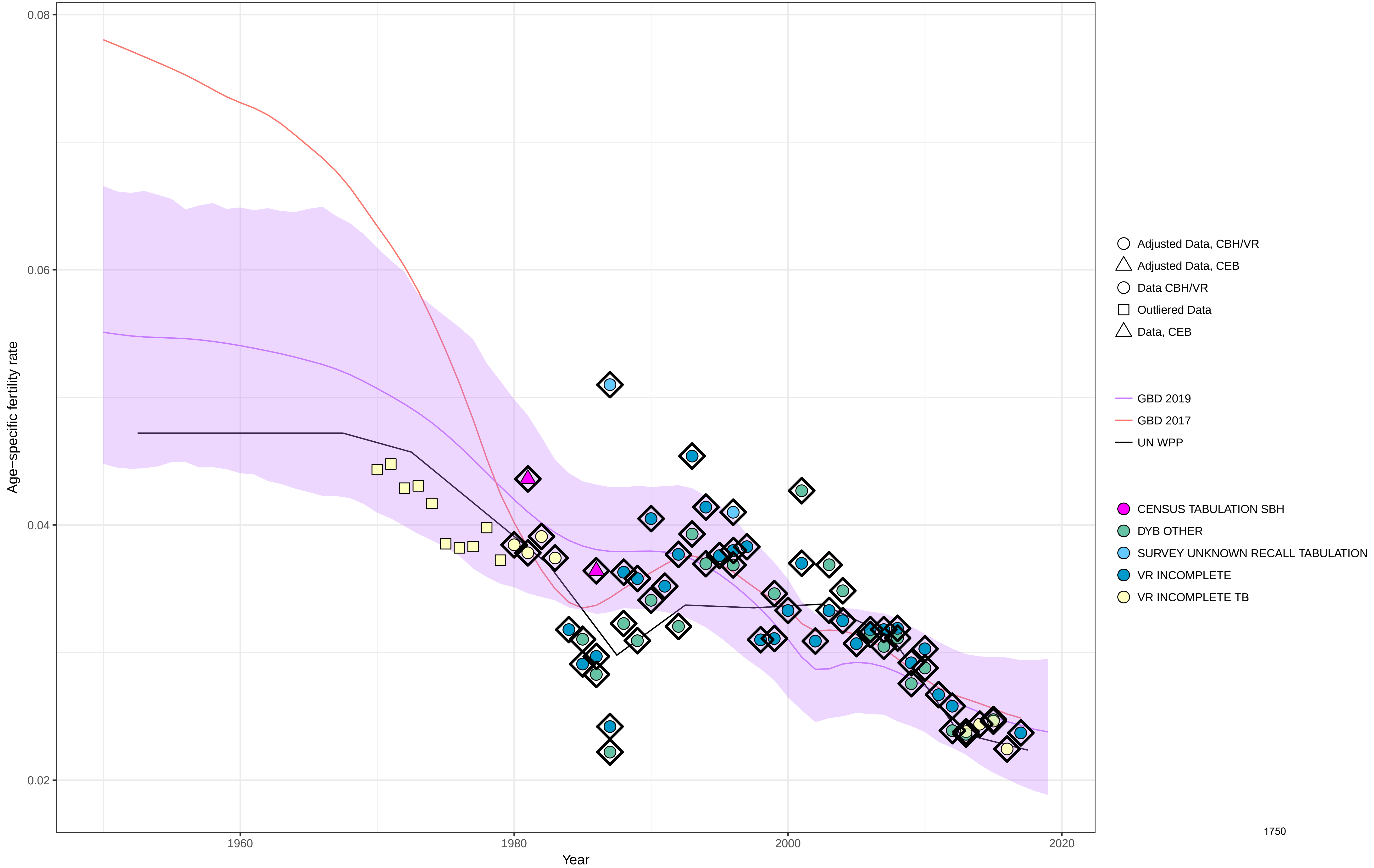

Age−specific fertility fitted trend lines, 1950−2019: Qatar, Ages: 45−49
Data points enclosed with a diamond are reference sources

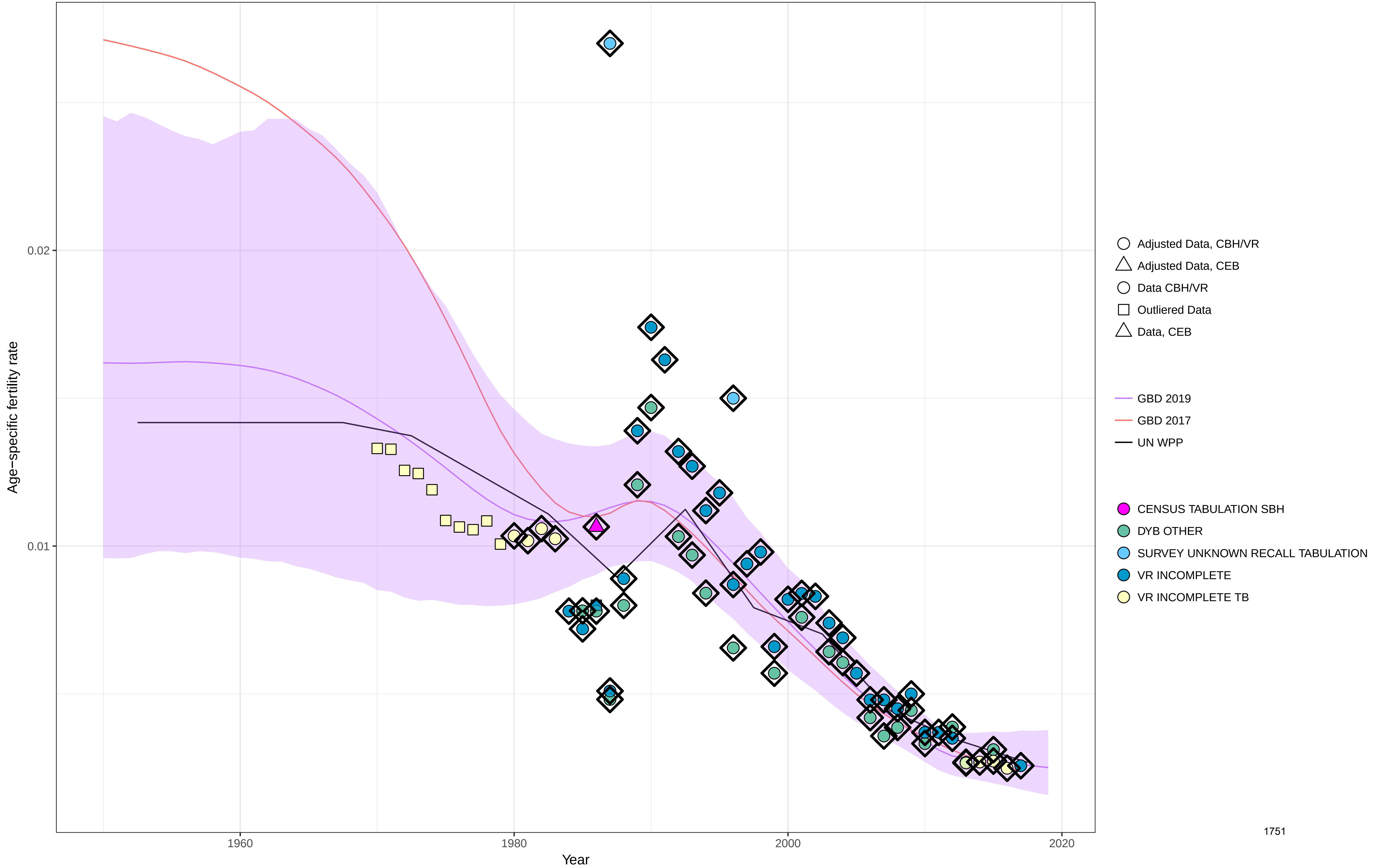

Age−specific fertility fitted trend lines, 1950−2019: Qatar, Ages: 50−54
Data points enclosed with a diamond are reference sources

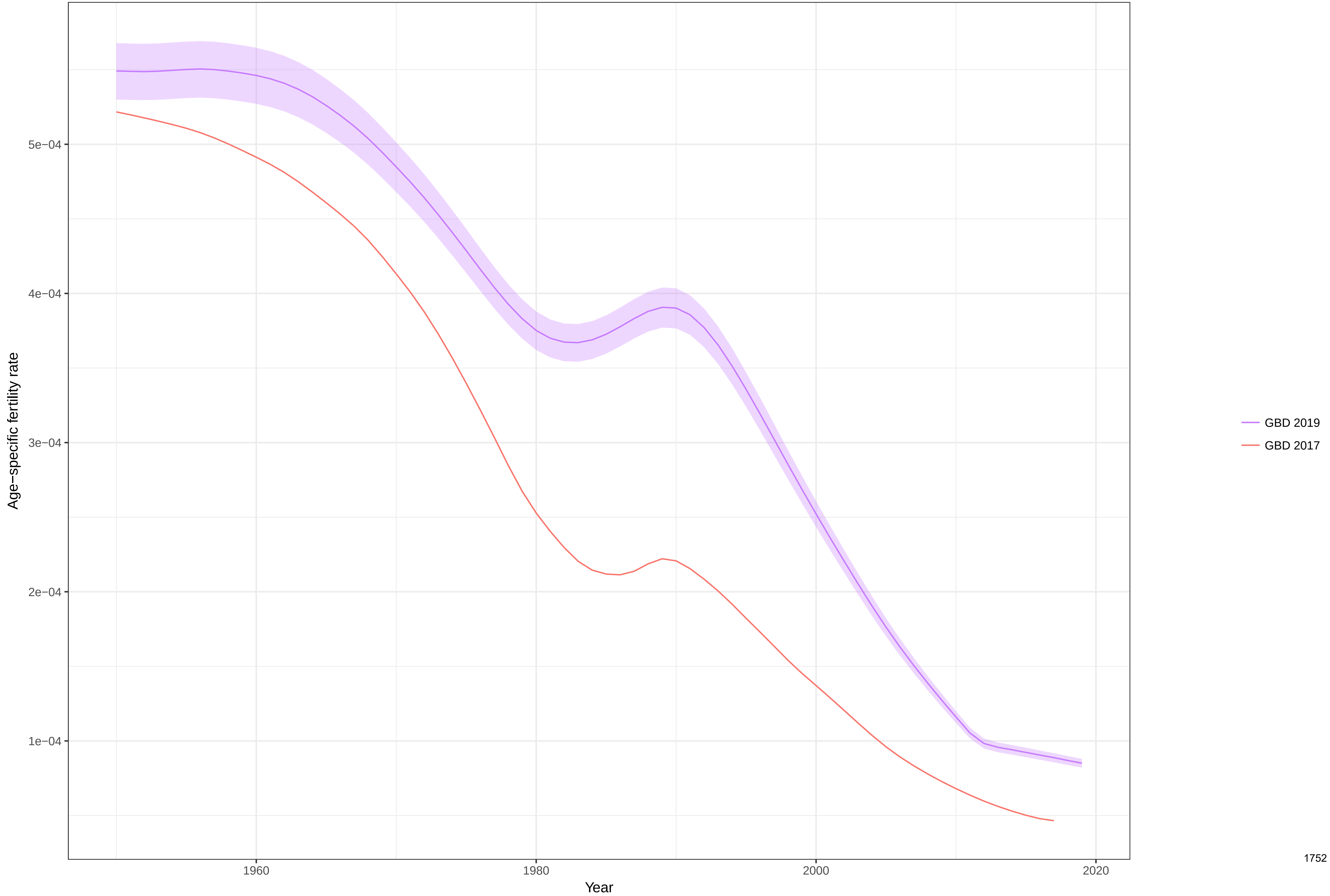

Total fertility rate fitted trend lines, 1950−2019: Qatar TFR
Data points enclosed with a diamond are reference sources

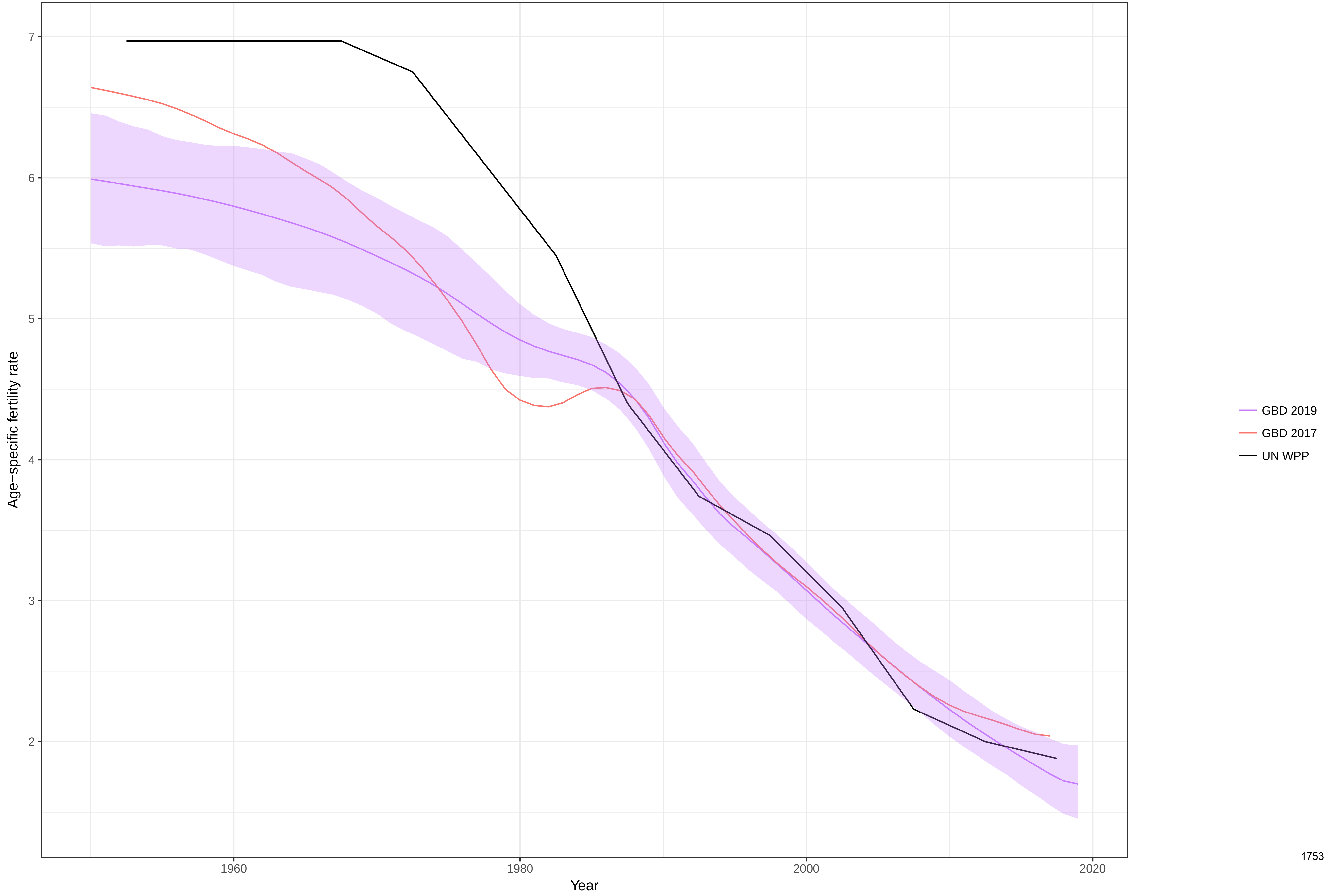

Age−specific fertility fitted trend lines, 1950−2019: Romania, Ages: 10−14
Data points enclosed with a diamond are reference sources

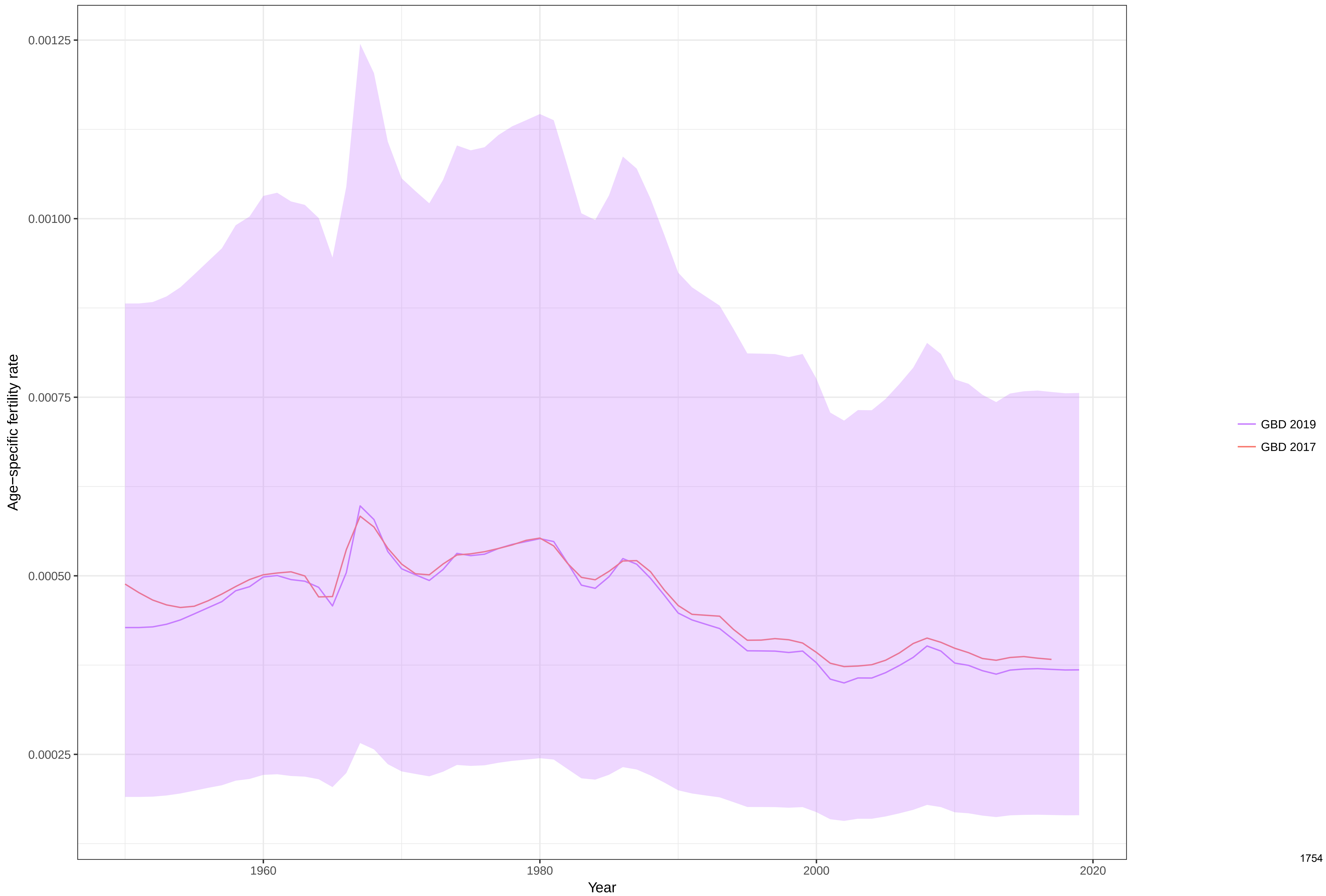

Age−specific fertility fitted trend lines, 1950−2019: Romania, Ages: 15−19
Data points enclosed with a diamond are reference sources

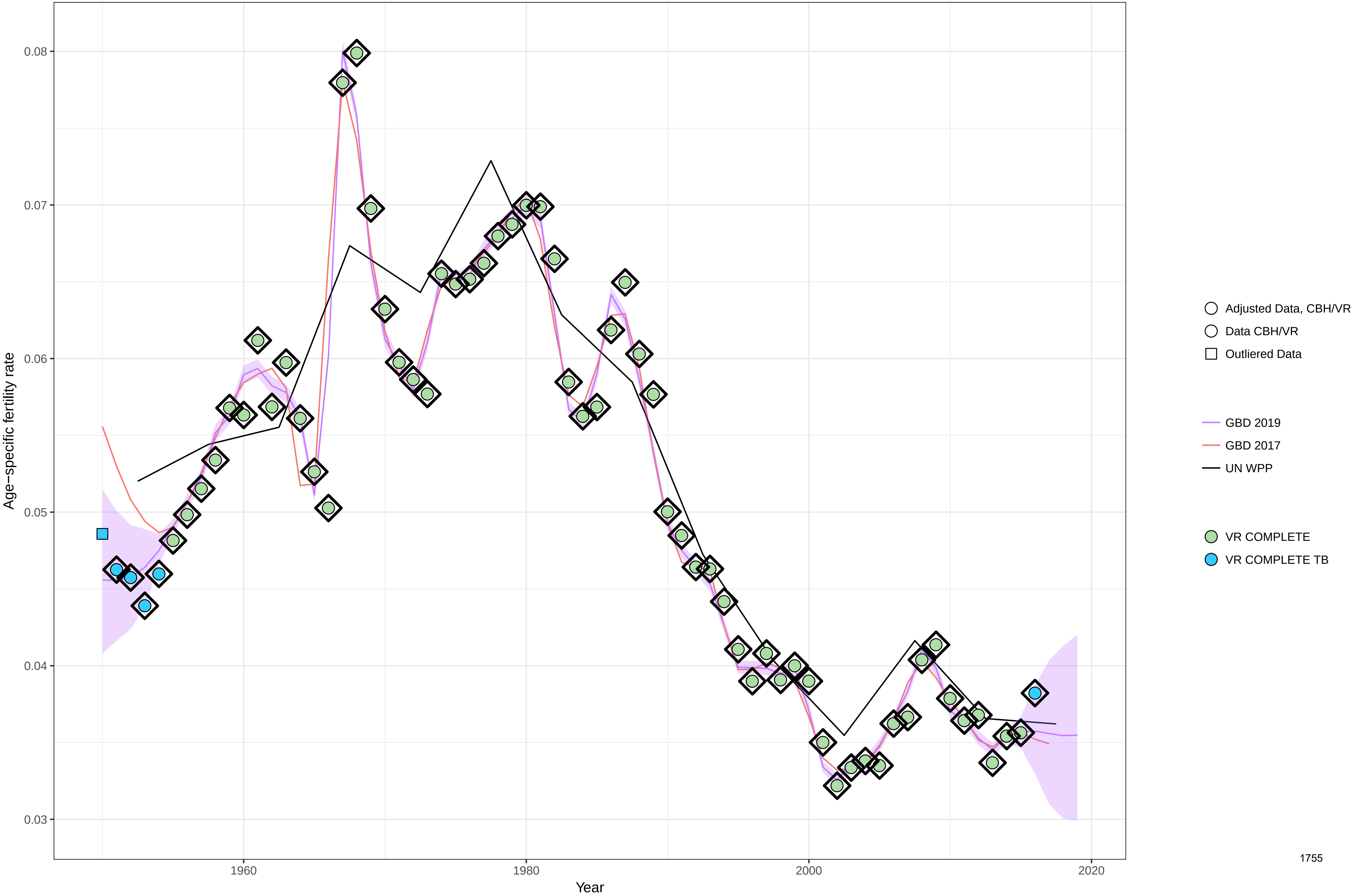

Age−specific fertility fitted trend lines, 1950−2019: Romania, Ages: 20−24
Data points enclosed with a diamond are reference sources

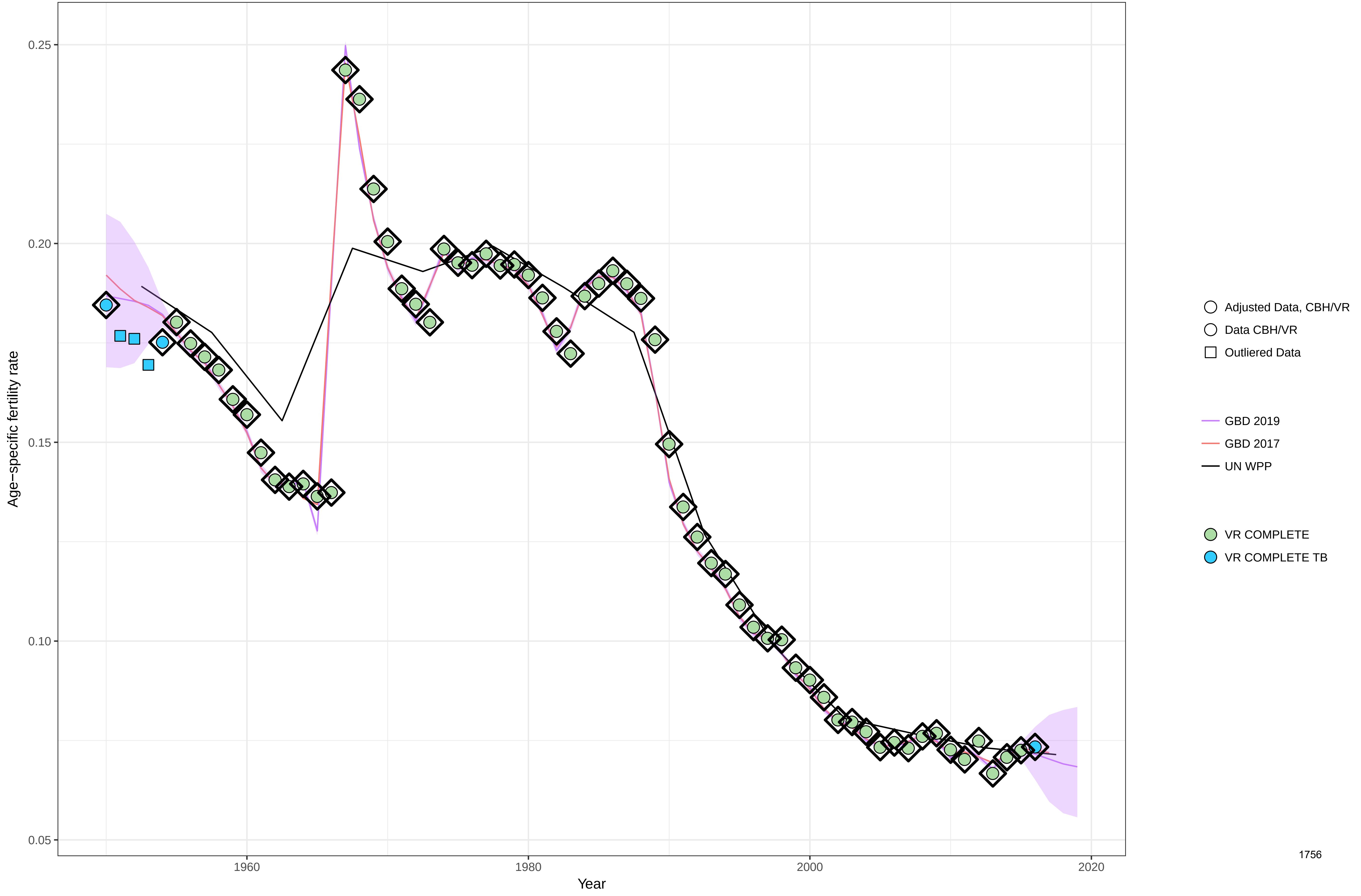

Age−specific fertility fitted trend lines, 1950−2019: Romania, Ages: 25−29
Data points enclosed with a diamond are reference sources

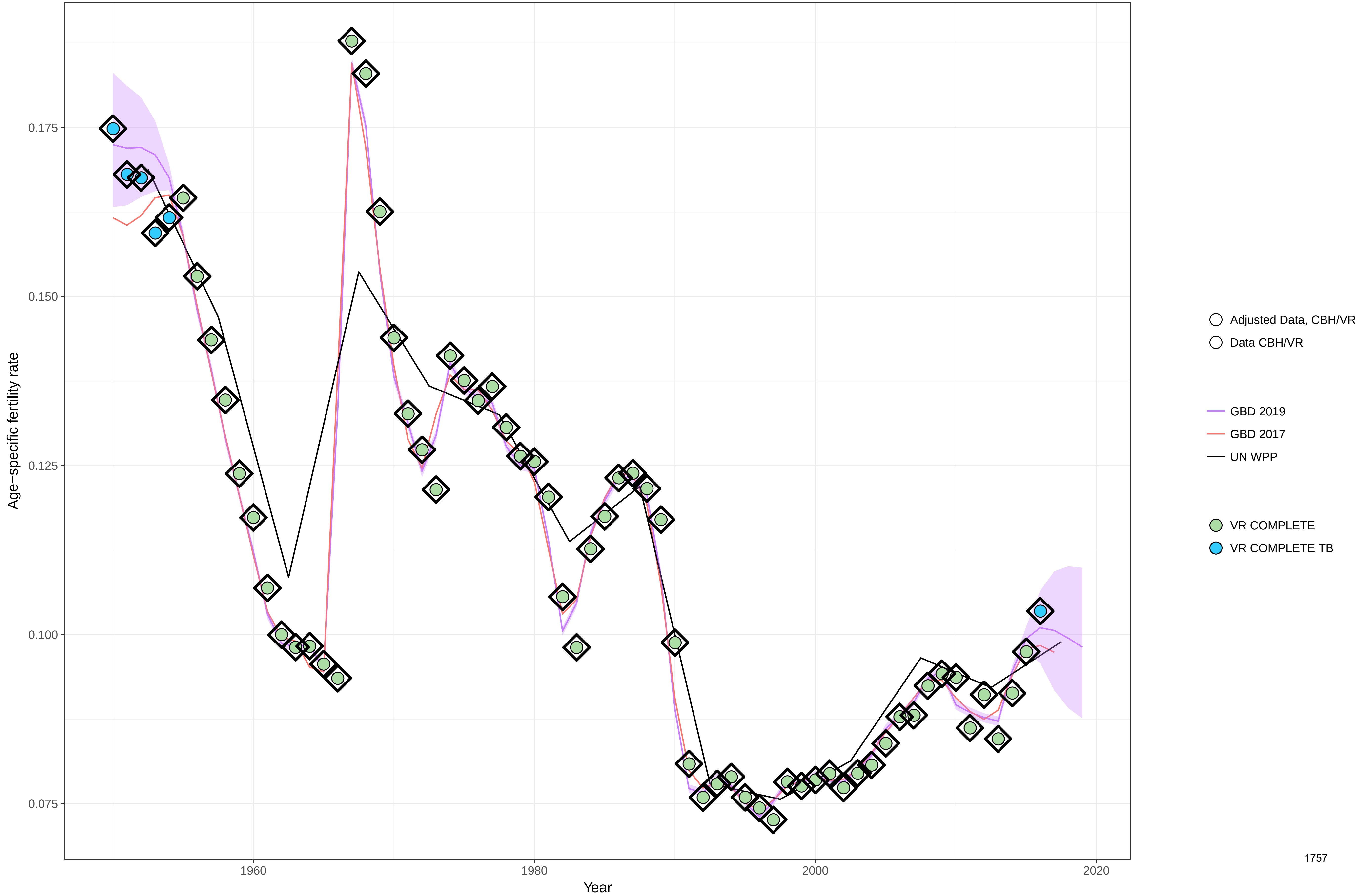

Age−specific fertility fitted trend lines, 1950−2019: Romania, Ages: 30−34
Data points enclosed with a diamond are reference sources

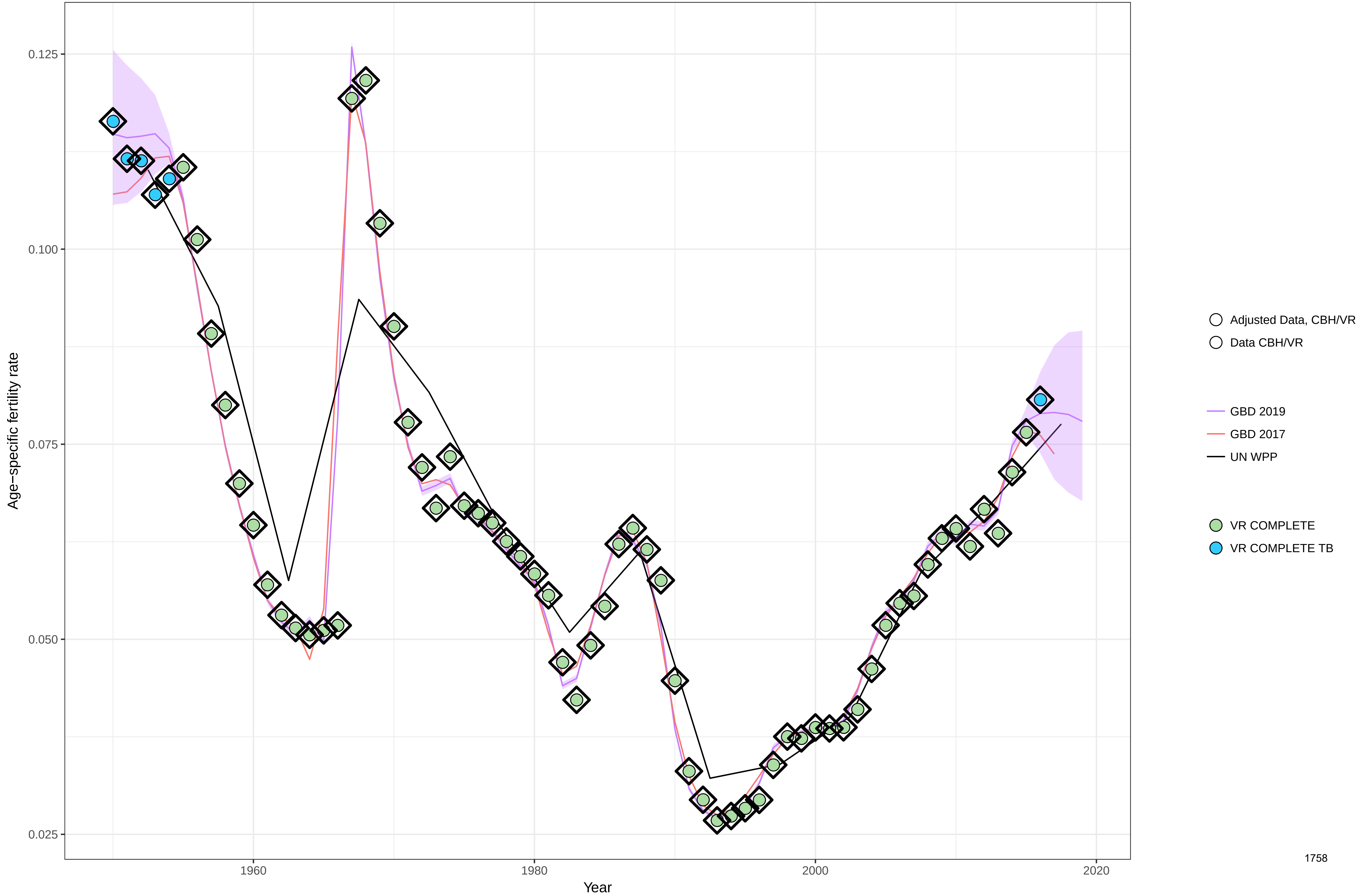

Age−specific fertility fitted trend lines, 1950−2019: Romania, Ages: 35−39
Data points enclosed with a diamond are reference sources

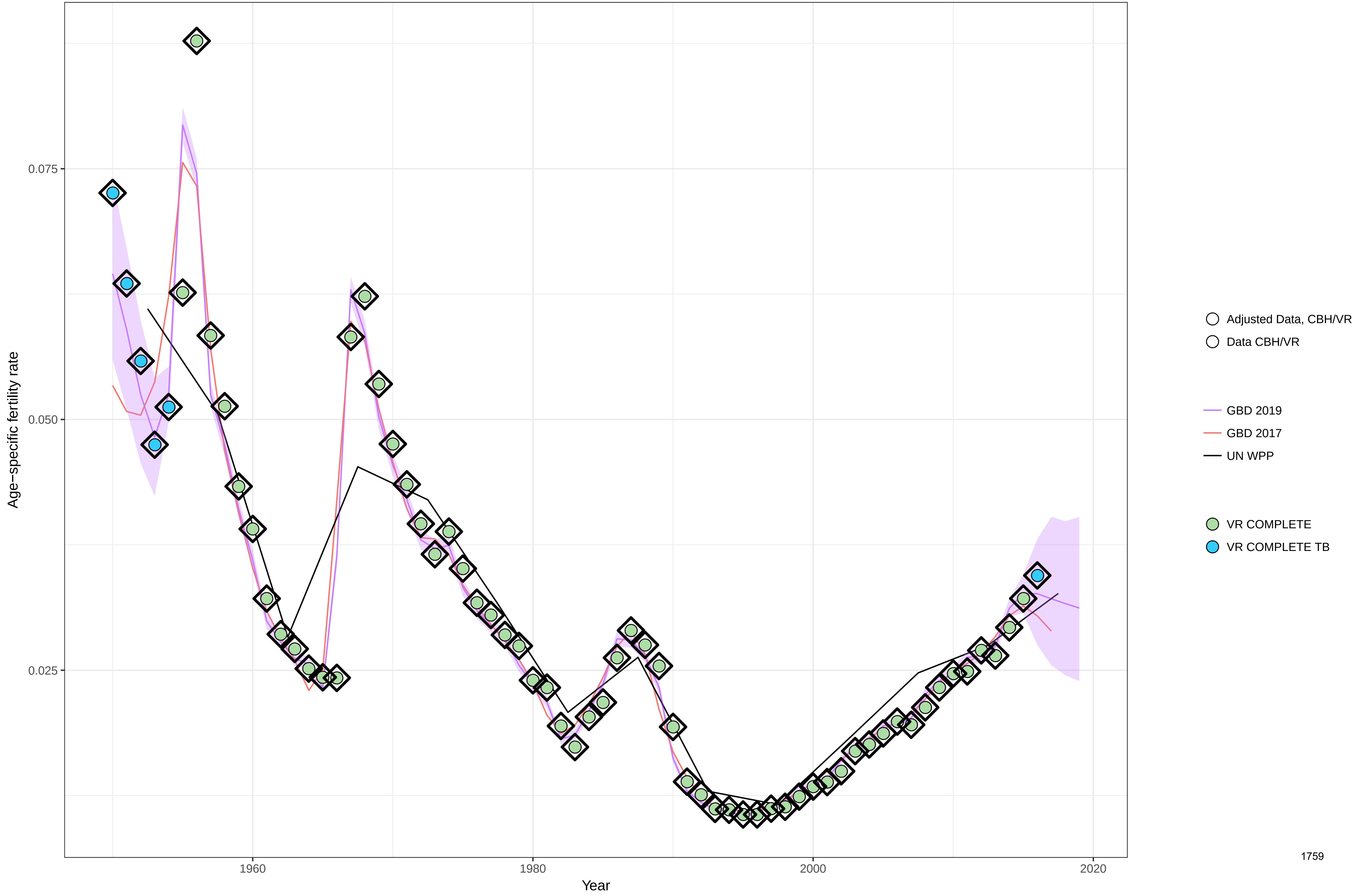

Age−specific fertility fitted trend lines, 1950−2019: Romania, Ages: 40−44
Data points enclosed with a diamond are reference sources

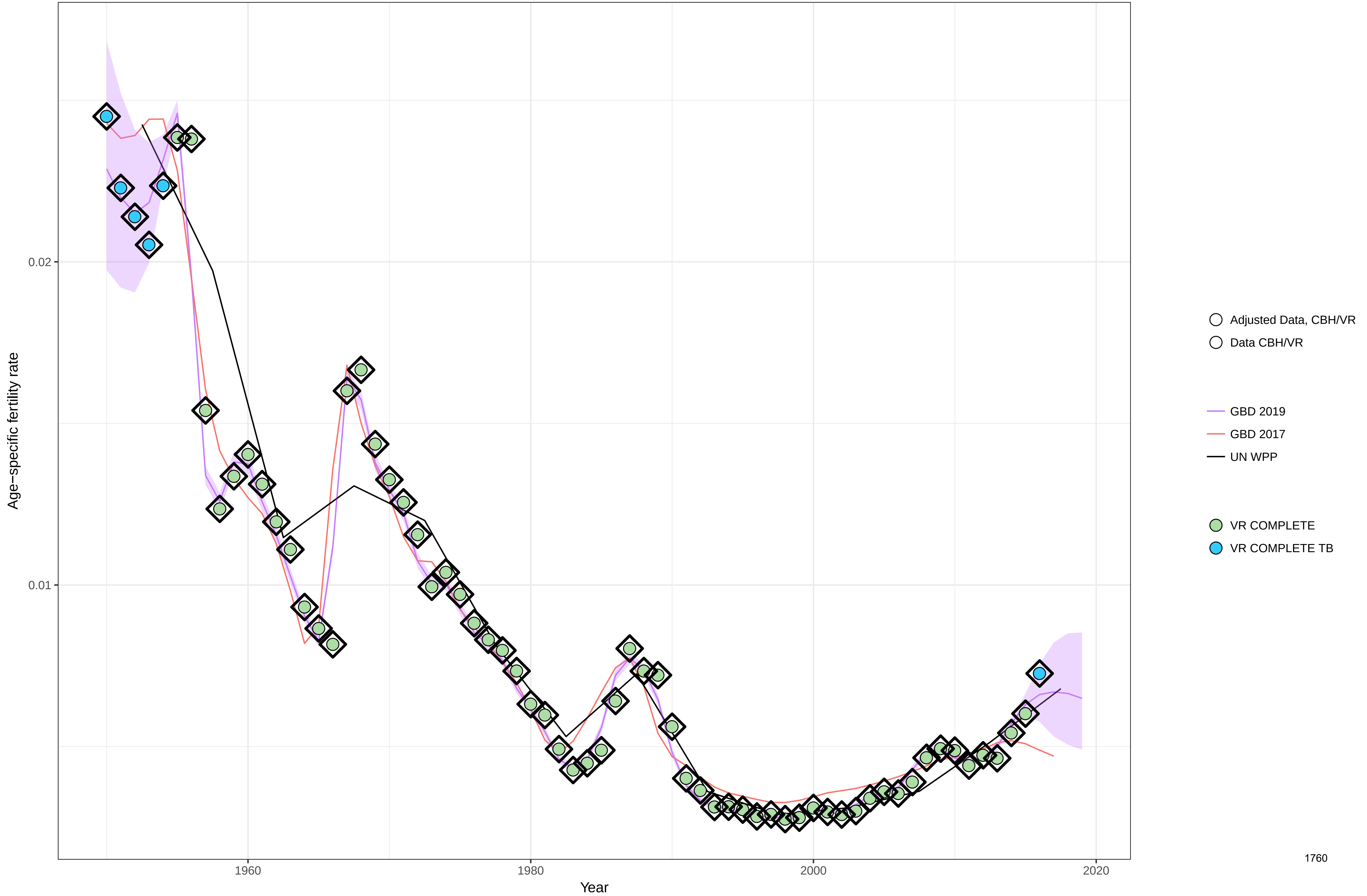

Age−specific fertility fitted trend lines, 1950−2019: Romania, Ages: 45−49
Data points enclosed with a diamond are reference sources

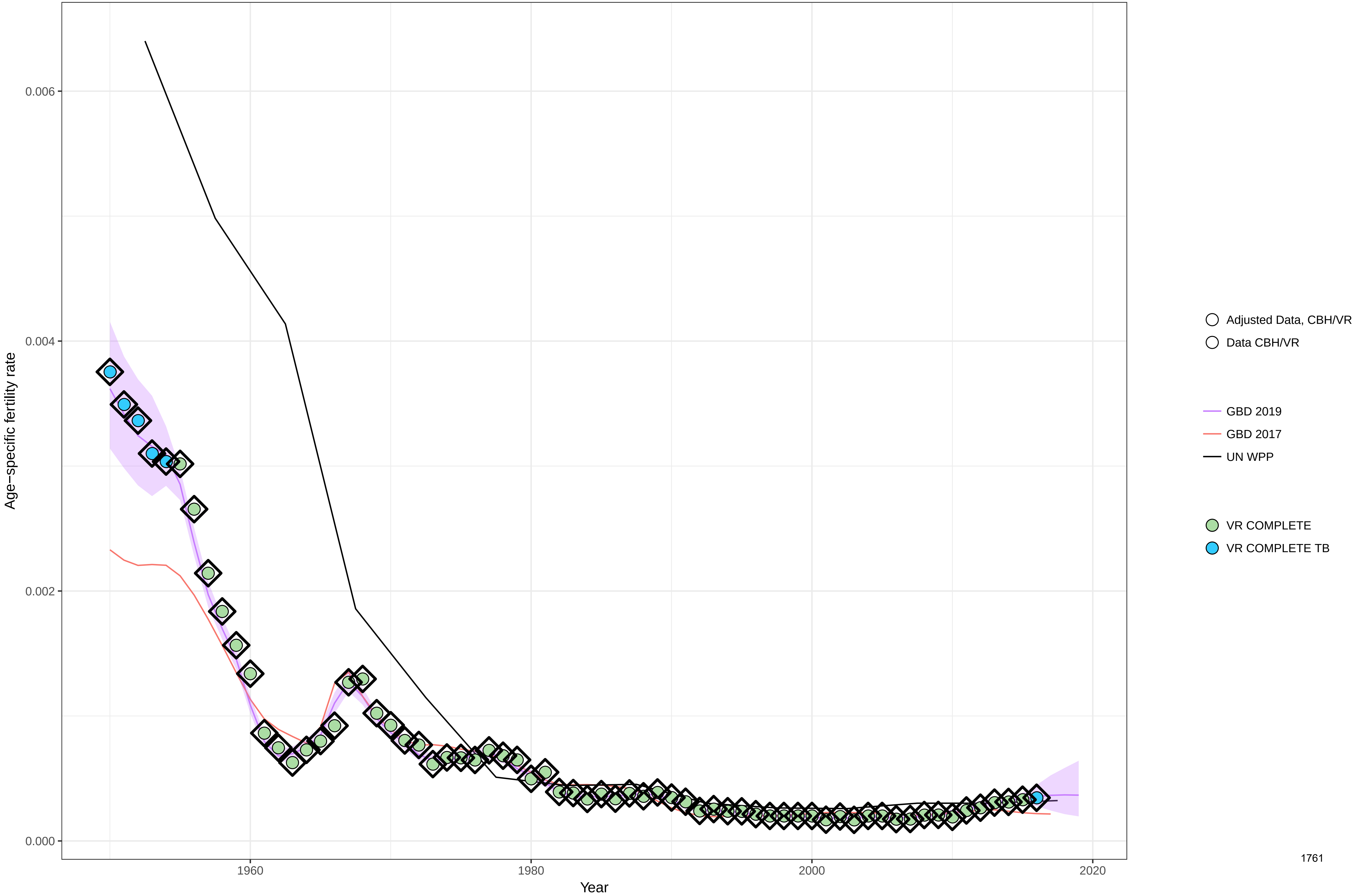

Age−specific fertility fitted trend lines, 1950−2019: Romania, Ages: 50−54
Data points enclosed with a diamond are reference sources

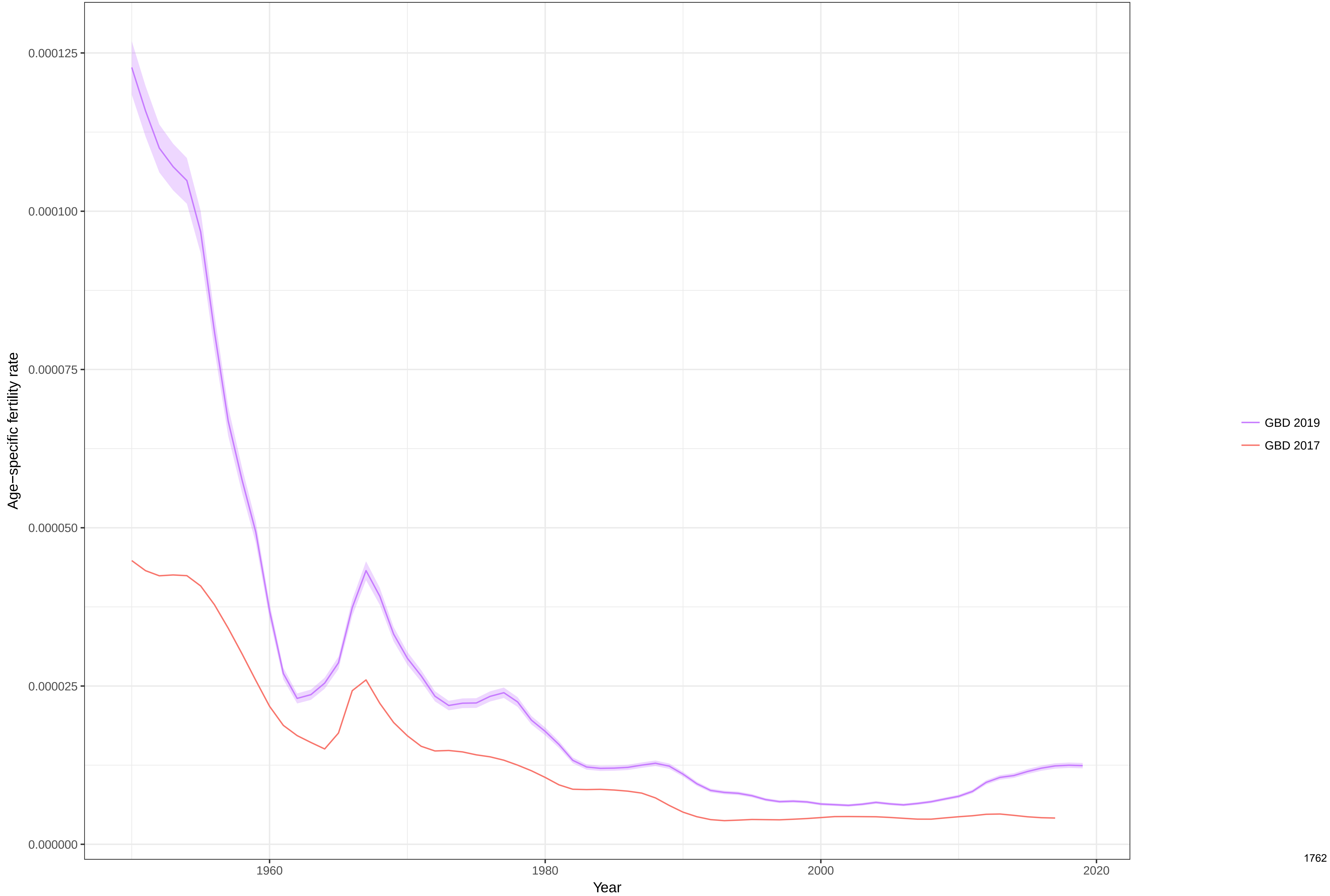

Total fertility rate fitted trend lines, 1950−2019: Romania TFR
Data points enclosed with a diamond are reference sources

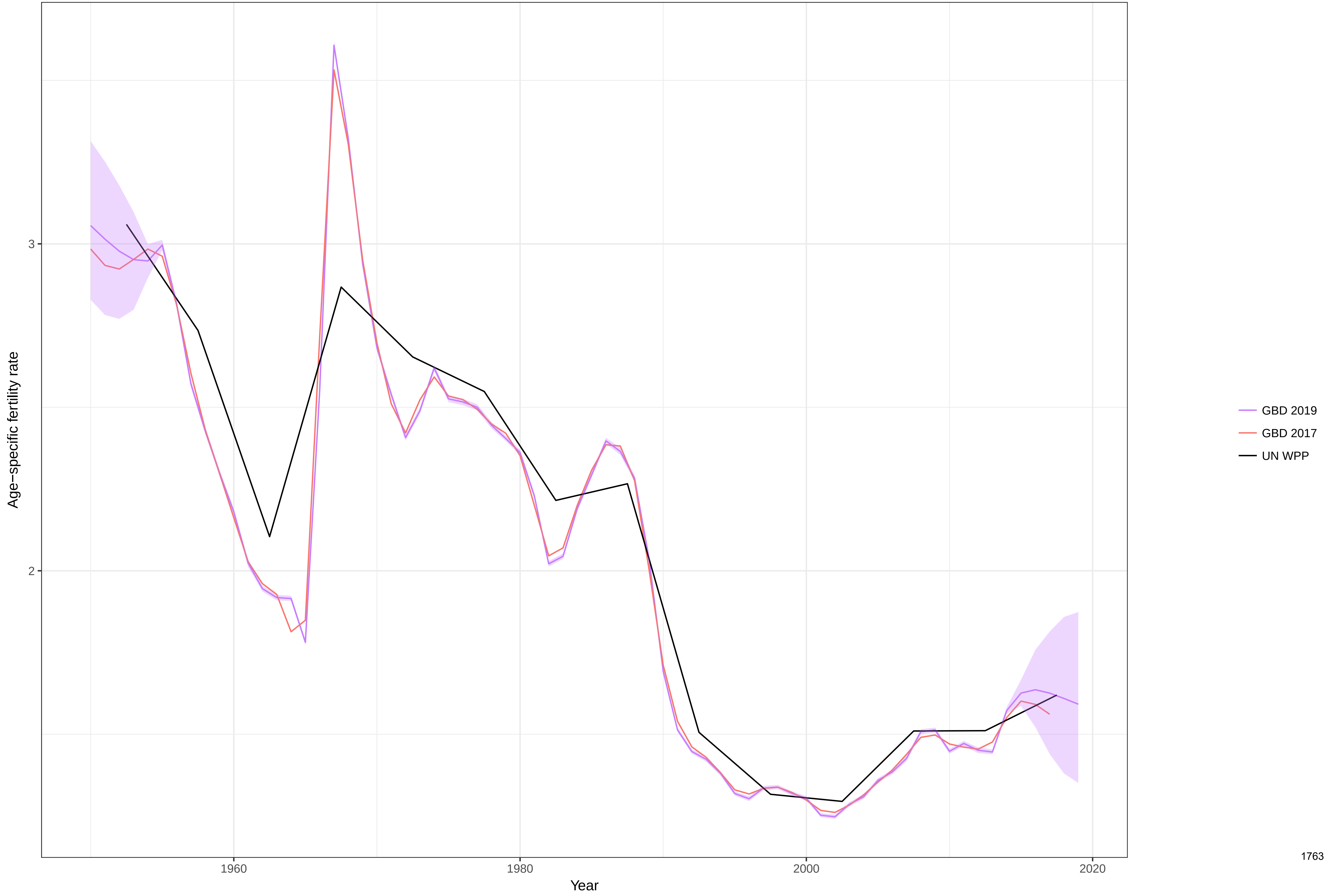

Age−specific fertility fitted trend lines, 1950−2019: Russian Federation, Ages: 10−14
Data points enclosed with a diamond are reference sources

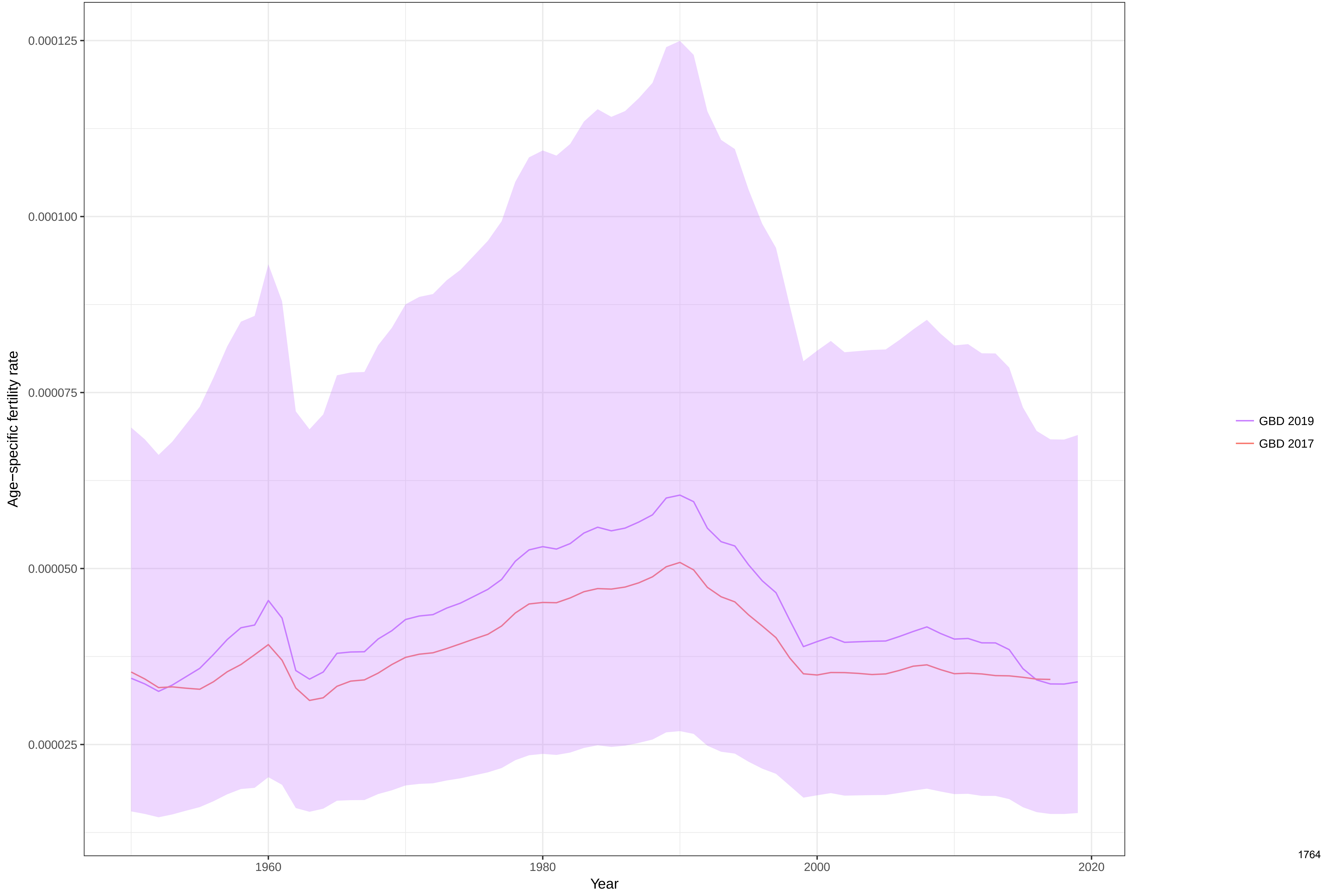

Age−specific fertility fitted trend lines, 1950−2019: Russian Federation, Ages: 15−19
Data points enclosed with a diamond are reference sources

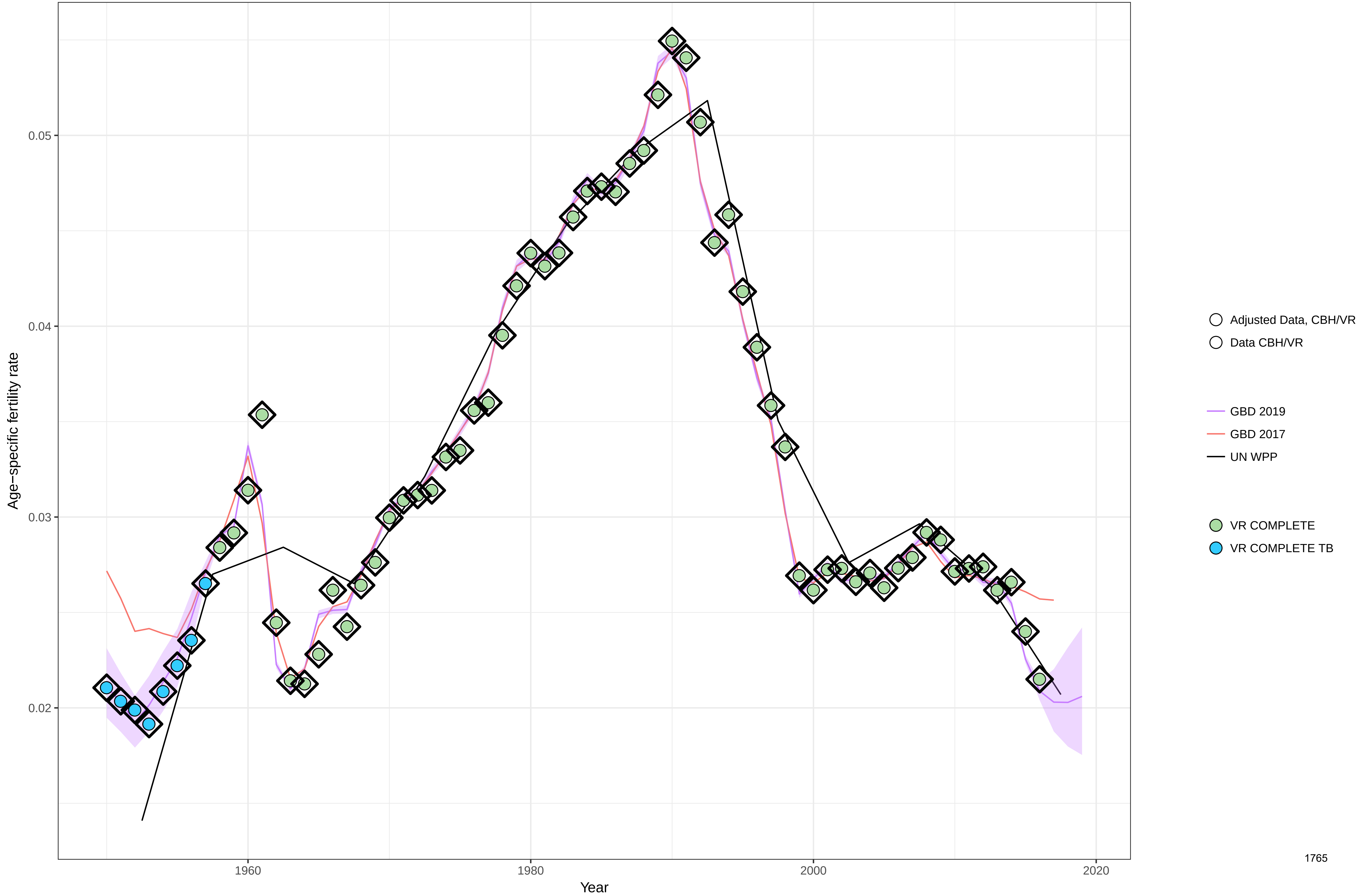

Age−specific fertility fitted trend lines, 1950−2019: Russian Federation, Ages: 20−24
Data points enclosed with a diamond are reference sources

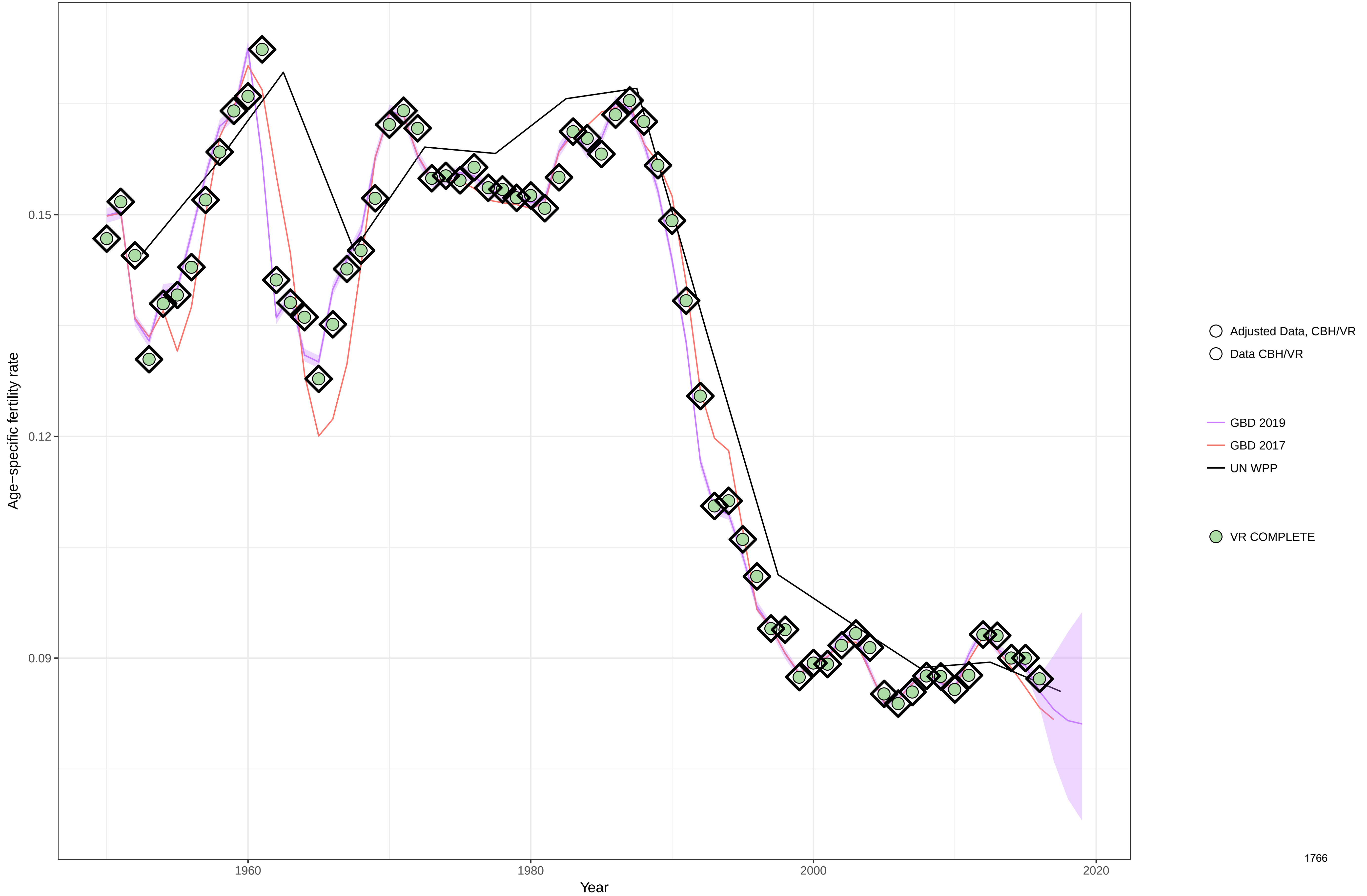

Age−specific fertility fitted trend lines, 1950−2019: Russian Federation, Ages: 25−29
Data points enclosed with a diamond are reference sources

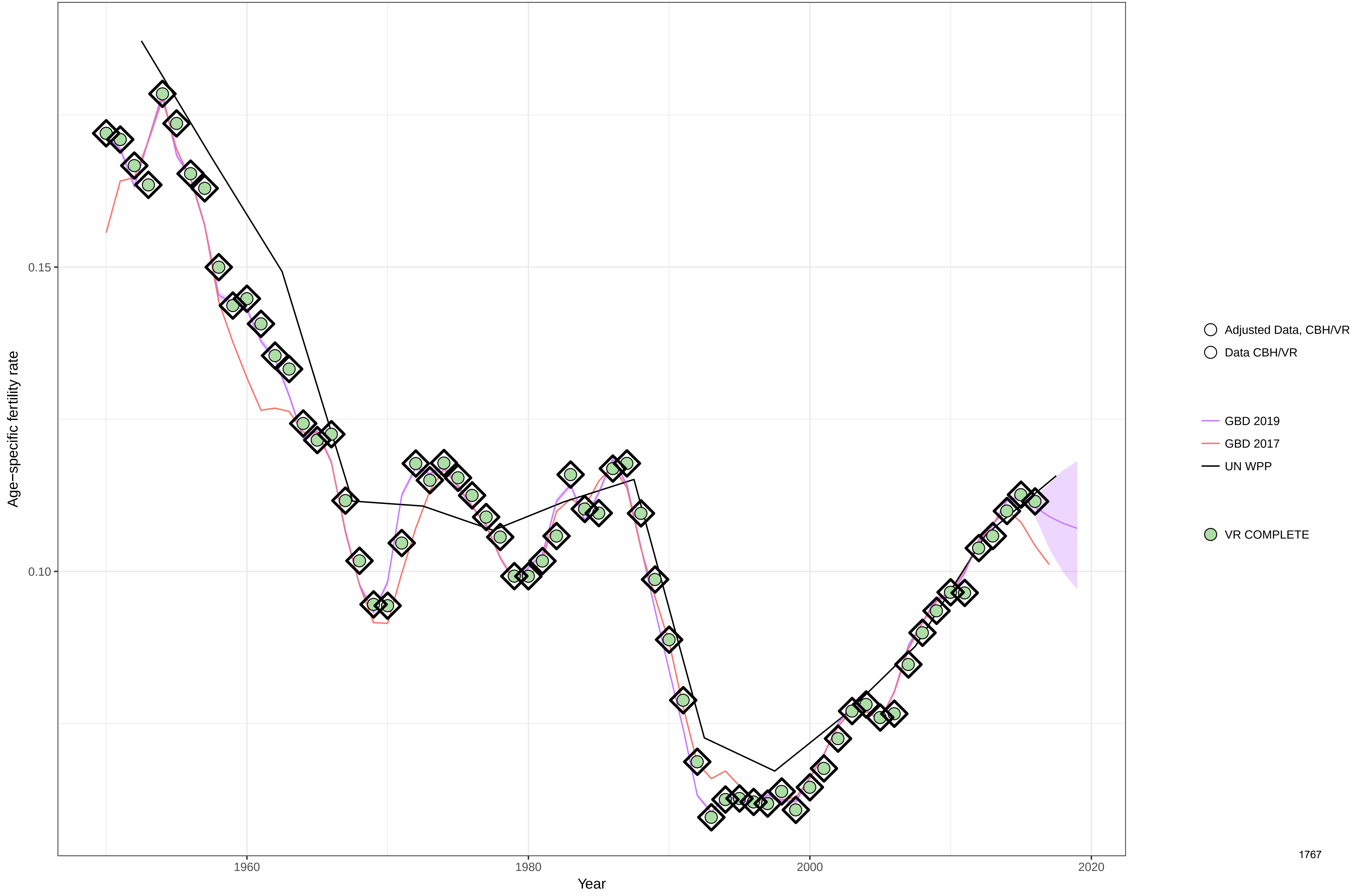

Age−specific fertility fitted trend lines, 1950−2019: Russian Federation, Ages: 30−34
Data points enclosed with a diamond are reference sources

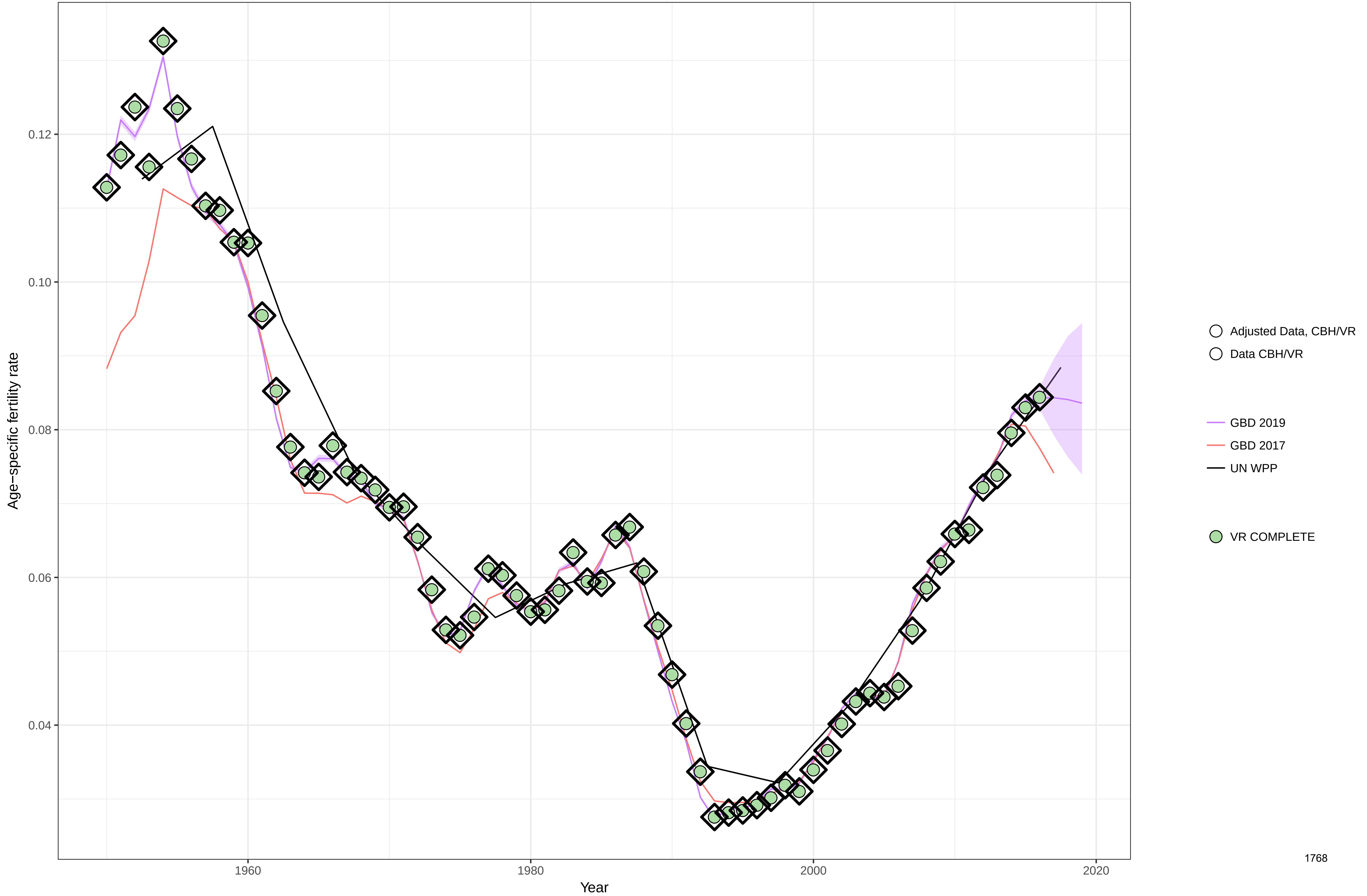

Age−specific fertility fitted trend lines, 1950−2019: Russian Federation, Ages: 35−39
Data points enclosed with a diamond are reference sources

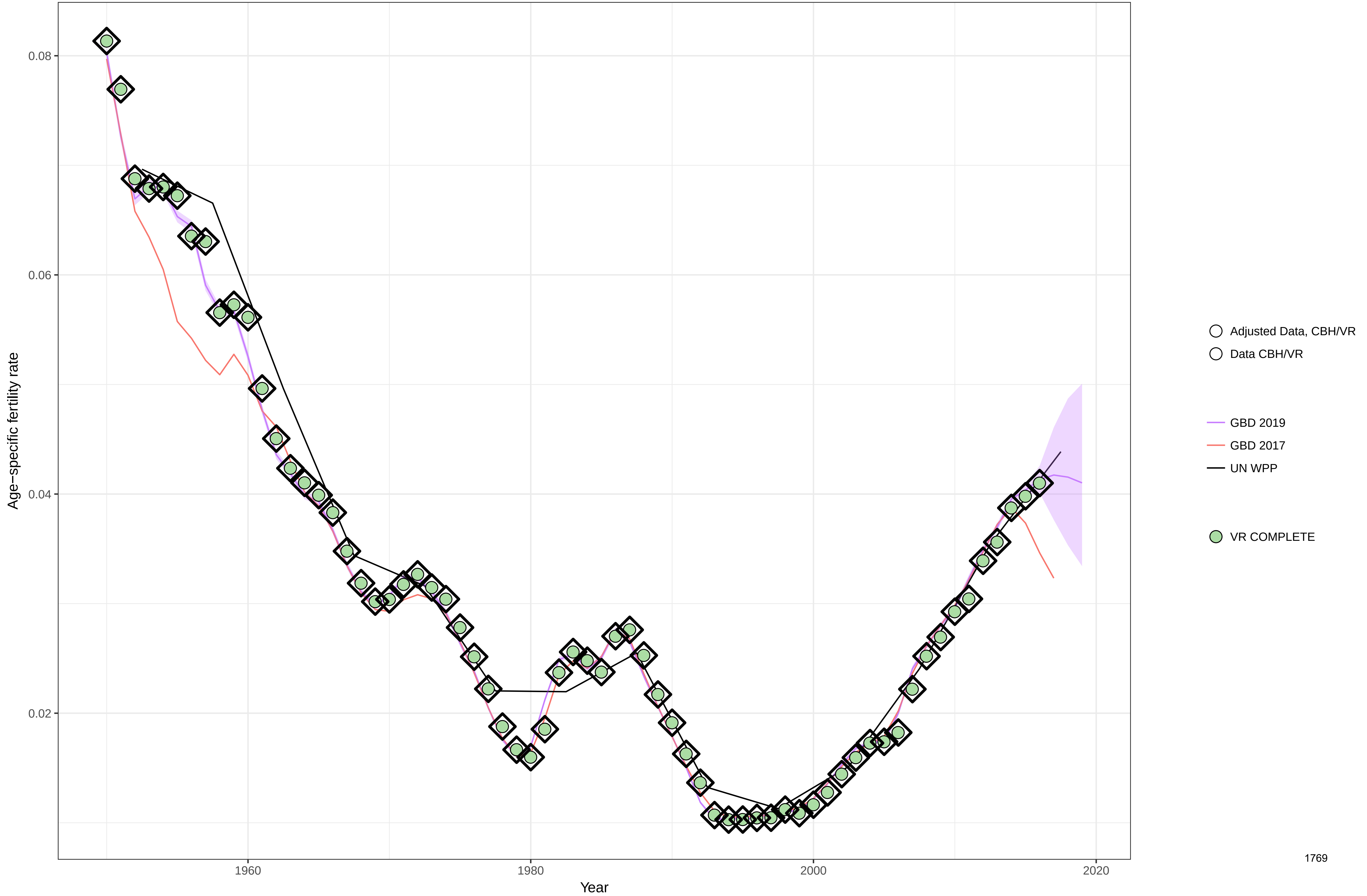

Age−specific fertility fitted trend lines, 1950−2019: Russian Federation, Ages: 40−44
Data points enclosed with a diamond are reference sources

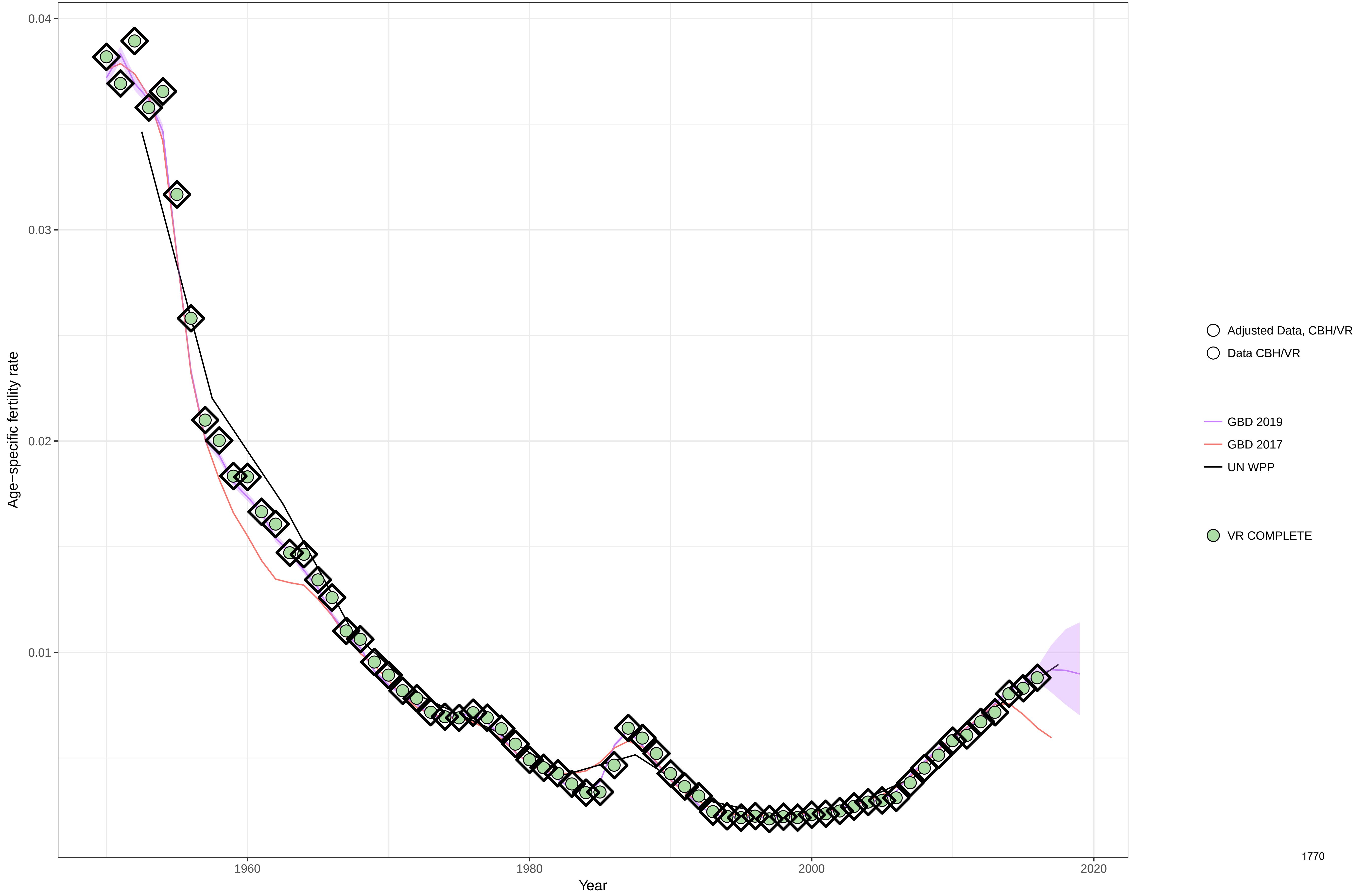

Age−specific fertility fitted trend lines, 1950−2019: Russian Federation, Ages: 45−49
Data points enclosed with a diamond are reference sources

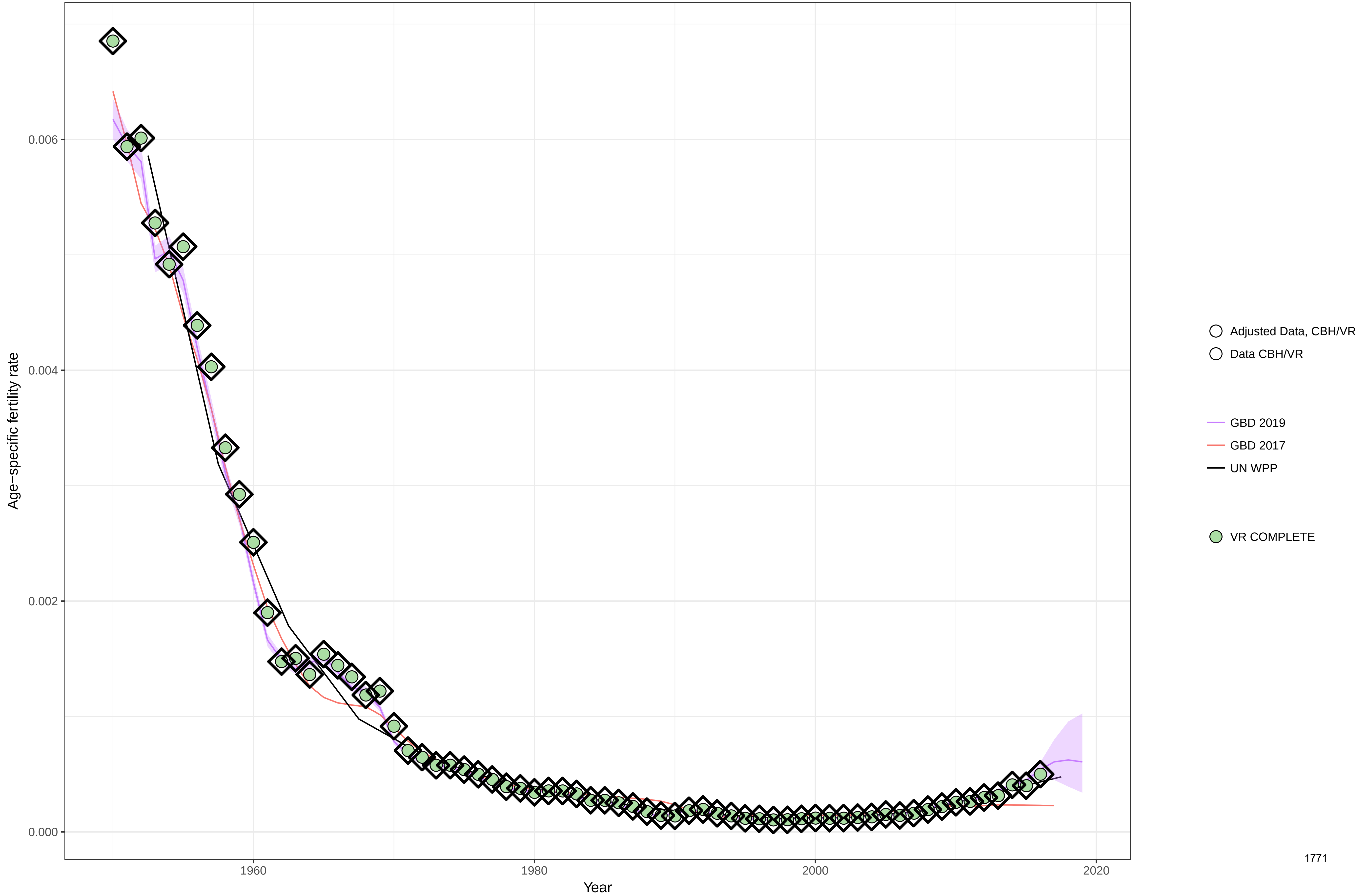

Age−specific fertility fitted trend lines, 1950−2019: Russian Federation, Ages: 50−54
Data points enclosed with a diamond are reference sources

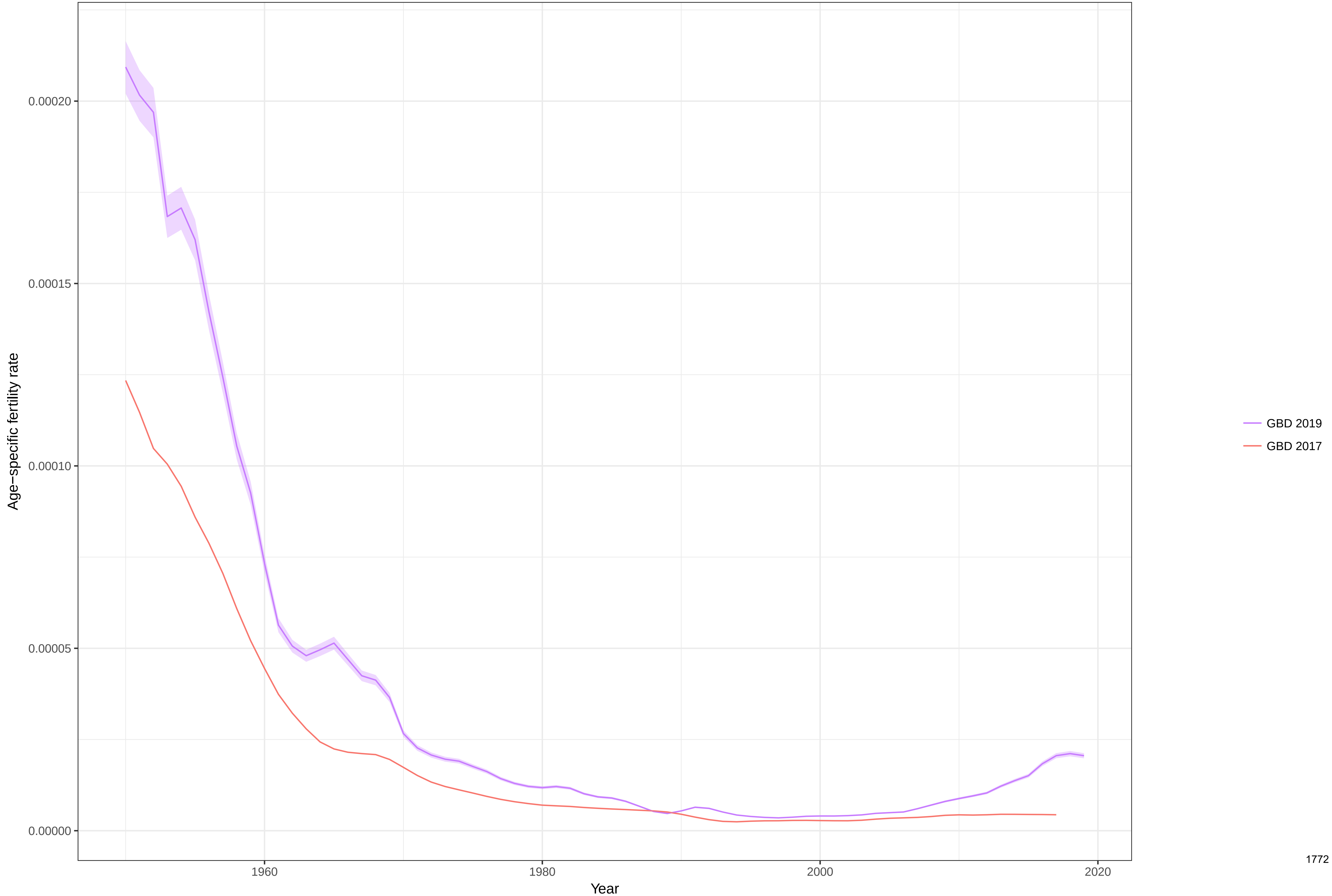

Total fertility rate fitted trend lines, 1950−2019: Russian Federation TFR
Data points enclosed with a diamond are reference sources

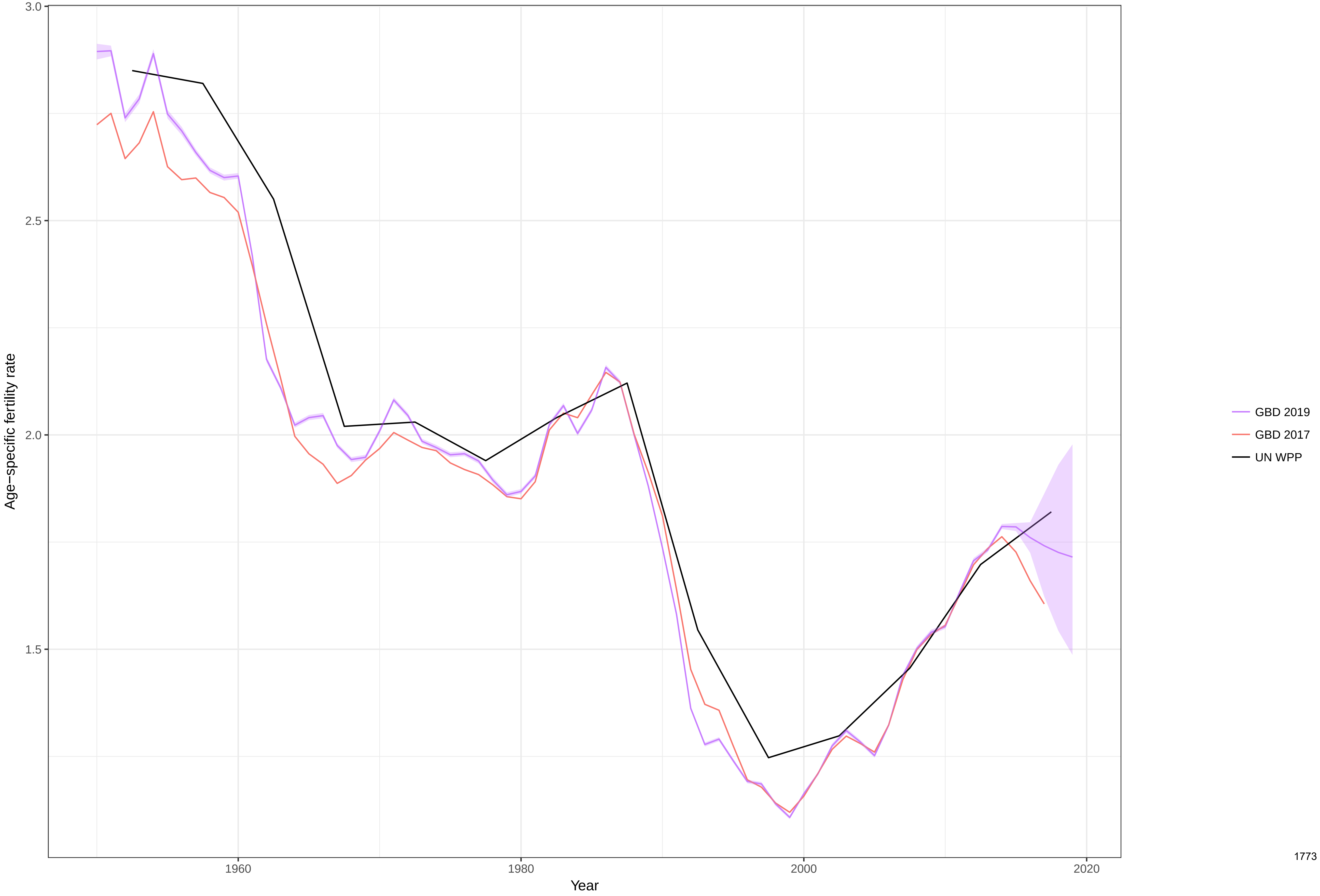

Age−specific fertility fitted trend lines, 1950−2019: Rwanda, Ages: 10−14
Data points enclosed with a diamond are reference sources

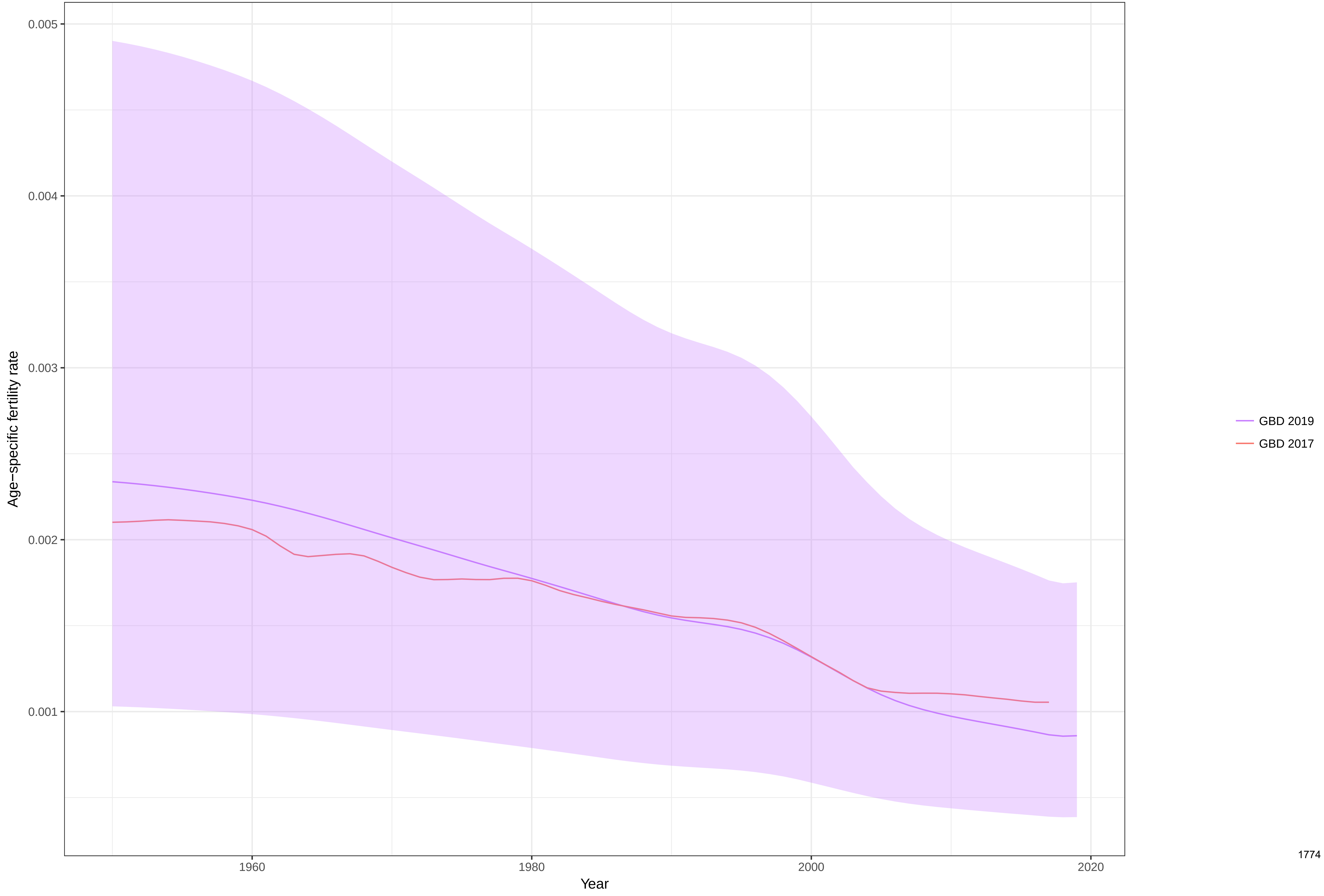

Age−specific fertility fitted trend lines, 1950−2019: Rwanda, Ages: 15−19
Data points enclosed with a diamond are reference sources

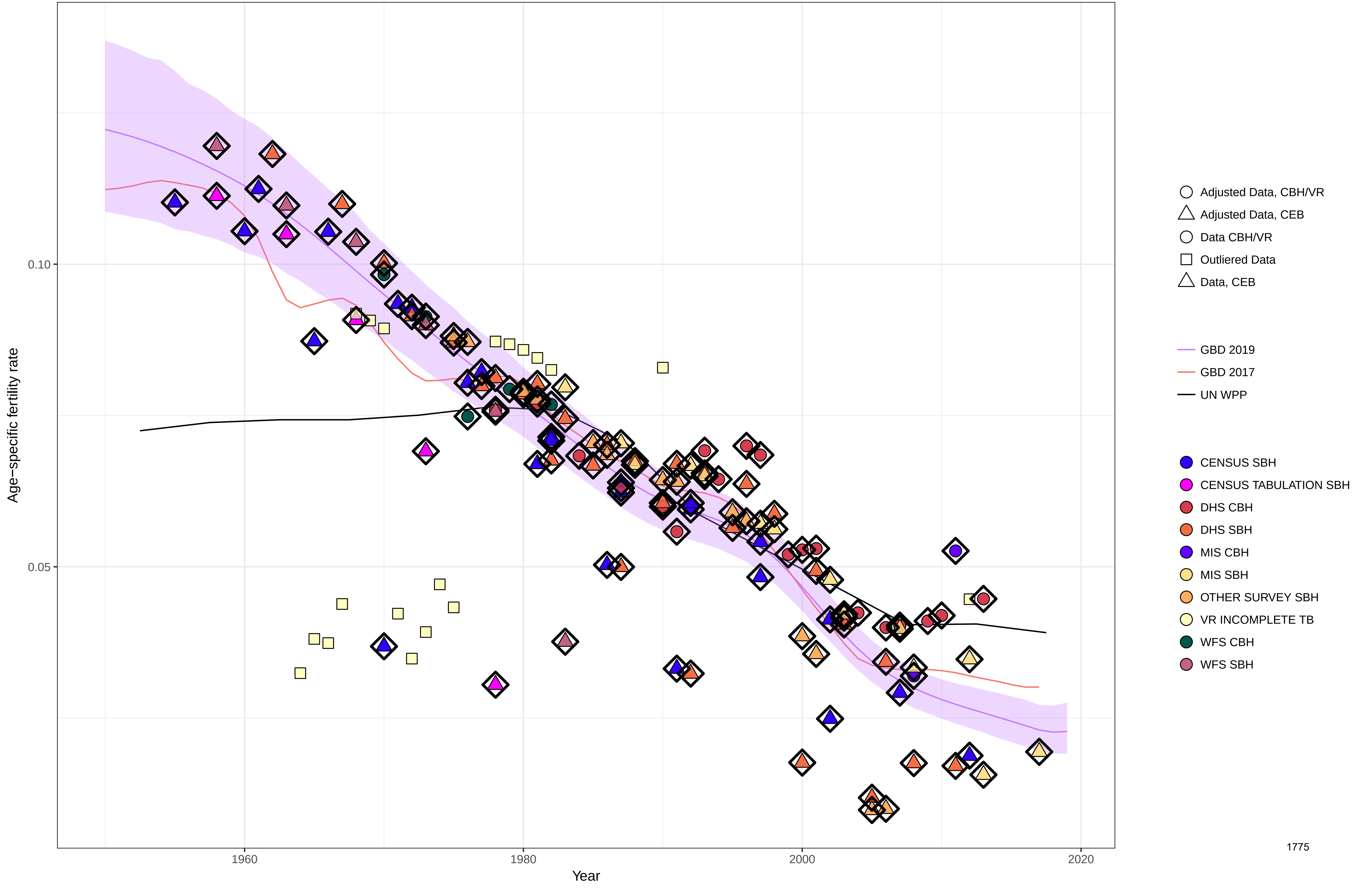

Age−specific fertility fitted trend lines, 1950−2019: Rwanda, Ages: 20−24
Data points enclosed with a diamond are reference sources

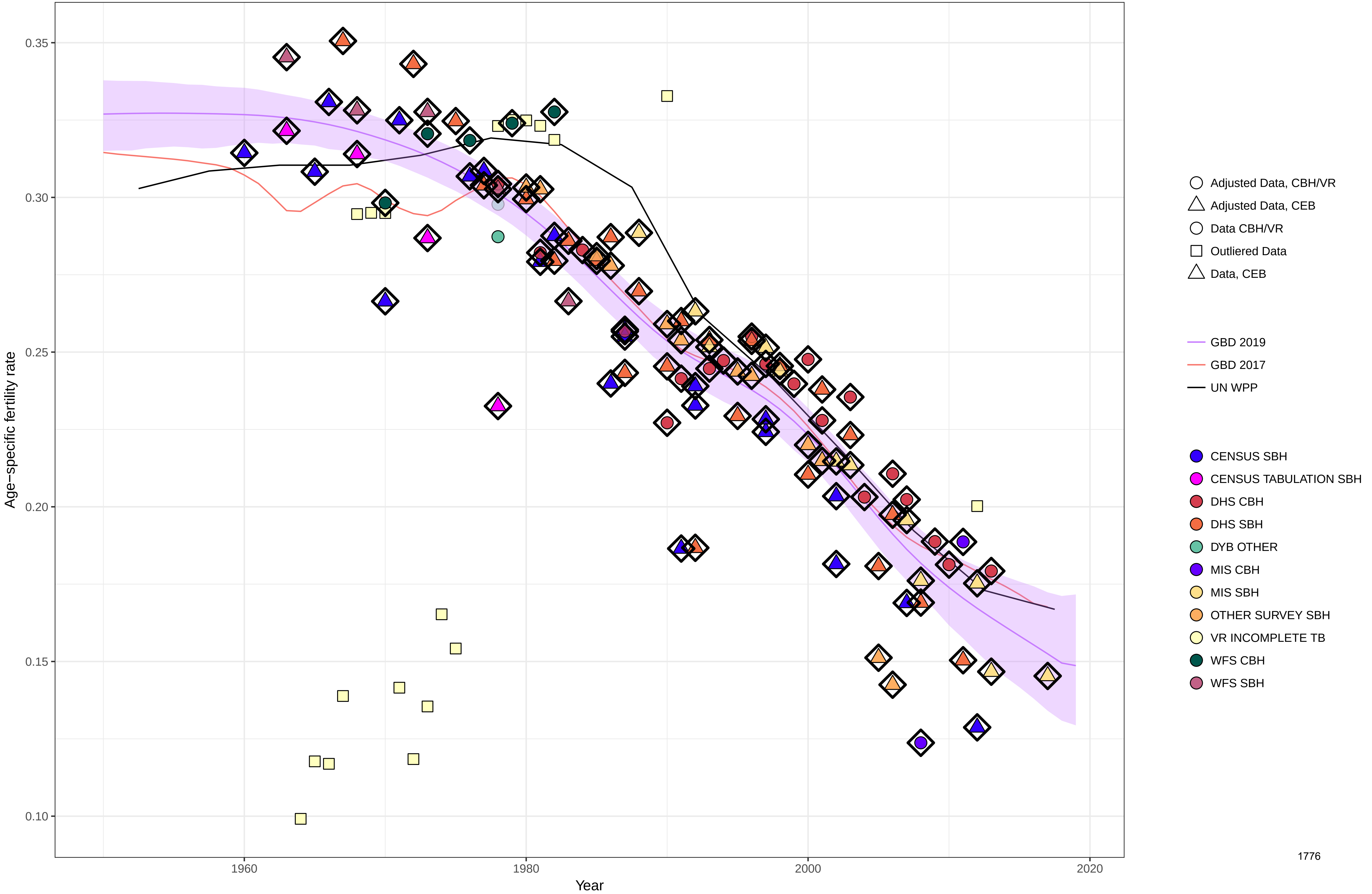

Age−specific fertility fitted trend lines, 1950−2019: Rwanda, Ages: 25−29
Data points enclosed with a diamond are reference sources

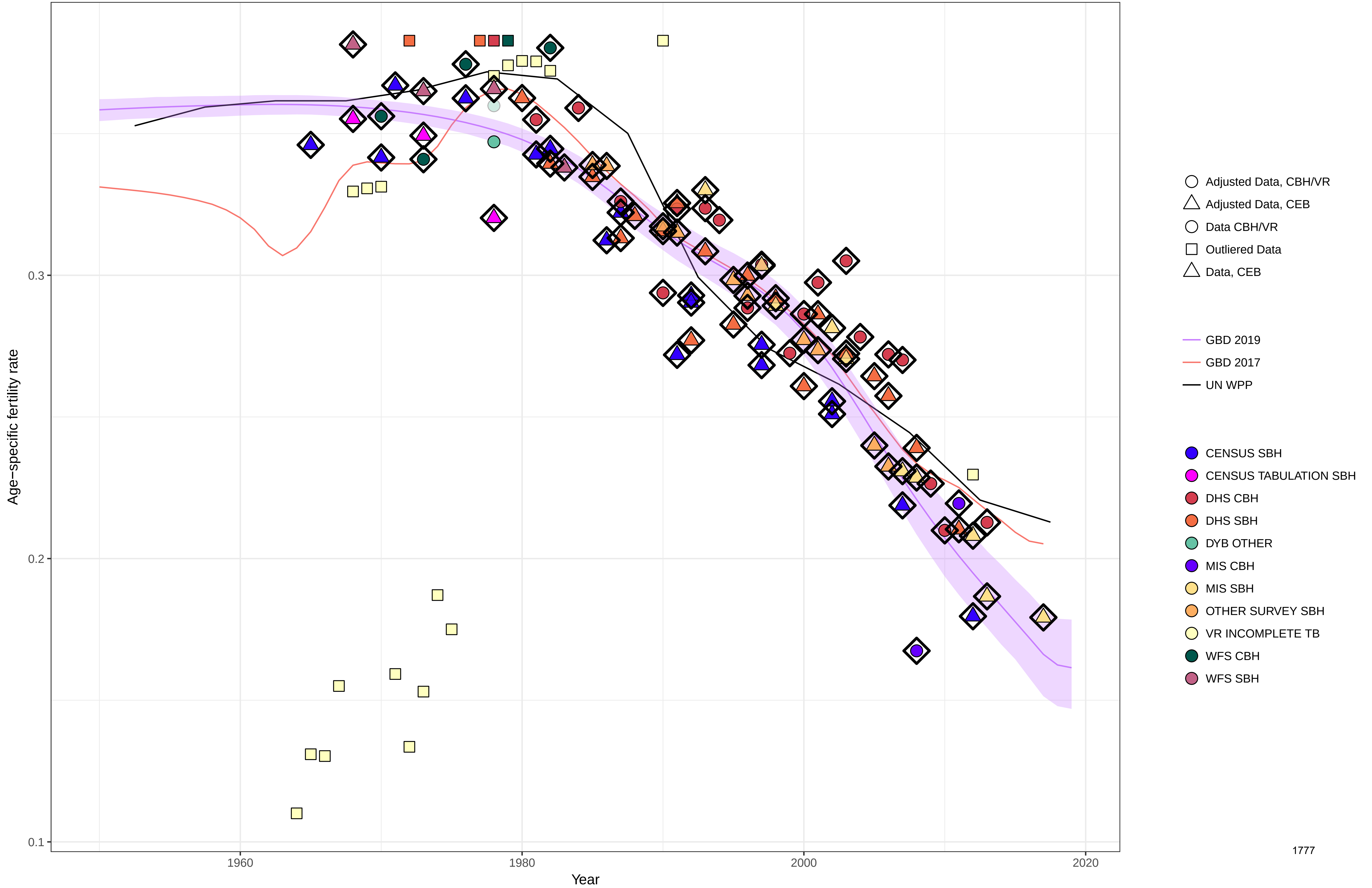

Age−specific fertility fitted trend lines, 1950−2019: Rwanda, Ages: 30−34
Data points enclosed with a diamond are reference sources

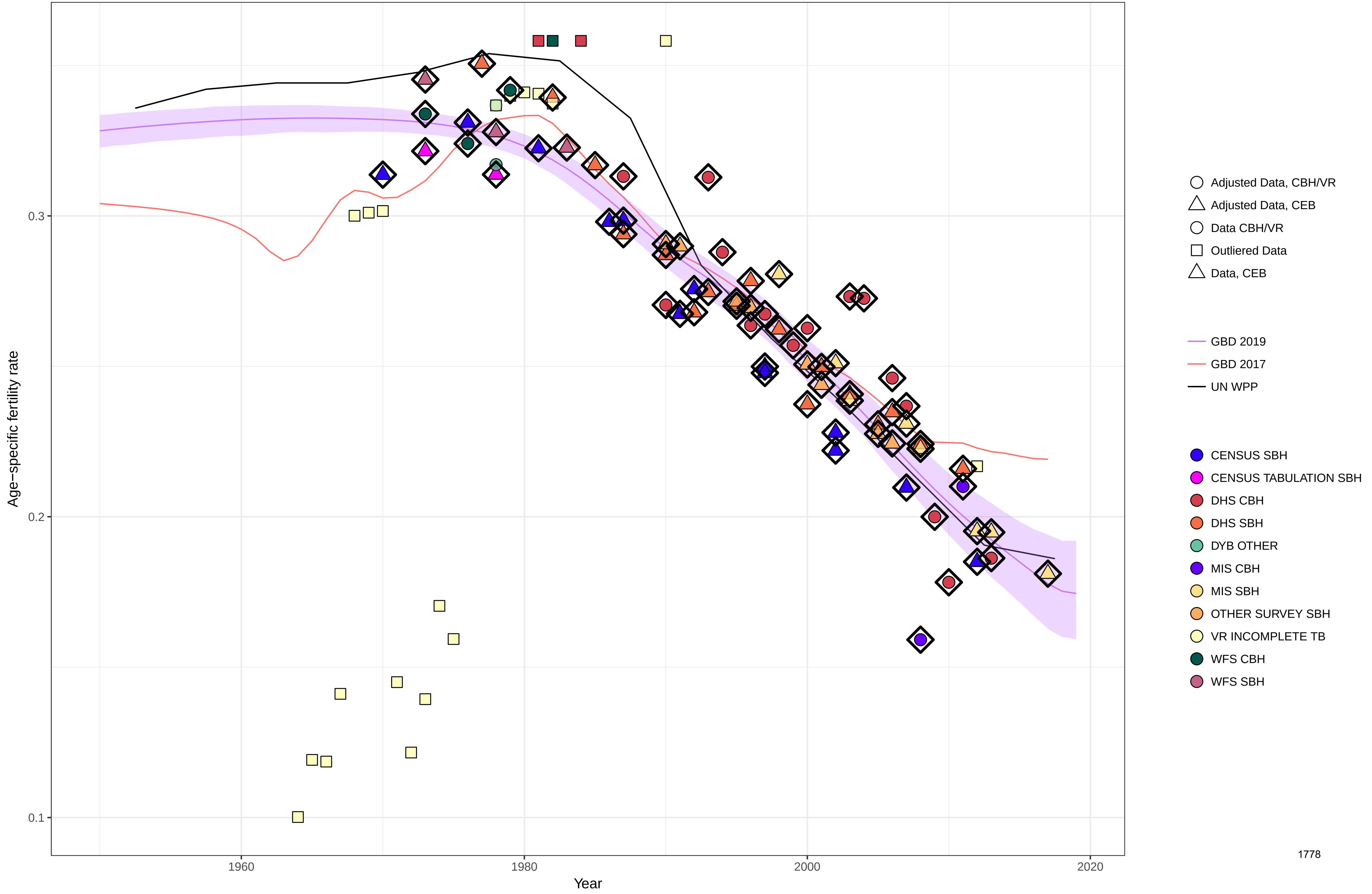

Age−specific fertility fitted trend lines, 1950−2019: Rwanda, Ages: 35−39
Data points enclosed with a diamond are reference sources

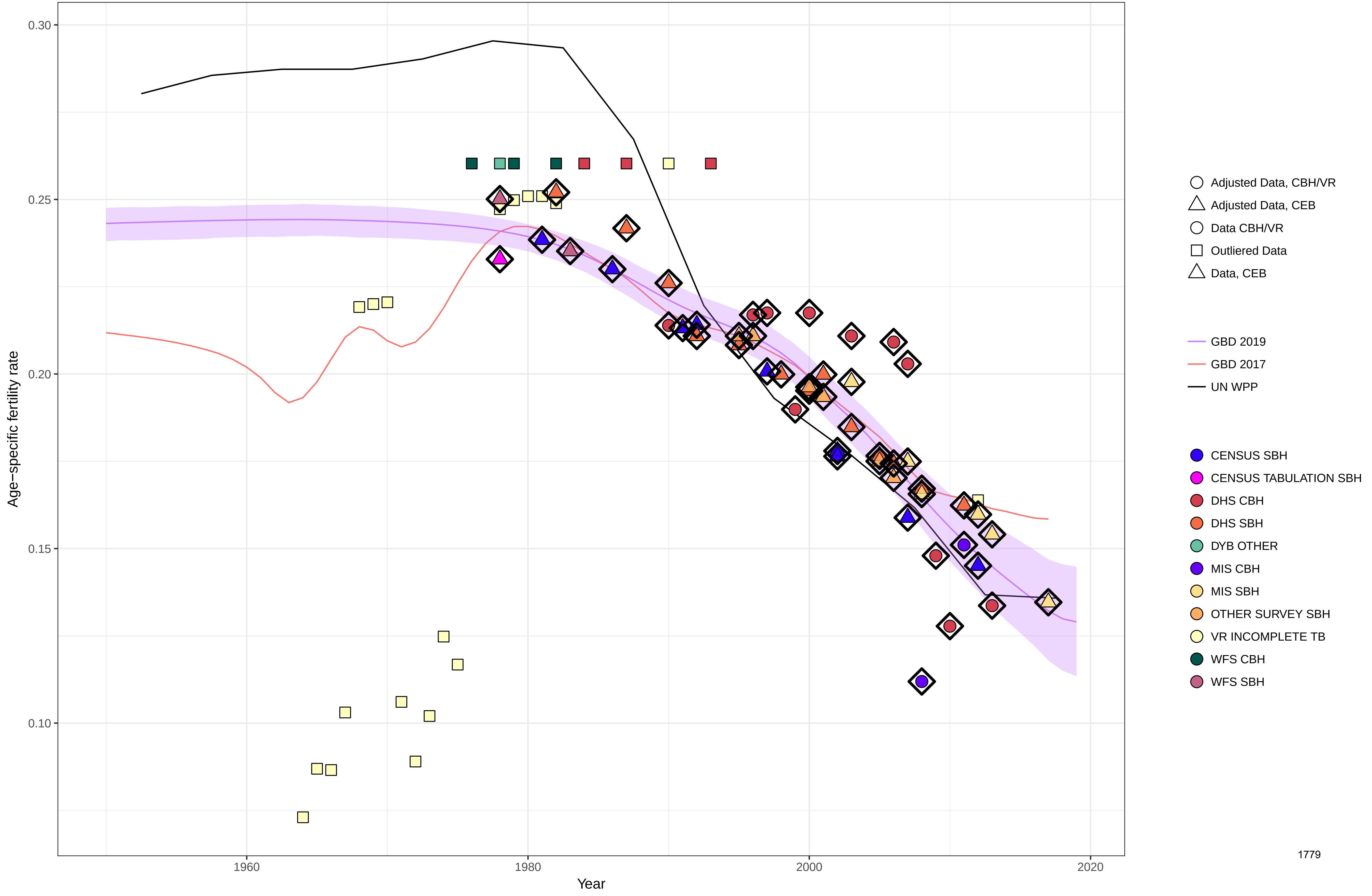

Age−specific fertility fitted trend lines, 1950−2019: Rwanda, Ages: 40−44
Data points enclosed with a diamond are reference sources

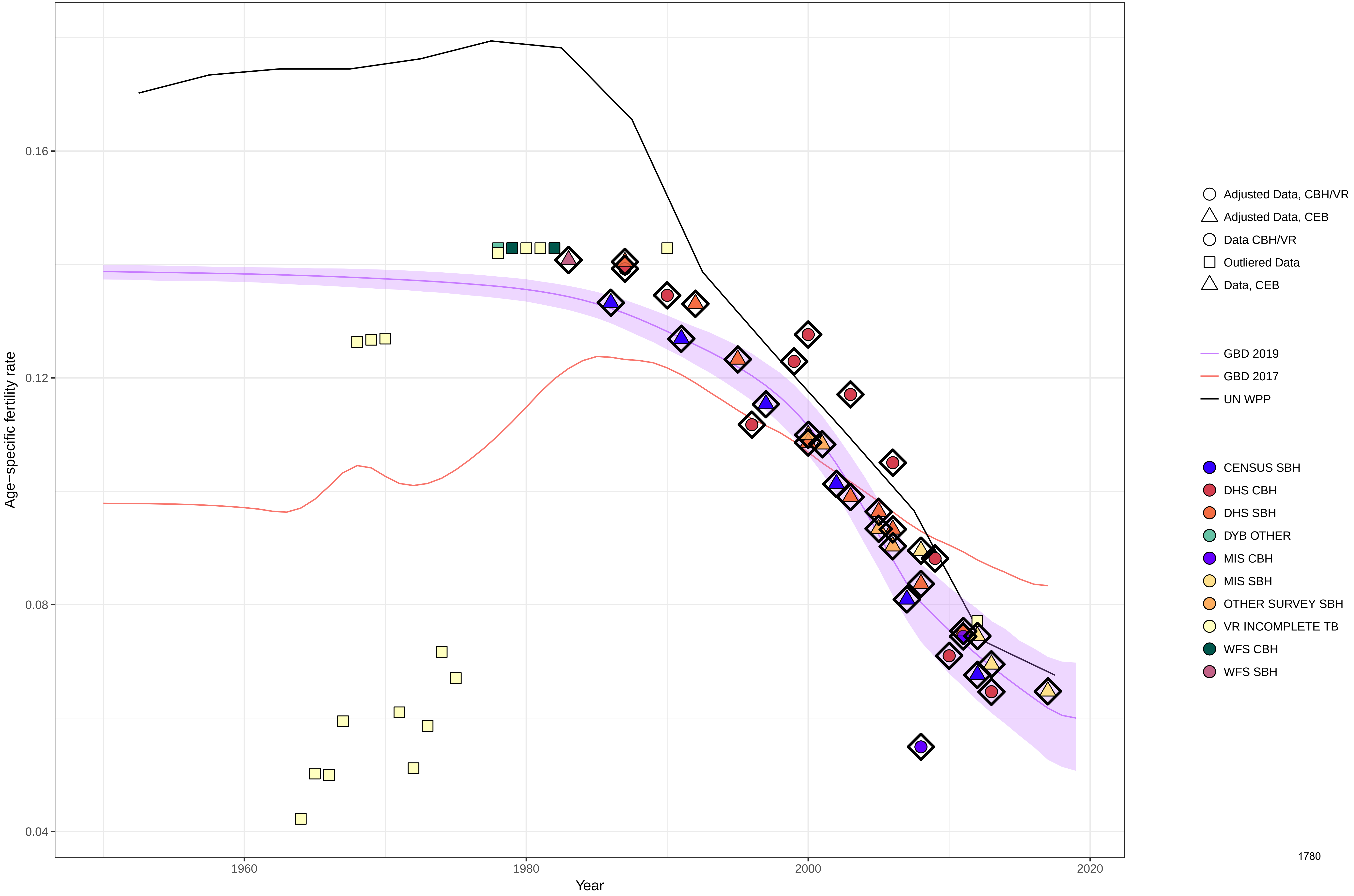

Age−specific fertility fitted trend lines, 1950−2019: Rwanda, Ages: 45−49
Data points enclosed with a diamond are reference sources

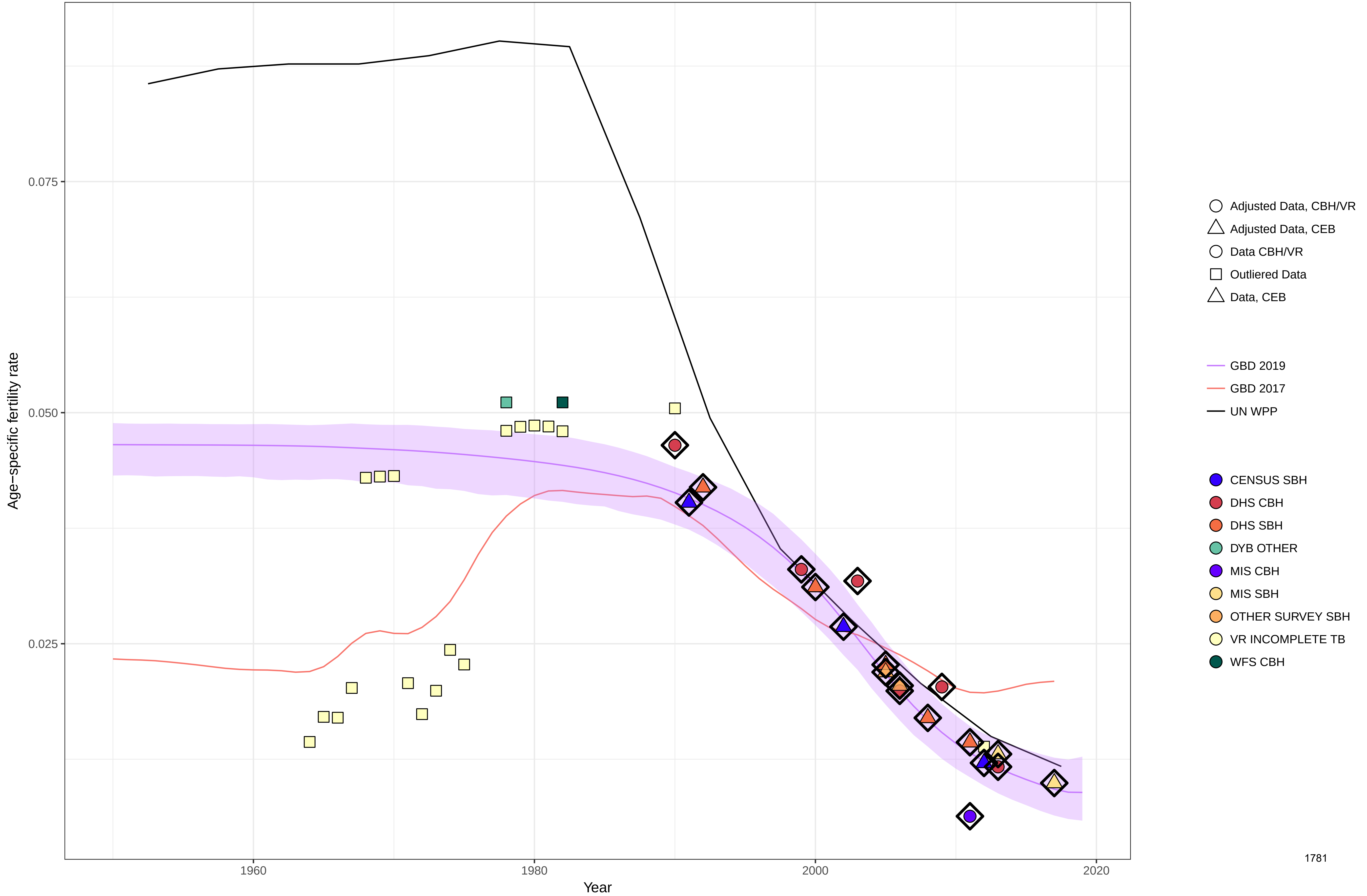

Age−specific fertility fitted trend lines, 1950−2019: Rwanda, Ages: 50−54
Data points enclosed with a diamond are reference sources

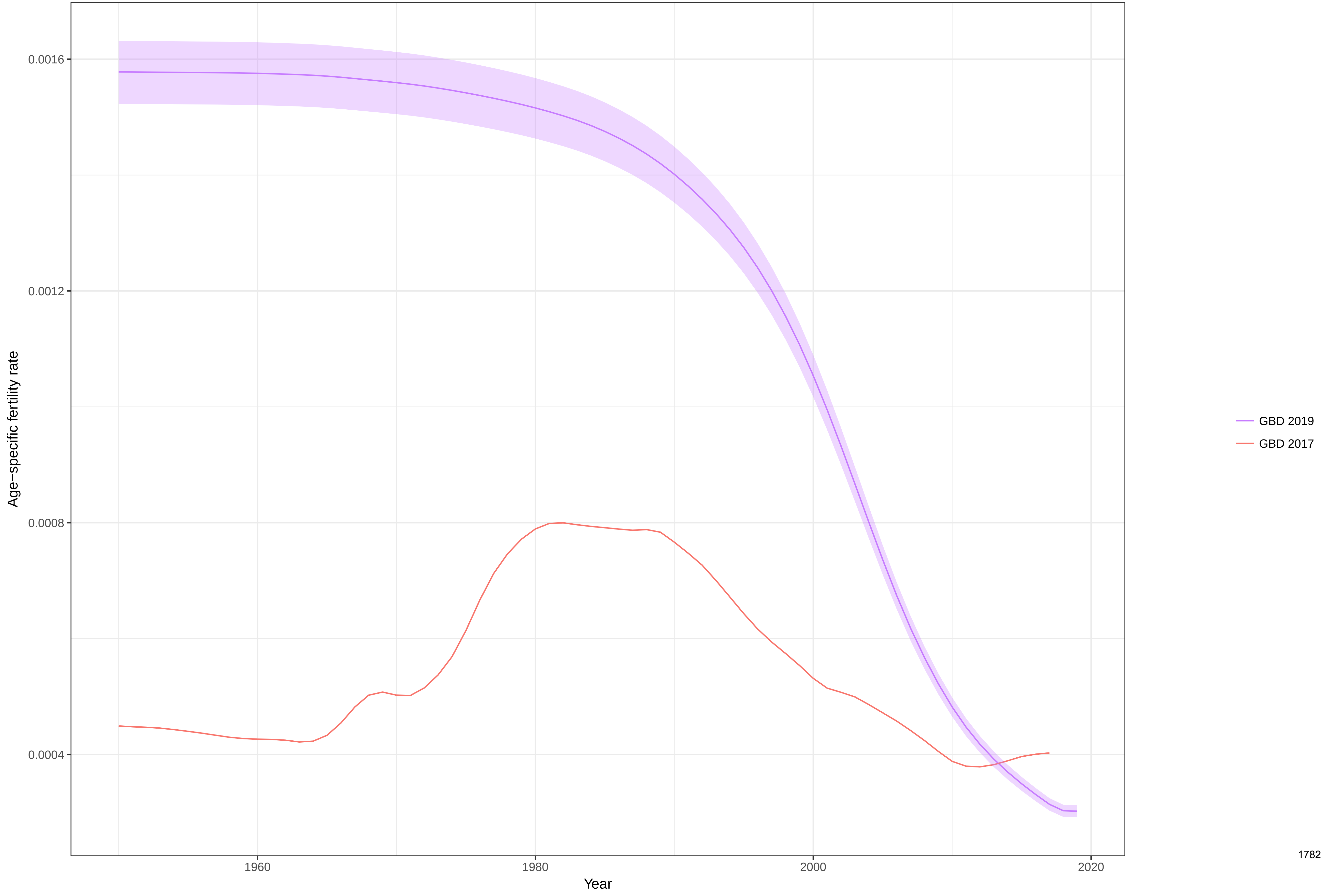

Total fertility rate fitted trend lines, 1950−2019: Rwanda TFR
Data points enclosed with a diamond are reference sources

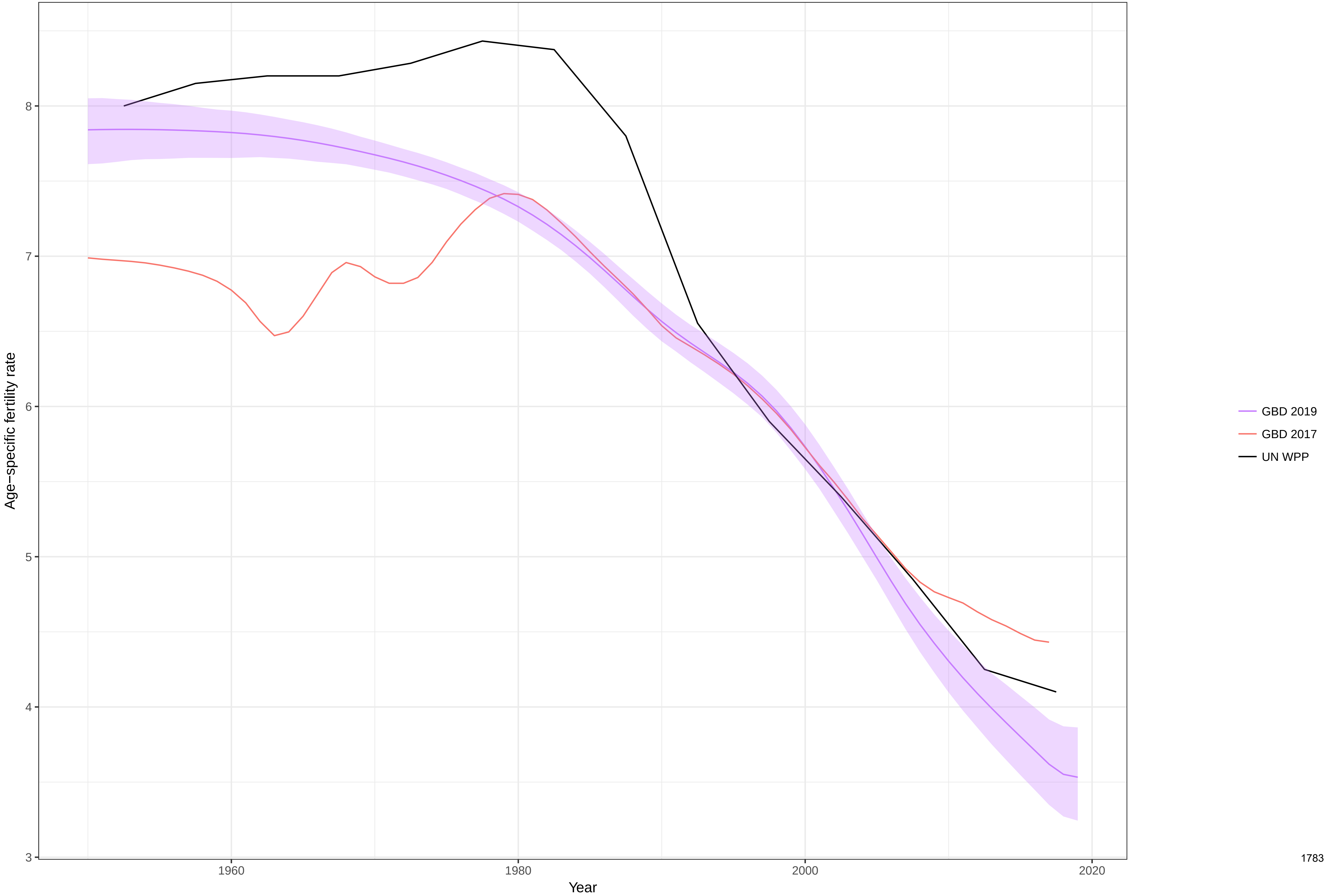

Age−specific fertility fitted trend lines, 1950−2019: Saudi Arabia, Ages: 10−14
Data points enclosed with a diamond are reference sources

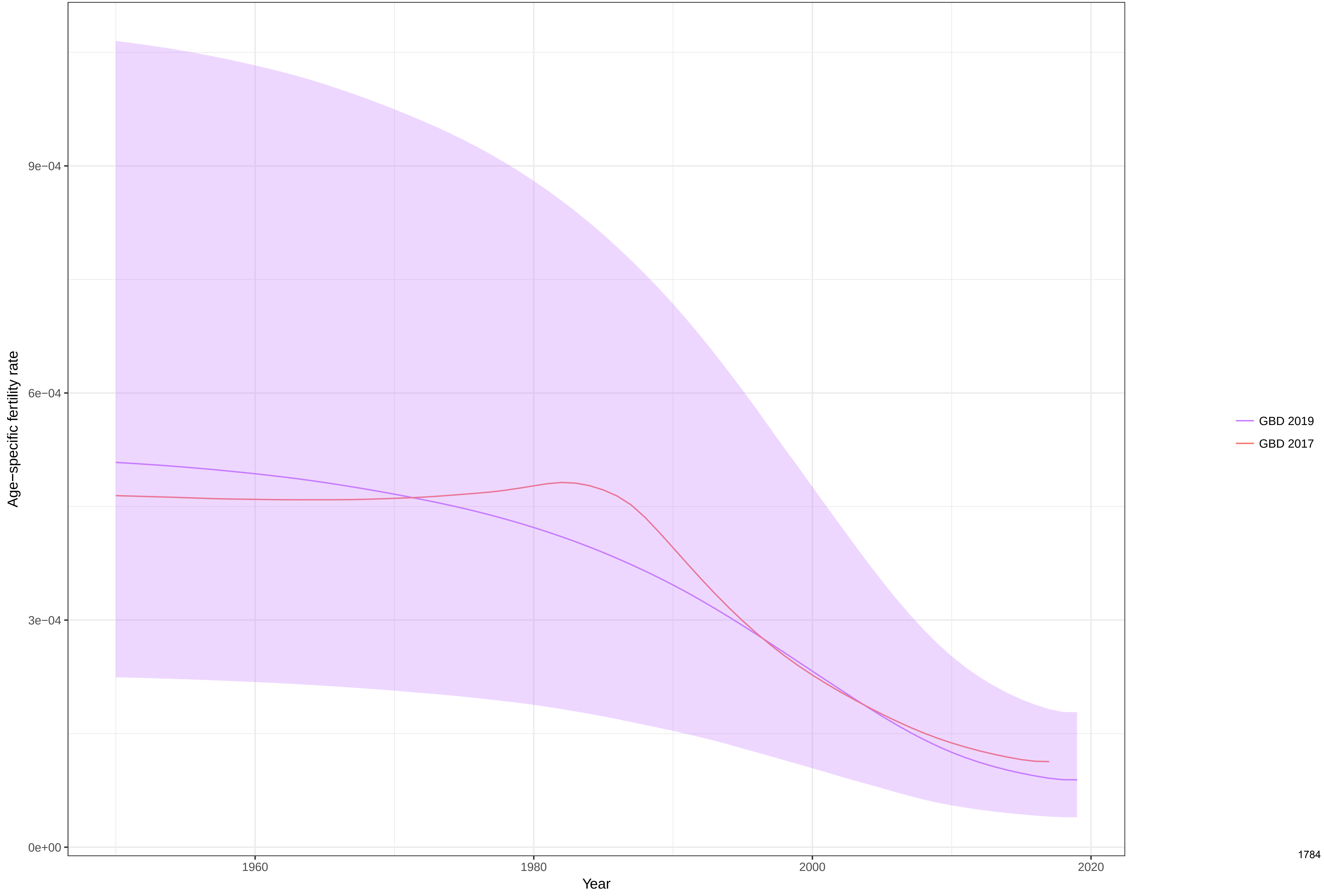

Age−specific fertility fitted trend lines, 1950−2019: Saudi Arabia, Ages: 15−19
Data points enclosed with a diamond are reference sources

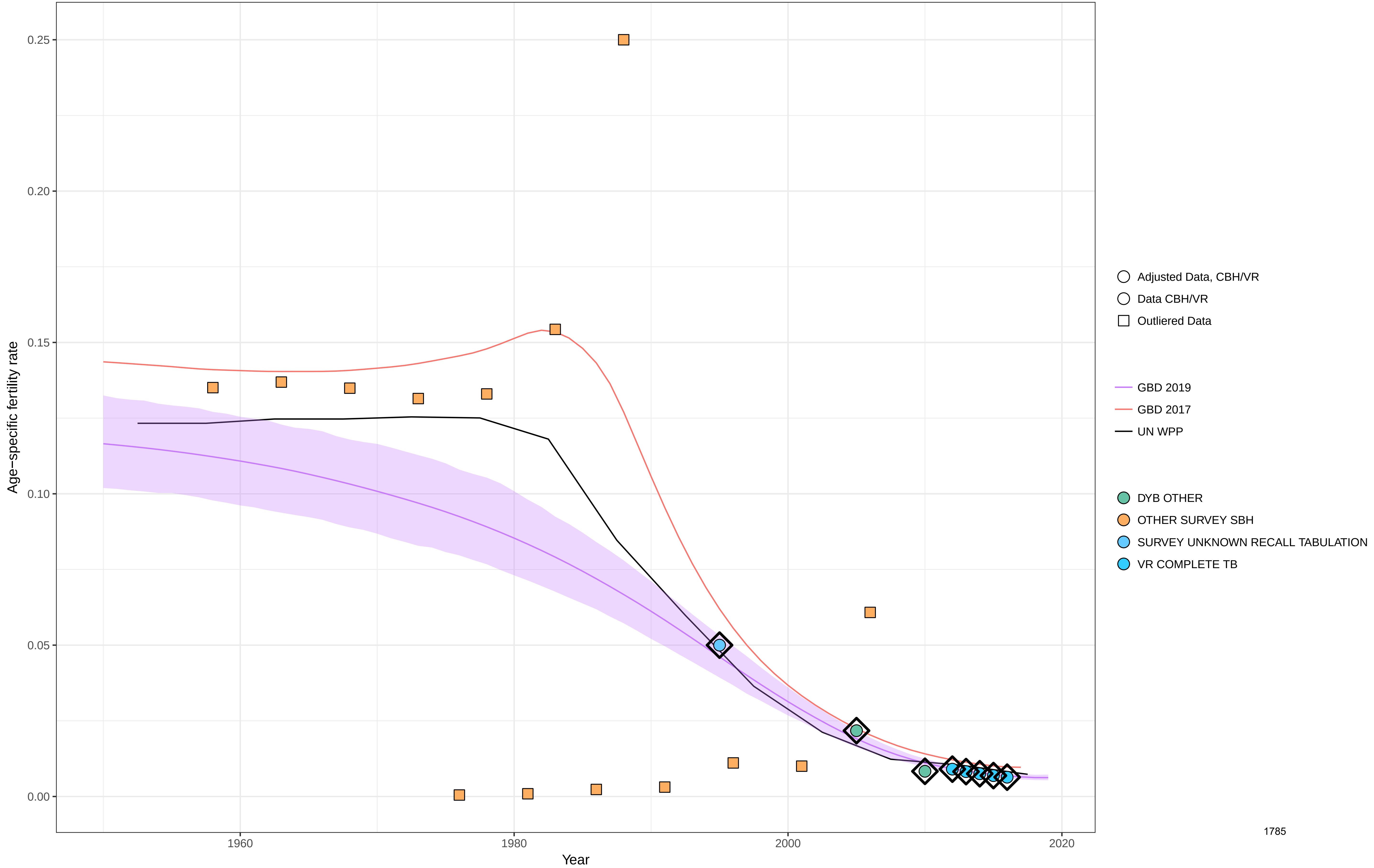

Age−specific fertility fitted trend lines, 1950−2019: Saudi Arabia, Ages: 20−24
Data points enclosed with a diamond are reference sources

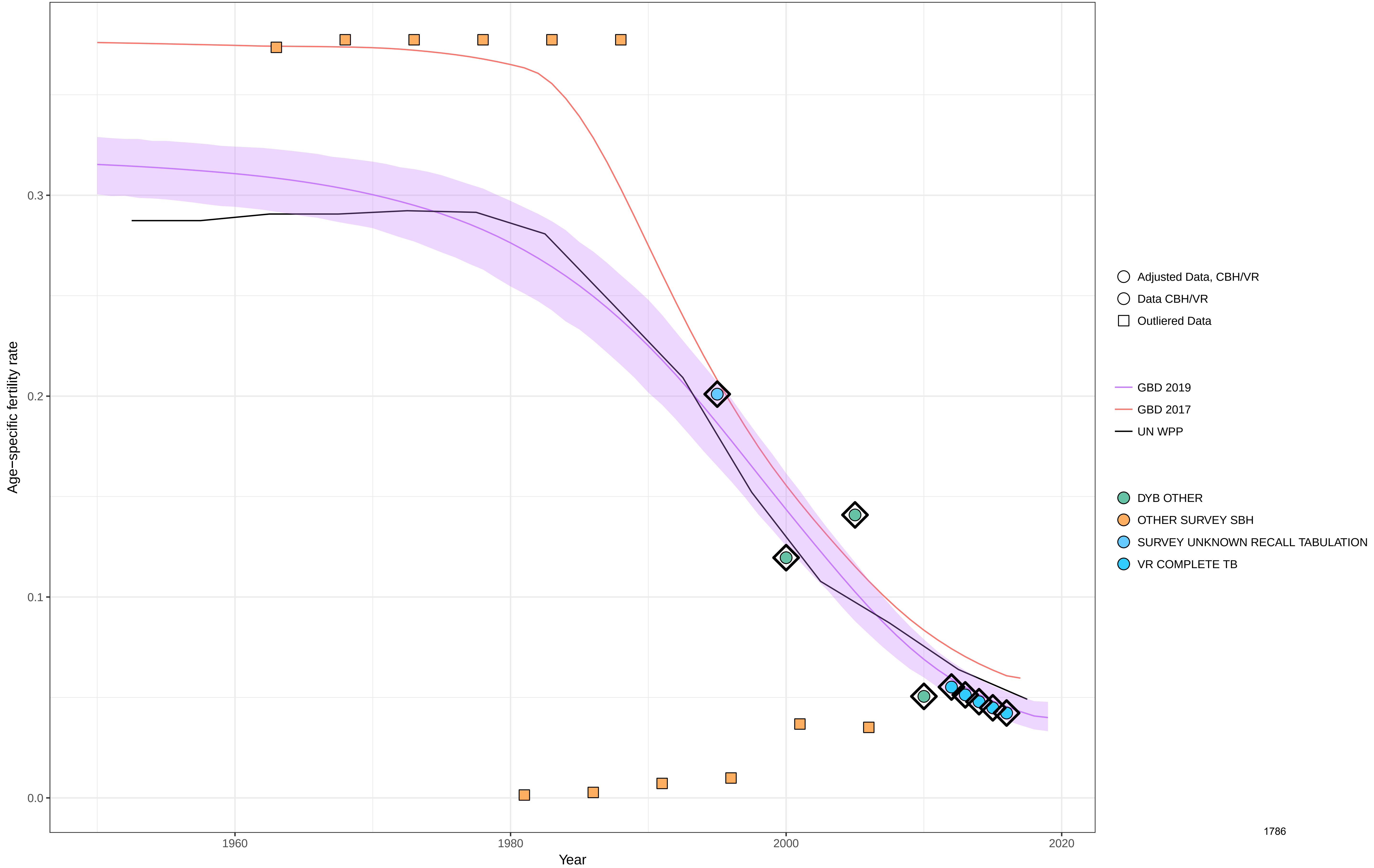

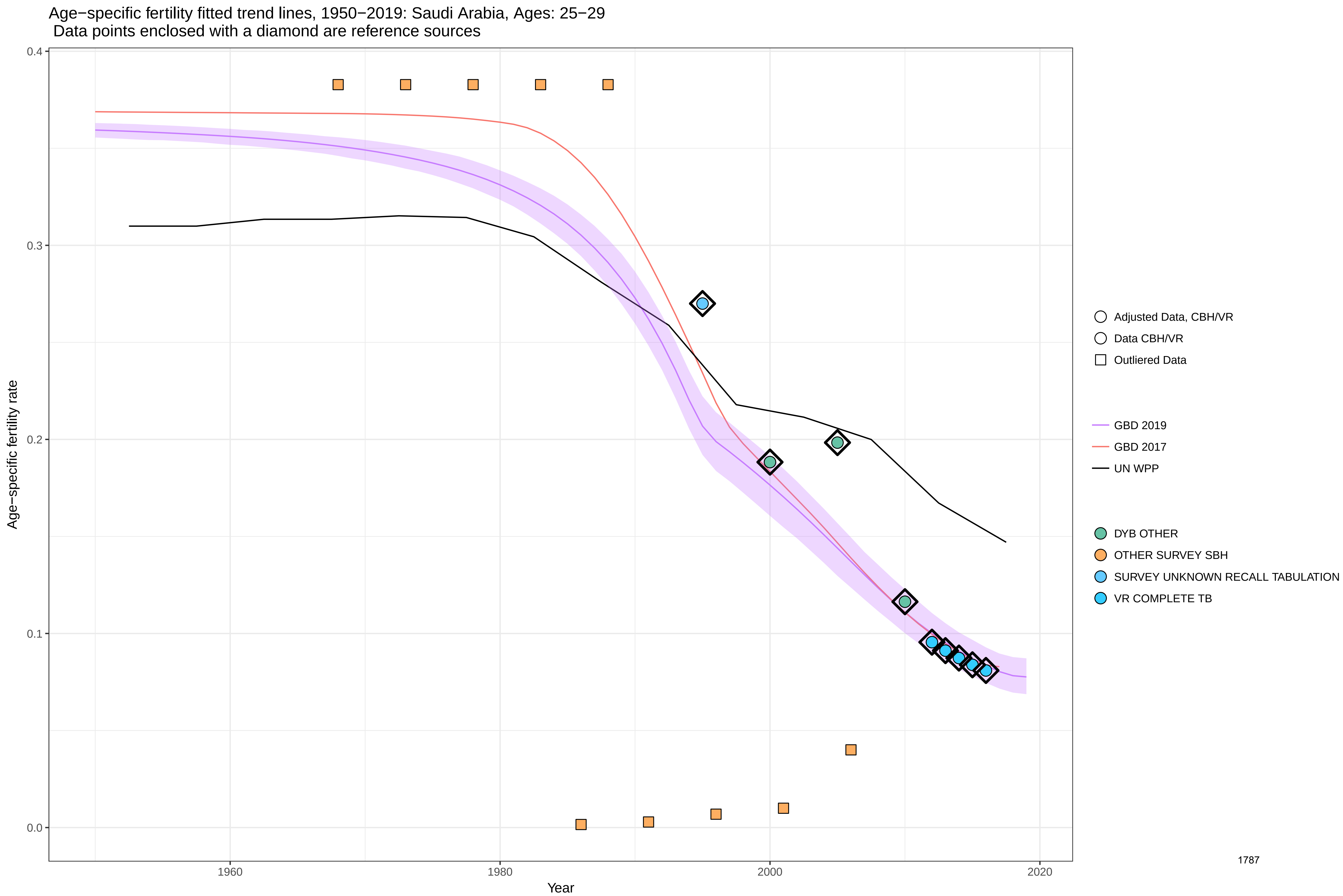

Age−specific fertility fitted trend lines, 1950−2019: Saudi Arabia, Ages: 25−29
Data points enclosed with a diamond are reference sources
Age−specific fertility rate
Year
0.0
0.1
0.2
0.3
0.4
1960
1980
2000
2020
Adjusted Data, CBH/VR
Data CBH/VR
Outliered Data
GBD 2019
GBD 2017
UN WPP
DYB OTHER
OTHER SURVEY SBH
SURVEY UNKNOWN RECALL TABULATION
VR COMPLETE TB

Age−specific fertility fitted trend lines, 1950−2019: Saudi Arabia, Ages: 30−34
Data points enclosed with a diamond are reference sources

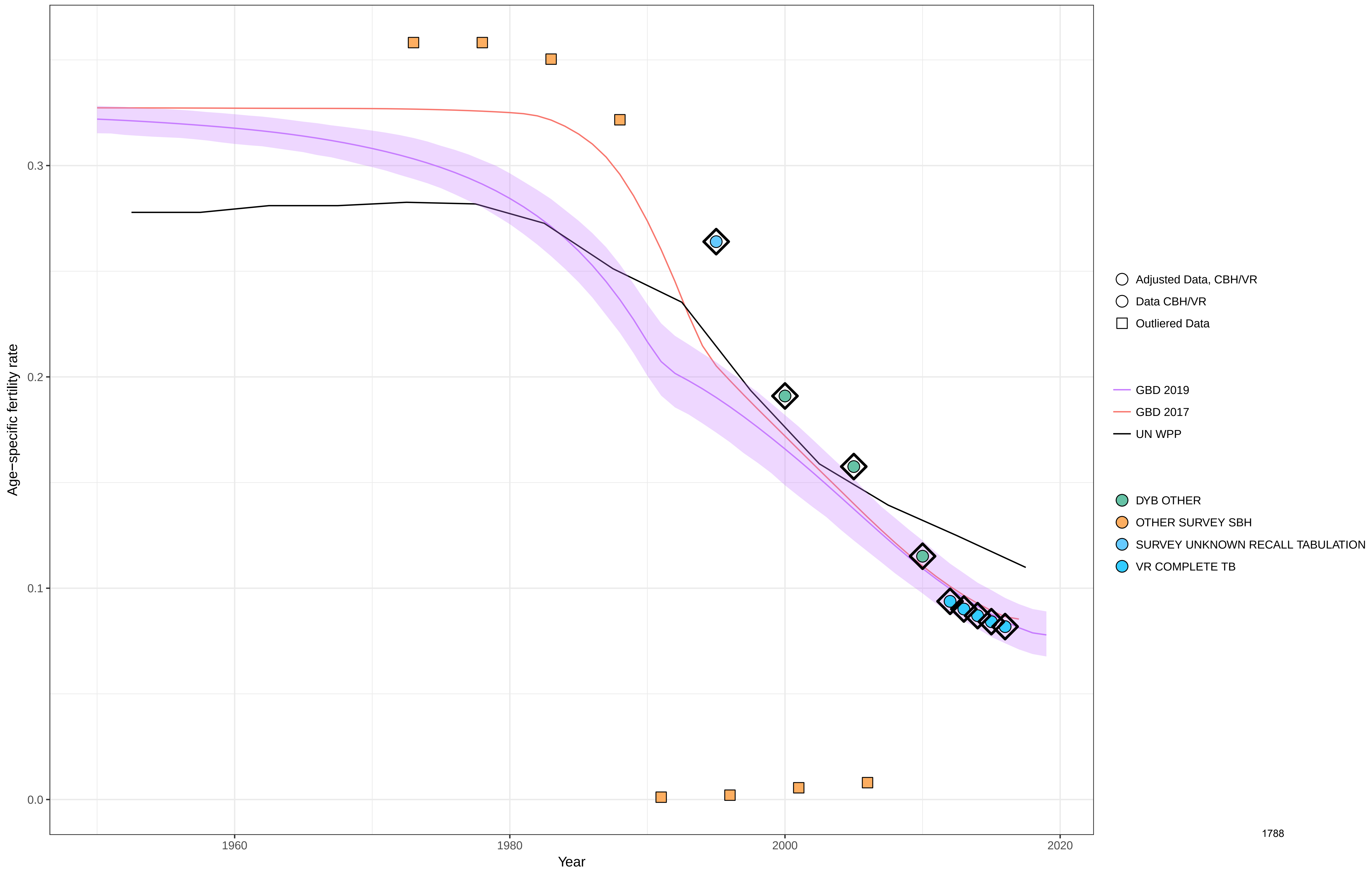

Age−specific fertility fitted trend lines, 1950−2019: Saudi Arabia, Ages: 35−39
Data points enclosed with a diamond are reference sources

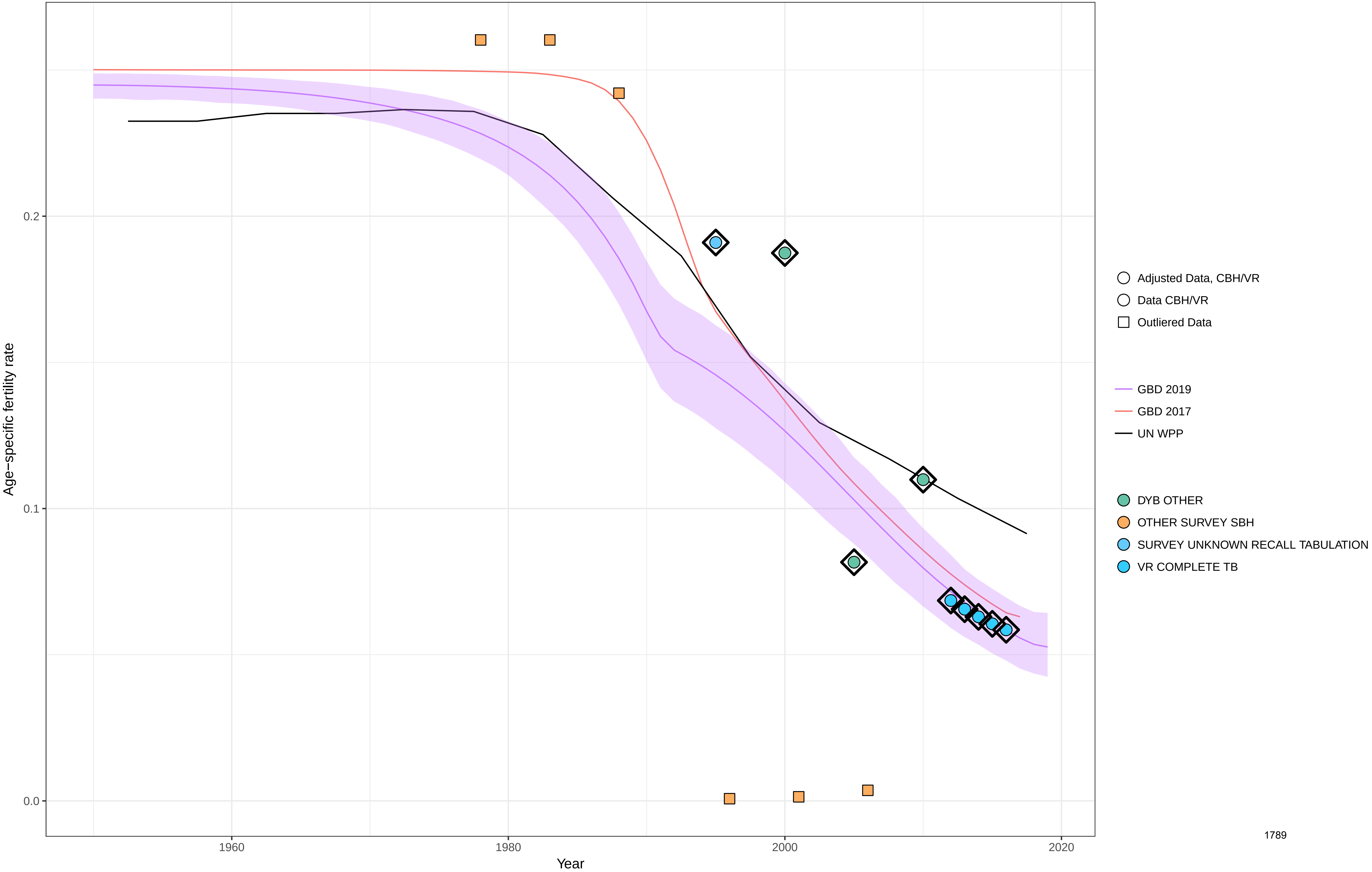

Age−specific fertility fitted trend lines, 1950−2019: Saudi Arabia, Ages: 40−44
Data points enclosed with a diamond are reference sources

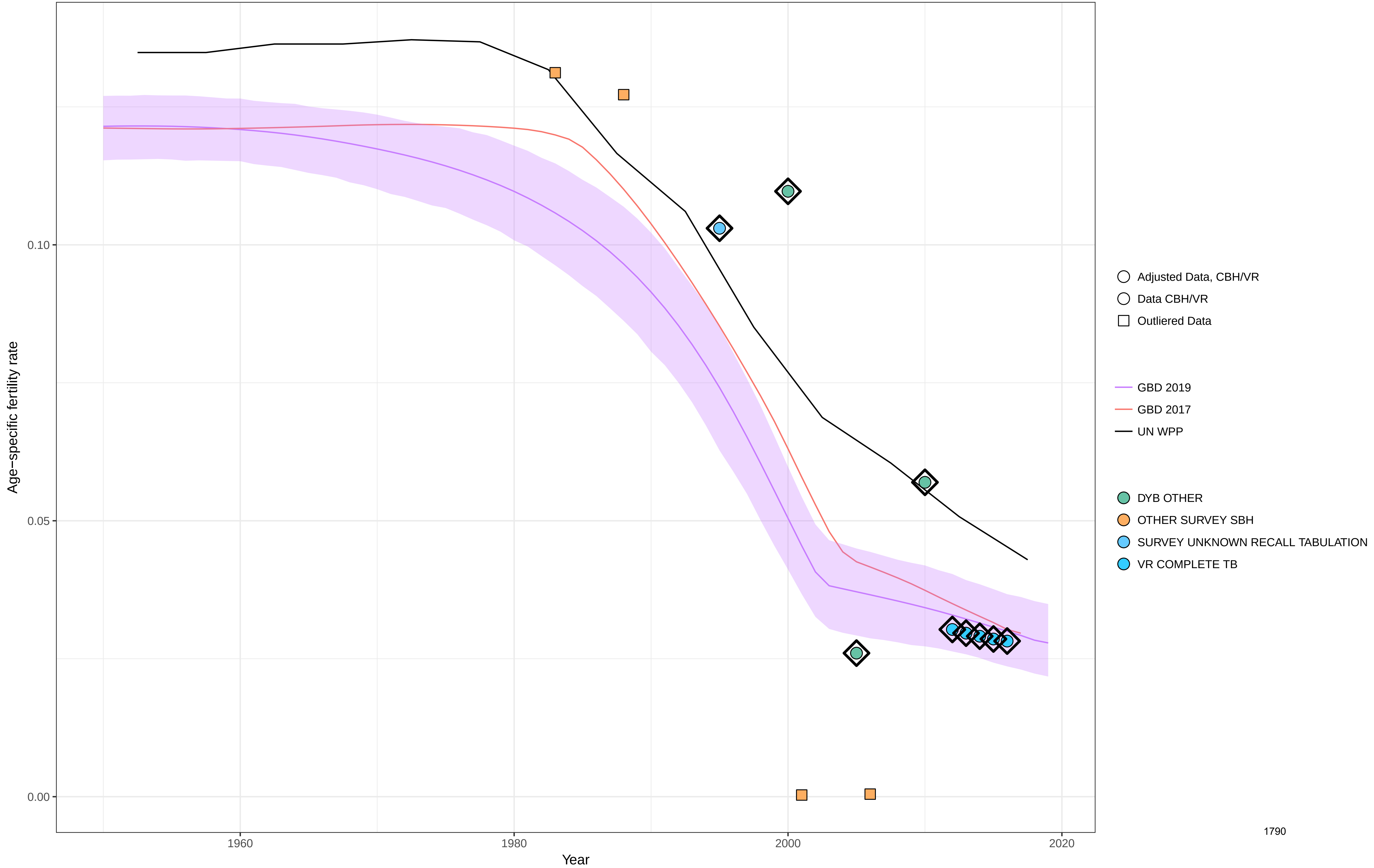

Age−specific fertility fitted trend lines, 1950−2019: Saudi Arabia, Ages: 45−49
Data points enclosed with a diamond are reference sources

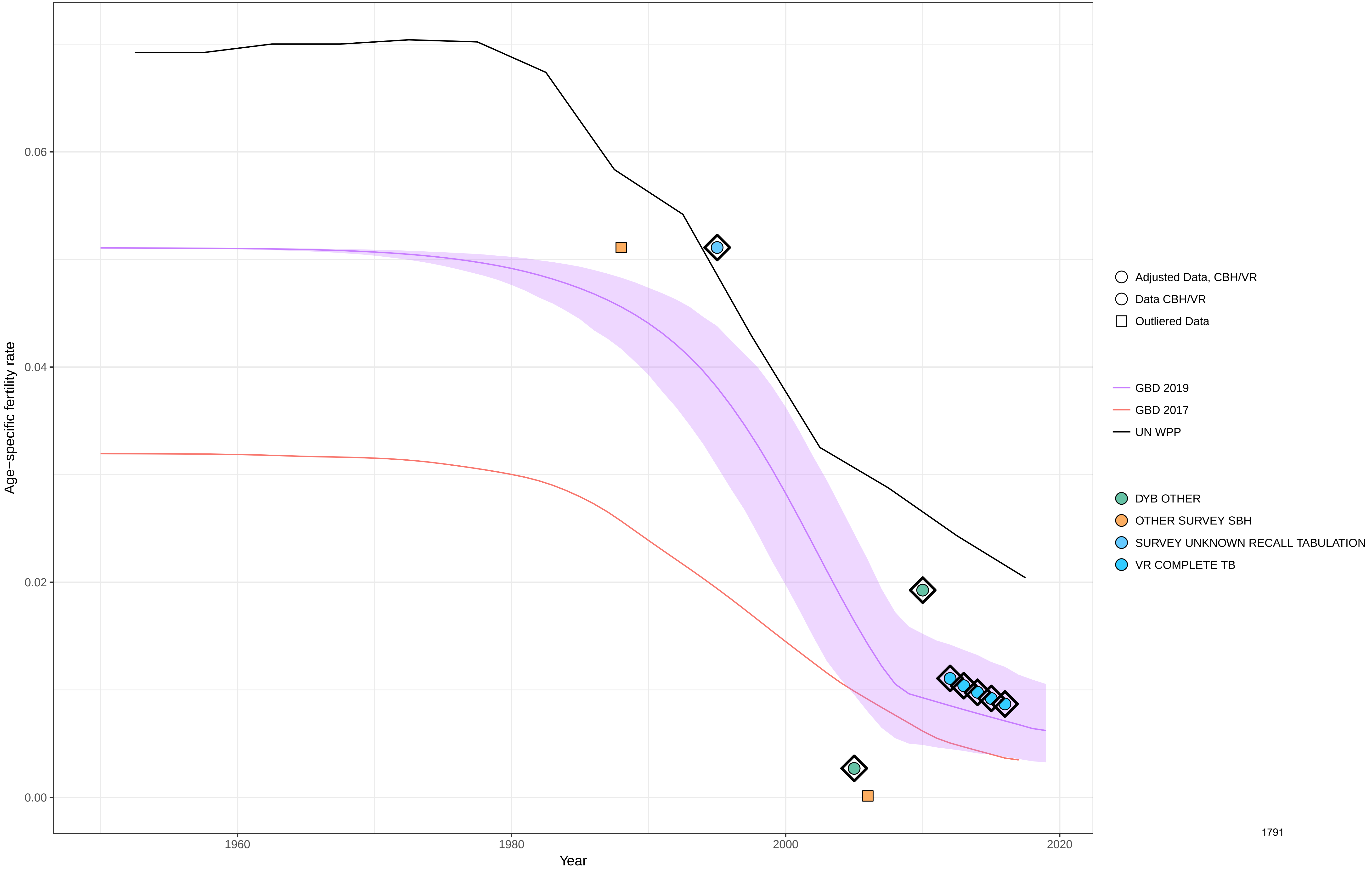

Age−specific fertility fitted trend lines, 1950−2019: Saudi Arabia, Ages: 50−54
Data points enclosed with a diamond are reference sources

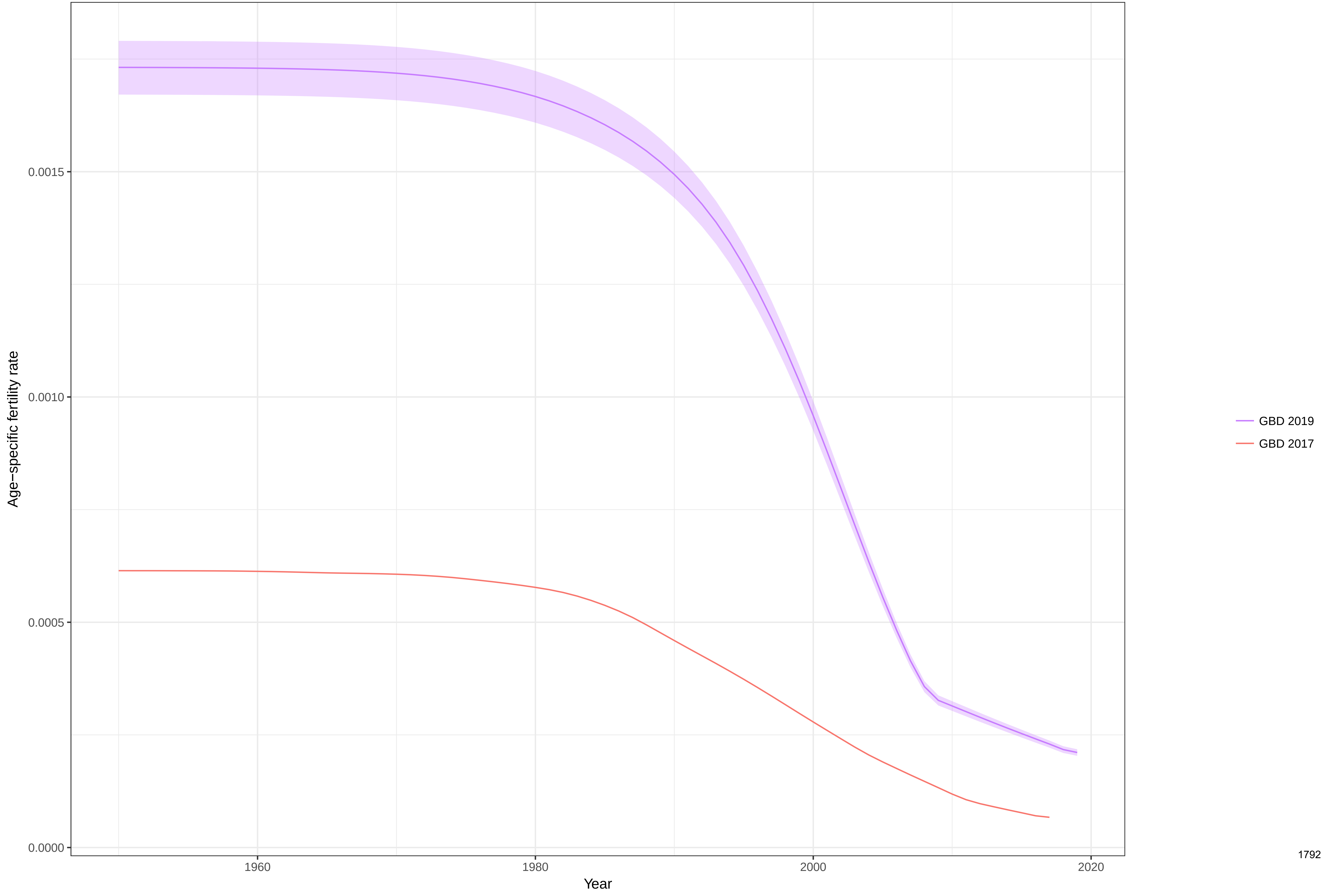

Total fertility rate fitted trend lines, 1950−2019: Saudi Arabia TFR
Data points enclosed with a diamond are reference sources

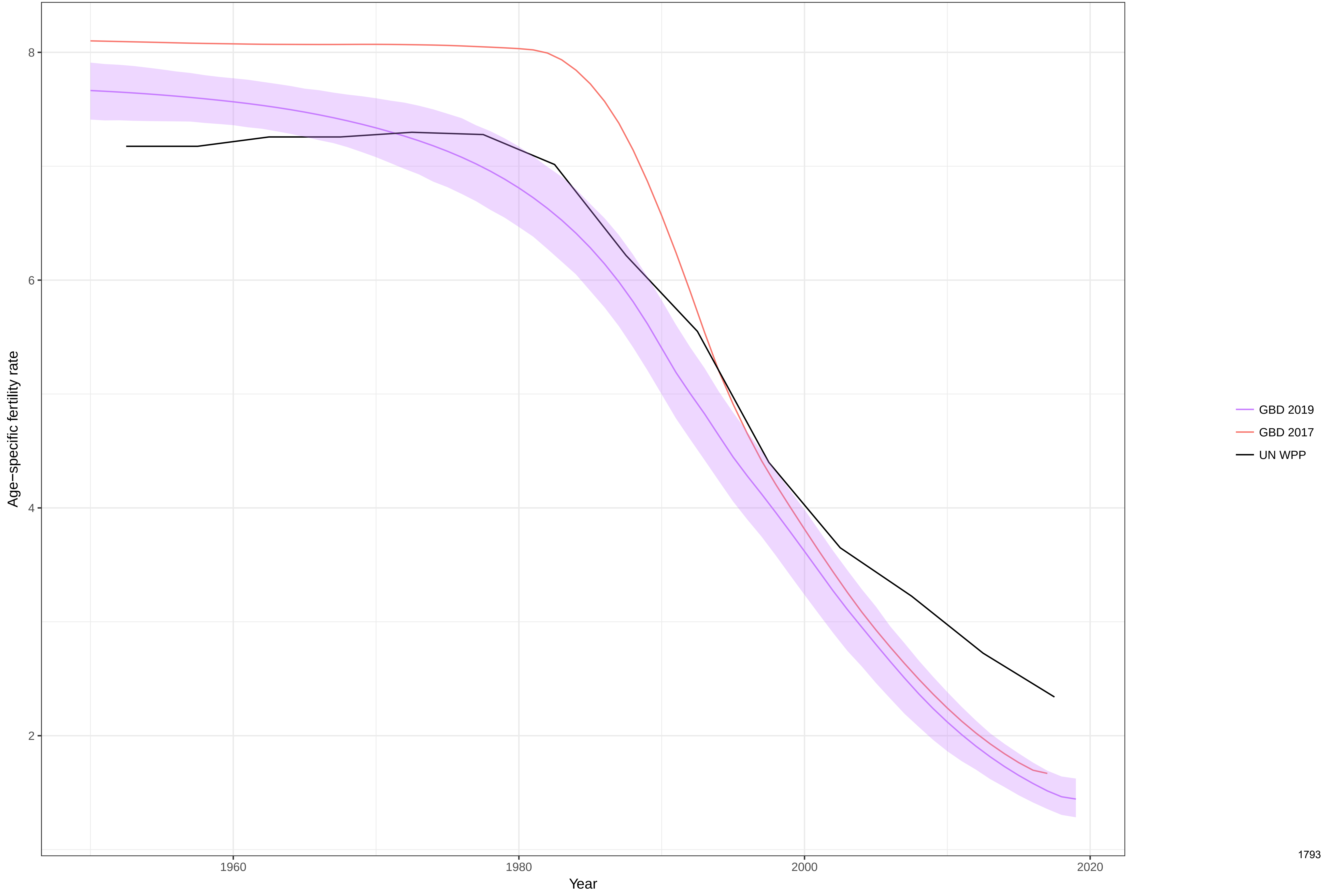

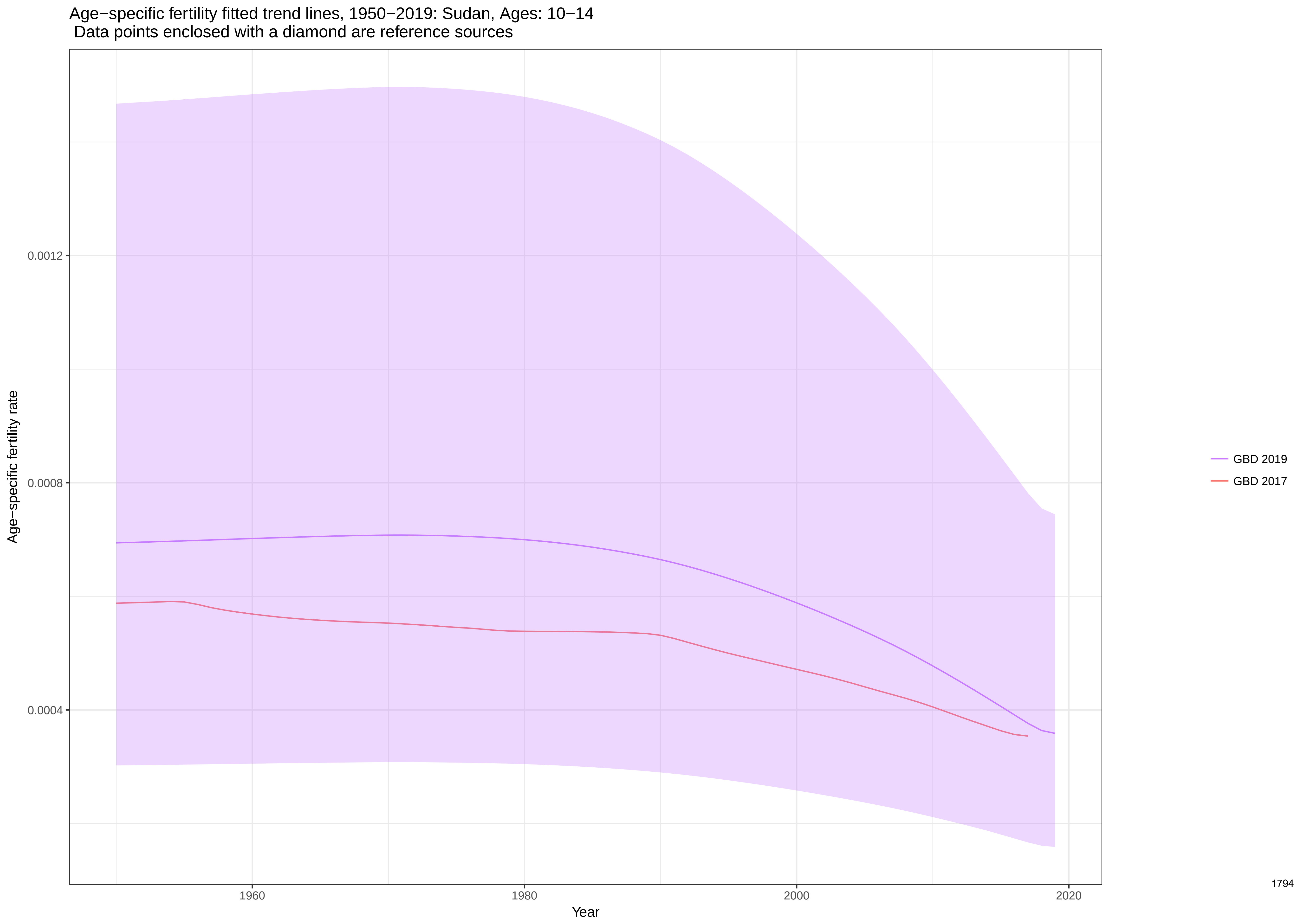

Age−specific fertility fitted trend lines, 1950−2019: Sudan, Ages: 10−14
Data points enclosed with a diamond are reference sources
Age−specific fertility rate
0.0012
0.0008
0.0004
1960
1980
2000
2020
Year
GBD 2019
GBD 2017

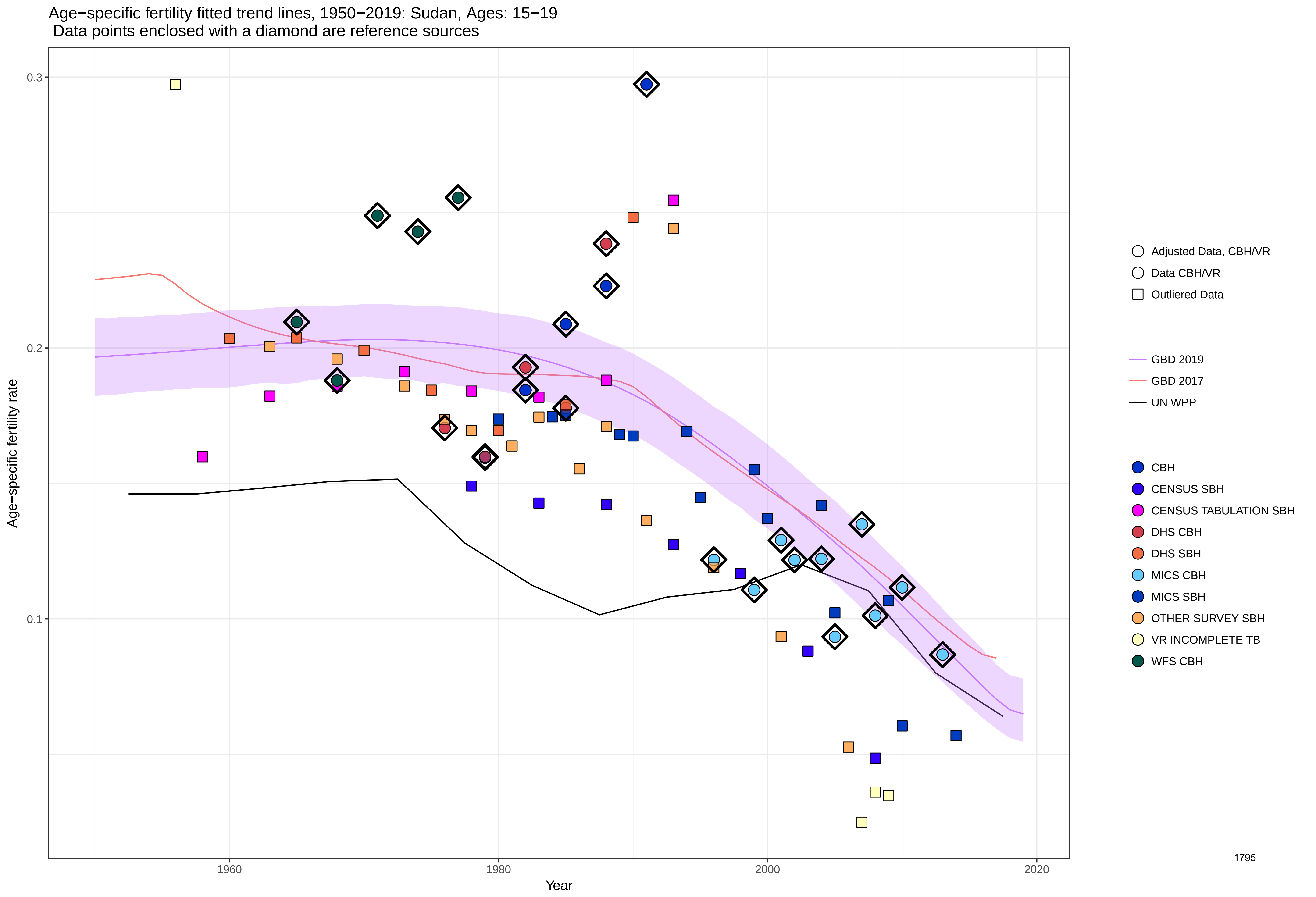

Age−specific fertility fitted trend lines, 1950−2019: Sudan, Ages: 15−19
Data points enclosed with a diamond are reference sources
Age−specific fertility rate
Year
0.1
0.2
0.3
1960
1980
2000
2020
Adjusted Data, CBH/VR
Data CBH/VR
Outliered Data
GBD 2019
GBD 2017
UN WPP
CBH
CENSUS SBH
CENSUS TABULATION SBH
DHS CBH
DHS SBH
MICS CBH
MICS SBH
OTHER SURVEY SBH
VR INCOMPLETE TB
WFS CBH

Age−specific fertility fitted trend lines, 1950−2019: Sudan, Ages: 20−24
Data points enclosed with a diamond are reference sources

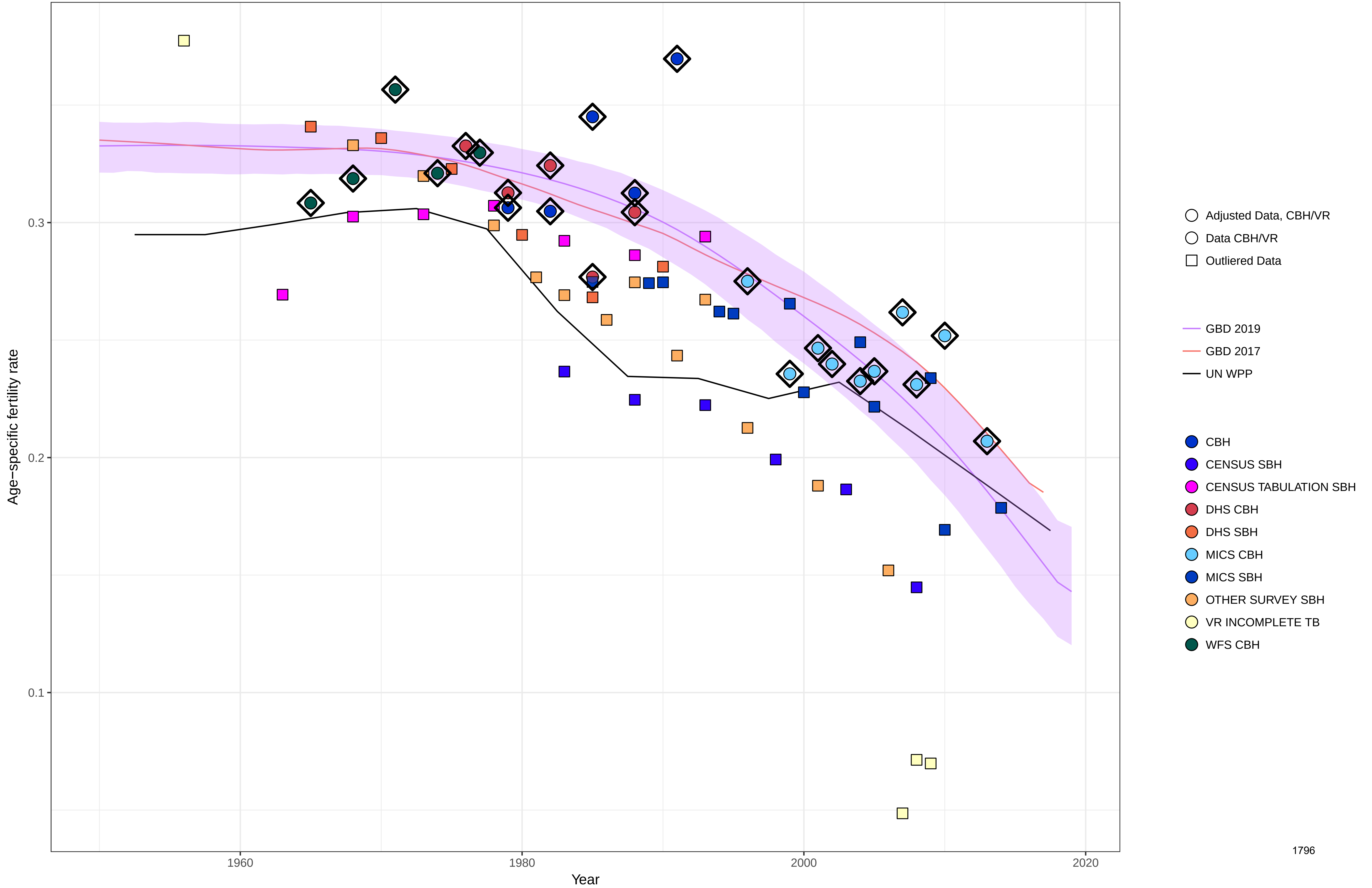

Age−specific fertility fitted trend lines, 1950−2019: Sudan, Ages: 25−29
Data points enclosed with a diamond are reference sources

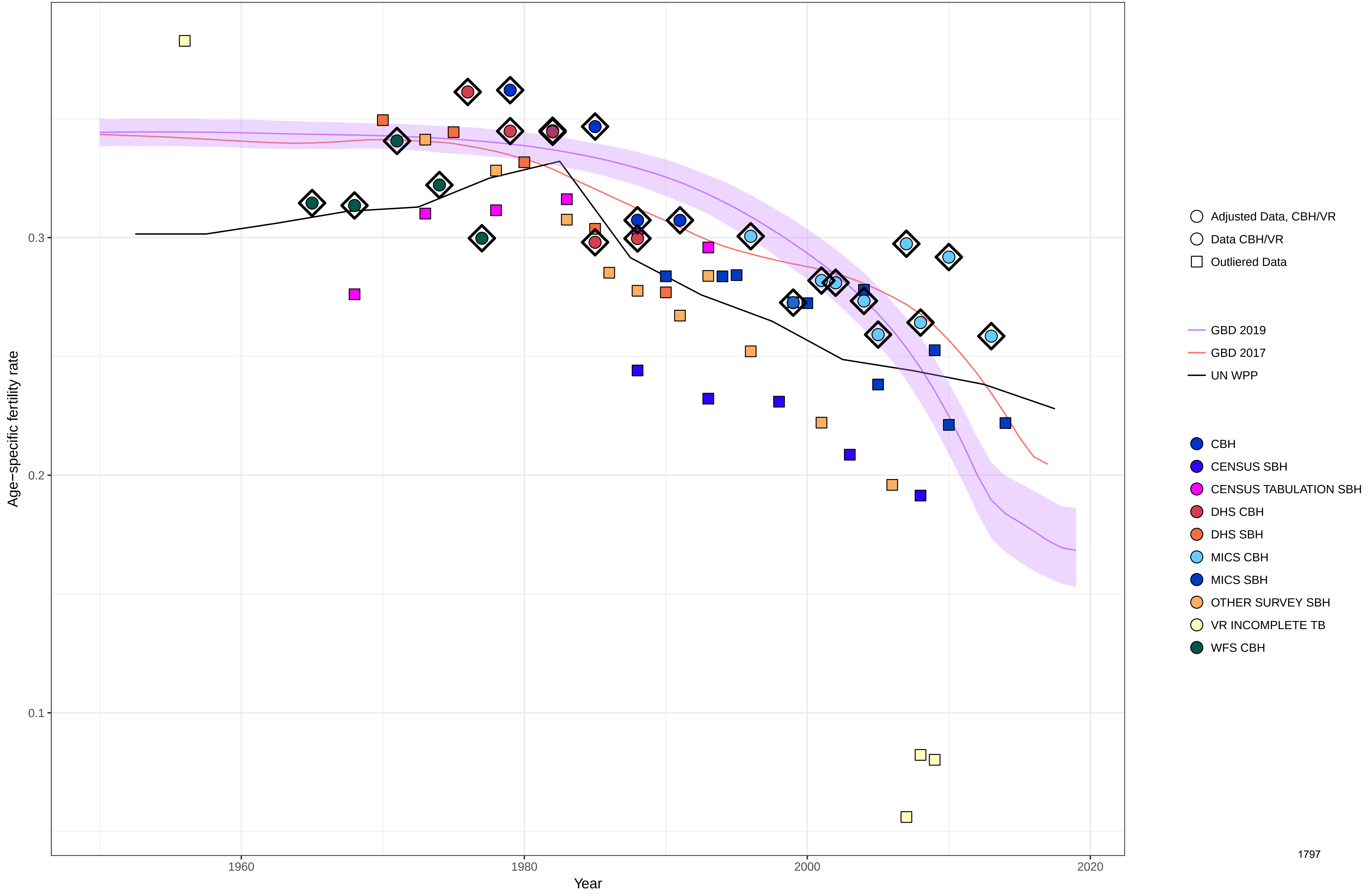

Age−specific fertility fitted trend lines, 1950−2019: Sudan, Ages: 30−34
Data points enclosed with a diamond are reference sources

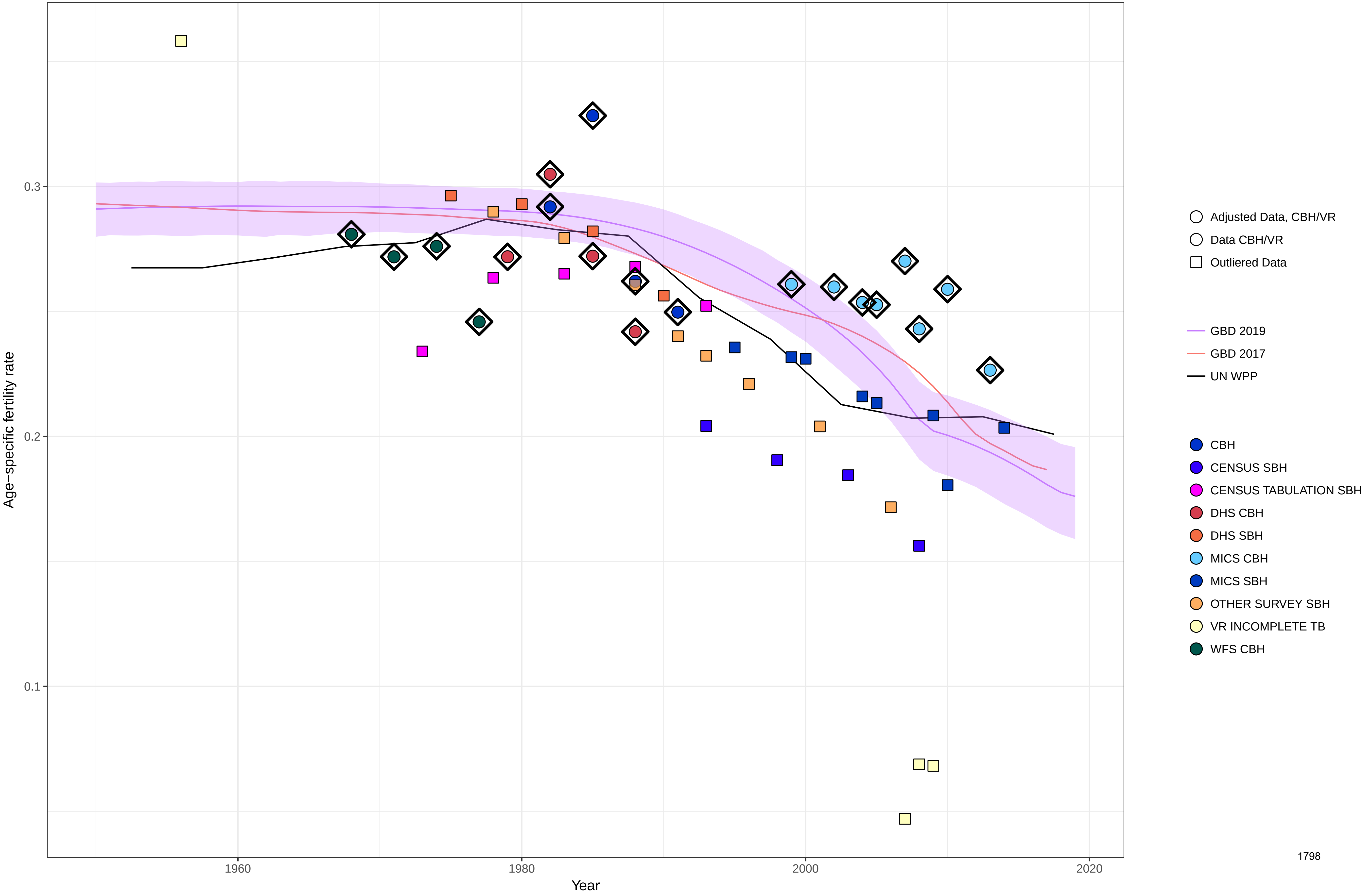

Age−specific fertility fitted trend lines, 1950−2019: Sudan, Ages: 35−39
Data points enclosed with a diamond are reference sources

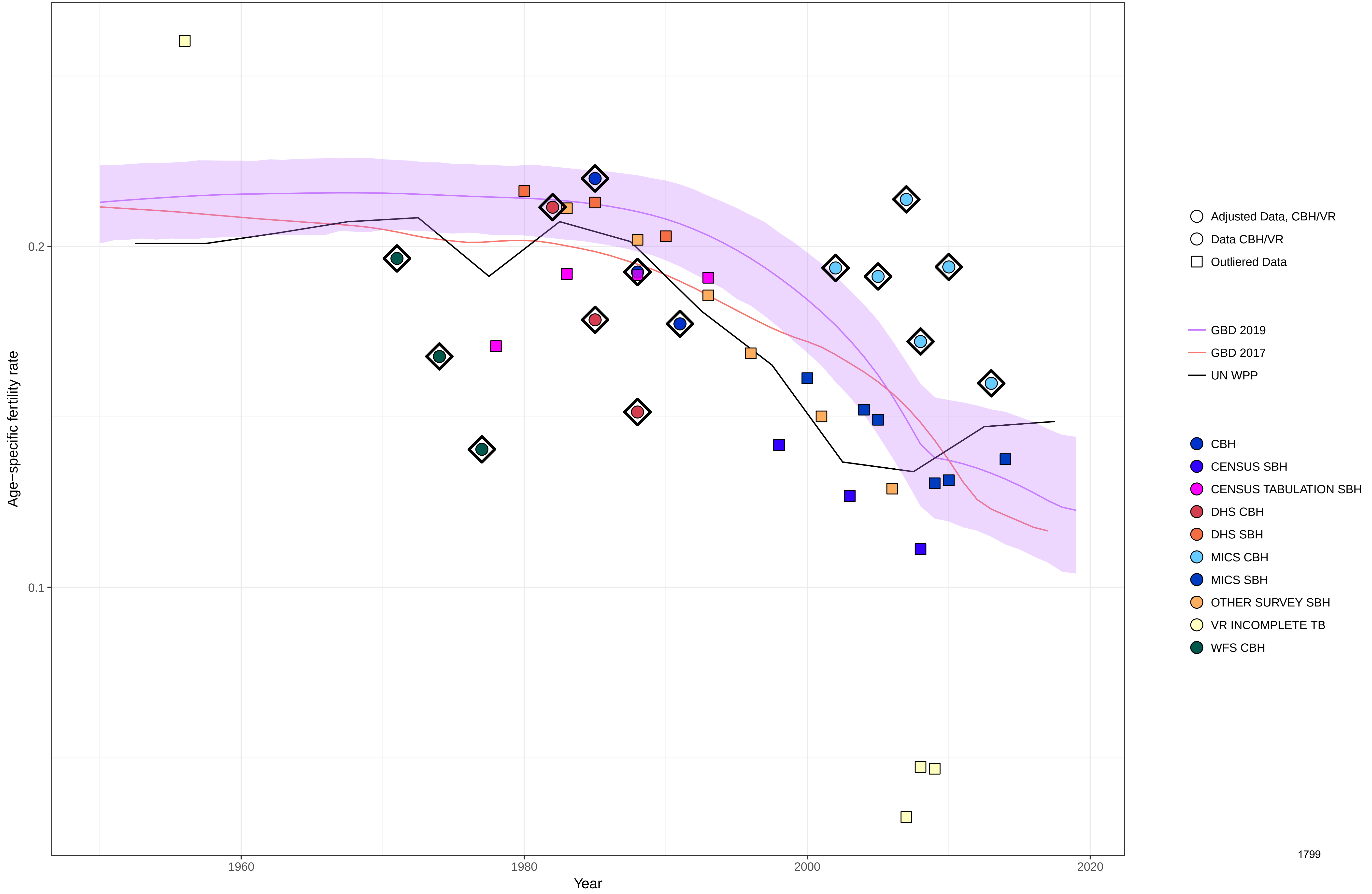

Age−specific fertility fitted trend lines, 1950−2019: Sudan, Ages: 40−44
Data points enclosed with a diamond are reference sources

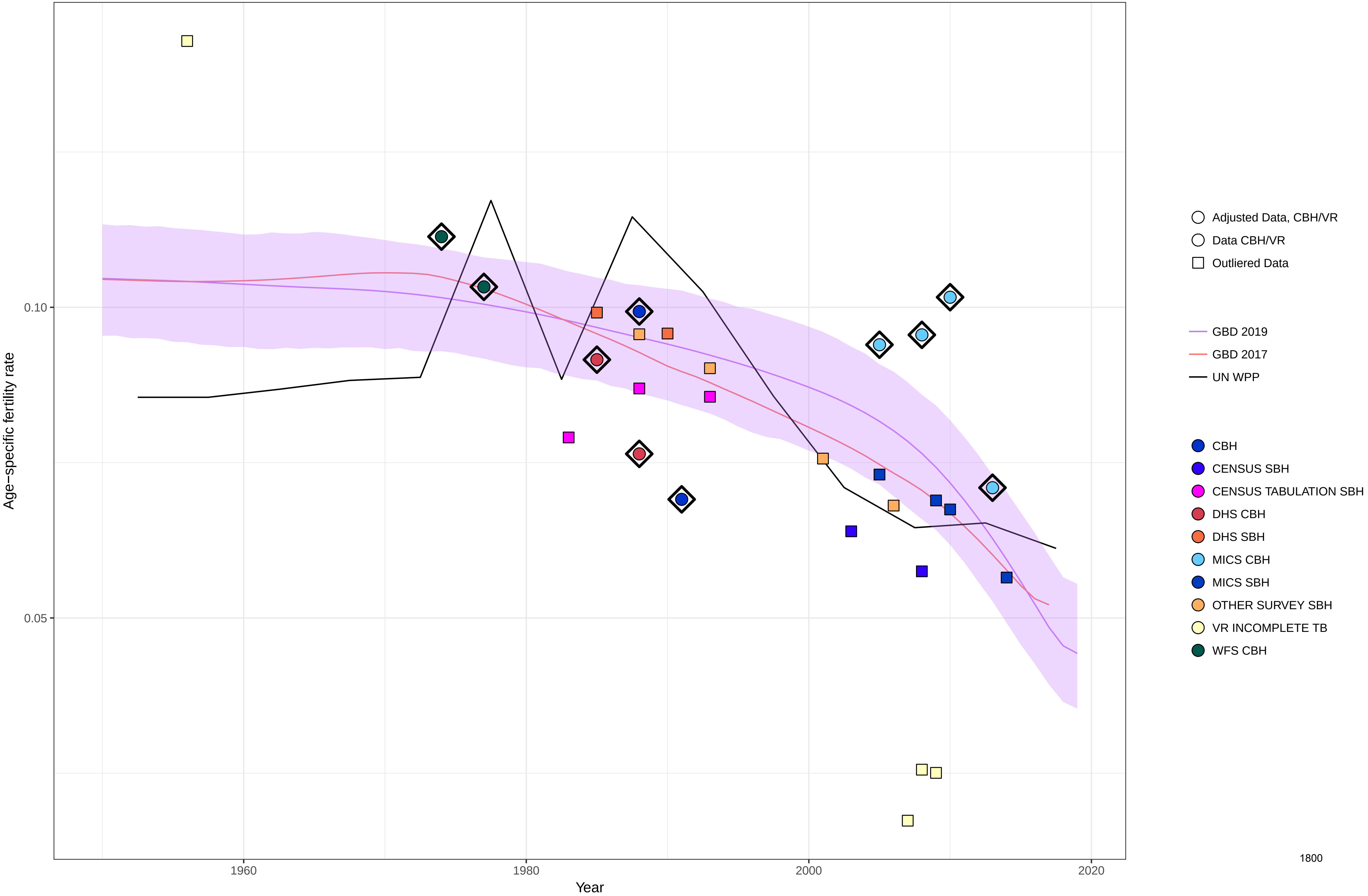

Age−specific fertility fitted trend lines, 1950−2019: Sudan, Ages: 45−49
Data points enclosed with a diamond are reference sources

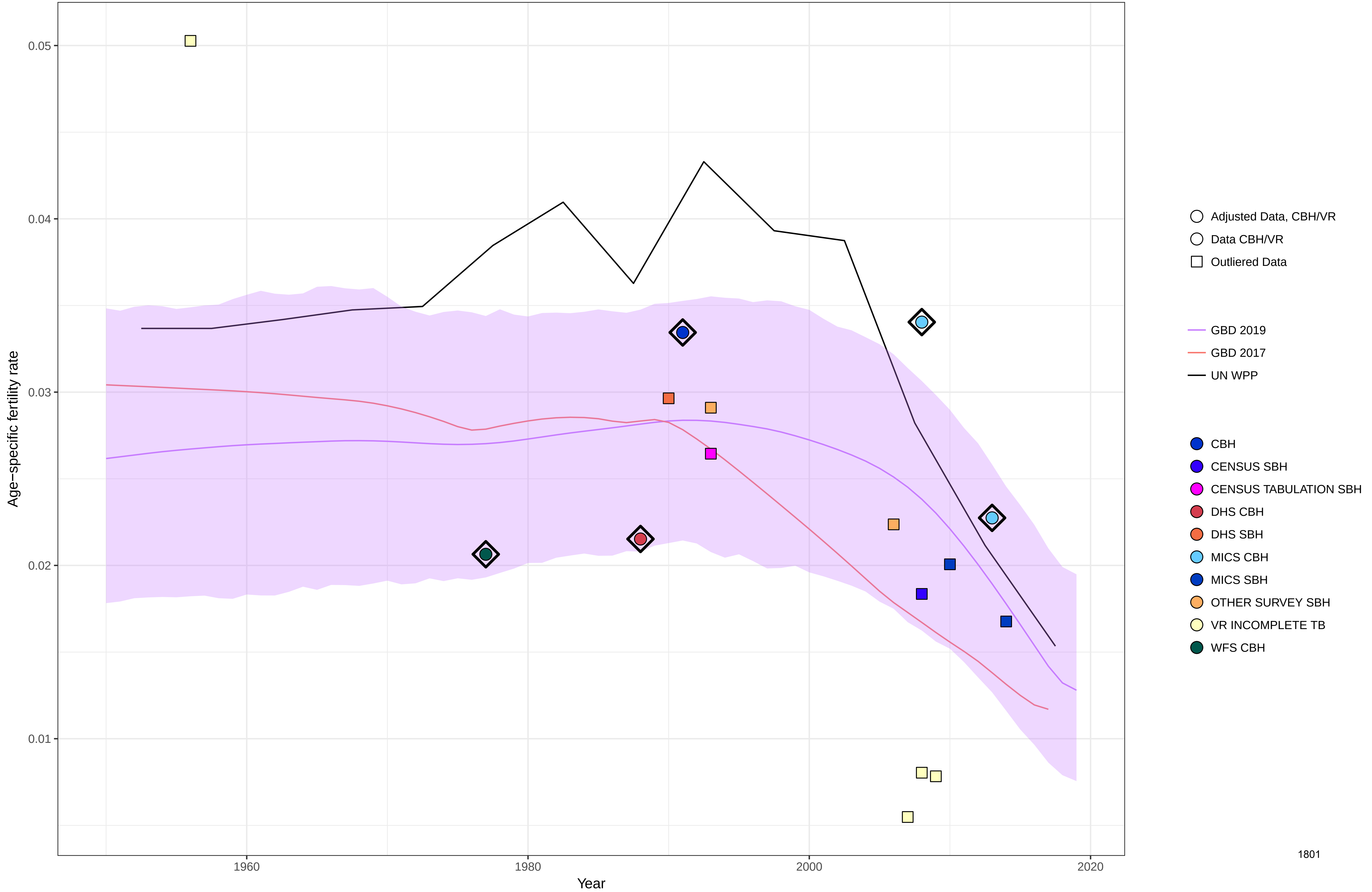

Age−specific fertility fitted trend lines, 1950−2019: Sudan, Ages: 50−54
Data points enclosed with a diamond are reference sources

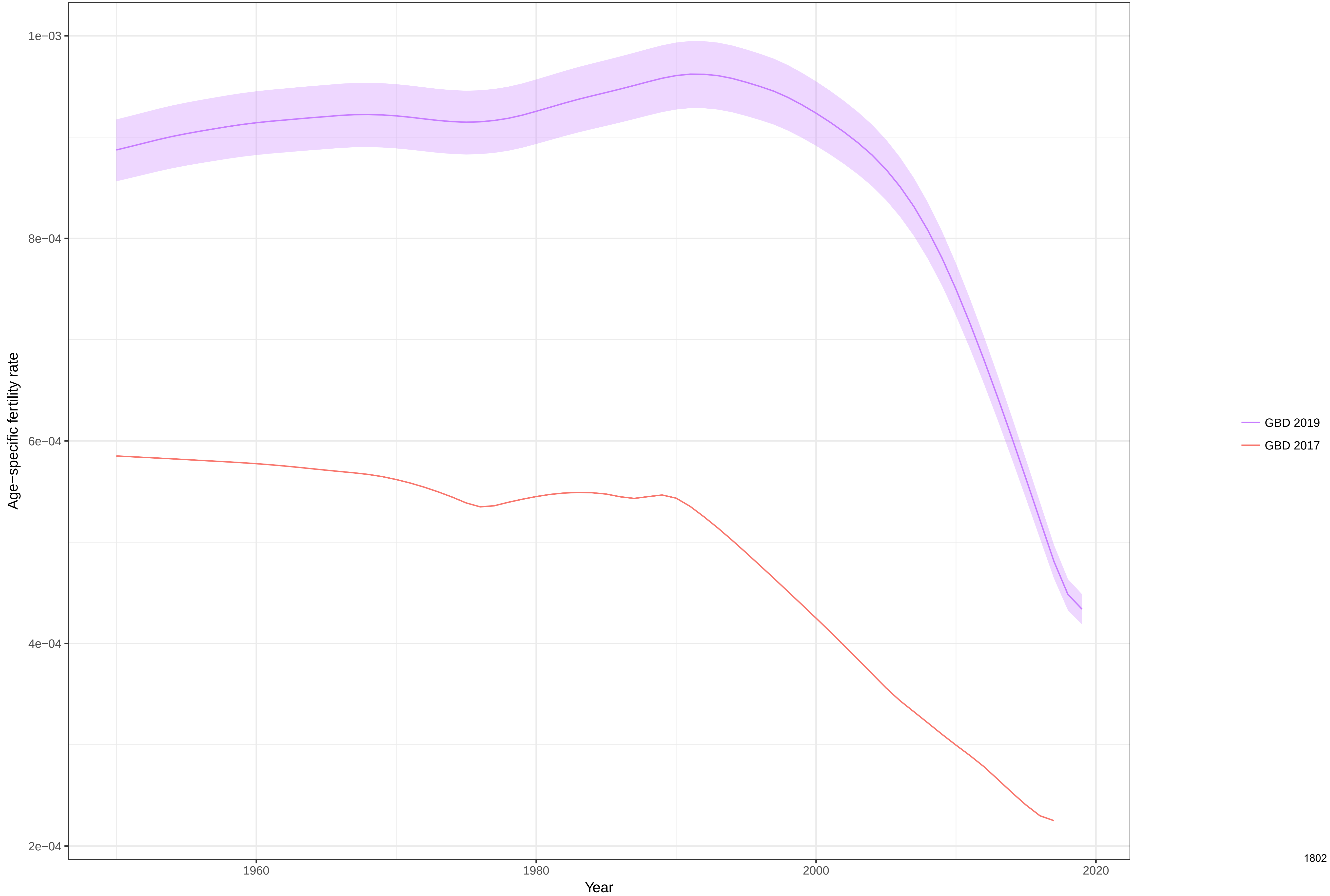

Total fertility rate fitted trend lines, 1950−2019: Sudan TFR
Data points enclosed with a diamond are reference sources

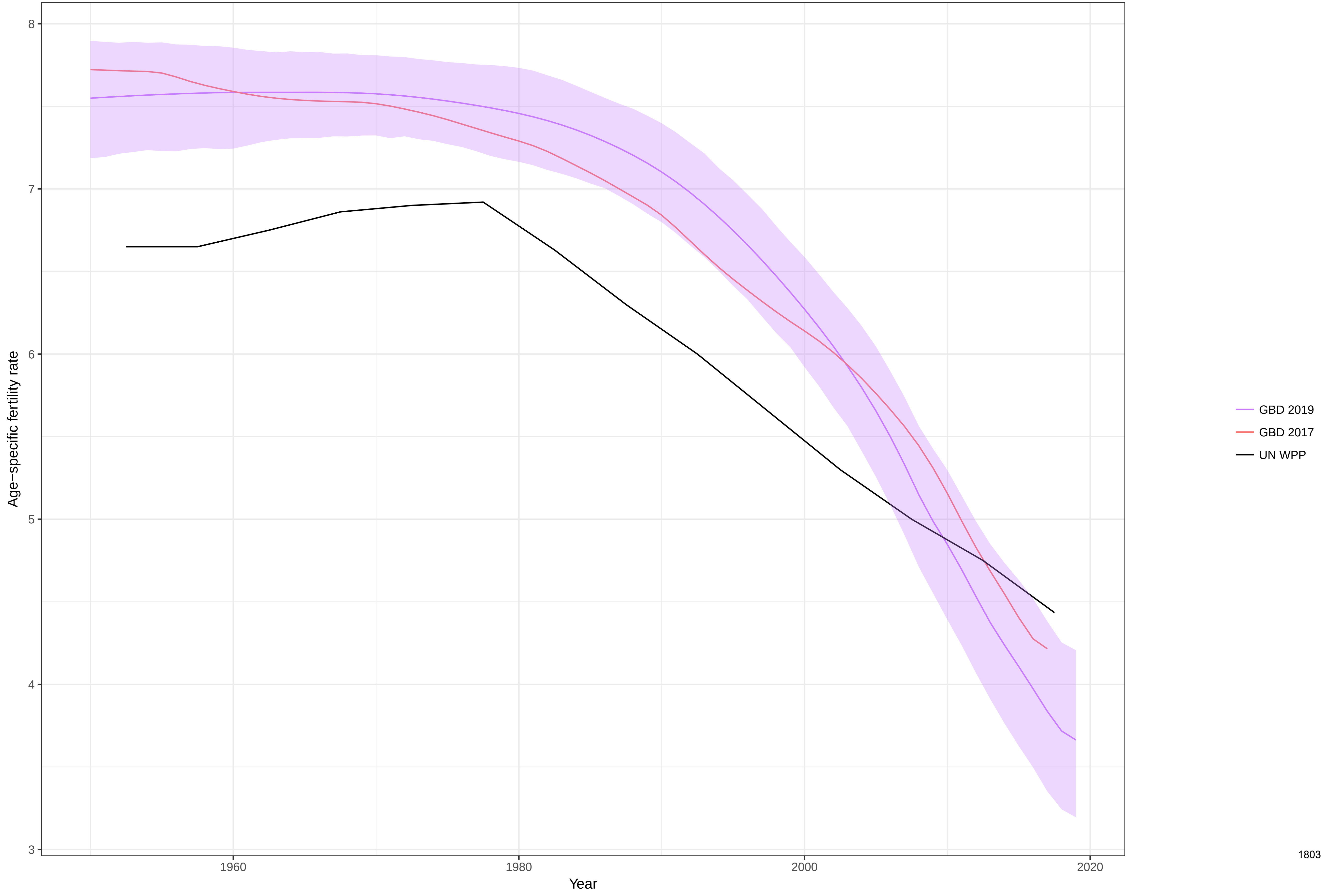

Age−specific fertility fitted trend lines, 1950−2019: Senegal, Ages: 10−14
Data points enclosed with a diamond are reference sources

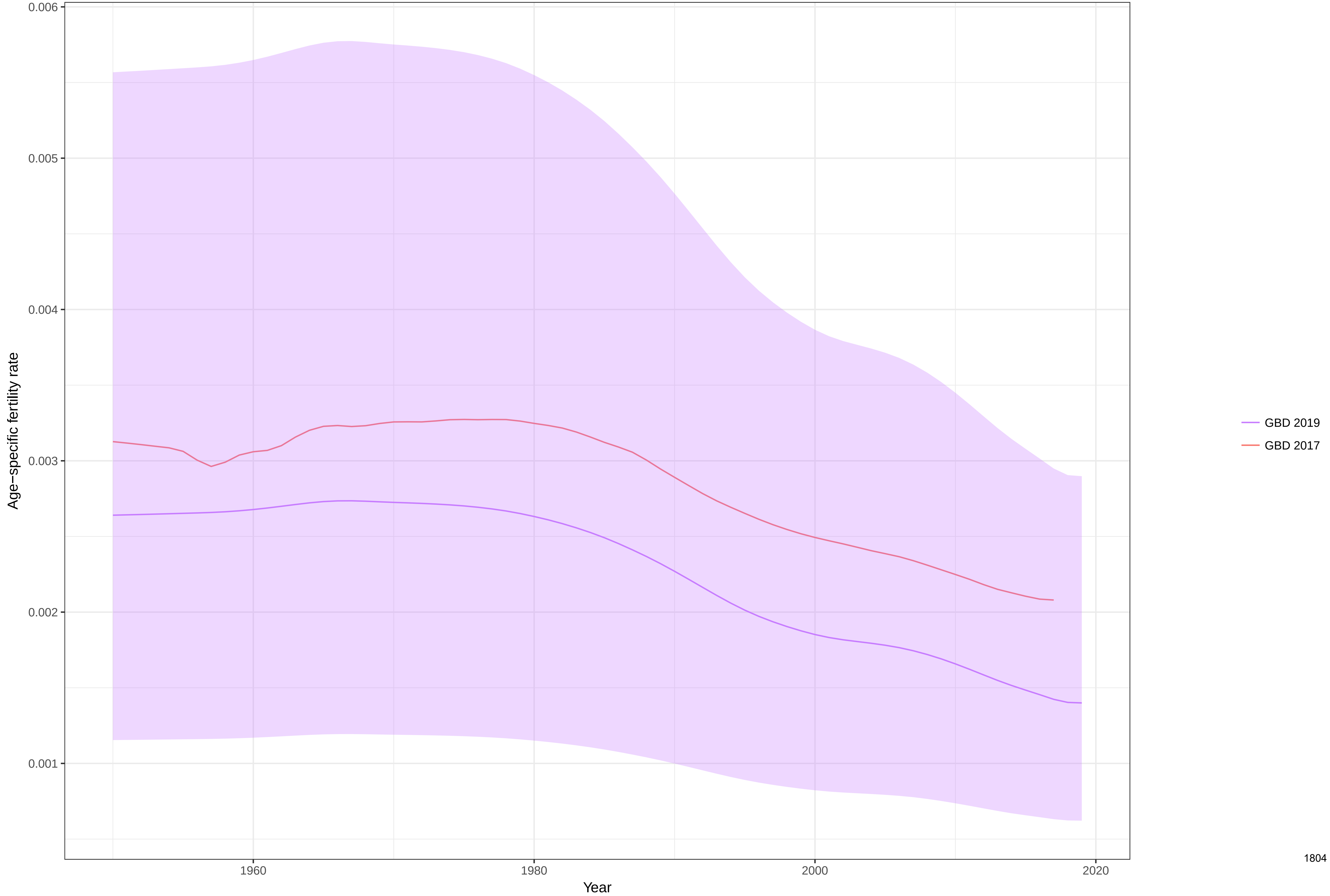

Age−specific fertility fitted trend lines, 1950−2019: Senegal, Ages: 15−19
Data points enclosed with a diamond are reference sources

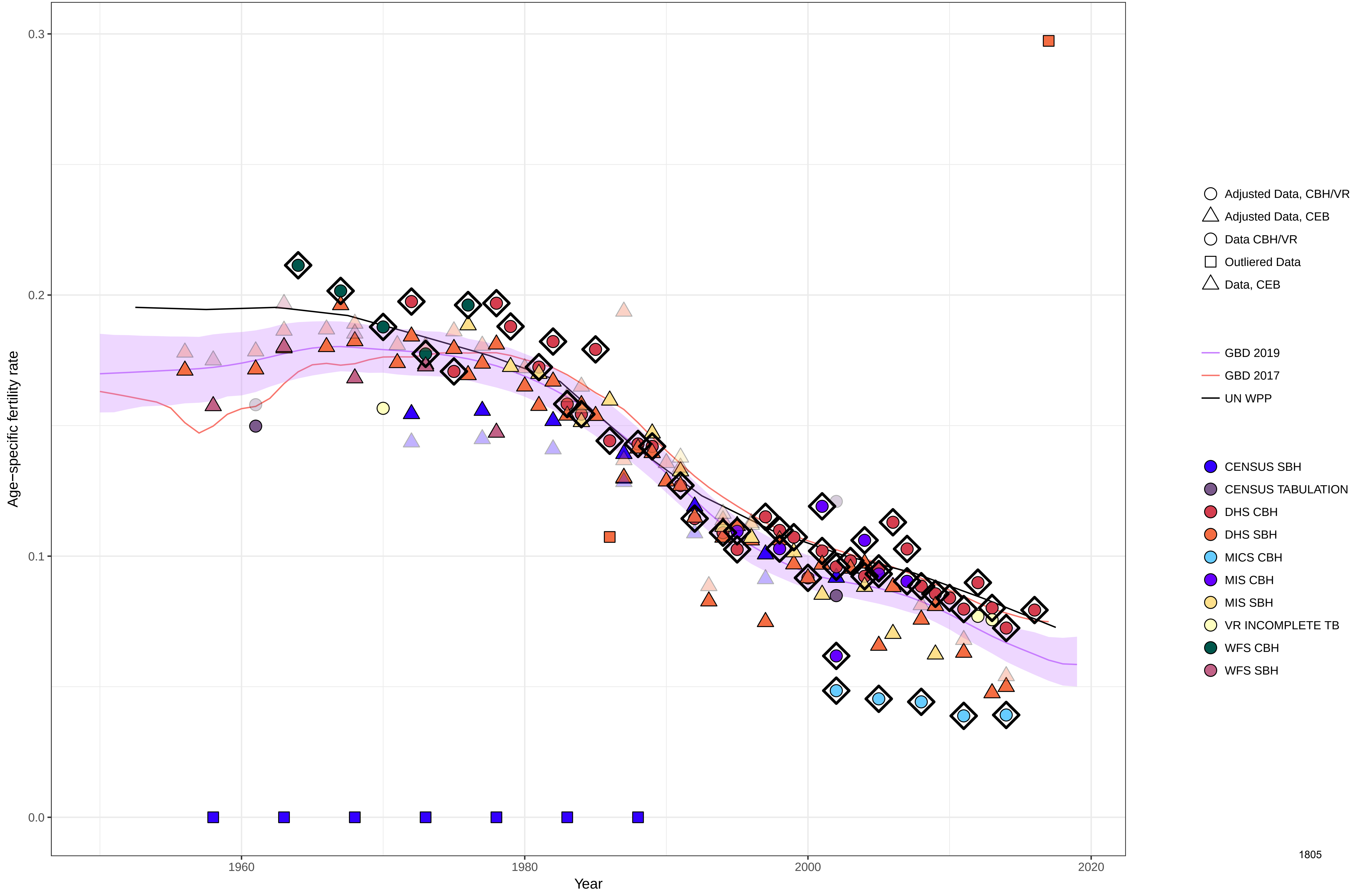

Age−specific fertility fitted trend lines, 1950−2019: Senegal, Ages: 20−24
Data points enclosed with a diamond are reference sources

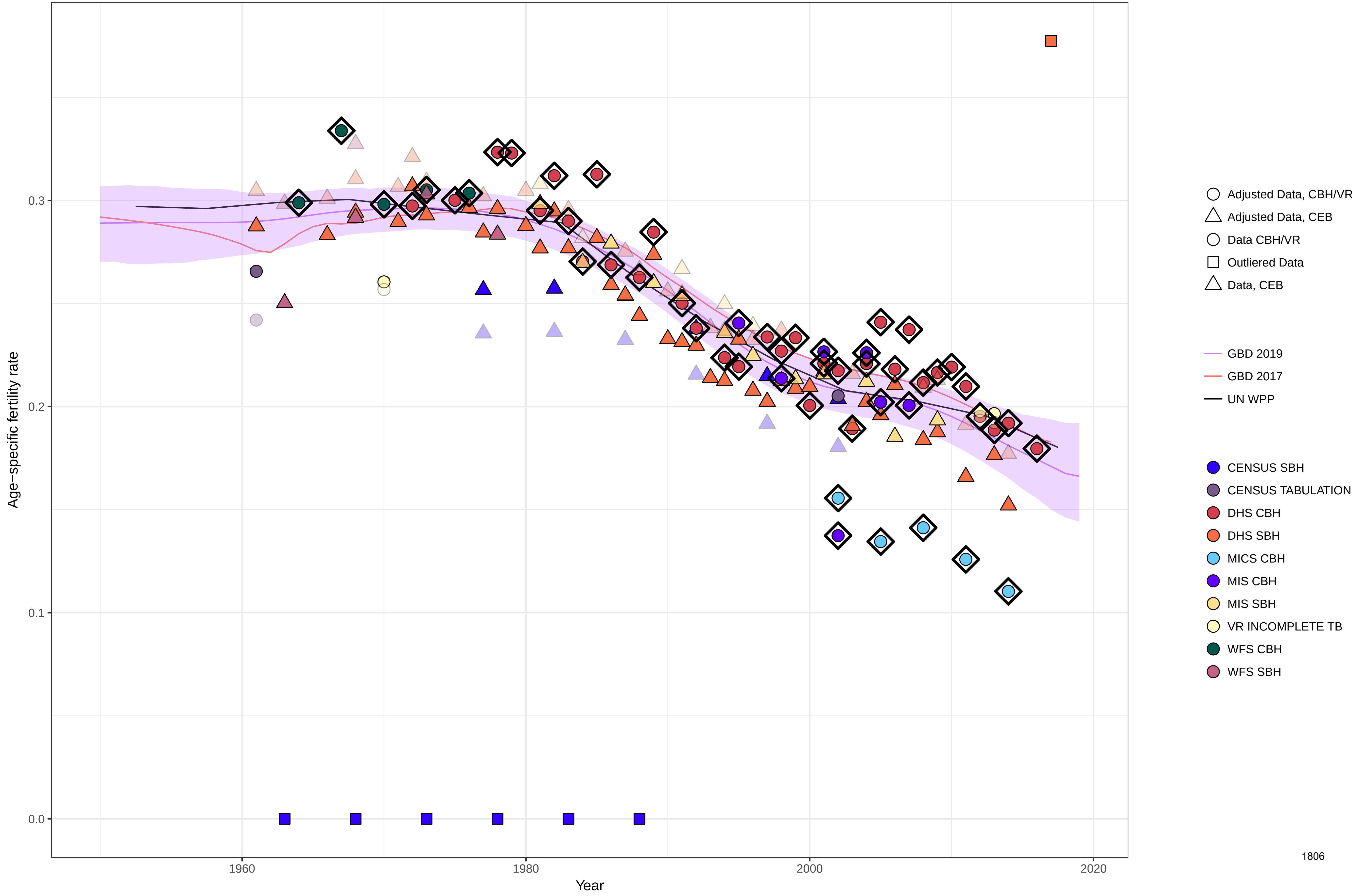

Age−specific fertility fitted trend lines, 1950−2019: Senegal, Ages: 25−29
Data points enclosed with a diamond are reference sources

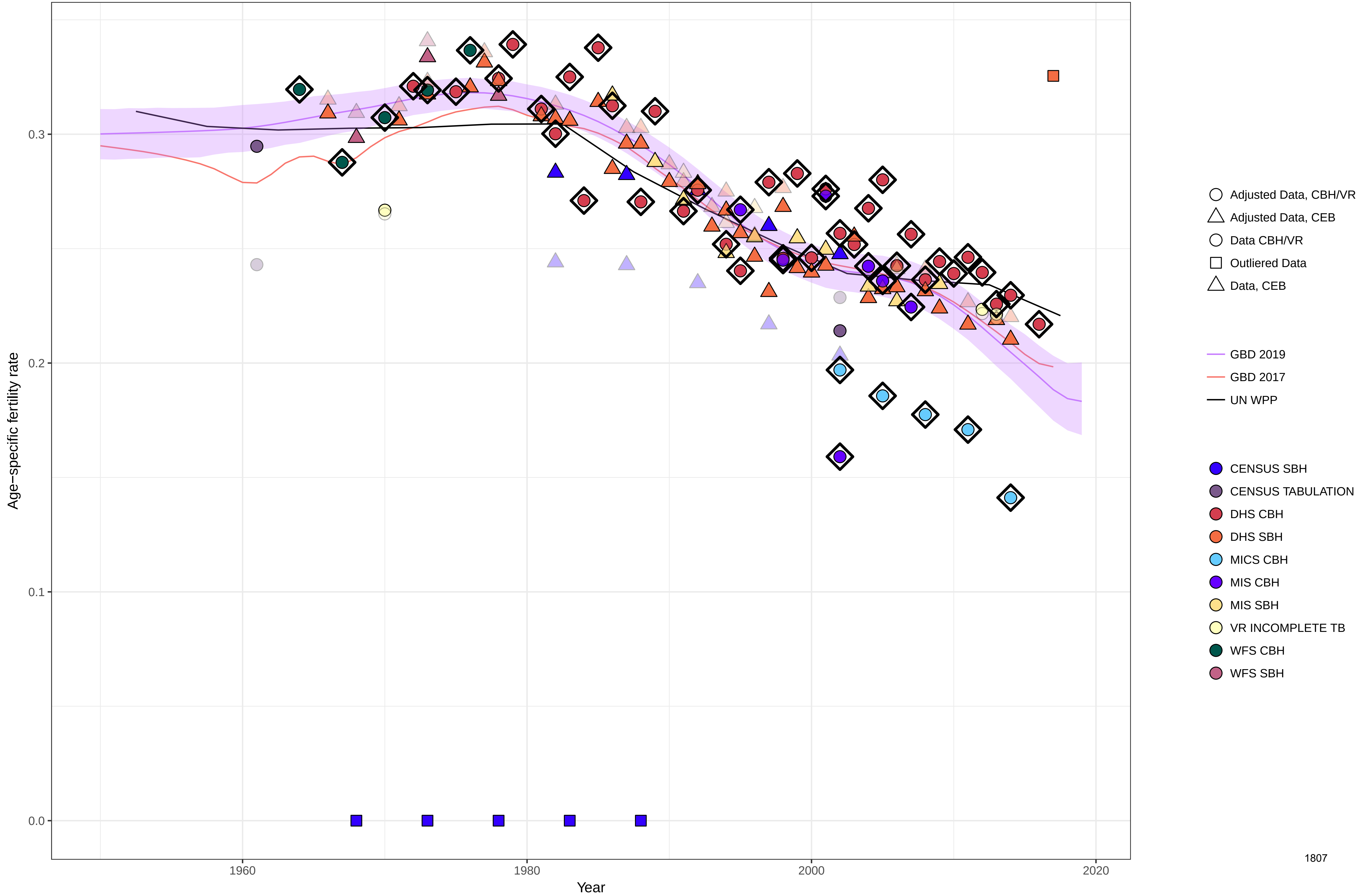

Age−specific fertility fitted trend lines, 1950−2019: Senegal, Ages: 30−34
Data points enclosed with a diamond are reference sources

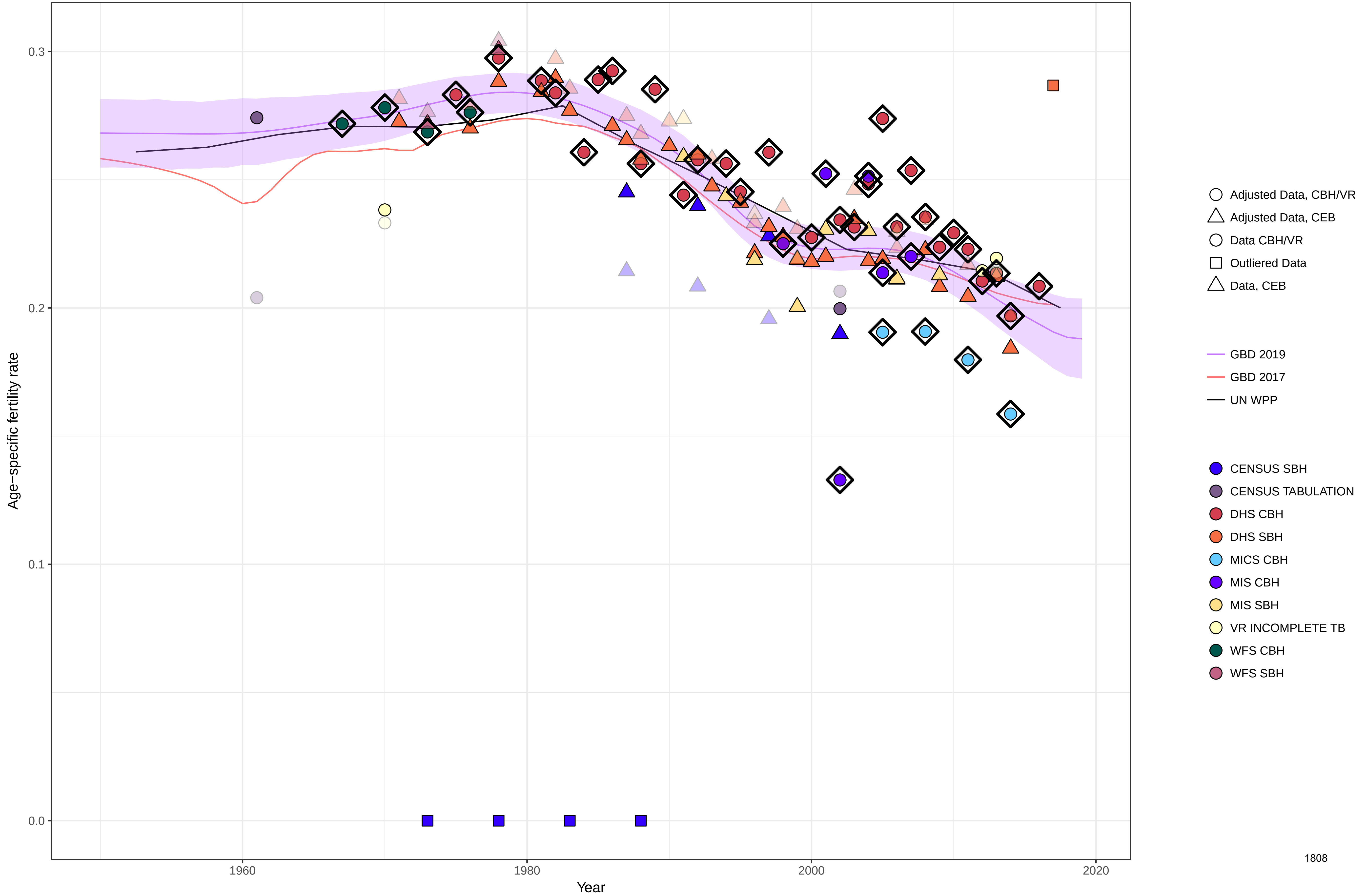

Age−specific fertility fitted trend lines, 1950−2019: Senegal, Ages: 35−39
Data points enclosed with a diamond are reference sources

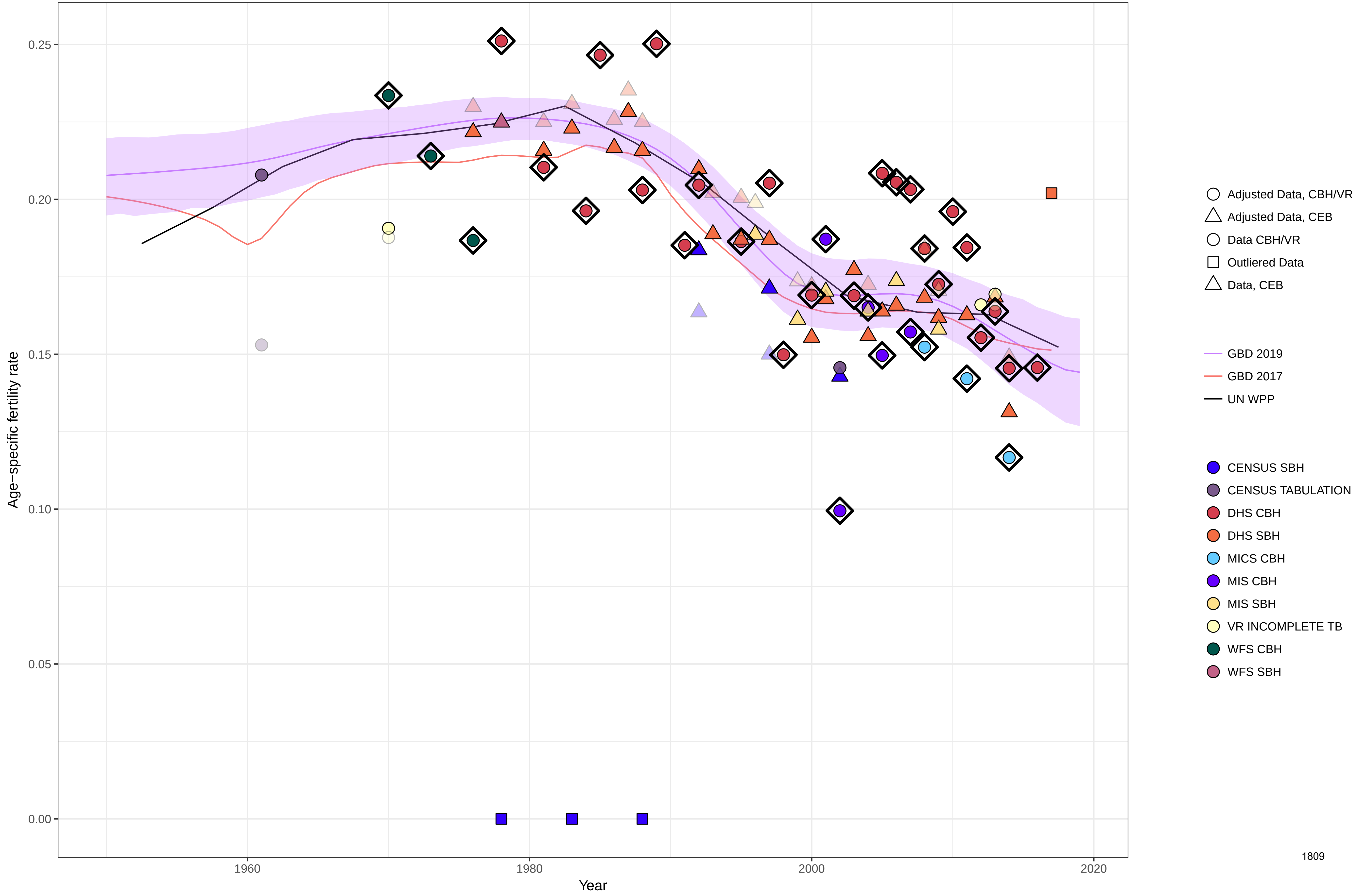

Age−specific fertility fitted trend lines, 1950−2019: Senegal, Ages: 40−44
Data points enclosed with a diamond are reference sources

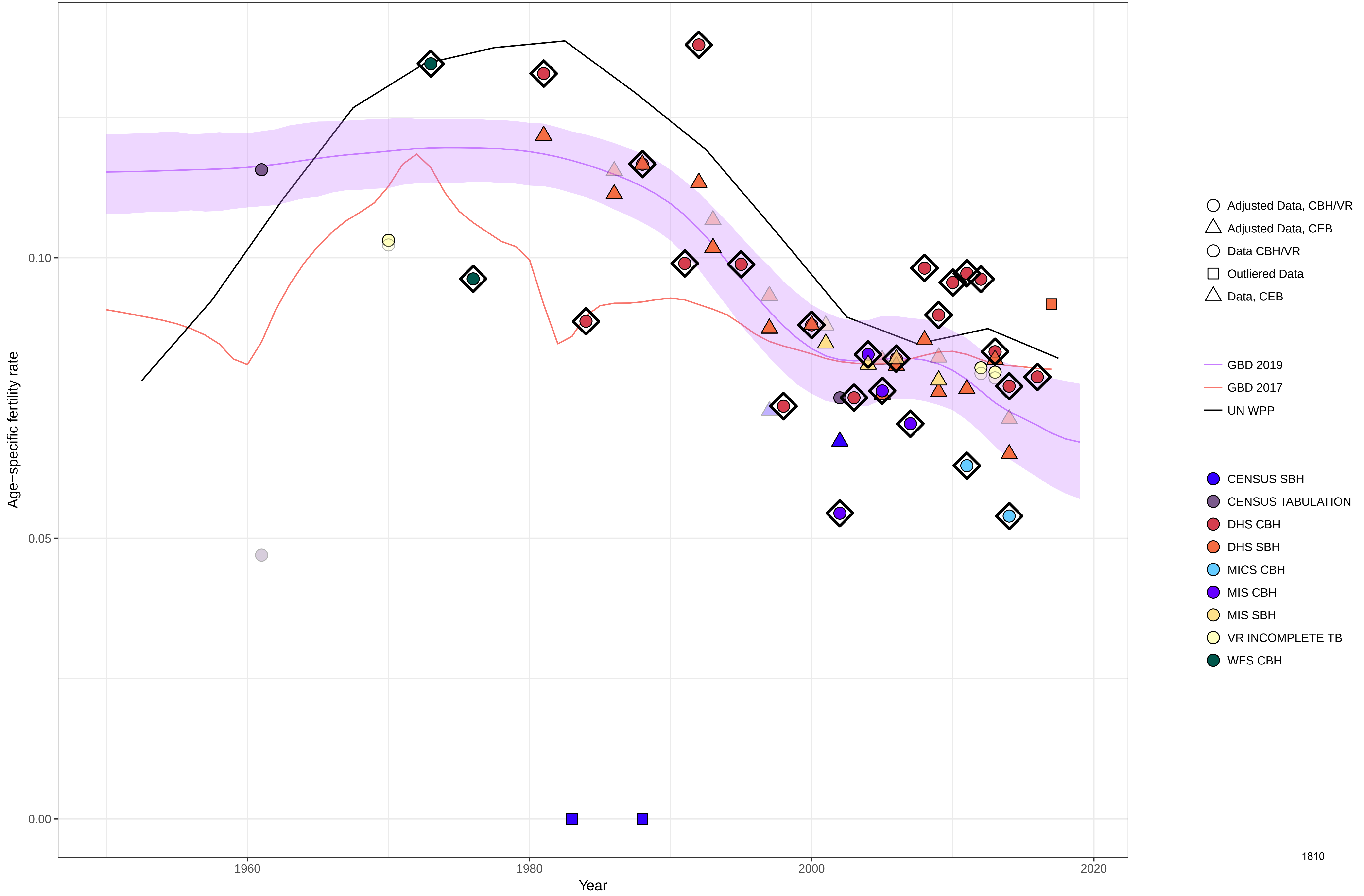

Age−specific fertility fitted trend lines, 1950−2019: Senegal, Ages: 45−49
Data points enclosed with a diamond are reference sources

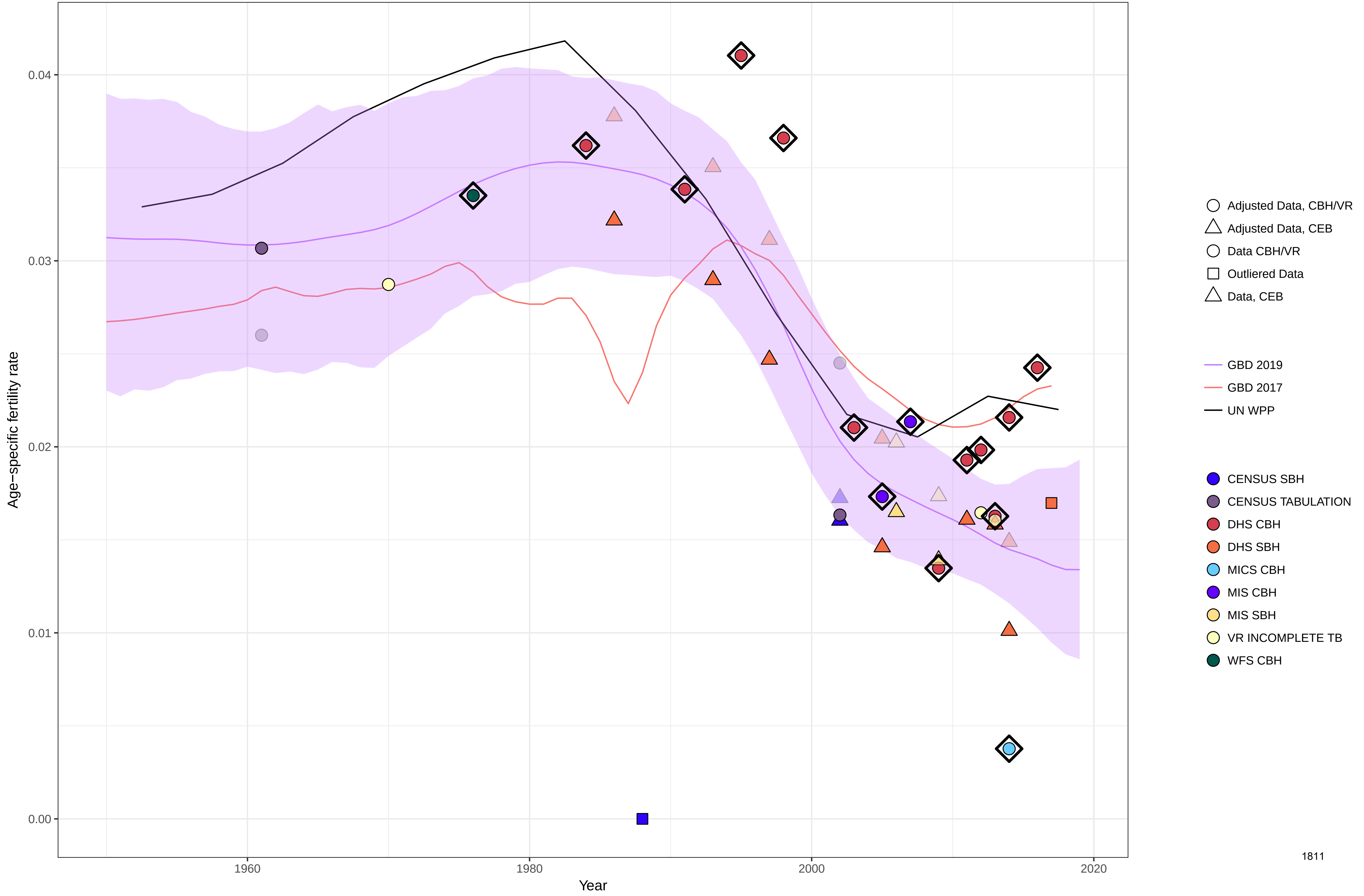

Age−specific fertility fitted trend lines, 1950−2019: Senegal, Ages: 50−54
Data points enclosed with a diamond are reference sources

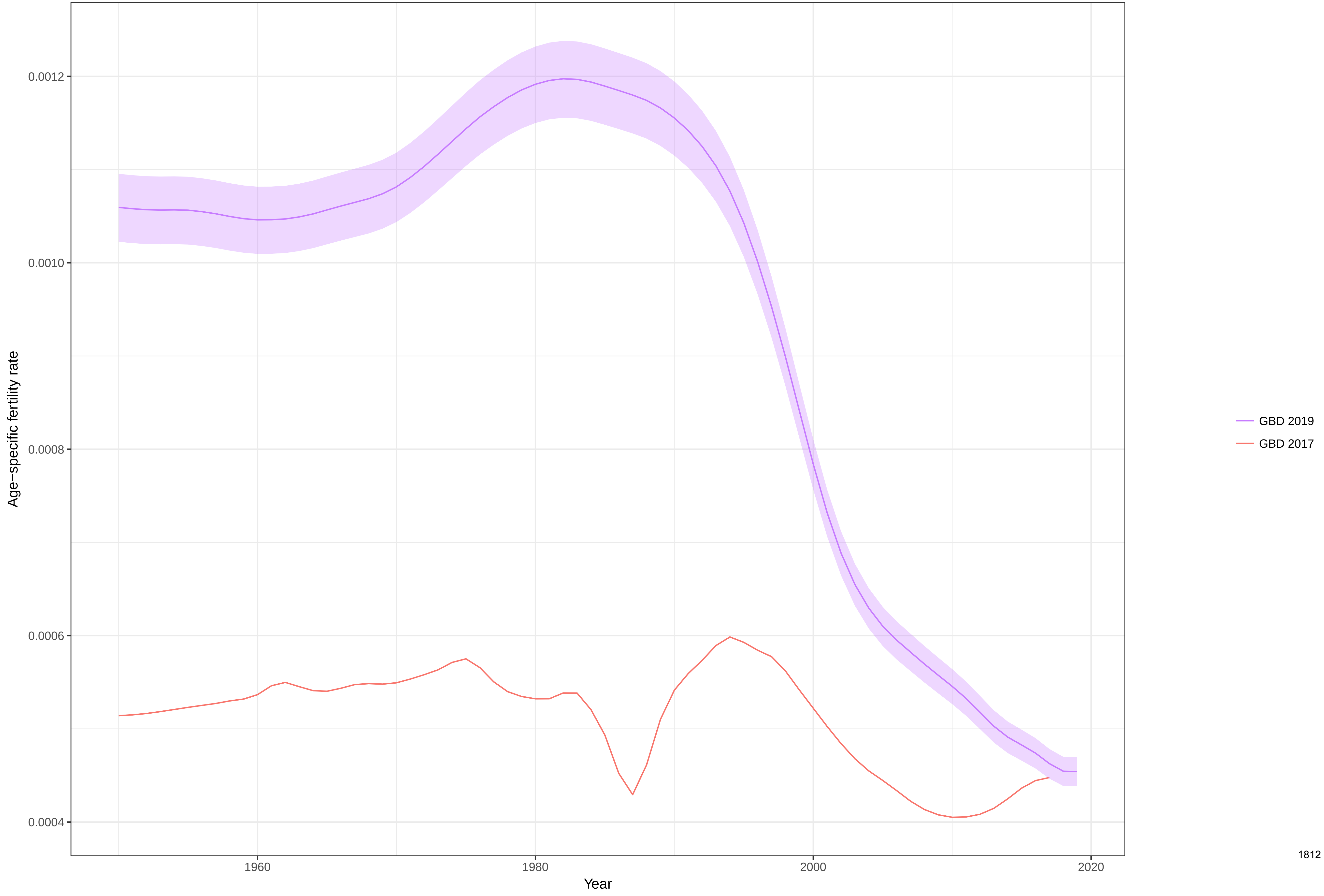

Total fertility rate fitted trend lines, 1950−2019: Senegal TFR
Data points enclosed with a diamond are reference sources

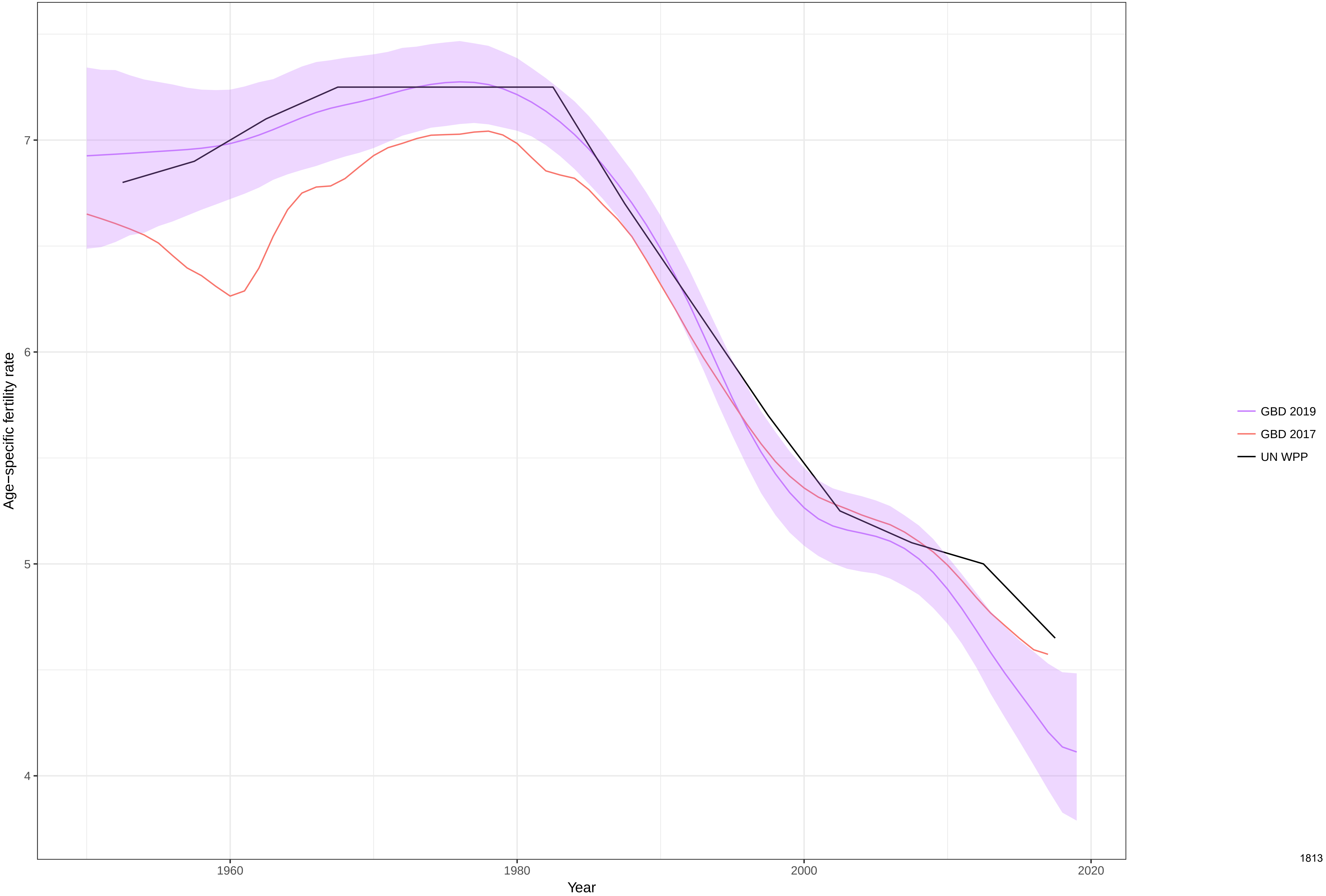

Age−specific fertility fitted trend lines, 1950−2019: Singapore, Ages: 10−14
Data points enclosed with a diamond are reference sources

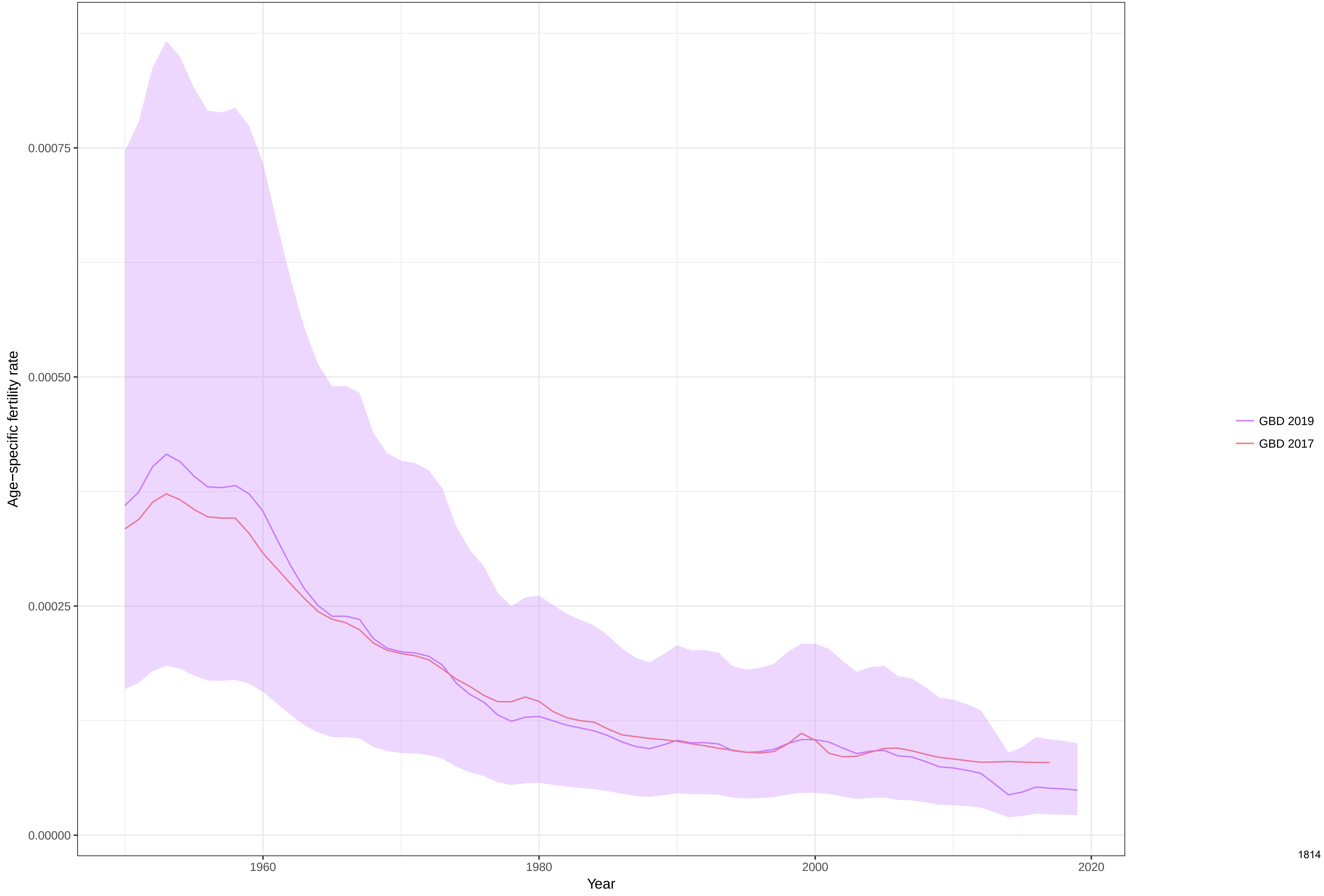

Age−specific fertility fitted trend lines, 1950−2019: Singapore, Ages: 15−19
Data points enclosed with a diamond are reference sources

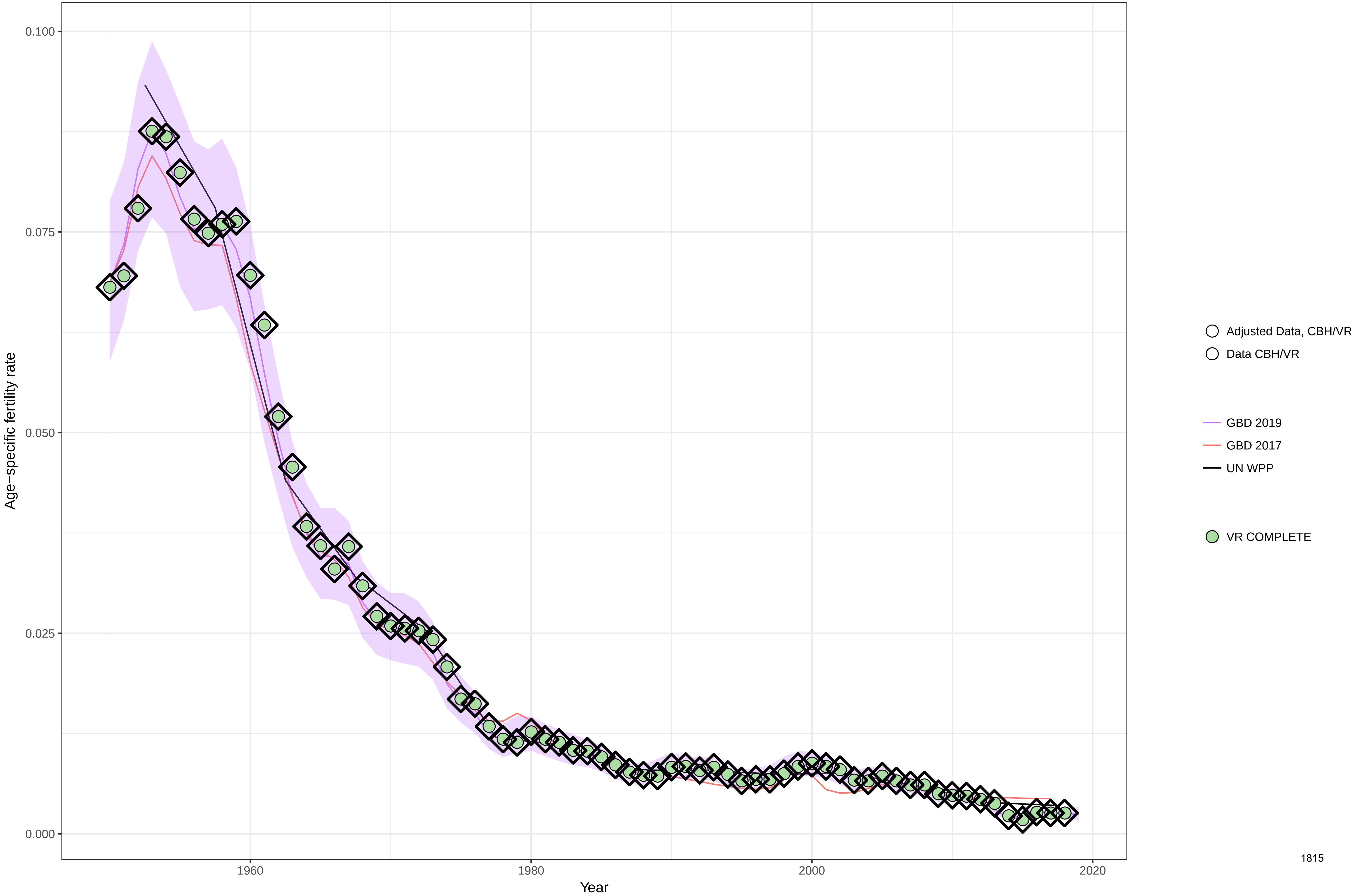

Age−specific fertility fitted trend lines, 1950−2019: Singapore, Ages: 20−24
Data points enclosed with a diamond are reference sources

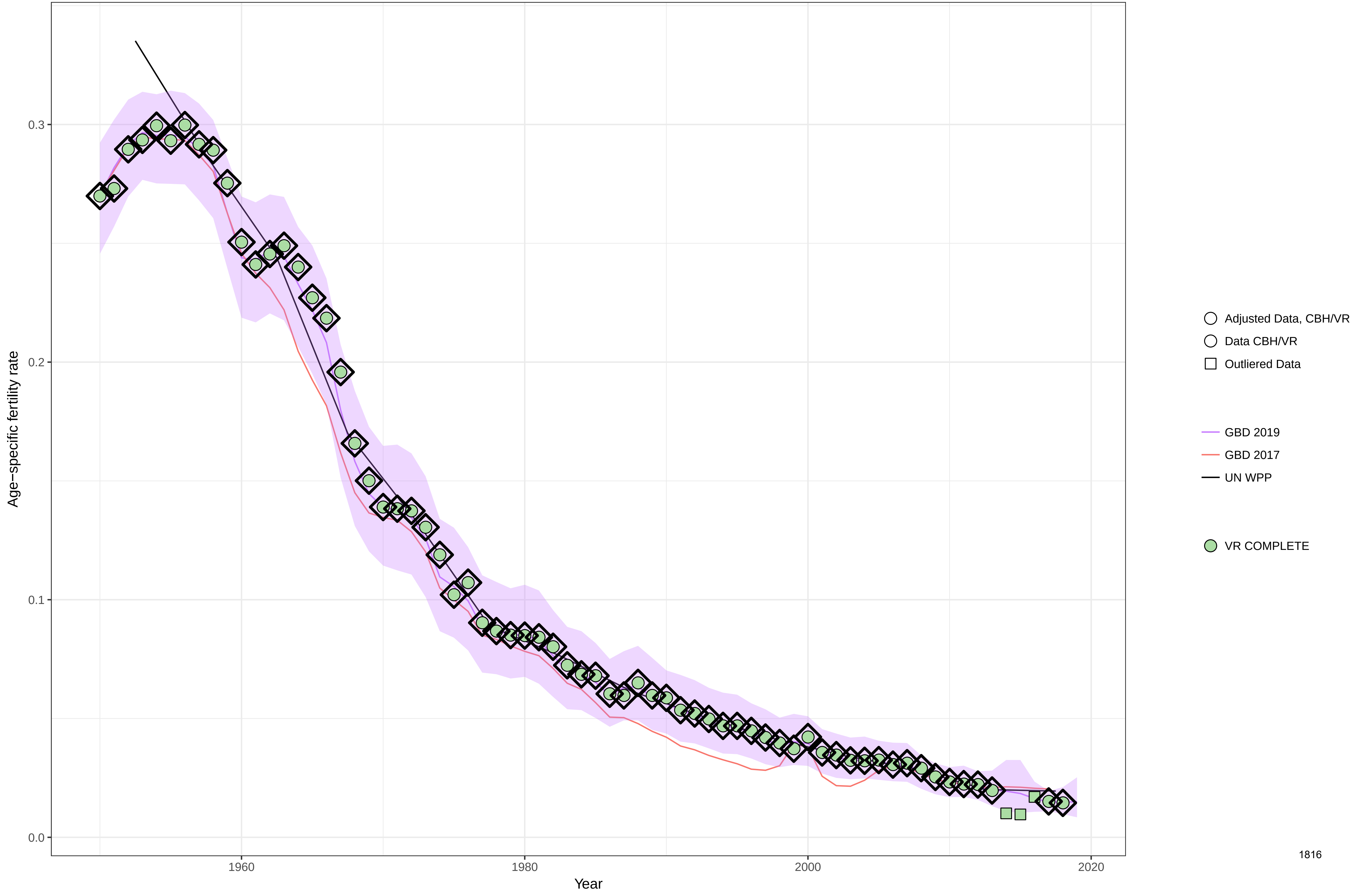

Age−specific fertility fitted trend lines, 1950−2019: Singapore, Ages: 25−29
Data points enclosed with a diamond are reference sources

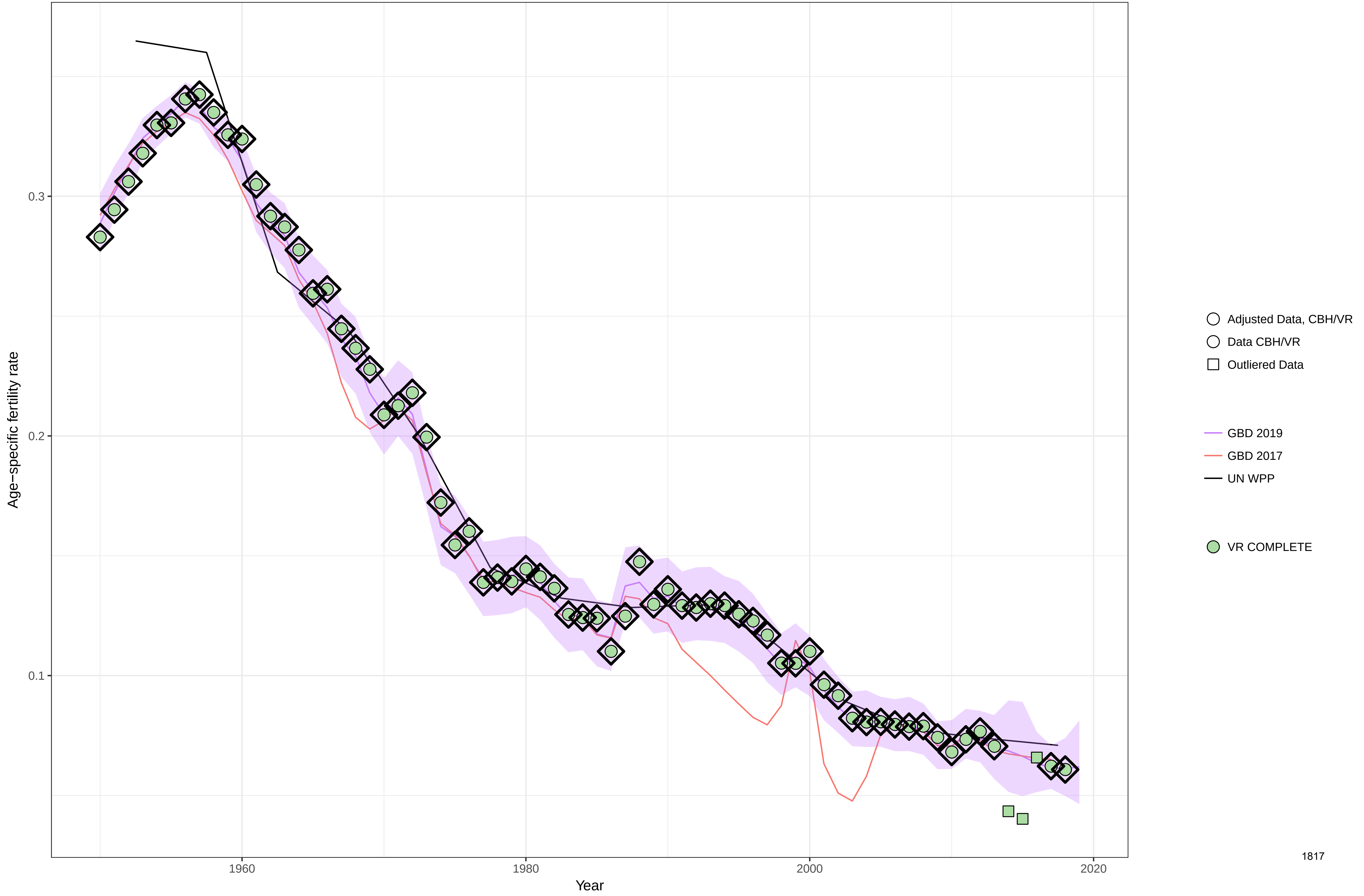

Age−specific fertility fitted trend lines, 1950−2019: Singapore, Ages: 30−34
Data points enclosed with a diamond are reference sources

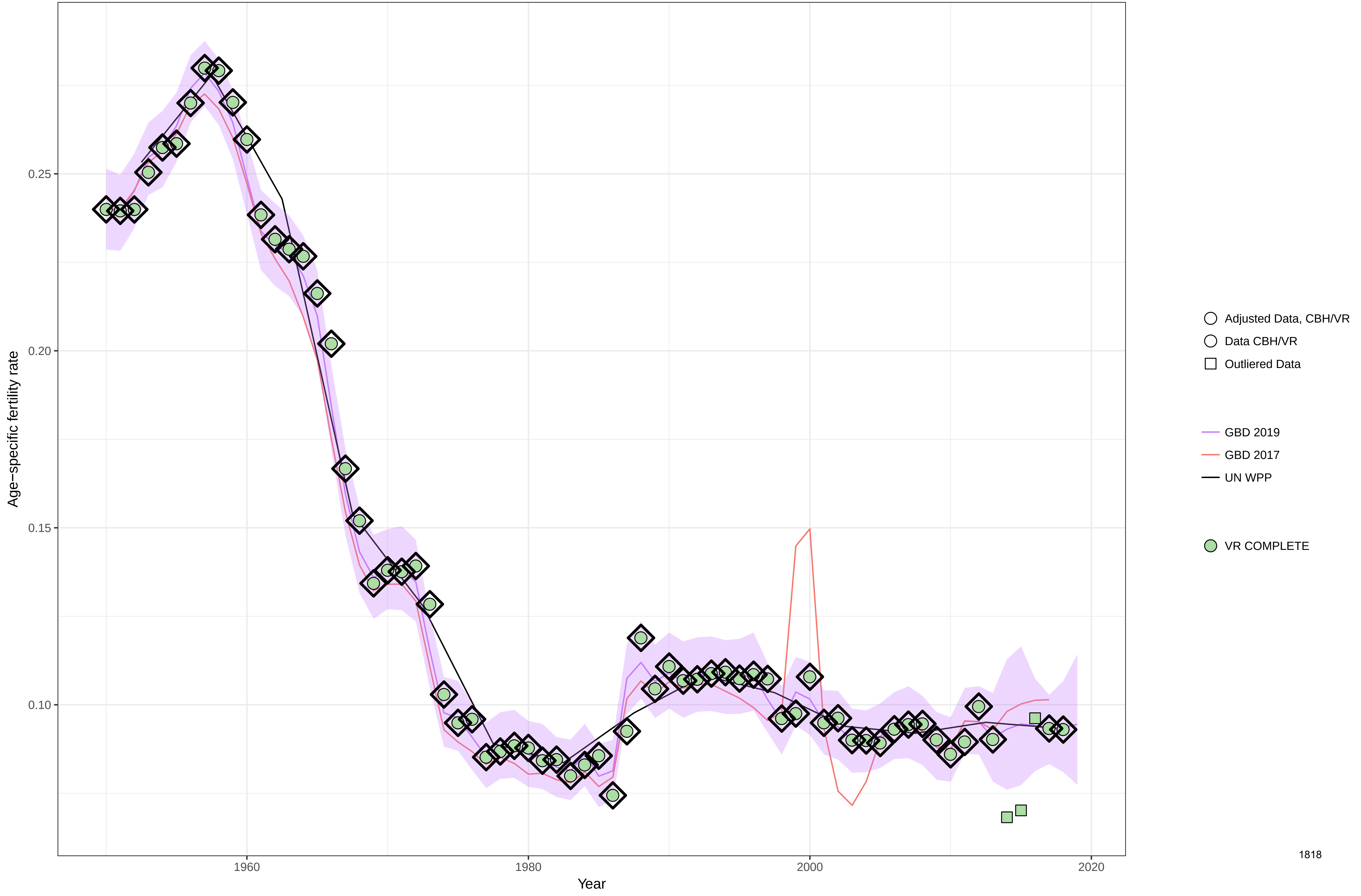

Age−specific fertility fitted trend lines, 1950−2019: Singapore, Ages: 35−39
Data points enclosed with a diamond are reference sources

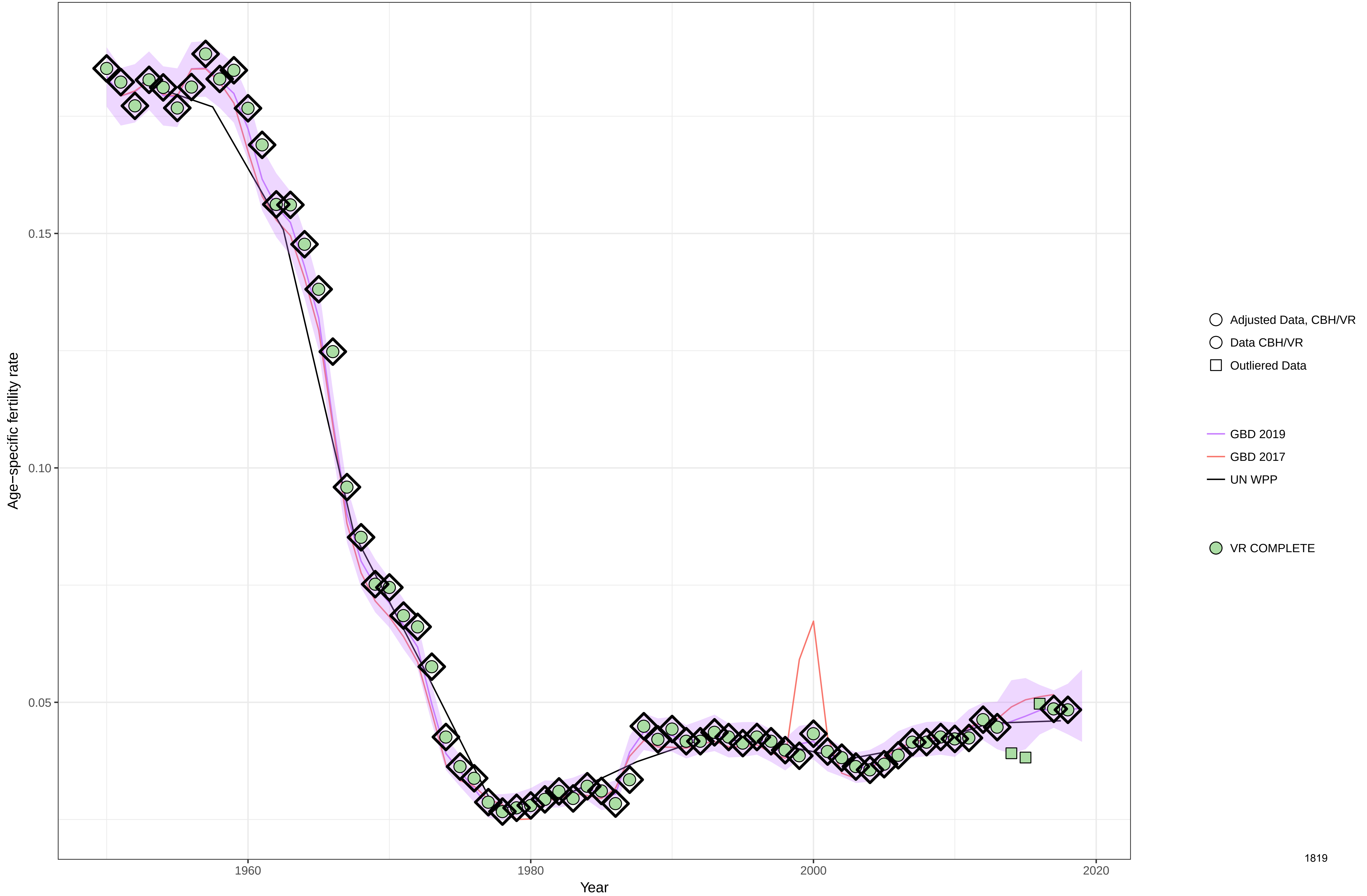

Age−specific fertility fitted trend lines, 1950−2019: Singapore, Ages: 40−44
Data points enclosed with a diamond are reference sources

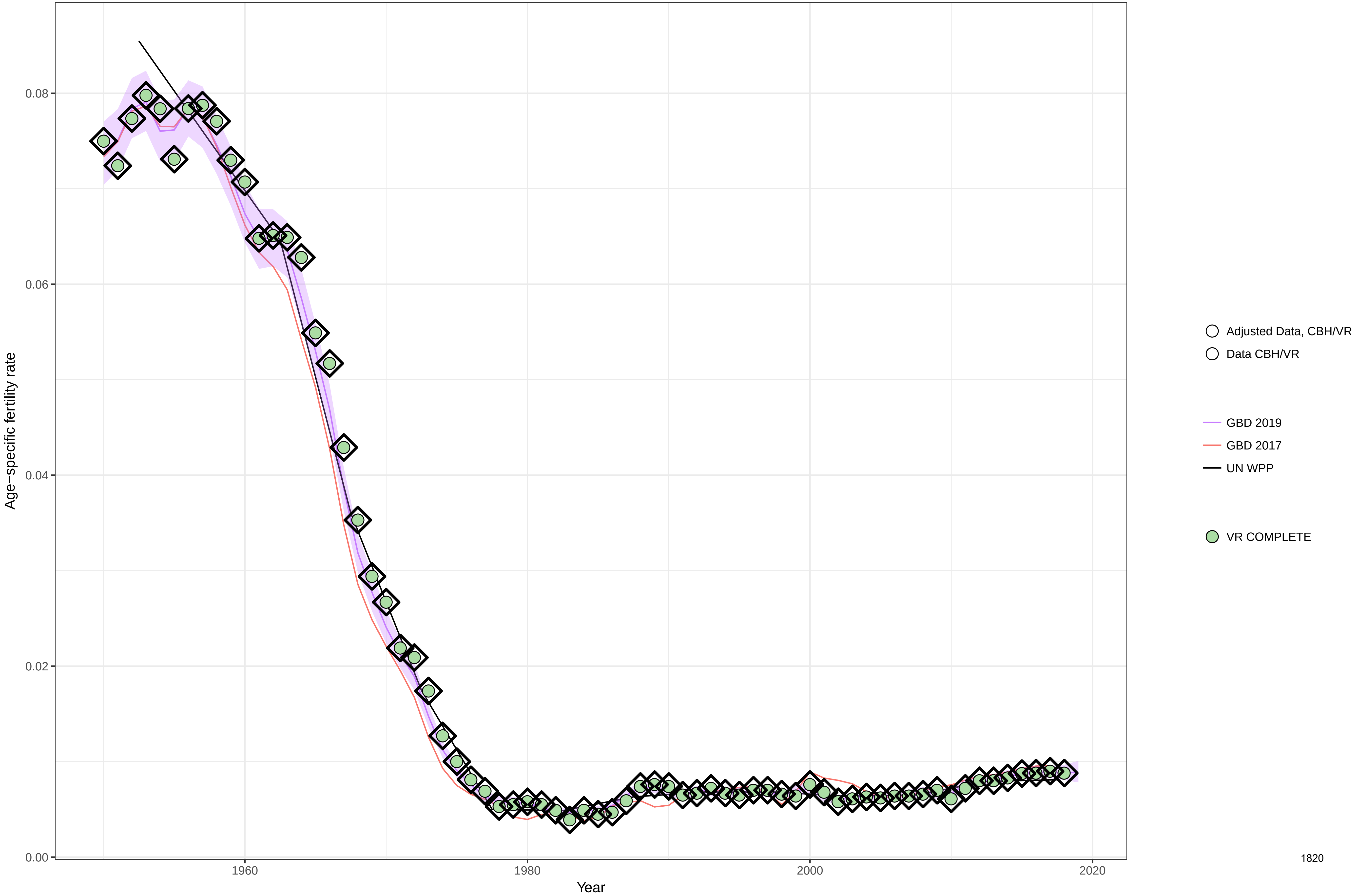

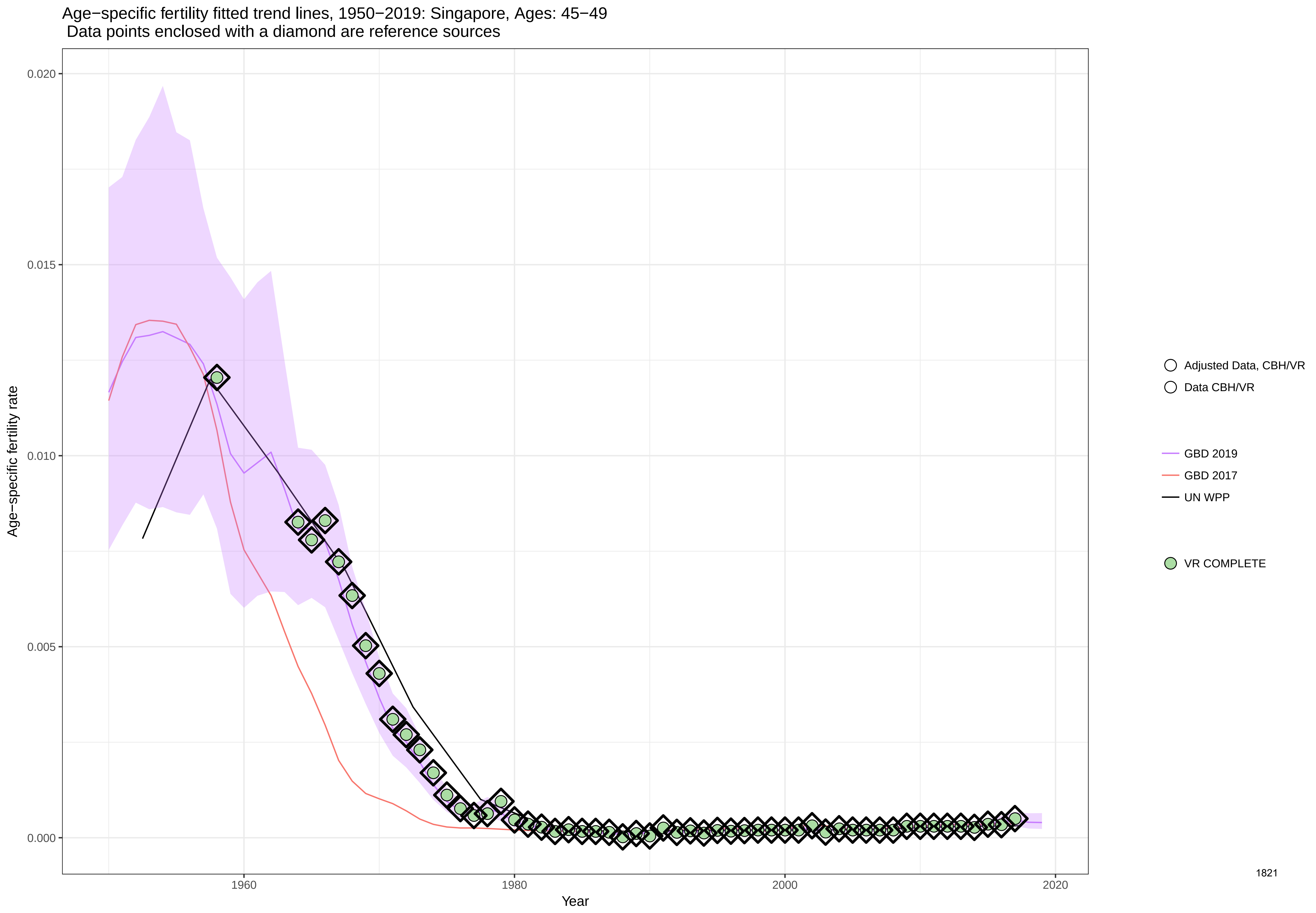
Age−specific fertility fitted trend lines, 1950−2019: Singapore, Ages: 45−49
Data points enclosed with a diamond are reference sources
Age−specific fertility rate
0.020
0.015
0.010
0.005
0.000
1960
1980
2000
2020
Year
Adjusted Data, CBH/VR
Data CBH/VR
GBD 2019
GBD 2017
UN WPP
VR COMPLETE

Age−specific fertility fitted trend lines, 1950−2019: Singapore, Ages: 50−54
Data points enclosed with a diamond are reference sources

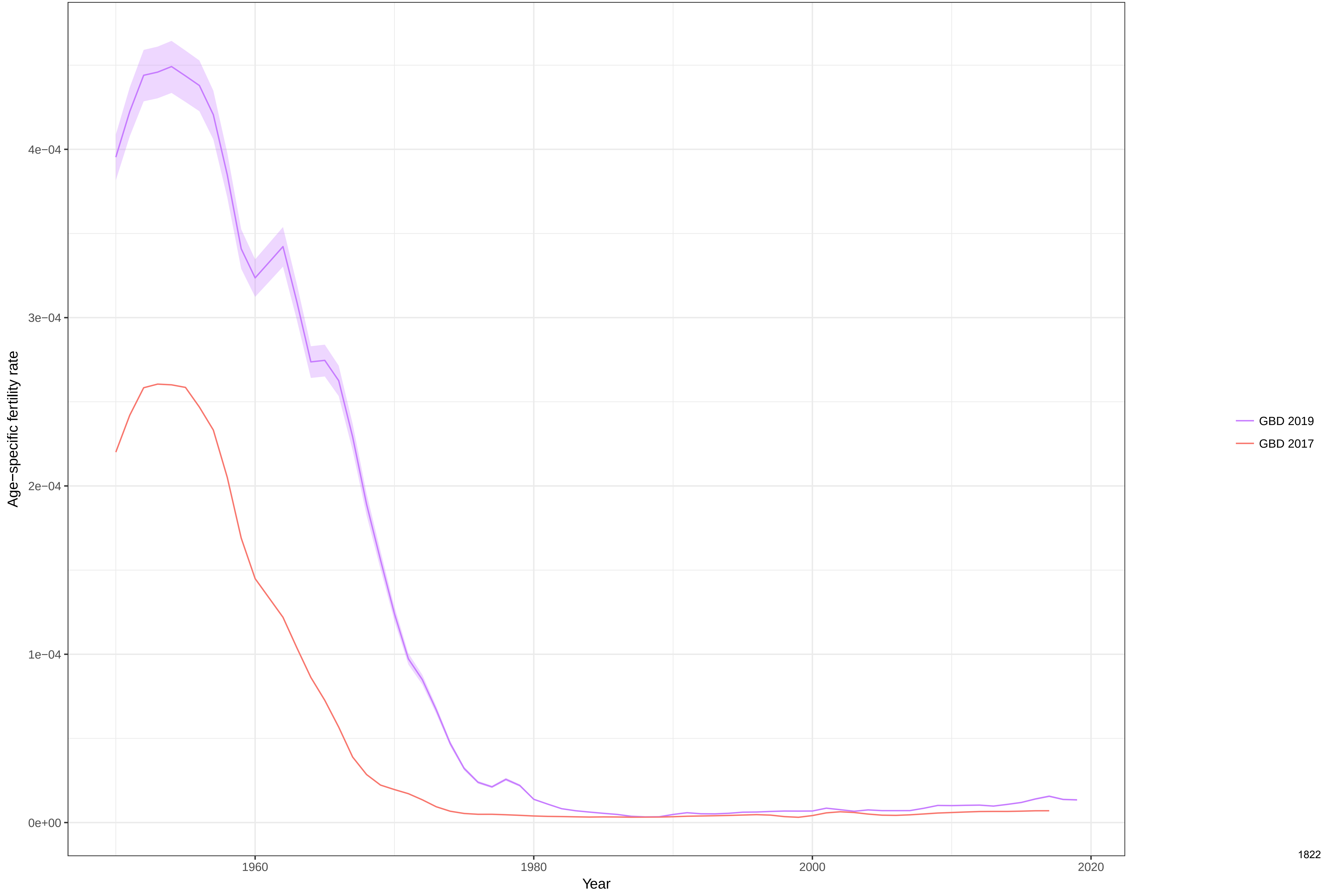

Total fertility rate fitted trend lines, 1950−2019: Singapore TFR
Data points enclosed with a diamond are reference sources

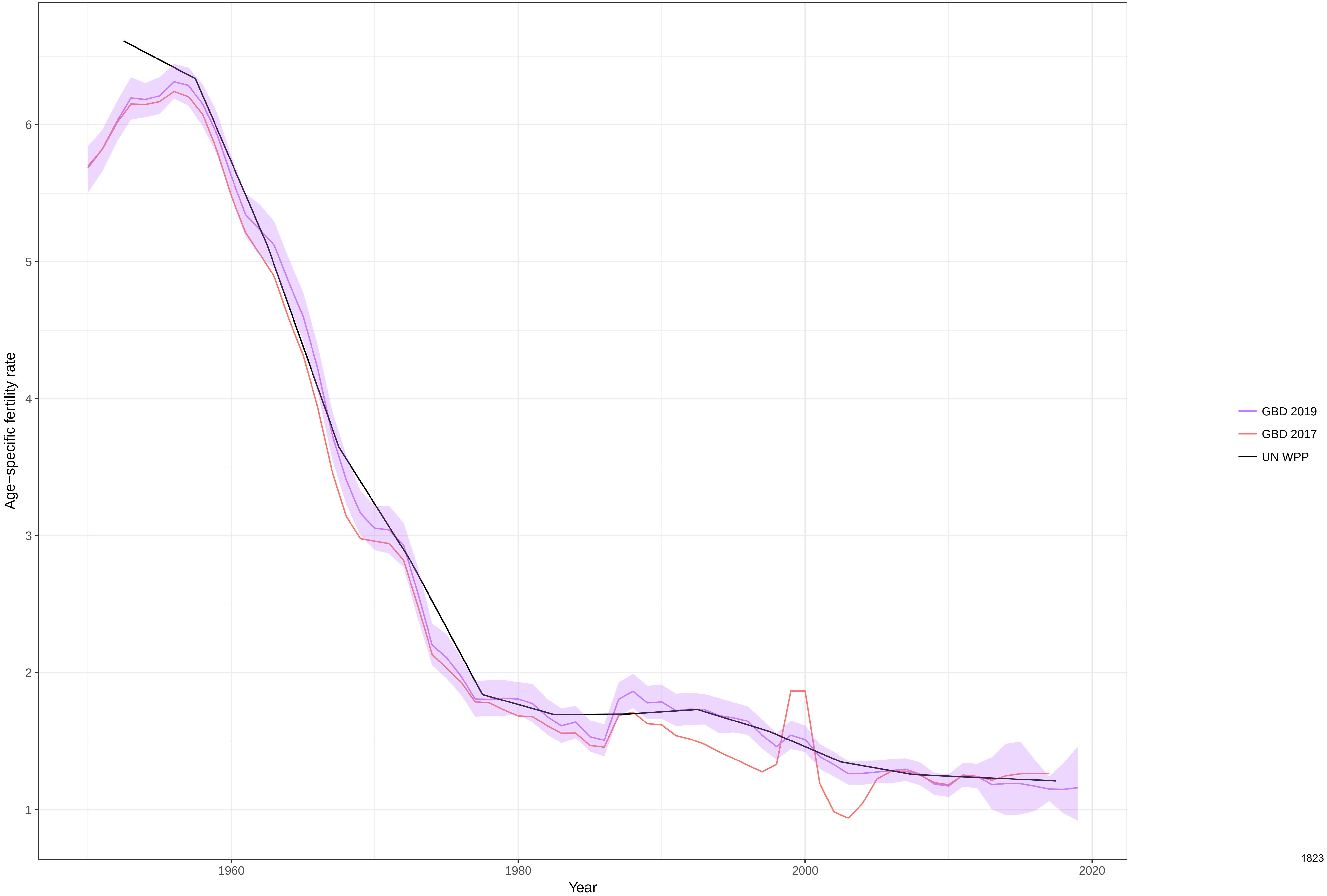

Age−specific fertility fitted trend lines, 1950−2019: Solomon Islands, Ages: 10−14
Data points enclosed with a diamond are reference sources

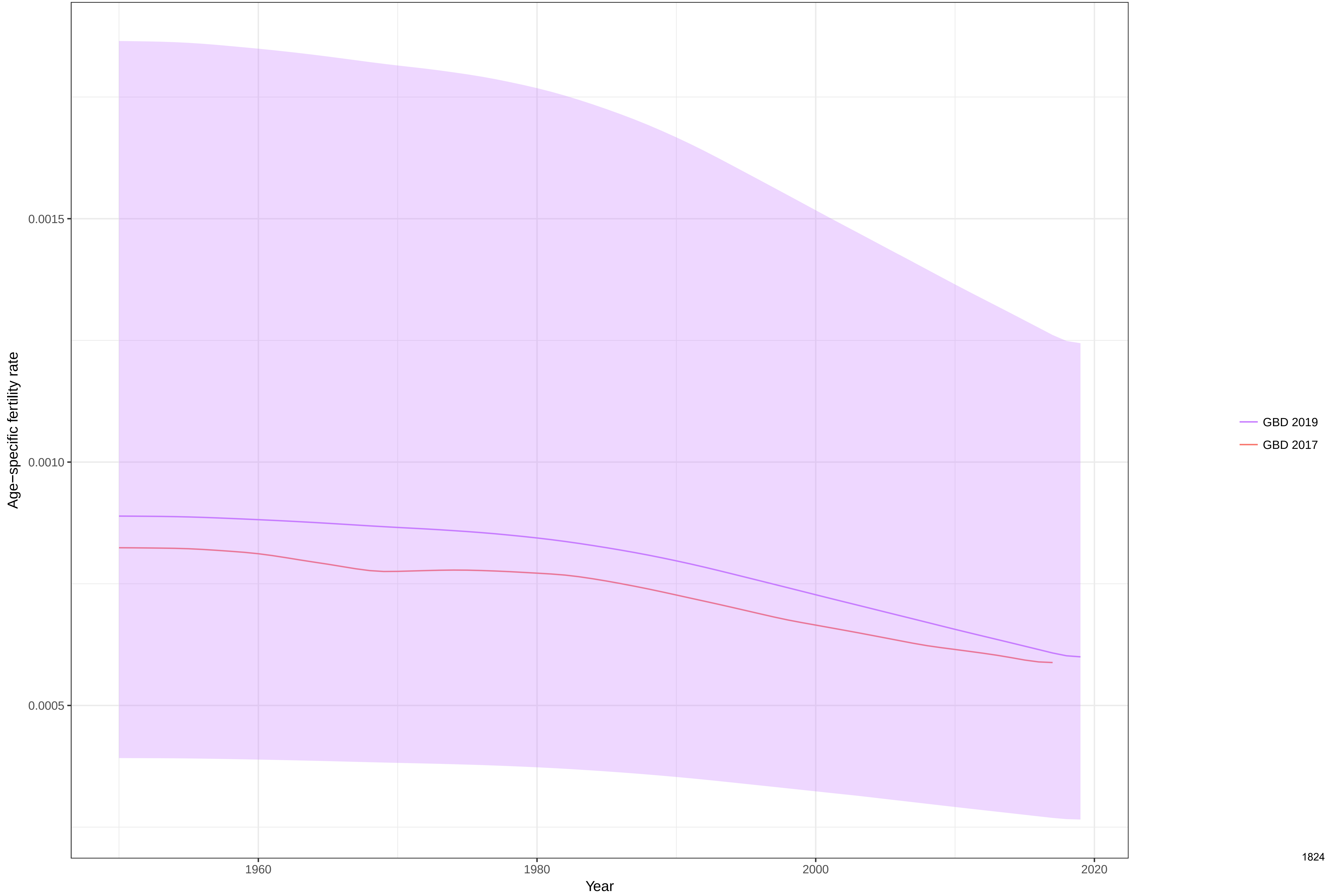

Age−specific fertility fitted trend lines, 1950−2019: Solomon Islands, Ages: 15−19
Data points enclosed with a diamond are reference sources

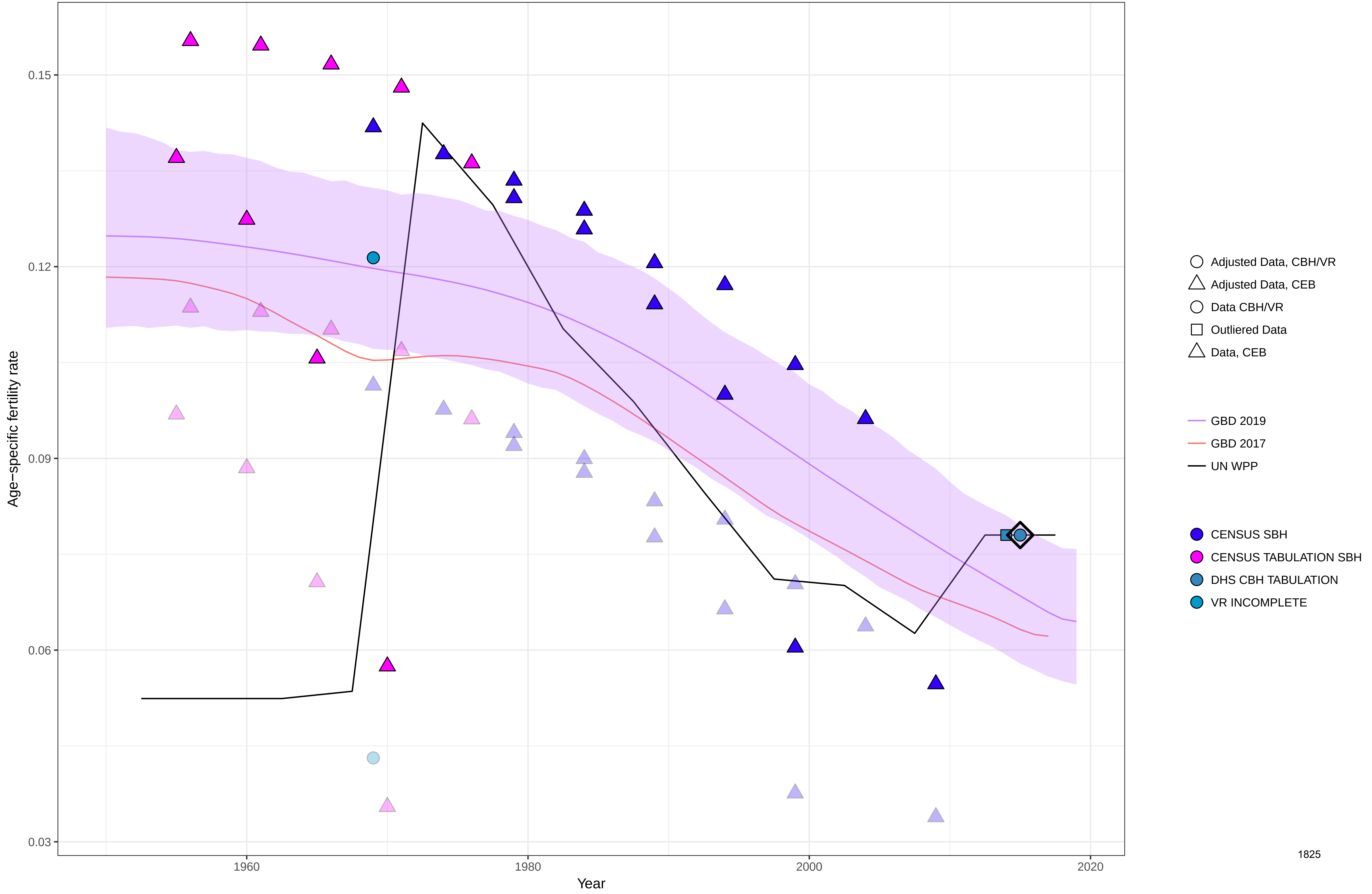

Age−specific fertility fitted trend lines, 1950−2019: Solomon Islands, Ages: 20−24
Data points enclosed with a diamond are reference sources

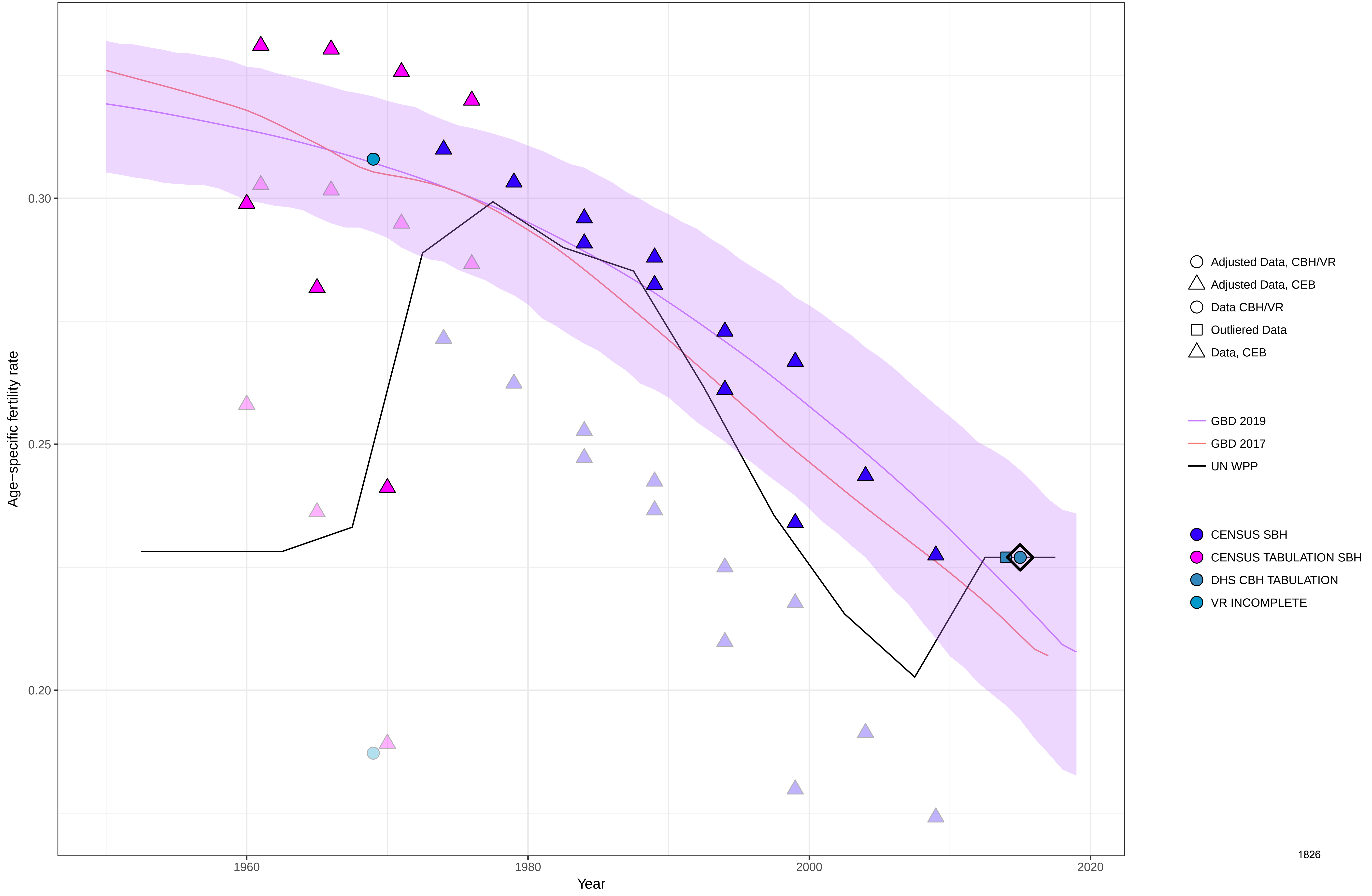

Age−specific fertility fitted trend lines, 1950−2019: Solomon Islands, Ages: 25−29
Data points enclosed with a diamond are reference sources

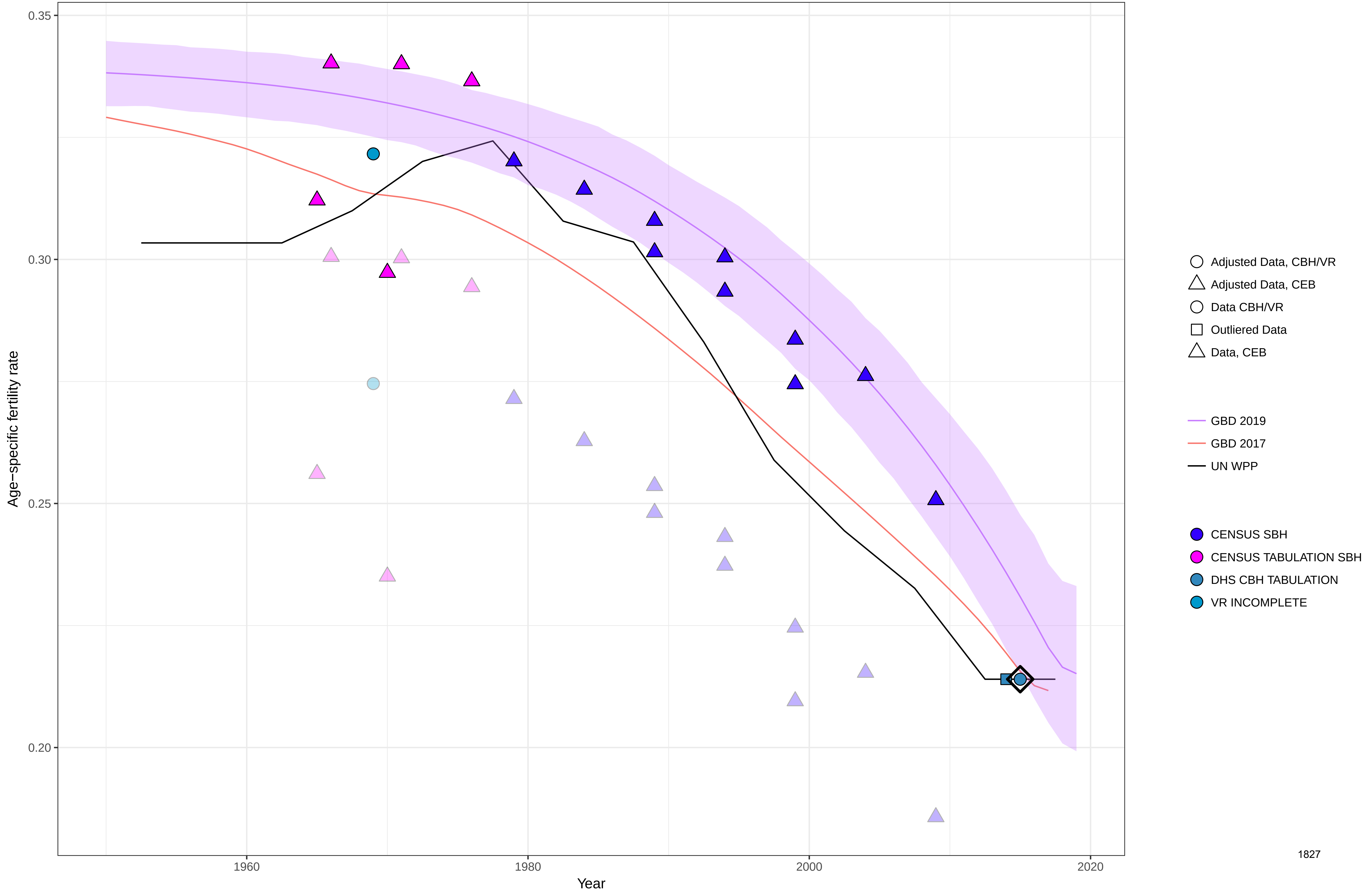

Age−specific fertility fitted trend lines, 1950−2019: Solomon Islands, Ages: 30−34
Data points enclosed with a diamond are reference sources

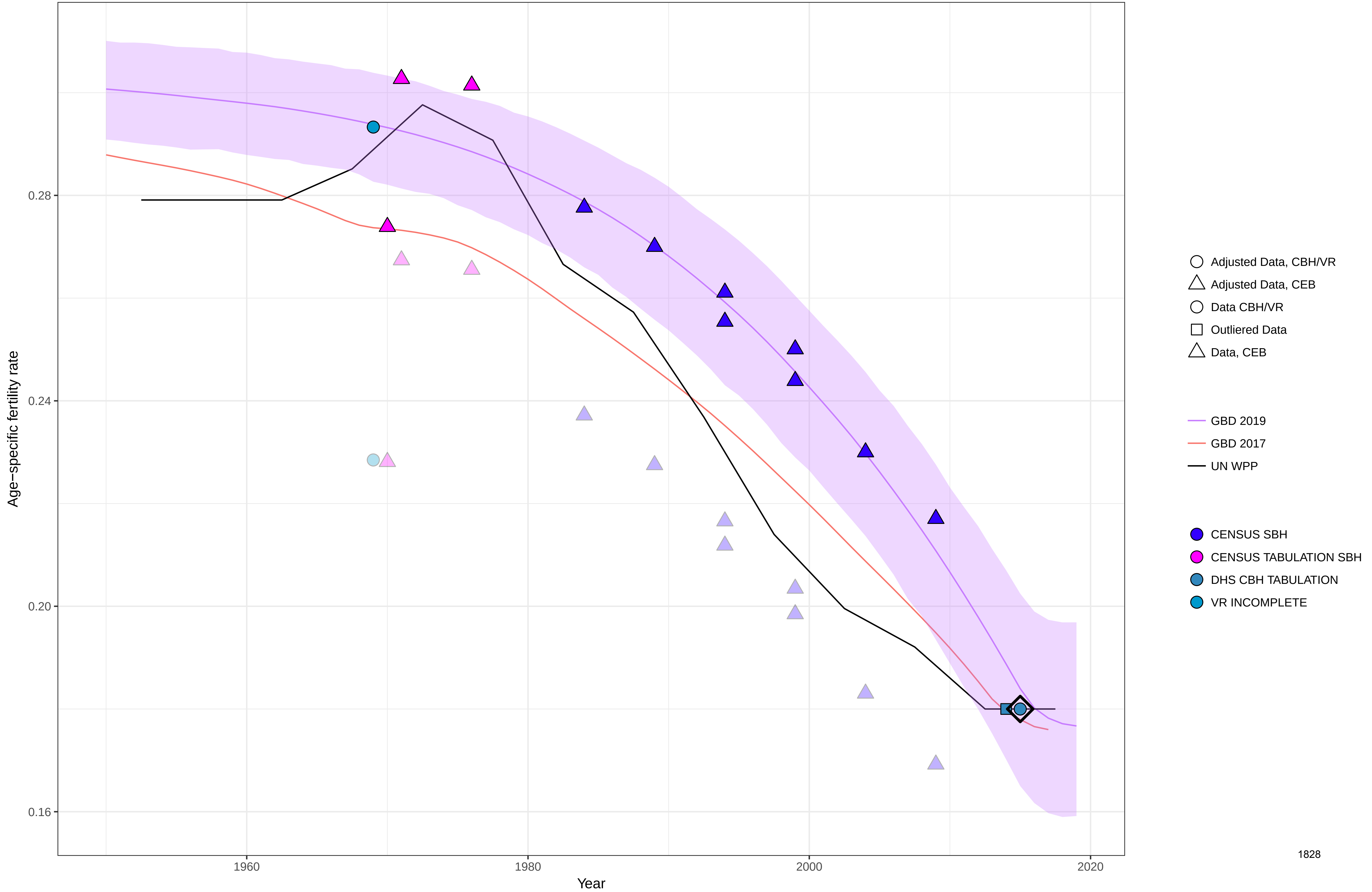

Age−specific fertility fitted trend lines, 1950−2019: Solomon Islands, Ages: 35−39
Data points enclosed with a diamond are reference sources

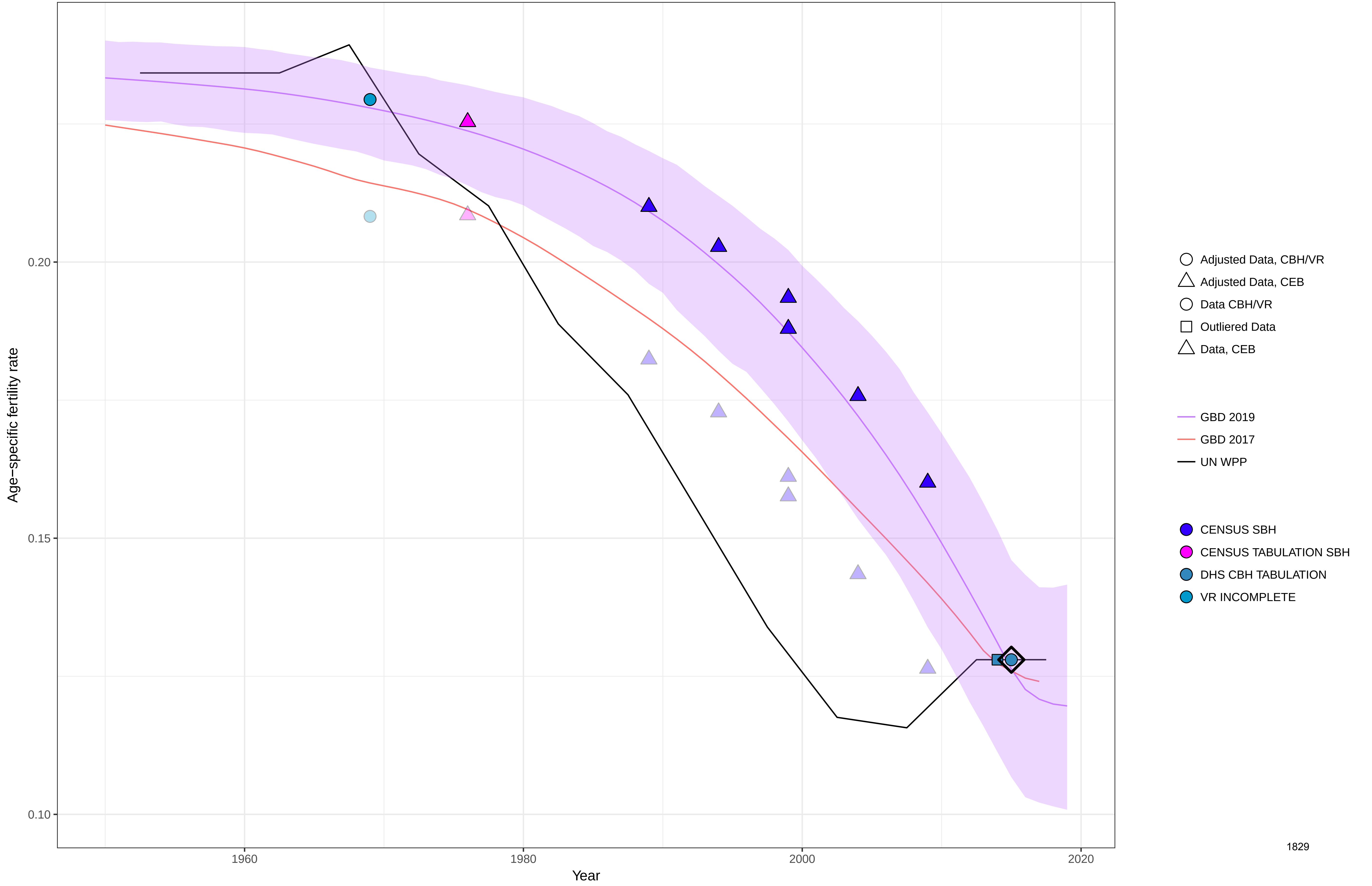

Age−specific fertility fitted trend lines, 1950−2019: Solomon Islands, Ages: 40−44
Data points enclosed with a diamond are reference sources

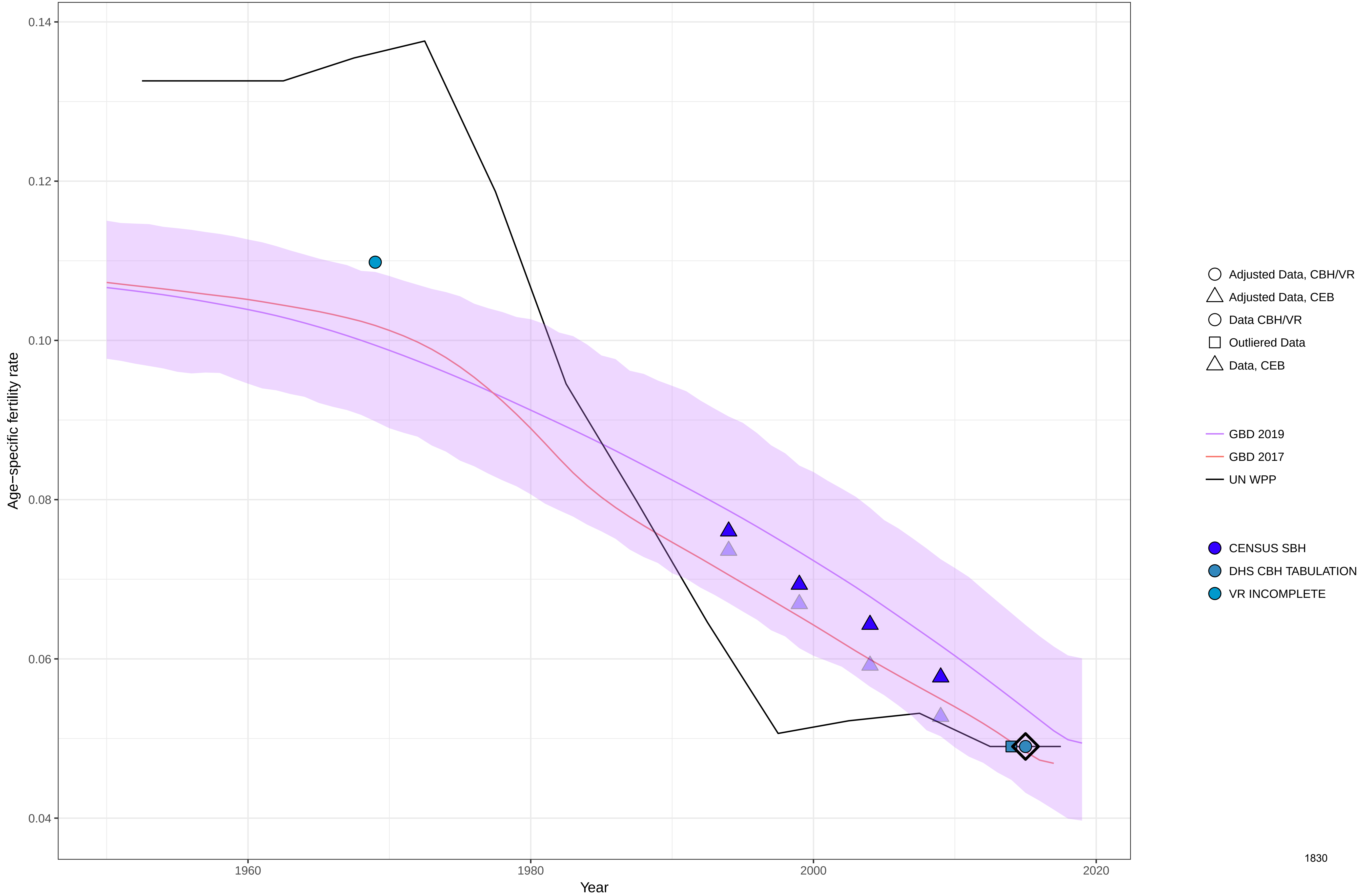

Age−specific fertility fitted trend lines, 1950−2019: Solomon Islands, Ages: 45−49
Data points enclosed with a diamond are reference sources

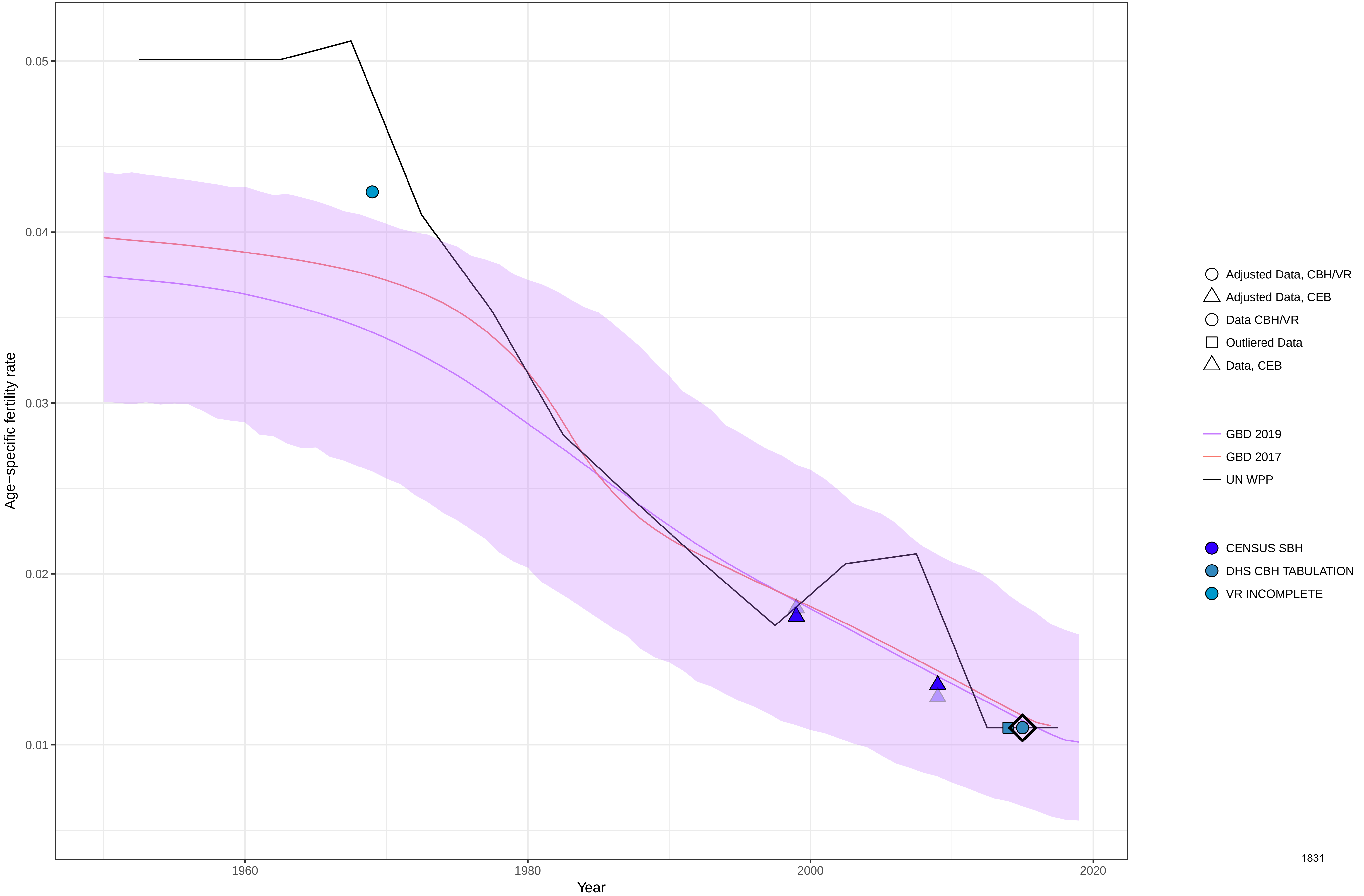

Age−specific fertility fitted trend lines, 1950−2019: Solomon Islands, Ages: 50−54
Data points enclosed with a diamond are reference sources

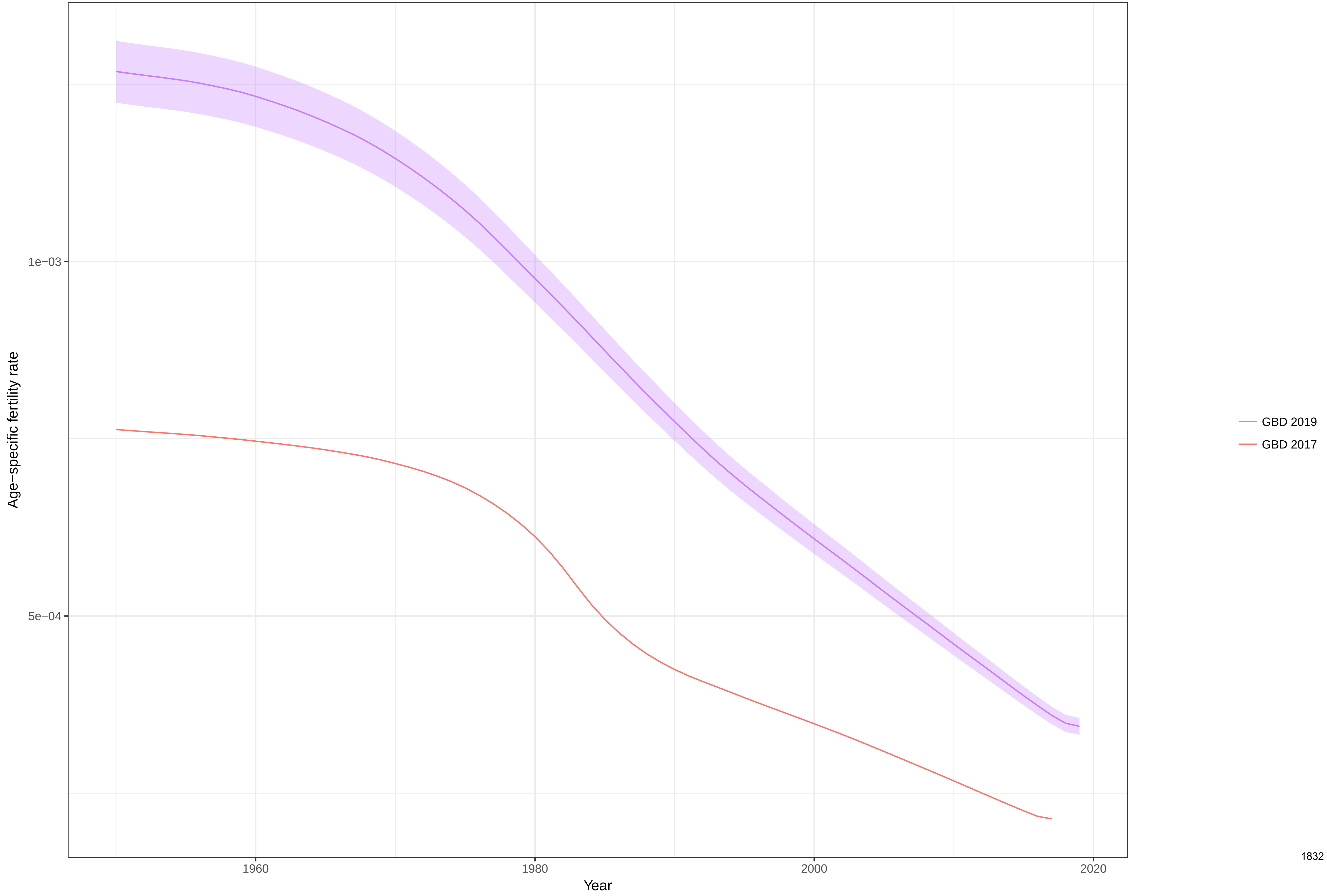

Total fertility rate fitted trend lines, 1950−2019: Solomon Islands TFR
Data points enclosed with a diamond are reference sources

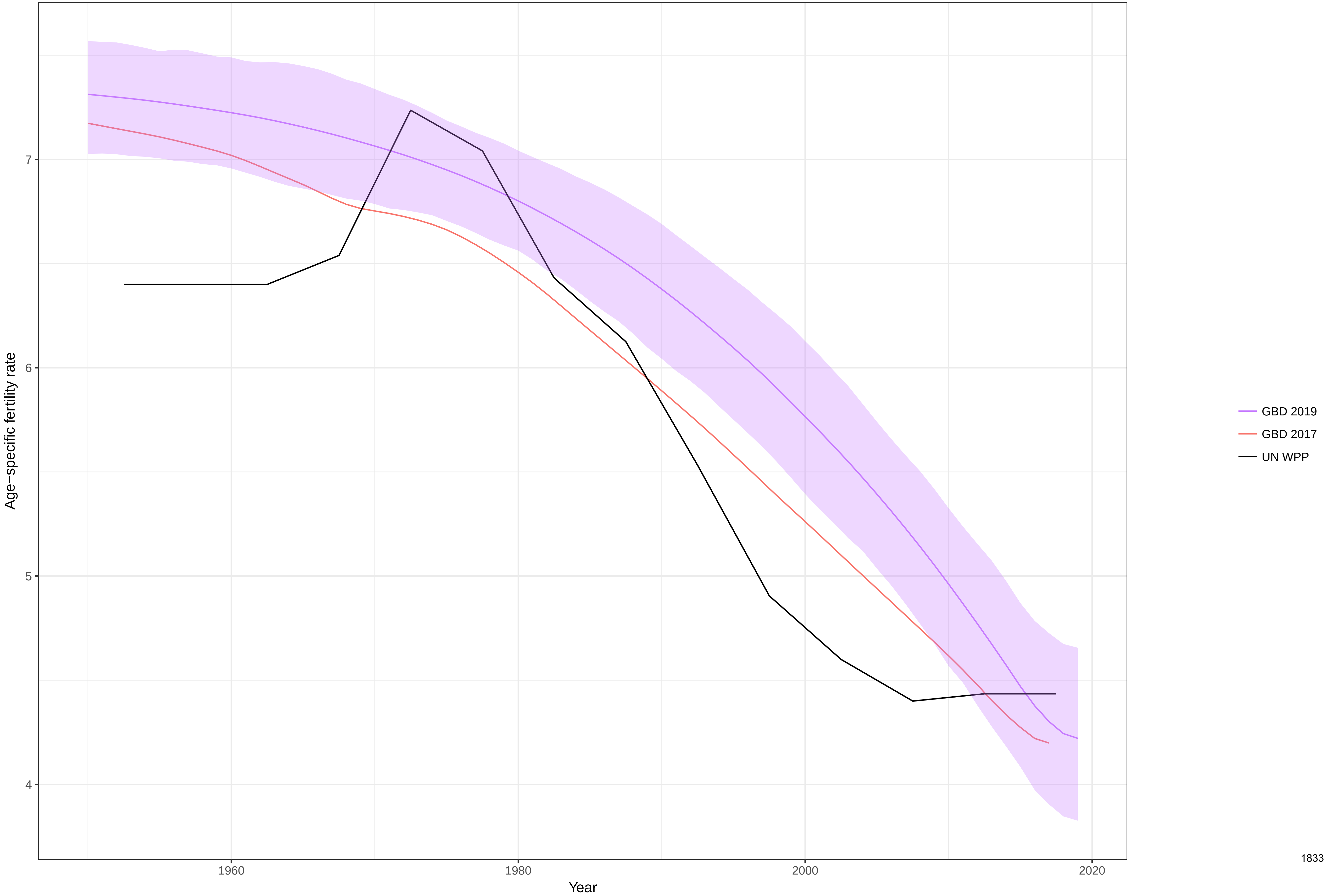

Age−specific fertility fitted trend lines, 1950−2019: Sierra Leone, Ages: 10−14
Data points enclosed with a diamond are reference sources

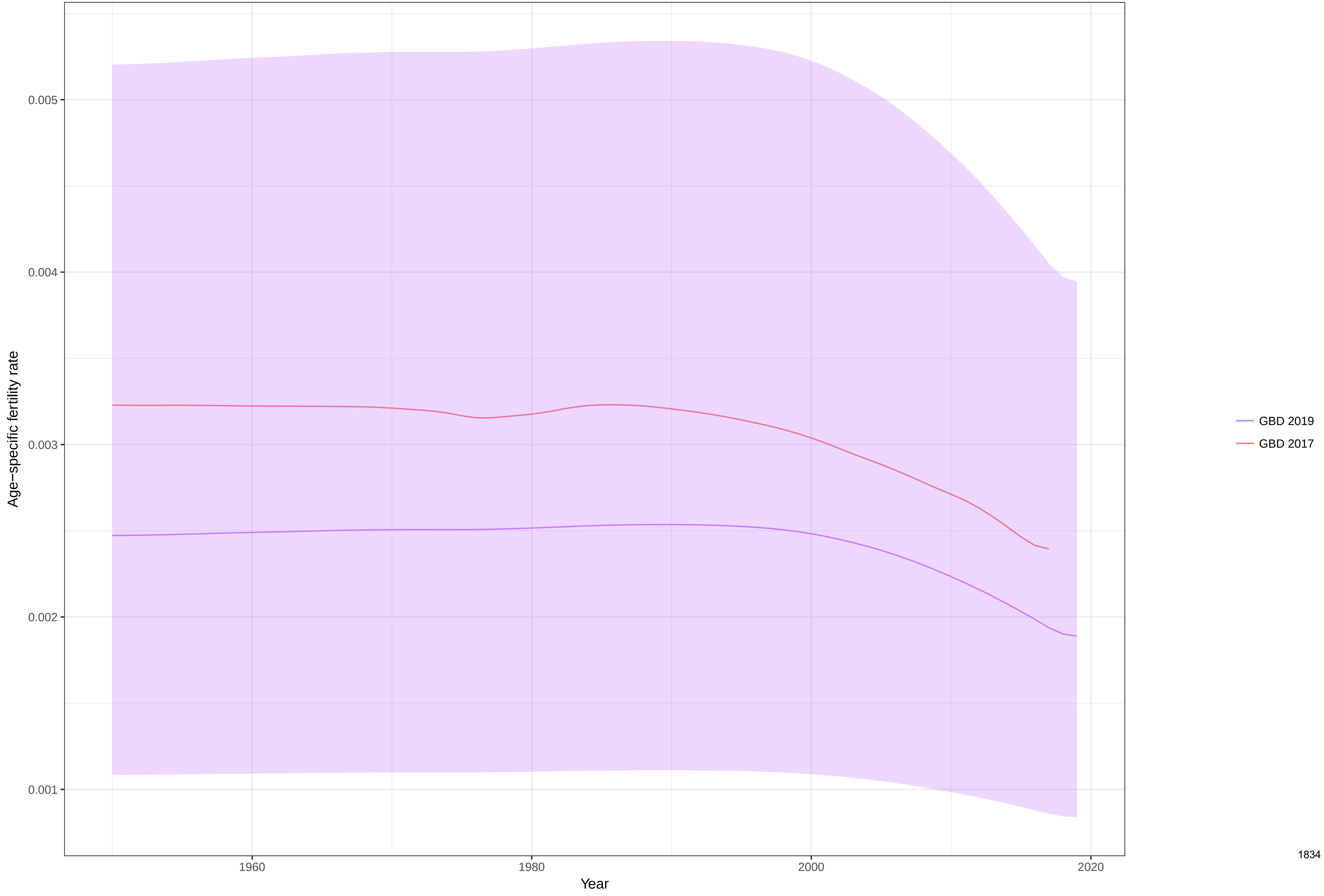

Age−specific fertility fitted trend lines, 1950−2019: Sierra Leone, Ages: 15−19
Data points enclosed with a diamond are reference sources

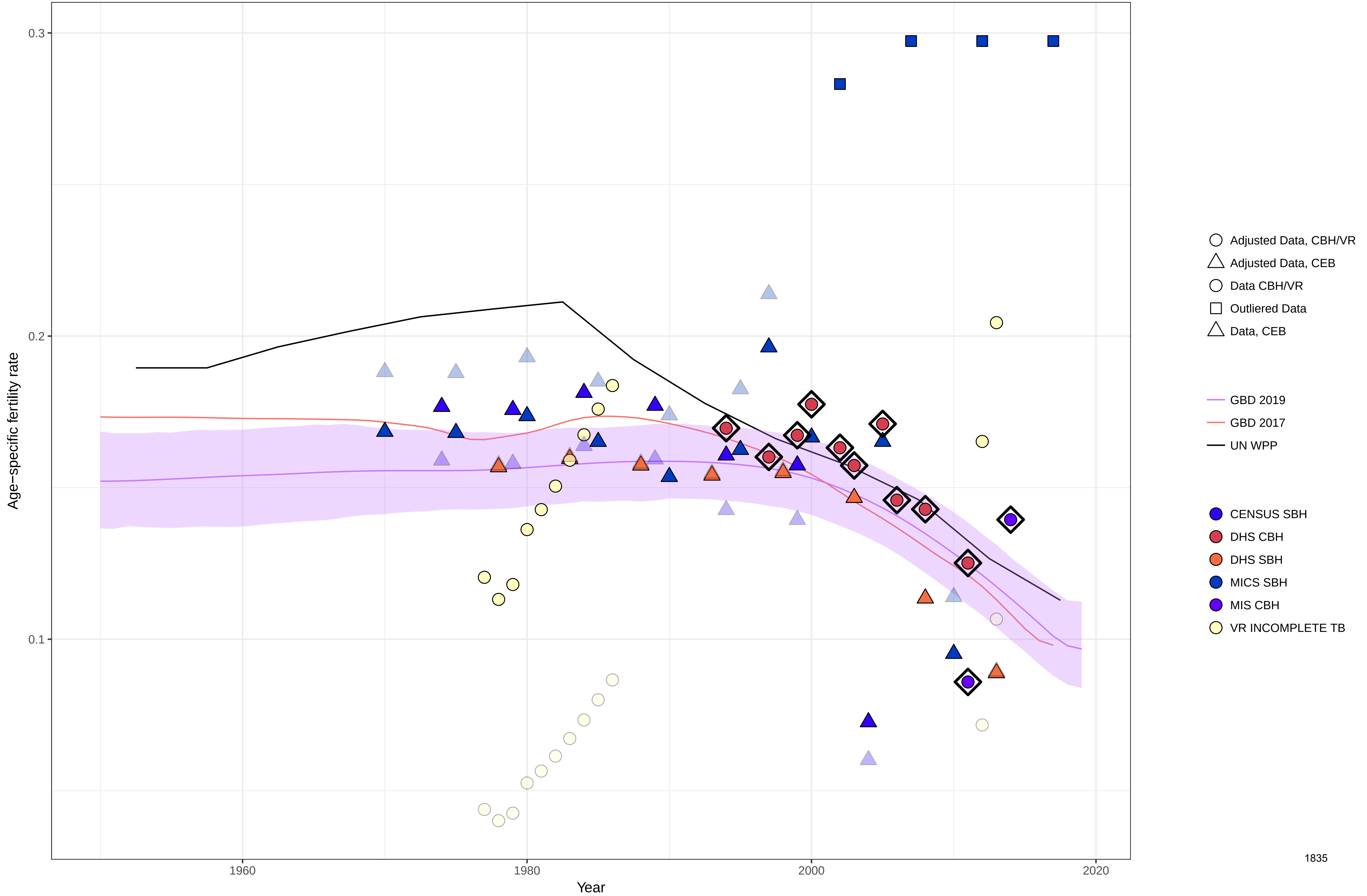

Age−specific fertility fitted trend lines, 1950−2019: Sierra Leone, Ages: 20−24
Data points enclosed with a diamond are reference sources

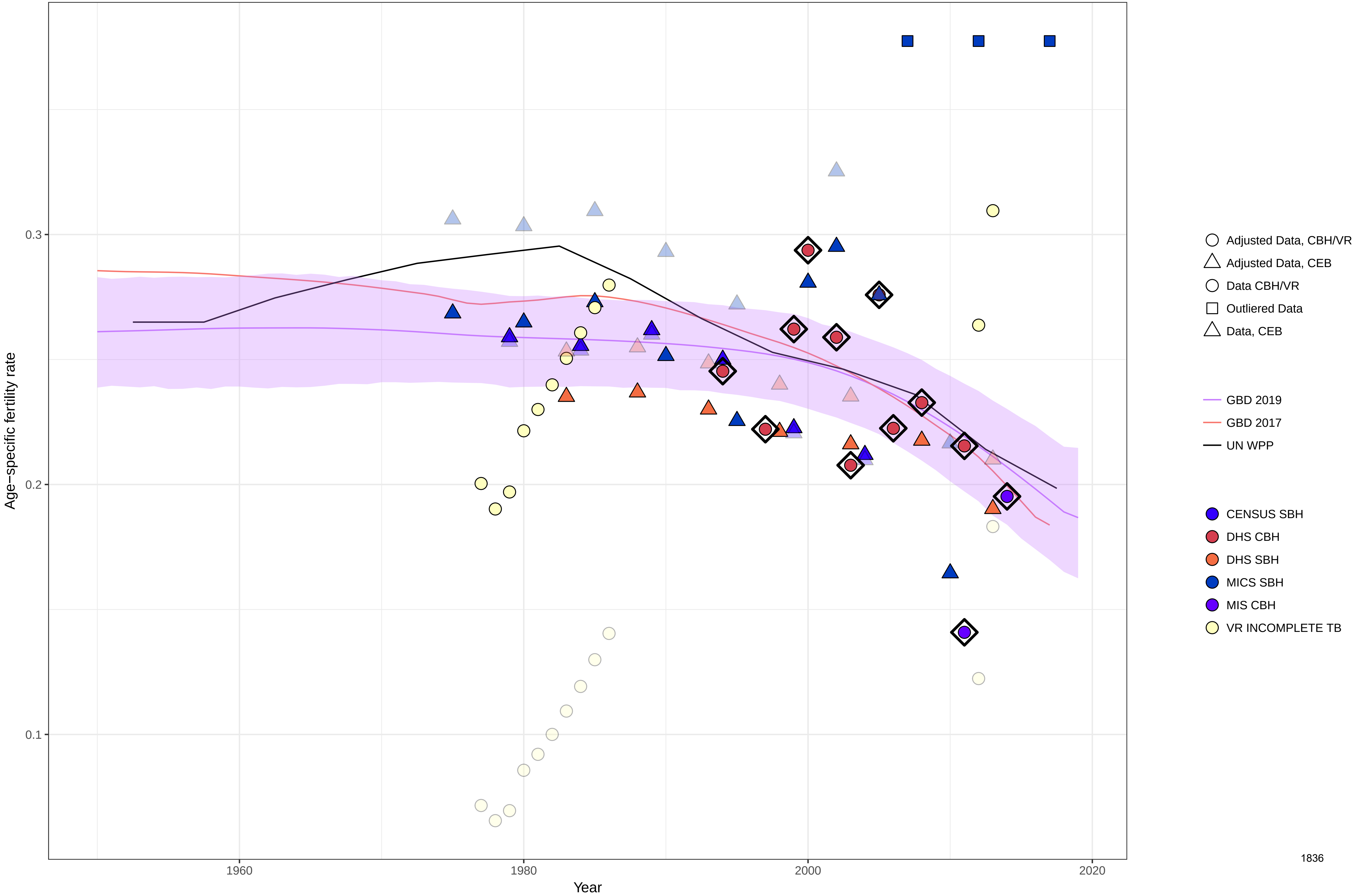

Age−specific fertility fitted trend lines, 1950−2019: Sierra Leone, Ages: 25−29
Data points enclosed with a diamond are reference sources

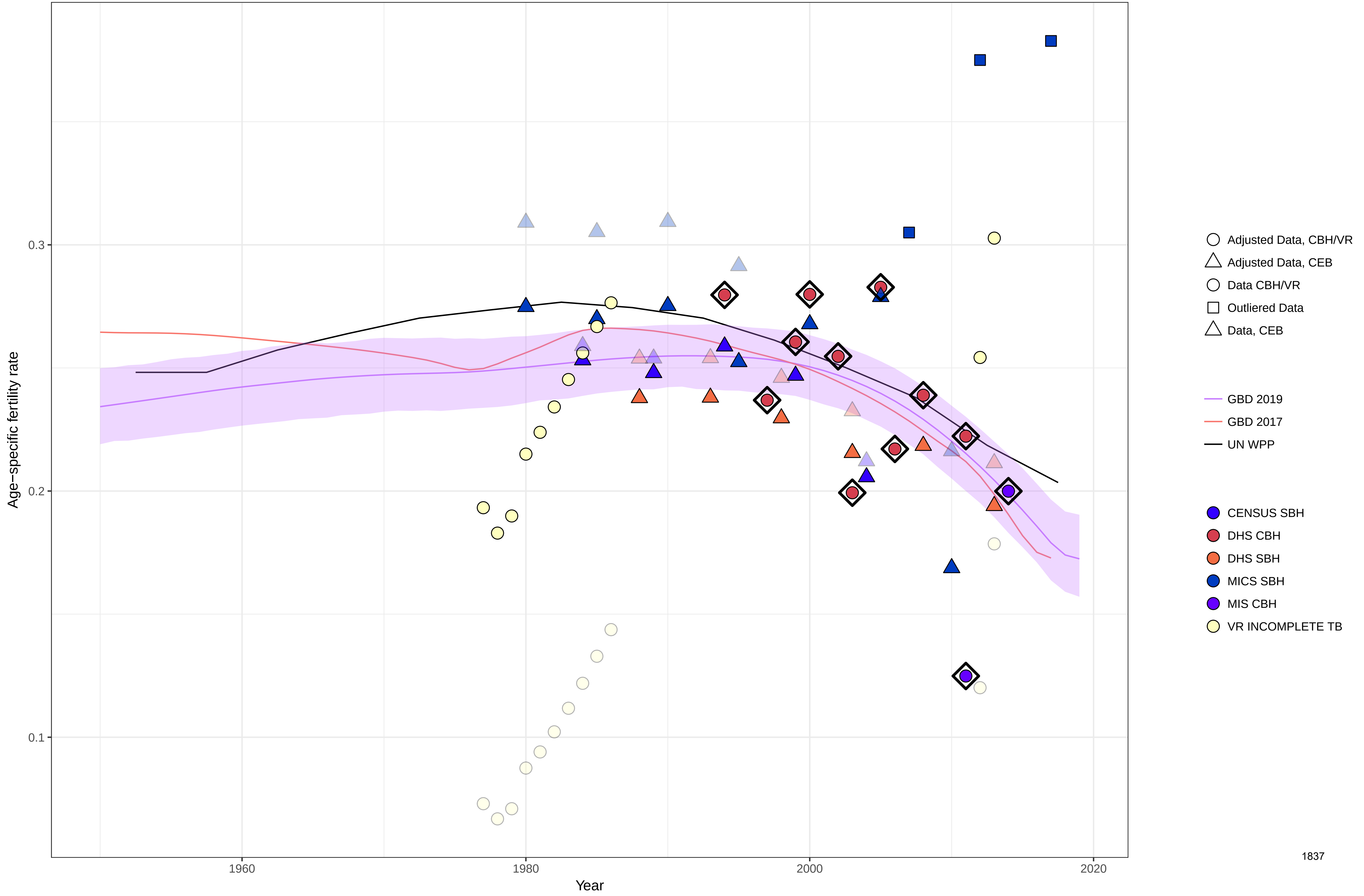

Age−specific fertility fitted trend lines, 1950−2019: Sierra Leone, Ages: 30−34
Data points enclosed with a diamond are reference sources

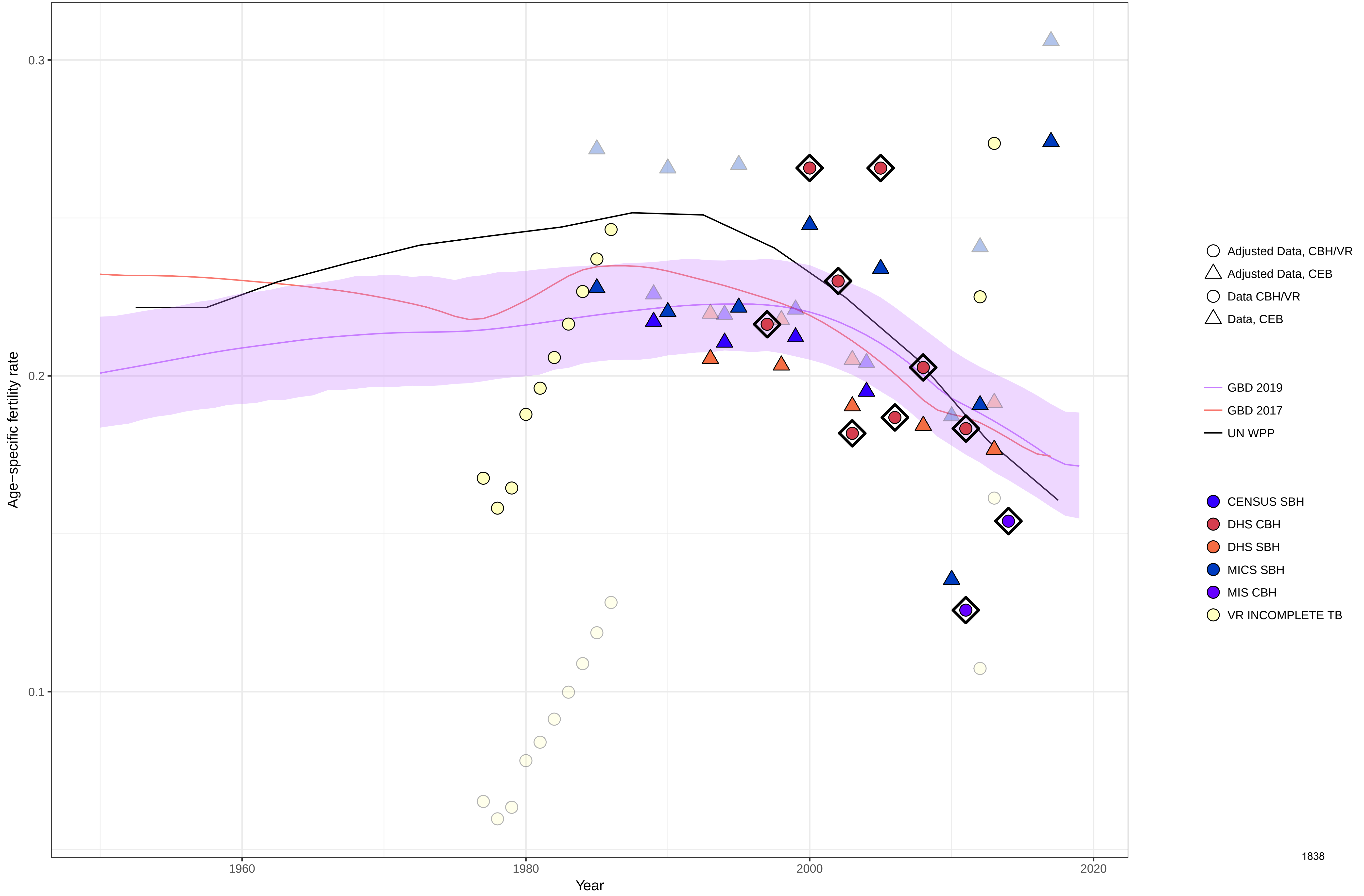

Age−specific fertility fitted trend lines, 1950−2019: Sierra Leone, Ages: 35−39
Data points enclosed with a diamond are reference sources

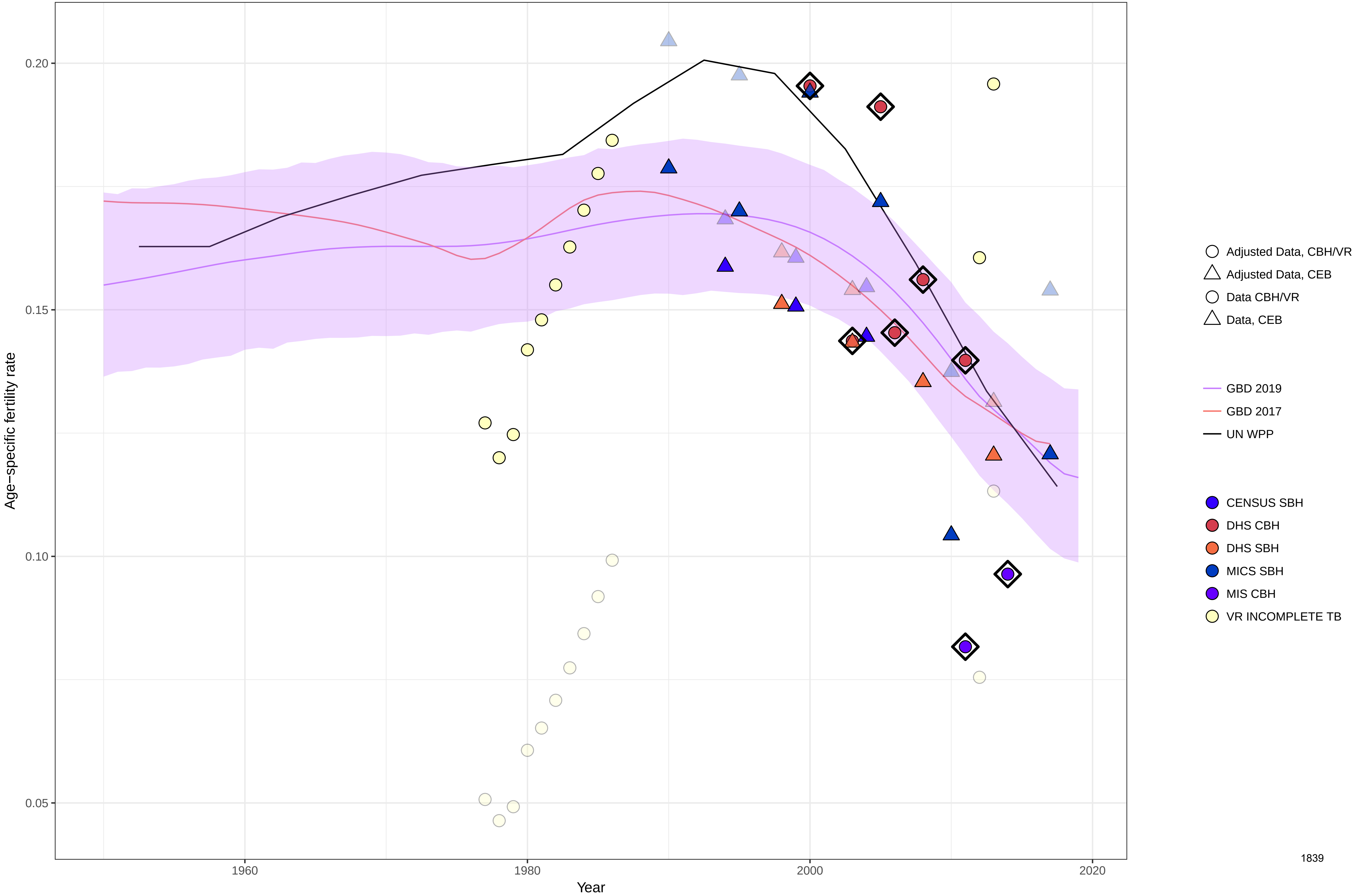

Age−specific fertility fitted trend lines, 1950−2019: Sierra Leone, Ages: 40−44
Data points enclosed with a diamond are reference sources

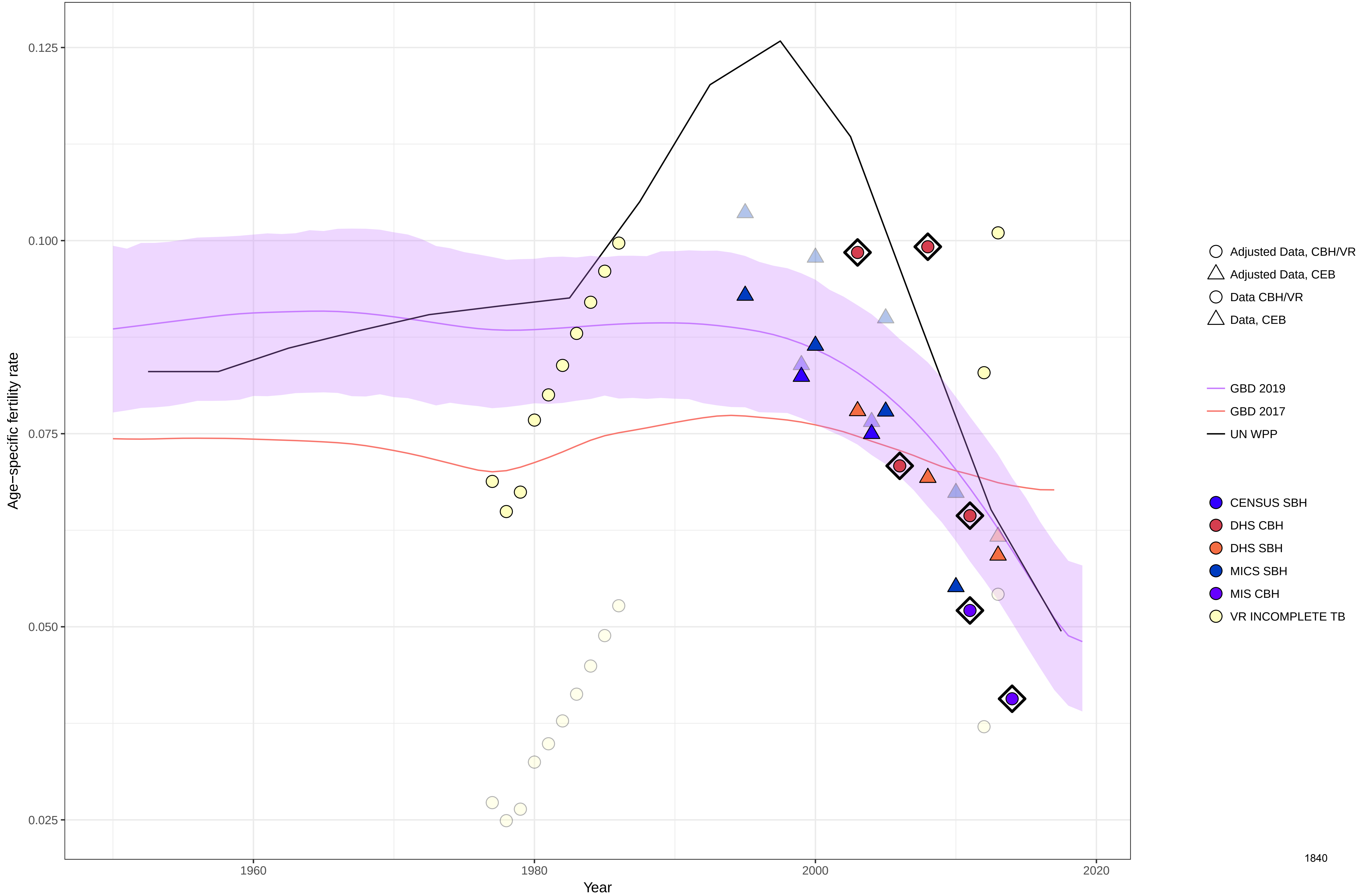

Age−specific fertility fitted trend lines, 1950−2019: Sierra Leone, Ages: 45−49
Data points enclosed with a diamond are reference sources

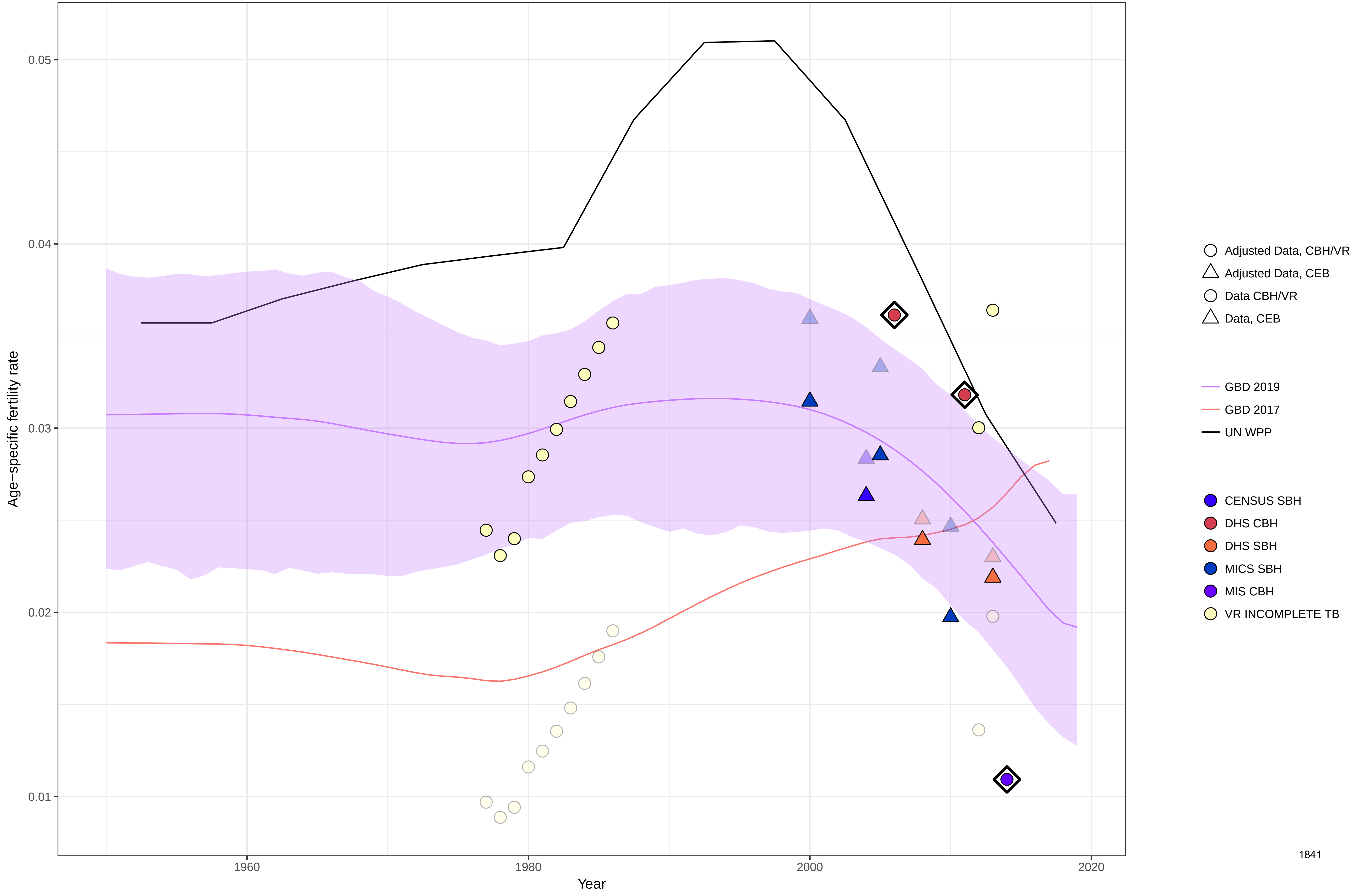

Age−specific fertility fitted trend lines, 1950−2019: Sierra Leone, Ages: 50−54
Data points enclosed with a diamond are reference sources

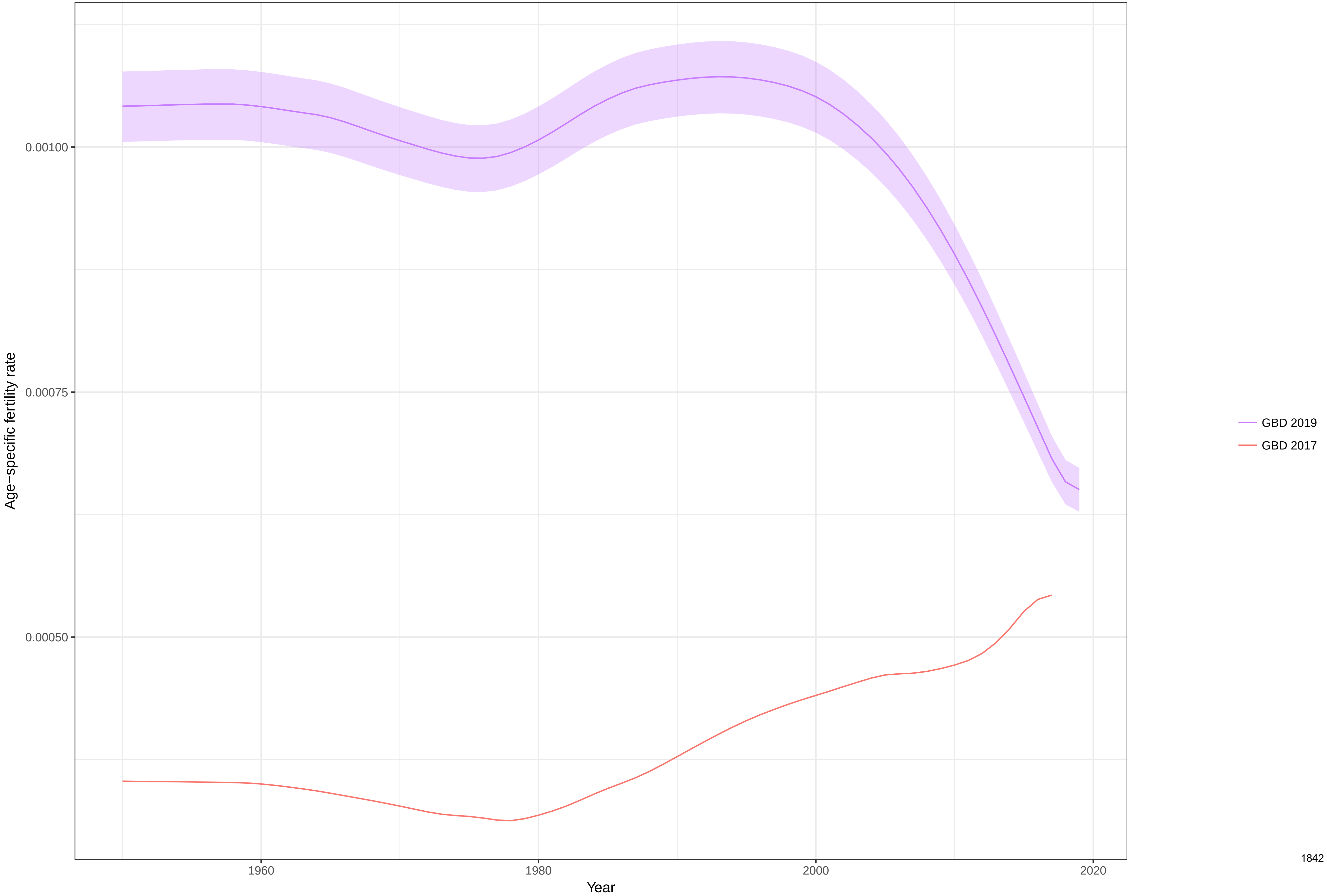

Total fertility rate fitted trend lines, 1950−2019: Sierra Leone TFR
Data points enclosed with a diamond are reference sources

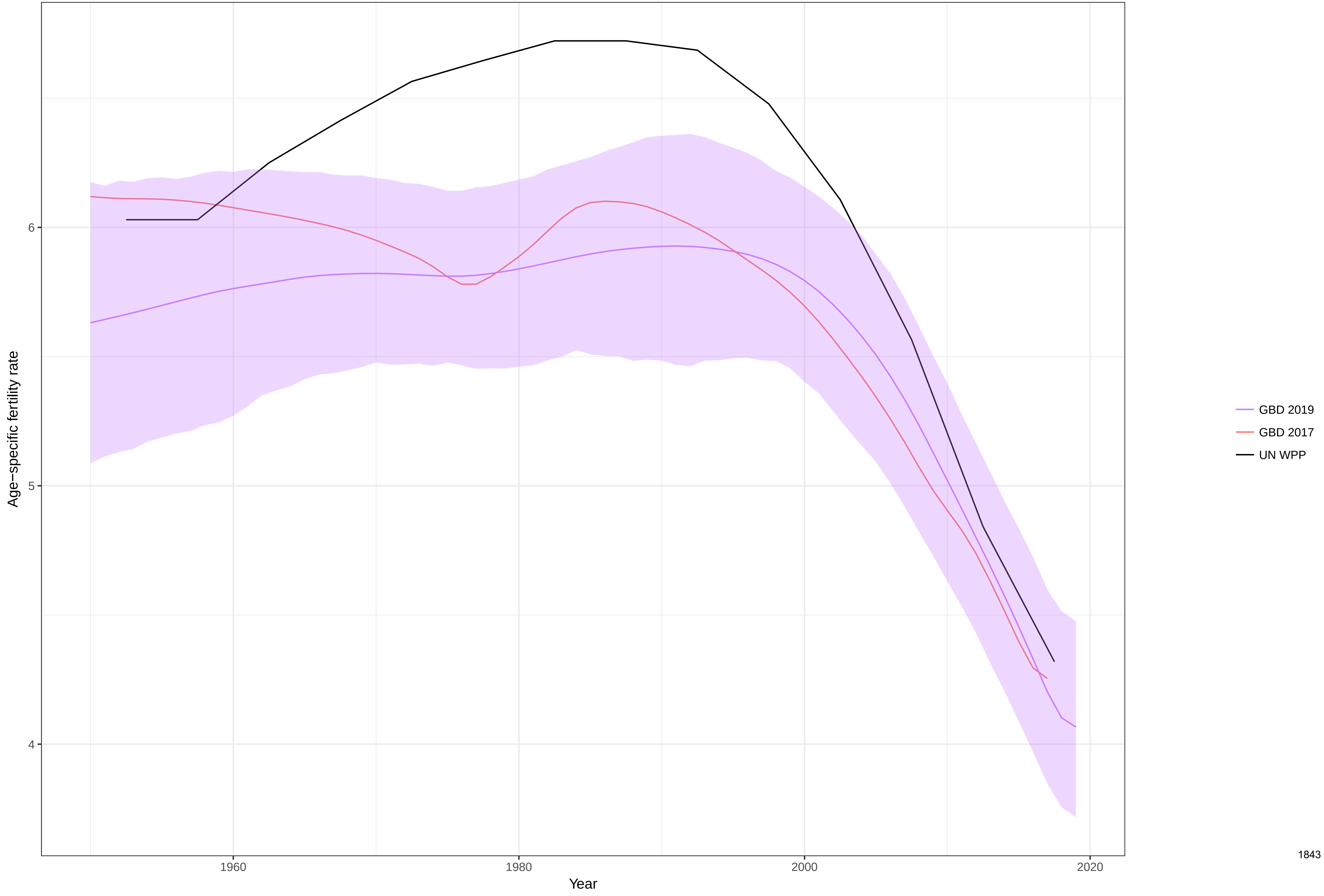

Age−specific fertility fitted trend lines, 1950−2019: El Salvador, Ages: 10−14
Data points enclosed with a diamond are reference sources

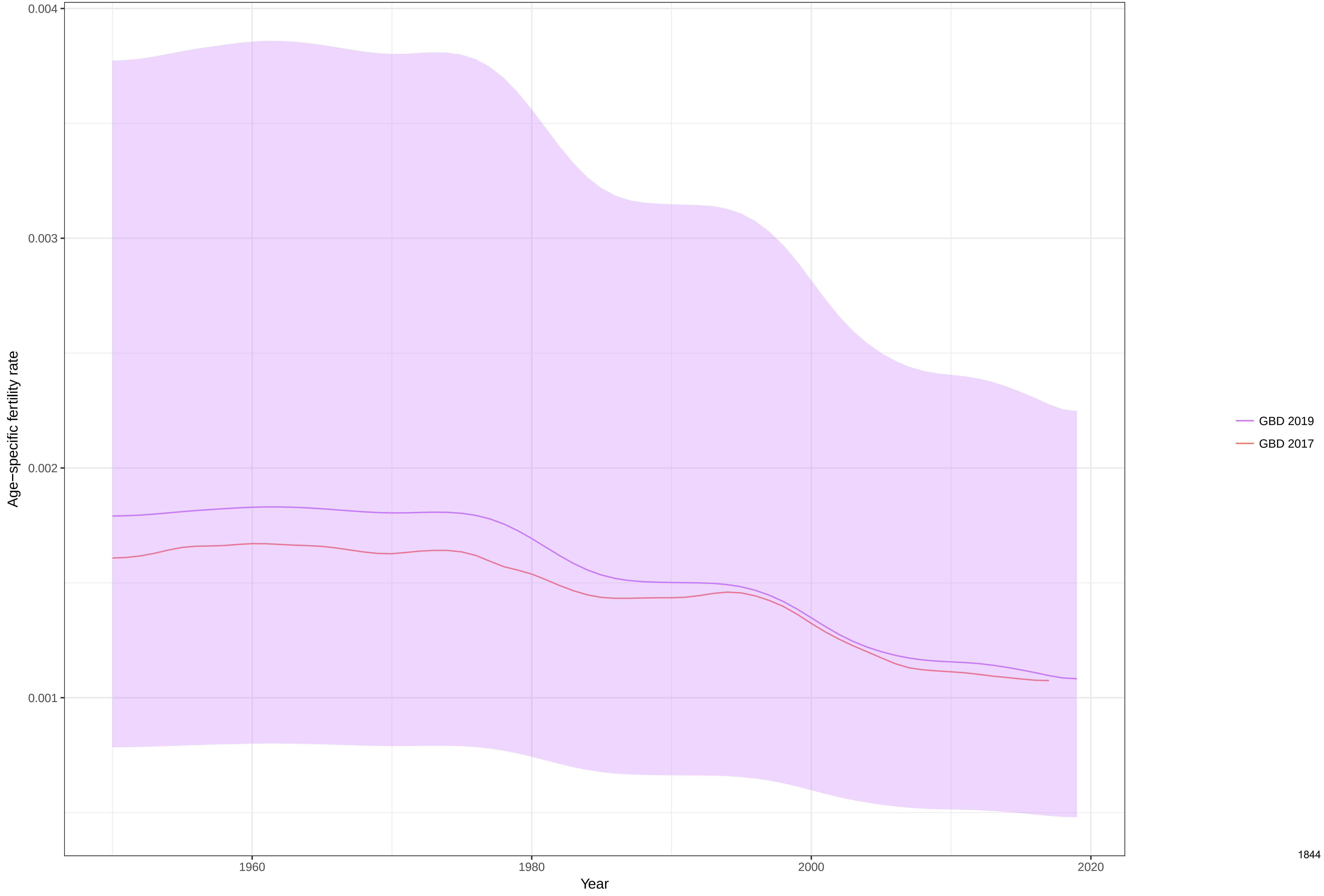

Age−specific fertility fitted trend lines, 1950−2019: El Salvador, Ages: 15−19
Data points enclosed with a diamond are reference sources

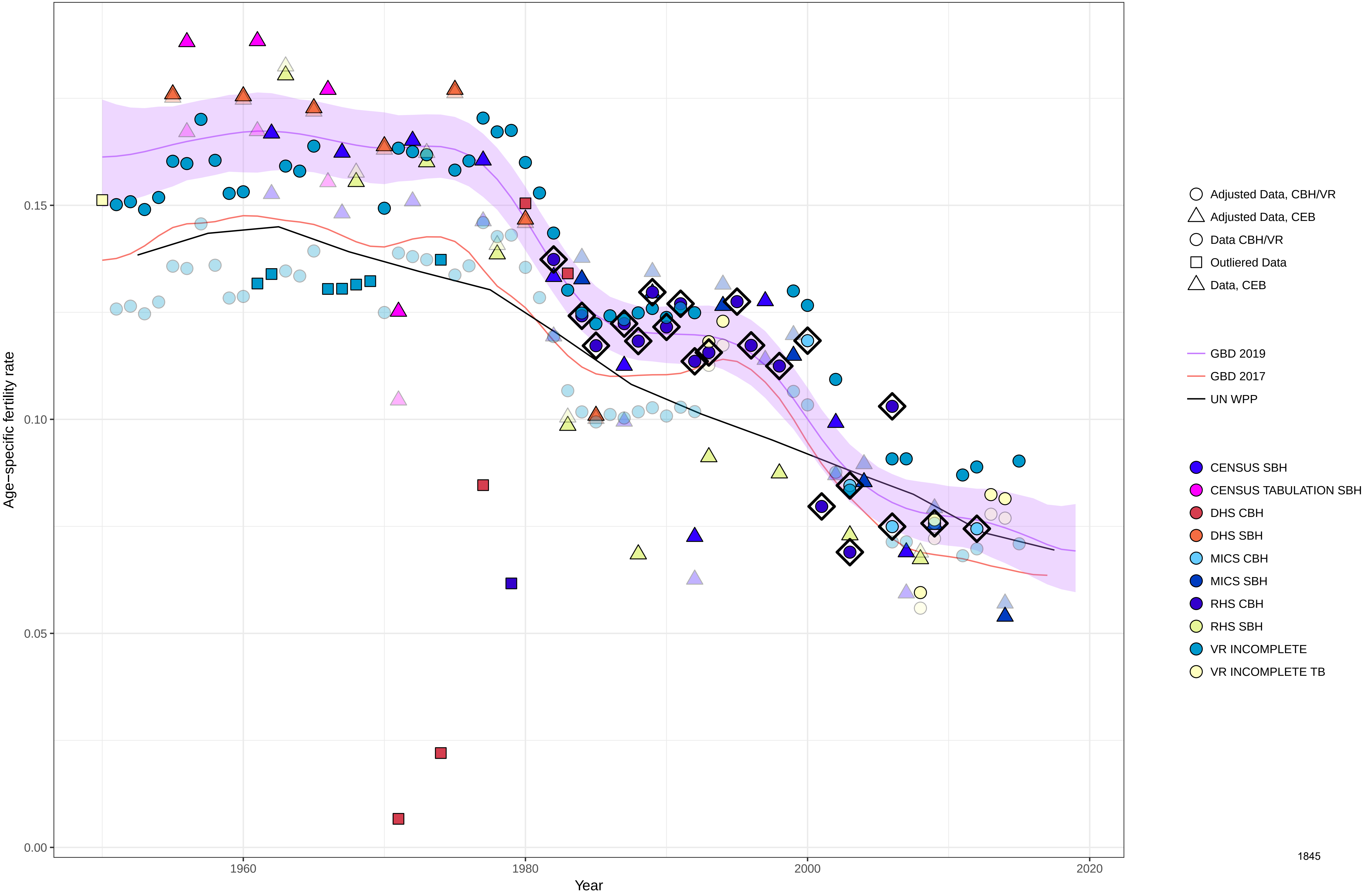

Age−specific fertility fitted trend lines, 1950−2019: El Salvador, Ages: 20−24
Data points enclosed with a diamond are reference sources

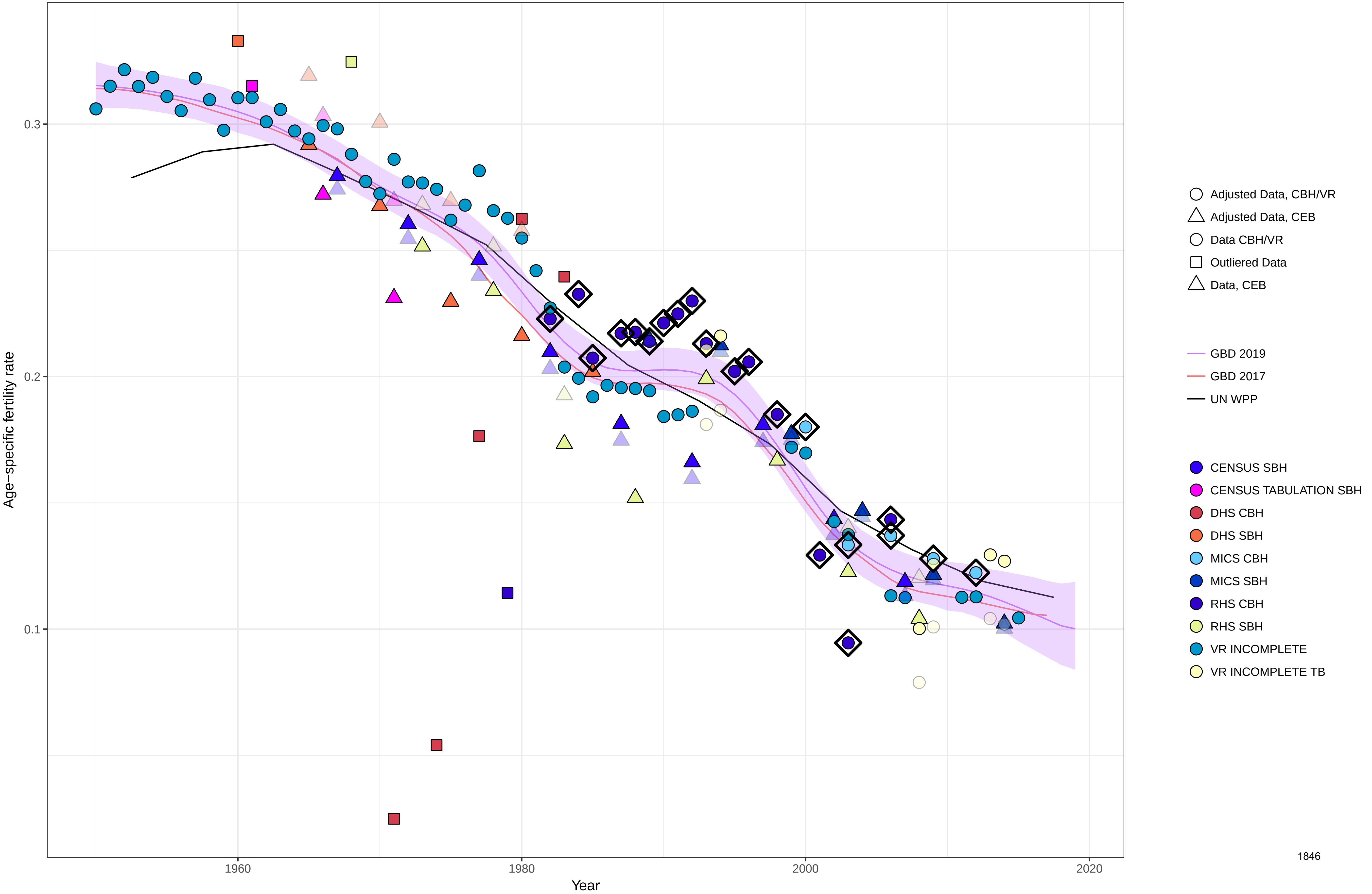

Age−specific fertility fitted trend lines, 1950−2019: El Salvador, Ages: 25−29
Data points enclosed with a diamond are reference sources

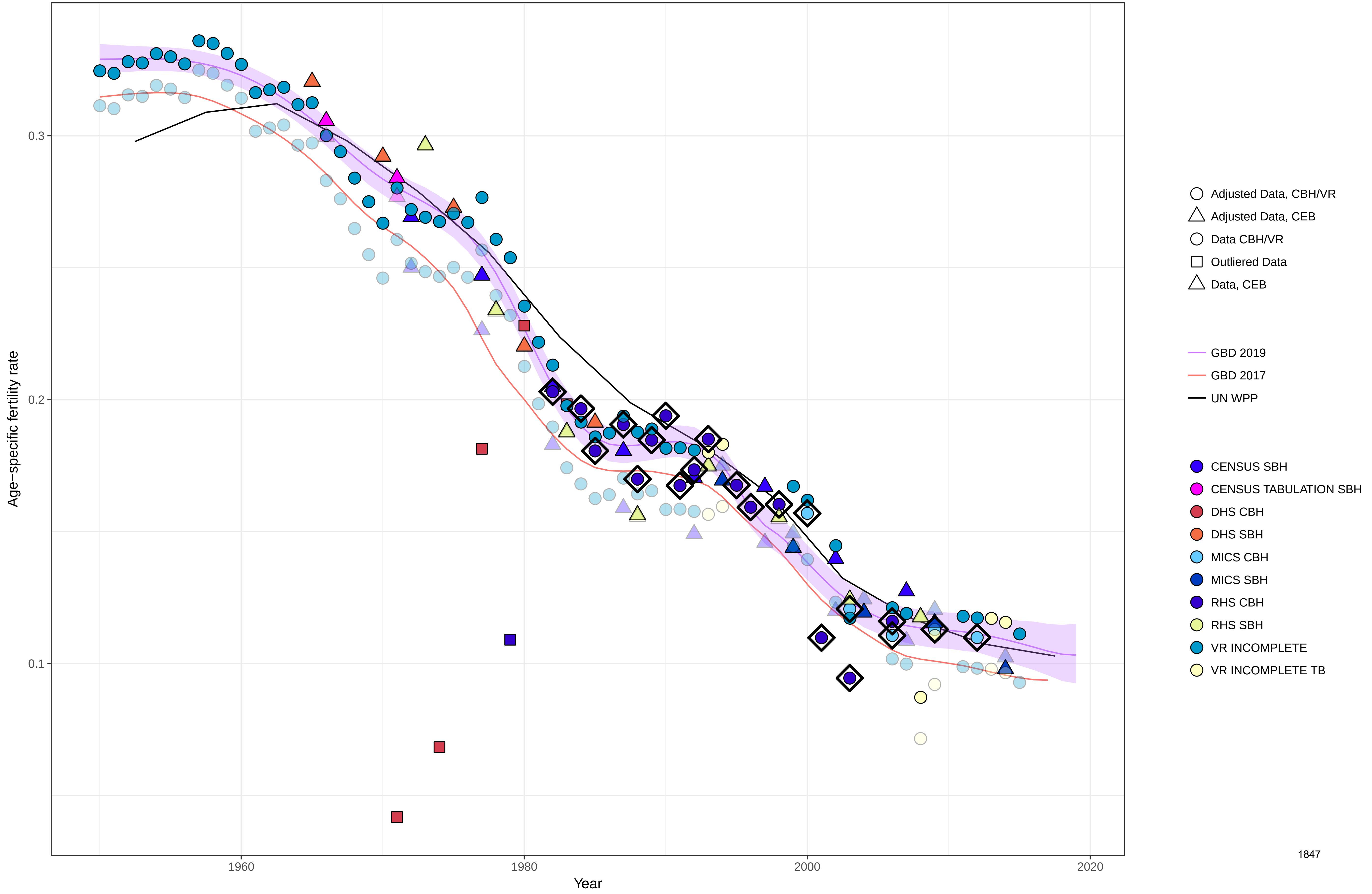

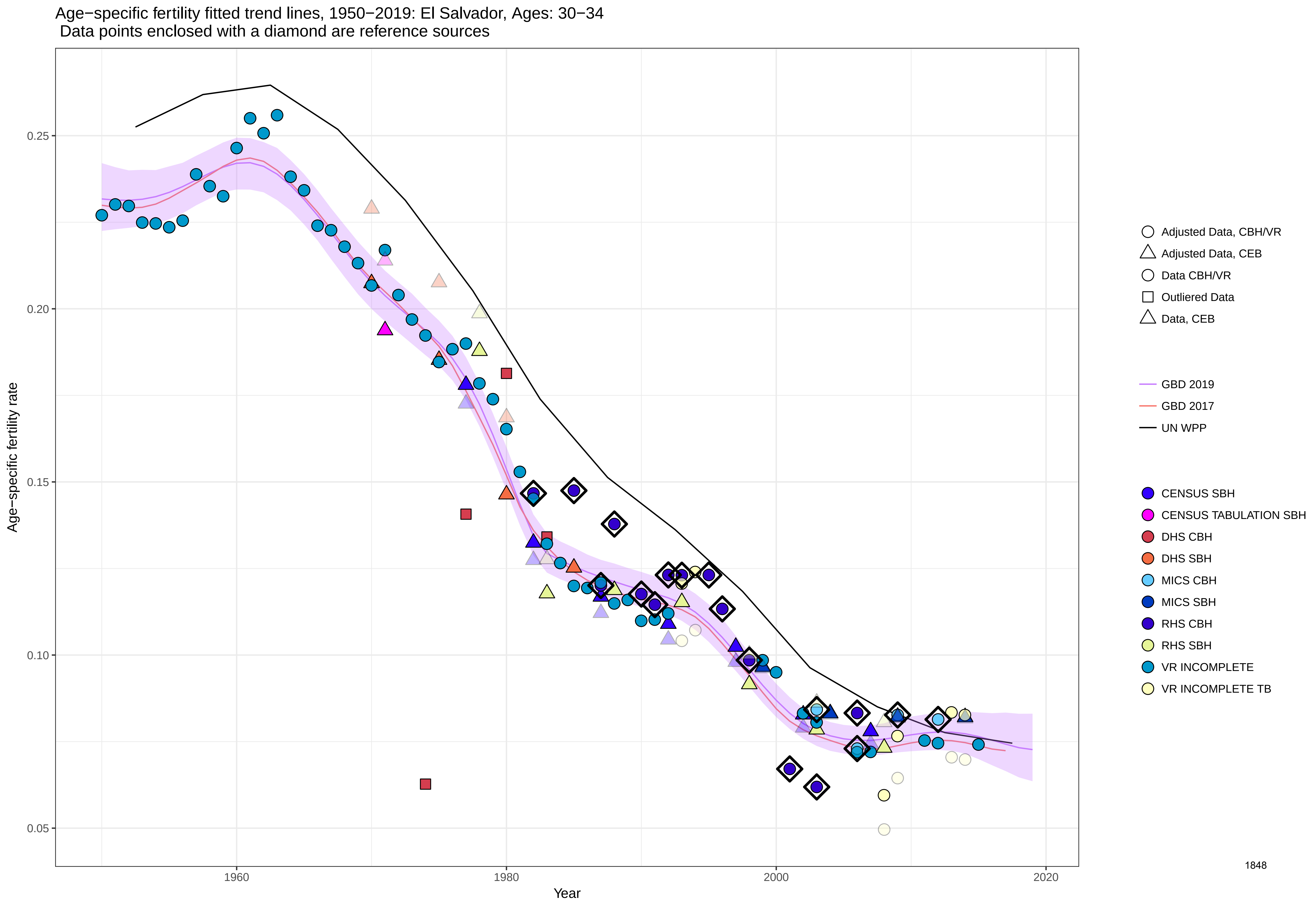
Age−specific fertility fitted trend lines, 1950−2019: El Salvador, Ages: 30−34
Data points enclosed with a diamond are reference sources
Age−specific fertility rate
Year
0.25
0.20
0.15
0.10
0.05
1960
1980
2000
2020
Adjusted Data, CBH/VR
Adjusted Data, CEB
Data CBH/VR
Outliered Data
Data, CEB
GBD 2019
GBD 2017
UN WPP
CENSUS SBH
CENSUS TABULATION SBH
DHS CBH
DHS SBH
MICS CBH
MICS SBH
RHS CBH
RHS SBH
VR INCOMPLETE
VR INCOMPLETE TB

Age−specific fertility fitted trend lines, 1950−2019: El Salvador, Ages: 35−39
Data points enclosed with a diamond are reference sources

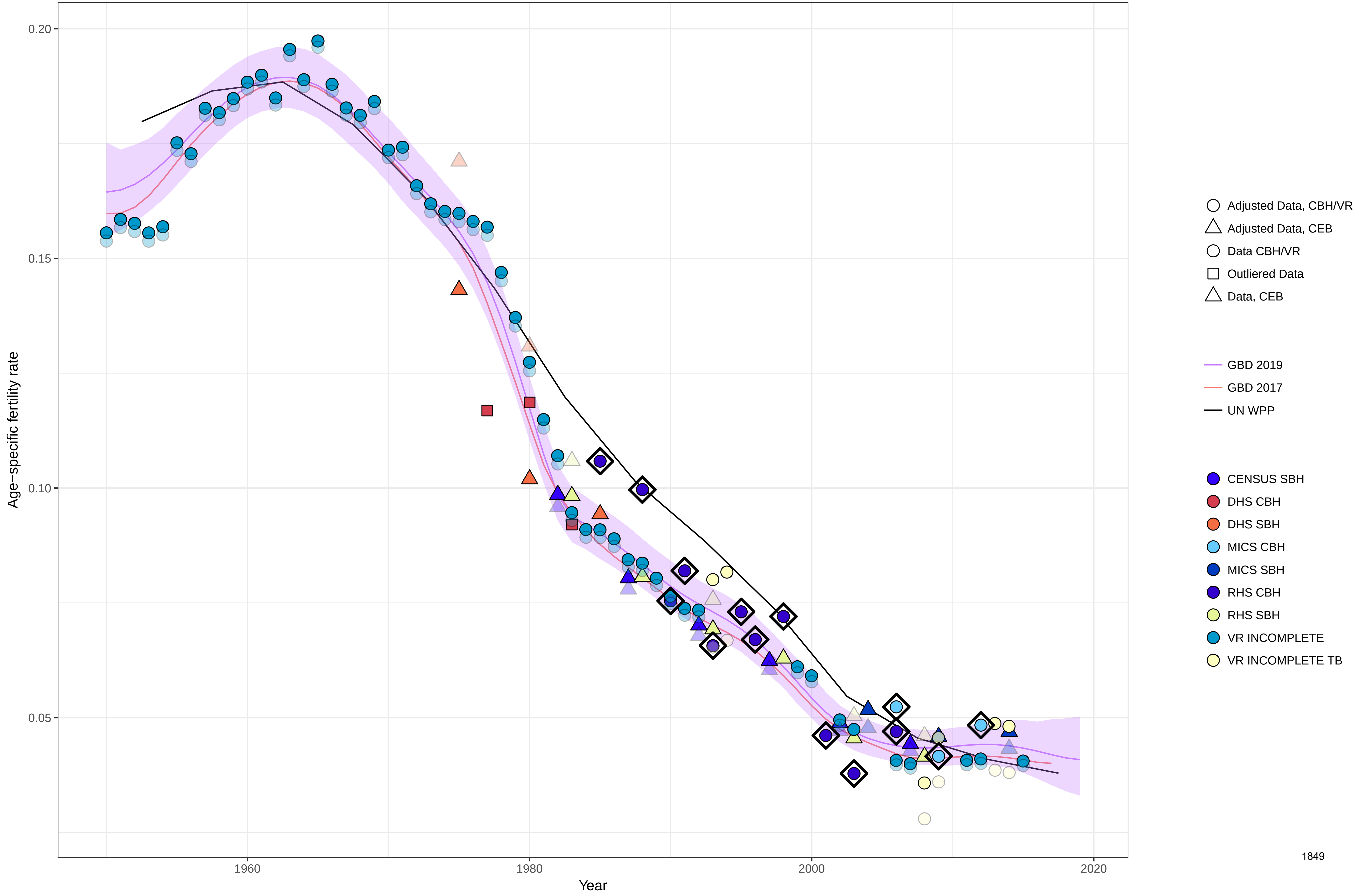

Age−specific fertility fitted trend lines, 1950−2019: El Salvador, Ages: 40−44
Data points enclosed with a diamond are reference sources

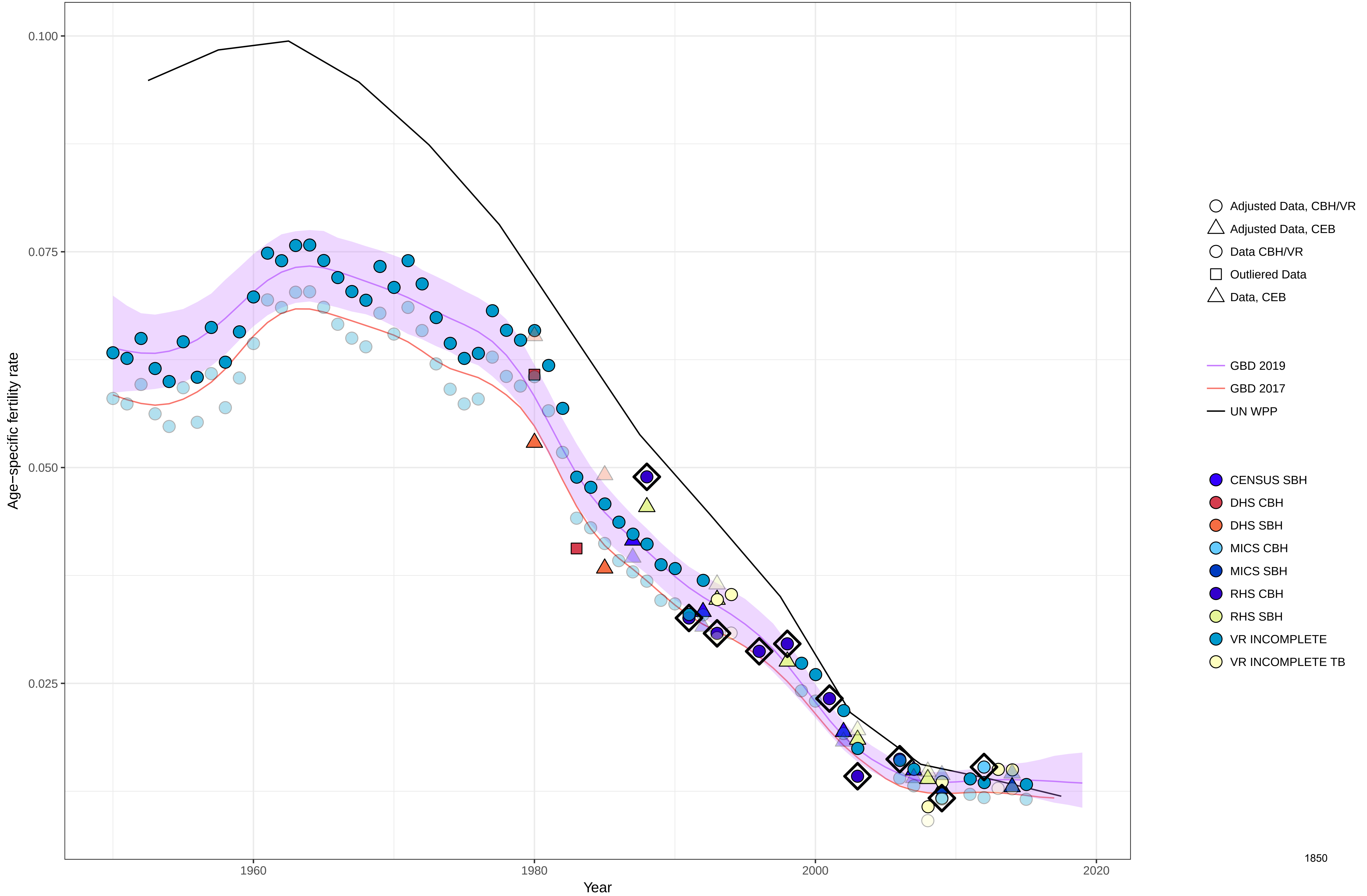

Age−specific fertility fitted trend lines, 1950−2019: El Salvador, Ages: 45−49
Data points enclosed with a diamond are reference sources

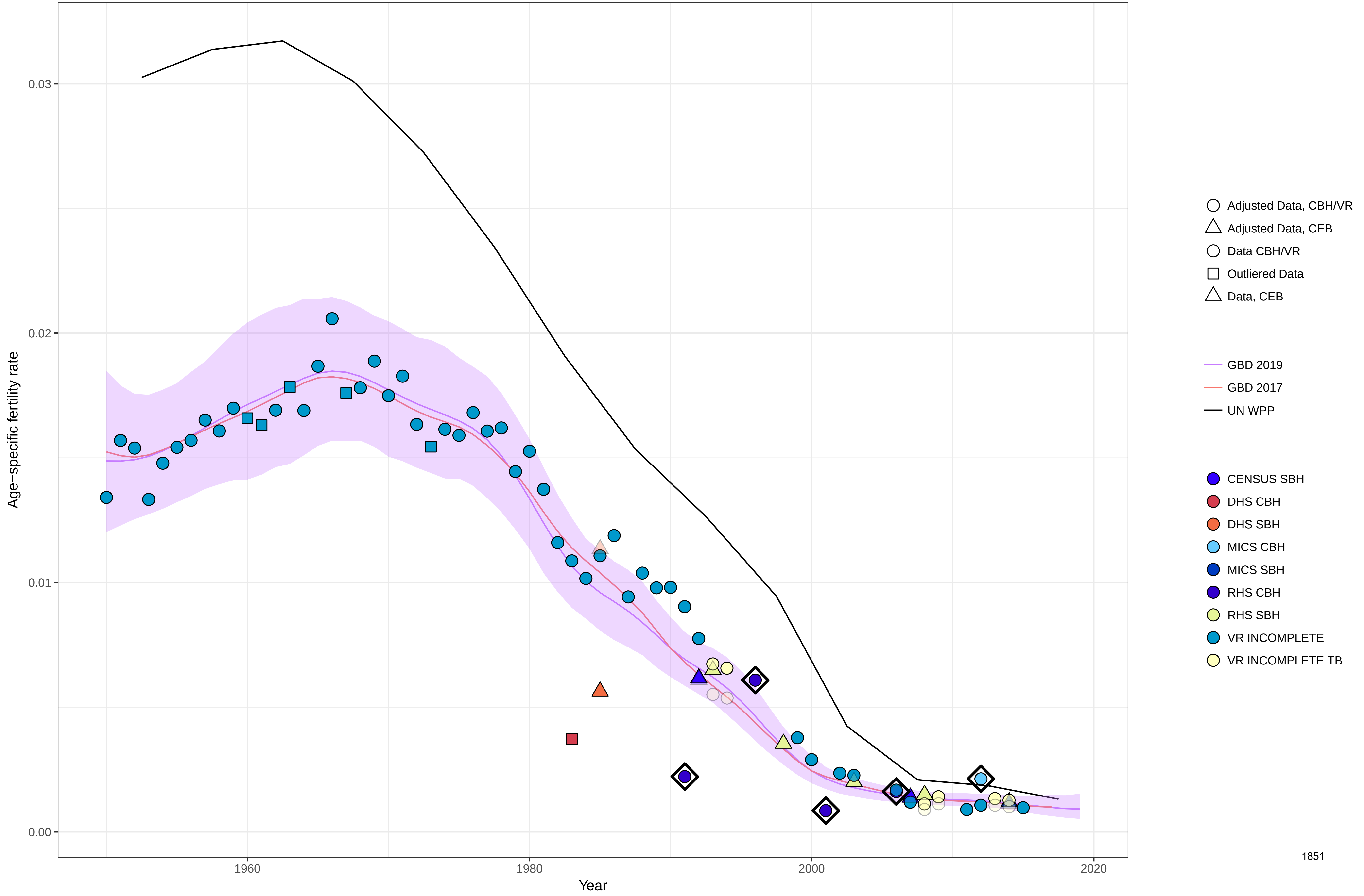

Age−specific fertility fitted trend lines, 1950−2019: El Salvador, Ages: 50−54
Data points enclosed with a diamond are reference sources

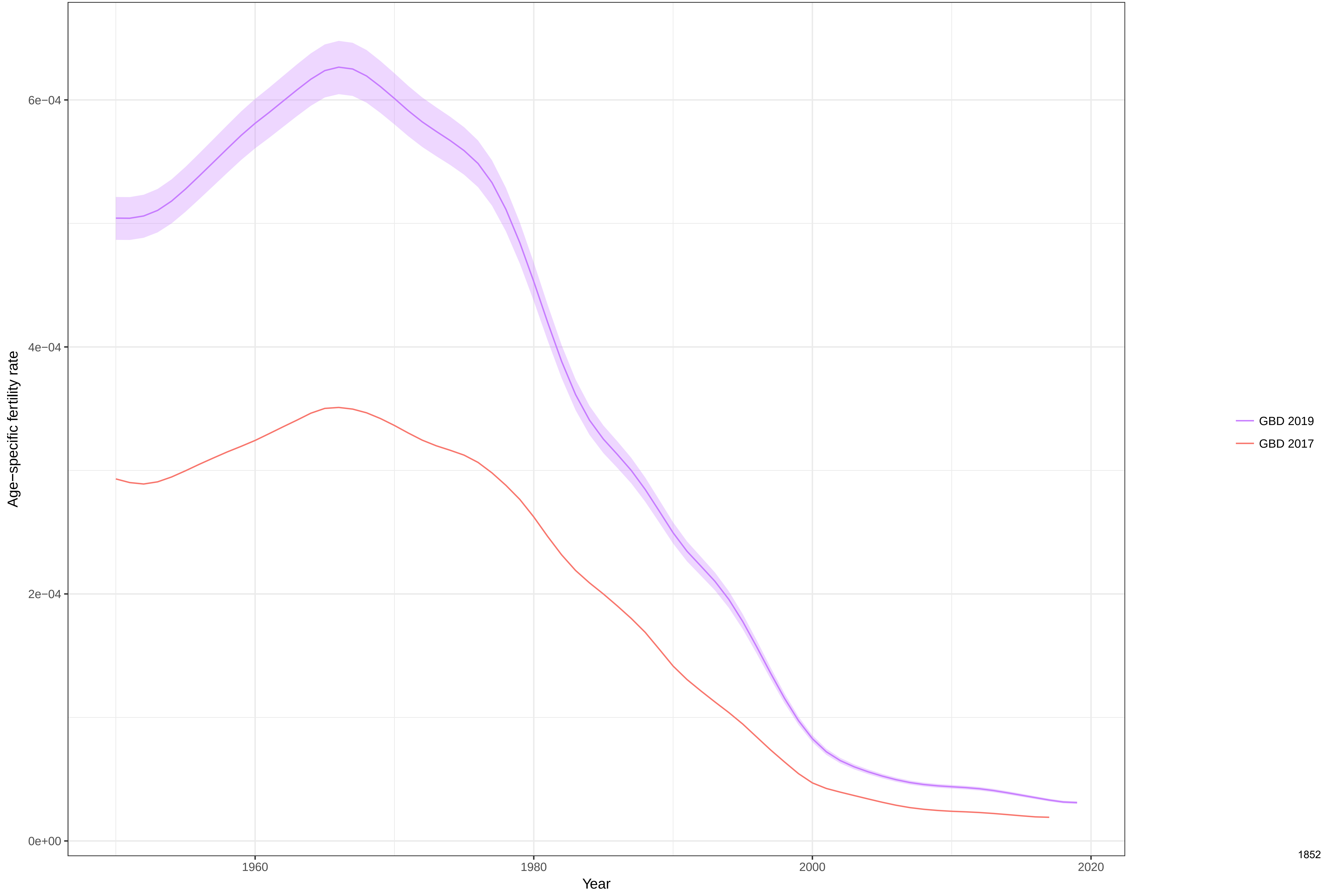

Total fertility rate fitted trend lines, 1950−2019: El Salvador TFR
Data points enclosed with a diamond are reference sources

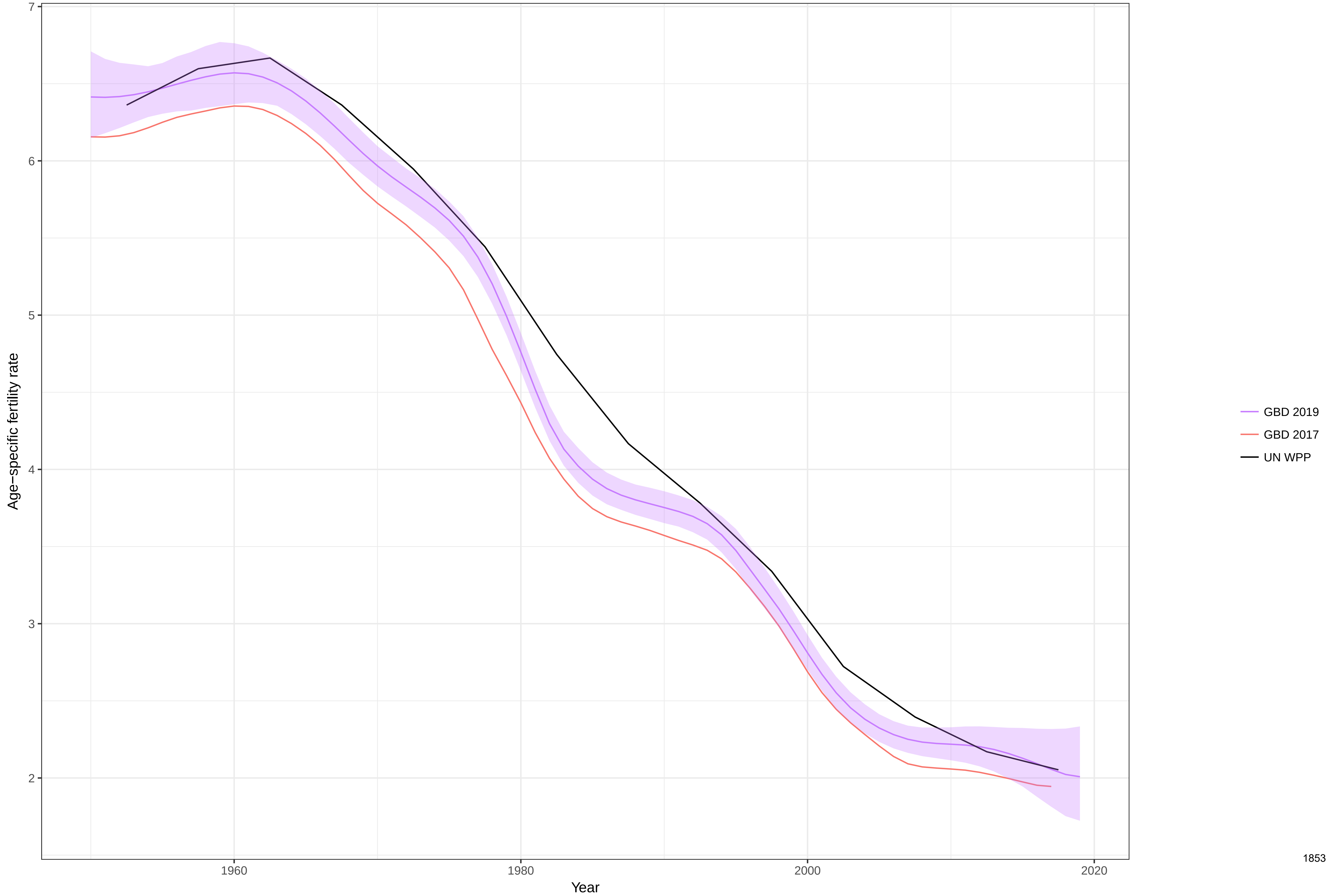

Age−specific fertility fitted trend lines, 1950−2019: San Marino, Ages: 10−14
Data points enclosed with a diamond are reference sources

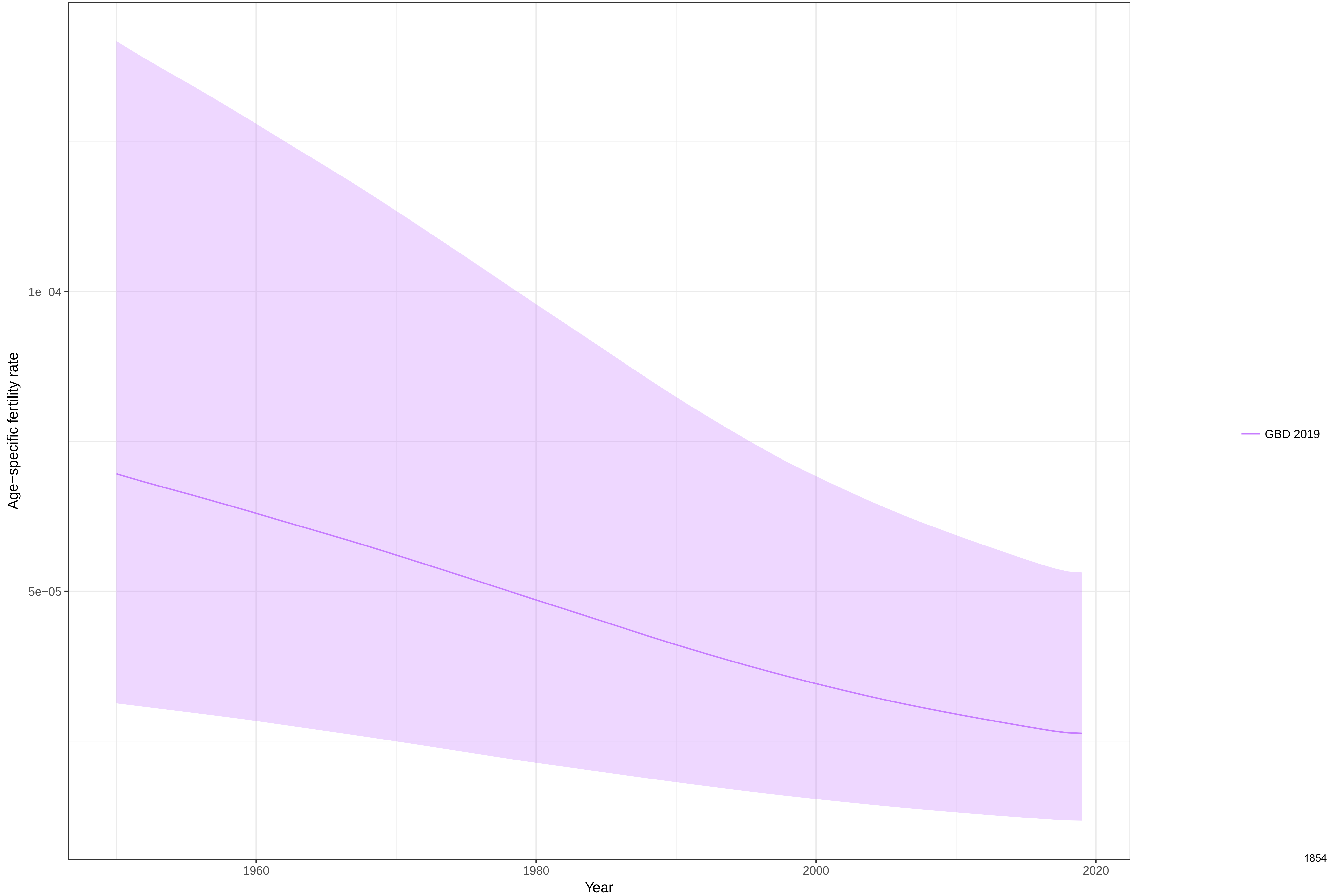

Age−specific fertility fitted trend lines, 1950−2019: San Marino, Ages: 15−19
Data points enclosed with a diamond are reference sources

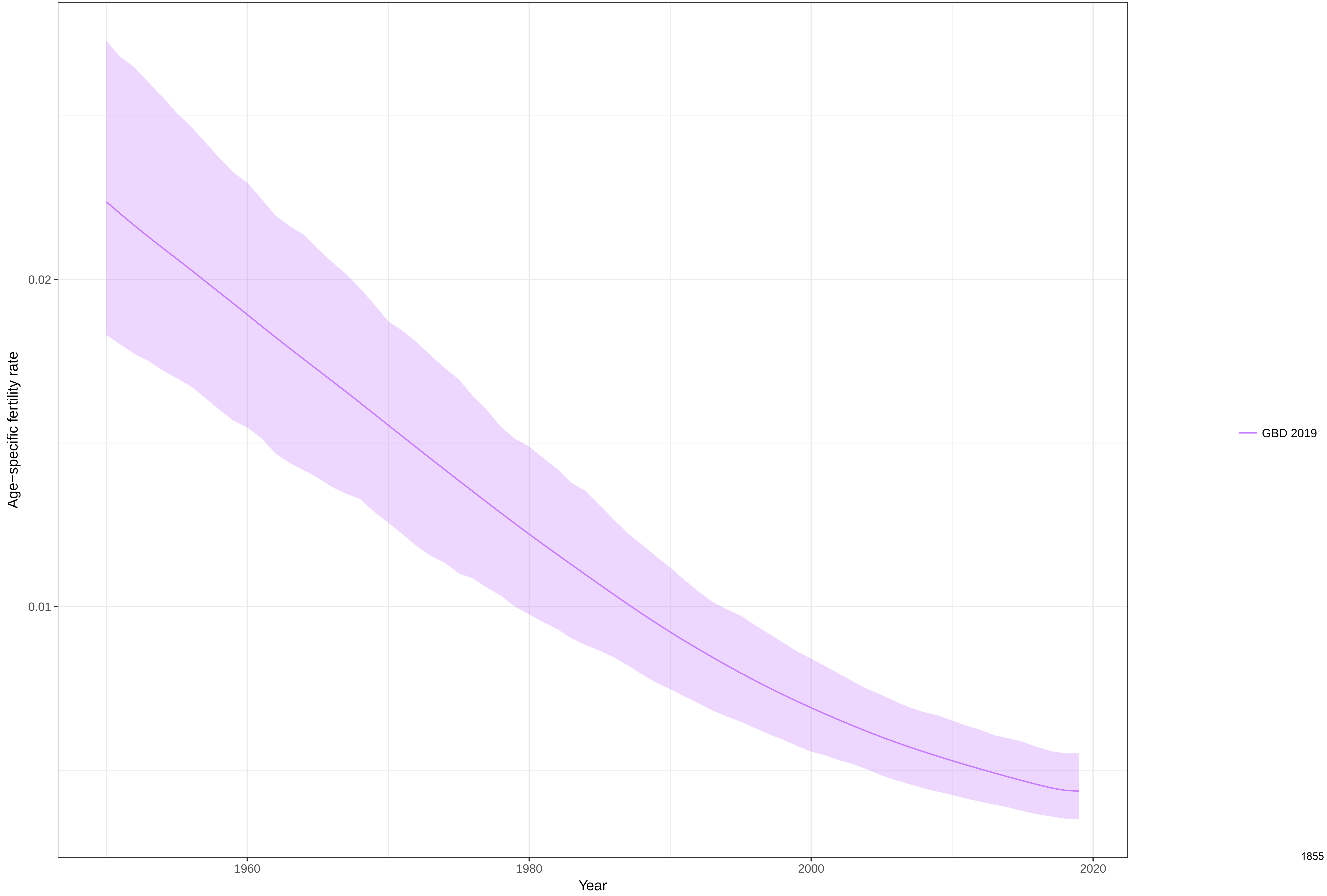

Age−specific fertility fitted trend lines, 1950−2019: San Marino, Ages: 20−24
 Data points enclosed with a diamond are reference sources

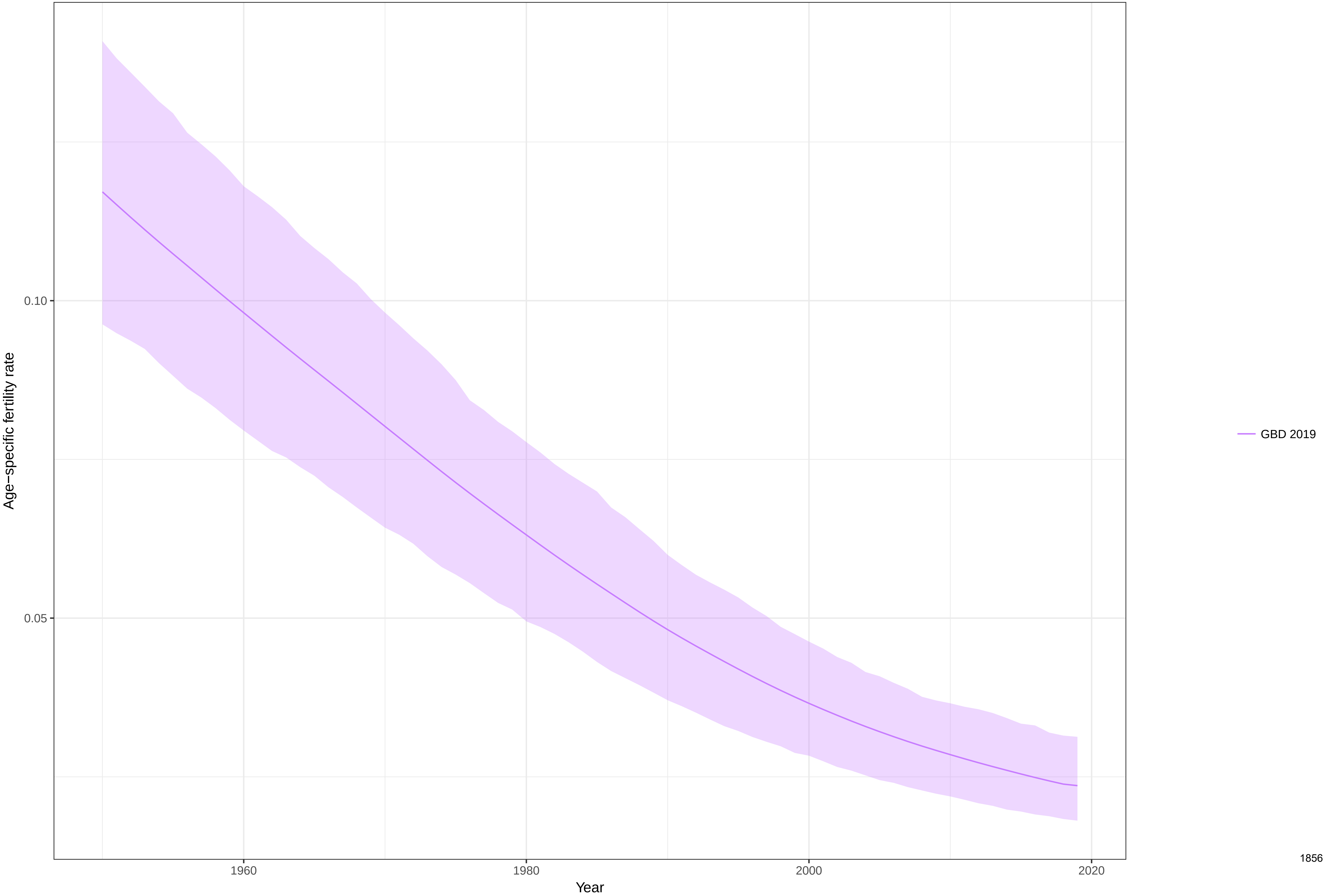

Age−specific fertility fitted trend lines, 1950−2019: San Marino, Ages: 25−29
Data points enclosed with a diamond are reference sources

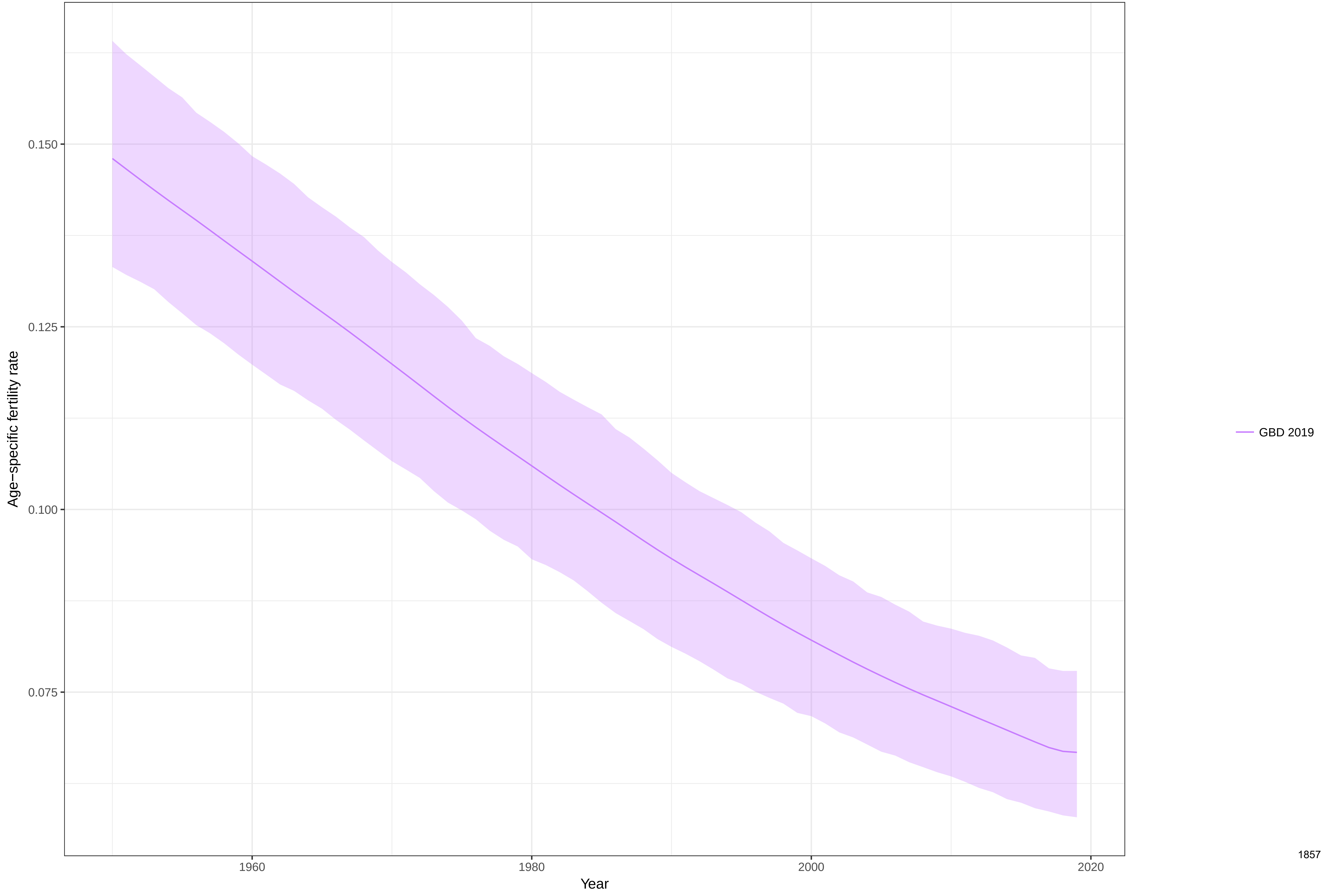

Age−specific fertility fitted trend lines, 1950−2019: San Marino, Ages: 30−34
Data points enclosed with a diamond are reference sources

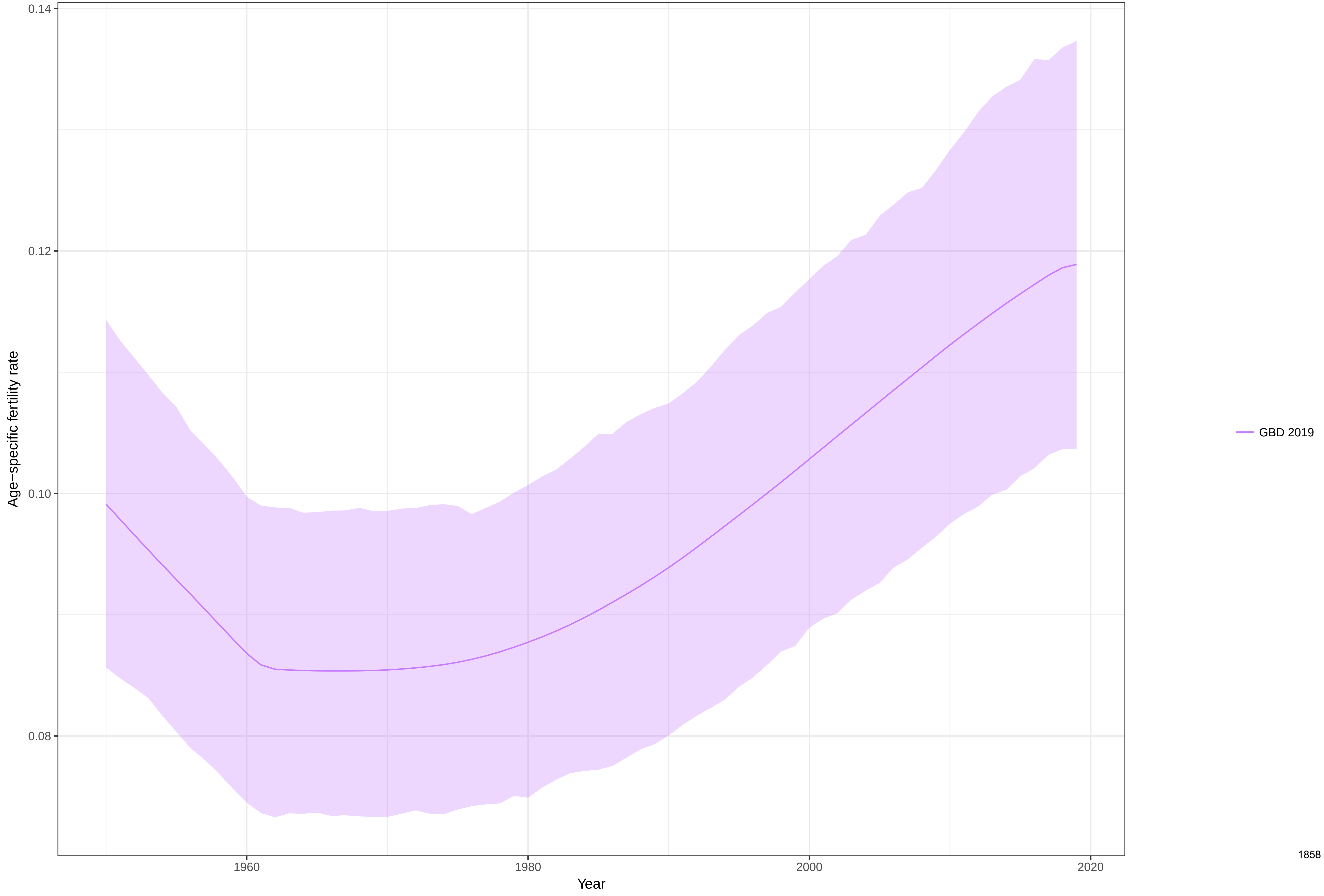

Age−specific fertility fitted trend lines, 1950−2019: San Marino, Ages: 35−39
Data points enclosed with a diamond are reference sources

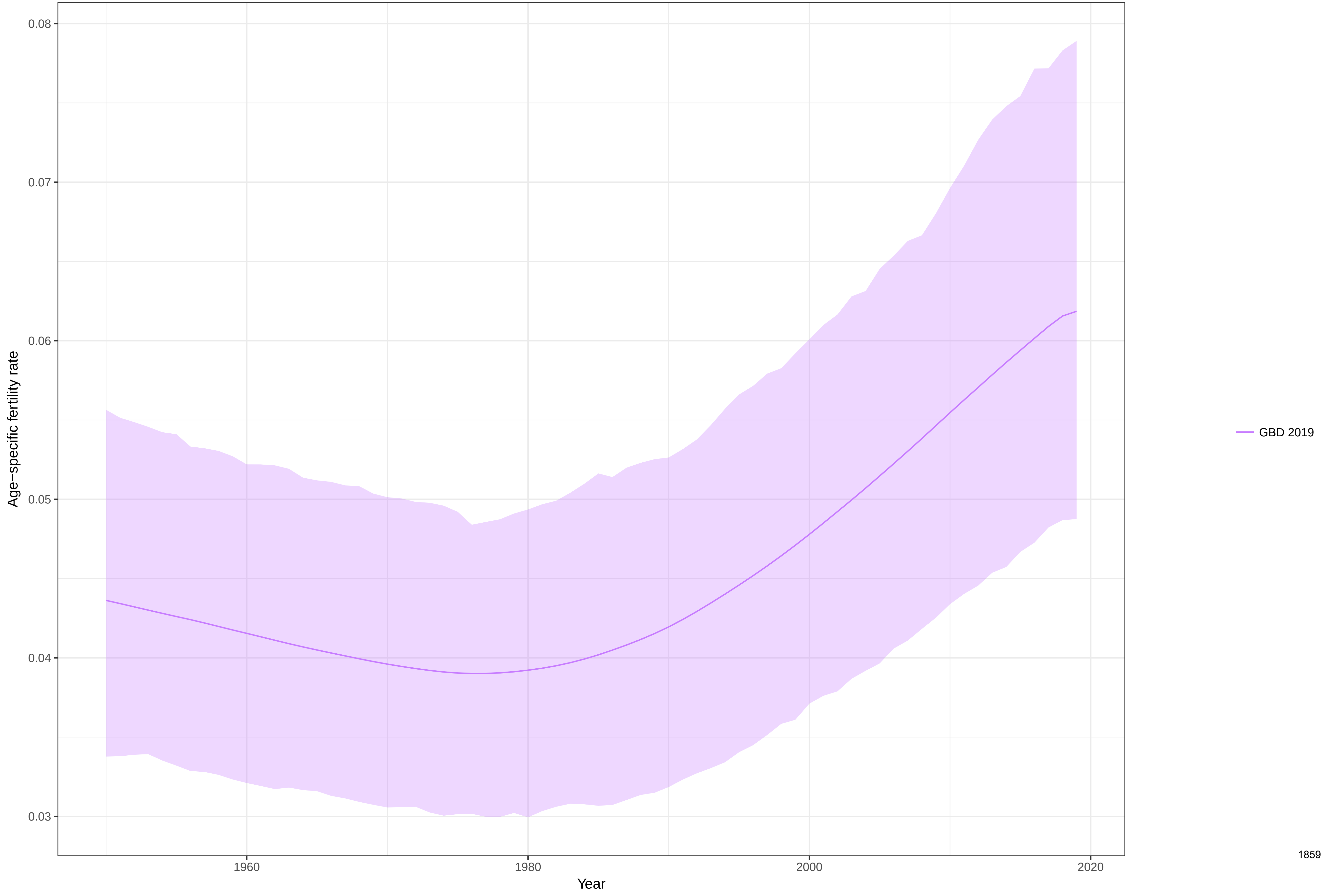

Age−specific fertility fitted trend lines, 1950−2019: San Marino, Ages: 40−44
Data points enclosed with a diamond are reference sources

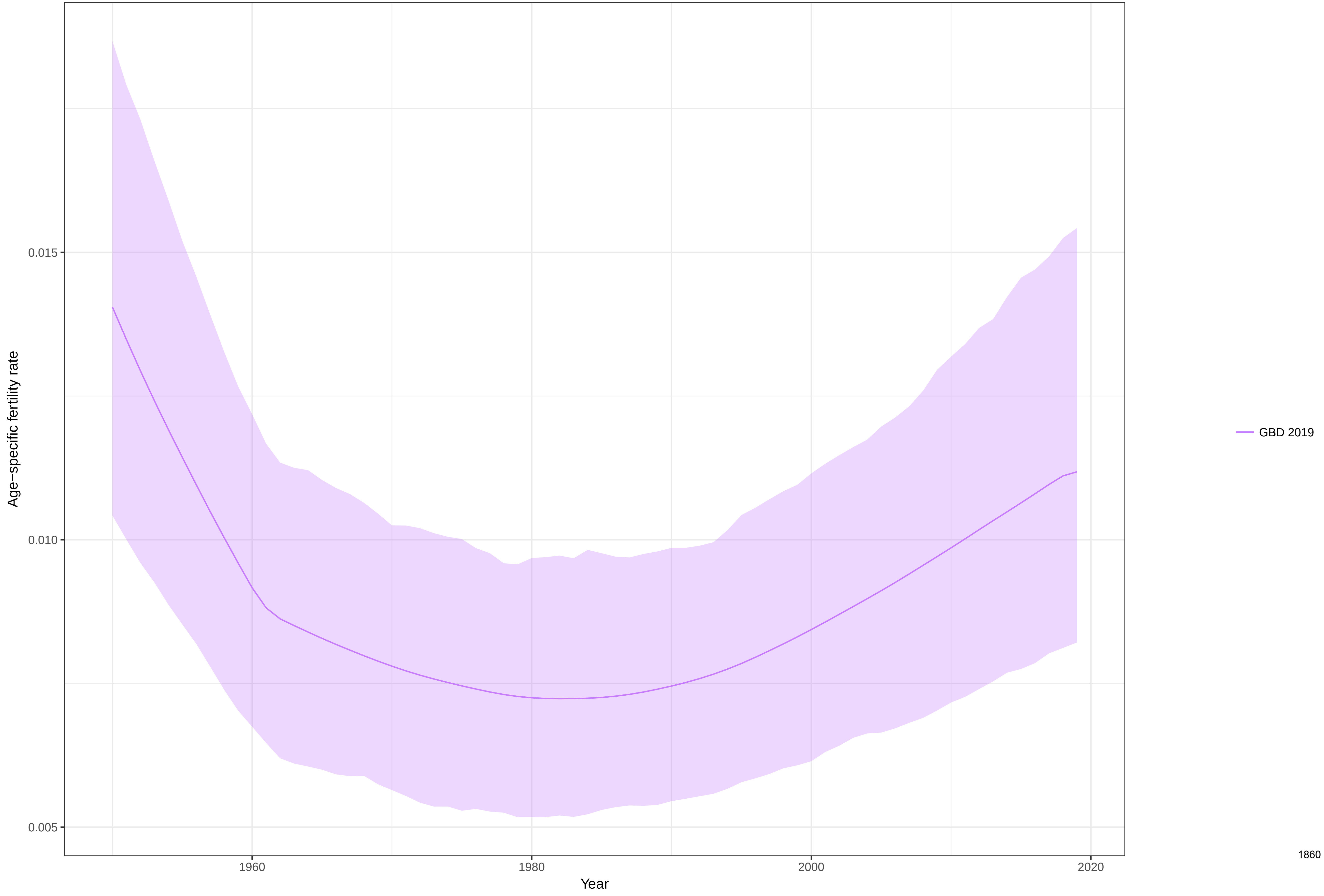

Age−specific fertility fitted trend lines, 1950−2019: San Marino, Ages: 45−49
Data points enclosed with a diamond are reference sources

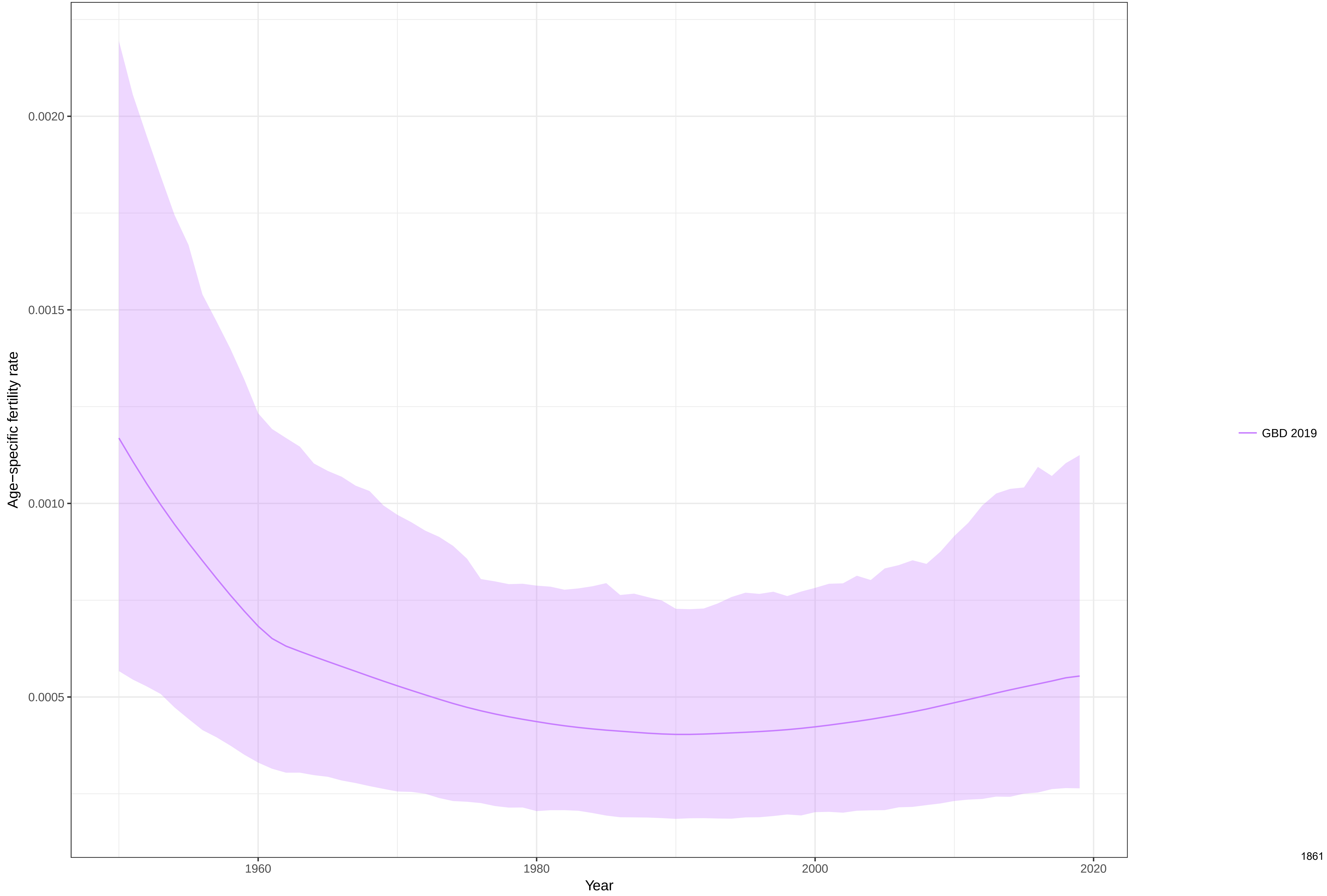

Age−specific fertility fitted trend lines, 1950−2019: San Marino, Ages: 50−54
Data points enclosed with a diamond are reference sources

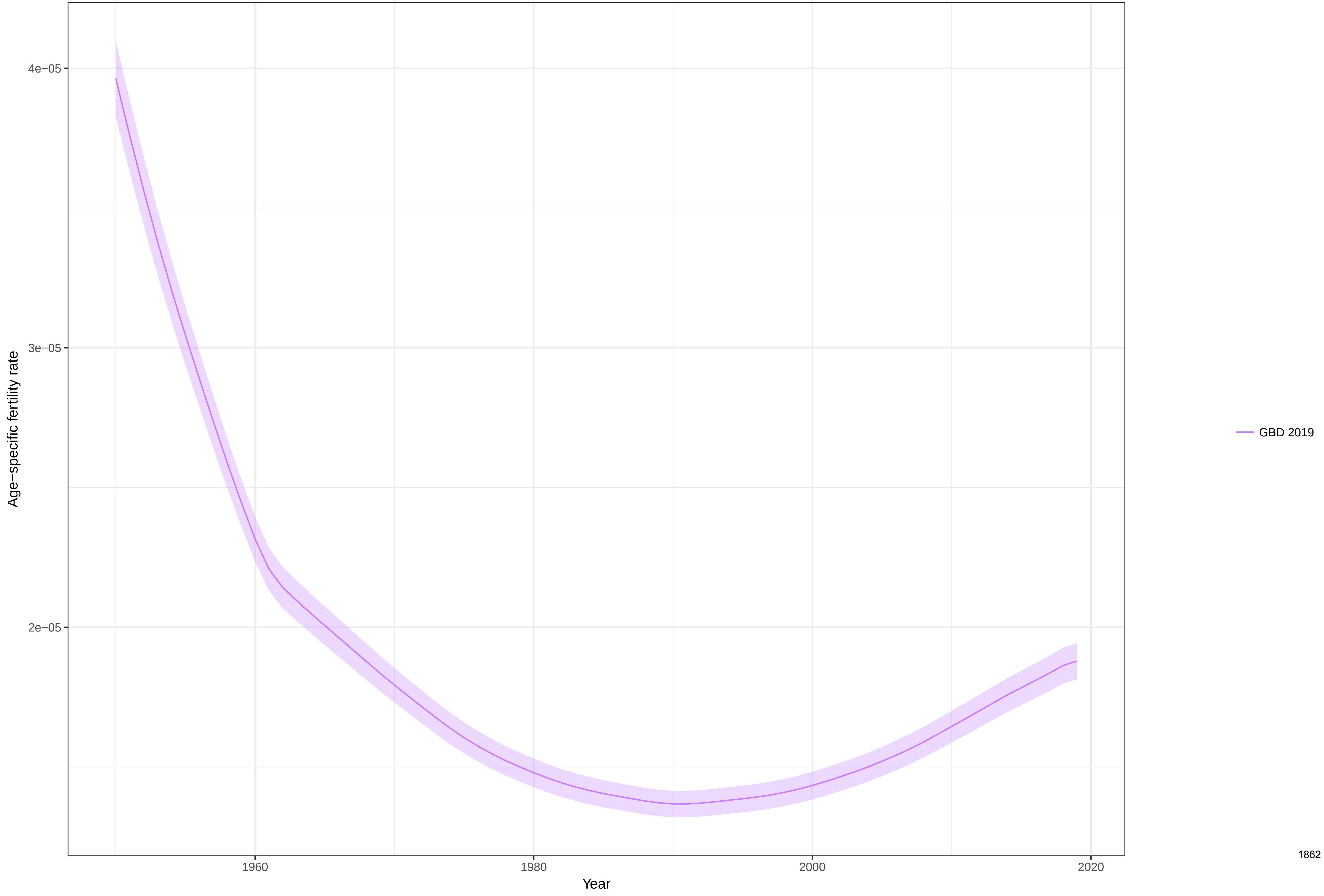

Total fertility rate fitted trend lines, 1950−2019: San Marino TFR
Data points enclosed with a diamond are reference sources

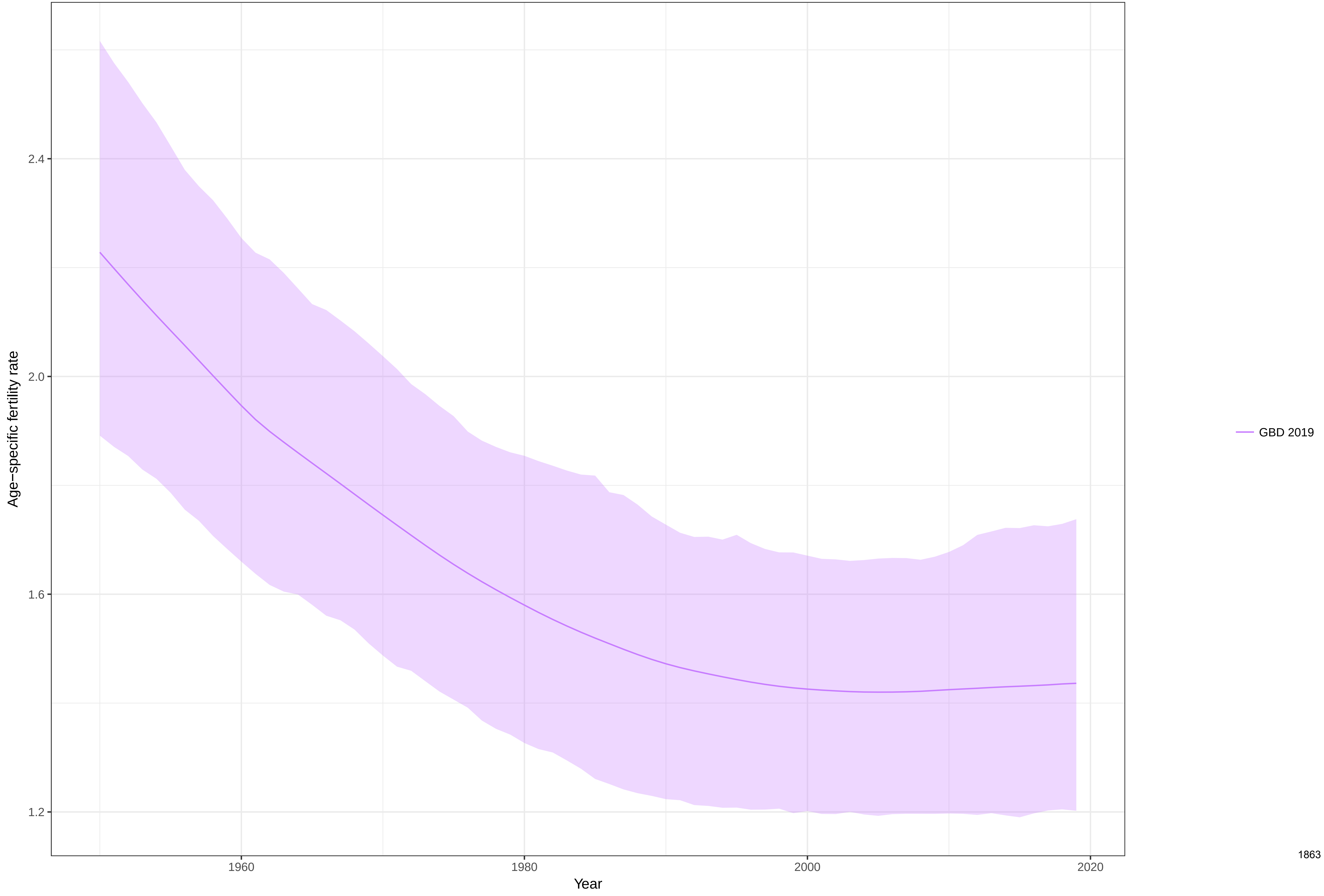

Age−specific fertility fitted trend lines, 1950−2019: Somalia, Ages: 10−14
Data points enclosed with a diamond are reference sources

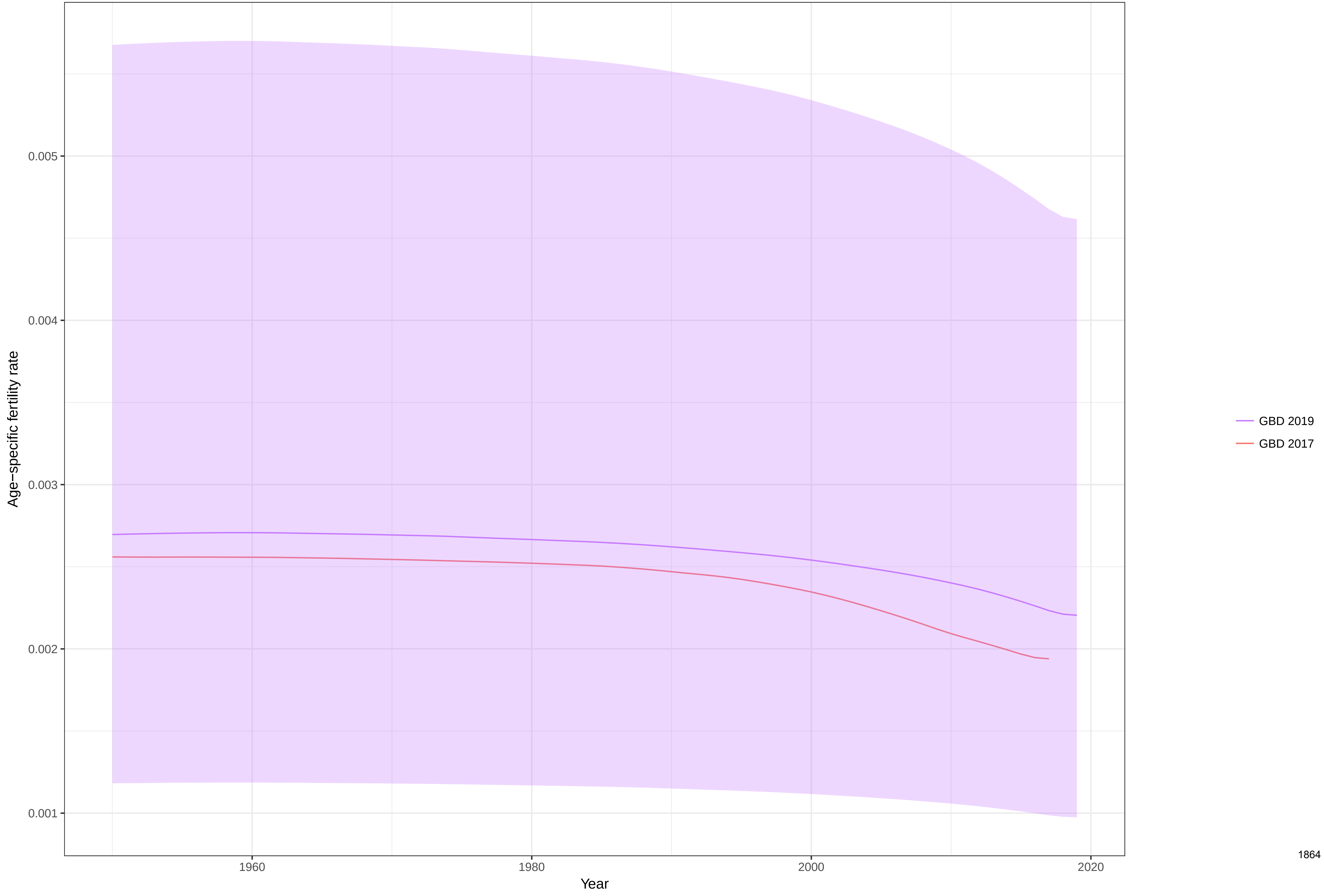

Age−specific fertility fitted trend lines, 1950−2019: Somalia, Ages: 15−19
Data points enclosed with a diamond are reference sources

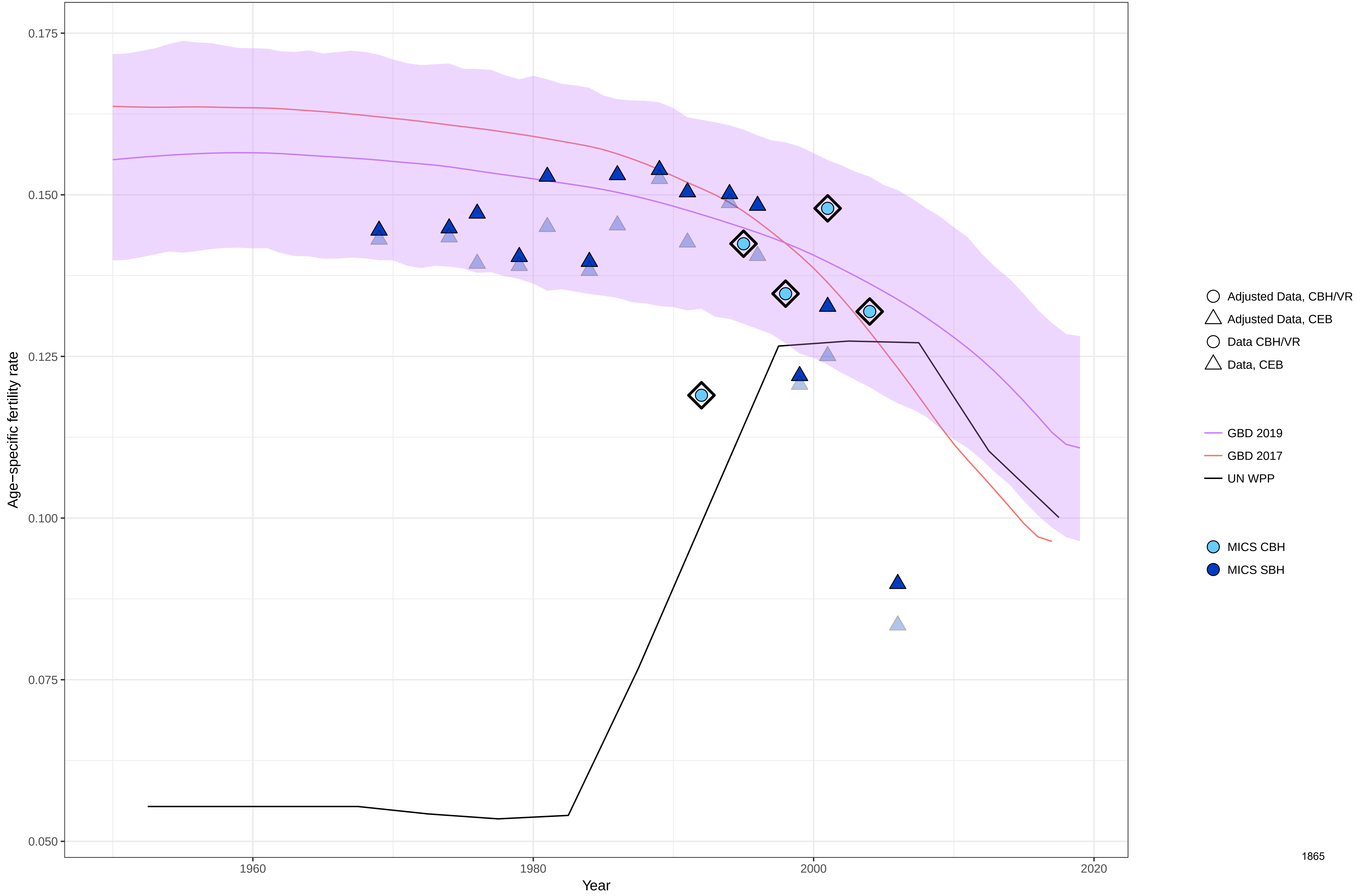

Age−specific fertility fitted trend lines, 1950−2019: Somalia, Ages: 20−24
Data points enclosed with a diamond are reference sources

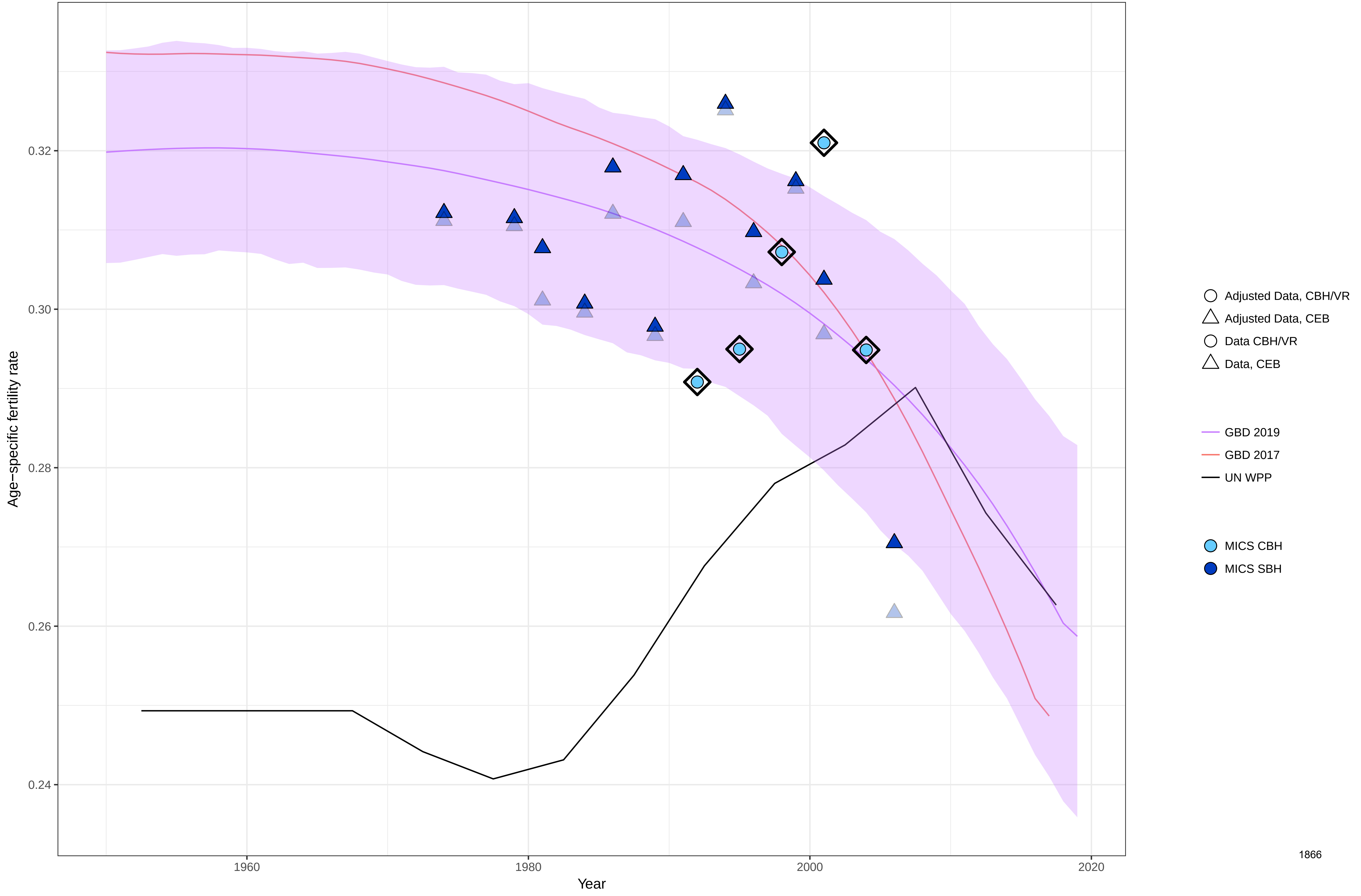

Age−specific fertility fitted trend lines, 1950−2019: Somalia, Ages: 25−29
Data points enclosed with a diamond are reference sources

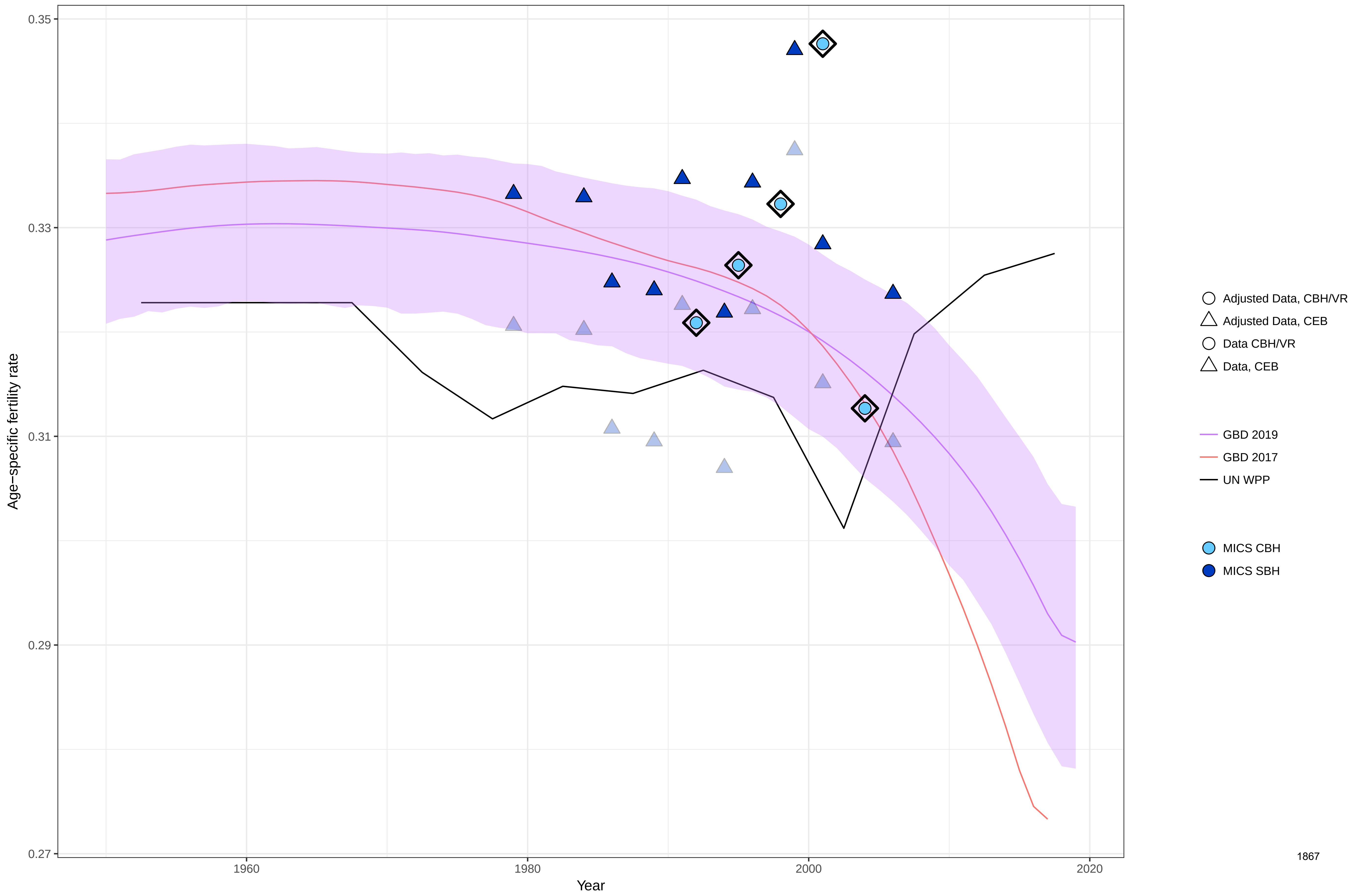

Age−specific fertility fitted trend lines, 1950−2019: Somalia, Ages: 30−34
Data points enclosed with a diamond are reference sources

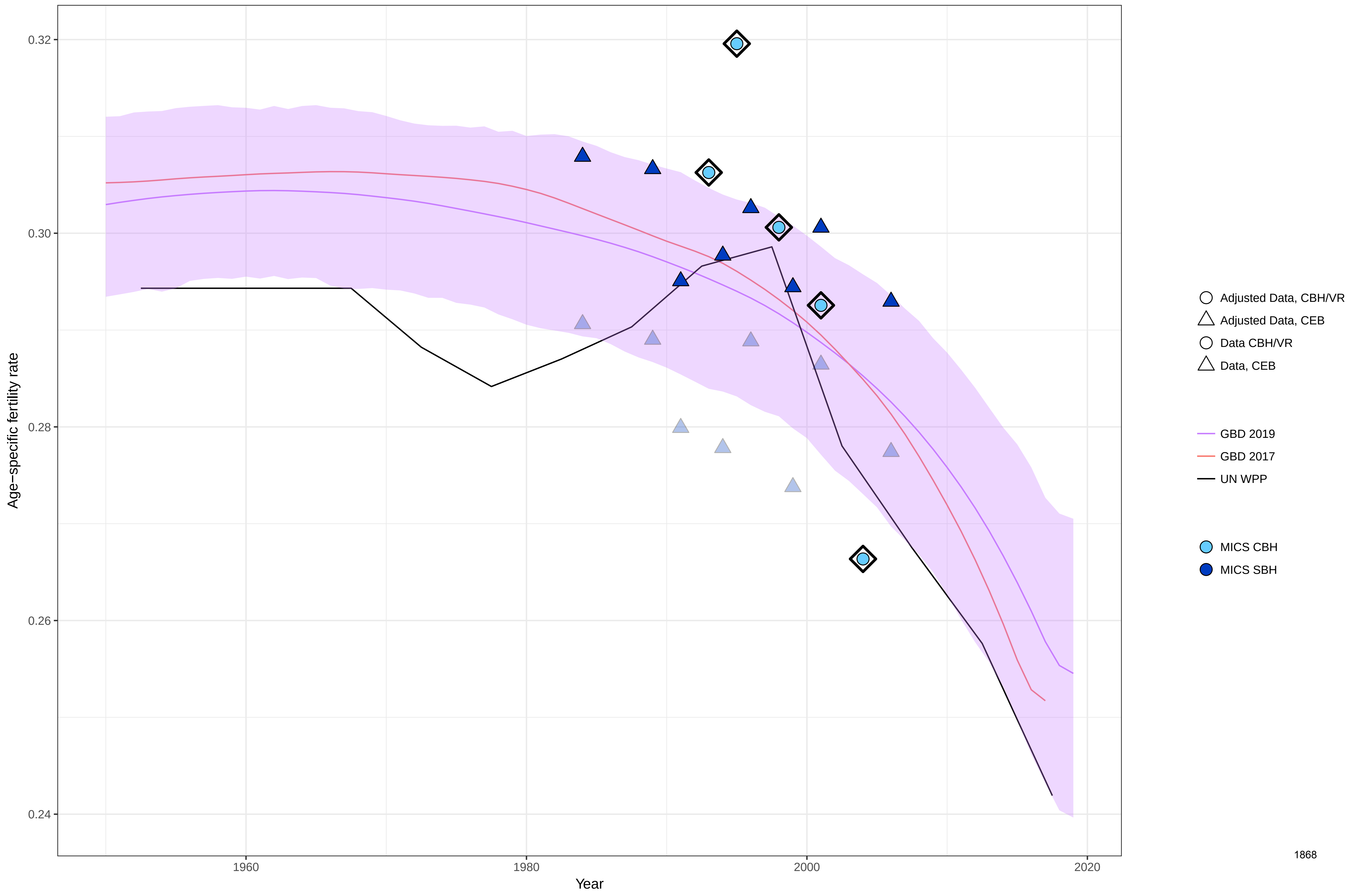

Age−specific fertility fitted trend lines, 1950−2019: Somalia, Ages: 35−39
Data points enclosed with a diamond are reference sources

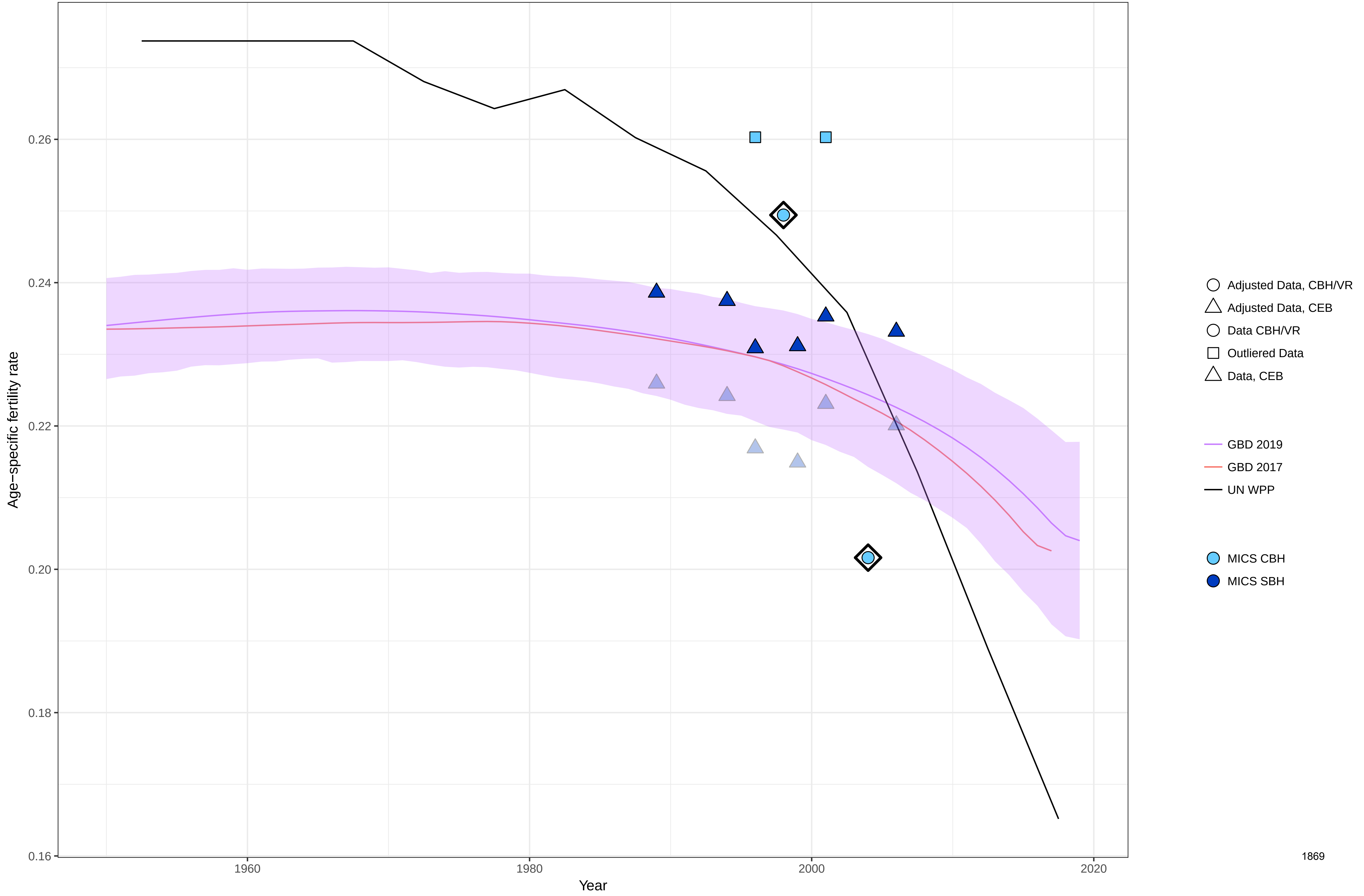

Age−specific fertility fitted trend lines, 1950−2019: Somalia, Ages: 40−44
Data points enclosed with a diamond are reference sources

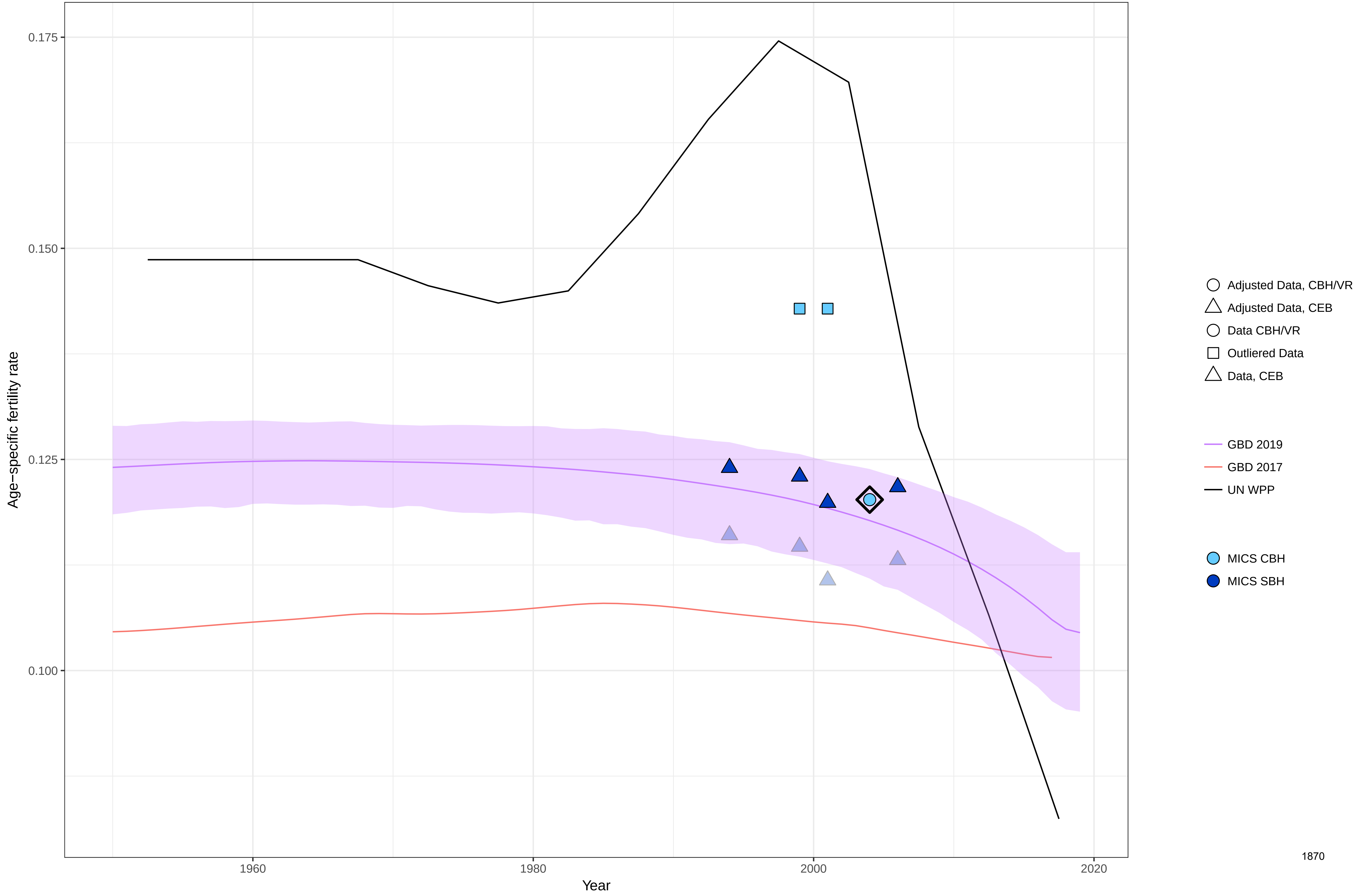

Age−specific fertility fitted trend lines, 1950−2019: Somalia, Ages: 45−49
Data points enclosed with a diamond are reference sources

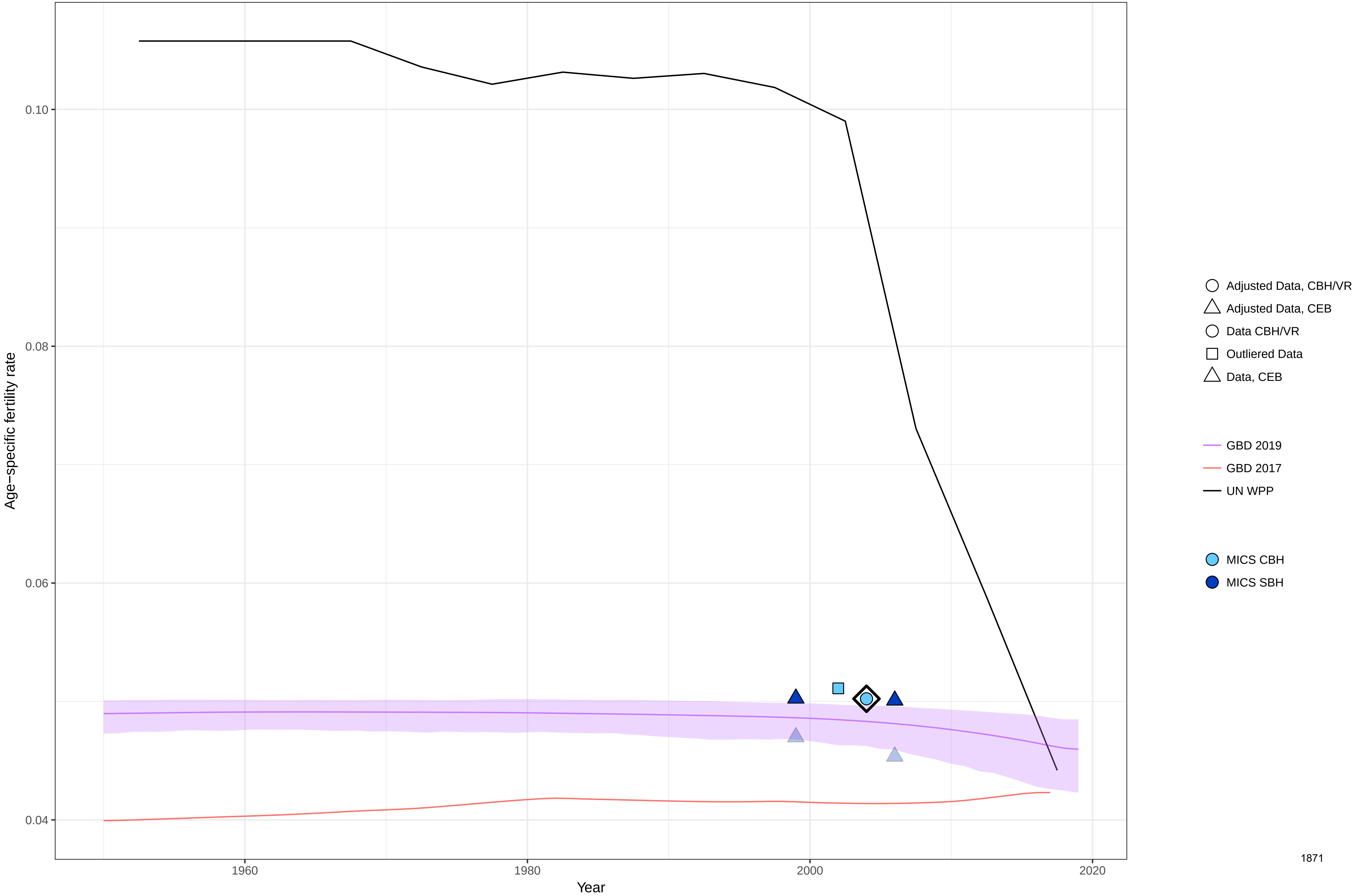

Age−specific fertility fitted trend lines, 1950−2019: Somalia, Ages: 50−54
Data points enclosed with a diamond are reference sources

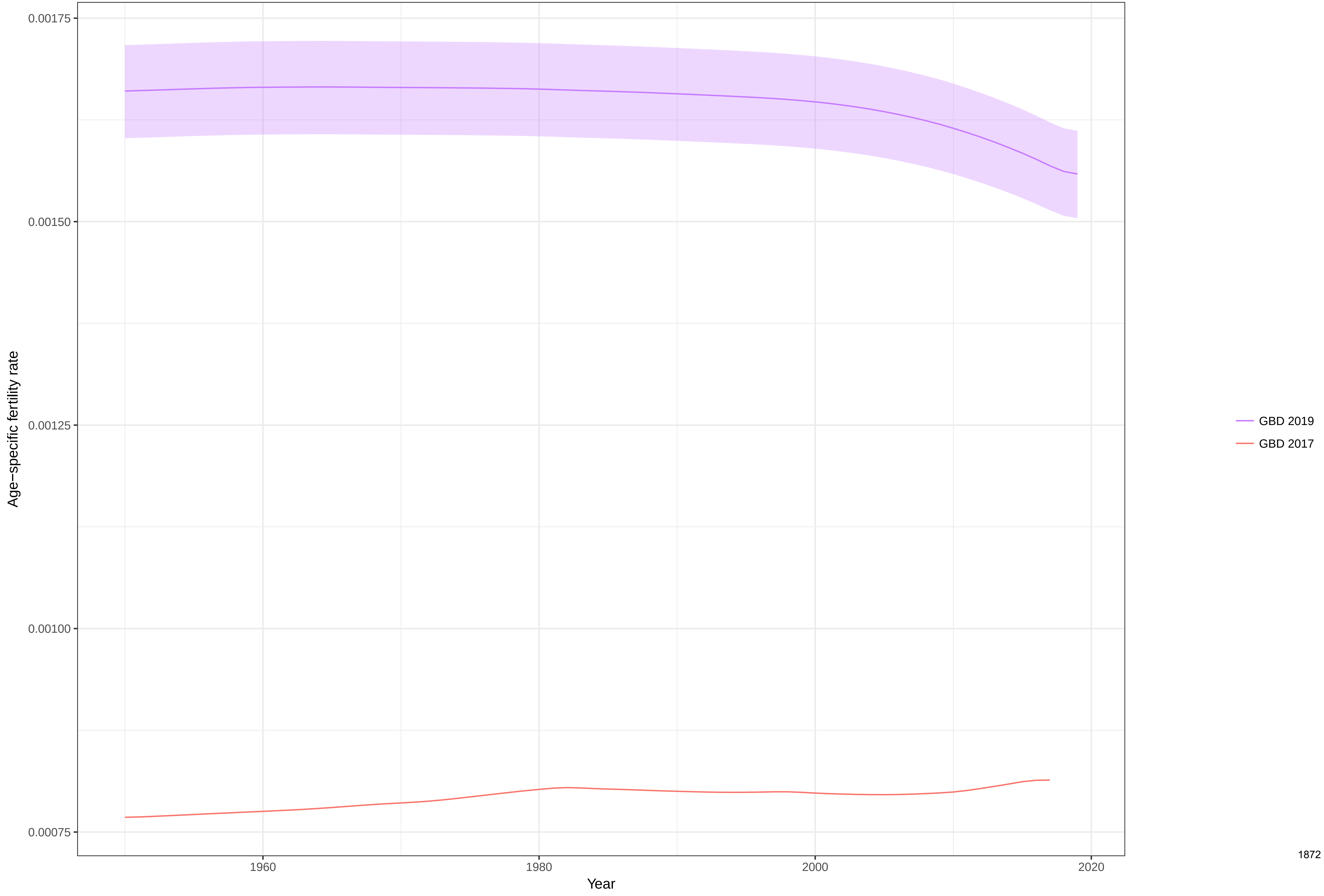

Total fertility rate fitted trend lines, 1950−2019: Somalia TFR
Data points enclosed with a diamond are reference sources

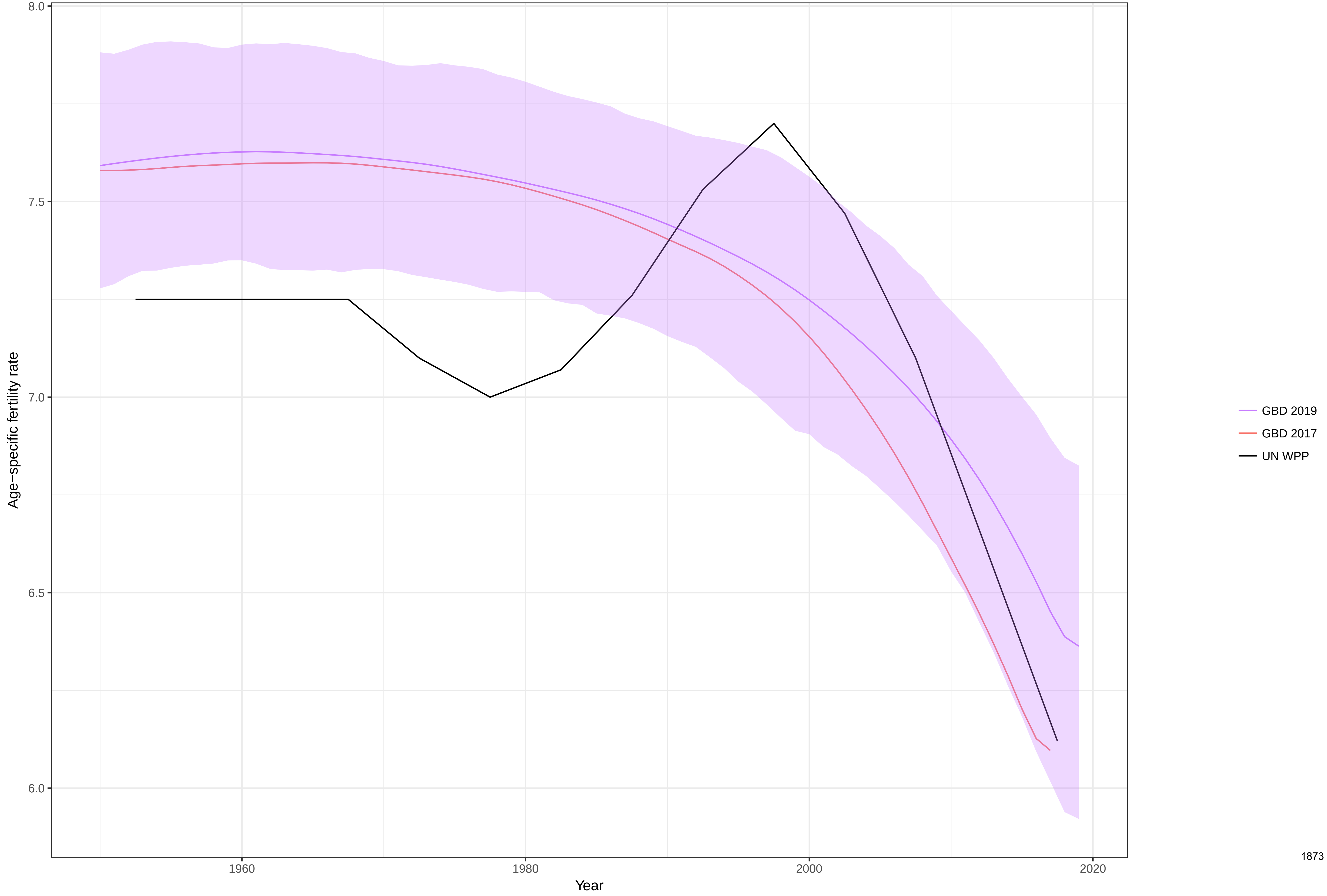

Age−specific fertility fitted trend lines, 1950−2019: Serbia, Ages: 10−14
Data points enclosed with a diamond are reference sources

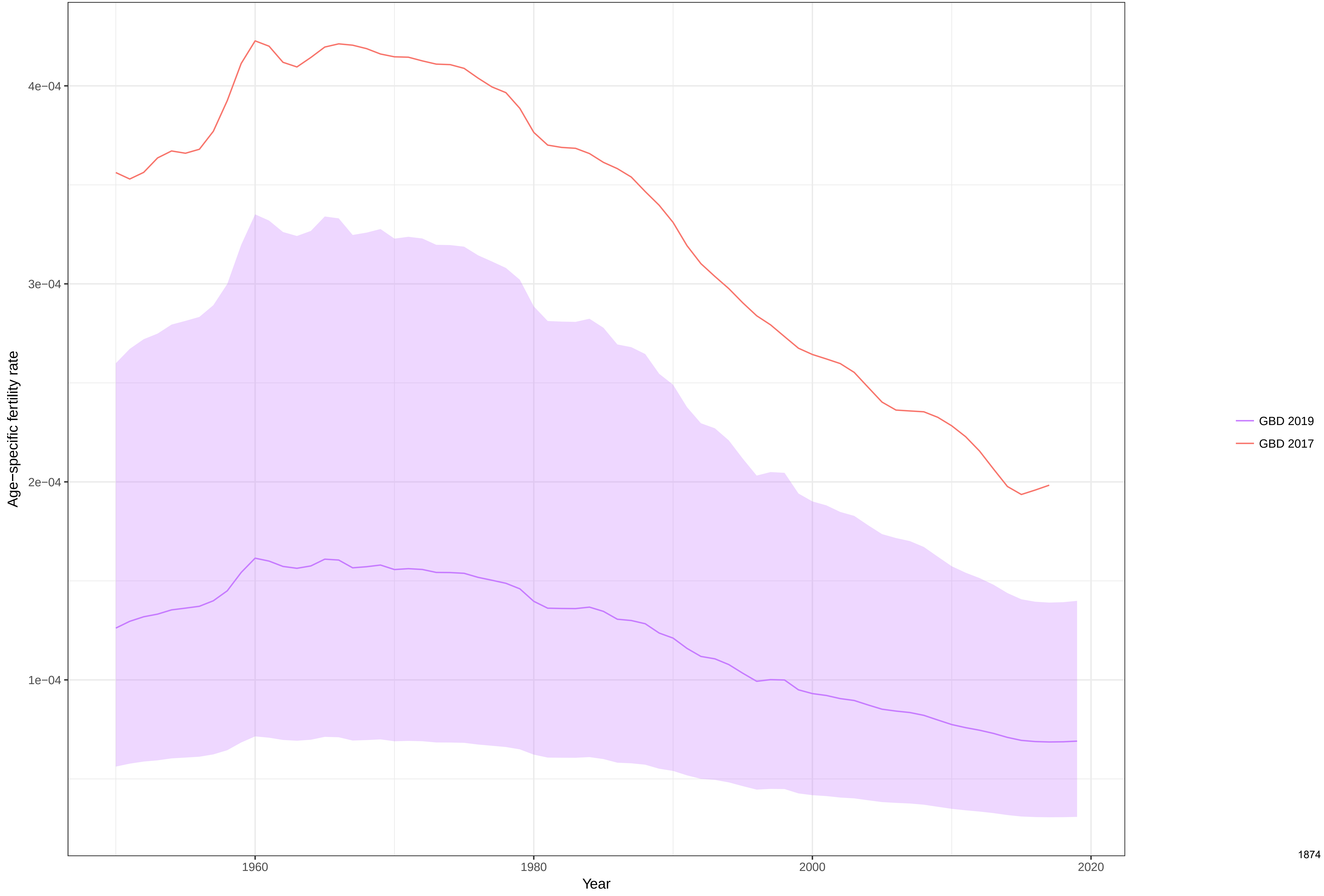

Age−specific fertility fitted trend lines, 1950−2019: Serbia, Ages: 15−19
Data points enclosed with a diamond are reference sources

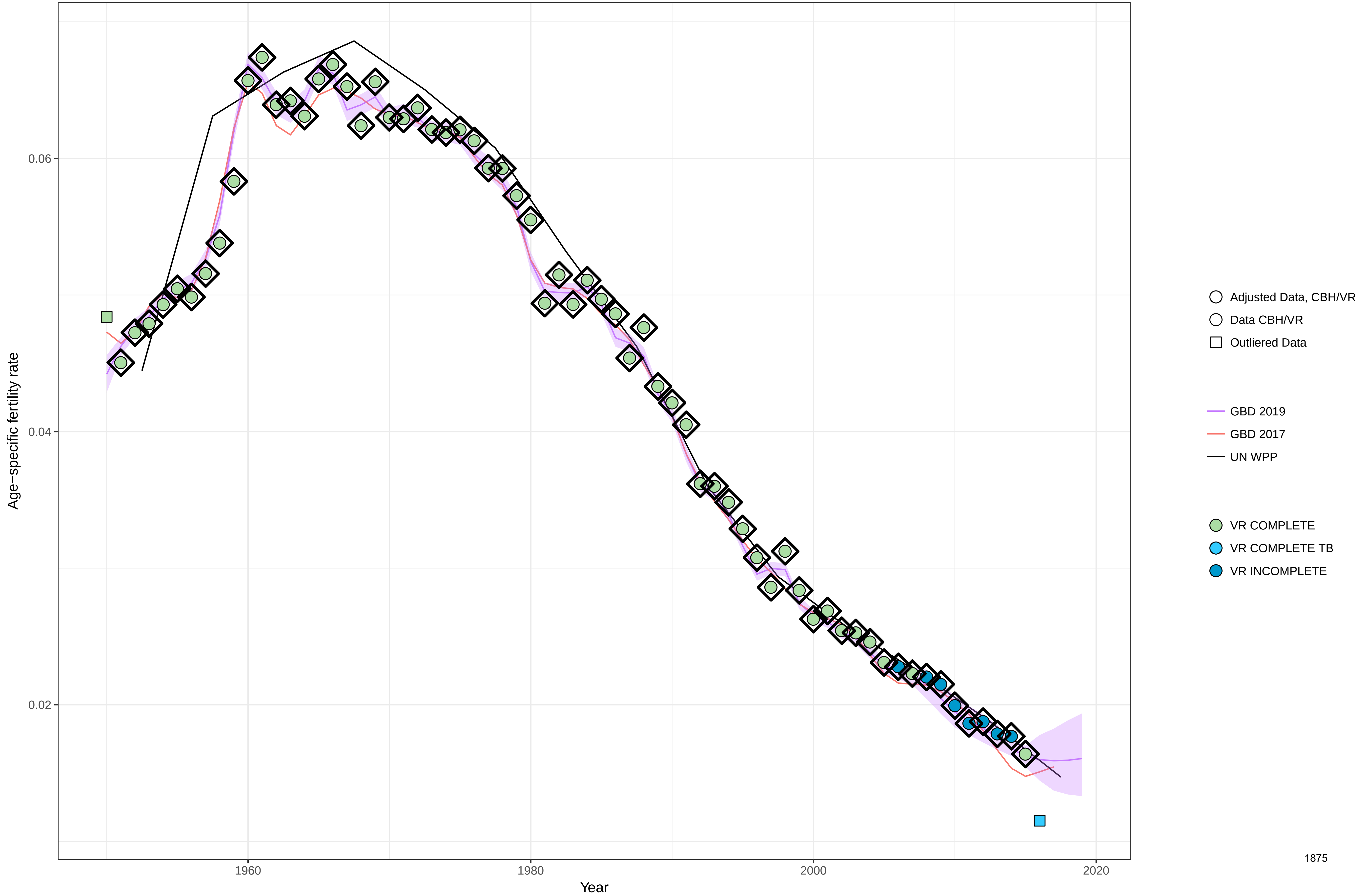

Age−specific fertility fitted trend lines, 1950−2019: Serbia, Ages: 20−24
Data points enclosed with a diamond are reference sources

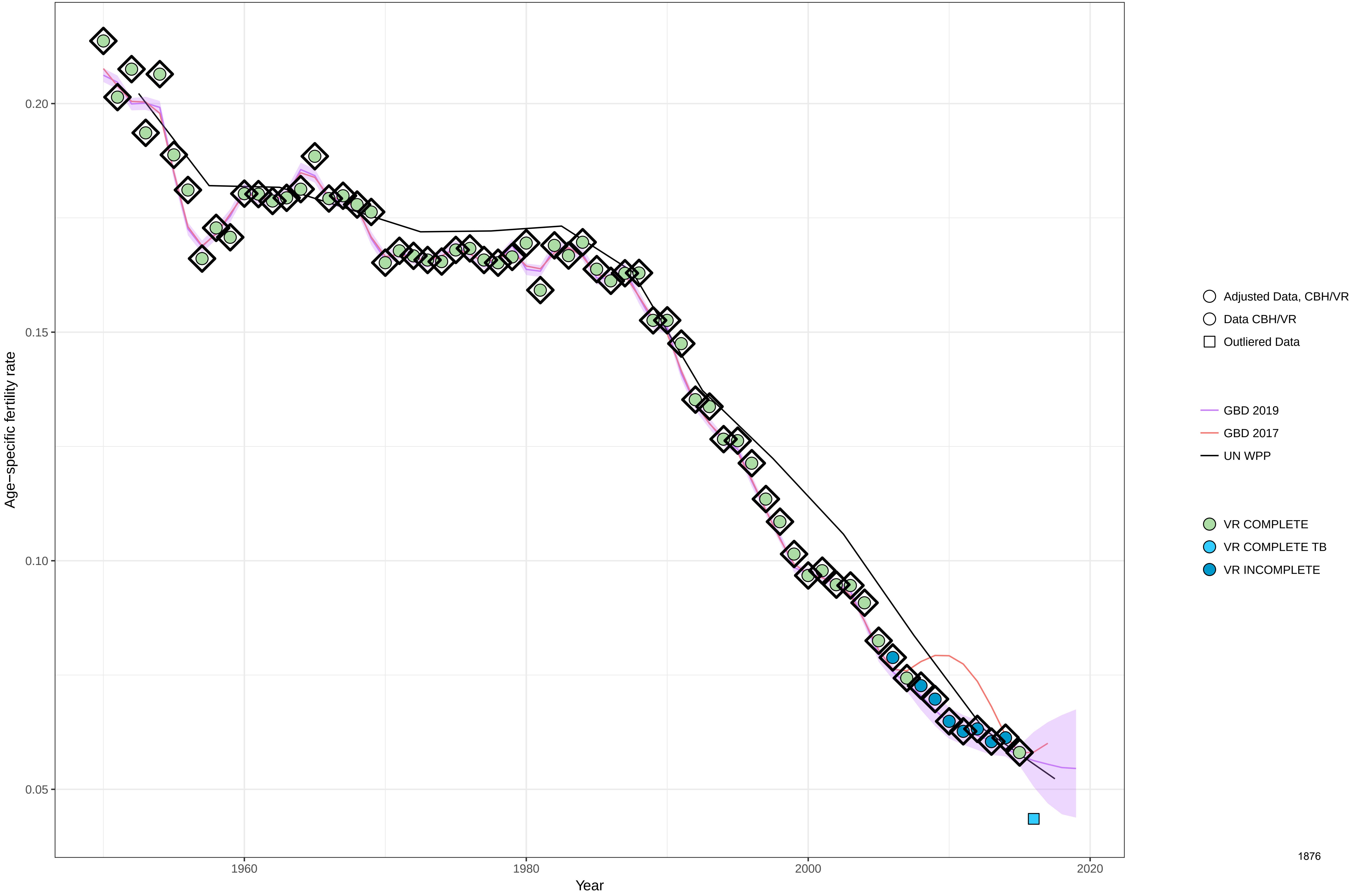

Age−specific fertility fitted trend lines, 1950−2019: Serbia, Ages: 25−29
Data points enclosed with a diamond are reference sources

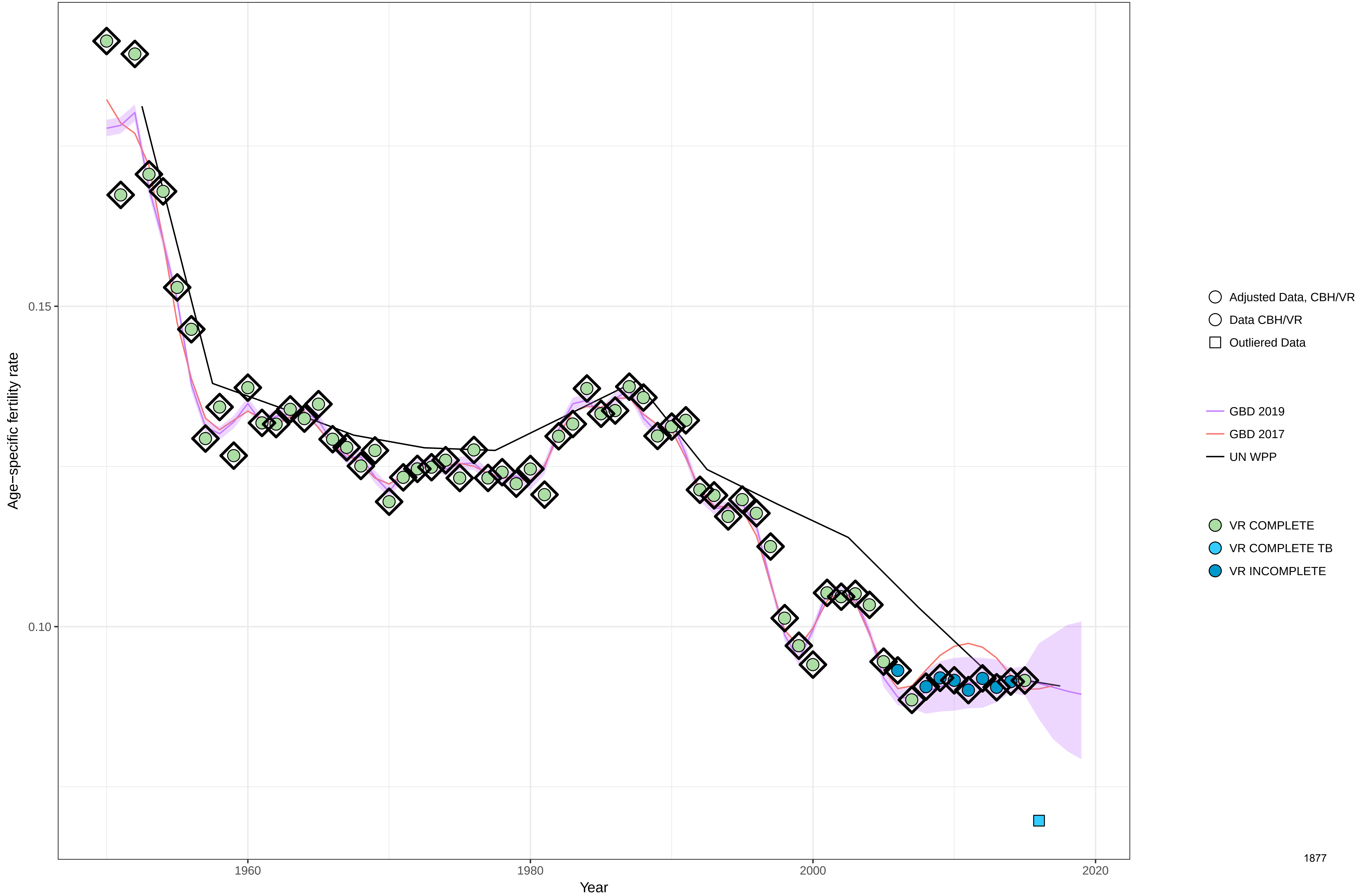

Age−specific fertility fitted trend lines, 1950−2019: Serbia, Ages: 30−34
Data points enclosed with a diamond are reference sources

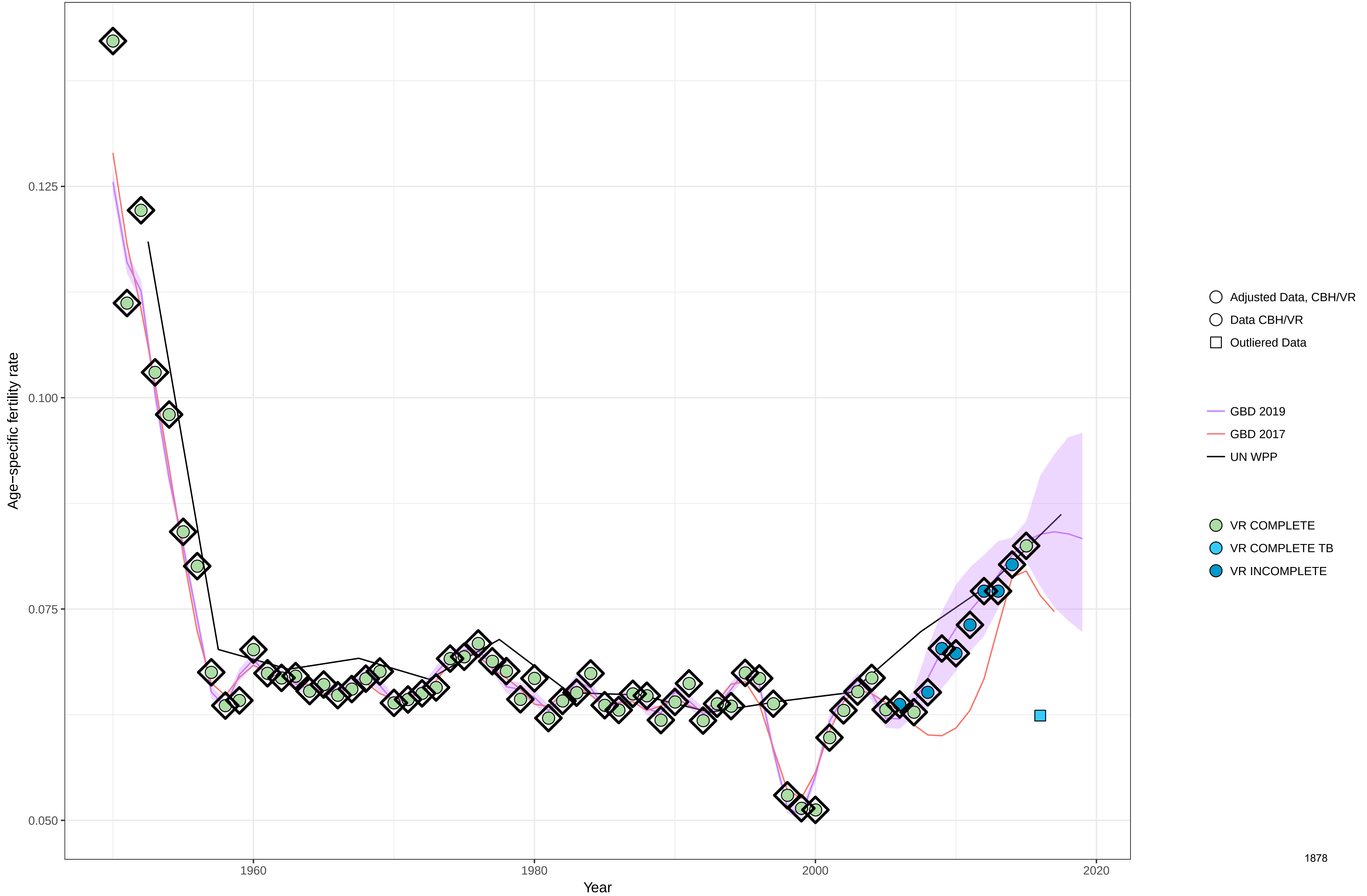

Age−specific fertility fitted trend lines, 1950−2019: Serbia, Ages: 35−39
Data points enclosed with a diamond are reference sources

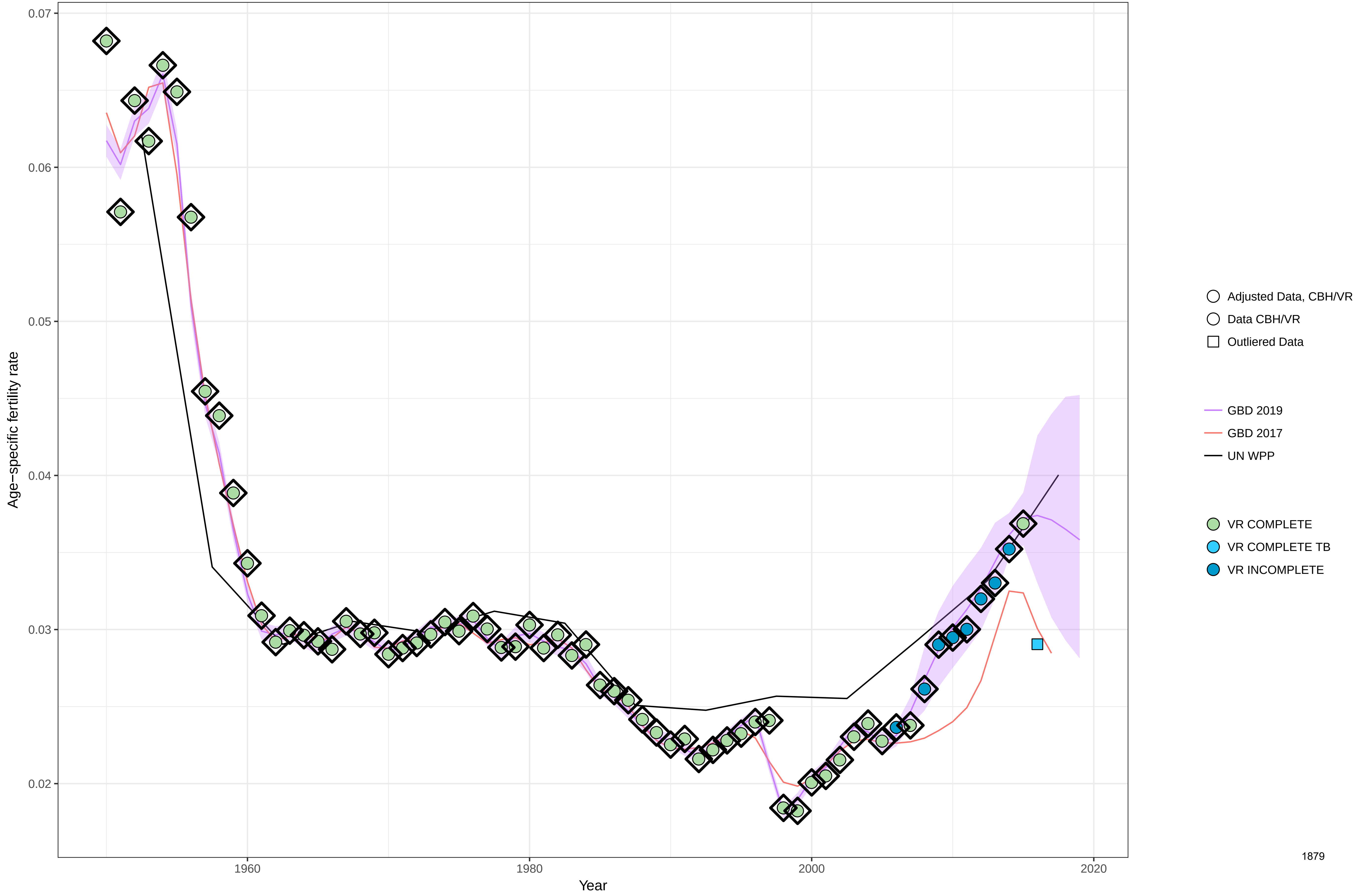

Age−specific fertility fitted trend lines, 1950−2019: Serbia, Ages: 40−44
Data points enclosed with a diamond are reference sources

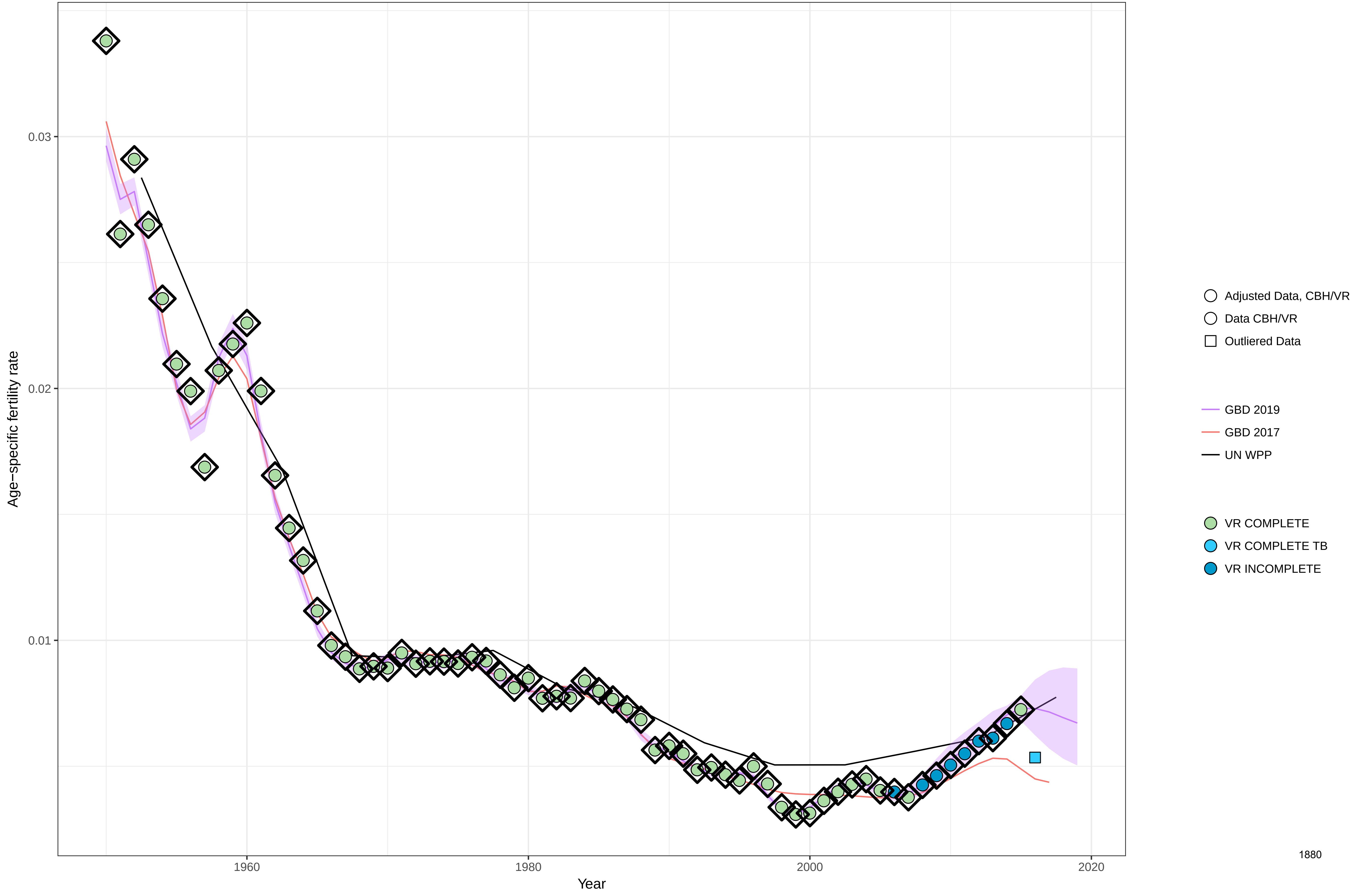

Age−specific fertility fitted trend lines, 1950−2019: Serbia, Ages: 45−49
Data points enclosed with a diamond are reference sources

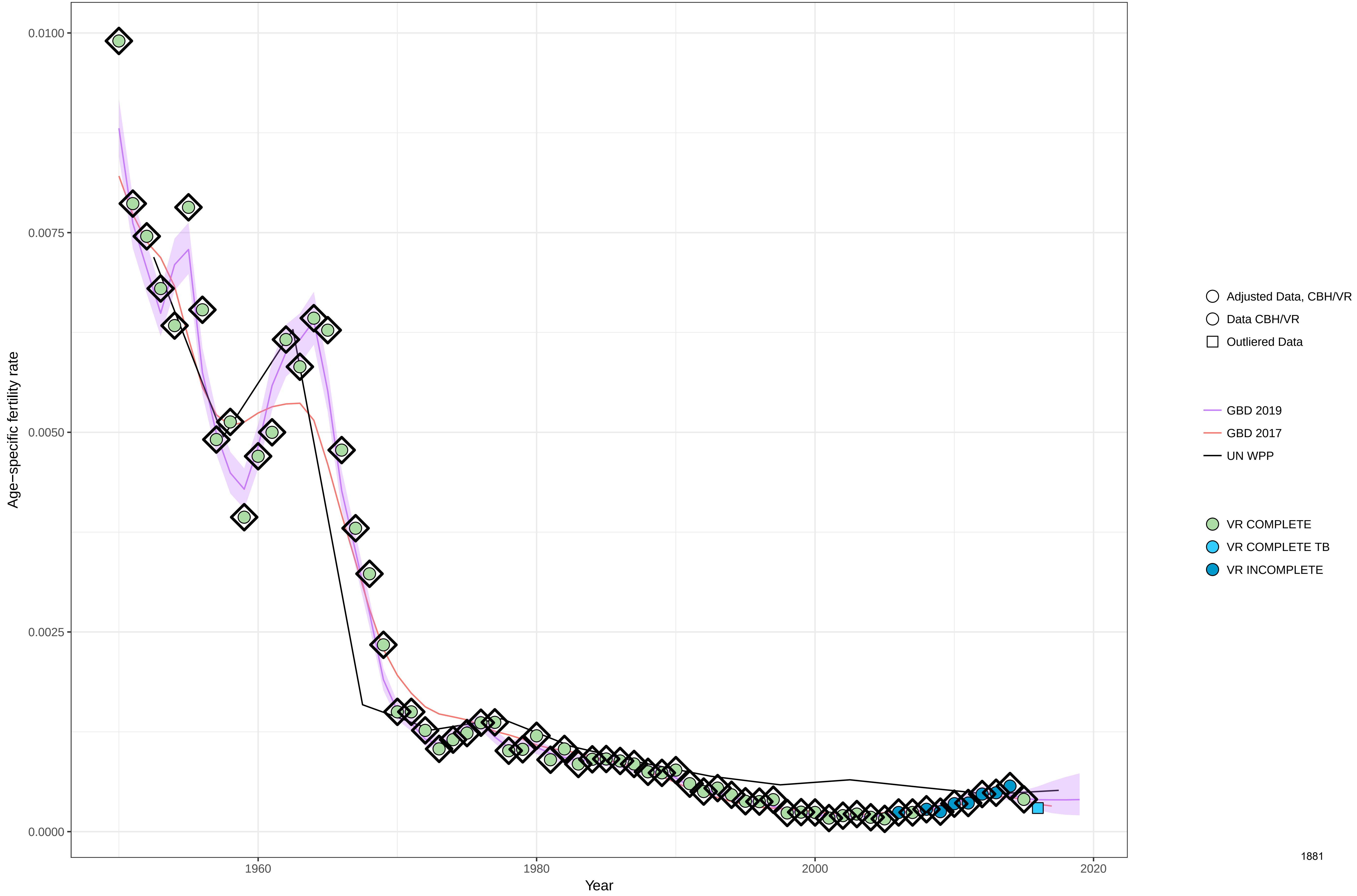

Age−specific fertility fitted trend lines, 1950−2019: Serbia, Ages: 50−54
Data points enclosed with a diamond are reference sources

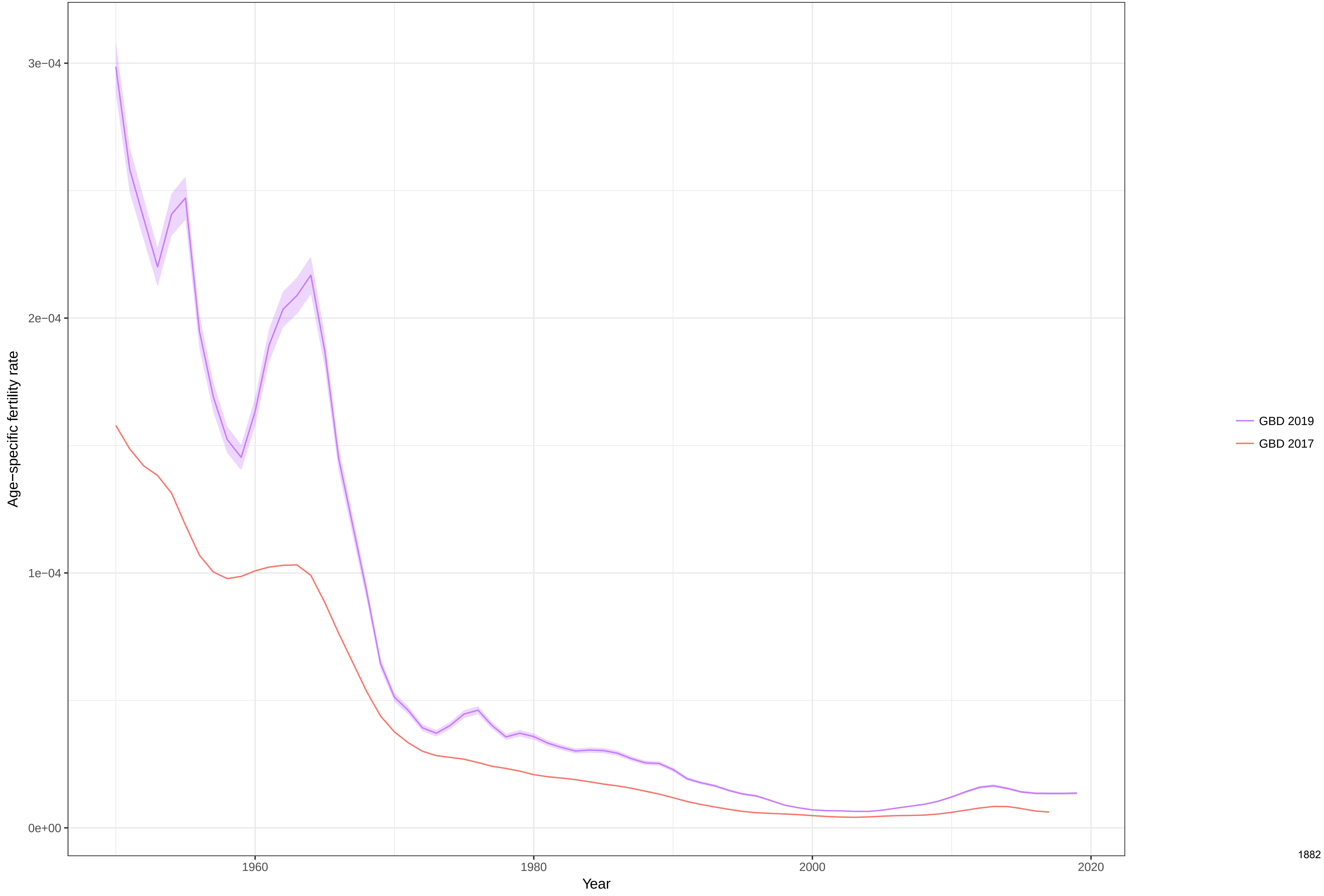

Total fertility rate fitted trend lines, 1950−2019: Serbia TFR
Data points enclosed with a diamond are reference sources

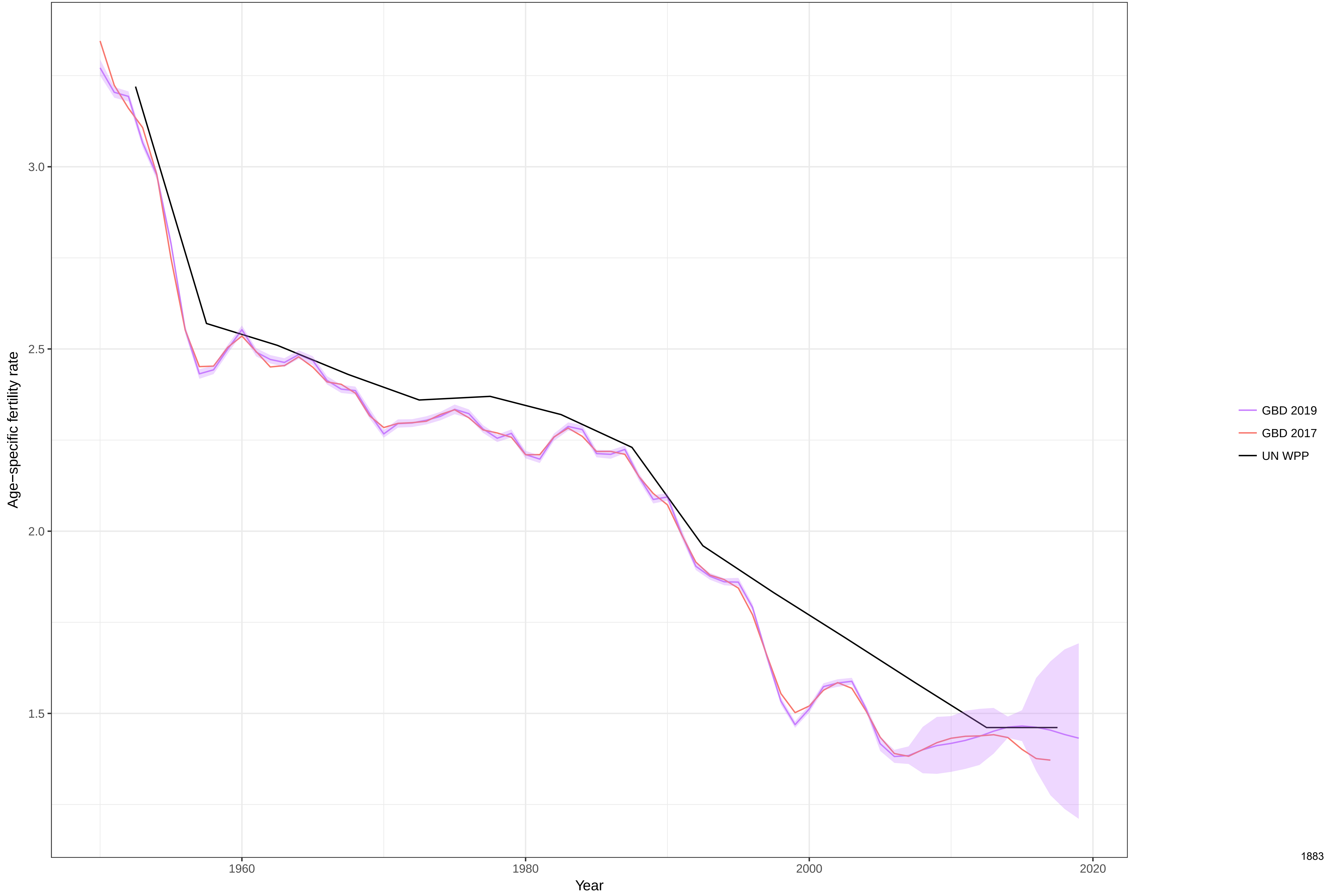

Age−specific fertility fitted trend lines, 1950−2019: South Sudan, Ages: 10−14
Data points enclosed with a diamond are reference sources

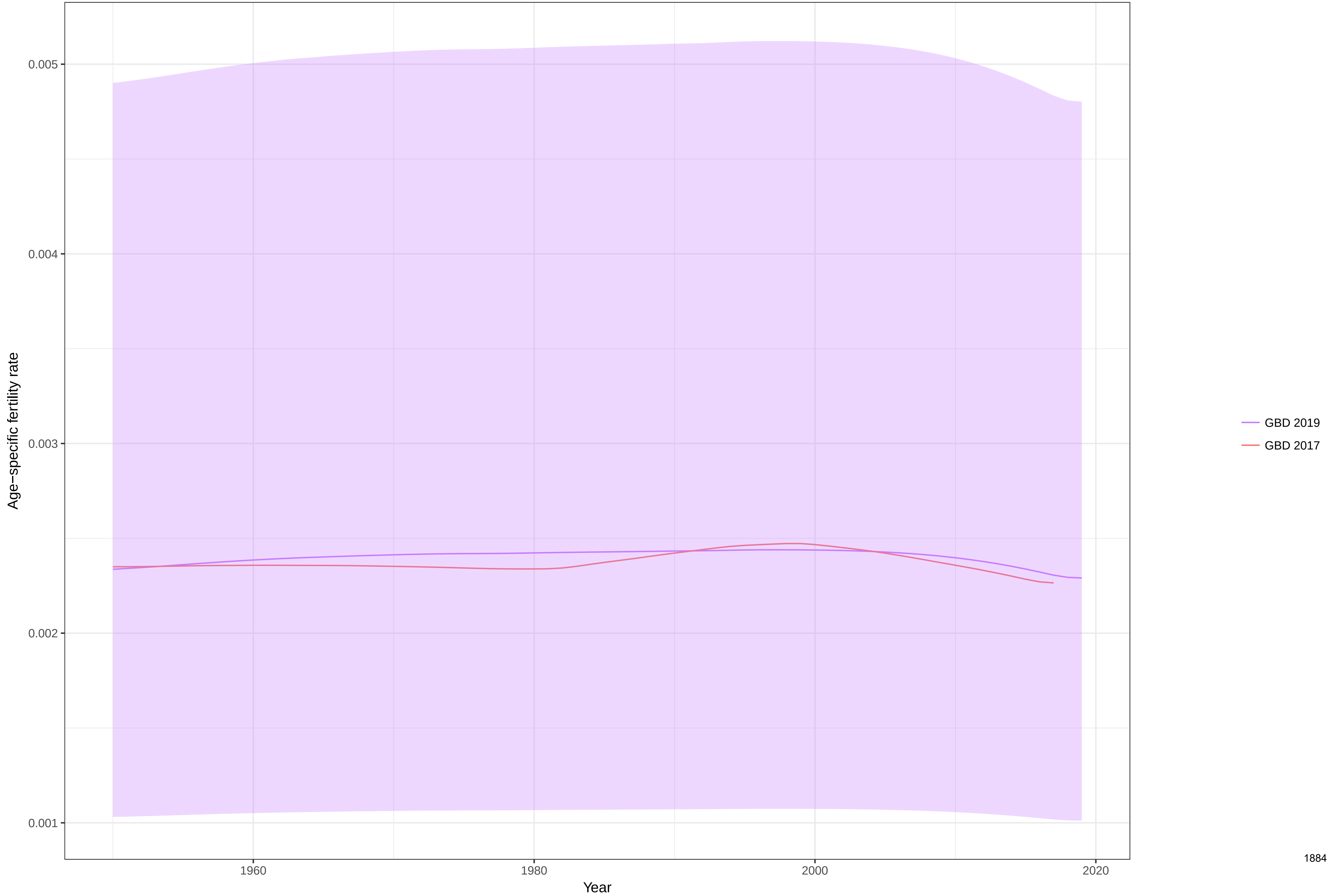

Age−specific fertility fitted trend lines, 1950−2019: South Sudan, Ages: 15−19
Data points enclosed with a diamond are reference sources

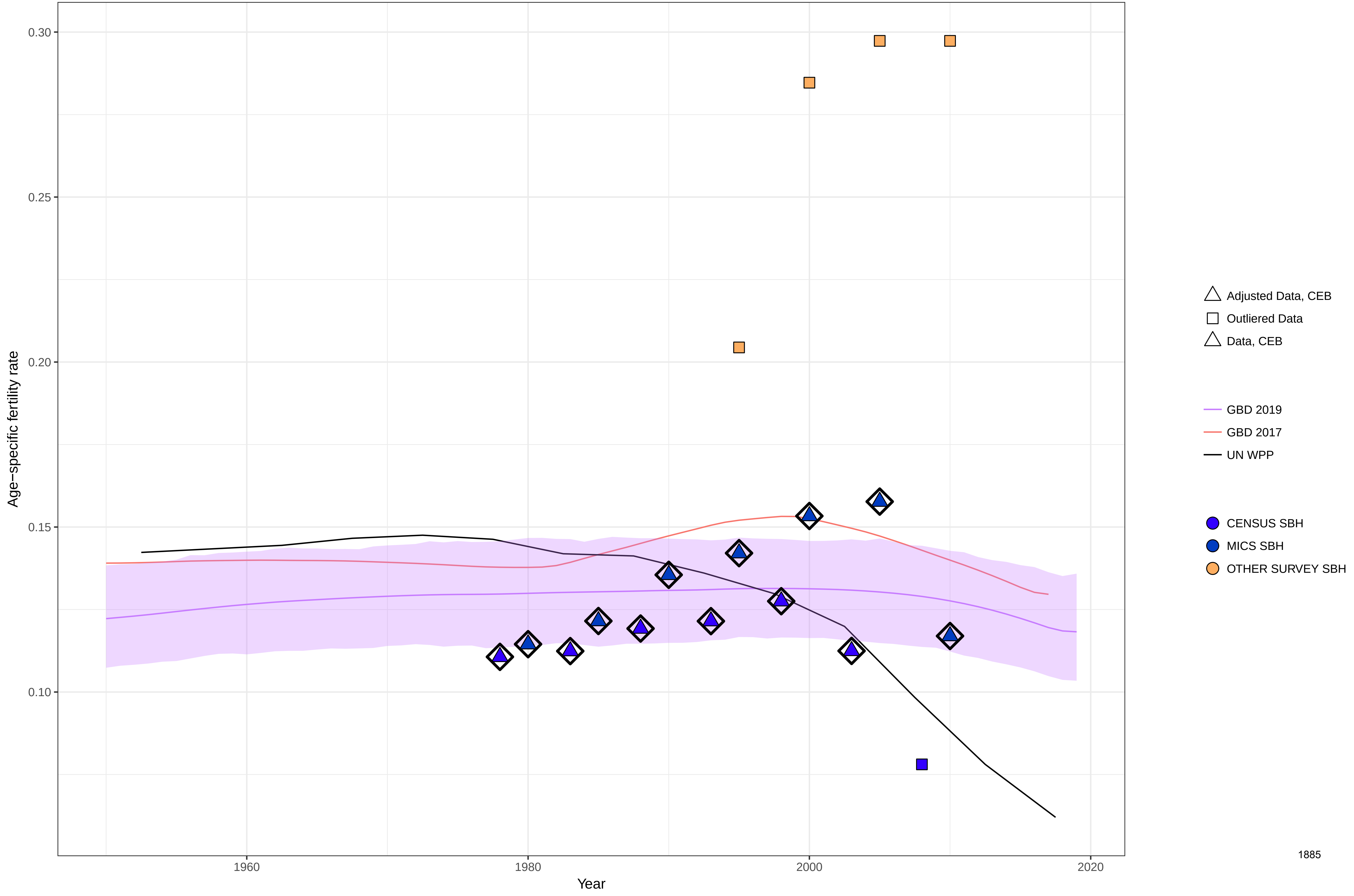

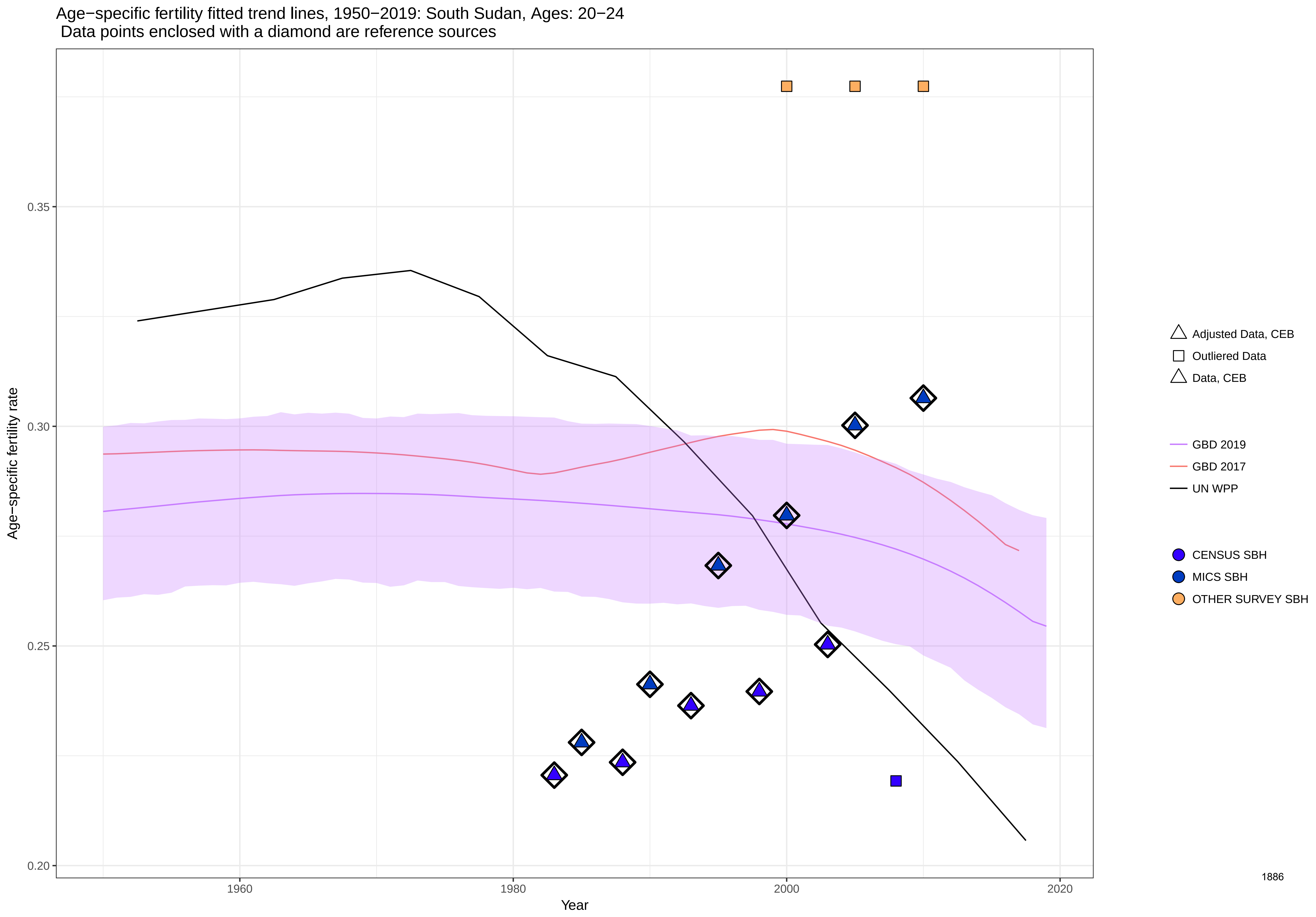

Age−specific fertility fitted trend lines, 1950−2019: South Sudan, Ages: 20−24
Data points enclosed with a diamond are reference sources
Age−specific fertility rate
0.35
0.30
0.25
0.20
1960
1980
2000
2020
Year
Adjusted Data, CEB
Outliered Data
Data, CEB
GBD 2019
GBD 2017
UN WPP
CENSUS SBH
MICS SBH
OTHER SURVEY SBH

Age−specific fertility fitted trend lines, 1950−2019: South Sudan, Ages: 25−29
Data points enclosed with a diamond are reference sources

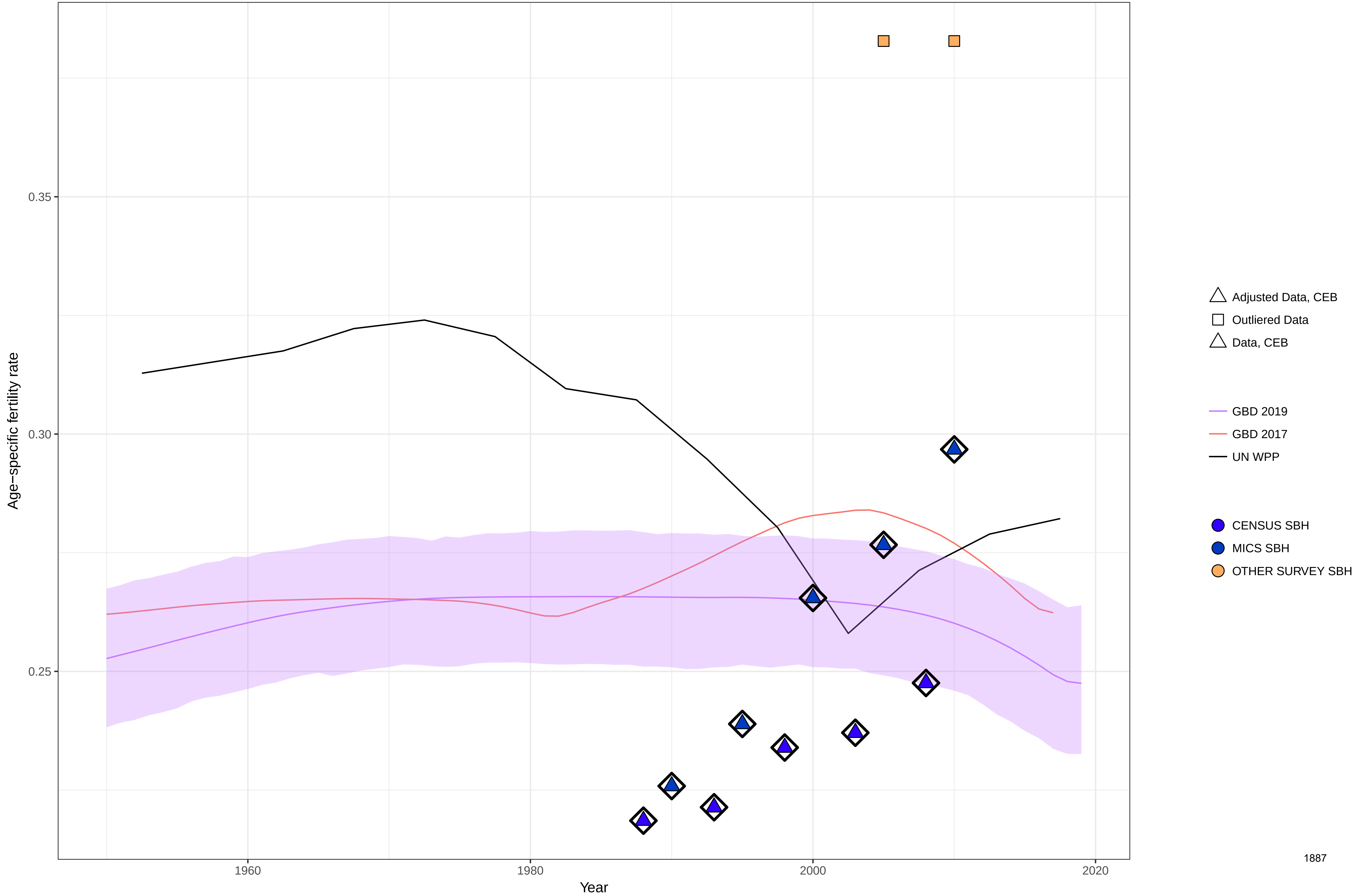

Age−specific fertility fitted trend lines, 1950−2019: South Sudan, Ages: 30−34
Data points enclosed with a diamond are reference sources

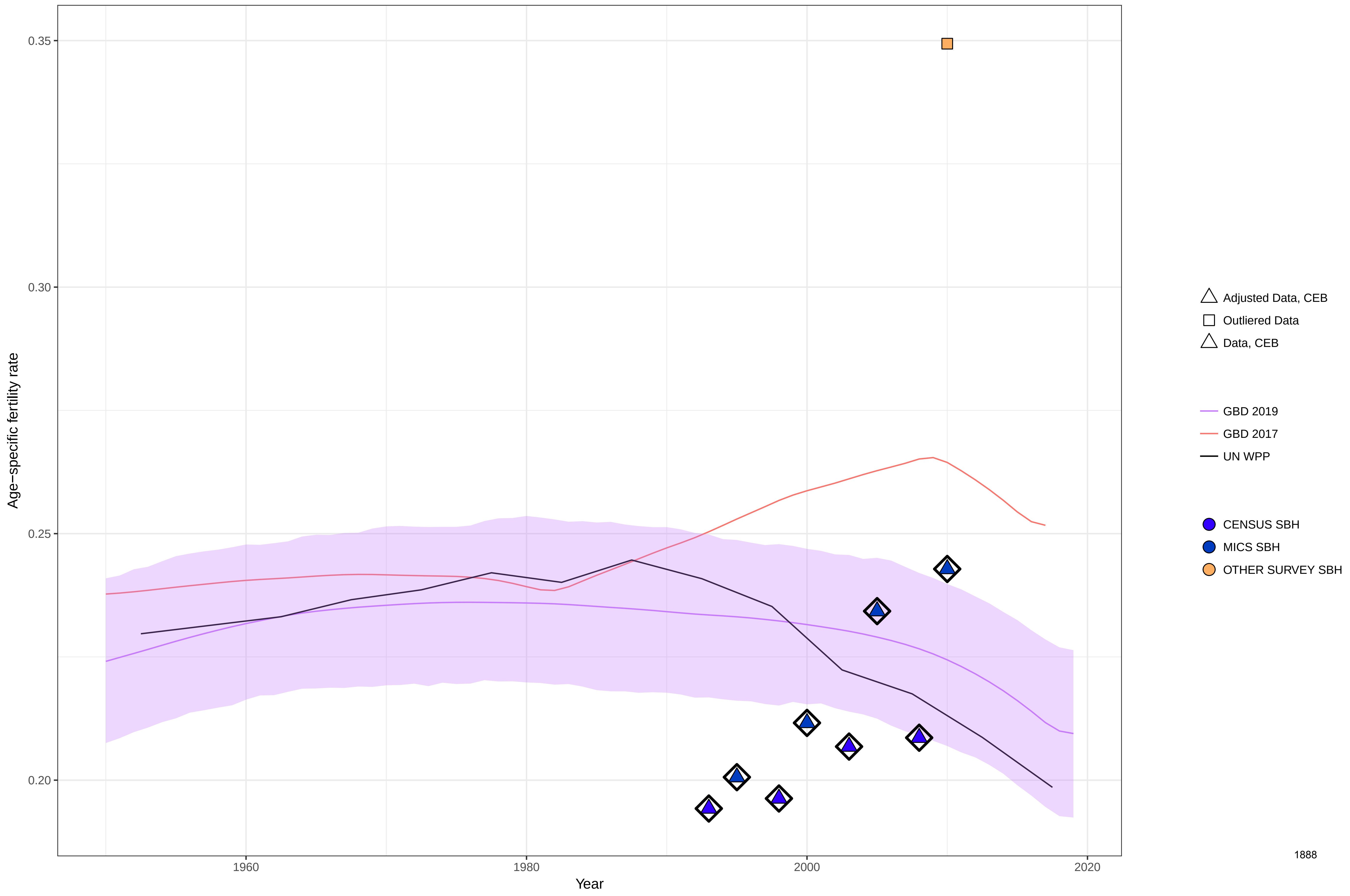

Age−specific fertility fitted trend lines, 1950−2019: South Sudan, Ages: 35−39
Data points enclosed with a diamond are reference sources

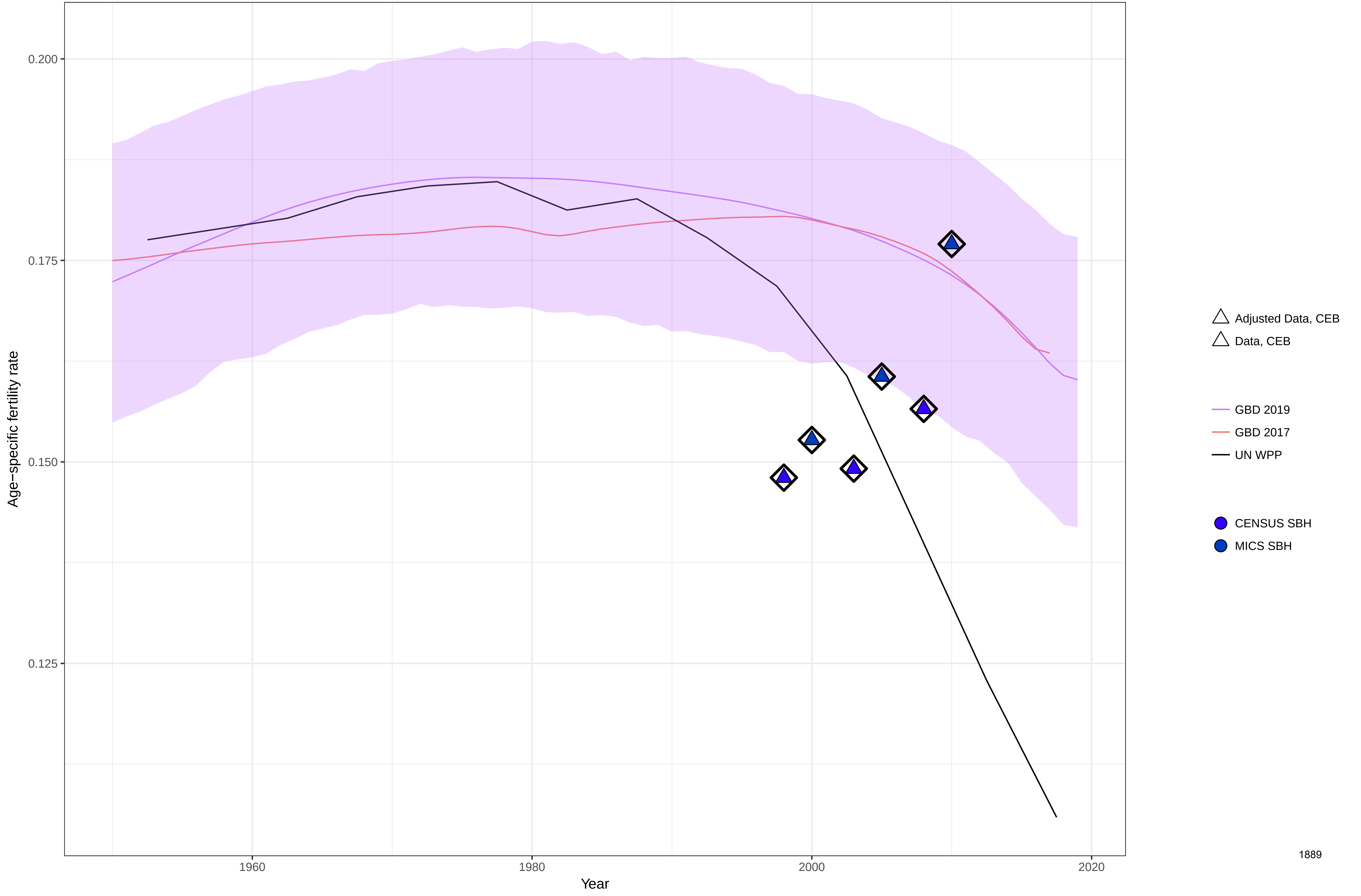

Age−specific fertility fitted trend lines, 1950−2019: South Sudan, Ages: 40−44
Data points enclosed with a diamond are reference sources

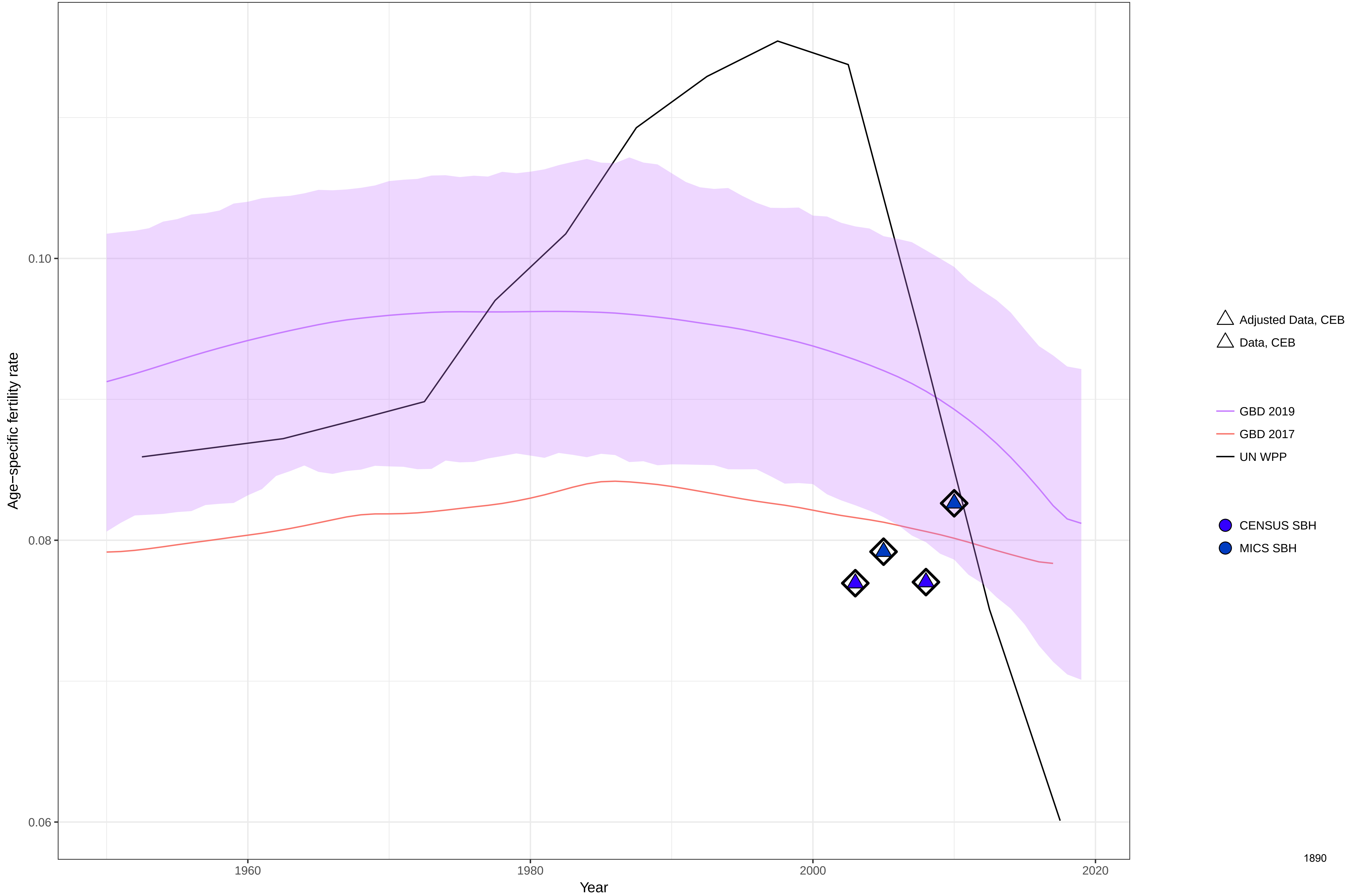

Age−specific fertility fitted trend lines, 1950−2019: South Sudan, Ages: 45−49
Data points enclosed with a diamond are reference sources

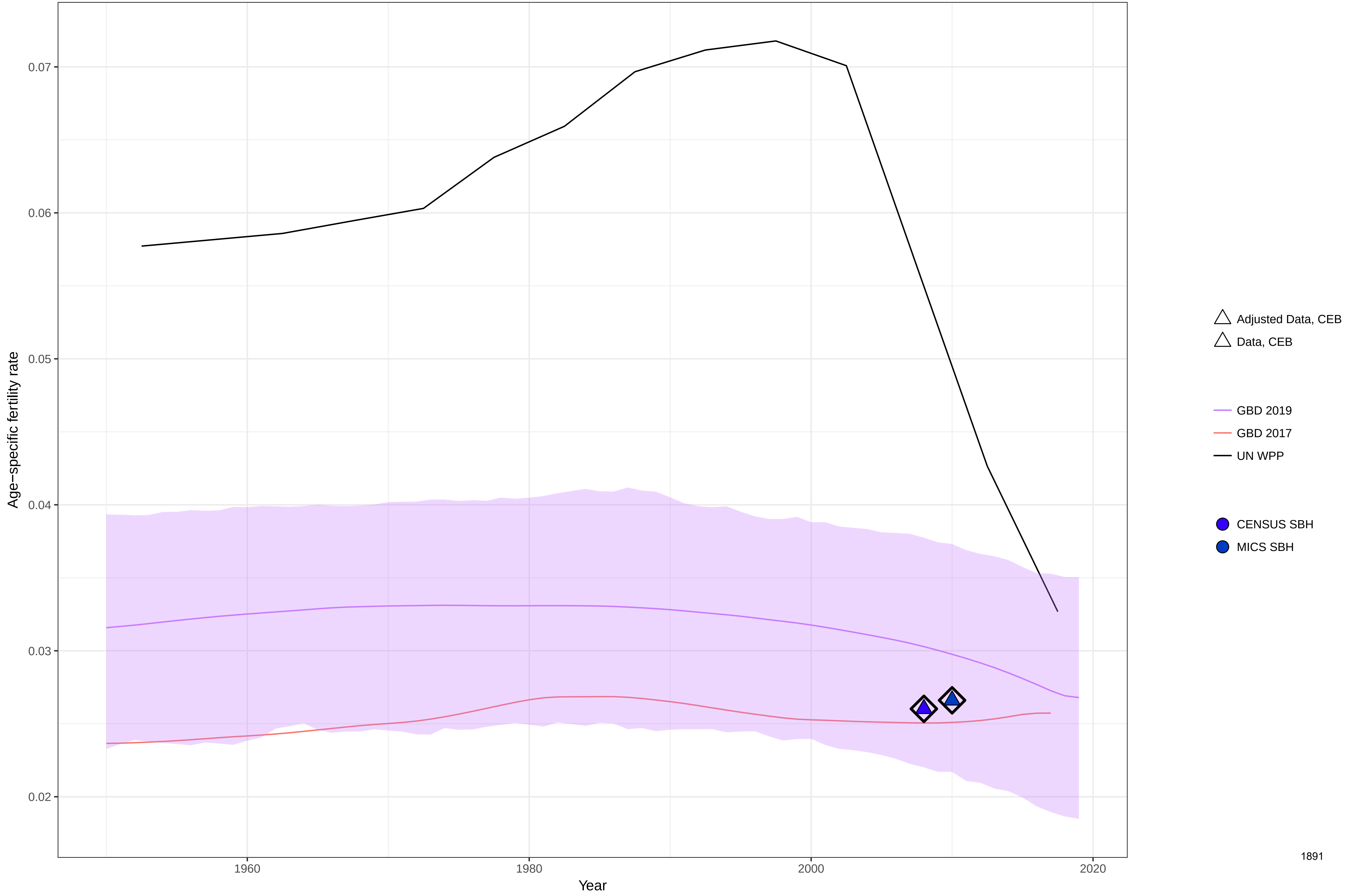

Age−specific fertility fitted trend lines, 1950−2019: South Sudan, Ages: 50−54
Data points enclosed with a diamond are reference sources

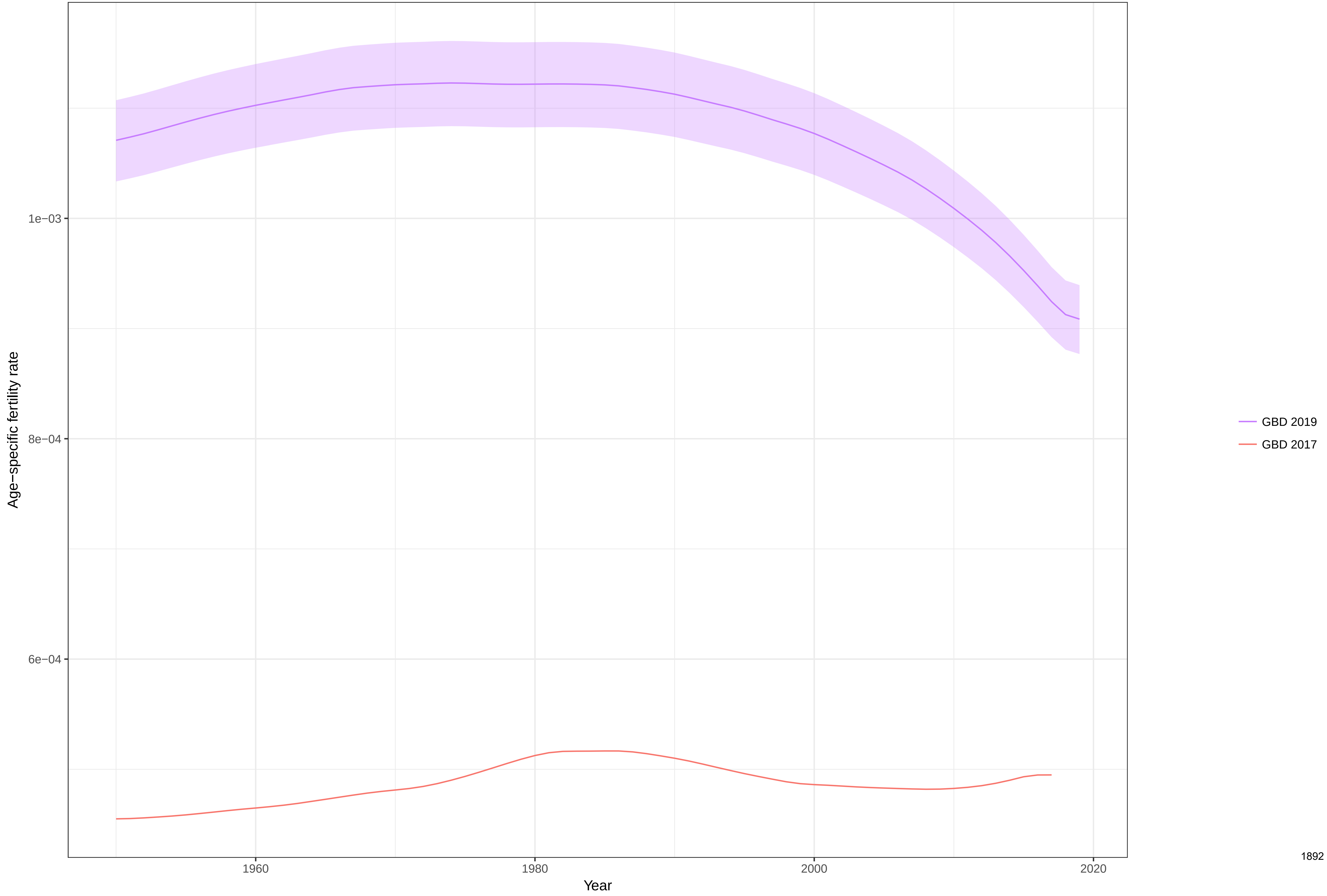

Total fertility rate fitted trend lines, 1950−2019: South Sudan TFR
Data points enclosed with a diamond are reference sources

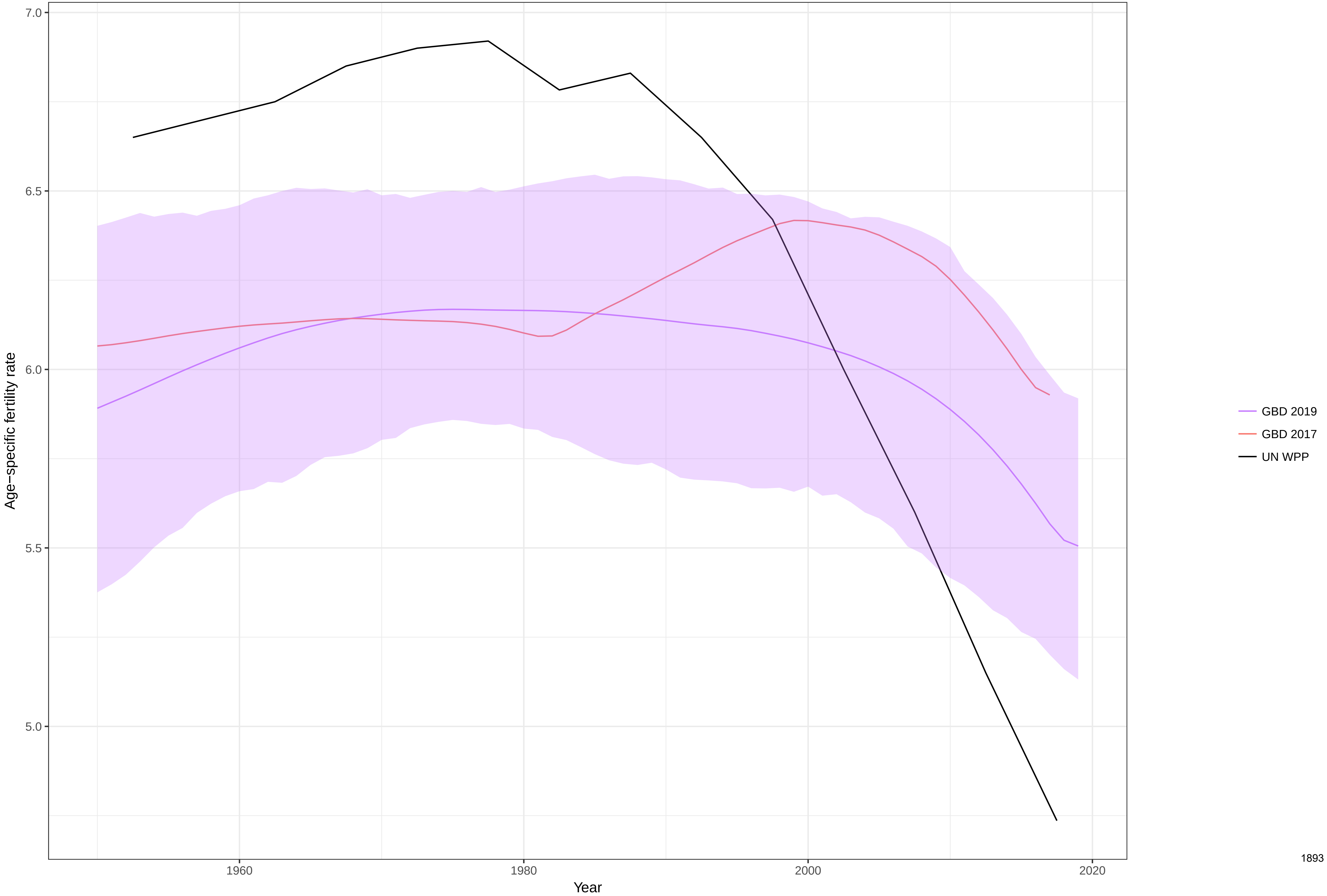

Age−specific fertility fitted trend lines, 1950−2019: Sao Tome and Principe, Ages: 10−14
Data points enclosed with a diamond are reference sources

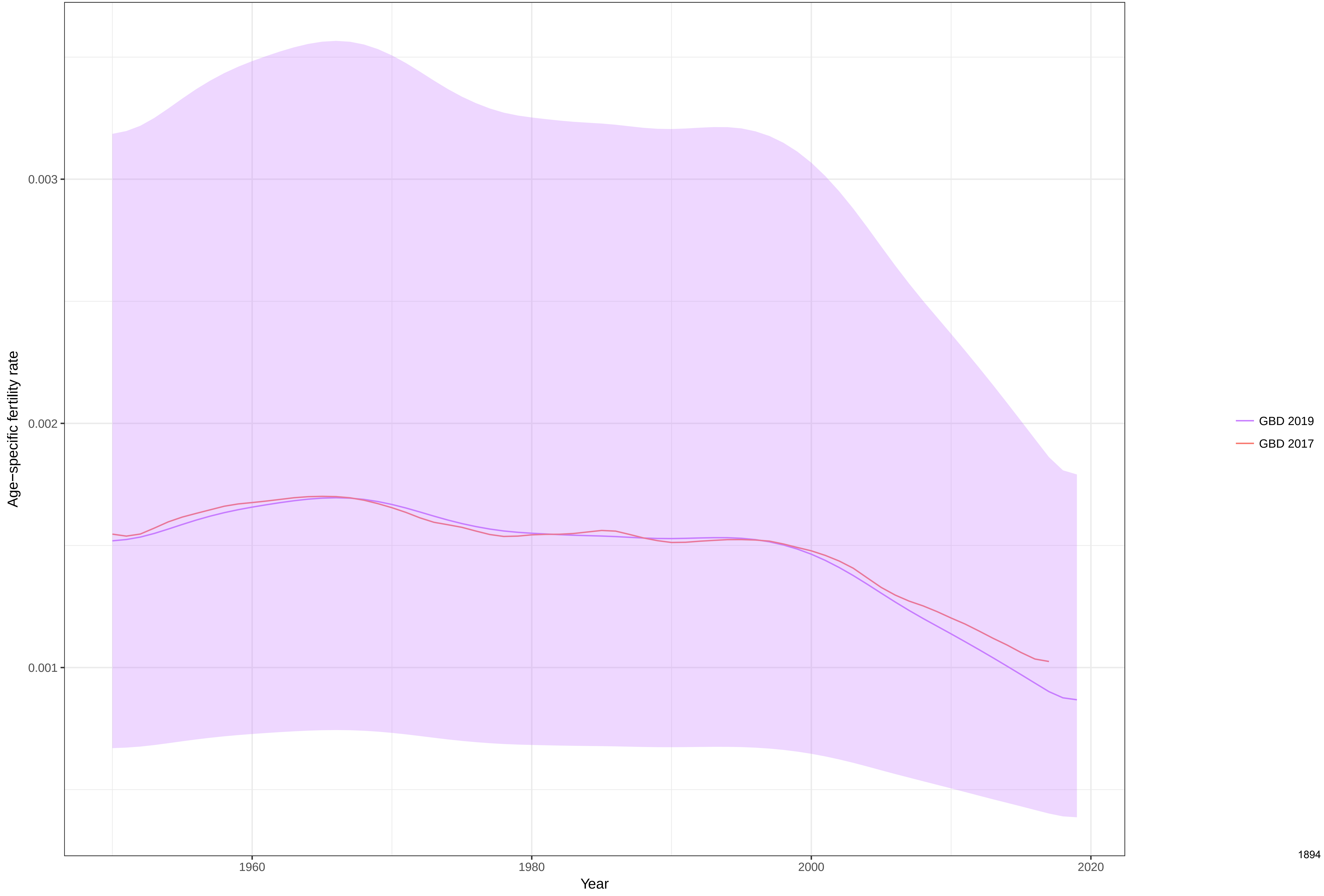

Age−specific fertility fitted trend lines, 1950−2019: Sao Tome and Principe, Ages: 15−19
Data points enclosed with a diamond are reference sources

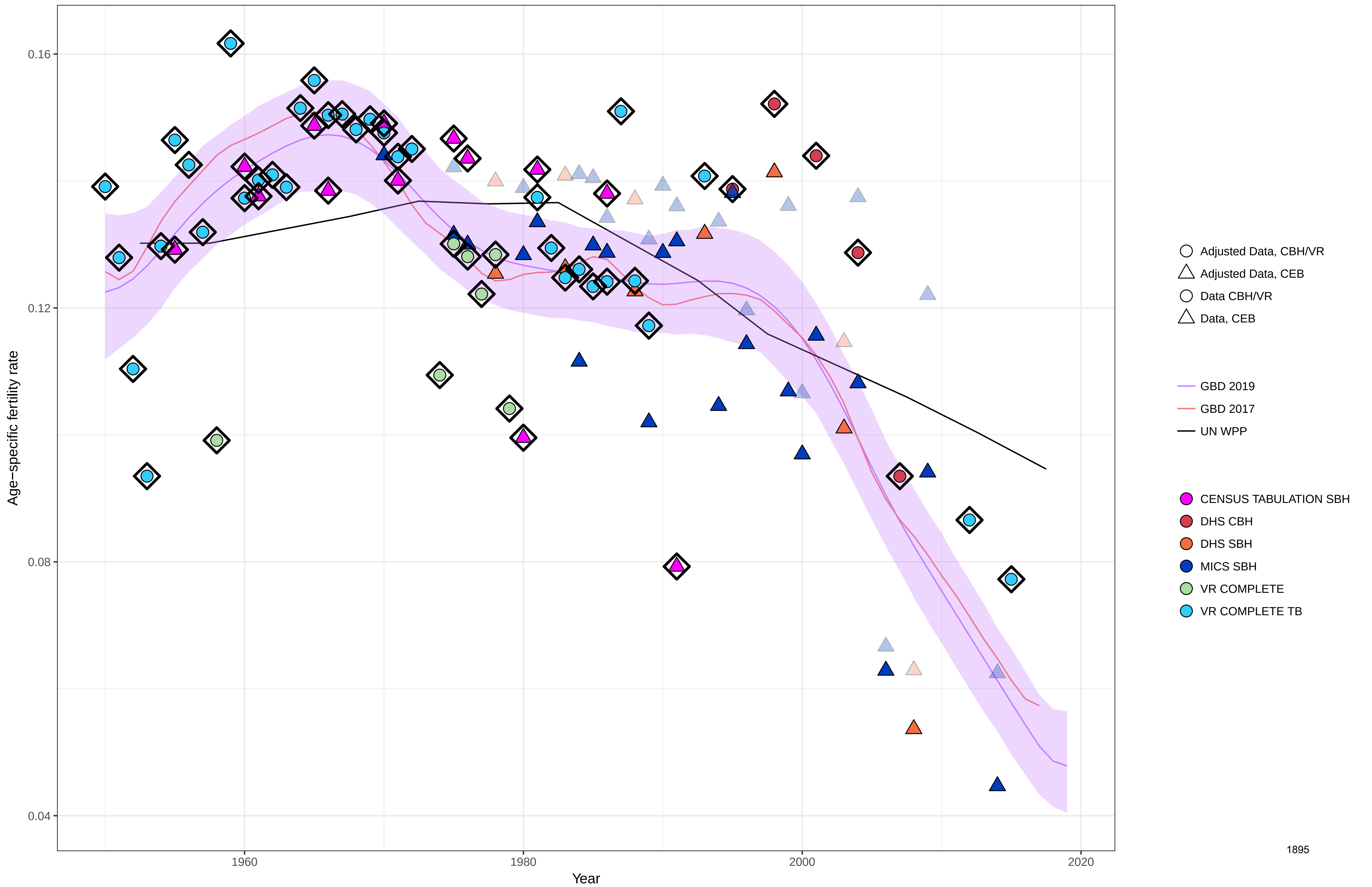

Age−specific fertility fitted trend lines, 1950−2019: Sao Tome and Principe, Ages: 20−24
Data points enclosed with a diamond are reference sources

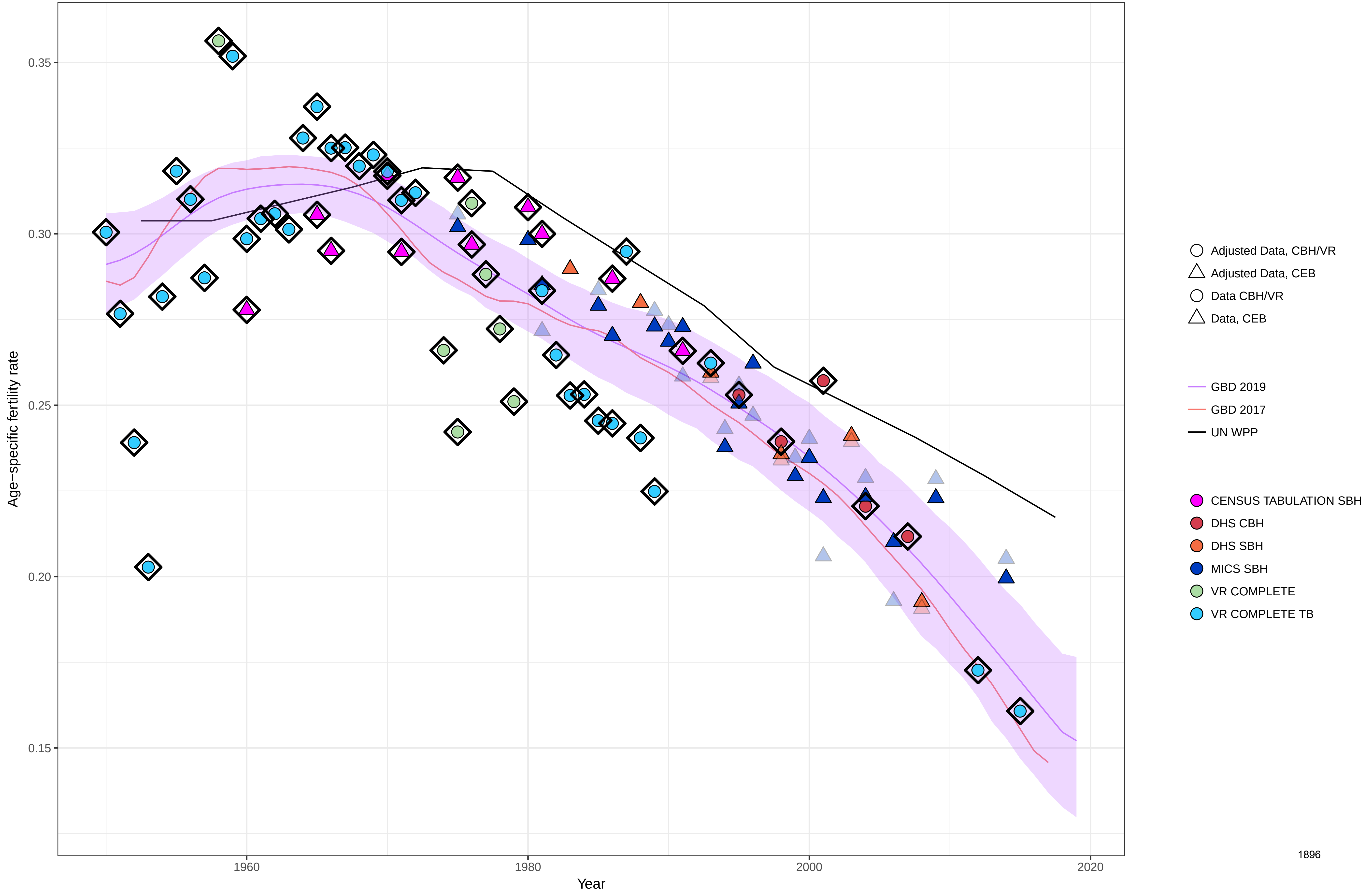

Age−specific fertility fitted trend lines, 1950−2019: Sao Tome and Principe, Ages: 25−29
Data points enclosed with a diamond are reference sources

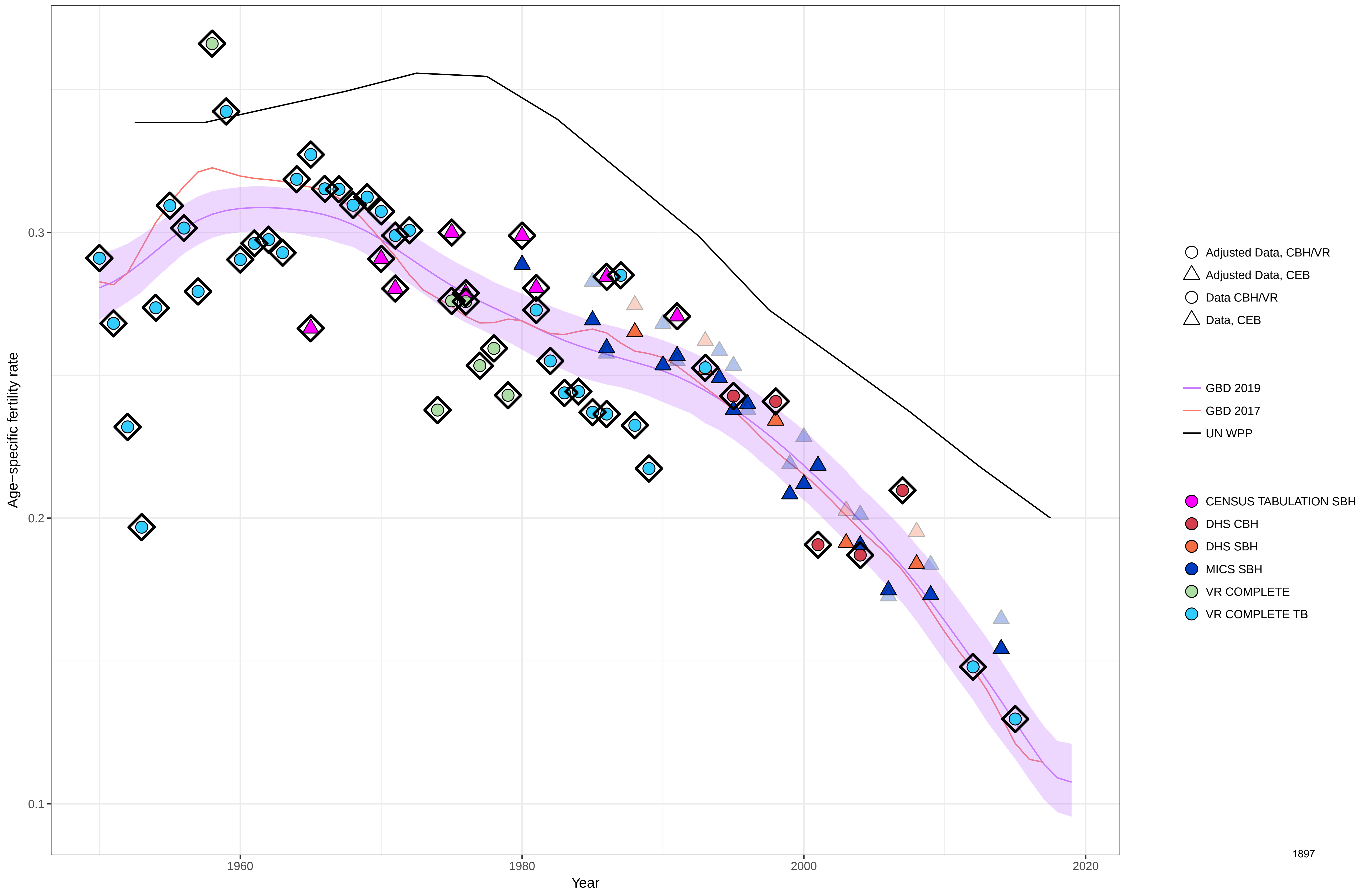

Age−specific fertility fitted trend lines, 1950−2019: Sao Tome and Principe, Ages: 30−34
Data points enclosed with a diamond are reference sources

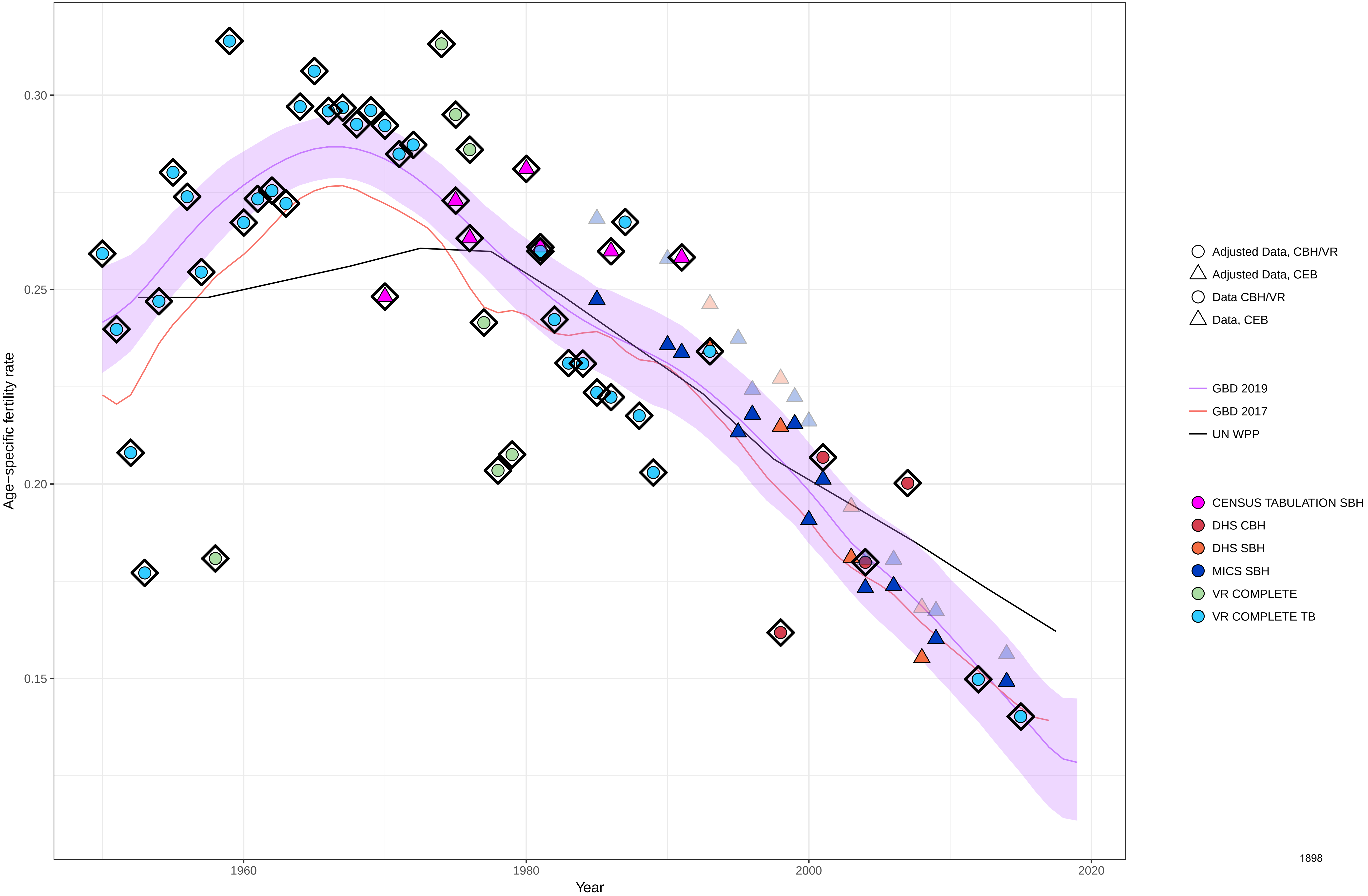

Age−specific fertility fitted trend lines, 1950−2019: Sao Tome and Principe, Ages: 35−39
Data points enclosed with a diamond are reference sources

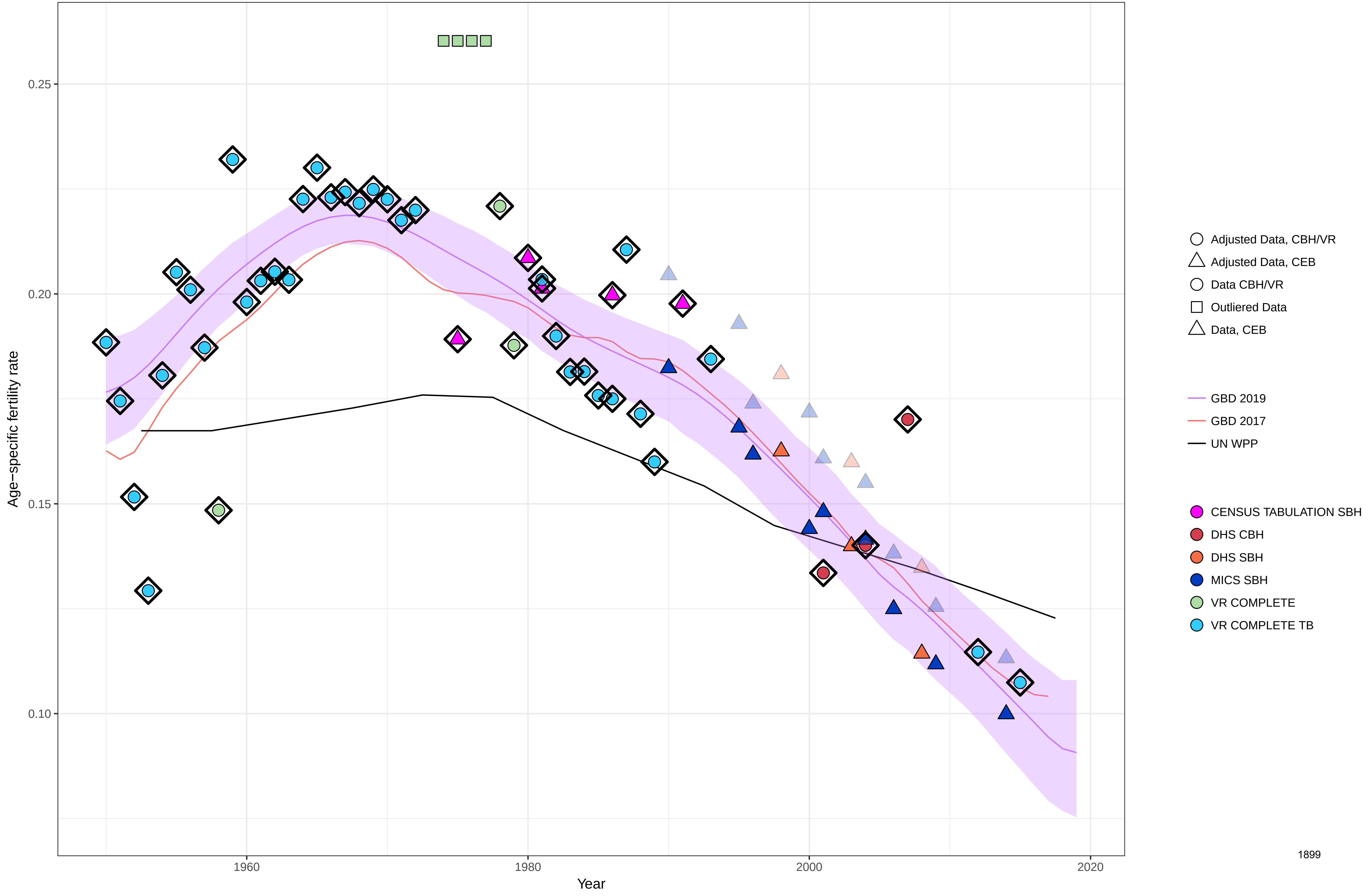

Age−specific fertility fitted trend lines, 1950−2019: Sao Tome and Principe, Ages: 40−44
Data points enclosed with a diamond are reference sources

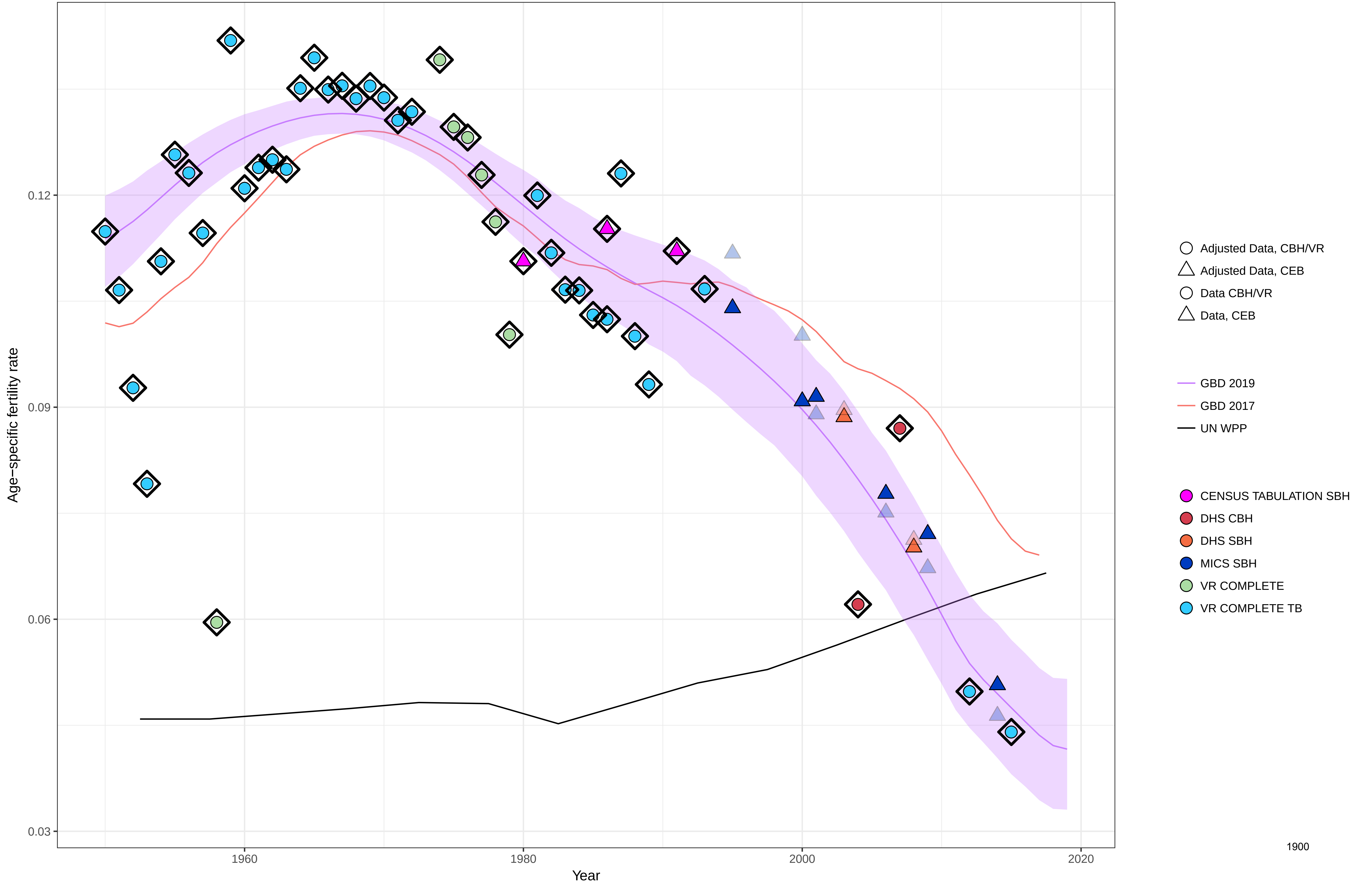

Age−specific fertility fitted trend lines, 1950−2019: Sao Tome and Principe, Ages: 45−49
Data points enclosed with a diamond are reference sources

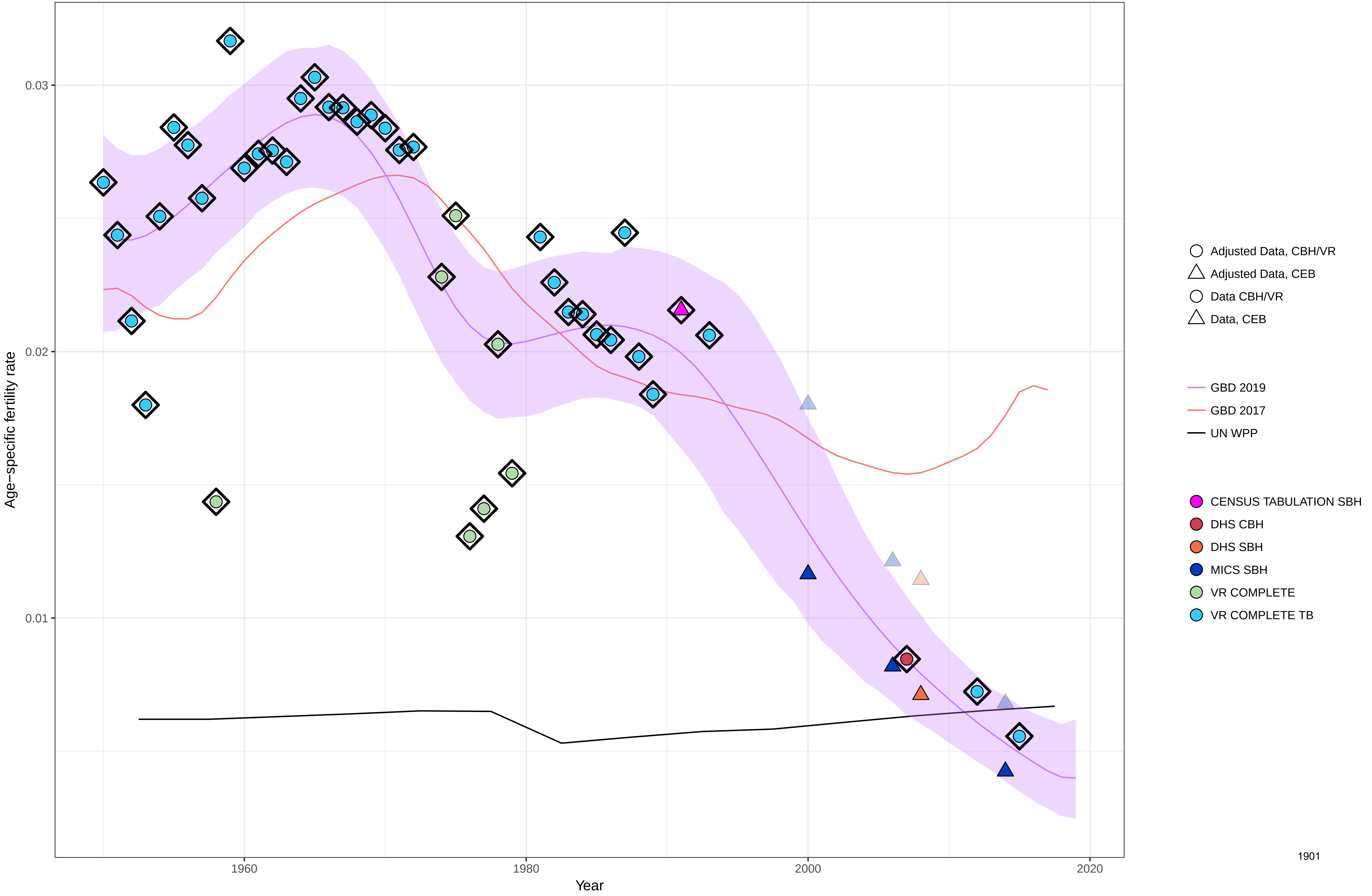

Age−specific fertility fitted trend lines, 1950−2019: Sao Tome and Principe, Ages: 50−54
Data points enclosed with a diamond are reference sources

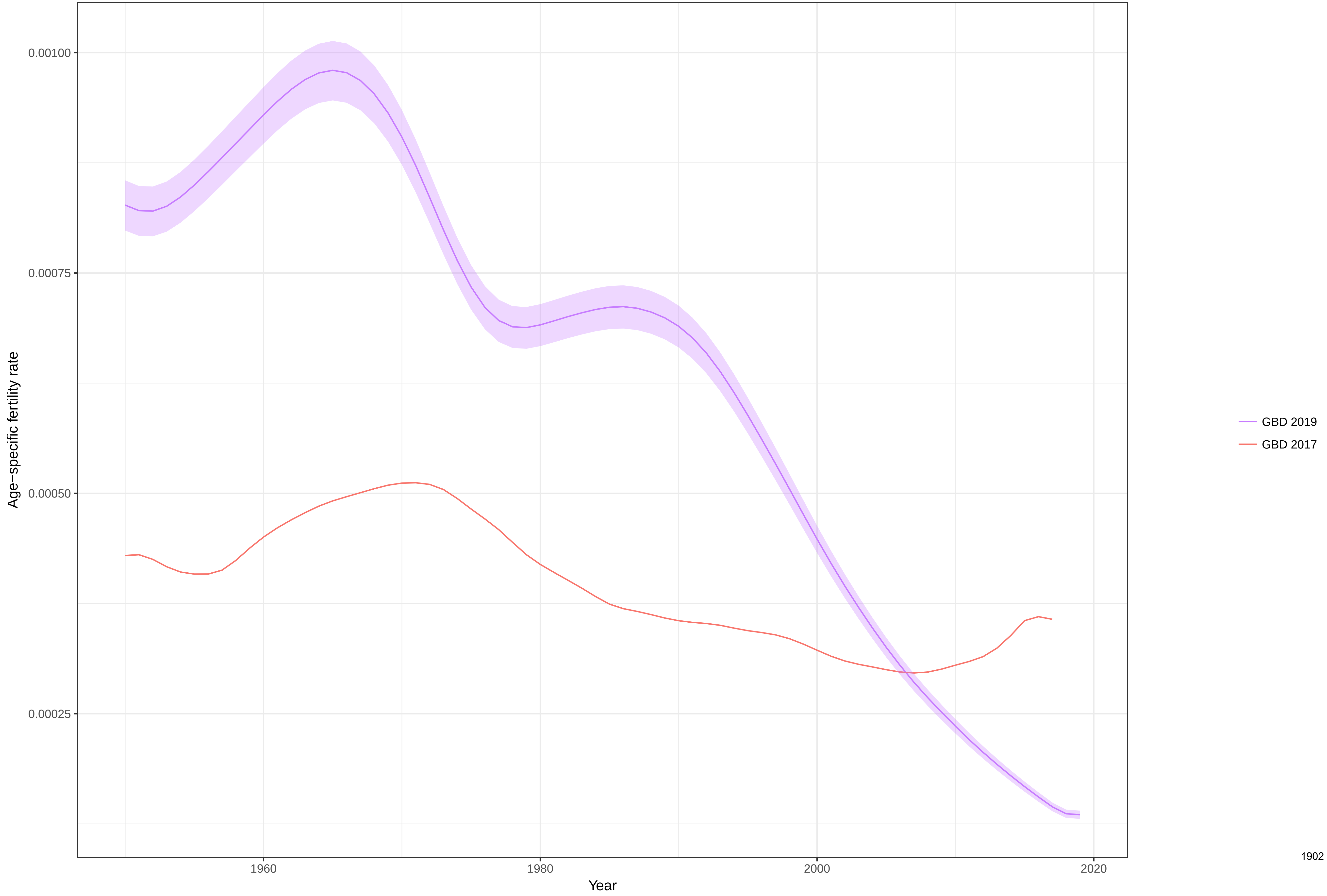

Total fertility rate fitted trend lines, 1950−2019: Sao Tome and Principe TFR
Data points enclosed with a diamond are reference sources

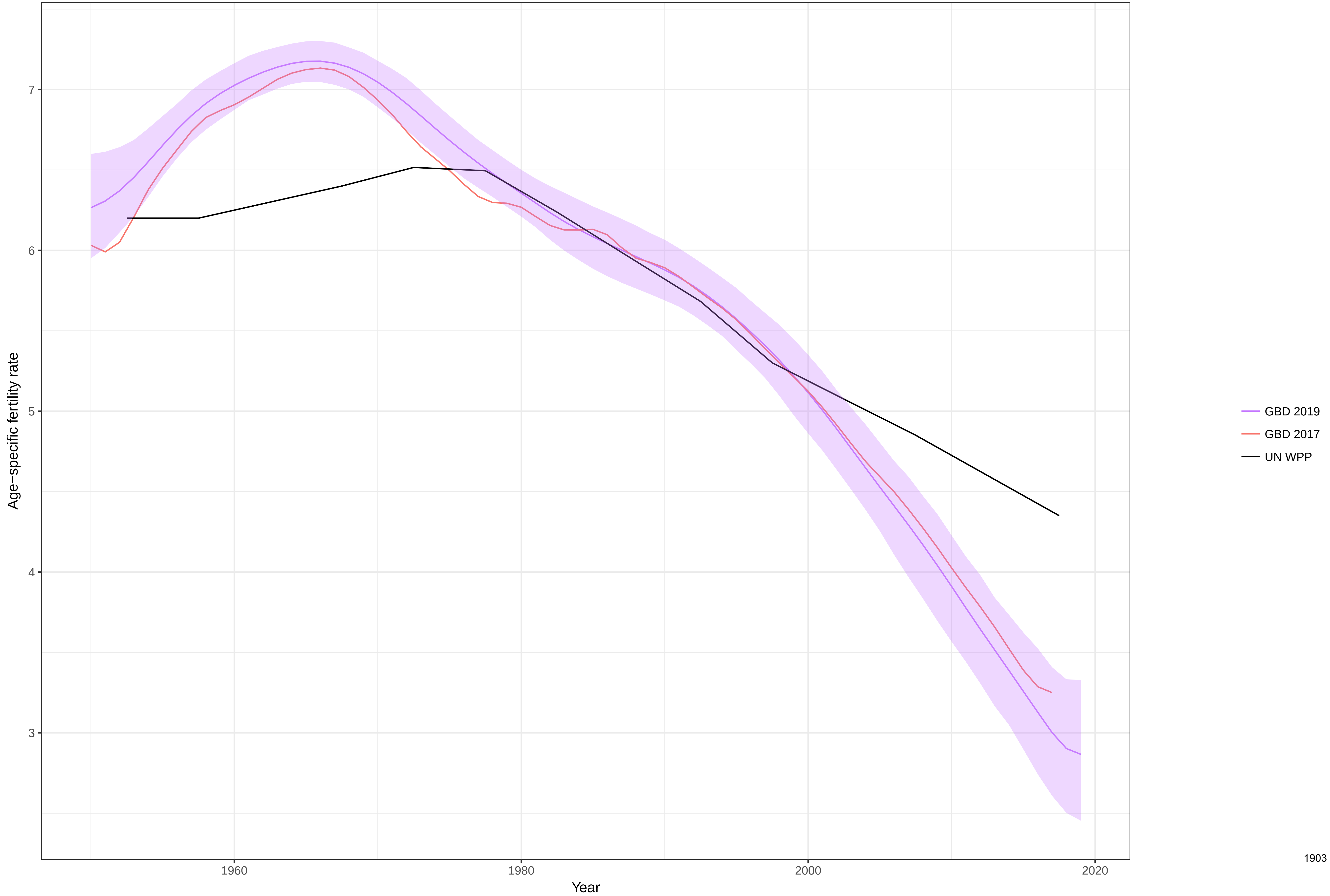

Age−specific fertility fitted trend lines, 1950−2019: Suriname, Ages: 10−14
Data points enclosed with a diamond are reference sources

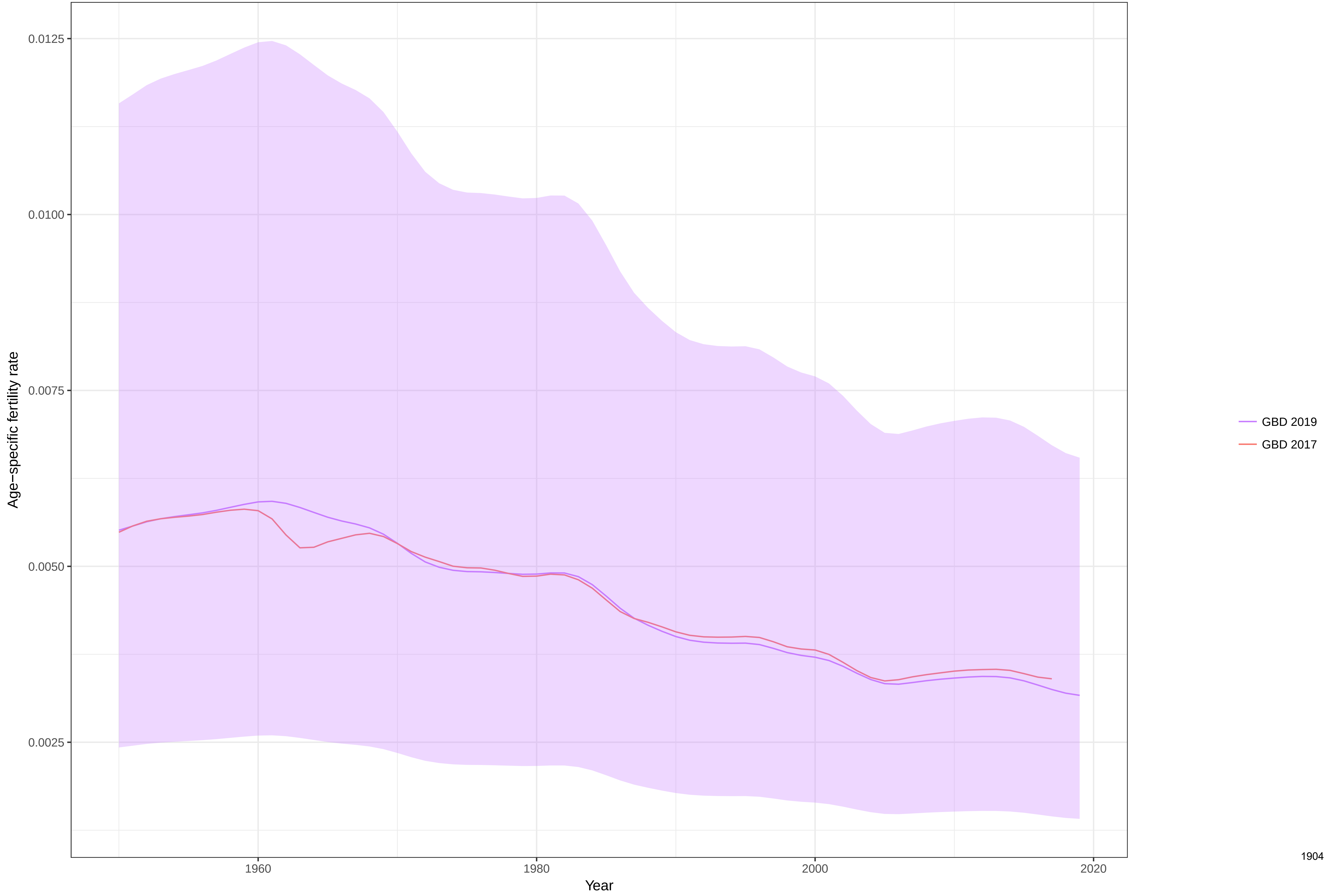

Age−specific fertility fitted trend lines, 1950−2019: Suriname, Ages: 15−19
Data points enclosed with a diamond are reference sources

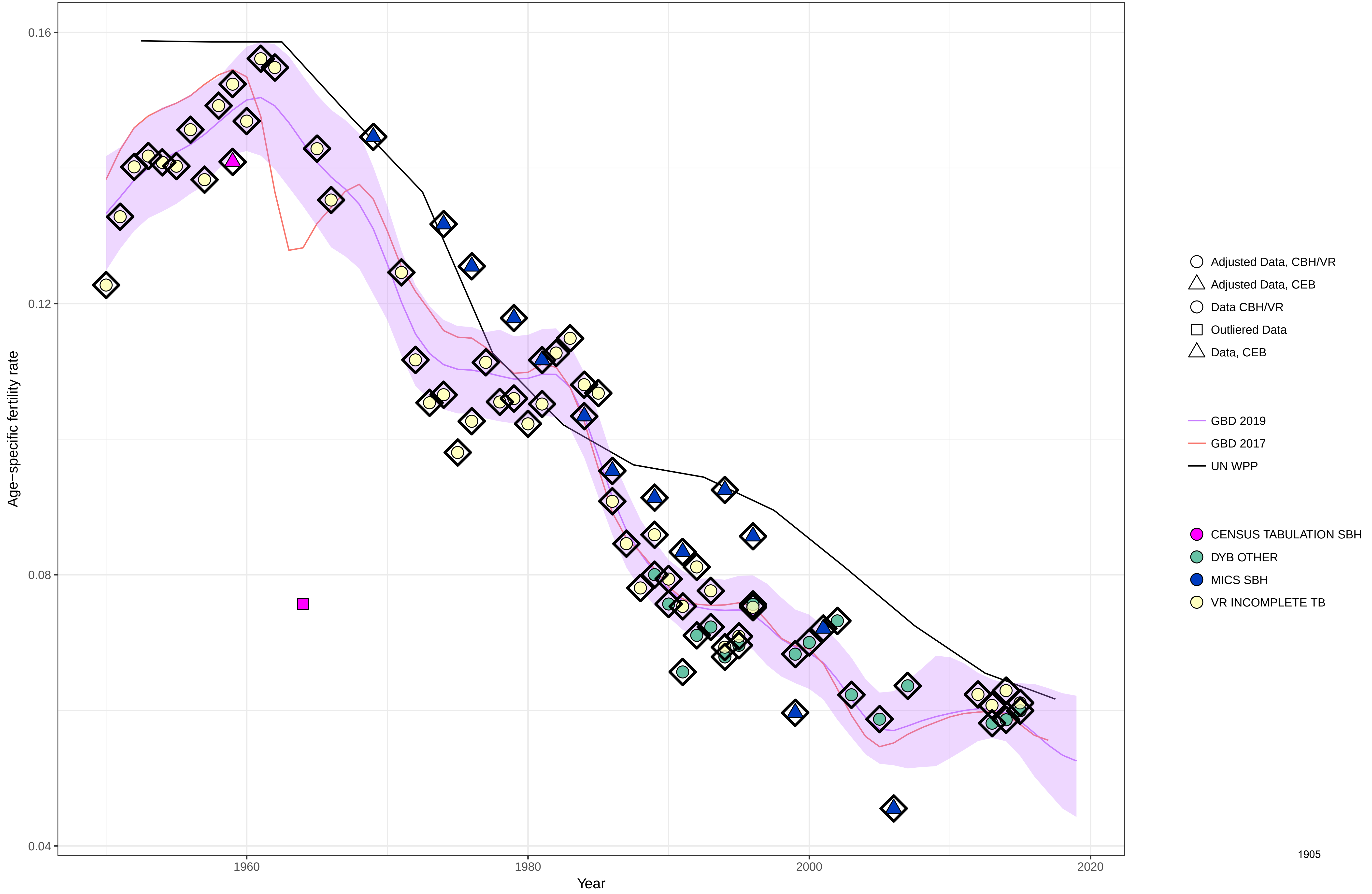

Age−specific fertility fitted trend lines, 1950−2019: Suriname, Ages: 20−24
Data points enclosed with a diamond are reference sources

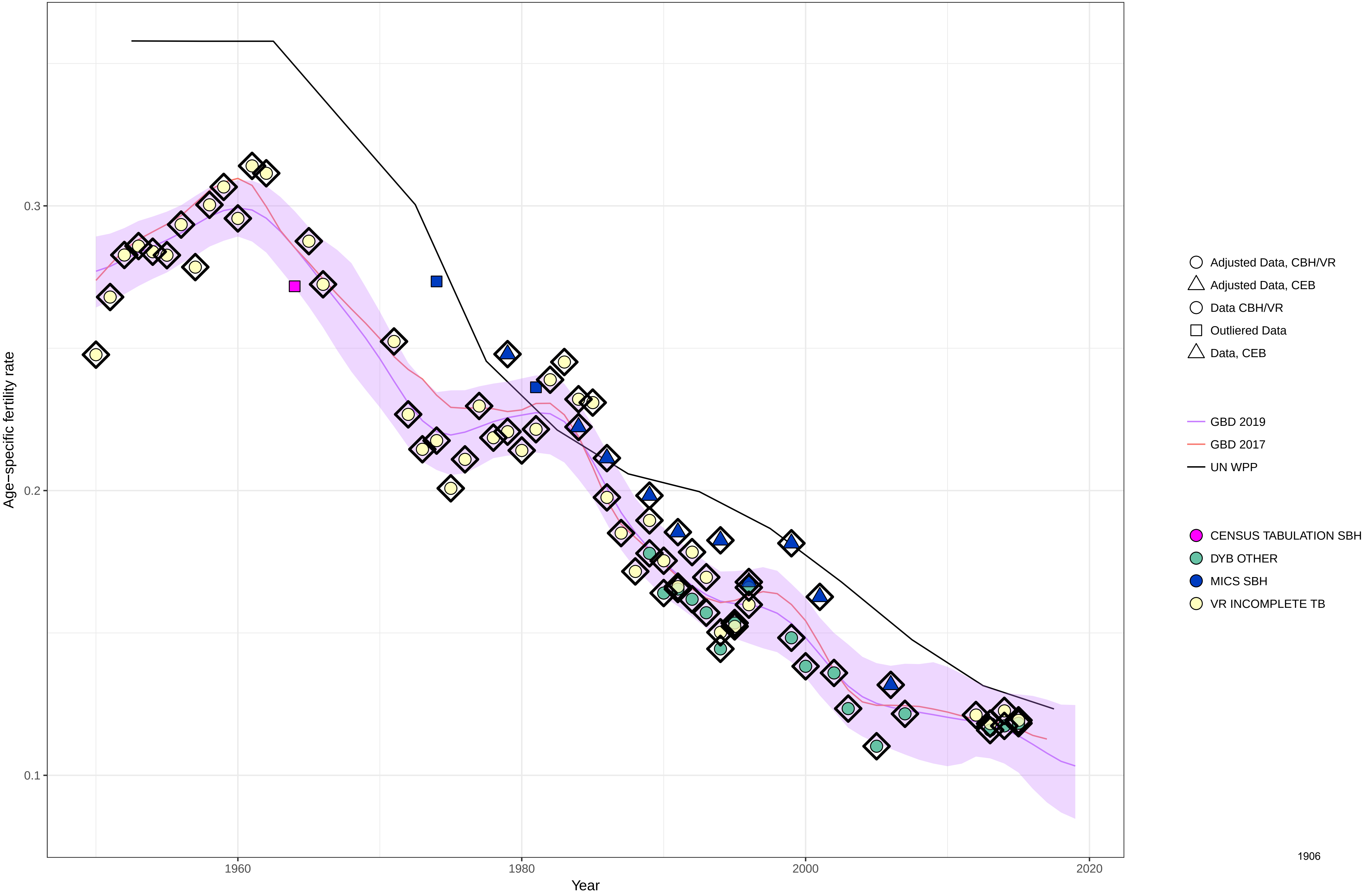

Age−specific fertility fitted trend lines, 1950−2019: Suriname, Ages: 25−29
Data points enclosed with a diamond are reference sources

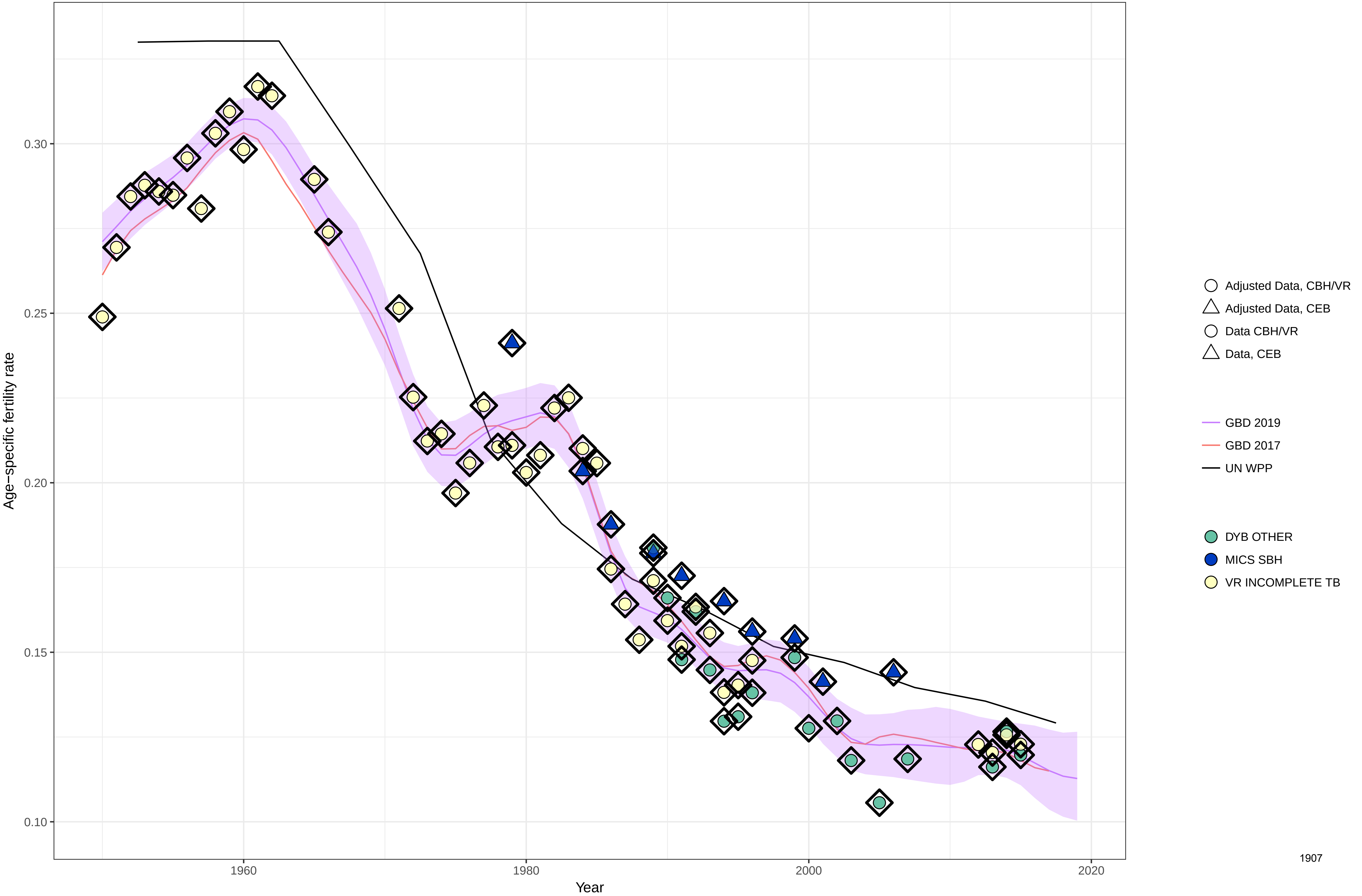

Age−specific fertility fitted trend lines, 1950−2019: Suriname, Ages: 30−34
Data points enclosed with a diamond are reference sources

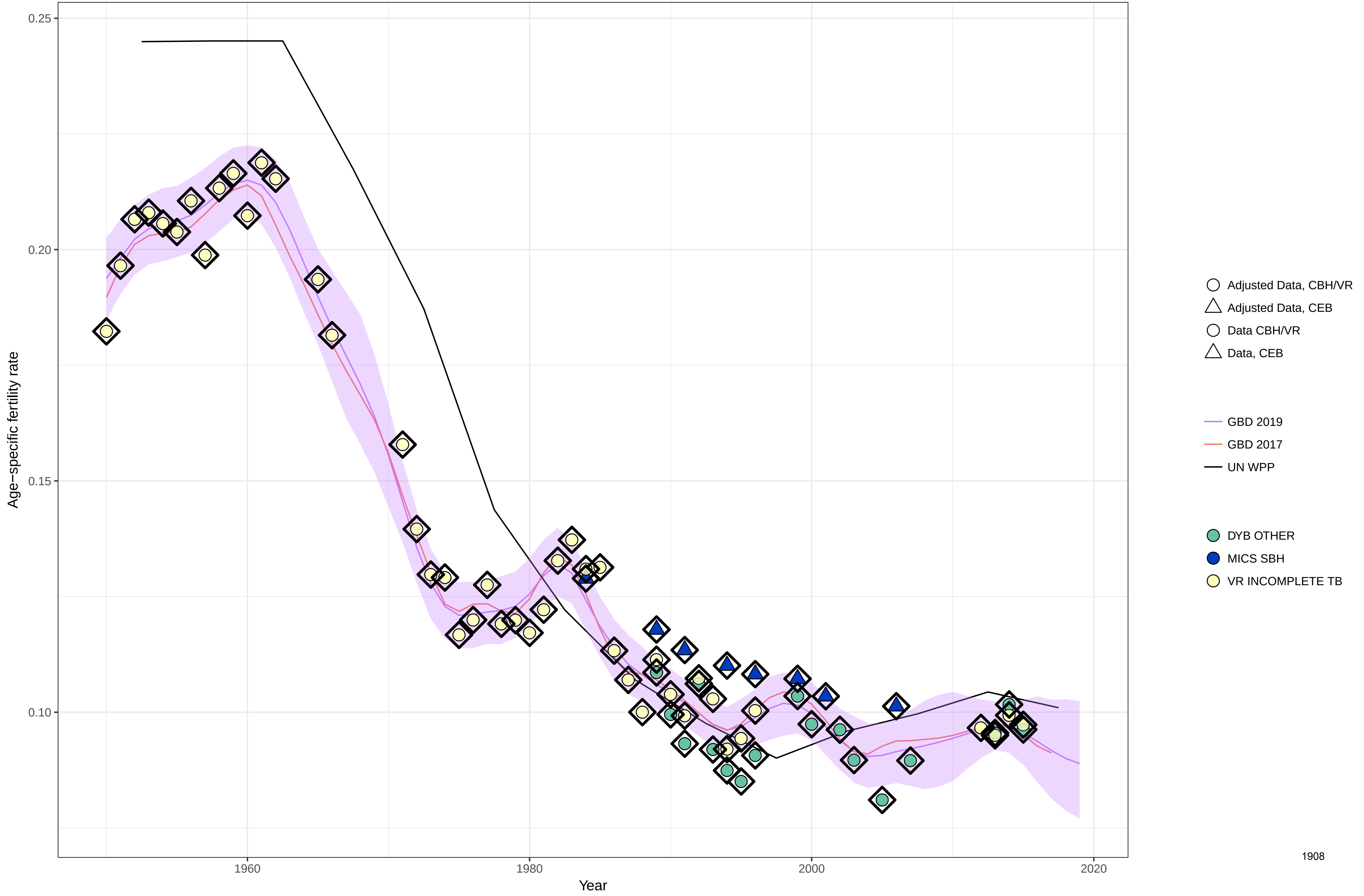

Age−specific fertility fitted trend lines, 1950−2019: Suriname, Ages: 35−39
Data points enclosed with a diamond are reference sources

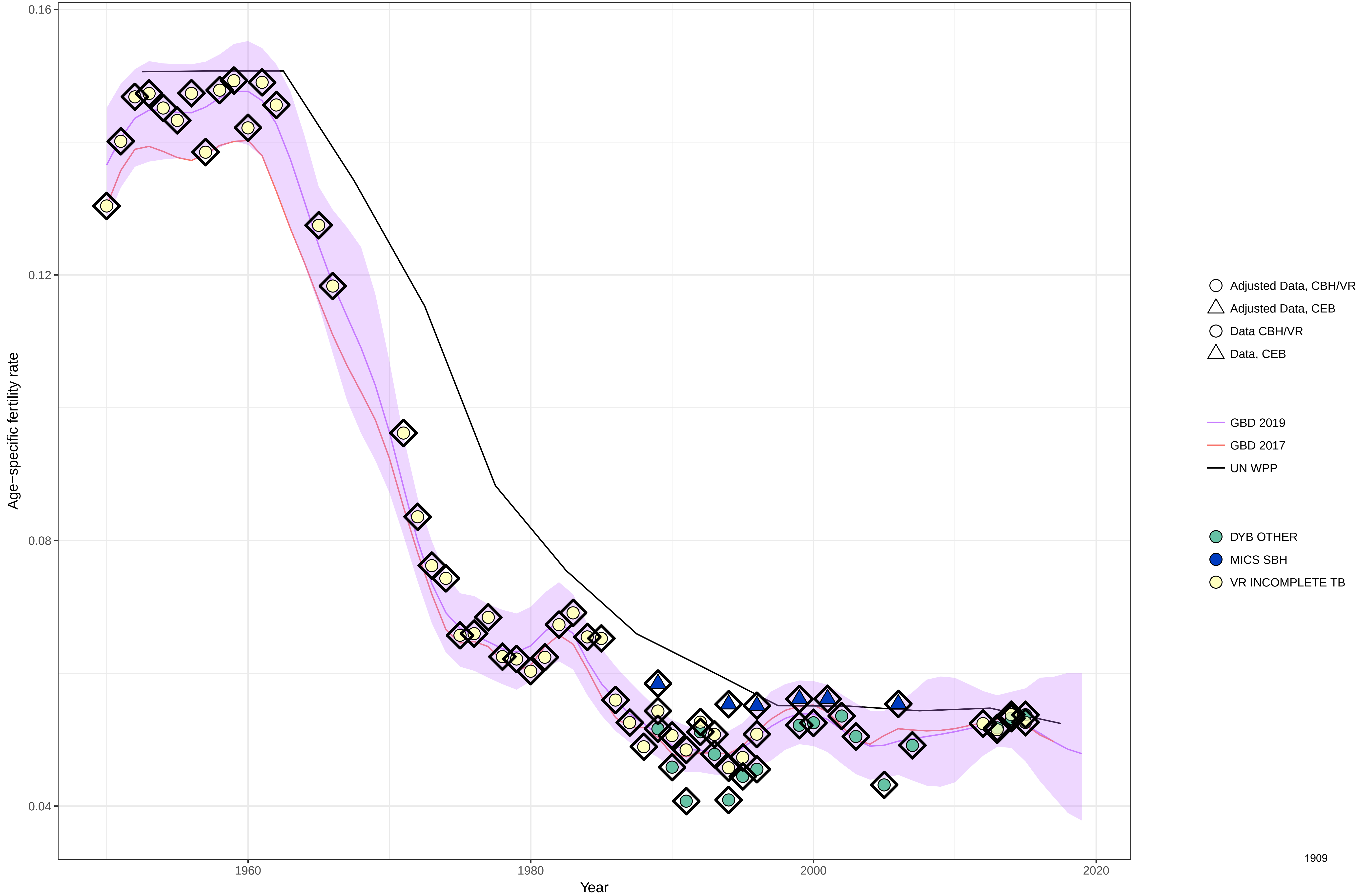

Age−specific fertility fitted trend lines, 1950−2019: Suriname, Ages: 40−44
Data points enclosed with a diamond are reference sources

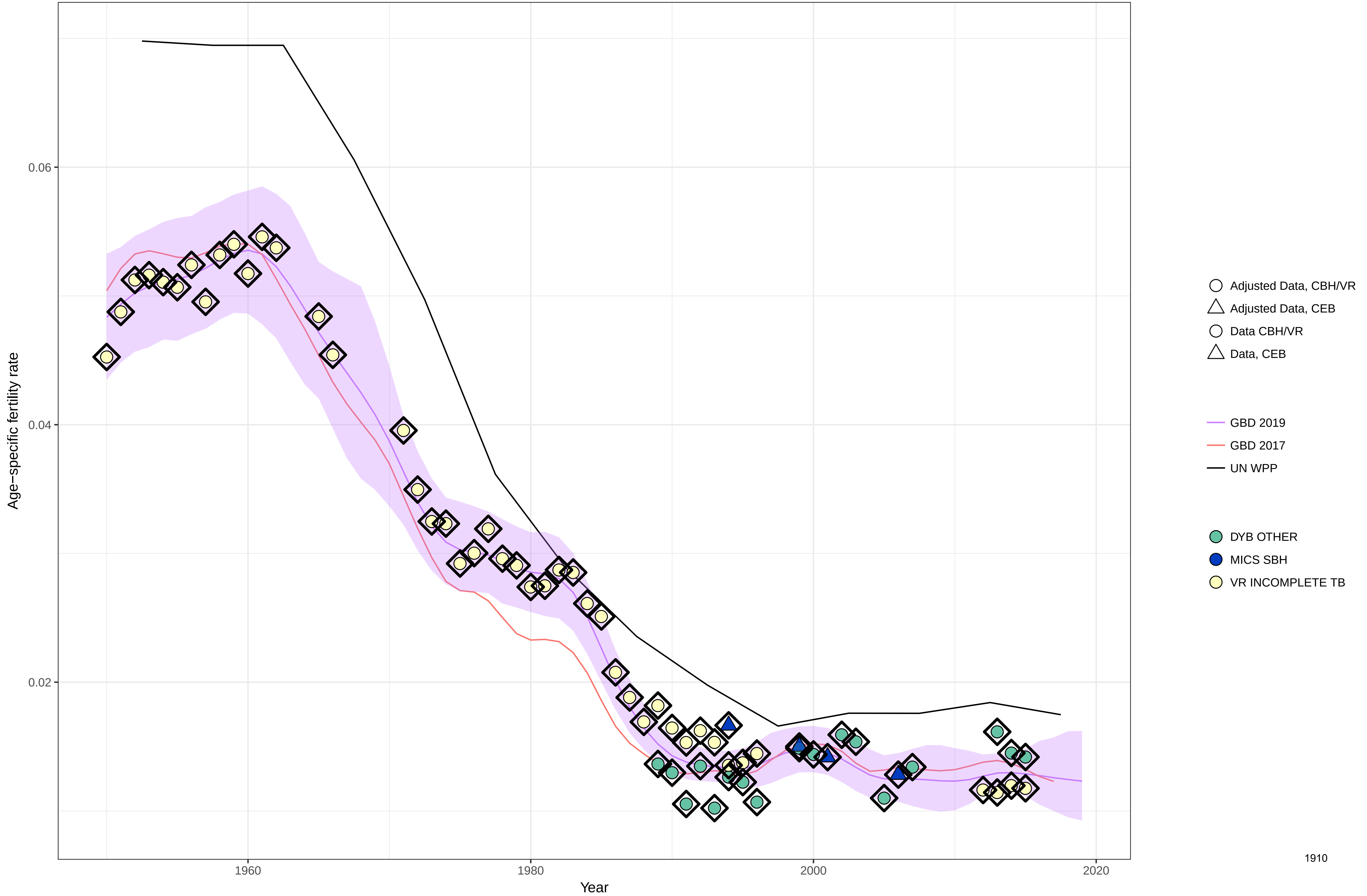

Age−specific fertility fitted trend lines, 1950−2019: Suriname, Ages: 45−49
Data points enclosed with a diamond are reference sources

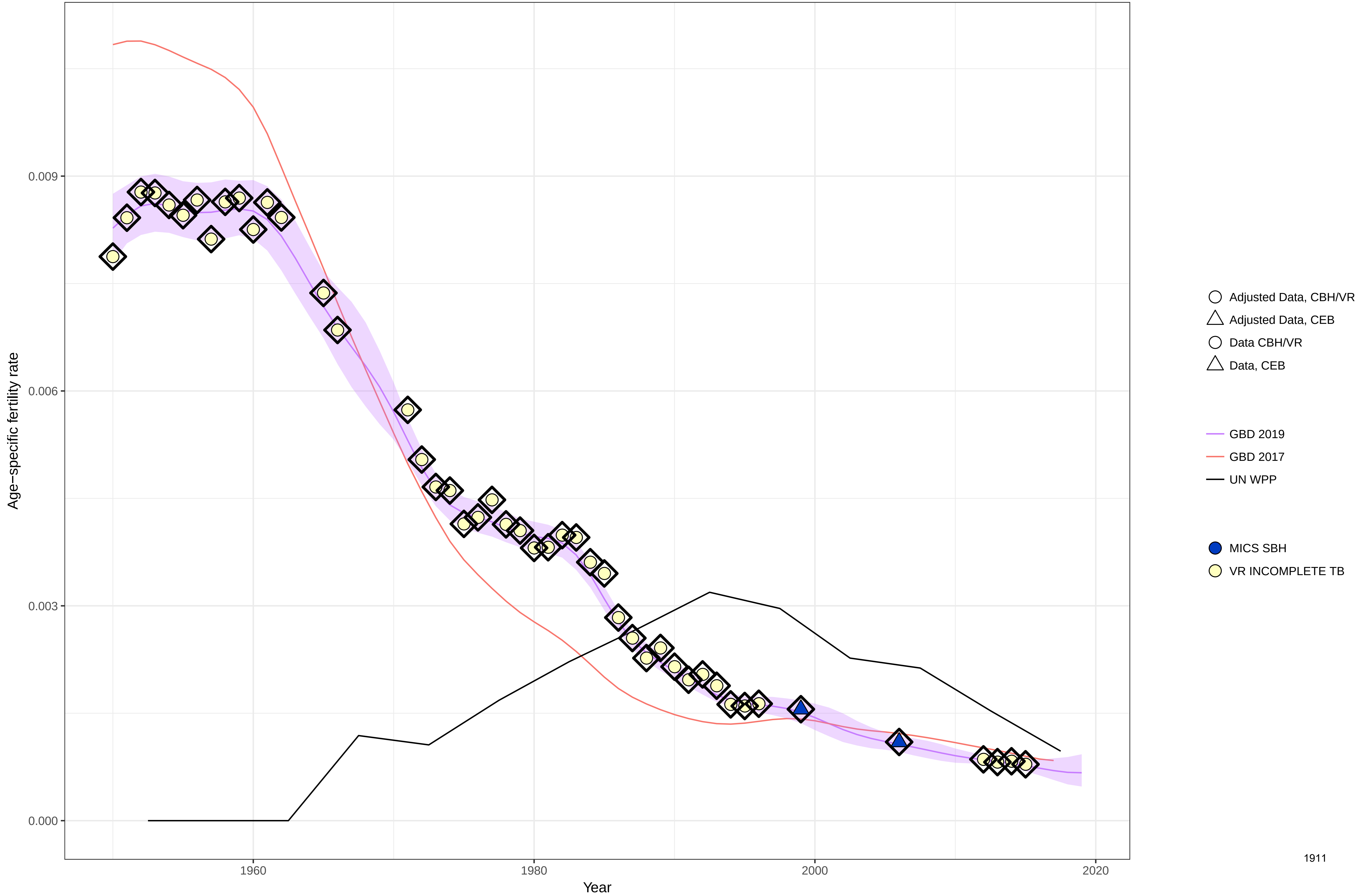

Age−specific fertility fitted trend lines, 1950−2019: Suriname, Ages: 50−54
Data points enclosed with a diamond are reference sources

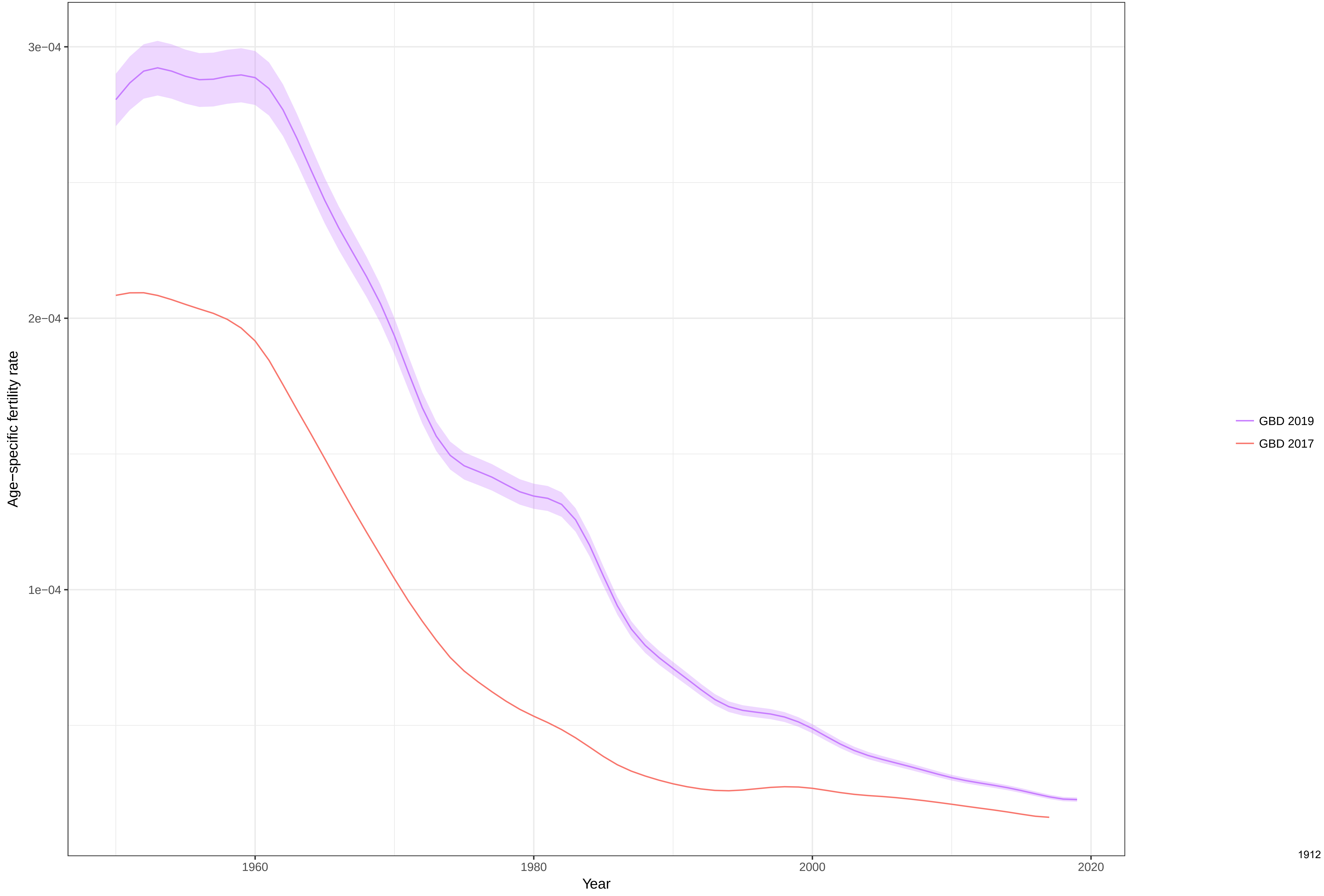

Total fertility rate fitted trend lines, 1950−2019: Suriname TFR
Data points enclosed with a diamond are reference sources

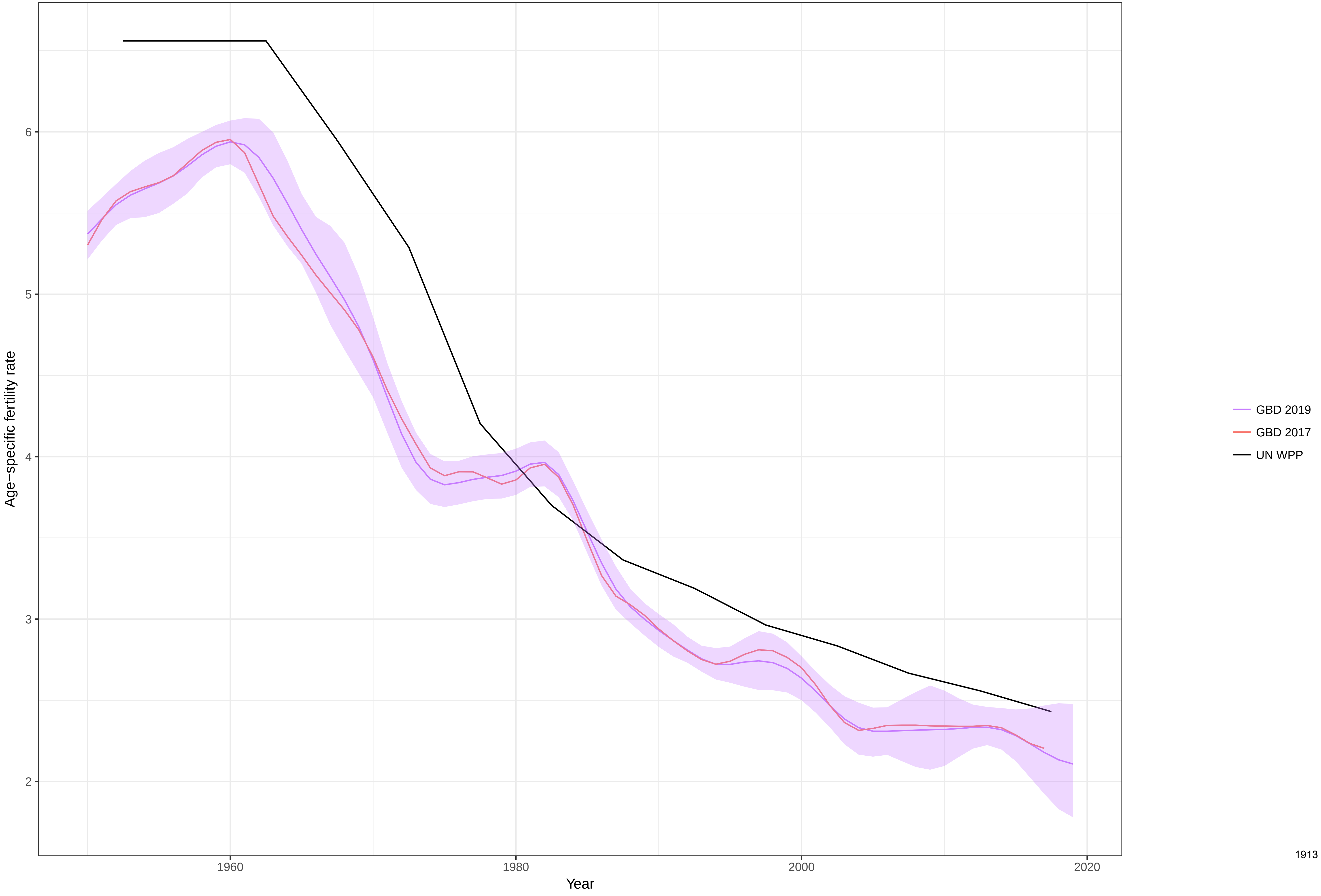

Age−specific fertility fitted trend lines, 1950−2019: Slovakia, Ages: 10−14
Data points enclosed with a diamond are reference sources

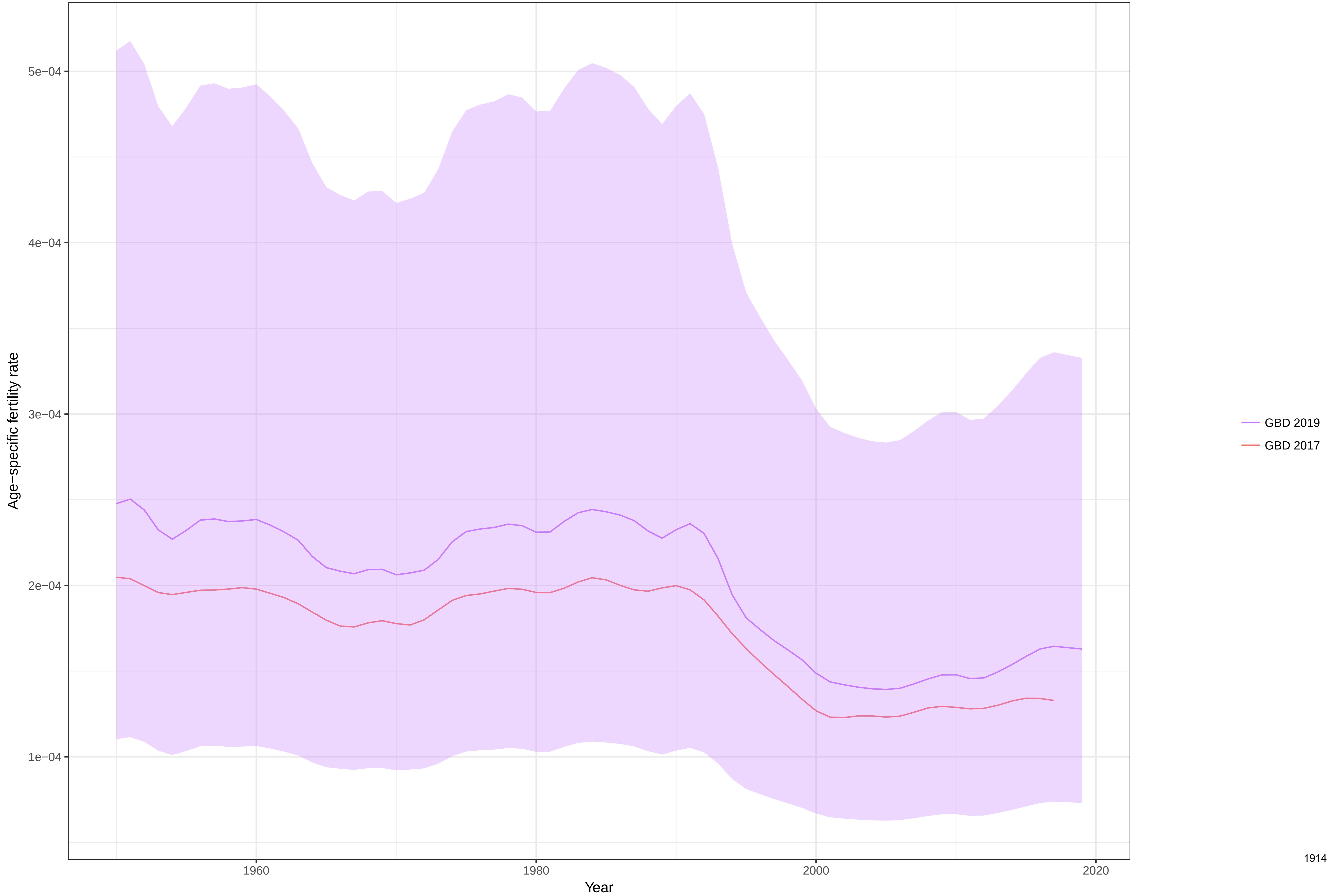

Age−specific fertility fitted trend lines, 1950−2019: Slovakia, Ages: 15−19
Data points enclosed with a diamond are reference sources

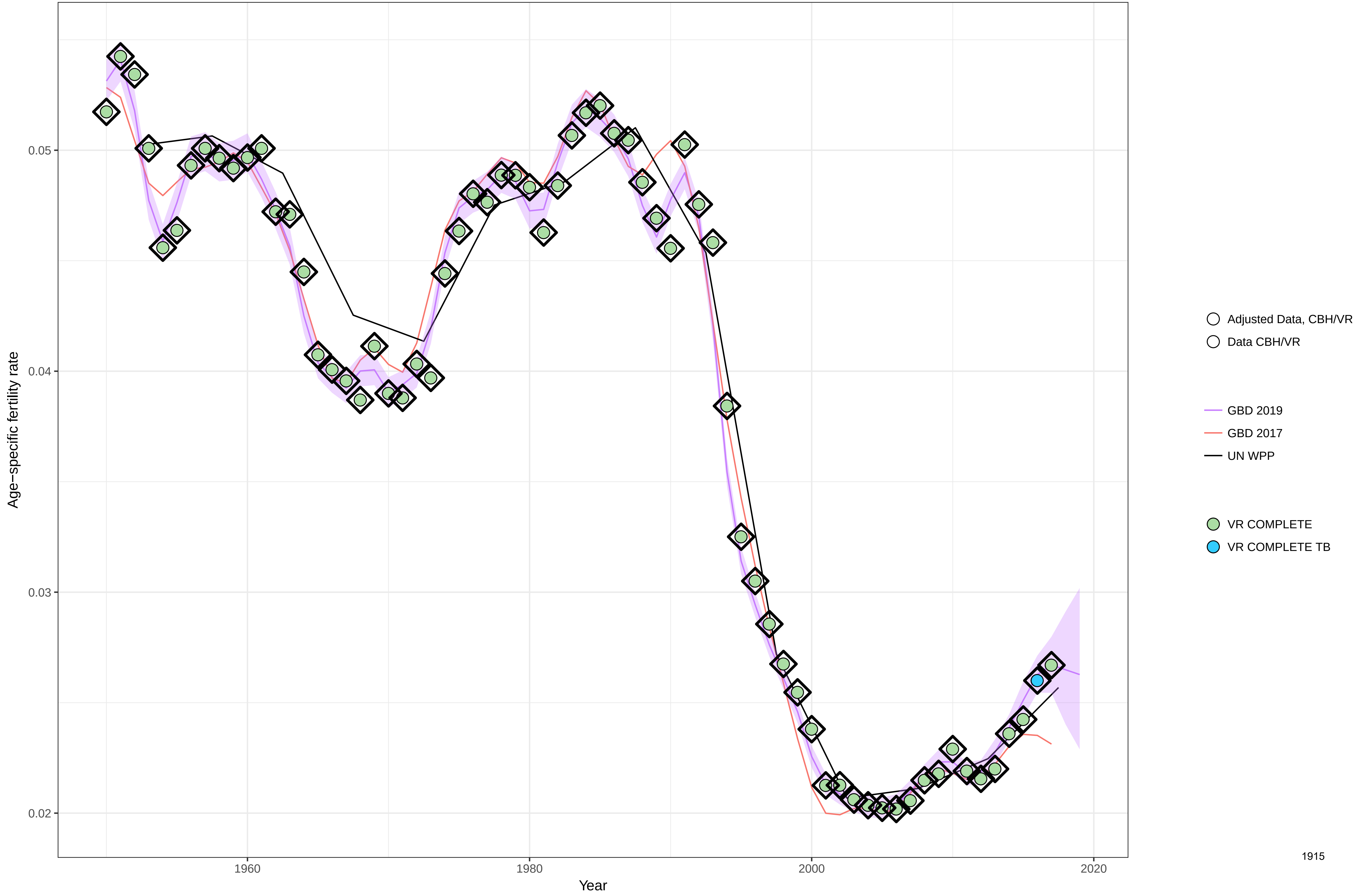

Age−specific fertility fitted trend lines, 1950−2019: Slovakia, Ages: 20−24
Data points enclosed with a diamond are reference sources

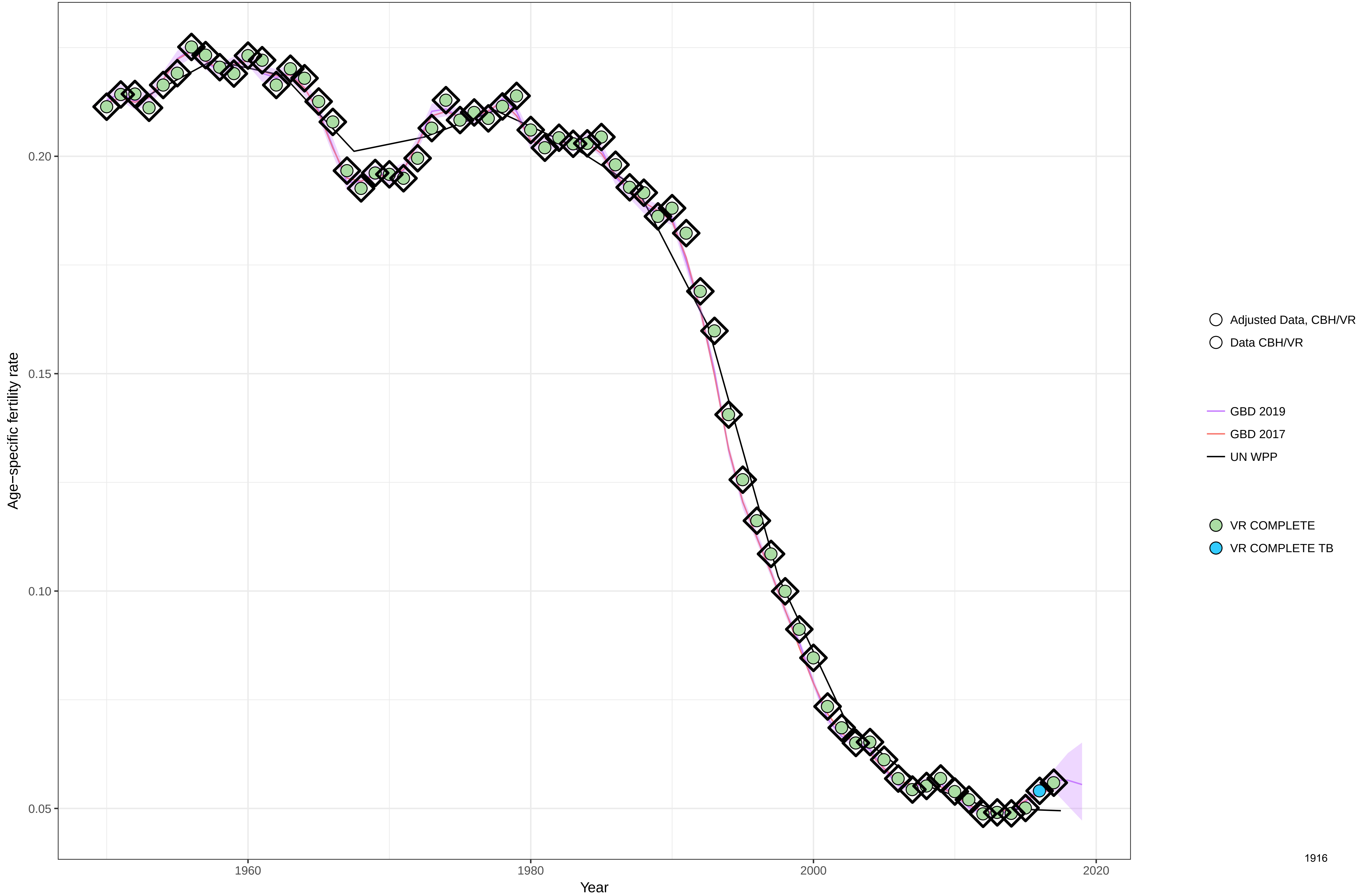

Age−specific fertility fitted trend lines, 1950−2019: Slovakia, Ages: 25−29
Data points enclosed with a diamond are reference sources

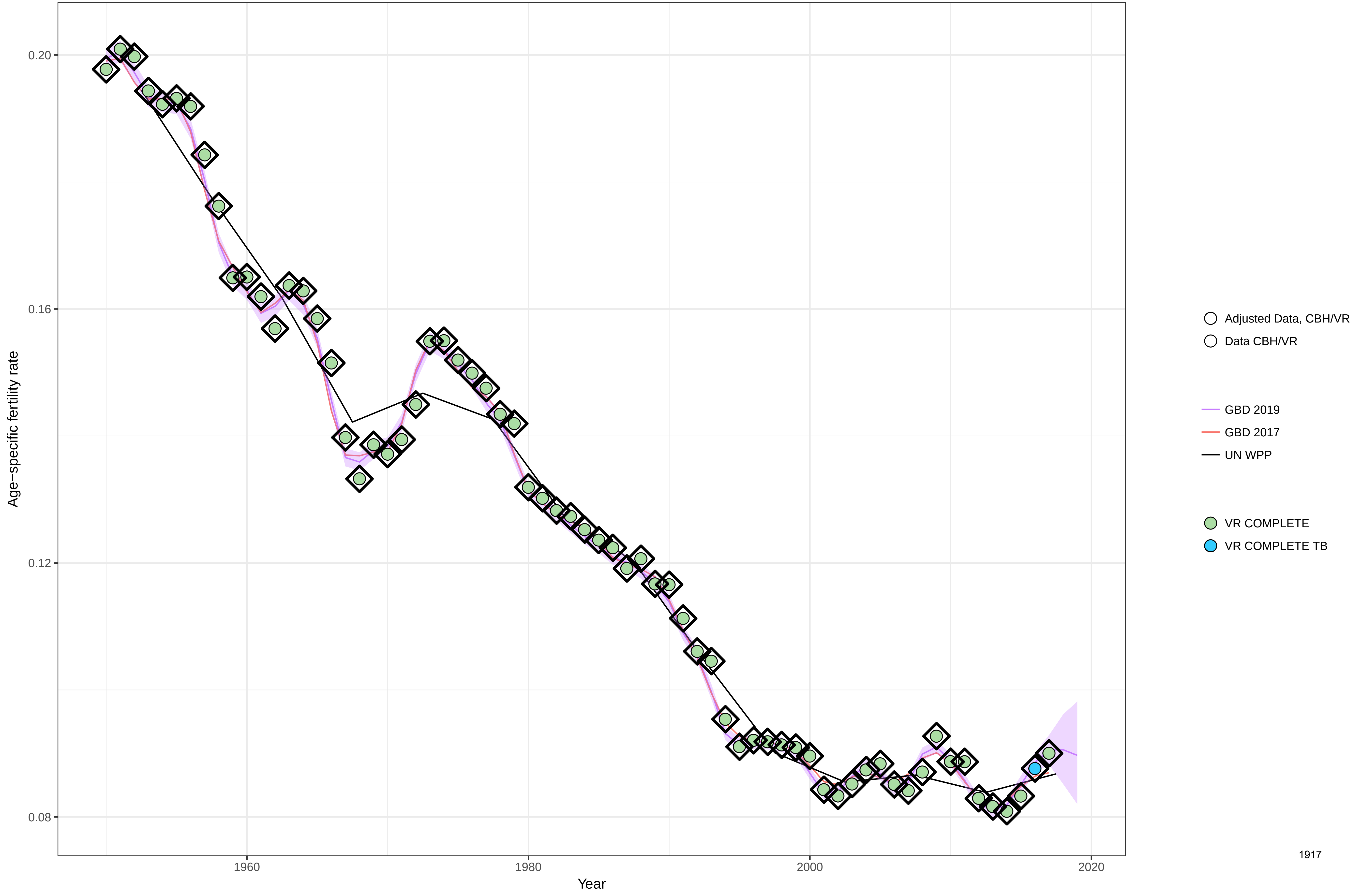

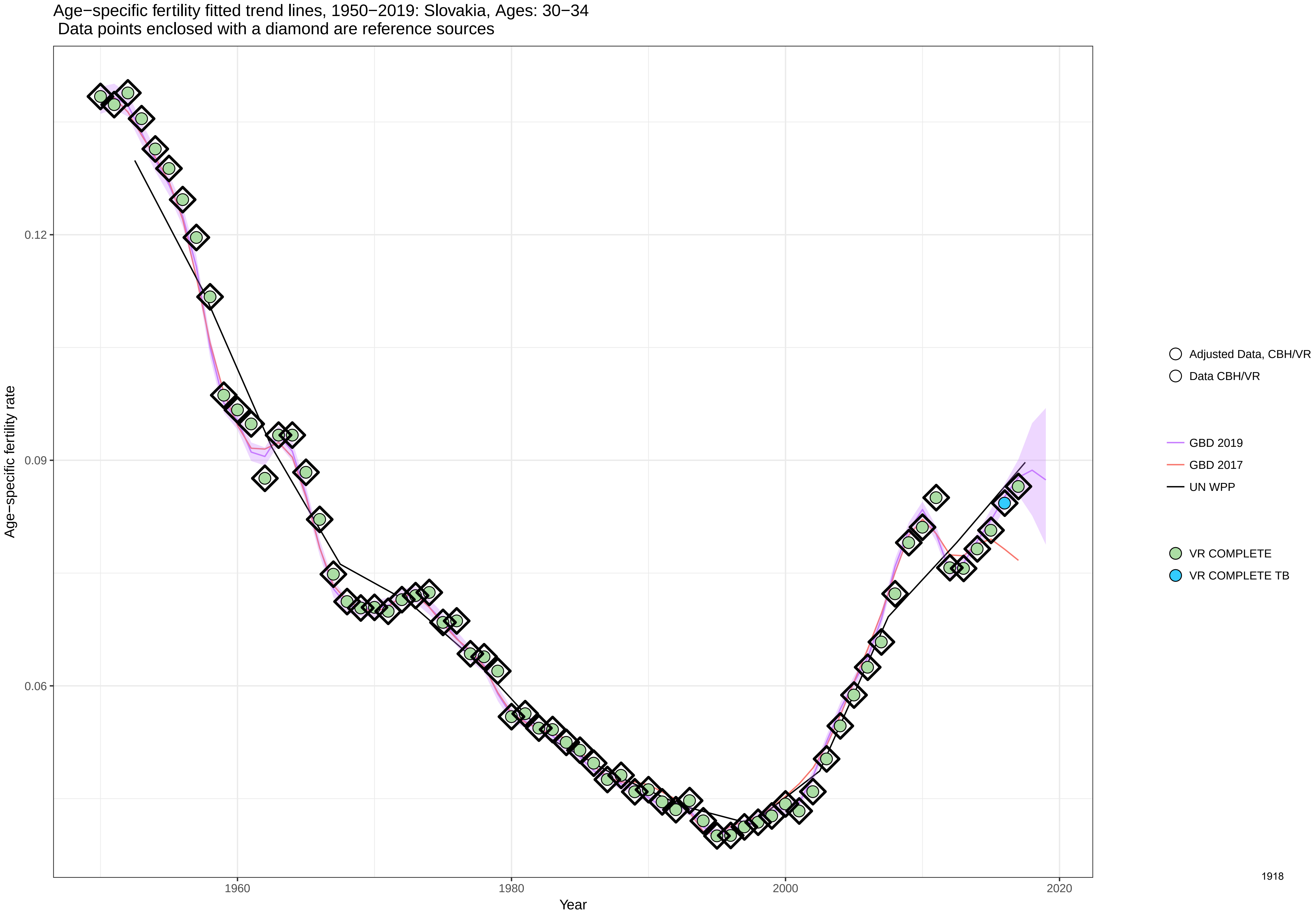
Age−specific fertility fitted trend lines, 1950−2019: Slovakia, Ages: 30−34
Data points enclosed with a diamond are reference sources
Age−specific fertility rate
0.12
0.09
0.06
1960
1980
2000
2020
Year
Adjusted Data, CBH/VR
Data CBH/VR
GBD 2019
GBD 2017
UN WPP
VR COMPLETE
VR COMPLETE TB

Age−specific fertility fitted trend lines, 1950−2019: Slovakia, Ages: 35−39
Data points enclosed with a diamond are reference sources

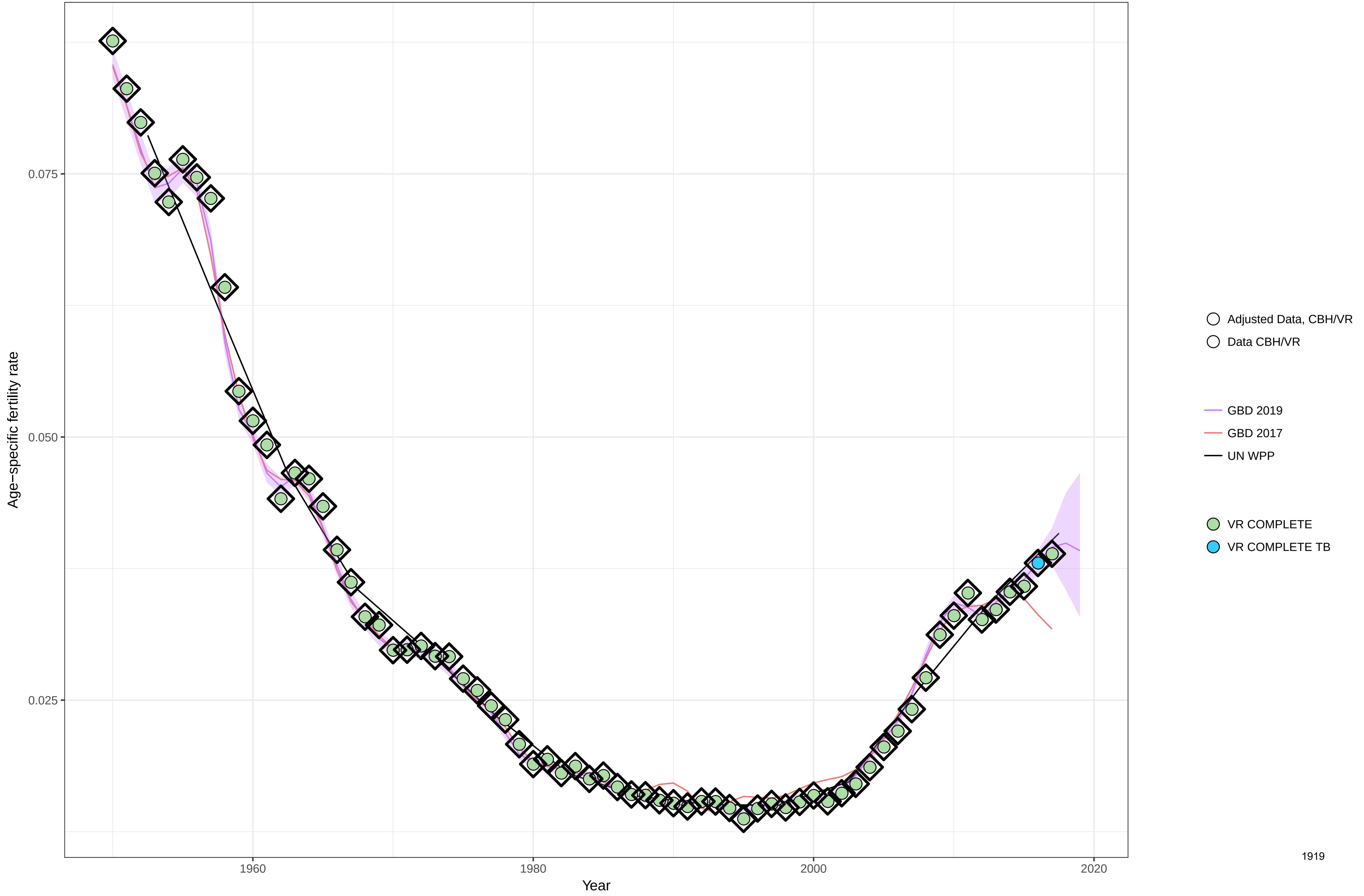

Age−specific fertility fitted trend lines, 1950−2019: Slovakia, Ages: 40−44
Data points enclosed with a diamond are reference sources

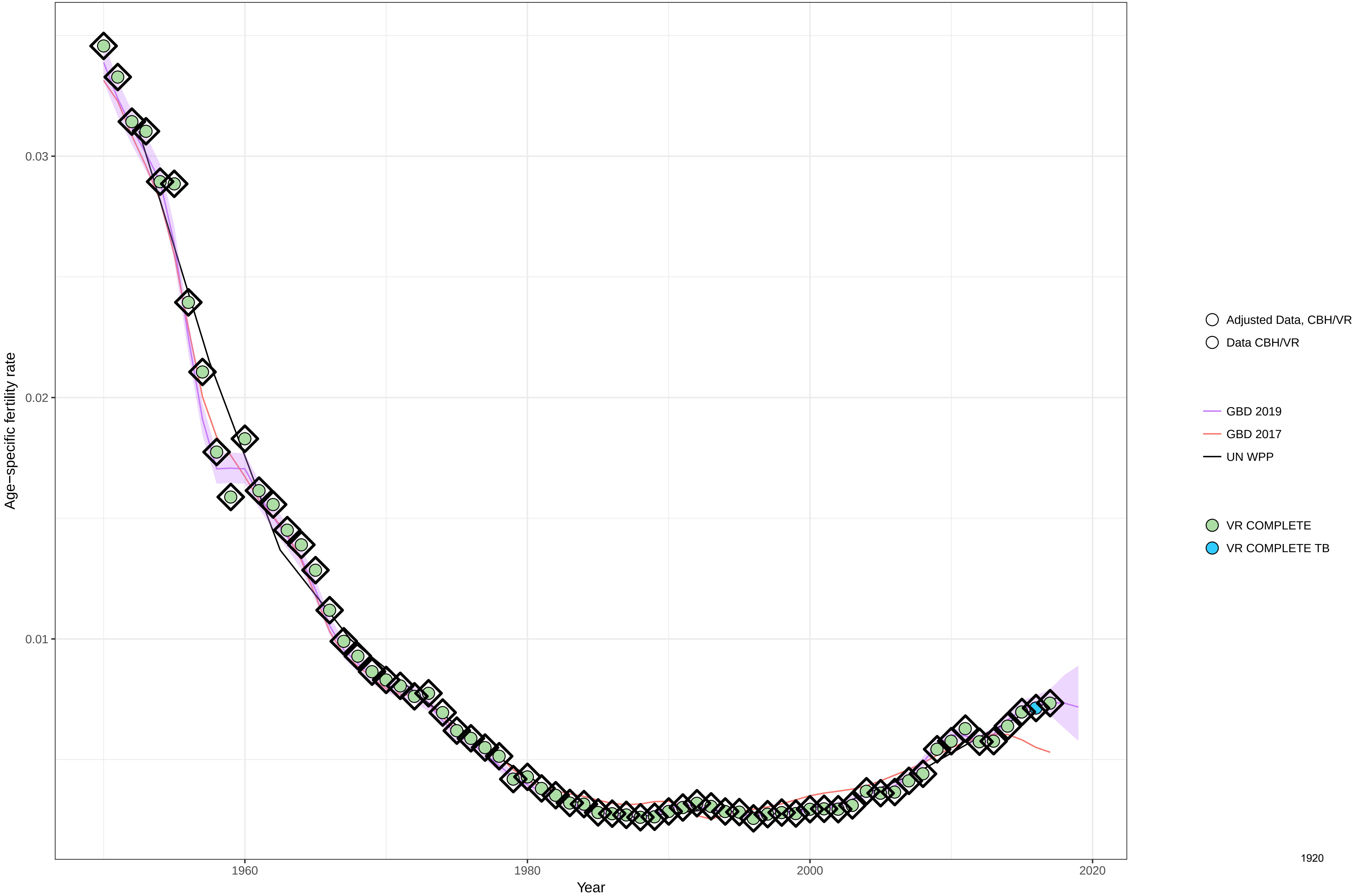

Age−specific fertility fitted trend lines, 1950−2019: Slovakia, Ages: 45−49
Data points enclosed with a diamond are reference sources

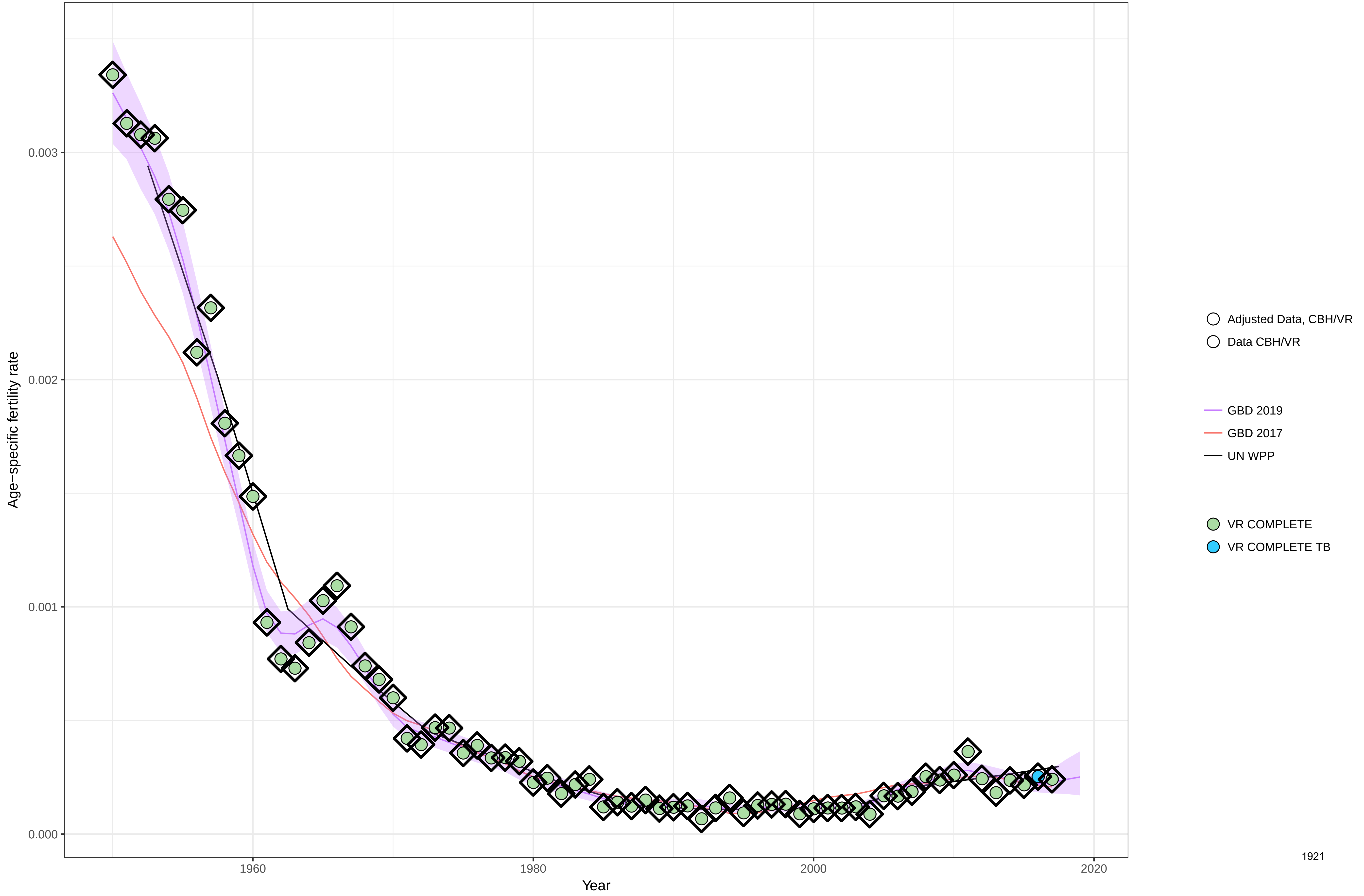

Age−specific fertility fitted trend lines, 1950−2019: Slovakia, Ages: 50−54
Data points enclosed with a diamond are reference sources

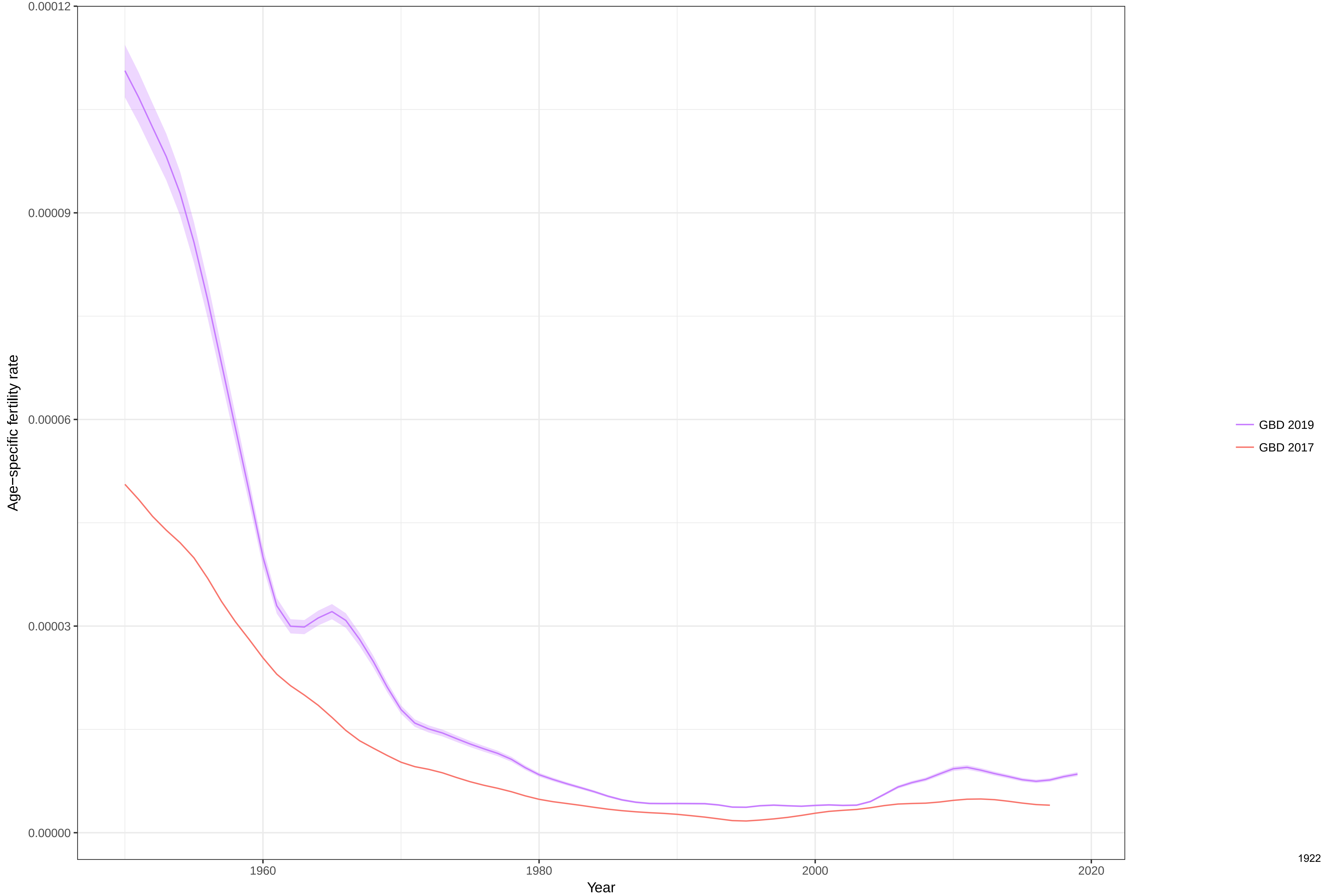

Total fertility rate fitted trend lines, 1950−2019: Slovakia TFR
Data points enclosed with a diamond are reference sources

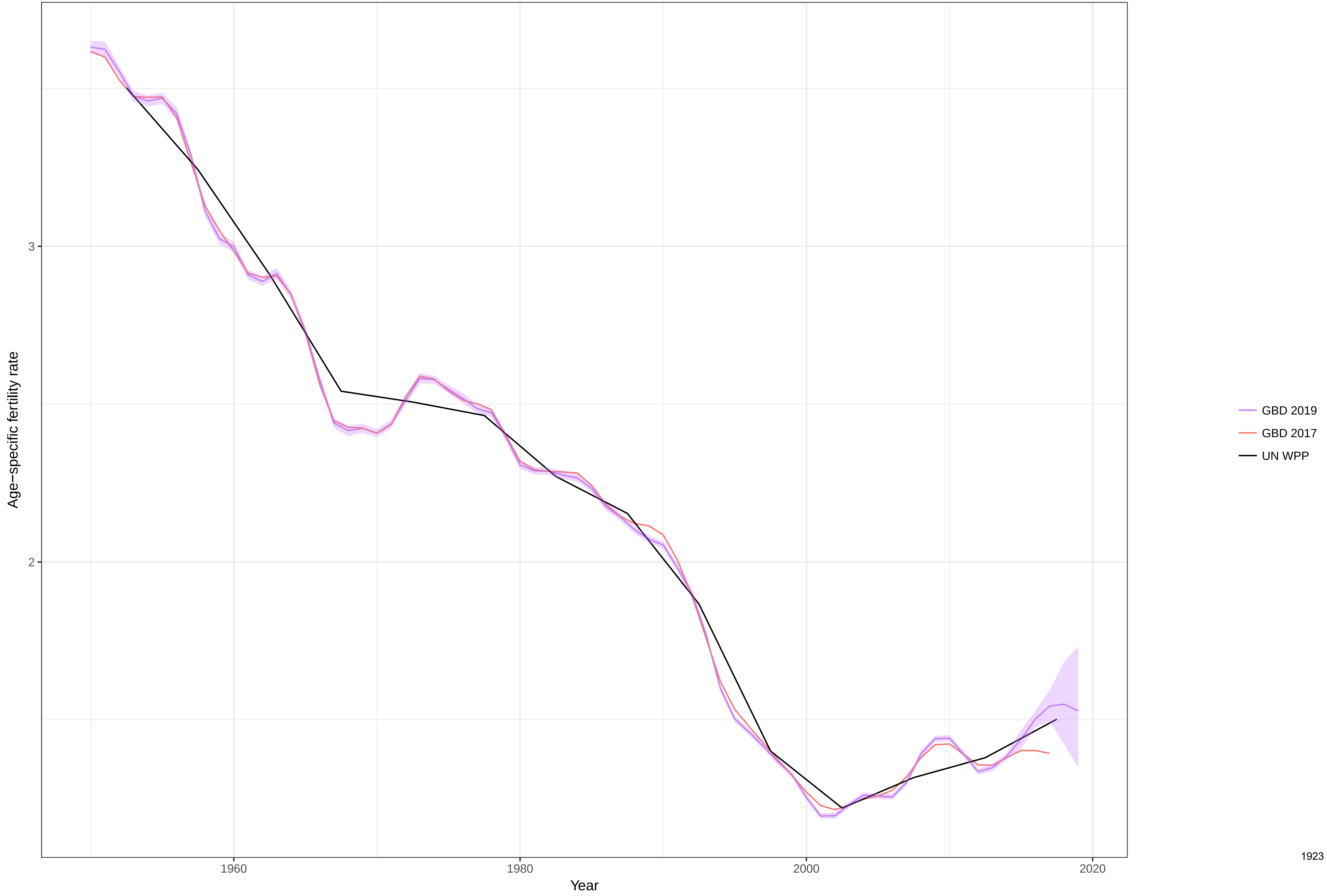

Age−specific fertility fitted trend lines, 1950−2019: Slovenia, Ages: 10−14
Data points enclosed with a diamond are reference sources

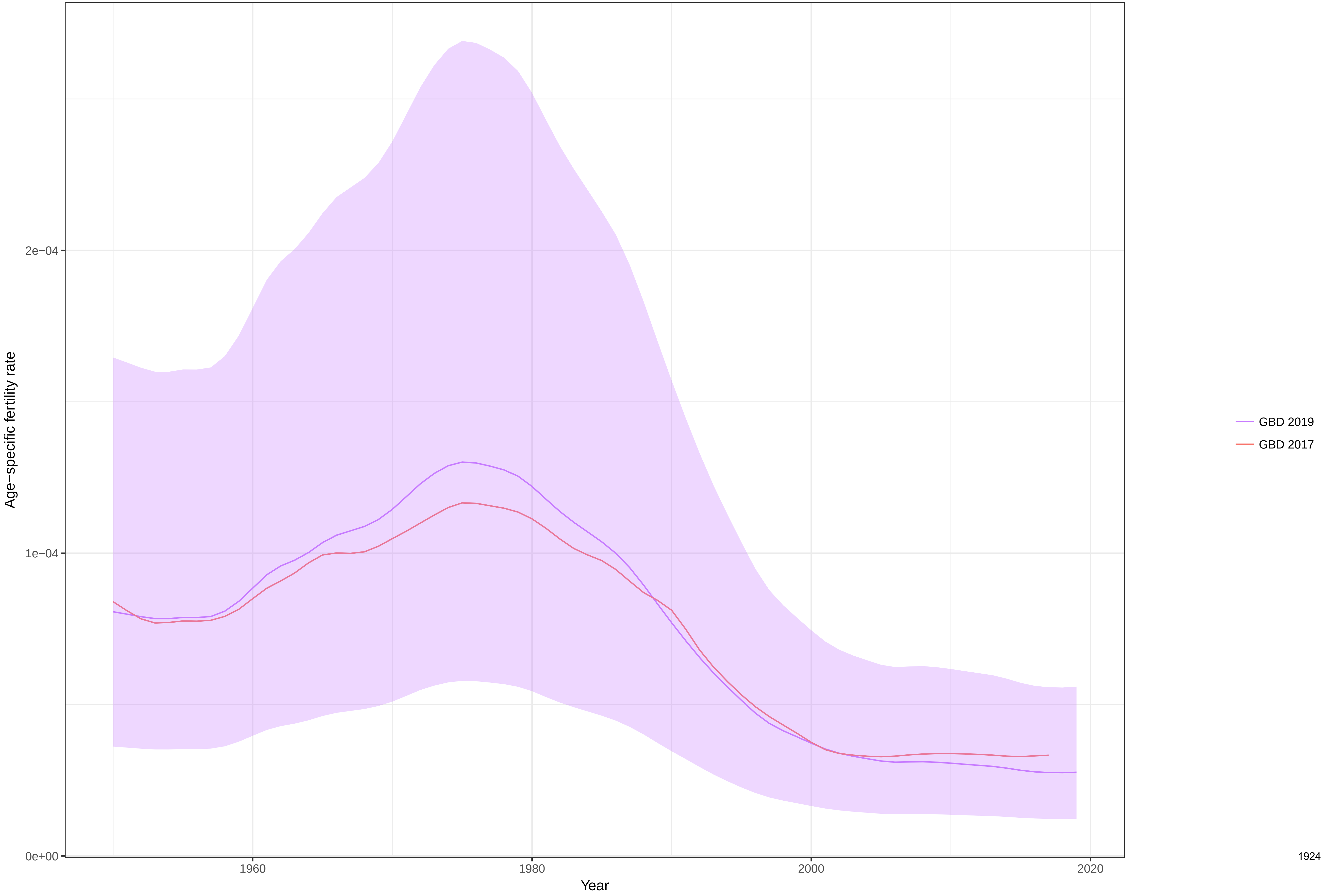

Age−specific fertility fitted trend lines, 1950−2019: Slovenia, Ages: 15−19
Data points enclosed with a diamond are reference sources

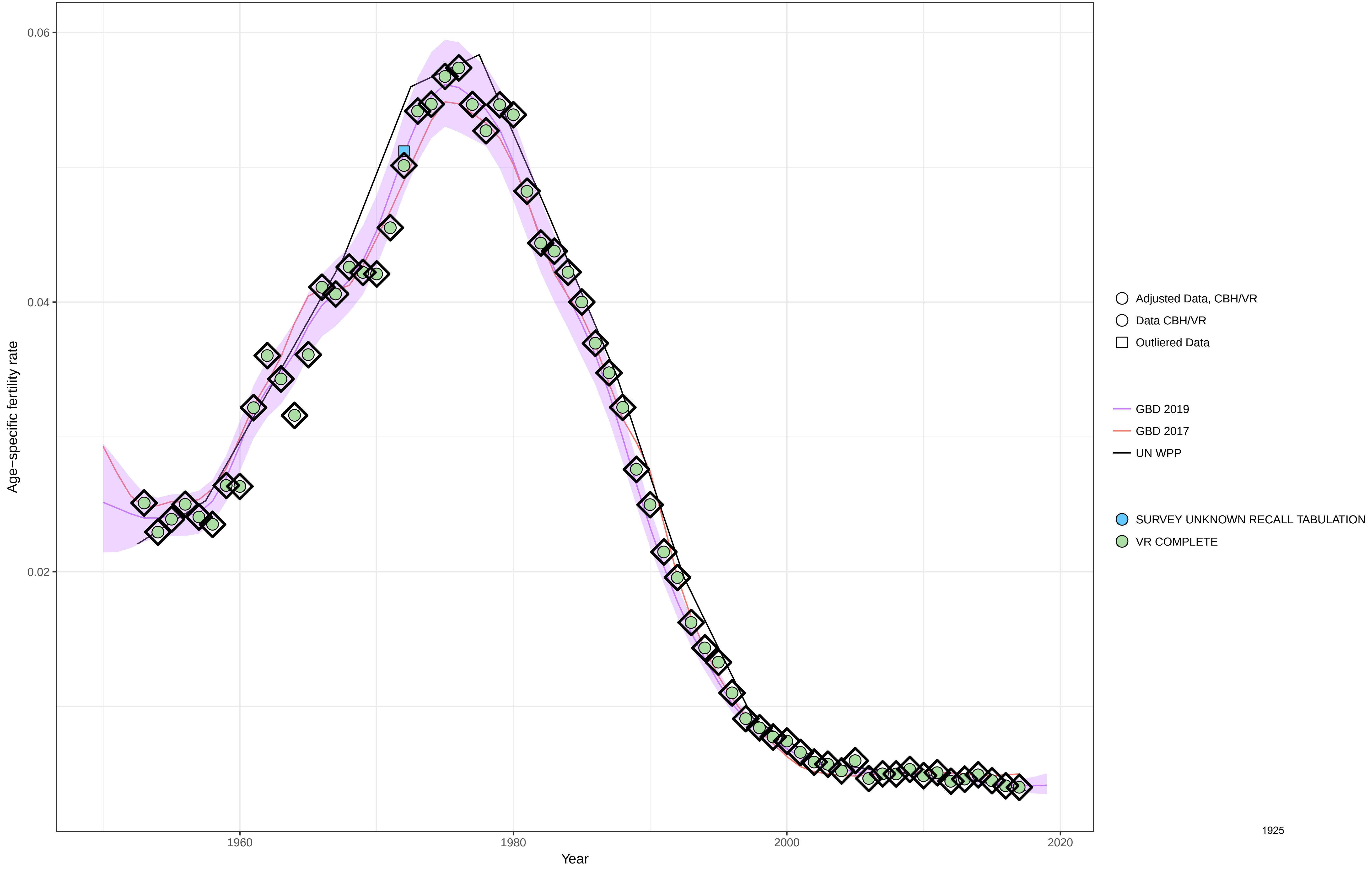

Age−specific fertility fitted trend lines, 1950−2019: Slovenia, Ages: 20−24
Data points enclosed with a diamond are reference sources

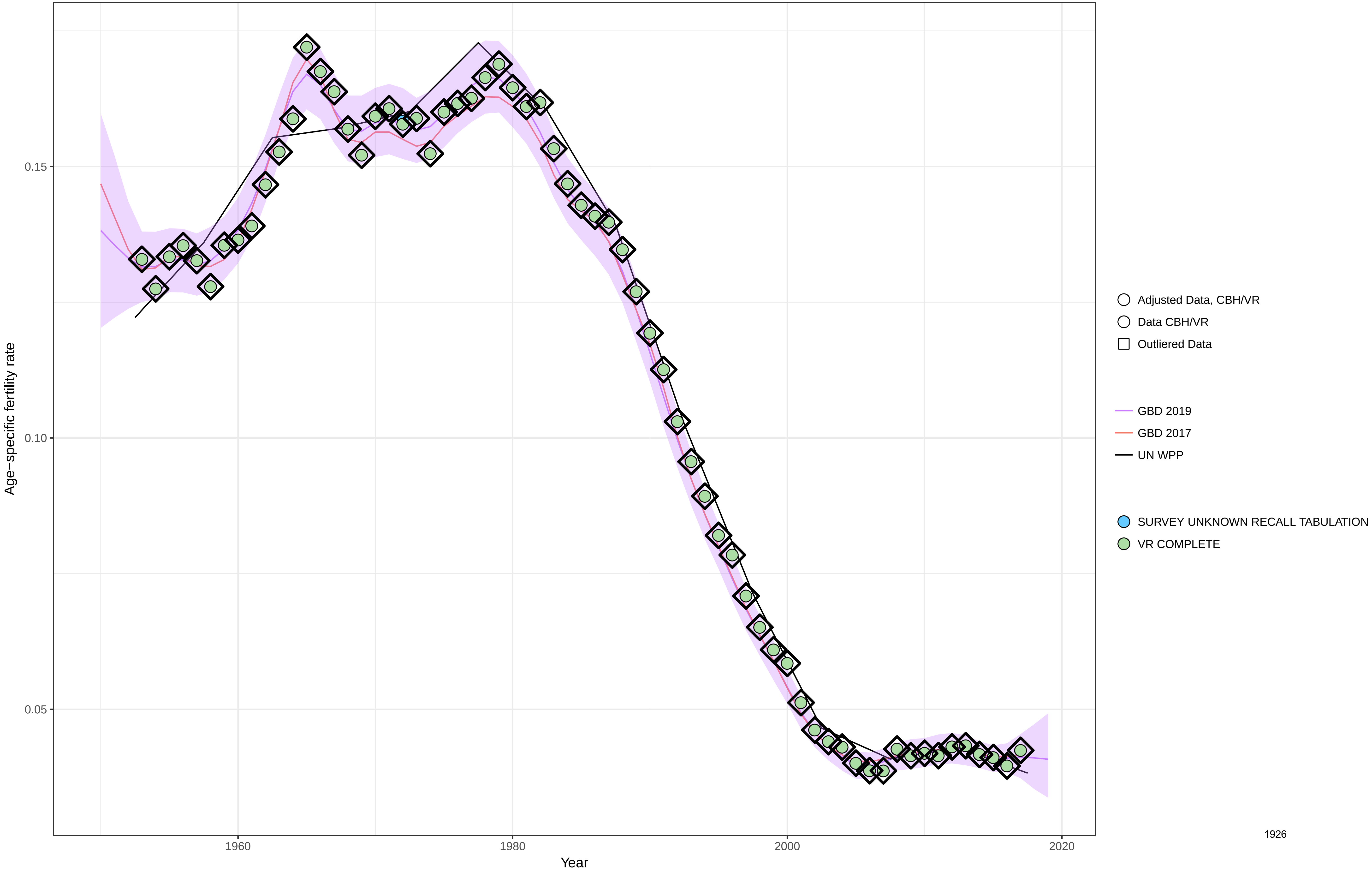

Age−specific fertility fitted trend lines, 1950−2019: Slovenia, Ages: 25−29
Data points enclosed with a diamond are reference sources

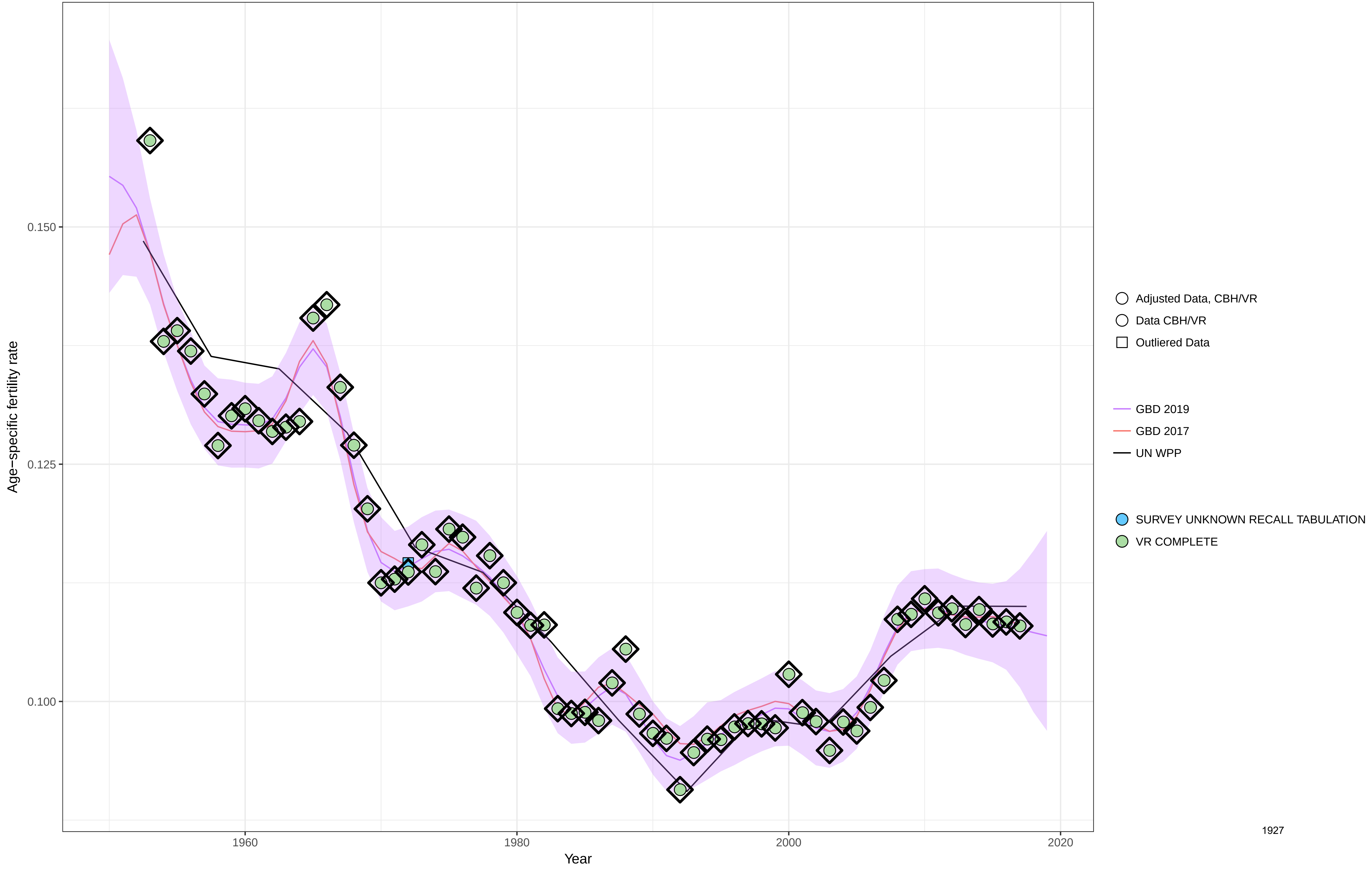

Age−specific fertility fitted trend lines, 1950−2019: Slovenia, Ages: 30−34
Data points enclosed with a diamond are reference sources

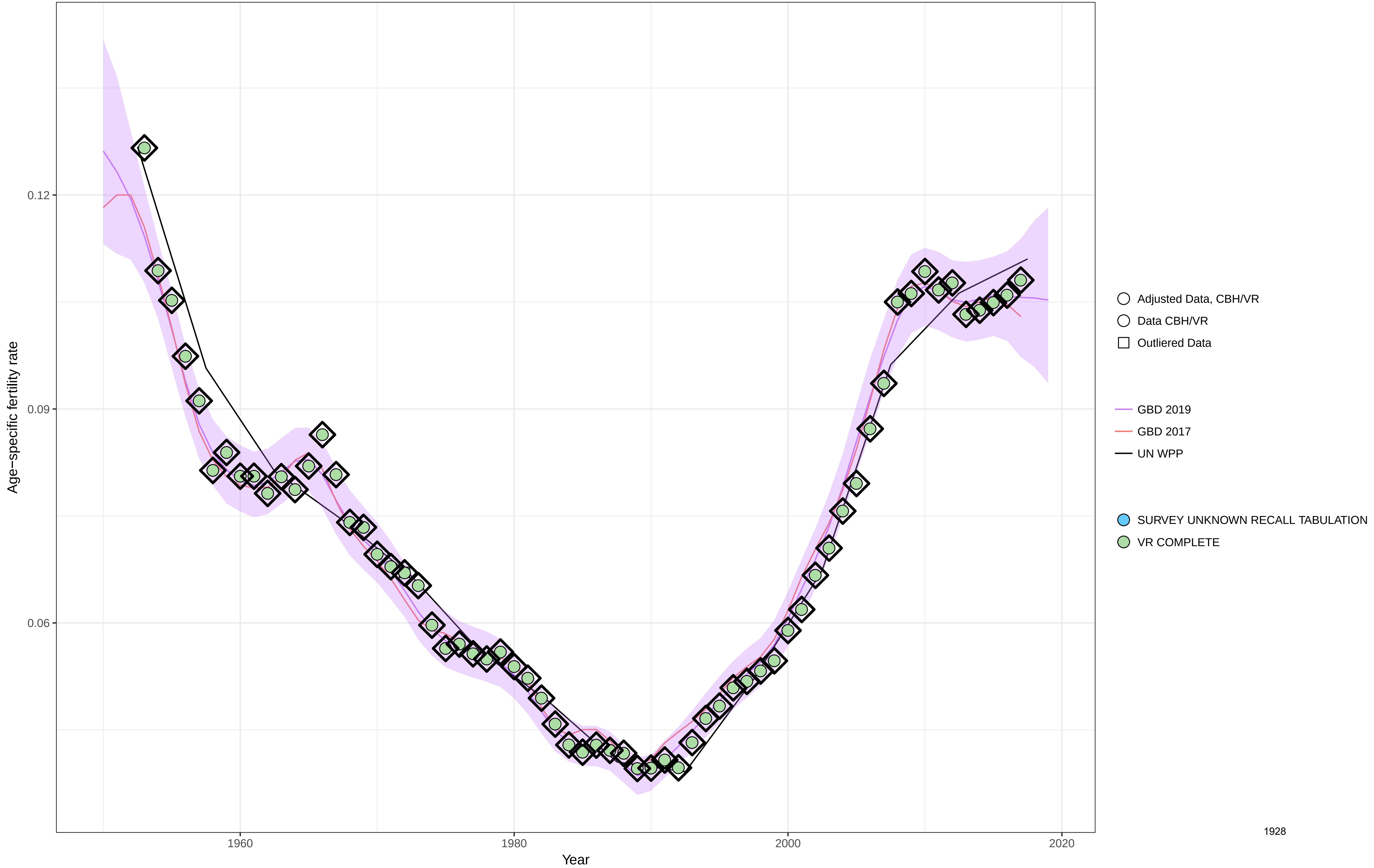

Age−specific fertility fitted trend lines, 1950−2019: Slovenia, Ages: 35−39
Data points enclosed with a diamond are reference sources

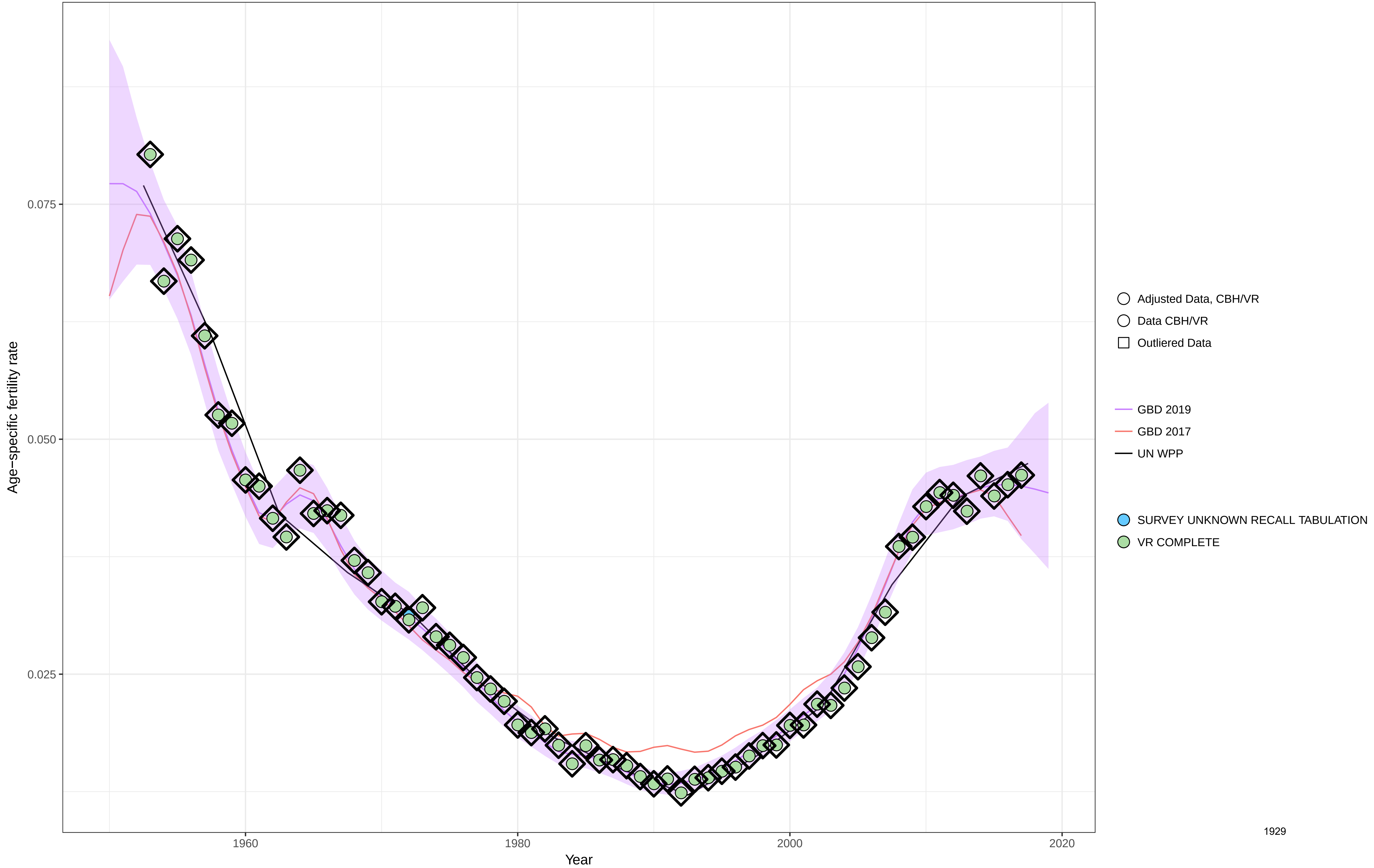

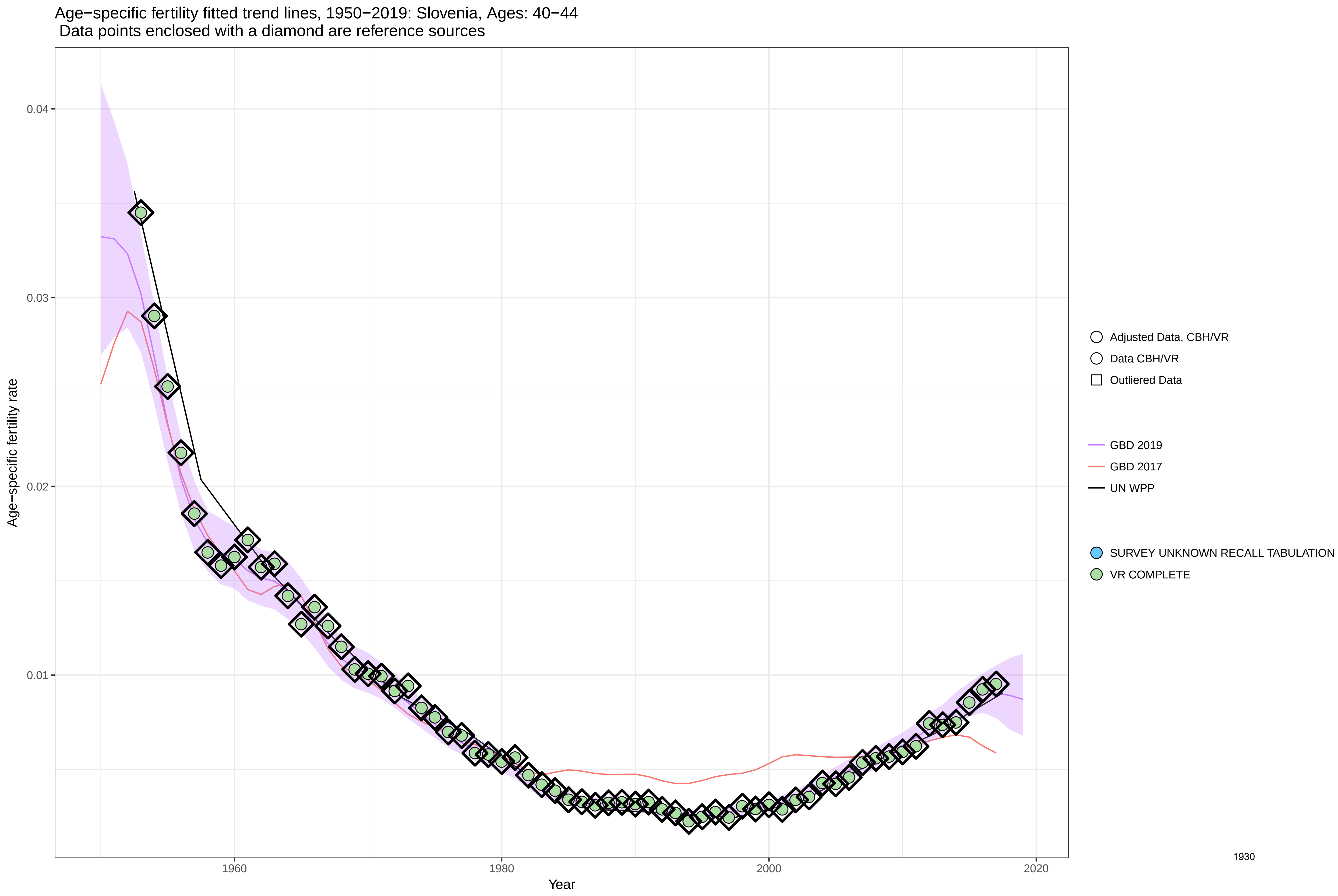
Age−specific fertility fitted trend lines, 1950−2019: Slovenia, Ages: 40−44
Data points enclosed with a diamond are reference sources
Age−specific fertility rate
0.04
0.03
0.02
0.01
1960
1980
2000
2020
Year
Adjusted Data, CBH/VR
Data CBH/VR
Outliered Data
GBD 2019
GBD 2017
UN WPP
SURVEY UNKNOWN RECALL TABULATION
VR COMPLETE

1930

Age−specific fertility fitted trend lines, 1950−2019: Slovenia, Ages: 45−49
Data points enclosed with a diamond are reference sources

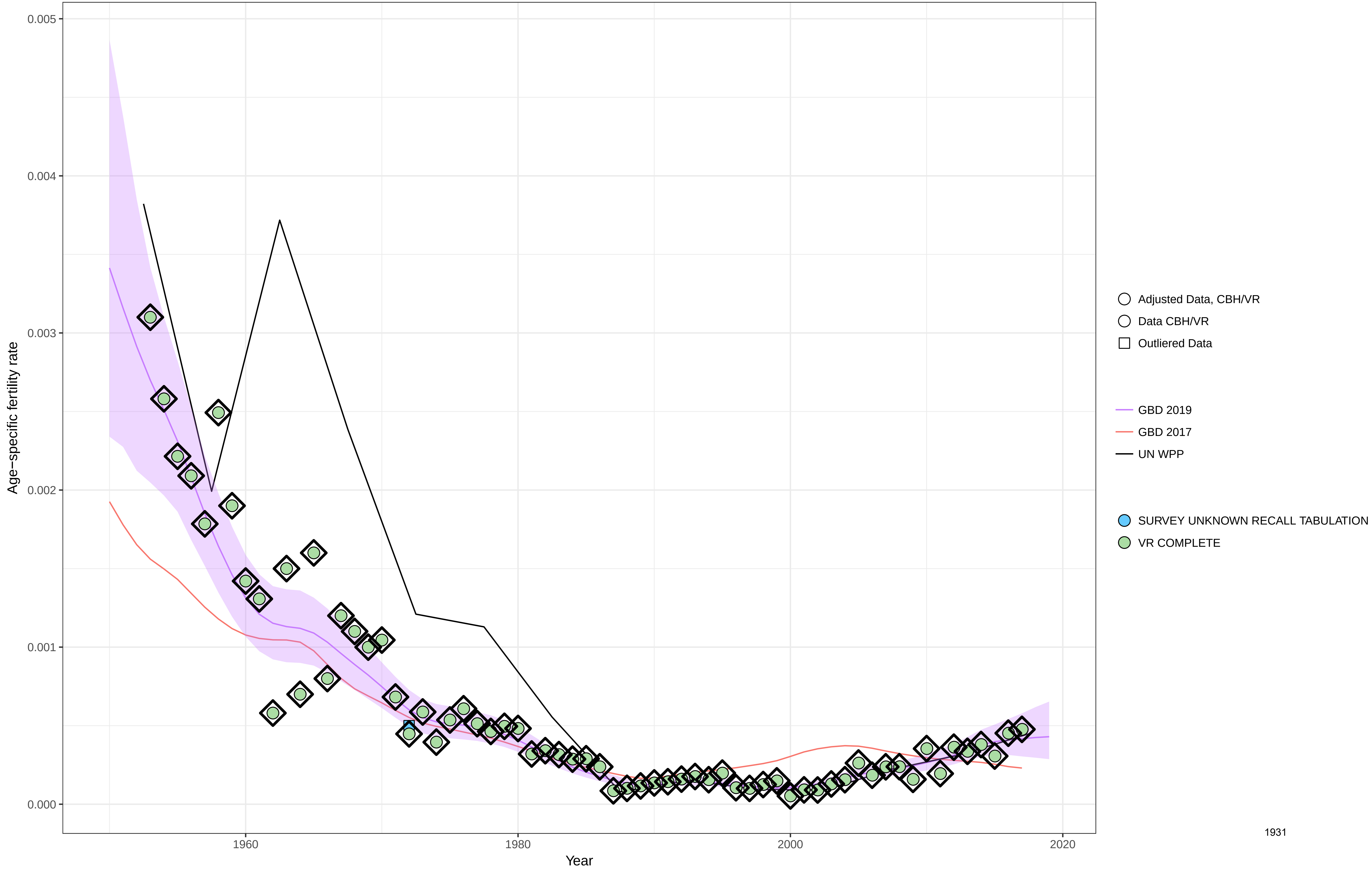

Age−specific fertility fitted trend lines, 1950−2019: Slovenia, Ages: 50−54
Data points enclosed with a diamond are reference sources

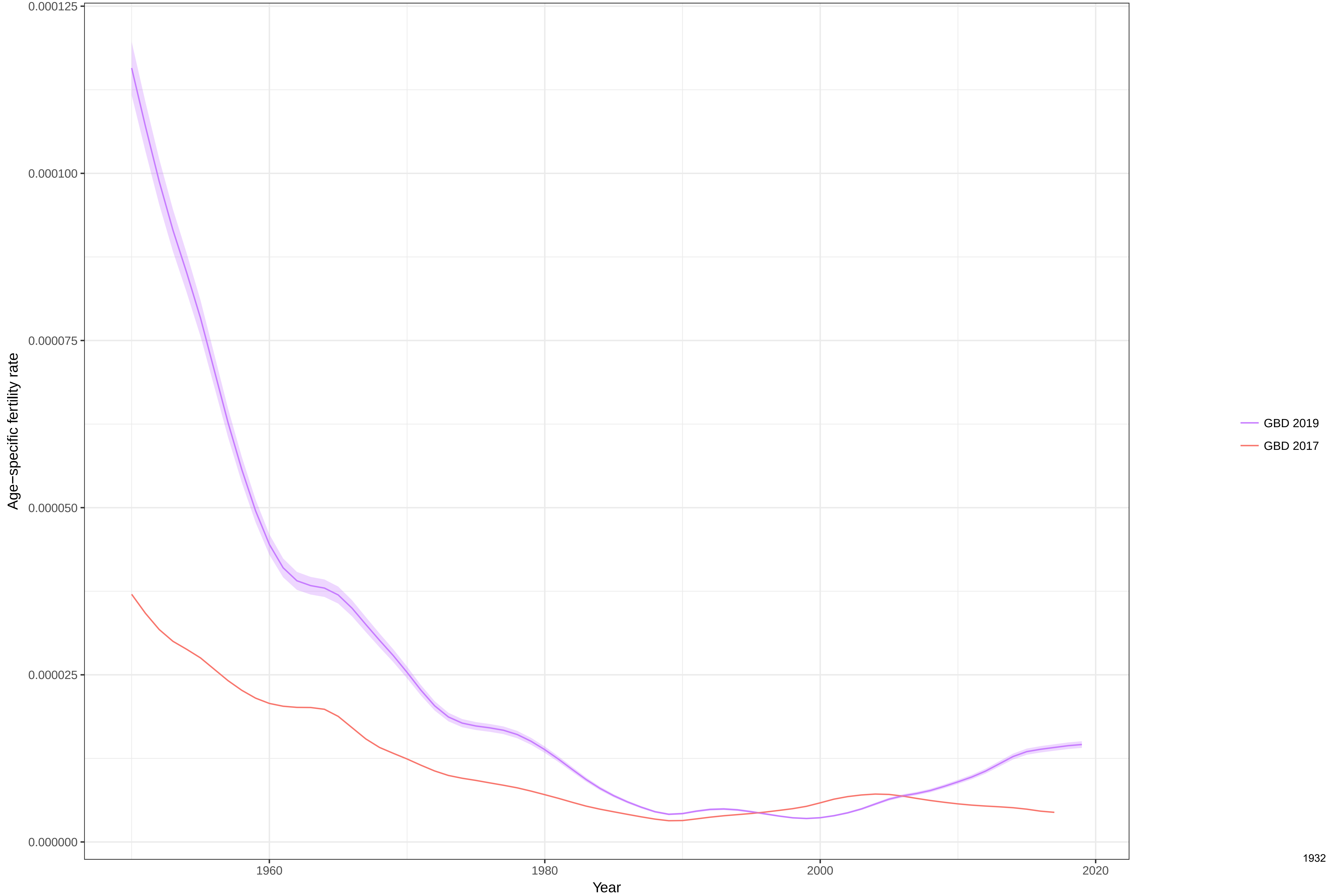

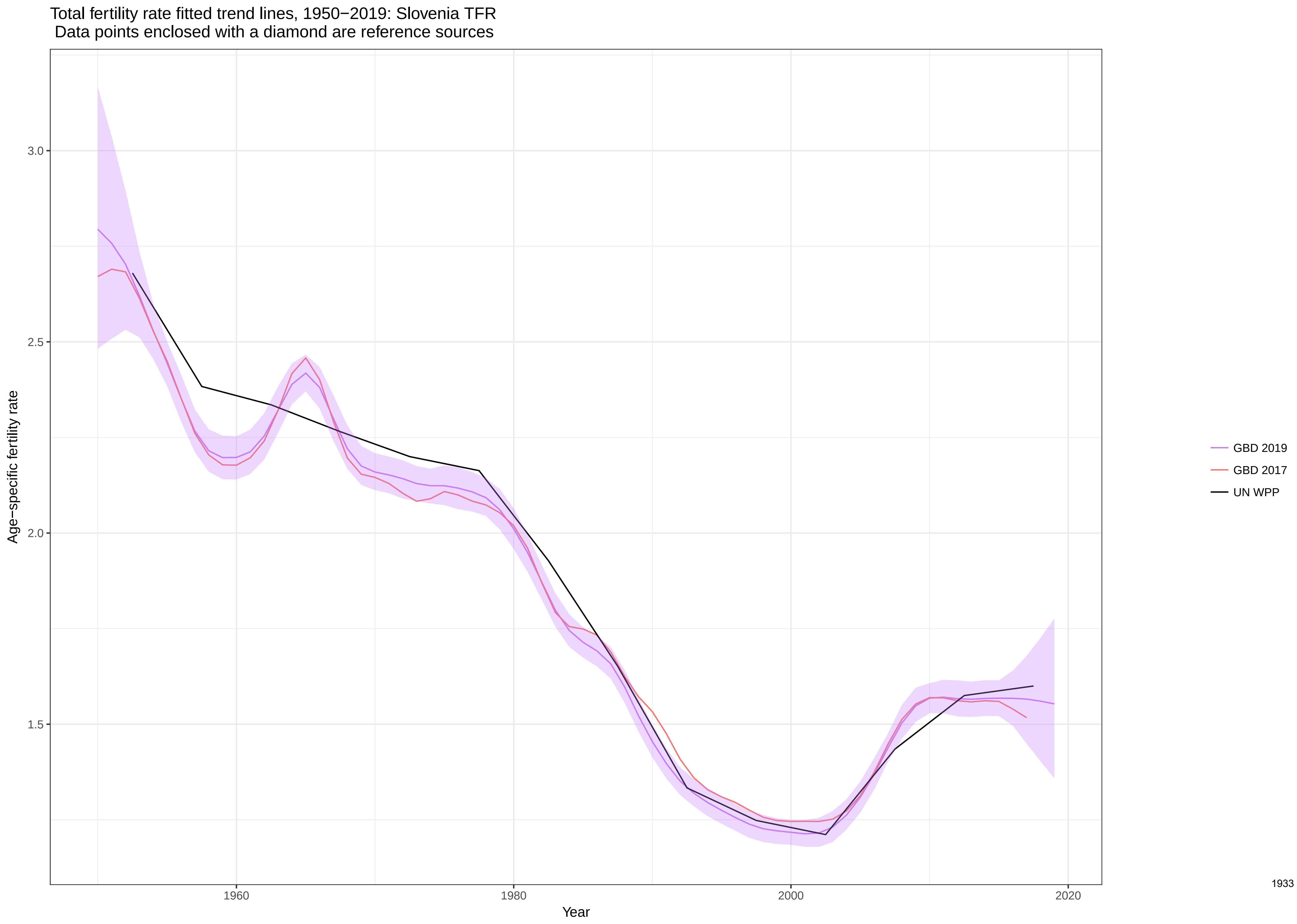

Total fertility rate fitted trend lines, 1950−2019: Slovenia TFR
Data points enclosed with a diamond are reference sources
Age−specific fertility rate
3.0
2.5
2.0
1.5
1960
1980
2000
2020
Year
GBD 2019
GBD 2017
UN WPP

Age−specific fertility fitted trend lines, 1950−2019: Sweden, Ages: 10−14
Data points enclosed with a diamond are reference sources

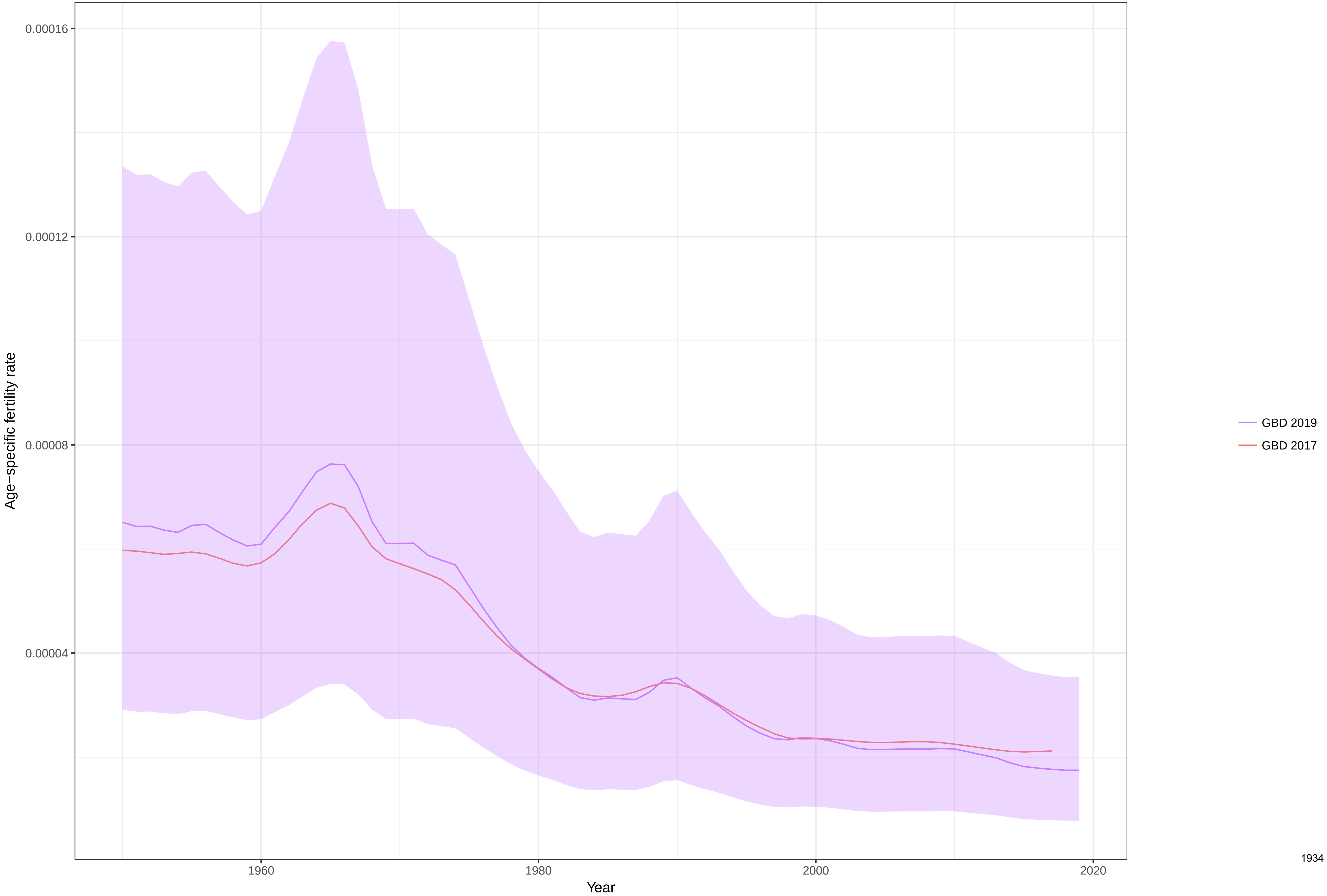

Age−specific fertility fitted trend lines, 1950−2019: Sweden, Ages: 15−19
Data points enclosed with a diamond are reference sources

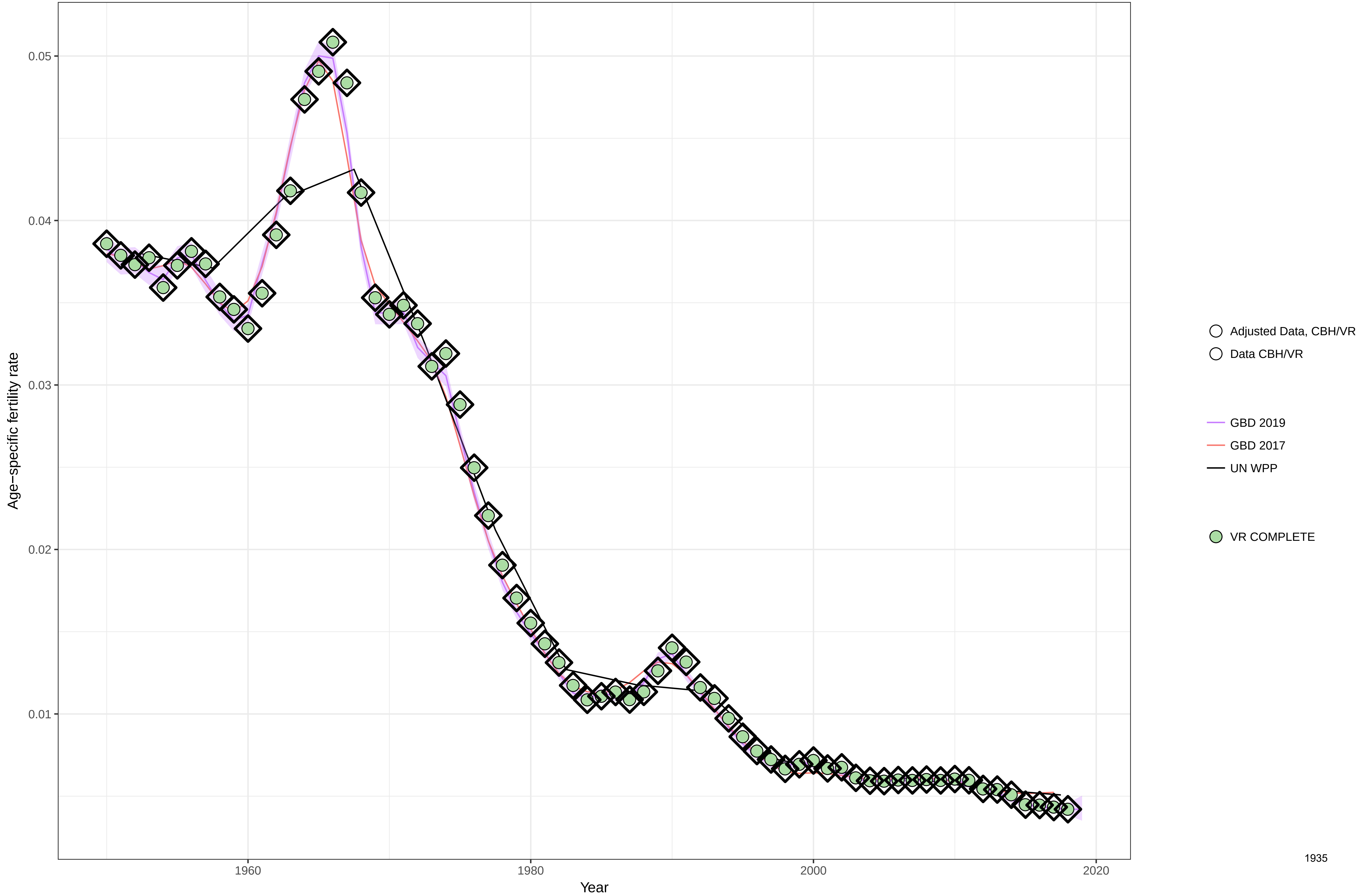

Age−specific fertility fitted trend lines, 1950−2019: Sweden, Ages: 20−24
Data points enclosed with a diamond are reference sources

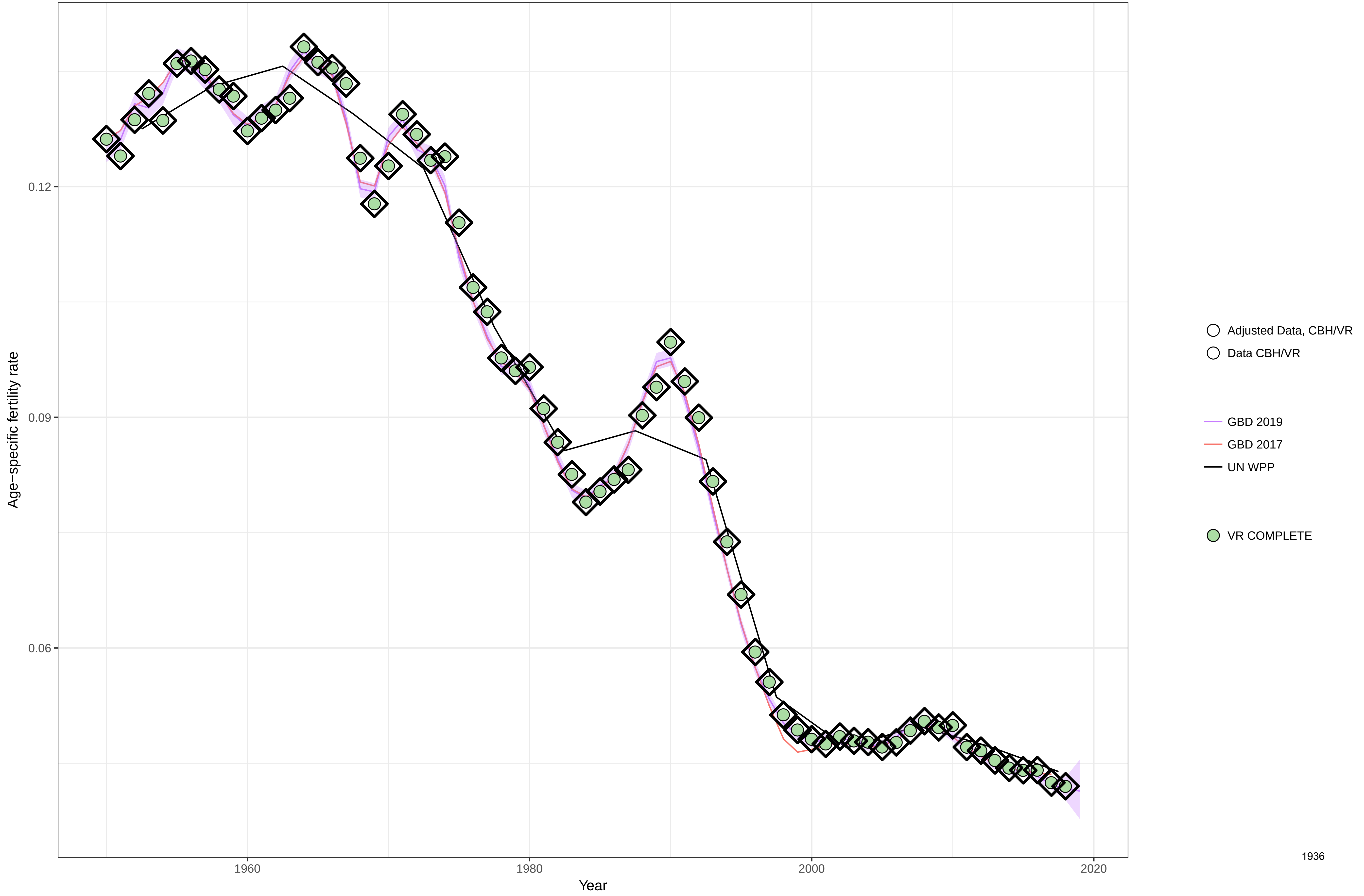

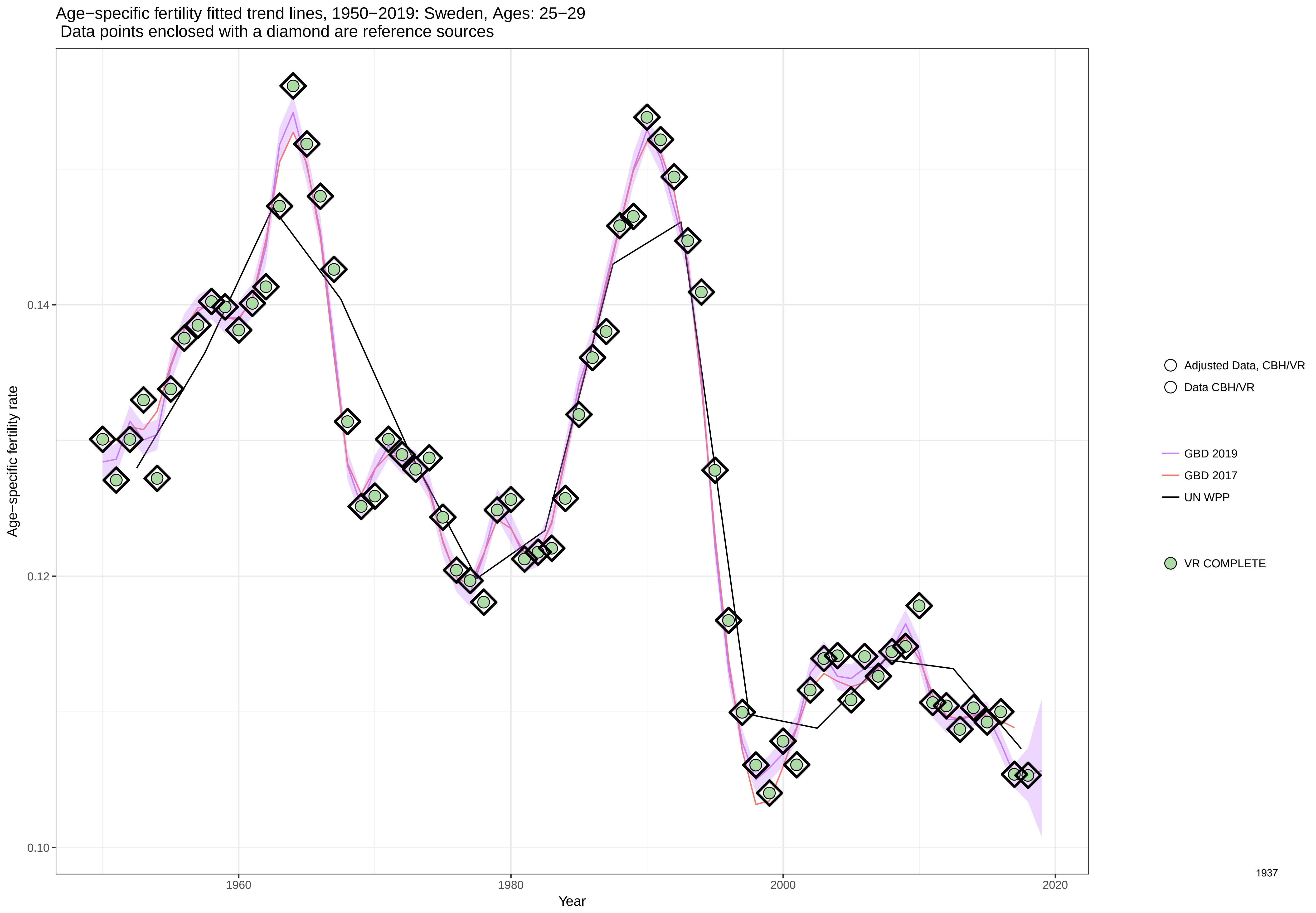
Age−specific fertility fitted trend lines, 1950−2019: Sweden, Ages: 25−29
Data points enclosed with a diamond are reference sources
Age−specific fertility rate
0.14
0.12
0.10
1960
1980
2000
2020
Year
Adjusted Data, CBH/VR
Data CBH/VR
GBD 2019
GBD 2017
UN WPP
VR COMPLETE

Age−specific fertility fitted trend lines, 1950−2019: Sweden, Ages: 30−34
Data points enclosed with a diamond are reference sources

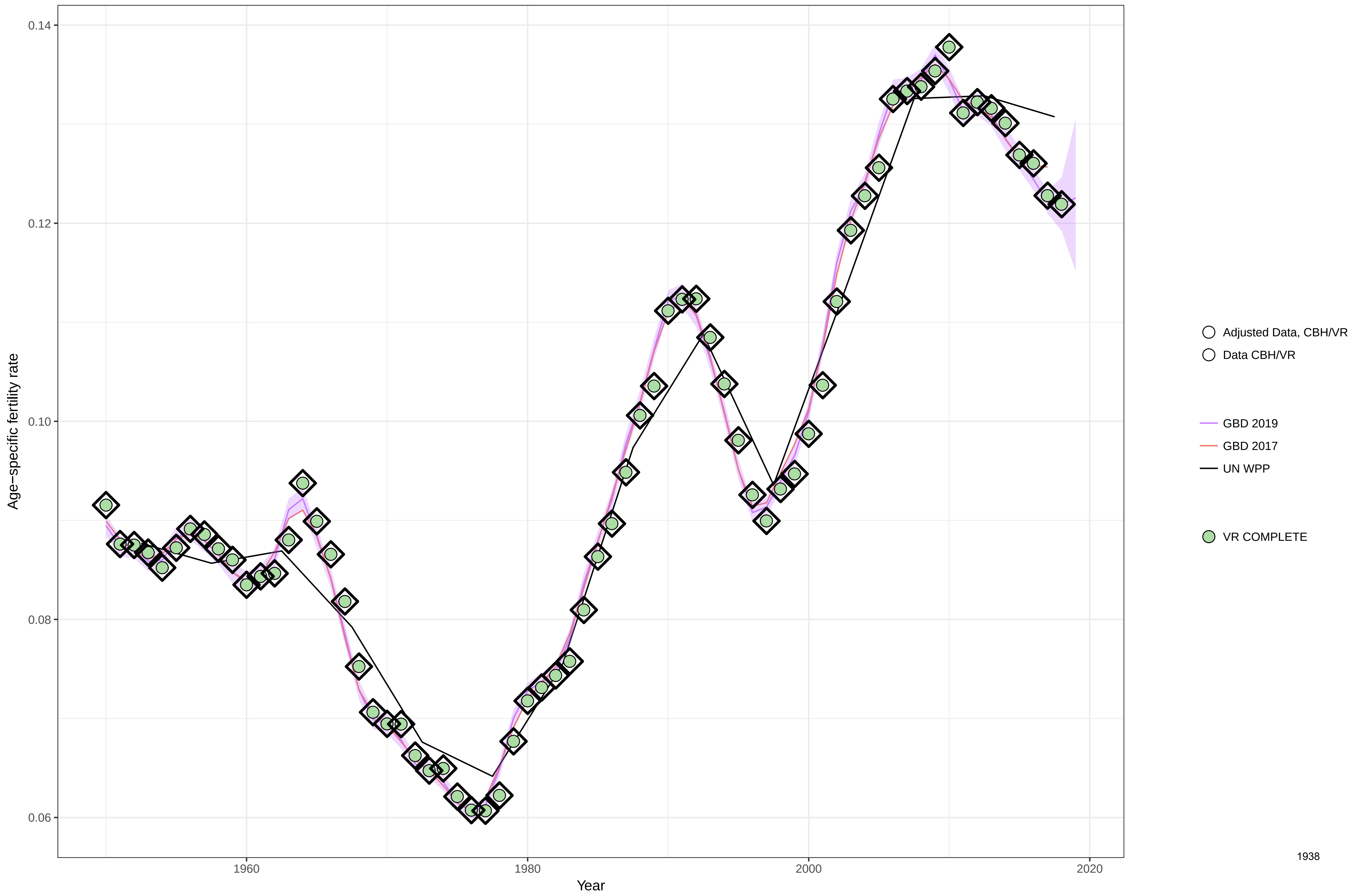

Age−specific fertility fitted trend lines, 1950−2019: Sweden, Ages: 35−39
Data points enclosed with a diamond are reference sources

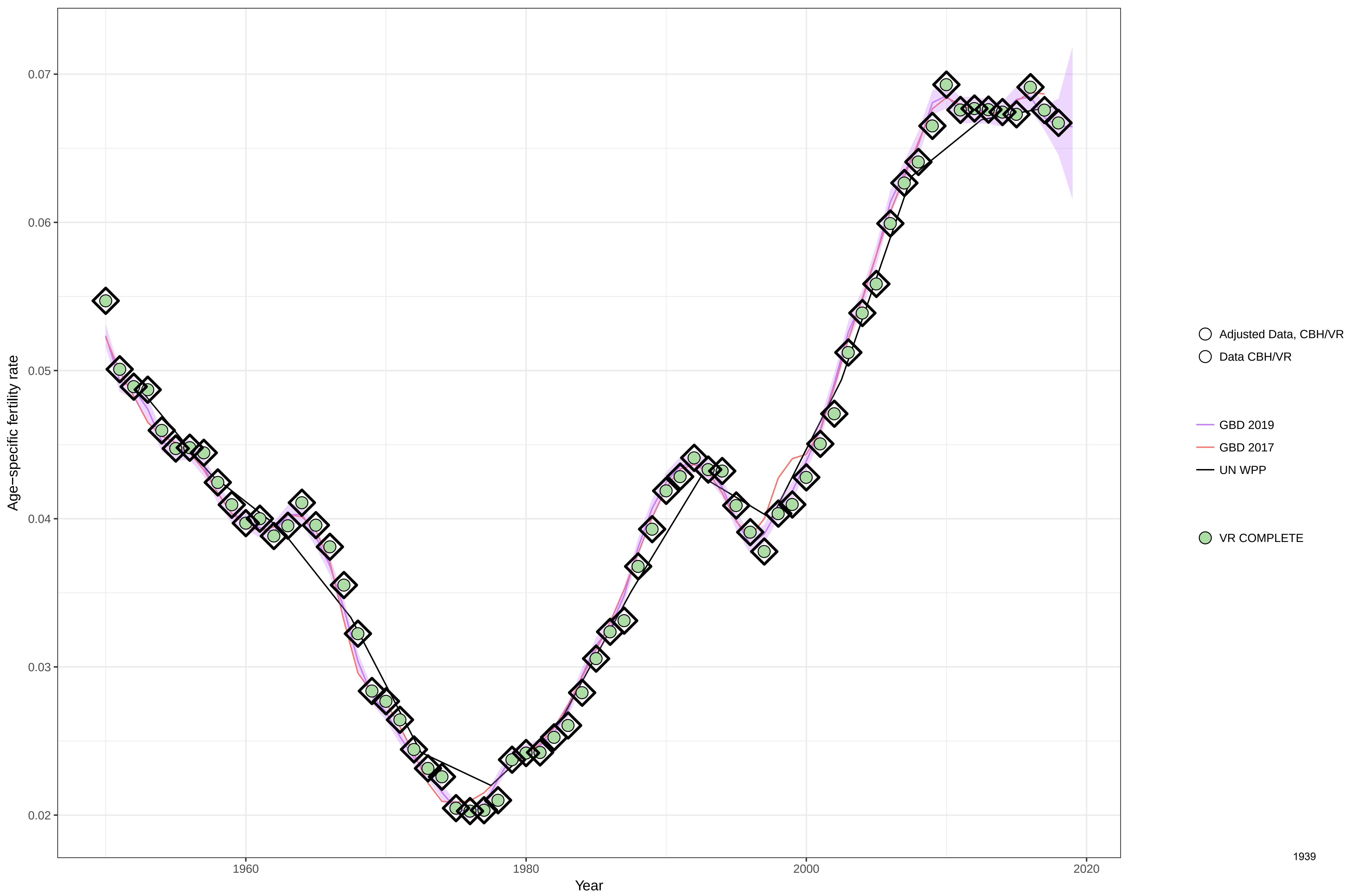

Age−specific fertility fitted trend lines, 1950−2019: Sweden, Ages: 40−44
Data points enclosed with a diamond are reference sources

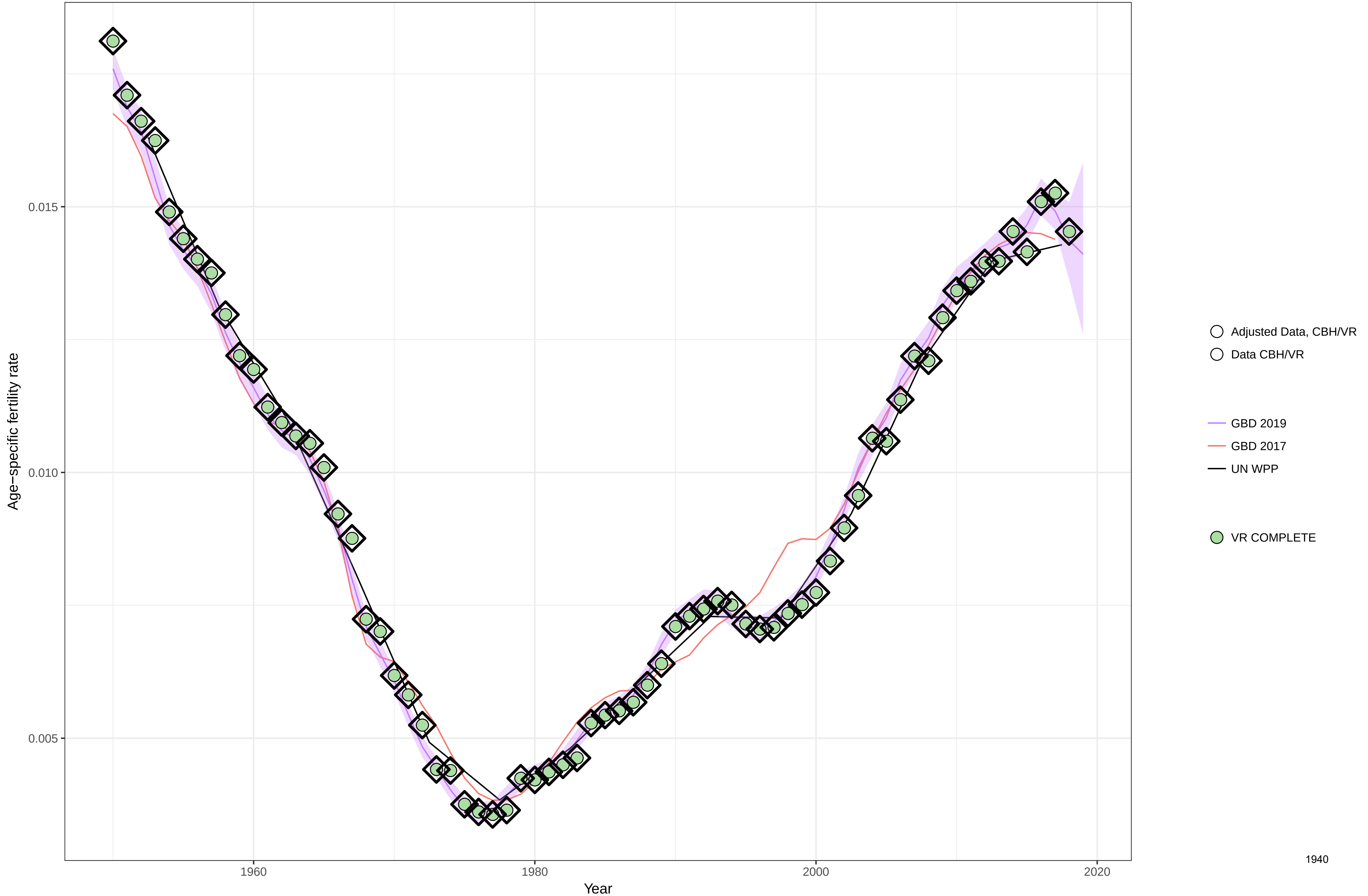

Age−specific fertility fitted trend lines, 1950−2019: Sweden, Ages: 45−49
Data points enclosed with a diamond are reference sources

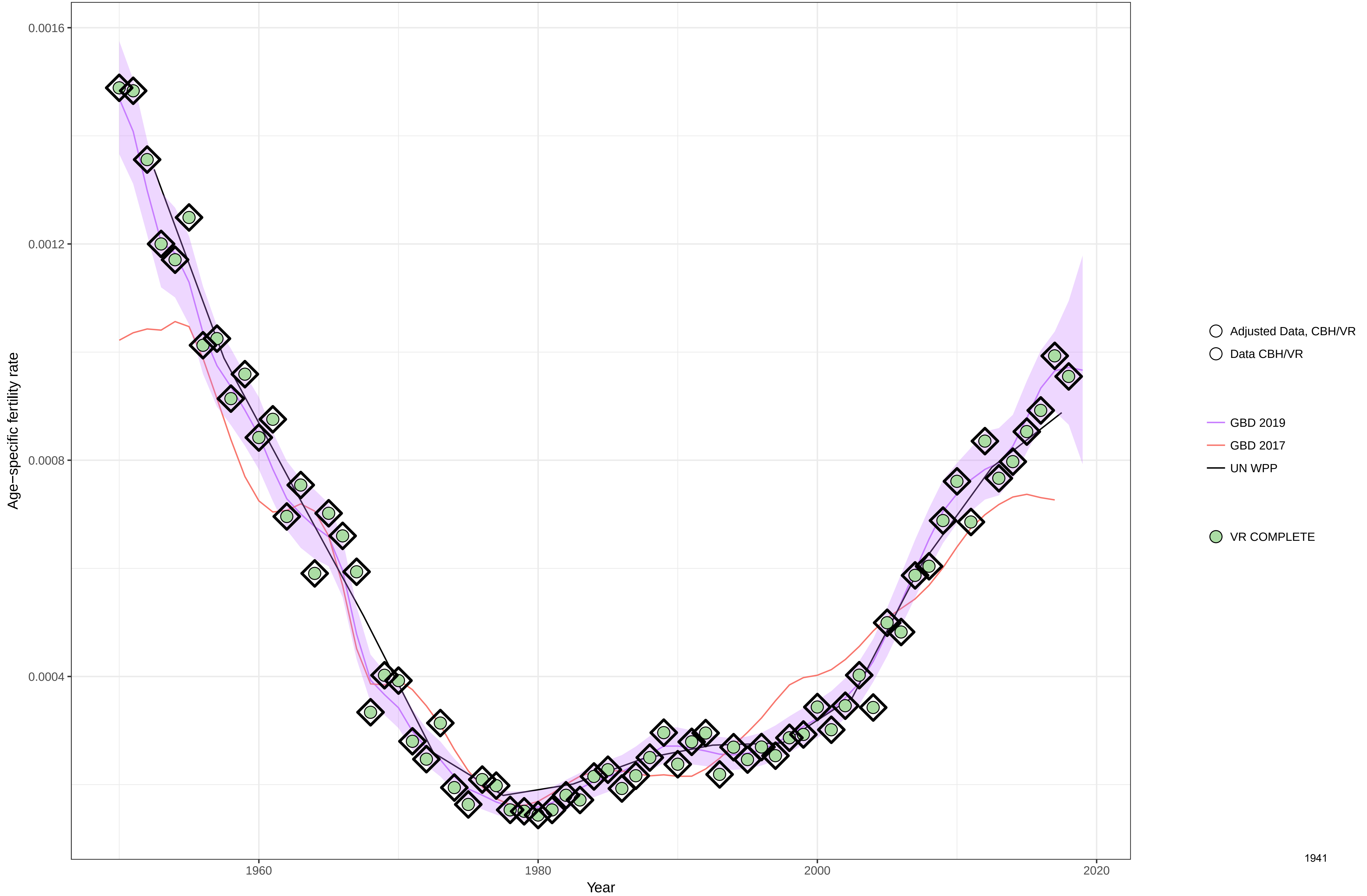

Age−specific fertility fitted trend lines, 1950−2019: Sweden, Ages: 50−54
Data points enclosed with a diamond are reference sources

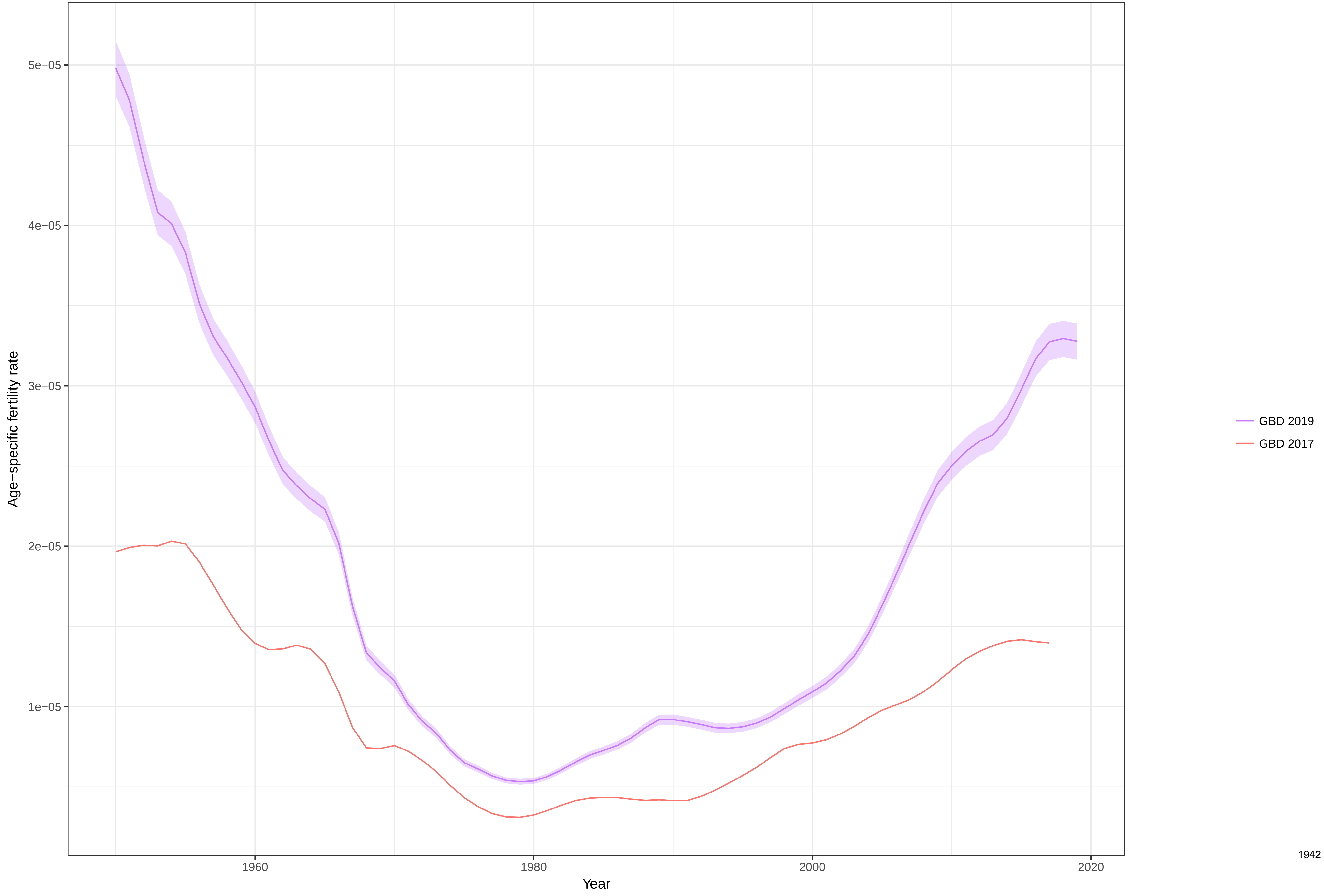

Total fertility rate fitted trend lines, 1950−2019: Sweden TFR
Data points enclosed with a diamond are reference sources

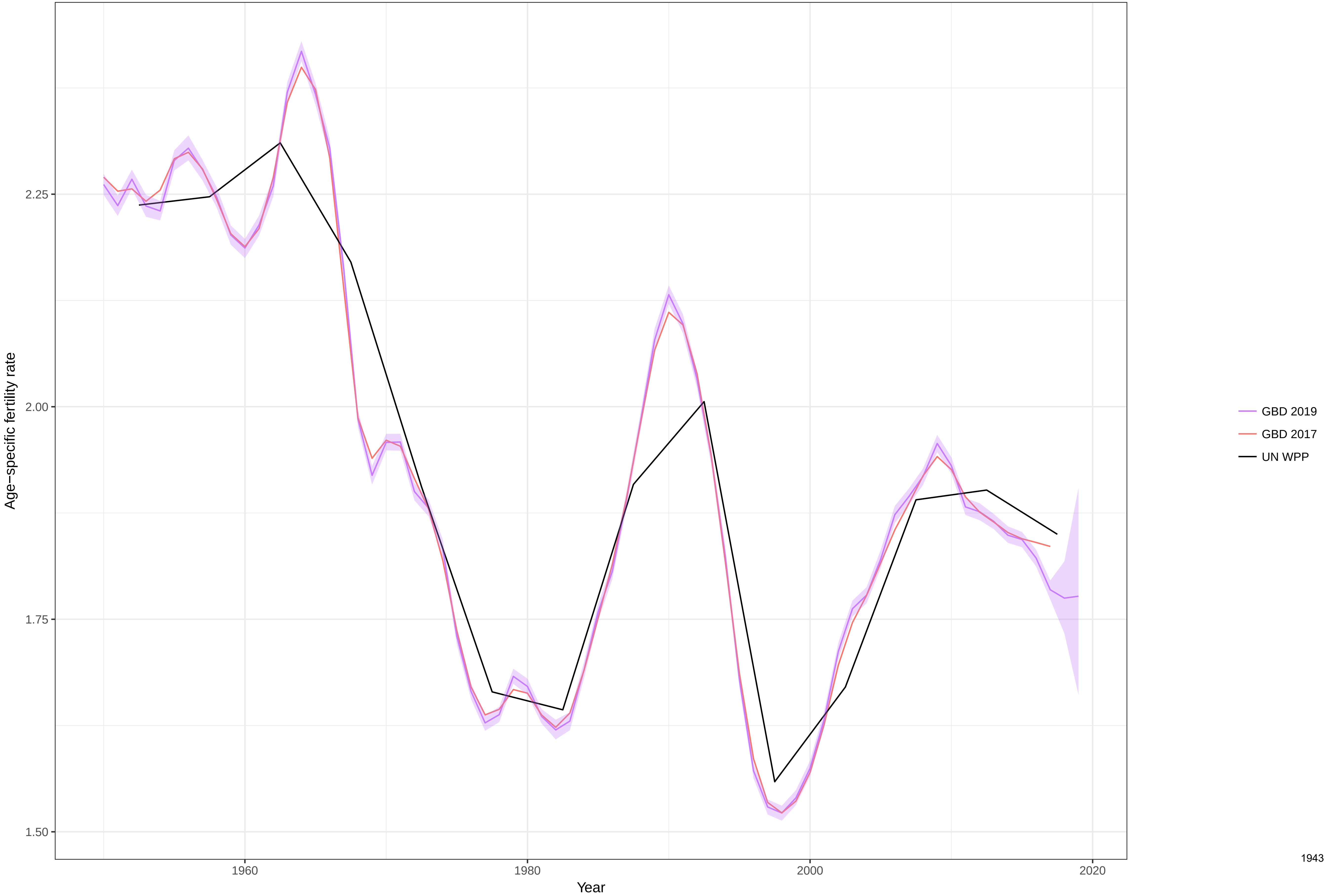

Age−specific fertility fitted trend lines, 1950−2019: Eswatini, Ages: 10−14
Data points enclosed with a diamond are reference sources

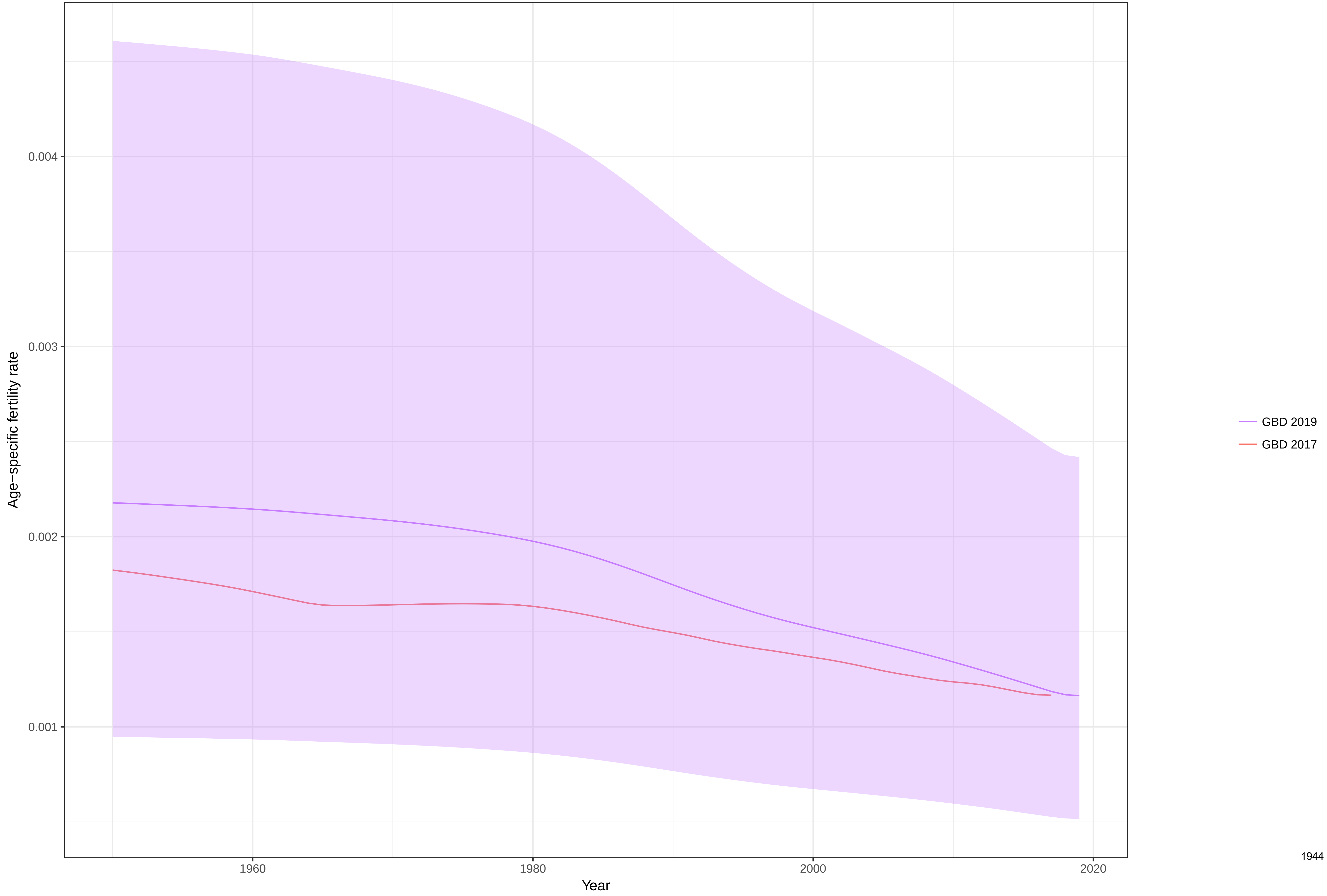

Age−specific fertility fitted trend lines, 1950−2019: Eswatini, Ages: 15−19
Data points enclosed with a diamond are reference sources

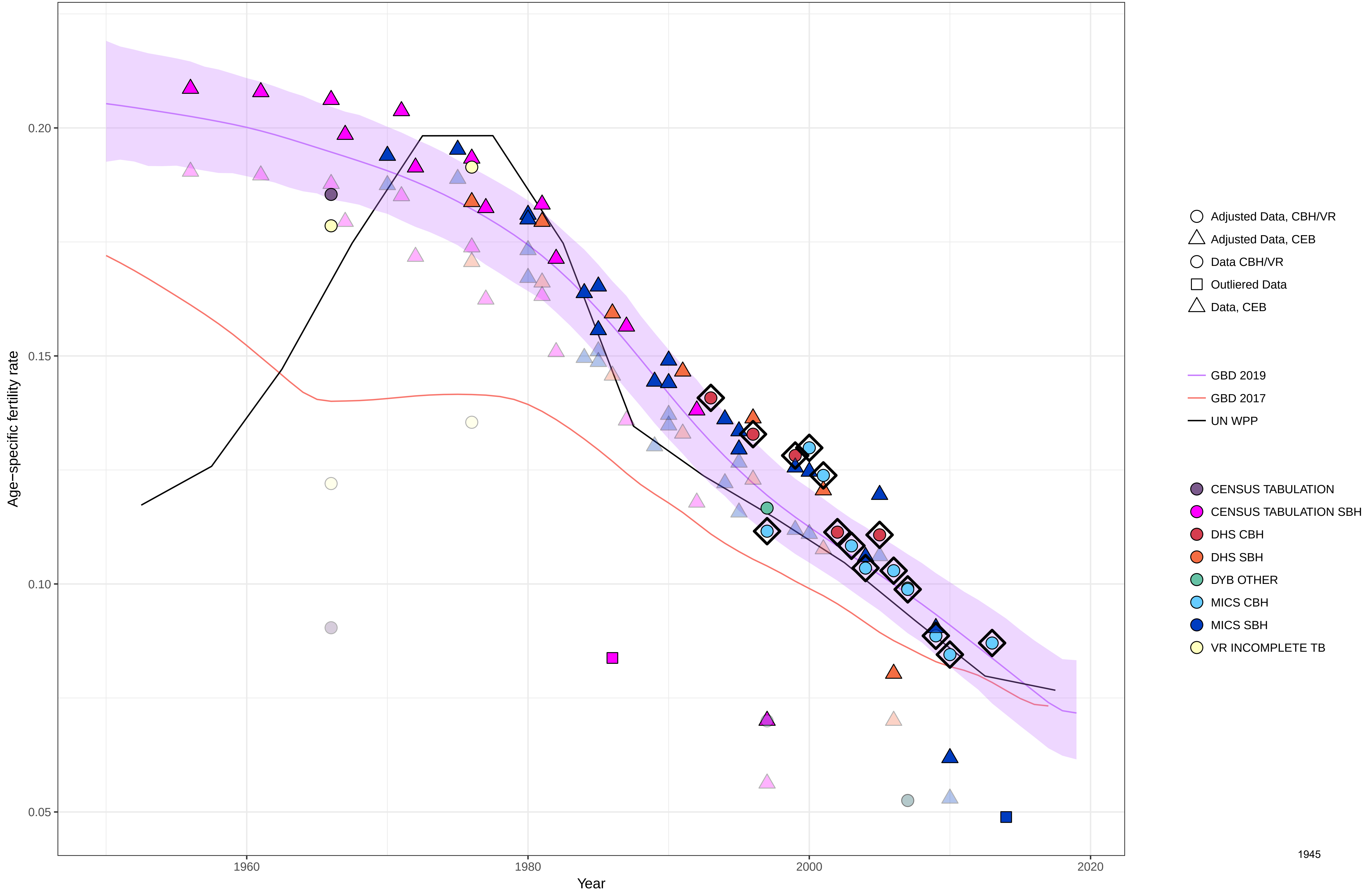

Age−specific fertility fitted trend lines, 1950−2019: Eswatini, Ages: 20−24
Data points enclosed with a diamond are reference sources

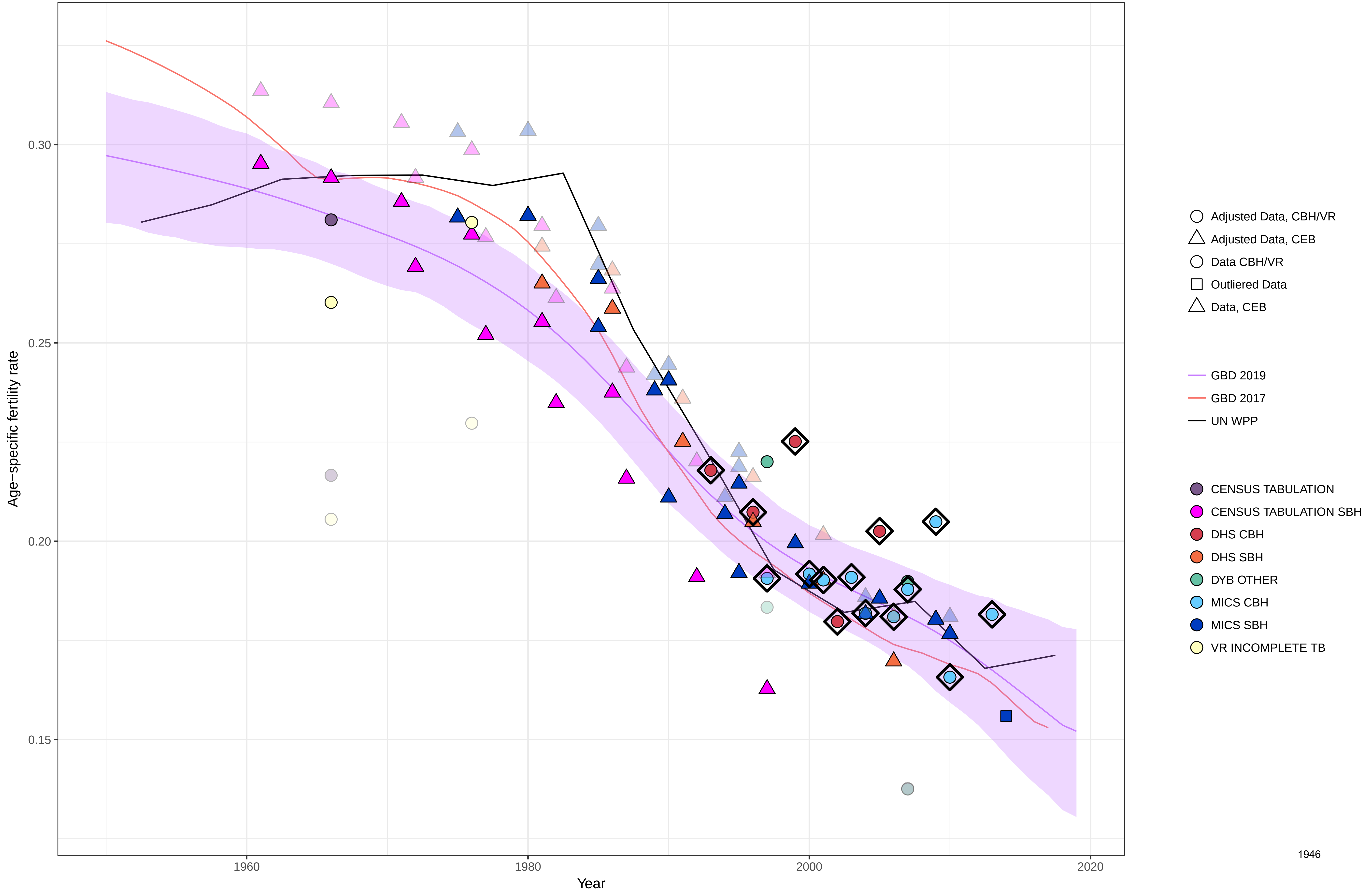

Age−specific fertility fitted trend lines, 1950−2019: Eswatini, Ages: 25−29
Data points enclosed with a diamond are reference sources

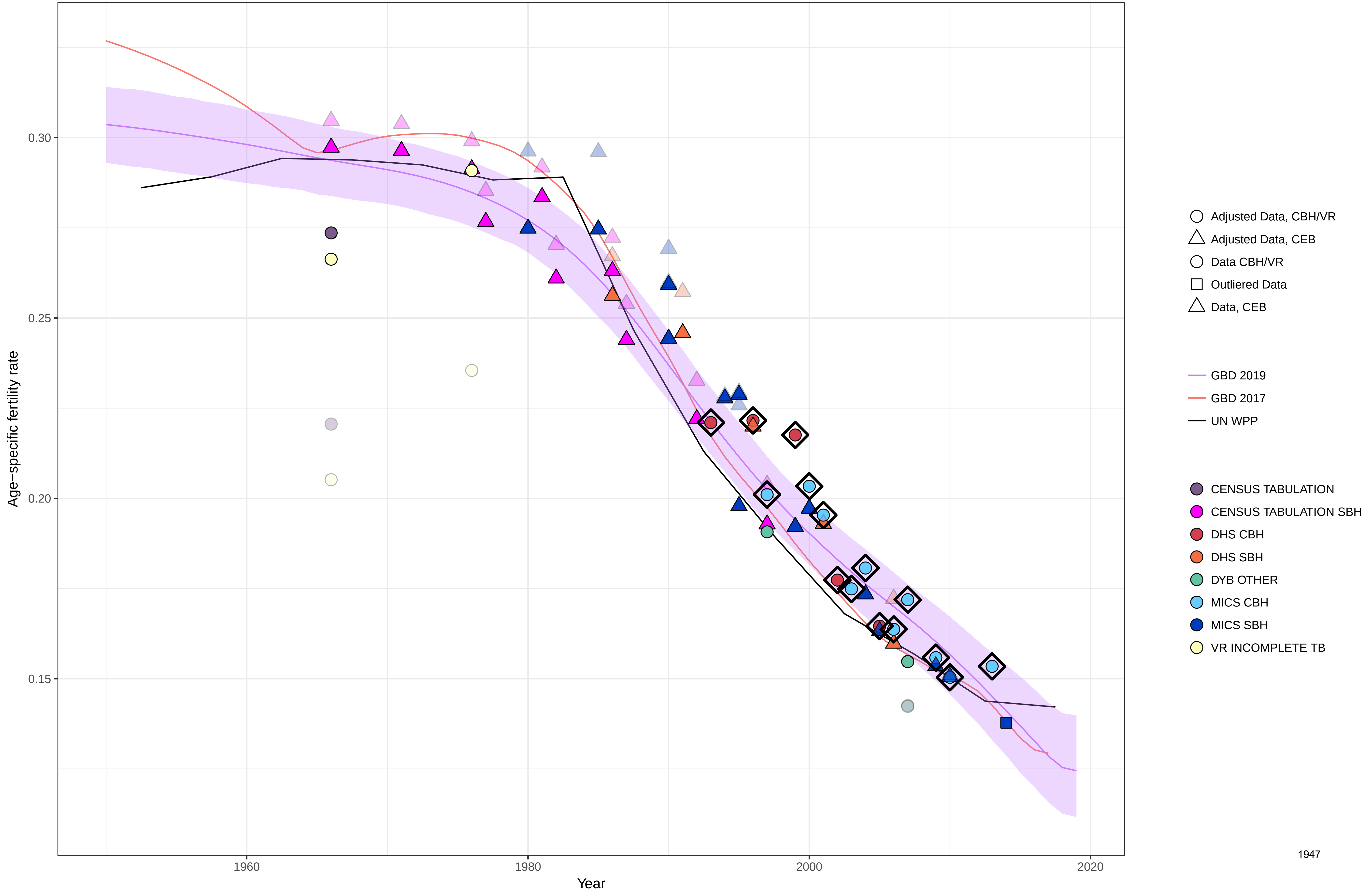

Age−specific fertility fitted trend lines, 1950−2019: Eswatini, Ages: 30−34
Data points enclosed with a diamond are reference sources

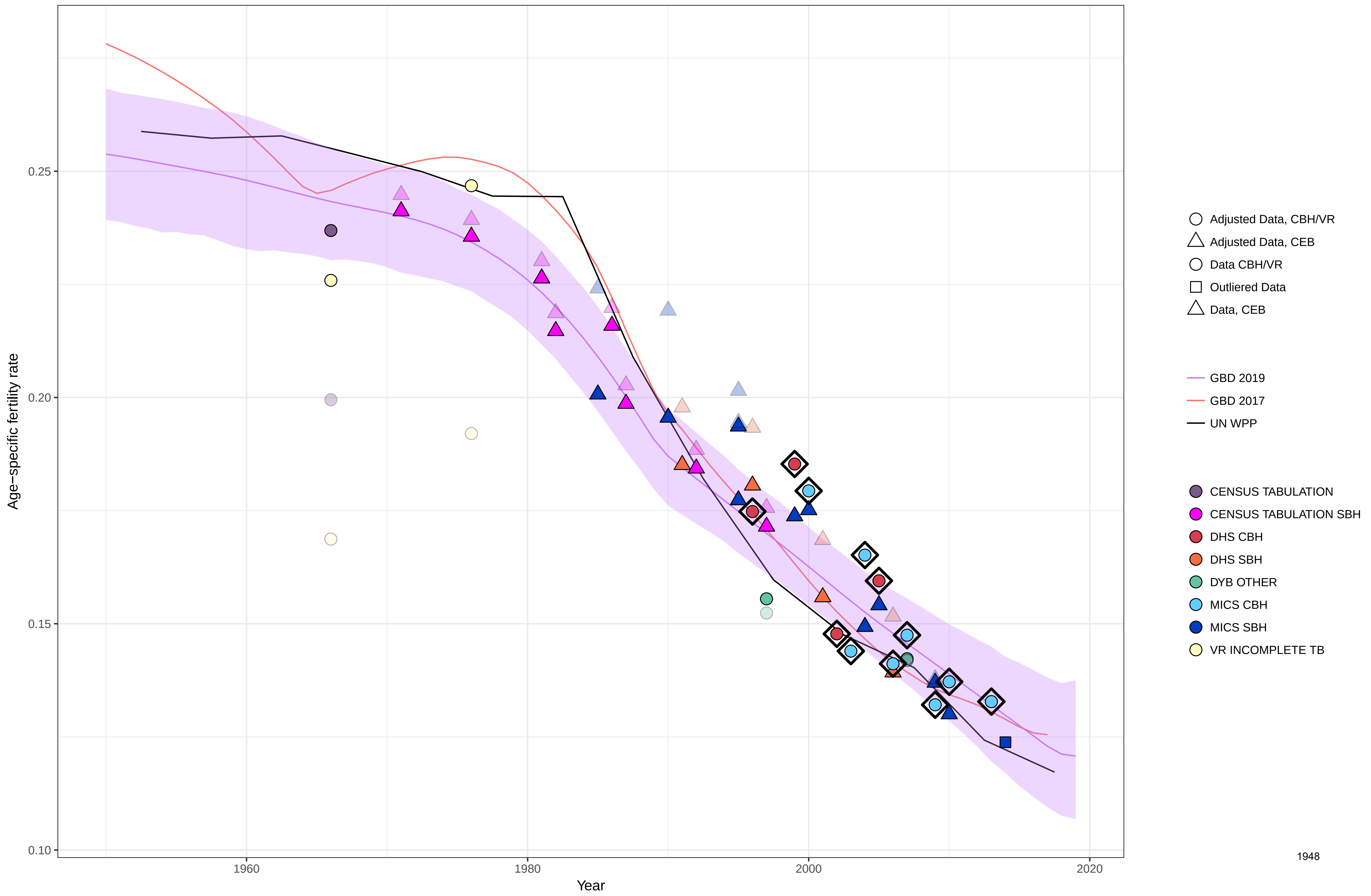

Age−specific fertility fitted trend lines, 1950−2019: Eswatini, Ages: 35−39
Data points enclosed with a diamond are reference sources

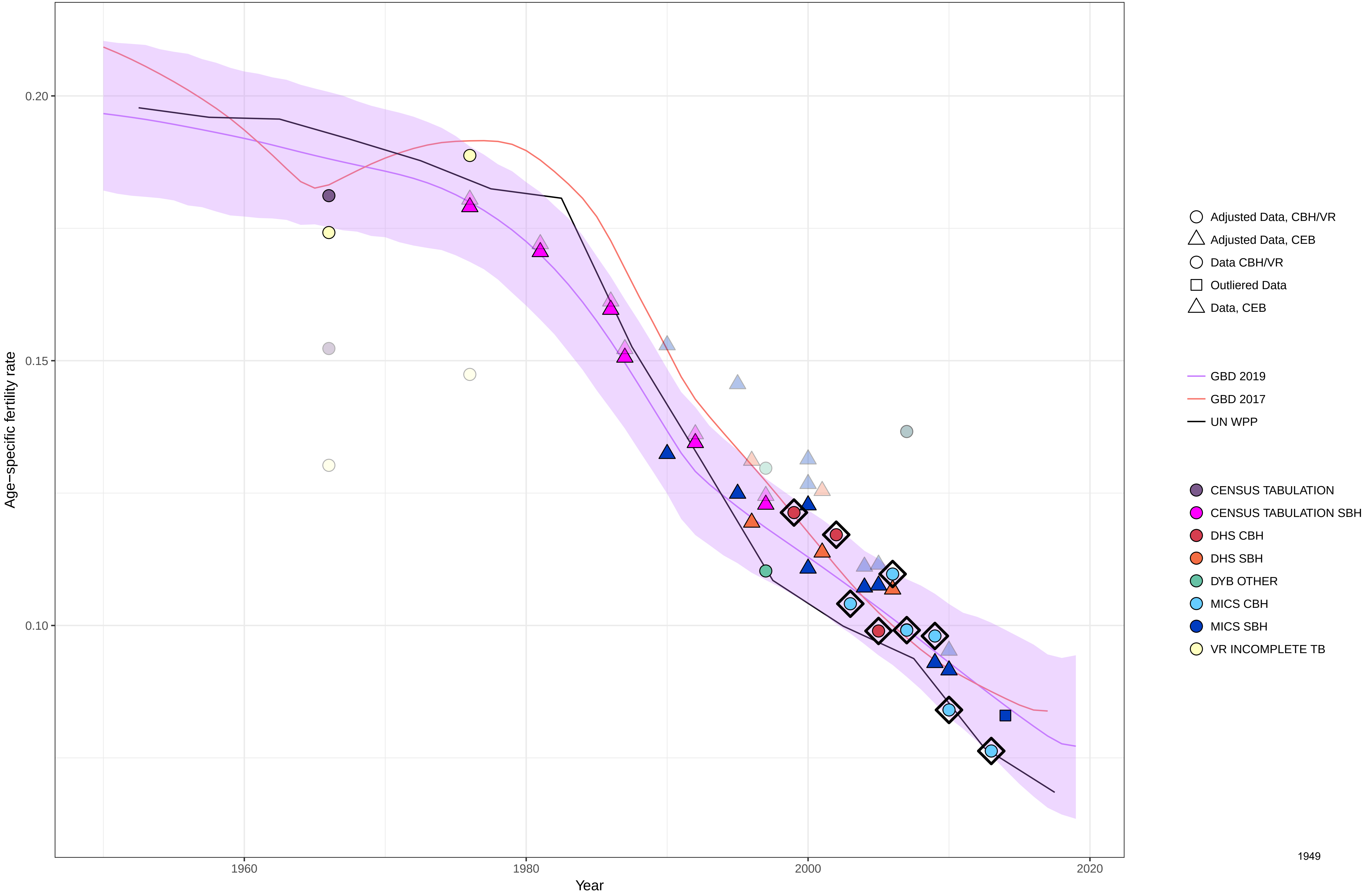

Age−specific fertility fitted trend lines, 1950−2019: Eswatini, Ages: 40−44
Data points enclosed with a diamond are reference sources

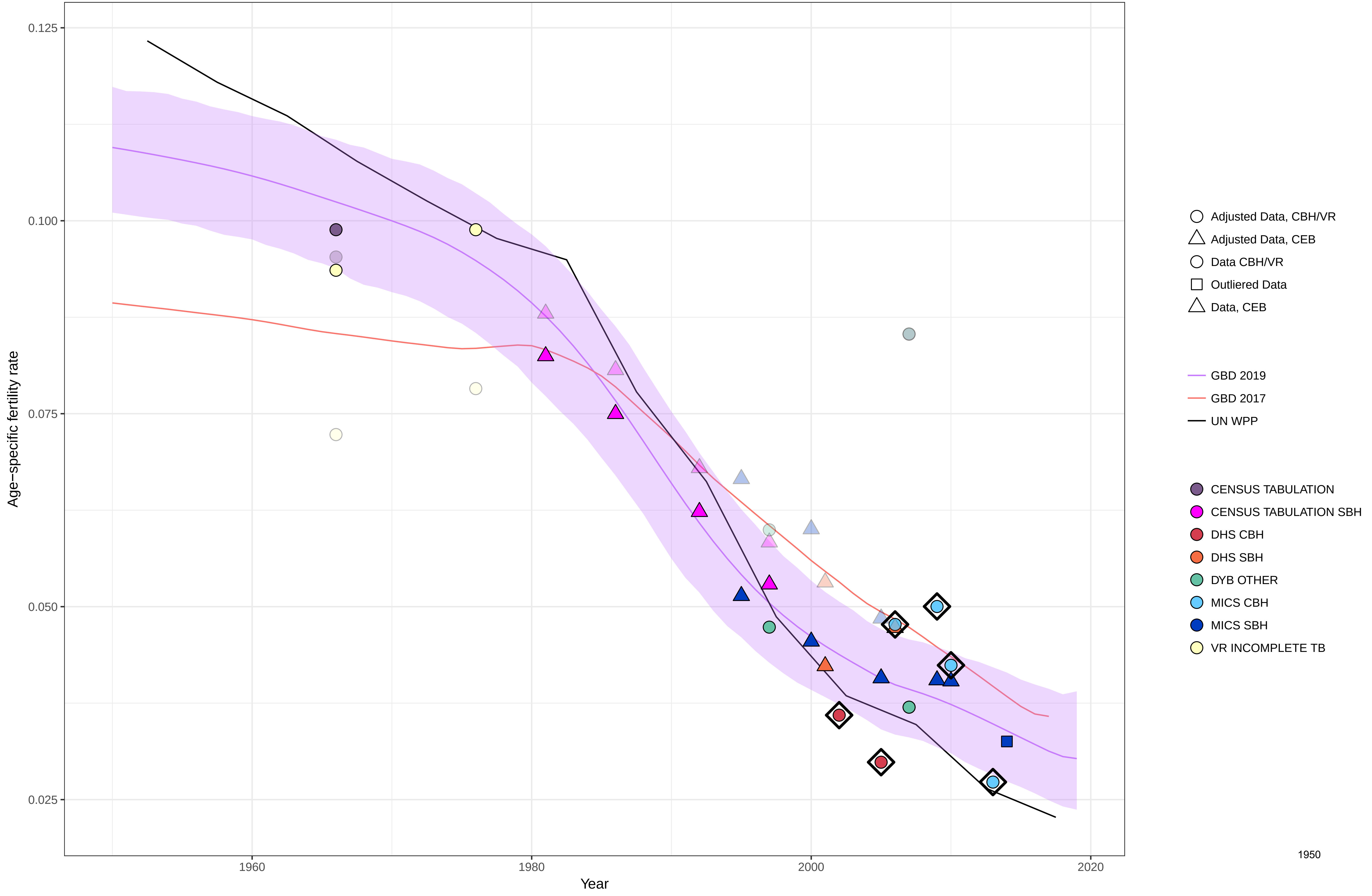

1950

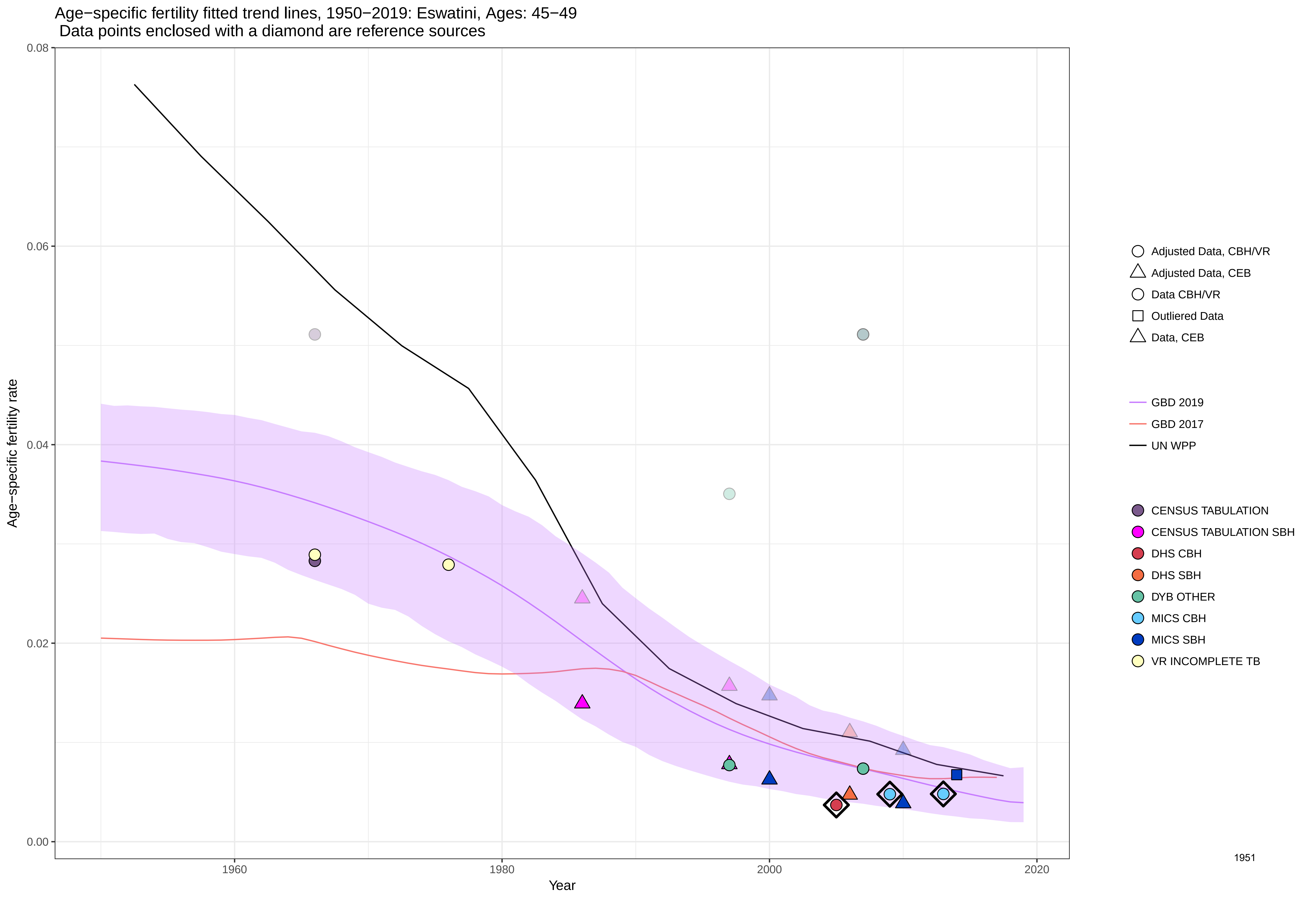

Age−specific fertility fitted trend lines, 1950−2019: Eswatini, Ages: 45−49
Data points enclosed with a diamond are reference sources
Age−specific fertility rate
Year
0.00
0.02
0.04
0.06
0.08
1960
1980
2000
2020
Adjusted Data, CBH/VR
Adjusted Data, CEB
Data CBH/VR
Outliered Data
Data, CEB
GBD 2019
GBD 2017
UN WPP
CENSUS TABULATION
CENSUS TABULATION SBH
DHS CBH
DHS SBH
DYB OTHER
MICS CBH
MICS SBH
VR INCOMPLETE TB

Age−specific fertility fitted trend lines, 1950−2019: Eswatini, Ages: 50−54
Data points enclosed with a diamond are reference sources

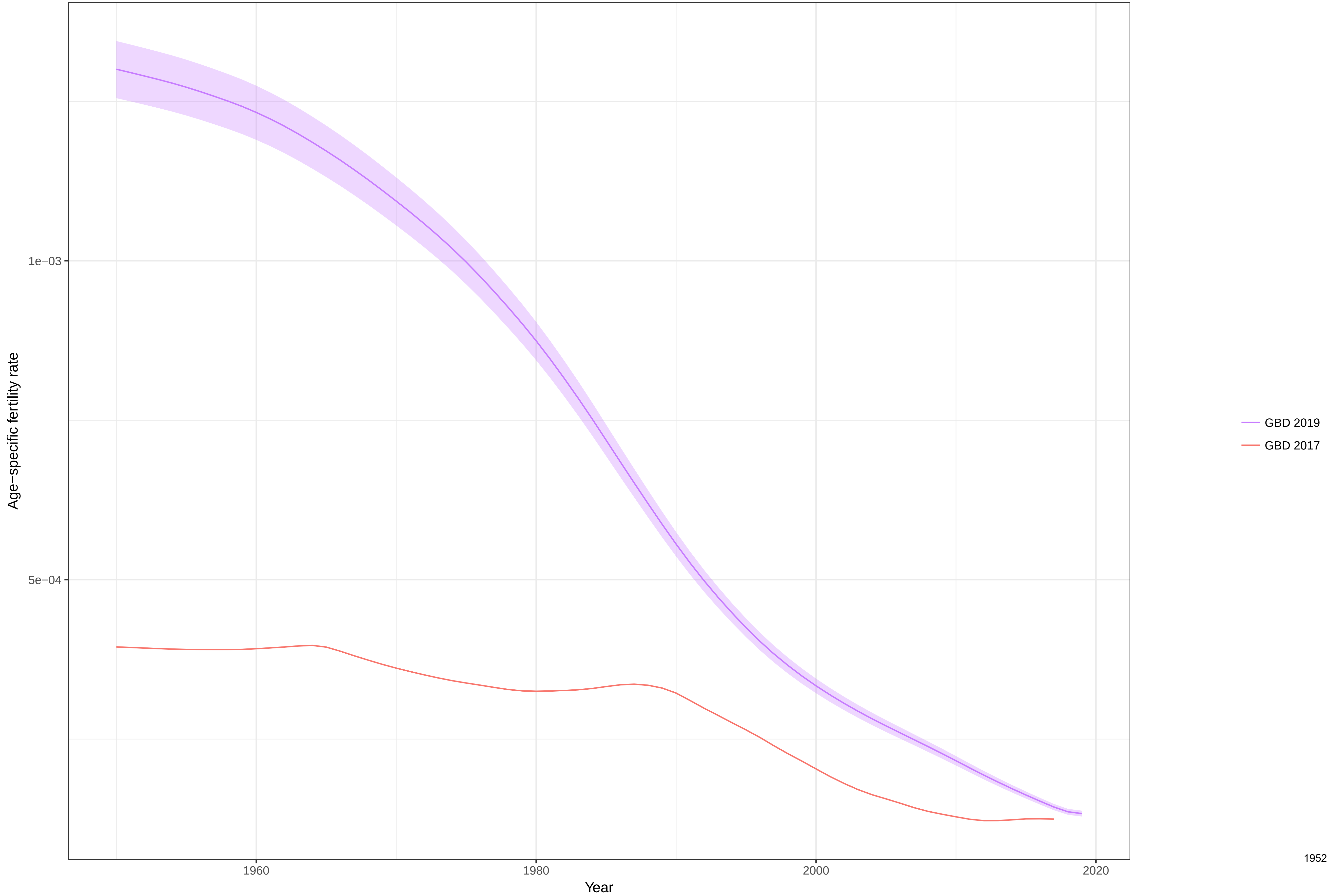

Total fertility rate fitted trend lines, 1950−2019: Eswatini TFR
Data points enclosed with a diamond are reference sources

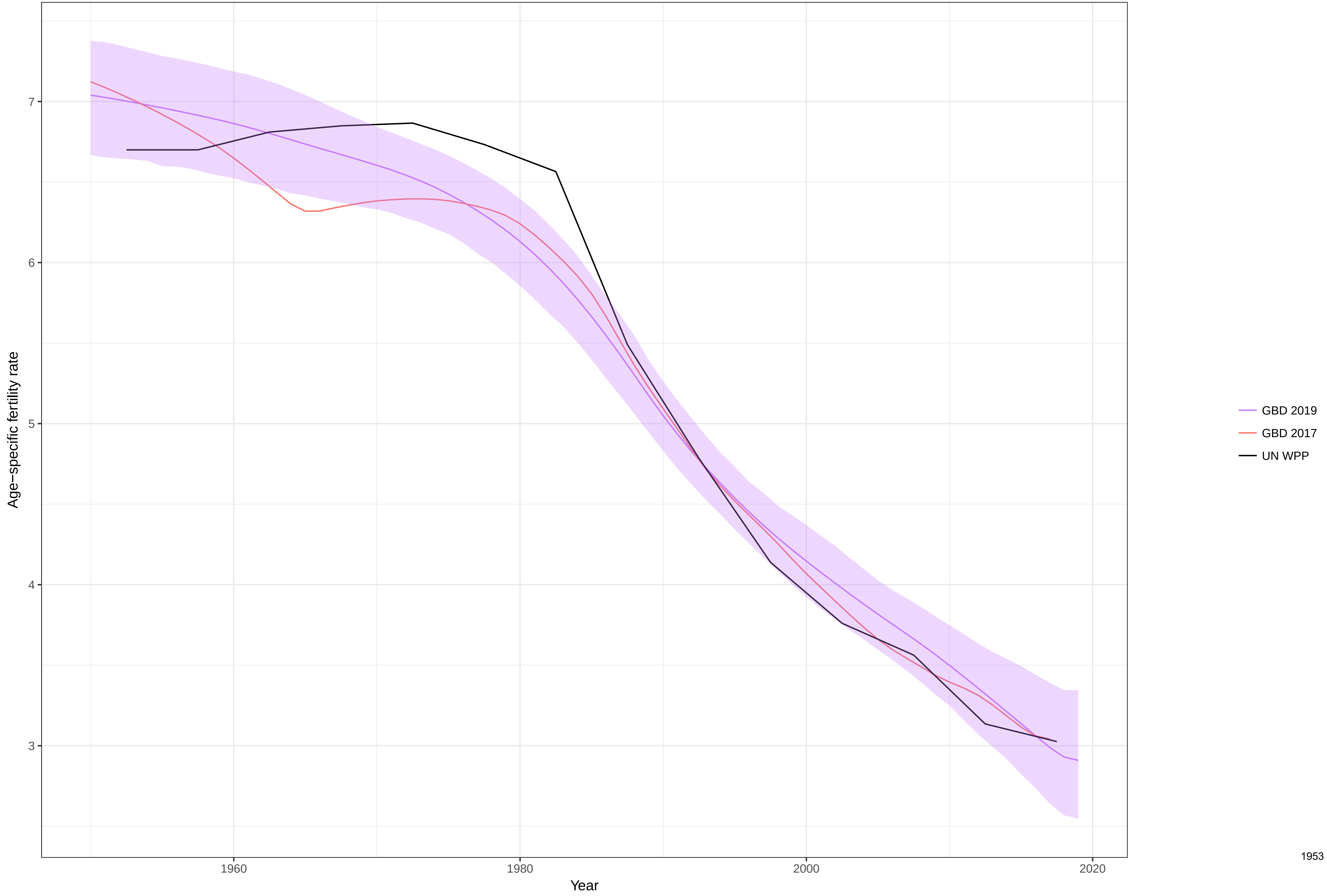

Age−specific fertility fitted trend lines, 1950−2019: Seychelles, Ages: 10−14
Data points enclosed with a diamond are reference sources

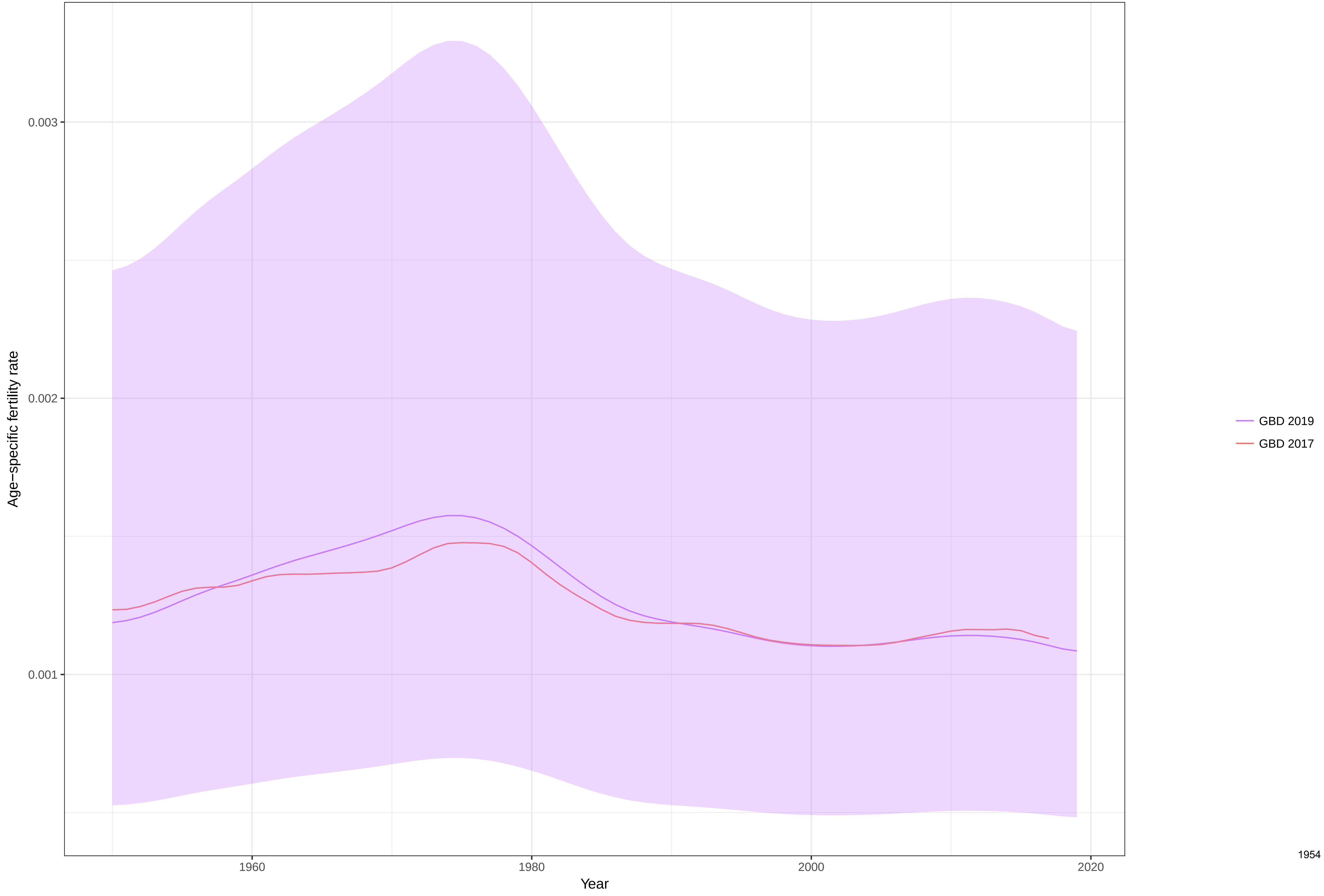

Age−specific fertility fitted trend lines, 1950−2019: Seychelles, Ages: 15−19
Data points enclosed with a diamond are reference sources

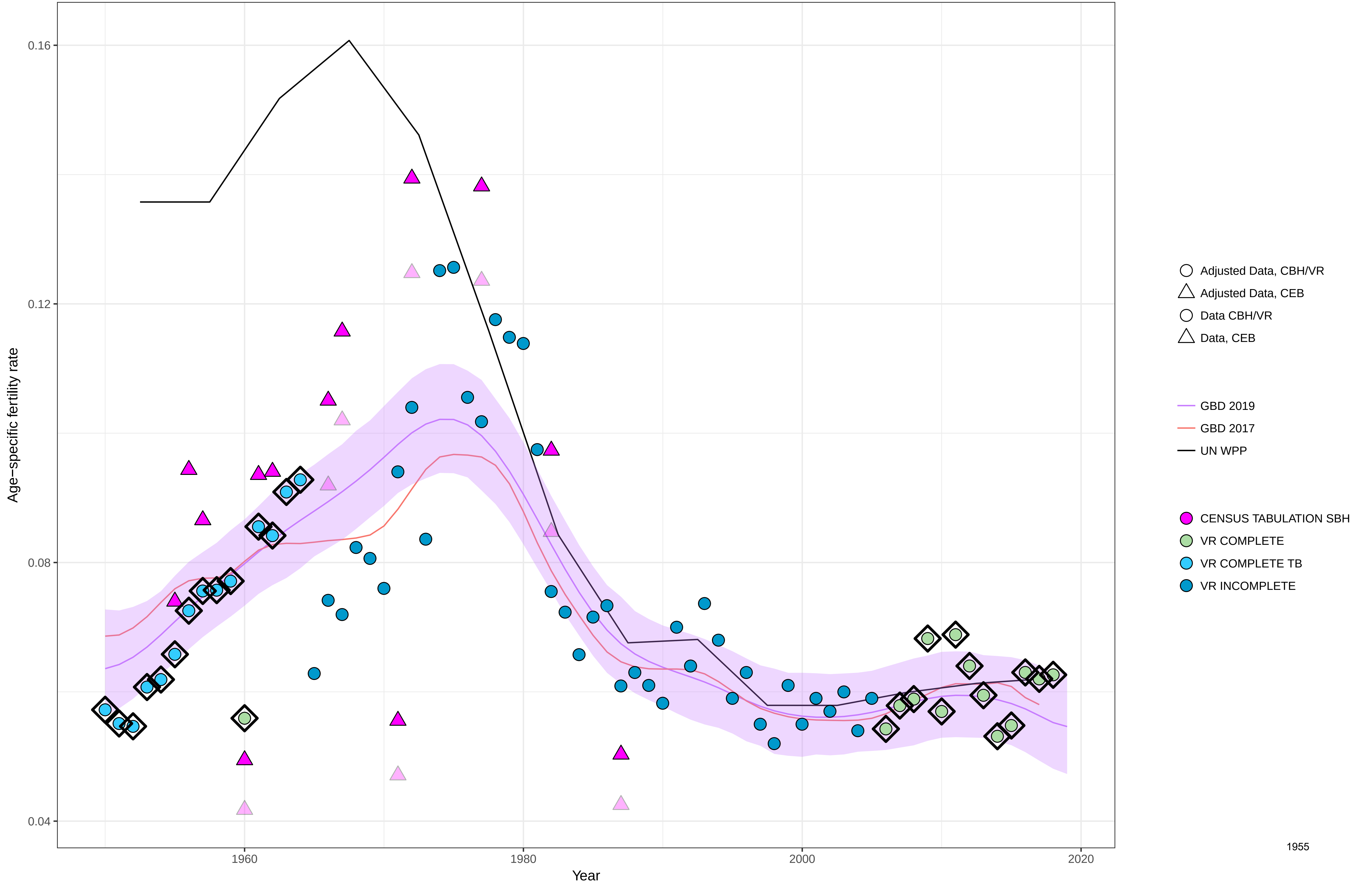

Age−specific fertility fitted trend lines, 1950−2019: Seychelles, Ages: 20−24
Data points enclosed with a diamond are reference sources

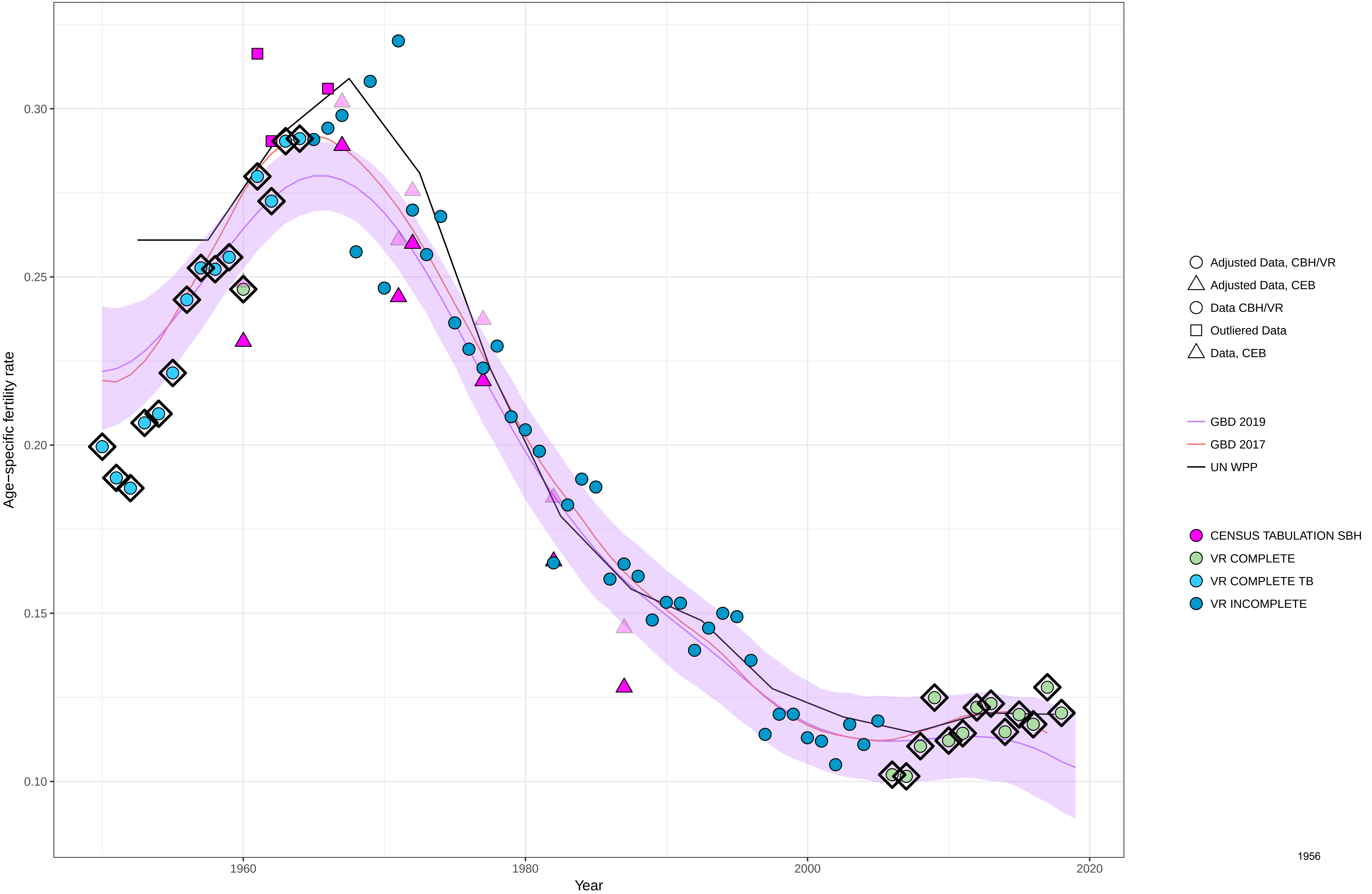

Age−specific fertility fitted trend lines, 1950−2019: Seychelles, Ages: 25−29
Data points enclosed with a diamond are reference sources

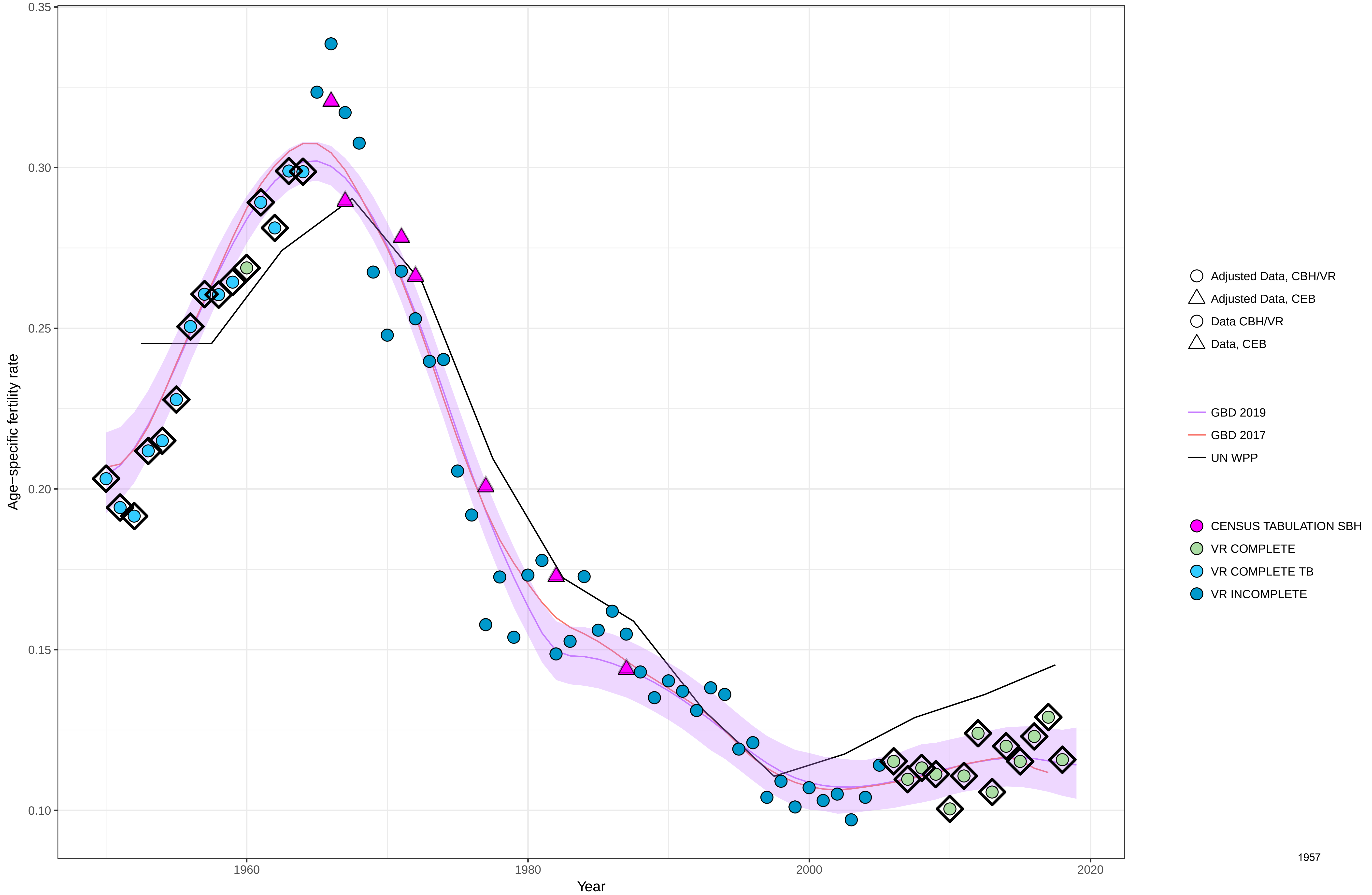

Age−specific fertility fitted trend lines, 1950−2019: Seychelles, Ages: 30−34
Data points enclosed with a diamond are reference sources

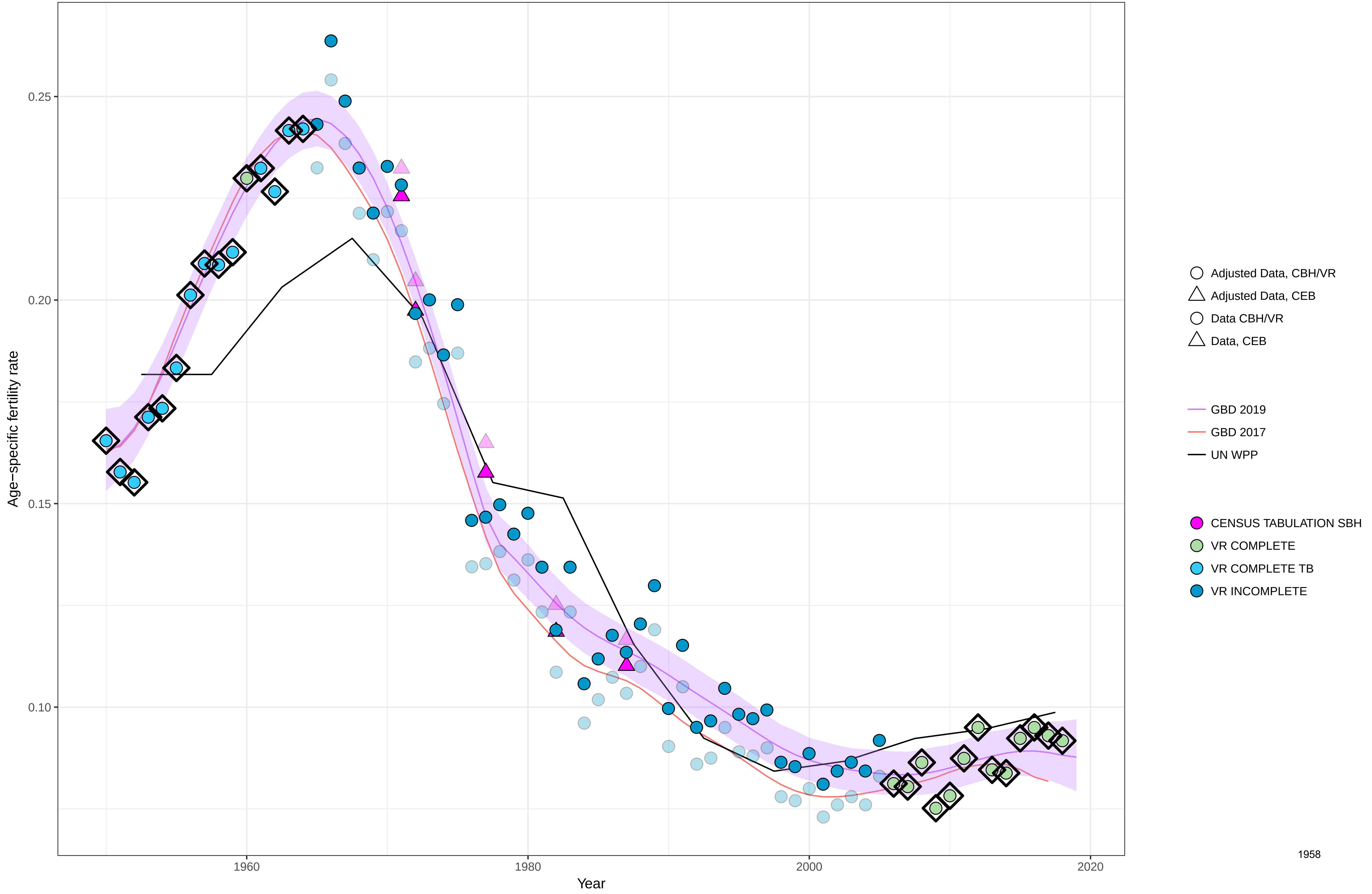

Age−specific fertility fitted trend lines, 1950−2019: Seychelles, Ages: 35−39
Data points enclosed with a diamond are reference sources

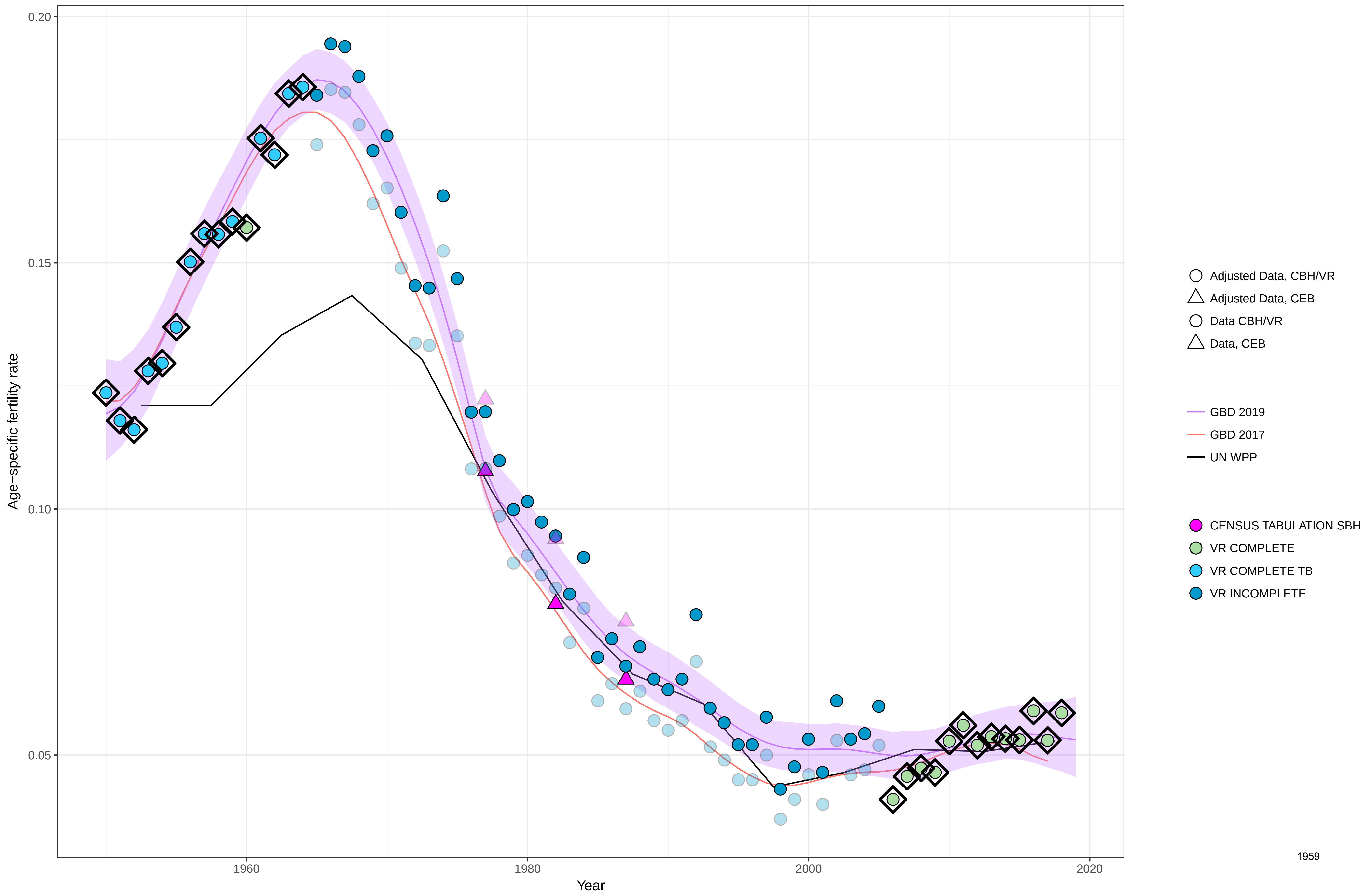

Age−specific fertility fitted trend lines, 1950−2019: Seychelles, Ages: 40−44
Data points enclosed with a diamond are reference sources

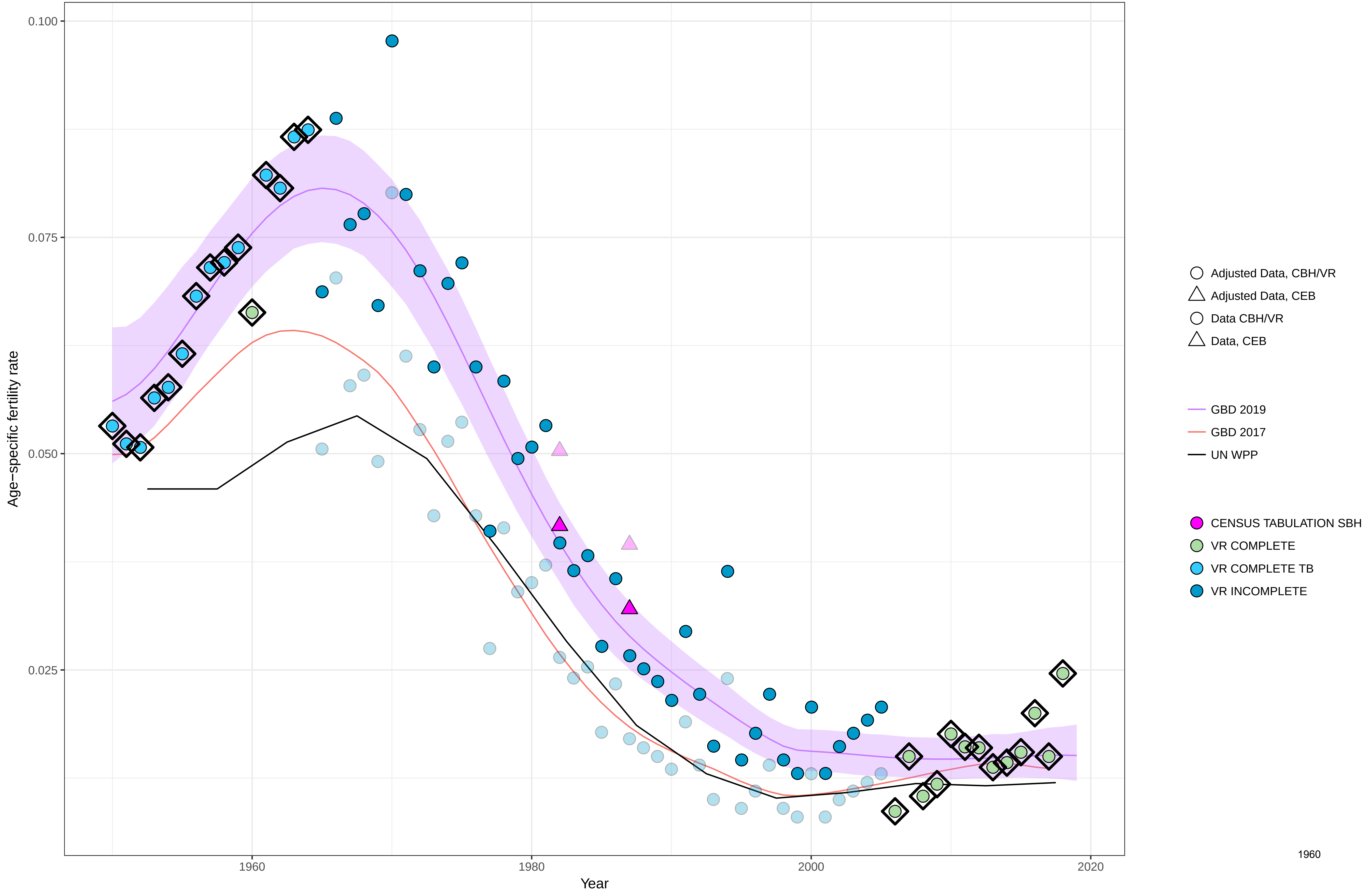

1960

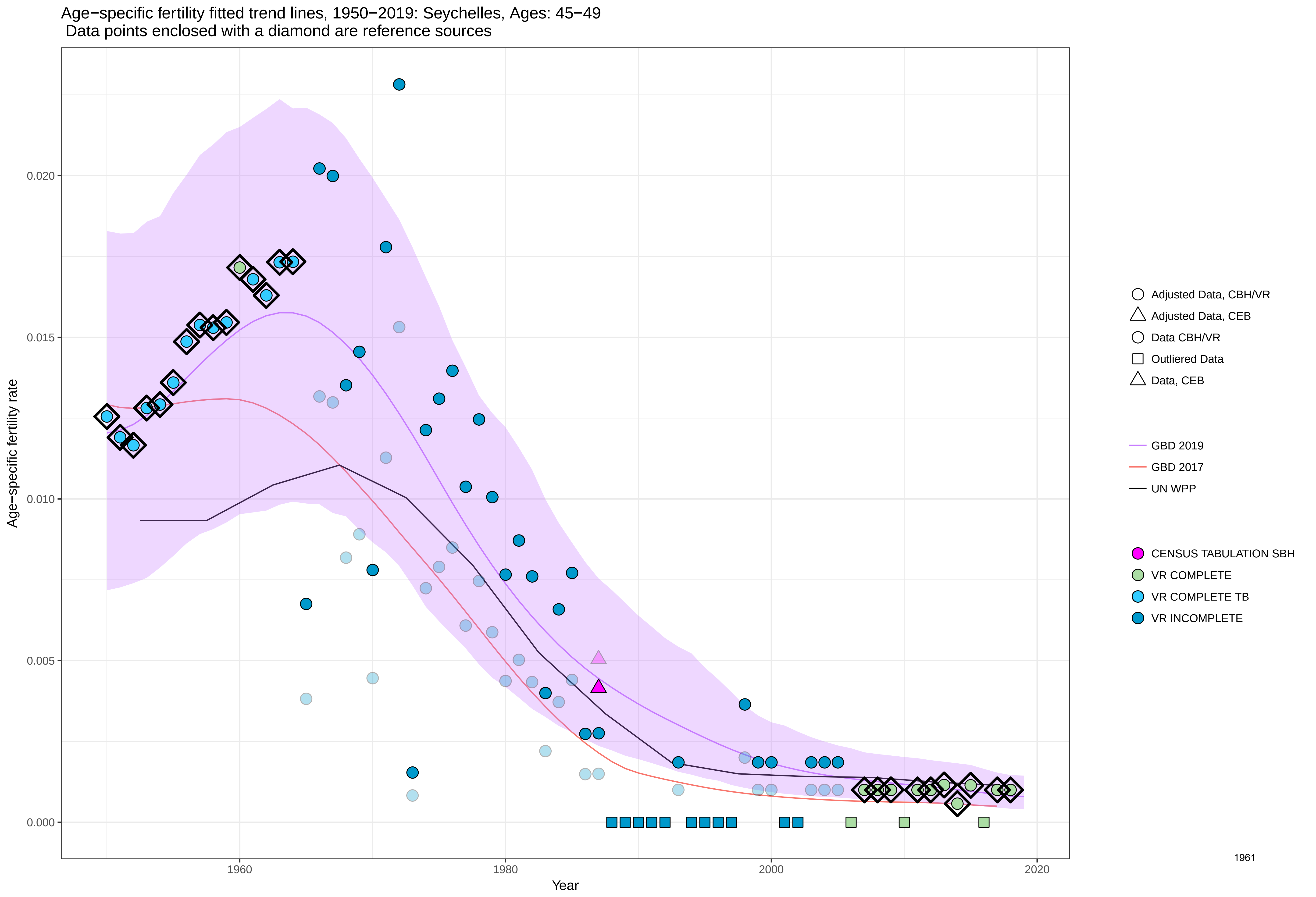
Age−specific fertility fitted trend lines, 1950−2019: Seychelles, Ages: 45−49
Data points enclosed with a diamond are reference sources
Age−specific fertility rate
Year
0.000
0.005
0.010
0.015
0.020
1960
1980
2000
2020
Adjusted Data, CBH/VR
Adjusted Data, CEB
Data CBH/VR
Outliered Data
Data, CEB
GBD 2019
GBD 2017
UN WPP
CENSUS TABULATION SBH
VR COMPLETE
VR COMPLETE TB
VR INCOMPLETE

Age−specific fertility fitted trend lines, 1950−2019: Seychelles, Ages: 50−54
Data points enclosed with a diamond are reference sources

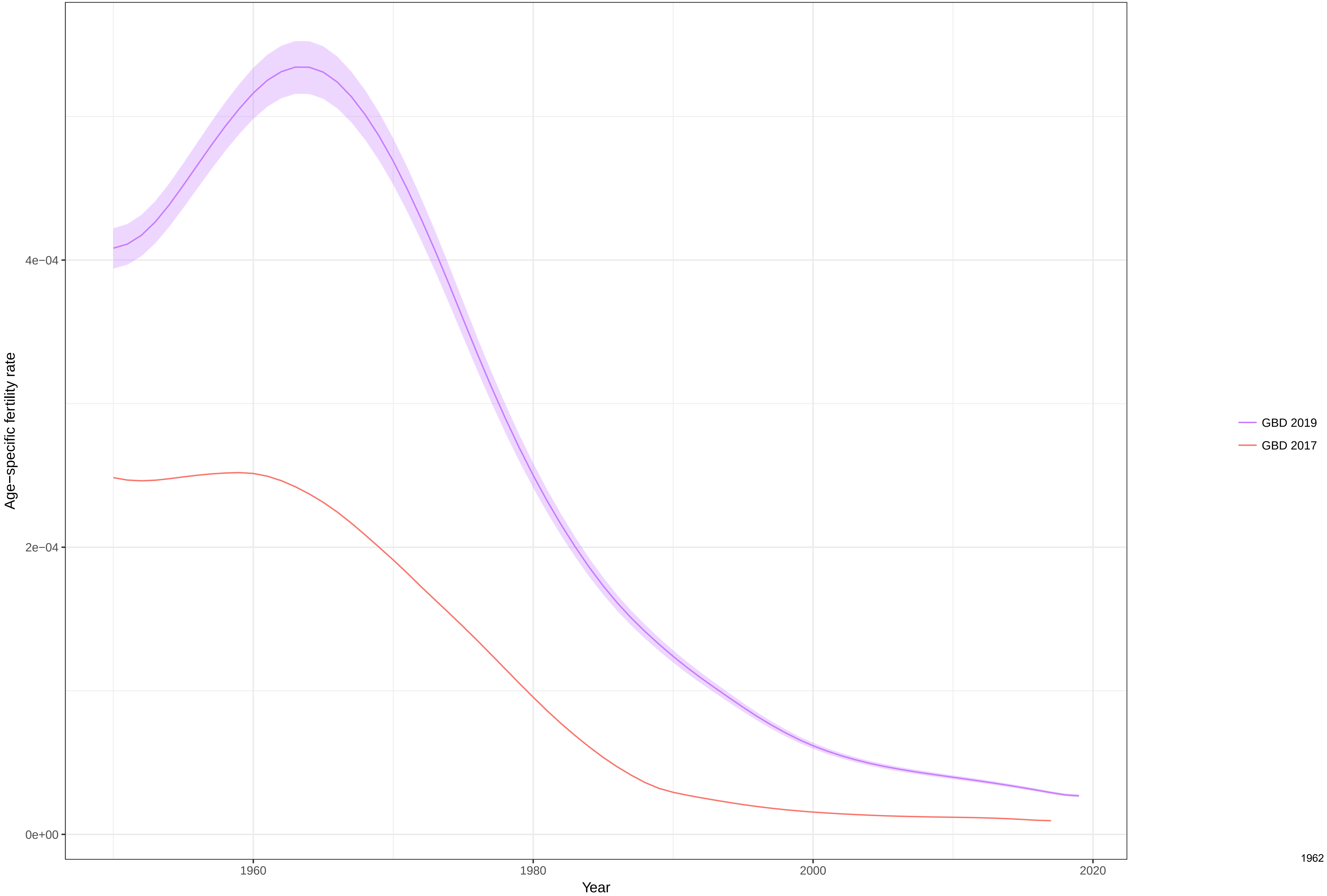

1962

Total fertility rate fitted trend lines, 1950−2019: Seychelles TFR
Data points enclosed with a diamond are reference sources

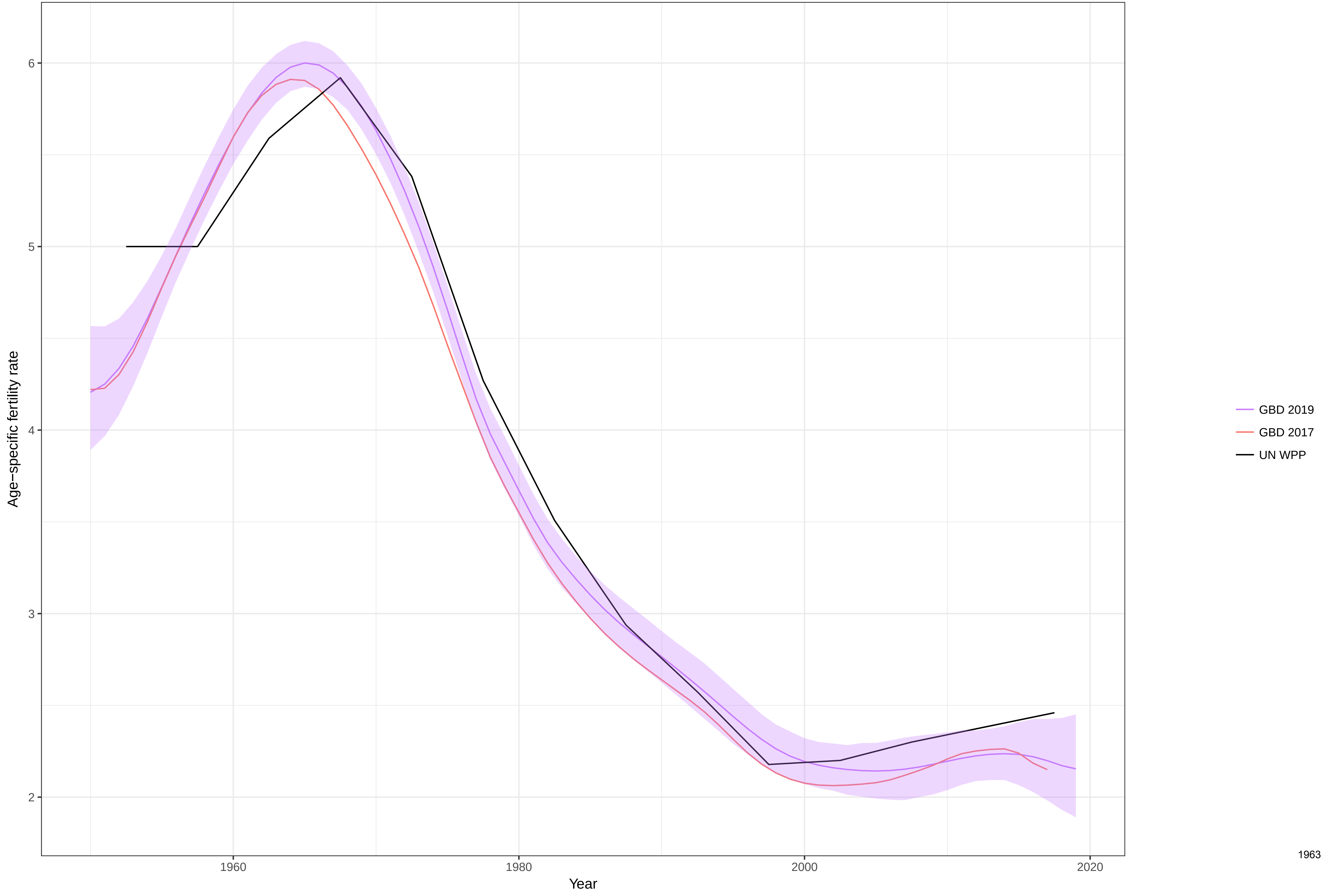

Age−specific fertility fitted trend lines, 1950−2019: Syrian Arab Republic, Ages: 10−14
Data points enclosed with a diamond are reference sources

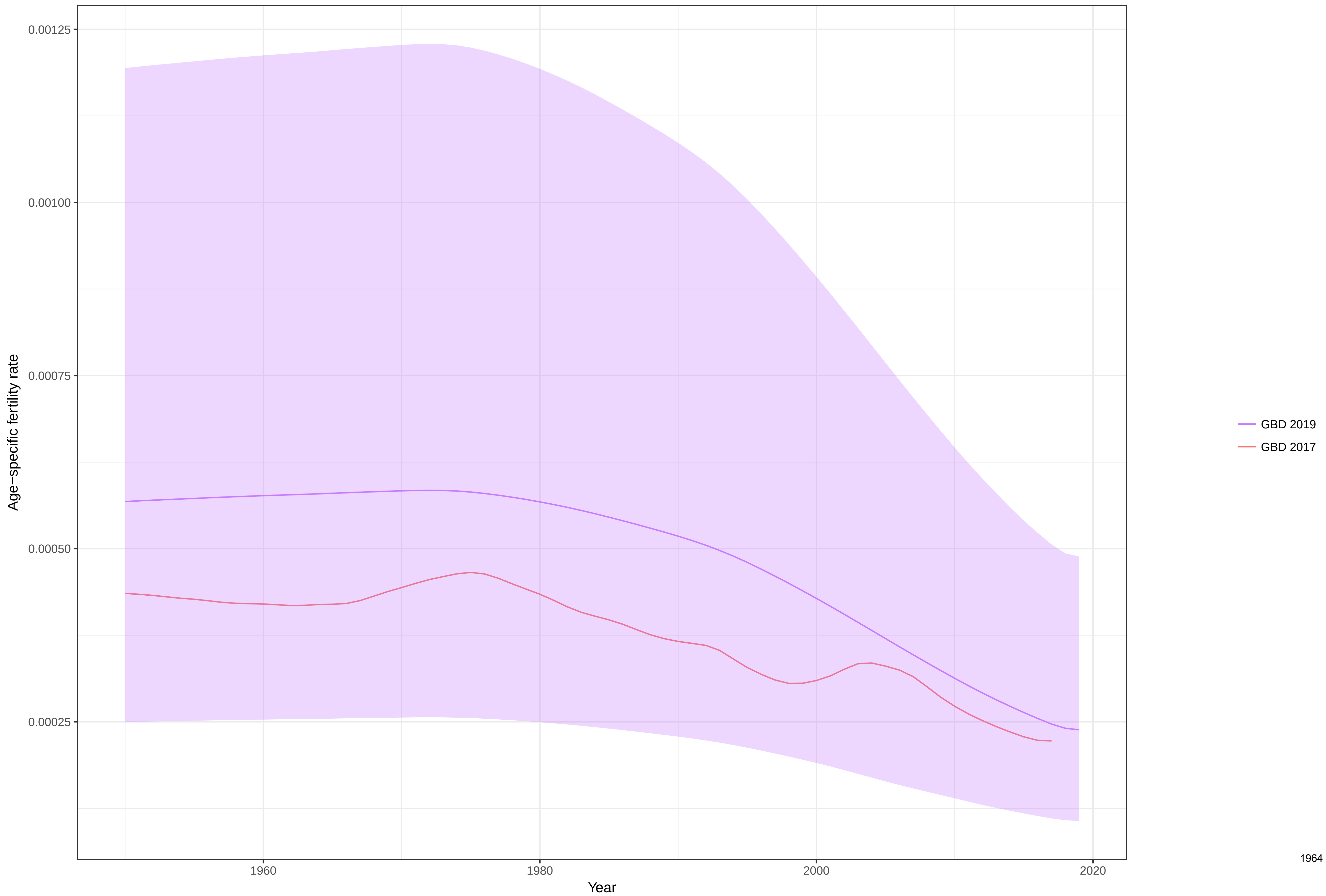

Age−specific fertility fitted trend lines, 1950−2019: Syrian Arab Republic, Ages: 15−19
Data points enclosed with a diamond are reference sources

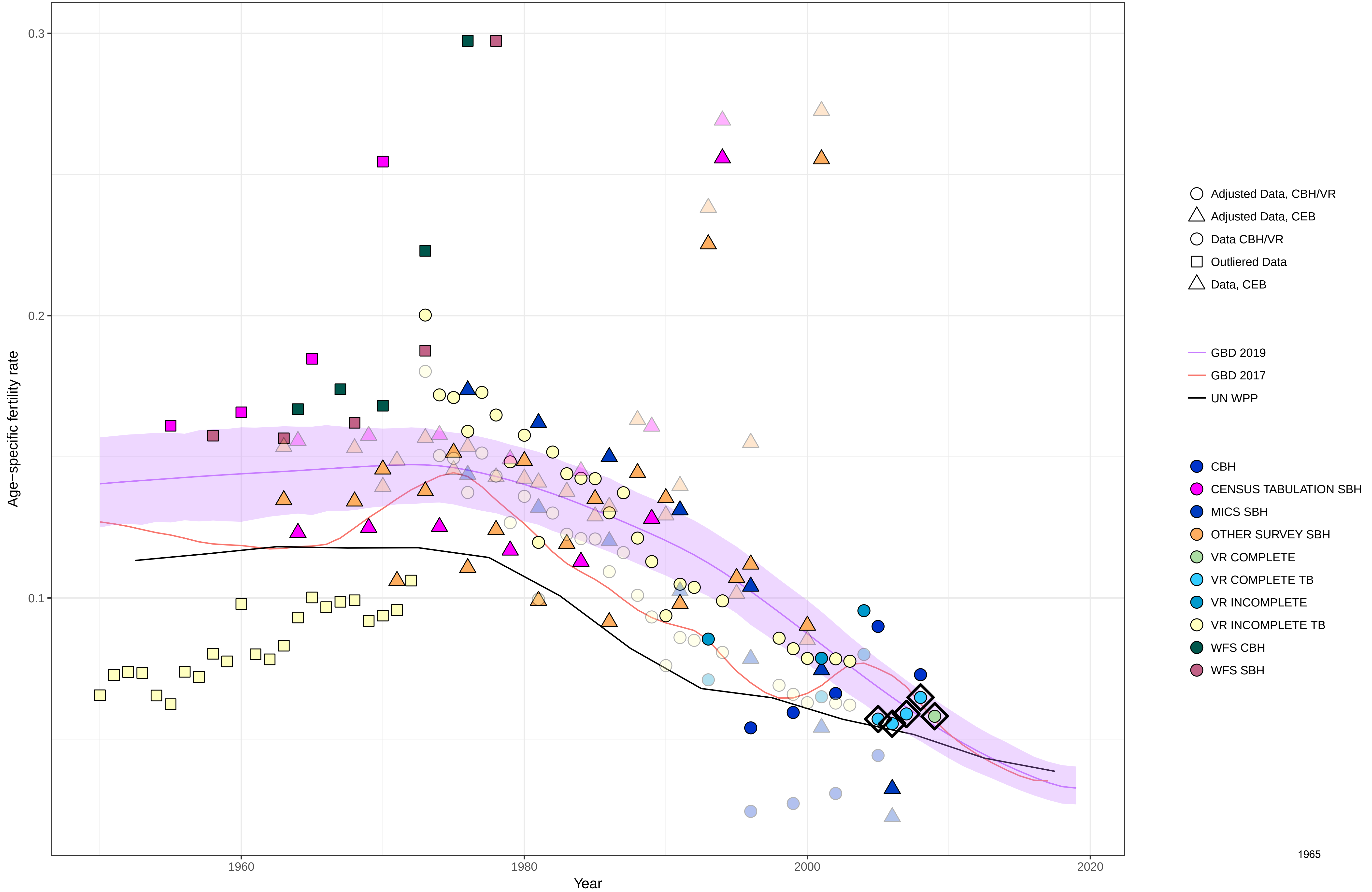

Age−specific fertility fitted trend lines, 1950−2019: Syrian Arab Republic, Ages: 20−24
Data points enclosed with a diamond are reference sources

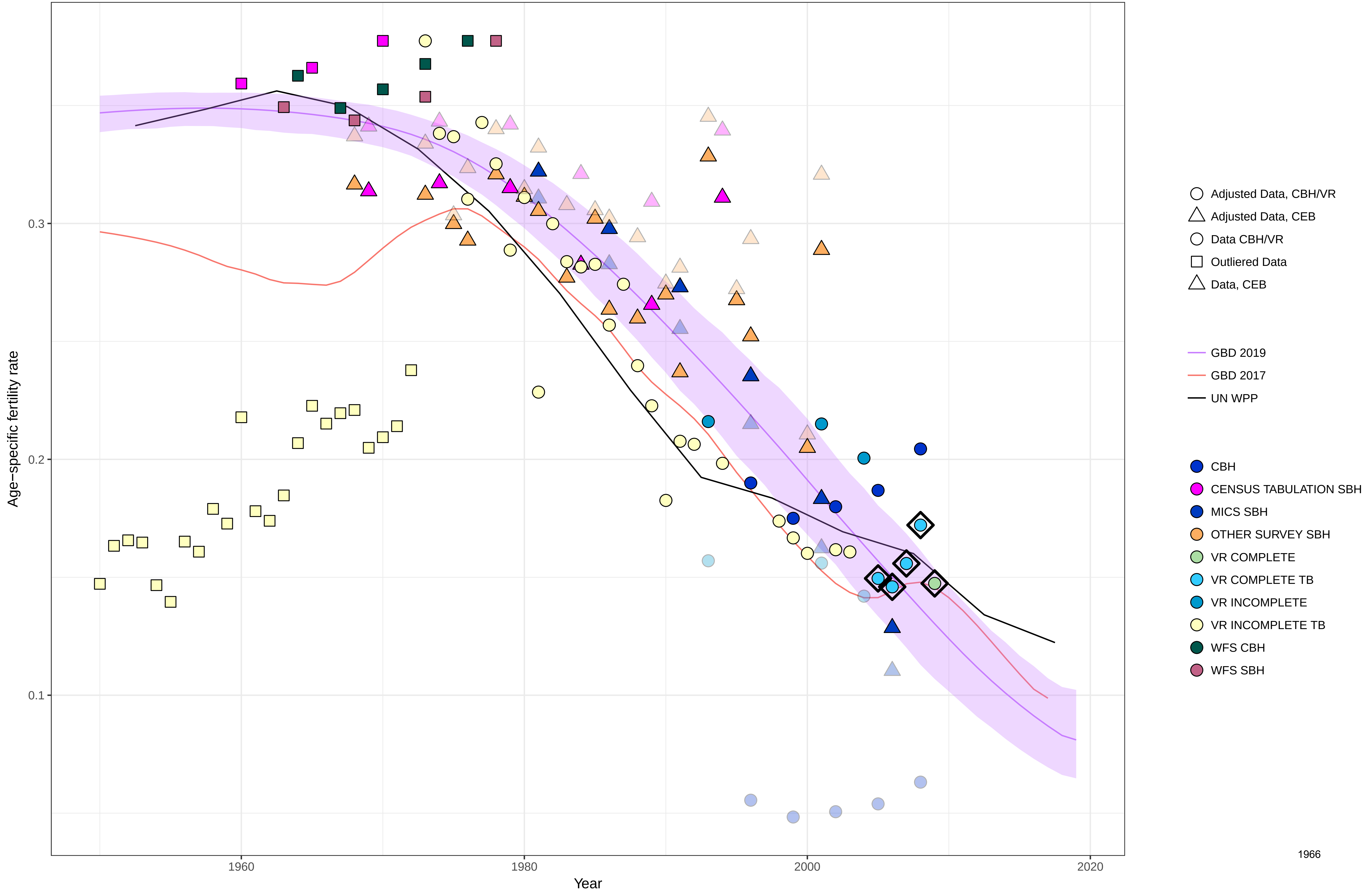

Age−specific fertility fitted trend lines, 1950−2019: Syrian Arab Republic, Ages: 25−29
Data points enclosed with a diamond are reference sources

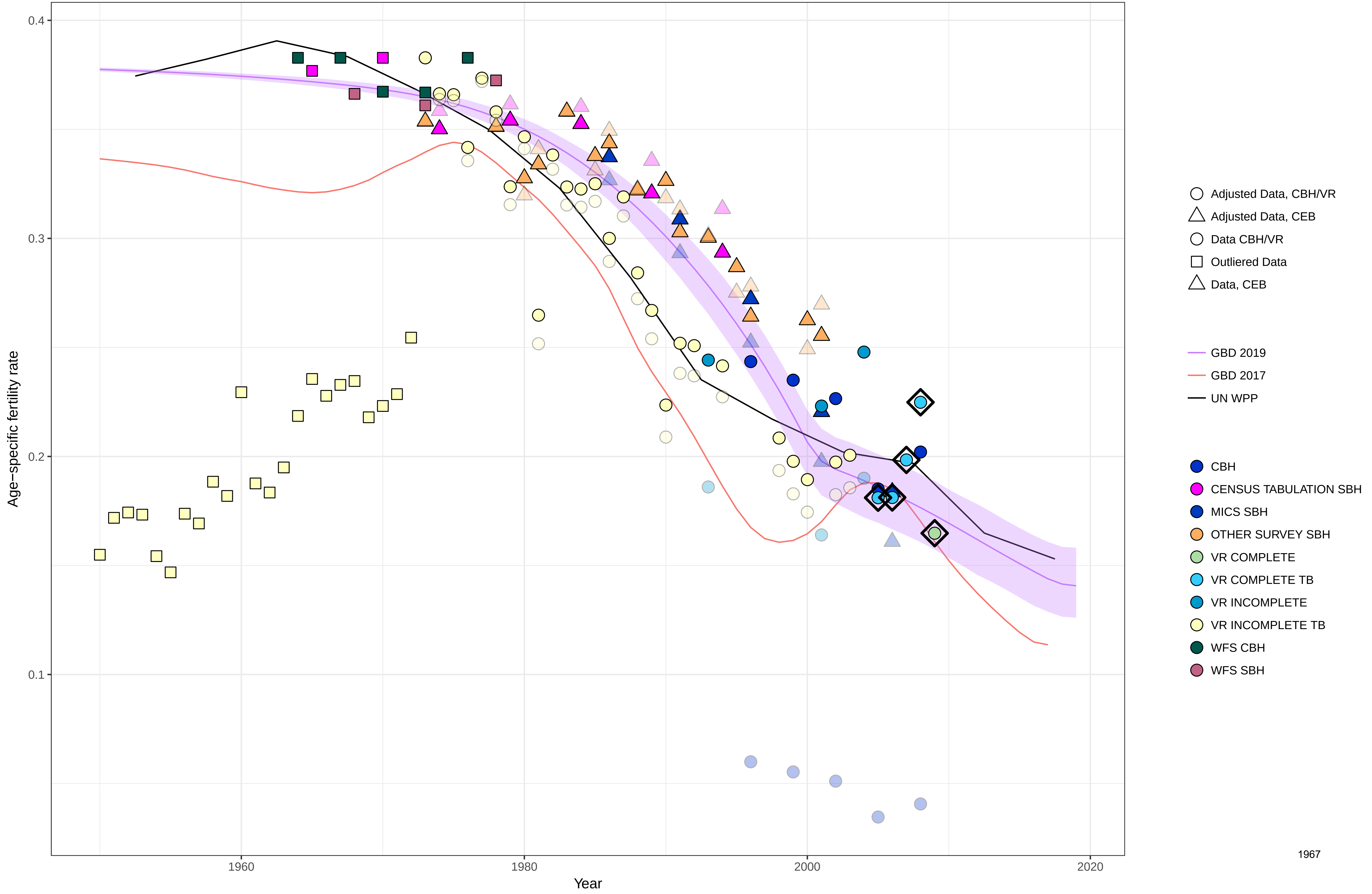

Age−specific fertility fitted trend lines, 1950−2019: Syrian Arab Republic, Ages: 30−34
Data points enclosed with a diamond are reference sources

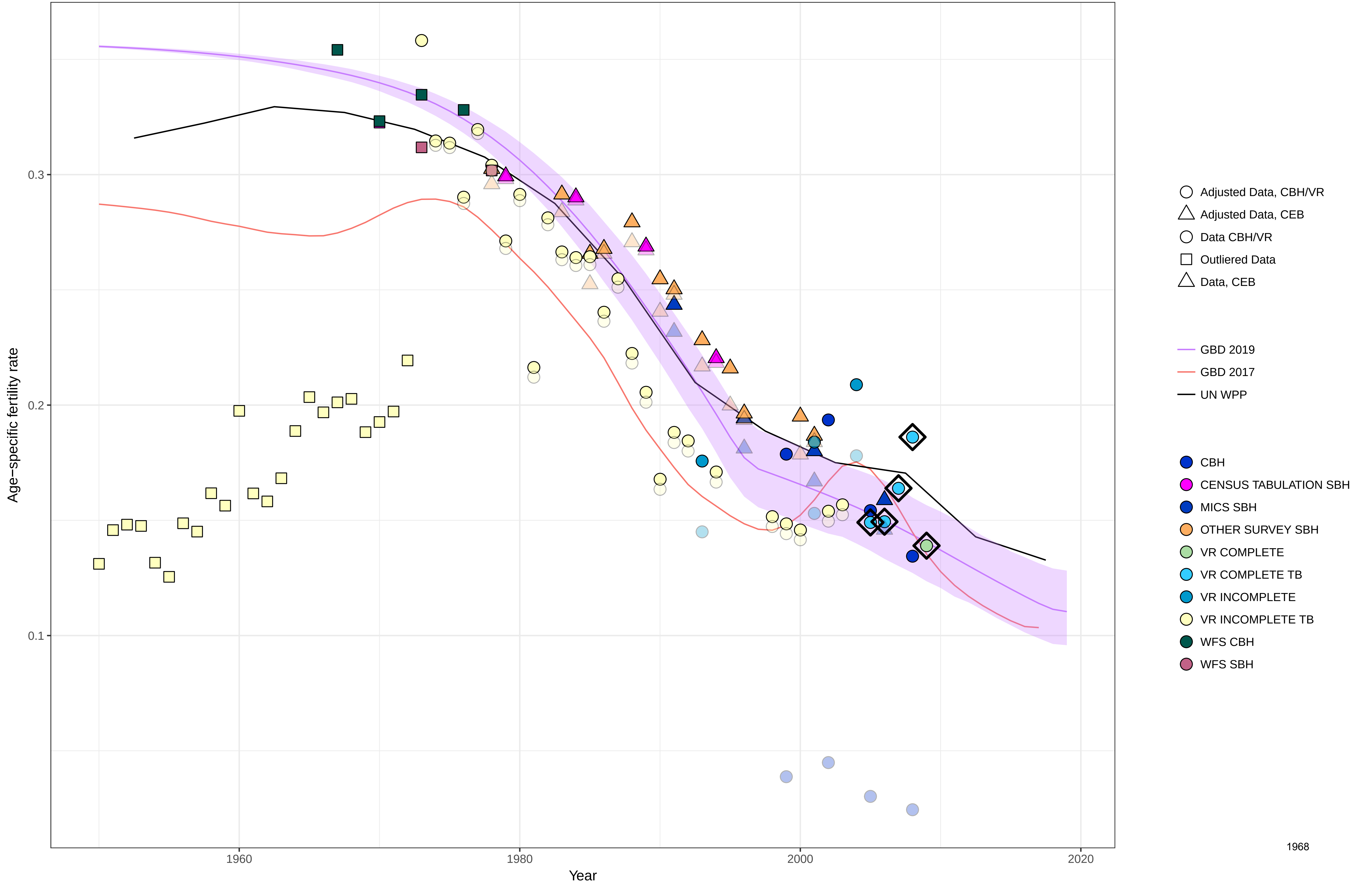

Age−specific fertility fitted trend lines, 1950−2019: Syrian Arab Republic, Ages: 35−39
Data points enclosed with a diamond are reference sources

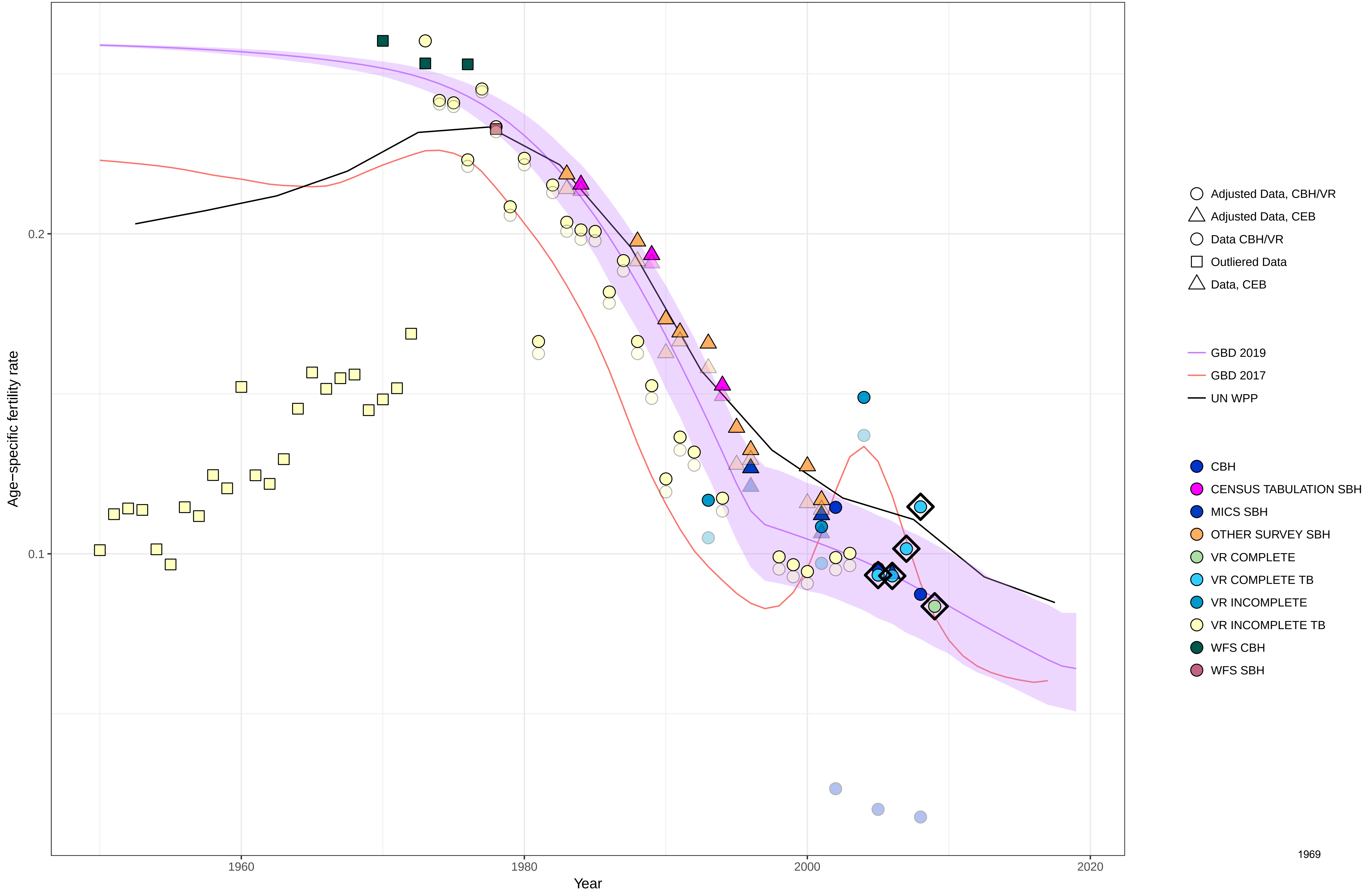

Age−specific fertility fitted trend lines, 1950−2019: Syrian Arab Republic, Ages: 40−44
Data points enclosed with a diamond are reference sources

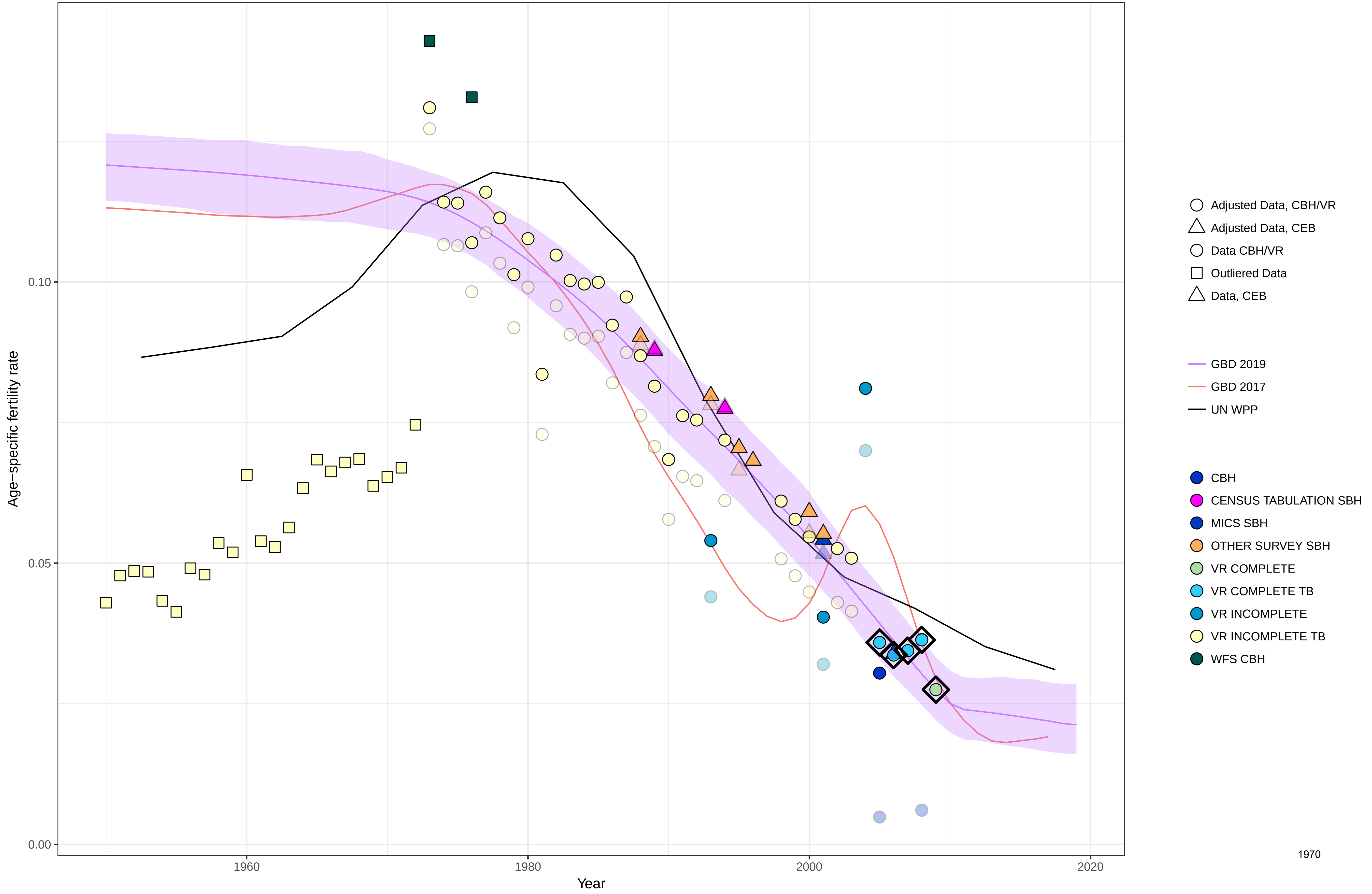

Age−specific fertility fitted trend lines, 1950−2019: Syrian Arab Republic, Ages: 45−49
Data points enclosed with a diamond are reference sources

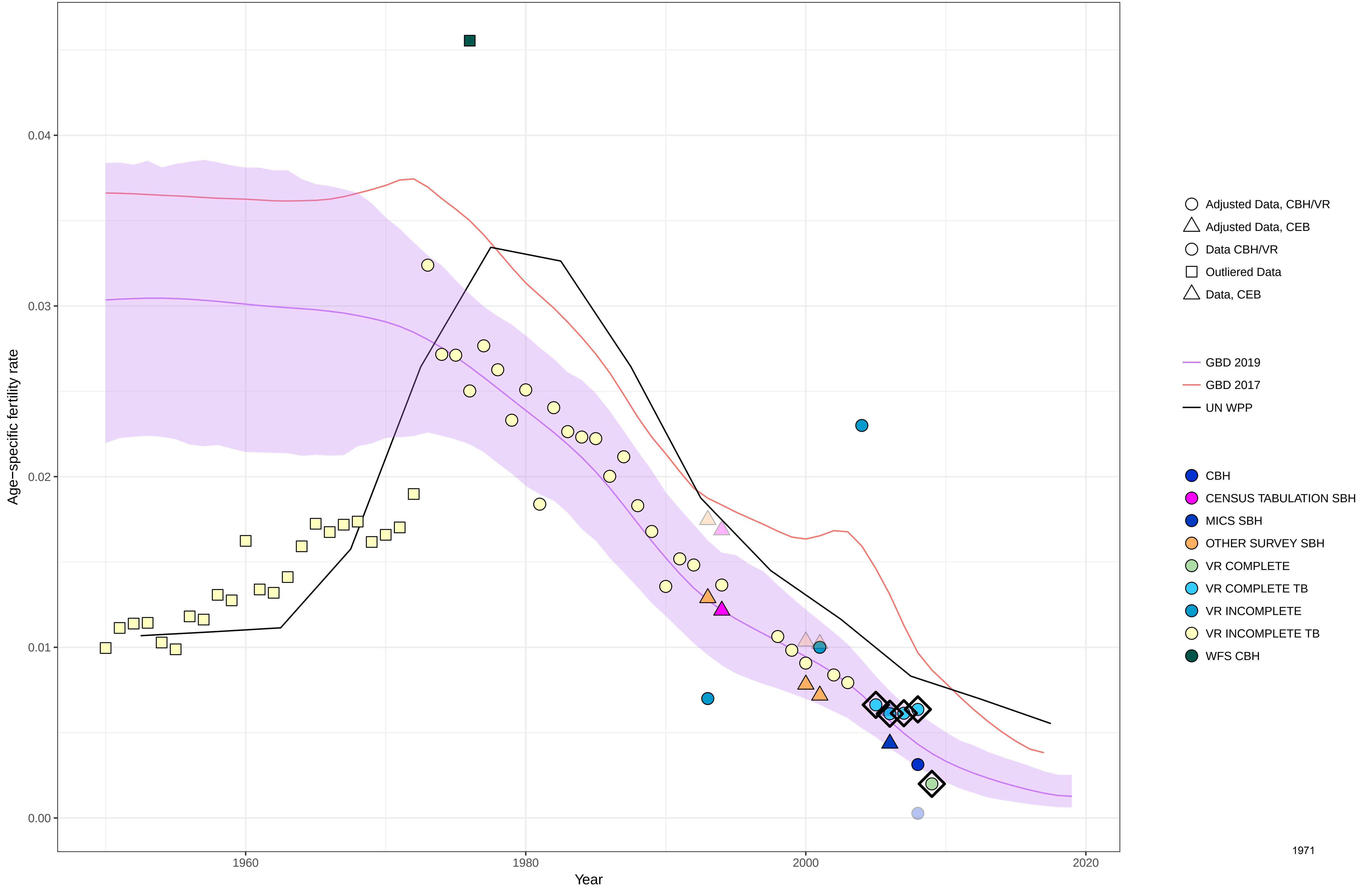

Age−specific fertility fitted trend lines, 1950−2019: Syrian Arab Republic, Ages: 50−54
Data points enclosed with a diamond are reference sources

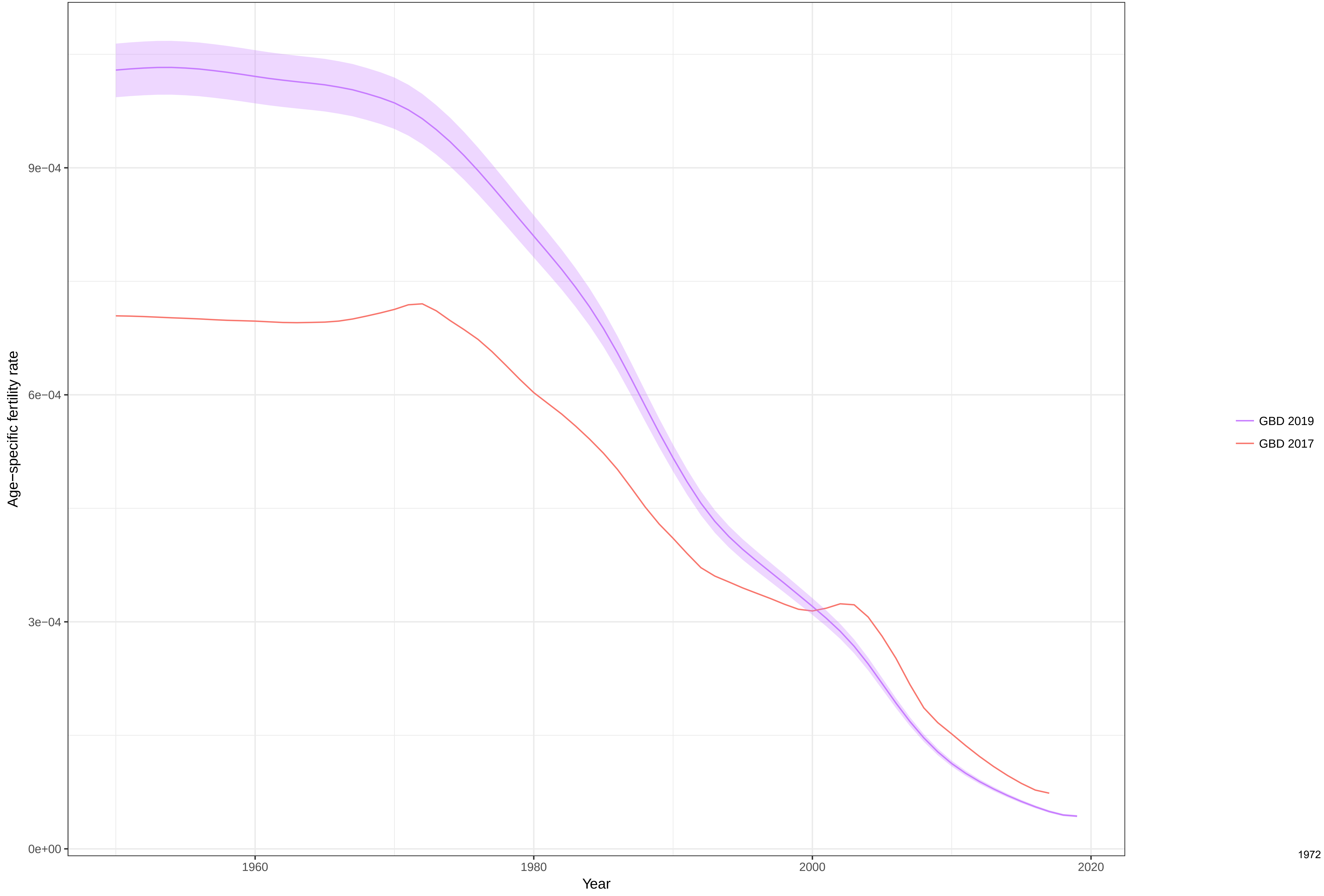

Total fertility rate fitted trend lines, 1950−2019: Syrian Arab Republic TFR
Data points enclosed with a diamond are reference sources

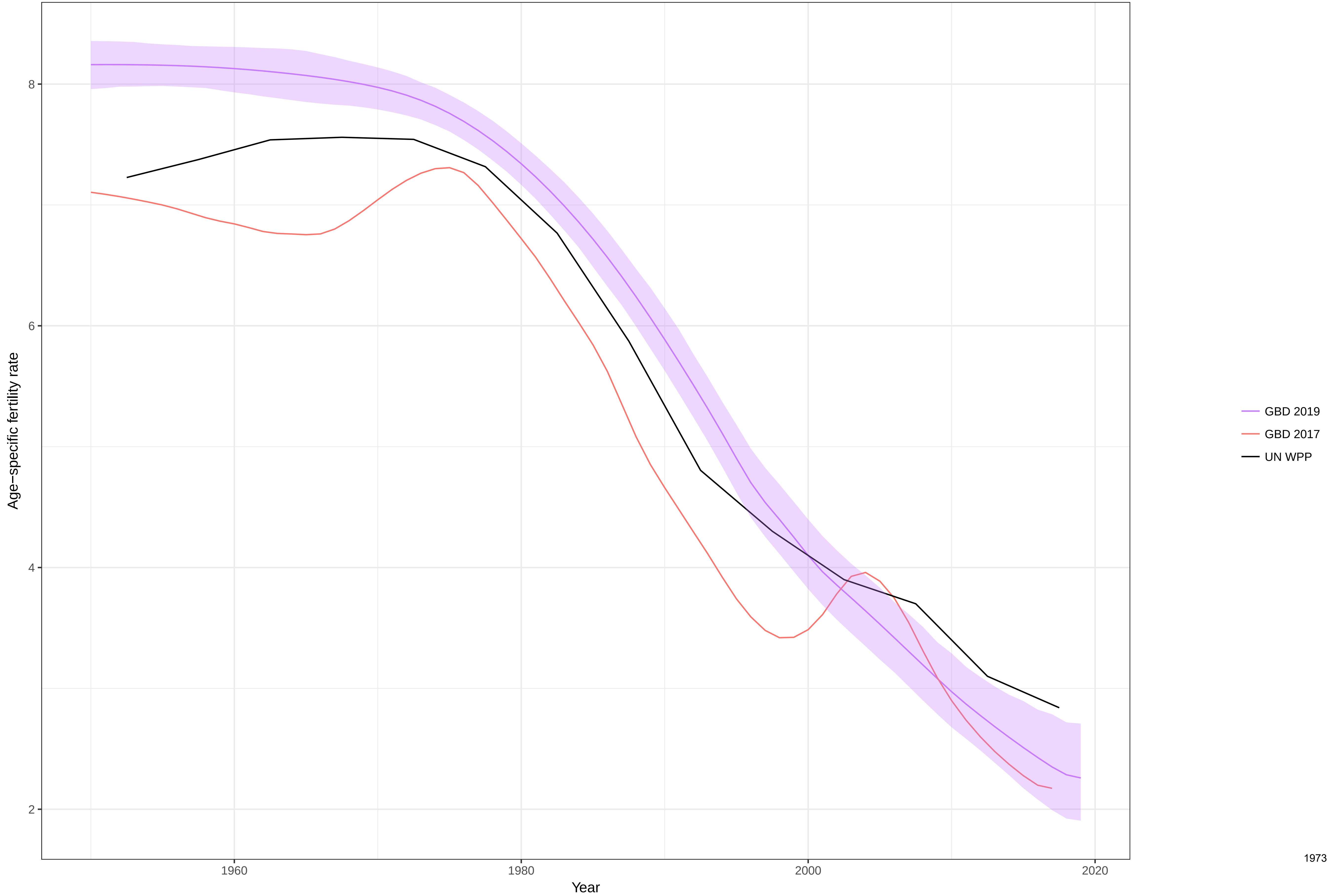

Age−specific fertility fitted trend lines, 1950−2019: Chad, Ages: 10−14
Data points enclosed with a diamond are reference sources

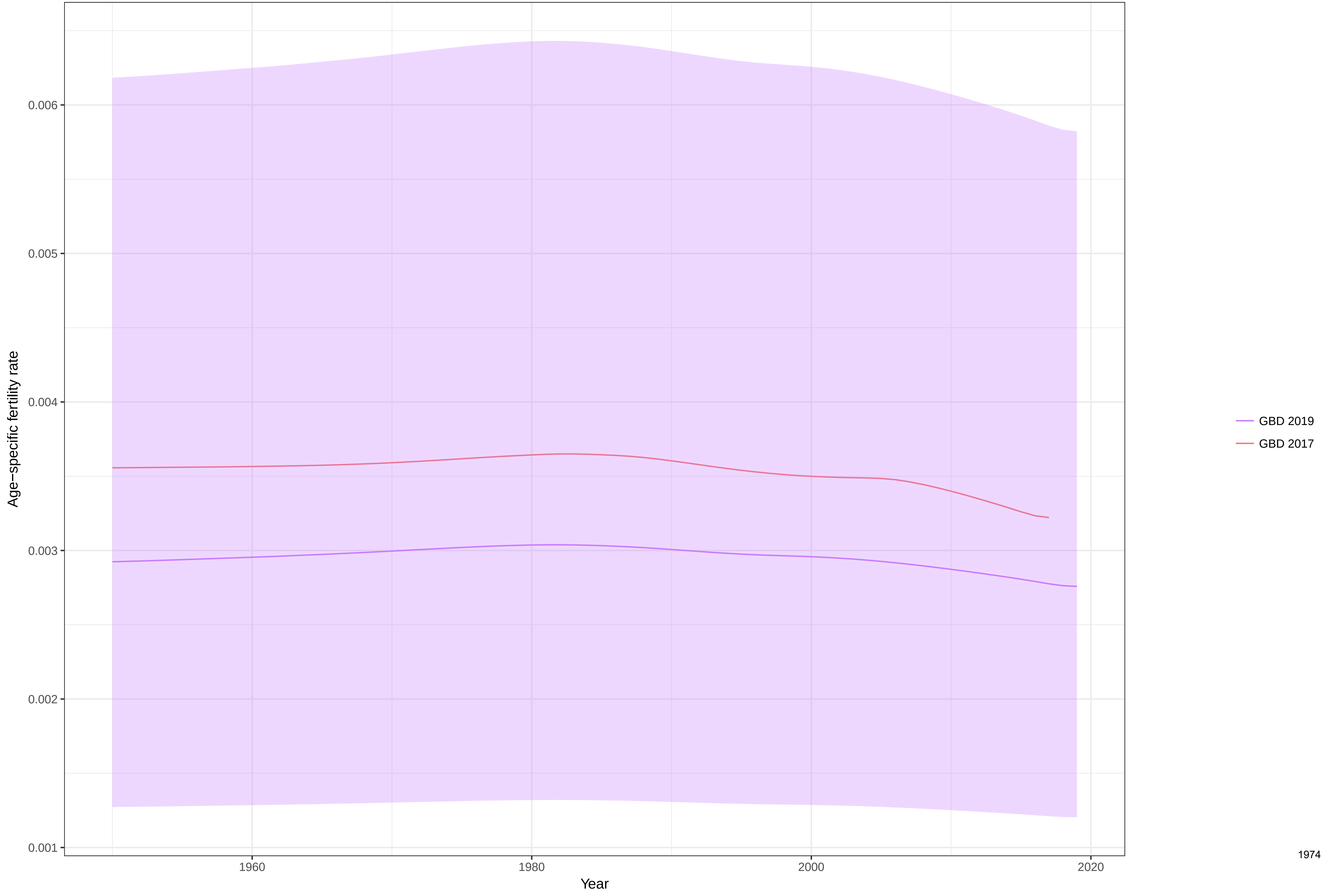

Age−specific fertility fitted trend lines, 1950−2019: Chad, Ages: 15−19
Data points enclosed with a diamond are reference sources

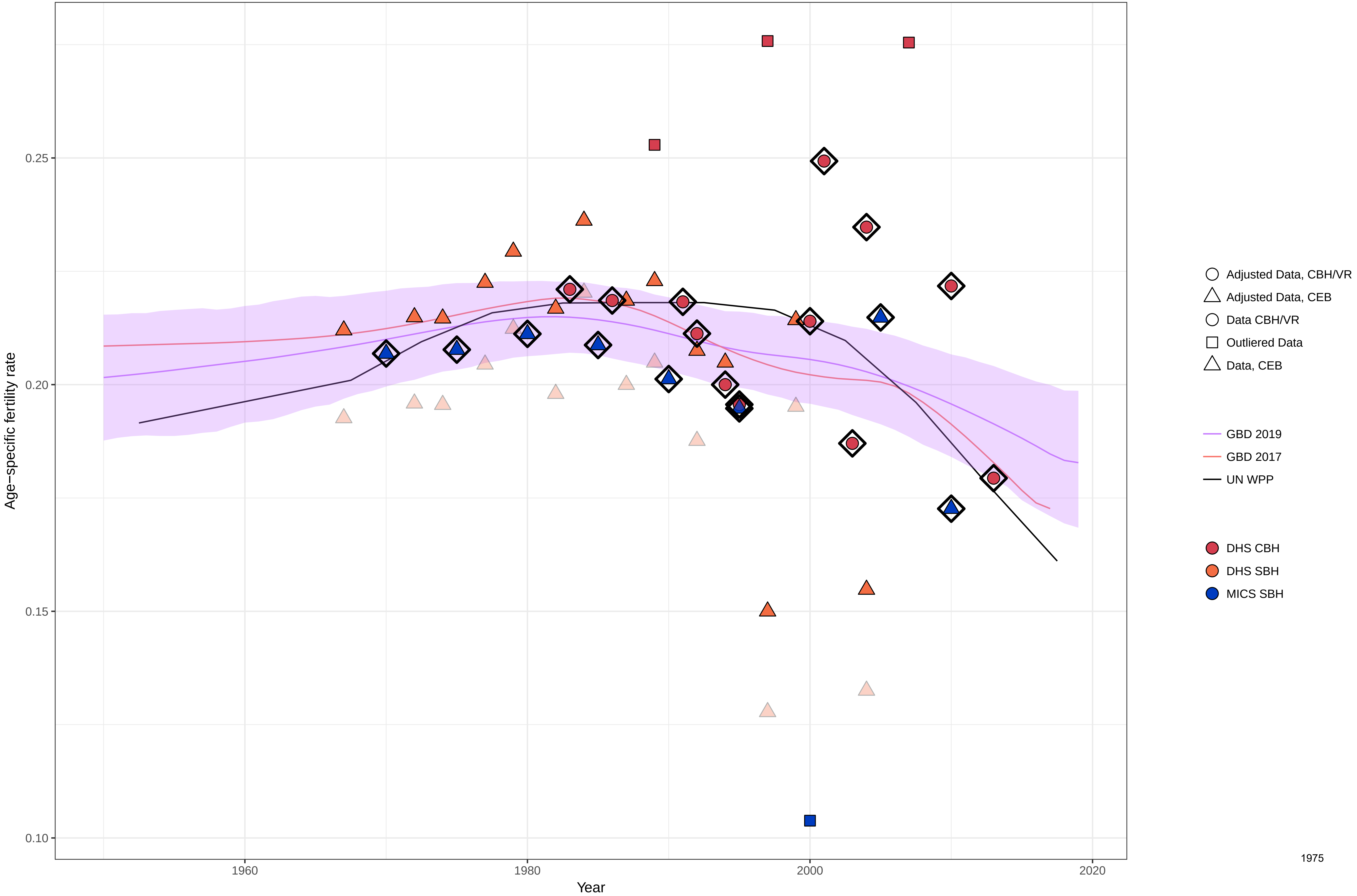

Age−specific fertility fitted trend lines, 1950−2019: Chad, Ages: 20−24
Data points enclosed with a diamond are reference sources

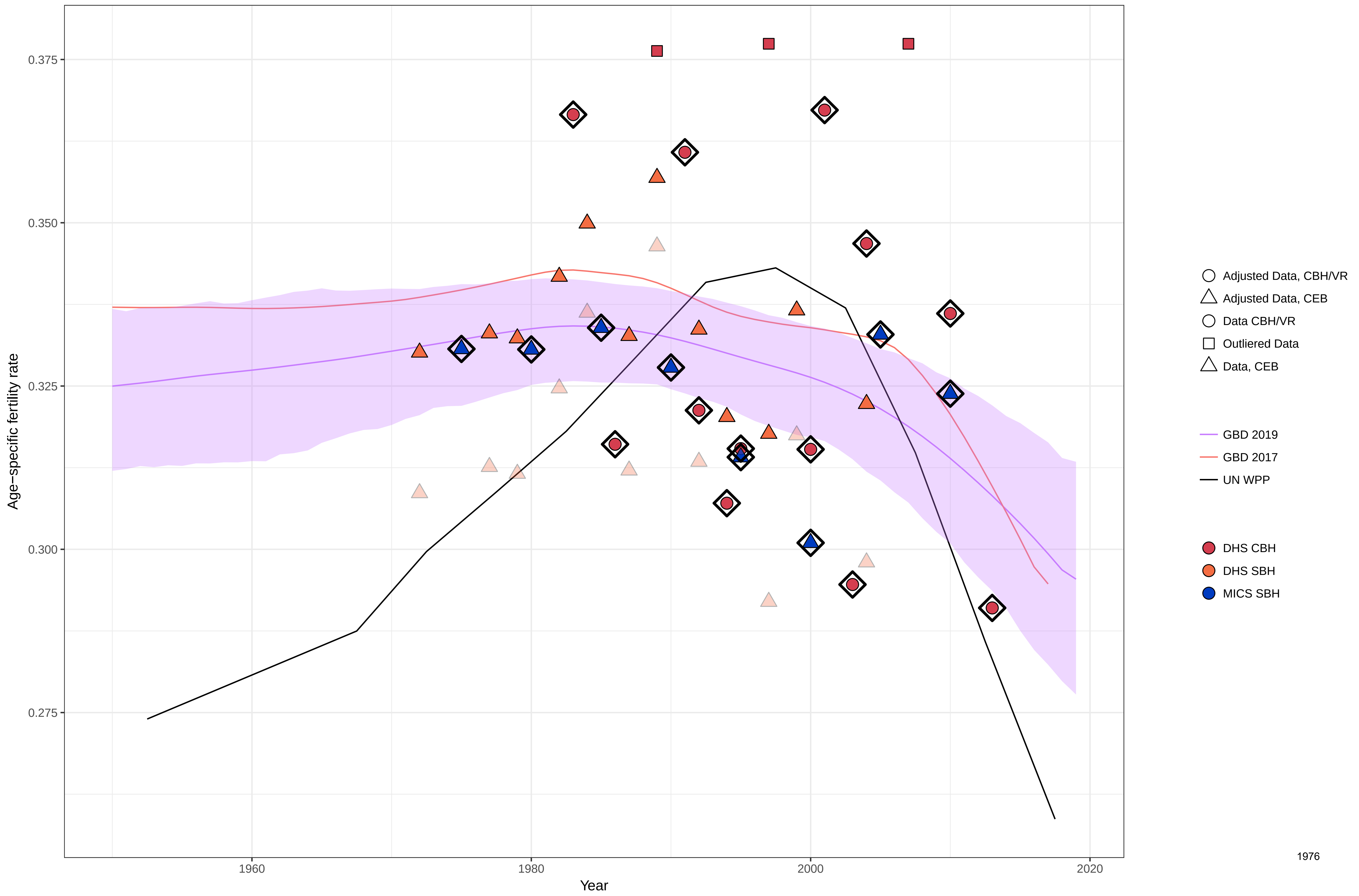

Age−specific fertility fitted trend lines, 1950−2019: Chad, Ages: 25−29
Data points enclosed with a diamond are reference sources

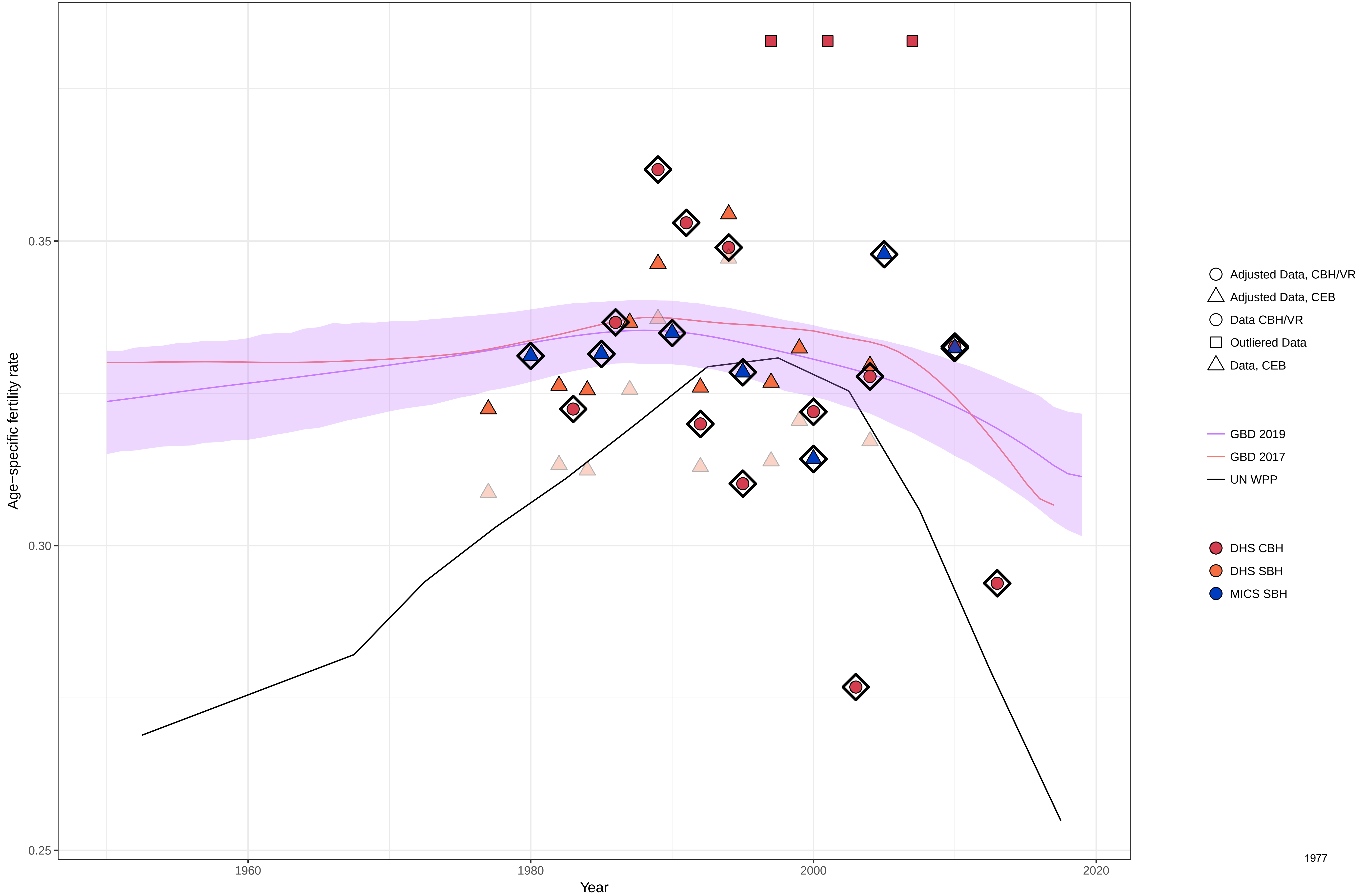

Age−specific fertility fitted trend lines, 1950−2019: Chad, Ages: 30−34
Data points enclosed with a diamond are reference sources

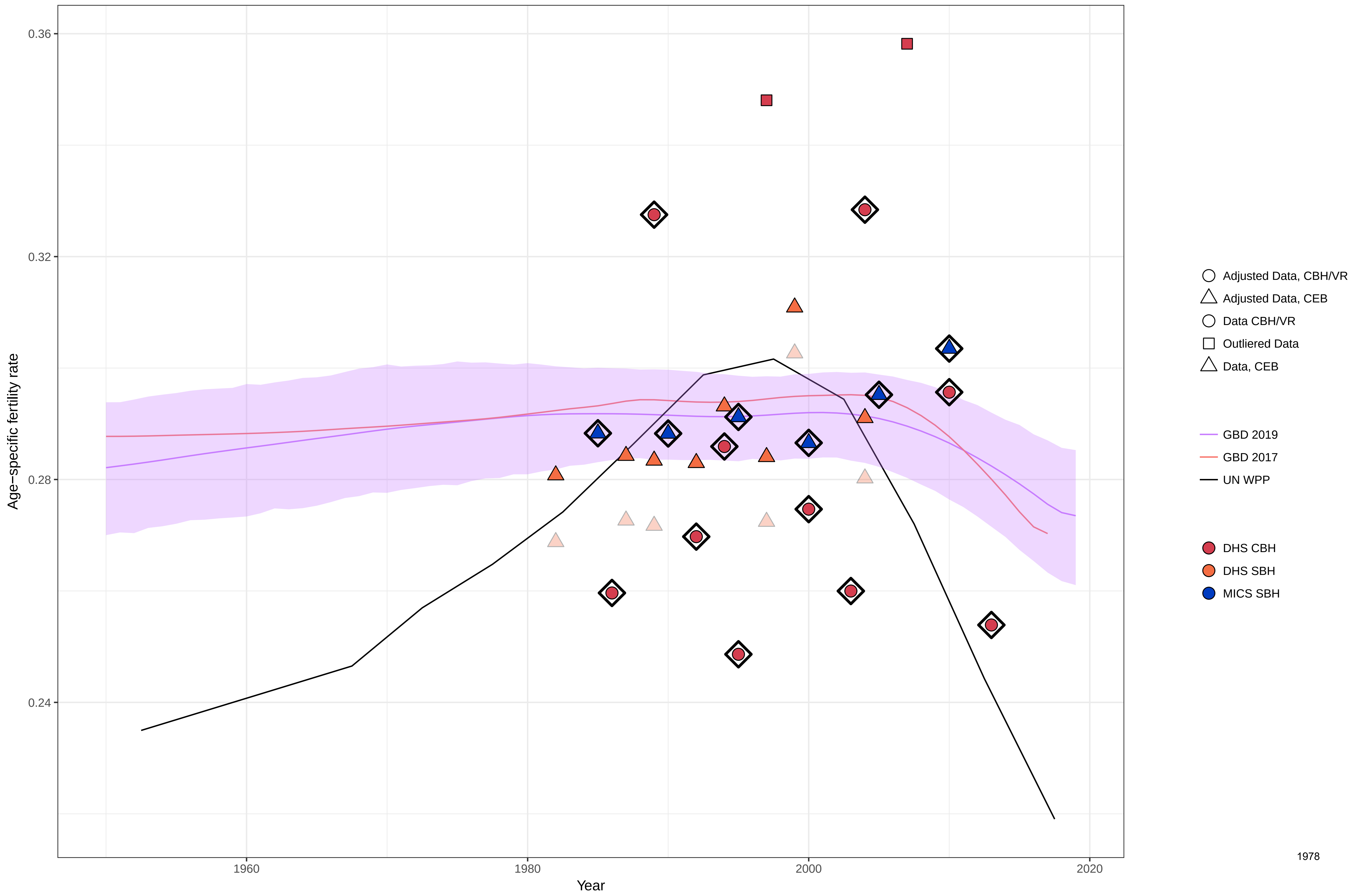

Age−specific fertility fitted trend lines, 1950−2019: Chad, Ages: 35−39
Data points enclosed with a diamond are reference sources

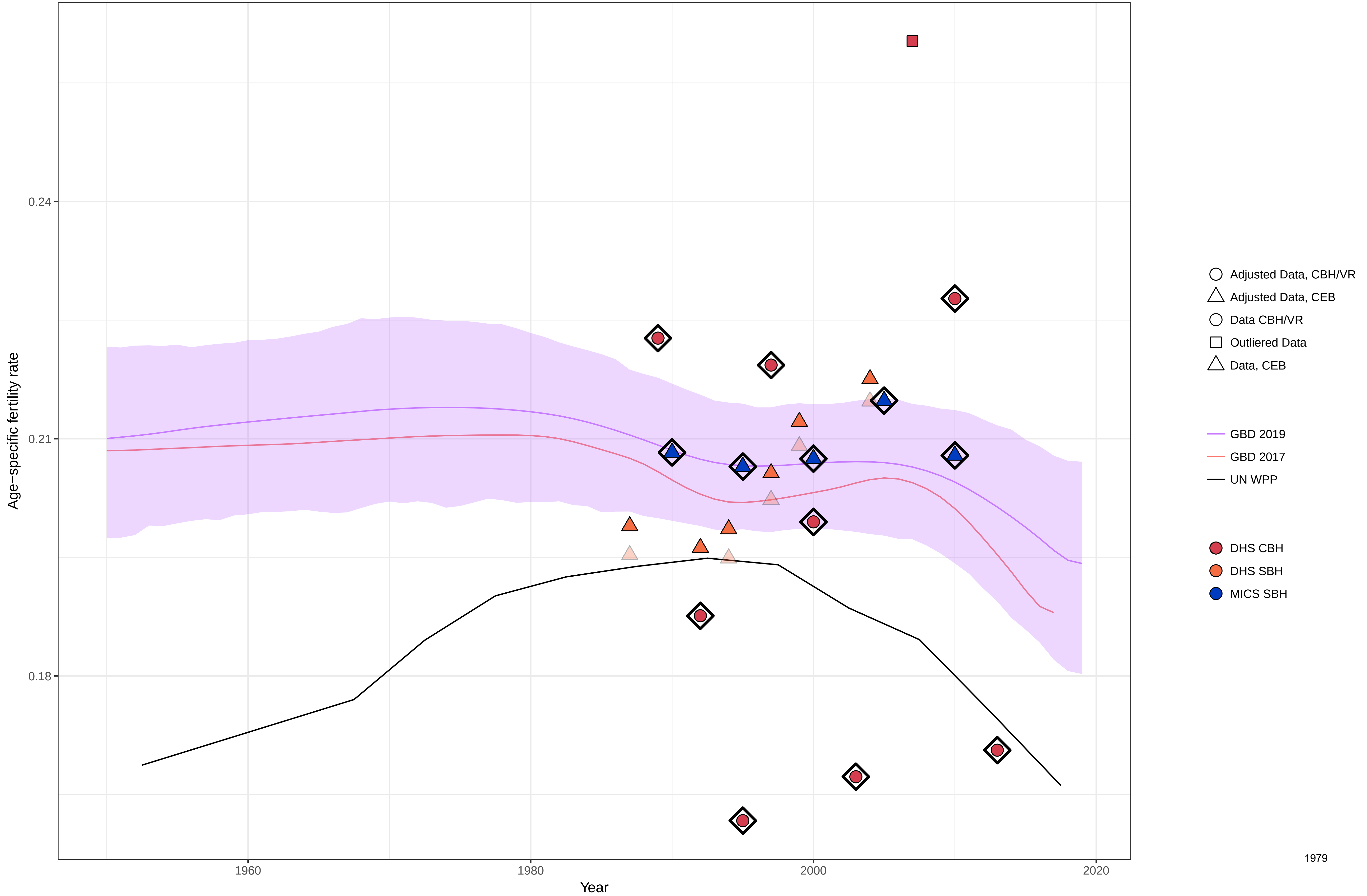

Age−specific fertility fitted trend lines, 1950−2019: Chad, Ages: 40−44
Data points enclosed with a diamond are reference sources

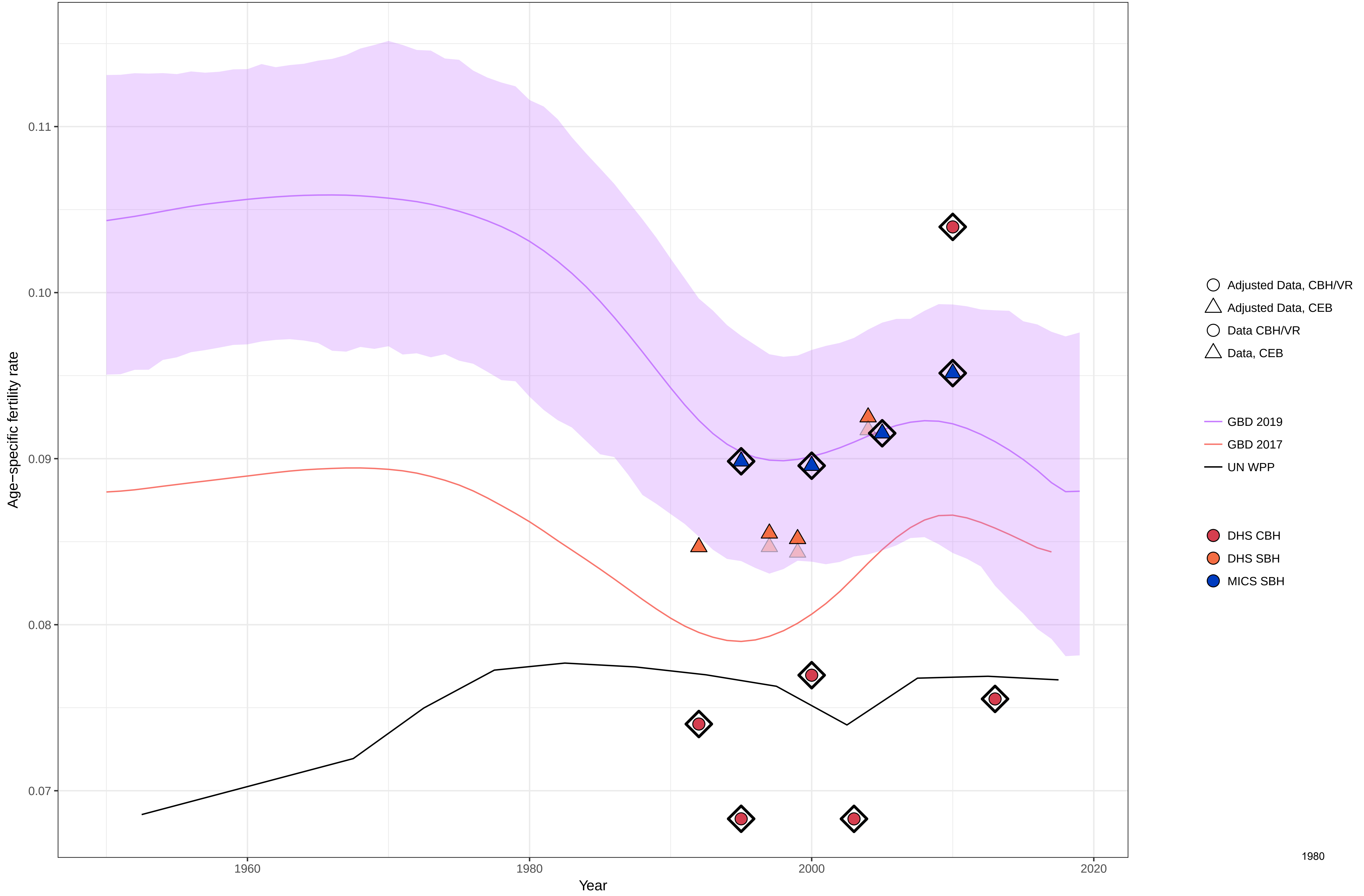

1980

Age−specific fertility fitted trend lines, 1950−2019: Chad, Ages: 45−49
Data points enclosed with a diamond are reference sources

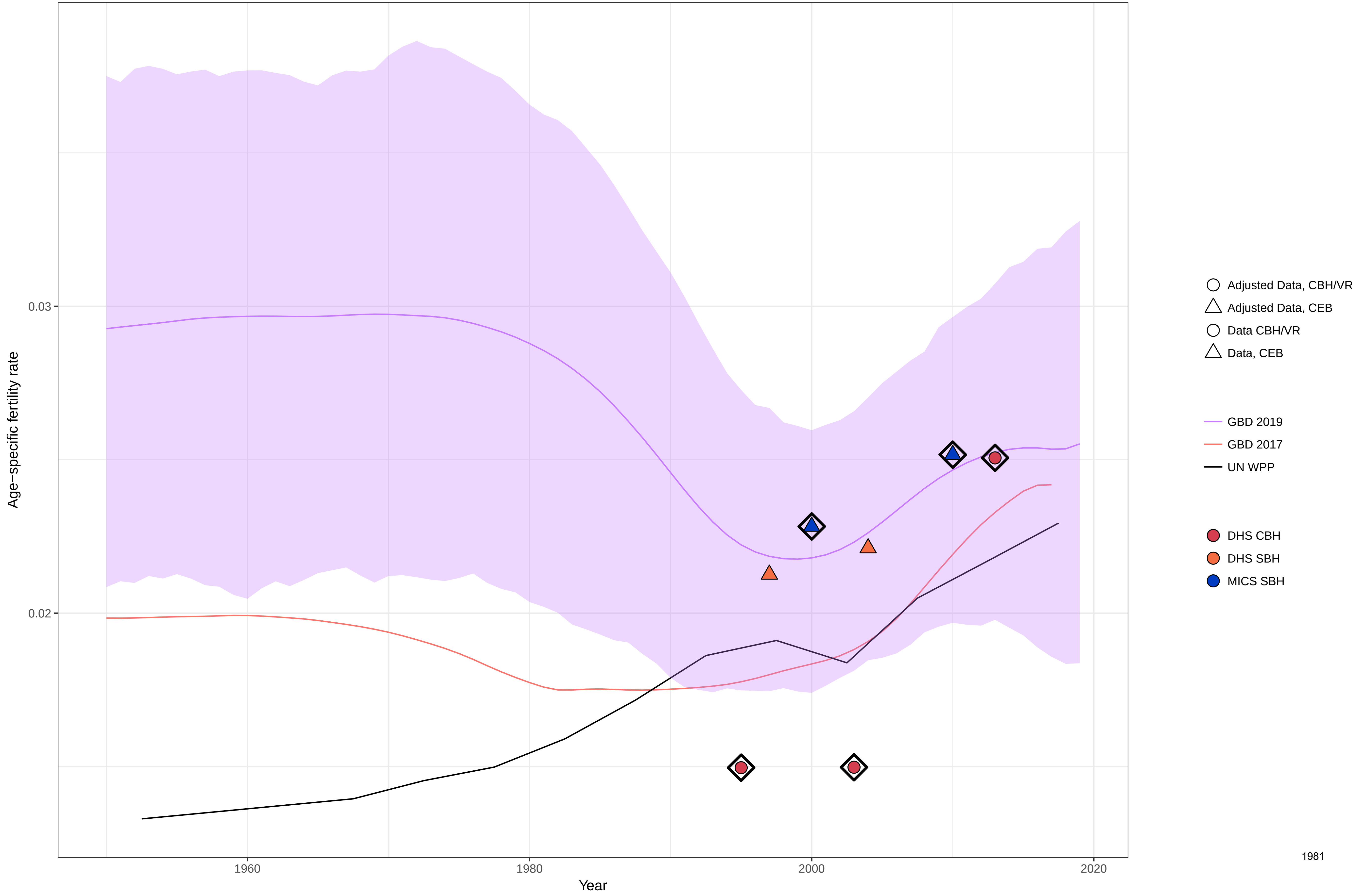

Age−specific fertility fitted trend lines, 1950−2019: Chad, Ages: 50−54
Data points enclosed with a diamond are reference sources

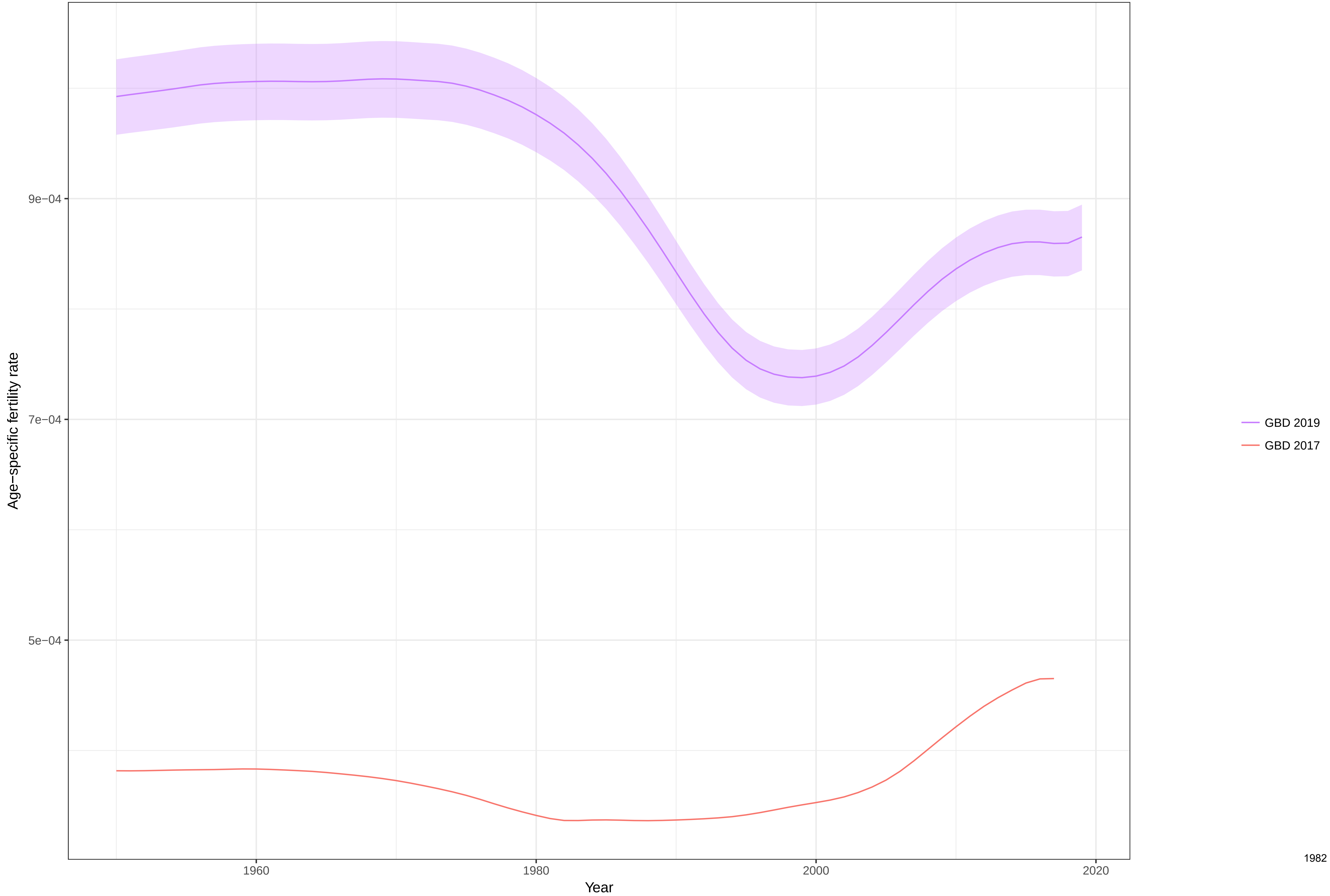

Total fertility rate fitted trend lines, 1950−2019: Chad TFR
Data points enclosed with a diamond are reference sources

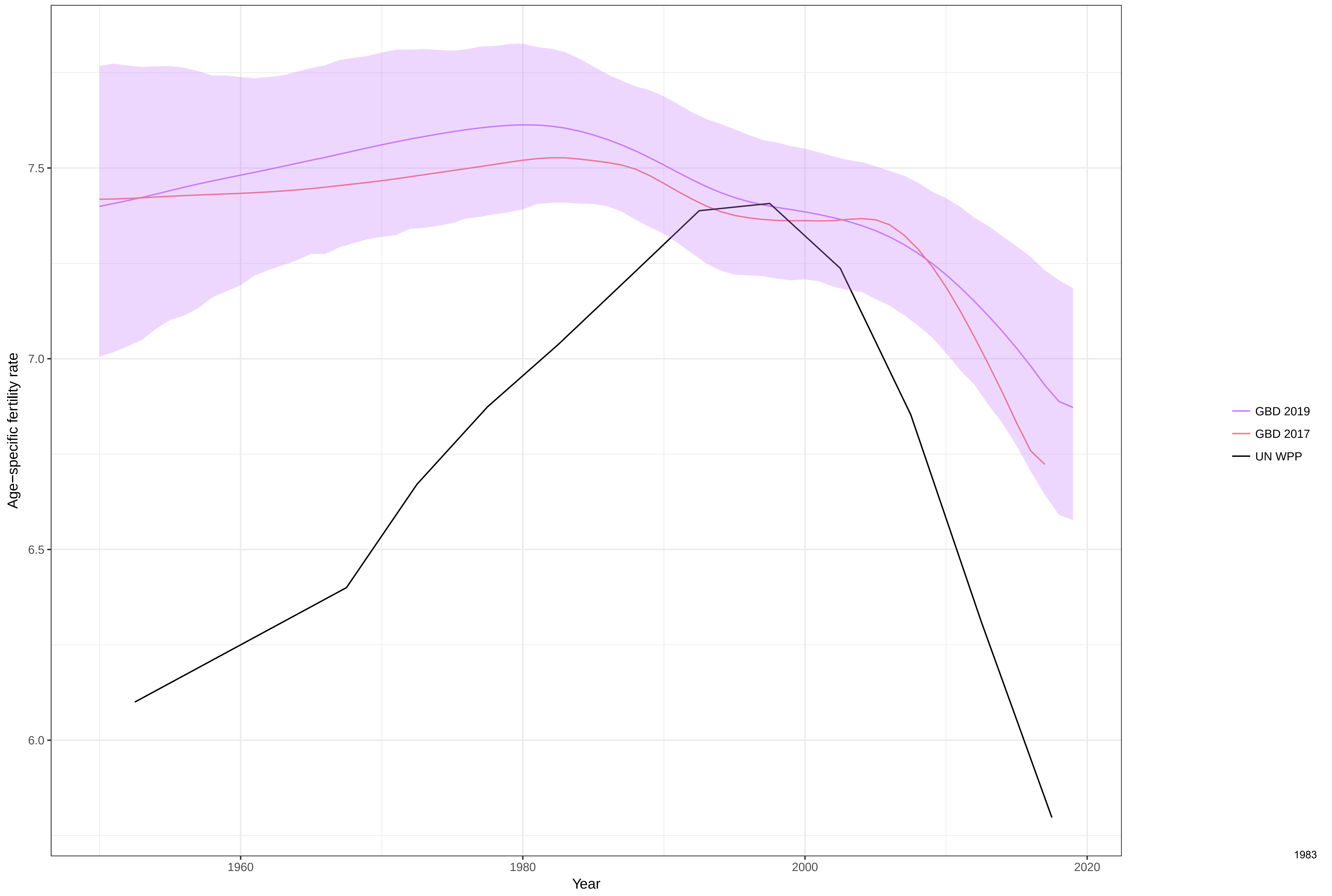

Age−specific fertility fitted trend lines, 1950−2019: Togo, Ages: 10−14
Data points enclosed with a diamond are reference sources

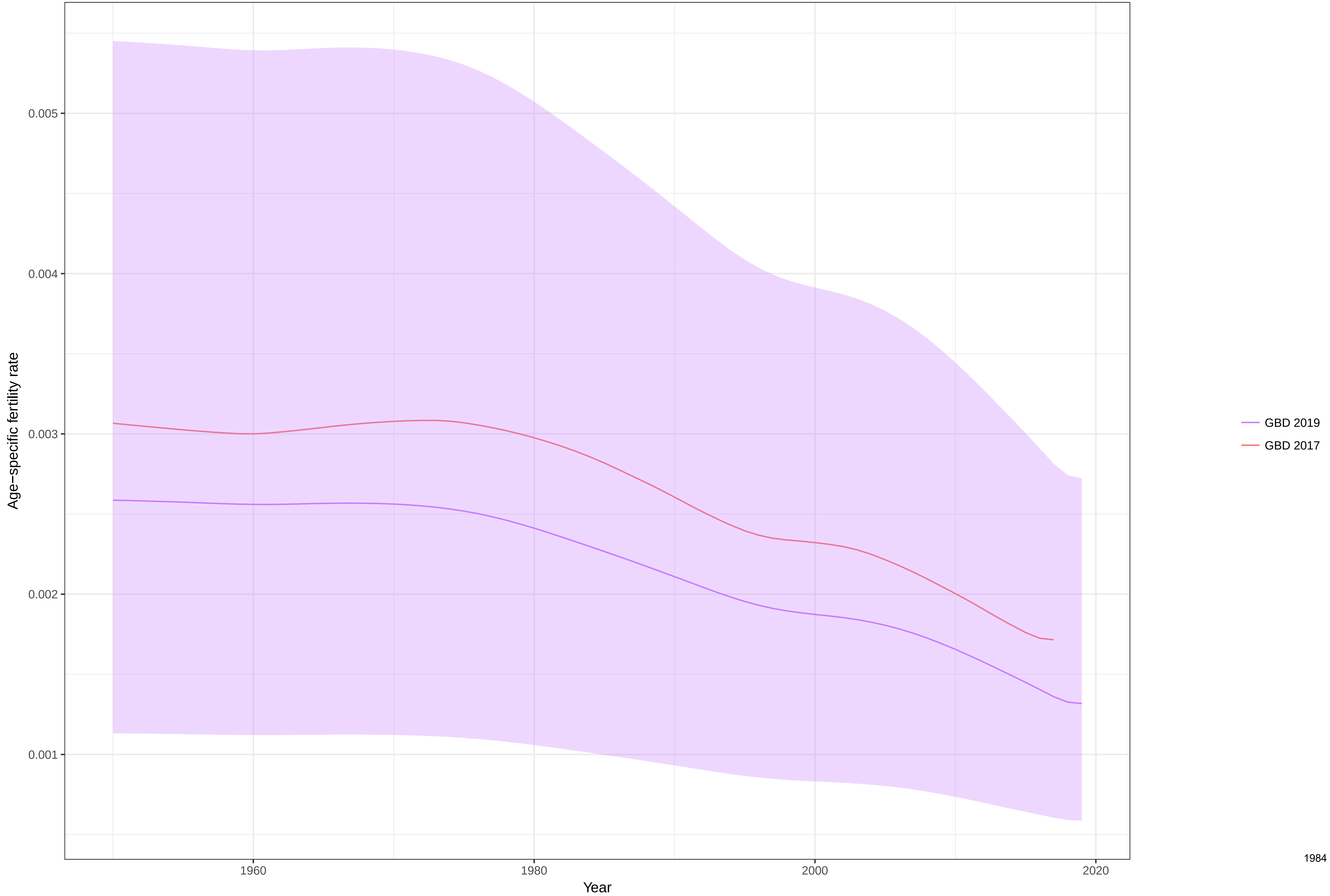

1984

Age−specific fertility fitted trend lines, 1950−2019: Togo, Ages: 15−19
Data points enclosed with a diamond are reference sources

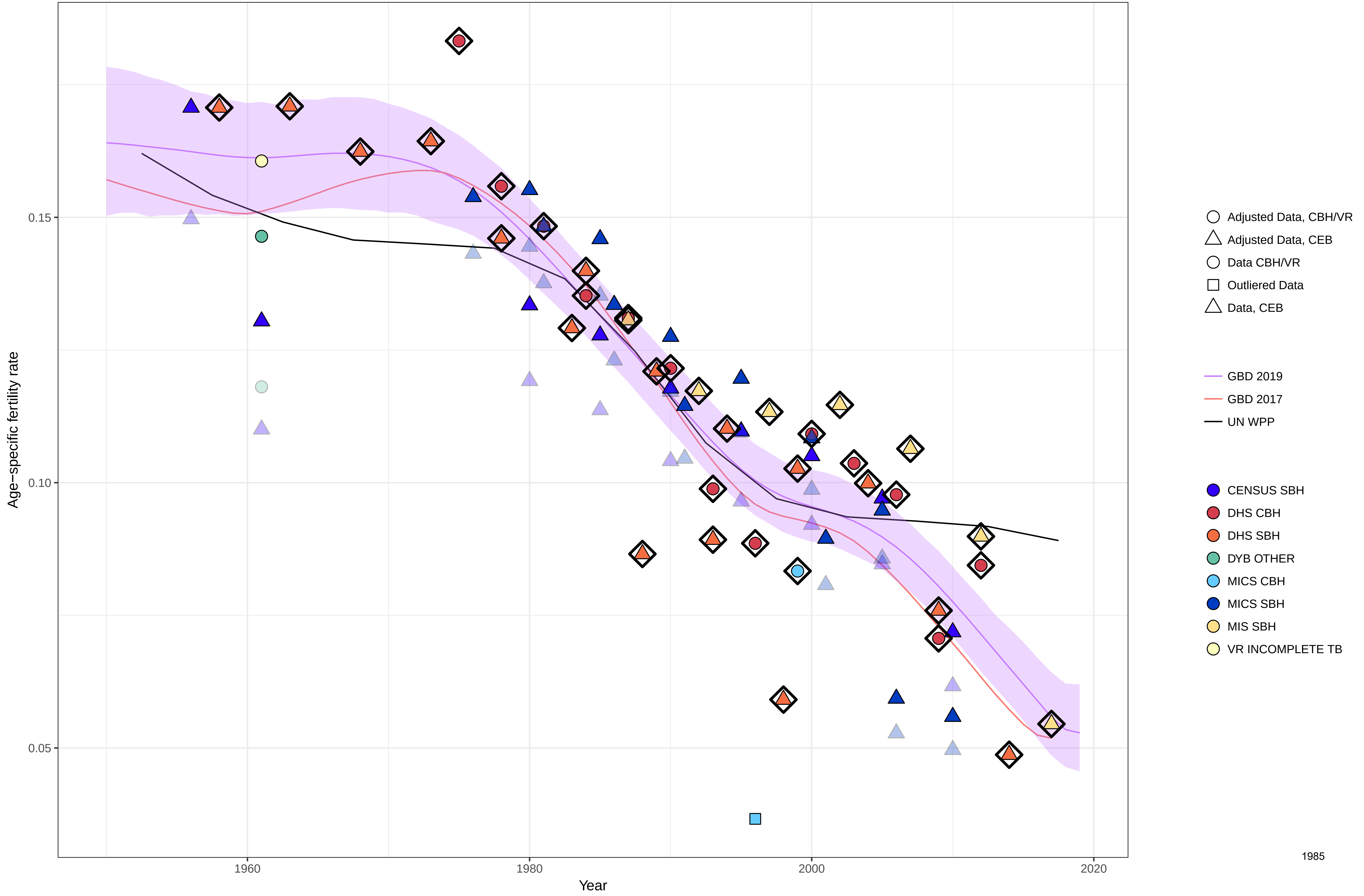

Age−specific fertility fitted trend lines, 1950−2019: Togo, Ages: 20−24
Data points enclosed with a diamond are reference sources

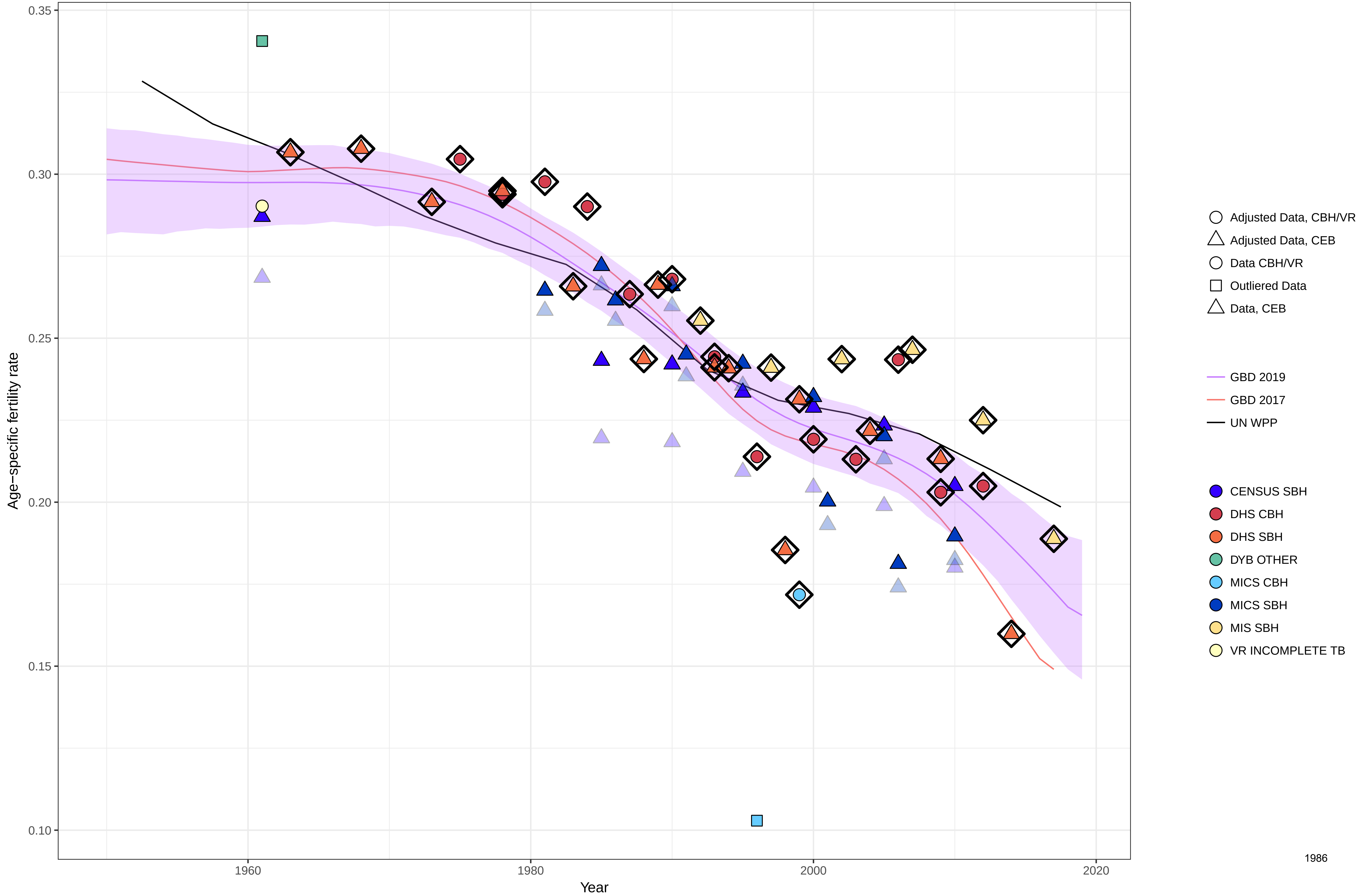

Age−specific fertility fitted trend lines, 1950−2019: Togo, Ages: 25−29
Data points enclosed with a diamond are reference sources

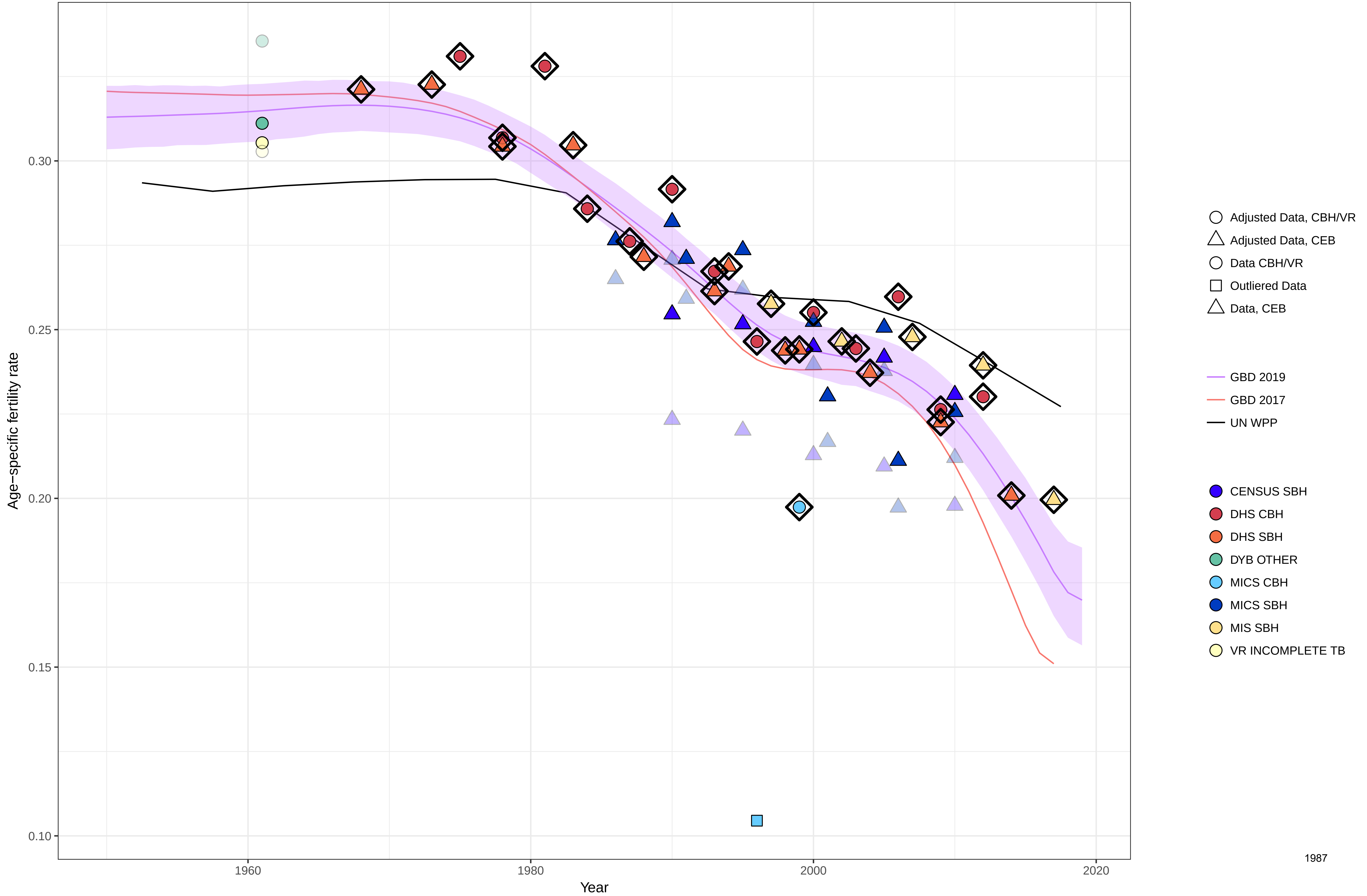

Age−specific fertility fitted trend lines, 1950−2019: Togo, Ages: 30−34
Data points enclosed with a diamond are reference sources

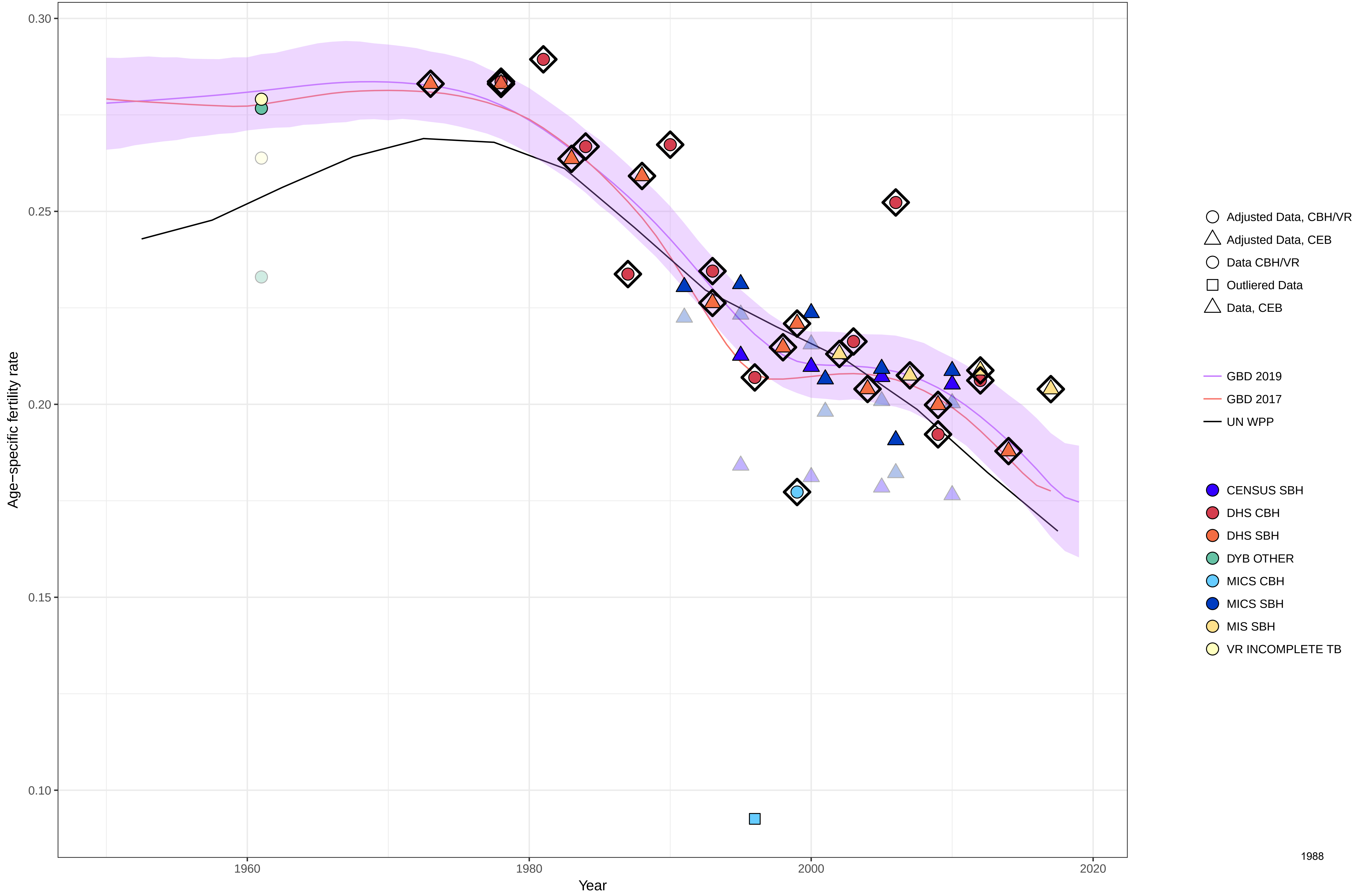

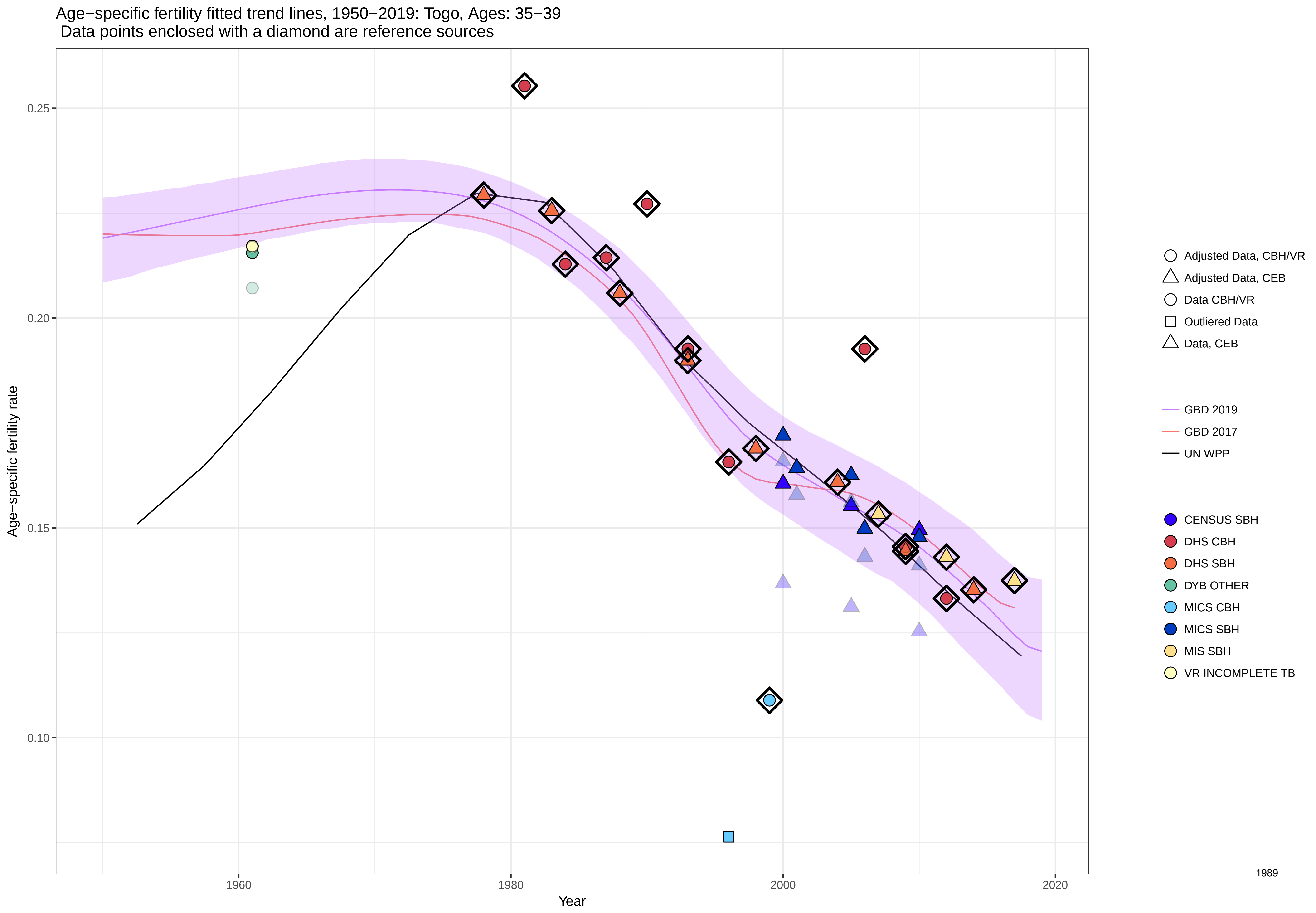

Age−specific fertility fitted trend lines, 1950−2019: Togo, Ages: 35−39
Data points enclosed with a diamond are reference sources
Age−specific fertility rate
Year
0.25
0.20
0.15
0.10
1960
1980
2000
2020
Adjusted Data, CBH/VR
Adjusted Data, CEB
Data CBH/VR
Outliered Data
Data, CEB
GBD 2019
GBD 2017
UN WPP
CENSUS SBH
DHS CBH
DHS SBH
DYB OTHER
MICS CBH
MICS SBH
MIS SBH
VR INCOMPLETE TB

Age−specific fertility fitted trend lines, 1950−2019: Togo, Ages: 40−44
Data points enclosed with a diamond are reference sources

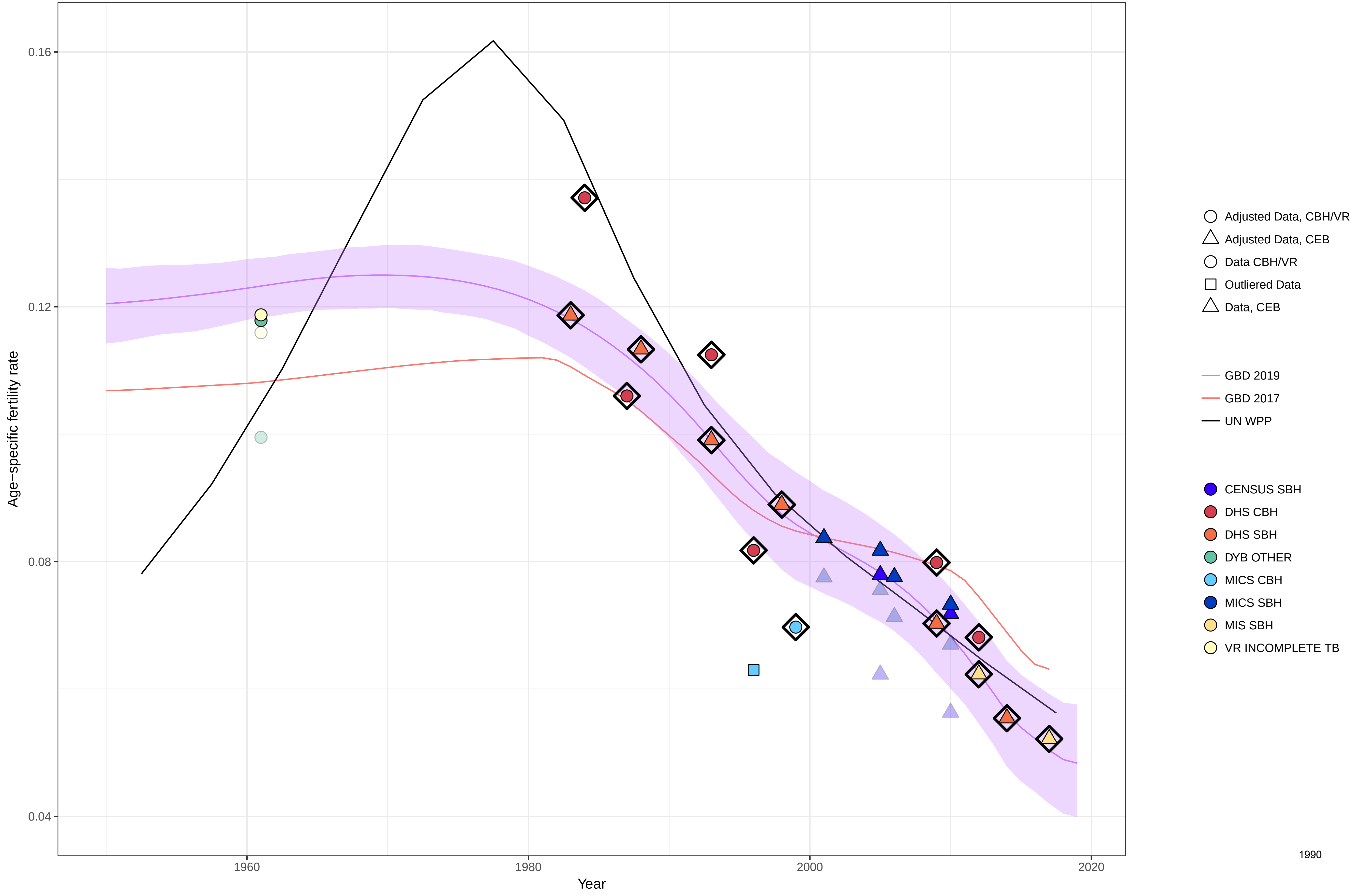

1990

Age−specific fertility fitted trend lines, 1950−2019: Togo, Ages: 45−49
Data points enclosed with a diamond are reference sources

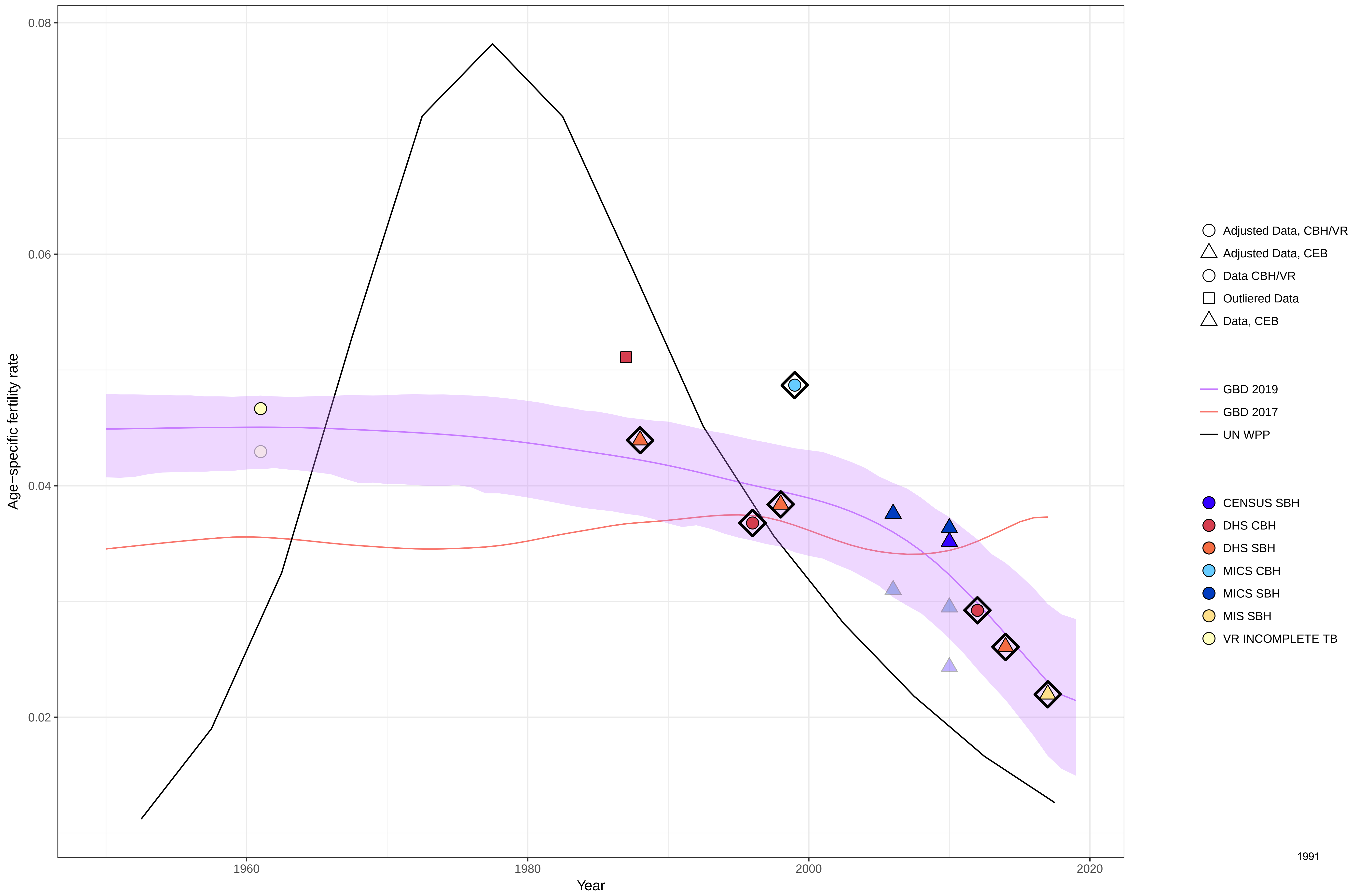

Age−specific fertility fitted trend lines, 1950−2019: Togo, Ages: 50−54
Data points enclosed with a diamond are reference sources

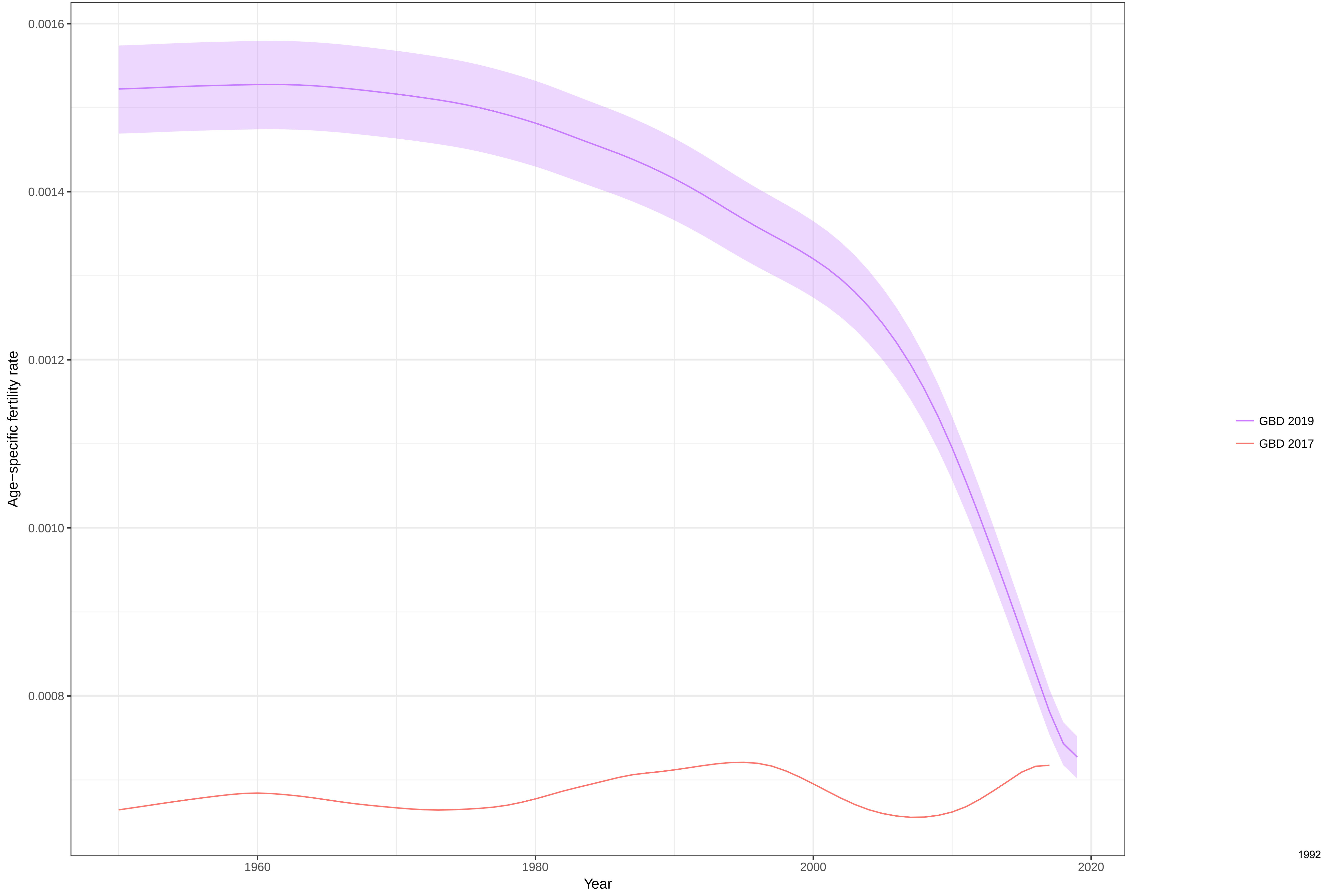

Total fertility rate fitted trend lines, 1950−2019: Togo TFR
Data points enclosed with a diamond are reference sources

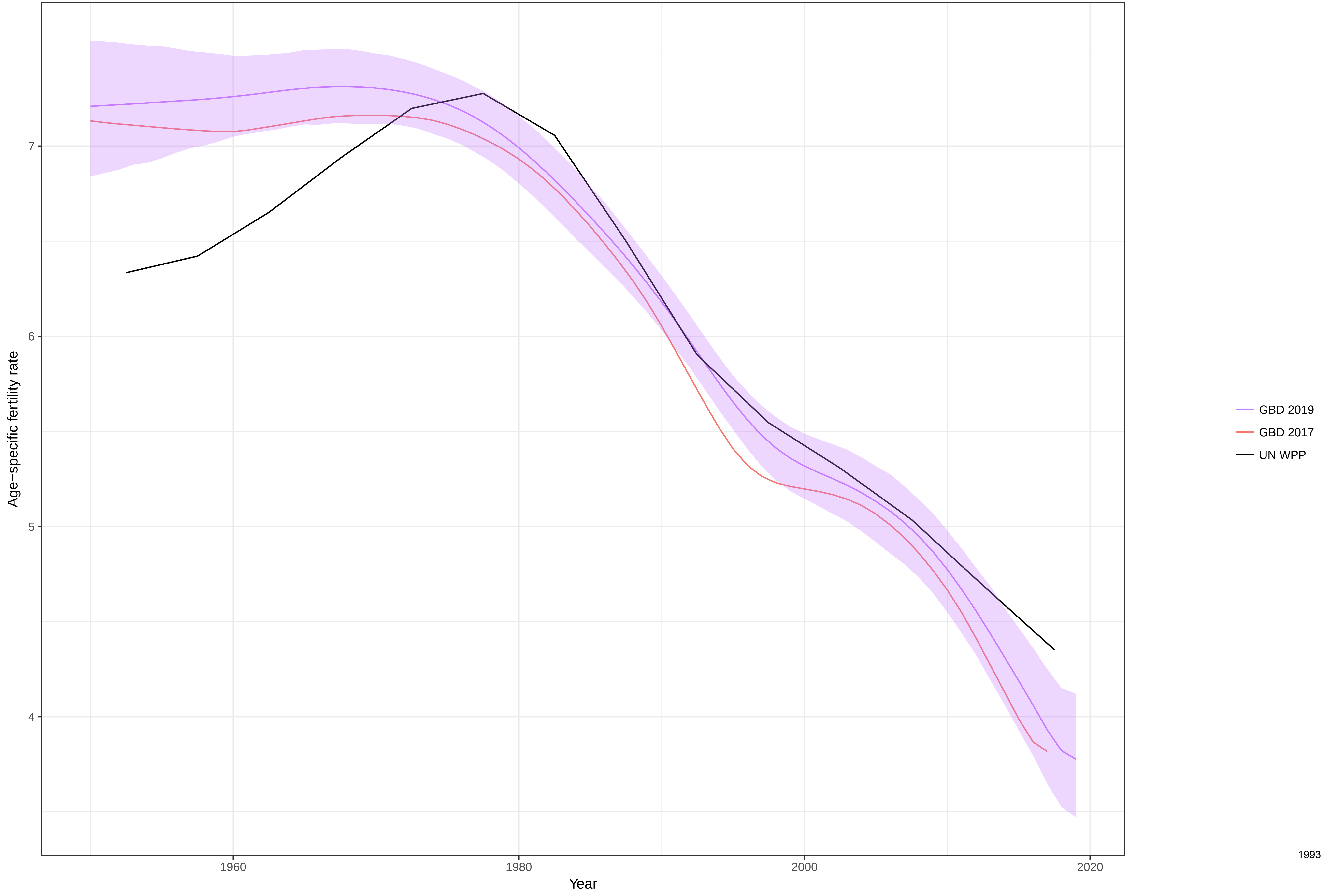

Age−specific fertility fitted trend lines, 1950−2019: Thailand, Ages: 10−14
Data points enclosed with a diamond are reference sources

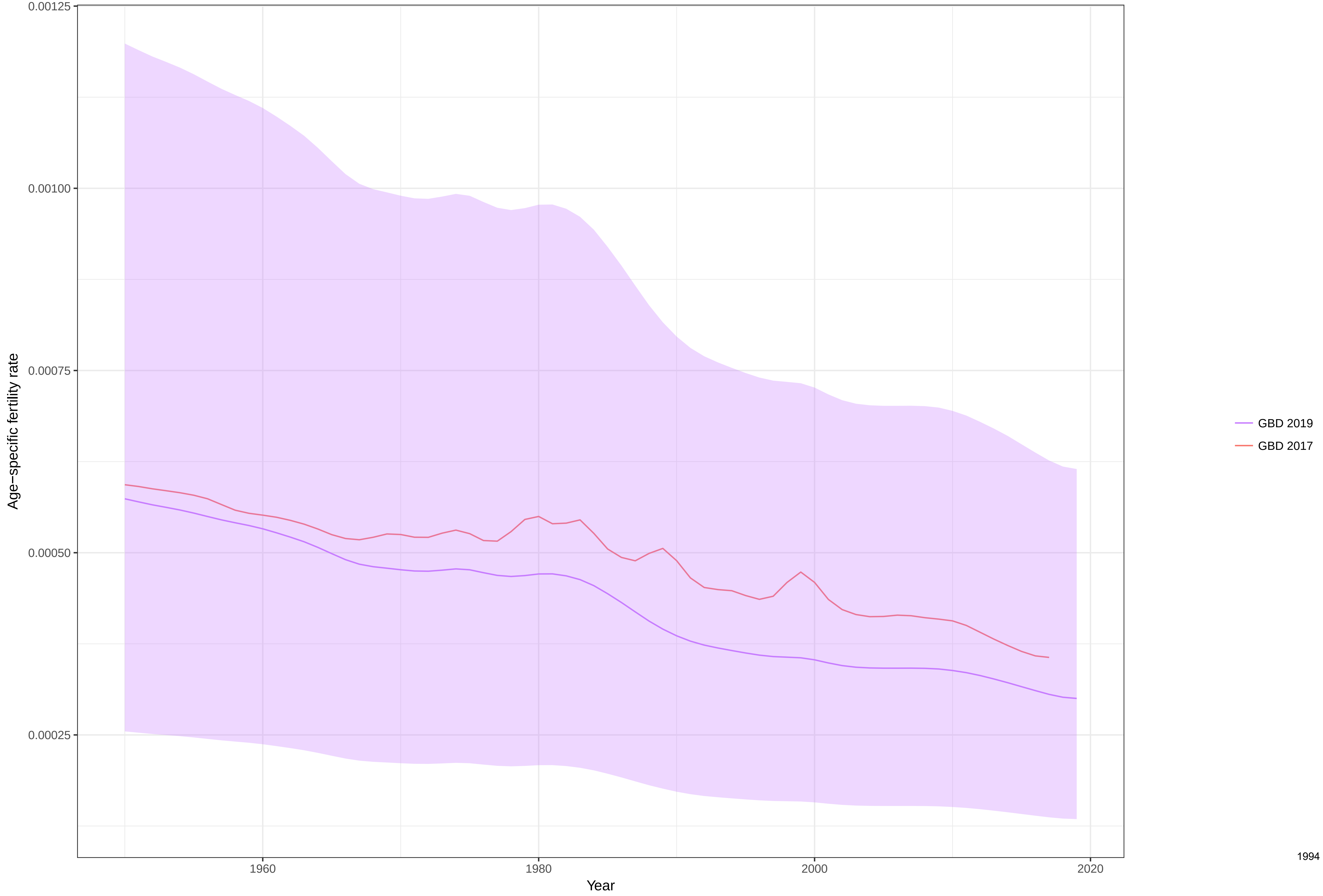

Age−specific fertility fitted trend lines, 1950−2019: Thailand, Ages: 15−19
Data points enclosed with a diamond are reference sources

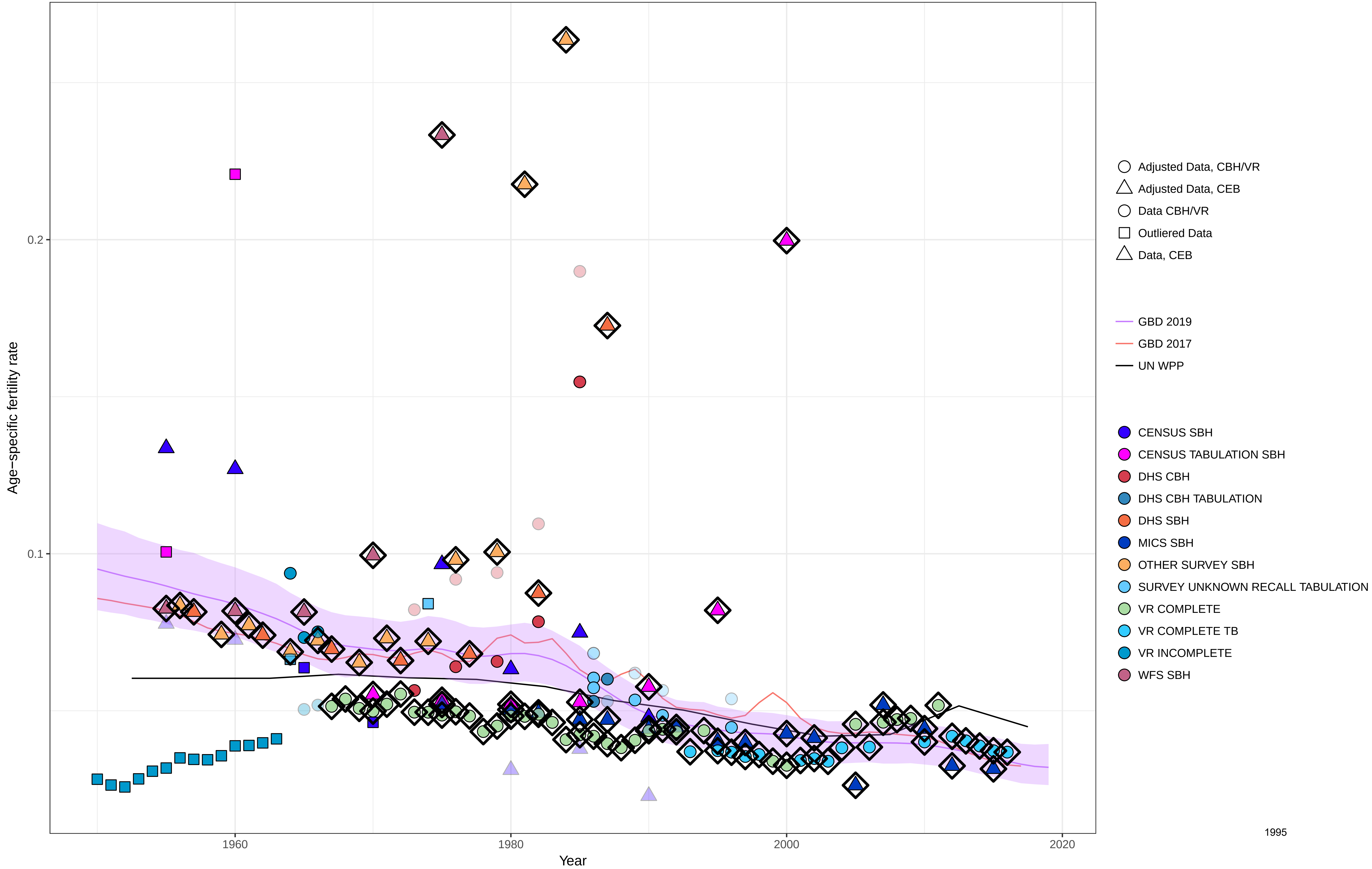

1995

Age−specific fertility fitted trend lines, 1950−2019: Thailand, Ages: 20−24
Data points enclosed with a diamond are reference sources

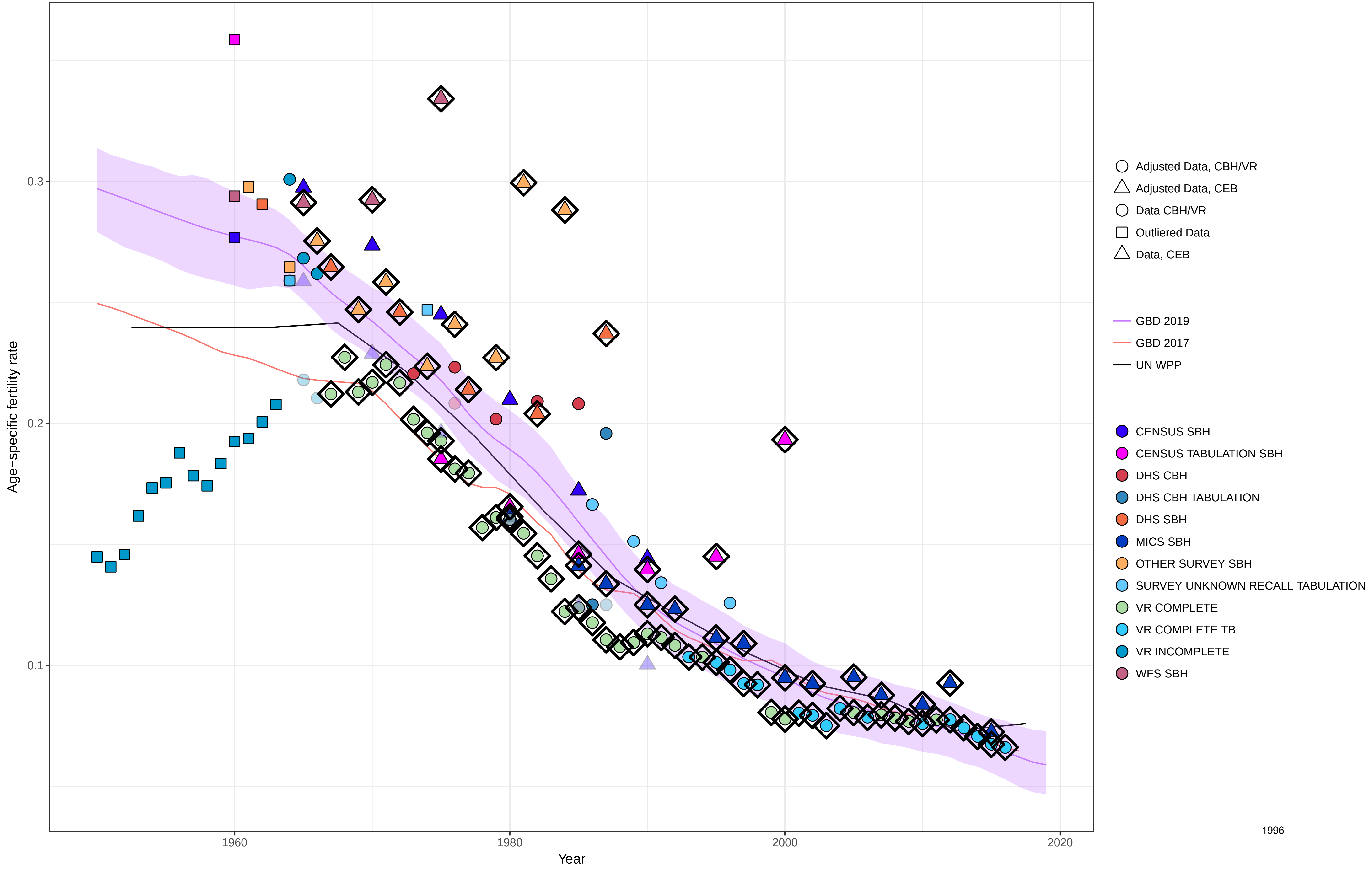

Age−specific fertility fitted trend lines, 1950−2019: Thailand, Ages: 25−29
Data points enclosed with a diamond are reference sources

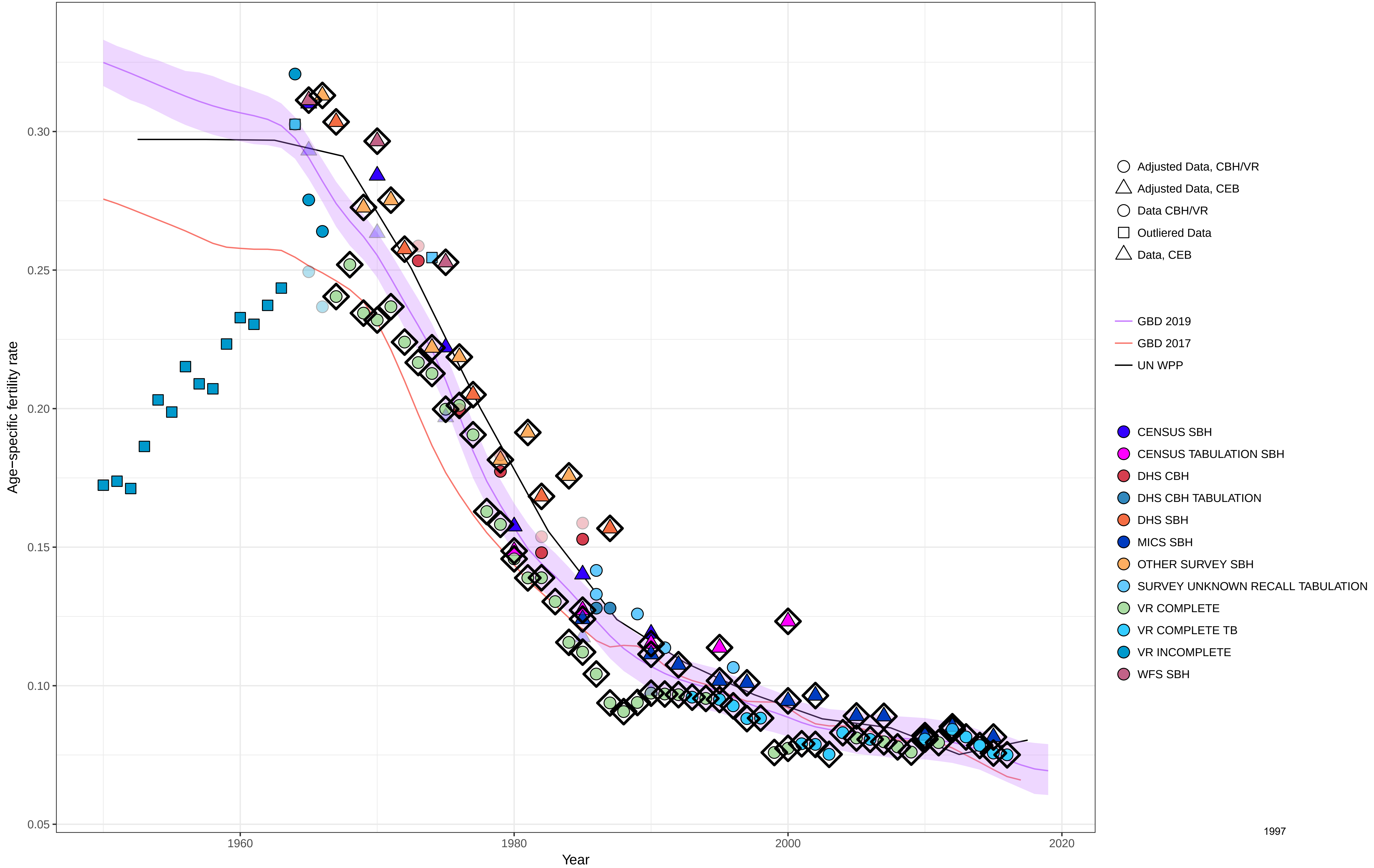

Age−specific fertility fitted trend lines, 1950−2019: Thailand, Ages: 30−34
Data points enclosed with a diamond are reference sources

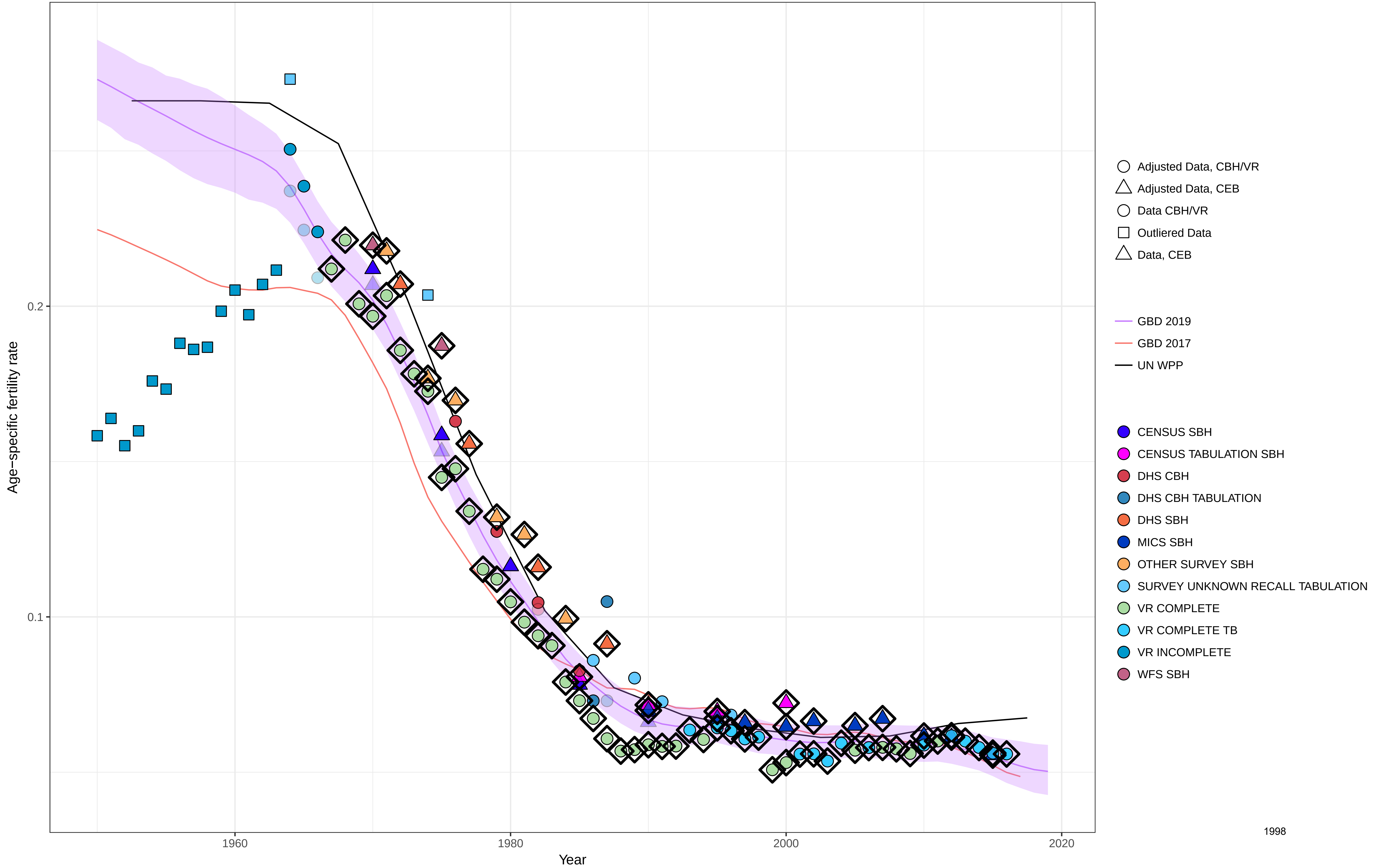

1998

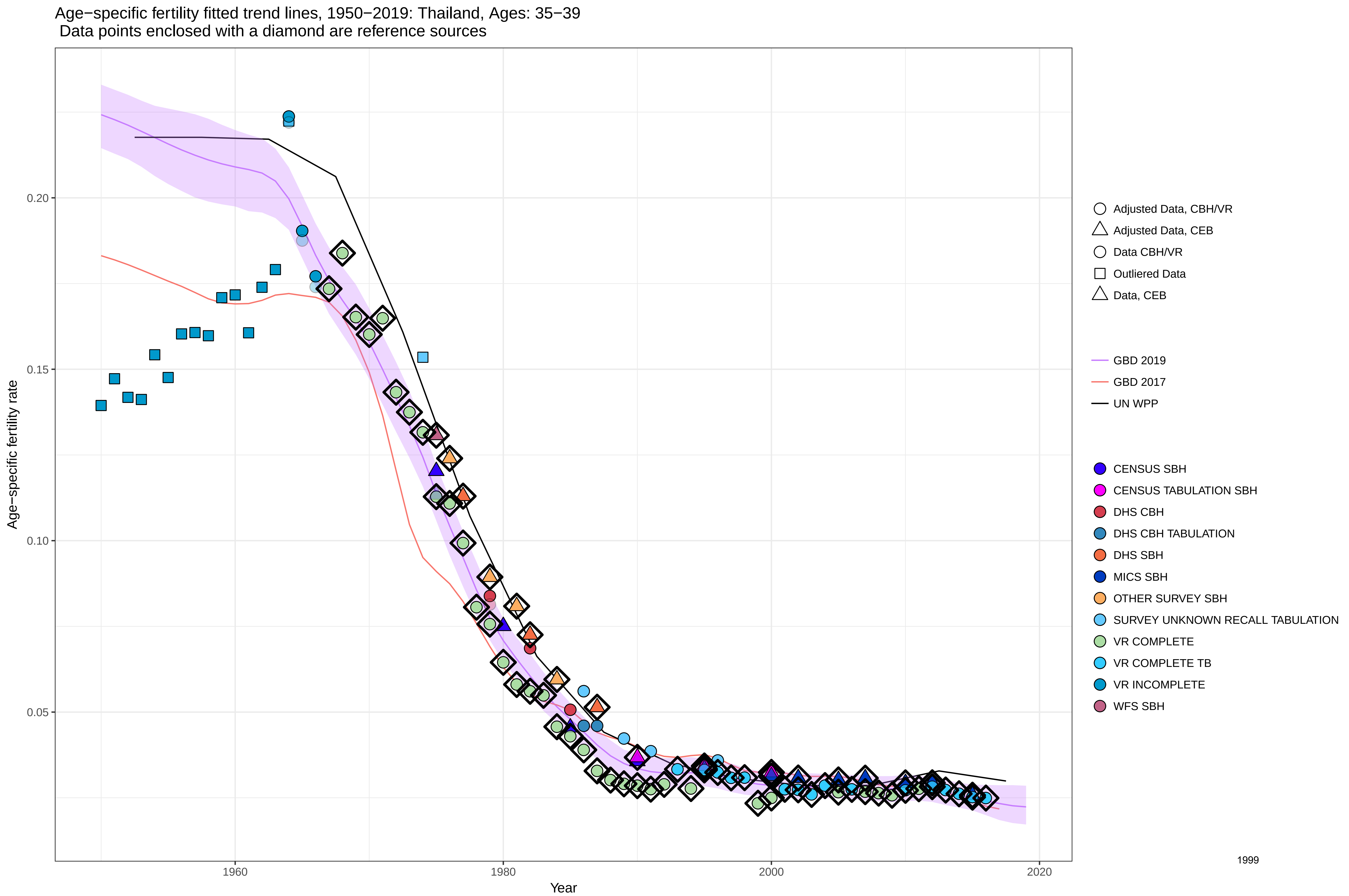
Age−specific fertility fitted trend lines, 1950−2019: Thailand, Ages: 35−39
Data points enclosed with a diamond are reference sources
Age−specific fertility rate
0.20
0.15
0.10
0.05
1960
1980
2000
2020
Year
Adjusted Data, CBH/VR
Adjusted Data, CEB
Data CBH/VR
Outliered Data
Data, CEB
GBD 2019
GBD 2017
UN WPP
CENSUS SBH
CENSUS TABULATION SBH
DHS CBH
DHS CBH TABULATION
DHS SBH
MICS SBH
OTHER SURVEY SBH
SURVEY UNKNOWN RECALL TABULATION
VR COMPLETE
VR COMPLETE TB
VR INCOMPLETE
WFS SBH

1999

Age−specific fertility fitted trend lines, 1950−2019: Thailand, Ages: 40−44
Data points enclosed with a diamond are reference sources

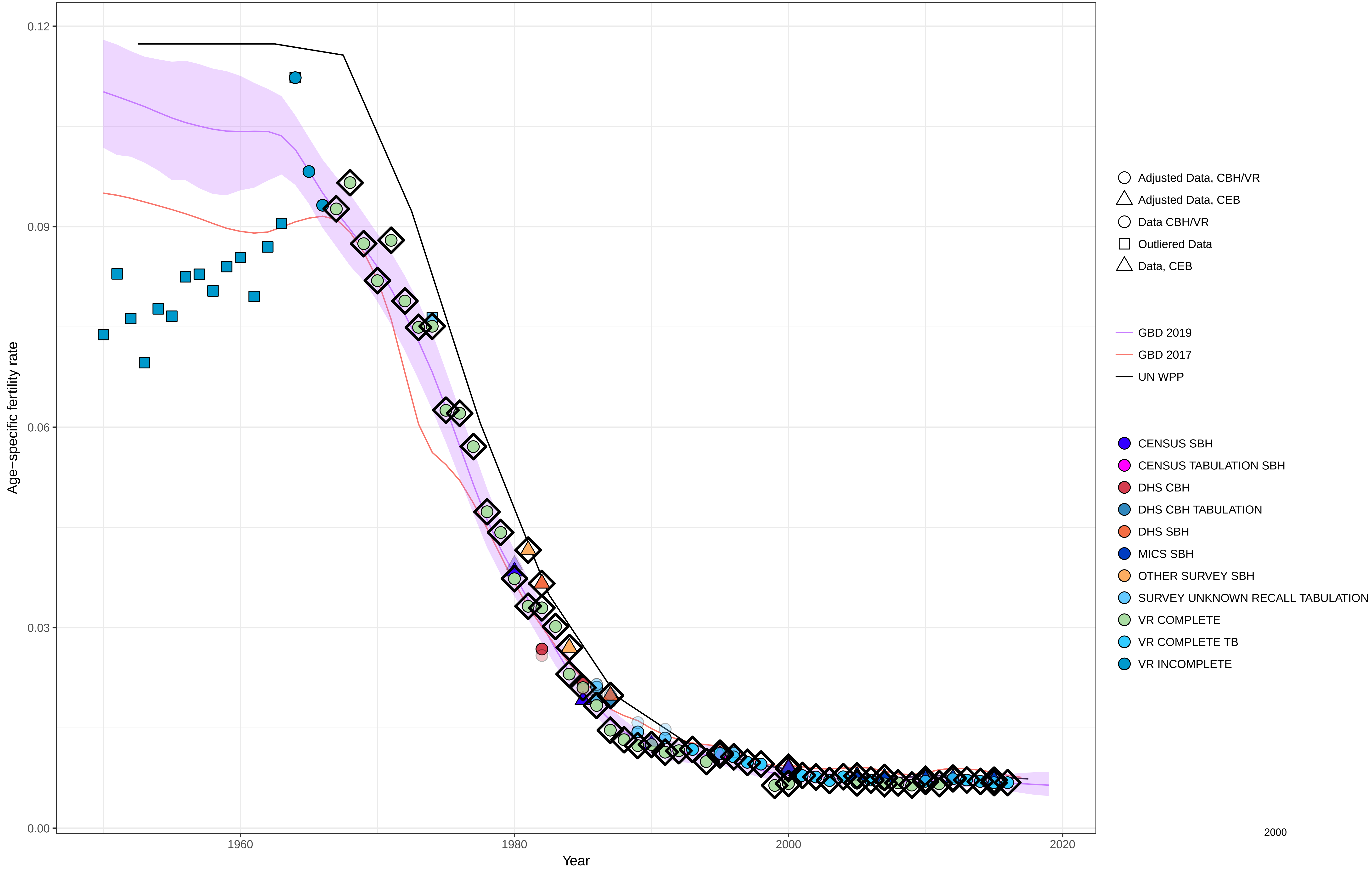

2000

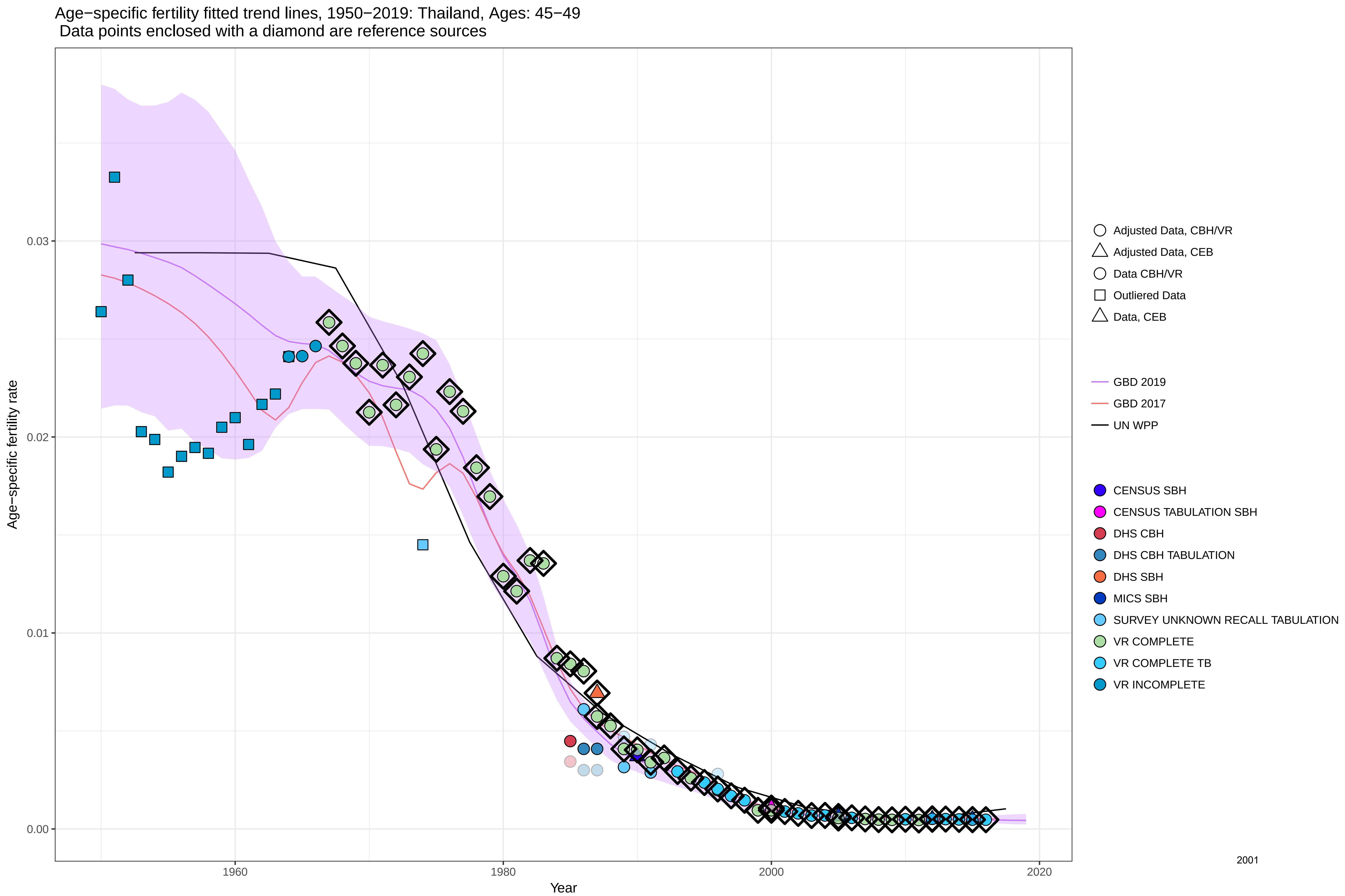

Age−specific fertility fitted trend lines, 1950−2019: Thailand, Ages: 45−49
Data points enclosed with a diamond are reference sources
Age−specific fertility rate
0.00
0.01
0.02
0.03
1960
1980
2000
2020
Year
Adjusted Data, CBH/VR
Adjusted Data, CEB
Data CBH/VR
Outliered Data
Data, CEB
GBD 2019
GBD 2017
UN WPP
CENSUS SBH
CENSUS TABULATION SBH
DHS CBH
DHS CBH TABULATION
DHS SBH
MICS SBH
SURVEY UNKNOWN RECALL TABULATION
VR COMPLETE
VR COMPLETE TB
VR INCOMPLETE

Age−specific fertility fitted trend lines, 1950−2019: Thailand, Ages: 50−54
Data points enclosed with a diamond are reference sources

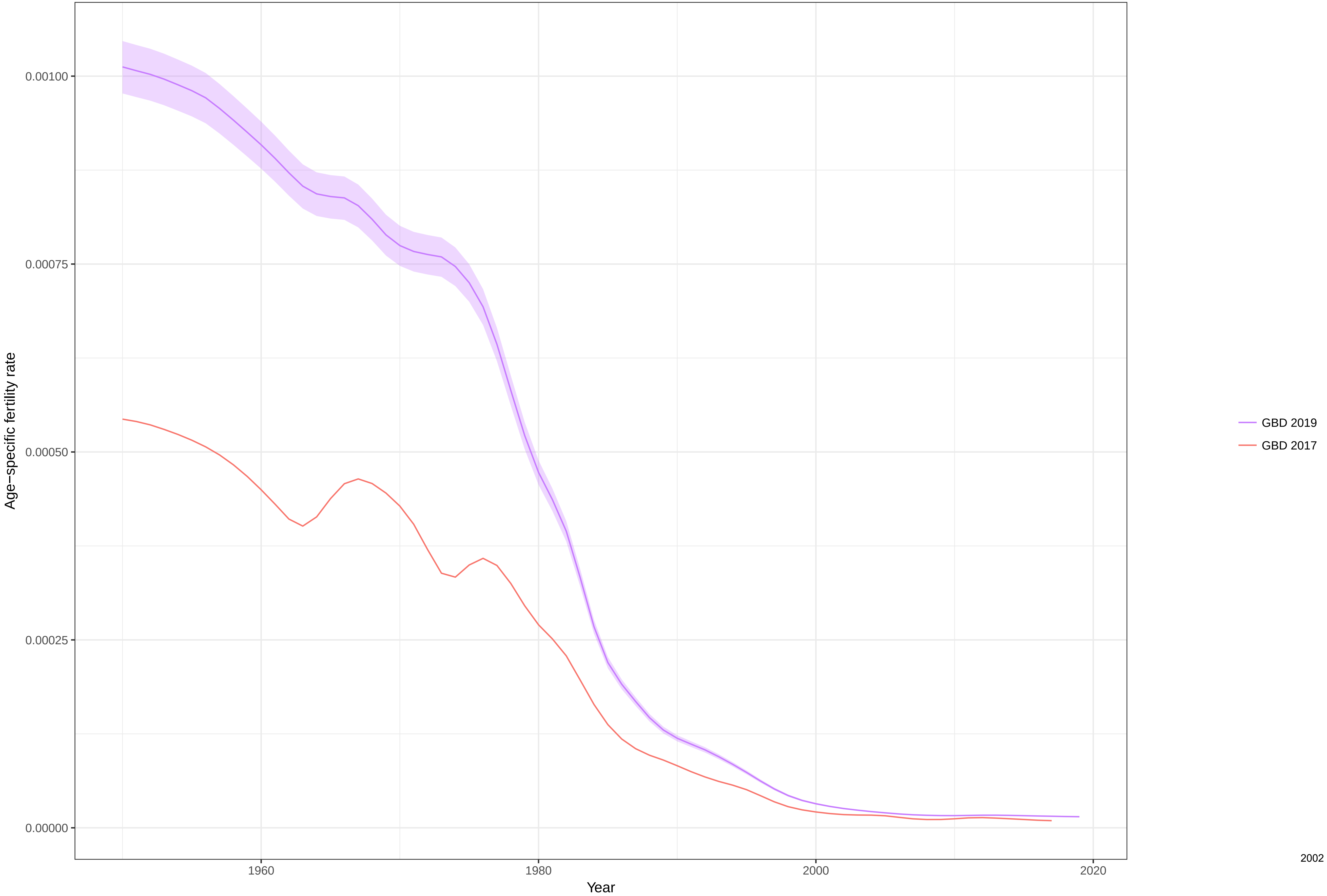

Total fertility rate fitted trend lines, 1950−2019: Thailand TFR
Data points enclosed with a diamond are reference sources

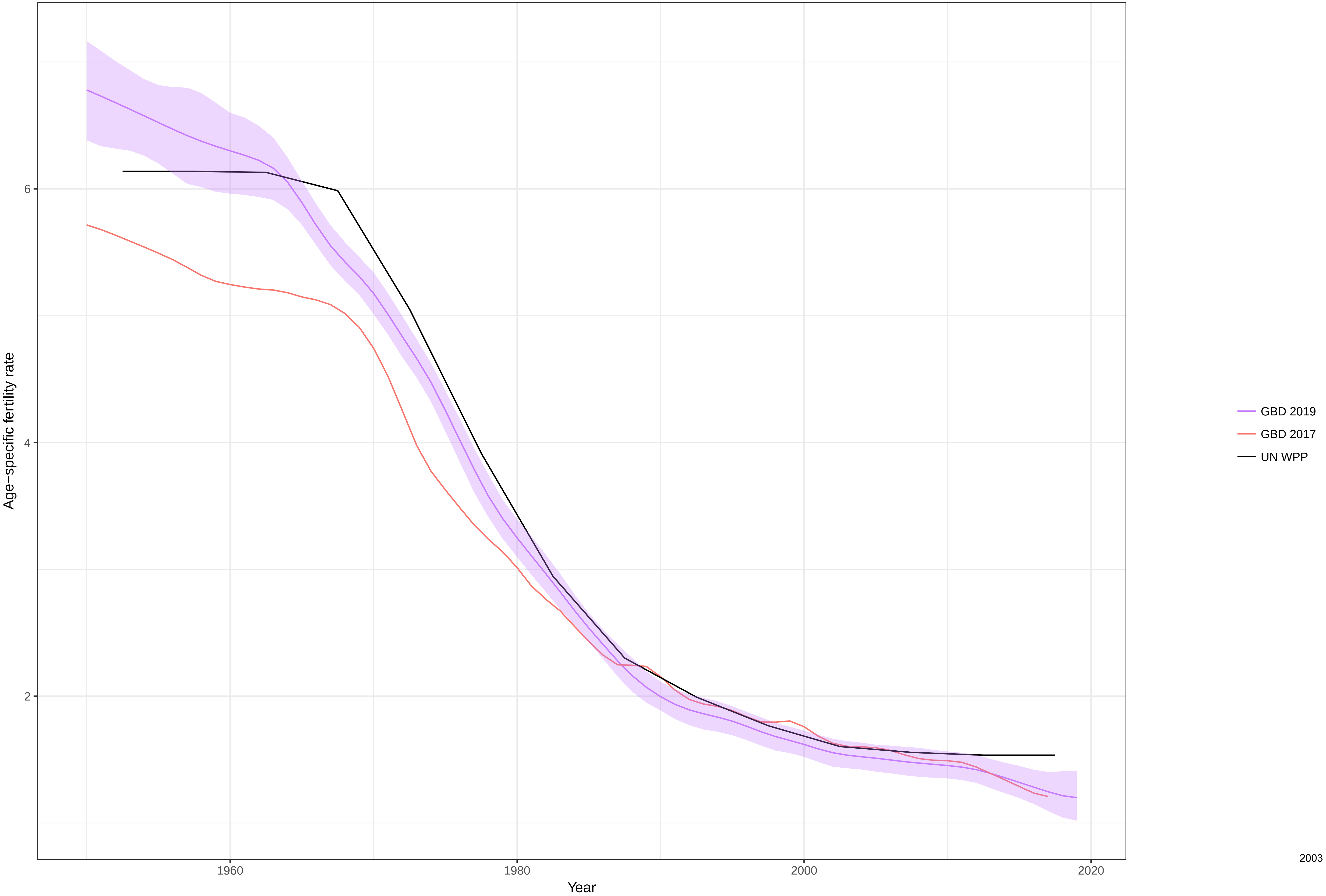

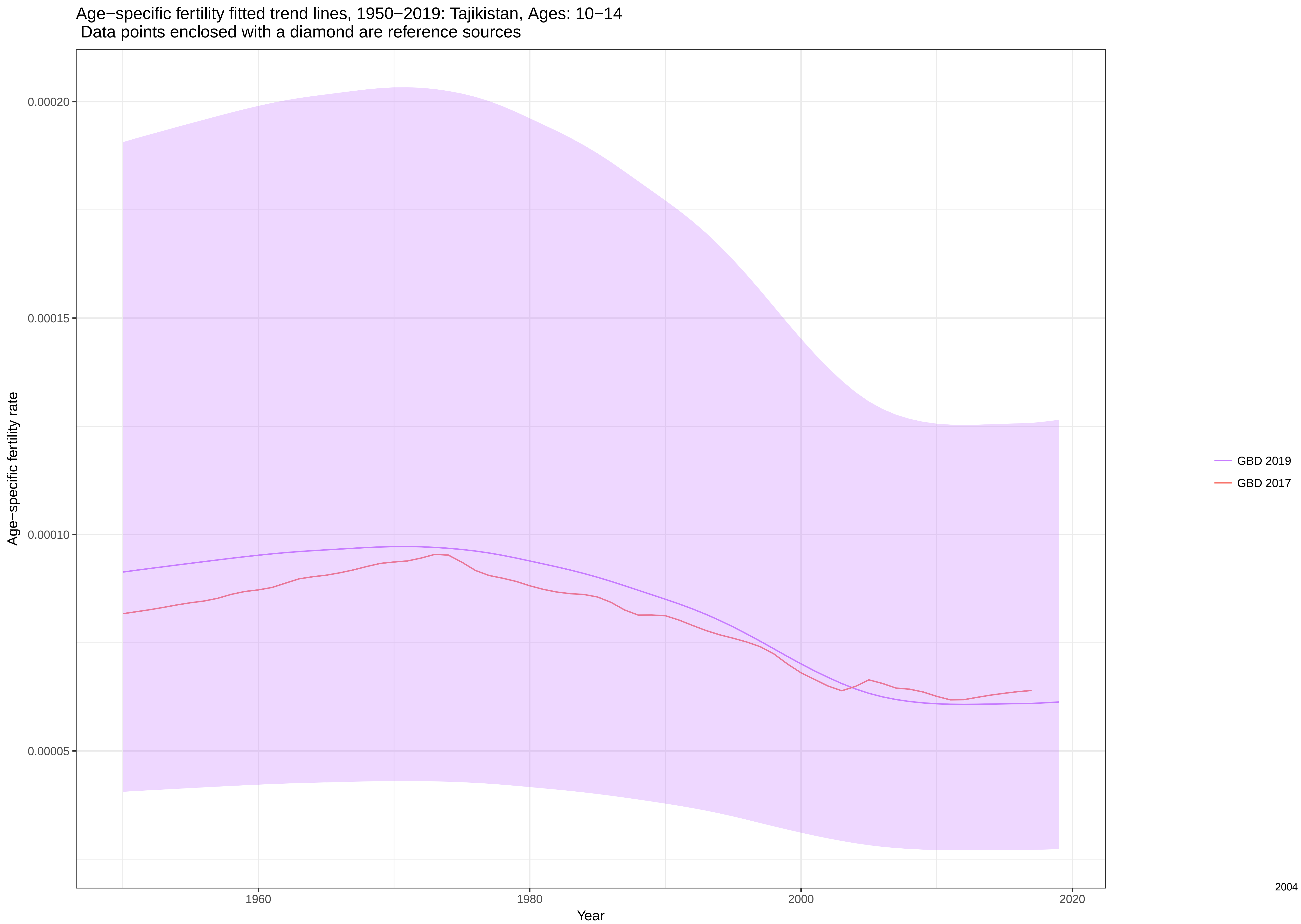

Age−specific fertility fitted trend lines, 1950−2019: Tajikistan, Ages: 10−14
Data points enclosed with a diamond are reference sources
0.00020
0.00015
0.00010
0.00005
Age−specific fertility rate
1960
1980
2000
2020
Year
GBD 2019
GBD 2017

Age−specific fertility fitted trend lines, 1950−2019: Tajikistan, Ages: 15−19
Data points enclosed with a diamond are reference sources

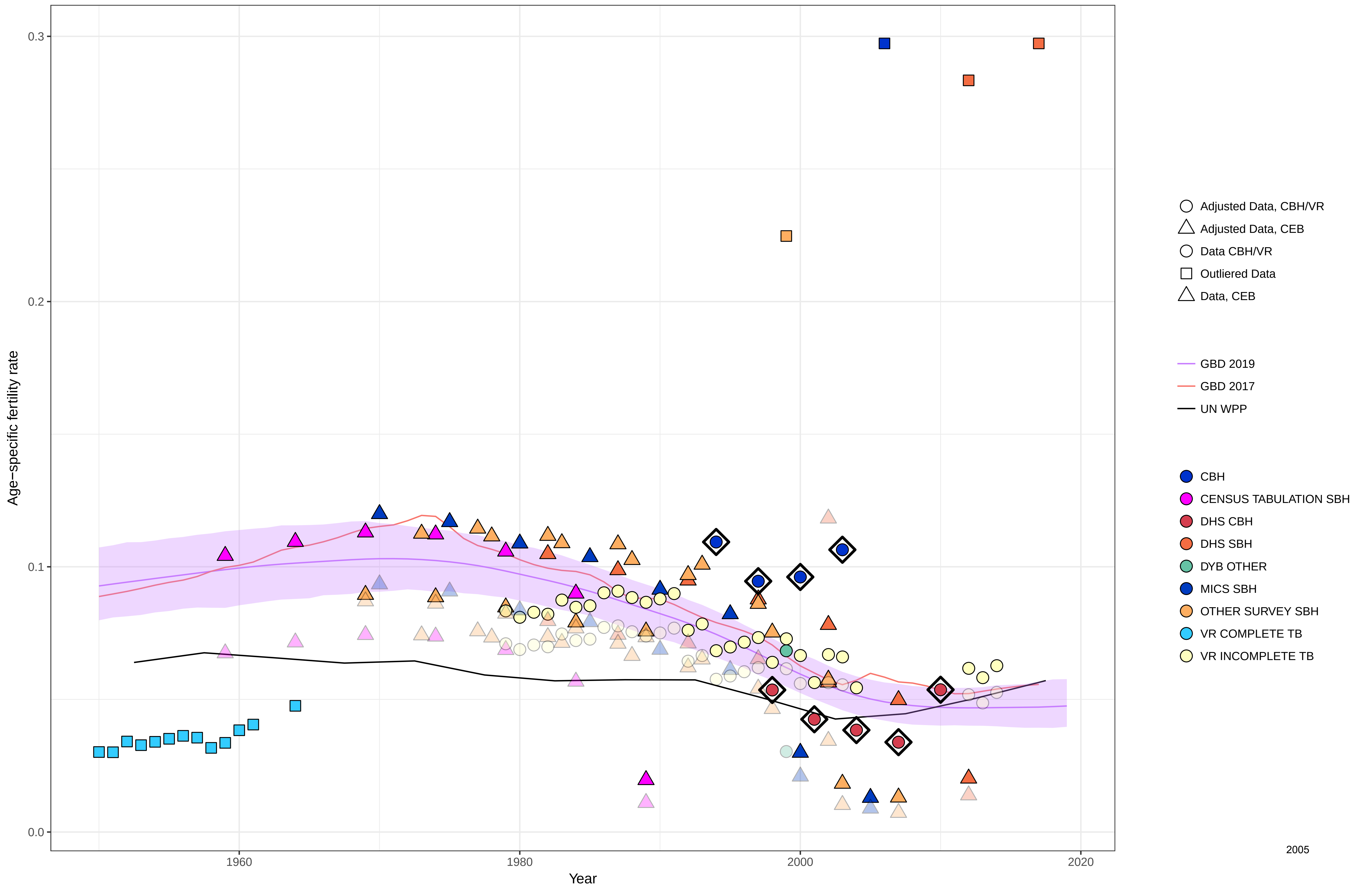

Age−specific fertility fitted trend lines, 1950−2019: Tajikistan, Ages: 20−24
Data points enclosed with a diamond are reference sources

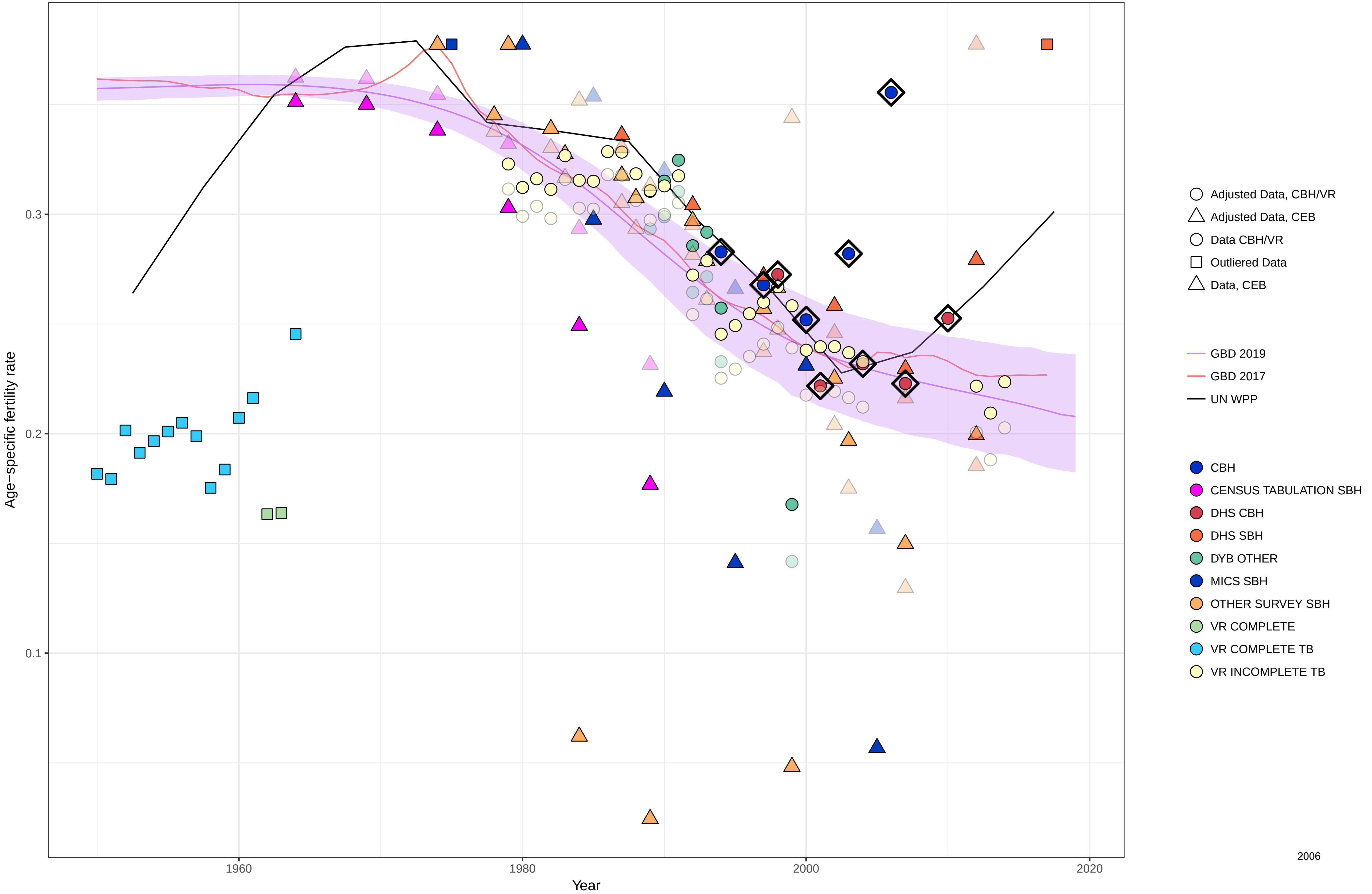

2006

Age−specific fertility fitted trend lines, 1950−2019: Tajikistan, Ages: 25−29
Data points enclosed with a diamond are reference sources

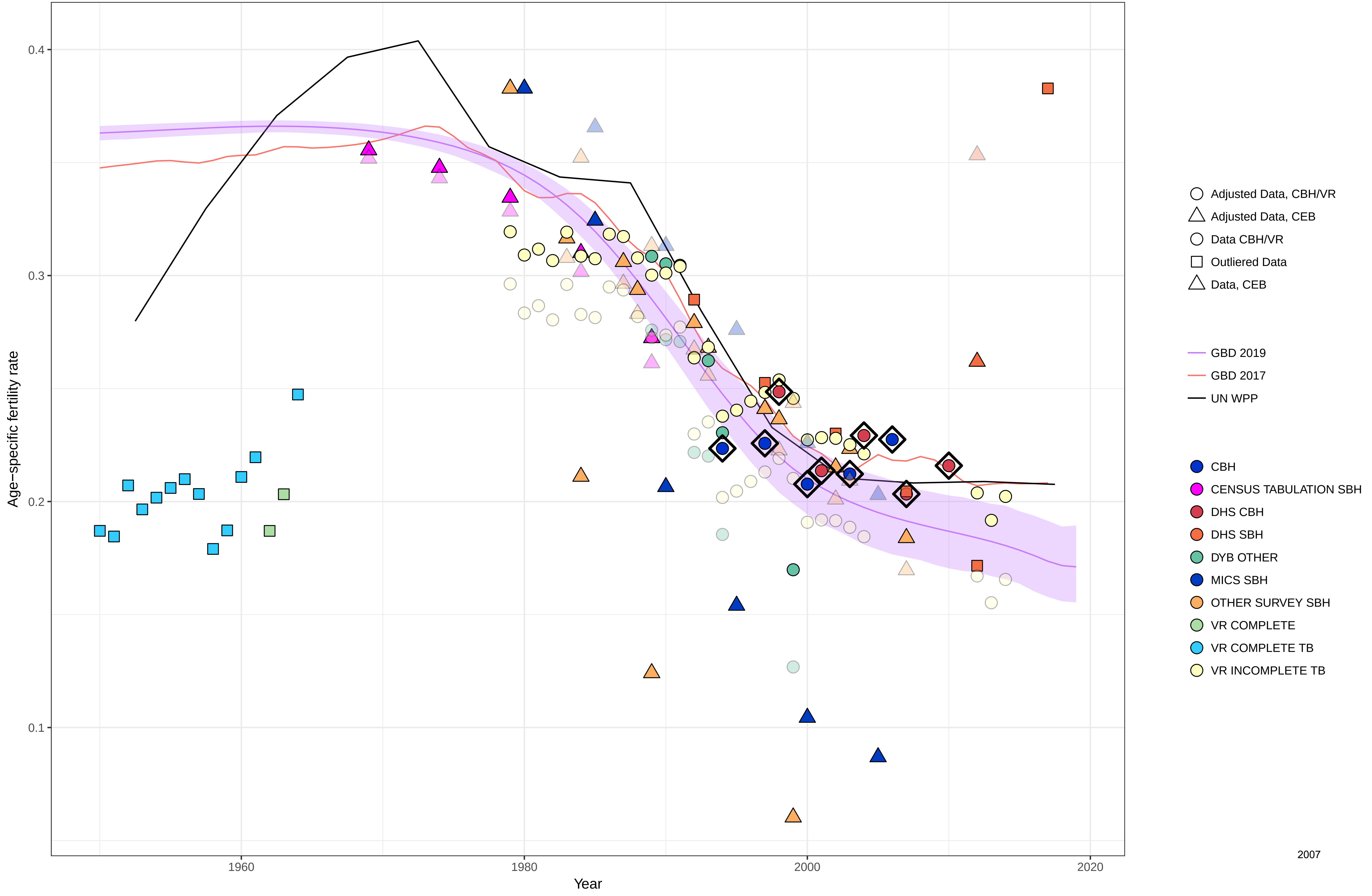

2007

Age−specific fertility fitted trend lines, 1950−2019: Tajikistan, Ages: 30−34
Data points enclosed with a diamond are reference sources

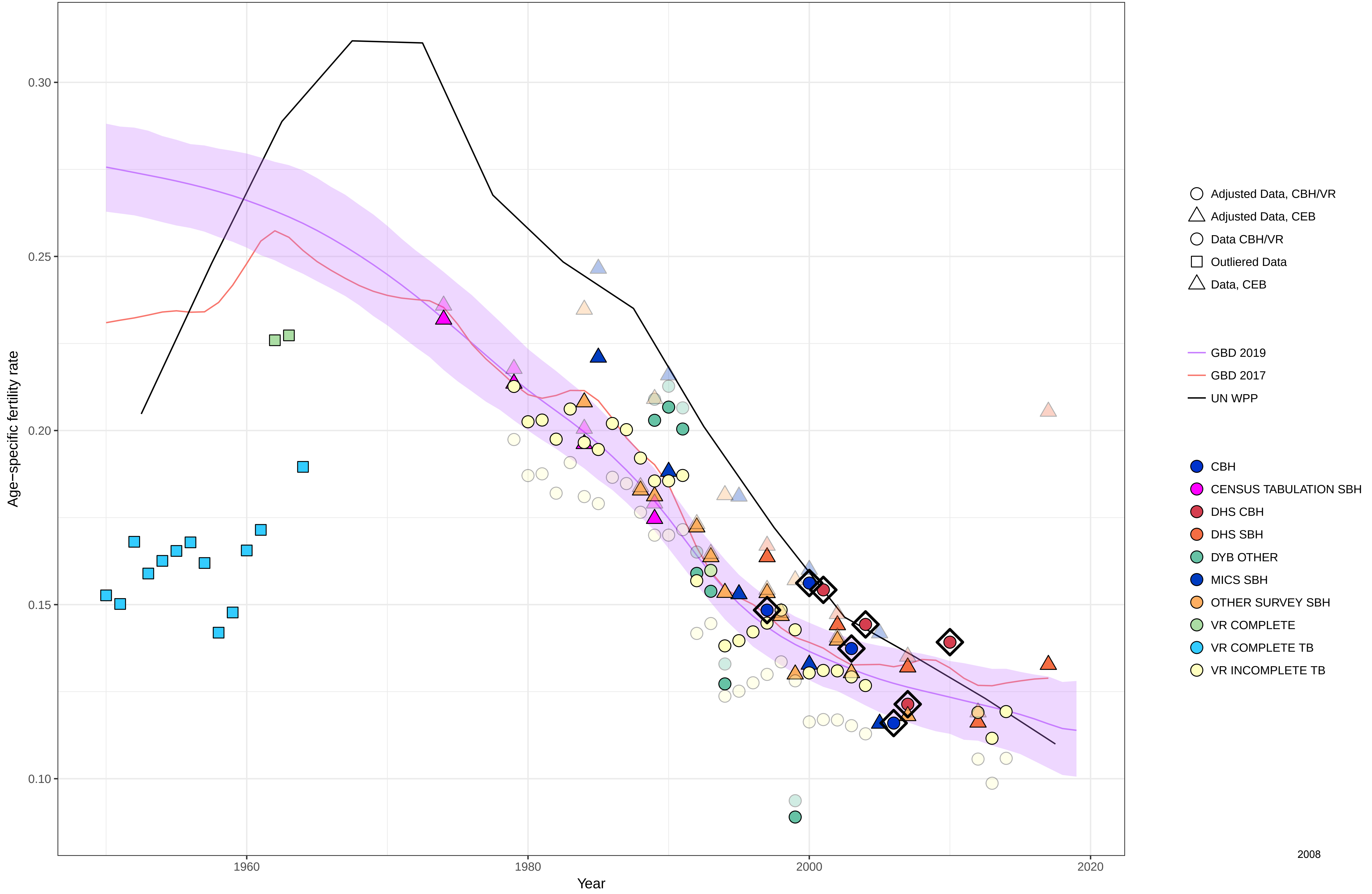

Age−specific fertility fitted trend lines, 1950−2019: Tajikistan, Ages: 35−39
Data points enclosed with a diamond are reference sources

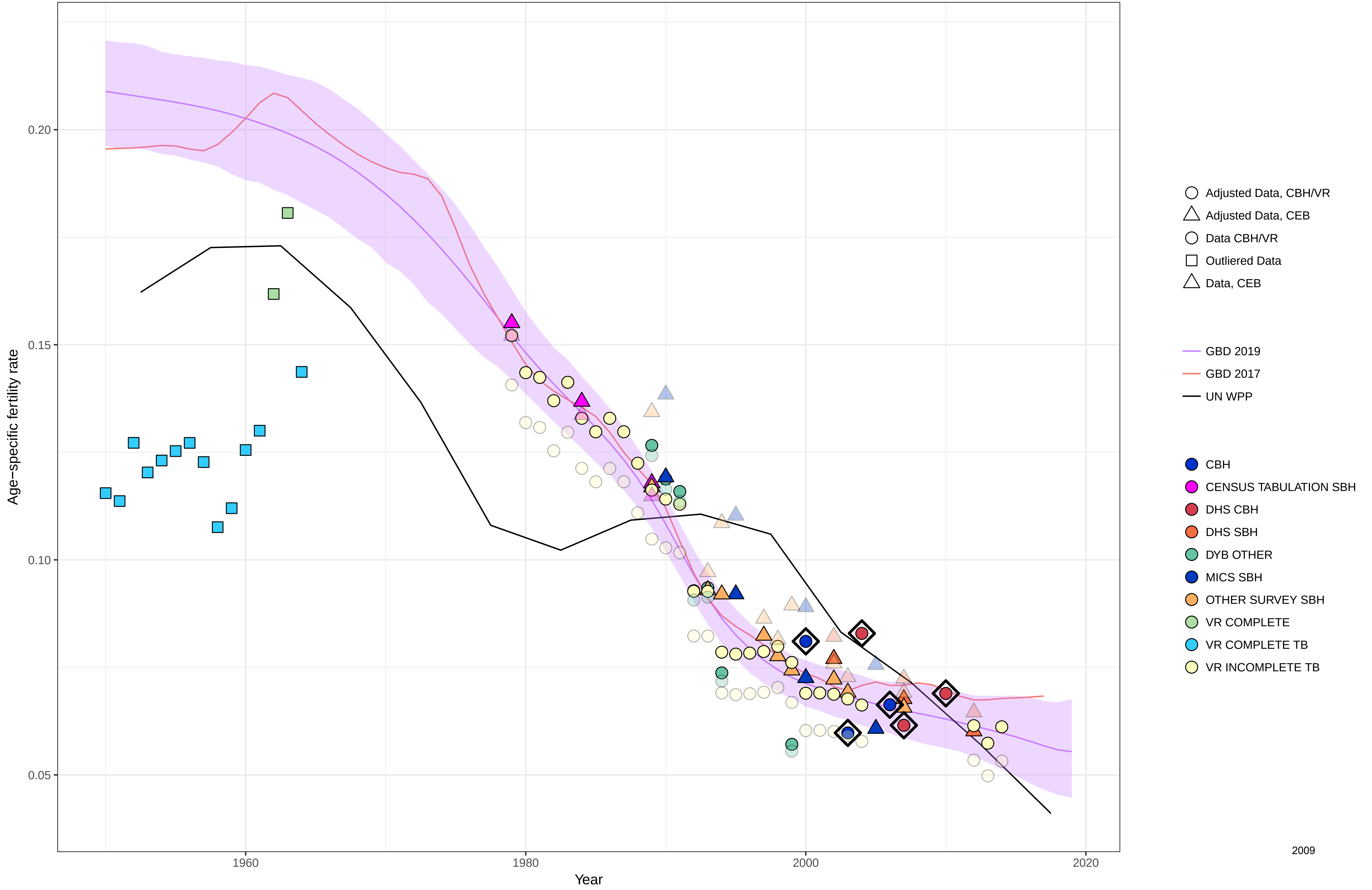

2009

Age−specific fertility fitted trend lines, 1950−2019: Tajikistan, Ages: 40−44
Data points enclosed with a diamond are reference sources

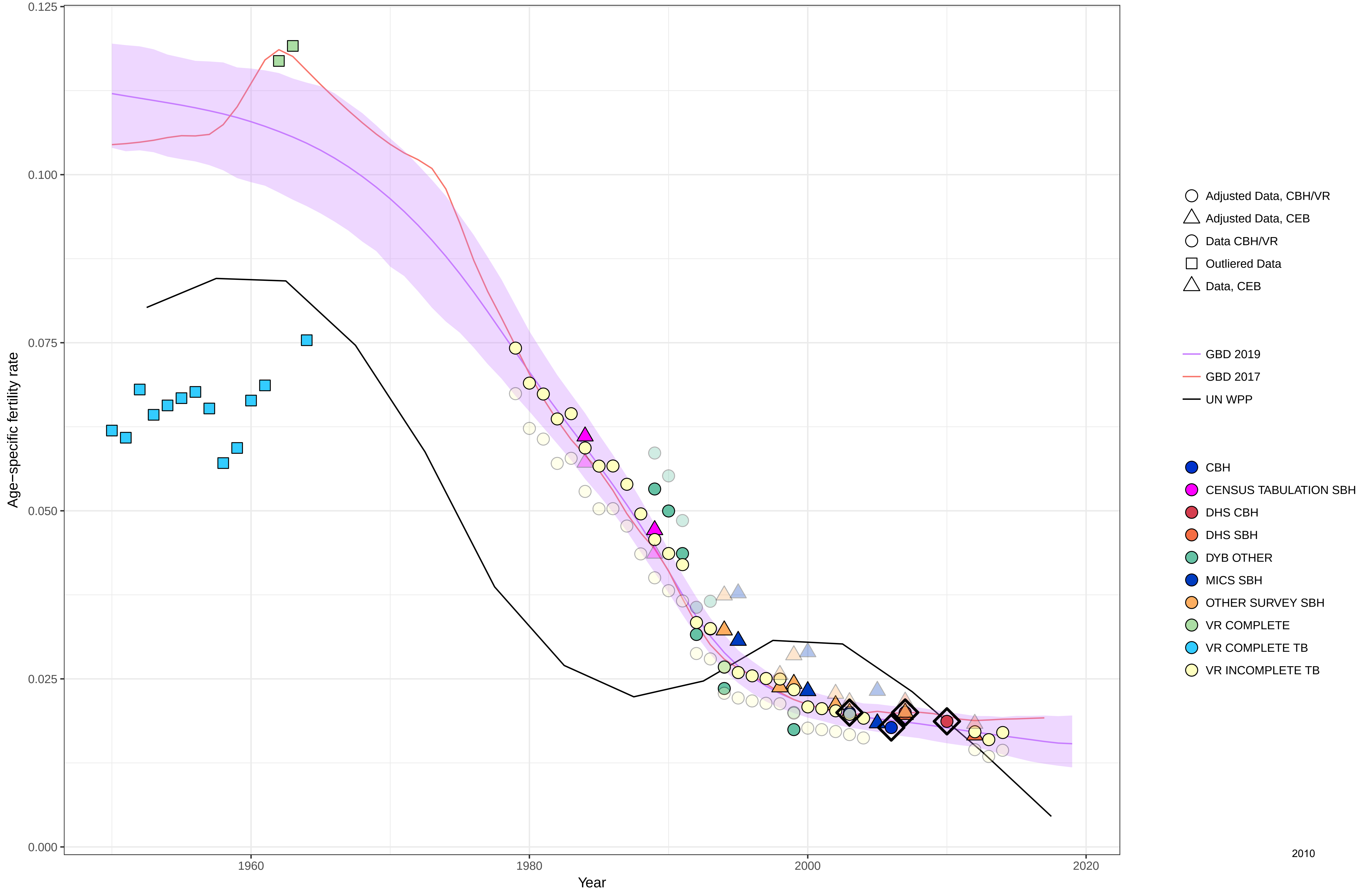

Age−specific fertility fitted trend lines, 1950−2019: Tajikistan, Ages: 45−49
Data points enclosed with a diamond are reference sources

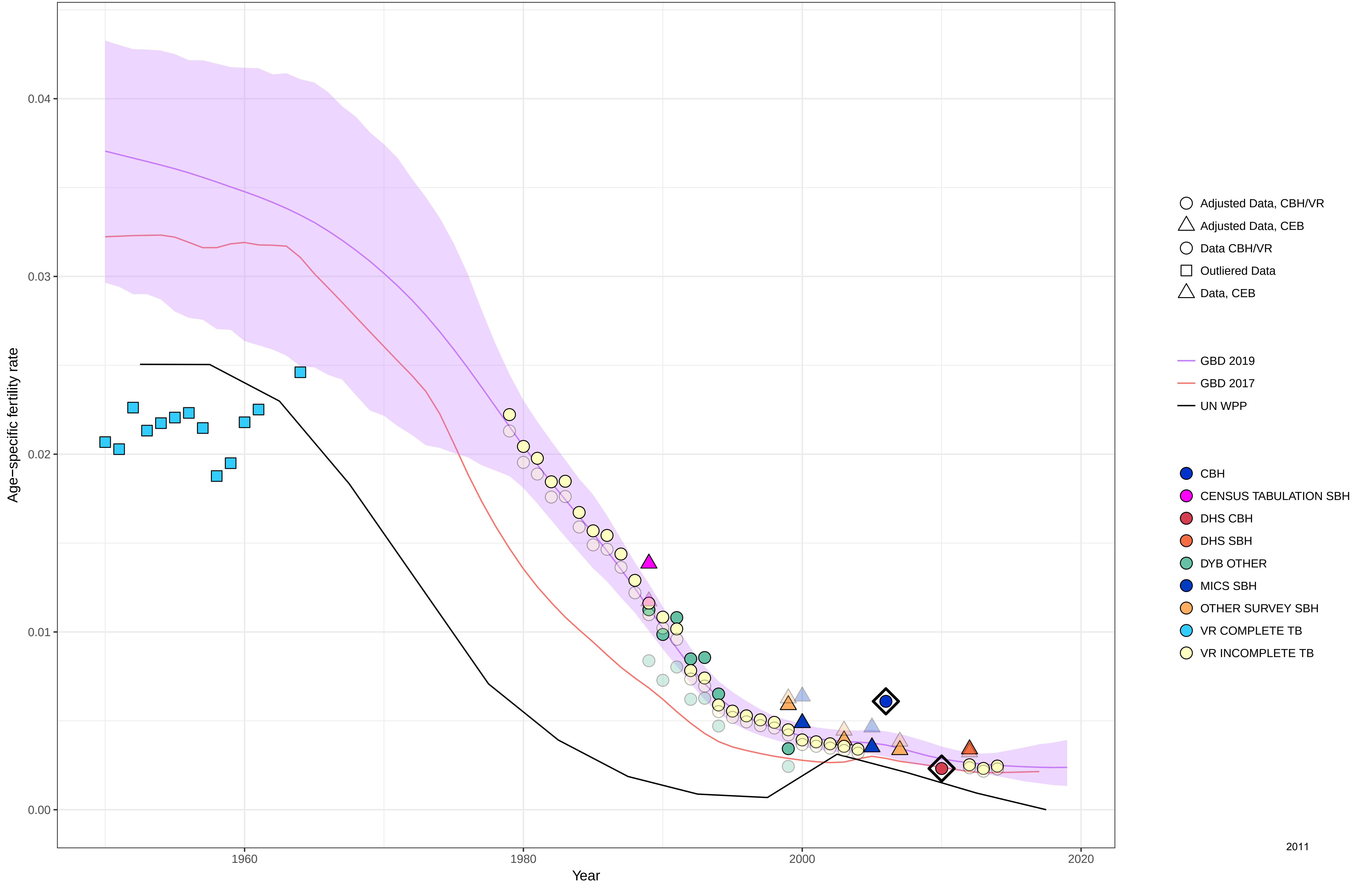

2011

Age−specific fertility fitted trend lines, 1950−2019: Tajikistan, Ages: 50−54
Data points enclosed with a diamond are reference sources

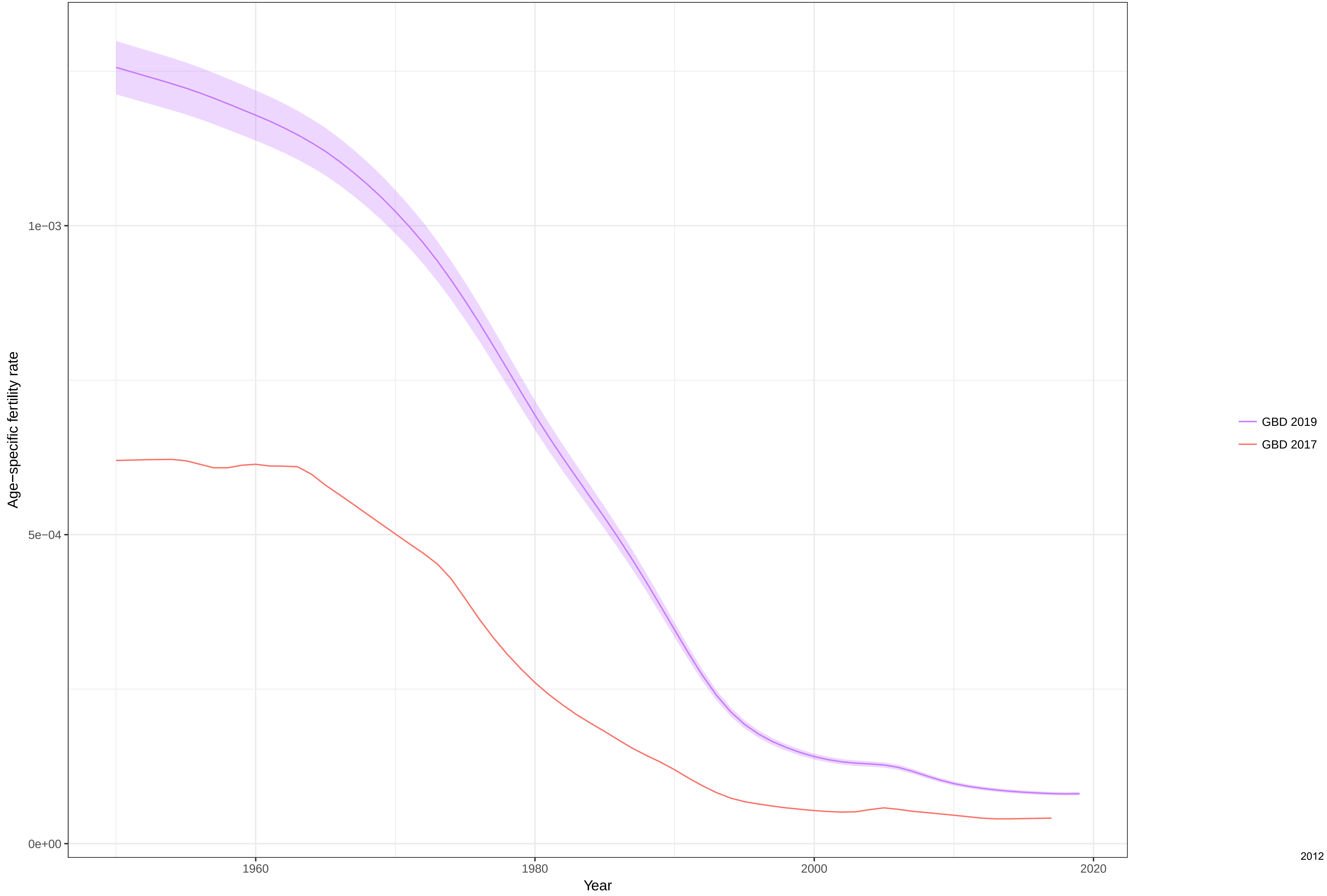

Total fertility rate fitted trend lines, 1950−2019: Tajikistan TFR
Data points enclosed with a diamond are reference sources

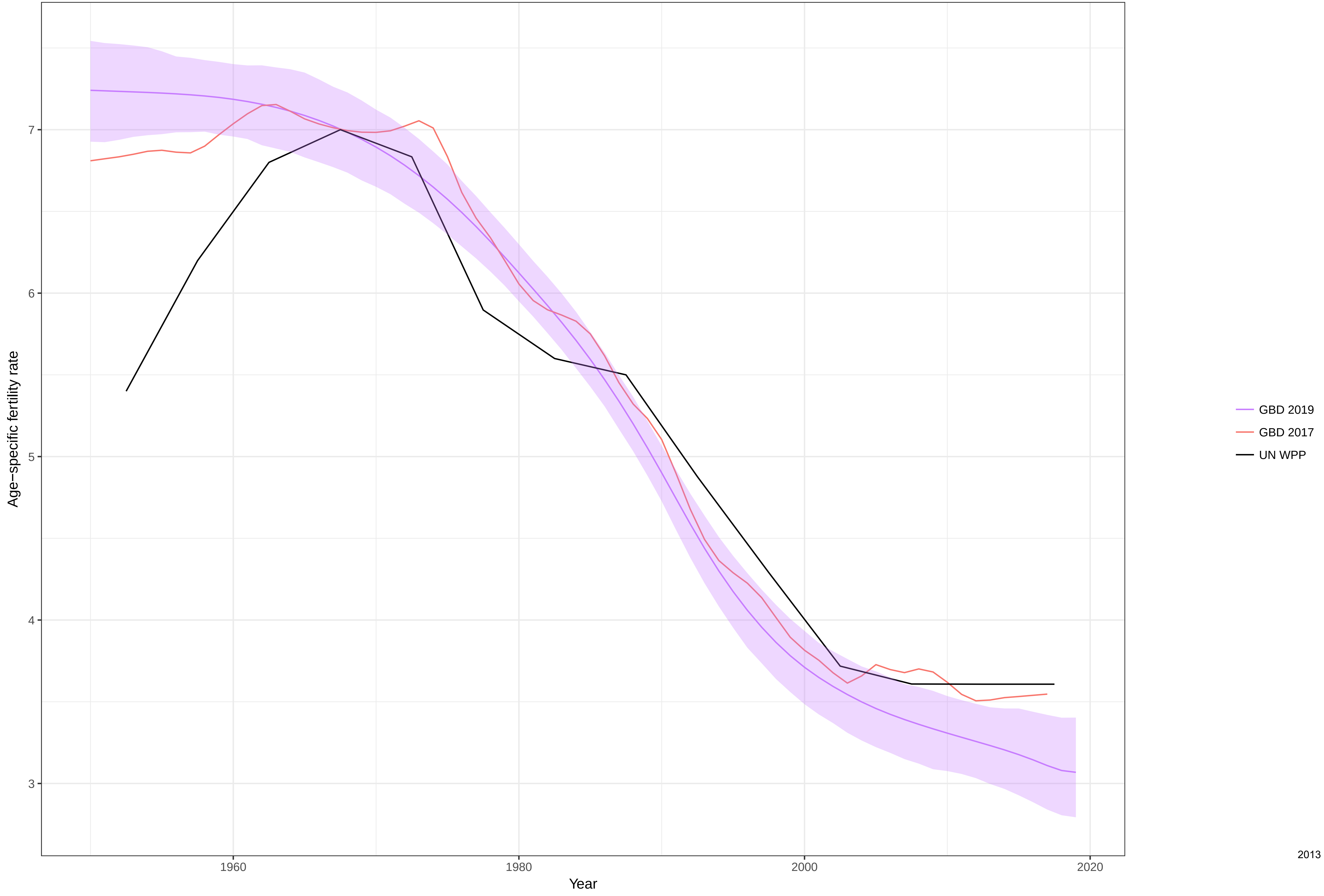

2013

Age−specific fertility fitted trend lines, 1950−2019: Tokelau, Ages: 10−14
Data points enclosed with a diamond are reference sources

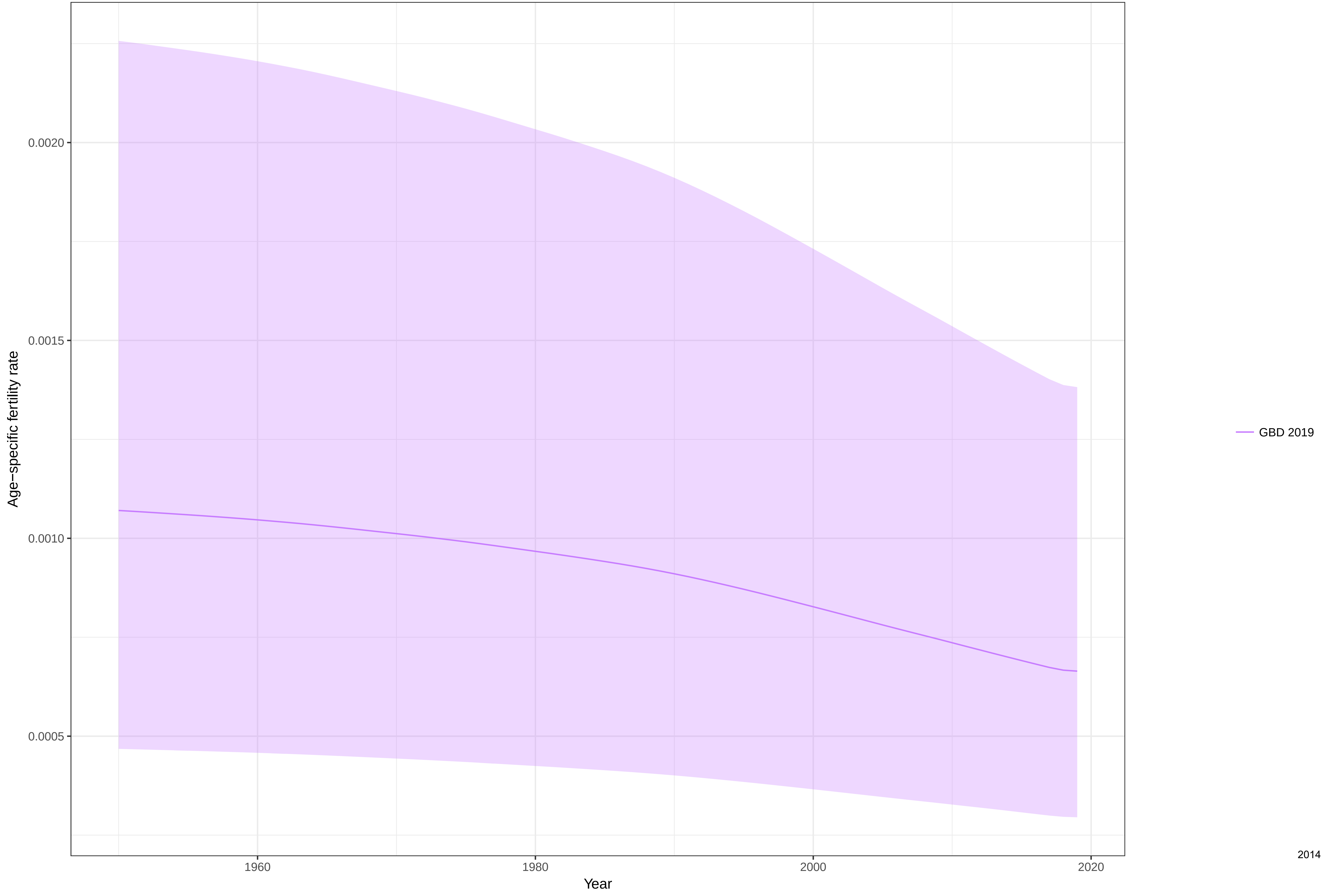

Age−specific fertility fitted trend lines, 1950−2019: Tokelau, Ages: 15−19
Data points enclosed with a diamond are reference sources

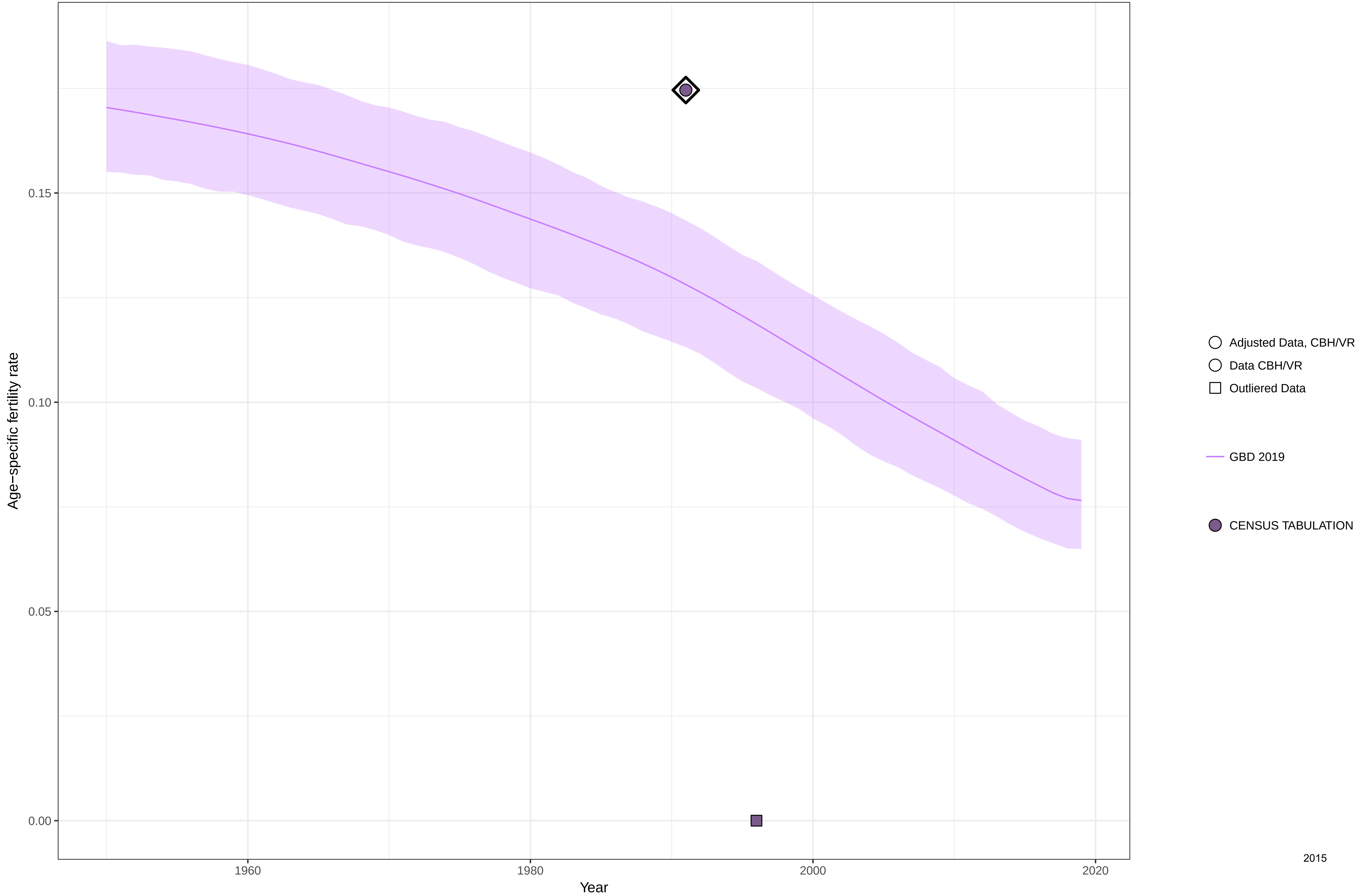

Age−specific fertility fitted trend lines, 1950−2019: Tokelau, Ages: 20−24
Data points enclosed with a diamond are reference sources

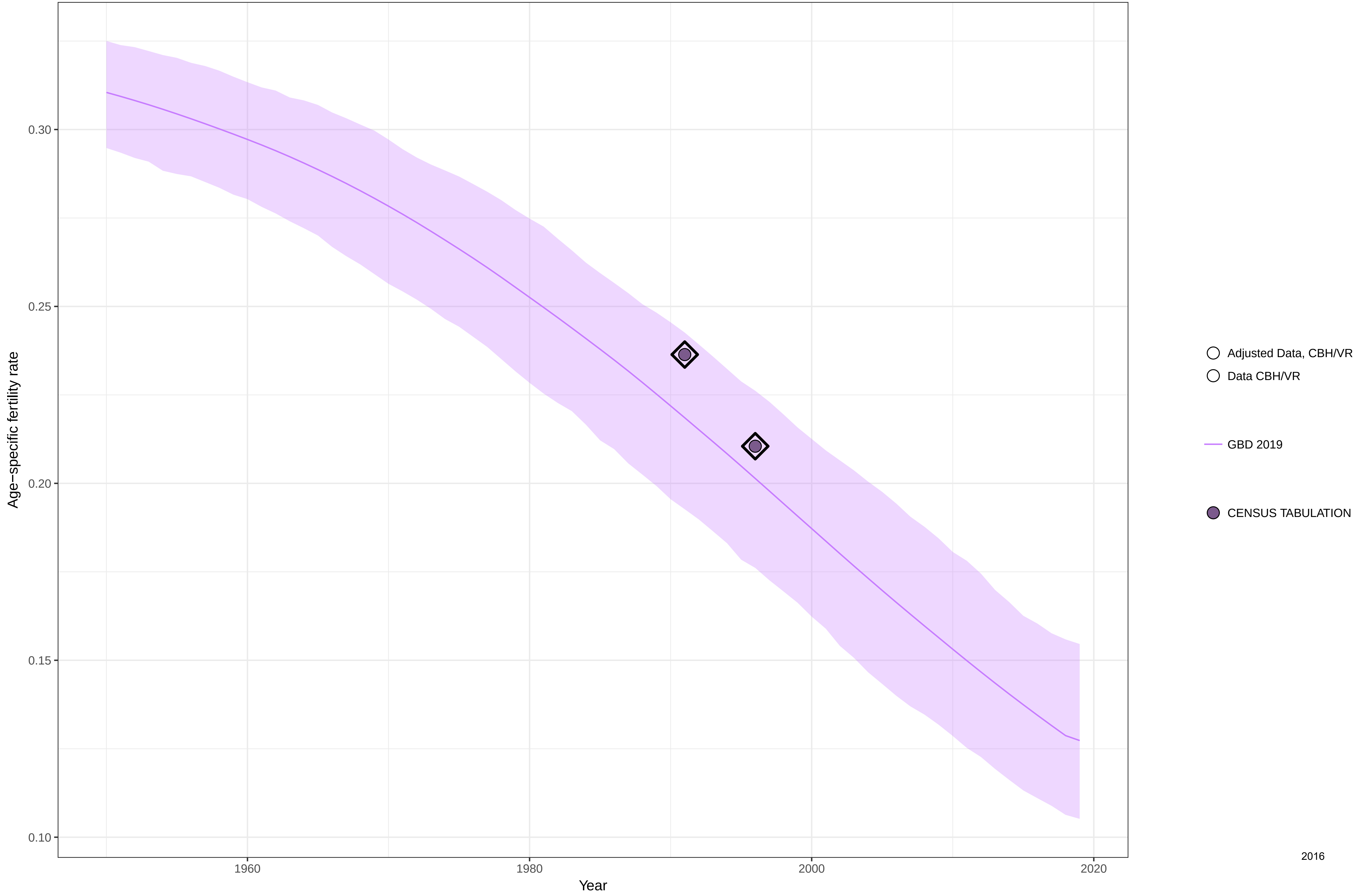

2016

Age−specific fertility fitted trend lines, 1950−2019: Tokelau, Ages: 25−29
Data points enclosed with a diamond are reference sources

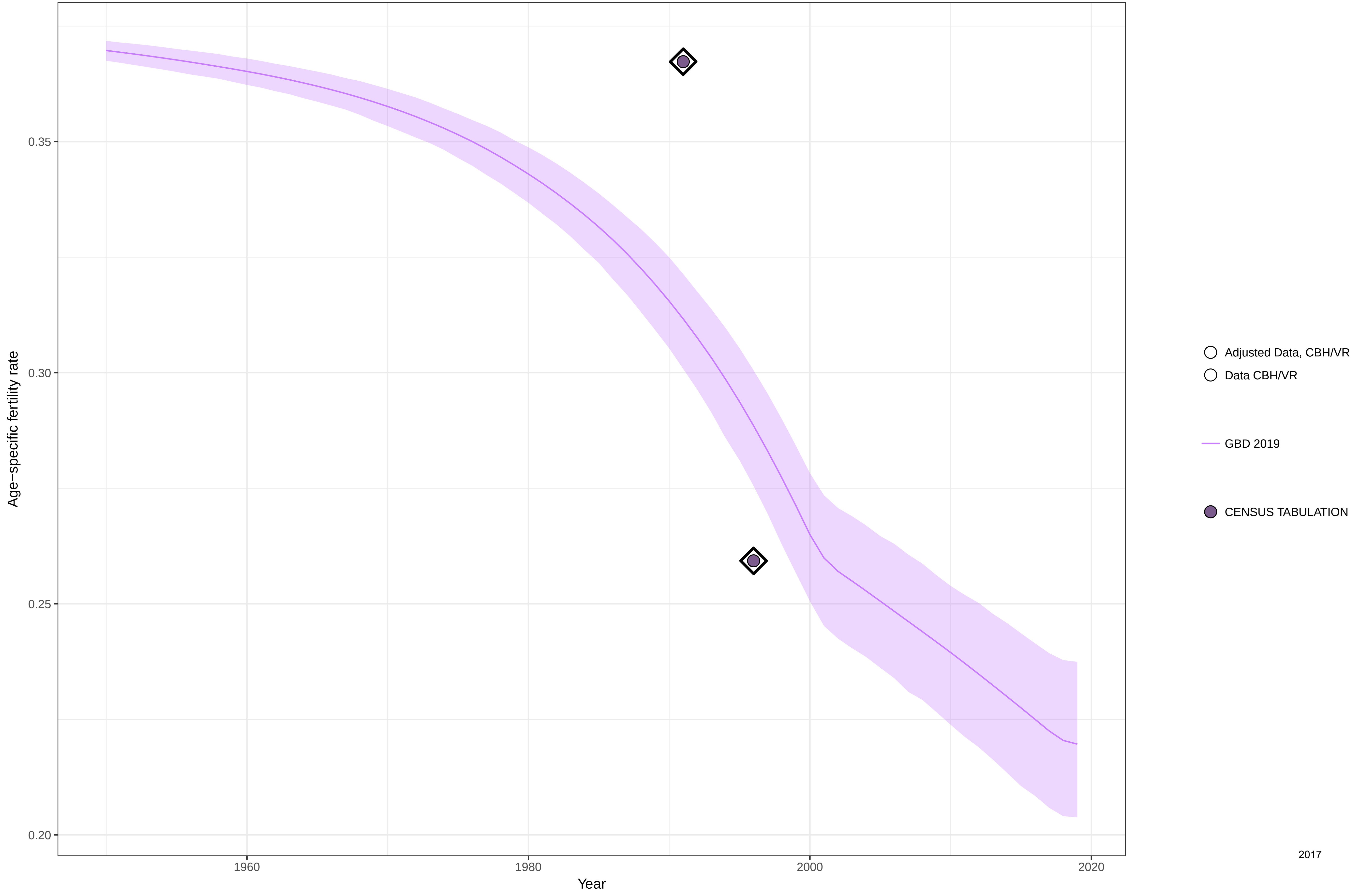

2017

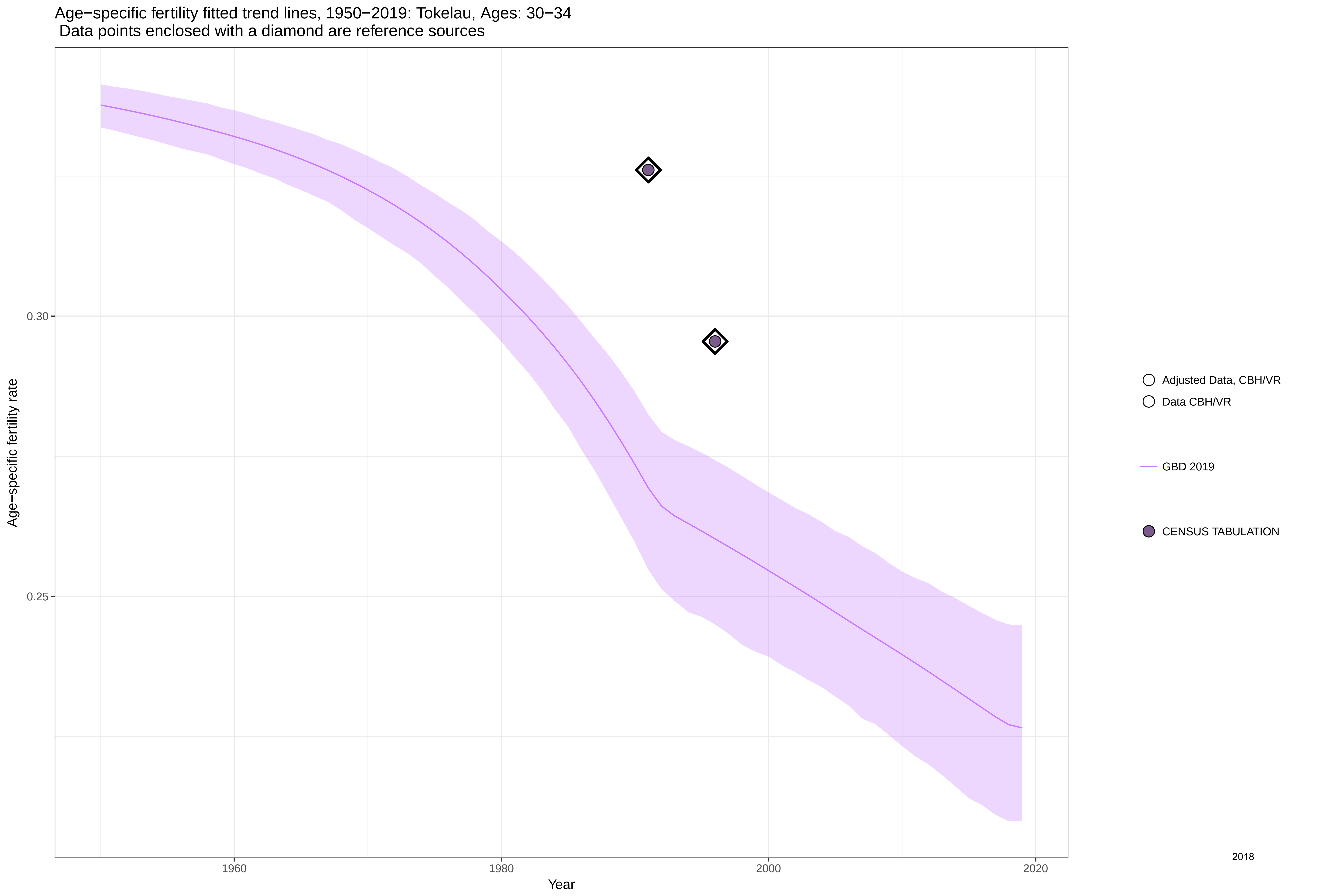
Age−specific fertility fitted trend lines, 1950−2019: Tokelau, Ages: 30−34
Data points enclosed with a diamond are reference sources
Age−specific fertility rate
0.30
0.25
1960
1980
2000
2020
Year
Adjusted Data, CBH/VR
Data CBH/VR
GBD 2019
CENSUS TABULATION
2018

Age−specific fertility fitted trend lines, 1950−2019: Tokelau, Ages: 35−39
Data points enclosed with a diamond are reference sources

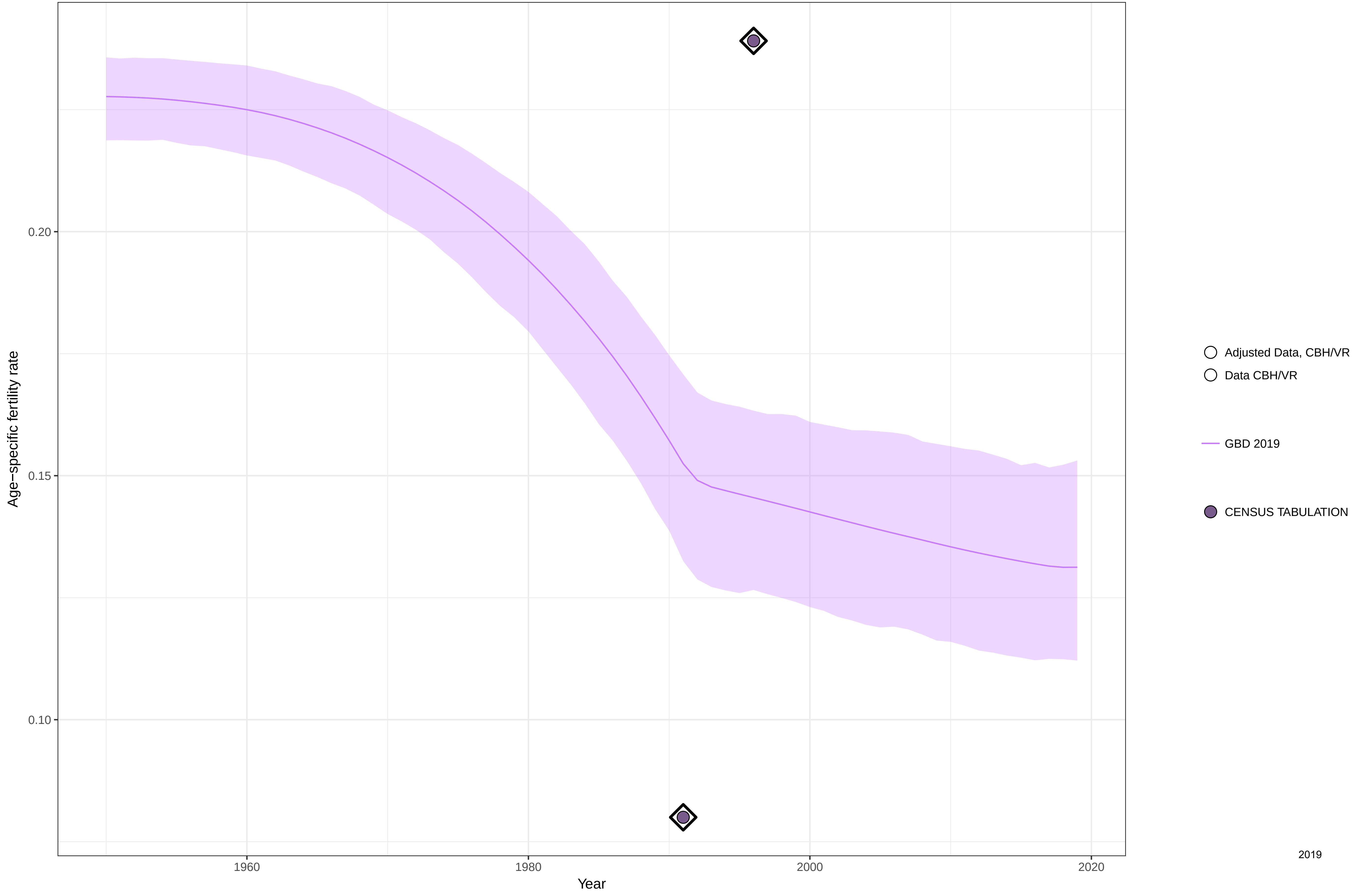

Age−specific fertility fitted trend lines, 1950−2019: Tokelau, Ages: 40−44
Data points enclosed with a diamond are reference sources

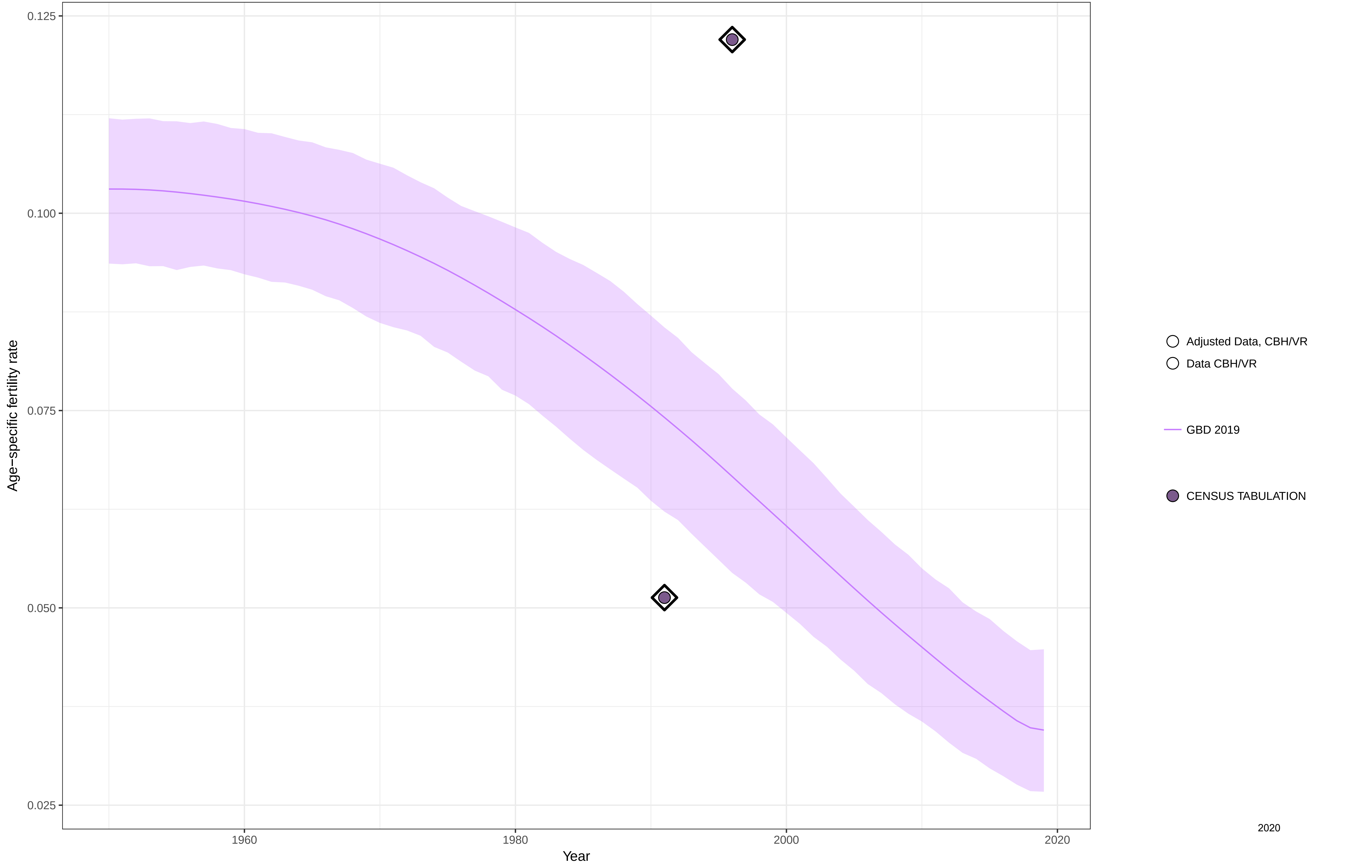

Age−specific fertility fitted trend lines, 1950−2019: Tokelau, Ages: 45−49
Data points enclosed with a diamond are reference sources

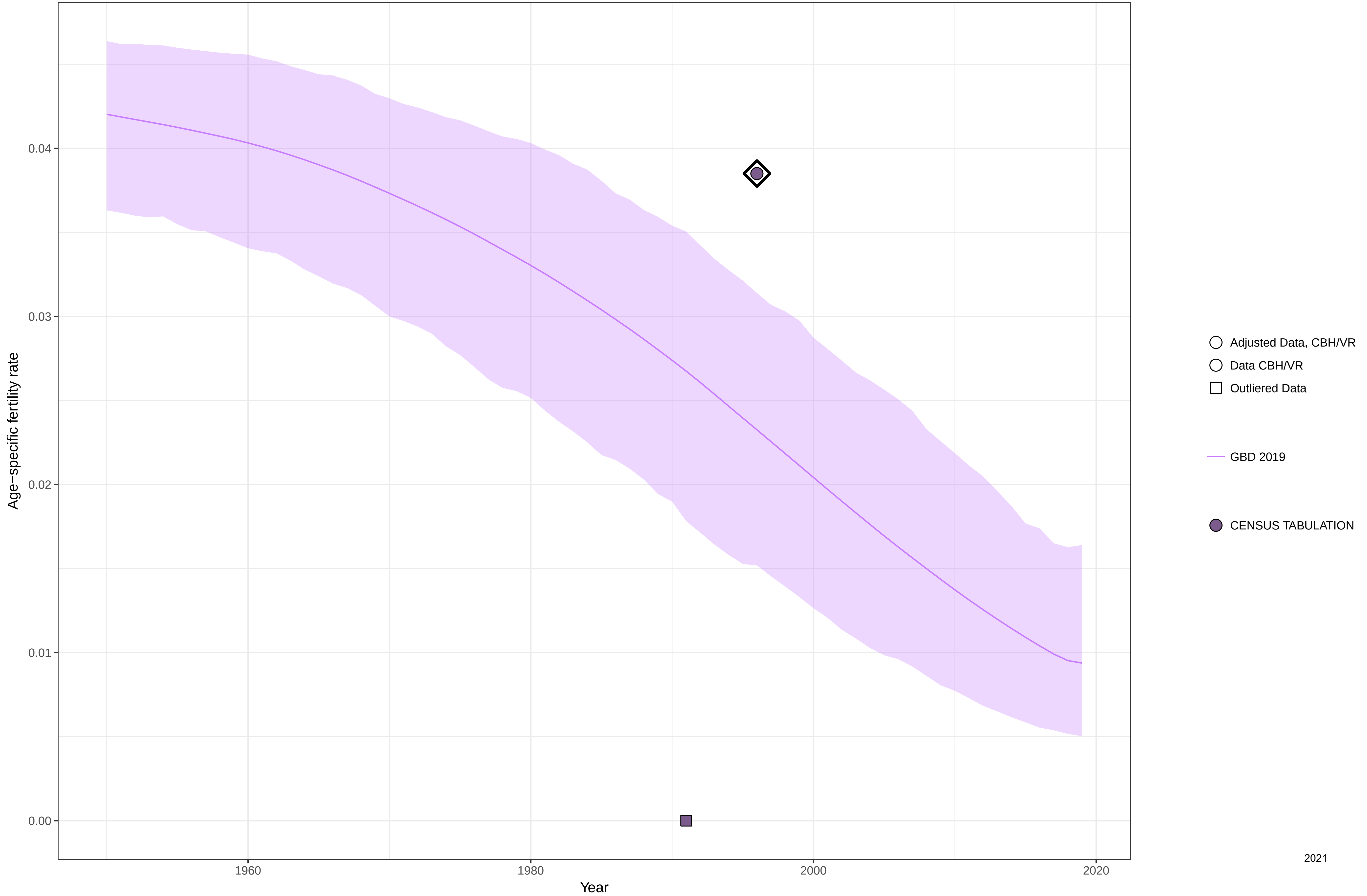

2021

Age−specific fertility fitted trend lines, 1950−2019: Tokelau, Ages: 50−54
Data points enclosed with a diamond are reference sources

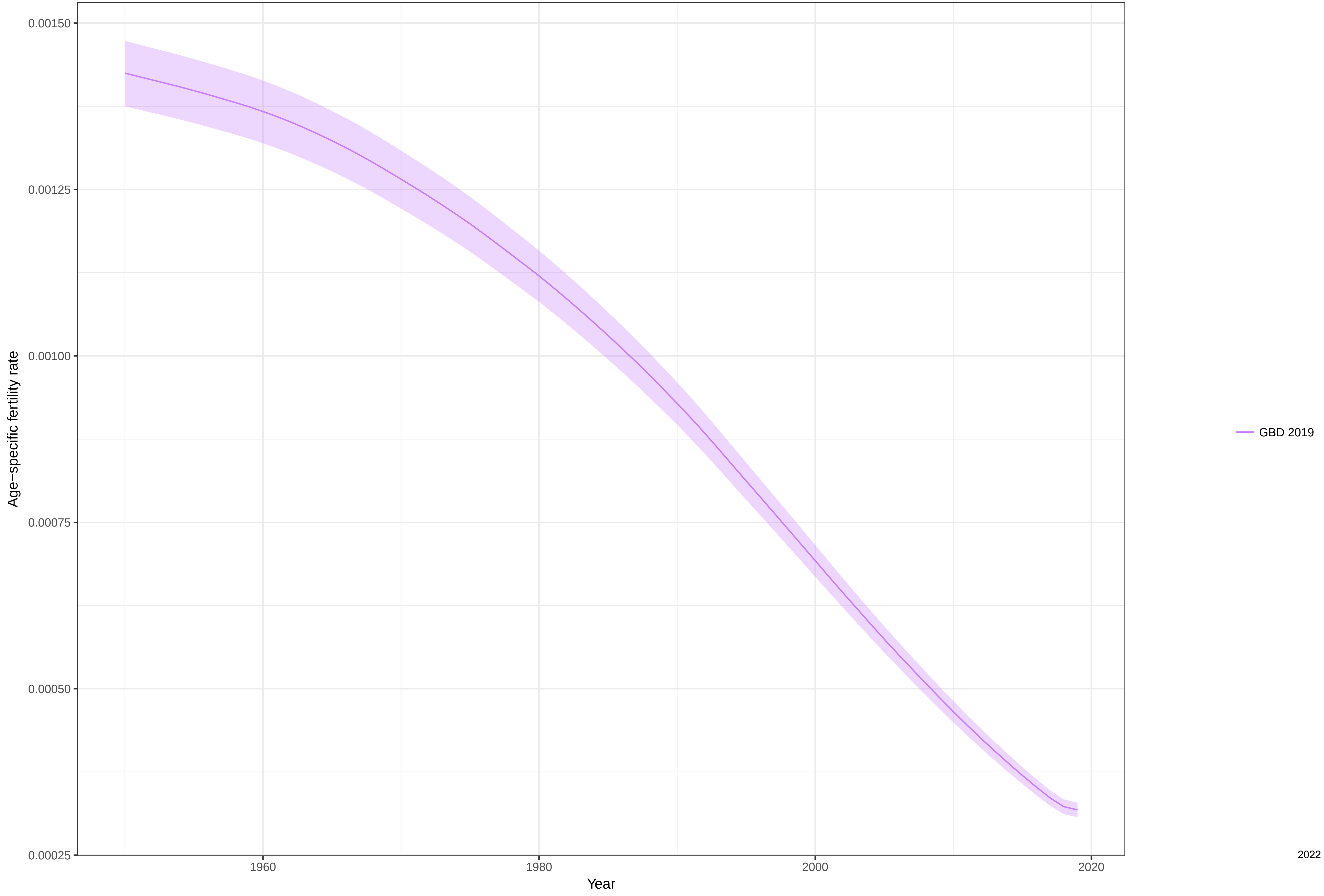

Total fertility rate fitted trend lines, 1950−2019: Tokelau TFR
Data points enclosed with a diamond are reference sources

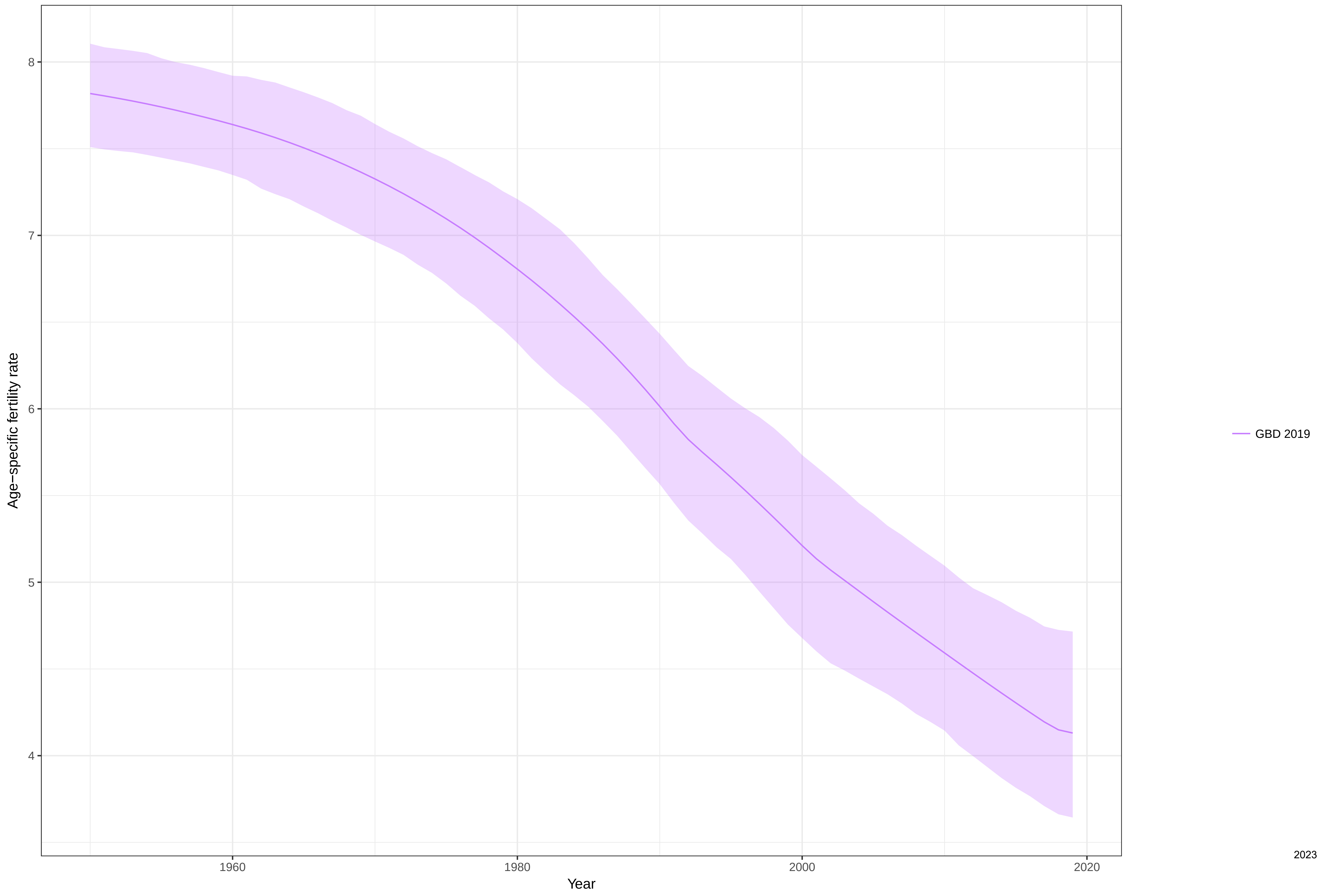

Age−specific fertility fitted trend lines, 1950−2019: Turkmenistan, Ages: 10−14
Data points enclosed with a diamond are reference sources

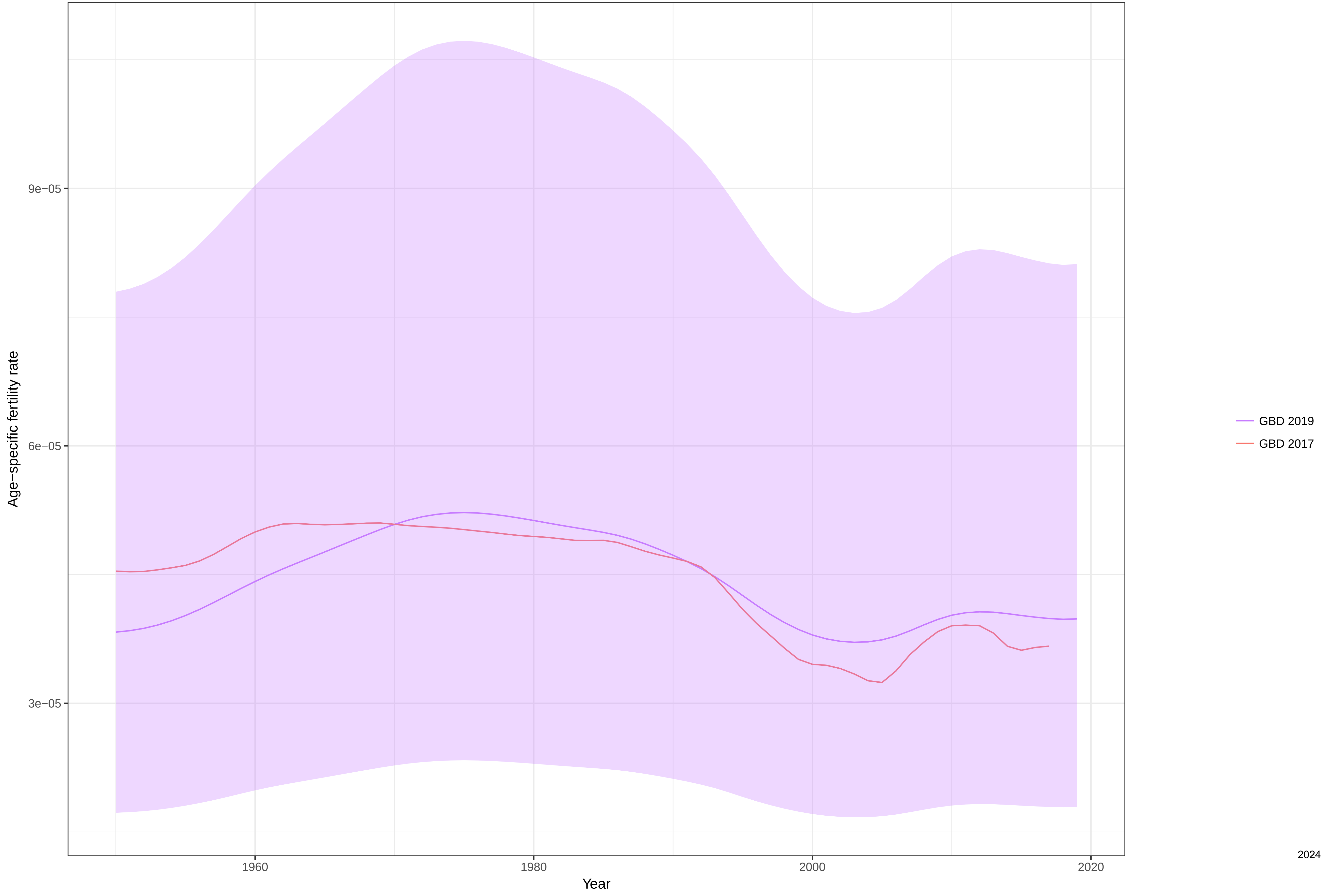

2024

Age−specific fertility fitted trend lines, 1950−2019: Turkmenistan, Ages: 15−19
Data points enclosed with a diamond are reference sources

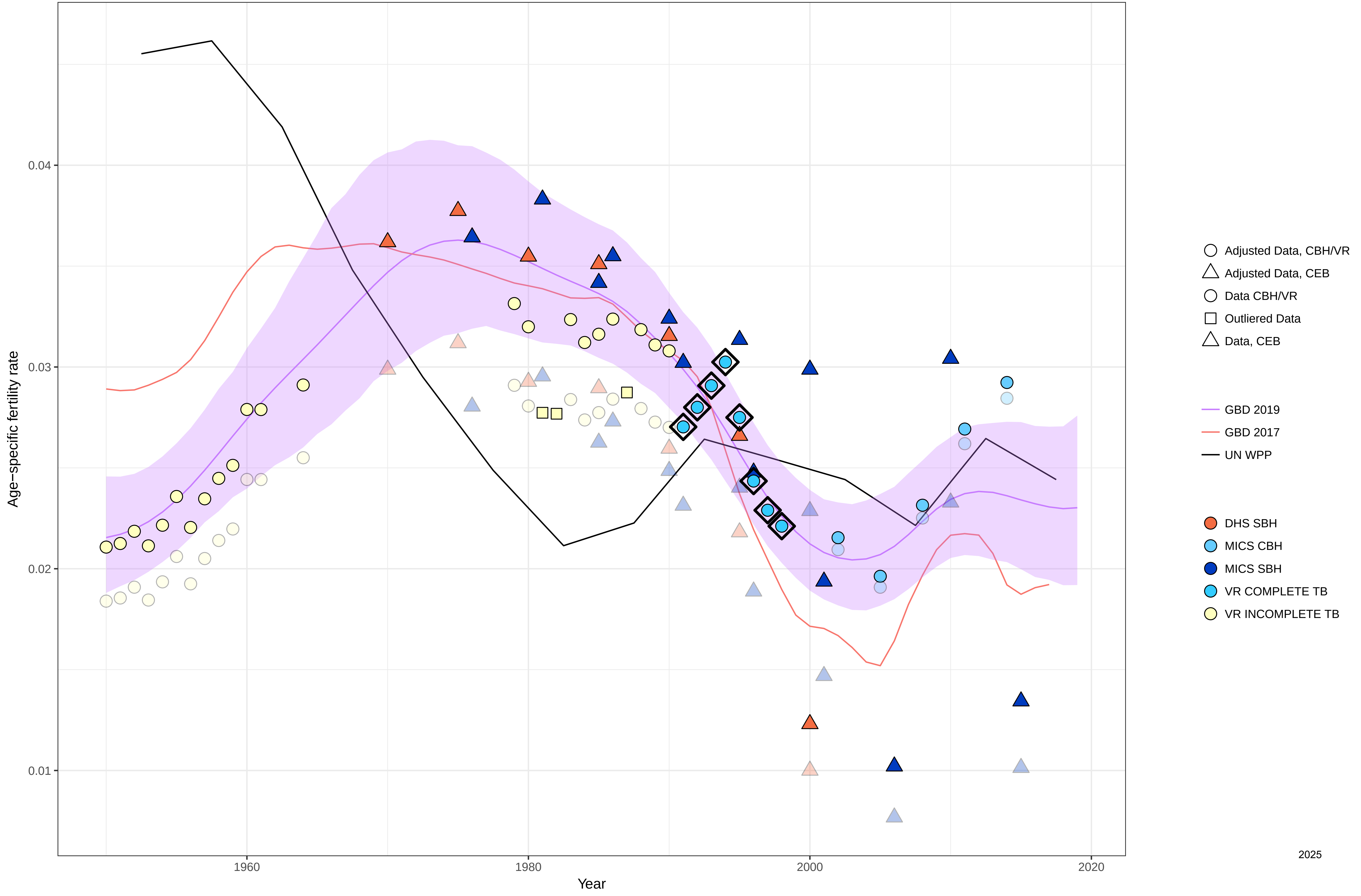

Age−specific fertility fitted trend lines, 1950−2019: Turkmenistan, Ages: 20−24
Data points enclosed with a diamond are reference sources

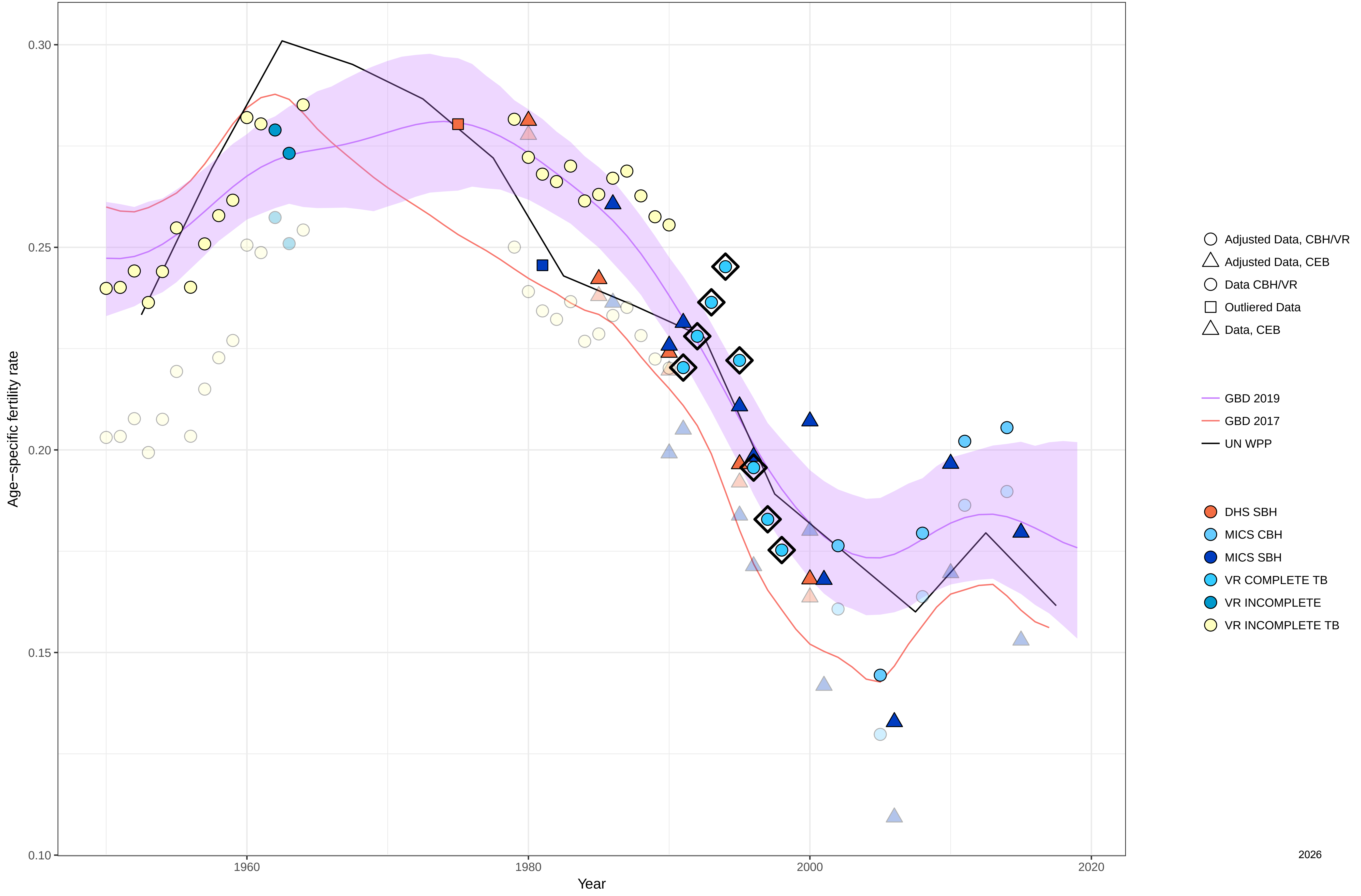

2026

Age−specific fertility fitted trend lines, 1950−2019: Turkmenistan, Ages: 25−29
Data points enclosed with a diamond are reference sources

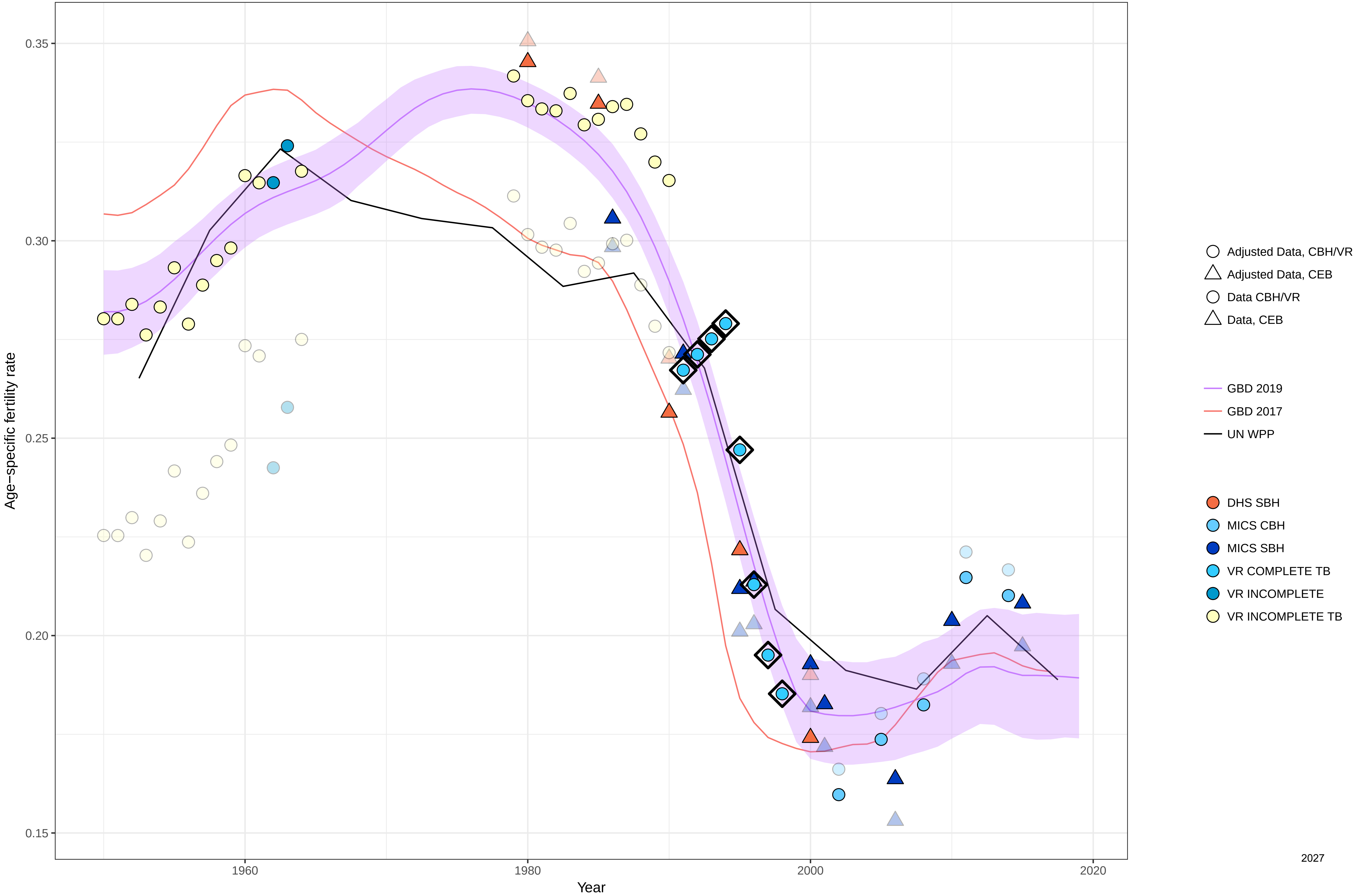

Age−specific fertility fitted trend lines, 1950−2019: Turkmenistan, Ages: 30−34
Data points enclosed with a diamond are reference sources

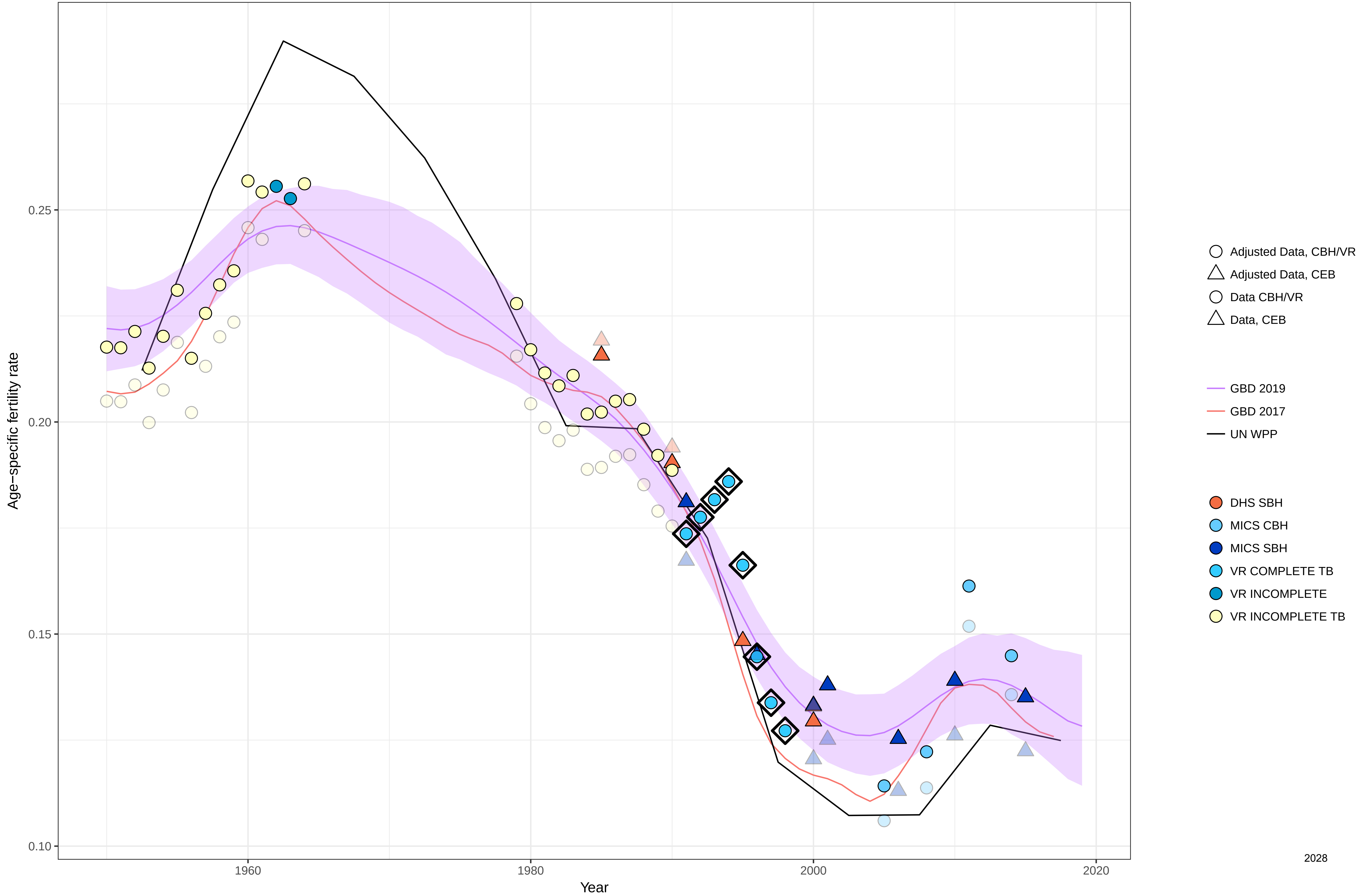

Age−specific fertility fitted trend lines, 1950−2019: Turkmenistan, Ages: 35−39
Data points enclosed with a diamond are reference sources

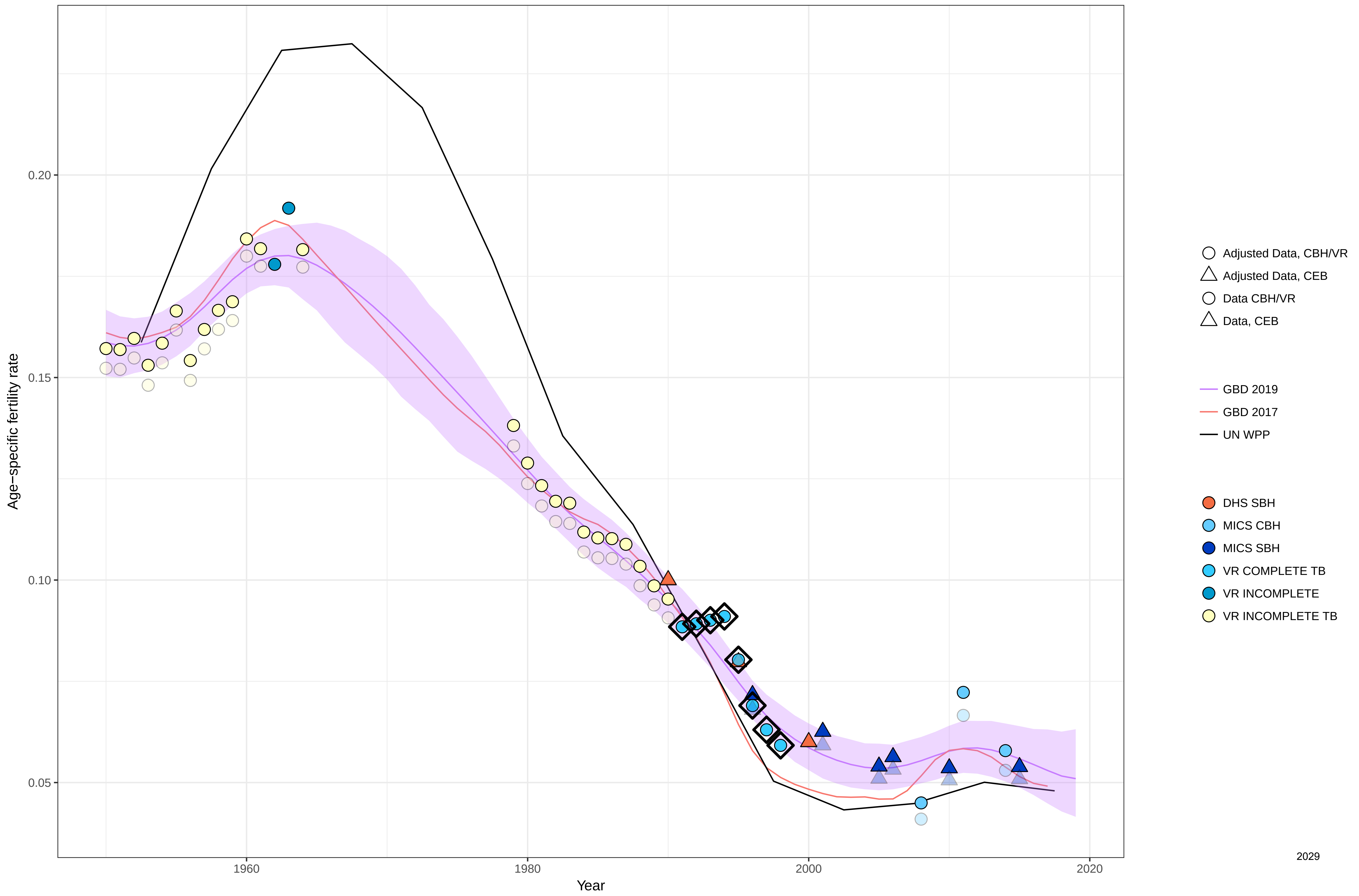

Age−specific fertility fitted trend lines, 1950−2019: Turkmenistan, Ages: 40−44
Data points enclosed with a diamond are reference sources

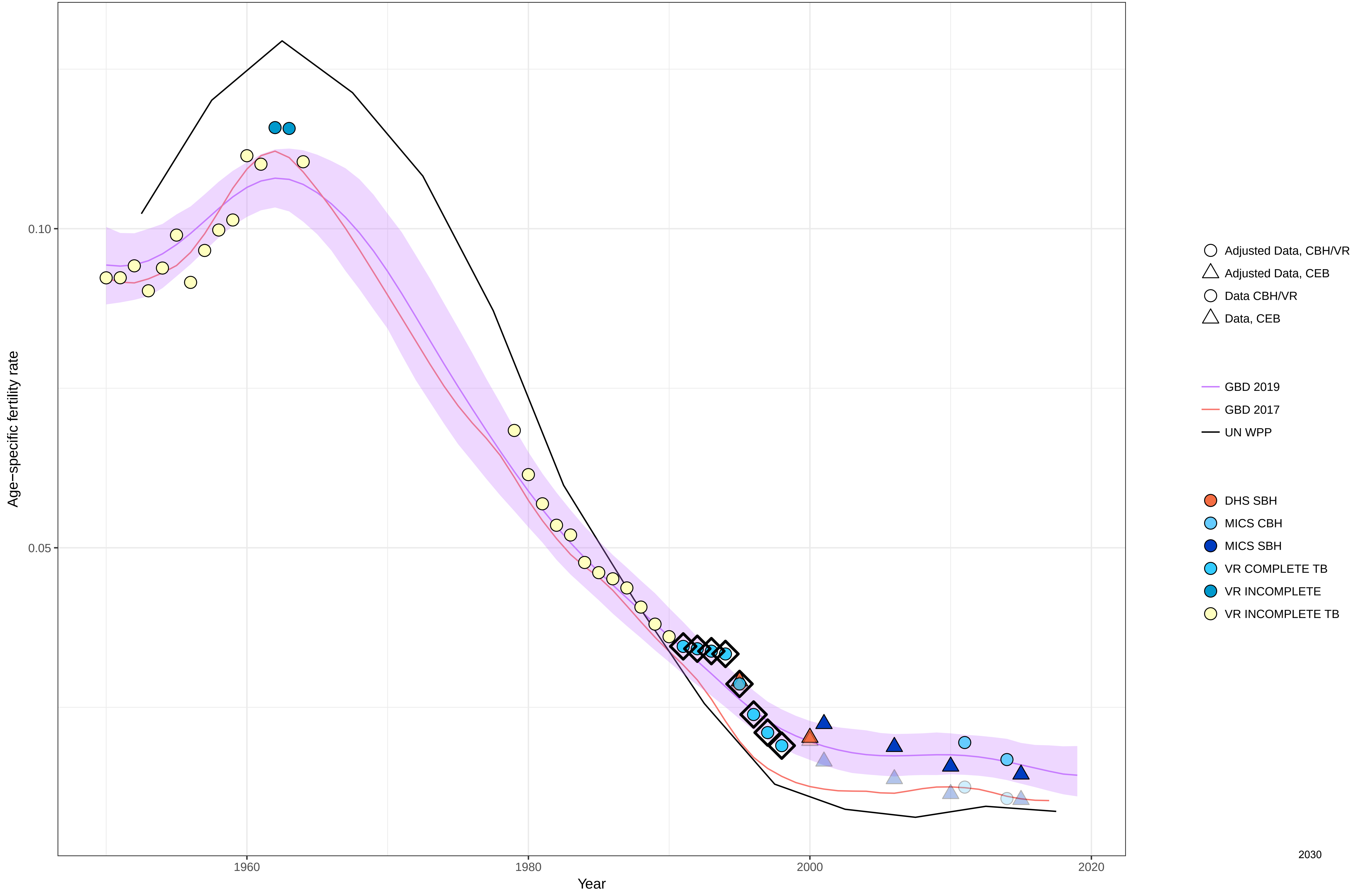

Age−specific fertility fitted trend lines, 1950−2019: Turkmenistan, Ages: 45−49
Data points enclosed with a diamond are reference sources

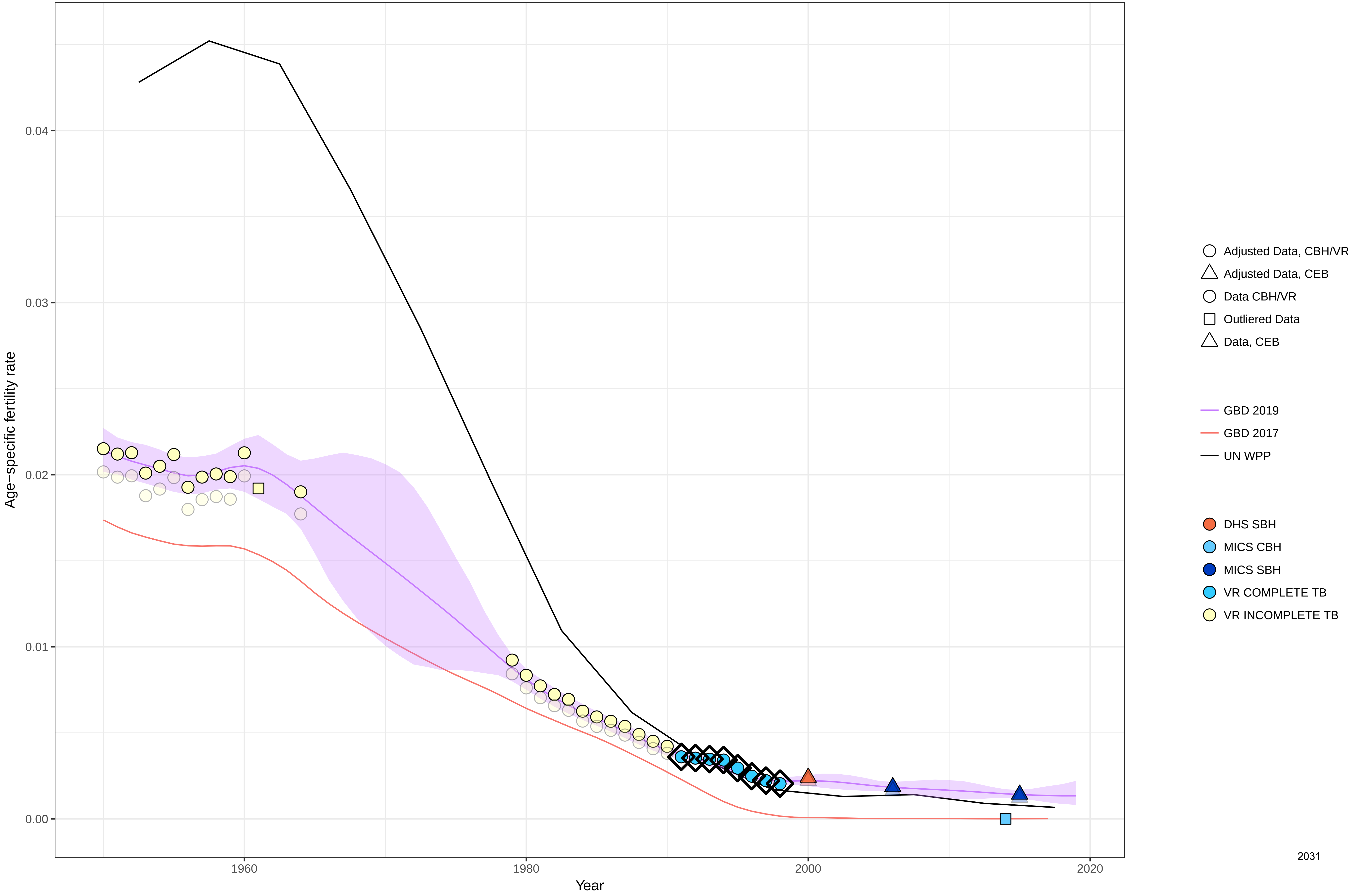

Age−specific fertility fitted trend lines, 1950−2019: Turkmenistan, Ages: 50−54
Data points enclosed with a diamond are reference sources

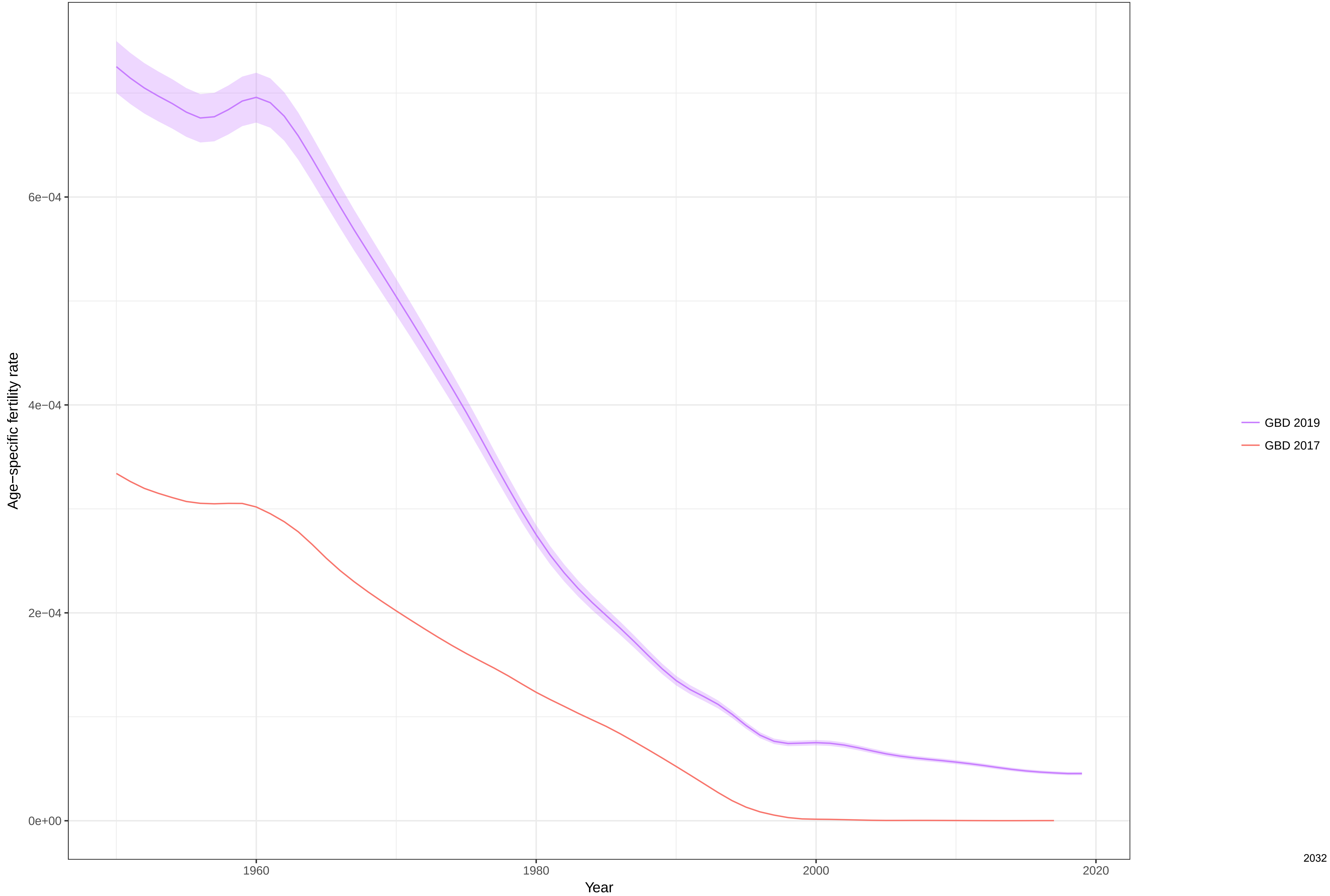

Total fertility rate fitted trend lines, 1950−2019: Turkmenistan TFR
Data points enclosed with a diamond are reference sources

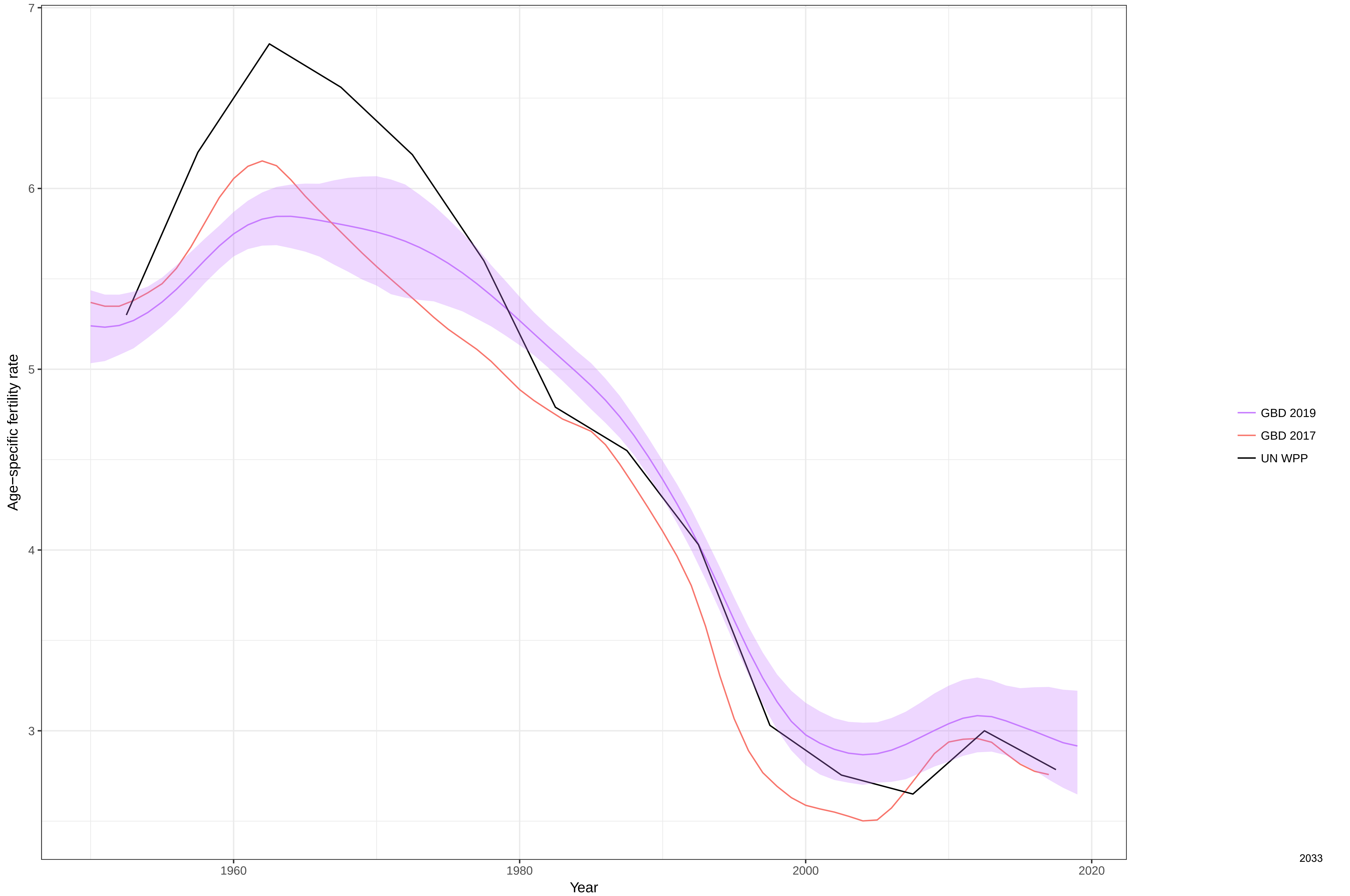

Age−specific fertility fitted trend lines, 1950−2019: Timor−Leste, Ages: 10−14
Data points enclosed with a diamond are reference sources

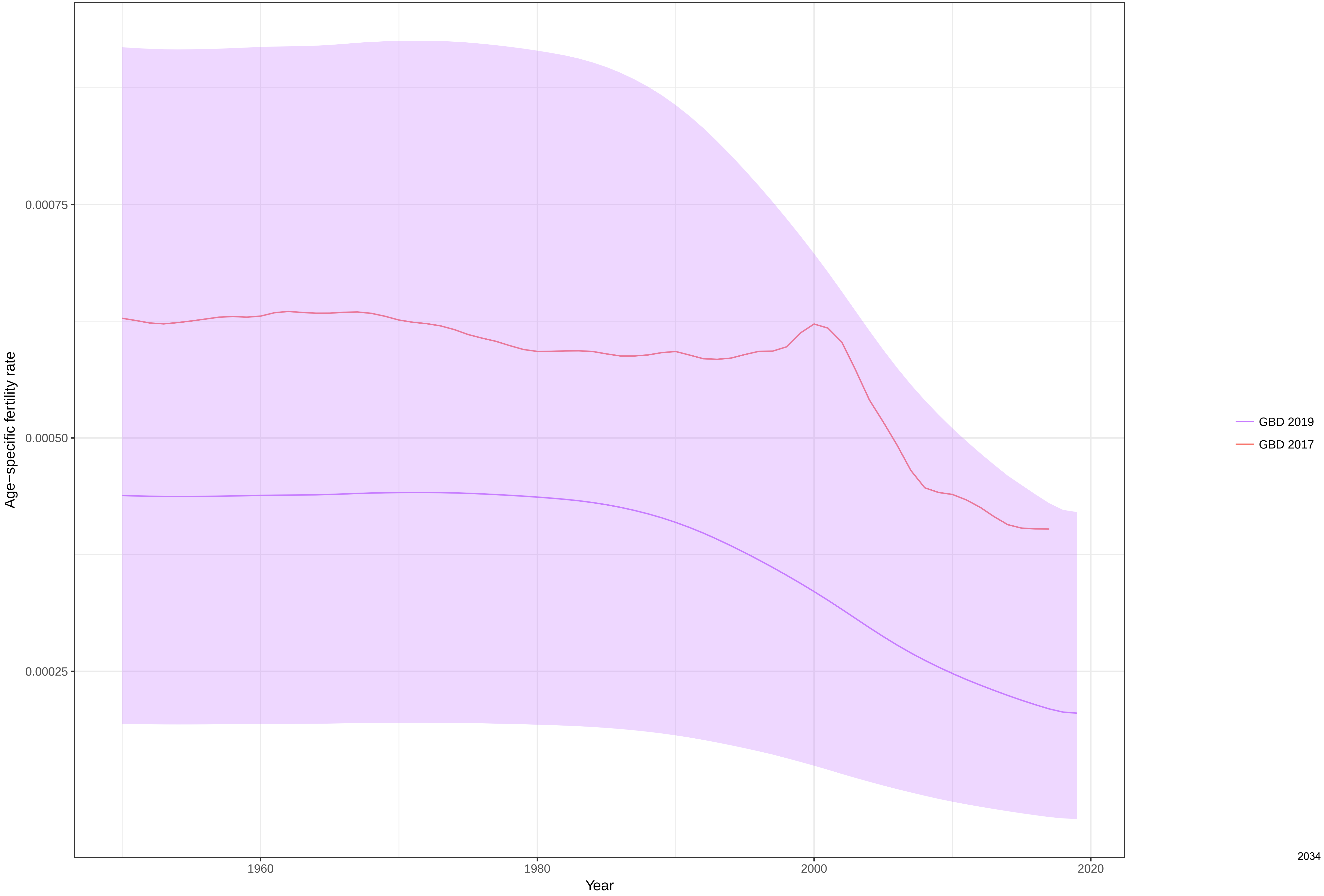

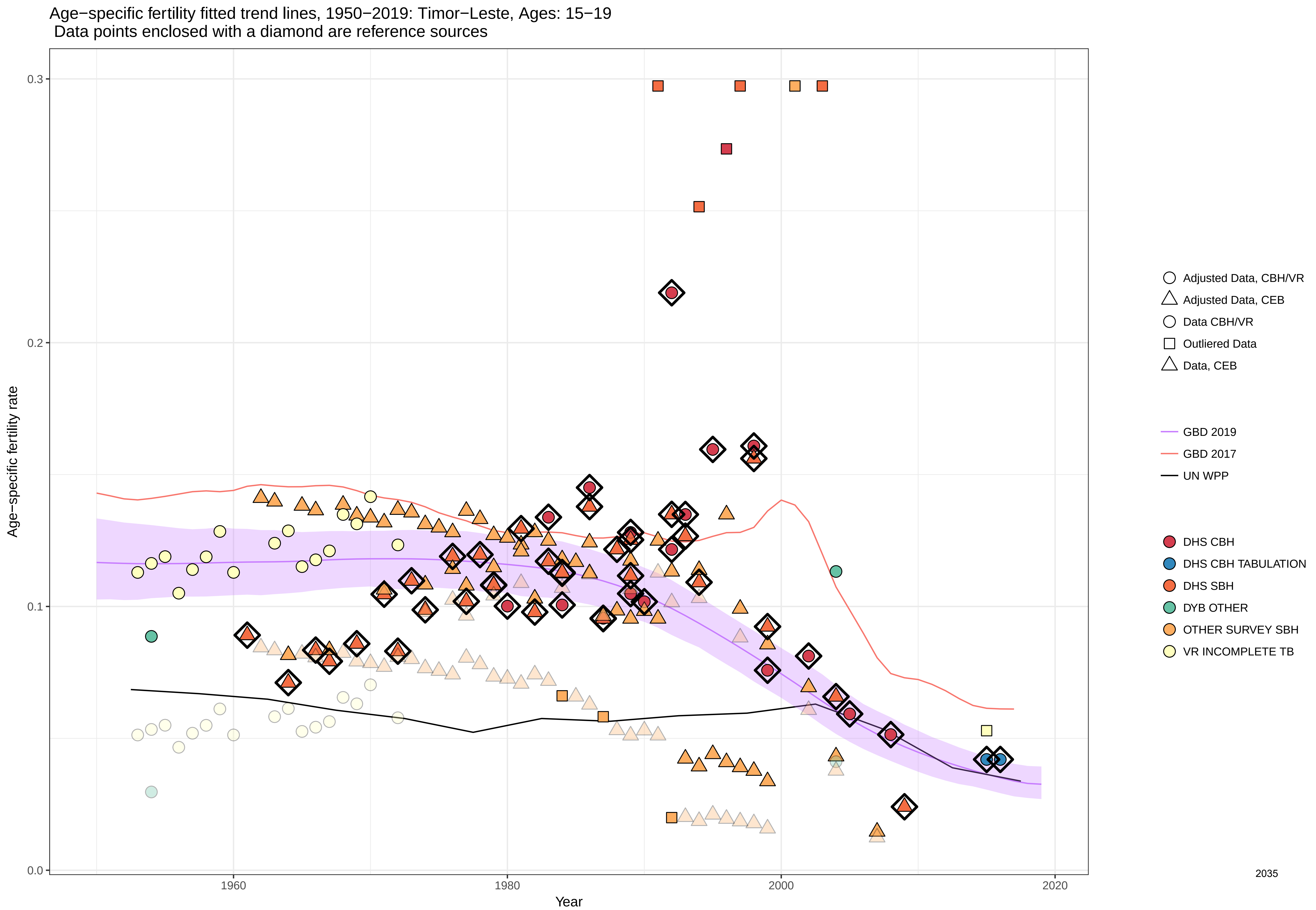

Age−specific fertility fitted trend lines, 1950−2019: Timor−Leste, Ages: 15−19
Data points enclosed with a diamond are reference sources
Age−specific fertility rate
Year
0.0
0.1
0.2
0.3
1960
1980
2000
2020
Adjusted Data, CBH/VR
Adjusted Data, CEB
Data CBH/VR
Outliered Data
Data, CEB
GBD 2019
GBD 2017
UN WPP
DHS CBH
DHS CBH TABULATION
DHS SBH
DYB OTHER
OTHER SURVEY SBH
VR INCOMPLETE TB

Age−specific fertility fitted trend lines, 1950−2019: Timor−Leste, Ages: 20−24
Data points enclosed with a diamond are reference sources

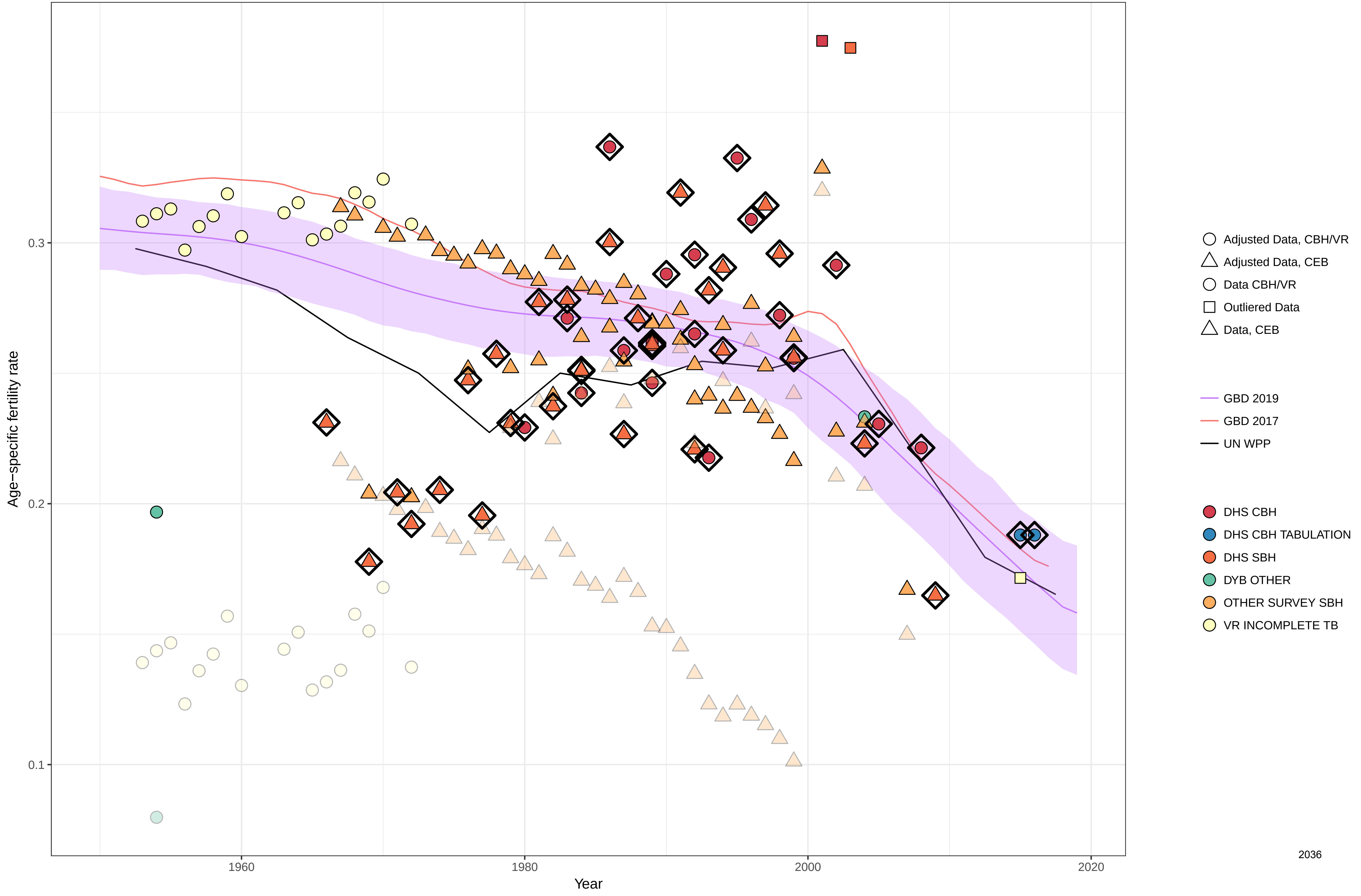

Age−specific fertility fitted trend lines, 1950−2019: Timor−Leste, Ages: 25−29
Data points enclosed with a diamond are reference sources

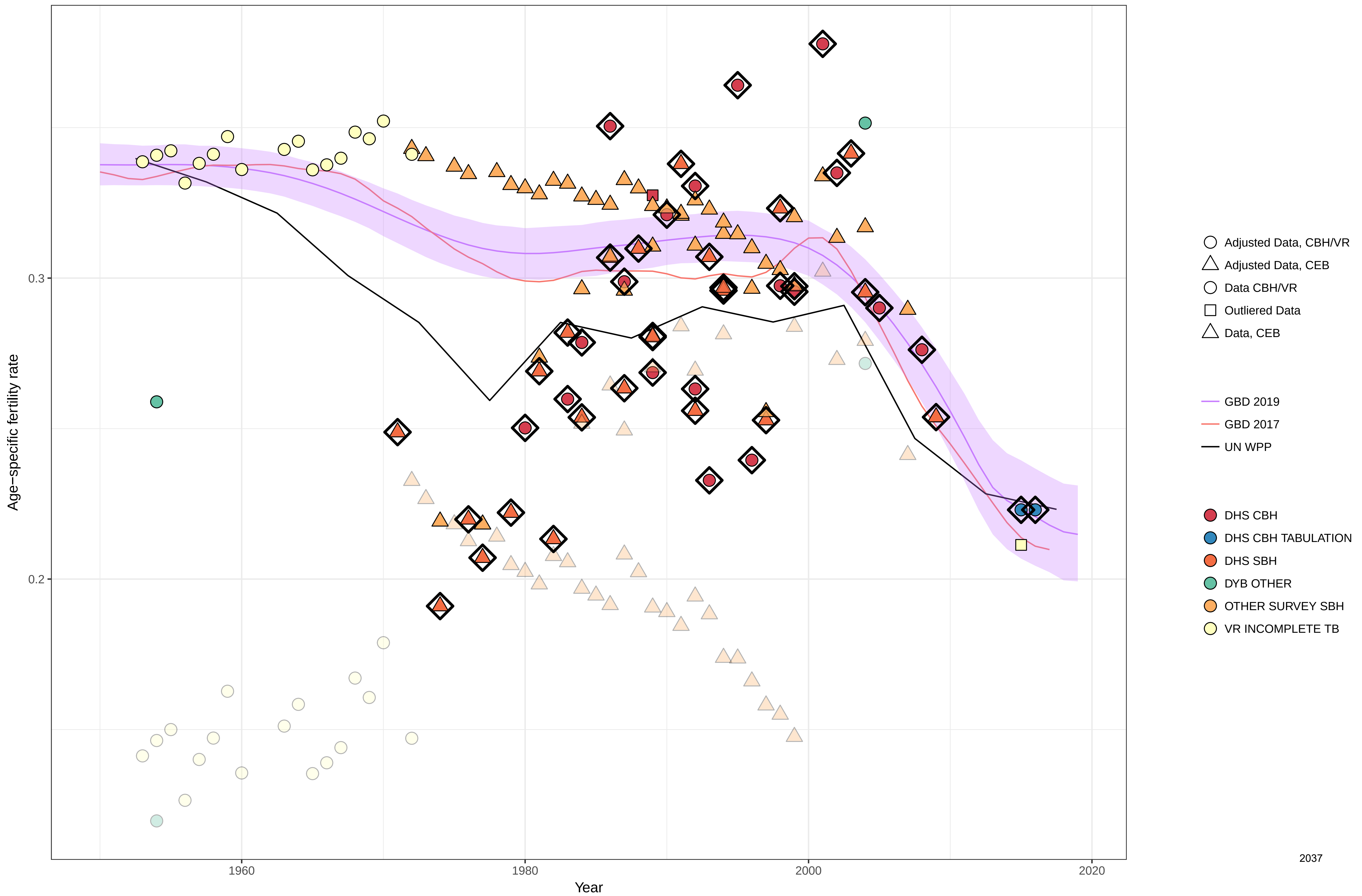

Age−specific fertility fitted trend lines, 1950−2019: Timor−Leste, Ages: 30−34
Data points enclosed with a diamond are reference sources

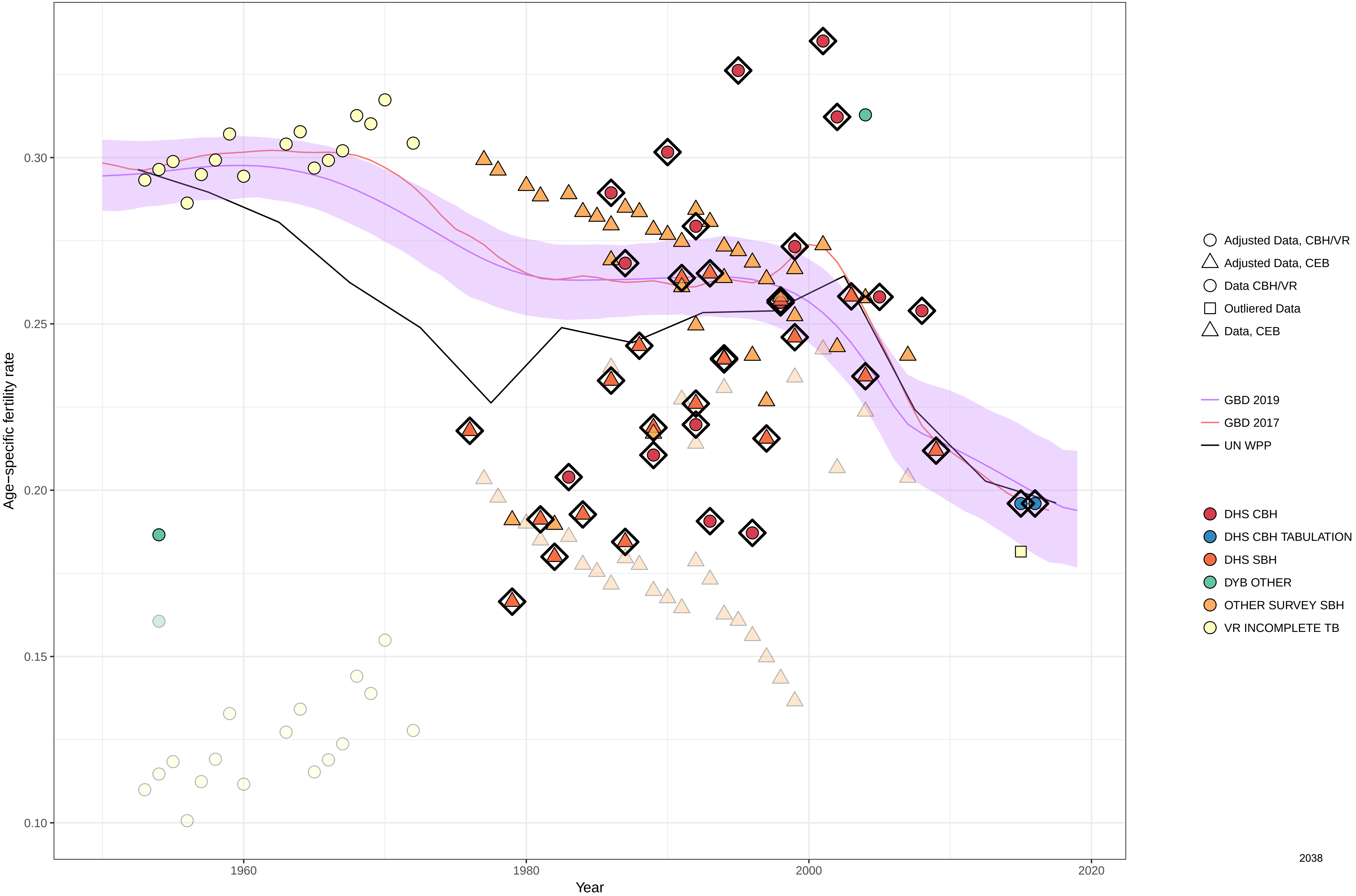

Age−specific fertility fitted trend lines, 1950−2019: Timor−Leste, Ages: 35−39
Data points enclosed with a diamond are reference sources

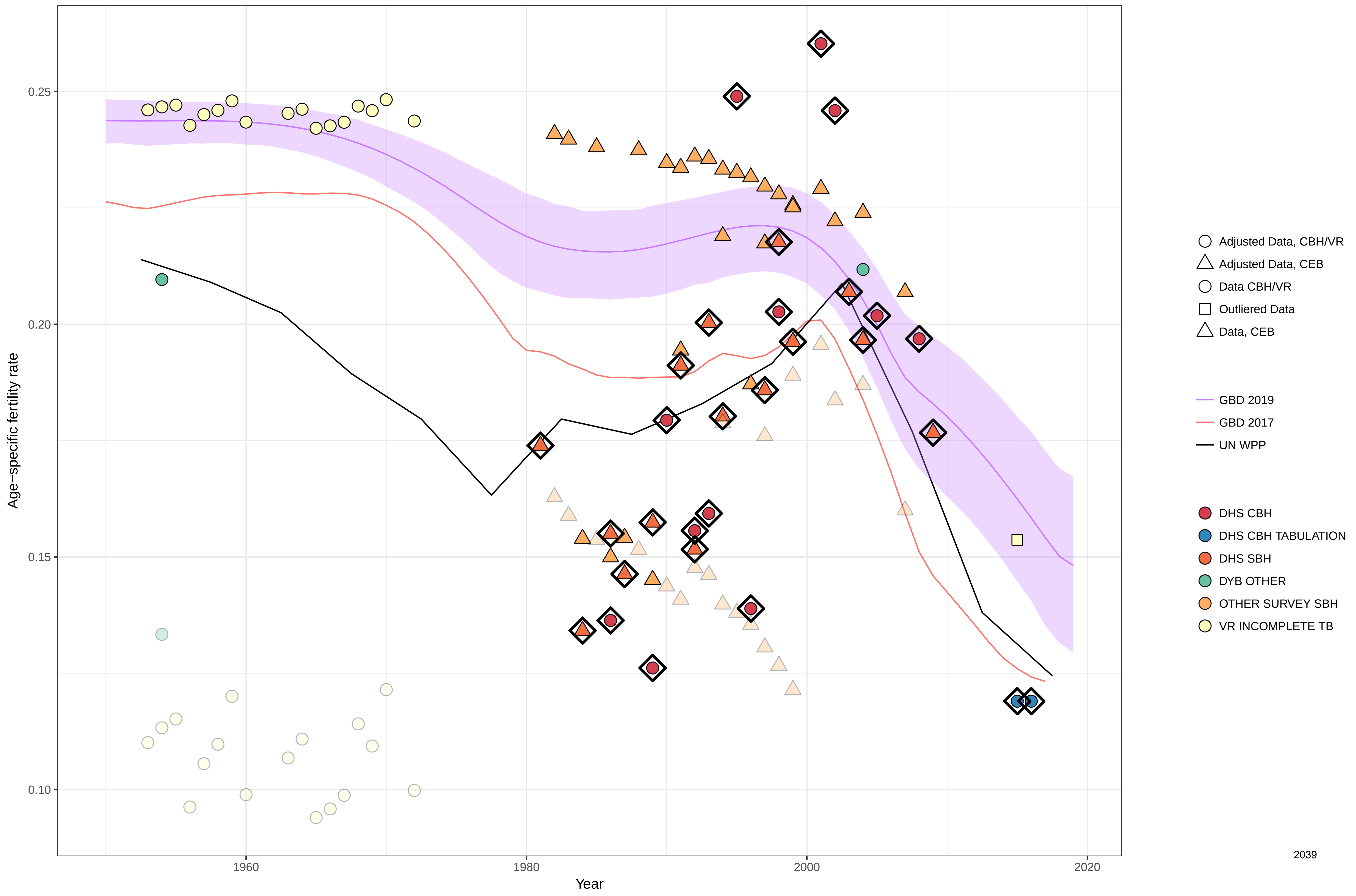

Age−specific fertility fitted trend lines, 1950−2019: Timor−Leste, Ages: 40−44
Data points enclosed with a diamond are reference sources

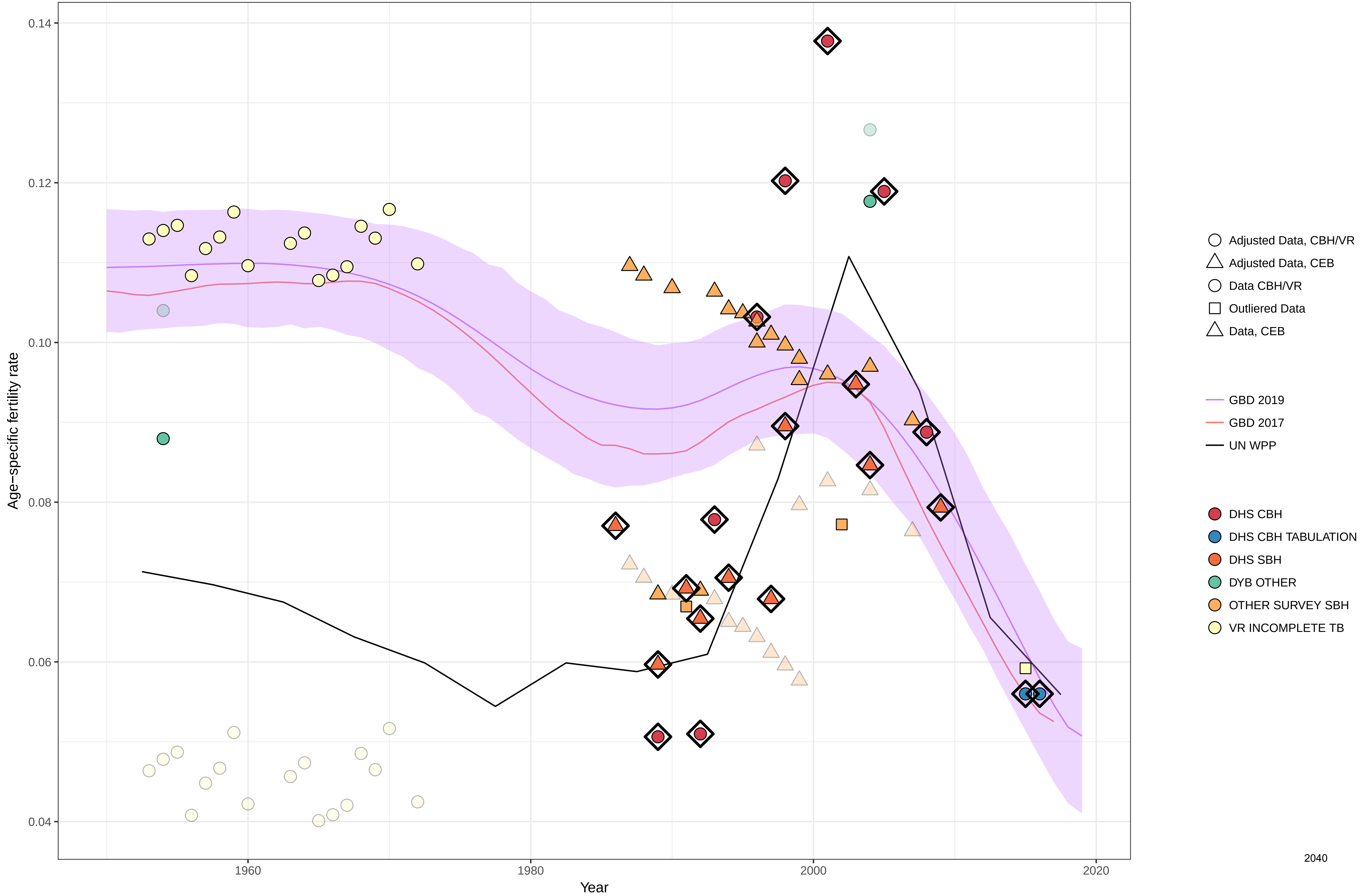

2040

Age−specific fertility fitted trend lines, 1950−2019: Timor−Leste, Ages: 45−49
Data points enclosed with a diamond are reference sources

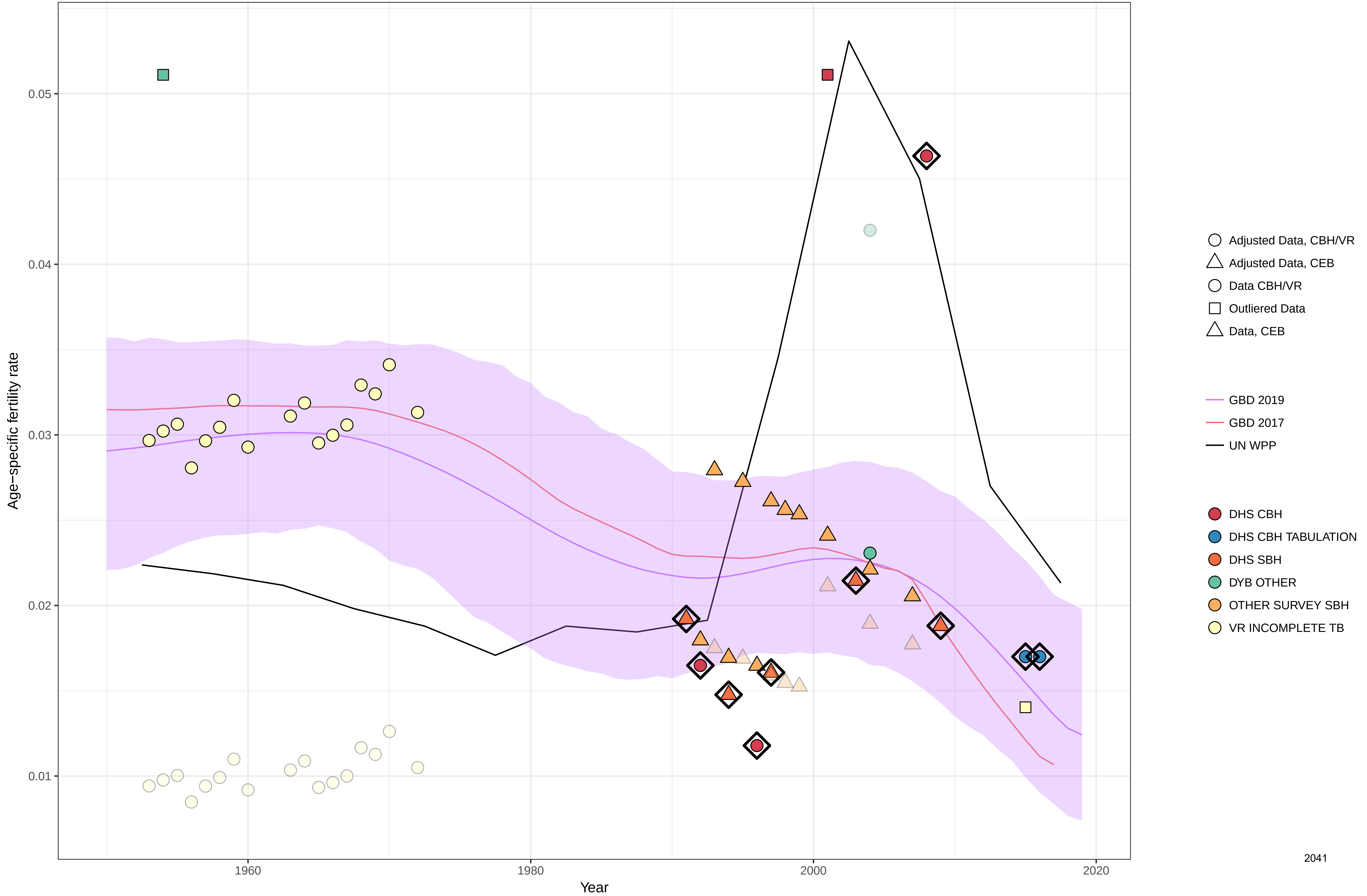

Age−specific fertility fitted trend lines, 1950−2019: Timor−Leste, Ages: 50−54
Data points enclosed with a diamond are reference sources

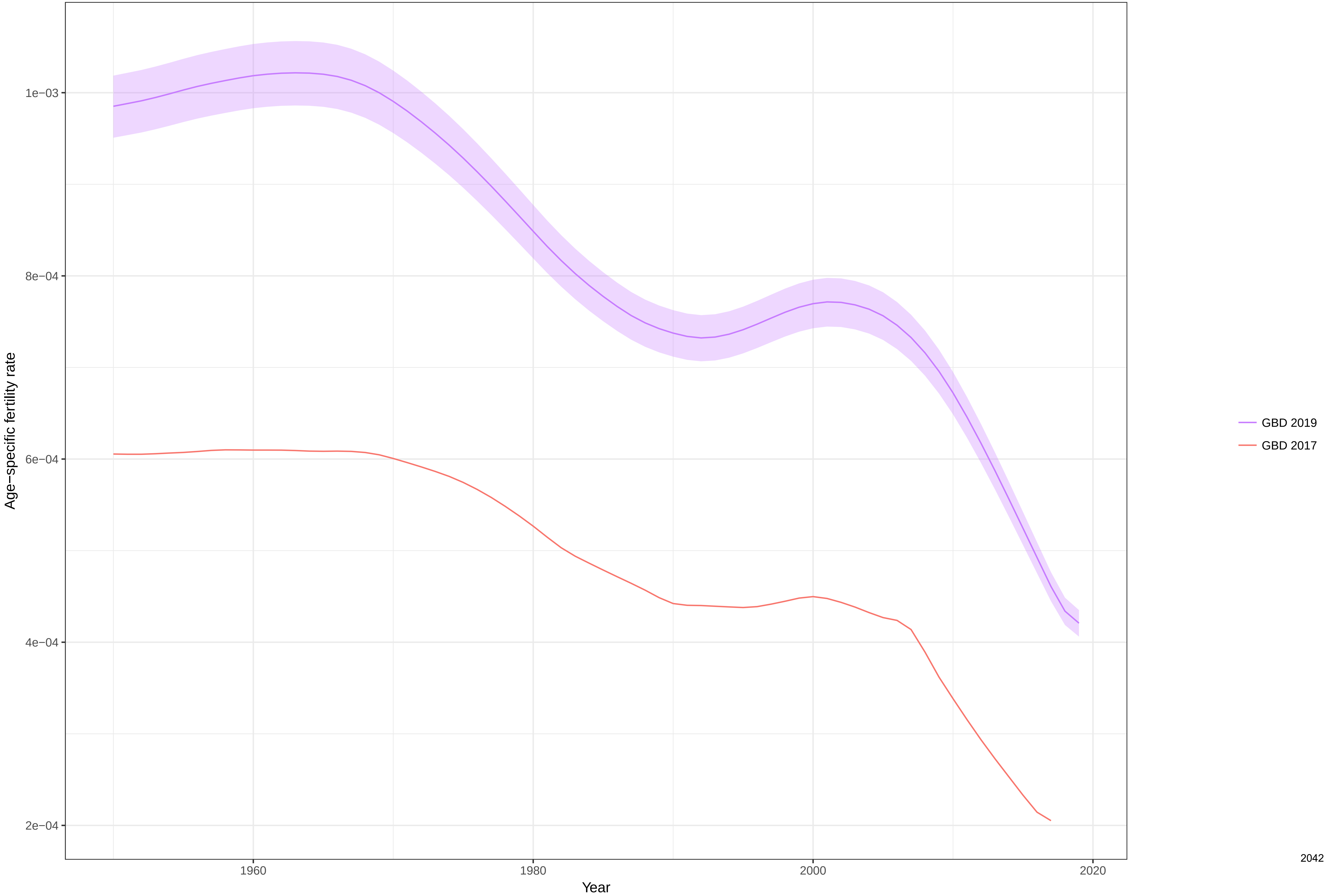

Total fertility rate fitted trend lines, 1950−2019: Timor−Leste TFR
Data points enclosed with a diamond are reference sources

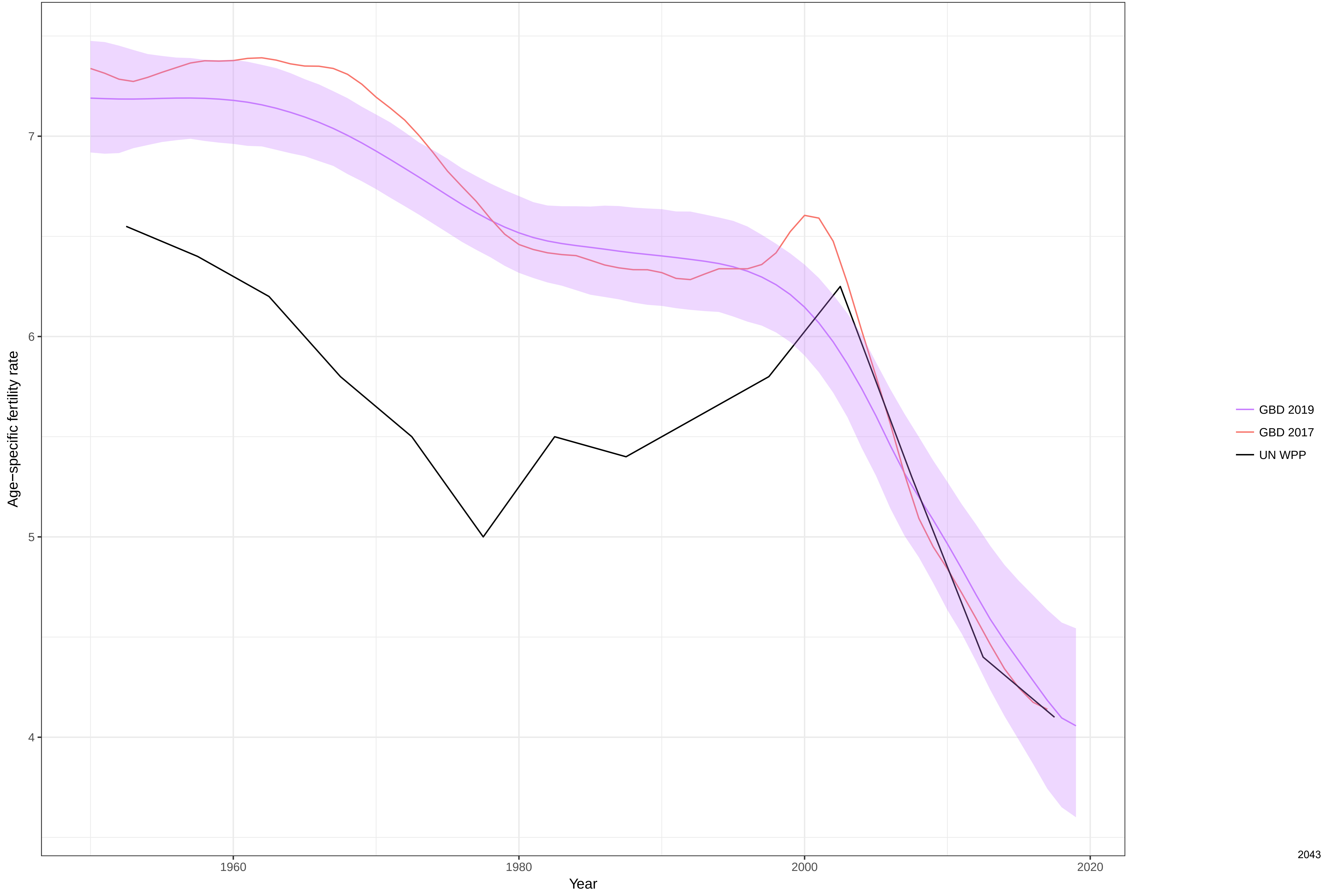

Age−specific fertility fitted trend lines, 1950−2019: Tonga, Ages: 10−14
Data points enclosed with a diamond are reference sources

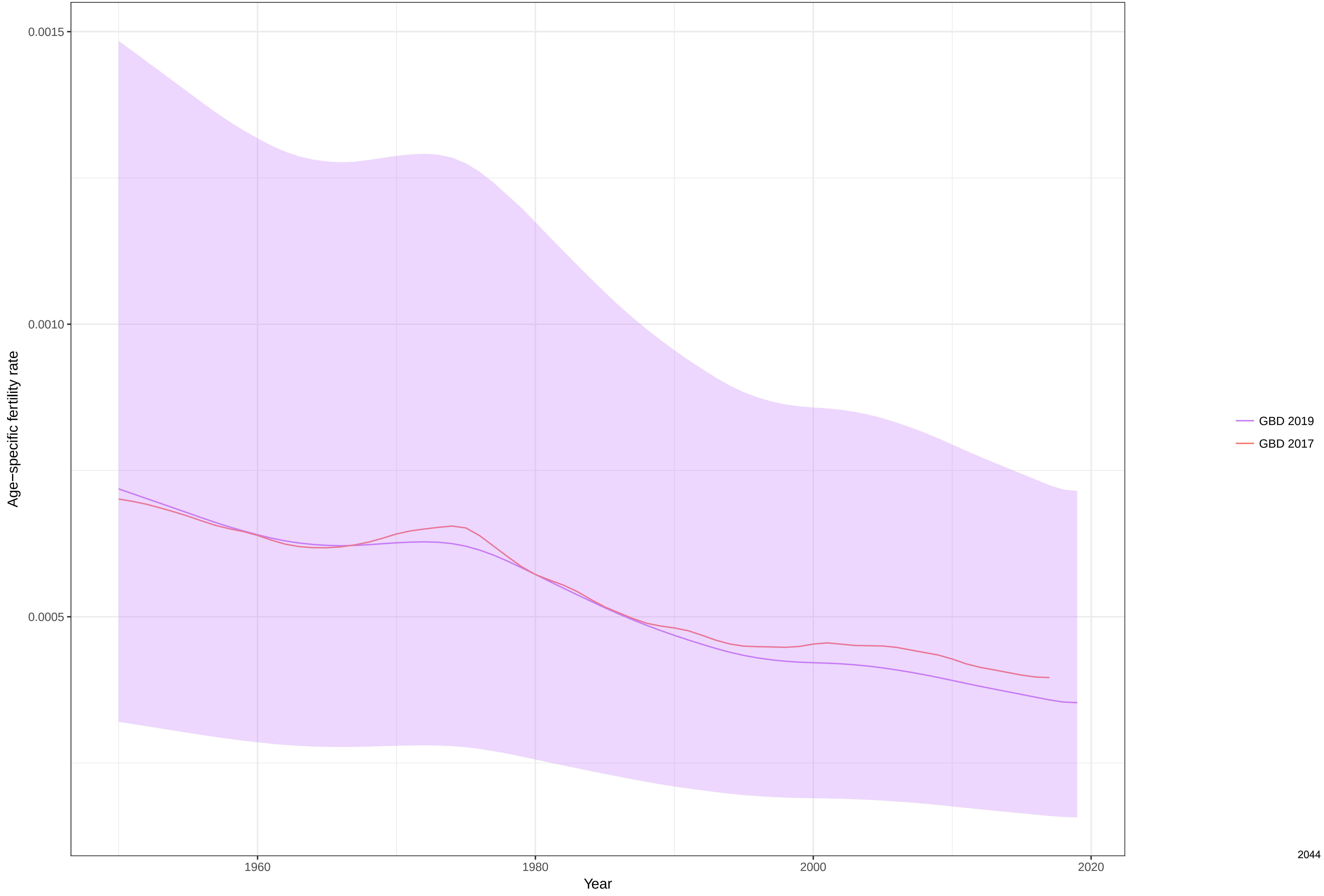

Age−specific fertility fitted trend lines, 1950−2019: Tonga, Ages: 15−19
Data points enclosed with a diamond are reference sources

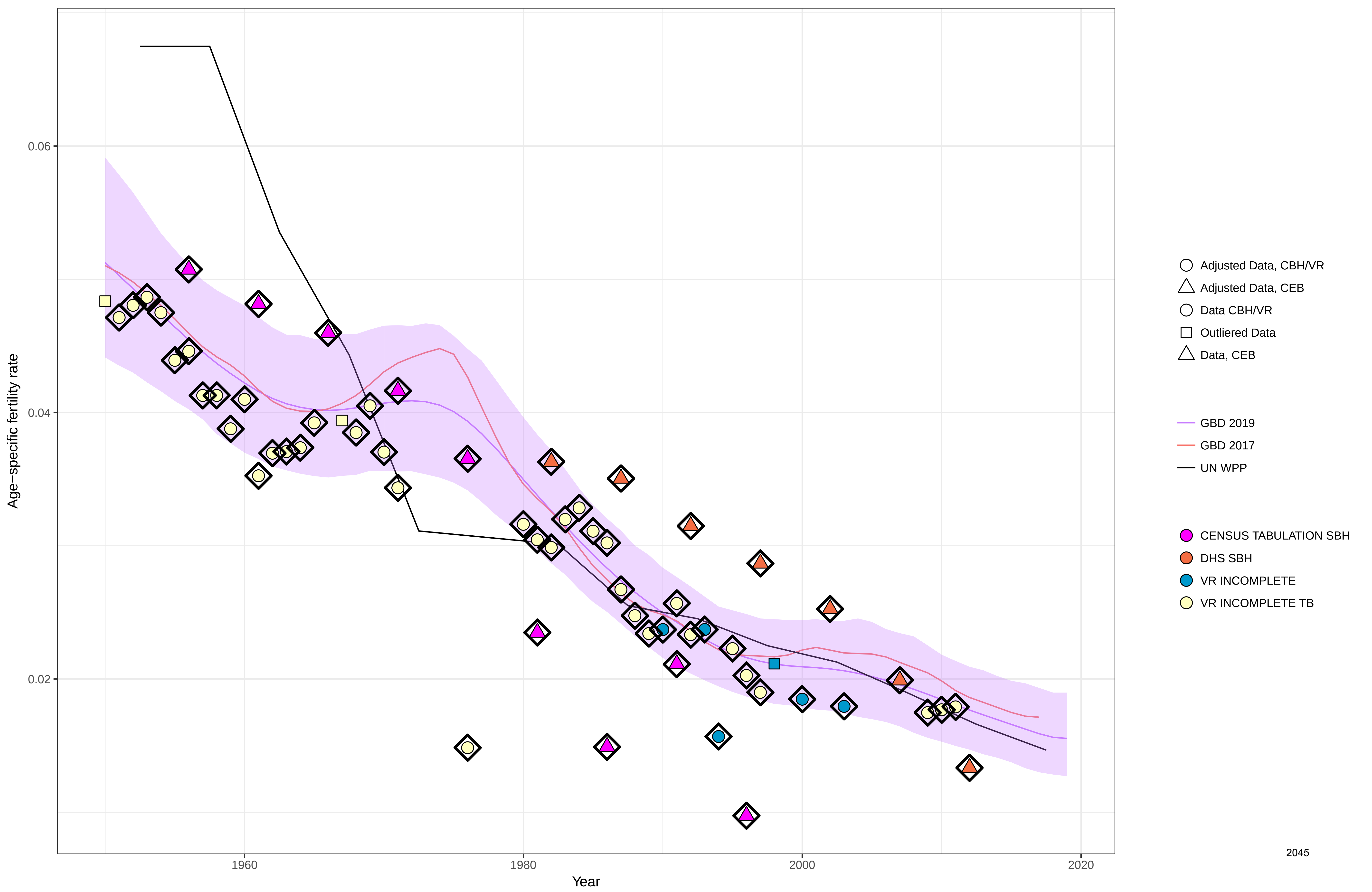

Age−specific fertility fitted trend lines, 1950−2019: Tonga, Ages: 20−24
Data points enclosed with a diamond are reference sources

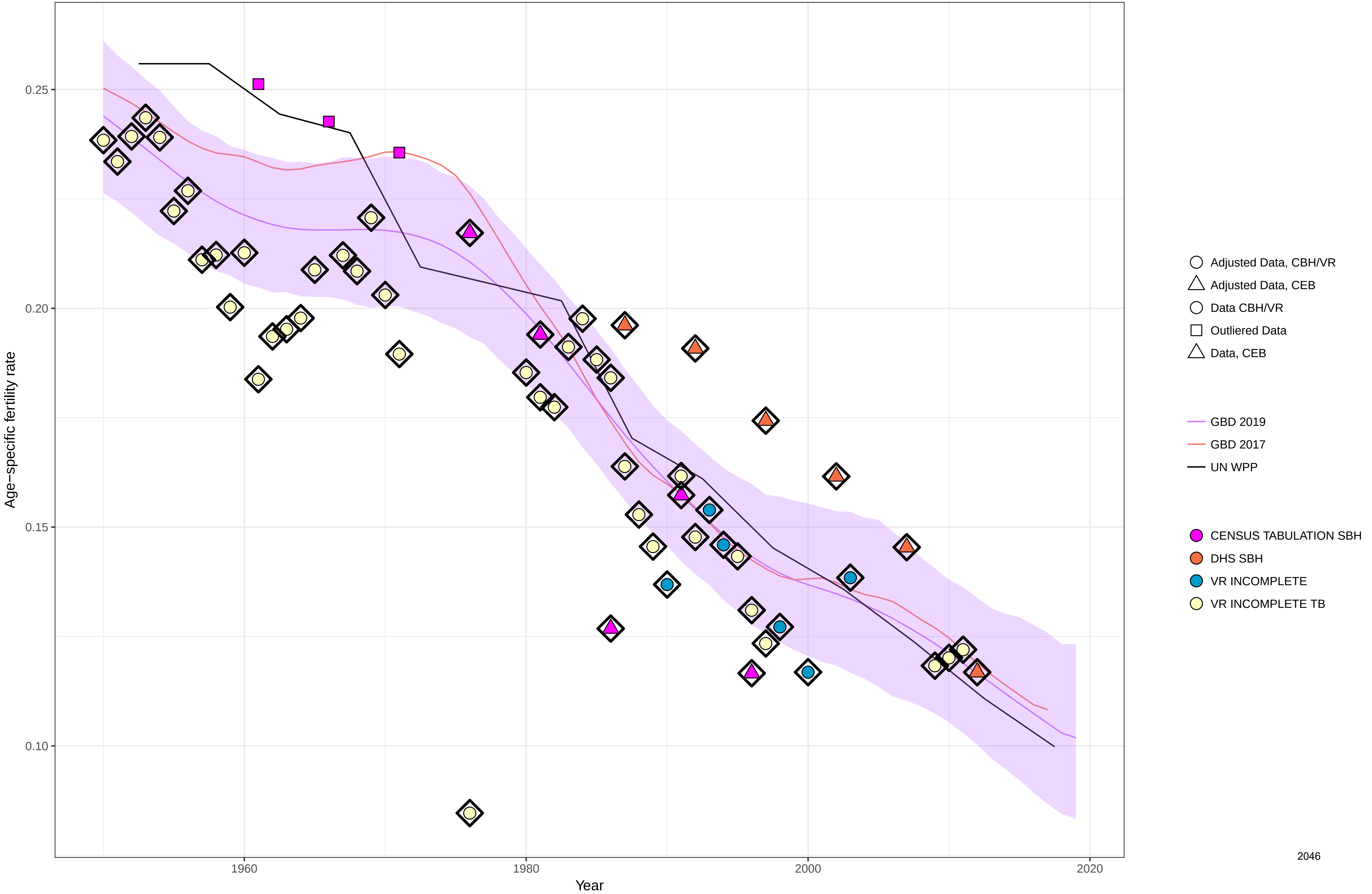

Age−specific fertility fitted trend lines, 1950−2019: Tonga, Ages: 25−29
Data points enclosed with a diamond are reference sources

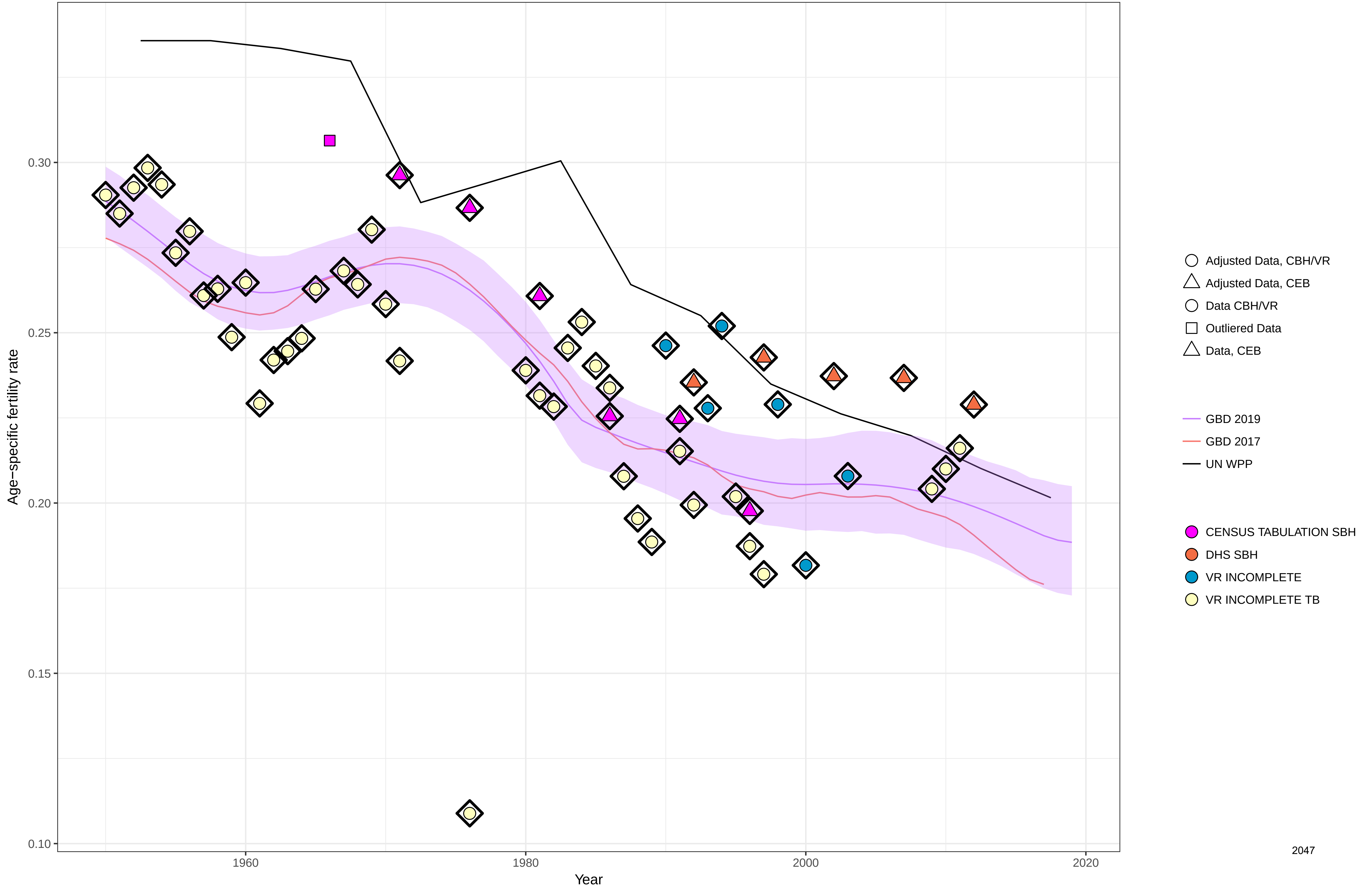

Age−specific fertility fitted trend lines, 1950−2019: Tonga, Ages: 30−34
Data points enclosed with a diamond are reference sources

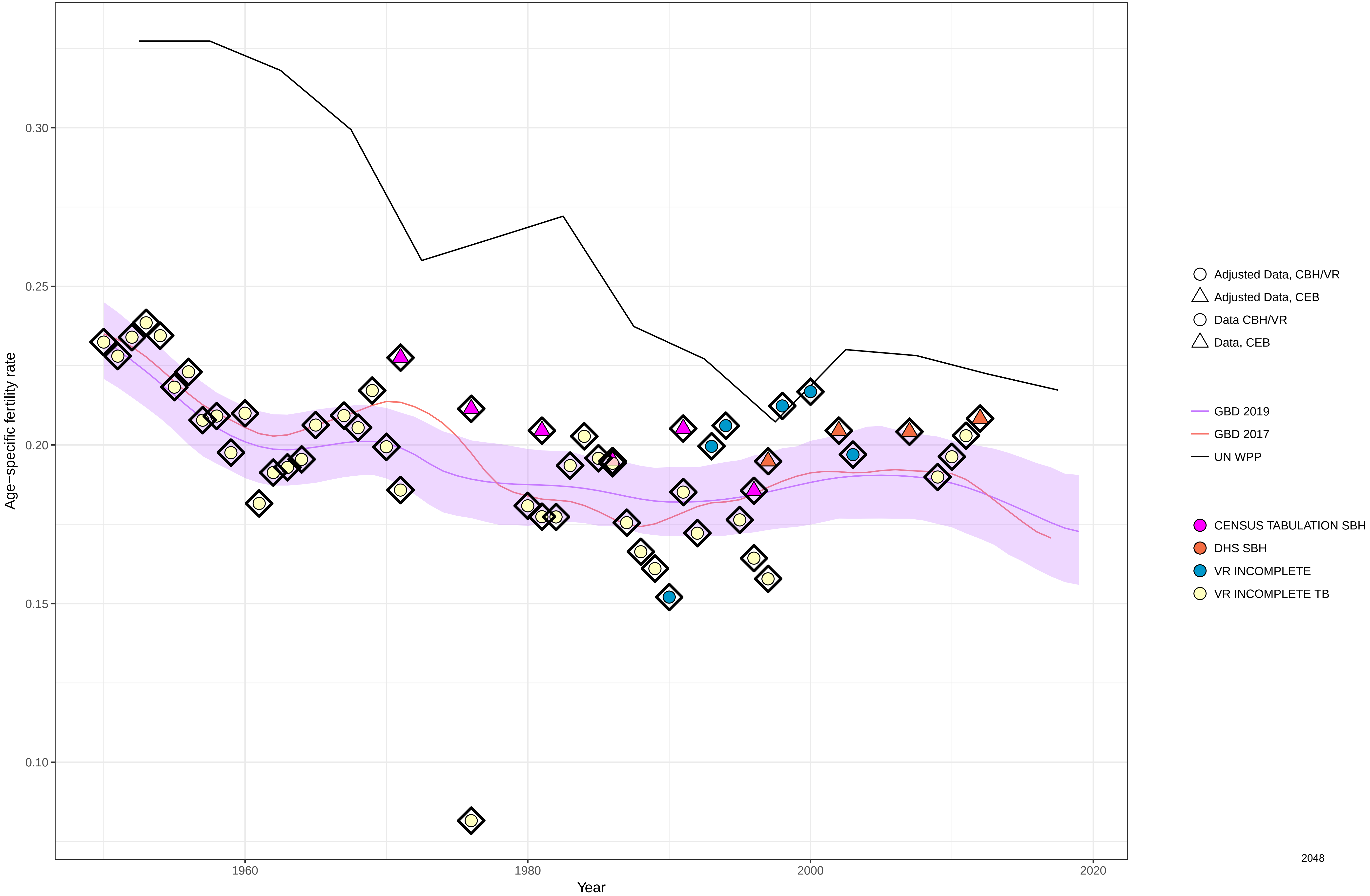

Age−specific fertility fitted trend lines, 1950−2019: Tonga, Ages: 35−39
Data points enclosed with a diamond are reference sources

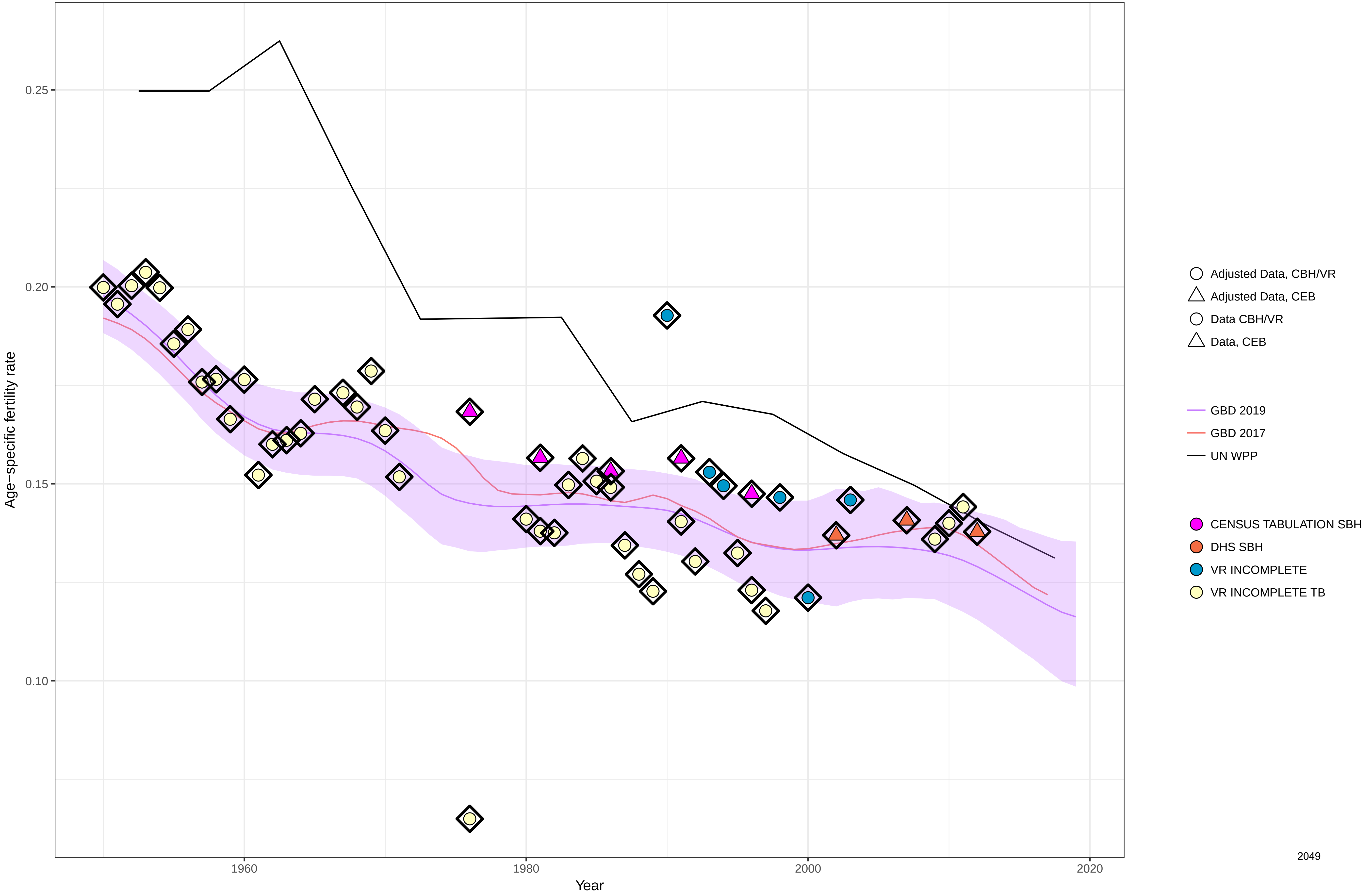

Age−specific fertility fitted trend lines, 1950−2019: Tonga, Ages: 40−44
Data points enclosed with a diamond are reference sources

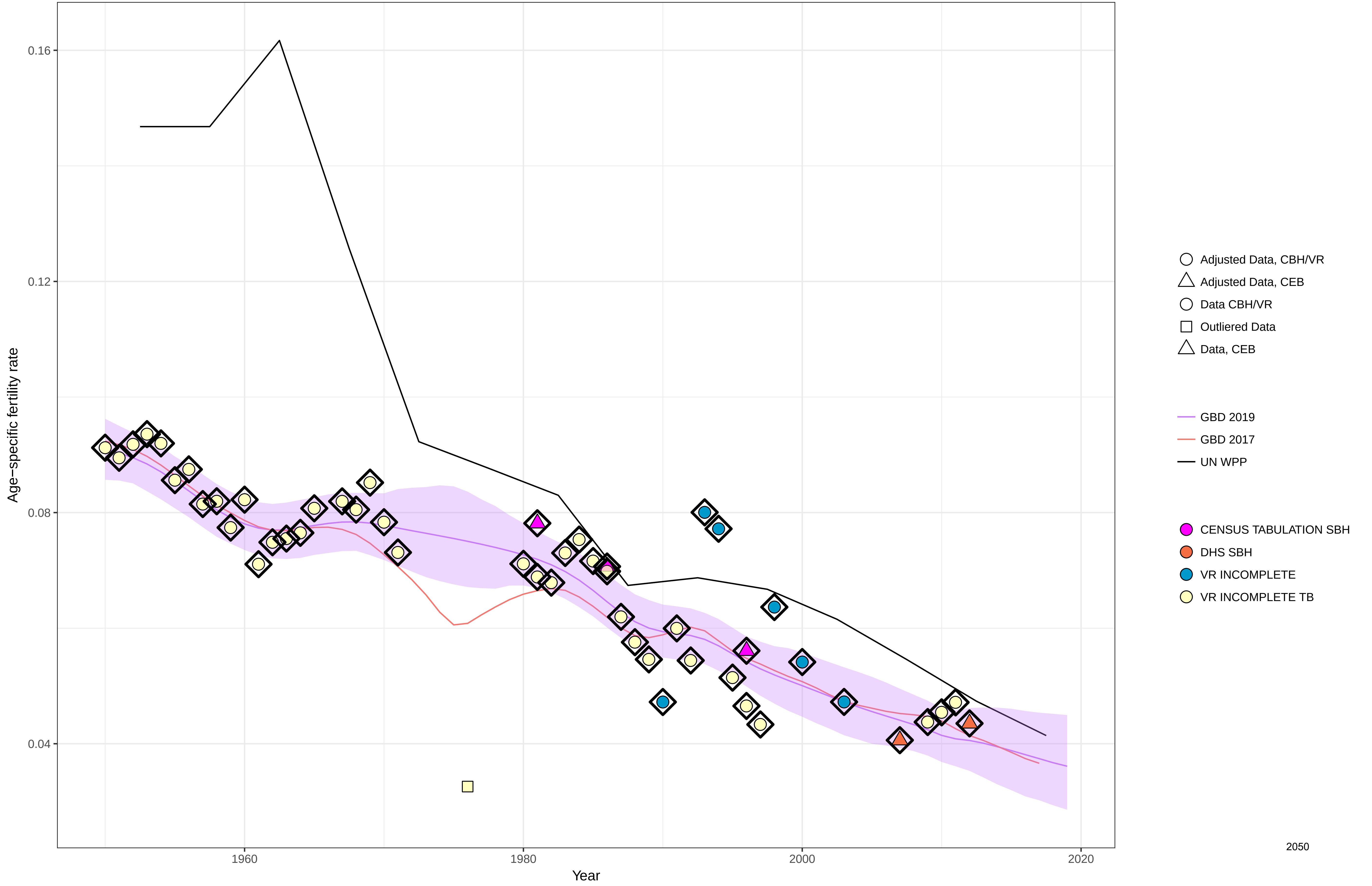

2050

Age−specific fertility fitted trend lines, 1950−2019: Tonga, Ages: 45−49
Data points enclosed with a diamond are reference sources

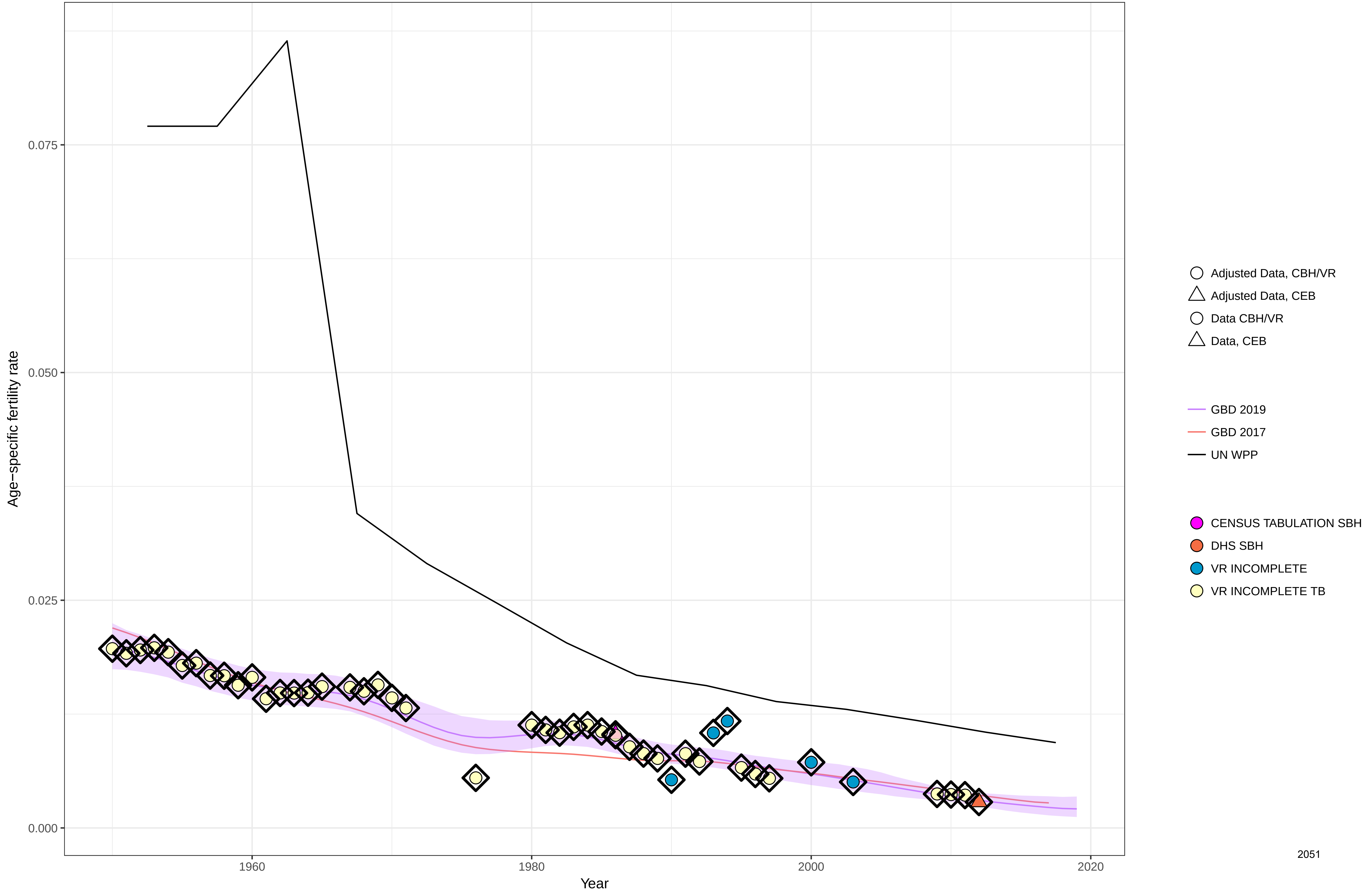

Age−specific fertility fitted trend lines, 1950−2019: Tonga, Ages: 50−54
Data points enclosed with a diamond are reference sources

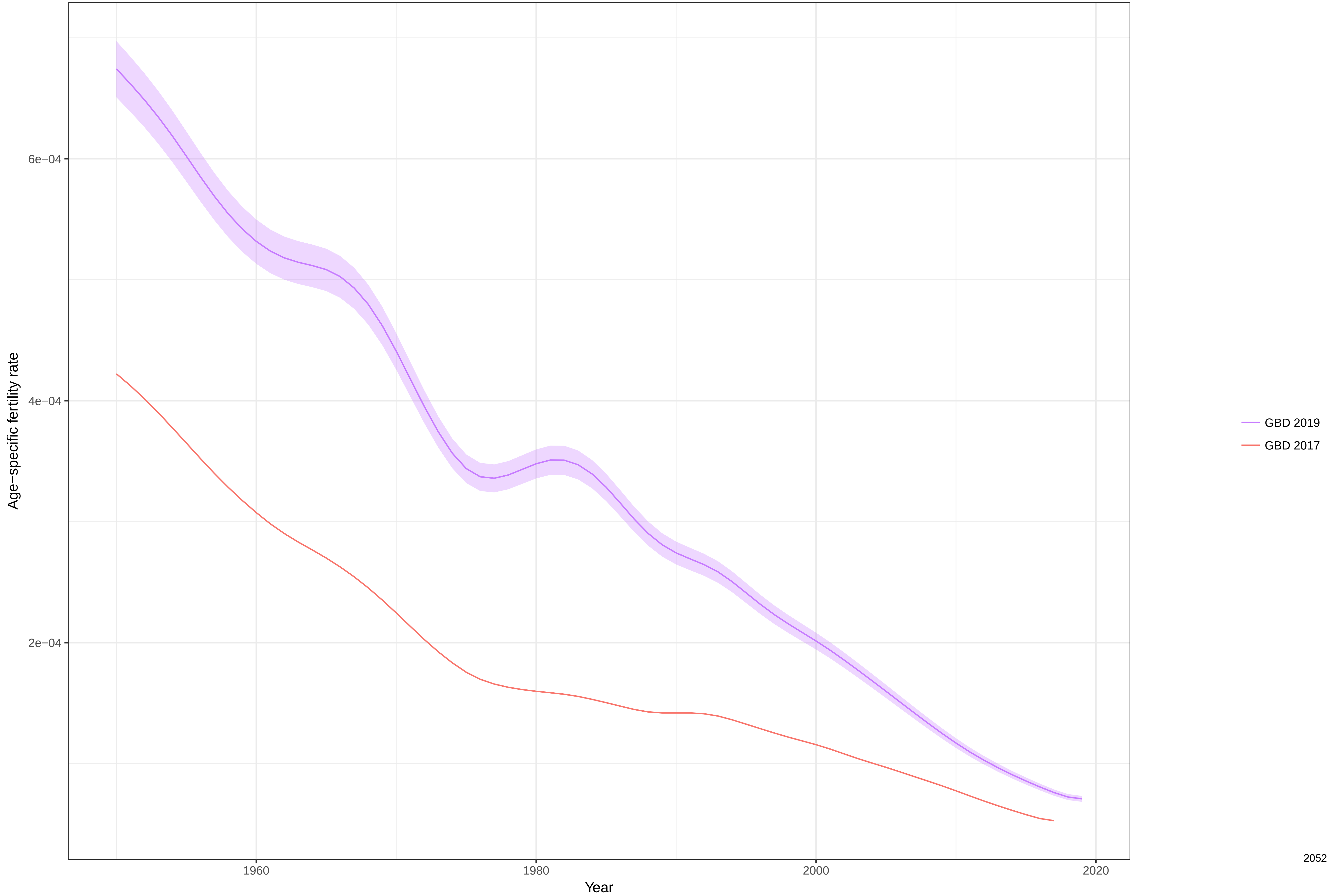

Total fertility rate fitted trend lines, 1950−2019: Tonga TFR
Data points enclosed with a diamond are reference sources

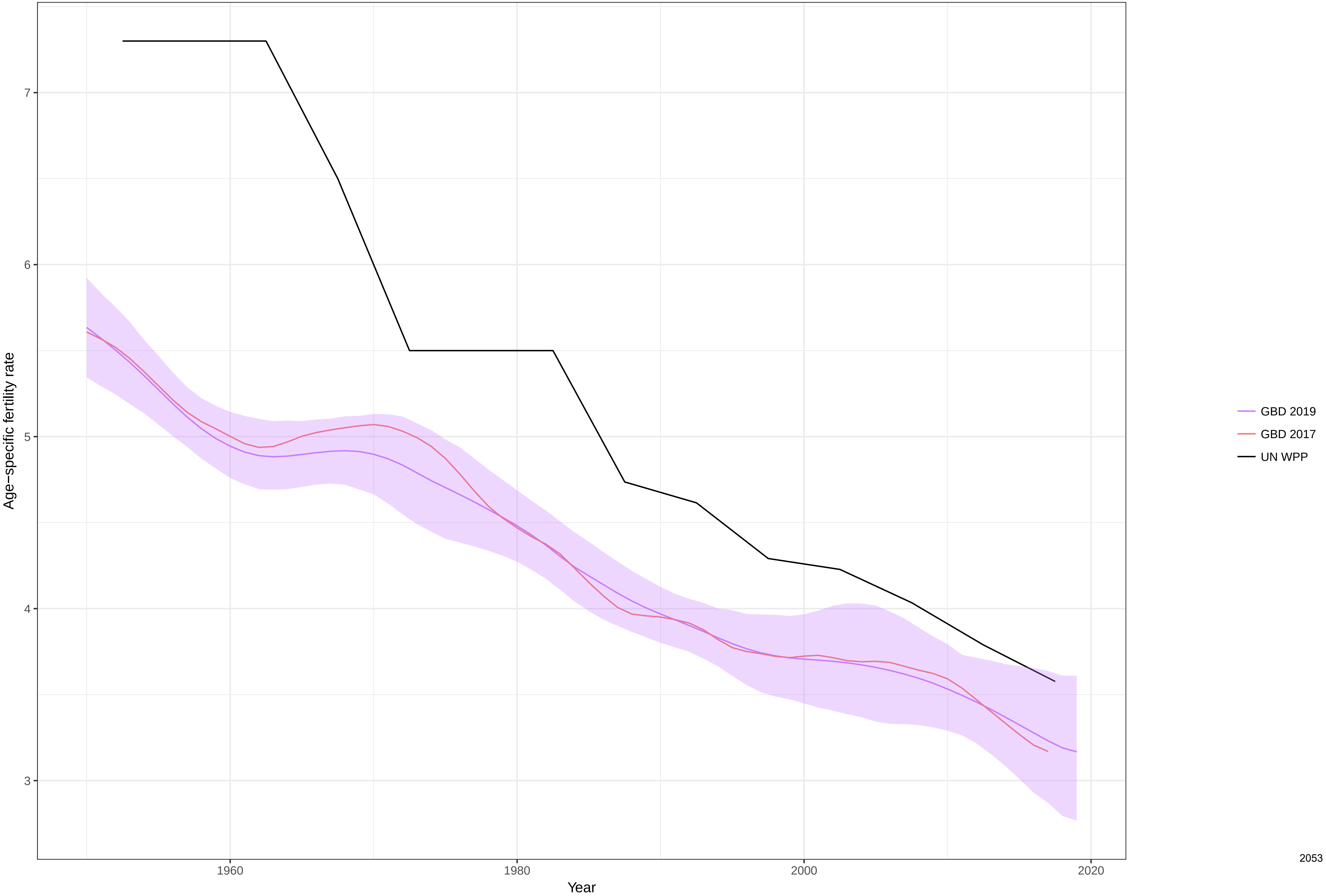

Age−specific fertility fitted trend lines, 1950−2019: Trinidad and Tobago, Ages: 10−14
Data points enclosed with a diamond are reference sources

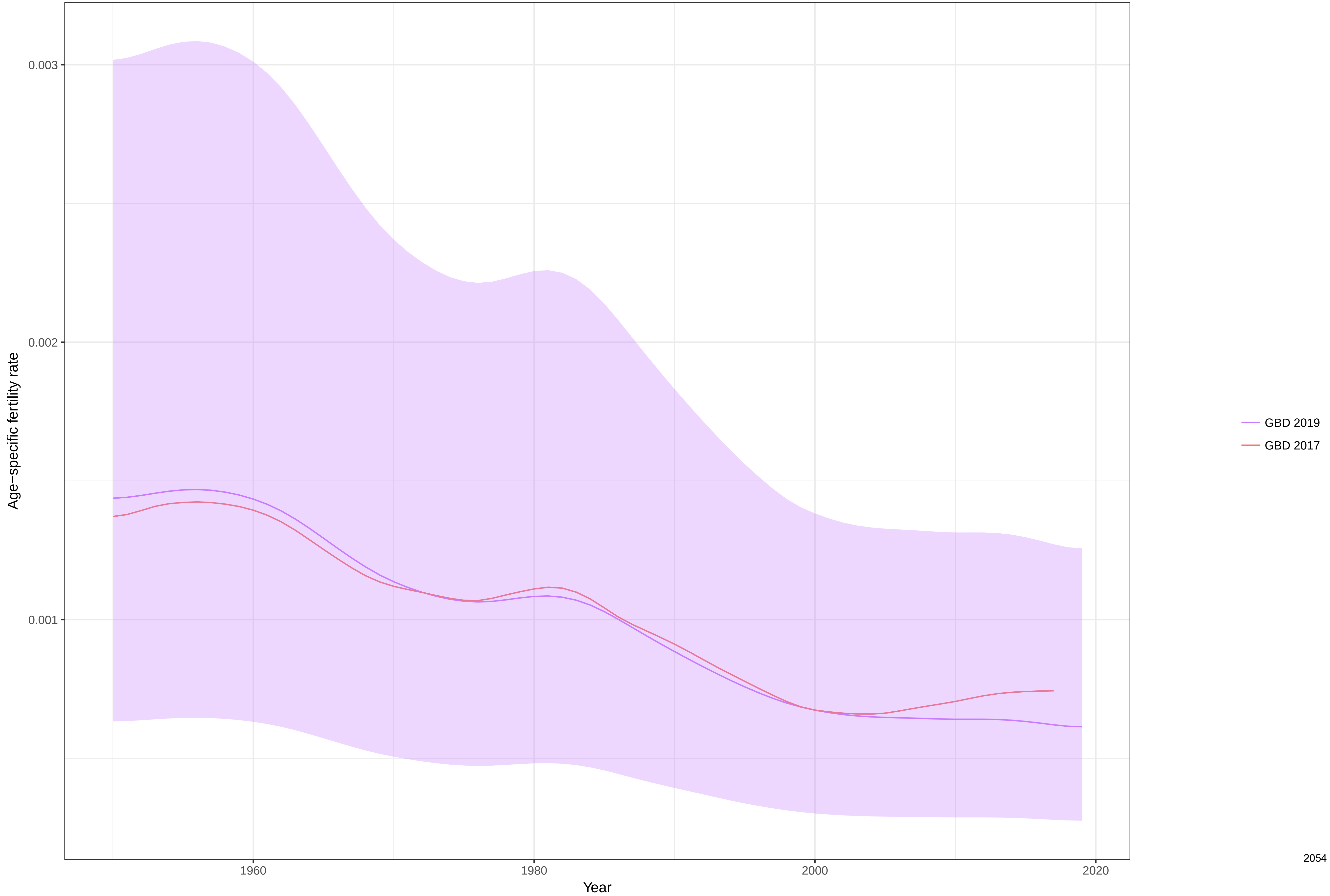

Age−specific fertility fitted trend lines, 1950−2019: Trinidad and Tobago, Ages: 15−19
Data points enclosed with a diamond are reference sources

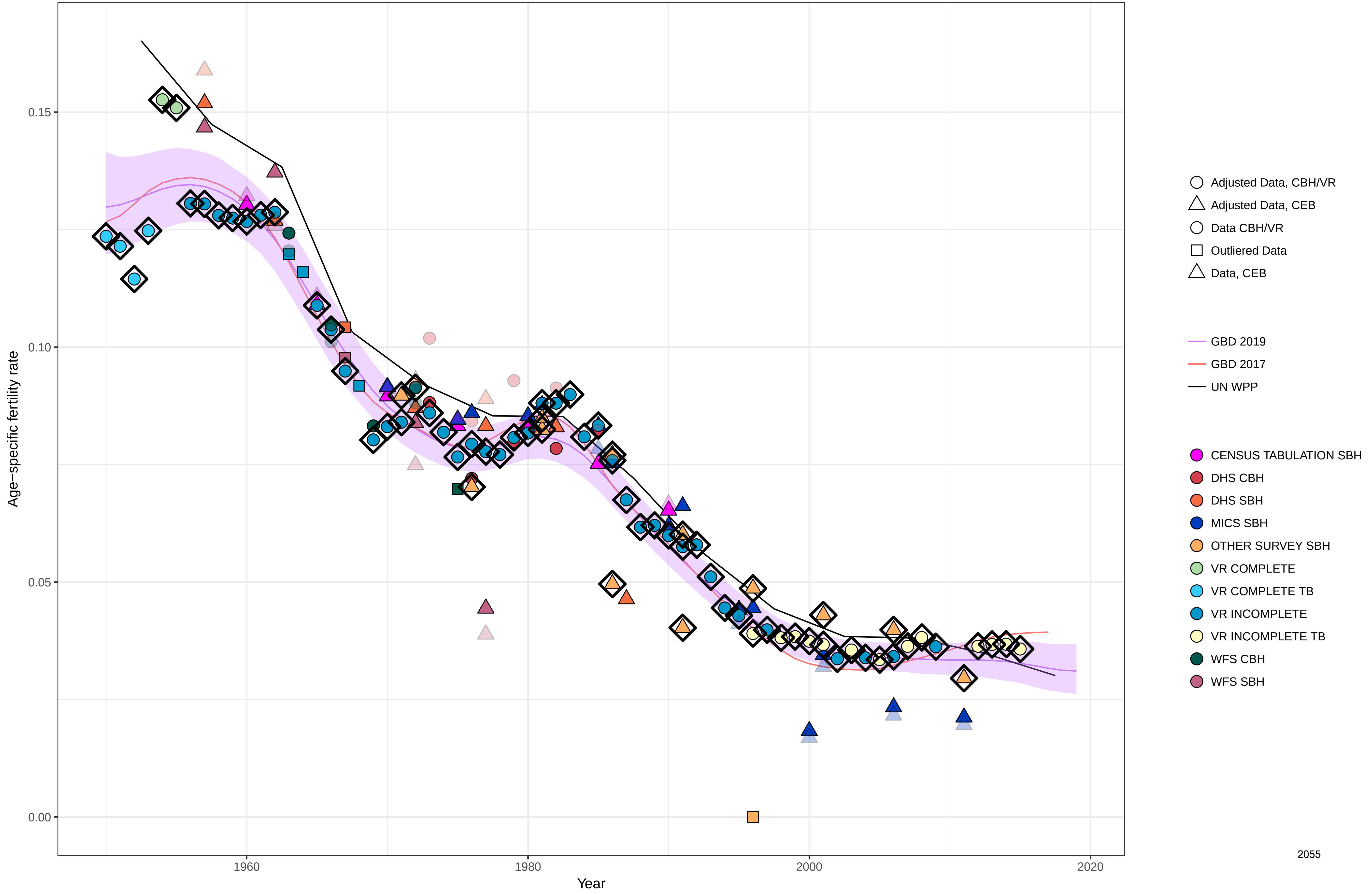

Age−specific fertility fitted trend lines, 1950−2019: Trinidad and Tobago, Ages: 20−24
Data points enclosed with a diamond are reference sources

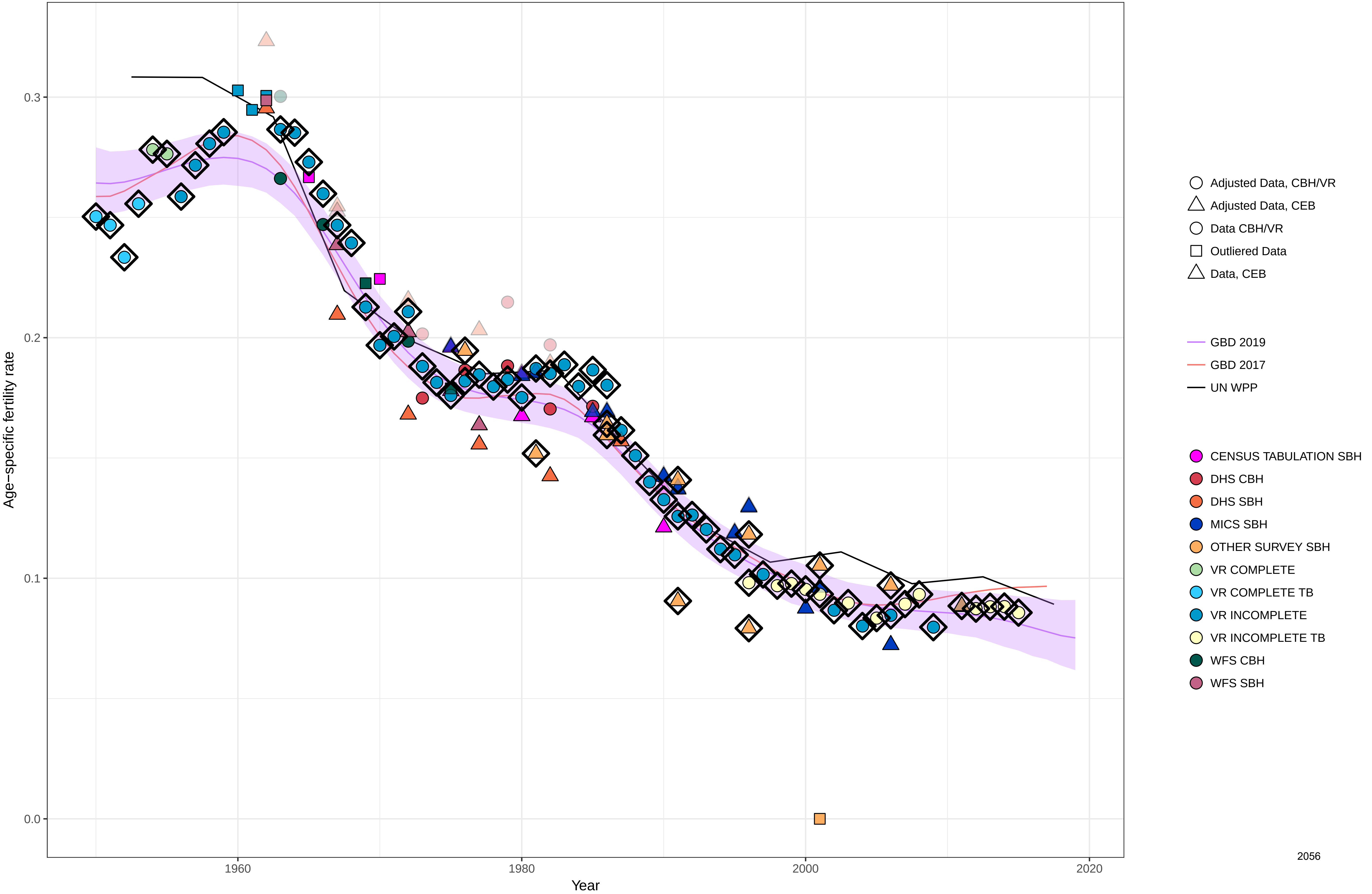

Age−specific fertility fitted trend lines, 1950−2019: Trinidad and Tobago, Ages: 25−29
Data points enclosed with a diamond are reference sources

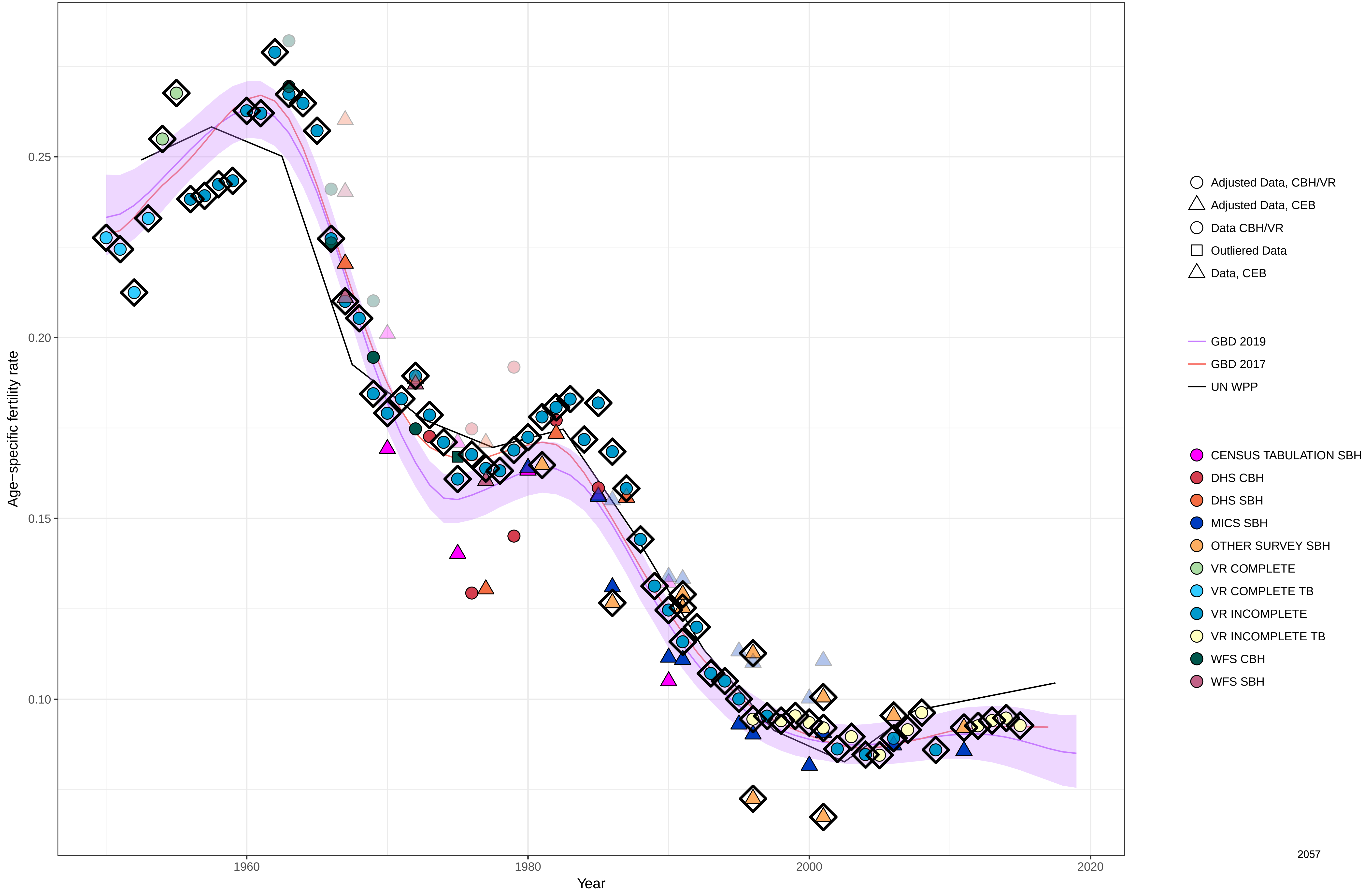

Age−specific fertility fitted trend lines, 1950−2019: Trinidad and Tobago, Ages: 30−34
Data points enclosed with a diamond are reference sources

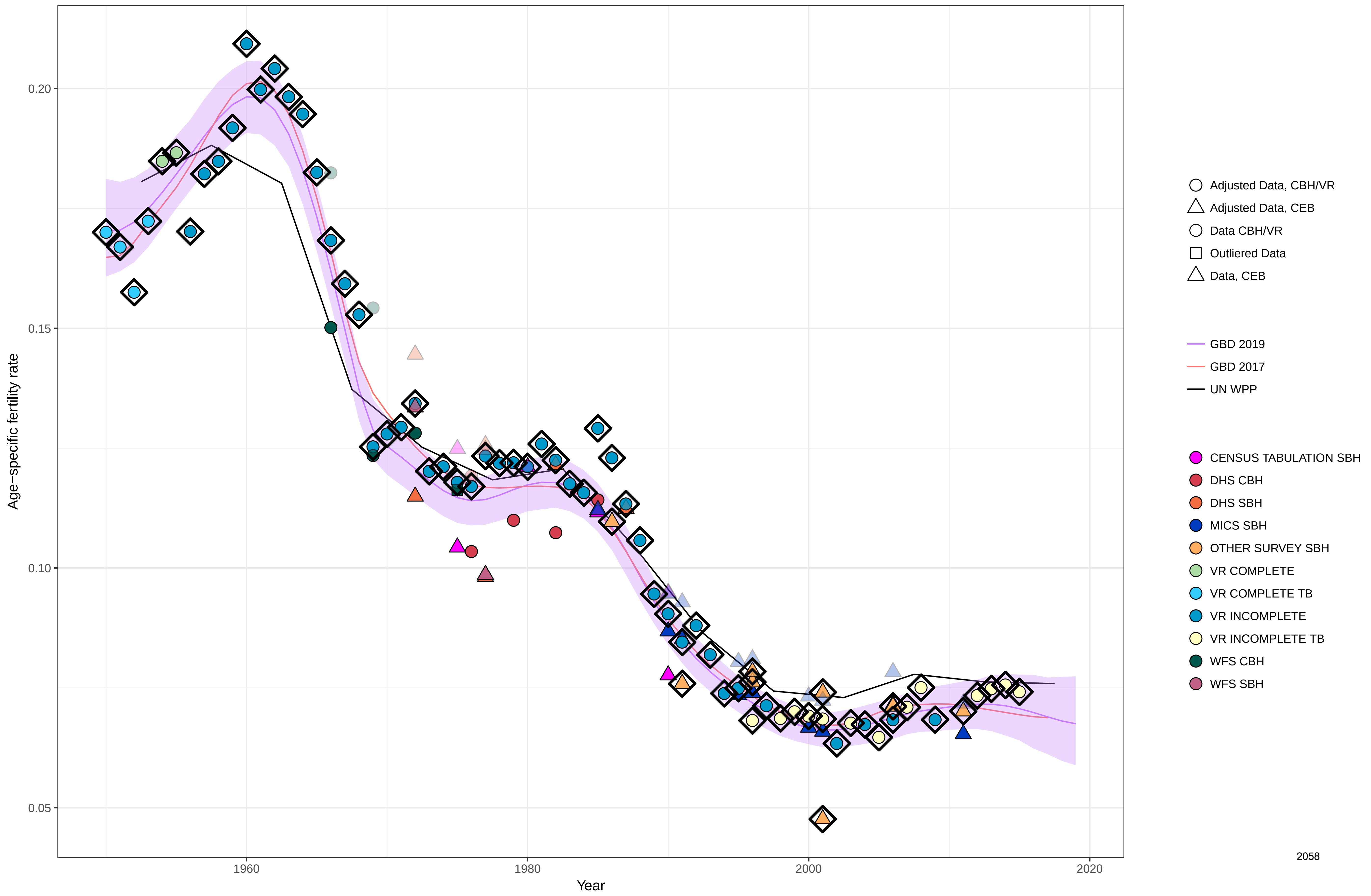

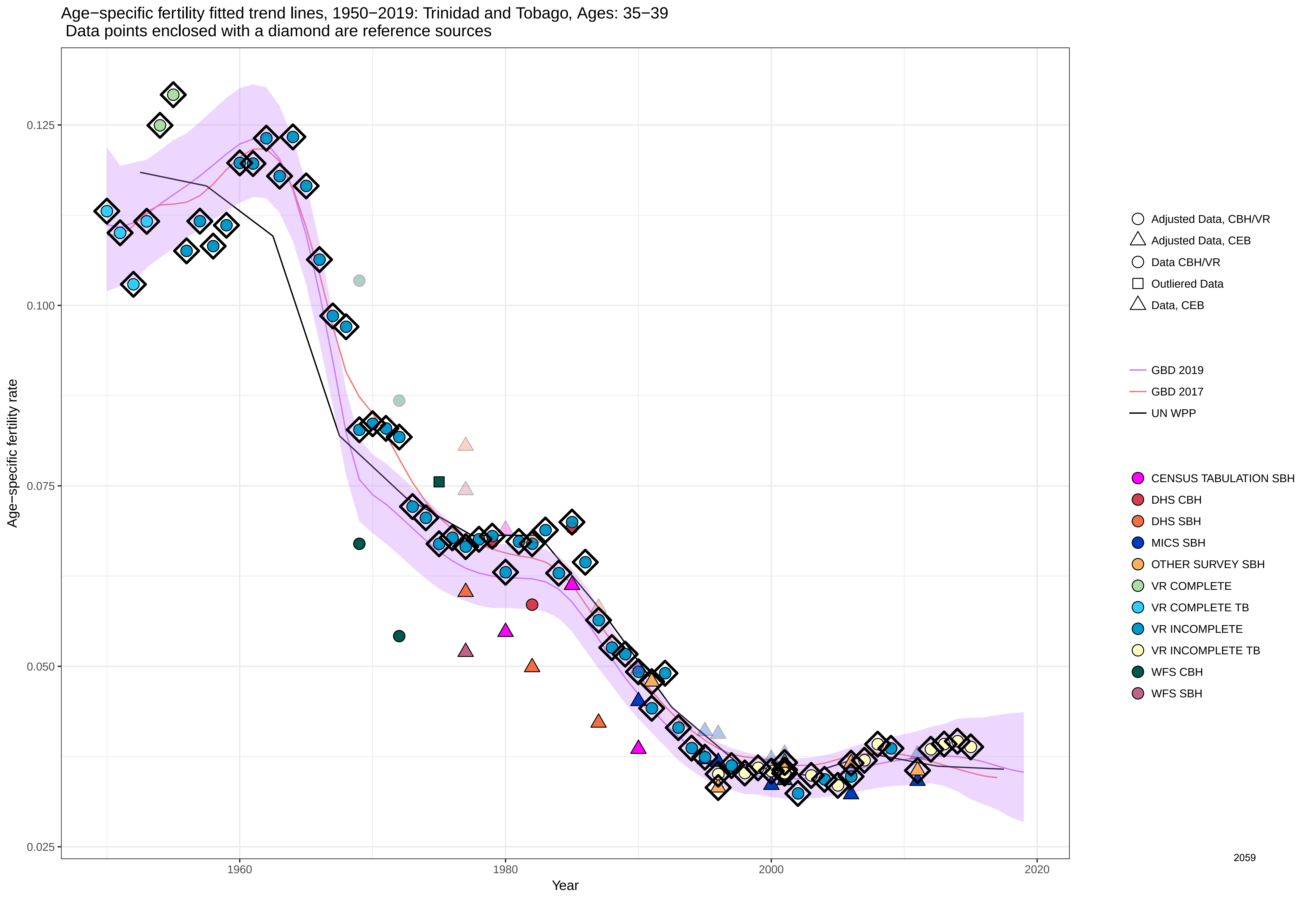

Age−specific fertility fitted trend lines, 1950−2019: Trinidad and Tobago, Ages: 35−39
Data points enclosed with a diamond are reference sources
Age−specific fertility rate
0.125
0.100
0.075
0.050
0.025
1960
1980
2000
2020
Year
Adjusted Data, CBH/VR
Adjusted Data, CEB
Data CBH/VR
Outliered Data
Data, CEB
GBD 2019
GBD 2017
UN WPP
CENSUS TABULATION SBH
DHS CBH
DHS SBH
MICS SBH
OTHER SURVEY SBH
VR COMPLETE
VR COMPLETE TB
VR INCOMPLETE
VR INCOMPLETE TB
WFS CBH
WFS SBH

Age−specific fertility fitted trend lines, 1950−2019: Trinidad and Tobago, Ages: 40−44
Data points enclosed with a diamond are reference sources

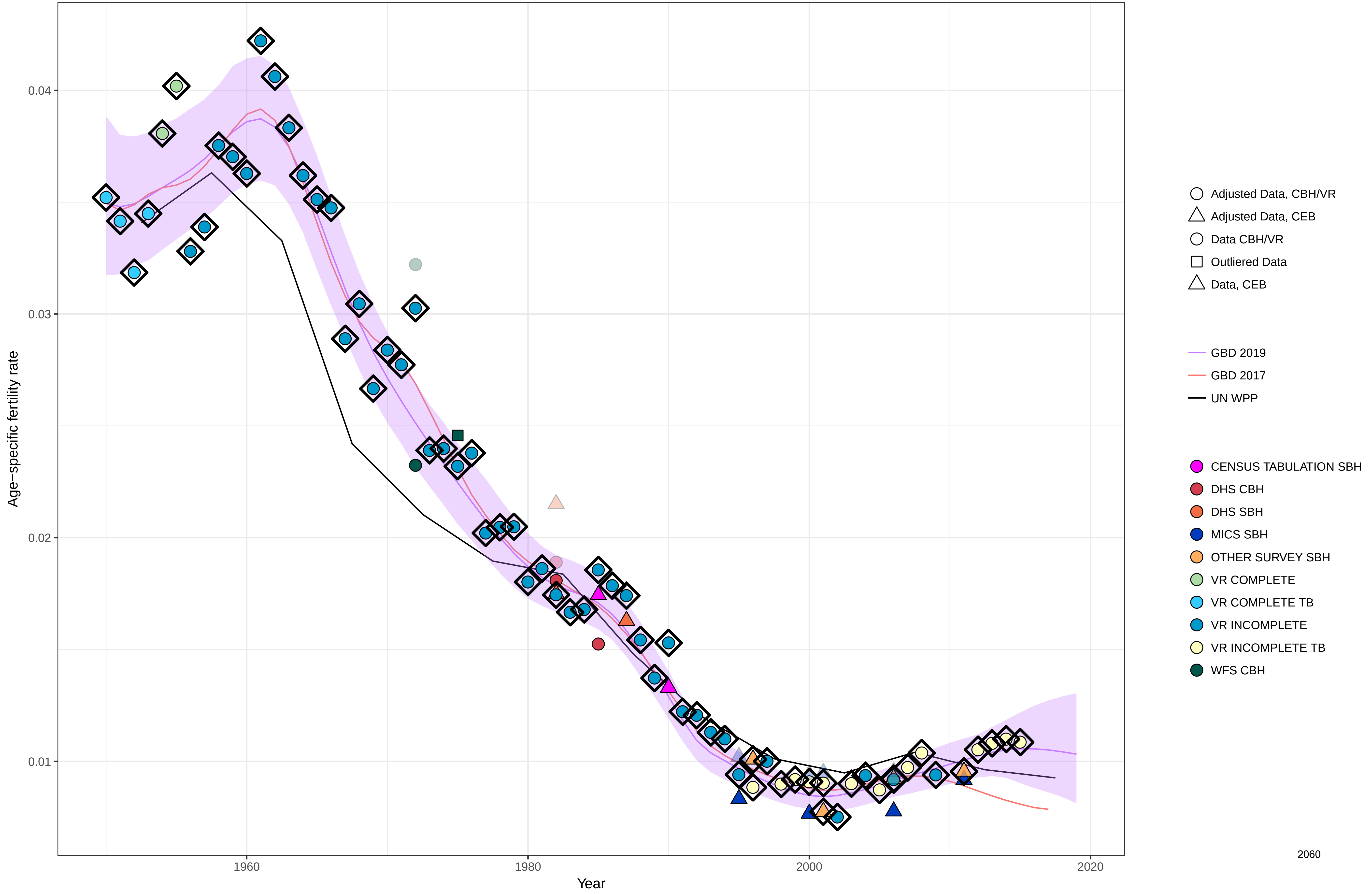

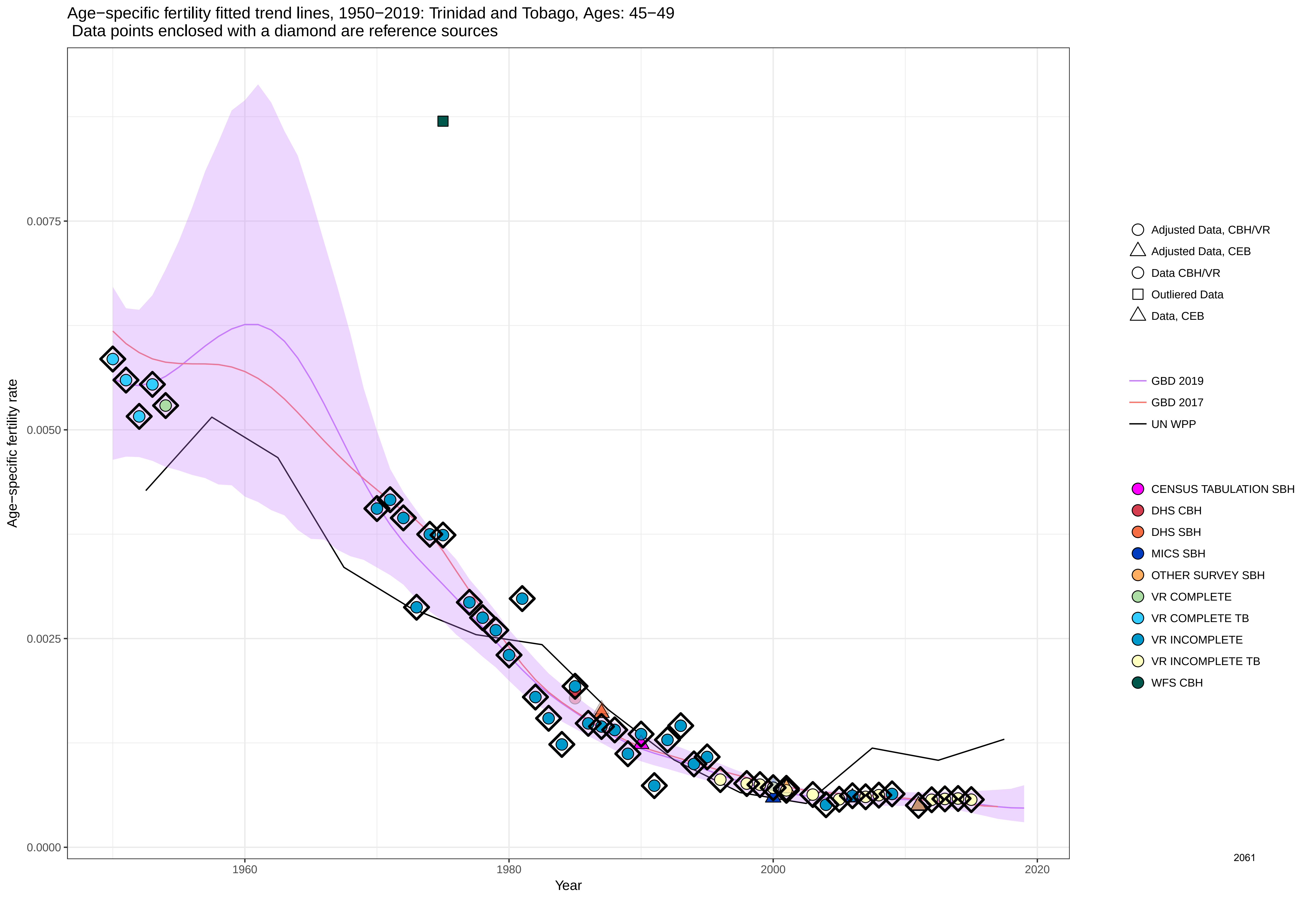
Age−specific fertility fitted trend lines, 1950−2019: Trinidad and Tobago, Ages: 45−49
Data points enclosed with a diamond are reference sources
Age−specific fertility rate
0.0000
0.0025
0.0050
0.0075
1960
1980
2000
2020
Year
Adjusted Data, CBH/VR
Adjusted Data, CEB
Data CBH/VR
Outliered Data
Data, CEB
GBD 2019
GBD 2017
UN WPP
CENSUS TABULATION SBH
DHS CBH
DHS SBH
MICS SBH
OTHER SURVEY SBH
VR COMPLETE
VR COMPLETE TB
VR INCOMPLETE
VR INCOMPLETE TB
WFS CBH

Age−specific fertility fitted trend lines, 1950−2019: Trinidad and Tobago, Ages: 50−54
Data points enclosed with a diamond are reference sources

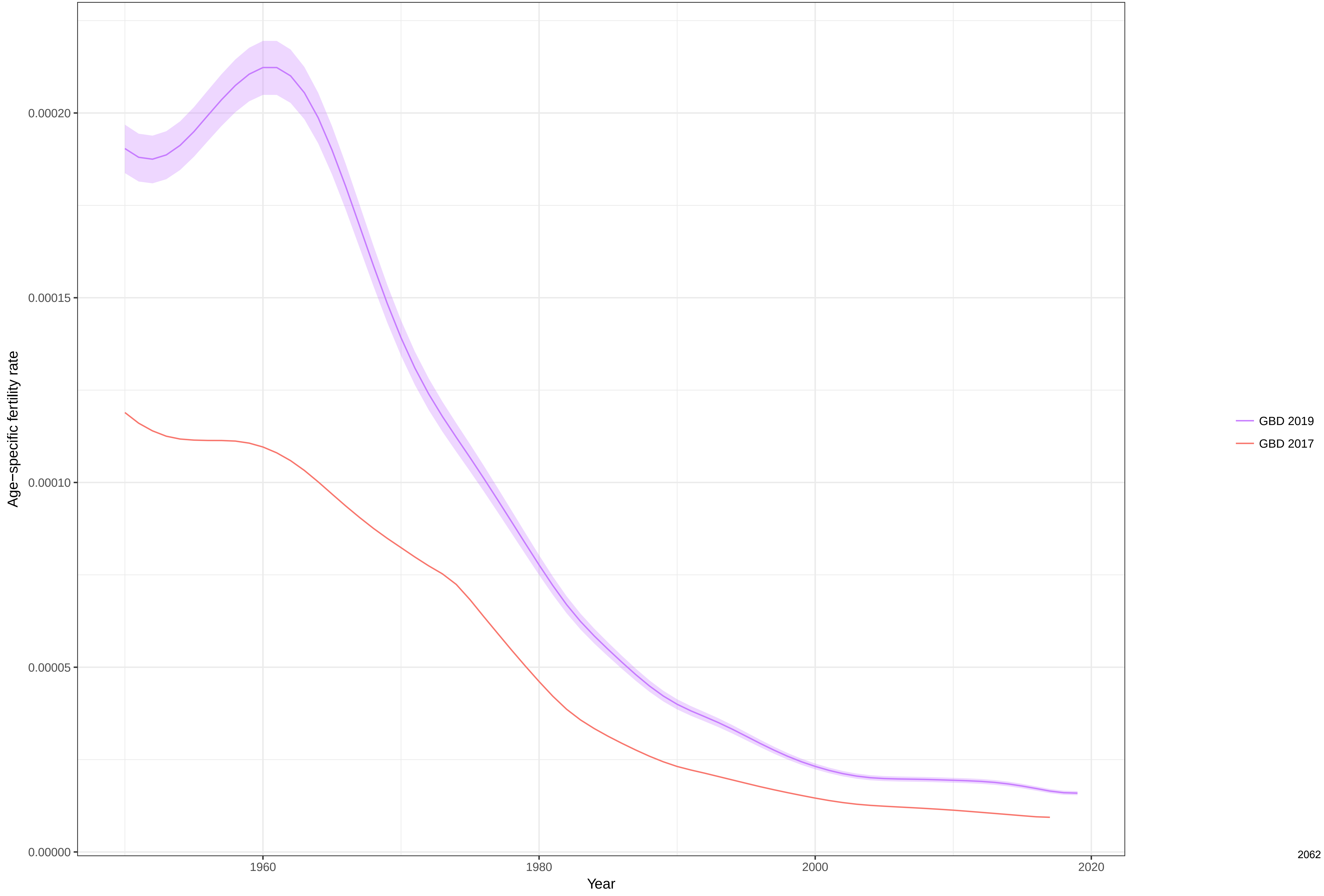

Total fertility rate fitted trend lines, 1950−2019: Trinidad and Tobago TFR
Data points enclosed with a diamond are reference sources

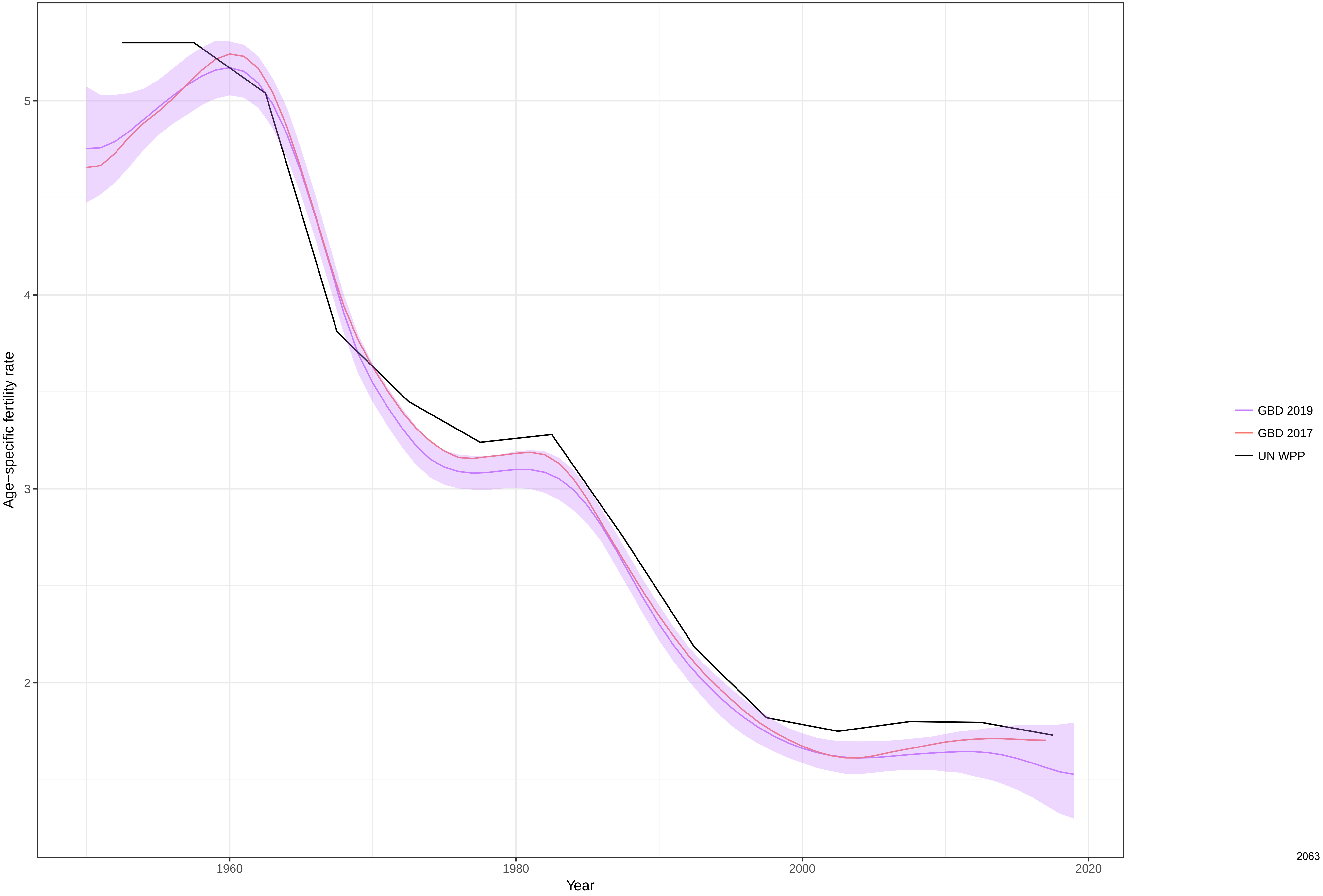

Age−specific fertility fitted trend lines, 1950−2019: Tunisia, Ages: 10−14
Data points enclosed with a diamond are reference sources

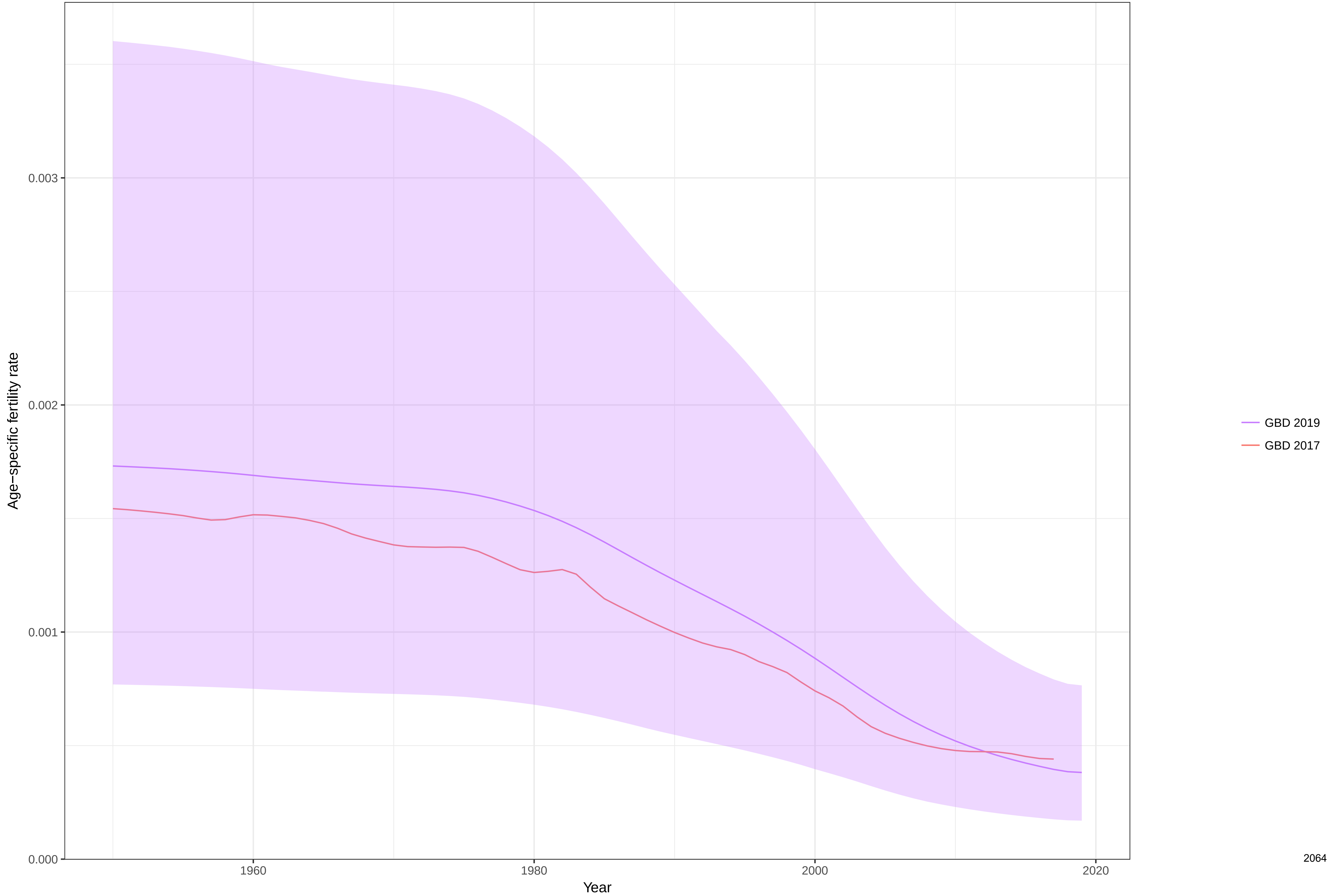

Age−specific fertility fitted trend lines, 1950−2019: Tunisia, Ages: 15−19
Data points enclosed with a diamond are reference sources

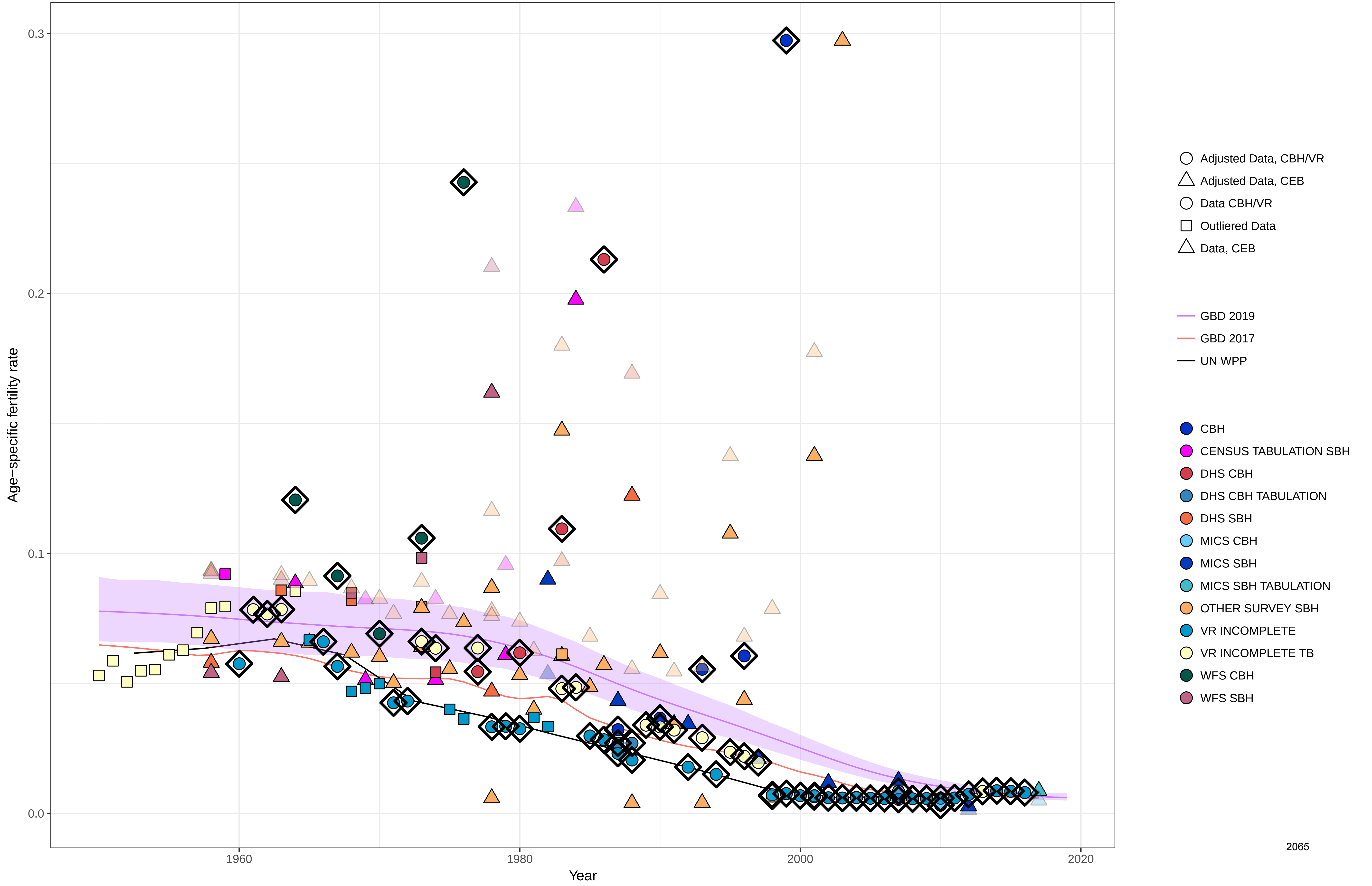

Age−specific fertility fitted trend lines, 1950−2019: Tunisia, Ages: 20−24
Data points enclosed with a diamond are reference sources

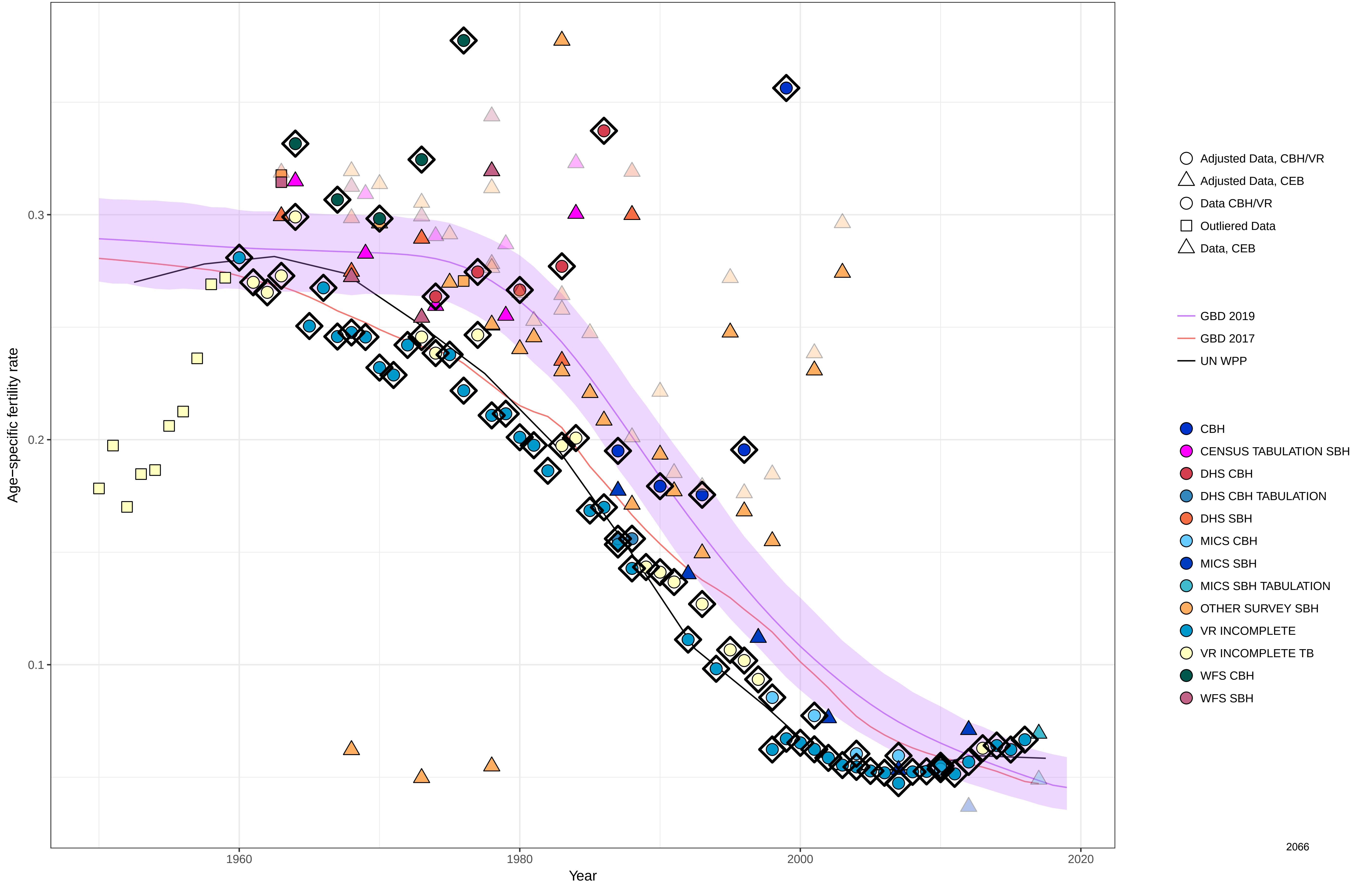

Age−specific fertility fitted trend lines, 1950−2019: Tunisia, Ages: 25−29
Data points enclosed with a diamond are reference sources

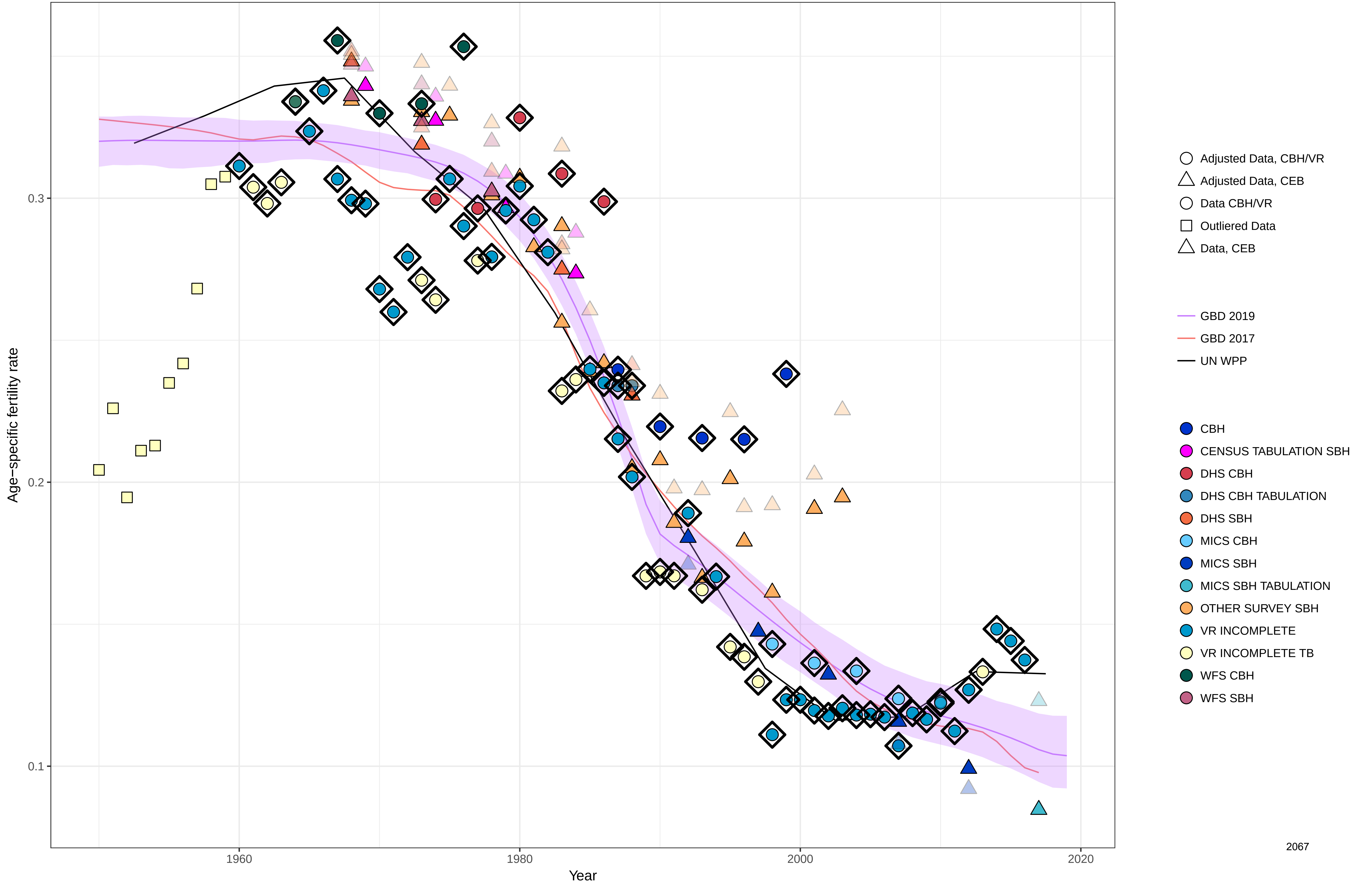

Age−specific fertility fitted trend lines, 1950−2019: Tunisia, Ages: 30−34
Data points enclosed with a diamond are reference sources

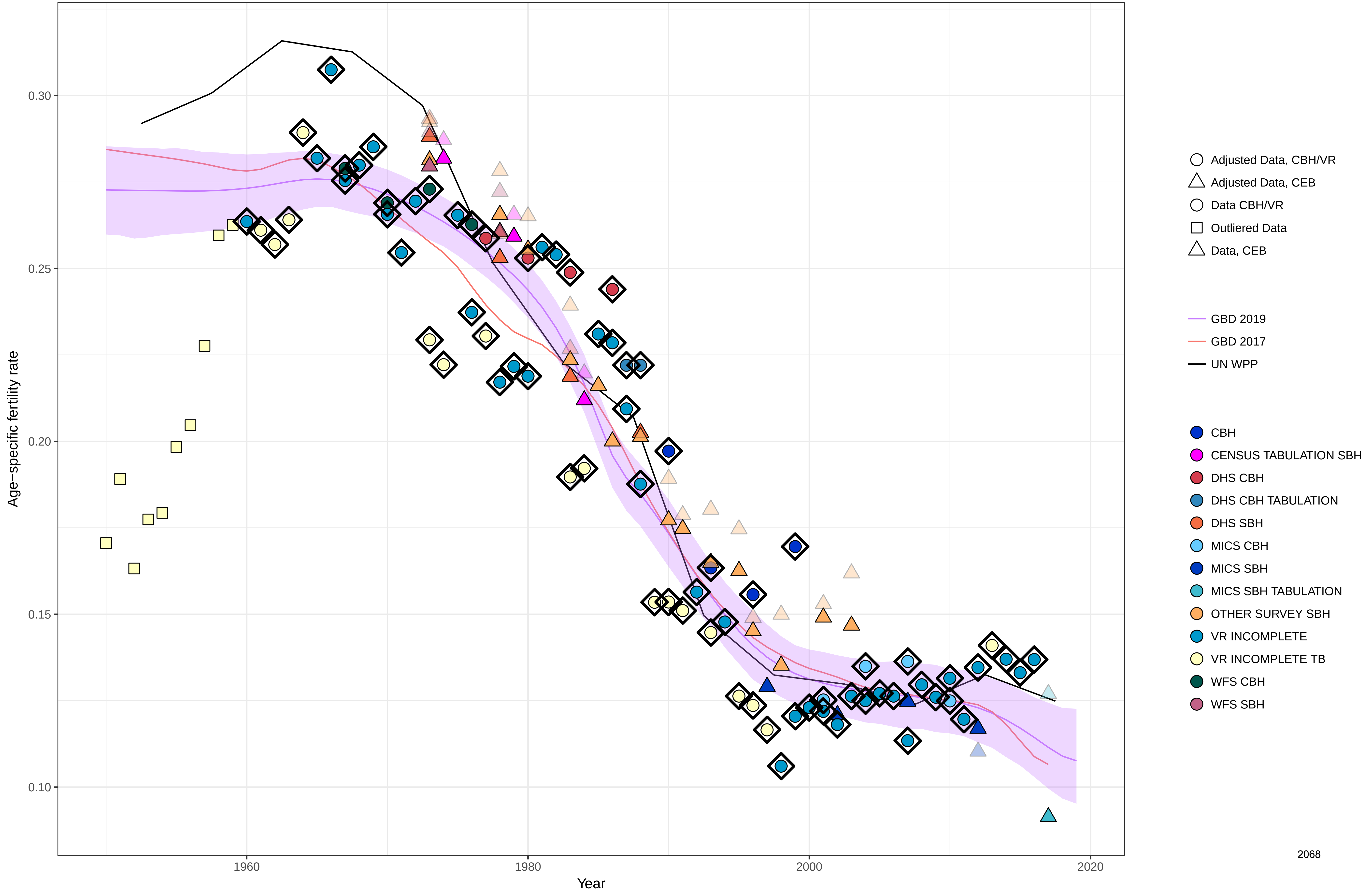

Age−specific fertility fitted trend lines, 1950−2019: Tunisia, Ages: 35−39
Data points enclosed with a diamond are reference sources

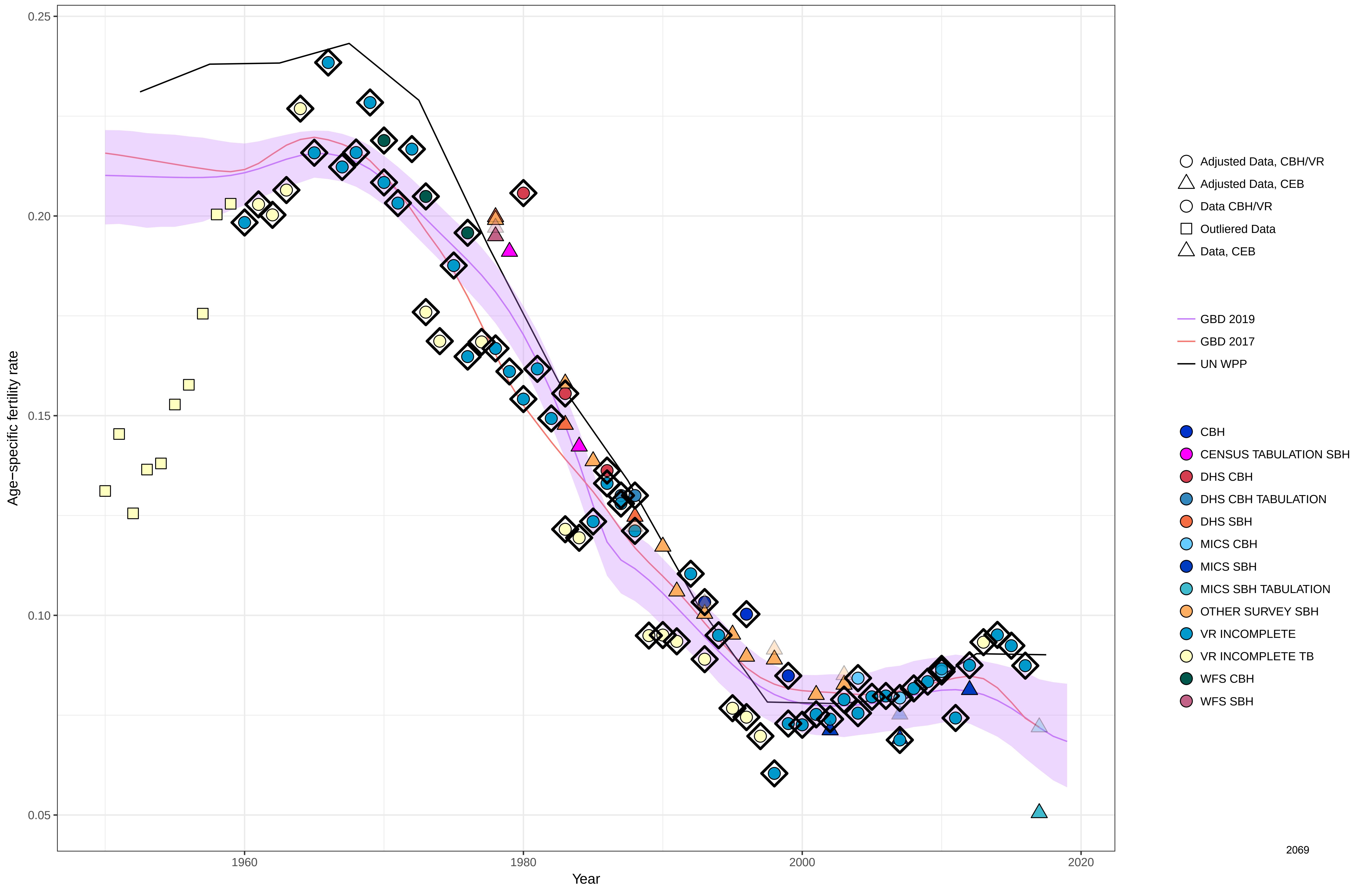

Age−specific fertility fitted trend lines, 1950−2019: Tunisia, Ages: 40−44
Data points enclosed with a diamond are reference sources

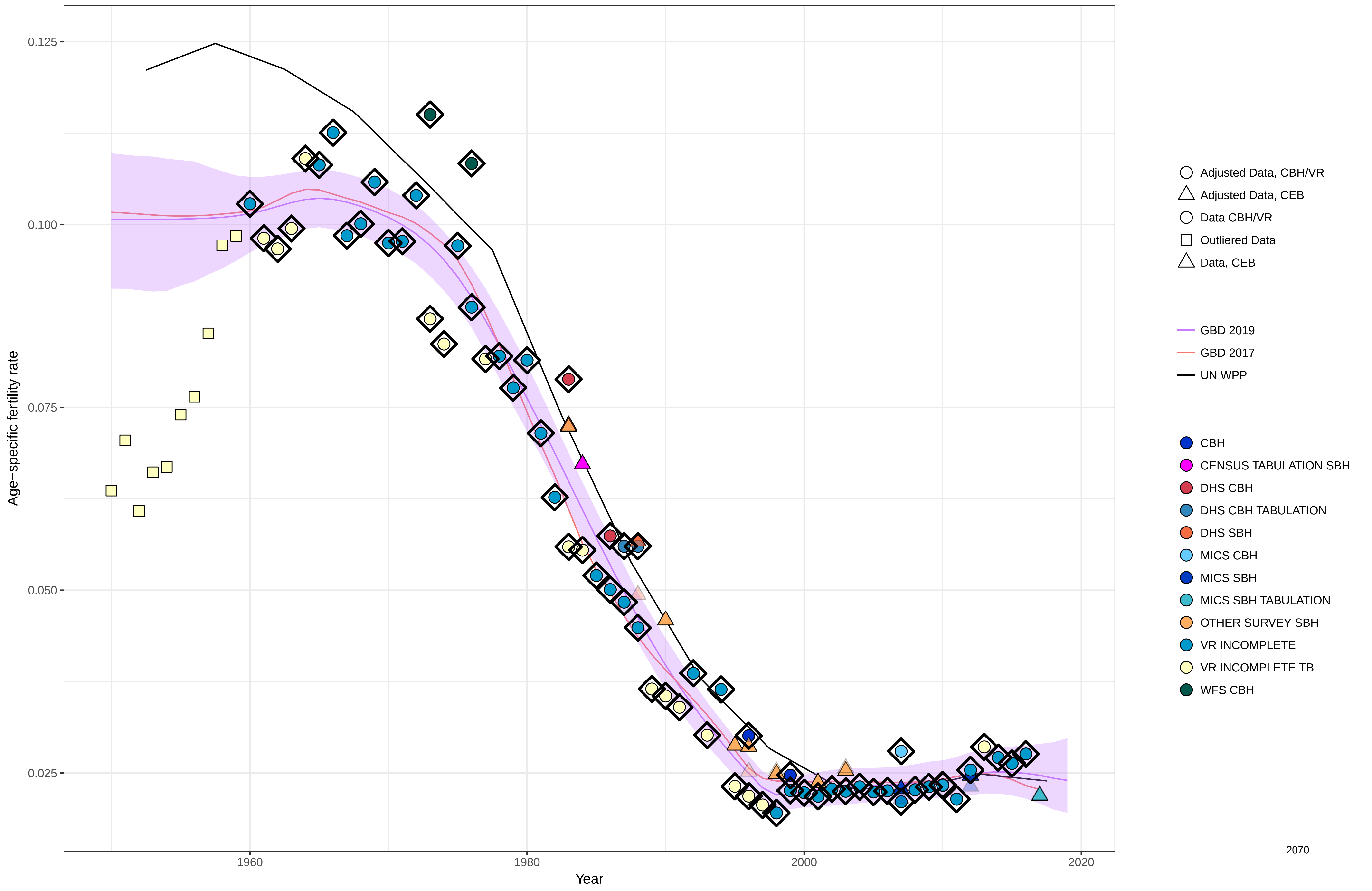

Age−specific fertility fitted trend lines, 1950−2019: Tunisia, Ages: 45−49
Data points enclosed with a diamond are reference sources

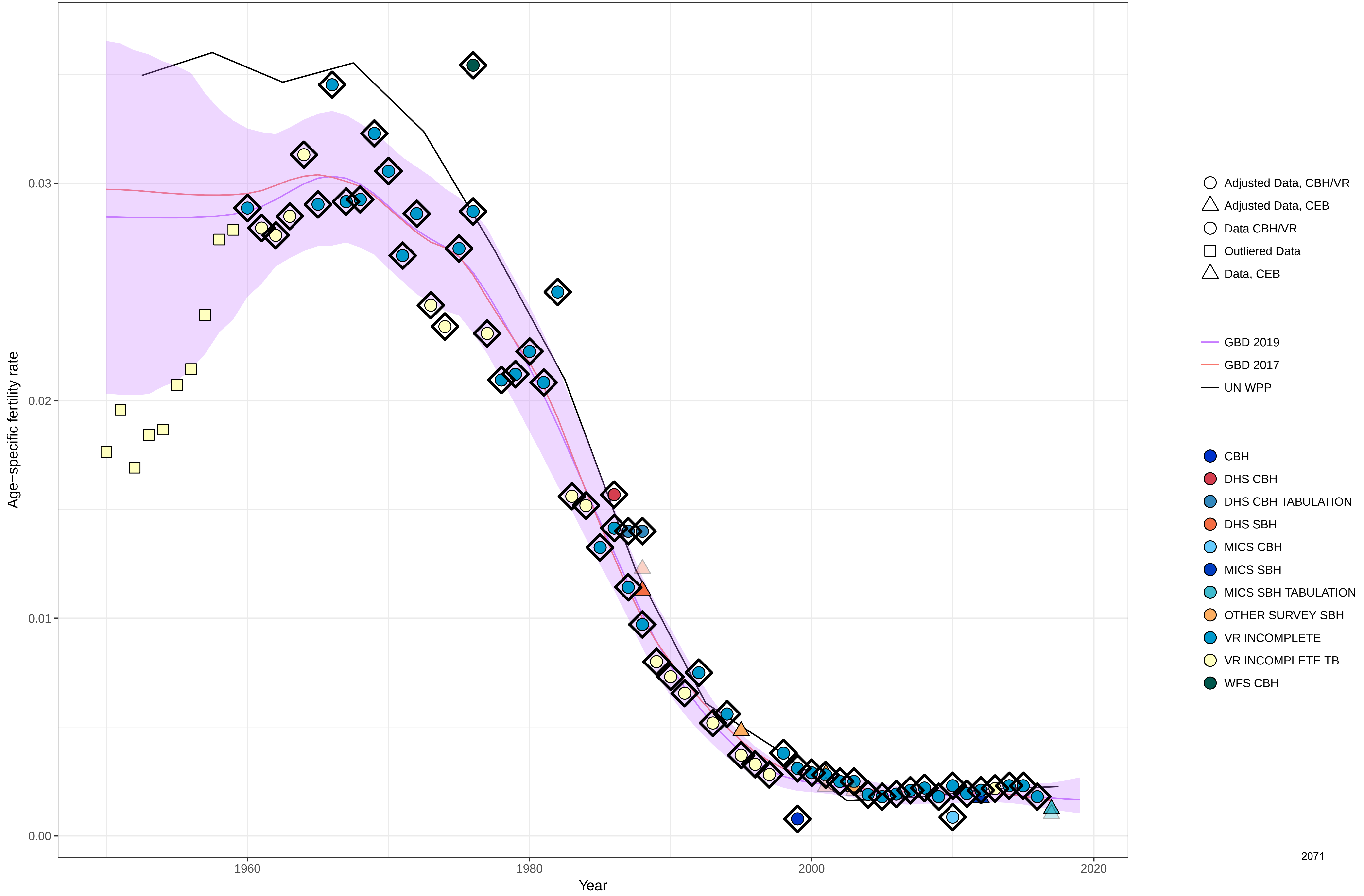

Age−specific fertility fitted trend lines, 1950−2019: Tunisia, Ages: 50−54
Data points enclosed with a diamond are reference sources

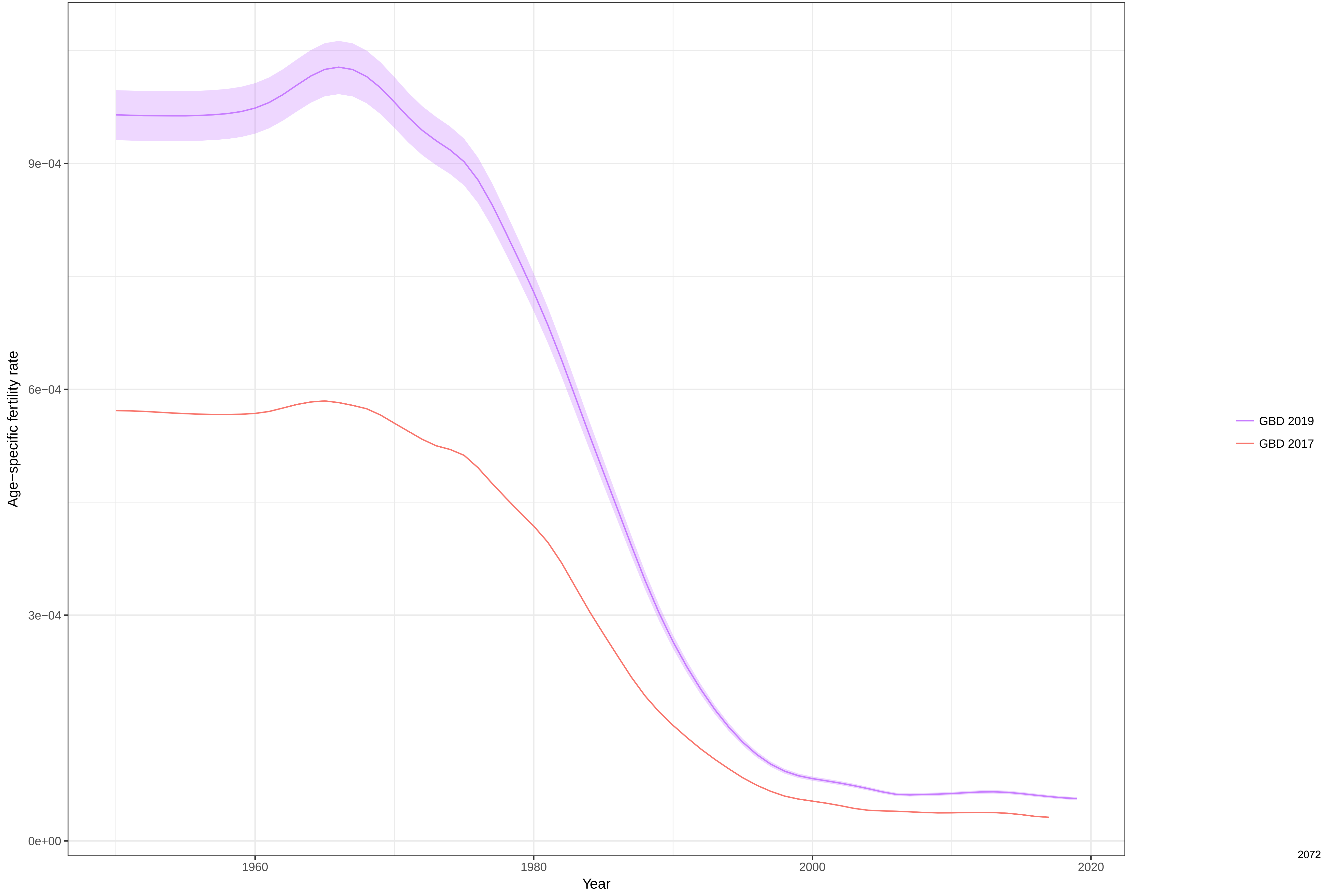

Total fertility rate fitted trend lines, 1950−2019: Tunisia TFR
Data points enclosed with a diamond are reference sources

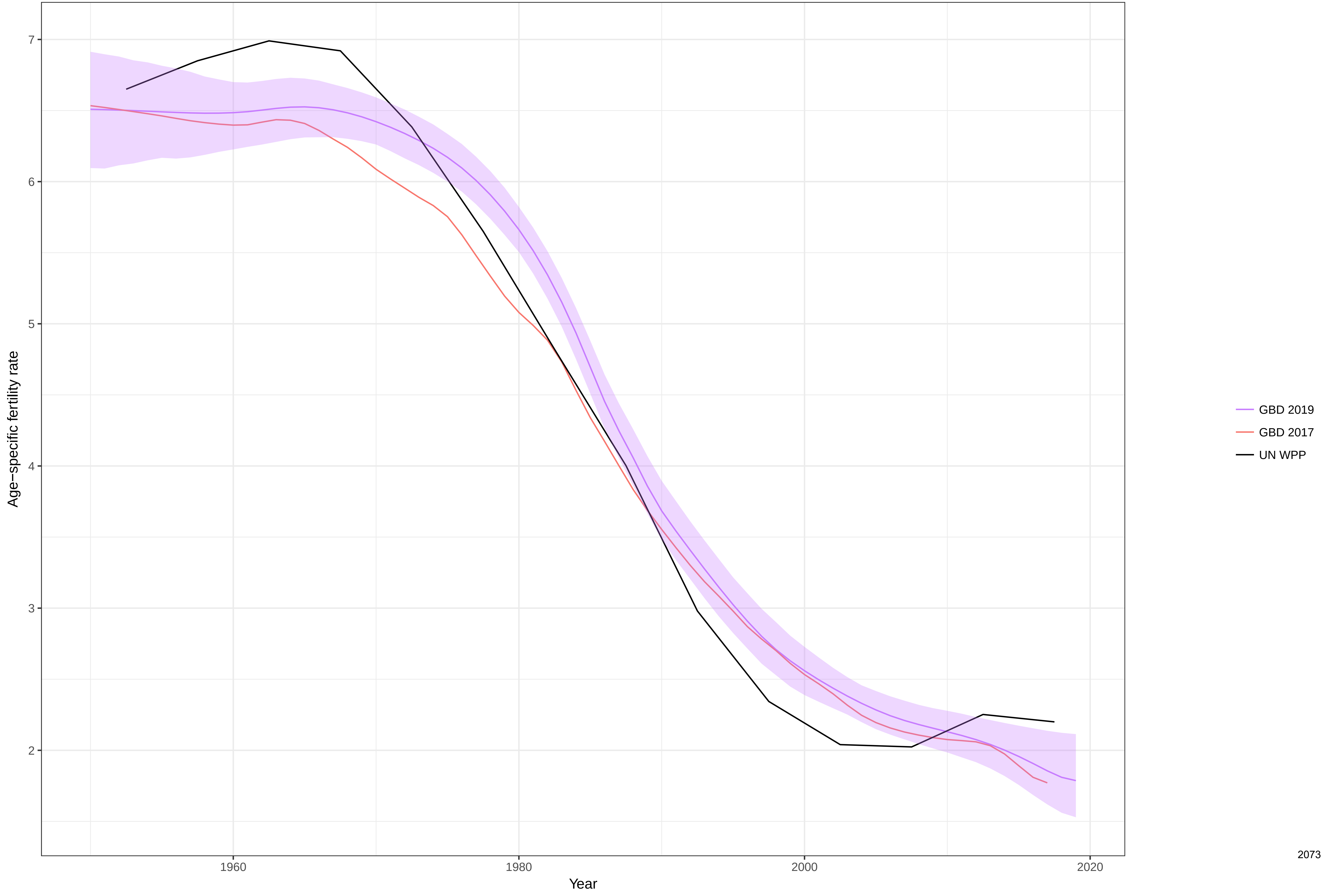

Age−specific fertility fitted trend lines, 1950−2019: Turkey, Ages: 10−14
Data points enclosed with a diamond are reference sources

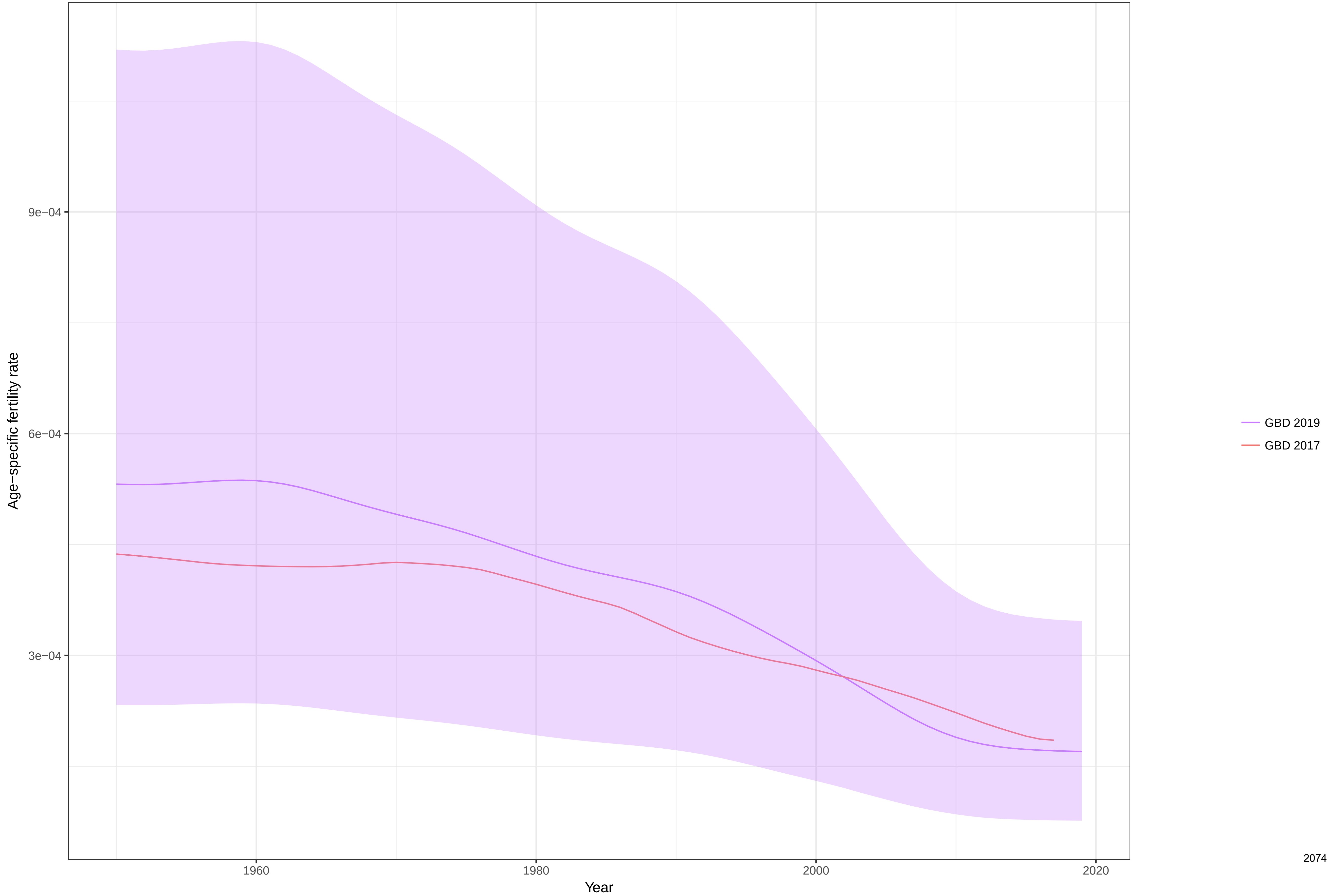

Age−specific fertility fitted trend lines, 1950−2019: Turkey, Ages: 15−19
Data points enclosed with a diamond are reference sources

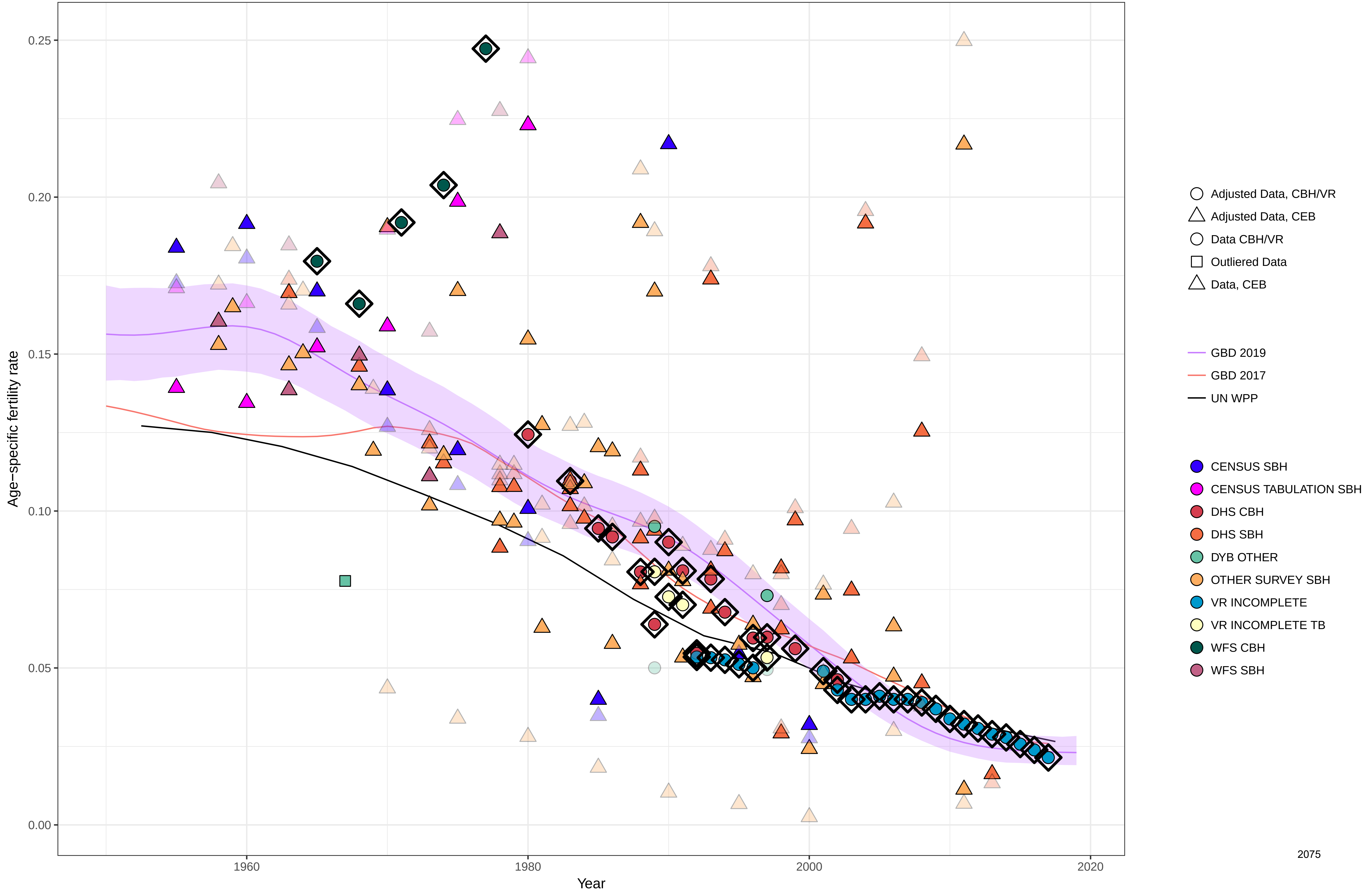

Age−specific fertility fitted trend lines, 1950−2019: Turkey, Ages: 20−24
Data points enclosed with a diamond are reference sources

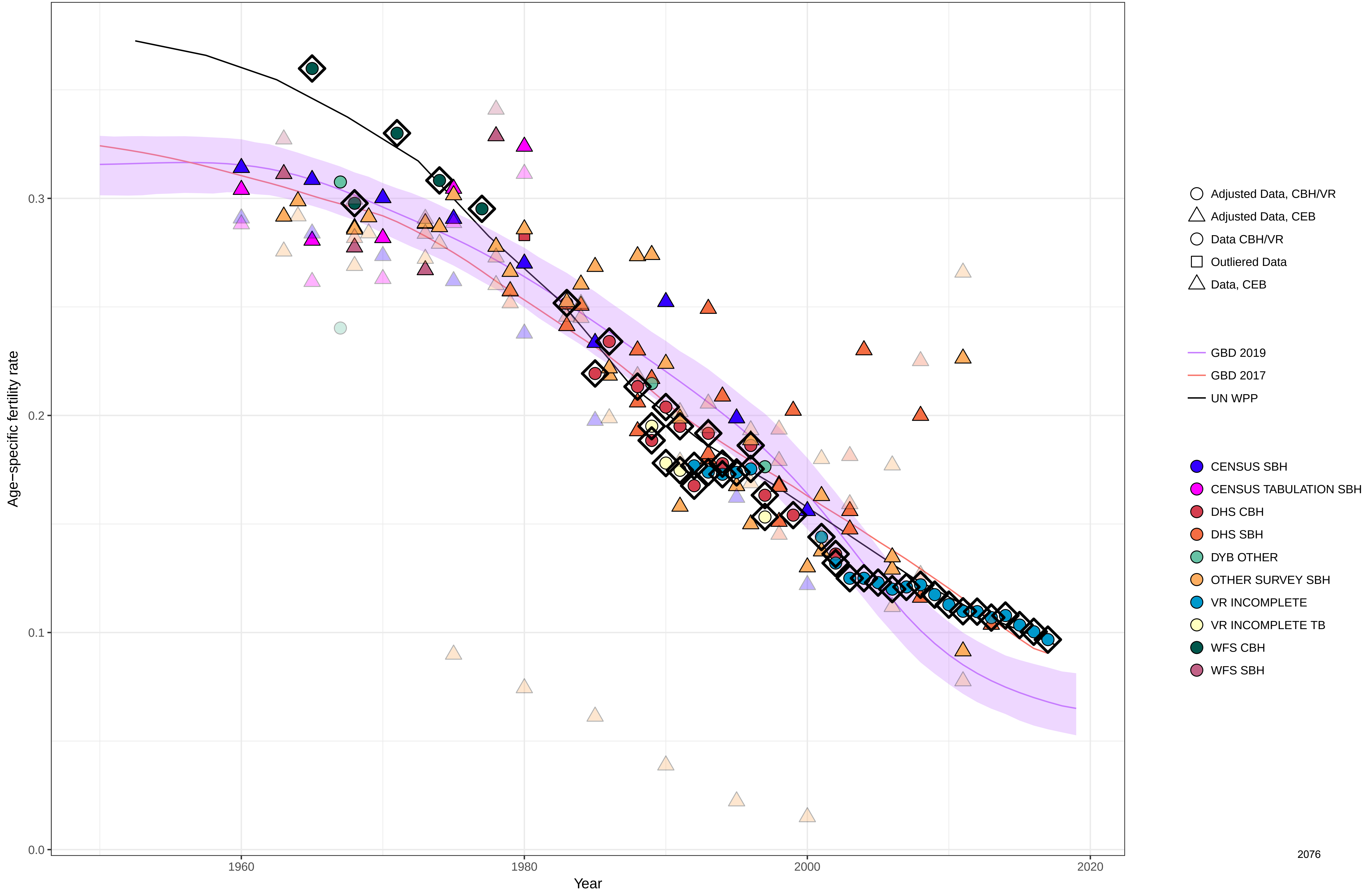

Age−specific fertility fitted trend lines, 1950−2019: Turkey, Ages: 25−29
Data points enclosed with a diamond are reference sources

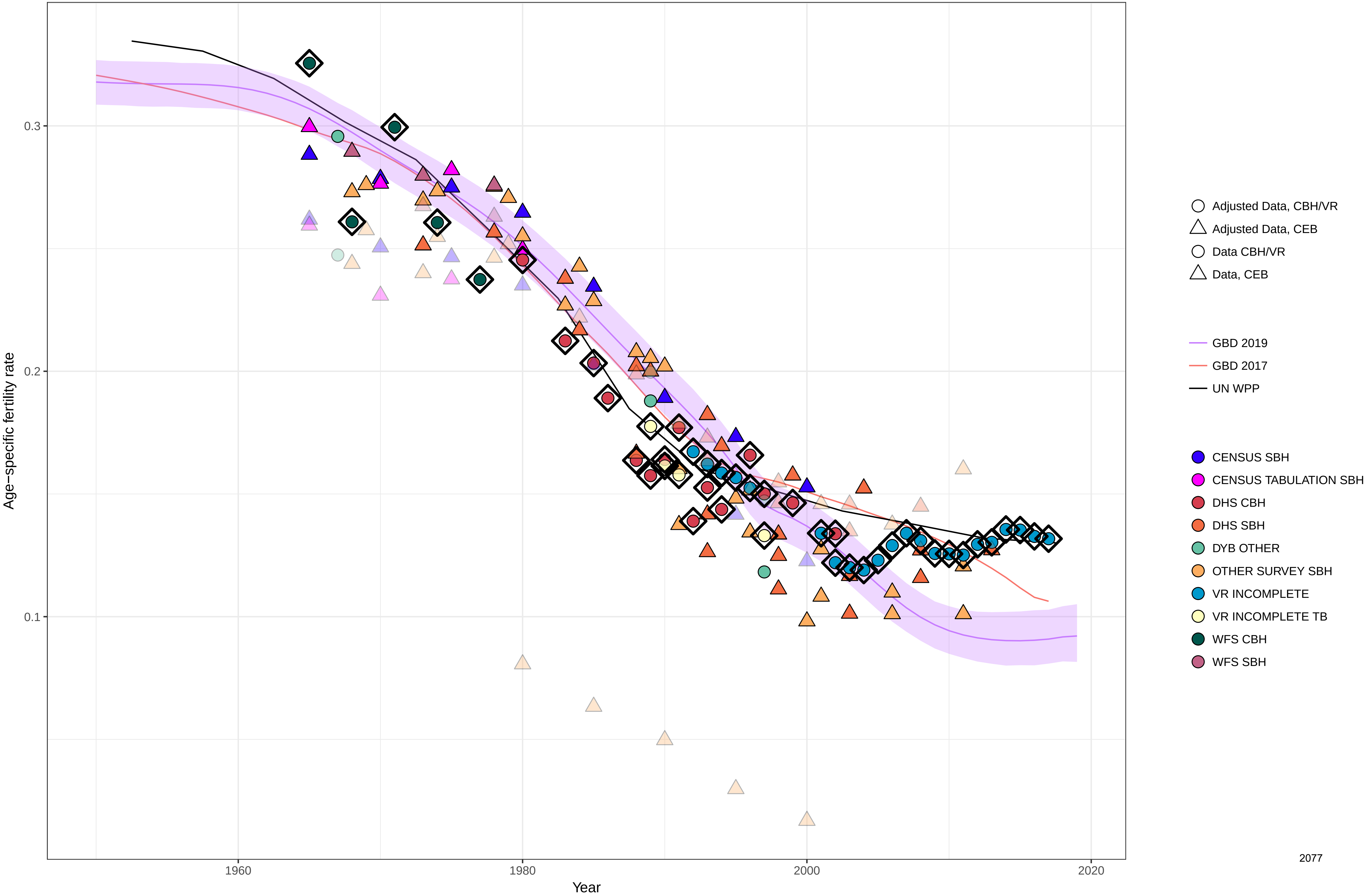

Age−specific fertility fitted trend lines, 1950−2019: Turkey, Ages: 30−34
Data points enclosed with a diamond are reference sources

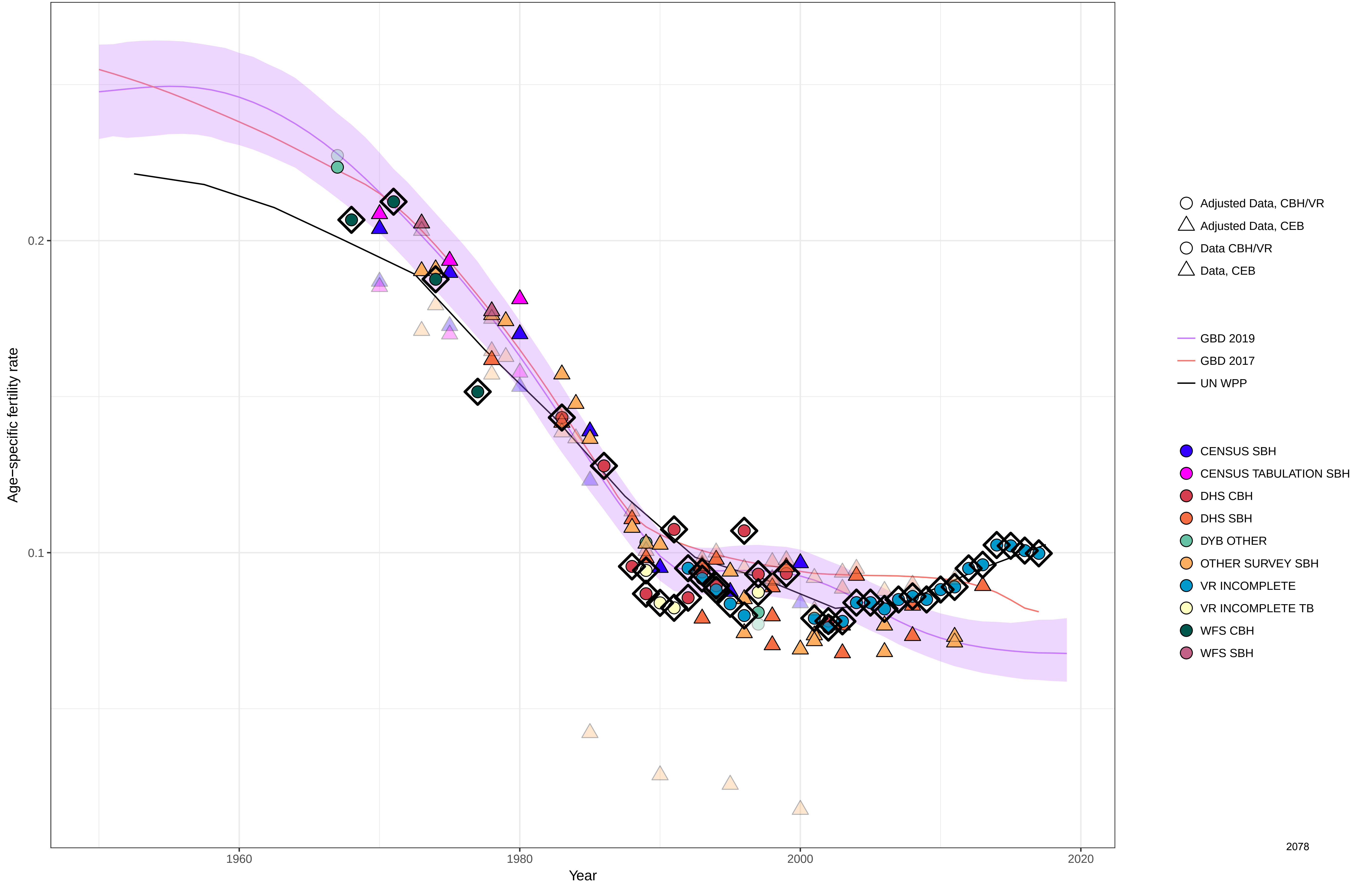

Age−specific fertility fitted trend lines, 1950−2019: Turkey, Ages: 35−39
Data points enclosed with a diamond are reference sources

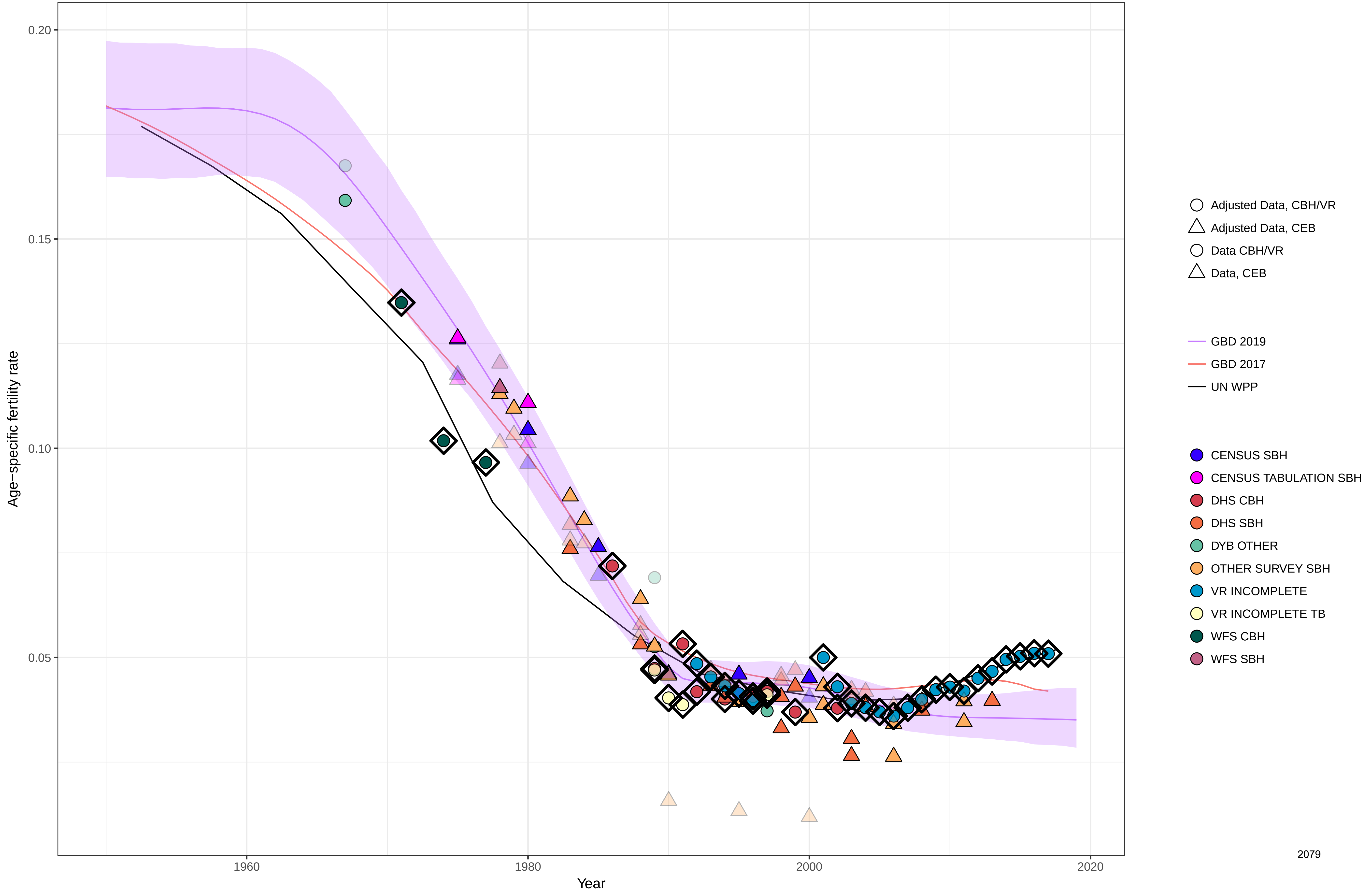

Age−specific fertility fitted trend lines, 1950−2019: Turkey, Ages: 40−44
Data points enclosed with a diamond are reference sources

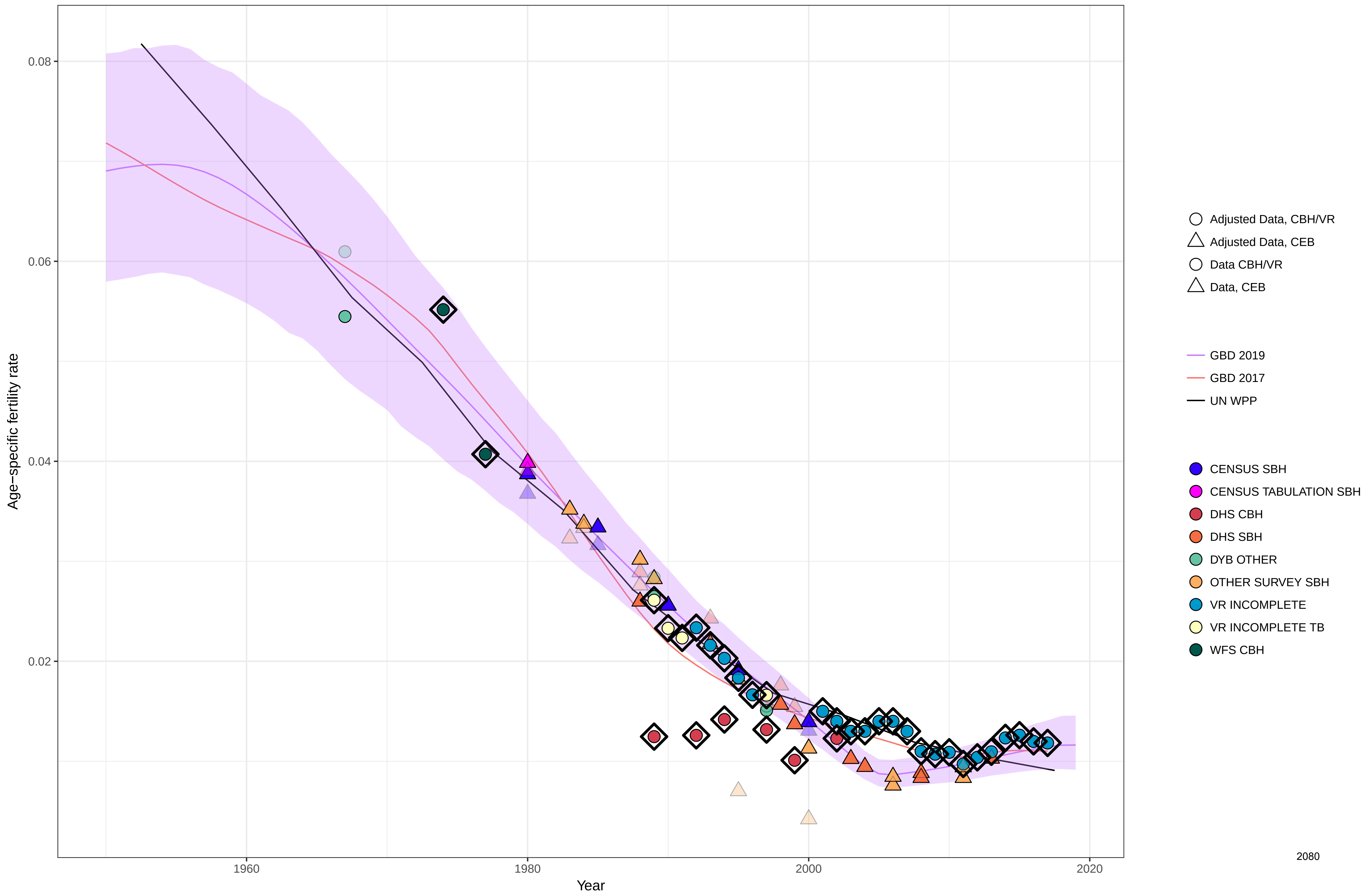

Age−specific fertility fitted trend lines, 1950−2019: Turkey, Ages: 45−49
Data points enclosed with a diamond are reference sources

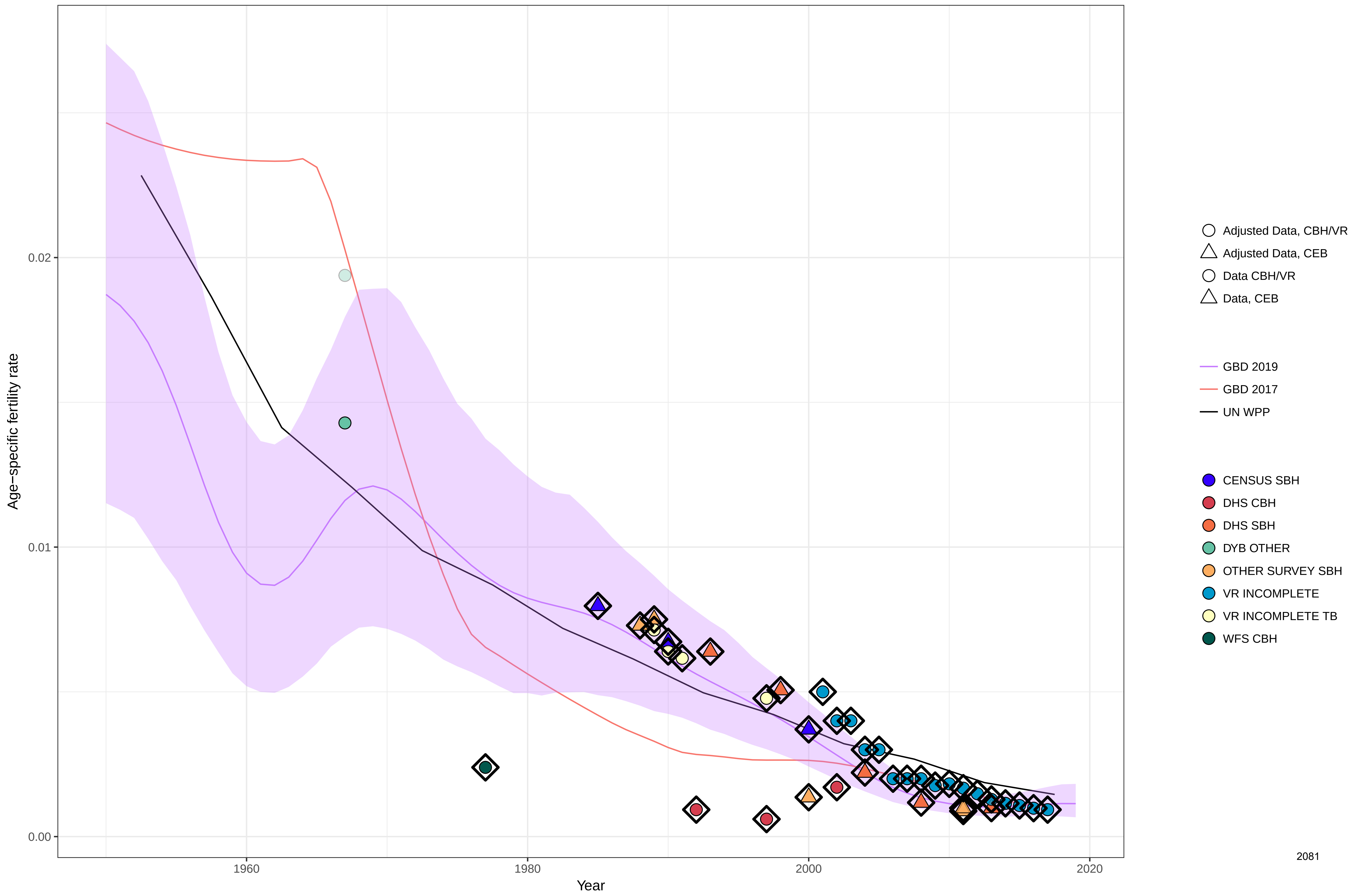

Age−specific fertility fitted trend lines, 1950−2019: Turkey, Ages: 50−54
Data points enclosed with a diamond are reference sources

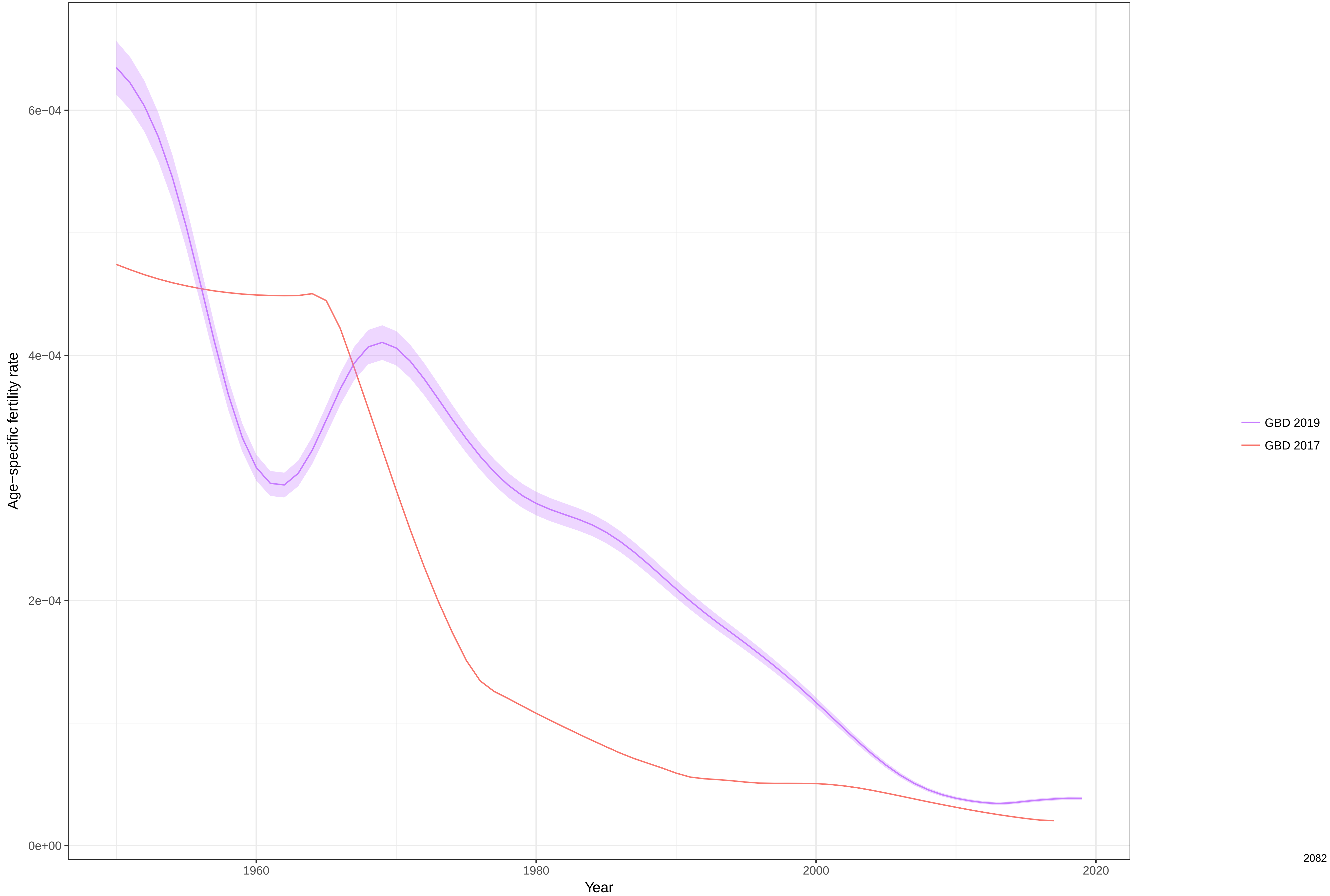

Total fertility rate fitted trend lines, 1950−2019: Turkey TFR
Data points enclosed with a diamond are reference sources

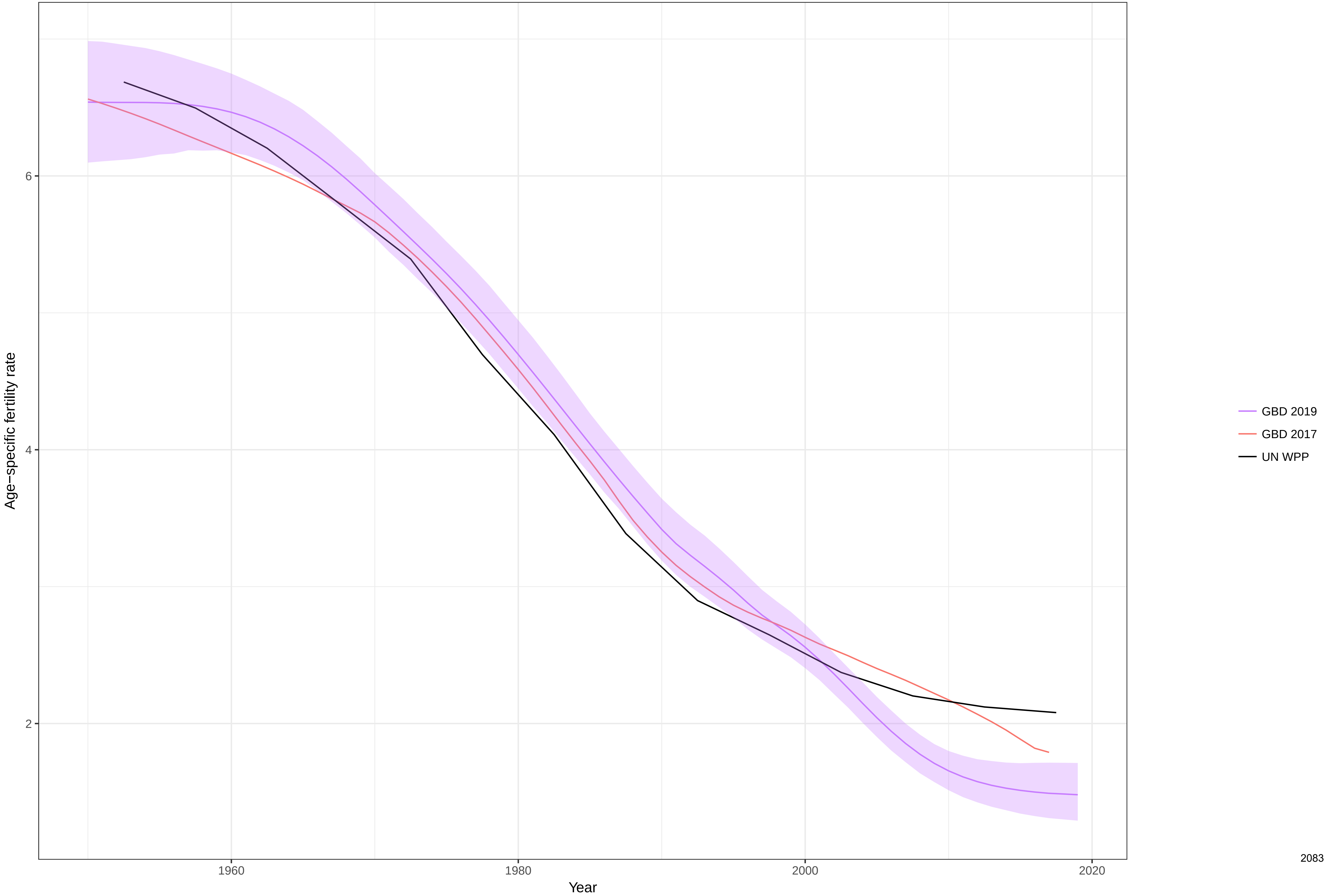

Age−specific fertility fitted trend lines, 1950−2019: Tuvalu, Ages: 10−14
Data points enclosed with a diamond are reference sources

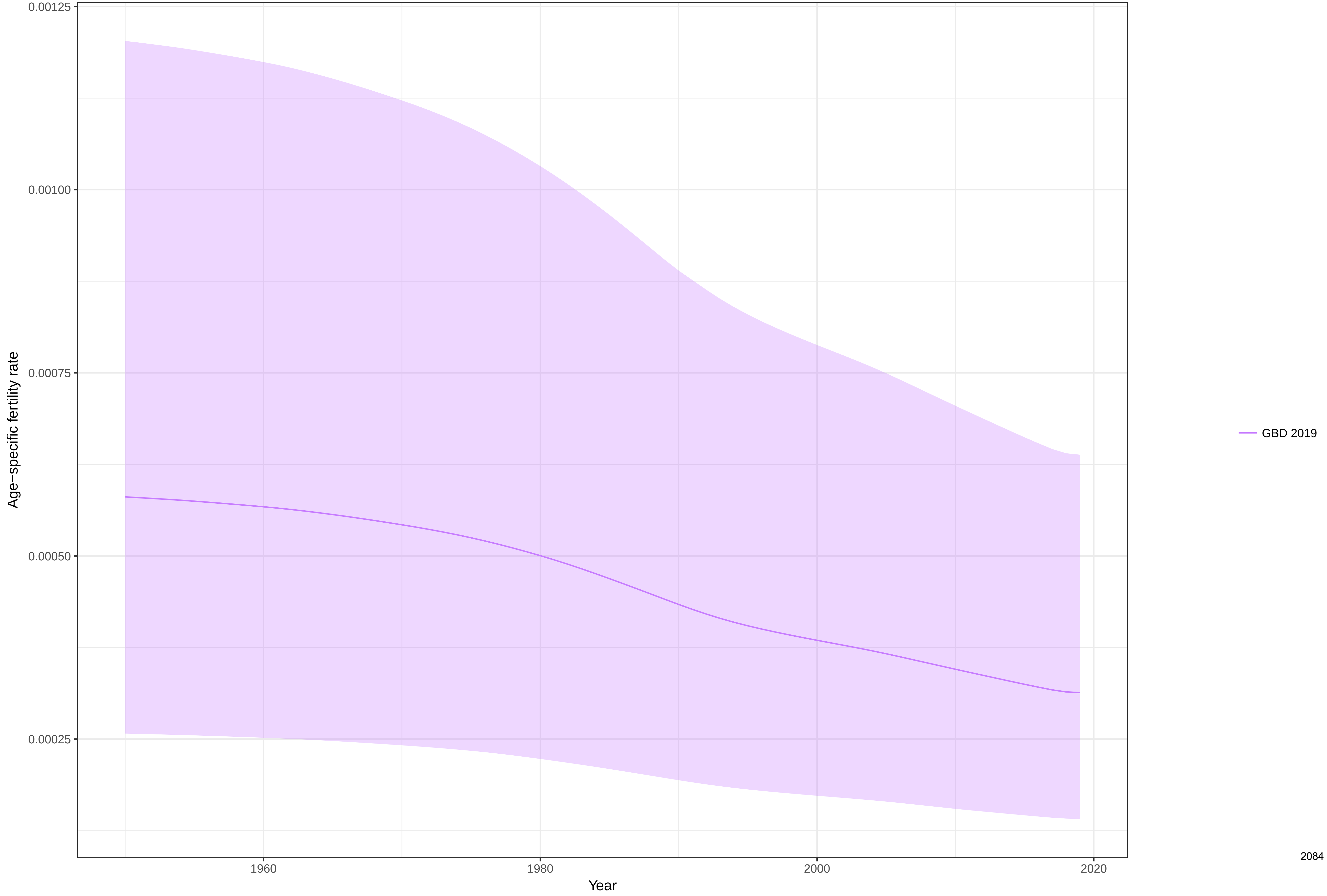

Age−specific fertility fitted trend lines, 1950−2019: Tuvalu, Ages: 15−19
Data points enclosed with a diamond are reference sources

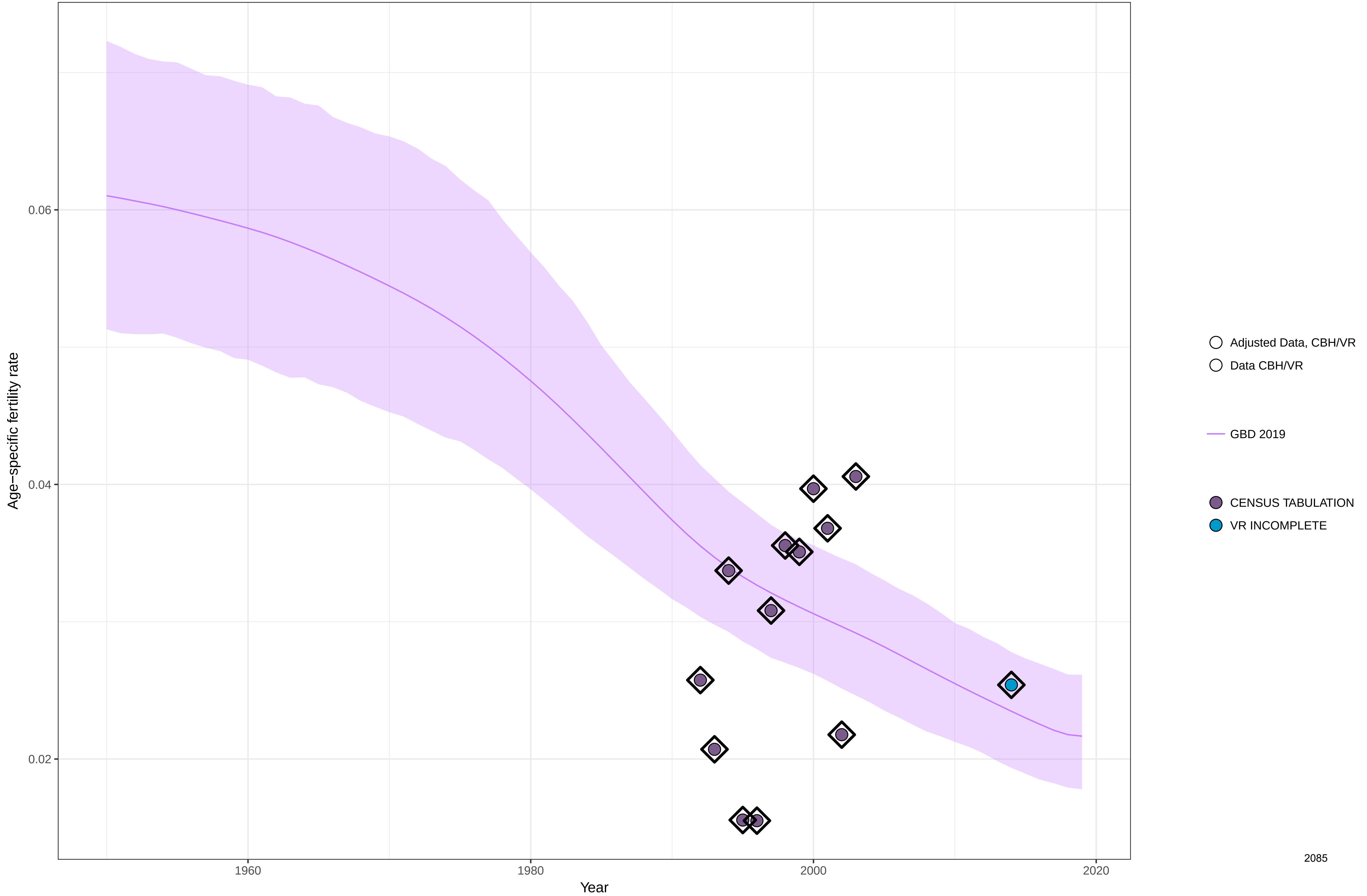

Age−specific fertility fitted trend lines, 1950−2019: Tuvalu, Ages: 20−24
Data points enclosed with a diamond are reference sources

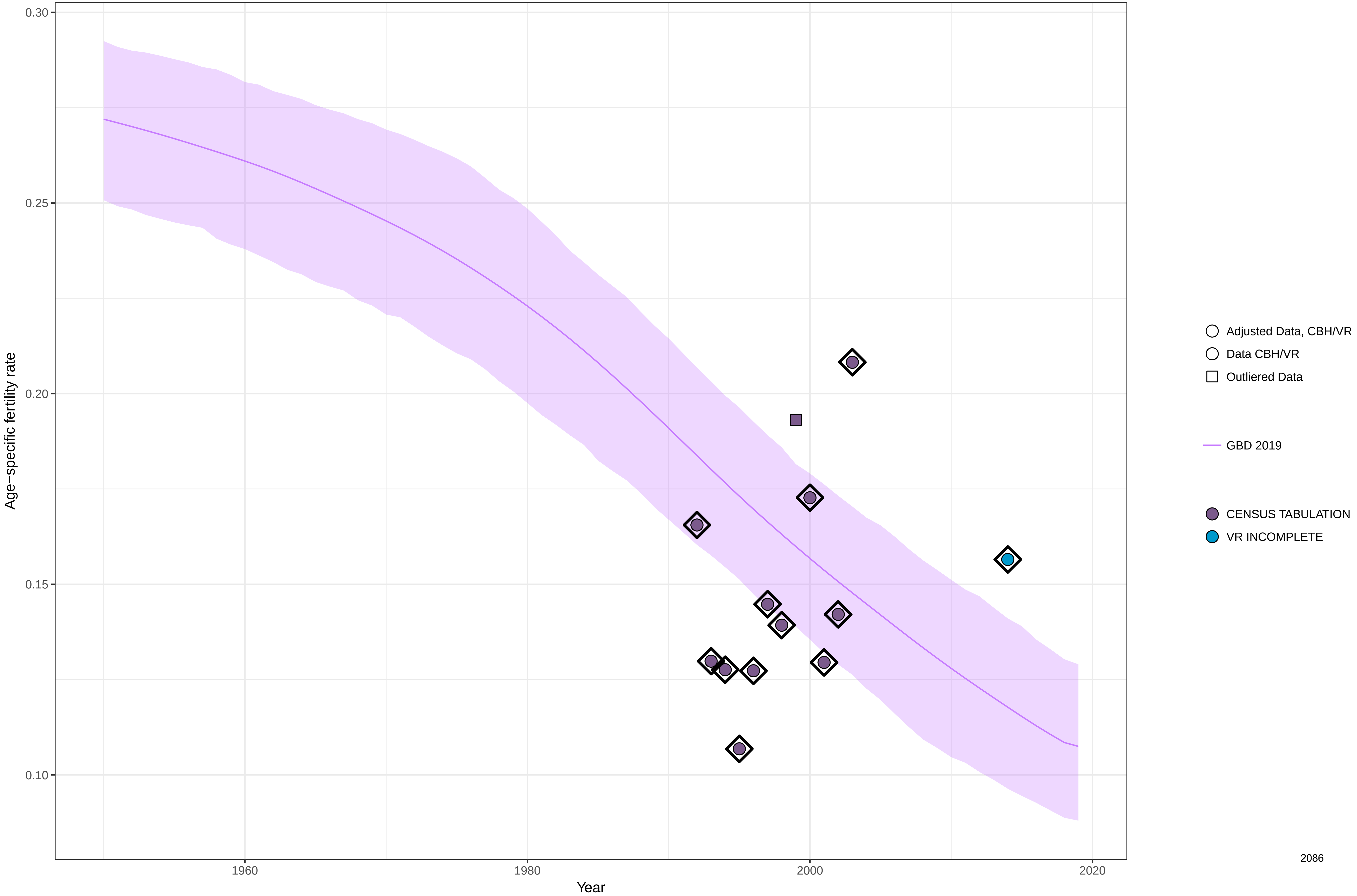

Age−specific fertility fitted trend lines, 1950−2019: Tuvalu, Ages: 25−29
Data points enclosed with a diamond are reference sources

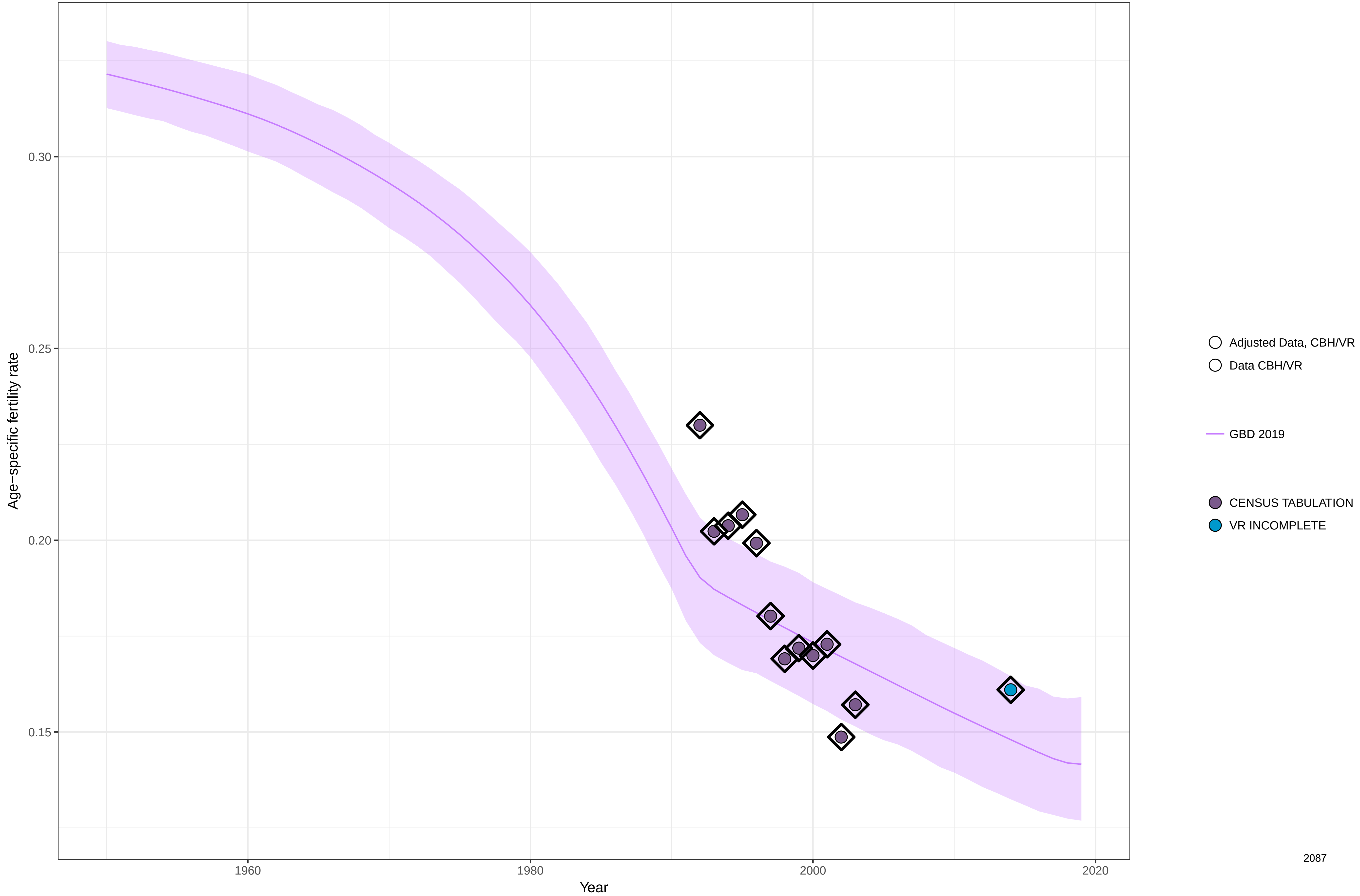

Age−specific fertility fitted trend lines, 1950−2019: Tuvalu, Ages: 30−34
Data points enclosed with a diamond are reference sources

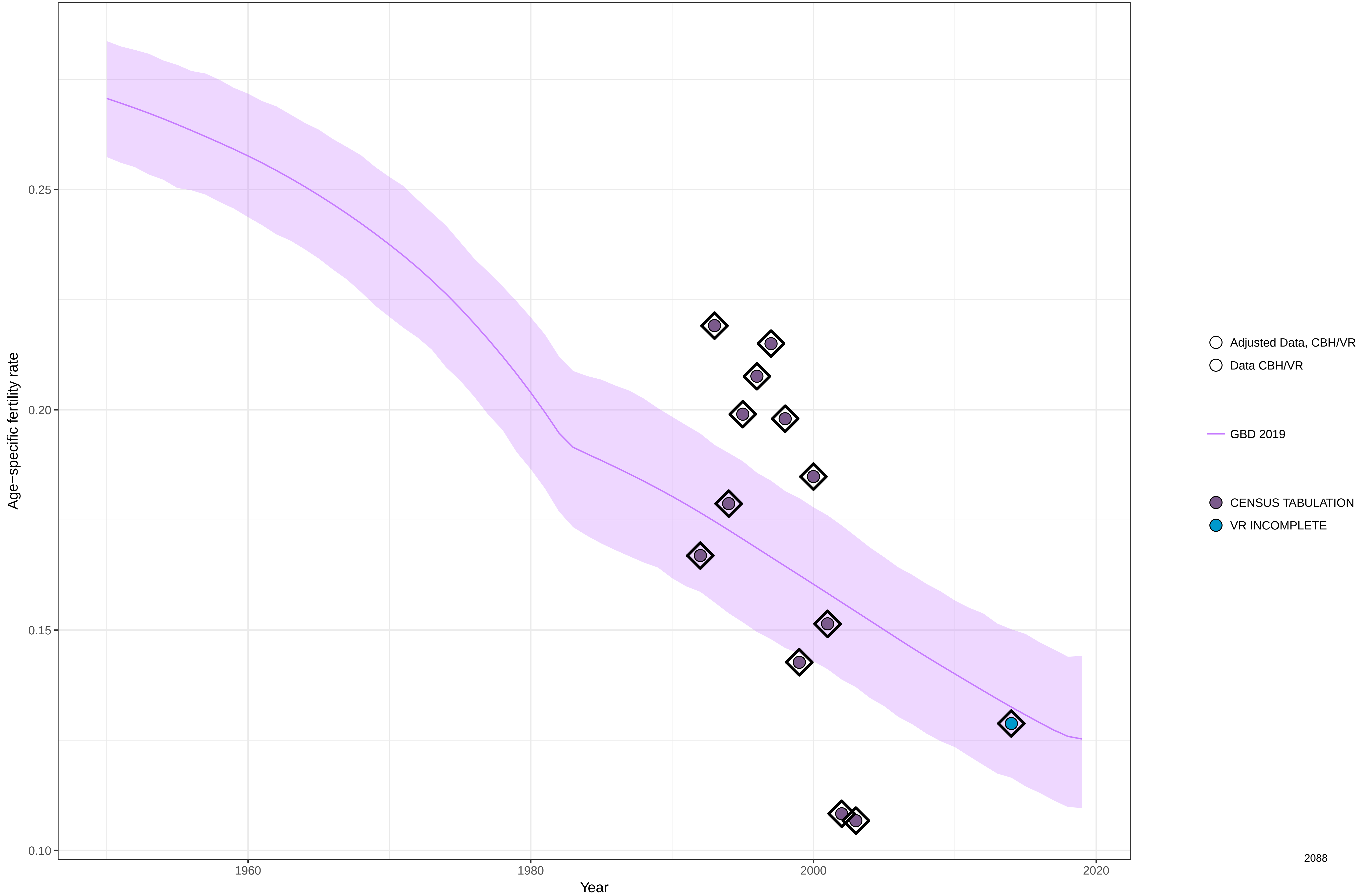

Age−specific fertility fitted trend lines, 1950−2019: Tuvalu, Ages: 35−39
Data points enclosed with a diamond are reference sources

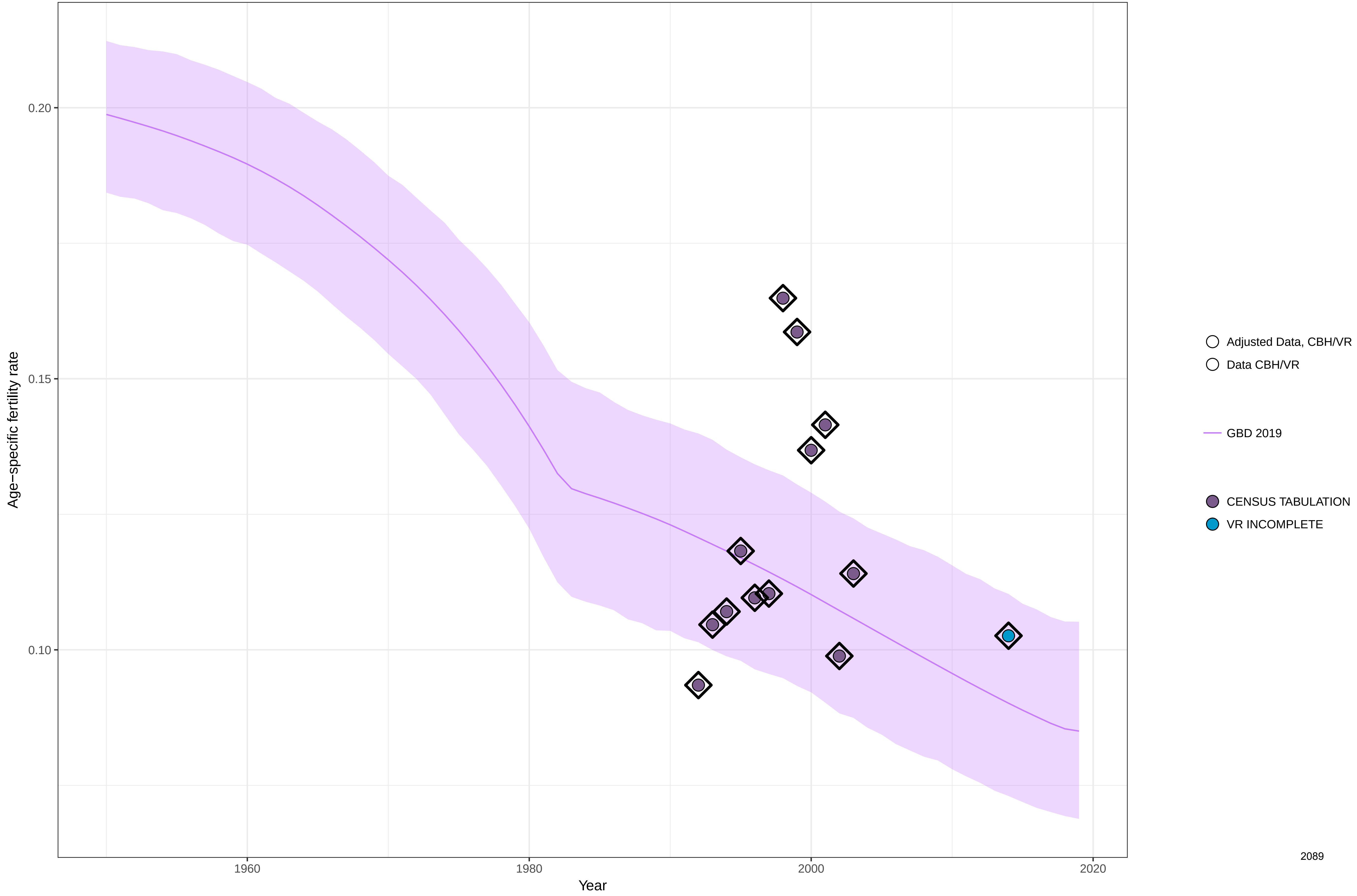

Age−specific fertility fitted trend lines, 1950−2019: Tuvalu, Ages: 40−44
Data points enclosed with a diamond are reference sources

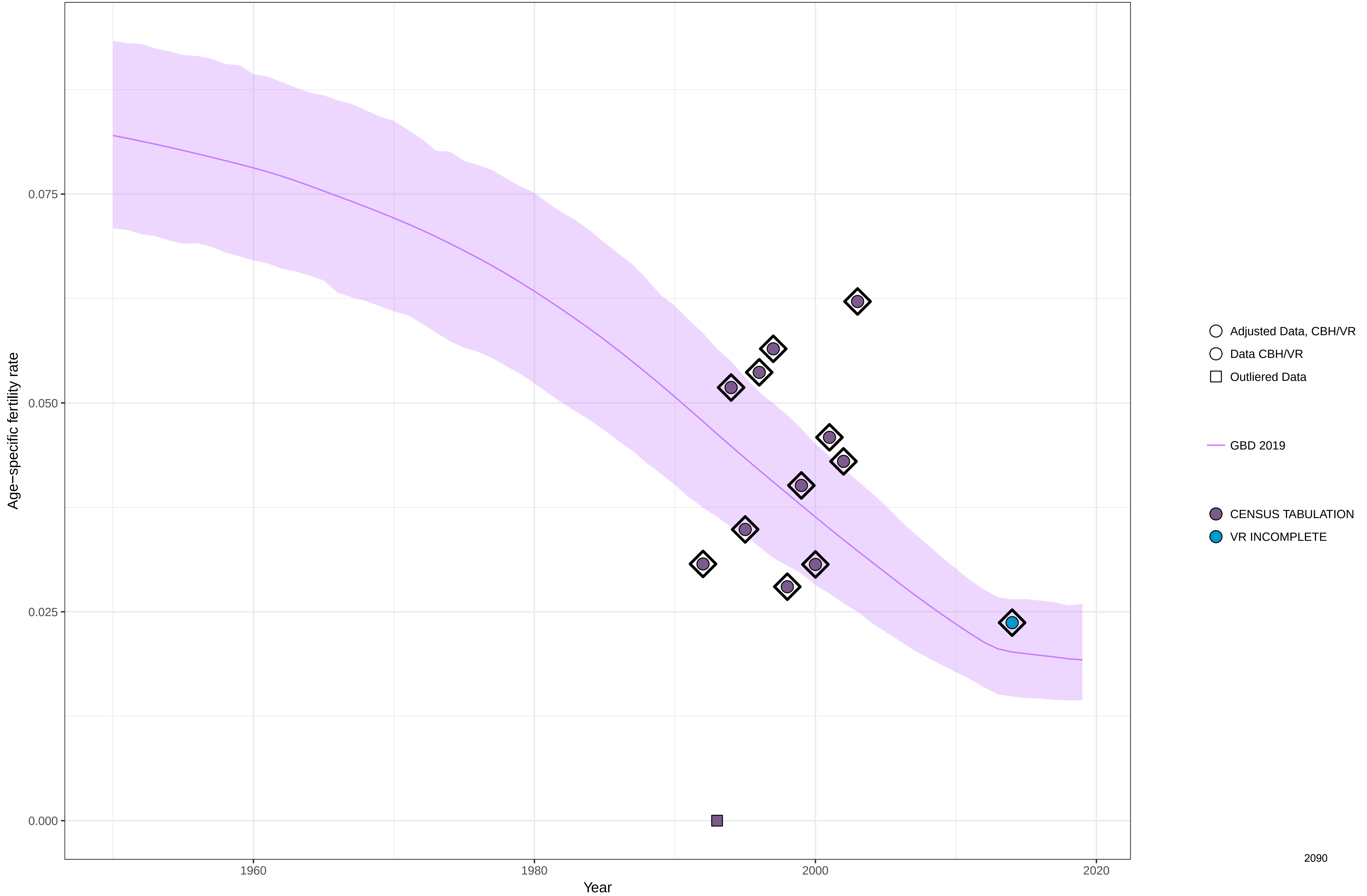

Age−specific fertility fitted trend lines, 1950−2019: Tuvalu, Ages: 45−49
Data points enclosed with a diamond are reference sources

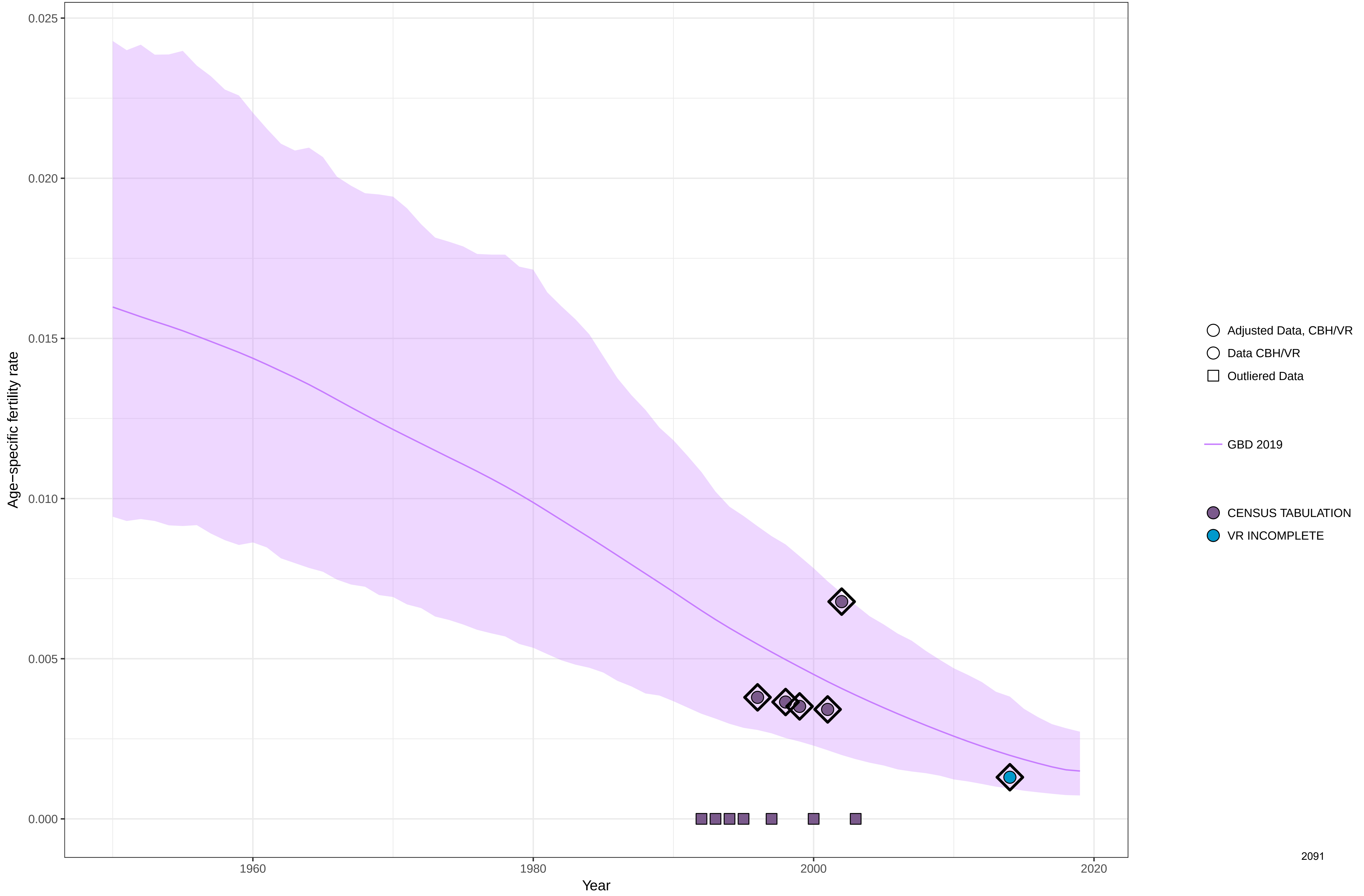

Age−specific fertility fitted trend lines, 1950−2019: Tuvalu, Ages: 50−54
Data points enclosed with a diamond are reference sources

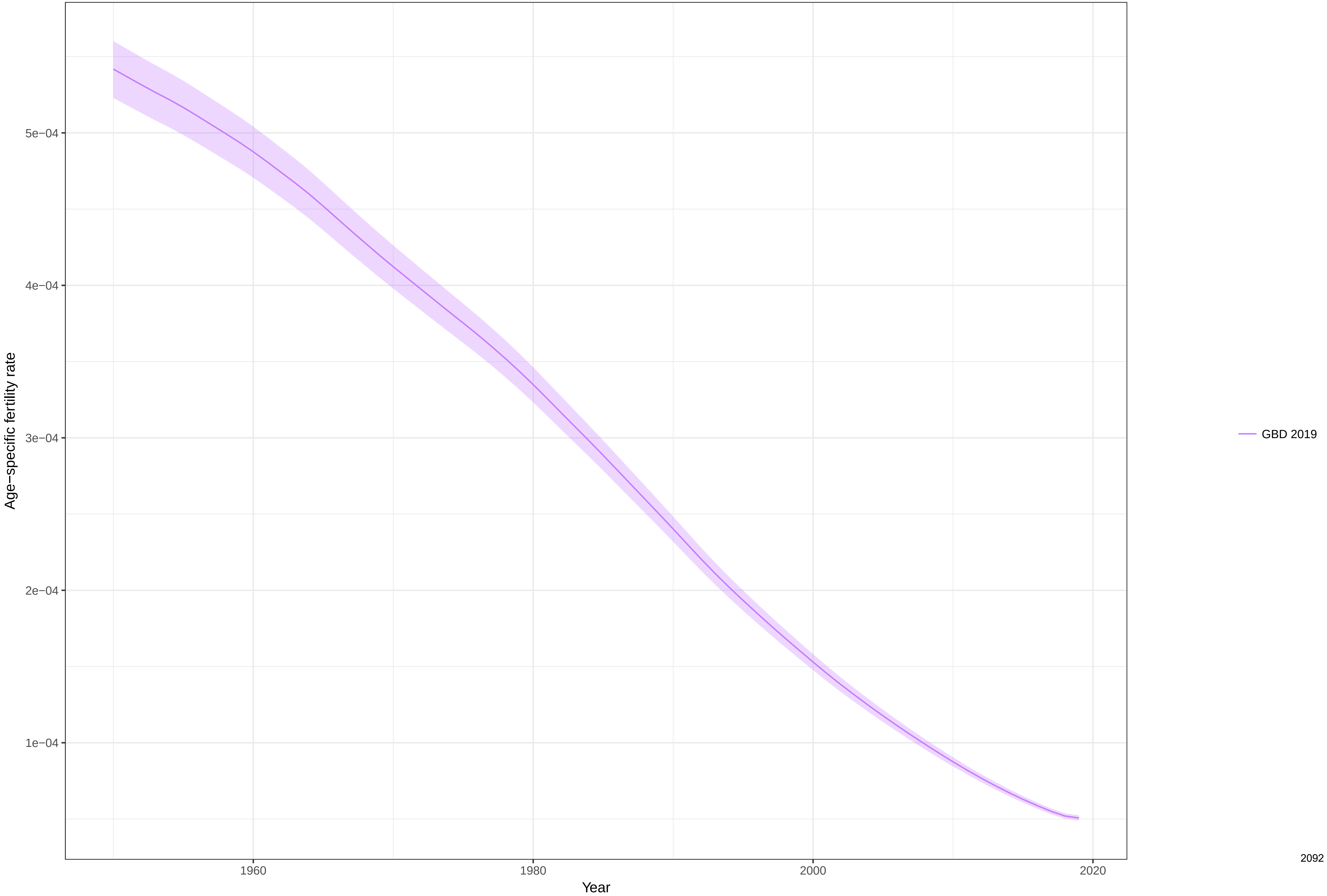

Total fertility rate fitted trend lines, 1950−2019: Tuvalu TFR
Data points enclosed with a diamond are reference sources

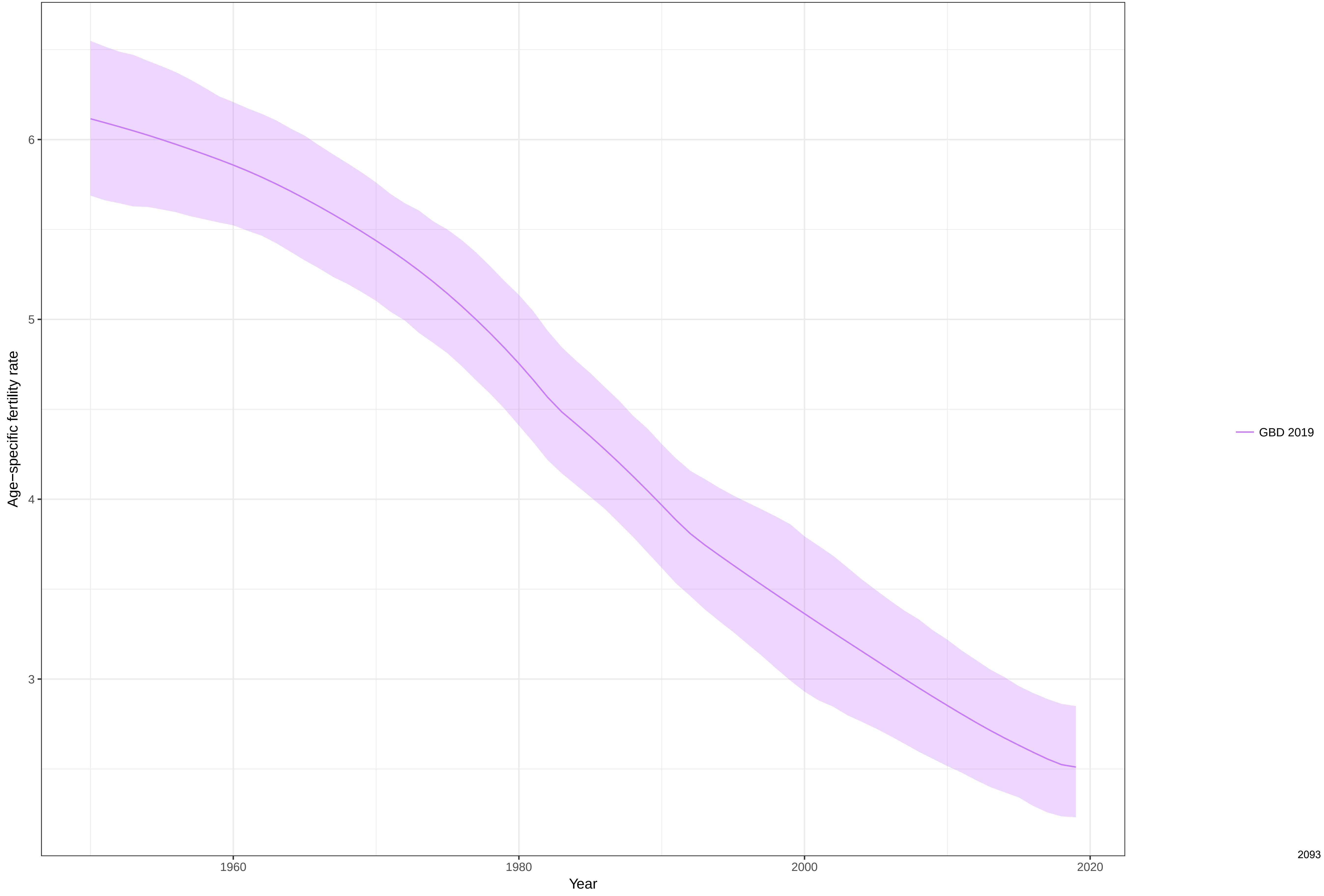

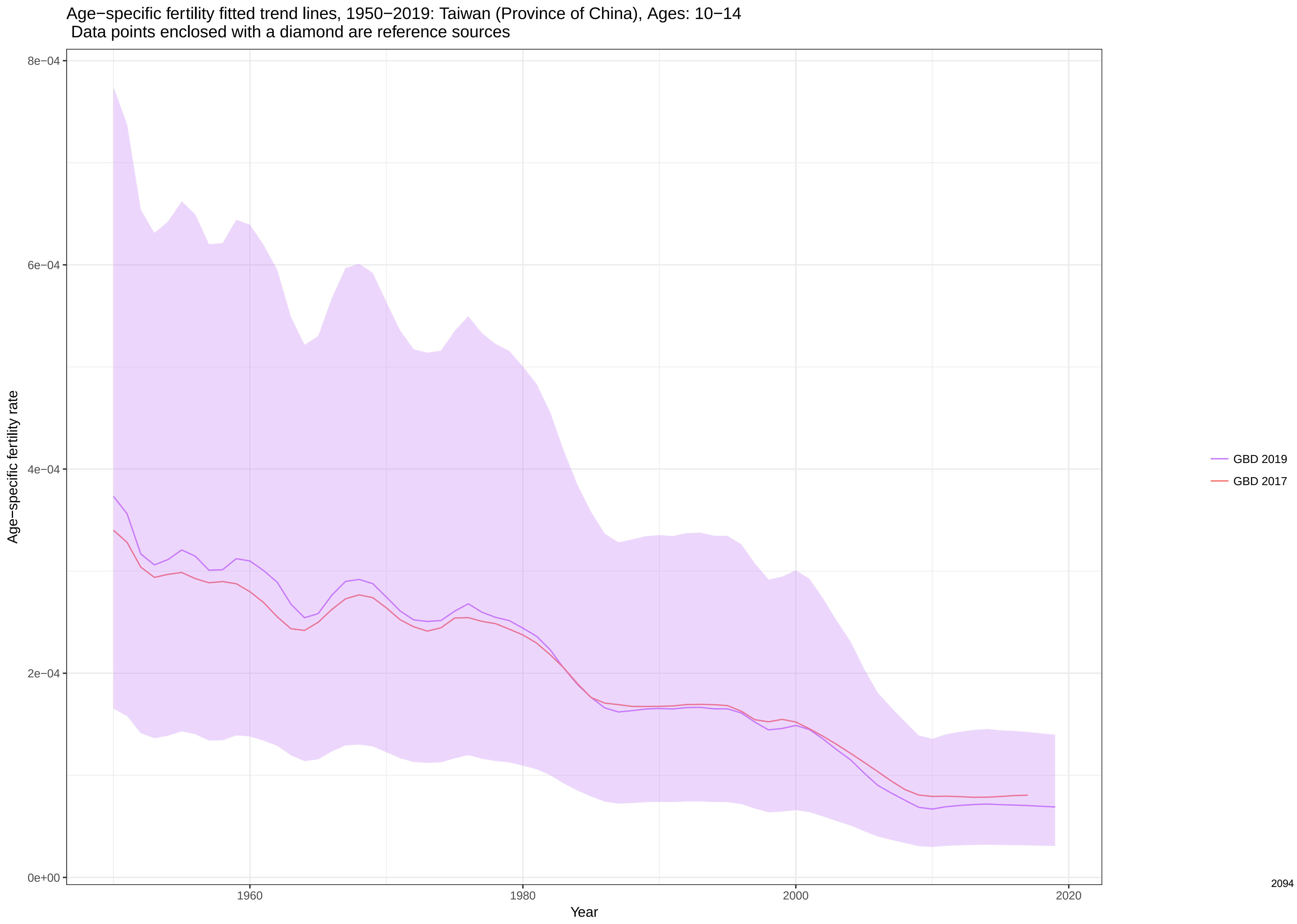
Age−specific fertility fitted trend lines, 1950−2019: Taiwan (Province of China), Ages: 10−14
Data points enclosed with a diamond are reference sources
8e−04
6e−04
4e−04
2e−04
0e+00
Age−specific fertility rate
1960
1980
2000
2020
Year
GBD 2019
GBD 2017

Age−specific fertility fitted trend lines, 1950−2019: Taiwan (Province of China), Ages: 15−19
Data points enclosed with a diamond are reference sources

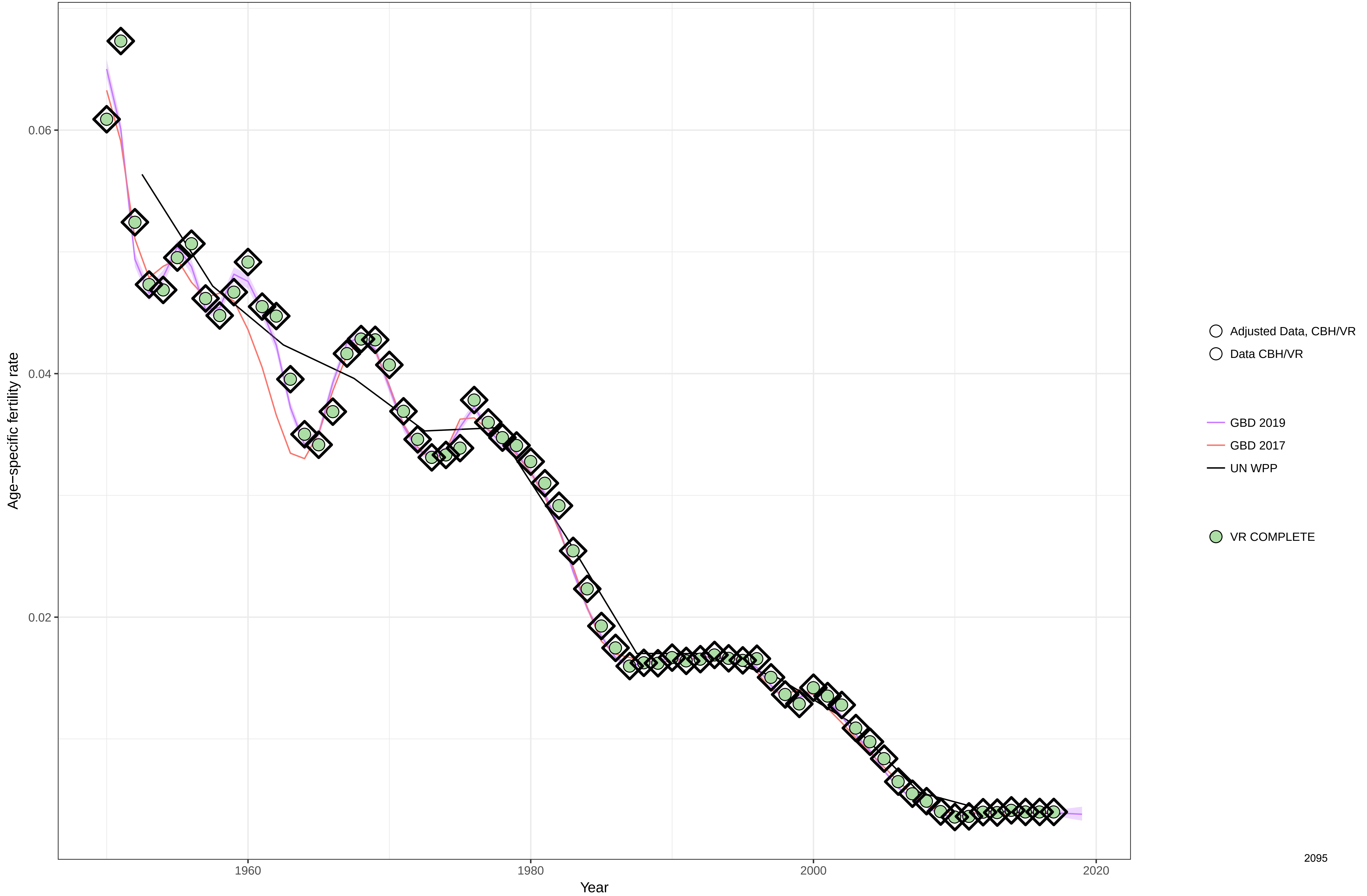

Age−specific fertility fitted trend lines, 1950−2019: Taiwan (Province of China), Ages: 20−24
Data points enclosed with a diamond are reference sources

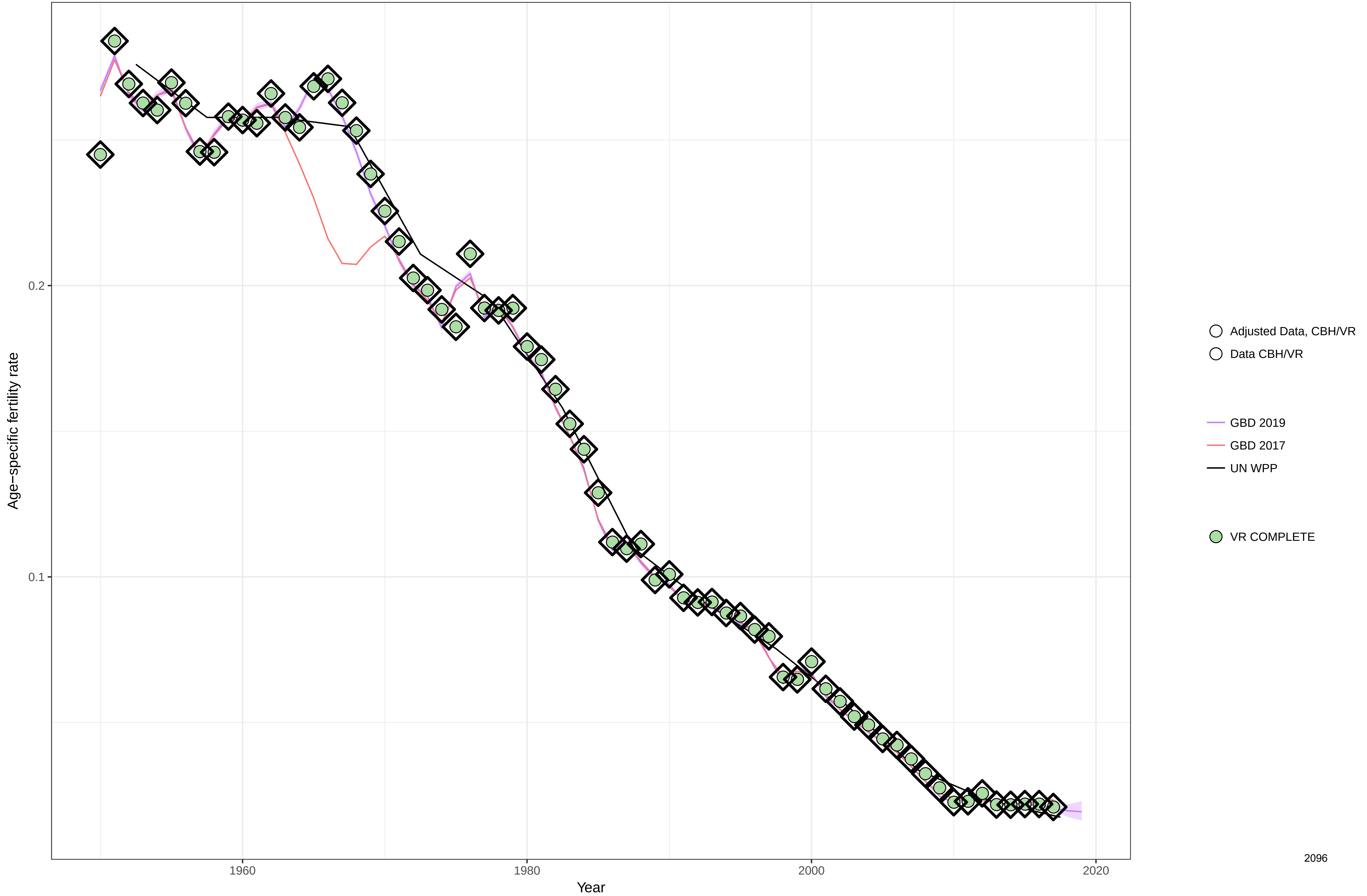

Age−specific fertility fitted trend lines, 1950−2019: Taiwan (Province of China), Ages: 25−29
Data points enclosed with a diamond are reference sources

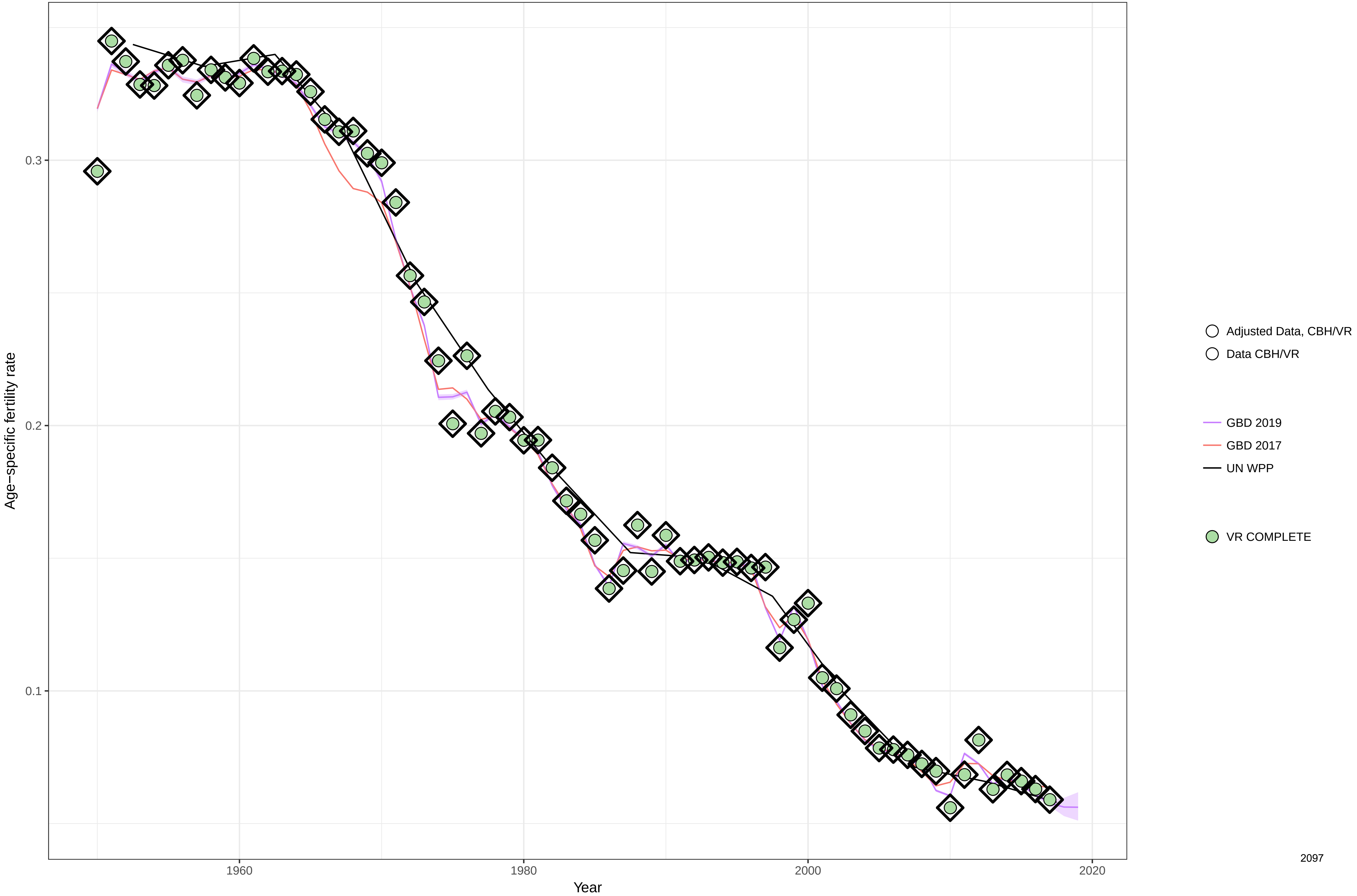

Age−specific fertility fitted trend lines, 1950−2019: Taiwan (Province of China), Ages: 30−34
Data points enclosed with a diamond are reference sources

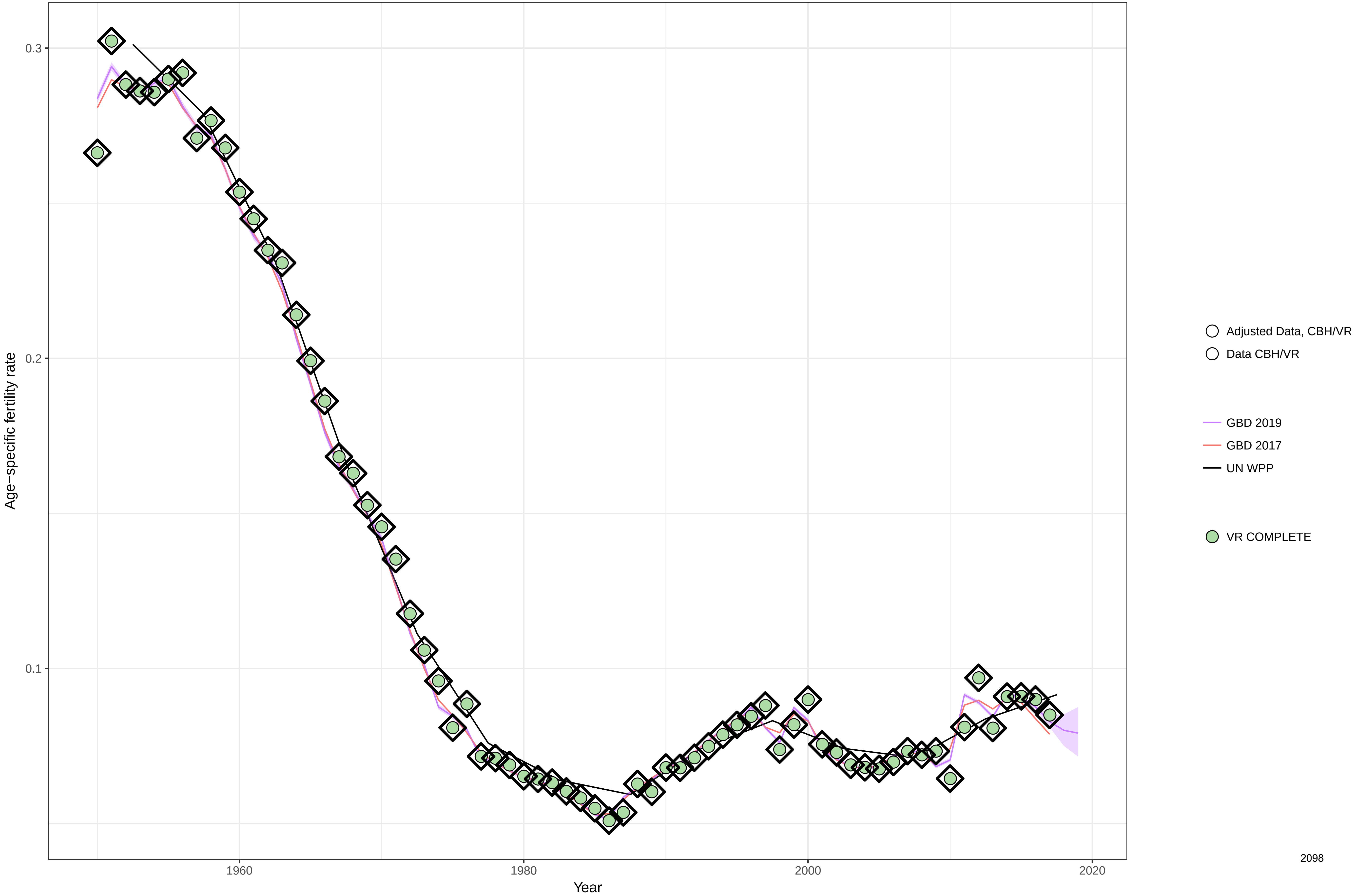

Age−specific fertility fitted trend lines, 1950−2019: Taiwan (Province of China), Ages: 35−39
Data points enclosed with a diamond are reference sources

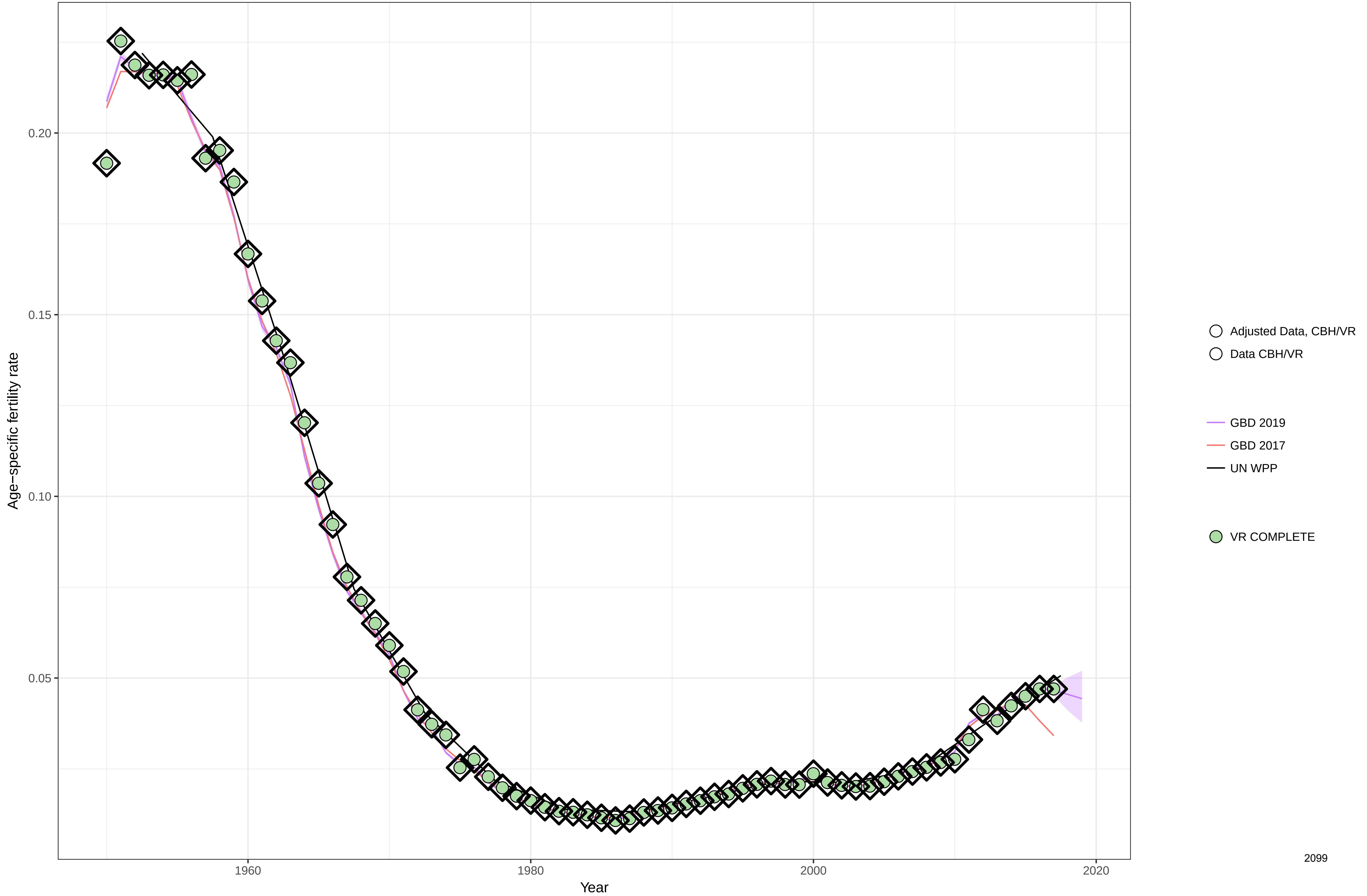

Age−specific fertility fitted trend lines, 1950−2019: Taiwan (Province of China), Ages: 40−44
Data points enclosed with a diamond are reference sources

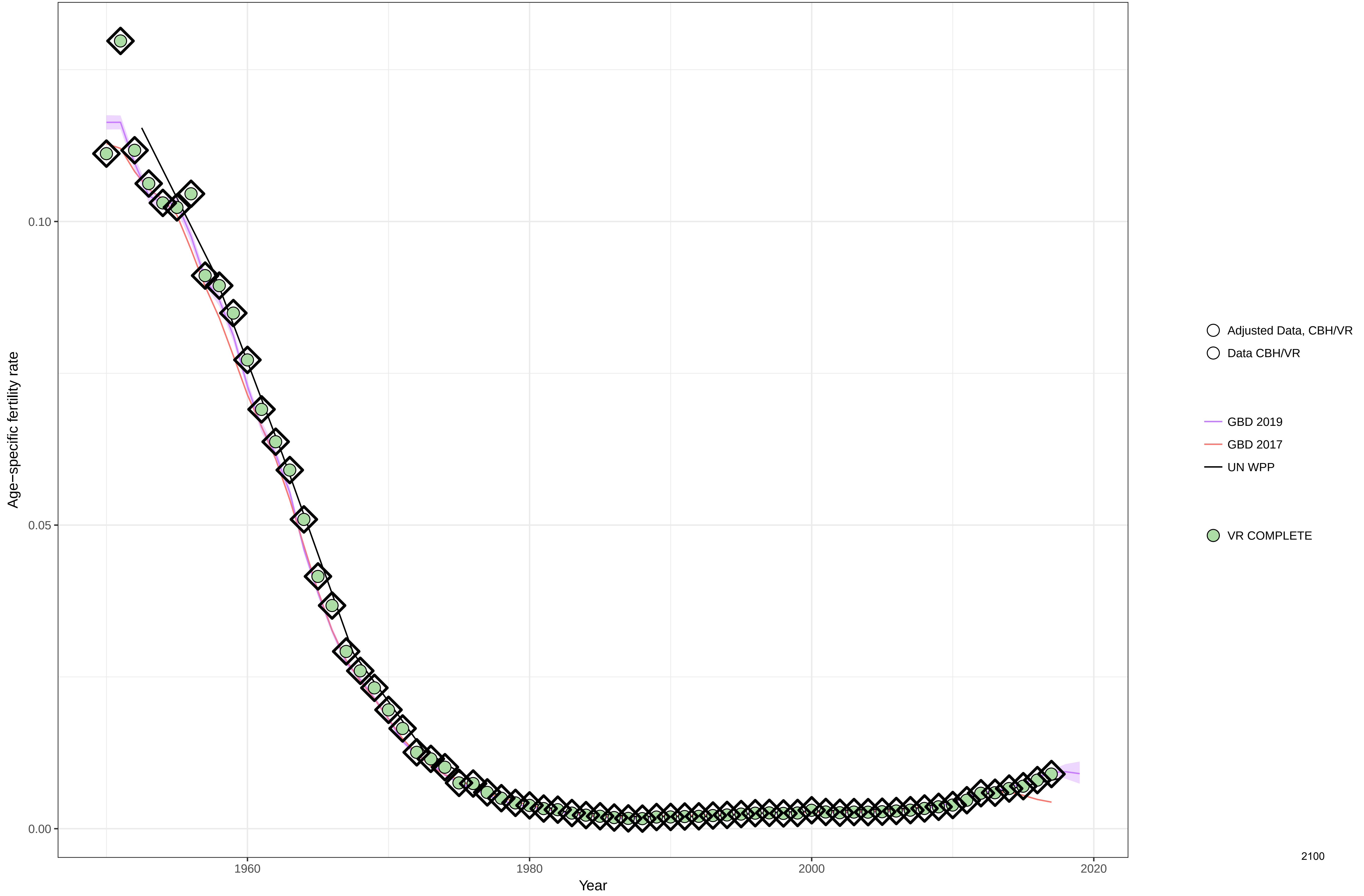

Age−specific fertility fitted trend lines, 1950−2019: Taiwan (Province of China), Ages: 45−49
Data points enclosed with a diamond are reference sources

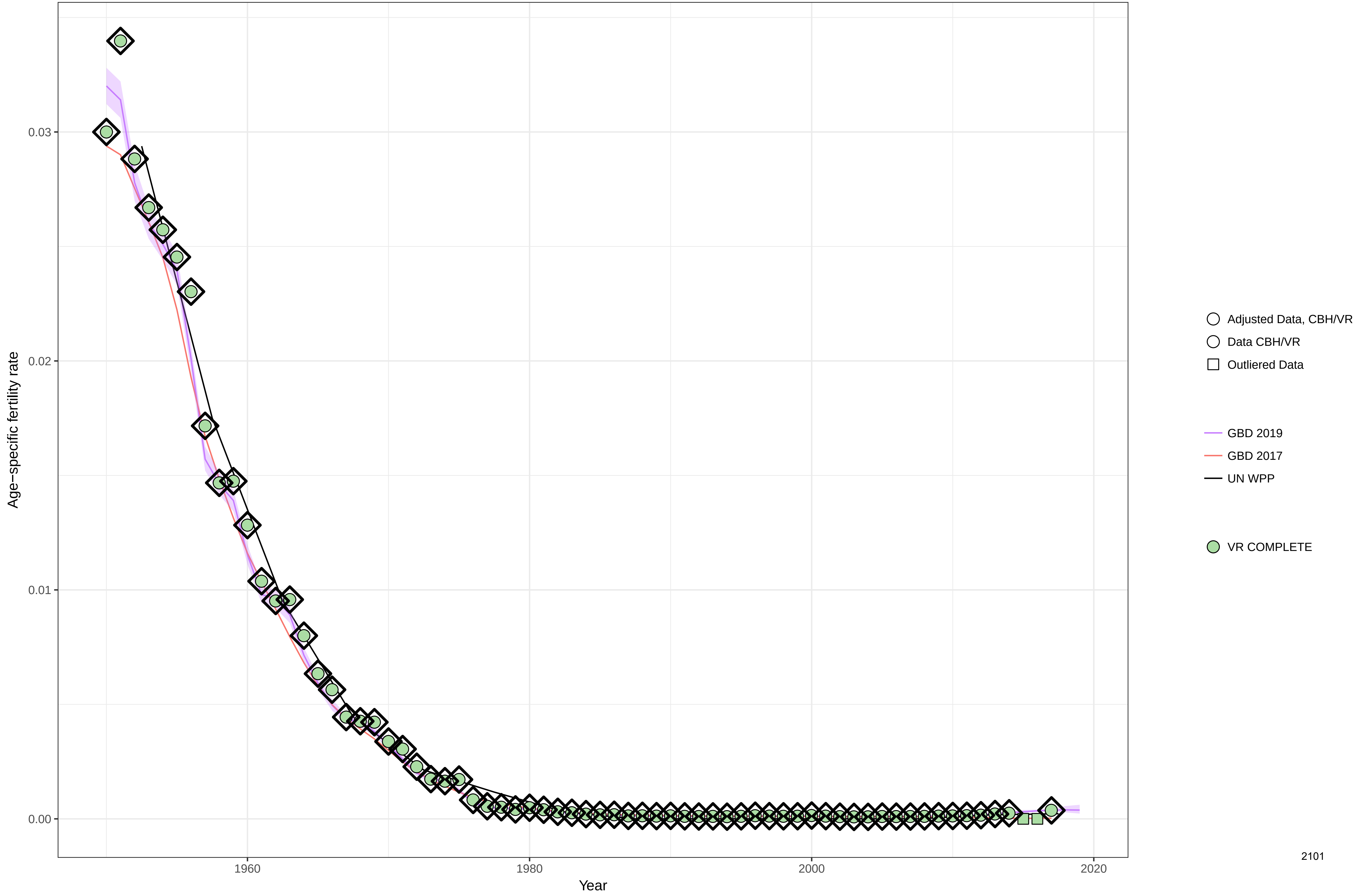

Age−specific fertility fitted trend lines, 1950−2019: Taiwan (Province of China), Ages: 50−54
Data points enclosed with a diamond are reference sources

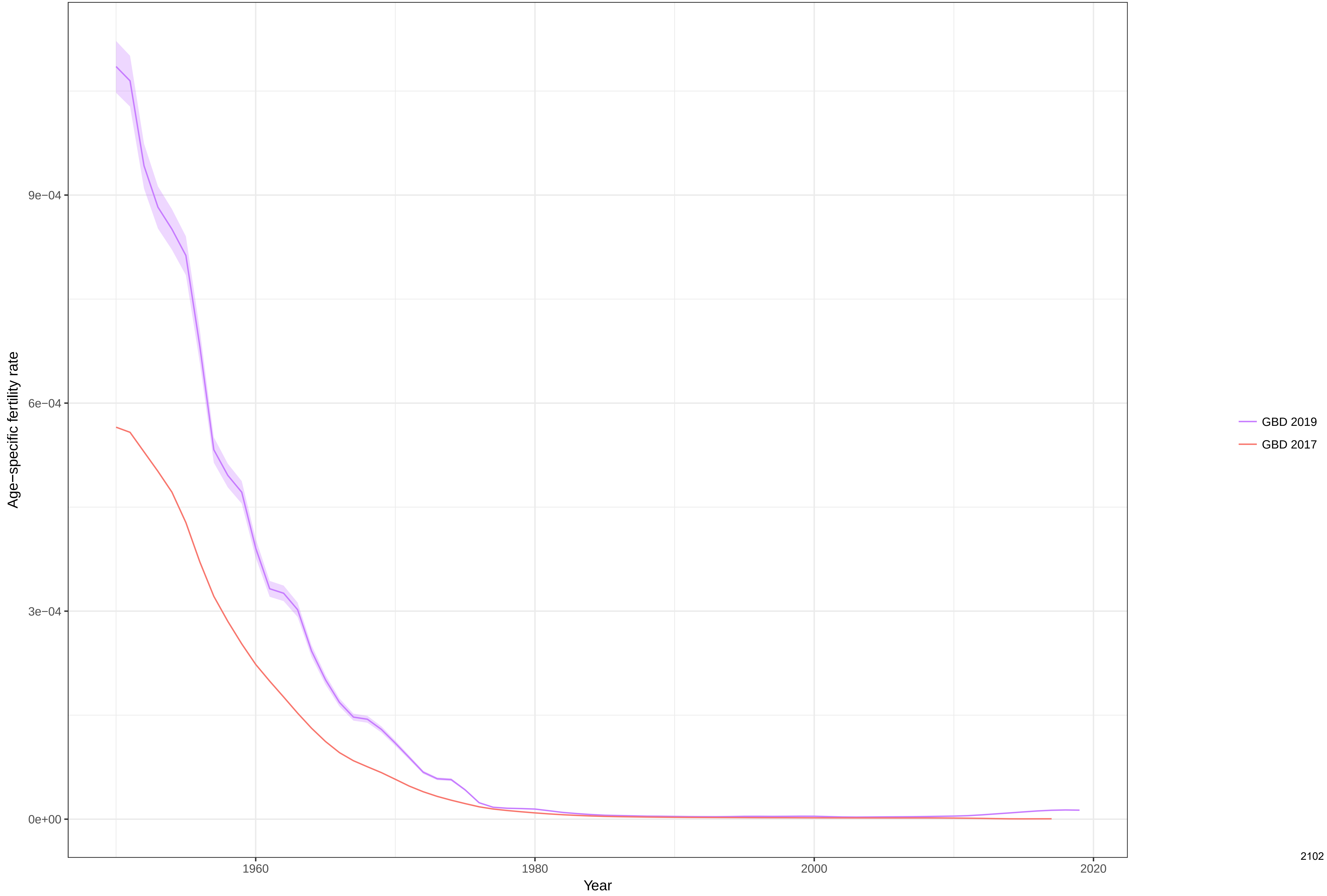

Total fertility rate fitted trend lines, 1950−2019: Taiwan (Province of China) TFR
Data points enclosed with a diamond are reference sources

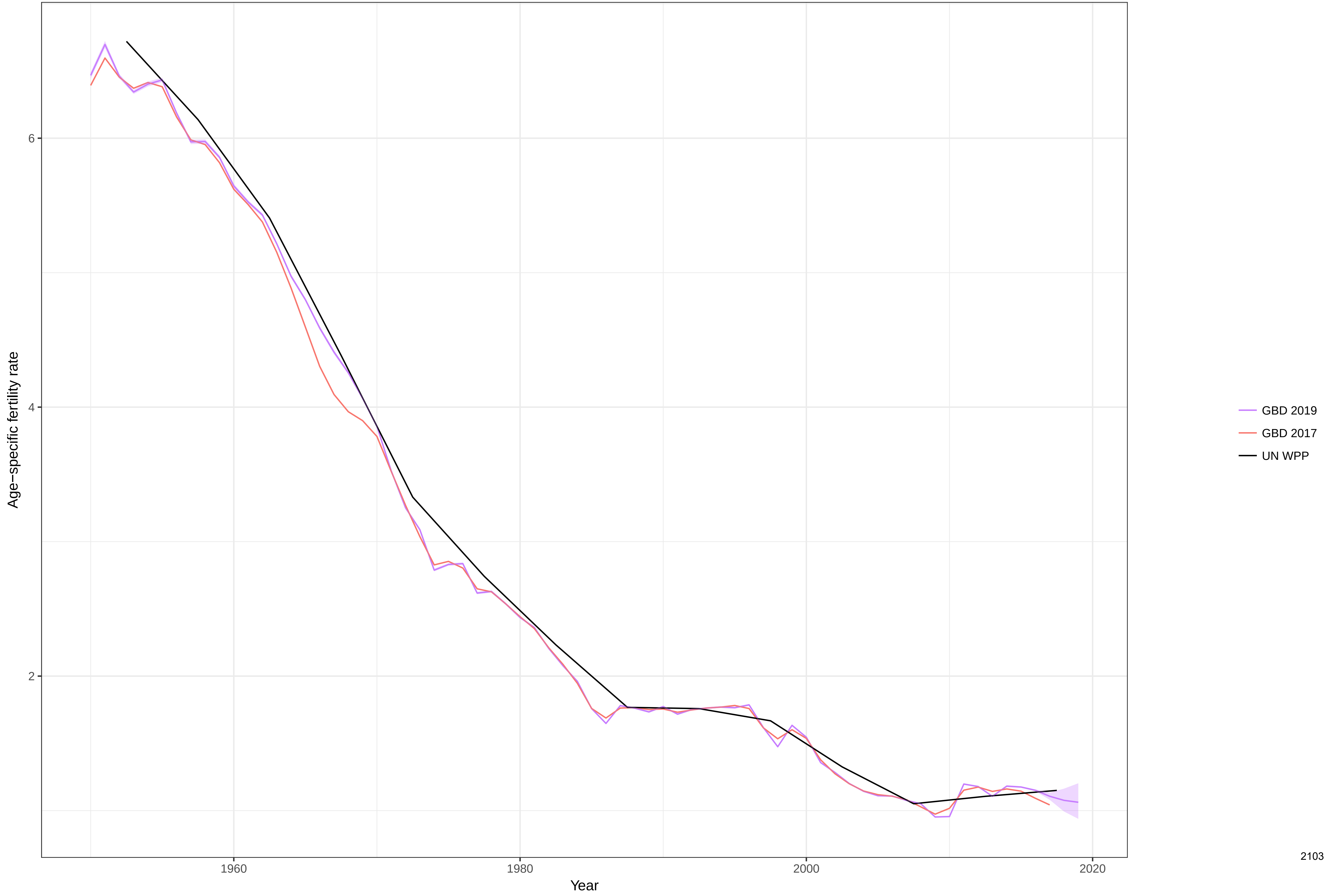

Age−specific fertility fitted trend lines, 1950−2019: United Republic of Tanzania, Ages: 10−14
Data points enclosed with a diamond are reference sources

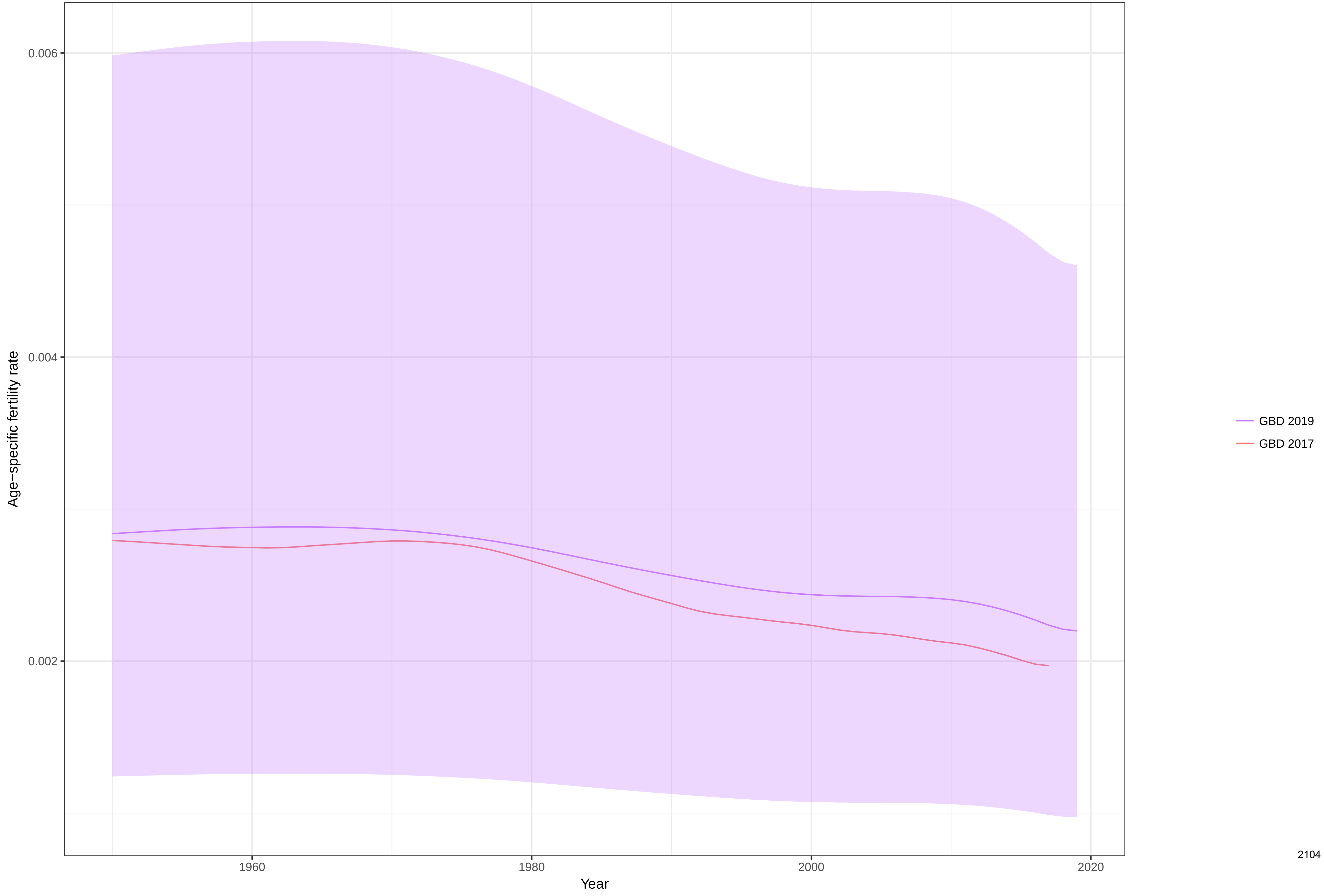

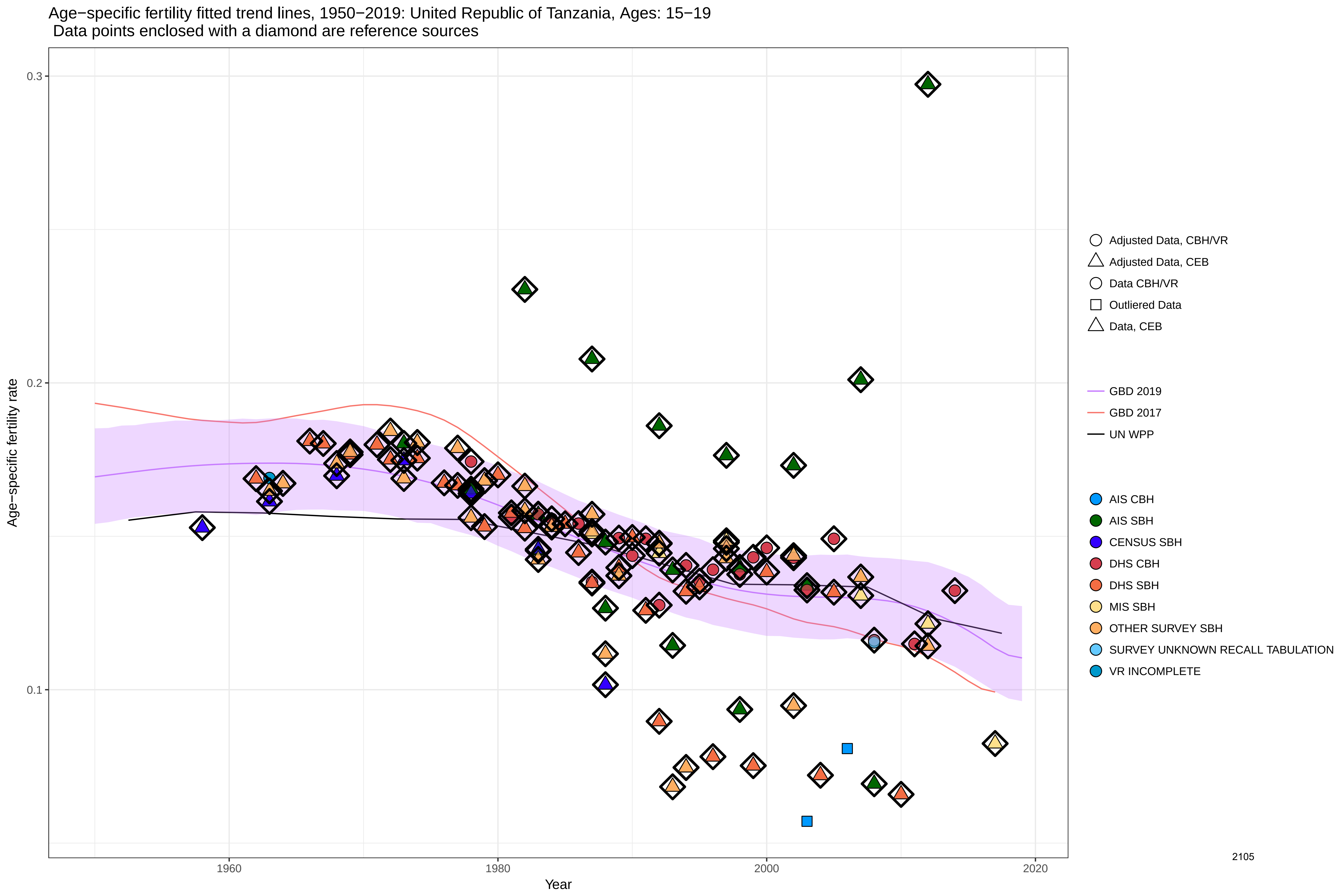

Age−specific fertility fitted trend lines, 1950−2019: United Republic of Tanzania, Ages: 15−19
Data points enclosed with a diamond are reference sources
Age−specific fertility rate
0.3
0.2
0.1
Year
1960
1980
2000
2020
Adjusted Data, CBH/VR
Adjusted Data, CEB
Data CBH/VR
Outliered Data
Data, CEB
GBD 2019
GBD 2017
UN WPP
AIS CBH
AIS SBH
CENSUS SBH
DHS CBH
DHS SBH
MIS SBH
OTHER SURVEY SBH
SURVEY UNKNOWN RECALL TABULATION
VR INCOMPLETE

Age−specific fertility fitted trend lines, 1950−2019: United Republic of Tanzania, Ages: 20−24
Data points enclosed with a diamond are reference sources

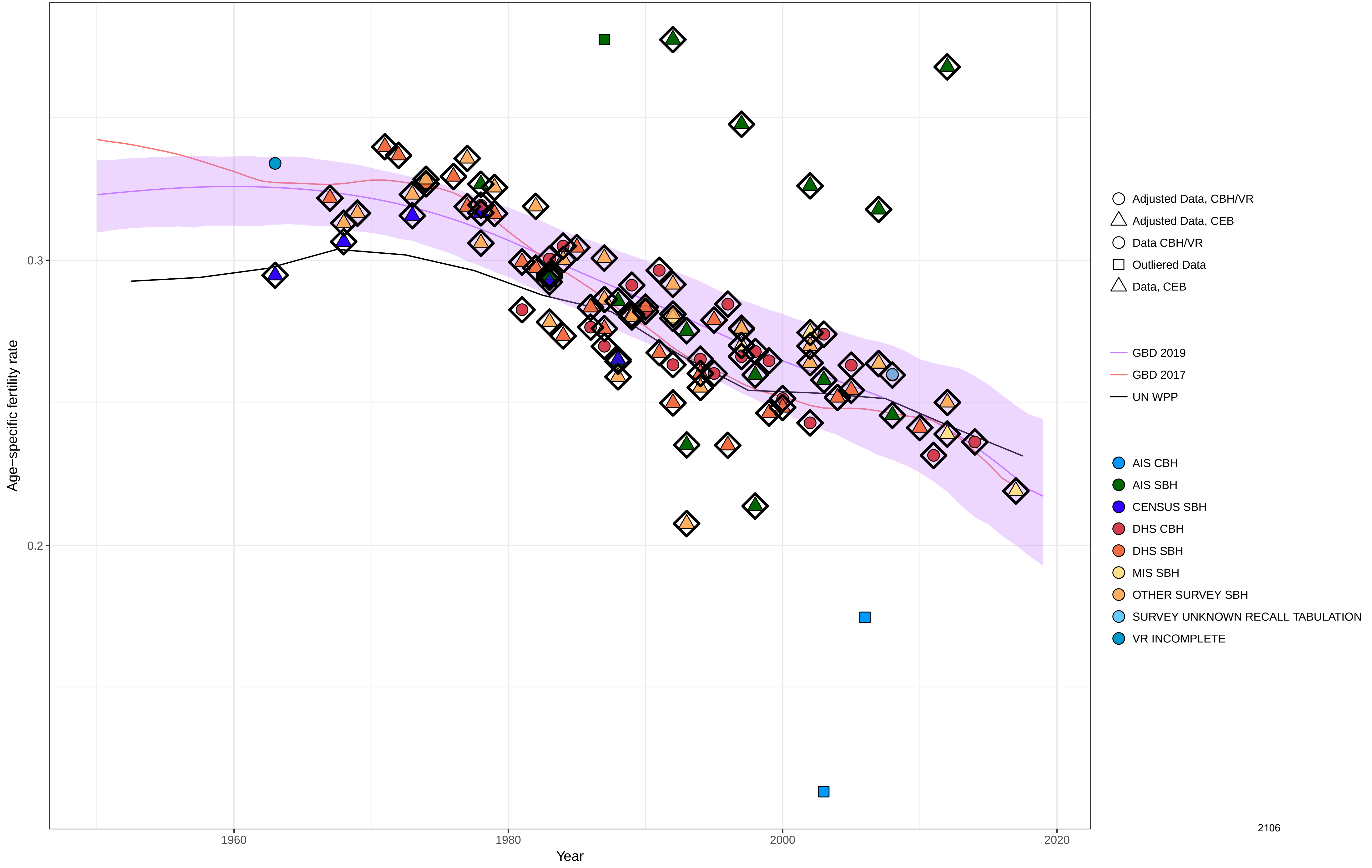

Age−specific fertility fitted trend lines, 1950−2019: United Republic of Tanzania, Ages: 25−29
Data points enclosed with a diamond are reference sources

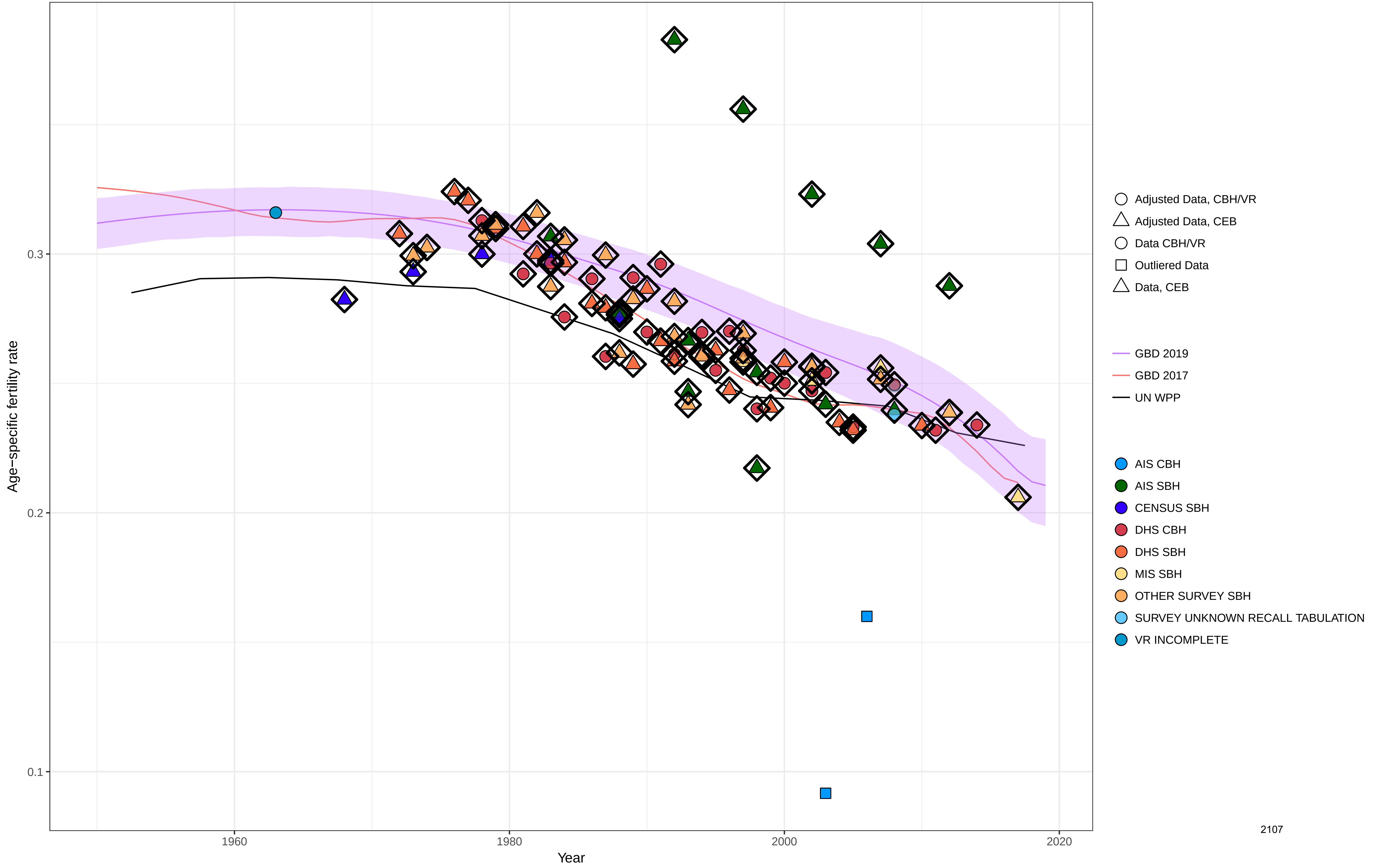

Age−specific fertility fitted trend lines, 1950−2019: United Republic of Tanzania, Ages: 30−34
Data points enclosed with a diamond are reference sources

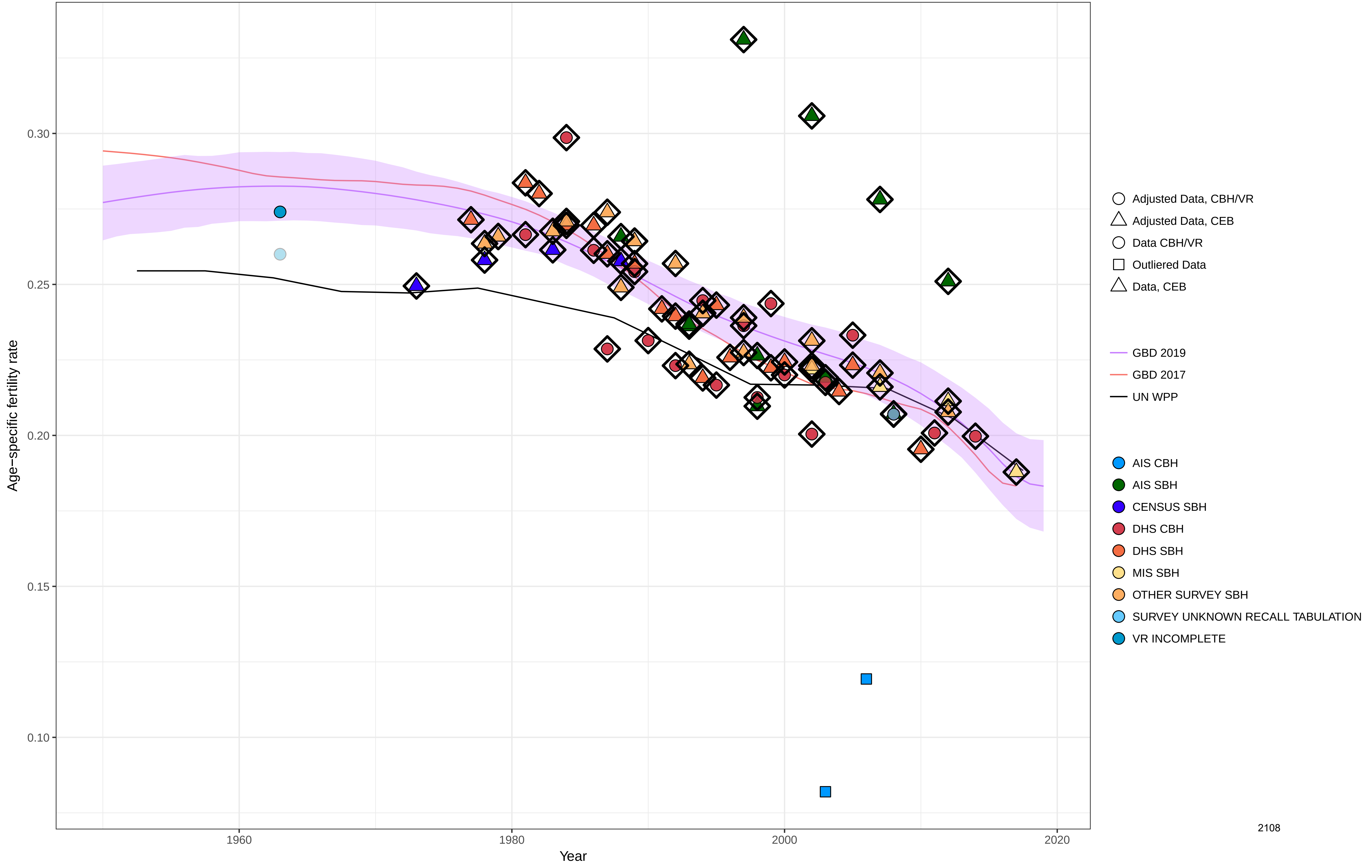

Age−specific fertility fitted trend lines, 1950−2019: United Republic of Tanzania, Ages: 35−39
Data points enclosed with a diamond are reference sources

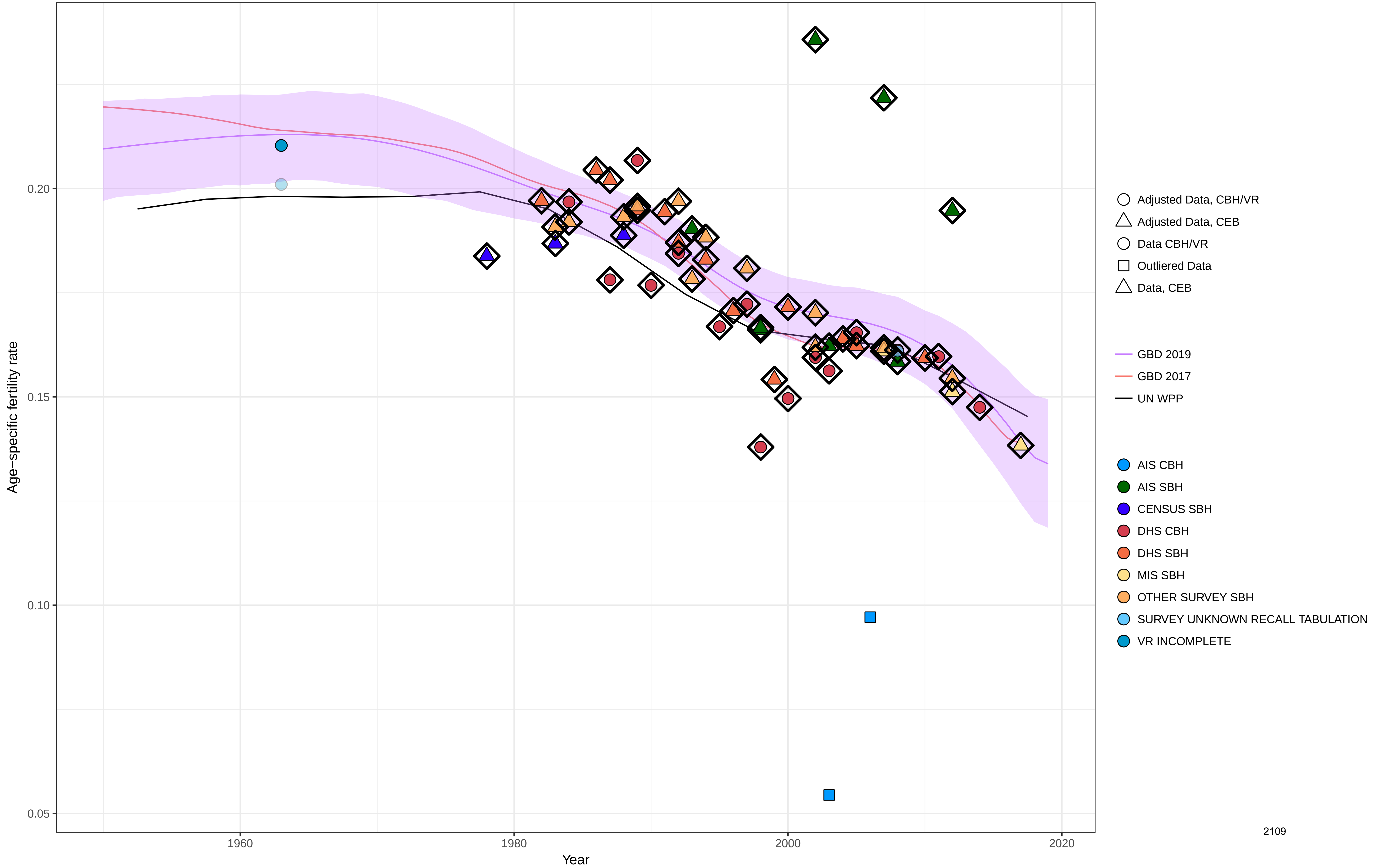

Age−specific fertility fitted trend lines, 1950−2019: United Republic of Tanzania, Ages: 40−44
Data points enclosed with a diamond are reference sources

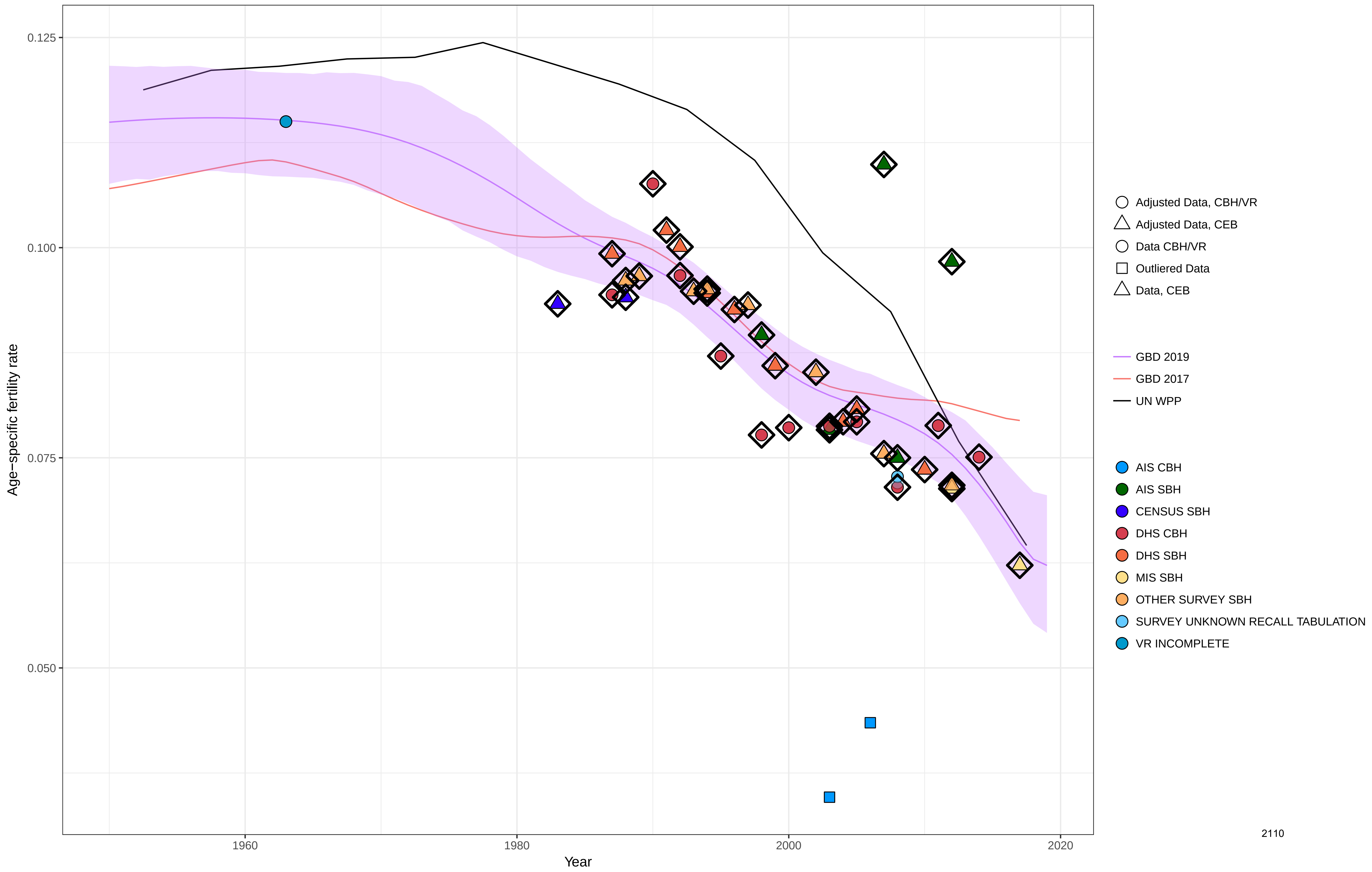

Age−specific fertility fitted trend lines, 1950−2019: United Republic of Tanzania, Ages: 45−49
Data points enclosed with a diamond are reference sources

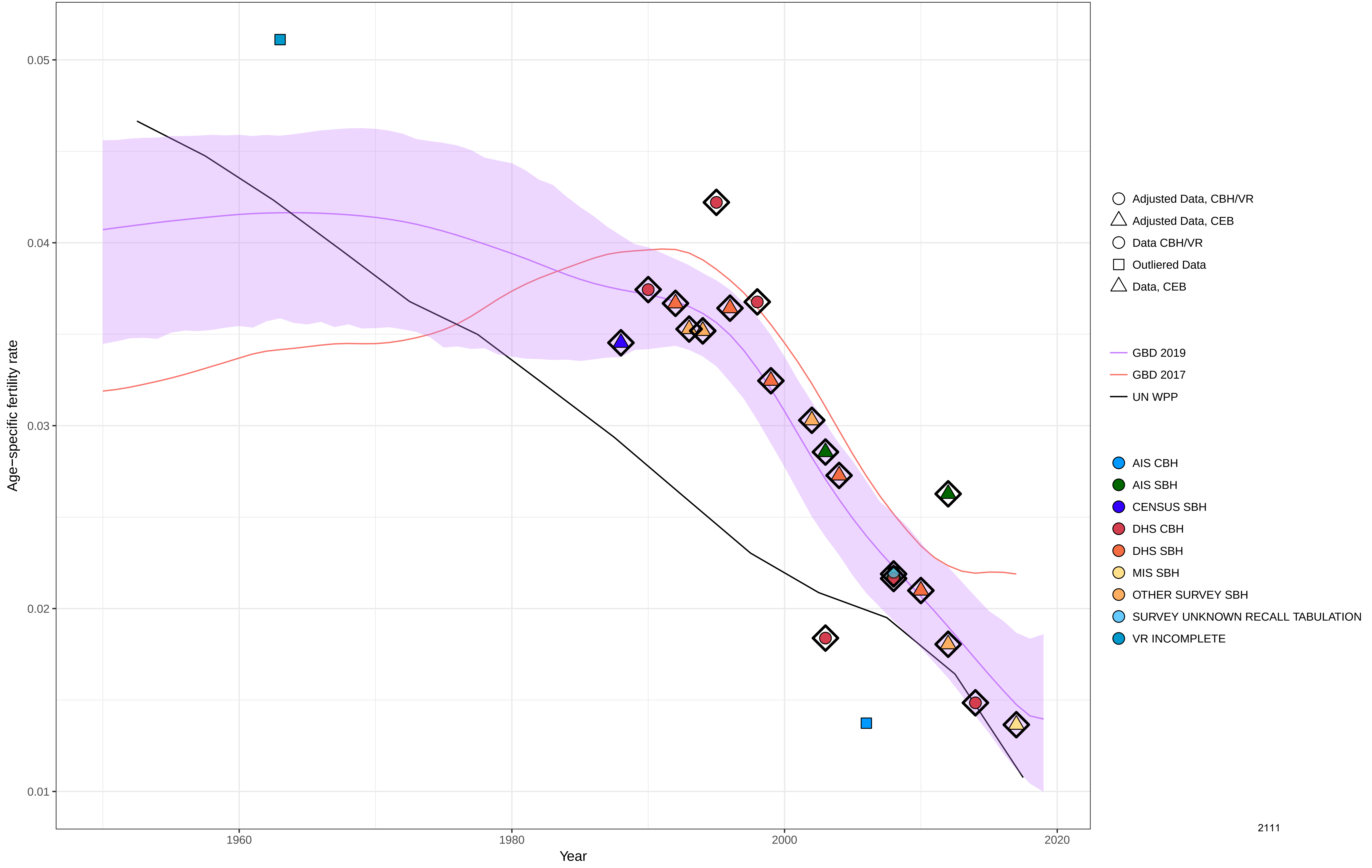

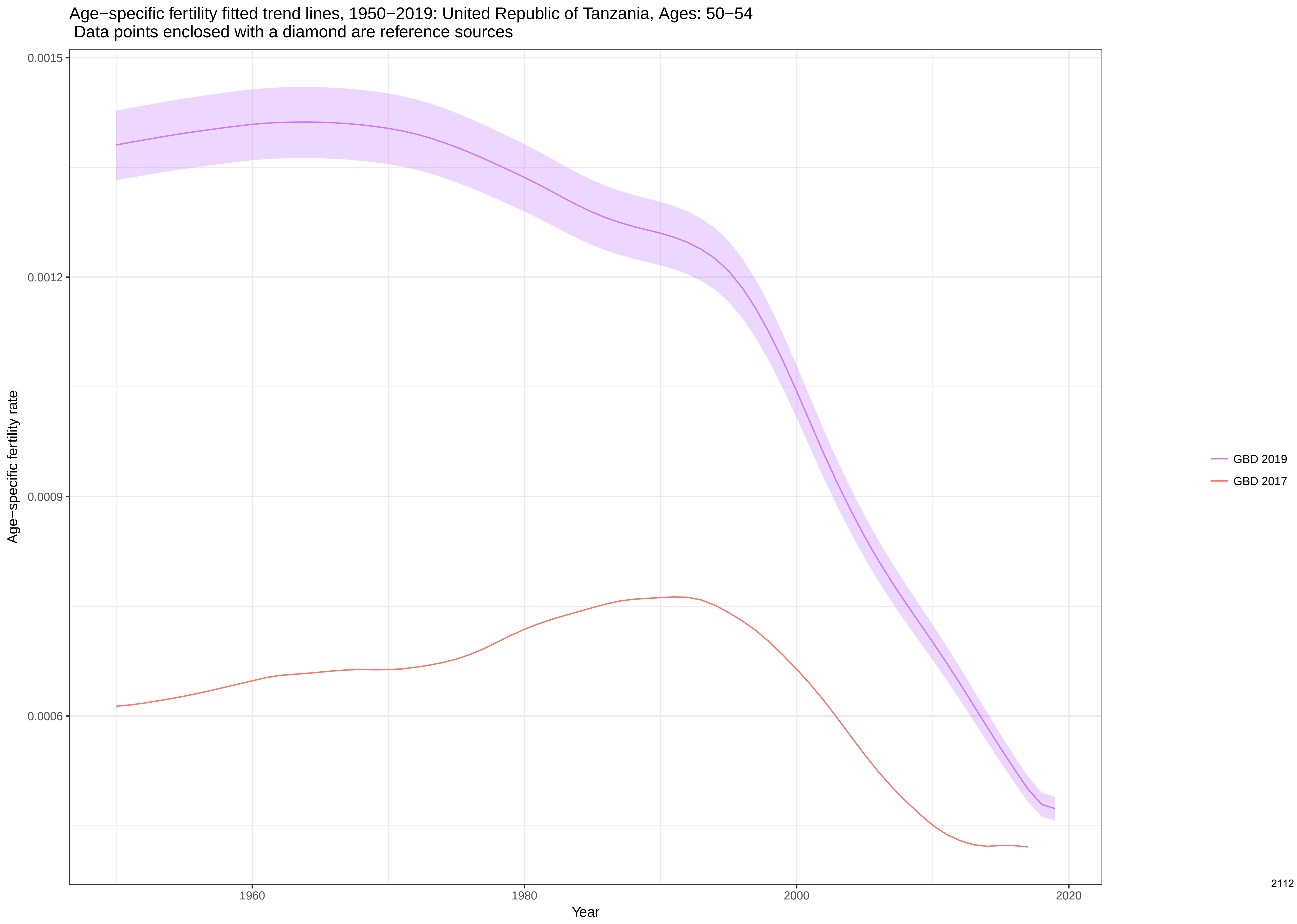
Age−specific fertility fitted trend lines, 1950−2019: United Republic of Tanzania, Ages: 50−54
Data points enclosed with a diamond are reference sources
0.0015
0.0012
0.0009
0.0006
Age−specific fertility rate
1960
1980
2000
2020
Year
GBD 2019
GBD 2017

Total fertility rate fitted trend lines, 1950−2019: United Republic of Tanzania TFR
Data points enclosed with a diamond are reference sources

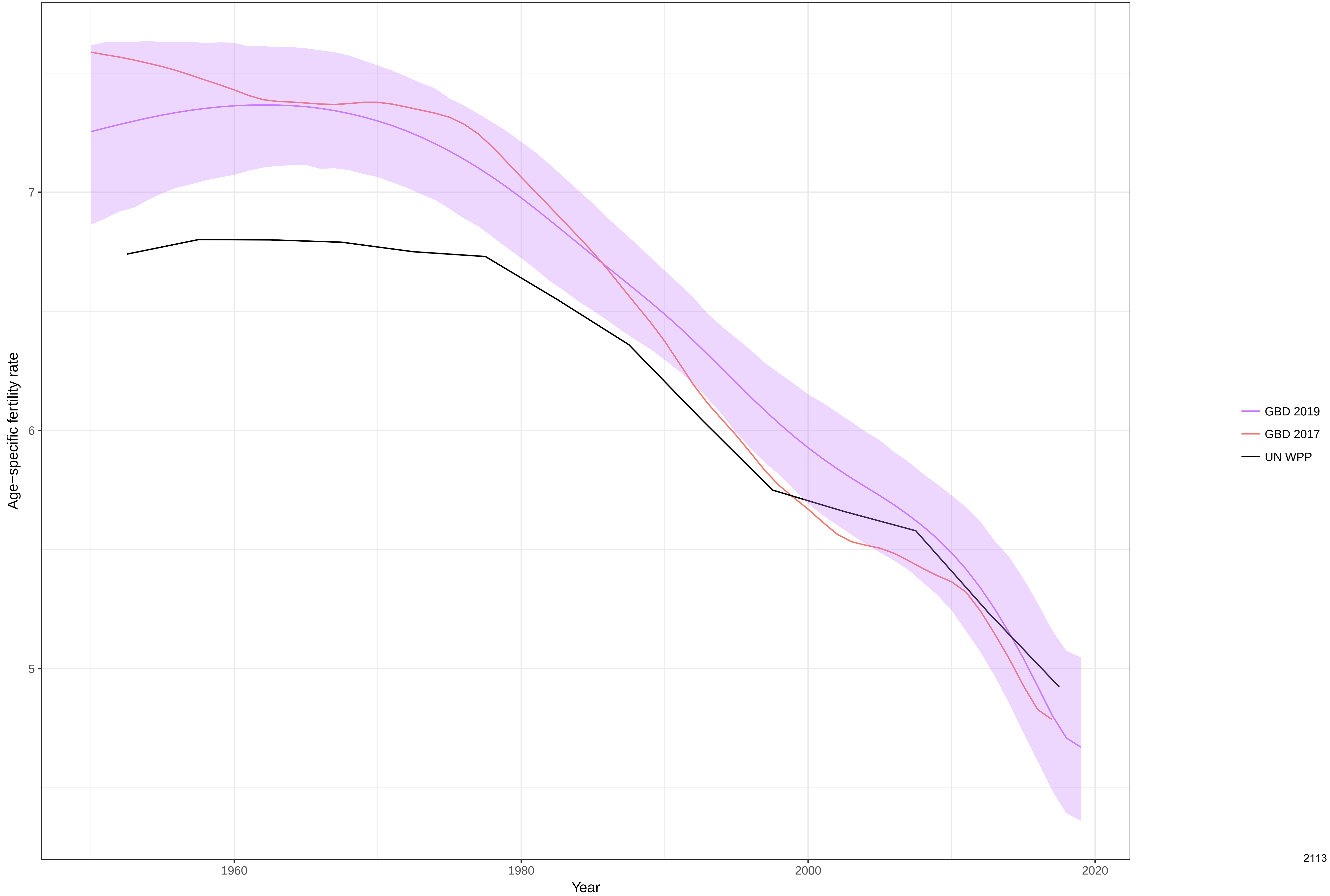

Age−specific fertility fitted trend lines, 1950−2019: Uganda, Ages: 10−14
Data points enclosed with a diamond are reference sources

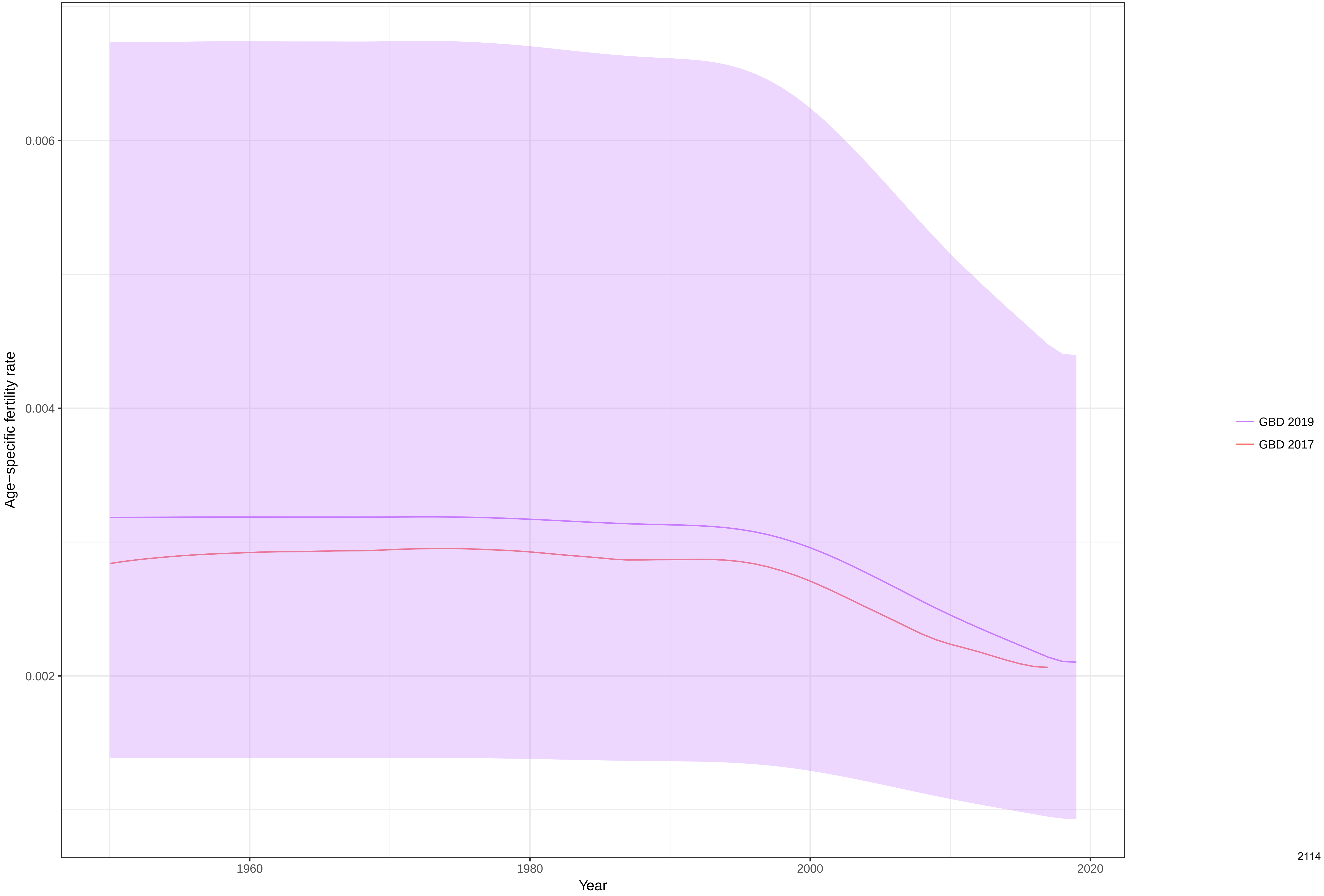

Age−specific fertility fitted trend lines, 1950−2019: Uganda, Ages: 15−19
Data points enclosed with a diamond are reference sources

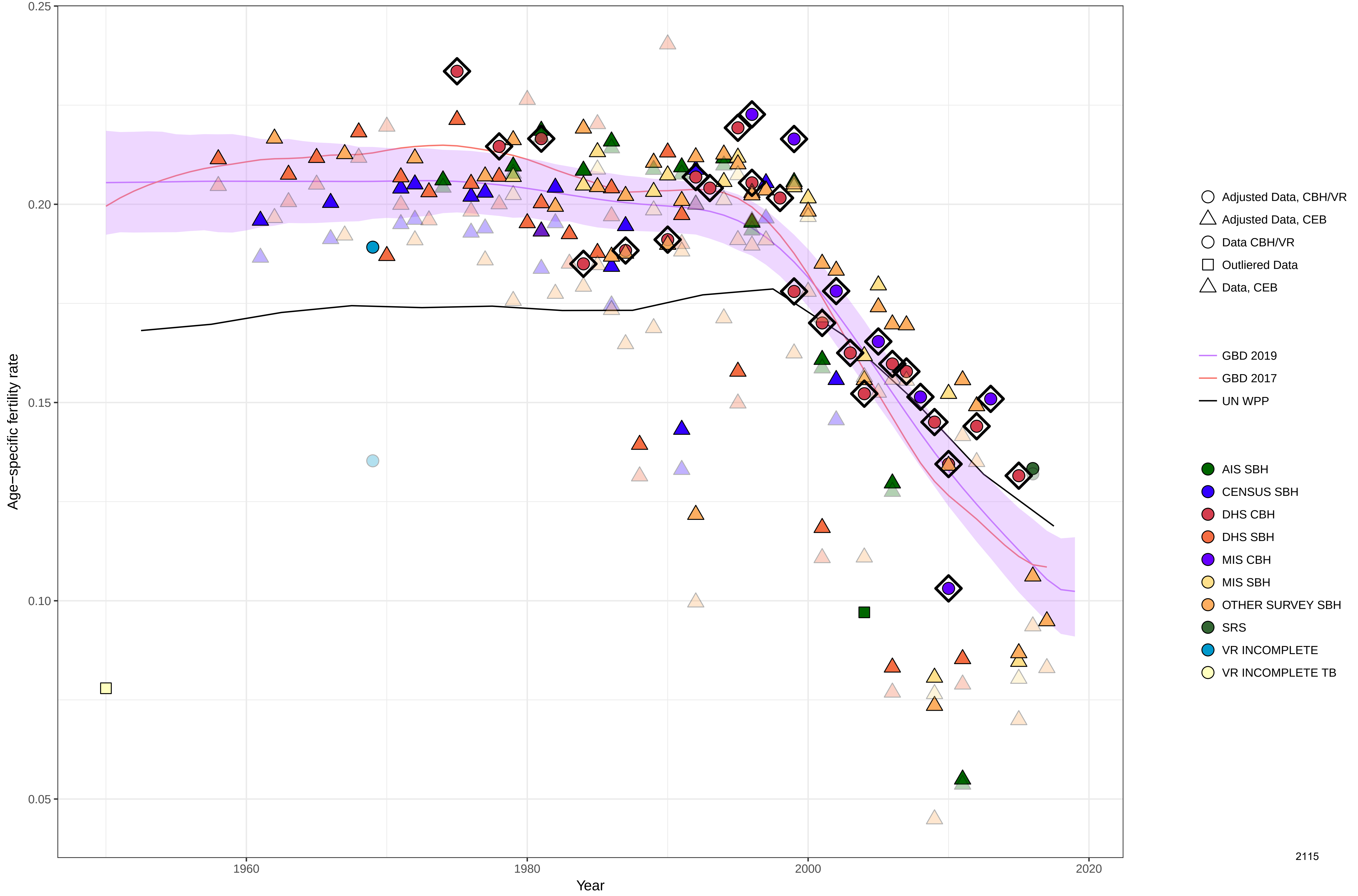

Age−specific fertility fitted trend lines, 1950−2019: Uganda, Ages: 20−24
Data points enclosed with a diamond are reference sources

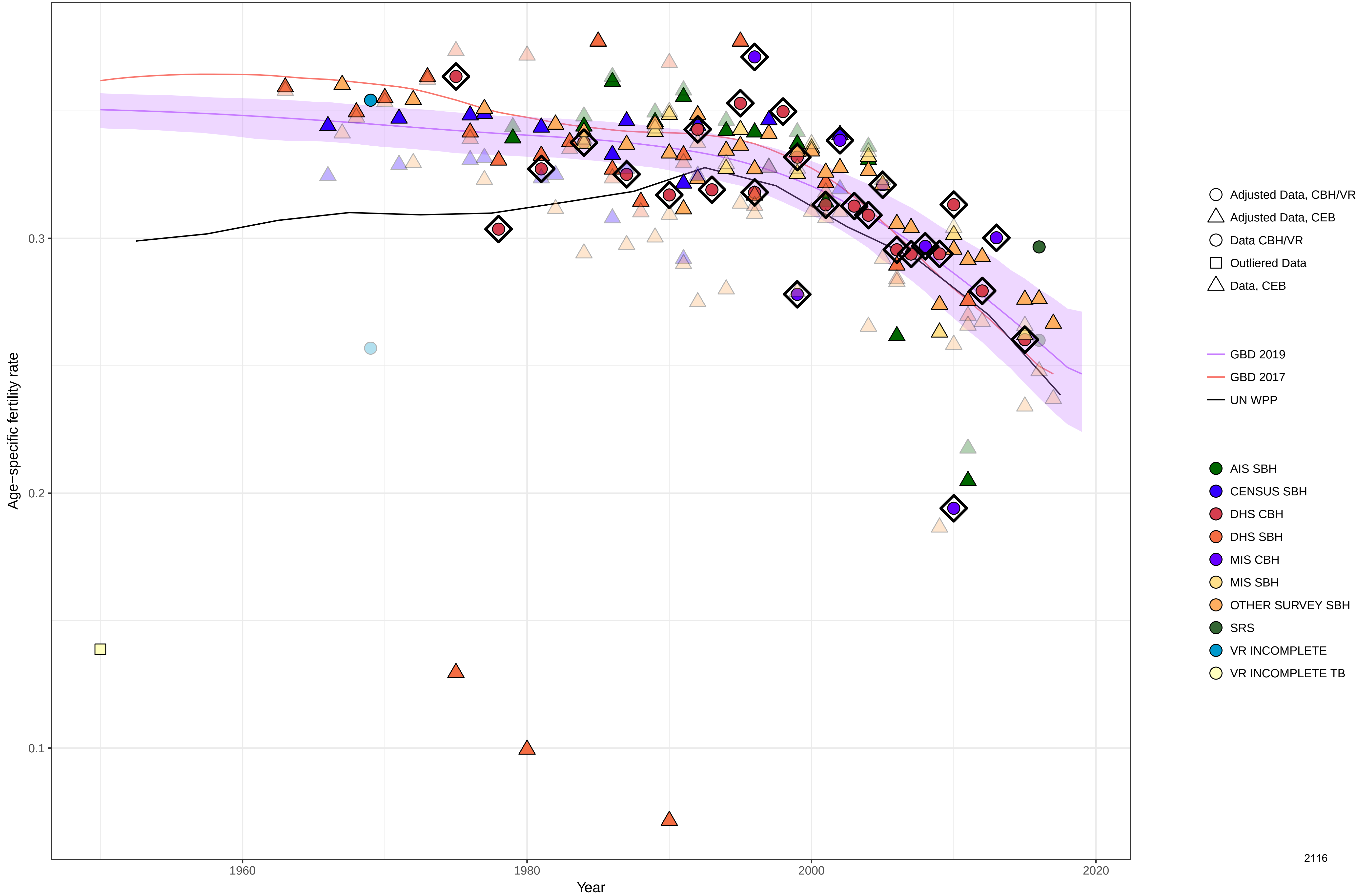

Age−specific fertility fitted trend lines, 1950−2019: Uganda, Ages: 25−29
Data points enclosed with a diamond are reference sources

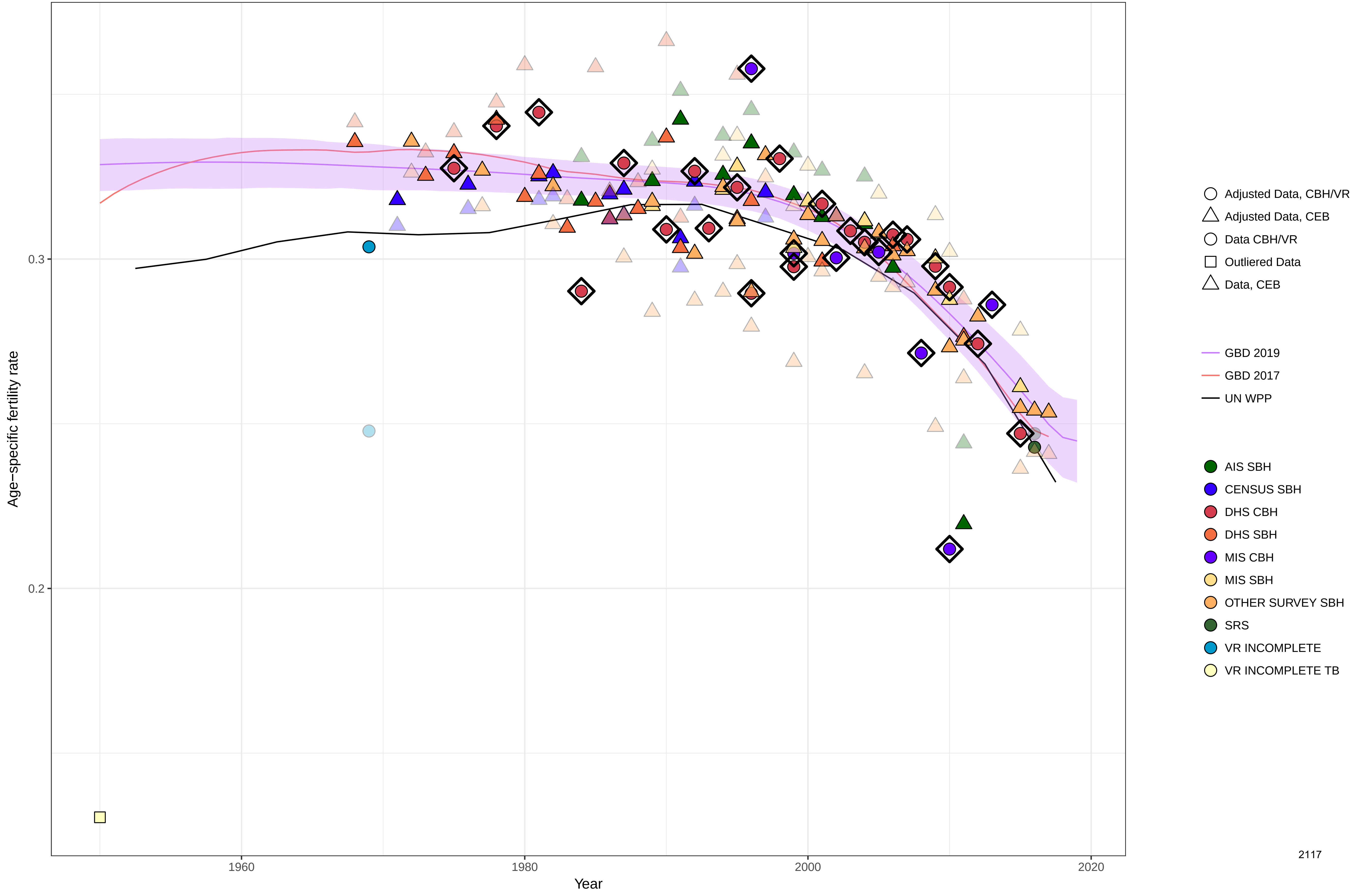

Age−specific fertility fitted trend lines, 1950−2019: Uganda, Ages: 30−34
Data points enclosed with a diamond are reference sources

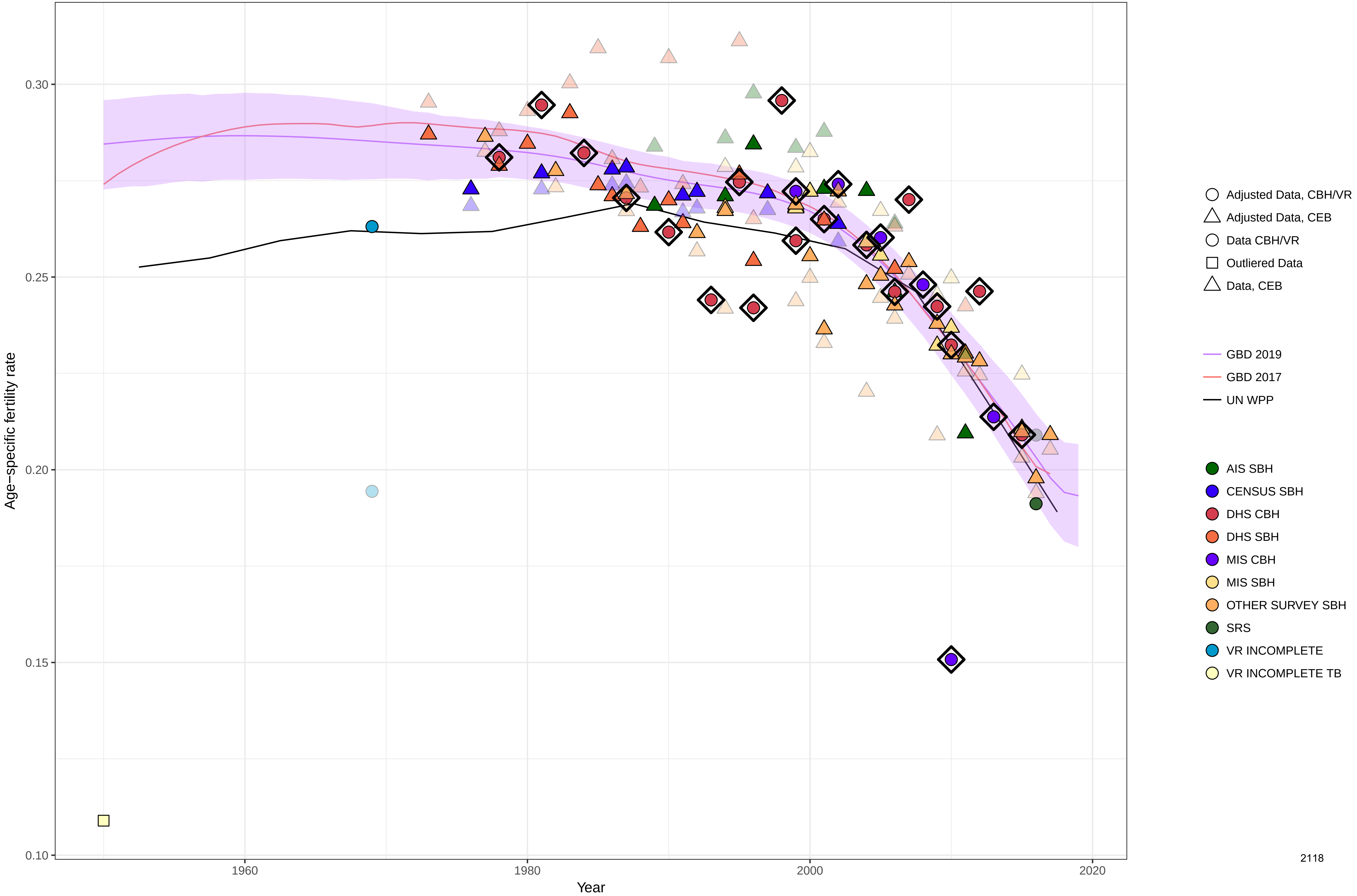

Age−specific fertility fitted trend lines, 1950−2019: Uganda, Ages: 35−39
Data points enclosed with a diamond are reference sources

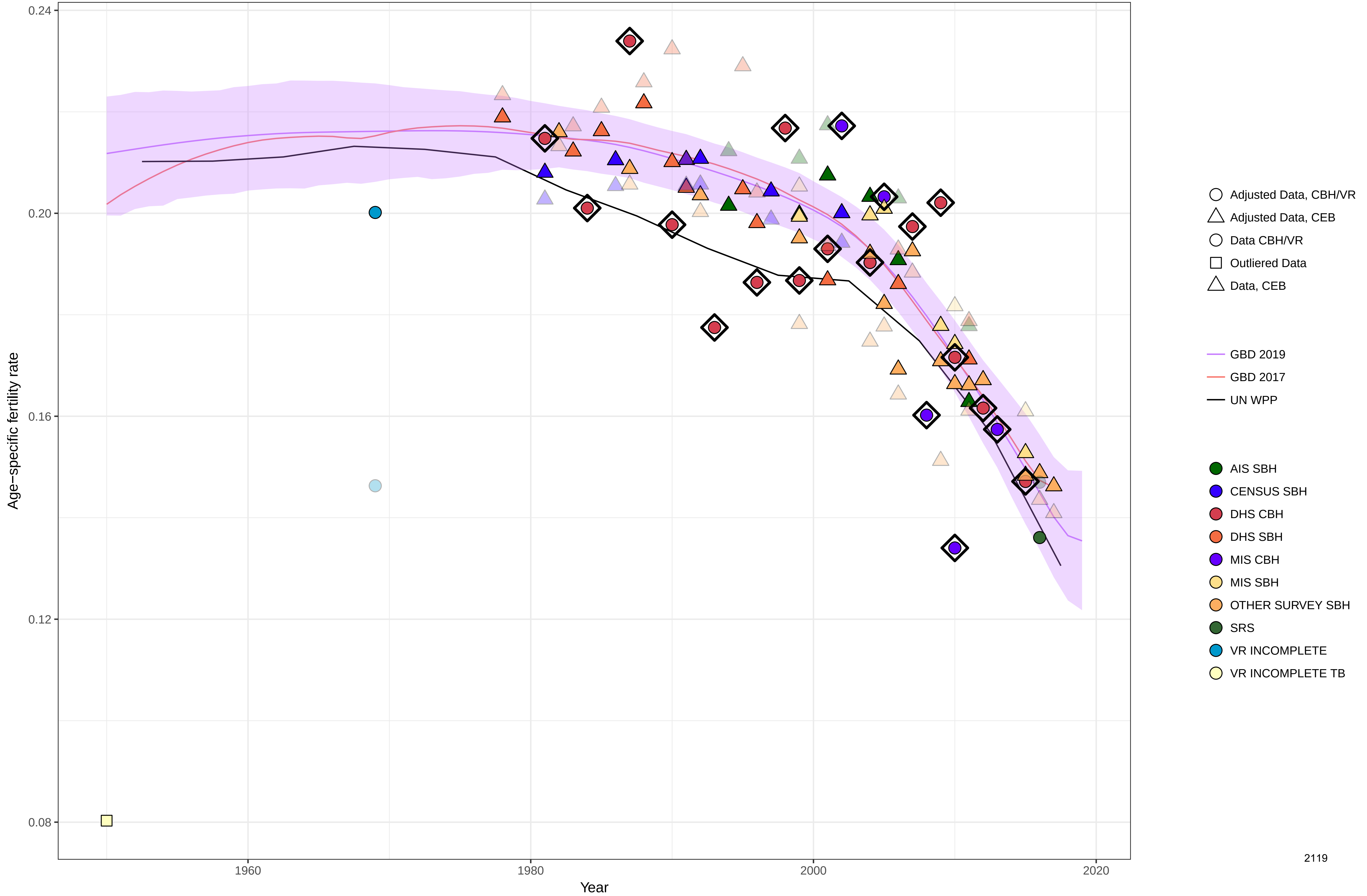

Age−specific fertility fitted trend lines, 1950−2019: Uganda, Ages: 40−44
Data points enclosed with a diamond are reference sources

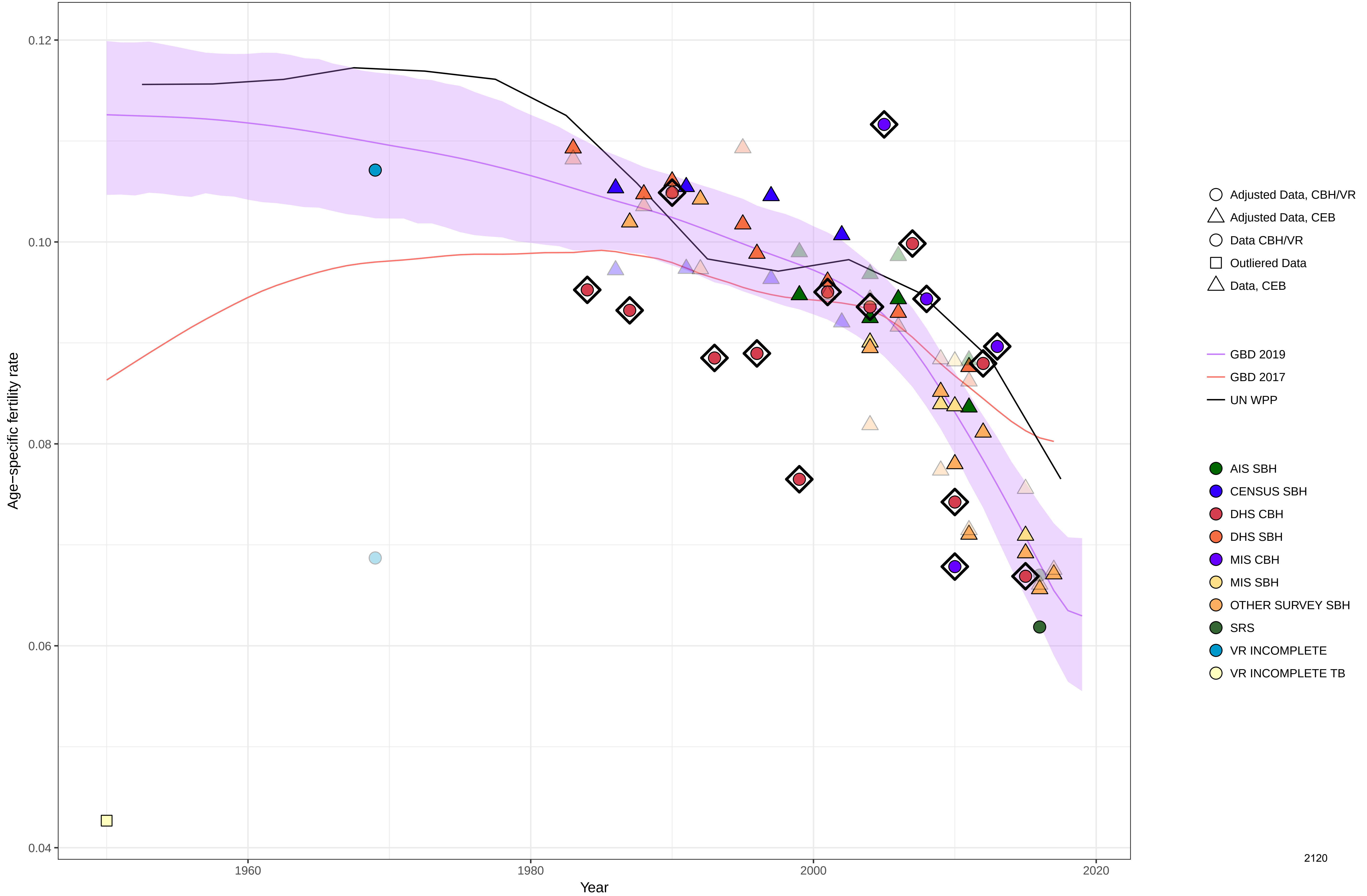

Age−specific fertility fitted trend lines, 1950−2019: Uganda, Ages: 45−49
Data points enclosed with a diamond are reference sources

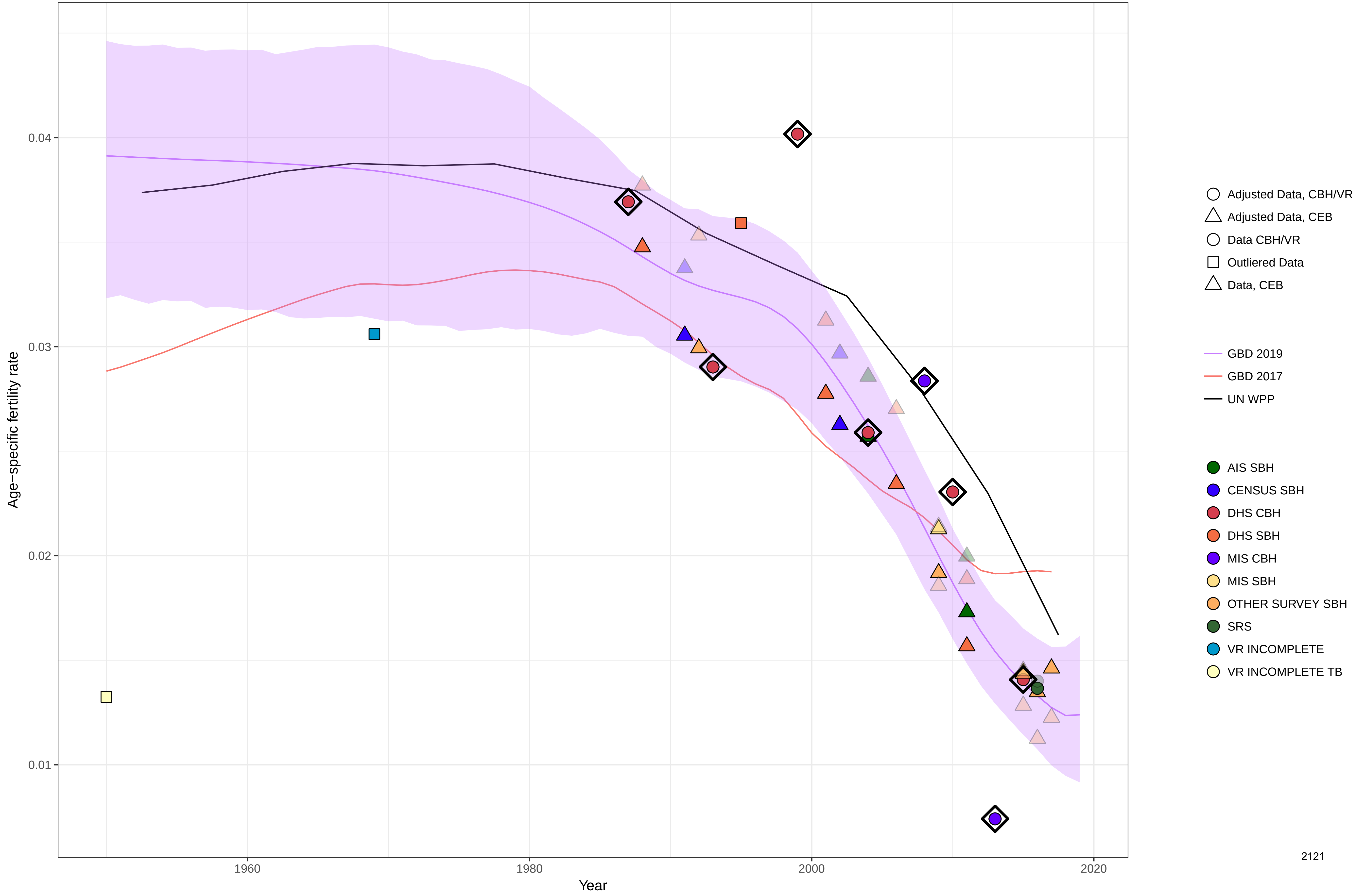

Age−specific fertility fitted trend lines, 1950−2019: Uganda, Ages: 50−54
Data points enclosed with a diamond are reference sources

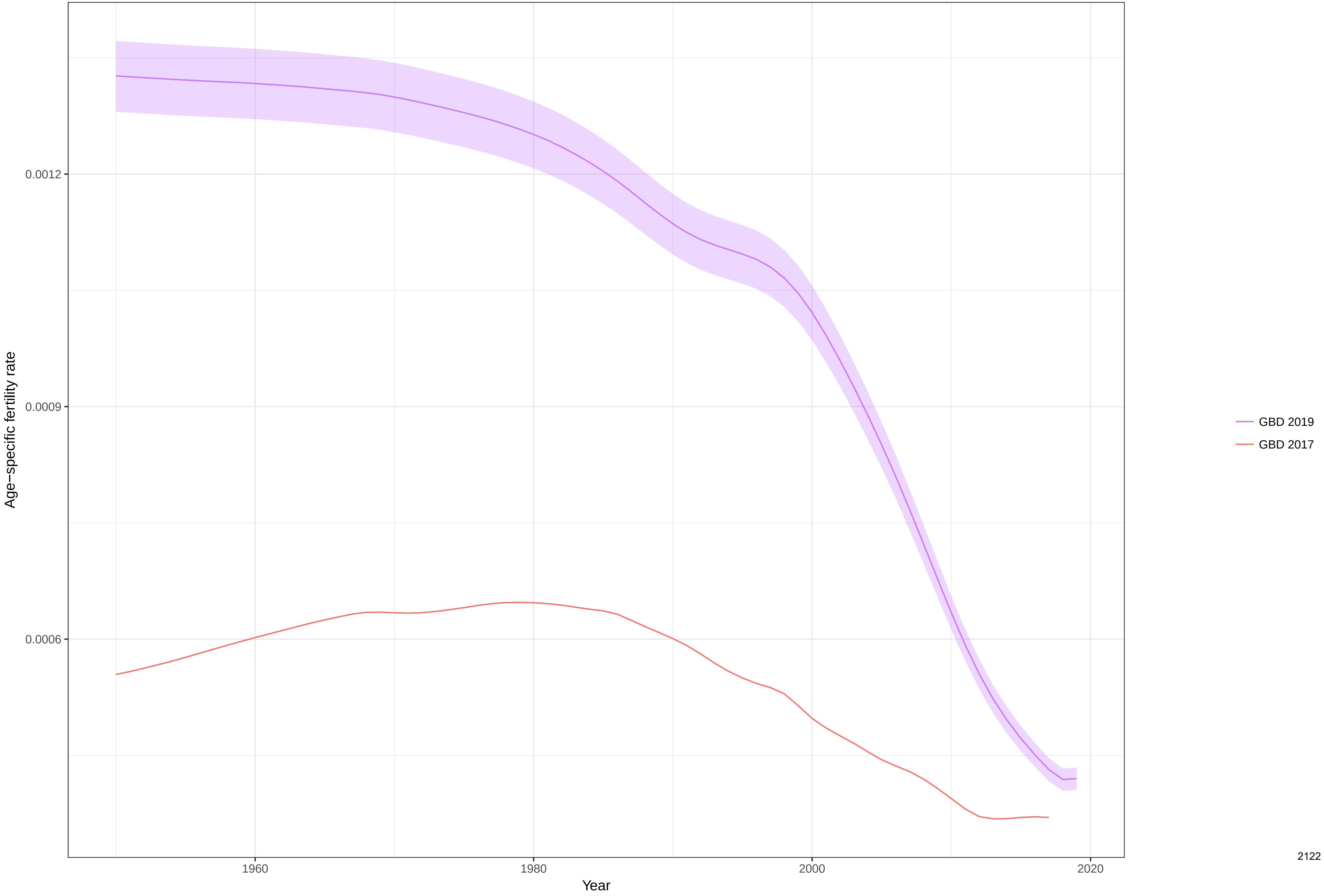

Total fertility rate fitted trend lines, 1950−2019: Uganda TFR
Data points enclosed with a diamond are reference sources

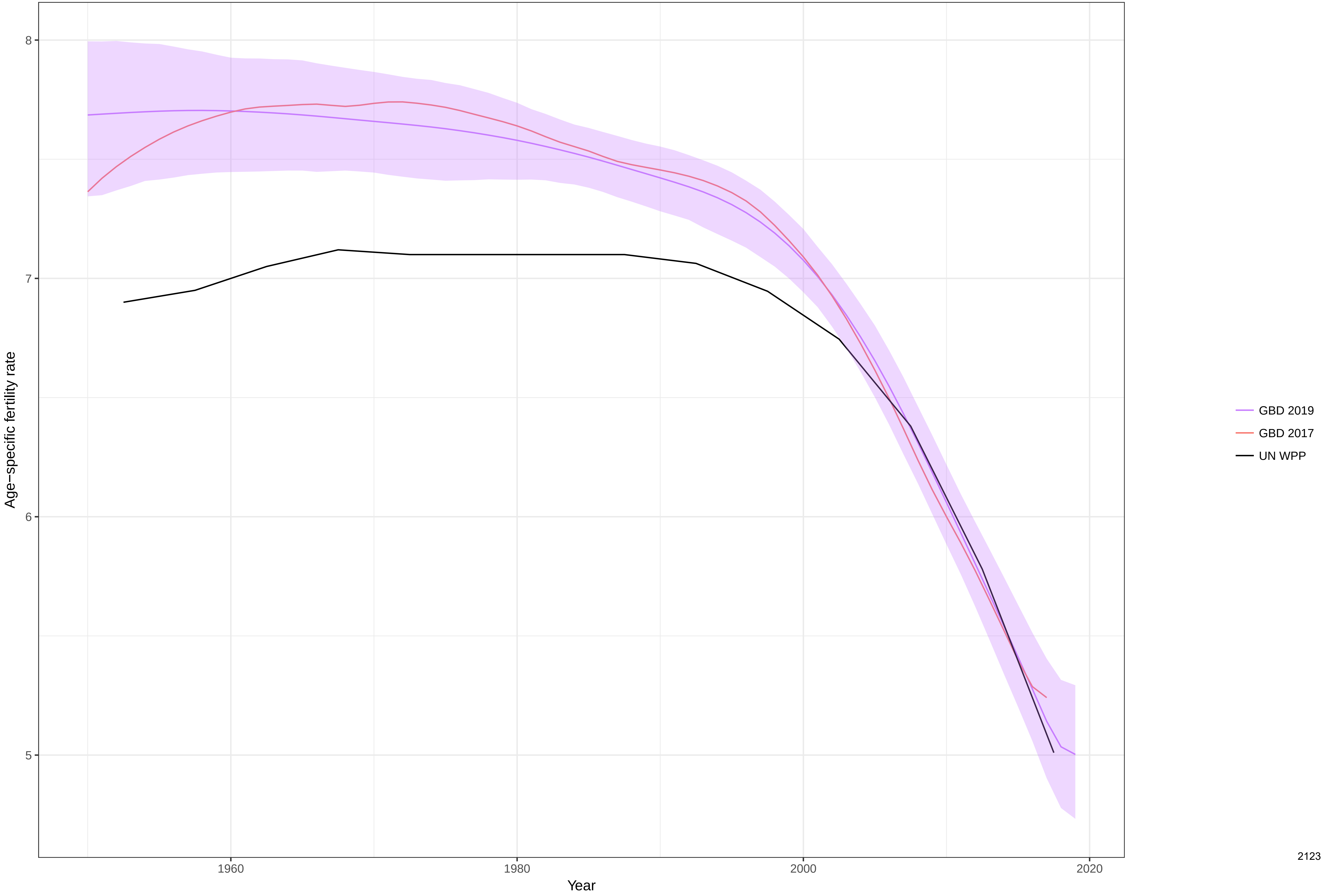

Age−specific fertility fitted trend lines, 1950−2019: Ukraine, Ages: 10−14
Data points enclosed with a diamond are reference sources

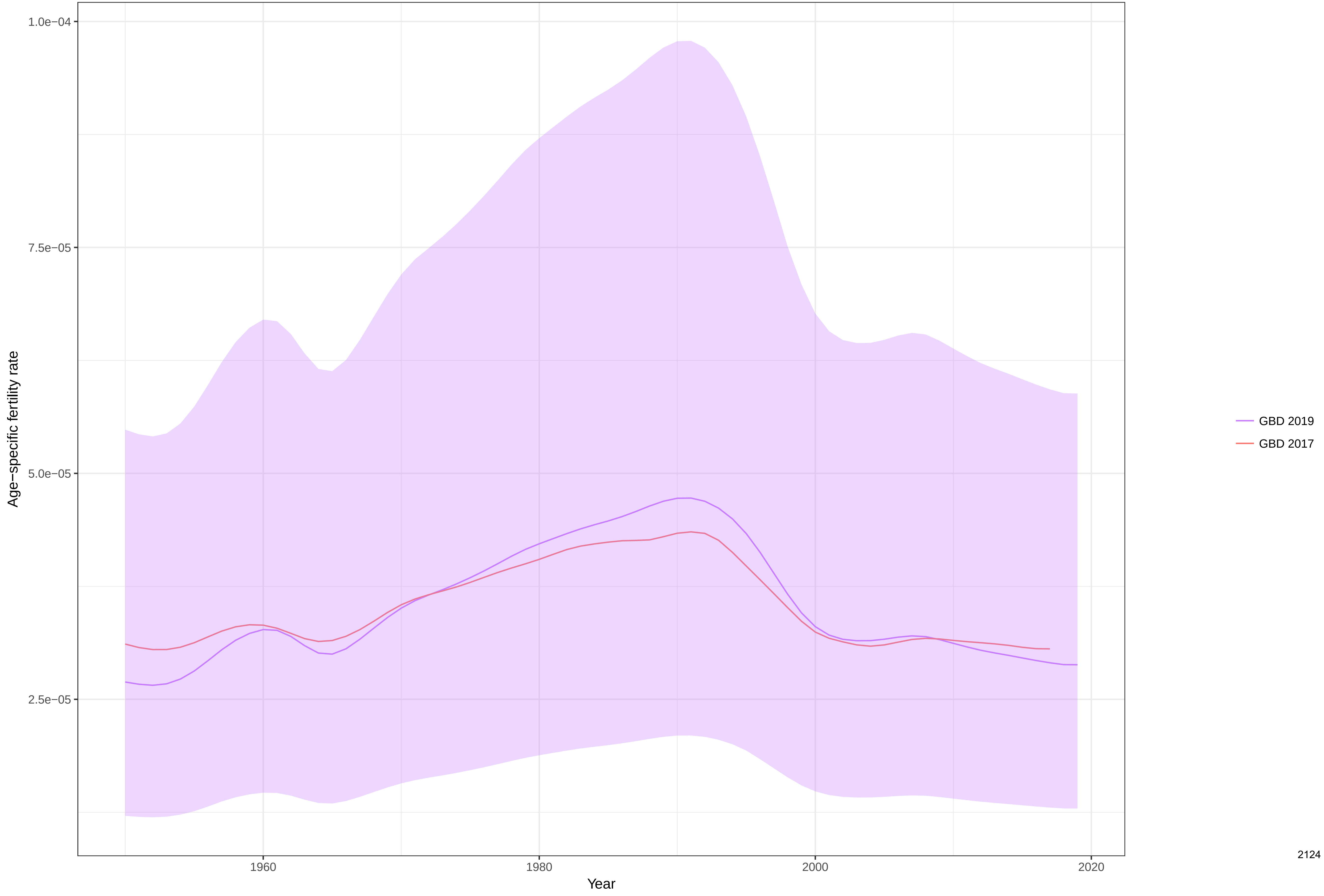

Age−specific fertility fitted trend lines, 1950−2019: Ukraine, Ages: 15−19
Data points enclosed with a diamond are reference sources

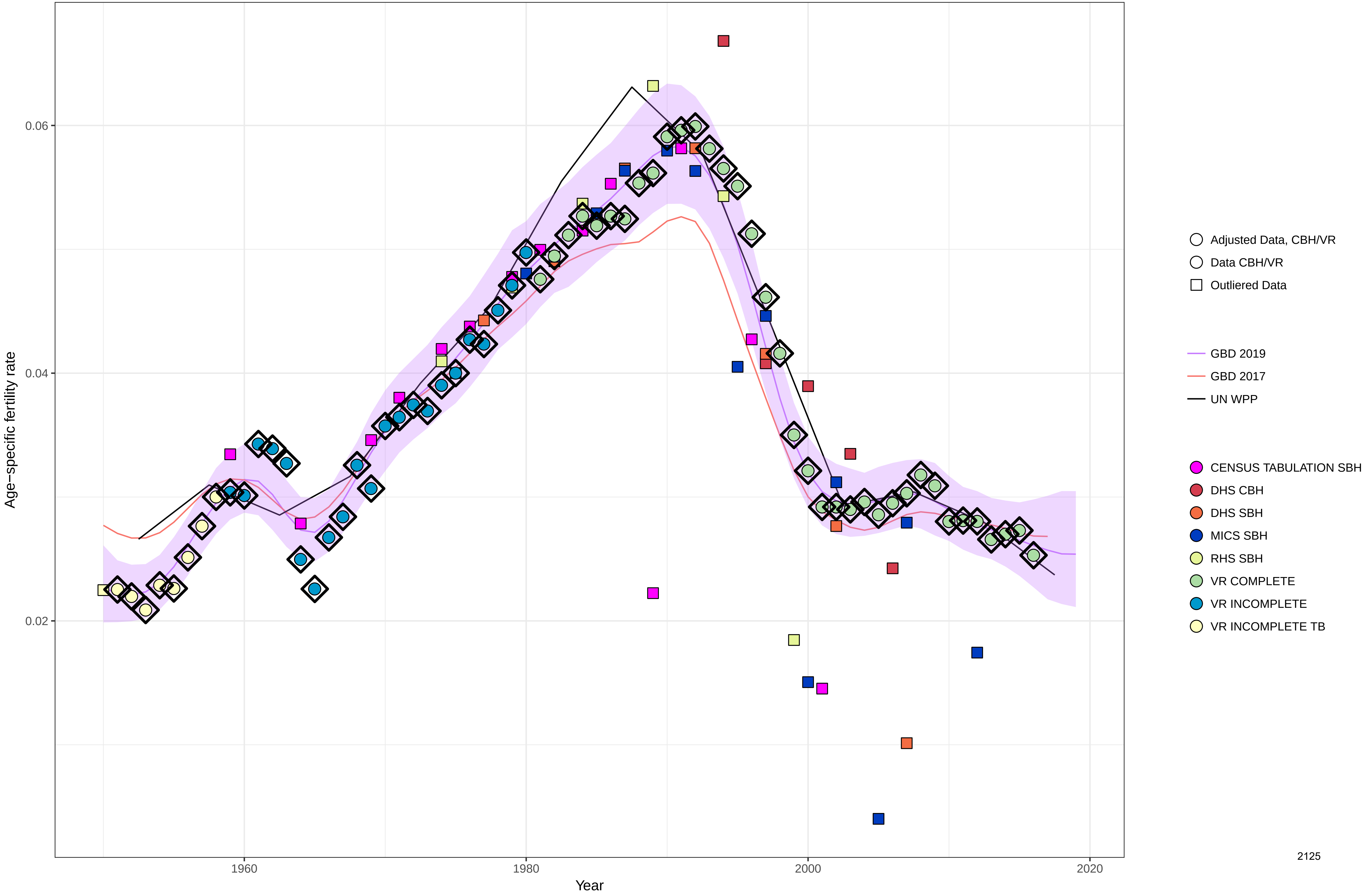

Age−specific fertility fitted trend lines, 1950−2019: Ukraine, Ages: 20−24
Data points enclosed with a diamond are reference sources

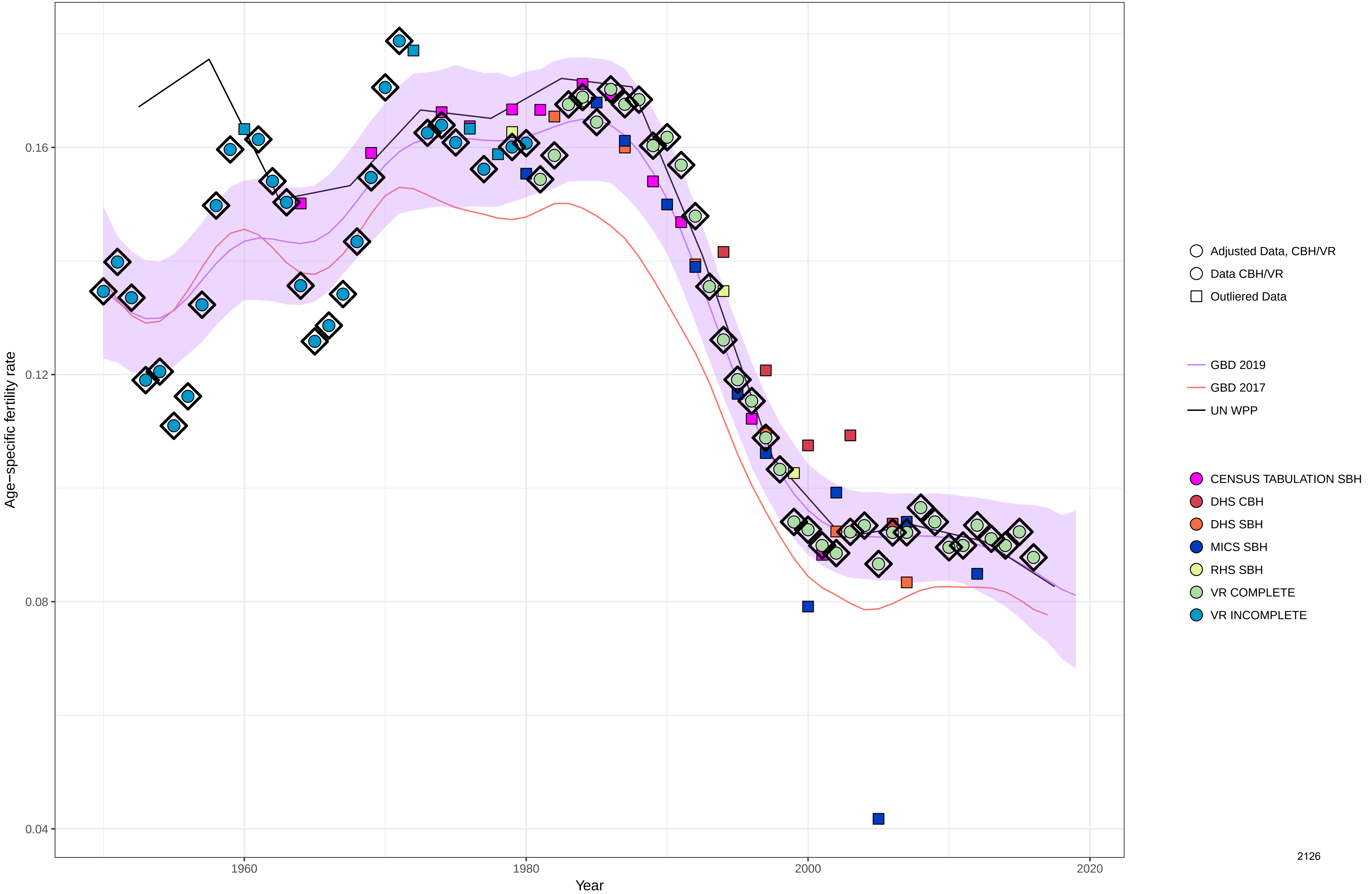

Age−specific fertility fitted trend lines, 1950−2019: Ukraine, Ages: 25−29
Data points enclosed with a diamond are reference sources

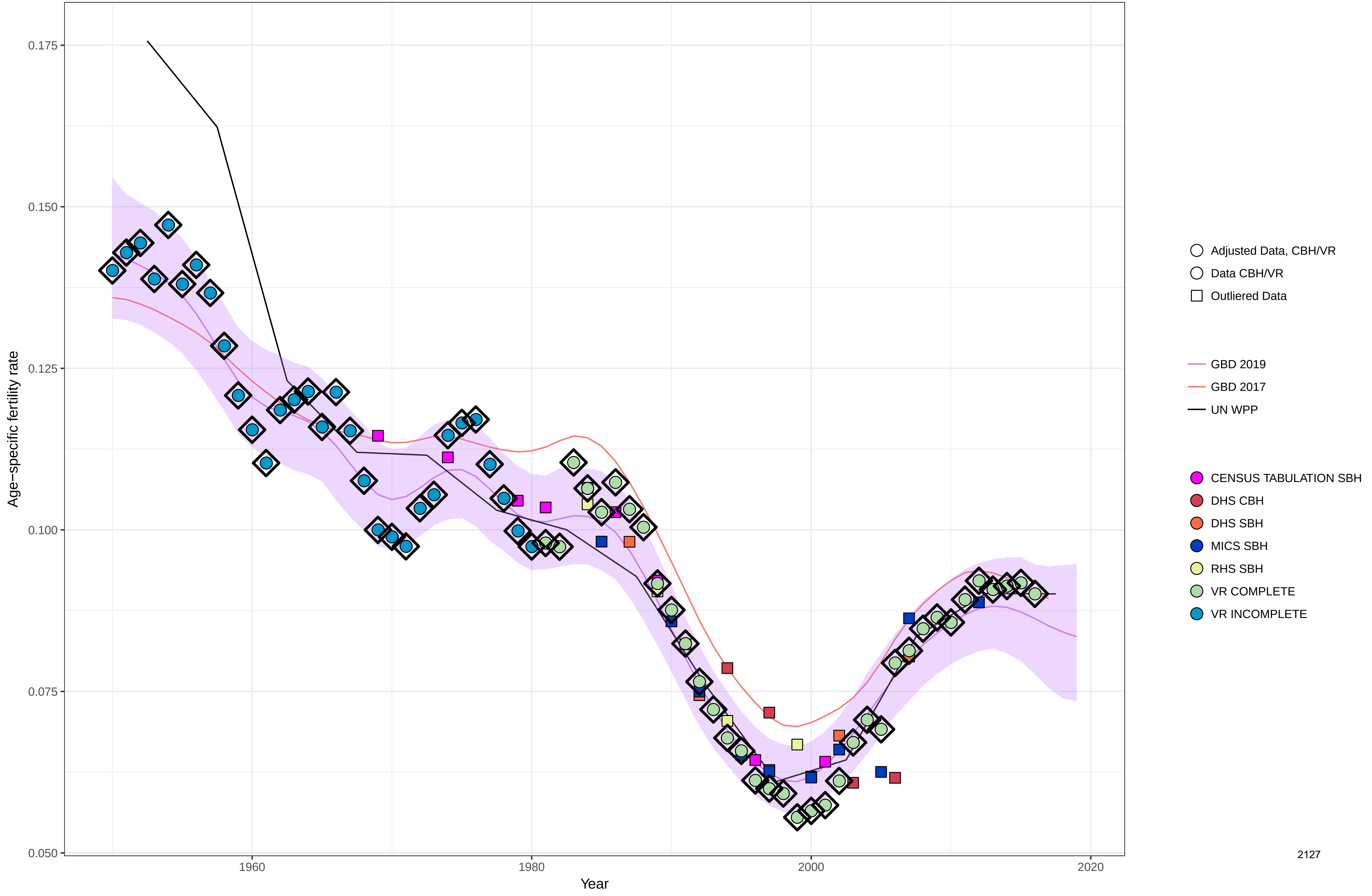

Age−specific fertility fitted trend lines, 1950−2019: Ukraine, Ages: 30−34
Data points enclosed with a diamond are reference sources

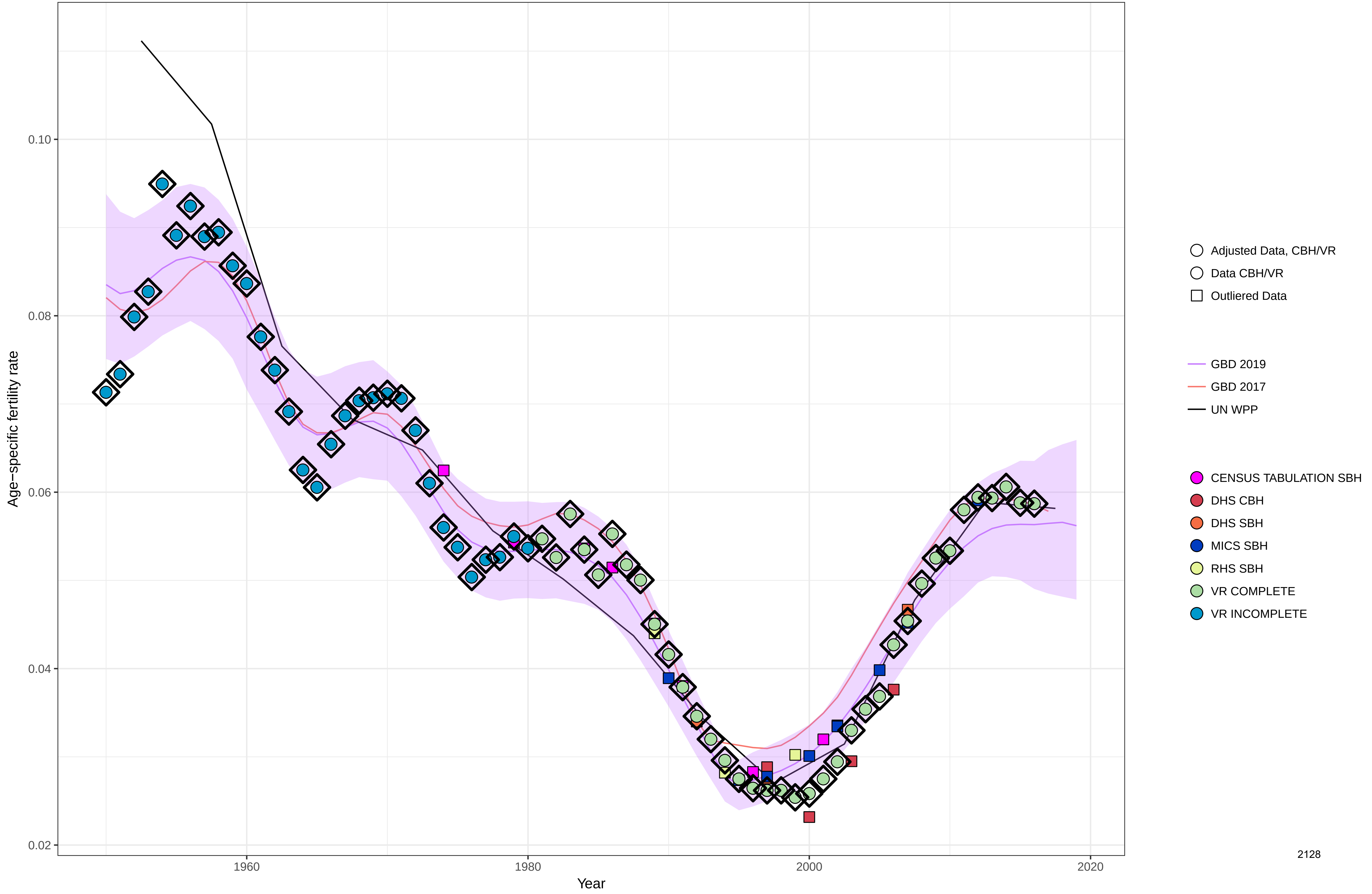

Age−specific fertility fitted trend lines, 1950−2019: Ukraine, Ages: 35−39
Data points enclosed with a diamond are reference sources

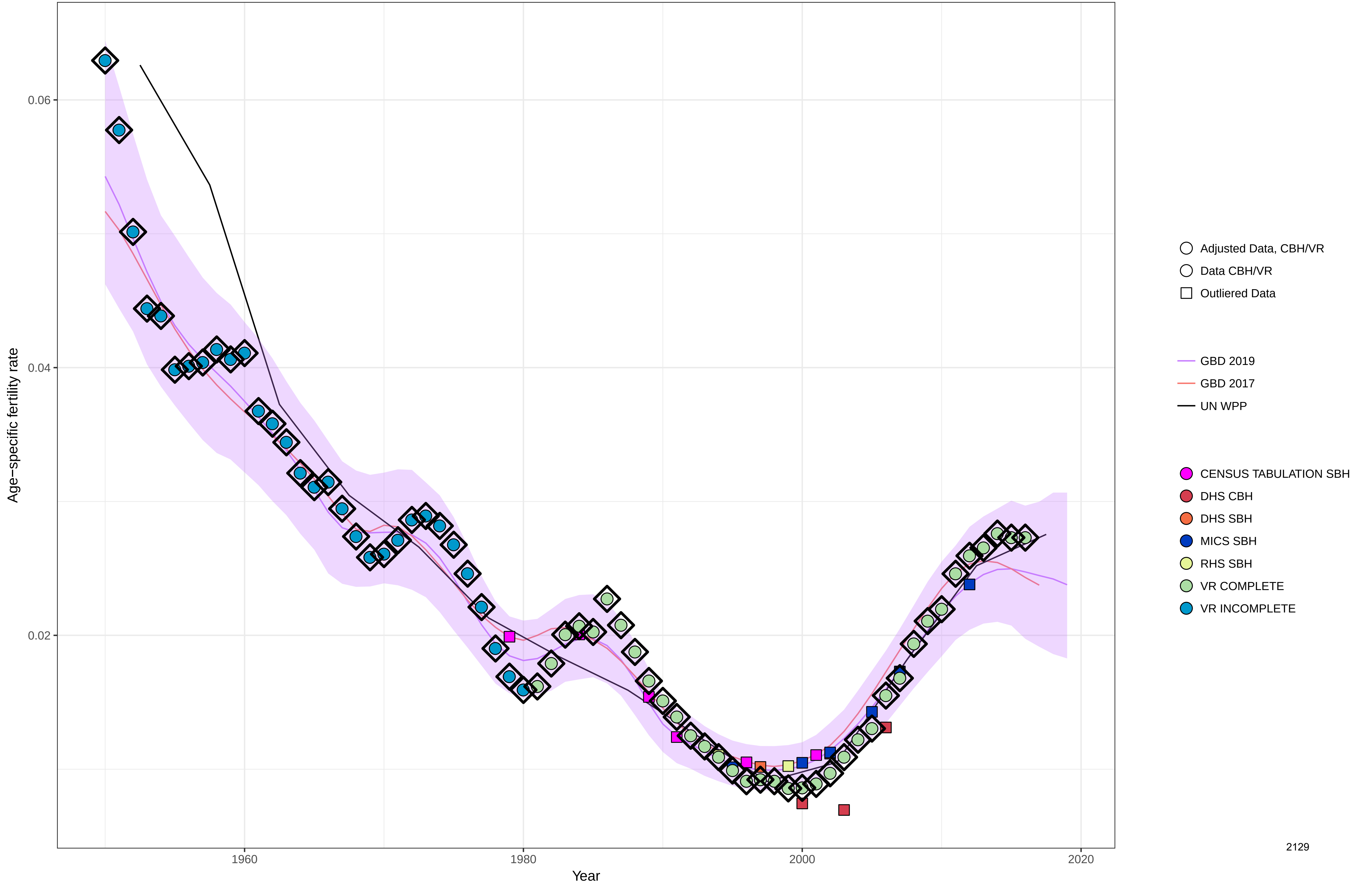

Age−specific fertility fitted trend lines, 1950−2019: Ukraine, Ages: 40−44
Data points enclosed with a diamond are reference sources

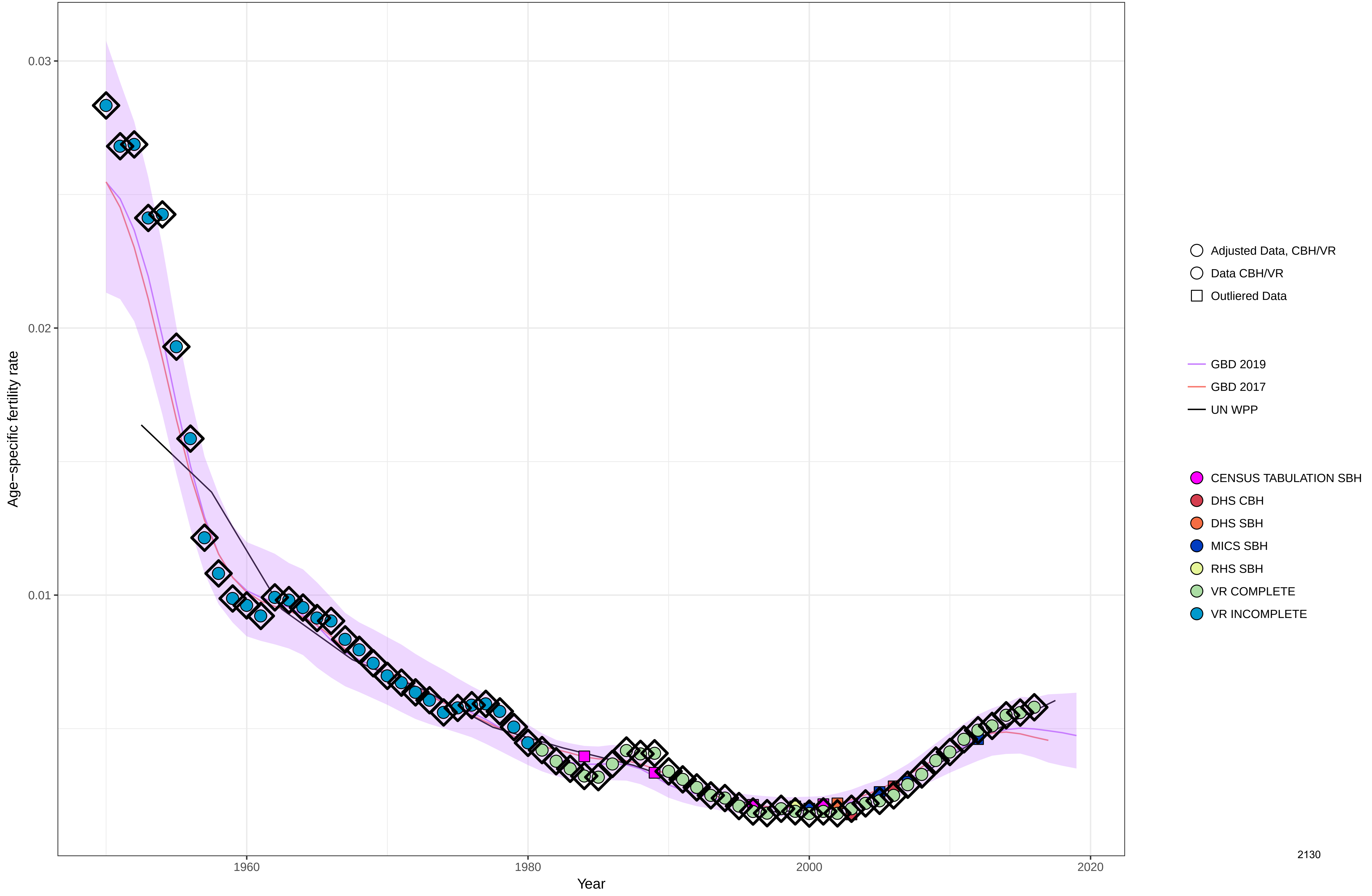

Age−specific fertility fitted trend lines, 1950−2019: Ukraine, Ages: 45−49
Data points enclosed with a diamond are reference sources

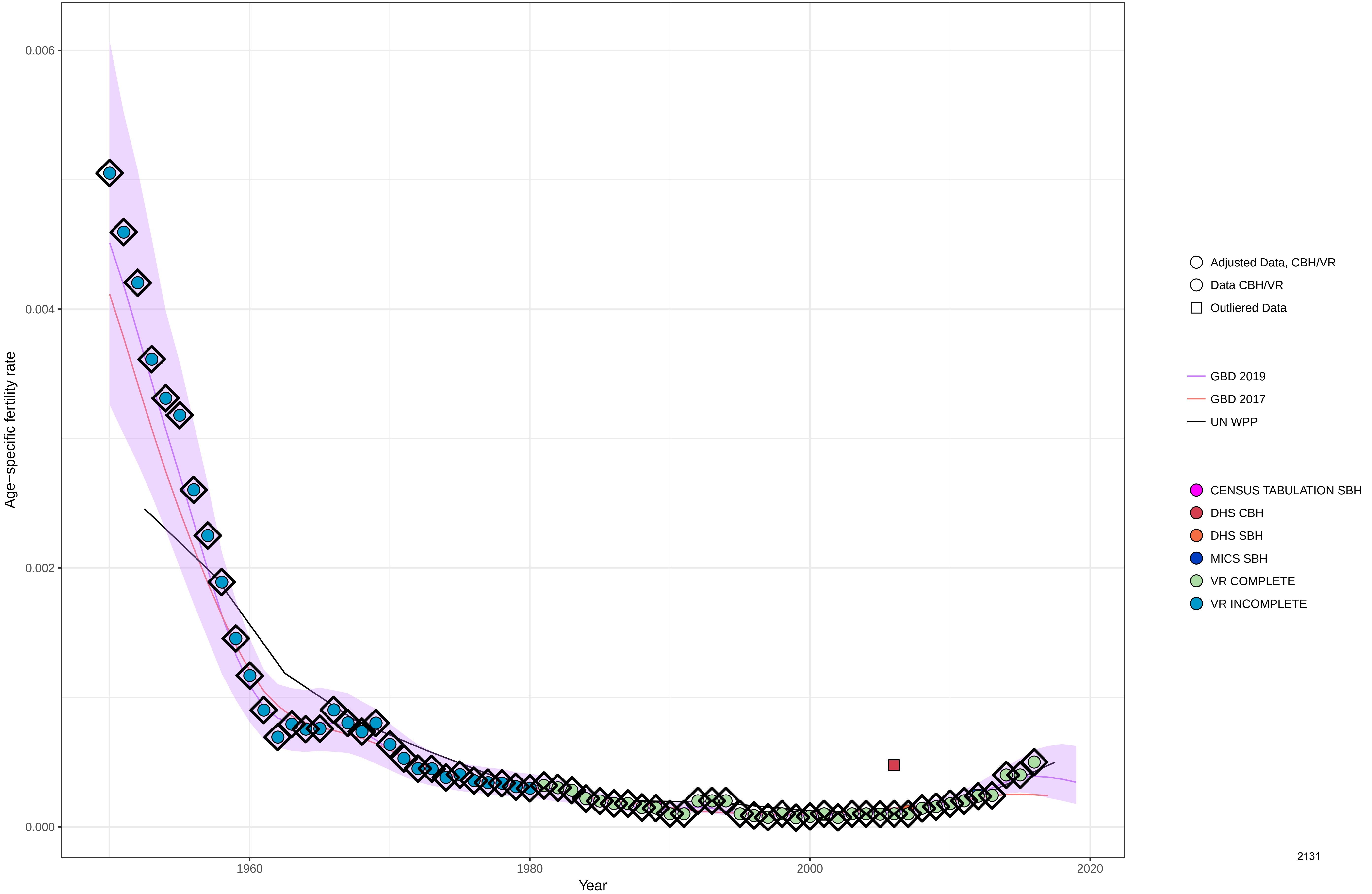

Age−specific fertility fitted trend lines, 1950−2019: Ukraine, Ages: 50−54
Data points enclosed with a diamond are reference sources

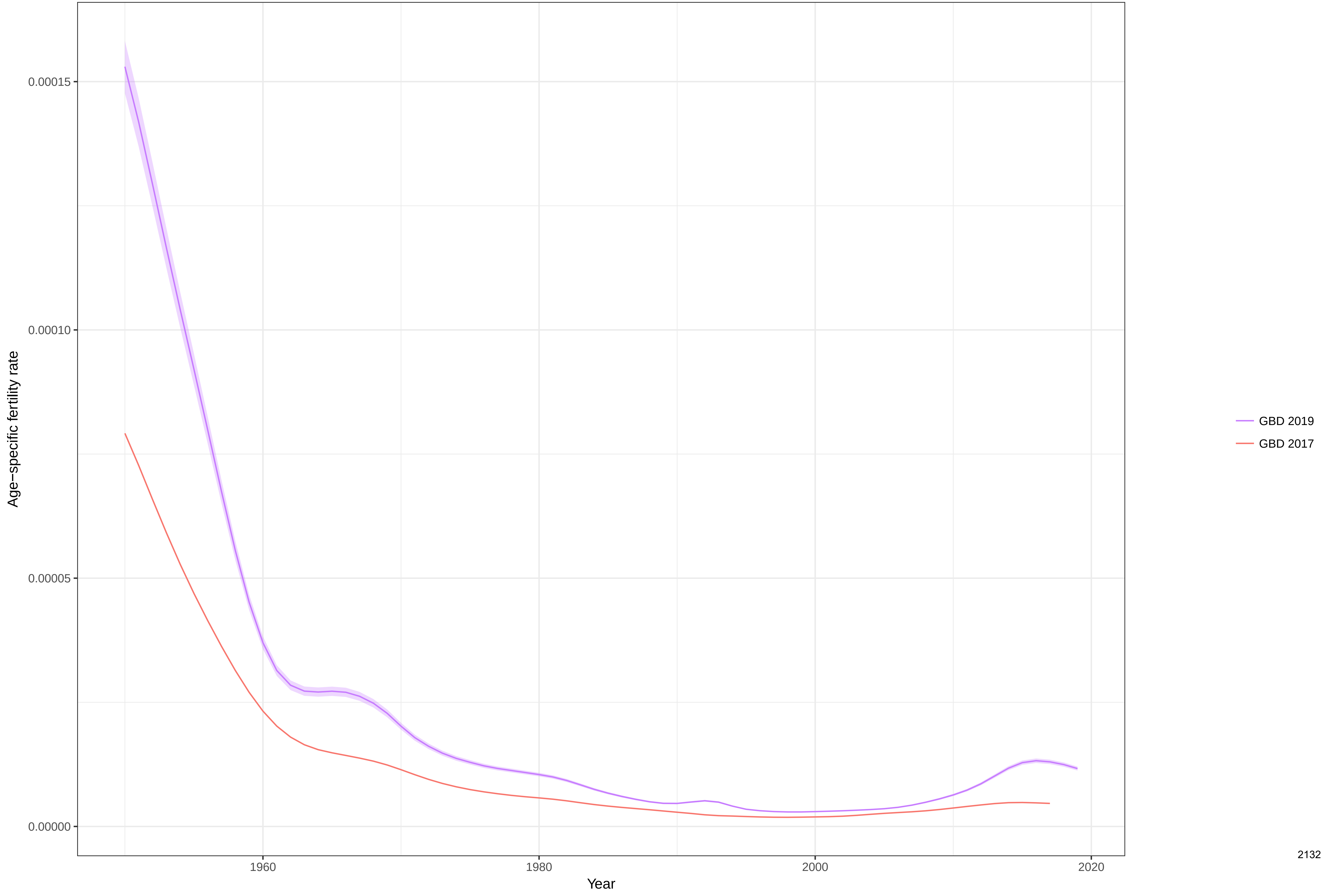

Total fertility rate fitted trend lines, 1950−2019: Ukraine TFR
Data points enclosed with a diamond are reference sources

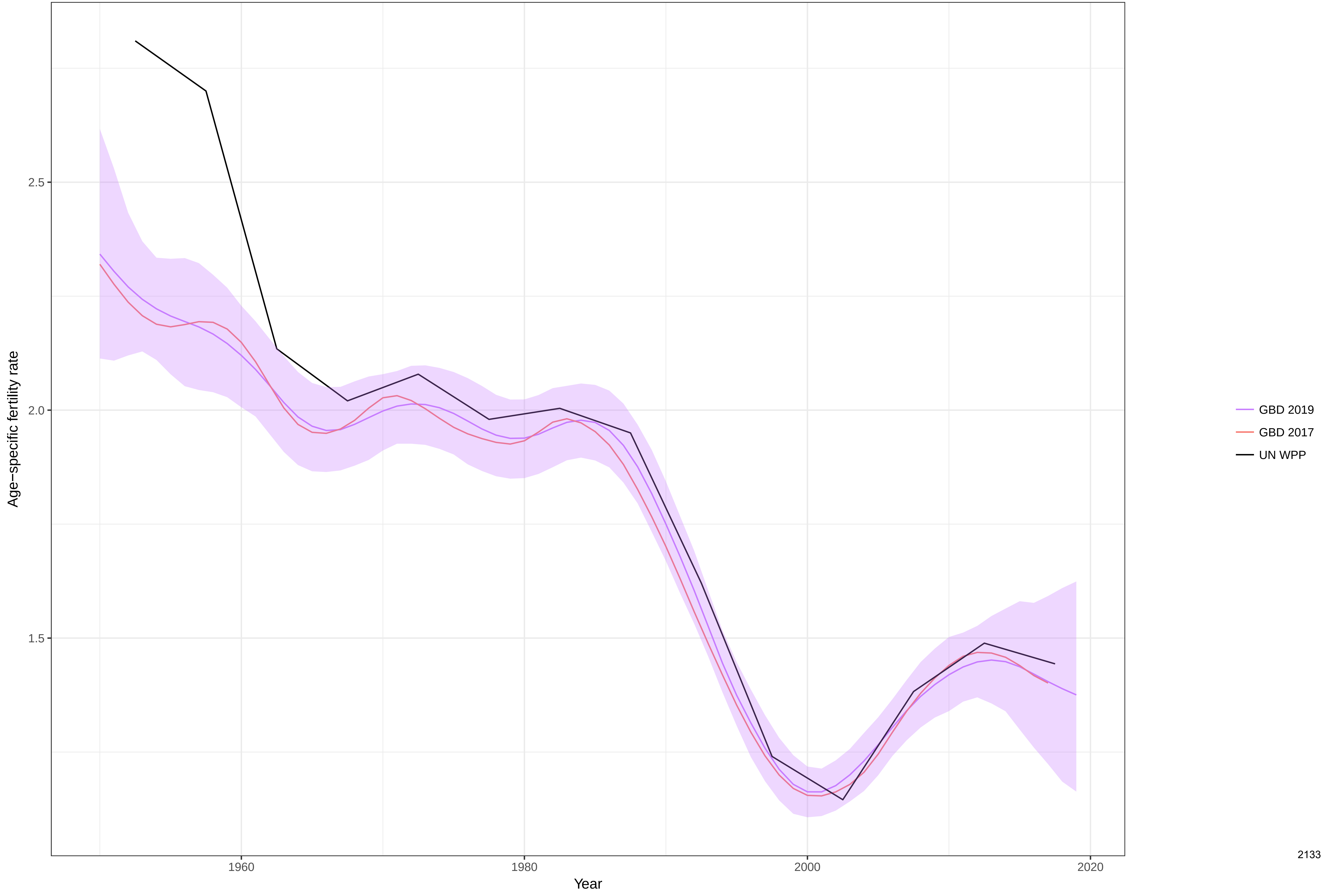

Age−specific fertility fitted trend lines, 1950−2019: Uruguay, Ages: 10−14
Data points enclosed with a diamond are reference sources

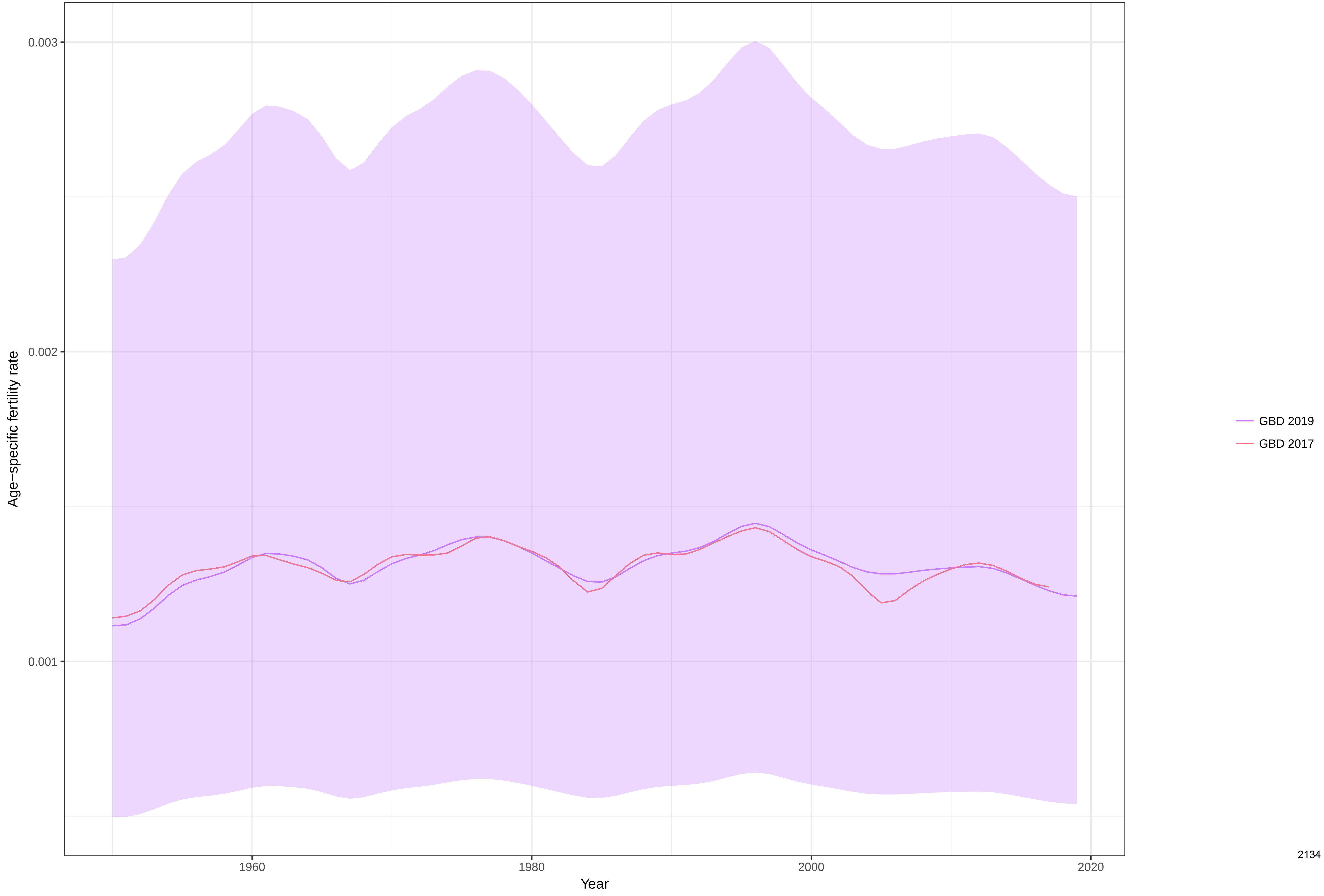

Age−specific fertility fitted trend lines, 1950−2019: Uruguay, Ages: 15−19
Data points enclosed with a diamond are reference sources

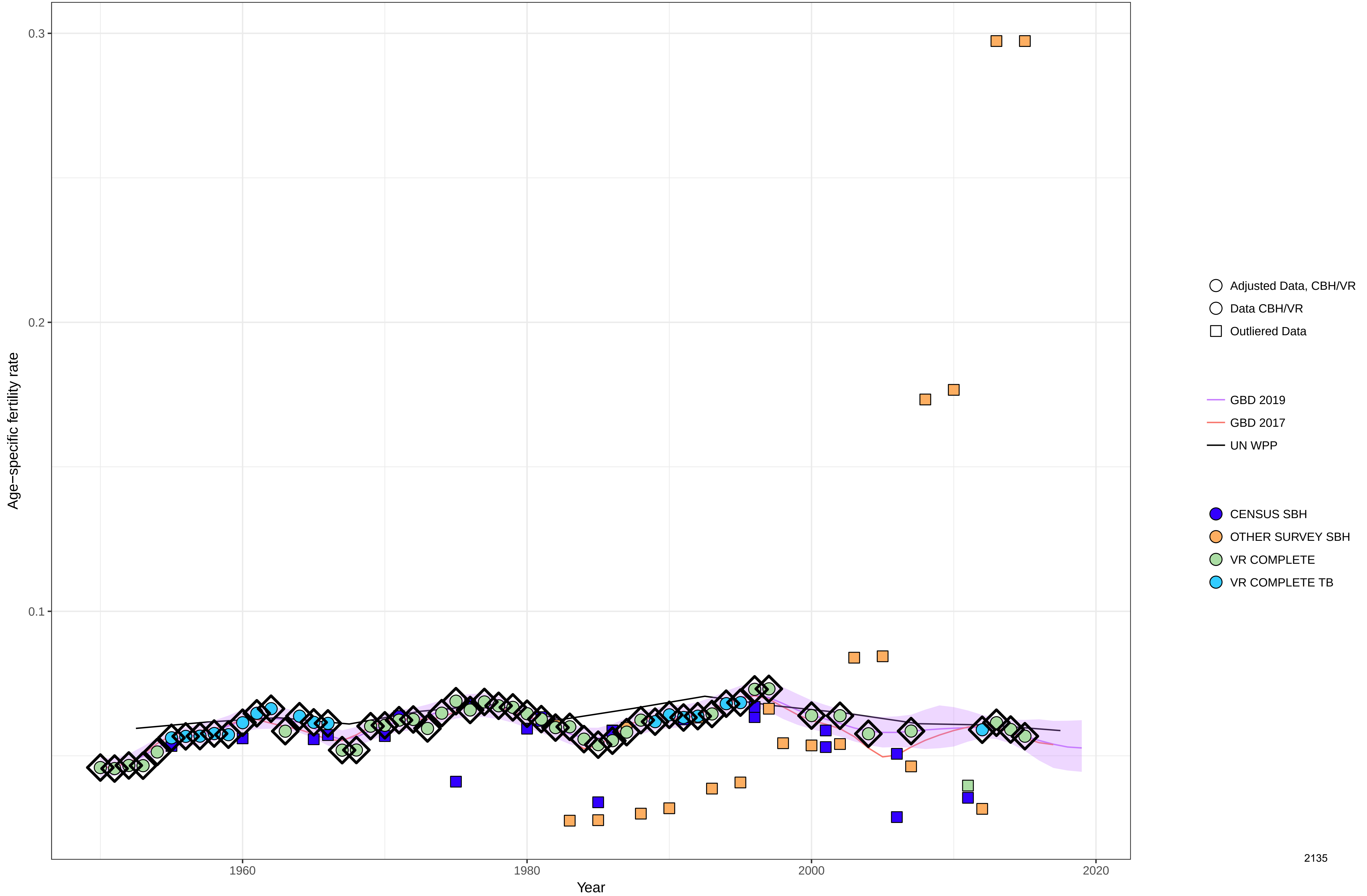

Age−specific fertility fitted trend lines, 1950−2019: Uruguay, Ages: 20−24
Data points enclosed with a diamond are reference sources

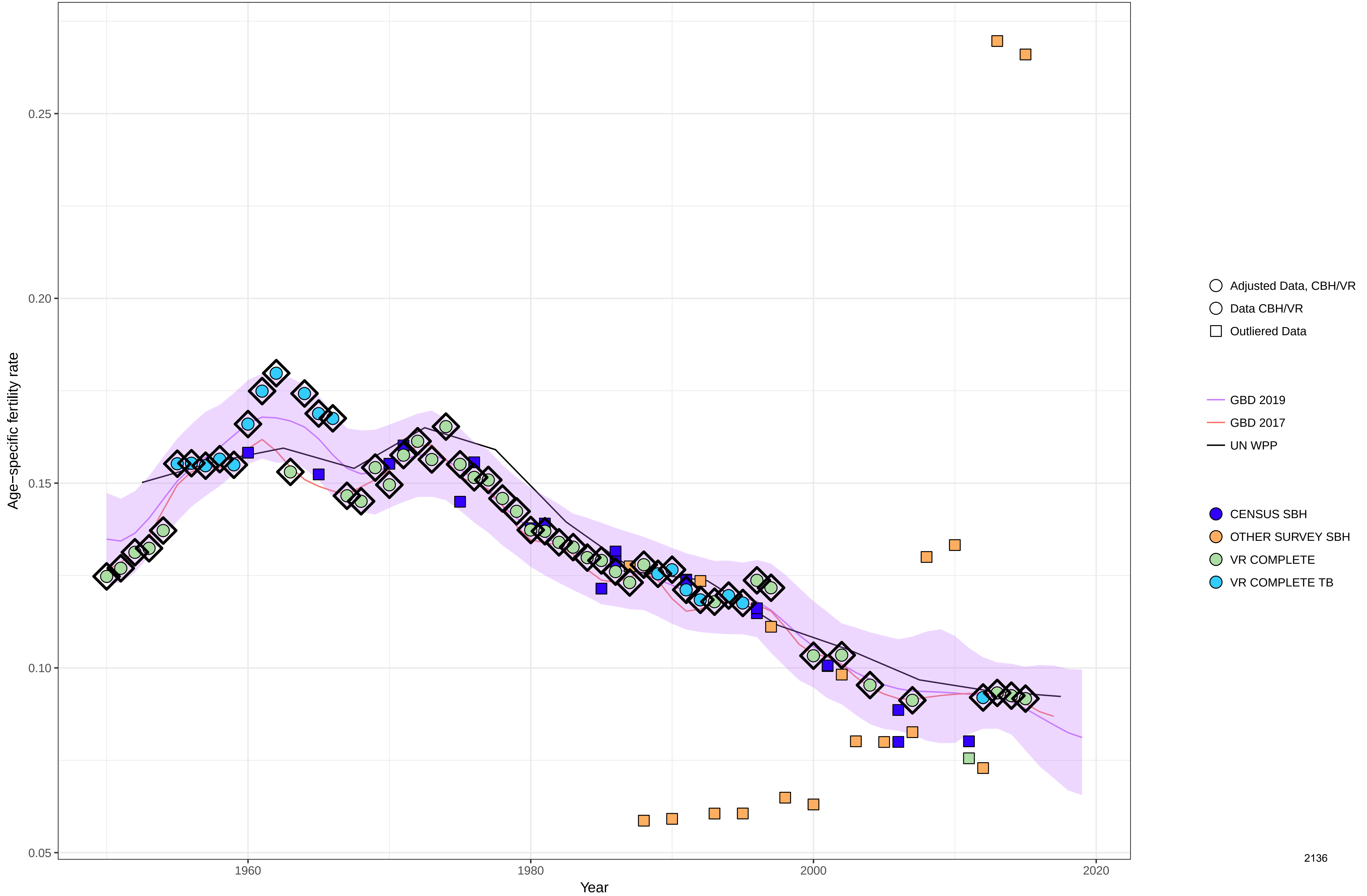

Age−specific fertility fitted trend lines, 1950−2019: Uruguay, Ages: 25−29
Data points enclosed with a diamond are reference sources

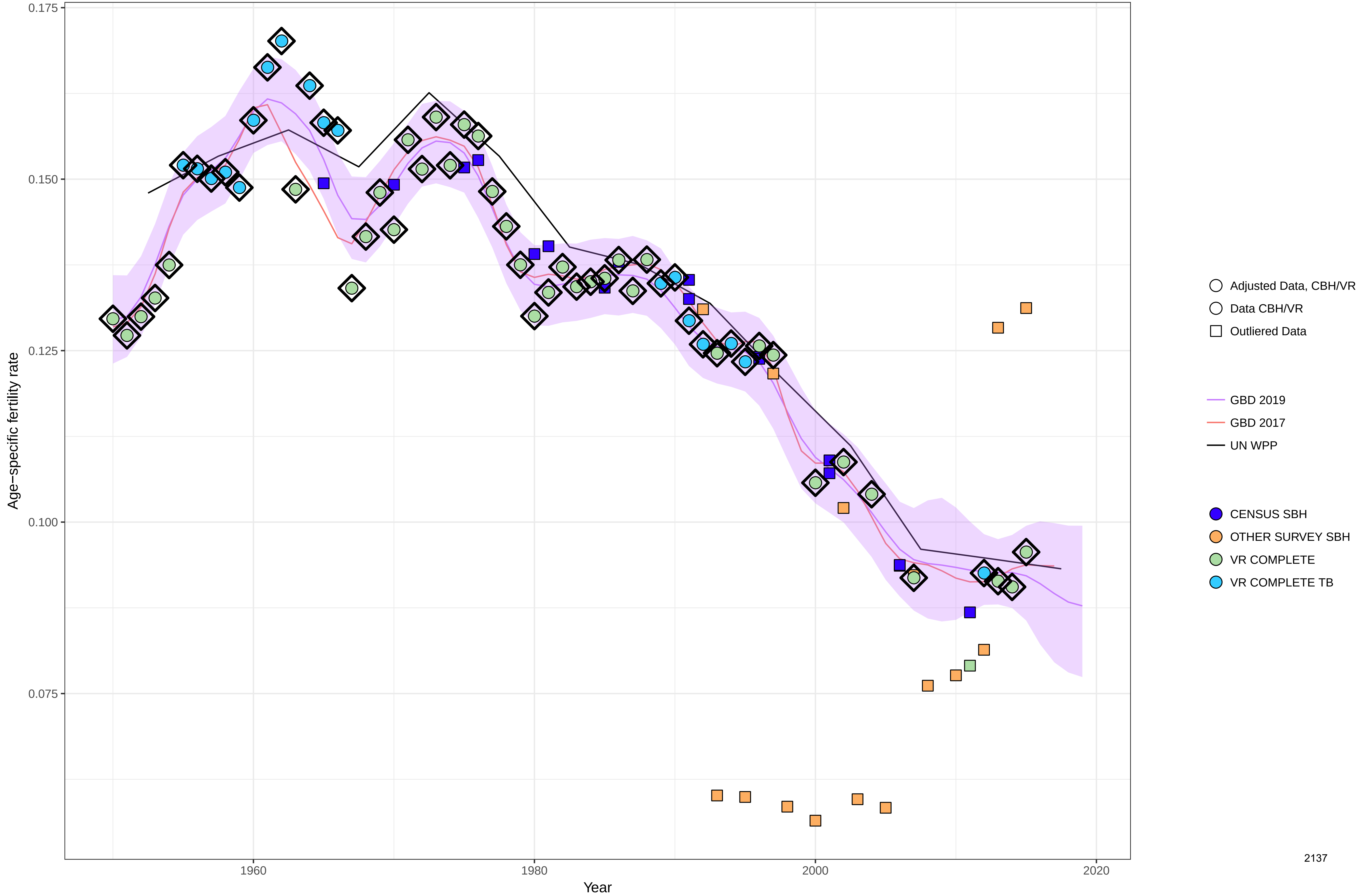

Age−specific fertility fitted trend lines, 1950−2019: Uruguay, Ages: 30−34
Data points enclosed with a diamond are reference sources

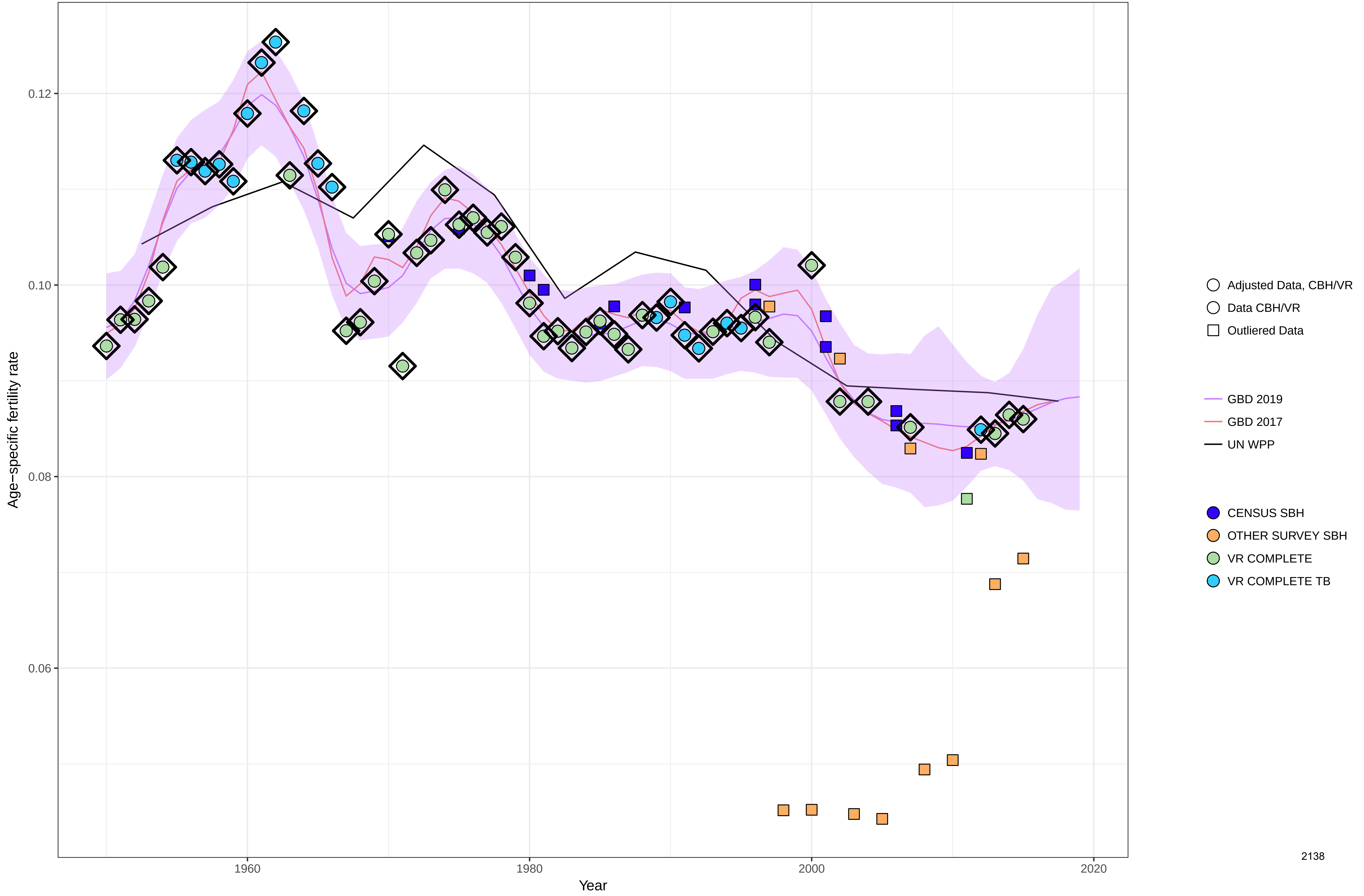

Age−specific fertility fitted trend lines, 1950−2019: Uruguay, Ages: 35−39
Data points enclosed with a diamond are reference sources

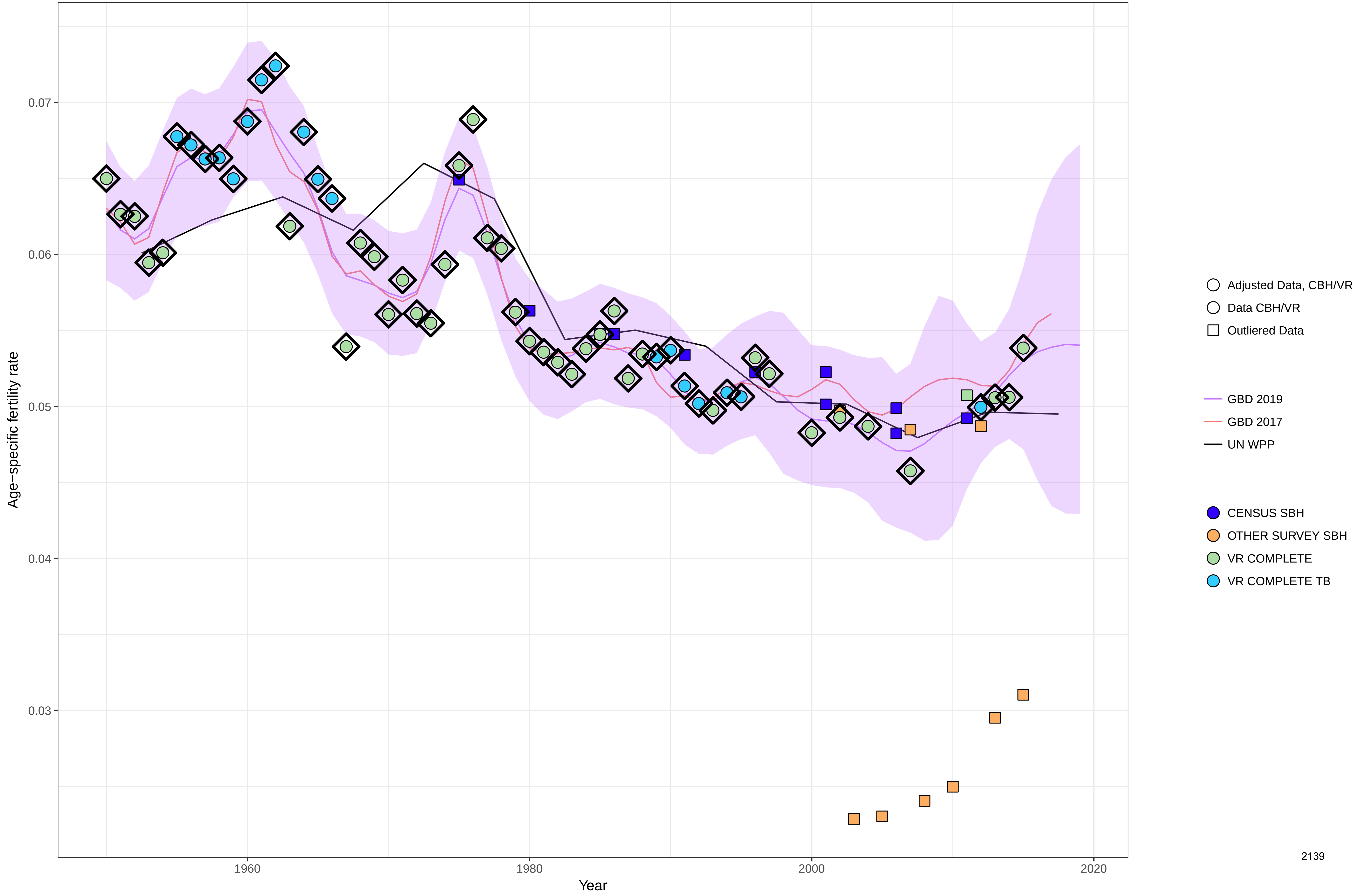

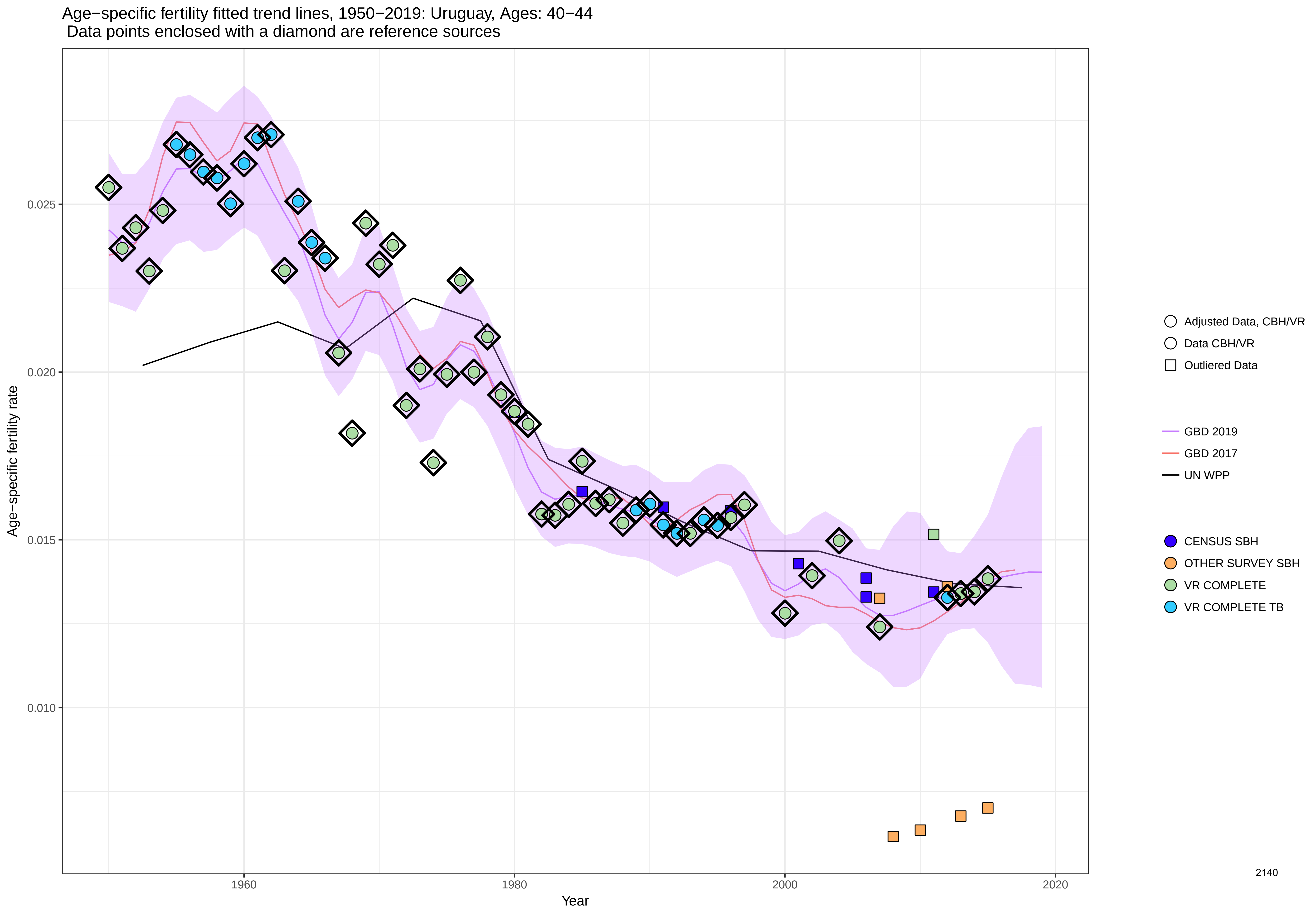
Age−specific fertility fitted trend lines, 1950−2019: Uruguay, Ages: 40−44
Data points enclosed with a diamond are reference sources
Age−specific fertility rate
0.025
0.020
0.015
0.010
1960
1980
2000
2020
Year
Adjusted Data, CBH/VR
Data CBH/VR
Outliered Data
GBD 2019
GBD 2017
UN WPP
CENSUS SBH
OTHER SURVEY SBH
VR COMPLETE
VR COMPLETE TB

Age−specific fertility fitted trend lines, 1950−2019: Uruguay, Ages: 45−49
Data points enclosed with a diamond are reference sources

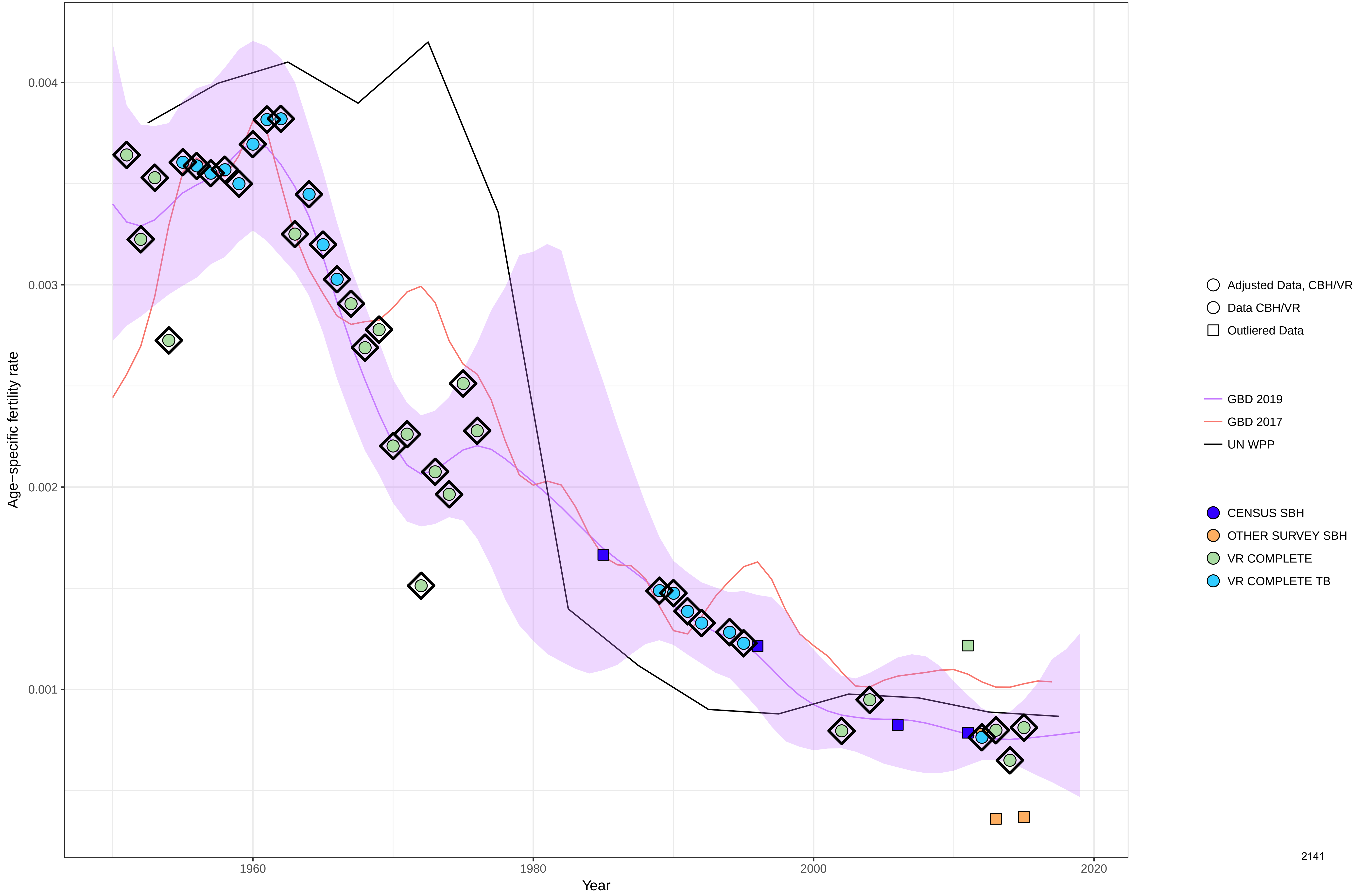

Age−specific fertility fitted trend lines, 1950−2019: Uruguay, Ages: 50−54
Data points enclosed with a diamond are reference sources

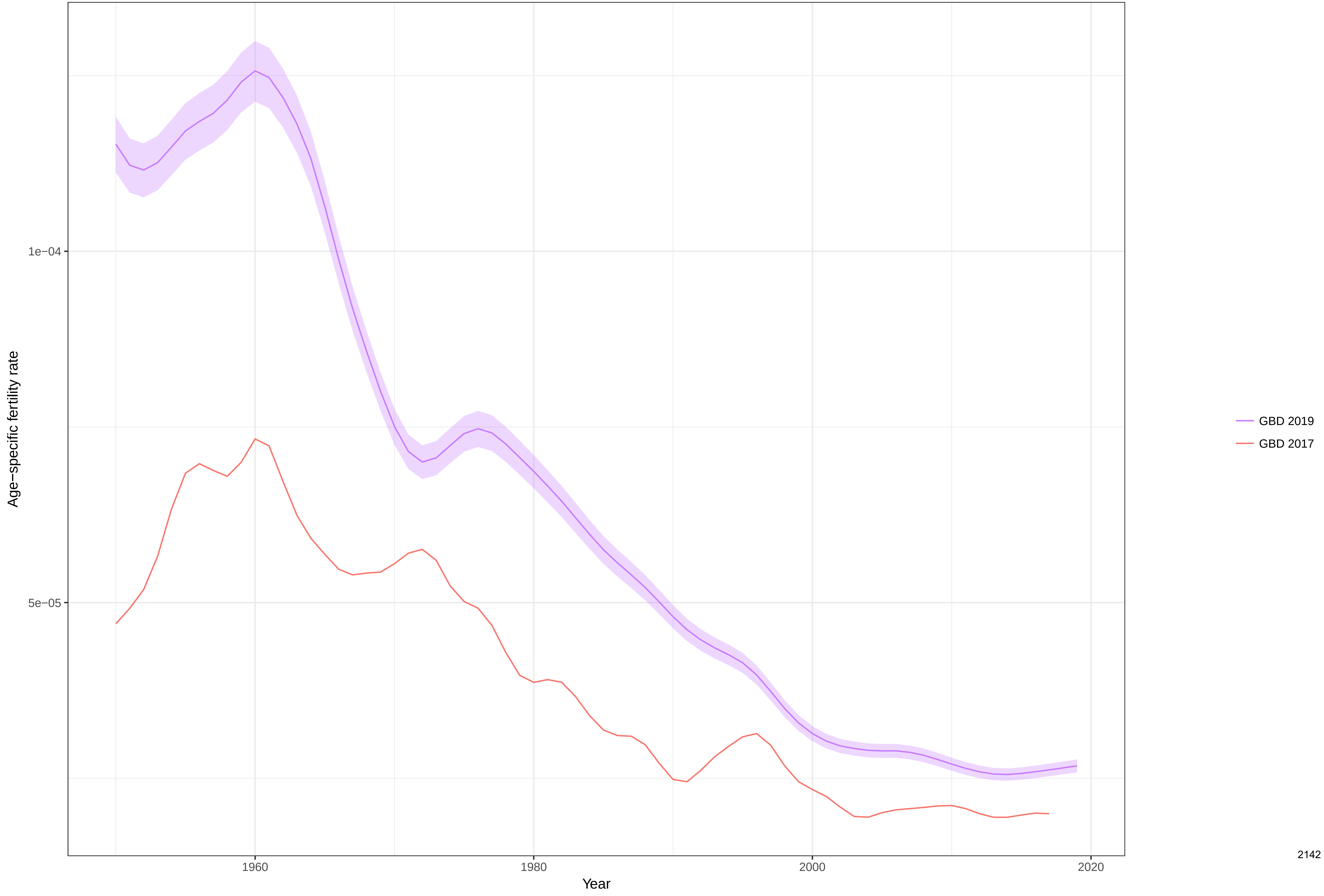

Total fertility rate fitted trend lines, 1950−2019: Uruguay TFR
Data points enclosed with a diamond are reference sources

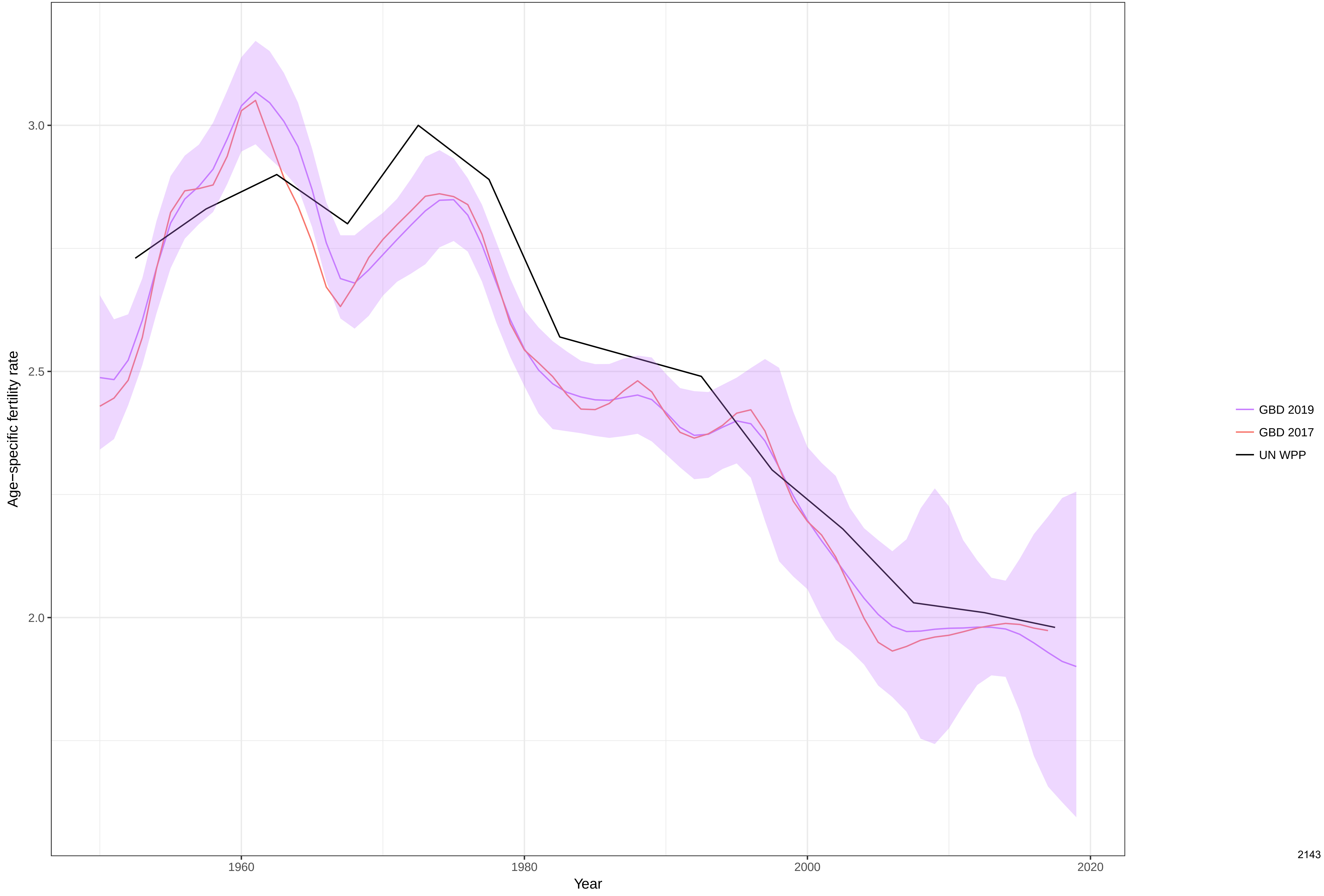

Age−specific fertility fitted trend lines, 1950−2019: United States of America, Ages: 10−14
Data points enclosed with a diamond are reference sources

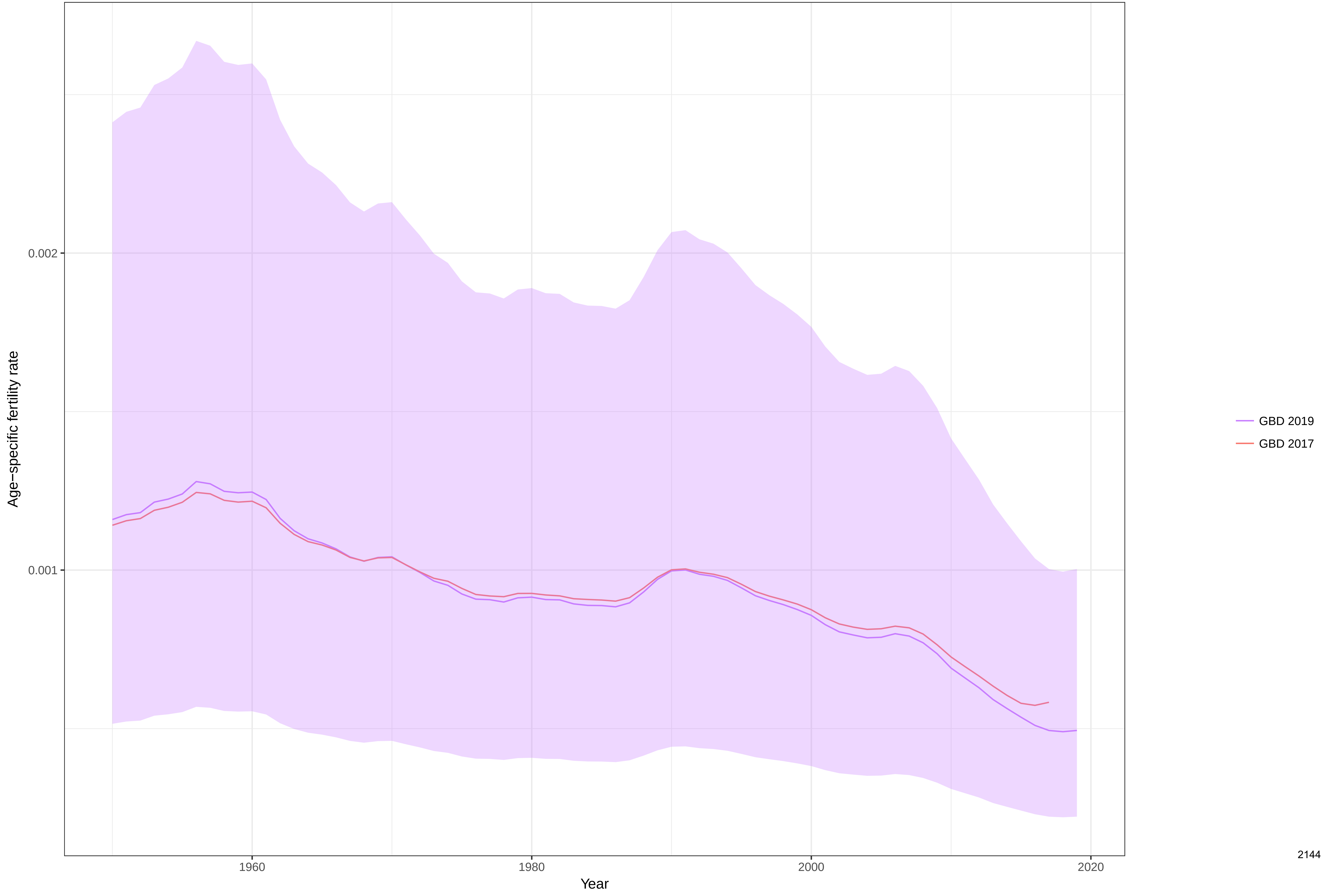

Age−specific fertility fitted trend lines, 1950−2019: United States of America, Ages: 15−19
Data points enclosed with a diamond are reference sources

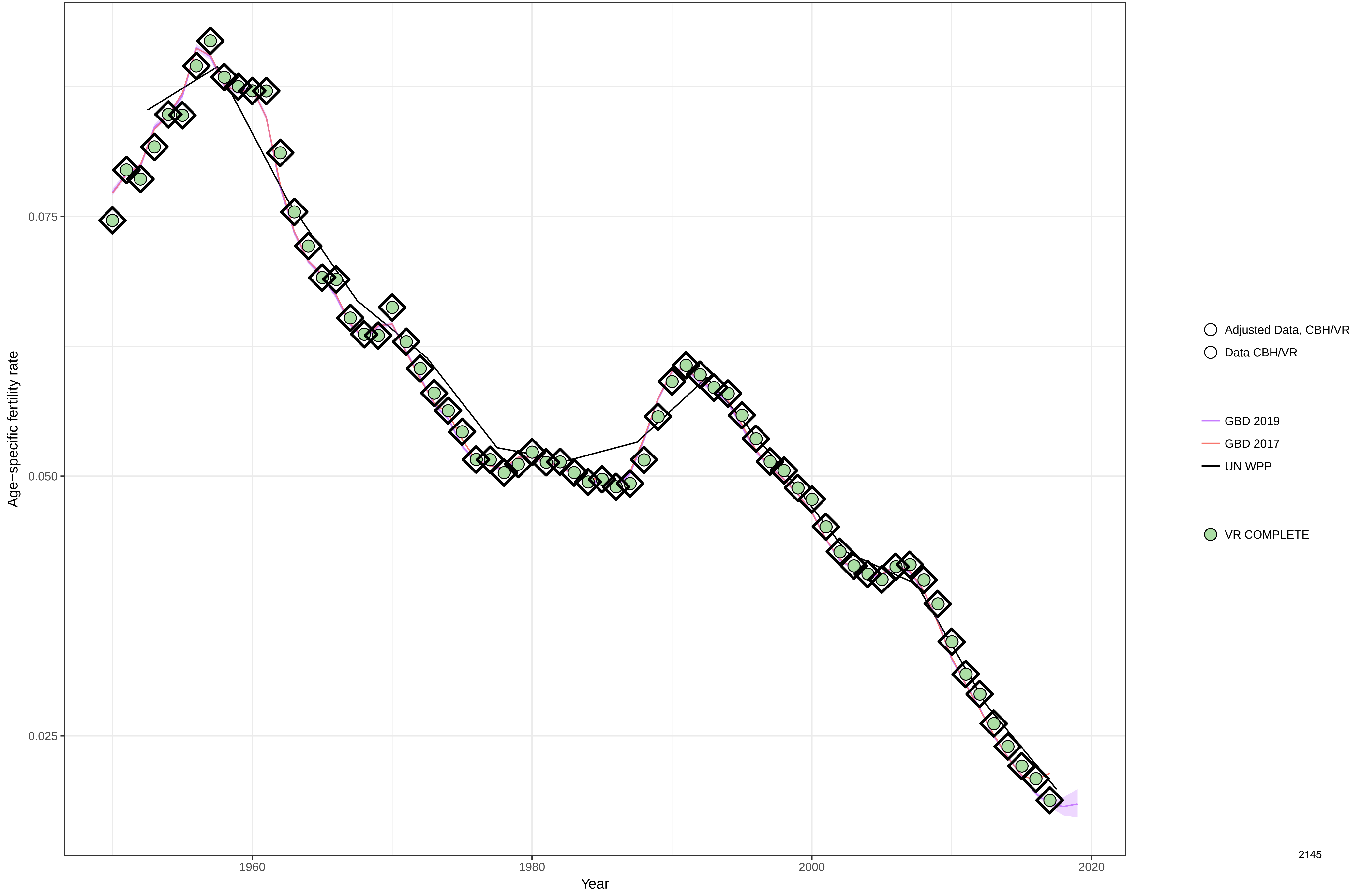

Age−specific fertility fitted trend lines, 1950−2019: United States of America, Ages: 20−24
Data points enclosed with a diamond are reference sources

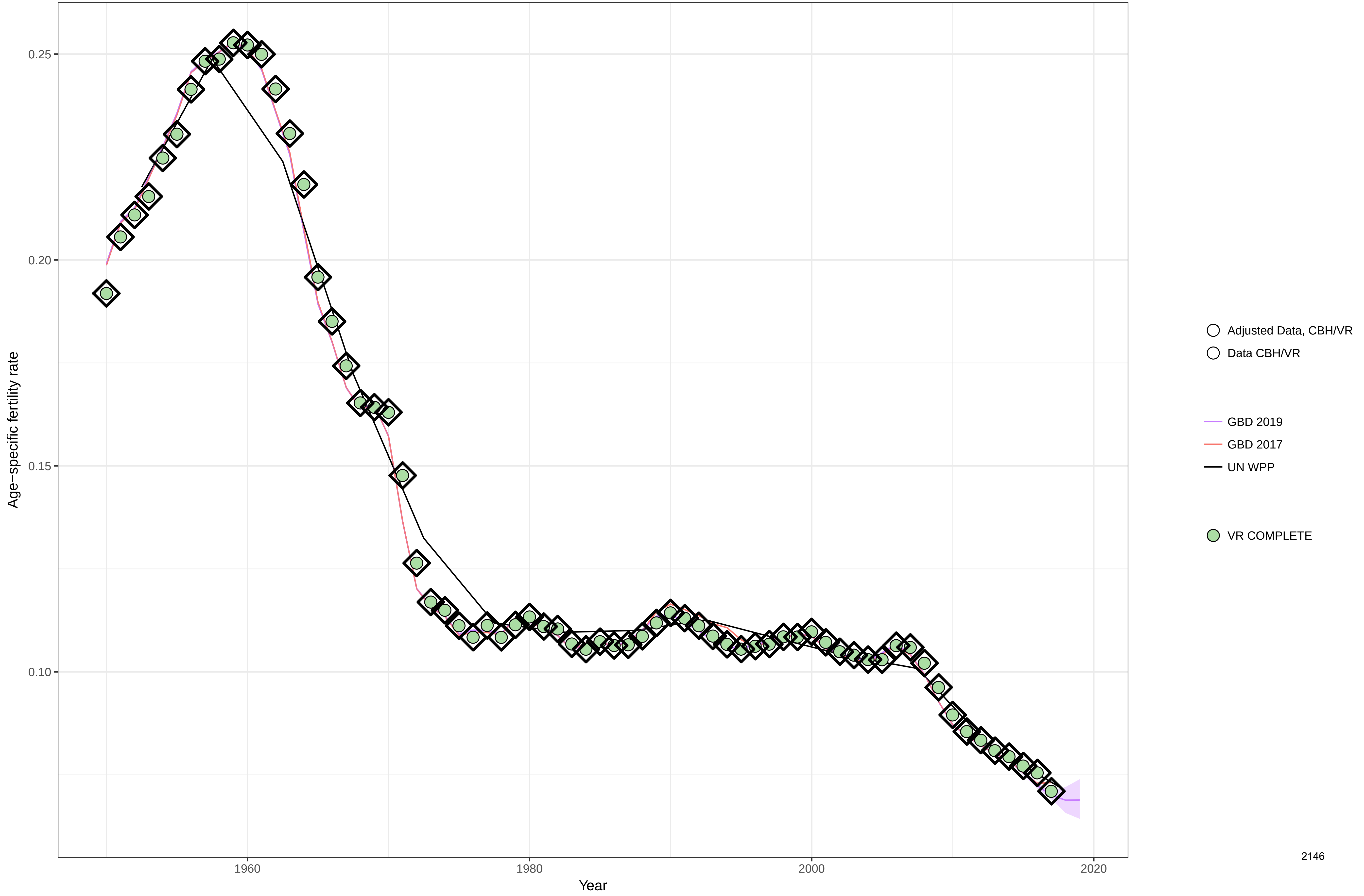

Age−specific fertility fitted trend lines, 1950−2019: United States of America, Ages: 25−29
Data points enclosed with a diamond are reference sources

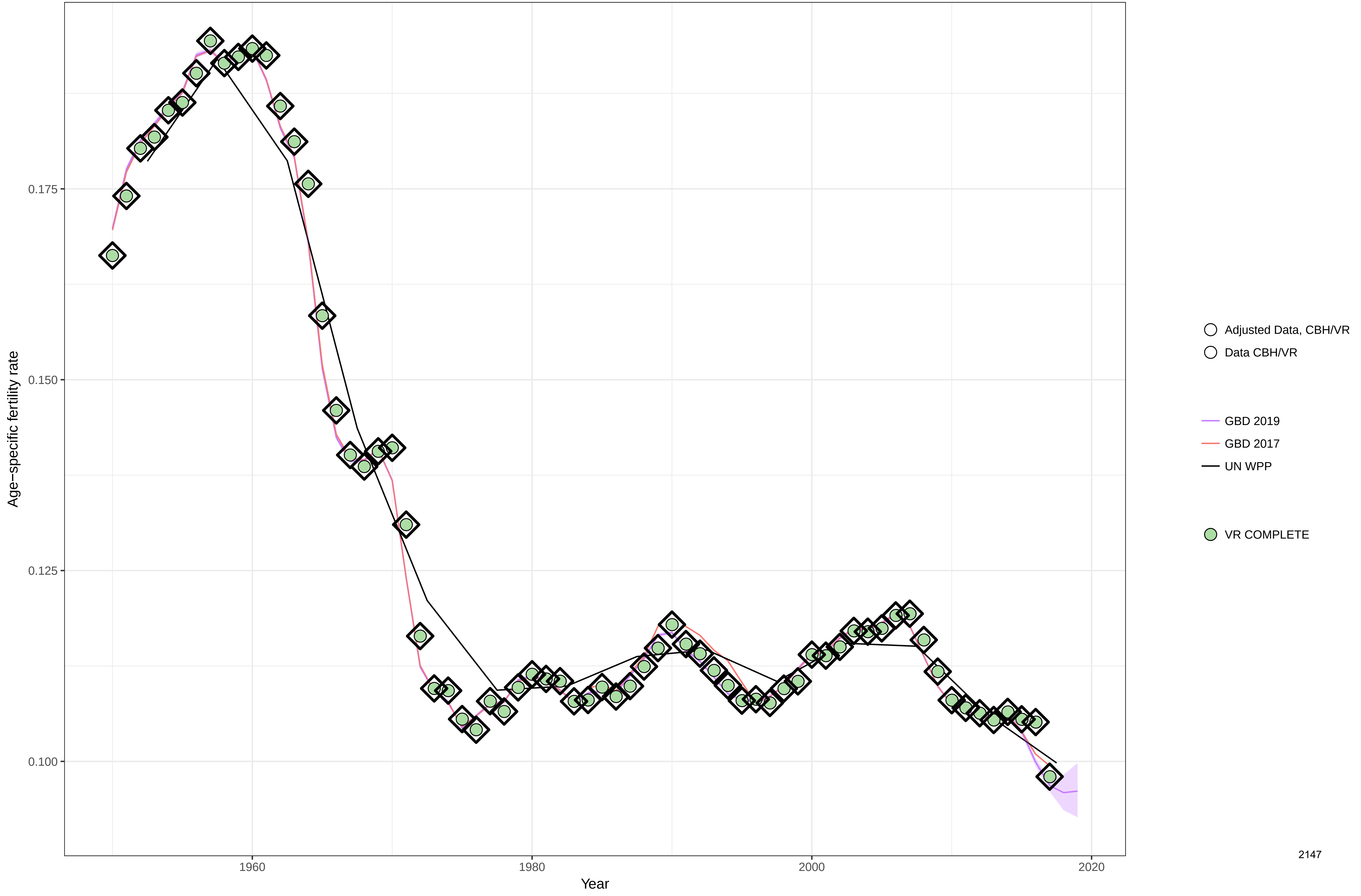

Age−specific fertility fitted trend lines, 1950−2019: United States of America, Ages: 30−34
Data points enclosed with a diamond are reference sources

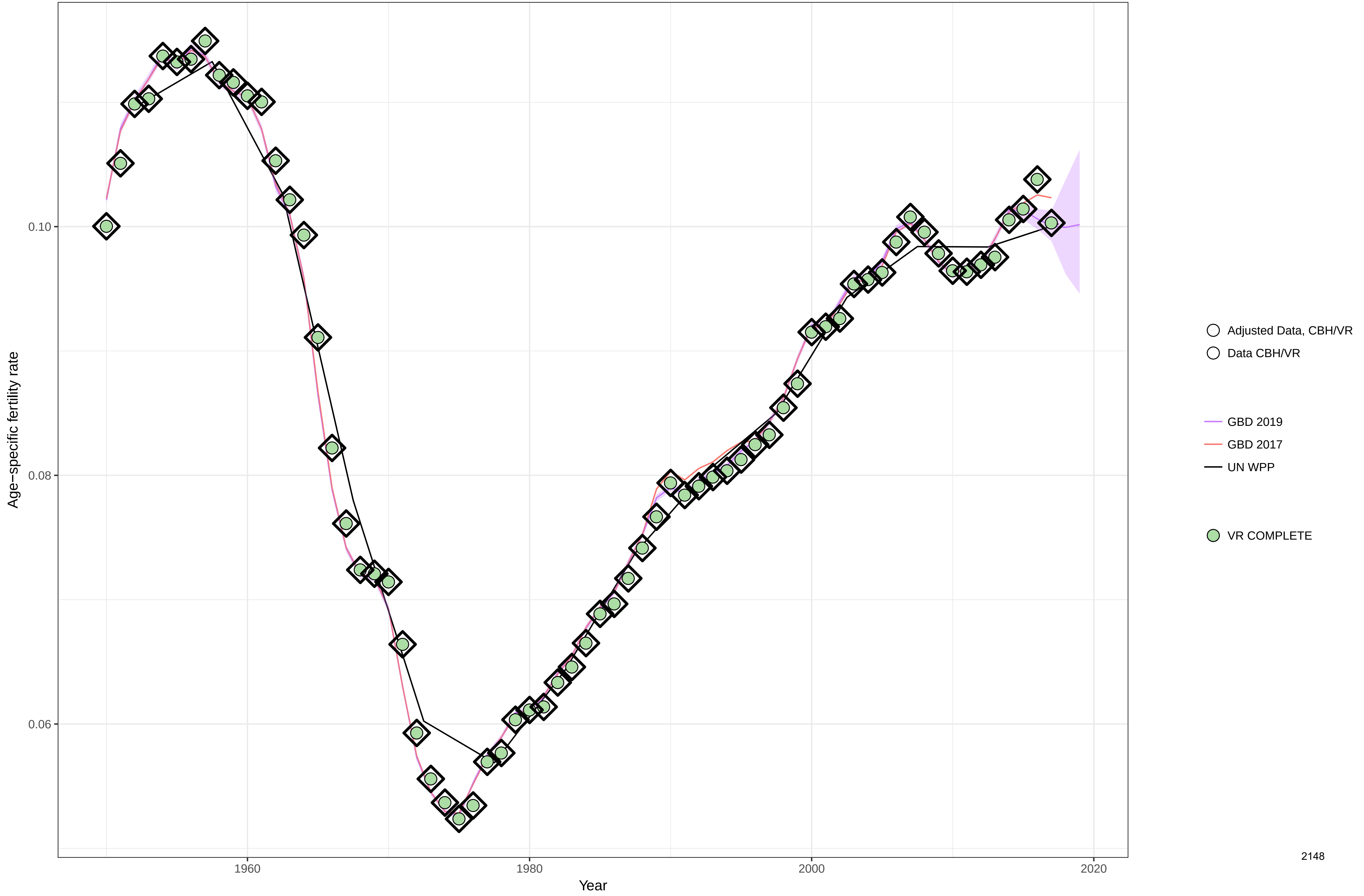

Age−specific fertility fitted trend lines, 1950−2019: United States of America, Ages: 35−39
Data points enclosed with a diamond are reference sources

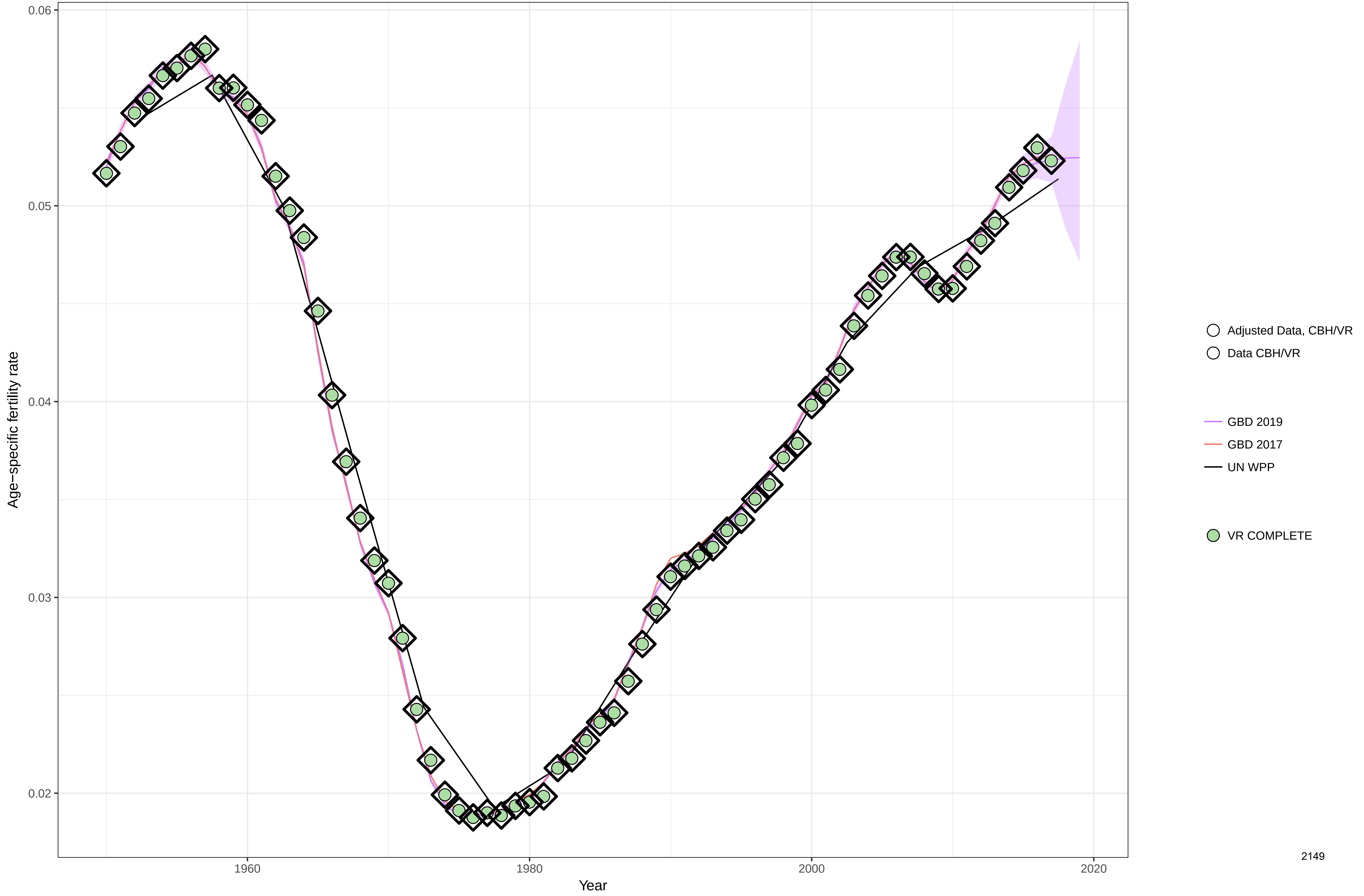

Age−specific fertility fitted trend lines, 1950−2019: United States of America, Ages: 40−44
Data points enclosed with a diamond are reference sources

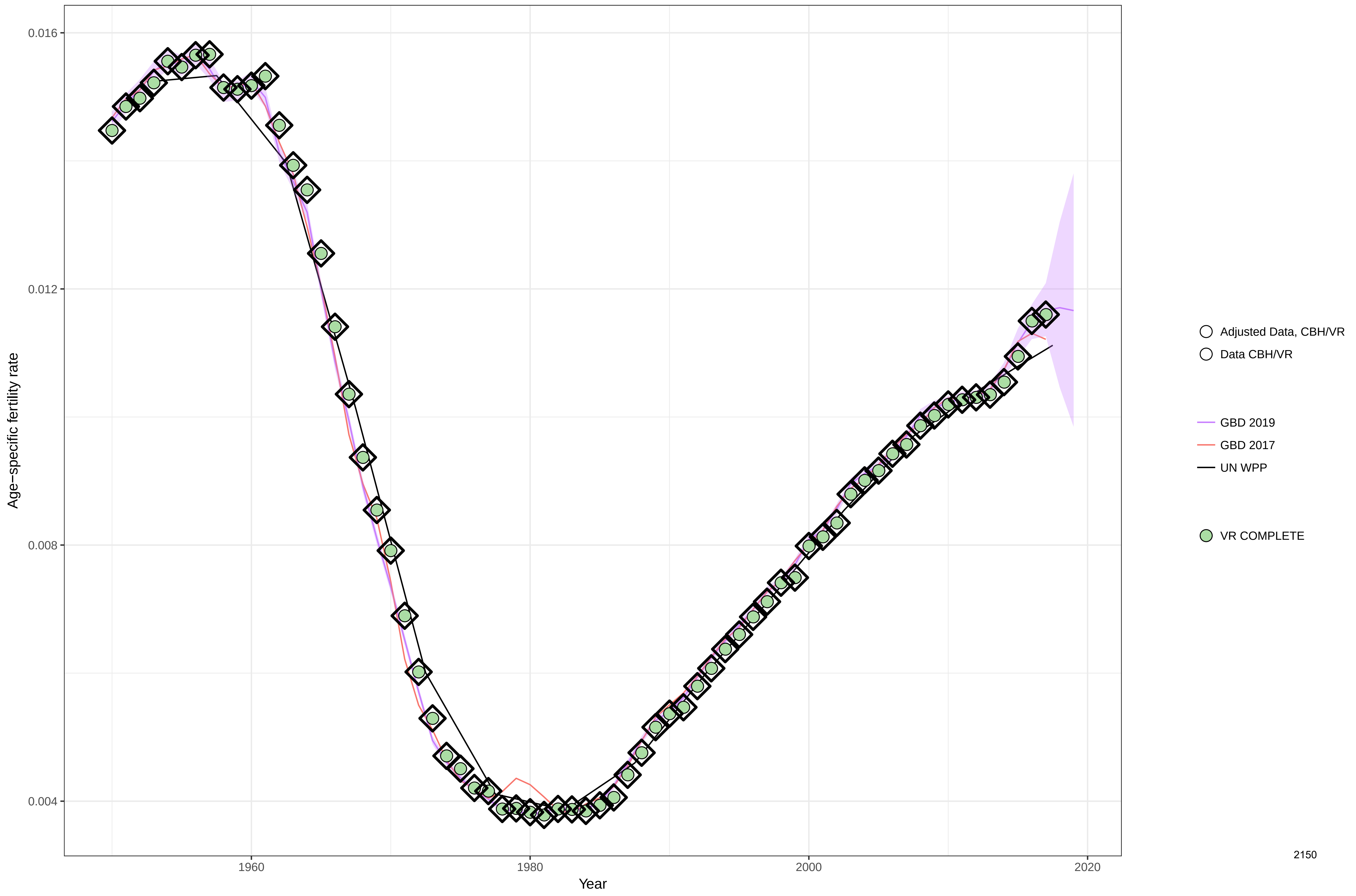

Age−specific fertility fitted trend lines, 1950−2019: United States of America, Ages: 45−49
Data points enclosed with a diamond are reference sources

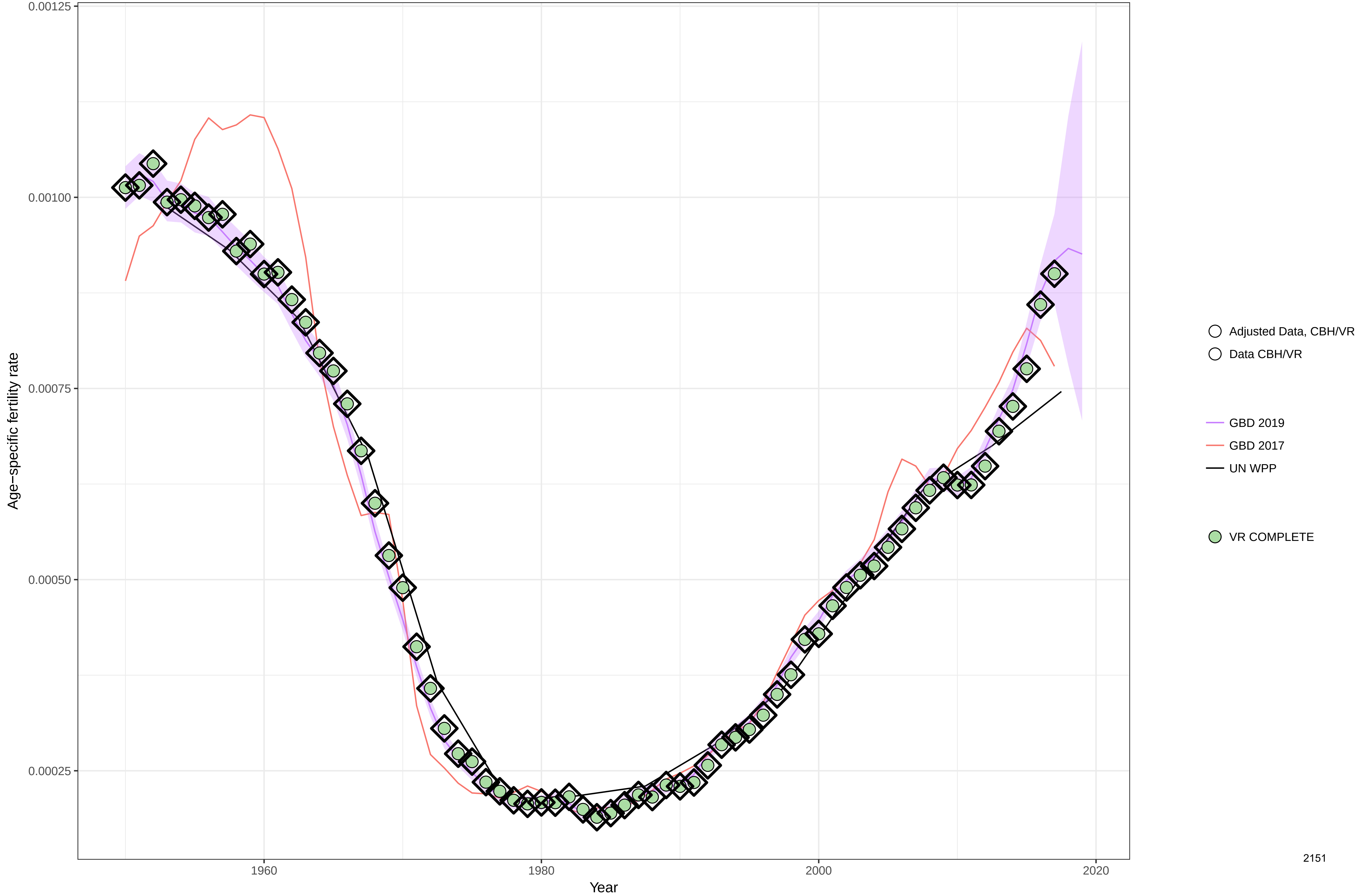

Age−specific fertility fitted trend lines, 1950−2019: United States of America, Ages: 50−54
Data points enclosed with a diamond are reference sources

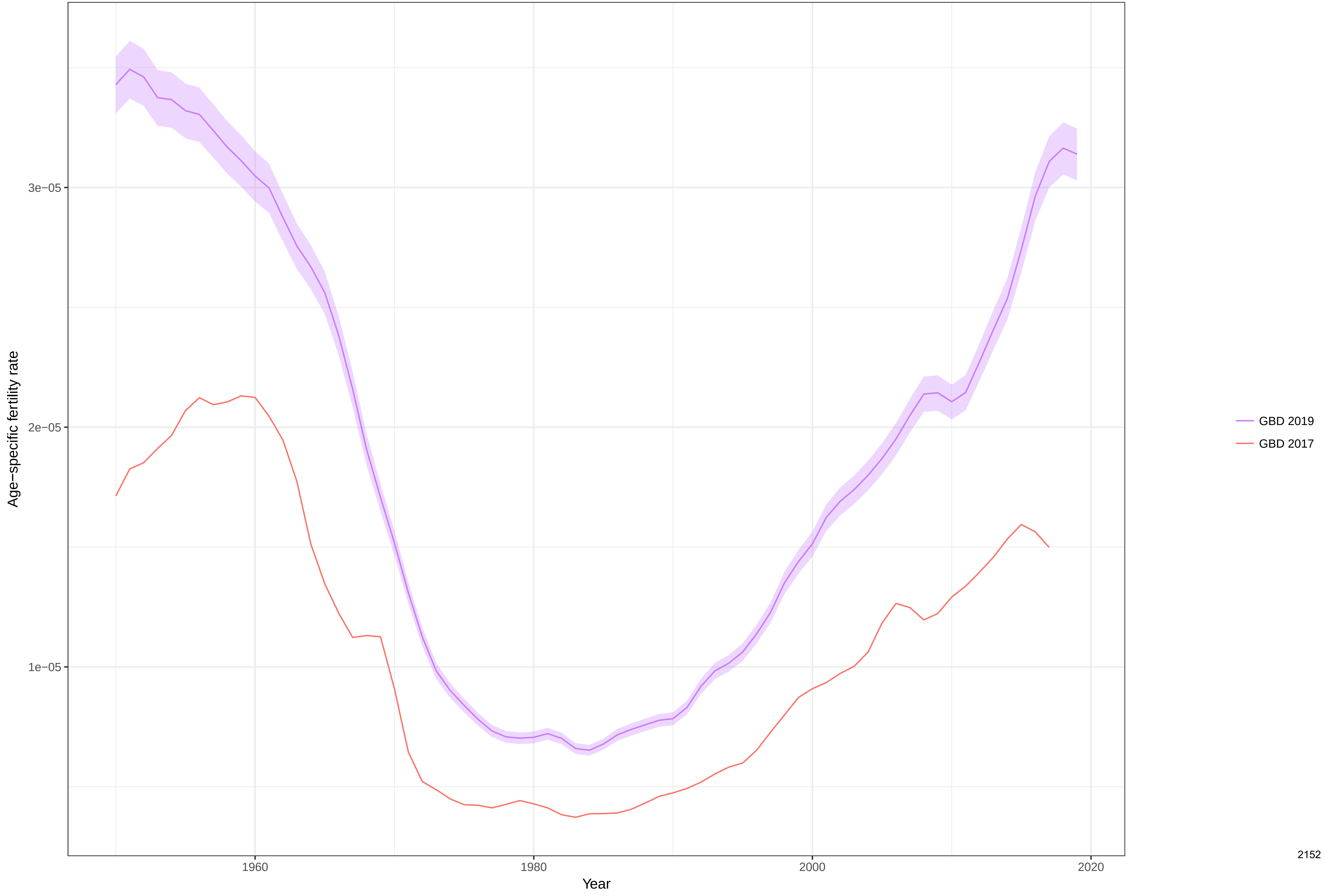

Total fertility rate fitted trend lines, 1950−2019: United States of America TFR
Data points enclosed with a diamond are reference sources

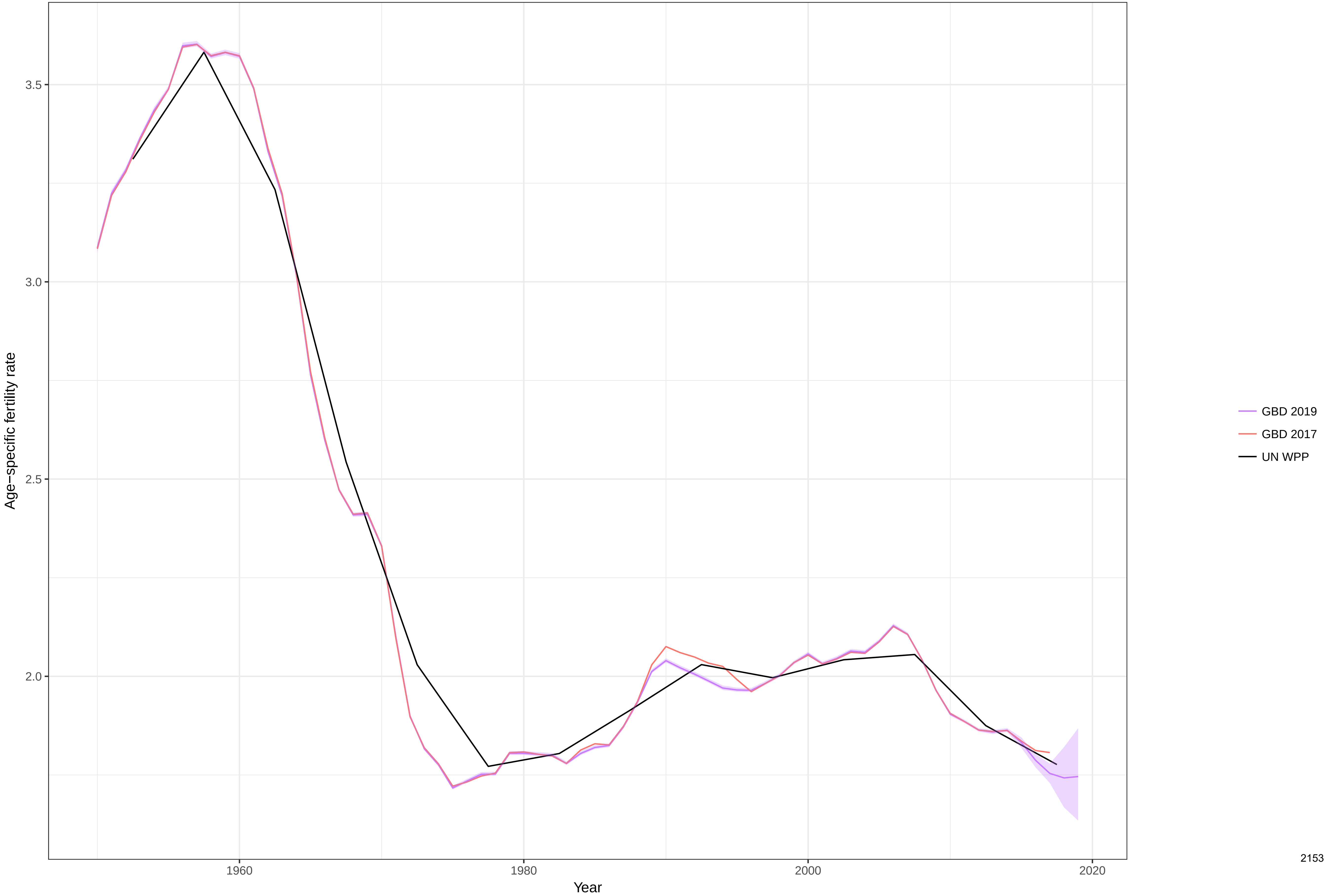

Age−specific fertility fitted trend lines, 1950−2019: Uzbekistan, Ages: 10−14
Data points enclosed with a diamond are reference sources

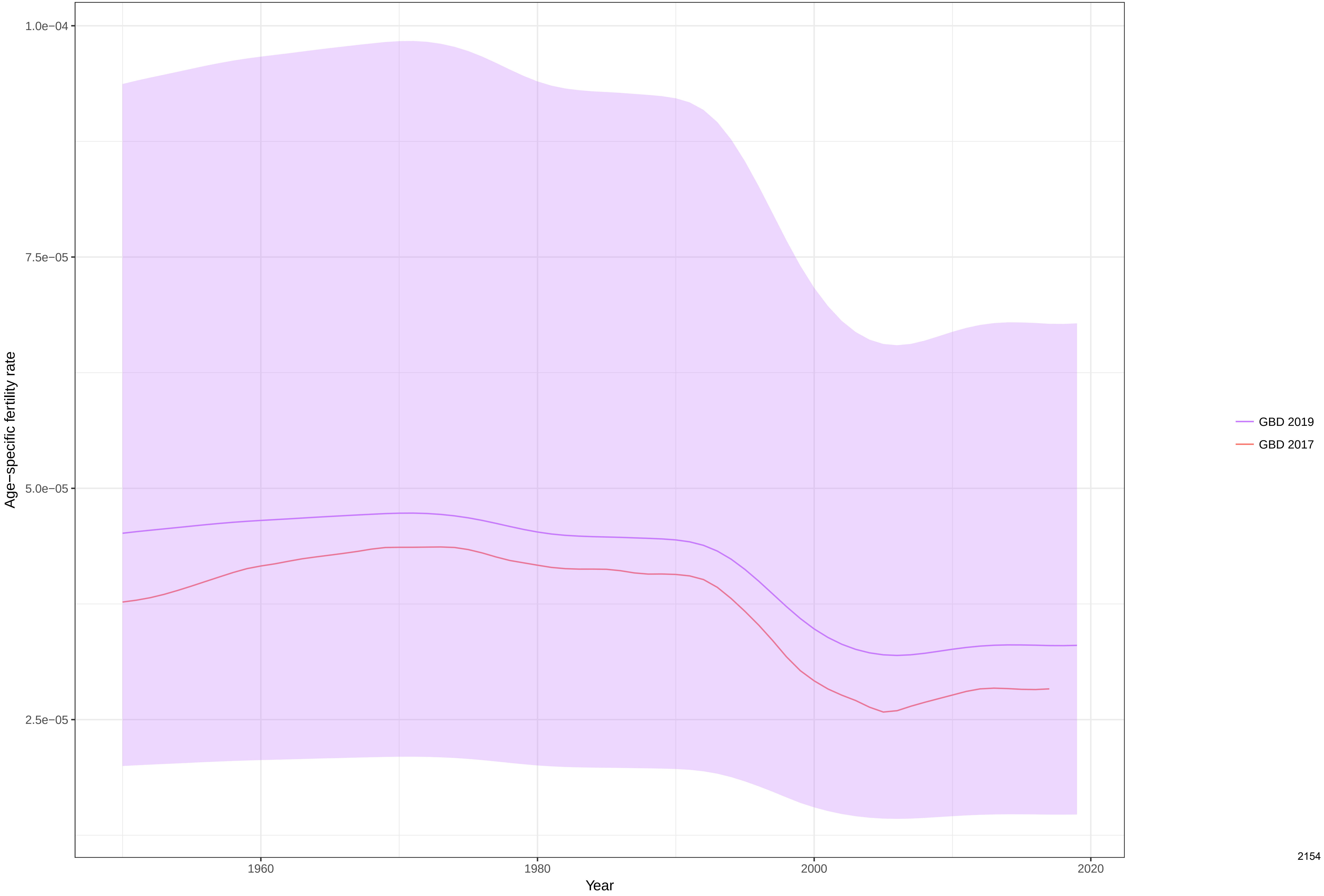

Age−specific fertility fitted trend lines, 1950−2019: Uzbekistan, Ages: 15−19
Data points enclosed with a diamond are reference sources

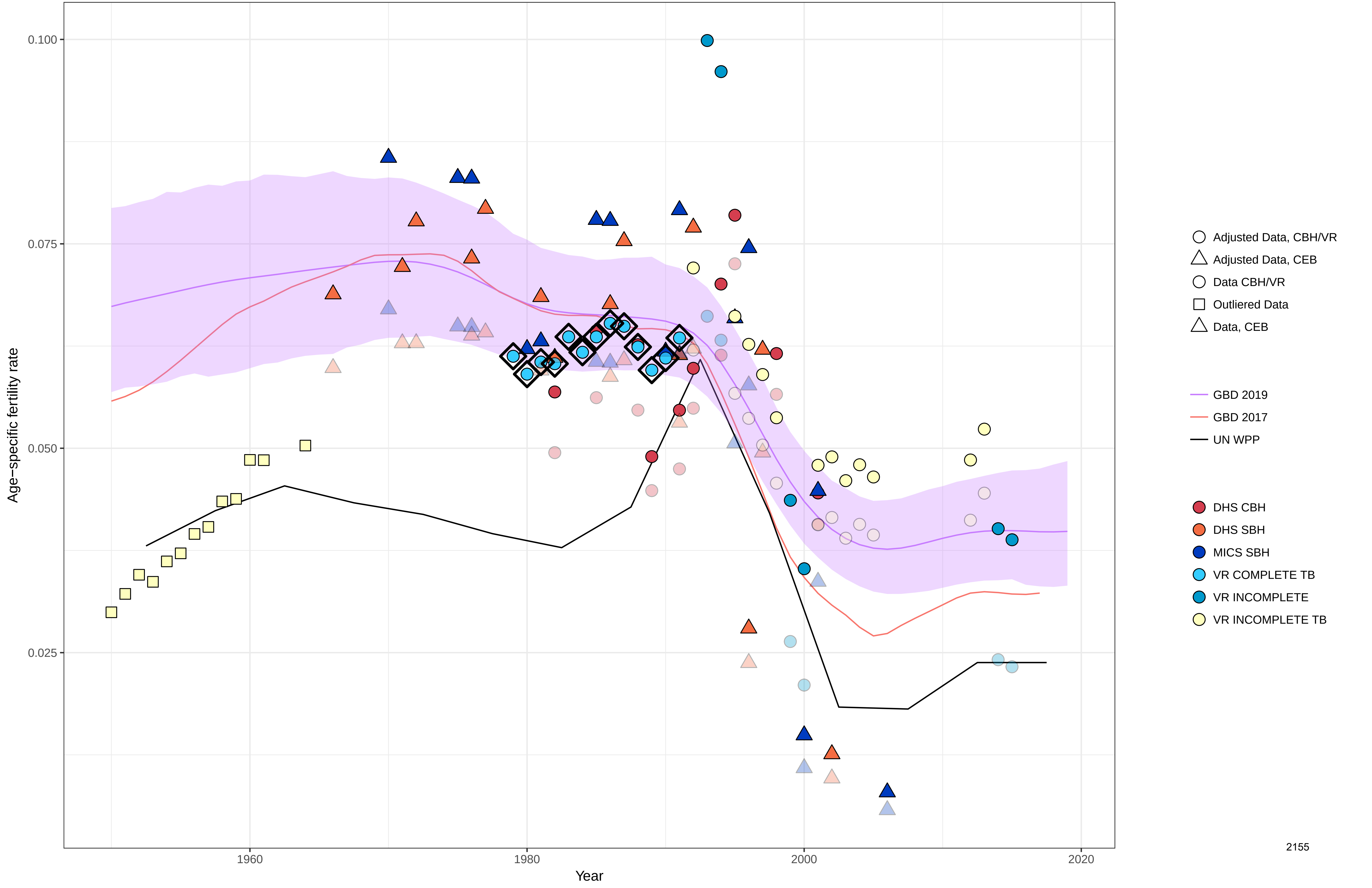

Age−specific fertility fitted trend lines, 1950−2019: Uzbekistan, Ages: 20−24
Data points enclosed with a diamond are reference sources

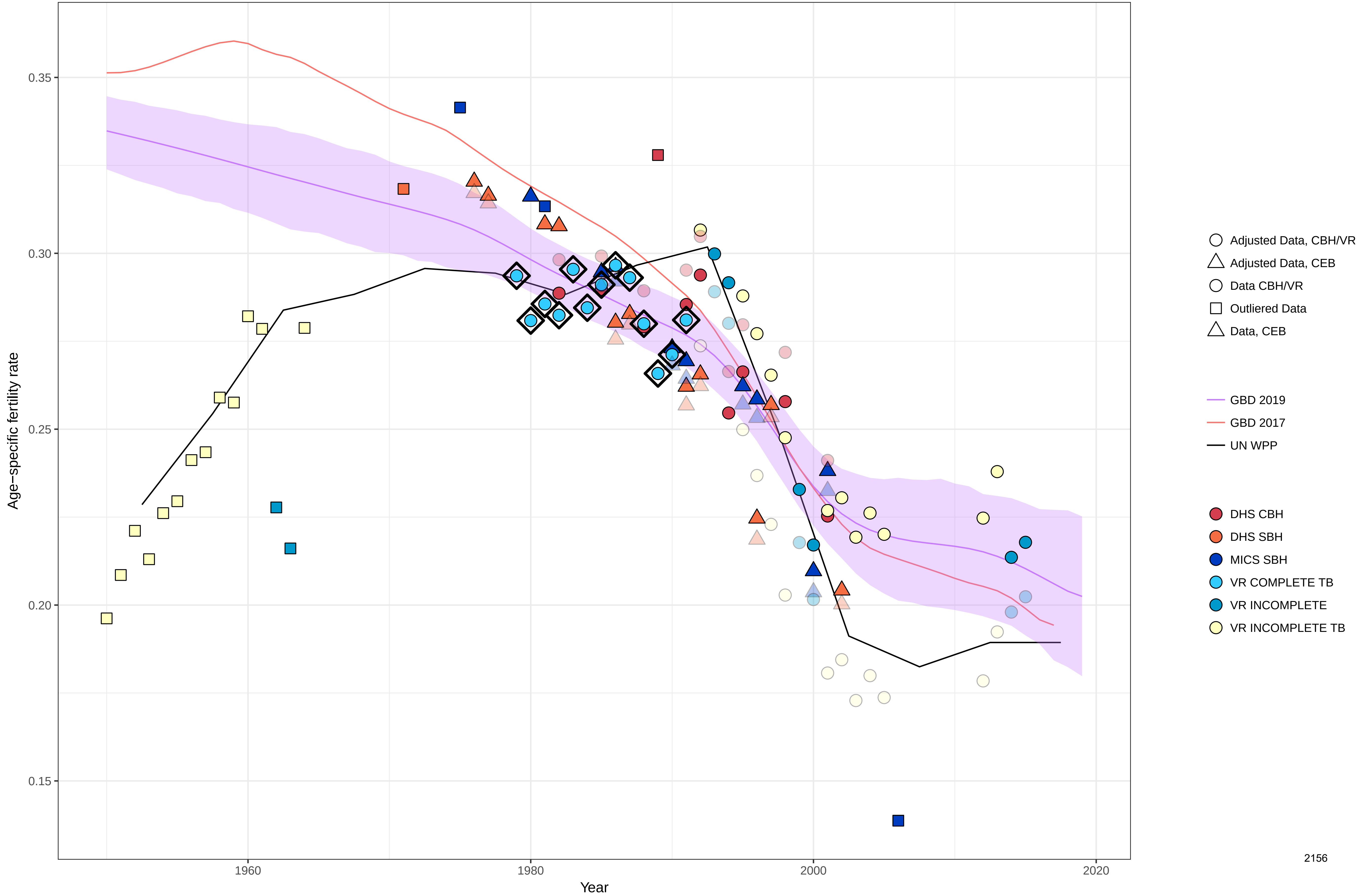

Age−specific fertility fitted trend lines, 1950−2019: Uzbekistan, Ages: 25−29
Data points enclosed with a diamond are reference sources

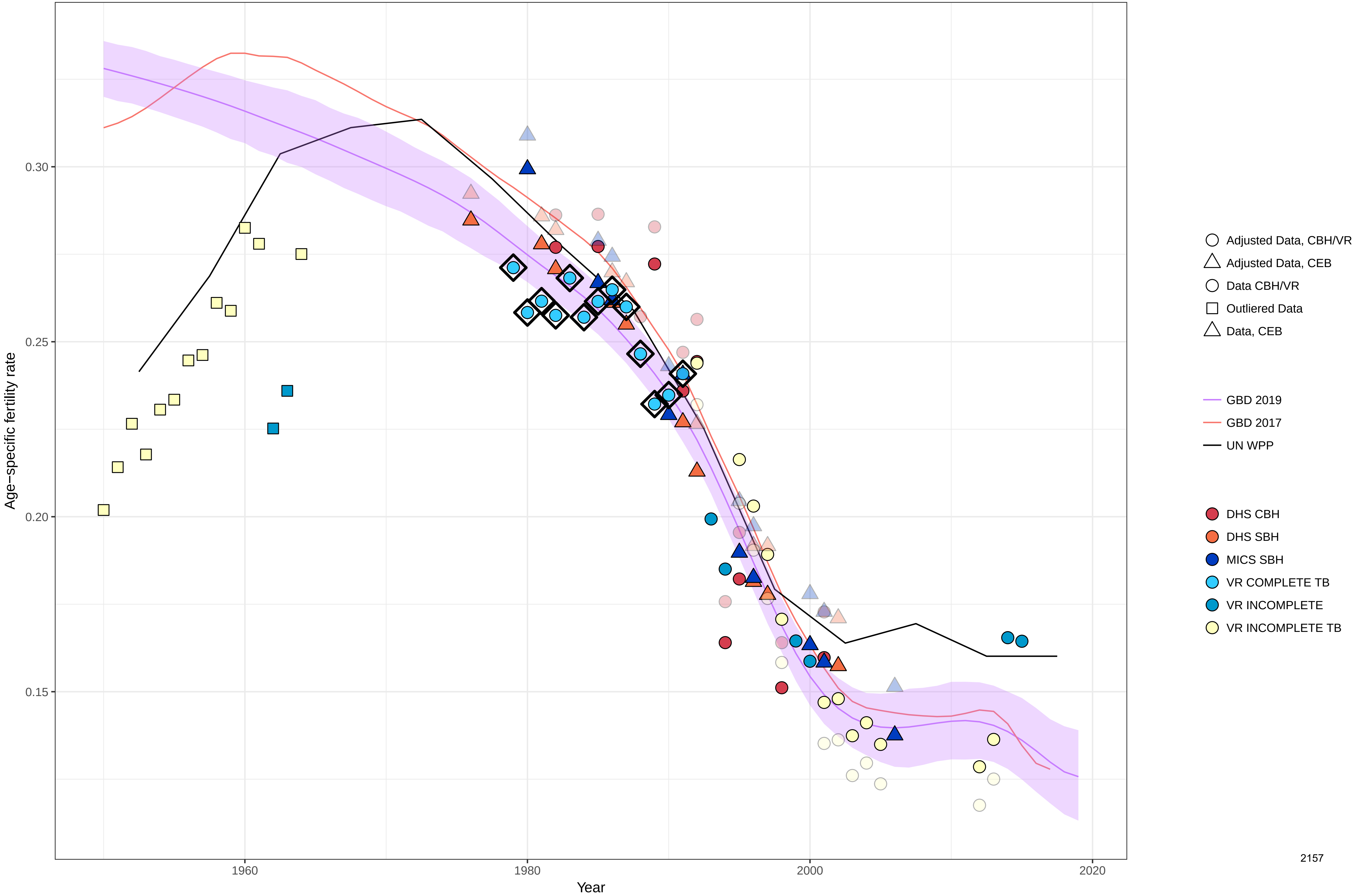

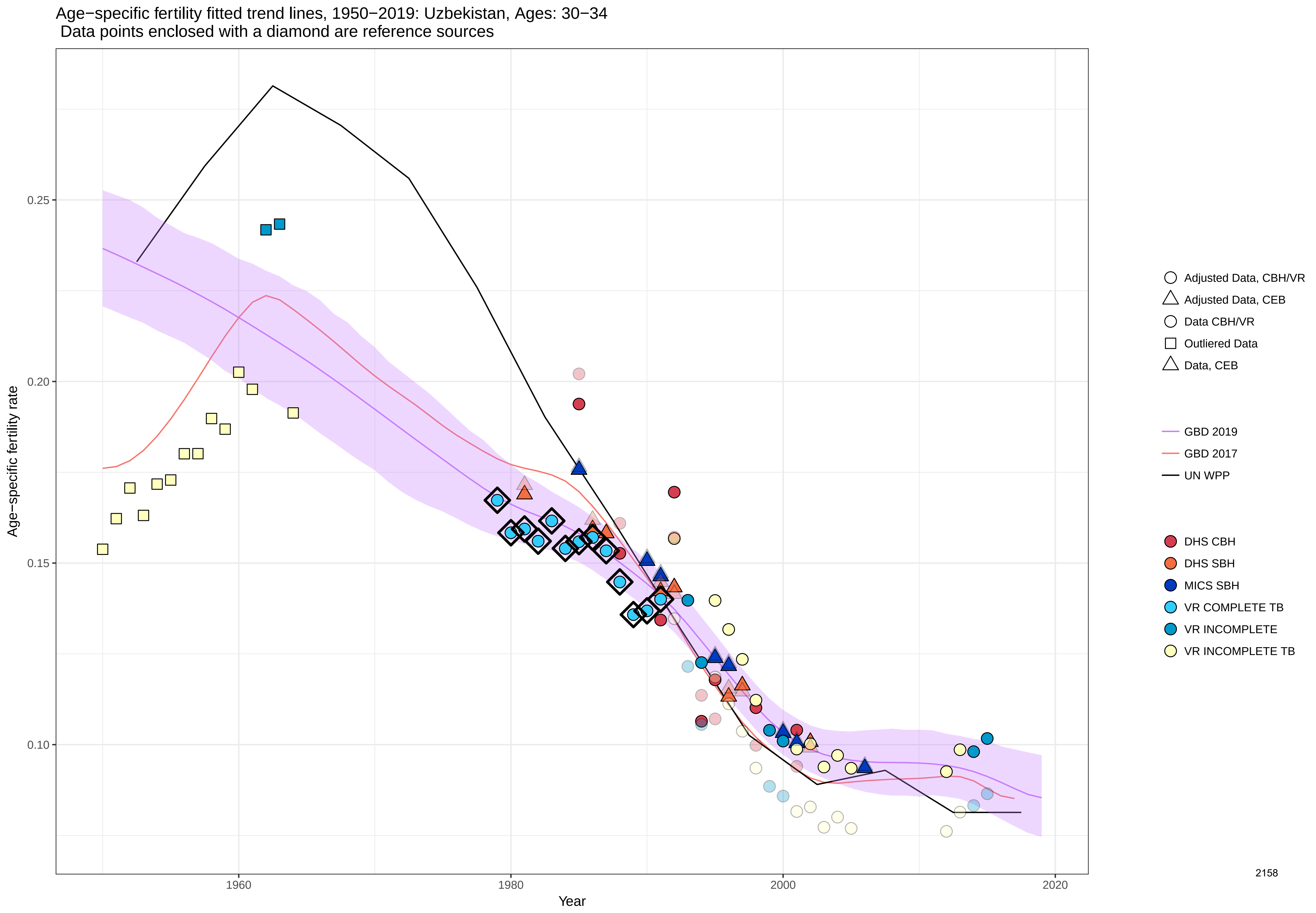
Age−specific fertility fitted trend lines, 1950−2019: Uzbekistan, Ages: 30−34
Data points enclosed with a diamond are reference sources
Age−specific fertility rate
0.25
0.20
0.15
0.10
1960
1980
2000
2020
Year
Adjusted Data, CBH/VR
Adjusted Data, CEB
Data CBH/VR
Outliered Data
Data, CEB
GBD 2019
GBD 2017
UN WPP
DHS CBH
DHS SBH
MICS SBH
VR COMPLETE TB
VR INCOMPLETE
VR INCOMPLETE TB

Age−specific fertility fitted trend lines, 1950−2019: Uzbekistan, Ages: 35−39
Data points enclosed with a diamond are reference sources

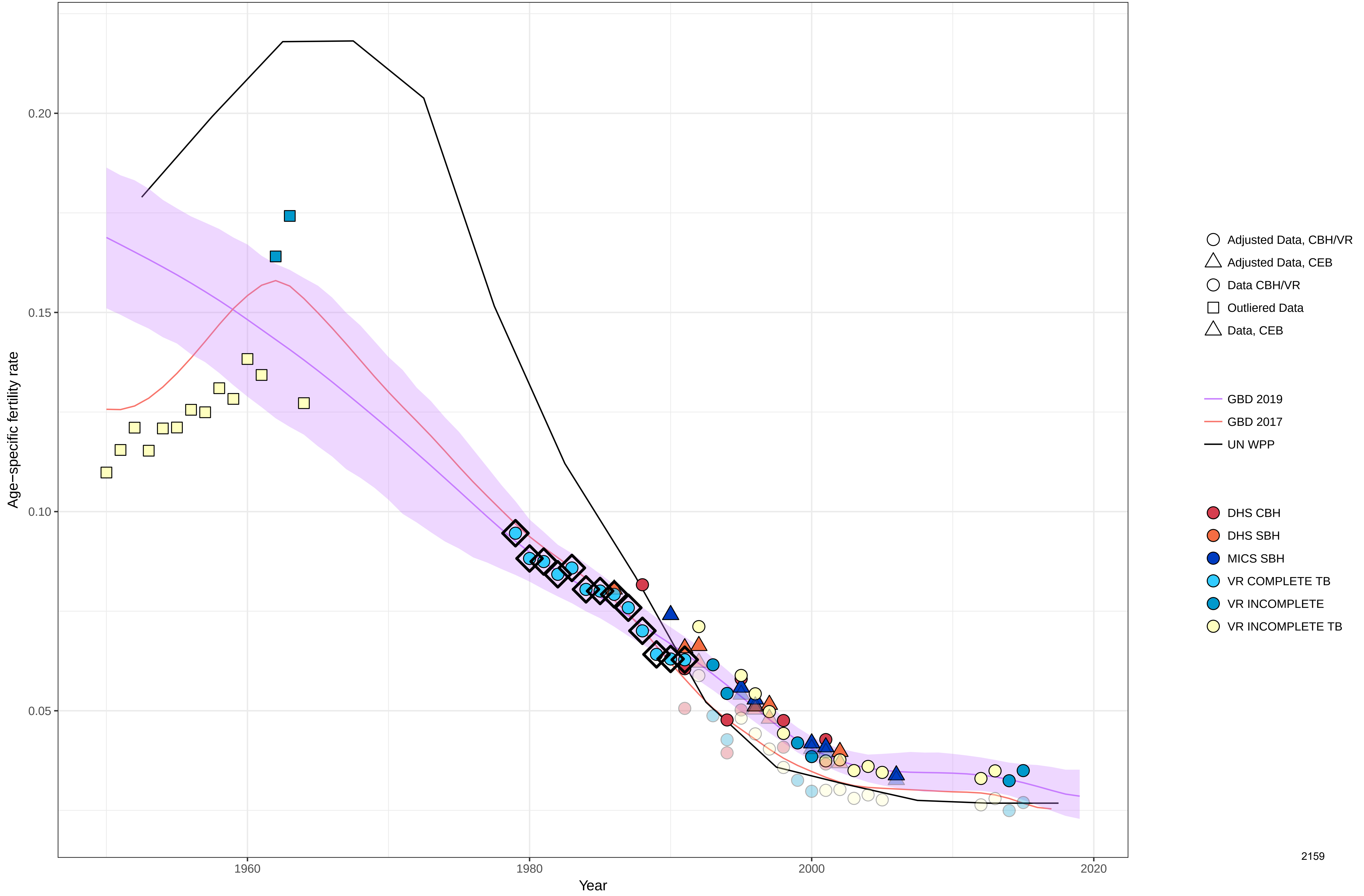

Age−specific fertility fitted trend lines, 1950−2019: Uzbekistan, Ages: 40−44
Data points enclosed with a diamond are reference sources

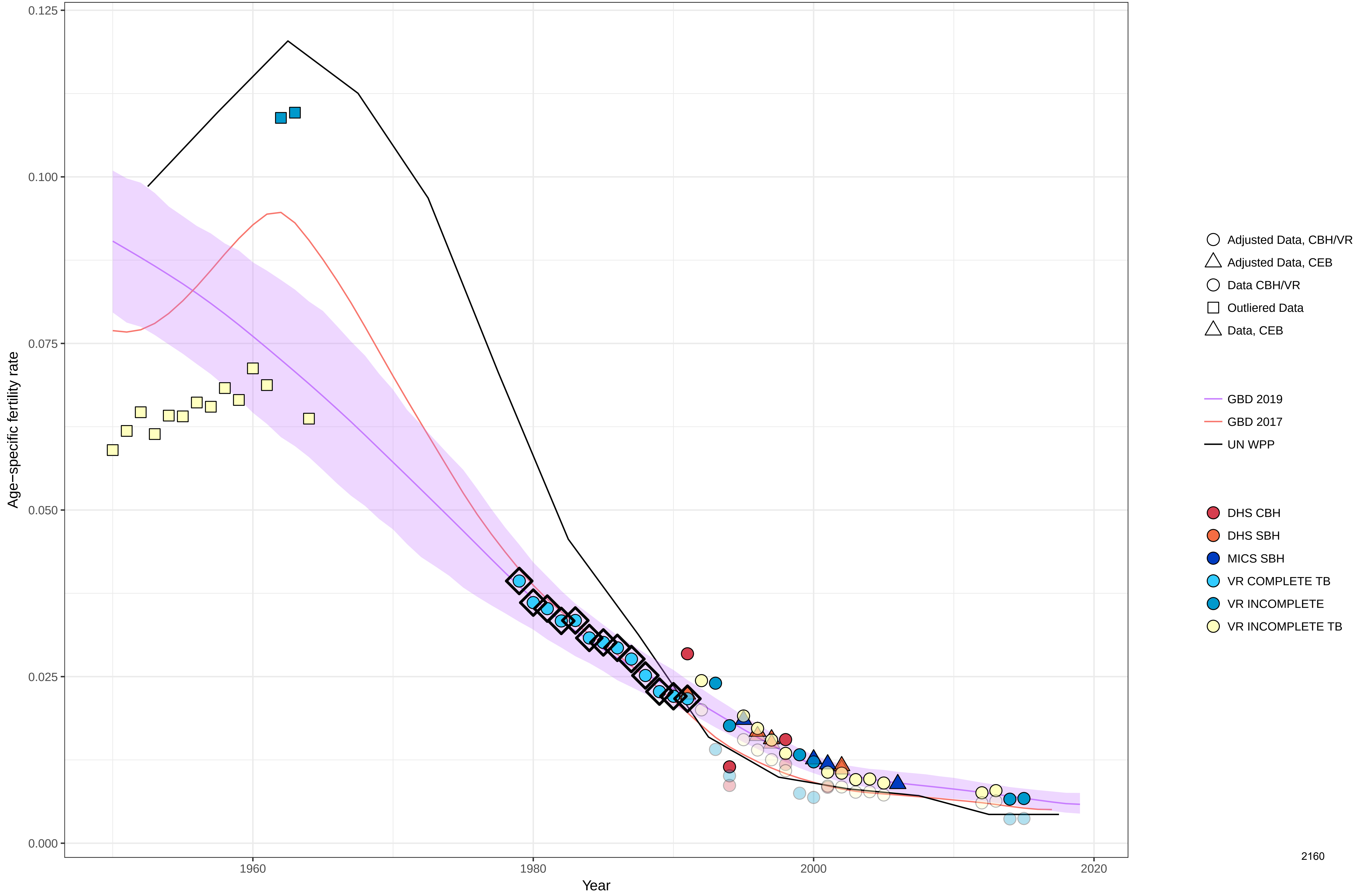

Age−specific fertility fitted trend lines, 1950−2019: Uzbekistan, Ages: 45−49
Data points enclosed with a diamond are reference sources

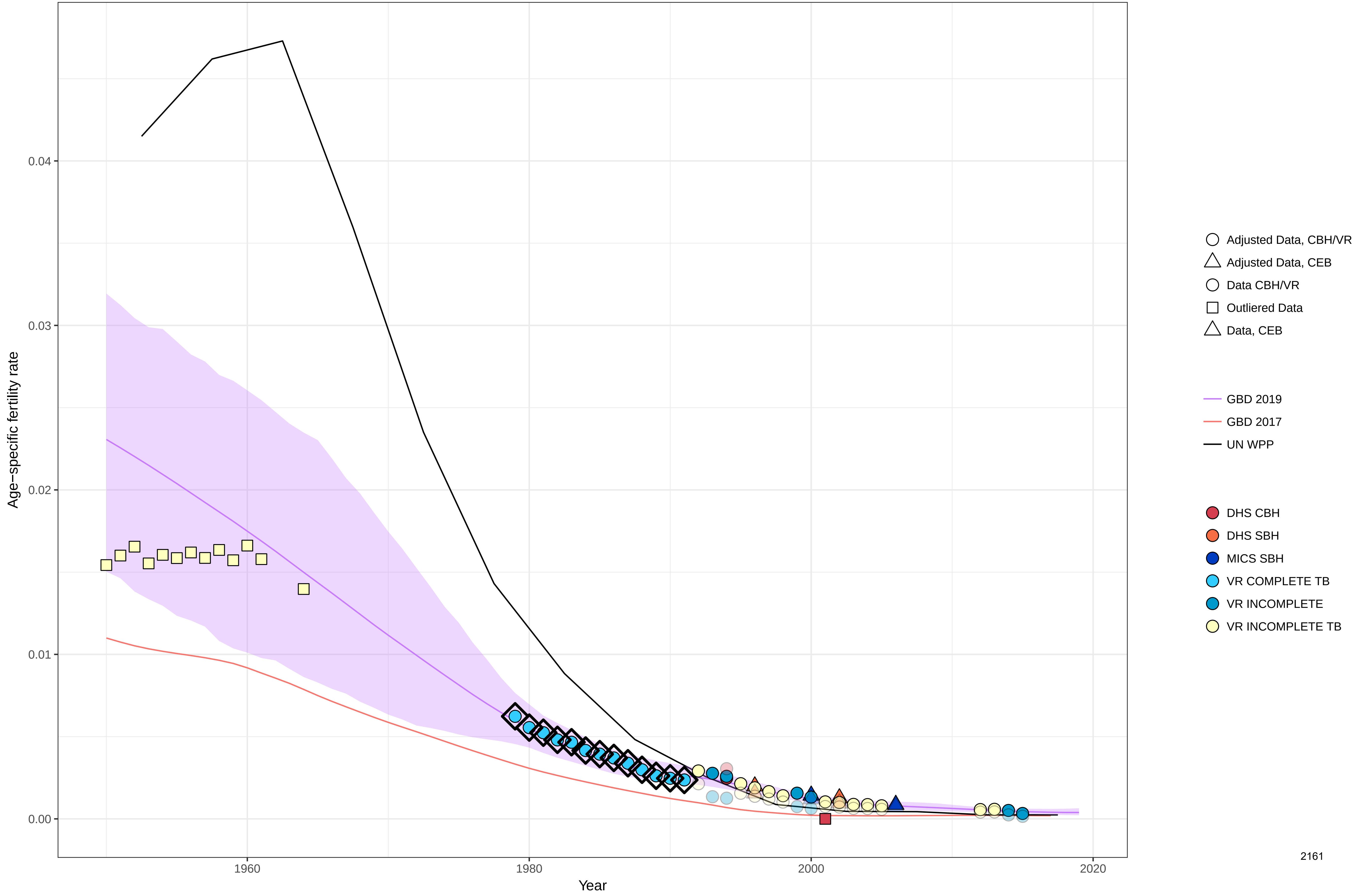

Age−specific fertility fitted trend lines, 1950−2019: Uzbekistan, Ages: 50−54
Data points enclosed with a diamond are reference sources

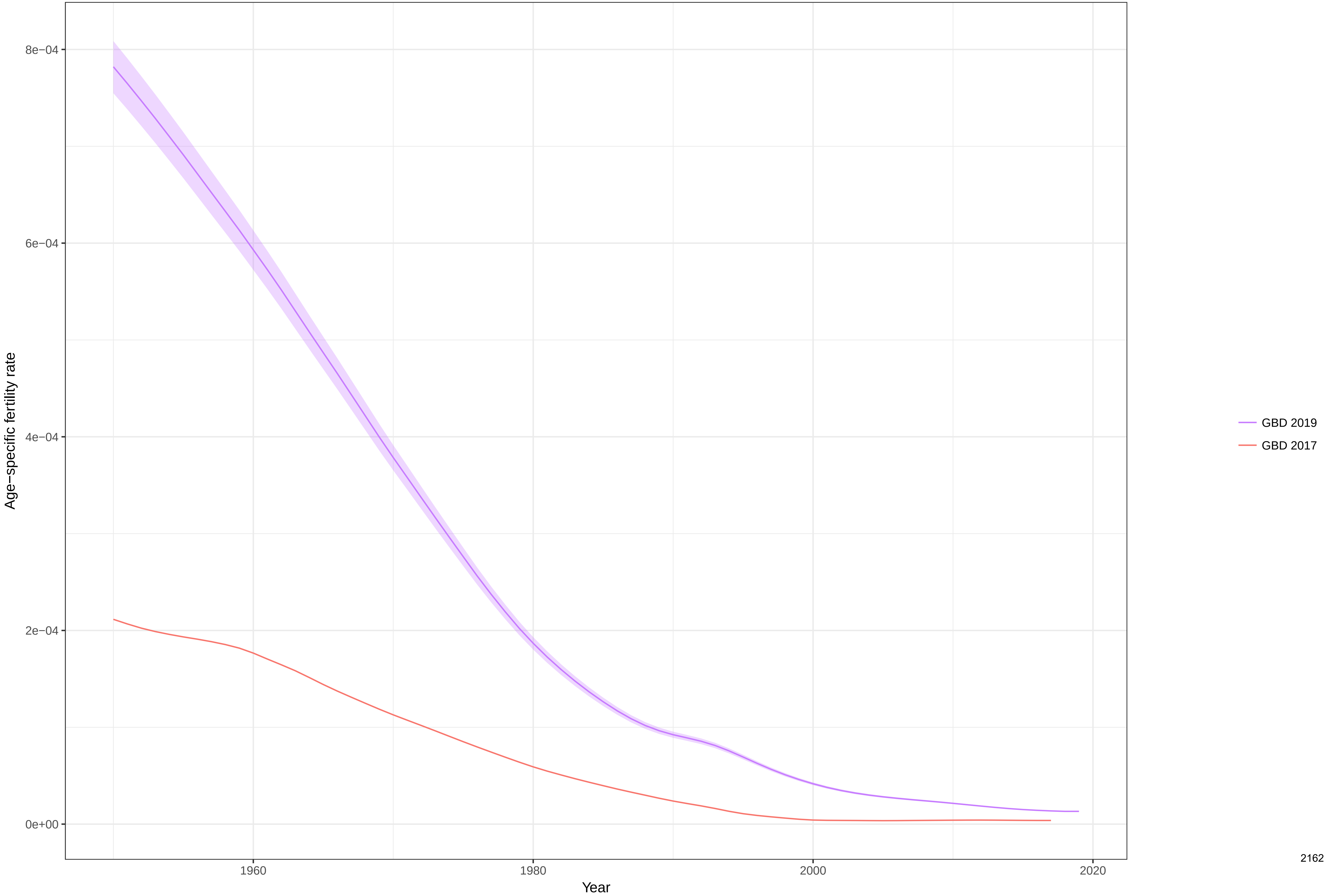

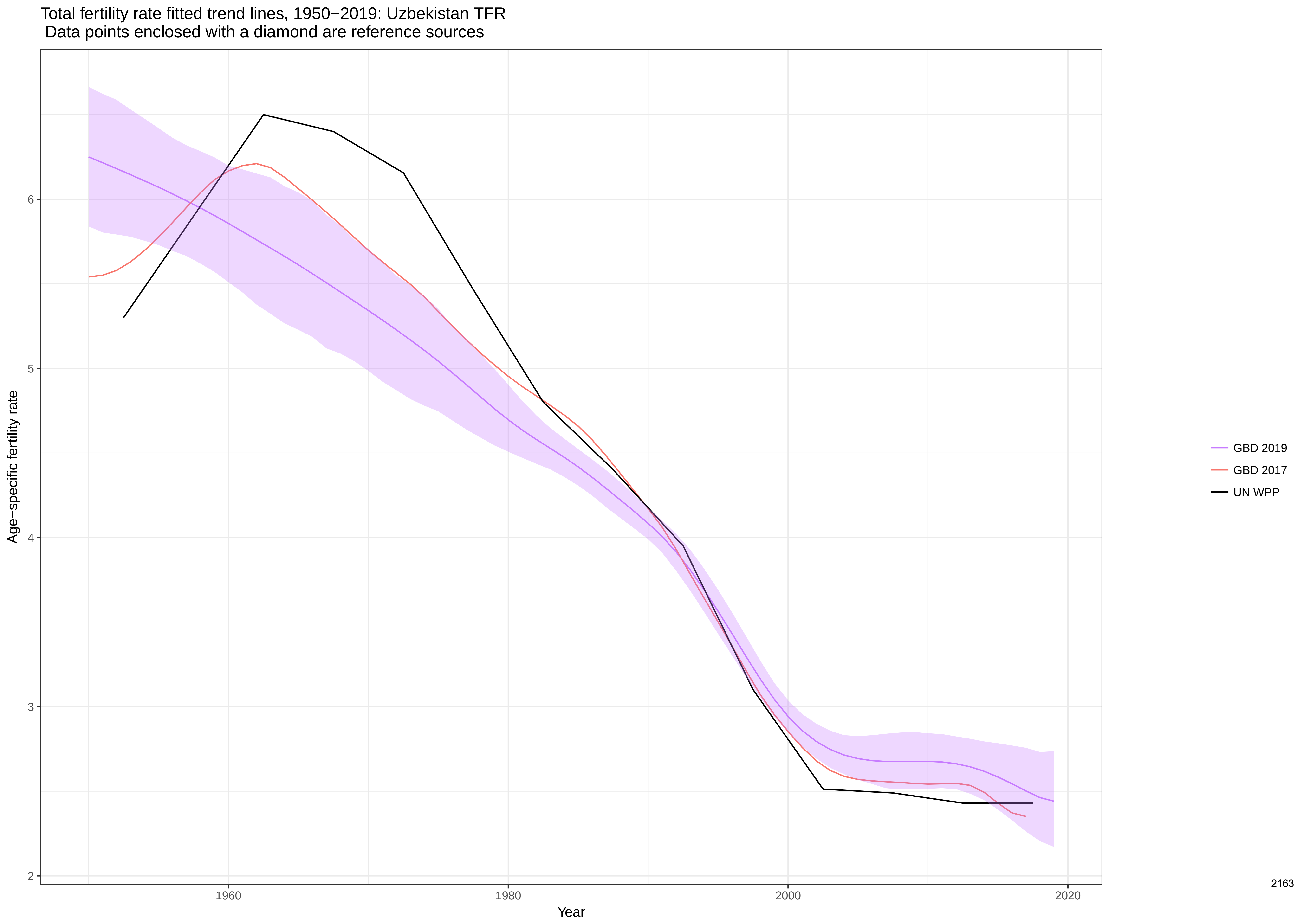

Total fertility rate fitted trend lines, 1950−2019: Uzbekistan TFR
Data points enclosed with a diamond are reference sources
Age−specific fertility rate
Year
2
3
4
5
6
1960
1980
2000
2020
GBD 2019
GBD 2017
UN WPP

Age−specific fertility fitted trend lines, 1950−2019: Saint Vincent and the Grenadines, Ages: 10−14
Data points enclosed with a diamond are reference sources

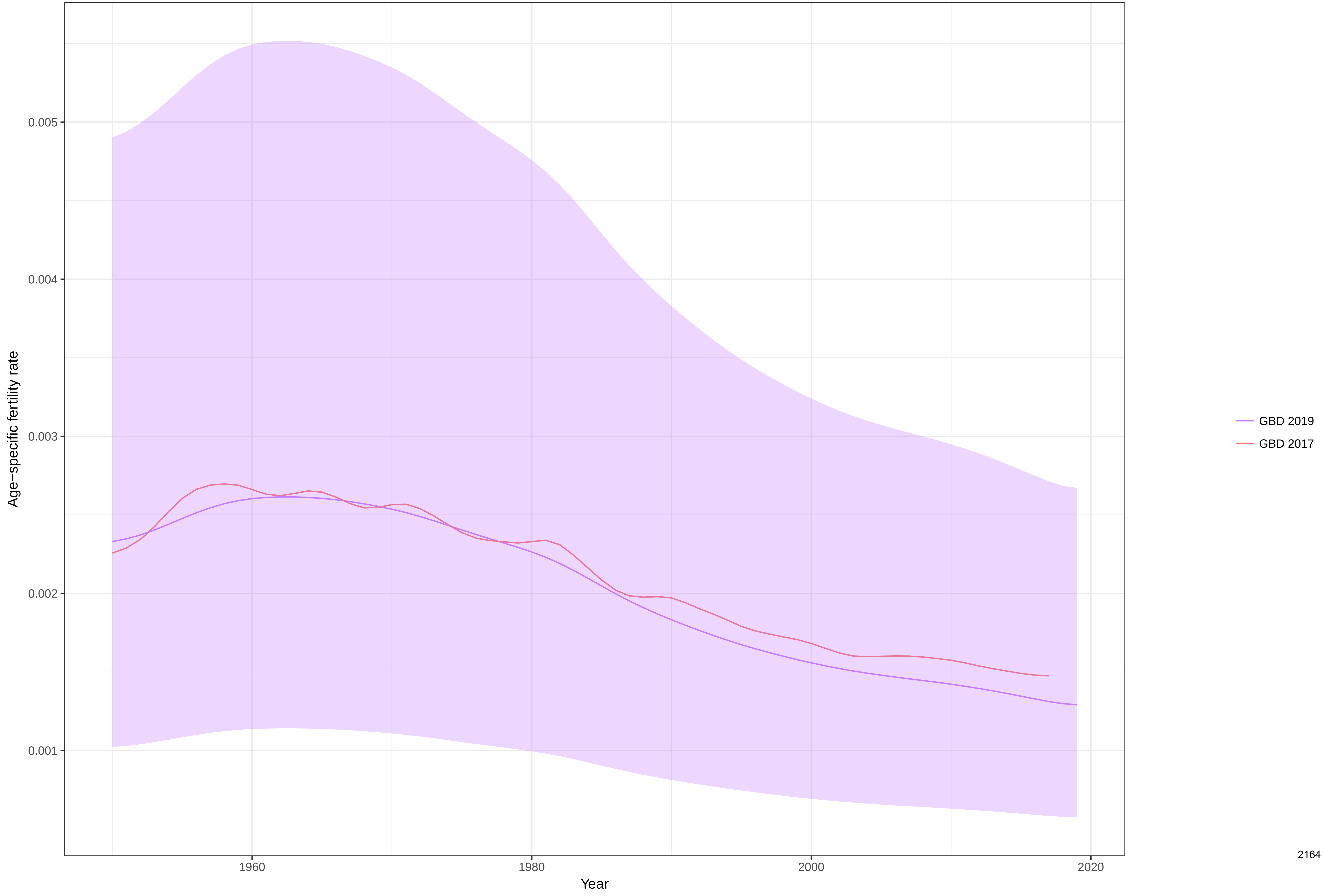

Age−specific fertility fitted trend lines, 1950−2019: Saint Vincent and the Grenadines, Ages: 15−19
Data points enclosed with a diamond are reference sources

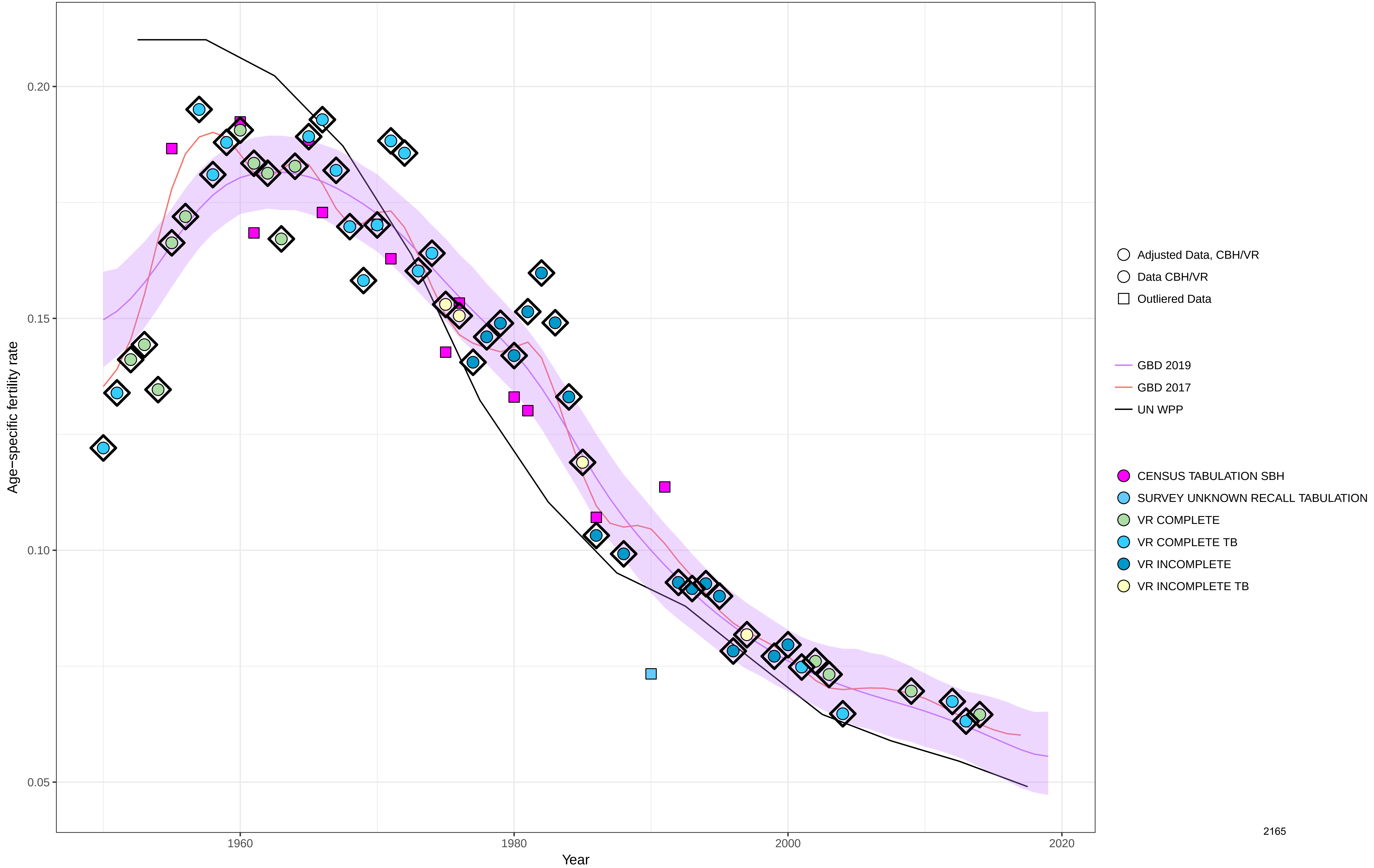

Age−specific fertility fitted trend lines, 1950−2019: Saint Vincent and the Grenadines, Ages: 20−24
Data points enclosed with a diamond are reference sources

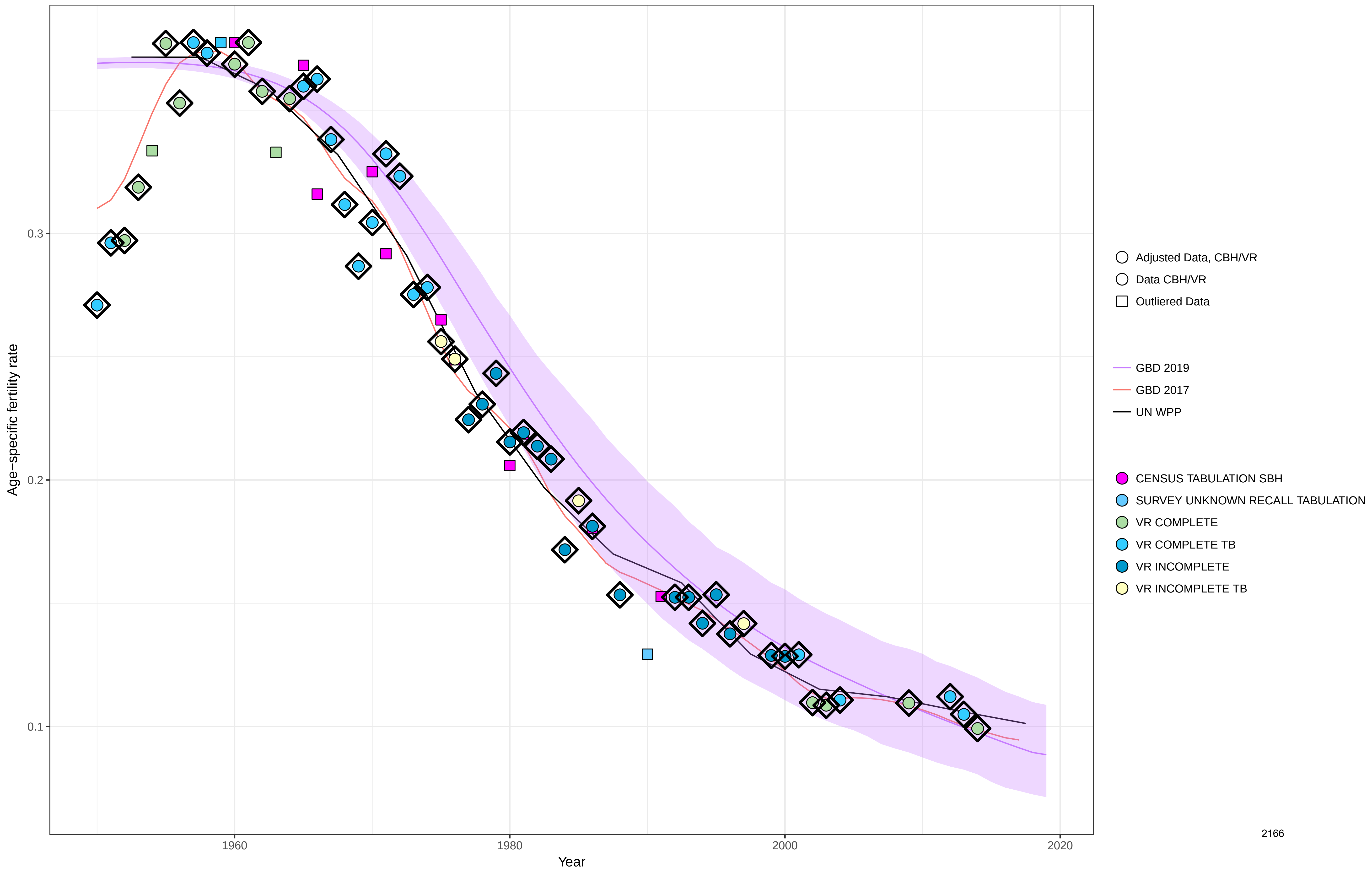

Age−specific fertility fitted trend lines, 1950−2019: Saint Vincent and the Grenadines, Ages: 25−29
Data points enclosed with a diamond are reference sources

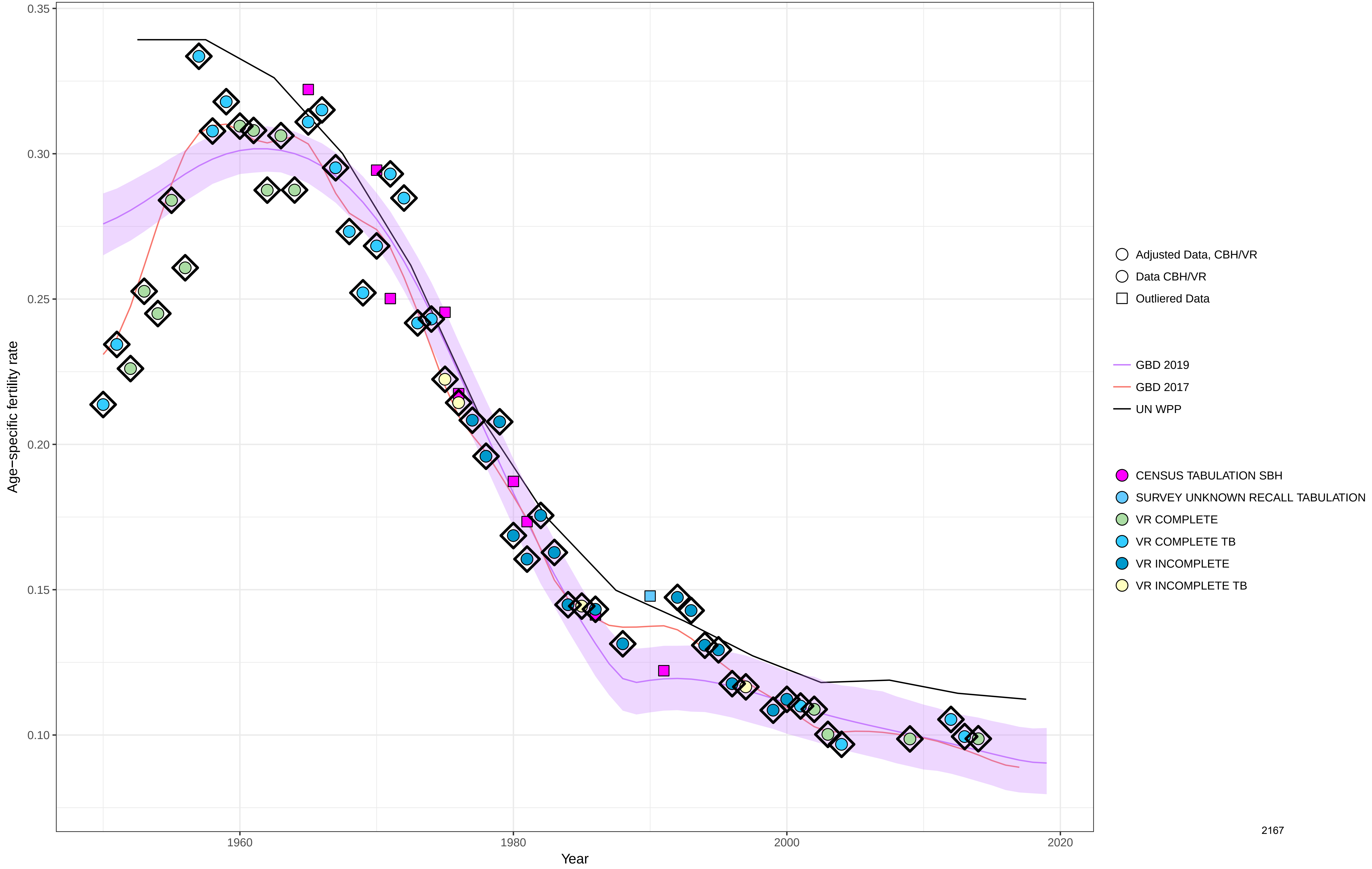

Age−specific fertility fitted trend lines, 1950−2019: Saint Vincent and the Grenadines, Ages: 30−34
Data points enclosed with a diamond are reference sources

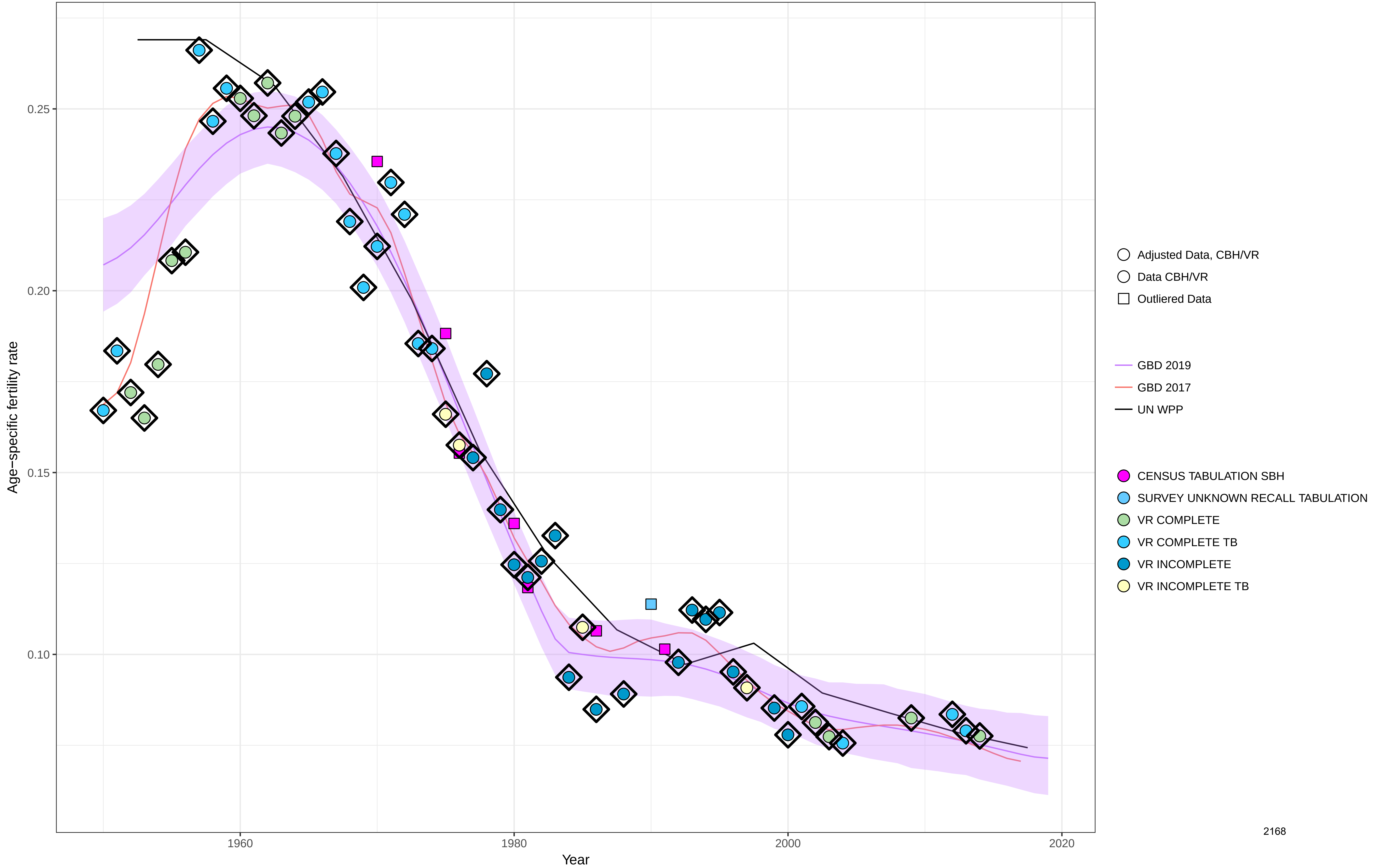

Age−specific fertility fitted trend lines, 1950−2019: Saint Vincent and the Grenadines, Ages: 35−39
Data points enclosed with a diamond are reference sources

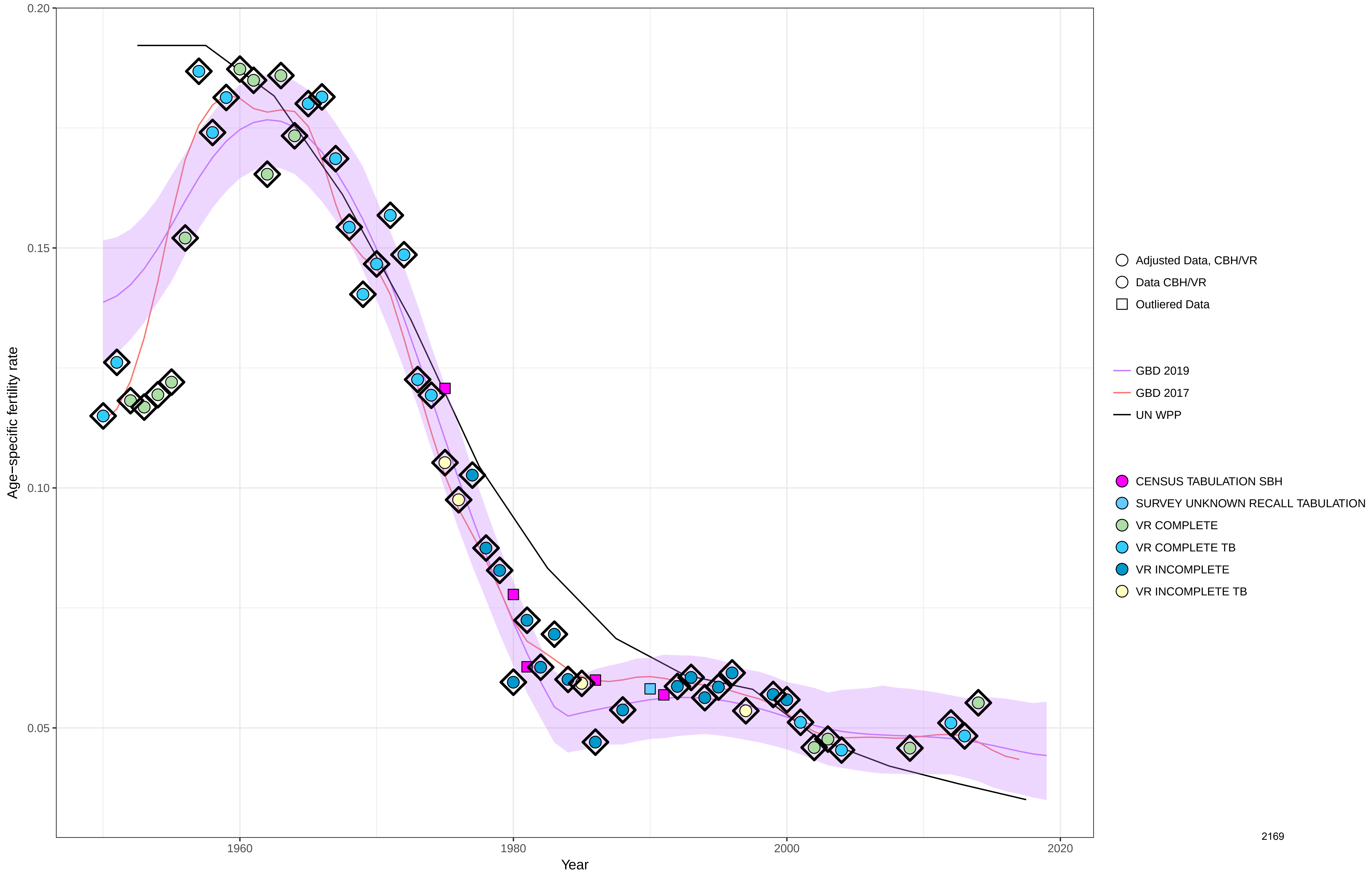

Age−specific fertility fitted trend lines, 1950−2019: Saint Vincent and the Grenadines, Ages: 40−44
Data points enclosed with a diamond are reference sources

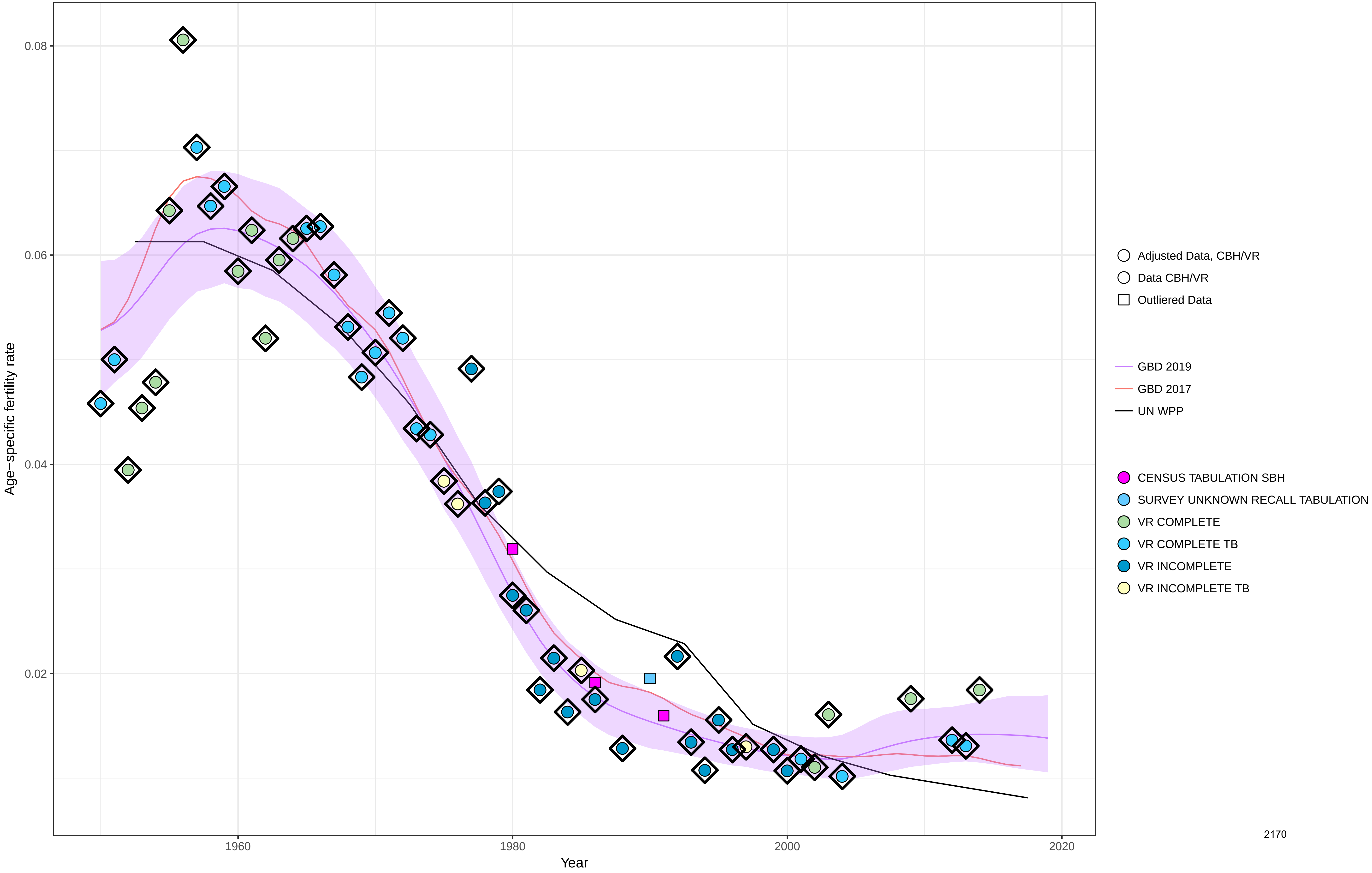

Age−specific fertility fitted trend lines, 1950−2019: Saint Vincent and the Grenadines, Ages: 45−49
Data points enclosed with a diamond are reference sources

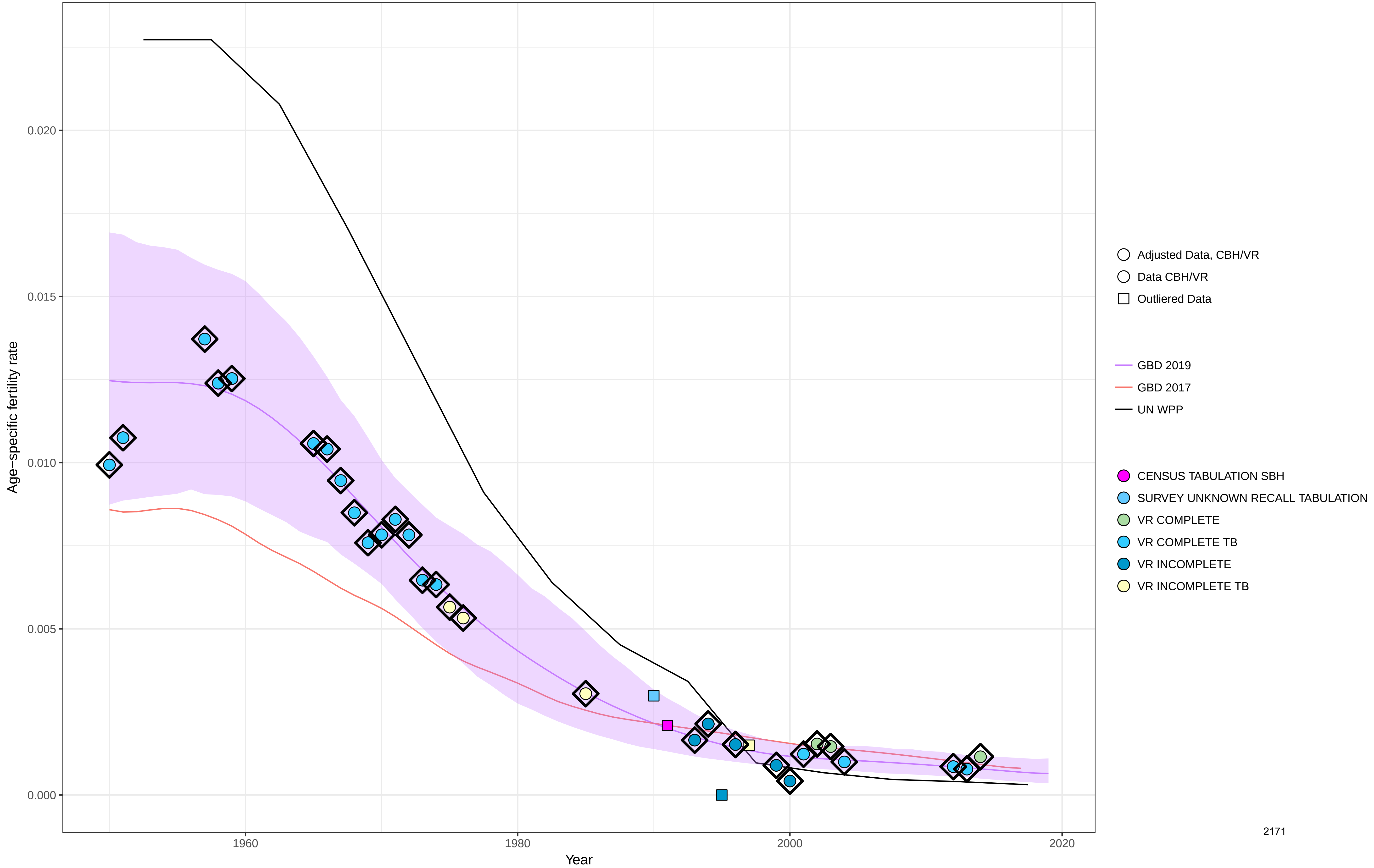

Age−specific fertility fitted trend lines, 1950−2019: Saint Vincent and the Grenadines, Ages: 50−54
Data points enclosed with a diamond are reference sources

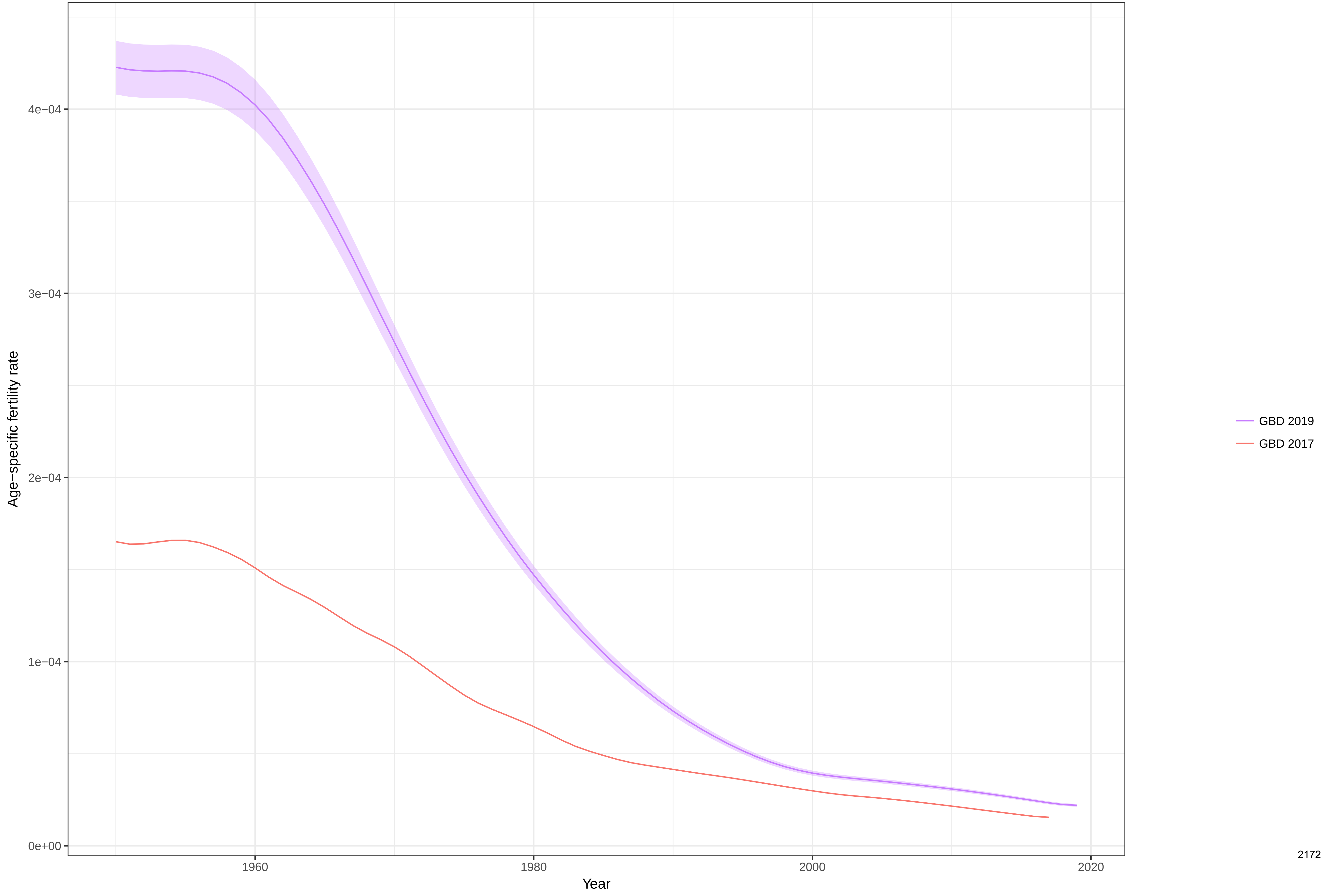

Total fertility rate fitted trend lines, 1950−2019: Saint Vincent and the Grenadines TFR
Data points enclosed with a diamond are reference sources

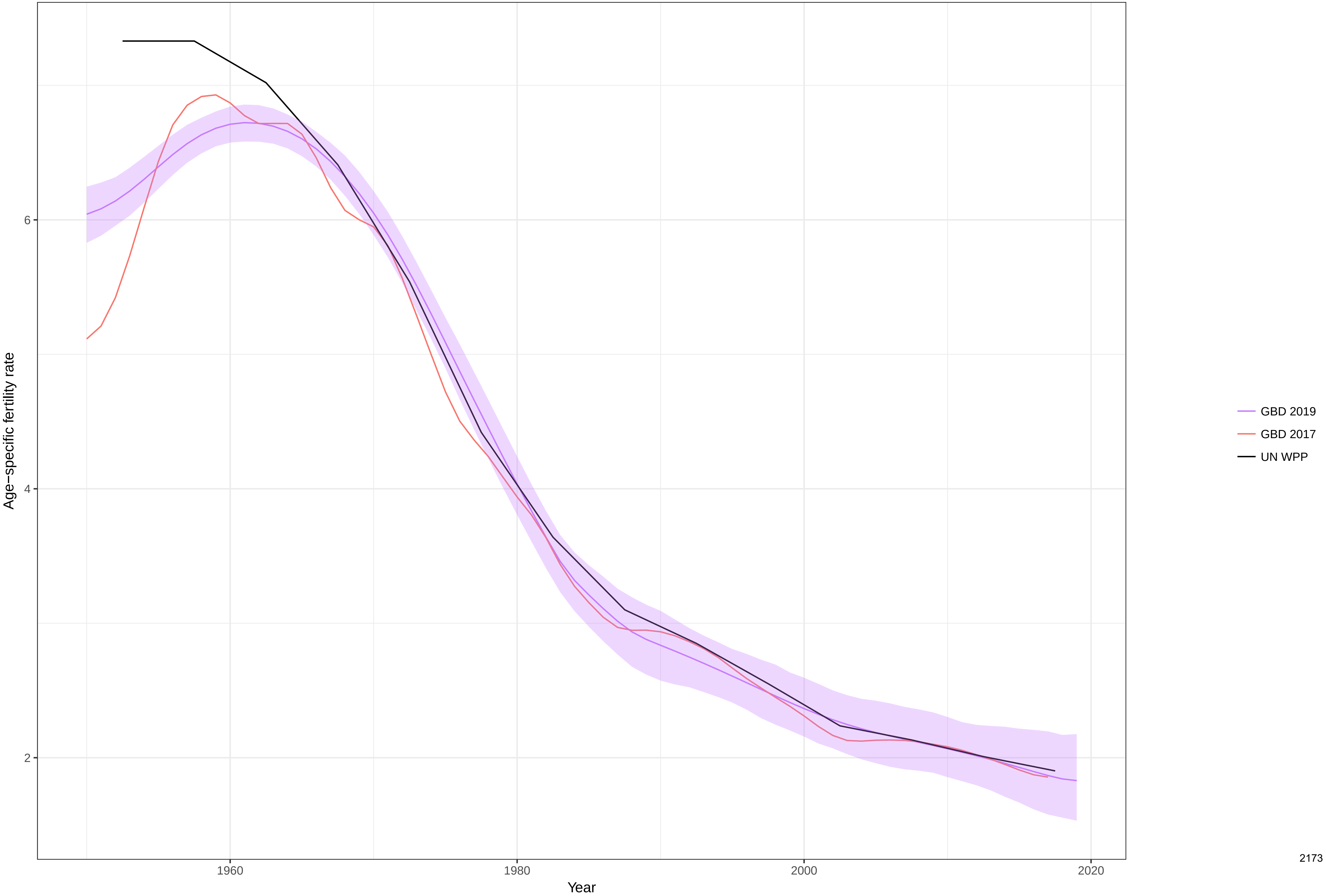

Age−specific fertility fitted trend lines, 1950−2019: Venezuela (Bolivarian Republic of), Ages: 10−14
Data points enclosed with a diamond are reference sources

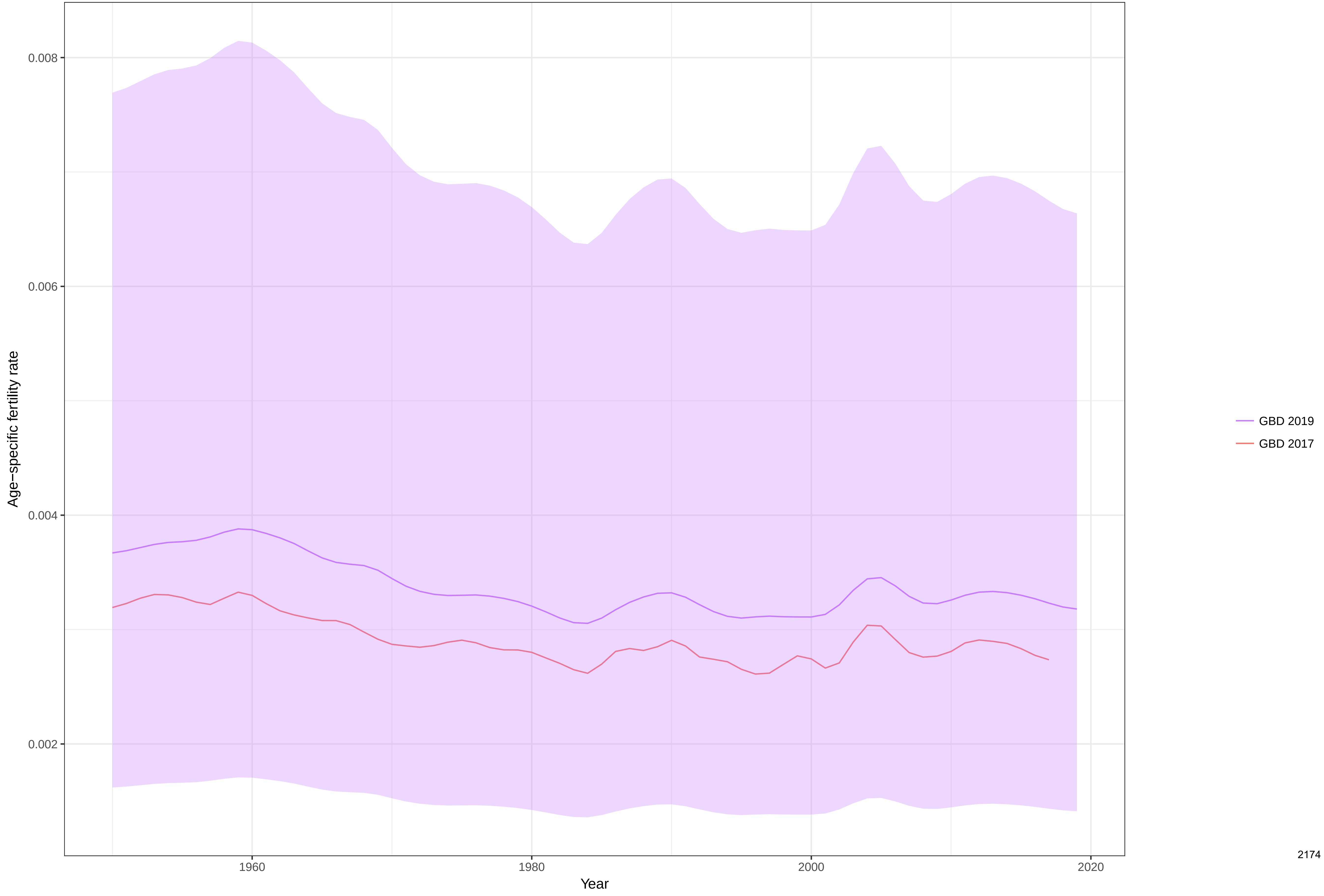

Age−specific fertility fitted trend lines, 1950−2019: Venezuela (Bolivarian Republic of), Ages: 15−19
Data points enclosed with a diamond are reference sources

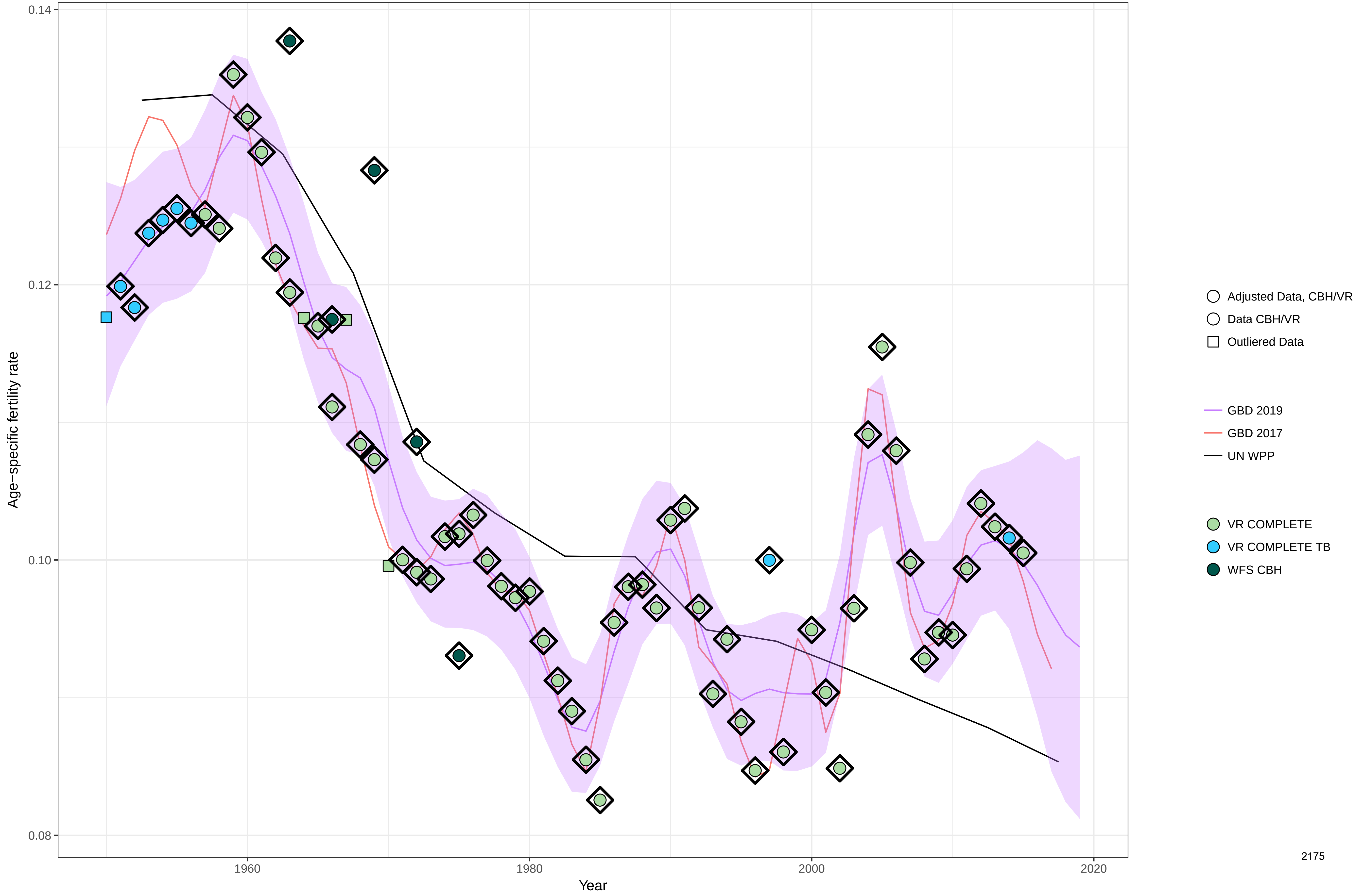

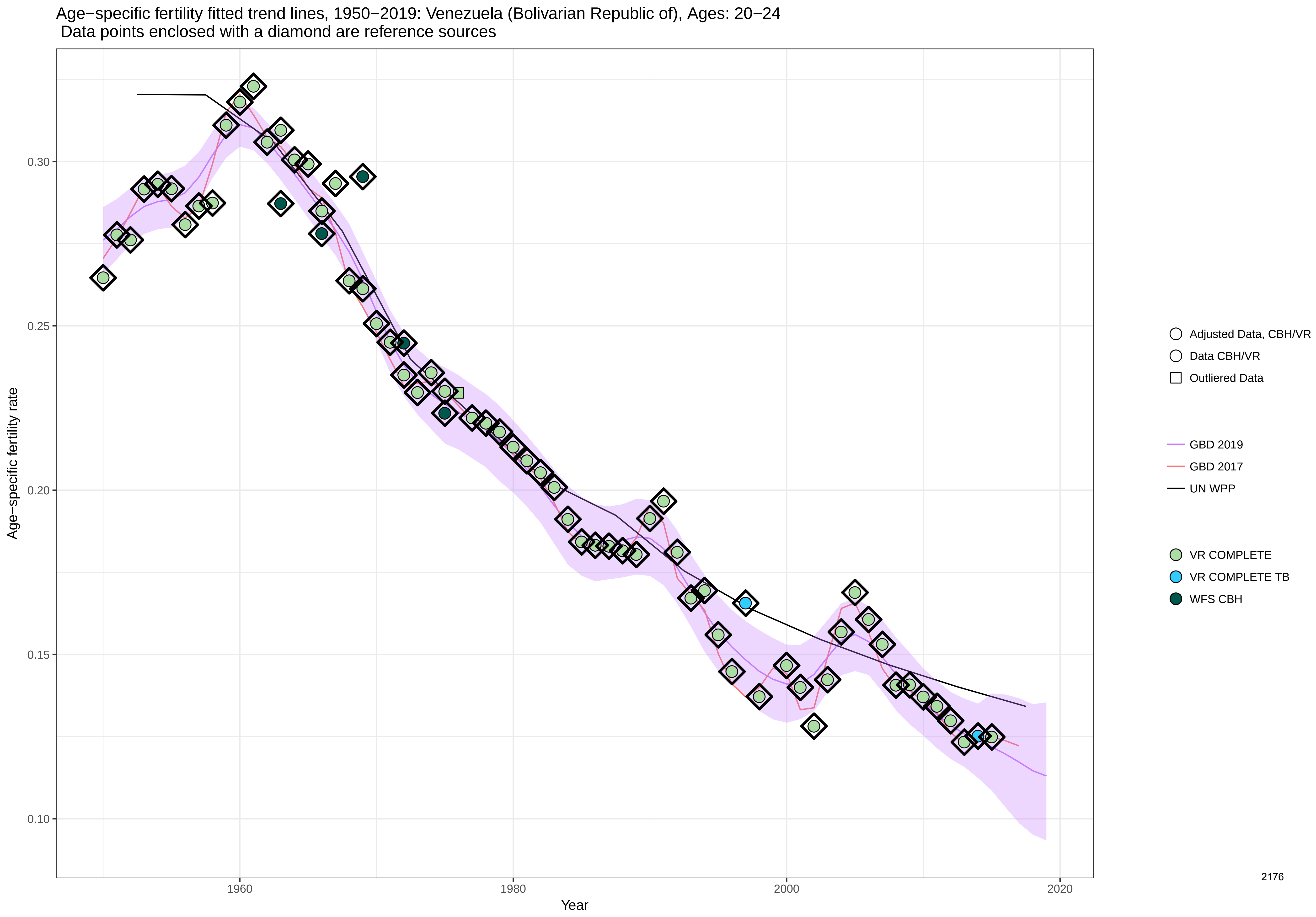

Age−specific fertility fitted trend lines, 1950−2019: Venezuela (Bolivarian Republic of), Ages: 20−24
Data points enclosed with a diamond are reference sources
Age−specific fertility rate
0.30
0.25
0.20
0.15
0.10
1960
1980
2000
2020
Year
Adjusted Data, CBH/VR
Data CBH/VR
Outliered Data
GBD 2019
GBD 2017
UN WPP
VR COMPLETE
VR COMPLETE TB
WFS CBH

Age−specific fertility fitted trend lines, 1950−2019: Venezuela (Bolivarian Republic of), Ages: 25−29
Data points enclosed with a diamond are reference sources

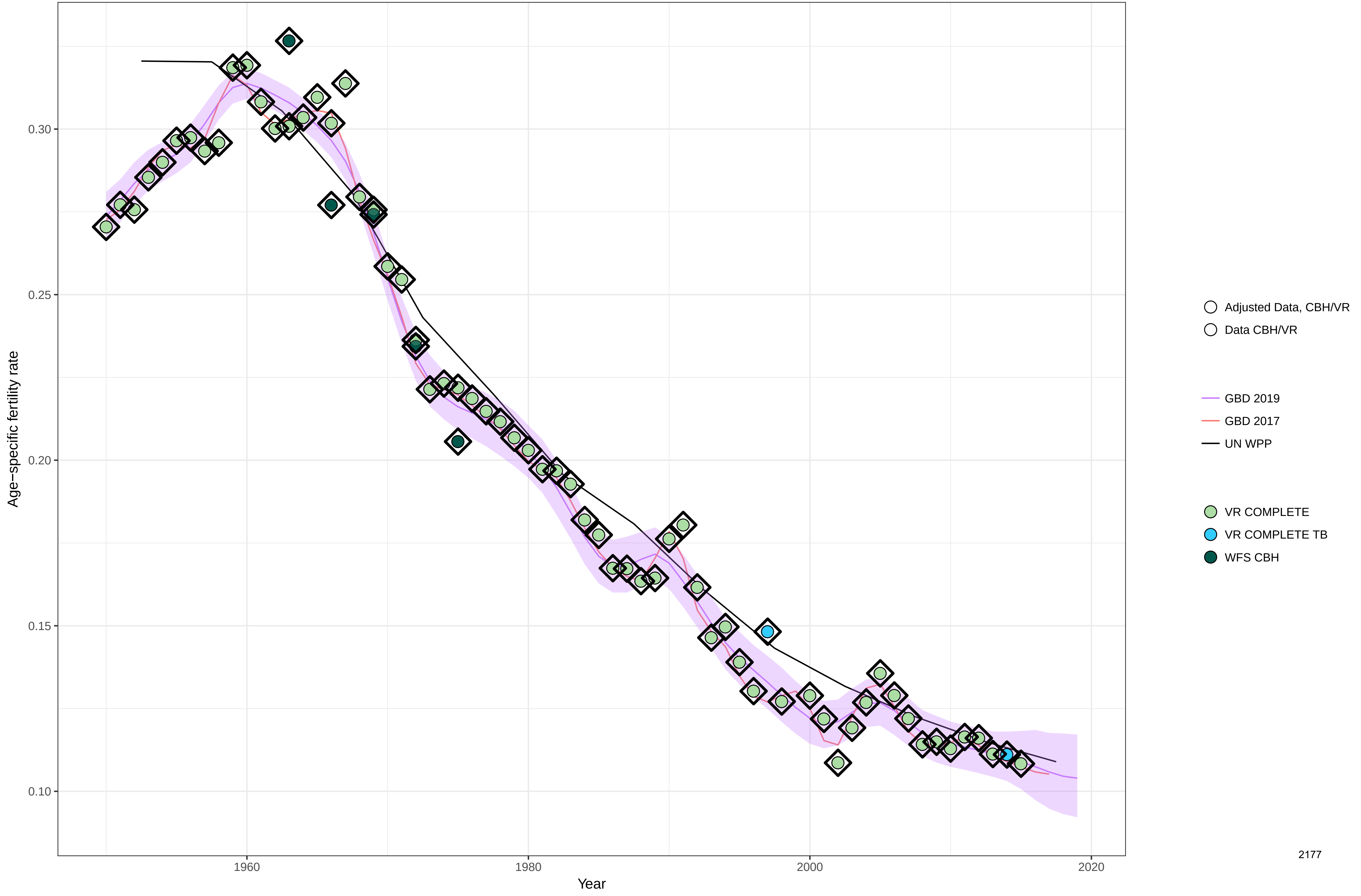

Age−specific fertility fitted trend lines, 1950−2019: Venezuela (Bolivarian Republic of), Ages: 30−34
Data points enclosed with a diamond are reference sources

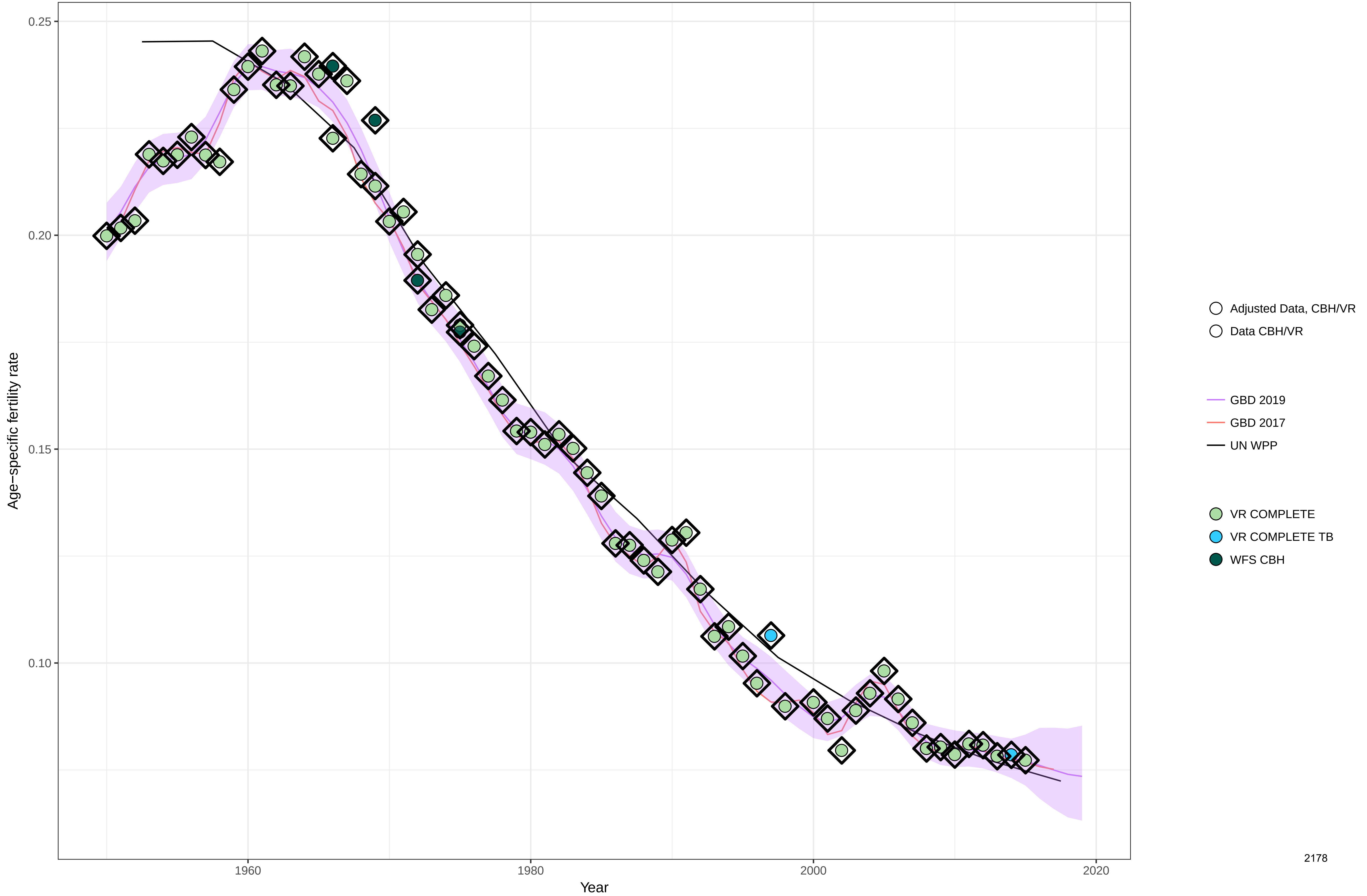

Age−specific fertility fitted trend lines, 1950−2019: Venezuela (Bolivarian Republic of), Ages: 35−39
Data points enclosed with a diamond are reference sources

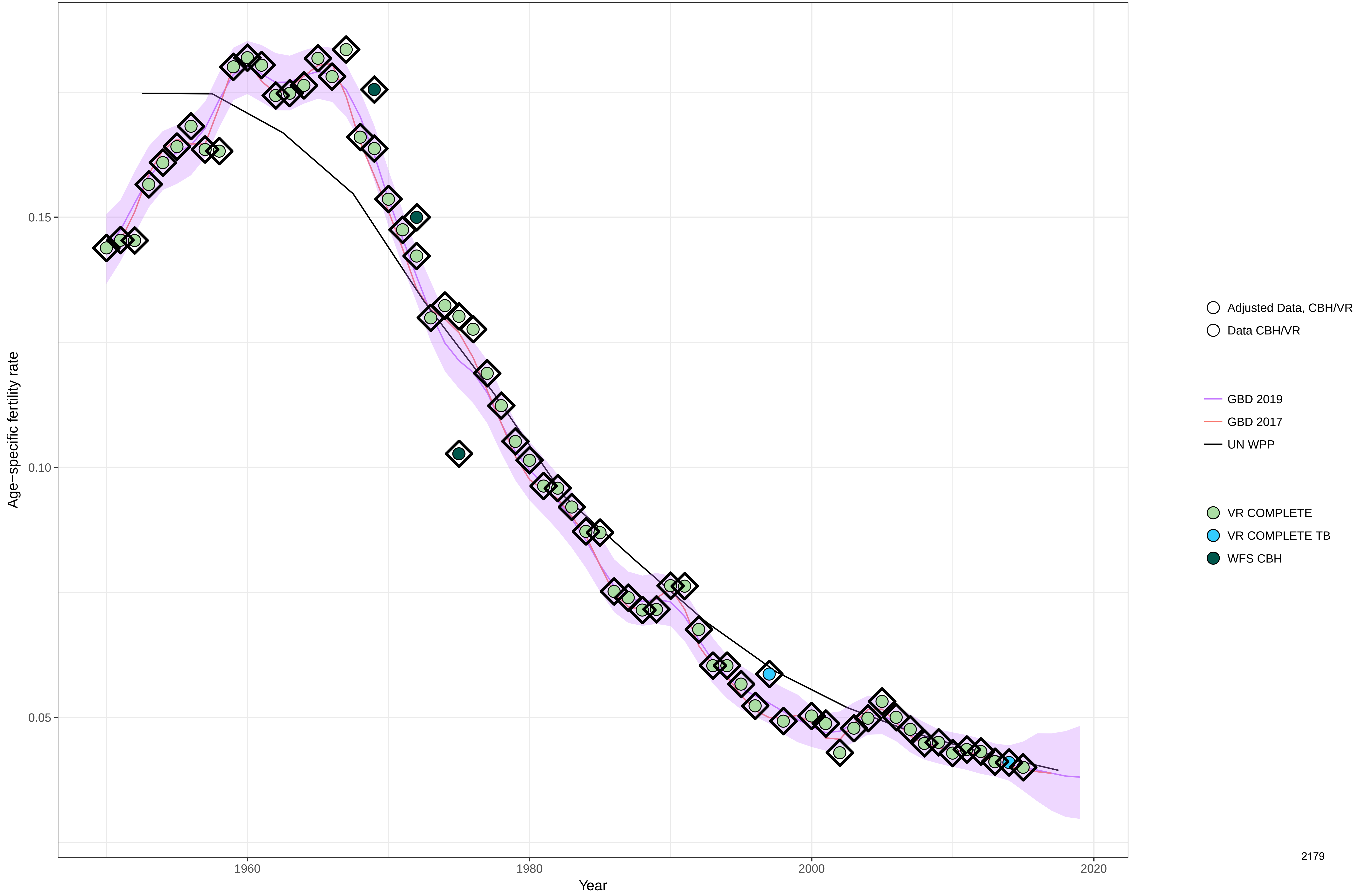

Age−specific fertility fitted trend lines, 1950−2019: Venezuela (Bolivarian Republic of), Ages: 40−44
Data points enclosed with a diamond are reference sources

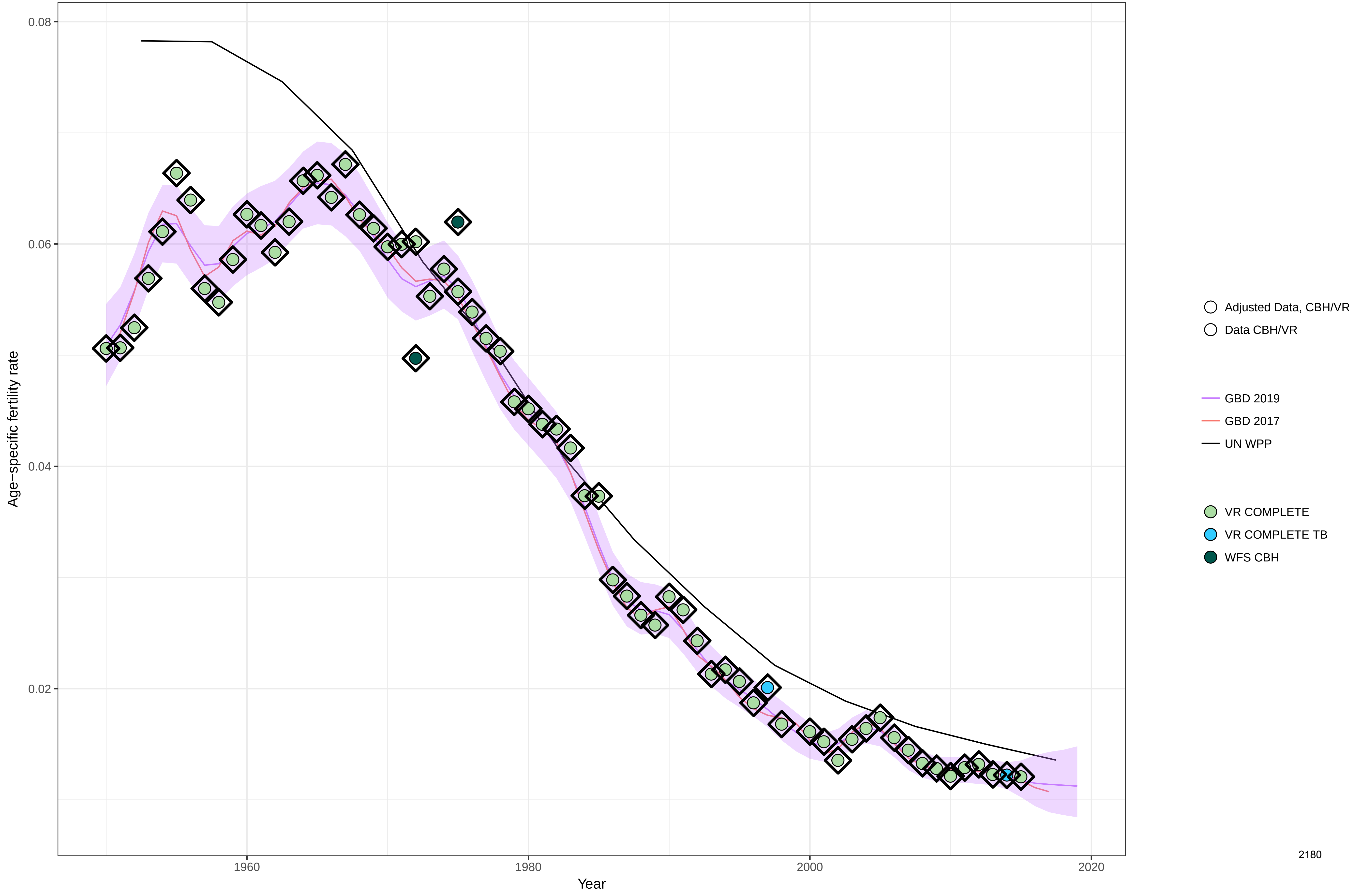

Age−specific fertility fitted trend lines, 1950−2019: Venezuela (Bolivarian Republic of), Ages: 45−49
Data points enclosed with a diamond are reference sources

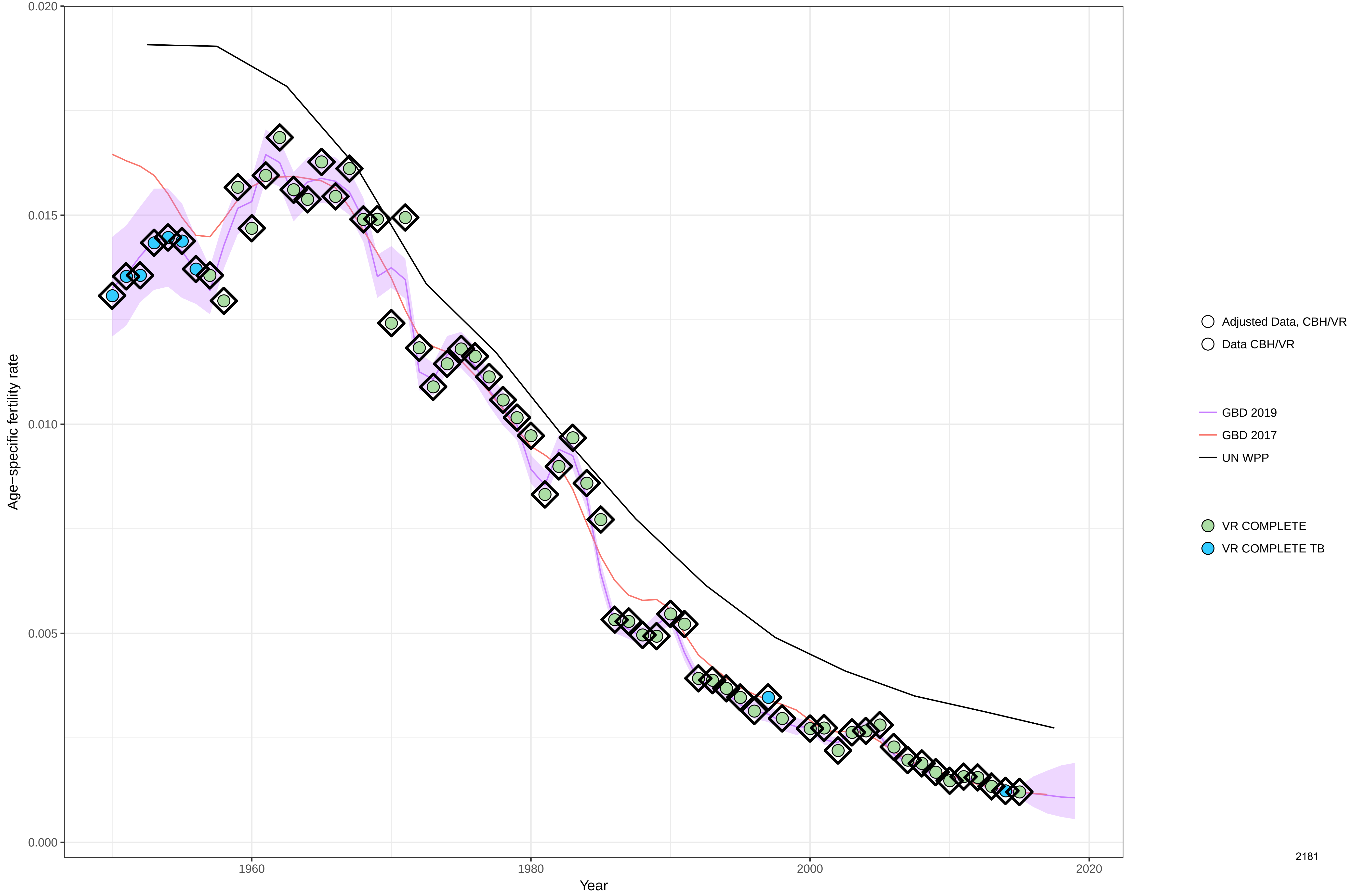

Age−specific fertility fitted trend lines, 1950−2019: Venezuela (Bolivarian Republic of), Ages: 50−54
Data points enclosed with a diamond are reference sources

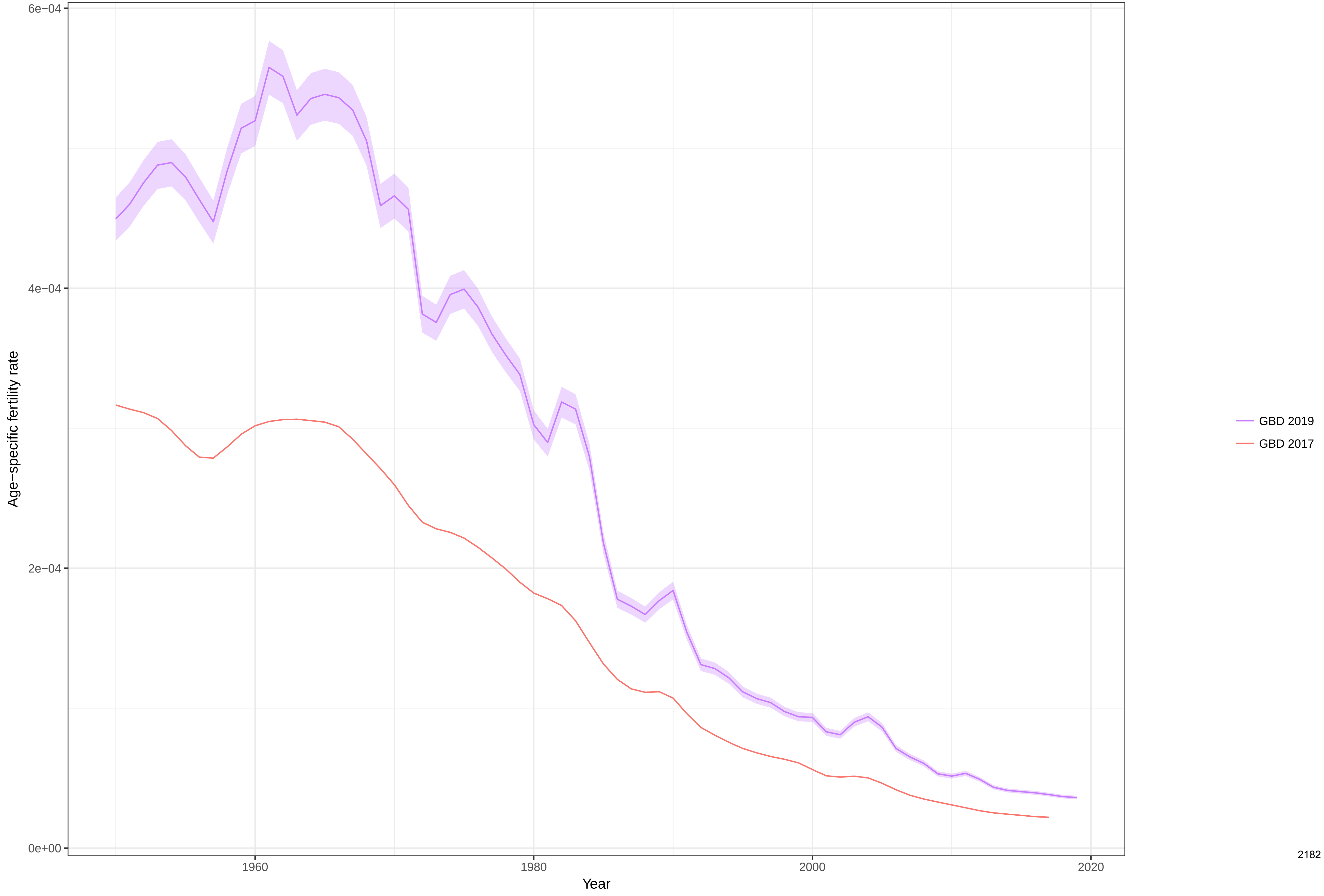

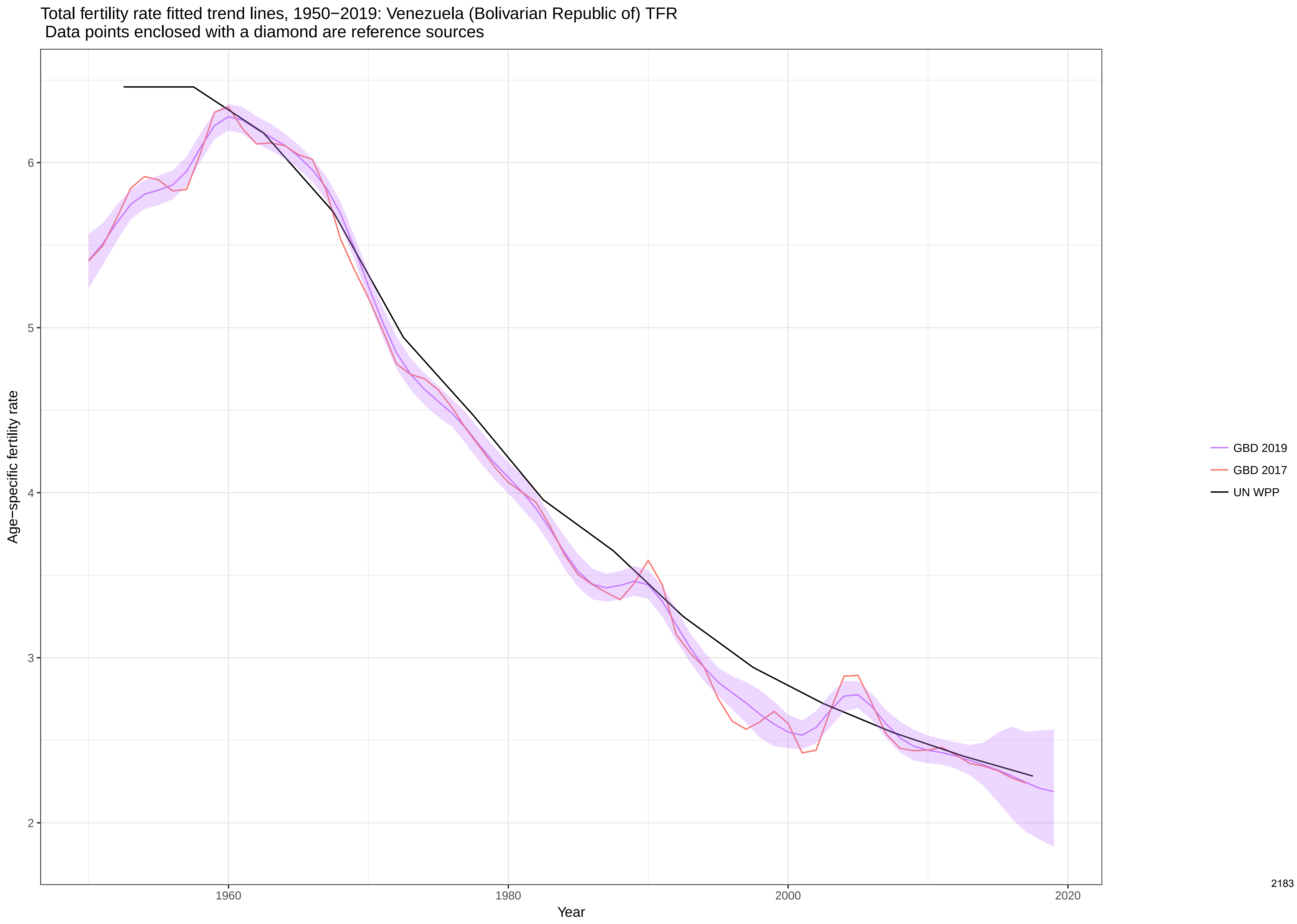
Total fertility rate fitted trend lines, 1950−2019: Venezuela (Bolivarian Republic of) TFR
Data points enclosed with a diamond are reference sources
Age−specific fertility rate
Year
2
3
4
5
6
1960
1980
2000
2020
GBD 2019
GBD 2017
UN WPP

Age−specific fertility fitted trend lines, 1950−2019: United States Virgin Islands, Ages: 10−14
Data points enclosed with a diamond are reference sources

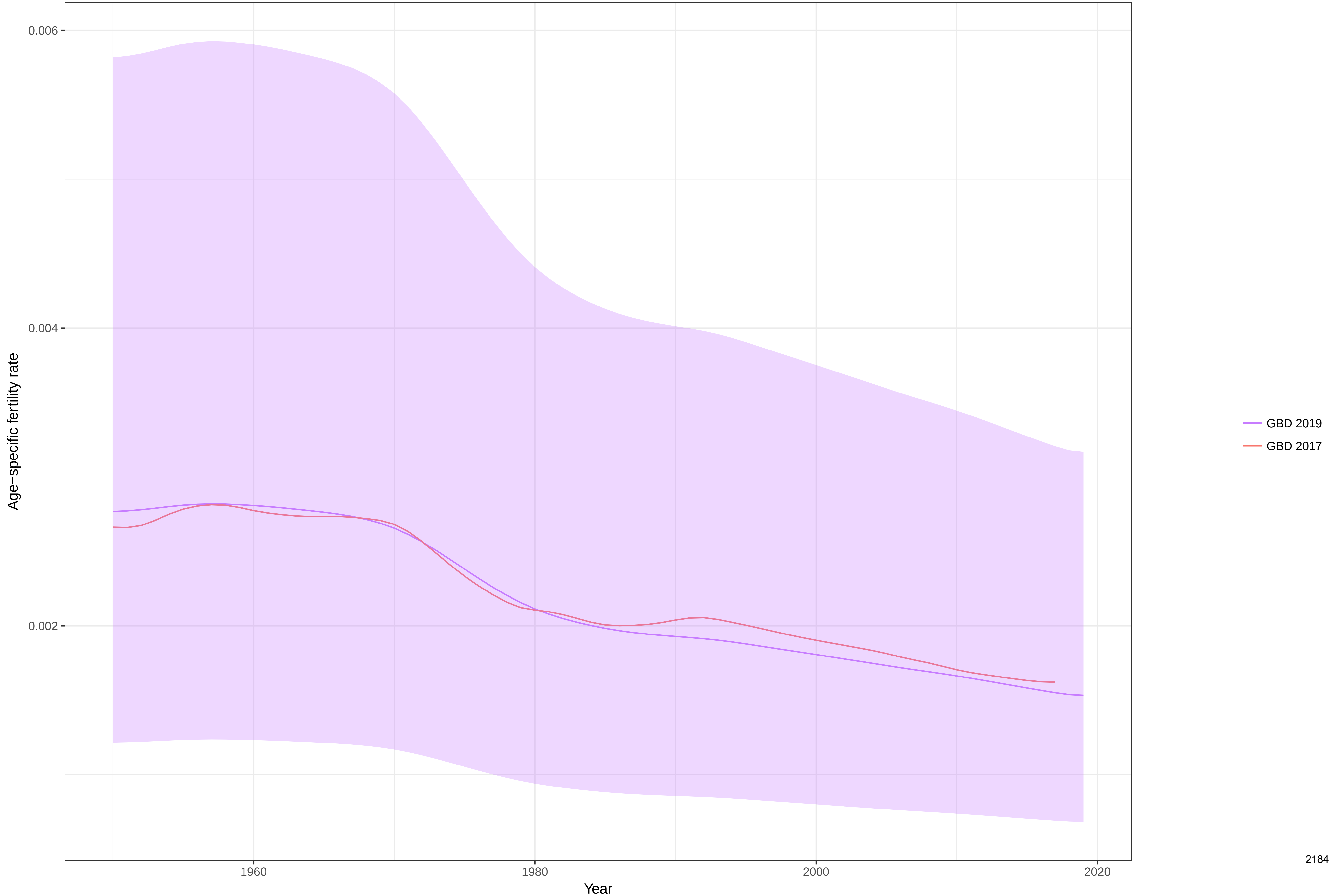

Age−specific fertility fitted trend lines, 1950−2019: United States Virgin Islands, Ages: 15−19
Data points enclosed with a diamond are reference sources

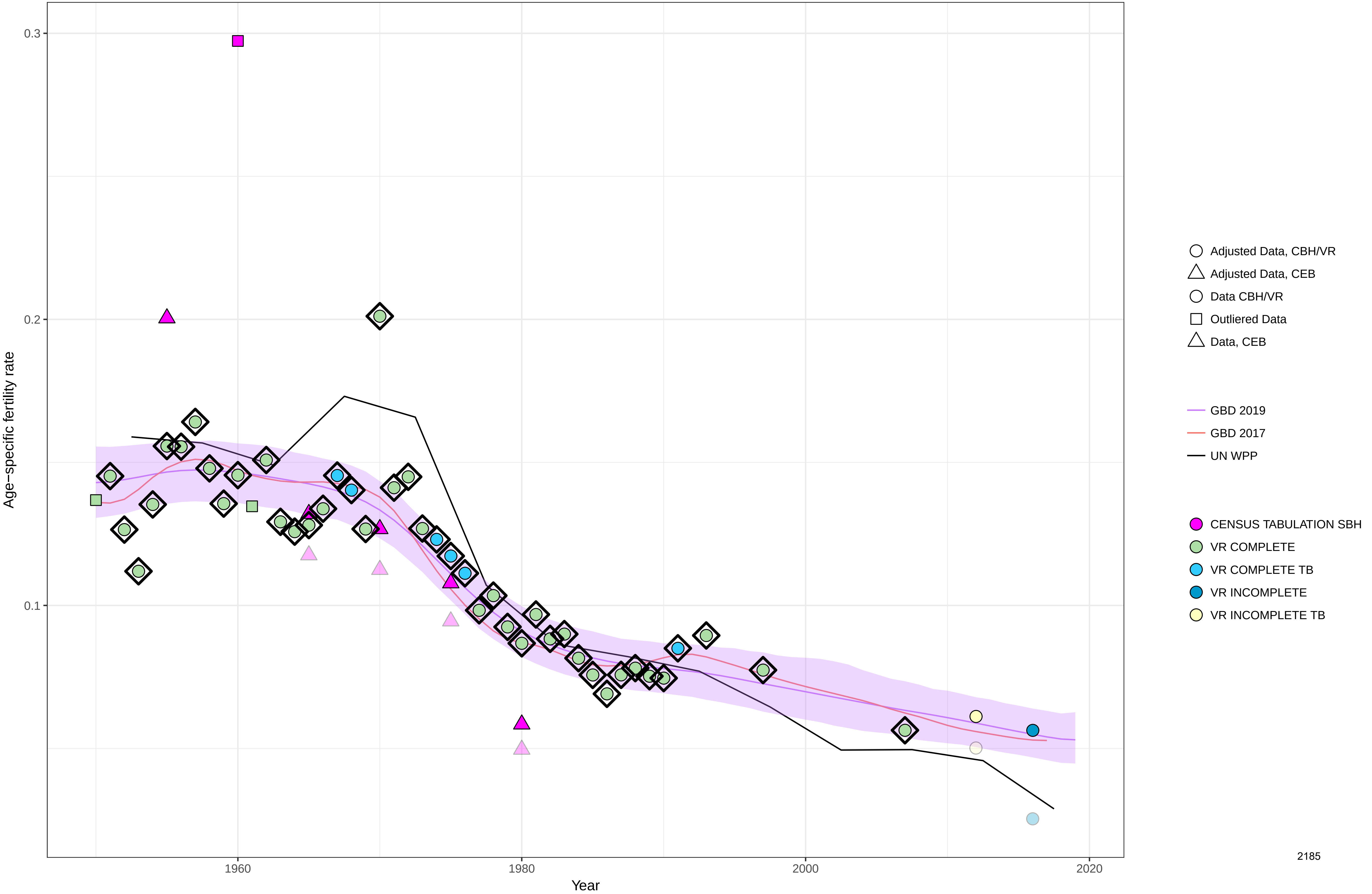

Age−specific fertility fitted trend lines, 1950−2019: United States Virgin Islands, Ages: 20−24
Data points enclosed with a diamond are reference sources

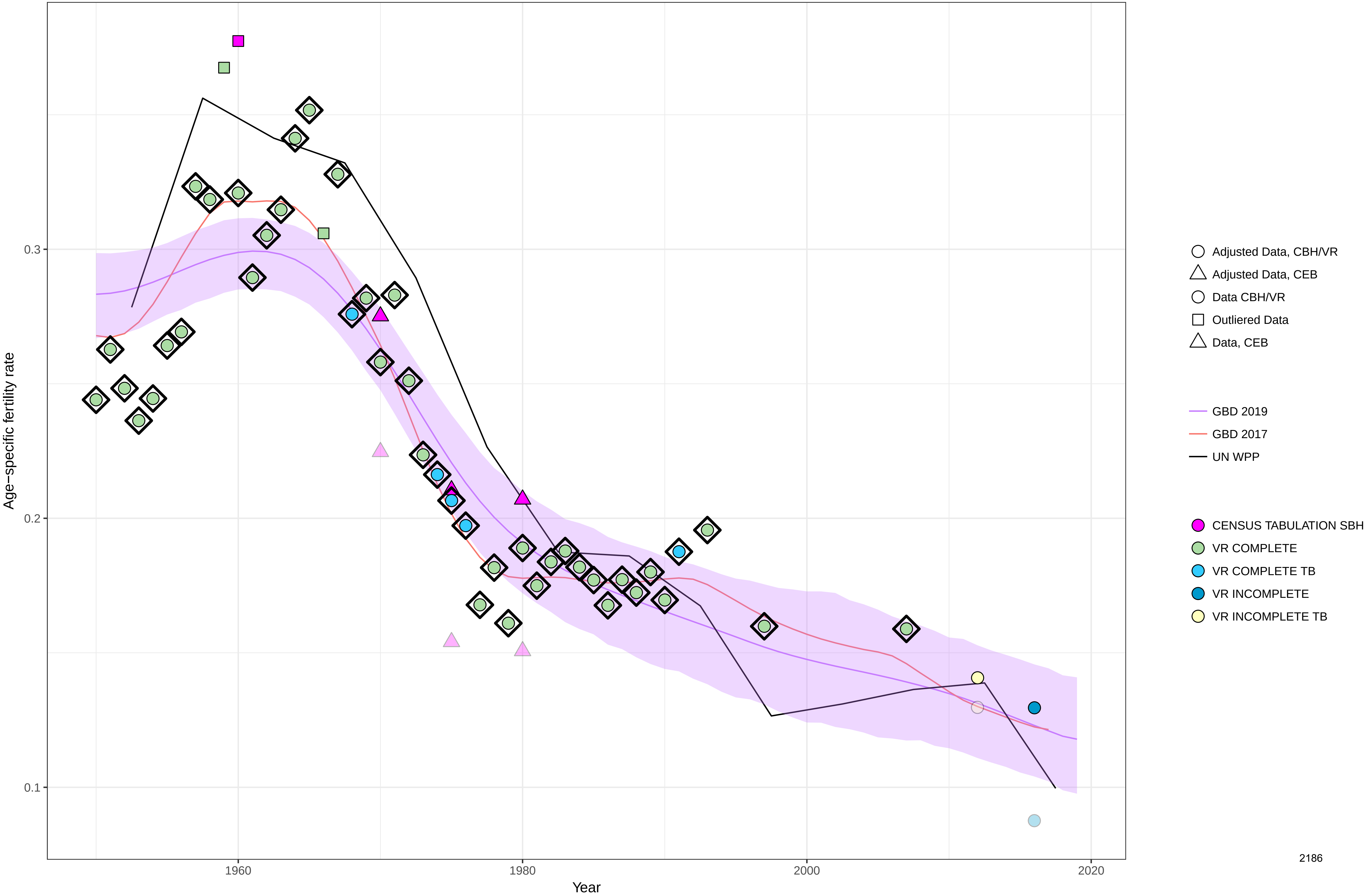

Age−specific fertility fitted trend lines, 1950−2019: United States Virgin Islands, Ages: 25−29
Data points enclosed with a diamond are reference sources

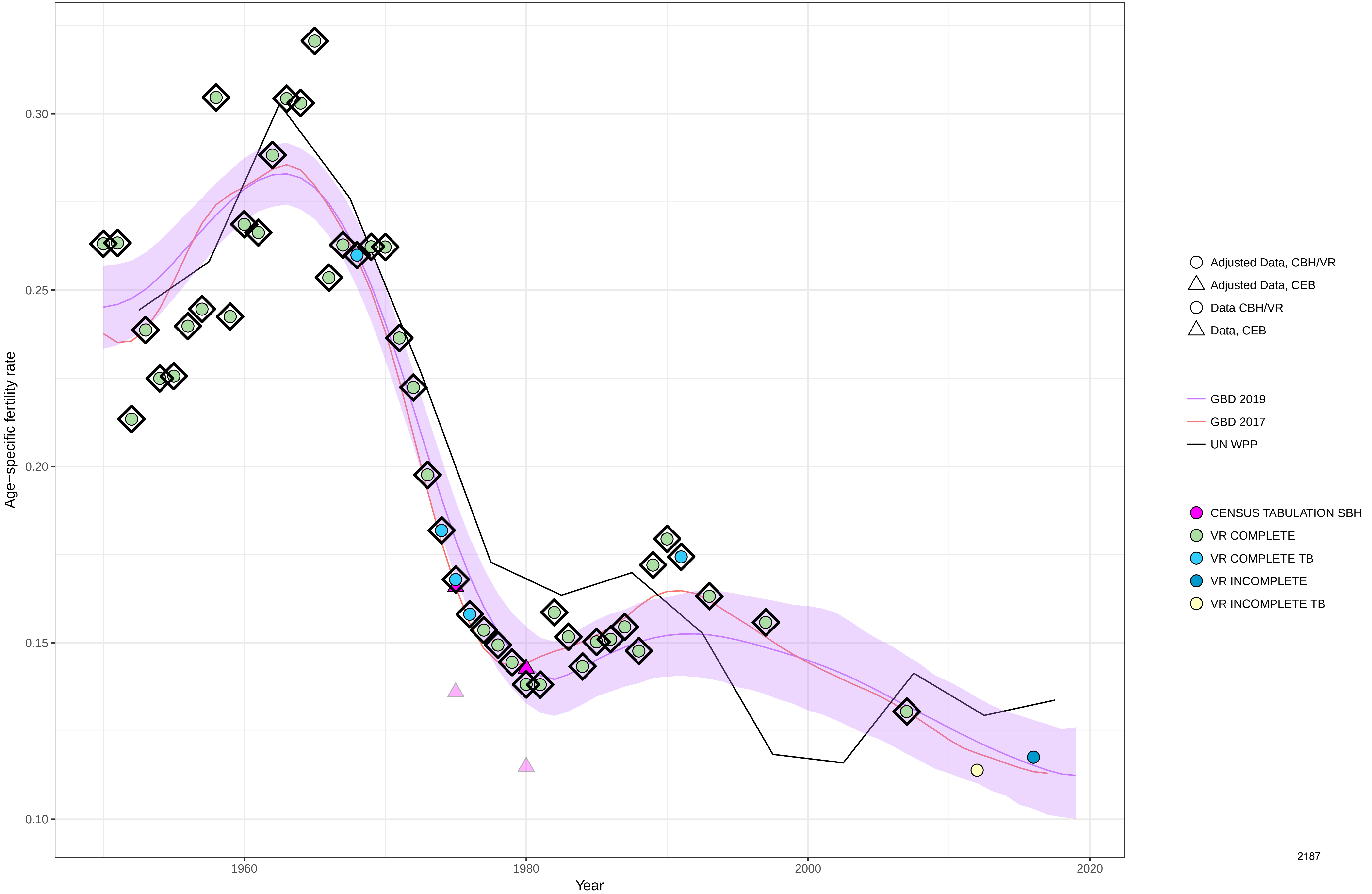

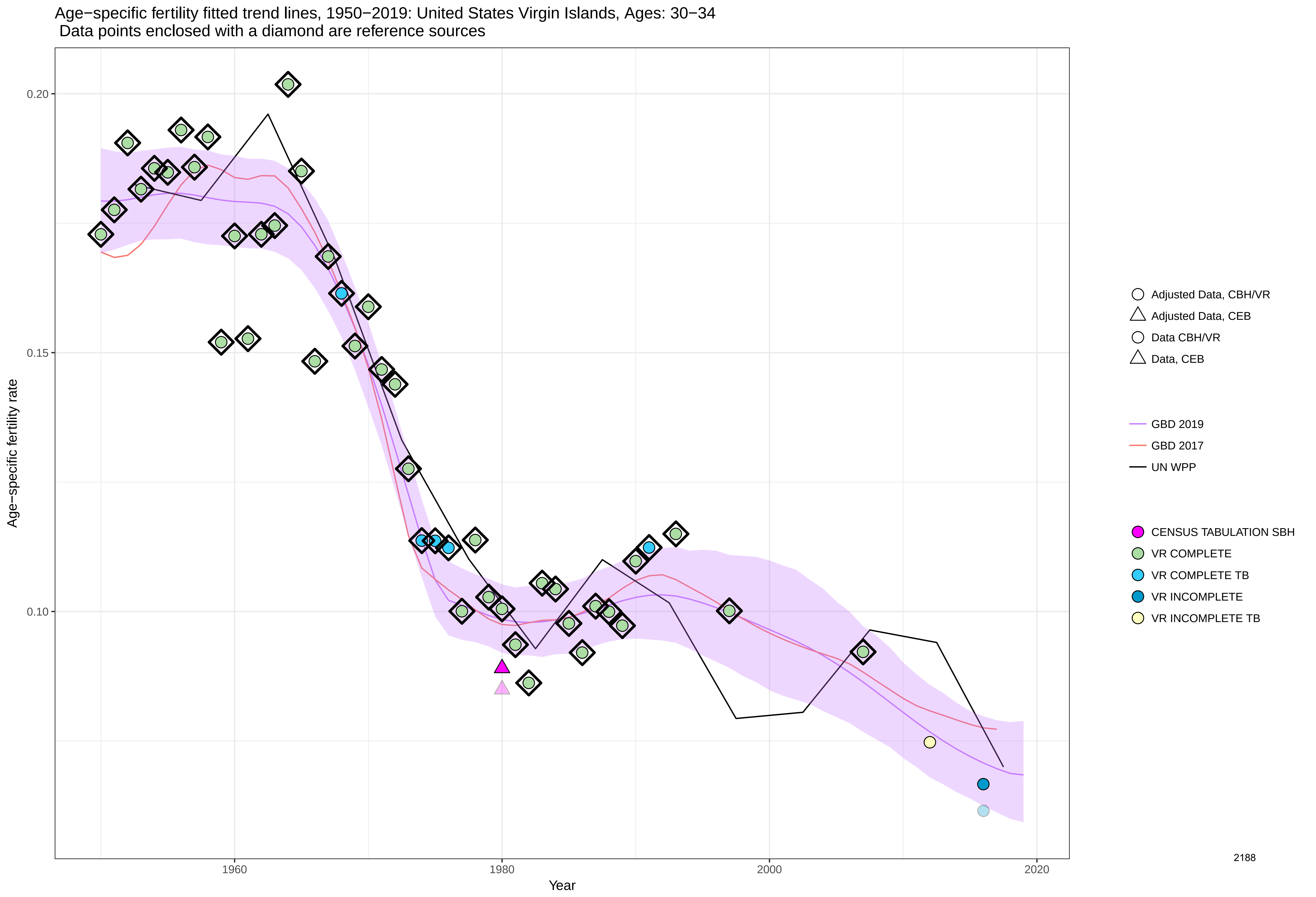

Age−specific fertility fitted trend lines, 1950−2019: United States Virgin Islands, Ages: 30−34
Data points enclosed with a diamond are reference sources
Age−specific fertility rate
Year
0.20
0.15
0.10
1960
1980
2000
2020
Adjusted Data, CBH/VR
Adjusted Data, CEB
Data CBH/VR
Data, CEB
GBD 2019
GBD 2017
UN WPP
CENSUS TABULATION SBH
VR COMPLETE
VR COMPLETE TB
VR INCOMPLETE
VR INCOMPLETE TB

Age−specific fertility fitted trend lines, 1950−2019: United States Virgin Islands, Ages: 35−39
Data points enclosed with a diamond are reference sources

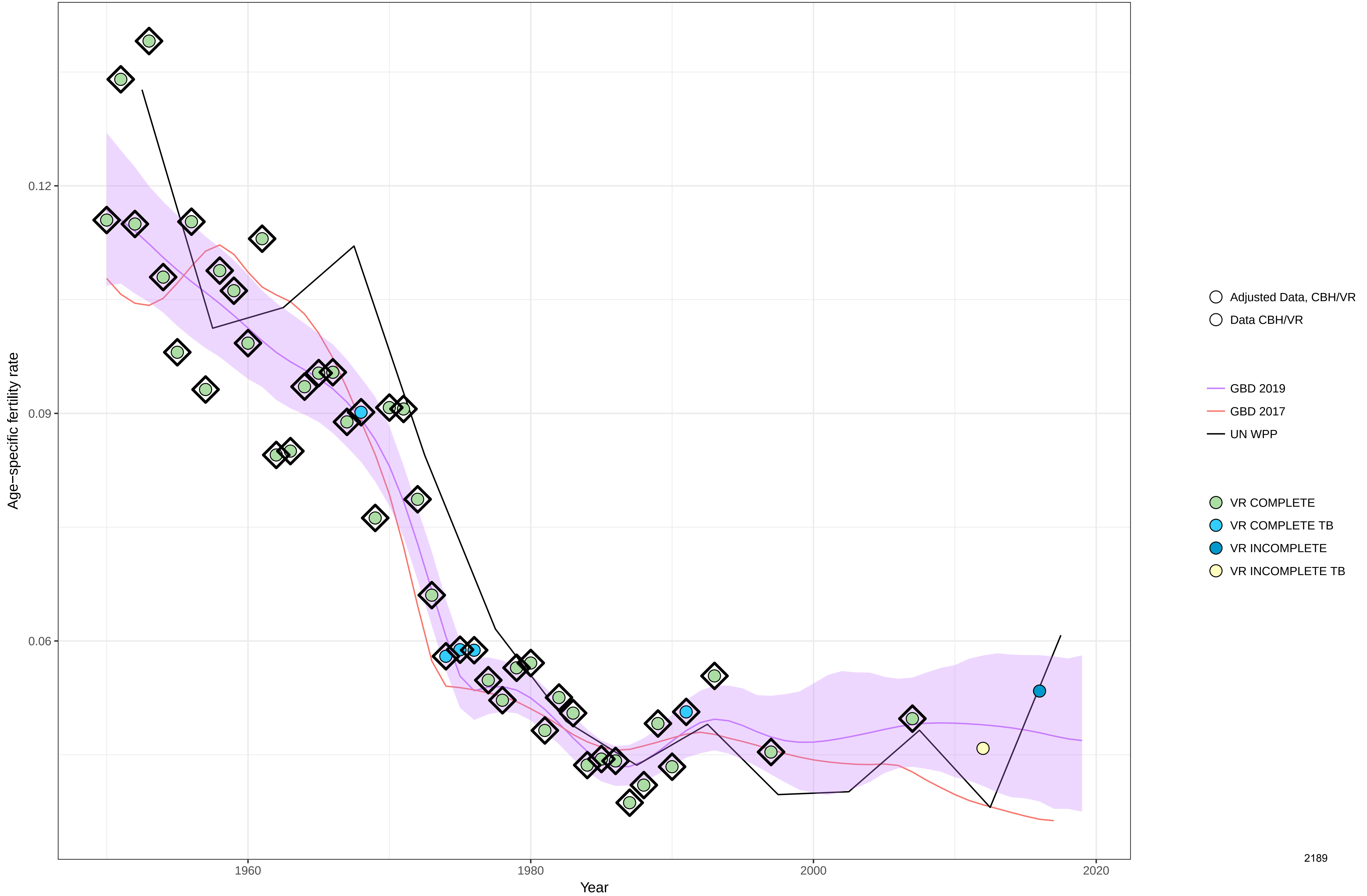

Age−specific fertility fitted trend lines, 1950−2019: United States Virgin Islands, Ages: 40−44
Data points enclosed with a diamond are reference sources

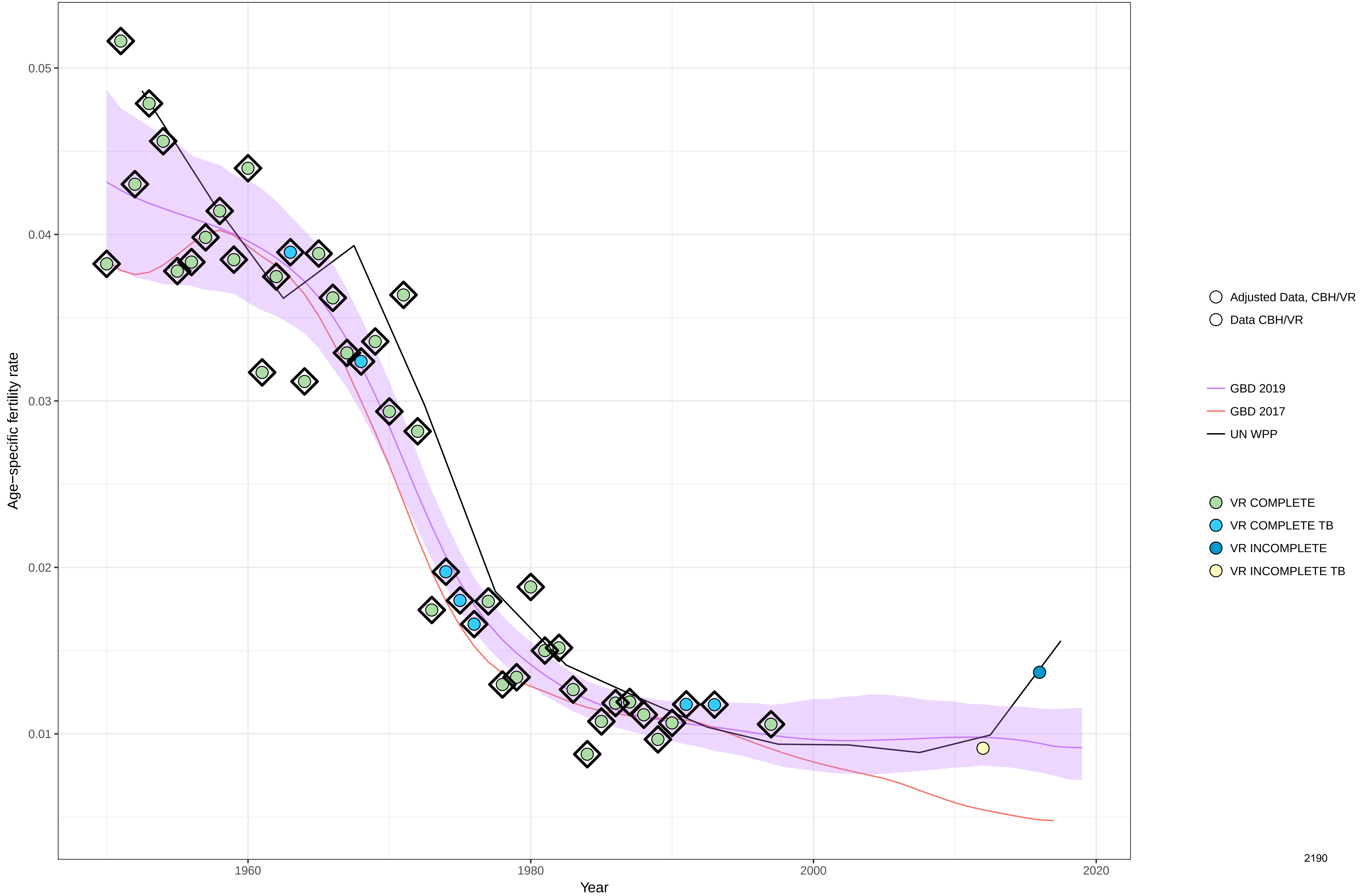

Age−specific fertility fitted trend lines, 1950−2019: United States Virgin Islands, Ages: 45−49
Data points enclosed with a diamond are reference sources

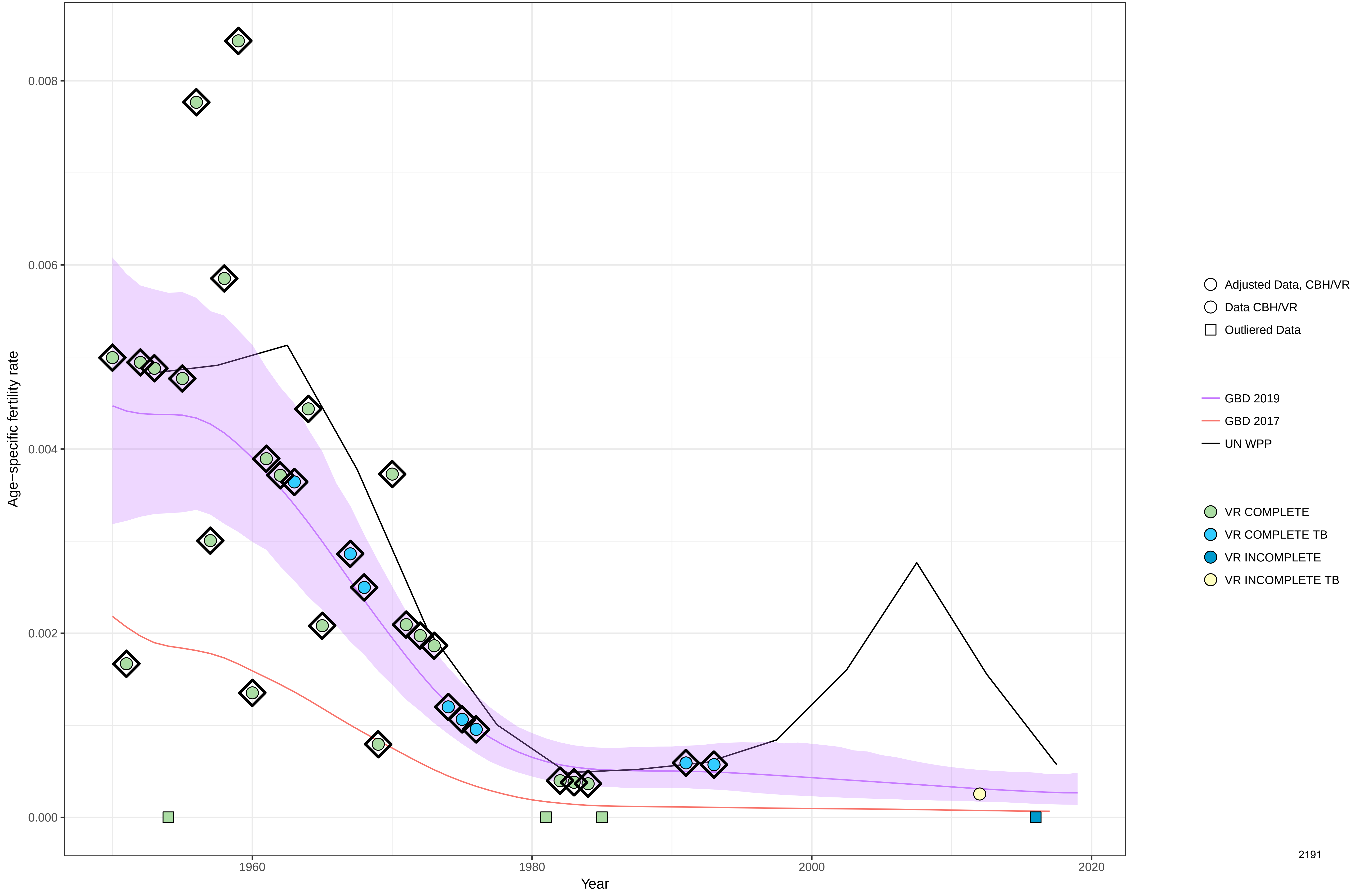

Age−specific fertility fitted trend lines, 1950−2019: United States Virgin Islands, Ages: 50−54
Data points enclosed with a diamond are reference sources

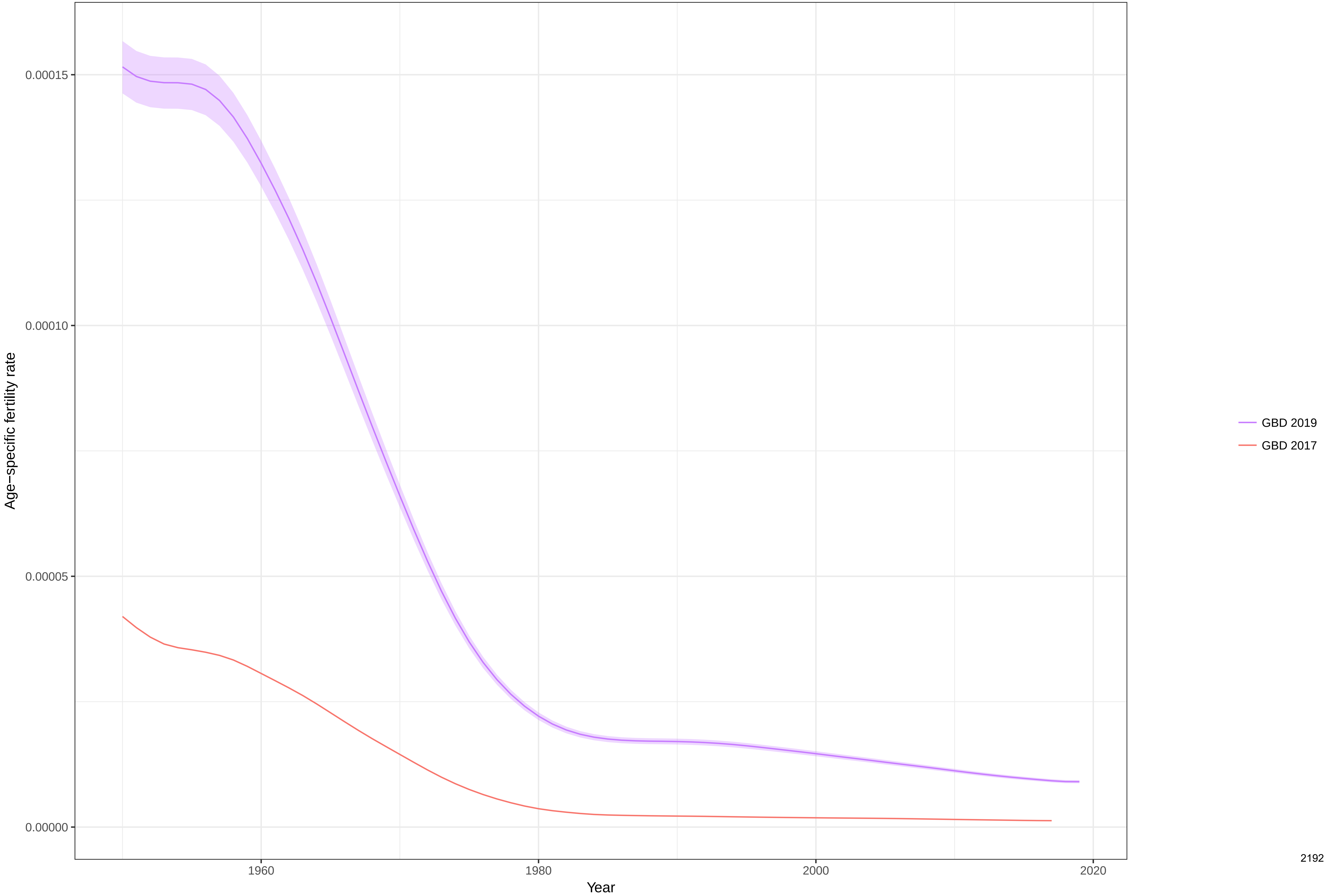

Total fertility rate fitted trend lines, 1950−2019: United States Virgin Islands TFR
Data points enclosed with a diamond are reference sources

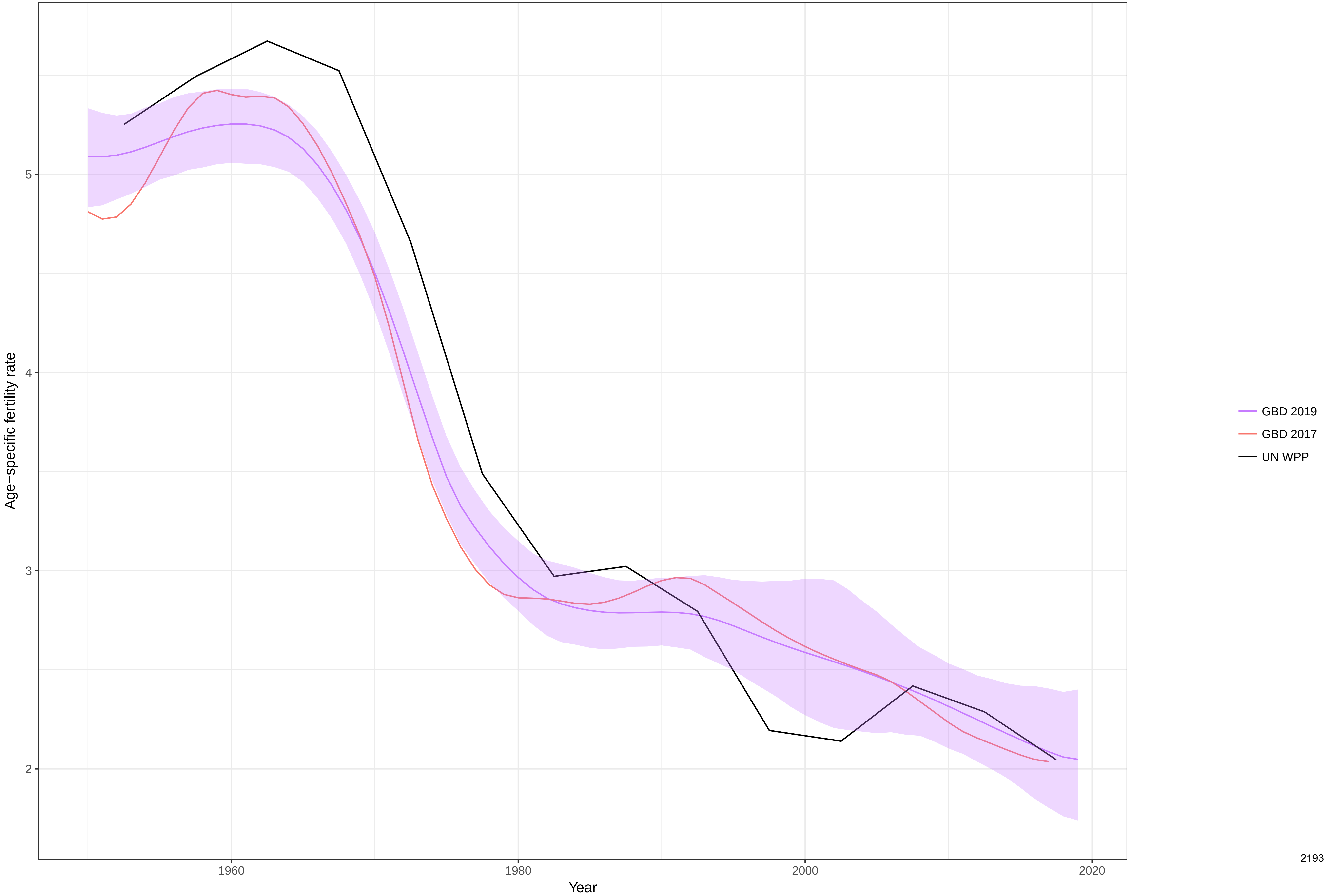

Age−specific fertility fitted trend lines, 1950−2019: Viet Nam, Ages: 10−14
Data points enclosed with a diamond are reference sources

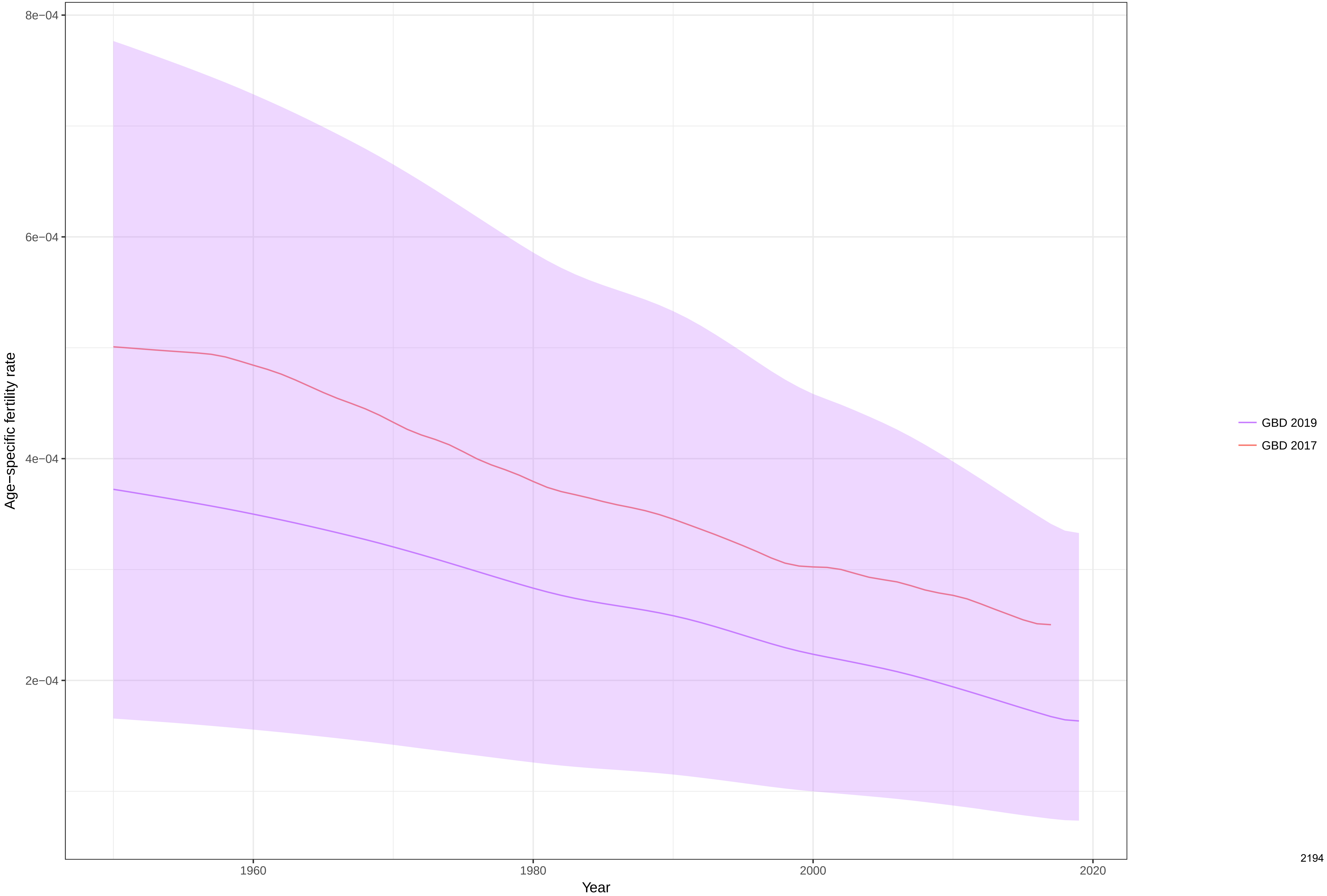

Age−specific fertility fitted trend lines, 1950−2019: Viet Nam, Ages: 15−19
Data points enclosed with a diamond are reference sources

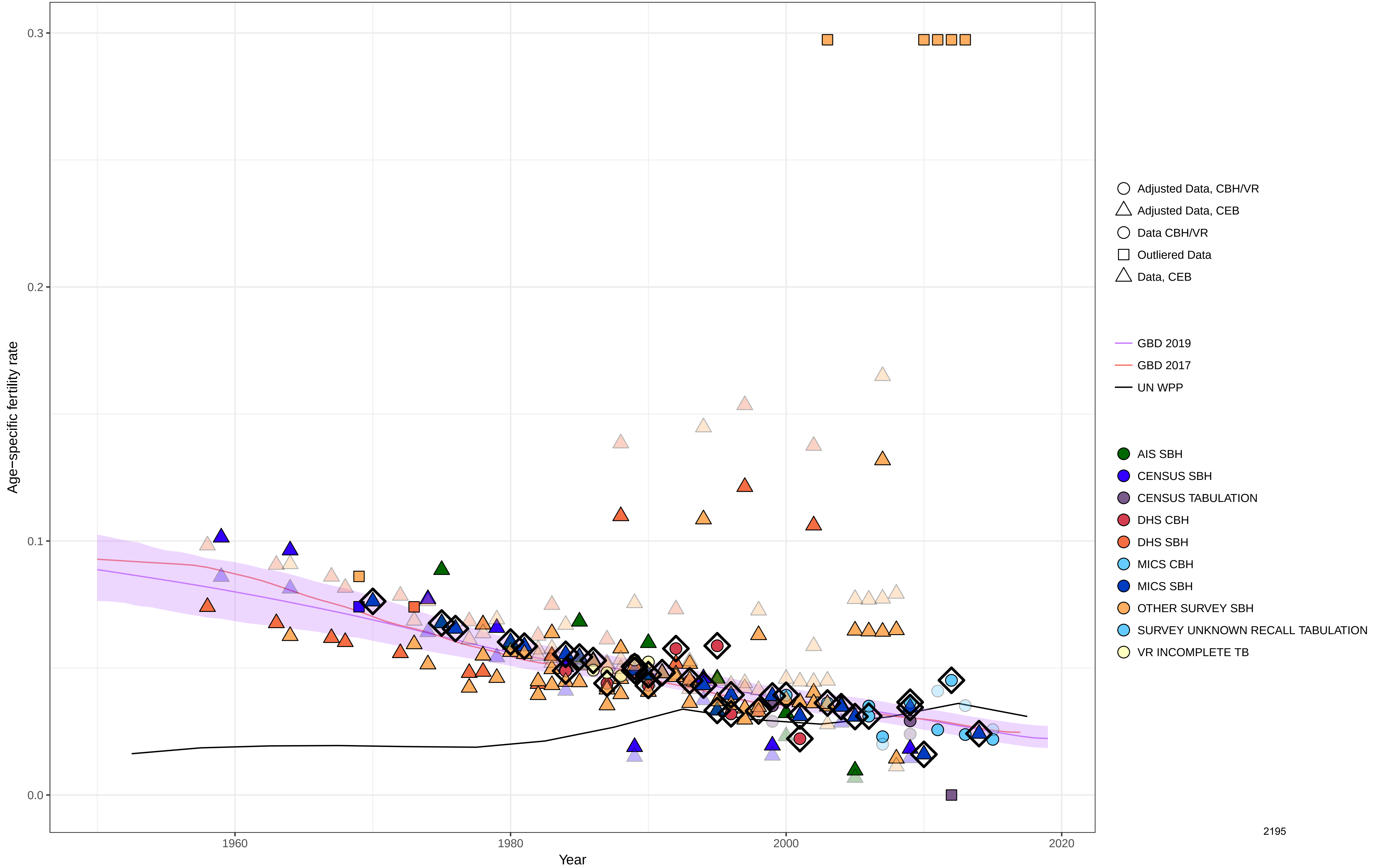

Age−specific fertility fitted trend lines, 1950−2019: Viet Nam, Ages: 20−24
Data points enclosed with a diamond are reference sources

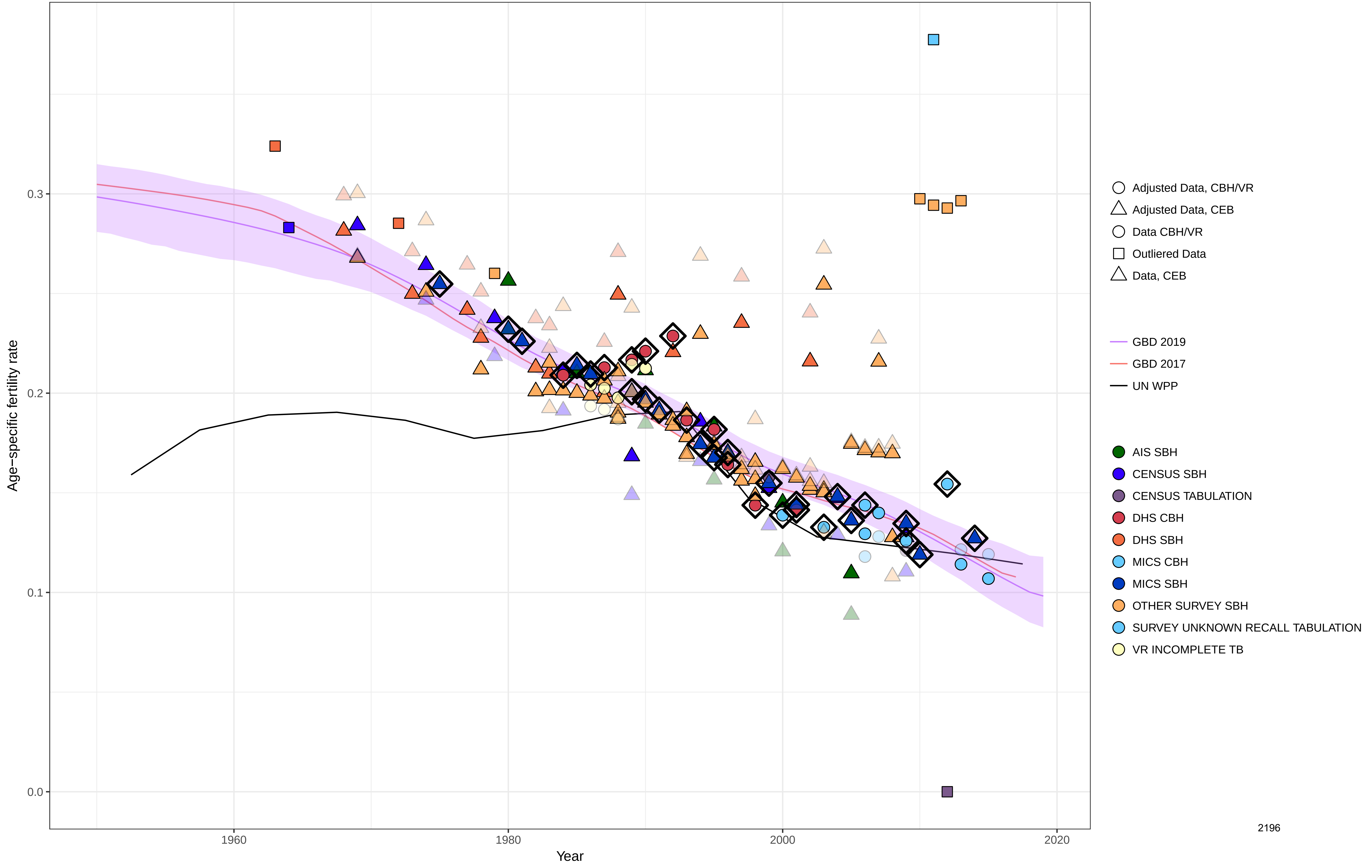

Age−specific fertility fitted trend lines, 1950−2019: Viet Nam, Ages: 25−29
Data points enclosed with a diamond are reference sources

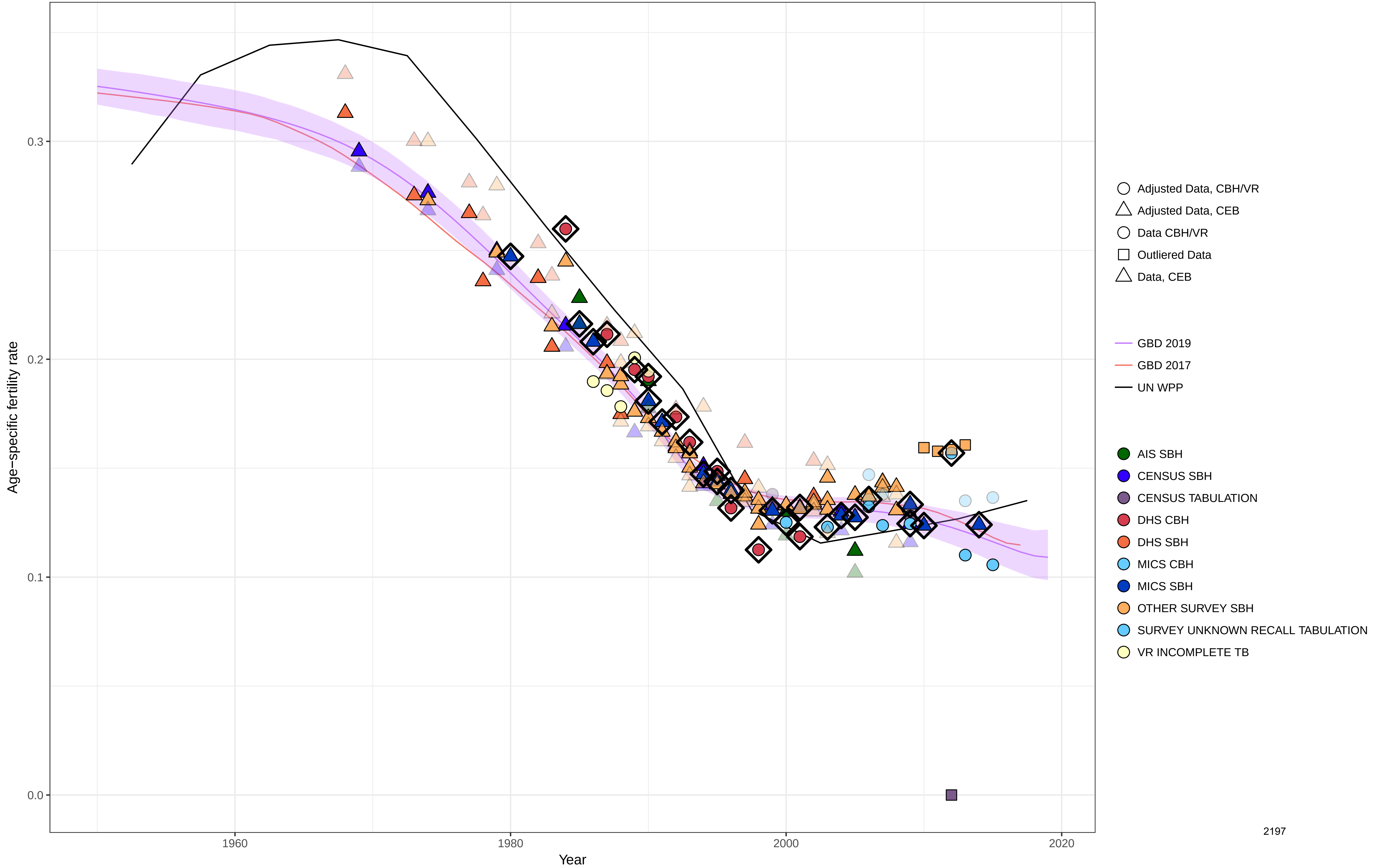

Age−specific fertility fitted trend lines, 1950−2019: Viet Nam, Ages: 30−34
Data points enclosed with a diamond are reference sources

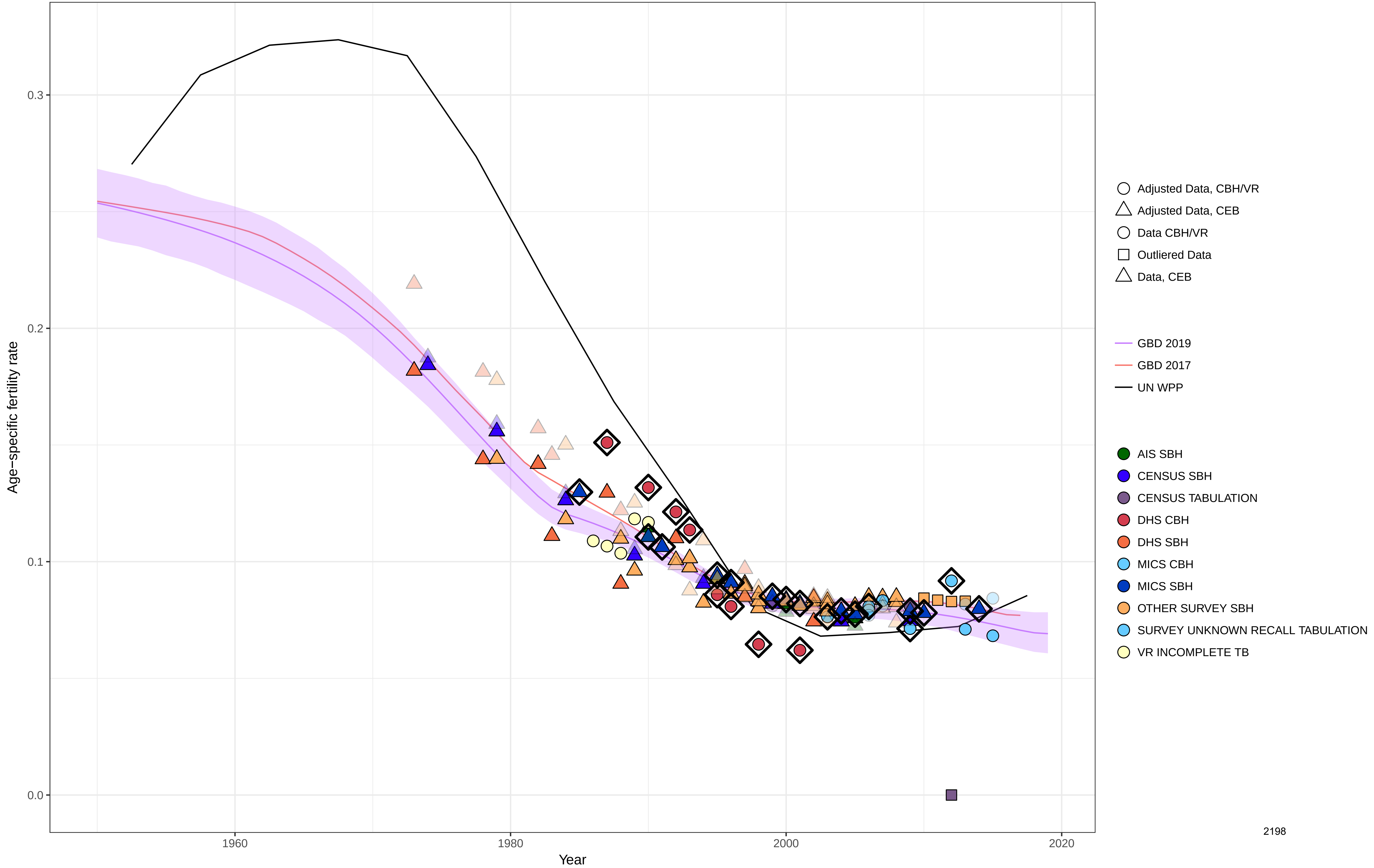

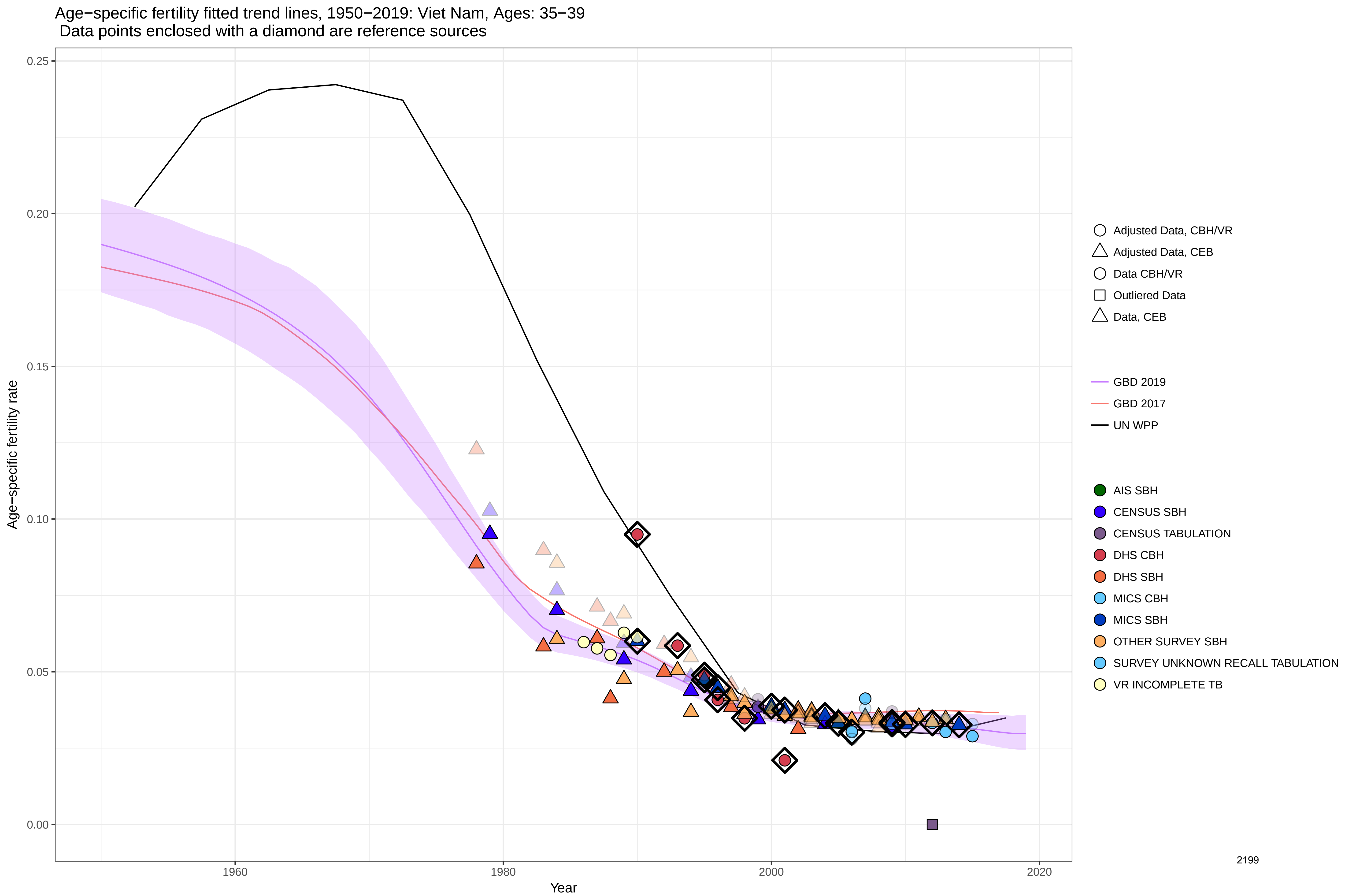

Age−specific fertility fitted trend lines, 1950−2019: Viet Nam, Ages: 35−39
Data points enclosed with a diamond are reference sources
Age−specific fertility rate
0.25
0.20
0.15
0.10
0.05
0.00
1960
1980
2000
2020
Year
Adjusted Data, CBH/VR
Adjusted Data, CEB
Data CBH/VR
Outliered Data
Data, CEB
GBD 2019
GBD 2017
UN WPP
AIS SBH
CENSUS SBH
CENSUS TABULATION
DHS CBH
DHS SBH
MICS CBH
MICS SBH
OTHER SURVEY SBH
SURVEY UNKNOWN RECALL TABULATION
VR INCOMPLETE TB

Age−specific fertility fitted trend lines, 1950−2019: Viet Nam, Ages: 40−44
Data points enclosed with a diamond are reference sources

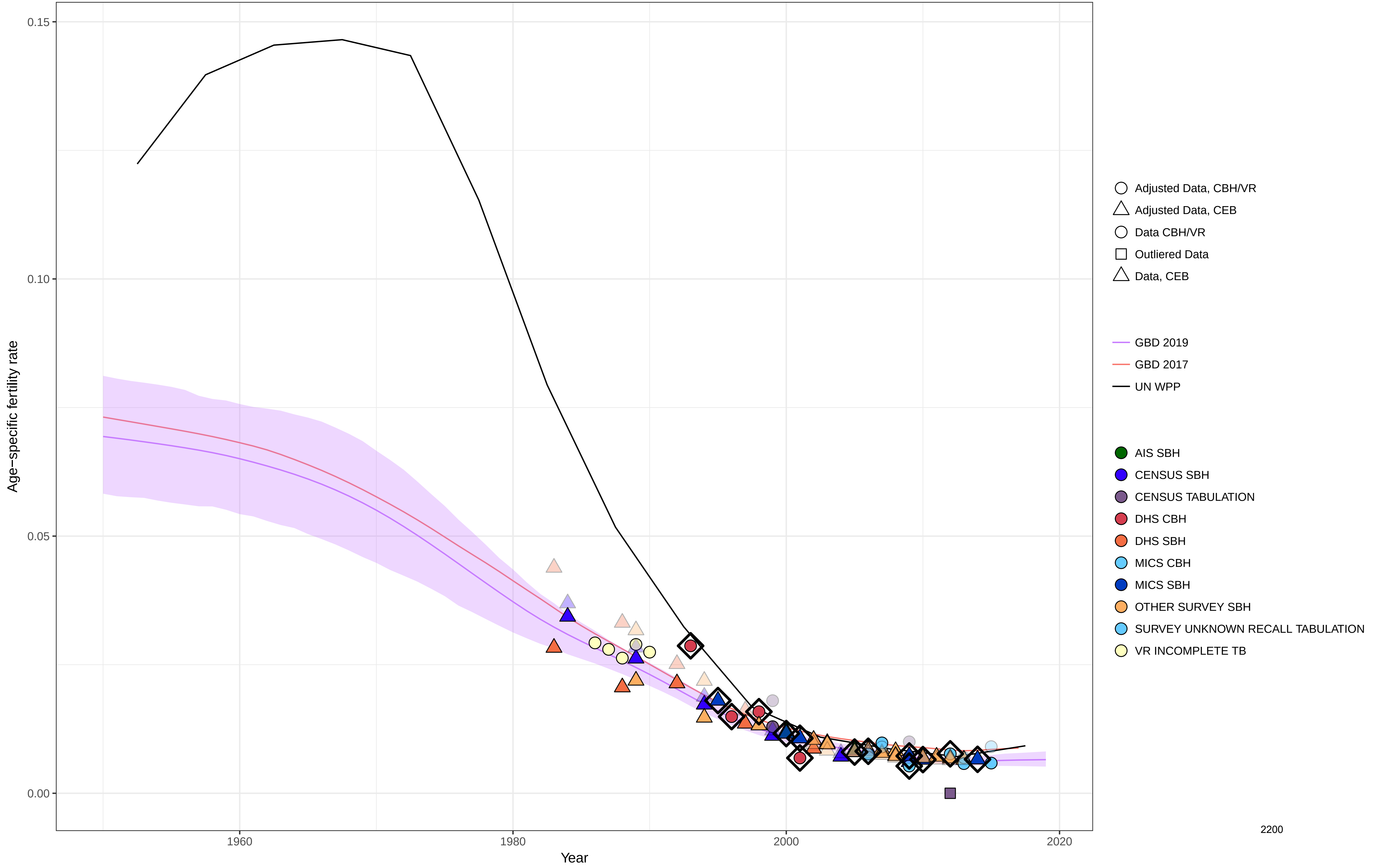

Age−specific fertility fitted trend lines, 1950−2019: Viet Nam, Ages: 45−49
Data points enclosed with a diamond are reference sources

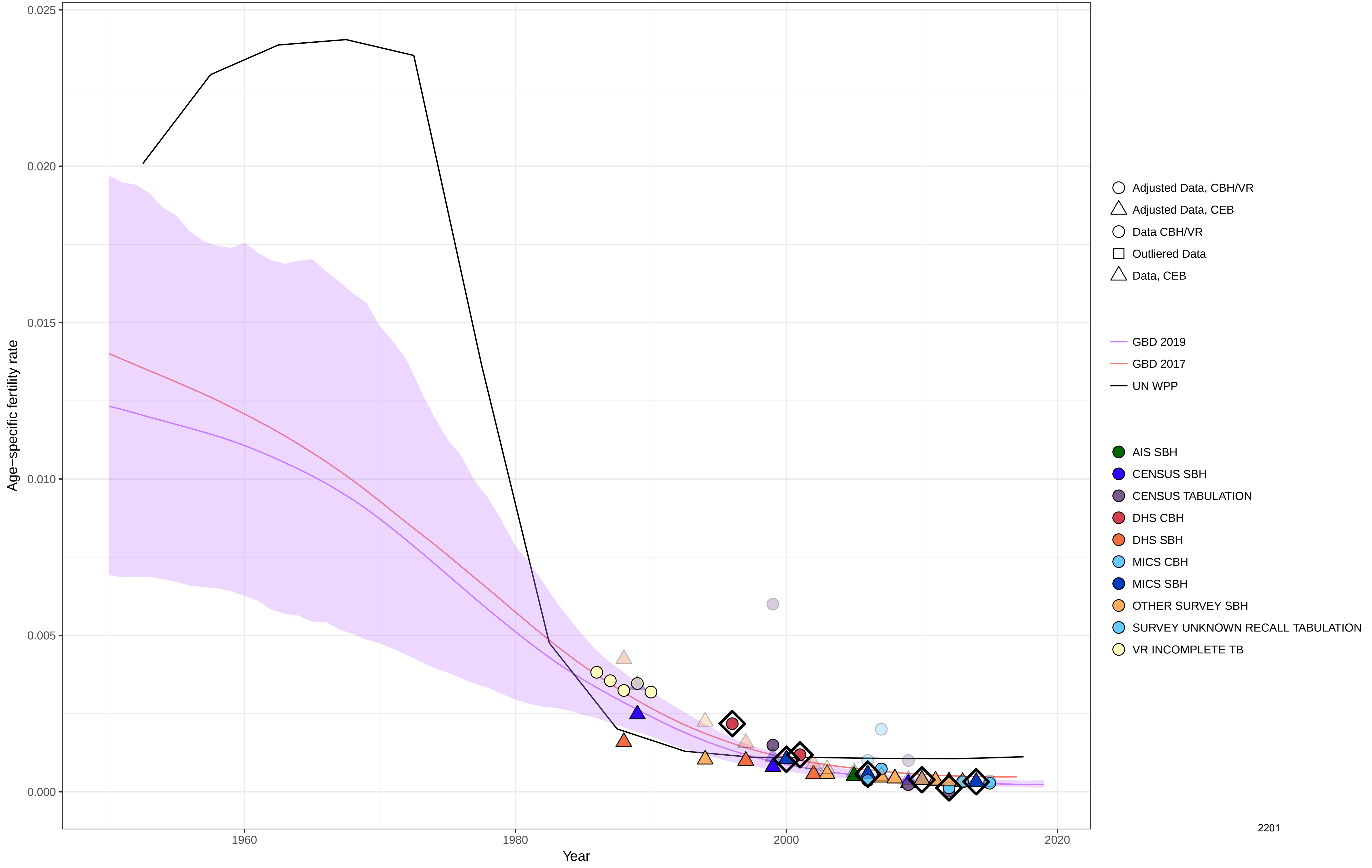

Age−specific fertility fitted trend lines, 1950−2019: Viet Nam, Ages: 50−54
Data points enclosed with a diamond are reference sources

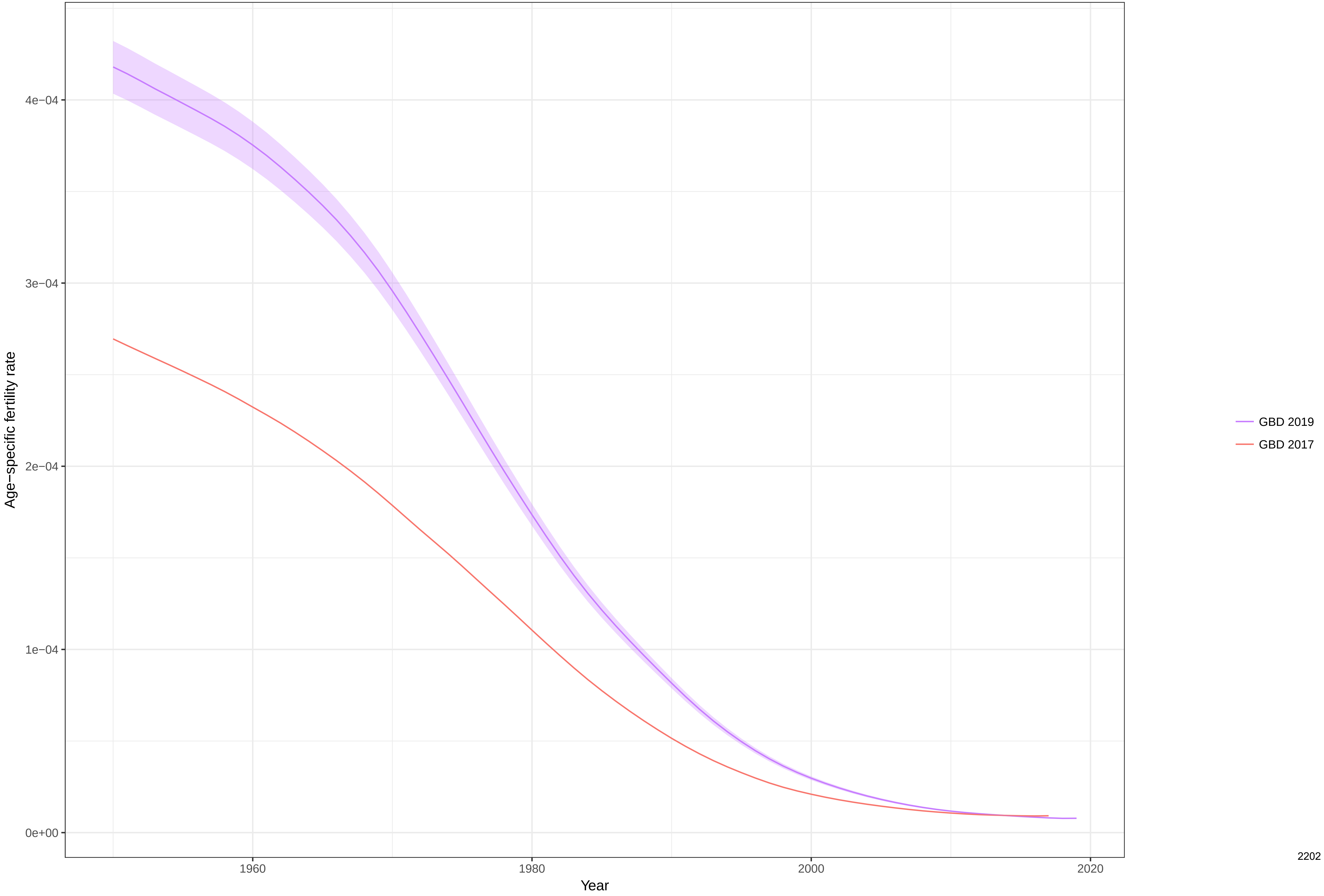

Total fertility rate fitted trend lines, 1950−2019: Viet Nam TFR
Data points enclosed with a diamond are reference sources

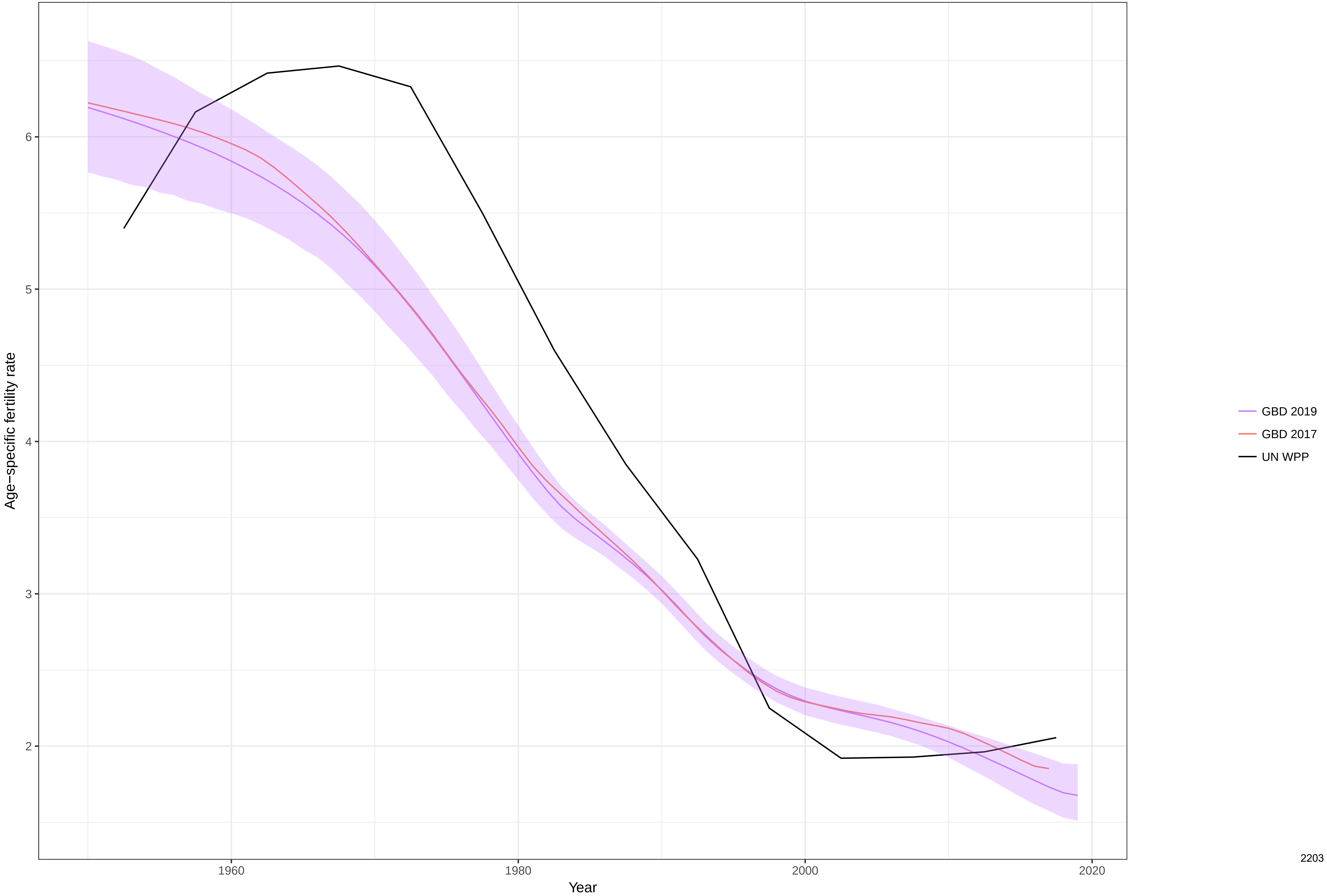

Age−specific fertility fitted trend lines, 1950−2019: Vanuatu, Ages: 10−14
Data points enclosed with a diamond are reference sources

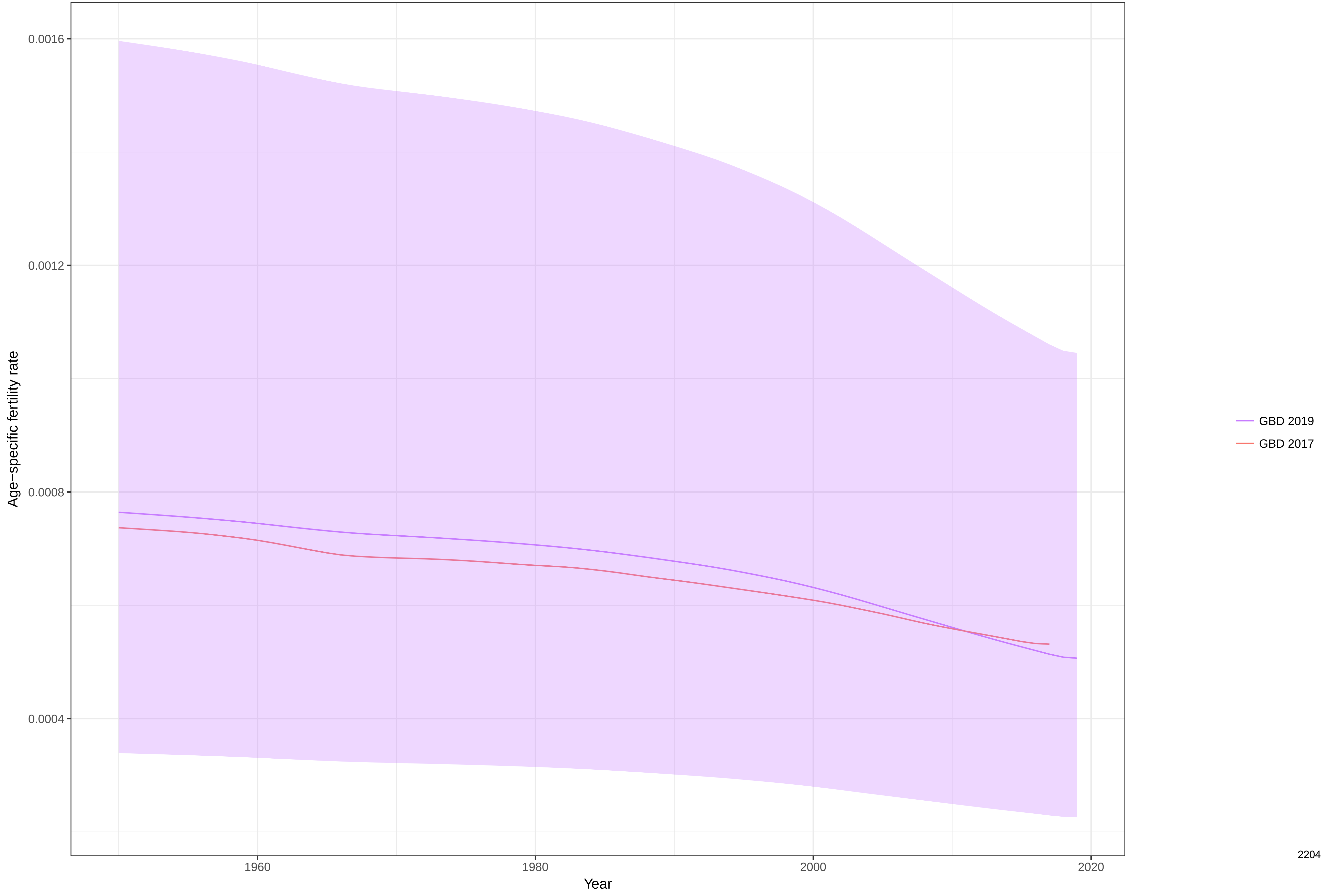

Age−specific fertility fitted trend lines, 1950−2019: Vanuatu, Ages: 15−19
Data points enclosed with a diamond are reference sources

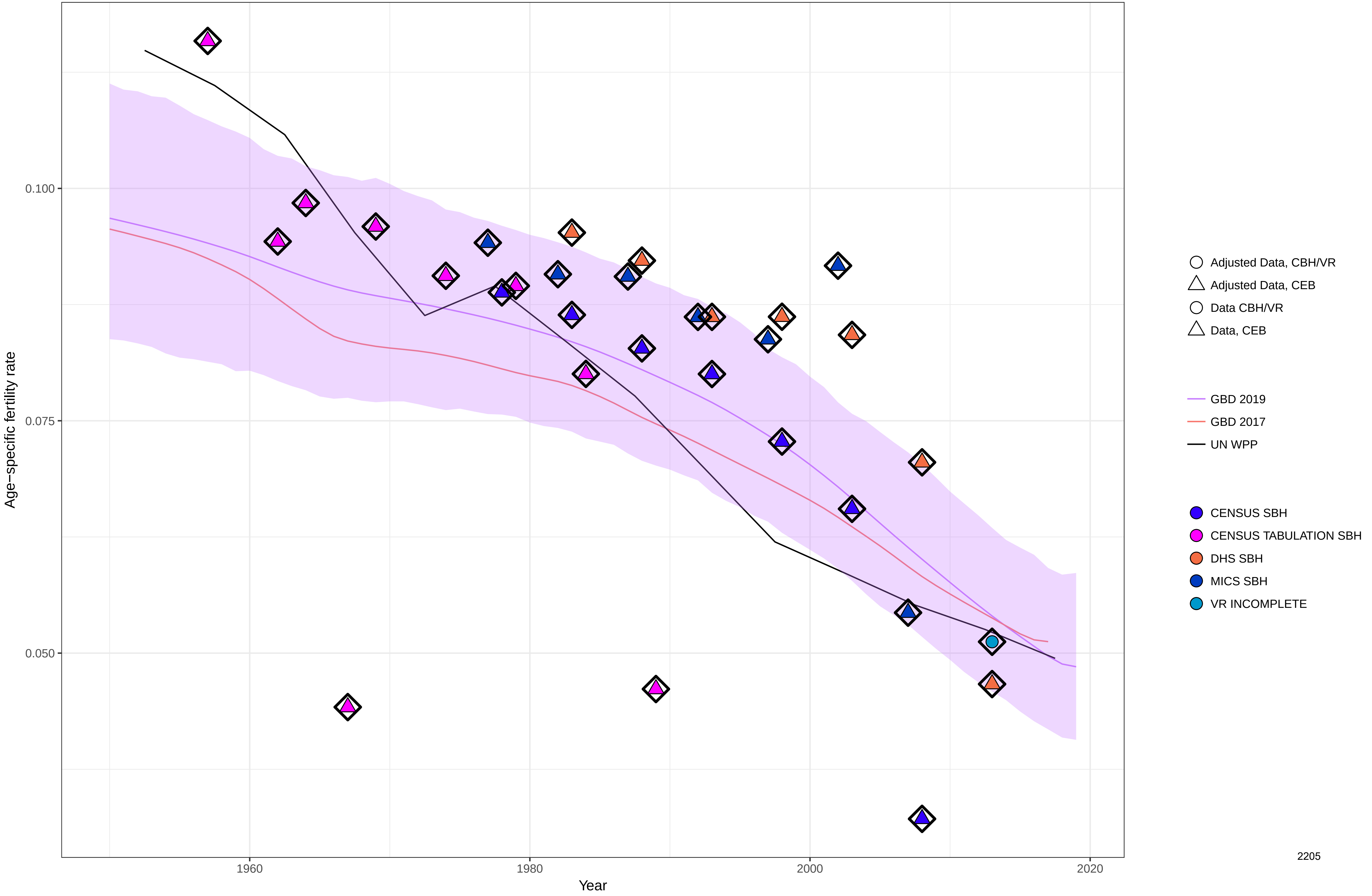

Age−specific fertility fitted trend lines, 1950−2019: Vanuatu, Ages: 20−24
Data points enclosed with a diamond are reference sources

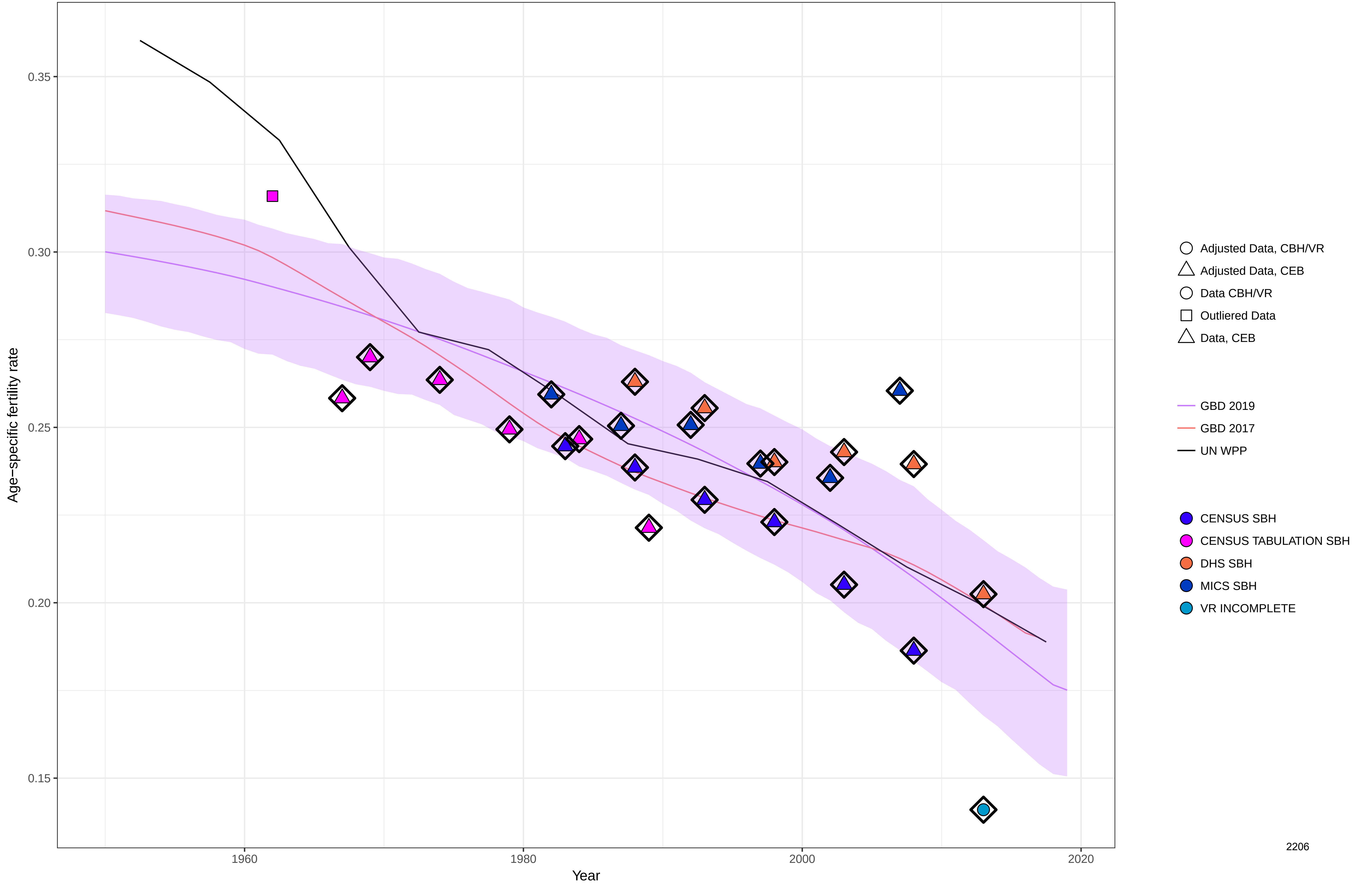

Age−specific fertility fitted trend lines, 1950−2019: Vanuatu, Ages: 25−29
Data points enclosed with a diamond are reference sources

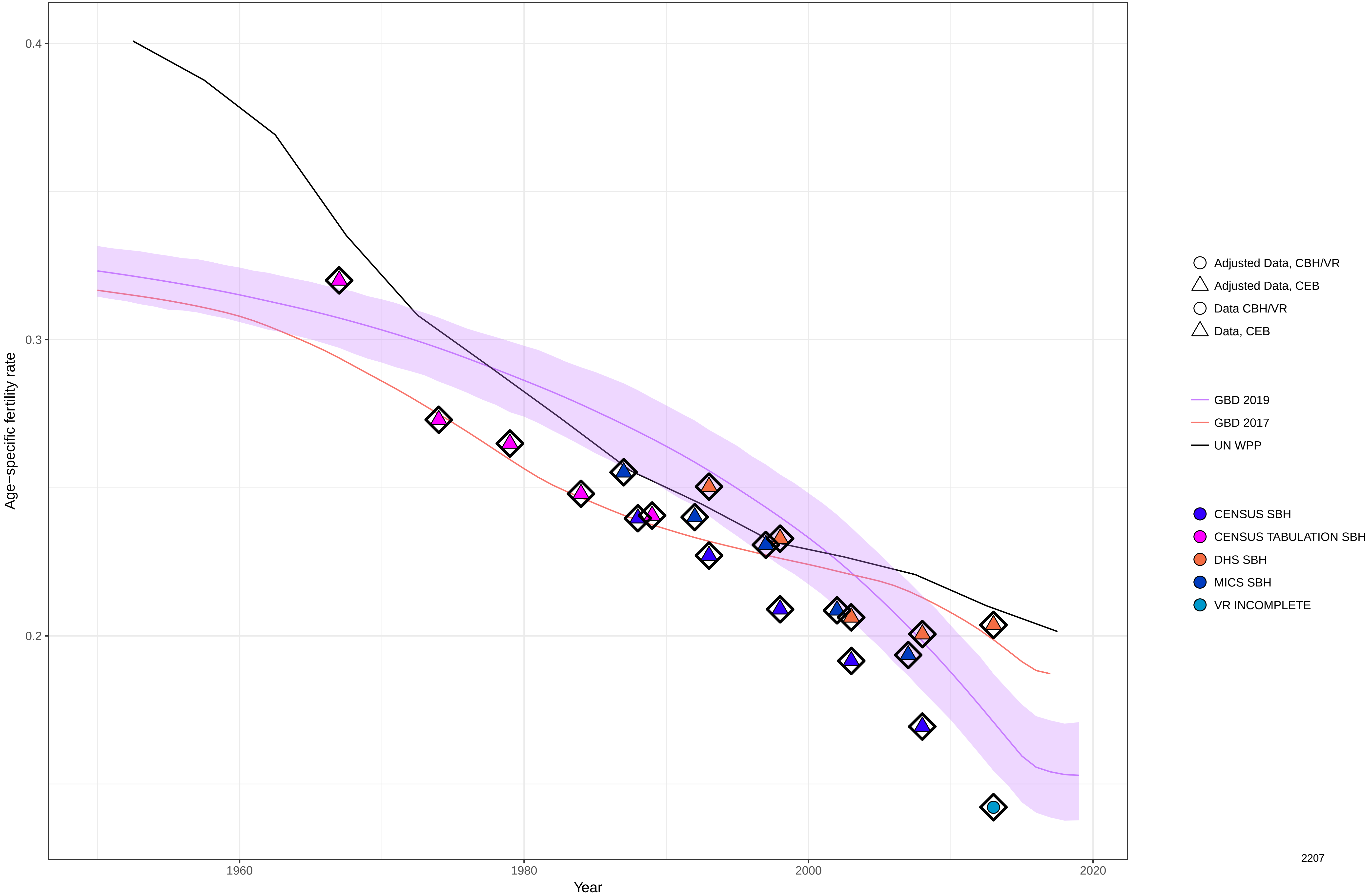

Age−specific fertility fitted trend lines, 1950−2019: Vanuatu, Ages: 30−34
Data points enclosed with a diamond are reference sources

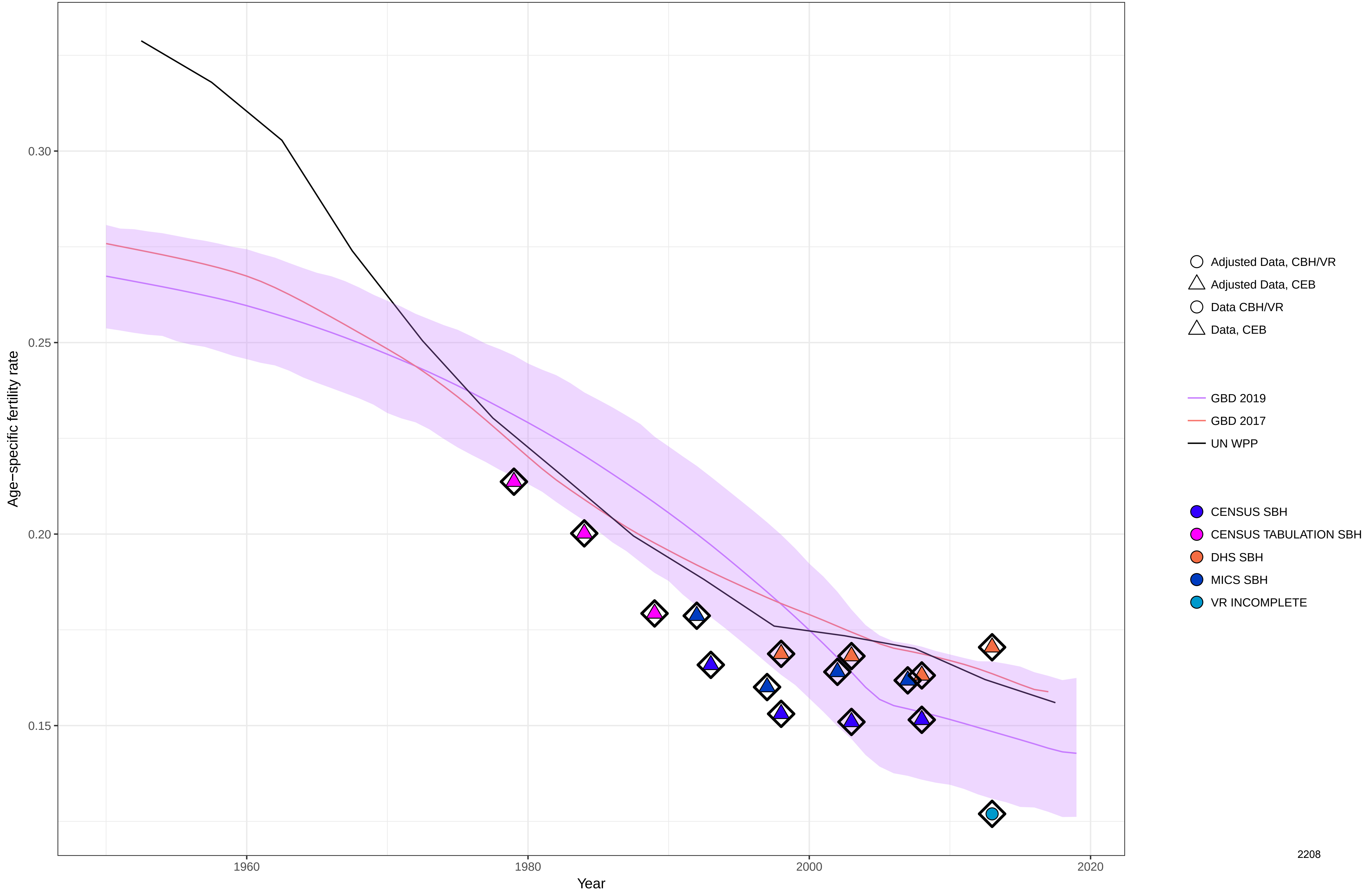

Age−specific fertility fitted trend lines, 1950−2019: Vanuatu, Ages: 35−39
Data points enclosed with a diamond are reference sources

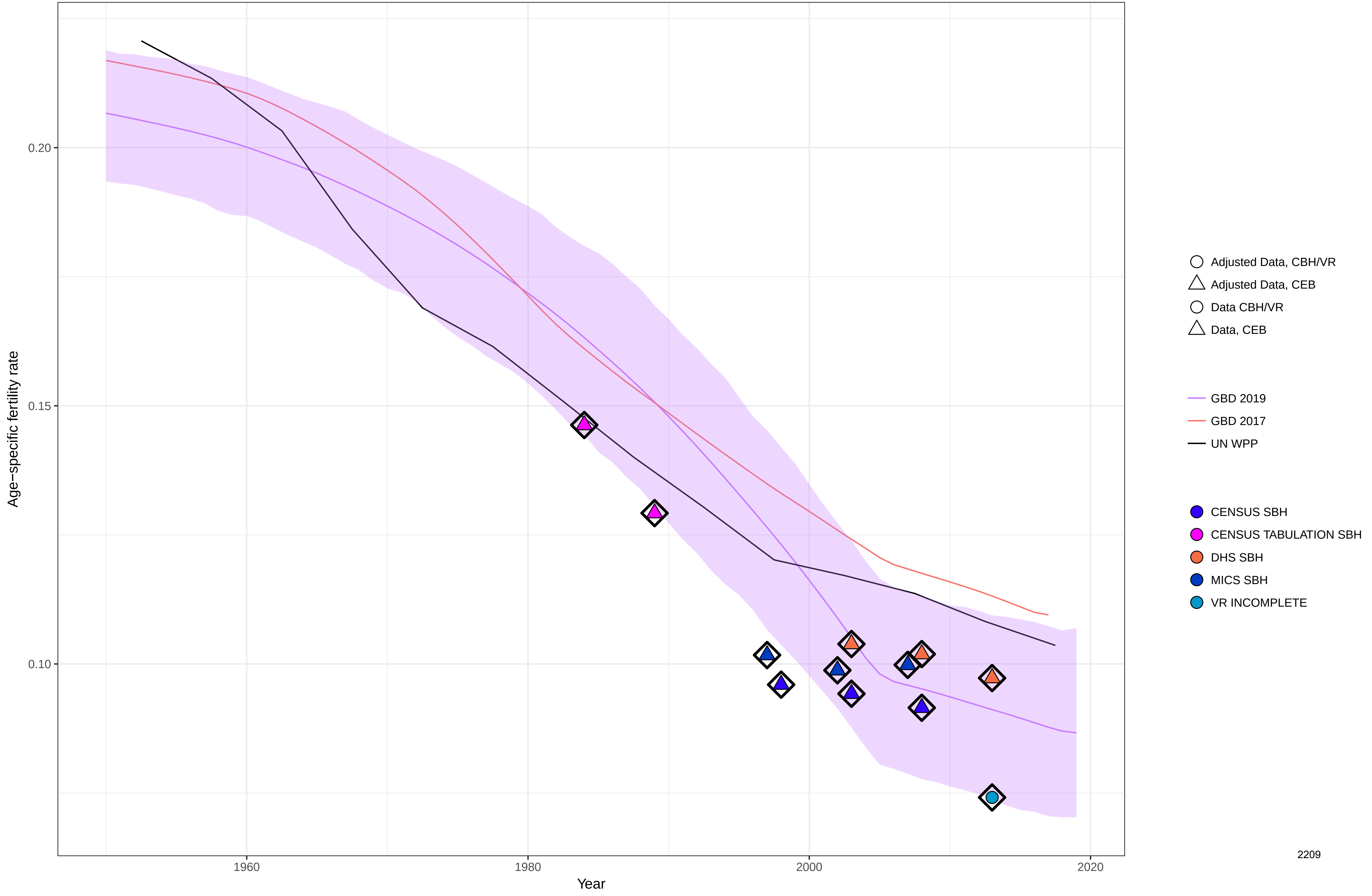

Age−specific fertility fitted trend lines, 1950−2019: Vanuatu, Ages: 40−44
Data points enclosed with a diamond are reference sources

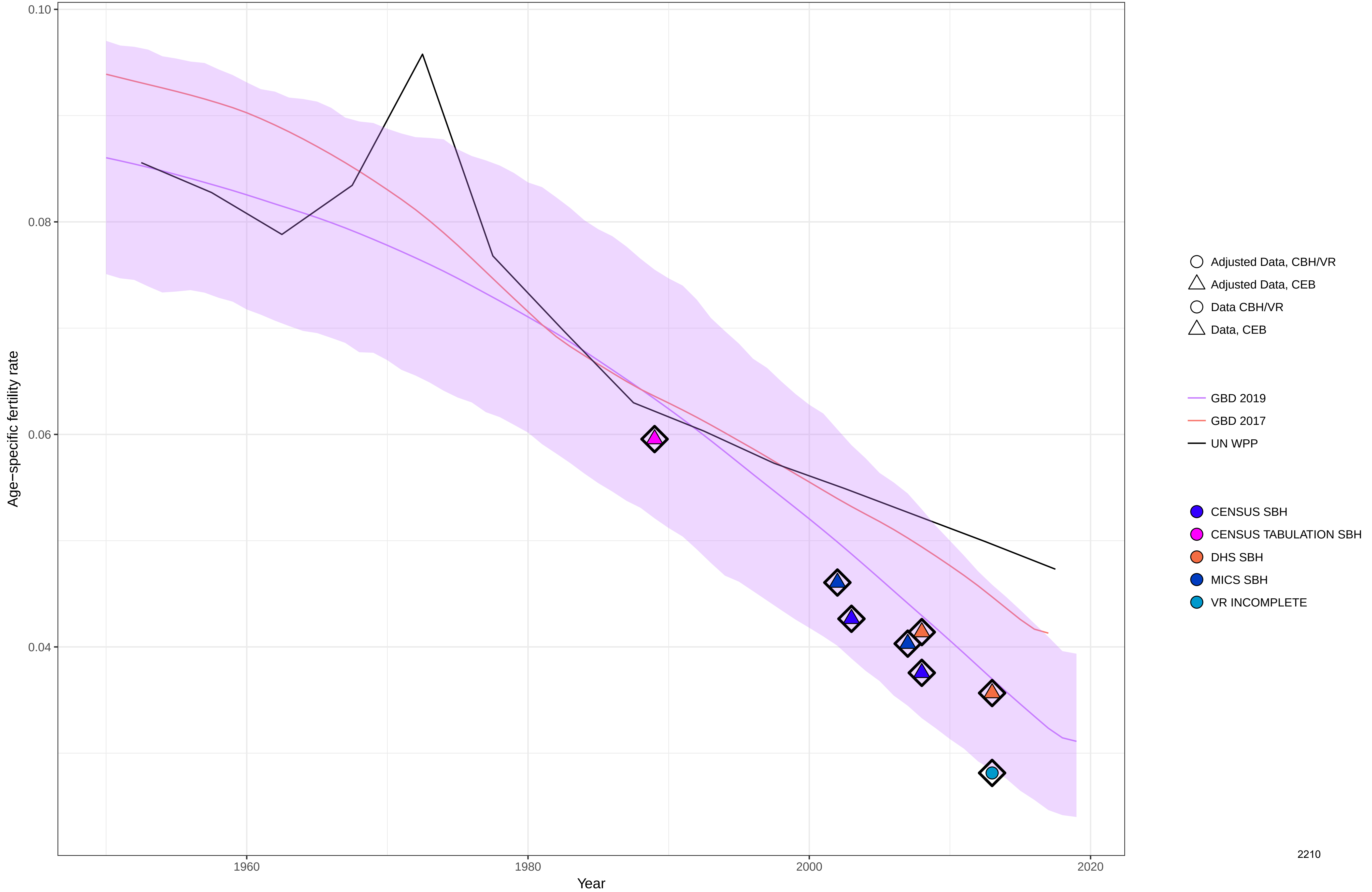

Age−specific fertility fitted trend lines, 1950−2019: Vanuatu, Ages: 45−49
Data points enclosed with a diamond are reference sources

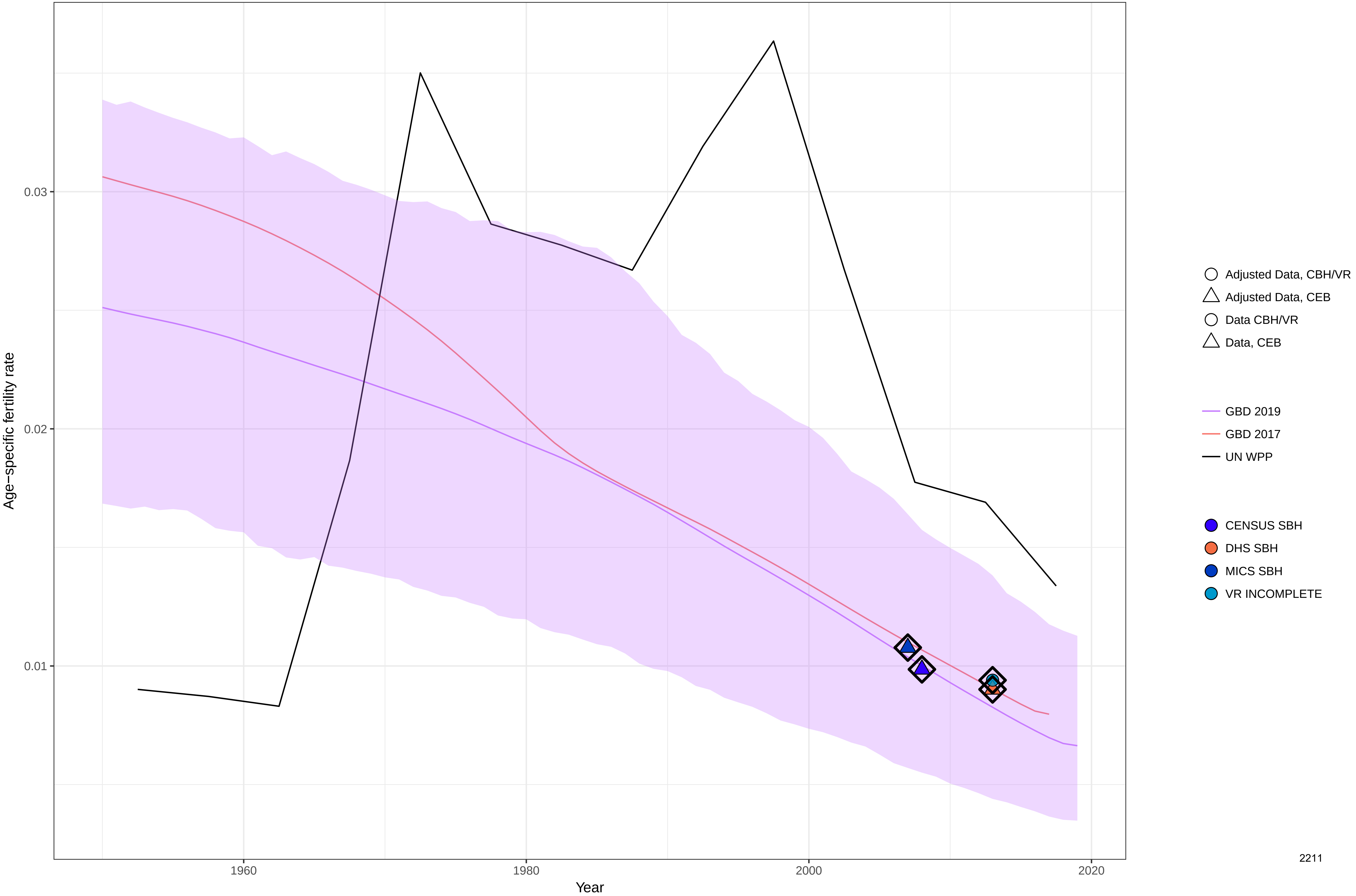

Age−specific fertility fitted trend lines, 1950−2019: Vanuatu, Ages: 50−54
Data points enclosed with a diamond are reference sources

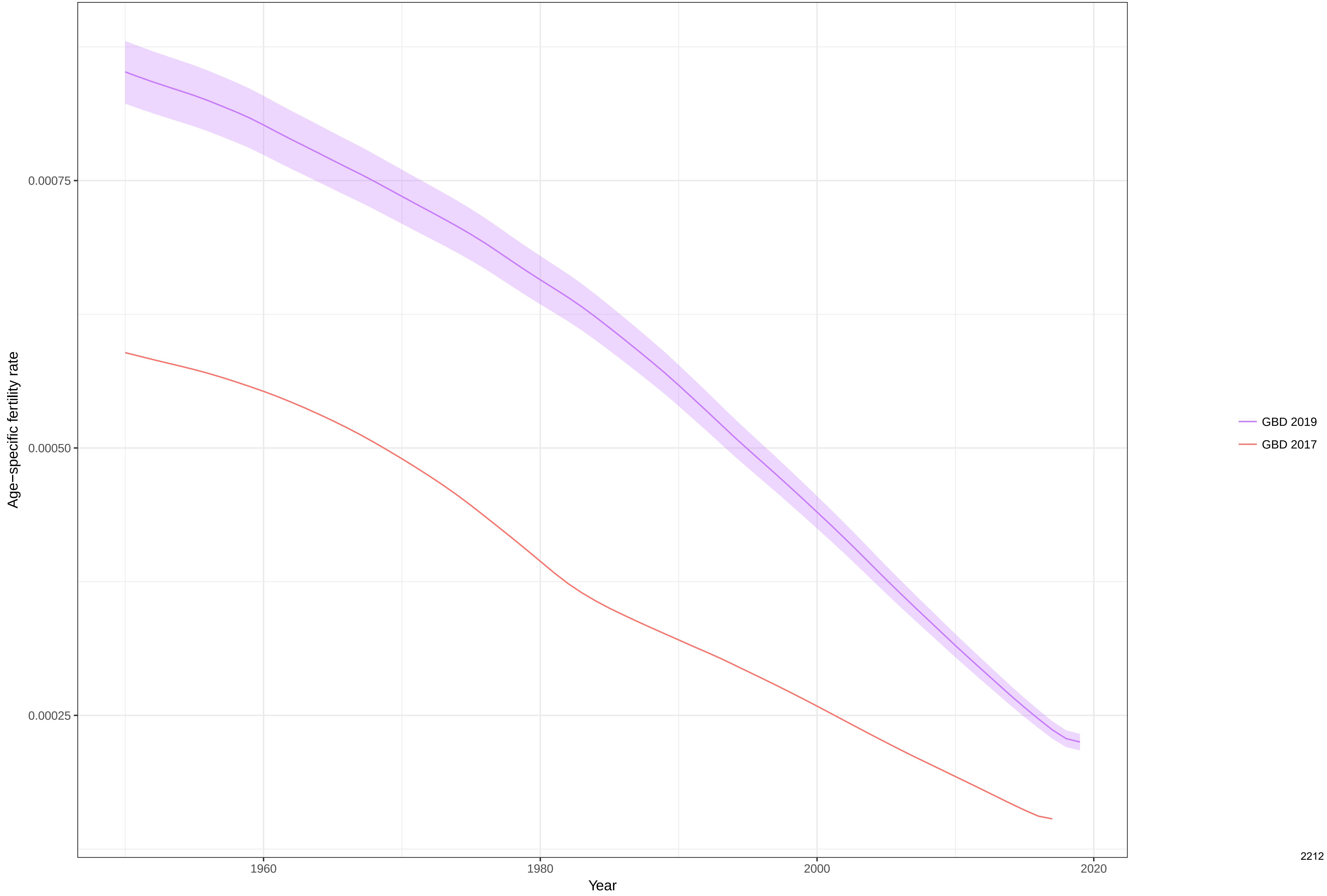

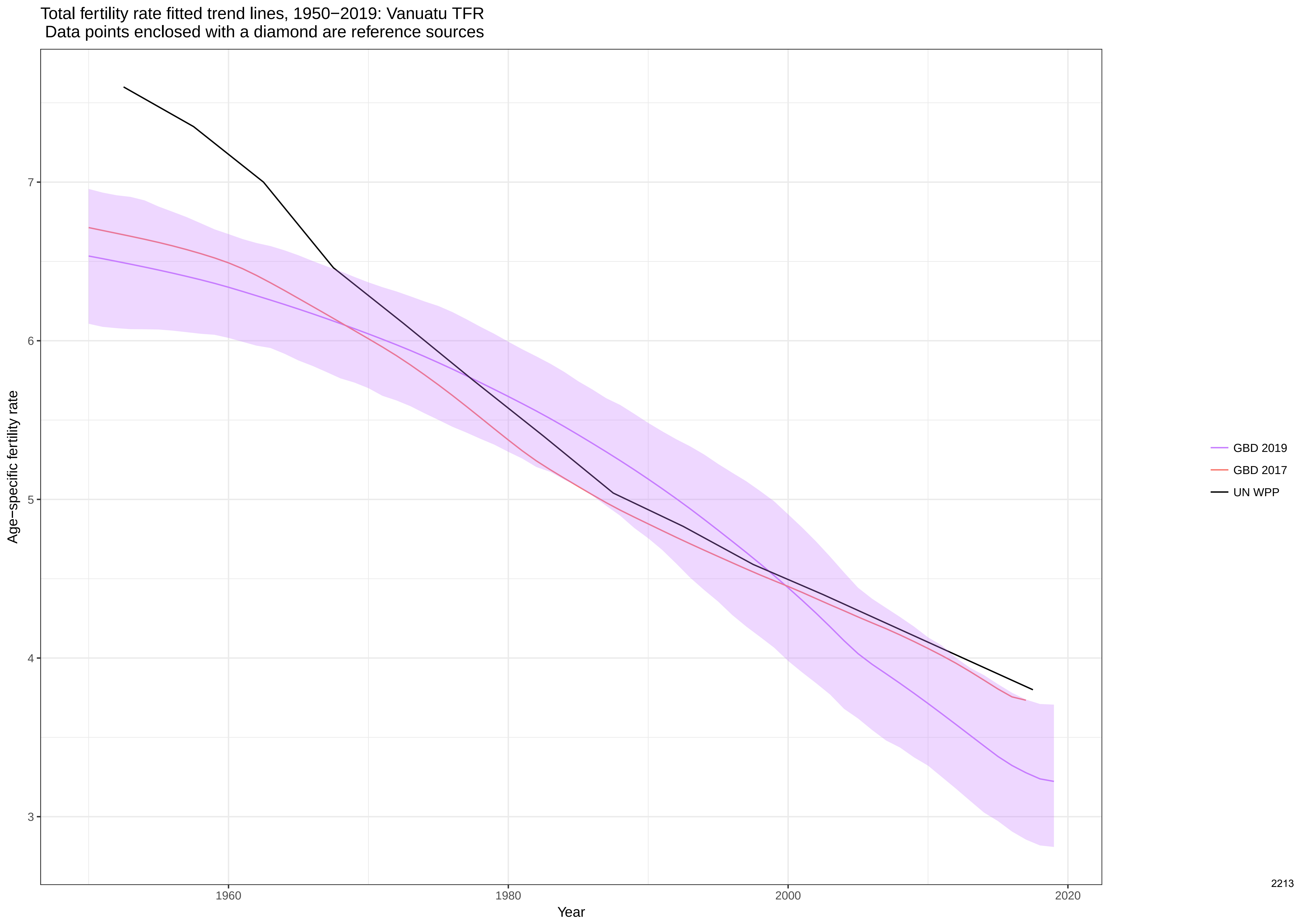
Total fertility rate fitted trend lines, 1950−2019: Vanuatu TFR
Data points enclosed with a diamond are reference sources
Age−specific fertility rate
Year
3
4
5
6
7
1960
1980
2000
2020
GBD 2019
GBD 2017
UN WPP

Age−specific fertility fitted trend lines, 1950−2019: Samoa, Ages: 10−14
Data points enclosed with a diamond are reference sources

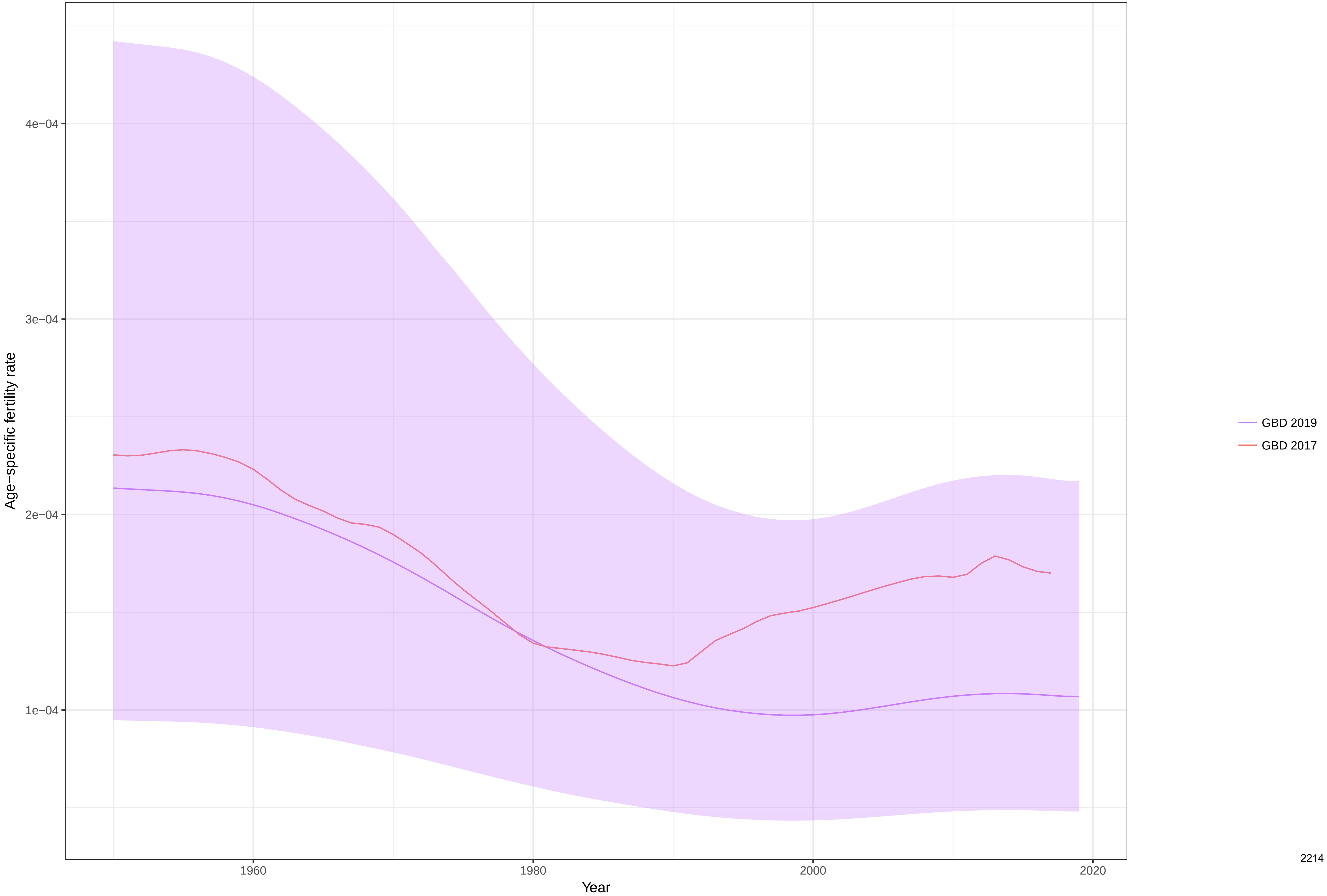

Age−specific fertility fitted trend lines, 1950−2019: Samoa, Ages: 15−19
Data points enclosed with a diamond are reference sources

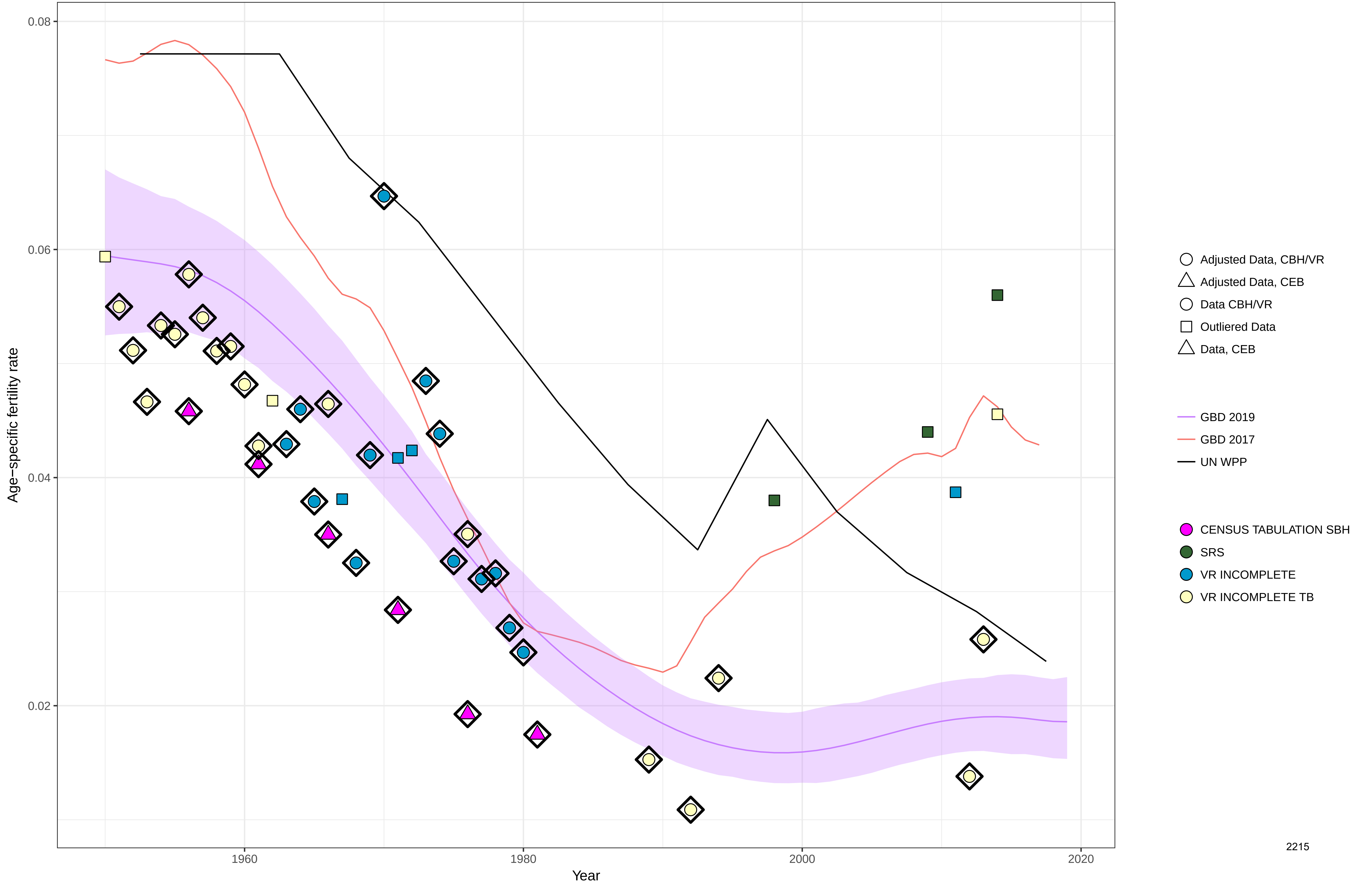

Age−specific fertility fitted trend lines, 1950−2019: Samoa, Ages: 20−24
Data points enclosed with a diamond are reference sources

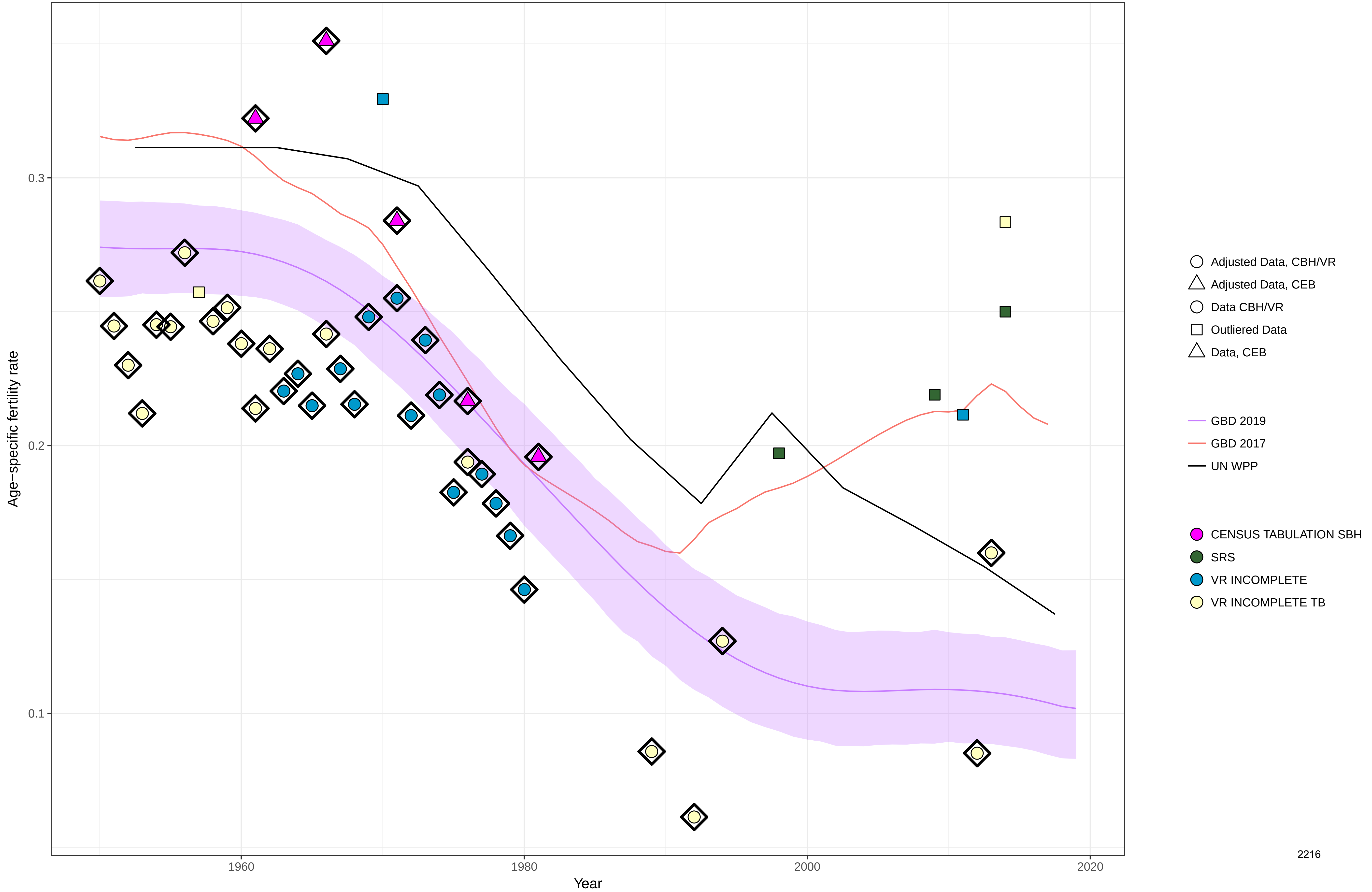

Age−specific fertility fitted trend lines, 1950−2019: Samoa, Ages: 25−29
Data points enclosed with a diamond are reference sources

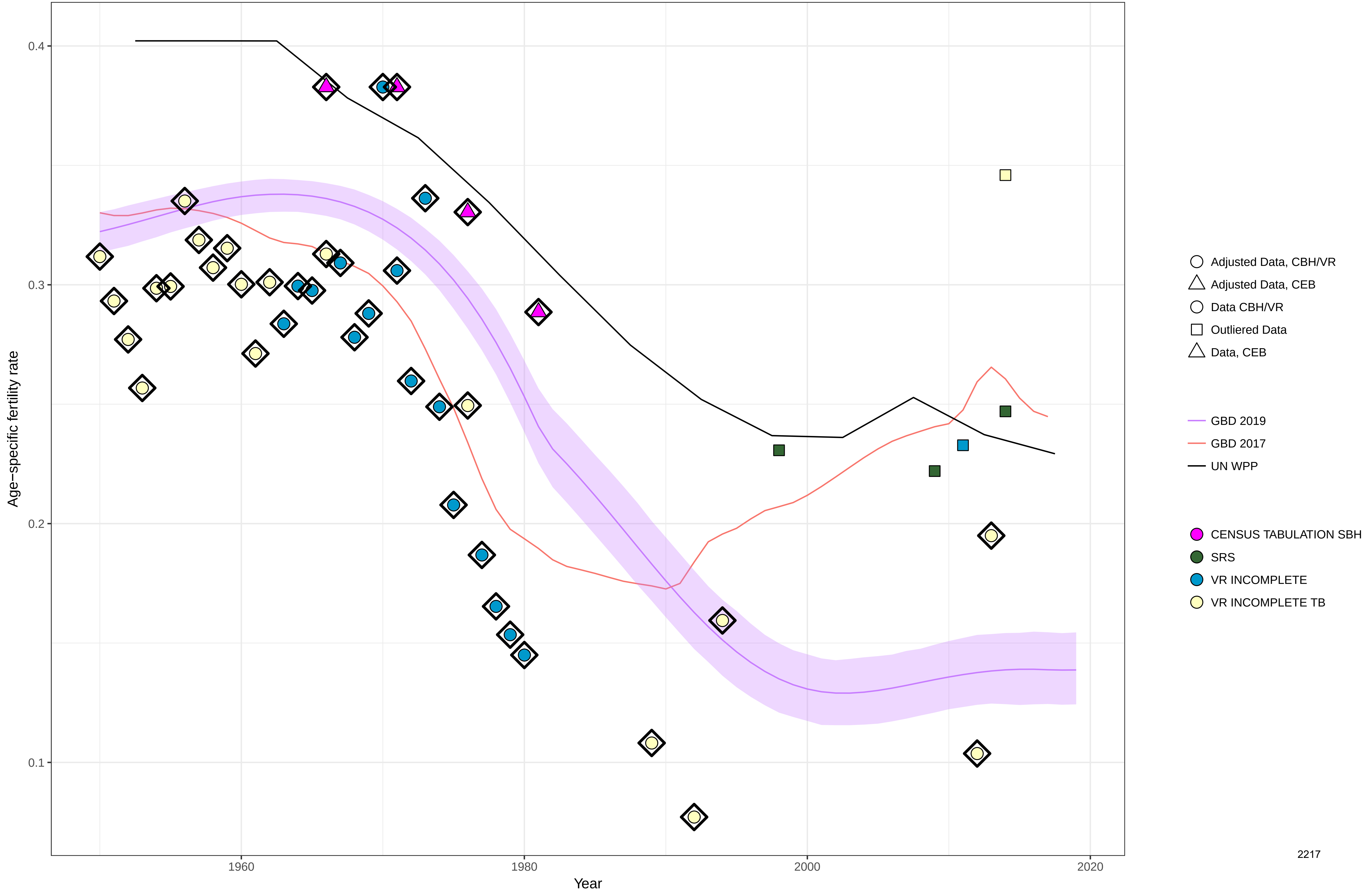

Age−specific fertility fitted trend lines, 1950−2019: Samoa, Ages: 30−34
Data points enclosed with a diamond are reference sources

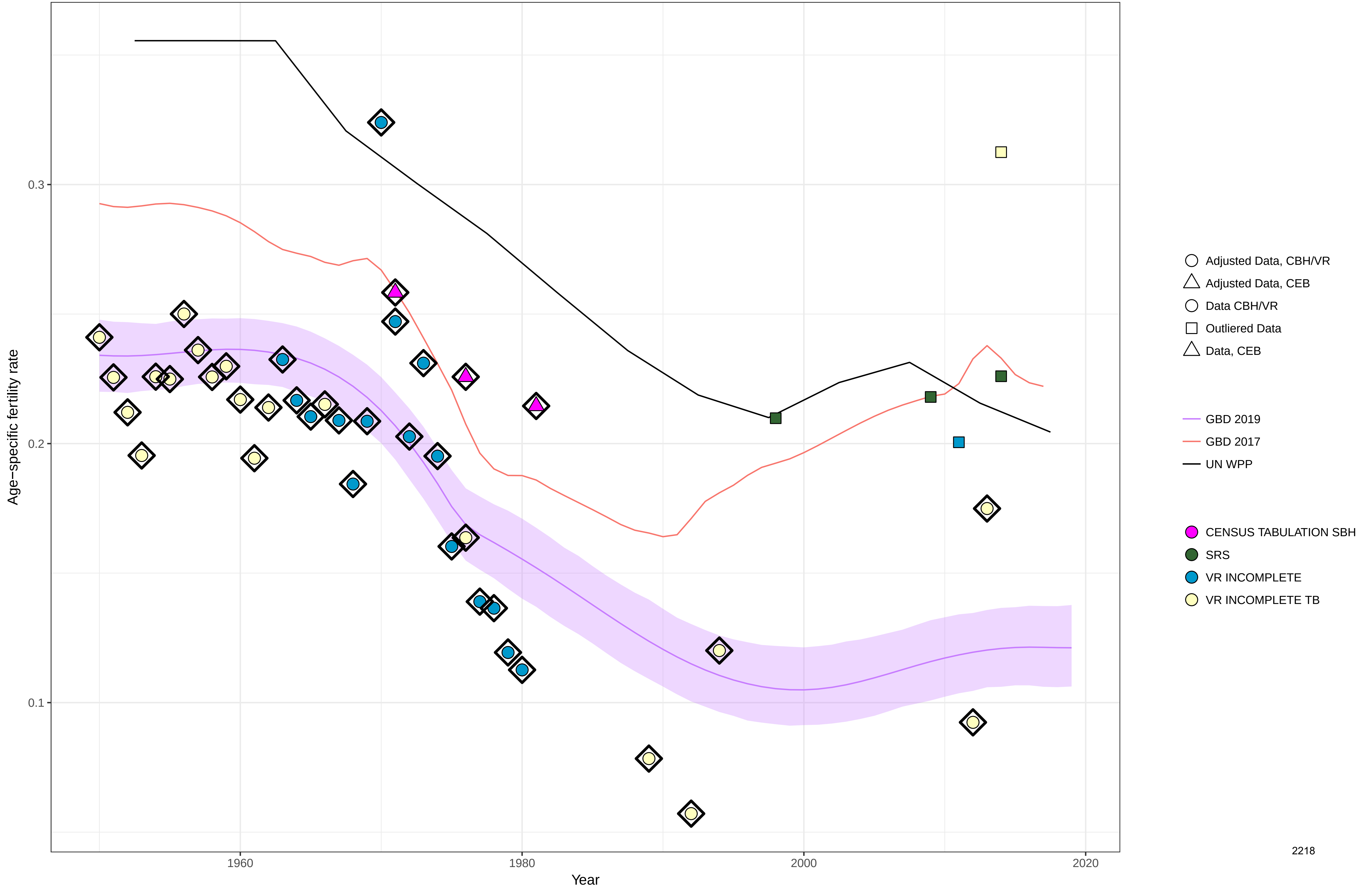

Age−specific fertility fitted trend lines, 1950−2019: Samoa, Ages: 35−39
Data points enclosed with a diamond are reference sources

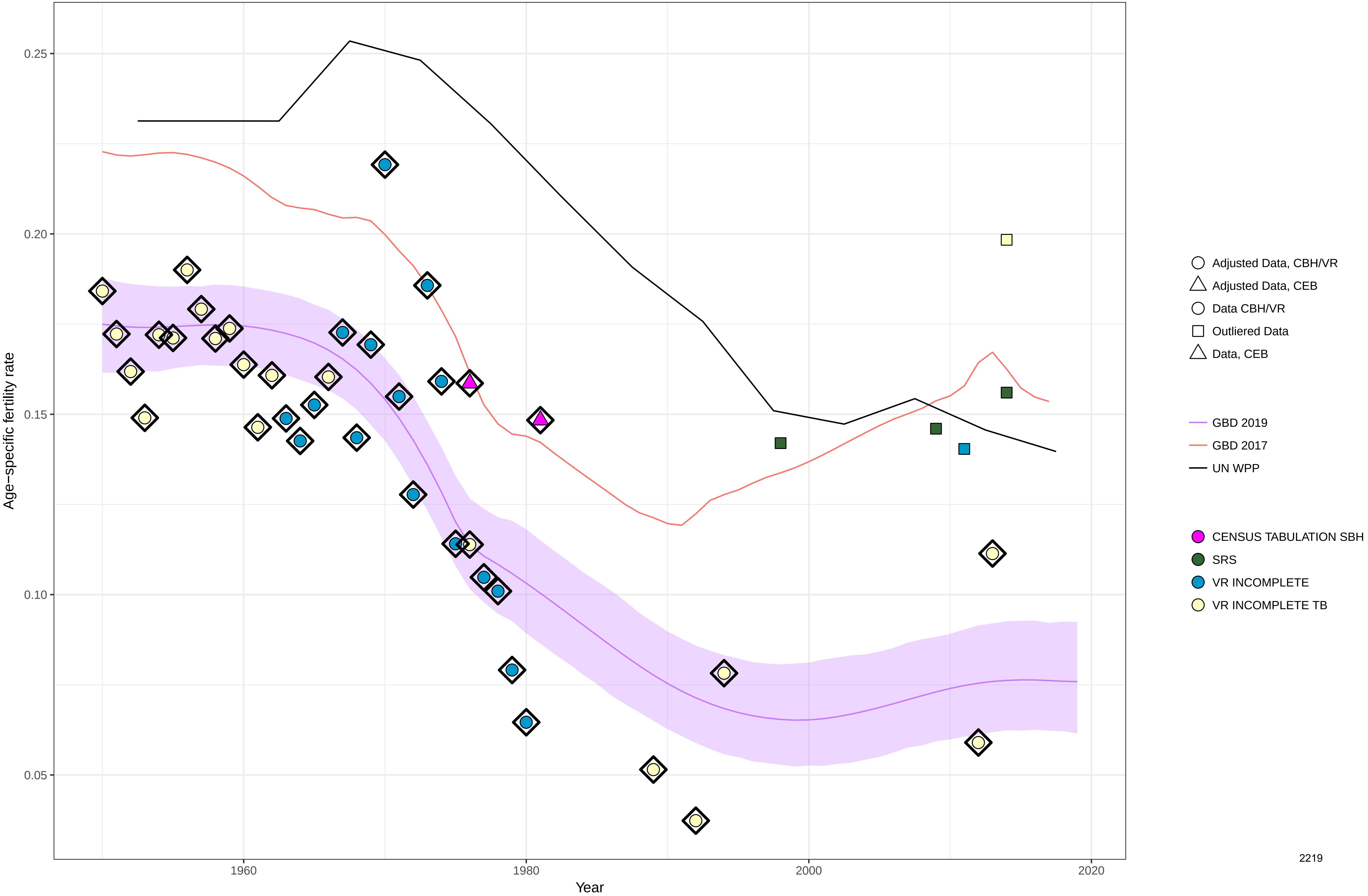

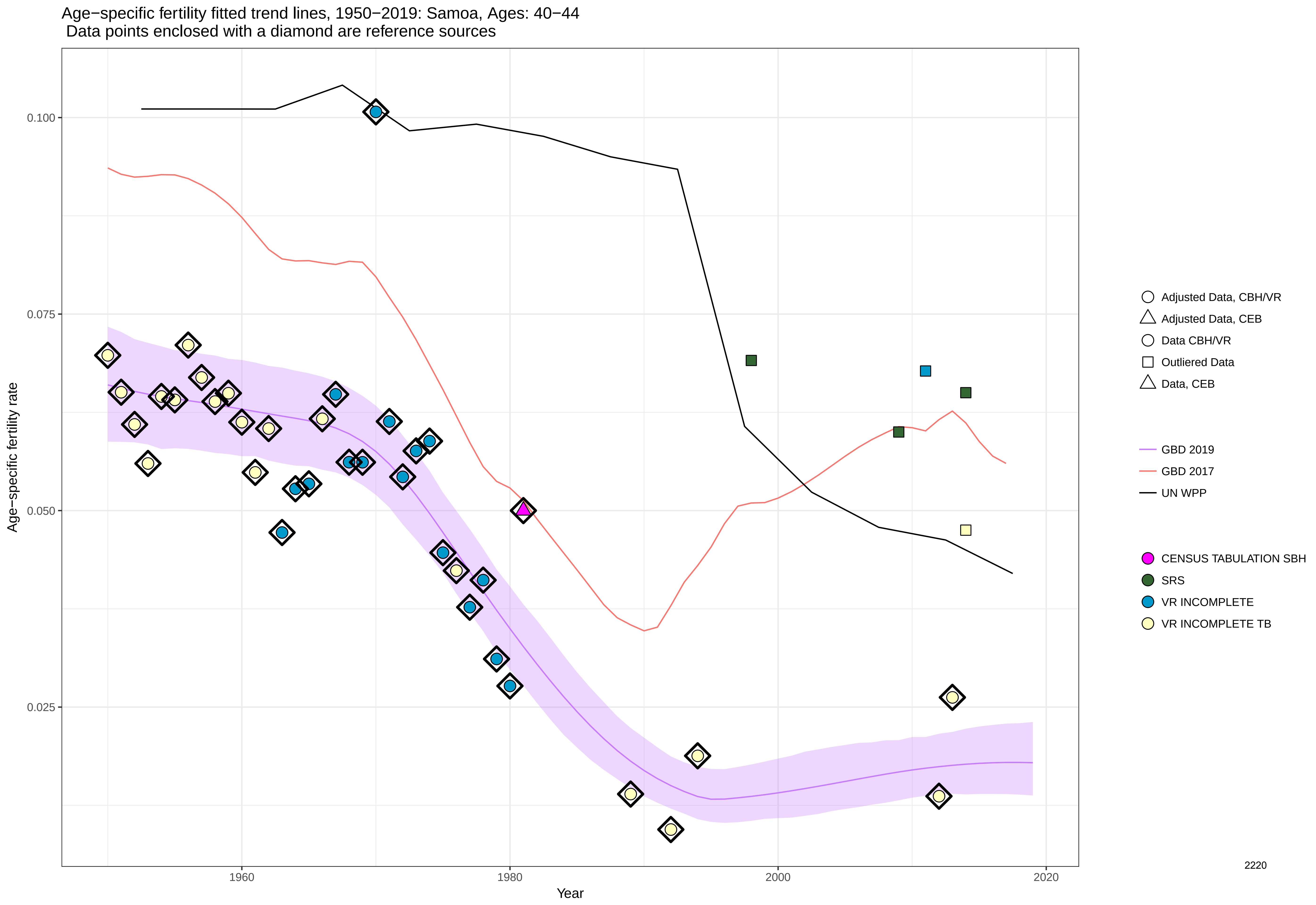
Age−specific fertility fitted trend lines, 1950−2019: Samoa, Ages: 40−44
Data points enclosed with a diamond are reference sources
0.100
0.075
0.050
0.025
Age−specific fertility rate
1960
1980
2000
2020
Year
Adjusted Data, CBH/VR
Adjusted Data, CEB
Data CBH/VR
Outliered Data
Data, CEB
GBD 2019
GBD 2017
UN WPP
CENSUS TABULATION SBH
SRS
VR INCOMPLETE
VR INCOMPLETE TB

Age−specific fertility fitted trend lines, 1950−2019: Samoa, Ages: 45−49
Data points enclosed with a diamond are reference sources

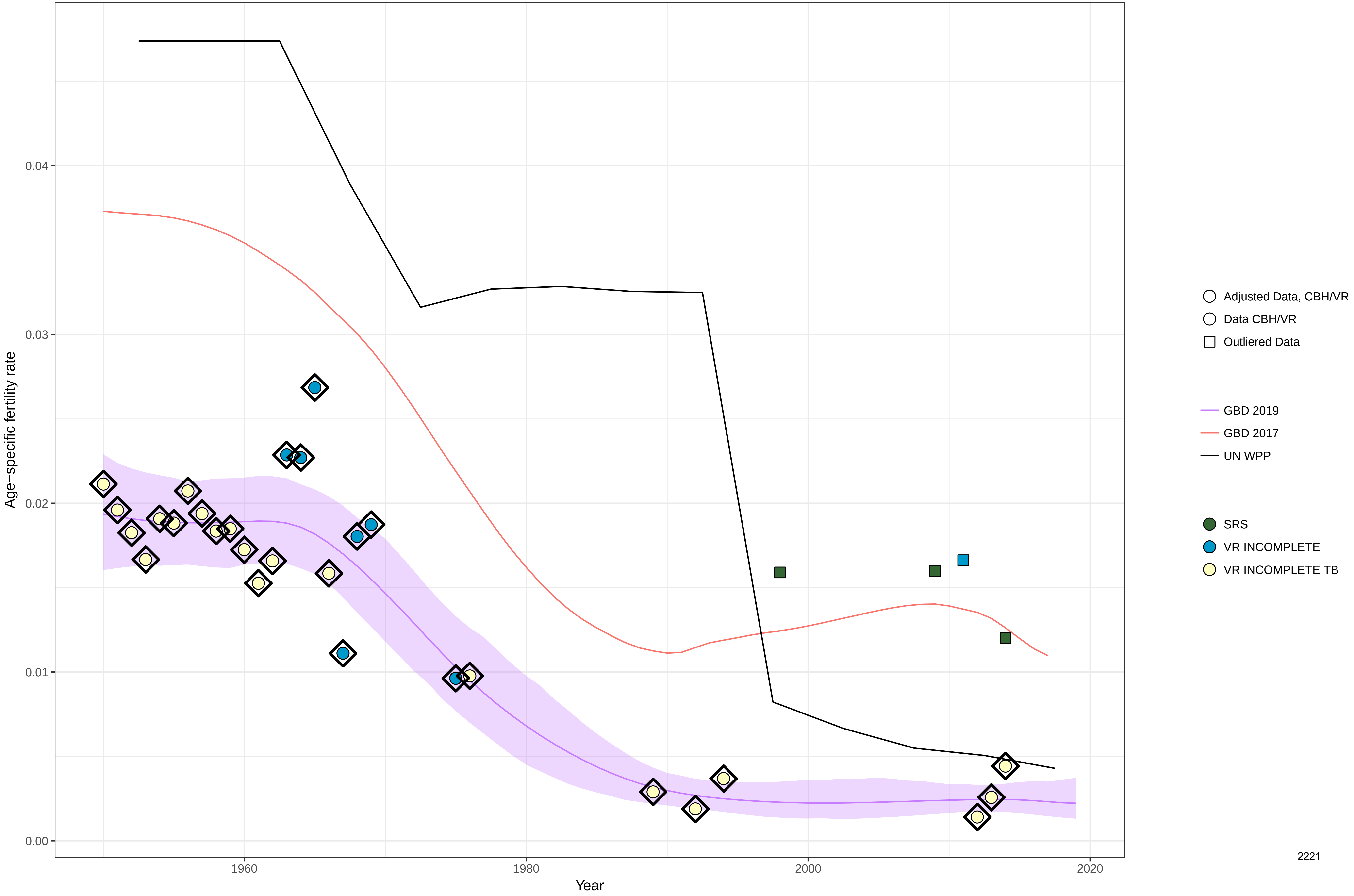

Age−specific fertility fitted trend lines, 1950−2019: Samoa, Ages: 50−54
 Data points enclosed with a diamond are reference sources

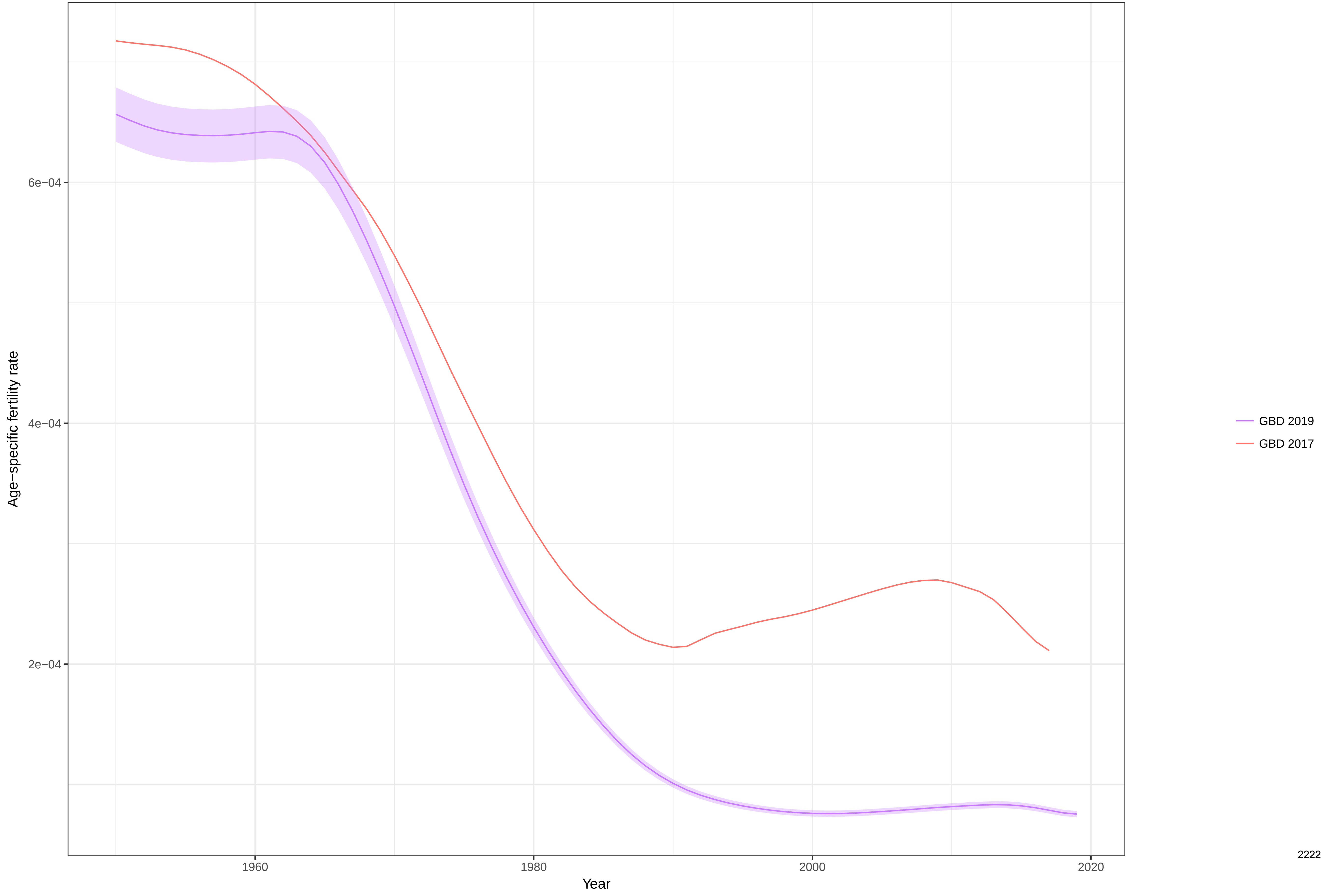

Total fertility rate fitted trend lines, 1950−2019: Samoa TFR
Data points enclosed with a diamond are reference sources

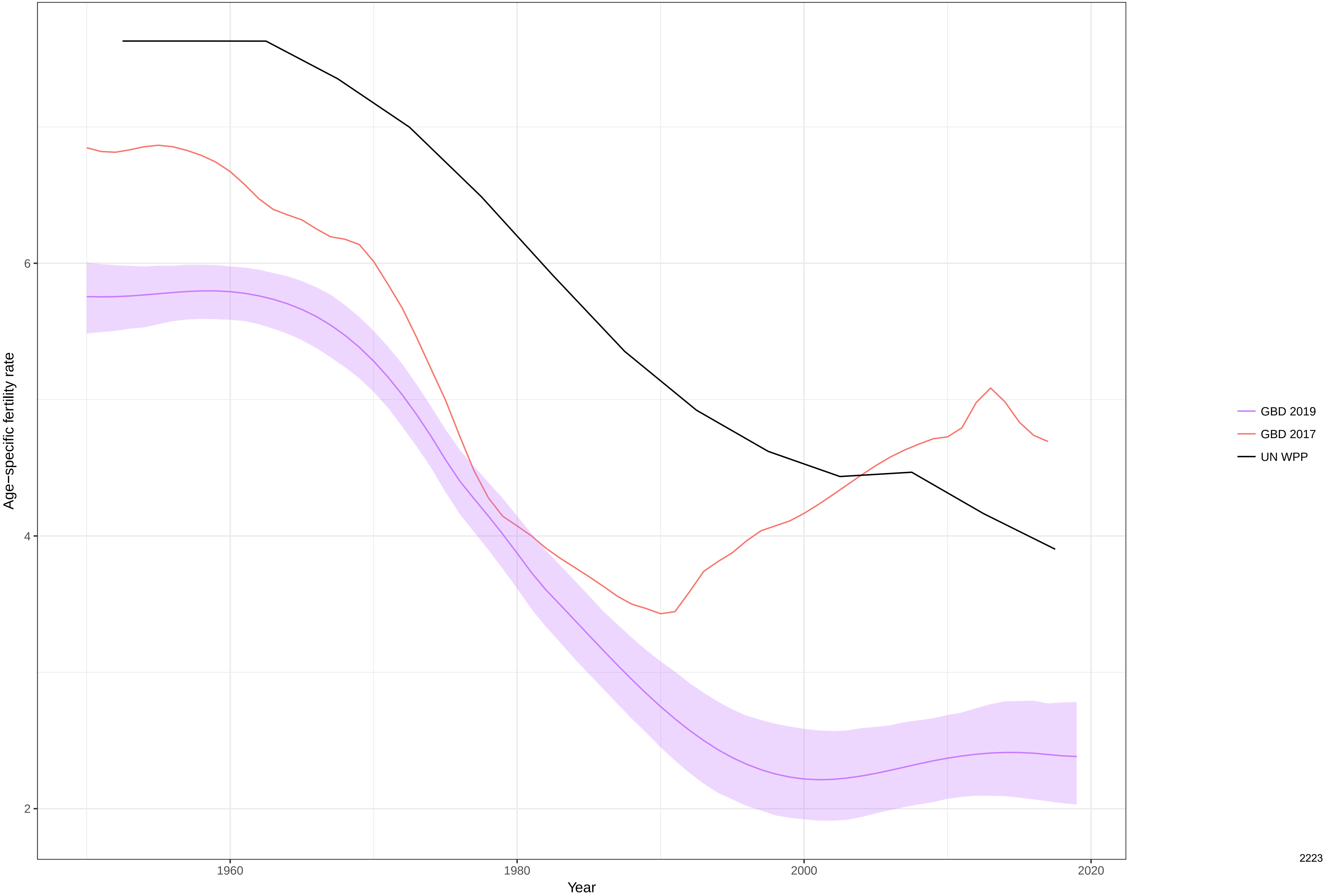

Age−specific fertility fitted trend lines, 1950−2019: Yemen, Ages: 10−14
Data points enclosed with a diamond are reference sources

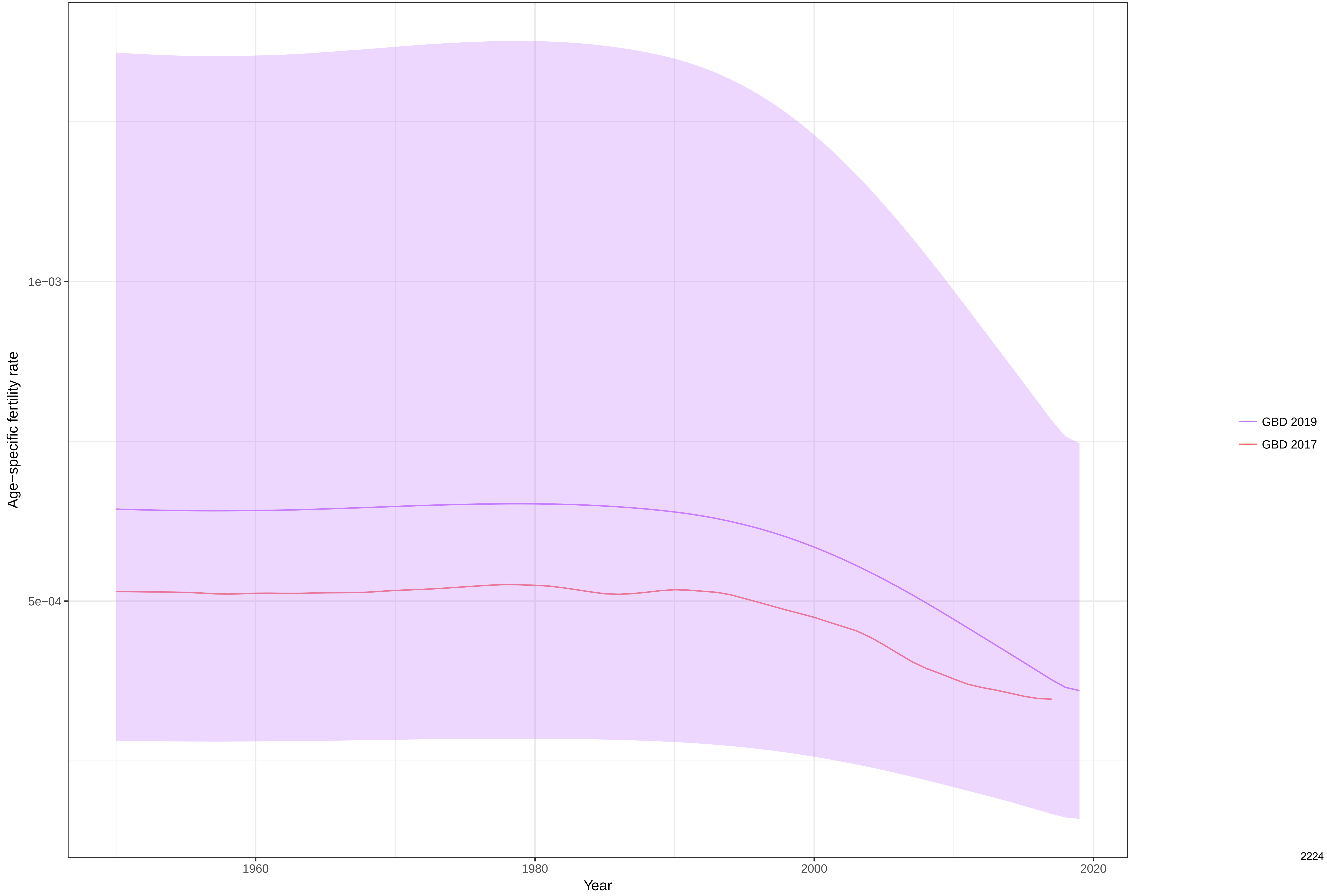

Age−specific fertility fitted trend lines, 1950−2019: Yemen, Ages: 15−19
Data points enclosed with a diamond are reference sources

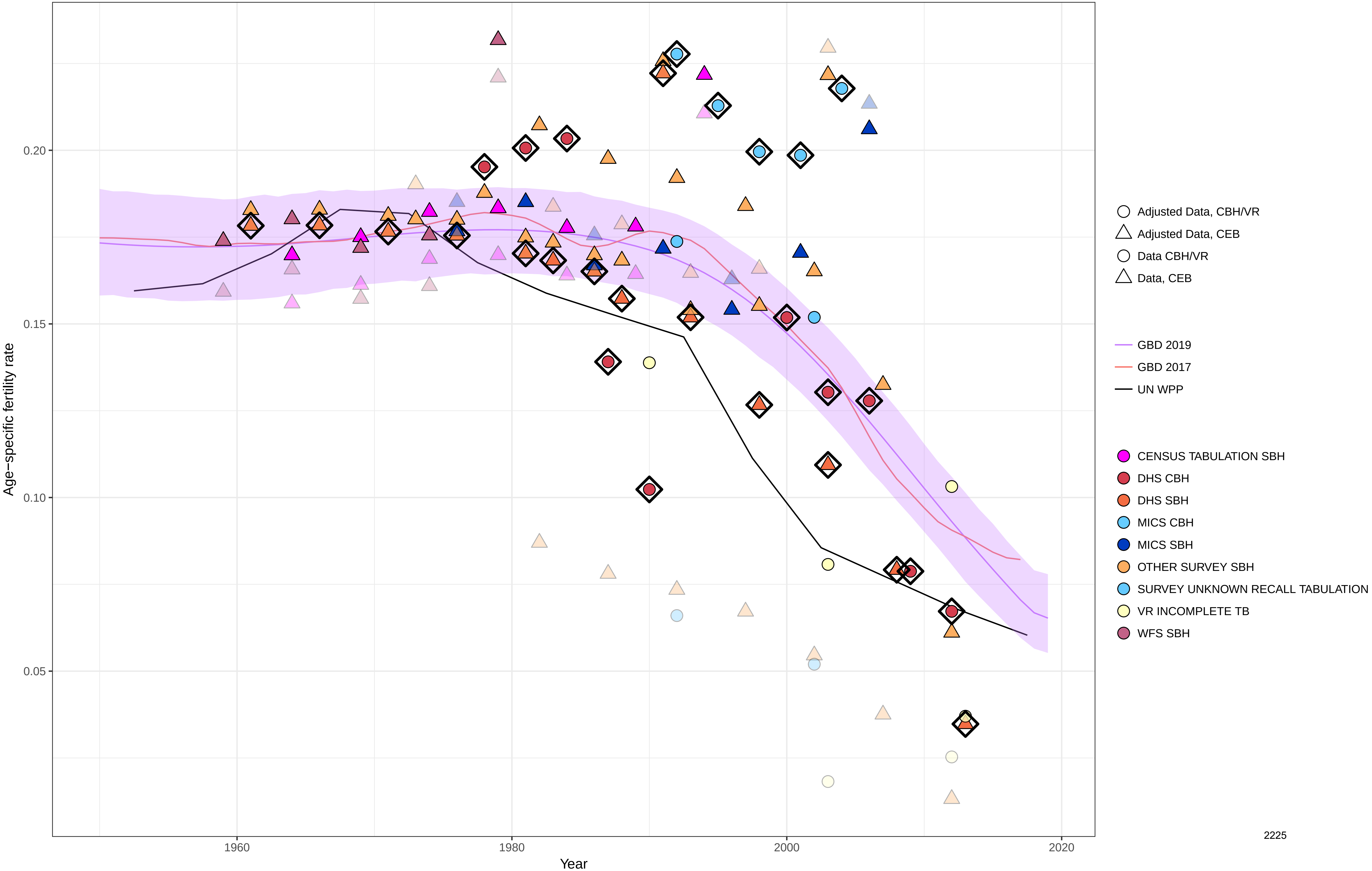

Age−specific fertility fitted trend lines, 1950−2019: Yemen, Ages: 20−24
Data points enclosed with a diamond are reference sources

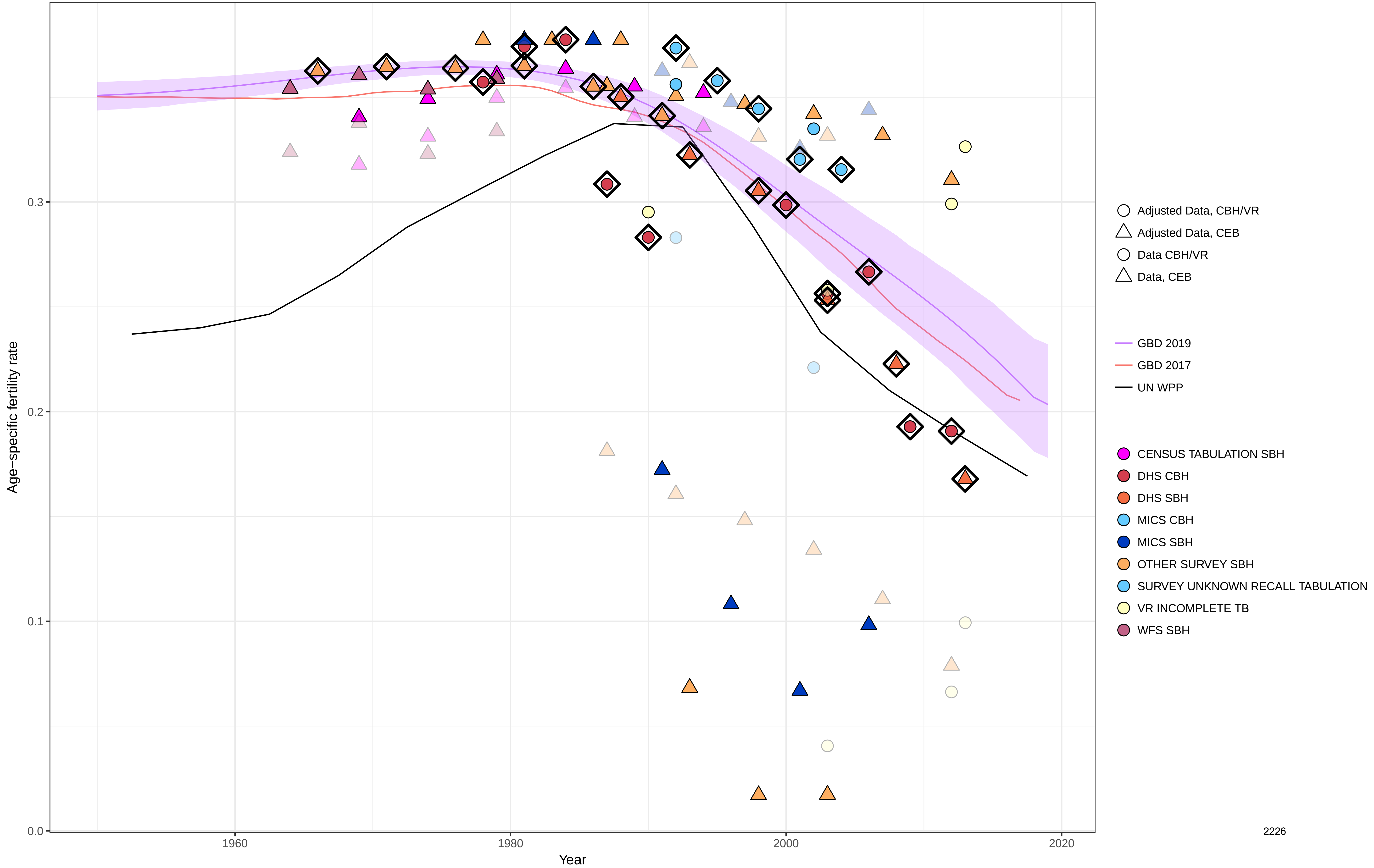

Age−specific fertility fitted trend lines, 1950−2019: Yemen, Ages: 25−29
Data points enclosed with a diamond are reference sources

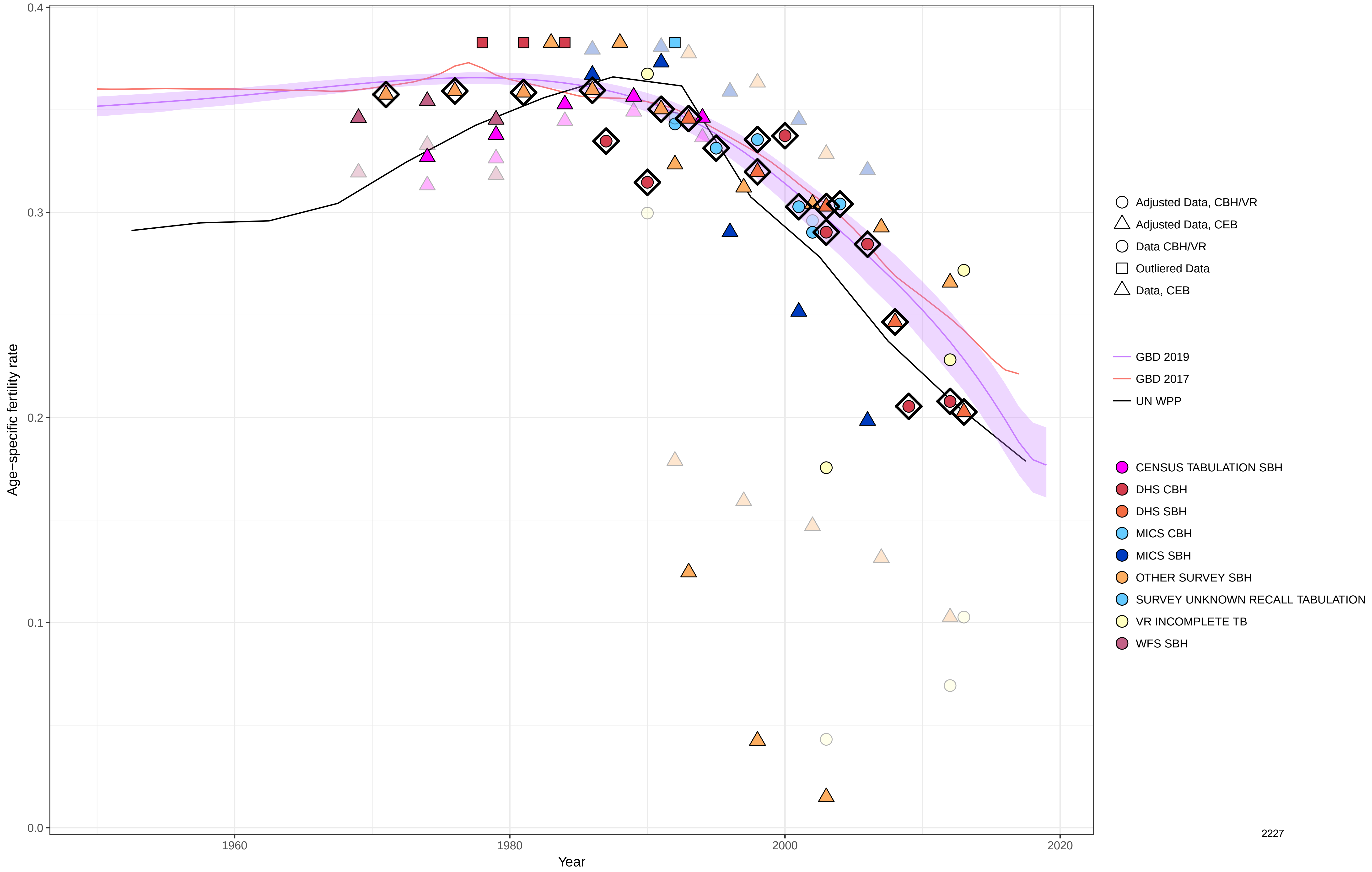

Age−specific fertility fitted trend lines, 1950−2019: Yemen, Ages: 30−34
Data points enclosed with a diamond are reference sources

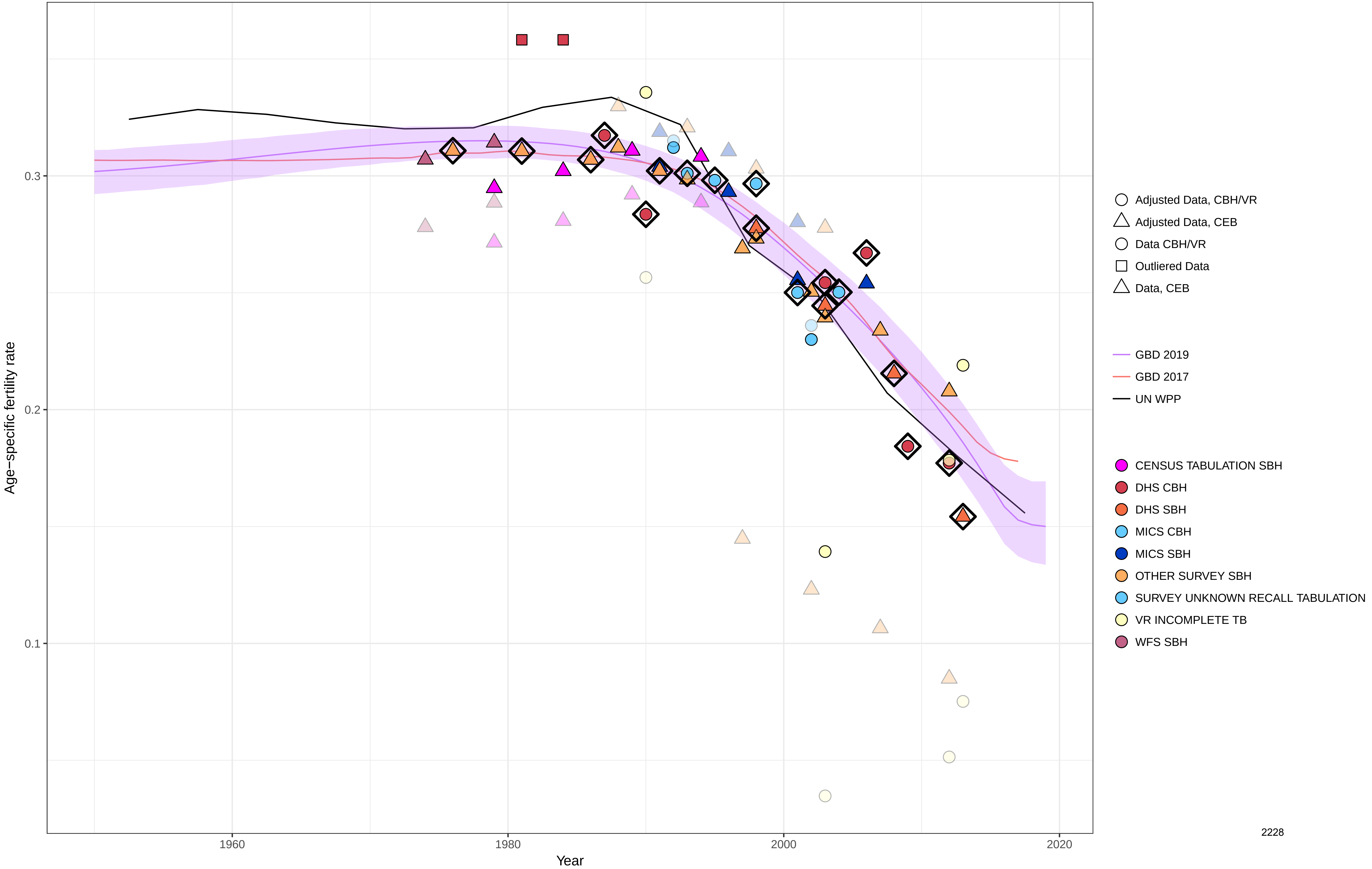

Age−specific fertility fitted trend lines, 1950−2019: Yemen, Ages: 35−39
Data points enclosed with a diamond are reference sources

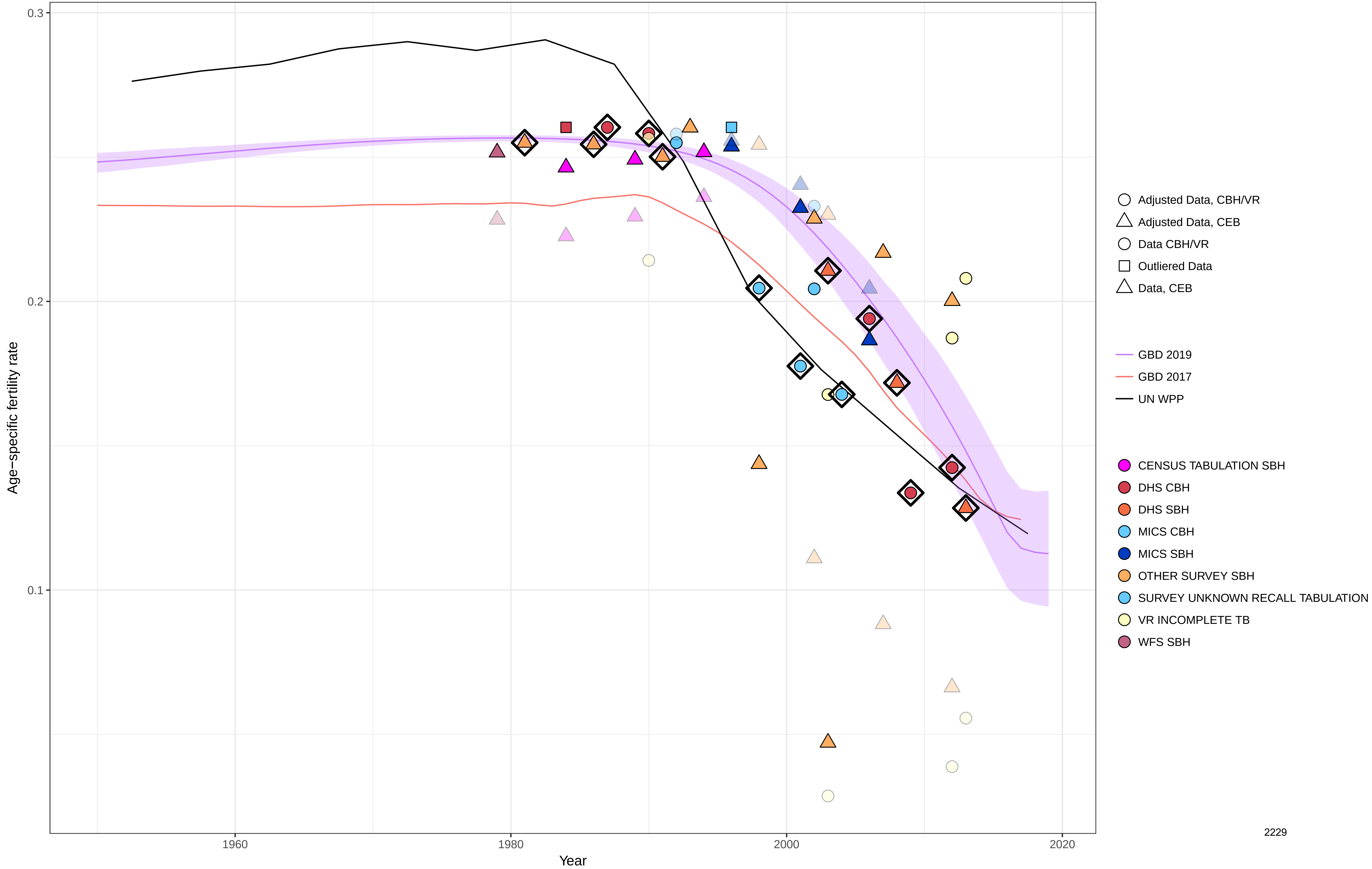

Age−specific fertility fitted trend lines, 1950−2019: Yemen, Ages: 40−44
Data points enclosed with a diamond are reference sources

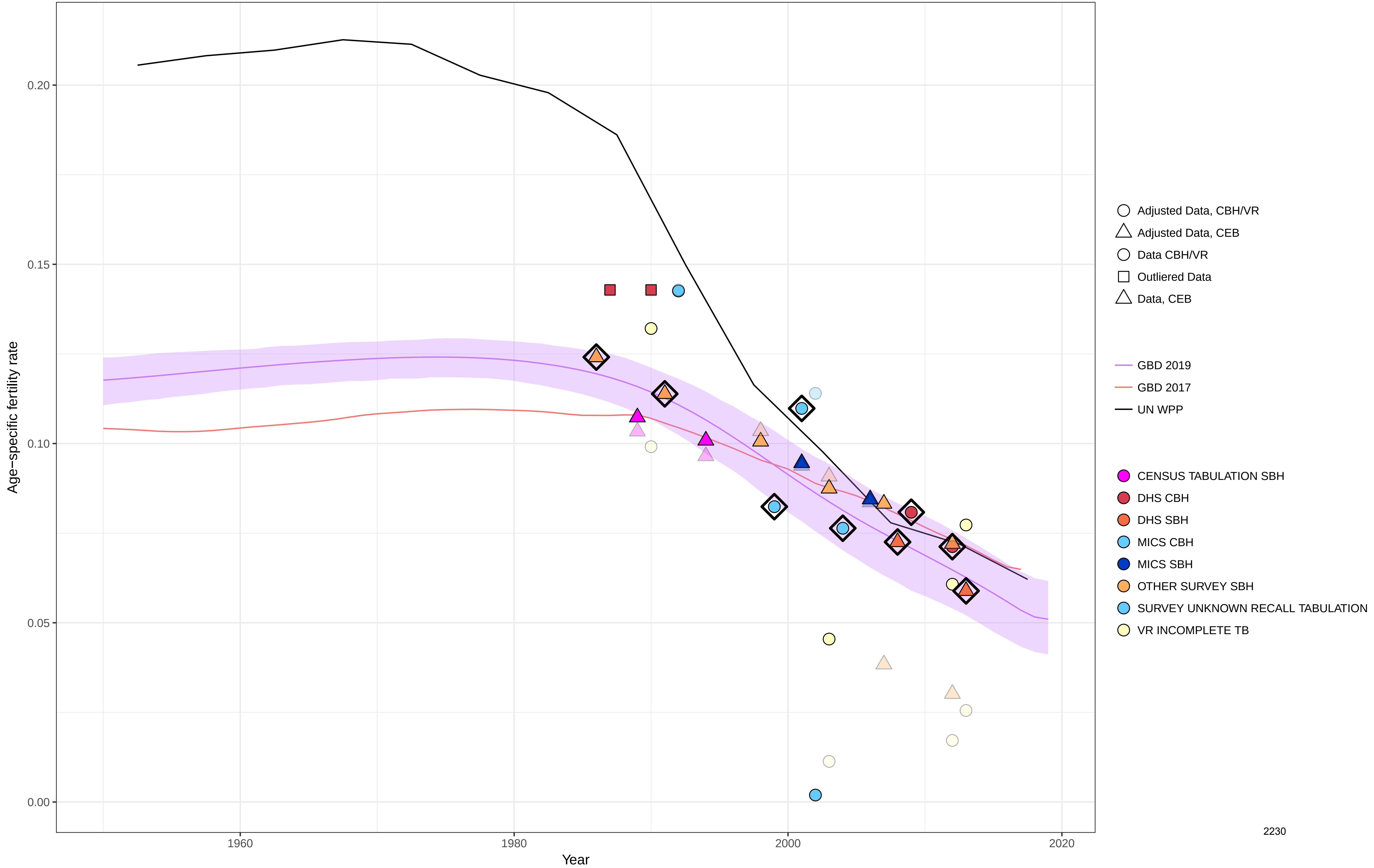

Age−specific fertility fitted trend lines, 1950−2019: Yemen, Ages: 45−49
Data points enclosed with a diamond are reference sources

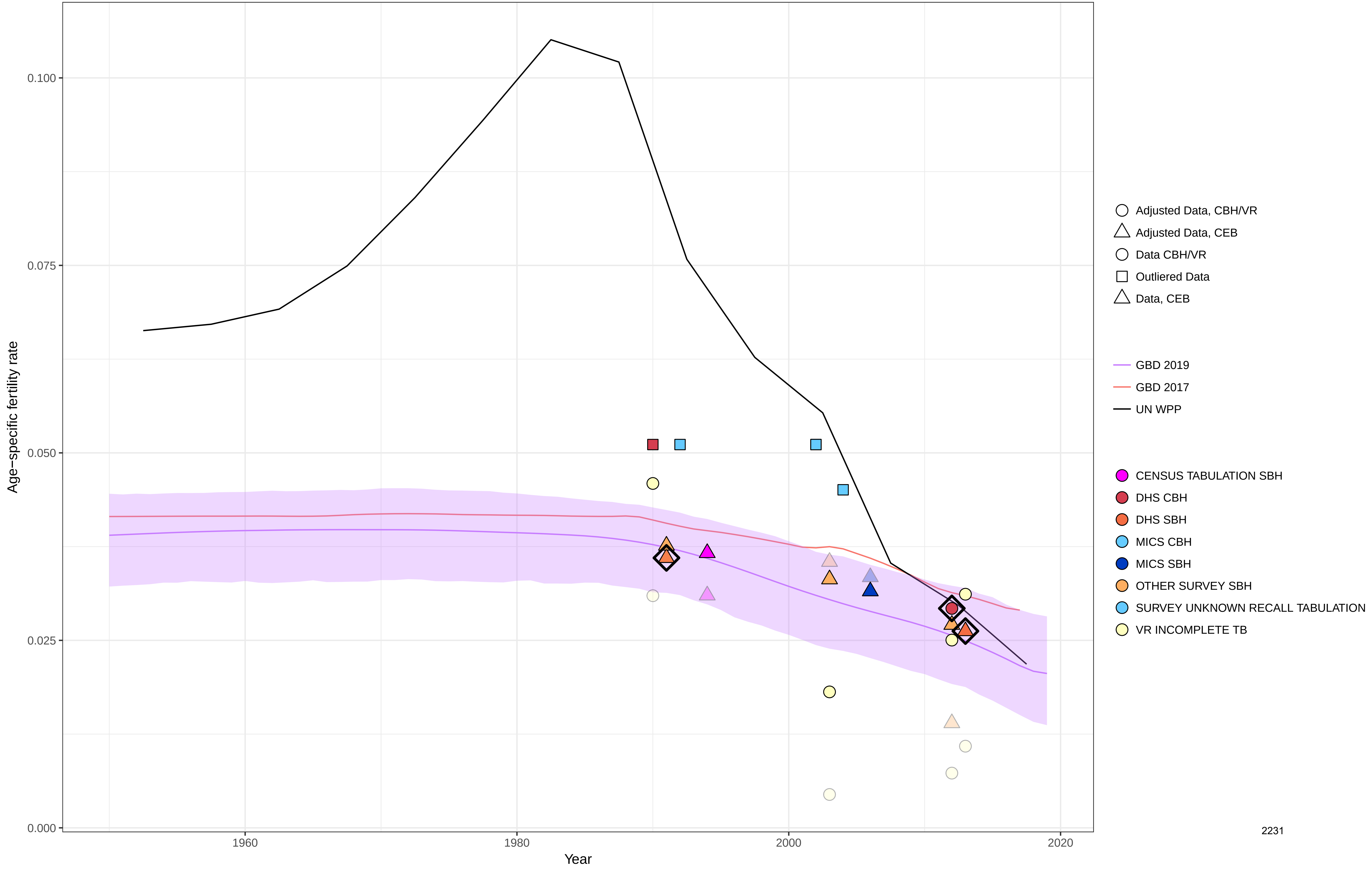

Age−specific fertility fitted trend lines, 1950−2019: Yemen, Ages: 50−54
Data points enclosed with a diamond are reference sources

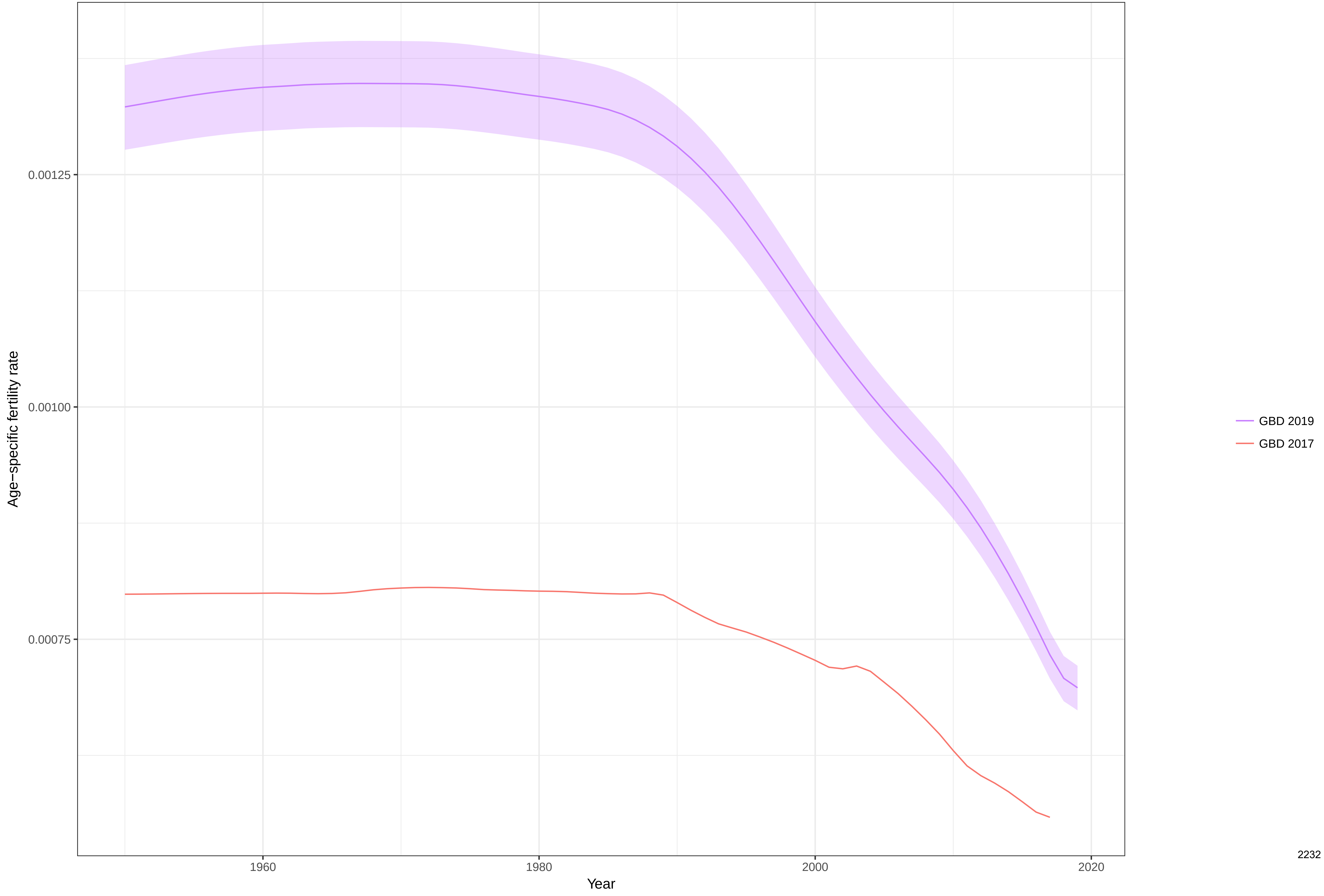

Total fertility rate fitted trend lines, 1950−2019: Yemen TFR
Data points enclosed with a diamond are reference sources

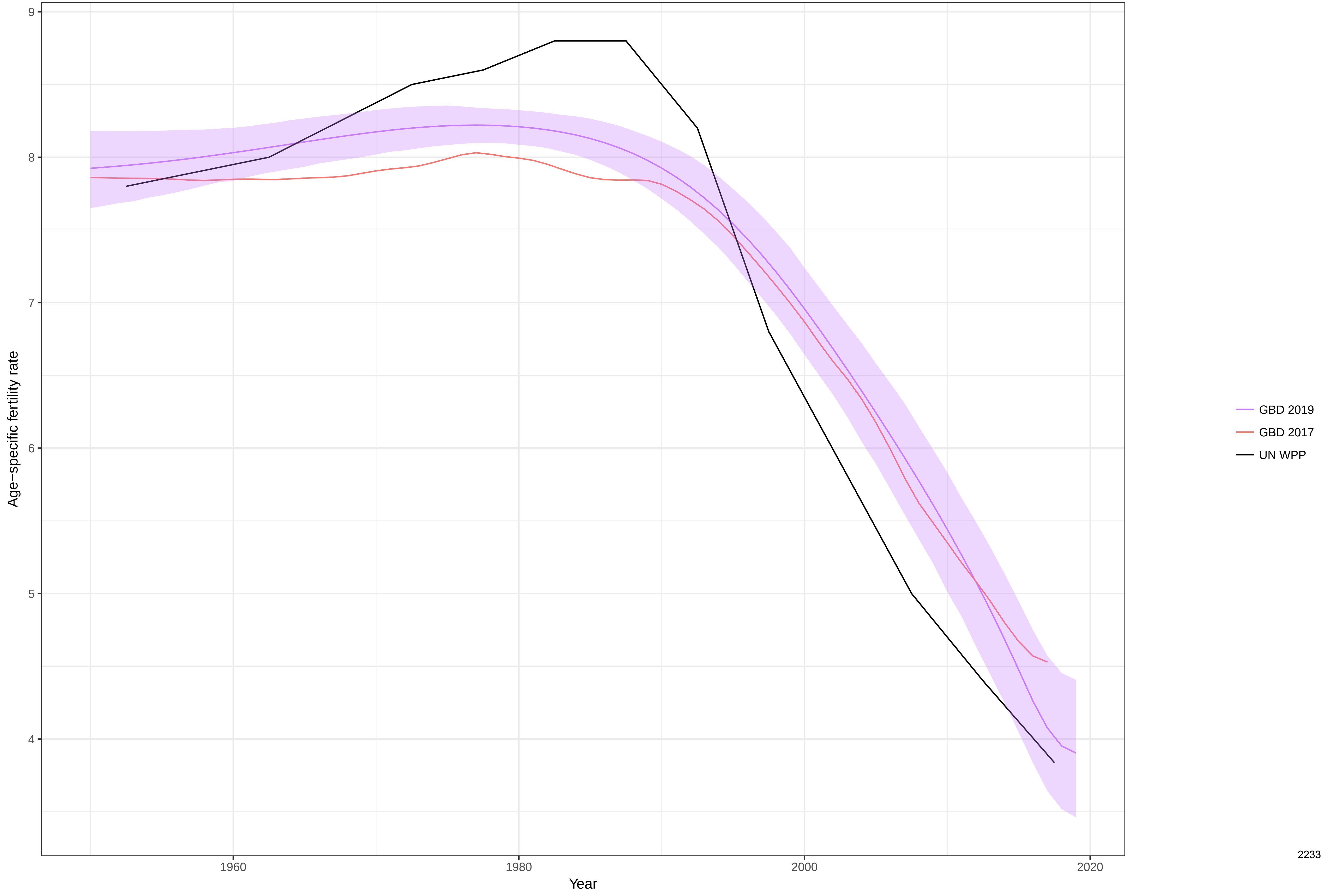

Age−specific fertility fitted trend lines, 1950−2019: South Africa, Ages: 10−14
Data points enclosed with a diamond are reference sources

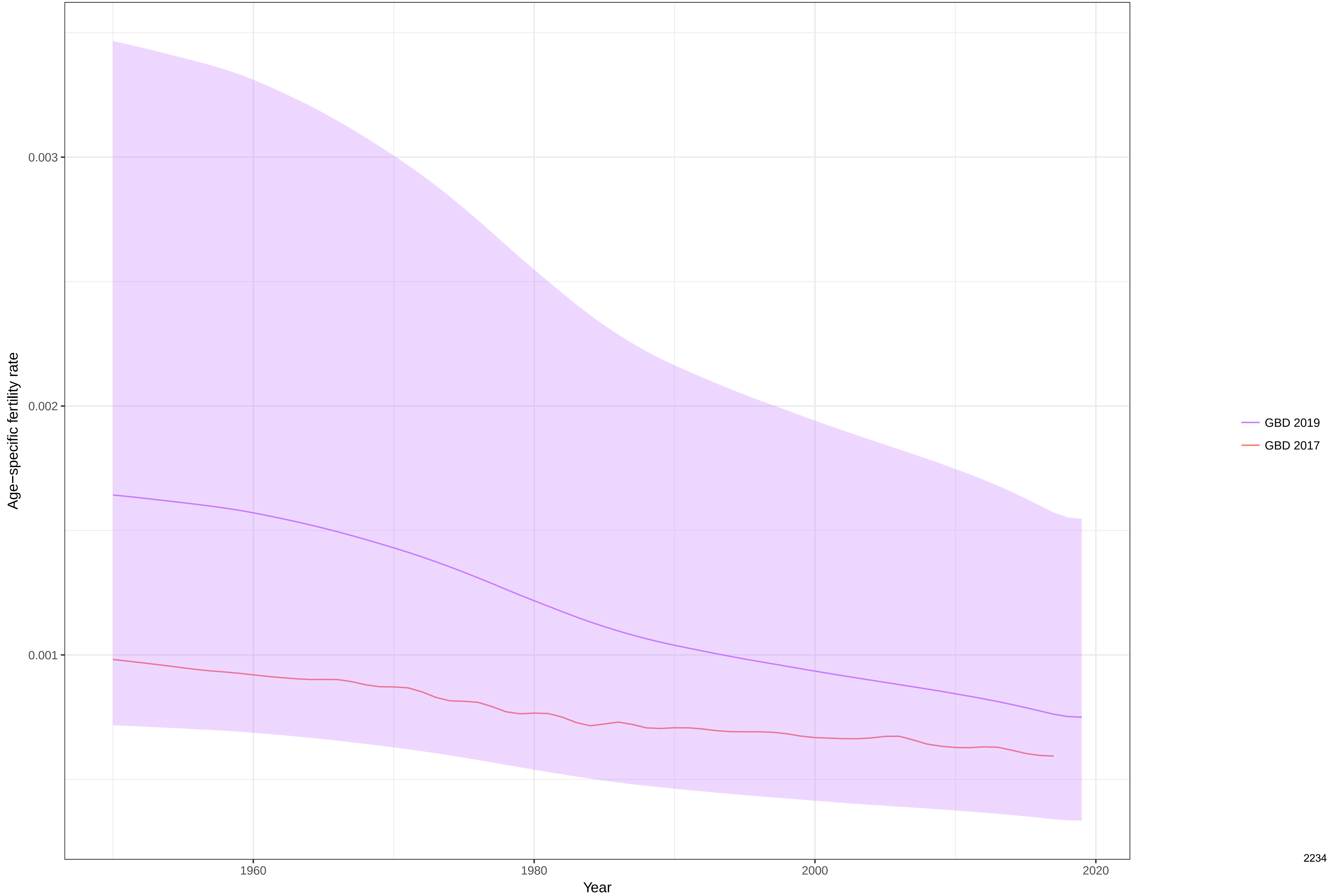

Age−specific fertility fitted trend lines, 1950−2019: South Africa, Ages: 15−19
Data points enclosed with a diamond are reference sources

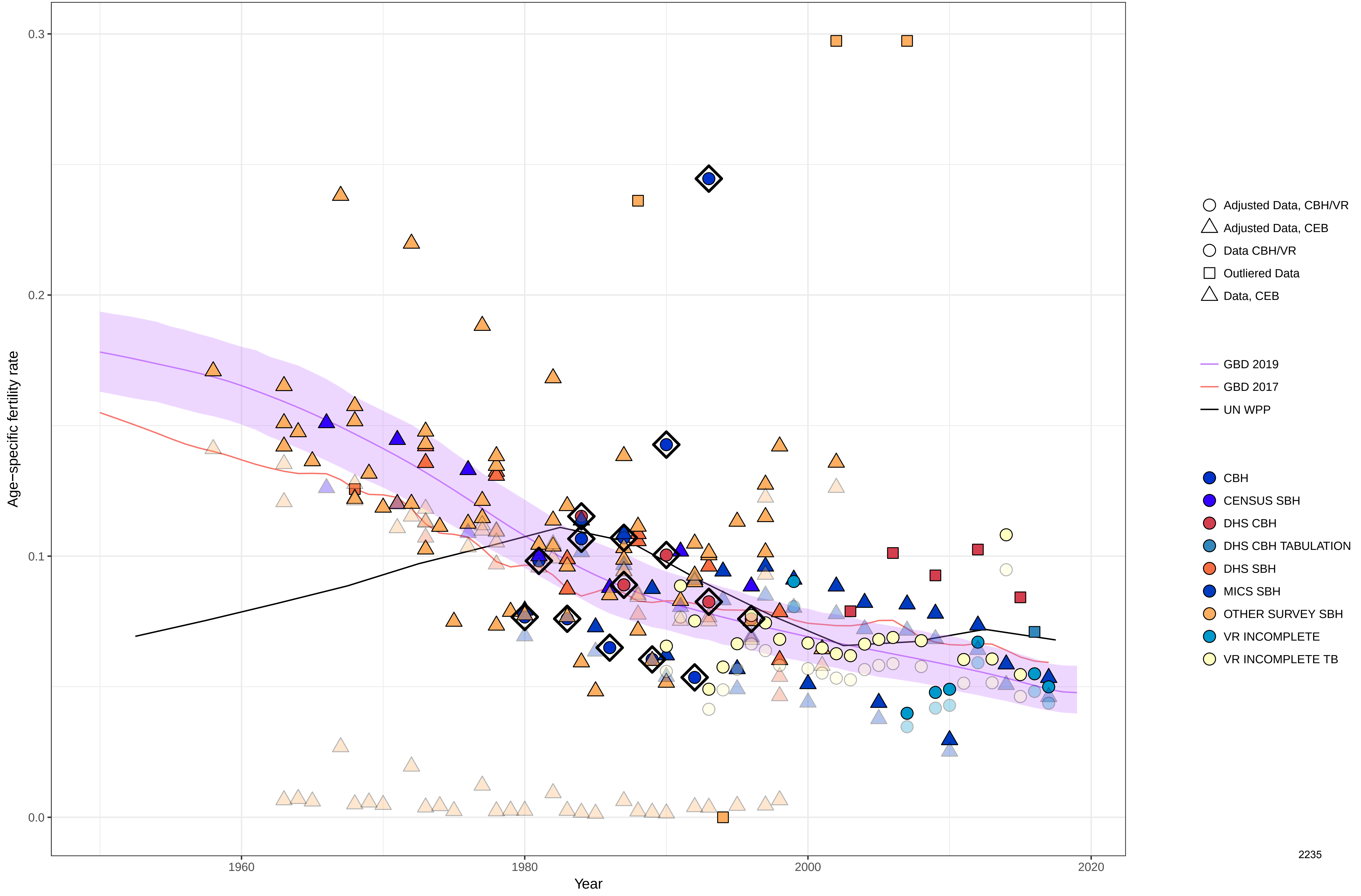

Age−specific fertility fitted trend lines, 1950−2019: South Africa, Ages: 20−24
Data points enclosed with a diamond are reference sources

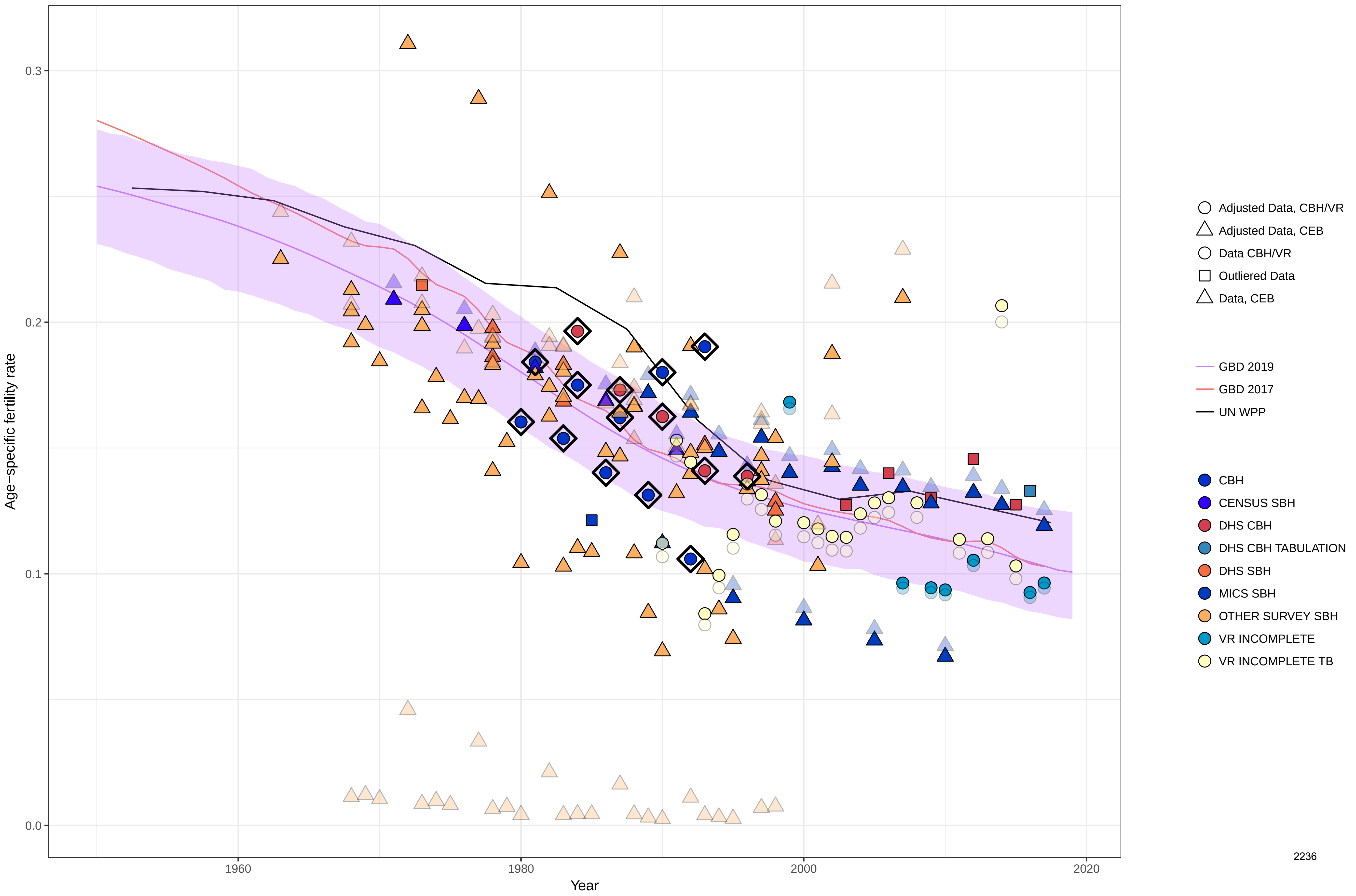

Age−specific fertility fitted trend lines, 1950−2019: South Africa, Ages: 25−29
Data points enclosed with a diamond are reference sources

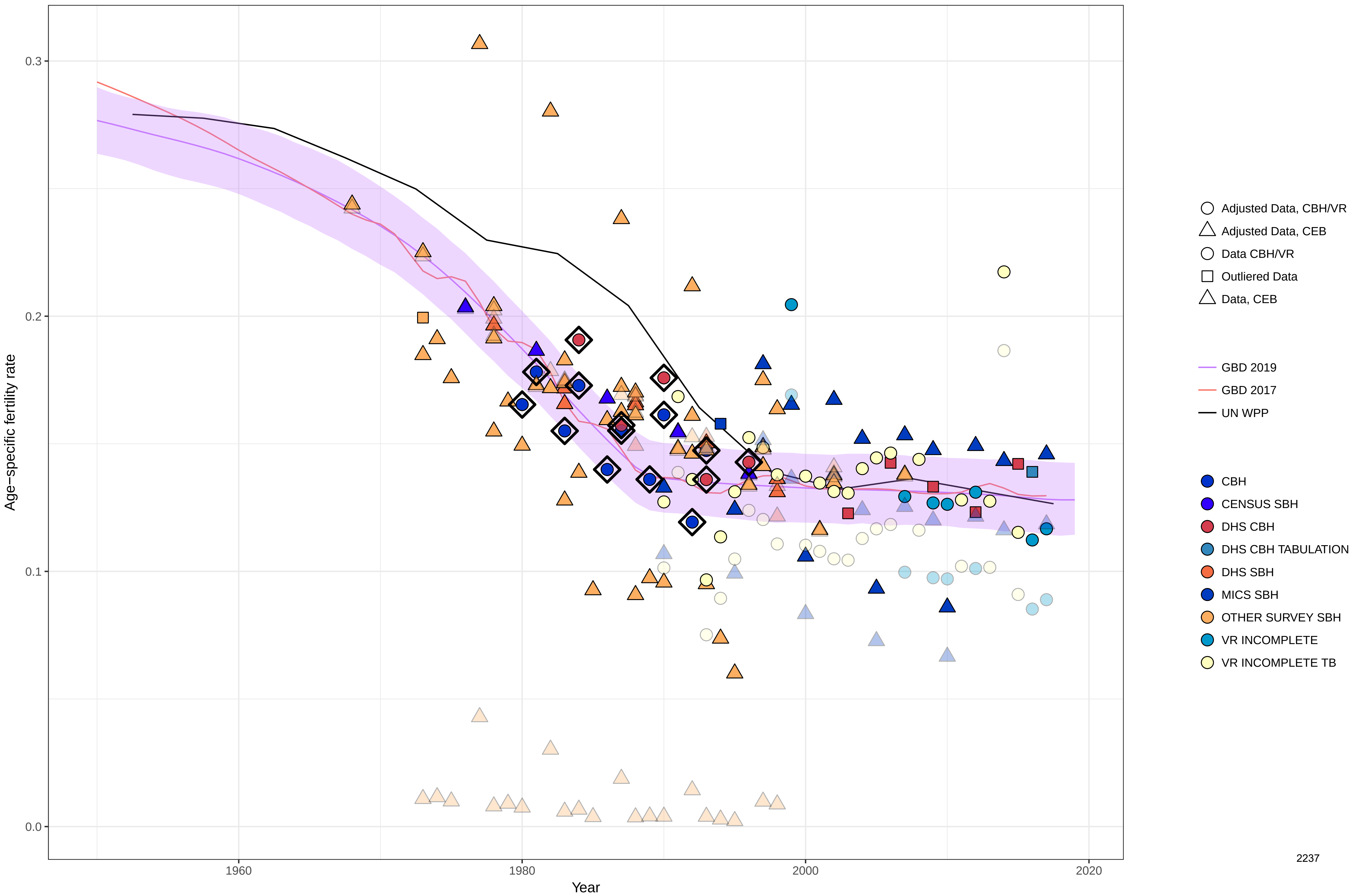

Age−specific fertility fitted trend lines, 1950−2019: South Africa, Ages: 30−34
Data points enclosed with a diamond are reference sources

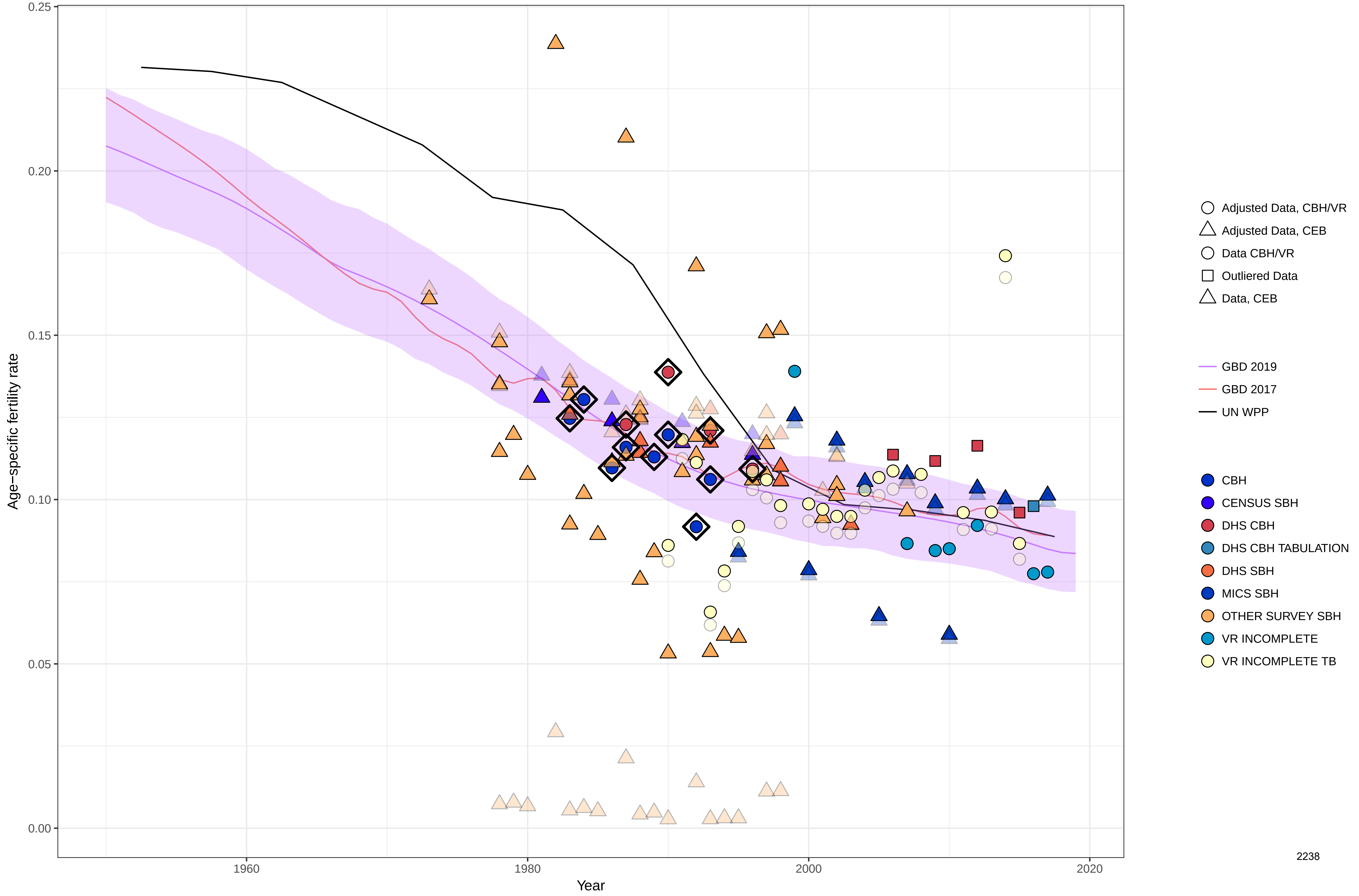

Age−specific fertility fitted trend lines, 1950−2019: South Africa, Ages: 35−39
Data points enclosed with a diamond are reference sources

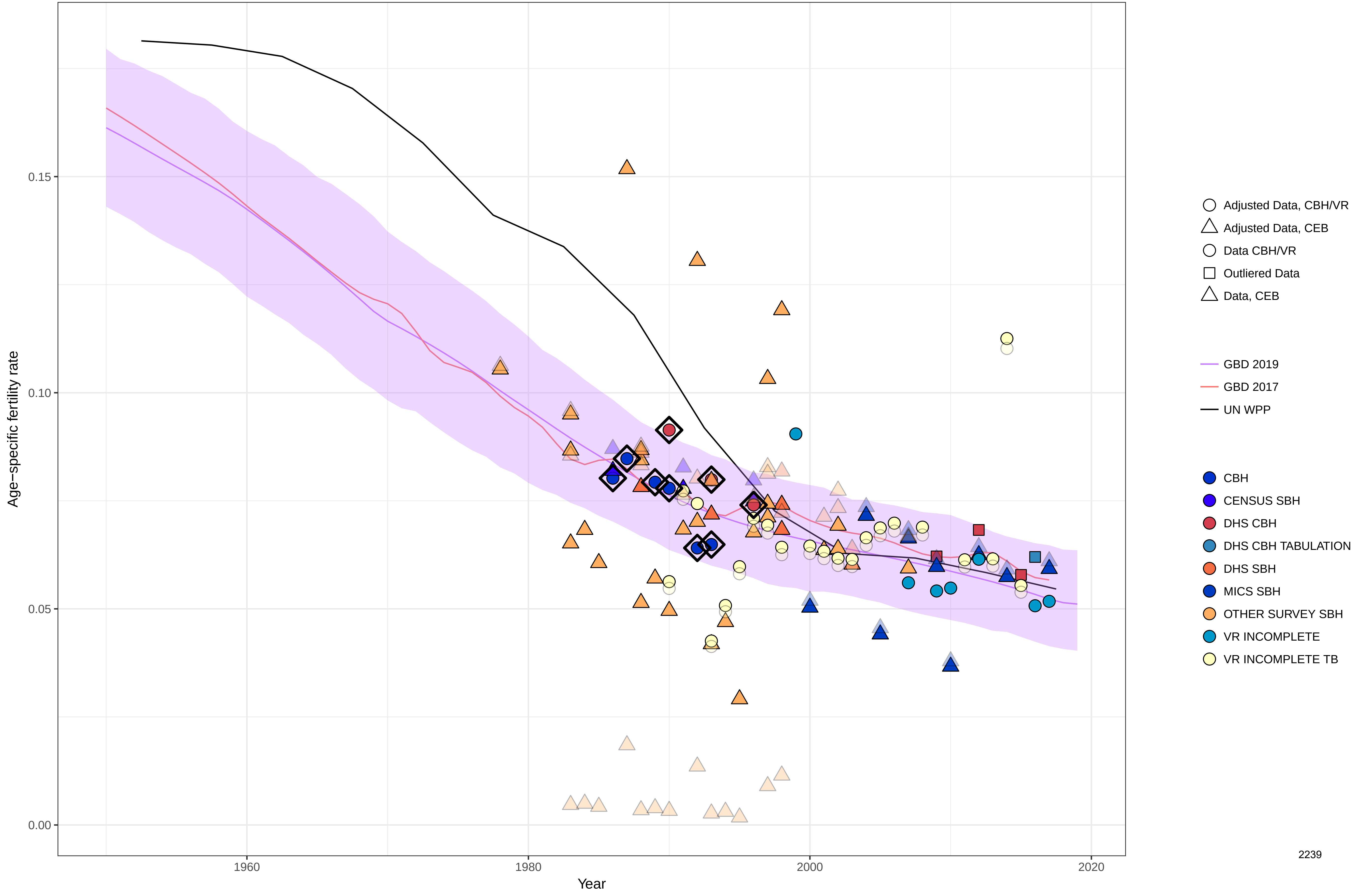

Age−specific fertility fitted trend lines, 1950−2019: South Africa, Ages: 40−44
Data points enclosed with a diamond are reference sources

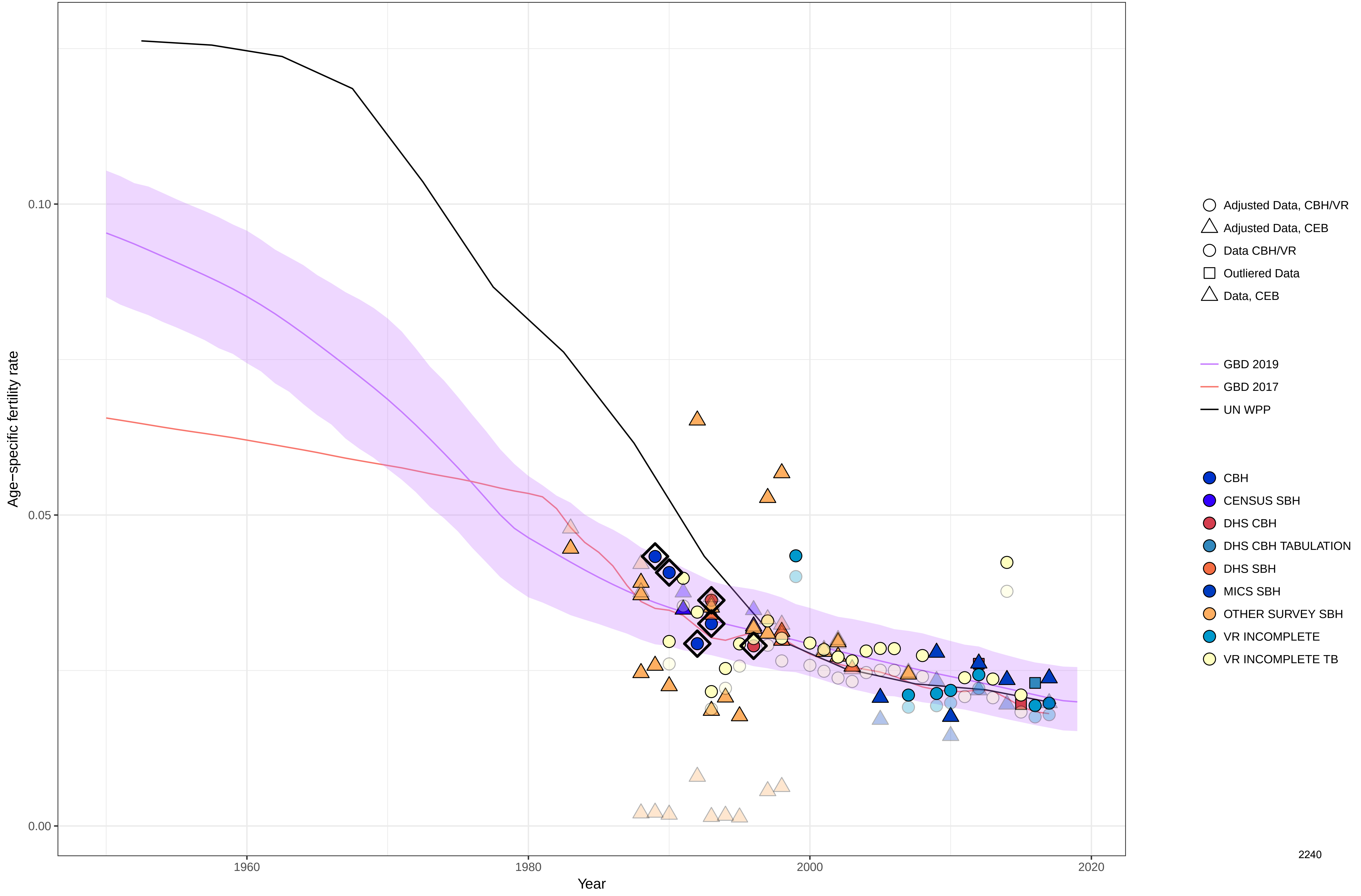

Age−specific fertility fitted trend lines, 1950−2019: South Africa, Ages: 45−49
Data points enclosed with a diamond are reference sources

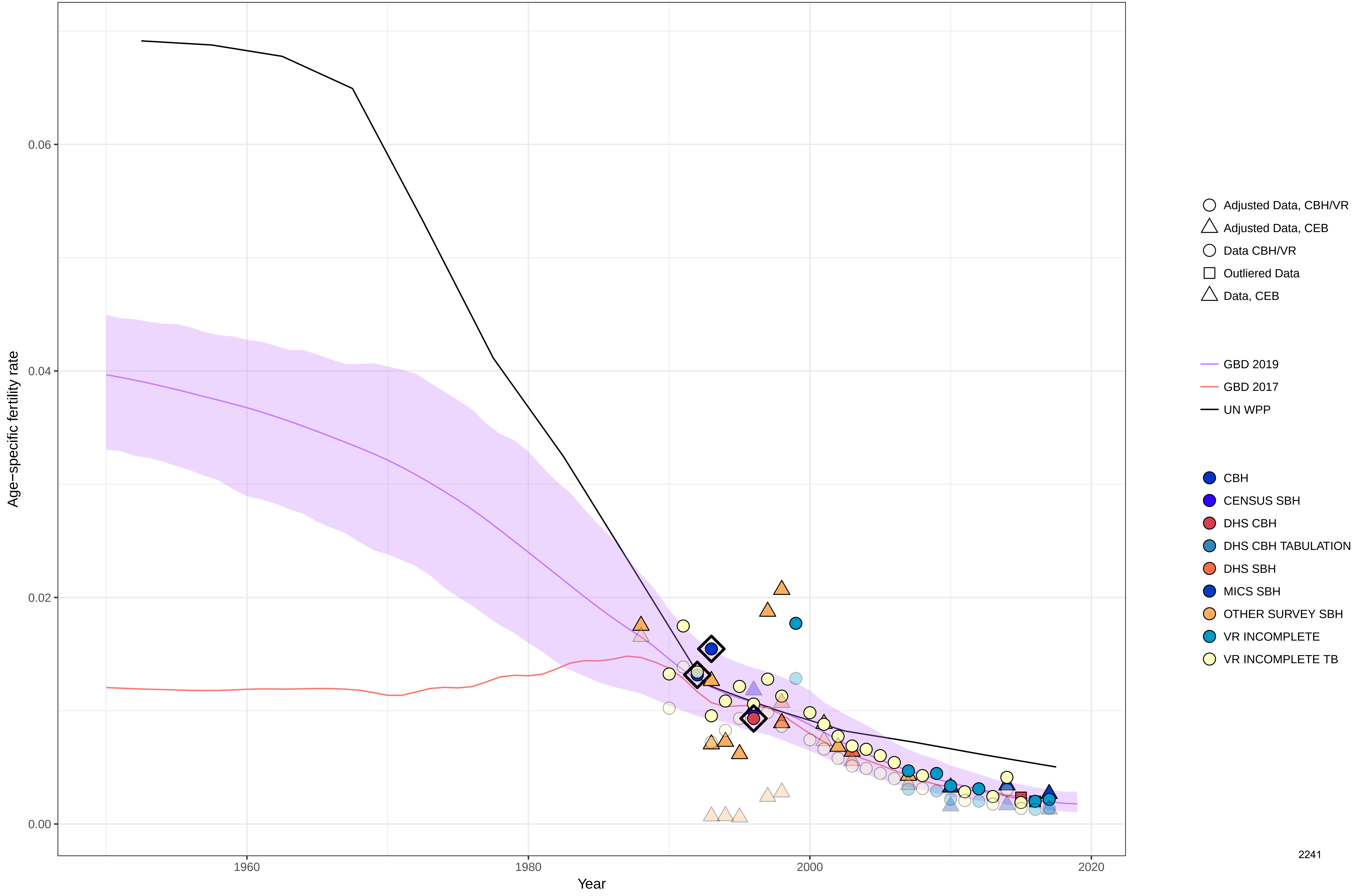

Age−specific fertility fitted trend lines, 1950−2019: South Africa, Ages: 50−54
Data points enclosed with a diamond are reference sources

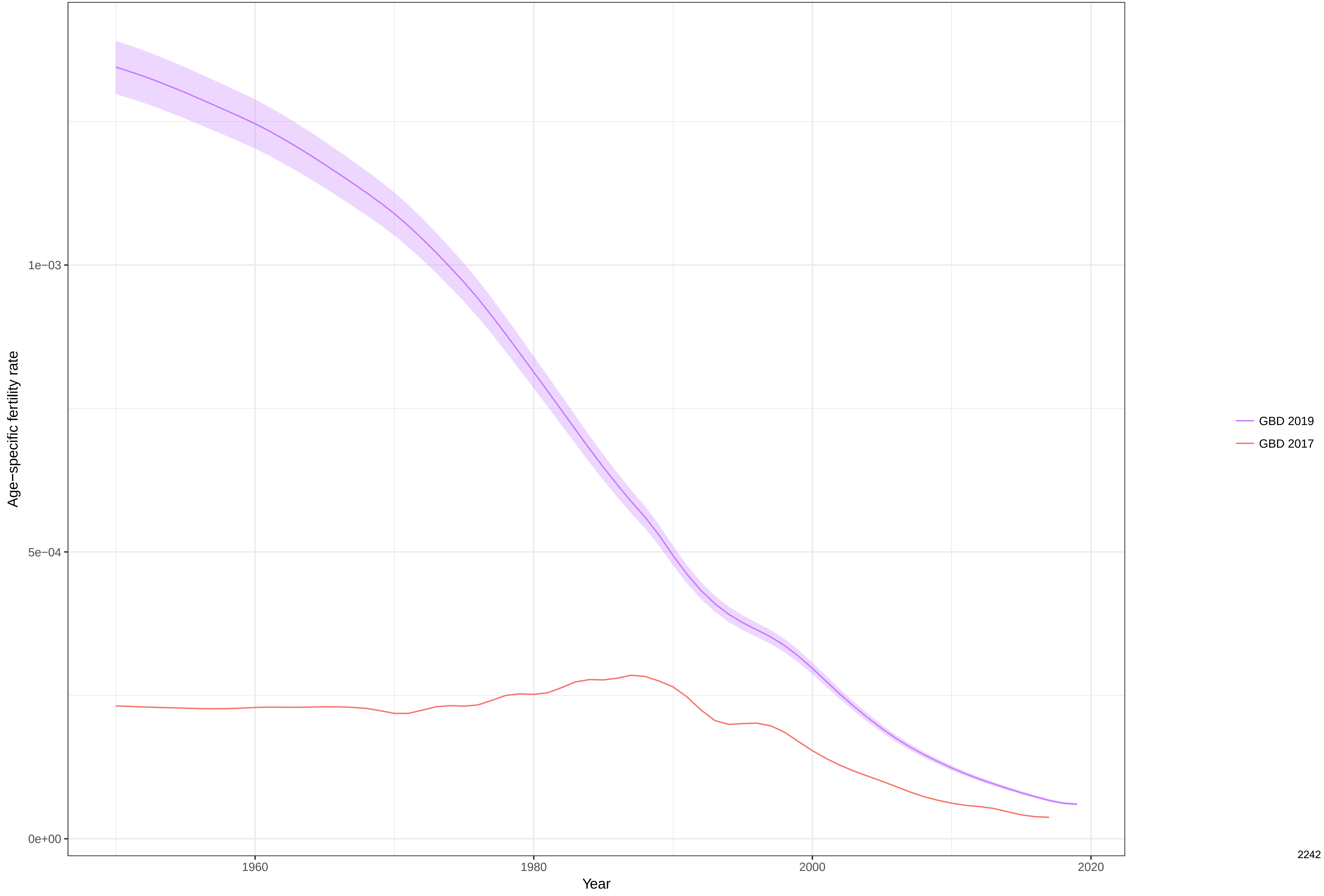

Total fertility rate fitted trend lines, 1950−2019: South Africa TFR
Data points enclosed with a diamond are reference sources

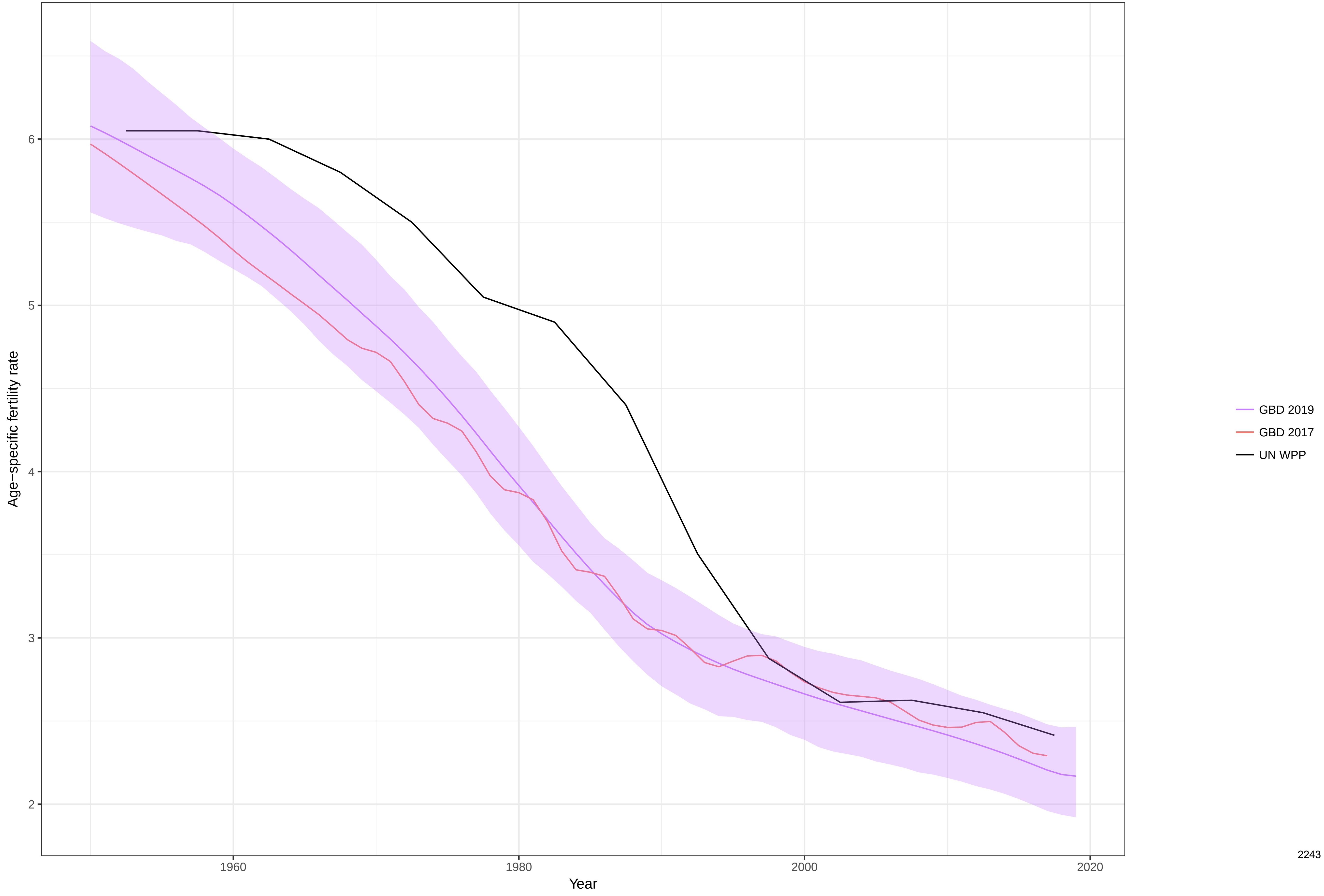

Age−specific fertility fitted trend lines, 1950−2019: Zambia, Ages: 10−14
Data points enclosed with a diamond are reference sources

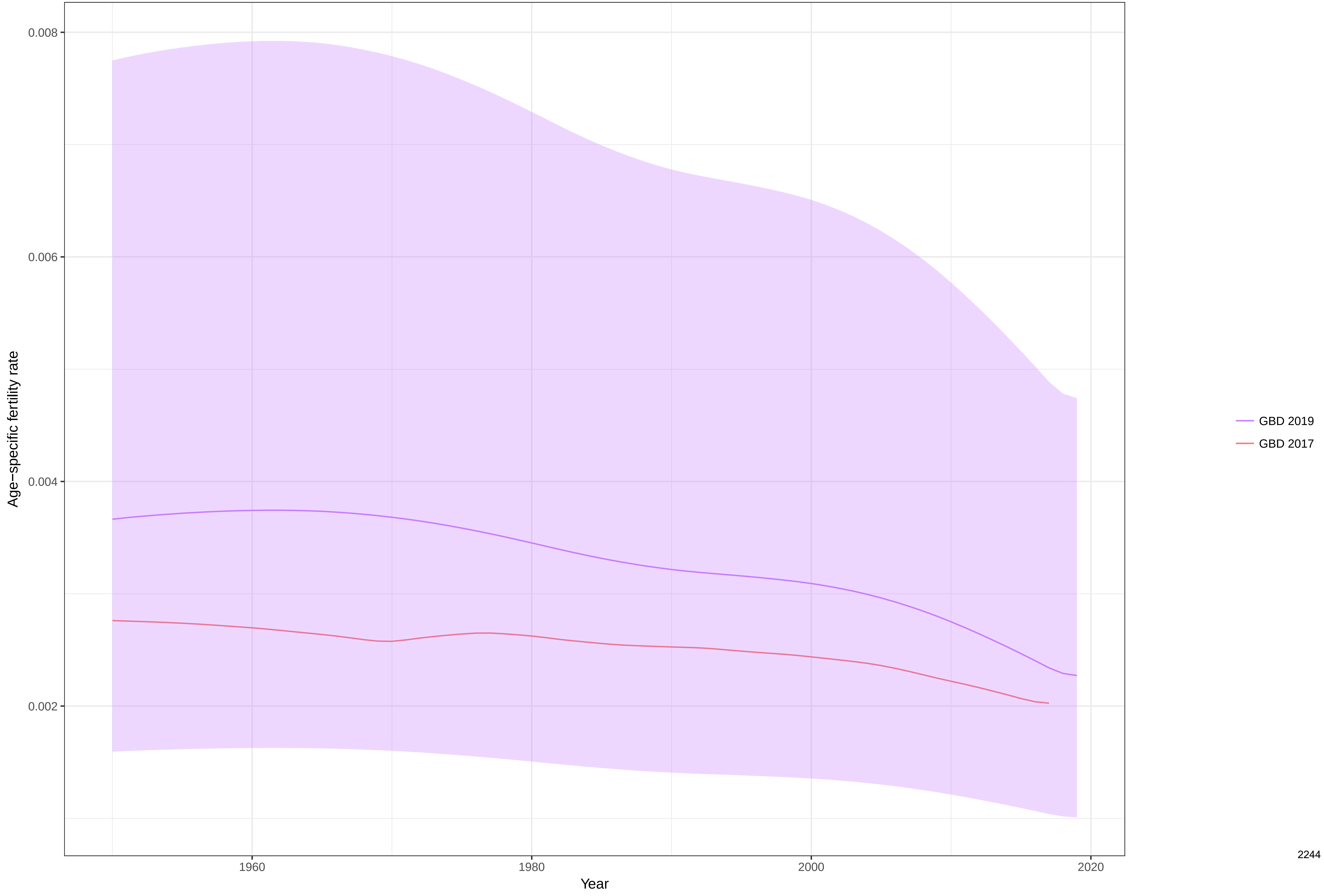

Age−specific fertility fitted trend lines, 1950−2019: Zambia, Ages: 15−19
Data points enclosed with a diamond are reference sources

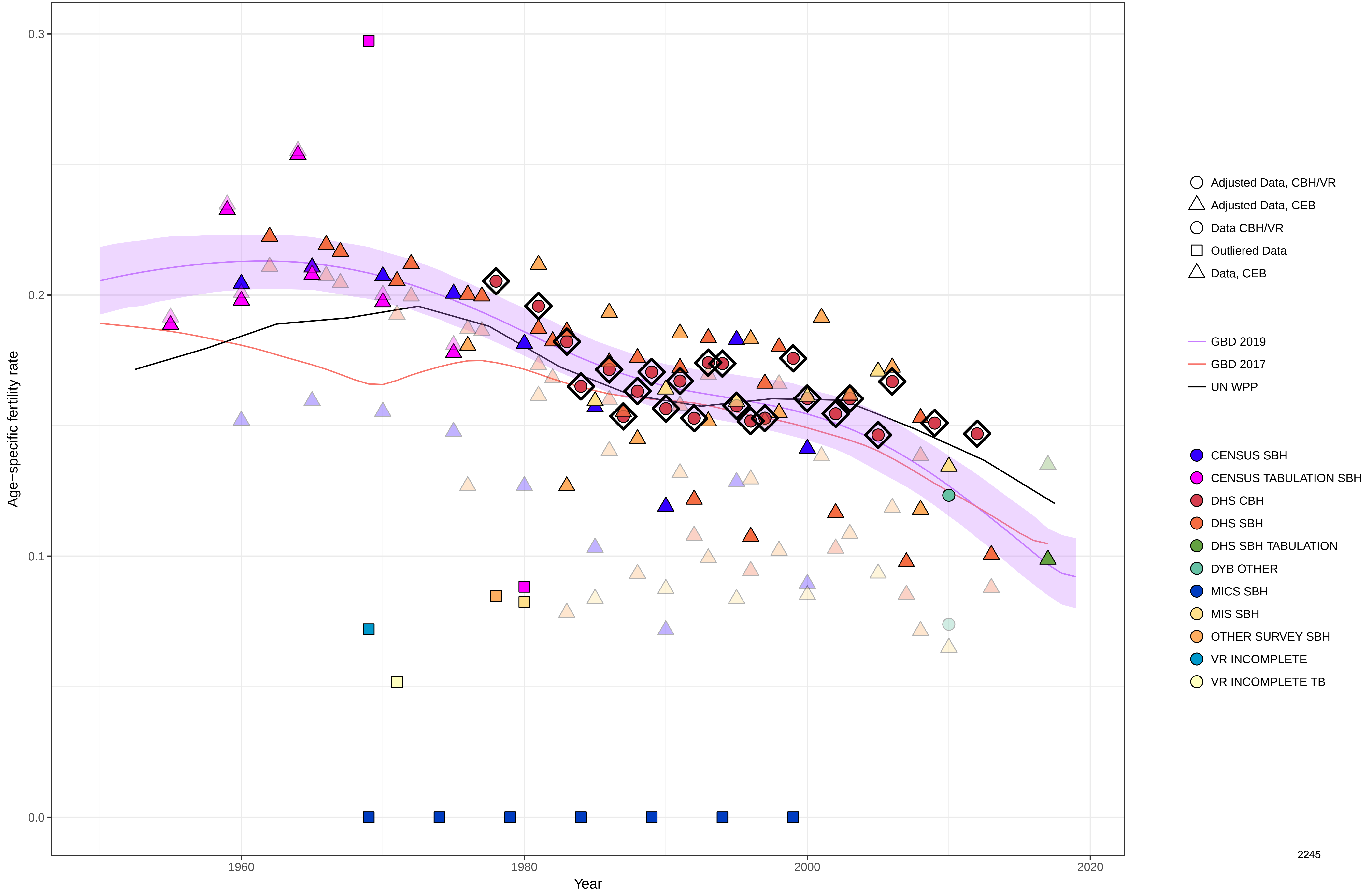

Age−specific fertility fitted trend lines, 1950−2019: Zambia, Ages: 20−24
Data points enclosed with a diamond are reference sources

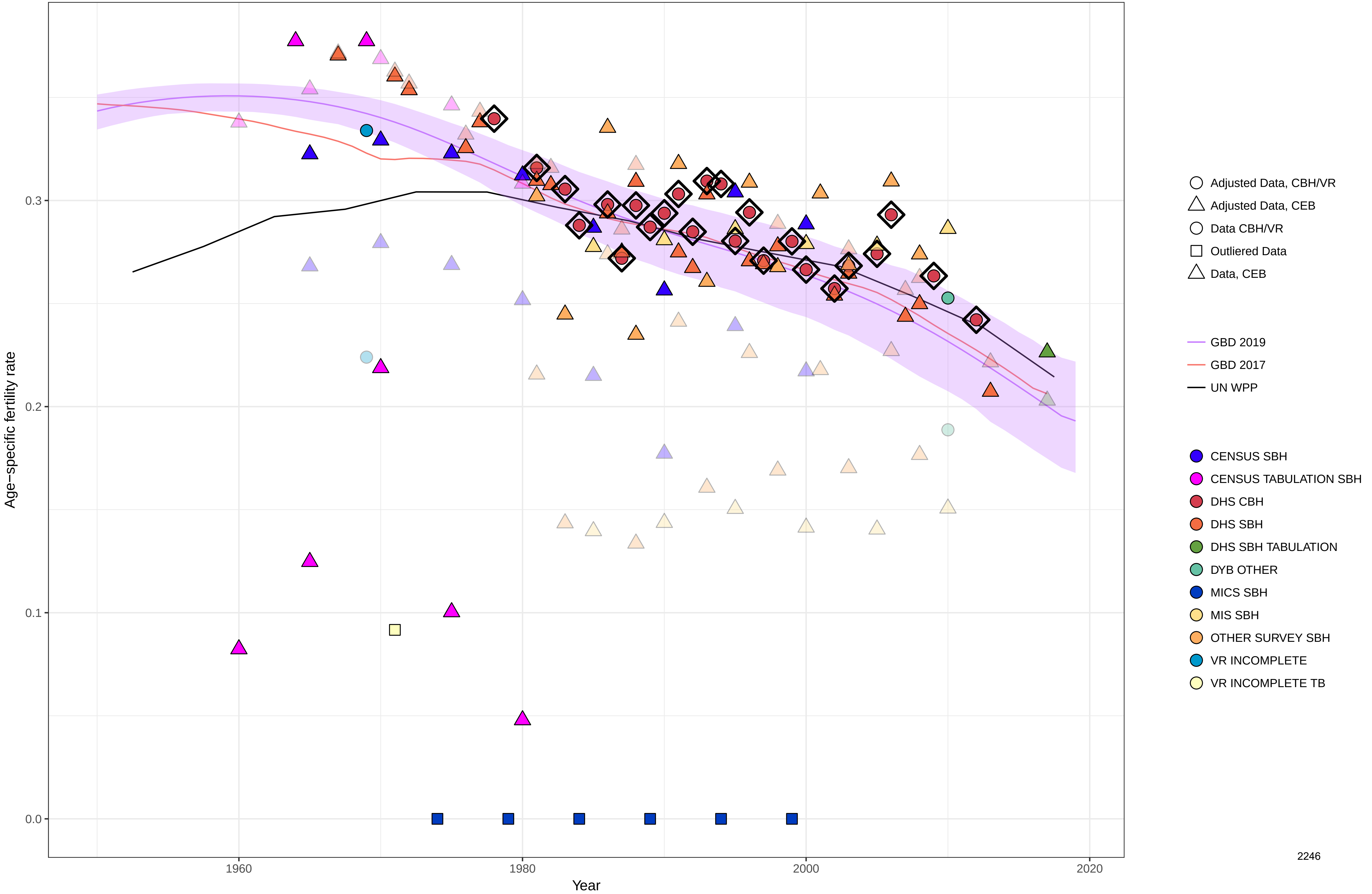

Age−specific fertility fitted trend lines, 1950−2019: Zambia, Ages: 25−29
Data points enclosed with a diamond are reference sources

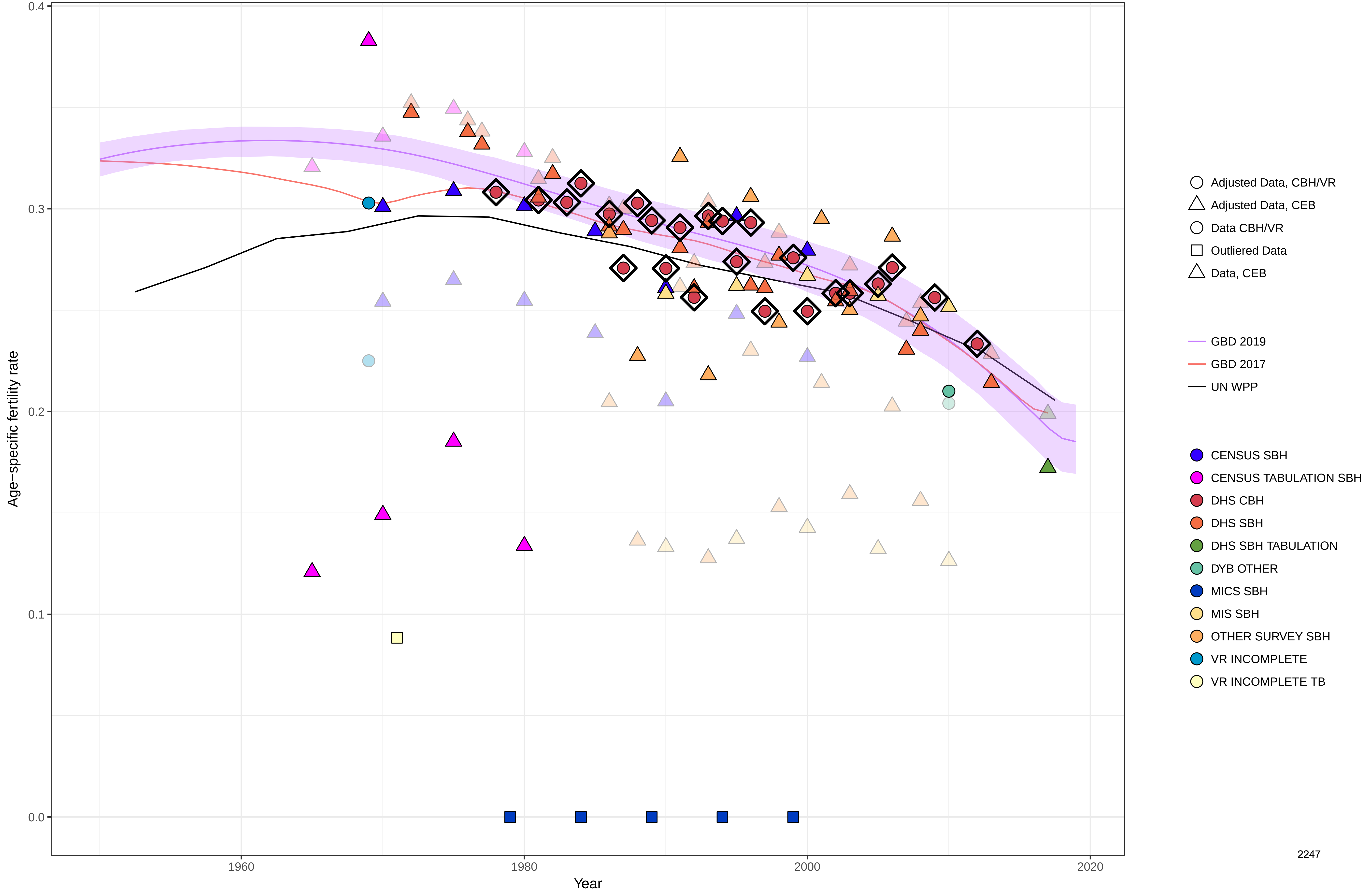

Age−specific fertility fitted trend lines, 1950−2019: Zambia, Ages: 30−34
Data points enclosed with a diamond are reference sources

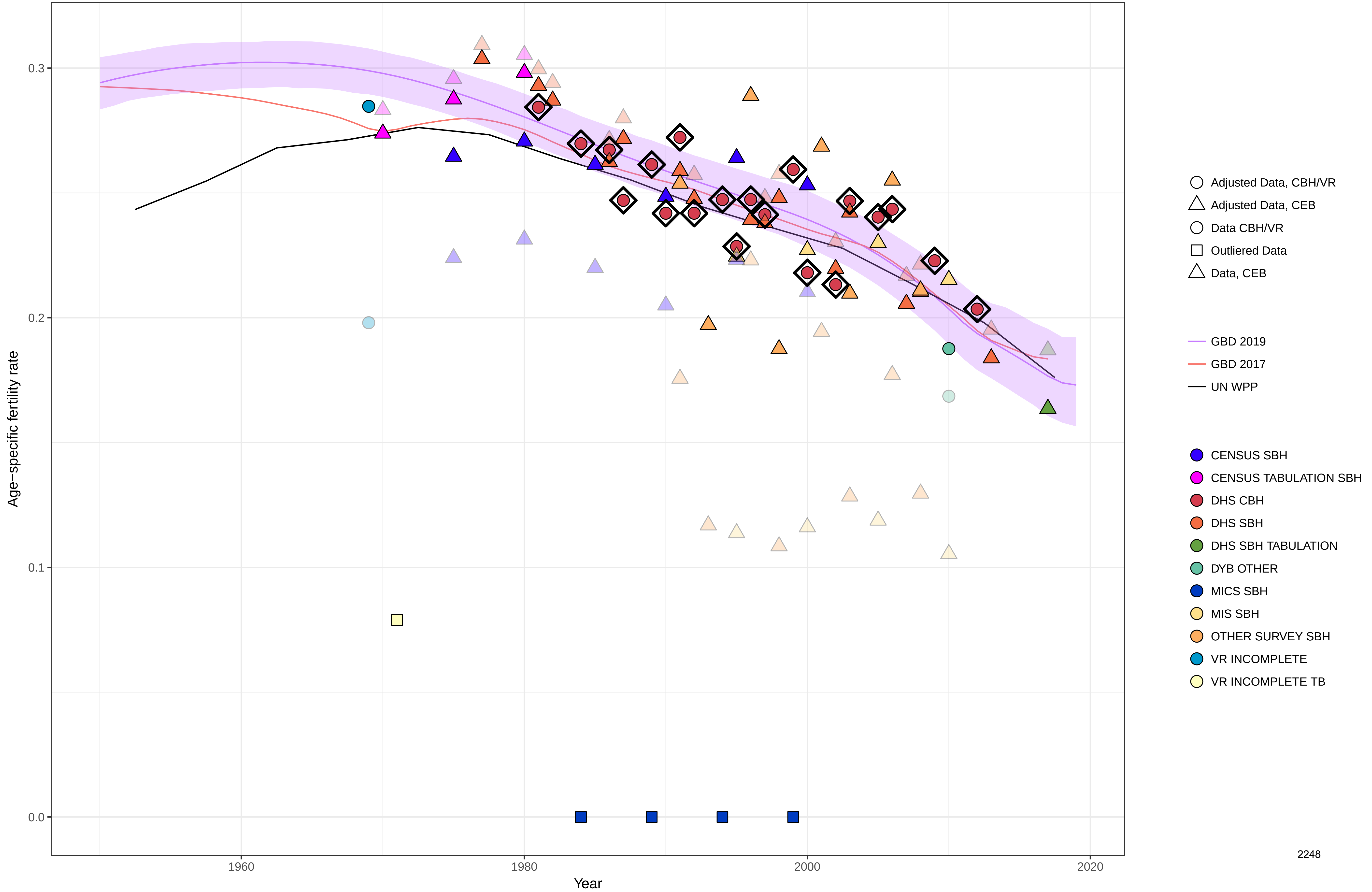

Age−specific fertility fitted trend lines, 1950−2019: Zambia, Ages: 35−39
Data points enclosed with a diamond are reference sources

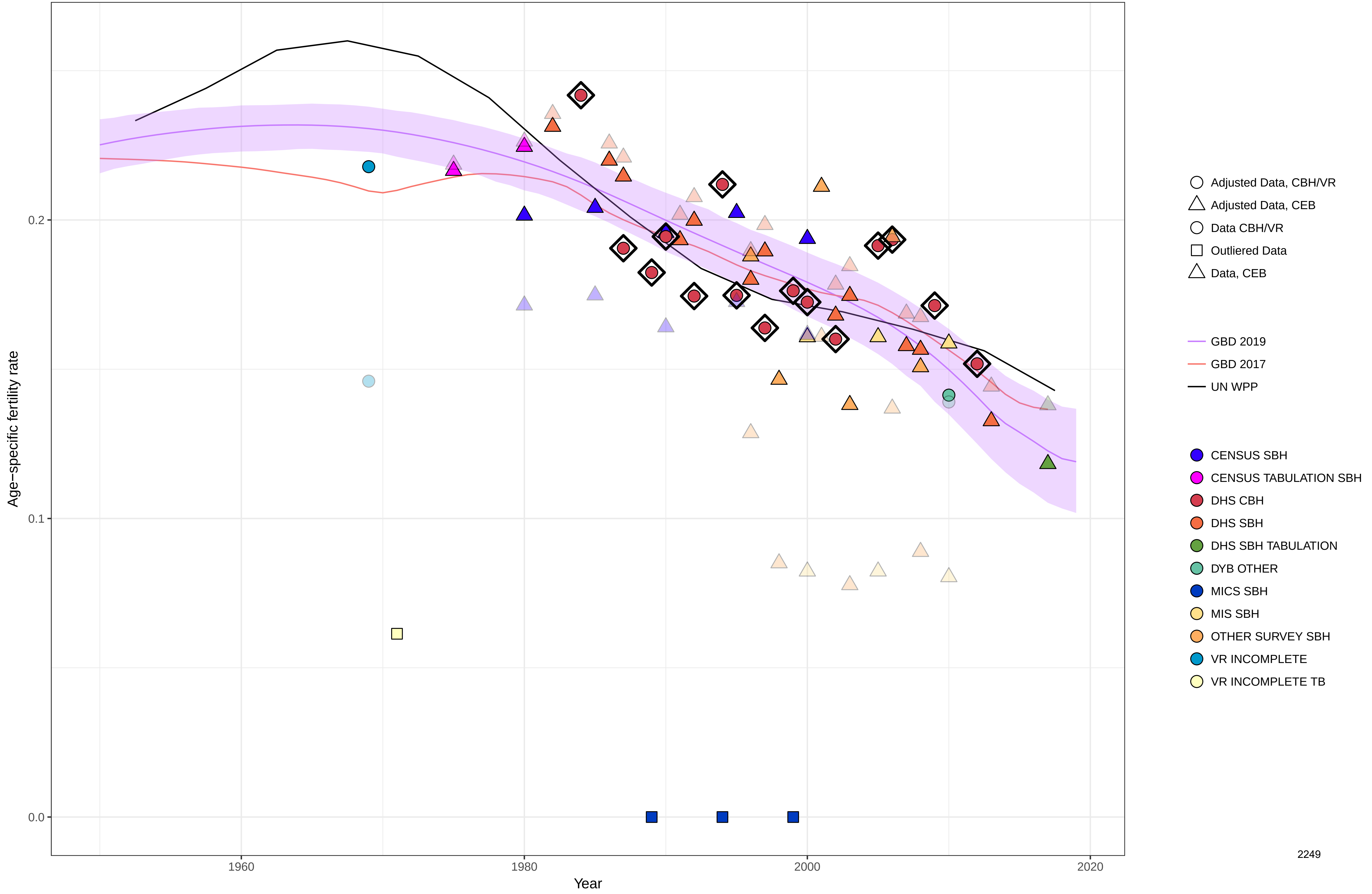

Age−specific fertility fitted trend lines, 1950−2019: Zambia, Ages: 40−44
Data points enclosed with a diamond are reference sources

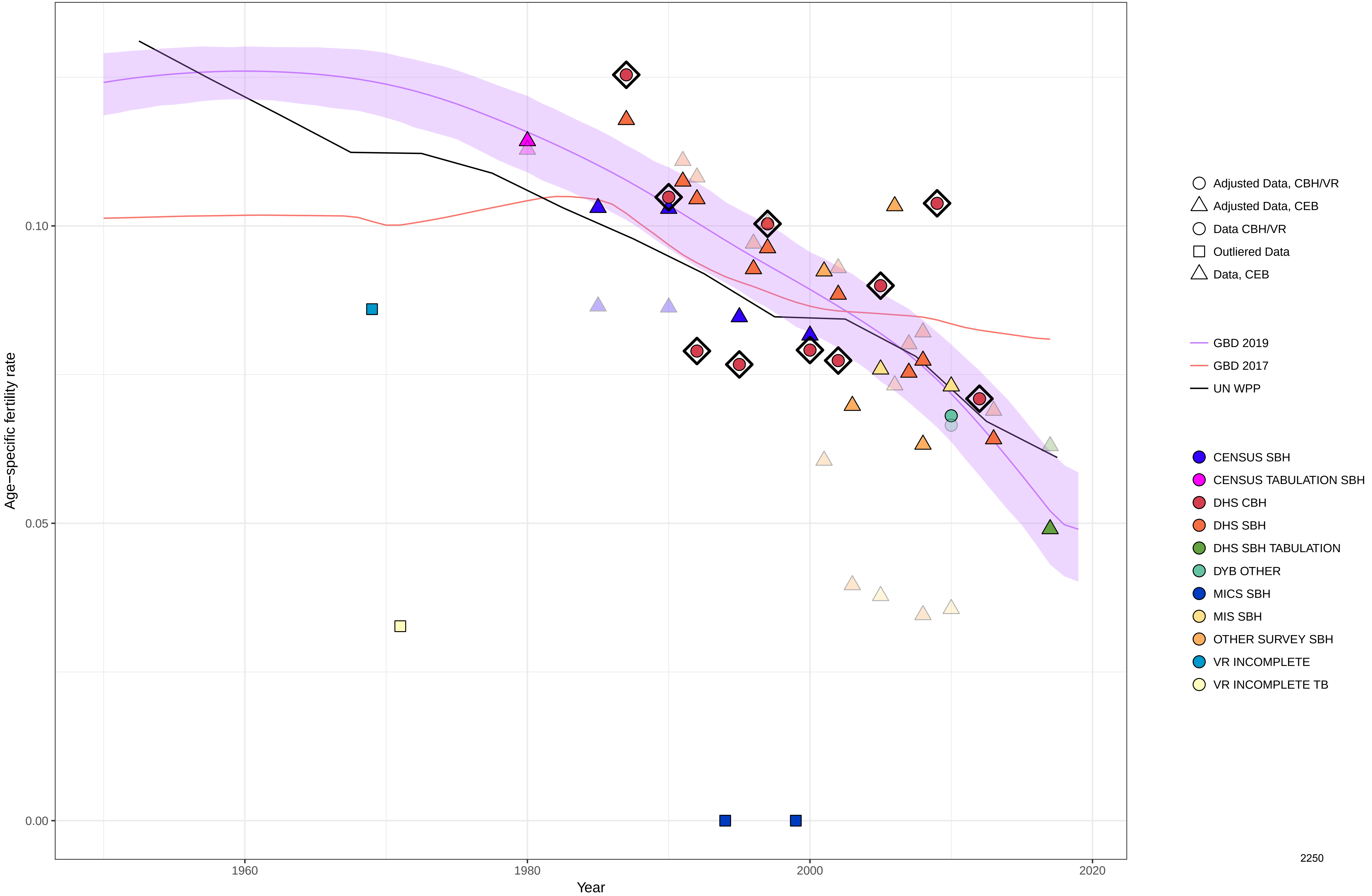

Age−specific fertility fitted trend lines, 1950−2019: Zambia, Ages: 45−49
Data points enclosed with a diamond are reference sources

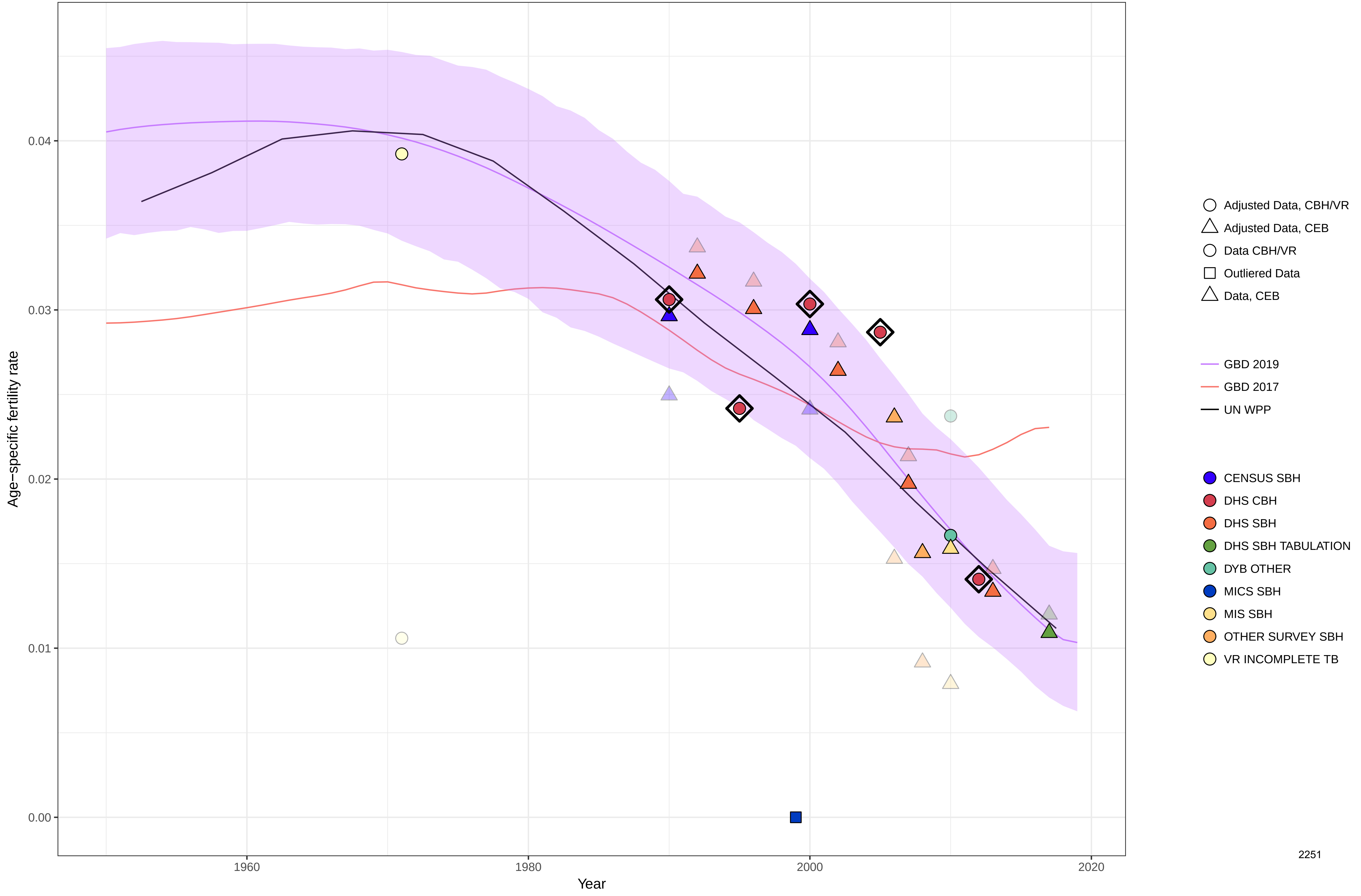

Age−specific fertility fitted trend lines, 1950−2019: Zambia, Ages: 50−54
Data points enclosed with a diamond are reference sources

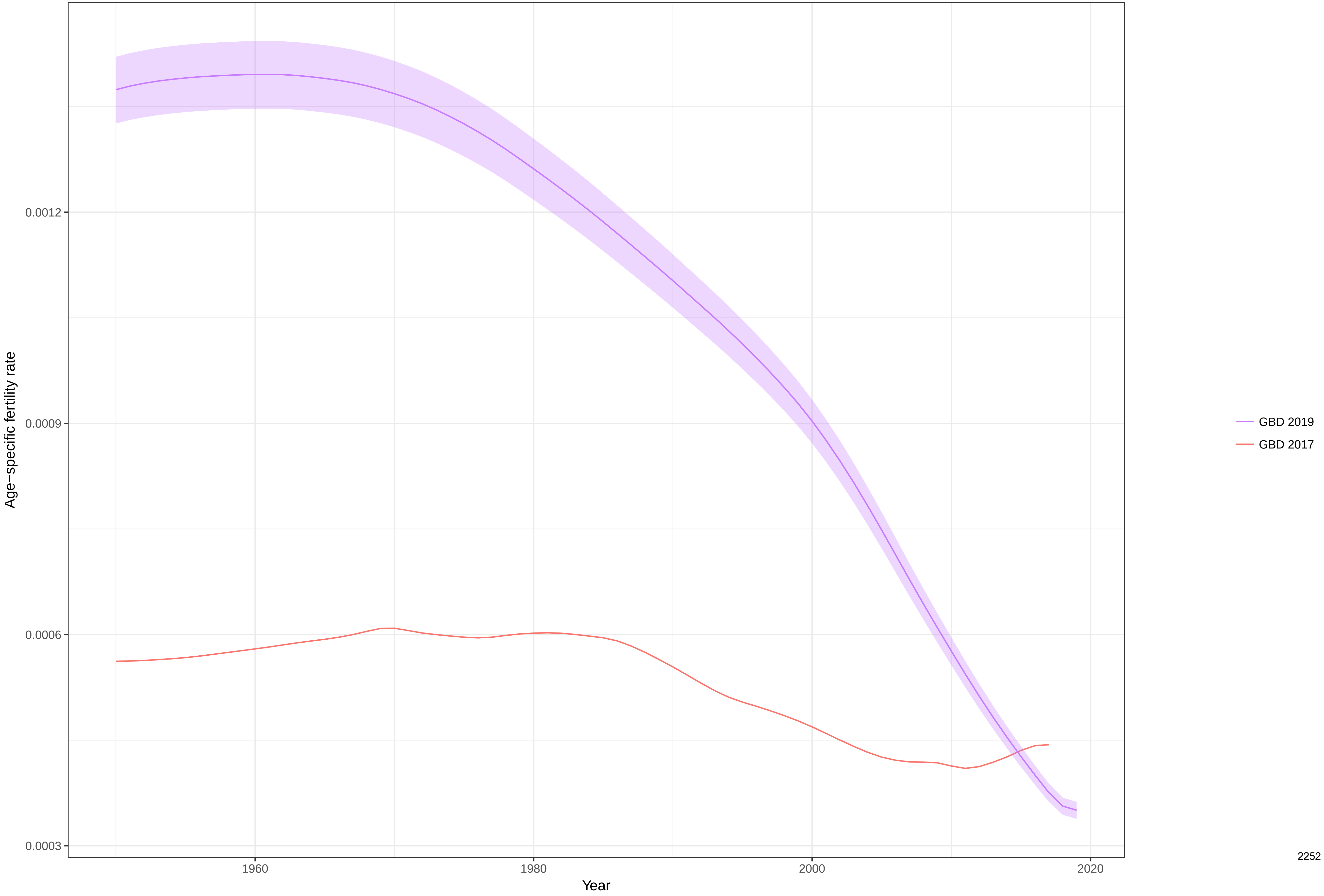

Total fertility rate fitted trend lines, 1950−2019: Zambia TFR
Data points enclosed with a diamond are reference sources

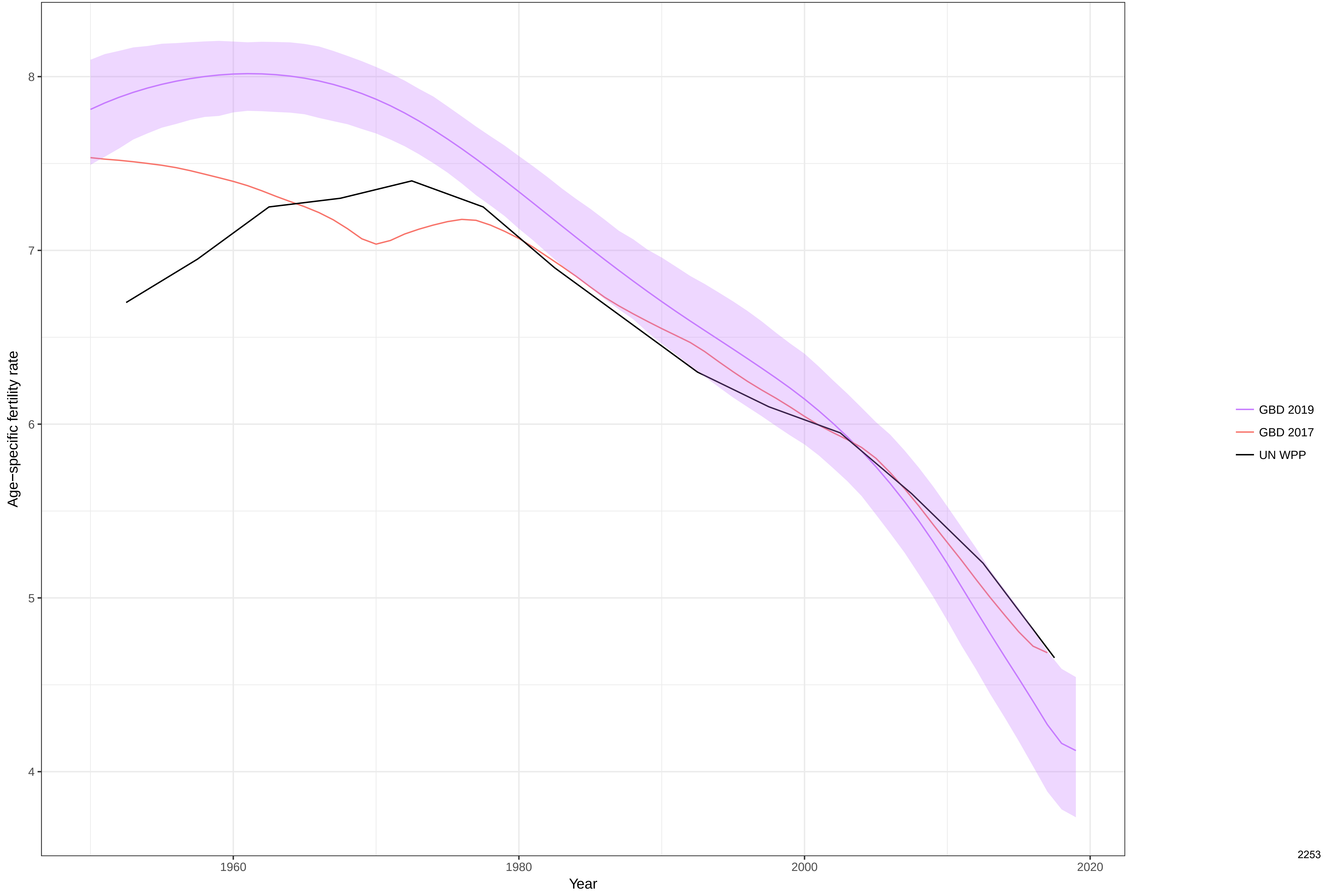

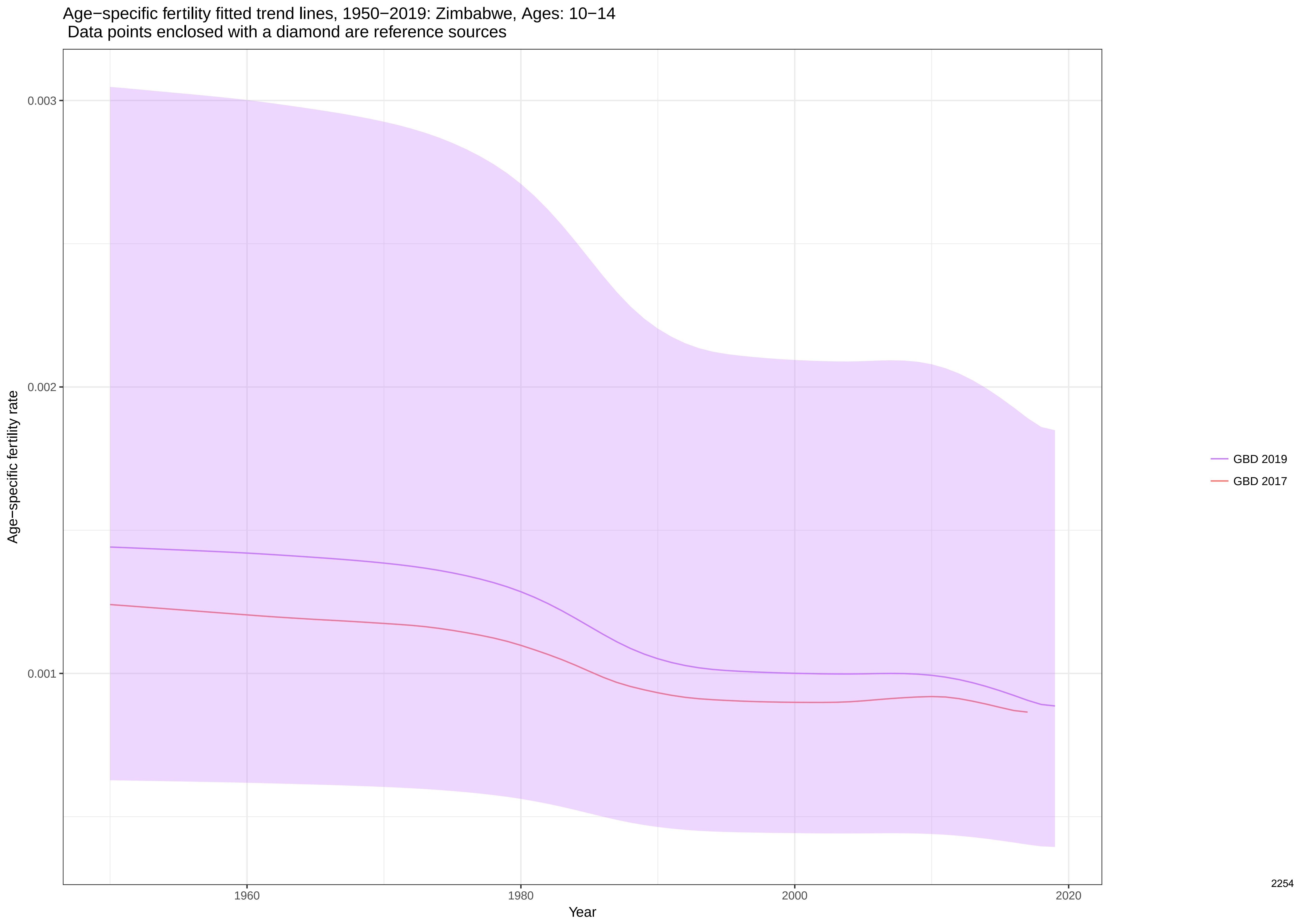

Age−specific fertility fitted trend lines, 1950−2019: Zimbabwe, Ages: 10−14
Data points enclosed with a diamond are reference sources
0.003
0.002
0.001
Age−specific fertility rate
1960
1980
2000
2020
Year
GBD 2019
GBD 2017

Age−specific fertility fitted trend lines, 1950−2019: Zimbabwe, Ages: 15−19
Data points enclosed with a diamond are reference sources

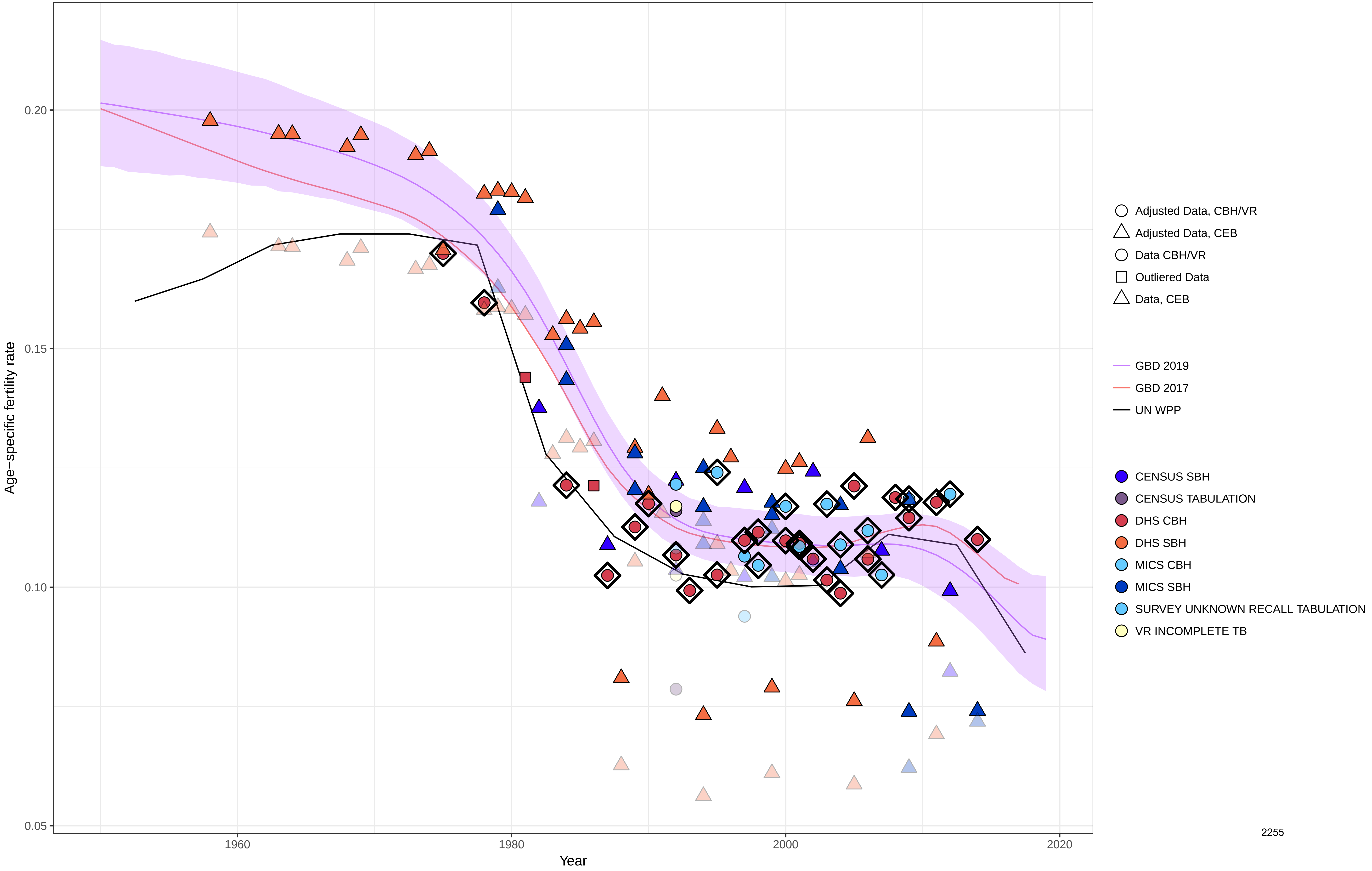

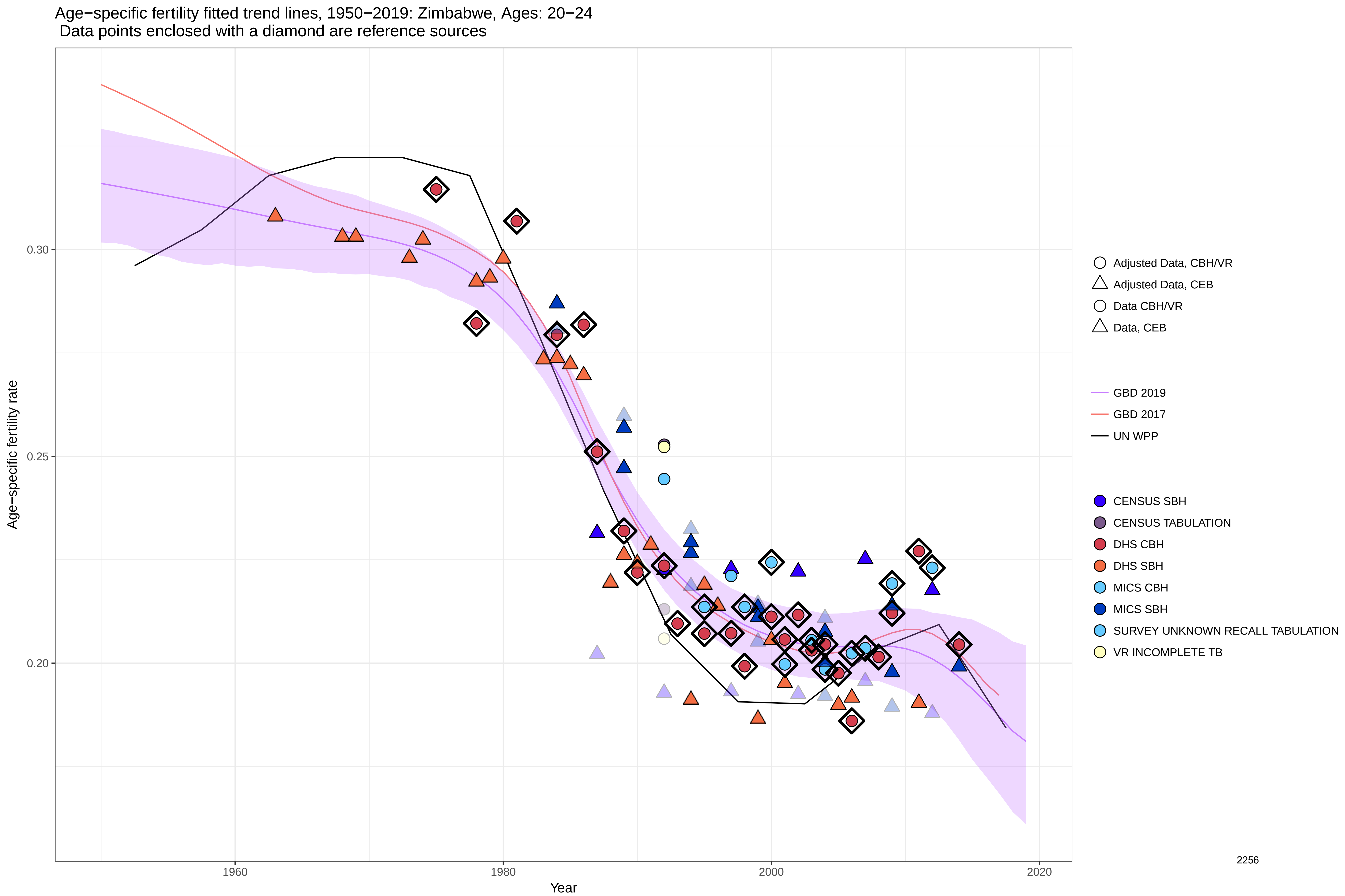
Age−specific fertility fitted trend lines, 1950−2019: Zimbabwe, Ages: 20−24
Data points enclosed with a diamond are reference sources
Age−specific fertility rate
0.30
0.25
0.20
Year
1960
1980
2000
2020
Adjusted Data, CBH/VR
Adjusted Data, CEB
Data CBH/VR
Data, CEB
GBD 2019
GBD 2017
UN WPP
CENSUS SBH
CENSUS TABULATION
DHS CBH
DHS SBH
MICS CBH
MICS SBH
SURVEY UNKNOWN RECALL TABULATION
VR INCOMPLETE TB

Age−specific fertility fitted trend lines, 1950−2019: Zimbabwe, Ages: 25−29
Data points enclosed with a diamond are reference sources

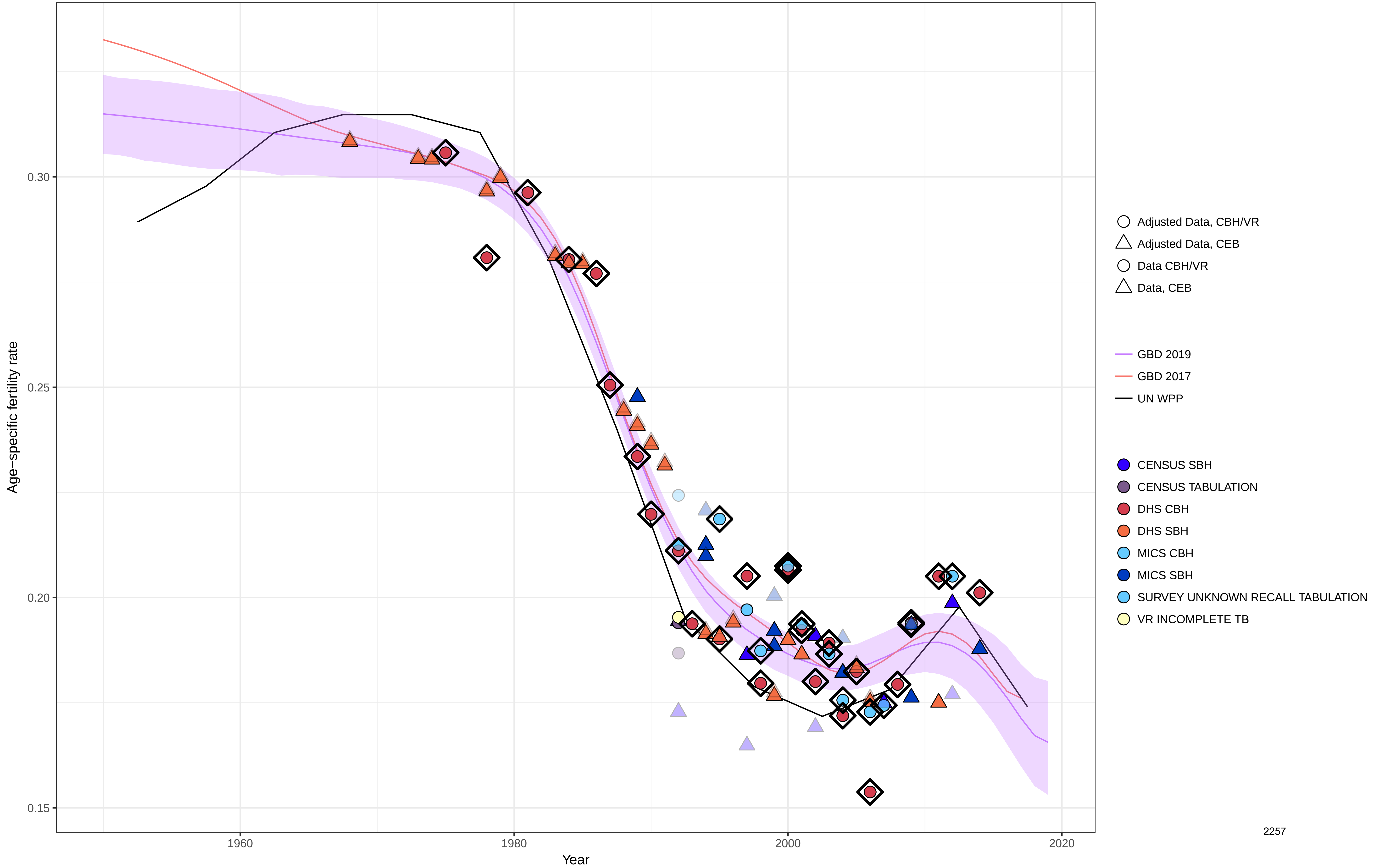

Age−specific fertility fitted trend lines, 1950−2019: Zimbabwe, Ages: 30−34
Data points enclosed with a diamond are reference sources

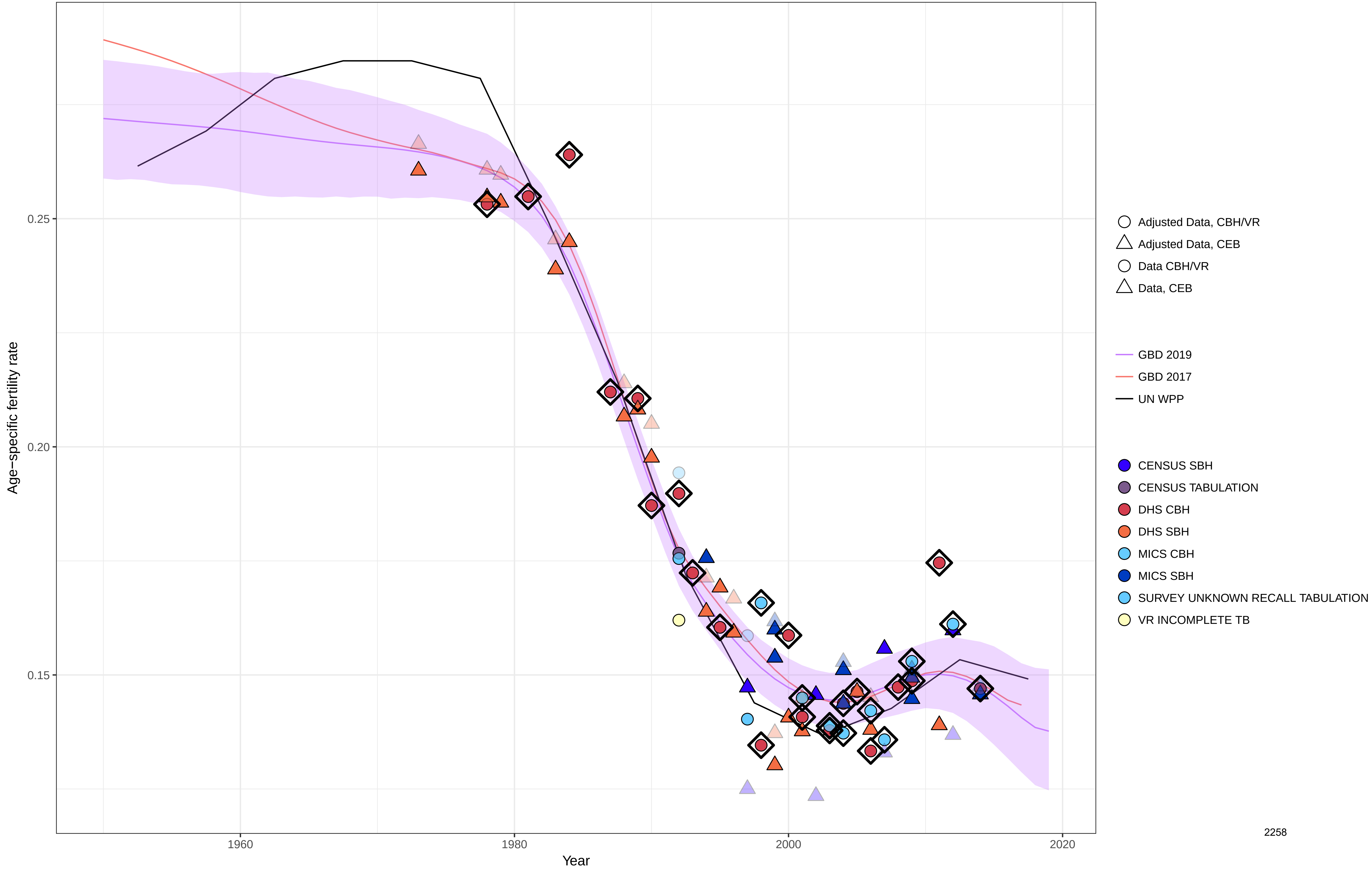

Age−specific fertility fitted trend lines, 1950−2019: Zimbabwe, Ages: 35−39
Data points enclosed with a diamond are reference sources

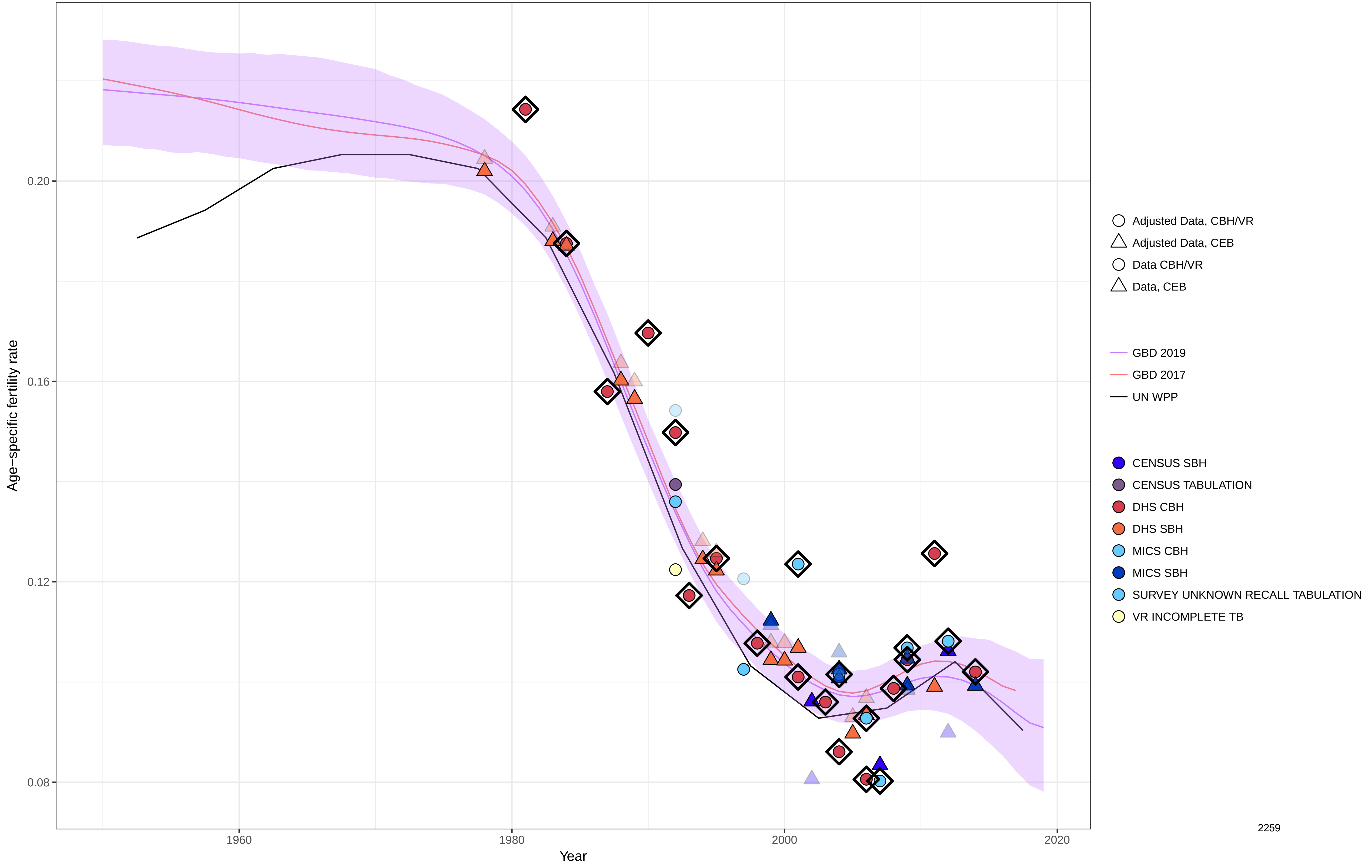

Age−specific fertility fitted trend lines, 1950−2019: Zimbabwe, Ages: 40−44
Data points enclosed with a diamond are reference sources

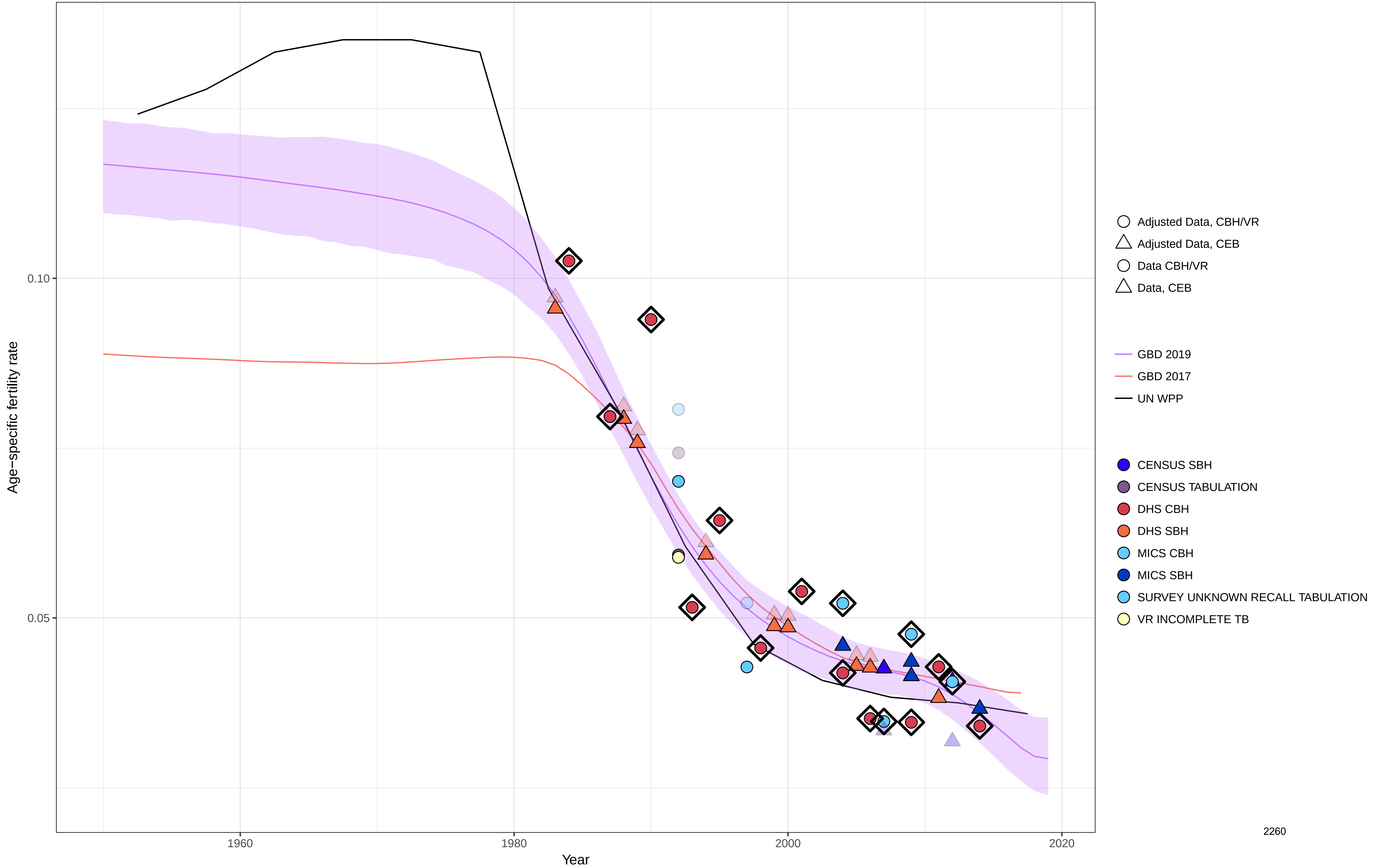

Age−specific fertility fitted trend lines, 1950−2019: Zimbabwe, Ages: 45−49
Data points enclosed with a diamond are reference sources

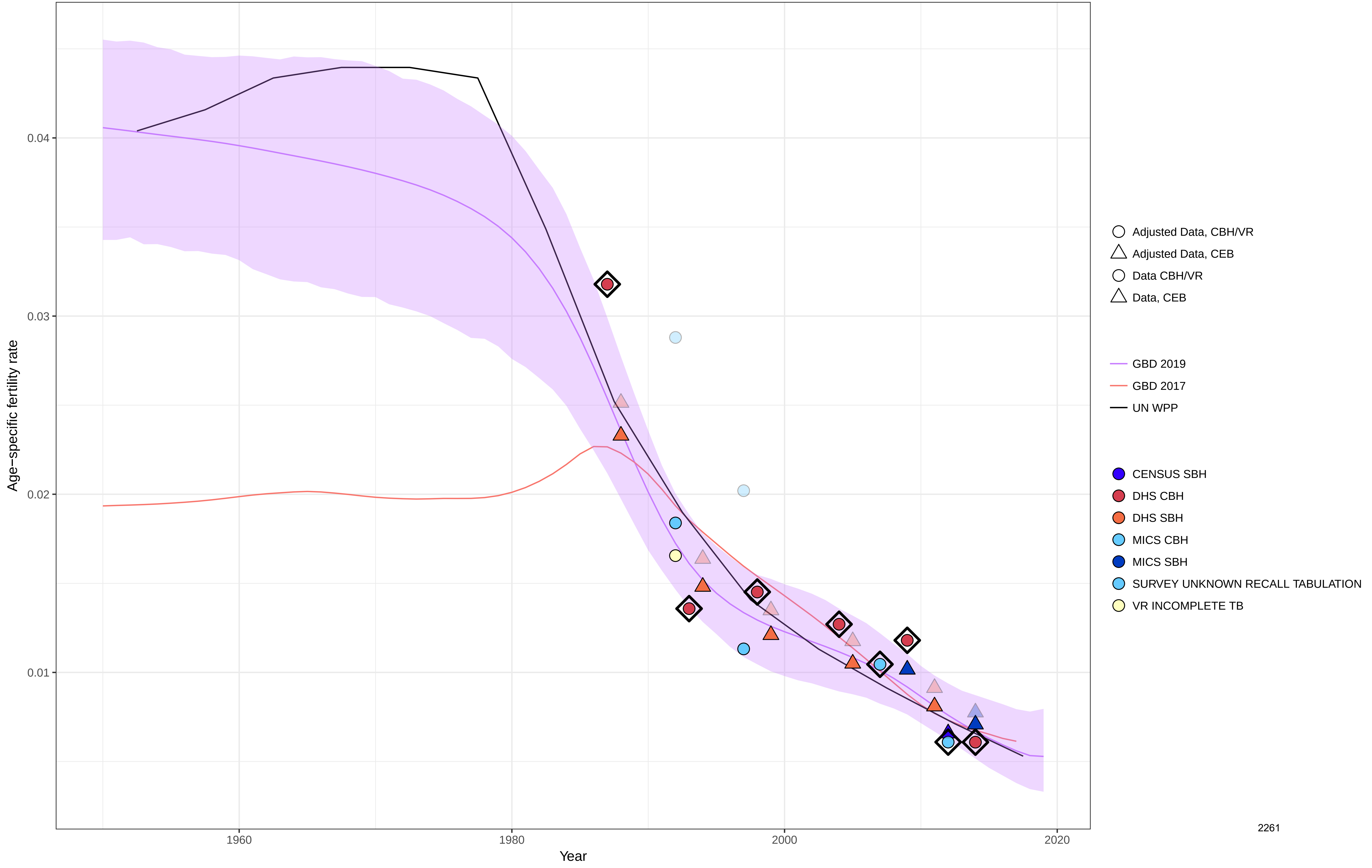

Age−specific fertility fitted trend lines, 1950−2019: Zimbabwe, Ages: 50−54
Data points enclosed with a diamond are reference sources

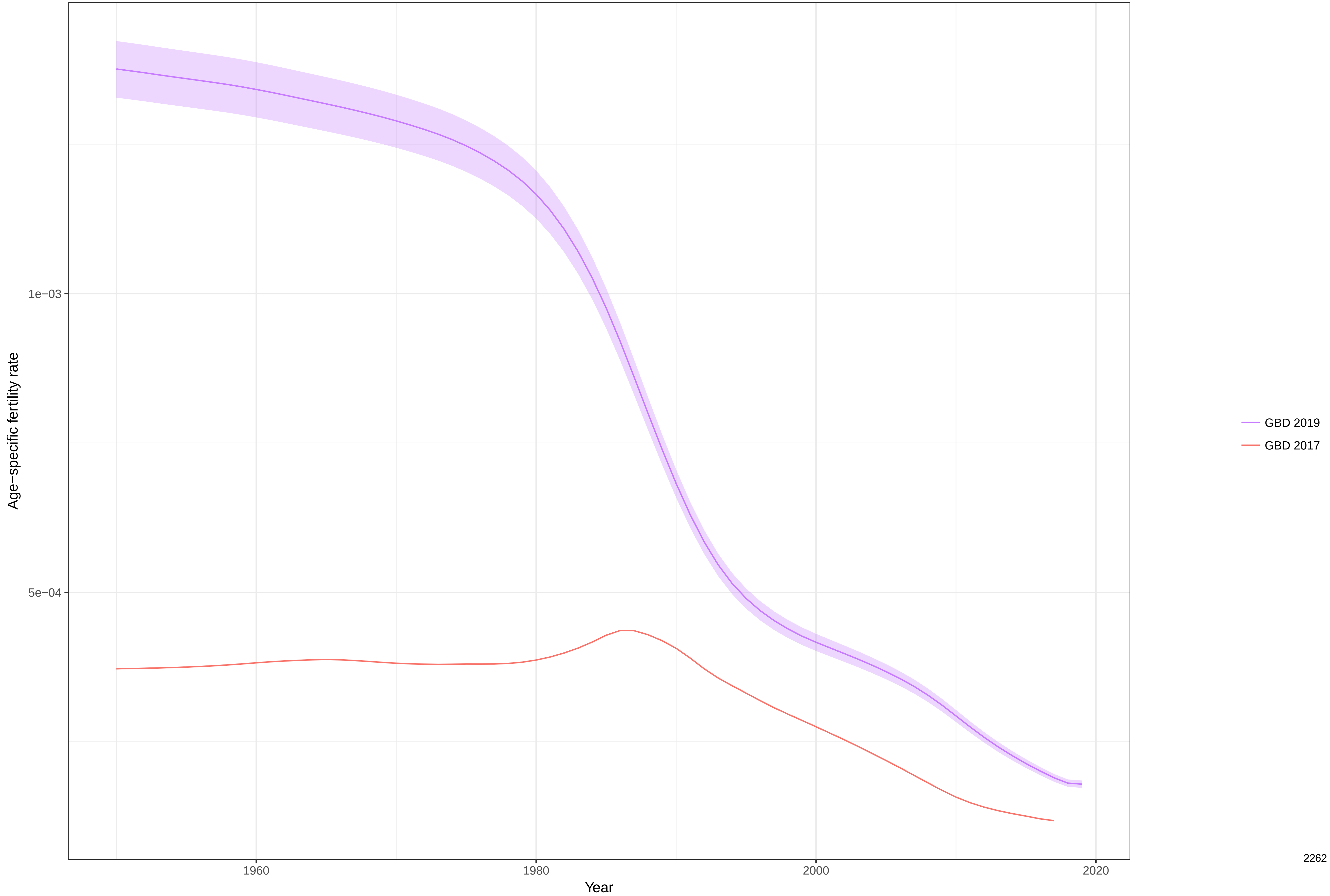

Total fertility rate fitted trend lines, 1950−2019: Zimbabwe TFR
Data points enclosed with a diamond are reference sources

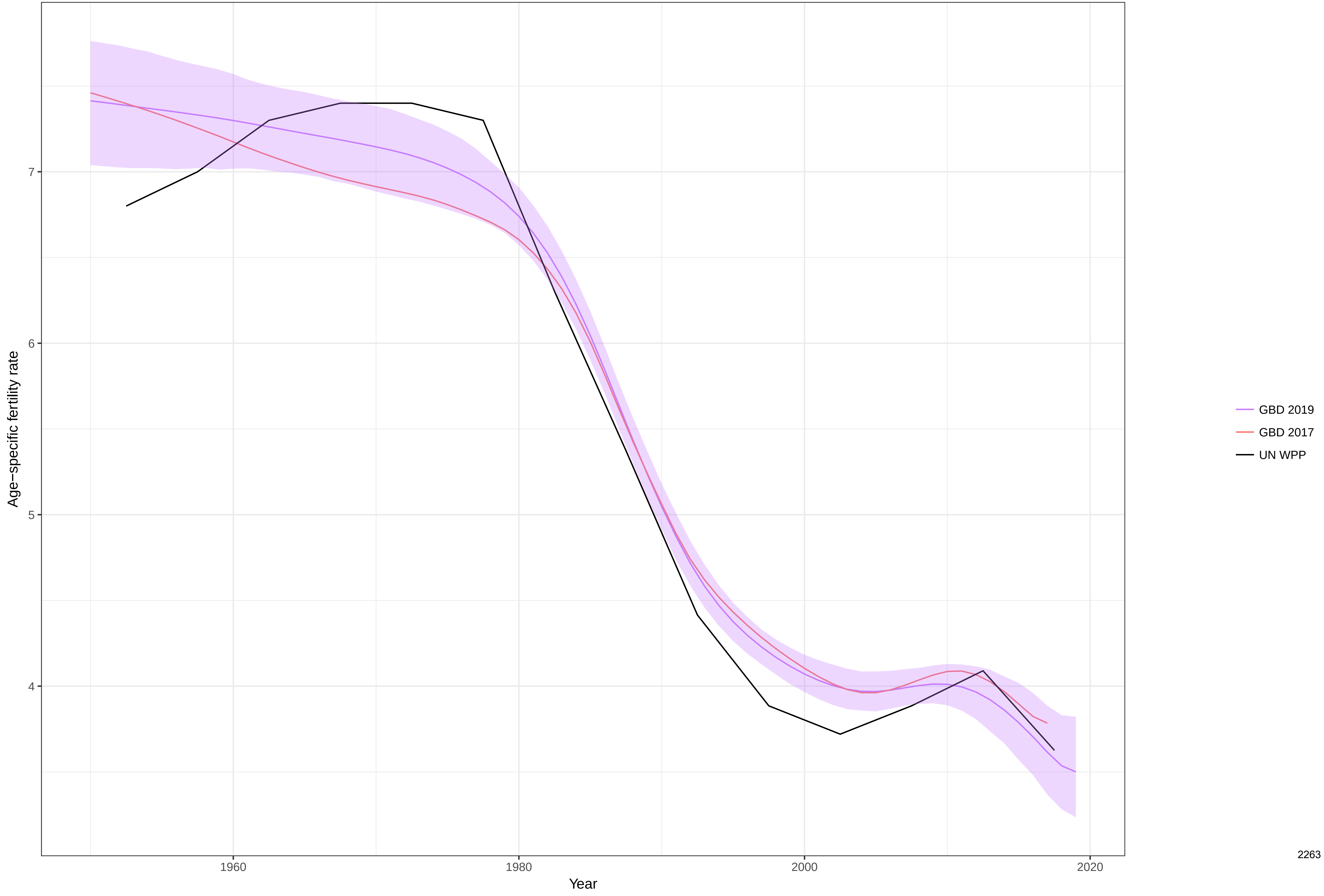

Supplemental Results Table 1a: Live births (in thousands) by location, both sexes, 1950, 1960, 1970, 1980, 1990, 2000, 2010, 2019

Location	1950	1960	1970	1980	1990	2000	2010	2019
Global	95940·2 (92550·3 to 99388·8)	108814·1 (105761·3 to 111855·8)	115559·7 (112558·6 to 118414·5)	130420·3 (127720·9 to 132974·6)	138138 (135211·6 to 141014·3)	134546·3 (131457·8 to 137767·7)	137611·5 (133217·8 to 141868·9)	135350 (127167·1 to 144081·2)
Central Europe, eastern Europe, and central Asia	7593·5 (7417·7 to 7782·2)	7561·8 (7471·2 to 7645·8)	6206·4 (6103 to 6314·7)	7173·5 (7094·2 to 7257)	6499·4 (6421·9 to 6584·2)	4629·3 (4558·3 to 4703·1)	5653·6 (5554·9 to 5759)	5206·6 (4672·8 to 5811·1)
Central Asia	1083 (1036·1 to 1128·5)	1408·7 (1362·6 to 1454·1)	1351·1 (1281·4 to 1425·9)	1756·2 (1706·6 to 1806·9)	1971·5 (1927·7 to 2016·2)	1530·2 (1483·6 to 1576·2)	1919·8 (1842·3 to 2004·8)	1895·5 (1755·2 to 2040·6)
Armenia	54·6 (51·4 to 57·8)	77·1 (73·8 to 80·3)	63·1 (59·2 to 67·5)	89·2 (84·7 to 93·2)	79·8 (77 to 82·9)	43·8 (41·7 to 45·9)	47·7 (45·3 to 50·1)	38·4 (34·5 to 42·5)
Azerbaijan	123·4 (115·5 to 131·4)	181·1 (174·7 to 187·9)	154 (145 to 164·2)	176·7 (168·8 to 184·5)	203·9 (193·7 to 214·1)	147·4 (139·6 to 155·5)	188·7 (174·5 to 202·8)	153·5 (133·2 to 176·4)
Georgia	86·5 (78·7 to 95)	100·7 (94·4 to 106·9)	87 (77·8 to 97·7)	92·6 (87·4 to 97·9)	81·6 (77·6 to 85·7)	49 (46·2 to 51·8)	55·2 (52·1 to 58·3)	46 (39·8 to 53·1)
Kazakhstan	257·9 (245·5 to 270·3)	346·9 (335·5 to 357·9)	330·3 (309·2 to 352·6)	368·7 (358·2 to 379·7)	354·1 (343·1 to 364·4)	232 (222·5 to 241·2)	345 (330·5 to 359·5)	350·6 (320·5 to 381·5)
Kyrgyzstan	58·5 (54·9 to 61·9)	76·8 (73·8 to 79·9)	80·8 (74 to 87·5)	113·5 (108·4 to 118·5)	123·5 (119·2 to 127·3)	98·4 (93·7 to 103·7)	137·6 (131·6 to 144·3)	143·9 (130·5 to 158·5)
Mongolia	31·7 (29·7 to 33·7)	38·1 (36 to 40)	47 (45·2 to 48·7)	63·3 (61·2 to 65·4)	74·6 (72·2 to 76·9)	58·2 (55·5 to 60·8)	82·2 (78·2 to 86·1)	83·1 (73·5 to 93·4)
Tajikistan	92·5 (88·7 to 96·2)	122·2 (119·1 to 125·2)	121·9 (117·6 to 126·2)	171·4 (166·5 to 176·5)	207·3 (199 to 215·1)	185·4 (173·2 to 197·3)	228·5 (210·7 to 245·5)	253 (229·8 to 279·9)
Turkmenistan	53·2 (51 to 55·4)	71·7 (70 to 73·2)	75 (71·2 to 78·9)	108·2 (105·3 to 111)	134·2 (131·1 to 137·1)	101·2 (95·3 to 107·3)	113·6 (105·7 to 121·7)	113·1 (103·2 to 124·4)
Uzbekistan	324·7 (304·7 to 345)	394·1 (373·6 to 414·6)	392 (366·4 to 417·5)	572·6 (552·4 to 593)	712·5 (694·7 to 729)	614·8 (595·5 to 635·1)	721·2 (678·2 to 765·6)	713·9 (635·4 to 799·1)
Central Europe	2299·3 (2240·9 to 2362·4)	1972·1 (1947·8 to 1994·9)	1884·5 (1863·3 to 1908·1)	2053 (2032·5 to 2075·8)	1663 (1645·1 to 1681·9)	1201·3 (1187·1 to 1215·2)	1209·1 (1194·7 to 1225·3)	1069·3 (940·4 to 1218·7)
Albania	50·2 (48·1 to 52·1)	67·1 (65·2 to 68·9)	70·8 (67·3 to 74)	73·4 (69·9 to 77)	88·9 (85·9 to 92·2)	59·7 (56·9 to 62·6)	42·6 (40·6 to 44·6)	37·4 (33·5 to 41·9)
Bosnia and Herzegovina	90·8 (79·9 to 102·7)	108·6 (98·3 to 119·3)	82·6 (70·6 to 96·5)	77·5 (69·8 to 85·8)	65·5 (60·5 to 70·6)	41 (39·2 to 42·9)	33·4 (31·4 to 35·3)	26·1 (23·8 to 28·5)
Bulgaria	166·8 (166 to 167·7)	139·2 (138·4 to 140·1)	137·2 (136·5 to 138)	127·3 (126·6 to 128)	99·3 (98·7 to 100)	71·3 (70·8 to 71·9)	74·5 (74·1 to 75)	60·1 (55·4 to 65·2)
Croatia	91·3 (90·8 to 91·8)	75·9 (75·4 to 76·4)	61·7 (61·3 to 62·2)	67·8 (67·4 to 68·3)	53·8 (53·4 to 54·2)	42·3 (42 to 42·7)	42·5 (42·2 to 42·9)	35·2 (31 to 39·9)
Czech Republic	188·9 (188·1 to 189·8)	128·4 (127·7 to 129)	151·1 (150·4 to 151·8)	149·5 (148·8 to 150·2)	128·3 (127·6 to 129)	91·4 (90·8 to 92)	114 (113·4 to 114·6)	108·9 (97·4 to 121·9)
Hungary	196·3 (195·4 to 197·2)	144·1 (143·4 to 144·8)	148·6 (147·9 to 149·4)	148·8 (147·9 to 149·6)	123·6 (123 to 124·3)	97·1 (96·5 to 97·7)	89·7 (89·2 to 90·2)	82·2 (72·3 to 93·6)
Montenegro	12·3 (11·4 to 13·3)	12·6 (11·8 to 13·3)	10·6 (9·9 to 11·3)	10·5 (9·8 to 11·2)	9·6 (9 to 10·3)	8·4 (8 to 8·8)	7·5 (7·2 to 7·7)	6·6 (6·1 to 7·1)
North Macedonia	39·5 (35·2 to 44·2)	39·5 (35 to 44·4)	36·9 (33·6 to 40·1)	39·8 (37·3 to 42·5)	35·3 (33·9 to 36·7)	24·9 (23·8 to 26)	23·7 (22·5 to 24·9)	22·3 (20·1 to 24·5)
Poland	730·2 (709·4 to 752·8)	633·3 (616·3 to 648·3)	525·3 (512·6 to 537·7)	678·1 (662·8 to 695·5)	531·3 (517·6 to 546·6)	363·4 (351·7 to 375·4)	403·8 (391·8 to 416·7)	363 (313·9 to 420)
Romania	418·8 (387·1 to 455)	343·2 (341·7 to 344·7)	406·8 (405·3 to 408·3)	398·8 (397·4 to 400·1)	289·1 (287·8 to 290·5)	226·9 (225·8 to 228·1)	206 (205 to 207·1)	173·3 (146·9 to 204·3)
Serbia	182·7 (181·4 to 184)	165·1 (164·3 to 165·8)	145·2 (144·5 to 145·9)	157·1 (156·4 to 157·9)	137·6 (136·9 to 138·3)	104 (103·3 to 104·7)	87·9 (83 to 92·5)	79·9 (67·6 to 94·5)
Slovakia	99·6 (99 to 100·1)	87·4 (86·9 to 87·9)	80·3 (79·8 to 80·7)	94·7 (94·2 to 95·2)	78·6 (78·2 to 79·1)	53 (52·6 to 53·3)	61·3 (60·8 to 61·7)	55·5 (49 to 62·9)
Slovenia	31·8 (28·2 to 36)	27·8 (27·1 to 28·5)	27·6 (27 to 28·2)	29·5 (28·8 to 30·3)	21·9 (21·3 to 22·6)	17·7 (17·2 to 18·1)	22·3 (21·7 to 22·8)	18·8 (16·4 to 21·6)
Eastern Europe	4211·2 (4107·3 to 4325·8)	4181 (4130·6 to 4227·8)	2970·8 (2937 to 3001·7)	3364·2 (3327·8 to 3401·9)	2864·9 (2833·7 to 2901·5)	1897·8 (1874·7 to 1920·3)	2524·7 (2494·6 to 2556)	2241·8 (1935 to 2596·5)
Belarus	194·2 (186·6 to 201·8)	198·3 (191·7 to 204·6)	145·6 (140·8 to 150·6)	156·5 (151·1 to 161·8)	138·2 (133·9 to 143·3)	92·1 (88·3 to 96·3)	110·4 (105·9 to 115·1)	102·3 (86 to 121·5)
Estonia	20·1 (19·9 to 20·3)	20·3 (20·1 to 20·5)	21·6 (21·3 to 21·8)	22·5 (22·3 to 22·7)	21 (20·8 to 21·2)	12·8 (12·6 to 13)	15·3 (15·1 to 15·5)	13·2 (11·7 to 15)
Latvia	32·7 (32 to 33·3)	36 (35·6 to 36·3)	35·2 (34·9 to 35·5)	35·6 (35·3 to 36)	36·3 (36 to 36·6)	20 (19·8 to 20·2)	18·6 (18·3 to 18·8)	19·2 (17 to 21·7)
Lithuania	57·9 (54·5 to 62)	61·5 (61·1 to 61·9)	54·8 (54·5 to 55·2)	49·9 (49·6 to 50·3)	55·6 (55·2 to 55·9)	32·2 (32 to 32·5)	32·2 (32 to 32·5)	27 (24 to 30·4)
Moldova	85·4 (80·9 to 89·9)	83·7 (79·5 to 88·1)	71·6 (65 to 79·3)	88·9 (84·2 to 93·7)	77 (73·7 to 80·3)	45·4 (42·9 to 48·2)	43·3 (40·6 to 46·1)	32·1 (28·2 to 36·5)
Russia	2962·2 (2941·9 to 2981·7)	2941·6 (2933·8 to 2950·2)	1943 (1936·5 to 1949·3)	2251·7 (2245·3 to 2258·8)	1897·2 (1892 to 1903·3)	1284·9 (1280·2 to 1289·8)	1802·8 (1796·9 to 1808·7)	1660·8 (1441·5 to 1913·3)
Ukraine	858·6 (775·8 to 957·7)	839·5 (794·4 to 882·9)	699 (669·3 to 728)	759·1 (727·4 to 792·3)	639·6 (610·3 to 674)	410·3 (390·7 to 430)	502 (473·8 to 531·3)	387·2 (327 to 458)
High income	13588·8 (13426·7 to 13752·3)	14449·9 (14357·3 to 14535·2)	13806·1 (13717·1 to 13892)	12482·3 (12409·5 to 12555·5)	12529·1 (12463·9 to 12594·2)	11848·2 (11787·7 to 11915·6)	11897·5 (11850·5 to 11943·9)	11186·1 (10206·9 to 12315·2)
Australasia	252·3 (250·9 to 253·7)	299·7 (298·2 to 301·3)	332·3 (331·3 to 333·3)	279·3 (278·2 to 280·3)	316·3 (314·9 to 317·5)	300 (298·8 to 301·1)	358·7 (357·4 to 359·9)	370·9 (334 to 412·2)
Australia	202·1 (201·1 to 203)	237·5 (236·7 to 238·3)	269 (268·1 to 269·9)	228 (226·9 to 229)	256·3 (255·1 to 257·3)	243·9 (242·9 to 244·8)	296·2 (295 to 297·4)	311·4 (281·1 to 345·2)
New Zealand	50·2 (49·2 to 51·3)	62·3 (61 to 63·5)	63·3 (63 to 63·7)	51·3 (50·9 to 51·6)	60 (59·4 to 60·6)	56·1 (55·7 to 56·5)	62·5 (62 to 62·9)	59·5 (52·8 to 67·1)
High-income Asia Pacific	3069·5 (2957·6 to 3180·8)	2625·8 (2545·6 to 2702·7)	2987·2 (2906·8 to 3064·2)	2445·5 (2376·5 to 2513·3)	1979·8 (1918·9 to 2041·9)	1828·3 (1773·5 to 1887·5)	1595·2 (1553·5 to 1640·6)	1376·6 (1276·3 to 1489·7)
Brunei	3·1 (3 to 3·2)	4·1 (4·1 to 4·2)	4·9 (4·8 to 5·1)	5·8 (5·5 to 6)	7 (6·7 to 7·3)	7·3 (7 to 7·6)	6·5 (5·8 to 7·1)	6·6 (5·7 to 7·5)
Japan	2217·7 (2121·9 to 2314·2)	1596·7 (1538·9 to 1656)	1943·8 (1881·7 to 2009·2)	1574·7 (1527·4 to 1623)	1210·7 (1174·1 to 1248·6)	1189·4 (1152·7 to 1228·3)	1075·1 (1048·2 to 1103·4)	900·4 (822·4 to 990·1)
Aichi	91·5 (87·5 to 95·6)	77·2 (73·9 to 80·6)	116 (111·7 to 120·2)	86·7 (83·9 to 89·6)	69·9 (67·6 to 72·2)	74·6 (72·3 to 77·1)	70·2 (68·5 to 72·1)	60·2 (55 to 66·2)
Akita	30·4 (29 to 31·9)	20·8 (19·9 to 21·8)	17 (16 to 17·9)	16·1 (15·6 to 16·7)	11·1 (10·7 to 11·4)	9 (8·7 to 9·2)	6·9 (6·7 to 7·1)	5 (4·5 to 5·5)
Aomori	32·8 (31·3 to 34·4)	23·6 (22·6 to 24·7)	23·4 (22·1 to 24·7)	21·4 (20·6 to 22·2)	14·9 (14·4 to 15·4)	13 (12·6 to 13·5)	9·7 (9·4 to 10)	7·7 (7 to 8·5)
Chiba	58·1 (55·7 to 60·5)	37·8 (36·3 to 39·4)	70·2 (67·7 to 72·7)	67·1 (65·3 to 69·1)	52·5 (50·9 to 54·1)	55·4 (53·8 to 57)	51·7 (50·4 to 53)	41·9 (38·3 to 45·9)
Ehime	38·5 (36·8 to 40·2)	23·4 (22·4 to 24·4)	23·4 (22·1 to 24·6)	19·7 (19·1 to 20·4)	14·6 (14·1 to 15)	13·2 (12·7 to 13·7)	11·5 (11·2 to 11·9)	8·7 (7·9 to 9·7)
Fukui	19 (18·2 to 19·9)	11·8 (11·2 to 12·5)	12 (11·1 to 13)	10·5 (10·2 to 10·9)	8·6 (8·3 to 8·9)	8 (7·8 to 8·3)	6·9 (6·7 to 7·1)	5·6 (5·1 to 6·2)
Fukuoka	94·2 (89·9 to 98·4)	67·3 (64·6 to 70·1)	70·9 (68·3 to 73·6)	64·8 (62·9 to 66·8)	48·1 (46·7 to 49·6)	47·3 (45·9 to 48·7)	47 (45·7 to 48·4)	40·6 (36·9 to 44·7)
Fukushima	56·2 (53·8 to 58·7)	33·9 (32·5 to 35·4)	29·4 (27·8 to 31)	29·3 (28·3 to 30·3)	22·8 (22·1 to 23·5)	20·3 (19·6 to 21)	15·7 (15·2 to 16·2)	12·1 (11 to 13·5)
Gifu	38 (36·4 to 39·7)	27·3 (26·1 to 28·4)	31·8 (30·1 to 33·5)	25·5 (24·7 to 26·4)	20 (19·3 to 20·7)	20·2 (19·5 to 20·9)	17·2 (16·7 to 17·7)	13·5 (12·3 to 14·9)
Gunma	43·2 (41·4 to 45·1)	25·8 (24·8 to 26·8)	29·5 (27·9 to 31·1)	25·3 (24·5 to 26·1)	19·5 (18·9 to 20·1)	19·4 (18·8 to 20·1)	16 (15·6 to 16·5)	12·6 (11·4 to 13·9)
Hiroshima	55·4 (53 to 57·8)	36·3 (34·8 to 37·8)	46·3 (44·4 to 48·1)	36·8 (35·6 to 38)	28·3 (27·3 to 29·3)	27·6 (26·7 to 28·5)	25·8 (25·1 to 26·5)	20·8 (19 to 23)
Hokkaidō	107·1 (101·9 to 112·2)	79·9 (76·4 to 83·5)	92·4 (88·8 to 96·1)	75·4 (73 to 77·8)	54 (52·3 to 55·7)	47 (45·5 to 48·6)	40·4 (39·3 to 41·4)	31·3 (28·5 to 34·6)

Hyōgo	89·8 (86 to 93·6)	67·7 (64·9 to 70·6)	91·5 (88·2 to 94·9)	68·3 (66·3 to 70·5)	52·9 (51·3 to 54·6)	54·1 (52·4 to 55·8)	48·2 (47·1 to 49·5)	39·9 (36·5 to 43·8)
Ibaraki	55·4 (53·1 to 57·8)	32·3 (31·1 to 33·5)	37·5 (36·1 to 38·9)	36·5 (35·4 to 37·7)	28·5 (27·6 to 29·5)	28·3 (27·4 to 29·2)	24 (23·4 to 24·6)	18·9 (17·2 to 20·8)
Ishikawa	23·6 (22·6 to 24·7)	15·2 (14·6 to 15·9)	18·3 (17·3 to 19·4)	14·9 (14·4 to 15·5)	11·3 (10·9 to 11·7)	11·5 (11·1 to 11·9)	9·8 (9·5 to 10·1)	8·2 (7·5 to 9)
Iwate	37·2 (35·5 to 38·8)	25·3 (24·2 to 26·4)	23·1 (21·8 to 24·3)	19·4 (18·7 to 20·1)	14·3 (13·8 to 14·7)	12·4 (11·9 to 12·8)	9·7 (9·4 to 10)	7·5 (6·8 to 8·3)
Kagawa	23·5 (22·4 to 24·5)	14·2 (13·6 to 14·8)	15·1 (14·2 to 16)	12·8 (12·4 to 13·3)	9·4 (9·1 to 9·8)	9·7 (9·4 to 10·1)	8·5 (8·2 to 8·7)	6·7 (6·1 to 7·4)
Kagoshima	51·5 (49·5 to 53·5)	31 (29·8 to 32·2)	24·6 (23·2 to 25·9)	24·7 (23·9 to 25·6)	18·9 (18·3 to 19·4)	16 (15·5 to 16·6)	15·3 (14·9 to 15·8)	12·2 (11·1 to 13·4)
Kanagawa	70·6 (67·6 to 73·5)	64·7 (62·1 to 67·4)	119·3 (114·2 to 124·8)	94·9 (92·4 to 97·6)	78·4 (76 to 80·7)	83·2 (80·8 to 85·5)	78·2 (76·3 to 80)	64·4 (59·1 to 70·4)
Kōchi	22·9 (21·9 to 23·9)	12·3 (11·7 to 13)	11·9 (10·9 to 13)	10·1 (9·7 to 10·4)	7·3 (7·1 to 7·6)	6·7 (6·5 to 7)	5·5 (5·3 to 5·7)	4·3 (3·9 to 4·8)
Kumamoto	47·2 (45·1 to 49·3)	29·1 (27·9 to 30·3)	25·5 (24·1 to 26·9)	24·5 (23·7 to 25·3)	19·1 (18·5 to 19·7)	17·2 (16·6 to 17·8)	16·4 (15·9 to 16·9)	13·6 (12·3 to 15)
Kyōto	46·5 (44·5 to 48·4)	32·3 (31 to 33·6)	40 (38·5 to 41·5)	31·9 (31·1 to 32·8)	23·6 (22·8 to 24·3)	23·8 (23 to 24·6)	21·1 (20·6 to 21·7)	18·2 (16·6 to 19·9)
Mie	36·4 (34·7 to 38)	24 (23 to 25·1)	26·5 (25·1 to 27·9)	21·1 (20·4 to 21·8)	17·6 (17 to 18·2)	17·6 (17 to 18·3)	15·4 (15 to 15·8)	12 (10·9 to 13·2)
Miyagi	43·2 (41·3 to 45·1)	29·3 (28·1 to 30·6)	29·6 (27·9 to 31·1)	31 (30 to 32·1)	23·2 (22·4 to 23·9)	22·1 (21·4 to 22·8)	19·1 (18·6 to 19·6)	15·8 (14·4 to 17·4)
Miyazaki	29·8 (28·5 to 31·1)	19 (18·2 to 19·8)	17·1 (16·2 to 18)	17·1 (16·6 to 17·7)	12·3 (11·9 to 12·7)	11 (10·6 to 11·4)	10·3 (10 to 10·6)	7·8 (7·1 to 8·6)
Nagano	54·3 (52·2 to 56·4)	33·2 (31·9 to 34·5)	33·2 (31·4 to 34·8)	26·7 (26 to 27·5)	21·3 (20·7 to 21·9)	21·1 (20·5 to 21·8)	17·4 (17 to 17·8)	13·6 (12·4 to 15)
Nagasaki	47·3 (45·5 to 49·1)	29·1 (28 to 30·3)	26·3 (24·8 to 27·7)	22·5 (21·8 to 23·2)	16·7 (16·2 to 17·2)	14 (13·5 to 14·5)	12·1 (11·7 to 12·4)	9·8 (8·9 to 10·8)
Nara	19 (18·1 to 19·9)	12·1 (11·6 to 12·6)	16·4 (15·5 to 17·2)	16 (15·5 to 16·5)	13 (12·6 to 13·5)	13·1 (12·8 to 13·5)	10·8 (10·5 to 11·1)	8·5 (7·8 to 9·3)
Niigata	63·8 (61·1 to 66·5)	39·3 (37·9 to 40·7)	37 (35·5 to 38·4)	32·7 (31·8 to 33·8)	24·1 (23·3 to 24·8)	21·6 (20·9 to 22·4)	18·1 (17·6 to 18·6)	14·3 (13 to 15·7)
Ōita	31·9 (30·5 to 33·3)	19·3 (18·5 to 20·1)	17·8 (16·8 to 18·8)	16·2 (15·6 to 16·7)	11·8 (11·4 to 12·1)	10·9 (10·5 to 11·3)	10·2 (9·9 to 10·5)	8·1 (7·3 to 8·9)
Okayama	41·8 (39·9 to 43·7)	26·7 (25·6 to 27·8)	30·4 (28·7 to 32)	24·2 (23·4 to 25)	18·7 (18·1 to 19·4)	19·1 (18·4 to 19·8)	16·8 (16·3 to 17·3)	14·4 (13 to 15·9)
Okinawa	27·4 (26·1 to 28·5)	17·6 (16·9 to 18·3)	20·3 (19·1 to 21·4)	19·8 (19·2 to 20·5)	17·4 (16·9 to 18)	16·9 (16·4 to 17·4)	17·2 (16·7 to 17·7)	15·6 (14·1 to 17·3)
Ōsaka	108·2 (103·4 to 112·9)	102 (98 to 106·4)	168·5 (161·4 to 176·3)	110·7 (107·5 to 114)	85·5 (82·8 to 88·4)	88·5 (85·8 to 91·3)	75·5 (73·6 to 77·5)	64·3 (58·5 to 70·9)
Saga	25·4 (24·4 to 26·5)	15·2 (14·5 to 16)	13·1 (12·1 to 14·1)	12·4 (12 to 12·8)	9·4 (9·1 to 9·7)	8·6 (8·3 to 8·9)	7·7 (7·4 to 7·9)	6·2 (5·6 to 6·8)
Saitama	59·2 (56·8 to 61·7)	42 (40·4 to 43·8)	89·4 (86·3 to 92·5)	75·9 (73·8 to 77·9)	62·8 (61 to 64·8)	66·7 (64·7 to 68·8)	59·4 (58 to 61)	50·3 (46·1 to 55·2)
Shiga	23·6 (22·6 to 24·6)	14·2 (13·6 to 14·8)	14·4 (13·6 to 15·2)	15·8 (15·3 to 16·3)	13·2 (12·8 to 13·6)	14·1 (13·6 to 14·6)	13·5 (13·2 to 13·9)	11 (10·1 to 12·1)
Shimane	23·6 (22·6 to 24·6)	14·1 (13·4 to 14·9)	11·5 (10·6 to 12·5)	9·9 (9·6 to 10·3)	7·5 (7·2 to 7·7)	6·5 (6·3 to 6·7)	5·7 (5·6 to 5·9)	4·8 (4·3 to 5·3)
Shizuoka	65·1 (62·3 to 68)	47·2 (45·3 to 49·2)	59·1 (57 to 61·1)	47·8 (46·2 to 49·4)	36·8 (35·6 to 38)	36 (34·8 to 37·1)	32·1 (31·3 to 32·9)	25·1 (23 to 27·6)
Tochigi	41·7 (40 to 43·5)	24·3 (23·3 to 25·3)	27·4 (25·9 to 28·9)	26 (25·2 to 26·9)	19·8 (19·1 to 20·4)	18·9 (18·2 to 19·7)	16·6 (16·1 to 17·1)	13·3 (12·2 to 14·7)
Tokushima	20·5 (19·5 to 21·4)	12·5 (11·8 to 13·2)	11·8 (10·8 to 12·8)	10·6 (10·2 to 11)	7·8 (7·5 to 8·1)	7·1 (6·9 to 7·4)	6 (5·8 to 6·2)	4·9 (4·4 to 5·4)
Tōkyō	165·1 (158 to 172·4)	186·3 (178·8 to 194·8)	227 (216·7 to 238·8)	139·9 (136·1 to 143·8)	102·6 (99·6 to 105·6)	100·1 (97·5 to 102·9)	108 (105·5 to 110·6)	109·1 (100·8 to 118·4)
Tottori	15·9 (15·2 to 16·6)	9·6 (9·1 to 10·1)	8·6 (7·9 to 9·3)	8·2 (8 to 8·5)	6·3 (6·1 to 6·5)	5·5 (5·4 to 5·7)	4·9 (4·8 to 5·1)	4 (3·6 to 4·5)
Toyama	23·2 (22·1 to 24·2)	15·6 (14·9 to 16·4)	17·5 (16·5 to 18·4)	13·4 (13 to 13·9)	9·9 (9·6 to 10·3)	10·2 (9·8 to 10·5)	8·2 (8 to 8·5)	6·6 (6·1 to 7·3)
Wakayama	25·8 (24·6 to 26·9)	16 (15·3 to 16·6)	18·3 (17·3 to 19·3)	13·6 (13·2 to 14·1)	10 (9·6 to 10·4)	9·6 (9·2 to 9·9)	7·6 (7·4 to 7·9)	6 (5·5 to 6·7)
Yamagata	34·6 (33·1 to 36·1)	21·5 (20·6 to 22·4)	16·8 (15·9 to 17·8)	16·6 (16·1 to 17·2)	12·6 (12·2 to 13)	10·9 (10·5 to 11·3)	8·7 (8·4 to 8·9)	6·9 (6·3 to 7·5)
Yamaguchi	38·4 (36·8 to 40·1)	25·2 (24·2 to 26·3)	25·2 (23·8 to 26·5)	19·7 (19·1 to 20·4)	13·8 (13·3 to 14·3)	13·2 (12·7 to 13·7)	11·5 (11·1 to 11·8)	8·9 (8 to 9·8)
Yamanashi	20·2 (19·4 to 21)	12·1 (11·5 to 12·8)	12 (11·1 to 12·9)	10·1 (9·8 to 10·4)	8·7 (8·4 to 9)	8·3 (8·1 to 8·6)	6·6 (6·4 to 6·8)	5·3 (4·8 to 5·8)
South Korea	803·7 (750·5 to 858)	966·4 (915·9 to 1015·5)	993·3 (934 to 1050·6)	821·9 (768·9 to 874·7)	707 (657·9 to 754·4)	571·2 (533·2 to 610·1)	458 (423·7 to 492·7)	412·5 (369·2 to 466·3)
Singapore	45 (43·4 to 46·4)	58·5 (56·7 to 60·3)	45 (42·2 to 47·9)	43·1 (40·1 to 46·3)	55·1 (51·2 to 59·2)	60·4 (56·6 to 64·7)	55·6 (51·7 to 59·7)	57·1 (45·7 to 71)
High-income North America	4015 (4001·9 to 4028·2)	4683·4 (4674·1 to 4696·3)	3948·7 (3940 to 3961·1)	3974·8 (3967 to 3984·4)	4579·5 (4572·2 to 4587·7)	4384·4 (4376·7 to 4393·6)	4330·2 (4322·9 to 4339·5)	4200·8 (3932·7 to 4495·2)
Canada	361·8 (360·7 to 362·9)	456·8 (455·6 to 457·9)	347·9 (346·7 to 349·1)	358·8 (357·6 to 360·1)	401·5 (400·3 to 402·8)	331·5 (330·3 to 332·8)	374·6 (373·5 to 375·8)	373·3 (347·5 to 401·7)
Greenland	1 (1 to 1)	1·5 (1·5 to 1·5)	1·1 (1 to 1·1)	1 (0·9 to 1)	1·3 (1·2 to 1·3)	0·9 (0·9 to 1)	0·8 (0·8 to 0·9)	0·8 (0·7 to 0·9)
USA	3652·1 (3639·4 to 3664·9)	4225 (4215·9 to 4237·5)	3599·7 (3591·6 to 3611·7)	3614·9 (3607·4 to 3623·9)	4176·6 (4169·5 to 4184·3)	4051·9 (4044·2 to 4061·2)	3954·7 (3947·5 to 3963·8)	3826·7 (3584·3 to 4092·5)
Alabama	76·3 (76 to 76·8)	82·2 (81 to 83·4)	63·9 (63·1 to 64·8)	62·6 (61·9 to 63·3)	63·9 (62·8 to 65)	62·2 (61·2 to 63·1)	57·7 (56·9 to 58·6)	61·8 (58·1 to 65·7)
Alaska	3·3 (3·3 to 3·4)	7·2 (7 to 7·3)	7·1 (7 to 7·2)	9·9 (9·7 to 10·1)	12 (11·9 to 12·2)	9·9 (9·7 to 10·1)	11 (10·8 to 11·2)	10·7 (9·8 to 11·7)
Arizona	20·4 (20·2 to 20·6)	35·4 (34·9 to 35·9)	37 (36·7 to 37·2)	49·7 (49·1 to 50·4)	69·1 (67·7 to 70·4)	84·6 (83·5 to 85·8)	87·9 (87·2 to 88·8)	84·1 (78·9 to 89·7)
Arkansas	44·7 (44·5 to 44·9)	41·7 (41·1 to 42·3)	35 (34·7 to 35·4)	36·2 (35·7 to 36·8)	36·3 (35·6 to 37)	37·8 (37 to 38·5)	37·1 (36·4 to 37·7)	38·2 (35·9 to 40·7)
California	250·9 (249·8 to 251·9)	357·3 (353 to 361·5)	345·6 (344·2 to 347)	406·3 (401 to 412·1)	609·6 (599·7 to 619·6)	530·4 (523·3 to 537·3)	505·5 (497·2 to 514·4)	453·9 (423·3 to 487·6)
Colorado	31·1 (30·9 to 31·2)	42·6 (42·1 to 43)	40·3 (40·1 to 40·6)	50·1 (49·5 to 50·7)	54·6 (53·7 to 55·4)	65·4 (64·8 to 66)	65·9 (65·1 to 66·7)	57·7 (53·9 to 61·7)
Connecticut	48·6 (48·4 to 48·8)	58·9 (58·2 to 59·7)	47·3 (47 to 47·6)	40 (39·4 to 40·6)	50·3 (49·3 to 51·3)	43·7 (42·6 to 44·8)	37·9 (37·1 to 38·6)	37·8 (35·3 to 40·5)
Delaware	8 (8 to 8·1)	11·9 (11·7 to 12·1)	9·9 (9·8 to 10·1)	9·2 (9 to 9·4)	11·1 (10·8 to 11·4)	11·1 (10·8 to 11·3)	11·5 (11·3 to 11·8)	11·3 (10·3 to 12·3)
Washington, DC	22·1 (17·8 to 26·9)	22·8 (20·3 to 25·3)	14·4 (14·2 to 14·5)	9·7 (9·5 to 9·9)	11·4 (11 to 11·7)	7·9 (7·4 to 8·4)	8·7 (7·3 to 10·1)	9·6 (7·5 to 12·2)
Florida	63·6 (63·1 to 64·1)	109·2 (108 to 110·3)	109·5 (108·2 to 111)	130·1 (128·9 to 131·5)	199·2 (196·2 to 202·2)	203·3 (200·5 to 206·3)	212·3 (209·6 to 215)	228·2 (213·7 to 243·4)
Georgia	84·9 (84·5 to 85·4)	102·9 (102·2 to 103·7)	94·5 (93·9 to 95·3)	90·4 (89·3 to 91·5)	112·9 (111·4 to 114·4)	132·5 (130·7 to 134·2)	133·6 (132·1 to 135)	133·9 (125·4 to 142·7)
Hawaii	14·5 (14·4 to 14·6)	18·5 (18·1 to 18·8)	15·9 (15·8 to 16·1)	18 (17·6 to 18·3)	20·4 (19·9 to 20·8)	17·4 (17·2 to 17·6)	18·6 (18·3 to 18·9)	17·8 (16·5 to 19·1)
Idaho	15·2 (15 to 15·4)	17·7 (17·4 to 18·1)	14·1 (13·9 to 14·2)	20 (19·7 to 20·2)	16·6 (16·3 to 17)	20·5 (20·2 to 20·8)	22·2 (21·9 to 22·5)	22·8 (21·2 to 24·6)
Illinois	209 (208·2 to 209·9)	225·3 (222·7 to 228·2)	200·4 (199·5 to 201·3)	190·1 (188·2 to 191·9)	197·1 (194 to 200)	185·5 (183·3 to 187·6)	158·8 (156·8 to 160·8)	151·6 (141·8 to 161·9)

Indiana	90 (89·6 to 90·4)	114·4 (113·6 to 115·2)	97·3 (96·7 to 97·9)	88·3 (87·4 to 89·3)	86·4 (84·9 to 87·8)	87·4 (86·4 to 88·5)	83·8 (83 to 84·7)	83·4 (78·4 to 88·7)
Iowa	60·3 (60 to 60·5)	64·3 (63·3 to 65·1)	46·3 (45·9 to 46·6)	49·1 (48·4 to 49·8)	39·6 (38·8 to 40·3)	38·2 (37·4 to 39)	38·6 (37·9 to 39·3)	37·7 (35·5 to 40·1)
Kansas	42·1 (41·9 to 42·2)	51·1 (50 to 52·2)	36·8 (36·5 to 37·1)	40·1 (39·5 to 40·6)	39·4 (38·7 to 40)	39·5 (38·9 to 40)	40·9 (40·3 to 41·6)	36·7 (34·5 to 39)
Kentucky	67·2 (66·8 to 67·7)	70·6 (69·6 to 71·7)	57·3 (56·5 to 58·1)	58·2 (57·5 to 59)	55·1 (54·1 to 56·2)	55·7 (55 to 56·4)	53·2 (52·6 to 53·8)	55·5 (52·1 to 59)
Louisiana	70·7 (70·3 to 71·1)	90 (88·5 to 91·4)	72·8 (72·3 to 73·4)	81·2 (80·1 to 82·2)	73·7 (72·3 to 75·2)	67 (65·8 to 68·3)	62·7 (61·9 to 63·5)	57·7 (54·2 to 61·4)
Maine	21·1 (21 to 21·2)	23·6 (23·4 to 23·9)	17·5 (17·3 to 17·8)	16·5 (16·2 to 16·8)	17·5 (17 to 17·9)	13·8 (13·5 to 14)	12·7 (12·4 to 13)	12·4 (11·5 to 13·4)
Maryland	58·1 (57·8 to 58·4)	75·1 (74·3 to 75·9)	66·7 (66·3 to 67·1)	60·6 (60 to 61·3)	80·7 (79·5 to 81·7)	73·3 (72·2 to 74·4)	70·1 (69·3 to 70·9)	74·1 (69·2 to 79·3)
Massachusetts	110·6 (110·1 to 111)	113 (112·1 to 113·8)	89·8 (89·3 to 90·4)	73·2 (72·3 to 74·1)	91·9 (89·6 to 94·2)	82·5 (80·4 to 84·8)	72·8 (70·8 to 75)	69·7 (65 to 74·9)
Michigan	160·9 (160·2 to 161·6)	186 (184·3 to 187·6)	166·3 (165·6 to 167·2)	152·3 (150·8 to 153·8)	152 (149·6 to 154·3)	135·2 (133·1 to 137·1)	112·3 (111·6 to 113·2)	107·1 (100·6 to 114)
Minnesota	71·5 (71·1 to 71·8)	83·6 (82·5 to 84·8)	63·5 (63·1 to 64)	67·6 (67 to 68·3)	68·8 (67·8 to 69·7)	67·9 (67·1 to 68·8)	68·1 (67·5 to 68·6)	67·1 (63 to 71·6)
Mississippi	61·7 (60·7 to 62·7)	60·6 (59·4 to 61·7)	48·2 (47·8 to 48·6)	45·5 (45 to 46·1)	43·8 (43·1 to 44·5)	43·4 (42·7 to 44·1)	39 (38·6 to 39·3)	37·9 (35·6 to 40·3)
Missouri	90·1 (89·8 to 90·5)	101·5 (100·1 to 102·8)	79·5 (79 to 79·9)	78·6 (77·4 to 79·7)	80 (78·6 to 81·4)	76·5 (75·6 to 77·4)	76·4 (75·4 to 77·5)	73·6 (69·1 to 78·3)
Montana	13·7 (13·6 to 13·7)	16·2 (15·9 to 16·5)	12 (11·8 to 12·1)	14·2 (14 to 14·5)	11·9 (11·6 to 12·2)	11 (10·8 to 11·2)	12 (11·8 to 12·3)	11·2 (10·2 to 12·4)
Nebraska	30·4 (30·3 to 30·6)	33·3 (32·7 to 33·9)	25·2 (24·9 to 25·4)	26·5 (26·2 to 26·9)	24·6 (24·2 to 25)	25 (24·7 to 25·4)	26·3 (26 to 26·5)	25·6 (24·1 to 27·2)
Nevada	3·6 (3·6 to 3·6)	6·8 (6·7 to 6·9)	8·4 (8·2 to 8·8)	13·3 (13 to 13·5)	21·2 (20·7 to 21·8)	30·5 (30 to 31)	35·4 (34·6 to 36·3)	39·8 (37·3 to 42·6)
New Hampshire	12·3 (12·3 to 12·4)	14·5 (14·2 to 14·7)	13 (12·9 to 13·1)	13·6 (13·3 to 14)	17·6 (17·2 to 17·9)	14·6 (14·3 to 14·8)	12·8 (12·6 to 13·1)	12·4 (11·3 to 13·6)
New Jersey	118·3 (117·9 to 118·8)	134·8 (133·3 to 136·2)	115·8 (115·3 to 116·4)	98·4 (97·5 to 99·4)	123·7 (121·7 to 125·6)	116·5 (114·7 to 118·2)	102·5 (101 to 104·2)	102·3 (95·4 to 109·6)
New Mexico	19 (18·8 to 19·2)	28·6 (28·2 to 29)	21·3 (21·1 to 21·5)	26·6 (26·3 to 27)	28·2 (27·7 to 28·6)	27·4 (27·1 to 27·8)	26·5 (26 to 27)	24·7 (23·2 to 26·4)
New York	356 (354·1 to 357·8)	361·2 (356·1 to 366·2)	303·5 (302·3 to 305)	238·4 (236 to 240·9)	298·2 (292 to 304·7)	258·4 (254·4 to 262·7)	241·3 (238·2 to 244)	228 (212·5 to 244·6)
North Carolina	98·1 (97·3 to 99)	111·2 (110 to 112·4)	92·8 (91·6 to 93·9)	83·9 (83·1 to 84·8)	104·8 (102·8 to 106·9)	118·7 (116·8 to 120·6)	121·9 (119·9 to 123·8)	126·2 (118·3 to 134·6)
North Dakota	15·6 (15·4 to 15·8)	16·7 (16·3 to 17)	10·5 (10·3 to 10·6)	12 (11·8 to 12·3)	9·5 (9·1 to 9·9)	8 (7·7 to 8·4)	10·9 (10·4 to 11·3)	9·6 (8·6 to 10·7)
Ohio	191·1 (190·4 to 191·7)	216·8 (214·4 to 219·2)	195·1 (194·2 to 196)	170·4 (169·1 to 172)	168·8 (165·7 to 171·9)	154·1 (151 to 157·7)	138·8 (136·2 to 141·5)	134·5 (126·2 to 143·1)
Oklahoma	47·9 (47·6 to 48·3)	50·7 (49·9 to 51·5)	42·8 (42·1 to 43·4)	51 (50·3 to 51·6)	48·9 (48·3 to 49·5)	50·5 (49·6 to 51·4)	51·3 (50·4 to 52·2)	48·7 (45·8 to 51·8)
Oregon	33·6 (33·5 to 33·7)	39·2 (38·7 to 39·7)	33·3 (33 to 33·5)	43·3 (42·8 to 43·8)	42·5 (41·7 to 43·4)	45·6 (44·8 to 46·4)	45·8 (45·2 to 46·4)	40·2 (37·6 to 43)
Pennsylvania	248·4 (246·9 to 249·9)	232·4 (229·2 to 235·8)	187·3 (186·6 to 188)	161·4 (159·6 to 163·3)	173·2 (170·1 to 176·4)	146·7 (144 to 149·5)	141·1 (138·9 to 143·3)	141 (132·2 to 150·3)
Rhode Island	19·5 (19·4 to 19·5)	20·1 (19·7 to 20·5)	15·1 (15 to 15·3)	12·1 (11·7 to 12·5)	15·1 (14·5 to 15·6)	12·9 (12·5 to 13·4)	11·9 (11·4 to 12·3)	10·8 (9·8 to 11·8)
South Carolina	53·4 (53·2 to 53·7)	60·8 (59·9 to 61·7)	50·8 (50·1 to 51·5)	50·8 (50·2 to 51·4)	58·1 (57·1 to 59·2)	55·9 (55 to 56·8)	54·8 (53·8 to 55·7)	58·5 (54·9 to 62·3)
South Dakota	16·4 (16·2 to 16·5)	17·4 (17 to 17·7)	11·2 (11·1 to 11·4)	13 (12·8 to 13·2)	11·4 (11·1 to 11·6)	10·6 (10·4 to 10·8)	12·4 (12·2 to 12·7)	11·8 (10·9 to 12·9)
Tennessee	77·1 (76·5 to 77·6)	87·9 (87 to 88·9)	71·6 (71 to 72·2)	68·8 (68·1 to 69·5)	75·3 (73·7 to 77·1)	79·6 (78·1 to 81·1)	84·5 (83·2 to 85·8)	81·2 (76·2 to 86·4)
Texas	198 (197·1 to 199)	261·5 (258·9 to 263·9)	228·8 (227·5 to 230·2)	271·9 (268·9 to 274·9)	323·2 (317·8 to 328·9)	363·4 (357 to 369·3)	385·4 (381·2 to 389·7)	358·9 (336·3 to 382·8)
Utah	20·4 (20 to 20·7)	27·6 (27 to 28·3)	26·4 (26·2 to 26·6)	41·2 (40·8 to 41·7)	35·8 (35·1 to 36·6)	47·6 (46·4 to 48·8)	53 (51·8 to 54·1)	46·7 (43·8 to 49·7)
Vermont	8·8 (8·8 to 8·8)	9·4 (9·3 to 9·6)	7·9 (7·9 to 8)	7·7 (7·5 to 7·9)	8·3 (8·1 to 8·6)	6·5 (6·4 to 6·7)	5·7 (5·6 to 5·9)	5·9 (5·3 to 6·6)
Virginia	82·5 (82·1 to 82·9)	98·3 (97·7 to 98·8)	84·2 (83·7 to 84·6)	79·5 (78·5 to 80·6)	100·1 (98·1 to 102·2)	99·2 (97·4 to 101)	99·8 (98·2 to 101·4)	103·7 (97·1 to 110·8)
Washington	54·5 (54·3 to 54·7)	66·4 (65·5 to 67·2)	56·7 (56·4 to 57·1)	68·2 (67·4 to 69·1)	79·5 (77·7 to 81·5)	80 (78·6 to 81·5)	86·2 (84·8 to 87·7)	80·2 (74·9 to 86)
West Virginia	47 (46·7 to 47·3)	42·4 (41·7 to 43)	29·6 (29·2 to 30·1)	28·9 (28·5 to 29·3)	22·8 (22·4 to 23·3)	21·1 (20·7 to 21·6)	20·8 (20·4 to 21·3)	19·2 (18 to 20·5)
Wisconsin	82·4 (82 to 82·7)	94·5 (93·6 to 95·5)	73·1 (72·7 to 73·7)	76·1 (75·4 to 76·7)	73·7 (72·1 to 75·4)	69·3 (68 to 70·7)	67·4 (66·4 to 68·5)	66 (62 to 70·3)
Wyoming	7·2 (7·1 to 7·2)	8·8 (8·6 to 9)	6 (5·9 to 6·1)	10·1 (9·9 to 10·3)	7 (6·8 to 7·2)	6·3 (6·1 to 6·4)	6·9 (6·7 to 7·1)	7·2 (6·5 to 8·1)
Southern Latin America	685·8 (668·5 to 704·8)	800·2 (795·2 to 805·7)	830·2 (823·9 to 836·8)	977·6 (973·6 to 982·7)	1023·5 (1019·3 to 1028·6)	985·3 (979·2 to 991·9)	1045·3 (1037·2 to 1053·5)	971·9 (817·9 to 1158·6)
Argentina	443·1 (428·5 to 459·4)	468·2 (464 to 472·6)	529·7 (524·3 to 535·4)	683·2 (680·1 to 686·8)	679·9 (676·8 to 683·5)	685 (680·7 to 689·4)	751·1 (746·2 to 756·4)	698·8 (578·3 to 846·1)
Chile	196·9 (194·5 to 199·3)	270·8 (269·5 to 272·1)	245·1 (244 to 246·4)	240·2 (239·1 to 241·6)	288·5 (287·1 to 290·1)	247·6 (246·5 to 249)	246 (244·8 to 247·3)	226·7 (200·7 to 257·4)
Uruguay	45·8 (43 to 48·9)	61·2 (59·3 to 63·2)	55·3 (53·5 to 57)	54·1 (52·5 to 55·9)	55·1 (53·1 to 56·9)	52·7 (49·3 to 56·3)	48·2 (43·3 to 54·3)	46·3 (38·8 to 55)
Western Europe	5566·2 (5491·3 to 5645·4)	6040·8 (6020·9 to 6060·3)	5707·8 (5686·7 to 5728·7)	4805·1 (4794·8 to 4816)	4630 (4619·8 to 4639·2)	4350·3 (4342·1 to 4358·6)	4568·2 (4560·3 to 4576·7)	4265·9 (3836 to 4761)
Andorra	0·1 (0·1 to 0·2)	0·2 (0·2 to 0·3)	0·4 (0·4 to 0·4)	0·5 (0·5 to 0·6)	0·6 (0·6 to 0·7)	0·7 (0·7 to 0·8)	0·8 (0·8 to 0·8)	0·6 (0·6 to 0·7)
Austria	105 (103·5 to 106·6)	128·1 (127·5 to 128·7)	110·1 (109·6 to 110·7)	91·5 (91 to 92)	93·1 (92·6 to 93·6)	77·6 (77·1 to 78)	78·3 (77·9 to 78·7)	87·7 (82·3 to 93·7)
Belgium	142·7 (142 to 143·5)	156·5 (155·8 to 157·2)	141·8 (141 to 142·6)	122·9 (122·2 to 123·5)	125·7 (124·9 to 126·4)	116·4 (115·7 to 117·1)	129 (128·3 to 129·7)	121·6 (106·3 to 138·9)
Cyprus	13·8 (13·3 to 14·4)	14·5 (14·1 to 14·9)	12·1 (11·7 to 12·5)	13·4 (13·1 to 13·8)	14·6 (14·2 to 15·1)	11·2 (10·7 to 11·6)	13·4 (12·9 to 13·9)	15·2 (12·9 to 17·9)
Denmark	78·4 (75·1 to 81·7)	74·7 (73·1 to 76·3)	73·1 (71·3 to 74·8)	55·3 (53·7 to 56·7)	64·1 (62·7 to 65·5)	66·5 (65 to 68·2)	60·9 (59·6 to 62·3)	62·8 (55·4 to 71·2)
Finland	95·4 (94·8 to 95·9)	81·1 (80·6 to 81·5)	62·8 (62·4 to 63·2)	63·5 (63·1 to 63·9)	65·9 (65·5 to 66·4)	56·5 (56·1 to 56·8)	60·4 (60 to 60·7)	49·9 (45·6 to 54·6)
France	840·7 (837·7 to 843·7)	831·3 (828·9 to 833·5)	850·9 (848·5 to 853·3)	803·7 (801·3 to 806·1)	762·3 (760·1 to 764·8)	779·1 (776·5 to 782)	799·5 (797·1 to 801·9)	718·7 (650·9 to 794·1)
Germany	1059·1 (1020·3 to 1099·5)	1241·4 (1238·5 to 1244·2)	1006·6 (1003·3 to 1009·8)	833·1 (830·7 to 835·4)	883·4 (881 to 885·7)	759·5 (757·6 to 761·3)	669·1 (667·3 to 670·8)	740·3 (681·9 to 806·3)
Greece	155·5 (153 to 158·1)	155·1 (154·3 to 155·9)	142·5 (141·7 to 143·4)	143·4 (142·5 to 144·3)	102 (101·3 to 102·7)	102·4 (101·7 to 103)	111·4 (110·4 to 112·5)	86 (76·3 to 97·9)
Iceland	4 (3·9 to 4·1)	4·6 (4·6 to 4·7)	4·1 (4·1 to 4·2)	4·4 (4·3 to 4·4)	4·6 (4·6 to 4·7)	4·1 (4 to 4·2)	4·8 (4·6 to 4·9)	4·3 (3·7 to 5)
Ireland	64·1 (61·4 to 66·9)	60·8 (59·8 to 61·9)	64·7 (63·4 to 65·9)	72 (70·4 to 73·4)	52·2 (50·9 to 53·5)	56·6 (55·1 to 58)	74 (72·3 to 75·9)	60·8 (52 to 70·9)
Israel	47·6 (45·8 to 49·6)	55·5 (55·1 to 56)	81·1 (80·4 to 81·8)	92·9 (92·2 to 93·6)	102·5 (101·9 to 103·1)	136·3 (135·4 to 137·1)	165·3 (164·2 to 166·4)	192·6 (160·1 to 229)
Italy	882·2 (877·1 to 886·9)	923 (919·6 to 926·4)	913·8 (909·6 to 917·8)	635·8 (632·7 to 639·1)	564·6 (562·2 to 567·2)	543·6 (541·8 to 545·3)	559·7 (558 to 561·4)	439·7 (401·5 to 485·6)

Luxembourg	4·4 (4·3 to 4·5)	4·9 (4·8 to 5)	4·3 (4·2 to 4·3)	4·2 (4·1 to 4·2)	4·9 (4·8 to 4·9)	5·6 (5·5 to 5·7)	5·7 (5·6 to 5·8)	6·4 (5·7 to 7·3)
Malta	9·8 (9·4 to 10·3)	8·2 (8·1 to 8·3)	5·4 (5·3 to 5·5)	5·7 (5·7 to 5·9)	5·5 (5·4 to 5·6)	4·1 (4 to 4·2)	4·1 (4·1 to 4·2)	4·2 (3·6 to 5)
Monaco	0·4 (0·3 to 0·5)	0·3 (0·3 to 0·4)	0·3 (0·3 to 0·4)	0·3 (0·3 to 0·4)	0·3 (0·3 to 0·4)	0·3 (0·3 to 0·3)	0·3 (0·2 to 0·3)	0·3 (0·2 to 0·3)
Netherlands	228·1 (226·8 to 229·4)	241·8 (240·9 to 242·8)	230 (228·8 to 231·1)	179·6 (178·2 to 181·1)	198·6 (197·5 to 199·6)	207·7 (206·6 to 208·7)	182·2 (181·3 to 183·1)	177·6 (152·8 to 206·4)
Norway	61·9 (61·4 to 62·3)	61·5 (61·1 to 62)	64·6 (64·2 to 65)	50·7 (50·4 to 51·1)	60·7 (60·3 to 61·1)	58·4 (58 to 58·7)	60·4 (60 to 60·8)	56·7 (51·4 to 62·6)
Portugal	205 (195·5 to 214·8)	217·1 (212·3 to 221·9)	179·8 (175·8 to 183·8)	154 (150 to 158·1)	116 (112·7 to 119·3)	116·3 (113·2 to 119·5)	98·4 (96·2 to 100·8)	79·6 (67 to 94·4)
San Marino	0·3 (0·2 to 0·3)	0·3 (0·2 to 0·3)	0·2 (0·2 to 0·3)	0·2 (0·2 to 0·3)	0·3 (0·2 to 0·3)	0·3 (0·3 to 0·4)	0·3 (0·3 to 0·4)	0·3 (0·3 to 0·4)
Spain	545·1 (541 to 549·2)	652·8 (649·7 to 655·8)	652·7 (649·1 to 656·4)	544·9 (541·4 to 548·6)	395·2 (393 to 397·4)	399·4 (397·7 to 401·2)	484·1 (482·2 to 485·9)	369·2 (329·4 to 418·8)
Sweden	113·4 (112·8 to 114)	101·8 (101·3 to 102·3)	112·5 (112 to 113·1)	96·2 (95·6 to 96·7)	124·2 (123·6 to 124·9)	92·2 (91·6 to 92·7)	112·8 (112·2 to 113·4)	117·4 (109·9 to 125·6)
Stockholm	17·1 (17 to 17·3)	14·6 (14·1 to 15·1)	21·9 (21·7 to 22·1)	18·6 (18·4 to 18·8)	25·1 (24·9 to 25·3)	22·2 (22 to 22·4)	28·4 (28·2 to 28·6)	28·5 (26·6 to 30·5)
Sweden except Stockholm	96·3 (95·7 to 96·8)	87·2 (86·6 to 87·9)	90·6 (90·2 to 91·1)	77·5 (77·1 to 78)	99·1 (98·6 to 99·7)	70 (69·6 to 70·4)	84·4 (84 to 84·9)	88·9 (83·2 to 95)
Switzerland	83·2 (82·7 to 83·8)	95·9 (95·4 to 96·4)	98 (97·5 to 98·6)	73·4 (73 to 73·8)	85·4 (84·9 to 85·8)	75·9 (75·5 to 76·3)	80·2 (79·8 to 80·6)	88·3 (82·1 to 95·2)
UK	822 (802·1 to 845·8)	925 (911·1 to 938·7)	891·6 (873·9 to 908·9)	759·7 (757·9 to 761·5)	799·6 (797·7 to 801·6)	676 (674·1 to 677·9)	809·1 (806·5 to 811·5)	782·1 (700·7 to 875·3)
England	678·9 (658·5 to 699·8)	741·7 (721·4 to 760·8)	719·2 (698·3 to 740·7)	622·1 (610·7 to 633·6)	659 (649·3 to 668·2)	570·7 (565·4 to 575·5)	686·3 (681·9 to 690·7)	671·1 (606 to 743·7)
East Midlands	54·1 (52·5 to 55·7)	58·9 (57·3 to 60·4)	58·6 (57 to 60·3)	51·8 (50·9 to 52·7)	54·1 (53·2 to 54·8)	45·8 (45·4 to 46·2)	54·5 (54·2 to 54·9)	53·9 (49 to 59·5)
Derby	3·6 (3·5 to 3·7)	4 (3·8 to 4·1)	3·8 (3·6 to 3·9)	3·2 (3·1 to 3·3)	3·4 (3·3 to 3·5)	2·9 (2·8 to 2·9)	3·6 (3·5 to 3·6)	3·3 (3 to 3·7)
Derbyshire	10·1 (9·8 to 10·4)	10·6 (10·1 to 11·1)	10·5 (10·1 to 10·9)	8·8 (8·5 to 9)	9·1 (8·8 to 9·3)	7·5 (7·4 to 7·7)	8·3 (8·1 to 8·4)	7·9 (7·1 to 8·7)
Leicester	4·7 (4·6 to 4·8)	4·9 (4·7 to 5·1)	4·9 (4·7 to 5·1)	4·8 (4·7 to 5)	4·8 (4·6 to 4·9)	4 (3·9 to 4·1)	5·2 (5·1 to 5·4)	5·4 (4·8 to 5·9)
Leicestershire	6·6 (6·4 to 6·9)	7·6 (7·3 to 7·9)	7·7 (7·3 to 8·1)	7 (6·8 to 7·2)	7·3 (7·1 to 7·4)	6·4 (6·3 to 6·5)	6·9 (6·8 to 7·1)	7 (6·4 to 7·8)
Lincolnshire	7·3 (7·1 to 7·5)	7·7 (7·3 to 8·1)	7·8 (7·5 to 8)	6·9 (6·7 to 7·1)	7·1 (6·9 to 7·3)	6·1 (6 to 6·3)	7·7 (7·6 to 7·9)	7·9 (7·2 to 8·7)
Northamptonshire	6·9 (6·7 to 7·1)	7·6 (7·3 to 7·9)	8·1 (7·8 to 8·4)	7·6 (7·4 to 7·8)	8·3 (8·1 to 8·4)	7·4 (7·3 to 7·6)	9·2 (9 to 9·3)	8·9 (8·1 to 9·9)
Nottingham	4·5 (4·4 to 4·7)	4·9 (4·7 to 5·1)	4·4 (4·3 to 4·6)	3·8 (3·7 to 4)	4·4 (4·3 to 4·5)	3·4 (3·3 to 3·5)	4·3 (4·3 to 4·4)	4·5 (4·1 to 5)
Nottinghamshire	9·9 (9·6 to 10·2)	11·2 (10·7 to 11·6)	11 (10·6 to 11·4)	9·3 (9·1 to 9·6)	9·4 (9·2 to 9·6)	7·7 (7·6 to 7·8)	8·9 (8·8 to 9·1)	8·6 (7·8 to 9·6)
Rutland	0·4 (0·4 to 0·4)	0·5 (0·4 to 0·5)	0·4 (0·4 to 0·5)	0·4 (0·4 to 0·4)	0·4 (0·4 to 0·4)	0·3 (0·3 to 0·3)	0·3 (0·3 to 0·4)	0·4 (0·3 to 0·4)
East of England	64·7 (62·8 to 66·7)	71·5 (69·6 to 73·4)	70·5 (68·5 to 72·6)	64·8 (63·7 to 66)	69·4 (68·4 to 70·3)	61·4 (60·9 to 61·9)	73·2 (72·8 to 73·7)	71·7 (64·7 to 79·4)
Bedford	1·9 (1·8 to 1·9)	2·2 (2·1 to 2·2)	2·1 (2 to 2·2)	1·9 (1·9 to 2)	1·8 (1·8 to 1·9)	1·8 (1·7 to 1·8)	2·1 (2·1 to 2·2)	2·1 (1·9 to 2·3)
Cambridgeshire	5 (4·9 to 5·2)	5·7 (5·4 to 5·9)	5·9 (5·7 to 6·2)	5·9 (5·7 to 6·1)	6·6 (6·5 to 6·8)	6 (6 to 6·1)	7·5 (7·4 to 7·6)	7·4 (6·6 to 8·2)
Central Bedfordshire	2·6 (2·6 to 2·7)	3·2 (3·1 to 3·3)	3·3 (3·2 to 3·4)	3·3 (3·2 to 3·4)	3·2 (3·1 to 3·3)	2·8 (2·7 to 2·8)	3·3 (3·2 to 3·3)	3·2 (2·9 to 3·6)
Essex	15·6 (15·1 to 16·1)	17·8 (17·3 to 18·3)	17·6 (16·9 to 18·3)	15·9 (15·6 to 16·3)	16·4 (16·1 to 16·7)	14·4 (14·2 to 14·6)	16·5 (16·3 to 16·7)	16·4 (14·8 to 18·1)
Hertfordshire	13·9 (13·5 to 14·4)	15·3 (14·8 to 15·9)	14 (13·4 to 14·5)	12·4 (12·2 to 12·6)	13·7 (13·4 to 13·9)	12·7 (12·5 to 12·8)	14·8 (14·6 to 15)	14·6 (13 to 16·3)
Luton	2·5 (2·5 to 2·6)	3·2 (3·1 to 3·3)	3·2 (3·1 to 3·3)	2·9 (2·8 to 2·9)	3·3 (3·3 to 3·4)	2·9 (2·9 to 2·9)	3·6 (3·5 to 3·7)	3·4 (3·1 to 3·8)
Norfolk	8·9 (8·6 to 9·1)	9·2 (8·8 to 9·6)	9·2 (8·8 to 9·7)	8·5 (8·2 to 8·8)	9 (8·8 to 9·2)	7·8 (7·7 to 7·9)	9·3 (9·2 to 9·4)	9·1 (8·3 to 10)
Peterborough	1·7 (1·7 to 1·8)	1·9 (1·9 to 2)	1·9 (1·9 to 2)	2·1 (2·1 to 2·2)	2·5 (2·4 to 2·6)	2·1 (2·1 to 2·2)	3·1 (3·1 to 3·2)	3·1 (2·8 to 3·5)
Southend-on-Sea	2·6 (2·5 to 2·7)	2·4 (2·3 to 2·4)	2·2 (2·1 to 2·3)	1·9 (1·9 to 2)	2·3 (2·2 to 2·3)	1·9 (1·9 to 1·9)	2·3 (2·2 to 2·3)	2·2 (2 to 2·5)
Suffolk	8 (7·8 to 8·2)	8·5 (8 to 9)	8·7 (8·3 to 9)	8 (7·8 to 8·2)	8·5 (8·3 to 8·7)	7·1 (7 to 7·3)	8·2 (8·1 to 8·4)	7·9 (7·2 to 8·8)
Thurrock	1·9 (1·9 to 2)	2·2 (2·1 to 2·2)	2·3 (2·2 to 2·4)	1·9 (1·9 to 2)	2·1 (2 to 2·1)	1·9 (1·9 to 2)	2·4 (2·4 to 2·5)	2·3 (2·1 to 2·6)
Greater London	99·3 (95·5 to 103·1)	111·7 (108 to 115·2)	108 (104 to 112)	89·4 (87·2 to 91·7)	103 (101·3 to 104·7)	102·6 (101·5 to 103·5)	134·7 (133·7 to 135·7)	135·8 (121·2 to 152)
Barking and Dagenham	2·7 (2·6 to 2·8)	2·7 (2·6 to 2·8)	2·5 (2·4 to 2·6)	2·1 (2 to 2·3)	2·5 (2·4 to 2·5)	2·4 (2·3 to 2·5)	3·8 (3·7 to 4)	3·9 (3·5 to 4·3)
Barnet	4 (3·9 to 4·2)	4·1 (4 to 4·3)	4·2 (3·9 to 4·4)	3·7 (3·6 to 3·8)	4·1 (4 to 4·1)	4 (4 to 4·1)	5·6 (5·4 to 5·7)	5·5 (4·9 to 6·1)
Bexley	3 (2·9 to 3·1)	3·4 (3·2 to 3·6)	3·2 (3·1 to 3·4)	2·6 (2·6 to 2·7)	3·1 (3 to 3·1)	2·6 (2·5 to 2·6)	3·1 (3 to 3·2)	3·3 (2·9 to 3·7)
Brent	3·8 (3·6 to 3·9)	4·7 (4·5 to 4·9)	4·6 (4·3 to 4·8)	3·7 (3·6 to 3·8)	3·9 (3·8 to 4)	3·9 (3·8 to 4)	5·4 (5·3 to 5·5)	5·4 (4·9 to 6)
Bromley	4·2 (4 to 4·3)	4·3 (4 to 4·5)	3·9 (3·7 to 4·2)	3·2 (3·1 to 3·3)	3·6 (3·5 to 3·7)	3·5 (3·4 to 3·5)	4·2 (4·1 to 4·3)	4·2 (3·7 to 4·7)
Camden	1·9 (1·7 to 2)	2·5 (2·3 to 2·7)	2·7 (2·5 to 2·9)	2·1 (2 to 2·2)	2·3 (2·2 to 2·4)	2·7 (2·6 to 2·7)	3·1 (3 to 3·2)	2·9 (2·6 to 3·3)
Croydon	4·8 (4·7 to 5)	5·4 (5·2 to 5·6)	5·2 (4·9 to 5·4)	4·3 (4·2 to 4·4)	4·8 (4·7 to 4·9)	4·4 (4·3 to 4·5)	5·7 (5·6 to 5·9)	6 (5·4 to 6·6)
Ealing	4·2 (4·1 to 4·4)	4·8 (4·5 to 5)	4·7 (4·5 to 4·9)	4·2 (4 to 4·3)	4·4 (4·3 to 4·5)	4·3 (4·2 to 4·4)	5·9 (5·8 to 5·9)	5·4 (4·8 to 6·1)
Enfield	3·8 (3·7 to 3·9)	3·9 (3·7 to 4·1)	3·8 (3·6 to 4)	3·2 (3·1 to 3·3)	3·8 (3·7 to 3·9)	3·8 (3·7 to 3·9)	5·1 (5 to 5·2)	5·2 (4·7 to 5·8)
Greenwich	3·2 (3·1 to 3·4)	3·3 (3·2 to 3·4)	3·3 (3·1 to 3·4)	3 (2·9 to 3·1)	3·4 (3·3 to 3·5)	3·2 (3·1 to 3·2)	4·7 (4·6 to 4·8)	4·8 (4·3 to 5·3)
Hackney	3·5 (3·4 to 3·6)	4·1 (4 to 4·2)	3·9 (3·8 to 4·1)	3·2 (3·1 to 3·3)	3·7 (3·6 to 3·8)	3·9 (3·8 to 4)	4·7 (4·6 to 4·8)	4·9 (4·4 to 5·5)
Hammersmith and Fulham	1·8 (1·7 to 1·9)	2·2 (2·1 to 2·4)	2·4 (2·2 to 2·5)	1·9 (1·8 to 1·9)	2·2 (2·1 to 2·2)	2·4 (2·3 to 2·4)	2·8 (2·7 to 2·9)	2·6 (2·3 to 3)
Haringey	2·9 (2·8 to 3)	3·8 (3·5 to 4)	3·8 (3·5 to 4·1)	2·9 (2·8 to 3)	3·4 (3·3 to 3·5)	3·6 (3·5 to 3·7)	4·4 (4·3 to 4·5)	4·1 (3·7 to 4·7)
Harrow	2·8 (2·7 to 2·9)	3 (2·9 to 3·1)	2·8 (2·7 to 3)	2·4 (2·4 to 2·5)	2·8 (2·7 to 2·8)	2·5 (2·5 to 2·6)	3·5 (3·4 to 3·6)	3·9 (3·5 to 4·3)
Havering	3·5 (3·4 to 3·6)	3·8 (3·5 to 4·2)	3·4 (3·1 to 3·7)	2·7 (2·6 to 2·9)	2·8 (2·7 to 2·9)	2·3 (2·3 to 2·4)	2·9 (2·8 to 3)	3·5 (3·1 to 3·8)
Hillingdon	3·3 (3·2 to 3·4)	3·7 (3·5 to 3·8)	3·4 (3·2 to 3·5)	2·9 (2·8 to 3)	3·5 (3·4 to 3·5)	3·2 (3·2 to 3·3)	4·4 (4·3 to 4·5)	4·7 (4·2 to 5·3)
Hounslow	2·8 (2·7 to 2·9)	3·1 (3 to 3·3)	3·1 (3 to 3·2)	2·8 (2·7 to 2·9)	3·1 (3 to 3·2)	3·1 (3·1 to 3·2)	4·6 (4·5 to 4·6)	4·5 (4 to 5)
Islington	2·3 (2·2 to 2·4)	2·9 (2·8 to 3·1)	2·8 (2·6 to 2·9)	2·2 (2·1 to 2·3)	2·6 (2·6 to 2·7)	2·5 (2·5 to 2·6)	3 (2·9 to 3·1)	3·3 (3 to 3·8)

Kensington and Chelsea	1·5 (1·4 to 1·6)	2·3 (2·2 to 2·5)	3 (2·8 to 3·2)	1·8 (1·8 to 1·9)	1·8 (1·8 to 1·9)	2 (2 to 2·1)	2·2 (2·1 to 2·2)	1·9 (1·6 to 2·1)
Kingston upon Thames	1·6 (1·6 to 1·7)	1·7 (1·6 to 1·8)	1·7 (1·6 to 1·7)	1·5 (1·5 to 1·6)	1·7 (1·7 to 1·8)	1·8 (1·8 to 1·8)	2·3 (2·3 to 2·4)	2·4 (2·1 to 2·7)
Lambeth	3·9 (3·8 to 4·1)	4·8 (4·6 to 5·1)	4·4 (4·2 to 4·7)	3·6 (3·4 to 3·7)	4·3 (4·2 to 4·4)	4·3 (4·2 to 4·4)	5 (4·9 to 5)	4·9 (4·3 to 5·5)
Lewisham	3·7 (3·5 to 3·8)	4·2 (4 to 4·4)	3·9 (3·7 to 4)	3·1 (3 to 3·2)	3·9 (3·8 to 4)	3·8 (3·7 to 3·9)	5·1 (4·9 to 5·2)	4·9 (4·4 to 5·5)
Merton	2·5 (2·4 to 2·6)	2·6 (2·5 to 2·7)	2·4 (2·3 to 2·5)	2 (2 to 2·1)	2·5 (2·4 to 2·6)	2·6 (2·5 to 2·7)	3·5 (3·4 to 3·6)	3·3 (2·9 to 3·8)
Newham	3·8 (3·7 to 3·9)	4·3 (4·1 to 4·4)	4·2 (4 to 4·4)	3·7 (3·6 to 3·8)	4·4 (4·3 to 4·5)	4·6 (4·6 to 4·7)	6·4 (6·3 to 6·5)	6·5 (5·9 to 7·2)
Redbridge	3·2 (3·1 to 3·3)	3·3 (3·1 to 3·4)	3·2 (3·1 to 3·3)	2·6 (2·5 to 2·7)	3·1 (3 to 3·1)	3·1 (3 to 3·2)	4·6 (4·5 to 4·7)	5 (4·5 to 5·5)
Richmond upon Thames	2 (1·9 to 2·1)	2·2 (2·1 to 2·3)	2·2 (2 to 2·3)	1·8 (1·8 to 1·9)	2·1 (2·1 to 2·2)	2·4 (2·3 to 2·4)	3 (2·9 to 3)	2·6 (2·3 to 3)
Southwark	3·8 (3·7 to 3·9)	4·3 (4·1 to 4·4)	3·6 (3·5 to 3·8)	3 (2·9 to 3·1)	3·9 (3·8 to 4)	4 (4 to 4·1)	5·1 (5 to 5·2)	5·1 (4·5 to 5·7)
Sutton	2·3 (2·3 to 2·4)	2·5 (2·3 to 2·6)	2·3 (2·2 to 2·5)	2 (2 to 2·1)	2·4 (2·3 to 2·5)	2·2 (2·1 to 2·2)	2·8 (2·7 to 2·8)	2·7 (2·4 to 3)
Tower Hamlets	3·1 (3 to 3·2)	3·4 (3·1 to 3·6)	2·8 (2·7 to 2·9)	2·5 (2·5 to 2·6)	3·2 (3·1 to 3·3)	3·5 (3·4 to 3·6)	4·7 (4·5 to 4·8)	5·3 (4·7 to 5·9)
Waltham Forest	3·4 (3·3 to 3·6)	3·6 (3·5 to 3·8)	3·8 (3·6 to 3·9)	3·1 (3 to 3·2)	3·7 (3·6 to 3·8)	3·5 (3·4 to 3·6)	4·8 (4·8 to 4·9)	5 (4·5 to 5·5)
Wandsworth	3·5 (3·4 to 3·7)	3·9 (3·6 to 4·2)	3·8 (3·6 to 4·1)	3·4 (3·2 to 3·5)	3·9 (3·7 to 4)	4 (3·9 to 4·1)	5·4 (5·3 to 5·5)	5·4 (4·7 to 6·1)
Westminster	2·3 (2·1 to 2·4)	2·9 (2·7 to 3·2)	3·1 (2·9 to 3·3)	2·1 (2 to 2·2)	2·3 (2·3 to 2·4)	2·5 (2·4 to 2·5)	3 (3 to 3·1)	2·8 (2·4 to 3·1)
North East England	44·3 (43·2 to 45·6)	49·2 (47·9 to 50·5)	47 (45·8 to 48·3)	39·1 (38·5 to 39·7)	35·8 (35·2 to 36·3)	26·4 (26·1 to 26·6)	29·8 (29·5 to 30)	28 (25·5 to 30·9)
County Durham	8·2 (8 to 8·5)	9 (8·7 to 9·3)	8·7 (8·4 to 8·9)	7·3 (7·1 to 7·4)	6·6 (6·5 to 6·7)	4·9 (4·9 to 5)	5·6 (5·5 to 5·7)	5·3 (4·8 to 5·8)
Darlington	1·7 (1·6 to 1·7)	1·8 (1·7 to 1·8)	1·8 (1·7 to 1·8)	1·4 (1·4 to 1·4)	1·4 (1·4 to 1·5)	1·1 (1·1 to 1·1)	1·3 (1·3 to 1·3)	1·2 (1·1 to 1·3)
Gateshead	3·8 (3·7 to 3·9)	4·3 (4·1 to 4·4)	4 (3·9 to 4·1)	3 (2·9 to 3)	2·7 (2·7 to 2·8)	2 (2 to 2)	2·3 (2·3 to 2·3)	2·2 (2 to 2·4)
Hartlepool	1·7 (1·6 to 1·7)	2 (2 to 2·1)	1·9 (1·8 to 2)	1·5 (1·5 to 1·5)	1·4 (1·3 to 1·4)	1 (1 to 1·1)	1·1 (1·1 to 1·2)	1 (0·9 to 1·1)
Middlesbrough	2·8 (2·7 to 2·9)	3·3 (3·2 to 3·3)	3 (3 to 3·1)	2·7 (2·7 to 2·7)	2·4 (2·3 to 2·4)	1·7 (1·7 to 1·8)	2 (2 to 2)	1·9 (1·7 to 2)
Newcastle upon Tyne	4·8 (4·7 to 4·9)	5·1 (4·9 to 5·3)	4·6 (4·5 to 4·8)	3·8 (3·8 to 3·9)	3·8 (3·7 to 3·9)	2·8 (2·8 to 2·9)	3·3 (3·3 to 3·4)	3·5 (3·2 to 3·9)
North Tyneside	3·5 (3·5 to 3·6)	3·7 (3·6 to 3·8)	3·4 (3·3 to 3·5)	2·7 (2·6 to 2·7)	2·5 (2·5 to 2·6)	2 (2 to 2)	2·3 (2·3 to 2·4)	2·2 (2 to 2·5)
Northumberland	4·7 (4·6 to 4·9)	4·9 (4·7 to 5)	4·8 (4·7 to 5)	4·2 (4·2 to 4·3)	3·7 (3·6 to 3·8)	2·8 (2·8 to 2·9)	3 (2·9 to 3)	2·8 (2·5 to 3·1)
Redcar and Cleveland	2·4 (2·3 to 2·4)	2·9 (2·8 to 3)	2·9 (2·8 to 3)	2·4 (2·3 to 2·4)	2·1 (2 to 2·1)	1·4 (1·4 to 1·4)	1·6 (1·5 to 1·6)	1·4 (1·3 to 1·5)
South Tyneside	3 (2·9 to 3·1)	3·3 (3·2 to 3·5)	3 (2·9 to 3·1)	2·2 (2·2 to 2·3)	2·2 (2·1 to 2·2)	1·5 (1·5 to 1·5)	1·7 (1·6 to 1·7)	1·5 (1·4 to 1·7)
Stockton-on-Tees	2·7 (2·7 to 2·8)	3·3 (3·1 to 3·4)	3·2 (3·1 to 3·3)	3 (2·9 to 3·1)	2·6 (2·6 to 2·7)	2 (1·9 to 2)	2·4 (2·4 to 2·4)	2·2 (2 to 2·4)
Sunderland	5 (4·9 to 5·1)	5·7 (5·5 to 5·8)	5·6 (5·5 to 5·8)	4·9 (4·8 to 5)	4·3 (4·1 to 4·4)	3 (2·9 to 3)	3·2 (3·1 to 3·2)	2·8 (2·5 to 3)
North West England	109 (105·9 to 112·2)	118 (114·8 to 121·1)	112·5 (109·4 to 115·7)	93·3 (91·6 to 94·9)	94·5 (93·1 to 95·9)	76·3 (75·6 to 77)	89·4 (88·9 to 89·9)	85·5 (77·6 to 94·3)
Blackburn with Darwen	2·5 (2·4 to 2·6)	2·5 (2·4 to 2·6)	2·6 (2·4 to 2·7)	2·4 (2·3 to 2·5)	2·4 (2·3 to 2·4)	2·1 (2 to 2·1)	2·4 (2·3 to 2·4)	2·1 (1·9 to 2·3)
Blackpool	2·3 (2·3 to 2·4)	2·1 (2 to 2·2)	1·9 (1·8 to 2)	1·6 (1·5 to 1·6)	1·9 (1·8 to 1·9)	1·5 (1·5 to 1·5)	1·8 (1·7 to 1·8)	1·7 (1·5 to 1·8)
Bolton	4·2 (4·1 to 4·4)	4·5 (4·3 to 4·6)	4·5 (4·4 to 4·6)	3·9 (3·8 to 3·9)	3·8 (3·7 to 3·9)	3·2 (3·1 to 3·3)	4 (3·9 to 4)	3·7 (3·4 to 4·1)
Bury	2·6 (2·5 to 2·7)	3 (2·9 to 3·1)	3 (2·9 to 3·1)	2·4 (2·3 to 2·4)	2·5 (2·5 to 2·6)	2·1 (2·1 to 2·2)	2·5 (2·4 to 2·6)	2·3 (2·1 to 2·6)
Cheshire East	4·5 (4·4 to 4·7)	4·9 (4·7 to 5·1)	4·7 (4·5 to 4·8)	3·8 (3·7 to 3·9)	4·1 (4 to 4·2)	3·6 (3·5 to 3·6)	4 (3·9 to 4·1)	3·8 (3·4 to 4·3)
Cheshire West and Chester	4·4 (4·3 to 4·6)	5 (4·9 to 5·2)	4·8 (4·6 to 4·9)	4 (3·9 to 4·1)	4 (3·9 to 4·1)	3·4 (3·4 to 3·5)	3·7 (3·7 to 3·8)	3·6 (3·2 to 4)
Cumbria	7·2 (6·9 to 7·4)	7·4 (7·1 to 7·6)	6·9 (6·7 to 7·2)	5·8 (5·7 to 5·9)	5·9 (5·8 to 6·1)	4·7 (4·6 to 4·8)	5·1 (5 to 5·2)	4·7 (4·2 to 5·1)
Halton	1·5 (1·5 to 1·6)	1·7 (1·6 to 1·7)	1·8 (1·7 to 1·8)	2 (1·9 to 2)	1·9 (1·9 to 2)	1·4 (1·4 to 1·5)	1·7 (1·6 to 1·7)	1·5 (1·3 to 1·6)
Knowsley	3 (2·9 to 3·1)	3·9 (3·6 to 4·1)	3·4 (3·2 to 3·6)	2·9 (2·8 to 3)	2·5 (2·5 to 2·6)	1·8 (1·8 to 1·9)	2 (1·9 to 2)	1·9 (1·7 to 2·1)
Lancashire	16·2 (15·7 to 16·6)	16·7 (16 to 17·4)	16·3 (15·7 to 16·8)	14·3 (13·9 to 14·6)	14·7 (14·3 to 15·1)	12·2 (11·9 to 12·4)	13·9 (13·7 to 14·2)	13 (11·8 to 14·4)
Liverpool	9·1 (8·9 to 9·4)	9·9 (9·5 to 10·3)	8·9 (8·5 to 9·2)	6·9 (6·8 to 7·1)	6·9 (6·7 to 7)	5 (4·9 to 5·2)	5·8 (5·7 to 5·9)	6·1 (5·5 to 6·7)
Manchester	8·2 (7·9 to 8·4)	9 (8·6 to 9·4)	8·1 (7·8 to 8·4)	6·5 (6·3 to 6·7)	7 (6·7 to 7·2)	5·6 (5·5 to 5·8)	8 (7·9 to 8·2)	8·5 (7·7 to 9·5)
Oldham	3·7 (3·6 to 3·8)	4 (3·9 to 4·2)	4·1 (3·9 to 4·3)	3·3 (3·3 to 3·4)	3·4 (3·3 to 3·4)	3 (2·9 to 3·1)	3·4 (3·3 to 3·5)	3·3 (3 to 3·6)
Rochdale	3·4 (3·3 to 3·5)	3·6 (3·5 to 3·7)	3·6 (3·5 to 3·6)	3·3 (3·2 to 3·3)	3·2 (3·1 to 3·3)	2·6 (2·5 to 2·7)	3·1 (3 to 3·2)	2·9 (2·7 to 3·2)
Salford	4·4 (4·3 to 4·5)	4·8 (4·7 to 5)	4·5 (4·4 to 4·7)	3·3 (3·2 to 3·4)	3·4 (3·4 to 3·5)	2·6 (2·5 to 2·6)	3·6 (3·5 to 3·6)	3·6 (3·3 to 4)
Sefton	4·7 (4·5 to 4·8)	5·1 (5 to 5·3)	4·5 (4·3 to 4·6)	3·6 (3·5 to 3·7)	3·6 (3·5 to 3·7)	2·7 (2·6 to 2·7)	2·8 (2·8 to 2·9)	2·7 (2·5 to 3)
St Helens	2·9 (2·8 to 2·9)	3·2 (3·1 to 3·3)	3·2 (3·1 to 3·3)	2·5 (2·5 to 2·6)	2·3 (2·3 to 2·4)	1·9 (1·8 to 2)	2·1 (2·1 to 2·2)	2 (1·8 to 2·2)
Stockport	4·5 (4·4 to 4·6)	5·2 (5 to 5·3)	4·8 (4·6 to 4·9)	3·8 (3·7 to 3·9)	3·7 (3·6 to 3·8)	3 (2·9 to 3·1)	3·5 (3·3 to 3·6)	3·3 (2·9 to 3·6)
Tameside	3·5 (3·4 to 3·6)	3·7 (3·6 to 3·8)	3·7 (3·6 to 3·8)	3 (2·9 to 3·1)	3·2 (3·1 to 3·3)	2·5 (2·4 to 2·5)	3·1 (3 to 3·2)	2·8 (2·6 to 3·1)
Trafford	3·6 (3·5 to 3·7)	3·9 (3·8 to 4·1)	3·6 (3·4 to 3·7)	2·9 (2·8 to 2·9)	2·9 (2·8 to 2·9)	2·4 (2·4 to 2·5)	2·9 (2·8 to 2·9)	2·7 (2·4 to 3·1)
Warrington	2·4 (2·4 to 2·5)	2·9 (2·8 to 3)	2·8 (2·7 to 2·9)	2·3 (2·2 to 2·3)	2·6 (2·5 to 2·6)	2·2 (2·2 to 2·3)	2·5 (2·4 to 2·5)	2·3 (2·1 to 2·5)
Wigan	4·5 (4·3 to 4·6)	5 (4·9 to 5·2)	5·4 (5·3 to 5·6)	4·3 (4·2 to 4·4)	4·2 (4·1 to 4·3)	3·4 (3·3 to 3·4)	3·9 (3·8 to 4)	3·6 (3·3 to 4)
Wirral	5·7 (5·5 to 5·9)	6·1 (5·9 to 6·3)	5·7 (5·5 to 5·8)	4·5 (4·4 to 4·6)	4·5 (4·4 to 4·6)	3·4 (3·3 to 3·4)	3·8 (3·7 to 3·8)	3·5 (3·1 to 3·8)
South East England	91·3 (88·2 to 94·4)	98·8 (95·9 to 101·6)	94·1 (91 to 97·2)	87·9 (86·2 to 89·8)	98·3 (96·8 to 99·7)	90·4 (89·6 to 91·2)	106·7 (105·9 to 107·4)	103 (92·6 to 114·4)
Bracknell Forest	0·8 (0·8 to 0·9)	1·1 (1·1 to 1·1)	1 (1 to 1·1)	1·2 (1·2 to 1·3)	1·5 (1·4 to 1·5)	1·4 (1·4 to 1·5)	1·6 (1·5 to 1·6)	1·4 (1·3 to 1·6)
Brighton and Hove	2·9 (2·8 to 3·1)	2·8 (2·6 to 2·9)	2·5 (2·4 to 2·7)	2·4 (2·3 to 2·5)	2·9 (2·9 to 3)	2·8 (2·8 to 2·9)	3·3 (3·2 to 3·3)	3·1 (2·7 to 3·5)
Buckinghamshire	5·6 (5·4 to 5·8)	6·5 (6·2 to 6·8)	5·9 (5·6 to 6·2)	5·4 (5·3 to 5·5)	5·9 (5·7 to 6)	5·6 (5·4 to 5·7)	6·2 (6 to 6·3)	5·9 (5·3 to 6·6)

East Sussex	5·5 (5·3 to 5·7)	5 (4·9 to 5·2)	4·8 (4·7 to 5)	4·3 (4·2 to 4·5)	5·2 (5·1 to 5·3)	4·7 (4·6 to 4·8)	5·3 (5·2 to 5·5)	5·2 (4·7 to 5·8)
Hampshire	13 (12·5 to 13·4)	15·3 (14·8 to 15·8)	15·1 (14·5 to 15·6)	13·8 (13·5 to 14·2)	15·2 (14·9 to 15·4)	13·3 (13 to 13·7)	15·1 (14·9 to 15·3)	14·3 (13 to 15·9)
Isle of Wight	1·6 (1·6 to 1·7)	1·5 (1·4 to 1·5)	1·4 (1·3 to 1·4)	1·2 (1·2 to 1·3)	1·3 (1·3 to 1·4)	1·1 (1·1 to 1·2)	1·3 (1·3 to 1·3)	1·3 (1·2 to 1·4)
Kent	16·6 (16·1 to 17·2)	17·6 (17·1 to 18·2)	16·9 (16·3 to 17·5)	14·9 (14·5 to 15·3)	16·5 (16·1 to 16·9)	14·9 (14·6 to 15·2)	17·6 (17·2 to 18)	18·1 (16·4 to 19·9)
Medway	3·2 (3·1 to 3·3)	3·9 (3·8 to 4·1)	4·2 (4·1 to 4·4)	3·8 (3·6 to 3·9)	3·8 (3·7 to 3·9)	3·1 (3 to 3·2)	3·6 (3·5 to 3·7)	3·7 (3·3 to 4)
Milton Keynes	1 (1 to 1·1)	1·1 (1 to 1·1)	1·3 (1·3 to 1·3)	2·3 (2·2 to 2·3)	2·9 (2·9 to 3)	2·9 (2·8 to 2·9)	3·9 (3·8 to 4)	3·7 (3·3 to 4·1)
Oxfordshire	5·8 (5·6 to 6·1)	7·1 (6·6 to 7·6)	6·9 (6·6 to 7·3)	6·7 (6·6 to 6·9)	7·7 (7·5 to 7·9)	7·2 (7 to 7·3)	8·4 (8·2 to 8·6)	7·9 (7 to 8·9)
Portsmouth	2·7 (2·7 to 2·8)	2·8 (2·7 to 2·9)	2·7 (2·6 to 2·8)	2·4 (2·3 to 2·5)	2·6 (2·6 to 2·7)	2·2 (2·2 to 2·3)	2·7 (2·7 to 2·8)	2·7 (2·5 to 3)
Reading	1·7 (1·7 to 1·8)	2·1 (2 to 2·2)	2·1 (2 to 2·2)	2 (1·9 to 2)	2 (2 to 2·1)	2 (1·9 to 2)	2·7 (2·6 to 2·7)	2·5 (2·3 to 2·8)
Slough	1·7 (1·7 to 1·8)	1·9 (1·8 to 1·9)	1·7 (1·7 to 1·8)	1·7 (1·7 to 1·7)	1·9 (1·8 to 2)	1·8 (1·8 to 1·9)	2·7 (2·7 to 2·8)	2·5 (2·3 to 2·8)
Southampton	2·9 (2·8 to 3)	3·1 (2·9 to 3·2)	3 (2·9 to 3·1)	2·8 (2·6 to 2·9)	2·9 (2·8 to 3)	2·5 (2·4 to 2·6)	3·4 (3·3 to 3·4)	3·3 (3 to 3·7)
Surrey	13·3 (12·8 to 13·9)	13·8 (13·3 to 14·4)	12·1 (11·6 to 12·6)	10·9 (10·7 to 11·2)	12·3 (12 to 12·8)	12·1 (11·6 to 12·5)	14·1 (13·6 to 14·6)	13·4 (11·9 to 15)
West Berkshire	1·5 (1·4 to 1·5)	1·7 (1·6 to 1·8)	1·7 (1·6 to 1·8)	1·7 (1·6 to 1·8)	1·9 (1·8 to 2)	1·7 (1·7 to 1·7)	1·9 (1·9 to 2)	1·7 (1·5 to 1·9)
West Sussex	8·4 (8·1 to 8·6)	8·1 (7·7 to 8·5)	7·5 (7·1 to 7·9)	7·4 (7·2 to 7·6)	8·2 (8 to 8·4)	7·8 (7·7 to 8)	9·1 (9 to 9·3)	8·9 (8 to 9·8)
Windsor and Maidenhead	1·7 (1·6 to 1·8)	1·8 (1·7 to 2)	1·7 (1·6 to 1·8)	1·6 (1·5 to 1·6)	1·6 (1·6 to 1·7)	1·6 (1·6 to 1·7)	1·8 (1·8 to 1·9)	1·7 (1·5 to 1·9)
Wokingham	1·2 (1·1 to 1·2)	1·6 (1·5 to 1·7)	1·5 (1·4 to 1·6)	1·4 (1·4 to 1·5)	1·9 (1·8 to 1·9)	1·7 (1·6 to 1·8)	1·9 (1·9 to 2)	1·6 (1·4 to 1·9)
South West England	58·8 (57 to 60·7)	60 (58·3 to 61·6)	58·1 (56·4 to 59·9)	53 (52 to 54)	58·2 (57·3 to 59)	50·6 (50·1 to 51·1)	59·2 (58·8 to 59·6)	59 (53·4 to 65·3)
Bath and North East Somerset	1·9 (1·9 to 2)	1·9 (1·9 to 2)	1·8 (1·7 to 1·8)	1·7 (1·7 to 1·8)	1·9 (1·8 to 1·9)	1·7 (1·6 to 1·7)	1·8 (1·7 to 1·8)	1·8 (1·6 to 2·1)
Bournemouth	2 (1·9 to 2)	1·7 (1·6 to 1·7)	1·5 (1·4 to 1·6)	1·3 (1·3 to 1·4)	1·7 (1·6 to 1·8)	1·6 (1·5 to 1·6)	2·2 (2·1 to 2·3)	2·4 (2·1 to 2·7)
Bristol, City of	5·9 (5·6 to 6·1)	6·1 (5·8 to 6·4)	5·8 (5·6 to 6·1)	5·2 (5·1 to 5·3)	5·6 (5·5 to 5·7)	4·8 (4·7 to 4·9)	6·4 (6·3 to 6·6)	6·5 (5·8 to 7·3)
Cornwall	6 (5·8 to 6·2)	5·7 (5·5 to 5·8)	5·6 (5·4 to 5·8)	5·2 (5 to 5·3)	5·5 (5·3 to 5·6)	4·7 (4·6 to 4·8)	5·5 (5·4 to 5·6)	5·5 (5 to 6)
Devon	7·7 (7·4 to 7·9)	7·2 (6·9 to 7·5)	6·9 (6·6 to 7·1)	6·4 (6·2 to 6·6)	7·4 (7·2 to 7·6)	6·5 (6·3 to 6·6)	7·3 (7·2 to 7·4)	7·2 (6·6 to 8)
Dorset	4·3 (4·1 to 4·4)	4·1 (4 to 4·2)	3·9 (3·8 to 4·1)	3·7 (3·6 to 3·8)	3·9 (3·9 to 4)	3·4 (3·3 to 3·5)	3·6 (3·5 to 3·7)	3·6 (3·2 to 3·9)
Gloucestershire	6·8 (6·6 to 7·1)	7·2 (6·9 to 7·5)	6·9 (6·6 to 7·3)	6·3 (6·1 to 6·4)	6·9 (6·7 to 7·1)	6 (5·9 to 6·2)	6·7 (6·6 to 6·9)	6·7 (6·1 to 7·4)
North Somerset	2·1 (2 to 2·1)	2·1 (2 to 2·1)	2·1 (2 to 2·1)	2 (1·9 to 2)	2 (1·9 to 2·1)	1·9 (1·8 to 1·9)	2·3 (2·2 to 2·3)	2·2 (2 to 2·5)
Plymouth	3·6 (3·5 to 3·7)	3·8 (3·7 to 3·9)	3·7 (3·6 to 3·8)	3·5 (3·4 to 3·6)	3·5 (3·4 to 3·6)	2·7 (2·6 to 2·7)	3·2 (3·2 to 3·3)	3·2 (2·9 to 3·5)
Poole	1·6 (1·6 to 1·7)	1·6 (1·6 to 1·7)	1·6 (1·5 to 1·6)	1·4 (1·4 to 1·4)	1·7 (1·6 to 1·7)	1·4 (1·4 to 1·4)	1·7 (1·6 to 1·7)	1·6 (1·4 to 1·8)
Somerset	5·8 (5·6 to 6)	5·8 (5·5 to 6·1)	5·7 (5·5 to 5·9)	5·3 (5·2 to 5·4)	5·7 (5·6 to 5·9)	4·9 (4·8 to 5)	5·6 (5·5 to 5·8)	5·6 (5·1 to 6·2)
South Gloucestershire	2·5 (2·4 to 2·5)	3·1 (2·9 to 3·3)	3 (2·8 to 3·2)	2·6 (2·5 to 2·7)	3 (3 to 3·1)	2·9 (2·8 to 3)	3·1 (3 to 3·2)	3·2 (2·9 to 3·5)
Swindon	2·1 (2·1 to 2·2)	2·5 (2·4 to 2·6)	2·4 (2·3 to 2·4)	2·3 (2·2 to 2·3)	2·7 (2·6 to 2·8)	2·3 (2·2 to 2·3)	2·9 (2·9 to 3)	3 (2·7 to 3·3)
Torbay	1·6 (1·5 to 1·6)	1·4 (1·3 to 1·5)	1·3 (1·3 to 1·4)	1·1 (1·1 to 1·2)	1·3 (1·3 to 1·4)	1·2 (1·2 to 1·2)	1·4 (1·4 to 1·5)	1·4 (1·2 to 1·5)
Wiltshire	5·1 (4·9 to 5·2)	5·8 (5·6 to 6)	6 (5·8 to 6·2)	5 (4·9 to 5·2)	5·3 (5·2 to 5·4)	4·8 (4·7 to 4·9)	5·3 (5·2 to 5·4)	5·1 (4·6 to 5·7)
West Midlands	82·7 (80·5 to 85)	92·6 (90·2 to 95)	92 (89·6 to 94·4)	75·9 (74·8 to 77)	76·5 (75·4 to 77·5)	61·2 (60·7 to 61·6)	72·3 (71·9 to 72·7)	70·2 (63·7 to 77·4)
Birmingham	19·4 (18·9 to 19·9)	20·8 (20 to 21·5)	20·1 (19·3 to 20·8)	16·9 (16·7 to 17·1)	17·3 (16·9 to 17·7)	14·3 (14 to 14·5)	17·4 (17·1 to 17·8)	17·3 (15·6 to 19)
Coventry	5·5 (5·3 to 5·6)	6·3 (6 to 6·5)	6·1 (5·9 to 6·4)	4·9 (4·8 to 5·1)	4·7 (4·6 to 4·8)	3·6 (3·5 to 3·7)	4·6 (4·6 to 4·7)	4·6 (4·1 to 5)
Dudley	4·6 (4·5 to 4·8)	5·5 (5·3 to 5·7)	5·5 (5·3 to 5·7)	4·2 (4·1 to 4·3)	4·3 (4·2 to 4·4)	3·4 (3·3 to 3·5)	3·8 (3·7 to 3·8)	3·7 (3·3 to 4)
Herefordshire, County of	2·2 (2·1 to 2·3)	2·3 (2·2 to 2·3)	2·2 (2·2 to 2·3)	2 (1·9 to 2)	2·1 (2 to 2·1)	1·6 (1·6 to 1·7)	1·8 (1·8 to 1·9)	1·8 (1·6 to 2)
Sandwell	5·9 (5·8 to 6·1)	6·3 (6 to 6·6)	6·1 (5·8 to 6·3)	4·7 (4·6 to 4·9)	4·6 (4·5 to 4·8)	3·7 (3·7 to 3·8)	4·9 (4·8 to 5)	4·7 (4·3 to 5·2)
Shropshire	3·5 (3·4 to 3·6)	4 (3·8 to 4·1)	4 (3·8 to 4·1)	3·2 (3·1 to 3·2)	3·3 (3·3 to 3·4)	2·7 (2·7 to 2·8)	2·9 (2·8 to 2·9)	2·9 (2·6 to 3·2)
Solihull	2·9 (2·8 to 3)	3·5 (3·4 to 3·6)	3·6 (3·4 to 3·7)	2·5 (2·5 to 2·6)	2·6 (2·6 to 2·7)	2 (1·9 to 2)	2·2 (2·2 to 2·3)	2·3 (2·1 to 2·6)
Staffordshire	10·4 (10·1 to 10·7)	12·4 (11·9 to 12·9)	12·9 (12·4 to 13·3)	10·9 (10·7 to 11·1)	10·6 (10·3 to 10·8)	8·1 (7·9 to 8·3)	8·9 (8·7 to 9·1)	8·5 (7·7 to 9·4)
Stoke-on-Trent	4·6 (4·5 to 4·8)	4·8 (4·6 to 4·9)	4·7 (4·5 to 4·8)	3·8 (3·7 to 3·9)	3·8 (3·7 to 3·9)	2·8 (2·8 to 2·9)	3·8 (3·7 to 3·8)	3·3 (3 to 3·6)
Telford and Wrekin	1·5 (1·5 to 1·5)	1·7 (1·6 to 1·8)	1·9 (1·9 to 2)	2·1 (2 to 2·1)	2·2 (2·2 to 2·3)	1·9 (1·9 to 1·9)	2·2 (2·2 to 2·3)	2·1 (1·9 to 2·2)
Walsall	4·5 (4·4 to 4·6)	5·5 (5·2 to 5·8)	5·3 (5 to 5·5)	3·9 (3·8 to 4·1)	3·9 (3·8 to 4)	3·2 (3·2 to 3·3)	3·8 (3·7 to 3·8)	3·8 (3·4 to 4·1)
Warwickshire	6·8 (6·6 to 7)	7·8 (7·5 to 8·1)	7·8 (7·5 to 8·2)	6·4 (6·2 to 6·5)	6·3 (6·2 to 6·5)	5·3 (5·2 to 5·4)	6·2 (6·1 to 6·3)	6·1 (5·4 to 6·7)
Wolverhampton	4·5 (4·4 to 4·6)	5 (4·7 to 5·3)	4·7 (4·5 to 4·9)	3·8 (3·7 to 4)	3·9 (3·8 to 4)	2·9 (2·9 to 3)	3·5 (3·4 to 3·6)	3·4 (3·1 to 3·8)
Worcestershire	6·3 (6·1 to 6·5)	6·9 (6·7 to 7·2)	7·2 (7 to 7·5)	6·6 (6·5 to 6·7)	6·7 (6·5 to 6·8)	5·7 (5·5 to 5·8)	6·2 (6 to 6·4)	5·9 (5·3 to 6·5)
Yorkshire and the Humber	74·7 (72·6 to 76·9)	80·9 (78·7 to 83)	78·4 (76·2 to 80·6)	67 (65·8 to 68·1)	69·3 (68·2 to 70·2)	56 (55·5 to 56·4)	66·5 (66·1 to 66·9)	64·1 (58·3 to 70·6)
Barnsley	3·5 (3·4 to 3·6)	3·9 (3·8 to 4)	3·8 (3·7 to 3·9)	3·1 (3 to 3·2)	3·1 (3·1 to 3·2)	2·3 (2·3 to 2·4)	2·9 (2·9 to 3)	2·7 (2·5 to 3)
Bradford	8·1 (7·9 to 8·3)	8·8 (8·6 to 9)	8·6 (8·4 to 8·8)	7·9 (7·8 to 8)	7·8 (7·7 to 8)	7 (6·9 to 7·1)	8·5 (8·4 to 8·6)	7·8 (7·1 to 8·6)
Calderdale	3·3 (3·2 to 3·4)	3·3 (3·2 to 3·4)	3·3 (3·2 to 3·3)	2·7 (2·7 to 2·8)	2·8 (2·8 to 2·9)	2·3 (2·2 to 2·3)	2·6 (2·6 to 2·7)	2·5 (2·2 to 2·7)
Doncaster	4·6 (4·4 to 4·7)	5·2 (5 to 5·4)	5 (4·8 to 5·1)	4·3 (4·2 to 4·3)	4·3 (4·2 to 4·4)	3·2 (3·1 to 3·3)	3·8 (3·7 to 3·9)	3·5 (3·2 to 3·8)
East Riding of Yorkshire	3·7 (3·5 to 3·8)	3·8 (3·7 to 4)	3·6 (3·5 to 3·8)	3·1 (3 to 3·2)	3·3 (3·2 to 3·4)	2·8 (2·8 to 2·9)	3·1 (3 to 3·1)	2·9 (2·7 to 3·2)
Kingston upon Hull, City of	4·5 (4·3 to 4·6)	4·9 (4·8 to 5·1)	4·9 (4·8 to 5)	4·1 (4 to 4·3)	4·3 (4·2 to 4·4)	3 (2·9 to 3)	3·8 (3·7 to 3·9)	3·4 (3·1 to 3·8)
Kirklees	6 (5·8 to 6·2)	6·4 (6·2 to 6·5)	6·4 (6·2 to 6·5)	5·6 (5·5 to 5·7)	5·6 (5·4 to 5·7)	5 (4·8 to 5·1)	5·8 (5·7 to 5·9)	5·3 (4·9 to 5·9)

Leeds	10·9 (10·6 to 11·3)	11·7 (11·3 to 12·2)	10·9 (10·5 to 11·4)	9·3 (9·1 to 9·5)	9·8 (9·6 to 10)	7·9 (7·8 to 8)	10·1 (9·9 to 10·3)	10·4 (9·4 to 11·5)
North East Lincolnshire	2·5 (2·5 to 2·6)	3 (2·9 to 3·1)	3 (2·9 to 3·1)	2·4 (2·4 to 2·5)	2·4 (2·4 to 2·5)	1·7 (1·7 to 1·7)	2 (2 to 2·1)	1·8 (1·7 to 2)
North Lincolnshire	2·2 (2·2 to 2·3)	2·5 (2·4 to 2·6)	2·5 (2·4 to 2·6)	2·1 (2·1 to 2·2)	2·1 (2 to 2·1)	1·6 (1·5 to 1·6)	1·9 (1·9 to 1·9)	1·9 (1·7 to 2)
North Yorkshire	6·8 (6·6 to 7)	7·2 (6·9 to 7·4)	6·8 (6·5 to 7·2)	5·9 (5·8 to 6)	6·5 (6·4 to 6·7)	5·6 (5·4 to 5·7)	5·9 (5·7 to 6)	5·7 (5·2 to 6·3)
Rotherham	3·8 (3·7 to 3·9)	4·4 (4·2 to 4·5)	4·5 (4·3 to 4·6)	3·7 (3·6 to 3·8)	3·7 (3·6 to 3·8)	2·8 (2·7 to 2·8)	3·2 (3·1 to 3·3)	3·1 (2·8 to 3·4)
Sheffield	8 (7·7 to 8·3)	8·3 (8 to 8·7)	7·7 (7·4 to 8·1)	6·3 (6·1 to 6·6)	6·8 (6·7 to 7)	5·7 (5·6 to 5·8)	6·7 (6·5 to 6·9)	7 (6·3 to 7·7)
Wakefield	4·7 (4·6 to 4·9)	5·3 (5·1 to 5·4)	5·2 (5·1 to 5·4)	4·5 (4·4 to 4·6)	4·5 (4·4 to 4·7)	3·4 (3·3 to 3·5)	4·2 (4·1 to 4·2)	3·9 (3·5 to 4·2)
York	2·1 (2 to 2·2)	2·2 (2·1 to 2·3)	2 (1·9 to 2·2)	2 (1·9 to 2)	2·1 (2·1 to 2·2)	1·8 (1·7 to 1·9)	2·1 (2 to 2·1)	2·1 (1·9 to 2·4)
Northern Ireland	25·5 (21·7 to 29·5)	33·3 (30·1 to 37·1)	34·4 (30·6 to 38·6)	27·5 (24 to 30·9)	28·2 (25·4 to 31)	21·5 (20·1 to 23·1)	26·3 (25·1 to 27·6)	24·3 (18·6 to 31·6)
Scotland	72·5 (63·6 to 82·5)	103·2 (94·1 to 113·9)	93·3 (82·7 to 104·8)	71 (62·5 to 79·6)	73·4 (66·3 to 80·7)	53 (49·8 to 56·8)	61·2 (58·5 to 64·1)	53·7 (44·2 to 65·5)
Wales	45·1 (43·9 to 46·4)	46·8 (45·6 to 47·9)	44·7 (43·6 to 45·8)	39·1 (38·3 to 39·8)	39 (38·2 to 39·8)	30·8 (30·5 to 31)	35·2 (34·5 to 35·8)	33·1 (30·1 to 36·4)
Latin America and Caribbean	6504·1 (6209·5 to 6799·1)	8212·8 (7924·1 to 8501·8)	9599·4 (9303·6 to 9875·7)	10773·7 (10520·3 to 11028)	10669·4 (10402·9 to 10922·3)	10645·2 (10329·9 to 10976·8)	10557 (10141·3 to 11020·3)	9793·7 (8950·1 to 10685·6)
Andean Latin America	719·7 (686·9 to 753·9)	910·5 (883·4 to 938·2)	1085·4 (1054·8 to 1113·4)	1231·6 (1193·9 to 1270·6)	1230·7 (1178·1 to 1281·6)	1253·7 (1187·7 to 1318·1)	1278·5 (1202·7 to 1364)	1329·8 (1139·7 to 1543·9)
Bolivia	165·6 (159·1 to 172·4)	178·2 (171·9 to 183·8)	192·1 (184·9 to 199·1)	229·7 (222·9 to 236·4)	245·4 (235·7 to 255·8)	262·6 (248·7 to 277·2)	298·5 (279·7 to 316·7)	326·9 (283·8 to 374·2)
Ecuador	161·5 (153·4 to 170·2)	224·4 (218·9 to 229·5)	272 (266 to 277·7)	293·2 (285 to 301·4)	302·8 (291·7 to 314·2)	326 (311·4 to 340·4)	332·5 (305·9 to 359)	349·1 (298·8 to 406·2)
Peru	392·6 (374·6 to 411·3)	507·9 (489·3 to 527·1)	621·3 (598 to 643·7)	708·7 (677·4 to 739·6)	682·6 (635·9 to 728·9)	665·1 (615·7 to 713·5)	647·5 (593·3 to 712·7)	653·8 (557·1 to 763·9)
Caribbean	687·9 (666 to 710·1)	830·5 (804·5 to 853·6)	883·5 (868·7 to 898·1)	820·3 (800·5 to 838·7)	912·9 (891·1 to 935·2)	872·4 (848 to 897·1)	855 (818·8 to 890·8)	819 (743·2 to 901·7)
Antigua and Barbuda	1·7 (1·6 to 1·8)	1·8 (1·8 to 1·9)	1·7 (1·6 to 1·7)	1·4 (1·4 to 1·5)	1·3 (1·3 to 1·4)	1·5 (1·3 to 1·7)	1·2 (1 to 1·3)	1 (0·8 to 1·2)
The Bahamas	2·6 (2·5 to 2·8)	3·5 (3·3 to 3·6)	4·3 (4·2 to 4·4)	5 (4·9 to 5·1)	5·3 (5·1 to 5·4)	5 (4·9 to 5·1)	4·7 (4·7 to 4·8)	4·1 (3·3 to 4·9)
Barbados	6·9 (6·4 to 7·5)	7·1 (6·8 to 7·3)	5 (4·9 to 5·2)	4·4 (4·2 to 4·6)	4·2 (4 to 4·4)	3·8 (3·7 to 4)	3·3 (2·9 to 3·7)	2·8 (2·3 to 3·4)
Belize	3 (2·9 to 3·2)	4·2 (4·1 to 4·3)	4·8 (4·7 to 4·9)	5·4 (5·3 to 5·6)	5·9 (5·7 to 6·1)	6·3 (6 to 6·6)	7 (6·6 to 7·5)	7·6 (6·5 to 8·8)
Bermuda	1·1 (1 to 1·2)	1·2 (1·1 to 1·2)	1 (1 to 1·1)	0·8 (0·8 to 0·9)	0·9 (0·9 to 0·9)	0·8 (0·8 to 0·9)	0·7 (0·7 to 0·8)	0·5 (0·4 to 0·6)
Cuba	154·9 (147·3 to 164)	221·4 (208 to 234·5)	249·3 (248 to 251)	129·8 (127·2 to 132·5)	180·6 (179·6 to 181·8)	146·3 (145·3 to 147·5)	131·6 (130·8 to 132·4)	104·3 (89·8 to 121)
Dominica	2·2 (2·1 to 2·2)	2·6 (2·6 to 2·7)	2·5 (2·4 to 2·6)	1·8 (1·7 to 1·9)	1·7 (1·6 to 1·8)	1·3 (1·1 to 1·5)	1 (0·9 to 1·1)	0·8 (0·7 to 1)
Dominican Republic	117·3 (111·3 to 123·8)	151·4 (145·8 to 156·5)	189·1 (183·7 to 194·5)	223·9 (213·6 to 234·2)	246·3 (236·5 to 256·9)	242·2 (230·8 to 254·4)	223·4 (210·4 to 237)	230·3 (198·3 to 266·3)
Grenada	3·8 (3·7 to 3·9)	3·8 (3·6 to 3·9)	2·9 (2·7 to 3)	2·7 (2·5 to 2·9)	1·9 (1·8 to 2·1)	2 (1·8 to 2·1)	1·6 (1·4 to 1·9)	1·4 (1·2 to 1·7)
Guyana	20·8 (19·9 to 21·7)	26·3 (25·8 to 26·8)	27·2 (26·2 to 28·4)	26·9 (25·4 to 28·4)	22·8 (21·2 to 24·4)	17·6 (16·4 to 18·9)	14·3 (12·5 to 16·1)	14·4 (12·1 to 16·9)
Haiti	171·1 (159·4 to 182·4)	181·4 (170 to 191·7)	184·2 (173·3 to 193·6)	212·9 (202·7 to 222·8)	248·3 (235·9 to 260·5)	276·4 (262·2 to 290·1)	319·6 (298·3 to 340·9)	333·3 (299·6 to 373)
Jamaica	50·5 (46·9 to 54·3)	66·1 (63·9 to 68·2)	61·7 (59·9 to 63·6)	56·5 (54 to 59·1)	55·4 (52·8 to 58·2)	46·5 (41·7 to 51·5)	40·8 (36·5 to 45·7)	35·8 (30·4 to 42·1)
Puerto Rico	85·2 (84·6 to 85·7)	74·4 (73·9 to 74·9)	68·4 (67·9 to 68·9)	71·3 (70·9 to 71·8)	65·3 (64·8 to 65·8)	58·1 (57·4 to 58·8)	41·8 (38·3 to 46)	25·3 (22·1 to 29)
Saint Kitts and Nevis	2·7 (2·4 to 2·9)	1·5 (1·3 to 1·6)	1 (0·9 to 1·1)	1·1 (1 to 1·2)	0·9 (0·8 to 0·9)	0·8 (0·8 to 0·9)	0·8 (0·7 to 0·9)	0·7 (0·6 to 0·8)
Saint Lucia	3·2 (3 to 3·4)	4 (3·9 to 4·1)	4·1 (4 to 4·2)	4·2 (4 to 4·4)	3·8 (3·5 to 4·1)	2·9 (2·6 to 3·2)	2·2 (1·9 to 2·6)	1·8 (1·5 to 2·1)
Saint Vincent and the Grenadines	3·4 (3·3 to 3·5)	3·8 (3·8 to 3·9)	3·4 (3·3 to 3·5)	3·3 (3·1 to 3·5)	2·7 (2·4 to 2·9)	2·2 (2 to 2·4)	1·8 (1·6 to 2)	1·5 (1·3 to 1·8)
Suriname	7·5 (7·3 to 7·7)	12·1 (11·8 to 12·4)	13·2 (12·6 to 13·9)	10·6 (10·1 to 10·9)	9·6 (9·2 to 9·9)	9·9 (9·4 to 10·4)	10 (9 to 11)	9·1 (7·7 to 10·7)
Trinidad and Tobago	24·4 (23 to 26)	31·6 (30·7 to 32·4)	25·4 (24·7 to 26·2)	29·2 (28·2 to 30·2)	23·3 (22·4 to 24·2)	17·7 (16·8 to 18·5)	18·5 (17·4 to 19·6)	15·3 (13 to 18)
Virgin Islands	0·9 (0·9 to 1)	1·2 (1·1 to 1·2)	2·6 (2·5 to 2·8)	2·5 (2·3 to 2·6)	2·3 (2·1 to 2·4)	1·9 (1·7 to 2·2)	1·6 (1·4 to 1·7)	1·3 (1·1 to 1·5)
Central Latin America	2473·4 (2381·3 to 2563·4)	3292·9 (3203·6 to 3375·9)	4109·1 (4003·5 to 4203·1)	4916·6 (4800·8 to 5027·2)	4971·9 (4827·2 to 5119·4)	4928 (4740·4 to 5128·2)	4753·5 (4556 to 4964·7)	4404·2 (3952·7 to 4878·9)
Colombia	537·1 (503·2 to 574·1)	727·8 (696·4 to 757·1)	845·3 (805 to 887·5)	891·3 (853·5 to 928·7)	941·5 (890·7 to 991·6)	960·8 (916·3 to 1006·7)	875·7 (810·2 to 947·3)	804·6 (682·6 to 943·2)
Costa Rica	38·1 (37·4 to 38·8)	58·9 (58·4 to 59·4)	55·1 (53·6 to 56·5)	69·7 (67·9 to 71·6)	80·6 (77·7 to 83·4)	73·5 (68·7 to 78·8)	72·5 (69·5 to 75·9)	66·3 (55·9 to 78·3)
El Salvador	96·5 (92·6 to 100·8)	126·5 (122·7 to 130·2)	154 (150·5 to 157·4)	166 (161·5 to 170·5)	165·5 (160·9 to 170·1)	142·3 (136·6 to 148·3)	116·6 (110·9 to 122·7)	112 (96·2 to 130)
Guatemala	147·5 (142·9 to 152·1)	185·3 (182·1 to 188·5)	219·3 (214·1 to 224·7)	307·5 (303·7 to 311·4)	341 (332·9 to 349·6)	367·8 (355·1 to 381)	387·8 (360·8 to 416·5)	409·1 (347·8 to 478·7)
Honduras	73·7 (70·4 to 76·9)	90·8 (88·2 to 93)	113·4 (111·5 to 115·4)	149·1 (145·5 to 152·7)	180·6 (175·5 to 185·1)	192·8 (185 to 200·2)	205·3 (191·1 to 220·6)	233·1 (206·9 to 264)
Mexico	1266·8 (1201·6 to 1331·2)	1647·7 (1589·5 to 1702)	2173·1 (2096·4 to 2243·4)	2662·4 (2574·7 to 2745)	2502·1 (2396·1 to 2610·9)	2484·1 (2334·6 to 2634)	2299·6 (2169·2 to 2445·1)	2095·8 (1848 to 2393·4)
Aguascalientes	9·4 (9 to 9·8)	11·5 (11·1 to 11·9)	15·1 (14·7 to 15·5)	19·9 (19·3 to 20·4)	21·7 (21 to 22·5)	23·7 (22·3 to 25·3)	23·5 (22·2 to 24·9)	22·9 (20·3 to 26)
Baja California	11·7 (11·2 to 12·2)	24·7 (23·8 to 25·6)	40 (38·6 to 41·3)	46·4 (45·1 to 47·9)	47·8 (45·8 to 50)	54·6 (51·1 to 58·7)	57·3 (53·8 to 61·2)	57·3 (50·6 to 65·5)
Baja California Sur	3 (2·9 to 3·1)	3·9 (3·7 to 4)	5·8 (5·6 to 5·9)	8·5 (8·2 to 8·8)	8·5 (8·1 to 9)	9·7 (9 to 10·4)	11·5 (10·6 to 12·3)	12 (10·5 to 13·8)
Campeche	5·8 (5·5 to 6·1)	7·8 (7·5 to 8·2)	11·5 (11·1 to 11·9)	16·9 (16·4 to 17·4)	15·9 (15·2 to 16·6)	15·8 (14·8 to 16·9)	16 (15 to 17·1)	15·2 (13·5 to 17·2)
Chiapas	41·7 (39·1 to 44·2)	56 (53·5 to 58·1)	71 (68 to 73·7)	88·7 (84·6 to 92·7)	112·7 (107 to 118·3)	115·4 (107·4 to 123·5)	125·5 (117·3 to 134·5)	132·1 (117·7 to 149·1)
Chihuahua	44 (42·1 to 46)	59·3 (57·1 to 61·4)	73·5 (71 to 75·8)	77·2 (74·4 to 79·8)	67·8 (64·5 to 71·1)	72·2 (67·9 to 76·8)	62·4 (58·5 to 66·8)	55 (48·7 to 62·5)
Coahuila	35·2 (33·4 to 37)	42 (40·6 to 43·3)	50 (48·2 to 51·8)	61·3 (59·6 to 62·9)	55·4 (53 to 57·8)	52·1 (49·1 to 55·4)	51·8 (48·9 to 54·9)	51·4 (45·9 to 57·9)
Colima	5·8 (5·5 to 6)	7·6 (7·3 to 7·9)	10·6 (10·3 to 10·9)	13·6 (13·2 to 14)	11·8 (11·4 to 12·3)	11·4 (10·7 to 12·1)	11·6 (10·8 to 12·4)	11 (9·8 to 12·4)
Durango	32·4 (31·1 to 33·6)	38·2 (36·8 to 39·6)	44·4 (43·2 to 45·6)	48·9 (47·4 to 50·5)	46·9 (44·8 to 48·9)	42·1 (39·4 to 45·2)	36·6 (34·4 to 39)	33·4 (29·9 to 37·4)
Guanajuato	65·4 (62·5 to 68·2)	80·2 (77·5 to 82·6)	99·3 (95·7 to 102·7)	126·5 (121·1 to 131·5)	136·7 (129·3 to 144·6)	134·8 (124·7 to 146)	119·3 (109·6 to 129)	106·1 (92·5 to 122·2)
Guerrero	43·5 (40·9 to 46·1)	56 (53·9 to 57·9)	71·6 (68·7 to 74·4)	88·1 (83·8 to 92·1)	98·4 (93·5 to 103·6)	102·1 (95·7 to 108·8)	86·6 (81 to 92·4)	76·9 (67·9 to 87·4)
Hidalgo	42·5 (40·6 to 44·3)	49·2 (47·5 to 50·7)	56·6 (54·7 to 58·4)	66·3 (64·7 to 67·9)	63·7 (61 to 66·5)	61·3 (57·2 to 65·8)	56·8 (53·1 to 60·8)	50·4 (44·7 to 57·3)

Jalisco	88·7 (84·3 to 92·9)	111·3 (107·5 to 115)	144·2 (139·2 to 148·9)	173·6 (169 to 178·3)	159·4 (153·4 to 165·6)	151·7 (143·3 to 161·2)	143·2 (136 to 150·9)	132·4 (117·7 to 149·8)
México	64·4 (61·2 to 67·6)	86·5 (83·4 to 89·5)	170·1 (164·3 to 175·7)	302·6 (293·2 to 311·6)	312 (296·2 to 327·1)	346·8 (324·5 to 370·5)	346·5 (323·8 to 372)	316·3 (280·8 to 358·5)
Mexico City	165·3 (155·8 to 174·6)	246·7 (238·2 to 254·6)	331·1 (317·5 to 343·4)	353·6 (335 to 371·2)	231·8 (217·3 to 246·1)	185·2 (170 to 201·1)	146·8 (136·5 to 158·8)	114·9 (100·4 to 132·6)
Michoacán de Ocampo	69·5 (65·8 to 73)	83·3 (80 to 86·2)	99·5 (95·6 to 102·9)	121·9 (116·7 to 126·8)	117·2 (110·2 to 124·2)	105·6 (98·1 to 113·6)	92·2 (85·7 to 99·1)	82 (72 to 93·9)
Morelos	13·1 (12·4 to 13·8)	18·2 (17·4 to 18·9)	27·4 (26·5 to 28·3)	37·8 (36·7 to 38·9)	34·1 (32·9 to 35·4)	33·6 (31·9 to 35·6)	33·2 (31 to 35·6)	30·6 (27·2 to 34·6)
Nayarit	14·8 (14·1 to 15·4)	18·5 (17·7 to 19·2)	24 (23·2 to 24·7)	26·7 (25·8 to 27·6)	23·4 (22·4 to 24·4)	21·2 (19·9 to 22·7)	21·1 (19·7 to 22·4)	20·1 (18 to 22·6)
Nuevo León	37·2 (35·7 to 38·8)	54·2 (52·4 to 55·9)	78·9 (76·2 to 81·5)	92·3 (88·7 to 95·9)	83·7 (79·2 to 88·9)	88·3 (81·8 to 94·8)	85·6 (79·6 to 92·2)	79·7 (70 to 91·5)
Oaxaca	66·5 (62·1 to 70·7)	79·6 (76·1 to 82·7)	89·1 (85·3 to 92·5)	94·2 (90·1 to 98·1)	92·6 (88·2 to 97·3)	96·3 (88·8 to 104·2)	85·8 (80·1 to 91·9)	74·8 (65·8 to 85·5)
Puebla	76·1 (71·5 to 80·5)	89·2 (85·1 to 92·7)	109·6 (104·6 to 114·3)	133·8 (127·8 to 139·7)	133·6 (125·4 to 142·1)	144 (133·5 to 155·2)	133·3 (122 to 145·2)	120 (102 to 141·6)
Querétaro	13·7 (13 to 14·3)	16·4 (15·8 to 17)	21·2 (20·5 to 21·7)	29·2 (28·4 to 29·9)	33·8 (32·3 to 35·2)	35·2 (33 to 37·7)	36·3 (34·2 to 38·6)	35·5 (31·5 to 40·4)
Quintana Roo	1·2 (1·2 to 1·3)	2·2 (2·2 to 2·3)	4 (3·9 to 4·1)	9·4 (9·1 to 9·6)	14·7 (14·1 to 15·4)	20·5 (19·4 to 21·8)	25·6 (24·2 to 27·1)	27·4 (24·3 to 31·1)
San Luis Potosí	41·1 (39·1 to 43·1)	48·6 (46·8 to 50·2)	56 (53·9 to 57·9)	65·6 (62·7 to 68·4)	64·3 (60·7 to 68·1)	61·1 (56·9 to 65·4)	52·7 (48·5 to 56·8)	46·8 (41 to 53·9)
Sinaloa	30·4 (29 to 31·8)	39·2 (37·8 to 40·7)	56·7 (55 to 58·2)	69 (66·9 to 71·3)	63·5 (60·9 to 66·1)	60·9 (57·1 to 65·1)	50·6 (47·6 to 53·6)	44·8 (39·8 to 50·6)
Sonora	26·5 (25·3 to 27·7)	36·9 (35·7 to 38·1)	49·1 (47·3 to 50·8)	58·5 (55·6 to 61·3)	52·4 (49·2 to 55·4)	53·9 (49·3 to 58·3)	49·2 (45·3 to 53·3)	45·3 (39·9 to 51·9)
Tabasco	16·6 (15·8 to 17·4)	23·8 (23·1 to 24·6)	34·9 (33·8 to 36·1)	43·7 (42·4 to 44·9)	46·3 (44·5 to 48·1)	46 (43·4 to 49)	44·2 (41·4 to 46·9)	40·6 (36·2 to 45·9)
Tamaulipas	35·4 (33·5 to 37·2)	48·4 (46·6 to 50)	63·4 (60·6 to 65·9)	73·1 (69·3 to 76·7)	66·6 (61·9 to 71·3)	70·8 (65·3 to 76·3)	62·9 (58·5 to 67·5)	54·2 (47·4 to 62·4)
Tlaxcala	13·1 (12·5 to 13·8)	15·2 (14·6 to 15·7)	17·4 (16·9 to 17·9)	21·4 (20·9 to 21·9)	23 (22·1 to 24)	24 (22·6 to 25·4)	23·2 (21·9 to 24·5)	21·7 (19·3 to 24·5)
Veracruz de Ignacio de la Llave	95·1 (89·3 to 100·9)	126·1 (120·9 to 130·9)	171·1 (164·4 to 177·6)	210·6 (204·9 to 216·7)	187·9 (180 to 196·3)	174·1 (164 to 185·1)	143·2 (134·2 to 153·4)	125·7 (111·2 to 142·7)
Yucatán	22·8 (21·3 to 24·2)	26·2 (25·1 to 27·2)	33·2 (31·8 to 34·5)	40·7 (39·6 to 41·8)	38·1 (36·5 to 39·6)	36·6 (34·6 to 38·8)	34·7 (32·7 to 36·7)	32·2 (28·5 to 36·6)
Zacatecas	34·9 (33·8 to 35·8)	40·6 (39·3 to 41·8)	42·7 (41·6 to 43·9)	42·4 (41 to 43·7)	36·3 (34·5 to 38)	33·1 (31 to 35·2)	30·5 (28·2 to 33·1)	27 (23·2 to 31·6)
Nicaragua	66·7 (64·2 to 69·1)	80·7 (78·5 to 83)	98·1 (95·4 to 100·7)	130·6 (126·9 to 133·9)	143·9 (137·1 to 150·7)	127·8 (120·7 to 135·1)	133·9 (123·6 to 144·4)	130·4 (115·9 to 147·5)
Panama	26·6 (25·7 to 27·6)	42·5 (41·7 to 43·3)	53·7 (52·5 to 54·9)	52·6 (50·9 to 54·3)	58·8 (56·8 to 61)	62·3 (59·6 to 65·3)	69·5 (67·2 to 71·9)	75·9 (65·8 to 86·9)
Venezuela	220·3 (213 to 227·1)	332·7 (328 to 337·1)	397·2 (390·5 to 404·9)	487·5 (475·5 to 498·9)	558·1 (543·5 to 573·1)	516·6 (495·6 to 538·7)	592·4 (573·4 to 614·2)	477 (404·3 to 558·4)
Tropical Latin America	2623·2 (2422·9 to 2820)	3179 (2973·1 to 3388·2)	3521·4 (3324·7 to 3719·6)	3805·2 (3695·7 to 3913·1)	3553·9 (3460·9 to 3651·2)	3591·2 (3477·3 to 3716·6)	3670 (3519·9 to 3828·2)	3240·7 (2890·4 to 3639·8)
Brazil	2558·1 (2358·2 to 2754·1)	3103·7 (2898 to 3311·9)	3435·4 (3241·7 to 3633)	3696·3 (3591·5 to 3802·8)	3426·8 (3331·8 to 3519·8)	3466·5 (3353·8 to 3591·6)	3540·4 (3389·2 to 3694·9)	3115·1 (2766·4 to 3503·6)
Acre	5·4 (4·9 to 5·9)	7 (6·5 to 7·4)	8·3 (7·9 to 8·7)	10·6 (10·2 to 11)	10·8 (10·4 to 11·2)	13·2 (12·8 to 13·7)	15·8 (15 to 16·7)	16·2 (14·4 to 18·3)
Alagoas	62·1 (58·3 to 65·7)	68·1 (64 to 72)	77·4 (73·8 to 81·3)	81·5 (79·2 to 83·8)	79·1 (76·4 to 82·1)	81·4 (77·8 to 85)	82·1 (78·2 to 86)	73 (65·1 to 81·7)
Amapá	1·8 (1·6 to 2)	2·9 (2·7 to 3·1)	3·9 (3·7 to 4·2)	4·8 (4·6 to 5·1)	5·8 (5·5 to 6)	9·3 (8·8 to 9·8)	12·2 (11·4 to 13·1)	12·9 (11·4 to 14·7)
Amazonas	28·2 (25·8 to 30·7)	35·4 (33·1 to 37·5)	40·1 (38·1 to 42)	49·5 (47·8 to 51·2)	50·7 (48·9 to 52·6)	63·5 (61·3 to 66)	76·3 (72·5 to 80·5)	73·7 (65·3 to 83·4)
Bahia	270·7 (254 to 286·7)	314·4 (295·2 to 332·7)	350·1 (332·3 to 368·2)	369·2 (357·5 to 381)	320·7 (311·7 to 329·8)	328·2 (316 to 340·6)	332·6 (317·3 to 348·7)	281·6 (249·4 to 318·2)
Ceará	143·9 (135·6 to 152)	172·5 (162·2 to 182·5)	192·3 (182·6 to 202·5)	200·7 (194·2 to 207)	180·3 (175·4 to 185)	194·7 (186·7 to 203·5)	211·3 (201·7 to 220·4)	196·7 (174·3 to 221)
Distrito Federal	1·9 (1·8 to 2·1)	4·8 (4·4 to 5·1)	19·9 (18·8 to 21)	37·4 (36·1 to 38·7)	38·6 (37 to 40)	42·2 (40·5 to 43·9)	43·2 (41·2 to 45·2)	38·6 (34·6 to 42·9)
Espírito Santo	41·2 (38·1 to 44·2)	52·1 (48·7 to 55·5)	56·5 (53·1 to 60)	62·7 (60·4 to 64·9)	61·7 (59·4 to 63·8)	61·6 (59·1 to 64·2)	62 (58·5 to 65·7)	53·8 (47·8 to 60·6)
Goiás	49·6 (45·7 to 53·4)	72·4 (67·7 to 77·1)	92·4 (87·7 to 97·4)	109 (105·8 to 112·1)	90·5 (87·1 to 93·9)	95·5 (91·3 to 99·8)	96 (91·6 to 100·4)	84·9 (75·2 to 96·1)
Maranhão	93·9 (87·1 to 101·1)	135·4 (127 to 142·9)	151·5 (144·5 to 158·7)	175·5 (170·5 to 180·4)	177·3 (170 to 184)	199·3 (190·8 to 208·6)	245·1 (234·8 to 255·2)	212·6 (188·5 to 238·7)
Mato Grosso	9·4 (8·7 to 10)	15·3 (14·2 to 16·3)	22·9 (21·6 to 24·2)	34·2 (33·2 to 35·1)	45·2 (43·3 to 47·1)	48·5 (46·5 to 50·7)	53·4 (50·8 to 56·1)	48·4 (43·1 to 54·4)
Mato Grosso do Sul	16·2 (14·7 to 17·7)	25·1 (23·3 to 26·8)	32·7 (30·7 to 34·7)	41·4 (40 to 42·8)	42·1 (40·4 to 43·6)	40·4 (38·8 to 42)	42·4 (39·9 to 44·9)	38·5 (34 to 43·7)
Minas Gerais	361 (331·6 to 390·8)	403·8 (375·4 to 432·7)	398·3 (374·5 to 423·6)	382·3 (373 to 392)	347·4 (338·4 to 356·6)	331·9 (319·5 to 345·3)	313 (297·2 to 328·9)	268·9 (238·9 to 303)
Pará	59·4 (54·7 to 64·4)	73·3 (68·6 to 77·7)	89·9 (85·3 to 94·5)	115·8 (111·7 to 119·5)	116·1 (112·4 to 119·5)	138·3 (132·9 to 144)	167·2 (158·9 to 175·3)	157·7 (139·3 to 178·8)
Paraíba	95·3 (89·5 to 100·8)	105·5 (99·2 to 111·5)	105·4 (100·1 to 110·8)	93 (89·5 to 96·5)	82·2 (79·9 to 84·8)	86·2 (82·5 to 90)	93·1 (88·8 to 97·5)	82·9 (73·6 to 93)
Paraná	99·3 (90·9 to 107·6)	172·6 (160·2 to 184·9)	237·6 (223·2 to 252·7)	202·2 (195·5 to 209·3)	186·7 (180·4 to 192·9)	173·6 (166·8 to 180·4)	158·7 (150·7 to 167·3)	139·2 (123·4 to 157·8)
Pernambuco	197·7 (186 to 208·8)	228 (214·7 to 241·2)	246·3 (234·4 to 259·1)	235·6 (227·9 to 243·6)	200·3 (194 to 206·4)	205 (196·1 to 214·6)	206·2 (197 to 215·6)	178·7 (158·1 to 202·1)
Piauí	56·7 (52·9 to 60·7)	65·2 (61·3 to 68·8)	75·7 (72 to 79·6)	81·5 (78·9 to 84·1)	75·9 (73·4 to 78·4)	80·1 (76·9 to 83·5)	86·3 (82·1 to 90·5)	75·1 (66·1 to 85·3)
Rio de Janeiro	207·6 (186·4 to 229·7)	245·6 (225·1 to 267·2)	260·7 (240·9 to 280·4)	270·3 (258·7 to 282·7)	240·5 (231·7 to 249)	229·1 (220·4 to 238·3)	223·3 (211·7 to 235·6)	197·9 (176·1 to 222·9)
Rio Grande do Norte	53·4 (50·1 to 56·4)	61·9 (58·2 to 65·4)	71·1 (67·4 to 74·8)	71·3 (69·1 to 73·3)	68·9 (66·9 to 70·9)	73·7 (70·8 to 76·7)	76 (72 to 80·1)	68·1 (60·2 to 77·1)
Rio Grande do Sul	170·8 (153·3 to 188·6)	190·5 (175·5 to 205·8)	189·2 (176·1 to 202·8)	192·4 (187 to 198)	170·9 (164·8 to 176·8)	153 (147·8 to 158·8)	133·8 (127·3 to 140·2)	115·9 (103·1 to 130·5)
Rondônia	1·8 (1·6 to 1·9)	2·9 (2·7 to 3·1)	4·3 (4 to 4·5)	15·7 (15·1 to 16·4)	25·7 (24·8 to 26·5)	27·6 (26·4 to 28·8)	27·5 (26·1 to 29)	24·1 (21·1 to 27·4)
Roraima	1 (0·9 to 1·1)	1·3 (1·3 to 1·4)	1·7 (1·6 to 1·8)	3·1 (3 to 3·2)	6·4 (6·2 to 6·6)	9·9 (9·5 to 10·3)	12·2 (11·7 to 12·8)	12·5 (11·2 to 14·1)
Santa Catarina	67·9 (62·3 to 73·4)	81·2 (75·3 to 87·4)	88·5 (82·9 to 94·1)	95·8 (92·9 to 98·5)	89·7 (86·4 to 92·8)	87·3 (83·7 to 90·9)	89·4 (84·8 to 94·3)	82·1 (73·3 to 91·9)
São Paulo	420·2 (382·9 to 458)	518·9 (481·8 to 557·3)	564·2 (528 to 601·1)	699·4 (677 to 722·3)	651·4 (627·4 to 674·6)	621·7 (599·6 to 645·5)	606·9 (577 to 638·5)	514·4 (457·5 to 579·3)
Sergipe	33·8 (31·4 to 36)	35·9 (33·6 to 38·1)	37·8 (35·8 to 39·8)	39 (37·7 to 40·3)	37·4 (35·7 to 39)	40·5 (38·8 to 42·2)	42·2 (40·2 to 44·3)	38 (33·7 to 42·7)
Tocantins	7·9 (7·3 to 8·6)	11·8 (11·1 to 12·5)	16·6 (15·7 to 17·5)	22·5 (21·6 to 23·3)	24·9 (24 to 25·7)	30·8 (29·6 to 32·1)	32 (30 to 33·9)	28·5 (25·1 to 32·4)
Paraguay	65 (61·1 to 69·2)	75·3 (71·7 to 78·8)	85·9 (83 to 89·1)	108·8 (105·1 to 112·4)	127·1 (121·3 to 133·1)	124·6 (118·4 to 131·3)	129·6 (118·4 to 141·5)	125·6 (107·6 to 146·1)
North Africa and Middle East	5617·1 (5392·9 to 5838)	6881·2 (6662·1 to 7089·4)	8551·5 (8328·4 to 8754)	11238·7 (10969·5 to 11490·4)	11948·5 (11523·2 to 12355·1)	12373·2 (11781·4 to 12966·1)	12830·4 (11960·5 to 13730·5)	12197·8 (11052 to 13533·2)
North Africa and Middle East	5617·1 (5392·9 to 5838)	6881·2 (6662·1 to 7089·4)	8551·5 (8328·4 to 8754)	11238·7 (10969·5 to 11490·4)	11948·5 (11523·2 to 12355·1)	12373·2 (11781·4 to 12966·1)	12830·4 (11960·5 to 13730·5)	12197·8 (11052 to 13533·2)

Afghanistan	372·1 (354·1 to 389·7)	448·5 (431·4 to 463·3)	557·4 (540·6 to 573·7)	524·6 (510·7 to 538·3)	528 (516·4 to 539·7)	921·6 (895·4 to 946·7)	1178·9 (1137·8 to 1218·6)	1496·2 (1417·3 to 1580·5)
Algeria	386·8 (362·7 to 411·1)	476 (455·6 to 495·9)	582·6 (563·2 to 601·4)	744·6 (718·2 to 768)	770·2 (724·4 to 817)	718·2 (649·8 to 789·8)	881·4 (795·1 to 974·6)	885·9 (794·9 to 993)
Bahrain	3·8 (3·6 to 4·1)	6·5 (6·2 to 6·9)	6·7 (6·3 to 7)	9·3 (8·6 to 9·9)	13·8 (12·7 to 14·9)	13·2 (12·1 to 14·3)	16·5 (14·6 to 18·7)	13 (10·7 to 16)
Egypt	1045·7 (1011·3 to 1081)	1239·5 (1195·7 to 1282·7)	1474·8 (1424·3 to 1525·8)	1884·4 (1832 to 1933·5)	1953·7 (1887·3 to 2022·8)	1916·7 (1808·1 to 2018·4)	2255·1 (2075·8 to 2435·7)	2107·7 (1879·6 to 2346)
Iran	822·5 (773·9 to 870·5)	953·2 (909·4 to 993·7)	1167·5 (1118·6 to 1212·4)	2082·9 (2056·8 to 2108·5)	1745·4 (1613·5 to 1870·7)	1441·6 (1295 to 1587·1)	1491·5 (1304·3 to 1670·1)	1349·8 (1135·5 to 1623·2)
Iraq	251·4 (241·4 to 260·9)	328·3 (314·9 to 340·6)	430·1 (410·3 to 448·8)	594·5 (573·5 to 614·2)	733·1 (695 to 771·3)	960·8 (904·3 to 1020·7)	921·8 (822·5 to 1024·4)	956·7 (832·7 to 1109·8)
Jordan	25·6 (25·5 to 25·7)	43·5 (43·3 to 43·8)	67·6 (66·8 to 68·3)	99·6 (97·5 to 101·8)	137·7 (131·4 to 144·1)	141·4 (127·8 to 154·8)	185·5 (167·1 to 204·5)	240·4 (209·7 to 277·7)
Kuwait	3 (2·7 to 3·2)	11·7 (11·5 to 11·9)	35·7 (35 to 36·2)	50·1 (48·3 to 51·8)	39·8 (36·4 to 43)	41·2 (38·5 to 44)	55·2 (51·6 to 59·2)	60·8 (50·3 to 74·5)
Lebanon	63·9 (60·8 to 66·9)	82·6 (79·5 to 85·7)	98 (93·7 to 102·1)	112·2 (105 to 119·2)	113·9 (104·6 to 122·1)	100·4 (90·5 to 110·3)	103·4 (90·9 to 116·3)	107·3 (92·9 to 124·7)
Libya	51·6 (50 to 53·1)	70·7 (68·2 to 73·1)	88·8 (86·6 to 91·1)	130·2 (125·9 to 134·3)	155·6 (146·8 to 163·9)	124·1 (112·3 to 136·5)	101·9 (89·5 to 115·3)	81·3 (68·1 to 98·5)
Morocco	463·2 (441·1 to 486·4)	569·6 (547·9 to 590·5)	684·2 (657·9 to 710·8)	788·8 (746·5 to 829·9)	807·4 (759·1 to 854·5)	836·8 (765·6 to 908·4)	778·3 (684 to 875·5)	608·2 (516·7 to 713·2)
Oman	25·7 (24·9 to 26·5)	33·8 (32·8 to 34·7)	42·6 (41·3 to 43·8)	60 (57·9 to 62·1)	66·3 (62·8 to 70)	61·9 (56·2 to 67·5)	66·7 (59 to 74·2)	77·8 (69·2 to 86·8)
Palestine	39·5 (38·2 to 40·8)	47·1 (45·9 to 48·3)	55·5 (54·1 to 56·9)	69·3 (67·1 to 71·4)	96 (92·2 to 99·8)	125·8 (120·2 to 131·8)	140·3 (130·2 to 150·3)	125·2 (112·1 to 140·2)
Qatar	1·1 (1 to 1·1)	2·2 (2 to 2·3)	4·4 (4·1 to 4·7)	7·9 (7·5 to 8·3)	10·6 (10 to 11·2)	11·2 (10·5 to 11·9)	22 (20·2 to 23·9)	26·8 (23·1 to 30·9)
Saudi Arabia	133·8 (128·6 to 138·8)	174·9 (168·9 to 180·7)	257·7 (246·9 to 268·8)	406·1 (384·3 to 430·4)	538·9 (497·1 to 582·6)	516·4 (462·1 to 568·4)	490 (433·9 to 548·6)	455·3 (403·8 to 510·7)
Sudan	279·2 (266·5 to 291·3)	326·6 (313·7 to 337·1)	543·7 (526·2 to 559·8)	793·4 (764·4 to 820·3)	1057·2 (1014·7 to 1096·1)	1267·8 (1205·5 to 1327·5)	1269·8 (1156·5 to 1380·4)	1206·7 (1051·7 to 1388·3)
Syria	177·6 (172·9 to 182·3)	242·2 (235·7 to 248·1)	317·3 (309·3 to 324·6)	412·4 (400·5 to 424)	517 (491·9 to 540·4)	522·8 (485·8 to 562·9)	509·4 (459·3 to 561·7)	231·9 (192·5 to 282·6)
Tunisia	168·4 (157·8 to 178·8)	191·4 (183·8 to 197·9)	207·8 (202 to 213·9)	253·8 (245·1 to 262·7)	241·3 (226·5 to 256·2)	209·8 (194·8 to 224·8)	199 (184·9 to 213·2)	167·6 (143·7 to 197·8)
Turkey	1055·8 (988·6 to 1123·1)	1334 (1277·4 to 1387·9)	1546·2 (1486 to 1604·3)	1640·7 (1550·4 to 1726·1)	1686·3 (1575·6 to 1796·8)	1515·9 (1421·2 to 1617)	1030 (941·4 to 1121·2)	981·7 (856·9 to 1135·2)
United Arab Emirates	3·5 (3·4 to 3·7)	4·3 (4·2 to 4·5)	9·4 (9 to 9·7)	35·5 (34·5 to 36·5)	46·5 (44·4 to 48·5)	49·7 (45·6 to 53·6)	93 (81·6 to 105·2)	56·2 (48·1 to 66·3)
Yemen	241·9 (233·6 to 249·8)	292·8 (285·5 to 299·5)	370 (361·9 to 377·8)	531·1 (522·7 to 539·3)	681·9 (664·8 to 697·3)	866·3 (825·3 to 903·8)	1027·8 (946·7 to 1101·4)	948·9 (838·1 to 1077)
South Asia	20216·6 (18733·9 to 21701·2)	22992·1 (21438·5 to 24590·8)	25970·9 (24387·3 to 27478·1)	33101·1 (31750·4 to 34432·9)	35823·2 (34350 to 37233·1)	36775·9 (35422·2 to 38103·6)	36166·9 (34802·3 to 37511·2)	33438·5 (30722 to 36348·4)
South Asia	20216·6 (18733·9 to 21701·2)	22992·1 (21438·5 to 24590·8)	25970·9 (24387·3 to 27478·1)	33101·1 (31750·4 to 34432·9)	35823·2 (34350 to 37233·1)	36775·9 (35422·2 to 38103·6)	36166·9 (34802·3 to 37511·2)	33438·5 (30722 to 36348·4)
Bangladesh	2220·8 (2145·9 to 2289·5)	2431·1 (2351·3 to 2507·4)	2933·6 (2835·7 to 3024·6)	3265·3 (3160·1 to 3376·7)	3787·5 (3676·7 to 3896·5)	3449 (3283·9 to 3617·6)	2989·8 (2830·7 to 3154·3)	2659·5 (2378·5 to 2945·6)
Bhutan	8·2 (7·7 to 8·8)	10·9 (10·2 to 11·5)	14·7 (13·9 to 15·4)	19·1 (17·9 to 20·4)	23 (20·9 to 25·1)	19·2 (17·2 to 21·3)	17 (14·5 to 19·3)	13·8 (11·8 to 16·1)
India	15887·5 (14589·5 to 17194·3)	18261·4 (16800·3 to 19747·9)	19640 (18212·9 to 21019·4)	25192·9 (24001·9 to 26356·9)	26294·8 (24911·4 to 27580)	26548·5 (25374·6 to 27677·8)	25925·8 (24765 to 27061·2)	23461 (21145·4 to 25772·6)
Andhra Pradesh	633·9 (569·4 to 701·7)	739·3 (676·9 to 803·4)	807 (726 to 897·8)	1043 (971·1 to 1115·9)	1048·4 (980 to 1115·1)	893·8 (836·5 to 955·4)	836·3 (786·1 to 888)	679·8 (605·9 to 758·5)
Arunachal Pradesh	11·3 (10·5 to 12·1)	13·3 (12·5 to 14·1)	19·4 (17·7 to 21·3)	26·2 (24·1 to 28·4)	25·2 (22·8 to 27·7)	26·6 (24·3 to 28·9)	32·1 (28·9 to 35·5)	35 (29 to 41·8)
Assam	365·8 (340·1 to 391·3)	444·6 (407·9 to 483·5)	524·2 (484·2 to 564)	696·8 (651·4 to 740·7)	721·5 (665·4 to 775·7)	629·6 (590·2 to 670·7)	659·8 (602·9 to 721·7)	660 (581·1 to 739·5)
Bihar	1355·8 (1242·7 to 1467·9)	1593·9 (1484·1 to 1699·8)	1776·7 (1619·6 to 1946)	2324·6 (2184 to 2463)	2536·6 (2378·6 to 2689·2)	2823·8 (2655·5 to 2985·4)	2917·6 (2776 to 3061·2)	2569·3 (2333·5 to 2801·8)
Chhattisgarh	275·3 (251·2 to 300·1)	333·5 (306·5 to 358·4)	354·5 (320 to 389·7)	503·8 (470·7 to 537·8)	574·5 (531 to 617·4)	573·2 (541·4 to 609·4)	609·2 (571·9 to 647·9)	604·6 (535·8 to 675·1)
Delhi	68·3 (61·9 to 74·8)	86·7 (77·3 to 97·4)	106·7 (94·9 to 118·2)	182·6 (170·3 to 195·4)	264 (246·8 to 281·5)	308·7 (286·5 to 331·6)	324·7 (306·3 to 344·2)	291·2 (247·3 to 339·1)
Goa	22 (20·3 to 23·7)	25·2 (23·2 to 27·3)	25·9 (23·3 to 28·9)	27·2 (24·3 to 30·2)	24·1 (22·5 to 25·8)	25·9 (24·2 to 27·6)	22·4 (20·7 to 24·3)	17·9 (15·1 to 21·2)
Gujarat	784·2 (732 to 835·6)	923·4 (845·6 to 1005·9)	1006·6 (937·6 to 1073·1)	1285·2 (1215·6 to 1355·9)	1167·1 (1093·7 to 1246·8)	1143·5 (1081·7 to 1210·5)	1232·8 (1176·1 to 1298·8)	1131·2 (1028·1 to 1233·8)
Haryana	260·1 (243·4 to 276·1)	311·2 (283·5 to 340·9)	367·8 (340·8 to 393·7)	443·9 (420·1 to 467·6)	490·8 (465·2 to 517·7)	540·3 (505·8 to 578·5)	560·4 (520·9 to 599·2)	463·6 (385·9 to 549·1)
Himachal Pradesh	86·9 (80·4 to 93·8)	101·5 (94·9 to 108·2)	117·4 (107·7 to 128·8)	145·5 (136·9 to 154·4)	144·7 (137·6 to 152·1)	126 (120·1 to 132·4)	120·4 (113·5 to 127·3)	94 (84·9 to 103·2)
Jammu and Kashmir	115·1 (105·8 to 124·5)	147·7 (133·6 to 163)	152·4 (136 to 168·1)	192·4 (180·6 to 204·4)	237·6 (220·4 to 255)	252·6 (235·3 to 269·8)	225·7 (204·6 to 246·6)	198·5 (164·4 to 236·2)
Jharkhand	493·6 (459·5 to 527·4)	553·3 (518·7 to 587·7)	604·7 (556·1 to 652·5)	783·3 (726·9 to 840)	812·2 (754·1 to 869·4)	822·8 (764·6 to 883·1)	748·4 (693·3 to 801·8)	695·2 (616·3 to 780·6)
Karnataka	724·5 (649·2 to 802·4)	838·1 (744 to 938·6)	889·9 (800·1 to 980·6)	1237·2 (1150·6 to 1331·1)	1149·5 (1074·3 to 1226)	1037·5 (962·8 to 1120·3)	1090·8 (1012·2 to 1177·2)	946·1 (829·7 to 1069·9)
Kerala	548·1 (499·5 to 598·2)	585 (518·3 to 661·1)	597·1 (545·9 to 646·3)	668·9 (625·5 to 713·5)	645·2 (605·3 to 686·2)	628·9 (590·4 to 674·3)	543·5 (514·8 to 573·6)	426·1 (385·7 to 469·4)
Madhya Pradesh	1032·5 (958·9 to 1104·5)	1190·1 (1082·9 to 1296·6)	1249·7 (1149·6 to 1348·3)	1523·7 (1422·2 to 1631·2)	1588·8 (1451·8 to 1734·1)	1711 (1601·2 to 1824·9)	1911 (1788·4 to 2038·8)	1628·9 (1433·4 to 1836·2)
Maharashtra	1293·1 (1172·1 to 1417·4)	1498·8 (1340·8 to 1674·2)	1515·9 (1380·1 to 1658·5)	2013·7 (1881·3 to 2147·7)	2001·6 (1891·2 to 2119·8)	1927·4 (1801 to 2063·4)	1899·5 (1785·3 to 2023·1)	1659·2 (1487·3 to 1841·7)
Manipur	32·7 (30·9 to 34·5)	38·9 (36·3 to 41·6)	45·8 (42·2 to 49·5)	55 (50·2 to 60·2)	55·2 (49·1 to 61·5)	54·8 (49·9 to 59·1)	58·4 (51·9 to 65·4)	60·1 (50·4 to 71)
Meghalaya	27·1 (25·5 to 28·6)	35·9 (33·3 to 38·4)	47·4 (44·1 to 50·8)	62·7 (58·1 to 67·4)	66·2 (59·7 to 72·6)	65·5 (59·4 to 71·1)	65·9 (57·9 to 74·2)	65·5 (54·1 to 78·4)
Mizoram	7·1 (6·7 to 7·5)	10·1 (9·4 to 10·8)	11·6 (10·6 to 12·8)	16·4 (14·9 to 17·9)	18·4 (16·5 to 20·4)	21·3 (19·4 to 23·2)	24·6 (22·1 to 27·1)	23·9 (20·4 to 27·7)
Nagaland	16·4 (15·6 to 17·1)	19 (17·8 to 20·2)	23 (21·4 to 24·7)	30·6 (28·4 to 32·9)	35·3 (32·1 to 38·5)	47·3 (43 to 51·8)	44·4 (40·2 to 48·6)	36 (29·7 to 43)
Odisha	713 (654·6 to 772·6)	763·6 (703·9 to 821·3)	790·4 (722·4 to 860·6)	950·1 (880·8 to 1021·6)	878·1 (829·6 to 930·4)	881·8 (832 to 937·2)	839·7 (796·6 to 887·4)	738·8 (646 to 836·2)
Punjab	335 (309·2 to 360·9)	374·5 (337·3 to 415·2)	419·9 (389·6 to 450·4)	504·1 (476·8 to 532·3)	562·3 (532·3 to 591·3)	511·2 (481·5 to 541·7)	480·2 (450·2 to 514·6)	401·9 (334·9 to 476·6)
Rajasthan	597·3 (539·3 to 657·3)	772·8 (702·5 to 846·5)	902·5 (821·8 to 984·6)	1333·6 (1254·1 to 1412·3)	1584·9 (1493·7 to 1674·7)	1757·2 (1666·4 to 1854·1)	1745·9 (1638 to 1856·7)	1539·2 (1352 to 1734·9)
Sikkim	6·2 (5·8 to 6·6)	7·8 (7·3 to 8·3)	9 (8·3 to 9·7)	12·5 (11·5 to 13·5)	11·6 (10·5 to 12·6)	9·8 (9 to 10·7)	8·7 (7·8 to 9·7)	7 (5·7 to 8·5)
Tamil Nadu	1157·9 (1044·8 to 1276·7)	1242 (1103·1 to 1395·9)	1221·5 (1140·2 to 1312·4)	1305·3 (1229·6 to 1380·5)	1259·7 (1196·1 to 1322·3)	1262 (1194 to 1335·7)	1210·2 (1134·5 to 1291·9)	973 (808·2 to 1157·4)
Telangana	493·1 (447·5 to 540·9)	575·8 (528·5 to 622)	615·8 (555 to 677)	786·2 (712·2 to 861·9)	792·5 (713·2 to 874·4)	718·3 (649·6 to 790·7)	663·7 (598·1 to 733·2)	543·5 (496·4 to 594·1)
Tripura	43·6 (40·6 to 46·5)	54·4 (50·6 to 58·1)	60·6 (54·8 to 66·4)	77·9 (70·3 to 85·8)	69·3 (64·1 to 74·6)	60·4 (55·4 to 65·3)	58·9 (53·6 to 64·1)	49·4 (40 to 60·2)
Union Territories other than Delhi	33·9 (32·4 to 35·3)	36·8 (35 to 38·6)	44·6 (41·9 to 47·3)	62·8 (57·9 to 67·8)	62·4 (54·8 to 70·1)	56·5 (49·8 to 63·3)	51 (43·1 to 59·1)	40·1 (32·3 to 49)
Uttar Pradesh	3016·8 (2810·8 to 3213)	3366·9 (3083·7 to 3641·7)	3616·7 (3368·4 to 3862·6)	4701·4 (4428·2 to 4992·7)	5398·6 (5015·4 to 5785·7)	5771·8 (5499·7 to 6039·1)	5269·6 (4950·6 to 5593·4)	5430·4 (4820·9 to 6065·8)

Uttarakhand	131·6 (123·6 to 139·6)	173·7 (163·7 to 183·3)	190·7 (177·7 to 203·7)	237·6 (222·7 to 253)	261·5 (244·3 to 279·1)	230·5 (214 to 246·9)	199·2 (183·6 to 214·3)	180·4 (163·3 to 198·4)
West Bengal	1205·4 (1105·6 to 1305·6)	1403·2 (1288·5 to 1515)	1524·4 (1386·6 to 1684·6)	1958·6 (1835·7 to 2082·6)	1807·2 (1706·4 to 1914·9)	1628·6 (1541·3 to 1722·3)	1470·5 (1379·6 to 1561·2)	1271·3 (1170·6 to 1378·8)
Nepal	429·8 (400·1 to 459·1)	471·7 (445·4 to 496·7)	552·4 (526·4 to 576·5)	724·1 (690·3 to 757·1)	830·8 (805·3 to 854·8)	803·4 (773·3 to 834·1)	669·8 (625·2 to 712·3)	618·1 (546·5 to 704·2)
Pakistan	1670·3 (1589·4 to 1748·8)	1817·1 (1739·4 to 1889·5)	2830·2 (2760·9 to 2898·3)	3899·7 (3770·3 to 4020·5)	4887·1 (4723·3 to 5031·6)	5955·8 (5697·8 to 6206)	6564·5 (6196·1 to 6952·7)	6686 (5942 to 7548·1)
Southeast Asia, east Asia, and Oceania	33486 (32623·7 to 34352·1)	37529·4 (36858·3 to 38230·5)	37564·3 (37023·1 to 38072·1)	37910·4 (37354·7 to 38445·7)	38299·7 (37697·4 to 38942·5)	30214·1 (29661·3 to 30794·6)	27022 (26089·1 to 27902·4)	26800·3 (25656·2 to 28075·6)
East Asia	24649·5 (24277·1 to 25036·8)	26394·9 (26095·9 to 26724·2)	25512·3 (25208·1 to 25824·9)	24963 (24514·5 to 25393)	25455·7 (24979·8 to 25939·2)	17524·6 (17117 to 17934·2)	14459·6 (13980·3 to 14968·4)	15448 (14999·4 to 15903·6)
China	23813·1 (23464·2 to 24175·5)	25341·5 (25051·1 to 25661·4)	24447 (24140·3 to 24757)	23907·6 (23459·9 to 24329·2)	24350·6 (23862·9 to 24825·3)	16634·2 (16224 to 17031·8)	13861 (13410·4 to 14359·7)	14921·8 (14467·4 to 15355·4)
North Korea	488·5 (464·2 to 513·1)	635·6 (604·5 to 665·4)	685 (648·8 to 720·6)	645·7 (599·7 to 695·1)	773·8 (717·9 to 826)	606·4 (546·7 to 668)	419·2 (368·2 to 472·8)	350·7 (304·4 to 400)
Taiwan (province of China)	348 (346·8 to 349·1)	417·7 (416·6 to 418·9)	380·3 (379·2 to 381·3)	409·6 (408·2 to 411·2)	331·3 (330·1 to 332·6)	284 (282·9 to 285·2)	179·4 (178·6 to 180·3)	175·5 (154·7 to 199·4)
Oceania	117·9 (113·4 to 122·2)	144·5 (139·2 to 149·2)	163·4 (158·2 to 168·3)	181·6 (176·5 to 186·6)	223·6 (216·8 to 230·2)	286·7 (275·3 to 298·2)	355·8 (330·6 to 380·6)	415·8 (380·9 to 452·6)
American Samoa	0·8 (0·8 to 0·9)	0·9 (0·8 to 0·9)	1·1 (1 to 1·1)	1·1 (1·1 to 1·2)	1·7 (1·6 to 1·8)	1·7 (1·5 to 1·8)	1·2 (1·1 to 1·3)	1·1 (0·9 to 1·2)
Cook Islands	0·7 (0·6 to 0·7)	0·7 (0·6 to 0·7)	0·6 (0·5 to 0·7)	0·4 (0·4 to 0·5)	0·5 (0·4 to 0·5)	0·4 (0·4 to 0·5)	0·3 (0·3 to 0·4)	0·3 (0·2 to 0·3)
Fiji	11·5 (10·9 to 12·3)	15·6 (15·1 to 16·1)	16·3 (15·7 to 17)	18·6 (17·4 to 19·8)	18·9 (17·9 to 20)	18·2 (16·7 to 19·9)	19·4 (17·6 to 21·2)	17·5 (15·2 to 20·2)
Guam	1·8 (1·7 to 1·8)	2·4 (2·4 to 2·5)	2·9 (2·8 to 2·9)	3 (2·9 to 3)	3·9 (3·8 to 4)	3·6 (3·4 to 3·7)	3·4 (3·3 to 3·6)	3·3 (3 to 3·6)
Kiribati	1·5 (1·4 to 1·5)	1·7 (1·6 to 1·7)	1·8 (1·7 to 1·9)	2·3 (2·2 to 2·4)	2·6 (2·4 to 2·8)	2·6 (2·4 to 2·7)	2·9 (2·6 to 3·2)	3 (2·6 to 3·4)
Marshall Islands	0·6 (0·5 to 0·6)	0·7 (0·6 to 0·7)	1 (0·9 to 1)	1·4 (1·3 to 1·5)	1·5 (1·5 to 1·6)	1·5 (1·4 to 1·6)	1·4 (1·3 to 1·5)	1·2 (1·1 to 1·4)
Federated States of Micronesia	1·8 (1·8 to 1·9)	2·1 (2·1 to 2·1)	2·6 (2·6 to 2·7)	3·5 (3·4 to 3·6)	3·5 (3·3 to 3·8)	3 (2·7 to 3·2)	2·3 (2 to 2·6)	1·9 (1·7 to 2·3)
Nauru	0·1 (0·1 to 0·1)	0·1 (0·1 to 0·1)	0·3 (0·3 to 0·3)	0·4 (0·4 to 0·4)	0·4 (0·4 to 0·5)	0·4 (0·4 to 0·4)	0·3 (0·3 to 0·4)	0·3 (0·3 to 0·3)
Niue	0·2 (0·2 to 0·2)	0·2 (0·2 to 0·2)	0·2 (0·2 to 0·2)	0·1 (0·1 to 0·1)	0·1 (0 to 0·1)	0 (0 to 0)	0 (0 to 0)	0 (0 to 0)
Northern Mariana Islands	0·2 (0·2 to 0·2)	0·2 (0·2 to 0·2)	0·3 (0·2 to 0·3)	0·4 (0·4 to 0·5)	1·3 (1·1 to 1·4)	2·5 (2·2 to 2·9)	0·8 (0·7 to 0·9)	0·5 (0·4 to 0·6)
Palau	0·3 (0·3 to 0·3)	0·3 (0·3 to 0·4)	0·3 (0·3 to 0·4)	0·3 (0·3 to 0·4)	0·4 (0·3 to 0·4)	0·4 (0·3 to 0·4)	0·3 (0·2 to 0·3)	0·2 (0·2 to 0·2)
Papua New Guinea	80 (76·7 to 83·1)	96·4 (91·8 to 100·2)	108·5 (104·3 to 112·3)	117·9 (114·1 to 121·4)	150 (145·3 to 154·7)	205·1 (197·1 to 212·8)	270·8 (251·5 to 290·2)	331·9 (304·5 to 361·6)
Samoa	3·5 (3·4 to 3·7)	4·3 (4·2 to 4·5)	4·5 (4·3 to 4·7)	3·8 (3·5 to 4)	3·1 (2·8 to 3·5)	2·8 (2·4 to 3·2)	3 (2·6 to 3·4)	3·5 (3 to 4·1)
Solomon Islands	5·4 (5·2 to 5·5)	6·6 (6·3 to 6·8)	8 (7·7 to 8·3)	10·2 (9·8 to 10·6)	14·7 (13·9 to 15·4)	19·2 (18 to 20·4)	21·2 (19·6 to 22·6)	21·2 (19·2 to 23·3)
Tokelau	0·1 (0·1 to 0·1)	0·1 (0·1 to 0·1)	0·1 (0·1 to 0·1)	0·1 (0·1 to 0·1)	0·1 (0·1 to 0·1)	0·1 (0 to 0·1)	0 (0 to 0)	0 (0 to 0)
Tonga	1·8 (1·7 to 1·9)	2·3 (2·2 to 2·4)	2·7 (2·6 to 2·9)	2·8 (2·6 to 2·9)	2·5 (2·4 to 2·6)	2·6 (2·4 to 2·8)	2·6 (2·4 to 2·8)	2·3 (2 to 2·6)
Tuvalu	0·2 (0·2 to 0·2)	0·2 (0·2 to 0·2)	0·2 (0·2 to 0·3)	0·3 (0·3 to 0·4)	0·3 (0·3 to 0·3)	0·2 (0·2 to 0·3)	0·2 (0·2 to 0·2)	0·2 (0·2 to 0·2)
Vanuatu	2 (1·8 to 2·1)	2·7 (2·6 to 2·9)	3·5 (3·3 to 3·7)	4·7 (4·4 to 5)	5·8 (5·4 to 6·1)	6·6 (5·9 to 7·2)	7·2 (6·5 to 8)	7·6 (6·7 to 8·8)
Southeast Asia	8718·7 (8229·6 to 9194·4)	10990 (10542·2 to 11417·8)	11888·7 (11499·9 to 12249·3)	12765·8 (12465·8 to 13058·8)	12620·4 (12291·9 to 12952·7)	12402·9 (12049·2 to 12759·8)	12206·5 (11726·7 to 12666·1)	10936·5 (9991·6 to 12064·5)
Cambodia	218·7 (204·6 to 232·6)	273·1 (259·4 to 285·9)	329·1 (315·9 to 342·2)	375·7 (365·2 to 386·1)	443·7 (431·5 to 455·2)	379·2 (364·8 to 393·8)	372·8 (349·4 to 398·2)	369·2 (333·7 to 413)
Indonesia	3701·3 (3507·3 to 3887·8)	4852 (4650·2 to 5048·4)	5180·6 (5036·4 to 5321·7)	5211·1 (5087·6 to 5330·1)	4770·6 (4625·4 to 4911·2)	4793·7 (4655·1 to 4926·2)	4606·6 (4352·6 to 4855·9)	3837·8 (3457·9 to 4287·3)
Aceh	59·9 (57·1 to 62·5)	81 (78·1 to 83·8)	94 (91·2 to 96·9)	108·1 (105·2 to 111·1)	105·6 (101·4 to 109·6)	109·4 (106·4 to 112·2)	107·3 (103 to 111·9)	90·3 (83·2 to 99)
Bali	70·9 (67·1 to 74·7)	85·6 (82 to 89)	94·2 (91·8 to 96·8)	86 (83·5 to 88·3)	68·7 (66·4 to 71·1)	69·6 (66·6 to 72·5)	63·9 (59·2 to 68·5)	51·5 (44·9 to 59·6)
Bangka-Belitung Islands	15·6 (14·7 to 16·5)	19·3 (18·3 to 20·3)	20·9 (19·7 to 22·3)	23·9 (22·9 to 24·8)	22·8 (22·2 to 23·4)	23·4 (22·6 to 24·2)	24·2 (23 to 25·2)	20 (18 to 22·1)
Banten	80·5 (76·3 to 84·6)	101·7 (97·3 to 105·9)	110·6 (105·9 to 115·5)	141·3 (135·7 to 146·8)	230·1 (221·2 to 238·3)	248·1 (239·9 to 256)	230·3 (223 to 237·2)	181·1 (167·7 to 196)
Bengkulu	18·2 (17·4 to 19)	23·3 (22·5 to 24)	26 (25·5 to 26·6)	33·5 (32·8 to 34·3)	40·2 (38·4 to 41·9)	40·5 (39 to 42)	39·1 (36·8 to 41·5)	32·5 (29·3 to 36·3)
Gorontalo	12·3 (11·7 to 13)	19·7 (18·7 to 20·6)	25 (23·6 to 26·5)	23·7 (22·7 to 24·8)	19·2 (18·7 to 19·7)	19·3 (18·8 to 19·9)	19·7 (18·8 to 20·6)	20·6 (18·7 to 22·6)
Jakarta	108·4 (103·6 to 113)	181·5 (174·7 to 187·7)	229 (223·6 to 234·6)	253·5 (246·2 to 260·3)	239·8 (232·4 to 247·7)	226·3 (217·2 to 234·6)	201·3 (186·9 to 215·5)	147 (128·4 to 169·6)
Jambi	29·7 (28·2 to 31·1)	41·5 (39·9 to 43)	46·2 (44·5 to 48·1)	53·5 (51·9 to 55·3)	55·1 (52·8 to 57·3)	59 (57·6 to 60·4)	61·2 (59·1 to 63·3)	51·7 (47·8 to 56·5)
West Java	591 (561·7 to 619·9)	797·2 (764·1 to 828·3)	874·9 (852 to 899·2)	870 (849·4 to 890·6)	748·8 (722·3 to 774·9)	800·9 (774·3 to 825·5)	823 (767·6 to 877·3)	725·1 (638 to 829·5)
Central Java	699·4 (663·1 to 734·5)	906·9 (866·3 to 945·6)	918·2 (891·9 to 945·2)	842·2 (820·7 to 863·1)	682·7 (659·1 to 706·1)	607·8 (583·2 to 632·1)	521·9 (484·2 to 558·3)	409·2 (357·5 to 471·9)
East Java	804·6 (755·9 to 853)	1011 (955·9 to 1062·9)	986·5 (951·7 to 1021·1)	848·8 (822·2 to 874·8)	659·9 (636·4 to 683·6)	600·1 (577·2 to 622)	526·1 (487·7 to 563·6)	405·7 (353·2 to 470·4)
West Kalimantan	57·9 (55·1 to 60·6)	79·1 (76·2 to 81·9)	92·4 (89·5 to 95·2)	107·9 (104·9 to 110·9)	99·2 (95·5 to 102·6)	100 (96·8 to 103·1)	97·4 (93·5 to 101·3)	81·9 (75·6 to 89·5)
South Kalimantan	53·1 (50·5 to 55·7)	72·4 (69·4 to 75·3)	75·9 (73·5 to 78·4)	75·6 (73·4 to 77·8)	72 (69 to 75·2)	75·4 (72·4 to 78·4)	72·3 (67·4 to 77·2)	59·7 (53·4 to 67·1)
Central Kalimantan	18·2 (17·3 to 19)	25·4 (24·4 to 26·3)	29·4 (28·4 to 30·6)	35·2 (34·1 to 36·4)	40 (38·4 to 41·4)	44·1 (42·5 to 45·6)	45·6 (43·9 to 47·3)	38·2 (35·2 to 41·7)
East Kalimantan	17·9 (17·1 to 18·7)	26·4 (25·5 to 27·3)	32·4 (31·3 to 33·6)	41·4 (39·9 to 42·9)	47·9 (46 to 49·5)	61·3 (59·5 to 63·1)	72·6 (70 to 75)	62·4 (57·7 to 68)
North Kalimantan	2·5 (2·4 to 2·6)	4·6 (4·4 to 4·7)	6·3 (6·1 to 6·5)	7·4 (7 to 7·7)	8 (7·2 to 8·8)	10·3 (9·2 to 11·4)	12 (10·7 to 13·2)	11·4 (10·3 to 12·7)
Riau Islands	7·6 (7·2 to 8)	9 (8·6 to 9·4)	9·9 (9·5 to 10·4)	15·3 (14·8 to 15·8)	27·7 (26·5 to 28·8)	36 (34·8 to 37·3)	45·9 (44·5 to 47·2)	32·8 (30·3 to 35·7)
Lampung	74·3 (70·6 to 77·9)	121·1 (116·2 to 125·9)	148·4 (144·3 to 152·5)	169·3 (164·9 to 173·5)	166·8 (160·4 to 173·5)	161·7 (155·6 to 167·6)	144·7 (135·2 to 154·2)	114·4 (102·5 to 128·2)
Maluku	26·7 (25·6 to 27·8)	31·7 (30·7 to 32·7)	33·2 (32·2 to 34·3)	37·5 (36·4 to 38·6)	40·1 (39 to 41)	42·7 (41·5 to 44)	41·8 (40·3 to 43·4)	34·5 (32 to 37·6)
North Maluku	13·6 (12·9 to 14·3)	16·1 (15·2 to 17)	16·6 (15·6 to 17·7)	18·2 (17·1 to 19·2)	21·1 (20·3 to 21·9)	25·1 (24·1 to 26)	24·6 (23·1 to 26·1)	20·5 (17·9 to 23·2)
West Nusa Tenggara	77·8 (73·6 to 81·9)	93·8 (89·8 to 97·5)	102·8 (100 to 105·9)	110·3 (107·2 to 113·2)	100·8 (97·2 to 104·6)	98·6 (94·6 to 102·7)	98·5 (91·9 to 105·1)	84·1 (75·5 to 94·1)
East Nusa Tenggara	83·3 (79·3 to 87)	100·5 (96·9 to 103·9)	105·8 (102·6 to 109·1)	104 (101·8 to 106·4)	106·1 (102·4 to 109·8)	116·9 (114·2 to 119·8)	118·6 (113·7 to 123)	104·9 (97·5 to 113·6)
Papua	10 (9·6 to 10·4)	17·5 (16·9 to 18)	22·6 (21·8 to 23·3)	28·6 (27·6 to 29·6)	36·2 (35·3 to 37)	55·9 (54·5 to 57·2)	83·7 (81·2 to 86·1)	88·7 (82·8 to 95·7)
West Papua	9·5 (9·1 to 9·9)	14·8 (14·2 to 15·3)	18·2 (17·6 to 18·7)	22·9 (22·2 to 23·7)	23·9 (23·3 to 24·4)	21·3 (20·7 to 21·8)	21·1 (20·4 to 21·9)	17·9 (16·6 to 19·5)

Riau	30·9 (29·4 to 32·3)	49 (47·3 to 50·6)	61·2 (59·5 to 63)	74·3 (72·2 to 76·5)	72·1 (69 to 74·9)	96·1 (93·7 to 98·4)	136·2 (129·4 to 142·7)	141·7 (131·1 to 154·5)
West Sulawesi	20·9 (20 to 21·8)	24·9 (24 to 25·8)	25·6 (24·7 to 26·6)	25·5 (24·8 to 26·2)	24·8 (24 to 25·4)	28 (27·2 to 28·7)	29·1 (28·1 to 30·1)	26·5 (24·4 to 28·8)
South Sulawesi	150·1 (142·3 to 157·7)	197·9 (190·1 to 205·3)	218 (212·2 to 224·3)	211·5 (205·7 to 217·4)	192·5 (185·3 to 199·9)	188·2 (181·2 to 194·6)	166 (155·7 to 176·9)	137·4 (123·5 to 153·8)
Central Sulawesi	29·7 (28·3 to 31)	38·9 (37·5 to 40·3)	45·2 (43·8 to 46·7)	52·7 (51·3 to 54)	51·8 (49·6 to 53·7)	58·6 (57 to 60·4)	57 (54·9 to 59·2)	48·2 (44·4 to 52·8)
Southeast Sulawesi	24·1 (23 to 25·2)	31·4 (30·3 to 32·3)	36·6 (35·5 to 37·7)	43·5 (42·2 to 44·7)	45·5 (44·2 to 46·8)	51·7 (50·1 to 53·5)	56·2 (53·6 to 58·8)	50·2 (46·3 to 54·9)
North Sulawesi	41·2 (39 to 43·3)	46·6 (44·6 to 48·5)	50·1 (48·7 to 51·7)	52·2 (50·5 to 53·9)	47·3 (44·9 to 49·4)	45·7 (44·4 to 47)	36·4 (34·9 to 38)	27·6 (24·9 to 30·7)
West Sumatra	111·6 (106·1 to 116·8)	124·1 (119·2 to 128·8)	119·8 (116·3 to 123·6)	126·4 (123·1 to 129·6)	117·4 (114·2 to 120·5)	116·7 (112·3 to 121·1)	103·2 (96·7 to 109·6)	83·6 (75·4 to 93·4)
South Sumatra	80·7 (76·9 to 84·4)	111·8 (107·5 to 115·9)	128·1 (124·8 to 131·6)	157·6 (153·7 to 161·5)	157·4 (152·2 to 163)	161·2 (154·7 to 168·2)	156·2 (145·3 to 167)	127·4 (114·4 to 142·7)
North Sumatra	184·7 (176·3 to 192·6)	251·8 (242·8 to 260·5)	287·2 (279·8 to 295·3)	332·7 (324·1 to 340·9)	339·6 (330·4 to 349·6)	340·4 (328·4 to 352·1)	320·7 (302·3 to 339·2)	268·9 (243·9 to 298)
Yogyakarta	84·4 (78·6 to 90)	94·7 (88·9 to 100·3)	89·3 (85·6 to 93)	76·4 (73·8 to 78·9)	59·5 (57·4 to 61·7)	53·5 (51·6 to 55·4)	48·8 (45·1 to 52·4)	40·2 (34·9 to 46·7)
Laos	83·8 (78·3 to 89·2)	102·4 (97 to 107·5)	121·7 (115·6 to 127·9)	144 (139·2 to 148·8)	165·6 (161·2 to 169·8)	184·2 (178·4 to 190·2)	173·7 (167·1 to 180·3)	173·7 (160·5 to 187·8)
Malaysia	265·7 (245 to 286·5)	309·2 (308·1 to 310·3)	353·7 (352·7 to 354·8)	379·9 (378·3 to 381·5)	493·9 (492·1 to 495·7)	528·7 (526·7 to 530·6)	480·6 (479 to 482·2)	538·7 (485·7 to 595·6)
Maldives	2·8 (2·6 to 2·9)	4·1 (3·9 to 4·4)	4·9 (4·7 to 5·1)	6·8 (6·6 to 7)	8·8 (8·5 to 9·2)	7·3 (6·8 to 7·9)	8·9 (8·1 to 9·8)	8·5 (7·4 to 9·9)
Mauritius	23·4 (22·7 to 24·2)	25·7 (25·2 to 26·1)	21·3 (20·7 to 21·9)	23·8 (23·6 to 24)	22·2 (22 to 22·4)	20 (19·8 to 20·2)	14·7 (14·5 to 14·9)	12·8 (11·2 to 14·6)
Myanmar	903·7 (844·4 to 963·1)	1054·3 (993·6 to 1112·3)	1128·2 (1059·1 to 1200)	1304·9 (1218·1 to 1388·8)	1346 (1264·2 to 1430·6)	1243·3 (1171·7 to 1310·9)	1151·4 (1085·8 to 1220)	1054·9 (930·5 to 1193·8)
Philippines	983·1 (930 to 1035·6)	1325·2 (1260·6 to 1388·3)	1610·4 (1521·5 to 1692)	1943·3 (1876·6 to 2011·5)	2088·6 (2006·8 to 2170·9)	2352·4 (2255·6 to 2449·7)	2585·8 (2463·7 to 2704·2)	2666·1 (2390·3 to 2962·9)
Seychelles	1·1 (1 to 1·2)	1·7 (1·7 to 1·7)	1·8 (1·7 to 1·8)	1·7 (1·6 to 1·8)	1·7 (1·6 to 1·8)	1·6 (1·5 to 1·6)	1·6 (1·5 to 1·7)	1·5 (1·3 to 1·7)
Sri Lanka	306·4 (292·9 to 321·8)	350·2 (338·8 to 362·5)	368·4 (350·6 to 385·2)	410·7 (388·4 to 432·3)	358·8 (336·5 to 380·9)	344·2 (323·2 to 364·9)	358·7 (337·5 to 380·9)	295·6 (249·8 to 349)
Thailand	984·2 (925·5 to 1042·1)	1238 (1171·3 to 1296·9)	1247·4 (1205·3 to 1290·1)	1231·1 (1166·1 to 1297)	1063·5 (1002·8 to 1130·3)	902 (847·6 to 961·4)	761·6 (710·4 to 817·1)	584 (493 to 691·2)
Timor-Leste	23·1 (22·1 to 24·1)	26·8 (26·1 to 27·6)	24·4 (23·7 to 25)	28·1 (27·2 to 29)	38·2 (36·8 to 39·6)	38·5 (37·1 to 39·8)	35·9 (33·4 to 38·3)	38·9 (34·5 to 43·7)
Vietnam	1208·8 (1128 to 1291·1)	1410·7 (1332·7 to 1488·5)	1478·1 (1395·4 to 1556·8)	1687·1 (1620·1 to 1754·1)	1802 (1748·2 to 1857·3)	1590·8 (1525·2 to 1653·7)	1637·7 (1551 to 1727·9)	1340·4 (1213·5 to 1498·6)
Sub-Saharan Africa	8934·2 (8474·2 to 9368·6)	11186·8 (10740·5 to 11588·5)	13861·1 (13424·3 to 14304·3)	17740·7 (17216·5 to 18236·3)	22368·7 (21601 to 23101·5)	28060·4 (27207·8 to 28966)	33484·1 (31981·5 to 34911·7)	36726·9 (34252·4 to 39559)
Central sub-Saharan Africa	1016·1 (979·1 to 1050·3)	1369·2 (1325·1 to 1411·7)	1680·3 (1620·3 to 1737·3)	2117·9 (2045·9 to 2183·3)	2696·8 (2612·4 to 2777·1)	3418·4 (3308·2 to 3530·6)	4264·3 (4108·8 to 4420·3)	4464·2 (4147·8 to 4782·4)
Angola	247·6 (238·9 to 256·9)	277·2 (266·1 to 287·3)	303·1 (292·2 to 312·3)	380·4 (367·3 to 393·1)	513·2 (487·2 to 537·3)	718·7 (675·8 to 757)	972·3 (896·5 to 1044·4)	1104·4 (999 to 1214·1)
Central African Republic	63 (59·4 to 66·4)	83·2 (77·9 to 88·2)	104 (98·4 to 109·1)	110·6 (104·5 to 116·6)	127·6 (119·9 to 135·4)	159·3 (148·5 to 169·6)	194 (177·6 to 209·7)	197·5 (182 to 213)
Congo (Brazzaville)	43·5 (41 to 45·8)	52·4 (50·4 to 54·2)	64·4 (62 to 66·5)	83·4 (80·5 to 86·3)	101·8 (98·2 to 105·1)	123·1 (120 to 126·4)	155·7 (150·9 to 160·5)	145·1 (133·1 to 157·6)
DR Congo	633 (604·7 to 659)	923·1 (888·6 to 954·1)	1170·6 (1122·6 to 1218·6)	1494·5 (1437·7 to 1546·8)	1890·9 (1831·5 to 1945·1)	2342·3 (2266·8 to 2420·8)	2861·5 (2746·6 to 2973·7)	2935·8 (2666·3 to 3206·1)
Equatorial Guinea	10·9 (10·5 to 11·2)	11·3 (11 to 11·7)	12·6 (12·2 to 13)	16·3 (15·7 to 16·8)	23·1 (22·1 to 23·9)	31·7 (29·4 to 33·8)	37·2 (33·9 to 40·9)	38·5 (33·5 to 44·2)
Gabon	18·2 (16·8 to 19·5)	22 (20·8 to 23·2)	25·6 (24·2 to 26·9)	32·6 (30·9 to 34·2)	40·3 (37·6 to 42·9)	43·4 (38·7 to 48·1)	43·5 (38·2 to 49·4)	42·9 (36·9 to 50·1)
Eastern sub-Saharan Africa	3417·6 (3250·6 to 3575·8)	4326·5 (4174·3 to 4465·9)	5403·2 (5244·3 to 5560·6)	7085·4 (6900·8 to 7258·6)	9021·9 (8751·4 to 9282·2)	11152·6 (10823·1 to 11475·9)	12929 (12332·3 to 13509·4)	14162·8 (13111·2 to 15323·9)
Burundi	130·9 (125·5 to 136)	154·2 (148·8 to 159·1)	175·1 (170·2 to 179·9)	223·3 (217·6 to 228·5)	265·1 (258·9 to 271·3)	265 (255·1 to 274·3)	379·1 (363·5 to 395·6)	466·2 (436·5 to 499·1)
Comoros	5·7 (5·1 to 6·3)	8·6 (8·1 to 9·1)	12·9 (12·4 to 13·4)	16·9 (16·3 to 17·5)	20·3 (19·3 to 21·2)	20·5 (18·7 to 22·3)	18·8 (16·7 to 20·8)	16·8 (14·4 to 19·5)
Djibouti	3·3 (3·1 to 3·4)	5·1 (5 to 5·3)	8·2 (7·9 to 8·5)	14·8 (14·2 to 15·3)	22·2 (20·9 to 23·4)	28·3 (26·4 to 30)	36·7 (33·7 to 39·5)	35·5 (30·8 to 40·6)
Eritrea	57·5 (54·4 to 60·4)	76 (72·8 to 79·4)	95 (90·4 to 99·3)	122·4 (116·5 to 128)	136·1 (128·7 to 143·4)	167·8 (153·6 to 181·2)	197·9 (176·2 to 220·6)	199·2 (174 to 228)
Ethiopia	911·5 (857·1 to 962·1)	1099·3 (1048·6 to 1145·7)	1344 (1293·4 to 1396)	1811·1 (1736·6 to 1880·3)	2467·4 (2379·8 to 2550·8)	2985·6 (2880·8 to 3085·9)	3313·8 (3102·8 to 3517·3)	3674·6 (3394·8 to 4002·6)
Kenya	319·2 (308·1 to 329·4)	468·2 (452·1 to 482)	611·3 (593·1 to 628·4)	813·5 (790·2 to 835·1)	1016·1 (966·2 to 1064·2)	1196·8 (1131·5 to 1260·8)	1328·9 (1216·5 to 1440·1)	1342·7 (1166·7 to 1554·7)
Baringo	5·1 (4·9 to 5·3)	7·6 (7·4 to 7·8)	9·5 (9·2 to 9·7)	11·5 (11·3 to 11·7)	15·8 (15·3 to 16·3)	19·4 (18·5 to 20·3)	20·9 (19·3 to 22·4)	19·7 (17·1 to 22·7)
Bomet	4·8 (4·7 to 4·9)	6·5 (6·3 to 6·7)	8·8 (8·6 to 9)	11·9 (11·7 to 12·1)	16·2 (15·6 to 16·7)	21·5 (20·6 to 22·3)	35·9 (33·2 to 38·5)	41·5 (36·3 to 47·4)
Bungoma	9·9 (9·7 to 10·1)	14·7 (14·3 to 15)	20·3 (19·7 to 20·8)	28·3 (27·6 to 29)	41·1 (39·7 to 42·5)	71·3 (67·6 to 74·9)	51·8 (47·8 to 56)	35·2 (30·8 to 40·5)
Busia	8·3 (8 to 8·5)	10·8 (10·5 to 11·1)	12·4 (11·9 to 12·8)	17·3 (16·6 to 17·9)	22·8 (21·8 to 23·7)	25 (23·3 to 26·6)	29 (26·4 to 31·8)	28·6 (24·7 to 33·4)
Elgeyo Marakwet	5·6 (5·4 to 5·8)	9 (8·7 to 9·2)	9·7 (9·4 to 10)	7·9 (7·7 to 8)	11·5 (11·1 to 11·9)	19·8 (18·9 to 20·9)	11·5 (10·6 to 12·5)	6·3 (5·4 to 7·4)
Embu	4·9 (4·7 to 5·1)	6·6 (6·3 to 6·8)	8·7 (8·4 to 9)	12·3 (11·9 to 12·7)	15·1 (14·2 to 16·1)	15·8 (14·7 to 16·9)	13·7 (12·3 to 15·2)	10·2 (8·5 to 12·3)
Garissa	1·5 (1·4 to 1·6)	2·4 (2·3 to 2·5)	3·8 (3·7 to 4)	5·9 (5·6 to 6·1)	5·9 (5·5 to 6·2)	13 (12·3 to 13·7)	27·1 (25·5 to 28·7)	42·6 (39·5 to 46·2)
Homa Bay	19·3 (18·6 to 19·9)	23·8 (23 to 24·4)	25·3 (24·5 to 26·1)	28·2 (27·4 to 28·9)	35·1 (33·7 to 36·5)	40·5 (38·3 to 42·6)	42·8 (39·8 to 45·8)	42 (37·1 to 47·5)
Isiolo	1 (1 to 1)	1·5 (1·4 to 1·5)	1·8 (1·7 to 1·9)	2·5 (2·4 to 2·5)	3·4 (3·2 to 3·6)	4·6 (4·3 to 4·9)	5·4 (4·9 to 6)	5·6 (4·8 to 6·5)
Kajiado	2·1 (2 to 2·2)	3·1 (2·9 to 3·2)	4·5 (4·3 to 4·7)	7·5 (7·2 to 7·8)	11·5 (10·8 to 12·1)	16·2 (15 to 17·3)	23·3 (20·6 to 25·9)	22·4 (18·8 to 27)
Kakamega	14·8 (14·4 to 15·1)	23·5 (22·8 to 24)	31·3 (30·4 to 32·1)	42·9 (41·8 to 43·8)	58·5 (56·3 to 60·5)	44·8 (42·7 to 46·9)	65·9 (60·5 to 71·4)	76·7 (66 to 89·7)
Kericho	5·6 (5·5 to 5·7)	11·3 (11·1 to 11·5)	17·3 (17 to 17·6)	23·3 (22·9 to 23·6)	31·2 (30·1 to 32·3)	34·5 (32·9 to 36·1)	21·5 (19·8 to 23·2)	12 (10·5 to 13·8)
Kiambu	11·6 (11·2 to 12)	15·7 (15·2 to 16·2)	23·4 (22·8 to 24)	33·8 (32·9 to 34·7)	39·4 (37 to 41·9)	51·7 (49 to 54·5)	48·9 (44·6 to 53·4)	37·6 (32·4 to 43·7)
Kilifi	10·3 (9·8 to 10·8)	15 (14·3 to 15·5)	18·5 (17·8 to 19·1)	25·2 (24·3 to 26·1)	28·5 (26·7 to 30·2)	33·2 (31·2 to 35·1)	38·2 (35·3 to 41·2)	38·2 (32·7 to 45·1)
Kirinyaga	6·2 (6 to 6·4)	9·2 (8·9 to 9·5)	11·7 (11·3 to 12)	13·8 (13·2 to 14·3)	15·5 (14·2 to 16·8)	16·1 (14·9 to 17·4)	14·2 (12·6 to 15·8)	10·5 (8·7 to 12·9)
Kisii	10·3 (10·1 to 10·5)	19·7 (19·1 to 20·1)	27·1 (26·3 to 27·7)	36·4 (35·2 to 37·5)	42·1 (40 to 44·2)	45·1 (42 to 48·1)	36·6 (32·8 to 40·6)	25·8 (21·4 to 31·4)
Kisumu	13·8 (13·4 to 14·2)	20·4 (19·6 to 21·1)	23·9 (23 to 24·7)	25·6 (24·7 to 26·5)	32·3 (30·3 to 34·2)	32·8 (30·4 to 35)	31·9 (28·2 to 35·6)	26·6 (22·3 to 32)
Kitui	12·4 (11·9 to 12·8)	17·2 (16·6 to 17·7)	20·9 (20·4 to 21·4)	25·6 (24·9 to 26·2)	33·3 (31·6 to 34·9)	37 (35·1 to 38·9)	35 (32 to 38)	31·6 (27·8 to 35·9)
Kwale	6·6 (6·3 to 6·9)	10·5 (10 to 10·9)	12·8 (12·1 to 13·4)	15·2 (14·6 to 15·9)	16·7 (15·6 to 17·8)	19·1 (17·8 to 20·5)	21·4 (19·2 to 23·6)	21·3 (17·8 to 25·6)

Laikipia	1·4 (1·4 to 1·5)	2 (2 to 2·1)	3·5 (3·4 to 3·6)	6·8 (6·6 to 7)	10 (9·4 to 10·5)	14·3 (13·4 to 15·1)	12·9 (11·7 to 14·2)	9·6 (8·2 to 11·4)
Lamu	0·9 (0·9 to 1)	1·1 (1 to 1·1)	1·3 (1·2 to 1·4)	1·9 (1·8 to 2)	2·4 (2·2 to 2·5)	2·7 (2·5 to 2·9)	3 (2·7 to 3·3)	2·9 (2·5 to 3·5)
Machakos	9·4 (9 to 9·7)	15·4 (14·9 to 16)	22·8 (22 to 23·5)	33·5 (32·2 to 34·7)	40·7 (38 to 43·3)	39·4 (36·5 to 42·2)	29·7 (26·4 to 33·1)	19·4 (16·3 to 23·4)
Makueni	9·9 (9·6 to 10·3)	11·7 (11·3 to 12·1)	14·1 (13·7 to 14·5)	17·5 (17·1 to 18)	21·9 (20·9 to 22·8)	25 (23·6 to 26·5)	31·1 (28 to 34·4)	33·1 (29 to 37·9)
Mandera	2·5 (2·3 to 2·6)	3·9 (3·7 to 4·1)	4·7 (4·4 to 4·9)	4·8 (4·5 to 5·1)	5·5 (5·2 to 5·9)	10·7 (10 to 11·5)	36·9 (34·4 to 39·5)	97·5 (88·4 to 107·9)
Marsabit	1·4 (1·3 to 1·4)	1·9 (1·8 to 1·9)	3·1 (3 to 3·2)	5·2 (5·1 to 5·4)	6·3 (5·9 to 6·6)	8·1 (7·6 to 8·5)	11·2 (10·3 to 12·1)	14·8 (13·2 to 16·6)
Meru	14·5 (14 to 15)	22·7 (21·8 to 23·4)	29·7 (28·7 to 30·5)	37·8 (36·7 to 38·8)	45 (42·6 to 47·2)	46·9 (44 to 49·8)	42·5 (37·9 to 47·4)	33·5 (28·1 to 40·5)
Migori	11·9 (11·4 to 12·3)	15·7 (15·1 to 16·1)	17·8 (17·2 to 18·4)	21·7 (21·1 to 22·2)	28·6 (27·3 to 29·8)	36·9 (34·9 to 39)	43·5 (40·5 to 46·5)	47·8 (42·6 to 53·6)
Mombasa	5·4 (5·1 to 5·7)	10 (9·4 to 10·5)	12·8 (12·1 to 13·4)	14·1 (13·3 to 14·8)	16·9 (15·5 to 18·3)	22 (20·3 to 23·7)	29·8 (26·5 to 33·1)	27·1 (22·2 to 33·5)
Murang'a	15·2 (14·7 to 15·7)	20 (19·4 to 20·5)	24·7 (24·1 to 25·2)	30·5 (29·6 to 31·2)	35·4 (33·6 to 37·1)	25·1 (23·5 to 26·6)	21·6 (19·6 to 23·6)	17·4 (14·8 to 20·3)
Nairobi	9·5 (9 to 9·9)	16·3 (15·4 to 17)	27 (25·7 to 28·3)	53·5 (50·4 to 56·4)	48·4 (44·2 to 52·5)	77·5 (71·4 to 83·9)	98·6 (88·1 to 109·3)	89·4 (75·6 to 106·9)
Nakuru	5·8 (5·6 to 6)	9·7 (9·4 to 10)	15·9 (15·5 to 16·3)	26·2 (25·4 to 26·9)	40·3 (38·4 to 42·2)	48·4 (45·2 to 51·5)	50·3 (45 to 55·5)	38·6 (32·5 to 46·3)
Nandi	5·8 (5·7 to 6)	8·8 (8·5 to 9)	12·3 (12 to 12·5)	15·5 (15·2 to 15·7)	20·8 (19·9 to 21·7)	24 (22·9 to 25·2)	24·4 (22·6 to 26·2)	20·1 (17·4 to 23·2)
Narok	3·9 (3·7 to 4)	5·4 (5·2 to 5·6)	7·4 (7·2 to 7·6)	12·5 (12·2 to 12·7)	21·3 (20·6 to 21·9)	18·7 (18 to 19·5)	40·2 (37·6 to 42·8)	63·7 (56·3 to 72·4)
Nyamira	6·3 (6·1 to 6·5)	8 (7·7 to 8·3)	9·3 (8·9 to 9·7)	10·7 (10·3 to 11·1)	12 (11·2 to 12·7)	13·8 (12·3 to 15·2)	16·5 (14·7 to 18·5)	15·4 (13·1 to 18·1)
Nyandarua	3·5 (3·4 to 3·7)	5·9 (5·7 to 6·1)	8·2 (8 to 8·5)	10·5 (10·1 to 10·8)	16·5 (15·4 to 17·5)	20 (18·7 to 21·4)	17·8 (16 to 19·6)	15·3 (12·9 to 18·3)
Nyeri	10·5 (10·1 to 10·8)	12·4 (11·9 to 12·8)	16 (15·5 to 16·5)	21·9 (21 to 22·8)	24·6 (22·7 to 26·5)	21·3 (19·4 to 23)	16·4 (14·2 to 18·6)	11·7 (9·7 to 14·4)
Samburu	2·2 (2·2 to 2·3)	3·8 (3·7 to 3·8)	4·5 (4·4 to 4·6)	4·7 (4·6 to 4·8)	6·7 (6·4 to 6·9)	7·8 (7·4 to 8·1)	9·9 (9·3 to 10·5)	11·6 (10·3 to 13·2)
Siaya	10·3 (10 to 10·6)	16·9 (16·3 to 17·4)	20·6 (20 to 21·2)	25 (24·3 to 25·7)	30·7 (29·2 to 32·3)	32·4 (30·6 to 34·3)	32 (29·4 to 34·6)	28·6 (25 to 32·6)
Taita Taveta	2·9 (2·8 to 3·1)	4·5 (4·3 to 4·7)	5·5 (5·2 to 5·7)	6·6 (6·3 to 6·9)	8·3 (7·7 to 8·9)	7·2 (6·7 to 7·9)	6·6 (5·9 to 7·4)	5·1 (4·2 to 6·3)
Tana River	1·6 (1·5 to 1·7)	2·2 (2·1 to 2·3)	3 (2·8 to 3·1)	5 (4·9 to 5·2)	5·9 (5·6 to 6·3)	7·7 (7·2 to 8·2)	8·9 (8·2 to 9·6)	10·2 (8·9 to 11·8)
Tharaka Nithi	3·4 (3·2 to 3·5)	3·4 (3·3 to 3·6)	3·4 (3·3 to 3·5)	3·5 (3·3 to 3·6)	3·8 (3·5 to 4)	3·8 (3·5 to 4·1)	3·6 (3·2 to 4·1)	3·2 (2·7 to 3·8)
Trans Nzoia	2·1 (2 to 2·2)	3·3 (3·2 to 3·4)	6·3 (6·1 to 6·5)	13·6 (13·3 to 13·9)	19·5 (18·7 to 20·4)	25·1 (23·8 to 26·4)	27·7 (25·1 to 30·3)	24·3 (20·6 to 28·7)
Turkana	4·3 (4·1 to 4·4)	8·1 (7·9 to 8·4)	10·7 (10·4 to 11)	9·5 (9·2 to 9·7)	12·5 (12 to 13)	23 (22 to 23·9)	40·1 (37·5 to 42·7)	57·1 (51·3 to 64·1)
Uasin Gishu	3·7 (3·6 to 3·8)	6·6 (6·4 to 6·8)	10·8 (10·5 to 11)	15·8 (15·4 to 16·3)	20·6 (19·6 to 21·5)	26·3 (24·6 to 27·8)	31 (27·8 to 34·2)	25 (21 to 30)
Vihiga	11·6 (11·2 to 11·9)	13·5 (13 to 13·9)	13·8 (13·3 to 14·3)	13·2 (12·7 to 13·7)	16·1 (15·3 to 16·9)	13·9 (12·9 to 14·9)	16·3 (14·7 to 18·1)	16·6 (14·2 to 19·6)
Wajir	2·4 (2·3 to 2·5)	3·2 (3 to 3·3)	4·9 (4·7 to 5·1)	7·2 (6·9 to 7·5)	6·3 (6 to 6·6)	16·5 (15·5 to 17·4)	28·2 (26·9 to 29·6)	40·9 (38 to 44·2)
West Pokot	2·6 (2·5 to 2·7)	3·6 (3·5 to 3·7)	5·5 (5·4 to 5·7)	9·9 (9·6 to 10·1)	13·2 (12·7 to 13·6)	17 (16·2 to 17·8)	23·1 (21·3 to 24·7)	28 (24·7 to 31·8)
Madagascar	219·9 (211·1 to 229·5)	272·5 (264·6 to 280·2)	349·7 (338·6 to 360·3)	442·3 (428·8 to 454·6)	540·2 (518·2 to 560·5)	678·7 (647·4 to 708·5)	798·3 (750·5 to 845·8)	861·8 (756·9 to 973·3)
Malawi	152·7 (143·3 to 161·5)	196·3 (187·9 to 204·1)	237·9 (229·9 to 246·1)	307·2 (297·4 to 316·2)	469·8 (458·1 to 481·9)	529 (510·8 to 546·3)	532·8 (503·5 to 562·1)	545·1 (498·5 to 600·7)
Mozambique	327·5 (305·5 to 348·2)	378·4 (361 to 395·2)	460·7 (439·9 to 480·7)	600·2 (580·5 to 619·5)	603·5 (588·7 to 617·4)	830·2 (804·6 to 855·5)	981 (949·7 to 1010·3)	1124·7 (1045·3 to 1206·2)
Rwanda	147·7 (143·1 to 152·1)	171 (167 to 174·5)	204 (200·9 to 207)	259·5 (255·3 to 263·5)	318·8 (312 to 324·7)	324·4 (316 to 332·9)	343 (325·2 to 359·9)	352·3 (321·2 to 387·9)
Somalia	105·2 (100·3 to 109·7)	140·1 (134·5 to 145·7)	189·7 (182 to 196·7)	316 (303·4 to 327·7)	335·3 (321·4 to 347·9)	509·9 (481·6 to 535)	674·3 (639·1 to 709·6)	871·2 (805·6 to 941·2)
South Sudan	105·3 (96·3 to 114)	134·4 (125·1 to 143·7)	170·8 (160·2 to 180·6)	203·7 (192·7 to 215·4)	252·4 (234·1 to 269·4)	324·4 (301·1 to 345·9)	409·2 (377·3 to 438·7)	364·1 (339·4 to 393·2)
Uganda	333·4 (319·8 to 345·8)	425·3 (412·4 to 436·5)	545·8 (532 to 558·7)	702·9 (688·1 to 717)	938·9 (921·7 to 955·4)	1253·2 (1227·7 to 1275·8)	1462·7 (1412 to 1509·1)	1593·7 (1505·2 to 1695·3)
Tanzania	461·6 (437·3 to 484)	591·7 (568·5 to 613·4)	739·2 (715 to 763·3)	944·9 (907·7 to 979·5)	1236·8 (1194·8 to 1278·8)	1570·2 (1496·6 to 1641)	1890·7 (1795·1 to 1989·3)	2087·5 (1926·2 to 2266)
Zambia	135 (129·7 to 139·9)	203·5 (197·9 to 208·1)	256 (249·4 to 262·1)	302·7 (293·2 to 311·4)	392·4 (378·4 to 406·9)	459·8 (439 to 478·6)	551·4 (515·3 to 590·3)	615·9 (556·3 to 684·1)
Southern sub-Saharan Africa	803·4 (742 to 863)	970·1 (911 to 1022)	1193·9 (1125·9 to 1263·3)	1427·7 (1330·4 to 1520·8)	1541·7 (1430·3 to 1659·6)	1671·6 (1541·4 to 1798·5)	1754·8 (1621·4 to 1894·3)	1670·8 (1504·6 to 1864·3)
Botswana	18·3 (17·1 to 19·6)	23·2 (21·7 to 24·6)	28·7 (26·9 to 30·5)	35 (32·3 to 37·6)	40·4 (36·3 to 44·5)	45·1 (39·9 to 50·6)	48·7 (43·6 to 54·3)	48·2 (42 to 55·5)
eSwatini	12·2 (11·6 to 12·7)	16·5 (15·7 to 17·2)	21·4 (20·6 to 22·1)	27·4 (26·2 to 28·6)	32·2 (30·9 to 33·4)	35·3 (33·4 to 37·2)	33·9 (31·4 to 36·3)	30 (26·4 to 34·1)
Lesotho	26 (23·6 to 28·4)	31·6 (28·6 to 34·4)	39 (36·1 to 42)	52·3 (47·1 to 57·8)	62·6 (57·8 to 67·2)	60·1 (57·2 to 62·8)	52 (48·9 to 55·1)	46·9 (40·5 to 53·8)
Namibia	21·9 (20·6 to 23·1)	27·9 (26·6 to 29·2)	33·3 (31·6 to 34·8)	39·7 (37·5 to 41·9)	48·2 (45·2 to 51·2)	55·2 (52 to 58·4)	60·3 (55·5 to 65·1)	62·8 (56·9 to 70·2)
South Africa	585·6 (536·8 to 633·5)	689·3 (641·9 to 732·4)	820·3 (762·1 to 878·4)	922·1 (833·5 to 1007·1)	968·3 (865·5 to 1078·5)	1061·4 (942·3 to 1179·8)	1106·6 (986 to 1235·8)	1035 (917·7 to 1172·5)
Zimbabwe	139·4 (132·4 to 145·8)	181·5 (174·5 to 188·1)	251·2 (242·6 to 259·1)	351·1 (343·5 to 359)	389·9 (379·1 to 400·6)	414·4 (403·4 to 426·4)	453·3 (438·6 to 466·6)	448 (413·1 to 489·5)
Western sub-Saharan Africa	3697 (3504·1 to 3879·6)	4521·1 (4324·8 to 4697·7)	5583·8 (5407·3 to 5759·4)	7109·7 (6883·8 to 7316·6)	9108·3 (8805 to 9390·6)	11817·8 (11436·8 to 12201·9)	14536·1 (13875·9 to 15183·7)	16429·1 (15275·3 to 17702·8)
Benin	99·5 (93·3 to 105·3)	112·8 (107·9 to 117·2)	137·1 (132·9 to 141·2)	175·3 (169·6 to 180·7)	236·9 (230 to 243·7)	307 (296·9 to 316·7)	416·5 (398·4 to 433·5)	505·2 (474·8 to 541·2)
Burkina Faso	185·5 (171·7 to 198·9)	210·9 (199·6 to 220·8)	259·1 (249·1 to 268·1)	348·6 (338·6 to 358·3)	457 (442 to 470·6)	572·7 (555·7 to 588·5)	744·9 (714 to 774·7)	937·3 (873·5 to 1007·7)
Cape Verde	7·1 (6·7 to 7·5)	9·2 (8·9 to 9·4)	9·2 (9 to 9·5)	10·4 (10 to 10·7)	11·6 (11·2 to 12·1)	12·6 (11·7 to 13·6)	12·3 (10·6 to 14·4)	10·8 (9·1 to 12·8)
Cameroon	211·9 (195·8 to 227·3)	267·2 (248·7 to 284·5)	323·1 (310·4 to 335·4)	381·6 (369·7 to 392·9)	464·6 (447·3 to 480·1)	636·4 (613·3 to 658)	864·6 (816·2 to 914·4)	903·8 (797·3 to 1027·7)
Chad	129·5 (122·8 to 135·6)	148·2 (142·5 to 153·1)	185·1 (179·7 to 190·6)	243·2 (237 to 248·9)	320·1 (312·8 to 327)	435·3 (424·9 to 444·6)	592·5 (574·4 to 608·8)	787·6 (753·5 to 823·7)
Côte d'Ivoire	122·4 (113·6 to 130·7)	199·8 (188·3 to 210·3)	279·9 (267·5 to 291·2)	397·7 (383·7 to 410·8)	574·9 (551·6 to 596·6)	751·1 (708·2 to 794·3)	862·3 (811·3 to 913·1)	892·3 (813·1 to 981·6)
The Gambia	11·3 (10·5 to 12·1)	14·6 (13·7 to 15·5)	23·1 (21·9 to 24·3)	35·6 (33·7 to 37·3)	50·1 (46·9 to 52·8)	63·8 (58·9 to 68·3)	70 (63·1 to 76·7)	72 (63 to 82·3)
Ghana	219·3 (201·1 to 238·8)	340·9 (323 to 358·2)	452·3 (438·1 to 466·4)	560·8 (541·9 to 578·8)	607·2 (577·7 to 638·7)	689·2 (650·1 to 727·8)	808·4 (758·9 to 860·6)	854·9 (734·9 to 987·7)
Guinea	129·7 (123·8 to 135)	160·4 (152·3 to 167·8)	202 (193·4 to 210·3)	251·9 (242·9 to 260·3)	308·5 (298·8 to 317·6)	350·6 (337·2 to 363·5)	414·1 (392·1 to 434·9)	477·3 (440·9 to 512·5)
Guinea-Bissau	31·7 (30·3 to 33)	29·9 (28·1 to 31·5)	30·7 (28·7 to 32·5)	37·7 (35·4 to 39·9)	46·6 (43·4 to 49·8)	53·5 (49·2 to 57·7)	61·3 (55 to 67·3)	62·8 (55·4 to 70·6)

Liberia	42·9 (39·9 to 45·8)	56·5 (53·3 to 59·2)	72·1 (69·2 to 74·9)	98·3 (94·4 to 101·7)	88·8 (84·5 to 92·7)	130·7 (124·8 to 137)	150·5 (138·8 to 161·3)	135·5 (118 to 156·2)
Mali	185 (175 to 194·1)	247·4 (237·4 to 255·9)	319 (310·1 to 327·5)	389·6 (379·9 to 398·1)	451·9 (440·8 to 462·6)	547·8 (532 to 563·4)	731·6 (701·8 to 758·1)	961·1 (902·4 to 1023·6)
Mauritania	31·1 (28·7 to 33·5)	40·4 (38 to 42·6)	53·3 (51·2 to 55·3)	72·3 (69·6 to 74·7)	90·5 (86·3 to 94·7)	101·9 (96·5 to 107·1)	111·3 (103·7 to 118·7)	109 (98·1 to 122)
Niger	140·4 (133·2 to 147·1)	194·6 (187·9 to 200·6)	267 (260·2 to 273·7)	349·7 (342·4 to 356·5)	444·9 (435·4 to 454·2)	640·6 (626·1 to 654)	845·9 (825·3 to 865·5)	1142·8 (1101·5 to 1186)
Nigeria	1863·4 (1767·1 to 1951·9)	2126·3 (2028·8 to 2211·8)	2520 (2422 to 2615·5)	3199·5 (3055·2 to 3329·8)	4277·4 (4096·6 to 4452·2)	5737·9 (5479·8 to 5982·3)	6891·2 (6497·9 to 7287·2)	7587·2 (7020·7 to 8238·2)
São Tomé and PrÍncipe	2·4 (2·3 to 2·6)	3·1 (3 to 3·2)	3·2 (3·1 to 3·2)	3·8 (3·7 to 3·9)	4·7 (4·5 to 4·8)	5·4 (5·1 to 5·6)	5·3 (4·8 to 5·7)	4·7 (4 to 5·4)
Senegal	130·6 (122·4 to 138·4)	174·1 (167·3 to 180·5)	233·8 (226·5 to 240·5)	294·4 (287·7 to 300·9)	344·4 (335·9 to 353)	386·3 (373·7 to 399·6)	455·4 (440 to 470·7)	466·5 (430·4 to 510·2)
Sierra Leone	79·4 (72·1 to 86·7)	98·4 (90·4 to 105·5)	113·6 (106·6 to 120·6)	130·7 (122 to 138·4)	164·9 (153·1 to 176·2)	196·4 (183·4 to 208·3)	254·9 (235·7 to 274·5)	280·7 (256·5 to 309·4)
Togo	73·6 (69·7 to 77·2)	86·3 (83·8 to 89)	99·9 (97·2 to 102·5)	128·5 (125·2 to 131·6)	163·1 (159·5 to 166·6)	198·4 (192 to 204·8)	242·7 (232·7 to 252·5)	237·5 (219·7 to 258·9)

Supplemental Results Table 1b: Live births (in thousands) by location, males, 1950, 1960, 1970, 1980, 1990, 2000, 2010, 2019

Location	1950	1960	1970	1980	1990	2000	2010	2019
Global	49027·1 (47298·8 to 50785·5)	55643·5 (54084·5 to 57196·7)	59272·9 (57740·4 to 60733)	67053·3 (65670 to 68363·9)	71371·3 (69859·7 to 72854·6)	69763·9 (68175·7 to 71421·3)	71279·1 (69017·3 to 73473·5)	70042·4 (65808·3 to 74556·9)
Central Europe, eastern Europe, and central Asia	3889·5 (3799·4 to 3986·2)	3865·9 (3819·5 to 3908·8)	3170·1 (3117·4 to 3225·3)	3671·7 (3631 to 3714·4)	3337·9 (3298 to 3381·5)	2389 (2352·2 to 2427·3)	2917·4 (2866·3 to 2972)	2688·2 (2412·7 to 3000·1)
Central Asia	551·6 (527·7 to 574·7)	717·2 (693·7 to 740·3)	686·6 (651·2 to 724·6)	896·4 (871·1 to 922·3)	1011·2 (988·6 to 1034·1)	792·3 (768 to 816·2)	995·8 (955·6 to 1040·1)	983·8 (910·8 to 1059·2)
Armenia	27·9 (26·3 to 29·6)	39·5 (37·8 to 41·1)	32·4 (30·4 to 34·6)	45·7 (43·4 to 47·8)	41·3 (39·9 to 42·9)	23·5 (22·4 to 24·6)	25·4 (24·2 to 26·7)	20·4 (18·4 to 22·6)
Azerbaijan	63 (59 to 67·1)	92·5 (89·2 to 95·9)	78·6 (74 to 83·7)	91·1 (87 to 95·1)	105·6 (100·3 to 110·8)	78·6 (74·4 to 82·9)	101·4 (93·7 to 109)	82·2 (71·3 to 94·4)
Georgia	44·1 (40·1 to 48·4)	51·3 (48·1 to 54·4)	44·3 (39·6 to 49·7)	47·4 (44·8 to 50·1)	42·2 (40·1 to 44·3)	26·2 (24·7 to 27·7)	28·9 (27·2 to 30·4)	23·8 (20·6 to 27·5)
Kazakhstan	131·4 (125 to 137·7)	176·6 (170·8 to 182·2)	167·7 (157 to 179)	187·7 (182·4 to 193·3)	181·8 (176·2 to 187·1)	119·4 (114·5 to 124·2)	177·6 (170·1 to 185)	180·7 (165·1 to 196·6)
Kyrgyzstan	29·7 (27·8 to 31·4)	38·9 (37·4 to 40·5)	40·9 (37·5 to 44·4)	58 (55·4 to 60·6)	63·4 (61·2 to 65·3)	50·5 (48 to 53·2)	70·7 (67·6 to 74·1)	74·3 (67·4 to 81·8)
Mongolia	16·2 (15·2 to 17·2)	19·4 (18·3 to 20·4)	24 (23·1 to 24·8)	32·3 (31·2 to 33·3)	38·1 (36·9 to 39·3)	29·8 (28·4 to 31·2)	42·1 (40·1 to 44·1)	42·6 (37·6 to 47·8)
Tajikistan	47·2 (45·2 to 49·1)	62·3 (60·7 to 63·8)	62·3 (60·1 to 64·5)	87·8 (85·3 to 90·4)	106·7 (102·5 to 110·7)	95·7 (89·4 to 101·9)	118 (108·8 to 126·8)	130·5 (118·5 to 144·3)
Turkmenistan	27·1 (25·9 to 28·2)	36·4 (35·6 to 37·2)	38·2 (36·2 to 40·2)	55·3 (53·8 to 56·7)	68·7 (67·1 to 70·2)	52 (48·9 to 55·1)	58·5 (54·4 to 62·6)	58·3 (53·2 to 64·1)
Uzbekistan	165·1 (154·9 to 175·4)	200·2 (189·8 to 210·6)	198·4 (185·4 to 211·3)	291·1 (280·8 to 301·4)	363·3 (354·2 to 371·7)	316·6 (306·7 to 327·1)	373·3 (351·1 to 396·3)	371·1 (330·3 to 415·4)
Central Europe	1184·3 (1154·3 to 1216·7)	1016 (1003·5 to 1027·8)	969·2 (958·3 to 981·3)	1054·8 (1044·3 to 1066·6)	854·4 (845·2 to 864·2)	619 (611·7 to 626·2)	622·4 (614·9 to 630·7)	550·1 (483·8 to 626·9)
Albania	25·8 (24·7 to 26·8)	34·5 (33·5 to 35·4)	36·5 (34·7 to 38·1)	38 (36·2 to 39·9)	46 (44·4 to 47·7)	31·3 (29·8 to 32·8)	22·4 (21·3 to 23·4)	19·5 (17·5 to 21·9)
Bosnia and Herzegovina	46·8 (41·1 to 52·9)	55·8 (50·6 to 61·3)	42·3 (36·2 to 49·5)	39·8 (35·9 to 44·1)	33·7 (31·1 to 36·3)	21·1 (20·2 to 22·1)	17·2 (16·2 to 18·2)	13·4 (12·3 to 14·7)
Bulgaria	86 (85·6 to 86·5)	71·6 (71·2 to 72·1)	70·6 (70·3 to 71)	65·4 (65 to 65·7)	51 (50·7 to 51·4)	36·7 (36·5 to 37)	38·3 (38·1 to 38·5)	30·9 (28·5 to 33·6)
Croatia	47 (46·8 to 47·3)	39 (38·8 to 39·3)	31·6 (31·4 to 31·9)	34·8 (34·6 to 35·1)	27·7 (27·5 to 27·9)	21·8 (21·6 to 21·9)	21·9 (21·8 to 22·1)	18·1 (16 to 20·5)
Czech Republic	97·2 (96·8 to 97·6)	65·8 (65·5 to 66·2)	77·4 (77 to 77·8)	76·8 (76·4 to 77·2)	65·9 (65·5 to 66·2)	47 (46·7 to 47·3)	58·4 (58·1 to 58·7)	55·9 (50 to 62·5)
Hungary	101·4 (101 to 101·9)	74·4 (74·1 to 74·8)	76·8 (76·4 to 77·2)	76·3 (75·9 to 76·8)	63·4 (63·1 to 63·7)	50 (49·7 to 50·3)	46·1 (45·8 to 46·4)	42·3 (37·1 to 48·1)
Montenegro	6·4 (5·9 to 6·9)	6·5 (6·1 to 6·8)	5·4 (5·1 to 5·8)	5·4 (5·1 to 5·8)	5 (4·7 to 5·3)	4·4 (4·1 to 4·6)	3·9 (3·8 to 4)	3·4 (3·2 to 3·7)
North Macedonia	20·3 (18·1 to 22·7)	20·3 (18 to 22·8)	18·9 (17·3 to 20·6)	20·6 (19·2 to 21·9)	18·4 (17·6 to 19·1)	12·9 (12·3 to 13·5)	12·3 (11·7 to 12·9)	11·5 (10·4 to 12·7)
Poland	377 (366·2 to 388·7)	327·1 (318·3 to 334·8)	270·6 (264·1 to 277·1)	348·7 (340·8 to 357·6)	272·8 (265·8 to 280·7)	187 (180·9 to 193·1)	207·7 (201·6 to 214·4)	186·5 (161·3 to 215·8)
Romania	214·8 (198·6 to 233·4)	176·8 (176·1 to 177·6)	209·1 (208·3 to 209·9)	204·7 (203·9 to 205·3)	148·2 (147·5 to 148·9)	116·9 (116·3 to 117·5)	106 (105·5 to 106·6)	89·1 (75·6 to 105·1)
Serbia	94·1 (93·4 to 94·8)	85 (84·6 to 85·3)	74·6 (74·3 to 75)	80·8 (80·4 to 81·1)	70·8 (70·5 to 71·2)	53·7 (53·3 to 54)	45·3 (42·8 to 47·7)	41·2 (34·9 to 48·7)
Slovakia	51·1 (50·8 to 51·4)	44·8 (44·6 to 45·1)	41·1 (40·8 to 41·3)	48·4 (48·1 to 48·6)	40·3 (40 to 40·5)	27·2 (27 to 27·4)	31·4 (31·2 to 31·6)	28·5 (25·1 to 32·2)
Slovenia	16·4 (14·5 to 18·6)	14·3 (14 to 14·7)	14·2 (13·9 to 14·5)	15·2 (14·8 to 15·6)	11·3 (11 to 11·7)	9·1 (8·9 to 9·4)	11·4 (11·2 to 11·7)	9·7 (8·4 to 11·1)
Eastern Europe	2153·6 (2100·4 to 2212·3)	2132·7 (2106·9 to 2156·6)	1514·3 (1497 to 1530·1)	1720·4 (1701·7 to 1739·7)	1472·2 (1456·2 to 1491·1)	977·7 (965·8 to 989·4)	1299·3 (1283·7 to 1315·4)	1154·2 (996·2 to 1336·9)
Belarus	99·4 (95·6 to 103·4)	101·4 (98 to 104·6)	74·3 (71·8 to 76·9)	80 (77·2 to 82·7)	71·2 (69 to 73·8)	47·5 (45·5 to 49·7)	56·9 (54·5 to 59·3)	52·8 (44·3 to 62·6)
Estonia	10·4 (10·3 to 10·5)	10·4 (10·3 to 10·5)	11 (10·9 to 11·1)	11·5 (11·4 to 11·6)	10·8 (10·7 to 10·9)	6·6 (6·5 to 6·7)	7·9 (7·8 to 8)	6·8 (6 to 7·7)
Latvia	16·7 (16·4 to 17·1)	18·4 (18·2 to 18·6)	18 (17·8 to 18·1)	18·3 (18·1 to 18·5)	18·6 (18·5 to 18·8)	10·2 (10·1 to 10·4)	9·5 (9·4 to 9·6)	9·9 (8·8 to 11·2)
Lithuania	29·6 (27·9 to 31·7)	31·3 (31·1 to 31·5)	27·9 (27·7 to 28·1)	25·6 (25·4 to 25·7)	28·5 (28·3 to 28·7)	16·6 (16·5 to 16·7)	16·5 (16·4 to 16·7)	13·9 (12·3 to 15·6)
Moldova	43·7 (41·4 to 46)	42·7 (40·6 to 45)	36·4 (33·1 to 40·3)	45·5 (43·1 to 48)	39·5 (37·9 to 41·2)	23·5 (22·2 to 24·9)	22·3 (20·9 to 23·8)	16·6 (14·5 to 18·8)
Russia	1513·6 (1503·2 to 1523·5)	1498·9 (1494·9 to 1503·3)	989·4 (986·1 to 992·6)	1150·4 (1147·1 to 1154)	973·9 (971·2 to 977)	661·5 (659·1 to 664·1)	926·6 (923·5 to 929·6)	854·2 (741·4 to 984·1)
Ukraine	440·1 (397·7 to 490·9)	429·5 (406·4 to 451·7)	357·1 (342 to 372)	389·2 (372·9 to 406·2)	329·7 (314·6 to 347·4)	211·8 (201·6 to 221·9)	259·6 (245 to 274·7)	200·1 (169 to 236·7)
High income	6970 (6886·7 to 7053·7)	7407 (7359·4 to 7450·8)	7090·8 (7044·9 to 7135)	6409·1 (6371·6 to 6446·7)	6434·3 (6400·2 to 6468·3)	6079·3 (6047·8 to 6114·3)	6094·8 (6070·6 to 6118·7)	5731·6 (5230 to 6310)
Australasia	129·4 (128·7 to 130·2)	153·8 (153 to 154·6)	170·3 (169·7 to 170·8)	143·4 (142·8 to 143·9)	162·3 (161·7 to 163)	153·9 (153·3 to 154·5)	184·2 (183·5 to 184·8)	190·5 (171·5 to 211·7)
Australia	103·7 (103·2 to 104·2)	121·9 (121·5 to 122·3)	137·8 (137·4 to 138·3)	117·1 (116·5 to 117·6)	131·6 (131 to 132·1)	125·1 (124·6 to 125·6)	152·2 (151·5 to 152·7)	159·9 (144·4 to 177·2)
New Zealand	25·8 (25·2 to 26·3)	31·9 (31·3 to 32·5)	32·4 (32·3 to 32·6)	26·3 (26·1 to 26·5)	30·8 (30·5 to 31·1)	28·8 (28·6 to 29)	32 (31·8 to 32·3)	30·6 (27·1 to 34·5)
High-income Asia Pacific	1575·4 (1517·9 to 1632·6)	1348·7 (1307·5 to 1388·2)	1539·3 (1497·9 to 1579)	1258·8 (1223·2 to 1293·7)	1027·7 (995·8 to 1060·4)	944·8 (916·5 to 975·4)	819·4 (798 to 842·7)	706·1 (654·6 to 764·1)
Brunei	1·6 (1·5 to 1·6)	2·1 (2·1 to 2·1)	2·5 (2·5 to 2·6)	3 (2·8 to 3·1)	3·6 (3·4 to 3·8)	3·8 (3·6 to 3·9)	3·3 (3 to 3·7)	3·4 (2·9 to 3·9)
Japan	1136·8 (1087·7 to 1186·3)	820·1 (790·4 to 850·5)	1002·5 (970·4 to 1036·2)	809·4 (785·1 to 834·3)	621·6 (602·8 to 641)	610·4 (591·6 to 630·4)	551·3 (537·5 to 565·8)	461·2 (421·3 to 507·1)
Aichi	46·9 (44·8 to 49)	39·6 (38 to 41·4)	59·8 (57·6 to 62)	44·5 (43·1 to 46·1)	35·9 (34·7 to 37)	38·3 (37·1 to 39·5)	36 (35·1 to 37)	30·8 (28·2 to 33·9)
Akita	15·6 (14·8 to 16·3)	10·7 (10·2 to 11·2)	8·7 (8·2 to 9·2)	8·3 (8 to 8·6)	5·7 (5·5 to 5·9)	4·6 (4·4 to 4·7)	3·5 (3·4 to 3·6)	2·5 (2·3 to 2·8)
Aomori	16·8 (16 to 17·6)	12·1 (11·6 to 12·7)	12·1 (11·4 to 12·7)	11 (10·6 to 11·4)	7·7 (7·4 to 7·9)	6·7 (6·4 to 6·9)	5 (4·8 to 5·1)	3·9 (3·6 to 4·3)
Chiba	29·8 (28·5 to 31)	19·4 (18·6 to 20·2)	36·2 (34·9 to 37·5)	34·5 (33·6 to 35·5)	27 (26·2 to 27·8)	28·4 (27·6 to 29·3)	26·5 (25·9 to 27·2)	21·4 (19·6 to 23·5)
Ehime	19·7 (18·9 to 20·6)	12 (11·5 to 12·5)	12 (11·4 to 12·7)	10·1 (9·8 to 10·5)	7·5 (7·3 to 7·7)	6·8 (6·5 to 7)	5·9 (5·8 to 6·1)	4·5 (4·1 to 5)
Fukui	9·7 (9·3 to 10·2)	6·1 (5·8 to 6·4)	6·2 (5·7 to 6·7)	5·4 (5·2 to 5·6)	4·4 (4·3 to 4·6)	4·1 (4 to 4·3)	3·6 (3·4 to 3·7)	2·9 (2·6 to 3·2)
Fukuoka	48·3 (46·1 to 50·5)	34·5 (33·2 to 36)	36·6 (35·2 to 38)	33·3 (32·3 to 34·3)	24·7 (24 to 25·5)	24·3 (23·6 to 25)	24·1 (23·4 to 24·8)	20·8 (18·9 to 22·9)
Fukushima	28·8 (27·6 to 30·1)	17·4 (16·7 to 18·2)	15·2 (14·3 to 16)	15·1 (14·6 to 15·6)	11·7 (11·4 to 12·1)	10·4 (10·1 to 10·8)	8·1 (7·8 to 8·3)	6·2 (5·6 to 6·9)
Gifu	19·5 (18·6 to 20·4)	14 (13·4 to 14·6)	16·4 (15·5 to 17·3)	13·1 (12·7 to 13·6)	10·3 (9·9 to 10·6)	10·4 (10 to 10·7)	8·8 (8·6 to 9·1)	6·9 (6·3 to 7·6)
Gunma	22·2 (21·2 to 23·1)	13·2 (12·7 to 13·8)	15·2 (14·4 to 16)	13 (12·6 to 13·4)	10 (9·7 to 10·3)	10 (9·6 to 10·3)	8·2 (8 to 8·5)	6·4 (5·9 to 7·1)
Hiroshima	28·4 (27·2 to 29·6)	18·6 (17·9 to 19·4)	23·9 (22·9 to 24·8)	18·9 (18·3 to 19·5)	14·5 (14 to 15)	14·2 (13·7 to 14·6)	13·2 (12·9 to 13·6)	10·7 (9·7 to 11·8)
Hokkaidō	54·9 (52·3 to 57·5)	41·1 (39·2 to 42·9)	47·7 (45·8 to 49·5)	38·7 (37·5 to 40)	27·7 (26·8 to 28·6)	24·1 (23·4 to 24·9)	20·7 (20·2 to 21·3)	16 (14·6 to 17·7)

Hyōgo	46 (44·1 to 48)	34·8 (33·3 to 36·2)	47·2 (45·5 to 48·9)	35·1 (34·1 to 36·2)	27·2 (26·4 to 28)	27·8 (26·9 to 28·6)	24·7 (24·1 to 25·4)	20·4 (18·7 to 22·4)
Ibaraki	28·4 (27·2 to 29·6)	16·6 (16 to 17·2)	19·4 (18·6 to 20·1)	18·8 (18·2 to 19·4)	14·6 (14·2 to 15·1)	14·5 (14·1 to 15)	12·3 (12 to 12·6)	9·7 (8·8 to 10·7)
Ishikawa	12·1 (11·6 to 12·6)	7·8 (7·5 to 8·2)	9·4 (8·9 to 10)	7·7 (7·4 to 8)	5·8 (5·6 to 6)	5·9 (5·7 to 6·1)	5 (4·9 to 5·2)	4·2 (3·8 to 4·6)
Iwate	19 (18·2 to 19·9)	13 (12·4 to 13·6)	11·9 (11·3 to 12·5)	10 (9·6 to 10·3)	7·3 (7·1 to 7·6)	6·3 (6·1 to 6·6)	5 (4·8 to 5·1)	3·9 (3·5 to 4·3)
Kagawa	12 (11·5 to 12·6)	7·3 (7 to 7·6)	7·8 (7·3 to 8·2)	6·6 (6·4 to 6·8)	4·9 (4·7 to 5)	5 (4·8 to 5·2)	4·4 (4·2 to 4·5)	3·4 (3·1 to 3·8)
Kagoshima	26·4 (25·4 to 27·4)	15·9 (15·3 to 16·6)	12·7 (12 to 13·4)	12·7 (12·3 to 13·1)	9·7 (9·4 to 10)	8·2 (8 to 8·5)	7·9 (7·6 to 8·1)	6·2 (5·7 to 6·9)
Kanagawa	36·2 (34·7 to 37·7)	33·2 (31·9 to 34·6)	61·5 (58·9 to 64·4)	48·8 (47·5 to 50·2)	40·2 (39 to 41·4)	42·7 (41·5 to 43·9)	40·1 (39·1 to 41)	33 (30·3 to 36·1)
Kōchi	11·7 (11·2 to 12·2)	6·3 (6 to 6·7)	6·2 (5·6 to 6·7)	5·2 (5 to 5·3)	3·8 (3·7 to 3·9)	3·5 (3·3 to 3·6)	2·8 (2·7 to 2·9)	2·2 (2 to 2·4)
Kumamoto	24·2 (23·1 to 25·3)	14·9 (14·3 to 15·6)	13·1 (12·4 to 13·9)	12·6 (12·2 to 13)	9·8 (9·5 to 10·1)	8·8 (8·5 to 9·2)	8·4 (8·2 to 8·7)	7 (6·3 to 7·7)
Kyōto	23·8 (22·8 to 24·8)	16·6 (15·9 to 17·2)	20·6 (19·8 to 21·4)	16·4 (16 to 16·9)	12·1 (11·7 to 12·5)	12·2 (11·8 to 12·6)	10·8 (10·6 to 11·1)	9·3 (8·5 to 10·2)
Mie	18·6 (17·8 to 19·5)	12·3 (11·8 to 12·9)	13·7 (12·9 to 14·4)	10·8 (10·5 to 11·2)	9 (8·7 to 9·4)	9 (8·7 to 9·4)	7·9 (7·7 to 8·1)	6·1 (5·6 to 6·8)
Miyagi	22·1 (21·2 to 23·1)	15·1 (14·4 to 15·7)	15·2 (14·4 to 16·1)	15·9 (15·4 to 16·5)	11·9 (11·5 to 12·3)	11·3 (11 to 11·7)	9·8 (9·5 to 10·1)	8·1 (7·4 to 8·9)
Miyazaki	15·3 (14·6 to 15·9)	9·7 (9·3 to 10·2)	8·8 (8·3 to 9·3)	8·8 (8·5 to 9·1)	6·3 (6·1 to 6·5)	5·6 (5·5 to 5·8)	5·3 (5·1 to 5·5)	4 (3·6 to 4·4)
Nagano	27·8 (26·7 to 28·9)	17 (16·4 to 17·7)	17·1 (16·2 to 17·9)	13·7 (13·4 to 14·1)	11 (10·6 to 11·3)	10·8 (10·5 to 11·2)	8·9 (8·7 to 9·2)	7 (6·4 to 7·7)
Nagasaki	24·2 (23·3 to 25·2)	15 (14·4 to 15·6)	13·6 (12·8 to 14·3)	11·6 (11·2 to 11·9)	8·6 (8·3 to 8·8)	7·2 (6·9 to 7·4)	6·2 (6 to 6·4)	5 (4·5 to 5·5)
Nara	9·7 (9·3 to 10·2)	6·2 (6 to 6·5)	8·5 (8 to 8·9)	8·2 (8 to 8·5)	6·7 (6·5 to 6·9)	6·7 (6·5 to 6·9)	5·5 (5·4 to 5·7)	4·4 (4 to 4·8)
Niigata	32·7 (31·3 to 34·1)	20·2 (19·4 to 20·9)	19·1 (18·3 to 19·8)	16·8 (16·3 to 17·4)	12·4 (12 to 12·7)	11·1 (10·7 to 11·5)	9·3 (9 to 9·5)	7·3 (6·7 to 8)
Ōita	16·3 (15·6 to 17·1)	9·9 (9·5 to 10·3)	9·2 (8·7 to 9·7)	8·3 (8 to 8·6)	6 (5·8 to 6·2)	5·6 (5·4 to 5·8)	5·2 (5·1 to 5·4)	4·1 (3·8 to 4·6)
Okayama	21·4 (20·5 to 22·4)	13·7 (13·1 to 14·3)	15·7 (14·8 to 16·5)	12·4 (12 to 12·8)	9·6 (9·3 to 9·9)	9·8 (9·4 to 10·1)	8·6 (8·4 to 8·9)	7·4 (6·7 to 8·1)
Okinawa	14 (13·4 to 14·6)	9·1 (8·7 to 9·4)	10·5 (9·9 to 11)	10·2 (9·9 to 10·5)	9 (8·7 to 9·2)	8·7 (8·4 to 9)	8·8 (8·6 to 9·1)	8 (7·2 to 8·8)
Ōsaka	55·4 (53 to 57·9)	52·4 (50·3 to 54·7)	86·9 (83·2 to 90·9)	56·9 (55·3 to 58·6)	43·9 (42·5 to 45·4)	45·4 (44 to 46·9)	38·7 (37·7 to 39·8)	32·9 (30 to 36·3)
Saga	13 (12·5 to 13·6)	7·8 (7·4 to 8·2)	6·8 (6·2 to 7·3)	6·4 (6·2 to 6·6)	4·8 (4·7 to 5)	4·4 (4·3 to 4·5)	3·9 (3·8 to 4·1)	3·2 (2·9 to 3·5)
Saitama	30·4 (29·1 to 31·6)	21·6 (20·7 to 22·5)	46·1 (44·5 to 47·7)	39 (38 to 40·1)	32·2 (31·3 to 33·3)	34·2 (33·2 to 35·3)	30·5 (29·7 to 31·3)	25·8 (23·6 to 28·3)
Shiga	12·1 (11·6 to 12·6)	7·3 (7 to 7·6)	7·4 (7 to 7·8)	8·1 (7·9 to 8·4)	6·8 (6·6 to 7)	7·2 (7 to 7·5)	6·9 (6·8 to 7·1)	5·6 (5·2 to 6·2)
Shimane	12·1 (11·6 to 12·6)	7·3 (6·9 to 7·6)	6 (5·5 to 6·4)	5·1 (4·9 to 5·3)	3·8 (3·7 to 4)	3·3 (3·2 to 3·4)	2·9 (2·8 to 3)	2·4 (2·2 to 2·7)
Shizuoka	33·4 (31·9 to 34·8)	24·3 (23·3 to 25·3)	30·5 (29·4 to 31·5)	24·6 (23·8 to 25·4)	18·9 (18·3 to 19·5)	18·5 (17·9 to 19·1)	16·5 (16·1 to 16·9)	12·9 (11·8 to 14·2)
Tochigi	21·4 (20·5 to 22·3)	12·5 (12 to 13)	14·1 (13·4 to 14·9)	13·4 (13 to 13·8)	10·1 (9·8 to 10·5)	9·7 (9·4 to 10·1)	8·5 (8·3 to 8·8)	6·8 (6·2 to 7·5)
Tokushima	10·5 (10 to 11)	6·4 (6·1 to 6·8)	6·1 (5·5 to 6·6)	5·4 (5·3 to 5·6)	4 (3·9 to 4·1)	3·7 (3·5 to 3·8)	3·1 (3 to 3·2)	2·5 (2·3 to 2·8)
Tōkyō	84·6 (81 to 88·4)	95·7 (91·8 to 100)	117 (111·8 to 123·2)	71·9 (70 to 73·9)	52·7 (51·2 to 54·2)	51·4 (50 to 52·8)	55·4 (54·1 to 56·7)	55·9 (51·6 to 60·6)
Tottori	8·1 (7·8 to 8·5)	4·9 (4·6 to 5·2)	4·4 (4·1 to 4·8)	4·2 (4·1 to 4·4)	3·2 (3·1 to 3·3)	2·8 (2·7 to 2·9)	2·5 (2·4 to 2·6)	2·1 (1·9 to 2·3)
Toyama	11·9 (11·3 to 12·4)	8 (7·7 to 8·4)	9 (8·5 to 9·5)	6·9 (6·7 to 7·2)	5·1 (4·9 to 5·3)	5·2 (5·1 to 5·4)	4·2 (4·1 to 4·3)	3·4 (3·1 to 3·7)
Wakayama	13·2 (12·6 to 13·8)	8·2 (7·9 to 8·5)	9·4 (8·9 to 10)	7 (6·8 to 7·3)	5·1 (4·9 to 5·3)	4·9 (4·7 to 5·1)	3·9 (3·8 to 4)	3·1 (2·8 to 3·4)
Yamagata	17·7 (17 to 18·5)	11 (10·6 to 11·5)	8·7 (8·2 to 9·2)	8·5 (8·3 to 8·8)	6·5 (6·3 to 6·7)	5·6 (5·4 to 5·8)	4·5 (4·3 to 4·6)	3·5 (3·2 to 3·9)
Yamaguchi	19·7 (18·8 to 20·6)	13 (12·4 to 13·5)	13 (12·3 to 13·6)	10·1 (9·8 to 10·5)	7·1 (6·8 to 7·3)	6·8 (6·5 to 7)	5·9 (5·7 to 6·1)	4·5 (4·1 to 5)
Yamanashi	10·4 (9·9 to 10·8)	6·2 (5·9 to 6·6)	6·2 (5·7 to 6·7)	5·2 (5·1 to 5·3)	4·5 (4·3 to 4·6)	4·3 (4·1 to 4·4)	3·4 (3·3 to 3·5)	2·7 (2·5 to 3)
South Korea	413·9 (386·5 to 441·8)	496·4 (470·4 to 521·6)	511·1 (480·6 to 540·5)	424 (396·7 to 451·2)	374 (348 to 399·1)	299·3 (279·4 to 319·6)	236 (218·4 to 253·9)	212 (189·7 to 239·6)
Singapore	23·2 (22·4 to 23·9)	30·1 (29·2 to 31·1)	23·2 (21·8 to 24·7)	22·3 (20·8 to 24)	28·5 (26·5 to 30·6)	31·3 (29·3 to 33·5)	28·7 (26·7 to 30·8)	29·5 (23·6 to 36·6)
High-income North America	2055·3 (2048·6 to 2062)	2395·9 (2391·2 to 2402·5)	2023·8 (2019·4 to 2030·2)	2037·2 (2033·2 to 2042·1)	2344·4 (2340·6 to 2348·6)	2243·9 (2240 to 2248·6)	2214·5 (2210·7 to 2219·2)	2150·4 (2013·1 to 2301·1)
Canada	185·7 (185·1 to 186·3)	234·4 (233·8 to 235)	178·8 (178·2 to 179·4)	184·3 (183·7 to 184·9)	205·9 (205·3 to 206·5)	170 (169·3 to 170·6)	191·9 (191·4 to 192·6)	191 (177·9 to 205·6)
Greenland	0·5 (0·5 to 0·5)	0·8 (0·8 to 0·8)	0·6 (0·5 to 0·6)	0·5 (0·5 to 0·5)	0·6 (0·6 to 0·7)	0·5 (0·5 to 0·5)	0·4 (0·4 to 0·5)	0·4 (0·4 to 0·5)
USA	1869 (1862·4 to 1875·5)	2160·7 (2156 to 2167)	1844·4 (1840·3 to 1850·6)	1852·4 (1848·5 to 1857)	2137·8 (2134·2 to 2141·7)	2073·4 (2069·5 to 2078·2)	2022 (2018·4 to 2026·7)	1958·9 (1834·8 to 2095)
Alabama	39·1 (38·9 to 39·3)	42·1 (41·4 to 42·7)	32·8 (32·3 to 33·2)	32·1 (31·7 to 32·4)	32·7 (32·1 to 33·3)	31·8 (31·3 to 32·3)	29·5 (29·1 to 30)	31·6 (29·7 to 33·6)
Alaska	1·7 (1·7 to 1·7)	3·7 (3·6 to 3·7)	3·6 (3·6 to 3·7)	5·1 (5 to 5·2)	6·2 (6·1 to 6·2)	5·1 (5 to 5·2)	5·6 (5·5 to 5·7)	5·5 (5 to 6)
Arizona	10·4 (10·3 to 10·5)	18·1 (17·9 to 18·4)	18·9 (18·8 to 19·1)	25·5 (25·2 to 25·8)	35·4 (34·7 to 36)	43·3 (42·7 to 43·9)	45 (44·6 to 45·4)	43·1 (40·4 to 45·9)
Arkansas	22·9 (22·8 to 23)	21·3 (21 to 21·7)	17·9 (17·8 to 18·1)	18·6 (18·3 to 18·8)	18·6 (18·2 to 18·9)	19·3 (19 to 19·7)	19 (18·6 to 19·3)	19·6 (18·4 to 20·8)
California	128·4 (127·8 to 128·9)	182·7 (180·5 to 184·8)	177·1 (176·4 to 177·8)	208·2 (205·5 to 211·1)	312 (307 to 317·1)	271·4 (267·8 to 275)	258·5 (254·2 to 263)	232·4 (216·7 to 249·6)
Colorado	15·9 (15·8 to 16)	21·8 (21·5 to 22)	20·7 (20·5 to 20·8)	25·7 (25·3 to 26)	27·9 (27·5 to 28·4)	33·5 (33·2 to 33·8)	33·7 (33·3 to 34·1)	29·5 (27·6 to 31·6)
Connecticut	24·9 (24·8 to 25)	30·1 (29·7 to 30·5)	24·2 (24·1 to 24·4)	20·5 (20·2 to 20·8)	25·8 (25·2 to 26·3)	22·3 (21·8 to 22·9)	19·4 (19 to 19·8)	19·3 (18·1 to 20·7)
Delaware	4·1 (4·1 to 4·1)	6·1 (6 to 6·2)	5·1 (5 to 5·2)	4·7 (4·6 to 4·8)	5·7 (5·5 to 5·9)	5·7 (5·6 to 5·8)	5·9 (5·8 to 6)	5·8 (5·3 to 6·3)
Washington, DC	11·3 (9·1 to 13·8)	11·6 (10·4 to 12·9)	7·4 (7·3 to 7·4)	5 (4·9 to 5·1)	5·8 (5·6 to 6)	4 (3·8 to 4·3)	4·4 (3·7 to 5·2)	4·9 (3·8 to 6·2)
Florida	32·5 (32·3 to 32·8)	55·8 (55·2 to 56·4)	56·1 (55·4 to 56·9)	66·7 (66 to 67·4)	102 (100·4 to 103·5)	104 (102·6 to 105·6)	108·5 (107·2 to 109·9)	116·8 (109·4 to 124·6)
Georgia	43·5 (43·3 to 43·7)	52·6 (52·3 to 53)	48·4 (48·1 to 48·9)	46·3 (45·8 to 46·9)	57·8 (57 to 58·6)	67·8 (66·9 to 68·7)	68·3 (67·6 to 69)	68·5 (64·2 to 73·1)
Hawaii	7·4 (7·4 to 7·5)	9·4 (9·3 to 9·6)	8·2 (8·1 to 8·2)	9·2 (9 to 9·4)	10·4 (10·2 to 10·7)	8·9 (8·8 to 9)	9·5 (9·3 to 9·6)	9·1 (8·5 to 9·8)
Idaho	7·8 (7·7 to 7·9)	9·1 (8·9 to 9·2)	7·2 (7·1 to 7·3)	10·2 (10·1 to 10·4)	8·5 (8·3 to 8·7)	10·5 (10·3 to 10·6)	11·4 (11·2 to 11·5)	11·7 (10·8 to 12·6)
Illinois	107 (106·5 to 107·4)	115·2 (113·9 to 116·7)	102·7 (102·2 to 103·2)	97·4 (96·4 to 98·3)	100·9 (99·3 to 102·4)	94·9 (93·8 to 96)	81·2 (80·2 to 82·2)	77·6 (72·6 to 82·9)

Indiana	46 (45·8 to 46·2)	58·5 (58·1 to 58·9)	49·9 (49·6 to 50·2)	45·3 (44·8 to 45·8)	44·2 (43·4 to 45)	44·7 (44·2 to 45·3)	42·9 (42·4 to 43·3)	42·7 (40·1 to 45·4)
Iowa	30·8 (30·7 to 31)	32·9 (32·4 to 33·3)	23·7 (23·5 to 23·9)	25·1 (24·8 to 25·5)	20·3 (19·8 to 20·7)	19·5 (19·1 to 20)	19·7 (19·4 to 20·1)	19·3 (18·2 to 20·5)
Kansas	21·5 (21·4 to 21·6)	26·1 (25·6 to 26·7)	18·9 (18·7 to 19)	20·5 (20·3 to 20·8)	20·2 (19·8 to 20·5)	20·2 (19·9 to 20·5)	20·9 (20·6 to 21·3)	18·8 (17·7 to 20)
Kentucky	34·4 (34·2 to 34·7)	36·1 (35·6 to 36·7)	29·4 (29 to 29·8)	29·8 (29·4 to 30·2)	28·2 (27·7 to 28·8)	28·5 (28·1 to 28·8)	27·2 (26·9 to 27·5)	28·4 (26·7 to 30·2)
Louisiana	36·2 (36 to 36·4)	46 (45·3 to 46·7)	37·3 (37 to 37·6)	41·6 (41·1 to 42·1)	37·7 (37 to 38·5)	34·3 (33·7 to 34·9)	32·1 (31·6 to 32·4)	29·6 (27·7 to 31·5)
Maine	10·8 (10·7 to 10·8)	12·1 (12 to 12·2)	9 (8·9 to 9·1)	8·5 (8·3 to 8·6)	8·9 (8·7 to 9·2)	7·1 (6·9 to 7·2)	6·5 (6·3 to 6·6)	6·4 (5·9 to 6·9)
Maryland	29·7 (29·6 to 29·9)	38·4 (38 to 38·8)	34·2 (34 to 34·4)	31·1 (30·7 to 31·4)	41·3 (40·7 to 41·8)	37·5 (36·9 to 38·1)	35·8 (35·5 to 36·2)	37·9 (35·4 to 40·6)
Massachusetts	56·6 (56·3 to 56·8)	57·8 (57·3 to 58·2)	46 (45·8 to 46·3)	37·5 (37 to 38)	47 (45·8 to 48·2)	42·2 (41·1 to 43·4)	37·2 (36·2 to 38·3)	35·7 (33·3 to 38·3)
Michigan	82·4 (82 to 82·7)	95·1 (94·2 to 96)	85·2 (84·8 to 85·7)	78 (77·3 to 78·8)	77·8 (76·6 to 79)	69·2 (68·1 to 70·2)	57·4 (57 to 57·9)	54·8 (51·5 to 58·3)
Minnesota	36·6 (36·4 to 36·7)	42·8 (42·2 to 43·3)	32·6 (32·3 to 32·8)	34·6 (34·3 to 35)	35·2 (34·7 to 35·7)	34·8 (34·3 to 35·2)	34·8 (34·5 to 35·1)	34·4 (32·2 to 36·6)
Mississippi	31·6 (31·1 to 32·1)	31 (30·4 to 31·6)	24·7 (24·5 to 24·9)	23·3 (23·1 to 23·6)	22·4 (22·1 to 22·8)	22·2 (21·9 to 22·6)	19·9 (19·8 to 20·1)	19·4 (18·2 to 20·6)
Missouri	46·1 (46 to 46·3)	51·9 (51·2 to 52·6)	40·7 (40·5 to 41)	40·3 (39·7 to 40·8)	40·9 (40·2 to 41·7)	39·2 (38·7 to 39·6)	39·1 (38·5 to 39·6)	37·7 (35·4 to 40·1)
Montana	7 (6·9 to 7)	8·3 (8·2 to 8·4)	6·1 (6·1 to 6·2)	7·3 (7·2 to 7·4)	6·1 (6 to 6·2)	5·6 (5·6 to 5·7)	6·1 (6 to 6·3)	5·8 (5·2 to 6·3)
Nebraska	15·6 (15·5 to 15·6)	17 (16·7 to 17·3)	12·9 (12·8 to 13)	13·6 (13·4 to 13·8)	12·6 (12·4 to 12·8)	12·8 (12·6 to 13)	13·4 (13·3 to 13·6)	13·1 (12·3 to 13·9)
Nevada	1·8 (1·8 to 1·8)	3·5 (3·4 to 3·5)	4·3 (4·2 to 4·5)	6·8 (6·7 to 6·9)	10·9 (10·6 to 11·2)	15·6 (15·4 to 15·9)	18·1 (17·7 to 18·5)	20·4 (19·1 to 21·8)
New Hampshire	6·3 (6·3 to 6·3)	7·4 (7·3 to 7·5)	6·7 (6·6 to 6·7)	7 (6·8 to 7·2)	9 (8·8 to 9·2)	7·5 (7·3 to 7·6)	6·6 (6·4 to 6·7)	6·3 (5·8 to 7)
New Jersey	60·6 (60·3 to 60·8)	68·9 (68·2 to 69·6)	59·3 (59·1 to 59·6)	50·4 (50 to 50·9)	63·3 (62·3 to 64·3)	59·6 (58·7 to 60·5)	52·4 (51·6 to 53·3)	52·3 (48·9 to 56·1)
New Mexico	9·7 (9·6 to 9·8)	14·6 (14·4 to 14·9)	10·9 (10·8 to 11)	13·6 (13·5 to 13·8)	14·4 (14·2 to 14·7)	14 (13·8 to 14·2)	13·5 (13·3 to 13·8)	12·6 (11·9 to 13·5)
New York	182·2 (181·2 to 183·1)	184·7 (182·1 to 187·3)	155·5 (154·9 to 156·3)	122·2 (120·9 to 123·5)	152·6 (149·5 to 156)	132·2 (130·2 to 134·4)	123·4 (121·8 to 124·8)	116·7 (108·8 to 125·2)
North Carolina	50·2 (49·8 to 50·6)	56·8 (56·2 to 57·5)	47·5 (46·9 to 48·1)	43 (42·6 to 43·5)	53·6 (52·6 to 54·7)	60·8 (59·8 to 61·7)	62·3 (61·3 to 63·3)	64·6 (60·5 to 68·9)
North Dakota	8 (7·9 to 8·1)	8·5 (8·4 to 8·7)	5·4 (5·3 to 5·4)	6·2 (6 to 6·3)	4·9 (4·6 to 5·1)	4·1 (3·9 to 4·3)	5·6 (5·3 to 5·8)	4·9 (4·4 to 5·5)
Ohio	97·8 (97·4 to 98·1)	110·9 (109·6 to 112·1)	99·9 (99·5 to 100·4)	87·3 (86·7 to 88·1)	86·4 (84·8 to 88)	78·8 (77·2 to 80·7)	71 (69·6 to 72·3)	68·8 (64·6 to 73·2)
Oklahoma	24·5 (24·4 to 24·7)	25·9 (25·5 to 26·4)	21·9 (21·6 to 22·3)	26·1 (25·8 to 26·4)	25 (24·7 to 25·4)	25·8 (25·4 to 26·3)	26·2 (25·8 to 26·7)	24·9 (23·4 to 26·5)
Oregon	17·2 (17·1 to 17·3)	20 (19·8 to 20·3)	17 (16·9 to 17·2)	22·2 (21·9 to 22·4)	21·8 (21·3 to 22·2)	23·3 (22·9 to 23·7)	23·4 (23·1 to 23·7)	20·6 (19·2 to 22)
Pennsylvania	127·1 (126·4 to 127·9)	118·9 (117·2 to 120·6)	96 (95·6 to 96·3)	82·7 (81·8 to 83·7)	88·6 (87·1 to 90·3)	75·1 (73·7 to 76·5)	72·1 (71 to 73·3)	72·2 (67·7 to 76·9)
Rhode Island	10 (9·9 to 10)	10·3 (10·1 to 10·5)	7·8 (7·7 to 7·8)	6·2 (6 to 6·4)	7·7 (7·4 to 8)	6·6 (6·4 to 6·9)	6·1 (5·8 to 6·3)	5·5 (5 to 6·1)
South Carolina	27·4 (27·2 to 27·5)	31·1 (30·6 to 31·5)	26 (25·7 to 26·4)	26·1 (25·7 to 26·4)	29·8 (29·2 to 30·3)	28·6 (28·1 to 29·1)	28 (27·5 to 28·5)	29·9 (28·1 to 31·9)
South Dakota	8·4 (8·3 to 8·5)	8·9 (8·7 to 9·1)	5·8 (5·7 to 5·8)	6·7 (6·6 to 6·8)	5·8 (5·7 to 5·9)	5·4 (5·3 to 5·6)	6·4 (6·2 to 6·5)	6·1 (5·6 to 6·6)
Tennessee	39·4 (39·2 to 39·7)	45 (44·5 to 45·5)	36·7 (36·4 to 37)	35·3 (34·9 to 35·6)	38·6 (37·7 to 39·5)	40·7 (40 to 41·5)	43·2 (42·5 to 43·9)	41·6 (39 to 44·2)
Texas	101·3 (100·9 to 101·8)	133·7 (132·4 to 135)	117·2 (116·6 to 118)	139·3 (137·8 to 140·9)	165·4 (162·7 to 168·4)	186 (182·7 to 189)	197 (194·9 to 199·3)	183·7 (172·2 to 195·9)
Utah	10·4 (10·2 to 10·6)	14·1 (13·8 to 14·5)	13·5 (13·4 to 13·6)	21·1 (20·9 to 21·3)	18·3 (18 to 18·7)	24·4 (23·7 to 25)	27·1 (26·5 to 27·7)	23·9 (22·4 to 25·4)
Vermont	4·5 (4·5 to 4·5)	4·8 (4·7 to 4·9)	4·1 (4 to 4·1)	4 (3·9 to 4·1)	4·3 (4·1 to 4·4)	3·3 (3·3 to 3·4)	2·9 (2·8 to 3)	3 (2·7 to 3·4)
Virginia	42·2 (42 to 42·4)	50·3 (50 to 50·5)	43·1 (42·9 to 43·4)	40·7 (40·2 to 41·3)	51·2 (50·2 to 52·3)	50·8 (49·8 to 51·7)	51 (50·2 to 51·8)	53·1 (49·7 to 56·7)
Washington	27·9 (27·8 to 28)	33·9 (33·5 to 34·3)	29·1 (28·9 to 29·2)	35 (34·5 to 35·4)	40·7 (39·8 to 41·7)	40·9 (40·2 to 41·7)	44·1 (43·3 to 44·8)	41·1 (38·3 to 44)
West Virginia	24·1 (23·9 to 24·2)	21·7 (21·3 to 22)	15·2 (15 to 15·4)	14·8 (14·6 to 15)	11·7 (11·5 to 11·9)	10·8 (10·6 to 11)	10·6 (10·4 to 10·9)	9·8 (9·2 to 10·5)
Wisconsin	42·2 (42 to 42·3)	48·3 (47·9 to 48·8)	37·5 (37·2 to 37·7)	39 (38·6 to 39·3)	37·7 (36·9 to 38·6)	35·5 (34·8 to 36·2)	34·5 (34 to 35)	33·8 (31·8 to 36)
Wyoming	3·7 (3·6 to 3·7)	4·5 (4·4 to 4·6)	3·1 (3 to 3·1)	5·2 (5·1 to 5·3)	3·6 (3·5 to 3·7)	3·2 (3·1 to 3·3)	3·5 (3·4 to 3·6)	3·7 (3·3 to 4·1)
Southern Latin America	349·2 (340·3 to 358·8)	407·6 (405·1 to 410·4)	424·4 (421·2 to 427·8)	498·7 (496·7 to 501·3)	522·2 (520 to 524·8)	503·3 (500·2 to 506·7)	532·9 (528·8 to 537·1)	495·6 (417·1 to 590·8)
Argentina	226 (218·6 to 234·3)	238·7 (236·6 to 240·9)	270·9 (268·2 to 273·9)	348·3 (346·8 to 350·2)	346·9 (345·3 to 348·7)	349·7 (347·6 to 352)	382·7 (380·2 to 385·4)	356·2 (294·8 to 431·3)
Chile	99·7 (98·5 to 101)	137·7 (137 to 138·4)	125·3 (124·7 to 125·9)	122·7 (122·1 to 123·4)	147·2 (146·5 to 148)	126·6 (126 to 127·3)	125·5 (124·9 to 126·2)	115·6 (102·3 to 131·2)
Uruguay	23·4 (21·9 to 24·9)	31·2 (30·2 to 32·2)	28·2 (27·3 to 29·1)	27·6 (26·8 to 28·6)	28·1 (27·1 to 29)	27 (25·2 to 28·8)	24·7 (22·2 to 27·8)	23·7 (19·9 to 28·2)
Western Europe	2860·7 (2822·2 to 2901·4)	3100·9 (3090·7 to 3110·9)	2933 (2922·1 to 2943·7)	2470·9 (2465·7 to 2476·5)	2377·7 (2372·5 to 2382·5)	2233·4 (2229·2 to 2237·7)	2343·8 (2339·8 to 2348·2)	2189·1 (1968·5 to 2443·1)
Andorra	0·1 (0 to 0·1)	0·1 (0·1 to 0·1)	0·2 (0·2 to 0·2)	0·3 (0·2 to 0·3)	0·3 (0·3 to 0·3)	0·4 (0·3 to 0·4)	0·4 (0·4 to 0·4)	0·3 (0·3 to 0·4)
Austria	53·9 (53·1 to 54·7)	65·7 (65·4 to 66·1)	56·6 (56·3 to 56·8)	46·9 (46·7 to 47·2)	47·8 (47·6 to 48·1)	39·8 (39·5 to 40)	40·2 (40 to 40·4)	45·2 (42·4 to 48·3)
Belgium	73·4 (73 to 73·7)	80·5 (80·1 to 80·8)	72·9 (72·5 to 73·3)	63·2 (62·9 to 63·6)	64·5 (64·1 to 64·8)	59·5 (59·2 to 59·9)	66 (65·7 to 66·4)	62·3 (54·5 to 71·1)
Cyprus	7·1 (6·8 to 7·4)	7·4 (7·2 to 7·6)	6·2 (6 to 6·5)	6·9 (6·7 to 7·1)	7·6 (7·3 to 7·8)	5·8 (5·5 to 6)	6·9 (6·6 to 7·1)	7·8 (6·7 to 9·2)
Denmark	40·2 (38·6 to 42)	38·4 (37·6 to 39·2)	37·6 (36·7 to 38·4)	28·3 (27·5 to 29·1)	32·9 (32·2 to 33·6)	34·1 (33·3 to 35)	31·2 (30·6 to 31·9)	32·2 (28·5 to 36·5)
Finland	48·9 (48·6 to 49·1)	41·5 (41·2 to 41·7)	32·2 (32 to 32·4)	32·5 (32·3 to 32·7)	33·7 (33·4 to 33·9)	28·9 (28·7 to 29·1)	30·9 (30·7 to 31)	25·5 (23·3 to 27·9)
France	430·5 (429 to 432·1)	424·9 (423·7 to 426)	436 (434·7 to 437·2)	412·2 (411 to 413·4)	390·6 (389·4 to 391·8)	399·2 (397·9 to 400·7)	408·9 (407·6 to 410·1)	367·7 (333 to 406·2)
Germany	544·3 (524·3 to 565)	637·3 (635·8 to 638·7)	517 (515·4 to 518·7)	428 (426·7 to 429·1)	453·5 (452·3 to 454·7)	390 (389 to 390·9)	343·1 (342·1 to 344)	379·6 (349·7 to 413·5)
Greece	80·4 (79·1 to 81·7)	80·1 (79·7 to 80·5)	73·6 (73·2 to 74·1)	74·3 (73·8 to 74·7)	52·6 (52·2 to 52·9)	52·8 (52·5 to 53·1)	57·4 (56·9 to 58)	44·2 (39·2 to 50·4)
Iceland	2·1 (2 to 2·1)	2·4 (2·3 to 2·4)	2·1 (2·1 to 2·2)	2·2 (2·2 to 2·3)	2·4 (2·3 to 2·4)	2·1 (2·1 to 2·1)	2·4 (2·4 to 2·5)	2·2 (1·9 to 2·5)
Ireland	32·9 (31·6 to 34·4)	31·1 (30·6 to 31·7)	33·2 (32·6 to 33·9)	37 (36·1 to 37·7)	26·9 (26·2 to 27·6)	29·1 (28·3 to 29·8)	37·9 (37 to 38·9)	31·2 (26·6 to 36·3)
Israel	24·5 (23·5 to 25·5)	28·5 (28·3 to 28·7)	41·7 (41·3 to 42)	47·7 (47·3 to 48)	52·6 (52·3 to 52·9)	70 (69·5 to 70·4)	84·8 (84·2 to 85·3)	99 (82·3 to 117·7)
Italy	452·8 (450·2 to 455·2)	473·8 (472·1 to 475·6)	469·7 (467·6 to 471·8)	327·1 (325·5 to 328·8)	290·9 (289·6 to 292·2)	279·6 (278·6 to 280·4)	287·9 (287 to 288·7)	226 (206·4 to 249·6)

Luxembourg	2·2 (2·2 to 2·3)	2·5 (2·5 to 2·6)	2·2 (2·1 to 2·2)	2·1 (2·1 to 2·2)	2·5 (2·5 to 2·5)	2·9 (2·8 to 3)	2·9 (2·9 to 3)	3·3 (2·9 to 3·7)
Malta	5 (4·8 to 5·3)	4·2 (4·1 to 4·3)	2·8 (2·7 to 2·8)	3 (2·9 to 3)	2·8 (2·8 to 2·9)	2·1 (2·1 to 2·2)	2·1 (2·1 to 2·2)	2·2 (1·9 to 2·6)
Monaco	0·2 (0·2 to 0·2)	0·2 (0·1 to 0·2)	0·2 (0·1 to 0·2)	0·2 (0·1 to 0·2)	0·2 (0·1 to 0·2)	0·2 (0·1 to 0·2)	0·1 (0·1 to 0·2)	0·1 (0·1 to 0·2)
Netherlands	117·5 (116·8 to 118·1)	124 (123·5 to 124·5)	117·8 (117·2 to 118·3)	92 (91·2 to 92·7)	101·5 (101 to 102·1)	106·4 (105·9 to 106·9)	93·3 (92·8 to 93·8)	91·1 (78·3 to 105·8)
Norway	31·9 (31·6 to 32·1)	31·6 (31·4 to 31·9)	33·3 (33·1 to 33·5)	26·1 (25·9 to 26·2)	31·2 (31 to 31·4)	30 (29·8 to 30·2)	31 (30·8 to 31·2)	29·1 (26·4 to 32·2)
Portugal	105·8 (100·9 to 110·9)	111·9 (109·5 to 114·4)	92·7 (90·7 to 94·8)	79·4 (77·3 to 81·5)	59·7 (58 to 61·4)	60 (58·4 to 61·7)	50·4 (49·2 to 51·6)	40·8 (34·3 to 48·3)
San Marino	0·1 (0·1 to 0·2)	0·1 (0·1 to 0·1)	0·1 (0·1 to 0·1)	0·1 (0·1 to 0·1)	0·1 (0·1 to 0·2)	0·2 (0·1 to 0·2)	0·2 (0·1 to 0·2)	0·2 (0·1 to 0·2)
Spain	280·7 (278·6 to 282·8)	334·9 (333·3 to 336·4)	335·6 (333·7 to 337·5)	282·2 (280·4 to 284·2)	204·2 (203·1 to 205·4)	205·7 (204·9 to 206·7)	249·8 (248·9 to 250·8)	190 (169·5 to 215·6)
Sweden	58·5 (58·2 to 58·8)	52·4 (52·2 to 52·7)	57·9 (57·7 to 58·2)	49·4 (49·1 to 49·7)	63·8 (63·5 to 64·1)	47·4 (47·1 to 47·7)	58 (57·7 to 58·3)	60·4 (56·6 to 64·6)
Stockholm	8·8 (8·8 to 8·9)	7·5 (7·3 to 7·8)	11·3 (11·2 to 11·4)	9·6 (9·5 to 9·7)	12·9 (12·8 to 13)	11·4 (11·3 to 11·5)	14·6 (14·5 to 14·7)	14·7 (13·7 to 15·7)
Sweden except Stockholm	49·7 (49·4 to 49·9)	44·9 (44·6 to 45·2)	46·7 (46·4 to 46·9)	39·8 (39·6 to 40·1)	50·9 (50·6 to 51·2)	36 (35·8 to 36·2)	43·4 (43·2 to 43·6)	45·8 (42·9 to 48·9)
Switzerland	42·8 (42·5 to 43)	49·1 (48·8 to 49·4)	50·4 (50·2 to 50·7)	37·6 (37·4 to 37·9)	43·7 (43·5 to 44)	39 (38·8 to 39·2)	41·2 (41 to 41·5)	45·4 (42·2 to 49)
UK	422·9 (412·7 to 435·1)	476 (468·9 to 483·1)	458·9 (449·8 to 467·8)	390·4 (389·4 to 391·3)	409·8 (408·8 to 410·8)	346·5 (345·5 to 347·5)	414·8 (413·5 to 416)	401·4 (359·6 to 449·2)
England	349·3 (338·8 to 360)	381·7 (371·2 to 391·5)	370·2 (359·4 to 381·2)	319·7 (313·8 to 325·5)	337·8 (332·8 to 342·5)	292·5 (289·8 to 295)	351·9 (349·5 to 354·1)	344·4 (311 to 381·6)
East Midlands	27·8 (27 to 28·7)	30·3 (29·5 to 31·1)	30·2 (29·3 to 31)	26·6 (26·1 to 27·1)	27·7 (27·3 to 28·1)	23·5 (23·3 to 23·7)	28 (27·8 to 28·1)	27·7 (25·1 to 30·5)
Derby	1·9 (1·8 to 1·9)	2 (2 to 2·1)	1·9 (1·9 to 2)	1·6 (1·6 to 1·7)	1·7 (1·7 to 1·8)	1·5 (1·4 to 1·5)	1·8 (1·8 to 1·9)	1·7 (1·5 to 1·9)
Derbyshire	5·2 (5 to 5·3)	5·5 (5·2 to 5·7)	5·4 (5·2 to 5·6)	4·5 (4·4 to 4·6)	4·6 (4·5 to 4·8)	3·9 (3·8 to 4)	4·2 (4·1 to 4·3)	4 (3·7 to 4·5)
Leicester	2·4 (2·3 to 2·5)	2·5 (2·4 to 2·6)	2·5 (2·4 to 2·6)	2·5 (2·4 to 2·6)	2·4 (2·4 to 2·5)	2·1 (2 to 2·1)	2·7 (2·6 to 2·7)	2·7 (2·5 to 3)
Leicestershire	3·4 (3·3 to 3·5)	3·9 (3·7 to 4·1)	4 (3·8 to 4·2)	3·6 (3·5 to 3·7)	3·7 (3·6 to 3·8)	3·3 (3·2 to 3·4)	3·6 (3·5 to 3·6)	3·6 (3·3 to 4)
Lincolnshire	3·7 (3·6 to 3·9)	4 (3·7 to 4·2)	4 (3·9 to 4·1)	3·5 (3·4 to 3·6)	3·6 (3·5 to 3·7)	3·2 (3·1 to 3·2)	4 (3·9 to 4)	4·1 (3·7 to 4·5)
Northamptonshire	3·6 (3·5 to 3·7)	3·9 (3·8 to 4·1)	4·2 (4 to 4·3)	3·9 (3·8 to 4)	4·2 (4·1 to 4·3)	3·8 (3·7 to 3·9)	4·7 (4·6 to 4·8)	4·6 (4·1 to 5·1)
Nottingham	2·3 (2·3 to 2·4)	2·5 (2·4 to 2·6)	2·3 (2·2 to 2·4)	2 (1·9 to 2)	2·3 (2·2 to 2·3)	1·7 (1·7 to 1·8)	2·2 (2·2 to 2·2)	2·3 (2·1 to 2·6)
Nottinghamshire	5·1 (5 to 5·3)	5·7 (5·5 to 6)	5·7 (5·5 to 5·9)	4·8 (4·7 to 4·9)	4·8 (4·7 to 4·9)	3·9 (3·9 to 4)	4·6 (4·5 to 4·7)	4·4 (4 to 4·9)
Rutland	0·2 (0·2 to 0·2)	0·2 (0·2 to 0·3)	0·2 (0·2 to 0·2)	0·2 (0·2 to 0·2)	0·2 (0·2 to 0·2)	0·2 (0·2 to 0·2)	0·2 (0·2 to 0·2)	0·2 (0·2 to 0·2)
East of England	33·3 (32·3 to 34·3)	36·8 (35·8 to 37·8)	36·3 (35·2 to 37·4)	33·3 (32·7 to 33·9)	35·6 (35·1 to 36)	31·5 (31·2 to 31·7)	37·5 (37·3 to 37·8)	36·8 (33·2 to 40·7)
Bedford	1 (0·9 to 1)	1·1 (1·1 to 1·2)	1·1 (1 to 1·1)	1 (1 to 1)	0·9 (0·9 to 1)	0·9 (0·9 to 0·9)	1·1 (1·1 to 1·1)	1·1 (1 to 1·2)
Cambridgeshire	2·6 (2·5 to 2·7)	2·9 (2·8 to 3·1)	3·1 (2·9 to 3·2)	3 (2·9 to 3·1)	3·4 (3·3 to 3·5)	3·1 (3·1 to 3·1)	3·8 (3·8 to 3·9)	3·8 (3·4 to 4·2)
Central Bedfordshire	1·4 (1·3 to 1·4)	1·7 (1·6 to 1·7)	1·7 (1·6 to 1·8)	1·7 (1·7 to 1·8)	1·6 (1·6 to 1·7)	1·4 (1·4 to 1·5)	1·7 (1·6 to 1·7)	1·6 (1·5 to 1·8)
Essex	8 (7·8 to 8·3)	9·2 (8·9 to 9·4)	9·1 (8·7 to 9·4)	8·2 (8 to 8·4)	8·4 (8·2 to 8·6)	7·4 (7·3 to 7·5)	8·5 (8·3 to 8·6)	8·4 (7·6 to 9·3)
Hertfordshire	7·2 (6·9 to 7·4)	7·9 (7·6 to 8·2)	7·2 (6·9 to 7·5)	6·4 (6·3 to 6·5)	7 (6·9 to 7·1)	6·5 (6·4 to 6·6)	7·6 (7·5 to 7·7)	7·5 (6·7 to 8·4)
Luton	1·3 (1·3 to 1·3)	1·7 (1·6 to 1·7)	1·6 (1·6 to 1·7)	1·5 (1·5 to 1·5)	1·7 (1·7 to 1·7)	1·5 (1·5 to 1·5)	1·8 (1·8 to 1·9)	1·8 (1·6 to 2)
Norfolk	4·6 (4·4 to 4·7)	4·7 (4·5 to 4·9)	4·8 (4·5 to 5)	4·4 (4·2 to 4·5)	4·6 (4·5 to 4·7)	4 (3·9 to 4·1)	4·8 (4·7 to 4·8)	4·7 (4·2 to 5·1)
Peterborough	0·9 (0·9 to 0·9)	1 (1 to 1)	1 (1 to 1)	1·1 (1·1 to 1·1)	1·3 (1·2 to 1·3)	1·1 (1·1 to 1·1)	1·6 (1·6 to 1·7)	1·6 (1·5 to 1·8)
Southend-on-Sea	1·3 (1·3 to 1·4)	1·2 (1·2 to 1·3)	1·1 (1·1 to 1·2)	1 (1 to 1)	1·2 (1·1 to 1·2)	1 (1 to 1)	1·2 (1·1 to 1·2)	1·1 (1 to 1·3)
Suffolk	4·1 (4 to 4·2)	4·4 (4·1 to 4·6)	4·5 (4·3 to 4·7)	4·1 (4 to 4·2)	4·4 (4·3 to 4·5)	3·7 (3·6 to 3·7)	4·2 (4·1 to 4·3)	4·1 (3·7 to 4·5)
Thurrock	1 (1 to 1)	1·1 (1·1 to 1·2)	1·2 (1·1 to 1·2)	1 (1 to 1)	1·1 (1 to 1·1)	1 (1 to 1)	1·3 (1·2 to 1·3)	1·2 (1·1 to 1·3)
Greater London	51·1 (49·1 to 53)	57·5 (55·6 to 59·3)	55·6 (53·5 to 57·7)	45·9 (44·8 to 47·1)	52·8 (51·9 to 53·6)	52·6 (52 to 53)	69·1 (68·5 to 69·6)	69·7 (62·2 to 78)
Barking and Dagenham	1·4 (1·4 to 1·5)	1·4 (1·3 to 1·4)	1·3 (1·2 to 1·3)	1·1 (1 to 1·2)	1·3 (1·2 to 1·3)	1·2 (1·2 to 1·3)	2 (1·9 to 2)	2 (1·8 to 2·2)
Barnet	2·1 (2 to 2·2)	2·1 (2·1 to 2·2)	2·1 (2 to 2·3)	1·9 (1·8 to 1·9)	2·1 (2 to 2·1)	2·1 (2 to 2·1)	2·8 (2·8 to 2·9)	2·8 (2·5 to 3·2)
Bexley	1·5 (1·5 to 1·6)	1·8 (1·7 to 1·8)	1·7 (1·6 to 1·7)	1·4 (1·3 to 1·4)	1·6 (1·5 to 1·6)	1·3 (1·3 to 1·4)	1·6 (1·6 to 1·6)	1·7 (1·5 to 1·9)
Brent	1·9 (1·9 to 2)	2·4 (2·3 to 2·5)	2·3 (2·2 to 2·5)	1·9 (1·8 to 1·9)	2 (1·9 to 2·1)	2 (1·9 to 2)	2·8 (2·7 to 2·8)	2·8 (2·5 to 3·1)
Bromley	2·1 (2 to 2·2)	2·2 (2·1 to 2·3)	2 (1·9 to 2·1)	1·7 (1·6 to 1·7)	1·9 (1·8 to 1·9)	1·8 (1·7 to 1·8)	2·1 (2·1 to 2·2)	2·2 (1·9 to 2·4)
Camden	1 (0·9 to 1)	1·3 (1·2 to 1·4)	1·4 (1·3 to 1·5)	1·1 (1 to 1·1)	1·2 (1·2 to 1·2)	1·4 (1·3 to 1·4)	1·6 (1·5 to 1·6)	1·5 (1·3 to 1·7)
Croydon	2·5 (2·4 to 2·6)	2·8 (2·7 to 2·9)	2·7 (2·5 to 2·8)	2·2 (2·1 to 2·3)	2·5 (2·4 to 2·5)	2·3 (2·2 to 2·3)	2·9 (2·9 to 3)	3·1 (2·7 to 3·4)
Ealing	2·2 (2·1 to 2·3)	2·4 (2·3 to 2·6)	2·4 (2·3 to 2·5)	2·1 (2·1 to 2·2)	2·3 (2·2 to 2·3)	2·2 (2·2 to 2·2)	3 (3 to 3)	2·8 (2·5 to 3·1)
Enfield	2 (1·9 to 2)	2 (1·9 to 2·1)	2 (1·9 to 2)	1·6 (1·6 to 1·7)	2 (1·9 to 2)	1·9 (1·9 to 2)	2·6 (2·6 to 2·7)	2·7 (2·4 to 3)
Greenwich	1·7 (1·6 to 1·7)	1·7 (1·7 to 1·8)	1·7 (1·6 to 1·7)	1·5 (1·5 to 1·6)	1·7 (1·7 to 1·8)	1·6 (1·6 to 1·7)	2·4 (2·3 to 2·4)	2·5 (2·2 to 2·7)
Hackney	1·8 (1·7 to 1·9)	2·1 (2·1 to 2·2)	2 (2 to 2·1)	1·6 (1·6 to 1·7)	1·9 (1·8 to 2)	2 (1·9 to 2·1)	2·4 (2·4 to 2·5)	2·5 (2·2 to 2·8)
Hammersmith and Fulham	0·9 (0·9 to 1)	1·1 (1·1 to 1·2)	1·2 (1·1 to 1·3)	1 (0·9 to 1)	1·1 (1·1 to 1·1)	1·2 (1·2 to 1·2)	1·4 (1·4 to 1·5)	1·3 (1·2 to 1·5)
Haringey	1·5 (1·4 to 1·5)	1·9 (1·8 to 2·1)	1·9 (1·8 to 2·1)	1·5 (1·4 to 1·5)	1·8 (1·7 to 1·8)	1·8 (1·8 to 1·9)	2·3 (2·2 to 2·3)	2·1 (1·9 to 2·4)
Harrow	1·5 (1·4 to 1·5)	1·5 (1·5 to 1·6)	1·5 (1·4 to 1·5)	1·3 (1·2 to 1·3)	1·4 (1·4 to 1·5)	1·3 (1·3 to 1·3)	1·8 (1·8 to 1·9)	2 (1·8 to 2·2)
Havering	1·8 (1·7 to 1·9)	2 (1·8 to 2·1)	1·8 (1·6 to 1·9)	1·4 (1·3 to 1·5)	1·4 (1·4 to 1·5)	1·2 (1·2 to 1·2)	1·5 (1·5 to 1·5)	1·8 (1·6 to 2)
Hillingdon	1·7 (1·6 to 1·8)	1·9 (1·8 to 2)	1·7 (1·7 to 1·8)	1·5 (1·5 to 1·6)	1·8 (1·7 to 1·8)	1·7 (1·6 to 1·7)	2·2 (2·2 to 2·3)	2·4 (2·2 to 2·7)
Hounslow	1·4 (1·4 to 1·5)	1·6 (1·5 to 1·7)	1·6 (1·5 to 1·7)	1·4 (1·4 to 1·5)	1·6 (1·6 to 1·6)	1·6 (1·6 to 1·6)	2·3 (2·3 to 2·4)	2·3 (2·1 to 2·6)
Islington	1·2 (1·1 to 1·2)	1·5 (1·4 to 1·6)	1·4 (1·4 to 1·5)	1·1 (1·1 to 1·2)	1·4 (1·3 to 1·4)	1·3 (1·3 to 1·3)	1·5 (1·5 to 1·6)	1·7 (1·5 to 1·9)

Kensington and Chelsea	0·8 (0·7 to 0·8)	1·2 (1·1 to 1·3)	1·6 (1·5 to 1·7)	0·9 (0·9 to 1)	0·9 (0·9 to 1)	1 (1 to 1·1)	1·1 (1·1 to 1·1)	1 (0·8 to 1·1)
Kingston upon Thames	0·8 (0·8 to 0·9)	0·9 (0·8 to 0·9)	0·9 (0·8 to 0·9)	0·8 (0·8 to 0·8)	0·9 (0·9 to 0·9)	0·9 (0·9 to 0·9)	1·2 (1·2 to 1·2)	1·2 (1·1 to 1·4)
Lambeth	2 (1·9 to 2·1)	2·5 (2·4 to 2·6)	2·3 (2·2 to 2·4)	1·8 (1·8 to 1·9)	2·2 (2·1 to 2·2)	2·2 (2·1 to 2·2)	2·5 (2·5 to 2·6)	2·5 (2·2 to 2·8)
Lewisham	1·9 (1·8 to 2)	2·2 (2·1 to 2·3)	2 (1·9 to 2·1)	1·6 (1·5 to 1·6)	2 (1·9 to 2)	1·9 (1·9 to 2)	2·6 (2·5 to 2·7)	2·5 (2·2 to 2·8)
Merton	1·3 (1·2 to 1·3)	1·3 (1·3 to 1·4)	1·2 (1·2 to 1·3)	1·1 (1 to 1·1)	1·3 (1·3 to 1·3)	1·3 (1·3 to 1·4)	1·8 (1·8 to 1·9)	1·7 (1·5 to 1·9)
Newham	2 (1·9 to 2)	2·2 (2·1 to 2·3)	2·2 (2·1 to 2·3)	1·9 (1·8 to 1·9)	2·2 (2·2 to 2·3)	2·4 (2·3 to 2·4)	3·3 (3·2 to 3·3)	3·4 (3 to 3·7)
Redbridge	1·6 (1·6 to 1·7)	1·7 (1·6 to 1·8)	1·6 (1·6 to 1·7)	1·3 (1·3 to 1·4)	1·6 (1·5 to 1·6)	1·6 (1·5 to 1·6)	2·4 (2·3 to 2·4)	2·6 (2·3 to 2·8)
Richmond upon Thames	1 (1 to 1·1)	1·1 (1·1 to 1·2)	1·1 (1·1 to 1·2)	0·9 (0·9 to 1)	1·1 (1·1 to 1·1)	1·2 (1·2 to 1·2)	1·5 (1·5 to 1·5)	1·3 (1·2 to 1·5)
Southwark	2 (1·9 to 2)	2·2 (2·1 to 2·3)	1·9 (1·8 to 2)	1·5 (1·5 to 1·6)	2 (2 to 2·1)	2·1 (2 to 2·1)	2·6 (2·5 to 2·7)	2·6 (2·3 to 2·9)
Sutton	1·2 (1·2 to 1·3)	1·3 (1·2 to 1·3)	1·2 (1·2 to 1·3)	1·1 (1 to 1·1)	1·2 (1·2 to 1·3)	1·1 (1·1 to 1·1)	1·4 (1·4 to 1·4)	1·4 (1·3 to 1·6)
Tower Hamlets	1·6 (1·5 to 1·6)	1·7 (1·6 to 1·8)	1·4 (1·4 to 1·5)	1·3 (1·3 to 1·4)	1·6 (1·6 to 1·7)	1·8 (1·7 to 1·8)	2·4 (2·3 to 2·5)	2·7 (2·4 to 3)
Waltham Forest	1·8 (1·7 to 1·8)	1·9 (1·8 to 1·9)	1·9 (1·9 to 2)	1·6 (1·5 to 1·6)	1·9 (1·8 to 2)	1·8 (1·7 to 1·8)	2·5 (2·4 to 2·5)	2·6 (2·3 to 2·8)
Wandsworth	1·8 (1·7 to 1·9)	2 (1·8 to 2·2)	2 (1·8 to 2·1)	1·7 (1·7 to 1·8)	2 (1·9 to 2·1)	2 (2 to 2·1)	2·8 (2·7 to 2·8)	2·8 (2·4 to 3·1)
Westminster	1·2 (1·1 to 1·2)	1·5 (1·4 to 1·6)	1·6 (1·5 to 1·7)	1·1 (1 to 1·1)	1·2 (1·2 to 1·2)	1·3 (1·2 to 1·3)	1·6 (1·5 to 1·6)	1·4 (1·2 to 1·6)
North East England	22·8 (22·2 to 23·5)	25·3 (24·7 to 26)	24·2 (23·6 to 24·9)	20·1 (19·8 to 20·4)	18·3 (18·1 to 18·6)	13·5 (13·4 to 13·6)	15·3 (15·1 to 15·4)	14·4 (13·1 to 15·8)
County Durham	4·2 (4·1 to 4·4)	4·6 (4·5 to 4·8)	4·5 (4·3 to 4·6)	3·7 (3·7 to 3·8)	3·4 (3·3 to 3·4)	2·5 (2·5 to 2·6)	2·9 (2·8 to 2·9)	2·7 (2·5 to 3)
Darlington	0·9 (0·8 to 0·9)	0·9 (0·9 to 0·9)	0·9 (0·9 to 0·9)	0·7 (0·7 to 0·7)	0·7 (0·7 to 0·8)	0·6 (0·6 to 0·6)	0·7 (0·7 to 0·7)	0·6 (0·6 to 0·7)
Gateshead	1·9 (1·9 to 2)	2·2 (2·1 to 2·3)	2·1 (2 to 2·1)	1·5 (1·5 to 1·6)	1·4 (1·4 to 1·4)	1 (1 to 1)	1·2 (1·2 to 1·2)	1·1 (1 to 1·3)
Hartlepool	0·9 (0·8 to 0·9)	1 (1 to 1·1)	1 (0·9 to 1)	0·8 (0·8 to 0·8)	0·7 (0·7 to 0·7)	0·5 (0·5 to 0·5)	0·6 (0·6 to 0·6)	0·5 (0·5 to 0·6)
Middlesbrough	1·4 (1·4 to 1·5)	1·7 (1·6 to 1·7)	1·6 (1·5 to 1·6)	1·4 (1·4 to 1·4)	1·2 (1·2 to 1·2)	0·9 (0·9 to 0·9)	1 (1 to 1·1)	1 (0·9 to 1)
Newcastle upon Tyne	2·5 (2·4 to 2·5)	2·6 (2·5 to 2·7)	2·4 (2·3 to 2·5)	2 (1·9 to 2)	2 (1·9 to 2)	1·5 (1·4 to 1·5)	1·7 (1·7 to 1·8)	1·8 (1·6 to 2)
North Tyneside	1·8 (1·8 to 1·9)	1·9 (1·9 to 2)	1·7 (1·7 to 1·8)	1·4 (1·3 to 1·4)	1·3 (1·3 to 1·3)	1 (1 to 1)	1·2 (1·2 to 1·2)	1·1 (1 to 1·3)
Northumberland	2·4 (2·4 to 2·5)	2·5 (2·4 to 2·6)	2·5 (2·4 to 2·6)	2·2 (2·1 to 2·2)	1·9 (1·9 to 2)	1·5 (1·4 to 1·5)	1·5 (1·5 to 1·5)	1·4 (1·3 to 1·6)
Redcar and Cleveland	1·2 (1·2 to 1·3)	1·5 (1·4 to 1·5)	1·5 (1·5 to 1·5)	1·2 (1·2 to 1·2)	1·1 (1 to 1·1)	0·7 (0·7 to 0·7)	0·8 (0·8 to 0·8)	0·7 (0·7 to 0·8)
South Tyneside	1·6 (1·5 to 1·6)	1·7 (1·7 to 1·8)	1·6 (1·5 to 1·6)	1·1 (1·1 to 1·2)	1·1 (1·1 to 1·1)	0·8 (0·8 to 0·8)	0·9 (0·8 to 0·9)	0·8 (0·7 to 0·9)
Stockton-on-Tees	1·4 (1·4 to 1·4)	1·7 (1·6 to 1·8)	1·6 (1·6 to 1·7)	1·5 (1·5 to 1·6)	1·4 (1·3 to 1·4)	1 (1 to 1)	1·2 (1·2 to 1·2)	1·1 (1 to 1·3)
Sunderland	2·6 (2·5 to 2·6)	2·9 (2·8 to 3)	2·9 (2·8 to 3)	2·5 (2·5 to 2·6)	2·2 (2·1 to 2·3)	1·5 (1·5 to 1·5)	1·6 (1·6 to 1·6)	1·4 (1·3 to 1·6)
North West England	56·1 (54·5 to 57·7)	60·7 (59·1 to 62·3)	57·9 (56·3 to 59·6)	47·9 (47·1 to 48·7)	48·5 (47·7 to 49·1)	39·1 (38·8 to 39·5)	45·8 (45·6 to 46·1)	43·9 (39·8 to 48·4)
Blackburn with Darwen	1·3 (1·3 to 1·3)	1·3 (1·3 to 1·4)	1·3 (1·3 to 1·4)	1·2 (1·2 to 1·3)	1·2 (1·2 to 1·2)	1·1 (1 to 1·1)	1·2 (1·2 to 1·2)	1·1 (1 to 1·2)
Blackpool	1·2 (1·2 to 1·2)	1·1 (1 to 1·1)	1 (0·9 to 1)	0·8 (0·8 to 0·8)	1 (0·9 to 1)	0·8 (0·7 to 0·8)	0·9 (0·9 to 0·9)	0·9 (0·8 to 0·9)
Bolton	2·2 (2·1 to 2·2)	2·3 (2·2 to 2·4)	2·3 (2·3 to 2·4)	2 (2 to 2)	1·9 (1·9 to 2)	1·6 (1·6 to 1·7)	2 (2 to 2·1)	1·9 (1·7 to 2·1)
Bury	1·3 (1·3 to 1·4)	1·5 (1·5 to 1·6)	1·6 (1·5 to 1·6)	1·2 (1·2 to 1·3)	1·3 (1·3 to 1·3)	1·1 (1·1 to 1·1)	1·3 (1·3 to 1·3)	1·2 (1·1 to 1·3)
Cheshire East	2·3 (2·3 to 2·4)	2·5 (2·4 to 2·6)	2·4 (2·3 to 2·5)	2 (1·9 to 2)	2·1 (2 to 2·1)	1·8 (1·8 to 1·9)	2 (2 to 2·1)	2 (1·8 to 2·2)
Cheshire West and Chester	2·3 (2·2 to 2·3)	2·6 (2·5 to 2·7)	2·5 (2·4 to 2·5)	2·1 (2 to 2·1)	2·1 (2 to 2·1)	1·8 (1·7 to 1·8)	1·9 (1·9 to 2)	1·8 (1·7 to 2)
Cumbria	3·7 (3·6 to 3·8)	3·8 (3·7 to 3·9)	3·6 (3·5 to 3·7)	3 (2·9 to 3)	3 (3 to 3·1)	2·4 (2·4 to 2·5)	2·6 (2·6 to 2·7)	2·4 (2·2 to 2·6)
Halton	0·8 (0·8 to 0·8)	0·8 (0·8 to 0·9)	0·9 (0·9 to 0·9)	1 (1 to 1)	1 (1 to 1)	0·7 (0·7 to 0·7)	0·9 (0·8 to 0·9)	0·8 (0·7 to 0·8)
Knowsley	1·5 (1·5 to 1·6)	2 (1·9 to 2·1)	1·7 (1·6 to 1·8)	1·5 (1·4 to 1·6)	1·3 (1·3 to 1·3)	0·9 (0·9 to 1)	1 (1 to 1)	1 (0·9 to 1·1)
Lancashire	8·3 (8·1 to 8·6)	8·6 (8·2 to 8·9)	8·4 (8·1 to 8·6)	7·3 (7·2 to 7·5)	7·5 (7·3 to 7·7)	6·2 (6·1 to 6·4)	7·1 (7 to 7·3)	6·7 (6·1 to 7·4)
Liverpool	4·7 (4·6 to 4·8)	5·1 (4·9 to 5·3)	4·6 (4·4 to 4·8)	3·6 (3·5 to 3·7)	3·5 (3·4 to 3·6)	2·6 (2·5 to 2·7)	3 (2·9 to 3)	3·1 (2·8 to 3·4)
Manchester	4·2 (4·1 to 4·3)	4·6 (4·4 to 4·8)	4·2 (4 to 4·3)	3·3 (3·2 to 3·5)	3·6 (3·5 to 3·7)	2·9 (2·8 to 2·9)	4·1 (4 to 4·2)	4·4 (4 to 4·9)
Oldham	1·9 (1·9 to 1·9)	2·1 (2 to 2·2)	2·1 (2 to 2·2)	1·7 (1·7 to 1·8)	1·7 (1·7 to 1·8)	1·5 (1·5 to 1·6)	1·7 (1·7 to 1·8)	1·7 (1·5 to 1·8)
Rochdale	1·8 (1·7 to 1·8)	1·8 (1·8 to 1·9)	1·8 (1·8 to 1·9)	1·7 (1·6 to 1·7)	1·6 (1·6 to 1·7)	1·3 (1·3 to 1·4)	1·6 (1·5 to 1·6)	1·5 (1·4 to 1·6)
Salford	2·2 (2·2 to 2·3)	2·5 (2·4 to 2·6)	2·3 (2·3 to 2·4)	1·7 (1·7 to 1·7)	1·8 (1·7 to 1·8)	1·3 (1·3 to 1·4)	1·8 (1·8 to 1·9)	1·9 (1·7 to 2·1)
Sefton	2·4 (2·3 to 2·5)	2·6 (2·6 to 2·7)	2·3 (2·2 to 2·4)	1·8 (1·8 to 1·9)	1·8 (1·8 to 1·9)	1·4 (1·3 to 1·4)	1·5 (1·4 to 1·5)	1·4 (1·3 to 1·5)
St Helens	1·5 (1·4 to 1·5)	1·6 (1·6 to 1·7)	1·6 (1·6 to 1·7)	1·3 (1·3 to 1·3)	1·2 (1·2 to 1·2)	1 (0·9 to 1)	1·1 (1·1 to 1·1)	1 (0·9 to 1·1)
Stockport	2·3 (2·2 to 2·4)	2·7 (2·6 to 2·7)	2·5 (2·4 to 2·5)	2 (1·9 to 2)	1·9 (1·8 to 1·9)	1·6 (1·5 to 1·6)	1·8 (1·7 to 1·8)	1·7 (1·5 to 1·9)
Tameside	1·8 (1·7 to 1·8)	1·9 (1·8 to 2)	1·9 (1·8 to 2)	1·5 (1·5 to 1·6)	1·7 (1·6 to 1·7)	1·3 (1·2 to 1·3)	1·6 (1·5 to 1·6)	1·5 (1·3 to 1·6)
Trafford	1·8 (1·8 to 1·9)	2 (2 to 2·1)	1·8 (1·8 to 1·9)	1·5 (1·4 to 1·5)	1·5 (1·4 to 1·5)	1·2 (1·2 to 1·3)	1·5 (1·5 to 1·5)	1·4 (1·2 to 1·6)
Warrington	1·3 (1·2 to 1·3)	1·5 (1·4 to 1·5)	1·4 (1·4 to 1·5)	1·2 (1·1 to 1·2)	1·3 (1·3 to 1·4)	1·1 (1·1 to 1·2)	1·3 (1·2 to 1·3)	1·2 (1·1 to 1·3)
Wigan	2·3 (2·2 to 2·4)	2·6 (2·5 to 2·7)	2·8 (2·7 to 2·9)	2·2 (2·2 to 2·2)	2·2 (2·1 to 2·2)	1·7 (1·7 to 1·8)	2 (2 to 2)	1·8 (1·7 to 2)
Wirral	2·9 (2·8 to 3)	3·1 (3·1 to 3·2)	2·9 (2·8 to 3)	2·3 (2·3 to 2·4)	2·3 (2·3 to 2·4)	1·7 (1·7 to 1·8)	1·9 (1·9 to 2)	1·8 (1·6 to 2)
South East England	47 (45·4 to 48·6)	50·9 (49·4 to 52·3)	48·4 (46·9 to 50)	45·2 (44·3 to 46·1)	50·4 (49·6 to 51·1)	46·3 (45·9 to 46·7)	54·7 (54·3 to 55)	52·8 (47·5 to 58·7)
Bracknell Forest	0·4 (0·4 to 0·5)	0·6 (0·5 to 0·6)	0·5 (0·5 to 0·6)	0·6 (0·6 to 0·7)	0·8 (0·7 to 0·8)	0·7 (0·7 to 0·8)	0·8 (0·8 to 0·8)	0·7 (0·7 to 0·8)
Brighton and Hove	1·5 (1·4 to 1·6)	1·4 (1·3 to 1·5)	1·3 (1·3 to 1·4)	1·2 (1·2 to 1·3)	1·5 (1·5 to 1·5)	1·4 (1·4 to 1·5)	1·7 (1·6 to 1·7)	1·6 (1·4 to 1·8)
Buckinghamshire	2·9 (2·8 to 3)	3·4 (3·2 to 3·5)	3 (2·9 to 3·2)	2·8 (2·7 to 2·8)	3 (2·9 to 3·1)	2·8 (2·7 to 2·9)	3·2 (3·1 to 3·2)	3 (2·7 to 3·4)

East Sussex	2·8 (2·7 to 2·9)	2·6 (2·5 to 2·7)	2·5 (2·4 to 2·6)	2·2 (2·2 to 2·3)	2·7 (2·6 to 2·7)	2·4 (2·4 to 2·4)	2·7 (2·7 to 2·8)	2·7 (2·4 to 3)
Hampshire	6·7 (6·5 to 6·9)	7·9 (7·6 to 8·1)	7·8 (7·5 to 8)	7·1 (6·9 to 7·3)	7·8 (7·6 to 7·9)	6·8 (6·7 to 7)	7·7 (7·6 to 7·9)	7·4 (6·7 to 8·1)
Isle of Wight	0·8 (0·8 to 0·9)	0·7 (0·7 to 0·8)	0·7 (0·7 to 0·7)	0·6 (0·6 to 0·7)	0·7 (0·7 to 0·7)	0·6 (0·6 to 0·6)	0·7 (0·7 to 0·7)	0·7 (0·6 to 0·7)
Kent	8·6 (8·3 to 8·8)	9·1 (8·8 to 9·4)	8·7 (8·4 to 9)	7·6 (7·4 to 7·8)	8·5 (8·3 to 8·6)	7·6 (7·5 to 7·8)	9 (8·8 to 9·3)	9·3 (8·4 to 10·2)
Medway	1·6 (1·6 to 1·7)	2 (2 to 2·1)	2·2 (2·1 to 2·3)	1·9 (1·9 to 2)	1·9 (1·9 to 2)	1·6 (1·6 to 1·6)	1·8 (1·8 to 1·9)	1·9 (1·7 to 2·1)
Milton Keynes	0·5 (0·5 to 0·5)	0·6 (0·5 to 0·6)	0·7 (0·6 to 0·7)	1·2 (1·1 to 1·2)	1·5 (1·5 to 1·5)	1·5 (1·4 to 1·5)	2 (2 to 2·1)	1·9 (1·7 to 2·1)
Oxfordshire	3 (2·9 to 3·1)	3·7 (3·4 to 3·9)	3·6 (3·4 to 3·8)	3·5 (3·4 to 3·6)	3·9 (3·8 to 4)	3·7 (3·6 to 3·8)	4·3 (4·2 to 4·4)	4 (3·6 to 4·6)
Portsmouth	1·4 (1·4 to 1·5)	1·5 (1·4 to 1·5)	1·4 (1·3 to 1·4)	1·2 (1·2 to 1·3)	1·4 (1·3 to 1·4)	1·1 (1·1 to 1·2)	1·4 (1·4 to 1·4)	1·4 (1·3 to 1·6)
Reading	0·9 (0·9 to 0·9)	1·1 (1 to 1·1)	1·1 (1 to 1·1)	1 (1 to 1)	1 (1 to 1·1)	1 (1 to 1)	1·4 (1·3 to 1·4)	1·3 (1·2 to 1·4)
Slough	0·9 (0·9 to 0·9)	1 (0·9 to 1)	0·9 (0·9 to 0·9)	0·9 (0·9 to 0·9)	1 (0·9 to 1)	0·9 (0·9 to 1)	1·4 (1·4 to 1·4)	1·3 (1·2 to 1·5)
Southampton	1·5 (1·4 to 1·5)	1·6 (1·5 to 1·7)	1·5 (1·5 to 1·6)	1·4 (1·3 to 1·5)	1·5 (1·4 to 1·5)	1·3 (1·2 to 1·3)	1·7 (1·7 to 1·8)	1·7 (1·5 to 1·9)
Surrey	6·9 (6·6 to 7·1)	7·1 (6·8 to 7·4)	6·2 (6 to 6·5)	5·6 (5·5 to 5·8)	6·3 (6·1 to 6·5)	6·2 (6 to 6·4)	7·2 (7 to 7·5)	6·9 (6·1 to 7·7)
West Berkshire	0·8 (0·7 to 0·8)	0·9 (0·8 to 0·9)	0·9 (0·8 to 0·9)	0·9 (0·8 to 0·9)	1 (0·9 to 1)	0·9 (0·9 to 0·9)	1 (1 to 1)	0·9 (0·8 to 1)
West Sussex	4·3 (4·2 to 4·4)	4·2 (4 to 4·4)	3·8 (3·6 to 4)	3·8 (3·7 to 3·9)	4·2 (4·1 to 4·3)	4 (3·9 to 4·1)	4·7 (4·6 to 4·8)	4·6 (4·1 to 5·1)
Windsor and Maidenhead	0·9 (0·8 to 0·9)	1 (0·9 to 1)	0·9 (0·8 to 0·9)	0·8 (0·8 to 0·8)	0·8 (0·8 to 0·9)	0·8 (0·8 to 0·9)	0·9 (0·9 to 1)	0·9 (0·8 to 1)
Wokingham	0·6 (0·6 to 0·6)	0·8 (0·8 to 0·9)	0·8 (0·7 to 0·8)	0·7 (0·7 to 0·8)	1 (0·9 to 1)	0·9 (0·8 to 0·9)	1 (1 to 1)	0·8 (0·7 to 1)
South West England	30·3 (29·3 to 31·2)	30·9 (30 to 31·7)	29·9 (29 to 30·8)	27·2 (26·7 to 27·8)	29·8 (29·4 to 30·3)	25·9 (25·7 to 26·2)	30·4 (30·1 to 30·6)	30·3 (27·4 to 33·5)
Bath and North East Somerset	1 (1 to 1)	1 (1 to 1)	0·9 (0·9 to 0·9)	0·9 (0·9 to 0·9)	1 (0·9 to 1)	0·9 (0·8 to 0·9)	0·9 (0·9 to 0·9)	0·9 (0·8 to 1·1)
Bournemouth	1 (1 to 1)	0·9 (0·8 to 0·9)	0·8 (0·7 to 0·8)	0·7 (0·7 to 0·7)	0·9 (0·8 to 0·9)	0·8 (0·8 to 0·8)	1·1 (1·1 to 1·2)	1·2 (1·1 to 1·4)
Bristol, City of	3 (2·9 to 3·1)	3·2 (3 to 3·3)	3 (2·9 to 3·1)	2·7 (2·6 to 2·7)	2·9 (2·8 to 2·9)	2·5 (2·4 to 2·5)	3·3 (3·2 to 3·4)	3·4 (3 to 3·8)
Cornwall	3·1 (3 to 3·2)	2·9 (2·8 to 3)	2·9 (2·8 to 3)	2·6 (2·6 to 2·7)	2·8 (2·7 to 2·9)	2·4 (2·3 to 2·4)	2·8 (2·8 to 2·9)	2·8 (2·6 to 3·1)
Devon	3·9 (3·8 to 4·1)	3·7 (3·6 to 3·8)	3·5 (3·4 to 3·7)	3·3 (3·2 to 3·4)	3·8 (3·7 to 3·9)	3·3 (3·2 to 3·4)	3·7 (3·7 to 3·8)	3·7 (3·4 to 4·1)
Dorset	2·2 (2·1 to 2·3)	2·1 (2 to 2·2)	2 (2 to 2·1)	1·9 (1·8 to 1·9)	2 (2 to 2·1)	1·7 (1·7 to 1·8)	1·9 (1·8 to 1·9)	1·8 (1·7 to 2)
Gloucestershire	3·5 (3·4 to 3·6)	3·7 (3·5 to 3·9)	3·6 (3·4 to 3·8)	3·2 (3·2 to 3·3)	3·5 (3·5 to 3·6)	3·1 (3 to 3·2)	3·4 (3·4 to 3·5)	3·4 (3·1 to 3·8)
North Somerset	1·1 (1 to 1·1)	1·1 (1 to 1·1)	1·1 (1 to 1·1)	1 (1 to 1)	1 (1 to 1·1)	1 (0·9 to 1)	1·2 (1·2 to 1·2)	1·1 (1 to 1·3)
Plymouth	1·8 (1·8 to 1·9)	1·9 (1·9 to 2)	1·9 (1·8 to 2)	1·8 (1·8 to 1·9)	1·8 (1·7 to 1·8)	1·4 (1·3 to 1·4)	1·7 (1·6 to 1·7)	1·6 (1·5 to 1·8)
Poole	0·8 (0·8 to 0·9)	0·8 (0·8 to 0·9)	0·8 (0·8 to 0·8)	0·7 (0·7 to 0·7)	0·9 (0·8 to 0·9)	0·7 (0·7 to 0·7)	0·9 (0·8 to 0·9)	0·8 (0·7 to 0·9)
Somerset	3 (2·9 to 3·1)	3 (2·8 to 3·1)	3 (2·9 to 3·1)	2·7 (2·6 to 2·8)	2·9 (2·9 to 3)	2·5 (2·5 to 2·6)	2·9 (2·8 to 3)	2·9 (2·6 to 3·2)
South Gloucestershire	1·3 (1·2 to 1·3)	1·6 (1·5 to 1·7)	1·5 (1·4 to 1·6)	1·4 (1·3 to 1·4)	1·6 (1·5 to 1·6)	1·5 (1·5 to 1·5)	1·6 (1·6 to 1·6)	1·6 (1·5 to 1·8)
Swindon	1·1 (1·1 to 1·1)	1·3 (1·2 to 1·3)	1·2 (1·2 to 1·3)	1·2 (1·1 to 1·2)	1·4 (1·3 to 1·4)	1·2 (1·1 to 1·2)	1·5 (1·5 to 1·5)	1·5 (1·4 to 1·7)
Torbay	0·8 (0·8 to 0·8)	0·7 (0·7 to 0·8)	0·7 (0·6 to 0·7)	0·6 (0·6 to 0·6)	0·7 (0·7 to 0·7)	0·6 (0·6 to 0·6)	0·7 (0·7 to 0·8)	0·7 (0·6 to 0·8)
Wiltshire	2·6 (2·5 to 2·7)	3 (2·9 to 3·1)	3·1 (3 to 3·2)	2·6 (2·5 to 2·7)	2·7 (2·7 to 2·8)	2·5 (2·4 to 2·5)	2·7 (2·7 to 2·8)	2·6 (2·4 to 2·9)
West Midlands	42·5 (41·4 to 43·7)	47·7 (46·4 to 48·9)	47·4 (46·1 to 48·6)	39 (38·4 to 39·5)	39·2 (38·6 to 39·7)	31·4 (31·1 to 31·6)	37·1 (36·9 to 37·3)	36 (32·7 to 39·7)
Birmingham	10 (9·7 to 10·2)	10·7 (10·3 to 11·1)	10·3 (9·9 to 10·7)	8·7 (8·6 to 8·8)	8·9 (8·7 to 9·1)	7·3 (7·2 to 7·4)	8·9 (8·8 to 9·1)	8·9 (8 to 9·8)
Coventry	2·8 (2·7 to 2·9)	3·2 (3·1 to 3·3)	3·2 (3 to 3·3)	2·5 (2·5 to 2·6)	2·4 (2·4 to 2·5)	1·9 (1·8 to 1·9)	2·4 (2·3 to 2·4)	2·3 (2·1 to 2·6)
Dudley	2·4 (2·3 to 2·5)	2·8 (2·7 to 2·9)	2·8 (2·7 to 2·9)	2·2 (2·1 to 2·2)	2·2 (2·1 to 2·2)	1·7 (1·7 to 1·8)	1·9 (1·9 to 2)	1·9 (1·7 to 2·1)
Herefordshire, County of	1·1 (1·1 to 1·2)	1·2 (1·1 to 1·2)	1·1 (1·1 to 1·2)	1 (1 to 1)	1·1 (1 to 1·1)	0·8 (0·8 to 0·9)	0·9 (0·9 to 1)	0·9 (0·8 to 1)
Sandwell	3 (3 to 3·1)	3·2 (3·1 to 3·4)	3·1 (3 to 3·2)	2·4 (2·4 to 2·5)	2·4 (2·3 to 2·4)	1·9 (1·9 to 1·9)	2·5 (2·5 to 2·5)	2·4 (2·2 to 2·7)
Shropshire	1·8 (1·8 to 1·9)	2 (2 to 2·1)	2 (2 to 2·1)	1·6 (1·6 to 1·6)	1·7 (1·7 to 1·8)	1·4 (1·4 to 1·4)	1·5 (1·5 to 1·5)	1·5 (1·3 to 1·6)
Solihull	1·5 (1·5 to 1·5)	1·8 (1·7 to 1·9)	1·8 (1·8 to 1·9)	1·3 (1·3 to 1·3)	1·3 (1·3 to 1·4)	1 (1 to 1)	1·1 (1·1 to 1·2)	1·2 (1·1 to 1·3)
Staffordshire	5·4 (5·2 to 5·5)	6·4 (6·1 to 6·7)	6·6 (6·4 to 6·9)	5·6 (5·5 to 5·7)	5·4 (5·3 to 5·5)	4·1 (4·1 to 4·2)	4·6 (4·5 to 4·7)	4·4 (4 to 4·8)
Stoke-on-Trent	2·4 (2·3 to 2·4)	2·4 (2·4 to 2·5)	2·4 (2·3 to 2·5)	2 (1·9 to 2)	1·9 (1·9 to 2)	1·5 (1·4 to 1·5)	1·9 (1·9 to 2)	1·7 (1·5 to 1·9)
Telford and Wrekin	0·8 (0·7 to 0·8)	0·9 (0·8 to 0·9)	1 (1 to 1)	1·1 (1 to 1·1)	1·1 (1·1 to 1·2)	1 (1 to 1)	1·1 (1·1 to 1·2)	1·1 (1 to 1·2)
Walsall	2·3 (2·3 to 2·4)	2·8 (2·7 to 3)	2·7 (2·6 to 2·8)	2 (2 to 2·1)	2 (2 to 2·1)	1·6 (1·6 to 1·7)	1·9 (1·9 to 1·9)	1·9 (1·8 to 2·1)
Warwickshire	3·5 (3·4 to 3·6)	4 (3·9 to 4·2)	4 (3·9 to 4·2)	3·3 (3·2 to 3·3)	3·2 (3·2 to 3·3)	2·7 (2·7 to 2·8)	3·2 (3·1 to 3·2)	3·1 (2·8 to 3·4)
Wolverhampton	2·3 (2·3 to 2·4)	2·6 (2·4 to 2·7)	2·4 (2·3 to 2·5)	2 (1·9 to 2)	2 (2 to 2·1)	1·5 (1·5 to 1·5)	1·8 (1·8 to 1·8)	1·8 (1·6 to 1·9)
Worcestershire	3·2 (3·2 to 3·3)	3·6 (3·4 to 3·7)	3·7 (3·6 to 3·9)	3·4 (3·3 to 3·4)	3·4 (3·3 to 3·5)	2·9 (2·8 to 3)	3·2 (3·1 to 3·3)	3 (2·7 to 3·3)
Yorkshire and the Humber	38·4 (37·4 to 39·5)	41·6 (40·5 to 42·7)	40·3 (39·2 to 41·5)	34·4 (33·8 to 35)	35·5 (35 to 36)	28·7 (28·4 to 28·9)	34·1 (33·9 to 34·3)	32·9 (29·9 to 36·2)
Barnsley	1·8 (1·8 to 1·9)	2 (1·9 to 2·1)	2 (1·9 to 2)	1·6 (1·6 to 1·6)	1·6 (1·6 to 1·6)	1·2 (1·2 to 1·2)	1·5 (1·5 to 1·5)	1·4 (1·3 to 1·5)
Bradford	4·2 (4·1 to 4·3)	4·5 (4·4 to 4·6)	4·4 (4·3 to 4·5)	4·1 (4 to 4·1)	4 (3·9 to 4·1)	3·6 (3·5 to 3·6)	4·4 (4·3 to 4·4)	4 (3·7 to 4·4)
Calderdale	1·7 (1·7 to 1·7)	1·7 (1·6 to 1·7)	1·7 (1·6 to 1·7)	1·4 (1·4 to 1·4)	1·4 (1·4 to 1·5)	1·2 (1·1 to 1·2)	1·4 (1·3 to 1·4)	1·3 (1·2 to 1·4)
Doncaster	2·3 (2·3 to 2·4)	2·7 (2·6 to 2·8)	2·6 (2·5 to 2·7)	2·2 (2·1 to 2·2)	2·2 (2·1 to 2·3)	1·6 (1·6 to 1·7)	2 (1·9 to 2)	1·8 (1·6 to 1·9)
East Riding of Yorkshire	1·9 (1·8 to 1·9)	2 (1·9 to 2)	1·9 (1·8 to 1·9)	1·6 (1·5 to 1·6)	1·7 (1·7 to 1·7)	1·4 (1·4 to 1·5)	1·6 (1·5 to 1·6)	1·5 (1·4 to 1·7)
Kingston upon Hull, City of	2·3 (2·2 to 2·4)	2·5 (2·5 to 2·6)	2·5 (2·4 to 2·6)	2·1 (2·1 to 2·2)	2·2 (2·2 to 2·3)	1·5 (1·5 to 1·5)	1·9 (1·9 to 2)	1·8 (1·6 to 1·9)
Kirklees	3·1 (3 to 3·2)	3·3 (3·2 to 3·3)	3·3 (3·2 to 3·4)	2·9 (2·8 to 2·9)	2·8 (2·8 to 2·9)	2·5 (2·5 to 2·6)	3 (2·9 to 3)	2·7 (2·5 to 3)

Leeds	5·6 (5·4 to 5·8)	6 (5·8 to 6·3)	5·6 (5·4 to 5·9)	4·8 (4·7 to 4·9)	5 (4·9 to 5·1)	4 (4 to 4·1)	5·2 (5·1 to 5·3)	5·3 (4·8 to 5·9)
North East Lincolnshire	1·3 (1·3 to 1·3)	1·5 (1·5 to 1·6)	1·5 (1·5 to 1·6)	1·2 (1·2 to 1·3)	1·2 (1·2 to 1·3)	0·9 (0·9 to 0·9)	1 (1 to 1·1)	0·9 (0·9 to 1)
North Lincolnshire	1·2 (1·1 to 1·2)	1·3 (1·3 to 1·4)	1·3 (1·2 to 1·3)	1·1 (1·1 to 1·1)	1·1 (1 to 1·1)	0·8 (0·8 to 0·8)	1 (1 to 1)	0·9 (0·9 to 1)
North Yorkshire	3·5 (3·4 to 3·6)	3·7 (3·5 to 3·8)	3·5 (3·4 to 3·7)	3 (3 to 3·1)	3·3 (3·3 to 3·4)	2·8 (2·8 to 2·9)	3 (2·9 to 3·1)	2·9 (2·7 to 3·2)
Rotherham	2 (1·9 to 2)	2·3 (2·2 to 2·3)	2·3 (2·2 to 2·4)	1·9 (1·9 to 1·9)	1·9 (1·9 to 1·9)	1·4 (1·4 to 1·5)	1·6 (1·6 to 1·7)	1·6 (1·4 to 1·7)
Sheffield	4·1 (4 to 4·3)	4·3 (4·1 to 4·5)	4 (3·8 to 4·2)	3·3 (3·1 to 3·4)	3·5 (3·4 to 3·6)	2·9 (2·9 to 3)	3·4 (3·3 to 3·5)	3·6 (3·2 to 4)
Wakefield	2·4 (2·4 to 2·5)	2·7 (2·6 to 2·8)	2·7 (2·6 to 2·8)	2·3 (2·2 to 2·4)	2·3 (2·3 to 2·4)	1·7 (1·7 to 1·8)	2·1 (2·1 to 2·2)	2 (1·8 to 2·2)
York	1·1 (1 to 1·1)	1·1 (1·1 to 1·2)	1·1 (1 to 1·1)	1 (1 to 1)	1·1 (1·1 to 1·1)	0·9 (0·9 to 1)	1·1 (1 to 1·1)	1·1 (1 to 1·2)
Northern Ireland	13·1 (11·2 to 15·2)	17·1 (15·5 to 19·1)	17·7 (15·7 to 19·9)	14·1 (12·3 to 15·9)	14·4 (13 to 15·9)	11 (10·3 to 11·9)	13·5 (12·9 to 14·1)	12·4 (9·5 to 16·2)
Scotland	37·3 (32·7 to 42·4)	53·1 (48·4 to 58·6)	48 (42·6 to 53·9)	36·5 (32·1 to 40·9)	37·6 (34 to 41·4)	27·2 (25·5 to 29·1)	31·4 (30 to 32·9)	27·6 (22·7 to 33·6)
Wales	23·2 (22·6 to 23·9)	24·1 (23·5 to 24·7)	23 (22·4 to 23·6)	20·1 (19·7 to 20·5)	20 (19·6 to 20·4)	15·8 (15·7 to 15·9)	18 (17·7 to 18·4)	17 (15·4 to 18·7)
Latin America and Caribbean	3309·5 (3160 to 3459·4)	4186·3 (4039·2 to 4333·5)	4893·7 (4743 to 5034·5)	5489·3 (5360·2 to 5618·8)	5436·5 (5300·9 to 5565·1)	5433·6 (5272·7 to 5602·3)	5397 (5184·6 to 5633·8)	5007·1 (4575·4 to 5462·7)
Andean Latin America	367·2 (350·5 to 384·7)	464·8 (451 to 478·9)	553·1 (537·6 to 567·4)	627·1 (607·9 to 647)	628 (601·2 to 654)	639·3 (605·7 to 672·2)	653 (614·3 to 696·6)	678·9 (581·9 to 788·2)
Bolivia	84·8 (81·4 to 88·3)	91 (87·8 to 93·9)	98 (94·3 to 101·6)	117·1 (113·6 to 120·5)	125·4 (120·5 to 130·8)	134 (126·9 to 141·5)	152·7 (143·1 to 162·1)	166·9 (144·9 to 191·1)
Ecuador	81·7 (77·7 to 86·1)	114·1 (111·3 to 116·7)	138 (135 to 140·9)	148·7 (144·6 to 152·8)	154·8 (149·2 to 160·7)	166·4 (158·9 to 173·7)	170 (156·4 to 183·6)	178·5 (152·7 to 207·6)
Peru	200·7 (191·5 to 210·3)	259·7 (250·2 to 269·5)	317·2 (305·3 to 328·6)	361·3 (345·3 to 377)	347·7 (324 to 371·4)	338·9 (313·7 to 363·6)	330·2 (302·5 to 363·4)	333·5 (284·2 to 389·7)
Caribbean	350·9 (339·8 to 362·3)	424·1 (410·8 to 436)	451·4 (443·8 to 458·8)	418·6 (408·5 to 428)	465·9 (454·8 to 477·3)	445·4 (432·9 to 457·9)	436·7 (418·3 to 455)	418·3 (379·6 to 460·5)
Antigua and Barbuda	0·9 (0·8 to 0·9)	0·9 (0·9 to 1)	0·9 (0·8 to 0·9)	0·7 (0·7 to 0·8)	0·7 (0·7 to 0·7)	0·7 (0·7 to 0·8)	0·6 (0·5 to 0·7)	0·5 (0·4 to 0·6)
The Bahamas	1·3 (1·3 to 1·4)	1·8 (1·7 to 1·8)	2·2 (2·1 to 2·2)	2·5 (2·5 to 2·6)	2·7 (2·6 to 2·7)	2·5 (2·5 to 2·6)	2·4 (2·4 to 2·4)	2·1 (1·7 to 2·5)
Barbados	3·5 (3·3 to 3·8)	3·6 (3·5 to 3·7)	2·6 (2·5 to 2·7)	2·2 (2·1 to 2·3)	2·1 (2 to 2·2)	2 (1·9 to 2)	1·7 (1·5 to 1·9)	1·4 (1·2 to 1·7)
Belize	1·5 (1·5 to 1·6)	2·2 (2·1 to 2·2)	2·4 (2·4 to 2·5)	2·8 (2·7 to 2·8)	3 (2·9 to 3·1)	3·2 (3 to 3·3)	3·6 (3·4 to 3·8)	3·9 (3·3 to 4·5)
Bermuda	0·6 (0·5 to 0·6)	0·6 (0·6 to 0·6)	0·5 (0·5 to 0·5)	0·4 (0·4 to 0·4)	0·5 (0·4 to 0·5)	0·4 (0·4 to 0·5)	0·4 (0·3 to 0·4)	0·3 (0·2 to 0·3)
Cuba	79·2 (75·4 to 83·9)	113·4 (106·6 to 120·2)	128 (127·3 to 128·8)	66·7 (65·4 to 68·1)	93·1 (92·6 to 93·7)	75·6 (75·1 to 76·2)	67·9 (67·5 to 68·3)	53·8 (46·3 to 62·4)
Dominica	1·1 (1 to 1·1)	1·3 (1·3 to 1·4)	1·3 (1·2 to 1·3)	0·9 (0·9 to 1)	0·9 (0·8 to 0·9)	0·7 (0·6 to 0·8)	0·5 (0·4 to 0·5)	0·4 (0·4 to 0·5)
Dominican Republic	60·1 (57 to 63·4)	77·5 (74·6 to 80·1)	96·7 (94 to 99·5)	114·4 (109·1 to 119·6)	125·6 (120·6 to 131)	123·5 (117·7 to 129·7)	114·2 (107·5 to 121·2)	117·8 (101·4 to 136·2)
Grenada	1·9 (1·9 to 2)	1·9 (1·9 to 2)	1·5 (1·4 to 1·5)	1·4 (1·3 to 1·5)	1 (0·9 to 1·1)	1 (0·9 to 1·1)	0·8 (0·7 to 1)	0·7 (0·6 to 0·9)
Guyana	10·6 (10·2 to 11·1)	13·4 (13·2 to 13·7)	13·9 (13·4 to 14·5)	13·8 (13 to 14·5)	11·6 (10·9 to 12·5)	9 (8·4 to 9·7)	7·3 (6·4 to 8·2)	7·3 (6·2 to 8·6)
Haiti	87 (81·1 to 92·8)	92·2 (86·5 to 97·5)	93·6 (88·1 to 98·4)	108·1 (102·9 to 113·1)	126 (119·7 to 132·2)	140·4 (133·2 to 147·3)	162·5 (151·7 to 173·4)	169·5 (152·3 to 189·7)
Jamaica	25·7 (23·8 to 27·6)	33·6 (32·5 to 34·7)	31·4 (30·4 to 32·3)	28·7 (27·4 to 30)	28·2 (26·9 to 29·6)	23·7 (21·2 to 26·2)	20·8 (18·6 to 23·3)	18·2 (15·5 to 21·4)
Puerto Rico	43·3 (43·1 to 43·6)	38·1 (37·8 to 38·3)	35 (34·7 to 35·2)	36·6 (36·3 to 36·8)	33·4 (33·1 to 33·7)	29·8 (29·5 to 30·2)	21·4 (19·7 to 23·6)	13 (11·4 to 14·9)
Saint Kitts and Nevis	1·4 (1·2 to 1·5)	0·7 (0·7 to 0·8)	0·5 (0·4 to 0·6)	0·5 (0·5 to 0·6)	0·5 (0·4 to 0·5)	0·4 (0·4 to 0·5)	0·4 (0·3 to 0·5)	0·4 (0·3 to 0·4)
Saint Lucia	1·6 (1·5 to 1·7)	2 (2 to 2·1)	2·1 (2 to 2·1)	2·1 (2·1 to 2·2)	1·9 (1·8 to 2·1)	1·5 (1·3 to 1·7)	1·1 (1 to 1·3)	0·9 (0·7 to 1·1)
Saint Vincent and the Grenadines	1·7 (1·7 to 1·8)	2 (1·9 to 2)	1·7 (1·7 to 1·8)	1·7 (1·6 to 1·8)	1·4 (1·2 to 1·5)	1·1 (1 to 1·2)	0·9 (0·8 to 1)	0·8 (0·6 to 0·9)
Suriname	3·8 (3·7 to 3·9)	6·1 (6 to 6·3)	6·7 (6·4 to 7·1)	5·4 (5·2 to 5·6)	4·9 (4·7 to 5·1)	5 (4·8 to 5·3)	5·1 (4·6 to 5·6)	4·6 (3·9 to 5·5)
Trinidad and Tobago	12·4 (11·7 to 13·3)	16·1 (15·7 to 16·6)	13 (12·6 to 13·4)	14·9 (14·4 to 15·4)	11·8 (11·4 to 12·3)	9 (8·5 to 9·4)	9·4 (8·9 to 10)	7·8 (6·6 to 9·2)
Virgin Islands	0·5 (0·4 to 0·5)	0·6 (0·6 to 0·6)	1·3 (1·3 to 1·4)	1·3 (1·2 to 1·3)	1·2 (1·1 to 1·2)	1 (0·9 to 1·1)	0·8 (0·7 to 0·9)	0·6 (0·5 to 0·8)
Central Latin America	1262·6 (1215·6 to 1308·5)	1682·9 (1637·3 to 1725·4)	2095·4 (2041·6 to 2143·4)	2500·2 (2441·3 to 2556·4)	2528·8 (2455 to 2603·7)	2511·3 (2415·9 to 2612·9)	2430·5 (2329·6 to 2538·6)	2250·5 (2019·6 to 2493)
Colombia	274 (256·7 to 292·8)	369·9 (354 to 384·8)	429·6 (409·1 to 451)	453·2 (434 to 472·2)	481·2 (455·3 to 506·8)	493·7 (470·7 to 517·2)	449·9 (416·2 to 486·6)	412·8 (350·2 to 483·9)
Costa Rica	19·5 (19·1 to 19·8)	30·1 (29·8 to 30·3)	28·1 (27·4 to 28·9)	35·7 (34·8 to 36·7)	41·2 (39·7 to 42·6)	37·5 (35·1 to 40·3)	37·1 (35·5 to 38·8)	33·8 (28·5 to 39·9)
El Salvador	49·3 (47·3 to 51·5)	64·3 (62·4 to 66·2)	78·2 (76·4 to 79·9)	84·5 (82·2 to 86·8)	84·3 (82 to 86·7)	73·4 (70·4 to 76·5)	60·2 (57·3 to 63·4)	57·5 (49·4 to 66·8)
Guatemala	75·5 (73·2 to 77·9)	94·9 (93·3 to 96·6)	112 (109·4 to 114·8)	156·7 (154·8 to 158·7)	173·7 (169·6 to 178·1)	186·7 (180·2 to 193·4)	196·9 (183·2 to 211·5)	207·5 (176·4 to 242·8)
Honduras	37·8 (36·1 to 39·4)	46·5 (45·2 to 47·7)	58·2 (57·2 to 59·2)	76·4 (74·6 to 78·3)	92·5 (89·9 to 94·9)	98·8 (94·8 to 102·6)	105·2 (97·9 to 113)	119·4 (106 to 135·2)
Mexico	646·3 (613 to 679·1)	844·5 (814·7 to 872·3)	1109·4 (1070·2 to 1145·3)	1351·2 (1306·7 to 1393·1)	1265·7 (1212·1 to 1320·7)	1255·8 (1180·2 to 1331·5)	1172 (1105·5 to 1246·1)	1068·8 (942·4 to 1220·6)
Aguascalientes	4·8 (4·6 to 5)	5·9 (5·7 to 6·1)	7·7 (7·5 to 7·9)	10·1 (9·8 to 10·4)	11 (10·6 to 11·4)	12 (11·3 to 12·8)	12 (11·3 to 12·7)	11·7 (10·4 to 13·2)
Baja California	6 (5·7 to 6·2)	12·7 (12·2 to 13·1)	20·4 (19·7 to 21·1)	23·6 (22·9 to 24·3)	24·2 (23·1 to 25·3)	27·6 (25·8 to 29·7)	29·2 (27·4 to 31·2)	29·2 (25·8 to 33·4)
Baja California Sur	1·5 (1·5 to 1·6)	2 (1·9 to 2)	2·9 (2·8 to 3)	4·3 (4·2 to 4·5)	4·3 (4·1 to 4·6)	4·9 (4·5 to 5·3)	5·8 (5·4 to 6·3)	6·1 (5·3 to 7)
Campeche	3 (2·8 to 3·1)	4 (3·8 to 4·2)	5·9 (5·7 to 6·1)	8·6 (8·3 to 8·8)	8 (7·7 to 8·4)	8 (7·5 to 8·6)	8·2 (7·7 to 8·7)	7·8 (6·9 to 8·8)
Chiapas	21·3 (20 to 22·5)	28·7 (27·4 to 29·8)	36·2 (34·7 to 37·6)	45 (42·9 to 47·1)	57 (54·1 to 59·9)	58·3 (54·3 to 62·4)	64 (59·8 to 68·5)	67·4 (60 to 76)
Chihuahua	22·5 (21·5 to 23·5)	30·4 (29·3 to 31·5)	37·5 (36·2 to 38·7)	39·2 (37·8 to 40·5)	34·3 (32·6 to 36)	36·5 (34·3 to 38·8)	31·8 (29·8 to 34)	28 (24·8 to 31·9)
Coahuila	18 (17·1 to 18·9)	21·5 (20·8 to 22·2)	25·5 (24·6 to 26·4)	31·1 (30·3 to 31·9)	28 (26·8 to 29·2)	26·3 (24·8 to 28)	26·4 (24·9 to 28)	26·2 (23·4 to 29·5)
Colima	2·9 (2·8 to 3·1)	3·9 (3·7 to 4)	5·4 (5·3 to 5·6)	6·9 (6·7 to 7·1)	6 (5·7 to 6·2)	5·8 (5·4 to 6·1)	5·9 (5·5 to 6·3)	5·6 (5 to 6·3)
Durango	16·5 (15·9 to 17·2)	19·6 (18·9 to 20·3)	22·7 (22 to 23·3)	24·8 (24·1 to 25·6)	23·7 (22·7 to 24·7)	21·3 (19·9 to 22·8)	18·6 (17·5 to 19·9)	17 (15·3 to 19·1)
Guanajuato	33·4 (31·9 to 34·8)	41·1 (39·7 to 42·4)	50·7 (48·8 to 52·4)	64·2 (61·5 to 66·8)	69·1 (65·4 to 73·1)	68·2 (63 to 73·8)	60·8 (55·8 to 65·8)	54·1 (47·2 to 62·3)
Guerrero	22·2 (20·9 to 23·5)	28·7 (27·6 to 29·7)	36·6 (35·1 to 38)	44·7 (42·5 to 46·7)	49·8 (47·3 to 52·4)	51·6 (48·4 to 55)	44·2 (41·3 to 47·1)	39·2 (34·7 to 44·6)
Hidalgo	21·7 (20·7 to 22·6)	25·2 (24·4 to 26)	28·9 (27·9 to 29·8)	33·7 (32·8 to 34·5)	32·2 (30·9 to 33·6)	31 (28·9 to 33·3)	28·9 (27·1 to 31)	25·7 (22·8 to 29·2)

Jalisco	45·2 (43 to 47·4)	57 (55·1 to 58·9)	73·6 (71·1 to 76)	88·1 (85·8 to 90·5)	80·7 (77·6 to 83·8)	76·7 (72·4 to 81·5)	73 (69·3 to 76·9)	67·5 (60 to 76·4)
México	32·9 (31·2 to 34·5)	44·3 (42·8 to 45·9)	86·9 (83·9 to 89·7)	153·6 (148·8 to 158·1)	157·8 (149·9 to 165·5)	175·3 (164 to 187·3)	176·6 (165 to 189·6)	161·3 (143·2 to 182·8)
Mexico City	84·3 (79·5 to 89·1)	126·5 (122·1 to 130·5)	169 (162·1 to 175·3)	179·5 (170 to 188·4)	117·2 (109·9 to 124·5)	93·6 (86 to 101·7)	74·8 (69·6 to 80·9)	58·6 (51·2 to 67·6)
Michoacán de Ocampo	35·4 (33·6 to 37·3)	42·7 (41 to 44·2)	50·8 (48·8 to 52·5)	61·9 (59·2 to 64·3)	59·3 (55·7 to 62·8)	53·4 (49·6 to 57·4)	47 (43·7 to 50·5)	41·8 (36·7 to 47·9)
Morelos	6·7 (6·3 to 7)	9·3 (8·9 to 9·7)	14 (13·5 to 14·4)	19·2 (18·6 to 19·7)	17·2 (16·6 to 17·9)	17 (16·1 to 18)	16·9 (15·8 to 18·1)	15·6 (13·9 to 17·6)
Nayarit	7·5 (7·2 to 7·8)	9·5 (9·1 to 9·9)	12·2 (11·9 to 12·6)	13·5 (13·1 to 14)	11·8 (11·4 to 12·4)	10·7 (10·1 to 11·5)	10·7 (10·1 to 11·4)	10·3 (9·2 to 11·5)
Nuevo León	19 (18·2 to 19·8)	27·8 (26·9 to 28·6)	40·3 (38·9 to 41·6)	46·8 (45 to 48·7)	42·3 (40 to 44·9)	44·6 (41·3 to 47·9)	43·6 (40·6 to 47)	40·7 (35·7 to 46·7)
Oaxaca	33·9 (31·7 to 36·1)	40·8 (39 to 42·4)	45·5 (43·6 to 47·2)	47·8 (45·7 to 49·8)	46·8 (44·6 to 49·2)	48·7 (44·9 to 52·7)	43·7 (40·8 to 46·9)	38·1 (33·5 to 43·6)
Puebla	38·8 (36·5 to 41·1)	45·7 (43·6 to 47·5)	56 (53·4 to 58·3)	67·9 (64·8 to 70·9)	67·6 (63·5 to 71·9)	72·8 (67·5 to 78·4)	67·9 (62·2 to 74)	61·2 (52 to 72·2)
Querétaro	7 (6·7 to 7·3)	8·4 (8·1 to 8·7)	10·8 (10·5 to 11·1)	14·8 (14·4 to 15·2)	17·1 (16·4 to 17·8)	17·8 (16·7 to 19·1)	18·5 (17·4 to 19·7)	18·1 (16·1 to 20·6)
Quintana Roo	0·6 (0·6 to 0·7)	1·2 (1·1 to 1·2)	2 (2 to 2·1)	4·8 (4·6 to 4·9)	7·4 (7·1 to 7·8)	10·4 (9·8 to 11)	13 (12·3 to 13·8)	14 (12·4 to 15·9)
San Luis Potosí	21 (19·9 to 22)	24·9 (24 to 25·7)	28·6 (27·5 to 29·6)	33·3 (31·8 to 34·7)	32·5 (30·7 to 34·4)	30·9 (28·8 to 33·1)	26·9 (24·7 to 29)	23·9 (20·9 to 27·5)
Sinaloa	15·5 (14·8 to 16·2)	20·1 (19·3 to 20·9)	28·9 (28·1 to 29·7)	35 (33·9 to 36·2)	32·1 (30·8 to 33·4)	30·8 (28·9 to 32·9)	25·8 (24·2 to 27·3)	22·8 (20·3 to 25·8)
Sonora	13·5 (12·9 to 14·1)	18·9 (18·3 to 19·5)	25·1 (24·1 to 26)	29·7 (28·2 to 31·1)	26·5 (24·9 to 28)	27·2 (24·9 to 29·5)	25·1 (23·1 to 27·2)	23·1 (20·3 to 26·5)
Tabasco	8·5 (8·1 to 8·9)	12·2 (11·8 to 12·6)	17·8 (17·2 to 18·4)	22·2 (21·5 to 22·8)	23·4 (22·5 to 24·3)	23·3 (21·9 to 24·8)	22·5 (21·1 to 23·9)	20·7 (18·4 to 23·4)
Tamaulipas	18·1 (17·1 to 19)	24·8 (23·9 to 25·6)	32·3 (30·9 to 33·6)	37·1 (35·2 to 38·9)	33·7 (31·3 to 36·1)	35·8 (33 to 38·6)	32·1 (29·8 to 34·4)	27·7 (24·2 to 31·8)
Tlaxcala	6·7 (6·4 to 7)	7·8 (7·5 to 8·1)	8·9 (8·6 to 9·1)	10·9 (10·6 to 11·1)	11·6 (11·2 to 12·1)	12·1 (11·4 to 12·9)	11·8 (11·1 to 12·5)	11·1 (9·8 to 12·5)
Veracruz de Ignacio de la Llave	48·5 (45·6 to 51·5)	64·7 (62 to 67·1)	87·4 (83·9 to 90·7)	106·9 (104 to 110)	95·1 (91 to 99·3)	88 (82·9 to 93·5)	73 (68·4 to 78·2)	64·1 (56·7 to 72·8)
Yucatán	11·6 (10·9 to 12·3)	13·4 (12·9 to 13·9)	16·9 (16·3 to 17·6)	20·7 (20·1 to 21·2)	19·3 (18·4 to 20)	18·5 (17·5 to 19·6)	17·7 (16·7 to 18·7)	16·4 (14·5 to 18·7)
Zacatecas	17·8 (17·3 to 18·3)	20·8 (20·1 to 21·4)	21·8 (21·2 to 22·4)	21·5 (20·8 to 22·2)	18·4 (17·4 to 19·2)	16·7 (15·7 to 17·8)	15·6 (14·4 to 16·9)	13·7 (11·8 to 16·1)
Nicaragua	34·4 (33 to 35·6)	41·6 (40·4 to 42·8)	50·4 (49 to 51·8)	66·7 (64·8 to 68·4)	73·4 (70 to 76·9)	65·6 (62 to 69·4)	68·6 (63·4 to 74)	66·6 (59·2 to 75·3)
Panama	13·6 (13·1 to 14·1)	21·6 (21·2 to 22)	27·4 (26·8 to 28)	26·9 (26 to 27·8)	30·1 (29·1 to 31·2)	32 (30·6 to 33·5)	35·6 (34·4 to 36·8)	38·9 (33·7 to 44·6)
Venezuela	112·3 (108·5 to 115·8)	169·4 (167·1 to 171·6)	202·2 (198·7 to 206)	248·8 (242·7 to 254·6)	286·5 (279 to 294·2)	267·9 (257·1 to 279·4)	305 (295·2 to 316·2)	245·1 (207·8 to 287)
Tropical Latin America	1328·8 (1227·4 to 1428·5)	1614·5 (1510 to 1720·7)	1793·8 (1693·6 to 1894·7)	1943·4 (1887·5 to 1998·5)	1813·8 (1766·4 to 1863·5)	1837·5 (1779·2 to 1901·7)	1876·9 (1800·1 to 1957·7)	1659·5 (1480·2 to 1863·8)
Brazil	1295·4 (1194·1 to 1394·6)	1575·8 (1471·4 to 1681·4)	1749·6 (1650·9 to 1850·2)	1887·4 (1833·8 to 1941·7)	1748·3 (1699·9 to 1795·8)	1773·2 (1715·6 to 1837·2)	1810 (1732·7 to 1888·9)	1594·7 (1416·2 to 1793·6)
Acre	2·7 (2·5 to 3)	3·6 (3·3 to 3·8)	4·2 (4 to 4·5)	5·4 (5·2 to 5·6)	5·5 (5·3 to 5·7)	6·8 (6·5 to 7)	8·1 (7·7 to 8·6)	8·3 (7·4 to 9·4)
Alagoas	31·4 (29·5 to 33·3)	34·6 (32·5 to 36·5)	39·4 (37·6 to 41·4)	41·6 (40·4 to 42·8)	40·4 (39 to 41·9)	41·6 (39·8 to 43·5)	42 (40 to 44)	37·4 (33·3 to 41·8)
Amapá	0·9 (0·8 to 1)	1·5 (1·4 to 1·6)	2 (1·9 to 2·1)	2·5 (2·3 to 2·6)	2·9 (2·8 to 3·1)	4·8 (4·5 to 5)	6·2 (5·8 to 6·7)	6·6 (5·8 to 7·5)
Amazonas	14·3 (13·1 to 15·5)	18 (16·8 to 19)	20·4 (19·4 to 21·4)	25·3 (24·4 to 26·1)	25·9 (25 to 26·8)	32·5 (31·4 to 33·8)	39 (37 to 41·1)	37·7 (33·4 to 42·7)
Bahia	137·1 (128·6 to 145·2)	159·6 (149·9 to 168·9)	178·3 (169·2 to 187·5)	188·5 (182·6 to 194·5)	163·6 (159 to 168·3)	167·9 (161·6 to 174·2)	170·1 (162·2 to 178·3)	144·2 (127·7 to 162·9)
Ceará	72·9 (68·7 to 76·9)	87·6 (82·3 to 92·7)	97·9 (93 to 103·1)	102·5 (99·2 to 105·7)	92 (89·5 to 94·4)	99·6 (95·5 to 104·1)	108 (103·1 to 112·7)	100·7 (89·3 to 113·2)
Distrito Federal	1 (0·9 to 1·1)	2·4 (2·2 to 2·6)	10·1 (9·6 to 10·7)	19·1 (18·4 to 19·8)	19·7 (18·9 to 20·4)	21·6 (20·7 to 22·5)	22·1 (21 to 23·1)	19·8 (17·7 to 22)
Espírito Santo	20·9 (19·3 to 22·4)	26·4 (24·7 to 28·2)	28·8 (27·1 to 30·6)	32 (30·8 to 33·1)	31·5 (30·3 to 32·6)	31·5 (30·2 to 32·8)	31·7 (29·9 to 33·6)	27·5 (24·5 to 31)
Goiás	25·1 (23·1 to 27)	36·8 (34·4 to 39·2)	47·1 (44·6 to 49·6)	55·7 (54 to 57·2)	46·2 (44·5 to 47·9)	48·9 (46·7 to 51)	49·1 (46·8 to 51·3)	43·5 (38·5 to 49·2)
Maranhão	47·5 (44·1 to 51·2)	68·7 (64·5 to 72·6)	77·2 (73·6 to 80·8)	89·6 (87 to 92·1)	90·4 (86·7 to 93·9)	102 (97·6 to 106·7)	125·3 (120 to 130·5)	108·9 (96·5 to 122·2)
Mato Grosso	4·7 (4·4 to 5·1)	7·8 (7·2 to 8·3)	11·7 (11 to 12·3)	17·4 (16·9 to 17·9)	23·1 (22·1 to 24)	24·8 (23·8 to 25·9)	27·3 (26 to 28·7)	24·8 (22 to 27·9)
Mato Grosso do Sul	8·2 (7·4 to 9)	12·7 (11·8 to 13·6)	16·7 (15·6 to 17·7)	21·1 (20·4 to 21·9)	21·5 (20·6 to 22·3)	20·6 (19·8 to 21·5)	21·7 (20·4 to 22·9)	19·7 (17·4 to 22·4)
Minas Gerais	182·8 (167·9 to 197·9)	205 (190·6 to 219·7)	202·8 (190·7 to 215·7)	195·2 (190·5 to 200·1)	177·3 (172·7 to 181·9)	169·8 (163·5 to 176·6)	160 (151·9 to 168·1)	137·7 (122·3 to 155·1)
Pará	30·1 (27·7 to 32·6)	37·2 (34·8 to 39·4)	45·8 (43·5 to 48·1)	59·1 (57 to 61)	59·2 (57·4 to 61)	70·8 (68 to 73·7)	85·5 (81·2 to 89·6)	80·7 (71·3 to 91·6)
Paraíba	48·2 (45·3 to 51·1)	53·6 (50·4 to 56·6)	53·7 (51 to 56·5)	47·5 (45·7 to 49·3)	42 (40·7 to 43·3)	44·1 (42·2 to 46·1)	47·6 (45·4 to 49·9)	42·4 (37·7 to 47·6)
Paraná	50·3 (46 to 54·5)	87·6 (81·4 to 93·9)	121 (113·7 to 128·7)	103·3 (99·8 to 106·9)	95·2 (92 to 98·4)	88·8 (85·3 to 92·3)	81·1 (77·1 to 85·5)	71·3 (63·1 to 80·8)
Pernambuco	100·1 (94·2 to 105·7)	115·8 (109 to 122·5)	125·4 (119·4 to 131·9)	120·3 (116·4 to 124·4)	102·2 (99 to 105·3)	104·9 (100·3 to 109·8)	105·4 (100·7 to 110·2)	91·5 (80·9 to 103·5)
Piauí	28·7 (26·8 to 30·8)	33·1 (31·1 to 34·9)	38·6 (36·7 to 40·5)	41·6 (40·3 to 43)	38·7 (37·4 to 40)	41 (39·3 to 42·7)	44·1 (42 to 46·3)	38·5 (33·8 to 43·6)
Rio de Janeiro	105·1 (94·4 to 116·3)	124·7 (114·3 to 135·6)	132·8 (122·7 to 142·8)	138 (132·1 to 144·4)	122·7 (118·2 to 127)	117·2 (112·8 to 121·9)	114·2 (108·2 to 120·5)	101·3 (90·1 to 114·1)
Rio Grande do Norte	27 (25·4 to 28·6)	31·4 (29·5 to 33·2)	36·2 (34·3 to 38·1)	36·4 (35·3 to 37·4)	35·1 (34·1 to 36·2)	37·7 (36·2 to 39·2)	38·9 (36·8 to 40·9)	34·9 (30·8 to 39·4)
Rio Grande do Sul	86·5 (77·6 to 95·5)	96·7 (89·1 to 104·5)	96·4 (89·7 to 103·3)	98·3 (95·5 to 101·1)	87·2 (84·1 to 90·2)	78·3 (75·6 to 81·2)	68·4 (65·1 to 71·6)	59·4 (52·8 to 66·8)
Rondônia	0·9 (0·8 to 1)	1·5 (1·4 to 1·6)	2·2 (2·1 to 2·3)	8 (7·7 to 8·4)	13·1 (12·7 to 13·5)	14·1 (13·5 to 14·7)	14·1 (13·3 to 14·8)	12·3 (10·8 to 14)
Roraima	0·5 (0·5 to 0·5)	0·7 (0·6 to 0·7)	0·9 (0·8 to 0·9)	1·6 (1·5 to 1·6)	3·2 (3·1 to 3·3)	5·1 (4·9 to 5·3)	6·3 (6 to 6·6)	6·4 (5·7 to 7·2)
Santa Catarina	34·4 (31·6 to 37·2)	41·2 (38·3 to 44·4)	45·1 (42·2 to 47·9)	48·9 (47·4 to 50·3)	45·8 (44·1 to 47·3)	44·7 (42·8 to 46·5)	45·7 (43·3 to 48·2)	42 (37·5 to 47·1)
São Paulo	212·8 (193·9 to 231·9)	263·4 (244·6 to 282·9)	287·3 (268·9 to 306·2)	357·1 (345·7 to 368·8)	332·3 (320·1 to 344·2)	318 (306·7 to 330·2)	310·3 (295 to 326·4)	263·4 (234·2 to 296·6)
Sergipe	17·1 (15·9 to 18·2)	18·2 (17·1 to 19·3)	19·3 (18·2 to 20·3)	19·9 (19·2 to 20·6)	19·1 (18·2 to 19·9)	20·7 (19·8 to 21·6)	21·6 (20·5 to 22·7)	19·4 (17·3 to 21·9)
Tocantins	4 (3·7 to 4·4)	6 (5·6 to 6·4)	8·5 (8 to 8·9)	11·5 (11 to 11·9)	12·7 (12·2 to 13·1)	15·8 (15·1 to 16·4)	16·4 (15·4 to 17·3)	14·6 (12·9 to 16·6)
Paraguay	33·4 (31·4 to 35·6)	38·7 (36·9 to 40·5)	44·2 (42·7 to 45·8)	56 (54·1 to 57·8)	65·5 (62·5 to 68·6)	64·3 (61·1 to 67·7)	66·9 (61·2 to 73·1)	64·8 (55·5 to 75·5)
North Africa and Middle East	2877·3 (2762·6 to 2990·4)	3525·8 (3413·6 to 3632·4)	4383·1 (4269 to 4486·7)	5761·4 (5623·5 to 5890·4)	6135 (5916·7 to 6343·8)	6362·9 (6058·5 to 6667·6)	6592·9 (6145·8 to 7055·3)	6268·4 (5679·8 to 6954·3)
North Africa and Middle East	2877·3 (2762·6 to 2990·4)	3525·8 (3413·6 to 3632·4)	4383·1 (4269 to 4486·7)	5761·4 (5623·5 to 5890·4)	6135 (5916·7 to 6343·8)	6362·9 (6058·5 to 6667·6)	6592·9 (6145·8 to 7055·3)	6268·4 (5679·8 to 6954·3)

Afghanistan	191·5 (182·2 to 200·5)	230·8 (222 to 238·4)	286·9 (278·3 to 295·3)	270·3 (263·1 to 277·3)	272·6 (266·6 to 278·6)	476·6 (463·1 to 489·6)	610 (588·7 to 630·5)	774 (733·2 to 817·6)
Algeria	198·2 (185·8 to 210·6)	243·9 (233·4 to 254·1)	298·6 (288·6 to 308·2)	381·8 (368·2 to 393·8)	395·3 (371·8 to 419·4)	369·1 (334 to 405·9)	453·3 (408·9 to 501·2)	455·6 (408·8 to 510·7)
Bahrain	2 (1·8 to 2·1)	3·3 (3·2 to 3·5)	3·4 (3·2 to 3·6)	4·7 (4·4 to 5·1)	7·1 (6·5 to 7·6)	6·7 (6·2 to 7·3)	8·4 (7·5 to 9·5)	6·7 (5·5 to 8·2)
Egypt	537·9 (520·1 to 556)	638·1 (615·5 to 660·3)	761 (734·9 to 787·3)	967·3 (940·4 to 992·5)	1003·5 (969·4 to 1038·9)	987 (931 to 1039·3)	1157·7 (1065·7 to 1250·5)	1081·9 (964·8 to 1204·2)
Iran	420·8 (396 to 445·4)	488·2 (465·7 to 508·9)	597·6 (572·5 to 620·6)	1065·6 (1052·3 to 1078·7)	894·9 (827·3 to 959·2)	741·1 (665·7 to 815·9)	766·3 (670·1 to 858)	693·7 (583·6 to 834·2)
Iraq	129·2 (124·1 to 134·1)	168·8 (161·9 to 175·1)	221·2 (211 to 230·8)	305·9 (295·1 to 316·1)	376·9 (357·3 to 396·5)	493·4 (464·4 to 524·2)	473·1 (422·2 to 525·8)	490·9 (427·3 to 569·5)
Jordan	13·1 (13·1 to 13·2)	22·3 (22·2 to 22·5)	34·7 (34·3 to 35·1)	51·2 (50·1 to 52·3)	70·8 (67·5 to 74)	72·6 (65·6 to 79·5)	95·1 (85·7 to 104·8)	123·2 (107·5 to 142·4)
Kuwait	1·5 (1·4 to 1·6)	6 (5·9 to 6·1)	18·2 (17·8 to 18·4)	25·5 (24·6 to 26·4)	20·3 (18·6 to 22)	21·1 (19·7 to 22·5)	28·4 (26·5 to 30·4)	31·3 (25·9 to 38·3)
Lebanon	32·7 (31·1 to 34·2)	42·3 (40·7 to 43·9)	50·1 (48 to 52·3)	57·4 (53·8 to 61)	58·4 (53·6 to 62·6)	51·6 (46·5 to 56·6)	53·1 (46·7 to 59·7)	55·1 (47·7 to 64)
Libya	26·2 (25·4 to 27)	36·1 (34·8 to 37·3)	45·3 (44·1 to 46·4)	66·5 (64·3 to 68·6)	79·9 (75·4 to 84·2)	63·9 (57·8 to 70·3)	52·4 (46 to 59·3)	41·8 (35 to 50·6)
Morocco	236·8 (225·5 to 248·7)	291·4 (280·3 to 302·2)	350·1 (336·6 to 363·7)	403·6 (381·9 to 424·6)	412·2 (387·5 to 436·2)	427·3 (391 to 463·9)	398·1 (349·9 to 447·8)	311·5 (264·6 to 365·2)
Oman	13·1 (12·7 to 13·5)	17·3 (16·8 to 17·8)	21·8 (21·2 to 22·4)	30·7 (29·7 to 31·8)	33·9 (32·1 to 35·8)	31·6 (28·7 to 34·5)	34 (30·1 to 37·9)	39·7 (35·3 to 44·3)
Palestine	20·3 (19·6 to 21)	24·2 (23·6 to 24·8)	28·5 (27·8 to 29·2)	35·6 (34·5 to 36·7)	49·4 (47·4 to 51·3)	64·6 (61·8 to 67·7)	72·1 (66·9 to 77·3)	64·5 (57·7 to 72·2)
Qatar	0·5 (0·5 to 0·6)	1·1 (1 to 1·2)	2·2 (2·1 to 2·4)	4 (3·8 to 4·2)	5·4 (5·1 to 5·7)	5·7 (5·4 to 6·1)	11·2 (10·3 to 12·2)	13·7 (11·8 to 15·8)
Saudi Arabia	68·6 (66 to 71·2)	89·7 (86·7 to 92·7)	132·2 (126·7 to 137·9)	208·4 (197·2 to 220·8)	276·7 (255·2 to 299·1)	265·3 (237·3 to 292)	251·9 (223 to 282)	234 (207·6 to 262·5)
Sudan	143·1 (136·6 to 149·3)	167·4 (160·8 to 172·8)	278·7 (269·7 to 286·9)	406·8 (391·9 to 420·6)	542·6 (520·8 to 562·6)	650·3 (618·4 to 680·9)	650·6 (592·5 to 707·2)	618·1 (538·7 to 711·1)
Syria	91·4 (88·9 to 93·8)	124·6 (121·2 to 127·6)	163·2 (159·1 to 167)	212·2 (206 to 218·2)	266·2 (253·3 to 278·2)	269·2 (250·2 to 289·9)	262·4 (236·6 to 289·3)	119·5 (99·2 to 145·6)
Tunisia	85·7 (80·3 to 91)	97·6 (93·7 to 100·9)	106·3 (103·3 to 109·4)	130·4 (125·9 to 134·9)	124·9 (117·2 to 132·6)	108·7 (101 to 116·5)	103·2 (95·9 to 110·6)	86·9 (74·5 to 102·5)
Turkey	538·3 (504 to 572·7)	679·4 (650·6 to 706·9)	786·8 (756·1 to 816·3)	839·2 (793 to 882·9)	866·1 (809·2 to 922·8)	781·5 (732·7 to 833·6)	529·8 (484·2 to 576·7)	504·6 (440·4 to 583·4)
United Arab Emirates	1·8 (1·7 to 1·9)	2·2 (2·1 to 2·3)	4·8 (4·6 to 5)	18·2 (17·7 to 18·7)	23·8 (22·8 to 24·9)	25·5 (23·4 to 27·5)	47·8 (41·9 to 54)	28·8 (24·7 to 34)
Yemen	124 (119·8 to 128·1)	150·1 (146·4 to 153·6)	189·7 (185·5 to 193·7)	272·4 (268·1 to 276·6)	350·1 (341·3 to 358)	444·9 (423·9 to 464·2)	527·4 (485·8 to 565·2)	486·7 (429·8 to 552·4)
South Asia	10283·9 (9530 to 11038·7)	11741·6 (10948·3 to 12557·9)	13297·1 (12486·5 to 14068·6)	17025·2 (16330·1 to 17710·7)	18526 (17762·4 to 19255·8)	19181·3 (18472·4 to 19874·2)	18876·5 (18163·5 to 19578·4)	17460·2 (16041·1 to 18980·2)
South Asia	10283·9 (9530 to 11038·7)	11741·6 (10948·3 to 12557·9)	13297·1 (12486·5 to 14068·6)	17025·2 (16330·1 to 17710·7)	18526 (17762·4 to 19255·8)	19181·3 (18472·4 to 19874·2)	18876·5 (18163·5 to 19578·4)	17460·2 (16041·1 to 18980·2)
Bangladesh	1134·8 (1096·5 to 1169·9)	1242·4 (1201·6 to 1281·4)	1499·8 (1449·8 to 1546·4)	1669·9 (1616·1 to 1726·8)	1935·5 (1878·8 to 1991·2)	1763·1 (1678·7 to 1849·2)	1529·3 (1447·9 to 1613·4)	1361·7 (1217·8 to 1508·2)
Bhutan	4·2 (3·9 to 4·5)	5·6 (5·2 to 5·9)	7·5 (7·1 to 7·9)	9·8 (9·2 to 10·4)	11·8 (10·7 to 12·9)	9·8 (8·8 to 10·9)	8·7 (7·4 to 9·9)	7·1 (6·1 to 8·3)
India	8071·4 (7411·9 to 8735·3)	9323·5 (8577·5 to 10082·4)	10053·2 (9322·7 to 10759·3)	12969·2 (12356·1 to 13568·5)	13627·8 (12910·8 to 14293·8)	13895·2 (13280·8 to 14486·3)	13577·4 (12969·4 to 14171·9)	12292·3 (11079 to 13503·5)
Andhra Pradesh	322 (289·3 to 356·5)	377·5 (345·6 to 410·2)	413·1 (371·6 to 459·6)	536·9 (499·9 to 574·5)	543·3 (507·9 to 577·9)	467·8 (437·8 to 500·1)	438 (411·7 to 465·1)	356·2 (317·4 to 397·4)
Arunachal Pradesh	5·7 (5·3 to 6·1)	6·8 (6·4 to 7·2)	9·9 (9·1 to 10·9)	13·5 (12·4 to 14·6)	13·1 (11·8 to 14·4)	13·9 (12·7 to 15·1)	16·8 (15·1 to 18·6)	18·4 (15·2 to 21·9)
Assam	185·8 (172·8 to 198·8)	227 (208·3 to 246·8)	268·3 (247·9 to 288·7)	358·7 (335·4 to 381·3)	373·9 (344·8 to 402)	329·5 (308·9 to 351·1)	345·5 (315·8 to 378)	345·8 (304·5 to 387·4)
Bihar	688·8 (631·3 to 745·8)	813·8 (757·7 to 867·9)	909·5 (829 to 996·1)	1196·7 (1124·3 to 1267·9)	1314·6 (1232·8 to 1393·7)	1478 (1389·9 to 1562·5)	1528 (1453·8 to 1603·1)	1346·2 (1222·6 to 1468)
Chhattisgarh	139·9 (127·6 to 152·5)	170·3 (156·5 to 183)	181·4 (163·8 to 199·5)	259·4 (242·3 to 276·9)	297·7 (275·2 to 320)	300 (283·4 to 318·9)	319·1 (299·5 to 339·3)	316·8 (280·7 to 353·7)
Delhi	34·7 (31·5 to 38)	44·3 (39·5 to 49·7)	54·6 (48·6 to 60·5)	94 (87·7 to 100·6)	136·8 (127·9 to 145·9)	161·6 (150 to 173·5)	170·1 (160·4 to 180·3)	152·6 (129·6 to 177·7)
Goa	11·2 (10·3 to 12·1)	12·9 (11·8 to 13·9)	13·3 (11·9 to 14·8)	14 (12·5 to 15·6)	12·5 (11·7 to 13·4)	13·6 (12·7 to 14·5)	11·8 (10·8 to 12·7)	9·4 (7·9 to 11·1)
Gujarat	398·4 (371·9 to 424·5)	471·5 (431·7 to 513·6)	515·3 (479·9 to 549·3)	661·6 (625·8 to 698)	604·9 (566·8 to 646·2)	598·5 (566·1 to 633·6)	645·6 (615·9 to 680·2)	592·7 (538·7 to 646·5)
Haryana	132·2 (123·7 to 140·3)	158·9 (144·7 to 174·1)	188·3 (174·4 to 201·5)	228·5 (216·3 to 240·7)	254·3 (241·1 to 268·3)	282·8 (264·7 to 302·8)	293·5 (272·8 to 313·8)	242·9 (202·2 to 287·7)
Himachal Pradesh	44·2 (40·8 to 47·7)	51·8 (48·4 to 55·2)	60·1 (55·1 to 65·9)	74·9 (70·5 to 79·5)	75 (71·3 to 78·8)	65·9 (62·9 to 69·3)	63·1 (59·4 to 66·7)	49·2 (44·5 to 54·1)
Jammu and Kashmir	58·5 (53·8 to 63·3)	75·4 (68·2 to 83·2)	78 (69·6 to 86·1)	99·1 (93 to 105·2)	123·2 (114·2 to 132·2)	132·2 (123·2 to 141·2)	118·2 (107·2 to 129·1)	104 (86·2 to 123·8)
Jharkhand	250·8 (233·4 to 267·9)	282·5 (264·8 to 300)	309·5 (284·6 to 334)	403·2 (374·2 to 432·4)	420·9 (390·8 to 450·6)	430·7 (400·2 to 462·2)	391·9 (363·1 to 419·9)	364·3 (322·9 to 409)
Karnataka	368 (329·8 to 407·6)	427·9 (379·9 to 479·2)	455·5 (409·6 to 501·9)	636·9 (592·3 to 685·3)	595·7 (556·8 to 635·4)	543 (503·9 to 586·4)	571·3 (530·1 to 616·5)	495·7 (434·7 to 560·6)
Kerala	278·5 (253·8 to 303·9)	298·7 (264·6 to 337·5)	305·6 (279·4 to 330·8)	344·3 (322 to 367·3)	334·4 (313·7 to 355·6)	329·1 (309 to 352·9)	284·6 (269·6 to 300·4)	223·2 (202·1 to 246)
Madhya Pradesh	524·5 (487·2 to 561·1)	607·6 (552·9 to 662)	639·7 (588·5 to 690·2)	784·4 (732·2 to 839·7)	823·4 (752·4 to 898·7)	895·5 (838·1 to 955·1)	1000·8 (936·6 to 1067·7)	853·5 (751 to 962)
Maharashtra	656·9 (595·4 to 720·1)	765·2 (684·6 to 854·8)	776 (706·4 to 849)	1036·6 (968·5 to 1105·6)	1037·4 (980·2 to 1098·6)	1008·8 (942·6 to 1079·9)	994·8 (935 to 1059·5)	869·3 (779·3 to 965)
Manipur	16·6 (15·7 to 17·5)	19·9 (18·5 to 21·3)	23·4 (21·6 to 25·4)	28·3 (25·8 to 31)	28·6 (25·4 to 31·9)	28·7 (26·1 to 30·9)	30·6 (27·2 to 34·3)	31·5 (26·4 to 37·2)
Meghalaya	13·8 (12·9 to 14·5)	18·3 (17 to 19·6)	24·3 (22·5 to 26)	32·3 (29·9 to 34·7)	34·3 (31 to 37·6)	34·3 (31·1 to 37·2)	34·5 (30·3 to 38·9)	34·3 (28·3 to 41·1)
Mizoram	3·6 (3·4 to 3·8)	5·2 (4·8 to 5·5)	6 (5·4 to 6·5)	8·4 (7·7 to 9·2)	9·6 (8·5 to 10·6)	11·1 (10·1 to 12·1)	12·9 (11·6 to 14·2)	12·5 (10·7 to 14·5)
Nagaland	8·3 (7·9 to 8·7)	9·7 (9·1 to 10·3)	11·8 (11 to 12·6)	15·8 (14·6 to 16·9)	18·3 (16·6 to 20)	24·8 (22·5 to 27·1)	23·2 (21·1 to 25·4)	18·8 (15·6 to 22·5)
Odisha	362·2 (332·5 to 392·5)	389·9 (359·4 to 419·3)	404·6 (369·8 to 440·5)	489·1 (453·5 to 525·9)	455·1 (429·9 to 482·2)	461·5 (435·5 to 490·5)	439·8 (417·2 to 464·7)	387·1 (338·5 to 438·1)
Punjab	170·2 (157·1 to 183·3)	191·2 (172·2 to 212)	214·9 (199·4 to 230·5)	259·5 (245·4 to 274)	291·4 (275·9 to 306·4)	267·6 (252 to 283·5)	251·5 (235·7 to 269·5)	210·6 (175·5 to 249·7)
Rajasthan	303·5 (274 to 333·9)	394·6 (358·7 to 432·2)	462 (420·6 to 504)	686·5 (645·6 to 727·1)	821·4 (774·1 to 868)	919·7 (872·2 to 970·4)	914·3 (857·8 to 972·3)	806·5 (708·4 to 909)
Sikkim	3·1 (2·9 to 3·3)	4 (3·7 to 4·3)	4·6 (4·2 to 5)	6·4 (5·9 to 6·9)	6 (5·5 to 6·5)	5·1 (4·7 to 5·6)	4·6 (4·1 to 5·1)	3·7 (3 to 4·5)
Tamil Nadu	588·3 (530·8 to 648·6)	634·1 (563·2 to 712·7)	625·3 (583·6 to 671·8)	672 (633 to 710·7)	652·8 (619·9 to 685·3)	660·5 (624·9 to 699·1)	633·8 (594·2 to 676·6)	509·8 (423·4 to 606·4)
Telangana	250·5 (227·3 to 274·8)	294 (269·8 to 317·6)	315·2 (284·1 to 346·5)	404·7 (366·6 to 443·7)	410·7 (369·6 to 453·2)	375·9 (340 to 413·9)	347·6 (313·2 to 384)	284·8 (260·1 to 311·3)
Tripura	22·1 (20·6 to 23·6)	27·8 (25·8 to 29·7)	31 (28 to 34)	40·1 (36·2 to 44·2)	35·9 (33·2 to 38·7)	31·6 (29 to 34·2)	30·8 (28·1 to 33·6)	25·9 (20·9 to 31·5)
Union Territories other than Delhi	17·2 (16·5 to 17·9)	18·8 (17·9 to 19·7)	22·8 (21·4 to 24·2)	32·3 (29·8 to 34·9)	32·3 (28·4 to 36·4)	29·6 (26·1 to 33·1)	26·7 (22·6 to 31)	21 (16·9 to 25·7)
Uttar Pradesh	1532·6 (1428 to 1632·3)	1719 (1574·4 to 1859·3)	1851·3 (1724·2 to 1977·2)	2420·3 (2279·6 to 2570·2)	2797·9 (2599·3 to 2998·5)	3020·9 (2878·5 to 3160·8)	2759·7 (2592·7 to 2929·2)	2845·2 (2525·9 to 3178·1)

Uttarakhand	66·9 (62·8 to 70·9)	88·7 (83·6 to 93·6)	97·6 (90·9 to 104·3)	122·3 (114·7 to 130·2)	135·5 (126·6 to 144·7)	120·7 (112 to 129·2)	104·3 (96·1 to 112·2)	94·5 (85·6 to 104)
West Bengal	612·4 (561·7 to 663·3)	716·4 (657·9 to 773·5)	780·3 (709·8 to 862·3)	1008·3 (945 to 1072·1)	936·6 (884·4 to 992·4)	852·4 (806·7 to 901·4)	770·1 (722·5 to 817·6)	666·1 (613·3 to 722·4)
Nepal	220·2 (205 to 235·2)	241·6 (228·2 to 254·5)	283·2 (269·8 to 295·5)	371·4 (354 to 388·3)	426·2 (413·1 to 438·5)	412·5 (397 to 428·3)	344·6 (321·6 to 366·4)	318·1 (281·3 to 362·5)
Pakistan	853·3 (812 to 893·4)	928·5 (888·8 to 965·5)	1453·4 (1417·7 to 1488·3)	2005 (1938·4 to 2067)	2524·8 (2440·1 to 2599·4)	3100·7 (2966·4 to 3231)	3416·6 (3224·8 to 3618·6)	3481 (3093·6 to 3929·8)
Southeast Asia, east Asia, and Oceania	17137·1 (16695·9 to 17580·4)	19208 (18865 to 19566·3)	19363·4 (19086 to 19624·6)	19642·9 (19354·5 to 19920·3)	20087·8 (19771·8 to 20420·9)	16009·3 (15719·5 to 16322·9)	14337·5 (13841·6 to 14802·4)	14167·5 (13574·3 to 14827·9)
East Asia	12619·6 (12428·9 to 12817·9)	13522·4 (13369·2 to 13691·1)	13206·6 (13049·1 to 13368·4)	13015·7 (12781·8 to 13239·8)	13495·4 (13242·9 to 13751·9)	9483·4 (9261·3 to 9705·4)	7859·3 (7598·2 to 8136·5)	8314·6 (8072·4 to 8559·5)
China	12190·6 (12012 to 12376·1)	12979·9 (12831·1 to 13143·7)	12656·9 (12498·1 to 12817·3)	12470·7 (12237·2 to 12690·6)	12923·9 (12665 to 13175·9)	9022·4 (8799·9 to 9238·1)	7550 (7304·5 to 7821·7)	8042·9 (7797·9 to 8276·6)
North Korea	251·6 (239·1 to 264·3)	327·6 (311·5 to 343)	353·2 (334·6 to 371·6)	333·1 (309·3 to 358·5)	399·1 (370·3 to 426)	312·7 (281·9 to 344·4)	216 (189·7 to 243·6)	180·7 (156·8 to 206·1)
Taiwan (province of China)	177·4 (176·8 to 178)	214·9 (214·3 to 215·5)	196·5 (195·9 to 197)	211·9 (211·1 to 212·7)	172·5 (171·8 to 173·1)	148·3 (147·7 to 149)	93·4 (92·9 to 93·8)	90·9 (80·2 to 103·3)
Oceania	60·6 (58·2 to 62·8)	74·2 (71·5 to 76·7)	84 (81·4 to 86·6)	93·5 (90·9 to 96·1)	115·4 (111·9 to 118·8)	148·2 (142·3 to 154·2)	184·1 (171·1 to 196·9)	215·1 (197·1 to 234·2)
American Samoa	0·4 (0·4 to 0·4)	0·4 (0·4 to 0·5)	0·6 (0·5 to 0·6)	0·6 (0·5 to 0·6)	0·9 (0·8 to 0·9)	0·9 (0·8 to 0·9)	0·6 (0·6 to 0·7)	0·5 (0·5 to 0·6)
Cook Islands	0·3 (0·3 to 0·4)	0·4 (0·3 to 0·4)	0·3 (0·3 to 0·3)	0·2 (0·2 to 0·3)	0·2 (0·2 to 0·3)	0·2 (0·2 to 0·2)	0·2 (0·2 to 0·2)	0·1 (0·1 to 0·2)
Fiji	5·9 (5·6 to 6·3)	8 (7·7 to 8·3)	8·4 (8·1 to 8·7)	9·6 (8·9 to 10·2)	9·7 (9·2 to 10·3)	9·4 (8·6 to 10·2)	10 (9·1 to 10·9)	9 (7·8 to 10·4)
Guam	0·9 (0·9 to 0·9)	1·3 (1·2 to 1·3)	1·5 (1·4 to 1·5)	1·5 (1·5 to 1·6)	2 (2 to 2)	1·9 (1·8 to 1·9)	1·8 (1·7 to 1·8)	1·7 (1·6 to 1·9)
Kiribati	0·8 (0·7 to 0·8)	0·8 (0·8 to 0·9)	0·9 (0·9 to 0·9)	1·2 (1·1 to 1·2)	1·3 (1·3 to 1·4)	1·3 (1·2 to 1·4)	1·5 (1·4 to 1·6)	1·6 (1·4 to 1·8)
Marshall Islands	0·3 (0·3 to 0·3)	0·3 (0·3 to 0·4)	0·5 (0·5 to 0·5)	0·7 (0·7 to 0·8)	0·8 (0·8 to 0·8)	0·8 (0·7 to 0·8)	0·7 (0·7 to 0·8)	0·6 (0·6 to 0·7)
Federated States of Micronesia	0·9 (0·9 to 1)	1·1 (1·1 to 1·1)	1·4 (1·3 to 1·4)	1·8 (1·8 to 1·9)	1·8 (1·7 to 1·9)	1·5 (1·4 to 1·7)	1·2 (1 to 1·3)	1 (0·9 to 1·2)
Nauru	0·1 (0·1 to 0·1)	0·1 (0·1 to 0·1)	0·2 (0·1 to 0·2)	0·2 (0·2 to 0·2)	0·2 (0·2 to 0·2)	0·2 (0·2 to 0·2)	0·2 (0·2 to 0·2)	0·2 (0·1 to 0·2)
Niue	0·1 (0·1 to 0·1)	0·1 (0·1 to 0·1)	0·1 (0·1 to 0·1)	0·1 (0 to 0·1)	0 (0 to 0)	0 (0 to 0)	0 (0 to 0)	0 (0 to 0)
Northern Mariana Islands	0·1 (0·1 to 0·1)	0·1 (0·1 to 0·1)	0·1 (0·1 to 0·2)	0·2 (0·2 to 0·2)	0·7 (0·6 to 0·7)	1·3 (1·2 to 1·5)	0·4 (0·4 to 0·5)	0·3 (0·2 to 0·3)
Palau	0·2 (0·1 to 0·2)	0·2 (0·2 to 0·2)	0·2 (0·1 to 0·2)	0·2 (0·1 to 0·2)	0·2 (0·2 to 0·2)	0·2 (0·2 to 0·2)	0·2 (0·1 to 0·2)	0·1 (0·1 to 0·1)
Papua New Guinea	41·1 (39·4 to 42·7)	49·5 (47·2 to 51·5)	55·8 (53·6 to 57·7)	60·7 (58·8 to 62·5)	77·4 (75 to 79·8)	106 (101·9 to 110)	140·1 (130·1 to 150·2)	171·7 (157·5 to 187·1)
Samoa	1·8 (1·7 to 1·9)	2·2 (2·2 to 2·3)	2·3 (2·2 to 2·4)	2 (1·8 to 2·1)	1·6 (1·4 to 1·8)	1·4 (1·3 to 1·7)	1·5 (1·4 to 1·8)	1·8 (1·6 to 2·1)
Solomon Islands	2·8 (2·7 to 2·9)	3·4 (3·3 to 3·5)	4·1 (4 to 4·3)	5·3 (5·1 to 5·5)	7·6 (7·2 to 8)	10 (9·3 to 10·6)	11 (10·2 to 11·7)	11 (10 to 12·1)
Tokelau	0 (0 to 0)	0·1 (0·1 to 0·1)	0 (0 to 0)	0 (0 to 0)	0 (0 to 0)	0 (0 to 0)	0 (0 to 0)	0 (0 to 0)
Tonga	0·9 (0·9 to 1)	1·2 (1·1 to 1·2)	1·4 (1·3 to 1·5)	1·4 (1·4 to 1·5)	1·3 (1·2 to 1·4)	1·3 (1·2 to 1·4)	1·3 (1·2 to 1·4)	1·2 (1 to 1·3)
Tuvalu	0·1 (0·1 to 0·1)	0·1 (0·1 to 0·1)	0·1 (0·1 to 0·1)	0·2 (0·2 to 0·2)	0·2 (0·1 to 0·2)	0·1 (0·1 to 0·1)	0·1 (0·1 to 0·1)	0·1 (0·1 to 0·1)
Vanuatu	1 (1 to 1·1)	1·4 (1·3 to 1·5)	1·8 (1·7 to 1·9)	2·4 (2·3 to 2·6)	3 (2·8 to 3·2)	3·4 (3·1 to 3·7)	3·7 (3·3 to 4·1)	3·9 (3·4 to 4·5)
Southeast Asia	4457 (4206·9 to 4700·3)	5611·4 (5382·8 to 5829·8)	6072·8 (5874·2 to 6257·2)	6533·6 (6380·2 to 6683·6)	6477 (6308·4 to 6647·3)	6377·8 (6195·9 to 6561·5)	6294 (6046·9 to 6530·7)	5637·8 (5150·5 to 6219·2)
Cambodia	111·8 (104·6 to 118·9)	139·6 (132·6 to 146·1)	168·3 (161·5 to 175)	192·1 (186·8 to 197·5)	226·8 (220·6 to 232·8)	193·9 (186·5 to 201·4)	190·7 (178·7 to 203·7)	188·9 (170·7 to 211·3)
Indonesia	1888·4 (1789·4 to 1983·6)	2471·8 (2369 to 2571·9)	2636·6 (2563·2 to 2708·4)	2662·5 (2599·4 to 2723·3)	2444·3 (2369·9 to 2516·3)	2461 (2389·8 to 2529)	2366·7 (2236·2 to 2494·8)	1972·2 (1777 to 2203·2)
Aceh	30·5 (29·1 to 31·9)	41·3 (39·8 to 42·7)	47·8 (46·4 to 49·3)	55·3 (53·8 to 56·8)	54·1 (52 to 56·2)	56·2 (54·6 to 57·6)	55·1 (52·9 to 57·5)	46·4 (42·7 to 50·8)
Bali	36·2 (34·2 to 38·1)	43·6 (41·8 to 45·3)	47·9 (46·7 to 49·2)	43·9 (42·7 to 45·1)	35·2 (34 to 36·4)	35·7 (34·2 to 37·2)	32·8 (30·4 to 35·2)	26·5 (23·1 to 30·6)
Bangka-Belitung Islands	8 (7·5 to 8·4)	9·8 (9·3 to 10·4)	10·7 (10 to 11·3)	12·2 (11·7 to 12·7)	11·7 (11·4 to 12)	12 (11·6 to 12·4)	12·4 (11·8 to 12·9)	10·3 (9·3 to 11·3)
Banten	41·1 (38·9 to 43·2)	51·8 (49·6 to 54)	56·3 (53·9 to 58·8)	72·2 (69·3 to 75)	117·9 (113·3 to 122·1)	127·4 (123·2 to 131·4)	118·3 (114·5 to 121·9)	93·1 (86·2 to 100·7)
Bengkulu	9·3 (8·9 to 9·7)	11·9 (11·5 to 12·2)	13·3 (13 to 13·6)	17·1 (16·8 to 17·5)	20·6 (19·7 to 21·5)	20·8 (20 to 21·6)	20·1 (18·9 to 21·3)	16·7 (15 to 18·6)
Gorontalo	6·3 (6 to 6·6)	10 (9·5 to 10·5)	12·7 (12 to 13·5)	12·1 (11·6 to 12·7)	9·8 (9·6 to 10·1)	9·9 (9·6 to 10·2)	10·1 (9·7 to 10·6)	10·6 (9·6 to 11·6)
Jakarta	55·3 (52·9 to 57·6)	92·5 (89 to 95·6)	116·6 (113·8 to 119·4)	129·5 (125·8 to 133)	122·9 (119·1 to 126·9)	116·2 (111·5 to 120·4)	103·4 (96 to 110·7)	75·6 (66 to 87·2)
Jambi	15·1 (14·4 to 15·9)	21·1 (20·3 to 21·9)	23·5 (22·6 to 24·5)	27·3 (26·5 to 28·2)	28·3 (27 to 29·4)	30·3 (29·6 to 31)	31·4 (30·4 to 32·5)	26·6 (24·6 to 29)
West Java	301·5 (286·6 to 316·3)	406·1 (389·3 to 422)	445·3 (433·6 to 457·6)	444·5 (434 to 455)	383·7 (370·1 to 397)	411·2 (397·5 to 423·8)	422·8 (394·4 to 450·7)	372·6 (327·8 to 426·3)
Central Java	356·8 (338·3 to 374·7)	462 (441·3 to 481·7)	467·3 (453·9 to 481·1)	430·3 (419·3 to 441)	349·8 (337·7 to 361·8)	312 (299·4 to 324·5)	268·1 (248·8 to 286·8)	210·3 (183·7 to 242·5)
East Java	410·5 (385·7 to 435·2)	515 (487 to 541·5)	502·1 (484·4 to 519·7)	433·7 (420·1 to 447)	338·1 (326·1 to 350·3)	308·1 (296·3 to 319·3)	270·3 (250·6 to 289·5)	208·5 (181·5 to 241·7)
West Kalimantan	29·5 (28·1 to 30·9)	40·3 (38·8 to 41·7)	47 (45·6 to 48·4)	55·1 (53·6 to 56·7)	50·8 (48·9 to 52·5)	51·3 (49·7 to 52·9)	50 (48 to 52)	42·1 (38·9 to 46)
South Kalimantan	27·1 (25·8 to 28·4)	36·9 (35·4 to 38·4)	38·6 (37·4 to 39·9)	38·6 (37·5 to 39·8)	36·9 (35·3 to 38·5)	38·7 (37·2 to 40·2)	37·1 (34·6 to 39·6)	30·7 (27·4 to 34·5)
Central Kalimantan	9·3 (8·8 to 9·7)	12·9 (12·5 to 13·4)	15 (14·4 to 15·5)	18 (17·4 to 18·6)	20·5 (19·7 to 21·2)	22·6 (21·8 to 23·4)	23·4 (22·6 to 24·3)	19·6 (18·1 to 21·4)
East Kalimantan	9·1 (8·7 to 9·5)	13·5 (13 to 13·9)	16·5 (15·9 to 17·1)	21·2 (20·4 to 21·9)	24·5 (23·6 to 25·4)	31·5 (30·6 to 32·4)	37·3 (36 to 38·5)	32 (29·7 to 34·9)
North Kalimantan	1·3 (1·2 to 1·3)	2·3 (2·2 to 2·4)	3·2 (3·1 to 3·3)	3·8 (3·6 to 3·9)	4·1 (3·7 to 4·5)	5·3 (4·7 to 5·8)	6·1 (5·5 to 6·8)	5·9 (5·3 to 6·5)
Riau Islands	3·9 (3·7 to 4·1)	4·6 (4·4 to 4·8)	5·1 (4·8 to 5·3)	7·8 (7·6 to 8·1)	14·2 (13·6 to 14·7)	18·5 (17·9 to 19·1)	23·6 (22·9 to 24·2)	16·9 (15·6 to 18·3)
Lampung	37·9 (36 to 39·8)	61·7 (59·2 to 64·2)	75·5 (73·5 to 77·6)	86·5 (84·2 to 88·6)	85·4 (82·2 to 88·9)	83 (79·9 to 86·1)	74·3 (69·4 to 79·2)	58·8 (52·7 to 65·9)
Maluku	13·6 (13 to 14·2)	16·1 (15·6 to 16·7)	16·9 (16·4 to 17·4)	19·1 (18·6 to 19·7)	20·5 (20 to 21)	21·9 (21·3 to 22·6)	21·5 (20·7 to 22·3)	17·7 (16·4 to 19·3)
North Maluku	7 (6·6 to 7·3)	8·2 (7·8 to 8·6)	8·5 (7·9 to 9)	9·3 (8·8 to 9·8)	10·8 (10·4 to 11·2)	12·9 (12·4 to 13·4)	12·6 (11·8 to 13·4)	10·5 (9·2 to 11·9)
West Nusa Tenggara	39·7 (37·5 to 41·8)	47·8 (45·8 to 49·7)	52·3 (50·9 to 53·9)	56·3 (54·8 to 57·8)	51·7 (49·8 to 53·6)	50·6 (48·6 to 52·7)	50·6 (47·2 to 54)	43·2 (38·8 to 48·4)
East Nusa Tenggara	42·5 (40·5 to 44·4)	51·2 (49·4 to 52·9)	53·8 (52·2 to 55·5)	53·1 (52 to 54·4)	54·4 (52·5 to 56·3)	60 (58·6 to 61·5)	60·9 (58·4 to 63·2)	53·9 (50·1 to 58·4)
Papua	5·1 (4·9 to 5·3)	8·9 (8·6 to 9·2)	11·5 (11·1 to 11·9)	14·6 (14·1 to 15·1)	18·6 (18·1 to 19)	28·7 (28 to 29·4)	43 (41·7 to 44·2)	45·6 (42·5 to 49·2)
West Papua	4·9 (4·6 to 5)	7·5 (7·3 to 7·8)	9·2 (9 to 9·5)	11·7 (11·3 to 12·1)	12·2 (12 to 12·5)	10·9 (10·6 to 11·2)	10·9 (10·5 to 11·2)	9·2 (8·6 to 10)

Riau	15·8 (15 to 16·5)	25 (24·1 to 25·8)	31·1 (30·3 to 32)	38 (36·9 to 39·1)	36·9 (35·3 to 38·4)	49·4 (48·1 to 50·5)	70 (66·5 to 73·3)	72·8 (67·4 to 79·4)
West Sulawesi	10·7 (10·2 to 11·1)	12·7 (12·2 to 13·2)	13 (12·6 to 13·5)	13 (12·7 to 13·4)	12·7 (12·3 to 13)	14·4 (14 to 14·7)	15 (14·4 to 15·4)	13·6 (12·5 to 14·8)
South Sulawesi	76·6 (72·6 to 80·4)	100·8 (96·8 to 104·6)	110·9 (108 to 114·2)	108·1 (105·1 to 111·1)	98·6 (95 to 102·4)	96·6 (93 to 99·9)	85·3 (80 to 90·9)	70·6 (63·4 to 79)
Central Sulawesi	15·1 (14·4 to 15·8)	19·8 (19·1 to 20·5)	23 (22·3 to 23·8)	26·9 (26·2 to 27·6)	26·5 (25·4 to 27·5)	30·1 (29·2 to 31)	29·3 (28·2 to 30·4)	24·8 (22·8 to 27·1)
Southeast Sulawesi	12·3 (11·8 to 12·9)	16 (15·4 to 16·5)	18·6 (18·1 to 19·2)	22·2 (21·6 to 22·8)	23·3 (22·7 to 24)	26·6 (25·7 to 27·5)	28·9 (27·5 to 30·2)	25·8 (23·8 to 28·2)
North Sulawesi	21 (19·9 to 22·1)	23·7 (22·7 to 24·7)	25·5 (24·8 to 26·3)	26·7 (25·8 to 27·5)	24·2 (23 to 25·3)	23·5 (22·8 to 24·1)	18·7 (17·9 to 19·5)	14·2 (12·8 to 15·8)
West Sumatra	56·9 (54·2 to 59·6)	63·2 (60·7 to 65·6)	61 (59·2 to 62·9)	64·6 (62·9 to 66·2)	60·1 (58·5 to 61·7)	59·9 (57·6 to 62·2)	53 (49·7 to 56·3)	42·9 (38·7 to 48)
South Sumatra	41·2 (39·2 to 43)	56·9 (54·8 to 59)	65·2 (63·5 to 67)	80·5 (78·5 to 82·5)	80·7 (78 to 83·5)	82·8 (79·4 to 86·4)	80·2 (74·6 to 85·8)	65·5 (58·8 to 73·3)
North Sumatra	94·2 (90 to 98·3)	128·3 (123·7 to 132·7)	146·2 (142·4 to 150·3)	170 (165·6 to 174·2)	174 (169·3 to 179·1)	174·8 (168·6 to 180·8)	164·8 (155·3 to 174·3)	138·2 (125·4 to 153·1)
Yogyakarta	43·1 (40·1 to 45·9)	48·2 (45·3 to 51·1)	45·4 (43·5 to 47·3)	39 (37·7 to 40·3)	30·5 (29·4 to 31·6)	27·4 (26·5 to 28·4)	25·1 (23·2 to 26·9)	20·7 (17·9 to 24)
Laos	42·8 (40 to 45·6)	52·4 (49·6 to 55)	62·3 (59·1 to 65·4)	73·7 (71·2 to 76·1)	84·7 (82·5 to 86·9)	94·2 (91·2 to 97·3)	88·8 (85·5 to 92·2)	88·9 (82·1 to 96·1)
Malaysia	135·8 (125·2 to 146·5)	158·2 (157·6 to 158·7)	181·5 (181 to 182)	195·3 (194·5 to 196·2)	254·1 (253·2 to 255·1)	272·4 (271·4 to 273·4)	248 (247·2 to 248·9)	278·2 (250·8 to 307·6)
Maldives	1·4 (1·3 to 1·5)	2·1 (2 to 2·2)	2·5 (2·4 to 2·6)	3·5 (3·4 to 3·6)	4·5 (4·4 to 4·7)	3·8 (3·5 to 4)	4·6 (4·2 to 5)	4·4 (3·8 to 5·1)
Mauritius	11·9 (11·5 to 12·3)	13·1 (12·8 to 13·3)	10·9 (10·5 to 11·2)	12·1 (12 to 12·2)	11·3 (11·2 to 11·4)	10·2 (10·1 to 10·3)	7·5 (7·4 to 7·6)	6·5 (5·7 to 7·4)
Myanmar	461·9 (431·5 to 492·2)	538·8 (507·8 to 568·5)	576·7 (541·4 to 613·4)	667·1 (622·8 to 710)	688·6 (646·7 to 731·9)	637·1 (600·4 to 671·7)	590·8 (557·1 to 625·9)	541·4 (477·6 to 612·7)
Philippines	507·6 (480·2 to 534·7)	681·1 (647·9 to 713·5)	827·1 (781·4 to 869)	999·5 (965·2 to 1034·6)	1081·7 (1039·3 to 1124·3)	1218·2 (1168 to 1268·5)	1342·1 (1278·7 to 1403·6)	1382 (1239 to 1535·9)
Seychelles	0·6 (0·5 to 0·6)	0·9 (0·8 to 0·9)	0·9 (0·9 to 0·9)	0·9 (0·8 to 0·9)	0·9 (0·8 to 0·9)	0·8 (0·8 to 0·8)	0·8 (0·7 to 0·9)	0·8 (0·7 to 0·9)
Sri Lanka	155·8 (148·9 to 163·6)	178·1 (172·2 to 184·3)	187·5 (178·5 to 196·1)	209·7 (198·2 to 220·7)	183·3 (171·9 to 194·6)	175·9 (165·1 to 186·4)	183·9 (173 to 195·3)	151·8 (128·3 to 179·2)
Thailand	503·3 (473·2 to 532·8)	633·5 (599·4 to 663·7)	640·8 (619·2 to 662·7)	630·2 (596·9 to 663·9)	545·6 (514·5 to 579·9)	464·5 (436·5 to 495·1)	391·6 (365·3 to 420·2)	300·4 (253·6 to 355·6)
Timor-Leste	11·8 (11·3 to 12·3)	13·7 (13·3 to 14·1)	12·5 (12·1 to 12·8)	14·4 (13·9 to 14·8)	19·6 (18·9 to 20·3)	19·7 (19 to 20·4)	18·4 (17·1 to 19·6)	19·9 (17·7 to 22·4)
Vietnam	617·5 (576·2 to 659·5)	719·6 (679·8 to 759·3)	755·8 (713·5 to 796·1)	863·7 (829·3 to 898)	923 (895·4 to 951·3)	817·4 (783·7 to 849·7)	851·6 (806·5 to 898·5)	695 (629·2 to 777)
Sub-Saharan Africa	4559·9 (4325·2 to 4781·5)	5709·1 (5481·3 to 5914·2)	7074·7 (6851·7 to 7300·7)	9053·8 (8785·7 to 9307)	11413·7 (11021·5 to 11787·6)	14308·4 (13872·9 to 14769·8)	17063 (16297 to 17790·9)	18719·4 (17457·6 to 20163)
Central sub-Saharan Africa	518·5 (499·6 to 535·9)	698·7 (676·2 to 720·4)	857·4 (826·9 to 886·5)	1080·8 (1044·1 to 1114·2)	1375·8 (1332·8 to 1416·8)	1740 (1683·9 to 1797·2)	2166 (2087 to 2245·3)	2267·9 (2107 to 2429·4)
Angola	126·7 (122·2 to 131·4)	141·8 (136·1 to 146·9)	155 (149·5 to 159·8)	194·7 (187·9 to 201·2)	262·7 (249·4 to 275·1)	367·2 (345·2 to 386·7)	496·1 (457·4 to 532·8)	563·6 (509·8 to 619·5)
Central African Republic	32 (30·2 to 33·7)	42·3 (39·6 to 44·8)	52·9 (50 to 55·4)	56·2 (53·1 to 59·3)	64·8 (60·9 to 68·8)	80·8 (75·4 to 86·1)	98·4 (90·1 to 106·4)	100·2 (92·3 to 108·1)
Congo (Brazzaville)	21·9 (20·7 to 23·1)	26·4 (25·4 to 27·3)	32·4 (31·3 to 33·5)	42 (40·5 to 43·5)	51·3 (49·5 to 53)	62 (60·5 to 63·7)	78·4 (76 to 80·8)	73·1 (67 to 79·4)
DR Congo	323·2 (308·7 to 336·5)	471·3 (453·7 to 487·1)	597·7 (573·2 to 622·2)	763·1 (734·1 to 789·8)	964·9 (934·5 to 992·5)	1191·9 (1153·5 to 1231·8)	1452·1 (1393·8 to 1509)	1489·7 (1353 to 1626·8)
Equatorial Guinea	5·5 (5·3 to 5·7)	5·8 (5·6 to 5·9)	6·4 (6·2 to 6·6)	8·3 (8 to 8·5)	11·7 (11·2 to 12·2)	16·1 (14·9 to 17·1)	18·9 (17·2 to 20·8)	19·5 (17 to 22·5)
Gabon	9·2 (8·5 to 9·9)	11·2 (10·6 to 11·8)	13 (12·3 to 13·7)	16·5 (15·7 to 17·4)	20·4 (19·1 to 21·8)	22 (19·6 to 24·4)	22·1 (19·4 to 25·1)	21·8 (18·7 to 25·5)
Eastern sub-Saharan Africa	1740·7 (1655·7 to 1821·3)	2203·5 (2125·9 to 2274·5)	2752·9 (2671·9 to 2833·2)	3609 (3514·9 to 3697·3)	4592·2 (4454·5 to 4724·7)	5673·2 (5505·5 to 5837·6)	6577·2 (6273·7 to 6872·8)	7207·8 (6672·8 to 7799)
Burundi	66·5 (63·8 to 69·1)	78·3 (75·6 to 80·8)	89 (86·5 to 91·4)	113·4 (110·6 to 116·1)	134·6 (131·4 to 137·7)	134·3 (129·3 to 139)	192·1 (184·1 to 200·4)	236·2 (221·2 to 252·9)
Comoros	2·9 (2·6 to 3·2)	4·4 (4·1 to 4·7)	6·6 (6·3 to 6·8)	8·6 (8·3 to 8·9)	10·4 (9·9 to 10·8)	10·5 (9·5 to 11·4)	9·6 (8·5 to 10·6)	8·6 (7·3 to 9·9)
Djibouti	1·7 (1·6 to 1·8)	2·6 (2·5 to 2·7)	4·2 (4·1 to 4·3)	7·6 (7·3 to 7·9)	11·4 (10·7 to 12)	14·5 (13·5 to 15·4)	18·8 (17·3 to 20·3)	18·2 (15·8 to 20·8)
Eritrea	29·3 (27·7 to 30·8)	38·8 (37·1 to 40·5)	48·5 (46·1 to 50·7)	62·5 (59·4 to 65·3)	69·4 (65·6 to 73·1)	85·4 (78·2 to 92·2)	100·7 (89·6 to 112·2)	101·4 (88·5 to 116)
Ethiopia	468·5 (440·5 to 494·5)	565·1 (539 to 588·9)	691 (665 to 717·7)	931·5 (893·2 to 967·1)	1268·4 (1223·3 to 1311·2)	1533·1 (1479·3 to 1584·6)	1701·8 (1593·4 to 1806·2)	1886·3 (1742·7 to 2054·7)
Kenya	161·9 (156·3 to 167·1)	237·7 (229·5 to 244·6)	311·1 (301·8 to 319·7)	412·6 (400·8 to 423·6)	514·8 (489·5 to 539·2)	608·2 (575 to 640·7)	676·4 (619·1 to 733)	685·2 (595·4 to 793·4)
Baringo	2·6 (2·5 to 2·7)	3·9 (3·7 to 4)	4·8 (4·7 to 5)	5·8 (5·7 to 5·9)	8 (7·8 to 8·3)	9·8 (9·4 to 10·3)	10·6 (9·8 to 11·4)	10 (8·7 to 11·6)
Bomet	2·4 (2·4 to 2·5)	3·3 (3·2 to 3·4)	4·5 (4·4 to 4·6)	6 (5·9 to 6·2)	8·2 (7·9 to 8·5)	10·9 (10·5 to 11·3)	18·3 (16·9 to 19·6)	21·2 (18·5 to 24·2)
Bungoma	5 (4·9 to 5·1)	7·5 (7·3 to 7·6)	10·3 (10 to 10·6)	14·4 (14 to 14·7)	20·8 (20·1 to 21·5)	36·2 (34·4 to 38·1)	26·4 (24·3 to 28·5)	17·9 (15·7 to 20·7)
Busia	4·2 (4·1 to 4·3)	5·5 (5·3 to 5·7)	6·3 (6 to 6·5)	8·8 (8·4 to 9·1)	11·5 (11 to 12)	12·7 (11·8 to 13·5)	14·8 (13·4 to 16·2)	14·6 (12·6 to 17)
Elgeyo Marakwet	2·8 (2·8 to 2·9)	4·6 (4·4 to 4·7)	4·9 (4·8 to 5·1)	4 (3·9 to 4·1)	5·8 (5·6 to 6)	10·1 (9·6 to 10·6)	5·9 (5·4 to 6·3)	3·2 (2·7 to 3·8)
Embu	2·5 (2·4 to 2·6)	3·3 (3·2 to 3·5)	4·4 (4·3 to 4·6)	6·2 (6 to 6·4)	7·7 (7·2 to 8·2)	8 (7·5 to 8·6)	7 (6·3 to 7·8)	5·2 (4·3 to 6·3)
Garissa	0·8 (0·7 to 0·8)	1·2 (1·2 to 1·3)	2 (1·9 to 2)	3 (2·8 to 3·1)	3 (2·8 to 3·1)	6·6 (6·2 to 7)	13·8 (13 to 14·6)	21·8 (20·1 to 23·6)
Homa Bay	9·8 (9·5 to 10·1)	12·1 (11·7 to 12·4)	12·9 (12·5 to 13·3)	14·3 (13·9 to 14·7)	17·8 (17·1 to 18·5)	20·6 (19·5 to 21·6)	21·8 (20·2 to 23·3)	21·5 (18·9 to 24·2)
Isiolo	0·5 (0·5 to 0·5)	0·8 (0·7 to 0·8)	0·9 (0·9 to 0·9)	1·2 (1·2 to 1·3)	1·7 (1·6 to 1·8)	2·3 (2·2 to 2·5)	2·8 (2·5 to 3)	2·8 (2·4 to 3·3)
Kajiado	1·1 (1 to 1·1)	1·6 (1·5 to 1·6)	2·3 (2·2 to 2·4)	3·8 (3·7 to 4)	5·8 (5·5 to 6·1)	8·2 (7·6 to 8·8)	11·9 (10·5 to 13·2)	11·5 (9·6 to 13·8)
Kakamega	7·5 (7·3 to 7·6)	11·9 (11·6 to 12·2)	15·9 (15·5 to 16·3)	21·7 (21·2 to 22·2)	29·6 (28·5 to 30·7)	22·8 (21·7 to 23·8)	33·5 (30·8 to 36·4)	39·1 (33·7 to 45·8)
Kericho	2·8 (2·8 to 2·9)	5·7 (5·6 to 5·9)	8·8 (8·7 to 9)	11·8 (11·6 to 12)	15·8 (15·3 to 16·4)	17·5 (16·7 to 18·4)	11 (10·1 to 11·8)	6·1 (5·4 to 7)
Kiambu	5·9 (5·7 to 6·1)	8 (7·7 to 8·2)	11·9 (11·6 to 12·2)	17·2 (16·7 to 17·6)	20 (18·7 to 21·2)	26·3 (24·9 to 27·7)	24·9 (22·7 to 27·2)	19·2 (16·5 to 22·3)
Kilifi	5·2 (5 to 5·5)	7·6 (7·3 to 7·9)	9·4 (9·1 to 9·7)	12·8 (12·3 to 13·2)	14·4 (13·5 to 15·3)	16·8 (15·8 to 17·8)	19·4 (18 to 21)	19·5 (16·7 to 23)
Kirinyaga	3·1 (3 to 3·2)	4·7 (4·5 to 4·8)	5·9 (5·7 to 6·1)	7 (6·7 to 7·2)	7·9 (7·2 to 8·5)	8·2 (7·6 to 8·8)	7·2 (6·4 to 8)	5·4 (4·4 to 6·6)
Kisii	5·2 (5·1 to 5·3)	10 (9·7 to 10·2)	13·8 (13·4 to 14·1)	18·5 (17·8 to 19)	21·3 (20·2 to 22·4)	22·9 (21·3 to 24·4)	18·6 (16·7 to 20·6)	13·2 (10·9 to 16)
Kisumu	7 (6·8 to 7·2)	10·3 (9·9 to 10·7)	12·1 (11·7 to 12·6)	13 (12·5 to 13·4)	16·4 (15·4 to 17·3)	16·6 (15·5 to 17·8)	16·2 (14·4 to 18·1)	13·6 (11·4 to 16·4)
Kitui	6·3 (6·1 to 6·5)	8·7 (8·4 to 9)	10·6 (10·4 to 10·9)	13 (12·6 to 13·3)	16·9 (16 to 17·7)	18·8 (17·8 to 19·8)	17·8 (16·3 to 19·3)	16·1 (14·2 to 18·3)
Kwale	3·3 (3·2 to 3·5)	5·3 (5·1 to 5·5)	6·5 (6·2 to 6·8)	7·7 (7·4 to 8·1)	8·4 (7·9 to 9)	9·7 (9 to 10·4)	10·9 (9·8 to 12)	10·9 (9·1 to 13·1)

Laikipia	0·7 (0·7 to 0·7)	1 (1 to 1·1)	1·8 (1·7 to 1·8)	3·5 (3·4 to 3·5)	5·1 (4·8 to 5·3)	7·3 (6·8 to 7·7)	6·6 (6 to 7·2)	4·9 (4·2 to 5·8)
Lamu	0·5 (0·5 to 0·5)	0·5 (0·5 to 0·6)	0·7 (0·6 to 0·7)	1 (0·9 to 1)	1·2 (1·1 to 1·3)	1·4 (1·3 to 1·5)	1·5 (1·4 to 1·7)	1·5 (1·3 to 1·8)
Machakos	4·8 (4·6 to 4·9)	7·8 (7·5 to 8·1)	11·6 (11·2 to 11·9)	17 (16·4 to 17·6)	20·6 (19·3 to 21·9)	20 (18·6 to 21·4)	15·1 (13·5 to 16·8)	9·9 (8·3 to 11·9)
Makueni	5 (4·9 to 5·2)	5·9 (5·7 to 6·1)	7·2 (6·9 to 7·4)	8·9 (8·7 to 9·1)	11·1 (10·6 to 11·6)	12·7 (12 to 13·5)	15·8 (14·2 to 17·5)	16·9 (14·8 to 19·3)
Mandera	1·3 (1·2 to 1·3)	2 (1·9 to 2·1)	2·4 (2·3 to 2·5)	2·4 (2·3 to 2·6)	2·8 (2·6 to 3)	5·4 (5·1 to 5·8)	18·8 (17·5 to 20·1)	49·8 (45·1 to 55·1)
Marsabit	0·7 (0·7 to 0·7)	1 (0·9 to 1)	1·6 (1·5 to 1·6)	2·7 (2·6 to 2·7)	3·2 (3 to 3·3)	4·1 (3·8 to 4·3)	5·7 (5·2 to 6·2)	7·6 (6·7 to 8·5)
Meru	7·4 (7·1 to 7·6)	11·5 (11·1 to 11·9)	15·1 (14·6 to 15·5)	19·2 (18·6 to 19·7)	22·8 (21·6 to 23·9)	23·9 (22·4 to 25·3)	21·6 (19·3 to 24·1)	17·1 (14·3 to 20·6)
Migori	6 (5·8 to 6·2)	7·9 (7·7 to 8·2)	9·1 (8·8 to 9·4)	11 (10·7 to 11·2)	14·5 (13·8 to 15·1)	18·8 (17·8 to 19·8)	22·1 (20·6 to 23·7)	24·4 (21·7 to 27·3)
Mombasa	2·7 (2·6 to 2·9)	5·1 (4·8 to 5·3)	6·5 (6·2 to 6·8)	7·1 (6·8 to 7·5)	8·5 (7·8 to 9·3)	11·2 (10·3 to 12·1)	15·1 (13·5 to 16·8)	13·8 (11·3 to 17·1)
Murang'a	7·7 (7·5 to 7·9)	10·2 (9·8 to 10·4)	12·5 (12·3 to 12·8)	15·5 (15 to 15·8)	18 (17 to 18·8)	12·8 (11·9 to 13·5)	11 (10 to 12)	8·9 (7·6 to 10·4)
Nairobi	4·8 (4·6 to 5)	8·3 (7·8 to 8·6)	13·8 (13·1 to 14·4)	27·1 (25·6 to 28·6)	24·5 (22·4 to 26·6)	39·4 (36·3 to 42·6)	50·2 (44·8 to 55·6)	45·6 (38·6 to 54·6)
Nakuru	2·9 (2·8 to 3)	4·9 (4·7 to 5·1)	8·1 (7·9 to 8·3)	13·3 (12·9 to 13·6)	20·4 (19·5 to 21·4)	24·6 (23 to 26·2)	25·6 (22·9 to 28·2)	19·7 (16·6 to 23·6)
Nandi	3 (2·9 to 3)	4·5 (4·3 to 4·6)	6·2 (6·1 to 6·4)	7·8 (7·7 to 8)	10·6 (10·1 to 11)	12·2 (11·6 to 12·8)	12·4 (11·5 to 13·3)	10·3 (8·9 to 11·9)
Narok	2 (1·9 to 2)	2·7 (2·6 to 2·8)	3·8 (3·7 to 3·8)	6·3 (6·2 to 6·4)	10·8 (10·4 to 11·1)	9·5 (9·1 to 9·9)	20·5 (19·1 to 21·8)	32·5 (28·8 to 37)
Nyamira	3·2 (3·1 to 3·3)	4·1 (3·9 to 4·2)	4·7 (4·6 to 4·9)	5·4 (5·2 to 5·6)	6·1 (5·7 to 6·4)	7 (6·2 to 7·7)	8·4 (7·5 to 9·4)	7·9 (6·7 to 9·2)
Nyandarua	1·8 (1·7 to 1·9)	3 (2·9 to 3·1)	4·2 (4·1 to 4·3)	5·3 (5·1 to 5·5)	8·3 (7·8 to 8·9)	10·2 (9·5 to 10·9)	9 (8·1 to 10)	7·8 (6·6 to 9·3)
Nyeri	5·3 (5·1 to 5·5)	6·3 (6·1 to 6·5)	8·1 (7·9 to 8·4)	11·1 (10·6 to 11·6)	12·5 (11·5 to 13·4)	10·8 (9·9 to 11·7)	8·3 (7·2 to 9·5)	6 (4·9 to 7·3)
Samburu	1·1 (1·1 to 1·2)	1·9 (1·9 to 2)	2·3 (2·3 to 2·4)	2·4 (2·3 to 2·4)	3·4 (3·3 to 3·5)	3·9 (3·8 to 4·1)	5 (4·7 to 5·4)	5·9 (5·2 to 6·7)
Siaya	5·2 (5·1 to 5·4)	8·6 (8·3 to 8·8)	10·5 (10·2 to 10·8)	12·7 (12·3 to 13)	15·6 (14·8 to 16·3)	16·5 (15·5 to 17·4)	16·3 (15 to 17·6)	14·6 (12·8 to 16·6)
Taita Taveta	1·5 (1·4 to 1·6)	2·3 (2·2 to 2·4)	2·8 (2·7 to 2·9)	3·4 (3·2 to 3·5)	4·2 (3·9 to 4·5)	3·7 (3·4 to 4)	3·4 (3 to 3·8)	2·6 (2·1 to 3·2)
Tana River	0·8 (0·8 to 0·9)	1·1 (1·1 to 1·1)	1·5 (1·4 to 1·6)	2·6 (2·5 to 2·6)	3 (2·8 to 3·2)	3·9 (3·6 to 4·2)	4·5 (4·2 to 4·9)	5·2 (4·5 to 6)
Tharaka Nithi	1·7 (1·6 to 1·8)	1·7 (1·7 to 1·8)	1·7 (1·7 to 1·8)	1·8 (1·7 to 1·8)	1·9 (1·8 to 2)	1·9 (1·8 to 2·1)	1·8 (1·6 to 2·1)	1·6 (1·4 to 1·9)
Trans Nzoia	1·1 (1 to 1·1)	1·7 (1·6 to 1·7)	3·2 (3·1 to 3·3)	6·9 (6·7 to 7)	9·9 (9·5 to 10·3)	12·7 (12·1 to 13·4)	14·1 (12·8 to 15·4)	12·4 (10·5 to 14·7)
Turkana	2·2 (2·1 to 2·2)	4·1 (4 to 4·3)	5·5 (5·3 to 5·6)	4·8 (4·7 to 4·9)	6·3 (6·1 to 6·6)	11·7 (11·2 to 12·1)	20·4 (19·1 to 21·8)	29·2 (26·2 to 32·7)
Uasin Gishu	1·9 (1·8 to 1·9)	3·4 (3·3 to 3·4)	5·5 (5·4 to 5·6)	8 (7·8 to 8·2)	10·5 (10 to 10·9)	13·4 (12·5 to 14·2)	15·8 (14·2 to 17·4)	12·8 (10·7 to 15·3)
Vihiga	5·9 (5·7 to 6)	6·8 (6·6 to 7)	7 (6·7 to 7·3)	6·7 (6·4 to 6·9)	8·2 (7·7 to 8·6)	7·1 (6·5 to 7·6)	8·3 (7·5 to 9·2)	8·5 (7·2 to 10)
Wajir	1·2 (1·1 to 1·3)	1·6 (1·5 to 1·7)	2·5 (2·4 to 2·6)	3·7 (3·5 to 3·8)	3·2 (3·1 to 3·3)	8·4 (7·9 to 8·8)	14·4 (13·7 to 15·1)	20·8 (19·4 to 22·5)
West Pokot	1·3 (1·3 to 1·4)	1·8 (1·8 to 1·9)	2·8 (2·7 to 2·9)	5 (4·9 to 5·1)	6·7 (6·4 to 6·9)	8·6 (8·3 to 9)	11·7 (10·9 to 12·6)	14·3 (12·6 to 16·2)
Madagascar	112·5 (108 to 117·4)	139·4 (135·4 to 143·4)	179 (173·2 to 184·4)	226·4 (219·5 to 232·7)	276 (264·8 to 286·4)	346·2 (330·3 to 361·5)	407·5 (383·1 to 431·8)	440·2 (386·6 to 497·1)
Malawi	77·3 (72·5 to 81·7)	99·3 (95·1 to 103·3)	120·4 (116·3 to 124·5)	155·4 (150·5 to 160)	237·5 (231·6 to 243·7)	266·9 (257·7 to 275·6)	268·4 (253·7 to 283·2)	274·7 (251·2 to 302·7)
Mozambique	166·2 (155 to 176·7)	192·1 (183·2 to 200·6)	233·8 (223·3 to 243·9)	304·4 (294·5 to 314·2)	305·7 (298·1 to 312·7)	419·9 (406·9 to 432·6)	496·4 (480·6 to 511·3)	569·6 (529·4 to 610·9)
Rwanda	74·9 (72·6 to 77·1)	86·7 (84·7 to 88·5)	103·4 (101·9 to 105)	131·5 (129·3 to 133·5)	161·4 (158 to 164·4)	164·3 (160·1 to 168·6)	173·7 (164·6 to 182·2)	178·4 (162·6 to 196·4)
Somalia	54·1 (51·6 to 56·4)	72 (69·1 to 74·9)	97·6 (93·6 to 101·2)	162·7 (156·2 to 168·7)	172·4 (165·3 to 178·9)	261·7 (247·2 to 274·5)	345·9 (327·8 to 364)	446·8 (413·1 to 482·7)
South Sudan	53·6 (49 to 58)	68·4 (63·6 to 73·1)	87 (81·5 to 91·9)	103·7 (98·1 to 109·6)	128·5 (119·2 to 137·1)	165·2 (153·3 to 176·1)	208·5 (192·3 to 223·6)	185·5 (172·9 to 200·3)
Uganda	168·6 (161·7 to 174·9)	215·1 (208·6 to 220·7)	276 (269 to 282·5)	355·4 (347·9 to 362·5)	474·8 (466·1 to 483·1)	633·3 (620·5 to 644·8)	739·2 (713·6 to 762·7)	805·8 (761 to 857·2)
Tanzania	233·9 (221·6 to 245·3)	299·9 (288·1 to 310·9)	374·7 (362·5 to 386·9)	478·5 (459·7 to 496·1)	626 (604·7 to 647·2)	793·4 (756·2 to 829·2)	955 (906·7 to 1004·8)	1054·7 (973·2 to 1144·9)
Zambia	68·2 (65·5 to 70·7)	102·8 (100 to 105·1)	129·3 (126 to 132·4)	152·8 (147·9 to 157·2)	197·8 (190·7 to 205·1)	231·8 (221·3 to 241·2)	278 (259·8 to 297·6)	310·6 (280·5 to 345)
Southern sub-Saharan Africa	406·2 (375·2 to 436·4)	490·5 (460·6 to 516·7)	603·6 (569·2 to 638·6)	721·6 (672·4 to 768·6)	779·9 (723·5 to 839·5)	846·6 (780·6 to 910·9)	887·9 (820·4 to 958·5)	845·3 (761·2 to 943·3)
Botswana	9·3 (8·7 to 9·9)	11·8 (11 to 12·4)	14·5 (13·6 to 15·4)	17·7 (16·4 to 19)	20·5 (18·4 to 22·5)	22·9 (20·3 to 25·7)	24·7 (22·1 to 27·6)	24·5 (21·3 to 28·2)
eSwatini	6·2 (5·9 to 6·4)	8·3 (7·9 to 8·7)	10·9 (10·4 to 11·2)	13·9 (13·3 to 14·5)	16·3 (15·7 to 16·9)	17·9 (16·9 to 18·9)	17·2 (15·9 to 18·4)	15·2 (13·4 to 17·3)
Lesotho	13·2 (12 to 14·4)	16 (14·5 to 17·5)	19·8 (18·3 to 21·3)	26·5 (23·9 to 29·3)	31·8 (29·3 to 34·1)	30·5 (29 to 31·9)	26·4 (24·8 to 27·9)	23·8 (20·5 to 27·2)
Namibia	11 (10·4 to 11·7)	14·1 (13·4 to 14·7)	16·7 (15·9 to 17·5)	20 (18·9 to 21·1)	24·2 (22·7 to 25·8)	27·7 (26·1 to 29·3)	30·3 (27·9 to 32·7)	31·5 (28·5 to 35·2)
South Africa	296·1 (271·4 to 320·3)	348·5 (324·5 to 370·2)	414·6 (385·2 to 444)	466 (421·2 to 508·9)	489·9 (437·9 to 545·6)	537·9 (477·5 to 597·9)	560·1 (499·1 to 625·5)	524 (464·5 to 593·6)
Zimbabwe	70·5 (67 to 73·7)	91·8 (88·2 to 95·1)	127 (122·7 to 131)	177·5 (173·7 to 181·5)	197·2 (191·7 to 202·6)	209·6 (204·1 to 215·7)	229·2 (221·8 to 236)	226·4 (208·8 to 247·4)
Western sub-Saharan Africa	1894·4 (1795·5 to 1987·9)	2316·5 (2215·9 to 2406·9)	2860·8 (2770·4 to 2950·8)	3642·4 (3526·6 to 3748·4)	4665·8 (4510·4 to 4810·5)	6048·6 (5853·3 to 6245·4)	7431·8 (7094·2 to 7763)	8398·3 (7808·5 to 9049·5)
Benin	50·9 (47·7 to 53·9)	57·7 (55·2 to 60)	70·2 (68 to 72·3)	89·7 (86·8 to 92·5)	121·2 (117·7 to 124·7)	156·9 (151·8 to 161·9)	212·9 (203·6 to 221·5)	258·3 (242·7 to 276·7)
Burkina Faso	94·7 (87·7 to 101·6)	107·7 (102 to 112·8)	132·3 (127·2 to 136·9)	177·9 (172·9 to 182·9)	233·3 (225·6 to 240·2)	292·3 (283·6 to 300·3)	380 (364·3 to 395·3)	478·2 (445·6 to 514·1)
Cape Verde	3·6 (3·5 to 3·8)	4·7 (4·6 to 4·8)	4·7 (4·6 to 4·9)	5·3 (5·1 to 5·5)	6 (5·7 to 6·2)	6·5 (6 to 6·9)	6·3 (5·4 to 7·3)	5·5 (4·6 to 6·5)
Cameroon	108·2 (99·9 to 116·1)	136·4 (127 to 145·3)	165 (158·5 to 171·3)	194·7 (188·6 to 200·4)	236·9 (228 to 244·8)	324·3 (312·5 to 335·2)	440·1 (415·5 to 465·5)	460 (405·8 to 523)
Chad	66·3 (62·9 to 69·4)	75·9 (73 to 78·4)	94·8 (92 to 97·6)	124·5 (121·4 to 127·5)	163·8 (160 to 167·3)	222·4 (217·2 to 227·2)	302·5 (293·3 to 310·8)	401·9 (384·5 to 420·3)
Côte d'Ivoire	62·5 (58 to 66·8)	102·1 (96·2 to 107·4)	142·9 (136·6 to 148·7)	203 (195·9 to 209·7)	293·4 (281·5 to 304·5)	383 (361·2 to 405·1)	439·7 (413·7 to 465·6)	455 (414·5 to 500·5)
The Gambia	5·8 (5·4 to 6·2)	7·5 (7 to 7·9)	11·8 (11·2 to 12·4)	18·2 (17·3 to 19·1)	25·6 (24 to 27)	32·6 (30·1 to 34·9)	35·7 (32·2 to 39·1)	36·7 (32·1 to 42)
Ghana	112·3 (103 to 122·3)	174·6 (165·4 to 183·4)	231·6 (224·3 to 238·8)	287·2 (277·5 to 296·4)	310·9 (295·8 to 327·1)	352·4 (332·4 to 372·2)	413 (387·7 to 439·6)	436·8 (375·5 to 504·6)
Guinea	66·4 (63·4 to 69·1)	82·1 (78 to 85·9)	103·4 (99 to 107·7)	129 (124·3 to 133·2)	157·8 (152·9 to 162·5)	179·2 (172·4 to 185·8)	211·4 (200·2 to 222·1)	243·5 (225 to 261·5)
Guinea-Bissau	16·2 (15·5 to 16·9)	15·3 (14·4 to 16·1)	15·7 (14·6 to 16·6)	19·3 (18·1 to 20·4)	23·8 (22·2 to 25·4)	27·3 (25·1 to 29·4)	31·3 (28·1 to 34·3)	32 (28·3 to 36)

Liberia	22 (20·5 to 23·5)	29 (27·4 to 30·4)	37 (35·6 to 38·5)	50·5 (48·5 to 52·3)	45·6 (43·4 to 47·6)	67·1 (64·1 to 70·3)	77·4 (71·4 to 82·9)	69·7 (60·7 to 80·3)
Mali	95·1 (90 to 99·8)	127·2 (122·1 to 131·6)	164 (159·5 to 168·4)	200·3 (195·4 to 204·8)	232·5 (226·7 to 237·9)	281·3 (273·2 to 289·3)	374·8 (359·5 to 388·4)	491·9 (461·8 to 523·9)
Mauritania	15·9 (14·7 to 17·2)	20·7 (19·5 to 21·8)	27·3 (26·2 to 28·3)	37 (35·7 to 38·3)	46·3 (44·2 to 48·4)	52·1 (49·3 to 54·7)	56·9 (53 to 60·6)	55·7 (50·1 to 62·3)
Niger	71·8 (68·2 to 75·3)	99·5 (96·1 to 102·6)	136·6 (133·1 to 140)	178·8 (175·1 to 182·3)	227·5 (222·6 to 232·2)	327·3 (319·8 to 334·1)	432·2 (421·6 to 442·1)	583·6 (562·5 to 605·7)
Nigeria	956·5 (907 to 1001·9)	1091·5 (1041·4 to 1135·3)	1293·7 (1243·4 to 1342·7)	1642·7 (1568·6 to 1709·7)	2196·2 (2103·4 to 2285·9)	2943 (2810·6 to 3068·3)	3529·6 (3328·2 to 3732·5)	3886·1 (3596 to 4219·6)
São Tomé and PrÍncipe	1·3 (1·2 to 1·3)	1·6 (1·6 to 1·6)	1·6 (1·6 to 1·7)	1·9 (1·9 to 2)	2·4 (2·3 to 2·5)	2·7 (2·6 to 2·9)	2·7 (2·5 to 2·9)	2·4 (2 to 2·8)
Senegal	66·5 (62·3 to 70·5)	88·7 (85·2 to 91·9)	119·1 (115·4 to 122·5)	149·8 (146·4 to 153·2)	175·3 (170·9 to 179·6)	196·7 (190·3 to 203·5)	231·9 (224 to 239·7)	237·4 (219 to 259·6)
Sierra Leone	40·6 (36·8 to 44·3)	50·3 (46·2 to 53·9)	58 (54·4 to 61·6)	66·7 (62·3 to 70·6)	84·2 (78·2 to 89·9)	100·1 (93·5 to 106·2)	129·7 (119·9 to 139·6)	142·7 (130·3 to 157·3)
Togo	37·5 (35·5 to 39·4)	44 (42·7 to 45·4)	50·9 (49·5 to 52·2)	65·5 (63·8 to 67·1)	83·2 (81·4 to 85)	101·2 (98 to 104·5)	123·7 (118·6 to 128·7)	121 (112 to 132)

Supplemental Results Table 1c: Live births (in thousands) by location, females, 1950, 1960, 1970, 1980, 1990, 2000, 2010, 2019

Location	1950	1960	1970	1980	1990	2000	2010	2019
Global	46913·1 (45251·5 to 48603·7)	53170·6 (51676·6 to 54658·7)	56286·9 (54818·3 to 57681·4)	63367 (62050·9 to 64610·7)	66766·8 (65344·1 to 68157·7)	64782·5 (63272·4 to 66346·4)	66332·4 (64200·6 to 68399·2)	65307·6 (61341·9 to 69524·2)
Central Europe, eastern Europe, and central Asia	3704 (3618·3 to 3796)	3696 (3651·7 to 3737)	3036·3 (2985·6 to 3089·4)	3501·8 (3463·2 to 3542·6)	3161·5 (3123·9 to 3202·8)	2240·2 (2206·1 to 2275·9)	2736·1 (2688·6 to 2787)	2518·5 (2260 to 2810·9)
Central Asia	531·4 (508·4 to 553·8)	691·5 (668·9 to 713·8)	664·5 (630·2 to 701·2)	859·8 (835·5 to 884·6)	960·3 (939·1 to 982·1)	737·9 (715·6 to 760)	923·9 (886·7 to 964·8)	911·7 (844·4 to 981·5)
Armenia	26·6 (25·1 to 28·2)	37·6 (36 to 39·2)	30·7 (28·8 to 32·8)	43·4 (41·3 to 45·4)	38·5 (37·1 to 40)	20·3 (19·4 to 21·3)	22·3 (21·2 to 23·4)	18 (16·2 to 19·9)
Azerbaijan	60·4 (56·5 to 64·3)	88·6 (85·5 to 91·9)	75·5 (71 to 80·4)	85·6 (81·8 to 89·4)	98·3 (93·4 to 103·2)	68·8 (65·1 to 72·5)	87·3 (80·7 to 93·8)	71·3 (61·9 to 82)
Georgia	42·4 (38·6 to 46·6)	49·4 (46·3 to 52·4)	42·7 (38·2 to 47·9)	45·2 (42·7 to 47·8)	39·4 (37·4 to 41·4)	22·8 (21·5 to 24·1)	26·4 (24·9 to 27·8)	22·2 (19·2 to 25·6)
Kazakhstan	126·6 (120·5 to 132·6)	170·3 (164·7 to 175·7)	162·7 (152·3 to 173·6)	181 (175·8 to 186·4)	172·3 (166·9 to 177·3)	112·6 (108 to 117·1)	167·4 (160·4 to 174·5)	170 (155·4 to 184·9)
Kyrgyzstan	28·8 (27 to 30·5)	37·9 (36·4 to 39·4)	39·8 (36·5 to 43·1)	55·5 (53 to 58)	60·1 (58 to 61·9)	48 (45·7 to 50·6)	66·9 (64 to 70·1)	69·6 (63·1 to 76·6)
Mongolia	15·5 (14·5 to 16·5)	18·6 (17·6 to 19·6)	23·1 (22·2 to 23·9)	31·1 (30 to 32·1)	36·5 (35·3 to 37·6)	28·3 (27 to 29·6)	40·1 (38·2 to 42)	40·6 (35·8 to 45·6)
Tajikistan	45·3 (43·4 to 47·1)	59·9 (58·4 to 61·3)	59·6 (57·5 to 61·7)	83·6 (81·2 to 86·1)	100·5 (96·5 to 104·3)	89·7 (83·8 to 95·5)	110·5 (101·9 to 118·8)	122·5 (111·3 to 135·6)
Turkmenistan	26·1 (25 to 27·2)	35·2 (34·4 to 36)	36·8 (35 to 38·7)	52·9 (51·5 to 54·3)	65·5 (64 to 66·9)	49·2 (46·3 to 52·2)	55·1 (51·3 to 59·1)	54·8 (50 to 60·3)
Uzbekistan	159·7 (149·8 to 169·7)	193·9 (183·8 to 204)	193·6 (181 to 206·2)	281·5 (271·6 to 291·5)	349·2 (340·5 to 357·3)	298·2 (288·8 to 308)	347·9 (327·1 to 369·3)	342·8 (305·1 to 383·7)
Central Europe	1115 (1086·7 to 1145·7)	956·1 (944·3 to 967·1)	915·3 (905 to 926·8)	998·2 (988·2 to 1009·2)	808·5 (799·9 to 817·7)	582·3 (575·4 to 589)	586·8 (579·8 to 594·6)	519·2 (456·6 to 591·8)
Albania	24·4 (23·4 to 25·3)	32·6 (31·7 to 33·5)	34·3 (32·7 to 35·9)	35·4 (33·7 to 37·1)	42·9 (41·4 to 44·5)	28·5 (27·1 to 29·8)	20·2 (19·3 to 21·2)	17·9 (16 to 20)
Bosnia and Herzegovina	44·1 (38·8 to 49·8)	52·8 (47·8 to 57·9)	40·2 (34·4 to 47)	37·7 (33·9 to 41·7)	31·8 (29·3 to 34·2)	19·9 (19 to 20·8)	16·1 (15·2 to 17·1)	12·6 (11·5 to 13·8)
Bulgaria	80·7 (80·3 to 81·2)	67·6 (67·2 to 68)	66·6 (66·2 to 67)	62 (61·6 to 62·3)	48·3 (48 to 48·6)	34·6 (34·3 to 34·9)	36·2 (36 to 36·5)	29·2 (26·9 to 31·7)
Croatia	44·3 (44 to 44·5)	36·9 (36·6 to 37·1)	30·1 (29·9 to 30·3)	33 (32·8 to 33·2)	26·1 (25·9 to 26·3)	20·6 (20·4 to 20·7)	20·6 (20·5 to 20·8)	17·1 (15·1 to 19·4)
Czech Republic	91·8 (91·3 to 92·2)	62·5 (62·2 to 62·9)	73·7 (73·4 to 74·1)	72·7 (72·4 to 73·1)	62·4 (62·1 to 62·8)	44·4 (44·1 to 44·6)	55·6 (55·3 to 55·9)	53·1 (47·5 to 59·4)
Hungary	94·9 (94·4 to 95·3)	69·7 (69·3 to 70)	71·9 (71·5 to 72·2)	72·4 (72 to 72·8)	60·3 (60 to 60·6)	47·1 (46·9 to 47·5)	43·6 (43·4 to 43·9)	40 (35·1 to 45·5)
Montenegro	6 (5·5 to 6·5)	6·1 (5·7 to 6·5)	5·1 (4·8 to 5·5)	5·1 (4·8 to 5·5)	4·7 (4·4 to 5)	4 (3·8 to 4·2)	3·6 (3·5 to 3·7)	3·2 (2·9 to 3·4)
North Macedonia	19·3 (17·1 to 21·5)	19·2 (17 to 21·6)	17·9 (16·4 to 19·5)	19·3 (18 to 20·6)	17 (16·3 to 17·6)	12 (11·5 to 12·5)	11·4 (10·9 to 12)	10·7 (9·7 to 11·8)
Poland	353·2 (343·1 to 364·2)	306·3 (298 to 313·5)	254·6 (248·5 to 260·7)	329·4 (322 to 337·9)	258·4 (251·8 to 265·9)	176·4 (170·7 to 182·3)	196·1 (190·3 to 202·3)	176·5 (152·6 to 204·2)
Romania	204 (188·5 to 221·6)	166·4 (165·7 to 167·1)	197·7 (197 to 198·4)	194·2 (193·5 to 194·8)	140·9 (140·3 to 141·6)	110 (109·5 to 110·6)	100 (99·5 to 100·5)	84·2 (71·3 to 99·2)
Serbia	88·6 (88 to 89·2)	80·1 (79·7 to 80·4)	70·5 (70·2 to 70·9)	76·4 (76 to 76·7)	66·8 (66·4 to 67·1)	50·4 (50 to 50·7)	42·5 (40·2 to 44·8)	38·7 (32·7 to 45·7)
Slovakia	48·5 (48·2 to 48·8)	42·5 (42·3 to 42·8)	39·2 (39 to 39·4)	46·3 (46·1 to 46·6)	38·4 (38·2 to 38·6)	25·8 (25·6 to 26)	29·9 (29·7 to 30·1)	27 (23·9 to 30·6)
Slovenia	15·4 (13·7 to 17·5)	13·5 (13·2 to 13·8)	13·4 (13·1 to 13·7)	14·3 (14 to 14·7)	10·6 (10·3 to 11)	8·6 (8·3 to 8·8)	10·8 (10·6 to 11·1)	9·2 (8 to 10·5)
Eastern Europe	2057·6 (2006·9 to 2113·5)	2048·3 (2023·7 to 2071·2)	1456·5 (1440 to 1471·7)	1643·8 (1626 to 1662·2)	1392·7 (1377·5 to 1410·4)	920 (908·9 to 931)	1225·4 (1210·9 to 1240·5)	1087·6 (938·7 to 1259·6)
Belarus	94·7 (91 to 98·5)	97 (93·7 to 100·1)	71·3 (68·9 to 73·7)	76·5 (73·8 to 79·1)	67 (65 to 69·5)	44·6 (42·8 to 46·7)	53·5 (51·4 to 55·8)	49·6 (41·6 to 58·8)
Estonia	9·7 (9·6 to 9·8)	9·9 (9·8 to 10)	10·5 (10·4 to 10·7)	11 (10·9 to 11·1)	10·2 (10·1 to 10·3)	6·2 (6·1 to 6·3)	7·4 (7·4 to 7·5)	6·4 (5·7 to 7·3)
Latvia	15·9 (15·6 to 16·3)	17·6 (17·4 to 17·8)	17·2 (17·1 to 17·4)	17·4 (17·2 to 17·5)	17·7 (17·5 to 17·8)	9·7 (9·6 to 9·9)	9·1 (8·9 to 9·2)	9·3 (8·2 to 10·5)
Lithuania	28·3 (26·7 to 30·3)	30·2 (30 to 30·4)	26·9 (26·7 to 27·1)	24·4 (24·2 to 24·5)	27 (26·9 to 27·2)	15·7 (15·5 to 15·8)	15·7 (15·6 to 15·9)	13·1 (11·6 to 14·8)
Moldova	41·7 (39·5 to 43·9)	41 (38·9 to 43·1)	35·2 (31·9 to 38·9)	43·4 (41·1 to 45·8)	37·5 (35·9 to 39·1)	22 (20·7 to 23·3)	21 (19·7 to 22·4)	15·6 (13·6 to 17·7)
Russia	1448·7 (1438·7 to 1458·2)	1442·7 (1438·9 to 1446·9)	953·6 (950·4 to 956·6)	1101·3 (1098·2 to 1104·8)	923·3 (920·8 to 926·2)	623·3 (621·1 to 625·7)	876·3 (873·4 to 879·1)	806·5 (700·1 to 929·2)
Ukraine	418·5 (378·1 to 466·8)	410 (387·9 to 431·2)	341·8 (327·3 to 356)	369·9 (354·4 to 386·1)	309·9 (295·7 to 326·6)	198·5 (189 to 208)	242·4 (228·8 to 256·6)	187·1 (158 to 221·4)
High income	6618·9 (6540 to 6698·5)	7042·9 (6997·9 to 7084·5)	6715·3 (6672·2 to 6757)	6073·3 (6037·9 to 6108·8)	6094·8 (6063·7 to 6125·9)	5768·9 (5739·6 to 5801·4)	5802·7 (5779·9 to 5825·2)	5454·5 (4976·9 to 6005·2)
Australasia	122·9 (122·2 to 123·6)	145·9 (145·2 to 146·7)	162 (161·5 to 162·5)	135·9 (135·4 to 136·4)	153·9 (153·3 to 154·5)	146·1 (145·5 to 146·6)	174·5 (173·9 to 175·1)	180·5 (162·5 to 200·5)
Australia	98·4 (97·9 to 98·9)	115·6 (115·2 to 116)	131·1 (130·7 to 131·6)	110·9 (110·4 to 111·4)	124·7 (124·1 to 125·2)	118·8 (118·3 to 119·2)	144·1 (143·5 to 144·7)	151·5 (136·8 to 167·9)
New Zealand	24·5 (24 to 25)	30·4 (29·7 to 30·9)	30·9 (30·7 to 31·1)	25 (24·8 to 25·2)	29·2 (28·9 to 29·5)	27·3 (27·1 to 27·5)	30·4 (30·2 to 30·6)	29 (25·7 to 32·7)
High-income Asia Pacific	1494·1 (1439·7 to 1548·2)	1277 (1238·1 to 1314·5)	1447·9 (1408·9 to 1485·2)	1186·7 (1153·3 to 1219·6)	952·2 (923·4 to 981·5)	883·5 (857 to 912·1)	775·8 (755·5 to 797·9)	670·5 (621·7 to 725·6)
Brunei	1·5 (1·5 to 1·5)	2 (2 to 2)	2·4 (2·4 to 2·5)	2·8 (2·7 to 2·9)	3·4 (3·2 to 3·5)	3·5 (3·4 to 3·7)	3·1 (2·8 to 3·4)	3·2 (2·7 to 3·6)
Japan	1080·9 (1034·2 to 1127·9)	776·6 (748·5 to 805·4)	941·4 (911·3 to 973)	765·3 (742·3 to 788·7)	589·2 (571·4 to 607·6)	578·9 (561·1 to 597·9)	523·8 (510·7 to 537·6)	439·2 (401·2 to 482·9)
Aichi	44·6 (42·6 to 46·6)	37·5 (36 to 39·2)	56·2 (54·1 to 58·2)	42·1 (40·8 to 43·6)	34 (32·9 to 35·1)	36·3 (35·2 to 37·5)	34·2 (33·4 to 35·1)	29·4 (26·8 to 32·3)
Akita	14·8 (14·1 to 15·5)	10·1 (9·7 to 10·6)	8·2 (7·7 to 8·7)	7·8 (7·6 to 8·1)	5·4 (5·2 to 5·6)	4·4 (4·2 to 4·5)	3·4 (3·2 to 3·5)	2·4 (2·2 to 2·7)
Aomori	16 (15·3 to 16·8)	11·5 (11 to 12)	11·3 (10·7 to 11·9)	10·4 (10 to 10·8)	7·3 (7 to 7·5)	6·3 (6·1 to 6·6)	4·7 (4·6 to 4·9)	3·7 (3·4 to 4·1)
Chiba	28·3 (27·1 to 29·5)	18·4 (17·7 to 19·1)	34 (32·8 to 35·2)	32·6 (31·7 to 33·6)	25·6 (24·8 to 26·3)	27 (26·2 to 27·7)	25·2 (24·6 to 25·8)	20·4 (18·7 to 22·4)
Ehime	18·8 (17·9 to 19·6)	11·4 (10·9 to 11·9)	11·3 (10·7 to 11·9)	9·6 (9·3 to 9·9)	7·1 (6·9 to 7·3)	6·4 (6·2 to 6·7)	5·6 (5·5 to 5·8)	4·3 (3·9 to 4·7)
Fukui	9·3 (8·9 to 9·7)	5·7 (5·5 to 6·1)	5·8 (5·4 to 6·3)	5·1 (4·9 to 5·3)	4·2 (4 to 4·3)	3·9 (3·8 to 4)	3·4 (3·3 to 3·5)	2·7 (2·5 to 3)
Fukuoka	45·9 (43·8 to 48)	32·7 (31·4 to 34·1)	34·3 (33·1 to 35·6)	31·5 (30·6 to 32·4)	23·4 (22·7 to 24·2)	23 (22·3 to 23·7)	22·9 (22·3 to 23·6)	19·8 (18 to 21·8)
Fukushima	27·4 (26·2 to 28·6)	16·5 (15·8 to 17·2)	14·2 (13·4 to 15)	14·2 (13·8 to 14·7)	11·1 (10·8 to 11·5)	9·9 (9·5 to 10·2)	7·7 (7·4 to 7·9)	5·9 (5·4 to 6·6)
Gifu	18·5 (17·7 to 19·4)	13·3 (12·7 to 13·8)	15·4 (14·6 to 16·2)	12·4 (12 to 12·8)	9·7 (9·4 to 10·1)	9·8 (9·5 to 10·2)	8·4 (8·1 to 8·6)	6·6 (6 to 7·3)
Gunma	21·1 (20·2 to 22)	12·5 (12 to 13)	14·3 (13·5 to 15·1)	12·3 (11·9 to 12·7)	9·5 (9·2 to 9·8)	9·4 (9·1 to 9·8)	7·8 (7·6 to 8)	6·1 (5·6 to 6·8)
Hiroshima	27 (25·8 to 28·2)	17·6 (16·9 to 18·4)	22·4 (21·5 to 23·3)	17·9 (17·3 to 18·5)	13·8 (13·3 to 14·2)	13·4 (13 to 13·9)	12·6 (12·2 to 12·9)	10·2 (9·3 to 11·2)
Hokkaidō	52·2 (49·7 to 54·7)	38·9 (37·1 to 40·6)	44·8 (43 to 46·5)	36·6 (35·5 to 37·8)	26·3 (25·4 to 27·1)	22·9 (22·2 to 23·6)	19·7 (19·1 to 20·2)	15·3 (13·9 to 16·9)

Hyōgo	43·8 (41·9 to 45·6)	32·9 (31·6 to 34·3)	44·3 (42·7 to 45·9)	33·2 (32·2 to 34·3)	25·8 (25 to 26·6)	26·3 (25·5 to 27·1)	23·5 (22·9 to 24·1)	19·5 (17·8 to 21·4)
Ibaraki	27 (25·9 to 28·2)	15·7 (15·1 to 16·3)	18·2 (17·5 to 18·8)	17·7 (17·2 to 18·3)	13·9 (13·4 to 14·4)	13·8 (13·3 to 14·2)	11·7 (11·4 to 12)	9·2 (8·4 to 10·2)
Ishikawa	11·5 (11 to 12)	7·4 (7·1 to 7·8)	8·9 (8·4 to 9·4)	7·3 (7 to 7·5)	5·5 (5·3 to 5·7)	5·6 (5·4 to 5·8)	4·8 (4·6 to 4·9)	4 (3·7 to 4·4)
Iwate	18·1 (17·3 to 18·9)	12·3 (11·8 to 12·8)	11·2 (10·6 to 11·8)	9·4 (9·1 to 9·7)	6·9 (6·7 to 7·2)	6 (5·8 to 6·2)	4·7 (4·6 to 4·9)	3·7 (3·3 to 4·1)
Kagawa	11·4 (10·9 to 12)	6·9 (6·6 to 7·2)	7·3 (6·9 to 7·7)	6·2 (6 to 6·4)	4·6 (4·4 to 4·8)	4·7 (4·6 to 4·9)	4·1 (4 to 4·3)	3·3 (3 to 3·6)
Kagoshima	25·1 (24·1 to 26·1)	15·1 (14·5 to 15·7)	11·9 (11·2 to 12·6)	12 (11·6 to 12·4)	9·2 (8·9 to 9·4)	7·8 (7·6 to 8·1)	7·5 (7·3 to 7·7)	5·9 (5·4 to 6·6)
Kanagawa	34·4 (33 to 35·8)	31·5 (30·2 to 32·8)	57·8 (55·3 to 60·4)	46·1 (44·9 to 47·4)	38·1 (37 to 39·3)	40·5 (39·4 to 41·6)	38·1 (37·2 to 39)	31·4 (28·8 to 34·4)
Kōchi	11·1 (10·7 to 11·6)	6 (5·7 to 6·3)	5·8 (5·3 to 6·3)	4·9 (4·7 to 5·1)	3·6 (3·5 to 3·7)	3·3 (3·1 to 3·4)	2·7 (2·6 to 2·8)	2·1 (1·9 to 2·3)
Kumamoto	23 (22 to 24)	14·1 (13·6 to 14·7)	12·3 (11·7 to 13)	11·9 (11·5 to 12·3)	9·3 (9 to 9·6)	8·4 (8·1 to 8·7)	8 (7·8 to 8·3)	6·6 (6 to 7·3)
Kyōto	22·7 (21·7 to 23·6)	15·7 (15·1 to 16·3)	19·4 (18·6 to 20·1)	15·5 (15·1 to 16)	11·5 (11·1 to 11·8)	11·6 (11·2 to 12)	10·3 (10·1 to 10·6)	8·9 (8·1 to 9·7)
Mie	17·7 (16·9 to 18·5)	11·7 (11·2 to 12·2)	12·8 (12·2 to 13·5)	10·2 (9·9 to 10·6)	8·6 (8·3 to 8·9)	8·6 (8·3 to 8·9)	7·5 (7·3 to 7·7)	5·9 (5·3 to 6·5)
Miyagi	21·1 (20·1 to 22)	14·3 (13·7 to 14·9)	14·3 (13·5 to 15·1)	15·1 (14·6 to 15·6)	11·3 (10·9 to 11·6)	10·7 (10·4 to 11·1)	9·3 (9 to 9·6)	7·7 (7 to 8·5)
Miyazaki	14·5 (13·9 to 15·1)	9·2 (8·8 to 9·6)	8·3 (7·8 to 8·7)	8·3 (8·1 to 8·6)	6 (5·8 to 6·2)	5·4 (5·2 to 5·5)	5 (4·9 to 5·2)	3·8 (3·4 to 4·2)
Nagano	26·5 (25·4 to 27·5)	16·1 (15·5 to 16·8)	16·1 (15·2 to 16·9)	13 (12·6 to 13·4)	10·4 (10·1 to 10·7)	10·3 (10 to 10·6)	8·5 (8·3 to 8·7)	6·6 (6·1 to 7·3)
Nagasaki	23 (22·2 to 24)	14·2 (13·6 to 14·7)	12·7 (12 to 13·4)	10·9 (10·6 to 11·3)	8·1 (7·9 to 8·4)	6·8 (6·6 to 7)	5·9 (5·7 to 6)	4·8 (4·3 to 5·3)
Nara	9·3 (8·8 to 9·7)	5·9 (5·6 to 6·1)	7·9 (7·5 to 8·3)	7·8 (7·6 to 8)	6·3 (6·1 to 6·5)	6·4 (6·2 to 6·6)	5·2 (5·1 to 5·4)	4·2 (3·8 to 4·6)
Niigata	31·1 (29·8 to 32·4)	19·1 (18·4 to 19·8)	17·9 (17·2 to 18·6)	15·9 (15·4 to 16·4)	11·7 (11·4 to 12·1)	10·5 (10·2 to 10·9)	8·8 (8·6 to 9·1)	7 (6·4 to 7·7)
Ōita	15·5 (14·9 to 16·2)	9·4 (9 to 9·8)	8·6 (8·2 to 9·1)	7·9 (7·6 to 8·1)	5·7 (5·5 to 5·9)	5·3 (5·1 to 5·5)	4·9 (4·8 to 5·1)	3·9 (3·6 to 4·4)
Okayama	20·4 (19·5 to 21·3)	13 (12·4 to 13·5)	14·7 (13·9 to 15·5)	11·7 (11·4 to 12·1)	9·1 (8·8 to 9·4)	9·3 (8·9 to 9·6)	8·2 (8 to 8·4)	7 (6·4 to 7·8)
Okinawa	13·3 (12·7 to 13·9)	8·6 (8·2 to 8·9)	9·8 (9·3 to 10·4)	9·6 (9·3 to 10)	8·5 (8·2 to 8·7)	8·2 (8 to 8·5)	8·4 (8·1 to 8·6)	7·6 (6·9 to 8·4)
Ōsaka	52·7 (50·4 to 55)	49·6 (47·6 to 51·8)	81·6 (78·2 to 85·4)	53·8 (52·3 to 55·4)	41·6 (40·3 to 43)	43·1 (41·8 to 44·4)	36·8 (35·9 to 37·8)	31·3 (28·5 to 34·6)
Saga	12·4 (11·9 to 12·9)	7·4 (7 to 7·8)	6·3 (5·8 to 6·9)	6 (5·8 to 6·2)	4·6 (4·4 to 4·7)	4·2 (4 to 4·3)	3·7 (3·6 to 3·9)	3 (2·7 to 3·3)
Saitama	28·9 (27·7 to 30·1)	20·5 (19·6 to 21·3)	43·3 (41·8 to 44·8)	36·9 (35·9 to 37·9)	30·5 (29·7 to 31·5)	32·4 (31·5 to 33·5)	29 (28·3 to 29·7)	24·5 (22·5 to 26·9)
Shiga	11·5 (11 to 12)	6·9 (6·6 to 7·2)	7 (6·6 to 7·4)	7·7 (7·4 to 7·9)	6·4 (6·2 to 6·6)	6·9 (6·6 to 7·1)	6·6 (6·4 to 6·8)	5·4 (4·9 to 5·9)
Shimane	11·5 (11 to 12)	6·9 (6·5 to 7·2)	5·6 (5·1 to 6)	4·8 (4·7 to 5)	3·6 (3·5 to 3·8)	3·2 (3·1 to 3·3)	2·8 (2·7 to 2·9)	2·3 (2·1 to 2·6)
Shizuoka	31·7 (30·4 to 33·1)	23 (22 to 23·9)	28·6 (27·6 to 29·6)	23·2 (22·5 to 24)	17·9 (17·3 to 18·5)	17·5 (17 to 18·1)	15·6 (15·3 to 16)	12·3 (11·2 to 13·5)
Tochigi	20·3 (19·5 to 21·2)	11·8 (11·3 to 12·3)	13·3 (12·6 to 14)	12·7 (12·3 to 13·1)	9·6 (9·3 to 9·9)	9·2 (8·9 to 9·6)	8·1 (7·9 to 8·3)	6·5 (5·9 to 7·1)
Tokushima	10 (9·5 to 10·4)	6·1 (5·8 to 6·4)	5·7 (5·2 to 6·2)	5·2 (5 to 5·3)	3·8 (3·7 to 3·9)	3·5 (3·3 to 3·6)	2·9 (2·8 to 3)	2·4 (2·2 to 2·6)
Tōkyō	80·5 (77 to 84)	90·6 (87 to 94·7)	109·9 (105 to 115·7)	68 (66·1 to 69·9)	49·9 (48·5 to 51·4)	48·7 (47·5 to 50·1)	52·6 (51·4 to 53·9)	53·2 (49·2 to 57·7)
Tottori	7·7 (7·4 to 8·1)	4·6 (4·4 to 4·9)	4·1 (3·8 to 4·5)	4 (3·9 to 4·1)	3·1 (3 to 3·2)	2·7 (2·6 to 2·8)	2·4 (2·3 to 2·5)	2 (1·8 to 2·2)
Toyama	11·3 (10·8 to 11·8)	7·6 (7·3 to 8)	8·5 (8 to 8·9)	6·5 (6·3 to 6·8)	4·8 (4·7 to 5)	5 (4·8 to 5·1)	4 (3·9 to 4·1)	3·2 (3 to 3·5)
Wakayama	12·6 (12 to 13·1)	7·8 (7·4 to 8·1)	8·8 (8·4 to 9·4)	6·6 (6·4 to 6·9)	4·9 (4·7 to 5)	4·7 (4·5 to 4·8)	3·7 (3·6 to 3·8)	3 (2·7 to 3·3)
Yamagata	16·9 (16·1 to 17·6)	10·5 (10 to 10·9)	8·2 (7·7 to 8·6)	8·1 (7·8 to 8·4)	6·1 (5·9 to 6·3)	5·3 (5·1 to 5·5)	4·2 (4·1 to 4·4)	3·3 (3·1 to 3·7)
Yamaguchi	18·7 (17·9 to 19·6)	12·3 (11·7 to 12·8)	12·2 (11·5 to 12·8)	9·6 (9·3 to 9·9)	6·7 (6·5 to 6·9)	6·4 (6·2 to 6·7)	5·6 (5·4 to 5·8)	4·3 (3·9 to 4·8)
Yamanashi	9·9 (9·4 to 10·3)	5·9 (5·6 to 6·2)	5·8 (5·4 to 6·3)	4·9 (4·8 to 5·1)	4·2 (4·1 to 4·4)	4 (3·9 to 4·2)	3·2 (3·1 to 3·3)	2·6 (2·3 to 2·8)
South Korea	389·9 (364·1 to 416·2)	470 (445·5 to 493·9)	482·2 (453·4 to 510)	397·9 (372·2 to 423·5)	333 (309·9 to 355·4)	271·9 (253·8 to 290·4)	222 (205·4 to 238·8)	200·5 (179·5 to 226·7)
Singapore	21·8 (21·1 to 22·5)	28·4 (27·5 to 29·3)	21·9 (20·5 to 23·2)	20·8 (19·3 to 22·3)	26·6 (24·7 to 28·5)	29·1 (27·2 to 31·2)	26·9 (25 to 28·9)	27·6 (22·1 to 34·3)
High-income North America	1959·8 (1953·4 to 1966·2)	2287·5 (2282·9 to 2293·8)	1924·9 (1920·7 to 1931)	1937·6 (1933·8 to 1942·3)	2235·1 (2231·6 to 2239·1)	2140·5 (2136·7 to 2145)	2115·7 (2112·1 to 2120·3)	2050·4 (1919·6 to 2194·1)
Canada	176·1 (175·5 to 176·6)	222·3 (221·7 to 222·9)	169·1 (168·5 to 169·6)	174·5 (173·9 to 175·1)	195·6 (195 to 196·2)	161·6 (160·9 to 162·2)	182·7 (182·1 to 183·3)	182·2 (169·6 to 196·1)
Greenland	0·5 (0·5 to 0·5)	0·7 (0·7 to 0·8)	0·5 (0·5 to 0·5)	0·5 (0·5 to 0·5)	0·6 (0·6 to 0·6)	0·5 (0·4 to 0·5)	0·4 (0·4 to 0·4)	0·4 (0·3 to 0·4)
USA	1783·2 (1776·9 to 1789·4)	2064·3 (2059·9 to 2070·4)	1755·3 (1751·3 to 1761·2)	1762·5 (1758·9 to 1766·9)	2038·8 (2035·4 to 2042·6)	1978·4 (1974·7 to 1983)	1932·6 (1929·1 to 1937·1)	1867·8 (1749·5 to 1997·6)
Alabama	37·3 (37·1 to 37·5)	40·2 (39·6 to 40·8)	31·2 (30·8 to 31·6)	30·5 (30·2 to 30·9)	31·2 (30·7 to 31·7)	30·4 (29·9 to 30·8)	28·2 (27·8 to 28·6)	30·2 (28·3 to 32·1)
Alaska	1·6 (1·6 to 1·6)	3·5 (3·4 to 3·6)	3·4 (3·4 to 3·5)	4·8 (4·7 to 4·9)	5·9 (5·8 to 5·9)	4·8 (4·7 to 4·9)	5·4 (5·3 to 5·5)	5·2 (4·8 to 5·7)
Arizona	10 (9·9 to 10)	17·3 (17·1 to 17·5)	18 (17·9 to 18·1)	24·3 (23·9 to 24·6)	33·7 (33·1 to 34·3)	41·3 (40·8 to 41·9)	43 (42·6 to 43·4)	41·1 (38·5 to 43·8)
Arkansas	21·8 (21·7 to 21·9)	20·4 (20·1 to 20·7)	17·1 (16·9 to 17·2)	17·7 (17·4 to 17·9)	17·7 (17·4 to 18·1)	18·4 (18·1 to 18·8)	18·1 (17·8 to 18·4)	18·7 (17·5 to 19·8)
California	122·5 (122 to 123)	174·6 (172·5 to 176·6)	168·5 (167·9 to 169·2)	198·1 (195·5 to 200·9)	297·6 (292·8 to 302·4)	259 (255·5 to 262·4)	247 (243 to 251·4)	221·6 (206·6 to 238)
Colorado	15·2 (15·1 to 15·2)	20·8 (20·6 to 21)	19·7 (19·5 to 19·8)	24·4 (24·1 to 24·7)	26·7 (26·2 to 27·1)	31·9 (31·6 to 32·2)	32·2 (31·8 to 32·6)	28·1 (26·3 to 30·1)
Connecticut	23·7 (23·6 to 23·8)	28·8 (28·4 to 29·2)	23·1 (22·9 to 23·2)	19·5 (19·2 to 19·8)	24·6 (24·1 to 25·1)	21·3 (20·8 to 21·9)	18·5 (18·1 to 18·9)	18·4 (17·2 to 19·7)
Delaware	3·9 (3·9 to 3·9)	5·8 (5·7 to 5·9)	4·9 (4·8 to 4·9)	4·5 (4·4 to 4·6)	5·4 (5·3 to 5·6)	5·4 (5·3 to 5·5)	5·6 (5·5 to 5·8)	5·5 (5 to 6)
Washington, DC	10·8 (8·7 to 13·1)	11·1 (9·9 to 12·4)	7 (6·9 to 7·1)	4·7 (4·6 to 4·8)	5·5 (5·4 to 5·7)	3·9 (3·6 to 4·1)	4·2 (3·5 to 5)	4·7 (3·7 to 6)
Florida	31 (30·8 to 31·3)	53·3 (52·8 to 53·9)	53·4 (52·8 to 54·1)	63·5 (62·8 to 64·1)	97·2 (95·8 to 98·7)	99·3 (97·9 to 100·8)	103·7 (102·4 to 105·1)	111·4 (104·3 to 118·8)
Georgia	41·5 (41·3 to 41·7)	50·3 (49·9 to 50·7)	46·1 (45·8 to 46·5)	44·1 (43·5 to 44·6)	55·1 (54·4 to 55·9)	64·7 (63·8 to 65·5)	65·3 (64·6 to 66)	65·4 (61·2 to 69·7)
Hawaii	7·1 (7 to 7·1)	9 (8·9 to 9·2)	7·8 (7·7 to 7·8)	8·8 (8·6 to 8·9)	10 (9·7 to 10·2)	8·5 (8·4 to 8·6)	9·1 (8·9 to 9·2)	8·7 (8·1 to 9·3)
Idaho	7·4 (7·3 to 7·5)	8·7 (8·5 to 8·8)	6·9 (6·8 to 6·9)	9·7 (9·6 to 9·9)	8·1 (7·9 to 8·3)	10 (9·9 to 10·2)	10·9 (10·7 to 11)	11·1 (10·3 to 12)
Illinois	102·1 (101·7 to 102·5)	110·1 (108·8 to 111·5)	97·7 (97·3 to 98·2)	92·7 (91·8 to 93·6)	96·2 (94·7 to 97·7)	90·6 (89·5 to 91·6)	77·6 (76·6 to 78·6)	74 (69·2 to 79)

Indiana	43·9 (43·7 to 44·1)	55·9 (55·5 to 56·3)	47·5 (47·2 to 47·8)	43·1 (42·6 to 43·5)	42·2 (41·4 to 42·9)	42·7 (42·2 to 43·2)	41 (40·6 to 41·4)	40·7 (38·3 to 43·3)
Iowa	29·4 (29·3 to 29·6)	31·4 (30·9 to 31·8)	22·6 (22·4 to 22·7)	23·9 (23·6 to 24·3)	19·3 (18·9 to 19·7)	18·6 (18·2 to 19·1)	18·9 (18·5 to 19·2)	18·4 (17·4 to 19·6)
Kansas	20·5 (20·4 to 20·6)	25 (24·4 to 25·5)	18 (17·8 to 18·1)	19·5 (19·3 to 19·8)	19·2 (18·9 to 19·5)	19·3 (19 to 19·5)	20 (19·7 to 20·3)	17·9 (16·8 to 19)
Kentucky	32·8 (32·6 to 33·1)	34·5 (34 to 35)	27·9 (27·6 to 28·3)	28·4 (28 to 28·8)	26·9 (26·4 to 27·4)	27·2 (26·8 to 27·5)	26 (25·7 to 26·3)	27·1 (25·4 to 28·8)
Louisiana	34·5 (34·3 to 34·7)	44 (43·3 to 44·6)	35·5 (35·3 to 35·8)	39·6 (39·1 to 40·1)	36 (35·3 to 36·7)	32·7 (32·1 to 33·3)	30·6 (30·2 to 31)	28·2 (26·4 to 30)
Maine	10·3 (10·2 to 10·3)	11·6 (11·4 to 11·7)	8·6 (8·4 to 8·7)	8 (7·9 to 8·2)	8·5 (8·3 to 8·7)	6·7 (6·6 to 6·9)	6·2 (6·1 to 6·3)	6·1 (5·6 to 6·6)
Maryland	28·4 (28·2 to 28·5)	36·7 (36·3 to 37·1)	32·5 (32·3 to 32·7)	29·5 (29·2 to 29·9)	39·4 (38·8 to 39·9)	35·8 (35·2 to 36·3)	34·3 (33·9 to 34·6)	36·2 (33·8 to 38·7)
Massachusetts	54 (53·8 to 54·2)	55·2 (54·8 to 55·6)	43·8 (43·6 to 44·1)	35·7 (35·3 to 36·1)	44·8 (43·7 to 46)	40·3 (39·3 to 41·4)	35·6 (34·6 to 36·6)	34 (31·7 to 36·6)
Michigan	78·6 (78·2 to 78·9)	90·9 (90 to 91·7)	81·1 (80·7 to 81·5)	74·2 (73·5 to 75)	74·2 (73 to 75·3)	66 (65 to 67)	54·9 (54·5 to 55·3)	52·3 (49·1 to 55·6)
Minnesota	34·9 (34·7 to 35)	40·9 (40·3 to 41·4)	31 (30·8 to 31·2)	33 (32·7 to 33·3)	33·6 (33·1 to 34)	33·2 (32·8 to 33·6)	33·3 (33 to 33·5)	32·8 (30·7 to 34·9)
Mississippi	30·1 (29·7 to 30·6)	29·6 (29 to 30·2)	23·5 (23·3 to 23·7)	22·2 (22 to 22·5)	21·4 (21 to 21·7)	21·2 (20·9 to 21·5)	19·1 (18·9 to 19·2)	18·5 (17·4 to 19·7)
Missouri	44 (43·8 to 44·2)	49·6 (48·9 to 50·2)	38·8 (38·5 to 39)	38·3 (37·7 to 38·8)	39 (38·4 to 39·7)	37·4 (36·9 to 37·8)	37·3 (36·8 to 37·9)	35·9 (33·7 to 38·2)
Montana	6·7 (6·6 to 6·7)	7·9 (7·8 to 8·1)	5·8 (5·8 to 5·9)	6·9 (6·8 to 7)	5·8 (5·7 to 5·9)	5·4 (5·3 to 5·5)	5·9 (5·8 to 6)	5·5 (5 to 6)
Nebraska	14·9 (14·8 to 14·9)	16·3 (16 to 16·6)	12·3 (12·2 to 12·4)	12·9 (12·8 to 13·1)	12 (11·8 to 12·2)	12·2 (12 to 12·4)	12·8 (12·7 to 13)	12·5 (11·8 to 13·3)
Nevada	1·7 (1·7 to 1·8)	3·3 (3·3 to 3·4)	4·1 (4 to 4·3)	6·5 (6·3 to 6·6)	10·4 (10·1 to 10·6)	14·9 (14·7 to 15·2)	17·3 (16·9 to 17·7)	19·4 (18·2 to 20·8)
New Hampshire	6 (6 to 6)	7·1 (7 to 7·2)	6·3 (6·3 to 6·4)	6·6 (6·5 to 6·8)	8·6 (8·4 to 8·7)	7·1 (7 to 7·2)	6·3 (6·2 to 6·4)	6·1 (5·5 to 6·6)
New Jersey	57·8 (57·5 to 58)	65·9 (65·2 to 66·5)	56·5 (56·2 to 56·8)	48 (47·5 to 48·5)	60·4 (59·4 to 61·3)	56·9 (56 to 57·7)	50·1 (49·3 to 50·9)	49·9 (46·6 to 53·5)
New Mexico	9·3 (9·2 to 9·4)	14 (13·8 to 14·2)	10·4 (10·3 to 10·5)	13 (12·8 to 13·2)	13·7 (13·5 to 14)	13·4 (13·2 to 13·6)	12·9 (12·7 to 13·2)	12 (11·3 to 12·9)
New York	173·8 (172·9 to 174·7)	176·5 (174 to 178·9)	148 (147·4 to 148·7)	116·3 (115·1 to 117·5)	145·6 (142·6 to 148·7)	126·2 (124·2 to 128·3)	117·9 (116·4 to 119·2)	111·3 (103·7 to 119·4)
North Carolina	47·9 (47·5 to 48·3)	54·3 (53·7 to 54·9)	45·2 (44·7 to 45·8)	40·9 (40·5 to 41·4)	51·1 (50·2 to 52·2)	58 (57 to 58·9)	59·6 (58·6 to 60·5)	61·6 (57·7 to 65·7)
North Dakota	7·6 (7·5 to 7·7)	8·2 (8 to 8·3)	5·1 (5 to 5·2)	5·9 (5·7 to 6)	4·6 (4·4 to 4·8)	3·9 (3·7 to 4·1)	5·3 (5·1 to 5·5)	4·7 (4·2 to 5·2)
Ohio	93·3 (93 to 93·6)	105·9 (104·7 to 107·1)	95·1 (94·7 to 95·6)	83·1 (82·5 to 83·8)	82·4 (80·9 to 83·9)	75·2 (73·7 to 77)	67·8 (66·5 to 69·1)	65·6 (61·6 to 69·8)
Oklahoma	23·4 (23·2 to 23·6)	24·8 (24·4 to 25·2)	20·9 (20·5 to 21·2)	24·8 (24·5 to 25·2)	23·9 (23·6 to 24·2)	24·6 (24·2 to 25·1)	25·1 (24·6 to 25·5)	23·8 (22·3 to 25·3)
Oregon	16·4 (16·3 to 16·5)	19·1 (18·9 to 19·4)	16·2 (16·1 to 16·3)	21·1 (20·8 to 21·4)	20·8 (20·3 to 21·2)	22·3 (21·9 to 22·7)	22·4 (22·1 to 22·7)	19·6 (18·3 to 21)
Pennsylvania	121·3 (120·6 to 122)	113·6 (112 to 115·2)	91·3 (91 to 91·7)	78·7 (77·8 to 79·6)	84·5 (83 to 86·1)	71·6 (70·3 to 73)	69 (67·9 to 70)	68·8 (64·5 to 73·4)
Rhode Island	9·5 (9·5 to 9·5)	9·8 (9·6 to 10)	7·4 (7·3 to 7·5)	5·9 (5·7 to 6·1)	7·3 (7·1 to 7·6)	6·3 (6·1 to 6·5)	5·8 (5·6 to 6)	5·3 (4·8 to 5·8)
South Carolina	26·1 (26 to 26·2)	29·7 (29·3 to 30·1)	24·8 (24·4 to 25·1)	24·8 (24·5 to 25·1)	28·4 (27·9 to 28·9)	27·3 (26·9 to 27·7)	26·8 (26·3 to 27·2)	28·5 (26·8 to 30·4)
South Dakota	8 (7·9 to 8·1)	8·5 (8·3 to 8·7)	5·5 (5·4 to 5·6)	6·4 (6·3 to 6·5)	5·5 (5·4 to 5·7)	5·2 (5·1 to 5·3)	6·1 (6 to 6·2)	5·8 (5·3 to 6·3)
Tennessee	37·6 (37·4 to 37·9)	43 (42·5 to 43·4)	34·9 (34·6 to 35·2)	33·6 (33·2 to 33·9)	36·8 (36 to 37·7)	38·9 (38·2 to 39·6)	41·3 (40·7 to 41·9)	39·6 (37·2 to 42·2)
Texas	96·7 (96·2 to 97·2)	127·8 (126·5 to 129)	111·6 (110·9 to 112·3)	132·6 (131·1 to 134)	157·8 (155·1 to 160·6)	177·4 (174·3 to 180·3)	188·3 (186·3 to 190·4)	175·2 (164·1 to 186·8)
Utah	9·9 (9·8 to 10·1)	13·5 (13·2 to 13·8)	12·9 (12·8 to 13)	20·1 (19·9 to 20·3)	17·5 (17·1 to 17·9)	23·2 (22·6 to 23·8)	25·9 (25·3 to 26·5)	22·8 (21·4 to 24·2)
Vermont	4·3 (4·3 to 4·3)	4·6 (4·5 to 4·7)	3·9 (3·8 to 3·9)	3·8 (3·7 to 3·9)	4·1 (3·9 to 4·2)	3·2 (3·1 to 3·3)	2·8 (2·7 to 2·9)	2·9 (2·6 to 3·2)
Virginia	40·3 (40·1 to 40·5)	48 (47·7 to 48·3)	41 (40·8 to 41·3)	38·8 (38·3 to 39·3)	48·9 (47·9 to 49·9)	48·4 (47·5 to 49·3)	48·8 (48 to 49·5)	50·6 (47·4 to 54·1)
Washington	26·6 (26·5 to 26·7)	32·4 (32 to 32·8)	27·7 (27·5 to 27·8)	33·3 (32·9 to 33·7)	38·8 (37·9 to 39·8)	39·1 (38·4 to 39·8)	42·1 (41·4 to 42·8)	39·2 (36·6 to 42)
West Virginia	23 (22·8 to 23·1)	20·7 (20·4 to 21)	14·4 (14·2 to 14·7)	14·1 (13·9 to 14·3)	11·2 (10·9 to 11·4)	10·3 (10·1 to 10·5)	10·2 (10 to 10·4)	9·4 (8·8 to 10)
Wisconsin	40·2 (40 to 40·4)	46·2 (45·7 to 46·7)	35·7 (35·4 to 35·9)	37·1 (36·8 to 37·4)	36 (35·2 to 36·8)	33·8 (33·2 to 34·5)	32·9 (32·5 to 33·5)	32·2 (30·3 to 34·3)
Wyoming	3·5 (3·5 to 3·5)	4·3 (4·2 to 4·4)	2·9 (2·9 to 3)	4·9 (4·8 to 5)	3·4 (3·3 to 3·5)	3·1 (3 to 3·1)	3·4 (3·3 to 3·5)	3·5 (3·2 to 3·9)
Southern Latin America	336·7 (328·2 to 346)	392·6 (390·1 to 395·3)	405·7 (402·6 to 409)	478·9 (476·9 to 481·4)	501·3 (499·3 to 503·8)	482 (479 to 485·2)	512·4 (508·4 to 516·4)	476·3 (400·8 to 567·8)
Argentina	217·1 (209·9 to 225)	229·5 (227·4 to 231·6)	258·7 (256·1 to 261·5)	334·9 (333·4 to 336·6)	333 (331·5 to 334·8)	335·2 (333·1 to 337·4)	368·4 (366 to 371)	342·6 (283·5 to 414·8)
Chile	97·1 (95·9 to 98·3)	133·1 (132·4 to 133·7)	119·9 (119·3 to 120·5)	117·5 (116·9 to 118·2)	141·3 (140·6 to 142·1)	121 (120·4 to 121·7)	120·4 (119·9 to 121·1)	111·1 (98·4 to 126·2)
Uruguay	22·5 (21·1 to 24)	30 (29·1 to 31)	27·1 (26·2 to 27·9)	26·5 (25·7 to 27·4)	27 (26 to 27·8)	25·8 (24·1 to 27·5)	23·5 (21·1 to 26·5)	22·6 (18·9 to 26·8)
Western Europe	2705·5 (2669·1 to 2744)	2939·9 (2930·2 to 2949·4)	2774·8 (2764·6 to 2785)	2334·2 (2329·2 to 2339·5)	2252·3 (2247·3 to 2256·8)	2116·9 (2112·9 to 2120·9)	2224·4 (2220·5 to 2228·5)	2076·8 (1867·5 to 2317·8)
Andorra	0·1 (0 to 0·1)	0·1 (0·1 to 0·1)	0·2 (0·2 to 0·2)	0·2 (0·2 to 0·3)	0·3 (0·3 to 0·3)	0·4 (0·3 to 0·4)	0·4 (0·4 to 0·4)	0·3 (0·3 to 0·3)
Austria	51·1 (50·4 to 51·9)	62·3 (62 to 62·6)	53·6 (53·3 to 53·8)	44·5 (44·3 to 44·8)	45·3 (45·1 to 45·5)	37·8 (37·6 to 38)	38·1 (37·9 to 38·3)	42·5 (39·9 to 45·4)
Belgium	69·4 (69 to 69·7)	76 (75·7 to 76·4)	68·9 (68·6 to 69·3)	59·6 (59·3 to 60)	61·2 (60·8 to 61·5)	56·8 (56·5 to 57·2)	63 (62·6 to 63·3)	59·3 (51·9 to 67·8)
Cyprus	6·7 (6·5 to 7)	7·1 (6·9 to 7·2)	5·9 (5·7 to 6·1)	6·5 (6·3 to 6·7)	7·1 (6·8 to 7·3)	5·4 (5·2 to 5·6)	6·5 (6·3 to 6·7)	7·3 (6·2 to 8·6)
Denmark	38·1 (36·5 to 39·8)	36·4 (35·6 to 37·1)	35·5 (34·7 to 36·3)	26·9 (26·2 to 27·7)	31·2 (30·5 to 31·9)	32·4 (31·6 to 33·2)	29·7 (29·1 to 30·3)	30·6 (27 to 34·6)
Finland	46·5 (46·3 to 46·8)	39·6 (39·4 to 39·8)	30·6 (30·4 to 30·8)	31 (30·8 to 31·2)	32·3 (32 to 32·5)	27·6 (27·4 to 27·8)	29·5 (29·3 to 29·7)	24·4 (22·3 to 26·7)
France	410·1 (408·7 to 411·6)	406·4 (405·3 to 407·5)	414·9 (413·8 to 416·1)	391·5 (390·3 to 392·6)	371·8 (370·7 to 372·9)	379·9 (378·6 to 381·3)	390·7 (389·5 to 391·8)	351 (317·9 to 387·9)
Germany	514·8 (495·9 to 534·4)	604·1 (602·7 to 605·5)	489·5 (487·9 to 491·1)	405·2 (404 to 406·3)	429·8 (428·7 to 431)	369·5 (368·6 to 370·4)	326 (325·2 to 326·9)	360·6 (332·2 to 392·8)
Greece	75·1 (73·9 to 76·4)	75 (74·6 to 75·4)	68·9 (68·5 to 69·3)	69·1 (68·7 to 69·5)	49·4 (49·1 to 49·8)	49·6 (49·3 to 49·9)	54 (53·5 to 54·6)	41·8 (37·1 to 47·6)
Iceland	1·9 (1·9 to 2)	2·3 (2·2 to 2·3)	2 (2 to 2·1)	2·1 (2·1 to 2·2)	2·3 (2·2 to 2·3)	2 (2 to 2·1)	2·3 (2·3 to 2·4)	2·1 (1·8 to 2·4)
Ireland	31·2 (29·9 to 32·5)	29·7 (29·2 to 30·2)	31·4 (30·8 to 32)	35 (34·2 to 35·7)	25·3 (24·6 to 25·9)	27·5 (26·8 to 28·2)	36·1 (35·3 to 37·1)	29·7 (25·4 to 34·6)
Israel	23·2 (22·3 to 24·1)	27 (26·8 to 27·3)	39·4 (39·1 to 39·8)	45·2 (44·9 to 45·6)	49·9 (49·6 to 50·2)	66·3 (65·8 to 66·7)	80·5 (80 to 81·1)	93·6 (77·8 to 111·3)
Italy	429·4 (426·9 to 431·7)	449·2 (447·5 to 450·8)	444·1 (442·1 to 446·1)	308·7 (307·2 to 310·3)	273·8 (272·6 to 275)	264 (263·2 to 264·9)	271·8 (271 to 272·7)	213·7 (195·1 to 236)

Luxembourg	2·1 (2·1 to 2·2)	2·4 (2·4 to 2·5)	2·1 (2 to 2·1)	2 (2 to 2·1)	2·4 (2·3 to 2·4)	2·7 (2·7 to 2·8)	2·8 (2·7 to 2·8)	3·1 (2·8 to 3·5)
Malta	4·8 (4·6 to 5)	4 (3·9 to 4·1)	2·6 (2·6 to 2·7)	2·8 (2·7 to 2·8)	2·7 (2·6 to 2·7)	2 (2 to 2)	2 (2 to 2)	2·1 (1·8 to 2·4)
Monaco	0·2 (0·2 to 0·2)	0·2 (0·1 to 0·2)	0·2 (0·1 to 0·2)	0·2 (0·1 to 0·2)	0·2 (0·1 to 0·2)	0·1 (0·1 to 0·2)	0·1 (0·1 to 0·2)	0·1 (0·1 to 0·2)
Netherlands	110·6 (110 to 111·2)	117·8 (117·4 to 118·3)	112·2 (111·6 to 112·7)	87·7 (87 to 88·4)	97 (96·5 to 97·5)	101·3 (100·8 to 101·8)	88·9 (88·5 to 89·4)	86·6 (74·5 to 100·6)
Norway	30 (29·8 to 30·3)	29·9 (29·7 to 30·1)	31·4 (31·2 to 31·6)	24·6 (24·5 to 24·8)	29·5 (29·3 to 29·7)	28·4 (28·2 to 28·6)	29·4 (29·2 to 29·6)	27·6 (25 to 30·5)
Portugal	99·2 (94·6 to 103·9)	105·2 (102·9 to 107·5)	87·1 (85·2 to 89)	74·7 (72·7 to 76·6)	56·3 (54·7 to 57·9)	56·3 (54·8 to 57·8)	48 (46·9 to 49·2)	38·8 (32·7 to 46·1)
San Marino	0·1 (0·1 to 0·1)	0·1 (0·1 to 0·1)	0·1 (0·1 to 0·1)	0·1 (0·1 to 0·1)	0·1 (0·1 to 0·1)	0·2 (0·1 to 0·2)	0·2 (0·1 to 0·2)	0·2 (0·1 to 0·2)
Spain	264·3 (262·4 to 266·3)	317·9 (316·4 to 319·4)	317·2 (315·4 to 319)	262·7 (261 to 264·5)	190·9 (189·9 to 192)	193·7 (192·9 to 194·5)	234·2 (233·3 to 235·1)	179·1 (159·8 to 203·2)
Sweden	54·9 (54·6 to 55·2)	49·4 (49·1 to 49·7)	54·6 (54·3 to 54·9)	46·8 (46·5 to 47)	60·4 (60·1 to 60·7)	44·8 (44·5 to 45)	54·8 (54·6 to 55·1)	56·9 (53·3 to 60·9)
Stockholm	8·3 (8·2 to 8·4)	7·1 (6·9 to 7·3)	10·6 (10·5 to 10·7)	9·1 (9 to 9·2)	12·2 (12·1 to 12·3)	10·8 (10·7 to 10·9)	13·8 (13·7 to 13·9)	13·8 (12·9 to 14·8)
Sweden except Stockholm	46·6 (46·3 to 46·9)	42·3 (42 to 42·6)	44 (43·8 to 44·2)	37·7 (37·5 to 37·9)	48·2 (47·9 to 48·5)	34 (33·8 to 34·2)	41 (40·8 to 41·3)	43·1 (40·4 to 46·1)
Switzerland	40·5 (40·2 to 40·7)	46·8 (46·6 to 47·1)	47·6 (47·4 to 47·9)	35·7 (35·5 to 35·9)	41·6 (41·4 to 41·9)	36·9 (36·7 to 37·1)	38·9 (38·7 to 39·1)	42·9 (39·9 to 46·2)
UK	399·1 (389·5 to 410·7)	449 (442·2 to 455·6)	432·7 (424·1 to 441·1)	369·4 (368·5 to 370·3)	389·8 (388·8 to 390·7)	329·5 (328·6 to 330·5)	394·3 (393·1 to 395·5)	380·8 (341·1 to 426·1)
England	329·7 (319·7 to 339·8)	360 (350·1 to 369·3)	349 (338·9 to 359·4)	302·5 (296·9 to 308)	321·3 (316·5 to 325·8)	278·2 (275·6 to 280·5)	334·5 (332·3 to 336·6)	326·7 (295 to 362)
East Midlands	26·3 (25·5 to 27·1)	28·6 (27·8 to 29·3)	28·4 (27·6 to 29·3)	25·2 (24·7 to 25·6)	26·4 (26 to 26·7)	22·3 (22·1 to 22·5)	26·6 (26·4 to 26·7)	26·3 (23·8 to 29)
Derby	1·8 (1·7 to 1·8)	1·9 (1·9 to 2)	1·8 (1·8 to 1·9)	1·5 (1·5 to 1·6)	1·7 (1·6 to 1·7)	1·4 (1·4 to 1·4)	1·7 (1·7 to 1·8)	1·6 (1·5 to 1·8)
Derbyshire	4·9 (4·7 to 5)	5·2 (4·9 to 5·4)	5·1 (4·9 to 5·3)	4·3 (4·2 to 4·4)	4·4 (4·3 to 4·5)	3·7 (3·6 to 3·8)	4 (3·9 to 4·1)	3·8 (3·5 to 4·2)
Leicester	2·3 (2·2 to 2·3)	2·4 (2·3 to 2·5)	2·4 (2·3 to 2·5)	2·3 (2·3 to 2·4)	2·3 (2·3 to 2·4)	2 (1·9 to 2)	2·6 (2·5 to 2·6)	2·6 (2·4 to 2·9)
Leicestershire	3·2 (3·1 to 3·3)	3·7 (3·5 to 3·8)	3·7 (3·6 to 3·9)	3·4 (3·3 to 3·5)	3·5 (3·4 to 3·6)	3·1 (3·1 to 3·2)	3·4 (3·3 to 3·5)	3·4 (3·1 to 3·8)
Lincolnshire	3·5 (3·4 to 3·6)	3·7 (3·5 to 3·9)	3·8 (3·6 to 3·9)	3·4 (3·3 to 3·4)	3·5 (3·4 to 3·5)	3 (2·9 to 3·1)	3·8 (3·7 to 3·8)	3·8 (3·5 to 4·2)
Northamptonshire	3·4 (3·3 to 3·5)	3·7 (3·5 to 3·8)	3·9 (3·8 to 4·1)	3·7 (3·6 to 3·8)	4 (3·9 to 4·1)	3·6 (3·5 to 3·7)	4·5 (4·4 to 4·6)	4·4 (3·9 to 4·8)
Nottingham	2·2 (2·1 to 2·3)	2·4 (2·3 to 2·5)	2·2 (2·1 to 2·2)	1·9 (1·8 to 1·9)	2·2 (2·1 to 2·2)	1·6 (1·6 to 1·7)	2·1 (2·1 to 2·1)	2·2 (2 to 2·4)
Nottinghamshire	4·8 (4·7 to 5)	5·4 (5·2 to 5·6)	5·3 (5·2 to 5·5)	4·5 (4·4 to 4·7)	4·6 (4·5 to 4·7)	3·7 (3·7 to 3·8)	4·4 (4·3 to 4·4)	4·2 (3·8 to 4·7)
Rutland	0·2 (0·2 to 0·2)	0·2 (0·2 to 0·2)	0·2 (0·2 to 0·2)	0·2 (0·2 to 0·2)	0·2 (0·2 to 0·2)	0·2 (0·1 to 0·2)	0·2 (0·2 to 0·2)	0·2 (0·2 to 0·2)
East of England	31·4 (30·5 to 32·4)	34·7 (33·8 to 35·6)	34·2 (33·2 to 35·2)	31·5 (30·9 to 32·1)	33·8 (33·3 to 34·3)	29·9 (29·7 to 30·2)	35·7 (35·5 to 35·9)	34·9 (31·5 to 38·6)
Bedford	0·9 (0·9 to 0·9)	1·1 (1 to 1·1)	1 (1 to 1·1)	0·9 (0·9 to 1)	0·9 (0·9 to 0·9)	0·9 (0·8 to 0·9)	1 (1 to 1·1)	1 (0·9 to 1·1)
Cambridgeshire	2·4 (2·4 to 2·5)	2·8 (2·6 to 2·9)	2·9 (2·7 to 3)	2·9 (2·8 to 3)	3·2 (3·2 to 3·3)	2·9 (2·9 to 3)	3·6 (3·6 to 3·7)	3·6 (3·2 to 4)
Central Bedfordshire	1·3 (1·2 to 1·3)	1·6 (1·5 to 1·6)	1·6 (1·5 to 1·7)	1·6 (1·6 to 1·7)	1·6 (1·5 to 1·6)	1·4 (1·3 to 1·4)	1·6 (1·6 to 1·6)	1·6 (1·4 to 1·7)
Essex	7·6 (7·3 to 7·8)	8·6 (8·4 to 8·9)	8·6 (8·2 to 8·9)	7·7 (7·6 to 7·9)	8 (7·8 to 8·1)	7 (6·9 to 7·1)	8 (7·9 to 8·1)	8 (7·2 to 8·8)
Hertfordshire	6·8 (6·5 to 7)	7·4 (7·2 to 7·7)	6·8 (6·5 to 7)	6 (5·9 to 6·1)	6·7 (6·6 to 6·8)	6·2 (6·1 to 6·3)	7·2 (7·1 to 7·3)	7·1 (6·3 to 7·9)
Luton	1·2 (1·2 to 1·3)	1·6 (1·5 to 1·6)	1·5 (1·5 to 1·6)	1·4 (1·4 to 1·4)	1·6 (1·6 to 1·7)	1·4 (1·4 to 1·4)	1·8 (1·7 to 1·8)	1·7 (1·5 to 1·9)
Norfolk	4·3 (4·2 to 4·4)	4·5 (4·3 to 4·7)	4·5 (4·3 to 4·7)	4·1 (4 to 4·3)	4·4 (4·3 to 4·5)	3·8 (3·7 to 3·9)	4·5 (4·5 to 4·6)	4·4 (4 to 4·8)
Peterborough	0·8 (0·8 to 0·9)	0·9 (0·9 to 1)	0·9 (0·9 to 1)	1 (1 to 1)	1·2 (1·2 to 1·2)	1 (1 to 1·1)	1·5 (1·5 to 1·6)	1·5 (1·4 to 1·7)
Southend-on-Sea	1·3 (1·2 to 1·3)	1·2 (1·1 to 1·2)	1·1 (1 to 1·1)	0·9 (0·9 to 1)	1·1 (1·1 to 1·1)	0·9 (0·9 to 1)	1·1 (1·1 to 1·1)	1·1 (1 to 1·2)
Suffolk	3·9 (3·8 to 4)	4·1 (3·9 to 4·4)	4·2 (4 to 4·4)	3·9 (3·8 to 4)	4·2 (4·1 to 4·2)	3·5 (3·4 to 3·5)	4 (3·9 to 4·1)	3·9 (3·5 to 4·3)
Thurrock	0·9 (0·9 to 1)	1 (1 to 1·1)	1·1 (1·1 to 1·2)	0·9 (0·9 to 1)	1 (1 to 1)	0·9 (0·9 to 1)	1·2 (1·2 to 1·2)	1·1 (1 to 1·3)
Greater London	48·2 (46·4 to 50)	54·2 (52·4 to 55·9)	52·4 (50·5 to 54·4)	43·4 (42·4 to 44·6)	50·2 (49·4 to 51)	50 (49·5 to 50·4)	65·7 (65·2 to 66·1)	66·1 (59 to 74)
Barking and Dagenham	1·3 (1·3 to 1·4)	1·3 (1·2 to 1·4)	1·2 (1·1 to 1·2)	1 (1 to 1·1)	1·2 (1·2 to 1·2)	1·2 (1·1 to 1·2)	1·9 (1·8 to 1·9)	1·9 (1·7 to 2·1)
Barnet	2 (1·9 to 2)	2 (1·9 to 2·1)	2 (1·9 to 2·1)	1·8 (1·7 to 1·8)	2 (1·9 to 2)	2 (1·9 to 2)	2·7 (2·7 to 2·8)	2·7 (2·4 to 3)
Bexley	1·5 (1·4 to 1·5)	1·7 (1·6 to 1·7)	1·6 (1·5 to 1·6)	1·3 (1·2 to 1·3)	1·5 (1·5 to 1·5)	1·3 (1·2 to 1·3)	1·5 (1·5 to 1·6)	1·6 (1·4 to 1·8)
Brent	1·8 (1·8 to 1·9)	2·3 (2·2 to 2·4)	2·2 (2·1 to 2·3)	1·8 (1·7 to 1·8)	1·9 (1·8 to 2)	1·9 (1·8 to 1·9)	2·6 (2·6 to 2·7)	2·6 (2·4 to 2·9)
Bromley	2 (1·9 to 2·1)	2·1 (2 to 2·2)	1·9 (1·8 to 2)	1·6 (1·5 to 1·6)	1·8 (1·7 to 1·8)	1·7 (1·6 to 1·7)	2 (2 to 2·1)	2 (1·8 to 2·3)
Camden	0·9 (0·8 to 1)	1·2 (1·1 to 1·3)	1·3 (1·2 to 1·4)	1 (1 to 1·1)	1·1 (1·1 to 1·2)	1·3 (1·3 to 1·3)	1·5 (1·5 to 1·6)	1·4 (1·2 to 1·6)
Croydon	2·3 (2·3 to 2·4)	2·6 (2·5 to 2·7)	2·5 (2·4 to 2·6)	2·1 (2 to 2·1)	2·3 (2·3 to 2·4)	2·1 (2·1 to 2·2)	2·8 (2·7 to 2·9)	2·9 (2·6 to 3·2)
Ealing	2·1 (2 to 2·1)	2·3 (2·2 to 2·4)	2·3 (2·2 to 2·4)	2 (2 to 2·1)	2·1 (2·1 to 2·2)	2·1 (2 to 2·1)	2·9 (2·8 to 2·9)	2·6 (2·4 to 2·9)
Enfield	1·8 (1·8 to 1·9)	1·9 (1·8 to 2)	1·8 (1·8 to 1·9)	1·6 (1·5 to 1·6)	1·9 (1·8 to 1·9)	1·8 (1·8 to 1·9)	2·5 (2·4 to 2·6)	2·5 (2·3 to 2·8)
Greenwich	1·6 (1·5 to 1·6)	1·6 (1·6 to 1·7)	1·6 (1·5 to 1·6)	1·5 (1·4 to 1·5)	1·6 (1·6 to 1·7)	1·6 (1·5 to 1·6)	2·3 (2·2 to 2·3)	2·3 (2·1 to 2·6)
Hackney	1·7 (1·6 to 1·7)	2 (1·9 to 2·1)	1·9 (1·8 to 2)	1·5 (1·5 to 1·6)	1·8 (1·8 to 1·9)	1·9 (1·8 to 2)	2·3 (2·2 to 2·4)	2·4 (2·1 to 2·7)
Hammersmith and Fulham	0·9 (0·8 to 0·9)	1·1 (1 to 1·2)	1·2 (1·1 to 1·2)	0·9 (0·9 to 0·9)	1 (1 to 1·1)	1·2 (1·1 to 1·2)	1·4 (1·3 to 1·4)	1·3 (1·1 to 1·5)
Haringey	1·4 (1·3 to 1·5)	1·8 (1·7 to 2)	1·8 (1·7 to 2)	1·4 (1·3 to 1·4)	1·7 (1·6 to 1·7)	1·8 (1·7 to 1·8)	2·2 (2·1 to 2·2)	2 (1·8 to 2·3)
Harrow	1·4 (1·3 to 1·4)	1·5 (1·4 to 1·5)	1·4 (1·3 to 1·4)	1·2 (1·2 to 1·2)	1·4 (1·3 to 1·4)	1·2 (1·2 to 1·3)	1·7 (1·7 to 1·8)	1·9 (1·7 to 2·1)
Havering	1·7 (1·6 to 1·8)	1·9 (1·7 to 2)	1·7 (1·5 to 1·8)	1·3 (1·2 to 1·4)	1·4 (1·3 to 1·4)	1·1 (1·1 to 1·2)	1·4 (1·4 to 1·5)	1·7 (1·5 to 1·9)
Hillingdon	1·6 (1·5 to 1·7)	1·8 (1·7 to 1·9)	1·6 (1·6 to 1·7)	1·4 (1·4 to 1·5)	1·7 (1·6 to 1·7)	1·6 (1·6 to 1·6)	2·1 (2·1 to 2·2)	2·3 (2 to 2·6)
Hounslow	1·3 (1·3 to 1·4)	1·5 (1·4 to 1·6)	1·5 (1·4 to 1·6)	1·4 (1·3 to 1·4)	1·5 (1·5 to 1·6)	1·5 (1·5 to 1·6)	2·2 (2·2 to 2·3)	2·2 (2 to 2·5)
Islington	1·1 (1·1 to 1·2)	1·4 (1·4 to 1·5)	1·3 (1·3 to 1·4)	1·1 (1 to 1·1)	1·3 (1·3 to 1·3)	1·2 (1·2 to 1·2)	1·5 (1·4 to 1·5)	1·6 (1·4 to 1·8)

Kensington and Chelsea	0·7 (0·7 to 0·8)	1·1 (1·1 to 1·2)	1·5 (1·4 to 1·6)	0·9 (0·9 to 0·9)	0·9 (0·9 to 0·9)	1 (1 to 1)	1·1 (1 to 1·1)	0·9 (0·8 to 1)
Kingston upon Thames	0·8 (0·8 to 0·8)	0·8 (0·8 to 0·9)	0·8 (0·8 to 0·8)	0·8 (0·7 to 0·8)	0·8 (0·8 to 0·9)	0·9 (0·9 to 0·9)	1·1 (1·1 to 1·2)	1·2 (1 to 1·3)
Lambeth	1·9 (1·8 to 2)	2·4 (2·2 to 2·5)	2·2 (2 to 2·3)	1·7 (1·7 to 1·8)	2·1 (2 to 2·1)	2·1 (2 to 2·1)	2·4 (2·4 to 2·5)	2·4 (2·1 to 2·7)
Lewisham	1·8 (1·7 to 1·9)	2 (1·9 to 2·1)	1·9 (1·8 to 2)	1·5 (1·4 to 1·5)	1·9 (1·8 to 1·9)	1·8 (1·8 to 1·9)	2·5 (2·4 to 2·5)	2·4 (2·1 to 2·7)
Merton	1·2 (1·2 to 1·3)	1·2 (1·2 to 1·3)	1·2 (1·1 to 1·2)	1 (1 to 1)	1·2 (1·2 to 1·3)	1·3 (1·2 to 1·3)	1·7 (1·7 to 1·8)	1·6 (1·4 to 1·8)
Newham	1·8 (1·8 to 1·9)	2·1 (2 to 2·1)	2·1 (2 to 2·1)	1·8 (1·7 to 1·8)	2·1 (2·1 to 2·2)	2·3 (2·2 to 2·3)	3·1 (3·1 to 3·2)	3·2 (2·9 to 3·5)
Redbridge	1·5 (1·5 to 1·6)	1·6 (1·5 to 1·7)	1·5 (1·5 to 1·6)	1·3 (1·2 to 1·3)	1·5 (1·5 to 1·5)	1·5 (1·5 to 1·6)	2·2 (2·2 to 2·3)	2·4 (2·2 to 2·7)
Richmond upon Thames	1 (0·9 to 1)	1·1 (1 to 1·1)	1 (1 to 1·1)	0·9 (0·9 to 0·9)	1 (1 to 1·1)	1·2 (1·1 to 1·2)	1·4 (1·4 to 1·5)	1·3 (1·1 to 1·4)
Southwark	1·8 (1·8 to 1·9)	2·1 (2 to 2·1)	1·8 (1·7 to 1·8)	1·5 (1·4 to 1·5)	1·9 (1·9 to 2)	2 (1·9 to 2)	2·5 (2·4 to 2·6)	2·5 (2·2 to 2·8)
Sutton	1·1 (1·1 to 1·2)	1·2 (1·1 to 1·2)	1·1 (1·1 to 1·2)	1 (1 to 1)	1·2 (1·1 to 1·2)	1·1 (1 to 1·1)	1·3 (1·3 to 1·4)	1·3 (1·2 to 1·5)
Tower Hamlets	1·5 (1·5 to 1·5)	1·6 (1·5 to 1·7)	1·4 (1·3 to 1·4)	1·2 (1·2 to 1·3)	1·5 (1·5 to 1·6)	1·7 (1·7 to 1·7)	2·3 (2·2 to 2·3)	2·6 (2·3 to 2·9)
Waltham Forest	1·7 (1·6 to 1·7)	1·8 (1·7 to 1·8)	1·8 (1·8 to 1·9)	1·5 (1·4 to 1·5)	1·8 (1·8 to 1·9)	1·7 (1·6 to 1·8)	2·4 (2·3 to 2·4)	2·4 (2·2 to 2·7)
Wandsworth	1·7 (1·6 to 1·8)	1·9 (1·7 to 2)	1·9 (1·7 to 2)	1·6 (1·6 to 1·7)	1·9 (1·8 to 2)	1·9 (1·9 to 2)	2·6 (2·6 to 2·7)	2·6 (2·3 to 3)
Westminster	1·1 (1 to 1·2)	1·4 (1·3 to 1·5)	1·5 (1·4 to 1·6)	1 (1 to 1·1)	1·1 (1·1 to 1·2)	1·2 (1·2 to 1·2)	1·5 (1·5 to 1·5)	1·3 (1·2 to 1·5)
North East England	21·5 (21 to 22·1)	23·9 (23·3 to 24·5)	22·8 (22·2 to 23·4)	19 (18·7 to 19·3)	17·4 (17·2 to 17·7)	12·9 (12·7 to 13)	14·5 (14·4 to 14·6)	13·6 (12·4 to 15)
County Durham	4 (3·9 to 4·1)	4·4 (4·2 to 4·5)	4·2 (4·1 to 4·3)	3·5 (3·5 to 3·6)	3·2 (3·1 to 3·3)	2·4 (2·4 to 2·4)	2·7 (2·7 to 2·8)	2·6 (2·3 to 2·8)
Darlington	0·8 (0·8 to 0·8)	0·9 (0·8 to 0·9)	0·9 (0·8 to 0·9)	0·7 (0·7 to 0·7)	0·7 (0·7 to 0·7)	0·5 (0·5 to 0·6)	0·6 (0·6 to 0·6)	0·6 (0·5 to 0·6)
Gateshead	1·8 (1·8 to 1·9)	2·1 (2 to 2·1)	1·9 (1·9 to 2)	1·4 (1·4 to 1·5)	1·3 (1·3 to 1·4)	1 (1 to 1)	1·1 (1·1 to 1·1)	1·1 (1 to 1·2)
Hartlepool	0·8 (0·8 to 0·8)	1 (1 to 1)	0·9 (0·9 to 1)	0·7 (0·7 to 0·7)	0·7 (0·7 to 0·7)	0·5 (0·5 to 0·5)	0·6 (0·5 to 0·6)	0·5 (0·5 to 0·5)
Middlesbrough	1·4 (1·3 to 1·4)	1·6 (1·5 to 1·6)	1·5 (1·4 to 1·5)	1·3 (1·3 to 1·3)	1·2 (1·1 to 1·2)	0·8 (0·8 to 0·9)	1 (1 to 1)	0·9 (0·8 to 1)
Newcastle upon Tyne	2·3 (2·3 to 2·4)	2·5 (2·4 to 2·6)	2·2 (2·2 to 2·3)	1·9 (1·8 to 1·9)	1·9 (1·8 to 1·9)	1·4 (1·4 to 1·4)	1·6 (1·6 to 1·7)	1·7 (1·5 to 1·9)
North Tyneside	1·7 (1·7 to 1·8)	1·8 (1·7 to 1·9)	1·6 (1·6 to 1·7)	1·3 (1·3 to 1·3)	1·2 (1·2 to 1·3)	1 (1 to 1)	1·1 (1·1 to 1·2)	1·1 (1 to 1·2)
Northumberland	2·3 (2·2 to 2·4)	2·4 (2·3 to 2·4)	2·3 (2·3 to 2·4)	2·1 (2 to 2·1)	1·8 (1·8 to 1·9)	1·4 (1·4 to 1·4)	1·4 (1·4 to 1·5)	1·4 (1·2 to 1·5)
Redcar and Cleveland	1·2 (1·1 to 1·2)	1·4 (1·4 to 1·4)	1·4 (1·4 to 1·5)	1·2 (1·1 to 1·2)	1 (1 to 1)	0·7 (0·7 to 0·7)	0·8 (0·7 to 0·8)	0·7 (0·6 to 0·7)
South Tyneside	1·5 (1·4 to 1·5)	1·6 (1·6 to 1·7)	1·5 (1·4 to 1·5)	1·1 (1·1 to 1·1)	1·1 (1 to 1·1)	0·7 (0·7 to 0·7)	0·8 (0·8 to 0·8)	0·8 (0·7 to 0·8)
Stockton-on-Tees	1·3 (1·3 to 1·4)	1·6 (1·5 to 1·7)	1·6 (1·5 to 1·6)	1·4 (1·4 to 1·5)	1·3 (1·3 to 1·3)	1 (0·9 to 1)	1·2 (1·2 to 1·2)	1·1 (1 to 1·2)
Sunderland	2·4 (2·4 to 2·5)	2·8 (2·7 to 2·8)	2·7 (2·7 to 2·8)	2·4 (2·3 to 2·4)	2·1 (2 to 2·1)	1·4 (1·4 to 1·5)	1·5 (1·5 to 1·6)	1·3 (1·2 to 1·5)
North West England	52·9 (51·4 to 54·5)	57·3 (55·7 to 58·8)	54·6 (53·1 to 56·2)	45·3 (44·5 to 46·1)	46·1 (45·4 to 46·7)	37·2 (36·9 to 37·5)	43·6 (43·3 to 43·8)	41·6 (37·8 to 45·9)
Blackburn with Darwen	1·2 (1·2 to 1·2)	1·2 (1·2 to 1·3)	1·2 (1·2 to 1·3)	1·2 (1·1 to 1·2)	1·2 (1·1 to 1·2)	1 (1 to 1)	1·2 (1·1 to 1·2)	1 (0·9 to 1·1)
Blackpool	1·1 (1·1 to 1·2)	1 (1 to 1·1)	0·9 (0·9 to 1)	0·8 (0·7 to 0·8)	0·9 (0·9 to 0·9)	0·7 (0·7 to 0·7)	0·9 (0·9 to 0·9)	0·8 (0·7 to 0·9)
Bolton	2·1 (2 to 2·1)	2·2 (2·1 to 2·2)	2·2 (2·1 to 2·3)	1·9 (1·8 to 1·9)	1·8 (1·8 to 1·9)	1·6 (1·5 to 1·6)	1·9 (1·9 to 2)	1·8 (1·7 to 2)
Bury	1·3 (1·2 to 1·3)	1·4 (1·4 to 1·5)	1·5 (1·4 to 1·5)	1·2 (1·1 to 1·2)	1·2 (1·2 to 1·3)	1 (1 to 1)	1·2 (1·2 to 1·2)	1·1 (1 to 1·2)
Cheshire East	2·2 (2·1 to 2·3)	2·4 (2·3 to 2·5)	2·3 (2·2 to 2·3)	1·9 (1·8 to 1·9)	2 (1·9 to 2)	1·7 (1·7 to 1·8)	1·9 (1·9 to 2)	1·9 (1·7 to 2·1)
Cheshire West and Chester	2·2 (2·1 to 2·2)	2·4 (2·4 to 2·5)	2·3 (2·2 to 2·4)	1·9 (1·9 to 2)	2 (1·9 to 2)	1·7 (1·6 to 1·7)	1·8 (1·8 to 1·9)	1·7 (1·6 to 1·9)
Cumbria	3·5 (3·4 to 3·6)	3·6 (3·5 to 3·7)	3·4 (3·3 to 3·5)	2·8 (2·8 to 2·9)	2·9 (2·8 to 3)	2·3 (2·2 to 2·4)	2·5 (2·4 to 2·5)	2·3 (2·1 to 2·5)
Halton	0·7 (0·7 to 0·8)	0·8 (0·8 to 0·8)	0·9 (0·8 to 0·9)	1 (0·9 to 1)	0·9 (0·9 to 1)	0·7 (0·7 to 0·7)	0·8 (0·8 to 0·8)	0·7 (0·7 to 0·8)
Knowsley	1·5 (1·4 to 1·5)	1·9 (1·7 to 2)	1·6 (1·5 to 1·7)	1·4 (1·4 to 1·5)	1·2 (1·2 to 1·3)	0·9 (0·9 to 0·9)	1 (0·9 to 1)	0·9 (0·8 to 1)
Lancashire	7·9 (7·6 to 8·1)	8·1 (7·8 to 8·4)	7·9 (7·6 to 8·1)	6·9 (6·8 to 7·1)	7·2 (7 to 7·4)	5·9 (5·8 to 6·1)	6·8 (6·7 to 6·9)	6·3 (5·8 to 7)
Liverpool	4·4 (4·3 to 4·6)	4·8 (4·6 to 5)	4·3 (4·1 to 4·5)	3·4 (3·3 to 3·5)	3·4 (3·3 to 3·4)	2·5 (2·4 to 2·5)	2·8 (2·8 to 2·9)	3 (2·7 to 3·2)
Manchester	4 (3·8 to 4·1)	4·4 (4·2 to 4·6)	3·9 (3·8 to 4·1)	3·2 (3 to 3·3)	3·4 (3·3 to 3·5)	2·7 (2·7 to 2·8)	3·9 (3·8 to 4)	4·1 (3·7 to 4·6)
Oldham	1·8 (1·7 to 1·8)	2 (1·9 to 2)	2 (1·9 to 2·1)	1·6 (1·6 to 1·7)	1·6 (1·6 to 1·7)	1·5 (1·4 to 1·5)	1·7 (1·6 to 1·7)	1·6 (1·5 to 1·8)
Rochdale	1·7 (1·6 to 1·7)	1·7 (1·7 to 1·8)	1·7 (1·7 to 1·8)	1·6 (1·6 to 1·6)	1·6 (1·5 to 1·6)	1·3 (1·2 to 1·3)	1·5 (1·5 to 1·5)	1·4 (1·3 to 1·6)
Salford	2·1 (2·1 to 2·2)	2·3 (2·3 to 2·4)	2·2 (2·1 to 2·3)	1·6 (1·6 to 1·6)	1·7 (1·6 to 1·7)	1·3 (1·2 to 1·3)	1·7 (1·7 to 1·8)	1·8 (1·6 to 1·9)
Sefton	2·3 (2·2 to 2·3)	2·5 (2·4 to 2·6)	2·2 (2·1 to 2·2)	1·7 (1·7 to 1·8)	1·7 (1·7 to 1·8)	1·3 (1·3 to 1·3)	1·4 (1·3 to 1·4)	1·3 (1·2 to 1·5)
St Helens	1·4 (1·4 to 1·4)	1·6 (1·5 to 1·6)	1·6 (1·5 to 1·6)	1·2 (1·2 to 1·3)	1·1 (1·1 to 1·2)	0·9 (0·9 to 1)	1 (1 to 1·1)	1 (0·9 to 1·1)
Stockport	2·2 (2·1 to 2·3)	2·5 (2·4 to 2·6)	2·3 (2·2 to 2·4)	1·9 (1·8 to 1·9)	1·8 (1·7 to 1·8)	1·5 (1·4 to 1·5)	1·7 (1·6 to 1·7)	1·6 (1·4 to 1·8)
Tameside	1·7 (1·7 to 1·7)	1·8 (1·7 to 1·8)	1·8 (1·7 to 1·9)	1·5 (1·4 to 1·5)	1·6 (1·5 to 1·6)	1·2 (1·2 to 1·2)	1·5 (1·5 to 1·6)	1·4 (1·3 to 1·5)
Trafford	1·7 (1·7 to 1·8)	1·9 (1·8 to 2)	1·7 (1·7 to 1·8)	1·4 (1·4 to 1·4)	1·4 (1·4 to 1·4)	1·2 (1·2 to 1·2)	1·4 (1·4 to 1·4)	1·3 (1·2 to 1·5)
Warrington	1·2 (1·2 to 1·2)	1·4 (1·3 to 1·4)	1·3 (1·3 to 1·4)	1·1 (1·1 to 1·1)	1·3 (1·2 to 1·3)	1·1 (1·1 to 1·1)	1·2 (1·2 to 1·2)	1·1 (1 to 1·2)
Wigan	2·2 (2·1 to 2·2)	2·5 (2·4 to 2·5)	2·6 (2·5 to 2·7)	2·1 (2 to 2·1)	2·1 (2 to 2·1)	1·6 (1·6 to 1·7)	1·9 (1·9 to 1·9)	1·8 (1·6 to 1·9)
Wirral	2·8 (2·7 to 2·8)	3 (2·9 to 3)	2·7 (2·7 to 2·8)	2·2 (2·2 to 2·2)	2·2 (2·1 to 2·2)	1·6 (1·6 to 1·7)	1·8 (1·8 to 1·9)	1·7 (1·5 to 1·9)
South East England	44·3 (42·8 to 45·8)	48 (46·5 to 49·3)	45·7 (44·2 to 47·2)	42·8 (41·9 to 43·6)	47·9 (47·2 to 48·6)	44·1 (43·7 to 44·4)	52 (51·6 to 52·3)	50·1 (45·1 to 55·7)
Bracknell Forest	0·4 (0·4 to 0·4)	0·5 (0·5 to 0·5)	0·5 (0·5 to 0·5)	0·6 (0·6 to 0·6)	0·7 (0·7 to 0·7)	0·7 (0·7 to 0·7)	0·8 (0·8 to 0·8)	0·7 (0·6 to 0·8)
Brighton and Hove	1·4 (1·4 to 1·5)	1·3 (1·3 to 1·4)	1·2 (1·2 to 1·3)	1·2 (1·1 to 1·2)	1·4 (1·4 to 1·4)	1·4 (1·3 to 1·4)	1·6 (1·6 to 1·6)	1·5 (1·3 to 1·7)
Buckinghamshire	2·7 (2·6 to 2·8)	3·2 (3 to 3·3)	2·9 (2·7 to 3)	2·6 (2·6 to 2·7)	2·9 (2·8 to 2·9)	2·7 (2·6 to 2·8)	3 (2·9 to 3·1)	2·9 (2·6 to 3·2)

East Sussex	2·7 (2·6 to 2·8)	2·4 (2·4 to 2·5)	2·3 (2·3 to 2·4)	2·1 (2·1 to 2·2)	2·5 (2·5 to 2·6)	2·3 (2·2 to 2·3)	2·6 (2·5 to 2·7)	2·5 (2·3 to 2·8)
Hampshire	6·3 (6·1 to 6·5)	7·4 (7·2 to 7·7)	7·3 (7·1 to 7·6)	6·7 (6·5 to 6·9)	7·4 (7·3 to 7·5)	6·5 (6·4 to 6·7)	7·4 (7·2 to 7·5)	7 (6·3 to 7·7)
Isle of Wight	0·8 (0·8 to 0·8)	0·7 (0·7 to 0·7)	0·7 (0·6 to 0·7)	0·6 (0·6 to 0·6)	0·7 (0·6 to 0·7)	0·6 (0·5 to 0·6)	0·6 (0·6 to 0·6)	0·6 (0·6 to 0·7)
Kent	8·1 (7·8 to 8·3)	8·6 (8·3 to 8·8)	8·2 (7·9 to 8·5)	7·2 (7 to 7·4)	8 (7·9 to 8·2)	7·3 (7·1 to 7·4)	8·6 (8·4 to 8·8)	8·8 (8 to 9·7)
Medway	1·5 (1·5 to 1·6)	1·9 (1·9 to 2)	2·1 (2 to 2·1)	1·8 (1·8 to 1·9)	1·8 (1·8 to 1·9)	1·5 (1·5 to 1·5)	1·8 (1·7 to 1·8)	1·8 (1·6 to 2)
Milton Keynes	0·5 (0·5 to 0·5)	0·5 (0·5 to 0·5)	0·6 (0·6 to 0·6)	1·1 (1·1 to 1·1)	1·4 (1·4 to 1·5)	1·4 (1·4 to 1·4)	1·9 (1·9 to 2)	1·8 (1·6 to 2)
Oxfordshire	2·8 (2·7 to 2·9)	3·5 (3·2 to 3·7)	3·4 (3·2 to 3·5)	3·3 (3·2 to 3·4)	3·7 (3·6 to 3·9)	3·5 (3·4 to 3·6)	4·1 (4 to 4·2)	3·8 (3·4 to 4·3)
Portsmouth	1·3 (1·3 to 1·4)	1·4 (1·3 to 1·4)	1·3 (1·2 to 1·3)	1·2 (1·1 to 1·2)	1·3 (1·3 to 1·3)	1·1 (1·1 to 1·1)	1·3 (1·3 to 1·3)	1·3 (1·2 to 1·5)
Reading	0·8 (0·8 to 0·9)	1 (1 to 1·1)	1 (1 to 1·1)	1 (0·9 to 1)	1 (1 to 1)	1 (0·9 to 1)	1·3 (1·3 to 1·3)	1·2 (1·1 to 1·4)
Slough	0·8 (0·8 to 0·9)	0·9 (0·9 to 0·9)	0·8 (0·8 to 0·9)	0·8 (0·8 to 0·9)	0·9 (0·9 to 1)	0·9 (0·9 to 0·9)	1·3 (1·3 to 1·4)	1·2 (1·1 to 1·4)
Southampton	1·4 (1·3 to 1·4)	1·5 (1·4 to 1·6)	1·5 (1·4 to 1·5)	1·3 (1·3 to 1·4)	1·4 (1·4 to 1·5)	1·2 (1·2 to 1·3)	1·6 (1·6 to 1·7)	1·6 (1·5 to 1·8)
Surrey	6·5 (6·2 to 6·7)	6·7 (6·5 to 7)	5·9 (5·6 to 6·1)	5·3 (5·2 to 5·4)	6 (5·8 to 6·2)	5·9 (5·7 to 6·1)	6·9 (6·6 to 7·1)	6·5 (5·8 to 7·3)
West Berkshire	0·7 (0·7 to 0·7)	0·8 (0·8 to 0·9)	0·8 (0·8 to 0·9)	0·8 (0·8 to 0·9)	0·9 (0·9 to 1)	0·8 (0·8 to 0·9)	0·9 (0·9 to 1)	0·8 (0·8 to 0·9)
West Sussex	4·1 (3·9 to 4·2)	3·9 (3·7 to 4·1)	3·6 (3·4 to 3·8)	3·6 (3·5 to 3·7)	4 (3·9 to 4·1)	3·8 (3·8 to 3·9)	4·5 (4·4 to 4·5)	4·3 (3·9 to 4·8)
Windsor and Maidenhead	0·8 (0·8 to 0·8)	0·9 (0·8 to 0·9)	0·8 (0·8 to 0·9)	0·8 (0·7 to 0·8)	0·8 (0·8 to 0·8)	0·8 (0·8 to 0·8)	0·9 (0·9 to 0·9)	0·8 (0·7 to 0·9)
Wokingham	0·6 (0·5 to 0·6)	0·8 (0·7 to 0·8)	0·7 (0·7 to 0·8)	0·7 (0·7 to 0·7)	0·9 (0·9 to 0·9)	0·8 (0·8 to 0·9)	0·9 (0·9 to 1)	0·8 (0·7 to 0·9)
South West England	28·6 (27·7 to 29·5)	29·1 (28·3 to 29·9)	28·2 (27·4 to 29·1)	25·8 (25·3 to 26·3)	28·4 (27·9 to 28·8)	24·7 (24·4 to 24·9)	28·9 (28·6 to 29·1)	28·7 (26 to 31·8)
Bath and North East Somerset	0·9 (0·9 to 1)	0·9 (0·9 to 1)	0·9 (0·8 to 0·9)	0·8 (0·8 to 0·9)	0·9 (0·9 to 0·9)	0·8 (0·8 to 0·8)	0·9 (0·8 to 0·9)	0·9 (0·8 to 1)
Bournemouth	1 (0·9 to 1)	0·8 (0·8 to 0·8)	0·7 (0·7 to 0·8)	0·6 (0·6 to 0·7)	0·8 (0·8 to 0·9)	0·8 (0·7 to 0·8)	1·1 (1 to 1·1)	1·2 (1 to 1·3)
Bristol, City of	2·8 (2·7 to 2·9)	3 (2·8 to 3·1)	2·8 (2·7 to 3)	2·5 (2·5 to 2·6)	2·7 (2·7 to 2·8)	2·3 (2·3 to 2·4)	3·1 (3·1 to 3·2)	3·2 (2·8 to 3·6)
Cornwall	2·9 (2·8 to 3)	2·8 (2·7 to 2·8)	2·7 (2·6 to 2·8)	2·5 (2·4 to 2·6)	2·7 (2·6 to 2·7)	2·3 (2·2 to 2·3)	2·7 (2·6 to 2·7)	2·7 (2·4 to 2·9)
Devon	3·7 (3·6 to 3·8)	3·5 (3·4 to 3·6)	3·3 (3·2 to 3·5)	3·1 (3 to 3·2)	3·6 (3·5 to 3·7)	3·1 (3·1 to 3·2)	3·6 (3·5 to 3·6)	3·5 (3·2 to 3·9)
Dorset	2·1 (2 to 2·1)	2 (1·9 to 2)	1·9 (1·8 to 2)	1·8 (1·7 to 1·8)	1·9 (1·9 to 2)	1·6 (1·6 to 1·7)	1·8 (1·7 to 1·8)	1·7 (1·6 to 1·9)
Gloucestershire	3·3 (3·2 to 3·4)	3·5 (3·3 to 3·6)	3·4 (3·2 to 3·5)	3 (3 to 3·1)	3·4 (3·3 to 3·4)	2·9 (2·9 to 3)	3·3 (3·2 to 3·3)	3·3 (2·9 to 3·6)
North Somerset	1 (1 to 1)	1 (1 to 1)	1 (1 to 1)	0·9 (0·9 to 1)	1 (0·9 to 1)	0·9 (0·9 to 0·9)	1·1 (1·1 to 1·1)	1·1 (1 to 1·2)
Plymouth	1·7 (1·7 to 1·8)	1·8 (1·8 to 1·9)	1·8 (1·7 to 1·9)	1·7 (1·7 to 1·8)	1·7 (1·7 to 1·7)	1·3 (1·3 to 1·3)	1·6 (1·5 to 1·6)	1·6 (1·4 to 1·7)
Poole	0·8 (0·8 to 0·8)	0·8 (0·8 to 0·8)	0·8 (0·7 to 0·8)	0·7 (0·7 to 0·7)	0·8 (0·8 to 0·8)	0·7 (0·7 to 0·7)	0·8 (0·8 to 0·8)	0·8 (0·7 to 0·9)
Somerset	2·8 (2·7 to 2·9)	2·8 (2·7 to 3)	2·8 (2·7 to 2·9)	2·6 (2·5 to 2·6)	2·8 (2·7 to 2·9)	2·4 (2·4 to 2·5)	2·8 (2·7 to 2·8)	2·7 (2·5 to 3)
South Gloucestershire	1·2 (1·2 to 1·2)	1·5 (1·4 to 1·6)	1·5 (1·4 to 1·5)	1·3 (1·2 to 1·3)	1·5 (1·4 to 1·5)	1·4 (1·4 to 1·4)	1·5 (1·5 to 1·5)	1·5 (1·4 to 1·7)
Swindon	1 (1 to 1·1)	1·2 (1·2 to 1·3)	1·2 (1·1 to 1·2)	1·1 (1·1 to 1·1)	1·3 (1·3 to 1·4)	1·1 (1·1 to 1·1)	1·4 (1·4 to 1·5)	1·4 (1·3 to 1·6)
Torbay	0·8 (0·7 to 0·8)	0·7 (0·6 to 0·7)	0·6 (0·6 to 0·7)	0·6 (0·5 to 0·6)	0·6 (0·6 to 0·7)	0·6 (0·6 to 0·6)	0·7 (0·7 to 0·7)	0·7 (0·6 to 0·7)
Wiltshire	2·5 (2·4 to 2·5)	2·8 (2·7 to 2·9)	2·9 (2·8 to 3)	2·5 (2·4 to 2·5)	2·6 (2·5 to 2·6)	2·3 (2·3 to 2·4)	2·6 (2·5 to 2·6)	2·5 (2·2 to 2·8)
West Midlands	40·1 (39·1 to 41·3)	45 (43·8 to 46·1)	44·7 (43·5 to 45·8)	36·9 (36·4 to 37·4)	37·3 (36·7 to 37·8)	29·8 (29·6 to 30)	35·2 (35·1 to 35·4)	34·2 (31 to 37·7)
Birmingham	9·4 (9·2 to 9·7)	10·1 (9·7 to 10·5)	9·7 (9·4 to 10·1)	8·2 (8·1 to 8·3)	8·4 (8·2 to 8·7)	6·9 (6·8 to 7·1)	8·5 (8·3 to 8·7)	8·4 (7·6 to 9·3)
Coventry	2·7 (2·6 to 2·7)	3·1 (2·9 to 3·2)	3 (2·9 to 3·1)	2·4 (2·3 to 2·5)	2·3 (2·2 to 2·4)	1·8 (1·7 to 1·8)	2·3 (2·2 to 2·3)	2·2 (2 to 2·5)
Dudley	2·3 (2·2 to 2·3)	2·7 (2·6 to 2·8)	2·7 (2·6 to 2·8)	2 (2 to 2·1)	2·1 (2 to 2·1)	1·6 (1·6 to 1·7)	1·8 (1·8 to 1·9)	1·8 (1·6 to 2)
Herefordshire, County of	1·1 (1 to 1·1)	1·1 (1·1 to 1·1)	1·1 (1 to 1·1)	0·9 (0·9 to 1)	1 (1 to 1)	0·8 (0·8 to 0·8)	0·9 (0·9 to 0·9)	0·9 (0·8 to 1)
Sandwell	2·9 (2·8 to 2·9)	3·1 (2·9 to 3·2)	2·9 (2·8 to 3·1)	2·3 (2·2 to 2·4)	2·3 (2·2 to 2·3)	1·8 (1·8 to 1·8)	2·4 (2·4 to 2·4)	2·3 (2·1 to 2·5)
Shropshire	1·7 (1·7 to 1·8)	1·9 (1·8 to 2)	1·9 (1·9 to 2)	1·5 (1·5 to 1·6)	1·6 (1·6 to 1·7)	1·3 (1·3 to 1·3)	1·4 (1·4 to 1·4)	1·4 (1·3 to 1·5)
Solihull	1·4 (1·4 to 1·5)	1·7 (1·6 to 1·7)	1·7 (1·7 to 1·8)	1·2 (1·2 to 1·3)	1·3 (1·2 to 1·3)	1 (0·9 to 1)	1·1 (1·1 to 1·1)	1·1 (1 to 1·2)
Staffordshire	5 (4·9 to 5·2)	6 (5·8 to 6·3)	6·2 (6 to 6·5)	5·3 (5·2 to 5·4)	5·2 (5 to 5·3)	3·9 (3·9 to 4)	4·4 (4·3 to 4·5)	4·2 (3·8 to 4·6)
Stoke-on-Trent	2·2 (2·2 to 2·3)	2·3 (2·2 to 2·4)	2·3 (2·2 to 2·3)	1·9 (1·8 to 1·9)	1·8 (1·8 to 1·9)	1·4 (1·4 to 1·4)	1·8 (1·8 to 1·9)	1·6 (1·5 to 1·8)
Telford and Wrekin	0·7 (0·7 to 0·7)	0·8 (0·8 to 0·9)	0·9 (0·9 to 1)	1 (1 to 1)	1·1 (1·1 to 1·1)	0·9 (0·9 to 0·9)	1·1 (1·1 to 1·1)	1 (0·9 to 1·1)
Walsall	2·2 (2·1 to 2·3)	2·7 (2·5 to 2·8)	2·6 (2·4 to 2·7)	1·9 (1·9 to 2)	1·9 (1·9 to 2)	1·6 (1·5 to 1·6)	1·8 (1·8 to 1·8)	1·8 (1·7 to 2)
Warwickshire	3·3 (3·2 to 3·4)	3·8 (3·6 to 3·9)	3·8 (3·7 to 4)	3·1 (3 to 3·1)	3·1 (3 to 3·2)	2·6 (2·5 to 2·6)	3 (3 to 3·1)	2·9 (2·7 to 3·3)
Wolverhampton	2·2 (2·1 to 2·3)	2·4 (2·3 to 2·6)	2·3 (2·2 to 2·4)	1·9 (1·8 to 1·9)	1·9 (1·9 to 2)	1·4 (1·4 to 1·4)	1·7 (1·7 to 1·8)	1·7 (1·5 to 1·8)
Worcestershire	3·1 (3 to 3·2)	3·4 (3·2 to 3·5)	3·5 (3·4 to 3·7)	3·2 (3·1 to 3·2)	3·2 (3·2 to 3·3)	2·8 (2·7 to 2·8)	3 (2·9 to 3·1)	2·9 (2·6 to 3·2)
Yorkshire and the Humber	36·3 (35·3 to 37·3)	39·3 (38·2 to 40·3)	38 (37 to 39·1)	32·6 (32 to 33·1)	33·8 (33·3 to 34·2)	27·3 (27 to 27·5)	32·4 (32·2 to 32·6)	31·2 (28·4 to 34·4)
Barnsley	1·7 (1·7 to 1·8)	1·9 (1·8 to 2)	1·9 (1·8 to 1·9)	1·5 (1·5 to 1·5)	1·5 (1·5 to 1·6)	1·1 (1·1 to 1·2)	1·4 (1·4 to 1·4)	1·3 (1·2 to 1·5)
Bradford	3·9 (3·8 to 4)	4·3 (4·2 to 4·3)	4·2 (4·1 to 4·3)	3·8 (3·8 to 3·9)	3·8 (3·7 to 3·9)	3·4 (3·4 to 3·5)	4·1 (4·1 to 4·2)	3·8 (3·5 to 4·2)
Calderdale	1·6 (1·6 to 1·7)	1·6 (1·5 to 1·6)	1·6 (1·5 to 1·6)	1·3 (1·3 to 1·3)	1·4 (1·3 to 1·4)	1·1 (1·1 to 1·1)	1·3 (1·3 to 1·3)	1·2 (1·1 to 1·3)
Doncaster	2·2 (2·2 to 2·3)	2·5 (2·4 to 2·6)	2·4 (2·3 to 2·5)	2·1 (2 to 2·1)	2·1 (2 to 2·1)	1·6 (1·5 to 1·6)	1·9 (1·8 to 1·9)	1·7 (1·5 to 1·8)
East Riding of Yorkshire	1·8 (1·7 to 1·8)	1·9 (1·8 to 1·9)	1·8 (1·7 to 1·8)	1·5 (1·5 to 1·5)	1·6 (1·6 to 1·7)	1·4 (1·3 to 1·4)	1·5 (1·5 to 1·5)	1·4 (1·3 to 1·6)
Kingston upon Hull, City of	2·2 (2·1 to 2·2)	2·4 (2·3 to 2·5)	2·4 (2·3 to 2·4)	2 (2 to 2·1)	2·1 (2·1 to 2·2)	1·4 (1·4 to 1·5)	1·8 (1·8 to 1·9)	1·7 (1·5 to 1·8)
Kirklees	2·9 (2·8 to 3)	3·1 (3 to 3·2)	3·1 (3 to 3·2)	2·7 (2·7 to 2·8)	2·7 (2·6 to 2·8)	2·4 (2·4 to 2·5)	2·8 (2·8 to 2·9)	2·6 (2·4 to 2·9)

Leeds	5·3 (5·1 to 5·5)	5·7 (5·5 to 5·9)	5·3 (5·1 to 5·5)	4·5 (4·4 to 4·6)	4·8 (4·7 to 4·9)	3·9 (3·8 to 3·9)	4·9 (4·8 to 5)	5·1 (4·6 to 5·6)
North East Lincolnshire	1·2 (1·2 to 1·3)	1·5 (1·4 to 1·5)	1·4 (1·4 to 1·5)	1·2 (1·2 to 1·2)	1·2 (1·2 to 1·2)	0·8 (0·8 to 0·9)	1 (1 to 1)	0·9 (0·8 to 1)
North Lincolnshire	1·1 (1·1 to 1·1)	1·2 (1·2 to 1·3)	1·2 (1·2 to 1·3)	1 (1 to 1)	1 (1 to 1)	0·8 (0·8 to 0·8)	0·9 (0·9 to 0·9)	0·9 (0·8 to 1)
North Yorkshire	3·3 (3·2 to 3·4)	3·5 (3·3 to 3·6)	3·3 (3·2 to 3·5)	2·9 (2·8 to 2·9)	3·2 (3·1 to 3·3)	2·7 (2·6 to 2·8)	2·9 (2·8 to 2·9)	2·8 (2·5 to 3·1)
Rotherham	1·8 (1·8 to 1·9)	2·1 (2·1 to 2·2)	2·2 (2·1 to 2·2)	1·8 (1·8 to 1·8)	1·8 (1·8 to 1·8)	1·4 (1·3 to 1·4)	1·6 (1·5 to 1·6)	1·5 (1·4 to 1·6)
Sheffield	3·9 (3·7 to 4)	4 (3·9 to 4·2)	3·7 (3·6 to 3·9)	3·1 (3 to 3·2)	3·3 (3·2 to 3·4)	2·8 (2·7 to 2·8)	3·3 (3·2 to 3·3)	3·4 (3·1 to 3·8)
Wakefield	2·3 (2·2 to 2·4)	2·6 (2·5 to 2·6)	2·5 (2·5 to 2·6)	2·2 (2·1 to 2·2)	2·2 (2·1 to 2·3)	1·7 (1·6 to 1·7)	2 (2 to 2·1)	1·9 (1·7 to 2·1)
York	1 (1 to 1)	1·1 (1 to 1·1)	1 (0·9 to 1)	1 (0·9 to 1)	1 (1 to 1)	0·9 (0·8 to 0·9)	1 (1 to 1)	1 (0·9 to 1·2)
Northern Ireland	12·4 (10·5 to 14·3)	16·1 (14·6 to 18)	16·7 (14·8 to 18·8)	13·4 (11·7 to 15)	13·7 (12·4 to 15·1)	10·5 (9·8 to 11·3)	12·8 (12·2 to 13·4)	11·8 (9·1 to 15·4)
Scotland	35·2 (30·9 to 40·1)	50·1 (45·7 to 55·3)	45·3 (40·1 to 50·8)	34·5 (30·4 to 38·7)	35·8 (32·3 to 39·3)	25·8 (24·3 to 27·7)	29·8 (28·5 to 31·2)	26·2 (21·5 to 31·9)
Wales	21·9 (21·3 to 22·5)	22·7 (22·1 to 23·3)	21·7 (21·1 to 22·2)	19 (18·6 to 19·4)	19 (18·6 to 19·4)	15 (14·9 to 15·1)	17·1 (16·8 to 17·5)	16·1 (14·6 to 17·7)
Latin America and Caribbean	3194·5 (3049·5 to 3339·7)	4026·5 (3885 to 4168·3)	4705·7 (4560·5 to 4841·1)	5284·5 (5160·1 to 5409·2)	5232·9 (5102 to 5357·2)	5211·7 (5056·9 to 5374·5)	5159·9 (4956·7 to 5386·5)	4786·6 (4374·7 to 5222·8)
Andean Latin America	352·4 (336·4 to 369·2)	445·7 (432·4 to 459·2)	532·2 (517·3 to 546)	604·5 (586 to 623·6)	602·7 (576·9 to 627·6)	614·4 (582 to 646)	625·5 (588·5 to 667·4)	650·9 (557·8 to 755·7)
Bolivia	80·8 (77·6 to 84·1)	87·2 (84·1 to 89·9)	94·1 (90·6 to 97·6)	112·6 (109·2 to 115·9)	119·9 (115·2 to 125)	128·6 (121·8 to 135·7)	145·7 (136·5 to 154·6)	160 (138·9 to 183·1)
Ecuador	79·7 (75·8 to 84·1)	110·3 (107·6 to 112·8)	134 (131·1 to 136·8)	144·5 (140·5 to 148·5)	147·9 (142·5 to 153·5)	159·6 (152·5 to 166·7)	162·5 (149·5 to 175·4)	170·7 (146·1 to 198·5)
Peru	191·9 (183·1 to 201)	248·2 (239·1 to 257·6)	304·1 (292·7 to 315)	347·4 (332·1 to 362·6)	334·8 (311·9 to 357·6)	326·2 (301·9 to 349·9)	317·3 (290·7 to 349·3)	320·3 (272·9 to 374·2)
Caribbean	336·9 (326·2 to 347·8)	406·3 (393·7 to 417·7)	432·2 (424·9 to 439·3)	401·7 (392 to 410·7)	447 (436·3 to 457·9)	427 (415 to 439·1)	418·3 (400·6 to 435·9)	400·8 (363·7 to 441·2)
Antigua and Barbuda	0·8 (0·8 to 0·9)	0·9 (0·9 to 0·9)	0·8 (0·8 to 0·9)	0·7 (0·7 to 0·7)	0·7 (0·6 to 0·7)	0·7 (0·6 to 0·8)	0·6 (0·5 to 0·7)	0·5 (0·4 to 0·6)
The Bahamas	1·3 (1·2 to 1·4)	1·7 (1·6 to 1·8)	2·1 (2·1 to 2·2)	2·5 (2·4 to 2·5)	2·6 (2·5 to 2·6)	2·5 (2·4 to 2·5)	2·3 (2·3 to 2·4)	2 (1·6 to 2·4)
Barbados	3·4 (3·2 to 3·7)	3·5 (3·3 to 3·6)	2·5 (2·4 to 2·6)	2·2 (2·1 to 2·2)	2·1 (2 to 2·2)	1·9 (1·8 to 2)	1·6 (1·4 to 1·8)	1·4 (1·1 to 1·7)
Belize	1·5 (1·4 to 1·6)	2·1 (2 to 2·1)	2·3 (2·3 to 2·4)	2·7 (2·6 to 2·7)	2·9 (2·8 to 3)	3·1 (2·9 to 3·2)	3·5 (3·3 to 3·7)	3·7 (3·2 to 4·3)
Bermuda	0·5 (0·5 to 0·6)	0·6 (0·5 to 0·6)	0·5 (0·5 to 0·5)	0·4 (0·4 to 0·4)	0·4 (0·4 to 0·5)	0·4 (0·4 to 0·4)	0·3 (0·3 to 0·4)	0·3 (0·2 to 0·3)
Cuba	75·6 (71·9 to 80·1)	107·9 (101·4 to 114·3)	121·3 (120·7 to 122·1)	63·1 (61·8 to 64·4)	87·5 (87 to 88·1)	70·7 (70·3 to 71·3)	63·7 (63·4 to 64·1)	50·5 (43·5 to 58·6)
Dominica	1·1 (1 to 1·1)	1·3 (1·3 to 1·3)	1·2 (1·2 to 1·3)	0·9 (0·8 to 0·9)	0·8 (0·8 to 0·9)	0·6 (0·6 to 0·7)	0·5 (0·4 to 0·5)	0·4 (0·3 to 0·5)
Dominican Republic	57·2 (54·3 to 60·4)	73·9 (71·2 to 76·4)	92·4 (89·8 to 95·1)	109·5 (104·5 to 114·6)	120·7 (115·9 to 125·9)	118·7 (113·1 to 124·6)	109·2 (102·8 to 115·9)	112·5 (96·9 to 130·1)
Grenada	1·9 (1·8 to 1·9)	1·8 (1·8 to 1·9)	1·4 (1·3 to 1·5)	1·3 (1·2 to 1·4)	1 (0·9 to 1)	1 (0·9 to 1·1)	0·8 (0·7 to 0·9)	0·7 (0·6 to 0·8)
Guyana	10·2 (9·8 to 10·6)	12·9 (12·6 to 13·1)	13·3 (12·8 to 13·9)	13·2 (12·4 to 13·9)	11·1 (10·4 to 11·9)	8·6 (8 to 9·3)	7 (6·1 to 7·9)	7 (5·9 to 8·3)
Haiti	84 (78·3 to 89·6)	89·1 (83·6 to 94·2)	90·6 (85·2 to 95·2)	104·8 (99·8 to 109·7)	122·3 (116·2 to 128·3)	136 (129 to 142·7)	157·1 (146·6 to 167·5)	163·8 (147·2 to 183·3)
Jamaica	24·8 (23 to 26·7)	32·5 (31·4 to 33·6)	30·4 (29·5 to 31·3)	27·8 (26·5 to 29)	27·2 (26 to 28·6)	22·8 (20·5 to 25·3)	20 (17·9 to 22·4)	17·6 (14·9 to 20·7)
Puerto Rico	41·8 (41·6 to 42·1)	36·3 (36·1 to 36·6)	33·4 (33·2 to 33·7)	34·8 (34·6 to 35)	31·9 (31·6 to 32·2)	28·3 (28 to 28·6)	20·4 (18·7 to 22·4)	12·3 (10·7 to 14·1)
Saint Kitts and Nevis	1·3 (1·2 to 1·4)	0·7 (0·6 to 0·8)	0·5 (0·4 to 0·5)	0·5 (0·5 to 0·6)	0·4 (0·4 to 0·5)	0·4 (0·4 to 0·4)	0·4 (0·3 to 0·4)	0·3 (0·3 to 0·4)
Saint Lucia	1·6 (1·5 to 1·7)	2 (1·9 to 2)	2 (2 to 2·1)	2·1 (2 to 2·1)	1·9 (1·7 to 2)	1·4 (1·3 to 1·6)	1·1 (0·9 to 1·3)	0·9 (0·7 to 1·1)
Saint Vincent and the Grenadines	1·7 (1·6 to 1·7)	1·9 (1·8 to 1·9)	1·7 (1·6 to 1·7)	1·6 (1·5 to 1·7)	1·3 (1·2 to 1·4)	1·1 (1 to 1·2)	0·9 (0·8 to 1)	0·7 (0·6 to 0·9)
Suriname	3·7 (3·6 to 3·8)	5·9 (5·8 to 6·1)	6·5 (6·2 to 6·8)	5·2 (5 to 5·4)	4·7 (4·5 to 4·9)	4·8 (4·6 to 5·1)	4·9 (4·4 to 5·4)	4·5 (3·8 to 5·3)
Trinidad and Tobago	12 (11·3 to 12·8)	15·5 (15 to 15·9)	12·5 (12·1 to 12·8)	14·3 (13·9 to 14·8)	11·5 (11 to 11·9)	8·7 (8·3 to 9·1)	9·1 (8·5 to 9·6)	7·5 (6·4 to 8·8)
Virgin Islands	0·5 (0·4 to 0·5)	0·6 (0·5 to 0·6)	1·3 (1·2 to 1·4)	1·2 (1·2 to 1·3)	1·1 (1 to 1·2)	1 (0·8 to 1·1)	0·8 (0·7 to 0·8)	0·6 (0·5 to 0·7)
Central Latin America	1210·8 (1165·7 to 1254·9)	1610 (1566·3 to 1650·5)	2013·7 (1961·9 to 2059·8)	2416·4 (2359·4 to 2470·9)	2443·2 (2372 to 2515·7)	2416·6 (2324·5 to 2515·2)	2323 (2226·4 to 2426·1)	2153·8 (1933·1 to 2386)
Colombia	263·1 (246·5 to 281·2)	357·9 (342·5 to 372·3)	415·7 (395·9 to 436·5)	438·1 (419·5 to 456·5)	460·2 (435·4 to 484·7)	467·2 (445·5 to 489·5)	425·8 (394 to 460·6)	391·8 (332·4 to 459·3)
Costa Rica	18·6 (18·3 to 19)	28·8 (28·6 to 29·1)	26·9 (26·2 to 27·6)	34 (33·1 to 34·9)	39·4 (38 to 40·7)	35·9 (33·6 to 38·6)	35·4 (33·9 to 37)	32·4 (27·4 to 38·3)
El Salvador	47·2 (45·3 to 49·3)	62·2 (60·3 to 64)	75·8 (74·1 to 77·5)	81·5 (79·3 to 83·7)	81·2 (78·9 to 83·4)	68·9 (66·1 to 71·8)	56·4 (53·6 to 59·3)	54·5 (46·8 to 63·3)
Guatemala	71·9 (69·7 to 74·2)	90·4 (88·8 to 91·9)	107·2 (104·7 to 109·9)	150·8 (148·9 to 152·7)	167·3 (163·3 to 171·5)	181·1 (174·9 to 187·6)	190·9 (177·6 to 205)	201·6 (171·4 to 235·9)
Honduras	35·9 (34·3 to 37·5)	44·2 (43 to 45·3)	55·3 (54·3 to 56·2)	72·7 (70·9 to 74·4)	88 (85·5 to 90·2)	94 (90·2 to 97·6)	100·1 (93·2 to 107·5)	113·7 (100·9 to 128·8)
Mexico	620·5 (588·5 to 652)	803·2 (774·8 to 829·7)	1063·7 (1026·2 to 1098·1)	1311·2 (1268 to 1351·8)	1236·4 (1184 to 1290·1)	1228·4 (1154·4 to 1302·5)	1127·7 (1063·7 to 1199)	1027 (905·6 to 1172·9)
Aguascalientes	4·6 (4·4 to 4·8)	5·6 (5·4 to 5·8)	7·4 (7·2 to 7·6)	9·8 (9·5 to 10·1)	10·7 (10·4 to 11·1)	11·7 (11 to 12·5)	11·5 (10·9 to 12·2)	11·2 (10 to 12·7)
Baja California	5·7 (5·5 to 6)	12 (11·6 to 12·5)	19·6 (18·9 to 20·2)	22·9 (22·2 to 23·6)	23·6 (22·6 to 24·7)	27 (25·3 to 29)	28·1 (26·4 to 30)	28·1 (24·8 to 32·1)
Baja California Sur	1·5 (1·4 to 1·5)	1·9 (1·8 to 1·9)	2·8 (2·7 to 2·9)	4·2 (4 to 4·3)	4·2 (4 to 4·5)	4·8 (4·4 to 5·1)	5·6 (5·2 to 6)	5·9 (5·1 to 6·8)
Campeche	2·8 (2·7 to 3)	3·8 (3·7 to 4)	5·6 (5·4 to 5·8)	8·3 (8·1 to 8·6)	7·8 (7·5 to 8·2)	7·8 (7·3 to 8·4)	7·9 (7·4 to 8·4)	7·5 (6·6 to 8·4)
Chiapas	20·4 (19·2 to 21·6)	27·3 (26·1 to 28·3)	34·7 (33·3 to 36·1)	43·7 (41·7 to 45·7)	55·7 (52·9 to 58·5)	57·1 (53·1 to 61·1)	61·6 (57·5 to 66)	64·8 (57·7 to 73·1)
Chihuahua	21·6 (20·6 to 22·5)	28·9 (27·8 to 30)	36 (34·7 to 37·1)	38 (36·6 to 39·3)	33·5 (31·9 to 35·2)	35·7 (33·6 to 38)	30·6 (28·7 to 32·7)	27 (23·8 to 30·6)
Coahuila	17·3 (16·4 to 18·1)	20·5 (19·8 to 21·1)	24·5 (23·6 to 25·3)	30·2 (29·4 to 31)	27·4 (26·2 to 28·6)	25·8 (24·3 to 27·4)	25·4 (24 to 26·9)	25·2 (22·5 to 28·4)
Colima	2·8 (2·7 to 2·9)	3·7 (3·6 to 3·8)	5·2 (5 to 5·3)	6·7 (6·5 to 6·9)	5·8 (5·6 to 6·1)	5·6 (5·3 to 6)	5·7 (5·3 to 6·1)	5·4 (4·8 to 6·1)
Durango	15·9 (15·2 to 16·5)	18·6 (17·9 to 19·3)	21·7 (21·1 to 22·3)	24·1 (23·4 to 24·9)	23·2 (22·1 to 24·2)	20·8 (19·5 to 22·3)	17·9 (16·9 to 19·1)	16·3 (14·7 to 18·3)
Guanajuato	32 (30·6 to 33·4)	39·1 (37·8 to 40·3)	48·6 (46·8 to 50·3)	62·3 (59·7 to 64·8)	67·5 (63·9 to 71·5)	66·7 (61·7 to 72·2)	58·5 (53·7 to 63·3)	52 (45·3 to 59·9)
Guerrero	21·3 (20 to 22·6)	27·3 (26·3 to 28·2)	35·1 (33·6 to 36·4)	43·4 (41·3 to 45·3)	48·6 (46·2 to 51·2)	50·5 (47·3 to 53·8)	42·5 (39·7 to 45·3)	37·7 (33·3 to 42·8)
Hidalgo	20·8 (19·9 to 21·7)	24 (23·2 to 24·7)	27·7 (26·8 to 28·6)	32·7 (31·8 to 33·5)	31·5 (30·2 to 32·9)	30·3 (28·3 to 32·6)	27·9 (26 to 29·8)	24·7 (21·9 to 28·1)

Jalisco	43·4 (41·3 to 45·5)	54·2 (52·4 to 56)	70·6 (68·2 to 72·9)	85·5 (83·2 to 87·8)	78·8 (75·8 to 81·8)	75 (70·8 to 79·7)	70·2 (66·7 to 74)	64·9 (57·7 to 73·4)
México	31·6 (30 to 33·1)	42·2 (40·7 to 43·6)	83·3 (80·4 to 86)	149 (144·4 to 153·5)	154·2 (146·4 to 161·6)	171·5 (160·5 to 183·2)	169·9 (158·8 to 182·4)	155 (137·6 to 175·7)
Mexico City	81 (76·3 to 85·5)	120·3 (116·1 to 124·1)	162·1 (155·4 to 168·1)	174·2 (165 to 182·8)	114·5 (107·4 to 121·6)	91·6 (84·1 to 99·4)	72 (66·9 to 77·9)	56·3 (49·2 to 65)
Michoacán de Ocampo	34 (32·2 to 35·8)	40·6 (39 to 42)	48·7 (46·8 to 50·4)	60 (57·5 to 62·4)	57·9 (54·4 to 61·4)	52·2 (48·5 to 56·2)	45·2 (42 to 48·6)	40·2 (35·3 to 46)
Morelos	6·4 (6·1 to 6·8)	8·9 (8·5 to 9·2)	13·4 (13 to 13·8)	18·6 (18·1 to 19·1)	16·8 (16·2 to 17·5)	16·6 (15·8 to 17·6)	16·3 (15·2 to 17·4)	15 (13·3 to 17)
Nayarit	7·2 (6·9 to 7·5)	9 (8·6 to 9·4)	11·7 (11·4 to 12·1)	13·1 (12·7 to 13·6)	11·6 (11·1 to 12·1)	10·5 (9·9 to 11·2)	10·3 (9·7 to 11)	9·9 (8·8 to 11·1)
Nuevo León	18·2 (17·5 to 19)	26·4 (25·6 to 27·2)	38·6 (37·3 to 39·9)	45·5 (43·7 to 47·3)	41·4 (39·1 to 43·9)	43·6 (40·4 to 46·9)	42 (39 to 45·2)	39·1 (34·3 to 44·8)
Oaxaca	32·6 (30·4 to 34·7)	38·8 (37·1 to 40·3)	43·6 (41·8 to 45·3)	46·4 (44·4 to 48·3)	45·8 (43·6 to 48·1)	47·6 (43·9 to 51·5)	42·1 (39·3 to 45·1)	36·6 (32·2 to 41·9)
Puebla	37·3 (35 to 39·4)	43·5 (41·5 to 45·2)	53·7 (51·2 to 55·9)	65·9 (62·9 to 68·8)	66 (62 to 70·2)	71·2 (66 to 76·7)	65·4 (59·8 to 71·2)	58·8 (50 to 69·4)
Querétaro	6·7 (6·4 to 7)	8 (7·7 to 8·3)	10·4 (10·1 to 10·6)	14·4 (14 to 14·7)	16·7 (16 to 17·4)	17·4 (16·3 to 18·6)	17·8 (16·8 to 18·9)	17·4 (15·4 to 19·8)
Quintana Roo	0·6 (0·6 to 0·6)	1·1 (1 to 1·1)	1·9 (1·9 to 2)	4·6 (4·5 to 4·7)	7·3 (7 to 7·6)	10·1 (9·6 to 10·8)	12·5 (11·9 to 13·3)	13·4 (11·9 to 15·3)
San Luis Potosí	20·1 (19·1 to 21·1)	23·7 (22·8 to 24·5)	27·4 (26·4 to 28·4)	32·3 (30·9 to 33·7)	31·8 (30 to 33·6)	30·2 (28·1 to 32·3)	25·8 (23·8 to 27·9)	23 (20·1 to 26·4)
Sinaloa	14·9 (14·2 to 15·6)	19·1 (18·4 to 19·9)	27·7 (26·9 to 28·5)	34 (32·9 to 35·1)	31·4 (30·1 to 32·7)	30·1 (28·2 to 32·2)	24·8 (23·3 to 26·3)	21·9 (19·5 to 24·8)
Sonora	13 (12·4 to 13·6)	18 (17·4 to 18·6)	24 (23·1 to 24·9)	28·8 (27·4 to 30·2)	25·9 (24·3 to 27·4)	26·6 (24·4 to 28·8)	24·1 (22·2 to 26·1)	22·2 (19·5 to 25·4)
Tabasco	8·2 (7·8 to 8·5)	11·6 (11·2 to 12)	17·1 (16·5 to 17·7)	21·5 (20·9 to 22·1)	22·9 (22 to 23·8)	22·8 (21·4 to 24·2)	21·7 (20·3 to 23)	19·9 (17·7 to 22·5)
Tamaulipas	17·3 (16·4 to 18·2)	23·6 (22·7 to 24·4)	31 (29·7 to 32·2)	36 (34·1 to 37·8)	32·9 (30·6 to 35·2)	35 (32·3 to 37·7)	30·9 (28·7 to 33·1)	26·6 (23·2 to 30·6)
Tlaxcala	6·4 (6·1 to 6·8)	7·4 (7·1 to 7·7)	8·5 (8·3 to 8·8)	10·5 (10·3 to 10·8)	11·4 (10·9 to 11·9)	11·9 (11·2 to 12·6)	11·4 (10·7 to 12)	10·6 (9·5 to 12)
Veracruz de Ignacio de la Llave	46·6 (43·8 to 49·4)	61·5 (58·9 to 63·8)	83·8 (80·5 to 86·9)	103·7 (100·9 to 106·7)	92·9 (88·9 to 97)	86·1 (81·1 to 91·5)	70·2 (65·8 to 75·2)	61·6 (54·5 to 70)
Yucatán	11·1 (10·4 to 11·9)	12·8 (12·2 to 13·3)	16·2 (15·6 to 16·9)	20·1 (19·5 to 20·6)	18·8 (18 to 19·6)	18·1 (17·1 to 19·2)	17 (16 to 18)	15·8 (14 to 17·9)
Zacatecas	17·1 (16·6 to 17·6)	19·8 (19·2 to 20·4)	20·9 (20·3 to 21·5)	20·9 (20·2 to 21·5)	17·9 (17 to 18·8)	16·4 (15·3 to 17·4)	15 (13·8 to 16·2)	13·2 (11·4 to 15·5)
Nicaragua	32·4 (31·1 to 33·5)	39·2 (38·1 to 40·3)	47·7 (46·4 to 49)	63·9 (62 to 65·5)	70·5 (67·1 to 73·8)	62·2 (58·7 to 65·7)	65·3 (60·3 to 70·4)	63·8 (56·7 to 72·2)
Panama	13 (12·6 to 13·5)	20·9 (20·5 to 21·3)	26·3 (25·7 to 26·9)	25·7 (24·9 to 26·5)	28·7 (27·7 to 29·7)	30·4 (29 to 31·8)	34 (32·8 to 35·1)	37 (32·1 to 42·4)
Venezuela	108 (104·4 to 111·4)	163·3 (161 to 165·4)	195·1 (191·8 to 198·8)	238·7 (232·8 to 244·3)	271·6 (264·5 to 278·9)	248·6 (238·6 to 259·3)	287·4 (278·2 to 298)	231·9 (196·6 to 271·4)
Tropical Latin America	1294·4 (1195·5 to 1391·5)	1564·5 (1463·2 to 1667·5)	1727·6 (1631·1 to 1824·8)	1861·8 (1808·2 to 1914·6)	1740 (1694·5 to 1787·7)	1753·6 (1698 to 1814·9)	1793·2 (1719·7 to 1870·5)	1581·2 (1410·2 to 1776)
Brazil	1262·8 (1164·1 to 1359·5)	1527·9 (1426·7 to 1630·4)	1685·8 (1590·8 to 1782·8)	1809 (1757·7 to 1861·1)	1678·4 (1631·9 to 1724)	1693·3 (1638·2 to 1754·4)	1730·5 (1656·6 to 1806)	1520·4 (1350·2 to 1710)
Acre	2·7 (2·4 to 2·9)	3·4 (3·2 to 3·7)	4·1 (3·9 to 4·3)	5·2 (5 to 5·4)	5·3 (5·1 to 5·5)	6·5 (6·2 to 6·7)	7·7 (7·3 to 8·2)	7·9 (7 to 8·9)
Alagoas	30·7 (28·8 to 32·4)	33·5 (31·5 to 35·4)	38 (36·2 to 39·9)	39·9 (38·8 to 41)	38·8 (37·4 to 40·2)	39·7 (38 to 41·5)	40·1 (38·2 to 42)	35·6 (31·8 to 39·9)
Amapá	0·9 (0·8 to 1)	1·4 (1·3 to 1·5)	1·9 (1·8 to 2)	2·4 (2·3 to 2·5)	2·8 (2·7 to 3)	4·5 (4·3 to 4·8)	6 (5·6 to 6·4)	6·3 (5·5 to 7·2)
Amazonas	13·9 (12·7 to 15·1)	17·4 (16·3 to 18·5)	19·7 (18·7 to 20·6)	24·2 (23·4 to 25)	24·8 (24 to 25·7)	31 (29·9 to 32·2)	37·3 (35·4 to 39·3)	36 (31·9 to 40·7)
Bahia	133·6 (125·4 to 141·5)	154·8 (145·3 to 163·8)	171·8 (163·1 to 180·7)	180·7 (175 to 186·5)	157·1 (152·7 to 161·5)	160·3 (154·4 to 166·4)	162·6 (155·1 to 170·4)	137·5 (121·7 to 155·3)
Ceará	71 (66·9 to 75)	84·9 (79·8 to 89·8)	94·4 (89·6 to 99·4)	98·2 (95 to 101·3)	88·3 (85·9 to 90·6)	95·1 (91·2 to 99·4)	103·3 (98·6 to 107·7)	96 (85·1 to 107·9)
Distrito Federal	1 (0·9 to 1)	2·3 (2·2 to 2·5)	9·8 (9·2 to 10·3)	18·3 (17·7 to 18·9)	18·9 (18·1 to 19·6)	20·6 (19·8 to 21·4)	21·1 (20·1 to 22·1)	18·8 (16·9 to 21)
Espírito Santo	20·3 (18·8 to 21·8)	25·6 (24 to 27·3)	27·7 (26·1 to 29·5)	30·7 (29·5 to 31·8)	30·2 (29·1 to 31·3)	30·1 (28·9 to 31·3)	30·3 (28·6 to 32·1)	26·3 (23·3 to 29·6)
Goiás	24·5 (22·6 to 26·3)	35·7 (33·3 to 38)	45·3 (43 to 47·8)	53·3 (51·8 to 54·9)	44·3 (42·7 to 46)	46·7 (44·6 to 48·7)	46·9 (44·8 to 49·1)	41·5 (36·7 to 46·9)
Maranhão	46·3 (43 to 49·9)	66·6 (62·5 to 70·4)	74·4 (70·9 to 77·9)	85·9 (83·4 to 88·3)	86·8 (83·3 to 90·1)	97·4 (93·2 to 101·9)	119·8 (114·8 to 124·7)	103·8 (92 to 116·5)
Mato Grosso	4·6 (4·3 to 4·9)	7·5 (7 to 8)	11·2 (10·6 to 11·9)	16·7 (16·2 to 17·2)	22·1 (21·2 to 23·1)	23·7 (22·7 to 24·8)	26·1 (24·8 to 27·4)	23·6 (21 to 26·6)
Mato Grosso do Sul	8 (7·3 to 8·7)	12·3 (11·5 to 13·2)	16·1 (15·1 to 17)	20·3 (19·6 to 21)	20·6 (19·8 to 21·4)	19·7 (18·9 to 20·5)	20·7 (19·5 to 21·9)	18·8 (16·6 to 21·4)
Minas Gerais	178·2 (163·7 to 192·9)	198·8 (184·8 to 213)	195·4 (183·8 to 207·9)	187·1 (182·5 to 191·8)	170·2 (165·8 to 174·7)	162·1 (156·1 to 168·6)	153 (145·3 to 160·7)	131·2 (116·6 to 147·9)
Pará	29·3 (27 to 31·8)	36·1 (33·8 to 38·2)	44·1 (41·9 to 46·4)	56·7 (54·7 to 58·5)	56·9 (55·1 to 58·5)	67·6 (64·9 to 70·3)	81·7 (77·7 to 85·7)	77 (68 to 87·3)
Paraíba	47 (44·2 to 49·8)	52 (48·8 to 54·9)	51·7 (49·1 to 54·4)	45·5 (43·8 to 47·2)	40·3 (39·1 to 41·5)	42·1 (40·3 to 44)	45·5 (43·4 to 47·7)	40·5 (35·9 to 45·4)
Paraná	49 (44·9 to 53·1)	85 (78·9 to 91)	116·6 (109·5 to 124)	99 (95·7 to 102·4)	91·4 (88·3 to 94·5)	84·8 (81·5 to 88·1)	77·6 (73·7 to 81·8)	68 (60·2 to 77)
Pernambuco	97·6 (91·8 to 103·1)	112·3 (105·7 to 118·8)	120·9 (115 to 127·1)	115·3 (111·5 to 119·2)	98·1 (95 to 101·1)	100·1 (95·8 to 104·8)	100·8 (96·3 to 105·4)	87·2 (77·2 to 98·7)
Piauí	28 (26·1 to 30)	32·1 (30·2 to 33·9)	37·2 (35·3 to 39·1)	39·9 (38·6 to 41·2)	37·2 (36 to 38·4)	39·1 (37·5 to 40·8)	42·2 (40·1 to 44·2)	36·7 (32·3 to 41·6)
Rio de Janeiro	102·5 (92 to 113·4)	120·9 (110·8 to 131·5)	127·9 (118·2 to 137·6)	132·3 (126·6 to 138·4)	117·8 (113·5 to 121·9)	111·9 (107·7 to 116·4)	109·2 (103·5 to 115·2)	96·6 (85·9 to 108·8)
Rio Grande do Norte	26·4 (24·8 to 27·8)	30·4 (28·6 to 32·2)	34·9 (33·1 to 36·7)	34·9 (33·8 to 35·9)	33·7 (32·8 to 34·7)	36 (34·6 to 37·5)	37·2 (35·2 to 39·1)	33·2 (29·4 to 37·6)
Rio Grande do Sul	84·3 (75·7 to 93·1)	93·8 (86·4 to 101·3)	92·8 (86·4 to 99·5)	94·2 (91·5 to 96·9)	83·7 (80·7 to 86·6)	74·7 (72·2 to 77·6)	65·4 (62·2 to 68·5)	56·6 (50·3 to 63·7)
Rondônia	0·9 (0·8 to 0·9)	1·4 (1·3 to 1·5)	2·1 (2 to 2·2)	7·7 (7·4 to 8)	12·6 (12·2 to 13)	13·5 (12·9 to 14)	13·4 (12·7 to 14·2)	11·7 (10·3 to 13·4)
Roraima	0·5 (0·5 to 0·5)	0·7 (0·6 to 0·7)	0·8 (0·8 to 0·9)	1·5 (1·5 to 1·6)	3·1 (3 to 3·2)	4·8 (4·6 to 5)	6 (5·7 to 6·3)	6·1 (5·5 to 6·9)
Santa Catarina	33·5 (30·8 to 36·2)	40 (37·1 to 43)	43·4 (40·7 to 46·2)	46·9 (45·5 to 48·2)	43·9 (42·3 to 45·4)	42·6 (40·9 to 44·4)	43·7 (41·4 to 46·1)	40·1 (35·8 to 44·9)
São Paulo	207·4 (189 to 226·1)	255·4 (237·2 to 274·3)	276·9 (259·1 to 295)	342·3 (331·3 to 353·5)	319 (307·3 to 330·4)	303·7 (292·9 to 315·3)	296·6 (282 to 312·1)	251·1 (223·3 to 282·7)
Sergipe	16·7 (15·5 to 17·8)	17·7 (16·5 to 18·8)	18·6 (17·6 to 19·6)	19·1 (18·5 to 19·7)	18·3 (17·5 to 19·1)	19·8 (18·9 to 20·6)	20·6 (19·6 to 21·7)	18·5 (16·5 to 20·9)
Tocantins	3·9 (3·6 to 4·3)	5·8 (5·5 to 6·2)	8·2 (7·7 to 8·6)	11 (10·6 to 11·4)	12·2 (11·8 to 12·6)	15·1 (14·4 to 15·7)	15·7 (14·7 to 16·6)	13·9 (12·3 to 15·8)
Paraguay	31·6 (29·7 to 33·6)	36·6 (34·9 to 38·3)	41·7 (40·3 to 43·3)	52·8 (51 to 54·5)	61·6 (58·8 to 64·5)	60·3 (57·3 to 63·6)	62·7 (57·3 to 68·4)	60·7 (52 to 70·7)
North Africa and Middle East	2739·8 (2630·3 to 2847·7)	3355·4 (3248·5 to 3457)	4168·4 (4059·4 to 4267·3)	5477·3 (5346 to 5600·1)	5813·5 (5606·5 to 6011·3)	6010·4 (5723 to 6298·5)	6237·6 (5814·7 to 6675·2)	5929·4 (5372·2 to 6578·9)
North Africa and Middle East	2739·8 (2630·3 to 2847·7)	3355·4 (3248·5 to 3457)	4168·4 (4059·4 to 4267·3)	5477·3 (5346 to 5600·1)	5813·5 (5606·5 to 6011·3)	6010·4 (5723 to 6298·5)	6237·6 (5814·7 to 6675·2)	5929·4 (5372·2 to 6578·9)

Afghanistan	180·6 (171·9 to 189·2)	217·7 (209·4 to 224·9)	270·5 (262·3 to 278·4)	254·3 (247·6 to 261)	255·4 (249·8 to 261·1)	444·9 (432·3 to 457)	568·9 (549·1 to 588·1)	722·2 (684·1 to 762·9)
Algeria	188·7 (176·9 to 200·5)	232·1 (222·2 to 241·8)	284 (274·6 to 293·2)	362·8 (350 to 374·2)	374·9 (352·6 to 397·7)	349·1 (315·9 to 383·9)	428·2 (386·2 to 473·4)	430·3 (386·1 to 482·3)
Bahrain	1·9 (1·7 to 2)	3·2 (3 to 3·4)	3·3 (3·1 to 3·4)	4·5 (4·2 to 4·8)	6·8 (6·2 to 7·3)	6·5 (5·9 to 7)	8·1 (7·2 to 9·1)	6·4 (5·2 to 7·8)
Egypt	507·8 (491·1 to 525)	601·4 (580·1 to 622·3)	713·8 (689·4 to 738·5)	917·1 (891·6 to 941)	950·2 (917·9 to 983·8)	929·8 (877·1 to 979·1)	1097·4 (1010·1 to 1185·3)	1025·8 (914·8 to 1141·8)
Iran	401·7 (377·9 to 425·1)	465·1 (443·7 to 484·8)	569·9 (546·1 to 591·9)	1017·3 (1004·5 to 1029·8)	850·5 (786·2 to 911·6)	700·5 (629·3 to 771·2)	725·3 (634·2 to 812·1)	656·1 (551·9 to 789)
Iraq	122·2 (117·3 to 126·8)	159·5 (153 to 165·5)	208·9 (199·3 to 218)	288·6 (278·4 to 298·2)	356·2 (337·7 to 374·8)	467·4 (439·9 to 496·5)	448·7 (400·4 to 498·7)	465·8 (405·4 to 540·3)
Jordan	12·5 (12·4 to 12·5)	21·2 (21·1 to 21·3)	32·9 (32·5 to 33·3)	48·5 (47·4 to 49·5)	67 (63·9 to 70·1)	68·8 (62·2 to 75·3)	90·4 (81·5 to 99·7)	117·1 (102·2 to 135·3)
Kuwait	1·5 (1·3 to 1·6)	5·7 (5·6 to 5·8)	17·5 (17·2 to 17·8)	24·6 (23·7 to 25·4)	19·5 (17·8 to 21·1)	20·1 (18·8 to 21·5)	26·8 (25·1 to 28·7)	29·5 (24·4 to 36·2)
Lebanon	31·2 (29·7 to 32·7)	40·3 (38·8 to 41·8)	47·8 (45·8 to 49·9)	54·8 (51·3 to 58·2)	55·5 (51 to 59·5)	48·9 (44 to 53·7)	50·3 (44·2 to 56·6)	52·2 (45·2 to 60·7)
Libya	25·4 (24·6 to 26·1)	34·6 (33·4 to 35·8)	43·6 (42·5 to 44·7)	63·7 (61·6 to 65·7)	75·6 (71·4 to 79·7)	60·1 (54·4 to 66·2)	49·5 (43·5 to 56)	39·5 (33·1 to 47·9)
Morocco	226·4 (215·6 to 237·7)	278·1 (267·6 to 288·4)	334·1 (321·2 to 347·1)	385·3 (364·6 to 405·3)	395·2 (371·6 to 418·2)	409·4 (374·6 to 444·5)	380·2 (334·1 to 427·7)	296·7 (252·1 to 348)
Oman	12·5 (12·2 to 12·9)	16·5 (16 to 16·9)	20·8 (20·2 to 21·4)	29·3 (28·3 to 30·3)	32·3 (30·7 to 34·1)	30·3 (27·5 to 33)	32·7 (28·9 to 36·4)	38·1 (33·9 to 42·5)
Palestine	19·2 (18·6 to 19·9)	22·9 (22·3 to 23·5)	27 (26·3 to 27·7)	33·7 (32·6 to 34·7)	46·7 (44·8 to 48·5)	61·2 (58·5 to 64·1)	68·1 (63·2 to 73)	60·8 (54·4 to 68)
Qatar	0·5 (0·5 to 0·6)	1·1 (1 to 1·1)	2·1 (2 to 2·3)	3·9 (3·7 to 4)	5·2 (4·9 to 5·5)	5·2 (5·1 to 5·8)	10·7 (9·9 to 11·7)	13·1 (11·3 to 15·1)
Saudi Arabia	65·2 (62·7 to 67·6)	85·2 (82·3 to 88)	125·5 (120·3 to 130·9)	197·7 (187·1 to 209·5)	262·2 (241·9 to 283·5)	251·2 (224·7 to 276·4)	238·1 (210·8 to 266·6)	221·2 (196·2 to 248·2)
Sudan	136·1 (129·9 to 142)	159·2 (152·9 to 164·3)	265 (256·5 to 272·9)	386·6 (372·5 to 399·7)	514·6 (493·9 to 533·5)	617·5 (587·1 to 646·6)	619·2 (564 to 673·2)	588·6 (513 to 677·2)
Syria	86·3 (84 to 88·6)	117·6 (114·5 to 120·5)	154·1 (150·2 to 157·6)	200·2 (194·4 to 205·9)	250·8 (238·7 to 262·2)	253·6 (235·6 to 273)	247 (222·7 to 272·4)	112·5 (93·3 to 137)
Tunisia	82·7 (77·5 to 87·8)	93·8 (90·1 to 97)	101·5 (98·6 to 104·5)	123·5 (119·2 to 127·8)	116·4 (109·2 to 123·6)	101 (93·8 to 108·3)	95·8 (89 to 102·6)	80·7 (69·2 to 95·3)
Turkey	517·5 (484·5 to 550·5)	654·6 (626·8 to 681·1)	759·4 (729·9 to 788)	801·5 (757·4 to 843·2)	820·2 (766·4 to 873·9)	734·4 (688·5 to 783·4)	500·2 (457·1 to 544·4)	477·2 (416·5 to 551·8)
United Arab Emirates	1·7 (1·6 to 1·8)	2·1 (2 to 2·2)	4·6 (4·4 to 4·7)	17·3 (16·8 to 17·8)	22·6 (21·6 to 23·6)	24·2 (22·2 to 26·1)	45·3 (39·7 to 51·2)	27·3 (23·4 to 32·2)
Yemen	117·9 (113·8 to 121·8)	142·7 (139·1 to 146)	180·3 (176·3 to 184·1)	258·7 (254·6 to 262·7)	331·8 (323·5 to 339·3)	421·4 (401·4 to 439·6)	500·4 (460·9 to 536·3)	462·2 (408·2 to 524·7)
South Asia	9932·7 (9203·8 to 10662·6)	11250·6 (10490·2 to 12033)	12673·8 (11900·8 to 13409·6)	16075·8 (15420·3 to 16722·2)	17297·2 (16587·6 to 17977·3)	17594·6 (16949·8 to 18229·3)	17290·4 (16639·4 to 17932·8)	15978·2 (14680·9 to 17367·6)
South Asia	9932·7 (9203·8 to 10662·6)	11250·6 (10490·2 to 12033)	12673·8 (11900·8 to 13409·6)	16075·8 (15420·3 to 16722·2)	17297·2 (16587·6 to 17977·3)	17594·6 (16949·8 to 18229·3)	17290·4 (16639·4 to 17932·8)	15978·2 (14680·9 to 17367·6)
Bangladesh	1086 (1049·4 to 1119·6)	1188·7 (1149·7 to 1226)	1433·8 (1386 to 1478·3)	1595·4 (1544 to 1649·8)	1852 (1797·8 to 1905·3)	1685·9 (1605·3 to 1768·3)	1460·5 (1382·8 to 1540·8)	1297·8 (1160·7 to 1437·4)
Bhutan	4 (3·8 to 4·3)	5·3 (5 to 5·6)	7·1 (6·8 to 7·5)	9·3 (8·7 to 9·9)	11·2 (10·2 to 12·2)	9·4 (8·4 to 10·4)	8·3 (7·1 to 9·4)	6·7 (5·8 to 7·8)
India	7816·2 (7177·6 to 8459·1)	8937·9 (8222·8 to 9665·5)	9586·8 (8890·2 to 10260·1)	12223·7 (11645·8 to 12788·5)	12667 (12000·6 to 13286·1)	12653·3 (12093·8 to 13191·5)	12348·5 (11795·6 to 12889·2)	11168·7 (10066·4 to 12269·2)
Andhra Pradesh	311·8 (280·1 to 345·2)	361·8 (331·3 to 393·2)	393·9 (354·4 to 438·2)	506·1 (471·2 to 541·5)	505 (472·1 to 537·2)	426 (398·7 to 455·4)	398·3 (374·4 to 423)	323·6 (288·4 to 361·1)
Arunachal Pradesh	5·6 (5·2 to 5·9)	6·5 (6·1 to 6·9)	9·5 (8·6 to 10·4)	12·7 (11·7 to 13·8)	12·1 (11 to 13·3)	12·7 (11·6 to 13·8)	15·3 (13·8 to 16·9)	16·7 (13·8 to 19·9)
Assam	179·9 (167·3 to 192·5)	217·6 (199·7 to 236·6)	255·9 (236·4 to 275·3)	338·1 (316·1 to 359·4)	347·6 (320·5 to 373·7)	300·1 (281·3 to 319·7)	314·2 (287·2 to 343·7)	314·2 (276·6 to 352)
Bihar	667 (611·4 to 722·2)	780·1 (726·4 to 832)	867·3 (790·5 to 949·9)	1127·9 (1059·7 to 1195)	1222 (1145·9 to 1295·5)	1345·9 (1265·6 to 1422·9)	1389·7 (1322·2 to 1458)	1223·1 (1110·9 to 1333·8)
Chhattisgarh	135·5 (123·6 to 147·6)	163·2 (150 to 175·4)	173 (156·2 to 190·2)	244·5 (228·4 to 260·9)	276·8 (255·8 to 297·4)	273·2 (258 to 290·4)	290·2 (272·4 to 308·6)	287·8 (255·1 to 321·4)
Delhi	33·6 (30·5 to 36·8)	42·4 (37·8 to 47·7)	52·1 (46·3 to 57·7)	88·6 (82·6 to 94·8)	127·2 (118·9 to 135·6)	147·1 (136·6 to 158)	154·7 (145·9 to 163·9)	138·6 (117·7 to 161·4)
Goa	10·8 (10 to 11·7)	12·3 (11·3 to 13·4)	12·7 (11·4 to 14·1)	13·2 (11·8 to 14·7)	11·6 (10·8 to 12·4)	12·4 (11·5 to 13·2)	10·7 (9·9 to 11·6)	8·5 (7·2 to 10·1)
Gujarat	385·8 (360·1 to 411·1)	452 (413·9 to 492·3)	491·4 (457·7 to 523·8)	623·6 (589·8 to 657·9)	562·2 (526·9 to 600·6)	545 (515·5 to 576·9)	587·2 (560·2 to 618·6)	538·5 (489·4 to 587·4)
Haryana	128 (119·8 to 135·8)	152·3 (138·7 to 166·9)	179·6 (166·3 to 192·2)	215·4 (203·9 to 226·9)	236·4 (224·1 to 249·4)	257·5 (241·1 to 275·7)	266·9 (248·1 to 285·4)	220·7 (183·7 to 261·4)
Himachal Pradesh	42·8 (39·5 to 46·2)	49·7 (46·4 to 52·9)	57·3 (52·6 to 62·9)	70·6 (66·4 to 74·9)	69·7 (66·3 to 73·3)	60·1 (57·3 to 63·1)	57·4 (54·1 to 60·7)	44·7 (40·4 to 49·1)
Jammu and Kashmir	56·6 (52·1 to 61·3)	72·3 (65·4 to 79·8)	74·4 (66·4 to 82·1)	93·4 (87·6 to 99·2)	114·5 (106·2 to 122·9)	120·4 (112·2 to 128·6)	107·5 (97·5 to 117·5)	94·5 (78·3 to 112·4)
Jharkhand	242·8 (226·1 to 259·5)	270·8 (253·9 to 287·6)	295·2 (271·4 to 318·5)	380 (352·7 to 407·6)	391·3 (363·3 to 418·8)	392·2 (364·4 to 420·9)	356·5 (330·2 to 381·9)	331 (293·4 to 371·6)
Karnataka	356·4 (319·4 to 394·7)	410·2 (364·1 to 459·4)	434·4 (390·6 to 478·6)	600·3 (558·3 to 645·9)	553·7 (517·5 to 590·6)	494·5 (458·9 to 534)	519·6 (482·1 to 560·7)	450·4 (395 to 509·3)
Kerala	269·7 (245·7 to 294·3)	286·3 (253·7 to 323·6)	291·4 (266·5 to 315·5)	324·6 (303·5 to 346·2)	310·8 (291·6 to 330·6)	299·7 (281·4 to 321·4)	258·9 (245·2 to 273·2)	202·8 (183·6 to 223·5)
Madhya Pradesh	507·9 (471·8 to 543·4)	582·5 (530 to 634·6)	610 (561·1 to 658·2)	739·3 (690·1 to 791·5)	765·4 (699·4 to 835·4)	815·5 (763·2 to 869·8)	910·2 (851·8 to 971·1)	775·4 (682·4 to 874·1)
Maharashtra	636·2 (576·6 to 697·3)	733·6 (656·3 to 819·4)	740 (673·6 to 809·6)	977 (912·8 to 1042·1)	964·3 (911·1 to 1021·2)	918·6 (858·4 to 983·4)	904·7 (850·4 to 963·6)	789·8 (708 to 876·8)
Manipur	16·1 (15·2 to 17)	19·1 (17·8 to 20·4)	22·3 (20·6 to 24·2)	26·7 (24·4 to 29·2)	26·6 (23·6 to 29·6)	26·1 (23·8 to 28·2)	27·8 (24·7 to 31·2)	28·6 (24 to 33·8)
Meghalaya	13·3 (12·5 to 14·1)	17·6 (16·3 to 18·8)	23·1 (21·5 to 24·8)	30·4 (28·2 to 32·7)	31·9 (28·8 to 35)	31·2 (28·3 to 33·9)	31·4 (27·6 to 35·3)	31·2 (25·7 to 37·3)
Mizoram	3·5 (3·3 to 3·7)	4·9 (4·6 to 5·3)	5·7 (5·2 to 6·2)	7·9 (7·2 to 8·7)	8·9 (7·9 to 9·9)	10·1 (9·2 to 11·1)	11·7 (10·5 to 12·9)	11·4 (9·7 to 13·2)
Nagaland	8·1 (7·7 to 8·4)	9·3 (8·7 to 9·9)	11·2 (10·4 to 12·1)	14·9 (13·8 to 16)	17 (15·5 to 18·6)	22·5 (20·5 to 24·7)	21·1 (19·2 to 23·1)	17·1 (14·2 to 20·4)
Odisha	350·8 (322 to 380·1)	373·8 (344·5 to 402)	385·8 (352·6 to 420·1)	461 (427·4 to 495·7)	423 (399·6 to 448·2)	420·3 (396·5 to 446·7)	400 (379·4 to 422·7)	351·7 (307·6 to 398·1)
Punjab	164·8 (152·1 to 177·5)	183·3 (165·1 to 203·2)	205 (190·2 to 219·8)	244·6 (231·3 to 258·3)	270·9 (256·4 to 284·8)	243·7 (229·5 to 258·2)	228·7 (214·4 to 245·1)	191·3 (159·4 to 226·9)
Rajasthan	293·9 (265·3 to 323·4)	378·2 (343·8 to 414·3)	440·5 (401·1 to 480·6)	647 (608·5 to 685·3)	763·5 (719·6 to 806·8)	837·5 (794·2 to 883·7)	831·6 (780·2 to 884·3)	732·7 (643·6 to 825·9)
Sikkim	3 (2·8 to 3·2)	3·8 (3·6 to 4·1)	4·4 (4 to 4·7)	6 (5·6 to 6·5)	5·6 (5·1 to 6·1)	4·7 (4·3 to 5·1)	4·2 (3·7 to 4·6)	3·3 (2·7 to 4·1)
Tamil Nadu	569·7 (514 to 628·1)	607·9 (539·9 to 683·2)	596·3 (556·5 to 640·6)	633·3 (596·6 to 669·8)	606·8 (576·2 to 637)	601·5 (569·1 to 636·6)	576·4 (540·4 to 615·3)	463·2 (384·7 to 551)
Telangana	242·6 (220·1 to 266·1)	281·8 (258·7 to 304·4)	300·6 (270·9 to 330·5)	381·5 (345·6 to 418·2)	381·8 (343·6 to 421·2)	342·3 (309·6 to 376·9)	316·1 (284·9 to 349·2)	258·7 (236·3 to 282·8)
Tripura	21·4 (20 to 22·9)	26·6 (24·8 to 28·5)	29·6 (26·7 to 32·4)	37·8 (34·1 to 41·7)	33·4 (30·9 to 36)	28·8 (26·4 to 31·1)	28·1 (25·5 to 30·5)	23·5 (19 to 28·7)
Union Territories other than Delhi	16·7 (15·9 to 17·4)	18 (17·1 to 18·9)	21·8 (20·4 to 23·1)	30·5 (28·1 to 32·9)	30 (26·4 to 33·8)	26·9 (23·7 to 30·2)	24·3 (20·5 to 28·2)	19·1 (15·4 to 23·3)
Uttar Pradesh	1484·2 (1382·8 to 1580·7)	1647·9 (1509·3 to 1782·4)	1765·4 (1644·2 to 1885·5)	2281·1 (2148·6 to 2422·5)	2600·7 (2416·1 to 2787·1)	2750·9 (2621·2 to 2878·3)	2509·9 (2358 to 2664·1)	2585·2 (2295 to 2887·6)

Uttarakhand	64·8 (60·8 to 68·7)	85 (80·1 to 89·7)	93·1 (86·7 to 99·4)	115·3 (108·1 to 122·8)	126 (117·7 to 134·5)	109·9 (102 to 117·7)	94·9 (87·4 to 102·1)	85·9 (77·7 to 94·5)
West Bengal	593 (543·9 to 642·3)	686·8 (630·7 to 741·5)	744·1 (676·8 to 822·3)	950·3 (890·7 to 1010·5)	870·6 (822 to 922·4)	776·2 (734·6 to 820·9)	700·4 (657·1 to 743·6)	605·2 (557·2 to 656·4)
Nepal	209·6 (195·1 to 223·9)	230·1 (217·2 to 242·3)	269·2 (256·5 to 280·9)	352·7 (336·2 to 368·8)	404·6 (392·2 to 416·3)	390·9 (376·2 to 405·9)	325·2 (303·6 to 345·9)	299·9 (265·2 to 341·7)
Pakistan	816·9 (777·4 to 855·3)	888·6 (850·6 to 924)	1376·9 (1343·1 to 1410)	1894·8 (1831·9 to 1953·4)	2362·4 (2283·2 to 2432·2)	2855·1 (2731·4 to 2975)	3147·9 (2971·2 to 3334·1)	3205 (2848·4 to 3618·3)
Southeast Asia, east Asia, and Oceania	16348·9 (15927·8 to 16771·8)	18321·4 (17993·2 to 18664·1)	18200·9 (17937·1 to 18447·4)	18267·5 (17999·4 to 18525·9)	18211·8 (17924·7 to 18520·5)	14204·8 (13947·4 to 14471·8)	12684·5 (12249·1 to 13099·8)	12632·9 (12082·9 to 13253·4)
East Asia	12029·9 (11848·2 to 12218·9)	12872·5 (12726·7 to 13033·1)	12305·7 (12158·9 to 12456·5)	11947·3 (11732·8 to 12153·2)	11960·3 (11736·9 to 12187·3)	8041·2 (7855·1 to 8228·8)	6600·3 (6382·1 to 6831·9)	7133·5 (6926·9 to 7343·8)
China	11622·5 (11452·3 to 11799·4)	12361·6 (12220 to 12517·7)	11790·1 (11642·2 to 11939·6)	11436·9 (11222·7 to 11638·6)	11426·7 (11197·8 to 11649·4)	7611·8 (7424·1 to 7793·8)	6311 (6105·9 to 6538)	6879 (6669·5 to 7078·8)
North Korea	236·8 (225·1 to 248·8)	308 (292·9 to 322·5)	331·7 (314·2 to 349)	312·7 (290·4 to 336·5)	374·7 (347·7 to 400)	293·8 (264·8 to 323·6)	203·3 (178·5 to 229·2)	170 (147·5 to 193·9)
Taiwan (province of China)	170·5 (170 to 171·1)	202·8 (202·3 to 203·4)	183·8 (183·3 to 184·3)	197·8 (197 to 198·5)	158·9 (158·2 to 159·5)	135·7 (135·1 to 136·2)	86 (85·6 to 86·5)	84·5 (74·5 to 96)
Oceania	57·3 (55·1 to 59·4)	70·2 (67·7 to 72·6)	79·4 (76·9 to 81·8)	88·1 (85·6 to 90·5)	108·2 (104·9 to 111·4)	138·5 (132·9 to 144)	171·7 (159·6 to 183·7)	200·7 (183·8 to 218·4)
American Samoa	0·4 (0·4 to 0·4)	0·4 (0·4 to 0·4)	0·5 (0·5 to 0·5)	0·5 (0·5 to 0·6)	0·8 (0·8 to 0·9)	0·8 (0·7 to 0·9)	0·6 (0·5 to 0·6)	0·5 (0·4 to 0·6)
Cook Islands	0·3 (0·3 to 0·3)	0·3 (0·3 to 0·4)	0·3 (0·3 to 0·3)	0·2 (0·2 to 0·2)	0·2 (0·2 to 0·3)	0·2 (0·2 to 0·2)	0·2 (0·1 to 0·2)	0·1 (0·1 to 0·2)
Fiji	5·6 (5·3 to 6)	7·6 (7·3 to 7·8)	8 (7·6 to 8·3)	9 (8·5 to 9·6)	9·2 (8·7 to 9·7)	8·8 (8·1 to 9·6)	9·4 (8·6 to 10·3)	8·5 (7·4 to 9·8)
Guam	0·9 (0·8 to 0·9)	1·2 (1·2 to 1·2)	1·4 (1·4 to 1·4)	1·4 (1·4 to 1·5)	1·9 (1·9 to 1·9)	1·7 (1·7 to 1·8)	1·6 (1·6 to 1·7)	1·6 (1·4 to 1·7)
Kiribati	0·7 (0·7 to 0·7)	0·8 (0·8 to 0·8)	0·9 (0·8 to 0·9)	1·1 (1·1 to 1·2)	1·3 (1·2 to 1·3)	1·2 (1·2 to 1·3)	1·4 (1·3 to 1·5)	1·4 (1·3 to 1·7)
Marshall Islands	0·3 (0·3 to 0·3)	0·3 (0·3 to 0·3)	0·5 (0·4 to 0·5)	0·7 (0·6 to 0·7)	0·7 (0·7 to 0·8)	0·7 (0·7 to 0·8)	0·7 (0·6 to 0·7)	0·6 (0·5 to 0·7)
Federated States of Micronesia	0·9 (0·9 to 0·9)	1 (1 to 1)	1·3 (1·2 to 1·3)	1·7 (1·7 to 1·8)	1·7 (1·6 to 1·8)	1·4 (1·3 to 1·6)	1·1 (1 to 1·2)	0·9 (0·8 to 1·1)
Nauru	0·1 (0·1 to 0·1)	0·1 (0·1 to 0·1)	0·1 (0·1 to 0·2)	0·2 (0·2 to 0·2)	0·2 (0·2 to 0·2)	0·2 (0·2 to 0·2)	0·2 (0·1 to 0·2)	0·1 (0·1 to 0·2)
Niue	0·1 (0·1 to 0·1)	0·1 (0·1 to 0·1)	0·1 (0·1 to 0·1)	0 (0 to 0·1)	0 (0 to 0)	0 (0 to 0)	0 (0 to 0)	0 (0 to 0)
Northern Mariana Islands	0·1 (0·1 to 0·1)	0·1 (0·1 to 0·1)	0·1 (0·1 to 0·1)	0·2 (0·2 to 0·2)	0·6 (0·5 to 0·7)	1·2 (1·1 to 1·4)	0·4 (0·4 to 0·5)	0·2 (0·2 to 0·3)
Palau	0·1 (0·1 to 0·2)	0·2 (0·1 to 0·2)	0·2 (0·1 to 0·2)	0·2 (0·1 to 0·2)	0·2 (0·2 to 0·2)	0·2 (0·2 to 0·2)	0·1 (0·1 to 0·2)	0·1 (0·1 to 0·1)
Papua New Guinea	38·9 (37·3 to 40·4)	46·9 (44·6 to 48·7)	52·7 (50·7 to 54·6)	57·2 (55·4 to 58·9)	72·6 (70·3 to 74·9)	99·1 (95·2 to 102·8)	130·7 (121·4 to 140·1)	160·2 (146·9 to 174·5)
Samoa	1·7 (1·6 to 1·8)	2·1 (2 to 2·2)	2·2 (2·1 to 2·3)	1·8 (1·7 to 1·9)	1·5 (1·3 to 1·7)	1·3 (1·2 to 1·5)	1·4 (1·2 to 1·6)	1·7 (1·4 to 2)
Solomon Islands	2·6 (2·5 to 2·7)	3·2 (3·1 to 3·3)	3·9 (3·7 to 4)	4·9 (4·8 to 5·1)	7·1 (6·7 to 7·4)	9·3 (8·7 to 9·8)	10·2 (9·4 to 10·9)	10·2 (9·3 to 11·2)
Tokelau	0 (0 to 0)	0 (0 to 0·1)	0 (0 to 0)	0 (0 to 0)	0 (0 to 0)	0 (0 to 0)	0 (0 to 0)	0 (0 to 0)
Tonga	0·9 (0·8 to 0·9)	1·1 (1·1 to 1·2)	1·3 (1·3 to 1·4)	1·3 (1·3 to 1·4)	1·2 (1·2 to 1·3)	1·2 (1·2 to 1·3)	1·3 (1·2 to 1·3)	1·1 (1 to 1·3)
Tuvalu	0·1 (0·1 to 0·1)	0·1 (0·1 to 0·1)	0·1 (0·1 to 0·1)	0·2 (0·2 to 0·2)	0·1 (0·1 to 0·2)	0·1 (0·1 to 0·1)	0·1 (0·1 to 0·1)	0·1 (0·1 to 0·1)
Vanuatu	1 (0·9 to 1)	1·3 (1·3 to 1·4)	1·7 (1·6 to 1·8)	2·3 (2·1 to 2·4)	2·8 (2·6 to 3)	3·2 (2·9 to 3·5)	3·5 (3·1 to 3·9)	3·7 (3·2 to 4·3)
Southeast Asia	4261·7 (4022·7 to 4494·2)	5378·7 (5159·4 to 5587·9)	5815·8 (5625·7 to 5992·1)	6232·1 (6085·6 to 6375·2)	6143·4 (5983·5 to 6305·1)	6025·1 (5853·4 to 6198·3)	5912·5 (5679·8 to 6134·7)	5298·7 (4841·1 to 5845·3)
Cambodia	106·9 (100 to 113·7)	133·5 (126·8 to 139·7)	160·8 (154·4 to 167·2)	183·6 (178·4 to 188·7)	216·8 (210·8 to 222·5)	185·3 (178·3 to 192·5)	182·1 (170·7 to 194·5)	180·4 (163 to 201·7)
Indonesia	1812·9 (1717·8 to 1904·2)	2380·1 (2281·1 to 2476·5)	2544 (2473·2 to 2613·3)	2548·6 (2488·2 to 2606·8)	2326·3 (2255·5 to 2394·8)	2332·7 (2265·2 to 2397·1)	2239·9 (2116·4 to 2361·2)	1865·6 (1680·9 to 2084·1)
Aceh	29·3 (28 to 30·6)	39·7 (38·3 to 41·1)	46·2 (44·8 to 47·6)	52·9 (51·5 to 54·3)	51·5 (49·4 to 53·5)	53·2 (51·8 to 54·6)	52·2 (50·1 to 54·4)	43·9 (40·4 to 48·1)
Bali	34·7 (32·8 to 36·6)	42 (40·2 to 43·7)	46·3 (45·1 to 47·5)	42·1 (40·8 to 43·2)	33·5 (32·4 to 34·7)	33·9 (32·4 to 35·3)	31·1 (28·8 to 33·3)	25 (21·8 to 29)
Bangka-Belitung Islands	7·6 (7·2 to 8·1)	9·5 (9 to 10)	10·3 (9·7 to 10·9)	11·7 (11·2 to 12·2)	11·1 (10·8 to 11·4)	11·4 (11 to 11·8)	11·7 (11·2 to 12·2)	9·7 (8·8 to 10·7)
Banten	39·5 (37·4 to 41·4)	49·9 (47·8 to 52)	54·3 (52 to 56·7)	69·1 (66·4 to 71·8)	112·2 (107·9 to 116·2)	120·7 (116·7 to 124·6)	112 (108·4 to 115·4)	88·1 (81·5 to 95·3)
Bengkulu	8·9 (8·5 to 9·3)	11·4 (11 to 11·8)	12·8 (12·5 to 13·1)	16·4 (16 to 16·8)	19·6 (18·7 to 20·4)	19·7 (19 to 20·4)	19 (17·9 to 20·2)	15·8 (14·2 to 17·6)
Gorontalo	6 (5·7 to 6·4)	9·6 (9·2 to 10·1)	12·3 (11·6 to 13)	11·6 (11·1 to 12·1)	9·4 (9·1 to 9·6)	9·4 (9·1 to 9·7)	9·6 (9·2 to 10)	10 (9·1 to 11)
Jakarta	53·1 (50·8 to 55·3)	89 (85·7 to 92·1)	112·5 (109·8 to 115·2)	124 (120·4 to 127·3)	116·9 (113·3 to 120·8)	110·1 (105·7 to 114·1)	97·9 (90·9 to 104·8)	71·5 (62·4 to 82·4)
Jambi	14·5 (13·8 to 15·2)	20·3 (19·6 to 21·1)	22·7 (21·8 to 23·6)	26·2 (25·4 to 27)	26·9 (25·7 to 27·9)	28·7 (28 to 29·4)	29·8 (28·7 to 30·8)	25·1 (23·2 to 27·5)
West Java	289·4 (275·1 to 303·6)	391·1 (374·8 to 406·3)	429·6 (418·4 to 441·6)	425·5 (415·4 to 435·6)	365·1 (352·2 to 377·9)	389·7 (376·8 to 401·7)	400·2 (373·2 to 426·6)	352·5 (310·1 to 403·2)
Central Java	342·6 (324·8 to 359·8)	444·9 (424·9 to 463·9)	450·9 (438 to 464·2)	411·9 (401·4 to 422·1)	332·9 (321·4 to 344·3)	295·8 (283·8 to 307·6)	253·8 (235·4 to 271·5)	198·9 (173·8 to 229·4)
East Java	394·1 (370·2 to 417·8)	495·9 (468·9 to 521·4)	484·4 (467·4 to 501·4)	415·1 (402·1 to 427·8)	321·8 (310·3 to 333·3)	292 (280·9 to 302·7)	255·8 (237·2 to 274)	197·2 (171·7 to 228·7)
West Kalimantan	28·4 (27 to 29·7)	38·8 (37·4 to 40·2)	45·4 (44 to 46·7)	52·8 (51·3 to 54·2)	48·4 (46·6 to 50)	48·7 (47·1 to 50·1)	47·4 (45·5 to 49·2)	39·8 (36·8 to 43·5)
South Kalimantan	26 (24·7 to 27·3)	35·5 (34 to 36·9)	37·3 (36·1 to 38·5)	37 (35·9 to 38·1)	35·1 (33·6 to 36·7)	36·7 (35·2 to 38·1)	35·1 (32·8 to 37·5)	29 (26 to 32·6)
Central Kalimantan	8·9 (8·5 to 9·3)	12·5 (12 to 12·9)	14·5 (13·9 to 15)	17·2 (16·7 to 17·8)	19·5 (18·7 to 20·2)	21·4 (20·7 to 22·2)	22·2 (21·3 to 23)	18·6 (17·1 to 20·3)
East Kalimantan	8·8 (8·4 to 9·1)	13 (12·5 to 13·4)	15·9 (15·4 to 16·5)	20·3 (19·5 to 21)	23·4 (22·4 to 24·2)	29·8 (29 to 30·7)	35·3 (34 to 36·5)	30·3 (28·1 to 33)
North Kalimantan	1·2 (1·2 to 1·3)	2·2 (2·2 to 2·3)	3·1 (3 to 3·2)	3·6 (3·4 to 3·8)	3·9 (3·5 to 4·3)	5 (4·5 to 5·5)	5·8 (5·2 to 6·4)	5·5 (5 to 6·2)
Riau Islands	3·7 (3·5 to 3·9)	4·4 (4·2 to 4·6)	4·9 (4·7 to 5·1)	7·5 (7·2 to 7·7)	13·5 (12·9 to 14)	17·5 (16·9 to 18·1)	22·3 (21·7 to 22·9)	16 (14·7 to 17·4)
Lampung	36·4 (34·6 to 38·2)	59·4 (57 to 61·8)	72·9 (70·9 to 74·9)	82·8 (80·6 to 84·8)	81·3 (78·2 to 84·6)	78·7 (75·7 to 81·6)	70·3 (65·7 to 75)	55·6 (49·8 to 62·3)
Maluku	13·1 (12·5 to 13·6)	15·5 (15 to 16)	16·3 (15·8 to 16·8)	18·3 (17·8 to 18·9)	19·5 (19 to 20)	20·8 (20·2 to 21·4)	20·3 (19·6 to 21·1)	16·8 (15·5 to 18·3)
North Maluku	6·7 (6·3 to 7)	7·9 (7·5 to 8·3)	8·2 (7·6 to 8·7)	8·9 (8·4 to 9·4)	10·3 (9·9 to 10·7)	12·2 (11·7 to 12·7)	12 (11·2 to 12·7)	9·9 (8·7 to 11·3)
West Nusa Tenggara	38·1 (36 to 40·1)	46 (44·1 to 47·9)	50·5 (49·1 to 52)	53·9 (52·4 to 55·4)	49·2 (47·4 to 51)	48 (46·1 to 50)	47·9 (44·7 to 51·1)	40·9 (36·7 to 45·8)
East Nusa Tenggara	40·8 (38·8 to 42·6)	49·3 (47·5 to 51)	51·9 (50·4 to 53·6)	50·9 (49·8 to 52)	51·8 (49·9 to 53·5)	56·9 (55·6 to 58·3)	57·7 (55·3 to 59·8)	51 (47·4 to 55·2)
Papua	4·9 (4·7 to 5·1)	8·6 (8·3 to 8·8)	11·1 (10·7 to 11·5)	14 (13·5 to 14·5)	17·7 (17·2 to 18·1)	27·2 (26·5 to 27·9)	40·7 (39·5 to 41·9)	43·1 (40·3 to 46·5)
West Papua	4·7 (4·5 to 4·8)	7·3 (7 to 7·5)	8·9 (8·7 to 9·2)	11·2 (10·8 to 11·6)	11·6 (11·4 to 11·9)	10·3 (10·1 to 10·6)	10·3 (9·9 to 10·6)	8·7 (8·1 to 9·5)

Riau	15·1 (14·4 to 15·8)	24 (23·2 to 24·8)	30 (29·2 to 30·9)	36·4 (35·3 to 37·4)	35·1 (33·6 to 36·5)	46·8 (45·6 to 47·9)	66·2 (62·9 to 69·4)	68·9 (63·7 to 75·1)
West Sulawesi	10·2 (9·8 to 10·7)	12·2 (11·7 to 12·7)	12·6 (12·1 to 13·1)	12·5 (12·1 to 12·8)	12·1 (11·7 to 12·4)	13·6 (13·3 to 14)	14·2 (13·7 to 14·6)	12·9 (11·9 to 14)
South Sulawesi	73·5 (69·7 to 77·2)	97·1 (93·3 to 100·7)	107 (104·2 to 110·1)	103·5 (100·6 to 106·3)	93·9 (90·4 to 97·5)	91·6 (88·2 to 94·7)	80·7 (75·7 to 86)	66·8 (60 to 74·8)
Central Sulawesi	14·5 (13·9 to 15·2)	19·1 (18·4 to 19·7)	22·2 (21·5 to 22·9)	25·8 (25·1 to 26·4)	25·2 (24·2 to 26·2)	28·5 (27·7 to 29·4)	27·7 (26·7 to 28·8)	23·4 (21·6 to 25·6)
Southeast Sulawesi	11·8 (11·3 to 12·3)	15·4 (14·9 to 15·9)	18 (17·4 to 18·5)	21·3 (20·6 to 21·9)	22·2 (21·6 to 22·8)	25·2 (24·4 to 26)	27·3 (26·1 to 28·6)	24·4 (22·5 to 26·7)
North Sulawesi	20·2 (19·1 to 21·2)	22·8 (21·9 to 23·8)	24·6 (23·9 to 25·4)	25·5 (24·7 to 26·3)	23·1 (21·9 to 24·1)	22·2 (21·6 to 22·9)	17·7 (17 to 18·5)	13·4 (12·1 to 14·9)
West Sumatra	54·7 (52 to 57·2)	60·9 (58·4 to 63·2)	58·8 (57·1 to 60·7)	61·8 (60·2 to 63·4)	57·2 (55·7 to 58·8)	56·8 (54·6 to 58·9)	50·2 (47 to 53·3)	40·6 (36·6 to 45·4)
South Sumatra	39·5 (37·6 to 41·3)	54·8 (52·7 to 56·8)	62·9 (61·3 to 64·6)	77·1 (75·2 to 79)	76·8 (74·2 to 79·5)	78·5 (75·3 to 81·9)	75·9 (70·6 to 81·2)	61·9 (55·6 to 69·4)
North Sumatra	90·4 (86·4 to 94·3)	123·5 (119·1 to 127·8)	141·1 (137·4 to 145)	162·7 (158·5 to 166·7)	165·6 (161·1 to 170·5)	165·6 (159·8 to 171·3)	155·9 (147 to 164·9)	130·7 (118·6 to 144·8)
Yogyakarta	41·3 (38·5 to 44·1)	46·5 (43·6 to 49·2)	43·8 (42 to 45·7)	37·3 (36·1 to 38·6)	29 (28 to 30·1)	26 (25·1 to 26·9)	23·7 (21·9 to 25·5)	19·5 (17 to 22·7)
Laos	41 (38·3 to 43·6)	50 (47·4 to 52·5)	59·5 (56·5 to 62·5)	70·3 (68 to 72·7)	80·9 (78·7 to 82·9)	90 (87·2 to 92·9)	84·8 (81·6 to 88·1)	84·9 (78·4 to 91·7)
Malaysia	129·9 (119·8 to 140·1)	151·1 (150·5 to 151·6)	172·2 (171·7 to 172·7)	184·5 (183·8 to 185·3)	239·7 (238·8 to 240·6)	256·2 (255·3 to 257·2)	232·6 (231·8 to 233·4)	260·5 (234·9 to 288)
Maldives	1·3 (1·3 to 1·4)	2 (1·9 to 2·1)	2·4 (2·3 to 2·5)	3·3 (3·2 to 3·4)	4·3 (4·1 to 4·5)	3·6 (3·3 to 3·8)	4·3 (3·9 to 4·7)	4·1 (3·6 to 4·8)
Mauritius	11·6 (11·2 to 11·9)	12·6 (12·4 to 12·8)	10·4 (10·1 to 10·7)	11·7 (11·6 to 11·8)	10·9 (10·8 to 11)	9·8 (9·7 to 9·9)	7·3 (7·2 to 7·3)	6·3 (5·5 to 7·2)
Myanmar	441·9 (412·8 to 470·9)	515·5 (485·8 to 543·9)	551·5 (517·7 to 586·6)	637·8 (595·4 to 678·8)	657·4 (617·5 to 698·8)	606·2 (571·3 to 639·2)	560·7 (528·7 to 594)	513·5 (452·9 to 581·1)
Philippines	475·5 (449·8 to 500·9)	644·1 (612·7 to 674·8)	783·4 (740·1 to 823)	943·8 (911·4 to 976·9)	1006·9 (967·4 to 1046·6)	1134·2 (1087·5 to 1181·1)	1243·7 (1184·9 to 1300·6)	1284·1 (1151·2 to 1427·1)
Seychelles	0·5 (0·5 to 0·6)	0·8 (0·8 to 0·9)	0·9 (0·8 to 0·9)	0·8 (0·8 to 0·9)	0·8 (0·8 to 0·9)	0·8 (0·7 to 0·8)	0·8 (0·7 to 0·8)	0·7 (0·6 to 0·8)
Sri Lanka	150·6 (144 to 158·2)	172·2 (166·5 to 178·2)	180·9 (172·1 to 189·1)	201·1 (190·1 to 211·7)	175·5 (164·6 to 186·4)	168·4 (158·1 to 178·5)	174·8 (164·5 to 185·6)	143·9 (121·6 to 169·8)
Thailand	481 (452·3 to 509·2)	604·4 (571·9 to 633·2)	606·6 (586·1 to 627·4)	600·9 (569·2 to 633·1)	517·9 (488·3 to 550·4)	437·5 (411·1 to 466·3)	369·9 (345·1 to 396·9)	283·5 (239·3 to 335·6)
Timor-Leste	11·3 (10·8 to 11·8)	13·1 (12·7 to 13·5)	11·9 (11·6 to 12·2)	13·7 (13·3 to 14·1)	18·6 (18 to 19·3)	18·8 (18·1 to 19·4)	17·5 (16·3 to 18·7)	19 (16·8 to 21·3)
Vietnam	591·3 (551·8 to 631·5)	691·1 (652·9 to 729·2)	722·3 (681·8 to 760·7)	823·4 (790·7 to 856·1)	879 (852·8 to 906)	773·4 (741·5 to 803·9)	786·1 (744·5 to 829·4)	645·4 (584·3 to 721·6)
Sub-Saharan Africa	4374·3 (4149 to 4587·1)	5477·7 (5259·3 to 5674·3)	6786·4 (6572·6 to 7003·6)	8686·8 (8430·8 to 8929·3)	10955 (10579·5 to 11313·9)	13752 (13333·8 to 14196·1)	16421·1 (15684·4 to 17120·8)	18007·5 (16794·8 to 19396·1)
Central sub-Saharan Africa	497·6 (479·5 to 514·4)	670·5 (648·9 to 691·3)	822·8 (793·5 to 850·8)	1037 (1001·8 to 1069·1)	1321 (1279·7 to 1360·3)	1678·4 (1624·3 to 1733·4)	2098·3 (2021·7 to 2175)	2196·3 (2040·8 to 2353)
Angola	121 (116·7 to 125·5)	135·4 (130 to 140·3)	148 (142·7 to 152·6)	185·8 (179·3 to 192)	250·5 (237·8 to 262·3)	351·5 (330·5 to 370·3)	476·3 (439·1 to 511·5)	540·9 (489·2 to 594·6)
Central African Republic	31 (29·2 to 32·6)	40·9 (38·3 to 43·4)	51·2 (48·4 to 53·6)	54·4 (51·4 to 57·4)	62·8 (59 to 66·6)	78·4 (73·1 to 83·5)	95·6 (87·5 to 103·3)	97·3 (89·6 to 104·9)
Congo (Brazzaville)	21·6 (20·4 to 22·7)	26 (25 to 26·9)	31·9 (30·8 to 33)	41·4 (39·9 to 42·8)	50·5 (48·7 to 52·1)	61·1 (59·5 to 62·7)	77·3 (74·9 to 79·7)	72 (66·1 to 78·2)
DR Congo	309·8 (296 to 322·5)	451·8 (434·9 to 467)	572·9 (549·4 to 596·4)	731·4 (703·6 to 757)	926 (896·9 to 952·6)	1150·4 (1113·3 to 1189)	1409·4 (1352·8 to 1464·7)	1446·1 (1313·4 to 1579·2)
Equatorial Guinea	5·4 (5·2 to 5·5)	5·6 (5·4 to 5·8)	6·2 (6 to 6·4)	8 (7·7 to 8·3)	11·4 (10·9 to 11·8)	15·6 (14·5 to 16·6)	18·3 (16·7 to 20·2)	18·9 (16·5 to 21·8)
Gabon	8·9 (8·3 to 9·6)	10·8 (10·2 to 11·4)	12·6 (11·9 to 13·2)	16 (15·2 to 16·8)	19·8 (18·5 to 21·1)	21·3 (19 to 23·7)	21·4 (18·8 to 24·3)	21·1 (18·1 to 24·6)
Eastern sub-Saharan Africa	1676·8 (1595 to 1754·5)	2123 (2048·4 to 2191·3)	2650·3 (2572·4 to 2727·5)	3476·4 (3385·9 to 3561·2)	4429·7 (4296·9 to 4557·5)	5479·4 (5317·6 to 5638·3)	6351·7 (6058·6 to 6636·6)	6954·9 (6438·4 to 7524·9)
Burundi	64·4 (61·7 to 66·9)	75·9 (73·2 to 78·3)	86·2 (83·7 to 88·5)	109·9 (107·1 to 112·4)	130·6 (127·5 to 133·6)	130·7 (125·8 to 135·2)	187 (179·3 to 195·2)	230 (215·3 to 246·2)
Comoros	2·8 (2·5 to 3·1)	4·2 (4 to 4·5)	6·3 (6·1 to 6·6)	8·3 (8 to 8·6)	9·9 (9·5 to 10·4)	10 (9·1 to 10·9)	9·2 (8·2 to 10·2)	8·2 (7 to 9·6)
Djibouti	1·6 (1·5 to 1·7)	2·5 (2·4 to 2·6)	4 (3·9 to 4·1)	7·2 (6·9 to 7·5)	10·8 (10·2 to 11·4)	13·8 (12·9 to 14·6)	17·9 (16·4 to 19·2)	17·3 (15 to 19·8)
Eritrea	28·2 (26·6 to 29·6)	37·3 (35·6 to 38·9)	46·5 (44·3 to 48·7)	60 (57 to 62·7)	66·7 (63·1 to 70·3)	82·3 (75·4 to 88·9)	97·2 (86·5 to 108·3)	97·9 (85·5 to 112)
Ethiopia	443 (416·6 to 467·6)	534·3 (509·6 to 556·8)	653 (628·4 to 678·2)	879·5 (843·4 to 913·1)	1199 (1156·5 to 1239·6)	1452·5 (1401·5 to 1501·3)	1612·1 (1509·4 to 1711)	1788·3 (1652·1 to 1947·9)
Kenya	157·3 (151·8 to 162·3)	230·6 (222·6 to 237·3)	300·3 (291·3 to 308·6)	400·9 (389·4 to 411·6)	501·3 (476·7 to 525)	588·6 (556·5 to 620·1)	652·6 (597·4 to 707·2)	657·5 (571·3 to 761·3)
Baringo	2·5 (2·4 to 2·6)	3·7 (3·6 to 3·8)	4·7 (4·5 to 4·8)	5·7 (5·5 to 5·8)	7·8 (7·6 to 8)	9·5 (9·1 to 10)	10·2 (9·5 to 11)	9·6 (8·4 to 11·1)
Bomet	2·4 (2·3 to 2·4)	3·2 (3·1 to 3·3)	4·3 (4·2 to 4·4)	5·9 (5·8 to 6)	8 (7·7 to 8·2)	10·6 (10·2 to 11)	17·6 (16·3 to 18·9)	20·3 (17·8 to 23·2)
Bungoma	4·9 (4·8 to 5)	7·2 (7·1 to 7·4)	10 (9·7 to 10·2)	14 (13·6 to 14·3)	20·3 (19·6 to 21)	35·1 (33·3 to 36·8)	25·4 (23·5 to 27·5)	17·2 (15·1 to 19·9)
Busia	4·1 (4 to 4·2)	5·3 (5·2 to 5·5)	6·1 (5·8 to 6·3)	8·5 (8·2 to 8·8)	11·2 (10·7 to 11·7)	12·3 (11·5 to 13·1)	14·3 (12·9 to 15·6)	14 (12·1 to 16·3)
Elgeyo Marakwet	2·8 (2·7 to 2·8)	4·4 (4·3 to 4·5)	4·8 (4·6 to 4·9)	3·9 (3·8 to 4)	5·7 (5·5 to 5·9)	9·8 (9·3 to 10·3)	5·7 (5·2 to 6·1)	3·1 (2·6 to 3·6)
Embu	2·4 (2·3 to 2·5)	3·2 (3·1 to 3·4)	4·3 (4·1 to 4·4)	6·1 (5·8 to 6·2)	7·5 (7 to 7·9)	7·8 (7·2 to 8·3)	6·7 (6 to 7·5)	5 (4·2 to 6)
Garissa	0·7 (0·7 to 0·8)	1·2 (1·1 to 1·2)	1·9 (1·8 to 2)	2·9 (2·8 to 3)	2·9 (2·7 to 3)	6·4 (6 to 6·7)	13·3 (12·5 to 14·1)	20·9 (19·3 to 22·6)
Homa Bay	9·5 (9·2 to 9·8)	11·7 (11·3 to 12)	12·4 (12 to 12·8)	13·9 (13·5 to 14·2)	17·3 (16·6 to 18)	19·9 (18·8 to 20·9)	21 (19·5 to 22·5)	20·6 (18·2 to 23·3)
Isiolo	0·5 (0·5 to 0·5)	0·7 (0·7 to 0·8)	0·9 (0·8 to 0·9)	1·2 (1·2 to 1·2)	1·7 (1·6 to 1·8)	2·2 (2·1 to 2·4)	2·7 (2·4 to 2·9)	2·7 (2·3 to 3·2)
Kajiado	1·1 (1 to 1·1)	1·5 (1·4 to 1·6)	2·2 (2·1 to 2·3)	3·7 (3·6 to 3·9)	5·7 (5·3 to 6)	7·9 (7·4 to 8·5)	11·4 (10·1 to 12·7)	11 (9·2 to 13·2)
Kakamega	7·3 (7·1 to 7·4)	11·6 (11·2 to 11·8)	15·4 (14·9 to 15·8)	21·1 (20·6 to 21·6)	28·9 (27·8 to 29·9)	22 (21 to 23·1)	32·4 (29·7 to 35·1)	37·6 (32·3 to 43·9)
Kericho	2·8 (2·7 to 2·8)	5·6 (5·4 to 5·7)	8·5 (8·4 to 8·7)	11·5 (11·3 to 11·6)	15·4 (14·9 to 15·9)	17 (16·2 to 17·8)	10·6 (9·7 to 11·4)	5·9 (5·1 to 6·8)
Kiambu	5·7 (5·5 to 5·9)	7·7 (7·5 to 8)	11·5 (11·2 to 11·8)	16·7 (16·2 to 17·1)	19·5 (18·2 to 20·7)	25·4 (24·1 to 26·8)	24 (21·9 to 26·2)	18·4 (15·9 to 21·4)
Kilifi	5·1 (4·8 to 5·3)	7·4 (7 to 7·7)	9·1 (8·8 to 9·4)	12·4 (12 to 12·8)	14·1 (13·2 to 14·9)	16·3 (15·3 to 17·3)	18·8 (17·4 to 20·2)	18·7 (16 to 22·1)
Kirinyaga	3 (2·9 to 3·1)	4·6 (4·4 to 4·7)	5·7 (5·5 to 5·9)	6·8 (6·5 to 7)	7·7 (7 to 8·3)	7·9 (7·3 to 8·6)	6·9 (6·2 to 7·8)	5·2 (4·3 to 6·3)
Kisii	5·1 (5 to 5·2)	9·7 (9·4 to 9·9)	13·3 (12·9 to 13·6)	17·9 (17·3 to 18·5)	20·8 (19·7 to 21·8)	22·2 (20·6 to 23·6)	18 (16·1 to 19·9)	12·6 (10·5 to 15·4)
Kisumu	6·8 (6·6 to 7)	10 (9·6 to 10·4)	11·7 (11·3 to 12·1)	12·6 (12·2 to 13·1)	15·9 (15 to 16·9)	16·1 (15 to 17·2)	15·7 (13·9 to 17·5)	13 (10·9 to 15·7)
Kitui	6·1 (5·9 to 6·3)	8·5 (8·2 to 8·7)	10·3 (10 to 10·5)	12·6 (12·3 to 12·9)	16·4 (15·6 to 17·2)	18·2 (17·3 to 19·1)	17·2 (15·7 to 18·6)	15·5 (13·6 to 17·6)
Kwale	3·3 (3·1 to 3·4)	5·2 (4·9 to 5·4)	6·3 (6 to 6·6)	7·5 (7·2 to 7·8)	8·2 (7·7 to 8·8)	9·4 (8·7 to 10·1)	10·5 (9·4 to 11·6)	10·4 (8·7 to 12·6)

Laikipia	0·7 (0·7 to 0·7)	1 (1 to 1)	1·7 (1·7 to 1·8)	3·4 (3·3 to 3·4)	4·9 (4·7 to 5·2)	7 (6·6 to 7·4)	6·4 (5·8 to 7)	4·7 (4 to 5·6)
Lamu	0·5 (0·4 to 0·5)	0·5 (0·5 to 0·5)	0·6 (0·6 to 0·7)	1 (0·9 to 1)	1·2 (1·1 to 1·3)	1·3 (1·2 to 1·4)	1·5 (1·3 to 1·6)	1·4 (1·2 to 1·7)
Machakos	4·6 (4·5 to 4·8)	7·6 (7·3 to 7·9)	11·2 (10·8 to 11·5)	16·5 (15·9 to 17·1)	20·1 (18·8 to 21·4)	19·4 (18 to 20·7)	14·6 (13 to 16·2)	9·5 (8 to 11·5)
Makueni	4·9 (4·7 to 5·1)	5·8 (5·6 to 5·9)	6·9 (6·7 to 7·1)	8·6 (8·4 to 8·9)	10·8 (10·3 to 11·3)	12·3 (11·6 to 13)	15·3 (13·7 to 16·9)	16·2 (14·2 to 18·5)
Mandera	1·2 (1·1 to 1·3)	1·9 (1·8 to 2)	2·3 (2·2 to 2·4)	2·4 (2·2 to 2·5)	2·7 (2·6 to 2·9)	5·3 (4·9 to 5·6)	18·1 (16·9 to 19·4)	47·8 (43·3 to 52·8)
Marsabit	0·7 (0·6 to 0·7)	0·9 (0·9 to 1)	1·5 (1·5 to 1·6)	2·6 (2·5 to 2·7)	3·1 (2·9 to 3·3)	4 (3·7 to 4·2)	5·5 (5·1 to 5·9)	7·3 (6·5 to 8·2)
Meru	7·2 (6·9 to 7·4)	11·2 (10·7 to 11·5)	14·6 (14·1 to 15)	18·6 (18·1 to 19·1)	22·2 (21 to 23·3)	23·1 (21·7 to 24·5)	20·9 (18·6 to 23·3)	16·4 (13·7 to 19·8)
Migori	5·9 (5·6 to 6·1)	7·7 (7·5 to 7·9)	8·8 (8·5 to 9)	10·7 (10·4 to 10·9)	14·1 (13·4 to 14·7)	18·2 (17·2 to 19·2)	21·4 (19·9 to 22·9)	23·4 (20·9 to 26·2)
Mombasa	2·7 (2·5 to 2·8)	4·9 (4·6 to 5·2)	6·3 (6 to 6·6)	6·9 (6·6 to 7·3)	8·3 (7·6 to 9)	10·8 (10 to 11·7)	14·6 (13 to 16·2)	13·3 (10·9 to 16·4)
Murang'a	7·5 (7·2 to 7·7)	9·9 (9·6 to 10·1)	12·1 (11·9 to 12·4)	15 (14·6 to 15·4)	17·5 (16·6 to 18·3)	12·3 (11·6 to 13·1)	10·6 (9·6 to 11·6)	8·5 (7·3 to 9·9)
Nairobi	4·7 (4·5 to 4·9)	8 (7·6 to 8·4)	13·3 (12·6 to 13·9)	26·4 (24·8 to 27·8)	23·9 (21·8 to 25·9)	38·1 (35·1 to 41·3)	48·4 (43·2 to 53·7)	43·8 (37 to 52·4)
Nakuru	2·9 (2·8 to 2·9)	4·8 (4·6 to 4·9)	7·8 (7·6 to 8)	12·9 (12·5 to 13·2)	19·9 (18·9 to 20·8)	23·8 (22·2 to 25·3)	24·7 (22·1 to 27·2)	18·9 (15·9 to 22·7)
Nandi	2·9 (2·8 to 2·9)	4·3 (4·2 to 4·4)	6 (5·9 to 6·1)	7·6 (7·5 to 7·8)	10·3 (9·8 to 10·7)	11·8 (11·2 to 12·4)	12 (11·1 to 12·9)	9·9 (8·5 to 11·4)
Narok	1·9 (1·8 to 2)	2·7 (2·6 to 2·7)	3·6 (3·5 to 3·7)	6·2 (6 to 6·3)	10·5 (10·2 to 10·8)	9·2 (8·9 to 9·6)	19·8 (18·4 to 21)	31·2 (27·6 to 35·5)
Nyamira	3·1 (3 to 3·2)	3·9 (3·8 to 4·1)	4·6 (4·4 to 4·7)	5·3 (5·1 to 5·5)	5·9 (5·5 to 6·3)	6·8 (6 to 7·5)	8·1 (7·2 to 9·1)	7·6 (6·4 to 8·9)
Nyandarua	1·7 (1·7 to 1·8)	2·9 (2·8 to 3)	4·1 (3·9 to 4·2)	5·2 (5 to 5·3)	8·1 (7·6 to 8·7)	9·9 (9·2 to 10·5)	8·7 (7·9 to 9·6)	7·5 (6·3 to 9)
Nyeri	5·2 (5 to 5·3)	6·1 (5·9 to 6·3)	7·9 (7·6 to 8·1)	10·8 (10·3 to 11·2)	12·1 (11·2 to 13·1)	10·5 (9·5 to 11·3)	8 (7 to 9·1)	5·8 (4·7 to 7)
Samburu	1·1 (1·1 to 1·1)	1·9 (1·8 to 1·9)	2·2 (2·2 to 2·3)	2·3 (2·2 to 2·4)	3·3 (3·2 to 3·4)	3·8 (3·6 to 4)	4·9 (4·5 to 5·2)	5·7 (5 to 6·4)
Siaya	5·1 (4·9 to 5·2)	8·3 (8 to 8·6)	10·1 (9·8 to 10·4)	12·3 (12 to 12·7)	15·2 (14·4 to 15·9)	15·9 (15 to 16·8)	15·7 (14·4 to 17)	14 (12·2 to 16)
Taita Taveta	1·4 (1·4 to 1·5)	2·2 (2·1 to 2·3)	2·7 (2·6 to 2·8)	3·3 (3·1 to 3·4)	4·1 (3·8 to 4·4)	3·6 (3·3 to 3·9)	3·2 (2·9 to 3·6)	2·5 (2 to 3·1)
Tana River	0·8 (0·8 to 0·8)	1·1 (1 to 1·1)	1·5 (1·4 to 1·5)	2·5 (2·4 to 2·6)	2·9 (2·8 to 3·1)	3·8 (3·5 to 4)	4·4 (4 to 4·7)	5 (4·4 to 5·8)
Tharaka Nithi	1·7 (1·6 to 1·7)	1·7 (1·6 to 1·8)	1·7 (1·6 to 1·7)	1·7 (1·6 to 1·8)	1·9 (1·7 to 2)	1·9 (1·7 to 2)	1·8 (1·6 to 2)	1·6 (1·3 to 1·8)
Trans Nzoia	1 (1 to 1·1)	1·6 (1·6 to 1·7)	3·1 (3 to 3·2)	6·7 (6·5 to 6·8)	9·6 (9·2 to 10)	12·3 (11·7 to 13)	13·6 (12·3 to 14·9)	11·9 (10·1 to 14·1)
Turkana	2·1 (2 to 2·2)	4 (3·9 to 4·1)	5·3 (5·1 to 5·4)	4·7 (4·5 to 4·8)	6·2 (5·9 to 6·4)	11·3 (10·8 to 11·8)	19·7 (18·4 to 21)	28 (25·1 to 31·4)
Uasin Gishu	1·8 (1·8 to 1·9)	3·3 (3·2 to 3·3)	5·3 (5·2 to 5·4)	7·8 (7·6 to 8)	10·2 (9·7 to 10·6)	12·9 (12·1 to 13·7)	15·2 (13·7 to 16·8)	12·3 (10·3 to 14·7)
Vihiga	5·7 (5·5 to 5·9)	6·6 (6·4 to 6·8)	6·8 (6·5 to 7)	6·5 (6·2 to 6·7)	8 (7·5 to 8·4)	6·8 (6·3 to 7·3)	8 (7·2 to 8·9)	8·1 (6·9 to 9·6)
Wajir	1·2 (1·1 to 1·2)	1·6 (1·5 to 1·6)	2·4 (2·3 to 2·5)	3·5 (3·4 to 3·7)	3·1 (3 to 3·3)	8·1 (7·6 to 8·5)	13·9 (13·2 to 14·5)	20 (18·6 to 21·6)
West Pokot	1·3 (1·2 to 1·3)	1·8 (1·7 to 1·8)	2·7 (2·6 to 2·8)	4·9 (4·7 to 5)	6·5 (6·3 to 6·7)	8·4 (8 to 8·7)	11·3 (10·5 to 12·1)	13·7 (12·1 to 15·6)
Madagascar	107·4 (103·1 to 112·1)	133·1 (129·2 to 136·9)	170·8 (165·3 to 175·9)	215·9 (209·3 to 221·9)	264·2 (253·5 to 274·1)	332·5 (317·1 to 347·1)	390·8 (367·4 to 414)	421·6 (370·3 to 476·2)
Malawi	75·4 (70·8 to 79·8)	96·9 (92·8 to 100·8)	117·5 (113·5 to 121·5)	151·7 (146·9 to 156·2)	232·3 (226·5 to 238·3)	262·1 (253·1 to 270·6)	264·3 (249·8 to 278·9)	270·4 (247·3 to 298)
Mozambique	161·3 (150·4 to 171·5)	186·4 (177·8 to 194·6)	226·9 (216·6 to 236·7)	295·8 (286·1 to 305·3)	297·9 (290·6 to 304·7)	410·4 (397·7 to 422·9)	484·5 (469·1 to 499)	555·1 (515·9 to 595·3)
Rwanda	72·8 (70·5 to 75)	84·3 (82·3 to 86)	100·5 (99 to 102)	128 (125·9 to 130)	157·3 (154 to 160·3)	160·1 (155·9 to 164·3)	169·3 (160·5 to 177·7)	173·9 (158·6 to 191·5)
Somalia	51·1 (48·7 to 53·3)	68 (65·3 to 70·8)	92·1 (88·4 to 95·5)	153·4 (147·2 to 159)	162·9 (156·1 to 169)	248·2 (234·5 to 260·4)	328·4 (311·3 to 345·6)	424·4 (392·5 to 458·5)
South Sudan	51·7 (47·3 to 56)	66 (61·4 to 70·6)	83·9 (78·7 to 88·7)	100 (94·6 to 105·7)	123·9 (115 to 132·3)	159·2 (147·8 to 169·8)	200·7 (185 to 215·2)	178·6 (166·5 to 192·9)
Uganda	164·8 (158·1 to 170·9)	210·2 (203·9 to 215·8)	269·8 (263 to 276·2)	347·5 (340·2 to 354·5)	464·1 (455·6 to 472·3)	619·8 (607·2 to 631)	723·4 (698·4 to 746·4)	787·9 (744·1 to 838·1)
Tanzania	227·7 (215·7 to 238·7)	291·8 (280·4 to 302·5)	364·5 (352·6 to 376·4)	466·3 (448 to 483·4)	610·8 (590·1 to 631·6)	776·8 (740·4 to 811·8)	935·7 (888·4 to 984·5)	1032·8 (953 to 1121·1)
Zambia	66·8 (64·2 to 69·2)	100·7 (97·9 to 103)	126·7 (123·4 to 129·7)	150 (145·2 to 154·3)	194·6 (187·6 to 201·8)	228·1 (217·8 to 237·4)	273·4 (255·5 to 292·7)	305·4 (275·8 to 339·2)
Southern sub-Saharan Africa	397·2 (366·8 to 426·6)	479·6 (450·4 to 505·3)	590·3 (556·7 to 624·6)	706·1 (658 to 752·1)	761·8 (706·8 to 820·1)	825 (760·8 to 887·6)	866·9 (801 to 935·8)	825·5 (743·3 to 921·1)
Botswana	9·1 (8·4 to 9·7)	11·5 (10·7 to 12·1)	14·2 (13·3 to 15·1)	17·3 (16 to 18·6)	19·9 (17·9 to 21·9)	22·2 (19·6 to 24·9)	24 (21·4 to 26·7)	23·7 (20·7 to 27·3)
eSwatini	6 (5·7 to 6·3)	8·1 (7·7 to 8·5)	10·6 (10·2 to 10·9)	13·5 (12·9 to 14·1)	15·9 (15·3 to 16·5)	17·4 (16·5 to 18·4)	16·7 (15·5 to 17·9)	14·7 (13 to 16·8)
Lesotho	12·8 (11·6 to 14)	15·5 (14·1 to 17)	19·2 (17·8 to 20·7)	25·7 (23·2 to 28·4)	30·9 (28·5 to 33·1)	29·6 (28·2 to 31)	25·6 (24·1 to 27·2)	23·1 (20 to 26·5)
Namibia	10·9 (10·3 to 11·5)	13·9 (13·2 to 14·5)	16·5 (15·7 to 17·3)	19·7 (18·6 to 20·8)	24 (22·5 to 25·5)	27·5 (25·9 to 29)	30 (27·6 to 32·4)	31·3 (28·3 to 35)
South Africa	289·5 (265·4 to 313·2)	340·9 (317·4 to 362·1)	405·7 (376·9 to 434·4)	456·2 (412·3 to 498·2)	478·4 (427·6 to 532·9)	523·5 (464·8 to 581·9)	546·4 (486·9 to 610·2)	511·1 (453·1 to 579)
Zimbabwe	68·9 (65·4 to 72)	89·7 (86·2 to 92·9)	124·1 (119·9 to 128·1)	173·6 (169·9 to 177·5)	192·7 (187·4 to 198)	204·7 (199·3 to 210·7)	224·1 (216·8 to 230·6)	221·6 (204·3 to 242·1)
Western sub-Saharan Africa	1802·7 (1708·5 to 1891·7)	2204·6 (2108·9 to 2290·8)	2723 (2636·9 to 2808·6)	3467·3 (3357·2 to 3568·2)	4442·5 (4294·6 to 4580·1)	5769·2 (5583·4 to 5956·5)	7104·3 (6781·7 to 7420·7)	8030·8 (7466·8 to 8653·3)
Benin	48·6 (45·5 to 51·4)	55·1 (52·7 to 57·2)	66·9 (64·9 to 69)	85·6 (82·8 to 88·2)	115·7 (112·3 to 119)	150 (145·1 to 154·8)	203·6 (194·8 to 211·9)	246·9 (232 to 264·5)
Burkina Faso	90·7 (84 to 97·3)	103·2 (97·7 to 108)	126·8 (121·9 to 131·2)	170·6 (165·8 to 175·4)	223·7 (216·4 to 230·3)	280·4 (272·1 to 288·1)	364·9 (349·7 to 379·5)	459·1 (427·9 to 493·6)
Cape Verde	3·5 (3·3 to 3·6)	4·5 (4·4 to 4·6)	4·5 (4·4 to 4·7)	5·1 (4·9 to 5·2)	5·7 (5·5 to 5·9)	6·2 (5·7 to 6·6)	6 (5·2 to 7)	5·3 (4·5 to 6·3)
Cameroon	103·7 (95·8 to 111·3)	130·8 (121·7 to 139·2)	158·1 (151·9 to 164·2)	186·9 (181·1 to 192·4)	227·8 (219·3 to 235·4)	312·2 (300·8 to 322·7)	424·4 (400·7 to 448·9)	443·8 (391·5 to 504·7)
Chad	63·2 (59·9 to 66·2)	72·3 (69·5 to 74·7)	90·3 (87·7 to 93)	118·7 (115·6 to 121·5)	156·3 (152·8 to 159·7)	212·8 (207·8 to 217·4)	290 (281·1 to 297·9)	385·7 (369 to 403·4)
Côte d'Ivoire	59·9 (55·5 to 64)	97·8 (92·1 to 102·9)	137 (130·9 to 142·5)	194·7 (187·8 to 201·1)	281·5 (270·1 to 292·1)	368 (347 to 389·2)	422·6 (397·6 to 447·5)	437·4 (398·5 to 481·2)
The Gambia	5·5 (5·1 to 5·9)	7·1 (6·7 to 7·6)	11·3 (10·7 to 11·9)	17·4 (16·5 to 18·2)	24·5 (22·9 to 25·8)	31·2 (28·9 to 33·4)	34·3 (30·9 to 37·6)	35·3 (30·9 to 40·3)
Ghana	107 (98·1 to 116·5)	166·4 (157·6 to 174·8)	220·7 (213·8 to 227·6)	273·6 (264·4 to 282·4)	296·3 (281·9 to 311·7)	336·8 (317·7 to 355·7)	395·4 (371·2 to 421)	418·1 (359·4 to 483·1)
Guinea	63·3 (60·4 to 65·9)	78·3 (74·3 to 81·9)	98·6 (94·4 to 102·7)	123 (118·5 to 127)	150·6 (145·9 to 155·1)	171·3 (164·8 to 177·7)	202·6 (191·9 to 212·9)	233·7 (215·9 to 251)
Guinea-Bissau	15·5 (14·8 to 16·1)	14·6 (13·7 to 15·4)	15 (14 to 15·9)	18·4 (17·3 to 19·5)	22·8 (21·2 to 24·4)	26·2 (24·1 to 28·2)	30 (27 to 33)	30·8 (27·2 to 34·6)

Liberia	20·9 (19·4 to 22·3)	27·4 (25·9 to 28·8)	35 (33·6 to 36·4)	47·8 (45·9 to 49·4)	43·2 (41·1 to 45·1)	63·6 (60·7 to 66·6)	73·2 (67·5 to 78·4)	65·8 (57·4 to 75·9)
Mali	89·9 (85 to 94·3)	120·2 (115·3 to 124·3)	155 (150·6 to 159·1)	189·2 (184·5 to 193·4)	219·5 (214·1 to 224·6)	266·5 (258·8 to 274·1)	356·8 (342·3 to 369·7)	469·2 (440·5 to 499·7)
Mauritania	15·2 (14 to 16·3)	19·7 (18·5 to 20·8)	26 (25 to 27)	35·3 (34 to 36·5)	44·2 (42·1 to 46·2)	49·8 (47·2 to 52·4)	54·5 (50·8 to 58·1)	53·3 (48 to 59·7)
Niger	68·6 (65·1 to 71·8)	95 (91·8 to 98)	130·4 (127·1 to 133·7)	170·9 (167·3 to 174·2)	217·4 (212·8 to 222)	313·3 (306·2 to 319·9)	413·8 (403·7 to 423·3)	559·2 (539 to 580·3)
Nigeria	906·9 (860·1 to 950)	1034·8 (987·4 to 1076·4)	1226·3 (1178·6 to 1272·8)	1556·8 (1486·5 to 1620·2)	2081·2 (1993·3 to 2166·3)	2794·9 (2669·2 to 2914)	3361·6 (3169·7 to 3554·8)	3701 (3424·7 to 4018·6)
São Tomé and PrÍncipe	1·2 (1·1 to 1·3)	1·5 (1·5 to 1·6)	1·6 (1·5 to 1·6)	1·9 (1·8 to 1·9)	2·3 (2·2 to 2·4)	2·6 (2·5 to 2·7)	2·6 (2·4 to 2·8)	2·3 (2 to 2·7)
Senegal	64·1 (60·1 to 67·9)	85·4 (82·1 to 88·6)	114·7 (111·1 to 118)	144·6 (141·3 to 147·7)	169·2 (165 to 173·4)	189·6 (183·4 to 196·1)	223·5 (216 to 231·1)	229·1 (211·4 to 250·6)
Sierra Leone	38·9 (35·3 to 42·4)	48·2 (44·3 to 51·6)	55·6 (52·2 to 59)	64 (59·8 to 67·8)	80·7 (75 to 86·3)	96·3 (89·9 to 102·1)	125·3 (115·8 to 134·9)	138 (126·1 to 152·1)
Togo	36·1 (34·2 to 37·9)	42·3 (41·1 to 43·6)	49 (47·7 to 50·2)	63 (61·4 to 64·5)	79·9 (78·1 to 81·6)	97·2 (94·1 to 100·3)	119 (114·1 to 123·8)	116·5 (107·7 to 127)

Supplemental Results Table 2a: Estimates of life expectancy at birth by location, both sexes combined, 1950, 1960, 1970, 1980, 1990, 2000, 2010, 2019

Location	1950	1960	1970	1980	1990	2000	2010	2019
Global	51·1 (49·4 to 52·7)	46·6 (42·2 to 49·9)	58·4 (57·5 to 59·2)	62·2 (61·7 to 62·8)	65·4 (65 to 65·8)	67·2 (66·8 to 67·6)	70·6 (70·2 to 71)	73·5 (72·8 to 74·3)
Low SDI	42·3 (39·3 to 45·1)	44·8 (42·7 to 47)	47·6 (46 to 49·3)	51·3 (50 to 52·4)	53·8 (52·9 to 54·6)	55·4 (54·5 to 56·4)	60·5 (59·5 to 61·5)	65·6 (64 to 66·9)
Low-middle SDI	41 (38·9 to 43·2)	40·9 (38·3 to 43·2)	48·9 (47·7 to 50·2)	55·3 (54·5 to 56·1)	59·7 (59 to 60·3)	62·4 (61·7 to 63)	66·5 (65·9 to 67·2)	69·7 (68·7 to 70·8)
Middle SDI	48·1 (46·3 to 50)	35·6 (28·5 to 41·3)	58·4 (57·3 to 59·5)	63·3 (62·4 to 64·1)	67 (66·4 to 67·6)	69·5 (69·1 to 69·9)	72·1 (71·7 to 72·5)	74·7 (73·9 to 75·5)
High-middle SDI	59·4 (58 to 60·7)	57·4 (53·9 to 59·8)	66 (65·2 to 66·7)	68·1 (67·5 to 68·6)	70·4 (70 to 70·8)	71·6 (71·3 to 71·8)	75 (74·7 to 75·3)	77·5 (76·9 to 78·2)
High SDI	64·4 (64·1 to 64·6)	69·1 (69 to 69·2)	71 (70·9 to 71·1)	73·7 (73·6 to 73·7)	76 (75·9 to 76)	78·1 (78 to 78·1)	80·4 (80·3 to 80·4)	81·2 (80·9 to 81·3)
Central Europe, eastern Europe, and central Asia	62·3 (60·6 to 63·8)	66·7 (66·3 to 67·1)	68·1 (67·8 to 68·3)	68·3 (68·1 to 68·4)	69·4 (69·3 to 69·6)	67·8 (67·7 to 68)	70·8 (70·7 to 70·9)	73·4 (72·5 to 74·3)
Central Asia	50·6 (46·4 to 54·8)	57·4 (55·4 to 59·4)	62·9 (61·7 to 64)	66·3 (65·8 to 66·8)	67·7 (67·3 to 68·1)	66 (65·6 to 66·4)	68·2 (67·9 to 68·5)	70·7 (69·6 to 71·8)
Armenia	48·2 (43·7 to 52·7)	59·2 (56·9 to 61·5)	65·9 (64·4 to 67·2)	70·2 (69·3 to 71·1)	70·6 (70·2 to 70·9)	72·4 (72·1 to 72·7)	73·9 (73·6 to 74·2)	75·7 (74·2 to 77·3)
Azerbaijan	37·8 (33·4 to 42·2)	48·6 (45·5 to 51·9)	59·9 (58·2 to 61·4)	66·2 (65·3 to 67·1)	66·9 (66 to 67·8)	66·5 (65·8 to 67·3)	69 (68·3 to 69·6)	71 (69·3 to 72·4)
Georgia	49·6 (45·2 to 53·9)	59·3 (57·2 to 61·5)	65·4 (63·9 to 66·8)	68·5 (67·2 to 69·9)	68·5 (67·6 to 69·5)	70·7 (69·8 to 71·5)	71·9 (71·1 to 72·7)	73·4 (71·7 to 75·2)
Kazakhstan	57·4 (53·6 to 61·2)	61·4 (59·7 to 63·1)	64·8 (63·7 to 65·8)	66·9 (66·3 to 67·4)	68·1 (67·6 to 68·6)	64·7 (64·2 to 65·1)	67·6 (67·2 to 68·1)	71·9 (70·5 to 73·3)
Kyrgyzstan	50 (45·7 to 54·1)	55·8 (52·9 to 58·6)	60·4 (58·5 to 62·1)	64·3 (63·4 to 65·1)	67·2 (66·6 to 67·9)	67·2 (66·8 to 67·7)	69·6 (69·5 to 69·8)	73·4 (72·2 to 74·7)
Mongolia	38·9 (34·6 to 43·2)	43·9 (40 to 47·8)	50·6 (47·3 to 54·2)	57·2 (54·5 to 60·1)	60·4 (58·6 to 62·4)	61 (59·2 to 62·8)	65·1 (63 to 67·1)	68·1 (64·9 to 71)
Tajikistan	46·2 (41·7 to 50·6)	52·5 (50·2 to 55)	60·4 (58·6 to 62·2)	65·3 (63·5 to 67·1)	66·9 (66 to 67·8)	66·6 (65·9 to 67·4)	68 (67 to 69)	69·6 (67·6 to 71·4)
Turkmenistan	48·4 (44·1 to 52·6)	55·1 (53·2 to 57)	60·4 (58·7 to 61·9)	63·6 (62·4 to 64·7)	66 (65·3 to 66·6)	66·3 (65·8 to 66·9)	69·5 (69 to 69·9)	70·8 (68·1 to 73·3)
Uzbekistan	56·3 (52·2 to 60·2)	61·1 (58·9 to 63·3)	64·8 (63·6 to 66)	67·5 (66·8 to 68·1)	69·3 (68·9 to 69·7)	65·1 (64·6 to 65·6)	66·5 (66 to 66·9)	68·4 (66·9 to 70)
Central Europe	59·1 (58·3 to 59·7)	66·6 (66·5 to 66·7)	68·9 (68·8 to 68·9)	70·1 (70·1 to 70·2)	70·8 (70·7 to 70·9)	72·8 (72·7 to 72·9)	75·4 (75·3 to 75·5)	77 (75·7 to 78·3)
Albania	52·2 (49·5 to 54·8)	62·6 (60·6 to 64·4)	67·8 (65·3 to 70·3)	71·2 (69·4 to 73·1)	72·8 (72·3 to 73·2)	75·4 (75 to 75·8)	78·1 (76·8 to 79·4)	78·5 (76·1 to 81)
Bosnia and Herzegovina	51·1 (50·8 to 51·3)	61·3 (61 to 61·5)	65·9 (65·7 to 66·2)	69·7 (69·6 to 69·9)	73·2 (73 to 73·4)	75·5 (74·6 to 76·3)	76·3 (76 to 76·6)	77 (74·7 to 79·1)
Bulgaria	61 (60·8 to 61·3)	69·4 (69·2 to 69·6)	71·5 (71·3 to 71·6)	71·2 (71·1 to 71·3)	70·1 (69·7 to 70·5)	70·2 (69·7 to 70·6)	72·3 (71·9 to 72·8)	73·3 (70·9 to 75·7)
Croatia	55 (54·6 to 55·3)	64·7 (64·5 to 64·9)	68·7 (68·4 to 68·9)	70·7 (70·2 to 71·1)	72·5 (71·9 to 73)	74·6 (74·2 to 75·1)	76·8 (76·6 to 77)	78·7 (76·5 to 80·8)
Czech Republic	64·3 (64·1 to 64·4)	70·4 (70·3 to 70·5)	69·7 (69·6 to 69·8)	70·5 (70·4 to 70·6)	71·6 (71·5 to 71·7)	75·2 (75·1 to 75·3)	77·8 (77·7 to 78)	79·5 (77·6 to 81·3)
Hungary	61·9 (61·7 to 62)	68·1 (68 to 68·3)	69·2 (69 to 69·4)	69·1 (69 to 69·2)	69·5 (69·4 to 69·6)	71·9 (71·8 to 72)	74·8 (74·7 to 74·9)	76·6 (74·6 to 78·6)
Montenegro	67·5 (66·9 to 68)	70·8 (70·2 to 71·4)	73·7 (73·1 to 74·3)	73·6 (73·2 to 74)	74·7 (74 to 75·3)	73·7 (73·3 to 74·1)	74·7 (74·3 to 75·1)	75·9 (74·2 to 77·4)
North Macedonia	50·2 (49·9 to 50·6)	57·5 (57·1 to 57·8)	65·3 (65 to 65·6)	70·1 (69·8 to 70·4)	70·5 (70·2 to 70·8)	71·5 (71·2 to 71·9)	73·4 (73·1 to 73·7)	74·4 (72·3 to 76·4)
Poland	59 (58·9 to 59·1)	67·9 (67·8 to 68)	70·2 (70·1 to 70·2)	70·5 (70·5 to 70·6)	71·1 (71 to 71·1)	73·9 (73·8 to 74)	76·5 (76·4 to 76·5)	78·1 (76·3 to 79·9)
Romania	60·5 (56·7 to 64·3)	65·8 (65·7 to 65·9)	68·3 (68·2 to 68·4)	69·6 (69·5 to 69·7)	69·8 (69·7 to 69·9)	71·2 (71·1 to 71·3)	73·8 (73·7 to 73·9)	75·5 (73·5 to 77·5)
Serbia	54·4 (54·2 to 54·6)	63·5 (63·4 to 63·7)	66·2 (66 to 66·3)	69·8 (69·7 to 69·9)	70·8 (70·7 to 71)	71·4 (71·2 to 71·6)	74·1 (73·9 to 74·2)	75·7 (73·6 to 77·7)
Slovakia	60·7 (60·4 to 61)	70·4 (70·2 to 70·6)	69·9 (69·7 to 70·1)	70·7 (70·5 to 70·9)	71·1 (70·9 to 71·2)	73·7 (73·5 to 73·9)	75·8 (75·6 to 76)	77·6 (75·2 to 79·9)
Slovenia	58·5 (58·1 to 58·8)	65·3 (65 to 65·6)	68·6 (68·3 to 68·8)	71·3 (71·1 to 71·6)	74 (71·1 to 76·7)	76·3 (76 to 76·5)	79·7 (79·4 to 80)	81·4 (78·9 to 83·7)
Eastern Europe	66·9 (65 to 68·9)	69·5 (69·3 to 69·6)	69·7 (69·5 to 69·8)	68·4 (68·3 to 68·5)	69·9 (69·8 to 69·9)	66·5 (66·5 to 66·6)	69·5 (69·5 to 69·5)	72·5 (71·3 to 73·7)
Belarus	68·2 (64·3 to 72)	71 (70·1 to 71·9)	72·1 (71·6 to 72·6)	71 (70·7 to 71·3)	71·3 (71 to 71·6)	69 (68·8 to 69·3)	70·6 (70·4 to 70·8)	74 (71 to 76·7)
Estonia	66·7 (63·3 to 70·1)	69·4 (69·1 to 69·7)	70·5 (70·2 to 70·8)	69·7 (69·4 to 69·9)	70 (69·7 to 70·3)	71·1 (70·8 to 71·4)	76·2 (75·9 to 76·6)	78 (75·6 to 80·5)
Latvia	68·4 (64·9 to 71·8)	70·3 (70 to 70·6)	70·5 (70·2 to 70·7)	69·2 (69 to 69·5)	69·8 (69·6 to 70·1)	70·6 (70·4 to 70·9)	73·3 (73 to 73·5)	75·9 (73·8 to 77·7)

Lithuania	67·1 (63·4 to 70·8)	69·9 (69·7 to 70·2)	71·3 (71 to 71·5)	70·6 (70·4 to 70·8)	71·4 (71·2 to 71·6)	72·2 (72 to 72·4)	73·2 (73 to 73·4)	76·2 (74·1 to 78·3)
Moldova	53·4 (49·4 to 57·3)	58·4 (56·3 to 60·5)	62·9 (61·3 to 64·4)	65·8 (65·2 to 66·3)	68·3 (68 to 68·6)	68·9 (68·6 to 69·1)	70 (69·7 to 70·2)	74·3 (73 to 75·7)
Russia	66·7 (64·4 to 69·1)	69 (69 to 69·1)	69·1 (69·1 to 69·2)	67·7 (67·7 to 67·7)	69·5 (69·5 to 69·6)	65·6 (65·6 to 65·7)	68·8 (68·8 to 68·9)	73 (71·3 to 74·5)
Ukraine	68·6 (66·4 to 70·8)	71·2 (70·7 to 71·7)	71 (70·6 to 71·3)	69·8 (69·5 to 70·1)	70·6 (70·4 to 70·8)	67·9 (67·9 to 68)	70·9 (70·8 to 70·9)	70·1 (68·2 to 72·1)
High income	64·5 (64·3 to 64·6)	69·1 (69·1 to 69·2)	71 (70·9 to 71)	73·9 (73·9 to 73·9)	76·3 (76·2 to 76·3)	78·4 (78·3 to 78·4)	80·6 (80·6 to 80·6)	81·3 (81·2 to 81·4)
Australasia	69·4 (69·3 to 69·5)	71·1 (71·1 to 71·2)	71·2 (71·2 to 71·3)	74·3 (74·2 to 74·4)	76·7 (76·7 to 76·8)	79·5 (79·4 to 79·6)	81·9 (81·9 to 82)	82·7 (82·5 to 82·9)
Australia	69·4 (69·3 to 69·5)	71·1 (71 to 71·2)	71·2 (71·1 to 71·2)	74·5 (74·4 to 74·6)	77 (76·9 to 77·1)	79·7 (79·7 to 79·8)	82·1 (82·1 to 82·2)	82·9 (82·7 to 83·1)
New Zealand	69·5 (69·3 to 69·7)	71·2 (71·1 to 71·4)	71·6 (71·5 to 71·8)	73·4 (73·3 to 73·5)	75·6 (75·4 to 75·7)	78·4 (78·3 to 78·5)	80·9 (80·8 to 81)	81·8 (81·6 to 82)
High-income Asia Pacific	54·9 (54·5 to 55·3)	65·9 (65·7 to 66·1)	70·5 (70·3 to 70·7)	74·5 (74·4 to 74·6)	77·8 (77·8 to 77·9)	80·6 (80·5 to 80·6)	82·9 (82·8 to 82·9)	84·4 (84·3 to 84·6)
Brunei	51·7 (50·5 to 52·9)	55·8 (54·4 to 57·2)	63 (62·1 to 64)	67·5 (66·5 to 68·5)	69·4 (68·6 to 70·3)	72·5 (71·8 to 73·3)	73·8 (73·1 to 74·5)	74·4 (73·2 to 75·4)
Japan	59·8 (59·7 to 59·8)	68·3 (68·3 to 68·3)	72·7 (72·6 to 72·7)	76·6 (76·5 to 76·6)	79·4 (79·4 to 79·4)	81·6 (81·5 to 81·6)	83·3 (83·3 to 83·4)	84·8 (84·7 to 84·9)
Aichi	61·4 (59·4 to 63·5)	69·3 (67·5 to 71·2)	73·2 (71·5 to 74·8)	76·6 (76·5 to 76·8)	79·4 (79·2 to 79·5)	81·4 (81·2 to 81·6)	83·3 (83·1 to 83·5)	85 (84·2 to 85·8)
Akita	59·5 (57·3 to 61·7)	67·8 (65·8 to 69·9)	72 (70·2 to 73·6)	75·8 (75·5 to 76)	78·8 (78·6 to 79·1)	81 (80·7 to 81·2)	82·5 (82·2 to 82·8)	83 (82·1 to 83·9)
Aomori	57·3 (55·2 to 59·4)	66·4 (64·4 to 68·5)	71·1 (69·3 to 73)	75·5 (75·2 to 75·7)	78·5 (78·2 to 78·7)	80·5 (80·2 to 80·7)	82 (81·7 to 82·3)	83·6 (82·7 to 84·6)
Chiba	59·8 (57·7 to 62)	68·5 (66·5 to 70·7)	73 (71·4 to 74·8)	76·8 (76·6 to 77)	79·8 (79·6 to 79·9)	81·5 (81·4 to 81·7)	83·3 (83·1 to 83·5)	84·5 (83·7 to 85·3)
Ehime	59·1 (57 to 61·3)	67·8 (65·9 to 69·8)	72·4 (70·6 to 74·2)	76·7 (76·4 to 76·9)	79·5 (79·2 to 79·7)	81·3 (81 to 81·6)	83·3 (83 to 83·6)	84·3 (83·4 to 85·2)
Fukui	61·5 (59·3 to 63·6)	69·3 (67·4 to 71·3)	73·6 (72·1 to 75·1)	77·9 (77·6 to 78·2)	80·2 (80 to 80·5)	82·6 (82·3 to 82·9)	84·2 (83·9 to 84·5)	85·7 (85 to 86·5)
Fukuoka	59·6 (57·5 to 61·8)	68 (66·1 to 69·9)	72·1 (70·4 to 73·9)	76·2 (76·1 to 76·4)	79·1 (78·9 to 79·3)	81·4 (81·3 to 81·6)	83·4 (83·2 to 83·6)	84·7 (83·9 to 85·5)
Fukushima	57·7 (55·5 to 59·8)	67·2 (65·2 to 69·2)	71·6 (70 to 73·4)	76·3 (76 to 76·5)	79·5 (79·2 to 79·7)	81·4 (81·1 to 81·7)	83 (82·8 to 83·3)	84·2 (83·3 to 85·2)
Gifu	60·5 (58·4 to 62·5)	68·6 (66·6 to 70·6)	72·7 (71 to 74·4)	76·5 (76·3 to 76·7)	79·6 (79·4 to 79·9)	81·5 (81·3 to 81·8)	83·5 (83·2 to 83·8)	84·7 (83·8 to 85·5)
Gunma	59·2 (57 to 61·5)	67·8 (65·9 to 69·9)	72·4 (70·7 to 74·2)	76·4 (76·2 to 76·6)	79·5 (79·3 to 79·8)	81·5 (81·3 to 81·8)	83 (82·7 to 83·2)	84·4 (83·4 to 85·2)
Hiroshima	58·8 (56·6 to 60·9)	68 (66 to 70·1)	72·6 (70·8 to 74·5)	76·7 (76·5 to 76·9)	79·6 (79·4 to 79·8)	81·9 (81·7 to 82·1)	83·7 (83·5 to 84)	85·2 (84·4 to 86·1)
Hokkaidō	59·2 (57·1 to 61·4)	67·9 (65·8 to 69·8)	71·9 (70·1 to 73·8)	76 (75·9 to 76·2)	79·1 (79 to 79·3)	81·5 (81·3 to 81·7)	83·2 (83 to 83·4)	84·6 (83·8 to 85·5)
Hyōgo	60·6 (58·5 to 62·9)	68·8 (66·7 to 70·6)	72·7 (70·9 to 74·3)	76·3 (76·1 to 76·4)	79 (78·8 to 79·1)	81·3 (81·1 to 81·5)	83·3 (83·1 to 83·5)	84·9 (84·2 to 85·7)
Ibaraki	57·2 (55·1 to 59·3)	66·9 (64·9 to 69)	71·6 (69·7 to 73·5)	75·9 (75·7 to 76·1)	78·9 (78·7 to 79·1)	81 (80·8 to 81·2)	82·6 (82·4 to 82·9)	84 (83·1 to 84·9)
Ishikawa	60·9 (58·6 to 63·1)	69 (67 to 71·1)	73·2 (71·6 to 74·7)	77·2 (76·9 to 77·4)	80 (79·7 to 80·2)	82·1 (81·8 to 82·3)	83·8 (83·5 to 84·1)	85 (84·3 to 85·8)
Iwate	57·8 (55·7 to 60)	67 (65 to 69)	71·7 (70 to 73·3)	75·9 (75·7 to 76·2)	79·1 (78·8 to 79·4)	81·4 (81·1 to 81·7)	82·6 (82·3 to 83)	83·7 (82·8 to 84·7)
Kagawa	60·1 (58 to 62·5)	68·6 (66·6 to 70·5)	73·2 (71·6 to 74·9)	77·3 (77 to 77·6)	79·6 (79·4 to 79·9)	81·7 (81·4 to 82)	83·3 (83 to 83·6)	85·2 (84·4 to 86)
Kagoshima	56·6 (54·5 to 58·9)	66·2 (64·2 to 68·3)	70·8 (69 to 72·7)	75·8 (75·5 to 76)	79·2 (78·9 to 79·5)	81·4 (81·1 to 81·6)	83·1 (82·8 to 83·4)	84·8 (83·9 to 85·7)
Kanagawa	62·3 (60·3 to 64·4)	70·1 (68·1 to 72·2)	74 (72·3 to 75·6)	77·2 (77·1 to 77·3)	79·7 (79·6 to 79·8)	81·8 (81·6 to 81·9)	83·6 (83·5 to 83·8)	85·3 (84·5 to 86)
Kōchi	57·3 (55 to 59·5)	66·4 (64·4 to 68·5)	71·1 (69·3 to 72·7)	75·8 (75·5 to 76·1)	79·3 (79 to 79·6)	81·3 (81 to 81·6)	83 (82·6 to 83·3)	84·7 (83·8 to 85·5)
Kumamoto	57·8 (55·6 to 59·9)	67 (65·1 to 69)	71·8 (70 to 73·6)	76·6 (76·4 to 76·9)	80 (79·8 to 80·2)	82·4 (82·2 to 82·7)	84·1 (83·8 to 84·4)	85·4 (84·5 to 86·3)
Kyōto	61·3 (59·1 to 63·5)	69·4 (67·6 to 71·5)	73·6 (71·8 to 75·4)	77·3 (77·1 to 77·5)	79·8 (79·6 to 80·1)	81·9 (81·7 to 82·2)	83·9 (83·7 to 84·2)	85·6 (84·7 to 86·5)
Mie	60·7 (58·4 to 62·8)	68·9 (66·9 to 70·8)	73·1 (71·3 to 74·8)	76·7 (76·5 to 77)	79·3 (79·1 to 79·6)	81·6 (81·3 to 81·8)	83·3 (83 to 83·6)	84·7 (83·9 to 85·5)
Miyagi	59·2 (57 to 61·5)	68·3 (66·1 to 70·3)	72·4 (70·6 to 74)	76·5 (76·3 to 76·7)	79·5 (79·3 to 79·7)	81·6 (81·4 to 81·9)	83·5 (83·2 to 83·8)	85·1 (84·3 to 86)
Miyazaki	57·8 (55·7 to 60·1)	66·9 (64·9 to 68·9)	71·7 (69·9 to 73·4)	76 (75·8 to 76·3)	79·3 (79 to 79·6)	81·7 (81·4 to 82)	83·5 (83·2 to 83·8)	84·5 (83·7 to 85·4)
Nagano	60·3 (58·1 to 62·5)	68·9 (66·9 to 71)	73·3 (71·5 to 75)	77·2 (77 to 77·5)	80·4 (80·2 to 80·7)	82·5 (82·2 to 82·7)	84·3 (84·1 to 84·6)	85·4 (84·6 to 86·2)

Nagasaki	59·2 (57 to 61·4)	67·4 (65·4 to 69·6)	71·5 (69·6 to 73·4)	75·9 (75·7 to 76·2)	79·2 (78·9 to 79·4)	81·5 (81·2 to 81·8)	83·1 (82·8 to 83·5)	84·6 (83·8 to 85·5)
Nara	60·5 (58·3 to 62·7)	68·7 (66·8 to 70·6)	72·6 (70·9 to 74·4)	76·4 (76·1 to 76·7)	79·4 (79·1 to 79·6)	82 (81·7 to 82·2)	83·9 (83·6 to 84·2)	85·2 (84·3 to 86)
Niigata	60·1 (58·1 to 62·3)	68·6 (66·8 to 70·5)	72·6 (70·8 to 74·3)	76·6 (76·4 to 76·8)	79·9 (79·7 to 80·2)	81·9 (81·7 to 82·2)	83·6 (83·4 to 83·9)	84·4 (83·6 to 85·4)
Ōita	58·4 (56·1 to 60·8)	67·4 (65·4 to 69·5)	72·1 (70·4 to 73·8)	76·2 (75·9 to 76·4)	79·4 (79·2 to 79·7)	81·7 (81·4 to 82·1)	83·9 (83·5 to 84·2)	85 (84·1 to 85·9)
Okayama	60·3 (58·3 to 62·5)	68·7 (66·7 to 70·8)	73·3 (71·5 to 75·1)	77·2 (77 to 77·4)	79·9 (79·6 to 80·1)	82·1 (81·8 to 82·4)	83·7 (83·4 to 84)	85·5 (84·7 to 86·3)
Okinawa	57·2 (55 to 59·6)	66·6 (64·5 to 68·7)	70·7 (68·8 to 72·5)	77·1 (76·9 to 77·4)	80·6 (80·3 to 80·9)	82·2 (81·9 to 82·5)	83·4 (83·1 to 83·7)	84·3 (83·3 to 85·3)
Ōsaka	60·5 (58·4 to 62·5)	68·6 (66·7 to 70·5)	72·5 (70·9 to 74·1)	75·8 (75·7 to 76)	78·3 (78·2 to 78·5)	80·8 (80·7 to 81)	82·7 (82·5 to 82·9)	84·6 (83·8 to 85·3)
Saga	58·7 (56·7 to 60·9)	67·5 (65·4 to 69·6)	72·2 (70·6 to 73·8)	76·6 (76·3 to 76·9)	79·4 (79·1 to 79·7)	81·6 (81·3 to 81·9)	83·5 (83·2 to 83·8)	85 (84·1 to 85·8)
Saitama	60·4 (58·1 to 62·6)	68·9 (67·1 to 70·8)	73·3 (71·5 to 75·1)	76·5 (76·4 to 76·7)	79·4 (79·2 to 79·5)	81·6 (81·4 to 81·7)	83·1 (82·9 to 83·3)	84·6 (83·7 to 85·4)
Shiga	60·4 (58·2 to 62·7)	68·6 (66·8 to 70·6)	72·6 (71 to 74·1)	76·8 (76·6 to 77·1)	79·5 (79·3 to 79·7)	82·2 (82 to 82·5)	84 (83·7 to 84·3)	85·8 (85·1 to 86·5)
Shimane	59·4 (57·3 to 61·6)	67·8 (65·7 to 69·8)	72·3 (70·7 to 74)	76·9 (76·6 to 77·2)	80·2 (79·9 to 80·6)	82·4 (82 to 82·7)	83·6 (83·2 to 83·9)	85·1 (84·3 to 85·9)
Shizuoka	61·2 (59 to 63·5)	69·4 (67·4 to 71·4)	73·5 (71·7 to 75·4)	77·1 (76·9 to 77·3)	79·9 (79·7 to 80·1)	81·9 (81·7 to 82·2)	83·5 (83·2 to 83·7)	84·8 (84 to 85·7)
Tochigi	58·7 (56·5 to 61)	67·4 (65·5 to 69·4)	72 (70·2 to 73·8)	75·8 (75·6 to 76)	78·6 (78·4 to 78·9)	81 (80·8 to 81·3)	82·6 (82·3 to 82·9)	83·9 (83 to 84·8)
Tokushima	58·2 (56 to 60·3)	67 (65·1 to 69)	71·5 (69·9 to 73·2)	75·8 (75·5 to 76·1)	79·1 (78·8 to 79·4)	81·5 (81·2 to 81·8)	83·3 (82·9 to 83·6)	84·5 (83·8 to 85·3)
Tōkyō	62·6 (60·6 to 64·7)	69·9 (68·1 to 71·7)	73·5 (71·9 to 75·3)	77·1 (77 to 77·3)	79·4 (79·2 to 79·5)	81·4 (81·3 to 81·5)	83·5 (83·3 to 83·6)	85·2 (84·4 to 86)
Tottori	58·8 (56·7 to 61·3)	67·7 (65·8 to 69·6)	72·3 (70·8 to 73·9)	76·7 (76·3 to 76·9)	79·3 (79 to 79·6)	81·6 (81·3 to 81·9)	83·1 (82·8 to 83·5)	84·3 (83·5 to 85·1)
Toyama	59·7 (57·5 to 61·8)	68·2 (66·2 to 70·2)	72·6 (71 to 74·2)	76·9 (76·6 to 77·1)	80 (79·7 to 80·3)	82·3 (82 to 82·6)	83·8 (83·5 to 84·1)	85·4 (84·5 to 86·1)
Wakayama	58·7 (56·6 to 60·9)	67·4 (65·3 to 69·5)	72 (70·4 to 73·7)	76 (75·7 to 76·3)	78·8 (78·5 to 79·1)	81·1 (80·8 to 81·4)	82·6 (82·3 to 83)	83·3 (82·4 to 84·3)
Yamagata	59·6 (57·4 to 61·7)	68·1 (66·2 to 70)	72·1 (70·5 to 73·8)	76·5 (76·2 to 76·8)	80 (79·7 to 80·3)	82 (81·7 to 82·3)	83·8 (83·4 to 84·1)	85·1 (84·1 to 86)
Yamaguchi	58·7 (56·6 to 61)	67·6 (65·7 to 69·6)	72·4 (70·4 to 74·1)	76·2 (76 to 76·4)	79·4 (79·2 to 79·7)	81·3 (81 to 81·6)	83 (82·7 to 83·3)	84·3 (83·4 to 85·2)
Yamanashi	59·4 (57·2 to 61·7)	67·9 (65·8 to 70)	72·5 (70·9 to 74·1)	76·6 (76·3 to 76·9)	79·6 (79·3 to 79·9)	81·7 (81·4 to 82)	83·6 (83·2 to 83·9)	85 (84·3 to 85·8)
South Korea	39·5 (38·2 to 40·8)	59·1 (58·3 to 59·9)	63·6 (62·9 to 64·2)	67·3 (67·1 to 67·5)	72·1 (72 to 72·2)	76·3 (76·3 to 76·4)	80·8 (80·8 to 80·9)	82·9 (82·6 to 83·3)
Singapore	57 (56·7 to 57·3)	65·5 (65·4 to 65·7)	69 (68·9 to 69·1)	71·7 (71·6 to 71·9)	75·6 (75·5 to 75·7)	79·4 (79·3 to 79·5)	82·8 (82·7 to 82·9)	84·9 (84·6 to 85·1)
High-income North America	68·3 (68·3 to 68·3)	70·1 (70·1 to 70·2)	71·1 (71·1 to 71·1)	74·1 (74 to 74·1)	75·9 (75·9 to 75·9)	77·3 (77·2 to 77·3)	79·2 (79·1 to 79·2)	79·3 (79·2 to 79·3)
Canada	68·5 (68·4 to 68·6)	71·2 (71·1 to 71·2)	72·8 (72·7 to 72·8)	75·2 (75·2 to 75·3)	77·4 (77·4 to 77·5)	79·4 (79·3 to 79·4)	81·6 (81·6 to 81·7)	82·2 (82 to 82·3)
Greenland	50·5 (47·8 to 52·8)	59·2 (58·1 to 60·3)	63 (61·9 to 64·1)	64·8 (63·1 to 66·5)	65·5 (63·8 to 67)	67·4 (65·9 to 68·7)	71·2 (69·7 to 72·6)	73·3 (70·9 to 75·8)
USA	68·3 (68·3 to 68·3)	70·1 (70 to 70·1)	70·9 (70·9 to 71)	74 (73·9 to 74)	75·7 (75·7 to 75·7)	77 (77 to 77·1)	78·9 (78·9 to 78·9)	78·9 (78·8 to 79)
Alabama	65·8 (63·6 to 68·1)	68·2 (68 to 68·4)	69·3 (69·1 to 69·6)	72·6 (72·4 to 72·9)	74 (73·8 to 74·3)	74·6 (74·4 to 74·8)	75·4 (75·2 to 75·6)	75·4 (73·5 to 77·3)
Alaska	63·4 (61·4 to 65·4)	67·2 (66·8 to 67·7)	69·4 (69 to 69·9)	72·1 (71·7 to 72·5)	74·6 (74·2 to 75)	76·2 (75·8 to 76·5)	78·2 (77·9 to 78·5)	78·1 (76·7 to 79·5)
Arizona	66·5 (64·3 to 68·8)	69·2 (68·8 to 69·5)	70·8 (70·5 to 71·2)	74·2 (74 to 74·4)	76·6 (76·3 to 76·8)	77·5 (77·2 to 77·7)	79·6 (79·4 to 79·8)	79·3 (77·5 to 81·1)
Arkansas	68 (65·8 to 70·3)	70·3 (70 to 70·6)	71 (70·7 to 71·3)	73·7 (73·4 to 73·9)	74·7 (74·4 to 75)	75·1 (74·8 to 75·3)	76·2 (75·9 to 76·5)	76 (74·1 to 78)
California	69·2 (67·3 to 71·2)	71 (70·9 to 71·1)	71·9 (71·8 to 72)	74·6 (74·5 to 74·7)	76·2 (76·1 to 76·3)	78·4 (78·4 to 78·5)	80·9 (80·8 to 81)	81·4 (79·8 to 83)
Colorado	68·7 (66·5 to 71)	71·2 (70·9 to 71·5)	72·3 (72 to 72·6)	75·5 (75·2 to 75·7)	77·4 (77·2 to 77·7)	78·4 (78·1 to 78·6)	80·1 (79·9 to 80·3)	79·9 (78·2 to 81·6)
Connecticut	69·8 (67·8 to 71·9)	71·3 (71·1 to 71·5)	72·8 (72·6 to 73)	75 (74·8 to 75·3)	77·5 (77·2 to 77·8)	78·5 (78·2 to 78·7)	80·8 (80·6 to 81·1)	81 (79·4 to 82·8)
Delaware	66·8 (64·9 to 68·8)	69·5 (69·1 to 69·8)	70·2 (69·8 to 70·5)	73·4 (73 to 73·7)	75 (74·7 to 75·4)	76·5 (76·2 to 76·8)	78·2 (77·9 to 78·5)	78·4 (76·9 to 79·8)
Washington, DC	65 (62·8 to 67·3)	66·6 (66·3 to 67)	66·1 (65·8 to 66·5)	69·9 (69·5 to 70·2)	68·4 (68 to 68·9)	72·9 (72·5 to 73·3)	76 (75·6 to 76·3)	77·2 (75·2 to 79·1)
Florida	67·7 (65·5 to 70)	69·9 (69·7 to 70·1)	70·8 (70·6 to 70·9)	74 (73·8 to 74·1)	76·2 (76 to 76·3)	77·5 (77·4 to 77·6)	79·5 (79·4 to 79·6)	79·4 (77·6 to 81·2)

Georgia	65·7 (63·5 to 67·9)	67·8 (67·6 to 68)	68·7 (68·5 to 68·9)	72·4 (72·2 to 72·6)	74 (73·8 to 74·1)	75·2 (75 to 75·3)	77·2 (77 to 77·3)	77·6 (75·9 to 79·3)
Hawaii	69 (66·9 to 71)	70·7 (70·3 to 71)	73·8 (73·4 to 74·1)	77·3 (76·9 to 77·6)	78·6 (78·2 to 79)	80·3 (79·9 to 80·6)	80·9 (80·6 to 81·2)	81·7 (80·1 to 83·4)
Idaho	69·2 (67·2 to 71·5)	71·4 (71 to 71·8)	72·1 (71·7 to 72·5)	75·4 (75 to 75·8)	77 (76·7 to 77·4)	78·2 (77·8 to 78·5)	79·2 (78·9 to 79·6)	79·4 (77·8 to 81)
Illinois	68·3 (66·3 to 70·4)	69·7 (69·6 to 69·9)	70·3 (70·2 to 70·4)	73·6 (73·5 to 73·7)	75·3 (75·2 to 75·4)	76·8 (76·7 to 77)	79·1 (79 to 79·3)	79·4 (77·6 to 81·2)
Indiana	69 (66·9 to 71)	70·5 (70·3 to 70·7)	71·1 (71 to 71·3)	74 (73·8 to 74·2)	75·7 (75·5 to 75·9)	76·2 (76·1 to 76·4)	77·6 (77·4 to 77·8)	77·2 (75·3 to 79)
Iowa	70·7 (68·7 to 72·9)	72·3 (72·1 to 72·6)	72·7 (72·5 to 73)	76 (75·8 to 76·3)	78 (77·7 to 78·3)	78·6 (78·3 to 78·8)	79·6 (79·4 to 79·9)	79·6 (77·8 to 81·4)
Kansas	70·6 (68·5 to 72·8)	72·1 (71·8 to 72·4)	73 (72·8 to 73·3)	75·5 (75·2 to 75·7)	77·4 (77·1 to 77·7)	77·6 (77·4 to 77·9)	78·6 (78·3 to 78·8)	78·8 (77·1 to 80·7)
Kentucky	67·9 (65·6 to 70)	69·8 (69·5 to 70)	70·1 (69·8 to 70·3)	73·1 (72·9 to 73·3)	74·8 (74·6 to 75)	75·3 (75·1 to 75·5)	75·9 (75·7 to 76·1)	75·4 (73·6 to 77·4)
Louisiana	66·1 (63·9 to 68·2)	68·2 (67·9 to 68·4)	69 (68·8 to 69·2)	71·9 (71·7 to 72·1)	73·5 (73·3 to 73·7)	74·2 (74 to 74·4)	75·9 (75·7 to 76·1)	75·8 (73·9 to 77·8)
Maine	68 (66 to 70·1)	70·2 (69·9 to 70·6)	71 (70·6 to 71·4)	74·7 (74·3 to 75)	76·9 (76·5 to 77·2)	77·6 (77·3 to 78)	79 (78·7 to 79·4)	78·4 (76·7 to 80·1)
Maryland	67·2 (65·3 to 69·2)	68·8 (68·6 to 69)	70·4 (70·2 to 70·6)	73·6 (73·4 to 73·8)	75·1 (74·9 to 75·3)	76·3 (76·1 to 76·5)	79·1 (78·9 to 79·3)	78·7 (77 to 80·5)
Massachusetts	69·5 (67·5 to 71·5)	70·9 (70·7 to 71)	72·1 (71·9 to 72·3)	75·1 (74·9 to 75·3)	77·1 (76·9 to 77·3)	78·5 (78·3 to 78·6)	80·7 (80·5 to 80·9)	80·4 (78·7 to 82·1)
Michigan	68·9 (66·9 to 70·9)	70·3 (70·2 to 70·5)	70·9 (70·7 to 71)	73·9 (73·8 to 74·1)	75·4 (75·3 to 75·6)	76·6 (76·4 to 76·7)	78·1 (77·9 to 78·2)	77·9 (76·2 to 79·8)
Minnesota	70·6 (68·5 to 72·7)	72·1 (71·9 to 72·3)	73·2 (73 to 73·4)	76·3 (76 to 76·5)	78 (77·8 to 78·3)	79 (78·8 to 79·2)	80·9 (80·7 to 81·1)	81 (79·3 to 82·7)
Mississippi	65 (62·9 to 67·4)	67·8 (67·5 to 68·1)	68·5 (68·2 to 68·7)	72 (71·7 to 72·2)	73·5 (73·3 to 73·8)	73·6 (73·4 to 73·9)	74·9 (74·7 to 75·2)	74·9 (73·1 to 76·7)
Missouri	68·9 (67 to 71)	70·5 (70·3 to 70·7)	70·9 (70·7 to 71·1)	73·9 (73·7 to 74·1)	75·6 (75·4 to 75·8)	76·2 (76·1 to 76·4)	77·5 (77·4 to 77·7)	77·5 (75·6 to 79·4)
Montana	67·4 (65·3 to 69·5)	69·7 (69·4 to 70·1)	71 (70·6 to 71·4)	74·1 (73·7 to 74·4)	76·7 (76·3 to 77·1)	77·3 (76·9 to 77·7)	78·6 (78·3 to 79)	78·4 (76·7 to 80·1)
Nebraska	70·7 (68·6 to 72·8)	72·2 (71·8 to 72·5)	72·7 (72·4 to 73)	75·5 (75·2 to 75·8)	77·3 (77 to 77·6)	78·5 (78·2 to 78·8)	79·8 (79·4 to 80·1)	79·9 (78·5 to 81·5)
Nevada	63·2 (61·2 to 65·2)	67·1 (66·7 to 67·6)	69·1 (68·7 to 69·5)	72 (71·6 to 72·3)	74 (73·7 to 74·3)	75·1 (74·9 to 75·4)	77·5 (77·3 to 77·8)	78 (76·3 to 79·6)
New Hampshire	68·2 (66·2 to 70·2)	70·9 (70·5 to 71·3)	71·5 (71·1 to 71·9)	75·3 (75 to 75·7)	77 (76·7 to 77·4)	78·2 (77·9 to 78·5)	80·1 (79·8 to 80·4)	79·1 (77·4 to 80·8)
New Jersey	68·5 (66·4 to 70·5)	70·1 (70 to 70·3)	71·1 (71 to 71·3)	74·1 (73·9 to 74·3)	75·8 (75·7 to 76)	77·6 (77·4 to 77·8)	80·2 (80 to 80·4)	80·3 (78·6 to 82)
New Mexico	66·7 (64·5 to 68·8)	69·5 (69·2 to 69·9)	70·6 (70·2 to 71)	73·9 (73·6 to 74·3)	76·1 (75·8 to 76·4)	77·3 (77 to 77·5)	78·4 (78·1 to 78·7)	77·9 (76 to 79·6)
New York	68·4 (66·5 to 70·2)	69·9 (69·8 to 70)	70·7 (70·6 to 70·8)	73·7 (73·6 to 73·8)	75·1 (75 to 75·2)	78 (77·9 to 78·1)	80·6 (80·4 to 80·7)	81 (79·3 to 82·7)
North Carolina	66·6 (64·4 to 68·9)	68·4 (68·2 to 68·6)	69·4 (69·2 to 69·6)	73·1 (72·9 to 73·3)	74·8 (74·7 to 75)	75·7 (75·5 to 75·9)	77·8 (77·7 to 78)	77·9 (76 to 79·6)
North Dakota	69·9 (68 to 71·7)	71·9 (71·5 to 72·2)	72·9 (72·5 to 73·2)	75·8 (75·4 to 76·1)	77·8 (77·4 to 78·1)	78·7 (78·3 to 79)	79·8 (79·5 to 80·2)	79·4 (78 to 80·7)
Ohio	68·8 (66·9 to 70·8)	70·4 (70·3 to 70·5)	71 (70·9 to 71·1)	73·6 (73·4 to 73·7)	75·6 (75·5 to 75·8)	76·5 (76·4 to 76·7)	77·8 (77·6 to 77·9)	77 (75·4 to 78·7)
Oklahoma	69·1 (67 to 71·3)	70·9 (70·7 to 71·2)	71·3 (71 to 71·6)	73·8 (73·5 to 74)	75·5 (75·2 to 75·7)	75·2 (75 to 75·5)	75·9 (75·7 to 76·1)	75·9 (74·1 to 77·9)
Oregon	69·4 (67·3 to 71·5)	71·1 (70·9 to 71·4)	72·4 (72·2 to 72·7)	75 (74·8 to 75·3)	76·6 (76·4 to 76·9)	78 (77·7 to 78·2)	79·5 (79·2 to 79·7)	79·7 (78 to 81·4)
Pennsylvania	68·1 (66·2 to 70)	69·7 (69·6 to 69·9)	70·6 (70·5 to 70·7)	73·7 (73·5 to 73·8)	75·7 (75·6 to 75·9)	76·9 (76·8 to 77)	78·6 (78·5 to 78·8)	78·3 (76·5 to 80·2)
Rhode Island	68·9 (67 to 70·9)	70·9 (70·5 to 71·2)	71·8 (71·5 to 72·2)	74·8 (74·5 to 75·2)	76·7 (76·4 to 77·1)	78·2 (77·9 to 78·6)	79·7 (79·3 to 80·1)	79·7 (78·1 to 81·3)
South Carolina	64·1 (62·1 to 66·5)	66·5 (66·2 to 66·7)	68·3 (68·1 to 68·5)	71·9 (71·7 to 72·1)	74 (73·7 to 74·2)	74·9 (74·7 to 75·1)	76·7 (76·5 to 76·9)	76·9 (75 to 78·7)
South Dakota	69·1 (67·1 to 71·2)	71 (70·7 to 71·4)	72·2 (71·8 to 72·5)	74·9 (74·6 to 75·3)	77·2 (76·9 to 77·6)	78·1 (77·8 to 78·5)	79·3 (79 to 79·7)	78·8 (77·3 to 80·2)
Tennessee	67·7 (65·6 to 69·9)	69·7 (69·5 to 69·9)	70·1 (69·9 to 70·3)	73·3 (73·1 to 73·5)	74·5 (74·3 to 74·7)	75 (74·8 to 75·1)	76·2 (76·1 to 76·4)	76·2 (74·2 to 78)
Texas	68·6 (66·4 to 70·8)	70·5 (70·4 to 70·7)	71·1 (71 to 71·2)	73·7 (73·6 to 73·8)	75·5 (75·4 to 75·6)	76·7 (76·6 to 76·8)	78·5 (78·4 to 78·5)	78·9 (77·3 to 80·6)
Utah	70 (67·8 to 72)	71·8 (71·4 to 72·2)	72·9 (72·5 to 73·2)	75·8 (75·5 to 76·1)	78 (77·7 to 78·3)	78·7 (78·4 to 78·9)	79·8 (79·6 to 80·1)	79·7 (78·1 to 81·2)
Vermont	67·8 (66·1 to 69·6)	70·6 (70·2 to 70·9)	71·9 (71·5 to 72·2)	74·8 (74·5 to 75·2)	76·7 (76·3 to 77)	78·2 (77·9 to 78·5)	80·3 (80 to 80·7)	79·6 (78·5 to 80·8)
Virginia	67 (65 to 69·3)	69 (68·9 to 69·2)	70·4 (70·2 to 70·6)	73·5 (73·3 to 73·7)	75·5 (75·3 to 75·7)	76·8 (76·7 to 77)	79·1 (78·9 to 79·2)	79·3 (77·6 to 81)

Washington	69·4 (67·3 to 71·6)	71·3 (71·1 to 71·6)	71·9 (71·7 to 72·1)	75·2 (75 to 75·4)	77 (76·8 to 77·2)	78·2 (78·1 to 78·4)	80 (79·8 to 80·2)	80·4 (78·8 to 82·1)
West Virginia	67·6 (65·5 to 69·8)	69·9 (69·6 to 70·1)	69·3 (69·1 to 69·6)	72·8 (72·5 to 73·1)	74·9 (74·6 to 75·2)	75·1 (74·8 to 75·4)	75·7 (75·4 to 76)	74·9 (72·9 to 76·9)
Wisconsin	69·9 (67·9 to 72)	71·3 (71·1 to 71·5)	72·6 (72·5 to 72·8)	75·5 (75·3 to 75·7)	77 (76·8 to 77·2)	78 (77·8 to 78·2)	79·7 (79·5 to 79·9)	79·4 (77·7 to 81·1)
Wyoming	67·3 (65·3 to 69·1)	69·9 (69·5 to 70·4)	70·8 (70·4 to 71·2)	73·9 (73·5 to 74·2)	76·4 (76 to 76·7)	77·1 (76·8 to 77·5)	78·3 (77·9 to 78·7)	78·8 (77·5 to 80)
Southern Latin America	59·9 (59·8 to 59·9)	62·2 (62·1 to 62·3)	65·3 (65·2 to 65·3)	70·1 (70 to 70·1)	72·8 (72·7 to 72·8)	74·9 (74·9 to 75)	76·7 (76·6 to 76·7)	77·6 (77·3 to 77·8)
Argentina	63·3 (63·2 to 63·4)	65·3 (65·2 to 65·3)	66·1 (66·1 to 66·2)	70·2 (70·2 to 70·3)	72·5 (72·4 to 72·5)	74·2 (74·2 to 74·3)	75·9 (75·9 to 76)	76·6 (76·3 to 76·8)
Chile	50·3 (50·1 to 50·4)	54·2 (54 to 54·4)	62 (61·9 to 62·1)	69·4 (69·3 to 69·5)	73·4 (73·3 to 73·5)	76·9 (76·9 to 77)	78·6 (78·5 to 78·7)	80·2 (79·8 to 80·5)
Uruguay	65·1 (64·9 to 65·3)	68·3 (68·2 to 68·5)	68·5 (68·3 to 68·6)	70·6 (70·4 to 70·8)	73·2 (73 to 73·3)	74·8 (74·6 to 74·9)	76·5 (76·2 to 76·8)	77·5 (77·1 to 77·9)
Western Europe	66·5 (66·4 to 66·6)	70·2 (70·2 to 70·2)	71·7 (71·7 to 71·7)	74·1 (74·1 to 74·1)	76·4 (76·4 to 76·4)	78·7 (78·7 to 78·7)	81·2 (81·1 to 81·2)	82·1 (82 to 82·2)
Andorra	74·6 (71·7 to 77·6)	75·7 (73·4 to 78·4)	77 (74·6 to 79·5)	78·3 (75·6 to 80·6)	79 (76·3 to 81·2)	80·8 (78·5 to 82·8)	81·8 (79·8 to 83·8)	82·2 (79·9 to 84·5)
Austria	64·8 (64·7 to 64·9)	69 (68·9 to 69·1)	70·2 (70·1 to 70·3)	72·8 (72·8 to 72·9)	76 (75·9 to 76·1)	78·5 (78·4 to 78·6)	80·8 (80·7 to 80·9)	82·2 (82 to 82·3)
Belgium	66·6 (66·5 to 66·7)	70·1 (70 to 70·2)	71·1 (71 to 71·2)	73·3 (73·3 to 73·4)	76·1 (76·1 to 76·2)	78 (77·9 to 78·1)	80·2 (80·1 to 80·3)	81·4 (81·2 to 81·6)
Cyprus	62·2 (58·4 to 65·5)	65 (62 to 67·6)	67·6 (66 to 69·2)	71·3 (71 to 71·5)	75·1 (74·9 to 75·3)	76·7 (76·4 to 76·9)	79·5 (79·3 to 79·8)	80·8 (80 to 81·6)
Denmark	70·7 (70·5 to 70·8)	72·5 (72·4 to 72·7)	73·6 (73·5 to 73·7)	74·4 (74·3 to 74·5)	75·2 (75·1 to 75·3)	77·2 (77·1 to 77·3)	79·5 (79·4 to 79·7)	81·1 (80·8 to 81·4)
Finland	64·5 (64·4 to 64·6)	69·2 (69·1 to 69·3)	70·7 (70·5 to 70·8)	74 (73·9 to 74·1)	75·4 (75·3 to 75·6)	78 (77·9 to 78·2)	80·4 (80·2 to 80·5)	81·9 (81·5 to 82·2)
France	67·1 (67·1 to 67·2)	71·2 (71·1 to 71·2)	72·6 (72·6 to 72·6)	74·7 (74·6 to 74·7)	77·2 (77·2 to 77·3)	79·2 (79·1 to 79·2)	81·6 (81·5 to 81·6)	82·9 (82·7 to 83·1)
Germany	66·5 (66·1 to 66·9)	69·4 (69·4 to 69·4)	70·6 (70·6 to 70·6)	73·3 (73·2 to 73·3)	75·7 (75·6 to 75·7)	78·4 (78·4 to 78·4)	80·5 (80·5 to 80·5)	81·2 (81 to 81·4)
Greece	69 (68·7 to 69·3)	72·4 (72·3 to 72·5)	73·8 (73·7 to 73·9)	75·3 (75·2 to 75·4)	77·2 (77·1 to 77·3)	78·4 (78·4 to 78·5)	80·4 (80·3 to 80·5)	80·9 (80·7 to 81·2)
Iceland	71·5 (70·7 to 72·2)	73·4 (73 to 73·7)	73·9 (73·5 to 74·4)	76·5 (76·1 to 76·9)	78·1 (77·7 to 78·5)	80·4 (79·9 to 80·8)	82·6 (82·1 to 83·2)	84·1 (83·2 to 84·9)
Ireland	66 (65·8 to 66·1)	70·1 (70 to 70·3)	71·2 (71·1 to 71·4)	72·7 (72·6 to 72·8)	75 (74·9 to 75·1)	76·8 (76·6 to 76·9)	80·8 (80·7 to 80·9)	82 (81·7 to 82·4)
Israel	73 (72·8 to 73·2)	71·8 (71·7 to 72)	71·7 (71·6 to 71·8)	74·2 (74 to 74·3)	77·3 (77·2 to 77·4)	78·9 (78·8 to 79)	81·9 (81·8 to 82)	83 (82·7 to 83·2)
Italy	65·9 (65·8 to 66)	69·9 (69·8 to 69·9)	71·8 (71·8 to 71·9)	74·2 (74·2 to 74·2)	77·2 (77·1 to 77·2)	79·6 (79·6 to 79·7)	82·1 (82 to 82·1)	83·1 (83 to 83·2)
Luxembourg	66·4 (65·8 to 67)	68·9 (68·5 to 69·4)	70·1 (69·8 to 70·5)	72·6 (72·3 to 72·9)	75·2 (75 to 75·5)	78 (77·6 to 78·4)	81 (80·6 to 81·4)	82·9 (81·8 to 83·9)
Malta	63·2 (62·7 to 63·8)	69·1 (68·8 to 69·4)	70·9 (70·6 to 71·2)	72·7 (72·4 to 73)	76·6 (76·2 to 77)	78·8 (78·5 to 79·2)	81·1 (80·6 to 81·5)	82·6 (81·6 to 83·5)
Monaco	68·1 (66·6 to 69·6)	70·9 (69·7 to 72)	73·6 (72·2 to 75·1)	76 (74·7 to 77·5)	78·1 (76·3 to 80·3)	78·8 (76·9 to 81·4)	79·2 (77·9 to 80·8)	80·1 (78·3 to 82·1)
Netherlands	71·6 (71·4 to 71·7)	73·5 (73·4 to 73·6)	73·7 (73·7 to 73·8)	75·9 (75·8 to 76)	77·2 (77·1 to 77·2)	78·3 (78·2 to 78·4)	80·9 (80·9 to 81)	81·7 (81·5 to 82)
Norway	71·9 (71·8 to 72·1)	73·4 (73·3 to 73·5)	74·3 (74·1 to 74·4)	76 (75·8 to 76·1)	76·9 (76·7 to 77)	78·9 (78·8 to 79)	81·3 (81·2 to 81·4)	82·9 (82·7 to 83·1)
Portugal	59·3 (59·1 to 59·4)	64·7 (64·6 to 64·8)	67·4 (67·3 to 67·5)	71·7 (71·6 to 71·8)	74·3 (74·2 to 74·4)	76·8 (76·8 to 76·9)	80·2 (80·1 to 80·3)	81·7 (81·5 to 82)
San Marino	72 (67·8 to 76)	72·9 (70·7 to 75·2)	74·1 (73·3 to 75·1)	76·4 (75·5 to 77·4)	79·7 (78·4 to 81)	81·5 (80·4 to 82·7)	81·9 (79·1 to 85·2)	82·2 (78·5 to 85·8)
Spain	61·8 (61·7 to 61·9)	69·5 (69·4 to 69·5)	72 (72 to 72·1)	75·5 (75·5 to 75·6)	77 (76·9 to 77)	79·5 (79·4 to 79·5)	82·1 (82 to 82·1)	83·1 (82·9 to 83·3)
Sweden	71·4 (71·3 to 71·5)	73·4 (73·3 to 73·5)	74·8 (74·7 to 74·9)	76·1 (76 to 76·2)	77·9 (77·9 to 78)	80 (79·9 to 80·1)	81·9 (81·8 to 81·9)	82·8 (82·7 to 83)
Stockholm	71·1 (69·1 to 73·3)	73 (71 to 75·2)	74·3 (72·1 to 76·4)	75·4 (75·1 to 75·7)	77·1 (76·8 to 77·4)	79·7 (79·5 to 80)	81·7 (81·5 to 82)	83 (82·1 to 83·9)
Sweden except Stockholm	71·4 (71 to 71·8)	73·5 (73·1 to 73·9)	75 (74·6 to 75·4)	76·2 (76·1 to 76·3)	78·1 (78 to 78·2)	80·1 (80 to 80·2)	81·9 (81·8 to 82)	82·8 (82·5 to 83)
Switzerland	69 (68·9 to 69·1)	71·8 (71·7 to 71·9)	73·3 (73·2 to 73·4)	76 (75·9 to 76·1)	77·9 (77·8 to 78)	80·3 (80·2 to 80·4)	82·8 (82·7 to 82·9)	84 (83·7 to 84·2)
UK	68·9 (68·8 to 68·9)	71·2 (71·1 to 71·2)	72·1 (72 to 72·1)	73·8 (73·7 to 73·8)	75·8 (75·8 to 75·9)	78 (77·9 to 78)	80·6 (80·5 to 80·6)	81·1 (81 to 81·2)
England	69·3 (69·3 to 69·4)	71·5 (71·5 to 71·6)	72·4 (72·3 to 72·4)	74·1 (74 to 74·1)	76·1 (76 to 76·1)	78·2 (78·2 to 78·3)	80·9 (80·8 to 80·9)	81·4 (81·3 to 81·4)
East Midlands	69·2 (67·3 to 71·1)	71·4 (69·6 to 73·2)	72·2 (70·5 to 74·1)	74 (73·4 to 74·6)	76·2 (76 to 76·4)	78·1 (78 to 78·3)	80·7 (80·5 to 80·9)	81·1 (80·3 to 81·8)

Derby	68·3 (65·8 to 71·1)	70·6 (68·2 to 72·9)	71·3 (68·8 to 73·8)	73·1 (72·3 to 74)	75·3 (74·9 to 75·8)	77·9 (77·5 to 78·3)	79·6 (79·2 to 80)	80·8 (79·7 to 81·9)
Derbyshire	69·2 (66·6 to 71·8)	71·4 (68·9 to 73·8)	72·1 (69·8 to 74·4)	73·8 (73·2 to 74·5)	76 (75·7 to 76·3)	78·2 (77·9 to 78·5)	81 (80·7 to 81·4)	81·3 (80·4 to 82·3)
Leicester	67·7 (65·2 to 70·2)	70 (67·6 to 72·4)	70·8 (68·5 to 73·3)	72·8 (72 to 73·6)	74·9 (74·5 to 75·3)	76·4 (76 to 76·8)	79·1 (78·7 to 79·5)	80·2 (79·1 to 81·3)
Leicestershire	70·7 (67·9 to 73·3)	72·8 (70·3 to 75·2)	73·6 (71·2 to 75·9)	75·2 (74·5 to 75·9)	77·4 (77·1 to 77·8)	79·4 (79·1 to 79·7)	81·8 (81·4 to 82·1)	82·5 (81·5 to 83·4)
Lincolnshire	69·4 (66·9 to 72)	71·6 (69·2 to 74·2)	72·5 (70·1 to 74·9)	74·2 (73·5 to 74·9)	76·4 (76 to 76·7)	78·2 (77·9 to 78·6)	81 (80·6 to 81·3)	80·8 (79·8 to 81·8)
Northamptonshire	69·7 (67·1 to 72·2)	71·9 (69·5 to 74·4)	72·8 (70·5 to 75·2)	74·5 (73·8 to 75·3)	76·4 (76 to 76·7)	78·5 (78·1 to 78·8)	80·7 (80·3 to 81)	80·9 (79·9 to 81·9)
Nottingham	67·7 (65·2 to 70·5)	69·9 (67·5 to 72·3)	70·6 (68·4 to 73)	72·5 (71·7 to 73·3)	74·4 (74·1 to 74·8)	75·1 (74·7 to 75·5)	78·6 (78·2 to 79)	79·7 (78·5 to 80·8)
Nottinghamshire	69·4 (67 to 72·2)	71·6 (69·1 to 74·1)	72·4 (70·1 to 74·8)	74·1 (73·4 to 74·9)	76·5 (76·2 to 76·8)	78·3 (78 to 78·6)	80·8 (80·5 to 81·1)	80·9 (80 to 81·9)
Rutland	71·6 (68·9 to 74·3)	73·8 (71·4 to 76·3)	74·6 (72·1 to 77·2)	76 (74·8 to 77·2)	77·6 (76·9 to 78·4)	80·2 (79·4 to 81)	82·6 (81·8 to 83·4)	83·5 (82·1 to 84·8)
East of England	70·9 (69 to 72·8)	73 (71·1 to 74·7)	73·7 (72 to 75·5)	75·1 (74·6 to 75·6)	77·1 (77 to 77·3)	79·1 (79 to 79·3)	81·6 (81·4 to 81·8)	82 (81·3 to 82·8)
Bedford	70·1 (67·4 to 72·8)	72·3 (69·7 to 75)	73·1 (70·7 to 75·5)	74·8 (73·9 to 75·8)	76·7 (76·2 to 77·2)	78·3 (77·8 to 78·8)	80·6 (80 to 81·1)	80·8 (79·7 to 82)
Cambridgeshire	71·7 (69·1 to 74·1)	73·7 (71·2 to 76·1)	74·4 (72·1 to 76·8)	75·6 (74·8 to 76·3)	77·6 (77·2 to 77·9)	79·4 (79·1 to 79·8)	82·4 (82·1 to 82·8)	82·8 (81·8 to 83·8)
Central Bedfordshire	70·8 (68·1 to 73·5)	72·9 (70·2 to 75·3)	73·7 (71·3 to 76·1)	75·3 (74·5 to 76·1)	77·1 (76·7 to 77·6)	79·5 (79·1 to 79·9)	82 (81·5 to 82·4)	83·1 (82 to 84·2)
Essex	71·1 (68·9 to 73·5)	73 (70·9 to 75·4)	73·6 (71·3 to 75·9)	74·9 (74·2 to 75·5)	77·2 (76·9 to 77·5)	79·4 (79·1 to 79·6)	81·5 (81·3 to 81·8)	81·9 (81·1 to 82·9)
Hertfordshire	71·6 (69·1 to 74)	73·5 (71·1 to 75·8)	74 (71·7 to 76·3)	75 (74·4 to 75·7)	77·3 (77 to 77·6)	79·2 (78·9 to 79·5)	81·9 (81·6 to 82·2)	82·7 (81·8 to 83·7)
Luton	69·3 (66·8 to 71·9)	71·4 (68·7 to 74·1)	72·2 (69·9 to 74·7)	73·9 (73·1 to 74·8)	75·6 (75·2 to 76·1)	77·3 (76·8 to 77·8)	80 (79·5 to 80·5)	80·1 (79 to 81·2)
Norfolk	70·7 (68·1 to 73·2)	72·9 (70·5 to 75·5)	74 (71·7 to 76·2)	75·6 (74·9 to 76·4)	77·2 (76·9 to 77·5)	79·3 (78·9 to 79·6)	81·4 (81·1 to 81·7)	81·8 (80·9 to 82·8)
Peterborough	69·6 (66·8 to 72·5)	71·7 (69·2 to 74·4)	72·3 (70 to 74·8)	74·3 (73·3 to 75·2)	75·9 (75·4 to 76·3)	77·7 (77·3 to 78·2)	79·9 (79·4 to 80·4)	80·5 (79·4 to 81·6)
Southend-on-Sea	69·6 (67 to 72·3)	72 (69·3 to 74·5)	73 (70·7 to 75·5)	74·9 (74 to 75·8)	76·5 (76 to 77)	78·2 (77·7 to 78·7)	80·7 (80·2 to 81·2)	80·8 (79·7 to 82)
Suffolk	70·9 (68·3 to 73·5)	73·1 (70·6 to 75·5)	74 (71·7 to 76·3)	75·6 (74·9 to 76·3)	77·6 (77·2 to 77·9)	79·4 (79·1 to 79·8)	82 (81·7 to 82·3)	82 (81 to 83)
Thurrock	69·6 (66·9 to 72·3)	71·5 (68·9 to 74·2)	72·1 (69·8 to 74·7)	73·8 (72·9 to 74·7)	76·1 (75·5 to 76·5)	78·7 (78·2 to 79·1)	81·1 (80·6 to 81·5)	81·9 (80·7 to 83)
Greater London	69·5 (67·8 to 71·3)	71·7 (70·1 to 73·5)	72·5 (70·9 to 74·2)	74·2 (73·7 to 74·7)	76 (75·8 to 76·1)	78 (77·9 to 78·2)	81·5 (81·4 to 81·7)	82·4 (81·6 to 83·2)
Barking and Dagenham	67·6 (64·9 to 70·2)	69·8 (67·5 to 72·3)	70·8 (68·5 to 73·1)	73 (72·1 to 73·8)	75·3 (74·8 to 75·7)	77 (76·6 to 77·5)	79·3 (78·9 to 79·8)	79·7 (78·5 to 80·9)
Barnet	71·2 (68·9 to 73·8)	73·4 (71·1 to 75·8)	74·4 (72·2 to 76·6)	76·2 (75·4 to 77)	77·8 (77·5 to 78·2)	79·9 (79·5 to 80·3)	82·4 (82 to 82·8)	83·8 (82·7 to 84·8)
Bexley	71·2 (68·9 to 73·6)	73·3 (71 to 75·7)	74·1 (72 to 76·4)	76 (75·2 to 76·8)	77·9 (77·5 to 78·3)	79·8 (79·4 to 80·2)	82 (81·6 to 82·5)	82·8 (81·7 to 83·8)
Brent	69·3 (66·9 to 71·8)	71·6 (69·2 to 74·1)	72·5 (70·3 to 75)	74·2 (73·5 to 75)	76 (75·6 to 76·4)	77·9 (77·5 to 78·3)	82·2 (81·8 to 82·6)	82·3 (81·3 to 83·3)
Bromley	71·7 (69·3 to 74·2)	73·7 (71·4 to 76·3)	74·5 (72·3 to 76·6)	76·2 (75·4 to 77)	78 (77·6 to 78·4)	79·9 (79·5 to 80·3)	83 (82·6 to 83·4)	83·8 (82·8 to 84·8)
Camden	69 (66·5 to 71·5)	71·1 (68·8 to 73·5)	71·6 (69·4 to 74)	72·6 (71·8 to 73·4)	74·6 (74·2 to 75·1)	75·8 (75·4 to 76·2)	80·6 (80·1 to 81)	82·1 (81·1 to 83·2)
Croydon	70·6 (68·2 to 73)	72·7 (70·4 to 75·3)	73·5 (71·4 to 75·8)	75·1 (74·3 to 75·8)	76·7 (76·3 to 77·1)	78·7 (78·3 to 79)	81·5 (81·1 to 81·8)	81·6 (80·5 to 82·6)
Ealing	69·5 (67·1 to 72·1)	71·6 (69·2 to 74·2)	72·3 (70·1 to 74·6)	73·8 (73 to 74·6)	75·9 (75·6 to 76·3)	78·1 (77·8 to 78·5)	81·9 (81·5 to 82·3)	82·9 (81·8 to 84)
Enfield	70·5 (68 to 73)	72·6 (70·2 to 75·1)	73·5 (71·4 to 75·9)	75·3 (74·4 to 76)	77·3 (76·9 to 77·7)	78·8 (78·4 to 79·1)	82·3 (81·9 to 82·7)	82·6 (81·5 to 83·7)
Greenwich	68·3 (65·8 to 70·8)	70·7 (68·5 to 73·1)	71·7 (69·6 to 74·1)	73·8 (72·9 to 74·5)	75·5 (75·1 to 75·9)	76·8 (76·3 to 77·2)	80·4 (80 to 80·9)	81·4 (80·2 to 82·5)
Hackney	68·6 (66·2 to 71·2)	70·6 (68·2 to 73·1)	70·9 (68·5 to 73·2)	72 (71·2 to 72·8)	73·7 (73·2 to 74·1)	76·3 (75·8 to 76·7)	79·6 (79·2 to 80)	80·3 (79·2 to 81·4)
Hammersmith and Fulham	68·6 (66·2 to 71·3)	70·8 (68·5 to 73·4)	71·3 (68·9 to 73·7)	72·4 (71·5 to 73·2)	73·5 (73·1 to 73·9)	76·5 (76·1 to 76·9)	80·7 (80·3 to 81·1)	82·4 (81·4 to 83·6)
Haringey	68·9 (66·4 to 71·4)	71·3 (69·1 to 73·8)	72·1 (70 to 74·3)	73·7 (72·8 to 74·5)	75·8 (75·4 to 76·2)	77 (76·6 to 77·4)	80·2 (79·7 to 80·6)	82·1 (81 to 83·2)
Harrow	71·4 (69 to 73·9)	73·4 (71·1 to 75·8)	74·2 (72 to 76·5)	76 (75·2 to 76·8)	78·1 (77·7 to 78·6)	80 (79·6 to 80·5)	83·9 (83·4 to 84·4)	84 (83 to 85·1)
Havering	71·1 (68·5 to 73·5)	73 (70·7 to 75·4)	73·8 (71·6 to 76·1)	75·5 (74·7 to 76·3)	77·5 (77·1 to 77·9)	79·6 (79·2 to 80·1)	81·9 (81·5 to 82·4)	82·5 (81·4 to 83·6)

Hillingdon	70·8 (68·3 to 73·2)	72·8 (70·4 to 75·3)	73·5 (71·2 to 75·9)	75·1 (74·3 to 76)	77 (76·6 to 77·4)	78·4 (78 to 78·8)	81·5 (81 to 81·9)	82·3 (81·2 to 83·4)
Hounslow	69·9 (67·5 to 72·5)	72 (69·7 to 74·4)	72·8 (70·6 to 75·1)	74·5 (73·6 to 75·2)	76 (75·6 to 76·4)	77·8 (77·4 to 78·2)	81·5 (81 to 81·9)	82·7 (81·6 to 83·8)
Islington	68·5 (66·2 to 71·1)	70·7 (68·1 to 73·4)	71·3 (69·1 to 73·6)	72·6 (71·7 to 73·5)	74·1 (73·7 to 74·5)	75·4 (75 to 75·8)	79·4 (79 to 79·8)	81·1 (80·1 to 82·2)
Kensington and Chelsea	69·6 (67 to 72·1)	71·7 (69·3 to 74)	72·2 (70·1 to 74·5)	73·1 (72·3 to 74)	75·1 (74·6 to 75·5)	79·6 (79·2 to 80·1)	83·1 (82·6 to 83·6)	83·3 (82·2 to 84·4)
Kingston upon Thames	71·4 (68·9 to 73·9)	73·5 (71·3 to 76)	74·3 (72·1 to 76·7)	76 (75·1 to 76·8)	77·7 (77·3 to 78·2)	79·9 (79·4 to 80·4)	83·7 (83·3 to 84·2)	83·9 (82·8 to 85)
Lambeth	67·2 (64·8 to 69·8)	69·6 (67·4 to 72)	70·2 (67·8 to 72·5)	72·2 (71·4 to 73)	74 (73·6 to 74·4)	75·8 (75·5 to 76·2)	79·5 (79·1 to 79·9)	81·5 (80·4 to 82·6)
Lewisham	68·7 (66·5 to 71·2)	71·1 (68·7 to 73·6)	72 (69·8 to 74·4)	73·9 (73·1 to 74·7)	75·1 (74·7 to 75·5)	76·3 (75·9 to 76·7)	80 (79·5 to 80·4)	81 (79·9 to 82·1)
Merton	70·3 (68 to 72·9)	72·5 (70·1 to 74·8)	73·4 (71·2 to 75·6)	75·2 (74·3 to 76·1)	76·9 (76·4 to 77·3)	79·4 (78·9 to 79·8)	82·4 (82 to 82·9)	82·9 (81·7 to 84)
Newham	68 (65·4 to 70·5)	70·3 (67·9 to 72·8)	71·1 (69 to 73·5)	73 (72·1 to 73·8)	74·6 (74·2 to 75)	75·5 (75 to 75·9)	79·6 (79·2 to 80)	81 (79·8 to 82·1)
Redbridge	70·8 (68·2 to 73·3)	72·9 (70·6 to 75·4)	73·7 (71·5 to 76)	75·6 (74·8 to 76·4)	77·5 (77·1 to 77·9)	79·5 (79·1 to 80)	82·4 (82 to 82·8)	83·4 (82·4 to 84·5)
Richmond upon Thames	71·1 (68·7 to 73·7)	73·1 (70·9 to 75·4)	73·7 (71·6 to 75·9)	75·2 (74·4 to 76·1)	77·9 (77·4 to 78·3)	80·4 (79·9 to 80·8)	84·1 (83·6 to 84·6)	84·5 (83·5 to 85·6)
Southwark	68·8 (66·4 to 71·4)	70·9 (68·7 to 73·3)	71·5 (69·4 to 73·7)	72·9 (72·1 to 73·8)	72·9 (72·5 to 73·3)	76·3 (75·9 to 76·7)	79·8 (79·4 to 80·2)	81·2 (80·2 to 82·3)
Sutton	70·8 (68·3 to 73·4)	73·1 (70·9 to 75·5)	74·2 (72·1 to 76·4)	76·1 (75·2 to 77)	77·5 (77·1 to 78)	79·3 (78·8 to 79·7)	82·6 (82 to 83·1)	82·7 (81·5 to 83·9)
Tower Hamlets	67·2 (64·9 to 69·9)	69·2 (66·9 to 71·9)	69·6 (67·2 to 71·9)	71 (70·2 to 71·9)	73·2 (72·8 to 73·7)	75 (74·6 to 75·4)	79·4 (79 to 79·8)	80·6 (79·5 to 81·7)
Waltham Forest	68·9 (66·5 to 71·3)	71·2 (68·8 to 73·6)	72·3 (70·1 to 74·5)	74·2 (73·4 to 75)	76·2 (75·8 to 76·6)	77·4 (77 to 77·8)	80·8 (80·4 to 81·2)	81·9 (80·8 to 83)
Wandsworth	69·1 (66·7 to 71·4)	71·2 (68·9 to 73·6)	71·8 (69·7 to 74·1)	73·4 (72·6 to 74·2)	74·7 (74·4 to 75·1)	77·5 (77·1 to 77·8)	81·6 (81·3 to 82)	82·8 (81·8 to 83·8)
Westminster	69 (66·4 to 71·6)	71·2 (68·9 to 73·6)	71·7 (69·5 to 73·9)	72·6 (71·7 to 73·5)	75 (74·6 to 75·4)	78·2 (77·7 to 78·6)	82·5 (82 to 82·9)	83 (82 to 84·1)
North East England	67·9 (66 to 69·7)	70·1 (68·3 to 72)	70·9 (69·1 to 72·8)	72·8 (72·2 to 73·4)	74·6 (74·4 to 74·8)	76·9 (76·7 to 77·1)	79·5 (79·3 to 79·8)	79·7 (78·8 to 80·6)
County Durham	67·8 (65·5 to 70·1)	70·1 (67·6 to 72·7)	70·9 (68·6 to 73·4)	72·8 (72·1 to 73·5)	74·7 (74·3 to 75)	76·9 (76·6 to 77·2)	79·4 (79 to 79·7)	79·6 (78·6 to 80·7)
Darlington	67·9 (65·2 to 70·6)	70·2 (67·6 to 72·7)	71 (68·6 to 73·4)	72·9 (72 to 73·9)	74·8 (74·2 to 75·3)	76·9 (76·3 to 77·5)	79·7 (79·1 to 80·2)	80·2 (78·9 to 81·5)
Gateshead	67·3 (64·6 to 70·1)	69·6 (67·2 to 72·1)	70·3 (67·9 to 72·8)	72·3 (71·5 to 73·1)	74·5 (74·1 to 74·9)	76·3 (75·9 to 76·8)	79·6 (79·2 to 80·1)	79·5 (78·4 to 80·6)
Hartlepool	66·5 (63·7 to 69·3)	68·9 (66·2 to 71·6)	69·7 (67·4 to 72·2)	71·7 (70·9 to 72·7)	74 (73·4 to 74·5)	75·9 (75·3 to 76·4)	78·7 (78·1 to 79·3)	78·5 (77·2 to 79·7)
Middlesbrough	66·6 (64 to 69·1)	68·9 (66·4 to 71·8)	69·6 (67·2 to 72)	71·6 (70·7 to 72·4)	73·5 (73 to 73·9)	75·4 (74·9 to 75·9)	78·1 (77·6 to 78·6)	77·7 (76·5 to 79·1)
Newcastle upon Tyne	68 (65·6 to 70·4)	70·1 (67·8 to 72·8)	70·9 (68·7 to 73·3)	72·7 (72 to 73·6)	73·9 (73·5 to 74·2)	76·4 (76 to 76·8)	79·3 (78·9 to 79·7)	79·6 (78·5 to 80·8)
North Tyneside	68·6 (66·1 to 71·3)	70·8 (68·5 to 73·3)	71·6 (69·4 to 74)	73·4 (72·6 to 74·3)	75 (74·6 to 75·4)	77·3 (76·8 to 77·7)	79·8 (79·3 to 80·3)	80·5 (79·3 to 81·7)
Northumberland	68·7 (66·1 to 71·3)	70·9 (68·4 to 73·6)	71·8 (69·4 to 74·1)	73·3 (72·6 to 74·1)	75·7 (75·3 to 76·1)	78·3 (77·9 to 78·7)	80·9 (80·5 to 81·3)	81 (80 to 82·1)
Redcar and Cleveland	67·6 (64·9 to 70·3)	69·9 (67·4 to 72·6)	70·7 (68·3 to 73·1)	72·7 (71·8 to 73·6)	74·8 (74·3 to 75·3)	77·1 (76·7 to 77·6)	79·8 (79·2 to 80·3)	79·2 (78 to 80·4)
South Tyneside	67·9 (65·5 to 70·7)	70·1 (67·6 to 72·6)	71 (68·6 to 73·3)	72·8 (72 to 73·7)	74·4 (74 to 74·9)	76·8 (76·3 to 77·2)	79·2 (78·7 to 79·7)	79·6 (78·4 to 80·8)
Stockton-on-Tees	68·4 (65·8 to 71·1)	70·6 (68 to 73·3)	71·4 (69 to 73·9)	73·4 (72·5 to 74·2)	74·8 (74·4 to 75·3)	77·6 (77·2 to 78)	80·1 (79·6 to 80·6)	79·6 (78·5 to 80·8)
Sunderland	67·9 (65·4 to 70·7)	70·1 (67·6 to 72·7)	71 (68·8 to 73·4)	73 (72·2 to 73·8)	74·3 (73·9 to 74·7)	76·4 (76 to 76·8)	78·8 (78·4 to 79·3)	79·1 (78 to 80·2)
North West England	68 (66·4 to 69·8)	70·3 (68·8 to 72)	71·2 (69·5 to 72·9)	72·9 (72·5 to 73·4)	74·9 (74·7 to 75)	76·9 (76·8 to 77·1)	79·5 (79·3 to 79·6)	80·1 (79·3 to 80·8)
Blackburn with Darwen	67·4 (65 to 70)	69·6 (67·4 to 72·1)	70·6 (68·2 to 72·9)	72·4 (71·6 to 73·3)	73·8 (73·3 to 74·3)	75·5 (75 to 76)	78·6 (78·1 to 79·1)	79·1 (77·9 to 80·3)
Blackpool	67·3 (64·8 to 69·9)	69·7 (67·3 to 72)	70·7 (68·3 to 73)	72·6 (71·8 to 73·5)	73·8 (73·3 to 74·3)	74·2 (73·8 to 74·7)	75·6 (75·2 to 76)	76·4 (75·1 to 77·6)
Bolton	67·5 (65·1 to 70·1)	69·8 (67·5 to 72·1)	70·7 (68·4 to 73·1)	72·5 (71·8 to 73·4)	74·7 (74·3 to 75·1)	76·5 (76·1 to 76·9)	79 (78·6 to 79·4)	80 (79 to 81·1)
Bury	68·5 (66 to 71·2)	70·9 (68·5 to 73·4)	71·9 (69·7 to 74·2)	73·7 (72·9 to 74·6)	75·3 (74·9 to 75·7)	77·7 (77·2 to 78·1)	80 (79·6 to 80·5)	80·6 (79·6 to 81·7)
Cheshire East	70·1 (67·6 to 72·6)	72·3 (70·1 to 74·6)	73·1 (71 to 75·6)	74·7 (74·1 to 75·4)	76·6 (76·2 to 76·9)	78·9 (78·6 to 79·3)	81·6 (81·2 to 81·9)	82·4 (81·4 to 83·3)
Cheshire West and Chester	69·8 (67·4 to 72·2)	71·8 (69·4 to 74)	72·4 (70·3 to 74·7)	73·9 (73·2 to 74·6)	76·1 (75·8 to 76·5)	78·7 (78·3 to 79·1)	81·1 (80·7 to 81·6)	81·5 (80·5 to 82·5)

Cumbria	68·5 (66·1 to 71·1)	70·8 (68·5 to 73·3)	71·7 (69·4 to 74·1)	73·5 (72·8 to 74·3)	75·4 (75·1 to 75·8)	78 (77·7 to 78·4)	80·6 (80·2 to 80·9)	80·5 (79·5 to 81·6)
Halton	67·9 (65·5 to 70·4)	69·9 (67·5 to 72·3)	70·6 (68·3 to 72·9)	72·3 (71·4 to 73·2)	74·6 (74·1 to 75)	76 (75·5 to 76·5)	78·9 (78·4 to 79·4)	79·5 (78·3 to 80·7)
Knowsley	67·5 (65 to 70·1)	69·6 (67·1 to 72)	70·2 (67·9 to 72·6)	72 (71·1 to 72·8)	74·1 (73·7 to 74·6)	75·7 (75·2 to 76·2)	78·4 (77·9 to 78·9)	79·3 (78·2 to 80·4)
Lancashire	68·3 (65·9 to 70·9)	70·7 (68·5 to 73·2)	71·8 (69·5 to 74·2)	73·2 (72·5 to 73·9)	75·1 (74·9 to 75·4)	77·5 (77·2 to 77·8)	80 (79·6 to 80·2)	80·3 (79·2 to 81·2)
Liverpool	67·2 (64·7 to 69·7)	69·4 (67·1 to 71·9)	70 (67·8 to 72·3)	72·1 (71·4 to 72·8)	73·8 (73·5 to 74·1)	74·9 (74·5 to 75·2)	77·8 (77·4 to 78·1)	78·1 (77 to 79·1)
Manchester	66·2 (63·7 to 68·6)	68·7 (66·3 to 71·1)	69·5 (67·4 to 71·9)	71·3 (70·6 to 72)	72·8 (72·4 to 73·2)	73·9 (73·6 to 74·3)	77 (76·6 to 77·3)	79·2 (78·2 to 80·3)
Oldham	67·2 (64·7 to 69·6)	69·6 (67·3 to 72)	70·5 (68·4 to 73)	72·5 (71·7 to 73·3)	74·3 (73·9 to 74·7)	76·3 (75·8 to 76·7)	78·7 (78·2 to 79·1)	79·5 (78·4 to 80·6)
Rochdale	67·2 (64·8 to 69·7)	69·6 (67·2 to 72·1)	70·6 (68·4 to 72·9)	72·6 (71·9 to 73·5)	74·1 (73·7 to 74·5)	76·2 (75·8 to 76·7)	78·4 (77·9 to 78·8)	78·9 (77·8 to 80)
Salford	66·9 (64·5 to 69·4)	69·2 (66·9 to 71·8)	69·9 (67·7 to 72·2)	71·5 (70·7 to 72·3)	73·5 (73·1 to 73·9)	75·6 (75·1 to 76)	78·1 (77·7 to 78·5)	79·3 (78·2 to 80·5)
Sefton	69·1 (66·8 to 71·5)	71·3 (68·9 to 73·7)	72 (69·7 to 74·2)	73·7 (72·9 to 74·5)	75·9 (75·5 to 76·3)	77·5 (77·1 to 77·9)	79·5 (79·1 to 79·9)	79·9 (78·8 to 81)
St Helens	68 (65·4 to 70·5)	70 (67·6 to 72·4)	70·7 (68·5 to 73)	72·1 (71·3 to 72·9)	74·8 (74·3 to 75·1)	76·9 (76·5 to 77·3)	78·9 (78·5 to 79·4)	79 (77·8 to 80·1)
Stockport	69·8 (67·3 to 72·4)	72 (69·5 to 74·4)	72·8 (70·5 to 75·2)	74·1 (73·4 to 74·9)	76 (75·7 to 76·4)	78·4 (78·1 to 78·8)	80·8 (80·4 to 81·3)	81·5 (80·4 to 82·5)
Tameside	67·6 (65·1 to 70)	69·8 (67·6 to 72·4)	70·7 (68·4 to 73·1)	72·6 (71·8 to 73·4)	74·5 (74·1 to 74·9)	76·3 (75·8 to 76·7)	78·8 (78·3 to 79·1)	80·2 (79·2 to 81·3)
Trafford	69·3 (67 to 72)	71·5 (69·2 to 73·9)	72·3 (70·1 to 74·8)	74 (73·3 to 74·8)	75·9 (75·4 to 76·3)	78·3 (77·9 to 78·8)	81·4 (80·9 to 81·8)	82·4 (81·4 to 83·5)
Warrington	69·1 (66·7 to 71·7)	71·1 (68·7 to 73·7)	71·8 (69·5 to 74·1)	73·3 (72·4 to 74·2)	75·1 (74·6 to 75·5)	78 (77·5 to 78·4)	80·4 (80 to 80·9)	80·8 (79·7 to 81·8)
Wigan	68·1 (65·6 to 70·5)	70·2 (68 to 72·7)	71·1 (68·9 to 73·3)	72·8 (72 to 73·5)	74·8 (74·4 to 75·2)	76·7 (76·3 to 77)	79·1 (78·7 to 79·4)	79·8 (78·8 to 80·8)
Wirral	68·3 (65·8 to 70·8)	70·6 (68·3 to 73·1)	71·5 (69·4 to 73·8)	73·4 (72·6 to 74·1)	75·2 (74·8 to 75·6)	77·2 (76·8 to 77·6)	79·7 (79·3 to 80·1)	80 (79 to 81·1)
South East England	70·6 (69 to 72·4)	72·9 (71·2 to 74·5)	73·7 (72 to 75·4)	75·1 (74·6 to 75·6)	77·2 (77·1 to 77·4)	79·2 (79·1 to 79·4)	81·8 (81·6 to 81·9)	82·3 (81·6 to 83)
Bracknell Forest	71·4 (69 to 73·9)	73·5 (71·2 to 75·9)	74·1 (71·7 to 76·7)	75·6 (74·6 to 76·6)	77·4 (76·9 to 78)	79·8 (79·3 to 80·4)	82·5 (81·9 to 83)	83·1 (82 to 84·2)
Brighton and Hove	69·4 (66·8 to 71·8)	71·9 (69·8 to 74·2)	72·7 (70·5 to 74·9)	74·3 (73·5 to 75·3)	75·3 (75 to 75·7)	77·2 (76·8 to 77·6)	79·9 (79·5 to 80·3)	80·9 (79·9 to 82)
Buckinghamshire	71·9 (69·7 to 74·5)	73·9 (71·4 to 76·3)	74·5 (72·3 to 76·8)	75·8 (75·1 to 76·5)	77·6 (77·3 to 78)	79·8 (79·5 to 80·2)	82·5 (82·1 to 82·8)	83·4 (82·5 to 84·4)
East Sussex	70·3 (67·9 to 72·7)	72·7 (70·2 to 75·1)	73·7 (71·6 to 75·9)	75·4 (74·7 to 76·2)	77·6 (77·3 to 78)	79·4 (79 to 79·8)	81·6 (81·2 to 81·9)	81·7 (80·7 to 82·7)
Hampshire	71 (68·4 to 73·5)	73·2 (70·8 to 75·5)	73·9 (71·5 to 76·2)	75·3 (74·7 to 76)	77·6 (77·3 to 77·9)	79·7 (79·4 to 80)	82·3 (82 to 82·6)	82·8 (81·8 to 83·7)
Isle of Wight	68·9 (66·4 to 71·5)	71·6 (69·2 to 74)	72·9 (70·5 to 75·5)	74·9 (74 to 75·9)	76·5 (76 to 77)	79 (78·5 to 79·5)	81 (80·5 to 81·5)	81·4 (80·3 to 82·6)
Kent	70·1 (67·7 to 72·4)	72·4 (70 to 74·6)	73·2 (71 to 75·6)	74·3 (73·6 to 75·1)	76·7 (76·4 to 77)	78·7 (78·4 to 79)	81·3 (81·1 to 81·6)	81·8 (80·7 to 82·7)
Medway	69·6 (67·1 to 72·2)	71·9 (69·5 to 74·4)	73 (70·7 to 75·2)	74·7 (73·9 to 75·5)	76·2 (75·8 to 76·6)	78 (77·6 to 78·4)	80·2 (79·8 to 80·7)	80·6 (79·5 to 81·6)
Milton Keynes	70·1 (67·5 to 72·7)	72·3 (69·9 to 74·7)	73·1 (70·8 to 75·7)	74·7 (73·8 to 75·6)	76·6 (76·2 to 77)	78·9 (78·5 to 79·4)	81·1 (80·7 to 81·5)	81·8 (80·8 to 82·8)
Oxfordshire	71·7 (69·2 to 74·1)	73·7 (71·4 to 76·1)	74·4 (72·1 to 76·7)	75·5 (74·8 to 76·3)	77·9 (77·6 to 78·2)	79·6 (79·3 to 79·9)	82·1 (81·8 to 82·4)	82·9 (82 to 83·9)
Portsmouth	68·8 (66·2 to 71·4)	71·2 (68·9 to 73·8)	72·2 (69·7 to 74·6)	73·8 (73 to 74·7)	75·2 (74·8 to 75·6)	76·8 (76·3 to 77·2)	79·7 (79·3 to 80·2)	80·6 (79·5 to 81·8)
Reading	70·4 (67·8 to 72·8)	72·5 (70·1 to 75·1)	73·2 (70·8 to 75·6)	74·7 (73·8 to 75·6)	76·2 (75·7 to 76·6)	77·9 (77·5 to 78·4)	80·3 (79·8 to 80·8)	80·6 (79·4 to 81·7)
Slough	69·6 (67·1 to 72·2)	71·6 (69·1 to 74·2)	72·2 (69·8 to 74·7)	73·7 (72·8 to 74·6)	75·1 (74·6 to 75·6)	77·2 (76·6 to 77·6)	80·1 (79·6 to 80·6)	80·8 (79·6 to 82)
Southampton	68·9 (66·3 to 71·4)	71·2 (68·6 to 73·8)	72·1 (69·7 to 74·5)	74 (73·1 to 74·8)	75·5 (75 to 75·9)	77·4 (77 to 77·8)	80·2 (79·8 to 80·6)	80·7 (79·6 to 81·8)
Surrey	71·7 (69·4 to 74·1)	73·8 (71·6 to 76·2)	74·4 (72·1 to 76·8)	75·6 (74·8 to 76·3)	77·9 (77·7 to 78·2)	80 (79·7 to 80·3)	82·8 (82·5 to 83·1)	83·1 (82·2 to 84·1)
West Berkshire	72 (69·6 to 74·6)	74 (71·7 to 76·4)	74·8 (72·3 to 77·2)	76·2 (75·2 to 77·1)	77·9 (77·4 to 78·4)	79·9 (79·4 to 80·4)	82·5 (82 to 83)	83·2 (82 to 84·3)
West Sussex	70·7 (68·2 to 73·1)	73·1 (70·7 to 75·5)	73·8 (71·3 to 76·3)	75·4 (74·7 to 76·2)	77·7 (77·4 to 78)	79·7 (79·3 to 80)	81·9 (81·6 to 82·2)	82·2 (81·3 to 83·1)
Windsor and Maidenhead	72·6 (70·1 to 75)	74·6 (72·4 to 77)	75·4 (73 to 77·6)	76·8 (75·9 to 77·7)	77·8 (77·4 to 78·4)	80·3 (79·8 to 80·8)	83·1 (82·5 to 83·7)	83·5 (82·4 to 84·6)
Wokingham	73 (70·4 to 75·5)	75 (72·8 to 77·4)	75·8 (73·6 to 78·2)	77·2 (76·3 to 78·2)	79·2 (78·7 to 79·7)	81·7 (81·2 to 82·2)	84·2 (83·7 to 84·7)	84·4 (83·3 to 85·6)

South West England	70·3 (68·5 to 72)	72·6 (70·8 to 74·5)	73·4 (71·6 to 75·1)	75 (74·5 to 75·6)	77·2 (77 to 77·3)	79·3 (79·2 to 79·5)	81·7 (81·5 to 81·9)	82 (81·2 to 82·8)
Bath and North East Somerset	71·4 (69 to 74)	73·8 (71·3 to 76·2)	74·7 (72·3 to 77·1)	76·5 (75·6 to 77·4)	77·9 (77·4 to 78·3)	80 (79·5 to 80·4)	82·6 (82·1 to 83·1)	82·7 (81·5 to 83·8)
Bournemouth	70·2 (67·7 to 72·8)	72·6 (70 to 75·2)	73·6 (71·4 to 76)	75·2 (74·3 to 76)	76·2 (75·8 to 76·7)	77·9 (77·4 to 78·3)	80·2 (79·7 to 80·6)	80·1 (79 to 81·3)
Bristol, City of	69·3 (66·8 to 71·8)	71·6 (69·4 to 74·2)	72·3 (69·8 to 74·6)	74 (73·2 to 74·8)	76 (75·6 to 76·4)	77·9 (77·6 to 78·3)	80·2 (79·8 to 80·6)	80·7 (79·7 to 81·8)
Cornwall	69·9 (67·1 to 72·4)	72·1 (69·7 to 74·7)	72·9 (70·6 to 75·4)	74·6 (73·8 to 75·4)	77 (76·6 to 77·3)	79·1 (78·8 to 79·5)	81·4 (81·1 to 81·8)	81·6 (80·6 to 82·7)
Devon	70·3 (68 to 72·8)	72·7 (70·1 to 75·1)	73·5 (71·1 to 75·9)	75·2 (74·5 to 75·9)	77·6 (77·3 to 77·9)	79·7 (79·4 to 80)	82·2 (81·8 to 82·5)	82·3 (81·4 to 83·3)
Dorset	70·6 (68·1 to 73·2)	72·9 (70·6 to 75·5)	73·9 (71·5 to 76·1)	75·7 (74·9 to 76·5)	78·1 (77·7 to 78·5)	80·5 (80·1 to 80·9)	83 (82·6 to 83·4)	82·9 (81·9 to 83·9)
Gloucestershire	70·8 (68·3 to 73·5)	73·1 (70·6 to 75·6)	73·8 (71·5 to 76·2)	75·1 (74·4 to 75·8)	77·1 (76·7 to 77·4)	79·4 (79·1 to 79·8)	81·7 (81·3 to 82·1)	82·3 (81·2 to 83·3)
North Somerset	70·9 (68·5 to 73·5)	73·4 (70·8 to 75·9)	74·4 (71·9 to 76·7)	76·1 (75·2 to 77)	77·8 (77·3 to 78·2)	79·7 (79·2 to 80·2)	81·3 (80·8 to 81·8)	81·5 (80·4 to 82·6)
Plymouth	69·1 (66·6 to 71·6)	71·3 (68·7 to 73·9)	72·1 (69·9 to 74·4)	73·7 (72·9 to 74·6)	75·9 (75·5 to 76·4)	77·8 (77·4 to 78·2)	80·1 (79·7 to 80·6)	80·7 (79·6 to 81·8)
Poole	70·5 (67·9 to 73·1)	72·7 (70·2 to 75·3)	73·6 (71·3 to 76)	75·2 (74·3 to 76·1)	77·2 (76·7 to 77·7)	79·9 (79·4 to 80·4)	82·2 (81·7 to 82·7)	82·9 (81·7 to 84)
Somerset	70·7 (68 to 73·2)	72·9 (70·2 to 75·4)	73·6 (71·2 to 75·9)	75·1 (74·4 to 75·8)	77·4 (77 to 77·8)	79·6 (79·2 to 79·9)	81·9 (81·5 to 82·2)	81·9 (80·9 to 82·9)
South Gloucestershire	71·6 (69 to 74·1)	73·6 (71·2 to 76·1)	74·2 (71·7 to 76·5)	76 (75·1 to 76·9)	78·1 (77·7 to 78·5)	80·4 (80 to 80·9)	82·9 (82·5 to 83·3)	83·7 (82·7 to 84·7)
Swindon	69·6 (66·9 to 72·1)	71·8 (69·2 to 74·4)	72·5 (70·1 to 74·8)	74·3 (73·4 to 75·2)	76·7 (76·2 to 77·2)	78·6 (78·2 to 79·1)	81·5 (81 to 81·9)	82·1 (81 to 83·3)
Torbay	69·9 (67·4 to 72·4)	72·2 (69·7 to 74·8)	73·3 (71 to 75·6)	75 (74·1 to 75·9)	76·7 (76·2 to 77·2)	77·7 (77·2 to 78·2)	79·7 (79·2 to 80·2)	80·1 (78·9 to 81·3)
Wiltshire	70·8 (68·3 to 73·3)	73 (70·6 to 75·6)	73·9 (71·7 to 76·1)	75·4 (74·7 to 76·2)	77·1 (76·7 to 77·4)	79·8 (79·4 to 80·1)	82·1 (81·7 to 82·5)	82·7 (81·7 to 83·6)
West Midlands	69 (67·1 to 70·8)	71·1 (69·4 to 72·9)	71·8 (70·1 to 73·7)	73·5 (73 to 74·1)	75·5 (75·4 to 75·7)	77·7 (77·6 to 77·9)	80·4 (80·3 to 80·6)	80·8 (80 to 81·6)
Birmingham	68·2 (65·9 to 70·5)	70·5 (68·2 to 72·9)	71·2 (69 to 73·4)	72·9 (72·3 to 73·6)	74·6 (74·3 to 74·9)	76·6 (76·3 to 76·9)	80 (79·6 to 80·3)	80·1 (79·1 to 81·1)
Coventry	68·3 (65·8 to 71·1)	70·5 (68·1 to 73)	71·2 (68·7 to 73·6)	73·1 (72·3 to 73·9)	74·9 (74·5 to 75·2)	77·2 (76·9 to 77·7)	79·7 (79·3 to 80·1)	80·1 (79 to 81·3)
Dudley	69·9 (67·4 to 72·3)	72 (69·6 to 74·7)	72·9 (70·5 to 75·2)	74·4 (73·7 to 75·2)	76·1 (75·7 to 76·5)	77·9 (77·5 to 78·3)	80·7 (80·3 to 81·1)	81·1 (80·1 to 82·2)
Herefordshire, County of	69·6 (66·9 to 72·4)	71·8 (69·5 to 74·5)	72·7 (70·4 to 75·1)	74·6 (73·6 to 75·5)	77·3 (76·9 to 77·8)	79·2 (78·8 to 79·7)	81·5 (81 to 81·9)	81·6 (80·5 to 82·7)
Sandwell	67·5 (64·8 to 70·1)	69·7 (67·2 to 72·3)	70·5 (68·2 to 72·9)	72·6 (71·8 to 73·4)	74·4 (74·1 to 74·8)	76·2 (75·8 to 76·6)	78·5 (78·1 to 78·9)	79·9 (78·8 to 81)
Shropshire	69·9 (67·4 to 72·6)	72 (69·5 to 74·4)	72·8 (70·5 to 75·2)	74·6 (73·9 to 75·4)	76·8 (76·4 to 77·2)	79 (78·6 to 79·4)	81·7 (81·3 to 82·1)	82·6 (81·5 to 83·6)
Solihull	71 (68·3 to 73·6)	73 (70·6 to 75·5)	73·7 (71·3 to 76)	75·5 (74·7 to 76·4)	77·6 (77·1 to 78)	79·2 (78·8 to 79·7)	82·4 (81·9 to 82·9)	82 (80·8 to 83·1)
Staffordshire	69·5 (67·1 to 72·2)	71·5 (69·3 to 74·1)	72·1 (69·9 to 74·5)	73·5 (72·8 to 74·3)	75·6 (75·3 to 75·9)	78·1 (77·7 to 78·4)	81 (80·6 to 81·2)	81·1 (80·1 to 82·1)
Stoke-on-Trent	67·1 (64·5 to 69·6)	69·2 (66·8 to 71·8)	69·9 (67·6 to 72·3)	71·6 (70·8 to 72·4)	74 (73·6 to 74·4)	76·2 (75·7 to 76·6)	78·3 (77·9 to 78·7)	79·2 (78 to 80·3)
Telford and Wrekin	68·7 (66 to 71·3)	70·8 (68·3 to 73·3)	71·5 (69·1 to 73·9)	73·2 (72·4 to 74·1)	75·7 (75·2 to 76·2)	77·8 (77·3 to 78·2)	80·2 (79·7 to 80·7)	80·5 (79·3 to 81·7)
Walsall	68·3 (65·7 to 71)	70·4 (68 to 73·1)	71 (68·6 to 73·4)	72·8 (72 to 73·7)	74·7 (74·3 to 75)	77·3 (76·9 to 77·8)	79·6 (79·2 to 80·1)	80 (79 to 81·1)
Warwickshire	70·3 (67·8 to 72·9)	72·2 (69·8 to 74·6)	72·8 (70·5 to 75·1)	74·3 (73·6 to 75)	76·3 (76 to 76·6)	78·8 (78·5 to 79·1)	81·4 (81·1 to 81·7)	81·7 (80·7 to 82·7)
Wolverhampton	68·4 (65·8 to 71)	70·5 (68 to 73·1)	71·2 (68·8 to 73·6)	73·1 (72·3 to 73·9)	74·6 (74·3 to 75)	76·6 (76·2 to 77)	78·9 (78·4 to 79·3)	79·3 (78·1 to 80·4)
Worcestershire	70·7 (68·2 to 73·3)	72·6 (70·4 to 75·3)	73·4 (71·1 to 75·5)	74·7 (74 to 75·5)	76·7 (76·4 to 77)	78·7 (78·4 to 79)	81·1 (80·8 to 81·5)	81·6 (80·6 to 82·6)
Yorkshire and the Humber	68·5 (66·7 to 70·2)	70·7 (69·1 to 72·4)	71·6 (69·9 to 73·3)	73·4 (72·9 to 74)	75·5 (75·3 to 75·7)	77·8 (77·6 to 78)	80·1 (79·9 to 80·3)	80·6 (79·8 to 81·4)
Barnsley	67·9 (65·3 to 70·4)	70·1 (67·6 to 72·7)	71·1 (68·8 to 73·5)	73 (72·2 to 73·7)	75·2 (74·8 to 75·6)	77·4 (77 to 77·8)	79·9 (79·5 to 80·3)	80·2 (79·1 to 81·3)
Bradford	67·4 (64·8 to 69·9)	69·8 (67·5 to 72·5)	70·8 (68·5 to 73·3)	72·8 (72·1 to 73·5)	74·7 (74·4 to 75·1)	76·8 (76·4 to 77·1)	79·4 (79 to 79·7)	79·9 (79 to 81)
Calderdale	67·9 (65·5 to 70·4)	70·2 (67·7 to 72·6)	71·1 (68·8 to 73·4)	72·9 (72·1 to 73·8)	74·8 (74·4 to 75·2)	77·7 (77·2 to 78·2)	79·7 (79·2 to 80·1)	80·4 (79·4 to 81·5)
Doncaster	68·5 (66 to 71·1)	70·7 (68·4 to 73·2)	71·7 (69·2 to 73·9)	73·6 (72·9 to 74·4)	75 (74·7 to 75·4)	77 (76·6 to 77·4)	79·3 (78·9 to 79·7)	79·7 (78·7 to 80·8)
East Riding of Yorkshire	70 (67·5 to 72·4)	72·2 (69·9 to 74·7)	73·1 (70·9 to 75·4)	74·8 (74 to 75·6)	76·8 (76·4 to 77·2)	78·9 (78·5 to 79·2)	81·2 (80·8 to 81·6)	81·9 (80·7 to 82·9)

Kingston upon Hull, City of	67·7 (65·1 to 70·2)	70·1 (67·8 to 72·4)	71·2 (69 to 73·5)	73·3 (72·5 to 74·1)	74·8 (74·5 to 75·2)	76·1 (75·7 to 76·5)	78·1 (77·7 to 78·5)	78 (76·9 to 79·2)
Kirklees	67·8 (65·1 to 70·4)	70·1 (67·8 to 72·4)	70·9 (68·7 to 73·3)	72·6 (71·8 to 73·4)	75·1 (74·8 to 75·5)	77·7 (77·4 to 78·2)	79·7 (79·4 to 80·1)	80·4 (79·2 to 81·5)
Leeds	68·5 (66 to 70·9)	70·7 (68·3 to 73·1)	71·5 (69 to 74)	73·3 (72·5 to 74)	75·5 (75·2 to 75·9)	77·9 (77·6 to 78·3)	80 (79·7 to 80·3)	80·6 (79·6 to 81·6)
North East Lincolnshire	68·7 (66·3 to 71·4)	71 (68·5 to 73·6)	72·1 (69·7 to 74·6)	74 (73·1 to 74·9)	74·9 (74·5 to 75·4)	77·3 (76·8 to 77·7)	79·6 (79·1 to 80·1)	79·9 (78·8 to 81)
North Lincolnshire	68·7 (66 to 71·3)	70·8 (68·2 to 73·4)	71·7 (69·5 to 74·1)	73·5 (72·7 to 74·4)	75 (74·6 to 75·5)	77·7 (77·3 to 78·2)	80·4 (79·9 to 80·9)	81·3 (80·2 to 82·4)
North Yorkshire	69·6 (67·3 to 72·1)	71·9 (69·5 to 74·3)	72·7 (70·5 to 75·1)	74·5 (73·8 to 75·2)	76·8 (76·5 to 77·1)	79·3 (79 to 79·6)	81·7 (81·4 to 82·1)	82·2 (81·2 to 83·2)
Rotherham	68·2 (65·6 to 70·7)	70·4 (67·9 to 72·9)	71·3 (68·9 to 73·7)	73·1 (72·3 to 73·9)	75·3 (74·9 to 75·7)	77·5 (77·2 to 78)	80 (79·6 to 80·4)	79·6 (78·5 to 80·7)
Sheffield	68·8 (66·4 to 71·2)	71 (68·6 to 73·4)	71·8 (69·4 to 74·2)	73·6 (72·9 to 74·3)	75·5 (75·2 to 75·8)	77·9 (77·5 to 78·2)	80·1 (79·7 to 80·4)	80·8 (79·8 to 81·7)
Wakefield	67·6 (65·1 to 70)	69·7 (67·4 to 72·2)	70·5 (68·2 to 72·9)	72·5 (71·7 to 73·2)	75·1 (74·7 to 75·4)	77·4 (77 to 77·8)	79·5 (79·1 to 79·9)	79·9 (78·9 to 81)
York	70 (67·5 to 72·7)	72·1 (69·9 to 74·5)	73 (70·6 to 75·2)	74·6 (73·8 to 75·5)	76·7 (76·2 to 77·1)	79·6 (79·1 to 80)	81·9 (81·5 to 82·4)	81·6 (80·5 to 82·8)
Northern Ireland	67·5 (67·2 to 67·7)	70·1 (70 to 70·3)	70·9 (70·7 to 71·1)	71·8 (71·6 to 71·9)	74·6 (74·4 to 74·8)	77·4 (77·3 to 77·6)	79·7 (79·6 to 79·9)	80·3 (80 to 80·7)
Scotland	66·5 (66·4 to 66·6)	69 (68·9 to 69·1)	70·3 (70·2 to 70·4)	72·1 (72 to 72·2)	74·1 (74 to 74·2)	76·1 (76 to 76·2)	78·6 (78·5 to 78·8)	79·1 (78·8 to 79·3)
Wales	67·6 (67·2 to 68·1)	70·2 (69·6 to 70·7)	71·3 (70·8 to 71·8)	73·5 (73·3 to 73·7)	75·8 (75·7 to 76)	77·5 (77·4 to 77·7)	80 (79·8 to 80·1)	80·5 (80·2 to 80·7)
Latin America and Caribbean	48·7 (46·8 to 50·4)	55 (54 to 55·9)	59·2 (58·6 to 59·8)	65 (64·6 to 65·4)	69·1 (68·8 to 69·3)	72·4 (72·2 to 72·7)	73·3 (73 to 73·7)	76 (74·8 to 77·1)
Andean Latin America	40·1 (37·3 to 42·7)	48·4 (46·8 to 49·9)	51·8 (50·7 to 53)	63·9 (62·7 to 65)	68·4 (67·5 to 69·3)	73 (72 to 73·9)	75·1 (74 to 76·2)	77·5 (75·3 to 79·6)
Bolivia	36·4 (33·6 to 39·4)	43 (40·5 to 45·6)	50·1 (48 to 52·2)	56·8 (55·1 to 58·4)	61·7 (60·3 to 63)	66·5 (65 to 67·9)	70·3 (68·2 to 72·3)	72 (69·9 to 74·2)
Ecuador	50·3 (47 to 53·9)	56·1 (55 to 57·1)	61·3 (60·6 to 62·1)	67 (66·5 to 67·5)	71·9 (71·6 to 72·3)	74·5 (74 to 74·9)	74·3 (73·2 to 75·3)	76·4 (73·8 to 78·7)
Peru	38·1 (33·7 to 42·1)	47·3 (45 to 49·4)	48·6 (47 to 50·3)	65·1 (63·3 to 67)	69·2 (67·8 to 70·7)	74·6 (73 to 76·1)	77·3 (75·7 to 79·1)	80·2 (77·1 to 83·2)
Caribbean	56·3 (54·5 to 58·1)	60·8 (59·9 to 61·8)	63·8 (62·9 to 64·5)	66·4 (65·7 to 67)	68·4 (67·8 to 68·9)	70·3 (69·7 to 71)	58·9 (57·5 to 60·2)	72·6 (70·7 to 74·6)
Antigua and Barbuda	58·1 (56·3 to 59·6)	63·8 (62·8 to 64·8)	68·3 (67·5 to 69·1)	72·1 (70·8 to 73·3)	74 (73·4 to 74·6)	74·8 (74·2 to 75·4)	75·9 (75·3 to 76·5)	76·3 (74·8 to 77·7)
The Bahamas	58 (54 to 62)	61·9 (59 to 65·1)	65·4 (64·3 to 66·6)	68·6 (67·8 to 69·5)	70·9 (70 to 71·8)	71·2 (70·5 to 72·1)	73·2 (72·6 to 73·9)	73·4 (70·7 to 75·7)
Barbados	58·8 (56·9 to 60·4)	66·1 (65·1 to 67·2)	69·2 (68·4 to 69·9)	73 (72·4 to 73·5)	73·9 (73·4 to 74·3)	74·7 (74·2 to 75·3)	76·2 (75·7 to 76·6)	76·2 (74·3 to 78·1)
Belize	55·4 (53·6 to 57·3)	63·9 (61·9 to 65·9)	67·5 (66·3 to 68·7)	70·9 (70 to 71·7)	72·8 (72 to 73·6)	70 (69·5 to 70·5)	73·6 (73·1 to 74)	74·5 (72·9 to 76)
Bermuda	63·4 (62·2 to 64·6)	67·1 (66·6 to 67·7)	70 (69·3 to 70·6)	73·2 (72·6 to 73·7)	73·3 (72·8 to 73·8)	77 (76·5 to 77·6)	80·5 (79·5 to 81·4)	81·3 (79·5 to 82·8)
Cuba	66·4 (62·8 to 69·8)	69·3 (69·1 to 69·4)	70·8 (70·6 to 70·9)	73·6 (73·4 to 73·7)	75 (74·9 to 75·1)	77 (76·9 to 77·1)	78·5 (78·3 to 78·6)	78·7 (76·6 to 80·7)
Dominica	49·3 (46·9 to 51·4)	60·2 (58·1 to 62·4)	67·2 (65·4 to 68·8)	71·8 (70·7 to 73)	71·1 (69·9 to 72·4)	72·1 (70·7 to 73·6)	72·4 (71 to 73·8)	72·3 (69·8 to 74·6)
Dominican Republic	56·2 (54·5 to 57·9)	61·9 (60·7 to 63·1)	64·9 (63·7 to 66)	69·3 (68·2 to 70·3)	72 (70·8 to 73·2)	73·7 (72·5 to 74·9)	73·5 (71·2 to 75·9)	73·2 (70·1 to 76·1)
Grenada	58·6 (57 to 60·3)	64·1 (62·1 to 66·2)	67 (64·9 to 68·9)	69·3 (66·9 to 71·6)	69·2 (68·1 to 70·3)	72 (71·5 to 72·5)	72·5 (72 to 73)	73·3 (72·3 to 74·3)
Guyana	51·8 (48·8 to 54·9)	58·8 (57·4 to 60·4)	61·7 (60 to 63·5)	62·4 (60·2 to 64·5)	62·4 (60·8 to 64·1)	64 (62·3 to 65·5)	65·7 (64·4 to 67·1)	67·3 (64 to 70·6)
Haiti	42·5 (39·7 to 45·5)	45·4 (42·9 to 48·1)	48 (45·6 to 50·5)	51·3 (49·5 to 53·3)	54·6 (53 to 56·2)	57·4 (55·5 to 59·4)	32·5 (30·8 to 34·3)	63·8 (60·8 to 66·7)
Jamaica	57·5 (56·5 to 58·6)	65·1 (64·6 to 65·7)	68·9 (68·4 to 69·4)	73·8 (73·4 to 74·3)	75·3 (74·9 to 75·7)	74·6 (74·3 to 75)	76·3 (76 to 76·7)	76·2 (73·6 to 78·8)
Puerto Rico	60·1 (59·7 to 60·4)	69·2 (68·9 to 69·4)	71·3 (71·1 to 71·5)	73·8 (73·6 to 74·1)	74·2 (74 to 74·4)	76·6 (76·3 to 76·8)	79·4 (79·1 to 79·6)	80·5 (77·8 to 83·2)
Saint Kitts and Nevis	60·8 (59·8 to 61·7)	58·2 (57·5 to 59)	62·6 (61·8 to 63·4)	63·8 (62·7 to 65)	65·9 (65·1 to 66·8)	69·9 (68·9 to 70·9)	72·2 (71·2 to 73·1)	72·1 (70·3 to 73·5)
Saint Lucia	51·8 (49·6 to 53·7)	59·2 (57·2 to 61)	65·4 (64 to 66·8)	68·7 (68 to 69·4)	70·2 (69·6 to 70·8)	73·4 (72·9 to 73·8)	75·6 (75 to 76·2)	75·1 (73·2 to 77)
Saint Vincent and the Grenadines	53·6 (51·4 to 55·5)	61 (59·2 to 62·8)	64·8 (63·4 to 66·1)	68·6 (67·4 to 69·7)	70·1 (69·3 to 70·9)	71·1 (70·5 to 71·7)	73·1 (72·4 to 73·8)	72·9 (71·3 to 74·5)
Suriname	57·7 (56·3 to 59)	63 (61·3 to 64·5)	65·9 (64·3 to 67·3)	66·8 (66 to 67·5)	68·9 (68·3 to 69·6)	69·8 (69·2 to 70·4)	72·3 (71·7 to 72·9)	72·7 (70·4 to 74·9)
Trinidad and Tobago	57·4 (56·3 to 58·3)	64·3 (63·6 to 64·9)	65·4 (64·9 to 65·8)	67·6 (67·2 to 68·1)	69·6 (69·2 to 70)	70·5 (70 to 70·9)	73·7 (73·3 to 74·1)	74·9 (71·4 to 78·3)

Virgin Islands	61·1 (59·1 to 62·9)	63·8 (62·5 to 65·3)	67·1 (65·5 to 68·5)	70·3 (69 to 71·4)	72·5 (71 to 74·2)	73·4 (72·2 to 74·6)	73·6 (72·7 to 74·5)	74·3 (72·6 to 76·1)
Central Latin America	50·1 (48·4 to 51·6)	57·5 (56·9 to 58·1)	62 (61·6 to 62·4)	66·7 (66·2 to 67·2)	70·8 (70·5 to 71)	73·8 (73·6 to 74)	75·6 (75·4 to 75·9)	76·4 (74·5 to 78·1)
Colombia	54·1 (52·6 to 55·3)	59·3 (58·4 to 60·1)	65·9 (65·4 to 66·5)	68·9 (66·9 to 70·9)	71·4 (71·2 to 71·7)	74·2 (74 to 74·5)	77·9 (77·6 to 78·1)	80·2 (77·3 to 83)
Costa Rica	57·5 (57·1 to 58)	65·3 (65 to 65·7)	67 (66·7 to 67·3)	74·7 (74·4 to 75)	76·9 (76·6 to 77·3)	77·9 (77·7 to 78·1)	79·5 (79·3 to 79·8)	80·1 (77·4 to 82·7)
El Salvador	46 (43·3 to 48·3)	53·5 (51·5 to 55·2)	57·8 (56·5 to 58·9)	63·8 (62·4 to 65·2)	69·8 (69·3 to 70·2)	73·1 (72·7 to 73·4)	75·2 (74·9 to 75·5)	75·7 (72·3 to 79·1)
Guatemala	42·7 (40·4 to 44·6)	47·9 (46·2 to 49·6)	49·9 (48·7 to 50·9)	58 (56·8 to 58·9)	61·4 (59·8 to 63)	66·7 (65·2 to 68·2)	70·3 (68·8 to 71·6)	72·6 (69·5 to 75·5)
Honduras	40·8 (38·3 to 43·6)	49·1 (47·1 to 50·9)	57·7 (56·3 to 59·1)	67·9 (66·5 to 69·3)	69·1 (67·5 to 70·8)	70·1 (67·5 to 72·7)	71·1 (68·9 to 73·6)	72·1 (69·5 to 73·9)
Mexico	48·6 (46·6 to 50·1)	57 (56·1 to 58)	61·4 (60·8 to 62)	66 (65·5 to 66·5)	71 (70·7 to 71·3)	74·3 (74 to 74·5)	75·5 (75·3 to 75·7)	75·7 (74·2 to 77·3)
Aguascalientes	52·8 (50·3 to 55·1)	59·9 (57·8 to 62·1)	63·3 (61·4 to 65·2)	67·5 (66·8 to 68·1)	72·5 (72 to 72·9)	75 (74·6 to 75·4)	76·3 (75·9 to 76·6)	77·2 (75·5 to 78·8)
Baja California	48·8 (46·3 to 51·5)	57·5 (55·2 to 59·8)	61·6 (59·5 to 63·8)	68 (67·5 to 68·5)	69·6 (69·3 to 70)	71·3 (71 to 71·5)	73·7 (73·4 to 74)	74·8 (72·7 to 76·9)
Baja California Sur	49·7 (47 to 52·2)	58·6 (56·5 to 60·8)	62·9 (61·1 to 64·7)	68·4 (67·8 to 69·1)	73·1 (72·7 to 73·5)	74·8 (74·4 to 75·2)	75·9 (75·5 to 76·3)	74·7 (72·8 to 76·7)
Campeche	49·1 (46·4 to 51·6)	57·6 (55·4 to 60)	61·9 (60 to 63·9)	66·4 (65·6 to 67·2)	71·5 (70·9 to 72·2)	75·5 (75 to 76)	76·2 (75·8 to 76·6)	76·4 (74·5 to 78·2)
Chiapas	46·3 (43·5 to 48·8)	54·6 (52·3 to 56·8)	59·2 (57·1 to 61·4)	63·5 (62·6 to 64·3)	69·8 (69·2 to 70·3)	73·3 (72·8 to 73·7)	75·3 (75 to 75·5)	75·5 (73·7 to 77·6)
Chihuahua	46·7 (44·1 to 49·2)	55·5 (53·3 to 57·8)	60 (57·8 to 62)	64·5 (63·8 to 65·2)	68·3 (67·9 to 68·7)	71·4 (70·9 to 71·7)	71·8 (71·5 to 72·1)	71·7 (69·7 to 73·8)
Coahuila	47 (44·6 to 49·7)	56·2 (54·1 to 58·4)	60·4 (58·3 to 62·3)	64·9 (64·3 to 65·6)	70·6 (70·2 to 71)	73·7 (73·3 to 74·1)	74·9 (74·7 to 75·3)	75·4 (73·6 to 77·2)
Colima	43·7 (41·1 to 46·4)	53·3 (51 to 55·7)	58·1 (56·2 to 60·1)	63·8 (62·7 to 64·8)	70·2 (69·6 to 70·7)	74·5 (74 to 74·9)	75·6 (75·3 to 76)	73·6 (71·5 to 75·7)
Durango	46·9 (44·3 to 49·4)	56·1 (53·7 to 58·4)	61·1 (59 to 63·3)	65·6 (64·9 to 66·4)	71·5 (71 to 71·9)	75·5 (75 to 75·9)	75·5 (75·1 to 75·9)	77·1 (75 to 79·2)
Guanajuato	46·7 (44·2 to 49·2)	55·2 (52·9 to 57·4)	59·8 (57·5 to 61·8)	65·9 (65·2 to 66·6)	70·8 (70·4 to 71·3)	74·9 (74·6 to 75·2)	76·4 (76·1 to 76·6)	75 (73·1 to 77·1)
Guerrero	47·1 (44·5 to 49·7)	54·5 (51·6 to 57·4)	57·3 (55 to 59·5)	61·5 (60·2 to 62·9)	68·8 (68·1 to 69·4)	71·6 (71·2 to 71·9)	72·8 (72·2 to 73·4)	73·8 (72·2 to 75·6)
Hidalgo	46·2 (43·8 to 48·8)	54·4 (52·3 to 56·8)	58·5 (56·2 to 60·9)	63·1 (62·3 to 63·8)	72·1 (71·6 to 72·5)	76·7 (76·3 to 77)	77·5 (77·2 to 77·8)	77·5 (75·6 to 79·5)
Jalisco	50·4 (47·9 to 52·7)	58·4 (56·2 to 60·7)	62·7 (60·5 to 64·6)	67·6 (67 to 68·2)	71·3 (70·9 to 71·6)	74·1 (73·7 to 74·5)	75·6 (75·3 to 75·8)	75·8 (74 to 77·9)
México	50·5 (48 to 53·1)	57·8 (55·5 to 60)	61·8 (59·7 to 64)	66·3 (64·8 to 67·9)	69·4 (69 to 69·8)	73·5 (73·3 to 73·8)	75·5 (75·2 to 75·7)	75·7 (74·1 to 77·5)
Mexico City	55·9 (53·6 to 58·2)	62·8 (60·9 to 64·9)	65·9 (64·2 to 67·7)	68·9 (68·5 to 69·4)	72·4 (72·1 to 72·6)	74·6 (74·4 to 74·9)	75·5 (75·3 to 75·8)	76·1 (74·4 to 77·8)
Michoacán de Ocampo	47·8 (45·4 to 50·2)	56·1 (53·8 to 58·6)	61·2 (59·2 to 63·4)	65·7 (65·1 to 66·3)	72·7 (72·3 to 73·1)	75·4 (75·1 to 75·8)	76·5 (76·2 to 76·8)	76·5 (74·5 to 78·4)
Morelos	45·6 (42·9 to 48·1)	55 (52·8 to 57·5)	60 (57·8 to 62·2)	65 (63·9 to 66·1)	71·5 (71 to 72)	75·3 (74·9 to 75·7)	76·3 (76 to 76·6)	75·6 (73·8 to 77·6)
Nayarit	46·8 (44·1 to 49·6)	56 (53·8 to 58·3)	61·8 (59·8 to 63·9)	67·7 (67 to 68·4)	71·9 (71·4 to 72·4)	76·3 (75·8 to 76·7)	76·8 (76·4 to 77·2)	76·4 (74·4 to 78·5)
Nuevo León	53·4 (50·9 to 56)	61·7 (59·7 to 63·8)	65·3 (63·3 to 67·3)	70·3 (69·7 to 70·9)	72·9 (72·5 to 73·3)	75·3 (75 to 75·7)	76·3 (76 to 76·5)	76·7 (74·9 to 78·6)
Oaxaca	44 (41·7 to 46·4)	52·1 (49·8 to 54·2)	56·5 (54·4 to 58·5)	61·1 (60·4 to 61·8)	69·4 (68·9 to 69·8)	74·4 (74 to 74·8)	76·3 (76·1 to 76·6)	77·3 (75·3 to 79·3)
Puebla	40·2 (37·5 to 42·7)	49·9 (47·7 to 52·2)	55·4 (53·3 to 57·5)	61·5 (60·7 to 62·2)	69·2 (68·8 to 69·7)	73·2 (72·8 to 73·6)	75·3 (75 to 75·6)	75·3 (73·5 to 77·2)
Querétaro	46·4 (43·8 to 49)	55·1 (53 to 57·4)	60·1 (57·8 to 62·2)	65 (63·6 to 66·4)	70·5 (70·1 to 70·9)	73·9 (73·6 to 74·3)	76·2 (75·8 to 76·5)	76·2 (74·4 to 77·9)
Quintana Roo	51·4 (48·7 to 54·1)	58·7 (56·3 to 61·1)	62·3 (60·3 to 64·3)	67 (66·2 to 67·8)	73·4 (72·9 to 73·9)	75·6 (75·1 to 76)	75·5 (75·2 to 75·8)	75·3 (73·5 to 77)
San Luis Potosí	47·9 (45 to 50·5)	56·7 (54·4 to 59)	61·2 (59 to 63·5)	68·6 (67·9 to 69·2)	72·9 (72·5 to 73·3)	76·3 (75·9 to 76·7)	77·5 (77·2 to 77·8)	76·7 (74·9 to 78·7)
Sinaloa	51·9 (49·3 to 54·4)	59·8 (57·6 to 62·2)	64 (61·8 to 66·2)	68·4 (67·8 to 69)	73·7 (73·3 to 74·1)	76·4 (76·1 to 76·8)	76·5 (76·3 to 76·8)	76·2 (74·2 to 78·3)
Sonora	52·7 (50·3 to 55·1)	60·1 (57·8 to 62·3)	63·2 (61 to 65·4)	66·9 (65·3 to 68·5)	71·1 (70·6 to 71·5)	73·3 (72·9 to 73·7)	74·2 (73·9 to 74·5)	75·3 (73·5 to 77·2)
Tabasco	47·6 (45·1 to 50·1)	55·6 (53·3 to 58)	59·4 (57·1 to 61·5)	63·2 (62·2 to 64·2)	71 (70·3 to 71·6)	74·5 (74·1 to 74·9)	74·6 (74·2 to 74·9)	75·4 (73·5 to 77·3)
Tamaulipas	50·8 (48·2 to 53·5)	58·9 (56·8 to 61·1)	62·9 (60·9 to 65)	66·7 (66 to 67·5)	72·2 (71·8 to 72·7)	75·6 (75·3 to 76)	75·6 (75·3 to 76)	75·7 (73·7 to 77·8)
Tlaxcala	46·4 (43·6 to 49·1)	55·7 (53·4 to 58)	61·1 (58·8 to 63·4)	66·3 (64·9 to 67·7)	71·3 (70·8 to 71·8)	75 (74·6 to 75·4)	76·6 (76·2 to 77)	76·9 (75·2 to 78·7)

Veracruz de Ignacio de la Llave	48·1 (45·6 to 50·5)	56·7 (54·4 to 58·9)	61·2 (59 to 63·3)	66·3 (65·5 to 67·1)	71·8 (71·2 to 72·4)	75·1 (74·7 to 75·4)	75·6 (75·3 to 75·8)	75·5 (73·5 to 77·4)
Yucatán	52·2 (49·7 to 54·7)	59·1 (57 to 61·2)	63·2 (61·1 to 65)	67·4 (66·8 to 68)	72·4 (72 to 72·7)	75·3 (75 to 75·6)	76 (75·6 to 76·3)	76·3 (74·6 to 78)
Zacatecas	51·6 (49·1 to 54·4)	59·4 (56·8 to 61·7)	63·7 (61·5 to 65·8)	69 (68·3 to 69·7)	73·6 (73·2 to 74·1)	76·2 (75·7 to 76·6)	76·5 (76·1 to 76·9)	76 (74·1 to 78)
Nicaragua	47·7 (45 to 49·9)	53·6 (51·7 to 55·3)	58·1 (56·5 to 59·6)	66·4 (64·5 to 68·2)	72·7 (72·2 to 73·2)	73·7 (73·4 to 74·1)	74·4 (73·9 to 74·8)	75·4 (73·7 to 77·2)
Panama	60·8 (59·7 to 61·9)	65·9 (65·2 to 66·6)	68·1 (67·5 to 68·7)	74·2 (73·7 to 74·7)	76·4 (75·9 to 76·9)	78 (77·5 to 78·4)	78·5 (78·1 to 78·8)	80 (77·2 to 82·8)
Venezuela	58·7 (58·5 to 58·9)	65·2 (65 to 65·3)	66·5 (66·4 to 66·6)	69·7 (69·5 to 69·8)	72·4 (72·3 to 72·6)	74·2 (74·1 to 74·3)	75·3 (75·2 to 75·4)	75 (71·6 to 78·4)
Tropical Latin America	47·8 (45·7 to 49·7)	52·8 (51·2 to 54·2)	57·2 (56·1 to 58·3)	63 (62·4 to 63·5)	67·5 (67·1 to 67·9)	71·3 (71 to 71·6)	73·9 (73·6 to 74·2)	75·9 (75·4 to 76·3)
Brazil	47·6 (45·4 to 49·4)	52·4 (50·9 to 53·9)	56·9 (55·8 to 58·1)	62·7 (62·1 to 63·3)	67·3 (66·9 to 67·7)	71·2 (70·8 to 71·5)	73·8 (73·5 to 74·2)	75·8 (75·4 to 76·3)
Acre	44·5 (41·1 to 48)	50·6 (47·4 to 53·9)	56·1 (53·8 to 58·3)	61·9 (60·3 to 63·3)	67·3 (66·5 to 68·1)	70·3 (69·6 to 71)	73·2 (72·2 to 74·2)	74·5 (73·5 to 75·6)
Alagoas	38·1 (34·4 to 41·4)	41·8 (39·3 to 44·4)	46·7 (44·3 to 49·1)	52·9 (51·3 to 54·5)	61·6 (60·5 to 62·6)	68·8 (67·9 to 69·7)	70·7 (69·8 to 71·5)	73·3 (71·7 to 74·8)
Amapá	41 (38·5 to 43·6)	48·7 (45·8 to 51·5)	58·3 (56·2 to 60·4)	70·6 (69·8 to 71·4)	73 (72·5 to 73·5)	72·5 (72·1 to 72·8)	76·1 (75·6 to 76·6)	75·8 (75 to 76·6)
Amazonas	52·1 (49·6 to 54·6)	56·8 (54·1 to 59·4)	60 (57·6 to 62·5)	64·8 (63·4 to 66·1)	69·6 (68·6 to 70·5)	72·7 (71·8 to 73·4)	75·3 (74·5 to 76·1)	76·5 (75·4 to 77·6)
Bahia	45·8 (42·5 to 49·1)	50 (47·5 to 52·4)	55 (52·5 to 57·3)	63·1 (61·7 to 64·6)	67·1 (65·7 to 68·4)	71·3 (70 to 72·8)	73 (71·6 to 74·5)	75 (73·1 to 76·9)
Ceará	34·9 (31·2 to 38·5)	40·7 (38 to 43·2)	48·1 (45·6 to 50·6)	57·9 (55·6 to 60·1)	67·6 (66 to 69·2)	71·5 (70 to 73·2)	73·6 (72 to 75·1)	74·8 (72·8 to 76·6)
Distrito Federal	47·1 (44·7 to 49·4)	53·1 (50·3 to 55·6)	58·5 (56·3 to 60·7)	64·5 (63·5 to 65·4)	68·6 (67·8 to 69·4)	72 (71·7 to 72·2)	74·8 (74·5 to 75·1)	77·3 (76·4 to 78·1)
Espírito Santo	51·7 (48·8 to 54·8)	55·7 (53·3 to 58·1)	59·7 (57·4 to 61·9)	66·2 (65·6 to 66·7)	69·7 (69·4 to 70)	72 (71·8 to 72·3)	73·3 (72·8 to 73·9)	75·1 (73·9 to 76·4)
Goiás	40·5 (38·4 to 42·7)	46 (43 to 48·7)	53·9 (51·7 to 56·2)	62·1 (60·2 to 63·9)	66·9 (65·6 to 68·2)	71·9 (70·8 to 73)	73·9 (72·8 to 74·9)	75·1 (73·3 to 76·9)
Maranhão	39·1 (35·7 to 42·5)	43·2 (41 to 45·6)	48 (45·8 to 50·6)	56·3 (53·9 to 58·6)	65·1 (63·4 to 67)	72·3 (70·6 to 74·1)	73·9 (72·2 to 75·5)	74·1 (72·2 to 76)
Mato Grosso	53 (50·7 to 55·5)	55·9 (53·2 to 58·7)	59·8 (57·4 to 62·2)	66·6 (65·3 to 68)	68·6 (67·5 to 69·9)	72 (70·9 to 73·1)	74·4 (74 to 74·7)	76·8 (75·7 to 77·8)
Mato Grosso do Sul	52·4 (50 to 55)	56·1 (53·4 to 58·7)	60·3 (58 to 62·5)	65·5 (64·7 to 66·3)	69·6 (69 to 70·2)	71·5 (71·2 to 71·8)	73·8 (73·4 to 74·2)	76·6 (75·4 to 77·6)
Minas Gerais	49·8 (47·1 to 52·8)	54·1 (52 to 56·3)	58·7 (56·4 to 60·8)	64·5 (63·9 to 65)	68·4 (67·8 to 69)	72·4 (71·8 to 73·1)	74·6 (74 to 75·2)	77·3 (76·2 to 78·3)
Pará	46·3 (42·9 to 49·5)	51·6 (49·3 to 54)	56·1 (53·8 to 58·4)	62·6 (60·9 to 64·3)	67·8 (66·4 to 69·2)	72 (70·7 to 73·4)	74·1 (72·8 to 75·4)	76·3 (75·2 to 77·5)
Paraíba	40·5 (37·1 to 44)	47·2 (44·6 to 49·7)	54·5 (52·1 to 56·7)	62·8 (61·6 to 64)	69 (68 to 69·9)	73·2 (72·4 to 73·9)	73·9 (73 to 74·7)	76·2 (74·7 to 77·6)
Paraná	53·2 (50·5 to 56)	57·2 (55 to 59·4)	60·9 (58·9 to 63·2)	64·7 (64·2 to 65·2)	68·9 (68·7 to 69·2)	71·7 (71·5 to 71·9)	73·6 (73·1 to 74)	75·8 (74·6 to 76·9)
Pernambuco	35·4 (31·7 to 39)	41·3 (38·8 to 43·8)	48·5 (46·1 to 50·8)	58·9 (57·8 to 59·8)	64·2 (63·4 to 64·9)	68·9 (68·2 to 69·6)	71·8 (71·2 to 72·4)	73·1 (71·9 to 74·4)
Piauí	45·5 (42·1 to 48·6)	49·8 (47·4 to 52·1)	53·5 (51·1 to 55·7)	61·6 (60·1 to 63·1)	68·4 (67·5 to 69·3)	72·8 (71·9 to 73·7)	75·7 (75·2 to 76·3)	77·2 (76·1 to 78·4)
Rio de Janeiro	52·7 (50·7 to 55)	57·1 (54·6 to 59·6)	59·8 (57·6 to 62·1)	63 (62·2 to 63·7)	65·8 (65·5 to 66)	69·8 (69·6 to 70)	72·8 (72·5 to 73·1)	74·9 (73·8 to 75·9)
Rio Grande do Norte	39·8 (36·2 to 43·2)	47·2 (44·7 to 49·8)	55 (52·7 to 57·3)	63·5 (61·9 to 64·9)	70·2 (69·1 to 71·4)	74 (72·8 to 75·1)	76·3 (75 to 77·6)	76·9 (75·1 to 78·8)
Rio Grande do Sul	55 (52·9 to 57·3)	59 (56·6 to 61·3)	63·3 (61·3 to 65·3)	67·5 (66·9 to 68)	70·4 (70·2 to 70·6)	72·3 (72·2 to 72·5)	74·8 (74·6 to 75·1)	76·2 (75·2 to 77·2)
Rondônia	39·4 (37·2 to 41·8)	44·2 (41·4 to 47)	50·5 (48·2 to 52·8)	58·3 (57·3 to 59·4)	64·7 (63·9 to 65·5)	70·8 (70 to 71·5)	74·1 (73·3 to 74·9)	75·6 (74·1 to 77)
Roraima	40·8 (38·6 to 43·2)	46·4 (43·8 to 49·1)	53·1 (50·9 to 55·1)	62 (60·9 to 63·1)	66·7 (66·1 to 67·4)	71·2 (70·7 to 71·7)	74·7 (74·2 to 75·1)	74·8 (74·1 to 75·6)
Santa Catarina	57·8 (55·4 to 60·3)	61 (58·9 to 63·2)	63·7 (61·6 to 65·8)	67·8 (67·3 to 68·2)	69·4 (68·8 to 69·9)	71·6 (71 to 72·1)	74·8 (74·3 to 75·3)	76·9 (75·8 to 78)
São Paulo	55·3 (53·1 to 57·6)	59 (56·5 to 61·5)	61·5 (59·4 to 63·6)	64·3 (63·6 to 64·8)	67·8 (67·4 to 68·4)	71 (70·9 to 71·1)	74·8 (74·5 to 75)	77 (76 to 77·9)
Sergipe	47·7 (44·7 to 50·6)	52·2 (49·5 to 54·7)	56·8 (54·5 to 59·1)	60·9 (59·2 to 62·5)	66·9 (65·9 to 67·9)	71·2 (70·7 to 71·8)	74·1 (73 to 75·2)	74·9 (73·2 to 76·5)
Tocantins	42 (39·5 to 44·7)	47·2 (43·1 to 51·4)	54·6 (52·2 to 57)	64 (61·6 to 66·2)	68·6 (67·3 to 69·8)	72·4 (71·5 to 73·5)	75·1 (73·9 to 76·2)	75·6 (74·2 to 77·1)
Paraguay	61·1 (57·8 to 64·1)	69·1 (67·8 to 70·4)	70·8 (69·6 to 72)	73·1 (72·2 to 74)	75·8 (74·6 to 76·9)	75·6 (74·7 to 76·5)	75·7 (74·9 to 76·6)	76·6 (73·6 to 79·6)
North Africa and Middle East	44·5 (42·1 to 47)	48·6 (46·8 to 50·4)	54·7 (53·2 to 56·1)	60·9 (59·7 to 61·9)	65·6 (64·9 to 66·4)	69·4 (68·6 to 70·1)	72 (71·2 to 72·9)	73·8 (72·5 to 75)

North Africa and Middle East	44·5 (42·1 to 47)	48·6 (46·8 to 50·4)	54·7 (53·2 to 56·1)	60·9 (59·7 to 61·9)	65·6 (64·9 to 66·4)	69·4 (68·6 to 70·1)	72 (71·2 to 72·9)	73·8 (72·5 to 75)
Afghanistan	42·7 (39·8 to 45·8)	45 (41·6 to 48·6)	47·1 (43·6 to 50·8)	43·7 (34·1 to 49·1)	53·8 (51·4 to 56·6)	54·7 (52·1 to 57·4)	60·5 (57·5 to 63·2)	63·3 (60·7 to 65·9)
Algeria	55·1 (53·3 to 56·9)	52·4 (50·6 to 54·1)	56·2 (53·9 to 58·5)	58·7 (56·6 to 60·9)	67·5 (65·6 to 69·3)	71 (69·1 to 72·7)	74·5 (73·1 to 75·8)	76·2 (74·5 to 77·8)
Bahrain	50·7 (46·5 to 54·9)	57·3 (54·4 to 60·2)	64·2 (61·1 to 67)	68·4 (67 to 70)	69·5 (68·5 to 70·6)	71·7 (70·7 to 72·7)	75 (74·2 to 75·8)	77 (75·5 to 78·6)
Egypt	41·3 (38·3 to 43·7)	46·9 (44·9 to 48·9)	51·2 (49·9 to 52·6)	58·5 (57·5 to 59·4)	63·9 (63·3 to 64·5)	68·5 (68·2 to 68·9)	69·4 (69·1 to 69·7)	71 (68·3 to 73·6)
Iran	39·4 (36·7 to 42·1)	44·5 (41·9 to 47)	54·5 (52·2 to 56·4)	64·7 (63·6 to 65·6)	66·7 (65·9 to 67·4)	71·9 (71·5 to 72·4)	75·5 (75·2 to 75·7)	77·8 (77·5 to 78)
Iraq	56 (53·1 to 59·2)	60·5 (58·1 to 62·9)	62·8 (59·3 to 65·4)	64·5 (62·5 to 66·6)	67 (65·3 to 68·7)	68·6 (65·8 to 71·5)	70·6 (68·1 to 73·3)	73·3 (71 to 75·7)
Jordan	56·9 (52·7 to 60·8)	59·4 (56·5 to 62·1)	63 (61·5 to 64·4)	67·1 (65·8 to 68·4)	71·2 (70 to 72·5)	72·9 (71·2 to 74·5)	76·9 (76 to 77·9)	78·2 (76·6 to 79·7)
Kuwait	65·4 (61·5 to 69·1)	69·2 (67·1 to 71·5)	70·5 (70·2 to 70·9)	72·5 (72·3 to 72·8)	74·9 (74·5 to 75·3)	78·1 (77·8 to 78·3)	80·1 (79·9 to 80·3)	81·5 (79·8 to 83)
Lebanon	58·8 (54·6 to 62·8)	64·9 (62 to 67·8)	67·6 (64·9 to 70·3)	69·4 (67·2 to 71·6)	69·9 (68·3 to 71·4)	74·8 (73·6 to 76)	75·6 (74·9 to 76·6)	76·6 (75·3 to 78)
Libya	47·3 (44·1 to 50·5)	52·8 (49·3 to 56·4)	64·6 (62·7 to 66·4)	69·8 (68·5 to 71·1)	72·6 (70·5 to 74·7)	74·9 (73·9 to 76)	76·2 (75·9 to 76·5)	75·9 (73·6 to 78·1)
Morocco	44·6 (41·7 to 47·6)	46·7 (44·3 to 49·1)	54·3 (52 to 56·6)	60·6 (59·1 to 62·2)	66·7 (65·6 to 67·8)	69·5 (67·9 to 71)	71·9 (69·7 to 74·1)	73·3 (71·5 to 75·6)
Oman	40·8 (36·6 to 45·1)	45·1 (41·1 to 49·4)	55·5 (51·9 to 58·9)	62 (58·7 to 65·1)	66·8 (64·3 to 69·5)	69·1 (67·7 to 70·4)	70·4 (70·1 to 70·8)	74 (73·2 to 74·8)
Palestine	54·4 (50·3 to 58·3)	58·8 (55·5 to 61·9)	57·8 (55·1 to 60·4)	66·7 (63·8 to 69·4)	69·1 (67·1 to 71·2)	70·2 (69·2 to 71·2)	73·7 (73·1 to 74·3)	74·8 (73·5 to 76·1)
Qatar	64·5 (60·8 to 68)	65·3 (62·7 to 67·9)	67 (64·1 to 69·9)	70·3 (67·9 to 72·8)	69·9 (68·3 to 71·6)	71·2 (69·7 to 72·7)	73·4 (72·1 to 74·7)	76·3 (74·5 to 78)
Saudi Arabia	50·9 (46·4 to 55·3)	54·3 (51·1 to 57·6)	59·8 (56·6 to 62·9)	66·1 (63·1 to 68·7)	68·1 (65·9 to 70·6)	70·4 (69·1 to 71·6)	71·8 (70·8 to 73)	74·5 (72·5 to 76·6)
Sudan	46·9 (44 to 50·1)	49·8 (47 to 52·6)	51·6 (49·4 to 53·9)	54·8 (52·8 to 57)	58·7 (56·9 to 60·5)	63·3 (60·8 to 65·7)	67·4 (64·8 to 70)	70·5 (68·1 to 72·6)
Syria	55 (52 to 58·1)	61 (58·2 to 63·8)	66·5 (64·6 to 68·3)	70·2 (68·1 to 72·4)	68·8 (66·8 to 70·9)	71·6 (70·4 to 72·9)	74·7 (73·2 to 76·2)	73·9 (71·3 to 76·2)
Tunisia	43·8 (39·4 to 47·8)	49·4 (47·4 to 51·4)	56·5 (55 to 58)	66·2 (65·2 to 67·3)	72·2 (71 to 73·4)	74·3 (72·5 to 76)	76·2 (73·6 to 79)	77·7 (74·9 to 80·4)
Turkey	45 (41·9 to 48·1)	49·5 (46·8 to 52·4)	57·1 (54·8 to 59·4)	64·1 (62·6 to 65·5)	68·7 (67·7 to 69·9)	74·7 (73·7 to 75·7)	76·7 (75·7 to 77·7)	78·4 (76·5 to 80·3)
United Arab Emirates	58·4 (54·2 to 62·4)	60·3 (57·2 to 63·5)	64 (61·2 to 66·8)	67·6 (65·3 to 70)	68·7 (67·2 to 70·2)	69·1 (67·8 to 70·4)	70·8 (69·3 to 72·3)	73·9 (71·7 to 76)
Yemen	31·9 (28·9 to 34·8)	36·5 (34·3 to 39·1)	41·9 (39 to 44·6)	53·2 (50·5 to 56·2)	59·1 (56·4 to 61·8)	63·5 (60·7 to 66·3)	67·6 (64·7 to 70·3)	67·7 (65 to 70)
South Asia	43 (40·8 to 45·3)	45·9 (43·7 to 47·9)	49·2 (47·8 to 50·6)	55·4 (54·5 to 56·3)	59·5 (58·8 to 60·3)	63 (62·3 to 63·6)	67 (66·3 to 67·7)	70·4 (69·2 to 71·7)
South Asia	43 (40·8 to 45·3)	45·9 (43·7 to 47·9)	49·2 (47·8 to 50·6)	55·4 (54·5 to 56·3)	59·5 (58·8 to 60·3)	63 (62·3 to 63·6)	67 (66·3 to 67·7)	70·4 (69·2 to 71·7)
Bangladesh	39·7 (37·1 to 42·5)	43·8 (41·2 to 46·6)	42·2 (40·2 to 44·3)	53·2 (51·4 to 55)	58·2 (57·1 to 59·2)	65·5 (64·4 to 66·4)	70 (69 to 71·1)	74·6 (72·4 to 76·7)
Bhutan	36·9 (32·6 to 41·4)	42·2 (38·4 to 46·4)	48·8 (44·3 to 52·9)	52·8 (49·3 to 56·4)	60·3 (57·7 to 62·9)	65·7 (64 to 67·6)	71 (69·2 to 73·1)	73·2 (70·9 to 75·6)
India	43·4 (40·7 to 46)	45·8 (43·2 to 48·2)	49·3 (47·6 to 51·1)	55·2 (54·1 to 56·2)	59·6 (58·7 to 60·5)	62·9 (62·1 to 63·7)	67·3 (66·4 to 68·1)	70·8 (69·3 to 72·2)
Andhra Pradesh	44·3 (41·2 to 47·5)	46·5 (43 to 49·8)	49·4 (46·7 to 52·1)	55·7 (53·3 to 57·9)	59·7 (57·6 to 62)	63·1 (60·9 to 65·4)	67·6 (65·5 to 70)	72·6 (70·2 to 75)
Arunachal Pradesh	44 (41·1 to 47·1)	45·9 (42·8 to 49·2)	49·3 (46·6 to 52)	57·4 (55·2 to 59·8)	60·9 (58·8 to 63·3)	64·5 (62·4 to 66·6)	69·4 (67·3 to 71·3)	72·6 (70·3 to 74·7)
Assam	41·9 (39 to 45)	44·2 (41·1 to 47·3)	47·9 (45·3 to 50·9)	54·6 (52·5 to 56·6)	57·9 (56·6 to 59·4)	58·6 (57·6 to 59·5)	63·4 (62·3 to 64·5)	68 (66·2 to 69·9)
Bihar	47·4 (44·4 to 50·6)	48·8 (45·8 to 51·8)	50·4 (47·7 to 53·2)	54·7 (52·5 to 56·8)	59·1 (57·1 to 61·2)	62·4 (60·9 to 64)	66·5 (65·1 to 67·9)	71·2 (69·5 to 73)
Chhattisgarh	43·4 (40·7 to 46·6)	44·9 (41·8 to 48·1)	47·7 (44·6 to 50·6)	52·7 (50 to 55·2)	57·3 (55·3 to 59·3)	60·3 (58·8 to 61·8)	63·6 (62·4 to 64·7)	67·7 (66·1 to 69·6)
Delhi	46·6 (43·6 to 49·9)	51·9 (48·9 to 54·9)	55·7 (53·3 to 58·1)	60·7 (58·5 to 62·8)	63·5 (61·7 to 65·5)	65·8 (64·6 to 67)	70·7 (69·9 to 71·4)	74·6 (73·1 to 76·1)
Goa	56·4 (53·5 to 59·4)	58·7 (55·8 to 61·3)	60·8 (58·5 to 63·2)	65·5 (63·5 to 67·5)	67·7 (65·9 to 69·5)	70·5 (68·7 to 72·4)	73·8 (71·9 to 75·7)	75·8 (73·7 to 78·2)
Gujarat	46·8 (43·8 to 49·9)	49·1 (46 to 52·1)	52·5 (50·1 to 54·9)	57·2 (55·2 to 59·3)	60·8 (59·3 to 62·3)	64·6 (63·5 to 65·7)	68·3 (67·4 to 69·2)	70·7 (68·7 to 72·7)
Haryana	46·8 (43·9 to 50·2)	48·8 (45·7 to 52)	52·5 (50 to 55)	59·6 (57·4 to 61·8)	63·5 (61·9 to 65·2)	65·2 (64·2 to 66·2)	68·6 (67·7 to 69·5)	72·5 (70·6 to 74·6)
Himachal Pradesh	51·7 (48·8 to 54·7)	53·3 (50·4 to 56·4)	56·1 (53·6 to 58·7)	61 (58·8 to 63·1)	64·1 (62·7 to 65·8)	67·7 (66·7 to 68·7)	71 (70·1 to 71·9)	72·9 (71·1 to 74·7)

Jammu and Kashmir	54·1 (51 to 57·3)	55·5 (52·4 to 58·6)	58·2 (55·7 to 60·9)	61·7 (59·5 to 63·8)	64·9 (62·9 to 66·7)	67·5 (66·3 to 68·8)	70·6 (69·8 to 71·5)	72·5 (70·7 to 74·1)
Jharkhand	47·9 (45·1 to 50·8)	49·5 (46·4 to 52·8)	52 (49 to 54·8)	55·7 (53·5 to 58)	57·1 (55·3 to 59)	60·3 (59 to 61·6)	65·6 (64·7 to 66·6)	73·5 (72·2 to 74·9)
Karnataka	44 (41 to 47)	46·5 (43·5 to 49·6)	50·3 (47·9 to 52·8)	57·6 (55·5 to 59·7)	62·3 (60·8 to 63·8)	64·9 (63·8 to 65·9)	68·6 (67·7 to 69·5)	70·5 (68·6 to 72·5)
Kerala	51·9 (49 to 55·1)	56·5 (53·5 to 59·3)	61·2 (58·7 to 63·5)	66·3 (64·4 to 68·2)	69 (67·5 to 70·6)	71 (70·2 to 71·8)	74·7 (74 to 75·5)	77·3 (75·5 to 79·1)
Madhya Pradesh	42·1 (39·1 to 45·3)	43·5 (40·3 to 46·6)	46·7 (43·8 to 49·7)	51·8 (49·6 to 53·9)	56·7 (54·8 to 58·8)	61 (59·5 to 62·4)	65·2 (63·8 to 66·3)	69·1 (67·4 to 70·9)
Maharashtra	46·2 (43·3 to 49·4)	48·9 (45·7 to 52·1)	52·2 (49·9 to 54·6)	58·9 (57 to 60·9)	63·1 (61·6 to 64·7)	65·4 (64·4 to 66·4)	70·8 (70 to 71·8)	73·4 (71·5 to 75·4)
Manipur	50·9 (48·1 to 53·9)	52·6 (49·5 to 55·5)	56·1 (53·7 to 58·6)	61·5 (59·5 to 63·8)	64·6 (62·7 to 66·6)	66·4 (64·5 to 68·5)	69·4 (67·4 to 71·7)	72·2 (70 to 74·5)
Meghalaya	55·6 (52·9 to 58·5)	56·7 (53·9 to 59·6)	58 (55·6 to 60·4)	59·4 (57·2 to 61·7)	61·8 (59·6 to 64)	65·1 (63·1 to 67·3)	68·9 (66·9 to 70·8)	71·6 (69·4 to 73·6)
Mizoram	58·7 (56 to 61·5)	60·2 (57·5 to 63)	62 (59·6 to 64·4)	63·6 (61·5 to 65·7)	65·8 (63·8 to 67·8)	67·6 (65·7 to 69·5)	69·1 (67 to 71·4)	72·2 (69·8 to 74·7)
Nagaland	48·5 (45·5 to 51·7)	50·9 (48·1 to 53·9)	55·2 (52·5 to 58)	61·1 (59 to 63·5)	64 (62 to 66·2)	65·9 (63·9 to 68·2)	68·5 (66·1 to 70·8)	71·8 (69·4 to 74·2)
Odisha	39·5 (36·5 to 42·6)	41·3 (38·3 to 44·6)	45 (42·7 to 47·5)	51·7 (49·6 to 53·8)	56·2 (54·5 to 58·1)	59·7 (58·4 to 60·9)	65·2 (64·1 to 66·3)	70·3 (68·1 to 72·5)
Punjab	52·6 (49·9 to 55·6)	54·8 (51·8 to 57·8)	58·4 (55·9 to 60·8)	62 (60·1 to 64·1)	64·8 (63·2 to 66·5)	67 (66·1 to 67·9)	70·4 (69·4 to 71·3)	73 (71 to 75·2)
Rajasthan	48·4 (45·6 to 51·4)	49·4 (46·6 to 52·3)	51·9 (49·1 to 54·7)	56 (53·7 to 58·1)	59·8 (58·2 to 61·4)	63·4 (62·3 to 64·4)	68 (67 to 69·1)	69·3 (67·4 to 71·1)
Sikkim	51·7 (48·9 to 54·9)	52·8 (49·8 to 55·7)	54·9 (52·4 to 57·6)	59·8 (57·6 to 62·1)	64 (62·1 to 66·1)	67·2 (65·3 to 69·2)	70·7 (68·8 to 72·8)	74·2 (72·1 to 76·4)
Tamil Nadu	41·6 (38·7 to 44·8)	44·7 (41·7 to 47·8)	48·8 (46·5 to 51·2)	55·1 (53·2 to 57·1)	60 (58·3 to 61·7)	64·1 (63 to 65·2)	68·1 (67·2 to 69·1)	72 (70·2 to 74)
Telangana	49·8 (46·4 to 53·2)	51·2 (47·8 to 54·8)	53·4 (50·6 to 56·1)	57·3 (54·9 to 59·9)	60·5 (58·1 to 63·1)	64 (61·4 to 66·7)	68·8 (66·7 to 71·1)	73·3 (70·6 to 75·7)
Tripura	41·4 (38·4 to 44·6)	45·1 (41·9 to 48·4)	49·8 (47·3 to 52·4)	57·1 (54·6 to 59·3)	61·4 (59·2 to 63·7)	65·6 (63·6 to 67·6)	69·6 (67·7 to 71·6)	72 (69·7 to 74·2)
Union Territories other than Delhi	53·9 (51·2 to 56·9)	56·6 (53·8 to 59·4)	59·8 (57·2 to 62·5)	64·6 (62·2 to 66·8)	66·9 (64·9 to 69)	69 (67·1 to 70·9)	71·9 (69·8 to 74·1)	74·3 (72·1 to 76·4)
Uttar Pradesh	34·6 (31·7 to 37·7)	36·8 (33·8 to 39·8)	41·6 (38·9 to 44·5)	49 (47·1 to 51·1)	55·3 (53·6 to 57)	60·2 (59·2 to 61·3)	64·3 (63·1 to 65·6)	66·9 (65·2 to 68·7)
Uttarakhand	48·6 (45·8 to 51·7)	50·4 (47·5 to 53·3)	53 (50·1 to 55·8)	56·8 (54·6 to 59)	59·1 (56·9 to 61·2)	61·6 (59·7 to 63·5)	65·3 (63·8 to 66·7)	69·2 (67·2 to 71·2)
West Bengal	43·7 (40·8 to 46·6)	46·9 (43·7 to 49·8)	50·6 (48·1 to 53)	56·7 (54·7 to 58·8)	61·4 (59·9 to 62·9)	64·7 (63·7 to 65·6)	69·7 (68·9 to 70·5)	73·7 (72 to 75·5)
Nepal	40·6 (38 to 43·4)	44·6 (42·1 to 47·2)	49·3 (46·7 to 51·9)	51·9 (49·3 to 54·3)	58·3 (56·3 to 60·3)	65·4 (63·9 to 66·9)	69 (67·5 to 70·5)	71·1 (69·4 to 73·2)
Pakistan	46 (43·6 to 48·6)	51·3 (49·4 to 53·5)	57·1 (55·6 to 58·7)	60·2 (58·9 to 61·6)	61·1 (60 to 62·1)	61 (60 to 62·1)	63·3 (61·7 to 65)	65·9 (63·8 to 67·8)
Southeast Asia, east Asia, and Oceania	49 (46·9 to 51·1)	33·4 (25·3 to 40·1)	59·4 (57·6 to 61)	63·5 (62·2 to 64·7)	67·3 (66·4 to 68·2)	70·2 (69·6 to 70·8)	73·5 (72·9 to 74·1)	76·1 (75·1 to 77·1)
East Asia	50·1 (47·5 to 52·4)	28·4 (19·5 to 36·3)	60·9 (58·6 to 63·1)	64·5 (62·9 to 66·1)	68·2 (67·1 to 69·4)	71·2 (70·5 to 72)	74·9 (74·2 to 75·7)	77·6 (76·3 to 78·9)
China	51 (48·4 to 53·4)	27·8 (18·8 to 35·8)	60·9 (58·5 to 63·1)	64·4 (62·8 to 66·1)	68·1 (67 to 69·4)	71·3 (70·6 to 72·1)	74·9 (74·2 to 75·7)	77·6 (76·3 to 79)
North Korea	20·3 (18·3 to 22·2)	47·6 (44·1 to 51·3)	57·1 (54·1 to 60·3)	64 (61 to 66·8)	68 (65·4 to 70·5)	63·9 (56·3 to 68·4)	70·9 (69·2 to 72·9)	73·1 (71 to 75·1)
Taiwan (province of China)	60·6 (57 to 64·1)	64·7 (64·6 to 64·8)	69·8 (69·7 to 69·9)	72·4 (72·3 to 72·5)	74·7 (74·6 to 74·8)	76·9 (76·8 to 77)	79·7 (79·6 to 79·8)	80·3 (77·5 to 82·7)
Oceania	50·9 (47·6 to 53·9)	55·1 (52·5 to 57·5)	59·4 (57·1 to 61·7)	62·5 (61·1 to 63·9)	63 (61·2 to 64·8)	63·3 (61·1 to 65·5)	63·4 (60·8 to 65·9)	64·9 (61·8 to 67·9)
American Samoa	62·1 (60·1 to 64·1)	66·5 (65·1 to 68)	66·5 (64·9 to 68)	69·4 (68·3 to 70·5)	70·6 (69·7 to 71·6)	71·2 (70·3 to 72·1)	71·9 (71 to 73)	72·2 (70 to 74·2)
Cook Islands	48·8 (47 to 50·9)	57·1 (55·5 to 58·7)	63·7 (62·1 to 65·2)	65·6 (64·5 to 66·7)	68·9 (67·4 to 70·2)	72·7 (71·2 to 74)	75 (73·8 to 76·1)	75·4 (73·3 to 77·3)
Fiji	59·3 (58·3 to 60·3)	64·6 (63·6 to 65·6)	65·2 (64·4 to 65·9)	66·5 (65·7 to 67·2)	66·3 (64·1 to 68·5)	65·7 (65·3 to 66·2)	67·6 (67·2 to 68·1)	68·4 (65·5 to 71·2)
Guam	68 (65·9 to 69·8)	67·7 (67 to 68·5)	67·3 (66·7 to 67·8)	71·4 (70·9 to 72)	73·3 (72·5 to 74·2)	75·6 (74·7 to 76·6)	77·1 (76 to 78·1)	76·4 (74·3 to 78·5)
Kiribati	44·7 (42 to 47·7)	48·2 (45·5 to 51·1)	51·7 (48·6 to 54·9)	54·8 (51·2 to 58·2)	54·6 (52 to 57·3)	56·4 (54·2 to 58·8)	58·8 (56·7 to 61·4)	60·8 (58 to 63·5)
Marshall Islands	52·1 (48·3 to 56)	56·3 (53·1 to 59·6)	59·4 (56 to 62·7)	62·9 (60·8 to 65)	62·9 (61·9 to 63·9)	62·7 (61 to 64·3)	64 (61·8 to 66·3)	65·5 (62·2 to 68·5)
Federated States of Micronesia	49·9 (47·3 to 52·7)	53 (50·1 to 56·2)	56·6 (53·5 to 59·9)	59·7 (56·7 to 62·6)	61·3 (58·7 to 64·1)	62·6 (61·1 to 64·2)	64 (61·8 to 66·4)	64·1 (61·4 to 66·9)
Nauru	58·5 (54·7 to 62·2)	60·6 (57·8 to 63·4)	62·1 (59·7 to 64·3)	62·6 (60·2 to 65)	62 (59·6 to 64·6)	59·1 (57 to 61·5)	60 (57·7 to 62·4)	63·4 (61·1 to 65·6)

Niue	55·6 (53·3 to 58·1)	63 (60·6 to 65·3)	66·1 (63·9 to 68·4)	68·2 (65·4 to 70·9)	68·3 (65·6 to 70·6)	68·2 (65·9 to 70·3)	69·7 (67·6 to 71·8)	70·6 (67·8 to 73·1)
Northern Mariana Islands	63·5 (59·9 to 66·8)	65·4 (62·6 to 68·2)	67·4 (64·6 to 69·9)	69·3 (66·5 to 71·6)	70·4 (68·4 to 72·2)	72 (70·3 to 73·4)	72·4 (71 to 74)	72·9 (71·1 to 74·7)
Palau	49 (45 to 53·1)	53·9 (50·8 to 57·2)	58·7 (56 to 61·7)	62·2 (59·1 to 65)	64·9 (61·8 to 67·8)	66·4 (63·8 to 69·1)	67 (64·5 to 69·7)	67·5 (64·7 to 70·4)
Papua New Guinea	49·6 (45·1 to 53·8)	53·7 (50·4 to 57)	59 (56·1 to 62·1)	62·1 (60·6 to 63·7)	62·6 (60·4 to 65)	63·1 (60·1 to 66·1)	62·8 (59·5 to 66)	64·7 (61·1 to 68)
Samoa	54·4 (50·8 to 58)	60·7 (58·2 to 63·3)	62·4 (59·9 to 64·8)	65·2 (62·5 to 67·9)	66·9 (63·8 to 69·4)	68·5 (65·6 to 70·9)	69·6 (67·2 to 72)	70·5 (67·6 to 73·1)
Solomon Islands	42·1 (39·5 to 44·9)	45·8 (43 to 48·8)	50·4 (47·7 to 53·3)	54·4 (51 to 57·8)	55·8 (52·9 to 59)	57 (54·6 to 59·6)	57·4 (55·3 to 59·7)	59·2 (56·6 to 61·8)
Tokelau	56·8 (53·7 to 59·6)	60 (56·9 to 63·2)	63·1 (59·9 to 66·3)	65·4 (62·7 to 68)	67·8 (65 to 70·4)	69·3 (66·4 to 72·1)	70·9 (68·2 to 73·4)	72·2 (69·1 to 74·9)
Tonga	61·5 (58·1 to 64·4)	64·6 (62·7 to 66·7)	66·5 (64·7 to 68·4)	68·4 (66·7 to 70)	71 (69·4 to 72·6)	70·7 (69·3 to 72·1)	72·2 (70·2 to 74·2)	73 (70·4 to 75·4)
Tuvalu	47 (43 to 51)	51·4 (48·1 to 55)	55·5 (51·9 to 59·3)	57·8 (54·7 to 61·2)	60·8 (58·7 to 63)	61·5 (59·8 to 63·1)	66·6 (64 to 69·3)	67·9 (64·4 to 71·1)
Vanuatu	47·4 (44·6 to 50·5)	51·4 (48·7 to 54·3)	56·7 (54·1 to 59·6)	62·1 (59·2 to 64·8)	63·1 (60·1 to 66·2)	63·6 (60·6 to 66·7)	64·4 (61·4 to 67·4)	65·5 (62·2 to 68·3)
Southeast Asia	46·1 (44·3 to 48)	52 (50·3 to 53·6)	55·5 (54 to 56·8)	61·4 (60·2 to 62·5)	65·3 (64·6 to 66·1)	68·1 (67·5 to 68·7)	70·8 (70·1 to 71·5)	72·9 (71·8 to 74·2)
Cambodia	46·2 (42 to 50·4)	49·4 (46·1 to 52·8)	44·5 (41·5 to 47·5)	52·2 (48·7 to 54·9)	57·8 (56·4 to 59·4)	60·4 (59 to 61·8)	66·9 (65·2 to 68·6)	69·9 (68 to 72·2)
Indonesia	43·2 (40·9 to 45·5)	48·6 (46·5 to 50·6)	54·1 (52·3 to 56)	59·7 (58·5 to 61)	64·1 (63·2 to 64·9)	67·2 (66·4 to 68·1)	69·3 (68·2 to 70·3)	71·4 (70 to 73)
Aceh	49 (46·2 to 52)	53·4 (50·6 to 56·2)	57·2 (54·6 to 59·8)	61·5 (59·3 to 63·7)	64·4 (62·6 to 66·1)	66·9 (65·4 to 68·4)	67·9 (66 to 69·5)	69·7 (67·6 to 72)
Bali	48·8 (46·2 to 51·7)	53·7 (50·9 to 56·7)	58 (55·6 to 60·8)	61·7 (59·8 to 63·7)	66·7 (65·2 to 68·1)	69·5 (68·3 to 70·5)	71·8 (70·5 to 73·1)	75·4 (73·1 to 77·8)
Bangka-Belitung Islands	40·3 (37·4 to 43·4)	45·1 (42·4 to 47·8)	50·7 (47·9 to 53·4)	56·7 (54·5 to 59·1)	60·5 (58·8 to 62·3)	64·2 (62·7 to 65·6)	66·2 (64·4 to 67·9)	68·1 (65·9 to 70·1)
Banten	31·7 (28·8 to 35)	38·3 (35·7 to 40·8)	45·5 (43 to 48·2)	53·5 (51·3 to 55·9)	61·3 (59·5 to 63)	66·7 (65·3 to 68·2)	68·4 (66·9 to 70·2)	70·4 (68·5 to 72·9)
Bengkulu	36·4 (33·7 to 39·2)	41·6 (38·9 to 44·2)	48 (45·6 to 50·4)	54·4 (52·4 to 56·6)	60·4 (58·6 to 62)	64·5 (63·1 to 66)	67·3 (65·6 to 68·9)	69·7 (67·7 to 71·8)
Gorontalo	39·5 (36·4 to 42·7)	44·7 (41·7 to 47·5)	50 (47·2 to 52·9)	55·6 (53·2 to 58)	59·7 (58 to 61·5)	61·9 (60·4 to 63·6)	64 (62·1 to 66·1)	67·4 (64·9 to 70·3)
Jakarta	48·9 (46 to 51·8)	54·5 (51·7 to 57·1)	60·2 (57·5 to 62·8)	65·2 (63·2 to 67·2)	68·2 (66·6 to 69·7)	69·5 (68·3 to 70·7)	71·1 (69·7 to 72·9)	73 (71 to 75·1)
Jambi	40·2 (37·6 to 43·3)	46 (43·5 to 48·7)	50·6 (48·1 to 53·3)	55·6 (53·8 to 57·8)	61 (59·5 to 62·8)	65·9 (64·6 to 67·3)	68·7 (67·1 to 70·1)	70·7 (68·9 to 73·1)
West Java	38·1 (35·3 to 40·8)	44·1 (41·6 to 46·5)	51 (48·5 to 53·5)	58·2 (56·4 to 60·1)	63·9 (62·6 to 65·3)	68·3 (67 to 69·5)	70·3 (68·9 to 71·9)	72·2 (70·3 to 74·3)
Central Java	48·1 (45·2 to 50·8)	52·7 (50 to 55·3)	57·9 (55·4 to 60·4)	63·4 (61·4 to 65·2)	67·7 (66·3 to 69)	69·4 (68·3 to 70·8)	71 (69·7 to 72·6)	72·9 (71·2 to 75)
East Java	46·8 (44·3 to 49·6)	51·4 (48·9 to 53·8)	56·2 (53·7 to 58·6)	61 (59·2 to 62·9)	64·7 (63·4 to 66)	68 (66·8 to 69·2)	70·2 (68·8 to 71·7)	72·2 (70·4 to 74·2)
West Kalimantan	44·3 (41·5 to 47·4)	47·1 (44·2 to 49·7)	50·7 (48·2 to 53·3)	55·4 (53·5 to 57·6)	60·6 (58·9 to 62·3)	64·7 (63·3 to 66·1)	68·2 (66·7 to 69·6)	71 (69·2 to 72·8)
South Kalimantan	35·3 (32·6 to 38·3)	39·4 (36·8 to 42)	46·2 (43·9 to 48·7)	53·1 (51·2 to 55·2)	57·9 (56·1 to 59·6)	61·8 (60·3 to 63·2)	65 (63·2 to 66·7)	67·8 (65·5 to 69·9)
Central Kalimantan	46·5 (43·6 to 49·4)	52·8 (49·9 to 55·6)	56·7 (54·2 to 59·2)	60·4 (58·3 to 62·7)	63·4 (61·8 to 64·9)	67·7 (66·3 to 69·1)	68·9 (67·4 to 70·3)	70·4 (68·6 to 72·7)
East Kalimantan	48·8 (46·1 to 51·8)	51·7 (48·6 to 54·8)	55·7 (53 to 58·6)	60·8 (58·8 to 63)	64 (62·5 to 65·7)	65·6 (64·1 to 67)	67·7 (65·8 to 69·3)	69·8 (67·6 to 71·8)
North Kalimantan	47·5 (44·4 to 50·8)	51·4 (48·7 to 54·1)	55·1 (52·6 to 57·7)	62 (59·5 to 64·4)	67·7 (65·5 to 69·9)	71·7 (69·8 to 73·6)	73·2 (71·3 to 75·4)	74·4 (71·8 to 77·3)
Riau Islands	47·9 (44·8 to 51·2)	52·4 (49·6 to 55·3)	57·1 (54·5 to 59·8)	60·8 (58·6 to 63·1)	64·3 (62·6 to 66)	69·6 (68·3 to 71)	71·1 (69·8 to 72·5)	72·5 (70·7 to 74·8)
Lampung	42·3 (39·7 to 45·2)	48·3 (45·5 to 51)	54 (51·5 to 56·8)	60·2 (58·1 to 62·2)	64·5 (62·9 to 66·1)	68·8 (67·4 to 70·2)	70·6 (69 to 72·3)	72·1 (69·9 to 74·4)
Maluku	34 (31·3 to 37)	40·7 (38·2 to 43·2)	46·7 (44 to 49·4)	52·3 (50·2 to 54·7)	57·3 (55·4 to 59·2)	61·1 (59·4 to 62·8)	63·4 (61·4 to 65·5)	67·2 (64·9 to 69·5)
North Maluku	36·9 (33·9 to 39·9)	41·7 (39·2 to 44·2)	46·6 (44·4 to 49)	50·8 (48·7 to 53)	56·9 (55 to 58·8)	60 (58·2 to 61·8)	63·1 (61·3 to 64·9)	66·5 (64·3 to 68·9)
West Nusa Tenggara	33·1 (30·6 to 35·7)	38·3 (35·9 to 40·8)	43·6 (41·2 to 46·2)	50 (48 to 52·1)	56·7 (55 to 58·4)	62·2 (60·7 to 63·7)	66·7 (65 to 68·3)	69·4 (67·4 to 71·5)
East Nusa Tenggara	38·4 (35·7 to 41·2)	43·9 (41·4 to 46·6)	49·5 (47 to 52·2)	55·2 (53 to 57·4)	60 (58·4 to 61·7)	63·8 (62·3 to 65·2)	67·4 (65·8 to 69·1)	70 (67·9 to 72·4)
Papua	48·7 (45·8 to 51·8)	51·4 (48·5 to 54·3)	54·4 (51·8 to 57)	57·6 (55·3 to 59·9)	59·8 (58 to 61·6)	61·5 (59·8 to 63·3)	62·5 (60·6 to 64·5)	65·2 (62·7 to 67·9)
West Papua	50·6 (47·6 to 53·8)	53·1 (50·1 to 56·2)	55·6 (53 to 58·3)	59·5 (57·2 to 62·1)	62·6 (60·6 to 64·7)	64·3 (62·8 to 65·9)	64·5 (62·9 to 66·2)	68·6 (66·3 to 70·9)

Riau	45·9 (42·9 to 48·6)	49·4 (46·8 to 52·1)	54·1 (51·5 to 56·8)	60·3 (58·2 to 62·4)	64·1 (62·5 to 65·7)	67·4 (66·1 to 69)	69·6 (68·1 to 71·3)	72·1 (70·1 to 74·6)
West Sulawesi	29·4 (26·7 to 32·5)	34·8 (32·3 to 37·1)	40·4 (38·1 to 43·2)	47·7 (45·7 to 50)	54·2 (52·3 to 56·2)	59·7 (58·1 to 61·1)	63·9 (62·1 to 65·8)	67·1 (64·9 to 69·6)
South Sulawesi	41 (38·5 to 43·8)	46 (43·5 to 48·6)	51·4 (48·9 to 53·9)	56·9 (55 to 59)	62·3 (60·6 to 64)	66·6 (65·2 to 68)	69 (67·6 to 70·6)	71·4 (69·6 to 73·7)
Central Sulawesi	33·8 (31·2 to 36·4)	42 (39·6 to 44·6)	48 (45·6 to 50·5)	53·5 (51·6 to 55·6)	57·7 (56·2 to 59·5)	61·4 (59·9 to 62·9)	65·1 (63·3 to 66·9)	68 (65·8 to 70·3)
Southeast Sulawesi	38·7 (36 to 41·5)	42·7 (40 to 45·4)	47·1 (44·7 to 49·7)	54·1 (51·9 to 56·6)	59·6 (57·9 to 61·3)	63 (61·5 to 64·5)	65·7 (64 to 67·5)	68 (65·7 to 70·1)
North Sulawesi	47·8 (45·3 to 50·9)	51·5 (48·9 to 54·1)	56·1 (53·8 to 58·8)	60·7 (58·6 to 63)	63·3 (61·8 to 65)	66·2 (64·9 to 67·5)	67·7 (66 to 69·2)	69·4 (67·4 to 71·7)
West Sumatra	35·9 (33·1 to 38·5)	44·2 (41·5 to 46·8)	51·6 (49·2 to 54)	58·8 (56·8 to 60·8)	63·3 (61·7 to 64·9)	65·9 (64·6 to 67·3)	68·1 (66·5 to 69·6)	71·2 (69·3 to 73·4)
South Sumatra	43·3 (40·5 to 46·3)	49·4 (46·7 to 52·1)	55 (52·3 to 57·6)	61·8 (60 to 63·8)	65 (63·4 to 66·6)	68·2 (66·9 to 69·5)	70·2 (68·6 to 71·7)	71·7 (69·5 to 73·7)
North Sumatra	46·9 (44·1 to 49·9)	51·4 (48·4 to 54)	55·8 (53·3 to 58·5)	60·4 (58·3 to 62·5)	63·3 (61·8 to 64·9)	65·4 (64·1 to 66·9)	67·3 (65·7 to 68·9)	69·1 (66·8 to 71·5)
Yogyakarta	56·4 (53·7 to 59·2)	60·4 (57·5 to 63·2)	63·6 (61 to 66·3)	66·8 (64·9 to 69·2)	68·7 (67·3 to 70·1)	70·2 (69 to 71·4)	70·9 (69·4 to 72·2)	71·9 (70·1 to 73·6)
Laos	38·9 (34·8 to 43·1)	40·2 (36·4 to 44·3)	42·4 (38·2 to 46·5)	47·3 (43·9 to 51)	52·9 (50·3 to 55·7)	58·4 (56·6 to 60·3)	64·9 (62·8 to 67·3)	69 (66·4 to 71·6)
Malaysia	53·5 (51·8 to 55)	62·8 (62·7 to 63)	67·3 (67·2 to 67·4)	70·5 (69·3 to 71·6)	71·9 (71·8 to 72)	73 (72·9 to 73·1)	74·4 (73·9 to 74·9)	75 (72·7 to 77·4)
Maldives	30·6 (26·8 to 34·6)	37·8 (34·6 to 41·2)	47·5 (45·2 to 49·8)	56 (54·6 to 57·2)	65·5 (64·8 to 66·2)	72·2 (71·7 to 72·7)	77·8 (77·2 to 78·3)	79·1 (77·6 to 80·6)
Mauritius	53·1 (52·5 to 53·7)	58·1 (57·7 to 58·5)	63·7 (63·3 to 64·1)	67·2 (66·9 to 67·6)	70·2 (69·9 to 70·5)	72·3 (72 to 72·7)	74·2 (73·9 to 74·6)	75·5 (73·1 to 77·8)
Myanmar	34·6 (30·6 to 38·7)	38·7 (35·3 to 42·2)	43·1 (39 to 47·3)	50·6 (47·2 to 54·1)	55·4 (52·9 to 58)	58·9 (56·8 to 61)	64·8 (62·6 to 66·8)	69·5 (66·9 to 71·5)
Philippines	55·1 (53·2 to 56·9)	61·3 (60 to 62·5)	62·7 (61·5 to 63·7)	65·7 (64·8 to 66·7)	68·4 (67·5 to 69·3)	70·3 (69·4 to 71·2)	70·6 (69·7 to 71·6)	71·8 (69·9 to 73·9)
Seychelles	60 (58·2 to 61·6)	63·6 (62·7 to 64·6)	65·7 (64·9 to 66·5)	71·1 (70·4 to 71·7)	70·6 (70 to 71·1)	72·2 (71·7 to 72·7)	73·2 (72·7 to 73·7)	73·6 (72·4 to 74·7)
Sri Lanka	56·3 (56·1 to 56·4)	64 (63·8 to 64·2)	66·1 (65·9 to 66·2)	69·3 (69·2 to 69·4)	69·6 (68·9 to 70·3)	71·5 (71·4 to 71·6)	74·1 (73·9 to 74·2)	77·3 (74·4 to 80·1)
Thailand	53·8 (51·1 to 56·6)	60·9 (59·5 to 62·2)	64·2 (62·9 to 65·6)	66·5 (65·4 to 67·5)	71·3 (70·2 to 72·4)	71·8 (70·4 to 73·3)	77 (75·8 to 78·3)	78·4 (75·3 to 81·5)
Timor-Leste	42·5 (38·1 to 46·9)	45·7 (42·1 to 49·5)	50·5 (47·4 to 53·7)	52 (49·2 to 54·5)	60·9 (58·8 to 62·9)	65·5 (63·8 to 67)	70 (68·6 to 71·4)	70·6 (69 to 72·7)
Vietnam	52·3 (48·4 to 56)	58·4 (55·1 to 61·6)	56 (53·4 to 58·5)	66·3 (63·6 to 68·7)	69·2 (67·2 to 71·1)	72·2 (70·8 to 73·6)	73·2 (71·9 to 74·4)	74·5 (72·8 to 76·4)
Sub-Saharan Africa	43·5 (40·3 to 46·6)	45·5 (43·2 to 47·9)	48·9 (47·1 to 50·6)	52·4 (51·1 to 53·6)	53·9 (52·9 to 54·8)	53 (51·5 to 54·4)	59 (57·8 to 60)	64·5 (62·8 to 65·9)
Central sub-Saharan Africa	45 (40·8 to 49·2)	46·9 (43·6 to 50·3)	48·8 (46 to 51·7)	51·3 (48·7 to 53·9)	53·1 (51·2 to 55)	53·1 (51·6 to 54·6)	58·6 (56·8 to 60·5)	64·4 (62 to 66·8)
Angola	45·6 (41·2 to 49·9)	46·3 (42·7 to 49·9)	46·6 (42·9 to 50·5)	48·1 (45·1 to 51·2)	49·7 (47·4 to 52·1)	52·8 (50·9 to 54·6)	60·2 (58 to 62·5)	65·1 (62·2 to 67·8)
Central African Republic	43·6 (39·7 to 47·7)	45·8 (42·2 to 49·8)	48·1 (45 to 51·1)	49·2 (46·9 to 51·5)	48·1 (46 to 50·2)	43·8 (41·4 to 46·6)	47·8 (44·6 to 51·3)	52·3 (48·2 to 56·2)
Congo (Brazzaville)	42 (38·1 to 46·1)	43·7 (40·4 to 47)	47·2 (43·1 to 51)	52·8 (50 to 55·6)	54 (51·8 to 56·2)	53 (50·9 to 55·5)	60·9 (58·5 to 63·2)	65·4 (62·5 to 68·1)
DR Congo	45·3 (41·2 to 49·6)	47·6 (43·9 to 51·1)	49·6 (46·7 to 52·8)	52·1 (49·4 to 55)	54·3 (52·1 to 56·6)	53·9 (52·3 to 55·6)	58·8 (56·9 to 60·7)	65 (62·7 to 67·5)
Equatorial Guinea	39·2 (35·4 to 43·2)	40·4 (36·9 to 44·2)	45·2 (41·8 to 49)	50·5 (47·4 to 53·7)	51·1 (48·3 to 54·2)	57·6 (53·9 to 61·5)	63·5 (59·7 to 67·8)	66 (62·1 to 69·9)
Gabon	48·7 (44·6 to 52·8)	50 (46·7 to 53·3)	53·2 (50·3 to 56·2)	58·1 (55·9 to 60·6)	60·4 (58·3 to 62·4)	59·6 (56·9 to 62·1)	63·3 (61·1 to 65·4)	67·8 (65·5 to 70·3)
Eastern sub-Saharan Africa	42 (38·8 to 45·1)	44·7 (42·3 to 47)	47·5 (45·8 to 49·2)	50·7 (49·4 to 51·9)	51·4 (50·2 to 52·4)	51·8 (50·1 to 53·4)	60·1 (58·9 to 61·2)	65·6 (64·1 to 66·9)
Burundi	40·2 (36·1 to 44·4)	43·9 (40·8 to 47·4)	46 (43·1 to 49·1)	48·5 (46 to 51·1)	48·7 (45·7 to 51·3)	44·7 (42·5 to 47)	58·6 (56·1 to 61·1)	63·8 (60·3 to 67)
Comoros	44·7 (41·5 to 48·2)	47·1 (44·1 to 50·3)	51·4 (48·3 to 54·5)	54·5 (52 to 57·2)	58·5 (56·2 to 60·9)	61·4 (59·4 to 63·5)	65·9 (63·8 to 68)	68·7 (66·4 to 70·8)
Djibouti	54·6 (50·4 to 58·8)	56·2 (52·4 to 60)	58·4 (54·8 to 62·1)	60·8 (57·7 to 63·9)	60·6 (57·6 to 63·7)	60·4 (57 to 64·2)	63·2 (59·5 to 67·1)	66·8 (63·2 to 70)
Eritrea	39·8 (35·8 to 43·9)	42·1 (38·6 to 46·2)	40·9 (26·6 to 47·4)	42·7 (40·5 to 44·8)	46·2 (44·4 to 48)	54·8 (52·1 to 57·5)	60 (56·5 to 63·2)	63·8 (59·9 to 67·1)
Ethiopia	39 (36·4 to 41·5)	40·8 (38·5 to 43·2)	42·7 (40·7 to 44·9)	44·7 (43·1 to 46·3)	46·9 (45·7 to 48·1)	51·1 (49·3 to 52·7)	63·1 (62 to 64)	68·8 (67·5 to 70·2)
Kenya	48·8 (46·5 to 51)	54·3 (52·3 to 56·2)	58·1 (56·4 to 59·9)	62·2 (60·7 to 63·8)	62·1 (60·6 to 63·5)	54·8 (52·3 to 57·6)	61·2 (60·2 to 62)	66·5 (65·1 to 67·9)
Baringo	48·8 (45·7 to 52·2)	51·6 (48·6 to 54·8)	52·2 (49·1 to 55·4)	54·8 (51·7 to 57·7)	56·4 (53·5 to 59·2)	55·8 (53·1 to 58·4)	60·9 (58·7 to 63·2)	66·3 (63·5 to 69)

Bomet	67·3 (64·6 to 70·2)	69 (66·3 to 71·9)	69·7 (66·9 to 72·5)	71·3 (68·6 to 74)	69 (64·7 to 71·8)	57·4 (52·3 to 62·5)	62·7 (59·1 to 65·8)	68 (65 to 70·6)
Bungoma	45·9 (42·9 to 49)	51·6 (48·6 to 54·8)	56·4 (53·4 to 59·7)	60·3 (57·6 to 63·1)	61·7 (59·2 to 64·5)	58·8 (55·4 to 62·1)	66·3 (64 to 68·6)	70·8 (68·6 to 72·9)
Busia	34·8 (32 to 37·8)	41·6 (38·6 to 44·7)	49 (46 to 52·1)	58·3 (55·5 to 61·1)	58·5 (55·3 to 61·8)	50·5 (46·2 to 55·1)	61 (58·1 to 64)	67·6 (64·6 to 70·4)
Elgeyo Marakwet	57 (53·8 to 60·3)	58·7 (55·7 to 62·1)	58·5 (55·6 to 61·6)	59·7 (56·9 to 62·8)	61·4 (58·6 to 64·3)	59·4 (56·4 to 62·5)	64·5 (62·5 to 66·6)	68·8 (66·2 to 71·1)
Embu	43·2 (40 to 46·3)	51 (47·9 to 54·3)	59·7 (56·6 to 62·7)	66·6 (63·8 to 69·5)	68·4 (65·9 to 71·1)	63·8 (58·7 to 68·2)	66·3 (63 to 69·6)	70 (67·1 to 72·5)
Garissa	48·8 (45·4 to 51·9)	52·9 (49·6 to 56·5)	55·8 (52·6 to 59·4)	58·6 (55·5 to 61·6)	60·2 (57·3 to 63·2)	60·1 (56·5 to 63·3)	63 (59·7 to 65·7)	66·7 (63·6 to 69·6)
Homa Bay	31·8 (29·1 to 34·6)	39·2 (36·6 to 42·1)	42·7 (39·9 to 45·7)	48·9 (46·4 to 51·7)	49·4 (45·7 to 52·9)	36·2 (32·4 to 40·8)	46·3 (43 to 49·5)	56·3 (52·5 to 60·6)
Isiolo	28·2 (25·3 to 31·2)	34·8 (31·5 to 38·1)	38·5 (35·2 to 42)	46·4 (43·1 to 50)	51·8 (48·8 to 54·8)	51·2 (46·6 to 55·6)	56·3 (52·4 to 60·1)	62·5 (58·6 to 65·8)
Kajiado	59·2 (56·1 to 62·6)	61·2 (58·2 to 64·6)	61·3 (58·2 to 64·5)	63·5 (60·5 to 66·4)	64·2 (61·3 to 67·3)	58·6 (54·9 to 62)	64·3 (61·7 to 66·4)	68·8 (66·2 to 71·2)
Kakamega	45·5 (42·6 to 48·5)	50·8 (48·1 to 53·7)	55·1 (52·1 to 58·1)	59·6 (56·8 to 62·7)	58·8 (56·1 to 61·6)	50·8 (47·2 to 54·9)	59·9 (57·4 to 62·8)	67 (64·1 to 69·6)
Kericho	63·9 (60·7 to 67·4)	65·5 (62·4 to 68·8)	66·1 (63 to 69·2)	67·2 (64·4 to 70)	67·2 (64 to 70·2)	60·4 (56 to 64·7)	65·9 (62·6 to 68·9)	70·8 (68·1 to 73·2)
Kiambu	57·4 (54·2 to 60·7)	61 (58 to 64·1)	63·9 (61·2 to 66·8)	66·1 (63·5 to 68·8)	65·3 (63 to 67·6)	57·6 (54·2 to 60·8)	62·8 (60·8 to 64·8)	67·5 (65·2 to 69·9)
Kilifi	38·6 (35·6 to 41·8)	46·6 (43·6 to 49·6)	51·6 (48·9 to 54·4)	57·4 (54·7 to 60·1)	59·8 (56·8 to 62·7)	56·5 (52·9 to 60·3)	62·9 (60·4 to 65·5)	68·4 (65·6 to 70·9)
Kirinyaga	47·7 (44·7 to 50·8)	53 (49·9 to 56·1)	57 (54 to 60)	65·9 (63 to 68·7)	67·1 (64·1 to 69·9)	61·1 (57·1 to 64·9)	65·1 (62·5 to 67·7)	68·4 (65·4 to 71·1)
Kisii	47·2 (44·1 to 50·1)	51·6 (48·6 to 54·7)	55·2 (52·2 to 58·4)	58·2 (55·1 to 61·3)	58·3 (55·8 to 60·9)	54·5 (51·5 to 57·5)	62·5 (60·6 to 64·5)	67 (64·5 to 69·3)
Kisumu	48·6 (45·8 to 51·6)	53·2 (50 to 56·3)	55·8 (53 to 58·8)	57·8 (54·9 to 60·9)	55·7 (52·5 to 58·8)	43·5 (39·7 to 47·7)	53·1 (49·8 to 56·3)	60·5 (57 to 64·2)
Kitui	46·9 (43·6 to 50)	51·7 (48·9 to 54·9)	56·7 (53·5 to 59·8)	62·3 (59·7 to 65·2)	64·1 (61·4 to 66·6)	58·9 (54·7 to 63·4)	64·8 (62·1 to 67·3)	69·6 (67·1 to 72)
Kwale	38·7 (35·8 to 41·9)	45·5 (42·3 to 48·6)	50·9 (47·9 to 53·9)	56·7 (53·7 to 59·6)	58·8 (55·7 to 61·8)	56·4 (52·9 to 60·2)	60·8 (58·6 to 63·2)	66·2 (63·4 to 69)
Laikipia	64·7 (61·5 to 68)	66·8 (63·7 to 69·7)	67·8 (64·7 to 70·7)	70·1 (67·4 to 72·7)	67·7 (64·5 to 70·7)	58·5 (54 to 63·1)	63·9 (60·8 to 67·2)	69·6 (66·5 to 72·3)
Lamu	34·6 (31·6 to 37·9)	43·5 (40·2 to 46·7)	50·9 (47·1 to 54·6)	62·3 (59·1 to 65·4)	61·2 (57·8 to 64·7)	58·1 (53·7 to 62·3)	62·6 (59 to 66·1)	67·4 (63·9 to 70·7)
Machakos	55·7 (52·5 to 58·8)	59·8 (56·7 to 62·9)	62·6 (59·4 to 65·6)	66·7 (64 to 69·4)	68·4 (65·9 to 70·8)	61·6 (56·7 to 65·8)	65·8 (62·7 to 68·4)	70·1 (67·6 to 72·4)
Makueni	53·8 (50·8 to 56·7)	58·2 (55·2 to 61·3)	61·3 (58·3 to 64·4)	65·4 (62·7 to 68·3)	65·5 (62 to 68·5)	54 (48·6 to 59·8)	59·6 (56·4 to 62·7)	66·1 (63·3 to 68·4)
Mandera	53·7 (50·7 to 56·8)	56·2 (53·2 to 59·6)	58 (54·7 to 61·4)	59·2 (56·1 to 62·5)	58·5 (55·8 to 61·6)	57·3 (53·9 to 60·5)	58·9 (56·3 to 61·5)	62·8 (60 to 65·8)
Marsabit	49·6 (46·1 to 53)	54·1 (50·5 to 57·5)	56·6 (53·3 to 59·7)	60·7 (57·5 to 63·9)	62·5 (59·7 to 65·4)	59·4 (54·7 to 63·7)	61 (57·9 to 64·2)	65·3 (61·8 to 68·6)
Meru	57·2 (54·2 to 60·2)	61 (58·2 to 63·9)	64·3 (61·3 to 67·2)	68·3 (65·5 to 70·8)	68·5 (66 to 70·7)	61·9 (57·8 to 66)	63·6 (60·9 to 66·2)	67·2 (64·1 to 69·9)
Migori	37·9 (34·8 to 40·7)	43·6 (40·4 to 46·8)	47·6 (44·8 to 50·7)	50 (47·3 to 52·7)	50·7 (47·5 to 54)	41·5 (36·9 to 46·3)	50·2 (46·1 to 53·9)	59·2 (55 to 63·4)
Mombasa	47 (43·9 to 50·2)	54·9 (51·8 to 58·2)	60·6 (57·5 to 63·5)	66·5 (63·5 to 69·4)	64·2 (60·7 to 67·4)	55·2 (50·9 to 59·8)	61·7 (59 to 64·6)	67·5 (64·5 to 70·3)
Murang'a	53·2 (50·4 to 56·3)	59 (56 to 61·9)	63 (60 to 65·8)	67·8 (65·2 to 70·6)	68·6 (66·3 to 70·8)	61·6 (57·1 to 65·5)	64·7 (62·1 to 67·2)	69·4 (66·8 to 71·8)
Nairobi	42·4 (39·6 to 45·4)	54·8 (51·8 to 57·6)	62·4 (59·5 to 65·2)	67·5 (65·1 to 69·9)	64·4 (61·6 to 67·4)	55·1 (52·1 to 58·5)	62·8 (61·1 to 64·5)	67·6 (65·6 to 69·5)
Nakuru	64·5 (61·3 to 67·7)	66·4 (63·4 to 69·3)	66·9 (63·9 to 69·6)	68 (65·2 to 70·6)	66·6 (63·6 to 69·4)	58 (54 to 61·9)	64·1 (61·2 to 66·7)	69·6 (66·8 to 72)
Nandi	59·5 (56·2 to 62·9)	61·8 (58·8 to 65·1)	63·4 (60·2 to 66·3)	64·8 (61·9 to 67·6)	64 (60·8 to 66·9)	57·7 (54·1 to 61·1)	63·6 (61 to 66·1)	68·9 (65·9 to 71·7)
Narok	63·4 (60·3 to 66·5)	63·8 (60·4 to 66·8)	62·6 (59·6 to 65·8)	60·9 (58 to 63·6)	60·5 (57·6 to 63·5)	56·4 (53·1 to 59·5)	62·3 (59·6 to 64·5)	66·6 (63·7 to 69·1)
Nyamira	58·5 (55·7 to 61·6)	61 (57·8 to 64·1)	63·4 (60·3 to 66·5)	64·9 (61·9 to 67·8)	64·3 (61·6 to 67)	53·8 (49·9 to 57·9)	57·6 (54·7 to 60·1)	63 (59·7 to 66·5)
Nyandarua	50·9 (47·7 to 54·3)	56·9 (53·6 to 60·2)	61·3 (58 to 64·6)	65·7 (62·8 to 68·7)	65·8 (63·2 to 68·3)	58·2 (54·2 to 61·9)	59·6 (57·1 to 62)	64·3 (61·4 to 67·3)
Nyeri	54·1 (51·1 to 57·3)	58·9 (55·7 to 62·1)	62·8 (59·6 to 66)	66·9 (63·8 to 69·7)	66·3 (63·7 to 68·6)	57·8 (53·9 to 61·5)	63·1 (60·6 to 65·5)	67·2 (64·4 to 70)
Samburu	63 (59·8 to 66·3)	64·2 (61·3 to 67·4)	64·6 (61·6 to 67·7)	65·3 (62·3 to 68)	62·7 (59 to 66·4)	55 (51·1 to 59·5)	59·7 (56·6 to 62·8)	65 (62 to 68·1)
Siaya	40·9 (37·9 to 44·2)	46·7 (43·7 to 49·8)	52 (49·3 to 54·9)	55·7 (52·9 to 58·4)	53 (49·7 to 56·1)	42 (37·9 to 47·1)	53·7 (50·3 to 57·4)	63·4 (59·6 to 67)

Taita Taveta	47·7 (44·6 to 50·8)	53·3 (50 to 56·5)	55·3 (52·1 to 58·4)	62·2 (58·8 to 65·6)	63·4 (60 to 66·6)	55·7 (51·9 to 60·1)	59·4 (56·9 to 62·3)	65·5 (62·3 to 68·7)
Tana River	49·2 (46·2 to 52·4)	52·9 (49·7 to 56·4)	54·9 (51·6 to 58·6)	57·3 (53·9 to 60·9)	57·4 (54·3 to 60·4)	51·3 (48 to 54·8)	55·3 (52·8 to 58)	61·5 (58·2 to 64·9)
Tharaka Nithi	56 (52·9 to 59·2)	58·7 (55·7 to 61·8)	60·2 (56·9 to 63·4)	63 (60·1 to 65·9)	62·4 (59·9 to 65·2)	57·3 (53·4 to 60·6)	63·3 (60·9 to 65·5)	67·3 (64·7 to 69·7)
Trans Nzoia	59·6 (56·3 to 63·1)	63·1 (59·7 to 66·3)	64·5 (61·2 to 67·6)	66·5 (63·6 to 69·6)	66·3 (63·1 to 69·2)	58·8 (53·9 to 63·6)	64 (60·6 to 67·2)	69·3 (66 to 72)
Turkana	56·6 (53·3 to 59·9)	58·6 (55·4 to 62)	58·3 (54·9 to 62)	58·2 (54·8 to 61·2)	57·3 (53·6 to 61)	48·7 (41·6 to 54·5)	56·7 (53 to 60·3)	63·6 (59·9 to 67·3)
Uasin Gishu	64·8 (61·4 to 68·3)	67·1 (63·9 to 70·2)	68·1 (65 to 71·2)	68·9 (66·2 to 71·4)	67·3 (64·3 to 70·1)	60·5 (56·9 to 64·2)	65·8 (63·5 to 68·1)	70·8 (68·3 to 72·8)
Vihiga	53·7 (50·7 to 56·7)	57·5 (54·4 to 60·6)	60·1 (57 to 63·3)	62·3 (59·4 to 65·5)	61 (57·9 to 64·1)	53·9 (50·4 to 57·8)	60·3 (57·5 to 63·2)	66 (63 to 69)
Wajir	40·7 (37·8 to 43·7)	46·4 (43·1 to 49·9)	50·4 (47·2 to 53·9)	55·3 (52·2 to 58·3)	56·2 (53·3 to 59·2)	55·9 (53·3 to 58·8)	59 (56·6 to 61·6)	63·4 (60·3 to 66·6)
West Pokot	55·8 (52·7 to 59·1)	57·9 (54·8 to 60·9)	57·7 (54·6 to 60·7)	58·8 (55·8 to 61·7)	59·2 (56·2 to 62·2)	58·3 (55·1 to 61·3)	62·7 (60·3 to 65·2)	67·4 (64·6 to 70)
Madagascar	41·3 (38·6 to 44·1)	44·8 (42·4 to 47·3)	48·4 (46·7 to 50·2)	54·9 (53 to 56·7)	55·9 (54·2 to 57·8)	59·5 (58·1 to 61)	62·6 (60·4 to 65)	65·5 (62·9 to 68·2)
Malawi	34·4 (30·3 to 38·5)	37·7 (34·6 to 41·1)	42·1 (39·6 to 44·8)	48·8 (46·9 to 50·9)	49·4 (47·4 to 51·1)	45·9 (43·1 to 48·8)	56·3 (54·5 to 58)	64·7 (62·4 to 66·8)
Mozambique	43 (38·5 to 47·4)	45·1 (41·4 to 48·7)	47·7 (44·7 to 50·7)	50·4 (47·9 to 52·9)	51·6 (50 to 53·2)	52·2 (49·9 to 54·3)	53·5 (51·2 to 55·7)	58·4 (55·7 to 60·9)
Rwanda	36·5 (32·6 to 40·6)	42·2 (39·5 to 45·1)	43·6 (41·1 to 46·1)	48·4 (46·4 to 50·7)	50 (48·5 to 51·6)	50·5 (48·5 to 52·5)	64·5 (62·9 to 66·2)	68·6 (66·4 to 70·5)
Somalia	44·2 (40·1 to 48·4)	45 (41·5 to 48·7)	46·7 (43·1 to 50·4)	48·9 (45·7 to 52·5)	49·3 (46·7 to 52·2)	51·4 (48·4 to 54·7)	53·9 (50·4 to 57·4)	58·5 (54·2 to 62·3)
South Sudan	47·2 (42·7 to 51·7)	48·6 (44·7 to 52·5)	50·1 (46·9 to 53·4)	53·1 (49·9 to 56·2)	54·5 (51·6 to 57·6)	57 (54 to 60·1)	60·6 (57·3 to 63·9)	63·7 (60·4 to 66·9)
Uganda	47·1 (42·7 to 51·4)	49 (45·7 to 52·4)	51·9 (49·3 to 54·7)	50·8 (49 to 52·7)	48·5 (45·5 to 51·4)	49·6 (47·7 to 51·5)	59·3 (57·7 to 61·1)	66·2 (64·1 to 68·2)
Tanzania	43·8 (39·7 to 48)	45·3 (42·2 to 48·8)	49·4 (47·2 to 52)	55·4 (53·5 to 57·3)	55·3 (53·1 to 57·1)	53·4 (51·2 to 55·8)	61·1 (59·5 to 62·6)	67·2 (65·2 to 69)
Zambia	47 (42·6 to 51·3)	49·6 (46·3 to 52·9)	53·5 (50·9 to 56)	55·6 (53·8 to 57·7)	51·9 (49·8 to 53·9)	45·3 (42·8 to 48)	56·5 (54·7 to 58·4)	63·2 (60·7 to 65·4)
Southern sub-Saharan Africa	52·1 (49·8 to 54·3)	54 (52·3 to 55·9)	56·8 (55·6 to 58·1)	59·7 (58·8 to 60·6)	63·6 (62·7 to 64·3)	53·5 (50·5 to 56·1)	55·9 (54·2 to 56·9)	63·8 (62·5 to 64·9)
Botswana	53·7 (49·9 to 57·4)	54·4 (51·4 to 57·8)	56·5 (54 to 59)	61 (58·5 to 63·5)	61·3 (58·3 to 64·2)	45·5 (41·8 to 49·9)	57 (54 to 60·1)	62·2 (59 to 65·3)
eSwatini	43·3 (39·3 to 47·4)	45·6 (42·3 to 49·2)	51·5 (48·7 to 54·4)	57·3 (54·7 to 60·4)	61·8 (59·9 to 63·9)	48·3 (43·8 to 53)	48 (44·9 to 51·2)	58·3 (55 to 61·5)
Lesotho	52 (49·4 to 54·8)	53·3 (50·8 to 55·9)	54·6 (51·8 to 57·3)	58·5 (55·8 to 61·3)	60·6 (58·5 to 62·7)	48·5 (44·9 to 52)	48·1 (45·6 to 50·5)	51·8 (49·1 to 54·8)
Namibia	54·8 (50·9 to 58·7)	56 (52·6 to 59·7)	57·9 (55·3 to 60·8)	58·9 (57·3 to 60·5)	62·4 (61·1 to 63·7)	53·9 (51·1 to 56·6)	60·9 (58·5 to 63·3)	65·2 (62 to 68·2)
South Africa	52·1 (50·3 to 53·8)	53·9 (52·3 to 55·6)	56·7 (55·4 to 58)	59·5 (58·6 to 60·5)	64·2 (63·5 to 64·9)	55·6 (53·2 to 57·5)	57·1 (55·2 to 58·2)	65·1 (63·9 to 66·1)
Zimbabwe	53·8 (49·8 to 57·6)	55·5 (52·5 to 58·6)	58 (55·4 to 60·7)	60·4 (58·6 to 62·2)	62·4 (59·3 to 64·1)	47·5 (43·1 to 52·6)	52·3 (50·6 to 54)	61 (58·6 to 63·4)
Western sub-Saharan Africa	42·8 (39·5 to 45·9)	43·9 (41·5 to 46·4)	48·4 (46·7 to 50·3)	52·8 (51·4 to 54·2)	54·6 (53·4 to 55·7)	54·6 (53·2 to 56)	59·3 (58 to 60·7)	63·8 (61·9 to 65·6)
Benin	43·6 (39·3 to 48)	44·8 (41·6 to 48·4)	48·5 (46·1 to 51·3)	52·7 (50·8 to 54·8)	55·7 (54·1 to 57·3)	57·4 (55·5 to 59·3)	61·1 (58·5 to 63·6)	64·5 (60·6 to 67·7)
Burkina Faso	36·1 (31·9 to 40·4)	39·5 (36·3 to 42·9)	44·1 (41·6 to 46·7)	48·6 (46·6 to 50·8)	50·7 (48·7 to 52·6)	52·5 (50·6 to 54·4)	57·9 (56·4 to 59·5)	61·7 (59·2 to 63·9)
Cape Verde	56·2 (54 to 58·6)	56·8 (55·5 to 58·1)	58·1 (56·9 to 59·2)	66·8 (66 to 67·6)	69·9 (69·3 to 70·6)	70·3 (68·4 to 72·2)	74·4 (73·7 to 75·1)	73·7 (72·2 to 75·1)
Cameroon	42·4 (39·7 to 45·3)	27·5 (25·8 to 29·2)	51·5 (49·1 to 54·1)	55·4 (53·5 to 57·5)	58·4 (56·9 to 59·9)	54·5 (52·6 to 56·4)	57·7 (55·5 to 60)	63·4 (60·6 to 66)
Chad	45 (40·4 to 49·6)	47·4 (43·8 to 51)	48·4 (45·5 to 51·5)	50·8 (48·2 to 53·4)	53·5 (51·8 to 55·3)	52·3 (50·5 to 54·1)	56·5 (54·6 to 58·5)	60·4 (57·7 to 63)
Côte d'Ivoire	41·8 (38·8 to 44·9)	45·6 (43·1 to 48·3)	50·9 (48·4 to 53·4)	56·7 (54·7 to 58·5)	55·7 (53·2 to 57·8)	51·1 (48·3 to 54)	57·4 (55·2 to 59·7)	64·3 (61·9 to 66·5)
The Gambia	51·8 (47·4 to 56·1)	53·8 (50·3 to 57·3)	56·6 (53·7 to 59·4)	58·2 (55·1 to 61·2)	60·6 (58 to 63·2)	61·6 (59·6 to 63·8)	63·7 (62 to 65·6)	66·7 (64·4 to 68·9)
Ghana	47·1 (44·3 to 50·1)	51 (48·4 to 53·6)	54·5 (51·9 to 57·2)	57·5 (55 to 60)	59·4 (57·3 to 61·3)	59·6 (57·9 to 61·5)	61·8 (60·6 to 63)	66·3 (64·2 to 68·3)
Guinea	38·4 (34 to 42·8)	41·1 (37·3 to 45·3)	43·9 (39·9 to 47·7)	47·3 (44·5 to 50)	51·9 (50·1 to 53·8)	54·3 (52·3 to 56·3)	57·6 (55 to 60·1)	61·2 (57·9 to 64·6)
Guinea-Bissau	33·3 (29·3 to 37·4)	37·4 (33·6 to 41·4)	39 (35·4 to 42·6)	45·3 (42·3 to 48·6)	48·6 (45·9 to 51·3)	51·5 (49·5 to 53·5)	56·2 (53·7 to 58·8)	61 (57·7 to 64·1)
Liberia	38 (33·7 to 42·4)	40·8 (37·6 to 44·6)	45·3 (42·2 to 48·5)	50·3 (47·9 to 52·7)	49·1 (47·3 to 50·9)	54·8 (53·1 to 56·6)	61·9 (60·1 to 63·9)	66·1 (63·5 to 68·5)

Mali	35·2 (31·1 to 39·5)	37·7 (33·8 to 41·8)	40·2 (37·5 to 43·3)	45·8 (43·9 to 47·8)	50·2 (48·7 to 51·7)	53·3 (51·6 to 54·9)	58·4 (56·5 to 60·5)	61·8 (57·9 to 65·2)
Mauritania	47·6 (43·5 to 51·8)	50·6 (47·4 to 53·8)	55 (52·4 to 57·8)	56·8 (54·8 to 59·2)	59·4 (57·8 to 61)	63·6 (61·9 to 65·4)	68·1 (66·1 to 69·8)	70·8 (67·8 to 73·1)
Niger	37·8 (33·4 to 42·3)	40·3 (36·2 to 44·7)	43·1 (39·9 to 46·1)	44·9 (43 to 47)	47·4 (45·7 to 49·2)	51·8 (49·7 to 53·9)	59 (56·8 to 61·3)	62·5 (58·5 to 65·9)
Nigeria	44·8 (41·9 to 47·6)	46·8 (44·1 to 49·6)	49·1 (46·8 to 51·4)	54·1 (52·1 to 56)	54·7 (52·9 to 56·6)	54·6 (52·4 to 56·8)	59·6 (57·5 to 61·8)	64·3 (62·2 to 66·6)
São Tomé and PrÍncipe	40·7 (37·2 to 43·9)	45 (43·8 to 46·3)	57 (55·9 to 58)	62·3 (61·1 to 63·5)	63·5 (62 to 64·9)	65 (63·5 to 66·8)	69 (67·6 to 70·3)	70·9 (69 to 73·1)
Senegal	45·2 (41·1 to 49·4)	46·9 (44·3 to 49·7)	47·9 (45·6 to 50·3)	52·9 (51·2 to 54·8)	58·6 (57·5 to 59·7)	60·6 (59·4 to 61·8)	65·7 (64·5 to 66·8)	68·5 (66·3 to 70·3)
Sierra Leone	40 (35·6 to 44·5)	42·7 (38·7 to 46·9)	45·7 (41·3 to 49·9)	48·4 (45·1 to 51·6)	51·1 (48·8 to 53·4)	51·6 (50 to 53·2)	56 (53·7 to 58·5)	61·8 (58·4 to 65)
Togo	44·8 (40·3 to 49·2)	46·9 (43·6 to 50·4)	51·4 (48·7 to 54·2)	55·8 (53·5 to 58)	58 (56·1 to 60)	56·4 (54·1 to 58·9)	59·5 (57·5 to 61·7)	65 (62·5 to 67·4)

Supplemental Results Table 2b: Estimates of life expectancy at birth by location, males, 1950, 1960, 1970, 1980, 1990, 2000, 2010, 2019

Location	1950	1960	1970	1980	1990	2000	2010	2019
Global	49 (47·1 to 50·9)	44·4 (40·1 to 47·7)	56·1 (55·1 to 57)	59·9 (59·2 to 60·6)	63 (62·5 to 63·5)	64·8 (64·3 to 65·3)	68 (67·5 to 68·5)	71 (70·1 to 71·9)
Low SDI	40·8 (37·5 to 43·9)	43·6 (41·2 to 46)	46·5 (44·8 to 48·2)	50 (48·5 to 51·3)	52·4 (51·4 to 53·5)	54·3 (53·3 to 55·3)	58·9 (57·8 to 60)	63·9 (62·2 to 65·4)
Low-middle SDI	39·6 (37·1 to 42·1)	39·8 (36·9 to 42·3)	47·9 (46·4 to 49·4)	54·2 (53·2 to 55·1)	58·4 (57·6 to 59·1)	60·9 (60·2 to 61·6)	64·5 (63·7 to 65·2)	67·8 (66·6 to 69·2)
Middle SDI	46·2 (44·1 to 48·3)	33·7 (26·8 to 39·5)	56·4 (54·8 to 57·8)	61·3 (60·1 to 62·5)	64·9 (64·1 to 65·8)	67·3 (66·7 to 67·9)	69·6 (69 to 70·1)	72·1 (71·1 to 73·2)
High-middle SDI	56·5 (54·9 to 58·2)	54·4 (50·9 to 57)	62·8 (61·8 to 63·8)	64·7 (63·9 to 65·5)	67 (66·4 to 67·6)	68·1 (67·7 to 68·4)	71·6 (71·2 to 72·1)	74·4 (73·5 to 75·4)
High SDI	61·6 (61·3 to 61·9)	66·2 (66·1 to 66·3)	67·7 (67·6 to 67·8)	70·3 (70·2 to 70·3)	72·6 (72·5 to 72·7)	75·1 (75 to 75·1)	77·6 (77·5 to 77·7)	78·6 (78·3 to 78·8)
Central Europe, eastern Europe, and central Asia	58·7 (56·9 to 60·6)	63·1 (62·6 to 63·5)	63·8 (63·5 to 64·1)	63·5 (63·4 to 63·7)	64·8 (64·7 to 64·9)	62·8 (62·6 to 62·9)	66 (65·9 to 66·1)	69·1 (67·9 to 70·3)
Central Asia	47·3 (42·9 to 51·6)	53·8 (51·7 to 56)	58·9 (57·7 to 60·1)	62·3 (61·7 to 62·9)	63·8 (63·3 to 64·2)	62·3 (61·8 to 62·7)	64·7 (64·3 to 65·1)	67·4 (66·2 to 68·6)
Armenia	45·3 (40·5 to 50·1)	56·2 (53·8 to 58·6)	62·5 (60·8 to 64·2)	67·3 (66·2 to 68·5)	67·3 (66·9 to 67·7)	69·4 (69 to 69·7)	70·5 (70·2 to 70·8)	72·5 (70·8 to 74·3)
Azerbaijan	35·3 (30·7 to 39·9)	45·7 (42·5 to 49·1)	56·2 (54·5 to 57·7)	62·6 (61·6 to 63·6)	62·8 (61·7 to 63·9)	63·5 (62·5 to 64·5)	66·3 (65·4 to 67·1)	68·3 (65·8 to 70·6)
Georgia	45·4 (40·7 to 50)	54·7 (52·4 to 57·1)	60·7 (59·2 to 62·2)	64·3 (62·8 to 65·9)	64·4 (63·2 to 65·5)	66·6 (65·5 to 67·7)	67·2 (66·1 to 68·4)	68·8 (67 to 70·7)
Kazakhstan	52·9 (49 to 56·8)	56·6 (54·9 to 58·4)	59·8 (58·6 to 61)	61·5 (60·8 to 62·3)	63·3 (62·7 to 63·8)	59·4 (58·8 to 60)	62·7 (62·1 to 63·2)	67·7 (66·2 to 69·2)
Kyrgyzstan	45·9 (41·5 to 50·2)	51·4 (48·4 to 54·4)	55·8 (53·8 to 57·6)	59·2 (58·3 to 60·1)	62·8 (62 to 63·4)	63·1 (62·5 to 63·7)	65·6 (65·4 to 65·8)	69·8 (68·5 to 71·2)
Mongolia	36·8 (32·3 to 41·4)	42 (37·9 to 46·1)	48·4 (45 to 52·2)	54·7 (52 to 57·6)	57·6 (55·7 to 59·7)	57·9 (56·1 to 59·9)	61·1 (59·1 to 63·4)	63·8 (60·5 to 66·9)
Tajikistan	44·7 (40·1 to 49·3)	50·8 (48·2 to 53·2)	58·1 (56·3 to 60)	63·4 (61·3 to 65·4)	64·1 (63 to 65·3)	64·6 (63·6 to 65·6)	66 (64·9 to 67)	67·6 (65·5 to 69·5)
Turkmenistan	45·3 (40·8 to 49·7)	51·8 (49·7 to 53·9)	57 (55 to 58·6)	60·1 (58·8 to 61·3)	62·6 (61·8 to 63·3)	62·6 (62 to 63·1)	66 (65·5 to 66·5)	67·4 (64·7 to 70)
Uzbekistan	53·3 (49 to 57·5)	58·1 (55·7 to 60·6)	61·7 (60·3 to 63·1)	64·4 (63·5 to 65·2)	66 (65·5 to 66·5)	62·2 (61·5 to 62·9)	63·9 (63·4 to 64·5)	65·9 (64·2 to 67·6)
Central Europe	56·9 (56·1 to 57·6)	64·3 (64·2 to 64·4)	66 (65·9 to 66·1)	66·7 (66·7 to 66·8)	67 (66·9 to 67·1)	69·2 (69·1 to 69·3)	71·8 (71·7 to 71·9)	73·7 (72·2 to 75·2)
Albania	51·5 (48·8 to 54)	62 (59·9 to 63·9)	66·1 (63·4 to 69)	68·5 (66·5 to 70·5)	69·7 (69·2 to 70·1)	72·3 (71·8 to 72·7)	75·8 (74·4 to 77·2)	75·8 (73·2 to 78·5)
Bosnia and Herzegovina	50 (49·6 to 50·3)	59·9 (59·6 to 60·2)	63·3 (63 to 63·5)	66·9 (66·7 to 67·2)	70·3 (70 to 70·5)	72·6 (71·4 to 73·9)	73·9 (73·6 to 74·2)	74·6 (72·1 to 76·9)
Bulgaria	59·3 (59·1 to 59·6)	67·5 (67·3 to 67·8)	69·4 (69·2 to 69·6)	68·7 (68·5 to 68·9)	66·8 (66·2 to 67·4)	66·7 (66·1 to 67·4)	68·8 (68·2 to 69·4)	69·9 (67·3 to 72·5)
Croatia	52·9 (52·5 to 53·3)	62·8 (62·5 to 63)	65·6 (65·4 to 65·9)	66·8 (66·2 to 67·5)	68·6 (67·8 to 69·4)	71 (70·4 to 71·6)	73·6 (73·4 to 73·9)	75·6 (73·2 to 77·9)
Czech Republic	61·9 (61·7 to 62)	67·4 (67·2 to 67·6)	66·1 (66 to 66·3)	66·9 (66·7 to 67)	67·6 (67·5 to 67·8)	71·8 (71·6 to 71·9)	74·6 (74·5 to 74·8)	76·6 (74·5 to 78·6)
Hungary	59·7 (59·5 to 60)	66 (65·7 to 66·1)	66·3 (66·1 to 66·5)	65·5 (65·4 to 65·7)	65·2 (65·1 to 65·4)	67·6 (67·5 to 67·8)	70·8 (70·7 to 71)	73·2 (71·1 to 75·3)
Montenegro	66·5 (65·7 to 67·3)	69·2 (68·4 to 69·9)	71·4 (70·7 to 72·1)	71·4 (70·7 to 72)	71·4 (70·7 to 72·1)	71 (70·4 to 71·6)	72·3 (71·7 to 72·9)	73·1 (71·2 to 74·9)
North Macedonia	50·7 (50·2 to 51·2)	57·9 (57·4 to 58·3)	64·5 (64·1 to 64·9)	68·7 (68·4 to 69·1)	68·6 (68·1 to 69)	69·3 (68·8 to 69·8)	71·4 (70·9 to 71·8)	72·5 (70·2 to 74·7)
Poland	55·9 (55·7 to 56)	64·8 (64·7 to 64·9)	66·6 (66·5 to 66·7)	66·3 (66·2 to 66·4)	66·6 (66·6 to 66·7)	69·7 (69·6 to 69·8)	72·2 (72·2 to 72·3)	74·2 (71·6 to 76·8)
Romania	58·7 (54·5 to 62·7)	63·8 (63·7 to 64)	66·1 (65·9 to 66·2)	67 (66·9 to 67·2)	66·6 (66·5 to 66·8)	67·8 (67·6 to 67·9)	70·1 (70 to 70·2)	72 (69·8 to 74·1)
Serbia	52·6 (52·3 to 52·9)	62 (61·7 to 62·3)	63·7 (63·5 to 63·9)	66·8 (66·6 to 67)	68·3 (68·2 to 68·5)	68·8 (68·6 to 69·1)	71·7 (71·5 to 71·9)	73·5 (71·2 to 75·7)
Slovakia	58·8 (58·4 to 59·1)	67·9 (67·6 to 68·2)	66·8 (66·5 to 67)	67 (66·8 to 67·2)	66·7 (66·6 to 66·9)	69·4 (69·2 to 69·6)	71·9 (71·7 to 72·1)	74·3 (71·7 to 76·8)
Slovenia	55·2 (54·8 to 55·7)	63 (62·7 to 63·4)	65·4 (65·1 to 65·8)	67·4 (67·1 to 67·8)	70·2 (67·2 to 73·2)	72·3 (72 to 72·7)	76·3 (75·9 to 76·6)	78·2 (75·5 to 80·5)
Eastern Europe	62·5 (60·1 to 65·2)	64·9 (64·7 to 65)	64·4 (64·3 to 64·5)	62·7 (62·6 to 62·7)	64·6 (64·5 to 64·7)	60·5 (60·4 to 60·5)	63·8 (63·8 to 63·9)	67·3 (65·5 to 69·1)
Belarus	64·3 (60·2 to 68·5)	66·8 (65·6 to 67·9)	67·6 (67 to 68·1)	66 (65·6 to 66·3)	66·4 (66 to 66·6)	63·4 (63·1 to 63·6)	64·7 (64·5 to 64·9)	68·9 (65·8 to 71·8)
Estonia	62·3 (58·6 to 66)	64·8 (64·3 to 65·2)	65·6 (65·2 to 66)	64·4 (64 to 64·7)	64·7 (64·4 to 65·1)	65·7 (65·3 to 66·1)	71·1 (70·7 to 71·5)	74 (71·3 to 76·7)
Latvia	64·2 (60·4 to 68)	66·2 (65·8 to 66·6)	66 (65·6 to 66·3)	63·8 (63·5 to 64·1)	64·6 (64·3 to 64·9)	65 (64·7 to 65·3)	68 (67·7 to 68·4)	71·5 (68·8 to 74·2)
Lithuania	63·6 (59·5 to 67·7)	66·4 (66·1 to 66·8)	66·9 (66·6 to 67·2)	65·4 (65·2 to 65·7)	66·3 (66 to 66·5)	66·7 (66·5 to 67)	67·5 (67·2 to 67·8)	71·5 (69·2 to 73·8)

Moldova	49·6 (45·4 to 53·8)	54·9 (52·6 to 57·2)	59·4 (57·7 to 60·9)	62·4 (61·8 to 63)	64·7 (64·3 to 65)	65 (64·6 to 65·3)	65·6 (65·3 to 65·9)	70·2 (68·8 to 71·7)
Russia	61·9 (58·8 to 65·2)	64 (64 to 64·1)	63·4 (63·4 to 63·5)	61·6 (61·6 to 61·7)	64 (64 to 64·1)	59·4 (59·3 to 59·4)	62·9 (62·9 to 63)	68 (65·6 to 70·4)
Ukraine	65·1 (61·9 to 68·3)	67·5 (66·9 to 68)	66·6 (66·2 to 67)	64·8 (64·4 to 65·1)	65·8 (65·5 to 66)	62·4 (62·3 to 62·5)	65·9 (65·8 to 65·9)	64·3 (61·6 to 67·1)
High income	61·8 (61·6 to 62)	66·3 (66·2 to 66·3)	67·7 (67·7 to 67·7)	70·5 (70·4 to 70·5)	72·9 (72·8 to 72·9)	75·3 (75·3 to 75·3)	77·8 (77·8 to 77·9)	78·8 (78·7 to 78·9)
Australasia	67·1 (67 to 67·2)	68·3 (68·2 to 68·4)	68·1 (68 to 68·2)	70·9 (70·9 to 71)	73·7 (73·6 to 73·8)	76·8 (76·8 to 76·9)	79·7 (79·6 to 79·8)	80·7 (80·5 to 80·9)
Australia	66·9 (66·8 to 67·1)	68·2 (68·1 to 68·3)	67·9 (67·8 to 68)	71·1 (71 to 71·2)	73·9 (73·8 to 74)	77 (76·9 to 77·1)	79·9 (79·8 to 80)	80·8 (80·6 to 81)
New Zealand	67·6 (67·4 to 67·8)	68·7 (68·6 to 68·9)	68·7 (68·5 to 68·8)	70·4 (70·3 to 70·6)	72·7 (72·5 to 72·8)	75·9 (75·7 to 76)	78·9 (78·8 to 79·1)	79·9 (79·6 to 80·1)
High-income Asia Pacific	51·8 (51·2 to 52·3)	63·2 (63 to 63·5)	67·6 (67·4 to 67·8)	71·5 (71·4 to 71·6)	74·5 (74·4 to 74·6)	77 (76·9 to 77)	79·4 (79·4 to 79·5)	81·5 (81·3 to 81·6)
Brunei	51·7 (50·1 to 53·2)	55·7 (54·4 to 57·1)	62·2 (61·3 to 63·2)	66·2 (65·2 to 67·2)	67 (66·2 to 67·9)	71 (70·2 to 71·8)	72 (71·2 to 72·8)	72·3 (70·9 to 73·7)
Japan	58·1 (58 to 58·2)	65·9 (65·9 to 66)	70 (69·9 to 70)	73·8 (73·8 to 73·9)	76·3 (76·3 to 76·3)	78·1 (78 to 78·1)	80 (79·9 to 80)	81·9 (81·7 to 82)
Aichi	60·2 (57·5 to 63·1)	67·5 (64·8 to 70·2)	71 (68·5 to 73·5)	74·3 (74·1 to 74·5)	76·7 (76·5 to 76·9)	78·2 (78 to 78·4)	80·2 (79·9 to 80·4)	82·3 (81·1 to 83·5)
Akita	56·9 (54 to 59·8)	64·9 (62·1 to 67·9)	68·7 (66·1 to 71·3)	72·6 (72·2 to 73)	75·3 (75 to 75·7)	77 (76·6 to 77·4)	78·5 (78 to 79)	79·5 (78·1 to 81)
Aomori	54·5 (51·7 to 57·2)	63·1 (60·5 to 66)	67·5 (64·8 to 70·2)	71·9 (71·6 to 72·3)	74·6 (74·2 to 75)	76·2 (75·8 to 76·6)	78 (77·5 to 78·4)	80·1 (78·6 to 81·7)
Chiba	57·9 (55·1 to 60·9)	66·1 (63·6 to 68·8)	70·5 (68 to 73·1)	74·2 (74 to 74·4)	76·8 (76·6 to 77)	78·3 (78·1 to 78·5)	80·2 (79·9 to 80·4)	81·9 (80·6 to 83)
Ehime	56·9 (54·1 to 59·9)	65 (62·3 to 68)	69·2 (66·5 to 72)	73·5 (73·2 to 73·9)	76·2 (75·8 to 76·5)	77·5 (77·1 to 77·9)	79·6 (79·1 to 80)	81 (79·7 to 82·6)
Fukui	59·9 (56·8 to 62·9)	67·1 (64·4 to 69·9)	71·1 (68·8 to 73·4)	75·5 (75·1 to 76)	77·2 (76·9 to 77·6)	79·1 (78·7 to 79·5)	80·8 (80·3 to 81·2)	83·1 (82 to 84·1)
Fukuoka	57·4 (54·7 to 60·3)	65·3 (62·6 to 68·3)	69·1 (66·4 to 71·7)	73 (72·8 to 73·2)	75·5 (75·2 to 75·7)	77·6 (77·4 to 77·9)	79·7 (79·4 to 80)	81·4 (80·2 to 82·6)
Fukushima	55·6 (52·9 to 58·4)	64·5 (61·8 to 67·5)	68·8 (66·2 to 71·5)	73·3 (73 to 73·6)	76·2 (75·8 to 76·5)	77·7 (77·3 to 78·1)	79·5 (79·2 to 80)	81·4 (80·1 to 82·9)
Gifu	59·5 (56·7 to 62·5)	67 (64·3 to 70)	70·9 (68·3 to 73·5)	74·3 (74 to 74·6)	77·1 (76·7 to 77·4)	78·4 (78 to 78·7)	80·4 (80 to 80·8)	81·7 (80·4 to 83·2)
Gunma	57·4 (54·7 to 60·4)	65·7 (62·9 to 68·6)	70 (67·4 to 72·6)	74·1 (73·8 to 74·4)	76·8 (76·4 to 77·1)	78·2 (77·8 to 78·6)	79·7 (79·3 to 80·1)	81·4 (80·1 to 82·8)
Hiroshima	56·8 (54 to 59·7)	65·4 (62·6 to 68·4)	69·7 (67·1 to 72·3)	73·9 (73·6 to 74·2)	76·4 (76·1 to 76·7)	78·1 (77·8 to 78·4)	80·2 (79·9 to 80·5)	82·1 (80·8 to 83·4)
Hokkaidō	57·3 (54·6 to 60·2)	65·5 (62·8 to 68·3)	69·2 (66·6 to 71·8)	73·3 (73·1 to 73·5)	75·9 (75·7 to 76·2)	77·7 (77·5 to 78)	79·6 (79·3 to 79·9)	81·4 (80·2 to 82·7)
Hyōgo	58·6 (55·9 to 61·5)	66·3 (63·7 to 69·2)	70 (67·4 to 72·6)	73·5 (73·2 to 73·7)	75·8 (75·6 to 76)	77·8 (77·6 to 78)	80 (79·7 to 80·3)	81·9 (80·7 to 83·1)
Ibaraki	55·1 (52·5 to 58)	64·4 (61·6 to 67·3)	68·8 (66·2 to 71·5)	73·1 (72·8 to 73·4)	75·9 (75·6 to 76·2)	77·6 (77·3 to 77·9)	79·4 (79·1 to 79·8)	81 (79·7 to 82·4)
Ishikawa	59·1 (56·3 to 62·2)	66·7 (64 to 69·6)	70·6 (68·1 to 73)	74·8 (74·5 to 75·2)	76·8 (76·5 to 77·2)	78·6 (78·2 to 79)	80·5 (80·1 to 80·9)	82·2 (81 to 83·4)
Iwate	55·6 (52·8 to 58·5)	64·3 (61·6 to 67·3)	68·8 (66 to 71·4)	73 (72·6 to 73·3)	75·8 (75·5 to 76·2)	77·6 (77·2 to 78)	78·8 (78·3 to 79·3)	80·7 (79·3 to 82·2)
Kagawa	58·2 (55·4 to 61·1)	66·2 (63·2 to 69·1)	70·5 (68·1 to 73·1)	74·5 (74·1 to 74·9)	76·4 (76 to 76·8)	78·3 (77·9 to 78·7)	80·1 (79·6 to 80·5)	82·5 (81·3 to 83·6)
Kagoshima	54·7 (51·9 to 57·6)	63·6 (60·8 to 66·4)	67·9 (65·1 to 70·5)	72·7 (72·4 to 73)	75·8 (75·5 to 76·2)	77·4 (77·1 to 77·8)	79·4 (79 to 79·8)	81·5 (80·2 to 83)
Kanagawa	60·7 (57·8 to 63·7)	68 (65·4 to 70·8)	71·6 (69 to 74·1)	74·7 (74·5 to 74·9)	76·9 (76·7 to 77·1)	78·5 (78·3 to 78·7)	80·7 (80·5 to 80·9)	82·7 (81·5 to 83·8)
Kōchi	55·2 (52·3 to 58·1)	63·6 (60·8 to 66·4)	67·8 (65·4 to 70·4)	72·5 (72·1 to 72·9)	75·7 (75·3 to 76·2)	77·4 (77 to 77·9)	79 (78·5 to 79·5)	81·3 (80·1 to 82·5)
Kumamoto	55·6 (52·9 to 58·5)	64·4 (61·6 to 67·2)	68·8 (66·2 to 71·7)	73·7 (73·3 to 74)	76·6 (76·2 to 77)	78·7 (78·3 to 79·2)	80·6 (80·2 to 81)	82·2 (80·9 to 83·6)
Kyōto	59·7 (56·8 to 62·7)	67·3 (64·6 to 70·3)	71·2 (68·6 to 73·9)	74·6 (74·2 to 74·9)	76·9 (76·6 to 77·2)	78·2 (77·9 to 78·5)	80·6 (80·2 to 80·9)	83·1 (81·8 to 84·5)
Mie	59 (56·1 to 61·9)	66·7 (64·1 to 69·6)	70·6 (68 to 73·2)	74 (73·7 to 74·4)	76·3 (75·9 to 76·6)	78·1 (77·7 to 78·5)	80 (79·6 to 80·4)	81·9 (80·6 to 83·3)
Miyagi	57·1 (54·3 to 60)	65·6 (62·9 to 68·6)	69·5 (66·8 to 72·1)	73·9 (73·5 to 74·2)	76·4 (76·1 to 76·7)	78·1 (77·7 to 78·4)	80·1 (79·7 to 80·5)	82·4 (81·2 to 83·8)
Miyazaki	55·6 (52·9 to 58·6)	64·2 (61·5 to 67·1)	68·7 (65·9 to 71·3)	72·8 (72·4 to 73·2)	75·7 (75·2 to 76·1)	77·7 (77·3 to 78·2)	79·9 (79·5 to 80·3)	81·3 (79·9 to 82·8)
Nagano	58·6 (55·9 to 61·5)	66·8 (64·1 to 69·6)	70·8 (68·1 to 73·4)	74·8 (74·4 to 75·1)	77·7 (77·3 to 78)	79·2 (78·8 to 79·5)	81·2 (80·8 to 81·6)	82·8 (81·5 to 84·2)
Nagasaki	56·9 (54·2 to 59·7)	64·6 (61·9 to 67·6)	68·3 (65·5 to 71)	72·8 (72·4 to 73·1)	75·6 (75·2 to 75·9)	77·6 (77·1 to 78)	79·3 (78·9 to 79·8)	81·6 (80·3 to 83·1)
Nara	58·7 (55·8 to 61·8)	66·5 (63·7 to 69·3)	70·1 (67·5 to 72·8)	73·8 (73·4 to 74·2)	76·5 (76·1 to 76·9)	78·8 (78·3 to 79·2)	81 (80·5 to 81·4)	82·7 (81·4 to 84·2)

Niigata	57·8 (55·1 to 60·7)	65·8 (63·2 to 68·8)	69·6 (67·1 to 72·1)	73·8 (73·5 to 74·1)	76·8 (76·5 to 77·1)	78·1 (77·8 to 78·4)	79·9 (79·6 to 80·3)	81·6 (80·3 to 82·9)
Ōita	56·7 (53·8 to 59·5)	65·1 (62·4 to 68·2)	69·6 (66·9 to 72·3)	73·3 (72·9 to 73·7)	76·3 (75·9 to 76·7)	78·2 (77·7 to 78·7)	80·3 (79·8 to 80·8)	81·7 (80·4 to 83·2)
Okayama	58·4 (55·6 to 61·4)	66·3 (63·5 to 69·2)	70·7 (67·9 to 73·3)	74·4 (74 to 74·7)	76·6 (76·2 to 76·9)	78·4 (78 to 78·8)	80·1 (79·7 to 80·5)	82·4 (81·1 to 83·7)
Okinawa	54·5 (51·7 to 57·5)	63·2 (60·4 to 66·3)	66·7 (63·8 to 69·5)	73·5 (73·1 to 74)	76·5 (76 to 76·9)	77·9 (77·4 to 78·3)	79·6 (79·1 to 80)	80·9 (79·5 to 82·4)
Ōsaka	58·8 (56·1 to 61·7)	66·3 (63·7 to 68·9)	70 (67·6 to 72·5)	73·1 (72·9 to 73·2)	75·1 (75 to 75·3)	77·3 (77·1 to 77·5)	79·3 (79 to 79·5)	81·4 (80·2 to 82·7)
Saga	56·5 (53·6 to 59·4)	64·8 (62 to 67·6)	69·1 (66·7 to 71·7)	73·4 (73 to 73·9)	75·9 (75·5 to 76·3)	77·5 (77·1 to 78)	79·8 (79·3 to 80·3)	82·4 (81·2 to 83·6)
Saitama	58·6 (55·9 to 61·5)	66·8 (64·2 to 69·6)	71·1 (68·6 to 73·6)	74·1 (73·9 to 74·3)	76·6 (76·4 to 76·8)	78·5 (78·3 to 78·7)	80·1 (79·9 to 80·3)	81·8 (80·6 to 83)
Shiga	58·7 (55·8 to 61·8)	66·4 (63·7 to 69·3)	70·1 (67·7 to 72·4)	74·5 (74·1 to 74·9)	76·7 (76·3 to 77)	78·9 (78·5 to 79·3)	80·9 (80·5 to 81·3)	83·1 (82 to 84·1)
Shimane	57·2 (54·3 to 60·2)	65·2 (62·3 to 68·1)	69·3 (66·8 to 72)	73·8 (73·3 to 74·2)	76·6 (76·2 to 77·1)	78·1 (77·6 to 78·6)	79·9 (79·3 to 80·4)	82 (80·7 to 83·2)
Shizuoka	59·2 (56·4 to 62·3)	67 (64·2 to 69·9)	70·9 (68·2 to 73·6)	74·4 (74·1 to 74·7)	77 (76·7 to 77·2)	78·5 (78·2 to 78·8)	80·3 (80 to 80·6)	81·8 (80·6 to 83·2)
Tochigi	56·8 (54·1 to 59·7)	65 (62·2 to 67·9)	69·2 (66·6 to 71·7)	73·1 (72·7 to 73·4)	75·7 (75·4 to 76)	77·7 (77·3 to 78)	79·3 (78·9 to 79·7)	81·1 (79·8 to 82·6)
Tokushima	56·1 (53·2 to 59)	64·4 (61·8 to 67·3)	68·7 (66·2 to 71·2)	72·8 (72·4 to 73·2)	75·7 (75·3 to 76·2)	77·7 (77·3 to 78·1)	79·7 (79·2 to 80·2)	81·5 (80·3 to 82·7)
Tōkyō	61·2 (58·5 to 64·1)	67·9 (65·3 to 70·4)	71·2 (68·7 to 73·6)	74·6 (74·5 to 74·8)	76·4 (76·2 to 76·5)	78·2 (78·1 to 78·4)	80·2 (80 to 80·4)	82·2 (81 to 83·3)
Tottori	56·4 (53·6 to 59·4)	64·8 (62 to 67·6)	69·2 (66·9 to 71·5)	73·4 (73 to 73·8)	75·7 (75·3 to 76·1)	77·6 (77·1 to 78)	79·3 (78·8 to 79·7)	81·2 (80 to 82·3)
Toyama	57·8 (54·9 to 60·7)	65·9 (63·2 to 68·8)	69·9 (67·3 to 72·4)	74 (73·6 to 74·4)	76·6 (76·3 to 77)	78·7 (78·2 to 79·1)	80·3 (79·9 to 80·8)	82 (80·8 to 83·2)
Wakayama	57·1 (54·4 to 60·1)	65·3 (62·4 to 68·2)	69·5 (66·8 to 72)	73·2 (72·8 to 73·6)	75·5 (75·1 to 75·9)	77·4 (76·9 to 77·9)	79·1 (78·6 to 79·6)	79·8 (78·4 to 81·3)
Yamagata	57·6 (54·8 to 60·6)	65·7 (62·7 to 68·5)	69·6 (66·9 to 72·2)	73·6 (73·2 to 73·9)	76·9 (76·4 to 77·3)	78·5 (78·1 to 79)	80·6 (80·1 to 81)	82·4 (80·9 to 83·9)
Yamaguchi	56·4 (53·7 to 59·3)	64·9 (62·1 to 67·8)	69·3 (66·6 to 72)	73 (72·6 to 73·3)	75·9 (75·5 to 76·2)	77·6 (77·1 to 77·9)	79·5 (79 to 79·9)	81 (79·6 to 82·5)
Yamanashi	57·9 (55 to 60·9)	65·5 (62·7 to 68·4)	69·6 (67·1 to 72·2)	73·7 (73·3 to 74·1)	76·4 (76 to 76·8)	78·3 (77·9 to 78·7)	80·2 (79·7 to 80·6)	82·1 (80·9 to 83·3)
South Korea	34·3 (33 to 35·5)	55·5 (54·7 to 56·4)	59·8 (59·1 to 60·5)	63·6 (63·3 to 63·8)	68·1 (68 to 68·3)	72·7 (72·6 to 72·7)	77·3 (77·2 to 77·4)	80 (79·7 to 80·4)
Singapore	54 (53·7 to 54·3)	62·7 (62·5 to 62·8)	66·1 (65·9 to 66·2)	69 (68·9 to 69·2)	72·9 (72·7 to 73)	77 (76·8 to 77·1)	80·5 (80·3 to 80·6)	82·9 (82·7 to 83·2)
High-income North America	65·6 (65·6 to 65·6)	67·1 (67 to 67·1)	67·5 (67·4 to 67·5)	70·4 (70·3 to 70·4)	72·4 (72·4 to 72·4)	74·5 (74·5 to 74·6)	76·7 (76·7 to 76·8)	76·7 (76·6 to 76·9)
Canada	66·5 (66·4 to 66·6)	68·5 (68·4 to 68·6)	69·5 (69·4 to 69·6)	71·7 (71·6 to 71·8)	74·2 (74·1 to 74·3)	76·7 (76·6 to 76·7)	79·4 (79·3 to 79·5)	80 (79·9 to 80·2)
Greenland	47·6 (44·7 to 50·1)	56·1 (54·5 to 57·7)	60 (58·4 to 61·6)	62·6 (60·8 to 64·3)	63·5 (61·6 to 65·2)	66·7 (64·9 to 68·2)	69 (67·1 to 70·7)	71·1 (68·6 to 73·7)
USA	65·5 (65·5 to 65·6)	66·9 (66·9 to 67)	67·3 (67·2 to 67·3)	70·2 (70·2 to 70·2)	72·2 (72·2 to 72·2)	74·3 (74·3 to 74·3)	76·4 (76·4 to 76·5)	76·4 (76·2 to 76·5)
Alabama	63·1 (60·1 to 66·2)	64·9 (64·6 to 65·2)	65·3 (65 to 65·6)	68·4 (68·1 to 68·7)	70 (69·7 to 70·3)	71·3 (71 to 71·5)	72·7 (72·4 to 73)	72·6 (69·9 to 75·5)
Alaska	61·3 (58·8 to 63·9)	64·9 (64·3 to 65·5)	66·3 (65·8 to 66·9)	68·7 (68·1 to 69·2)	71·9 (71·4 to 72·4)	74·1 (73·6 to 74·6)	76·3 (75·8 to 76·8)	76·2 (73·7 to 78·5)
Arizona	63·2 (60·1 to 66·4)	65·4 (65 to 65·8)	66·9 (66·6 to 67·4)	70·6 (70·3 to 70·9)	73·2 (72·9 to 73·5)	74·7 (74·5 to 75)	77·2 (76·9 to 77·4)	76·7 (73·9 to 79·5)
Arkansas	65·2 (62·2 to 68·4)	67 (66·6 to 67·4)	67·1 (66·7 to 67·4)	69·5 (69·2 to 69·9)	70·9 (70·6 to 71·3)	72 (71·7 to 72·3)	73·4 (73·1 to 73·8)	73·5 (70·8 to 76·4)
California	66·4 (63·5 to 69·4)	67·7 (67·6 to 67·8)	68·4 (68·3 to 68·6)	71·1 (71 to 71·2)	72·9 (72·7 to 73)	76 (75·9 to 76·1)	78·7 (78·6 to 78·8)	79 (76·6 to 81·4)
Colorado	65·8 (62·8 to 69)	68·1 (67·7 to 68·5)	68·7 (68·3 to 69·1)	72 (71·7 to 72·4)	74·3 (74 to 74·6)	76·2 (75·9 to 76·5)	78 (77·8 to 78·3)	77·4 (74·6 to 80)
Connecticut	67·2 (64·3 to 70·2)	68·3 (68 to 68·6)	69·4 (69·1 to 69·7)	71·4 (71·1 to 71·7)	74·3 (74 to 74·7)	75·7 (75·4 to 76·1)	78·3 (78 to 78·6)	78·5 (75·9 to 81·1)
Delaware	63·7 (61·2 to 66·3)	66·3 (65·8 to 66·8)	66·6 (66·1 to 67·1)	69·8 (69·3 to 70·3)	71·9 (71·4 to 72·4)	73·9 (73·5 to 74·4)	75·8 (75·3 to 76·4)	75·4 (73·1 to 77·5)
Washington, DC	61·5 (58·3 to 64·8)	63 (62·5 to 63·4)	61·5 (61 to 62)	65 (64·5 to 65·6)	62·4 (61·8 to 63)	68·2 (67·6 to 68·8)	74·5 (73·8 to 75·1)	74·5 (71·5 to 77·6)
Florida	64·6 (61·5 to 67·8)	66·4 (66·1 to 66·6)	66·7 (66·5 to 67)	70·1 (69·9 to 70·3)	72·4 (72·2 to 72·6)	74·5 (74·4 to 74·7)	76·8 (76·6 to 76·9)	76·6 (74·1 to 79·2)
Georgia	62·7 (59·7 to 65·9)	64·3 (64 to 64·5)	64·5 (64·2 to 64·7)	68·3 (68 to 68·5)	70·1 (69·9 to 70·3)	72·3 (72·1 to 72·5)	74·7 (74·5 to 74·9)	75 (72·3 to 77·7)
Hawaii	67·4 (64·6 to 70·2)	68·6 (68·1 to 69·1)	71·5 (70·9 to 72)	74·2 (73·7 to 74·8)	75·7 (75·1 to 76·3)	77·6 (77 to 78·1)	77·4 (76·9 to 77·9)	78·7 (76 to 81·4)
Idaho	66·4 (63·4 to 69·5)	68·2 (67·6 to 68·8)	68·4 (67·8 to 69)	71·9 (71·4 to 72·4)	73·9 (73·3 to 74·4)	76 (75·5 to 76·5)	77·2 (76·7 to 77·7)	77·5 (74·8 to 80·1)

Illinois	65·6 (62·9 to 68·5)	66·7 (66·6 to 66·9)	66·7 (66·5 to 66·8)	69·8 (69·6 to 69·9)	71·7 (71·6 to 71·9)	74 (73·9 to 74·2)	76·6 (76·4 to 76·8)	76·7 (74·1 to 79·3)
Indiana	66·3 (63·5 to 69·3)	67·5 (67·2 to 67·7)	67·6 (67·4 to 67·9)	70·3 (70·1 to 70·5)	72·3 (72·1 to 72·6)	73·5 (73·2 to 73·7)	75·1 (74·9 to 75·3)	74·4 (71·8 to 77·2)
Iowa	67·7 (64·7 to 70·7)	69·1 (68·8 to 69·5)	69·1 (68·8 to 69·5)	72·1 (71·8 to 72·4)	74·6 (74·3 to 74·9)	76 (75·6 to 76·3)	77·3 (76·9 to 77·6)	77 (74·5 to 79·7)
Kansas	67·7 (64·8 to 70·8)	68·9 (68·5 to 69·2)	69·4 (69 to 69·7)	71·7 (71·3 to 72·1)	73·8 (73·4 to 74·2)	75 (74·6 to 75·3)	76·1 (75·7 to 76·4)	76·3 (73·7 to 79)
Kentucky	65·1 (62·1 to 68·2)	66·5 (66·2 to 66·8)	66·2 (65·9 to 66·4)	69·2 (68·9 to 69·4)	71·2 (70·9 to 71·5)	72·4 (72·1 to 72·6)	73·4 (73·2 to 73·7)	72·8 (70 to 75·7)
Louisiana	63·2 (60·2 to 66·3)	64·9 (64·6 to 65·2)	65·1 (64·8 to 65·4)	67·8 (67·5 to 68·1)	69·6 (69·3 to 69·9)	71·1 (70·8 to 71·3)	73 (72·7 to 73·3)	73 (70·1 to 75·9)
Maine	65·1 (62·2 to 68)	67·1 (66·6 to 67·5)	67·3 (66·9 to 67·8)	70·8 (70·3 to 71·3)	73·8 (73·3 to 74·3)	75·1 (74·6 to 75·6)	76·7 (76·2 to 77·2)	76·2 (73·6 to 78·9)
Maryland	64·5 (61·7 to 67·5)	65·8 (65·5 to 66·1)	66·8 (66·5 to 67)	69·9 (69·7 to 70·2)	71·7 (71·4 to 71·9)	73·6 (73·3 to 73·9)	76·7 (76·4 to 77)	75·8 (73·2 to 78·7)
Massachusetts	66·7 (63·8 to 69·7)	67·6 (67·4 to 67·8)	68·3 (68·1 to 68·5)	71·4 (71·2 to 71·6)	73·8 (73·5 to 74)	75·9 (75·7 to 76·2)	78·1 (77·9 to 78·4)	77·6 (75·1 to 80·3)
Michigan	66·5 (63·7 to 69·4)	67·5 (67·3 to 67·6)	67·4 (67·3 to 67·6)	70·3 (70·1 to 70·5)	72·1 (71·9 to 72·3)	74 (73·8 to 74·2)	75·7 (75·5 to 75·9)	75·3 (72·7 to 78)
Minnesota	67·8 (64·9 to 70·8)	69·1 (68·8 to 69·4)	69·7 (69·4 to 69·9)	72·6 (72·3 to 72·9)	74·8 (74·5 to 75·1)	76·6 (76·3 to 76·9)	78·6 (78·4 to 78·9)	78·6 (76·2 to 81·2)
Mississippi	62·4 (59·3 to 65·4)	64·8 (64·4 to 65·1)	64·3 (64 to 64·7)	67·8 (67·4 to 68·1)	69·5 (69·2 to 69·9)	70·4 (70·1 to 70·7)	72 (71·7 to 72·4)	71·9 (69·2 to 74·8)
Missouri	66 (63·2 to 69·1)	67·3 (67·1 to 67·6)	67·1 (66·9 to 67·4)	70·1 (69·9 to 70·4)	72 (71·7 to 72·3)	73·5 (73·3 to 73·8)	75 (74·7 to 75·2)	74·8 (72 to 77·7)
Montana	64·3 (61·2 to 67·3)	66·4 (65·9 to 67)	67·4 (66·9 to 68·1)	70·5 (69·9 to 71)	73·8 (73·2 to 74·4)	74·7 (74·1 to 75·3)	76·5 (75·9 to 77)	76·5 (73·8 to 79·3)
Nebraska	67·6 (64·7 to 70·6)	69·1 (68·6 to 69·5)	69 (68·6 to 69·4)	71·8 (71·4 to 72·2)	73·8 (73·4 to 74·3)	75·9 (75·4 to 76·3)	77·4 (76·9 to 77·8)	77·7 (75·4 to 80·2)
Nevada	60·2 (57·5 to 62·9)	63·9 (63·3 to 64·5)	65·7 (65·1 to 66·3)	68·8 (68·3 to 69·2)	71 (70·6 to 71·4)	72·8 (72·4 to 73·1)	75·5 (75·2 to 75·9)	75·8 (73·3 to 78·5)
New Hampshire	64·9 (62·2 to 67·7)	67·5 (66·9 to 68)	67·8 (67·3 to 68·4)	72 (71·5 to 72·6)	73·9 (73·3 to 74·4)	76 (75·5 to 76·5)	77·9 (77·4 to 78·4)	76·4 (73·7 to 79·2)
New Jersey	66 (63·1 to 68·9)	67·3 (67·1 to 67·5)	67·7 (67·5 to 67·9)	70·6 (70·4 to 70·8)	72·6 (72·4 to 72·8)	74·9 (74·7 to 75·2)	77·8 (77·5 to 78)	77·9 (75·3 to 80·5)
New Mexico	63·8 (60·7 to 67)	66·5 (66 to 67)	66·9 (66·4 to 67·5)	69·8 (69·3 to 70·3)	72·7 (72·2 to 73·1)	74·5 (74 to 74·9)	75·8 (75·4 to 76·3)	75 (72 to 78)
New York	65·8 (63·2 to 68·6)	66·9 (66·8 to 67·1)	67·1 (67 to 67·3)	70·1 (69·9 to 70·2)	71·3 (71·1 to 71·4)	75·2 (75·1 to 75·4)	78·1 (77·9 to 78·2)	78·4 (75·9 to 81)
North Carolina	63·3 (60·3 to 66·5)	64·8 (64·5 to 65)	65·2 (64·9 to 65·4)	68·8 (68·6 to 69)	71 (70·8 to 71·2)	72·7 (72·5 to 73)	75·3 (75·1 to 75·5)	75·3 (72·7 to 77·9)
North Dakota	67·3 (64·5 to 70·2)	69 (68·5 to 69·5)	69·4 (68·8 to 69·9)	72·3 (71·8 to 72·8)	74·4 (73·9 to 74·9)	75·8 (75·3 to 76·4)	77·2 (76·6 to 77·7)	76·8 (74·4 to 79·1)
Ohio	66·3 (63·5 to 69·3)	67·4 (67·3 to 67·6)	67·5 (67·3 to 67·7)	70 (69·8 to 70·1)	72·3 (72·1 to 72·5)	73·9 (73·7 to 74)	75·3 (75·2 to 75·5)	74·3 (71·8 to 77·1)
Oklahoma	66·1 (63·2 to 69·2)	67·4 (67·1 to 67·7)	67·3 (66·9 to 67·6)	69·7 (69·4 to 70)	72·2 (71·9 to 72·5)	72·5 (72·2 to 72·8)	73·3 (73 to 73·6)	73·5 (70·8 to 76·4)
Oregon	66·3 (63·3 to 69·5)	67·8 (67·5 to 68·2)	68·8 (68·5 to 69·2)	71·4 (71·1 to 71·7)	73·4 (73·1 to 73·7)	75·7 (75·4 to 76)	77·4 (77·1 to 77·7)	77·4 (74·8 to 80·1)
Pennsylvania	65·6 (62·9 to 68·4)	66·8 (66·7 to 67)	67·1 (67 to 67·3)	70 (69·8 to 70·2)	72·4 (72·2 to 72·5)	74 (73·8 to 74·2)	76 (75·8 to 76·2)	75·5 (72·9 to 78·2)
Rhode Island	66 (63·3 to 69)	67·8 (67·4 to 68·3)	68·3 (67·9 to 68·8)	70·9 (70·4 to 71·4)	73·3 (72·8 to 73·8)	75·6 (75 to 76·1)	77 (76·4 to 77·5)	77·4 (74·8 to 80·1)
South Carolina	61·3 (58·4 to 64·4)	62·9 (62·6 to 63·2)	64·1 (63·8 to 64·4)	67·7 (67·4 to 67·9)	70·2 (69·9 to 70·5)	71·7 (71·4 to 72)	73·9 (73·7 to 74·2)	74 (71·2 to 76·9)
South Dakota	66 (63·2 to 69)	67·8 (67·3 to 68·3)	68·7 (68·2 to 69·2)	70·9 (70·4 to 71·5)	73·6 (73·1 to 74·2)	75·2 (74·7 to 75·7)	76·7 (76·2 to 77·3)	76·1 (73·7 to 78·3)
Tennessee	65 (62·1 to 68·2)	66·5 (66·2 to 66·7)	66·2 (65·9 to 66·5)	69·2 (68·9 to 69·4)	70·6 (70·3 to 70·8)	71·8 (71·6 to 72·1)	73·6 (73·3 to 73·8)	73·5 (70·6 to 76·3)
Texas	65·7 (62·7 to 68·8)	67·2 (67·1 to 67·4)	67·3 (67·1 to 67·4)	69·9 (69·7 to 70)	71·7 (71·6 to 71·9)	74·1 (73·9 to 74·2)	76·2 (76 to 76·3)	76·5 (74·2 to 79·1)
Utah	67·2 (64·2 to 70·3)	68·6 (68 to 69·1)	69·4 (68·9 to 69·9)	72·7 (72·2 to 73·1)	75·1 (74·7 to 75·6)	76·6 (76·2 to 77)	77·9 (77·5 to 78·3)	77·7 (75·1 to 80·3)
Vermont	64·4 (62 to 67)	67·2 (66·7 to 67·7)	68·1 (67·7 to 68·6)	71·1 (70·6 to 71·6)	73·4 (73 to 73·9)	75·8 (75·3 to 76·2)	77·8 (77·3 to 78·3)	77 (75·2 to 78·7)
Virginia	64·2 (61·4 to 67·2)	65·8 (65·6 to 66·1)	66·6 (66·3 to 66·8)	69·7 (69·5 to 69·9)	72 (71·8 to 72·3)	74·3 (74 to 74·5)	76·8 (76·5 to 77)	76·9 (74·4 to 79·4)
Washington	66·5 (63·7 to 69·6)	68·2 (67·9 to 68·4)	68·4 (68·1 to 68·7)	71·8 (71·5 to 72·1)	73·8 (73·6 to 74·1)	75·8 (75·6 to 76·1)	77·9 (77·7 to 78·2)	78·3 (75·7 to 80·8)
West Virginia	64·5 (61·6 to 67·6)	66·4 (66 to 66·7)	65·6 (65·3 to 66)	68·8 (68·5 to 69·2)	71·2 (70·8 to 71·6)	72·3 (71·9 to 72·7)	73 (72·6 to 73·4)	72·3 (69·5 to 75·3)
Wisconsin	67·3 (64·5 to 70·3)	68·4 (68·2 to 68·7)	69·3 (69·1 to 69·6)	72 (71·7 to 72·3)	73·8 (73·6 to 74·1)	75·4 (75·1 to 75·6)	77·4 (77·1 to 77·7)	77·1 (74·6 to 79·7)
Wyoming	64·2 (61·5 to 66·8)	66·6 (66·1 to 67·2)	66·9 (66·3 to 67·4)	70·1 (69·7 to 70·6)	73·3 (72·8 to 73·8)	74·6 (74·1 to 75·1)	76·3 (75·8 to 76·8)	76·9 (74·9 to 78·7)

Southern Latin America	57·6 (57·5 to 57·7)	59·4 (59·2 to 59·5)	61·9 (61·9 to 62)	66·8 (66·7 to 66·8)	69·3 (69·2 to 69·4)	71·4 (71·3 to 71·5)	73·5 (73·4 to 73·5)	74·6 (74·3 to 74·9)
Argentina	61 (60·8 to 61·1)	62·4 (62·3 to 62·5)	62·7 (62·6 to 62·8)	66·8 (66·8 to 66·9)	69 (68·9 to 69·1)	70·5 (70·5 to 70·6)	72·7 (72·6 to 72·7)	73·5 (73·3 to 73·8)
Chile	48·1 (47·8 to 48·4)	51·5 (51·2 to 51·7)	59·2 (59 to 59·4)	66·5 (66·4 to 66·7)	70·3 (70·2 to 70·4)	74 (73·9 to 74·2)	75·9 (75·8 to 76)	77·7 (77·3 to 78·1)
Uruguay	62·7 (62·4 to 62·9)	65·5 (65·2 to 65·7)	65·2 (65 to 65·4)	67·1 (66·9 to 67·3)	69·5 (69·3 to 69·7)	71 (70·8 to 71·2)	72·8 (72·6 to 73·1)	74·1 (73·6 to 74·5)
Western Europe	64·1 (64 to 64·2)	67·4 (67·4 to 67·5)	68·6 (68·5 to 68·6)	70·7 (70·7 to 70·8)	73·1 (73 to 73·1)	75·7 (75·6 to 75·7)	78·5 (78·5 to 78·6)	79·8 (79·6 to 79·9)
Andorra	72·5 (69·4 to 75·7)	73·5 (70·8 to 76·4)	74·6 (71·8 to 77·4)	75·8 (72·9 to 78·3)	76·1 (73·5 to 78·4)	77·8 (75·8 to 79·9)	79·1 (77·2 to 81)	79·8 (77·6 to 82)
Austria	62·1 (61·9 to 62·3)	65·6 (65·5 to 65·8)	66·6 (66·4 to 66·7)	69·1 (69 to 69·2)	72·5 (72·3 to 72·6)	75·4 (75·2 to 75·5)	78 (77·9 to 78·2)	79·8 (79·6 to 80)
Belgium	64·1 (64 to 64·3)	67·2 (67·1 to 67·3)	67·9 (67·8 to 68·1)	69·9 (69·8 to 70)	72·8 (72·7 to 72·9)	74·8 (74·7 to 75)	77·5 (77·4 to 77·6)	79 (78·7 to 79·2)
Cyprus	59·4 (55·3 to 63)	62·3 (59·1 to 65·3)	64·7 (63 to 66·5)	68·9 (68·6 to 69·2)	73·5 (73·2 to 73·8)	74·5 (74·1 to 74·8)	77·3 (77 to 77·6)	78·9 (78 to 79·9)
Denmark	69·4 (69·2 to 69·6)	70·7 (70·5 to 70·9)	71·1 (70·9 to 71·2)	71·4 (71·2 to 71·5)	72·4 (72·3 to 72·6)	74·8 (74·6 to 74·9)	77·4 (77·3 to 77·6)	79·2 (78·8 to 79·5)
Finland	60·7 (60·6 to 60·9)	65·7 (65·5 to 65·8)	66·3 (66·2 to 66·5)	69·5 (69·4 to 69·7)	71·3 (71·1 to 71·4)	74·5 (74·3 to 74·6)	77·1 (77 to 77·3)	79·1 (78·7 to 79·5)
France	64·4 (64·3 to 64·5)	67·9 (67·8 to 68)	68·9 (68·8 to 69)	70·6 (70·6 to 70·7)	73·1 (73 to 73·2)	75·5 (75·4 to 75·5)	78·2 (78·2 to 78·3)	79·9 (79·7 to 80·1)
Germany	63·5 (63·1 to 64)	66·5 (66·4 to 66·5)	67·4 (67·3 to 67·4)	69·8 (69·7 to 69·8)	72·2 (72·2 to 72·3)	75·3 (75·3 to 75·4)	78 (77·9 to 78)	78·9 (78·7 to 79·1)
Greece	67·4 (67 to 67·7)	70·6 (70·5 to 70·8)	71·6 (71·5 to 71·8)	72·9 (72·7 to 73)	74·9 (74·7 to 75)	76 (75·8 to 76·1)	78 (77·8 to 78·1)	78·5 (78·2 to 78·8)
Iceland	69·2 (68·2 to 70)	71·3 (70·8 to 71·8)	71·4 (71 to 72)	73·6 (73·1 to 74·1)	75·8 (75·3 to 76·2)	78·4 (77·9 to 78·9)	80 (79·5 to 80·5)	81·5 (80·5 to 82·3)
Ireland	64·9 (64·6 to 65·1)	68·5 (68·3 to 68·7)	69 (68·8 to 69·2)	70·1 (69·9 to 70·2)	72·3 (72·2 to 72·5)	74·1 (74 to 74·3)	78·6 (78·4 to 78·8)	80·2 (79·8 to 80·5)
Israel	72·4 (72·1 to 72·7)	70·8 (70·6 to 71)	70 (69·8 to 70·1)	72·5 (72·3 to 72·6)	75·6 (75·4 to 75·7)	76·8 (76·7 to 77)	80·1 (80 to 80·2)	81·3 (81 to 81·5)
Italy	64·2 (64 to 64·3)	67·4 (67·3 to 67·5)	68·9 (68·8 to 68·9)	70·8 (70·8 to 70·9)	73·8 (73·8 to 73·9)	76·6 (76·5 to 76·6)	79·5 (79·4 to 79·5)	80·8 (80·7 to 80·9)
Luxembourg	64·1 (63·4 to 64·7)	66·2 (65·7 to 66·6)	66·6 (66·2 to 67)	69·2 (68·8 to 69·6)	71·6 (71·2 to 72·1)	75 (74·6 to 75·5)	78·7 (78·2 to 79·2)	80·8 (79·6 to 81·9)
Malta	61·7 (61 to 62·4)	67·1 (66·7 to 67·5)	68·7 (68·3 to 69·1)	70·6 (70·2 to 70·9)	74·1 (73·6 to 74·5)	76·5 (76·1 to 76·9)	78·3 (77·8 to 78·7)	80·2 (79·2 to 81·1)
Monaco	66·1 (64·7 to 67·5)	68·2 (66·9 to 69·6)	70·9 (69·5 to 72·4)	72·7 (71·2 to 74·2)	75 (73·3 to 77)	76·2 (74·3 to 78·5)	76·9 (75·6 to 78·5)	77·9 (76·3 to 79·7)
Netherlands	70·4 (70·3 to 70·6)	71·5 (71·4 to 71·7)	70·9 (70·8 to 71)	72·5 (72·5 to 72·6)	73·9 (73·8 to 74)	75·6 (75·5 to 75·7)	78·9 (78·8 to 79)	80 (79·8 to 80·3)
Norway	70·3 (70·1 to 70·5)	71·1 (71 to 71·3)	71·2 (71·1 to 71·4)	72·6 (72·5 to 72·8)	73·6 (73·5 to 73·8)	76·1 (76 to 76·3)	79·1 (78·9 to 79·2)	81·1 (80·9 to 81·3)
Portugal	56·7 (56·5 to 56·9)	61·9 (61·8 to 62·1)	64·2 (64·1 to 64·4)	68·3 (68·2 to 68·4)	70·8 (70·7 to 70·9)	73·4 (73·3 to 73·5)	77·1 (77 to 77·2)	78·7 (78·5 to 79)
San Marino	68·8 (64·3 to 72·9)	69·7 (67·4 to 72)	70·9 (69·7 to 72·1)	73·2 (71·9 to 74·5)	76·9 (75·5 to 78·3)	78·7 (77·3 to 80)	79·9 (77·2 to 83·3)	80 (76·4 to 83·9)
Spain	59·3 (59·2 to 59·5)	66·9 (66·8 to 67)	69·2 (69·2 to 69·3)	72·4 (72·3 to 72·5)	73·4 (73·3 to 73·4)	76 (75·9 to 76·1)	79 (78·9 to 79·1)	80·4 (80·1 to 80·6)
Sweden	70 (69·9 to 70·2)	71·6 (71·5 to 71·7)	72·3 (72·2 to 72·4)	73 (72·9 to 73·2)	75·1 (75 to 75·2)	77·6 (77·5 to 77·7)	79·9 (79·8 to 80)	81·1 (80·9 to 81·3)
Stockholm	69·6 (66·5 to 72·7)	70·9 (67·9 to 74·3)	71·4 (68·2 to 74·6)	71·8 (71·4 to 72·2)	73·8 (73·4 to 74·2)	77 (76·7 to 77·4)	79·5 (79·2 to 79·9)	81·1 (79·7 to 82·6)
Sweden except Stockholm	70·2 (69·7 to 70·8)	71·8 (71·2 to 72·4)	72·6 (72 to 73·3)	73·3 (73·2 to 73·5)	75·4 (75·2 to 75·5)	77·7 (77·6 to 77·9)	80 (79·8 to 80·1)	81·1 (80·7 to 81·5)
Switzerland	66·7 (66·6 to 66·9)	69 (68·9 to 69·2)	70·3 (70·1 to 70·4)	72·6 (72·5 to 72·7)	74·4 (74·3 to 74·6)	77·4 (77·2 to 77·5)	80·5 (80·4 to 80·6)	82 (81·8 to 82·3)
UK	66·5 (66·4 to 66·5)	68·2 (68·2 to 68·3)	68·9 (68·9 to 69)	70·7 (70·7 to 70·8)	73 (73 to 73·1)	75·6 (75·5 to 75·6)	78·5 (78·5 to 78·6)	79·2 (79·1 to 79·4)
England	66·8 (66·7 to 66·9)	68·5 (68·5 to 68·6)	69·2 (69·1 to 69·3)	71 (71 to 71·1)	73·3 (73·2 to 73·3)	75·9 (75·8 to 75·9)	78·9 (78·8 to 78·9)	79·6 (79·4 to 79·7)
East Midlands	66·8 (64·2 to 69·4)	68·5 (66 to 71)	69·2 (66·6 to 71·9)	71·1 (70·4 to 71·9)	73·4 (73·1 to 73·6)	75·8 (75·5 to 76)	78·7 (78·4 to 79)	79·4 (78·2 to 80·5)
Derby	66 (62·2 to 70)	67·7 (64·2 to 71·3)	68·4 (65·1 to 72)	70·3 (69·1 to 71·5)	72·6 (72·1 to 73·2)	75·5 (74·8 to 76·1)	77·4 (76·8 to 78·1)	79·1 (77·4 to 80·7)
Derbyshire	67·1 (63·6 to 70·6)	68·6 (65·5 to 72·3)	69·2 (66 to 72·5)	71 (70 to 72)	73·2 (72·7 to 73·6)	76 (75·5 to 76·4)	79·1 (78·7 to 79·6)	79·7 (78·2 to 81·1)
Leicester	65·3 (61·9 to 68·8)	67·1 (63·7 to 70·8)	67·9 (64·4 to 71·4)	70 (68·9 to 71·1)	71·7 (71·2 to 72·2)	73·7 (73·2 to 74·3)	76·6 (76·1 to 77·2)	78 (76·4 to 79·6)
Leicestershire	68·5 (65 to 72·4)	70·1 (66·7 to 73·7)	70·7 (67·3 to 74·2)	72·3 (71·3 to 73·4)	74·8 (74·3 to 75·3)	77·3 (76·9 to 77·8)	79·6 (79·1 to 80·1)	80·9 (79·5 to 82·4)
Lincolnshire	67·2 (63·8 to 70·8)	68·9 (65·5 to 72·4)	69·6 (66·4 to 73·1)	71·4 (70·4 to 72·4)	73·7 (73·3 to 74·2)	75·9 (75·5 to 76·4)	79 (78·5 to 79·4)	78·7 (77·3 to 80·2)

Northamptonshire	67·4 (64 to 71·1)	69·1 (65·6 to 72·9)	69·9 (66·5 to 73·4)	71·7 (70·5 to 72·7)	73·6 (73·2 to 74·1)	76 (75·6 to 76·5)	78·7 (78·2 to 79·1)	79·5 (78 to 80·9)
Nottingham	65·2 (61·6 to 69·3)	66·8 (63·4 to 70·5)	67·3 (64·2 to 70·8)	69·3 (68·2 to 70·4)	71·3 (70·7 to 71·8)	72·5 (72 to 73)	76·4 (75·8 to 76·9)	77·5 (75·7 to 79·1)
Nottinghamshire	67·2 (63·6 to 70·9)	68·8 (65·5 to 72·5)	69·5 (66·3 to 72·9)	71·3 (70·3 to 72·2)	73·9 (73·4 to 74·3)	76 (75·5 to 76·4)	79 (78·5 to 79·5)	79·6 (78·1 to 81·1)
Rutland	69·5 (65·6 to 73·4)	71·3 (67·6 to 75·1)	72·1 (68·6 to 75·7)	73·8 (72·3 to 75·4)	75·4 (74·4 to 76·4)	78·1 (77 to 79·2)	80·8 (79·6 to 81·9)	82·2 (80·1 to 84·2)
East of England	68·7 (66 to 71·3)	70·3 (67·7 to 72·8)	70·8 (68·2 to 73·5)	72·3 (71·6 to 73·1)	74·6 (74·3 to 74·8)	76·9 (76·7 to 77·1)	79·7 (79·4 to 79·9)	80·3 (79·2 to 81·5)
Bedford	67·6 (63·8 to 71·4)	69·2 (65·6 to 73·1)	69·7 (66·2 to 73·2)	71·6 (70·3 to 72·9)	74·1 (73·5 to 74·8)	75·8 (75·1 to 76·5)	78·4 (77·7 to 79·1)	78·9 (77·2 to 80·7)
Cambridgeshire	69·5 (66 to 73·3)	70·9 (67·3 to 74·6)	71·3 (67·9 to 74·8)	72·4 (71·4 to 73·5)	74·9 (74·4 to 75·4)	77·3 (76·8 to 77·8)	80·5 (79·9 to 81)	81·1 (79·6 to 82·7)
Central Bedfordshire	69 (65·2 to 73·1)	70·6 (67 to 74·5)	71·3 (67·8 to 74·9)	73 (71·9 to 74·2)	75·2 (74·6 to 75·8)	77·6 (77 to 78·2)	80·3 (79·6 to 80·9)	81·9 (80·3 to 83·5)
Essex	69 (65·6 to 72·9)	70·4 (67 to 74)	70·9 (67·6 to 74·3)	72·1 (71·2 to 73·1)	74·7 (74·3 to 75·1)	77·1 (76·7 to 77·5)	79·6 (79·2 to 80)	80·6 (79·1 to 82·1)
Hertfordshire	69·4 (66·2 to 73)	70·7 (67·3 to 74·3)	71·1 (67·7 to 74·7)	72·3 (71·4 to 73·3)	74·8 (74·4 to 75·2)	77·1 (76·7 to 77·6)	80·1 (79·6 to 80·5)	81·2 (79·8 to 82·7)
Luton	67·1 (63·6 to 71)	68·9 (65·3 to 72·8)	69·7 (66·2 to 73·3)	71·6 (70·4 to 72·8)	72·9 (72·4 to 73·5)	74·9 (74·3 to 75·5)	78·2 (77·5 to 78·8)	78·1 (76·4 to 79·7)
Norfolk	68·7 (65·2 to 72·2)	70·3 (66·8 to 74·3)	71·1 (68 to 74·5)	72·8 (71·8 to 73·8)	74·5 (74·1 to 75)	76·9 (76·5 to 77·3)	79·3 (78·8 to 79·7)	79·6 (78·2 to 81·1)
Peterborough	67·1 (63·3 to 71·4)	68·6 (65·2 to 72·5)	69 (65·9 to 72·6)	71·4 (70·1 to 72·7)	73·2 (72·6 to 73·8)	75·4 (74·7 to 76·1)	77·7 (77 to 78·3)	78·3 (76·5 to 80)
Southend-on-Sea	67·6 (63·9 to 71·5)	69·5 (65·9 to 73)	70·3 (67 to 74)	72·4 (71·2 to 73·7)	74 (73·4 to 74·7)	75·7 (75·1 to 76·4)	79 (78·3 to 79·7)	79·2 (77·5 to 80·9)
Suffolk	68·8 (65 to 72·7)	70·5 (67 to 74)	71·2 (68 to 74·9)	73 (71·9 to 74)	75 (74·5 to 75·4)	77·2 (76·7 to 77·6)	80·2 (79·7 to 80·7)	80·2 (78·6 to 81·7)
Thurrock	67·5 (63·8 to 71·5)	68·8 (65·4 to 72·6)	69·2 (65·8 to 72·7)	71 (69·7 to 72·2)	73·5 (72·8 to 74·1)	76·6 (75·9 to 77·3)	79·4 (78·7 to 80)	80·5 (78·8 to 82·2)
Greater London	66·9 (64·6 to 69·3)	68·5 (66·3 to 71·1)	69·2 (66·9 to 71·5)	71 (70·3 to 71·7)	73 (72·8 to 73·2)	75·6 (75·4 to 75·8)	79·3 (79·1 to 79·5)	80·6 (79·4 to 81·7)
Barking and Dagenham	65·2 (61·8 to 69·1)	66·8 (63·7 to 70·3)	67·5 (64·2 to 70·8)	69·6 (68·5 to 70·7)	72·3 (71·7 to 72·8)	74·4 (73·8 to 75·1)	76·7 (76·1 to 77·3)	77·4 (75·9 to 79·1)
Barnet	69·1 (65·8 to 72·8)	70·7 (67·5 to 74·1)	71·5 (68·3 to 74·7)	73·3 (72·2 to 74·4)	75·3 (74·8 to 75·8)	77·9 (77·3 to 78·4)	80·7 (80·2 to 81·2)	82 (80·6 to 83·5)
Bexley	69·1 (65·7 to 72·6)	70·5 (67·3 to 74·3)	71·1 (68·2 to 74·5)	73 (71·9 to 74·1)	75·4 (74·8 to 75·9)	77·7 (77·1 to 78·3)	79·3 (78·7 to 80)	81·4 (79·8 to 83)
Brent	66·8 (63·1 to 70·5)	68·6 (65·3 to 72·3)	69·5 (66·4 to 72·8)	71·3 (70·2 to 72·4)	73·2 (72·7 to 73·7)	75·5 (75 to 76)	79·5 (78·9 to 80·1)	79·8 (78·2 to 81·4)
Bromley	69·4 (66 to 73)	70·9 (67·6 to 74·6)	71·6 (68·6 to 74·6)	73·3 (72·2 to 74·4)	75·1 (74·6 to 75·6)	77·8 (77·2 to 78·3)	81·3 (80·7 to 81·9)	82·2 (80·6 to 83·8)
Camden	66·1 (62·8 to 69·9)	67·6 (64·4 to 71·3)	67·9 (64·7 to 70·9)	69 (67·9 to 70·2)	71·4 (70·8 to 72)	72·4 (71·8 to 72·9)	78·2 (77·7 to 78·8)	81 (79·5 to 82·6)
Croydon	68·3 (64·8 to 72)	70 (66·8 to 73·4)	70·8 (67·7 to 74·1)	72·5 (71·5 to 73·6)	74·3 (73·8 to 74·8)	76·6 (76·1 to 77·1)	79·2 (78·7 to 79·8)	80 (78·3 to 81·5)
Ealing	67 (63·7 to 70·8)	68·5 (65·2 to 72·3)	68·9 (65·9 to 72·3)	70·5 (69·4 to 71·6)	72·9 (72·4 to 73·4)	75·5 (75 to 76·1)	79·4 (78·9 to 80·1)	80·8 (79·2 to 82·2)
Enfield	67·9 (64·5 to 71·7)	69·5 (66·2 to 72·9)	70·2 (67·3 to 73·5)	72 (70·9 to 73)	74·3 (73·8 to 74·9)	76·7 (76·2 to 77·3)	80·4 (79·8 to 81)	80·1 (78·6 to 81·7)
Greenwich	65·8 (62·3 to 69·5)	67·6 (64·6 to 71·1)	68·5 (65·5 to 71·7)	70·5 (69·5 to 71·5)	72·5 (72 to 73·1)	74·3 (73·7 to 74·8)	78 (77·4 to 78·6)	79·6 (78 to 81·2)
Hackney	66·2 (62·8 to 70)	67·5 (64·4 to 71·3)	67·6 (64·4 to 70·7)	68·8 (67·7 to 70)	70·7 (70·1 to 71·2)	74·1 (73·5 to 74·6)	77·6 (77·1 to 78·2)	78·3 (76·7 to 79·8)
Hammersmith and Fulham	65·9 (62·6 to 69·5)	67·4 (64·2 to 71·2)	67·8 (64·4 to 71·2)	68·9 (67·8 to 70·1)	69·7 (69·1 to 70·3)	73·8 (73·2 to 74·3)	78·1 (77·4 to 78·7)	80·5 (78·8 to 82·1)
Haringey	66·4 (62·9 to 70·5)	68·1 (65 to 71·6)	68·7 (65·8 to 71·9)	70·5 (69·3 to 71·6)	73 (72·5 to 73·6)	74·2 (73·7 to 74·7)	77·4 (76·8 to 77·9)	80·4 (78·9 to 82)
Harrow	69·3 (65·9 to 73)	70·9 (67·5 to 74·5)	71·6 (68·4 to 74·7)	73·5 (72·4 to 74·7)	75·3 (74·7 to 75·9)	77·9 (77·3 to 78·5)	82·5 (81·8 to 83·2)	82·7 (81·1 to 84·4)
Havering	68·7 (65·3 to 72·6)	70·2 (66·9 to 74·1)	70·9 (67·5 to 74)	72·7 (71·7 to 73·8)	74·7 (74·2 to 75·3)	77·5 (76·9 to 78·1)	79·8 (79·1 to 80·4)	80·1 (78·5 to 81·7)
Hillingdon	68·5 (65 to 72·3)	69·9 (66·8 to 73·5)	70·4 (67·2 to 73·6)	72·1 (71 to 73·2)	74·5 (74 to 75·1)	76·1 (75·6 to 76·7)	79·6 (79 to 80·1)	81 (79·4 to 82·5)
Hounslow	67·6 (64 to 71·3)	69·2 (65·8 to 72·8)	69·8 (66·6 to 73·1)	71·6 (70·6 to 72·7)	73·2 (72·6 to 73·7)	75·3 (74·8 to 75·9)	79·4 (78·8 to 80)	80·7 (79·1 to 82·3)
Islington	66 (62·6 to 69·8)	67·7 (64·6 to 71·3)	68 (65 to 71·3)	69·5 (68·3 to 70·6)	70·6 (70·1 to 71·2)	72·5 (71·9 to 73)	76·5 (75·9 to 77·1)	79·3 (77·7 to 80·9)
Kensington and Chelsea	67 (63·5 to 70·5)	68·4 (65·1 to 71·9)	68·7 (65·5 to 71·8)	69·8 (68·7 to 70·9)	71·3 (70·7 to 71·9)	76·9 (76·3 to 77·5)	81·1 (80·4 to 81·8)	81·9 (80·2 to 83·5)
Kingston upon Thames	68·9 (65·5 to 72·6)	70·4 (67·1 to 73·9)	70·9 (67·9 to 74·5)	72·6 (71·3 to 73·8)	74·8 (74·2 to 75·5)	77·9 (77·3 to 78·5)	81·9 (81·2 to 82·7)	82·6 (81 to 84·3)
Lambeth	64·3 (60·8 to 68)	66 (62·9 to 69·8)	66·4 (63·4 to 69·6)	68·6 (67·5 to 69·8)	70·5 (70 to 71)	73·4 (72·9 to 73·9)	76·8 (76·3 to 77·4)	79·3 (77·8 to 80·9)

Lewisham	66·1 (62·8 to 69·8)	67·9 (64·8 to 71·6)	68·7 (65·5 to 71·8)	70·7 (69·6 to 71·7)	72·1 (71·6 to 72·6)	73·7 (73·2 to 74·2)	77·7 (77·2 to 78·3)	78·7 (77 to 80·3)
Merton	68 (64·6 to 71·7)	69·7 (66·5 to 73·3)	70·5 (67·3 to 73·8)	72·4 (71·2 to 73·6)	74·1 (73·5 to 74·7)	77·4 (76·9 to 78)	80·4 (79·8 to 81)	81 (79·3 to 82·7)
Newham	65·5 (62 to 69·2)	67·2 (64 to 70·8)	68 (64·8 to 71·4)	70 (68·9 to 71)	71·4 (70·9 to 71·9)	72·7 (72·1 to 73·2)	77·1 (76·6 to 77·6)	79 (77·6 to 80·7)
Redbridge	68·4 (65 to 72·1)	69·9 (66·8 to 73·7)	70·6 (67·5 to 74)	72·5 (71·5 to 73·7)	74·5 (74 to 75·1)	77·3 (76·7 to 77·8)	80·2 (79·5 to 80·8)	81·8 (80·2 to 83·4)
Richmond upon Thames	68·6 (65·3 to 72·3)	70 (66·7 to 73·6)	70·4 (67·2 to 73·6)	71·9 (70·8 to 73·1)	75·1 (74·5 to 75·7)	78·8 (78·2 to 79·5)	82·5 (81·8 to 83·2)	83·4 (81·8 to 85·1)
Southwark	66 (62·6 to 69·7)	67·5 (64·4 to 71·3)	68 (64·9 to 71·2)	69·5 (68·4 to 70·6)	69·5 (69 to 69·9)	73·8 (73·3 to 74·4)	77·5 (76·9 to 78)	78·8 (77·3 to 80·4)
Sutton	68·7 (65·2 to 72·4)	70·5 (67·1 to 73·9)	71·4 (68·3 to 74·8)	73·4 (72·2 to 74·7)	75·2 (74·6 to 75·8)	77·4 (76·8 to 78)	80·5 (79·8 to 81·1)	81·2 (79·6 to 82·8)
Tower Hamlets	64·5 (61·3 to 68·5)	65·8 (62·7 to 69·4)	65·9 (62·7 to 69)	67·3 (66·2 to 68·4)	69·7 (69·2 to 70·2)	72 (71·5 to 72·6)	76·8 (76·2 to 77·3)	79 (77·4 to 80·6)
Waltham Forest	66·7 (63·4 to 70·2)	68·4 (65·1 to 72)	69·2 (65·8 to 72·7)	71·2 (70·1 to 72·3)	73·7 (73 to 74·2)	75·2 (74·6 to 75·7)	78·4 (77·8 to 79)	80·1 (78·5 to 81·6)
Wandsworth	66·7 (63·4 to 70·2)	68·2 (65·2 to 71·7)	68·7 (65·7 to 72·2)	70·3 (69·3 to 71·3)	71·4 (70·9 to 71·9)	75·2 (74·7 to 75·7)	79·4 (78·9 to 79·9)	81·9 (80·4 to 83·4)
Westminster	66·2 (62·4 to 69·8)	67·8 (64·7 to 71·1)	68 (65 to 71·3)	69 (67·9 to 70)	71·8 (71·2 to 72·3)	75·5 (74·9 to 76)	80·6 (80 to 81·3)	81·3 (79·8 to 82·9)
North East England	65·5 (63 to 68·2)	67·2 (64·7 to 70)	67·9 (65·3 to 70·6)	69·8 (69·1 to 70·7)	71·8 (71·5 to 72·1)	74·5 (74·2 to 74·8)	77·6 (77·3 to 77·9)	77·7 (76·4 to 79)
County Durham	65·5 (62·5 to 69·1)	67·2 (64 to 71)	67·9 (64·8 to 71·1)	69·9 (69 to 70·9)	72·1 (71·7 to 72·5)	74·8 (74·3 to 75·3)	77·4 (76·9 to 78)	78 (76·5 to 79·6)
Darlington	65·7 (62·1 to 69·5)	67·5 (64·2 to 71·4)	68·1 (64·7 to 71·6)	70·2 (68·9 to 71·5)	72·2 (71·5 to 72·9)	74·6 (73·8 to 75·4)	77·7 (76·9 to 78·5)	78·3 (76·5 to 80·1)
Gateshead	65 (61·3 to 68·8)	66·6 (63·1 to 70·4)	67 (63·8 to 70·4)	69·1 (67·9 to 70·2)	71·4 (70·9 to 72)	73·5 (73 to 74·1)	77·7 (77 to 78·3)	77·5 (75·9 to 79·2)
Hartlepool	64·3 (60·7 to 68·4)	66·2 (62·9 to 69·9)	66·9 (63·5 to 70·3)	68·9 (67·7 to 70·3)	71·3 (70·6 to 71·9)	73·4 (72·6 to 74·1)	76·9 (76·1 to 77·7)	76·3 (74·5 to 78·2)
Middlesbrough	64·5 (60·7 to 68·4)	66·2 (63·1 to 69·9)	66·9 (63·6 to 70·3)	69 (67·9 to 70·3)	71 (70·5 to 71·7)	73·1 (72·5 to 73·7)	76·1 (75·3 to 76·8)	75·4 (73·6 to 77·4)
Newcastle upon Tyne	65·3 (61·7 to 69)	66·9 (63·7 to 70·6)	67·5 (64·5 to 71)	69·4 (68·3 to 70·4)	70·8 (70·3 to 71·3)	73·9 (73·3 to 74·5)	77·3 (76·8 to 78)	77·9 (76·3 to 79·4)
North Tyneside	66·4 (62·9 to 70·3)	68·1 (64·8 to 71·7)	68·7 (65·4 to 72·1)	70·6 (69·4 to 71·7)	72·4 (71·8 to 73)	74·9 (74·3 to 75·5)	77·7 (77 to 78·3)	78·4 (76·6 to 80)
Northumberland	66·6 (62·8 to 70·3)	68·4 (64·9 to 72·3)	69·2 (66 to 72·5)	70·6 (69·5 to 71·7)	73·2 (72·7 to 73·7)	76·2 (75·7 to 76·7)	79 (78·4 to 79·6)	78·9 (77·2 to 80·5)
Redcar and Cleveland	65·3 (61·7 to 69·1)	67 (63·7 to 70·8)	67·7 (64·5 to 71·1)	69·8 (68·6 to 71)	71·7 (71·1 to 72·3)	74·9 (74·2 to 75·5)	78·2 (77·5 to 79)	77·5 (75·8 to 79·3)
South Tyneside	65·7 (62 to 69·5)	67·3 (63·7 to 71·1)	67·9 (64·6 to 71·2)	69·8 (68·6 to 71)	71·6 (71·1 to 72·2)	74·1 (73·4 to 74·7)	77·2 (76·5 to 78)	77·3 (75·6 to 79·2)
Stockton-on-Tees	66 (62·6 to 69·9)	67·7 (64·3 to 71·5)	68·4 (65·1 to 71·7)	70·5 (69·3 to 71·6)	72 (71·4 to 72·6)	75·3 (74·6 to 75·9)	78·3 (77·6 to 79)	78 (76·3 to 79·7)
Sunderland	65·6 (62·2 to 69·4)	67·3 (63·9 to 71)	68 (64·9 to 71·5)	70 (68·9 to 71·1)	71·4 (70·9 to 72)	74 (73·5 to 74·6)	76·7 (76·2 to 77·3)	76·8 (75·2 to 78·5)
North West England	65·5 (63·3 to 67·9)	67·2 (65·1 to 69·6)	67·9 (65·6 to 70·3)	69·8 (69·2 to 70·5)	72 (71·8 to 72·1)	74·4 (74·2 to 74·6)	77·4 (77·2 to 77·6)	78·2 (77·1 to 79·3)
Blackburn with Darwen	65 (61·6 to 68·7)	66·7 (63·6 to 70·3)	67·4 (64·3 to 70·7)	69·4 (68·2 to 70·6)	70·7 (70·1 to 71·3)	72·7 (72·1 to 73·4)	76·2 (75·5 to 76·9)	77·1 (75·4 to 78·8)
Blackpool	64·8 (61·3 to 68·4)	66·7 (63·4 to 70·2)	67·5 (64·2 to 70·8)	69·6 (68·5 to 70·8)	70·5 (69·9 to 71·1)	71·1 (70·4 to 71·7)	72·6 (72 to 73·2)	73·6 (71·9 to 75·2)
Bolton	64·9 (61·6 to 68·6)	66·7 (63·2 to 70)	67·3 (64·2 to 70·4)	69·3 (68·3 to 70·4)	71·8 (71·3 to 72·3)	73·9 (73·3 to 74·4)	76·9 (76·3 to 77·5)	77·8 (76·3 to 79·3)
Bury	66·2 (62·6 to 69·8)	67·9 (64·6 to 71·4)	68·6 (65·4 to 72·1)	70·6 (69·4 to 71·8)	72·5 (71·9 to 73)	75·3 (74·6 to 75·9)	78·2 (77·5 to 78·8)	79·2 (77·7 to 80·8)
Cheshire East	67·8 (64·2 to 71·4)	69·3 (66·2 to 72·9)	69·8 (66·8 to 73)	71·6 (70·6 to 72·6)	73·9 (73·4 to 74·4)	76·5 (76 to 77)	79·8 (79·3 to 80·4)	80·4 (79 to 81·8)
Cheshire West and Chester	67·5 (64·1 to 71·1)	69 (65·5 to 72·8)	69·5 (66·5 to 72·7)	71·2 (70·1 to 72·2)	73·5 (73 to 74)	76·4 (75·9 to 76·9)	79·2 (78·6 to 79·8)	79·7 (78 to 81·3)
Cumbria	66·2 (63 to 69·7)	68 (64·5 to 71·7)	68·7 (65·5 to 72·1)	70·6 (69·5 to 71·6)	72·6 (72·2 to 73·1)	75·7 (75·2 to 76·2)	78·9 (78·4 to 79·5)	78·5 (77 to 80)
Halton	65·8 (62·5 to 69·6)	67·3 (64 to 70·8)	67·7 (64·7 to 71·1)	69·5 (68·4 to 70·6)	71·9 (71·3 to 72·6)	73·7 (73 to 74·3)	77·2 (76·5 to 77·9)	77·3 (75·6 to 79)
Knowsley	65·1 (61·8 to 68·8)	66·7 (63·4 to 70·2)	67·2 (64·1 to 70·4)	69·1 (68 to 70·2)	71·2 (70·7 to 71·8)	72·8 (72·2 to 73·4)	75·9 (75·3 to 76·7)	77·3 (75·7 to 78·9)
Lancashire	65·7 (62·5 to 69·2)	67·6 (64·5 to 70·9)	68·5 (65·4 to 71·9)	70·1 (69·1 to 71·2)	72·3 (71·9 to 72·7)	75 (74·5 to 75·4)	78 (77·5 to 78·4)	78·6 (77·1 to 80·1)
Liverpool	64·6 (61·2 to 68)	66·3 (63·1 to 69·8)	66·9 (63·8 to 70·3)	69·1 (68·1 to 70·1)	70·8 (70·3 to 71·2)	72·3 (71·8 to 72·8)	75·6 (75·1 to 76·1)	75·9 (74·4 to 77·4)
Manchester	63·4 (60 to 66·9)	65·2 (62·2 to 68·7)	65·9 (62·8 to 69·3)	67·9 (67 to 68·9)	69·4 (69 to 69·9)	71·1 (70·7 to 71·6)	74·7 (74·2 to 75·1)	77·5 (75·9 to 79)
Oldham	64·8 (61·4 to 68·3)	66·6 (63·3 to 70·1)	67·4 (64·5 to 70·6)	69·4 (68·3 to 70·5)	71·2 (70·7 to 71·8)	73·6 (73 to 74·1)	76·5 (75·9 to 77·1)	77·7 (76 to 79·3)

Rochdale	64·9 (61·4 to 68·3)	66·7 (63·3 to 70·3)	67·4 (64·4 to 70·6)	69·6 (68·5 to 70·7)	71·7 (71·2 to 72·2)	73·9 (73·3 to 74·5)	76·3 (75·6 to 76·9)	77·2 (75·6 to 78·9)
Salford	64·1 (60·7 to 67·7)	65·8 (62·7 to 69·4)	66·2 (63·4 to 69·5)	67·9 (66·8 to 68·9)	70·4 (69·9 to 70·9)	73·1 (72·5 to 73·6)	75·6 (75·1 to 76·2)	77·2 (75·6 to 78·8)
Sefton	66·6 (63·1 to 70·2)	68·2 (64·9 to 71·6)	68·8 (65·8 to 72·3)	70·7 (69·6 to 71·7)	73 (72·5 to 73·5)	75 (74·5 to 75·5)	77·1 (76·5 to 77·7)	77·8 (76·2 to 79·3)
St Helens	65·7 (62·2 to 69·3)	67·1 (63·8 to 70·7)	67·5 (64·4 to 70·9)	69·2 (68·1 to 70·3)	71·9 (71·4 to 72·5)	74·4 (73·8 to 75)	76·9 (76·2 to 77·5)	77 (75·4 to 78·6)
Stockport	67·3 (63·9 to 71)	68·9 (65·7 to 72·5)	69·5 (66·5 to 72·6)	71 (70 to 72)	73·1 (72·7 to 73·6)	76·4 (75·8 to 76·9)	79 (78·5 to 79·6)	79·7 (78·2 to 81·3)
Tameside	65·2 (61·8 to 68·8)	67 (63·8 to 70·3)	67·8 (64·7 to 71)	69·8 (68·7 to 70·9)	71·8 (71·2 to 72·3)	73·3 (72·7 to 73·8)	76·5 (75·9 to 77·1)	78·6 (77·1 to 80·3)
Trafford	67·2 (63·9 to 71)	69 (65·8 to 72·5)	69·6 (66·5 to 72·8)	71·5 (70·4 to 72·6)	73 (72·4 to 73·5)	76·4 (75·8 to 76·9)	79·2 (78·6 to 79·8)	80·6 (79·1 to 82·2)
Warrington	67 (63·6 to 70·9)	68·5 (65·1 to 72·2)	68·9 (65·8 to 72·1)	70·5 (69·4 to 71·6)	72·5 (71·9 to 73)	75·6 (75 to 76·2)	78·8 (78·2 to 79·4)	79·2 (77·5 to 80·7)
Wigan	65·6 (62·3 to 69·2)	67·2 (63·9 to 70·6)	67·8 (64·8 to 71)	69·5 (68·5 to 70·5)	71·8 (71·4 to 72·3)	74·1 (73·6 to 74·6)	76·9 (76·3 to 77·4)	78 (76·5 to 79·4)
Wirral	65·9 (62·4 to 69·6)	67·7 (64·4 to 71·1)	68·4 (65·2 to 71·3)	70·3 (69·2 to 71·3)	72·2 (71·7 to 72·7)	74·6 (74 to 75·1)	77·9 (77·4 to 78·5)	78·4 (76·9 to 79·9)
South East England	68·2 (65·7 to 70·7)	69·9 (67·7 to 72·4)	70·6 (68·2 to 73·1)	72·1 (71·3 to 72·7)	74·5 (74·3 to 74·7)	77 (76·9 to 77·2)	79·9 (79·7 to 80·1)	80·6 (79·5 to 81·7)
Bracknell Forest	68·9 (65·1 to 72·6)	70·6 (67·1 to 74·1)	70·9 (67·7 to 74·5)	72·5 (71·2 to 73·8)	74·6 (73·9 to 75·2)	77·6 (76·9 to 78·3)	81·2 (80·5 to 82)	81·5 (79·9 to 83·3)
Brighton and Hove	67 (63·3 to 70·7)	68·8 (65·6 to 72·6)	69·5 (66·2 to 73)	71·4 (70·3 to 72·5)	71·7 (71·2 to 72·2)	74·5 (73·9 to 75)	77·6 (77 to 78·2)	79 (77·5 to 80·6)
Buckinghamshire	70 (66·6 to 74·1)	71·4 (67·9 to 75·2)	71·9 (68·6 to 75·2)	73·3 (72·3 to 74·3)	75·2 (74·7 to 75·7)	78 (77·5 to 78·5)	80·7 (80·2 to 81·1)	81·9 (80·5 to 83·3)
East Sussex	67·9 (64·3 to 71·5)	69·7 (66·4 to 73·4)	70·4 (67·2 to 73·7)	72·2 (71·2 to 73·3)	75 (74·5 to 75·5)	77·1 (76·6 to 77·6)	79·5 (79 to 80)	79·8 (78·3 to 81·4)
Hampshire	68·9 (65·3 to 72·8)	70·6 (67·3 to 74·1)	71·1 (67·6 to 74·6)	72·4 (71·4 to 73·4)	75 (74·6 to 75·4)	77·6 (77·3 to 78·1)	80·5 (80·1 to 80·9)	81·1 (79·8 to 82·5)
Isle of Wight	66·4 (62·6 to 70·1)	68·5 (65·2 to 72·2)	69·5 (65·8 to 73·1)	71·6 (70·3 to 72·9)	73·3 (72·6 to 74)	76·4 (75·7 to 77·1)	78·8 (78·1 to 79·5)	79·3 (77·7 to 81)
Kent	67·7 (64·7 to 71·1)	69·5 (66·2 to 72·6)	70 (67 to 73·3)	71·2 (70·1 to 72·1)	73·9 (73·5 to 74·3)	76·6 (76·1 to 77)	79·4 (79 to 79·9)	80 (78·4 to 81·6)
Medway	67·6 (64·1 to 71·4)	69·4 (66 to 73·2)	70·2 (66·9 to 73·8)	71·9 (70·8 to 73)	73·4 (72·9 to 73·9)	75·5 (75 to 76·1)	78·4 (77·8 to 79)	78·9 (77·3 to 80·4)
Milton Keynes	67·8 (64 to 71·9)	69·4 (66·1 to 73·1)	69·8 (66·3 to 73·4)	71·4 (70·2 to 72·7)	74·1 (73·5 to 74·7)	76·8 (76·2 to 77·5)	79·5 (78·9 to 80·2)	80·2 (78·7 to 81·7)
Oxfordshire	69·7 (66 to 73·2)	71·2 (67·9 to 74·9)	71·7 (68·4 to 75·3)	72·9 (71·9 to 73·8)	75·4 (74·9 to 75·8)	77·5 (77·1 to 78)	80·3 (79·8 to 80·7)	81·6 (80·1 to 83)
Portsmouth	66·4 (62·8 to 70·2)	68·2 (64·8 to 72)	68·8 (65·5 to 72·3)	70·5 (69·4 to 71·7)	72 (71·4 to 72·5)	74·3 (73·7 to 74·9)	77·5 (76·8 to 78·1)	79 (77·5 to 80·6)
Reading	67·9 (64·2 to 71·7)	69·3 (65·9 to 73·1)	69·5 (66 to 73·1)	71 (69·8 to 72·2)	73 (72·4 to 73·6)	75·5 (74·9 to 76·2)	78·1 (77·4 to 78·7)	78·4 (76·7 to 80·1)
Slough	67·4 (63·6 to 71·2)	68·9 (65·4 to 72·8)	69·4 (66·1 to 72·9)	71·1 (69·9 to 72·5)	72·4 (71·7 to 73·1)	74·9 (74·2 to 75·5)	77·6 (76·9 to 78·3)	78·6 (76·7 to 80·3)
Southampton	66·4 (62·8 to 70·3)	68·1 (64·8 to 71·7)	68·7 (65·5 to 72·1)	70·6 (69·5 to 71·8)	72·3 (71·8 to 72·9)	75·1 (74·5 to 75·6)	78·4 (77·8 to 79)	78·6 (76·9 to 80·2)
Surrey	69·6 (66·2 to 73·1)	71·1 (67·8 to 74·6)	71·5 (68·1 to 75·2)	72·8 (71·7 to 73·8)	75·5 (75·1 to 75·9)	77·7 (77·3 to 78·1)	80·9 (80·5 to 81·4)	81·8 (80·4 to 83·2)
West Berkshire	69·8 (66·2 to 73·5)	71·2 (67·7 to 74·8)	71·5 (68·1 to 75)	73 (71·7 to 74·2)	75·2 (74·5 to 75·8)	78 (77·3 to 78·7)	81 (80·3 to 81·7)	81·6 (79·9 to 83·2)
West Sussex	68·2 (64·7 to 71·6)	69·9 (66·5 to 73·7)	70·4 (67 to 73·8)	72·1 (71·1 to 73·1)	74·9 (74·5 to 75·3)	77·5 (77 to 77·9)	80·1 (79·6 to 80·5)	80·2 (78·8 to 81·7)
Windsor and Maidenhead	70·8 (67·4 to 74·6)	72·4 (69·1 to 75·9)	73·2 (69·9 to 76·6)	74·9 (73·7 to 76·2)	75·7 (75 to 76·4)	77·8 (77·1 to 78·5)	81·6 (80·8 to 82·3)	82·2 (80·5 to 83·8)
Wokingham	71 (67·5 to 74·5)	72·5 (69·1 to 76·1)	73 (69·6 to 76·8)	74·6 (73·2 to 75·9)	77·3 (76·6 to 77·9)	80·3 (79·6 to 81)	82·9 (82·2 to 83·7)	82·9 (81·4 to 84·5)
South West England	67·9 (65·3 to 70·5)	69·6 (67 to 72·3)	70·2 (67·5 to 72·8)	72·1 (71·3 to 72·8)	74·4 (74·1 to 74·6)	77 (76·8 to 77·3)	79·7 (79·5 to 79·9)	80·2 (79 to 81·3)
Bath and North East Somerset	69·1 (65·5 to 72·8)	71 (67·5 to 74·5)	71·7 (68·1 to 75·4)	73·8 (72·5 to 75·1)	75 (74·3 to 75·6)	77·7 (77·1 to 78·4)	80·4 (79·8 to 81·2)	80·7 (79 to 82·4)
Bournemouth	68·1 (64·7 to 71·9)	69·9 (66·2 to 73·8)	70·7 (67·6 to 74·3)	72·5 (71·3 to 73·9)	73·2 (72·5 to 73·8)	75·2 (74·5 to 75·9)	77·7 (77·1 to 78·3)	78·4 (76·8 to 80)
Bristol, City of	66·8 (63·4 to 70·4)	68·5 (65·1 to 72·2)	69·1 (65·6 to 72·4)	70·9 (69·7 to 72·1)	72·9 (72·4 to 73·4)	75·4 (74·9 to 76)	78·2 (77·6 to 78·7)	78·7 (77·1 to 80·3)
Cornwall	67·6 (63·9 to 71·1)	69·2 (65·8 to 73)	69·7 (66·5 to 73)	71·5 (70·4 to 72·6)	74·3 (73·8 to 74·8)	77 (76·5 to 77·5)	79·5 (78·9 to 80)	79·8 (78·3 to 81·4)
Devon	68·1 (64·8 to 71·7)	69·8 (66·3 to 73·4)	70·3 (67·1 to 73·7)	72·2 (71·1 to 73·3)	74·9 (74·4 to 75·3)	77·4 (77 to 77·8)	80·1 (79·7 to 80·6)	80·4 (79 to 81·8)
Dorset	68·6 (65·1 to 72·3)	70·3 (67 to 73·9)	71 (67·6 to 74·3)	73 (71·9 to 74·1)	75·6 (75·1 to 76·2)	78·1 (77·5 to 78·6)	80·9 (80·4 to 81·5)	81·1 (79·6 to 82·6)
Gloucestershire	68·7 (65·1 to 72·5)	70·3 (66·7 to 73·7)	70·9 (67·5 to 74·2)	72·1 (71·1 to 73·2)	74·4 (73·9 to 74·8)	77·2 (76·7 to 77·7)	80 (79·5 to 80·5)	80·6 (79·1 to 82·2)

North Somerset	68·9 (65·2 to 72·6)	70·7 (66·9 to 74·4)	71·4 (68·1 to 74·8)	73·3 (72·1 to 74·7)	75·2 (74·6 to 75·9)	77·4 (76·7 to 78)	79·2 (78·5 to 79·9)	79·4 (77·8 to 81)
Plymouth	66·6 (62·8 to 70·2)	68·2 (64·8 to 71·8)	68·6 (65·6 to 71·7)	70·4 (69·3 to 71·5)	72·9 (72·4 to 73·5)	75 (74·5 to 75·6)	78 (77·4 to 78·6)	78·6 (76·9 to 80·2)
Poole	67·9 (64·3 to 71·9)	69·6 (66·2 to 73·4)	70·2 (67 to 73·7)	71·9 (70·7 to 73·2)	74·6 (74 to 75·3)	77·6 (76·8 to 78·3)	80·4 (79·7 to 81·1)	81·6 (80 to 83·2)
Somerset	68·5 (64·8 to 72·1)	70·1 (66·3 to 73·9)	70·7 (67·4 to 74)	72·3 (71·3 to 73·4)	74·8 (74·3 to 75·4)	77·5 (77 to 78)	79·9 (79·4 to 80·5)	80·3 (78·7 to 81·8)
South Gloucestershire	69·5 (65·7 to 73·1)	70·9 (67·6 to 74·5)	71·2 (67·9 to 74·8)	73 (71·7 to 74·1)	75·9 (75·3 to 76·5)	78·1 (77·5 to 78·7)	80·6 (80 to 81·2)	82·2 (80·7 to 83·8)
Swindon	67·4 (63·8 to 71)	69·2 (65·8 to 72·9)	69·6 (66·3 to 73·1)	71·6 (70·4 to 72·9)	73·9 (73·2 to 74·5)	76·5 (75·9 to 77·2)	79·8 (79·2 to 80·4)	80·7 (79 to 82·3)
Torbay	67·6 (64·2 to 71·3)	69·3 (65·6 to 73·4)	70·1 (67 to 73·8)	72 (70·7 to 73·3)	73·6 (72·9 to 74·2)	74·9 (74·3 to 75·6)	77·4 (76·7 to 78·1)	77·8 (76 to 79·6)
Wiltshire	68·6 (65·1 to 72·3)	70·4 (66·9 to 74·1)	71·1 (68 to 74·2)	72·6 (71·4 to 73·7)	74·4 (73·9 to 74·9)	77·6 (77·1 to 78·1)	80·4 (79·9 to 80·9)	81 (79·6 to 82·5)
West Midlands	66·5 (64·1 to 69·2)	68·1 (65·8 to 70·6)	68·6 (66·2 to 71·2)	70·4 (69·7 to 71·1)	72·7 (72·5 to 72·9)	75·2 (75 to 75·5)	78·4 (78·1 to 78·6)	78·9 (77·7 to 80·1)
Birmingham	65·5 (62·5 to 68·8)	67·2 (64·1 to 70·8)	67·9 (64·7 to 71·1)	69·8 (68·8 to 70·6)	71·6 (71·2 to 72)	73·7 (73·3 to 74·1)	77·6 (77·1 to 78)	77·7 (76·1 to 79·2)
Coventry	65·9 (62·4 to 69·7)	67·6 (64·4 to 71·2)	68·2 (64·9 to 71·5)	70·1 (69 to 71·1)	71·9 (71·4 to 72·4)	74·9 (74·4 to 75·4)	77·2 (76·6 to 77·7)	77·9 (76·3 to 79·4)
Dudley	67·5 (63·8 to 71·1)	69 (65·6 to 72·7)	69·6 (66·6 to 73·1)	71·2 (70·2 to 72·3)	73 (72·5 to 73·5)	75·3 (74·7 to 75·8)	78·4 (77·8 to 79)	79·4 (77·8 to 80·9)
Herefordshire, County of	67·6 (64 to 71·5)	69·4 (66·2 to 73·4)	70·1 (66·7 to 73·6)	72·2 (70·9 to 73·4)	75·1 (74·4 to 75·7)	76·8 (76·2 to 77·4)	79·6 (79 to 80·3)	80 (78·4 to 81·7)
Sandwell	65 (61·5 to 68·9)	66·7 (63·2 to 70·4)	67·4 (64·1 to 70·9)	69·4 (68·4 to 70·5)	71·1 (70·6 to 71·6)	73·5 (73 to 74)	76·3 (75·8 to 76·9)	78·2 (76·5 to 79·8)
Shropshire	67·7 (64·2 to 71·5)	69·2 (65·8 to 72·9)	69·6 (66·4 to 73·1)	71·6 (70·7 to 72·7)	74·1 (73·6 to 74·6)	76·5 (76 to 77)	79·8 (79·2 to 80·3)	81·1 (79·5 to 82·6)
Solihull	68·4 (64·9 to 72·3)	69·9 (66·4 to 73·7)	70·4 (67·1 to 73·5)	72·2 (71·1 to 73·4)	75 (74·4 to 75·6)	77·2 (76·5 to 77·8)	80·7 (80·1 to 81·4)	80·5 (78·8 to 82·1)
Staffordshire	67·2 (63·7 to 70·9)	68·6 (65·4 to 72·4)	69 (65·9 to 72·5)	70·4 (69·5 to 71·5)	73 (72·5 to 73·4)	75·9 (75·4 to 76·3)	79·2 (78·7 to 79·6)	79·4 (77·9 to 80·9)
Stoke-on-Trent	64·6 (61 to 68·4)	66·1 (62·8 to 69·7)	66·6 (63·8 to 69·7)	68·4 (67·3 to 69·5)	71 (70·5 to 71·5)	73·4 (72·8 to 74)	76·3 (75·7 to 76·9)	77·1 (75·5 to 78·9)
Telford and Wrekin	66·5 (62·9 to 70·1)	68 (64·6 to 71·7)	68·3 (65 to 71·8)	70 (68·9 to 71·3)	72·7 (72·1 to 73·3)	75·8 (75·1 to 76·4)	78·4 (77·7 to 79·1)	78·8 (77·1 to 80·5)
Walsall	66 (62·4 to 69·8)	67·5 (64 to 71·1)	67·9 (64·7 to 71·3)	69·7 (68·7 to 70·8)	71·9 (71·4 to 72·4)	74·6 (74·1 to 75·2)	77 (76·4 to 77·6)	78·1 (76·5 to 79·6)
Warwickshire	68·1 (64·7 to 71·9)	69·4 (66·1 to 73·3)	69·9 (66·5 to 73·2)	71·4 (70·4 to 72·4)	73·8 (73·4 to 74·3)	76·5 (76 to 77)	79·4 (78·9 to 79·9)	80·1 (78·6 to 81·6)
Wolverhampton	66 (62·6 to 69·8)	67·5 (64 to 71·4)	68 (64·8 to 71·4)	69·9 (68·9 to 71)	71·8 (71·3 to 72·3)	73·9 (73·4 to 74·5)	76·7 (76·1 to 77·3)	77·3 (75·6 to 78·8)
Worcestershire	68·4 (64·7 to 72·2)	69·8 (66·5 to 73·5)	70·5 (67·3 to 73·7)	71·9 (70·9 to 73)	74·1 (73·7 to 74·6)	76·6 (76·1 to 77·1)	79·3 (78·8 to 79·8)	79·6 (77·9 to 81·1)
Yorkshire and the Humber	65·9 (63·4 to 68·6)	67·6 (65·3 to 70·2)	68·3 (65·9 to 70·8)	70·3 (69·6 to 71)	72·7 (72·5 to 72·9)	75·5 (75·2 to 75·7)	78·1 (77·8 to 78·3)	78·7 (77·6 to 80)
Barnsley	65·7 (62·1 to 69·5)	67·4 (64 to 71)	68·1 (65 to 71·5)	70 (69 to 71·2)	72·5 (71·9 to 73)	75·2 (74·6 to 75·7)	78·1 (77·5 to 78·7)	78·4 (76·9 to 80·1)
Bradford	64·9 (61·5 to 68·5)	66·8 (63·3 to 70·2)	67·7 (64·6 to 71)	69·8 (68·7 to 70·8)	71·8 (71·3 to 72·3)	74·2 (73·7 to 74·7)	77·3 (76·8 to 77·8)	78·3 (76·9 to 79·8)
Calderdale	65·4 (61·9 to 69·2)	67·1 (63·7 to 70·7)	67·7 (64·7 to 71·1)	69·6 (68·5 to 70·8)	71·8 (71·2 to 72·4)	75·3 (74·6 to 75·9)	77·7 (77·1 to 78·3)	78·9 (77·2 to 80·5)
Doncaster	66·4 (63 to 70·3)	68 (64·8 to 71·8)	68·8 (65·7 to 72·1)	70·8 (69·8 to 71·9)	72·8 (72·4 to 73·3)	74·7 (74·2 to 75·2)	77 (76·5 to 77·6)	78·2 (76·6 to 79·8)
East Riding of Yorkshire	68 (64·5 to 71·7)	69·5 (66·2 to 73·4)	70·3 (67·1 to 73·5)	72·1 (71 to 73·2)	74·2 (73·7 to 74·7)	76·7 (76·1 to 77·2)	79·3 (78·8 to 79·9)	79·9 (78·3 to 81·5)
Kingston upon Hull, City of	65·1 (61·7 to 68·9)	67 (63·7 to 70·6)	68 (64·9 to 71·1)	70·1 (69 to 71·2)	71·9 (71·4 to 72·4)	73·5 (72·9 to 74)	75·7 (75·1 to 76·2)	75·3 (73·7 to 77)
Kirklees	65·1 (61·3 to 68·9)	66·8 (63·4 to 70·5)	67·5 (64·4 to 70·7)	69·3 (68·3 to 70·5)	72·4 (71·9 to 72·8)	75·3 (74·8 to 75·9)	77·6 (77·1 to 78·1)	78·3 (76·8 to 79·9)
Leeds	65·8 (62·5 to 69·4)	67·5 (64·3 to 71·2)	68·1 (64·9 to 71·4)	70 (69 to 71)	72·6 (72·1 to 73·1)	75·4 (74·9 to 75·9)	77·9 (77·4 to 78·3)	78·6 (77·1 to 80·2)
North East Lincolnshire	66·5 (63 to 70·5)	68·4 (64·8 to 72·2)	69·3 (66·3 to 72·7)	71·4 (70·2 to 72·6)	72·3 (71·7 to 72·9)	74·7 (74 to 75·4)	77·3 (76·6 to 77·9)	77·7 (76·1 to 79·4)
North Lincolnshire	66·5 (62·7 to 70·1)	68 (64·6 to 71·8)	68·5 (65·4 to 72·1)	70·4 (69·2 to 71·7)	72·3 (71·6 to 72·9)	75·5 (74·8 to 76·2)	78·5 (77·8 to 79·2)	79·6 (77·9 to 81·3)
North Yorkshire	67·2 (63·8 to 70·9)	68·9 (65·4 to 72·8)	69·6 (66·4 to 72·9)	71·5 (70·5 to 72·5)	74·1 (73·6 to 74·5)	77·3 (76·8 to 77·8)	79·9 (79·4 to 80·4)	80·7 (79·2 to 82·2)
Rotherham	65·9 (62·4 to 69·4)	67·5 (64 to 71)	68 (64·7 to 71·3)	69·7 (68·7 to 70·8)	72·4 (71·9 to 72·9)	75·1 (74·6 to 75·6)	78·2 (77·7 to 78·8)	77·6 (76 to 79·2)
Sheffield	66·4 (63·1 to 70·1)	68 (64·9 to 72)	68·7 (65·6 to 72·1)	70·6 (69·6 to 71·6)	72·8 (72·3 to 73·2)	75·5 (75 to 75·9)	78·1 (77·6 to 78·6)	79·3 (77·9 to 80·8)
Wakefield	65·3 (62 to 69·1)	66·8 (63·7 to 70·2)	67·2 (63·9 to 70·4)	69·1 (68·2 to 70)	72·4 (71·9 to 72·9)	75·2 (74·6 to 75·7)	77·3 (76·8 to 77·8)	77·9 (76·3 to 79·4)

York	67·7 (64·2 to 71·6)	69·3 (65·9 to 73·2)	70 (66·8 to 73·1)	71·8 (70·6 to 73·1)	73·8 (73·2 to 74·4)	77·1 (76·5 to 77·7)	80 (79·3 to 80·7)	80·1 (78·5 to 81·7)
Northern Ireland	65·8 (65·5 to 66·1)	68·1 (67·8 to 68·3)	68 (67·8 to 68·2)	68·5 (68·3 to 68·7)	71·6 (71·4 to 71·9)	75 (74·7 to 75·2)	77·5 (77·2 to 77·8)	78·5 (78 to 79)
Scotland	64·5 (64·3 to 64·6)	66·4 (66·2 to 66·5)	67·2 (67·1 to 67·3)	69 (68·9 to 69·1)	71·2 (71·1 to 71·3)	73·4 (73·2 to 73·5)	76·3 (76·1 to 76·5)	76·9 (76·5 to 77·3)
Wales	65·3 (64·7 to 65·9)	67·3 (66·5 to 68)	68·2 (67·5 to 68·9)	70·4 (70·1 to 70·7)	73 (72·8 to 73·1)	75·1 (74·9 to 75·3)	77·9 (77·7 to 78·1)	78·6 (78·2 to 78·9)
Latin America and Caribbean	46·3 (44·2 to 48·2)	52·5 (51·4 to 53·6)	56·5 (55·8 to 57·3)	62·1 (61·6 to 62·6)	66 (65·7 to 66·3)	69·3 (69·1 to 69·6)	70·1 (69·7 to 70·5)	72·9 (71·5 to 74·3)
Andean Latin America	38·9 (35·7 to 41·8)	47 (45·2 to 48·7)	49·4 (48·2 to 50·6)	62·5 (61·3 to 63·7)	66·5 (65·5 to 67·4)	71·3 (70·2 to 72·3)	73·3 (72·1 to 74·5)	75·9 (73·6 to 78·3)
Bolivia	35·3 (32·5 to 38·2)	41·4 (38·7 to 44)	48·1 (46 to 50·3)	55·1 (53·4 to 56·9)	60·4 (59 to 61·9)	65·4 (63·6 to 66·9)	69·5 (67·3 to 71·6)	71·1 (68·8 to 73·7)
Ecuador	49·5 (45·9 to 53·4)	55 (53·9 to 56)	60·3 (59·5 to 61·1)	64·9 (64·4 to 65·5)	70 (69·6 to 70·4)	72 (71·5 to 72·4)	71·5 (70 to 73·1)	74·2 (71·2 to 76·8)
Peru	36·8 (32 to 41·2)	45·9 (43·6 to 48·3)	45·8 (44·1 to 47·5)	64 (62·1 to 66·1)	67 (65·5 to 68·6)	73·1 (71·3 to 74·7)	75·6 (73·8 to 77·6)	78·7 (75·3 to 82)
Caribbean	54·5 (52·5 to 56·5)	59·1 (58·1 to 60·1)	61·9 (61 to 62·7)	64·4 (63·7 to 65·2)	66·4 (65·7 to 66·9)	68·2 (67·5 to 68·9)	55·2 (53·7 to 56·7)	70·2 (68·1 to 72·4)
Antigua and Barbuda	54·9 (53·1 to 56·4)	61 (59·8 to 62·2)	65·4 (64·5 to 66·3)	69·1 (67·7 to 70·3)	70·4 (69·7 to 71·1)	72·2 (71·4 to 73·1)	73·5 (72·6 to 74·3)	74·6 (72·9 to 76·3)
The Bahamas	55·1 (51 to 59·3)	59·1 (56·2 to 62·4)	62·2 (61·1 to 63·5)	65·1 (64·2 to 66·1)	67·3 (66·3 to 68·3)	67·8 (66·8 to 68·9)	69·8 (68·9 to 70·8)	69·9 (67·1 to 72·4)
Barbados	56 (53·8 to 57·8)	63·3 (62·1 to 64·6)	66·4 (65·6 to 67·2)	70·4 (69·8 to 71·1)	71·4 (70·8 to 72)	72·4 (71·7 to 73·1)	74·8 (74·2 to 75·4)	74·5 (72·4 to 76·6)
Belize	53·7 (51·7 to 55·7)	62 (59·9 to 64·1)	66·4 (65 to 67·9)	69·2 (68·1 to 70·5)	71·4 (70·4 to 72·4)	66·8 (66·2 to 67·5)	71 (70·4 to 71·7)	71·3 (69·6 to 73)
Bermuda	61 (59·8 to 62·2)	64·4 (63·6 to 65·1)	66·6 (65·9 to 67·3)	69·1 (68·4 to 69·8)	69·4 (68·9 to 70)	73·6 (72·9 to 74·3)	76·5 (75·7 to 77·3)	77·5 (75·7 to 79)
Cuba	64·7 (61 to 68·3)	67·8 (67·6 to 68)	69·3 (69·1 to 69·5)	71·9 (71·7 to 72·1)	73·2 (73 to 73·3)	75 (74·8 to 75·2)	76·4 (76·2 to 76·6)	76·3 (74·1 to 78·5)
Dominica	47·2 (44·4 to 49·5)	58 (55·8 to 60·2)	65·4 (63·4 to 67·4)	71·1 (69·3 to 72·9)	68·5 (67 to 69·9)	69·5 (67·9 to 71·1)	70 (68·1 to 71·6)	70 (67·3 to 72·4)
Dominican Republic	54·9 (52·8 to 56·8)	60·9 (59·6 to 62·1)	63·2 (61·8 to 64·8)	67·7 (66·3 to 69)	69·8 (68·3 to 71·2)	70·3 (68·7 to 71·9)	70·6 (68·1 to 73·3)	70 (66·6 to 73·3)
Grenada	56·7 (54·8 to 58·5)	61·3 (59·2 to 63·6)	64·2 (62 to 66·2)	66·6 (64 to 69·1)	66·3 (65·2 to 67·5)	68·4 (67·7 to 69)	69·2 (68·6 to 69·8)	71 (70·1 to 72)
Guyana	50·2 (47·4 to 53)	56·9 (55·1 to 58·6)	59·1 (57·1 to 61·3)	59·7 (57·4 to 62)	59·3 (57·5 to 61·4)	61·2 (59·2 to 63·2)	62·7 (60·7 to 64·6)	64·1 (60·7 to 67·6)
Haiti	40·1 (37·1 to 43·2)	43·2 (40·7 to 45·9)	46 (43·5 to 48·7)	49·6 (47·5 to 51·8)	53·8 (52·1 to 55·5)	57·4 (55·1 to 59·6)	28·8 (27·1 to 30·5)	62·9 (59·8 to 66)
Jamaica	55·8 (54·5 to 57)	63·2 (62·5 to 63·8)	66·9 (66·3 to 67·4)	72·1 (71·6 to 72·6)	74 (73·5 to 74·5)	72·4 (72 to 72·9)	73·7 (73·3 to 74·2)	74·6 (71·9 to 77·2)
Puerto Rico	59·1 (58·6 to 59·5)	67·4 (67 to 67·7)	68·5 (68·2 to 68·8)	70·5 (70·2 to 70·8)	70 (69·7 to 70·3)	72·7 (72·4 to 73·1)	75·7 (75·3 to 76)	77·2 (74·1 to 80·2)
Saint Kitts and Nevis	58·3 (57·2 to 59·6)	56·4 (55·4 to 57·5)	59·7 (58·7 to 60·7)	61·9 (60·6 to 63·1)	64·3 (63·2 to 65·4)	67·4 (66·2 to 68·5)	69·3 (68·2 to 70·4)	68·9 (67·1 to 70·2)
Saint Lucia	50·3 (47·7 to 52·4)	57·2 (55·1 to 59·1)	63·3 (61·7 to 64·8)	66·3 (65·4 to 67·1)	67·7 (67 to 68·4)	70·4 (69·8 to 71)	72·2 (71·5 to 72·9)	72·1 (70·1 to 74)
Saint Vincent and the Grenadines	52·3 (49·9 to 54·3)	59·4 (57·6 to 61·3)	62·6 (61·2 to 64·1)	66·1 (64·8 to 67·4)	68·3 (67·4 to 69·2)	68·7 (68 to 69·4)	71·2 (70·5 to 72)	70·9 (69·2 to 72·5)
Suriname	55·9 (54·2 to 57·4)	61·5 (59·6 to 63·1)	63·4 (61·9 to 64·8)	63·9 (63 to 64·8)	66·4 (65·6 to 67·4)	66·7 (66 to 67·5)	69 (68·3 to 69·8)	69·8 (67·3 to 72·3)
Trinidad and Tobago	55·9 (54·7 to 57)	62 (61·2 to 62·7)	63·3 (62·7 to 64)	65·4 (64·8 to 65·9)	67·2 (66·7 to 67·7)	68 (67·5 to 68·6)	70·7 (70·2 to 71·2)	72·1 (68·4 to 75·7)
Virgin Islands	58 (55·9 to 59·9)	60·8 (59·2 to 62·4)	63·7 (61·9 to 65·5)	67·3 (65·5 to 69·1)	69·3 (67·5 to 71·2)	69·8 (68·2 to 71·5)	69·5 (68·3 to 70·6)	69·9 (67·9 to 71·8)
Central Latin America	48·8 (47 to 50·3)	56·1 (55·4 to 56·7)	60·1 (59·7 to 60·6)	63·9 (63·3 to 64·5)	68 (67·7 to 68·3)	70·9 (70·6 to 71·1)	72·7 (72·4 to 72·9)	73·4 (71·2 to 75·4)
Colombia	52·6 (51 to 53·9)	58·1 (57·1 to 58·9)	64·6 (64 to 65·2)	66·8 (64·6 to 69)	68·2 (67·9 to 68·5)	70·5 (70·2 to 70·8)	74·7 (74·4 to 74·9)	77·5 (74·1 to 80·7)
Costa Rica	56·4 (55·8 to 57)	64·2 (63·7 to 64·7)	65·6 (65·2 to 66·1)	72·2 (71·8 to 72·6)	74·7 (74·4 to 75·1)	75·5 (75·2 to 75·8)	76·6 (76·3 to 76·9)	77·1 (74·1 to 80)
El Salvador	44·5 (41·3 to 47)	51·8 (49·7 to 53·8)	55·4 (53·9 to 56·6)	59·6 (57·9 to 61·4)	65·4 (64·9 to 65·9)	68·2 (67·8 to 68·6)	70·7 (70·3 to 71·1)	71·1 (67·3 to 74·9)
Guatemala	42·8 (40·2 to 45)	47·9 (46·1 to 49·6)	48·7 (47·5 to 49·8)	54·8 (53·5 to 55·9)	58·7 (56·7 to 60·9)	63·3 (61·3 to 65·5)	66·8 (64·7 to 68·9)	69·5 (66·1 to 72·8)
Honduras	39·6 (36·7 to 42·5)	47·4 (45·2 to 49·4)	56·2 (54·5 to 57·8)	66·3 (64·4 to 68·3)	66·9 (64·8 to 69)	69·2 (65·9 to 72·5)	70·2 (67·4 to 73·2)	70·8 (68·3 to 72·6)
Mexico	47 (45·2 to 48·7)	55·4 (54·3 to 56·3)	59·3 (58·7 to 60)	63·1 (62·5 to 63·6)	68·4 (68 to 68·7)	71·9 (71·6 to 72·2)	73 (72·7 to 73·2)	72·8 (70·3 to 75·2)
Aguascalientes	50·8 (47·7 to 53·9)	58 (55·1 to 60·9)	60·9 (58·2 to 63·8)	64·7 (63·8 to 65·5)	70·3 (69·7 to 70·9)	73·1 (72·6 to 73·7)	74·6 (74·1 to 75·1)	75·1 (72·5 to 77·5)
Baja California	46·5 (43·5 to 49·8)	55·1 (52·3 to 58·3)	58·7 (55·8 to 61·7)	64·4 (63·8 to 65)	66·4 (65·9 to 66·9)	67·8 (67·4 to 68·2)	70·5 (70·1 to 70·9)	71·5 (68·7 to 74·7)

Baja California Sur	47·9 (44·7 to 50·8)	56·6 (53·8 to 59·6)	60·3 (58 to 62·7)	65 (64·1 to 65·8)	70·7 (70·1 to 71·3)	72·2 (71·6 to 72·7)	73·7 (73·2 to 74·2)	70·9 (68·2 to 73·7)
Campeche	47·1 (43·9 to 50·5)	55·8 (52·8 to 59)	60·1 (57·4 to 62·9)	64·1 (63·1 to 65·1)	69·3 (68·5 to 70·1)	73·8 (73·1 to 74·5)	74·5 (74 to 75)	74·6 (72 to 77·4)
Chiapas	44·7 (41·6 to 47·8)	53·1 (50·5 to 56·1)	57·5 (54·7 to 60·3)	61·3 (60·3 to 62·3)	68 (67·4 to 68·6)	71·5 (70·9 to 72)	73·6 (73·3 to 74)	74·1 (71·2 to 77)
Chihuahua	45·5 (42·4 to 48·6)	54·3 (51·5 to 57·1)	58·5 (55·6 to 61·3)	61·9 (61 to 62·7)	65·3 (64·8 to 65·8)	68·5 (68 to 69)	68·3 (67·9 to 68·7)	68·1 (65·1 to 71·2)
Coahuila	45·9 (42·6 to 49·2)	55 (52·4 to 57·9)	58·8 (56·1 to 61·5)	62·5 (61·7 to 63·3)	68·4 (67·9 to 68·8)	71·7 (71·3 to 72·2)	72·8 (72·5 to 73·2)	73·4 (70·5 to 76·1)
Colima	41·7 (38·7 to 44·9)	51 (48 to 53·8)	55·1 (52·6 to 57·7)	60·1 (58·8 to 61·2)	67·5 (66·8 to 68·2)	72·3 (71·6 to 72·9)	73·3 (72·8 to 73·8)	69·2 (66·1 to 72·2)
Durango	46·1 (43·1 to 49·2)	55·1 (52·1 to 58·2)	59·6 (56·5 to 62·5)	63·1 (62·3 to 63·9)	68·9 (68·3 to 69·4)	73·2 (72·6 to 73·7)	72·8 (72·2 to 73·3)	74·6 (71·7 to 77·9)
Guanajuato	43·9 (40·8 to 47·1)	52·7 (49·9 to 55·6)	57·1 (54·3 to 59·9)	62·7 (62 to 63·6)	68·1 (67·6 to 68·6)	72·6 (72·2 to 72·9)	74·2 (73·8 to 74·5)	71·5 (68·5 to 74·6)
Guerrero	45·4 (42·6 to 48·4)	52·7 (49·5 to 56·2)	55·3 (52·4 to 58·1)	58·1 (56·8 to 59·5)	65·5 (64·8 to 66·2)	68·7 (68·3 to 69·2)	68·3 (67·7 to 69)	68·7 (66·5 to 70·9)
Hidalgo	44·6 (41·8 to 47·8)	52·8 (49·9 to 55·8)	56·5 (53·3 to 59·5)	59·6 (58·7 to 60·5)	69·3 (68·7 to 69·9)	74·2 (73·7 to 74·7)	74·9 (74·5 to 75·3)	74·7 (71·8 to 77·7)
Jalisco	48·9 (45·9 to 52·1)	56·9 (54 to 60)	60·8 (57·8 to 63·7)	65·3 (64·6 to 66)	68·8 (68·4 to 69·2)	71·9 (71·5 to 72·3)	73·2 (72·8 to 73·4)	72·7 (69·8 to 75·8)
México	48·8 (45·6 to 52·2)	56 (53·3 to 59)	59·6 (56·9 to 62·5)	63·2 (60·9 to 65·6)	66·5 (66 to 67)	71·4 (71·1 to 71·7)	73·3 (73 to 73·6)	73·3 (70·6 to 76·1)
Mexico City	54·1 (51·2 to 57·1)	61 (58·5 to 63·8)	63·7 (61·2 to 66·4)	65·8 (65·3 to 66·4)	69·5 (69·2 to 69·8)	72·1 (71·8 to 72·4)	73 (72·7 to 73·3)	73·4 (70·5 to 76·1)
Michoacán de Ocampo	45·1 (42·2 to 48·1)	53·6 (50·7 to 56·7)	58·5 (55·5 to 61·6)	62 (61·3 to 62·7)	69·7 (69·3 to 70·2)	72·9 (72·5 to 73·3)	73·7 (73·4 to 74·1)	72·8 (69·6 to 75·9)
Morelos	41·1 (38·1 to 44·2)	51·1 (48·1 to 54·3)	56·2 (53·2 to 59·2)	60·5 (59·2 to 61·8)	68·4 (67·8 to 69·1)	73 (72·4 to 73·5)	74 (73·5 to 74·4)	72·8 (69·8 to 75·7)
Nayarit	43·7 (40·4 to 46·8)	53·4 (50·6 to 56·3)	59·4 (56·6 to 62·3)	64·8 (63·9 to 65·7)	69 (68·3 to 69·7)	74·1 (73·5 to 74·7)	74·1 (73·5 to 74·6)	73 (69·9 to 76·1)
Nuevo León	52·3 (49·4 to 55·6)	60·6 (57·7 to 63·2)	64 (61·1 to 66·8)	68·4 (67·7 to 69·1)	70·8 (70·3 to 71·2)	73·4 (73 to 73·8)	73·8 (73·5 to 74·1)	74·3 (71·6 to 77·1)
Oaxaca	42·3 (39·4 to 45·4)	50·4 (47·8 to 53·4)	54·5 (51·7 to 57·3)	57·9 (57·1 to 58·7)	66·6 (66·1 to 67·1)	71·9 (71·4 to 72·3)	73·6 (73·2 to 73·9)	74·5 (71·4 to 77·7)
Puebla	38 (35·2 to 40·9)	47·8 (45·1 to 50·6)	53·1 (50·3 to 56)	57·7 (56·7 to 58·6)	66·4 (65·8 to 66·9)	70·4 (69·9 to 70·9)	73 (72·7 to 73·4)	72·8 (69·8 to 75·7)
Querétaro	44·3 (41·2 to 47·4)	53 (50·3 to 56·2)	57·7 (54·5 to 60·7)	61·7 (59·6 to 63·8)	67·5 (67 to 68·1)	71·2 (70·7 to 71·6)	73·8 (73·3 to 74·2)	73·5 (70·7 to 76·2)
Quintana Roo	49·6 (46·3 to 52·9)	57·1 (54·2 to 60·2)	60·6 (57·9 to 63·3)	64·4 (63·4 to 65·3)	71 (70·3 to 71·7)	73·9 (73·3 to 74·4)	73·6 (73·1 to 74)	73·1 (70·4 to 75·8)
San Luis Potosí	46·4 (43·3 to 49·5)	55·2 (52·3 to 58·2)	59·3 (56·3 to 62·3)	66·6 (65·7 to 67·4)	70·7 (70·2 to 71·2)	74·2 (73·7 to 74·7)	75·4 (75 to 75·8)	73·9 (70·8 to 76·9)
Sinaloa	49·8 (46·6 to 53)	57·6 (54·6 to 60·7)	61·5 (58·4 to 64·5)	65·3 (64·6 to 66·1)	71 (70·5 to 71·5)	73·8 (73·4 to 74·2)	73·2 (72·8 to 73·6)	72·1 (69 to 75·3)
Sonora	50·7 (47·6 to 53·7)	58·3 (55·4 to 61·3)	61·2 (58·4 to 64·3)	64·3 (62·1 to 66·7)	68·4 (67·9 to 68·9)	70·8 (70·3 to 71·2)	71·6 (71·2 to 72)	72·2 (69·4 to 74·9)
Tabasco	45·3 (42 to 48·5)	53·4 (50·5 to 56·5)	57 (53·8 to 60)	59·9 (58·8 to 61)	68·9 (68·1 to 69·6)	72·5 (72 to 73)	72·2 (71·7 to 72·6)	73·1 (70·1 to 76·1)
Tamaulipas	49·1 (46 to 52·3)	57·2 (54·5 to 60·2)	61·2 (58·1 to 64)	63·7 (62·9 to 64·5)	69·7 (69·2 to 70·2)	73·5 (73·1 to 73·9)	72·5 (72·1 to 72·9)	72·5 (69·6 to 75·6)
Tlaxcala	44·7 (41·7 to 47·9)	54·1 (51·3 to 57·2)	59·3 (56·1 to 62·4)	63·9 (61·8 to 66·2)	69·3 (68·6 to 69·9)	73 (72·4 to 73·5)	74·9 (74·3 to 75·5)	74·4 (71·7 to 77·1)
Veracruz de Ignacio de la Llave	45·9 (42·7 to 48·9)	54·5 (51·6 to 57·3)	58·6 (55·6 to 61·6)	62·9 (61·9 to 63·8)	69·2 (68·5 to 69·9)	72·4 (71·9 to 72·8)	73·3 (73 to 73·6)	72·7 (69·6 to 75·7)
Yucatán	51·6 (48·6 to 54·8)	58·7 (55·8 to 61·5)	62·7 (60 to 65·4)	66·1 (65·4 to 66·9)	70·7 (70·3 to 71·2)	73·9 (73·5 to 74·3)	74·3 (73·9 to 74·7)	74·3 (71·6 to 76·9)
Zacatecas	49·8 (46·7 to 53·1)	57·6 (54·4 to 60·7)	61·5 (58·5 to 64·5)	66·2 (65·3 to 67·1)	71·5 (70·9 to 72·2)	74·2 (73·6 to 74·8)	74·1 (73·6 to 74·6)	72·7 (69·6 to 75·8)
Nicaragua	46·1 (42·9 to 48·7)	51·8 (49·7 to 53·6)	55·7 (54 to 57·4)	63·4 (61·4 to 65·5)	68·7 (68·2 to 69·2)	71·3 (70·9 to 71·6)	71·8 (71·3 to 72·2)	72·4 (70·4 to 74·6)
Panama	59·7 (58·5 to 60·8)	64·7 (63·9 to 65·6)	67 (66·4 to 67·7)	72·2 (71·5 to 72·8)	74·2 (73·6 to 74·8)	75·4 (74·9 to 75·9)	75·5 (75 to 75·9)	77·5 (74·3 to 80·6)
Venezuela	57·3 (57 to 57·6)	63·6 (63·3 to 63·8)	64·4 (64·2 to 64·6)	66·9 (66·7 to 67)	69·6 (69·5 to 69·8)	70·1 (70 to 70·2)	70·9 (70·8 to 71·1)	71·4 (67·7 to 75)
Tropical Latin America	44·2 (41·7 to 46·4)	49·1 (47·4 to 50·8)	53·6 (52·3 to 54·9)	59·5 (58·8 to 60·2)	63·8 (63·3 to 64·2)	67·6 (67·2 to 68)	70·2 (69·8 to 70·6)	72·4 (71·7 to 73)
Brazil	44 (41·5 to 46·1)	48·8 (47 to 50·5)	53·3 (52 to 54·6)	59·3 (58·5 to 59·9)	63·6 (63·1 to 64)	67·4 (67 to 67·8)	70·1 (69·7 to 70·5)	72·3 (71·7 to 72·9)
Acre	41 (37·5 to 44·8)	47 (43·4 to 51)	52·4 (49·5 to 55·2)	58·2 (56·3 to 59·8)	63·9 (62·9 to 64·8)	67·3 (66·4 to 68·1)	69·9 (68·7 to 70·9)	71 (69·7 to 72·5)
Alagoas	35·4 (31·2 to 39·4)	39·2 (36·2 to 42·3)	44 (41·3 to 47)	49·6 (47·7 to 51·3)	58·1 (56·8 to 59·4)	65·6 (64·3 to 66·8)	66·2 (64·9 to 67·4)	69·5 (67·2 to 71·9)
Amapá	38·3 (35·2 to 41·6)	46 (42·7 to 49·2)	55·8 (53·1 to 58·5)	68·5 (67·5 to 69·4)	69·8 (69·2 to 70·4)	69 (68·5 to 69·5)	72·5 (71·8 to 73·1)	72·3 (71·1 to 73·5)

Amazonas	50 (47·1 to 53·1)	54·6 (51·5 to 57·9)	57·7 (54·8 to 60·8)	62 (60·4 to 63·6)	66·9 (65·5 to 68·2)	70 (68·7 to 71·2)	72·2 (71 to 73·3)	73·3 (71·6 to 74·9)
Bahia	41·8 (38·2 to 45·8)	46·1 (43·3 to 49·3)	51·3 (48·3 to 54·3)	59·6 (57·6 to 61·5)	63·9 (61·9 to 65·8)	68 (66·2 to 70·1)	68·6 (66·7 to 70·6)	70·4 (67·8 to 73·1)
Ceará	29·1 (25·1 to 33·2)	35 (31·9 to 37·9)	42·7 (39·8 to 46)	52·9 (50 to 55·8)	63·4 (61·3 to 65·7)	67·5 (65·2 to 69·9)	69·4 (67·1 to 72)	70·5 (67·6 to 73·7)
Distrito Federal	45·5 (42·7 to 48·3)	51·2 (48·1 to 54·2)	56 (53·2 to 58·9)	61·6 (60·3 to 62·9)	65·1 (64 to 66·3)	68·6 (68·2 to 68·9)	71·3 (70·9 to 71·7)	74·4 (72·9 to 75·7)
Espírito Santo	48·4 (45 to 51·9)	52·3 (49·3 to 55·4)	56·5 (53·6 to 59·4)	63·1 (62·5 to 63·6)	66·3 (65·9 to 66·7)	68·1 (67·7 to 68·4)	69·2 (68·4 to 70)	71·2 (69·3 to 73·1)
Goiás	37 (34·6 to 39·8)	42·5 (39·3 to 45·5)	50·5 (47·8 to 53·3)	58·9 (56·3 to 61·5)	63·8 (62 to 65·7)	69 (67·3 to 70·6)	70·6 (68·9 to 72·3)	71·7 (69·1 to 74·4)
Maranhão	33·8 (30·1 to 37·5)	37·7 (35·2 to 40·4)	42·4 (39·8 to 45·1)	50·2 (47·5 to 53·1)	58·7 (56·5 to 61·3)	68·4 (66 to 70·9)	70·3 (67·8 to 72·8)	70·1 (67·5 to 72·7)
Mato Grosso	51·8 (48·9 to 54·8)	54·6 (51·4 to 58·1)	58·5 (55·3 to 61·6)	64·4 (62·6 to 66·3)	65·4 (63·8 to 67·1)	68·9 (67·4 to 70·5)	71·2 (70·7 to 71·6)	73·9 (72·2 to 75·6)
Mato Grosso do Sul	49·9 (46·7 to 53)	53·4 (50·3 to 56·5)	57·7 (54·5 to 60·8)	63 (61·9 to 64·2)	66·8 (66 to 67·7)	68·3 (67·9 to 68·6)	70·5 (70 to 71)	73·7 (72 to 75·3)
Minas Gerais	46·7 (43·3 to 50·1)	50·9 (48·1 to 53·9)	55·5 (52·6 to 58·5)	61·6 (61 to 62·1)	65·2 (64·2 to 66)	69·1 (68·2 to 69·9)	71·2 (70·4 to 72·1)	74·1 (72·5 to 75·7)
Pará	42·7 (39·1 to 46·3)	48·1 (45·4 to 51·4)	52·7 (49·8 to 55·6)	59·4 (57·1 to 61·6)	64·8 (63 to 66·7)	69·1 (67·1 to 71)	70·6 (68·6 to 72·7)	72·8 (71 to 74·6)
Paraíba	38·1 (33·9 to 42·2)	44·7 (41·7 to 47·9)	52 (48·9 to 55·1)	60·2 (58·7 to 61·8)	66·4 (65·2 to 67·6)	69·9 (68·8 to 71)	69·7 (68·5 to 71)	72·6 (70·3 to 74·9)
Paraná	50·2 (47·1 to 53·5)	54·3 (51·5 to 57·1)	58·1 (55·3 to 61·1)	62 (61·5 to 62·5)	66 (65·7 to 66·3)	68·6 (68·3 to 68·9)	70 (69·3 to 70·6)	72·5 (70·6 to 74·4)
Pernambuco	32 (27·8 to 35·9)	37·9 (34·9 to 41)	45·3 (42·6 to 48·1)	55·7 (54·6 to 56·8)	60·5 (59·6 to 61·2)	64·8 (63·9 to 65·6)	67·7 (66·9 to 68·5)	68·7 (66·9 to 70·7)
Piauí	42·4 (38·5 to 46·2)	46·6 (43·7 to 49·6)	50·4 (47·5 to 53·3)	58·6 (56·8 to 60·8)	65·3 (64·1 to 66·4)	69·4 (68·3 to 70·6)	72·4 (71·8 to 72·9)	74·1 (72·4 to 75·8)
Rio de Janeiro	48·7 (46·1 to 51·8)	52·9 (49·9 to 56·2)	55·6 (52·7 to 58·6)	58·9 (57·9 to 59·8)	60·9 (60·6 to 61·1)	65·3 (65·1 to 65·5)	68·8 (68·5 to 69·1)	71·2 (69·6 to 72·7)
Rio Grande do Norte	36 (32·2 to 39·5)	43·5 (40·7 to 46·6)	51·5 (48·5 to 54·7)	60·2 (58·2 to 62·3)	67·1 (65·5 to 68·7)	70·8 (69 to 72·7)	72·5 (70·5 to 74·4)	72·7 (70 to 75·5)
Rio Grande do Sul	51·5 (48·9 to 54·4)	55·5 (52·5 to 58·5)	59·8 (57 to 62·7)	64 (63·3 to 64·8)	66·6 (66·4 to 66·8)	68·6 (68·4 to 68·8)	71·2 (70·9 to 71·5)	72·9 (71·3 to 74·4)
Rondônia	36·6 (34 to 39·5)	41·2 (38·1 to 44·5)	47·2 (44·3 to 50·2)	56·5 (54·9 to 57·9)	61·5 (60·3 to 62·8)	67·8 (66·7 to 68·9)	71·1 (69·7 to 72·3)	72·4 (70·2 to 74·7)
Roraima	38 (35·3 to 40·8)	43·5 (40·5 to 46·4)	50·1 (47·5 to 52·6)	59 (57·7 to 60·4)	63·6 (62·8 to 64·5)	68·3 (67·6 to 68·9)	72 (71·4 to 72·6)	72·1 (70·9 to 73·2)
Santa Catarina	54·6 (51·4 to 57·8)	57·8 (55·2 to 61)	60·6 (57·8 to 63·5)	64·8 (64·3 to 65·3)	66·1 (65·4 to 66·9)	68·1 (67·4 to 68·9)	71·3 (70·7 to 72)	74 (72·4 to 75·5)
São Paulo	51·6 (48·7 to 54·5)	55·3 (52·2 to 58·6)	57·8 (55 to 60·8)	60·7 (60 to 61·5)	63·7 (63 to 64·4)	66·7 (66·5 to 66·9)	71·2 (71 to 71·5)	74 (72·5 to 75·4)
Sergipe	44·2 (40·7 to 47·9)	48·8 (45·3 to 52·1)	53·6 (50·5 to 56·5)	57·9 (55·8 to 59·8)	63·7 (62·3 to 65·1)	68·1 (67·4 to 68·8)	70·2 (68·5 to 71·8)	70·8 (68·2 to 73·4)
Tocantins	39·2 (36·2 to 42·5)	44·3 (39·8 to 48·9)	51·6 (48·7 to 54·6)	61·2 (58 to 64·1)	66·1 (64·4 to 67·7)	70·3 (68·8 to 71·7)	72 (70·5 to 73·7)	72·2 (70·1 to 74·3)
Paraguay	58 (54·9 to 60·9)	67·1 (65·5 to 68·7)	69·4 (68 to 70·8)	71·6 (70·3 to 72·9)	74 (72·5 to 75·4)	73·3 (72 to 74·7)	73·2 (71·8 to 74·4)	73·8 (70·5 to 77)
North Africa and Middle East	42·3 (39·7 to 44·9)	46·5 (44·5 to 48·4)	52·7 (51·1 to 54·3)	58·8 (57·4 to 60)	63·8 (62·9 to 64·6)	67·6 (66·8 to 68·5)	70·5 (69·5 to 71·4)	72·3 (70·9 to 73·6)
North Africa and Middle East	42·3 (39·7 to 44·9)	46·5 (44·5 to 48·4)	52·7 (51·1 to 54·3)	58·8 (57·4 to 60)	63·8 (62·9 to 64·6)	67·6 (66·8 to 68·5)	70·5 (69·5 to 71·4)	72·3 (70·9 to 73·6)
Afghanistan	43·5 (40·6 to 46·6)	45·8 (42·4 to 49·3)	47·8 (44·5 to 51·5)	42·6 (31·1 to 49·3)	53·9 (51·4 to 56·8)	54·5 (51·9 to 57·2)	60·2 (57·3 to 63)	63·5 (60·9 to 66)
Algeria	53·3 (51·4 to 55·2)	49·4 (47·7 to 51·2)	54·7 (52·3 to 57)	57·2 (54·9 to 59·6)	66 (64 to 67·9)	69·6 (67·6 to 71·6)	73·8 (72·2 to 75·4)	75·6 (73·7 to 77·5)
Bahrain	49·1 (44·7 to 53·3)	55·5 (52·4 to 58·5)	62·4 (59·2 to 65·2)	66·8 (65·2 to 68·5)	68·3 (67·2 to 69·5)	70·7 (69·5 to 72)	74·2 (73·2 to 75·2)	76·6 (74·9 to 78·3)
Egypt	39·8 (36·4 to 42·6)	46 (44 to 47·8)	50·1 (48·9 to 51·5)	57 (56 to 57·9)	62·9 (62·3 to 63·5)	67·4 (67 to 67·8)	68·6 (68·2 to 68·9)	70·1 (67·1 to 73·1)
Iran	36·8 (33·8 to 39·7)	41·7 (39·1 to 44·2)	51·8 (49·4 to 53·9)	61·9 (60·8 to 63·1)	64·1 (63·2 to 65)	69·9 (69·3 to 70·5)	73·3 (72·9 to 73·6)	76·1 (75·6 to 76·5)
Iraq	52·9 (49·8 to 56·1)	57·5 (54·9 to 60·3)	60·1 (55·8 to 63)	61·9 (59·8 to 64·1)	64·4 (62·3 to 66·8)	66·4 (63·4 to 69·4)	68·3 (65·5 to 71·4)	71 (68·6 to 73·7)
Jordan	54·7 (50·5 to 58·8)	57·2 (54·2 to 60·2)	60·6 (58·9 to 62·2)	64·8 (63·3 to 66·6)	71·3 (69·7 to 73)	73·3 (71·4 to 75·5)	76 (74·7 to 77·1)	77·5 (75·2 to 79·7)
Kuwait	63·8 (59·6 to 67·8)	67·6 (65·2 to 70)	68·5 (68·1 to 69)	71·1 (70·8 to 71·5)	73·9 (73·4 to 74·6)	76·6 (76·3 to 77)	79 (78·7 to 79·3)	79·5 (77·4 to 81·6)
Lebanon	57 (52·7 to 61·2)	63 (59·9 to 66·1)	65·6 (62·8 to 68·5)	67·3 (64·8 to 69·6)	67·1 (65·2 to 68·8)	73·3 (71·8 to 74·8)	73·3 (72·3 to 74·4)	74 (72·6 to 75·6)
Libya	45·8 (42·6 to 49·2)	51·4 (47·8 to 55)	63·4 (61·5 to 65·3)	68·5 (67 to 69·9)	71·2 (69 to 73·5)	73·3 (72·2 to 74·5)	75 (74·7 to 75·4)	74·5 (72 to 76·8)
Morocco	42·2 (39 to 45·5)	44·2 (41·9 to 46·6)	52·2 (49·9 to 54·6)	58·7 (57 to 60·5)	65·3 (64 to 66·7)	68·1 (66·1 to 70·1)	71·2 (68·6 to 74·1)	72 (70·2 to 74·6)

Oman	39·5 (35·2 to 43·7)	43·5 (39·6 to 47·6)	53·5 (49·7 to 57·3)	60·3 (56·8 to 63·7)	65·1 (62·4 to 67·9)	67·4 (65·8 to 69·1)	69 (68·6 to 69·4)	73 (71·8 to 74·1)
Palestine	51·5 (47·1 to 55·7)	55·8 (52·4 to 59·3)	53·1 (50·1 to 55·8)	64·4 (61·1 to 67·3)	66·9 (64·8 to 69·2)	67·4 (66·3 to 68·5)	71·5 (70·8 to 72·3)	73·2 (71·9 to 74·5)
Qatar	61·9 (58 to 65·7)	62·9 (60 to 66)	65 (61·9 to 68·2)	68 (65·4 to 70·8)	69 (67·1 to 70·9)	70·9 (68·9 to 73)	74 (72·2 to 75·9)	76·7 (74·8 to 78·6)
Saudi Arabia	49·6 (45 to 54·2)	53 (49·6 to 56·3)	58·6 (55·1 to 61·8)	65 (61·9 to 67·9)	67·2 (64·8 to 69·9)	69·5 (68·2 to 70·8)	70·5 (69·5 to 71·6)	73·3 (71·5 to 75·3)
Sudan	45·2 (42 to 48·6)	48·2 (45·3 to 51·2)	50 (47·8 to 52·5)	53·4 (51·4 to 55·6)	56·6 (54·8 to 58·6)	61·2 (58·6 to 63·8)	65·7 (62·9 to 68·5)	69 (66·4 to 71·4)
Syria	53·5 (50·4 to 56·7)	59·2 (56·3 to 62)	64·4 (62·1 to 66·7)	68·1 (65·1 to 70·9)	67·3 (65 to 69·8)	70·5 (68·5 to 72·5)	73·5 (71·6 to 75·6)	72·4 (69·6 to 75·1)
Tunisia	41·3 (36·7 to 45·7)	47·6 (45·5 to 49·7)	55·5 (54 to 57·1)	64·9 (63·6 to 66)	70·4 (69 to 71·7)	72·1 (70·1 to 74·1)	74 (71·3 to 77)	75·5 (72·5 to 78·5)
Turkey	40·4 (37·2 to 43·7)	46·1 (43·3 to 49)	54·1 (51·5 to 56·9)	60·9 (59·2 to 62·7)	65·9 (64·4 to 67·5)	72·1 (70·7 to 73·4)	74·4 (73·2 to 75·7)	76·2 (74·1 to 78·3)
United Arab Emirates	56·3 (51·9 to 60·6)	58·5 (54·5 to 62·5)	62·6 (59·6 to 65·6)	66·7 (64·1 to 69·3)	67·7 (65·9 to 69·8)	68·2 (66·8 to 69·6)	71·4 (69·8 to 73·2)	72·9 (70·6 to 75)
Yemen	29·3 (26 to 32·4)	33·8 (31·4 to 36·5)	39·1 (36·1 to 41·8)	50·9 (48·1 to 54)	57·3 (54·5 to 60·2)	61·9 (58·9 to 64·9)	66 (62·9 to 68·9)	65·7 (62·9 to 68·1)
South Asia	42·2 (39·5 to 44·9)	45·3 (42·8 to 47·9)	49·2 (47·6 to 50·9)	55·1 (54·1 to 56·2)	59 (58 to 59·9)	62 (61·2 to 62·8)	65·5 (64·5 to 66·4)	69·1 (67·3 to 71)
South Asia	42·2 (39·5 to 44·9)	45·3 (42·8 to 47·9)	49·2 (47·6 to 50·9)	55·1 (54·1 to 56·2)	59 (58 to 59·9)	62 (61·2 to 62·8)	65·5 (64·5 to 66·4)	69·1 (67·3 to 71)
Bangladesh	38·3 (35·5 to 41·3)	42·6 (39·7 to 46·1)	40·3 (38·3 to 42·3)	53·1 (51·1 to 55·2)	57·1 (55·8 to 58·3)	64·1 (62·7 to 65·3)	68·5 (67·4 to 69·8)	73·4 (71·1 to 75·6)
Bhutan	35·8 (31·3 to 40·4)	41·1 (37·2 to 45·5)	47·9 (43·4 to 52·2)	52·3 (48·7 to 56)	60·1 (57·3 to 62·8)	65·2 (63·3 to 67·2)	70 (68·1 to 72·4)	72·2 (69·7 to 74·8)
India	42·6 (39·6 to 45·7)	45·2 (42·4 to 48·3)	49·6 (47·6 to 51·7)	54·9 (53·8 to 56·1)	59·1 (58 to 60·3)	62·1 (61·1 to 63)	65·7 (64·6 to 66·8)	69·5 (67·3 to 71·8)
Andhra Pradesh	44·3 (40·5 to 48·5)	46·9 (42·9 to 50·9)	50·2 (46·9 to 53·8)	55·7 (52·9 to 58·7)	59·1 (56·3 to 62·4)	61·6 (58·7 to 65·1)	65·8 (62·6 to 69·4)	71·6 (67·8 to 75·5)
Arunachal Pradesh	43·6 (39·7 to 47·4)	45·6 (41·9 to 49·8)	49·3 (46 to 52·7)	56·9 (53·9 to 60)	60·1 (57·1 to 63·2)	62·9 (59·9 to 66·2)	67·4 (64·4 to 70·5)	70·6 (67·3 to 74·1)
Assam	41·1 (37·6 to 44·8)	43·7 (40·3 to 47·6)	47·8 (44·6 to 51·4)	53·9 (51·2 to 56·8)	57·3 (55·3 to 59·4)	58·8 (57·7 to 59·8)	62·4 (61 to 63·8)	66·5 (63·8 to 69·3)
Bihar	46·1 (42·6 to 50·1)	48 (44·4 to 51·8)	50·5 (47·2 to 53·9)	54·7 (51·9 to 57·3)	59·1 (56·5 to 62·1)	62 (60 to 64·3)	65·7 (63·9 to 67·5)	71 (68·4 to 73·9)
Chhattisgarh	41·9 (38·2 to 45·7)	43·7 (40 to 47·5)	46·9 (43·3 to 50·4)	51·4 (48·2 to 54·4)	56·3 (53·5 to 59·1)	60·1 (58·1 to 62·4)	61·6 (60 to 63·2)	65·6 (63 to 68·5)
Delhi	46·6 (42·8 to 50·7)	52 (48·3 to 56)	55·9 (52·7 to 59·3)	60·1 (57·3 to 63·1)	62·5 (60 to 65·2)	64·2 (62·6 to 65·9)	68·6 (67·5 to 69·7)	73·1 (70·7 to 75·7)
Goa	56·3 (52·6 to 60·3)	58·8 (55 to 62·9)	61·5 (58·3 to 64·9)	65 (62 to 68)	66·8 (64·1 to 69·8)	68·7 (66·1 to 71·7)	71·8 (68·9 to 74·8)	73·7 (70·6 to 77·2)
Gujarat	45·6 (41·8 to 49·4)	48·2 (44·5 to 52)	51·9 (48·8 to 55·3)	56·3 (53·7 to 59·1)	59·9 (57·8 to 62)	62·9 (61·6 to 64·3)	66·2 (65 to 67·4)	69·3 (66·7 to 72)
Haryana	46·3 (42·6 to 50·2)	48·5 (44·7 to 52·5)	52·3 (49·1 to 55·6)	59·3 (56·7 to 62·3)	63·1 (61·1 to 65·6)	64·1 (62·8 to 65·3)	66·4 (65·2 to 67·7)	71·5 (68·6 to 74·5)
Himachal Pradesh	51 (47·3 to 54·8)	52·9 (49·2 to 56·8)	56·1 (53·1 to 59·5)	60·7 (57·8 to 63·6)	63·6 (61·3 to 66)	66·4 (65·1 to 67·8)	68·3 (66·9 to 69·7)	69·7 (67·1 to 72·5)
Jammu and Kashmir	53·4 (49·7 to 57·4)	55 (51·2 to 59·2)	58·2 (54·9 to 61·7)	61 (58 to 64)	64·1 (61·6 to 67)	66·3 (64·6 to 68·3)	69·8 (68·6 to 70·9)	71 (68·7 to 73·6)
Jharkhand	47·5 (44·1 to 51·2)	49·4 (45·7 to 53·6)	52·2 (48·5 to 55·7)	55·2 (52·3 to 58·3)	56·3 (53·9 to 58·9)	58·8 (57·1 to 60·3)	64·5 (63·3 to 65·7)	75·2 (72·9 to 77·6)
Karnataka	42·8 (39·2 to 46·6)	45·6 (42·1 to 49·5)	49·8 (46·8 to 53·1)	56·4 (53·8 to 59·3)	61 (58·9 to 63·3)	62·6 (61·4 to 63·9)	66·2 (64·9 to 67·4)	68·8 (66 to 71·7)
Kerala	49·1 (45·6 to 52·9)	54 (50·2 to 57·8)	59·2 (55·9 to 62·6)	64 (61·2 to 66·9)	66·5 (64·3 to 68·7)	68·4 (67·3 to 69·5)	71·6 (70·5 to 72·8)	74·4 (71·9 to 76·9)
Madhya Pradesh	42 (38·1 to 45·7)	43·6 (40 to 47·7)	47·1 (43·7 to 50·6)	51·4 (48·9 to 54·2)	56·1 (53·6 to 59)	60·1 (58·3 to 62·2)	62·9 (61·4 to 64·4)	66·8 (64·2 to 69·6)
Maharashtra	46·1 (42·5 to 49·8)	48·8 (45·3 to 52·4)	52·1 (49 to 55·3)	57·8 (55·3 to 60·6)	61·8 (59·8 to 64)	63·9 (62·7 to 65·1)	68·8 (67·6 to 70·2)	72·4 (69·5 to 75·6)
Manipur	50·7 (47·1 to 54·9)	52·7 (49·1 to 56·5)	56·6 (53·5 to 59·9)	61·1 (58·3 to 64·2)	63·8 (60·9 to 67)	64·5 (61·6 to 67·8)	67·3 (64·3 to 70·6)	70 (66·7 to 73·6)
Meghalaya	55·3 (51·6 to 59·3)	56·6 (52·9 to 60·9)	58·2 (54·9 to 61·5)	58·8 (56 to 61·8)	60·5 (57·5 to 63·6)	63·1 (60·4 to 66·3)	66·6 (63·6 to 69·7)	69·3 (66 to 72·6)
Mizoram	59·2 (55·5 to 62·9)	60·9 (57·4 to 64·8)	62·8 (59·6 to 66·2)	63·5 (60·7 to 66·6)	65·1 (62·2 to 68·2)	66 (63·3 to 69)	67·2 (64·1 to 70·7)	70·3 (66·9 to 74·3)
Nagaland	48·5 (44·7 to 52·5)	51·2 (47·8 to 55·1)	55·7 (52·4 to 59·7)	60·9 (57·9 to 63·9)	63·3 (60·3 to 66·4)	64·5 (61·7 to 67·8)	66·8 (63·5 to 70·4)	70 (66·5 to 73·8)
Odisha	38·6 (35 to 42·5)	40·8 (37·4 to 45)	45·1 (42·1 to 48·4)	51·5 (48·8 to 54·3)	55·8 (53·5 to 58·3)	58·9 (57·2 to 60·6)	64·2 (62·6 to 65·7)	69·1 (66·1 to 72·3)
Punjab	52·3 (48·9 to 56·1)	54·6 (50·8 to 58·7)	58·4 (55·1 to 61·7)	61·4 (58·6 to 64·2)	64 (61·5 to 66·5)	65·9 (64·7 to 67·3)	68·6 (67·2 to 70)	72·7 (69·3 to 76·4)
Rajasthan	47·8 (44·2 to 51·5)	49 (45·5 to 52·6)	51·7 (48·7 to 55·3)	55·1 (52·3 to 57·9)	58·8 (56·6 to 61)	62·1 (60·9 to 63·4)	65·9 (64·7 to 67·1)	67·1 (64·6 to 69·9)

Sikkim	51·5 (47·7 to 55·4)	52·9 (49·4 to 56·8)	55·2 (51·7 to 58·8)	59·4 (56·5 to 62·5)	63·3 (60·4 to 66·4)	65·6 (62·9 to 68·6)	68·9 (66·2 to 71·9)	72·4 (69·3 to 75·7)
Tamil Nadu	40·5 (37 to 44·2)	43·9 (40·5 to 47·5)	48·5 (45·2 to 51·6)	54·6 (52 to 57·2)	59·2 (56·9 to 61·6)	63·2 (61·8 to 64·6)	66·3 (65 to 67·8)	70·4 (67·9 to 73·4)
Telangana	49·5 (45·4 to 53·8)	51·2 (47·1 to 55·7)	53·7 (50·4 to 57·3)	56·8 (53·4 to 60·2)	59·7 (56·3 to 63·4)	62·5 (59·1 to 66·2)	67·4 (64·2 to 70·7)	71·9 (68·3 to 75·6)
Tripura	41·4 (37·7 to 45·4)	45·4 (41·8 to 49·4)	50·3 (47·1 to 53·8)	56·9 (54·1 to 59·9)	60·2 (57·3 to 63·4)	63·9 (61·2 to 66·8)	67·6 (64·7 to 70·5)	69·9 (66·9 to 73·2)
Union Territories other than Delhi	53 (49·2 to 56·9)	56·1 (52·7 to 60·1)	59·8 (56·3 to 63·4)	63·9 (60·8 to 67·2)	65·9 (63·1 to 69·1)	67·2 (64·5 to 70·2)	69·8 (66·9 to 73)	72·1 (69 to 75·5)
Uttar Pradesh	34·6 (31 to 38·3)	37·2 (34 to 40·7)	42·5 (39·6 to 45·9)	49·6 (47·4 to 52·2)	55·5 (53·3 to 57·8)	60 (58·7 to 61·4)	62·9 (61·4 to 64·5)	65·6 (63·1 to 68·1)
Uttarakhand	47·1 (43·6 to 50·8)	49 (45·3 to 52·8)	51·8 (48·2 to 55·5)	54·9 (52 to 57·8)	56·9 (53·9 to 60·1)	58·9 (56 to 61·6)	62·6 (60·5 to 64·7)	66·2 (63·5 to 69·2)
West Bengal	43·8 (40·3 to 47·6)	47·1 (43·5 to 50·7)	51·1 (48 to 54·3)	56·4 (53·8 to 59·2)	61 (59 to 63·1)	64·1 (62·9 to 65·2)	68·5 (67·4 to 69·6)	73·5 (71·1 to 76·1)
Nepal	39·2 (36·5 to 42·1)	43·3 (40·8 to 46)	48·1 (45·5 to 50·8)	51·1 (48·5 to 53·6)	57·7 (55·6 to 59·7)	64·1 (62·4 to 65·7)	67·5 (65·8 to 69)	69·2 (67·4 to 71·5)
Pakistan	45·6 (42·7 to 48·9)	51·5 (49 to 54·5)	57·9 (56·1 to 59·9)	60 (58·2 to 61·6)	60·5 (59·3 to 61·7)	60·4 (59 to 61·8)	62·3 (60·1 to 64·8)	65 (61·9 to 68)
Southeast Asia, east Asia, and Oceania	47·1 (44·8 to 49·6)	31·7 (23·7 to 38·5)	57·2 (54·9 to 59·3)	61·6 (59·8 to 63·3)	65·2 (64 to 66·6)	67·9 (67 to 68·7)	70·8 (69·9 to 71·6)	73·2 (71·8 to 74·7)
East Asia	48·5 (45·7 to 51·4)	27·1 (18·2 to 35)	59·1 (56·1 to 61·9)	62·8 (60·5 to 65·1)	66·2 (64·7 to 68)	69 (67·9 to 70)	72·2 (71·2 to 73·2)	74·7 (72·8 to 76·6)
China	49·6 (46·8 to 52·6)	26·5 (17·6 to 34·6)	59·1 (56 to 61·9)	62·8 (60·4 to 65·1)	66·2 (64·6 to 68)	69·1 (68 to 70·2)	72·2 (71·2 to 73·3)	74·7 (72·8 to 76·7)
North Korea	16·9 (15·3 to 18·5)	44·8 (41·3 to 48·5)	53·9 (50·7 to 57·2)	60·8 (57·6 to 63·8)	64·9 (62·1 to 67·7)	60·8 (53·2 to 65·4)	67·7 (66 to 69·8)	69·8 (67·9 to 71·6)
Taiwan (province of China)	58·7 (55 to 62·3)	62·7 (62·6 to 62·9)	67·7 (67·6 to 67·9)	70·2 (70·1 to 70·4)	72·3 (72·2 to 72·5)	74·3 (74·2 to 74·4)	76·8 (76·7 to 76·9)	77·1 (74·1 to 79·9)
Oceania	49 (45·7 to 52)	53 (50·3 to 55·5)	57·2 (54·9 to 59·5)	60·2 (58·7 to 61·7)	60·7 (58·7 to 62·9)	61·3 (59 to 63·5)	61·6 (58·8 to 64·2)	63·1 (60 to 66·1)
American Samoa	60·6 (58·5 to 62·6)	65·8 (64 to 67·7)	64 (62·3 to 65·8)	66·7 (65·1 to 68·4)	67·6 (66·5 to 68·7)	68·4 (67·1 to 69·9)	69·3 (68·1 to 70·6)	69·9 (67·8 to 71·9)
Cook Islands	47·7 (45·7 to 50)	56·3 (54·6 to 58·1)	62·5 (60·8 to 64·2)	63·4 (62 to 64·8)	66·4 (64·9 to 67·7)	69·9 (68·2 to 71·5)	71·8 (70·4 to 73·3)	72·2 (70 to 74·1)
Fiji	59·3 (58·2 to 60·3)	63·2 (61·9 to 64·5)	62·7 (61·7 to 63·6)	64·1 (63·2 to 65·2)	64·1 (61·8 to 66·2)	63·5 (63 to 64)	65·9 (65·2 to 66·5)	66·3 (63·4 to 69·1)
Guam	67 (64·9 to 68·9)	65·8 (64·8 to 66·9)	65·1 (64·4 to 65·7)	69·2 (68·6 to 69·9)	71·1 (70 to 72·2)	73·3 (71·9 to 74·7)	73·4 (72·3 to 74·5)	73·5 (71·3 to 75·7)
Kiribati	42·9 (40·4 to 45·7)	45·9 (43·3 to 48·6)	49·1 (46·2 to 52·2)	52 (48·4 to 55·3)	51·7 (49 to 54·8)	53·4 (50·8 to 56·2)	55·8 (53·2 to 59)	57·6 (55 to 60·5)
Marshall Islands	49·2 (45·2 to 53)	53·1 (49·8 to 56·6)	56 (52·6 to 59·3)	59·5 (57·2 to 61·7)	59·8 (58·7 to 60·9)	61·1 (59·4 to 62·8)	62·4 (60·3 to 64·8)	63·9 (60·4 to 67)
Federated States of Micronesia	47·6 (45 to 50·5)	50·6 (47·8 to 53·7)	54 (51 to 57·4)	57·1 (54·1 to 60)	58·7 (56 to 61·5)	59·9 (58·2 to 61·6)	61·5 (59·3 to 64)	61·5 (58·8 to 64·4)
Nauru	58·8 (55 to 62·6)	61 (57·9 to 63·9)	61·7 (58·9 to 64·3)	60·7 (57·5 to 63·5)	59·1 (56·6 to 62·1)	56·2 (54 to 58·8)	57 (54·5 to 59·6)	60·3 (57·5 to 63·2)
Niue	54 (51·5 to 56·6)	60·9 (58·4 to 63·4)	62 (59·6 to 64·6)	64·1 (61·1 to 66·8)	65 (62·2 to 67·3)	65·1 (62·9 to 67·1)	67 (64·9 to 69·1)	67·8 (65·2 to 70·2)
Northern Mariana Islands	61·5 (57·9 to 65)	63·5 (60·6 to 66·5)	65·6 (62·8 to 68·3)	67·8 (65·1 to 70)	69·1 (67·2 to 70·7)	69·8 (68 to 71·4)	69·6 (68 to 71·2)	70·2 (68·5 to 71·9)
Palau	46 (42 to 50·1)	50·8 (47·8 to 54·1)	55·6 (52·8 to 58·5)	59 (56 to 61·9)	61·8 (58·7 to 64·7)	63·6 (61 to 66·4)	64·1 (61·6 to 66·7)	64·8 (61·8 to 67·6)
Papua New Guinea	47·5 (43·1 to 51·7)	51·5 (48·1 to 54·9)	56·8 (53·9 to 60)	60 (58·1 to 61·9)	60·4 (57·7 to 63·3)	61·2 (58·2 to 64·4)	61·1 (57·6 to 64·6)	63 (59·3 to 66·4)
Samoa	52·9 (49 to 56·7)	59·4 (56·8 to 62·1)	59·7 (57·1 to 62·5)	62·2 (59·6 to 65)	64·2 (61·1 to 66·9)	66·4 (63·6 to 68·7)	68·1 (65·7 to 70·3)	69·2 (66·4 to 71·5)
Solomon Islands	40·7 (38·1 to 43·6)	44·2 (41·5 to 47·2)	48·6 (46 to 51·4)	52·5 (49·3 to 55·9)	54·1 (51·3 to 57·2)	55·3 (52·9 to 57·9)	55·7 (53·7 to 58)	57·4 (54·9 to 60·1)
Tokelau	55·7 (52·6 to 58·6)	59·3 (55·9 to 62·6)	62·9 (59·7 to 65·9)	65·9 (62·9 to 69)	68·8 (66·2 to 71·1)	70·3 (67·7 to 72·8)	71·9 (69·1 to 74·4)	73·3 (70·4 to 75·6)
Tonga	59·6 (56·3 to 62·5)	62·9 (61 to 65·1)	65 (63·2 to 66·9)	66·2 (64·5 to 67·8)	68·5 (66·9 to 70·1)	67·6 (66·2 to 68·9)	68·9 (66·9 to 70·8)	69·8 (67·3 to 72·3)
Tuvalu	44·7 (40·6 to 48·7)	49 (45·7 to 52·5)	53·1 (49·5 to 56·8)	55·4 (52·3 to 58·8)	58·6 (56·5 to 60·8)	60·3 (58·6 to 61·9)	64·8 (62 to 67·4)	66·1 (62·4 to 69·3)
Vanuatu	46·2 (43·3 to 49·4)	49·9 (47·1 to 52·9)	54·7 (52 to 57·7)	59·7 (56·7 to 62·5)	60·6 (57·5 to 63·7)	61·1 (58 to 64·3)	61·8 (58·7 to 64·8)	62·9 (59·5 to 65·8)
Southeast Asia	43·3 (41·3 to 45·5)	49·3 (47·4 to 51·1)	52·4 (50·8 to 53·9)	58·7 (57·3 to 60·1)	62·7 (61·8 to 63·7)	65·4 (64·6 to 66·3)	68 (67·2 to 68·9)	70·1 (68·7 to 71·6)
Cambodia	44·4 (40 to 48·8)	47·7 (44·3 to 51·2)	40·2 (37·2 to 43·3)	48·9 (44·8 to 51·9)	55·3 (53·8 to 56·9)	57·6 (56·1 to 59·1)	63·8 (62 to 65·6)	67 (65 to 69·4)
Indonesia	41 (38·3 to 43·9)	46·4 (43·9 to 49)	52 (49·7 to 54·5)	58·1 (56·4 to 60)	62·5 (61·3 to 63·7)	65·9 (64·6 to 67·1)	67·5 (66 to 69·2)	69·4 (67·2 to 71·6)
Aceh	49·5 (46 to 53·2)	53·7 (49·8 to 57·6)	57·5 (54·1 to 61·3)	61·9 (59 to 65·2)	64·2 (61·7 to 66·6)	66·2 (63·8 to 68·5)	66·9 (64 to 69·7)	68·6 (65·2 to 72)

Bali	47·6 (44 to 51·1)	52·5 (48·8 to 56·6)	57·1 (53·7 to 61)	61·4 (58·6 to 64·6)	65·5 (63·5 to 67·8)	68·3 (66·5 to 70·2)	70·4 (68·1 to 73·1)	74·4 (70·9 to 77·9)
Bangka-Belitung Islands	39·7 (36 to 43·7)	44·4 (41·3 to 47·9)	50·1 (46·6 to 53·8)	56·5 (53·4 to 59·7)	60·3 (57·8 to 62·9)	63·9 (61·9 to 66·1)	65·4 (62·8 to 67·9)	67 (63·6 to 70·5)
Banten	29·7 (26·3 to 33·7)	36·2 (33·1 to 39·3)	43·4 (40·3 to 46·8)	51·6 (48·5 to 54·7)	59·1 (56·7 to 61·5)	65·5 (63·5 to 67·7)	66·7 (64·4 to 69·4)	68·5 (65·4 to 71·9)
Bengkulu	35·7 (32·5 to 38·9)	41·2 (37·9 to 44·7)	48·2 (45·2 to 51·7)	55·3 (52·6 to 58·5)	61·4 (59·2 to 63·8)	64·8 (62·8 to 66·9)	67·1 (64·5 to 69·8)	69·3 (66·1 to 72·6)
Gorontalo	38 (34·2 to 42·1)	43·7 (40·3 to 47·6)	49·8 (46·2 to 53·7)	56·3 (53·1 to 59·5)	62 (59·6 to 64·5)	66·4 (64·1 to 68·7)	68·1 (65·6 to 71)	70·4 (66·9 to 74·1)
Jakarta	48·9 (45·4 to 52·6)	54·6 (50·8 to 58·3)	60·7 (57·3 to 64·5)	66·1 (63·1 to 69·2)	68·2 (66 to 70·5)	68·1 (66·2 to 70)	69·4 (67·2 to 71·9)	71·2 (68·2 to 74·4)
Jambi	41·2 (38 to 44·7)	46·8 (43·6 to 50·2)	51·4 (48·2 to 55·2)	56·7 (54·1 to 59·7)	61·3 (59 to 63·6)	65·6 (63·5 to 67·7)	68 (65·5 to 70·4)	69·7 (66·8 to 72·9)
West Java	35·4 (32·1 to 38·8)	41·4 (38·3 to 44·3)	48·5 (45·5 to 51·6)	56 (53·6 to 58·7)	62·2 (60·3 to 64·2)	67·3 (65·5 to 69·2)	68·9 (66·9 to 71·5)	70·5 (67·6 to 73·6)
Central Java	45·9 (42·5 to 49·7)	50·4 (47·1 to 54)	55·7 (52·5 to 59)	61·5 (58·8 to 64·4)	66·3 (64·2 to 68·4)	67·9 (66·2 to 69·8)	69·3 (67·1 to 71·7)	71 (68·4 to 74)
East Java	45·3 (42·1 to 48·5)	49·4 (46·2 to 52·6)	53·9 (50·9 to 57·3)	58·3 (56 to 61)	62 (60·1 to 63·9)	65·6 (63·9 to 67·5)	67·4 (65·2 to 69·5)	69·3 (66·5 to 72·3)
West Kalimantan	44·5 (41·3 to 48·2)	47·4 (43·9 to 51)	51·5 (48·2 to 55·2)	56·8 (54·2 to 60)	61 (58·7 to 63·3)	64·4 (62·3 to 66·5)	67·4 (65 to 70)	70 (66·9 to 73·3)
South Kalimantan	32·8 (29·7 to 35·9)	37·1 (34·2 to 40·2)	44·3 (41·3 to 47·6)	51·7 (49·1 to 54·8)	56·4 (54·2 to 58·6)	59·9 (57·9 to 62)	62·6 (60·1 to 65·4)	65·4 (61·9 to 68·9)
Central Kalimantan	45·9 (42·5 to 49·3)	52·2 (48·8 to 56)	56·5 (53·1 to 60)	60·6 (57·8 to 63·9)	63·3 (61 to 65·7)	66·9 (64·7 to 69·1)	67·6 (65·2 to 70·1)	68·9 (66 to 72·1)
East Kalimantan	47·9 (44·5 to 51·7)	50·9 (47·2 to 55)	55·3 (52 to 59·3)	60·9 (58·2 to 64·2)	64·2 (62·1 to 66·5)	64·7 (62·7 to 66·9)	66·4 (63·7 to 69)	68·4 (64·8 to 71·8)
North Kalimantan	45·3 (41·4 to 49·4)	49 (45·6 to 52·6)	52·6 (49·5 to 56·1)	59·6 (56·4 to 63)	65·3 (62·4 to 68·3)	69·7 (67·1 to 72·5)	70·8 (68·1 to 73·8)	72 (68·5 to 75·8)
Riau Islands	46·9 (43·1 to 50·9)	51·3 (47·8 to 55)	56·2 (52·6 to 59·9)	60·1 (56·9 to 63·4)	63·6 (61·3 to 66·4)	70·3 (68·1 to 72·6)	71·8 (69·4 to 74·3)	72·7 (69·7 to 76·2)
Lampung	42·2 (39·3 to 45·9)	47·6 (44·1 to 51·3)	53 (49·8 to 56·6)	58·7 (55·9 to 62)	62·5 (60·4 to 64·7)	67·5 (65·4 to 69·8)	69·4 (66·9 to 72)	70·6 (67·4 to 74·1)
Maluku	32·3 (29·3 to 35·6)	39·4 (36·4 to 42·6)	46 (42·6 to 49·6)	52·3 (49·3 to 55·8)	57·5 (55 to 60·3)	61·6 (59·1 to 64·3)	63·7 (60·7 to 66·8)	67·2 (63·7 to 71·1)
North Maluku	37 (33·4 to 40·7)	41·8 (38·7 to 45·1)	46·9 (43·9 to 50·5)	51·4 (48·6 to 54·4)	59 (56·4 to 61·9)	62·8 (60·6 to 65·3)	66·5 (63·8 to 69·3)	69·4 (65·9 to 72·9)
West Nusa Tenggara	32·5 (29·6 to 35·6)	37·7 (34·7 to 40·8)	43·3 (40·3 to 46·5)	50·2 (47·6 to 53·2)	56·1 (53·8 to 58·5)	61·3 (59·2 to 63·7)	65·4 (63·1 to 68·3)	68·1 (64·6 to 71·5)
East Nusa Tenggara	35·4 (32·3 to 38·6)	41·3 (38·4 to 44·7)	47·4 (44·3 to 50·7)	53·7 (51 to 56·7)	59·2 (57·1 to 61·4)	63·1 (61·1 to 65·3)	66·3 (63·5 to 69·1)	68·7 (65·5 to 72·1)
Papua	49·6 (46·1 to 53·5)	52·5 (48·6 to 56·2)	55·9 (52·2 to 59·6)	59·6 (56·3 to 63·2)	61·2 (58·9 to 64)	61·8 (59·6 to 64·2)	62·2 (59·6 to 65·2)	64·5 (60·9 to 68·2)
West Papua	51·6 (48 to 55·9)	54·4 (50·7 to 58·4)	57·4 (53·8 to 61·4)	62·2 (59 to 65·6)	65·5 (62·9 to 68·5)	67·4 (65·3 to 69·7)	66·7 (64·2 to 69·2)	70·6 (67·2 to 74·1)
Riau	43·8 (40·3 to 47·5)	47·2 (44 to 51)	52 (48·6 to 55·8)	58·4 (55·6 to 61·5)	63·2 (61·1 to 65·3)	66·8 (64·8 to 69·1)	68·2 (65·8 to 70·8)	70·4 (67·2 to 73·8)
West Sulawesi	29·4 (26 to 32·9)	35·1 (31·9 to 38·4)	41·3 (38·2 to 44·8)	49·2 (46·2 to 52·2)	55·1 (52·9 to 57·7)	60 (57·8 to 62·1)	64·2 (61·4 to 67·2)	67·2 (63·7 to 70·7)
South Sulawesi	40·3 (37·2 to 43·7)	45·1 (41·7 to 48·5)	50·6 (47·4 to 53·9)	56·3 (53·6 to 59·2)	61·3 (59 to 63·7)	65·5 (63·4 to 67·6)	67·5 (65·3 to 69·9)	69·5 (66·6 to 72·6)
Central Sulawesi	32·3 (29·4 to 35·4)	40·7 (37·8 to 44)	47 (43·8 to 50·5)	52·9 (50·1 to 55·9)	57·8 (55·7 to 60·2)	61·4 (59·3 to 63·8)	65 (62·2 to 67·8)	67·5 (64 to 71·2)
Southeast Sulawesi	38·2 (35·1 to 41·5)	42·7 (39·4 to 46)	47·8 (44·6 to 51·3)	56·1 (52·9 to 59·5)	61·9 (59·5 to 64·2)	63·9 (61·8 to 65·9)	65·3 (62·7 to 68·2)	67·1 (63·8 to 70·7)
North Sulawesi	47·6 (44·4 to 51·3)	51·2 (47·8 to 54·8)	56 (52·5 to 59·7)	60·9 (58 to 64·3)	62·9 (60·7 to 65·2)	65·3 (63·5 to 67·4)	66·7 (64·1 to 69·2)	68·3 (65·2 to 71·5)
West Sumatra	34·2 (31·1 to 37·2)	42·5 (39·2 to 45·9)	50·4 (47 to 53·9)	58·3 (55·5 to 61·5)	62 (59·8 to 64·4)	63·9 (61·9 to 66·1)	65·6 (63·1 to 68·1)	68·6 (65·7 to 72)
South Sumatra	40·4 (37·1 to 44·1)	46·7 (43·1 to 50·4)	52·6 (49·1 to 56·3)	60 (57·3 to 63·1)	63·5 (61·3 to 65·9)	67·1 (65·1 to 69·2)	68·9 (66·5 to 71·3)	70·2 (67 to 73·4)
North Sumatra	45·5 (42·3 to 49·1)	49·8 (46·6 to 53·6)	54·5 (51 to 58)	59·2 (56·4 to 62·2)	61·6 (59·4 to 63·8)	63·5 (61·4 to 65·8)	64·7 (62·2 to 67·3)	66·3 (62·8 to 69·7)
Yogyakarta	55·6 (52·1 to 59·5)	59·7 (55·5 to 63·7)	63·2 (59·6 to 66·9)	66·8 (63·6 to 70)	68·9 (66·7 to 71·1)	70·6 (68·5 to 72·6)	70·1 (67·7 to 72·8)	70·7 (67·7 to 74)
Laos	36·7 (32·4 to 41·1)	37·9 (34·1 to 41·8)	39·9 (35·6 to 43·9)	45·2 (41·8 to 48·9)	50·7 (48·1 to 53·6)	56·2 (54·4 to 58·2)	62·5 (60·3 to 65)	66·7 (64 to 69·6)
Malaysia	51·3 (49·6 to 52·8)	61 (60·8 to 61·2)	64·5 (64·3 to 64·6)	68 (66·8 to 69·3)	69·7 (69·5 to 69·8)	70·7 (70·6 to 70·8)	72·4 (71·8 to 73·1)	72·9 (70·3 to 75·5)
Maldives	29·8 (25·5 to 34·1)	36·9 (33·3 to 40·7)	46·6 (44·1 to 49)	56·7 (55·2 to 58)	65·5 (64·7 to 66·2)	71·8 (71·2 to 72·3)	76·8 (76 to 77·4)	78 (76·5 to 79·6)
Mauritius	52·1 (51·5 to 52·7)	56·5 (56 to 57)	61·5 (61 to 62·1)	63·8 (63·3 to 64·3)	66·5 (66·1 to 67)	69·1 (68·7 to 69·6)	70·8 (70·4 to 71·2)	72·5 (69·9 to 75)
Myanmar	31·2 (27·1 to 35·4)	35·4 (31·9 to 38·9)	39·7 (35·4 to 43·9)	47·4 (43·9 to 50·9)	52·5 (50 to 55·2)	56·2 (54·1 to 58·3)	61·6 (59·3 to 63·8)	66·2 (63·5 to 68·6)

Philippines	54·5 (51·9 to 56·7)	59·8 (58·3 to 61·5)	60·2 (58·6 to 61·6)	62·9 (61·6 to 64·3)	65·3 (63·9 to 66·7)	67·1 (65·8 to 68·4)	67·4 (66·1 to 68·7)	68·8 (65·9 to 71·9)
Seychelles	57·3 (55·7 to 59)	60·9 (60 to 61·9)	62·1 (61·3 to 63)	67·5 (66·6 to 68·4)	66·2 (65·5 to 66·8)	68·2 (67·4 to 68·9)	69·8 (69·2 to 70·4)	70·3 (68·7 to 71·8)
Sri Lanka	56·7 (56·4 to 56·9)	64 (63·8 to 64·2)	64·8 (64·6 to 65)	67·3 (67·2 to 67·5)	65·9 (65 to 66·9)	67·3 (67·1 to 67·4)	70·5 (70·3 to 70·6)	74·3 (71·1 to 77·4)
Thailand	51·4 (48·5 to 54·2)	58·2 (56·6 to 59·8)	61·3 (59·7 to 63·1)	63 (61·4 to 64·5)	67·8 (66·4 to 69·3)	68 (66·1 to 70·1)	73·9 (72·4 to 75·6)	74·9 (71·5 to 78·5)
Timor-Leste	41·7 (37·1 to 46·3)	45 (41·2 to 48·8)	49·9 (46·9 to 53·1)	50·5 (47·7 to 53·2)	60·5 (58·3 to 62·4)	64·9 (63·2 to 66·6)	68·8 (67·3 to 70·4)	69 (67·2 to 71·3)
Vietnam	47·2 (43·3 to 51)	53·9 (50·6 to 57·3)	50·1 (47·7 to 52·7)	62·3 (59·4 to 65·1)	65·3 (63 to 67·4)	68·1 (66·4 to 69·7)	68·9 (67·6 to 70)	70·2 (68·5 to 72·3)
Sub-Saharan Africa	41·6 (38·1 to 44·9)	43·5 (41·1 to 46)	46·8 (45 to 48·8)	50·4 (49 to 51·8)	51·8 (50·7 to 52·9)	51·4 (50·1 to 52·7)	57·1 (55·9 to 58·3)	62·2 (60·4 to 63·7)
Central sub-Saharan Africa	42·5 (38·2 to 46·9)	44·4 (41 to 47·7)	46·1 (43·2 to 49)	48·5 (46 to 51·2)	50·7 (48·8 to 52·6)	51·1 (49·7 to 52·6)	56·7 (54·9 to 58·5)	62·1 (59·7 to 64·5)
Angola	42·4 (37·9 to 46·9)	43·2 (39·7 to 46·9)	43·3 (39·4 to 47·3)	45 (41·9 to 48·2)	47 (44·8 to 49·4)	50·4 (48·4 to 52·3)	57·8 (55·7 to 60·1)	62·6 (59·7 to 65·5)
Central African Republic	41 (37·2 to 44·7)	42·9 (39·7 to 46·6)	45·2 (42·2 to 48)	46·1 (43·9 to 48·5)	45·2 (43·2 to 47·3)	42·5 (40·2 to 45)	45·7 (42·7 to 49·1)	49·3 (45·2 to 52·9)
Congo (Brazzaville)	38·5 (34·4 to 42·5)	40·2 (36·9 to 43·4)	43·8 (39·9 to 47·4)	49·3 (46·7 to 52·1)	51·7 (49·8 to 53·8)	52·4 (50·3 to 54·6)	60·7 (58·4 to 63·1)	64·5 (61·6 to 67)
DR Congo	43·2 (38·9 to 47·6)	45·3 (41·6 to 49)	47·3 (44·2 to 50·5)	49·6 (46·9 to 52·5)	52·1 (50 to 54·5)	51·9 (50·3 to 53·6)	56·8 (55 to 58·7)	62·7 (60·3 to 65·2)
Equatorial Guinea	36·3 (32·4 to 40·3)	37·5 (34 to 41·4)	42·2 (38·9 to 46)	47·3 (44·2 to 50·6)	47·8 (44·9 to 51)	55·3 (51·8 to 59·1)	64 (60·3 to 67·8)	64·5 (60·9 to 68·1)
Gabon	44·3 (40·2 to 48·4)	45·7 (42·4 to 49)	49 (46·2 to 51·9)	54·1 (51·9 to 56·5)	56·6 (54·5 to 58·6)	57·5 (55·3 to 59·6)	61·1 (59·3 to 63)	64·8 (62·6 to 67·3)
Eastern sub-Saharan Africa	40·2 (36·8 to 43·5)	42·9 (40·4 to 45·4)	45·6 (43·7 to 47·5)	48·7 (47·4 to 50·1)	49·4 (48·2 to 50·5)	50·4 (48·9 to 51·8)	58·1 (57 to 59·3)	63·3 (61·7 to 64·7)
Burundi	39 (34·7 to 43·4)	42·7 (39·5 to 46·3)	44·6 (41·8 to 47·7)	46·5 (44 to 49·3)	46·9 (44·3 to 49·3)	42·7 (40·7 to 44·8)	56·9 (54·5 to 59·5)	61·7 (58·1 to 65·1)
Comoros	43·7 (40·3 to 47·3)	46·1 (42·9 to 49·4)	50·1 (47 to 53·4)	52·8 (50·4 to 55·7)	56·8 (54·6 to 59·4)	60·2 (58·1 to 62·4)	64·7 (62·5 to 66·9)	67·4 (64·9 to 69·6)
Djibouti	52·7 (48·1 to 57·1)	54·4 (50·4 to 58·5)	56·5 (52·6 to 60·4)	58·9 (55·7 to 62·3)	58·3 (55·2 to 61·8)	58·5 (55·1 to 62·3)	61·7 (58 to 65·6)	65·2 (61·5 to 68·4)
Eritrea	37·2 (33·3 to 41·1)	39·4 (36 to 43·3)	37·2 (22·3 to 44·4)	38·6 (36·4 to 40·8)	42 (40·1 to 43·7)	51·3 (48·8 to 53·8)	56·8 (53·5 to 60)	60·9 (57 to 64·1)
Ethiopia	36·6 (33·6 to 39·7)	38·4 (35·7 to 41·2)	40·4 (38·1 to 43)	42·7 (40·8 to 44·7)	44·7 (43·3 to 46·1)	50·1 (48·1 to 51·7)	61·5 (60·2 to 62·9)	67·1 (65·1 to 69·2)
Kenya	46·8 (44 to 49·7)	52·3 (49·8 to 54·9)	56·5 (53·9 to 59·1)	60·8 (58·8 to 63)	60·6 (58·9 to 62·3)	53·8 (51·2 to 56·3)	59·3 (57·9 to 60·6)	64·2 (62·3 to 66·3)
Baringo	46·3 (42·6 to 50·5)	49·1 (44·9 to 53·3)	49·9 (46·1 to 54·2)	52·8 (48·9 to 56·7)	54·1 (50·6 to 57·7)	53·4 (50·4 to 56·5)	58·2 (55·2 to 61)	63·8 (59·8 to 67·7)
Bomet	65 (60·9 to 69·6)	66·7 (62·8 to 70·9)	67·7 (63·5 to 71·9)	69·6 (65·9 to 73·6)	67·4 (62·9 to 70·4)	56·7 (51·5 to 61·8)	61·3 (57·4 to 65)	66·6 (63 to 69·6)
Bungoma	42·2 (38·6 to 46·2)	48·2 (44·5 to 52·2)	53·4 (49·4 to 58·1)	58·1 (54·3 to 62·2)	59·6 (56·3 to 63·5)	57 (53·2 to 60·8)	64·8 (61·9 to 67·7)	69·3 (65·9 to 72·4)
Busia	31·3 (27·9 to 34·9)	38·5 (35·1 to 42·1)	46 (42·3 to 50·1)	55·9 (52·2 to 59·6)	56 (52·2 to 60·2)	48·4 (44·2 to 53·4)	58·5 (55 to 62·6)	64·8 (60·9 to 69·3)
Elgeyo Marakwet	55·4 (51·3 to 59·9)	57·2 (53 to 61·4)	57·3 (53·5 to 61·4)	58·8 (54·7 to 63·1)	60·5 (56·6 to 64·4)	58·5 (55·1 to 62·2)	63·4 (60·6 to 66)	68·1 (64·6 to 72)
Embu	43 (39·5 to 47·1)	50·8 (46·6 to 55·3)	59·5 (55·4 to 63·8)	66·5 (62·3 to 71)	67·2 (63·6 to 70·4)	62·5 (57·1 to 67·1)	64 (60·1 to 68)	67·2 (63·2 to 70·8)
Garissa	48·3 (44 to 52·3)	52·2 (48·1 to 57·2)	55·2 (50·8 to 60)	58·2 (54 to 62·5)	59·3 (55·5 to 63·2)	58·9 (55·1 to 63)	62·1 (58·3 to 65·9)	66·1 (62·1 to 70·3)
Homa Bay	28·6 (25·6 to 31·7)	35·9 (32·9 to 39·4)	39·5 (36·1 to 43·3)	46 (42·9 to 49·6)	47 (43·2 to 51)	35·7 (32·5 to 39·8)	44·7 (40·9 to 48·4)	52·5 (48·2 to 58)
Isiolo	27·3 (24·1 to 30·7)	34 (30·3 to 37·9)	37·8 (33·9 to 42·1)	45·6 (41·6 to 50·4)	50·4 (46·5 to 54·6)	49·5 (45·2 to 54·2)	53·9 (50 to 58·1)	59·7 (55·1 to 64·6)
Kajiado	58·9 (54·8 to 63·4)	61 (57 to 65·5)	61·3 (57·2 to 65·9)	63·7 (59·6 to 68·1)	64·4 (60·7 to 68·8)	58·9 (54·9 to 63·2)	63·6 (60·8 to 66·8)	68 (64·6 to 71·4)
Kakamega	43·2 (39·8 to 47·1)	48·8 (45·2 to 52·5)	53 (49·1 to 57·1)	58·2 (54·4 to 62·1)	57·1 (53·6 to 61·1)	49·2 (45·3 to 53·6)	58·1 (55 to 61·6)	64·6 (61 to 68·3)
Kericho	62·9 (58·9 to 67·5)	64·5 (60·5 to 69)	65·1 (61·1 to 69·6)	66·4 (62·5 to 70·4)	66·3 (62·3 to 70·2)	59·5 (54·9 to 64·1)	63·5 (59·7 to 67·4)	68·2 (64·5 to 71·7)
Kiambu	54·4 (50·4 to 58·8)	58·2 (54 to 62·5)	61·3 (57·4 to 65·2)	63·7 (60·1 to 67·9)	62·8 (59·8 to 66)	55·6 (52·4 to 58·7)	59·3 (57 to 61·7)	63·6 (60·3 to 66·9)
Kilifi	40·2 (36·9 to 43·8)	47·9 (44·2 to 52)	53 (49·2 to 56·8)	58·8 (55·1 to 62·7)	61·4 (57·4 to 65·1)	58·4 (54·1 to 62·8)	62·3 (59·4 to 65·5)	66·9 (63·1 to 70·5)
Kirinyaga	47·1 (43·2 to 51·2)	52·3 (48·1 to 56·5)	56·3 (52·3 to 60·3)	65·1 (61·1 to 69·5)	65·3 (61·7 to 69·5)	59 (54·9 to 63·6)	62 (58·6 to 65·4)	64·8 (60·9 to 69)
Kisii	46·5 (42·9 to 50·5)	51·1 (47·4 to 55·2)	55·1 (51 to 59·7)	58·4 (54·6 to 62·8)	58·2 (54·9 to 61·7)	54·9 (51·6 to 58·4)	63·1 (60·5 to 65·7)	67 (63·4 to 70·9)
Kisumu	45·5 (42·3 to 49·2)	49·6 (45·9 to 53·9)	52 (48·3 to 56·3)	54 (50·4 to 58·1)	51·6 (48·2 to 55·3)	40·9 (37·8 to 44·3)	49·6 (46·5 to 52·7)	55·3 (51·3 to 60)

Kitui	43·2 (39·4 to 47)	48·5 (44·8 to 52·6)	53·9 (49·7 to 58·4)	60·3 (56·3 to 64·7)	62·3 (58·6 to 66·1)	57·1 (52·8 to 62·1)	60·7 (57·7 to 63·8)	65·4 (61·7 to 69·2)
Kwale	37·5 (34·2 to 41·1)	44·5 (40·7 to 48·7)	50·4 (46·5 to 54·6)	56·7 (53 to 60·9)	59·5 (55·3 to 63·7)	58 (53·9 to 62·6)	59·4 (56·6 to 62·6)	63·9 (60·1 to 68·5)
Laikipia	63·4 (59·1 to 67·9)	65·6 (61·3 to 70·2)	66·7 (62·6 to 71·2)	69·2 (65·2 to 73·2)	66·7 (62·8 to 70·2)	57·9 (53·3 to 62·8)	62·4 (58·7 to 66·3)	67·6 (63·8 to 70·8)
Lamu	36·4 (33·1 to 40·2)	45·1 (41·3 to 49·2)	52·3 (47·9 to 57·2)	63·6 (58·9 to 68·2)	62·4 (58·4 to 66·5)	59·4 (54·6 to 63·9)	61·5 (57·5 to 65·8)	65·8 (61·5 to 70·3)
Machakos	54·2 (50·2 to 58·4)	58·5 (54·3 to 62·6)	61·4 (57·4 to 65·6)	65·7 (61·9 to 69·7)	67·5 (63·9 to 70·8)	61·3 (55·6 to 66·1)	64 (60·7 to 67)	67·5 (63·9 to 71)
Makueni	52·7 (49 to 56·7)	56·8 (52·7 to 61·2)	59·8 (55·5 to 64·3)	63·9 (60 to 68)	63·8 (59·6 to 67·9)	53·9 (48·5 to 59·9)	58·5 (54·8 to 62·5)	64·4 (60·7 to 67·5)
Mandera	52·4 (48·4 to 56·5)	54·9 (51·2 to 59·2)	56·8 (52·8 to 61·4)	58·3 (54·1 to 63·1)	57·2 (53·5 to 61·2)	55·8 (52·2 to 60)	58 (55·2 to 61·5)	62·2 (58·7 to 66·2)
Marsabit	48·4 (44·4 to 52·5)	53 (48·7 to 57·5)	55·5 (51·4 to 60)	59·7 (55·5 to 64·2)	60·9 (57·1 to 65·2)	57·8 (53·2 to 62·8)	58·8 (55·2 to 63)	62·9 (58·7 to 67·5)
Meru	56·8 (52·9 to 60·9)	60·7 (56·6 to 65·2)	64·4 (60·3 to 69·1)	68·8 (64·9 to 73)	68·1 (64·6 to 71·3)	61·6 (57 to 66·1)	62·2 (58·8 to 66)	65·2 (61·3 to 69·1)
Migori	35·1 (31·9 to 38·7)	40·9 (37·4 to 44·8)	45 (41·6 to 49)	47·6 (44·4 to 51·3)	48·6 (44·7 to 52·8)	40·8 (36·3 to 45·3)	48·9 (45·1 to 52·6)	56·4 (51·9 to 61·8)
Mombasa	47 (43·2 to 51·2)	55·1 (50·9 to 59·5)	60·8 (56·7 to 65·4)	67 (62·8 to 71·3)	64·9 (60·8 to 68·5)	56·8 (52·3 to 61·8)	61 (57·7 to 64·6)	65·9 (62·1 to 69·6)
Murang'a	51 (47·7 to 55)	56·7 (52·8 to 60·7)	60·9 (56·6 to 65·3)	66 (62·3 to 70)	66·3 (63·2 to 69·8)	59·3 (54·9 to 63·5)	61·8 (59 to 64·4)	66·7 (63·2 to 69·8)
Nairobi	41·2 (37·9 to 44·9)	53·6 (50 to 57·5)	61·4 (57·9 to 64·9)	66·8 (63·4 to 70·5)	64 (60·8 to 67·9)	55·8 (52·6 to 59·5)	62·1 (60 to 64·3)	66·4 (63·6 to 69·4)
Nakuru	62·4 (58·2 to 66·7)	64·2 (59·9 to 68·3)	64·8 (60·8 to 68·9)	65·8 (62 to 69·8)	64·2 (60·6 to 67·7)	56·2 (52·4 to 60·3)	62·2 (59·2 to 65·2)	67·6 (64·2 to 70·6)
Nandi	56·2 (52·1 to 60·7)	58·2 (54·3 to 62·6)	59·6 (55·3 to 63·6)	61·1 (57·1 to 65)	59·8 (56·2 to 63·5)	53·8 (50·5 to 57·3)	58·5 (55·5 to 61·3)	64·1 (60·6 to 67·9)
Narok	61·7 (57·4 to 66)	62 (57·4 to 66·1)	60·9 (56·5 to 65·6)	59·3 (55·2 to 63·6)	58·9 (55·4 to 62·6)	55·1 (51·6 to 58·6)	60 (57·1 to 62·9)	64·2 (60·7 to 67·8)
Nyamira	57·3 (53·3 to 61·6)	59·6 (55·2 to 64·2)	62·1 (57·8 to 66·9)	63·7 (59·6 to 68·1)	62·8 (59 to 66·7)	52·9 (48·9 to 57)	56·6 (53·3 to 59·9)	61·5 (57·2 to 66·7)
Nyandarua	48·2 (44·2 to 52·6)	54·1 (49·9 to 58·4)	58·7 (54·1 to 63·2)	63·3 (59·2 to 67·4)	62·9 (59·3 to 66·9)	55·8 (51·9 to 59·5)	56·3 (53·3 to 59·1)	59·8 (56·2 to 63·9)
Nyeri	54·3 (50·6 to 58·6)	59·2 (55·2 to 64·1)	63·5 (59·4 to 68·2)	67·8 (63·6 to 71·9)	67·1 (63·6 to 70·7)	59·8 (55·5 to 64·6)	63·9 (60·9 to 67·3)	66·9 (63·3 to 70)
Samburu	61·8 (57·4 to 66·6)	63 (58·7 to 67·8)	63·4 (59·2 to 67·8)	64·2 (60 to 68·4)	61·3 (56·9 to 66)	53·3 (49 to 58·4)	57·3 (53·5 to 61·2)	62·5 (58·5 to 66·9)
Siaya	40·2 (36·9 to 44·1)	46 (42·3 to 49·8)	51·3 (47·6 to 55·1)	55 (51·5 to 58·8)	52·4 (48·3 to 56·4)	42 (37·7 to 47·2)	53·2 (48·7 to 57·7)	61·5 (56·9 to 65·5)
Taita Taveta	45·9 (42·4 to 50)	51·7 (47·7 to 56·4)	53·8 (49·8 to 58·3)	61·1 (56·3 to 65·6)	62·9 (58·8 to 67·5)	56·4 (51·6 to 61·6)	59 (55·5 to 63)	64·8 (60·3 to 69·2)
Tana River	47·8 (44·1 to 52·2)	51·5 (47·2 to 56·2)	53·5 (48·9 to 58·5)	56·2 (51·6 to 61·1)	56·6 (52·5 to 60·7)	51·1 (47·6 to 55·1)	53·7 (50·7 to 57·1)	59·4 (55 to 63·8)
Tharaka Nithi	53·6 (49·5 to 57·9)	56·3 (51·9 to 60·9)	58 (53·4 to 63)	61·2 (57 to 65·7)	60·2 (56·3 to 64·3)	55·4 (51·4 to 59·3)	59·2 (56·3 to 62·1)	63·2 (59·2 to 67)
Trans Nzoia	58·9 (54·6 to 63·3)	62·4 (58·3 to 66·6)	63·7 (59·8 to 68·2)	65·8 (61·9 to 70·3)	65·4 (61·3 to 69·2)	58·1 (53·3 to 63·5)	62·1 (58·1 to 66·2)	67·1 (63·3 to 70·4)
Turkana	55·1 (50·8 to 59·5)	56·9 (52·7 to 61·2)	56·5 (52·4 to 61)	56·1 (52 to 60)	55·3 (51·1 to 59·9)	46·8 (37·7 to 53·6)	54·8 (51·4 to 58·4)	61·5 (57·3 to 66·1)
Uasin Gishu	64·8 (60·5 to 69·5)	67·1 (62·8 to 71·3)	68·3 (64·1 to 72·6)	69·3 (65·6 to 73·4)	67·6 (63·9 to 71·1)	60·6 (57 to 64·4)	64·5 (61·8 to 67·1)	69·1 (66·3 to 72·1)
Vihiga	52·1 (48·2 to 56·5)	55·8 (52 to 60·1)	58·6 (54·7 to 63·4)	61·2 (57 to 65·5)	60·1 (56·3 to 64·2)	53·7 (49·9 to 58·1)	59·9 (56·7 to 63·6)	64·8 (60·9 to 69·2)
Wajir	39·9 (36·4 to 43·6)	45·2 (41·5 to 49·9)	49·3 (45·2 to 53·7)	54·4 (50·6 to 58·5)	54·8 (51·1 to 58·8)	53·9 (51 to 57·7)	57·7 (54·6 to 61·2)	62·5 (58·2 to 66·6)
West Pokot	56·7 (52·7 to 61·3)	58·8 (54·5 to 63·2)	59·1 (55·1 to 63·4)	60·8 (56·5 to 64·9)	60·7 (56·9 to 65·1)	59 (55·3 to 63·2)	62·1 (58·9 to 65·6)	66·4 (62·8 to 70·8)
Madagascar	41·2 (38·3 to 44·3)	44·7 (42·2 to 47·2)	47·7 (45·8 to 49·6)	53·6 (51·7 to 55·5)	54·4 (52·6 to 56·3)	58·3 (56·7 to 59·8)	61·3 (59 to 63·9)	64·3 (61·6 to 67·2)
Malawi	32·7 (28·4 to 37·2)	36·2 (32·9 to 39·6)	40·6 (38·1 to 43·5)	47·5 (45·5 to 49·8)	47·9 (46·2 to 49·6)	45·1 (42·6 to 47·5)	53·9 (52 to 55·7)	61·4 (59·1 to 63·6)
Mozambique	40·8 (36·1 to 45·4)	43·1 (39·3 to 46·9)	45·7 (42·7 to 48·8)	49 (46·4 to 51·5)	49·3 (47·7 to 50·9)	49·9 (47·9 to 51·8)	50·8 (48·7 to 52·9)	54·8 (52·1 to 57·3)
Rwanda	36·4 (32·5 to 40·4)	41·2 (38·4 to 44)	41 (38·5 to 43·4)	46·7 (44·5 to 49)	47·9 (46·4 to 49·4)	48·7 (47 to 50·4)	62·3 (60·6 to 64)	66·3 (64 to 68·4)
Somalia	42·7 (38·3 to 47)	43·4 (39·9 to 47·2)	45 (41·2 to 48·8)	47 (43·7 to 50·7)	46·8 (44·3 to 49·7)	48·9 (45·8 to 52·2)	51 (47·5 to 54·6)	55·8 (51·4 to 59·6)
South Sudan	45·4 (40·5 to 50·2)	46·8 (42·6 to 51)	47·9 (44·5 to 51·4)	51·2 (47·8 to 54·5)	52·3 (49·4 to 55·6)	54·9 (51·9 to 58)	58·7 (55·3 to 62)	61·7 (58·3 to 65)
Uganda	44·9 (40·4 to 49·5)	46·9 (43·5 to 50·4)	49·3 (46·7 to 52·2)	48 (46·2 to 49·9)	46·5 (43·6 to 49·1)	47·6 (45·7 to 49·3)	56·7 (54·9 to 58·5)	62·7 (60·4 to 64·8)
Tanzania	42·5 (38·2 to 46·8)	43·9 (40·8 to 47·5)	47·9 (45·7 to 50·5)	53·6 (51·6 to 55·6)	53·6 (51·6 to 55·6)	52·4 (50·4 to 54·5)	59·8 (58 to 61·4)	65·3 (63·1 to 67·3)

Zambia	45·5 (40·9 to 50)	48 (44·6 to 51·5)	51·9 (49·3 to 54·4)	54·2 (52·3 to 56·3)	50·8 (48·8 to 52·7)	44·7 (42·3 to 47)	54·3 (52·4 to 56·1)	60·4 (57·9 to 62·8)
Southern sub-Saharan Africa	49·4 (46·9 to 51·7)	51·2 (49·2 to 53·3)	53·9 (52·4 to 55·4)	56·6 (55·5 to 57·7)	59·6 (58·7 to 60·4)	50·7 (48·4 to 52·7)	53·5 (51·7 to 54·6)	60·5 (59 to 61·8)
Botswana	50·6 (46·8 to 54·4)	51·2 (48·2 to 54·6)	53·1 (50·6 to 55·7)	57·4 (54·9 to 59·9)	57·5 (54·5 to 60·4)	43·7 (40·7 to 47·2)	53·9 (51 to 57)	59 (55·8 to 62·3)
eSwatini	39·7 (35·6 to 43·8)	41·9 (38·5 to 45·4)	47·3 (44·6 to 50·3)	52·9 (50·4 to 56·1)	57·4 (55·5 to 59·6)	45·9 (42·3 to 49·6)	45 (42·2 to 48)	53·5 (50·3 to 56·4)
Lesotho	47 (44·5 to 49·7)	48·3 (45·8 to 50·9)	50 (47·4 to 52·6)	54·2 (51·6 to 57)	56·1 (54 to 58·2)	45·9 (42·9 to 48·6)	45 (42·6 to 47·4)	48·6 (46·1 to 51·1)
Namibia	52·1 (48·1 to 56·1)	53·3 (49·9 to 57)	55 (52·3 to 58)	55·8 (54·1 to 57·5)	58·9 (57·6 to 60·2)	51·6 (49·1 to 53·9)	57·2 (54·7 to 59·4)	61·3 (58·2 to 64·4)
South Africa	49·5 (47·4 to 51·6)	51·3 (49·3 to 53·3)	54 (52·3 to 55·5)	56·6 (55·3 to 57·8)	60 (59·1 to 60·9)	52·4 (50·5 to 54)	54·7 (52·8 to 56)	61·9 (60·3 to 63·2)
Zimbabwe	50·8 (46·9 to 54·7)	52·6 (49·7 to 55·6)	55·1 (52·5 to 57·7)	57·5 (55·7 to 59·4)	59·7 (56·7 to 61·5)	46·1 (42·3 to 50·2)	50·4 (48·3 to 52·2)	57·9 (55·5 to 60·2)
Western sub-Saharan Africa	41·1 (37·5 to 44·5)	42·1 (39·5 to 44·8)	46·6 (44·7 to 48·8)	51·3 (49·6 to 52·9)	53·1 (51·7 to 54·7)	53·3 (51·7 to 55)	57·8 (56 to 59·4)	62 (59·8 to 64)
Benin	42·4 (37·9 to 47)	43·5 (40·1 to 47·3)	47·1 (44·4 to 50·1)	51 (49 to 53·2)	53·7 (52 to 55·3)	55·1 (53·2 to 57·1)	58·6 (56 to 61·3)	62·2 (58 to 65·6)
Burkina Faso	34·8 (30·4 to 39·3)	38·2 (34·7 to 41·7)	42·4 (39·8 to 45·1)	46·8 (44·8 to 49)	49 (47·1 to 50·9)	50·9 (48·6 to 53·1)	55·7 (53·9 to 57·5)	59·1 (56·5 to 61·4)
Cape Verde	54·4 (52 to 57·1)	55·3 (53·8 to 56·8)	56·9 (55·4 to 58·3)	64·8 (64 to 65·7)	66·7 (66 to 67·5)	66·1 (64·1 to 68·2)	71 (70·1 to 71·8)	69·7 (68·2 to 71·1)
Cameroon	40·2 (37·2 to 43·3)	25 (23·4 to 26·5)	50·1 (47·6 to 52·9)	54·1 (52·1 to 56·4)	56·7 (55·2 to 58·3)	53·1 (51·4 to 54·8)	56·4 (54·2 to 58·6)	61·4 (58·7 to 64·1)
Chad	42·6 (37·7 to 47·5)	45·1 (41·2 to 49)	46·1 (43·1 to 49·4)	48·9 (46·3 to 51·6)	52·1 (50·3 to 54·1)	50·6 (48·7 to 52·8)	55 (53 to 57)	58·9 (56 to 61·6)
Côte d'Ivoire	39·5 (36·2 to 42·8)	43·4 (40·8 to 46·1)	48·8 (46·4 to 51·3)	54·8 (52·7 to 56·7)	53·5 (51·4 to 55·4)	49·9 (47·5 to 52·1)	55·7 (53·6 to 57·9)	62 (59·5 to 64·4)
The Gambia	48·7 (44·2 to 53·3)	50·7 (47·1 to 54·3)	53·6 (50·6 to 56·8)	55·4 (52·4 to 58·5)	58·3 (55·5 to 61)	59·5 (57·5 to 61·7)	61·9 (60 to 64·1)	64·8 (62·5 to 67·1)
Ghana	45·1 (42·2 to 48·3)	49 (46·3 to 51·8)	52·5 (49·8 to 55·3)	55·8 (53·1 to 58·4)	57·8 (55·7 to 59·9)	58·2 (56·5 to 60)	59·5 (58·2 to 60·8)	63·6 (61·4 to 65·6)
Guinea	37·7 (33 to 42·4)	40·6 (36·6 to 44·8)	43·5 (39·5 to 47·5)	47·1 (44·1 to 50·1)	51·8 (49·9 to 53·8)	53·2 (51·1 to 55·1)	56·2 (53·8 to 58·8)	59·8 (56·4 to 63·2)
Guinea-Bissau	29·8 (25·6 to 34·1)	33·9 (30·1 to 38)	35 (31·5 to 38·4)	41·9 (38·9 to 45·2)	45·5 (42·7 to 48·2)	48·8 (46·8 to 50·8)	53·7 (51·4 to 56·3)	58·1 (54·8 to 61·2)
Liberia	34·8 (30·6 to 39·3)	37·9 (34·6 to 41·8)	43·1 (40·1 to 46·4)	49 (46·6 to 51·6)	46·7 (44·9 to 48·5)	54·1 (52·4 to 55·9)	61·5 (59·6 to 63·5)	65·4 (62·8 to 67·8)
Mali	34·1 (29·6 to 38·7)	36·7 (32·6 to 41)	39·3 (36·5 to 42·6)	45·1 (43·2 to 47·2)	50 (48·5 to 51·7)	53·3 (51·6 to 55)	57·8 (55·7 to 60)	61 (56·8 to 64·7)
Mauritania	45·6 (41·5 to 49·8)	49·1 (46 to 52·4)	53·9 (51·3 to 56·9)	55·9 (53·8 to 58·4)	58·7 (57·1 to 60·4)	63·4 (61·6 to 65·3)	68 (65·9 to 69·9)	70·6 (67·5 to 73)
Niger	35 (30·3 to 39·9)	37·8 (33·5 to 42·6)	41·3 (38 to 44·6)	43·9 (41·9 to 46·1)	46·6 (44·8 to 48·5)	51·2 (49 to 53·4)	57·8 (55·3 to 60·2)	61·2 (57 to 64·9)
Nigeria	43·3 (40 to 46·9)	45·3 (42·2 to 48·4)	47·2 (44·6 to 50·1)	52·3 (49·6 to 55)	53·2 (50·8 to 55·9)	53·3 (50·7 to 56·3)	58·1 (55·2 to 61·2)	62·8 (59·5 to 65·8)
São Tomé and PrÍncipe	39·9 (36·2 to 43·3)	44·1 (42·7 to 45·5)	55·2 (54·1 to 56·3)	60·5 (59·2 to 61·8)	62·1 (60·3 to 63·8)	64·3 (62·8 to 66·2)	67·8 (66·4 to 69·2)	69·5 (67·5 to 71·7)
Senegal	42·8 (38·5 to 47·2)	45·1 (42·4 to 48·1)	46·3 (43·9 to 48·7)	51·2 (49·4 to 53·3)	56·6 (55·2 to 58)	58·9 (57·6 to 60·1)	63·9 (62·4 to 65·5)	66·8 (64·6 to 68·8)
Sierra Leone	38·3 (33·7 to 43)	41 (36·9 to 45·3)	44 (39·4 to 48·4)	46·6 (43·2 to 50)	49·2 (46·7 to 51·7)	49·8 (48·2 to 51·4)	54·8 (52·4 to 57·4)	60·6 (57 to 64)
Togo	42·1 (37·4 to 46·8)	44·4 (41 to 48)	49·3 (46·5 to 52·1)	54·2 (51·8 to 56·6)	56·2 (54·2 to 58·3)	54·2 (52·2 to 56·6)	57 (55·1 to 59·1)	62 (59·5 to 64·5)

Supplemental Results Table 2c: Estimates of life expectancy at birth by location, females, 1950, 1960, 1970, 1980, 1990, 2000, 2010, 2019

Location	1950	1960	1970	1980	1990	2000	2010	2019
Global	53·5 (51·8 to 55·1)	49 (44·6 to 52·1)	60·7 (59·8 to 61·6)	64·7 (64 to 65·3)	68 (67·5 to 68·4)	69·8 (69·4 to 70·2)	73·4 (72·9 to 73·8)	76·1 (75·4 to 76·9)
Low SDI	43·9 (41·2 to 46·6)	46·2 (44·1 to 48·3)	48·9 (47·3 to 50·6)	52·8 (51·6 to 53·9)	55·3 (54·4 to 56·1)	56·8 (55·7 to 57·9)	62·3 (61·3 to 63·3)	67·3 (65·8 to 68·6)
Low-middle SDI	42·8 (40·7 to 44·8)	42·2 (39·7 to 44·5)	50·2 (48·8 to 51·5)	56·6 (55·7 to 57·4)	61·2 (60·4 to 61·8)	64 (63·3 to 64·8)	68·7 (68·1 to 69·4)	71·7 (70·5 to 72·8)
Middle SDI	50·3 (48·4 to 52·2)	37·6 (30·4 to 43·4)	60·6 (59·2 to 61·8)	65·3 (64·2 to 66·3)	69·2 (68·5 to 70)	71·9 (71·4 to 72·3)	74·8 (74·4 to 75·2)	77·4 (76·5 to 78·3)
High-middle SDI	62·2 (60·8 to 63·5)	60·3 (56·9 to 62·7)	69·1 (68·1 to 69·8)	71·4 (70·7 to 72)	73·9 (73·4 to 74·4)	75·3 (75 to 75·6)	78·4 (78·1 to 78·8)	80·6 (79·8 to 81·3)
High SDI	67·3 (67·1 to 67·5)	72 (72 to 72·1)	74·3 (74·2 to 74·3)	77 (77 to 77·1)	79·3 (79·2 to 79·3)	81 (81 to 81·1)	83·1 (83 to 83·1)	83·8 (83·6 to 83·9)
Central Europe, eastern Europe, and central Asia	65·3 (63·6 to 66·8)	69·8 (69·4 to 70·1)	71·8 (71·6 to 72·1)	72·7 (72·6 to 72·8)	73·9 (73·8 to 74)	73·2 (73·1 to 73·3)	75·6 (75·5 to 75·7)	77·7 (76·8 to 78·6)
Central Asia	54·1 (50·2 to 57·9)	60·8 (58·9 to 62·6)	66·6 (65·5 to 67·6)	70 (69·5 to 70·4)	71·5 (71·1 to 71·8)	69·9 (69·5 to 70·2)	71·9 (71·6 to 72·2)	74 (73 to 75)
Armenia	51·2 (47·1 to 55·2)	62 (59·5 to 64·6)	68·9 (67·6 to 70·2)	72·7 (71·9 to 73·6)	73·7 (73·3 to 74·1)	75·2 (74·9 to 75·6)	77·2 (76·9 to 77·5)	78·8 (77·5 to 80·2)
Azerbaijan	40·3 (36·3 to 44·4)	51·2 (48·1 to 54·2)	63·2 (61·4 to 64·9)	69·4 (68·4 to 70·4)	70·8 (69·9 to 71·7)	69·7 (68·9 to 70·5)	71·8 (71·1 to 72·5)	73·7 (72·2 to 75·1)
Georgia	54·1 (50·2 to 58)	63·6 (61·9 to 65·7)	69·8 (68·4 to 71·2)	72·3 (71·3 to 73·4)	72·5 (71·7 to 73·3)	74·6 (73·8 to 75·4)	76·6 (75·8 to 77·4)	78·1 (76·6 to 79·6)
Kazakhstan	62 (58·4 to 65·5)	65·9 (64·3 to 67·4)	69·4 (68·3 to 70·5)	71·8 (71·2 to 72·5)	72·7 (72·2 to 73·1)	70·3 (69·8 to 70·8)	72·7 (72·3 to 73·1)	76 (74·8 to 77·2)
Kyrgyzstan	54·3 (50·3 to 58·2)	59·8 (57·1 to 62·3)	64·8 (63 to 66·5)	69·1 (68·2 to 70)	71·6 (71 to 72·1)	71·5 (71 to 72)	73·7 (73·5 to 74)	76·9 (75·8 to 78)
Mongolia	41·1 (37·1 to 45·1)	46 (42·2 to 49·8)	52·8 (49·8 to 56·3)	59·9 (57·3 to 62·8)	63·5 (61·6 to 65·5)	64·4 (62·7 to 66·2)	69·4 (67·5 to 71·3)	72·7 (69·8 to 75·3)
Tajikistan	47·6 (43·4 to 51·7)	54·2 (51·9 to 56·4)	62·5 (60·8 to 64·2)	67·1 (65·5 to 68·8)	69·7 (68·7 to 70·6)	68·7 (67·9 to 69·6)	70·3 (69·3 to 71·3)	71·7 (69·8 to 73·5)
Turkmenistan	51·8 (47·9 to 55·7)	58·4 (56·5 to 60·2)	63·7 (62·3 to 65·1)	66·9 (65·9 to 67·8)	69·2 (68·6 to 69·8)	70·2 (69·7 to 70·7)	72·9 (72·5 to 73·3)	74·4 (71·8 to 76·7)
Uzbekistan	59·3 (55·6 to 62·9)	63·9 (61·9 to 66)	67·7 (66·5 to 68·8)	70·3 (69·5 to 71)	72·4 (72 to 72·8)	68·1 (67·6 to 68·6)	69·1 (68·7 to 69·6)	71 (69·5 to 72·4)
Central Europe	61·2 (60·5 to 61·8)	68·7 (68·6 to 68·8)	71·7 (71·6 to 71·8)	73·6 (73·5 to 73·7)	74·7 (74·6 to 74·8)	76·5 (76·5 to 76·6)	79 (78·9 to 79·1)	80·4 (79·2 to 81·5)
Albania	52·9 (50 to 55·5)	63·2 (61·3 to 65)	69·5 (67·2 to 71·9)	74·4 (72·7 to 76·1)	76·3 (75·9 to 76·7)	78·7 (78·2 to 79)	80·5 (79·3 to 81·6)	81·4 (79·3 to 83·5)
Bosnia and Herzegovina	52·1 (51·7 to 52·5)	62·5 (62·2 to 62·9)	68·5 (68·1 to 68·9)	72·4 (72·2 to 72·8)	76 (75·8 to 76·3)	78·4 (77·2 to 79·6)	78·6 (78·3 to 78·9)	79·3 (77·4 to 81·2)
Bulgaria	62·8 (62·4 to 63·1)	71·3 (71 to 71·5)	73·6 (73·4 to 73·8)	73·9 (73·7 to 74·1)	73·7 (73·2 to 74·1)	73·8 (73·4 to 74·3)	76·1 (75·6 to 76·5)	77 (74·9 to 79)
Croatia	57 (56·6 to 57·4)	66·5 (66·2 to 66·8)	71·5 (71·2 to 71·8)	74·4 (73·8 to 74·9)	76·2 (75·7 to 76·8)	78·2 (77·7 to 78·7)	79·9 (79·6 to 80·2)	81·7 (79·9 to 83·5)
Czech Republic	66·6 (66·4 to 66·8)	73·3 (73·1 to 73·5)	73·3 (73·1 to 73·5)	74·2 (74 to 74·4)	75·7 (75·5 to 75·8)	78·5 (78·3 to 78·7)	81 (80·8 to 81·2)	82·3 (80·7 to 83·9)
Hungary	64 (63·8 to 64·2)	70·3 (70·1 to 70·4)	72·1 (71·9 to 72·3)	72·8 (72·6 to 73)	73·9 (73·8 to 74·1)	76·2 (76 to 76·3)	78·5 (78·4 to 78·7)	79·8 (78 to 81·6)
Montenegro	68·3 (67·7 to 69)	72·2 (71·7 to 72·7)	75·7 (75·2 to 76·2)	75·6 (75·2 to 76·1)	77·9 (77·3 to 78·5)	76·4 (76 to 76·9)	77·2 (76·7 to 77·6)	78·7 (77·5 to 79·8)
North Macedonia	49·7 (49·2 to 50·1)	57·1 (56·6 to 57·5)	66·1 (65·7 to 66·6)	71·5 (71·1 to 71·9)	72·5 (72·1 to 72·9)	73·9 (73·5 to 74·4)	75·6 (75·1 to 75·9)	76·4 (74·6 to 78·2)
Poland	61·9 (61·7 to 62·1)	70·8 (70·7 to 70·9)	73·6 (73·4 to 73·7)	74·9 (74·8 to 75)	75·7 (75·6 to 75·8)	78·1 (78 to 78·2)	80·7 (80·6 to 80·8)	81·9 (80 to 83·9)
Romania	62·3 (58·7 to 65·7)	67·6 (67·4 to 67·7)	70·5 (70·3 to 70·6)	72·2 (72·1 to 72·3)	73·2 (73 to 73·3)	74·8 (74·7 to 75)	77·6 (77·5 to 77·8)	79·2 (77·5 to 80·9)
Serbia	56·2 (55·8 to 56·5)	65 (64·8 to 65·3)	68·7 (68·5 to 68·9)	72·9 (72·7 to 73·1)	73·4 (73·2 to 73·6)	74·1 (73·9 to 74·3)	76·5 (76·3 to 76·6)	77·9 (76·1 to 79·6)
Slovakia	62·6 (62·2 to 63)	72·8 (72·5 to 73·1)	73·3 (73 to 73·5)	74·6 (74·4 to 74·9)	75·6 (75·4 to 75·9)	77·9 (77·6 to 78·2)	79·6 (79·3 to 79·8)	80·8 (78·8 to 82·7)
Slovenia	61·5 (61 to 62)	67·4 (67 to 67·7)	71·6 (71·2 to 72)	75·1 (74·8 to 75·5)	77·5 (75·1 to 79·8)	80 (79·6 to 80·4)	83 (82·5 to 83·4)	84·6 (82·4 to 86·6)
Eastern Europe	70·2 (68·1 to 72·4)	72·8 (72·7 to 73)	73·9 (73·8 to 74)	73·5 (73·4 to 73·5)	74·7 (74·7 to 74·8)	73 (72·9 to 73)	75·2 (75·2 to 75·3)	77·6 (76·1 to 78·9)
Belarus	71·2 (67·7 to 74·5)	74·3 (73·4 to 75·3)	75·8 (75·3 to 76·2)	75·5 (75·1 to 75·8)	75·9 (75·6 to 76·1)	74·8 (74·6 to 75·1)	76·7 (76·5 to 76·9)	78·9 (76·4 to 81·1)
Estonia	70·2 (67·1 to 73·2)	73 (72·6 to 73·5)	74·6 (74·1 to 75)	74·5 (74·1 to 74·9)	75 (74·6 to 75·4)	76·3 (75·9 to 76·7)	80·9 (80·4 to 81·4)	81·8 (79·8 to 83·7)
Latvia	71·6 (68·5 to 74·6)	73·6 (73·2 to 74)	74·3 (74 to 74·7)	74·3 (73·9 to 74·6)	74·8 (74·5 to 75·1)	76·1 (75·8 to 76·4)	78·1 (77·8 to 78·5)	79·9 (77·3 to 82·2)

Lithuania	70·1 (66·7 to 73·3)	72·8 (72·5 to 73·2)	75·3 (75 to 75·6)	75·5 (75·2 to 75·8)	76·3 (76 to 76·6)	77·6 (77·3 to 77·9)	78·8 (78·5 to 79·1)	80·7 (79 to 82·3)
Moldova	56·9 (53·3 to 60·5)	61·7 (59·7 to 63·6)	66·1 (64·6 to 67·5)	68·9 (68·4 to 69·4)	71·7 (71·3 to 72)	72·6 (72·3 to 72·9)	74·4 (74·1 to 74·6)	78·2 (77 to 79·4)
Russia	70·2 (67·5 to 73)	72·6 (72·6 to 72·7)	73·7 (73·7 to 73·8)	73·2 (73·1 to 73·2)	74·6 (74·6 to 74·7)	72·5 (72·4 to 72·5)	74·9 (74·8 to 74·9)	77·8 (75·9 to 79·6)
Ukraine	71·3 (68·5 to 73·9)	74·1 (73·6 to 74·5)	74·4 (74·1 to 74·7)	74·1 (73·9 to 74·4)	75 (74·8 to 75·2)	73·7 (73·6 to 73·7)	75·7 (75·6 to 75·8)	76·2 (74 to 78·3)
High income	67·2 (67·1 to 67·2)	72 (72 to 72)	74·2 (74·2 to 74·3)	77·2 (77·2 to 77·3)	79·5 (79·5 to 79·6)	81·3 (81·3 to 81·3)	83·2 (83·2 to 83·2)	83·8 (83·8 to 83·9)
Australasia	72 (71·9 to 72·1)	74·2 (74·1 to 74·3)	74·7 (74·6 to 74·8)	77·8 (77·7 to 77·9)	79·8 (79·8 to 79·9)	82·1 (82 to 82·2)	84·1 (84 to 84·2)	84·8 (84·6 to 84·9)
Australia	72·1 (72 to 72·2)	74·3 (74·2 to 74·4)	74·6 (74·6 to 74·7)	78·1 (78 to 78·2)	80·1 (80·1 to 80·2)	82·4 (82·3 to 82·5)	84·3 (84·3 to 84·4)	85 (84·8 to 85·1)
New Zealand	71·6 (71·3 to 71·8)	73·9 (73·7 to 74)	74·8 (74·7 to 75)	76·4 (76·2 to 76·5)	78·4 (78·3 to 78·6)	80·8 (80·7 to 81)	82·8 (82·7 to 83)	83·6 (83·4 to 83·8)
High-income Asia Pacific	58·3 (58 to 58·5)	68·7 (68·4 to 68·9)	73·5 (73·4 to 73·7)	77·4 (77·4 to 77·5)	81 (81 to 81·1)	84 (83·9 to 84)	86·1 (86·1 to 86·2)	87·3 (87·2 to 87·4)
Brunei	51·3 (49·8 to 52·9)	55·8 (54·3 to 57·3)	63·8 (62·8 to 64·8)	68·9 (67·9 to 70)	72·3 (71·3 to 73·2)	74·2 (73·5 to 74·9)	75·8 (75·1 to 76·4)	76·4 (75·4 to 77·4)
Japan	61·5 (61·3 to 61·6)	70·7 (70·7 to 70·8)	75·3 (75·3 to 75·4)	79·2 (79·1 to 79·2)	82·3 (82·3 to 82·4)	84·9 (84·8 to 84·9)	86·6 (86·5 to 86·6)	87·7 (87·5 to 87·8)
Aichi	62·6 (59·6 to 65·6)	71·1 (68·5 to 73·7)	75·3 (72·9 to 77·4)	78·8 (78·6 to 79·1)	82 (81·7 to 82·2)	84·5 (84·3 to 84·7)	86·4 (86·2 to 86·7)	87·7 (86·7 to 88·7)
Akita	62·1 (59·1 to 65·3)	70·9 (68·3 to 73·4)	75·1 (73·2 to 77)	78·8 (78·5 to 79·1)	82·1 (81·7 to 82·4)	84·6 (84·2 to 85)	86·2 (85·8 to 86·6)	86·3 (85·4 to 87·2)
Aomori	60·4 (57·2 to 63·4)	69·9 (67·2 to 72·5)	74·8 (72·7 to 77)	78·9 (78·6 to 79·3)	82·1 (81·7 to 82·4)	84·4 (84·1 to 84·8)	85·9 (85·5 to 86·2)	87 (86 to 88)
Chiba	61·7 (58·6 to 64·7)	70·8 (68·2 to 73·6)	75·5 (73·2 to 77·7)	79·3 (79·1 to 79·5)	82·6 (82·4 to 82·9)	84·7 (84·5 to 85)	86·3 (86·1 to 86·6)	87·2 (86·3 to 88·2)
Ehime	61·5 (58·2 to 64·6)	70·7 (68 to 73·5)	75·7 (73·6 to 77·8)	79·6 (79·3 to 80)	82·5 (82·2 to 82·9)	84·8 (84·4 to 85·2)	86·7 (86·3 to 87·1)	87·4 (86·4 to 88·4)
Fukui	63 (60 to 66·1)	71·3 (68·8 to 73·8)	76 (74·1 to 77·8)	80 (79·6 to 80·4)	83 (82·6 to 83·3)	85·8 (85·4 to 86·2)	87·4 (87·1 to 87·8)	88·3 (87·4 to 89·2)
Fukuoka	61·8 (58·7 to 64·9)	70·9 (68·2 to 73·5)	75·2 (72·8 to 77·4)	79·2 (79 to 79·5)	82·4 (82·2 to 82·7)	84·9 (84·6 to 85·1)	86·7 (86·4 to 87)	87·6 (86·6 to 88·6)
Fukushima	59·9 (56·9 to 63)	69·8 (67·3 to 72·5)	74·5 (72·3 to 76·6)	79·1 (78·7 to 79·4)	82·6 (82·3 to 82·9)	84·9 (84·6 to 85·3)	86·4 (86·1 to 86·8)	87·2 (86·2 to 88·1)
Gifu	61·5 (58·4 to 64·7)	70·3 (67·7 to 72·8)	74·6 (72·5 to 76·7)	78·6 (78·3 to 78·9)	82 (81·7 to 82·3)	84·5 (84·2 to 84·9)	86·5 (86·1 to 86·9)	87·5 (86·7 to 88·4)
Gunma	61 (57·8 to 64·1)	70 (67·5 to 72·8)	74·7 (72·5 to 76·8)	78·7 (78·4 to 78·9)	82·1 (81·8 to 82·4)	84·8 (84·5 to 85·1)	86·2 (85·8 to 86·5)	87·3 (86·4 to 88·2)
Hiroshima	61 (57·8 to 63·9)	70·6 (67·9 to 73·4)	75·6 (73·2 to 77·9)	79·4 (79·1 to 79·7)	82·5 (82·3 to 82·8)	85·5 (85·1 to 85·8)	87·1 (86·7 to 87·4)	88·2 (87·2 to 89·3)
Hokkaidō	61·4 (58·3 to 64·4)	70·6 (68 to 73·3)	74·9 (72·5 to 77·1)	78·7 (78·5 to 78·9)	82·2 (82 to 82·5)	85·1 (84·8 to 85·3)	86·5 (86·3 to 86·8)	87·5 (86·5 to 88·5)
Hyōgo	62·7 (59·5 to 65·8)	71·2 (68·6 to 73·9)	75·3 (73 to 77·6)	78·9 (78·7 to 79·1)	81·9 (81·7 to 82·1)	84·5 (84·3 to 84·8)	86·5 (86·2 to 86·7)	87·8 (86·9 to 88·8)
Ibaraki	59·2 (56·2 to 62·4)	69·5 (66·8 to 72·2)	74·4 (72·1 to 76·7)	78·7 (78·4 to 79)	81·8 (81·5 to 82·1)	84·3 (84 to 84·6)	85·9 (85·5 to 86·2)	87·2 (86·2 to 88·3)
Ishikawa	62·6 (59·4 to 65·6)	71·1 (68·5 to 73·7)	75·7 (73·7 to 77·6)	79·3 (78·9 to 79·6)	82·8 (82·5 to 83·2)	85·2 (84·8 to 85·6)	87 (86·6 to 87·3)	87·8 (86·9 to 88·6)
Iwate	60·1 (57 to 63·1)	69·8 (67·2 to 72·5)	74·6 (72·7 to 76·6)	78·8 (78·5 to 79·1)	82·1 (81·8 to 82·5)	84·9 (84·6 to 85·2)	86·3 (85·9 to 86·7)	86·7 (85·8 to 87·5)
Kagawa	62·1 (58·8 to 65·2)	71 (68·4 to 73·6)	75·8 (73·8 to 77·8)	79·8 (79·4 to 80·2)	82·6 (82·3 to 83)	84·8 (84·4 to 85·2)	86·4 (86 to 86·8)	87·9 (87 to 88·8)
Kagoshima	58·5 (55·3 to 61·7)	68·6 (66 to 71·2)	73·6 (71·3 to 75·8)	78·5 (78·2 to 78·8)	82·2 (81·8 to 82·5)	84·9 (84·6 to 85·2)	86·5 (86·1 to 86·8)	87·9 (87 to 88·8)
Kanagawa	63·8 (60·8 to 66·9)	72·3 (69·7 to 74·9)	76·4 (74 to 78·6)	79·7 (79·5 to 79·9)	82·5 (82·3 to 82·7)	85 (84·7 to 85·2)	86·6 (86·4 to 86·8)	87·9 (86·9 to 88·8)
Kōchi	59·5 (56·4 to 62·7)	69·3 (66·7 to 72)	74·4 (72·4 to 76·4)	79·1 (78·7 to 79·4)	82·5 (82·1 to 82·9)	84·8 (84·4 to 85·2)	86·7 (86·3 to 87·2)	87·9 (86·9 to 88·8)
Kumamoto	60 (57 to 63·2)	69·8 (67·1 to 72·4)	74·7 (72·5 to 76·7)	79·4 (79·1 to 79·7)	83 (82·7 to 83·3)	85·7 (85·3 to 86·1)	87·2 (86·8 to 87·5)	88·3 (87·4 to 89·2)
Kyōto	62·9 (59·7 to 66·1)	71·5 (68·9 to 74·3)	75·9 (73·5 to 78·2)	79·8 (79·5 to 80·1)	82·5 (82·2 to 82·8)	85·3 (85 to 85·7)	87 (86·7 to 87·4)	88 (87 to 89·2)
Mie	62·3 (59·2 to 65·4)	71 (68·3 to 73·7)	75·5 (73·4 to 77·6)	79·2 (78·9 to 79·5)	82·2 (81·9 to 82·5)	84·8 (84·4 to 85·1)	86·5 (86·1 to 86·8)	87·4 (86·5 to 88·3)
Miyagi	61·4 (58·4 to 64·4)	70·9 (68·4 to 73·5)	75·3 (73·1 to 77·3)	79 (78·7 to 79·3)	82·4 (82·1 to 82·7)	85 (84·7 to 85·3)	86·7 (86·4 to 87·1)	87·7 (86·9 to 88·6)
Miyazaki	60·2 (57·1 to 63·3)	69·7 (67 to 72·2)	74·6 (72·6 to 76·6)	79·1 (78·7 to 79·5)	82·6 (82·2 to 82·9)	85·3 (84·9 to 85·7)	86·7 (86·4 to 87·1)	87·5 (86·6 to 88·4)
Nagano	62 (58·8 to 65)	71 (68·5 to 73·6)	75·7 (73·5 to 77·8)	79·6 (79·3 to 79·9)	82·9 (82·6 to 83·2)	85·6 (85·3 to 85·9)	87·3 (86·9 to 87·6)	87·9 (87 to 88·8)

Nagasaki	61·7 (58·6 to 64·7)	70·4 (67·7 to 73)	74·8 (72·6 to 77)	78·9 (78·5 to 79·2)	82·4 (82·1 to 82·8)	85 (84·7 to 85·3)	86·6 (86·2 to 87)	87·4 (86·5 to 88·4)
Nara	62·2 (58·9 to 65·3)	70·9 (68·3 to 73·5)	75·1 (73·2 to 77)	78·8 (78·5 to 79·2)	82 (81·7 to 82·3)	84·9 (84·6 to 85·3)	86·6 (86·2 to 86·9)	87·5 (86·7 to 88·3)
Niigata	62·4 (59·3 to 65·3)	71·3 (68·7 to 73·9)	75·5 (73·2 to 77·6)	79·3 (79 to 79·6)	82·8 (82·5 to 83·1)	85·5 (85·1 to 85·9)	87·2 (86·8 to 87·6)	87·2 (86·2 to 88·3)
Ōita	60·1 (57·1 to 63·1)	69·7 (67 to 72·3)	74·4 (72·5 to 76·4)	78·9 (78·5 to 79·2)	82·3 (82 to 82·7)	84·9 (84·6 to 85·3)	87·1 (86·7 to 87·5)	88 (87·2 to 88·8)
Okayama	62·3 (59·2 to 65·3)	71·2 (68·6 to 73·9)	76 (74 to 78·1)	79·9 (79·6 to 80·2)	83 (82·6 to 83·3)	85·5 (85·2 to 85·8)	87·2 (86·8 to 87·5)	88·5 (87·6 to 89·4)
Okinawa	59·9 (56·5 to 63·1)	69·6 (67 to 72·4)	74·5 (72·4 to 76·7)	80·2 (79·9 to 80·6)	84·2 (83·9 to 84·6)	86·2 (85·9 to 86·6)	87·1 (86·7 to 87·4)	87·8 (86·9 to 88·6)
Ōsaka	62·2 (59·2 to 65·4)	70·9 (68·3 to 73·5)	75·1 (72·8 to 77·2)	78·5 (78·3 to 78·7)	81·3 (81·1 to 81·5)	84·2 (84 to 84·5)	86 (85·8 to 86·2)	87·5 (86·6 to 88·5)
Saga	60·9 (57·8 to 64·1)	70·3 (67·5 to 72·9)	75·2 (73·1 to 77·2)	79·5 (79·1 to 79·9)	82·6 (82·2 to 83)	85·3 (84·9 to 85·7)	86·9 (86·5 to 87·4)	87·4 (86·5 to 88·3)
Saitama	62·1 (59·2 to 65·1)	71·1 (68·3 to 73·8)	75·5 (73·2 to 77·6)	79 (78·7 to 79·2)	82 (81·8 to 82·2)	84·5 (84·3 to 84·8)	86·1 (85·9 to 86·4)	87·4 (86·3 to 88·4)
Shiga	62·1 (58·8 to 65·1)	70·6 (68 to 73·1)	75 (73·1 to 77)	78·9 (78·6 to 79·3)	82·1 (81·8 to 82·4)	85·4 (85 to 85·7)	87 (86·6 to 87·3)	88·4 (87·6 to 89·3)
Shimane	61·7 (58·6 to 64·8)	70·6 (68·1 to 73·1)	75·3 (73·5 to 77·2)	79·9 (79·5 to 80·3)	83·6 (83·2 to 84)	86·3 (85·9 to 86·7)	87·1 (86·7 to 87·6)	88·1 (87·2 to 89)
Shizuoka	63·2 (60 to 66·3)	71·8 (69·1 to 74·5)	76·1 (73·8 to 78·3)	79·7 (79·5 to 80)	82·7 (82·4 to 83)	85·2 (84·9 to 85·5)	86·6 (86·3 to 86·9)	87·9 (86·9 to 89)
Tochigi	60·5 (57·4 to 63·7)	69·8 (67·2 to 72·6)	74·7 (72·7 to 76·8)	78·4 (78·1 to 78·7)	81·4 (81·1 to 81·8)	84·3 (84 to 84·6)	85·8 (85·5 to 86·2)	86·6 (85·7 to 87·5)
Tokushima	60·4 (57·2 to 63·5)	69·7 (67·2 to 72·4)	74·5 (72·5 to 76·4)	78·7 (78·3 to 79·1)	82·2 (81·8 to 82·5)	85 (84·6 to 85·4)	86·6 (86·2 to 87)	87·4 (86·5 to 88·4)
Tōkyō	64 (61 to 67)	72 (69·6 to 74·4)	76 (73·8 to 78·1)	79·5 (79·4 to 79·7)	82·2 (82·1 to 82·4)	84·5 (84·3 to 84·7)	86·6 (86·5 to 86·8)	88·1 (87·2 to 89)
Tottori	61·3 (58·2 to 64·3)	70·6 (68·1 to 73·2)	75·4 (73·6 to 77·3)	79·7 (79·3 to 80·1)	82·6 (82·2 to 83)	85·3 (84·9 to 85·7)	86·8 (86·3 to 87·2)	87·3 (86·4 to 88·1)
Toyama	61·4 (58·4 to 64·5)	70·6 (68 to 73·3)	75·3 (73·4 to 77·3)	79·6 (79·2 to 79·9)	83 (82·7 to 83·4)	85·7 (85·3 to 86·1)	87·2 (86·8 to 87·6)	88·6 (87·7 to 89·5)
Wakayama	60·3 (57·2 to 63·3)	69·7 (67·1 to 72·2)	74·4 (72·5 to 76·3)	78·6 (78·3 to 78·9)	81·9 (81·5 to 82·2)	84·5 (84·1 to 84·9)	86 (85·6 to 86·4)	86·8 (85·9 to 87·7)
Yamagata	61·5 (58·4 to 64·6)	70·4 (67·9 to 73)	74·6 (72·7 to 76·5)	79·2 (78·9 to 79·6)	82·8 (82·5 to 83·2)	85·1 (84·8 to 85·5)	86·8 (86·4 to 87·2)	87·7 (86·8 to 88·6)
Yamaguchi	61·2 (58·1 to 64·2)	70·6 (67·8 to 73·3)	75·5 (73·4 to 77·7)	79·3 (79 to 79·6)	82·6 (82·3 to 83)	84·8 (84·4 to 85·1)	86·2 (85·9 to 86·6)	87·4 (86·4 to 88·2)
Yamanashi	60·9 (57·7 to 63·9)	70·3 (67·7 to 73)	75·4 (73·4 to 77·3)	79·3 (78·9 to 79·7)	82·6 (82·2 to 82·9)	85 (84·6 to 85·4)	86·9 (86·5 to 87·3)	88 (87·1 to 88·9)
South Korea	47 (45·7 to 48·1)	63·3 (62·5 to 64·1)	67·7 (67 to 68·3)	71·2 (71 to 71·4)	76·1 (76 to 76·2)	79·9 (79·8 to 79·9)	84·1 (84 to 84·1)	85·6 (85·4 to 85·9)
Singapore	60·9 (60·5 to 61·2)	68·9 (68·7 to 69·2)	72·4 (72·3 to 72·6)	74·7 (74·5 to 74·9)	78·4 (78·2 to 78·5)	81·8 (81·6 to 81·9)	84·9 (84·8 to 85·1)	86·7 (86·5 to 86·9)
High-income North America	71·3 (71·2 to 71·3)	73·5 (73·5 to 73·6)	74·9 (74·9 to 74·9)	77·8 (77·8 to 77·8)	79·3 (79·2 to 79·3)	79·9 (79·8 to 79·9)	81·5 (81·5 to 81·5)	81·8 (81·7 to 81·9)
Canada	70·8 (70·7 to 70·9)	74·2 (74·1 to 74·3)	76·4 (76·3 to 76·4)	78·9 (78·8 to 79)	80·6 (80·6 to 80·7)	81·9 (81·9 to 82)	83·8 (83·7 to 83·8)	84·3 (84·1 to 84·4)
Greenland	53·5 (51 to 55·7)	62·5 (61·3 to 63·8)	66·4 (65·4 to 67·6)	67·5 (65·6 to 69·3)	67·9 (66·3 to 69·6)	68·4 (67·1 to 69·8)	74·1 (72·7 to 75·4)	76·3 (73·9 to 78·4)
USA	71·3 (71·3 to 71·4)	73·5 (73·4 to 73·5)	74·8 (74·7 to 74·8)	77·7 (77·7 to 77·7)	79·1 (79·1 to 79·2)	79·6 (79·6 to 79·7)	81·3 (81·2 to 81·3)	81·5 (81·4 to 81·6)
Alabama	68·6 (65·7 to 71·6)	71·8 (71·5 to 72·1)	73·6 (73·3 to 73·9)	76·9 (76·6 to 77·2)	77·9 (77·6 to 78·3)	77·9 (77·6 to 78·2)	78·1 (77·9 to 78·4)	78·3 (75·9 to 80·7)
Alaska	67·8 (65·8 to 69·7)	71·2 (70·5 to 71·8)	74·1 (73·6 to 74·6)	76·6 (76·1 to 77·1)	77·8 (77·4 to 78·2)	78·5 (78·1 to 78·9)	80·4 (80 to 80·8)	80·3 (78·8 to 81·7)
Arizona	70·6 (67·6 to 73·5)	73·9 (73·4 to 74·3)	75·2 (74·8 to 75·6)	78 (77·6 to 78·3)	80 (79·7 to 80·4)	80·2 (79·9 to 80·5)	82·1 (81·8 to 82·4)	82 (79·8 to 84·3)
Arkansas	71·2 (68·4 to 74·1)	73·9 (73·5 to 74·4)	75·3 (74·9 to 75·7)	78 (77·6 to 78·4)	78·4 (78 to 78·8)	78·1 (77·8 to 78·5)	79 (78·6 to 79·3)	78·6 (76·1 to 81)
California	72·3 (69·6 to 74·9)	74·6 (74·4 to 74·7)	75·5 (75·3 to 75·6)	78 (77·9 to 78·2)	79·5 (79·4 to 79·6)	80·8 (80·7 to 80·9)	83·1 (82·9 to 83·2)	83·8 (81·9 to 85·7)
Colorado	72·1 (69·4 to 74·9)	74·6 (74·2 to 75)	76·1 (75·8 to 76·6)	78·9 (78·6 to 79·2)	80·4 (80·1 to 80·8)	80·5 (80·2 to 80·8)	82·1 (81·8 to 82·4)	82·5 (80·4 to 84·6)
Connecticut	72·6 (69·9 to 75·4)	74·5 (74·1 to 74·8)	76·2 (75·9 to 76·5)	78·5 (78·2 to 78·9)	80·4 (80 to 80·7)	81 (80·7 to 81·4)	83·2 (82·8 to 83·5)	83·5 (81·3 to 85·6)
Delaware	70·2 (67·8 to 72·6)	72·9 (72·5 to 73·4)	73·9 (73·5 to 74·4)	76·8 (76·4 to 77·3)	78 (77·6 to 78·5)	78·9 (78·5 to 79·3)	80·5 (80·1 to 80·9)	81·3 (79·7 to 82·7)
Washington, DC	68·4 (65·3 to 71·4)	70·2 (69·8 to 70·7)	70·7 (70·3 to 71·2)	74·5 (74 to 75·1)	74·6 (74·1 to 75·1)	77·3 (76·8 to 77·9)	77·4 (76·9 to 77·9)	79·8 (77·5 to 81·8)
Florida	71·2 (68·3 to 74·2)	73·8 (73·6 to 74·1)	75·1 (74·8 to 75·3)	78 (77·8 to 78·2)	79·9 (79·8 to 80·1)	80·4 (80·3 to 80·6)	82·2 (82 to 82·4)	82·3 (80·1 to 84·5)

Georgia	68·9 (66 to 71·9)	71·5 (71·3 to 71·8)	73·1 (72·9 to 73·4)	76·5 (76·2 to 76·7)	77·7 (77·5 to 78)	78 (77·7 to 78·2)	79·6 (79·4 to 79·8)	80·1 (77·9 to 82·3)
Hawaii	71·2 (68·9 to 73·6)	73·4 (73 to 73·8)	76·7 (76·3 to 77)	80·8 (80·4 to 81·2)	81·8 (81·4 to 82·2)	83·1 (82·6 to 83·5)	84·6 (84·2 to 85)	84·8 (83·3 to 86·2)
Idaho	72·8 (70·2 to 75·4)	75·4 (74·9 to 75·9)	76·3 (75·8 to 76·8)	79·2 (78·7 to 79·7)	80·3 (79·8 to 80·8)	80·3 (79·9 to 80·8)	81·3 (80·9 to 81·7)	81·5 (79·7 to 83)
Illinois	71·2 (68·5 to 73·9)	73 (72·8 to 73·2)	74·1 (73·9 to 74·3)	77·3 (77·2 to 77·5)	78·7 (78·5 to 78·9)	79·5 (79·3 to 79·7)	81·6 (81·4 to 81·7)	82 (79·9 to 84·1)
Indiana	72 (69·3 to 74·7)	73·8 (73·5 to 74)	74·8 (74·5 to 75)	77·7 (77·5 to 78)	79 (78·7 to 79·2)	78·9 (78·6 to 79·1)	80 (79·8 to 80·3)	79·9 (77·6 to 82·2)
Iowa	74·2 (71·5 to 76·8)	75·8 (75·5 to 76·2)	76·5 (76·2 to 76·8)	79·9 (79·6 to 80·2)	81·2 (80·8 to 81·6)	81 (80·6 to 81·4)	82 (81·6 to 82·3)	82·2 (79·9 to 84·3)
Kansas	73·9 (71·1 to 76·7)	75·6 (75·2 to 76)	76·8 (76·4 to 77·2)	79·2 (78·8 to 79·6)	80·9 (80·5 to 81·3)	80·2 (79·8 to 80·6)	81·1 (80·8 to 81·5)	81·4 (79·2 to 83·6)
Kentucky	71·1 (68·2 to 73·8)	73·4 (73·1 to 73·7)	74·3 (74 to 74·6)	77·2 (76·9 to 77·6)	78·4 (78·1 to 78·7)	78·2 (77·9 to 78·5)	78·4 (78·1 to 78·7)	78 (75·7 to 80·3)
Louisiana	69·1 (66·2 to 72·1)	71·6 (71·3 to 71·9)	73·1 (72·8 to 73·4)	76·1 (75·8 to 76·4)	77·3 (77 to 77·6)	77·2 (77 to 77·5)	78·8 (78·5 to 79)	78·8 (76·4 to 81·2)
Maine	71·2 (68·6 to 73·8)	73·7 (73·3 to 74·2)	74·9 (74·4 to 75·3)	78·5 (78·1 to 79)	79·8 (79·4 to 80·2)	80 (79·6 to 80·4)	81·3 (80·9 to 81·7)	80·6 (78·7 to 82·3)
Maryland	70·2 (67·4 to 73)	72·1 (71·8 to 72·4)	74·2 (73·9 to 74·4)	77·1 (76·8 to 77·4)	78·4 (78·1 to 78·7)	78·8 (78·5 to 79·1)	81·3 (81·1 to 81·6)	81·4 (79·2 to 83·6)
Massachusetts	72·3 (69·6 to 75)	74·2 (74 to 74·5)	75·9 (75·6 to 76·1)	78·5 (78·2 to 78·7)	80·2 (79·9 to 80·4)	80·8 (80·5 to 81)	83 (82·7 to 83·3)	83 (80·9 to 85)
Michigan	71·7 (69 to 74·4)	73·6 (73·4 to 73·8)	74·6 (74·4 to 74·8)	77·5 (77·3 to 77·8)	78·6 (78·4 to 78·8)	79·1 (78·9 to 79·3)	80·4 (80·2 to 80·6)	80·6 (78·4 to 82·8)
Minnesota	73·8 (71·1 to 76·4)	75·3 (75 to 75·6)	76·9 (76·6 to 77·3)	80 (79·6 to 80·3)	81·1 (80·8 to 81·4)	81·3 (81 to 81·6)	83·1 (82·8 to 83·4)	83·4 (81·3 to 85·4)
Mississippi	68 (65·1 to 70·9)	71 (70·6 to 71·3)	72·9 (72·5 to 73·3)	76·3 (75·9 to 76·6)	77·5 (77·1 to 77·9)	76·8 (76·4 to 77·1)	77·8 (77·4 to 78·1)	78 (75·5 to 80·4)
Missouri	72·1 (69·3 to 74·9)	73·9 (73·7 to 74·2)	74·8 (74·5 to 75·1)	77·7 (77·4 to 77·9)	79 (78·7 to 79·3)	78·8 (78·6 to 79·1)	80·1 (79·8 to 80·3)	80·3 (78 to 82·6)
Montana	71·6 (69·2 to 74)	73·9 (73·4 to 74·4)	75·2 (74·7 to 75·6)	78 (77·5 to 78·4)	79·5 (79·1 to 80)	80 (79·6 to 80·5)	80·8 (80·4 to 81·3)	80·5 (78·9 to 82)
Nebraska	74·2 (71·7 to 76·6)	75·6 (75·2 to 76)	76·6 (76·3 to 77)	79·3 (78·9 to 79·7)	80·7 (80·3 to 81·1)	81·1 (80·7 to 81·4)	82·1 (81·7 to 82·4)	82·1 (80·4 to 83·8)
Nevada	67·6 (65·1 to 70·1)	71·8 (71·2 to 72·4)	73·4 (72·9 to 73·9)	75·6 (75·1 to 76)	77·3 (76·9 to 77·6)	77·7 (77·4 to 78·1)	79·7 (79·4 to 80)	80·3 (78·7 to 81·9)
New Hampshire	71·8 (69·2 to 74·2)	74·5 (74 to 75·1)	75·4 (75 to 75·9)	78·5 (78 to 78·9)	80·1 (79·6 to 80·5)	80·2 (79·8 to 80·7)	82·2 (81·8 to 82·6)	81·9 (80·1 to 83·6)
New Jersey	71·1 (68·3 to 73·7)	73·1 (72·9 to 73·3)	74·6 (74·4 to 74·8)	77·5 (77·2 to 77·7)	78·9 (78·7 to 79·1)	80 (79·8 to 80·3)	82·5 (82·3 to 82·7)	82·7 (80·6 to 84·8)
New Mexico	70·3 (67·5 to 73)	73 (72·6 to 73·4)	74·6 (74·2 to 75)	78·3 (77·9 to 78·8)	79·6 (79·2 to 80)	80·1 (79·7 to 80·4)	81 (80·6 to 81·4)	80·8 (78·9 to 82·6)
New York	71 (68·6 to 73·5)	73 (72·8 to 73·1)	74·2 (74·1 to 74·3)	77·2 (77 to 77·3)	78·7 (78·5 to 78·8)	80·5 (80·4 to 80·7)	82·8 (82·7 to 83)	83·4 (81·4 to 85·5)
North Carolina	70·1 (67·3 to 72·9)	72·3 (72·1 to 72·6)	74 (73·7 to 74·2)	77·5 (77·2 to 77·7)	78·5 (78·3 to 78·8)	78·5 (78·3 to 78·7)	80·3 (80·1 to 80·5)	80·4 (78·2 to 82·5)
North Dakota	73·1 (71 to 75·2)	75·4 (74·9 to 75·8)	77 (76·6 to 77·4)	79·6 (79·2 to 80)	81·3 (80·8 to 81·7)	81·5 (81·1 to 81·9)	82·6 (82·1 to 83·1)	82·2 (80·7 to 83·6)
Ohio	71·6 (69·1 to 74·2)	73·6 (73·4 to 73·7)	74·6 (74·4 to 74·8)	77·1 (76·9 to 77·3)	78·7 (78·5 to 78·9)	79 (78·8 to 79·2)	80·1 (79·9 to 80·3)	79·8 (77·5 to 82)
Oklahoma	72·7 (69·8 to 75·4)	74·8 (74·4 to 75·1)	75·6 (75·3 to 76)	77·9 (77·5 to 78·2)	78·7 (78·3 to 79)	77·9 (77·6 to 78·2)	78·5 (78·1 to 78·8)	78·4 (75·9 to 80·7)
Oregon	73·1 (70·5 to 75·8)	75 (74·6 to 75·4)	76·4 (76 to 76·8)	78·8 (78·5 to 79·2)	79·8 (79·5 to 80·2)	80·2 (79·8 to 80·5)	81·5 (81·2 to 81·9)	82 (79·8 to 84·1)
Pennsylvania	70·8 (68·3 to 73·4)	72·9 (72·7 to 73)	74·2 (74 to 74·3)	77·2 (77·1 to 77·4)	78·9 (78·7 to 79)	79·6 (79·4 to 79·8)	81·1 (81 to 81·3)	81·2 (79 to 83·3)
Rhode Island	71·9 (69·5 to 74·5)	74 (73·6 to 74·5)	75·3 (74·9 to 75·8)	78·6 (78·2 to 79)	79·9 (79·4 to 80·3)	80·6 (80·2 to 81·1)	82·2 (81·8 to 82·7)	81·8 (80 to 83·5)
South Carolina	67·1 (64·2 to 70·1)	70·3 (70 to 70·7)	72·8 (72·5 to 73·1)	76·2 (75·9 to 76·5)	77·6 (77·3 to 77·9)	78 (77·7 to 78·3)	79·4 (79·1 to 79·7)	79·7 (77·3 to 81·9)
South Dakota	73 (70·6 to 75·4)	74·9 (74·4 to 75·3)	76·1 (75·7 to 76·6)	79·2 (78·7 to 79·7)	80·9 (80·4 to 81·4)	81·1 (80·6 to 81·6)	82 (81·5 to 82·4)	81·6 (80 to 83·1)
Tennessee	70·6 (67·8 to 73·4)	73·1 (72·8 to 73·3)	74·3 (74 to 74·6)	77·5 (77·2 to 77·7)	78·3 (78 to 78·6)	78 (77·8 to 78·3)	78·9 (78·6 to 79·1)	78·8 (76·6 to 81·1)
Texas	71·8 (69·2 to 74·8)	74·2 (74 to 74·4)	75·1 (75 to 75·3)	77·7 (77·5 to 77·9)	79·2 (79·1 to 79·4)	79·3 (79·1 to 79·4)	80·7 (80·6 to 80·8)	81·3 (79·2 to 83·4)
Utah	73 (70·5 to 75·4)	75·4 (75 to 75·9)	76·7 (76·3 to 77·1)	79 (78·6 to 79·4)	80·7 (80·3 to 81·1)	80·7 (80·3 to 81)	81·8 (81·5 to 82·1)	81·8 (80·1 to 83·3)
Vermont	71·6 (69·5 to 73·8)	74·2 (73·7 to 74·7)	75·8 (75·4 to 76·2)	78·5 (78·1 to 78·9)	79·8 (79·4 to 80·3)	80·4 (80 to 80·8)	82·9 (82·4 to 83·3)	82·4 (81 to 83·6)
Virginia	70·2 (67·4 to 72·9)	72·5 (72·2 to 72·8)	74·5 (74·2 to 74·7)	77·4 (77·1 to 77·7)	78·9 (78·6 to 79·1)	79·2 (79 to 79·5)	81·3 (81 to 81·5)	81·6 (79·6 to 83·7)

Washington	73 (70·2 to 75·6)	75·1 (74·7 to 75·4)	75·8 (75·5 to 76·1)	78·6 (78·3 to 78·9)	80·1 (79·8 to 80·4)	80·6 (80·3 to 80·9)	82·1 (81·9 to 82·4)	82·6 (80·6 to 84·7)
West Virginia	71·2 (68·3 to 74)	73·9 (73·5 to 74·3)	73·4 (73 to 73·7)	76·9 (76·5 to 77·3)	78·6 (78·1 to 79)	77·8 (77·4 to 78·3)	78·5 (78 to 78·9)	77·7 (75·2 to 80·1)
Wisconsin	72·9 (70·3 to 75·5)	74·5 (74·3 to 74·8)	76·2 (75·9 to 76·5)	79·1 (78·8 to 79·3)	80·1 (79·8 to 80·4)	80·6 (80·3 to 80·9)	82 (81·7 to 82·2)	81·8 (79·7 to 83·8)
Wyoming	71·5 (69·2 to 73·6)	74·1 (73·5 to 74·7)	75·6 (75·1 to 76·1)	78·1 (77·6 to 78·5)	79·4 (78·9 to 80)	79·8 (79·3 to 80·2)	80·5 (80 to 80·9)	80·9 (79·5 to 82·2)
Southern Latin America	62·5 (62·4 to 62·6)	65·4 (65·3 to 65·5)	68·9 (68·9 to 69)	73·6 (73·6 to 73·7)	76·3 (76·3 to 76·4)	78·5 (78·4 to 78·5)	79·8 (79·7 to 79·8)	80·4 (80·2 to 80·7)
Argentina	66·1 (66 to 66·3)	68·7 (68·5 to 68·7)	70·1 (70 to 70·2)	73·9 (73·8 to 73·9)	76·1 (76 to 76·1)	77·9 (77·9 to 78)	79·2 (79·1 to 79·2)	79·6 (79·4 to 79·8)
Chile	52·6 (52·5 to 52·7)	57·1 (56·8 to 57·3)	64·9 (64·8 to 65·1)	72·4 (72·3 to 72·5)	76·6 (76·5 to 76·7)	79·8 (79·7 to 79·9)	81·3 (81·2 to 81·4)	82·6 (82·2 to 82·9)
Uruguay	67·8 (67·5 to 68·1)	71·5 (71·3 to 71·7)	72·1 (71·9 to 72·3)	74·3 (74·1 to 74·5)	77 (76·8 to 77·1)	78·6 (78·5 to 78·8)	80·1 (79·9 to 80·4)	80·8 (80·5 to 81·1)
Western Europe	68·9 (68·8 to 69)	72·8 (72·8 to 72·9)	74·7 (74·7 to 74·7)	77·4 (77·3 to 77·4)	79·6 (79·6 to 79·7)	81·6 (81·6 to 81·7)	83·7 (83·7 to 83·7)	84·4 (84·3 to 84·5)
Andorra	78·7 (76 to 81·4)	79·4 (77·2 to 81·8)	80·2 (78·3 to 82·4)	81·6 (79·1 to 83·6)	82·7 (79·9 to 84·8)	84·5 (81·8 to 86·7)	85·2 (82·8 to 87·1)	84·9 (82·6 to 87·4)
Austria	67·3 (67·2 to 67·5)	72·1 (71·9 to 72·2)	73·6 (73·5 to 73·7)	76·2 (76·1 to 76·4)	79·1 (79 to 79·2)	81·3 (81·2 to 81·5)	83·4 (83·3 to 83·5)	84·5 (84·3 to 84·6)
Belgium	69·2 (69 to 69·3)	73·1 (72·9 to 73·2)	74·3 (74·2 to 74·4)	76·8 (76·7 to 76·9)	79·4 (79·3 to 79·5)	81·1 (81 to 81·3)	82·8 (82·7 to 82·9)	83·8 (83·6 to 84)
Cyprus	65·3 (62 to 68·3)	67·8 (65·1 to 70·2)	70·8 (69·4 to 72·1)	73·7 (73·4 to 74)	76·7 (76·5 to 76·9)	78·9 (78·6 to 79·1)	81·8 (81·5 to 82·1)	82·8 (82·1 to 83·4)
Denmark	71·9 (71·7 to 72·1)	74·4 (74·2 to 74·5)	76·1 (76 to 76·3)	77·5 (77·3 to 77·6)	78 (77·9 to 78·2)	79·4 (79·3 to 79·6)	81·6 (81·4 to 81·7)	83·1 (82·8 to 83·4)
Finland	68·1 (67·9 to 68·3)	72·6 (72·4 to 72·7)	74·9 (74·7 to 75·1)	78·3 (78·2 to 78·5)	79·4 (79·3 to 79·6)	81·4 (81·3 to 81·6)	83·6 (83·5 to 83·8)	84·7 (84·4 to 85)
France	69·8 (69·7 to 69·9)	74·4 (74·3 to 74·4)	76·3 (76·2 to 76·3)	78·8 (78·7 to 78·8)	81·3 (81·3 to 81·4)	82·9 (82·8 to 82·9)	84·8 (84·7 to 84·9)	85·7 (85·6 to 85·9)
Germany	69·4 (69·2 to 69·7)	72·1 (72 to 72·1)	73·5 (73·5 to 73·6)	76·3 (76·3 to 76·4)	78·7 (78·7 to 78·8)	81·2 (81·2 to 81·3)	83 (82·9 to 83)	83·5 (83·3 to 83·6)
Greece	70·6 (70·3 to 70·8)	74·1 (74 to 74·3)	76 (75·8 to 76·1)	77·7 (77·6 to 77·9)	79·6 (79·5 to 79·7)	81 (80·9 to 81·1)	82·9 (82·8 to 83)	83·3 (83·1 to 83·5)
Iceland	73·9 (73·2 to 74·5)	75·5 (75·1 to 76)	76·6 (76·1 to 77·1)	79·5 (79·1 to 80)	80·5 (80 to 81)	82·2 (81·7 to 82·8)	85·4 (84·8 to 86)	86·9 (86·1 to 87·6)
Ireland	67·1 (66·9 to 67·3)	71·9 (71·7 to 72)	73·7 (73·5 to 73·9)	75·6 (75·4 to 75·8)	77·8 (77·6 to 77·9)	79·5 (79·3 to 79·6)	82·9 (82·8 to 83·1)	83·9 (83·6 to 84·2)
Israel	73·7 (73·5 to 73·8)	72·9 (72·7 to 73·1)	73·5 (73·4 to 73·7)	75·9 (75·7 to 76)	78·9 (78·8 to 79·1)	80·8 (80·6 to 80·9)	83·7 (83·5 to 83·8)	84·6 (84·4 to 84·8)
Italy	67·6 (67·5 to 67·7)	72·3 (72·2 to 72·4)	74·7 (74·7 to 74·8)	77·6 (77·5 to 77·6)	80·4 (80·4 to 80·5)	82·5 (82·5 to 82·6)	84·5 (84·4 to 84·5)	85·3 (85·2 to 85·4)
Luxembourg	68·8 (68·3 to 69·3)	71·8 (71·4 to 72·2)	73·9 (73·5 to 74·3)	76 (75·7 to 76·4)	78·8 (78·4 to 79·1)	80·8 (80·4 to 81·2)	83·1 (82·6 to 83·6)	85 (84 to 85·9)
Malta	64·8 (64·3 to 65·2)	71 (70·6 to 71·4)	73·2 (72·8 to 73·5)	74·9 (74·6 to 75·3)	79 (78·7 to 79·4)	81 (80·5 to 81·4)	83·8 (83·3 to 84·3)	84·9 (84 to 85·9)
Monaco	69·8 (67·9 to 71·5)	73·2 (71·8 to 74·6)	76 (74·4 to 77·6)	79·1 (77·9 to 80·6)	81·2 (79·3 to 83·4)	81·5 (79·5 to 84·3)	81·4 (80·1 to 83·3)	82·3 (80·5 to 84·6)
Netherlands	72·7 (72·6 to 72·9)	75·5 (75·4 to 75·6)	76·6 (76·5 to 76·7)	79·3 (79·2 to 79·4)	80·3 (80·2 to 80·4)	80·7 (80·6 to 80·8)	82·9 (82·8 to 83)	83·4 (83·2 to 83·6)
Norway	73·5 (73·3 to 73·7)	75·7 (75·6 to 75·9)	77·4 (77·3 to 77·6)	79·5 (79·3 to 79·6)	80·2 (80 to 80·3)	81·6 (81·5 to 81·8)	83·4 (83·3 to 83·6)	84·7 (84·4 to 84·9)
Portugal	61·8 (61·5 to 62)	67·4 (67·2 to 67·6)	70·5 (70·3 to 70·6)	75·1 (75 to 75·2)	77·8 (77·7 to 77·9)	80·3 (80·2 to 80·4)	83·3 (83·2 to 83·4)	84·5 (84·3 to 84·7)
San Marino	75·7 (72·1 to 79·4)	76·5 (74·6 to 78·7)	77·8 (76·7 to 79·1)	79·8 (78·4 to 81·3)	82·5 (81·3 to 83·8)	84·4 (82·6 to 86)	83·8 (81·1 to 86·8)	84·3 (80·7 to 87·6)
Spain	64·2 (64·1 to 64·4)	71·8 (71·7 to 71·9)	74·7 (74·6 to 74·8)	78·5 (78·5 to 78·6)	80·5 (80·5 to 80·6)	82·9 (82·9 to 83)	85·1 (85 to 85·1)	85·7 (85·6 to 85·9)
Sweden	72·7 (72·6 to 72·8)	75·3 (75·2 to 75·4)	77·5 (77·3 to 77·6)	79·2 (79·1 to 79·4)	80·8 (80·7 to 80·9)	82·3 (82·2 to 82·4)	83·8 (83·7 to 83·9)	84·6 (84·4 to 84·7)
Stockholm	72·4 (69·6 to 75·6)	74·9 (72·3 to 77·7)	76·9 (74·3 to 79·5)	78·8 (78·4 to 79·2)	80·3 (79·9 to 80·7)	82·2 (81·8 to 82·6)	83·8 (83·5 to 84·2)	84·8 (83·7 to 85·9)
Sweden except Stockholm	72·7 (72·1 to 73·3)	75·3 (74·8 to 75·9)	77·5 (77 to 78·1)	79·3 (79·2 to 79·5)	80·9 (80·8 to 81·1)	82·3 (82·2 to 82·5)	83·8 (83·6 to 83·9)	84·5 (84·2 to 84·9)
Switzerland	71·2 (71·1 to 71·4)	74·4 (74·3 to 74·5)	76·4 (76·2 to 76·5)	79·2 (79·1 to 79·4)	81·3 (81·2 to 81·5)	83 (82·9 to 83·2)	84·9 (84·8 to 85)	85·8 (85·6 to 86)
UK	71·2 (71·1 to 71·2)	74 (73·9 to 74)	75·1 (75·1 to 75·2)	76·7 (76·7 to 76·8)	78·5 (78·5 to 78·6)	80·2 (80·2 to 80·3)	82·5 (82·5 to 82·6)	82·9 (82·8 to 83)
England	71·7 (71·6 to 71·7)	74·4 (74·3 to 74·5)	75·4 (75·4 to 75·5)	77 (76·9 to 77·1)	78·8 (78·7 to 78·8)	80·4 (80·4 to 80·5)	82·8 (82·7 to 82·8)	83·1 (83 to 83·3)
East Midlands	71·6 (69 to 74)	74·3 (71·9 to 76·7)	75·3 (72·9 to 77·6)	76·9 (76·2 to 77·6)	78·9 (78·6 to 79·2)	80·4 (80·2 to 80·7)	82·7 (82·4 to 82·9)	82·8 (81·8 to 83·7)

Derby	70·4 (67 to 74)	73·2 (70·3 to 76·6)	74·2 (70·9 to 77·3)	75·9 (74·7 to 77·1)	78 (77·4 to 78·6)	80·3 (79·7 to 80·8)	81·8 (81·2 to 82·4)	82·5 (81·2 to 84)
Derbyshire	71·5 (68·3 to 75)	74·3 (71 to 77·7)	75·2 (72·2 to 78·2)	76·7 (75·8 to 77·7)	78·8 (78·3 to 79·2)	80·4 (80 to 80·9)	82·9 (82·4 to 83·3)	83 (81·7 to 84·2)
Leicester	69·9 (66·3 to 73·4)	72·6 (69·4 to 76)	73·7 (70·5 to 77)	75·4 (74·2 to 76·5)	78·1 (77·5 to 78·7)	79 (78·4 to 79·6)	81·5 (81 to 82·1)	82·5 (81·1 to 83·9)
Leicestershire	72·8 (69·5 to 76·4)	75·5 (72·4 to 78·6)	76·4 (73·4 to 79·4)	78 (77 to 79)	79·9 (79·5 to 80·4)	81·4 (81 to 81·9)	83·8 (83·4 to 84·3)	83·9 (82·7 to 85·2)
Lincolnshire	71·8 (68·4 to 75·6)	74·5 (71·4 to 77·8)	75·4 (72·2 to 78·5)	77·1 (76·1 to 78·1)	79 (78·5 to 79·5)	80·5 (80 to 81)	82·9 (82·4 to 83·3)	82·9 (81·6 to 84·1)
Northamptonshire	71·8 (68·4 to 75·6)	74·6 (71·5 to 77·7)	75·6 (72·6 to 78·6)	77·3 (76·3 to 78·3)	79 (78·5 to 79·4)	80·9 (80·4 to 81·3)	82·7 (82·2 to 83·1)	82·3 (81 to 83·5)
Nottingham	70·1 (66·6 to 73·7)	72·9 (69·8 to 76·1)	74 (70·9 to 77)	75·6 (74·5 to 76·8)	77·6 (77·1 to 78·2)	77·8 (77·3 to 78·4)	80·8 (80·2 to 81·4)	82 (80·5 to 83·4)
Nottinghamshire	71·8 (68·4 to 75·2)	74·5 (71·4 to 77·9)	75·5 (72·7 to 78·4)	77 (76 to 77·9)	79 (78·6 to 79·4)	80·5 (80 to 80·9)	82·5 (82·1 to 83)	82·3 (81 to 83·5)
Rutland	73·5 (69·8 to 77)	76·1 (73 to 79·6)	77 (73·8 to 80·2)	78·2 (76·5 to 79·9)	79·8 (78·6 to 80·8)	82·2 (81·2 to 83·3)	84·4 (83·3 to 85·6)	84·8 (83 to 86·7)
East of England	72·9 (70·4 to 75·4)	75·5 (73·3 to 77·9)	76·5 (74·1 to 78·8)	77·8 (77·1 to 78·6)	79·6 (79·3 to 79·8)	81·3 (81 to 81·5)	83·4 (83·2 to 83·7)	83·7 (82·7 to 84·7)
Bedford	72·5 (69·1 to 76)	75·3 (72 to 78·7)	76·5 (73·5 to 79·6)	78·1 (76·9 to 79·4)	79·1 (78·5 to 79·8)	80·8 (80·2 to 81·5)	82·6 (81·9 to 83·3)	82·7 (81·3 to 84·3)
Cambridgeshire	73·9 (70·4 to 77·2)	76·5 (73·3 to 79·7)	77·6 (74·7 to 80·7)	78·8 (77·8 to 79·8)	80·1 (79·6 to 80·6)	81·4 (80·9 to 81·8)	84·3 (83·8 to 84·8)	84·4 (83·1 to 85·8)
Central Bedfordshire	72·3 (68·9 to 76)	75 (71·7 to 78·3)	76·1 (72·8 to 79·2)	77·5 (76·3 to 78·7)	79 (78·3 to 79·5)	81·3 (80·7 to 81·9)	83·5 (82·9 to 84·1)	84·3 (82·8 to 85·8)
Essex	72·9 (69·7 to 76)	75·4 (72·5 to 78·5)	76·2 (73·3 to 79)	77·5 (76·7 to 78·4)	79·6 (79·2 to 79·9)	81·5 (81·1 to 81·8)	83·3 (83 to 83·7)	83·2 (82·1 to 84·3)
Hertfordshire	73·4 (70·3 to 76·6)	75·9 (72·8 to 79·2)	76·7 (73·7 to 79·7)	77·7 (76·8 to 78·6)	79·7 (79·2 to 80)	81·2 (80·7 to 81·6)	83·6 (83·2 to 84)	84·1 (83 to 85·3)
Luton	71·2 (67·8 to 74·5)	73·8 (70·6 to 77·3)	74·7 (71·6 to 77·9)	76·2 (75·1 to 77·4)	78·4 (77·7 to 79)	79·9 (79·2 to 80·5)	81·9 (81·2 to 82·5)	82·2 (80·8 to 83·7)
Norfolk	72·6 (69·1 to 75·9)	75·5 (72·4 to 78·7)	76·8 (74 to 79·9)	78·5 (77·5 to 79·4)	79·8 (79·3 to 80·2)	81·5 (81·1 to 81·9)	83·4 (83 to 83·9)	84 (82·7 to 85·3)
Peterborough	72·2 (68·7 to 75·8)	74·8 (71·7 to 78·3)	75·8 (72·8 to 79)	77·2 (76 to 78·5)	78·5 (77·8 to 79·1)	80 (79·4 to 80·7)	82 (81·3 to 82·7)	82·7 (81·2 to 84·3)
Southend-on-Sea	71·2 (67·8 to 74·9)	74·1 (70·7 to 77·5)	75·3 (72·3 to 78·4)	77 (75·9 to 78·3)	78·7 (78·1 to 79·4)	80·4 (79·7 to 81·1)	82·4 (81·7 to 83·1)	82·4 (80·8 to 83·9)
Suffolk	73 (69·5 to 76·3)	75·6 (72·6 to 78·8)	76·7 (73·7 to 79·6)	78·1 (77·2 to 79·2)	80 (79·6 to 80·5)	81·6 (81·1 to 82)	83·7 (83·3 to 84·2)	83·9 (82·7 to 85·2)
Thurrock	71·7 (68·1 to 75·2)	74·3 (71·1 to 77·8)	75·3 (72·4 to 78·4)	76·7 (75·5 to 77·9)	78·6 (77·9 to 79·3)	80·5 (79·8 to 81·1)	82·6 (81·9 to 83·2)	83·2 (81·7 to 84·7)
Greater London	71·9 (69·7 to 74·2)	74·6 (72·4 to 76·7)	75·6 (73·4 to 77·7)	77·2 (76·6 to 77·9)	78·9 (78·7 to 79·1)	80·3 (80·1 to 80·5)	83·6 (83·4 to 83·8)	84·1 (83·1 to 85·2)
Barking and Dagenham	70·2 (66·8 to 73·7)	73 (69·8 to 76·3)	74·3 (71·6 to 77·1)	76·4 (75·2 to 77·5)	78·1 (77·5 to 78·8)	79·4 (78·8 to 80)	81·8 (81·2 to 82·4)	81·9 (80·4 to 83·4)
Barnet	72·9 (69·6 to 76·3)	75·7 (72·6 to 78·7)	76·9 (74·1 to 79·8)	78·6 (77·6 to 79·7)	80·1 (79·6 to 80·6)	81·7 (81·2 to 82·2)	83·9 (83·4 to 84·5)	85·4 (84 to 86·7)
Bexley	73·3 (69·8 to 76·6)	75·9 (72·9 to 78·9)	77 (74·1 to 79·9)	78·8 (77·7 to 79·9)	80·2 (79·6 to 80·7)	81·7 (81·1 to 82·2)	84·6 (83·9 to 85·2)	84·1 (82·7 to 85·5)
Brent	71·6 (68·5 to 74·7)	74·4 (71·4 to 77·6)	75·4 (72·4 to 78·2)	77·1 (76·1 to 78·2)	78·8 (78·2 to 79·3)	80·4 (79·8 to 80·9)	85 (84·4 to 85·6)	84·9 (83·5 to 86·3)
Bromley	73·5 (70·5 to 76·8)	76 (73·1 to 79·2)	77 (74·4 to 79·8)	78·7 (77·7 to 79·8)	80·5 (79·9 to 81)	81·6 (81·1 to 82·2)	84·5 (83·9 to 85·1)	85·3 (83·8 to 86·7)
Camden	71·5 (68·1 to 74·7)	74·2 (71·3 to 77·4)	75 (71·9 to 77·9)	76·1 (74·9 to 77·3)	77·8 (77·2 to 78·4)	79·5 (78·9 to 80)	82·8 (82·1 to 83·4)	83·3 (81·8 to 84·7)
Croydon	72·3 (68·9 to 75·7)	74·9 (72 to 78·3)	75·8 (72·9 to 78·7)	77·3 (76·3 to 78·4)	78·8 (78·3 to 79·3)	80·6 (80·1 to 81·1)	83·6 (83 to 84·1)	83·1 (81·6 to 84·5)
Ealing	71·9 (68·7 to 75·2)	74·6 (71·5 to 77·8)	75·6 (72·5 to 78·6)	77·2 (76·1 to 78·2)	78·9 (78·4 to 79·4)	80·7 (80·1 to 81·2)	84·4 (83·8 to 84·9)	84·9 (83·5 to 86·5)
Enfield	72·6 (69·5 to 76)	75·3 (72·3 to 78·5)	76·5 (73·9 to 79·2)	78·4 (77·3 to 79·5)	79·9 (79·4 to 80·5)	80·5 (80 to 81·1)	84·1 (83·5 to 84·6)	84·9 (83·5 to 86·2)
Greenwich	70·7 (67·3 to 74·1)	73·7 (70·7 to 76·7)	75 (72 to 77·9)	76·8 (75·7 to 78)	78·3 (77·8 to 78·9)	79 (78·4 to 79·5)	82·7 (82·1 to 83·3)	83·1 (81·7 to 84·6)
Hackney	70·8 (67·4 to 74·2)	73·4 (70·1 to 76·9)	74·1 (71·1 to 77·1)	75·3 (74·1 to 76·4)	76·7 (76·1 to 77·3)	78·5 (78 to 79)	81·7 (81·1 to 82·2)	82·4 (80·9 to 83·9)
Hammersmith and Fulham	71·1 (67·8 to 74·4)	73·8 (70·7 to 76·9)	74·7 (71·4 to 77·8)	75·8 (74·6 to 77·1)	77·3 (76·7 to 77·9)	79·2 (78·6 to 79·7)	83·2 (82·6 to 83·8)	84·3 (82·8 to 85·7)
Haringey	71·2 (68·1 to 74·4)	74·1 (71·2 to 77·2)	75·2 (72·3 to 78·1)	76·7 (75·6 to 77·9)	78·5 (77·9 to 79)	79·7 (79·1 to 80·3)	82·9 (82·3 to 83·4)	83·8 (82·3 to 85·2)
Harrow	73·2 (69·8 to 76·5)	75·6 (72·7 to 78·7)	76·5 (73·6 to 79·4)	78·1 (77 to 79·2)	80·6 (80 to 81·2)	81·9 (81·3 to 82·5)	85·2 (84·6 to 85·9)	85·3 (83·9 to 86·7)
Havering	73·2 (69·9 to 76·3)	75·6 (72·6 to 78·6)	76·5 (73·6 to 79·4)	78·2 (77·2 to 79·3)	80 (79·4 to 80·5)	81·5 (80·9 to 82·1)	83·8 (83·2 to 84·4)	84·7 (83·1 to 86·1)

Hillingdon	72·9 (69·6 to 76·2)	75·5 (72·5 to 78·6)	76·4 (73·5 to 79·3)	78·1 (77·1 to 79·2)	79·3 (78·7 to 79·9)	80·5 (79·9 to 81·1)	83·3 (82·7 to 83·9)	83·6 (82·3 to 85)
Hounslow	71·9 (68·5 to 75·2)	74·6 (71·5 to 77·7)	75·6 (72·5 to 78·7)	77·1 (76 to 78·2)	78·7 (78·2 to 79·3)	80·1 (79·6 to 80·7)	83·4 (82·8 to 84)	84·7 (83·4 to 86·1)
Islington	70·7 (67·4 to 74·1)	73·5 (70·5 to 76·8)	74·3 (71·3 to 77·2)	75·6 (74·5 to 76·8)	77·6 (77 to 78·2)	78·4 (77·9 to 79)	82·3 (81·7 to 82·9)	83 (81·6 to 84·4)
Kensington and Chelsea	71·5 (68 to 74·9)	74·2 (71·1 to 77·4)	75·1 (72·1 to 78·2)	76·2 (75·1 to 77·3)	78·9 (78·3 to 79·5)	82·3 (81·6 to 82·9)	85·1 (84·4 to 85·8)	84·7 (83·2 to 86·2)
Kingston upon Thames	73·6 (70·3 to 76·9)	76·2 (73·3 to 79·5)	77·4 (74·4 to 80·4)	79·1 (77·8 to 80·2)	80·3 (79·6 to 81)	81·6 (80·9 to 82·2)	85·3 (84·6 to 86)	85·1 (83·6 to 86·6)
Lambeth	70·1 (66·7 to 73·4)	73 (69·9 to 76·3)	74 (71·2 to 77)	75·6 (74·6 to 76·8)	77·6 (77 to 78·1)	78·4 (77·9 to 78·9)	82·2 (81·7 to 82·8)	83·7 (82·3 to 85)
Lewisham	71 (67·7 to 74·5)	74 (70·9 to 77·2)	75·1 (72·3 to 78)	76·9 (75·9 to 78)	78 (77·5 to 78·6)	78·8 (78·3 to 79·3)	82·1 (81·5 to 82·6)	83·3 (81·9 to 84·7)
Merton	72·2 (69 to 75·5)	74·8 (71·7 to 77·9)	75·9 (73·1 to 78·9)	77·6 (76·4 to 78·8)	79·3 (78·7 to 79·9)	81 (80·4 to 81·6)	84·4 (83·7 to 85)	84·6 (83·2 to 86·1)
Newham	70·3 (67 to 73·7)	73·1 (69·9 to 76·4)	74·1 (71·2 to 77·1)	75·9 (74·7 to 76·9)	77·8 (77·2 to 78·4)	78·3 (77·8 to 78·9)	82·3 (81·7 to 82·8)	83 (81·6 to 84·5)
Redbridge	72·7 (69·4 to 75·9)	75·3 (72·1 to 78·4)	76·4 (73·5 to 79·4)	78·4 (77·2 to 79·5)	80·2 (79·7 to 80·8)	81·6 (81 to 82·2)	84·5 (83·9 to 85·1)	85 (83·7 to 86·4)
Richmond upon Thames	73·3 (70·1 to 76·4)	75·8 (72·8 to 78·8)	76·7 (74 to 79·6)	78·2 (77 to 79·4)	80·3 (79·6 to 80·9)	81·7 (81·1 to 82·4)	85·4 (84·7 to 86)	85·4 (84 to 86·8)
Southwark	71·3 (68·2 to 74·6)	74 (70·8 to 77·2)	74·9 (72 to 78)	76·3 (75·2 to 77·4)	76·6 (76·1 to 77·2)	78·8 (78·2 to 79·3)	82·1 (81·5 to 82·7)	83·6 (82·3 to 85)
Sutton	72·5 (69·2 to 75·7)	75·3 (72·3 to 78·4)	76·5 (73·7 to 79·3)	78·3 (77·2 to 79·5)	79·5 (78·8 to 80)	80·8 (80·2 to 81·4)	84·5 (83·8 to 85·1)	84·1 (82·6 to 85·6)
Tower Hamlets	70·2 (67 to 73·7)	72·9 (69·8 to 76·2)	73·7 (70·6 to 76·7)	75·3 (74·1 to 76·6)	77·3 (76·7 to 77·9)	78·4 (77·8 to 78·9)	82·3 (81·7 to 82·9)	82·4 (81 to 83·8)
Waltham Forest	70·8 (67·6 to 74·2)	73·8 (70·8 to 77)	75·1 (72·4 to 77·9)	77 (75·9 to 78·2)	78·5 (78 to 79·1)	79·4 (78·8 to 80)	83·1 (82·5 to 83·7)	83·7 (82·3 to 85·1)
Wandsworth	70·9 (67·8 to 74·2)	73·7 (70·6 to 76·8)	74·6 (71·8 to 77·4)	76·2 (75·3 to 77·3)	77·9 (77·4 to 78·4)	79·5 (79 to 80·1)	83·6 (83·1 to 84·2)	83·6 (82·2 to 84·9)
Westminster	71·5 (68·3 to 74·7)	74·2 (71·3 to 77·3)	75·2 (72·3 to 78)	76·2 (75·1 to 77·4)	78·2 (77·6 to 78·8)	80·8 (80·2 to 81·4)	84·4 (83·8 to 85)	84·8 (83·4 to 86·2)
North East England	70·3 (67·8 to 72·9)	73·1 (70·4 to 75·6)	74 (71·6 to 76·4)	75·7 (75 to 76·5)	77·3 (76·9 to 77·6)	79·1 (78·8 to 79·4)	81·4 (81·1 to 81·7)	81·6 (80·5 to 82·7)
County Durham	70·5 (67·3 to 74·1)	73·3 (69·9 to 76·4)	74·2 (71·2 to 77)	75·7 (74·7 to 76·6)	77·1 (76·7 to 77·6)	78·8 (78·3 to 79·3)	81·2 (80·8 to 81·7)	81·2 (79·9 to 82·5)
Darlington	70 (66·4 to 73·8)	72·8 (69·2 to 76·6)	73·8 (70·4 to 76·9)	75·5 (74·3 to 76·9)	77·2 (76·4 to 77·9)	79 (78·2 to 79·8)	81·5 (80·8 to 82·2)	82·1 (80·5 to 83·7)
Gateshead	70 (66·2 to 73·6)	72·7 (69·6 to 76·1)	73·7 (70·7 to 77)	75·5 (74·4 to 76·6)	77·6 (76·9 to 78·2)	79·1 (78·5 to 79·7)	81·5 (80·8 to 82·1)	81·5 (79·9 to 83)
Hartlepool	68·7 (65 to 72·4)	71·7 (68 to 75·5)	72·7 (69·6 to 75·8)	74·5 (73·3 to 75·8)	76·6 (75·9 to 77·3)	78·3 (77·5 to 79·1)	80·5 (79·7 to 81·3)	80·7 (79 to 82·4)
Middlesbrough	68·6 (65 to 71·9)	71·4 (68·2 to 74·8)	72·3 (69·1 to 75·4)	74 (72·9 to 75·2)	75·8 (75·2 to 76·5)	77·6 (77 to 78·3)	80·1 (79·4 to 80·8)	80·1 (78·6 to 81·9)
Newcastle upon Tyne	70·7 (67·3 to 74·1)	73·3 (70 to 76·7)	74·3 (71·1 to 77·4)	76 (74·9 to 77·1)	76·8 (76·2 to 77·4)	78·9 (78·3 to 79·5)	81·2 (80·6 to 81·8)	81·5 (80 to 83)
North Tyneside	70·7 (67·2 to 74·4)	73·4 (70·3 to 76·7)	74·3 (71·2 to 77·5)	76·1 (74·9 to 77·3)	77·4 (76·8 to 78)	79·4 (78·8 to 80)	81·8 (81·2 to 82·4)	82·6 (81·2 to 84)
Northumberland	70·8 (67·3 to 74·5)	73·5 (70·4 to 76·7)	74·5 (71·3 to 77·7)	76·1 (75 to 77·1)	78 (77·5 to 78·5)	80·2 (79·7 to 80·8)	82·7 (82·1 to 83·3)	83·2 (81·8 to 84·6)
Redcar and Cleveland	70·1 (66·3 to 73·6)	72·9 (69·7 to 76·2)	73·9 (70·6 to 76·9)	75·7 (74·5 to 77)	77·8 (77·2 to 78·5)	79·2 (78·6 to 79·9)	81·2 (80·6 to 81·9)	80·8 (79·3 to 82·4)
South Tyneside	70·3 (66·9 to 73·9)	73 (69·6 to 76·4)	74·1 (70·9 to 77·2)	75·8 (74·6 to 77)	77·1 (76·4 to 77·8)	79·4 (78·8 to 80·1)	81 (80·3 to 81·7)	81·8 (80·3 to 83·4)
Stockton-on-Tees	70·8 (67·5 to 74·3)	73·5 (70·2 to 76·9)	74·5 (71·4 to 77·6)	76·3 (75·1 to 77·5)	77·6 (77 to 78·3)	79·8 (79·1 to 80·4)	81·8 (81·1 to 82·4)	81·2 (79·7 to 82·7)
Sunderland	70·3 (66·8 to 73·7)	73 (69·8 to 76·4)	74·1 (71·1 to 77·2)	75·8 (74·7 to 76·9)	77·1 (76·5 to 77·6)	78·6 (78 to 79·1)	80·8 (80·2 to 81·4)	81·4 (79·9 to 82·7)
North West England	70·4 (68·3 to 72·7)	73·3 (71 to 75·4)	74·3 (72 to 76·6)	75·9 (75·3 to 76·5)	77·7 (77·5 to 77·9)	79·3 (79·1 to 79·5)	81·4 (81·2 to 81·6)	82 (81 to 82·9)
Blackburn with Darwen	69·5 (66·4 to 72·7)	72·3 (69·2 to 75·5)	73·5 (70·6 to 76·6)	75·2 (74·1 to 76·5)	76·8 (76·2 to 77·4)	78·3 (77·7 to 79)	81 (80·3 to 81·6)	81·3 (79·8 to 82·8)
Blackpool	69·6 (66·2 to 73)	72·4 (69·3 to 75·5)	73·5 (70·6 to 76·6)	75·3 (74·3 to 76·6)	77·1 (76·4 to 77·7)	77·6 (76·9 to 78·2)	78·9 (78·3 to 79·7)	79·5 (78 to 81)
Bolton	70 (66·7 to 73·4)	72·8 (69·9 to 76)	74 (71 to 77)	75·7 (74·7 to 76·7)	77·5 (76·9 to 78)	79 (78·5 to 79·6)	81·1 (80·6 to 81·7)	82·4 (80·9 to 83·7)
Bury	70·6 (67·2 to 73·9)	73·6 (70·5 to 76·8)	74·9 (71·9 to 78·1)	76·7 (75·6 to 77·9)	78 (77·4 to 78·6)	79·9 (79·3 to 80·6)	81·8 (81·2 to 82·4)	81·9 (80·5 to 83·3)
Cheshire East	72·3 (69·1 to 75·4)	75·1 (72 to 78·4)	76·3 (73·4 to 79·2)	77·7 (76·7 to 78·7)	79 (78·5 to 79·5)	81·2 (80·7 to 81·7)	83·1 (82·6 to 83·7)	84·3 (83 to 85·6)
Cheshire West and Chester	71·9 (68·6 to 75·1)	74·4 (71·5 to 77·5)	75·2 (72·4 to 78·3)	76·5 (75·5 to 77·5)	78·7 (78·1 to 79·1)	80·8 (80·3 to 81·3)	82·9 (82·5 to 83·5)	83·2 (82 to 84·6)

Cumbria	70·9 (67·8 to 74·2)	73·7 (70·6 to 77)	74·7 (71·9 to 77·6)	76·4 (75·6 to 77·4)	78·1 (77·7 to 78·6)	80·3 (79·8 to 80·7)	82·1 (81·6 to 82·6)	82·5 (81·2 to 83·8)
Halton	70 (66·5 to 73·4)	72·6 (69·6 to 75·8)	73·5 (70·3 to 76·5)	75 (73·8 to 76·2)	77·1 (76·4 to 77·7)	78·3 (77·7 to 79)	80·5 (79·8 to 81·3)	81·7 (80·1 to 83·2)
Knowsley	69·6 (66·1 to 73·1)	72·3 (69·1 to 75·5)	73·1 (70·2 to 76·3)	74·7 (73·5 to 75·7)	76·9 (76·3 to 77·5)	78·5 (77·8 to 79·1)	80·7 (80 to 81·4)	81·3 (79·8 to 82·8)
Lancashire	70·9 (67·7 to 74·1)	73·8 (71 to 77)	75 (72·1 to 78)	76·2 (75·3 to 77·1)	77·8 (77·5 to 78·2)	79·8 (79·5 to 80·2)	81·8 (81·4 to 82·2)	81·9 (80·6 to 83·2)
Liverpool	69·5 (66·2 to 72·7)	72·2 (69·2 to 75·5)	72·9 (70 to 75·8)	74·9 (73·9 to 75·8)	76·7 (76·3 to 77·1)	77·4 (76·9 to 77·8)	79·9 (79·5 to 80·4)	80·3 (79 to 81·7)
Manchester	69 (65·7 to 72·3)	72·1 (68·7 to 75·3)	73·1 (70·3 to 76·1)	74·7 (73·7 to 75·6)	76·3 (75·8 to 76·7)	76·8 (76·4 to 77·3)	79·4 (78·9 to 79·9)	81 (79·6 to 82·4)
Oldham	69·5 (66·3 to 72·9)	72·4 (69·3 to 75·5)	73·6 (70·7 to 76·6)	75·5 (74·3 to 76·6)	77·3 (76·7 to 77·8)	78·9 (78·3 to 79·5)	80·7 (80·1 to 81·3)	81·4 (80 to 82·7)
Rochdale	69·3 (66 to 72·6)	72·3 (69·2 to 75·5)	73·6 (70·5 to 76·6)	75·6 (74·6 to 76·7)	76·4 (75·8 to 76·9)	78·4 (77·9 to 79)	80·4 (79·8 to 81)	80·5 (79 to 82)
Salford	69·7 (66·4 to 72·9)	72·6 (69·4 to 75·9)	73·6 (70·7 to 76·7)	75·3 (74·3 to 76·4)	76·5 (76 to 77·1)	78 (77·4 to 78·6)	80·6 (80 to 81·2)	81·6 (80·2 to 83)
Sefton	71·1 (67·9 to 74·4)	73·8 (70·8 to 77·2)	74·7 (71·7 to 77·5)	76·3 (75·3 to 77·4)	78·5 (78 to 79)	79·7 (79·2 to 80·2)	81·7 (81·2 to 82·3)	81·9 (80·5 to 83·2)
St Helens	70·4 (67 to 73·9)	73·1 (70 to 76·3)	74·1 (71·3 to 77)	75 (73·8 to 76·1)	77·5 (76·9 to 78)	79·3 (78·7 to 79·9)	80·9 (80·3 to 81·5)	81·1 (79·6 to 82·5)
Stockport	72 (68·6 to 75·4)	74·7 (71·6 to 77·9)	75·8 (72·9 to 78·8)	77 (76·1 to 78)	78·7 (78·2 to 79·2)	80·2 (79·7 to 80·8)	82·4 (81·9 to 83)	83·2 (81·8 to 84·6)
Tameside	69·6 (66·4 to 72·9)	72·4 (69·4 to 75·6)	73·5 (70·5 to 76·5)	75·2 (74·2 to 76·3)	77·1 (76·6 to 77·6)	79·2 (78·6 to 79·7)	80·9 (80·4 to 81·6)	81·8 (80·4 to 83·2)
Trafford	71 (67·7 to 74·4)	73·7 (70·6 to 76·7)	74·7 (71·6 to 77·6)	76·3 (75·3 to 77·5)	78·6 (78 to 79·1)	80·1 (79·5 to 80·6)	83·4 (82·7 to 84)	84·2 (82·9 to 85·6)
Warrington	71·1 (67·8 to 74·6)	73·7 (70·7 to 76·9)	74·6 (71·6 to 77·7)	76 (74·9 to 77·1)	77·5 (76·9 to 78·1)	80·3 (79·7 to 80·8)	82 (81·4 to 82·6)	82·4 (81 to 83·7)
Wigan	70·6 (67·5 to 73·8)	73·3 (70·3 to 76·3)	74·3 (71·7 to 77·3)	75·9 (74·9 to 77)	77·6 (77·1 to 78·1)	79·1 (78·6 to 79·6)	81·2 (80·7 to 81·7)	81·7 (80·3 to 83·1)
Wirral	70·4 (67·1 to 73·6)	73·3 (70·3 to 76·6)	74·4 (71·3 to 77·4)	76·2 (75·2 to 77·3)	78 (77·5 to 78·5)	79·5 (79 to 80)	81·2 (80·7 to 81·8)	81·6 (80·3 to 83)
South East England	72·8 (70·7 to 75·1)	75·6 (73·4 to 77·8)	76·6 (74·3 to 78·9)	78 (77·4 to 78·7)	79·8 (79·6 to 80)	81·3 (81·1 to 81·5)	83·6 (83·4 to 83·8)	83·9 (83 to 84·8)
Bracknell Forest	73·7 (70·3 to 76·9)	76·2 (73·4 to 79·2)	77·2 (74 to 80·2)	78·5 (77·1 to 80)	80·1 (79·4 to 80·9)	81·9 (81·1 to 82·6)	83·6 (82·9 to 84·3)	84·5 (83 to 86·1)
Brighton and Hove	71·3 (68·1 to 74·6)	74·2 (71·4 to 77·2)	75·4 (72·7 to 78·3)	76·9 (75·8 to 78)	78·9 (78·3 to 79·4)	79·9 (79·3 to 80·4)	82·2 (81·6 to 82·7)	82·9 (81·5 to 84·2)
Buckinghamshire	73·6 (70·2 to 77)	76·1 (73·3 to 79·3)	77 (74 to 80)	78·2 (77·2 to 79·3)	79·9 (79·4 to 80·4)	81·5 (81 to 82)	84·1 (83·6 to 84·6)	84·8 (83·5 to 86·1)
East Sussex	72·3 (69·2 to 75·8)	75·2 (72·3 to 78·4)	76·5 (73·6 to 79·4)	78·2 (77·2 to 79·2)	79·9 (79·4 to 80·3)	81·4 (80·9 to 81·9)	83·5 (83 to 84)	83·5 (82·2 to 84·7)
Hampshire	73 (69·7 to 76·2)	75·7 (72·4 to 78·6)	76·5 (73·5 to 79·5)	78·2 (77·3 to 79·1)	80·1 (79·7 to 80·5)	81·7 (81·3 to 82)	84 (83·6 to 84·4)	84·3 (83·2 to 85·5)
Isle of Wight	71·4 (68·2 to 74·9)	74·6 (71·5 to 77·7)	76·2 (73·2 to 79·1)	78·1 (76·8 to 79·4)	79·5 (78·8 to 80·2)	81·3 (80·6 to 82·1)	83·1 (82·4 to 83·8)	83·5 (82 to 85)
Kent	72·3 (69·1 to 75·6)	75·1 (72·2 to 78·4)	76·1 (73·2 to 78·9)	77·4 (76·5 to 78·3)	79·3 (78·9 to 79·7)	80·6 (80·3 to 81)	83·2 (82·8 to 83·5)	83·5 (82·3 to 84·7)
Medway	71·5 (68·1 to 74·8)	74·4 (71·1 to 77·6)	75·8 (73 to 78·8)	77·4 (76·3 to 78·6)	78·9 (78·3 to 79·4)	80·4 (79·8 to 80·9)	82 (81·4 to 82·6)	82·2 (80·8 to 83·6)
Milton Keynes	72·5 (69·3 to 75·8)	75·3 (72·3 to 78·6)	76·6 (73·8 to 79·5)	78·1 (76·9 to 79·3)	78·9 (78·3 to 79·5)	80·9 (80·3 to 81·5)	82·6 (82·1 to 83·2)	83·3 (81·8 to 84·7)
Oxfordshire	73·5 (70·1 to 76·8)	76 (73 to 79·1)	76·9 (73·9 to 79·8)	78·2 (77·2 to 79·1)	80·2 (79·8 to 80·7)	81·6 (81·2 to 82)	83·8 (83·4 to 84·2)	84·2 (83 to 85·3)
Portsmouth	71·1 (68 to 74·5)	74·1 (71·1 to 77·5)	75·4 (72·5 to 78·4)	77 (75·9 to 78·2)	78·4 (77·8 to 79)	79·1 (78·5 to 79·7)	82 (81·3 to 82·5)	82·3 (80·9 to 83·8)
Reading	72·8 (69·7 to 76·1)	75·7 (72·5 to 79·1)	76·9 (74 to 79·9)	78·4 (77·2 to 79·5)	79·3 (78·7 to 80)	80·3 (79·7 to 80·9)	82·5 (81·8 to 83·1)	82·8 (81·3 to 84·3)
Slough	71·7 (68·5 to 75)	74·2 (71 to 77·5)	75·1 (72·1 to 78·1)	76·3 (75·1 to 77·5)	77·9 (77·1 to 78·6)	79·5 (78·8 to 80·1)	82·7 (82 to 83·3)	83 (81·6 to 84·5)
Southampton	71·5 (68·3 to 74·8)	74·4 (71·2 to 77·4)	75·7 (72·8 to 78·7)	77·4 (76·2 to 78·5)	78·6 (77·9 to 79·2)	79·8 (79·2 to 80·4)	82 (81·4 to 82·5)	82·7 (81·3 to 84)
Surrey	73·4 (70·2 to 76·6)	76 (73·3 to 79·1)	77 (74·1 to 79·8)	78·1 (77·3 to 79)	80·1 (79·8 to 80·5)	82·1 (81·7 to 82·5)	84·5 (84·1 to 84·9)	84·4 (83·2 to 85·5)
West Berkshire	74·4 (70·9 to 77·7)	76·9 (73·8 to 79·9)	78 (75·1 to 81)	79·4 (78·1 to 80·7)	80·5 (79·7 to 81·1)	81·6 (80·9 to 82·2)	83·8 (83·1 to 84·5)	84·8 (83·4 to 86·4)
West Sussex	72·9 (69·7 to 76·3)	75·8 (72·6 to 79·1)	76·9 (74 to 79·7)	78·5 (77·5 to 79·4)	80·1 (79·7 to 80·5)	81·6 (81·2 to 82)	83·5 (83·1 to 84)	84·1 (83 to 85·3)
Windsor and Maidenhead	73·8 (70·5 to 77·1)	76·2 (73·3 to 79·6)	77·2 (74·1 to 80·2)	78·4 (77·2 to 79·6)	79·8 (79·1 to 80·4)	82·6 (81·9 to 83·3)	84·4 (83·7 to 85·2)	84·8 (83·4 to 86·3)
Wokingham	74·8 (71·5 to 78·2)	77·3 (74·3 to 80·3)	78·4 (75·4 to 81·2)	79·6 (78·4 to 80·9)	80·9 (80·2 to 81·5)	82·9 (82·2 to 83·6)	85·3 (84·7 to 86·1)	85·9 (84·4 to 87·3)

South West England	72·6 (70·3 to 75)	75·4 (73·2 to 77·7)	76·4 (74 to 78·7)	77·9 (77·2 to 78·6)	79·8 (79·5 to 80)	81·5 (81·3 to 81·7)	83·6 (83·4 to 83·9)	83·8 (82·8 to 84·8)
Bath and North East Somerset	73·4 (70·2 to 76·9)	76·2 (73 to 79·4)	77·3 (74·3 to 80·3)	78·9 (77·7 to 80·1)	80·5 (79·9 to 81·1)	82 (81·3 to 82·6)	84·6 (84 to 85·4)	84·6 (83·1 to 86·1)
Bournemouth	71·9 (68·4 to 75·3)	74·7 (71·6 to 78)	75·8 (72·9 to 78·9)	77·3 (76 to 78·5)	79 (78·4 to 79·6)	80·3 (79·7 to 81)	82·7 (82·1 to 83·4)	82 (80·6 to 83·5)
Bristol, City of	71·5 (68 to 74·9)	74·4 (71·3 to 77·5)	75·3 (72·4 to 78·2)	77·1 (76·1 to 78·1)	79 (78·5 to 79·5)	80·3 (79·8 to 80·8)	82·2 (81·6 to 82·7)	82·8 (81·5 to 84·1)
Cornwall	72·2 (68·8 to 75·6)	75 (71·9 to 78)	76·1 (73·3 to 78·9)	77·6 (76·6 to 78·7)	79·5 (79 to 80)	81·1 (80·6 to 81·5)	83·3 (82·8 to 83·8)	83·3 (82 to 84·6)
Devon	72·6 (69·7 to 75·9)	75·5 (72·4 to 78·6)	76·5 (73·4 to 79·5)	77·9 (77 to 78·9)	80·2 (79·7 to 80·6)	81·8 (81·4 to 82·2)	84·1 (83·7 to 84·5)	84·2 (83 to 85·5)
Dorset	72·6 (69·3 to 75·8)	75·4 (72·4 to 78·6)	76·6 (73·6 to 79·7)	78·2 (77·1 to 79·2)	80·4 (79·9 to 80·9)	82·7 (82·2 to 83·2)	85 (84·5 to 85·5)	84·6 (83·4 to 86)
Gloucestershire	72·7 (69·3 to 76·1)	75·6 (72·6 to 78·9)	76·6 (73·6 to 79·6)	77·9 (77 to 78·9)	79·6 (79·1 to 80·1)	81·5 (81 to 81·9)	83·3 (82·9 to 83·8)	83·9 (82·6 to 85·1)
North Somerset	72·7 (69·5 to 76·1)	75·7 (72·6 to 78·9)	76·9 (73·8 to 79·8)	78·5 (77·3 to 79·6)	80·1 (79·5 to 80·7)	81·9 (81·3 to 82·5)	83·3 (82·7 to 83·9)	83·6 (82·2 to 85)
Plymouth	71·8 (68·4 to 75·2)	74·6 (71·1 to 77·7)	75·6 (72·5 to 78·8)	77·1 (76 to 78·2)	78·8 (78·3 to 79·4)	80·5 (79·9 to 81)	82·2 (81·7 to 82·8)	82·8 (81·5 to 84·1)
Poole	72·9 (69·6 to 76·2)	75·7 (72·6 to 78·8)	76·8 (73·8 to 79·7)	78·2 (77 to 79·4)	79·5 (78·8 to 80·2)	82·1 (81·3 to 82·8)	83·8 (83·1 to 84·6)	84 (82·5 to 85·6)
Somerset	72·7 (69·5 to 75·9)	75·4 (72·2 to 78·6)	76·4 (73·3 to 79·4)	77·8 (76·8 to 78·8)	79·8 (79·3 to 80·2)	81·5 (81 to 81·9)	83·6 (83·2 to 84·1)	83·5 (82·3 to 84·8)
South Gloucestershire	73·5 (70·2 to 77·1)	76·1 (73 to 79·2)	77·1 (74 to 80)	78·9 (77·7 to 80·1)	80·1 (79·5 to 80·7)	82·6 (82 to 83·2)	85·1 (84·4 to 85·7)	85·1 (83·6 to 86·4)
Swindon	71·8 (68·5 to 75·4)	74·6 (71·4 to 77·8)	75·6 (72·4 to 78·6)	77 (75·8 to 78·3)	79·5 (78·9 to 80·1)	80·6 (80 to 81·3)	83·1 (82·4 to 83·7)	83·6 (82·2 to 84·9)
Torbay	72 (68·6 to 75·5)	74·8 (71·6 to 78·5)	76 (73·1 to 79·2)	77·6 (76·4 to 78·9)	79·5 (78·9 to 80·2)	80·4 (79·7 to 81·1)	81·9 (81·2 to 82·7)	82·4 (80·8 to 83·9)
Wiltshire	72·9 (69·5 to 76·3)	75·6 (72·7 to 78·9)	76·7 (73·8 to 79·6)	78·2 (77·2 to 79·3)	79·7 (79·2 to 80·2)	81·8 (81·3 to 82·3)	83·7 (83·2 to 84·2)	84·3 (83 to 85·5)
West Midlands	71·4 (69 to 73·9)	74 (71·8 to 76·3)	75 (72·8 to 77·4)	76·6 (76 to 77·3)	78·3 (78·1 to 78·5)	80·1 (79·9 to 80·4)	82·5 (82·2 to 82·7)	82·7 (81·7 to 83·8)
Birmingham	70·8 (67·6 to 73·8)	73·5 (70·6 to 76·6)	74·5 (71·8 to 77·7)	76·1 (75·3 to 77)	77·6 (77·2 to 78·1)	79·5 (79·1 to 79·9)	82·3 (81·8 to 82·7)	82·5 (81·2 to 83·8)
Coventry	70·8 (67·1 to 74·4)	73·5 (70·3 to 76·8)	74·4 (71·5 to 77·6)	76·3 (75·3 to 77·3)	77·8 (77·3 to 78·3)	79·6 (79 to 80·1)	82·2 (81·7 to 82·8)	82·4 (80·9 to 83·8)
Dudley	72·3 (69 to 75·6)	74·9 (71·7 to 78·3)	76 (72·6 to 79·1)	77·6 (76·5 to 78·7)	79·1 (78·6 to 79·6)	80·4 (79·9 to 80·9)	82·9 (82·3 to 83·4)	82·9 (81·5 to 84·2)
Herefordshire, County of	71·5 (68·1 to 74·9)	74·2 (71·1 to 77·5)	75·2 (72·1 to 78·1)	76·9 (75·7 to 78·1)	79·5 (78·8 to 80·2)	81·6 (80·9 to 82·3)	83·2 (82·5 to 83·9)	83·1 (81·7 to 84·6)
Sandwell	69·9 (66·5 to 73·3)	72·7 (69·5 to 75·9)	73·8 (70·8 to 76·8)	75·7 (74·6 to 76·7)	77·8 (77·3 to 78·3)	78·7 (78·2 to 79·2)	80·6 (80·1 to 81·2)	81·7 (80·2 to 83)
Shropshire	72·3 (69 to 75·8)	74·9 (71·8 to 78·3)	76 (72·9 to 79)	77·7 (76·6 to 78·8)	79·4 (78·9 to 79·9)	81·4 (80·9 to 82)	83·5 (82·9 to 84·1)	84 (82·6 to 85·4)
Solihull	73·3 (69·9 to 76·9)	75·8 (72·8 to 79·1)	76·9 (74 to 79·9)	78·6 (77·5 to 79·9)	79·9 (79·3 to 80·5)	81·1 (80·5 to 81·7)	83·9 (83·2 to 84·5)	83·4 (81·9 to 84·9)
Staffordshire	71·9 (68·6 to 75·3)	74·5 (71·4 to 77·7)	75·4 (72·6 to 78·5)	76·8 (75·8 to 77·7)	78·3 (77·9 to 78·7)	80·2 (79·8 to 80·6)	82·6 (82·3 to 83·1)	82·7 (81·5 to 84)
Stoke-on-Trent	69·6 (66·2 to 73·1)	72·3 (68·9 to 75·7)	73·2 (70 to 76·4)	74·8 (73·8 to 75·9)	76·9 (76·4 to 77·5)	78·9 (78·4 to 79·4)	80·3 (79·7 to 80·8)	81·4 (79·9 to 82·9)
Telford and Wrekin	71·3 (68 to 74·9)	74 (70·6 to 77·2)	75 (71·8 to 78)	76·6 (75·4 to 77·9)	78·7 (78·1 to 79·4)	79·6 (79 to 80·3)	82 (81·3 to 82·6)	82·2 (80·8 to 83·7)
Walsall	70·6 (67·1 to 74·3)	73·3 (69·9 to 76·6)	74·3 (71·1 to 77·2)	75·9 (74·9 to 77·1)	77·4 (76·9 to 77·9)	79·9 (79·4 to 80·5)	82·2 (81·6 to 82·8)	82 (80·5 to 83·5)
Warwickshire	72·4 (69 to 76)	75 (71·9 to 78·1)	75·8 (72·8 to 78·8)	77·2 (76·3 to 78·2)	78·7 (78·2 to 79·2)	81 (80·5 to 81·5)	83·3 (82·8 to 83·8)	83·3 (81·9 to 84·5)
Wolverhampton	71 (67·3 to 74·4)	73·6 (70·4 to 77)	74·6 (71·6 to 77·7)	76·3 (75·2 to 77·4)	77·5 (77 to 78·1)	79·3 (78·7 to 79·9)	81 (80·5 to 81·6)	81·3 (79·9 to 82·8)
Worcestershire	72·7 (69·4 to 76·1)	75·2 (72·3 to 78·4)	76·2 (73·4 to 79·1)	77·4 (76·5 to 78·4)	79·2 (78·8 to 79·6)	80·7 (80·2 to 81·1)	82·9 (82·5 to 83·4)	83·7 (82·5 to 84·9)
Yorkshire and the Humber	71 (68·5 to 73·3)	73·7 (71·4 to 76)	74·8 (72·6 to 77·1)	76·5 (75·8 to 77·3)	78·2 (77·9 to 78·4)	80 (79·8 to 80·3)	82·1 (81·8 to 82·3)	82·4 (81·4 to 83·4)
Barnsley	70·4 (66·8 to 73·8)	73·1 (69·9 to 76·4)	74·3 (71·2 to 77·5)	76·1 (75 to 77·2)	77·8 (77·2 to 78·3)	79·5 (79 to 80·1)	81·5 (80·9 to 82·1)	82 (80·6 to 83·5)
Bradford	69·7 (66·1 to 73·1)	72·6 (69·5 to 75·9)	73·7 (70·9 to 76·6)	75·6 (74·6 to 76·6)	77·5 (77 to 77·9)	79·2 (78·8 to 79·7)	81·3 (80·8 to 81·8)	81·5 (80·2 to 82·9)
Calderdale	70·2 (66·9 to 73·6)	73 (69·9 to 76·2)	74·3 (71·4 to 77·4)	76·1 (75 to 77·3)	77·7 (77·1 to 78·2)	79·9 (79·4 to 80·6)	81·5 (80·9 to 82·1)	82 (80·5 to 83·4)
Doncaster	70·9 (67·4 to 74·6)	73·6 (70·6 to 76·8)	74·8 (71·9 to 77·8)	76·6 (75·5 to 77·7)	77·1 (76·6 to 77·7)	79·2 (78·7 to 79·8)	81·6 (81·1 to 82·2)	81·3 (79·9 to 82·8)
East Riding of Yorkshire	72 (68·6 to 75·3)	74·7 (71·6 to 77·8)	75·8 (72·9 to 78·9)	77·4 (76·4 to 78·5)	79·3 (78·8 to 79·8)	80·9 (80·4 to 81·4)	83 (82·5 to 83·6)	83·8 (82·5 to 85)

Kingston upon Hull, City of	70·2 (66·9 to 73·7)	73 (69·8 to 76·4)	74·3 (71·1 to 77·3)	76·3 (75·2 to 77·4)	77·5 (77 to 78·1)	78·7 (78·2 to 79·3)	80·6 (80 to 81·1)	80·9 (79·5 to 82·3)
Kirklees	70·4 (66·9 to 73·9)	73·2 (70·3 to 76·5)	74·3 (71·3 to 77·4)	75·7 (74·8 to 76·8)	77·8 (77·3 to 78·2)	80 (79·4 to 80·5)	81·8 (81·3 to 82·3)	82·5 (81·1 to 83·8)
Leeds	71 (67·6 to 74·2)	73·7 (70·6 to 77·1)	74·7 (71·8 to 77·7)	76·4 (75·5 to 77·4)	78·3 (77·9 to 78·7)	80·3 (79·8 to 80·7)	82·1 (81·6 to 82·5)	82·4 (81·1 to 83·8)
North East Lincolnshire	70·7 (67·3 to 74·2)	73·5 (70·2 to 77)	74·7 (71·8 to 77·9)	76·5 (75·3 to 77·6)	77·5 (76·8 to 78·1)	79·7 (79·1 to 80·4)	81·9 (81·2 to 82·5)	82·2 (80·8 to 83·6)
North Lincolnshire	71·4 (68·1 to 74·9)	74 (70·9 to 77·4)	75·3 (72·1 to 78·5)	77 (75·8 to 78·1)	77·9 (77·2 to 78·5)	79·7 (79·1 to 80·4)	82·2 (81·5 to 82·9)	83 (81·5 to 84·5)
North Yorkshire	72 (68·8 to 75·3)	74·8 (71·9 to 77·8)	75·8 (72·9 to 78·7)	77·4 (76·5 to 78·3)	79·4 (79 to 79·9)	81 (80·6 to 81·5)	83·4 (82·9 to 83·8)	83·7 (82·4 to 84·9)
Rotherham	70·9 (67·3 to 74·5)	73·7 (70·5 to 77)	74·9 (71·9 to 78·1)	76·8 (75·7 to 77·8)	78·1 (77·5 to 78·7)	79·8 (79·3 to 80·4)	81·6 (81 to 82·1)	81·7 (80·3 to 83·2)
Sheffield	71·1 (67·9 to 74·7)	73·9 (70·8 to 77·2)	74·9 (72·3 to 77·8)	76·5 (75·6 to 77·5)	78·1 (77·7 to 78·5)	80·1 (79·6 to 80·6)	82 (81·5 to 82·5)	82·2 (81 to 83·4)
Wakefield	70·3 (66·8 to 73·7)	73·1 (70 to 76·4)	74·2 (71·3 to 77·5)	76 (75 to 77·1)	77·7 (77·2 to 78·2)	79·5 (79 to 80)	81·7 (81·1 to 82·2)	82 (80·7 to 83·3)
York	72 (68·3 to 75·4)	74·6 (71·6 to 78)	75·7 (72·8 to 78·7)	77·3 (76·1 to 78·5)	79·4 (78·8 to 80·1)	81·8 (81·1 to 82·4)	83·7 (83 to 84·3)	83·1 (81·7 to 84·6)
Northern Ireland	69·1 (68·9 to 69·4)	72·2 (71·9 to 72·4)	73·8 (73·6 to 74)	75 (74·8 to 75·3)	77·4 (77·1 to 77·6)	79·7 (79·5 to 80)	81·9 (81·6 to 82·1)	82·1 (81·7 to 82·6)
Scotland	68·4 (68·3 to 68·6)	71·5 (71·4 to 71·7)	73·3 (73·2 to 73·4)	75 (74·9 to 75·2)	76·9 (76·7 to 77)	78·7 (78·5 to 78·9)	80·8 (80·7 to 81)	81·2 (80·9 to 81·5)
Wales	70·1 (69·5 to 70·7)	73·2 (72·5 to 73·8)	74·4 (73·8 to 75)	76·4 (76·2 to 76·7)	78·6 (78·4 to 78·7)	79·9 (79·7 to 80)	82 (81·8 to 82·1)	82·4 (82 to 82·7)
Latin America and Caribbean	51·4 (49·7 to 52·8)	57·7 (56·9 to 58·5)	62·2 (61·6 to 62·7)	68 (67·7 to 68·4)	72·3 (72 to 72·5)	75·6 (75·3 to 75·8)	76·6 (76·3 to 76·9)	79 (78 to 80)
Andean Latin America	41·4 (39 to 43·7)	49·8 (48·2 to 51·2)	54·4 (53·3 to 55·4)	65·4 (64·3 to 66·5)	70·5 (69·7 to 71·3)	74·7 (73·8 to 75·5)	76·9 (75·9 to 77·9)	79·1 (77·1 to 81)
Bolivia	37·7 (34·9 to 40·7)	44·7 (42 to 47·2)	52·1 (50 to 54·2)	58·4 (56·8 to 59·9)	62·9 (61·6 to 64·2)	67·7 (66·1 to 69)	71·1 (69·1 to 72·9)	72·9 (71 to 74·8)
Ecuador	51·1 (48 to 54·5)	57·3 (56·1 to 58·3)	62·5 (61·7 to 63·1)	69·3 (68·7 to 69·8)	74 (73·6 to 74·4)	77 (76·7 to 77·4)	77·1 (76·2 to 78·1)	78·7 (76·4 to 80·6)
Peru	39·4 (35·6 to 43)	48·6 (46·3 to 50·9)	51·8 (50·2 to 53·4)	66·2 (64·6 to 68)	71·6 (70·2 to 73)	76·2 (74·8 to 77·5)	79 (77·6 to 80·6)	81·8 (79 to 84·4)
Caribbean	58·3 (56·7 to 59·8)	62·7 (61·8 to 63·6)	65·8 (65 to 66·5)	68·5 (67·8 to 69·2)	70·5 (69·9 to 71·1)	72·5 (71·7 to 73·2)	62·9 (61·6 to 64·1)	75·1 (73·3 to 76·8)
Antigua and Barbuda	60·9 (59·1 to 62·2)	65·8 (64·7 to 66·9)	70·6 (69·7 to 71·6)	74·8 (73·6 to 75·9)	77·7 (76·8 to 78·6)	77·3 (76·5 to 77·9)	78·3 (77·3 to 79·2)	77·9 (76·7 to 79·1)
The Bahamas	60·8 (56·9 to 64·6)	64·6 (61·6 to 67·5)	68·6 (67·5 to 69·7)	72·1 (71·2 to 73)	74·5 (73·6 to 75·3)	74·6 (73·7 to 75·5)	76·6 (75·8 to 77·4)	76·9 (74·5 to 79)
Barbados	60·8 (59·1 to 62·2)	68·1 (67 to 69·1)	71·5 (70·7 to 72·2)	75·1 (74·5 to 75·8)	76·2 (75·6 to 76·7)	77 (76·2 to 77·7)	77·4 (76·8 to 77·9)	77·8 (76 to 79·6)
Belize	57·5 (55·8 to 59·3)	66·1 (64 to 68·1)	68·6 (67·5 to 69·7)	72·6 (71·6 to 73·6)	74·3 (73·4 to 75·2)	73·8 (73 to 74·5)	76·4 (75·9 to 76·9)	78 (76·7 to 79·2)
Bermuda	65·9 (64·6 to 67·1)	70·1 (69·3 to 71)	73·6 (72·7 to 74·6)	77·4 (76·7 to 78·2)	77·4 (76·9 to 78·1)	80·4 (79·8 to 81·1)	84·5 (83·4 to 85·5)	85·3 (83·5 to 86·7)
Cuba	68·5 (65·2 to 71·6)	71 (70·8 to 71·2)	72·6 (72·4 to 72·7)	75·5 (75·3 to 75·6)	77 (76·8 to 77·2)	79·1 (78·9 to 79·3)	80·6 (80·4 to 80·8)	81·1 (79·3 to 82·9)
Dominica	51·4 (49·3 to 53·2)	62·2 (60·1 to 64·3)	68·5 (67 to 70)	72·7 (71·6 to 74)	73·5 (72·3 to 74·8)	74·7 (73·3 to 76·3)	75·1 (73·7 to 76·6)	75 (72·8 to 76·9)
Dominican Republic	57·8 (56·4 to 59·1)	63 (61·9 to 64·2)	66·7 (65·3 to 68·1)	71 (69·8 to 72·3)	74·3 (73 to 75·4)	77·5 (76·2 to 78·8)	76·7 (74·7 to 78·7)	76·7 (74·1 to 79·1)
Grenada	60·1 (58·6 to 61·7)	66·3 (64·4 to 68·3)	69·3 (67·4 to 71)	71·7 (69·5 to 73·8)	72·1 (70·9 to 73·2)	75·9 (75·2 to 76·7)	75·6 (74·9 to 76·3)	75·8 (74·8 to 76·8)
Guyana	53·5 (50·4 to 56·7)	60·9 (59 to 62·8)	64·5 (62·7 to 66·4)	65·3 (63·2 to 67·4)	65·8 (64·2 to 67·5)	67 (65·2 to 68·7)	69·1 (67·6 to 70·8)	70·8 (67·7 to 73·7)
Haiti	45·2 (42·5 to 48)	47·8 (45·2 to 50·5)	50·1 (47·8 to 52·5)	53 (51·1 to 55·3)	55·4 (53·7 to 57·3)	57·6 (55·3 to 59·8)	36·9 (35·1 to 38·7)	64·6 (61·8 to 67·4)
Jamaica	59·3 (58·3 to 60·1)	67 (66·4 to 67·6)	70·8 (70·3 to 71·4)	75·5 (75 to 76)	76·6 (76·2 to 77)	76·9 (76·5 to 77·3)	79·2 (78·8 to 79·6)	78 (75·5 to 80·4)
Puerto Rico	61·4 (60·9 to 61·8)	71·1 (70·8 to 71·5)	74·3 (74 to 74·6)	77·3 (77 to 77·7)	78·5 (78·2 to 78·8)	80·4 (80·1 to 80·7)	82·9 (82·5 to 83·2)	83·7 (81·4 to 86)
Saint Kitts and Nevis	62·6 (61·5 to 63·8)	59·7 (58·8 to 60·7)	65·1 (64·1 to 66)	65·7 (64·7 to 66·8)	67·5 (66·5 to 68·5)	72·6 (71·5 to 73·5)	75·3 (74·2 to 76·2)	75·8 (74 to 77·2)
Saint Lucia	53·3 (51·4 to 54·9)	60·9 (59 to 62·8)	67·3 (65·9 to 68·5)	71 (70·1 to 71·8)	72·7 (72·1 to 73·3)	76·4 (75·8 to 77)	79·2 (78·6 to 80)	78·4 (76·7 to 80·1)
Saint Vincent and the Grenadines	54·6 (52·5 to 56·4)	62 (60·2 to 63·8)	66·5 (65·2 to 67·8)	70·5 (69·4 to 71·6)	71·9 (71·1 to 72·6)	73·6 (72·9 to 74·4)	75·2 (74·5 to 75·9)	75·3 (73·7 to 76·7)
Suriname	59·8 (58·5 to 61)	64·5 (62·9 to 65·9)	68·5 (66·8 to 70·1)	69·8 (69·1 to 70·5)	71·5 (70·9 to 72·2)	73·2 (72·5 to 73·8)	75·8 (75·1 to 76·4)	75·7 (73·7 to 77·5)
Trinidad and Tobago	58·9 (57·8 to 59·9)	66·8 (66·1 to 67·5)	67·5 (66·9 to 68)	70·1 (69·6 to 70·6)	72·2 (71·6 to 72·6)	73·1 (72·6 to 73·7)	77 (76·5 to 77·6)	77·9 (74·8 to 80·9)

Virgin Islands	64·5 (62·5 to 66)	67·2 (65·9 to 68·5)	70·9 (69·3 to 72·2)	73·2 (72·1 to 74)	75·7 (74·4 to 77)	76·9 (76 to 77·7)	78·1 (77·4 to 78·9)	79·1 (77·5 to 80·5)
Central Latin America	51·6 (50 to 52·9)	59 (58·5 to 59·6)	63·9 (63·6 to 64·3)	69·6 (69·2 to 70)	73·7 (73·5 to 73·9)	76·8 (76·6 to 77)	78·6 (78·4 to 78·9)	79·4 (77·8 to 80·9)
Colombia	55·7 (54·2 to 56·9)	60·5 (59·6 to 61·3)	67·2 (66·7 to 67·7)	71·1 (69·3 to 72·8)	74·8 (74·6 to 75·1)	78·1 (77·9 to 78·3)	81·1 (80·9 to 81·3)	82·9 (80·4 to 85·2)
Costa Rica	58·7 (58·1 to 59·3)	66·6 (66·1 to 67)	68·5 (68·1 to 68·9)	77·3 (76·9 to 77·8)	79·2 (78·9 to 79·6)	80·4 (80·1 to 80·7)	82·6 (82·2 to 82·9)	83 (80·8 to 85·2)
El Salvador	47·7 (45·6 to 49·6)	55·1 (53·5 to 56·7)	60·4 (59·2 to 61·4)	68·4 (67 to 69·7)	74·3 (73·8 to 74·8)	77·9 (77·5 to 78·3)	79·4 (79·1 to 79·7)	79·9 (77 to 82·7)
Guatemala	42·6 (40·7 to 44·2)	47·9 (46·3 to 49·6)	51·1 (49·9 to 52·2)	61·4 (60·4 to 62·3)	64·2 (62·4 to 66·2)	70·2 (68·6 to 71·7)	73·6 (72·1 to 75)	75·5 (72·8 to 78)
Honduras	42·1 (39·8 to 44·6)	50·9 (49 to 52·7)	59·2 (57·8 to 60·6)	69·4 (68·2 to 70·7)	71·6 (70·1 to 73·4)	70·9 (68·2 to 73·6)	72 (69·3 to 74·6)	73·3 (70·6 to 75·3)
Mexico	50·2 (48·3 to 51·7)	58·8 (57·9 to 59·7)	63·6 (63 to 64·2)	69·2 (68·7 to 69·7)	73·7 (73·4 to 74)	76·7 (76·4 to 76·9)	78·1 (77·9 to 78·2)	78·6 (76·8 to 80·4)
Aguascalientes	54·9 (51·7 to 58)	62 (59·1 to 64·7)	65·7 (63·4 to 68)	70·3 (69·6 to 70·9)	74·5 (74·1 to 75)	76·8 (76·4 to 77·2)	77·9 (77·5 to 78·3)	79·2 (77·4 to 81·1)
Baja California	51·6 (48·3 to 54·9)	60·2 (57·3 to 63·2)	64·8 (62·1 to 67·5)	72 (71·5 to 72·5)	73·2 (72·8 to 73·6)	75·1 (74·8 to 75·5)	77·3 (76·9 to 77·6)	78·4 (76·3 to 80·6)
Baja California Sur	51·7 (48·6 to 55)	61 (58·3 to 63·9)	66 (63·7 to 68·2)	72·5 (71·7 to 73·2)	75·9 (75·4 to 76·4)	77·8 (77·3 to 78·2)	78·4 (78 to 78·8)	79·1 (77·2 to 81)
Campeche	51·4 (48·1 to 54·7)	59·5 (56·6 to 62·3)	64 (61·6 to 66·4)	68·9 (68·1 to 69·7)	73·8 (73·1 to 74·4)	77·2 (76·7 to 77·7)	78 (77·5 to 78·4)	78·1 (75·9 to 80·1)
Chiapas	48 (44·9 to 51·2)	56·2 (53·2 to 59·2)	61·1 (58·2 to 64·1)	65·8 (65 to 66·6)	71·5 (71 to 72)	75 (74·5 to 75·5)	76·8 (76·5 to 77·1)	76·9 (74·5 to 79·2)
Chihuahua	48 (44·6 to 51·5)	56·8 (53·9 to 59·6)	61·5 (58·6 to 64·1)	67·3 (66·6 to 68)	71·6 (71·1 to 72)	74·4 (73·9 to 74·8)	75·5 (75·1 to 75·8)	75·7 (73·4 to 78)
Coahuila	48·2 (45·4 to 51·4)	57·5 (54·5 to 60·4)	62 (59·4 to 64·6)	67·5 (66·8 to 68·2)	73 (72·5 to 73·4)	75·7 (75·3 to 76·1)	77·1 (76·8 to 77·5)	77·5 (75·4 to 79·8)
Colima	45·9 (42·4 to 49·3)	55·9 (53·3 to 58·7)	61·7 (59·5 to 64·1)	68·1 (67 to 69·2)	73·1 (72·5 to 73·6)	76·8 (76·3 to 77·2)	78·1 (77·6 to 78·5)	78·6 (76·7 to 80·6)
Durango	47·7 (44·4 to 50·9)	57·2 (54·3 to 60·2)	62·7 (60·2 to 65·3)	68·3 (67·5 to 69·1)	74·1 (73·5 to 74·6)	77·9 (77·3 to 78·4)	78·4 (78 to 78·8)	79·6 (77·3 to 82)
Guanajuato	49·8 (46·8 to 52·9)	57·9 (55 to 60·8)	62·7 (59·7 to 65·2)	69·1 (68·5 to 69·8)	73·5 (73·2 to 73·9)	77·1 (76·9 to 77·4)	78·5 (78·2 to 78·8)	78·6 (76·4 to 80·6)
Guerrero	49 (45·6 to 52·3)	56·5 (52·9 to 60·2)	59·7 (56·7 to 62·6)	65·5 (64·1 to 66·9)	72·4 (71·8 to 73·1)	74·5 (74·1 to 74·9)	78·6 (78 to 79·1)	79·7 (77·3 to 82·3)
Hidalgo	47·9 (44·7 to 51·1)	56·1 (52·9 to 59·6)	60·7 (57·5 to 63·7)	67 (66·2 to 67·8)	74·9 (74·4 to 75·4)	79 (78·6 to 79·5)	80 (79·6 to 80·4)	80·2 (78 to 82·4)
Jalisco	51·8 (48·6 to 55·1)	59·9 (57·2 to 62·7)	64·5 (61·9 to 67·1)	70 (69·4 to 70·5)	73·6 (73·2 to 73·9)	76·2 (75·9 to 76·6)	78 (77·7 to 78·3)	79 (76·9 to 81)
México	52·4 (49·5 to 55·3)	59·7 (56·9 to 62·4)	64·3 (61·5 to 67)	69·7 (67·8 to 71·6)	72·3 (72 to 72·7)	75·6 (75·4 to 75·9)	77·6 (77·3 to 77·8)	78·2 (76·1 to 80·2)
Mexico City	57·5 (54·6 to 60·4)	64·5 (61·8 to 67·2)	68 (65·6 to 70·5)	71·8 (71·4 to 72·3)	75 (74·7 to 75·3)	76·9 (76·7 to 77·2)	77·9 (77·6 to 78·1)	78·6 (76·5 to 80·6)
Michoacán de Ocampo	50·7 (47·6 to 53·9)	58·8 (55·9 to 61·7)	64·2 (61·2 to 67)	69·6 (69 to 70·1)	75·6 (75·2 to 76)	77·8 (77·5 to 78·2)	79·2 (78·9 to 79·5)	80·2 (78 to 82·4)
Morelos	50·9 (47·7 to 54·2)	59·3 (56·3 to 62·4)	64·2 (61·4 to 66·7)	69·9 (68·9 to 70·8)	74·6 (74·1 to 75)	77·6 (77·2 to 78)	78·5 (78·1 to 78·8)	78·4 (76·4 to 80·6)
Nayarit	50·2 (47·1 to 53·5)	59 (55·8 to 61·9)	64·5 (62·1 to 66·9)	70·9 (70·2 to 71·6)	75 (74·5 to 75·5)	78·5 (78 to 78·9)	79·8 (79·3 to 80·2)	80·1 (78 to 82·2)
Nuevo León	54·8 (52 to 57·8)	62·9 (60·3 to 65·7)	66·7 (64·1 to 69·2)	72·3 (71·8 to 72·8)	75·1 (74·7 to 75·5)	77·3 (76·9 to 77·6)	78·9 (78·6 to 79·2)	79·2 (77·1 to 81·2)
Oaxaca	45·8 (42·5 to 49·1)	53·9 (51 to 57)	58·7 (55·8 to 61·4)	64·7 (64 to 65·3)	72·1 (71·6 to 72·5)	76·8 (76·4 to 77·2)	78·9 (78·6 to 79·3)	79·8 (77·4 to 82)
Puebla	42·6 (39·5 to 45·7)	52·1 (49·1 to 55)	57·7 (54·8 to 60·6)	65·5 (64·8 to 66·2)	72 (71·6 to 72·5)	75·9 (75·4 to 76·3)	77·5 (77·2 to 77·8)	77·7 (75·5 to 79·9)
Querétaro	48·8 (45·5 to 51·9)	57·4 (54·3 to 60·4)	62·7 (59·8 to 65·6)	68·5 (67 to 70)	73·6 (73·1 to 74·1)	76·7 (76·3 to 77·1)	78·5 (78·1 to 78·8)	78·8 (76·9 to 80·8)
Quintana Roo	53·6 (50·4 to 57·1)	60·8 (58 to 63·7)	64·5 (62·1 to 66·8)	70·6 (69·7 to 71·4)	76·3 (75·7 to 76·8)	77·4 (76·9 to 77·8)	77·6 (77·2 to 78)	77·5 (75·5 to 79·4)
San Luis Potosí	49·5 (46·1 to 52·7)	58·3 (55·2 to 61·3)	63·3 (60·5 to 66·1)	70·8 (70·1 to 71·5)	75·1 (74·7 to 75·5)	78·4 (78 to 78·8)	79·6 (79·2 to 80)	79·6 (77·4 to 81·8)
Sinaloa	54·2 (51 to 57·2)	62·1 (59·2 to 65·1)	66·7 (63·9 to 69·4)	71·9 (71·3 to 72·4)	76·7 (76·2 to 77·1)	79·2 (78·9 to 79·6)	80·2 (79·8 to 80·5)	80·8 (78·5 to 83)
Sonora	54·8 (51·5 to 58)	62 (59 to 65)	65·5 (62·6 to 68·1)	69·8 (67·9 to 71·6)	74 (73·6 to 74·4)	76·1 (75·7 to 76·5)	77·1 (76·7 to 77·4)	78·7 (76·5 to 80·8)
Tabasco	50·2 (47·2 to 53·4)	58·1 (54·9 to 61·1)	62·1 (59·2 to 64·8)	67 (66 to 68)	73·2 (72·6 to 73·8)	76·5 (76·1 to 77)	77 (76·6 to 77·4)	77·7 (75·6 to 79·9)
Tamaulipas	52·6 (49·6 to 55·6)	60·6 (57·7 to 63·5)	64·9 (62 to 67·5)	69·9 (69·1 to 70·7)	74·8 (74·3 to 75·3)	77·7 (77·3 to 78·1)	79 (78·6 to 79·4)	79·1 (76·7 to 81·3)
Tlaxcala	48·1 (44·8 to 51·7)	57·4 (54·3 to 60·4)	63 (60·1 to 65·8)	68·9 (67·4 to 70·5)	73·4 (72·9 to 73·9)	76·9 (76·5 to 77·3)	78·2 (77·8 to 78·6)	79·3 (77·4 to 81·3)

Veracruz de Ignacio de la Llave	50·6 (47·6 to 53·6)	59·1 (56 to 62)	63·9 (61·1 to 66·5)	70·1 (69·3 to 70·8)	74·5 (73·8 to 75·1)	77·8 (77·4 to 78·1)	77·8 (77·4 to 78)	78·2 (76 to 80·5)
Yucatán	52·8 (49·5 to 56)	59·5 (56·8 to 62·3)	63·6 (61·1 to 66·1)	68·7 (68·1 to 69·3)	74 (73·6 to 74·4)	76·7 (76·4 to 77·1)	77·6 (77·3 to 78)	78·2 (76·2 to 80·1)
Zacatecas	53·7 (50·4 to 56·9)	61·3 (58·2 to 64·3)	66·2 (63·5 to 68·8)	71·9 (71·2 to 72·6)	75·7 (75·3 to 76·1)	78·1 (77·6 to 78·5)	78·8 (78·4 to 79·3)	79·5 (77·4 to 81·5)
Nicaragua	49·3 (47·2 to 51·1)	55·5 (53·7 to 57·1)	60·6 (58·7 to 62·4)	69·5 (67·7 to 71·3)	77·3 (76·7 to 77·8)	76·2 (75·9 to 76·6)	77 (76·6 to 77·5)	78·4 (77·1 to 79·7)
Panama	62·1 (61 to 63·2)	67·2 (66·4 to 68)	69·3 (68·6 to 69·9)	76·5 (75·9 to 77·1)	78·9 (78·3 to 79·5)	80·8 (80·3 to 81·3)	81·8 (81·3 to 82·2)	82·7 (80·2 to 85·1)
Venezuela	60 (59·7 to 60·3)	66·8 (66·6 to 67)	68·7 (68·5 to 68·9)	72·6 (72·4 to 72·8)	75·4 (75·2 to 75·5)	78·5 (78·4 to 78·7)	80 (79·9 to 80·1)	78·9 (75·9 to 81·7)
Tropical Latin America	52·4 (50·6 to 53·9)	57·2 (55·7 to 58·6)	61·5 (60·3 to 62·6)	66·8 (66·3 to 67·3)	71·5 (71·1 to 71·8)	75·2 (74·8 to 75·5)	77·7 (77·4 to 78)	79·4 (78·9 to 79·9)
Brazil	52·1 (50·3 to 53·6)	56·9 (55·4 to 58·3)	61·2 (60 to 62·4)	66·6 (66·1 to 67·1)	71·3 (71 to 71·7)	75·1 (74·8 to 75·4)	77·6 (77·3 to 78)	79·4 (78·9 to 79·8)
Acre	50·4 (46·9 to 54·1)	56·2 (52·7 to 59·5)	61·3 (58·8 to 63·9)	66·6 (65·2 to 68)	71·6 (70·7 to 72·4)	73·8 (73·1 to 74·5)	77·1 (76 to 78·1)	78·4 (77·3 to 79·5)
Alagoas	41·1 (37·9 to 44·3)	44·9 (42·1 to 48)	49·7 (47·1 to 52·5)	56·6 (55·1 to 58·2)	65·3 (64·2 to 66·3)	72·1 (71·2 to 73)	75·3 (74·4 to 76·1)	77·1 (75·3 to 78·8)
Amapá	44·7 (42·1 to 47·3)	52·1 (49 to 55·1)	61·1 (58·8 to 63·6)	72·9 (71·9 to 73·8)	76·5 (76 to 77·1)	76·3 (75·8 to 76·7)	80 (79·4 to 80·5)	79·6 (78·6 to 80·5)
Amazonas	54·9 (52 to 58)	59·8 (56·9 to 62·7)	62·8 (59·9 to 65·8)	68 (66·7 to 69·4)	72·6 (71·6 to 73·7)	75·6 (74·6 to 76·5)	78·7 (77·9 to 79·6)	80·1 (78·7 to 81·3)
Bahia	50·4 (47·1 to 53·6)	54·4 (51·5 to 57·3)	59 (56·1 to 61·7)	67·1 (65·4 to 68·8)	70·5 (69·1 to 72)	74·8 (73·2 to 76·3)	77·7 (76·1 to 79·2)	79·7 (77·6 to 81·9)
Ceará	42·4 (38·9 to 45·6)	47·8 (45 to 50·8)	54·6 (51·7 to 57·3)	63·4 (61 to 66·1)	71·9 (70·3 to 73·7)	75·8 (74·1 to 77·5)	77·8 (76·1 to 79·4)	79 (77 to 81·1)
Distrito Federal	50·6 (48 to 53·3)	56·6 (53·7 to 59·7)	61·4 (58·9 to 64·1)	67·6 (66·3 to 68·8)	71·9 (71 to 72·8)	75·3 (75 to 75·7)	78·1 (77·8 to 78·5)	80 (79 to 81)
Espírito Santo	56·2 (53 to 59·6)	60 (57·1 to 62·9)	63·7 (61 to 66·2)	69·8 (69·2 to 70·3)	73·5 (73·1 to 73·9)	76·3 (76 to 76·6)	77·7 (77·1 to 78·3)	79·1 (77·7 to 80·6)
Goiás	45·1 (42·5 to 47·6)	50·6 (47·3 to 53·8)	58·3 (55·3 to 61·3)	65·8 (63·6 to 68·3)	70·5 (69 to 72)	75 (73·9 to 76·2)	77·3 (76·2 to 78·6)	78·7 (76·8 to 80·5)
Maranhão	47·1 (43·6 to 50·6)	51·3 (48·5 to 54·2)	56·2 (53·3 to 59·1)	64·3 (61·7 to 66·9)	72·8 (70·8 to 74·7)	76·2 (74·1 to 78·2)	77·6 (75·8 to 79·4)	78·5 (76·4 to 80·4)
Mato Grosso	55·1 (52·4 to 58)	58·1 (54·8 to 61·4)	62·1 (59·1 to 64·9)	69·7 (68·3 to 71·1)	72·8 (71·6 to 74·2)	75·8 (74·6 to 77)	78·1 (77·8 to 78·5)	80·1 (78·7 to 81·4)
Mato Grosso do Sul	56·2 (53·4 to 59)	59·8 (56·7 to 63)	64 (61·1 to 66·7)	68·6 (67·7 to 69·7)	72·8 (72·1 to 73·5)	75·3 (74·9 to 75·7)	77·4 (76·9 to 77·8)	79·6 (78·2 to 80·9)
Minas Gerais	53·8 (50·7 to 56·9)	58 (55·2 to 60·7)	62·3 (59·6 to 65)	67·7 (67·1 to 68·2)	71·9 (71·2 to 72·5)	75·9 (75·2 to 76·5)	78·1 (77·5 to 78·8)	80·5 (79·2 to 81·7)
Pará	50·5 (46·9 to 54·1)	55·7 (52·9 to 58·6)	60 (57·2 to 62·8)	66·2 (64·4 to 68)	71·1 (69·6 to 72·6)	75·3 (73·8 to 76·8)	78 (76·5 to 79·5)	80·2 (78·8 to 81·6)
Paraíba	43·3 (40·1 to 46·7)	50 (46·8 to 53)	57·2 (54·4 to 59·9)	65·4 (64·1 to 66·6)	71·5 (70·5 to 72·4)	76·5 (75·5 to 77·4)	78·1 (77·2 to 78·9)	79·7 (78·1 to 81·3)
Paraná	57·3 (54·1 to 60·6)	61·1 (58·4 to 63·8)	64·6 (61·8 to 67·2)	68 (67·4 to 68·5)	72·2 (71·9 to 72·4)	74·9 (74·7 to 75·2)	77·3 (76·8 to 77·8)	79·2 (77·8 to 80·5)
Pernambuco	39·4 (36·3 to 42·7)	45·1 (42·4 to 48)	51·9 (49·2 to 54·8)	62·1 (61 to 63)	67·9 (67·2 to 68·6)	73·1 (72·4 to 73·8)	75·8 (75·3 to 76·4)	77·5 (76·1 to 78·9)
Piauí	49·3 (46·2 to 52·6)	53·6 (50·8 to 56·4)	57·3 (54·3 to 60·1)	65 (63·1 to 66·8)	71·8 (70·8 to 72·7)	76·5 (75·6 to 77·5)	79·2 (78·6 to 79·7)	80·3 (78·9 to 81·7)
Rio de Janeiro	57·7 (54·8 to 60·4)	61·9 (58·8 to 64·8)	64·4 (61·7 to 67)	67·3 (66·6 to 68·1)	71 (70·7 to 71·3)	74·3 (74·1 to 74·5)	76·6 (76·4 to 76·9)	78·5 (77·2 to 79·7)
Rio Grande do Norte	44·4 (40·5 to 48·2)	51·6 (48·8 to 54·5)	59 (56·3 to 61·6)	67·1 (65·3 to 68·7)	73·5 (72·3 to 74·9)	77·2 (75·8 to 78·5)	80·2 (78·8 to 81·5)	81·1 (79·1 to 83)
Rio Grande do Sul	59·5 (56·8 to 62·1)	63·3 (60·4 to 66·1)	67·3 (64·7 to 69·8)	71·2 (70·6 to 71·8)	74·3 (74 to 74·5)	76·1 (75·9 to 76·3)	78·4 (78·1 to 78·7)	79·5 (78·3 to 80·7)
Rondônia	44·4 (41·8 to 47·1)	49·1 (45·8 to 52·3)	54·7 (52·2 to 57·1)	61 (60 to 62·2)	68·9 (68·2 to 69·7)	74·5 (73·7 to 75·3)	77·7 (76·9 to 78·5)	79·1 (77·7 to 80·6)
Roraima	45·5 (42·8 to 48·2)	51 (48·1 to 54·1)	57·5 (55·1 to 59·9)	65·9 (64·5 to 67·3)	71·4 (70·7 to 72·2)	74·7 (74·2 to 75·2)	77·8 (77·3 to 78·2)	78 (77·1 to 78·8)
Santa Catarina	62·2 (59·4 to 64·9)	65·1 (62·4 to 67·6)	67·4 (65 to 69·9)	71·2 (70·7 to 71·6)	73 (72·4 to 73·6)	75·2 (74·6 to 75·8)	78·4 (77·9 to 78·9)	79·9 (78·7 to 81·1)
São Paulo	60·2 (57·6 to 62·8)	63·6 (60·7 to 66·4)	65·8 (63·2 to 68·3)	68·2 (67·6 to 68·9)	72·3 (71·8 to 72·9)	75·5 (75·4 to 75·7)	78·3 (78 to 78·5)	79·9 (78·8 to 81)
Sergipe	51·6 (48·6 to 54·7)	55·9 (53 to 58·8)	60·4 (57·8 to 63)	64·1 (62·4 to 65·9)	70·1 (69 to 71·2)	74·3 (73·8 to 74·9)	78 (76·8 to 79·1)	79 (77·1 to 80·8)
Tocantins	45·8 (43 to 48·6)	51·1 (46·6 to 55·7)	58·4 (55·5 to 61·2)	67·4 (64·9 to 69·7)	71·6 (70·3 to 72·9)	74·9 (73·8 to 75·9)	78·8 (77·5 to 80·1)	79·8 (78·1 to 81·4)
Paraguay	65·5 (61·8 to 68·5)	70·8 (69·4 to 72·2)	72·2 (70·7 to 73·7)	74·5 (73·3 to 75·6)	77·6 (76·4 to 78·8)	78 (76·8 to 79·1)	78·5 (77·4 to 79·6)	79·7 (77 to 82·3)
North Africa and Middle East	47·4 (45·3 to 49·6)	51·2 (49·5 to 52·9)	57 (55·7 to 58·3)	63·3 (62·4 to 64·2)	67·7 (67 to 68·4)	71·3 (70·6 to 71·9)	73·7 (73 to 74·5)	75·5 (74·3 to 76·6)

North Africa and Middle East	47·4 (45·3 to 49·6)	51·2 (49·5 to 52·9)	57 (55·7 to 58·3)	63·3 (62·4 to 64·2)	67·7 (67 to 68·4)	71·3 (70·6 to 71·9)	73·7 (73 to 74·5)	75·5 (74·3 to 76·6)
Afghanistan	42 (39 to 45·2)	44·4 (40·7 to 47·9)	46·4 (42·7 to 50·2)	44·8 (37·6 to 49·2)	53·8 (51·5 to 56·5)	55 (52·3 to 57·7)	60·7 (57·7 to 63·4)	63·2 (60·6 to 65·8)
Algeria	57·4 (55·7 to 59)	55·7 (54 to 57·4)	58 (55·8 to 60)	60·3 (58·5 to 62·2)	69·1 (67·3 to 70·7)	72·3 (70·6 to 73·9)	75·2 (74 to 76·3)	76·8 (75·4 to 78·2)
Bahrain	53·2 (49·2 to 57·1)	59·8 (56·9 to 62·6)	66·7 (63·8 to 69·3)	70·7 (69·3 to 72·1)	71·2 (70·2 to 72·2)	73·1 (72·1 to 74·1)	76 (75·1 to 76·8)	77·6 (76·2 to 78·9)
Egypt	42·9 (40·5 to 45)	47·8 (45·6 to 49·9)	52·5 (51 to 54)	60·1 (59 to 61·2)	65 (64·5 to 65·6)	69·9 (69·5 to 70·2)	70·4 (70·1 to 70·6)	71·9 (69·6 to 74·1)
Iran	43·3 (40·9 to 45·7)	48·2 (45·5 to 50·8)	58·2 (55·9 to 60·2)	68·2 (67·1 to 69·2)	69·7 (69 to 70·4)	74·3 (73·8 to 74·7)	77·8 (77·5 to 78·1)	79·6 (79·2 to 79·9)
Iraq	59·8 (56·9 to 62·8)	63·8 (61·6 to 66)	65·9 (63·2 to 68)	67·4 (65·4 to 69·4)	69·9 (68·1 to 71·7)	71·2 (68·4 to 73·9)	73·2 (70·6 to 75·8)	75·9 (73·7 to 77·9)
Jordan	59·9 (55·9 to 63·6)	62·3 (58·7 to 65·6)	66·1 (64·5 to 67·5)	70·1 (68·7 to 71·4)	71·3 (70 to 72·7)	72·6 (71·1 to 74)	78 (77 to 79·1)	79·1 (77·1 to 80·9)
Kuwait	67·8 (64·2 to 71·1)	71·5 (69·6 to 73·5)	73·1 (72·7 to 73·6)	74·5 (74·1 to 74·9)	76·5 (76 to 76·9)	80·6 (80·2 to 81)	81·8 (81·5 to 82·1)	84·7 (83·2 to 86·1)
Lebanon	61·2 (57·3 to 64·9)	67·3 (64·5 to 69·9)	69·9 (67·5 to 72·4)	71·8 (69·9 to 73·8)	73 (71·4 to 74·4)	76·4 (75·4 to 77·3)	78·2 (77·4 to 79)	79·3 (78·1 to 80·4)
Libya	49·3 (46·1 to 52·3)	54·6 (51·1 to 58·1)	65·9 (64·1 to 67·7)	71·4 (70·2 to 72·6)	74·3 (72·3 to 76·2)	76·8 (75·9 to 77·8)	77·5 (77·1 to 77·8)	77·6 (75·4 to 79·6)
Morocco	47·8 (45·1 to 50·5)	49·6 (47 to 52·1)	56·7 (54·5 to 58·9)	62·8 (61·3 to 64·3)	68·1 (66·9 to 69·4)	70·9 (69·4 to 72·3)	72·5 (70·7 to 74·3)	74·7 (72·8 to 76·7)
Oman	42·7 (38·4 to 47·1)	47 (42·9 to 51·3)	57·7 (54·4 to 60·8)	64·3 (61·3 to 67·2)	69·3 (66·9 to 71·8)	71·6 (70·2 to 73·1)	72·6 (72·2 to 73)	75·4 (74·4 to 76·3)
Palestine	58·3 (54·4 to 62)	62·3 (59·4 to 65·2)	62·7 (60·3 to 65·1)	69·1 (66·5 to 71·6)	71·3 (69·3 to 73·4)	73·1 (72·2 to 74·1)	75·8 (75·1 to 76·5)	76·5 (75·3 to 77·6)
Qatar	67·4 (64·1 to 70·7)	68·2 (65·5 to 71·1)	70 (67·6 to 72·4)	74·3 (72 to 76·6)	71·4 (69·7 to 73·3)	71·9 (70·3 to 73·4)	72·6 (71·4 to 73·8)	75·3 (74 to 76·6)
Saudi Arabia	52·9 (48·7 to 57)	56·2 (53 to 59·2)	61·4 (58·5 to 64·3)	67·4 (64·7 to 69·8)	69·4 (67·4 to 71·7)	71·5 (70·3 to 72·8)	73·8 (72·5 to 75)	76·4 (74·2 to 78·6)
Sudan	49·5 (46·7 to 52·4)	52·2 (49·5 to 54·9)	53·6 (51·7 to 55·7)	56·5 (54·5 to 58·5)	61 (59·3 to 62·7)	65·8 (63·6 to 67·9)	69·6 (67·1 to 71·8)	72·3 (70·1 to 74)
Syria	56·9 (54·1 to 59·9)	63·2 (60·5 to 65·9)	68·9 (66·7 to 71)	72·7 (70·2 to 75·1)	70·5 (68·5 to 72·6)	72·8 (71·4 to 74·3)	75·9 (74·5 to 77·3)	75·3 (73·1 to 77·3)
Tunisia	46·5 (42·5 to 50·1)	51·3 (49·3 to 53·3)	57·8 (56·4 to 59·3)	67·9 (66·8 to 69)	74·4 (73·3 to 75·4)	76·8 (75·2 to 78·4)	78·5 (76·2 to 81·1)	80 (77·6 to 82·4)
Turkey	49·9 (47 to 52·7)	53·5 (50·6 to 56·3)	60·7 (58·3 to 63·3)	67·6 (66·1 to 69·1)	72 (70·9 to 73·1)	77·5 (76·4 to 78·6)	79·1 (78·1 to 80)	80·6 (79 to 82·3)
United Arab Emirates	60·7 (56·9 to 64·3)	62·5 (59·9 to 65·2)	66·2 (63·6 to 68·7)	69·4 (67·2 to 71·5)	70·3 (68·8 to 72)	71 (69·9 to 72)	70·7 (69·6 to 71·9)	76·5 (74·4 to 78·4)
Yemen	35·5 (33 to 38·1)	39·6 (37·2 to 42)	45·1 (42·2 to 47·7)	55·8 (53·1 to 58·5)	61 (58·6 to 63·5)	65·4 (62·8 to 67·9)	69·4 (66·8 to 71·8)	69·9 (67·3 to 72)
South Asia	44 (41·6 to 46·6)	46·6 (44·1 to 49)	49·2 (47·6 to 50·9)	55·8 (54·7 to 56·7)	60·2 (59·3 to 61·1)	64 (63·3 to 64·8)	68·8 (68 to 69·6)	71·7 (70·1 to 73·4)
South Asia	44 (41·6 to 46·6)	46·6 (44·1 to 49)	49·2 (47·6 to 50·9)	55·8 (54·7 to 56·7)	60·2 (59·3 to 61·1)	64 (63·3 to 64·8)	68·8 (68 to 69·6)	71·7 (70·1 to 73·4)
Bangladesh	41·4 (39 to 44)	45·4 (43 to 47·9)	44·6 (42·5 to 46·8)	53·5 (51·4 to 55·5)	59·7 (58·4 to 61)	67·3 (66·2 to 68·3)	71·9 (70·9 to 73)	75·9 (73·9 to 77·9)
Bhutan	37·9 (33·9 to 42·1)	43·1 (39·4 to 47·1)	49·5 (45 to 53·6)	53·3 (49·7 to 56·9)	60·5 (58·1 to 63)	66·4 (64·8 to 68·1)	72·1 (70·5 to 74)	74·3 (72·3 to 76·4)
India	44·3 (41·5 to 47·5)	46·4 (43·5 to 49·2)	49·1 (47·1 to 51·1)	55·5 (54·4 to 56·8)	60·2 (59·1 to 61·2)	63·9 (63 to 64·9)	69·1 (68 to 70·1)	72·1 (70·2 to 74)
Andhra Pradesh	44·3 (40·6 to 48·3)	46·1 (42·1 to 50·4)	48·5 (44·9 to 52·2)	55·6 (52·5 to 58·9)	60·3 (57·5 to 63·3)	64·7 (61·9 to 67·8)	69·5 (66·4 to 72·6)	73·6 (70·7 to 76·4)
Arunachal Pradesh	44·4 (40·8 to 48·4)	46 (42·1 to 50·2)	49 (45·8 to 52·6)	58 (55·2 to 61)	62·1 (59·3 to 64·8)	66·7 (64 to 69·3)	71·8 (69·1 to 74·2)	74·9 (72·2 to 77·5)
Assam	42·6 (38·8 to 46·6)	44·6 (40·8 to 48·8)	48 (44·7 to 51·4)	55·5 (52·5 to 58·4)	58·7 (56·6 to 60·7)	58·5 (57·3 to 59·6)	64·6 (63·4 to 65·8)	69·7 (67·4 to 71·9)
Bihar	48·8 (45 to 52·6)	49·7 (45·9 to 53·4)	50·3 (46·9 to 53·9)	54·8 (52 to 57·6)	59·2 (56·6 to 61·8)	62·8 (60·7 to 64·8)	67·4 (65·8 to 68·9)	71·4 (69·1 to 73·7)
Chhattisgarh	45 (41·5 to 48·8)	46·2 (42·4 to 50·2)	48·5 (44·9 to 52·3)	53·9 (50·8 to 57·1)	58·3 (55·5 to 61·1)	60·6 (58·5 to 62·5)	65·7 (64·3 to 67·1)	69·9 (67·8 to 72)
Delhi	46·5 (42·8 to 50·1)	51·8 (47·9 to 55·9)	55·5 (52·3 to 58·6)	61·5 (58·7 to 64·4)	65 (62·7 to 67·3)	67·8 (66·2 to 69·3)	73·2 (72·3 to 74)	76·2 (74·5 to 77·9)
Goa	56·7 (53 to 60·5)	58·6 (54·8 to 62·2)	60·4 (57·2 to 63·7)	65·9 (63·2 to 68·6)	68·6 (66 to 71)	72·2 (70 to 74·6)	75·8 (73·4 to 78·2)	77·9 (75·3 to 80·6)
Gujarat	48·1 (44·5 to 51·8)	50·1 (46·3 to 53·9)	53·2 (49·8 to 56·4)	58·2 (55·5 to 61·3)	61·7 (59·7 to 63·8)	66·5 (65·4 to 67·8)	70·6 (69·5 to 71·7)	72·1 (69·7 to 74·7)
Haryana	48 (44·5 to 52)	49·7 (45·9 to 53·5)	52·9 (49·6 to 56·1)	60·3 (57·4 to 62·9)	63·9 (61·8 to 65·8)	66·6 (65·5 to 67·8)	71·2 (70·2 to 72·3)	73·4 (70·9 to 76·2)
Himachal Pradesh	52·7 (48·8 to 56·5)	53·9 (49·8 to 57·9)	56 (52·8 to 59·4)	61·5 (58·6 to 64·4)	64·8 (62·7 to 66·9)	69·1 (68 to 70·2)	74 (73 to 75)	76·5 (74·6 to 78·6)

Jammu and Kashmir	55·1 (51·5 to 58·9)	56·2 (52·4 to 60·3)	58·3 (55 to 61·6)	62·7 (59·8 to 65·4)	65·8 (63·3 to 68·2)	69 (67·4 to 70·5)	71·7 (70·8 to 72·5)	74·1 (71·9 to 76·1)
Jharkhand	48·3 (44·5 to 52·3)	49·7 (45·7 to 53·5)	51·8 (48·2 to 55·4)	56·3 (53·3 to 59·2)	58·1 (55·7 to 60·7)	62·2 (60·6 to 63·7)	66·9 (65·9 to 67·9)	72·1 (70·4 to 74·1)
Karnataka	45·3 (41·6 to 49·1)	47·6 (43·6 to 51·6)	51 (47·8 to 54·1)	58·9 (56·1 to 61·8)	63·8 (61·8 to 65·8)	67·5 (66·2 to 68·8)	71·3 (70·2 to 72·4)	72·3 (69·6 to 75)
Kerala	55·1 (51·6 to 58·8)	59·2 (55·3 to 62·7)	63·4 (60·3 to 66·3)	68·9 (66·5 to 71·4)	71·7 (69·8 to 73·6)	73·7 (72·8 to 74·5)	77·7 (76·9 to 78·7)	80 (77·5 to 82·4)
Madhya Pradesh	42·2 (38·9 to 46)	43·5 (39·3 to 47·4)	46·3 (43 to 49·9)	52·3 (49·4 to 55·2)	57·4 (54·7 to 59·9)	62 (60·1 to 63·9)	67·7 (66·3 to 69)	71·6 (69·6 to 73·7)
Maharashtra	46·4 (42·7 to 50·3)	49 (45·1 to 52·9)	52·4 (49·3 to 55·5)	60·1 (57·7 to 62·9)	64·5 (62·5 to 66·5)	67·2 (66·1 to 68·3)	73 (72 to 74·2)	74·5 (72·2 to 77·1)
Manipur	51 (47·3 to 54·9)	52·5 (48·7 to 56·6)	55·6 (52·4 to 58·8)	61·9 (59·1 to 64·7)	65·6 (63·1 to 68·3)	68·6 (66 to 71·2)	71·8 (69 to 74·8)	74·6 (71·5 to 77·5)
Meghalaya	55·8 (52 to 59·6)	56·8 (52·9 to 60·5)	57·8 (54·5 to 61·2)	60·2 (57·2 to 63·1)	63·4 (60·6 to 66·3)	67·6 (65 to 70·2)	71·4 (68·7 to 73·9)	73·9 (71·1 to 76·5)
Mizoram	58 (54·2 to 61·8)	59·4 (55·5 to 63·2)	61 (57·8 to 64·1)	63·8 (61 to 66·6)	66·6 (63·9 to 69·1)	69·4 (67·1 to 71·9)	71·2 (68·3 to 74·2)	74·2 (70·9 to 77·7)
Nagaland	48·3 (44·5 to 52·4)	50·6 (46·8 to 54·5)	54·5 (51·1 to 58·1)	61·4 (58·5 to 64·4)	65 (62·3 to 67·7)	67·9 (65·3 to 70·6)	70·6 (67·6 to 74)	73·8 (70·7 to 77·1)
Odisha	40·4 (37 to 44·1)	41·9 (38·1 to 45·9)	45 (41·9 to 48·3)	52 (49·1 to 54·8)	56·6 (54·4 to 59·1)	60·6 (59·1 to 62·1)	66·4 (65 to 67·7)	71·6 (68·8 to 74·4)
Punjab	53·2 (49·5 to 57)	55·2 (51·2 to 59·3)	58·5 (55·3 to 61·6)	63 (60·2 to 65·7)	65·8 (63·8 to 67·6)	68·2 (67·2 to 69·3)	72·3 (71·3 to 73·4)	73·3 (71·2 to 75·8)
Rajasthan	49·3 (45·7 to 53·1)	50·1 (46·5 to 53·9)	52·3 (48·9 to 55·7)	57 (54·2 to 59·9)	61·1 (58·9 to 63·1)	64·8 (63·6 to 65·9)	70·4 (69·2 to 71·7)	71·6 (69·2 to 74·2)
Sikkim	51·8 (47·8 to 55·6)	52·6 (48·7 to 56·7)	54·4 (51·1 to 57·8)	60·3 (57·2 to 63·2)	65·1 (62·4 to 67·8)	69·3 (66·8 to 71·8)	73·1 (70·5 to 75·5)	76·5 (73·6 to 79)
Tamil Nadu	43 (39·4 to 46·8)	45·6 (42 to 49·8)	49·2 (46·3 to 52·3)	55·7 (52·9 to 58·4)	60·9 (58·7 to 63·2)	65·1 (63·8 to 66·5)	70·1 (68·9 to 71·3)	73·6 (71·3 to 76)
Telangana	50 (45·7 to 54·3)	51·3 (46·8 to 56)	53·1 (49·3 to 56·9)	57·8 (54·4 to 61·4)	61·5 (58·2 to 64·9)	65·7 (62·3 to 69)	70·3 (67·5 to 73·2)	74·7 (71·5 to 77·7)
Tripura	41·4 (37·9 to 45·3)	44·9 (40·9 to 49·1)	49·2 (45·9 to 52·6)	57·3 (54·3 to 60·1)	62·8 (60 to 65·6)	67·5 (65 to 70)	71·9 (69·3 to 74·4)	74·2 (71·4 to 77)
Union Territories other than Delhi	54·9 (51 to 58·7)	57·1 (53·6 to 60·8)	59·9 (56·6 to 63·3)	65·4 (62·5 to 68·2)	68·2 (65·5 to 70·8)	71·2 (68·8 to 73·6)	74·3 (71·7 to 76·8)	76·6 (73·6 to 79·2)
Uttar Pradesh	34·8 (31·6 to 38·1)	36·5 (33·1 to 40·6)	40·6 (37·4 to 44)	48·5 (46 to 51·2)	55·1 (53 to 57·3)	60·5 (59·3 to 61·8)	66 (64·5 to 67·4)	68·3 (65·9 to 70·7)
Uttarakhand	50·6 (46·8 to 54·6)	52·2 (48·2 to 56·1)	54·4 (51·1 to 57·9)	59·1 (56 to 62·2)	61·6 (58·5 to 64·3)	64·9 (62·2 to 67·3)	68·5 (66·7 to 70·2)	72·6 (70·6 to 74·8)
West Bengal	43·5 (39·9 to 47·2)	46·5 (42·7 to 50·5)	50·1 (46·9 to 53·4)	57 (54·2 to 59·8)	61·9 (60·1 to 63·9)	65·4 (64·4 to 66·5)	71 (70·1 to 71·9)	73·9 (71·7 to 76·4)
Nepal	42·1 (39·7 to 44·7)	46 (43·4 to 48·4)	50·5 (47·9 to 53·1)	52·8 (50·3 to 55·3)	59·1 (57·1 to 60·9)	66·9 (65·5 to 68·2)	70·7 (69·3 to 72)	73 (71·3 to 74·8)
Pakistan	46·5 (43·9 to 49·1)	51 (48·6 to 53·7)	56·1 (54·1 to 58·2)	60·6 (59·2 to 62)	61·8 (60·5 to 63·1)	61·8 (60·5 to 63·2)	64·4 (62·4 to 66·5)	66·8 (63·9 to 69·5)
Southeast Asia, east Asia, and Oceania	51·2 (48·8 to 53·7)	35·3 (27·1 to 42·1)	61·7 (59·4 to 63·5)	65·6 (63·9 to 67)	69·6 (68·5 to 70·8)	72·8 (72·1 to 73·5)	76·6 (75·9 to 77·3)	79·2 (77·9 to 80·4)
East Asia	51·8 (48·8 to 54·8)	30 (20·9 to 38)	62·8 (59·9 to 65·3)	66·3 (64·2 to 68·2)	70·3 (68·9 to 71·9)	73·8 (72·8 to 74·7)	78·1 (77·2 to 78·9)	80·7 (79·1 to 82·4)
China	52·6 (49·6 to 55·6)	29·3 (20·2 to 37·4)	62·7 (59·8 to 65·3)	66·2 (64 to 68·2)	70·2 (68·8 to 71·9)	73·9 (72·9 to 74·8)	78·1 (77·2 to 79)	80·8 (79·1 to 82·5)
North Korea	25·5 (23·2 to 27·9)	50·2 (46·9 to 53·6)	59·9 (57·1 to 62·7)	66·7 (63·9 to 69·2)	70·4 (68·1 to 72·7)	66·8 (59·5 to 71)	73·8 (72·2 to 75·5)	76·2 (73·9 to 78)
Taiwan (province of China)	62·6 (59·1 to 66)	66·7 (66·6 to 66·9)	72·3 (72·1 to 72·4)	75·1 (74·9 to 75·2)	77·5 (77·4 to 77·7)	80 (79·9 to 80·2)	82·9 (82·7 to 83)	83·6 (81·3 to 85·7)
Oceania	53·6 (50·2 to 56·6)	57·8 (55·1 to 60·4)	62·3 (60 to 64·7)	65·3 (63·8 to 66·7)	65·7 (64·1 to 67·3)	65·8 (63·7 to 67·9)	65·5 (62·9 to 67·9)	67·1 (64 to 69·9)
American Samoa	63·9 (62 to 65·8)	67·6 (66·4 to 68·9)	69·4 (67·9 to 70·8)	72·6 (71·3 to 73·7)	74·4 (73·4 to 75·4)	74·5 (73·4 to 75·4)	74·9 (73·7 to 75·9)	74·6 (72·4 to 76·6)
Cook Islands	50 (48·2 to 51·9)	57·7 (56·3 to 59·4)	65 (63·4 to 66·6)	68·5 (66·9 to 70)	72·2 (70·4 to 73·7)	76·4 (74·8 to 77·8)	78·7 (77·2 to 79·9)	79 (76·8 to 81)
Fiji	59·2 (58·2 to 60·2)	66·6 (65·7 to 67·4)	68·6 (67·6 to 69·5)	69·2 (68·3 to 70·1)	69 (66·7 to 71·2)	68·3 (67·8 to 68·8)	69·5 (68·9 to 70·2)	70·6 (67·8 to 73·4)
Guam	69·4 (67·3 to 71·2)	70·1 (69·1 to 71·1)	70·3 (69·5 to 71)	74·1 (73·4 to 74·8)	76·1 (75·3 to 77)	78·2 (77·3 to 79·1)	81·3 (80·3 to 82·3)	79·7 (77·7 to 81·5)
Kiribati	47 (44·2 to 50·3)	50·7 (47·9 to 53·9)	54·6 (51·4 to 58)	57·9 (54·2 to 61·3)	57·7 (55·2 to 60·3)	59·7 (57·5 to 62·1)	62 (59·7 to 64·5)	63·9 (61·3 to 66·6)
Marshall Islands	56·6 (52·8 to 60·4)	60·7 (56·9 to 64·2)	63·7 (60·3 to 66·9)	67 (64·5 to 69·7)	66·7 (65·5 to 67·8)	64·5 (62·7 to 66·1)	65·6 (63·6 to 67·9)	67·3 (64·2 to 70·2)
Federated States of Micronesia	53·4 (50·8 to 56·2)	56·5 (53·4 to 59·9)	60·1 (57 to 63·5)	63·1 (60 to 65·9)	64·4 (61·8 to 67·2)	65·6 (63·8 to 67·3)	66·9 (64·7 to 69·1)	67·1 (64·4 to 69·7)
Nauru	57·6 (53·9 to 61·2)	59·8 (56·8 to 62·5)	62·8 (60 to 65·6)	65·4 (62·5 to 68·3)	65·7 (63·4 to 68·2)	63 (61 to 65·1)	63·5 (61 to 65·9)	66·7 (63·6 to 69·5)

Niue	57·3 (55·1 to 59·6)	65·1 (62·8 to 67·3)	70·4 (68·2 to 72·4)	72·6 (70·1 to 74·9)	71·8 (69·1 to 74·2)	71·7 (69·3 to 74)	72·6 (70 to 75·1)	73·6 (70·6 to 76·2)
Northern Mariana Islands	65·7 (62·3 to 68·6)	67·5 (64·7 to 70·1)	69·4 (66·6 to 71·9)	71·2 (68·1 to 73·7)	72·3 (70·1 to 74·5)	74·6 (72·8 to 76·6)	76·1 (74·3 to 77·9)	76·3 (74·1 to 78·1)
Palau	52·4 (48·4 to 56·3)	57·5 (54·4 to 60·8)	62·6 (59·8 to 65·5)	66 (63 to 68·8)	68·6 (65·6 to 71·5)	70·2 (67·8 to 72·8)	70·6 (68·3 to 73·3)	71·2 (68·6 to 74·1)
Papua New Guinea	52·6 (48 to 56·8)	56·6 (53·3 to 60)	61·8 (58·8 to 64·9)	64·9 (63 to 66·5)	65·3 (63·2 to 67·5)	65·6 (62·6 to 68·6)	64·9 (61·5 to 68·1)	66·8 (63·1 to 70·1)
Samoa	56·3 (53 to 59·6)	62 (59·7 to 64·6)	65·6 (63 to 67·8)	68·9 (66·3 to 71·5)	70·1 (67·1 to 72·4)	70·8 (67·8 to 73·4)	71·3 (68·7 to 73·8)	71·9 (69 to 74·7)
Solomon Islands	44·4 (41·8 to 47·3)	48·2 (45·2 to 51·4)	52·8 (49·9 to 55·9)	56·7 (53·3 to 60·2)	58 (55·1 to 61·2)	59 (56·5 to 61·6)	59·3 (57·2 to 61·7)	61·2 (58·5 to 63·7)
Tokelau	57·8 (54·8 to 60·6)	60·7 (57·6 to 63·9)	63·3 (60 to 66·7)	65·2 (62·2 to 67·9)	67·1 (64·2 to 69·9)	68·6 (65·6 to 71·5)	70·1 (67 to 73)	71·3 (68·1 to 74·2)
Tonga	63·6 (60·1 to 66·4)	66·4 (64·7 to 68·3)	68·3 (66·4 to 70·1)	70·9 (69·2 to 72·5)	73·8 (72·2 to 75·4)	74·6 (73·1 to 76)	76 (74 to 78)	76·4 (73·7 to 78·8)
Tuvalu	49·1 (45·1 to 53·1)	53·6 (50·2 to 57·2)	57·7 (54·2 to 61·5)	59·9 (57 to 63·3)	62·9 (60·8 to 65·1)	62·5 (60·9 to 64·2)	68·5 (66 to 71·3)	70 (66·6 to 73·1)
Vanuatu	49·7 (46·9 to 52·9)	54 (51·3 to 56·8)	60 (57·3 to 62·8)	65·5 (62·7 to 68·3)	66·3 (63·4 to 69·6)	66·6 (63·7 to 69·8)	67·4 (64·6 to 70·5)	68·5 (65·3 to 71·4)
Southeast Asia	49·3 (47·6 to 51·2)	55·1 (53·3 to 56·7)	58·9 (57·5 to 60·3)	64·2 (63 to 65·2)	68·1 (67·3 to 68·8)	70·8 (70·2 to 71·4)	73·7 (72·9 to 74·6)	75·9 (74·6 to 77·2)
Cambodia	48·3 (44·3 to 52·3)	51·4 (48·2 to 54·6)	49·7 (46·6 to 52·7)	55·4 (52·6 to 57·9)	60·3 (58·9 to 61·8)	63 (61·7 to 64·3)	69·8 (68·2 to 71·3)	72·6 (70·9 to 74·6)
Indonesia	45·9 (43·2 to 48·5)	51·3 (48·5 to 53·8)	56·4 (53·9 to 58·9)	61·4 (59·8 to 63·2)	65·7 (64·5 to 66·8)	68·6 (67·6 to 69·8)	71·2 (69·9 to 72·7)	73·5 (71·6 to 75·6)
Aceh	48·5 (44·8 to 52·3)	53·2 (49·6 to 56·8)	56·9 (53·3 to 60·6)	61 (58·1 to 64·1)	64·5 (62·2 to 66·8)	67·6 (65·7 to 69·5)	68·9 (66·7 to 71·1)	70·7 (67·7 to 73·9)
Bali	50·5 (46·9 to 54·3)	55·2 (51·4 to 59·2)	59 (55·4 to 62·8)	62 (59·1 to 65·1)	67·8 (65·9 to 69·8)	70·8 (69·1 to 72·4)	73·2 (71·3 to 75·3)	76·5 (73·1 to 79·7)
Bangka-Belitung Islands	41·2 (37·8 to 44·8)	46 (42·6 to 49·7)	51·3 (47·6 to 55·2)	57 (53·9 to 60·3)	60·7 (58·3 to 63·1)	64·4 (62·3 to 66·7)	67·1 (64·6 to 69·7)	69·3 (66·2 to 72·5)
Banten	34·6 (31·5 to 38)	41 (37·8 to 44·4)	48·1 (44·8 to 51·5)	55·8 (52·8 to 58·6)	63·7 (61·4 to 65·9)	68 (66·1 to 69·9)	70·3 (68·2 to 72·7)	72·5 (69·8 to 75·5)
Bengkulu	37·1 (33·7 to 40·8)	42 (38·6 to 45·7)	47·9 (44·6 to 51·6)	53·5 (50·7 to 56·7)	59·3 (57 to 61·6)	64·2 (62·4 to 66·2)	67·4 (65·1 to 69·8)	70·1 (67·1 to 73)
Gorontalo	40·9 (37·5 to 44·7)	45·7 (42·2 to 49·5)	50·3 (46·7 to 54·3)	55·1 (51·9 to 58·4)	58·1 (55·7 to 60·7)	59 (56·9 to 61·4)	61·1 (58·7 to 64)	65 (61·7 to 68·5)
Jakarta	48·7 (45·1 to 52·4)	54·3 (50·5 to 57·7)	59·7 (56·1 to 63·1)	64·5 (61·6 to 67)	68·3 (66·3 to 70·2)	71 (69·4 to 72·7)	73 (70·8 to 75·3)	74·8 (72·1 to 77·3)
Jambi	39·6 (36 to 43·4)	45·4 (42·1 to 48·9)	49·7 (46·2 to 53·3)	54·5 (51·6 to 57·5)	60·8 (58·7 to 62·9)	66·2 (64·3 to 68·1)	69·4 (67·3 to 71·4)	71·8 (69·1 to 74·8)
West Java	41·7 (38·7 to 44·9)	47·5 (44·4 to 51)	54 (50·8 to 57·4)	60·6 (58·2 to 63·2)	65·8 (64·1 to 67·5)	69·4 (67·7 to 70·9)	71·8 (69·9 to 73·8)	74 (71·4 to 76·4)
Central Java	50·8 (47·6 to 54·1)	55·3 (51·9 to 58·6)	60·3 (57·1 to 63·4)	65·3 (62·9 to 67·6)	69·1 (67·5 to 70·9)	70·9 (69·5 to 72·5)	72·8 (71 to 74·9)	75 (72·6 to 77·5)
East Java	49 (45·9 to 52·1)	53·8 (50·6 to 56·8)	58·6 (55·3 to 61·8)	63·8 (61·4 to 66·1)	67·5 (65·7 to 69)	70·3 (68·8 to 71·7)	73·2 (71·4 to 75·1)	75·3 (72·8 to 77·8)
West Kalimantan	44·1 (40·5 to 47·9)	46·7 (43 to 50·3)	49·9 (46·7 to 53·5)	54·1 (51·5 to 57·1)	60·1 (57·8 to 62·3)	65 (63·2 to 67·1)	69·1 (66·9 to 71·4)	72 (69·2 to 75)
South Kalimantan	38·1 (34·7 to 41·8)	42 (38·6 to 45·6)	48·4 (44·9 to 51·7)	54·5 (51·7 to 57·2)	59·4 (57·3 to 61·8)	63·7 (61·8 to 65·8)	67·6 (65 to 70)	70·4 (67·3 to 73·2)
Central Kalimantan	47·2 (43·3 to 51·2)	53·4 (49·6 to 57·3)	57 (53·7 to 60·7)	60·1 (57·1 to 63·3)	63·4 (61·3 to 65·7)	68·6 (66·9 to 70·6)	70·4 (68·1 to 72·5)	72·2 (69·5 to 75·2)
East Kalimantan	49·8 (46·1 to 53·8)	52·5 (48·7 to 56·4)	56·1 (52·5 to 59·9)	60·5 (57·7 to 63·6)	63·8 (61·6 to 66)	66·6 (64·7 to 68·7)	69·2 (66·6 to 71·7)	71·6 (68·3 to 74·5)
North Kalimantan	51·5 (47·9 to 55·2)	55·2 (51·5 to 58·4)	58·6 (55·4 to 62·1)	65·1 (62·1 to 68)	70·8 (68·6 to 73·3)	74·3 (72·3 to 76·5)	76·5 (74 to 79)	77·7 (74·7 to 81·2)
Riau Islands	49·3 (45·7 to 53·2)	53·7 (49·8 to 57·7)	58·2 (54·5 to 61·9)	61·5 (58·4 to 64·7)	65 (62·7 to 67·6)	68·9 (67·1 to 70·7)	70·3 (68·1 to 72·4)	72·2 (69·7 to 75·2)
Lampung	43·6 (39·9 to 47·5)	49·8 (46·2 to 53·3)	55·8 (52·4 to 59·1)	62·1 (59·4 to 64·8)	67·2 (65·3 to 69)	70·4 (68·7 to 71·9)	72 (69·8 to 74·1)	73·8 (71·1 to 76·2)
Maluku	35·9 (32·6 to 39·5)	42·1 (38·8 to 45·5)	47·5 (43·8 to 51·1)	52·4 (49·5 to 55·7)	57·1 (54·6 to 59·7)	60·7 (58·5 to 63)	63·2 (60·5 to 66·2)	67·2 (63·9 to 70·6)
North Maluku	37 (33·7 to 40·6)	41·7 (38·3 to 45·4)	46·4 (43·2 to 49·7)	50·2 (47·5 to 53·3)	54·9 (52·4 to 57·6)	57·6 (55·3 to 60)	60·1 (57·6 to 62·9)	64 (60·7 to 67·3)
West Nusa Tenggara	34 (30·8 to 37·5)	39·1 (35·9 to 42·3)	44 (40·9 to 47·6)	49·9 (47·2 to 52·9)	57·2 (54·8 to 59·8)	63·1 (61 to 65·2)	68 (65·6 to 70·3)	70·7 (67·9 to 73·6)
East Nusa Tenggara	41·7 (38·4 to 45·6)	46·9 (43·5 to 50·5)	51·8 (48·1 to 55·2)	56·8 (53·9 to 60)	60·8 (58·5 to 63·1)	64·4 (62·4 to 66·5)	68·6 (66·3 to 70·8)	71·3 (68·3 to 74·6)
Papua	47·9 (44·1 to 51·7)	50·4 (46·9 to 54·1)	52·9 (49·1 to 56·6)	55·5 (52·3 to 58·8)	58·1 (55·8 to 60·6)	61·1 (58·9 to 63·3)	63 (60·4 to 65·6)	66·1 (62·9 to 69·5)
West Papua	49·4 (45·6 to 53·4)	51·7 (47·8 to 55·8)	53·6 (50·2 to 57·4)	56·9 (53·8 to 60·4)	59·9 (57·2 to 62·7)	61·4 (59·3 to 63·8)	62·3 (59·6 to 65)	66·4 (62·6 to 69·8)

Riau	48·8 (45·2 to 52·5)	52·2 (48·6 to 55·8)	56·7 (53·1 to 60·3)	62·4 (59·7 to 65·3)	65 (63 to 67·3)	68·1 (66·1 to 69·9)	71·2 (69·1 to 73·4)	74·1 (71·2 to 76·7)
West Sulawesi	29·5 (26·6 to 32·8)	34·6 (31·4 to 37·9)	39·9 (36·9 to 43)	46·6 (43·7 to 49·9)	53·5 (50·9 to 56·4)	59·4 (57·2 to 61·8)	63·6 (61 to 66·2)	67 (63·8 to 70·2)
South Sulawesi	42·1 (38·6 to 45·9)	47·1 (43·5 to 50·6)	52·2 (48·8 to 55·9)	57·4 (54·8 to 60·3)	63·2 (61 to 65·4)	67·5 (65·7 to 69·5)	70·4 (68·3 to 72·6)	73·2 (70·6 to 76·1)
Central Sulawesi	35·6 (32·4 to 39·4)	43·6 (40·2 to 47·2)	49·2 (45·8 to 52·7)	54·1 (51·3 to 57·2)	57·6 (55·4 to 60)	61·3 (59·3 to 63·5)	65·3 (62·7 to 67·8)	68·5 (65·4 to 71·9)
Southeast Sulawesi	39·3 (35·9 to 43·1)	43 (39·1 to 46·8)	46·8 (43·4 to 50·3)	52·7 (49·7 to 55·8)	57·7 (55·4 to 60)	62·3 (60·3 to 64·4)	66·1 (63·5 to 68·7)	68·9 (65·7 to 72)
North Sulawesi	48·3 (44·7 to 52·4)	51·9 (48·3 to 55·9)	56·3 (52·7 to 60·1)	60·5 (57·5 to 63·6)	63·8 (61·5 to 66·3)	67 (65·1 to 69)	68·8 (66·5 to 71·1)	70·6 (67·9 to 74)
West Sumatra	37·4 (34·2 to 41·1)	45·7 (42·3 to 49·1)	52·9 (49·5 to 56·2)	59·3 (56·4 to 62)	64·5 (62·5 to 66·7)	67·9 (66·1 to 69·7)	70·6 (68·3 to 72·7)	73·8 (71·1 to 76·8)
South Sumatra	46·7 (43·2 to 50·3)	52·6 (49 to 56·2)	57·7 (54·1 to 61·1)	63·7 (61·5 to 66·3)	66·7 (64·7 to 68·6)	69·4 (67·6 to 70·9)	71·5 (69·4 to 73·5)	73·2 (70·4 to 76)
North Sumatra	49 (45·2 to 52·8)	53·3 (49·3 to 57·2)	57·5 (53·9 to 61·2)	61·7 (59 to 64·5)	65·1 (63 to 67·2)	67·5 (65·7 to 69·3)	70·1 (67·9 to 72·2)	72 (69·2 to 74·9)
Yogyakarta	57·2 (53·5 to 61)	61·1 (57·5 to 65·1)	64 (60·3 to 67·7)	67 (64·2 to 70)	68·6 (66·6 to 70·5)	69·9 (68 to 71·8)	71·7 (69·3 to 73·9)	73·2 (70·5 to 76·1)
Laos	41·4 (37·5 to 45·6)	42·9 (39 to 47)	45·2 (41·1 to 49·2)	49·7 (46·3 to 53·3)	55·2 (52·7 to 57·9)	60·7 (59·1 to 62·5)	67·5 (65·5 to 69·8)	71·4 (69·1 to 73·8)
Malaysia	56·4 (54·7 to 57·9)	65 (64·7 to 65·2)	70·9 (70·7 to 71·1)	73·2 (72·2 to 74·3)	74·3 (74·1 to 74·4)	75·5 (75·4 to 75·7)	76·7 (76·2 to 77·2)	77·4 (75·4 to 79·4)
Maldives	32 (28·5 to 35·7)	39·3 (36·3 to 42·4)	49 (46·6 to 51·5)	55 (53·7 to 56·2)	65·4 (64·7 to 66·1)	72·6 (72 to 73·2)	79 (78·3 to 79·7)	80·5 (79·1 to 82)
Mauritius	54·2 (53·5 to 54·8)	59·8 (59·3 to 60·3)	66 (65·5 to 66·5)	71·1 (70·6 to 71·6)	74·2 (73·8 to 74·7)	75·6 (75·2 to 76·1)	77·8 (77·2 to 78·2)	78·5 (76·4 to 80·6)
Myanmar	38·9 (35·1 to 42·8)	42·9 (39·6 to 46·2)	47·3 (43·4 to 51·4)	54·3 (51 to 57·7)	58·7 (56·2 to 61·1)	61·9 (59·8 to 63·9)	68 (65·9 to 69·9)	72·6 (70·5 to 74·4)
Philippines	56 (54·3 to 57·7)	62·8 (61·5 to 64·1)	65·3 (64·1 to 66·5)	68·8 (67·8 to 69·9)	71·8 (70·9 to 72·7)	73·9 (72·9 to 74·8)	74 (73 to 75)	75·1 (72·6 to 77·5)
Seychelles	62·6 (60·3 to 64·6)	66·3 (65·3 to 67·2)	69·6 (68·8 to 70·3)	74·7 (73·8 to 75·5)	75·6 (74·9 to 76·4)	76·6 (75·8 to 77·3)	77 (76·3 to 77·7)	77·4 (76 to 78·8)
Sri Lanka	55·9 (55·7 to 56·2)	64·1 (63·9 to 64·3)	67·7 (67·5 to 67·8)	71·7 (71·5 to 71·9)	74 (73·2 to 74·7)	76·2 (76·1 to 76·4)	77·7 (77·6 to 77·9)	80·2 (77·7 to 82·6)
Thailand	56·4 (53·8 to 59·1)	63·6 (61·9 to 65·4)	67·2 (65·8 to 68·7)	70·3 (69 to 71·6)	74·9 (73·7 to 76)	75·8 (74·4 to 77·2)	80·1 (79·1 to 81·2)	81·9 (79·4 to 84·5)
Timor-Leste	43·1 (38·8 to 47·4)	46·3 (42·9 to 50)	51 (47·9 to 54·4)	53·6 (50·8 to 56·1)	61·4 (59·3 to 63·4)	66·1 (64·4 to 67·6)	71·3 (70 to 72·5)	72·4 (70·8 to 74·3)
Vietnam	57·9 (54·1 to 61·4)	63 (59·9 to 65·9)	62·2 (59·7 to 64·4)	70·2 (67·8 to 72·3)	73 (71·4 to 74·7)	76·3 (74·9 to 77·4)	77·6 (76·3 to 78·9)	78·8 (77·3 to 80·6)
Sub-Saharan Africa	45·8 (42·8 to 48·7)	47·7 (45·6 to 50)	51·2 (49·6 to 52·8)	54·6 (53·4 to 55·8)	56·1 (55·1 to 57·1)	54·8 (53 to 56·4)	61 (59·7 to 62·1)	66·8 (65·1 to 68·1)
Central sub-Saharan Africa	49 (44·8 to 53·1)	50·8 (47·5 to 54)	52·5 (49·7 to 55·3)	54·7 (52·1 to 57·2)	55·6 (53·8 to 57·6)	55·1 (53·5 to 56·7)	60·5 (58·7 to 62·4)	66·8 (64·4 to 69·1)
Angola	49·6 (45·4 to 53·7)	50·2 (46·7 to 53·7)	51 (47·3 to 54·7)	52·2 (49·1 to 55·1)	52·9 (50·6 to 55·2)	55·5 (53·7 to 57·3)	62·8 (60·6 to 65)	67·5 (64·6 to 70·1)
Central African Republic	47·1 (42·9 to 51·3)	49·2 (45·5 to 53·5)	51·6 (48·4 to 54·9)	52·8 (50·4 to 55·1)	51·4 (49·3 to 53·7)	45·3 (42·5 to 48·6)	50·1 (46·7 to 54)	55·8 (51·6 to 59·9)
Congo (Brazzaville)	46·4 (42·6 to 50·4)	47·9 (44·5 to 51·2)	51·2 (46·8 to 55·2)	56·5 (53·7 to 59·4)	56·3 (53·9 to 58·8)	53·6 (51·2 to 56·3)	60·9 (58·5 to 63·3)	66·4 (63·3 to 69·2)
DR Congo	49·5 (45·3 to 53·6)	51·5 (47·9 to 55)	53·2 (50·2 to 56·2)	55·3 (52·4 to 58)	56·5 (54·4 to 58·8)	55·9 (54·1 to 57·6)	60·6 (58·7 to 62·5)	67·4 (65 to 69·7)
Equatorial Guinea	42·9 (39·1 to 46·9)	44 (40·6 to 47·8)	49 (45·6 to 52·8)	54·2 (51·2 to 57·3)	54·6 (51·9 to 57·6)	59·8 (56 to 63·7)	63·2 (59·1 to 67·9)	67·1 (62·8 to 71·3)
Gabon	53·6 (49·6 to 57·5)	54·8 (51·6 to 58)	58 (55·1 to 60·9)	62·7 (60·5 to 65)	64·8 (62·5 to 66·5)	61·8 (58·5 to 64·7)	65·6 (63·1 to 68·1)	71·1 (68·5 to 73·6)
Eastern sub-Saharan Africa	44 (41 to 46·8)	46·6 (44·4 to 48·9)	49·7 (47·9 to 51·3)	52·8 (51·6 to 54·1)	53·7 (52·5 to 54·8)	53·4 (51·5 to 55·3)	62·2 (61 to 63·3)	68·1 (66·6 to 69·3)
Burundi	41·4 (37·5 to 45·4)	45·2 (42·1 to 48·6)	47·4 (44·6 to 50·4)	50·5 (48·1 to 52·9)	50·7 (47 to 53·5)	46·8 (44·2 to 49·5)	60·5 (58 to 63·1)	66·3 (63·1 to 69·4)
Comoros	45·8 (42·8 to 49)	48·2 (45·3 to 51·3)	52·8 (49·8 to 55·8)	56·4 (53·8 to 58·9)	60·2 (58·1 to 62·5)	62·6 (60·7 to 64·5)	67 (65·1 to 68·9)	70 (67·7 to 71·9)
Djibouti	56·5 (52·5 to 60·4)	58·1 (54·4 to 61·7)	60·5 (57·1 to 63·9)	63 (60·1 to 65·7)	63·2 (60·5 to 66·1)	62·7 (59·4 to 66·4)	65·2 (61·6 to 69·1)	68·9 (65·2 to 72)
Eritrea	42·8 (38·7 to 46·9)	45 (41·4 to 49·1)	45·2 (33·3 to 50·5)	47·5 (45·3 to 49·5)	51·1 (49·3 to 53)	58·5 (55·6 to 61·6)	63 (59·6 to 66·4)	66·7 (62·7 to 70)
Ethiopia	41·7 (39·1 to 44·6)	43·4 (40·7 to 46·2)	45·2 (42·7 to 47·7)	47·1 (45 to 49·3)	49·5 (48 to 51)	52·4 (50·4 to 54·4)	64·9 (63·7 to 66)	70·8 (69·2 to 72·3)
Kenya	51·4 (48·7 to 54)	56·7 (54·3 to 59·1)	60 (57·5 to 62·4)	63·7 (61·8 to 65·7)	63·8 (61·9 to 65·4)	56·1 (52·8 to 59·7)	63·2 (61·9 to 64·6)	69 (66·9 to 71·2)
Baringo	52·1 (47·7 to 56·2)	54·7 (50·6 to 59)	55 (50·3 to 59·2)	57·1 (52·9 to 60·8)	58·8 (55 to 62·8)	58·8 (55 to 62·4)	64·1 (61·2 to 67)	69 (65·3 to 72·2)

Bomet	70 (66·4 to 73·5)	71·4 (67·9 to 74·8)	71·8 (68·2 to 75·4)	73 (69·6 to 76·4)	70·7 (66·2 to 73·5)	58 (52 to 64·4)	64·1 (59·8 to 67·8)	69·4 (65·8 to 72·4)
Bungoma	50·4 (46·7 to 54·2)	55·5 (51·7 to 59·4)	59·6 (55·6 to 63·7)	62·6 (58·9 to 66·5)	63·9 (60·3 to 67·7)	60·7 (56·5 to 65·2)	67·8 (64·7 to 70·9)	72·1 (68·8 to 75·2)
Busia	39 (35·3 to 43)	45·6 (41·6 to 49·8)	52·3 (48·3 to 56·3)	61 (57 to 64·8)	61·3 (56·9 to 66·2)	53·1 (47·5 to 59·1)	63·4 (59·3 to 68)	70·1 (65·9 to 73·8)
Elgeyo Marakwet	58·8 (54·7 to 62·9)	60·4 (56 to 64·6)	59·9 (55·6 to 64·2)	60·7 (56·9 to 64·7)	62·4 (58·6 to 66·4)	60·5 (56·6 to 64·7)	65·8 (62·7 to 68·9)	69·5 (65·7 to 73)
Embu	43·3 (39·4 to 47·5)	51·2 (47·2 to 55·1)	60 (55·9 to 63·9)	66·8 (63·1 to 70·3)	69·6 (66·1 to 73·3)	65·2 (59·2 to 70·5)	68·6 (64·2 to 72·4)	72·8 (69 to 75·4)
Garissa	49·5 (45·1 to 53·8)	53·8 (49·3 to 58·3)	56·6 (51·9 to 61·4)	59·3 (55 to 63·5)	61·3 (57·2 to 65·3)	61·8 (57·1 to 66·4)	64·2 (60 to 68·1)	67·4 (62·8 to 71·8)
Homa Bay	36·5 (33 to 40·1)	43·3 (40 to 47·2)	46·4 (42·7 to 50·1)	52·2 (48·8 to 55·7)	51·9 (47·4 to 56·7)	37·1 (32·8 to 43·1)	48·1 (44·2 to 53·2)	60·1 (54·7 to 65·4)
Isiolo	29·3 (25·7 to 33·5)	35·7 (31·5 to 40·1)	39·5 (35·5 to 43·8)	47·1 (42·9 to 51·4)	53·4 (49·4 to 57·3)	53·6 (47·6 to 59·2)	59·4 (54·3 to 64·8)	66 (61 to 70·4)
Kajiado	59·8 (55·5 to 63·9)	61·8 (57·6 to 66·1)	61·5 (57·1 to 65·5)	63·3 (59·3 to 67·1)	64 (60 to 68·2)	58·3 (53·7 to 63·4)	65 (61·5 to 68·7)	69·7 (65·7 to 73·8)
Kakamega	48·5 (44·5 to 52·3)	53·6 (49·7 to 57·4)	57·6 (53·6 to 61·3)	61·3 (57·7 to 65)	60·8 (57·1 to 64·8)	52·9 (47·9 to 58·5)	61·8 (58·3 to 65·6)	69·3 (65·3 to 73·1)
Kericho	65·5 (61·4 to 69·3)	66·9 (62·8 to 70·6)	67·4 (63 to 71·2)	68·3 (64·7 to 71·8)	68·5 (64·7 to 72·6)	61·8 (56·6 to 67·4)	68·8 (64·6 to 72·9)	74 (70·5 to 76·3)
Kiambu	61·5 (57·4 to 65·4)	64·6 (60·4 to 68·6)	67 (62·7 to 70·8)	68·7 (65 to 72)	68 (64·7 to 71·4)	60 (55·7 to 64·8)	67·1 (64·4 to 70·1)	72·1 (68·9 to 75·2)
Kilifi	37·2 (33·3 to 41)	45·5 (42 to 48·8)	50·3 (46·4 to 53·8)	56·1 (52·3 to 59·4)	58·4 (54·6 to 62·2)	55·1 (50·5 to 59·9)	63·5 (60 to 67·3)	69·8 (66 to 73·7)
Kirinyaga	48·6 (44·4 to 52·6)	54 (49·7 to 58·2)	57·9 (53·7 to 61·9)	66·7 (62·9 to 70·5)	68·9 (65·1 to 72·8)	63·4 (58·5 to 68·3)	68·6 (65 to 72)	72·3 (68·5 to 75·1)
Kisii	47·8 (43·5 to 51·9)	52·1 (47·8 to 56·4)	55·3 (50·9 to 59·8)	58 (54 to 62·2)	58·6 (55·2 to 62)	54·4 (50·6 to 58·3)	62·1 (59·6 to 65)	67·1 (63·5 to 70·8)
Kisumu	54 (50·1 to 57·9)	58·4 (54·5 to 62·2)	60·7 (57 to 64·4)	62·8 (59·4 to 66·1)	61·2 (57·1 to 65·5)	47·1 (41·8 to 53·8)	57·4 (52·9 to 62·4)	67 (61·9 to 71·9)
Kitui	50·9 (47 to 54·9)	55·4 (51·4 to 59·5)	59·6 (55·3 to 63·5)	64·4 (60·6 to 68)	65·8 (62·3 to 69·4)	60·7 (55·5 to 66·3)	68·9 (65·2 to 72·2)	73·6 (70·2 to 75·8)
Kwale	40 (36 to 44·1)	46·6 (42·3 to 50·8)	51·7 (47·7 to 55·8)	56·7 (52·9 to 61)	58·2 (54·1 to 62·6)	55·1 (50·8 to 59·7)	62·6 (59·4 to 65·9)	68·7 (64·8 to 72·5)
Laikipia	66·4 (62·3 to 70·3)	68·3 (64 to 72)	69·2 (65·2 to 72·9)	71·2 (67·8 to 74·4)	69 (64·4 to 73·1)	59·4 (53·6 to 65·6)	65·6 (61·4 to 70)	71·6 (67·6 to 75·2)
Lamu	33·1 (29 to 37·2)	42 (38 to 46·3)	49·5 (45 to 54)	60·9 (56·5 to 65·3)	60 (55·4 to 64·8)	56·8 (50·7 to 62·7)	64·2 (59·7 to 68·8)	69·5 (65·1 to 73·5)
Machakos	57·9 (53·8 to 61·9)	61·8 (57·7 to 65·7)	64 (59·9 to 67·8)	67·8 (64 to 71·2)	69·3 (66·2 to 72·7)	62 (56·2 to 67·4)	67·7 (63·9 to 71·2)	72·9 (69·5 to 75·3)
Makueni	55·2 (51·4 to 59·3)	59·7 (55·9 to 63·7)	62·7 (59·1 to 66·3)	66·8 (63·2 to 70·1)	66·9 (63 to 70·7)	54·1 (48 to 61·1)	60·5 (56·5 to 64·7)	67·6 (63·9 to 70·9)
Mandera	54·9 (50·7 to 58·8)	57·6 (53·2 to 61·9)	59·3 (55 to 63·4)	60·3 (55·9 to 64·7)	60·1 (56·1 to 64·1)	59·4 (54·8 to 64)	60·6 (57 to 64·6)	63·9 (60·1 to 68·1)
Marsabit	51·2 (46·9 to 55·6)	55·5 (51·1 to 59·6)	57·9 (53·4 to 62·1)	61·8 (57·8 to 65·7)	64·2 (60·4 to 68·2)	61·3 (55·6 to 66·9)	63·8 (59·5 to 68·7)	68·3 (64 to 72·1)
Meru	57·7 (53·6 to 61·8)	61·4 (57·4 to 65·3)	64·3 (60·3 to 68·2)	67·8 (64·3 to 71)	68·8 (65·8 to 72·2)	62·2 (57·2 to 67·8)	65 (61·5 to 68·9)	69·2 (65·3 to 72·5)
Migori	41·1 (37·4 to 45)	46·6 (42·6 to 50·8)	50·3 (46·4 to 54·1)	52·4 (48·7 to 56)	52·8 (48·4 to 57)	42·4 (37 to 48·6)	51·5 (46·8 to 56·7)	61·9 (56·4 to 67·9)
Mombasa	46·8 (42·7 to 50·8)	54·6 (50·5 to 58·4)	60 (55·4 to 63·8)	65·6 (61·9 to 69·7)	63·2 (58·6 to 68·3)	53·1 (47·9 to 59·5)	62·9 (58·8 to 67·1)	69·6 (65·2 to 73·1)
Murang'a	55·6 (51·4 to 59·5)	61·1 (56·9 to 64·9)	64·8 (60·9 to 68·4)	69·7 (66·3 to 73)	70·8 (67·6 to 73·8)	64·2 (58·6 to 69·3)	67·8 (64·3 to 71)	72·1 (68·4 to 75·1)
Nairobi	44·5 (40·8 to 48·3)	56·6 (52·8 to 60·1)	63·7 (60 to 67·1)	68·3 (65·2 to 71·1)	64·9 (61·2 to 68·8)	54·3 (50·1 to 59)	63·8 (61·5 to 66·3)	69·2 (66 to 72·2)
Nakuru	68·4 (64·7 to 71·9)	70·1 (66·6 to 73·8)	70·3 (66·2 to 73·7)	71·2 (67·8 to 74·3)	69·6 (65·7 to 73·4)	60·4 (55·1 to 65·7)	66·1 (62·5 to 69·7)	71·7 (68·3 to 74·6)
Nandi	65·5 (61·8 to 69·3)	67·5 (63·8 to 71·2)	68·7 (65·1 to 72·1)	69·9 (66·3 to 73·6)	69·6 (66 to 73·2)	63·6 (58·3 to 68·7)	70·6 (66·8 to 73·8)	74·4 (70·8 to 77·1)
Narok	65·8 (61·1 to 70·1)	66·1 (61·6 to 70·2)	64·8 (60·1 to 69)	62·8 (58·4 to 66·6)	62·4 (58·3 to 66·5)	57·9 (53·5 to 62·7)	64·9 (61·4 to 68·5)	69·4 (65·5 to 73)
Nyamira	59·6 (55·3 to 63·7)	62·2 (58·1 to 66·1)	64·6 (60·2 to 68·2)	66 (62 to 69·6)	65·8 (61·9 to 69·4)	55 (50 to 60)	58·6 (54·9 to 62·3)	64·4 (60 to 69·4)
Nyandarua	55·4 (51·1 to 59·7)	60·9 (56·5 to 65·3)	64·8 (60·4 to 68·8)	68·5 (64·5 to 72·2)	68·9 (65·1 to 72·3)	61 (56 to 66)	63·3 (59·8 to 66·7)	69 (64·7 to 73·5)
Nyeri	53·8 (49·5 to 58·2)	58·6 (54·2 to 62·8)	62·2 (58 to 66·3)	66·2 (62 to 69·7)	65·6 (62·1 to 69·3)	56·1 (51·7 to 60·6)	62·4 (58·9 to 65·9)	67·5 (62·9 to 72·5)
Samburu	64·3 (59·8 to 68·3)	65·5 (61·3 to 69·2)	65·8 (61·3 to 69·6)	66·4 (62·5 to 70)	64·3 (59·6 to 69·2)	57·3 (51·6 to 63·1)	62·5 (58·1 to 66·8)	67·7 (63·5 to 71·5)
Siaya	42·5 (38·6 to 46·5)	48·1 (44·4 to 51·9)	53 (49 to 56·8)	56·5 (53 to 59·9)	53·9 (49·8 to 58)	42·3 (37·4 to 48·3)	53·9 (49·8 to 58·4)	64·9 (59·8 to 69·7)

Taita Taveta	49·8 (45·8 to 54·1)	55·4 (51 to 59·6)	57 (52·5 to 61·1)	63·3 (59·1 to 67·6)	64 (59·9 to 68·3)	55·1 (50·4 to 60·6)	59·9 (56·6 to 63·6)	66·2 (61·8 to 71·5)
Tana River	51·7 (47 to 56·4)	55·3 (50·8 to 59·5)	56·8 (51·9 to 61·5)	58·6 (54·2 to 63·8)	58·4 (54·1 to 63·1)	51·7 (47·4 to 56·6)	57·3 (53·7 to 61)	64 (59·6 to 68·7)
Tharaka Nithi	58·2 (53·8 to 62·6)	60·8 (56·6 to 64·9)	62·1 (57·6 to 66·4)	64·7 (60·2 to 68·6)	64·6 (60·9 to 67·9)	59·2 (54·5 to 63·9)	67·5 (64·5 to 70·2)	71·2 (67·8 to 74·5)
Trans Nzoia	60·9 (56·8 to 65·1)	64·3 (60·1 to 68·3)	65·6 (61·7 to 69·5)	67·3 (63·8 to 70·9)	67·5 (63·3 to 71·8)	59·9 (54·2 to 66·1)	66·2 (61·6 to 70·8)	71·8 (67·5 to 75)
Turkana	59·2 (54·9 to 63·2)	61 (56·8 to 64·8)	61 (56·5 to 65)	61 (56·9 to 64·9)	59·6 (54·6 to 64·7)	51·2 (45·3 to 58·1)	59·2 (54·1 to 64·4)	66·2 (60·9 to 70·8)
Uasin Gishu	65·2 (61·1 to 69·1)	67·5 (63·9 to 71·1)	68 (64·1 to 71·6)	68·3 (64·2 to 71·6)	67 (62·8 to 71)	60·6 (55·8 to 65·9)	67·4 (64 to 70·9)	72·5 (68·9 to 75·1)
Vihiga	55·2 (51·3 to 59·3)	59 (55 to 62·9)	61·3 (57·2 to 65·4)	63·2 (59 to 67·2)	61·8 (57·6 to 65·8)	54·3 (49·5 to 59·4)	60·6 (57·1 to 64·6)	67·1 (62·9 to 71·4)
Wajir	42·2 (38·2 to 45·9)	47·8 (43·6 to 52·2)	51·6 (47·3 to 56)	56·3 (52 to 60·2)	57·8 (53·7 to 61·7)	58·9 (55·2 to 62·8)	61·3 (57·8 to 64·9)	64·8 (60·7 to 69·1)
West Pokot	55·1 (50·7 to 59·6)	57·1 (53·1 to 61·5)	56·4 (52·2 to 60·6)	56·7 (52·6 to 60·7)	57·7 (53·7 to 61·7)	57·8 (53·7 to 61·8)	63·2 (59·9 to 66·4)	68·2 (64·5 to 71·5)
Madagascar	41·3 (38·8 to 43·9)	45 (42·6 to 47·3)	49·4 (47·8 to 51)	56·3 (54·6 to 58)	57·7 (56 to 59·4)	60·9 (59·5 to 62·3)	63·9 (61·8 to 66·2)	66·7 (64·1 to 69·3)
Malawi	36·3 (32·5 to 40·2)	39·5 (36·6 to 42·5)	43·6 (41·2 to 46·2)	50·2 (48·3 to 52·2)	50·9 (48·8 to 52·6)	46·8 (43·5 to 50·4)	58·9 (56·7 to 60·9)	68·2 (66 to 70·1)
Mozambique	45·1 (41 to 49·2)	47·1 (43·7 to 50·6)	49·6 (46·9 to 52·6)	52 (49·4 to 54·5)	54 (52·5 to 55·6)	54·8 (52·3 to 57·2)	56·7 (54 to 59·2)	62·2 (59·3 to 65·1)
Rwanda	36·8 (32·9 to 41)	43·3 (40·6 to 46·1)	46·4 (43·8 to 49)	50·2 (48·1 to 52·4)	52·1 (50·4 to 53·7)	52·3 (50 to 54·5)	66·5 (64·9 to 68·1)	70·7 (68·6 to 72·4)
Somalia	46·2 (42·3 to 50·2)	47 (43·7 to 50·6)	49 (45·4 to 52·6)	51·2 (48·1 to 54·7)	52·1 (49·5 to 55)	54·2 (51·2 to 57·4)	56·8 (53·3 to 60·3)	61·3 (57·1 to 64·8)
South Sudan	49·2 (45 to 53·4)	50·6 (46·9 to 54·3)	52·6 (49·6 to 55·7)	55·5 (52·6 to 58·3)	57·2 (54·6 to 60)	59·9 (57 to 62·9)	63·1 (59·9 to 66·2)	66·2 (63 to 69)
Uganda	50·1 (46 to 54·2)	51·6 (48·4 to 54·7)	55·1 (52·5 to 57·7)	54·1 (52·2 to 55·9)	51 (47·6 to 54·5)	51·9 (49·7 to 54·2)	62·1 (60·2 to 64·2)	69·8 (67·8 to 71·7)
Tanzania	45·5 (41·5 to 49·5)	46·8 (43·8 to 50·2)	51·1 (48·8 to 53·6)	57·3 (55·5 to 59)	57 (54·8 to 59)	54·6 (52 to 57·6)	62·4 (60·9 to 64)	69·1 (67·4 to 70·8)
Zambia	49·2 (45 to 53·3)	51·6 (48·4 to 54·8)	55·5 (53·1 to 58)	57·4 (55·5 to 59·2)	53·3 (51·1 to 55·3)	46·3 (43·3 to 49·4)	59·2 (57 to 61·4)	66·4 (63·8 to 68·5)
Southern sub-Saharan Africa	55·5 (53·4 to 57·6)	57·3 (55·6 to 59·1)	60 (58·7 to 61·3)	62·9 (61·9 to 63·9)	67·7 (66·8 to 68·5)	56·3 (52·5 to 59·7)	58·3 (56 to 59·6)	67·1 (65·7 to 68·3)
Botswana	57·8 (54 to 61·6)	58·3 (55·3 to 61·7)	60·4 (57·9 to 62·8)	64·8 (62·3 to 67·2)	65·4 (62·2 to 68·3)	47·8 (42·8 to 53·5)	60·2 (56·6 to 63·8)	65·6 (62·2 to 68·7)
eSwatini	48·1 (44·1 to 52·2)	50·5 (47·1 to 54)	56·4 (53·7 to 59·1)	62·1 (59·6 to 65)	66·4 (64·7 to 68·4)	51·1 (45·3 to 56·8)	51·5 (47·6 to 55·6)	63·6 (60·2 to 67·5)
Lesotho	56·8 (54·2 to 59·4)	57·7 (55·3 to 60·2)	59·2 (56·3 to 61·8)	63·4 (60·9 to 65·9)	65·8 (63·7 to 67·6)	51·5 (46·8 to 56·1)	51·6 (48·5 to 54·7)	55·4 (51·7 to 59·3)
Namibia	58·5 (54·6 to 62·2)	59·4 (56 to 62·9)	61·3 (58·7 to 64)	62·4 (60·9 to 64)	66·2 (64·9 to 67·5)	56·4 (52·8 to 59·8)	64·9 (62·3 to 67·7)	69·3 (65·9 to 72·3)
South Africa	55·2 (53·4 to 57)	57 (55·3 to 58·8)	59·6 (58·1 to 61·1)	62·5 (61·4 to 63·7)	68·4 (67·6 to 69·2)	58·7 (55·6 to 61·3)	59·3 (57 to 60·9)	68·3 (66·9 to 69·5)
Zimbabwe	57·2 (53·3 to 60·8)	58·9 (56 to 61·7)	61·4 (58·8 to 64)	63·7 (62 to 65·3)	65·5 (61·8 to 67·7)	49·3 (43·7 to 55·8)	54·2 (51·8 to 56·4)	64 (61·5 to 66·6)
Western sub-Saharan Africa	44·7 (41·7 to 47·6)	45·8 (43·4 to 48·3)	50·2 (48·5 to 52)	54·5 (53 to 56)	56·2 (54·9 to 57·5)	56·2 (54·5 to 57·8)	61·1 (59·4 to 62·6)	65·7 (63·6 to 67·6)
Benin	44·8 (40·7 to 48·9)	46·1 (43·1 to 49·5)	50·1 (48 to 52·7)	54·5 (52·6 to 56·4)	57·9 (56·4 to 59·3)	59·8 (58 to 61·7)	63·7 (61·3 to 65·9)	66·9 (63·3 to 69·8)
Burkina Faso	38·2 (34·2 to 42·2)	41·6 (38·5 to 44·9)	46·3 (43·8 to 48·9)	50·8 (48·7 to 52·9)	52·4 (50·4 to 54·4)	54·2 (52·4 to 56·1)	60·3 (58·7 to 61·9)	64·3 (62 to 66·3)
Cape Verde	57·5 (55·4 to 59·7)	58 (56·9 to 59·2)	59·3 (58·1 to 60·5)	68·5 (67·8 to 69·3)	72·5 (71·8 to 73·3)	74·1 (72·3 to 75·9)	77·6 (76·9 to 78·3)	78 (76·6 to 79·1)
Cameroon	44·7 (42·2 to 47·4)	30·4 (28·7 to 32·1)	53·1 (50·9 to 55·4)	56·8 (54·9 to 58·9)	60·3 (58·8 to 61·6)	56·1 (53·8 to 58·3)	59·2 (56·9 to 61·7)	65·5 (62·7 to 68·1)
Chad	47·7 (43·5 to 51·8)	49·8 (46·5 to 53·1)	50·8 (48 to 53·7)	52·7 (50·2 to 55·2)	55 (53·3 to 56·7)	54·1 (52·4 to 55·8)	58·3 (56·3 to 60·2)	62·2 (59·7 to 64·6)
Côte d'Ivoire	44·6 (41·9 to 47·4)	48·3 (45·9 to 50·8)	53·5 (51·1 to 55·8)	59 (57·1 to 60·7)	58·8 (55·4 to 60·9)	52·9 (49·3 to 56·6)	59·5 (57·1 to 62)	67 (64·5 to 69·2)
The Gambia	55·6 (51·5 to 59·5)	57·3 (54 to 60·5)	59·9 (57·2 to 62·7)	61·4 (58·5 to 64·1)	63·5 (61 to 65·9)	64·1 (62·1 to 66·3)	65·7 (63·8 to 67·7)	68·9 (66·5 to 71)
Ghana	49·7 (47·1 to 52·5)	53·5 (51 to 55·9)	56·7 (54·1 to 59·5)	59·4 (56·9 to 61·7)	61 (59·1 to 62·8)	61·1 (59·3 to 63·1)	64·2 (62·7 to 65·8)	69 (66·9 to 70·9)
Guinea	39·1 (35 to 43·2)	41·8 (37·9 to 45·8)	44·3 (40·2 to 48·1)	47·5 (45 to 50)	52·1 (50·3 to 54)	55·6 (53·6 to 57·6)	59·1 (56·6 to 61·6)	62·8 (59·6 to 66·1)
Guinea-Bissau	38 (34·3 to 41·9)	42 (38·3 to 45·9)	44·1 (40·4 to 47·9)	49·5 (46·3 to 52·8)	52·3 (49·6 to 54·8)	54·4 (52·5 to 56·5)	58·8 (56·3 to 61·4)	64 (60·7 to 67·1)
Liberia	41·8 (37·4 to 46·1)	44·4 (41·1 to 47·9)	48 (45·1 to 51·2)	51·8 (49·3 to 54·1)	51·6 (49·7 to 53·2)	55·7 (54 to 57·4)	62·4 (60·6 to 64·4)	66·9 (64·4 to 69·2)

Mali	36·4 (32·6 to 40·4)	38·7 (35·2 to 42·6)	41·2 (38·7 to 44·1)	46·5 (44·7 to 48·5)	50·4 (48·9 to 51·9)	53·3 (51·7 to 54·8)	59·2 (57·3 to 61·1)	62·7 (59 to 65·6)
Mauritania	49·9 (45·8 to 53·9)	52·1 (49 to 55·2)	56·1 (53·6 to 58·7)	57·8 (55·8 to 59·9)	60·1 (58·5 to 61·5)	63·8 (62·2 to 65·5)	68·2 (66·4 to 69·9)	71 (68·1 to 73·1)
Niger	40·4 (36·4 to 44·4)	42·8 (39 to 46·8)	45 (42·1 to 47·9)	46·2 (44·3 to 48·1)	48·4 (46·7 to 50·1)	52·5 (50·6 to 54·4)	60·4 (58·3 to 62·5)	63·8 (60·1 to 66·9)
Nigeria	46·1 (43 to 49·1)	48·2 (45·5 to 51·4)	50·9 (48·3 to 53·6)	55·9 (53·4 to 58·4)	56·5 (54 to 59)	56·3 (53·4 to 59·4)	61·3 (58·4 to 64·2)	65·9 (62·9 to 68·7)
São Tomé and PrÍncipe	41·6 (38·4 to 44·6)	46·1 (45 to 47·2)	58·9 (57·7 to 60·1)	64·1 (63 to 65·2)	64·9 (63·6 to 66·2)	65·7 (64·2 to 67·3)	70·2 (68·9 to 71·5)	72·5 (70·6 to 74·4)
Senegal	47·8 (44 to 51·6)	49 (46·5 to 51·6)	49·8 (47·5 to 52·2)	54·9 (53·3 to 56·6)	60·8 (59·4 to 62·2)	62·6 (61·3 to 63·7)	67·5 (66 to 68·8)	70·1 (68·1 to 71·8)
Sierra Leone	42·2 (38 to 46·4)	44·9 (41 to 49)	48 (43·9 to 51·8)	50·5 (47·5 to 53·5)	53·3 (51·3 to 55·4)	53·6 (52 to 55·1)	57·2 (55 to 59·7)	63·1 (59·8 to 66)
Togo	48 (43·9 to 52)	49·8 (46·6 to 52·9)	53·7 (51 to 56·4)	57·4 (55·3 to 59·4)	59·9 (58·1 to 61·7)	58·6 (56 to 61·3)	62 (59·9 to 64·2)	67·9 (65·5 to 70·1)

Supplemental Results Table 3a: Age-standardised mortality rates by location, both sexes, 1950, 1960, 1970, 1980, 1990, 2000, 2010, 2019

Location	1950	1960	1970	1980	1990	2000	2010	2019
Global	1975·99 (1854·46 to 2107·27)	2080·16 (1888·47 to 2360·98)	1459·62 (1400·25 to 1523·58)	1272·39 (1231·64 to 1316·66)	1114·35 (1083·05 to 1148·15)	1019·93 (992·73 to 1049·08)	855·06 (829·93 to 881·78)	735·73 (693·05 to 779·01)
Central Europe, eastern Europe, and central Asia	1371·46 (1263·78 to 1482·17)	1076·52 (1038·26 to 1116·14)	1055·36 (1024·98 to 1087·31)	1076·92 (1041·62 to 1111·9)	1018·16 (985·62 to 1054·3)	1126·41 (1088·43 to 1165·81)	947·26 (919 to 978·31)	800·93 (738·63 to 865·49)
Central Asia	2064·46 (1739·53 to 2431·71)	1514·37 (1389·68 to 1637·53)	1239·47 (1176·25 to 1310·61)	1082·15 (1040·56 to 1123·2)	1031·88 (995·81 to 1070·15)	1222·06 (1166·57 to 1276·79)	1153·05 (1098·19 to 1213·48)	998·47 (909·13 to 1103·69)
Armenia	2049·22 (1709·4 to 2479·51)	1308·03 (1156·31 to 1463·57)	1099·08 (1005·12 to 1202·37)	905·79 (818·69 to 995·84)	948·74 (876·58 to 1024·29)	903·19 (840·34 to 975·38)	825·16 (763·54 to 891·68)	728·7 (606·85 to 872·55)
Azerbaijan	2780·75 (2353·78 to 3256·75)	1943·61 (1716·47 to 2176·94)	1336·5 (1229·02 to 1462·16)	1079·52 (995·06 to 1171·74)	1038·99 (955·36 to 1131·36)	1158·64 (1067·19 to 1270·72)	1193·03 (1103·26 to 1302·94)	1106·98 (942·8 to 1311·26)
Georgia	1930·14 (1599·86 to 2303·03)	1349·72 (1195·18 to 1515·13)	1116·28 (1018·5 to 1221·58)	1065·04 (945·91 to 1188·73)	1098·9 (999·68 to 1212·97)	919·99 (828·29 to 1017·19)	885·57 (794·1 to 984·35)	816·17 (683·96 to 954·39)
Kazakhstan	1654·93 (1378·2 to 1975·83)	1378·33 (1226·31 to 1541·31)	1186·8 (1092·32 to 1298·83)	1069·03 (990·29 to 1162·63)	1051·32 (972·98 to 1145·81)	1314·09 (1216·37 to 1420·98)	1170·74 (1144·35 to 1198·75)	925·49 (805·11 to 1051·99)
Kyrgyzstan	1995·84 (1670·05 to 2401·02)	1558·95 (1364·44 to 1774·57)	1369·37 (1236·59 to 1520·01)	1184·86 (1083·71 to 1294·13)	1014·24 (932·77 to 1108·5)	1099·74 (1017·31 to 1194·54)	1029·07 (960·74 to 1101·84)	813·4 (704·11 to 955·35)
Mongolia	3209·91 (2673·02 to 3792·05)	2452·41 (2065·77 to 2881·49)	2076·68 (1753·82 to 2419·18)	1663·25 (1398·59 to 1950·98)	1555·44 (1357·25 to 1775·17)	1713·93 (1509·03 to 1942·25)	1412·47 (1211·31 to 1630·14)	1203·89 (954·87 to 1490·65)
Tajikistan	2493·59 (2047·84 to 3005·33)	1818·63 (1621·06 to 2024·78)	1299·44 (1178·09 to 1438·38)	1055·67 (943·41 to 1185·88)	989·93 (910·95 to 1081·94)	1149·17 (1056·97 to 1245·91)	1207·96 (1079·51 to 1330·8)	1145·56 (937·96 to 1398·45)
Turkmenistan	2299·29 (1919·81 to 2740·93)	1681·46 (1524·8 to 1859·81)	1361·78 (1240·24 to 1493·2)	1208·5 (1102·94 to 1315·53)	1129·67 (1042·13 to 1224·73)	1130·97 (993·32 to 1314·71)	951·42 (857·31 to 1055·65)	909·24 (740·67 to 1130·17)
Uzbekistan	1967·39 (1586·37 to 2403·33)	1354·11 (1199·69 to 1514·54)	1160·99 (1057·31 to 1271·03)	1017·42 (940·13 to 1103·47)	938·32 (871·92 to 1015·59)	1442·03 (1243·04 to 1667·81)	1614·04 (1332·41 to 2005·91)	1398·67 (1063·6 to 1865·16)
Central Europe	1586·79 (1523·01 to 1656·92)	1140·8 (1106·81 to 1175·87)	1077·62 (1044·76 to 1111·08)	1039·05 (1004·19 to 1073·94)	995·07 (962·44 to 1025·93)	878·53 (849·39 to 908·64)	736·03 (723·04 to 749·33)	651·73 (578·55 to 727·58)
Albania	1978·81 (1760·92 to 2226·47)	1147·81 (1038·55 to 1262·24)	973·26 (815·36 to 1153·79)	882·51 (753·02 to 1029·15)	832·68 (767·14 to 900·76)	698·81 (644·88 to 756·16)	589·42 (513·69 to 679·25)	577·67 (447·59 to 729·56)
Bosnia and Herzegovina	2103·35 (1935·03 to 2296·17)	1357·61 (1251·08 to 1474·29)	1169·85 (1087·9 to 1264·13)	1041·83 (967·69 to 1125·12)	841·67 (785·25 to 906·09)	715·03 (616·31 to 823·65)	708·78 (646·44 to 778·29)	685·26 (550·84 to 852·85)
Bulgaria	1417·08 (1311·11 to 1527·98)	970·73 (878·18 to 1069·29)	940·59 (847·38 to 1039·25)	1024·77 (932·92 to 1141·4)	1125·93 (1035·03 to 1226·59)	1144·71 (1060·13 to 1236·79)	963·57 (935·22 to 996·93)	897·43 (730·42 to 1084·36)
Croatia	2158·6 (2008·28 to 2332·26)	1408·19 (1307·53 to 1524·49)	1150·83 (1067·18 to 1246·09)	1028·14 (950·7 to 1117·8)	932·42 (854·09 to 1018·85)	796·44 (736·34 to 869·63)	691·09 (640·71 to 742·91)	594·15 (467·76 to 738·9)
Czech Republic	1344·48 (1254·44 to 1448·88)	1081·73 (1005·92 to 1167·05)	1125·46 (1049·6 to 1208·21)	1085·21 (1010·69 to 1169·67)	1000·97 (927·63 to 1086·02)	764·94 (755·31 to 774·73)	616·04 (604·16 to 627·09)	542·28 (449·91 to 646·24)
Hungary	1425·4 (1331·6 to 1529·48)	1163·13 (1088·77 to 1247·57)	1107·68 (1036·04 to 1186·91)	1136·05 (1060·36 to 1212·36)	1085·71 (1012·25 to 1169·15)	925·75 (860·38 to 998·86)	769·73 (716·07 to 832·79)	670·66 (548·65 to 823·26)
Montenegro	1020·54 (943·12 to 1105·21)	899·2 (824·51 to 979·72)	740·54 (680 to 806·76)	778·81 (722·42 to 843·25)	750·47 (688·24 to 819·38)	808·79 (736·91 to 884·92)	809·57 (746·18 to 882·25)	754·39 (633·2 to 906·02)
North Macedonia	1933·57 (1769·57 to 2112·23)	1495·83 (1382·97 to 1627·02)	1130·9 (1046·68 to 1219·05)	929·77 (861·02 to 1008·69)	1038·25 (941·16 to 1136·79)	1084·33 (995·34 to 1177·18)	1046·06 (931·01 to 1183·17)	936·64 (737·12 to 1182·46)
Poland	1605·38 (1495·93 to 1726·93)	1050·6 (983·55 to 1122·83)	989·64 (923·88 to 1064·21)	987·26 (909·12 to 1075·57)	960·06 (889·34 to 1041·53)	807·27 (746·27 to 879·14)	658·2 (652·43 to 663·94)	585·3 (495·58 to 687·33)
Romania	1516·13 (1267·37 to 1797·67)	1166·96 (1063·79 to 1283·96)	1120·45 (1028·99 to 1227·65)	1103·15 (1011·56 to 1203·32)	1050·71 (970·07 to 1139·31)	962·6 (887·49 to 1045·26)	818·31 (757·81 to 882·6)	719·93 (588·49 to 876·15)
Serbia	2010·44 (1871·96 to 2158·66)	1373·86 (1280·86 to 1487·67)	1272·41 (1174·11 to 1379·71)	1039·63 (970·34 to 1123·79)	1008·2 (936·23 to 1095·53)	1020·73 (939·65 to 1108·28)	911·55 (848·93 to 980·98)	821·21 (671·56 to 1010·49)
Slovakia	1317·61 (1297·26 to 1338·85)	996·98 (981·68 to 1011·35)	1049·96 (1035·32 to 1064·74)	1012·95 (999·85 to 1026·38)	977·23 (964·29 to 989·34)	821·51 (809·2 to 834·62)	714·67 (704·2 to 726·03)	625·8 (503·26 to 778·05)
Slovenia	1501·86 (1402·41 to 1613·27)	1307·69 (1211·17 to 1416·02)	1125·92 (1040·2 to 1214·59)	964·5 (885·99 to 1044·74)	811·92 (635·32 to 1024·46)	679·62 (663·96 to 695·29)	521·67 (508·39 to 536·81)	448·49 (358·6 to 565·15)
Eastern Europe	1110·31 (985·54 to 1243·79)	943·66 (896·93 to 993·23)	984·63 (943·69 to 1031·8)	1074·97 (1024·48 to 1132·63)	1005·21 (955·87 to 1063·77)	1224·47 (1160·74 to 1298·51)	1021·86 (969·52 to 1081·09)	844·7 (748·69 to 947·66)
Belarus	965·03 (766·46 to 1209·83)	818·42 (754·16 to 892)	807·27 (753·99 to 868·1)	910·78 (849·23 to 983·05)	909·94 (845·13 to 983·49)	1051 (974·75 to 1134·03)	968·36 (895·67 to 1049·65)	791·28 (617·55 to 1022·94)
Estonia	1193·6 (972·68 to 1457·83)	1033·54 (1010·89 to 1056·71)	1005·95 (984·54 to 1028·49)	1035·99 (1014·45 to 1057·46)	1015·46 (994·38 to 1036·57)	936·49 (916·72 to 956·71)	670·51 (653·23 to 688·77)	585·79 (470·9 to 717·58)
Latvia	1041·83 (829·1 to 1300·15)	955·46 (886·87 to 1034·76)	953·56 (883·42 to 1022·58)	1036·22 (964·86 to 1113·09)	1004·42 (931·47 to 1086·16)	945·84 (881·1 to 1022·06)	813·77 (798·24 to 830·46)	686·97 (581·77 to 810·8)
Lithuania	1070·64 (835·79 to 1332·12)	904·7 (840·72 to 970·92)	889·62 (831·09 to 953·54)	942·08 (872·63 to 1012·68)	905·23 (839·56 to 977·72)	854·82 (793·35 to 925·18)	818·95 (762·17 to 887·22)	668·06 (552·46 to 805·56)
Moldova	1973·68 (1643·01 to 2346·59)	1716·16 (1535·86 to 1912·8)	1401·85 (1282·21 to 1533·3)	1223·1 (1133·95 to 1316·98)	1113·33 (1037·26 to 1201·32)	1061·15 (969·41 to 1172·22)	994·3 (895·05 to 1113·24)	750·87 (615·75 to 923·35)
Russia	1128·45 (961·78 to 1314·71)	966·73 (894·66 to 1040·2)	1015·79 (949·42 to 1089·67)	1109·38 (1027·92 to 1195·84)	1021·72 (947·5 to 1107·31)	1275·94 (1177·15 to 1383·77)	1050·39 (981·4 to 1127·06)	815·15 (700·14 to 954·08)
Ukraine	1035·21 (885·89 to 1203·3)	869·88 (807·54 to 940·16)	931·58 (872·25 to 993·58)	1019·94 (949·04 to 1105·05)	981·25 (913·32 to 1059·13)	1184·05 (1103·54 to 1273·25)	988·3 (877·2 to 1120·26)	997·51 (797·62 to 1248·4)
High income	1241·94 (1207·32 to 1275·89)	1046·84 (1021·52 to 1072·4)	955·9 (932·76 to 979·37)	792·66 (773·27 to 813·13)	667·07 (652·15 to 683)	569·01 (556·43 to 582·71)	471·81 (461·75 to 482·5)	443·12 (425·15 to 461·68)
Australasia	1073·4 (1008·68 to 1141·49)	976·27 (920·22 to 1042·01)	972·11 (912·46 to 1037·39)	779·46 (729·01 to 829·4)	649·26 (607·59 to 693·08)	523·98 (492·19 to 558·96)	425·11 (399·8 to 453·34)	396·54 (366·17 to 428·31)
Australia	1078·46 (1002·43 to 1161·36)	977·59 (910·16 to 1059·06)	977·17 (908 to 1057)	767·3 (710·76 to 826·91)	639·17 (593·14 to 689·8)	514·94 (478·45 to 556·2)	418·17 (390·27 to 450·37)	390·37 (356·4 to 428·11)
New Zealand	1060·27 (990·23 to 1138·36)	970·32 (903·87 to 1044·29)	950·99 (885·9 to 1025·12)	836·29 (780·52 to 900·14)	700·42 (650·53 to 758·06)	571·67 (529·91 to 616·26)	462·61 (422·08 to 506·1)	431·27 (388·3 to 483·23)
High-income Asia Pacific	1778·27 (1681·66 to 1893·86)	1240·87 (1170·89 to 1319·11)	1010·03 (951 to 1075·77)	778·05 (734·27 to 827·56)	598·13 (565·14 to 635·61)	473·44 (447·34 to 502·27)	387·6 (366·4 to 412·9)	335·75 (310·86 to 364·62)
Brunei	2404·93 (2205·18 to 2630·15)	1909·38 (1731·95 to 2109)	1584·27 (1434·31 to 1735·89)	1297·35 (1175·65 to 1443·03)	1236·9 (1122·49 to 1358·88)	1059·29 (969·71 to 1170·02)	936·44 (857·23 to 1027·25)	871·81 (751·88 to 1011·18)
Japan	1499·9 (1406·86 to 1610·37)	1138·87 (1069·51 to 1219·02)	916·24 (854·32 to 988·09)	690·14 (644·89 to 739·74)	535·8 (502·37 to 575·67)	435·6 (407·39 to 468·24)	371·4 (346·85 to 400·66)	324·39 (296·63 to 357·59)
Aichi	1413·04 (1192·44 to 1660·72)	1095·99 (925·87 to 1285·05)	907·51 (773·39 to 1072·14)	704·01 (641·03 to 780·58)	548·04 (500·68 to 608·32)	445·47 (404·47 to 494·89)	374·13 (339·21 to 416·7)	319·93 (273·42 to 376·75)
Akita	1513·73 (1268·95 to 1785·65)	1181·72 (991·79 to 1401·48)	979·77 (832·8 to 1149·76)	757·24 (686·56 to 830·79)	573·15 (517·86 to 630·37)	463·66 (420·94 to 512·69)	400·83 (362·44 to 442·26)	387·88 (332·06 to 462·18)
Aomori	1634·83 (1393·46 to 1914·27)	1234·01 (1029·42 to 1451·73)	976·75 (817·33 to 1150·99)	719·86 (655·61 to 788·3)	567·31 (515·71 to 625·14)	476·21 (433·17 to 524·56)	416·02 (376·22 to 460·26)	362·45 (310·23 to 423·98)
Chiba	1502·99 (1253·63 to 1768·55)	1133·42 (960·45 to 1327·18)	905·58 (755 to 1068·13)	692·24 (628·46 to 762·64)	524·79 (475·43 to 579·04)	438·32 (397·58 to 484·05)	374·71 (339·04 to 413·6)	333·96 (288·23 to 390·26)
Ehime	1532·65 (1310·03 to 1784·13)	1160·43 (973·17 to 1370·87)	911·24 (773·74 to 1077·53)	674·63 (612·59 to 742·28)	529·2 (482·22 to 585·56)	444·17 (403·56 to 493)	372·72 (338·97 to 416·37)	340·67 (290·22 to 400·72)
Fukui	1364·19 (1147·06 to 1617·59)	1066·06 (892·58 to 1271·94)	842·42 (719·45 to 977·37)	606·24 (546·8 to 671·51)	496·52 (447·71 to 550·99)	397·33 (357·92 to 440·74)	343·69 (309·58 to 381·59)	297·74 (254·65 to 345·37)
Fukuoka	1530·23 (1308·01 to 1787·5)	1164·25 (983·55 to 1378·57)	939·61 (790·24 to 1104·64)	701·31 (636·36 to 777·51)	546·33 (495·63 to 606·03)	439·01 (398·11 to 483·41)	369·68 (334·98 to 410·82)	329·8 (283·97 to 389·44)
Fukushima	1598·82 (1369·93 to 1862·48)	1182·95 (994·75 to 1393·73)	952·84 (801·62 to 1117·93)	686·18 (623·66 to 756·66)	524·3 (478·31 to 574·73)	440·39 (400·5 to 483·47)	381·66 (346·5 to 421·26)	343·63 (287·4 to 404·08)
Gifu	1461·84 (1249·66 to 1711·5)	1131·48 (950·43 to 1330·77)	934·72 (789·65 to 1104·47)	718·67 (650·6 to 793·49)	534·11 (485·23 to 587·86)	439·54 (396·6 to 484·72)	367·07 (332·43 to 403·29)	329·45 (281·87 to 385·44)
Gunma	1547·01 (1310·17 to 1819·72)	1178·22 (989·86 to 1393·35)	946·78 (803·18 to 1105·91)	715·93 (652·05 to 787·87)	537·23 (488·41 to 591·25)	437·53 (396·46 to 481·42)	384·57 (348·89 to 424·44)	339·57 (286·93 to 394·83)
Hiroshima	1553·55 (1321·06 to 1817·66)	1164·02 (973·63 to 1377·74)	908·66 (758·18 to 1082·57)	680·96 (616·26 to 752·62)	527·95 (476·7 to 584·89)	423·11 (382·98 to 467·78)	358·44 (323·81 to 398·8)	312·4 (265·99 to 366·55)
Hokkaidō	1514·58 (1284·17 to 1770·07)	1157·19 (974·35 to 1371·96)	953·88 (804·19 to 1120·65)	718·14 (652·72 to 792·69)	545·78 (495·57 to 604·32)	436·6 (395·99 to 484·06)	376·41 (340 to 416·95)	330·55 (282·25 to 387·82)
Hyōgo	1471·36 (1238·35 to 1714·51)	1129·84 (944·29 to 1340·52)	928·3 (784·02 to 1088·79)	715·15 (650·15 to 788·05)	561·78 (509·11 to 620·01)	448·91 (406·72 to 494·1)	373·51 (338·17 to 412·4)	321·36 (275·69 to 375·39)
Ibaraki	1660·78 (1425·88 to 1905·01)	1221·05 (1028·94 to 1426·2)	978·15 (829·84 to 1151·5)	725·91 (660·69 to 795·73)	564·83 (512·22 to 622·77)	459·53 (417·22 to 506·01)	396·58 (360·2 to 436·64)	349·18 (299·39 to 404·44)
Ishikawa	1385·84 (1169·73 to 1624·64)	1080·8 (900·36 to 1262·3)	867·7 (743·8 to 1019·17)	654·53 (592·99 to 720·89)	507·94 (460·09 to 558·61)	417·78 (378·53 to 460·29)	354·68 (319·58 to 391·8)	318·92 (271·4 to 372·02)
Iwate	1602·68 (1367·04 to 1861·04)	1212·84 (1015·61 to 1421·58)	965·35 (818·86 to 1135·07)	719·47 (647·81 to 792·99)	551·55 (499·27 to 609·95)	442·52 (401·19 to 487·42)	394·81 (355·55 to 438·69)	360·85 (309·05 to 420·69)
Kagawa	1460·73 (1230·26 to 1709·83)	1120·84 (939·28 to 1316·43)	875·56 (745·52 to 1029·57)	645·67 (588·24 to 712·86)	524·71 (478·27 to 581·75)	432·19 (391·72 to 477·57)	371·65 (336·46 to 411·32)	312·01 (266·77 to 363·63)
Kagoshima	1722·88 (1477·29 to 1992·46)	1262·15 (1070·57 to 1478·66)	1012·1 (850·54 to 1194·69)	721·38 (657·47 to 795·41)	541·43 (492·92 to 596·33)	441·05 (402·3 to 485·25)	378·15 (342·41 to 419·62)	324·05 (278·75 to 377·8)
Kanagawa	1343·42 (1136·86 to 1572·67)	1029·13 (862·64 to 1220·68)	839·62 (704·16 to 988·9)	660·6 (600·67 to 720·49)	526·1 (479·41 to 575·24)	430·38 (391·95 to 470·5)	362·61 (330·13 to 397·58)	310·98 (265·6 to 365·28)
Kōchi	1680·89 (1432·2 to 1968·63)	1267·16 (1075·29 to 1494·7)	995·46 (845·76 to 1160·38)	707·57 (642·21 to 779·73)	530·23 (483·25 to 586·04)	443·62 (403·67 to 490·8)	381·58 (348·06 to 421·09)	328·4 (284·42 to 378·74)
Kumamoto	1597·56 (1364·57 to 1851·62)	1196·56 (997·23 to 1412·73)	951·73 (801·11 to 1124·82)	678·6 (612·27 to 745·48)	505·13 (458·27 to 555·17)	401·81 (365·33 to 442·1)	345·89 (312·69 to 383·07)	305·96 (262·85 to 355·05)
Kyōto	1393·82 (1176·24 to 1635·19)	1063·05 (887·55 to 1247·83)	842·6 (709·8 to 1001·41)	638·09 (577·61 to 707·85)	511·22 (464·88 to 565·57)	422·56 (382·9 to 467·97)	353·07 (319·53 to 390·49)	300·47 (252·3 to 349·51)
Mie	1442·63 (1224·67 to 1704·57)	1107·68 (935·08 to 1293·84)	895·18 (756·57 to 1055·28)	688·44 (624·17 to 760·53)	546·35 (496·95 to 603·93)	437·27 (396·81 to 484·54)	374·77 (339·61 to 416·24)	329·2 (279·64 to 383·02)
Miyagi	1530·52 (1301·08 to 1795·32)	1137·61 (949·84 to 1358·71)	946·77 (801·74 to 1111·2)	709 (642·08 to 784·04)	538·8 (490·38 to 592·81)	435·56 (395·06 to 481·7)	366·51 (331·14 to 405·24)	314·77 (267·2 to 365·47)
Miyazaki	1620·64 (1363·36 to 1898·01)	1225·13 (1022·37 to 1443·48)	964 (824·1 to 1132·16)	709·07 (642·08 to 783·03)	534·85 (484·73 to 591·61)	427·08 (385·42 to 472·91)	366·93 (330·67 to 404·89)	332·93 (282·9 to 387·81)
Nagano	1470·37 (1235·19 to 1727·52)	1102·59 (916·26 to 1300·96)	888·54 (752·24 to 1055·37)	664·03 (601·57 to 728·17)	496·16 (448·96 to 545·27)	404·11 (365·09 to 444·73)	338·82 (305·42 to 373·86)	306·72 (261·79 to 357·07)
Nagasaki	1568·33 (1311·27 to 1825·23)	1198·22 (1007·97 to 1403·24)	982·32 (828·89 to 1161·61)	725·5 (658·08 to 801·56)	543·45 (493·81 to 599·63)	436·93 (396·49 to 484·9)	377·14 (340·64 to 419·81)	329·66 (279·74 to 385·27)
Nara	1467·5 (1238·64 to 1725·49)	1124·54 (953·79 to 1318·63)	928·79 (788·31 to 1081·36)	707·65 (637·92 to 781·5)	546·62 (491·82 to 604·28)	424·23 (380·96 to 470·1)	355·12 (320·77 to 394·71)	313·71 (268·54 to 366·05)
Niigata	1468·83 (1249·71 to 1711·09)	1119·73 (941·69 to 1323·49)	936·07 (791·58 to 1098·56)	709·84 (643·95 to 788·48)	519·12 (472·14 to 575·78)	423·39 (383·52 to 466·41)	360·96 (326·53 to 401·07)	336·9 (286·57 to 395·04)
Ōita	1585·13 (1329·51 to 1872·98)	1196·96 (1000·92 to 1414·17)	948·45 (803·84 to 1113·4)	704·16 (641·15 to 778·41)	536·66 (487·07 to 595·01)	431·27 (390·22 to 477·32)	354·47 (321·57 to 394·38)	318·83 (271·68 to 375·59)
Okayama	1468·82 (1249·43 to 1714·17)	1120·95 (941·31 to 1327·63)	878·04 (750·74 to 1031·15)	662·09 (599·11 to 733·16)	514·33 (465·9 to 567·51)	415·71 (377·37 to 459·34)	358·29 (324·61 to 397·35)	303·5 (259·38 to 355·64)
Okinawa	1722·85 (1450·59 to 2010·68)	1220·28 (1004·88 to 1489·73)	975·82 (829·63 to 1145·25)	632 (575·62 to 694·62)	465·2 (423·39 to 514·83)	405·99 (367·38 to 448·47)	368·44 (333·89 to 409·16)	341·26 (289·02 to 399·87)
Ōsaka	1495·3 (1256·06 to 1753·63)	1149·5 (963·08 to 1353·04)	943·06 (796·4 to 1106·83)	744·38 (678·39 to 817·97)	591·81 (539·56 to 648·43)	466·38 (423·18 to 513·04)	395·11 (359·56 to 436·21)	333·62 (288·49 to 384·63)

Supplemental Results Table 3a: Age-standardised mortality rates by location, both sexes, 1950, 1960, 1970, 1980, 1990, 2000, 2010, 2019

Location	1950	1960	1970	1980	1990	2000	2010	2019
Saga	1567·93<br>(1337·82 to 1838·02)	1173·62<br>(983·41 to 1387·91)	921·1<br>(785·22 to 1072·44)	669·43<br>(608·05 to 743·66)	527·73<br>(479·78 to 587·18)	432·42<br>(391·65 to 481·1)	365·36<br>(329·73 to 405·08)	319·72<br>(274·9 to 373·97)
Saitama	1462·58<br>(1227·3 to 1721·1)	1103·9<br>(932·28 to 1299·93)	892·52<br>(746·76 to 1061·04)	706·28<br>(640·31 to 776·61)	544·11<br>(493·88 to 600·55)	438·83<br>(397·83 to 484·82)	381·11<br>(344·49 to 422·53)	332·71<br>(285·33 to 388·49)
Shiga	1465·77<br>(1247·56 to 1740·2)	1136·49<br>(963·08 to 1338·64)	944·25<br>(810·91 to 1097·37)	692·81<br>(624·9 to 765·66)	545·29<br>(492·01 to 603·86)	413·33<br>(373·72 to 455·75)	350·35<br>(316·02 to 388·86)	293·91<br>(250·09 to 343·48)
Shimane	1508·92<br>(1273·96 to 1774·91)	1166·54<br>(987·59 to 1392·52)	915·25<br>(784·47 to 1072·5)	652·53<br>(592·27 to 725·04)	488·77<br>(443·76 to 540·32)	402·37<br>(365·16 to 445·42)	362·8<br>(328·92 to 403·47)	316·08<br>(271·43 to 370·63)
Shizuoka	1407·35<br>(1199·35 to 1645·28)	1071·74<br>(898·66 to 1269·6)	868·29<br>(728·69 to 1028·01)	664·96<br>(603·66 to 729·28)	514·77<br>(467·22 to 566·04)	423·3<br>(384·33 to 465·08)	367·96<br>(333·87 to 405·14)	323·53<br>(274·34 to 374·41)
Tochigi	1582·68<br>(1317·55 to 1849·11)	1197·95<br>(1010·27 to 1397·28)	964·01<br>(808·09 to 1126·84)	742·26<br>(677·26 to 815·43)	575·96<br>(524·48 to 635·44)	457·02<br>(415·06 to 503·73)	399·17<br>(364·2 to 440·7)	355·83<br>(306·31 to 415·13)
Tokushima	1609·92<br>(1363·25 to 1891·18)	1236·97<br>(1038·25 to 1466·09)	986·89<br>(838·03 to 1151·04)	727·96<br>(660·18 to 809·05)	548·12<br>(496·78 to 606·82)	439<br>(396·82 to 486·25)	373·17<br>(337·5 to 415·45)	332·7<br>(286·1 to 383·15)
Tōkyō	1342·85<br>(1139·65 to 1587·32)	1056·46<br>(892·89 to 1234·86)	868·64<br>(736·83 to 1019·45)	663·37<br>(603·12 to 730·37)	542·84<br>(494·65 to 597·6)	443·58<br>(403·61 to 486·58)	368·88<br>(336 to 406·94)	312·77<br>(268·51 to 361·08)
Tottori	1553·02<br>(1311·2 to 1825·57)	1176·77<br>(999·77 to 1382·27)	927·77<br>(793·27 to 1077·48)	680·5<br>(614·19 to 749·57)	540·33<br>(488·74 to 597·08)	433·62<br>(389·99 to 478·63)	377·45<br>(340·12 to 417·33)	341·48<br>(292·18 to 396·64)
Toyama	1445·92<br>(1231·51 to 1694·46)	1119·14<br>(952·63 to 1317·37)	896·01<br>(759·27 to 1047·46)	657·64<br>(598·99 to 728·01)	502·71<br>(458·19 to 556·13)	406·8<br>(370·72 to 449·43)	353·56<br>(320·63 to 392·3)	308·23<br>(265·61 to 355·44)
Wakayama	1576·86<br>(1350 to 1847·1)	1202·16<br>(998·59 to 1418·3)	967·19<br>(817·22 to 1125·39)	726·33<br>(658·03 to 803·89)	567·46<br>(513·54 to 626·88)	455·77<br>(410·58 to 506·47)	397·64<br>(360·02 to 442·47)	374·33<br>(318·26 to 440·46)
Yamagata	1499·04<br>(1288·65 to 1752·34)	1141·81<br>(962·8 to 1355·33)	939·61<br>(795·13 to 1090·93)	679·23<br>(616·26 to 747·25)	508·56<br>(459·52 to 561·84)	421·96<br>(382·31 to 469·31)	356·39<br>(324·02 to 394·79)	316·41<br>(270·98 to 368·32)
Yamaguchi	1547·82<br>(1318·81 to 1806·53)	1178·6<br>(1003·56 to 1391·36)	926·18<br>(784·55 to 1090·84)	710·34<br>(643·74 to 781·26)	532·6<br>(482·55 to 586·8)	444·32<br>(402·74 to 489·98)	383·48<br>(346·46 to 424·87)	341·19<br>(294·46 to 399·31)
Yamanashi	1541·32<br>(1304·35 to 1805·44)	1165·83<br>(981·67 to 1370·47)	917·15<br>(778·53 to 1069·54)	683·63<br>(619·17 to 757·3)	528·11<br>(479·03 to 585·28)	430·83<br>(389·79 to 479·1)	363·99<br>(327·78 to 405·43)	317·94<br>(272·91 to 369·8)
South Korea	3056·92<br>(2831·91 to 3327·59)	1656·38<br>(1520·73 to 1793·88)	1426·1<br>(1317·95 to 1542·37)	1236·99<br>(1159·14 to 1332·17)	930·43<br>(874·43 to 1000·4)	697·88<br>(653·75 to 750·4)	472·27<br>(440·75 to 507·85)	392·42<br>(354·67 to 432·12)
Singapore	1688·9<br>(1578·7 to 1817·21)	1290·17<br>(1184·72 to 1413·17)	1102·75<br>(1033·39 to 1185·97)	928·56<br>(870·62 to 991·06)	720·5<br>(673·03 to 770·06)	534·32<br>(499·86 to 573·34)	396·85<br>(370·74 to 426·67)	324·84<br>(302·67 to 352·22)
High-income North America	1078·41<br>(1015·67 to 1146)	984·86<br>(928·99 to 1050·83)	918·07<br>(863·03 to 978·4)	754·05<br>(708·66 to 806·45)	664·22<br>(624·11 to 708·15)	610·62<br>(574·04 to 651·43)	522·15<br>(491·29 to 555·2)	517·93<br>(462·62 to 583·22)
Canada	1063·75<br>(995·69 to 1137·95)	937·64<br>(871·59 to 1011·68)	834·55<br>(777·98 to 898·17)	708·46<br>(659·99 to 759·97)	612·79<br>(566·71 to 661·52)	529·54<br>(494·61 to 570·19)	431·26<br>(402·09 to 466·96)	411·5<br>(377·97 to 449·79)
Greenland	2255·29<br>(2005·24 to 2529·17)	1585·08<br>(1453·32 to 1731·42)	1373·19<br>(1263·16 to 1492·91)	1323·63<br>(1216·01 to 1436·64)	1296·44<br>(1190·6 to 1430·03)	1212·6<br>(1128·84 to 1320·25)	924·91<br>(849·43 to 1007·7)	807·93<br>(669·42 to 957·01)
USA	1079·24<br>(1010·85 to 1153·35)	989·33<br>(928·54 to 1061·96)	926·09<br>(866·67 to 992·85)	758·82<br>(708·07 to 817)	670·18<br>(626·01 to 720·09)	619·9<br>(578·59 to 665·56)	532·87<br>(497·88 to 571·8)	530·94<br>(468·79 to 606·8)
Alabama	1195·41<br>(1001·87 to 1415·42)	1055·78<br>(956·93 to 1167·31)	990·9<br>(897·59 to 1103·86)	816·02<br>(738·92 to 907·61)	743·96<br>(673·36 to 822·76)	724·85<br>(656·42 to 803·76)	683·78<br>(617·52 to 754·47)	684·06<br>(551·28 to 863·93)
Alaska	1420·4<br>(1210·03 to 1678·7)	1190·63<br>(1078·63 to 1315·5)	1024·69<br>(930·39 to 1137·44)	857·67<br>(777·01 to 945·62)	778·26<br>(704·8 to 861·56)	688·72<br>(624·28 to 763·25)	555·97<br>(505·88 to 611·96)	559·51<br>(450·05 to 698·13)
Arizona	1112·06<br>(933·98 to 1323·23)	975·67<br>(886·45 to 1077·87)	909·47<br>(826·97 to 1004·8)	740·24<br>(674·06 to 815·53)	625·6<br>(569·5 to 691·8)	591·31<br>(537·51 to 651·8)	497·7<br>(453·23 to 548·31)	513·21<br>(408·72 to 645·78)
Arkansas	1052·82<br>(877·74 to 1250·4)	941·41<br>(853·48 to 1037·21)	899·76<br>(814·47 to 996·07)	766·41<br>(692·43 to 849·6)	714·48<br>(648·51 to 786·97)	704·95<br>(640·18 to 778·29)	643·07<br>(583·08 to 712·17)	654·78<br>(513·04 to 812·62)
California	1013·99<br>(854·69 to 1190·22)	932·07<br>(853·8 to 1020·75)	873·57<br>(800·82 to 956·61)	729·21<br>(666·14 to 802·74)	648·77<br>(595·76 to 709·67)	559·25<br>(511·25 to 614·13)	455·35<br>(417·41 to 498·99)	439·08<br>(349·58 to 536·47)
Colorado	1026·69<br>(864·19 to 1220·32)	909·07<br>(823·23 to 1007·8)	839·88<br>(762·35 to 927·75)	692·04<br>(627·92 to 768·94)	598·8<br>(544·1 to 662·02)	569·51<br>(515·12 to 632·36)	491·36<br>(445·3 to 543·64)	498·82<br>(393·42 to 624·44)
Connecticut	1024·06<br>(855·92 to 1210·37)	952·4<br>(861·11 to 1051·87)	844·63<br>(762·69 to 933·83)	716·8<br>(647·14 to 793·21)	594·67<br>(536·85 to 654·23)	557·82<br>(502·79 to 618·02)	459·29<br>(414·1 to 507·82)	453·65<br>(350·44 to 565·69)
Delaware	1184·46<br>(1009·1 to 1372·55)	1055·63<br>(958·92 to 1166·08)	1001·23<br>(911·49 to 1104·04)	800·35<br>(728·71 to 881·55)	718·45<br>(650·2 to 793·37)	650·49<br>(589·32 to 718·93)	563·31<br>(512·88 to 617·88)	557<br>(451·86 to 689·7)
Washington, DC	1220·19<br>(1021·78 to 1426·04)	1145·48<br>(1034·9 to 1268·34)	1118·08<br>(1009·43 to 1235·2)	918·4<br>(828·69 to 1021·38)	963·25<br>(871·31 to 1063·14)	768·27<br>(688·4 to 851·97)	649·46<br>(583·79 to 719·71)	591·3<br>(464·52 to 740·32)
Florida	1039·92<br>(864·23 to 1248·22)	928·58<br>(840·49 to 1028·04)	885·4<br>(795·66 to 979·45)	734·39<br>(661·44 to 814·19)	634·51<br>(572·96 to 698·91)	585·43<br>(528·08 to 648·13)	499·51<br>(451·37 to 550·08)	505·71<br>(402·06 to 635·71)
Georgia	1189·65<br>(1002·18 to 1405·86)	1066·27<br>(964·37 to 1173·19)	1015·16<br>(915·74 to 1119·81)	824·07<br>(742·91 to 912·69)	749·13<br>(675·62 to 823·43)	715·94<br>(646·53 to 788·84)	619·21<br>(559·72 to 680·71)	596·69<br>(474·15 to 748·07)
Hawaii	1067·65<br>(887·24 to 1280·64)	1103·39<br>(997·84 to 1220)	824·03<br>(744·08 to 910·23)	597·17<br>(539·83 to 662·26)	542·69<br>(490·95 to 600·75)	474·17<br>(426·78 to 524·38)	449·49<br>(404·2 to 497·65)	423·25<br>(334·72 to 527·78)
Idaho	1008·09<br>(825·9 to 1191·38)	909·77<br>(822·41 to 1009·23)	861·24<br>(775·7 to 954·01)	687·28<br>(623·12 to 767·11)	617<br>(557·27 to 682·66)	576·16<br>(521·31 to 641·34)	528·87<br>(479·84 to 585·41)	522·38<br>(414·67 to 656·53)
Illinois	1101·69<br>(920·77 to 1306·89)	1024·08<br>(933·95 to 1131·05)	971·75<br>(884·58 to 1075·29)	780·25<br>(708·34 to 864·34)	690·41<br>(625·94 to 763·51)	627·23<br>(569·88 to 694·94)	523·17<br>(473·37 to 577·69)	517·26<br>(408·69 to 651·49)
Indiana	1054·77<br>(886·42 to 1266·55)	985·54<br>(897·55 to 1082·8)	931·1<br>(845·87 to 1023·13)	766·29<br>(697·41 to 841·87)	680·34<br>(618·02 to 747·06)	661·17<br>(598·59 to 725·46)	589·5<br>(536·84 to 649·37)	610·41<br>(486·46 to 756·57)
Iowa	953·15<br>(795·46 to 1120·72)	881·17<br>(799·47 to 971·45)	839·56<br>(755·54 to 924·26)	665·67<br>(603·72 to 734·24)	574·56<br>(518·77 to 634·75)	556·07<br>(504·13 to 611·57)	507·03<br>(458·73 to 559·5)	511·07<br>(402·51 to 643·38)
Kansas	939·73<br>(778·9 to 1117·67)	880·31<br>(799·4 to 970·97)	815·15<br>(738·94 to 895·84)	685·5<br>(619·24 to 757·15)	594·72<br>(540·93 to 651·52)	593·08<br>(536·47 to 656·7)	547·16<br>(495·57 to 601·22)	540·56<br>(425·45 to 670·74)
Kentucky	1089·37<br>(918·08 to 1299·51)	996·63<br>(903·9 to 1100·3)	985·33<br>(891·1 to 1090·84)	807·06<br>(731·03 to 893·19)	721·85<br>(651·58 to 798·2)	708·21<br>(645·71 to 783·05)	665·89<br>(603·7 to 735·37)	692·25<br>(552·88 to 862·27)
Louisiana	1174·06<br>(985·01 to 1388·33)	1057·5<br>(960·94 to 1163·18)	1009·06<br>(912·55 to 1119·05)	851·02<br>(768·45 to 942·86)	773·67<br>(704·28 to 849·36)	746·07<br>(675·37 to 824·38)	657·57<br>(598·09 to 724·3)	660·42<br>(524·85 to 829·2)
Maine	1103·39<br>(932·19 to 1322·49)	997·25<br>(904·3 to 1100·81)	935·96<br>(846·13 to 1040·62)	739·28<br>(668·08 to 821·83)	633·44<br>(574·05 to 699·8)	609·26<br>(548·21 to 675·78)	536·12<br>(482·32 to 593·72)	566·07<br>(452·92 to 710·46)
Maryland	1161·81<br>(982·17 to 1371·07)	1081·21<br>(980·74 to 1195·43)	968·66<br>(876·72 to 1070·19)	785·56<br>(711·6 to 869·46)	705·91<br>(640·47 to 780·44)	658·58<br>(595·46 to 729·35)	525·19<br>(476·53 to 579·69)	544·27<br>(429·26 to 676·21)
Massachusetts	1043·07<br>(881·7 to 1225·96)	977·17<br>(893·34 to 1077·02)	880·11<br>(800·63 to 971·92)	717·02<br>(651·86 to 792·03)	615·49<br>(560·72 to 680·22)	568·16<br>(518·73 to 628·77)	468·36<br>(426·82 to 514·83)	480·57<br>(384·14 to 599·11)
Michigan	1070·04<br>(901·42 to 1259·12)	993·99<br>(904·52 to 1098·35)	945·61<br>(855·93 to 1050·37)	769·44<br>(698·71 to 854·46)	686·87<br>(621·29 to 761·92)	642·9<br>(580·16 to 714·24)	566·52<br>(509·91 to 627·88)	576·55<br>(456·99 to 731·39)
Minnesota	961·12<br>(796·61 to 1136·48)	889·97<br>(811·39 to 982·15)	816·72<br>(740·19 to 901·78)	652·74<br>(590·19 to 723·82)	578·37<br>(525·54 to 637·23)	540·98<br>(491·09 to 597·9)	460·36<br>(418·86 to 511·31)	457·49<br>(359·72 to 577·81)
Mississippi	1210·22<br>(1015·01 to 1433·77)	1063·71<br>(962·79 to 1177·12)	1033·88<br>(934·31 to 1142·01)	846·71<br>(764·68 to 935·08)	764·58<br>(688·91 to 839·87)	768·94<br>(694·4 to 847·68)	700·3<br>(634·43 to 772·14)	705·73<br>(554·49 to 886·17)
Missouri	1045·63<br>(880·35 to 1237·74)	969·49<br>(883·8 to 1067·92)	928·6<br>(841·92 to 1022·74)	760·26<br>(690·87 to 842·18)	679·04<br>(618·48 to 746·41)	657·42<br>(595·4 to 726·34)	589·21<br>(534·01 to 649·85)	590·62<br>(461·71 to 750·31)
Montana	1113·74<br>(920·47 to 1314·03)	996·23<br>(901·59 to 1107·61)	912·71<br>(825·53 to 1010·63)	749·79<br>(676·18 to 840·52)	632·51<br>(570·27 to 700·57)	607·35<br>(549·04 to 671·73)	542·97<br>(490·07 to 601·2)	553·18<br>(438·44 to 692·73)
Nebraska	940·08<br>(787·84 to 1107·44)	880·13<br>(800·46 to 979·09)	845·2<br>(770·92 to 937·86)	684·82<br>(623·92 to 761·38)	604·75<br>(549·78 to 667·57)	555·04<br>(504·48 to 614·66)	504·04<br>(456·25 to 560·67)	498·19<br>(398·95 to 627·69)
Nevada	1468·48<br>(1255·59 to 1712·08)	1212·08<br>(1104·71 to 1342·98)	1090·5<br>(992·16 to 1213·01)	928·35<br>(841·23 to 1035·11)	843·13<br>(768·22 to 934·24)	779·76<br>(710·21 to 863·21)	621·63<br>(566·05 to 687·99)	588·2<br>(470·96 to 739·65)
New Hampshire	1103·01<br>(934·68 to 1298·09)	968·81<br>(874·02 to 1077·25)	919·84<br>(831·66 to 1013·29)	710·65<br>(640·84 to 790·38)	629·07<br>(571·29 to 694·84)	590·42<br>(535·55 to 652·33)	495·63<br>(446·96 to 547·61)	535·29<br>(425·93 to 671·5)
New Jersey	1108·99<br>(926·83 to 1312·42)	1016·14<br>(922·79 to 1120·42)	941·58<br>(852·36 to 1042·61)	767·91<br>(696·37 to 852·67)	670·86<br>(609·66 to 739·33)	600·85<br>(545·38 to 661·78)	483·56<br>(438·55 to 532·27)	480·03<br>(378·03 to 597·49)
New Mexico	1093·53<br>(926·39 to 1296·49)	959·98<br>(873·53 to 1062·18)	909·94<br>(822·47 to 1005·79)	745·9<br>(675·09 to 825·62)	644·37<br>(586·31 to 713·62)	599·95<br>(545·18 to 662·22)	542·75<br>(493·4 to 599·69)	566·27<br>(455·78 to 707·42)
New York	1110·78<br>(944·95 to 1299·34)	1034·44<br>(948·36 to 1137·76)	961·6<br>(876·54 to 1053·24)	780·42<br>(713·12 to 858·47)	693·91<br>(632·71 to 759·65)	575·98<br>(524·81 to 632·17)	468·56<br>(427·37 to 515·33)	453·92<br>(360·6 to 563·47)
North Carolina	1153·14<br>(954·92 to 1371·02)	1060·15<br>(961·39 to 1167·86)	989·33<br>(893·75 to 1094·91)	793·95<br>(715·7 to 880·46)	712·52<br>(646·33 to 786·6)	686·98<br>(621·45 to 761·29)	582·56<br>(527·1 to 643·03)	582·04<br>(465·33 to 733·2)
North Dakota	983·7<br>(830·08 to 1156·31)	892·53<br>(805·88 to 992·79)	847·11<br>(766·47 to 936·54)	678·41<br>(612·62 to 755·16)	584·82<br>(527·21 to 644·54)	549·36<br>(495·81 to 610·37)	494<br>(446·37 to 544·73)	510·41<br>(409·66 to 630·27)
Ohio	1080·42<br>(906·27 to 1269·12)	995·67<br>(907·7 to 1089·39)	944·32<br>(859·51 to 1033·79)	789·66<br>(716·11 to 869·95)	685·68<br>(623·77 to 749·98)	651·63<br>(590·49 to 714·17)	583·74<br>(531·3 to 637·43)	614·88<br>(493·62 to 758·75)
Oklahoma	1003·36<br>(834·93 to 1197·35)	921·9<br>(836·28 to 1018·94)	894·11<br>(811·29 to 981·84)	761·68<br>(692·87 to 840·62)	683·1<br>(620·45 to 750·36)	705·53<br>(637·66 to 779·01)	662·14<br>(597·51 to 728·82)	662·82<br>(521·97 to 818·15)
Oregon	1011·84<br>(853·83 to 1196·3)	923·74<br>(839·43 to 1016·56)	849·93<br>(771·29 to 945·81)	709·1<br>(643·56 to 786·43)	638·4<br>(577·41 to 710·99)	588·18<br>(532·23 to 652·6)	518·36<br>(470·04 to 572·87)	509·1<br>(400·81 to 643·88)
Pennsylvania	1139·83<br>(966·87 to 1341·85)	1042·77<br>(951·64 to 1146·1)	969·15<br>(879·95 to 1067·43)	787·22<br>(714·95 to 868·4)	678·97<br>(617·2 to 743·02)	629·46<br>(574·33 to 692·27)	544·08<br>(493·96 to 596·92)	557·69<br>(437·19 to 690·78)
Rhode Island	1076·06<br>(896·45 to 1293·86)	982·81<br>(886·04 to 1094·1)	913·51<br>(826·17 to 1015·42)	726·88<br>(654·61 to 807·76)	633·58<br>(571·87 to 701·71)	571·77<br>(514·63 to 635·72)	505·97<br>(455·69 to 557·67)	509·69<br>(405·15 to 638·23)
South Carolina	1289·49<br>(1072·81 to 1529·74)	1134·99<br>(1023·59 to 1252·89)	1034·47<br>(931·91 to 1133·22)	850·68<br>(768·68 to 937·53)	746·62<br>(670·28 to 821·38)	715·9<br>(643·42 to 789·53)	628·17<br>(565·53 to 688·68)	621·34<br>(488·48 to 772·97)
South Dakota	1005·24<br>(835·61 to 1199·76)	927<br>(842·25 to 1032·78)	857·64<br>(776·2 to 949·66)	703·91<br>(634·42 to 783·48)	598·27<br>(541·32 to 664·57)	562·84<br>(508·12 to 625·92)	510·74<br>(462·42 to 568·57)	534·89<br>(432·29 to 660·01)
Tennessee	1097·42<br>(915·19 to 1292·06)	1001·26<br>(906·06 to 1108·02)	963·94<br>(871·97 to 1062·26)	789·06<br>(712·14 to 874·84)	730·98<br>(657·41 to 808·26)	716·4<br>(649·34 to 790·26)	650·4<br>(590·18 to 717·29)	655·46<br>(517·07 to 815·13)
Texas	1024·26<br>(852 to 1227·62)	928<br>(847·3 to 1020·73)	894·68<br>(814·75 to 987·12)	763·57<br>(693·06 to 846·22)	682·18<br>(621·84 to 750·23)	642·33<br>(585·84 to 707·63)	555·55<br>(504·95 to 610·54)	537·38<br>(427·89 to 673·02)
Utah	992·48<br>(830·8 to 1176·24)	899·39<br>(820·05 to 990·16)	834·44<br>(760·46 to 919·17)	683·47<br>(620·96 to 750·66)	582·83<br>(529·72 to 642·19)	560·6<br>(509·51 to 616·4)	505·65<br>(461·4 to 556·16)	510·87<br>(414·69 to 634·55)
Vermont	1107·6<br>(946·77 to 1283·87)	988·59<br>(897·08 to 1090·61)	898·08<br>(811·99 to 995·92)	734·73<br>(660·3 to 812·5)	647·27<br>(583·66 to 712·72)	585·63<br>(528·44 to 646·21)	479·15<br>(430·89 to 530·78)	510·32<br>(416·19 to 631·63)
Virginia	1159·6<br>(965·11 to 1366·89)	1043·75<br>(952·15 to 1150·04)	952·78<br>(865·6 to 1048·87)	786·88<br>(713·32 to 871·01)	690·43<br>(628·39 to 763·62)	642·31<br>(582·8 to 711·6)	532·91<br>(484·38 to 591·7)	525·44<br>(418·78 to 661·88)
Washington	1020·12<br>(845·49 to 1224·86)	927·98<br>(840·68 to 1030·06)	877·03<br>(793·24 to 965·14)	704·23<br>(638·56 to 778·17)	622·99<br>(562·6 to 686·31)	574·79<br>(519·5 to 633·32)	494·34<br>(448·31 to 543·02)	479·8<br>(377·85 to 601·25)
West Virginia	1126·79<br>(936 to 1322·05)	1003·46<br>(912·16 to 1108·68)	1028·09<br>(921·09 to 1141·09)	821·01<br>(740·14 to 911·54)	713·64<br>(642·48 to 786·87)	716·2<br>(648·95 to 790·28)	671·63<br>(605·28 to 739·84)	710·76<br>(558·55 to 895·62)
Wisconsin	1008·44<br>(847·93 to 1208·98)	945·13<br>(856·56 to 1042·6)	859·72<br>(777·98 to 952·71)	695·68<br>(628·37 to 772·18)	625·52<br>(566·33 to 690·66)	582·45<br>(525·57 to 642·45)	506·82<br>(459·09 to 558·88)	520·21<br>(407·05 to 657·12)
Wyoming	1098·89<br>(930·71 to 1303·98)	981·29<br>(886·92 to 1086·62)	915·37<br>(826·64 to 1017·2)	763·8<br>(691·35 to 852·18)	648·4<br>(586·87 to 719·71)	613·42<br>(551·76 to 681·74)	552·13<br>(498·99 to 613·07)	534·74<br>(435·26 to 659·04)
Southern Latin America	1368·2<br>(1301·48 to 1432·62)	1296·28<br>(1234·62 to 1364·03)	1169·59<br>(1110·84 to 1232·73)	954·21<br>(907·23 to 1005·21)	821·33<br>(778·53 to 867·01)	717·09<br>(677·65 to 755·82)	636·22<br>(602·16 to 669·59)	601·97<br>(548·78 to 657·99)
Argentina	1244·65<br>(1163·79 to 1329·95)	1168·95<br>(1095·27 to 1256·24)	1126·33<br>(1054·33 to 1208·46)	944·81<br>(885·43 to 1012·28)	831·88<br>(776·42 to 895·04)	748·27<br>(694·34 to 803·97)	668·54<br>(625·83 to 712·63)	652·52<br>(576·31 to 741·68)
Chile	1810·23<br>(1697·56 to 1941·92)	1782·01<br>(1663·75 to 1908·63)	1435·15<br>(1340·97 to 1541·1)	1017·31<br>(936·78 to 1107·85)	804·08<br>(743·5 to 870·03)	631·09<br>(583·06 to 686·39)	557·26<br>(500·69 to 625·59)	490·31<br>(451·95 to 533·84)

Supplemental Results Table 3a: Age-standardised mortality rates by location, both sexes, 1950, 1960, 1970, 1980, 1990, 2000, 2010, 2019								
Location	1950	1960	1970	1980	1990	2000	2010	2019
Uruguay	1165·3 (1088·75 to 1252·35)	1002·49 (914·85 to 1096·86)	1015·72 (925·1 to 1119·39)	918·55 (833·79 to 1015·6)	804·24 (723·36 to 894·88)	724·29 (660·14 to 798·54)	637·41 (591·91 to 688·11)	600·19 (530·39 to 681·66)
Western Europe	1170·54 (1129·68 to 1214·04)	1016·04 (988·45 to 1043·48)	944·18 (920·73 to 968·77)	807·51 (787 to 829·07)	679·15 (666·38 to 692·17)	566·27 (555·74 to 577·09)	458·77 (452·34 to 465·68)	423·7 (408·7 to 438·5)
Andorra	739·49 (575·86 to 933·39)	717·43 (562·16 to 896·55)	666·3 (527·72 to 812·95)	591·74 (468·86 to 763·81)	548·91 (453·57 to 696·67)	471·25 (392·53 to 577·56)	429·06 (358·6 to 517·58)	418·43 (327·22 to 518·14)
Austria	1258·05 (1181·42 to 1345·69)	1087·23 (1011·97 to 1172·46)	1049·77 (974·6 to 1132·15)	893·4 (829·06 to 959·95)	708·36 (660·98 to 761·2)	581·65 (541·06 to 626·3)	474·09 (468·11 to 480·5)	421·98 (387·04 to 459·92)
Belgium	1160·98 (1089·25 to 1242·29)	1057·09 (979·36 to 1138·54)	1000·1 (935·42 to 1069·52)	860·83 (796·26 to 931·09)	696·4 (645·03 to 755·68)	599·3 (555·92 to 645·44)	496·87 (491·76 to 501·72)	450·71 (417·17 to 490·07)
Cyprus	1368·57 (1170·33 to 1585·61)	1270·1 (1094·84 to 1447·94)	1272·14 (1092·31 to 1513·9)	1172·89 (1075·06 to 1276·29)	924·56 (856·32 to 1001·56)	785·34 (733·03 to 849·11)	608·67 (564·58 to 658·25)	521·77 (454·87 to 602·97)
Denmark	1014·92 (950·02 to 1093·2)	901·3 (841·89 to 966·9)	829·67 (777·17 to 889·79)	780·99 (771·07 to 791·02)	735·86 (727·14 to 744·3)	639·23 (631 to 646·74)	533·53 (526·21 to 540·71)	463·65 (426·97 to 506·77)
Finland	1388·05 (1293·35 to 1493·45)	1116·41 (1045·44 to 1196·46)	1021·51 (1011·34 to 1031·45)	804·25 (795·21 to 812·95)	723·13 (713·36 to 733·22)	595·92 (588·8 to 602·96)	485·06 (477·5 to 493·07)	429·62 (394·14 to 470·97)
France	1147·96 (1074·43 to 1231·47)	1006·84 (931·02 to 1091·82)	901·59 (832·53 to 975·99)	769·32 (712·49 to 834·13)	626·72 (588·49 to 674·74)	535·83 (498·11 to 577·53)	434·97 (407·5 to 465·68)	388·76 (353·59 to 428·21)
Germany	1174·12 (1028·86 to 1355·15)	1081·25 (1008·32 to 1163·15)	1045·66 (983·45 to 1123·41)	867·87 (810·96 to 927·26)	726·02 (720·22 to 731·56)	581·75 (576·56 to 587·29)	488·18 (483·59 to 492·66)	463·94 (424·91 to 506·65)
Greece	925·21 (864·26 to 989·44)	828·63 (771·4 to 896·02)	776·72 (720·45 to 838·46)	746·35 (694·41 to 803·34)	656·56 (609·78 to 708·55)	606·02 (563·23 to 653·28)	497·56 (464·36 to 534·44)	475·13 (424·51 to 540·53)
Iceland	897·52 (849·63 to 953·01)	829·95 (808·09 to 853·13)	797·4 (772·86 to 819·53)	657·63 (637·15 to 679·96)	588·66 (570·41 to 607·78)	494·52 (474·88 to 515·42)	401·65 (381·13 to 423·75)	348·93 (310·06 to 390·53)
Ireland	1265·62 (1186·22 to 1352·43)	1076·5 (995·65 to 1161·74)	1018·82 (951·32 to 1093·49)	940·2 (871·03 to 1018·75)	797·9 (741·17 to 858·31)	686·41 (636·11 to 746·35)	478·03 (441·54 to 518·05)	431·88 (391·59 to 476·06)
Israel	832·38 (776·96 to 894·96)	854·9 (793·13 to 926·33)	927·41 (858·1 to 1004·4)	822·05 (755·74 to 893·29)	641·13 (580·23 to 710·39)	567·3 (511·03 to 628·03)	430·93 (396·92 to 470·01)	391·93 (342·89 to 448·51)
Italy	1152·16 (1080·48 to 1235·39)	987·3 (916·23 to 1072·55)	895·63 (832·85 to 966·64)	806·11 (751·26 to 870·78)	642·12 (599·65 to 690·08)	526·23 (490·04 to 567·74)	426·42 (423·05 to 430·06)	387·91 (358·29 to 421·16)
Luxembourg	1304·86 (1211·34 to 1410·72)	1131·6 (1051·16 to 1218·85)	1060·37 (991·72 to 1136·79)	913·56 (843·83 to 988·61)	747·68 (694·42 to 810·28)	605·93 (557·59 to 658·1)	468·15 (449·19 to 486·61)	390·19 (343·88 to 444·26)
Malta	1287·49 (1193·06 to 1399·55)	1127·56 (1031·44 to 1229·13)	1044·61 (952·59 to 1151·58)	960·48 (863·9 to 1059·76)	690 (621·35 to 768·46)	571·02 (515·61 to 634·49)	462·04 (422·64 to 505·27)	402·29 (345·47 to 467·82)
Monaco	1182·67 (1057·53 to 1327·95)	1016·04 (897·03 to 1140·35)	845·5 (742·67 to 955)	712·67 (621·88 to 807·02)	595·39 (484·87 to 697·55)	557·97 (441·84 to 663·97)	545·07 (452·34 to 639·67)	503·62 (407·09 to 594·5)
Netherlands	963·97 (953·03 to 974·64)	858·88 (851·93 to 865·62)	835·4 (828·77 to 842·33)	708·5 (702·31 to 714·9)	647·54 (641·57 to 654·06)	598·69 (592·66 to 604·67)	474·82 (468·99 to 480·21)	444·33 (409·22 to 487·76)
Norway	889·52 (831·16 to 955·48)	834·55 (779·52 to 892)	795·56 (742·87 to 854·22)	697·33 (650·39 to 744·2)	652·45 (609·54 to 699·55)	562·78 (555·65 to 570·29)	456·63 (450·68 to 462·93)	395·07 (365·72 to 428·33)
Portugal	1457·68 (1359·57 to 1565·97)	1187·08 (1106·71 to 1272·26)	1138·63 (1062·2 to 1223·54)	934·02 (870·47 to 1007·97)	808·28 (751·43 to 873·42)	664·44 (618·24 to 714·96)	500·94 (467·67 to 538·95)	441·97 (393·31 to 499·77)
San Marino	871·16 (639·09 to 1102·38)	858·25 (690·86 to 1030·57)	801·28 (711·82 to 911·22)	682·89 (608·11 to 764·7)	521 (430·76 to 630·34)	442·59 (372·47 to 524·6)	430·13 (309·7 to 558·49)	419·43 (290·27 to 577·59)
Spain	1357·88 (1268·76 to 1458·85)	998·17 (931·31 to 1067·11)	917·84 (855·87 to 980·36)	721·18 (671·76 to 775·65)	647·54 (601·81 to 693·51)	529·57 (525·4 to 533·65)	423·81 (420·21 to 427·12)	387·06 (355·64 to 424·42)
Sweden	995·97 (929·33 to 1069·14)	871·36 (814·63 to 932·89)	769·34 (762·42 to 775·97)	705·03 (698·56 to 711·65)	605·48 (599·53 to 611·63)	515·31 (509·54 to 520·29)	435·55 (430·97 to 440·8)	399·12 (366·33 to 437·54)
Stockholm	1018·06 (828·16 to 1205·94)	897·88 (726·15 to 1089·25)	798·72 (681·09 to 933·07)	739·52 (720·2 to 759·4)	643·57 (626·55 to 661·93)	524·28 (509·88 to 537·13)	442·62 (429·92 to 454·43)	396·91 (335·71 to 460·33)
Sweden except Stockholm	992·41 (920·49 to 1069·24)	867·08 (807·07 to 934·16)	763·56 (737·69 to 787·67)	697·89 (690·27 to 705·25)	597·5 (590·98 to 604·24)	513·32 (507·24 to 519·2)	434·34 (429·02 to 439·77)	400·45 (365·34 to 439·65)
Switzerland	1092·34 (1018·38 to 1170·63)	951·31 (888·82 to 1021·08)	857·64 (803·75 to 919·66)	700·44 (653·29 to 749·09)	592·71 (554·05 to 633·84)	493·02 (462·52 to 528·08)	395·35 (390·18 to 400·43)	354·3 (324·07 to 389·05)
UK	1146·11 (1082·97 to 1213·69)	1008·82 (948·39 to 1076·24)	938·22 (881·12 to 995·43)	833·74 (785·36 to 890·09)	718·53 (673·89 to 766·59)	608·14 (572·1 to 647·38)	481·6 (451·68 to 513·75)	467·56 (424·07 to 518·39)
England	1117·47 (1045·28 to 1193·62)	978·88 (910·49 to 1053·51)	917·58 (854·61 to 983·48)	817·9 (763·49 to 885·49)	705·9 (654·96 to 762·34)	596·68 (555·4 to 644·06)	470·54 (436·5 to 508·66)	457·17 (407·71 to 519·23)
East Midlands	1135·79 (949·29 to 1338·15)	999·84 (810·4 to 1231·4)	933·39 (778·92 to 1109·1)	827·08 (739·8 to 930·26)	703·49 (635·69 to 781·43)	603·74 (543·58 to 669·22)	479·27 (429·43 to 532·19)	472·85 (395·82 to 565·83)
Derby	1200·52 (950 to 1478·28)	1055·95 (801·28 to 1367·8)	988·12 (785·81 to 1225·12)	875·4 (758·76 to 1008·76)	747·85 (656·18 to 855·43)	609·81 (532·36 to 691·93)	525·48 (461·64 to 601·97)	480·25 (384·3 to 593·87)
Derbyshire	1145·99 (922·81 to 1417·78)	1013·19 (780·18 to 1302·64)	945·96 (766·06 to 1146·01)	839·2 (741·95 to 954·28)	722·62 (637·49 to 818·05)	603·12 (531·4 to 679·51)	470·66 (412·26 to 534·97)	465·18 (373·39 to 572·12)
Leicester	1232·26 (980·03 to 1495·79)	1095·98 (825·49 to 1414·52)	1023·74 (818·69 to 1262·15)	900·02 (779·91 to 1031·37)	768·46 (667·7 to 877·53)	690·64 (601·45 to 783·73)	542·99 (471·59 to 618·41)	503·69 (397·46 to 625·74)
Leicestershire	1031·64 (831·94 to 1305·13)	913·69 (694·66 to 1209·66)	849·43 (678·28 to 1046·61)	760·79 (658·45 to 876·87)	641·07 (562·61 to 730·71)	547·32 (481·1 to 624·69)	437·63 (384·93 to 501·95)	416·38 (329·86 to 521·24)
Lincolnshire	1122·83 (895·48 to 1386·97)	988·53 (743·06 to 1285·44)	921·02 (741·71 to 1121·41)	818·71 (713·07 to 938·84)	689·82 (602·68 to 778·72)	597·05 (523·05 to 674·45)	469·19 (409·81 to 530·14)	486·17 (390·87 to 607·69)
Northamptonshire	1093·58 (883·1 to 1345·83)	960·73 (721 to 1251·33)	892·02 (709·56 to 1102·43)	793·05 (689·32 to 905·17)	688·64 (603·71 to 784·65)	586·05 (517·15 to 667·81)	481·66 (423·23 to 547·65)	487·36 (392·36 to 606·98)
Nottingham	1237·63 (970·43 to 1540·17)	1103·13 (838·72 to 1440·91)	1035·43 (824·21 to 1261·82)	920·13 (794·14 to 1070·7)	790·33 (688·87 to 903·93)	759·91 (661·43 to 867·19)	570·35 (494·48 to 654·79)	530·33 (414·18 to 671·6)
Nottinghamshire	1128·5 (891·39 to 1380·53)	993·91 (745·97 to 1302·98)	923·62 (741·92 to 1142·58)	818·48 (717·87 to 939·1)	693·58 (613·98 to 782·51)	600·52 (530·5 to 679·47)	480·12 (425·67 to 543·17)	490 (394·11 to 602·67)
Rutland	1001·78 (783·25 to 1259·52)	871·53 (652·35 to 1148·11)	804·8 (623·19 to 1024·94)	716·26 (602·24 to 830·65)	633·09 (548·04 to 733·31)	504·99 (430·31 to 588·1)	402·73 (344·59 to 469·49)	376·83 (295·24 to 482·1)
East of England	1020 (858·11 to 1209·62)	899·48 (730·23 to 1092·56)	841·45 (705·76 to 996·84)	767·35 (688·43 to 862·36)	659·5 (596·11 to 733·47)	558·75 (504·54 to 617·73)	444·63 (401·96 to 494·08)	433·57 (361·59 to 525·17)
Bedford	1071·64 (842·25 to 1342·9)	940·45 (701·47 to 1236·52)	870·53 (690·47 to 1084·47)	770·3 (655·18 to 894·41)	674·4 (586·23 to 776·61)	594·14 (517·75 to 677·31)	483·4 (421·57 to 553·35)	481·05 (374·99 to 610·01)
Cambridgeshire	968·38 (766·16 to 1210·79)	859·7 (644·96 to 1108·94)	800·06 (627·52 to 1005·13)	743·94 (644·2 to 859·93)	640·52 (559·32 to 729·22)	548·3 (481·94 to 625·8)	411·65 (361·76 to 470·4)	403·85 (321·99 to 512·74)
Central Bedfordshire	1047·09 (835·39 to 1299·73)	920·34 (689·26 to 1197·74)	852·22 (680·13 to 1064·22)	756·23 (648·58 to 878·23)	658·48 (578 to 755·48)	540·5 (472·44 to 620·74)	429·49 (377·53 to 494·12)	390·89 (306·48 to 495·87)
Essex	1010·68 (808·78 to 1244·76)	902·35 (682·76 to 1160·62)	851·71 (686·17 to 1059·53)	792·85 (692·55 to 902·75)	657·13 (580·21 to 741·66)	550·72 (488·26 to 620·06)	448·08 (395·02 to 505·14)	444·58 (357·29 to 554·12)
Hertfordshire	979·4 (775·01 to 1205·21)	873 (665·81 to 1116·76)	827·68 (663·88 to 1011·57)	782·32 (687·59 to 894·01)	657·7 (584·76 to 747·84)	564·41 (501·41 to 638·56)	435·42 (385·55 to 492·63)	411·07 (330·9 to 510·97)
Luton	1134·46 (902·44 to 1386·85)	1005·7 (754·52 to 1322·57)	939·8 (747·81 to 1162·05)	836·99 (724·33 to 973·21)	737·83 (645·85 to 840·07)	643·06 (559·53 to 736·51)	503·14 (442·13 to 575·86)	509·1 (409·59 to 634·82)
Norfolk	1022·59 (819·54 to 1270·66)	893·49 (680·99 to 1178·99)	819·42 (655·89 to 1006·42)	729·2 (636·52 to 835·63)	659·02 (584·17 to 740·76)	550·44 (487·65 to 624·63)	453·22 (399·25 to 511·89)	442·35 (356·78 to 548·69)
Peterborough	1127·99 (893·64 to 1433·48)	995·48 (744·8 to 1287·64)	933·06 (745·94 to 1163·4)	807·04 (693·72 to 934·19)	725·84 (640·67 to 827·19)	623·25 (547·75 to 712·9)	512·16 (449·38 to 589·55)	493·53 (392·78 to 622·21)
Southend-on-Sea	1115·37 (884·94 to 1393·85)	967·25 (722·07 to 1266·82)	886·47 (699·84 to 1094·6)	776·36 (667·27 to 897·84)	689·7 (600·14 to 790·96)	603·76 (528·3 to 687)	477·67 (413·55 to 548)	485·04 (384·66 to 612·29)
Suffolk	1017·56 (804·05 to 1264·06)	893·44 (674·33 to 1164·78)	826·19 (662·32 to 1009·77)	738·91 (646·93 to 844·79)	638·11 (565·34 to 721·32)	545·73 (482·1 to 614·7)	429·84 (380·64 to 484·46)	434·01 (350 to 541·76)
Thurrock	1125·94 (881·1 to 1413·14)	1010·63 (758·33 to 1336·54)	949·71 (752·83 to 1172·27)	848·37 (729·42 to 982·94)	717·21 (626·43 to 813·89)	578·46 (504·12 to 654·7)	465·11 (402·29 to 529·54)	437·73 (349·11 to 539·96)
Greater London	1079·36 (916·13 to 1253·57)	955·97 (785·61 to 1157·82)	900·17 (765·78 to 1050·94)	797·69 (714·09 to 886·46)	699·72 (628·49 to 775·02)	600·3 (541·61 to 664·69)	440·76 (395·67 to 493·42)	413·6 (344·51 to 492·67)
Barking and Dagenham	1223·78 (977·94 to 1501·52)	1078·12 (828·15 to 1388·25)	1001·75 (807·78 to 1237·76)	866·41 (753·41 to 1005·76)	748·15 (650·21 to 848·74)	659·29 (578·96 to 746·26)	535·04 (468·18 to 610·56)	534·69 (418·69 to 674·87)
Barnet	977·15 (753·41 to 1198·12)	856·6 (642·8 to 1108·77)	790·46 (636·69 to 982·78)	688·84 (589·06 to 794·45)	614·55 (533·21 to 699·44)	515·85 (452·13 to 588·73)	410·04 (355·14 to 470·73)	365·29 (288·31 to 456·04)
Bexley	1004·66 (799·29 to 1244·05)	879·23 (666·44 to 1141·42)	813·54 (650·28 to 995)	704·44 (606·35 to 814·85)	610·08 (528·46 to 691·33)	521·88 (453·45 to 592·65)	424·94 (368·88 to 484·38)	401·81 (317·21 to 506·27)
Brent	1089·5 (870·01 to 1321·7)	964·5 (729·61 to 1250·02)	899·19 (719·26 to 1100·97)	794·75 (684·48 to 912·8)	691·11 (602·41 to 785·01)	602·37 (529·09 to 687·1)	412·48 (360·51 to 471·47)	416·31 (329·09 to 521·9)
Bromley	965·99 (766·04 to 1206·93)	845·19 (635·74 to 1099·54)	786·03 (639·36 to 993·43)	692·32 (600·24 to 802·17)	607·2 (532·93 to 693·67)	517·64 (457·85 to 591·18)	387·04 (338·36 to 445·17)	363·79 (291·43 to 459·02)
Camden	1097·28 (880·91 to 1356·01)	1006·98 (772·88 to 1308·83)	973·08 (774·58 to 1190·58)	904·78 (779·58 to 1036·79)	763·23 (665·96 to 865·26)	725·76 (640·57 to 822·72)	483·91 (420·83 to 547·96)	428·18 (338·96 to 534·65)
Croydon	1025·14 (812·48 to 1274·84)	900·59 (685·45 to 1165·74)	842·22 (672·07 to 1040·38)	759·57 (653·97 to 867·81)	674·94 (588·98 to 772·42)	568·97 (500·48 to 654·08)	442·25 (388·08 to 509·78)	446·98 (353 to 559·37)
Ealing	1095·11 (872·32 to 1360·11)	972·96 (731·96 to 1272·92)	918·75 (746·36 to 1119·98)	821·13 (716·11 to 940·1)	704·5 (618·48 to 799·04)	592·58 (520·82 to 669·17)	424·72 (372·46 to 481·39)	396·21 (315·9 to 497·07)
Enfield	1033·83 (821·19 to 1283·2)	903·83 (691·28 to 1173·21)	837·4 (662·12 to 1032·64)	738·83 (640·5 to 852·09)	638·48 (555·51 to 724·95)	570·56 (498·35 to 649·32)	410·02 (358·76 to 467·54)	409·85 (322·16 to 512·77)
Greenwich	1147·42 (921·35 to 1420·54)	1006·18 (764·34 to 1293·89)	931·04 (756·99 to 1139·56)	813·79 (705·9 to 940·17)	729·38 (637·4 to 836·46)	669·68 (586·5 to 766·13)	486·94 (425·4 to 560·98)	456·95 (359·28 to 572·61)
Hackney	1146·08 (922·97 to 1411·22)	1033·41 (784·1 to 1350·23)	1006·1 (802·82 to 1253·08)	936·93 (811·32 to 1069·71)	827·82 (725·07 to 938·7)	686·02 (602·43 to 776·13)	517·62 (453·98 to 591·56)	502·74 (401·19 to 626·77)
Hammersmith and Fulham	1138·37 (900·17 to 1416·46)	1032·96 (792·19 to 1368·42)	988·69 (792·52 to 1219·05)	923·46 (802·9 to 1079·44)	830·83 (720·92 to 959·34)	687·87 (603·62 to 788·7)	476·56 (415·74 to 552·95)	414·96 (325·95 to 525·72)
Haringey	1109·74 (893·44 to 1371·94)	988·32 (753·26 to 1260·45)	926·88 (748·79 to 1127·2)	829·26 (719·16 to 956·37)	705·42 (613·93 to 800·2)	655·4 (575·3 to 743·93)	498·36 (435·35 to 568·07)	428·52 (339·01 to 534·53)
Harrow	984·71 (772·62 to 1238·31)	865·37 (657·68 to 1127·73)	807·5 (639·62 to 999·1)	705·66 (610·4 to 819·69)	594·52 (515·77 to 679·86)	506·47 (442·89 to 577·87)	354·64 (308·8 to 409·11)	353·96 (275·29 to 445·72)
Havering	1007·72 (807·19 to 1229·15)	885·84 (669·63 to 1151·69)	824·7 (664·41 to 1023·97)	736·59 (642·37 to 842·81)	633·98 (557·65 to 720·94)	528·83 (467·35 to 597·15)	428·21 (373·92 to 491·37)	412·66 (329·15 to 522·56)
Hillingdon	1015·2 (825·86 to 1253·65)	897·13 (677·51 to 1166·24)	844·69 (677·54 to 1056·82)	747·15 (644·03 to 858·37)	653·89 (573·51 to 743·39)	587·67 (520·52 to 668·65)	445·45 (389·79 to 512·63)	418·73 (335·02 to 523·43)
Hounslow	1070·18 (856·09 to 1315·02)	949·38 (721·78 to 1209·39)	890·47 (710·56 to 1092·48)	788·32 (686·05 to 908·02)	703·81 (617·58 to 801·07)	619·57 (544·72 to 702·84)	444·66 (388·82 to 511·58)	401·66 (320·55 to 499·82)
Islington	1131·06 (905·76 to 1385·33)	1020·26 (764·38 to 1336·98)	977·79 (778·21 to 1196·51)	891·19 (771·06 to 1022·86)	798·18 (698·27 to 906·02)	752·35 (660·6 to 854·45)	536·06 (465·48 to 615·94)	471·18 (370·72 to 580·71)
Kensington and Chelsea	1042·28 (828·4 to 1295·1)	970·74 (742·96 to 1262·81)	938·04 (751·98 to 1144·34)	874·74 (761·19 to 1004·24)	760·67 (666·56 to 873·53)	525·85 (460·95 to 598·35)	383·1 (335·54 to 440·72)	383·33 (307·03 to 475·52)
Kingston upon Thames	969·3 (771·15 to 1206·06)	851·85 (634·24 to 1118·91)	791·19 (627·71 to 975·76)	701·06 (606·11 to 814·19)	616·38 (541·11 to 700·29)	518·74 (456·87 to 591)	362·48 (317·65 to 416·23)	359·29 (282·37 to 449·97)
Lambeth	1210·4 (973·25 to 1483·72)	1086·6 (844·43 to 1397·6)	1034·06 (832·69 to 1273·22)	901·27 (784·68 to 1038·19)	781·42 (688·61 to 889·8)	705·38 (624·94 to 800·59)	526·31 (459·99 to 601·8)	449·92 (355·79 to 562·49)
Lewisham	1132·63 (913·98 to 1387·21)	994·03 (756·79 to 1291·03)	925·11 (735·41 to 1129·71)	813·3 (705·11 to 934·42)	745·78 (651·78 to 843·84)	691·52 (607·17 to 786·02)	507·09 (441·39 to 577·94)	470·89 (380·65 to 581·99)

Supplemental Results Table 3a: Age-standardised mortality rates by location, both sexes, 1950, 1960, 1970, 1980, 1990, 2000, 2010, 2019

Location	1950	1960	1970	1980	1990	2000	2010	2019
Merton	1035·93 (824·33 to 1270·08)	911·98 (694·18 to 1189·58)	848·3 (678·25 to 1039·4)	742·71 (643·77 to 862·48)	658·63 (572·92 to 753·22)	541·62 (474·77 to 616·84)	407·82 (357·62 to 468·03)	398·07 (313·29 to 502·32)
Newham	1186·46 (947·25 to 1456·7)	1048·55 (795·37 to 1345·24)	985·1 (790·36 to 1217·82)	872·23 (759·24 to 1005·78)	769·8 (674·69 to 876·14)	732·18 (643·97 to 834·63)	520·77 (455·45 to 592·69)	472·35 (376·94 to 590·71)
Redbridge	1012·27 (803·75 to 1249·66)	891·55 (673·02 to 1147·9)	833·2 (656·48 to 1022·89)	719·59 (620·17 to 832·26)	625 (545·96 to 706·57)	529·7 (466·48 to 601·81)	407·75 (357·64 to 468·4)	377·35 (298·26 to 471·03)
Richmond upon Thames	987·53 (803·29 to 1217·05)	882·28 (678·23 to 1125·44)	832·51 (661·27 to 1023·71)	746·33 (645·91 to 862·52)	609·84 (533·34 to 691·78)	496·46 (433·98 to 565·52)	351·11 (305·9 to 405·05)	340·5 (271·28 to 428·04)
Southwark	1147·71 (930·84 to 1409·49)	1017·72 (765·83 to 1291·9)	966·24 (785·04 to 1186·1)	867·31 (757·5 to 1000·28)	890·03 (783·78 to 1017·46)	682·13 (604·69 to 773·81)	511·41 (448·05 to 585·9)	464·24 (370·85 to 578·91)
Sutton	1010·19 (800·94 to 1265·77)	874·61 (656·88 to 1155·43)	800·64 (640·27 to 990·31)	692·55 (592·99 to 803·81)	628·35 (544·25 to 715·49)	546·47 (476·38 to 622·45)	402·85 (351·61 to 460·69)	404·44 (317·65 to 506·91)
Tower Hamlets	1262·25 (1028·57 to 1541·34)	1122·44 (843·35 to 1434·53)	1087·19 (872·89 to 1322·44)	998·24 (859·8 to 1141·51)	865·17 (756·9 to 983·79)	774·91 (679·55 to 880·49)	529·49 (464·69 to 605·49)	490·36 (392·36 to 610·26)
Waltham Forest	1119·93 (898·67 to 1383·49)	984·2 (756·72 to 1269·16)	909·24 (733·48 to 1117·36)	795·92 (690·14 to 926·34)	690·91 (605·75 to 793·57)	635·9 (555·72 to 725·88)	469·22 (410·59 to 540·97)	436·76 (345 to 546·47)
Wandsworth	1106·61 (892·14 to 1342·65)	994·27 (746 to 1295·92)	944·29 (757·89 to 1153·99)	844 (732·83 to 969·93)	780·51 (686·15 to 887·14)	638·29 (556·18 to 730·93)	439·81 (384·47 to 503·06)	402·94 (317·24 to 504·27)
Westminster	1061·67 (854·88 to 1313·68)	985·91 (756·57 to 1264·57)	956·94 (774·56 to 1176·23)	894·15 (769·33 to 1024·79)	743·22 (646·45 to 848·23)	597·07 (524·02 to 681·66)	404·16 (351·58 to 464·33)	392·75 (307·74 to 500·37)
North East England	1263·88 (1079·1 to 1481·17)	1109·75 (892·59 to 1347·08)	1035·58 (872·88 to 1230·73)	908·24 (810·32 to 1019·8)	799·87 (716·56 to 884·35)	669·32 (602·71 to 740·03)	530·82 (476·98 to 588·52)	535·06 (441·77 to 646·37)
County Durham	1268·08 (1031·58 to 1531·47)	1113·75 (852·58 to 1447·04)	1035·58 (827·83 to 1271·56)	909·1 (794·52 to 1043·59)	798·33 (701 to 906·84)	670·52 (591·34 to 759·19)	542·35 (478·21 to 617·6)	540·69 (426·4 to 670·56)
Darlington	1250·64 (1003·39 to 1558·77)	1103·22 (834·93 to 1431·68)	1030·79 (825·21 to 1288·37)	894·64 (772·55 to 1036·73)	790·27 (695·67 to 897·77)	670·43 (587·38 to 761·1)	525·27 (459·19 to 599·7)	514·49 (413·08 to 654·39)
Gateshead	1299·27 (1048·93 to 1615·86)	1150·25 (872·44 to 1485·7)	1076·09 (849·64 to 1343·32)	937·33 (811·28 to 1082·76)	799·03 (701·43 to 901·54)	701·12 (610·29 to 793·65)	526·69 (460·6 to 601·78)	545·48 (434·01 to 676·39)
Hartlepool	1351·31 (1071·6 to 1674·59)	1194 (898·5 to 1586·88)	1113·1 (882·21 to 1364·16)	971·27 (836·91 to 1126·99)	840·05 (730·29 to 961·48)	730·08 (633·2 to 840·64)	569·72 (492·71 to 655·85)	597·73 (465·68 to 749·64)
Middlesbrough	1352·13 (1096·81 to 1659·22)	1198·9 (899·36 to 1563·77)	1128·64 (899·25 to 1384·41)	993·56 (868·18 to 1141·3)	866·72 (759·96 to 985·87)	754·57 (657·44 to 861·49)	598·94 (519 to 685·32)	633·54 (498·12 to 794·66)
Newcastle upon Tyne	1257·68 (1026·07 to 1545·8)	1115·67 (852·95 to 1418·93)	1041·91 (842·66 to 1284·25)	915·02 (795·55 to 1053·82)	842·86 (738·19 to 956·98)	687·19 (600·08 to 782·09)	542·08 (475·95 to 617·45)	536·26 (424·16 to 673·5)
North Tyneside	1224·55 (977·57 to 1513·33)	1071·83 (831·01 to 1400·33)	997·2 (785·19 to 1222·63)	869·77 (753·73 to 1010·73)	778·14 (677·49 to 886·17)	653·56 (571·28 to 741·45)	520·18 (454·31 to 597·03)	498·92 (394·58 to 622·79)
Northumberland	1212·69 (971·84 to 1491·04)	1062·05 (793·33 to 1414·07)	983·25 (790·53 to 1216·03)	890·12 (769·97 to 1025·88)	738·32 (646·85 to 840·91)	596·59 (522·07 to 681·29)	470·27 (411·93 to 534·78)	475·09 (381·4 to 592·58)
Redcar and Cleveland	1270·03 (1014·84 to 1588·12)	1120·8 (849·95 to 1481·32)	1044·24 (822·55 to 1300·2)	907·57 (786·35 to 1054·47)	787·98 (687·46 to 904·76)	660·57 (577·45 to 756·36)	522·38 (456·6 to 600·74)	563·38 (450·06 to 708·93)
South Tyneside	1286·69 (1023·38 to 1579·94)	1128·14 (858·63 to 1487)	1045·26 (836·51 to 1290·31)	912·26 (782·83 to 1052·67)	817·12 (712·27 to 931·95)	677·53 (587·25 to 774·5)	548·9 (474·62 to 629·68)	541·46 (424·11 to 682·36)
Stockton-on-Tees	1223·02 (964·46 to 1510·94)	1075·75 (799·7 to 1418·4)	1000·33 (788·57 to 1235·95)	863·19 (742·34 to 993·52)	785·03 (686·64 to 890·63)	629·28 (548·43 to 713·61)	505·3 (440·26 to 577·05)	540·6 (425·8 to 675·06)
Sunderland	1263·03 (1001·87 to 1540·6)	1111·24 (847·61 to 1438·36)	1028·77 (816·94 to 1258·16)	898·27 (777·28 to 1029·29)	821·89 (717·01 to 936·52)	703·52 (616·43 to 794·51)	567·04 (496·54 to 644·6)	564·06 (446·15 to 699·79)
North West England	1226·26 (1056·06 to 1413·67)	1073·22 (904·07 to 1298·46)	1001·99 (847·77 to 1172·95)	886·89 (803·12 to 995·35)	775·8 (703·99 to 866·76)	660·6 (601·28 to 736·82)	530·81 (483·12 to 594·18)	512·29 (431·41 to 607·83)
Blackburn with Darwen	1286·76 (1023·16 to 1565·22)	1130·47 (878·93 to 1457·59)	1048·64 (832·51 to 1295·87)	925·92 (805·51 to 1064·22)	839·2 (738·2 to 955·24)	730·97 (645·57 to 835·77)	567·68 (500·28 to 654·25)	555·95 (448·54 to 698·66)
Blackpool	1305·93 (1058·56 to 1595·77)	1137·22 (888 to 1458·83)	1049·66 (857·56 to 1317·82)	915·16 (797·02 to 1056·34)	846·82 (747·23 to 967·64)	821·18 (724·83 to 938·64)	727·89 (641·31 to 833·53)	701·45 (568·55 to 885·49)
Bolton	1267·7 (1022·59 to 1548·88)	1114·95 (875·2 to 1436·25)	1037·23 (843·69 to 1264·53)	910·19 (799·15 to 1036·08)	783·05 (696·73 to 884·32)	682·68 (607·68 to 769·82)	550·48 (489·02 to 625·99)	513·76 (418·6 to 636·1)
Bury	1180·2 (939·93 to 1435·33)	1036·55 (787·73 to 1344·61)	954·57 (754·28 to 1189·14)	830·82 (724·95 to 957·25)	752·51 (661·08 to 857·62)	624·48 (548·85 to 716·12)	507·47 (446·87 to 585·18)	493·73 (390·53 to 618·19)
Cheshire East	1079·69 (855·1 to 1331·96)	942·49 (712·11 to 1211·37)	872·28 (704·58 to 1060·1)	779·42 (682·09 to 888·7)	690·97 (606·1 to 783·51)	566·53 (498·63 to 641·84)	443·83 (390·66 to 501·55)	416·89 (334·84 to 508·61)
Cheshire West and Chester	1123·46 (906·7 to 1377·63)	991·43 (772·56 to 1292·15)	932·28 (743·36 to 1158·57)	837·48 (734·57 to 970·99)	712·36 (631·62 to 812·36)	575·74 (509·75 to 656·75)	460·75 (407·03 to 526·46)	453·72 (366·94 to 558·72)
Cumbria	1186·36 (960·53 to 1437·44)	1038·49 (779·77 to 1329·2)	967·31 (767·28 to 1203·82)	849·54 (744·77 to 976·72)	745·42 (656·91 to 843·09)	603·09 (531·12 to 683·25)	485·42 (425·75 to 550·18)	497·97 (400·49 to 618·57)
Halton	1258·34 (1017·7 to 1551·93)	1127·74 (883·63 to 1437·85)	1062·74 (857·74 to 1291·55)	942·4 (816·65 to 1082·31)	803·19 (700·92 to 919·27)	718·1 (628·87 to 809·83)	558·7 (484·36 to 642·21)	543·78 (430·8 to 681·35)
Knowsley	1283·35 (1036·24 to 1557·39)	1142·08 (884·75 to 1501·83)	1079·16 (857·13 to 1334·66)	959·99 (828·12 to 1111·75)	835·02 (728·87 to 967·5)	735·06 (645·36 to 838·47)	584·47 (509·47 to 674·84)	552·4 (439·25 to 688·83)
Lancashire	1195·33 (983·17 to 1470·13)	1038·34 (827·44 to 1328·81)	956·04 (759·75 to 1178·86)	876·61 (769·99 to 1001·17)	760·38 (672·72 to 858·21)	633·16 (560·7 to 717·29)	508·5 (449·49 to 577·74)	507·76 (410·92 to 630·57)
Liverpool	1296·09 (1044·28 to 1579·24)	1150·43 (882·07 to 1473·7)	1094·76 (870·92 to 1336·21)	926·34 (808·86 to 1074·45)	838·55 (742·8 to 950·94)	774·26 (684·6 to 879·79)	614·46 (540·71 to 706·32)	610·38 (490·36 to 755·76)
Manchester	1336·63 (1083·8 to 1635·02)	1176·72 (907·98 to 1532·64)	1105·84 (897·03 to 1349·57)	986·28 (862·43 to 1127·67)	878·52 (775 to 1000·67)	806·46 (713·67 to 914·03)	642·09 (568·26 to 726·47)	543·21 (438·61 to 680·44)
Oldham	1287·04 (1048·36 to 1575·56)	1133·19 (866·41 to 1487·88)	1047·46 (854·1 to 1291·65)	916·51 (793·96 to 1047·9)	811·9 (709·24 to 922·95)	692·36 (608·48 to 785·25)	568·19 (497·27 to 648·12)	536·47 (432·19 to 664·16)
Rochdale	1279·02 (1038·41 to 1562·12)	1121·15 (859·77 to 1458·29)	1036·02 (832·81 to 1272·61)	899·29 (784·23 to 1042·38)	833·41 (735·27 to 949·63)	696·44 (617·23 to 794·83)	584·23 (511·39 to 664·11)	570·59 (454·98 to 705·09)
Salford	1308·55 (1053·11 to 1590·18)	1158·78 (898·11 to 1489·53)	1091·18 (871·92 to 1329·14)	977·3 (849·9 to 1124·64)	851·01 (747·61 to 964·76)	727·36 (638·73 to 817·05)	594·85 (524·7 to 682·21)	544·58 (433·01 to 676·21)
Sefton	1176·78 (951·05 to 1431·72)	1024·53 (786·55 to 1332·43)	959·14 (764·34 to 1189·99)	849·57 (741·95 to 978·79)	713·33 (632·11 to 813·79)	630·19 (555·57 to 719·46)	530·88 (464·95 to 613·75)	524·5 (421·06 to 663)
St Helens	1263·35 (1019·11 to 1555·7)	1124·72 (869·53 to 1446·11)	1052·06 (854·33 to 1278·64)	967·04 (839·29 to 1119·9)	793·13 (696·17 to 912·02)	665·44 (583·7 to 760·08)	560·15 (493·51 to 639·38)	566·73 (445·81 to 719·59)
Stockport	1107·17 (889·34 to 1350·52)	968·81 (741·6 to 1251·2)	901·21 (724·12 to 1102·3)	821·54 (713·04 to 955·29)	720·78 (636·08 to 815·82)	588·43 (520·9 to 667·88)	474·2 (416·91 to 542·11)	452·55 (362·14 to 569·67)
Tameside	1263·89 (1018·02 to 1544·02)	1117·69 (843·32 to 1462·64)	1040·09 (828·71 to 1278·02)	913·08 (795·23 to 1049·43)	803·99 (705·21 to 919·31)	702·94 (617·08 to 799·91)	567·08 (500·19 to 650·98)	510·71 (407·72 to 630·33)
Trafford	1128·4 (899·34 to 1375·33)	992·43 (749·64 to 1263·34)	926·75 (740·45 to 1153·32)	816·86 (711·55 to 947·35)	719·55 (629·19 to 823·14)	594·35 (523·17 to 676·08)	451·51 (395·32 to 518·22)	414·58 (329·86 to 521·4)
Warrington	1175·19 (927·64 to 1464)	1046·2 (790·74 to 1344·81)	982·84 (795·53 to 1206·66)	874·64 (756·58 to 1011·34)	772·11 (678·98 to 888·06)	612·35 (534·83 to 699·15)	490·36 (429·99 to 559·52)	492·24 (391·52 to 614·06)
Wigan	1245·86 (1004·93 to 1522·6)	1106·93 (861·78 to 1409·47)	1029 (824·35 to 1272·1)	909·23 (794·63 to 1053·75)	794·67 (694·74 to 905·35)	685·95 (603·02 to 777·07)	555·04 (488·18 to 630·74)	532·66 (428·73 to 663·94)
Wirral	1210·07 (975·79 to 1487·6)	1052·92 (813·32 to 1354·17)	978·18 (796·27 to 1206·76)	859·64 (751·91 to 995·94)	754·95 (664·98 to 858·66)	644·61 (568·56 to 731·77)	520·74 (456·21 to 595·44)	516·77 (410·33 to 642·96)
South East England	1018·84 (865·44 to 1176·89)	892·82 (738·95 to 1080·68)	836·64 (705·68 to 979·64)	759·07 (688·4 to 848·3)	649·52 (588·49 to 715·75)	552·63 (499·99 to 610·07)	435·65 (393·96 to 483·72)	422·87 (358·65 to 505·38)
Bracknell Forest	989·12 (781·53 to 1215·33)	871·09 (649·87 to 1143·78)	818·22 (643·22 to 1031·5)	732·37 (628·14 to 847·09)	635·93 (557·03 to 731·86)	523·63 (460·18 to 594·82)	408·11 (353·16 to 467·97)	391·78 (308·25 to 489·03)
Brighton and Hove	1092·19 (877·39 to 1349·8)	963·04 (736·58 to 1257·17)	895·18 (724·68 to 1097·18)	802·18 (698·64 to 925·31)	746·49 (660·22 to 853·79)	642·41 (566·8 to 733·78)	513·03 (451·65 to 591·05)	476·69 (386·44 to 593·34)
Buckinghamshire	955·12 (749·38 to 1169·76)	843·6 (637·88 to 1087·98)	794·16 (623·49 to 974·23)	726·6 (631·93 to 834·83)	636·69 (560·56 to 724·47)	525·54 (464·51 to 596·55)	408·69 (361·68 to 465·93)	378·35 (304·6 to 468·71)
East Sussex	1063·7 (849·91 to 1301·84)	915·99 (696·21 to 1174)	842·37 (671·27 to 1025·12)	739·7 (642·63 to 849·84)	622·76 (548·31 to 706·8)	538·5 (474·47 to 609·22)	441·57 (388·28 to 504·55)	448·32 (359·74 to 553·49)
Hampshire	978·26 (780·16 to 1198·5)	870·25 (668·13 to 1134·27)	821·81 (656·71 to 1014·62)	745·77 (653·09 to 856·42)	635·19 (561·85 to 719·98)	534·28 (473·08 to 604·58)	418·72 (367·68 to 474·1)	406·46 (325·74 to 511·11)
Isle of Wight	1130·47 (902·66 to 1374·87)	965·09 (735·6 to 1250·72)	872·29 (693·5 to 1077·96)	759·68 (655·47 to 874·07)	684·07 (603·67 to 776·59)	563·87 (495·64 to 641·96)	468·19 (410·23 to 537·89)	458·15 (369·03 to 567·19)
Kent	1052·09 (866·15 to 1285·82)	924·25 (713·95 to 1190·76)	869·35 (694·17 to 1058·81)	812·42 (720·44 to 923·26)	678·1 (604·98 to 763·01)	583·22 (520·91 to 657·29)	455·45 (405·71 to 514·24)	447·62 (362·46 to 549·27)
Medway	1091·75 (858·92 to 1357·11)	961·88 (725·51 to 1258·9)	880·47 (702·4 to 1095·04)	780·82 (680·86 to 905·94)	701·18 (615·49 to 797·84)	612·5 (541·06 to 696·1)	500·5 (434·08 to 575·05)	496·77 (401·83 to 627·88)
Milton Keynes	1061·06 (844·39 to 1310·6)	933·45 (703·89 to 1211·34)	871·19 (690·07 to 1073·38)	776·52 (670·54 to 899·54)	680·13 (599·85 to 775·75)	562·65 (493·29 to 638·73)	459·92 (403·18 to 523·89)	438·49 (349·86 to 541·21)
Oxfordshire	957·62 (760·24 to 1184·09)	854·23 (650·48 to 1108·66)	806·68 (634·29 to 981·79)	740·68 (647·35 to 853·31)	620·7 (549·25 to 707·39)	535·7 (473·53 to 609·95)	427·22 (374·62 to 487·7)	402·91 (324·37 to 504·22)
Portsmouth	1136·18 (912·59 to 1387·22)	1005·87 (766 to 1309·16)	932·22 (751·51 to 1151·67)	835·22 (722·08 to 964·46)	755·45 (661·91 to 855·89)	679·52 (594 to 771·68)	521·49 (454·47 to 597·8)	490·82 (388·36 to 616·81)
Reading	1034·59 (819·48 to 1275·91)	919·72 (687·62 to 1198·04)	861·42 (681·19 to 1066·24)	778·53 (674·35 to 903·25)	702·83 (612·56 to 800·47)	611·19 (536·64 to 692·01)	495·31 (429·92 to 571·34)	493·5 (389·38 to 620·34)
Slough	1108·39 (879·88 to 1358·91)	986·69 (742·15 to 1306·13)	934·5 (744·87 to 1146·3)	846·79 (732·65 to 976·27)	767·84 (670·77 to 870·75)	657·87 (575·48 to 752·11)	501·56 (438·95 to 575·62)	479·18 (383·31 to 602·25)
Southampton	1124·15 (888·64 to 1374·82)	999·34 (757·37 to 1297·24)	928·31 (748·68 to 1152·01)	816·72 (713·86 to 926·17)	736·36 (648·92 to 838·48)	639·02 (566·13 to 726·08)	500·01 (442·61 to 576·07)	488·71 (390·52 to 609·84)
Surrey	943·09 (735·2 to 1160·92)	833·92 (627·21 to 1085·09)	788·11 (630·91 to 972·46)	735·49 (646·49 to 837·05)	619·03 (550·52 to 696·71)	524·66 (465·42 to 590·22)	398·95 (352·57 to 450·67)	395·61 (320·49 to 486·8)
West Berkshire	961·87 (752·88 to 1181·57)	844·92 (622·62 to 1135·03)	783·81 (625·55 to 968·64)	703·63 (601·54 to 815·2)	614·7 (532·85 to 707·57)	522·59 (454·95 to 593·41)	407·77 (355·48 to 468·14)	385·02 (308·79 to 480·55)
West Sussex	1018·99 (821·26 to 1257·38)	880·14 (673·8 to 1118·41)	823·98 (653·91 to 1023·15)	737·72 (642·83 to 845·14)	624·38 (552·57 to 703·16)	533·48 (467·8 to 601·95)	434·34 (380·05 to 494·01)	425·49 (338·92 to 527·44)
Windsor and Maidenhead	921·9 (726·32 to 1149·46)	808·09 (596·06 to 1079·17)	751·28 (585·23 to 930·85)	672·85 (579·91 to 771·39)	618·65 (542·38 to 702·01)	498·7 (436·32 to 565·53)	384·55 (336·29 to 442·75)	372·92 (293·77 to 467·61)
Wokingham	889·98 (708·38 to 1110·72)	781·72 (593·12 to 1013·85)	721·81 (572·31 to 887·8)	644·5 (549·45 to 745·88)	550·61 (486·77 to 626·14)	442·69 (387·72 to 503·44)	345·87 (302·88 to 393·93)	339·82 (272·92 to 423·14)
South West England	1051·06 (893·47 to 1226·66)	915·39 (745·48 to 1135·03)	854·49 (715·48 to 1017·72)	762·71 (684·01 to 856·83)	650·86 (589·52 to 718·36)	546·58 (494·24 to 605·3)	438·45 (396·36 to 485·29)	433·29 (367·01 to 514·22)
Bath and North East Somerset	962·59 (759·83 to 1191·7)	836·12 (642·87 to 1113·57)	768·44 (605·68 to 969·3)	673·74 (579·93 to 779·29)	613·85 (539·59 to 698·74)	517·18 (452·74 to 591·33)	403·19 (351·06 to 460·06)	407·02 (323·62 to 520·1)
Bournemouth	1069·93 (857·59 to 1325·85)	929·29 (699·25 to 1219·23)	855·02 (671·98 to 1071·97)	763·3 (659·07 to 882·7)	705·78 (617·78 to 799·52)	620·45 (546·18 to 705·8)	502·1 (440·43 to 572·43)	518·04 (416·77 to 643·22)
Bristol, City of	1099·6 (876·29 to 1350·98)	975·79 (726·87 to 1274·43)	920·47 (743·04 to 1149·99)	818·12 (706·82 to 943·25)	706·82 (619·78 to 802·89)	613·55 (541·49 to 696)	501·64 (438·47 to 575·71)	487·62 (393·68 to 606·32)
Cornwall	1098·96 (882·83 to 1368·71)	957·05 (730·32 to 1282·28)	891·71 (710·46 to 1105·11)	792·07 (689·72 to 908·93)	663·38 (589 to 755·05)	556·74 (492·46 to 631·37)	448·75 (393·95 to 513·66)	450·72 (366·52 to 556·68)
Devon	1036·02 (831·74 to 1259·75)	901·16 (672·64 to 1154·24)	845·64 (668·61 to 1053·43)	756·36 (662·13 to 867·7)	628·04 (551·28 to 709·05)	532·78 (471·02 to 598·04)	421·97 (368·66 to 476·13)	423·95 (339·21 to 521·35)
Dorset	1040·55 (838·4 to 1281·22)	900·96 (671·97 to 1170·57)	830·42 (663·99 to 1037·2)	727·18 (628·52 to 836·79)	601·48 (524·78 to 679·51)	493·31 (427·99 to 560·27)	388·33 (338·88 to 443·26)	397·77 (318·4 to 488·8)

Supplemental Results Table 3a: Age-standardised mortality rates by location, both sexes, 1950, 1960, 1970, 1980, 1990, 2000, 2010, 2019

Location	1950	1960	1970	1980	1990	2000	2010	2019
Gloucestershire	1012·03 (805·34 to 1256·64)	882·73 (664·63 to 1142·07)	824·37 (651·16 to 1010·37)	771·55 (673·54 to 886·25)	655·45 (578·02 to 740·8)	543·99 (479·66 to 613·36)	443·22 (390·43 to 498·81)	431·44 (351·77 to 534·91)
North Somerset	1011·9 (800·8 to 1248·13)	870·62 (648·52 to 1164·3)	795·16 (625·13 to 997·14)	698·07 (601·18 to 807·46)	618·85 (541·08 to 703·85)	525·24 (459·28 to 601)	454·88 (399·34 to 523·21)	455·83 (360·28 to 571·58)
Plymouth	1134·05 (913·43 to 1395·81)	1002·31 (758·12 to 1285·66)	937·5 (758·95 to 1148·84)	839·7 (731·46 to 957·48)	716·92 (630·78 to 803·59)	619·97 (547·16 to 700·72)	503·05 (440·17 to 569·88)	491·06 (397·1 to 597·37)
Poole	1053·58 (834·69 to 1298·81)	916·61 (681·86 to 1205·1)	845·8 (675·69 to 1058·27)	753·98 (646·5 to 883·5)	652·85 (569·39 to 746)	517·87 (453·22 to 590·37)	418·34 (363·64 to 480·74)	401·21 (323·57 to 507·06)
Somerset	1040·65 (836·94 to 1293·31)	909·17 (684·93 to 1214·6)	851·07 (677·62 to 1065·96)	764·05 (667·1 to 882·24)	643·97 (567·49 to 731·01)	542·9 (476·36 to 613·47)	434·96 (383·58 to 494·4)	439·69 (349·43 to 543·05)
South Gloucestershire	979·79 (770·42 to 1218·71)	864·42 (649·84 to 1151·7)	812·38 (643·36 to 1008·33)	703 (605·53 to 819·66)	602·57 (533·04 to 682·12)	499·04 (440·73 to 568·09)	394·45 (343·66 to 450·32)	369·49 (294·82 to 464·76)
Swindon	1087·45 (872·5 to 1346·28)	957·66 (717·21 to 1262·39)	899·54 (718·01 to 1121·94)	801·59 (689·65 to 923·64)	675·95 (597·67 to 769·35)	580·28 (508·71 to 657·63)	448 (392·13 to 514·5)	429·73 (348·5 to 532·59)
Torbay	1110 (893·03 to 1358·18)	956·51 (714·2 to 1288·85)	874·53 (693·88 to 1099·16)	770·87 (667·08 to 897·88)	676·29 (591·11 to 768·28)	628·3 (545·23 to 714·71)	523·12 (459 to 598·25)	519·08 (412·45 to 649·7)
Wiltshire	1015·03 (821·89 to 1254·75)	892·33 (669·12 to 1163)	827·1 (658·5 to 1030·4)	743·91 (641·11 to 856·38)	664·54 (582·98 to 762·98)	530·73 (468·53 to 607·49)	424·03 (371·44 to 483·94)	407·72 (326·19 to 502·94)
West Midlands	1156·47 (980·8 to 1363·16)	1021·68 (830·13 to 1243·37)	957·74 (804·74 to 1138·77)	860·24 (776·51 to 962·09)	738·33 (667·26 to 825·35)	620·91 (564·05 to 689·03)	486·96 (441·34 to 544·01)	478·9 (401·41 to 577·52)
Birmingham	1190·48 (970·63 to 1456·02)	1047·24 (805·08 to 1315·99)	983·82 (795·22 to 1204·32)	899·26 (794·73 to 1022·09)	776·19 (688·01 to 883·97)	657·94 (584·61 to 747·02)	499·07 (441·57 to 568·89)	504·47 (405·53 to 627·23)
Coventry	1196·46 (958·19 to 1474·99)	1064·15 (807·72 to 1373·42)	998·49 (788·17 to 1219·3)	873·33 (761·72 to 1005·43)	764·67 (673·94 to 875·74)	637·41 (566·75 to 725)	515·85 (452·21 to 589·87)	510·47 (405·13 to 641·19)
Dudley	1109·79 (894·14 to 1381·44)	976·84 (735·61 to 1276·29)	899·02 (724·41 to 1121·46)	807·72 (704·2 to 936·02)	708·7 (627·04 to 808·97)	617·59 (546·24 to 706·86)	479·45 (423·05 to 549·78)	468·93 (371·97 to 592·84)
Herefordshire, County of	1115·63 (887·6 to 1399·83)	977·81 (730·39 to 1282·58)	908·24 (719·41 to 1131·39)	794·14 (687·97 to 930·93)	642·98 (559·4 to 736·64)	551·74 (483·09 to 627)	447·5 (391·07 to 512·96)	450·15 (358·28 to 566·53)
Sandwell	1258·5 (1007·59 to 1548·62)	1112·79 (850·86 to 1444·01)	1034·41 (825·79 to 1281·78)	905·84 (795·61 to 1045·2)	790·15 (689·51 to 911·69)	704·48 (623 to 810·4)	575·54 (506·35 to 668·9)	516·93 (413·36 to 658·87)
Shropshire	1108·32 (871·52 to 1380·8)	980·11 (733·51 to 1277·58)	910·43 (720·7 to 1138·94)	794·44 (694·96 to 912·85)	673·48 (593·81 to 768·65)	563·4 (495·72 to 644·01)	438·85 (382·84 to 505·39)	410·58 (326·44 to 510·72)
Solihull	1019·45 (800·14 to 1275·58)	898·77 (679·75 to 1182·91)	832·6 (656·71 to 1040·44)	729·26 (636·56 to 846·92)	627·18 (550·9 to 724·44)	550·51 (485·39 to 631·66)	409·59 (358·67 to 468·43)	432·34 (344·25 to 553·14)
Staffordshire	1123·77 (899·76 to 1391·58)	1004·41 (762·48 to 1292·89)	945·58 (755·59 to 1169·36)	869·43 (763·06 to 989·71)	748·6 (658·73 to 851·77)	617·71 (546·22 to 699·8)	473·82 (418·39 to 538·19)	479·09 (389·21 to 598·63)
Stoke-on-Trent	1301·55 (1037·87 to 1613·68)	1165·38 (880·58 to 1510·41)	1097·11 (869·47 to 1359·63)	985·88 (854·2 to 1136·18)	833·52 (728·57 to 959·51)	699·1 (615·66 to 797·93)	585·77 (513·37 to 673·12)	552·67 (438 to 694·91)
Telford and Wrekin	1187·41 (937·33 to 1486·88)	1060·71 (811·1 to 1377·37)	989·42 (782·6 to 1228·68)	878·71 (764·94 to 1019·28)	730·56 (639·37 to 834·81)	622·84 (544·68 to 716·27)	497·29 (436·32 to 570·14)	494·45 (390·74 to 629·34)
Walsall	1220·26 (975·48 to 1527·52)	1083·85 (811·89 to 1421·05)	1015·42 (809·73 to 1268·66)	901·45 (787·19 to 1037·68)	795·75 (692·97 to 912·69)	638·33 (562·78 to 732·09)	518·94 (452·64 to 595·45)	512·71 (406·9 to 639·38)
Warwickshire	1083·85 (859·35 to 1334·05)	963·34 (742·95 to 1257·13)	905·54 (718·93 to 1135·36)	820·37 (715·39 to 939·9)	703·4 (619·02 to 806·44)	579·29 (512·22 to 661·81)	454·62 (401·13 to 518·02)	448·59 (358·25 to 565)
Wolverhampton	1213·78 (982·72 to 1497·53)	1075·3 (811·02 to 1400·96)	1004·11 (802·45 to 1237·47)	887·24 (776·23 to 1024)	786·12 (694·45 to 898·59)	674·85 (596·26 to 773·47)	559·74 (489·92 to 639·34)	551·42 (440·59 to 691·48)
Worcestershire	1048·81 (831·84 to 1290·83)	932·43 (698·42 to 1207·11)	869·44 (698·36 to 1068·43)	799·62 (696·26 to 914·49)	686·86 (603·17 to 783·52)	586·48 (516·54 to 663·91)	465·36 (409·66 to 527·56)	452·74 (362·6 to 561·62)
Yorkshire and the Humber	1186·03 (995·66 to 1392·33)	1041·92 (849·05 to 1267·91)	969·34 (813·43 to 1143·03)	856·98 (766·77 to 965·35)	738·63 (667·8 to 812·16)	613·27 (552·29 to 678·97)	502·59 (453·38 to 557·57)	492·88 (412·39 to 584·12)
Barnsley	1254·14 (1002·98 to 1542·28)	1106·64 (849·27 to 1426·97)	1019·19 (808·91 to 1241·05)	896·18 (770·88 to 1030·47)	763·91 (665·84 to 866·77)	641·86 (561·69 to 736·92)	515·98 (452·68 to 587·3)	511·24 (406·06 to 638·87)
Bradford	1242·72 (995·14 to 1531·86)	1087·73 (830·15 to 1378·14)	1009·35 (805·36 to 1243·15)	887·48 (776·53 to 1016·75)	770·72 (680·02 to 869·25)	657·38 (583·63 to 745·28)	531·17 (469·83 to 601·08)	522·78 (419·7 to 646·58)
Calderdale	1231·32 (991·31 to 1508·88)	1080·18 (811·78 to 1401·89)	1000·85 (807·69 to 1220·98)	885·9 (766·54 to 1018·31)	773·25 (673·72 to 884·78)	617·47 (538·74 to 702·99)	520·62 (453·34 to 598·09)	499·15 (395·98 to 617·12)
Doncaster	1216·75 (950·66 to 1503·3)	1068·32 (812·42 to 1385·02)	979·48 (791·03 to 1209·85)	855·97 (744·23 to 974·45)	774·79 (680·39 to 878·25)	660·34 (572·93 to 749·09)	539·69 (471·9 to 612·41)	538·49 (424·7 to 669·89)
East Riding of Yorkshire	1098·49 (875·92 to 1365·4)	960·69 (718·12 to 1239·49)	886·25 (714·61 to 1088·57)	782·25 (680·59 to 897·19)	677·18 (596·46 to 763·77)	571·27 (504·58 to 649·69)	459·34 (403·31 to 520·34)	441 (355·36 to 544·6)
Kingston upon Hull, City of	1232·94 (995·7 to 1519·19)	1082·82 (826·36 to 1386·03)	991·13 (790·52 to 1212·68)	862·23 (746·15 to 996·98)	768·97 (677·46 to 869·12)	707·33 (620·18 to 806·96)	595·68 (522·1 to 675·4)	617·9 (495·16 to 766·79)
Kirklees	1232·54 (985·85 to 1548·79)	1086·9 (820·12 to 1416·43)	1011·72 (800·82 to 1253·12)	921·82 (798·4 to 1070·5)	764·81 (672·76 to 871·73)	618·8 (547·19 to 710·3)	520·05 (456·21 to 601·04)	501·65 (408·01 to 634·42)
Leeds	1167·76 (938·78 to 1429·53)	1029·54 (783·79 to 1320·08)	967·85 (766·18 to 1203·12)	856·64 (747·07 to 975·56)	726·11 (639·31 to 818·86)	604·04 (530·52 to 682·32)	508·7 (448·73 to 577·57)	496·4 (397·89 to 614·06)
North East Lincolnshire	1189·59 (943·26 to 1461·24)	1040·32 (778·33 to 1353·78)	948·12 (755·05 to 1181·75)	827·55 (711·12 to 958·08)	780·95 (679·78 to 888·8)	637·42 (552·05 to 725·29)	526·51 (456·14 to 604·79)	522·54 (412·72 to 649·67)
North Lincolnshire	1200·15 (941·99 to 1493·24)	1060·26 (795·91 to 1397·97)	978·75 (778·28 to 1205·75)	859·4 (741·5 to 984·34)	769·02 (670·96 to 865·56)	621·78 (541·3 to 704·3)	491·71 (428·14 to 559·87)	463·54 (370·33 to 572·81)
North Yorkshire	1102·17 (872·79 to 1359·84)	968·99 (729·71 to 1249·86)	899·39 (722·15 to 1102·25)	794·21 (693·4 to 911·01)	674·89 (592 to 761·08)	549·85 (484·27 to 621·42)	438·95 (387·22 to 497·37)	428·95 (342·62 to 527·6)
Rotherham	1226·32 (978·34 to 1514·41)	1085·05 (818·42 to 1410·11)	999·19 (798·23 to 1242·89)	883·14 (754·95 to 1012·08)	758·96 (654·88 to 857·99)	634·97 (552·95 to 718·12)	513·5 (446·79 to 582·09)	538 (421·89 to 674·78)
Sheffield	1161·84 (939·38 to 1425·82)	1029·8 (771·19 to 1312·74)	958·38 (766·08 to 1177·77)	854·25 (740·72 to 979·17)	744·92 (656·64 to 840·79)	613·29 (537·76 to 692·78)	507·56 (448·54 to 573·69)	490·21 (387·55 to 601·54)
Wakefield	1265·25 (1024·33 to 1539·68)	1126·7 (865·57 to 1455·25)	1050·62 (848·05 to 1287·44)	927·34 (808·27 to 1064·53)	773·68 (678·52 to 874·53)	637·13 (561·58 to 723·85)	530·08 (464·65 to 602·48)	523·87 (418·48 to 649·21)
York	1086·41 (845·44 to 1358·93)	959·95 (731·86 to 1229·8)	887·92 (708·47 to 1110·13)	788·61 (677·45 to 916·14)	675·77 (587·6 to 771·02)	531·27 (460·79 to 602·18)	427·61 (372·12 to 488·96)	448·73 (351·84 to 562·8)
Northern Ireland	1165·21 (1089·73 to 1249·28)	1092·94 (1019·04 to 1181·31)	1019·26 (942·86 to 1101·36)	970·8 (900·56 to 1043·45)	809·83 (751·82 to 873·43)	647·98 (598·29 to 701·35)	514·16 (478·4 to 552·63)	496·56 (440·7 to 560·73)
Scotland	1366·3 (1271·73 to 1467·05)	1305·66 (1213·12 to 1412·33)	1130·95 (1046·69 to 1224·45)	946·42 (877·64 to 1020·86)	819·73 (761·86 to 887·15)	698·62 (649·2 to 754·91)	564·38 (523·3 to 609·74)	549·74 (484·55 to 619·22)
Wales	1246·21 (1154·32 to 1346·17)	1077·27 (990·24 to 1162·16)	989·67 (912 to 1073·22)	854·56 (792·58 to 925·21)	721·33 (669·75 to 778·65)	629·74 (581·81 to 679·8)	508·42 (470·42 to 549·36)	493·37 (441·93 to 561·57)
Latin America and Caribbean	2054·48 (1940·21 to 2173·95)	1639·24 (1566·01 to 1713·41)	1422·16 (1367·32 to 1478·78)	1123·48 (1079·72 to 1164·29)	930·93 (896·44 to 964·17)	772·72 (744·97 to 799·47)	728·33 (699·15 to 755·92)	632·85 (577·29 to 700·8)
Andean Latin America	2452·44 (2214·58 to 2723·44)	1919·02 (1769·76 to 2079·79)	1759·08 (1623·61 to 1898·56)	1111·63 (1023·54 to 1201·64)	928·06 (858·03 to 999·49)	744·02 (681·21 to 816·46)	671·4 (609·27 to 737·12)	577·12 (477·82 to 685·48)
Bolivia	3079·55 (2734·86 to 3444·8)	2412·73 (2054·72 to 2842·06)	1967·76 (1702·06 to 2250·02)	1599·88 (1410·49 to 1826·46)	1337·33 (1201·12 to 1490·88)	1098·93 (988·1 to 1229·67)	946·02 (817·55 to 1088·51)	900·54 (724·99 to 1104·55)
Ecuador	1804·86 (1506·89 to 2113·62)	1518·96 (1397·62 to 1657·29)	1390·01 (1262·56 to 1540)	1034·4 (953·63 to 1122·73)	804·5 (749·25 to 866·84)	680·14 (631·71 to 733·26)	740·92 (674·33 to 809·58)	658·71 (525·49 to 820·02)
Peru	2474·82 (2119·75 to 2887·18)	1932·22 (1730·36 to 2161·26)	1888·77 (1691·59 to 2112·62)	1028·7 (900·68 to 1155·22)	875·4 (768·18 to 988·42)	675·68 (578·3 to 792·39)	574·95 (490·69 to 664·18)	466·69 (356·36 to 597·71)
Caribbean	1700·8 (1538·15 to 1870·29)	1353·7 (1269·87 to 1445·67)	1228·12 (1164·01 to 1291·74)	1059·97 (1012·34 to 1110·88)	948·62 (895·96 to 1010·26)	850·3 (810·31 to 898·25)	1293·08 (1211·86 to 1390·42)	757·92 (657·68 to 874)
Antigua and Barbuda	1602·62 (1460·17 to 1760·72)	1230·17 (1134·24 to 1338·93)	1026·05 (946·7 to 1111·6)	841·82 (730·67 to 980·23)	763·76 (700·52 to 835·52)	728·13 (669·15 to 795·49)	696·36 (637·24 to 762·95)	685·76 (585·51 to 808·31)
The Bahamas	1523·92 (1249·4 to 1832·5)	1308·26 (1090·39 to 1537·96)	1168·73 (1063·07 to 1281·3)	997·86 (912·88 to 1092·3)	888·81 (815·99 to 970·43)	858·71 (787·6 to 931·24)	762·68 (703·19 to 831·26)	758·84 (620·48 to 929·82)
Barbados	1523·17 (1394·15 to 1681·34)	1074·32 (999·19 to 1158·3)	1037·24 (962·41 to 1124·73)	850·97 (787·64 to 920·04)	777·58 (722·93 to 837·16)	729·09 (676·84 to 790·84)	665·65 (620·44 to 718·58)	673·9 (556·94 to 817·02)
Belize	1682·29 (1504·12 to 1873·14)	1070·48 (958·85 to 1196·82)	943·85 (862·3 to 1027·85)	823·58 (760·44 to 893·5)	759·32 (693·61 to 832·14)	939·22 (869·62 to 1015·3)	733·1 (683·94 to 788·48)	698·79 (591·84 to 836·61)
Bermuda	1461·68 (1317·23 to 1613·56)	1254·65 (1162·24 to 1351·4)	1019·45 (943·95 to 1100·32)	836·37 (778·15 to 905·89)	849·59 (782·2 to 926·07)	637·71 (590·6 to 691·19)	479·29 (429·19 to 535·34)	445·9 (378·8 to 537·23)
Cuba	1224·54 (996·52 to 1498·89)	1108·23 (995·93 to 1236·14)	1042·92 (976·21 to 1117·18)	842·22 (781·77 to 911·12)	712·24 (634·99 to 805·48)	622·26 (575·37 to 677·03)	561·6 (519·15 to 608·84)	555·41 (448·29 to 688·11)
Dominica	2042·61 (1849·67 to 2280·87)	1315·75 (1172·17 to 1473·18)	1060·94 (941·65 to 1185·01)	877·48 (796·2 to 961·99)	947·85 (851·63 to 1058·32)	869·26 (767·23 to 981·75)	852·6 (751·49 to 970·32)	861·3 (694·85 to 1071·48)
Dominican Republic	1471·01 (1327·14 to 1649·46)	1125·97 (1044·98 to 1213·87)	1046·25 (953·67 to 1148·38)	888·15 (811·59 to 972·09)	765·47 (683·2 to 856·59)	694·39 (624·67 to 776·3)	742·1 (625·25 to 874·68)	769·96 (605·28 to 984·81)
Grenada	1455·13 (1317·7 to 1603·29)	1124·7 (1002·6 to 1256·99)	1043·32 (913·02 to 1195·63)	923·9 (793·92 to 1077·7)	1050·04 (951·01 to 1165·44)	904·52 (835·14 to 983·89)	884·73 (816·42 to 964·42)	828·46 (723·43 to 953·33)
Guyana	2107·75 (1839·96 to 2412·69)	1574·41 (1420·69 to 1758·26)	1453·74 (1294·76 to 1636·62)	1478·41 (1277·27 to 1709·16)	1486·71 (1309·78 to 1670·25)	1312·4 (1152·27 to 1479·91)	1212·14 (1080·3 to 1356·61)	1133·89 (893·43 to 1428·99)
Haiti	2834·18 (2399·46 to 3288·59)	2385·08 (1941·71 to 2869·25)	2207·06 (1908·65 to 2530·73)	1991·26 (1759·35 to 2243·18)	1800·04 (1563·88 to 2070·76)	1617·11 (1421·44 to 1832·46)	3830·97 (3337·2 to 4375·29)	1277·58 (982·49 to 1645·15)
Jamaica	1644·14 (1513·94 to 1777·65)	1160·77 (1082·17 to 1250·58)	934·27 (871·18 to 1000·09)	724·03 (665·69 to 787·81)	670·19 (622·51 to 726·22)	693·38 (641·23 to 746·61)	625·49 (577·51 to 677·24)	639·45 (506·66 to 790·65)
Puerto Rico	1439·91 (1341·24 to 1547·64)	1056·11 (988·98 to 1128·13)	1020·27 (951·24 to 1094·38)	791·92 (741·08 to 846·8)	737·36 (684·37 to 794·8)	617·01 (576·89 to 663·38)	502·15 (469 to 538·54)	461·17 (350·93 to 583·61)
Saint Kitts and Nevis	1325·57 (1096·64 to 1621·24)	1290·34 (1120·85 to 1485·41)	1227·49 (1135·55 to 1327·87)	1374·36 (1256·41 to 1500·93)	1364·73 (1244·32 to 1487·02)	1027·09 (929·31 to 1125·57)	884·96 (769·75 to 1019·28)	900·12 (732·05 to 1119·33)
Saint Lucia	1800·29 (1632·75 to 1992·09)	1399·92 (1277·18 to 1538·88)	1137·79 (1029·16 to 1255·07)	1009·53 (937·76 to 1083·48)	1040·1 (961·02 to 1129·28)	813·66 (751·31 to 880·52)	659·23 (608·54 to 713·5)	713·22 (595·74 to 855·73)
Saint Vincent and the Grenadines	1752·15 (1573·87 to 1950·47)	1295·18 (1176·3 to 1420·38)	1157·11 (1060·78 to 1263·47)	1009·17 (925·01 to 1106·19)	983·5 (907·07 to 1075·45)	904·61 (834·6 to 980·07)	817·91 (744·35 to 900·28)	843·78 (716·57 to 982·62)
Suriname	1734·96 (1606·33 to 1880·58)	1264·46 (1121·06 to 1418·91)	1109·91 (991·83 to 1233·72)	1083·78 (955·21 to 1239·08)	951·35 (842·62 to 1081·63)	902·67 (840·1 to 973·61)	781·08 (719·77 to 856·45)	781·07 (641·94 to 940·07)
Trinidad and Tobago	1729·46 (1613·07 to 1862·66)	1300·54 (1212·99 to 1396·09)	1370·6 (1282·51 to 1473·35)	1216·76 (1138·03 to 1307·56)	1024·04 (954·91 to 1106·69)	913·58 (852·06 to 985·85)	736·74 (683·02 to 799·65)	693·19 (528·26 to 894·08)
Virgin Islands	1481·34 (1316·36 to 1690·58)	1458·9 (1317·22 to 1619·15)	1179·64 (1054·28 to 1338·92)	955·09 (874·78 to 1048·59)	833·43 (731·5 to 938·89)	784·9 (705·75 to 875·98)	821·58 (748·44 to 896·93)	781·67 (655·99 to 923·62)
Central Latin America	1975·37 (1870·25 to 2084·63)	1529·94 (1461·74 to 1600·97)	1298·19 (1243·98 to 1353·96)	1054·02 (998·74 to 1111·23)	856·7 (821·33 to 896·16)	716·38 (685·15 to 747·65)	644·83 (616·69 to 673·62)	621·29 (544·57 to 713·81)
Colombia	1736·34 (1600·37 to 1901·25)	1554·78 (1415·96 to 1721·53)	1079·53 (990·82 to 1181·4)	954·73 (813·27 to 1127·34)	818·85 (749·73 to 895·87)	684·21 (620·5 to 761·08)	545·58 (495·86 to 601·68)	464·82 (366·31 to 582·41)
Costa Rica	1597·76 (1481·8 to 1717·37)	1114·81 (1022·1 to 1220·71)	1053·12 (967·6 to 1149·24)	703·71 (642·16 to 776·82)	608·06 (547·3 to 675·22)	575·19 (537·16 to 618·03)	500·24 (463·28 to 538·75)	481·42 (370·07 to 611·36)
El Salvador	2194·17 (1973·61 to 2461·99)	1599·16 (1466·98 to 1749·45)	1450·58 (1341·3 to 1572·09)	1075·05 (937·7 to 1233·44)	836·03 (769·81 to 911·99)	726·76 (647·32 to 809·13)	651·41 (591·16 to 715·32)	644·28 (486·01 to 833·31)
Guatemala	2566·46 (2328·86 to 2830·51)	2219·23 (2017·58 to 2450·44)	2032·49 (1860 to 2224·27)	1458·07 (1350·65 to 1581·65)	1434·78 (1258·32 to 1624·06)	1182·01 (1051·89 to 1323·64)	940·48 (809·15 to 1089·11)	835 (635·57 to 1074·05)
Honduras	2570·56 (2271·93 to 2929·8)	1975·79 (1785·13 to 2177·98)	1506·45 (1352·64 to 1674·94)	913·74 (813·08 to 1036·28)	887·62 (782·53 to 1008·06)	919·73 (763·11 to 1113·14)	915·42 (770·87 to 1072·37)	903·25 (785·57 to 1063·28)

Supplemental Results Table 3a: Age-standardised mortality rates by location, both sexes, 1950, 1960, 1970, 1980, 1990, 2000, 2010, 2019								
Location	1950	1960	1970	1980	1990	2000	2010	2019
Mexico	2110·93 (1931·79 to 2301·92)	1544·17 (1442·4 to 1665·47)	1351·75 (1263·12 to 1452·28)	1120·31 (1049·37 to 1204·42)	871·84 (812·46 to 938·9)	710·75 (660·74 to 761·38)	659·98 (617·25 to 708·21)	663·72 (553·12 to 793·46)
Aguascalientes	1906·56 (1655·09 to 2178·7)	1445·37 (1251·39 to 1671·73)	1316·39 (1131·94 to 1506·1)	1096·13 (987·71 to 1211·04)	882·82 (800·2 to 974·77)	728·2 (658·53 to 804·41)	646·31 (583·24 to 710·46)	616·9 (488·44 to 757·72)
Baja California	2204·69 (1895·36 to 2557·97)	1598·06 (1381·22 to 1835·84)	1447·85 (1239·32 to 1675·55)	1129·27 (1022·9 to 1244·83)	1054·12 (947·09 to 1161·65)	912·25 (820·13 to 1001·98)	755·77 (681·47 to 831·21)	721·57 (572·74 to 906·06)
Baja California Sur	2036·23 (1745·54 to 2403·68)	1457·15 (1273·95 to 1679·62)	1301·14 (1140·21 to 1482·46)	1043·43 (945·84 to 1156·31)	840·02 (759·53 to 931·51)	721·23 (651·89 to 796·51)	658·34 (597·27 to 727·87)	729·96 (570·33 to 915·89)
Campeche	2013·15 (1739·76 to 2310·94)	1483·83 (1270·86 to 1719·14)	1303·93 (1127·8 to 1486·75)	1085·71 (972·4 to 1199·28)	847·16 (768·32 to 937·86)	645·96 (580·99 to 716·91)	631·35 (568·77 to 700·69)	638·67 (503·6 to 796·63)
Chiapas	2160·73 (1860·9 to 2506·85)	1631·35 (1418·32 to 1860·16)	1412·33 (1210·43 to 1647·6)	1179·11 (1062·39 to 1312·89)	895·5 (807·99 to 996·06)	728·56 (655·35 to 804·96)	665·23 (600·46 to 734·4)	670·86 (527·13 to 836·73)
Chihuahua	2247·97 (1922·05 to 2572·16)	1673·58 (1454·39 to 1914·52)	1487·15 (1293·13 to 1718·74)	1248·94 (1131·64 to 1390·41)	1097·87 (996·44 to 1218·05)	903·21 (811·05 to 998)	833·48 (757·62 to 918·79)	863·6 (684·23 to 1081·61)
Coahuila	2282·5 (1968·93 to 2637·03)	1675·48 (1451·28 to 1917·86)	1505·55 (1315·14 to 1733·77)	1273·52 (1157 to 1396·25)	988·49 (894·51 to 1085·26)	776·44 (700·85 to 853·95)	702·96 (636·75 to 772·64)	689·08 (544·19 to 862·32)
Colima	2484·53 (2144·34 to 2859·19)	1767·48 (1524·47 to 2024·78)	1558·4 (1352·08 to 1793·37)	1266·37 (1138·47 to 1403·48)	956·29 (864·97 to 1053·97)	719·35 (648·92 to 793·81)	670·74 (605·87 to 736·7)	787·15 (624·88 to 993·02)
Durango	2171·03 (1864·47 to 2520·39)	1548·57 (1338·96 to 1794·12)	1312·7 (1122·17 to 1535·75)	1092·35 (983·83 to 1218·23)	816·37 (735·98 to 910·52)	641·76 (579·16 to 710·25)	654·82 (593·96 to 728·16)	595·58 (466·9 to 760·59)
Guanajuato	2205·23 (1915·24 to 2520·94)	1655·51 (1437·15 to 1905·19)	1466·57 (1279·86 to 1682·68)	1118·39 (1014·44 to 1236·77)	976·13 (888·79 to 1079·04)	748·8 (680·03 to 826·45)	629·6 (573·32 to 692·33)	711·95 (559·83 to 901·29)
Guerrero	2194·7 (1909·43 to 2544·37)	1675·47 (1447·68 to 1917·8)	1551·88 (1341·1 to 1788·79)	1354·51 (1226·12 to 1508·53)	958·38 (871·48 to 1059·4)	851·26 (773·24 to 939·85)	796·17 (718·15 to 883·73)	749·85 (610·27 to 922·08)
Hidalgo	2325·49 (2013·66 to 2716·81)	1702·33 (1469·56 to 1947·53)	1495·1 (1290·87 to 1724·94)	1278·95 (1160·32 to 1425·4)	795·24 (720·69 to 880·23)	592·4 (535·96 to 652·64)	571·58 (518·8 to 633·35)	581·17 (465·12 to 734·79)
Jalisco	2014·25 (1761·77 to 2318·19)	1498·94 (1300·13 to 1717·86)	1321·28 (1146·81 to 1524·71)	1061·72 (966·12 to 1179·05)	913·59 (832·24 to 1015·05)	750·46 (684·83 to 827·09)	669·06 (612·15 to 737·93)	671·02 (536·69 to 838·7)
México	2016·08 (1752·66 to 2311·65)	1521·09 (1319·56 to 1761·83)	1364·09 (1164·09 to 1576·26)	1171·52 (1027·8 to 1328·91)	988·39 (898·7 to 1086·61)	765·09 (695·99 to 836·98)	665·13 (608·73 to 725·73)	669·77 (537·76 to 820·24)
Mexico City	1741·33 (1502·15 to 2008·43)	1260·51 (1086·89 to 1452·15)	1135·87 (987·4 to 1302·46)	1020·37 (934·14 to 1120·79)	815·47 (744·8 to 896·91)	708·59 (647·21 to 779·34)	670·55 (612·56 to 737·69)	657·85 (528·07 to 819·88)
Michoacán de Ocampo	2135·7 (1833·82 to 2453·67)	1581·51 (1363·79 to 1829·75)	1355·11 (1157·7 to 1572·08)	1130·16 (1021·24 to 1247·95)	780·35 (705·09 to 865·92)	655·32 (591·7 to 725·67)	607·36 (549·99 to 672·35)	619·43 (492·3 to 781·88)
Morelos	2266·82 (1952·86 to 2602·02)	1622·33 (1406·03 to 1861·67)	1405·28 (1209·3 to 1620·81)	1160·99 (1053·19 to 1289·35)	855·54 (771·68 to 947·26)	672·17 (606·42 to 742·76)	625·99 (568·86 to 688·11)	679·2 (543·22 to 838·9)
Nayarit	2204·38 (1883·05 to 2552·35)	1564·11 (1368·82 to 1800·52)	1303·66 (1128·55 to 1520·14)	1029·66 (930·7 to 1148·88)	807·42 (728·87 to 894·86)	613·66 (550·1 to 679·16)	601·65 (543·47 to 664·88)	625·54 (498·76 to 800·31)
Nuevo León	1831·55 (1568·28 to 2117)	1347·92 (1173·02 to 1560·16)	1214·32 (1038·79 to 1417·1)	957·46 (866·9 to 1060·39)	836·96 (762·64 to 925·16)	702·19 (639·31 to 778·44)	643·25 (588·19 to 705·12)	631·66 (501·92 to 789·05)
Oaxaca	2550·99 (2219 to 2915·96)	1910·04 (1668·55 to 2195·65)	1686·45 (1460·7 to 1951·38)	1421·12 (1281·13 to 1563·3)	966·31 (874·29 to 1067·39)	690·89 (625·34 to 758·88)	617·06 (559·77 to 680·6)	588·16 (466·52 to 739·42)
Puebla	2678·51 (2324·37 to 3083·77)	1940·27 (1689·43 to 2200·72)	1662·15 (1446·08 to 1914·52)	1315·05 (1192·86 to 1458·36)	947·28 (861·87 to 1049·48)	753·53 (683·88 to 828·3)	663·14 (604·05 to 732·29)	682·68 (545·16 to 850·29)
Querétaro	2258·64 (1932·15 to 2648·47)	1658·09 (1444·77 to 1902·69)	1437·9 (1237·49 to 1662·21)	1186·47 (1051·47 to 1328·82)	951·46 (860·91 to 1046·89)	781·44 (707·44 to 865·48)	650·22 (589·52 to 720·15)	677·2 (538·47 to 847·38)
Quintana Roo	1838·64 (1577·44 to 2124·03)	1438·05 (1234·94 to 1666·43)	1290·8 (1110·95 to 1482·75)	1048·97 (952·3 to 1167·75)	747·81 (677·56 to 829·71)	657·15 (595·74 to 731·25)	678·98 (616·46 to 752·92)	714·2 (571·15 to 895·72)
San Luis Potosí	2130·66 (1824·39 to 2459·1)	1535·39 (1344·98 to 1765·35)	1333·35 (1149·84 to 1547·55)	937·56 (847·59 to 1032·97)	772·81 (696·85 to 849·71)	619·11 (559·15 to 675·98)	576·06 (521·53 to 632·64)	621·5 (486·01 to 778·86)
Sinaloa	1884·39 (1599·71 to 2200·65)	1382·2 (1194·38 to 1593·73)	1209·44 (1020·98 to 1425·45)	1024·18 (931·79 to 1131·12)	738·41 (668·35 to 815·88)	626·24 (567·86 to 687·72)	614·45 (556·03 to 678·71)	635·9 (499·07 to 802·45)
Sonora	1899·55 (1632·3 to 2188·44)	1451·94 (1244·36 to 1679·96)	1350·85 (1137·97 to 1590·88)	1176 (1022·59 to 1367·15)	955·91 (862·05 to 1063·74)	800·49 (720·94 to 887·13)	746·53 (673·68 to 824·32)	701·84 (554·48 to 875·59)
Tabasco	2130·16 (1808·69 to 2506·88)	1611·88 (1384·75 to 1852·6)	1458·19 (1258·71 to 1705·37)	1252·21 (1128·81 to 1392·38)	854·84 (772·81 to 953·94)	699·09 (630·69 to 776·12)	699·88 (636·23 to 774·57)	671·96 (532·06 to 852·11)
Tamaulipas	1957·6 (1676·09 to 2295·01)	1453·37 (1258·03 to 1675·59)	1293·79 (1102·7 to 1501·01)	1125·23 (1018·39 to 1236·64)	828·73 (750·62 to 913·26)	652·58 (590·58 to 716·61)	651·59 (592·73 to 715·22)	656·5 (513·99 to 820·52)
Tlaxcala	2177·59 (1879·68 to 2514·36)	1567·73 (1364·94 to 1825·34)	1336·07 (1156·69 to 1565·97)	1082·41 (959·13 to 1226·34)	852·56 (773·13 to 949·37)	692·32 (628·52 to 769·36)	610·04 (555·05 to 674·1)	612·21 (485·73 to 775·26)
Veracruz de Ignacio de la Llave	2095·7 (1793·38 to 2454·07)	1510·86 (1316·53 to 1728·55)	1295·68 (1109·87 to 1506·55)	1045·87 (952·03 to 1155·3)	788·69 (716·41 to 874·09)	662·98 (600·31 to 731·04)	650·08 (589·66 to 716·46)	670·89 (531·83 to 851·29)
Yucatán	1943·11 (1672·27 to 2260·08)	1544·62 (1334·97 to 1776·65)	1409·11 (1227·16 to 1623·68)	1198·74 (1082·96 to 1318·03)	954·33 (859·64 to 1049·94)	719·55 (647·89 to 792·7)	684·89 (622·36 to 752·94)	679·47 (539·62 to 844·29)
Zacatecas	1850·12 (1605·63 to 2139·34)	1371·01 (1187·28 to 1598·43)	1182·51 (1008·97 to 1369·68)	911·92 (828·99 to 1003·46)	733·35 (666·78 to 807·22)	627·46 (565·8 to 692·9)	614·27 (557·13 to 673·16)	645·35 (509·09 to 811·91)
Nicaragua	2110·17 (1904·7 to 2338·86)	1663·19 (1507·39 to 1841·39)	1478·43 (1345·66 to 1625·66)	1030·06 (871·25 to 1236·44)	721·99 (653·03 to 802·89)	765·94 (696·08 to 843·65)	780·99 (702·9 to 870·72)	730·21 (592·29 to 883·48)
Panama	1287·85 (1182·34 to 1402·72)	1036·93 (966·27 to 1114·78)	990·62 (925·01 to 1072·26)	687·23 (640·63 to 739·02)	601·35 (559·27 to 653·51)	542·18 (503·57 to 583·99)	521·59 (489·22 to 561·02)	471·33 (366·29 to 605·13)
Venezuela	1479·59 (1382·21 to 1586·45)	1170·65 (1087·66 to 1265·92)	1123·54 (1045·69 to 1212·39)	949·86 (882·55 to 1022·59)	787·19 (732·75 to 847·75)	702·55 (652·78 to 760·83)	654·95 (609·18 to 703·35)	675·69 (518·96 to 871·66)
Tropical Latin America	2142·36 (1959·19 to 2371·59)	1782·27 (1635·5 to 1941·78)	1529·44 (1410·86 to 1647·54)	1224·99 (1144·45 to 1312·32)	1010·14 (939·9 to 1085·84)	821·05 (766·34 to 878·79)	712·47 (662·36 to 764·95)	635·32 (562·18 to 724·48)
Brazil	2161·82 (1976·64 to 2396·27)	1812·42 (1662·52 to 1977·71)	1549·89 (1425·81 to 1673·64)	1239·26 (1155·16 to 1330·33)	1022 (949·72 to 1100·76)	826·14 (770·61 to 885·06)	714·53 (664·1 to 767·63)	636·23 (562·57 to 726·08)
Acre	2324·4 (1963·07 to 2700·09)	1868·6 (1592·87 to 2183·34)	1531·25 (1316·85 to 1769·44)	1204·35 (1072·37 to 1346·8)	988·84 (886·13 to 1114·18)	867·56 (777·08 to 958·37)	717·28 (639·85 to 798·9)	693·18 (564·52 to 837·25)
Alagoas	2739·65 (2363·8 to 3173·77)	2351·23 (2040·35 to 2698·66)	1995·87 (1744·75 to 2273·65)	1566·71 (1400·67 to 1741·63)	1181·27 (1054·77 to 1328·8)	873·23 (782·59 to 970·31)	814·68 (727·24 to 908·69)	742·47 (596·65 to 905·11)
Amapá	2463·81 (2080·81 to 2917·6)	1849·27 (1605·45 to 2129·13)	1338·86 (1165·49 to 1546·71)	822·93 (741·56 to 913·66)	753·75 (675·53 to 845·11)	816·26 (739·39 to 902·57)	596·32 (537·51 to 662·71)	618·95 (512·82 to 748·95)
Amazonas	1950·42 (1631·01 to 2341·04)	1587·15 (1347·8 to 1859·8)	1404·11 (1197·54 to 1651·93)	1116·42 (988·33 to 1251·53)	894·6 (793·08 to 1007·67)	744·35 (664·29 to 832·09)	635·41 (570·34 to 709·72)	596·37 (492·29 to 723·12)
Bahia	2199·39 (1890·86 to 2554·95)	1860·34 (1604·36 to 2158·95)	1561·72 (1359·18 to 1809·94)	1111·28 (987·69 to 1251·74)	947·99 (839·99 to 1069·1)	778·12 (691·08 to 873·04)	732·53 (641·61 to 833·99)	667·88 (544·22 to 817)
Ceará	2788·19 (2403·38 to 3225·31)	2305·63 (2012·91 to 2637·07)	1816·33 (1555·17 to 2075·54)	1288·75 (1106·57 to 1488·94)	880·84 (764·49 to 1009·61)	754·56 (655·04 to 852·73)	701·16 (607·55 to 809·98)	679·22 (533·36 to 840·59)
Distrito Federal	2154·42 (1833·61 to 2530·64)	1852·77 (1589·63 to 2145·33)	1618·42 (1381·91 to 1870·39)	1373·31 (1222·1 to 1545·07)	1174·29 (1040·97 to 1320·02)	907·76 (822·82 to 1003·12)	766·93 (692·33 to 845·7)	645·76 (531·04 to 773·31)
Espírito Santo	1964·95 (1681·82 to 2288·25)	1681·21 (1429·67 to 1959·22)	1468·12 (1259·58 to 1695·51)	1160·36 (1047·41 to 1287·12)	954·47 (854·63 to 1062·6)	795·16 (718·28 to 874·68)	737·98 (662·65 to 819·14)	672·19 (552·86 to 816·21)
Goiás	2861·61 (2450·52 to 3356·31)	2418·81 (2087·09 to 2801·57)	1903·87 (1631·4 to 2194·91)	1444·06 (1253·5 to 1673·79)	1134·89 (996·74 to 1296·89)	845·17 (743·83 to 962·59)	732·34 (640·46 to 827·2)	670·74 (540·47 to 842·17)
Maranhão	2763·57 (2405·19 to 3171·03)	2340·89 (2055·06 to 2679·26)	1976·52 (1701·3 to 2269·47)	1475·06 (1273·82 to 1704·86)	1000·47 (866·64 to 1149·96)	719·25 (624·23 to 825·74)	691·14 (594·87 to 798·48)	729·29 (587·65 to 902·09)
Mato Grosso	1701·08 (1434·76 to 1993·12)	1521·09 (1280·99 to 1780·97)	1311·91 (1117·87 to 1550·51)	1012·32 (882·54 to 1140·41)	944·26 (831·52 to 1074·04)	792·66 (704·12 to 893·26)	688·5 (622·74 to 763·25)	591·41 (482·28 to 723·13)
Mato Grosso do Sul	1955·18 (1652·41 to 2290·5)	1687·66 (1434·91 to 1971·81)	1470·28 (1257·05 to 1714·11)	1207·81 (1092·06 to 1343·76)	962·1 (863·69 to 1068·11)	826·5 (751·86 to 911·64)	727·71 (659·96 to 805·17)	610·27 (500·01 to 738·97)
Minas Gerais	2099·53 (1787·81 to 2432·91)	1785·82 (1552·99 to 2058·22)	1531·63 (1316·03 to 1777·53)	1214·82 (1102·61 to 1334·26)	1025·45 (921·81 to 1135·72)	770·06 (698·93 to 845·09)	679·79 (612·71 to 751·58)	576·15 (479·53 to 695·52)
Pará	2239·13 (1900·07 to 2593·02)	1810·17 (1568·53 to 2080·31)	1549·46 (1328·84 to 1809·25)	1201·68 (1048·68 to 1370·05)	974·11 (850·12 to 1108·63)	757·92 (664·45 to 856·21)	674·61 (586·02 to 763·63)	607·09 (503·1 to 736·88)
Paraíba	2481·7 (2141·37 to 2843·06)	1957·31 (1696·86 to 2251·1)	1533·8 (1334·8 to 1777·75)	1110·97 (994·81 to 1243·84)	863·62 (771·82 to 962·12)	708·18 (637·36 to 794·39)	696·15 (621·06 to 775·92)	618·83 (502·98 to 750·68)
Paraná	1868·99 (1585·97 to 2214·37)	1629·54 (1400·54 to 1897·79)	1446·49 (1239·19 to 1673·06)	1245·64 (1131·5 to 1364·9)	1038·79 (939·79 to 1145·07)	864·99 (788·32 to 947·91)	745·66 (676·79 to 822·83)	653·13 (541·28 to 798·96)
Pernambuco	2939·73 (2550·04 to 3386·56)	2380·1 (2065·97 to 2722·33)	1881·55 (1656·96 to 2135·88)	1310·19 (1187·8 to 1444·77)	1129·58 (1020·95 to 1253·26)	896·14 (812·18 to 988·29)	797·42 (719·85 to 877·99)	767·69 (636·39 to 935·66)
Piauí	2265·39 (1958·99 to 2591·96)	1936·07 (1669·2 to 2207·59)	1702·91 (1468·18 to 1951·15)	1259·35 (1106·12 to 1406·4)	935·36 (828·1 to 1048·99)	727·5 (650·34 to 809·16)	617 (555·8 to 685·18)	571·01 (467·28 to 684·15)
Rio de Janeiro	1938·87 (1646·89 to 2264·03)	1635·63 (1399·51 to 1881·02)	1486·43 (1270·44 to 1709·89)	1325·26 (1201·34 to 1469·98)	1158·57 (1045·07 to 1289·54)	915·9 (833·09 to 1010·49)	779·64 (706·27 to 860·44)	682·58 (568·25 to 828·9)
Rio Grande do Norte	2522·93 (2164·3 to 2926·29)	1955·61 (1688·8 to 2258·59)	1516·3 (1307·05 to 1751·14)	1093·56 (961·52 to 1238·13)	817·3 (718·63 to 925·45)	682·27 (601·58 to 770·74)	598·58 (527·76 to 679·28)	587·91 (472·12 to 728·32)
Rio Grande do Sul	1795·47 (1528·62 to 2078·34)	1532·7 (1312·25 to 1777·58)	1309·71 (1132·65 to 1519·75)	1089·76 (979·91 to 1200·12)	949·38 (858·31 to 1043·15)	830·34 (753·09 to 910·77)	695·12 (629·89 to 763·62)	632·91 (522·58 to 772·69)
Rondônia	3149·39 (2700·14 to 3639·53)	2523·94 (2198·1 to 2909·8)	2048·98 (1796·3 to 2350·71)	1730·46 (1549·08 to 1925·37)	1316·25 (1167·93 to 1467·67)	939·92 (844·97 to 1039·88)	699·5 (621·71 to 779·21)	653·93 (532·9 to 795·68)
Roraima	2799·37 (2422·89 to 3209·46)	2238·15 (1934·03 to 2613·23)	1828·24 (1592·72 to 2081·75)	1341·22 (1188·47 to 1495·5)	1176·26 (1048·67 to 1321·52)	925·64 (835·33 to 1023·64)	715·29 (642·9 to 794·54)	714·25 (587·07 to 859·35)
Santa Catarina	1641·21 (1403·3 to 1914·87)	1422·15 (1215·31 to 1653·96)	1269·59 (1081·79 to 1482·76)	1048·58 (947·2 to 1155·72)	1013·63 (908·11 to 1128·29)	893·81 (807·31 to 994·15)	690·07 (618·86 to 766·24)	605·9 (498·52 to 734·49)
São Paulo	1801·87 (1553·3 to 2089·66)	1564·96 (1340·38 to 1813·5)	1425·84 (1233·96 to 1645·52)	1284·51 (1174·29 to 1407·84)	1080·97 (983·17 to 1200·73)	883·99 (811·81 to 966·66)	708·42 (648·16 to 776·14)	606·17 (507·18 to 732·21)
Sergipe	2132·11 (1820·1 to 2489·56)	1797·87 (1551·77 to 2078·32)	1530·36 (1310·76 to 1773·93)	1292·8 (1151·03 to 1465·47)	1040·95 (922·86 to 1163·03)	792·37 (719·36 to 874·59)	678·73 (598·24 to 766·12)	665·26 (538·58 to 825·39)
Tocantins	2457·89 (2070·36 to 2888·15)	2061·2 (1756·56 to 2385·09)	1625·72 (1394·64 to 1877·15)	1125·69 (931·71 to 1362·4)	988·69 (866·73 to 1122·8)	780·8 (698·44 to 871·62)	641·41 (565·2 to 721·45)	656·04 (530·26 to 803·38)
Paraguay	1406·7 (1179·6 to 1668·79)	855·02 (765·67 to 949·81)	828·74 (745·68 to 917·63)	734·61 (666·86 to 810·9)	626·74 (565·45 to 699·43)	638·86 (574·29 to 711·8)	648·06 (566·3 to 741·92)	621·07 (462·88 to 821·45)
North Africa and Middle East	2409·11 (2204·37 to 2633·9)	1977·88 (1823·46 to 2130·97)	1641·27 (1531·33 to 1763·2)	1333·28 (1260·92 to 1411·26)	1147·81 (1091·93 to 1207·57)	955·28 (900·68 to 1018·63)	850·24 (798·65 to 906·83)	775·33 (707·85 to 853·88)
North Africa and Middle East	2409·11 (2204·37 to 2633·9)	1977·88 (1823·46 to 2130·97)	1641·27 (1531·33 to 1763·2)	1333·28 (1260·92 to 1411·26)	1147·81 (1091·93 to 1207·57)	955·28 (900·68 to 1018·63)	850·24 (798·65 to 906·83)	775·33 (707·85 to 853·88)
Afghanistan	3009·72 (2286·69 to 4105·23)	2460·6 (1964·04 to 3159·14)	2309·63 (1919·51 to 2817·76)	2668·12 (2195·07 to 3554·46)	1895·2 (1559·47 to 2259·25)	1896·17 (1501·51 to 2435·55)	1552·85 (1131·18 to 2118·69)	1474·33 (1043·95 to 2112·68)
Algeria	1765·07 (1579·11 to 1959·84)	1911·73 (1664·74 to 2173·84)	1710·9 (1478·15 to 1956·48)	1634·88 (1433·64 to 1851·35)	1231·27 (1073·53 to 1412·5)	995·8 (856·14 to 1162·69)	815·47 (711·57 to 927·48)	738·12 (612·64 to 881·2)
Bahrain	1953·47 (1619·66 to 2344·92)	1689·29 (1423·2 to 1982·26)	1397·65 (1159·06 to 1663·77)	1291·46 (1126·24 to 1468·96)	1241·79 (1114·33 to 1375·6)	1030·24 (921·37 to 1143·33)	916·45 (829·16 to 1005·69)	735·42 (602·17 to 895·29)
Egypt	2625·3 (2345·16 to 2922·55)	2019·47 (1873·69 to 2191·01)	1899·67 (1726·68 to 2092·46)	1499·66 (1366·91 to 1660·47)	1340·6 (1222·76 to 1465·1)	1097·85 (1006·28 to 1199·92)	1103·84 (1008·33 to 1216·76)	1025·52 (835·23 to 1257·03)
Iran	2756·38 (2463·18 to 3099·28)	2212·5 (1976·42 to 2485·22)	1609·84 (1444·56 to 1796·2)	1137·43 (1037·44 to 1259·19)	1068·06 (971·94 to 1177·08)	847·46 (771·8 to 927·83)	676·76 (628·66 to 731·14)	596·07 (544·16 to 654·05)
Iraq	1564·19 (1343·25 to 1809·7)	1259·15 (1101·19 to 1436·53)	1135·67 (966·35 to 1325·86)	1100·54 (956·37 to 1266·86)	1082·4 (925·95 to 1244·63)	999·06 (787·34 to 1261·01)	926·08 (690·11 to 1239·92)	836·07 (607·62 to 1140·44)

Supplemental Results Table 3a: Age-standardised mortality rates by location, both sexes, 1950, 1960, 1970, 1980, 1990, 2000, 2010, 2019

Location	1950	1960	1970	1980	1990	2000	2010	2019
Jordan	1779·65 (1318·33 to 2405·18)	1510·31 (1222·49 to 1873·74)	1295·28 (1125·75 to 1495·87)	1106·14 (992·18 to 1229·54)	945·89 (836·65 to 1077·15)	849·06 (726·81 to 992·53)	642·43 (572·09 to 729·81)	574·97 (488·93 to 672·13)
Kuwait	1199·38 (945·58 to 1484·5)	863·63 (749·85 to 989)	848·11 (792·23 to 907·66)	804·54 (748·63 to 862·77)	680·26 (615·57 to 751·03)	564·36 (509·09 to 628·69)	487·73 (451·92 to 527·38)	438·38 (367·67 to 528·19)
Lebanon	1593·73 (1301·62 to 1942·82)	1187·2 (953·63 to 1433·8)	1066·03 (890·01 to 1251·35)	1015·31 (844·48 to 1227·38)	1003·15 (829·55 to 1243·93)	759·35 (661·59 to 871·49)	730·42 (654·31 to 810·47)	669·31 (561·57 to 794·82)
Libya	2285·62 (1925·72 to 2644·18)	1758·8 (1455·74 to 2093·16)	1070·85 (950·79 to 1205·05)	883·55 (788·45 to 995·09)	773·66 (656·06 to 914·41)	678·53 (601·47 to 764·17)	639·42 (586·31 to 705·61)	655·27 (530·91 to 837·99)
Morocco	2550·01 (2211·42 to 2921·7)	2184·33 (1902·31 to 2515·05)	1704·72 (1474·63 to 1951·93)	1376·74 (1225·09 to 1552·14)	1089·41 (972·08 to 1221·49)	965·04 (843·14 to 1101·98)	867·74 (725·68 to 1033·36)	854·37 (704·67 to 991·1)
Oman	3172·36 (2189·58 to 4706·17)	2439·44 (1792·66 to 3366·74)	1711·47 (1326·82 to 2229·78)	1441·19 (1141·94 to 1802·75)	1278·84 (1049·81 to 1524·8)	1167·01 (1026·95 to 1331·9)	1089·82 (1015·58 to 1167·89)	916·5 (824·99 to 1020·52)
Palestine	1893·01 (1467·91 to 2418·38)	1519·69 (1235·41 to 1835·31)	1517·9 (1289·94 to 1781·68)	1145·87 (908·72 to 1410·28)	1077·47 (884·26 to 1294·32)	992·68 (881·42 to 1117·32)	823·28 (747·56 to 910·35)	798·62 (695·98 to 916·68)
Qatar	1220·78 (849·48 to 1756·76)	1180·32 (888·66 to 1595·64)	1090·77 (847·63 to 1392·41)	934 (756·99 to 1125·22)	1157·37 (995·22 to 1361·22)	1103·33 (962·5 to 1272·64)	1017·14 (887·14 to 1152·7)	879·28 (729·86 to 1062·35)
Saudi Arabia	1819·92 (1491·26 to 2208·36)	1759·32 (1440·17 to 2100·01)	1437·97 (1175·99 to 1714·07)	1123·95 (927·39 to 1373·34)	1091·37 (920·51 to 1290·86)	1014·42 (904·87 to 1134·06)	916·16 (825·01 to 1005·96)	763·03 (626·13 to 928·97)
Sudan	2258·59 (1941·96 to 2616·53)	2035·69 (1731·2 to 2383·05)	1911·53 (1650·21 to 2179·93)	1749·1 (1531·66 to 1978·81)	1511·92 (1330·07 to 1709·1)	1270·51 (1058·13 to 1500·85)	1060·87 (861·4 to 1261·56)	941·15 (780·54 to 1156·06)
Syria	1704·17 (1430·62 to 2011·25)	1262·99 (1057·99 to 1515·32)	991·4 (874·69 to 1125·54)	821·73 (706·94 to 946·14)	1038·75 (869·69 to 1233·74)	973·66 (852·92 to 1121·4)	825·27 (697·94 to 976·5)	878·29 (692·7 to 1119·1)
Tunisia	2557·25 (2189·66 to 2974·68)	2099·16 (1848·06 to 2384·96)	1712·51 (1531·77 to 1929·62)	1212·28 (1087·06 to 1348·59)	820·07 (726·4 to 926·28)	761·36 (641·39 to 898·51)	681·18 (522·15 to 846·47)	610·26 (470·4 to 788·41)
Turkey	2348·1 (1994·13 to 2720·92)	1885·34 (1603·29 to 2207·52)	1457·4 (1257·21 to 1695·47)	1126·75 (1022·44 to 1247·63)	955·13 (868·55 to 1048·12)	671·89 (585·58 to 778·75)	637·37 (569·32 to 714·89)	566·25 (456·73 to 684·67)
United Arab Emirates	1507·32 (1153·57 to 1956·49)	1434·73 (1184·27 to 1743·1)	1269·41 (1064·93 to 1543)	1195·67 (995·55 to 1411·97)	1272·97 (1110·28 to 1461·54)	1369·08 (1197·94 to 1571·43)	1184·47 (1013·43 to 1377·7)	841·18 (678·96 to 1057·57)
Yemen	3816·59 (2640·98 to 5747·61)	3025·86 (2285·69 to 4207·68)	2568·79 (2034·14 to 3292·43)	1787·68 (1466·2 to 2187·59)	1476·01 (1245·9 to 1757·29)	1265·65 (1054·07 to 1523·12)	1071 (889·9 to 1299·32)	1077·33 (856·03 to 1367·85)
South Asia	2673·94 (2391·24 to 2985·8)	2411·83 (2184·28 to 2670·46)	2170·41 (2001·74 to 2344·49)	1753·6 (1646·91 to 1880·61)	1544·84 (1443·05 to 1654·07)	1358·11 (1268·26 to 1454·92)	1072·62 (999·11 to 1152·88)	918·88 (806·62 to 1051·47)
South Asia	2673·94 (2391·24 to 2985·8)	2411·83 (2184·28 to 2670·46)	2170·41 (2001·74 to 2344·49)	1753·6 (1646·91 to 1880·61)	1544·84 (1443·05 to 1654·07)	1358·11 (1268·26 to 1454·92)	1072·62 (999·11 to 1152·88)	918·88 (806·62 to 1051·47)
Bangladesh	2745·64 (2350·93 to 3137·29)	2360·61 (2035·12 to 2710·37)	2525·21 (2190·39 to 2916·15)	1743·71 (1547·6 to 1955·44)	1512·29 (1383·63 to 1666·35)	1145·46 (1045·75 to 1262·21)	946·31 (853·72 to 1049·51)	717·76 (574·4 to 874·49)
Bhutan	3022·57 (2511·54 to 3568·56)	2512·89 (2022·91 to 3039·45)	2037·75 (1570·34 to 2609·54)	1805·85 (1372·86 to 2391·17)	1400·9 (1127·4 to 1768·09)	1122·34 (962·63 to 1291·22)	899·74 (747·86 to 1041·82)	819·77 (677·09 to 946·75)
India	2720·77 (2393·91 to 3107·25)	2521·74 (2239·68 to 2825·08)	2285·85 (2078 to 2514·63)	1863·63 (1725·63 to 2036·77)	1597·2 (1468·47 to 1738·62)	1382·51 (1270·96 to 1510·86)	1058·4 (972·73 to 1156·74)	910·42 (777·39 to 1064·28)
Andhra Pradesh	2553·4 (2132·05 to 3024·85)	2427·28 (2039·2 to 2869·67)	2241·67 (1893·49 to 2594·81)	1800·32 (1539·73 to 2107·55)	1571·94 (1326·18 to 1843·36)	1351·45 (1148·32 to 1575·51)	1045·77 (880·19 to 1232·99)	830·62 (652·51 to 1043·25)
Arunachal Pradesh	2667·84 (2218·77 to 3186·57)	2509·73 (2082·66 to 2962·91)	2304·58 (1942·8 to 2680·84)	1726·99 (1460·48 to 2033·38)	1502·92 (1262·62 to 1759·79)	1285·02 (1095·53 to 1499·87)	991·92 (839·42 to 1170·34)	853·57 (673·32 to 1061·17)
Assam	3001·02 (2518·8 to 3530·52)	2776·48 (2347·81 to 3305·52)	2515·79 (2131·05 to 2947·96)	1994·28 (1672·89 to 2400·91)	1745·42 (1529·97 to 1979·27)	1705·96 (1515·88 to 1909·77)	1344·77 (1198·1 to 1499·94)	1057·02 (847·53 to 1294·71)
Bihar	2427·49 (2030·34 to 2868·85)	2289·95 (1947·32 to 2657·79)	2194 (1884·02 to 2524·03)	1850·17 (1594·69 to 2138·38)	1581·43 (1352·31 to 1828·72)	1395·28 (1208·68 to 1594·3)	1111·1 (980·7 to 1258·41)	881·72 (716·72 to 1088·43)
Chhattisgarh	2739·25 (2324·95 to 3219·84)	2652·84 (2243·21 to 3119·54)	2457·91 (2091·69 to 2863·66)	2044·82 (1752·72 to 2407·92)	1752·57 (1484·14 to 2042·34)	1617·83 (1420·16 to 1853·36)	1284·59 (1143·17 to 1450·54)	1101·08 (900·49 to 1342·04)
Delhi	2316·67 (1927·6 to 2770·91)	2034·94 (1690·44 to 2421·77)	1829·23 (1558·73 to 2162·25)	1514·85 (1290·08 to 1783·47)	1373·78 (1176·57 to 1590·5)	1248·09 (1093·75 to 1422·46)	930·4 (825·4 to 1050·83)	741·25 (602·84 to 916·72)
Goa	1768·43 (1467·65 to 2136·39)	1649·09 (1334·47 to 2041·49)	1548·24 (1288·18 to 1837·11)	1256·74 (1068·08 to 1479·23)	1167·65 (988·7 to 1359·12)	1035·73 (865·72 to 1228·58)	788·53 (661·14 to 952·45)	695·63 (530·92 to 879·57)
Gujarat	2464·92 (2072·51 to 2928·76)	2282·93 (1948·61 to 2737·48)	2080·9 (1765·51 to 2425·57)	1775·24 (1518·4 to 2094·15)	1558·8 (1351·01 to 1789·57)	1274·41 (1129·41 to 1440·85)	1009·55 (901·26 to 1136·78)	933·21 (739·98 to 1145·14)
Haryana	2431·55 (2029·1 to 2927·11)	2283·55 (1866·18 to 2855·52)	2037·75 (1724·76 to 2385·3)	1537·73 (1307·05 to 1796·17)	1285·87 (1111·41 to 1471·69)	1197·66 (1061·78 to 1351·63)	953·52 (848·63 to 1077·71)	793·42 (629·04 to 978·69)
Himachal Pradesh	2150·27 (1806·64 to 2561·61)	2037·89 (1639·99 to 2470·61)	1872·24 (1586·26 to 2191·4)	1541·8 (1309·09 to 1814·1)	1356·27 (1169·05 to 1548·45)	1172·63 (1043·3 to 1331·04)	900·08 (802·55 to 1015·22)	830·76 (661·86 to 1017·97)
Jammu and Kashmir	1906·93 (1590·44 to 2295·41)	1838·08 (1519·18 to 2191·01)	1681·29 (1399·47 to 1994·66)	1434·47 (1221·45 to 1697·76)	1271·21 (1049·69 to 1528·49)	1111·25 (976·44 to 1264·73)	929·66 (826·31 to 1040·88)	855·53 (688·71 to 1062·87)
Jharkhand	2523·98 (2119·22 to 2987·02)	2360·54 (1977·7 to 2803·28)	2210·77 (1883·48 to 2561·05)	1900·08 (1635·28 to 2209·1)	1776·91 (1538·35 to 2053·77)	1578·37 (1388·24 to 1783·68)	1157·15 (1029·57 to 1294·98)	765·52 (629·48 to 924·97)
Karnataka	2600·06 (2172·72 to 3066·55)	2434·73 (2059·06 to 2873·9)	2179·56 (1863·12 to 2561·4)	1652·7 (1418·68 to 1934·12)	1378·26 (1199·72 to 1588·62)	1205·99 (1064·27 to 1357·3)	990·38 (882·58 to 1111·83)	939·76 (757·84 to 1143·98)
Kerala	2049·42 (1706·33 to 2455·57)	1778·76 (1497·57 to 2112·68)	1555·62 (1321·85 to 1826·36)	1318·03 (1117·16 to 1543·17)	1174·38 (1002·49 to 1346·81)	1047·31 (925 to 1173·03)	764·99 (677·27 to 862·88)	616·22 (494·56 to 766·58)
Madhya Pradesh	2961·35 (2477·93 to 3531·18)	2715·07 (2309·42 to 3203·08)	2474 (2104·56 to 2880·38)	2040·42 (1758·21 to 2342·07)	1719·28 (1477·87 to 1971·96)	1447·84 (1268·99 to 1643·15)	1160·14 (1031·47 to 1301·52)	993·9 (814·27 to 1196·74)
Maharashtra	2507·96 (2090·57 to 2933·75)	2334·11 (1971·73 to 2741·99)	2149·01 (1830·39 to 2491·87)	1691·09 (1462·12 to 1967·02)	1444·8 (1238·52 to 1665·84)	1297·64 (1158·07 to 1455·67)	876·02 (779·55 to 984·12)	776·3 (623·15 to 945·55)
Manipur	2273·21 (1898·97 to 2691·41)	2099·29 (1768·73 to 2500·99)	1893·47 (1578·7 to 2239·46)	1507·06 (1275·9 to 1766·52)	1330·01 (1133·8 to 1573·5)	1227·02 (1029·63 to 1453·78)	1003·23 (831 to 1175·67)	873·83 (686·56 to 1088·49)
Meghalaya	1912·21 (1564·83 to 2252·55)	1832·35 (1517·73 to 2161·7)	1772·94 (1511·91 to 2071·59)	1628·88 (1375·07 to 1912·96)	1467·46 (1226·56 to 1713·11)	1260·35 (1065·73 to 1478·49)	996·02 (845·16 to 1166·58)	887·94 (698·3 to 1117·17)
Mizoram	1700·3 (1428·47 to 2029·28)	1627·79 (1345·13 to 1956·96)	1550 (1298·91 to 1854·75)	1396·56 (1174·68 to 1636·39)	1268·22 (1081·48 to 1499·72)	1162·69 (975·1 to 1371·64)	984·83 (819·79 to 1160·21)	847·19 (663·83 to 1069·58)
Nagaland	2430·85 (2019·75 to 2894·72)	2220·4 (1843·9 to 2627·04)	1939·84 (1631·4 to 2267·71)	1519·17 (1284·4 to 1789·33)	1330·03 (1119·68 to 1575·82)	1218·34 (1024·35 to 1438·25)	1012·91 (834·76 to 1220·19)	869·26 (683·72 to 1079)
Odisha	3095·57 (2623·08 to 3667·33)	2859·72 (2434·17 to 3360·32)	2589·23 (2233·17 to 2986·62)	2071·3 (1775·43 to 2393·3)	1815·75 (1590·71 to 2103·61)	1630·57 (1450·36 to 1843·74)	1189·79 (1055·18 to 1350·73)	918·34 (735·33 to 1141·12)
Punjab	2070·46 (1719·87 to 2461·58)	1938·53 (1534·85 to 2404·38)	1735·57 (1463·93 to 2018·01)	1479·27 (1259·78 to 1723·89)	1325·43 (1144·76 to 1521·18)	1167·84 (1035·85 to 1316·49)	891·07 (787·78 to 1008·67)	772·2 (620·74 to 950·91)
Rajasthan	2245·35 (1862·32 to 2665·95)	2210·33 (1875·76 to 2622·02)	2063·56 (1757·21 to 2400·85)	1779·31 (1528·53 to 2058·88)	1545·14 (1344·36 to 1780·19)	1326·19 (1184·78 to 1493·32)	994·25 (891·86 to 1115·95)	987·62 (801·27 to 1204·06)
Sikkim	2130·22 (1768·58 to 2514·41)	2086·84 (1744·52 to 2499·51)	1959·39 (1637·47 to 2289·01)	1600·24 (1356·58 to 1861·6)	1363·6 (1159·72 to 1607·52)	1202·66 (1008·98 to 1416·7)	937·09 (784·01 to 1103·98)	772·31 (608·73 to 976·83)
Tamil Nadu	2950·68 (2509·5 to 3473·36)	2696·15 (2273·59 to 3145·12)	2407·17 (2063·15 to 2780·74)	2037·68 (1742·7 to 2344·95)	1759·56 (1527·29 to 2010·61)	1456·55 (1296·19 to 1638·2)	1156·73 (1025·08 to 1305·32)	930·45 (758·8 to 1140·12)
Telangana	2219·28 (1798·18 to 2674·51)	2120·61 (1749·38 to 2516·84)	2008 (1665·3 to 2370·43)	1714·76 (1439·28 to 2034·43)	1592·85 (1320·26 to 1898·96)	1414·64 (1168·9 to 1708·62)	1108·75 (908·4 to 1323·42)	842·52 (643·68 to 1091·82)
Tripura	2867·84 (2383·29 to 3397·47)	2548·24 (2138·09 to 2994·47)	2231·44 (1904·85 to 2598·24)	1725·32 (1455·15 to 2020·76)	1477·69 (1242·23 to 1735·21)	1245·34 (1059·56 to 1449·42)	986·47 (835·38 to 1154·08)	882·77 (698·65 to 1121·25)
Union Territories other than Delhi	2001·66 (1674·21 to 2375·49)	1776·7 (1444·21 to 2173·63)	1607·24 (1345·01 to 1913·04)	1302·76 (1095·11 to 1543·43)	1193·63 (1022·9 to 1413·98)	1093·88 (929·65 to 1274·76)	869·09 (718·76 to 1039·38)	768·54 (612·21 to 970·92)
Uttar Pradesh	3515·59 (2996·01 to 4131·77)	3277·54 (2831·07 to 3771·17)	2865·42 (2494·59 to 3256·96)	2249·15 (1964·12 to 2549·09)	1839·96 (1620 to 2097·05)	1510·74 (1356·05 to 1704·45)	1207·35 (1087·42 to 1352·5)	1186·05 (977·37 to 1441·86)
Uttarakhand	2466·11 (2053·23 to 2912·95)	2397·82 (2010·89 to 2858·78)	2239·58 (1870·25 to 2669·72)	1920·54 (1639·25 to 2217·48)	1782·62 (1519·33 to 2083·71)	1605·22 (1358·32 to 1872·96)	1248·89 (1081·26 to 1430·01)	1067·62 (848·85 to 1314·69)
West Bengal	2657·15 (2236·1 to 3130·75)	2395·98 (2010·31 to 2819·09)	2159·95 (1864·5 to 2494·36)	1794·19 (1545·84 to 2077·48)	1528·45 (1333·92 to 1755·43)	1360·43 (1215·62 to 1524·65)	957·88 (855·79 to 1069·56)	774·18 (621·3 to 950·38)
Nepal	2802·55 (2375·66 to 3251·26)	2401·11 (2066·22 to 2755·06)	2070·43 (1795·52 to 2393·74)	1911·27 (1648·77 to 2215·67)	1542·39 (1362·1 to 1782·54)	1156·11 (1039·07 to 1283·73)	1003·83 (906·66 to 1118·5)	964·49 (786·01 to 1146·14)
Pakistan	2173·02 (1927·66 to 2456·36)	1790·08 (1589·76 to 2011·24)	1473·51 (1321·28 to 1637·65)	1351·11 (1232·96 to 1492·42)	1378·73 (1223·29 to 1556·37)	1446·53 (1311·69 to 1592·49)	1309·63 (1126·78 to 1513·13)	1152·39 (1003·38 to 1326·02)
Southeast Asia, east Asia, and Oceania	2194·06 (1983·66 to 2424·76)	3145·22 (2533·82 to 4034·79)	1433·54 (1284·3 to 1606·18)	1311·01 (1196·53 to 1440·07)	1127·7 (1045·91 to 1222·21)	979·45 (915·29 to 1049·51)	824·29 (771·86 to 882·32)	686·67 (598·81 to 775·51)
East Asia	2153·19 (1908·84 to 2445·22)	3629·16 (2791·12 to 4858)	1369·27 (1184·09 to 1596·81)	1302·45 (1154·69 to 1471·33)	1120·2 (1015·14 to 1241·01)	959·47 (874·85 to 1051·26)	782·32 (719·07 to 855·87)	636·01 (533·2 to 749·94)
China	2099·69 (1855·64 to 2381)	3692·08 (2825·79 to 4956·33)	1370·54 (1179·24 to 1604·22)	1311·42 (1159·03 to 1484·19)	1129·6 (1022·13 to 1255·36)	958·3 (870·68 to 1053·71)	787·07 (721·98 to 863·15)	637·56 (530·27 to 757·59)
North Korea	5635·86 (3985·38 to 8304·72)	2205·15 (1618·59 to 3058·45)	1578·35 (1214·46 to 2069·62)	1218·99 (982·69 to 1518·85)	1056·46 (889·62 to 1251·55)	1469·32 (1065·88 to 2300·07)	960·15 (828·64 to 1094·41)	824·63 (684·91 to 1013·92)
Taiwan (province of China)	1689·19 (1458·21 to 1953·08)	1336·38 (1324·31 to 1347·95)	1050·93 (1041·96 to 1060·58)	875·5 (868·13 to 882·56)	739·96 (732·98 to 746·91)	625·4 (619·37 to 631·37)	504·21 (498·72 to 510·4)	482·09 (387·52 to 606·61)
Oceania	2206·97 (1877·04 to 2620·54)	1812·95 (1586·92 to 2059·3)	1537·78 (1364·26 to 1725·88)	1366·9 (1260·52 to 1481·53)	1367·66 (1210·41 to 1556·13)	1356·55 (1200·82 to 1534·11)	1349·21 (1177·4 to 1553·17)	1260·47 (1059·43 to 1521·01)
American Samoa	1411·78 (1241·53 to 1612·04)	1074·41 (963·98 to 1201·93)	1148·39 (1020·6 to 1297·85)	1055·19 (953·5 to 1163·19)	989·11 (905·31 to 1078·41)	947·22 (867·34 to 1039·52)	901·45 (827·34 to 988·66)	890·75 (745·09 to 1074·71)
Cook Islands	2419·82 (2139·92 to 2724·47)	1681·42 (1484·61 to 1891·7)	1322·59 (1169·17 to 1489·14)	1267·39 (1155·78 to 1398·64)	1096·99 (981·04 to 1224·32)	865·37 (771·24 to 968·78)	737·56 (660·54 to 829·48)	716·08 (608·64 to 844·25)
Fiji	1628·64 (1501·99 to 1762·77)	1319·14 (1187·01 to 1454·35)	1299·85 (1165·32 to 1452·18)	1204·77 (1096·29 to 1332·64)	1286·18 (1088·26 to 1511·32)	1399·89 (1270·81 to 1548·5)	1206·79 (1104·99 to 1332·6)	1160·35 (931·21 to 1414·72)
Guam	1047·57 (915·93 to 1190·68)	1170·53 (1073·84 to 1281·63)	1250·01 (1160·44 to 1355·86)	994·22 (924·2 to 1070·68)	866·69 (792·51 to 948·84)	670·28 (606·06 to 737·58)	583·37 (529·54 to 646·57)	617·08 (507·05 to 744·9)
Kiribati	2957·64 (2587·86 to 3372·46)	2537·75 (2166·03 to 2958·91)	2298·53 (1951·63 to 2690·23)	2093·08 (1747·65 to 2501·58)	2165·42 (1858·07 to 2495·45)	2038·47 (1753·48 to 2316·79)	1857·8 (1602·77 to 2123·1)	1725·86 (1427·64 to 2049·34)
Marshall Islands	2228·69 (1548·88 to 3235·86)	1855·09 (1387·39 to 2451·87)	1655·83 (1307·22 to 2104·98)	1451·71 (1223·61 to 1694·12)	1547·96 (1394·72 to 1718·13)	1516·32 (1349·67 to 1713·12)	1448·85 (1224·27 to 1676·06)	1344·71 (1073·75 to 1670·21)
Federated States of Micronesia	2550·12 (2200·63 to 2954·83)	2144·41 (1752·12 to 2596·47)	1920·63 (1485·6 to 2509·79)	1721·85 (1381·85 to 2152·96)	1633·56 (1351·43 to 1928·49)	1548·45 (1381·33 to 1720·79)	1469·62 (1246·06 to 1699·87)	1487·82 (1192·33 to 1805·26)
Nauru	1701·05 (1402·35 to 2045·61)	1559·32 (1327·14 to 1824·87)	1511·65 (1300·81 to 1756·76)	1531·54 (1264·5 to 1874·06)	1618·58 (1354·47 to 1898·98)	1866·51 (1595·98 to 2162·67)	1786·13 (1536·74 to 2056·01)	1523·51 (1270·79 to 1818·46)
Niue	1886·05 (1651·24 to 2151·07)	1361·39 (1169·98 to 1575·2)	1205·85 (1039·19 to 1418·04)	1103·91 (943·31 to 1323·7)	1117·16 (943·75 to 1351·1)	1122·37 (963·93 to 1330·77)	1033·12 (896·17 to 1197·83)	976·7 (796·08 to 1193·29)
Northern Mariana Islands	1323·42 (1088 to 1587·96)	1271 (1039·17 to 1540·19)	1172·63 (1001·29 to 1389·4)	1056·97 (901·98 to 1283·42)	998·25 (880·84 to 1168·05)	908·7 (803·45 to 1044·19)	878·07 (775·41 to 984·6)	849·51 (708·95 to 1009·08)
Palau	2208·96 (1832·79 to 2665·46)	1891·56 (1588·89 to 2275·27)	1631·54 (1359·5 to 1929·42)	1485·17 (1239·28 to 1782·48)	1352 (1095·79 to 1667·64)	1275·86 (1049·25 to 1519·76)	1229·52 (1000·82 to 1470·03)	1192·31 (960·71 to 1466·09)
Papua New Guinea	2296·05 (1828·19 to 2892·64)	1868·62 (1554·92 to 2218·87)	1516·7 (1285·42 to 1760·12)	1342·54 (1216·23 to 1481·62)	1346·77 (1127·28 to 1595·48)	1332·3 (1112·47 to 1576·97)	1376·38 (1155·95 to 1655·21)	1274·18 (1028·45 to 1585·08)
Samoa	1723·03 (1457·58 to 2034·02)	1319·49 (1163·77 to 1493·35)	1290·92 (1131·36 to 1475·48)	1232·57 (1033·32 to 1451·87)	1186·19 (1019·7 to 1404·07)	1091·7 (937·87 to 1320·42)	1027·51 (876·05 to 1214·24)	986·61 (825·68 to 1212·48)
Solomon Islands	3312·71 (2888·1 to 3790·72)	2902·42 (2509·52 to 3368·14)	2534·87 (2162·79 to 2936·59)	2256·09 (1885·04 to 2695·67)	2176·17 (1830·15 to 2581·39)	2095·53 (1785·36 to 2420·28)	2079·27 (1801·02 to 2396·31)	1929·93 (1581·1 to 2323·64)
Tokelau	1927·44 (1645·96 to 2265·83)	1663·92 (1402·61 to 1965·99)	1461·68 (1221·15 to 1769·6)	1302·32 (1114·21 to 1533·55)	1148·89 (983·6 to 1358·78)	1065·52 (891·79 to 1273·34)	972·64 (821·28 to 1169·72)	894·19 (738·36 to 1101·18)

Supplemental Results Table 3a: Age-standardised mortality rates by location, both sexes, 1950, 1960, 1970, 1980, 1990, 2000, 2010, 2019

Location	1950	1960	1970	1980	1990	2000	2010	2019
Tonga	1500·54 (1274·67 to 1768·09)	1323·62 (1155·73 to 1508·2)	1209·07 (1050·03 to 1390·18)	1087·86 (946·86 to 1240·29)	919·16 (796·24 to 1053·65)	954·3 (841·46 to 1090·55)	859·28 (736·6 to 987·57)	828·16 (681·84 to 1006·34)
Tuvalu	2558·2 (2117·01 to 3089·65)	2108·24 (1738·98 to 2530·47)	1852·49 (1485·69 to 2237·75)	1738·86 (1423·73 to 2048·95)	1562·34 (1344·34 to 1809·64)	1613·82 (1428·47 to 1825·7)	1239·4 (1018·41 to 1496·77)	1166·14 (908·86 to 1470·41)
Vanuatu	2479·97 (2106·66 to 2897·1)	2139·07 (1770·26 to 2565·64)	1816·2 (1504·03 to 2183·48)	1523·93 (1280·42 to 1807·38)	1492·8 (1222·1 to 1809·14)	1463·64 (1201·6 to 1769·45)	1400·31 (1150·12 to 1698·58)	1335·66 (1091·56 to 1659·79)
Southeast Asia	2345·05 (2140·72 to 2573·65)	1811·58 (1665·02 to 1983·57)	1614·25 (1493·11 to 1744·44)	1322·92 (1241·89 to 1414·44)	1147·27 (1084·44 to 1210·88)	1029·6 (960·23 to 1101·78)	914·91 (871 to 961·7)	812·44 (732·16 to 893·2)
Cambodia	2423·4 (2012·82 to 2885·62)	2102·54 (1762·65 to 2469·9)	2383·56 (2005·47 to 2838·09)	1926·24 (1559·84 to 2428·08)	1603·63 (1400·24 to 1840·24)	1464·28 (1310·42 to 1624·63)	1138·79 (1000·02 to 1283·75)	1016·08 (826·47 to 1205·73)
Indonesia	2605·16 (2307·34 to 2912·91)	2096·92 (1862·71 to 2392·26)	1736·04 (1539·44 to 1942·84)	1405·28 (1282·62 to 1543·21)	1213·28 (1119·55 to 1320·77)	1100·51 (964·99 to 1265·79)	1044·89 (956·94 to 1137·31)	956·05 (800·87 to 1123·74)
Aceh	2181·07 (1819·9 to 2594·43)	1805·5 (1468·88 to 2188·63)	1573·47 (1254·61 to 1913·59)	1322·38 (1126·69 to 1544·65)	1203·86 (1048·63 to 1385·47)	1116·25 (900·18 to 1357·82)	1138·79 (994·58 to 1302·13)	1073·83 (845·78 to 1327·92)
Bali	2351·5 (1982·07 to 2796·85)	1896·11 (1529·32 to 2322)	1634·08 (1312·84 to 2027·16)	1405·17 (1188·1 to 1638·6)	1194·45 (1041·44 to 1370·12)	1057·08 (878·36 to 1295·3)	936·99 (822·87 to 1068·58)	735·14 (560·89 to 956·43)
Bangka-Belitung Islands	2959·94 (2499·79 to 3466·62)	2433·61 (1977·29 to 2991·38)	2038·46 (1653·93 to 2479·64)	1652·28 (1397·84 to 1936·08)	1469·89 (1276·13 to 1704·08)	1351·97 (1120·95 to 1620·04)	1260·91 (1105·1 to 1440·84)	1164·25 (936·04 to 1437·81)
Banten	3278·28 (2796·87 to 3810·56)	2804·68 (2337·01 to 3439·78)	2201·22 (1811·8 to 2664·35)	1657·18 (1430·46 to 1931·77)	1304·47 (1141·68 to 1487·4)	1100·65 (904·03 to 1340·14)	1098·86 (949·48 to 1240·49)	1032·56 (794·16 to 1275·96)
Bengkulu	3285·1 (2774·33 to 3811·39)	2679·94 (2222·55 to 3262·27)	2185·04 (1766·87 to 2626·42)	1742·08 (1490·47 to 2009·63)	1437·1 (1253·18 to 1646·76)	1260·61 (1022·4 to 1554·77)	1174·86 (1020·15 to 1337·34)	1072·95 (840·78 to 1331·2)
Gorontalo	2733·52 (2303·98 to 3235·53)	2362·04 (1902·88 to 2891·51)	1989·84 (1593·15 to 2445·23)	1641·62 (1397·7 to 1932·11)	1438·96 (1246·29 to 1652·82)	1347·38 (1100·13 to 1654·19)	1300·69 (1115·13 to 1504·07)	1151·32 (871·45 to 1456·4)
Jakarta	1945·96 (1635·75 to 2312·65)	1671·9 (1380·16 to 2055·39)	1368·26 (1122·65 to 1677·2)	1116·04 (966·73 to 1298·53)	1021·2 (881·67 to 1175·58)	1054·22 (863·56 to 1304·61)	986·2 (833·91 to 1129·33)	893·49 (694·05 to 1114·84)
Jambi	2958·62 (2499·73 to 3442·55)	2428·57 (1990·24 to 2991)	2100·2 (1710·62 to 2611·32)	1755·9 (1512·06 to 2059·48)	1442·57 (1263·58 to 1659·01)	1203·3 (978·47 to 1479·7)	1104·09 (976·93 to 1256·06)	1024·23 (809·92 to 1249·22)
West Java	2969·12 (2552·77 to 3433·25)	2379·63 (1982·07 to 2858·69)	1893·32 (1552·39 to 2306·15)	1461·01 (1281·56 to 1664·98)	1198·89 (1063·08 to 1351·68)	1020·04 (845·28 to 1240·22)	985·51 (852·6 to 1134·27)	924·09 (728·16 to 1150·93)
Central Java	2313·67 (1975·33 to 2692·2)	1842·12 (1524·09 to 2203·85)	1522·86 (1262·92 to 1831·93)	1218·65 (1069·67 to 1395·38)	1035·27 (920·89 to 1157·82)	1032·16 (850·18 to 1244·35)	986·51 (861·85 to 1113·12)	891·76 (711·25 to 1094·86)
East Java	2360·04 (2031·38 to 2722·66)	1919·35 (1603·94 to 2281·56)	1615·94 (1347·77 to 1934·39)	1335·7 (1178·24 to 1520·75)	1177·07 (1060·03 to 1323·24)	1065·24 (889·88 to 1305·9)	999·5 (875·93 to 1141·48)	916·46 (733·39 to 1132·82)
West Kalimantan	2562·38 (2166·62 to 3034·63)	2286·49 (1848·22 to 2797·51)	2027·37 (1669·48 to 2448·54)	1698·96 (1458·12 to 1958·95)	1433·72 (1238·91 to 1640·24)	1262·26 (1013·64 to 1549·6)	1132·14 (990·5 to 1288·33)	1001·8 (798·84 to 1237·59)
South Kalimantan	3203·15 (2720·89 to 3763·26)	2741·99 (2261·25 to 3316·59)	2196·91 (1804·61 to 2677·71)	1721·53 (1500·13 to 1985·01)	1540·03 (1344·86 to 1762·7)	1430·71 (1168·05 to 1770·39)	1310·52 (1140·32 to 1513·45)	1171·51 (951·81 to 1467·38)
Central Kalimantan	2409·36 (2047·59 to 2833·11)	1938·97 (1579·05 to 2416·83)	1692·21 (1370·13 to 2146·19)	1457·25 (1234·49 to 1729·88)	1301·21 (1130·75 to 1492·95)	1080·02 (883·77 to 1327·93)	1097·52 (971·04 to 1240·83)	1039·73 (835·19 to 1290·42)
East Kalimantan	2094·44 (1737·41 to 2500·09)	1915·14 (1535·02 to 2362·61)	1669·08 (1334·19 to 2050·6)	1374·3 (1176·02 to 1592·59)	1263·69 (1101·31 to 1447·15)	1284·43 (1051·95 to 1567·65)	1166·78 (1023·17 to 1342·16)	1058·91 (831·73 to 1306·77)
North Kalimantan	1950·03 (1621·99 to 2332·97)	1822·12 (1492·12 to 2248·52)	1607·36 (1306·79 to 1943·92)	1236·79 (1057·17 to 1444·28)	978·93 (836·99 to 1143·28)	808·48 (628·66 to 1004·05)	779·54 (639·49 to 929·47)	743·61 (548·09 to 973·84)
Riau Islands	2193·3 (1830·79 to 2603·38)	1915·83 (1544·53 to 2330·86)	1630·45 (1316·05 to 2019·14)	1400·26 (1179·01 to 1672·64)	1246·18 (1073·56 to 1448·7)	978·86 (795·37 to 1215·5)	975·61 (847·24 to 1102·11)	920·93 (716·51 to 1149·3)
Lampung	2539·12 (2129·55 to 3012·99)	2114·36 (1717·62 to 2625·77)	1733·71 (1416·67 to 2112·05)	1380·14 (1200·78 to 1594·66)	1199·06 (1049·94 to 1369·64)	1011·35 (815·87 to 1248·73)	968·77 (828·02 to 1124·54)	918·44 (713·82 to 1168·04)
Maluku	3588·61 (3061·31 to 4165·84)	2791·16 (2306·89 to 3399·81)	2310·13 (1890·42 to 2801·17)	1893·88 (1628·04 to 2184·46)	1620·83 (1412·11 to 1857·75)	1415·77 (1139·89 to 1715·38)	1382·73 (1182·16 to 1610·63)	1208·82 (965 to 1510·04)
North Maluku	3460·97 (2916·25 to 4057·39)	2866·82 (2325·97 to 3484·43)	2455·6 (1967·64 to 3008·29)	2097·01 (1795·51 to 2441·35)	1667·52 (1435·53 to 1941·98)	1495·7 (1210·53 to 1821·04)	1394·21 (1211·88 to 1603·81)	1224·52 (955·55 to 1517·27)
West Nusa Tenggara	3546·37 (3049·74 to 4138·41)	2946·14 (2449·69 to 3575·47)	2478·23 (2039·74 to 2997·04)	1973·3 (1697·86 to 2273)	1615·37 (1389·86 to 1858·79)	1363·2 (1114·74 to 1677·77)	1188·08 (1026·06 to 1355·93)	1089·44 (877·21 to 1344·48)
East Nusa Tenggara	3178·33 (2695·55 to 3748·16)	2537·54 (2068·61 to 3091·02)	2113·33 (1718·49 to 2597·47)	1708·31 (1464·61 to 1959·49)	1461·24 (1281·75 to 1662·32)	1273·6 (1043·8 to 1578·41)	1111·59 (962·37 to 1279·82)	1046·21 (810·43 to 1297·81)
Papua	2060·48 (1718·43 to 2441·82)	1964·13 (1577·57 to 2463·99)	1767·56 (1419·62 to 2175·13)	1549·61 (1316·9 to 1807·95)	1454·62 (1256·19 to 1666·07)	1431·97 (1166·08 to 1768·09)	1429·86 (1210·8 to 1661·3)	1286·89 (986·68 to 1610·21)
West Papua	2073·3 (1725·11 to 2493·61)	1915·96 (1543·73 to 2402·74)	1757·34 (1408·75 to 2164·47)	1513·26 (1262·84 to 1784·19)	1369·44 (1163·17 to 1597·59)	1294·11 (1065·57 to 1586·87)	1329·26 (1162·83 to 1508·44)	1098·33 (877·1 to 1380·2)
Riau	2266·48 (1897·63 to 2683·79)	2026·84 (1648·35 to 2509·48)	1735·52 (1410·69 to 2138·6)	1389·59 (1188·4 to 1607·34)	1219·22 (1055·89 to 1395·14)	1087·58 (881·87 to 1341·51)	1036·93 (885·95 to 1187·54)	941·14 (725·88 to 1180·14)
West Sulawesi	4143·6 (3543·55 to 4833·6)	3336·24 (2753·89 to 4063·7)	2788·73 (2265·19 to 3401·09)	2197·96 (1898·53 to 2553·53)	1829·45 (1567·72 to 2090·23)	1550·64 (1243·99 to 1920·62)	1330·28 (1147·78 to 1530·34)	1194·56 (927·92 to 1522·63)
South Sulawesi	2837·26 (2424·61 to 3309·47)	2349·28 (1934·22 to 2824·07)	1966·21 (1607·44 to 2419·23)	1603·1 (1383·44 to 1848·36)	1329·81 (1160·2 to 1525·17)	1141·33 (933·83 to 1406·7)	1077·88 (938·95 to 1217)	976·81 (758·89 to 1227·76)
Central Sulawesi	3512·9 (3008·27 to 4103·61)	2703·91 (2241·05 to 3319)	2241·86 (1839·01 to 2759·8)	1848·4 (1589·14 to 2124·17)	1642·86 (1420·14 to 1887·81)	1488·5 (1208·94 to 1838·69)	1296·98 (1106·99 to 1505·93)	1158·09 (913·33 to 1475·46)
Southeast Sulawesi	3017·64 (2560·32 to 3539·29)	2584·04 (2097·49 to 3154·1)	2234·61 (1831·69 to 2717·63)	1747·17 (1493·63 to 2026·57)	1468 (1266·44 to 1675·36)	1351·12 (1084·25 to 1644·82)	1255·59 (1082·08 to 1441·78)	1150·74 (904·71 to 1445·24)
North Sulawesi	2462·6 (2068·9 to 2933·57)	2054·98 (1681·25 to 2525·76)	1765·5 (1418·31 to 2221·5)	1496·88 (1249·3 to 1753·82)	1375·68 (1191·07 to 1585·83)	1228·75 (1008·12 to 1513·49)	1152·49 (1009·69 to 1319·51)	1079·26 (848·04 to 1340·49)
West Sumatra	3470·52 (2956·33 to 4064·36)	2374·3 (1947·36 to 2880·82)	1862·84 (1530·11 to 2236·35)	1438·15 (1239·98 to 1662·55)	1255·19 (1091·75 to 1431·97)	1168·53 (955·06 to 1418·07)	1123·5 (991·7 to 1264·13)	981·37 (788·78 to 1209·45)
South Sumatra	2498·87 (2123·76 to 2921)	1983·97 (1623·86 to 2442·13)	1634·98 (1334·29 to 2025·3)	1269·8 (1106·78 to 1442·61)	1150·64 (1005·95 to 1309·4)	1033·27 (842·77 to 1261·45)	989·38 (854·54 to 1136·11)	939·83 (731·33 to 1181·08)
North Sumatra	2420·32 (2040·62 to 2819·5)	1988·2 (1621·48 to 2448·58)	1694·9 (1364·48 to 2071·52)	1417·11 (1208·23 to 1647·4)	1294·91 (1134·02 to 1479·42)	1237·02 (1009·25 to 1524·9)	1165·15 (1027·57 to 1327·28)	1080·04 (857·79 to 1345·51)
Yogyakarta	1852·42 (1550·74 to 2215·41)	1475·86 (1204·63 to 1837·29)	1305·4 (1023·67 to 1645·94)	1140·44 (942·85 to 1344·58)	1091·22 (956·57 to 1233·38)	1002·81 (813·08 to 1216·91)	973·15 (859·77 to 1104·76)	938·08 (763·55 to 1146·29)
Laos	3064·41 (2205·49 to 4361·57)	2826·32 (2127·49 to 3742·62)	2588·28 (2048·99 to 3203·42)	2212·52 (1834·8 to 2635·29)	1874·5 (1590·76 to 2179·32)	1575·4 (1359·97 to 1792·34)	1220·9 (1036·03 to 1418·61)	1040·08 (854·92 to 1235·41)
Malaysia	1835·19 (1554·92 to 2141·81)	1129·85 (1060·25 to 1212·96)	1021·61 (957·38 to 1095·25)	926·84 (837·01 to 1022·5)	870·19 (808·54 to 941·46)	854·83 (796·65 to 915·36)	790·51 (732·1 to 852·41)	746·43 (595·19 to 917·06)
Maldives	3512·93 (2968·27 to 4105·5)	2852·39 (2445·11 to 3278·37)	2096·27 (1852·35 to 2356·98)	1627·86 (1483·56 to 1794·01)	1234·47 (1145·02 to 1330·31)	880·62 (814·72 to 948·87)	595·82 (534·25 to 663·43)	538·78 (457·16 to 632·57)
Mauritius	2137·65 (1983·07 to 2307·6)	1733·43 (1589·52 to 1880·19)	1329·58 (1226·93 to 1443·76)	1170·11 (1067·82 to 1289·42)	985·07 (917·95 to 1057·27)	852·37 (789·26 to 921·35)	741·3 (687·22 to 796·89)	682·09 (545·25 to 839·62)
Myanmar	3612·94 (3010·84 to 4302·87)	2923·93 (2424·73 to 3511·22)	2529·19 (2094·36 to 3032·75)	2010·75 (1672·64 to 2374·14)	1739·24 (1462·5 to 2055·91)	1554·93 (1283·3 to 1874·33)	1221·32 (1060·72 to 1410·01)	1001·92 (848·39 to 1194·96)
Philippines	1481·81 (1348·4 to 1645·04)	1210·54 (1107·88 to 1325·1)	1238·18 (1142·48 to 1349·6)	1151·32 (1062·04 to 1252·19)	1023·44 (940·19 to 1115·2)	920·01 (844·01 to 1004·78)	929·96 (846·31 to 1014·06)	869·61 (732·79 to 1027·11)
Seychelles	1426·38 (1285·15 to 1579·39)	1197·56 (1104·74 to 1299·44)	1104·87 (1013·04 to 1206·38)	851·67 (773·18 to 941·95)	954·5 (867·43 to 1053·57)	869·36 (794·41 to 947·68)	833·5 (772·46 to 899·35)	810·06 (701·16 to 942·5)
Sri Lanka	1633·12 (1517·08 to 1753·15)	1234·22 (1132·99 to 1350·83)	1182·09 (1096·76 to 1274·93)	1001·2 (931·46 to 1079·73)	1011·94 (884·07 to 1153·95)	893·64 (783·64 to 1030·95)	781·98 (724·54 to 845·32)	622·72 (478·27 to 807·22)
Thailand	1754·86 (1530·9 to 1987·8)	1227·43 (1124·76 to 1347·91)	1134·92 (1028·73 to 1243·38)	1064·56 (972·05 to 1171·45)	848·65 (766·71 to 936·79)	804·01 (709·04 to 905·49)	589·48 (525·5 to 654·97)	533·97 (403·53 to 684·57)
Timor-Leste	2691·31 (2164·3 to 3301·83)	2262·71 (1928·55 to 2637·55)	1948·06 (1617·8 to 2306·94)	1828·4 (1614·12 to 2109·27)	1350·54 (1205·92 to 1533·38)	1146·3 (1018·21 to 1308·06)	973·38 (863·3 to 1090·76)	981·51 (819·61 to 1126·06)
Vietnam	2077·28 (1720·57 to 2532·65)	1479·16 (1210·88 to 1816·84)	1572·94 (1342·67 to 1874·97)	1106·98 (958·77 to 1300·09)	1002·45 (866·66 to 1152·27)	868·63 (782·65 to 975·36)	827·79 (747·35 to 921·96)	772·28 (637·93 to 917·83)
Sub-Saharan Africa	2571·02 (2229·77 to 2927·99)	2332·52 (2092·84 to 2572·72)	2074·71 (1908·29 to 2249·64)	1823·77 (1703·28 to 1948·77)	1739·5 (1656 to 1828·79)	1839·45 (1717·78 to 1957·39)	1482·6 (1395·57 to 1582·13)	1193·34 (1101·78 to 1305·83)
Central sub-Saharan Africa	2719·21 (2286·91 to 3201·76)	2363·11 (2006·19 to 2779·44)	2196·93 (1908·26 to 2501·26)	2015·88 (1752·18 to 2304·53)	1877·69 (1652·81 to 2115·57)	1882·08 (1630·21 to 2155·21)	1564·96 (1297·77 to 1874·62)	1266·87 (996·09 to 1631·56)
Angola	2588·2 (2161·23 to 3058·36)	2412·55 (2040 to 2801·89)	2345·52 (1965·75 to 2741·68)	2210·49 (1822·19 to 2666·17)	2103·6 (1667·85 to 2668·9)	1901·4 (1600·05 to 2277·08)	1451·54 (1242·69 to 1700·99)	1208·68 (1011·83 to 1468·44)
Central African Republic	2789·63 (2105·62 to 3650·6)	2529·84 (2014·7 to 3136·91)	2345·01 (1960·64 to 2756·46)	2264·19 (1931·42 to 2597·59)	2364·12 (2068·83 to 2684·53)	2807·66 (2357·03 to 3270·63)	2426·9 (1958·57 to 2986·72)	2107·64 (1626·38 to 2716·19)
Congo (Brazzaville)	3175·24 (2391·17 to 4173·93)	2712·01 (2240·96 to 3302·19)	2430·16 (2054·51 to 2851·02)	2057·18 (1754·67 to 2423·63)	2010·2 (1711·36 to 2332·52)	2036·64 (1749·44 to 2366·97)	1496·26 (1275·68 to 1738·1)	1263·7 (1031·32 to 1531·91)
DR Congo	2729·43 (2244·11 to 3262·25)	2310·55 (1909·68 to 2830·01)	2132·84 (1805·58 to 2471·52)	1957·09 (1667·08 to 2303·55)	1789·85 (1522·83 to 2089·64)	1821·36 (1500·21 to 2205·94)	1564·24 (1211·69 to 2028·33)	1251·53 (901·51 to 1789·02)
Equatorial Guinea	3848·75 (3223·44 to 4540·45)	3059·1 (2511·18 to 3707·95)	2593·91 (2088·97 to 3175·39)	2162·87 (1796·43 to 2569·77)	2108·49 (1726 to 2554·5)	1581·43 (1207·1 to 2045·73)	1240·39 (952·86 to 1560·82)	1160·22 (920·95 to 1447)
Gabon	2744·59 (2204·19 to 3378·36)	2135·05 (1810·04 to 2491·92)	1917·12 (1571·24 to 2357·08)	1615·39 (1325·88 to 1965·57)	1512·11 (1318·34 to 1741·09)	1574·78 (1319·19 to 1840·96)	1336·58 (1144·12 to 1535·59)	1114·65 (914·73 to 1313·15)
Eastern sub-Saharan Africa	2856·52 (2473·53 to 3262·79)	2473·12 (2226·14 to 2743·51)	2246·38 (2070·76 to 2436·74)	1990·54 (1873·62 to 2116·86)	1965·9 (1860·1 to 2080·18)	1969·3 (1842·89 to 2108·64)	1439·93 (1351·01 to 1525·27)	1162·1 (1064·64 to 1269·89)
Burundi	2920·14 (2181 to 3895·52)	2502·51 (1995·43 to 3095·45)	2392·7 (2003·92 to 2807·05)	2226·56 (1940·28 to 2555·85)	2262·77 (1959·9 to 2621·85)	2588·46 (2218·03 to 2999·53)	1574·42 (1337·53 to 1810·29)	1294·59 (1031·37 to 1629·15)
Comoros	2495·24 (1956·18 to 3131·97)	2277·85 (1883·53 to 2695·85)	1970·22 (1634·33 to 2354·91)	1786·58 (1537·06 to 2085·17)	1560·78 (1341·94 to 1810·48)	1382·98 (1201·85 to 1586·33)	1135·03 (966·28 to 1330·18)	1014·5 (822·21 to 1266·99)
Djibouti	1803·5 (1335·16 to 2493·95)	1672·64 (1257·36 to 2212·13)	1514·08 (1175·83 to 1892·85)	1359·73 (1130·69 to 1619·51)	1397·17 (1137·92 to 1694·55)	1451·63 (1135·2 to 1802·61)	1290·6 (1025·13 to 1597·87)	1119·48 (916·56 to 1418·14)
Eritrea	3212·57 (2338·07 to 4451·98)	2832·53 (2147·34 to 3639·32)	2881·22 (2141·59 to 4458·34)	2710·24 (2382·74 to 3078·48)	2496·55 (2167·95 to 2857·38)	1917·45 (1518·68 to 2364·25)	1609·26 (1187·23 to 2175·17)	1396·71 (982·91 to 1987·37)
Ethiopia	3336·55 (2480·95 to 4782·98)	2905·91 (2298·3 to 3810·97)	2745·62 (2319·27 to 3303·97)	2584·45 (2290·29 to 2894·84)	2393·41 (2165·95 to 2651·38)	2051·3 (1823·69 to 2307·93)	1264·73 (1156·16 to 1399·5)	999·26 (859·42 to 1175·95)
Kenya	2209·01 (1953·68 to 2457·75)	1777·26 (1569·26 to 2006·33)	1531·06 (1362·57 to 1742·61)	1304·27 (1169·65 to 1455·36)	1303·71 (1181·16 to 1440·6)	1751·83 (1542·46 to 1963·47)	1409·15 (1297·62 to 1528·22)	1142·44 (970·22 to 1343·15)
Baringo	2347·39 (1916·16 to 2791·92)	2054·11 (1600·47 to 2616·83)	1955·5 (1606·65 to 2372·19)	1769·07 (1469·03 to 2113·5)	1658·74 (1386·06 to 1994·44)	1707·91 (1420·61 to 2019·96)	1438·09 (1208·95 to 1700·69)	1165·08 (887·19 to 1502·53)
Bomet	1106 (884·5 to 1360·57)	971·33 (753·81 to 1244·56)	917·07 (724·59 to 1140·35)	837·01 (670·06 to 1016·21)	936·85 (770·39 to 1163·36)	1560·74 (1220·46 to 1982·87)	1283·06 (1055·18 to 1551·44)	1065·46 (830·2 to 1358·25)
Bungoma	2422·44 (2000·95 to 2935·81)	1910·56 (1519·03 to 2425·33)	1598·99 (1289·92 to 1948·83)	1384·16 (1154·35 to 1669·79)	1268·43 (1053·21 to 1522·53)	1451·4 (1190·2 to 1746·52)	1119·53 (933·76 to 1331·86)	950·38 (728·67 to 1232·23)
Busia	3895·2 (3223·79 to 4680·95)	2646·38 (2079·54 to 3369·72)	2025·35 (1664·68 to 2449·63)	1466·43 (1207·55 to 1771·97)	1452·04 (1166·69 to 1786·65)	1987·31 (1568·94 to 2472·43)	1375·05 (1121·44 to 1672·49)	1066·08 (781·39 to 1402·71)
Elgeyo Marakwet	1864·44 (1498·44 to 2263·97)	1633·05 (1283·43 to 2055·02)	1616·64 (1318·58 to 1953·84)	1505·19 (1225·11 to 1833·88)	1379·91 (1120·39 to 1676·77)	1549·32 (1278·16 to 1844·26)	1303·51 (1114·31 to 1533·97)	1096·2 (854·8 to 1418·51)
Embu	2577·1 (2133·52 to 3101·29)	1903·65 (1492·81 to 2406·03)	1384·43 (1131·22 to 1692·83)	1051·28 (859·47 to 1293·33)	978·25 (803·93 to 1192·91)	1199·08 (931·57 to 1554·48)	1116·34 (894·29 to 1372·89)	978·35 (744·86 to 1281·31)
Garissa	2087·25 (1717·84 to 2560·21)	1906·01 (1473·09 to 2405·57)	1692·98 (1354·26 to 2078·12)	1561·17 (1267·88 to 1919·86)	1454·25 (1183·7 to 1781·51)	1513·26 (1225·68 to 1884·96)	1419·9 (1168·88 to 1723·89)	1226·39 (939·56 to 1599·26)
Homa Bay	3979·12 (3387·73 to 4597·76)	2836·68 (2311·38 to 3414·37)	2469·82 (2082 to 2950)	1993·27 (1696·63 to 2334·09)	1968·04 (1626·21 to 2369·98)	3550·2 (2771·15 to 4449·56)	2454·34 (2019·67 to 2960·2)	1708·03 (1254·87 to 2341·16)

Supplemental Results Table 3a: Age-standardised mortality rates by location, both sexes, 1950, 1960, 1970, 1980, 1990, 2000, 2010, 2019

Location	1950	1960	1970	1980	1990	2000	2010	2019
Isiolo	3529·63 (2946·08 to 4171·24)	3096·35 (2451·25 to 3847·92)	2706·64 (2275·75 to 3242·62)	2068·34 (1701·32 to 2494·72)	1736·32 (1444·92 to 2073·16)	1803·36 (1399·67 to 2285·04)	1600·24 (1264·41 to 1975·64)	1290·9 (962·97 to 1711·82)
Kajiado	1610·1 (1284·47 to 1943·52)	1458·72 (1126·56 to 1846·12)	1400·74 (1112·73 to 1723·93)	1273·58 (1016·86 to 1559·87)	1242·9 (985·48 to 1536·67)	1561·25 (1272·65 to 1891·88)	1283·94 (1090·39 to 1504·05)	1077·76 (845·87 to 1366·18)
Kakamega	2557·62 (2122·43 to 3032·9)	2007·81 (1584·07 to 2494·73)	1687·74 (1398·91 to 2061·01)	1392·16 (1145·32 to 1675·7)	1452·76 (1209·92 to 1741·57)	1949·63 (1558·93 to 2396·06)	1424·14 (1176·28 to 1696·7)	1088·75 (839·97 to 1429·71)
Kericho	1283·66 (999·53 to 1618·59)	1233·19 (939·29 to 1569·86)	1160·43 (922·46 to 1440·71)	1064·53 (855·35 to 1321·36)	1078·85 (856·31 to 1354·76)	1445·74 (1148·75 to 1806·78)	1178·01 (945·94 to 1457·14)	965·27 (731·43 to 1277·58)
Kiambu	1776·45 (1437·39 to 2160·85)	1499·19 (1168·52 to 1903·45)	1327·15 (1083·01 to 1624·78)	1218·69 (996·94 to 1469·88)	1255·65 (1057·41 to 1481·01)	1718·41 (1416·98 to 2081·05)	1396·97 (1196·97 to 1633·67)	1138·54 (878·37 to 1441·22)
Kilifi	2856·15 (2381·98 to 3404·42)	2212·16 (1772·6 to 2758·25)	1838·07 (1528·3 to 2215·39)	1494·08 (1246·39 to 1782·03)	1346·61 (1116·24 to 1619·71)	1545·04 (1232·76 to 1903·27)	1277·6 (1054·95 to 1535·62)	1035·56 (783·79 to 1355·35)
Kirinyaga	2397·32 (1959·24 to 2908·45)	1894·29 (1481·57 to 2383·5)	1586·82 (1290·8 to 1933·99)	1127·8 (918·87 to 1373·63)	1097·61 (882·12 to 1364·55)	1395·34 (1090·09 to 1750·04)	1207·4 (981·97 to 1468·73)	1046·24 (783·26 to 1363·21)
Kisii	2251·02 (1862·85 to 2674·68)	2019·62 (1586·95 to 2528·45)	1764·16 (1415·17 to 2191·41)	1564·83 (1264·24 to 1925·71)	1558·68 (1277·57 to 1871)	1866·46 (1545·77 to 2252·52)	1419·52 (1220·08 to 1677·27)	1194·35 (919·93 to 1560·43)
Kisumu	2370·44 (1969·41 to 2836·91)	1935·59 (1520·61 to 2409·35)	1723·66 (1410·95 to 2089·17)	1553·12 (1274·37 to 1870·35)	1671·91 (1361·3 to 2017·33)	2803·51 (2199·21 to 3488·45)	2009·62 (1648·03 to 2416·37)	1538·68 (1112·73 to 2091·8)
Kitui	2636·6 (2176·75 to 3146·44)	1972·47 (1559·89 to 2477·84)	1624·49 (1354·81 to 1988·74)	1315·05 (1088·49 to 1602·11)	1222·47 (1006·99 to 1474·65)	1490·03 (1184·56 to 1835·9)	1208·66 (1018·15 to 1442·42)	1009·83 (762·1 to 1308·54)
Kwale	2866·38 (2366·93 to 3451·59)	2355·69 (1846·9 to 2988·23)	1933·07 (1580·15 to 2374·84)	1570·86 (1295·56 to 1897·26)	1442·25 (1169·58 to 1783·54)	1616·04 (1286·47 to 1983·33)	1454·51 (1215·19 to 1720·78)	1185·56 (884·46 to 1559·25)
Laikipia	1262·69 (991·75 to 1572·05)	1146·44 (878·38 to 1470·35)	1053·17 (837·6 to 1335·64)	934·22 (743·46 to 1165·04)	1050·02 (849·43 to 1286·99)	1561·57 (1214·73 to 1944·69)	1284·15 (1040·26 to 1532·45)	1015·89 (772·84 to 1292·2)
Lamu	3529·88 (2884·05 to 4233·27)	2482·49 (1932·09 to 3138·51)	1843·47 (1500·97 to 2297·59)	1242·64 (996·22 to 1527·37)	1277·68 (1026·22 to 1594·05)	1448·41 (1127 to 1861·62)	1278·4 (1008·12 to 1630·42)	1063·38 (776·34 to 1429·96)
Machakos	1692·89 (1373·69 to 2091·94)	1437·52 (1105·38 to 1847·45)	1262·7 (1022·42 to 1545·45)	1062·85 (859·03 to 1292·39)	989·23 (812·78 to 1188·14)	1320·71 (1040·55 to 1713·55)	1161·17 (965·83 to 1398·18)	988·3 (762·13 to 1269·26)
Makueni	2107·04 (1712·21 to 2564·65)	1504·91 (1173·06 to 1884·78)	1311·14 (1062·6 to 1602·73)	1107·19 (917·66 to 1337·86)	1095·91 (885·19 to 1321·98)	1799·17 (1344·4 to 2316·52)	1499·5 (1241·89 to 1804·41)	1165·57 (911·35 to 1482·3)
Mandera	1855·58 (1516·2 to 2248·85)	1723·15 (1336·44 to 2167·4)	1611·44 (1298·51 to 1982·14)	1520·75 (1232·1 to 1874·58)	1526·78 (1247·3 to 1851·4)	1629·57 (1318·32 to 2021·54)	1602·93 (1317·44 to 1919·18)	1389·18 (1032·18 to 1810·13)
Marsabit	2037·53 (1634·43 to 2479·32)	1851·78 (1422·16 to 2339·98)	1601·8 (1304·73 to 1984·21)	1345·27 (1086·46 to 1648·4)	1253·75 (1013·46 to 1508·82)	1400·34 (1081·31 to 1793·61)	1374·41 (1094·67 to 1683·06)	1179·29 (883·4 to 1569·19)
Meru	1690·8 (1383·62 to 2029·4)	1399·59 (1106·24 to 1729·09)	1208·84 (994·41 to 1466·48)	1013·01 (834·08 to 1228·2)	1009·24 (840·77 to 1203·17)	1324·16 (1055·07 to 1628·61)	1259·88 (1043·25 to 1495·49)	1088·16 (840·6 to 1417·19)
Migori	3283·86 (2675·94 to 3897·36)	2456·9 (1951·02 to 3071·22)	2098·21 (1739·18 to 2514·89)	1889·74 (1598 to 2232·12)	1830·62 (1507·13 to 2206·46)	2661·1 (2044·67 to 3368·66)	2109·09 (1699·47 to 2617·31)	1528·71 (1090·11 to 2056·62)
Mombasa	1900·76 (1547·14 to 2315·89)	1712·8 (1305·71 to 2183·7)	1362·91 (1106·35 to 1706·81)	1053·56 (845·74 to 1297·05)	1154·22 (940·68 to 1422·24)	1707·38 (1315·72 to 2182·87)	1368·35 (1111·47 to 1638·15)	1099·47 (826·61 to 1448·85)
Murang'a	2138·27 (1790·11 to 2555·62)	1512·36 (1183·92 to 1870·73)	1262·71 (1034·84 to 1547·42)	1045·11 (853·47 to 1248·66)	1047·31 (871·77 to 1241·57)	1390·67 (1113·3 to 1745·5)	1239·45 (1035·39 to 1488·95)	1013·2 (776·82 to 1306·75)
Nairobi	2173·2 (1815·86 to 2608·65)	1753·51 (1415·21 to 2185·34)	1323·65 (1098·16 to 1607·49)	1081·82 (902·11 to 1278·92)	1206·43 (991·24 to 1437·83)	1822·16 (1501·97 to 2170·13)	1430·89 (1255·37 to 1630·41)	1157·19 (935·87 to 1434·91)
Nakuru	1275·86 (1021·85 to 1557·78)	1191·15 (922·86 to 1530·86)	1133·98 (915·22 to 1410·82)	1060·25 (862·39 to 1291·34)	1124·14 (917·36 to 1370·12)	1600·02 (1285·61 to 1961·98)	1243·91 (1036·17 to 1482·14)	992·72 (768·01 to 1286·96)
Nandi	1676·53 (1328·29 to 2053·16)	1447·61 (1125·03 to 1836·71)	1329·29 (1065·47 to 1665·81)	1231·55 (1020·6 to 1522·7)	1265·78 (1028·63 to 1562·27)	1671·43 (1369·34 to 2045·44)	1308·53 (1092·28 to 1568·84)	1052·87 (785·95 to 1369·56)
Narok	1509·72 (1230·2 to 1839·82)	1429·15 (1121·03 to 1819·95)	1452·7 (1160·14 to 1801·68)	1491·18 (1209·54 to 1852·69)	1535·58 (1232·76 to 1881·22)	1825·4 (1500·01 to 2259·97)	1460·31 (1222·6 to 1775·29)	1222·23 (941·86 to 1590·46)
Nyamira	1787·09 (1468·31 to 2159·73)	1494·11 (1161·87 to 1898·26)	1348 (1083·22 to 1678·12)	1250·49 (1010·49 to 1547·33)	1290·91 (1059·07 to 1556·3)	2001·32 (1599·26 to 2506·58)	1874·1 (1553·98 to 2263·45)	1500·12 (1066·8 to 2003·15)
Nyandarua	2059·06 (1652·8 to 2524·12)	1716·79 (1313·24 to 2204·02)	1442·64 (1154·88 to 1780·41)	1237·05 (1000·1 to 1535·12)	1248·81 (1036·12 to 1504·34)	1709·21 (1367·17 to 2135·64)	1643·33 (1364·78 to 1955·15)	1338·42 (991·26 to 1749·36)
Nyeri	2096·9 (1694·3 to 2533·58)	1567·29 (1226·21 to 1985·29)	1335·31 (1056·17 to 1667·49)	1160·3 (949·55 to 1425·57)	1202·12 (1000·83 to 1445·01)	1677·22 (1361·2 to 2066·81)	1366·32 (1142·69 to 1636·24)	1151·1 (858·75 to 1502·19)
Samburu	1443·08 (1153·5 to 1764·19)	1311·01 (1004·02 to 1654·45)	1255·36 (1021·14 to 1553·73)	1175·7 (954·31 to 1429·96)	1268·8 (993·99 to 1580·62)	1689·77 (1309·8 to 2097·36)	1457·02 (1180·97 to 1781·96)	1200·08 (891·74 to 1564·86)
Siaya	2749·79 (2260·64 to 3289·76)	2236·35 (1776·8 to 2785·19)	1824·88 (1486·62 to 2199·79)	1569·87 (1288·32 to 1870·78)	1709·24 (1420 to 2070·06)	2702·89 (2081·86 to 3375·04)	1763·98 (1414·78 to 2163·45)	1229·46 (916·56 to 1609·45)
Taita Taveta	2191·63 (1783·33 to 2656·24)	1813·8 (1399·53 to 2286·4)	1643·81 (1340·55 to 2025·86)	1283·76 (1047·45 to 1591·65)	1226·5 (976·68 to 1504·58)	1682·61 (1311·74 to 2135·7)	1579·49 (1281·87 to 1884·84)	1253·45 (928·19 to 1679·62)
Tana River	2467·66 (1982·34 to 2994·23)	2025·7 (1525·34 to 2612·22)	1832·14 (1446·64 to 2310·8)	1672·86 (1352·25 to 2107·8)	1655·66 (1310·22 to 2070·04)	2113·51 (1701·41 to 2631·23)	1920·52 (1563·14 to 2313·5)	1533·83 (1109·65 to 2080·54)
Tharaka Nithi	2268·52 (1846·5 to 2695·17)	1632·56 (1263·05 to 2050·26)	1516·39 (1242·13 to 1855·43)	1360·94 (1107·15 to 1643·54)	1376·38 (1143·77 to 1638·85)	1709·91 (1366·88 to 2084·98)	1387·94 (1153·7 to 1639·42)	1172·19 (900·92 to 1536·08)
Trans Nzoia	1411·57 (1105·35 to 1786·45)	1290·44 (996·87 to 1650·39)	1176·95 (934·88 to 1495·4)	1062·4 (845·68 to 1316·58)	1078·37 (865·54 to 1325·22)	1501·01 (1138·95 to 1903·19)	1258·61 (1017·03 to 1533·82)	1025·28 (775·06 to 1357·92)
Turkana	1673·58 (1331·1 to 2033·64)	1621·94 (1269·85 to 2084·06)	1629·03 (1308·52 to 1987·85)	1597·05 (1307·17 to 1950·7)	1569·3 (1265·26 to 1937·2)	2219·81 (1657·03 to 2964·38)	1686·01 (1346·02 to 2087·83)	1302·82 (971·51 to 1783·81)
Uasin Gishu	1218·87 (957·42 to 1524·29)	1132·14 (851·79 to 1462·04)	1059·49 (830·67 to 1318·18)	999·84 (817·92 to 1243·59)	1065·64 (870·01 to 1307·14)	1442·37 (1150·37 to 1738·28)	1174·32 (989·48 to 1389·99)	960·21 (753·53 to 1245·85)
Vihiga	2411·98 (2000·57 to 2896·95)	1647·59 (1298·14 to 2058·62)	1473·32 (1201·27 to 1784·57)	1329·34 (1086·61 to 1621·12)	1366·73 (1131·59 to 1668·02)	1786·58 (1427·05 to 2195·95)	1446·16 (1206·27 to 1740·63)	1174·76 (875·32 to 1581·25)
Wajir	2659·35 (2192·26 to 3190·36)	2346·78 (1859·41 to 2958·28)	2019·6 (1654·12 to 2442·65)	1770·16 (1451·5 to 2132·4)	1646·99 (1339·4 to 1969·09)	1719·49 (1421·96 to 2053·95)	1609·77 (1323·93 to 1920·32)	1373·28 (1014·33 to 1797·84)
West Pokot	1845·92 (1503·69 to 2231·74)	1619·34 (1249·23 to 2041·61)	1560·74 (1269·05 to 1885·77)	1480·7 (1213·34 to 1820·38)	1451·19 (1178·92 to 1771·27)	1519·31 (1237·67 to 1852·43)	1321·8 (1092·45 to 1579·83)	1096·73 (824·25 to 1450·59)
Madagascar	2739·74 (2404·21 to 3140·07)	2341·07 (2016·41 to 2719·91)	2154·18 (1914·94 to 2446·44)	1601·69 (1401·84 to 1838·32)	1646·9 (1454·96 to 1852·74)	1531·58 (1351·95 to 1746·27)	1367·38 (1093·82 to 1701·19)	1223·08 (908·76 to 1650·68)
Malawi	3307·14 (2762·27 to 3923·43)	2907·8 (2449·75 to 3416·83)	2496·39 (2159·83 to 2879·55)	1976·37 (1749·35 to 2226·25)	2017·03 (1805·02 to 2288·75)	2446·01 (2129·47 to 2810·11)	1658·21 (1494·55 to 1867·26)	1206·22 (1047·75 to 1390·21)
Mozambique	2654·76 (2191·99 to 3210·71)	2367·53 (2000·86 to 2763·27)	2134·67 (1849·4 to 2447·95)	1932·94 (1692 to 2186·62)	1897·88 (1674·06 to 2154·14)	1871·65 (1658·31 to 2118·25)	1865·43 (1624·55 to 2126·13)	1593 (1340·54 to 1894)
Rwanda	3562·29 (2989·13 to 4183·17)	2672·57 (2244·3 to 3206·5)	2555·22 (2175·83 to 2987·54)	2205·49 (1923·24 to 2509·06)	2180·66 (1949·36 to 2444·19)	2141·86 (1908·8 to 2394·53)	1193·04 (1071·35 to 1335·4)	1020·72 (865·53 to 1195·13)
Somalia	2642·7 (2214·94 to 3117·63)	2591·91 (2128·91 to 3124·48)	2430·09 (2009·1 to 2891·61)	2239·49 (1857·07 to 2646·83)	2258·63 (1910·08 to 2621·43)	2091·42 (1692·31 to 2630·64)	1938·52 (1463·28 to 2627·34)	1650·31 (1189·54 to 2398·26)
South Sudan	2090·34 (1672·25 to 2594·16)	2050·86 (1667·06 to 2468·54)	1932·76 (1581·63 to 2336·29)	1724·71 (1459·18 to 2014·52)	1680·42 (1385·92 to 1990·87)	1563·49 (1271·25 to 1897·97)	1364·98 (1111·22 to 1635·08)	1213·08 (963·93 to 1505·53)
Uganda	2649·09 (2160·17 to 3200·6)	2153·35 (1842·01 to 2495·58)	1920·63 (1655·04 to 2225·98)	1968·63 (1664·47 to 2336·55)	2143·28 (1873·73 to 2470·89)	2153·57 (1930·04 to 2427·71)	1471·97 (1303·23 to 1661·33)	1125·16 (959·24 to 1306·67)
Tanzania	2805·51 (2311·96 to 3338·4)	2502·22 (2112·19 to 2890·06)	2128·24 (1840·59 to 2441·55)	1659·18 (1455·44 to 1886·81)	1669·25 (1483·71 to 1884·39)	1818·3 (1610·84 to 2040·11)	1367·99 (1227·49 to 1523·96)	1070·13 (911·01 to 1247·26)
Zambia	2386 (1965·62 to 2875·83)	2114·66 (1749·79 to 2516·37)	1820·1 (1576·62 to 2103·33)	1689·31 (1507·66 to 1903·92)	1978·63 (1761·85 to 2203·77)	2630·12 (2289·96 to 2955·1)	1730·7 (1547·95 to 1931·26)	1346·28 (1122·85 to 1623·71)
Southern sub-Saharan Africa	1933·42 (1735·56 to 2160·95)	1748·26 (1597·33 to 1905·48)	1574·86 (1455·63 to 1702·69)	1396·26 (1245·14 to 1557·88)	1163·52 (1063·74 to 1278)	1879·64 (1674·27 to 2118·21)	1727·59 (1603·11 to 1878·62)	1225·66 (1095·7 to 1376·57)
Botswana	2120·01 (1725·12 to 2535·88)	1976·54 (1624·27 to 2372·65)	1810·99 (1501·53 to 2208·21)	1503·91 (1267·39 to 1789·87)	1457·48 (1224·68 to 1755·41)	2760·46 (2148·24 to 3406·59)	1730·92 (1413·19 to 2098·6)	1410·29 (1143·16 to 1787·71)
eSwatini	2739·62 (2288·88 to 3270·07)	2486·06 (2053·63 to 2977·01)	2041·5 (1718·67 to 2429·19)	1665·45 (1377·56 to 1957·76)	1430·86 (1219 to 1655·17)	2409·11 (1897·1 to 2971·61)	2513·91 (2075·39 to 3015·37)	1680·03 (1358·72 to 2095·2)
Lesotho	2039·14 (1723·02 to 2413·38)	1905·06 (1624·32 to 2247·59)	1790·28 (1508·88 to 2153)	1556·03 (1303·07 to 1857·51)	1439·85 (1255·77 to 1656·72)	2353·56 (1956·75 to 2809·75)	2555·69 (2180·43 to 2963·15)	2243·95 (1848·87 to 2674)
Namibia	1961·41 (1613·08 to 2365·92)	1786·95 (1494·14 to 2123·08)	1639·87 (1390·68 to 1889·58)	1587·45 (1352·88 to 1863·03)	1405·05 (1256·88 to 1560·59)	1979·56 (1702·56 to 2279·92)	1452·3 (1244·46 to 1667·9)	1229·29 (1002·03 to 1502·98)
South Africa	1884·85 (1701·98 to 2091·13)	1709·7 (1564·71 to 1871·48)	1541·32 (1416·44 to 1681·34)	1368·18 (1182·42 to 1575·24)	1101·57 (983·16 to 1226·14)	1713·51 (1535·45 to 1918·96)	1629·95 (1497·42 to 1791·14)	1141·42 (995·7 to 1308·36)
Zimbabwe	2045·23 (1503·78 to 2840·35)	1816·96 (1422·37 to 2299·92)	1655·82 (1366·53 to 2020·11)	1503·6 (1332·41 to 1707·51)	1354·57 (1198·41 to 1559·64)	2455·47 (1988·51 to 3015·42)	2135 (1918·6 to 2381·7)	1511·39 (1275·65 to 1807·91)
Western sub-Saharan Africa	2485·92 (2046·16 to 2975·78)	2385·5 (2035·04 to 2777·29)	2053·1 (1819·84 to 2320·89)	1758·31 (1596·83 to 1936·9)	1672·79 (1560·92 to 1788·92)	1691·52 (1559·65 to 1837·17)	1425·49 (1317·07 to 1556·15)	1195·97 (1077·76 to 1330·02)
Benin	2721·23 (1985·86 to 3717·63)	2329·4 (1835·6 to 2945·37)	2049·01 (1697·5 to 2431·66)	1788·26 (1574·03 to 2023·03)	1614·82 (1447·27 to 1787·1)	1555·55 (1377·66 to 1759·85)	1361·3 (1172·88 to 1600·66)	1189·24 (977·09 to 1464·77)
Burkina Faso	3124·99 (2347·02 to 4241·03)	2774·22 (2251·58 to 3459·59)	2361·15 (2028·98 to 2757·4)	2020·96 (1771·39 to 2325·75)	1903·83 (1682·38 to 2164·07)	1830·37 (1631·53 to 2080·5)	1527·81 (1365·33 to 1709·65)	1332·74 (1115·16 to 1605·26)
Cape Verde	1854·17 (1660·23 to 2067·41)	1513·17 (1374·06 to 1677·79)	1377·47 (1251·45 to 1519·41)	960·84 (880·71 to 1042·11)	845·75 (779·52 to 921·09)	856·85 (737·27 to 985·9)	690·79 (637·28 to 749·23)	809·5 (687·97 to 936·66)
Cameroon	2609·18 (1974·12 to 3438·2)	4233·16 (3548·2 to 5041·99)	1928·59 (1610·39 to 2267·55)	1686·2 (1458·49 to 1928·94)	1506·61 (1340·7 to 1674·59)	1792·51 (1588·57 to 2054·36)	1589·55 (1361·98 to 1853·4)	1270·46 (1023·07 to 1584·24)
Chad	2558·72 (1752·6 to 3713·43)	2147·59 (1574·6 to 3003·15)	2056·9 (1606·7 to 2718·83)	1893·63 (1579·36 to 2251·08)	1737·88 (1559·08 to 1948·73)	1876·78 (1644·13 to 2157·63)	1623·77 (1438·43 to 1822·72)	1406·26 (1164·91 to 1696·3)
Côte d'Ivoire	2405·9 (2049·09 to 2803·54)	2244·58 (1867·52 to 2674·15)	1894·36 (1615·5 to 2206·98)	1572·39 (1376·35 to 1797·7)	1677·62 (1486·21 to 1932·49)	2032·71 (1775·68 to 2321·13)	1613·99 (1408·3 to 1868·21)	1221·04 (1040·05 to 1449·19)
The Gambia	1871·69 (1535·46 to 2297·8)	1842·13 (1536·06 to 2203·65)	1658·04 (1400·51 to 1975·95)	1558·3 (1317·42 to 1865·4)	1431·04 (1214·76 to 1695·87)	1401·87 (1208·34 to 1623·42)	1306·85 (1125·72 to 1493·5)	1175·82 (989·91 to 1392·41)
Ghana	2264·83 (1946·59 to 2611·7)	1941·12 (1670·4 to 2253·35)	1737·75 (1502·74 to 2011·97)	1573·13 (1342·49 to 1858·2)	1485 (1271·72 to 1730·92)	1481·52 (1313·82 to 1665)	1381 (1247·23 to 1523·29)	1144·65 (974·63 to 1361·29)
Guinea	2781·61 (2307·51 to 3301·76)	2600·54 (2129·85 to 3128·5)	2409·71 (2022·3 to 2857·86)	2124·14 (1838·73 to 2461·1)	1777·63 (1542·64 to 2039·89)	1719·46 (1529·77 to 1928·99)	1576·52 (1358·11 to 1822·57)	1377·49 (1119·72 to 1684·83)
Guinea-Bissau	3924·22 (3400·89 to 4534·82)	3187·06 (2737·21 to 3688·6)	2935·43 (2442·53 to 3513·33)	2415·39 (2048·89 to 2826·21)	2203·4 (1885·78 to 2552·11)	2043·9 (1737·45 to 2410·77)	1768·26 (1529·27 to 2044·41)	1492·26 (1199·94 to 1838·56)
Liberia	3251·34 (2536·29 to 4106·38)	2601·83 (2191·87 to 3038·99)	2247·52 (1939·22 to 2628·19)	1883·85 (1643·54 to 2182·92)	2056·64 (1807·1 to 2345·14)	1673·79 (1448·82 to 1924·12)	1339·31 (1153·47 to 1533·64)	1128·74 (931·26 to 1404·06)
Mali	3145·04 (2598·85 to 3694·25)	2892·31 (2359·73 to 3496·36)	2662·15 (2265·92 to 3139·89)	2234·18 (1976·52 to 2543·3)	1934·48 (1720·86 to 2161·39)	1715·27 (1546·81 to 1915·61)	1442·77 (1282·54 to 1629·28)	1279·94 (1053·04 to 1569·13)
Mauritania	2385·81 (1750·38 to 3222·63)	2071·2 (1640·18 to 2598·64)	1764·44 (1460·67 to 2104·77)	1665·92 (1410·59 to 1931·8)	1524·39 (1346·06 to 1735·05)	1241·73 (1095·43 to 1405·76)	1004·73 (873·9 to 1171·85)	901·16 (756·62 to 1081·66)
Niger	2660·98 (1994·94 to 3567·07)	2580·5 (2082·03 to 3227·11)	2365·47 (2025·64 to 2757·34)	2214·25 (1981·38 to 2477·95)	2028·85 (1828·34 to 2248·47)	1791·76 (1610·77 to 1993·72)	1435·65 (1271·25 to 1636·53)	1268·55 (1055·37 to 1559·6)
Nigeria	2367·38 (1717·37 to 3405)	2185·33 (1644·16 to 2942·48)	2000·63 (1608·5 to 2522·52)	1670·76 (1410·88 to 1959·98)	1653·36 (1469·38 to 1851·17)	1672·8 (1437·21 to 1929·89)	1374·74 (1192·71 to 1600·67)	1146·36 (981·78 to 1345·39)
São Tomé and PrÍncipe	3057·62 (2670·9 to 3473·49)	2414·48 (2213·53 to 2624·82)	1607·5 (1481·3 to 1753·41)	1281·8 (1162·03 to 1417·39)	1234·38 (1114·87 to 1375·99)	1205·84 (1076·87 to 1358·77)	1056·67 (950·2 to 1180·56)	988·58 (835·94 to 1147·98)
Senegal	2329·64 (1748·67 to 3125·69)	2209·27 (1792·54 to 2751·17)	2146·59 (1832·39 to 2505·88)	1831·76 (1623·29 to 2061·74)	1505·18 (1360·98 to 1660·91)	1392·93 (1269·69 to 1541·79)	1150·59 (1027·45 to 1288·98)	1024·47 (880·9 to 1205·48)
Sierra Leone	2662·33 (2090·31 to 3406·56)	2442·16 (2027·51 to 2886·1)	2202·76 (1830·93 to 2635·2)	2017·69 (1695·1 to 2383·5)	1863·47 (1584·85 to 2145·41)	1823·24 (1609·09 to 2045·29)	1640·57 (1419·98 to 1918·86)	1316·66 (1108·13 to 1595·57)

Supplemental Results Table 3a: Age-standardised mortality rates by location, both sexes, 1950, 1960, 1970, 1980, 1990, 2000, 2010, 2019

Location	1950	1960	1970	1980	1990	2000	2010	2019
Togo	2440·38 (1992·2 to 2993·53)	2201·92 (1872·26 to 2554·99)	1864·5 (1610·64 to 2150·73)	1615·58 (1415·96 to 1841·93)	1525·27 (1317·43 to 1765·53)	1649·96 (1395·06 to 1948·74)	1486·01 (1308·89 to 1694·52)	1191·04 (984·36 to 1445·27)

Supplemental Results Table 3b: Age-standardised mortality rates by location, males, 1950, 1960, 1970, 1980, 1990, 2000, 2010, 2019								
Location	1950	1960	1970	1980	1990	2000	2010	2019
Global	2215·92 (2073·22 to 2361·81)	2336·78 (2123·14 to 2631·32)	1678·98 (1603·21 to 1759·96)	1480·47 (1422·4 to 1545·92)	1305·98 (1261·38 to 1351·68)	1196·53 (1159·7 to 1234·78)	1017·65 (986·5 to 1052·28)	874·68 (816·35 to 933·24)
Central Europe, eastern Europe, and central Asia	1637·69 (1496·67 to 1780·64)	1330·08 (1285·7 to 1374·27)	1362·31 (1325·75 to 1398·56)	1422·8 (1380·99 to 1465·07)	1349·79 (1310·57 to 1393·03)	1501·27 (1454·01 to 1550·25)	1268·19 (1233·74 to 1307·09)	1057·12 (969·84 to 1159·82)
Central Asia	2418·52 (2032·46 to 2850·96)	1807·9 (1659·97 to 1952·33)	1543 (1466·91 to 1631·65)	1340·74 (1287·55 to 1397·15)	1302·1 (1254·47 to 1350·84)	1520·57 (1450·4 to 1595·67)	1439·49 (1367·92 to 1523·28)	1248·14 (1134·48 to 1382·73)
Armenia	2330·48 (1925·25 to 2842·08)	1498·69 (1321·36 to 1690·18)	1305·73 (1186·76 to 1438·6)	1043·41 (935·36 to 1151·97)	1146·92 (1057·55 to 1237·99)	1080·48 (1003·72 to 1168·8)	1019·18 (944·03 to 1099·73)	901·46 (749·71 to 1081·91)
Azerbaijan	3180·52 (2673·29 to 3738·69)	2235·31 (1965·37 to 2518·89)	1658·69 (1524·8 to 1821·59)	1310·53 (1206·87 to 1428·6)	1325·73 (1211·11 to 1451·41)	1380·26 (1257·4 to 1521·95)	1355·45 (1242·4 to 1496·18)	1272·88 (1041·74 to 1554·09)
Georgia	2356·38 (1948·71 to 2803·99)	1723·05 (1528 to 1944·1)	1518·06 (1386·35 to 1658·2)	1363·08 (1207·15 to 1527·12)	1423·39 (1286·07 to 1587·67)	1176·69 (1046·81 to 1308·77)	1229·42 (1097·3 to 1378·64)	1124·31 (945·28 to 1312·15)
Kazakhstan	2078·57 (1730·11 to 2474·31)	1796·34 (1602·15 to 2011·03)	1564·58 (1430·02 to 1714·91)	1451·39 (1332·82 to 1581·91)	1406·24 (1300·03 to 1535·84)	1777·04 (1640·48 to 1927·69)	1552·5 (1501·13 to 1605·87)	1218·75 (1057·11 to 1386·18)
Kyrgyzstan	2439·08 (2036·41 to 2917·21)	1946·12 (1698·91 to 2212·62)	1792·39 (1612·36 to 1990·92)	1673·48 (1528·14 to 1825·82)	1319·93 (1213·99 to 1444·52)	1385·12 (1276·95 to 1505·06)	1298·85 (1209·77 to 1392·85)	1024·57 (886·78 to 1204·48)
Mongolia	3598·77 (2977·61 to 4263·75)	2736·52 (2304·28 to 3225·72)	2342·55 (1972·25 to 2725·96)	1965·59 (1648·55 to 2293·03)	1916·78 (1650·62 to 2190·36)	2106·46 (1865·25 to 2384·23)	1784·17 (1531·27 to 2068·43)	1595·29 (1274·6 to 1957·5)
Tajikistan	2690·06 (2191·65 to 3237·73)	2008·07 (1782·52 to 2242·89)	1474·11 (1337·08 to 1631·59)	1153·33 (1021·8 to 1305·32)	1170·78 (1061·21 to 1283·31)	1259·45 (1148·01 to 1377·16)	1417·59 (1269·06 to 1561·92)	1347 (1102·15 to 1644)
Turkmenistan	2622·52 (2185·56 to 3125·47)	1974·11 (1781·73 to 2191·45)	1596·48 (1451·71 to 1751·68)	1439·97 (1314·63 to 1567·5)	1376·34 (1271·09 to 1495·92)	1424·33 (1250·22 to 1657·86)	1194·39 (1074·25 to 1326·98)	1146·44 (935·31 to 1417·22)
Uzbekistan	2228·5 (1787·69 to 2732·27)	1523·6 (1342·88 to 1716·34)	1360·13 (1229·49 to 1500·28)	1193·54 (1091·05 to 1298·31)	1143·2 (1062·64 to 1236·97)	1759·56 (1516·98 to 2038·17)	1824·69 (1500·97 to 2263·7)	1598·69 (1211·03 to 2133·51)
Central Europe	1751·56 (1678·65 to 1828·92)	1301·54 (1263·69 to 1340·49)	1287·74 (1248·4 to 1327·31)	1289·94 (1247·36 to 1333·45)	1261·12 (1219·36 to 1300·72)	1118·17 (1081·42 to 1155·97)	950·76 (932·5 to 969·83)	836·75 (736·22 to 938·4)
Albania	2110·89 (1875·11 to 2376·05)	1165·89 (1049·96 to 1283·9)	1095·8 (900·64 to 1324·93)	1108·35 (938·97 to 1297·83)	1106·38 (1016·44 to 1201·09)	911·13 (839·91 to 984·74)	720·55 (626·47 to 831·41)	729·6 (561·65 to 926·76)
Bosnia and Herzegovina	2195·32 (2017·47 to 2406·29)	1471·13 (1354·08 to 1602·37)	1418·53 (1318·57 to 1532·96)	1286·59 (1196·09 to 1385·26)	1005·98 (935·01 to 1087·67)	894·11 (757·47 to 1050·9)	836·11 (763·38 to 919·67)	814·18 (650·34 to 1017·01)
Bulgaria	1529·85 (1415·87 to 1645·79)	1080·73 (979·25 to 1189·37)	1064·31 (960·98 to 1175·81)	1183·68 (1075·95 to 1318·7)	1369·61 (1247·09 to 1499·71)	1401·29 (1289·24 to 1522·05)	1212·35 (1160·72 to 1267·46)	1132·57 (922·09 to 1366·45)
Croatia	2309·02 (2143·27 to 2493·08)	1526·41 (1417·78 to 1653·27)	1405·54 (1302·96 to 1525·37)	1326·91 (1214·64 to 1452·52)	1207·39 (1101·46 to 1322·02)	1044·63 (958·52 to 1147·48)	879·4 (817·38 to 946·22)	759·24 (596·5 to 944·8)
Czech Republic	1502·41 (1403·83 to 1618·54)	1309·69 (1217·37 to 1412·76)	1422·74 (1325·6 to 1526·06)	1401·2 (1302·86 to 1509·05)	1323·43 (1224·63 to 1436·44)	978·35 (964·18 to 992·53)	791·05 (777·94 to 803·17)	686·47 (568·47 to 818·95)
Hungary	1545·92 (1444·19 to 1659·87)	1314·99 (1229·38 to 1413·24)	1326·12 (1240·1 to 1420·13)	1431·38 (1335·57 to 1530·41)	1421·41 (1324·45 to 1528·65)	1225·41 (1139·05 to 1323·85)	1022·45 (950·79 to 1105·04)	865·92 (709·08 to 1061·03)
Montenegro	1083·11 (993·5 to 1179·15)	1042·6 (952·01 to 1141·53)	897·23 (822·69 to 977·28)	934·37 (856·15 to 1017·76)	958·19 (878·32 to 1048·11)	963·74 (875·39 to 1060·77)	940·31 (860·05 to 1030·44)	940·58 (776·98 to 1143·9)
North Macedonia	1872·58 (1714·86 to 2051·37)	1496·4 (1379·98 to 1625·21)	1200·7 (1112·52 to 1299·6)	1050·54 (972·78 to 1141·63)	1145·57 (1040·74 to 1257·42)	1251·47 (1147·41 to 1356·93)	1191·82 (1057·16 to 1352·19)	1054·23 (827·42 to 1333·29)
Poland	1871·5 (1745·03 to 2013·87)	1269·38 (1187·7 to 1359·04)	1243·01 (1157·78 to 1334·15)	1299·24 (1197·24 to 1417·66)	1277·86 (1185·63 to 1383·73)	1072·55 (991·99 to 1167·18)	892·65 (884·6 to 900·95)	784·64 (637·83 to 966·55)
Romania	1651·24 (1363·79 to 1968·99)	1287·3 (1172·15 to 1414·39)	1270·82 (1170·3 to 1386·69)	1262·27 (1156·88 to 1374·39)	1245·49 (1149·8 to 1350·5)	1177·21 (1083·32 to 1280·21)	1038·64 (961·53 to 1118·89)	920·44 (751·36 to 1120·47)
Serbia	2168·71 (2014·61 to 2330·76)	1478·38 (1377·36 to 1602·59)	1469·81 (1357·74 to 1598·98)	1267·51 (1181·38 to 1369·63)	1140·45 (1057·26 to 1241·63)	1155·44 (1063·15 to 1254·35)	1032·15 (962 to 1112·72)	934·51 (759·33 to 1156·38)
Slovakia	1502·77 (1472·44 to 1537·06)	1197·65 (1175·91 to 1218·8)	1262·63 (1241·8 to 1283·69)	1276·22 (1256·83 to 1295·17)	1288·37 (1269·44 to 1305·75)	1095·8 (1077·52 to 1114·36)	948·77 (933·65 to 965·39)	801·93 (644·5 to 997·47)
Slovenia	1824·52 (1698·95 to 1960·76)	1493·04 (1383·41 to 1622·49)	1342·54 (1236·23 to 1450·72)	1208·85 (1110·61 to 1310·44)	1047·67 (818·33 to 1322·14)	917·42 (893·64 to 942·54)	697·53 (676·07 to 720·01)	602·25 (484·39 to 754·45)
Eastern Europe	1400·16 (1196·54 to 1616·37)	1234·91 (1175·74 to 1299·07)	1347·97 (1294·37 to 1411·55)	1504·05 (1435·26 to 1582·72)	1389·58 (1324·64 to 1467·19)	1714·06 (1627·05 to 1815·23)	1427·85 (1354·77 to 1509·96)	1156·8 (1001·23 to 1341·7)
Belarus	1178·02 (927·6 to 1486·39)	1053·58 (961·1 to 1159·22)	1061·1 (992·11 to 1139·78)	1230·55 (1149·07 to 1326·23)	1227·09 (1138·71 to 1326·16)	1470·22 (1364·59 to 1585·22)	1420·95 (1315·43 to 1538·42)	1120·67 (874·54 to 1448·44)
Estonia	1504·4 (1216·28 to 1836·19)	1357·24 (1319·91 to 1398·84)	1343·97 (1308·45 to 1377·6)	1440·29 (1406·03 to 1473·03)	1400·04 (1368·31 to 1430·32)	1315·91 (1282·59 to 1347·72)	976·1 (945·75 to 1008·61)	792·59 (635·53 to 972·28)
Latvia	1298·01 (1026·85 to 1625·93)	1221·69 (1135·52 to 1324·52)	1240·05 (1151·02 to 1329·78)	1412·8 (1308·12 to 1520·7)	1374·41 (1275·97 to 1485·18)	1336·17 (1246·5 to 1442·8)	1153·05 (1126·9 to 1179·38)	928·55 (747·06 to 1129·3)
Lithuania	1270·6 (982·69 to 1594·99)	1109·45 (1031·63 to 1193·07)	1149·96 (1074·15 to 1230·88)	1248·34 (1158·97 to 1343·18)	1220·78 (1127·7 to 1320·63)	1196·76 (1110·62 to 1294·32)	1183·84 (1102·36 to 1284·92)	926·78 (765·27 to 1117·52)
Moldova	2316·66 (1927·42 to 2759·96)	1980·53 (1772·54 to 2206·28)	1645·31 (1506·26 to 1798·58)	1462·46 (1354·05 to 1577·68)	1357·43 (1261·71 to 1462·48)	1342·52 (1225·79 to 1482·36)	1281·62 (1152·05 to 1432·45)	980·96 (804·79 to 1206·23)
Russia	1453·65 (1180·56 to 1770·27)	1292·92 (1198·4 to 1392·74)	1427·85 (1333 to 1530·94)	1598·82 (1481·35 to 1722·2)	1442·23 (1337·04 to 1564·39)	1808·76 (1669·09 to 1963·46)	1473·42 (1377·3 to 1579·59)	1102·46 (910·09 to 1326·91)
Ukraine	1255·66 (1017·86 to 1541·09)	1102·97 (1025·02 to 1193·44)	1235·01 (1153·61 to 1317·97)	1374·4 (1279·13 to 1488·91)	1322·61 (1230·96 to 1427·04)	1619·83 (1508·12 to 1742·25)	1354·1 (1201·38 to 1534·33)	1405·34 (1086·87 to 1811·08)
High income	1440·84 (1400·18 to 1481·37)	1250·77 (1220·25 to 1281·72)	1185·61 (1155·78 to 1215·18)	1017·42 (992·63 to 1044·12)	864·52 (845·32 to 885·34)	726·36 (710·13 to 744·02)	593·59 (580·76 to 607·07)	550·36 (527·71 to 573·29)
Australasia	1253·37 (1178·09 to 1331·16)	1184·88 (1116·39 to 1263·11)	1225·05 (1148·86 to 1305·77)	1010·43 (944·78 to 1075·44)	829·17 (776·29 to 884·97)	654·4 (615·35 to 698·37)	515·86 (485·29 to 550·35)	476·28 (439·76 to 514·19)
Australia	1269·42 (1180 to 1366·89)	1195·77 (1112·13 to 1293·96)	1236·26 (1148·34 to 1336·13)	1000·13 (926·47 to 1077·86)	821·4 (762·87 to 887·59)	644·98 (599·57 to 696·45)	510·15 (475·94 to 549·75)	470·67 (429·73 to 516·18)
New Zealand	1194·27 (1114·99 to 1283·97)	1140·45 (1060·89 to 1226·33)	1178·13 (1098·26 to 1267·06)	1058·66 (985·67 to 1138·23)	870·98 (808·51 to 942·67)	704·94 (652·84 to 761·01)	547·3 (498·5 to 598·95)	508·32 (457·31 to 568·99)
High-income Asia Pacific	2099·88 (1982·07 to 2240·81)	1484·37 (1401·43 to 1577·39)	1233·97 (1160·35 to 1315·06)	965·21 (910·77 to 1025·74)	782·14 (738·76 to 831·35)	644·14 (607·96 to 684·32)	529·45 (500·08 to 564·53)	444·3 (411·1 to 482·19)
Brunei	2431·22 (2187·03 to 2715·83)	1991·17 (1797·06 to 2208·39)	1747·21 (1581·8 to 1915·17)	1575·44 (1434·32 to 1746·17)	1675·96 (1529·08 to 1838·42)	1123·55 (1022·65 to 1250·14)	1135·24 (1031·39 to 1253·34)	1192·38 (1008·89 to 1399·72)
Japan	1698·65 (1594·25 to 1818·37)	1347·23 (1264·51 to 1442·19)	1112·56 (1036·95 to 1199·98)	855·98 (800·13 to 918·34)	701·61 (657·86 to 754·8)	596·36 (557·7 to 640·37)	507·72 (473·9 to 547·72)	430·48 (392·65 to 474·96)
Aichi	1537·41 (1222·86 to 1905·35)	1249·17 (1004·53 to 1537·4)	1068·72 (861·12 to 1336·09)	850·16 (771·81 to 941·84)	701·83 (638·31 to 778·66)	598·59 (545·72 to 664·58)	505·39 (457·7 to 562·73)	417·98 (352·79 to 500·1)
Akita	1806·7 (1454·25 to 2204·8)	1469·57 (1176·77 to 1810·41)	1269·31 (1022·54 to 1565·34)	989·66 (896·78 to 1087·77)	779·72 (704·59 to 864·3)	666·35 (602·3 to 737·72)	576·11 (518·08 to 638·96)	534·66 (443·76 to 646·78)
Aomori	1942·41 (1576·81 to 2346·48)	1538·78 (1205·11 to 1881·78)	1250·14 (986·49 to 1540·91)	930·18 (844·24 to 1024·27)	768·17 (695·38 to 846)	677·8 (619·21 to 750·38)	590·24 (535·24 to 653·48)	498·23 (416·1 to 599·57)
Chiba	1698 (1365·87 to 2057·18)	1337·86 (1078·87 to 1633·21)	1090·96 (880·13 to 1367·19)	851·48 (773·6 to 938·99)	686·15 (622·41 to 758·52)	587·57 (532·91 to 648·85)	501·02 (450·86 to 553·99)	431·39 (363·01 to 512·06)
Ehime	1770·2 (1437·21 to 2152·59)	1405·78 (1122·54 to 1753·67)	1142·29 (909·22 to 1412·11)	855·2 (778·04 to 943)	701·7 (636·13 to 779·77)	617·33 (559·64 to 686·58)	522·12 (472·91 to 582·28)	462·13 (384·01 to 555·66)
Fukui	1515·3 (1196·34 to 1898·03)	1236·66 (982·61 to 1528·51)	1004·04 (817·56 to 1210·81)	736·03 (663·42 to 819·19)	646·1 (582·33 to 722·47)	544·35 (489·17 to 603·74)	474·72 (427·24 to 528·59)	386 (322·2 to 455·41)
Fukuoka	1778·24 (1449·7 to 2179·47)	1419·26 (1138·35 to 1747·36)	1175·77 (948·2 to 1452·75)	899·06 (815·12 to 997·34)	740·79 (671·94 to 822·19)	614·54 (555·02 to 677·18)	520·05 (471·8 to 576·65)	451·12 (376·08 to 538·92)
Fukushima	1821·2 (1476·86 to 2217·47)	1413·54 (1128·69 to 1758·81)	1165·61 (940·08 to 1439·34)	859·96 (779·93 to 953·14)	691·85 (629·46 to 763·28)	605·97 (546·85 to 667·82)	519·84 (471·35 to 576·52)	445·84 (365·16 to 538·96)
Gifu	1570·65 (1256·29 to 1916·8)	1262·43 (1001·21 to 1558·68)	1064·86 (842·41 to 1326·85)	848·5 (768·3 to 938·65)	669·05 (602·54 to 738·84)	588·53 (530·71 to 652)	495·17 (448·47 to 548·21)	438·12 (363·45 to 523·64)
Gunma	1710·27 (1392·23 to 2073·62)	1356·77 (1088·44 to 1673·44)	1116·35 (895·63 to 1385·64)	847·76 (769·6 to 933·5)	679·1 (616·89 to 748·69)	592·73 (536·01 to 652·17)	520·31 (470·36 to 573·91)	449·15 (372·12 to 536·02)
Hiroshima	1766·2 (1423·81 to 2144·94)	1387·99 (1095·17 to 1702·79)	1121·3 (889·14 to 1407·54)	850·2 (769·95 to 943·75)	690·1 (624·41 to 767·6)	592·28 (534·79 to 655·46)	498·77 (450·36 to 552·91)	423·73 (350·9 to 507·36)
Hokkaidō	1710·82 (1394·66 to 2092·92)	1365·34 (1099·21 to 1688·27)	1158·73 (930·92 to 1428·34)	877·82 (795·79 to 970·29)	714·41 (648·8 to 790·99)	605·09 (547·62 to 668·73)	522·04 (470·65 to 580·46)	446·3 (374·51 to 528·67)
Hyōgo	1663·48 (1342·11 to 2057·37)	1335·44 (1073·96 to 1644·31)	1127·33 (904·25 to 1407·14)	885·53 (803·86 to 977·14)	735·36 (663·28 to 812·71)	614·88 (557·41 to 677·22)	509·64 (459·79 to 564·76)	432·85 (366·33 to 511·12)
Ibaraki	1879·13 (1542·42 to 2274·04)	1448·57 (1146·61 to 1780·02)	1188·66 (953·48 to 1451·16)	903·3 (820·69 to 991·5)	726·53 (657·49 to 802·36)	621·1 (562·02 to 681·78)	532·46 (483·89 to 589·13)	465·79 (389·85 to 547·07)
Ishikawa	1561·27 (1252·5 to 1937·34)	1250·43 (995·67 to 1576·52)	1050·73 (847·05 to 1302·51)	775·43 (698·23 to 856·64)	667·3 (602·89 to 735·41)	564·7 (509·95 to 625·5)	484·9 (435·79 to 536·15)	419·47 (349·64 to 497·12)
Iwate	1846·7 (1502·3 to 2240·08)	1451·16 (1150·67 to 1778·31)	1184·89 (951·76 to 1474·89)	904·19 (814·37 to 997·15)	726·04 (656·04 to 800·98)	617·88 (555·1 to 683·56)	558·29 (500·09 to 620·7)	477·91 (397·15 to 572·21)
Kagawa	1643·22 (1322·45 to 2010·61)	1316·49 (1053·25 to 1605·33)	1062·77 (860·4 to 1310·66)	808·31 (732·51 to 896·72)	693·87 (629·16 to 768·44)	585·7 (530·19 to 647·9)	502·42 (454·29 to 557·32)	406·34 (338·66 to 482·3)
Kagoshima	1957·25 (1609·15 to 2365·34)	1505·98 (1198·59 to 1837·09)	1246·39 (992·01 to 1538·38)	912·2 (831·24 to 1007·53)	720·8 (654·69 to 800·67)	621·38 (563·85 to 684·74)	529·64 (479·96 to 588·49)	441·58 (365·98 to 526·17)
Kanagawa	1491·63 (1186·6 to 1843·83)	1200·57 (945·89 to 1482·45)	1006·21 (801·07 to 1271·07)	816·8 (740·1 to 891·97)	673·74 (614·29 to 736·27)	576·69 (524·84 to 630·8)	477·92 (435·25 to 525·74)	399 (333·22 to 474·8)
Kōchi	1934·17 (1584·79 to 2324·99)	1534·3 (1254·57 to 1892·39)	1236·64 (998·05 to 1513·36)	901·81 (816·55 to 995·58)	708·69 (644·11 to 781·97)	619·87 (560·27 to 689·13)	546·92 (496·75 to 606·34)	451·75 (381·37 to 534·08)
Kumamoto	1836·79 (1490·47 to 2213·49)	1436·23 (1137·24 to 1759·86)	1170·1 (932·71 to 1451·76)	849·85 (770·99 to 936·31)	674·7 (610·95 to 743·71)	560·98 (507·79 to 619·85)	480·94 (434·69 to 533·08)	416·84 (345·27 to 496·71)
Kyōto	1538·41 (1221·36 to 1895·92)	1228·1 (974·83 to 1535·09)	1006 (804·04 to 1265·81)	795·53 (721·01 to 885·79)	656·57 (595·09 to 727·82)	587·97 (532·46 to 652·77)	481·35 (434·88 to 534·67)	385·47 (318·03 to 454·21)
Mie	1608·81 (1287·21 to 1980·72)	1290·75 (1027·09 to 1578·42)	1072·27 (848·63 to 1350·34)	855·27 (775·38 to 943·59)	715·66 (648·13 to 792·74)	602·42 (543·44 to 671·56)	512·94 (463·62 to 570·46)	430·6 (356·57 to 508·85)
Miyagi	1743·78 (1427·41 to 2118·56)	1372·41 (1086·35 to 1719·27)	1163·93 (932·43 to 1434·52)	870·93 (788·88 to 964·78)	708·93 (644·47 to 782·23)	601·42 (542·59 to 666·34)	504·46 (454·53 to 561·02)	408·86 (337·66 to 484·87)
Miyazaki	1859·1 (1495·01 to 2281·06)	1461·47 (1144·21 to 1815·06)	1180·03 (949·17 to 1458·82)	895·33 (807·52 to 991·91)	726·98 (656·19 to 809·39)	609·43 (545·27 to 678·53)	511·22 (458·68 to 566·6)	450·41 (374·27 to 539·27)
Nagano	1631·22 (1310·64 to 2007·1)	1279·26 (1011·59 to 1576·42)	1072·46 (862·42 to 1344·32)	815·97 (736·47 to 898·55)	638·85 (576·76 to 707·31)	547·81 (494·42 to 604·96)	456·98 (410·83 to 504·8)	396·56 (327·76 to 472·63)
Nagasaki	1828·58 (1466·47 to 2217·35)	1461·72 (1169·6 to 1787·37)	1242·2 (991·08 to 1539·15)	928·64 (842·81 to 1026·03)	743·54 (670·81 to 824·14)	619·66 (558·44 to 688·66)	536·47 (483·16 to 599·66)	440·46 (361·03 to 529·69)
Nara	1637·24 (1313·42 to 1997·41)	1303·34 (1033·34 to 1619·31)	1103·02 (877·29 to 1363·31)	851·91 (768·02 to 938·88)	691·19 (621·4 to 765·18)	567·61 (508·84 to 629·13)	465·43 (419·29 to 518·05)	398·17 (330·09 to 475·69)
Niigata	1705·93 (1386·26 to 2067·86)	1359·45 (1085·03 to 1690·97)	1159·59 (934·08 to 1440·71)	875·05 (789·35 to 968·87)	684·6 (621·86 to 760·48)	595·54 (539·77 to 657·72)	511·62 (463·98 to 569·02)	442·05 (368·01 to 530·49)
Ōita	1781·03 (1439·22 to 2179·06)	1396·25 (1121·24 to 1715·89)	1122·99 (895·07 to 1426·45)	877·45 (796·64 to 976·15)	703·12 (635·3 to 781·51)	594·61 (534·57 to 662·37)	495·14 (445·9 to 552·1)	436·24 (360·3 to 525·62)
Okayama	1668·17 (1344·34 to 2039·21)	1327·42 (1060·65 to 1649·05)	1065·76 (855·68 to 1332·61)	826·07 (746·97 to 915·09)	682·31 (618·12 to 755·93)	580·31 (526·25 to 644·21)	503·41 (453·22 to 558·83)	412·91 (344·95 to 487·13)
Okinawa	2058·46 (1679·69 to 2467·21)	1537·76 (1199·06 to 1942·64)	1303·74 (1041·8 to 1616·8)	836·05 (758·52 to 923·48)	662·1 (600·41 to 735·06)	582·48 (526·33 to 647·55)	510·86 (461·39 to 566·74)	462·45 (382·64 to 554·96)
Ōsaka	1689·53 (1373·24 to 2081·03)	1361·39 (1101·3 to 1672·92)	1137·9 (918·93 to 1404·09)	922·37 (837·81 to 1012·47)	777·58 (706·73 to 854·76)	641·59 (582·39 to 706·15)	541·07 (489·79 to 596·68)	448·54 (379·08 to 526·88)
Saga	1810·2 (1463·44 to 2223·33)	1423·16 (1123·89 to 1752·87)	1151·11 (931·32 to 1425·86)	854·34 (772·58 to 950·95)	712·44 (645·91 to 794·81)	615·48 (555·46 to 688·17)	512·71 (464·06 to 573·99)	409·13 (342·27 to 486·48)
Saitama	1622·47 (1301·07 to 2000·96)	1279·98 (1020·67 to 1566·31)	1055·57 (831·97 to 1319·06)	861·73 (783·5 to 947·09)	698·31 (633·57 to 770·69)	582·16 (526·39 to 643·39)	504·61 (455·65 to 559·04)	434·9 (364·61 to 520·13)

Supplemental Results Table 3b: Age-standardised mortality rates by location, males, 1950, 1960, 1970, 1980, 1990, 2000, 2010, 2019

Location	1950	1960	1970	1980	1990	2000	2010	2019
Shiga	1633·09 (1312·72 to 2037·16)	1320·44 (1051·9 to 1647·44)	1139·81 (922·26 to 1391·47)	832·55 (749·76 to 925·76)	700·46 (629·18 to 778·35)	568·7 (511·81 to 630·34)	474·91 (428·43 to 527·65)	387·9 (325·25 to 460·09)
Shimane	1744·33 (1407·42 to 2129·42)	1406·64 (1122·88 to 1752·72)	1132·8 (917·14 to 1373·69)	827·61 (751·79 to 918·41)	666·27 (601·6 to 739·61)	580·99 (524·68 to 643·57)	513·58 (466·09 to 573·54)	425·86 (357·9 to 507·24)
Shizuoka	1591·88 (1282·22 to 1941·42)	1267·91 (1009·99 to 1579·69)	1057·63 (836·78 to 1308·51)	828·69 (752·63 to 911·08)	670·35 (608·58 to 738·34)	581·01 (527·47 to 637·14)	499·11 (452·84 to 551·53)	434·48 (360·34 to 513·92)
Tochigi	1788·55 (1435·82 to 2159·02)	1416·6 (1132·52 to 1746·59)	1174·17 (944·37 to 1457·6)	911·45 (829·25 to 1001·78)	740·28 (671·11 to 817·23)	616·7 (558·69 to 680·68)	539·63 (490·61 to 597·05)	460·29 (383·98 to 549·59)
Tokushima	1834·59 (1488 to 2248·27)	1467·06 (1173·13 to 1794·69)	1196·63 (972·68 to 1448·48)	903·06 (813·41 to 1002·97)	729·24 (661·63 to 806·55)	613·7 (553·18 to 680·07)	517·01 (467·28 to 574·16)	444·61 (372·94 to 523·34)
Tōkyō	1486·12 (1200·65 to 1805·11)	1227·5 (987·61 to 1495·91)	1041·52 (837·51 to 1276·86)	810·48 (735·75 to 888·46)	705·59 (643·29 to 777·31)	589·44 (536·85 to 647·33)	499·51 (454·73 to 549·84)	419·98 (353·87 to 495·5)
Tottori	1822·66 (1485·4 to 2216·11)	1446·65 (1152·09 to 1783·64)	1163·35 (950·54 to 1407·35)	875·16 (791·3 to 968·86)	734·65 (661·82 to 815·82)	618·46 (554·77 to 685·16)	541·08 (486·21 to 603·9)	456·97 (386·54 to 540·26)
Toyama	1643·3 (1321·67 to 2023·55)	1326·79 (1062·93 to 1624·51)	1101·88 (887·49 to 1360·24)	833·57 (755·34 to 924·39)	669·11 (609·76 to 741·3)	560·84 (508·29 to 621·08)	488·5 (440·91 to 543·95)	422·43 (358·63 to 496·89)
Wakayama	1775·52 (1444·3 to 2177·53)	1407·01 (1118·48 to 1739·33)	1154·6 (928·88 to 1430)	896·42 (814·38 to 995·9)	751·24 (677·44 to 832·07)	630·8 (566·91 to 699·88)	551·37 (497·66 to 614·68)	522·68 (430·29 to 633·35)
Yamagata	1700·92 (1381·89 to 2058·13)	1351·65 (1075·01 to 1689·53)	1124·44 (894·36 to 1397·36)	848·03 (768·61 to 930·6)	664·68 (598·47 to 735·25)	568·77 (515·29 to 634·91)	479·49 (433·2 to 534·13)	408·5 (338·35 to 486·41)
Yamaguchi	1797·05 (1457·36 to 2196·41)	1429·67 (1150·76 to 1751·9)	1161·79 (934·72 to 1441·52)	921·57 (833·38 to 1015·23)	723·07 (653·03 to 797·3)	620·99 (560·08 to 689·44)	534·37 (480·87 to 594·9)	466·26 (393·09 to 557·22)
Yamanashi	1758·47 (1402·54 to 2136·93)	1400·61 (1129·33 to 1720·25)	1136·32 (919·57 to 1392·61)	854·66 (768·07 to 951·4)	689·38 (622·73 to 765·08)	587·04 (528·68 to 649·91)	498·13 (448·3 to 557·34)	422·24 (353·77 to 495·92)
South Korea	3969·22 (3677·15 to 4323·13)	2111·08 (1947·21 to 2282·61)	1815·83 (1683·05 to 1958·4)	1577·25 (1477·7 to 1696·89)	1245·67 (1169·17 to 1340·79)	935·1 (876·75 to 1006·79)	640·92 (598·4 to 688·41)	513·35 (463·86 to 565·18)
Singapore	2162·16 (2021·39 to 2327·79)	1646·02 (1513·44 to 1804·35)	1371·95 (1285·55 to 1476·33)	1119·62 (1049·32 to 1194·27)	913·93 (854·55 to 977·58)	656·83 (614·91 to 706·45)	491·64 (458·43 to 528·56)	392·13 (365·39 to 425·27)
High-income North America	1259·76 (1186·35 to 1338·98)	1188·44 (1120·88 to 1267·19)	1154·53 (1086·64 to 1229·81)	981·86 (923·12 to 1049·68)	856·94 (805·15 to 913·83)	750·6 (705·43 to 799·88)	628·97 (591·69 to 668·41)	628·73 (561·28 to 707·91)
Canada	1191·3 (1113·99 to 1275·5)	1097·55 (1021·17 to 1184·18)	1040·96 (968·81 to 1121)	923 (859·59 to 991·14)	797·12 (737·12 to 860·16)	668·72 (624·2 to 719·02)	524·47 (488·59 to 567·63)	495·79 (455·49 to 541·86)
Greenland	2690·04 (2377·88 to 3026·2)	1937·17 (1720·29 to 2157·85)	1627·74 (1460·42 to 1825·86)	1510·16 (1389·54 to 1640·38)	1442·05 (1329·8 to 1583·7)	1195·92 (1115·81 to 1329·86)	1065·69 (966·5 to 1177·69)	945·92 (782·81 to 1108·74)
USA	1265·59 (1185·55 to 1353·26)	1197·39 (1124·54 to 1284·37)	1165·82 (1091·46 to 1249·42)	988·36 (922·5 to 1064·97)	864·25 (807·56 to 928·9)	760·46 (709·98 to 816·91)	641·41 (598·17 to 687·59)	645·13 (569·77 to 737·97)
Alabama	1386·84 (1114·46 to 1712·03)	1300·91 (1178·95 to 1442·16)	1257·78 (1138·76 to 1402·42)	1085·64 (982·37 to 1211·21)	981·14 (885·44 to 1086·46)	918·07 (830·14 to 1016·58)	826·96 (749·94 to 913·7)	829·47 (630·19 to 1075·92)
Alaska	1567·75 (1292·9 to 1898·85)	1337·05 (1206·16 to 1481·86)	1229·45 (1111·17 to 1362·79)	1084·08 (981·72 to 1199·08)	937·68 (846·86 to 1037·1)	770·77 (695·62 to 857·89)	633·93 (575·7 to 701·61)	639·38 (497·03 to 822·16)
Arizona	1326·67 (1055·17 to 1651·68)	1212·21 (1102·02 to 1338·93)	1152·15 (1042·45 to 1272·84)	940·43 (856·97 to 1035·48)	791·46 (719·84 to 873·82)	712·28 (646·89 to 785·68)	594·6 (541·56 to 654·1)	621·5 (464·66 to 809·85)
Arkansas	1224·91 (964·16 to 1528·29)	1146·68 (1036·1 to 1265·69)	1143·75 (1037·84 to 1265·61)	1011·8 (914·71 to 1126·02)	926·05 (838·03 to 1022·31)	878·85 (795·77 to 969·53)	777·92 (706·14 to 859·01)	776·86 (584·25 to 1013·07)
California	1200·64 (949·15 to 1491·26)	1145·51 (1048·62 to 1256·47)	1091·94 (1000·92 to 1196·54)	935·35 (855·71 to 1026·18)	821·27 (752·73 to 897·99)	675·5 (617·64 to 742·15)	544·58 (500·07 to 595·75)	533·19 (407·59 to 676·33)
Colorado	1214·13 (964·29 to 1502·74)	1092·7 (987·67 to 1208·25)	1058·02 (959·06 to 1167·58)	893·39 (807·27 to 992·89)	761·39 (691·72 to 842·4)	679·55 (616·01 to 754·81)	575·52 (523·48 to 636·09)	608·52 (453·42 to 803·38)
Connecticut	1203·19 (948·21 to 1496·97)	1152·36 (1040·42 to 1270·12)	1063·15 (961·23 to 1177·83)	941·86 (850·24 to 1041·7)	759·87 (686·95 to 839·78)	693·73 (623·58 to 768·83)	564·12 (507·85 to 623·07)	560·22 (416·05 to 737·45)
Delaware	1404·71 (1135·33 to 1726·63)	1279·96 (1155·7 to 1416·53)	1255·76 (1138·56 to 1392·29)	1053·3 (953·8 to 1162·98)	922·9 (833·52 to 1017·32)	788·06 (709·95 to 872·83)	663·53 (599·52 to 730·56)	690·23 (545·31 to 890·98)
Washington, DC	1467·27 (1160·38 to 1818·46)	1415·12 (1274·04 to 1565·18)	1440·22 (1298·89 to 1594·86)	1231·6 (1103·86 to 1371·26)	1326·4 (1195·99 to 1468·83)	1040·66 (933·02 to 1156·85)	702·77 (630·39 to 785·75)	707·77 (523·93 to 931·58)
Florida	1229·02 (985·57 to 1556·25)	1132·29 (1023·13 to 1253·33)	1123·85 (1010·68 to 1243·31)	944·72 (850·26 to 1046·96)	816·06 (738·49 to 898·45)	720·05 (649·28 to 797·18)	608·51 (549·99 to 671·34)	620·39 (469·87 to 803·44)
Georgia	1413·32 (1114·29 to 1767·04)	1327·09 (1202·03 to 1457·74)	1324·28 (1195·85 to 1459·52)	1097 (988·33 to 1217)	983·91 (887·01 to 1081·57)	888·34 (802·13 to 979·18)	748·67 (675·86 to 824·61)	725·35 (546·31 to 941·39)
Hawaii	1170·72 (921·74 to 1470·79)	1383·47 (1247·82 to 1534·47)	952·13 (860·94 to 1058·56)	743·26 (666·34 to 825·9)	670·21 (601·7 to 746·22)	584·55 (526·33 to 648·36)	597·85 (538·79 to 666·38)	541·35 (402·74 to 697·46)
Idaho	1178·26 (924·47 to 1461·33)	1109·01 (995·14 to 1232·12)	1086·46 (973·86 to 1206·56)	884·87 (796·84 to 991·24)	785·09 (706·72 to 872·93)	683·63 (615·3 to 758·24)	622 (561·98 to 690·38)	608·82 (456·98 to 790·91)
Illinois	1290·37 (1029·37 to 1568·72)	1234·38 (1127·85 to 1364·59)	1223·43 (1116·76 to 1356·79)	1023·84 (932·14 to 1135·89)	897·68 (817·9 to 993·06)	778·18 (706·74 to 860·56)	636·3 (577·32 to 703·77)	636·31 (479·03 to 828·78)
Indiana	1240·12 (993·16 to 1555·03)	1187·5 (1081·83 to 1301·61)	1164·17 (1058·21 to 1278·48)	1006·37 (913·88 to 1107·21)	883·95 (803·67 to 968·66)	817·69 (739·89 to 899·53)	711·57 (645·31 to 784·36)	748·2 (570·34 to 963·47)
Iowa	1151·11 (904·83 to 1442·94)	1081·99 (979·29 to 1192·72)	1066·46 (960·6 to 1175·8)	894·04 (806·09 to 988·34)	758·17 (683·89 to 840)	689·47 (621·36 to 764·74)	614·85 (552·6 to 681·54)	627·92 (471·47 to 808·71)
Kansas	1121·6 (884·29 to 1425·37)	1074·37 (973·19 to 1180·61)	1032·96 (933·07 to 1135·94)	906·01 (817·54 to 998·23)	790·97 (719·33 to 868·18)	733·04 (659·49 to 810·85)	663·85 (602·6 to 729·66)	658·39 (504·35 to 857·45)
Kentucky	1273·09 (1016·36 to 1578·8)	1203·06 (1095·81 to 1331·86)	1234·89 (1115·52 to 1363·51)	1055·67 (955·65 to 1169·97)	941·51 (850·07 to 1044·41)	885·34 (801·65 to 980·72)	796·84 (722·05 to 882·66)	829·78 (635·86 to 1073·38)
Louisiana	1372·33 (1087·2 to 1698·3)	1273·47 (1154·39 to 1403·28)	1279·07 (1157·46 to 1416·83)	1112·75 (1007·04 to 1234·67)	1002·49 (910·01 to 1104·09)	924·9 (835·82 to 1024·14)	799·97 (724·83 to 881·01)	803·19 (602·51 to 1060·94)
Maine	1308·83 (1045·25 to 1640·48)	1215·74 (1100·42 to 1344·95)	1187·86 (1069·18 to 1324·22)	994·04 (893·4 to 1109·06)	813·41 (730·67 to 899·79)	744·96 (669·69 to 827·29)	644·22 (577·44 to 717·63)	665 (502·31 to 878·44)
Maryland	1364·98 (1086·11 to 1686·12)	1311·19 (1185·9 to 1454·84)	1220·47 (1104·88 to 1352·31)	1027·49 (930·48 to 1135·57)	904·79 (819·61 to 999·43)	799·59 (724·19 to 883·85)	627·63 (568·94 to 692·29)	675·29 (504·86 to 896·47)
Massachusetts	1235·86 (994·28 to 1534·71)	1209·62 (1105·14 to 1333·93)	1141·26 (1041·93 to 1260·23)	944·04 (859·59 to 1046·21)	798·24 (724·87 to 884·91)	699·92 (637·28 to 776·75)	577·29 (524·68 to 636·36)	598·78 (456·25 to 780·1)
Michigan	1238·06 (982·66 to 1516·23)	1193·01 (1084·11 to 1317·59)	1184·43 (1072·8 to 1315)	1000·59 (906·42 to 1114·99)	878·96 (791·7 to 973·66)	783·94 (707·12 to 869·76)	678·87 (609·86 to 751·91)	704·92 (537·2 to 937·25)
Minnesota	1139·4 (905·79 to 1419·89)	1065·43 (971·95 to 1175·73)	1030·74 (930·2 to 1142·92)	861·6 (780·11 to 955·06)	753·7 (686·36 to 831·71)	664·07 (603·47 to 736·07)	556·8 (504·63 to 621·09)	557·91 (420·09 to 731·42)
Mississippi	1392·36 (1115·72 to 1708·12)	1260·12 (1142·35 to 1396·99)	1308·89 (1177·16 to 1443·5)	1109·78 (999·22 to 1233·21)	1005·98 (908·61 to 1104·48)	961·77 (867·57 to 1063·26)	854·45 (772·75 to 942·39)	873·86 (651·23 to 1145·81)
Missouri	1239·61 (979·91 to 1541·59)	1173·96 (1070·22 to 1292·79)	1170·62 (1059·9 to 1291·03)	993·35 (902·99 to 1102·05)	880·84 (798·88 to 969·81)	805·24 (727·89 to 890·57)	708·74 (640·39 to 783·61)	720·73 (541·98 to 950·13)
Montana	1323·28 (1046 to 1645·26)	1209·44 (1089·96 to 1348·23)	1140·73 (1028·78 to 1270·86)	961·87 (866·86 to 1077·94)	792·06 (710·03 to 880·2)	741·12 (664·17 to 827·66)	635·73 (572·93 to 710·03)	638·14 (480·64 to 833·83)
Nebraska	1129·99 (894·45 to 1406·09)	1068·55 (972·3 to 1188·6)	1071·33 (973·19 to 1187·25)	898·88 (819·12 to 1003·96)	802·25 (722·62 to 886·6)	690·33 (625·63 to 768·73)	615·32 (556·88 to 684·92)	598·57 (444·5 to 790·8)
Nevada	1711·84 (1400·72 to 2069·56)	1480·61 (1343·88 to 1648·12)	1320·84 (1195·66 to 1471·5)	1138·48 (1028·72 to 1273·25)	1036·79 (945·63 to 1149·28)	905·54 (826·57 to 1008·58)	708·01 (642·86 to 784·97)	684·78 (517·56 to 894·1)
New Hampshire	1337·42 (1076·5 to 1650·75)	1211·09 (1091·58 to 1348·43)	1168·96 (1047·74 to 1297·2)	911·67 (821·53 to 1017·54)	810·92 (732·49 to 898·37)	707·96 (637·18 to 785·84)	597·23 (537·9 to 666·82)	665·11 (494·02 to 882·04)
New Jersey	1287·19 (1020·14 to 1598·22)	1214·08 (1103·22 to 1343·48)	1174·99 (1066·43 to 1296·26)	989·2 (895·21 to 1101·79)	853·97 (775·89 to 944·07)	737·95 (668·78 to 815·23)	589·18 (534·59 to 649·38)	583·14 (433·76 to 761·28)
New Mexico	1268·53 (1011·09 to 1604·13)	1133·62 (1028·61 to 1258·26)	1124·32 (1014·68 to 1244·81)	977·34 (882·65 to 1088·49)	815·66 (740·81 to 906·53)	725·3 (657·86 to 803·35)	647·23 (586·57 to 714·1)	690·11 (525·83 to 899·2)
New York	1283·97 (1040 to 1556·79)	1235·9 (1132·18 to 1361·83)	1207·7 (1101·53 to 1327·32)	1011·62 (923·58 to 1112·31)	901·82 (821·46 to 986·77)	714·48 (650·27 to 784·6)	572·25 (522·72 to 628·08)	558·44 (423·74 to 716·82)
North Carolina	1379·66 (1093·83 to 1713·14)	1316·14 (1192·78 to 1451·77)	1277·12 (1155·62 to 1414·55)	1067·89 (961·52 to 1181·22)	935·22 (844·65 to 1035·17)	851·51 (768·12 to 947·5)	703·83 (638·03 to 777·59)	707·39 (541·65 to 926·08)
North Dakota	1132·75 (903·26 to 1409·38)	1059·05 (960·57 to 1181·8)	1064·31 (958·49 to 1179)	876·14 (789·67 to 982·9)	766·75 (690·98 to 848·32)	693·75 (624·5 to 769·8)	611·35 (549·37 to 683·24)	621·8 (478·03 to 792·45)
Ohio	1261·22 (1002·65 to 1551·86)	1192·34 (1084·5 to 1302·45)	1187·48 (1080·37 to 1299·08)	1029·27 (932·42 to 1132·26)	885·2 (802·92 to 966·9)	800·56 (724·92 to 878·36)	699·41 (635·56 to 766·03)	750·64 (576·2 to 960·79)
Oklahoma	1195·71 (930·69 to 1511·04)	1140·33 (1032·98 to 1262·03)	1152·41 (1045·48 to 1266·35)	1005·66 (909·66 to 1108·46)	866·68 (784·82 to 953·92)	859·21 (778·38 to 950·37)	792·05 (717·68 to 872·59)	781·3 (593·11 to 1006·61)
Oregon	1212·78 (964·35 to 1515·94)	1133·43 (1028·01 to 1249·39)	1073·32 (969·72 to 1189·8)	926·68 (840·1 to 1033·74)	814·68 (737·16 to 904·92)	702·04 (637·93 to 778·54)	611·61 (552·89 to 678·11)	612·7 (461·15 to 793·71)
Pennsylvania	1323·13 (1063·68 to 1604·98)	1251·41 (1143·6 to 1376·09)	1212·32 (1100·81 to 1332·59)	1024·53 (930·07 to 1129·11)	872·64 (793·26 to 954·14)	784·56 (712·82 to 862·32)	664·07 (603·97 to 730·81)	689·18 (517·83 to 890·45)
Rhode Island	1281·1 (1008·4 to 1596·97)	1186·59 (1069·2 to 1319·69)	1151·33 (1037·64 to 1279·27)	975·36 (879·68 to 1089·97)	823·47 (741·1 to 911)	708·29 (632·51 to 789·44)	630·64 (563·54 to 697·82)	607·37 (452·71 to 799·74)
South Carolina	1515·69 (1202·32 to 1887·58)	1424·97 (1285·65 to 1570·01)	1343·71 (1209·27 to 1475·89)	1142 (1032·19 to 1261·56)	966·76 (869·34 to 1066·92)	895·04 (805·78 to 988·14)	761·6 (687·75 to 835·47)	756·16 (579·34 to 993·02)
South Dakota	1196·93 (944·49 to 1479·08)	1118·36 (1012·66 to 1240·73)	1071·71 (964·18 to 1188·47)	933·07 (839·7 to 1045·67)	789·59 (711·2 to 874·85)	705·52 (633·87 to 786·69)	624·32 (558·01 to 694·2)	655·32 (510·47 to 835·19)
Tennessee	1271·1 (991·96 to 1570·77)	1209·9 (1094·88 to 1339·13)	1217·89 (1101·38 to 1341·94)	1045·2 (943·17 to 1157·76)	967·98 (870·31 to 1069·21)	897·34 (811·3 to 990·46)	784·57 (711·05 to 865·99)	789·23 (603·83 to 1013·37)
Texas	1212·5 (961·49 to 1501·92)	1142·62 (1044·39 to 1256·32)	1129·3 (1026·23 to 1247·02)	990·14 (896·96 to 1094·55)	889·46 (811·81 to 978·77)	780·48 (711·61 to 860·58)	658·34 (598·08 to 723·16)	644·73 (492·4 to 827·11)
Utah	1155·33 (909·62 to 1444·59)	1092·96 (993·83 to 1205·22)	1044·46 (949·55 to 1156·76)	851·78 (771·41 to 939·09)	727·08 (659·1 to 803·41)	657·59 (595·44 to 726·03)	587·19 (534·34 to 649·01)	597·41 (453·42 to 791·41)
Vermont	1343·67 (1095·95 to 1627·53)	1219·21 (1106·05 to 1350·48)	1160·24 (1045·09 to 1282·96)	992·29 (890·46 to 1101·18)	836·44 (750·09 to 925·43)	715·89 (639·55 to 794·77)	595·86 (535·1 to 661)	634·18 (505·03 to 797·21)
Virginia	1365·82 (1090·14 to 1683·15)	1266·88 (1154·9 to 1395·53)	1210·39 (1098·7 to 1333·16)	1040·98 (945·71 to 1150·6)	901·43 (816·76 to 997·01)	788·22 (715·26 to 873·98)	641·38 (583·5 to 711·03)	634·69 (483 to 828·37)
Washington	1215·41 (966·57 to 1523·8)	1129·77 (1024·06 to 1250·87)	1099·94 (996·38 to 1212·18)	903·11 (817·41 to 994·97)	793·17 (719·22 to 875·56)	695·79 (629·51 to 771·89)	587·47 (532·08 to 646·2)	573·4 (431·33 to 744·71)
West Virginia	1328·3 (1044·89 to 1624·36)	1224·93 (1112·27 to 1354·41)	1271·9 (1142·78 to 1416·03)	1077·42 (968·73 to 1198·4)	939·47 (849·14 to 1039·09)	883·78 (799·71 to 976·4)	810·15 (729·18 to 898·22)	845·7 (631·6 to 1101·34)
Wisconsin	1182·83 (925·01 to 1483·84)	1127·75 (1019·23 to 1243·63)	1074·04 (969·57 to 1192·35)	908·45 (818·23 to 1013·33)	809·24 (734·7 to 894·94)	723·49 (652·93 to 800·58)	612·72 (554·2 to 680·23)	628·94 (472·79 to 815·96)
Wyoming	1296·31 (1058·07 to 1592·44)	1177·23 (1061·14 to 1312·64)	1166·78 (1051·27 to 1299·51)	999·61 (901·31 to 1117·01)	836·29 (749·57 to 931·33)	759·78 (684·2 to 848·27)	639·39 (573·59 to 710·58)	614·92 (484·31 to 784·23)
Southern Latin America	1565·44 (1487·25 to 1641·11)	1522·45 (1448·79 to 1601·75)	1424·04 (1353·09 to 1499·83)	1185·9 (1126·95 to 1248·67)	1032·54 (978·67 to 1088·6)	913·17 (863·11 to 962·63)	800·21 (757·22 to 842·42)	747·92 (682·01 to 816·78)
Argentina	1448·14 (1355·56 to 1548·94)	1409·56 (1318·55 to 1515·9)	1403·3 (1312·42 to 1504·3)	1191·84 (1117·58 to 1277·55)	1055·28 (984·56 to 1134·53)	962·19 (892·48 to 1034·59)	846·9 (793·03 to 902·74)	818·78 (723·11 to 930·57)
Chile	2015·03 (1886·62 to 2160·82)	1958·63 (1828·83 to 2097·9)	1585·26 (1480·45 to 1700·77)	1177·39 (1084·5 to 1278·65)	961·79 (889·3 to 1042·07)	772·08 (712·72 to 840·36)	678·97 (610·58 to 761·66)	591·59 (545·24 to 644·21)
Uruguay	1354·54 (1263·32 to 1454·81)	1195·42 (1093·01 to 1307·72)	1235·73 (1126·1 to 1360·4)	1155·32 (1046·9 to 1273·66)	1038·07 (932·42 to 1154·06)	943·11 (860·49 to 1042·02)	833·01 (775·44 to 899·39)	775·13 (684·89 to 880·26)
Western Europe	1357·64 (1308·71 to 1408·75)	1217·72 (1185·65 to 1250·06)	1175·27 (1146·3 to 1205·42)	1044·92 (1019·64 to 1071·62)	885·21 (868·33 to 902·43)	727·68 (714·12 to 741·59)	577·37 (569·15 to 586·05)	523·23 (504·86 to 541·88)
Andorra	843·53 (656·42 to 1066·09)	833·29 (646·69 to 1040·65)	793·17 (620·59 to 985·73)	727·54 (572·26 to 931·01)	703·23 (586·54 to 870·98)	611·55 (513·16 to 720·94)	551·64 (469·87 to 647·11)	520·42 (418·52 to 628·97)
Austria	1440·79 (1351·56 to 1541·3)	1324·47 (1233·66 to 1427·61)	1323·55 (1229·53 to 1427·8)	1156·05 (1074·73 to 1242·66)	923·62 (861·57 to 992·77)	747·01 (695·58 to 805·11)	599·29 (591·65 to 607·34)	522 (478·81 to 568·94)

Supplemental Results Table 3b: Age-standardised mortality rates by location, males, 1950, 1960, 1970, 1980, 1990, 2000, 2010, 2019								
Location	1950	1960	1970	1980	1990	2000	2010	2019
Belgium	1320·18<br>(1237·46 to 1414·89)	1265·78<br>(1173·88 to 1361·78)	1239·9<br>(1159·15 to 1326·64)	1117·06<br>(1032·72 to 1208·37)	925·48<br>(858·19 to 1003·24)	784·64<br>(727·23 to 847·46)	628·87<br>(621·47 to 636·08)	560·09<br>(518·42 to 608·98)
Cyprus	1582·23<br>(1344·94 to 1832·16)	1447·96<br>(1242·36 to 1662·94)	1522·35<br>(1303·1 to 1810·63)	1352·54<br>(1238·57 to 1475·14)	921·42<br>(850·8 to 1001·06)	905·9<br>(843·14 to 983·47)	796·43<br>(736·85 to 861·53)	585<br>(507 to 677·47)
Denmark	1075·87<br>(1005·12 to 1160·54)	1005·46<br>(938·51 to 1078·32)	991·07<br>(928·68 to 1063·3)	989·54<br>(976·12 to 1002·8)	931·05<br>(918·39 to 943·9)	786·53<br>(774·53 to 797·62)	646·73<br>(636·66 to 656·22)	556·9<br>(512·91 to 608·5)
Finland	1697·21<br>(1581·97 to 1827·77)	1355·32<br>(1272·87 to 1452·78)	1351·68<br>(1336·67 to 1367·04)	1114·17<br>(1100·84 to 1127·86)	985·67<br>(973·08 to 998·51)	791·07<br>(779·5 to 802·2)	632·52<br>(622·68 to 642·95)	545·38<br>(500·32 to 597·98)
France	1384·96<br>(1294·08 to 1484·97)	1275·48<br>(1178·17 to 1379·84)	1181·03<br>(1090·08 to 1278·23)	1046·63<br>(970·23 to 1135·79)	864·94<br>(811·21 to 931·25)	724·95<br>(674·14 to 780·68)	579·99<br>(543·54 to 620·7)	507·36<br>(461·42 to 558·8)
Germany	1420·4<br>(1243·82 to 1640·66)	1312·29<br>(1224·3 to 1412·21)	1332·04<br>(1252·55 to 1429·87)	1177·27<br>(1100·18 to 1258·42)	954·47<br>(946·68 to 962·21)	746·89<br>(739·9 to 754·19)	603·08<br>(597·11 to 608·98)	566·18<br>(518·6 to 618·35)
Greece	1005·92<br>(940·31 to 1075·87)	926·34<br>(858·95 to 1001·84)	903·85<br>(837·97 to 976·28)	890·76<br>(828·26 to 959·02)	769·34<br>(714·22 to 831·99)	711·64<br>(662·47 to 766·22)	588·62<br>(549·28 to 631·65)	566·83<br>(506·71 to 644·86)
Iceland	1019·86<br>(963·4 to 1085·66)	938·91<br>(905·11 to 973·44)	926·88<br>(891·94 to 957·77)	811·51<br>(783·4 to 845·73)	708·58<br>(680·23 to 738·33)	588·32<br>(562·7 to 613·33)	520·25<br>(496·47 to 543·7)	448·86<br>(399·27 to 501·78)
Ireland	1352·94<br>(1266·12 to 1452·45)	1201·41<br>(1113·79 to 1297·58)	1199·73<br>(1119·24 to 1287·67)	1151·68<br>(1068·89 to 1251·05)	1001·86<br>(930·18 to 1078·45)	861·15<br>(799·58 to 937·09)	581·35<br>(533·63 to 628·98)	514·51<br>(466·41 to 567·22)
Israel	844·62<br>(788·73 to 906·69)	907·29<br>(842·96 to 983·9)	1013·42<br>(935·42 to 1096·66)	912·4<br>(839 to 990·95)	723·61<br>(656·28 to 802·27)	655·77<br>(590·55 to 727·14)	502·26<br>(462·36 to 547·57)	456·14<br>(399·01 to 521·96)
Italy	1254·19<br>(1176·02 to 1344·27)	1145·5<br>(1063·18 to 1243·97)	1084·49<br>(1007·91 to 1171·32)	1032·7<br>(962·26 to 1116·83)	832·18<br>(776·4 to 893·79)	681·44<br>(633·99 to 735·89)	544·55<br>(539·83 to 549·78)	483·85<br>(446·8 to 526·35)
Luxembourg	1460·12<br>(1354·5 to 1579·63)	1312·93<br>(1220·25 to 1414·93)	1340·19<br>(1254·05 to 1442·28)	1169·5<br>(1081·39 to 1268·16)	994·72<br>(920·61 to 1077·82)	776·13<br>(717·63 to 844·67)	583·73<br>(559·82 to 608·96)	475·29<br>(418·57 to 541·47)
Malta	1370·34<br>(1268·58 to 1491·68)	1276·39<br>(1163·9 to 1395·55)	1200·81<br>(1091·39 to 1322·92)	1129·63<br>(1021·2 to 1244·39)	864·83<br>(779·33 to 965·38)	698·66<br>(630·84 to 776·3)	600·16<br>(551·39 to 655·72)	502·86<br>(435·25 to 581·16)
Monaco	1359·53<br>(1207·3 to 1528·71)	1214·91<br>(1067·43 to 1370·66)	1039·12<br>(913·02 to 1172·74)	923·67<br>(810·62 to 1043·17)	773·88<br>(643·5 to 892·22)	701·2<br>(573·64 to 819·1)	657·23<br>(555·91 to 764·32)	606·44<br>(510·59 to 700·84)
Netherlands	1024·04<br>(1012·23 to 1035·86)	967·28<br>(957·7 to 976·59)	1010·4<br>(1000·35 to 1020·21)	925·44<br>(915·6 to 935·04)	863·72<br>(853·99 to 873·23)	764·97<br>(756·86 to 774·51)	583·46<br>(576·14 to 590·81)	526·51<br>(484·88 to 578·01)
Norway	973·39<br>(907·04 to 1045·52)	957·16<br>(894·29 to 1023·95)	981·4<br>(916·16 to 1052·48)	896·1<br>(838·03 to 956·04)	851·36<br>(794·13 to 914·26)	723·15<br>(711·96 to 734·26)	564·13<br>(555·51 to 573·81)	470·91<br>(433·31 to 512·11)
Portugal	1637·55<br>(1525·27 to 1757·89)	1378·06<br>(1285·79 to 1474·91)	1357·44<br>(1267·11 to 1461·04)	1161·24<br>(1081·06 to 1253·58)	1027·72<br>(955·27 to 1112·22)	852·81<br>(792·6 to 919·63)	649·07<br>(605·64 to 698·47)	572<br>(509·01 to 646·72)
San Marino	1102·21<br>(822·52 to 1384·18)	1083·11<br>(879·22 to 1296·51)	1023·66<br>(901·76 to 1172·1)	879·74<br>(774·1 to 997·08)	664·42<br>(550·33 to 804·96)	574·45<br>(482·55 to 688·94)	518·87<br>(371·84 to 666·81)	512·04<br>(354·03 to 692·26)
Spain	1554·97<br>(1455·32 to 1670·68)	1173·71<br>(1094·12 to 1254·05)	1094·2<br>(1021·04 to 1170·13)	896·3<br>(834·57 to 964·47)	842·24<br>(782·45 to 902·92)	700·19<br>(694·28 to 706·31)	558·52<br>(553·83 to 563·46)	498·81<br>(458·3 to 546·91)
Sweden	1073·25<br>(1002·32 to 1152·35)	979·52<br>(916·45 to 1049·99)	922·87<br>(912·49 to 932·63)	902·79<br>(893·01 to 912·75)	775·98<br>(766·73 to 785·32)	643·56<br>(635·05 to 651·96)	523·9<br>(517·16 to 530·93)	470·86<br>(431·19 to 515·78)
Stockholm	1128·57<br>(873·13 to 1449·5)	1048·5<br>(770·42 to 1356·89)	997·87<br>(781·5 to 1268·48)	1011·44<br>(978·92 to 1043·53)	866·73<br>(838·41 to 897·38)	678·05<br>(655·33 to 701·52)	547·81<br>(528·15 to 568·21)	477·38<br>(391·1 to 570·82)
Sweden except Stockholm	1065·59<br>(985·89 to 1156·84)	970·2<br>(896·93 to 1052·33)	910·99<br>(868·7 to 947·64)	885·15<br>(874·31 to 896·03)	759·91<br>(749·6 to 769·9)	637·08<br>(627·68 to 646·5)	519·76<br>(512·19 to 528·16)	470·82<br>(426·63 to 517·87)
Switzerland	1247·77<br>(1166·06 to 1335·6)	1126·27<br>(1051·29 to 1209·05)	1059·44<br>(993·67 to 1137·84)	909·95<br>(847·78 to 973·19)	789·64<br>(739·27 to 844·03)	634·27<br>(594·52 to 679·22)	490·33<br>(482·65 to 497·86)	427·93<br>(391·39 to 469·99)
UK	1369·12<br>(1293·41 to 1449·49)	1256·13<br>(1181·85 to 1338·39)	1210·2<br>(1137·22 to 1284·19)	1080·45<br>(1018·23 to 1153·32)	916·91<br>(858·84 to 978·21)	749·37<br>(703·85 to 798·64)	576·45<br>(540·48 to 615·62)	551·83<br>(500·13 to 612·89)
England	1343·91<br>(1255·41 to 1436·82)	1229·22<br>(1143·1 to 1324·07)	1186·43<br>(1101·68 to 1270·34)	1060·41<br>(990·77 to 1147·63)	900·97<br>(835·27 to 973·49)	734·35<br>(683·62 to 793·22)	562·93<br>(522·93 to 609·31)	538·6<br>(478·42 to 611·73)
East Midlands	1351·16<br>(1082·32 to 1648·6)	1241·06<br>(965·48 to 1565·79)	1189·81<br>(936·98 to 1458·55)	1058·68<br>(938·81 to 1195·14)	894·73<br>(806·53 to 992·96)	744·04<br>(670·08 to 825·62)	575·48<br>(514·7 to 640·05)	553·03<br>(454·42 to 677·41)
Derby	1442·21<br>(1061·09 to 1879·99)	1314·11<br>(936·66 to 1851·13)	1259·59<br>(941·47 to 1648·65)	1122<br>(958·21 to 1311·71)	953·9<br>(838·42 to 1095·81)	752·17<br>(660·09 to 860·77)	644·53<br>(563·37 to 744·87)	562·82<br>(433·79 to 714·9)
Derbyshire	1359·42<br>(1017·36 to 1754·65)	1257·67<br>(915·42 to 1716·9)	1211<br>(905·5 to 1548·21)	1078·08<br>(940·3 to 1244·15)	918·6<br>(807·33 to 1040·06)	740·41<br>(651·97 to 840·26)	561·24<br>(492·38 to 640·4)	548·28<br>(436·19 to 693·08)
Leicester	1486·84<br>(1114·29 to 1926·27)	1366·28<br>(964·11 to 1868·98)	1303·27<br>(957·6 to 1685·23)	1130·74<br>(962·38 to 1308·74)	1002·33<br>(870·09 to 1148·6)	851·31<br>(739·17 to 967·48)	667·52<br>(579·85 to 765·32)	612·18<br>(472·26 to 768·55)
Leicestershire	1227·53<br>(891·86 to 1634·26)	1136·59<br>(799·99 to 1575·44)	1086·48<br>(804·13 to 1421)	985·02<br>(839·52 to 1149·45)	818·14<br>(717·88 to 939·08)	665·56<br>(584·46 to 762·78)	539·53<br>(473·01 to 623·23)	487·56<br>(382·53 to 626·5)
Lincolnshire	1325·76<br>(987·5 to 1719·32)	1219·2<br>(877·41 to 1662·64)	1163·76<br>(868 to 1489·31)	1036·45<br>(903·08 to 1202·74)	873·55<br>(764·13 to 986·3)	736·01<br>(643·41 to 835·75)	558·72<br>(486·58 to 634·01)	585·82<br>(464·29 to 755·72)
Northamptonshire	1301·02<br>(958·07 to 1678·07)	1191·12<br>(841·68 to 1615·08)	1130·4<br>(847·27 to 1480·94)	1013·81<br>(872·74 to 1172·49)	871·54<br>(769·3 to 996·59)	732·25<br>(643·47 to 835·76)	581·86<br>(510·96 to 669·52)	552·04<br>(432·63 to 702·47)
Nottingham	1502·16<br>(1108·33 to 1950·17)	1402·03<br>(1002·97 to 1938·42)	1361·34<br>(1018·95 to 1778·3)	1207·45<br>(1024·44 to 1416·53)	1055·65<br>(918·73 to 1209·78)	945·8<br>(826·21 to 1084·56)	686·52<br>(597·21 to 790·94)	646·92<br>(506·82 to 835·74)
Nottinghamshire	1334·55<br>(1001·65 to 1731·23)	1228·1<br>(859·43 to 1660·51)	1173·7<br>(872·86 to 1546·81)	1052·25<br>(910·7 to 1212·33)	864·65<br>(763·41 to 980·56)	743·56<br>(658·52 to 840·51)	565·45<br>(498·17 to 638·4)	550·19<br>(433·29 to 690·27)
Rutland	1181·23<br>(855·3 to 1598·89)	1063·12<br>(729·63 to 1483·17)	999·82<br>(720·02 to 1349·82)	873·58<br>(726·24 to 1045·24)	768·17<br>(655·81 to 895·39)	613·57<br>(520·23 to 725·15)	478·56<br>(404·08 to 564·16)	429·13<br>(318·87 to 567·73)
East of England	1203·47<br>(955·57 to 1483·3)	1108·52<br>(865·88 to 1401·79)	1067·89<br>(845·91 to 1315·23)	981·75<br>(875·53 to 1112·36)	830·73<br>(751·42 to 924)	686·13<br>(618·78 to 757·86)	530·87<br>(478·21 to 591·52)	508·96<br>(418·58 to 624·17)
Bedford	1301·61<br>(966·07 to 1744·48)	1193·63<br>(837·33 to 1652·36)	1150·26<br>(859·86 to 1535·08)	1019·35<br>(865·67 to 1202·07)	845·1<br>(732·37 to 971·06)	750·72<br>(647·95 to 862·54)	583·58<br>(507·05 to 673·66)	571·71<br>(436·73 to 744·72)
Cambridgeshire	1149·52<br>(843·87 to 1531·86)	1074·25<br>(741·25 to 1472·05)	1043·92<br>(785·2 to 1392·94)	985·31<br>(841·01 to 1146·32)	817·39<br>(714·18 to 932·65)	659·33<br>(576·57 to 753·13)	500·42<br>(438·54 to 575·15)	479·02<br>(369·14 to 618·84)
Central Bedfordshire	1213·14<br>(881·83 to 1621·92)	1099·91<br>(785·33 to 1516·42)	1048·32<br>(787·42 to 1390·17)	924·21<br>(787·62 to 1075·91)	781·01<br>(682·36 to 892·04)	646·04<br>(563·29 to 738·1)	503·27<br>(436·1 to 580·88)	439·31<br>(334·55 to 567·39)
Essex	1185·06<br>(870·61 to 1534·31)	1108·36<br>(802·26 to 1495·87)	1073·62<br>(803·53 to 1404·27)	1024·05<br>(886·59 to 1181·35)	824·12<br>(725·04 to 935·35)	681·55<br>(597·83 to 765·58)	536·27<br>(468·45 to 607·8)	504·03<br>(396·74 to 639·93)
Hertfordshire	1160·5<br>(852·39 to 1506·1)	1087·55<br>(766·08 to 1462·36)	1058·04<br>(787·22 to 1372·59)	1003·89<br>(867·32 to 1163·63)	831·11<br>(732·16 to 944·27)	689·06<br>(609·68 to 782·65)	517·81<br>(457·55 to 587·93)	475·89<br>(373·39 to 605·11)
Luton	1335·74<br>(979·07 to 1768·15)	1210·55<br>(861·53 to 1661·59)	1149·15<br>(849·28 to 1500·01)	1015·84<br>(867·83 to 1197·61)	944·11<br>(822·41 to 1072·4)	790·48<br>(688·5 to 908·98)	584·95<br>(508·63 to 669·55)	610·66<br>(475·64 to 778·24)
Norfolk	1208·54<br>(894·31 to 1584·77)	1104·34<br>(773·83 to 1528·07)	1048·06<br>(785·58 to 1365·99)	941·29<br>(814·92 to 1100·02)	836·81<br>(737·97 to 943·66)	680·11<br>(598·75 to 770·4)	547·83<br>(481·85 to 622·5)	545·25<br>(429·84 to 681·11)
Peterborough	1357·52<br>(989·85 to 1817·09)	1250·52<br>(870·6 to 1716·55)	1219·64<br>(905·56 to 1572·08)	1014·21<br>(855·54 to 1200·47)	910·7<br>(797·79 to 1042·88)	761·86<br>(661·74 to 876·21)	619·3<br>(541·91 to 714·11)	601·84<br>(470·36 to 776·09)
Southend-on-Sea	1314·4<br>(966·91 to 1741·39)	1179·73<br>(826·7 to 1638·01)	1111·94<br>(811·14 to 1468·51)	965·93<br>(809·5 to 1137·03)	861<br>(746·56 to 997·69)	751·68<br>(650·22 to 861·83)	559·61<br>(483·86 to 642·67)	561·94<br>(437·09 to 723·1)
Suffolk	1194·38<br>(875·43 to 1576·94)	1091·32<br>(764·22 to 1507·89)	1034<br>(766·06 to 1345·55)	925·83<br>(799·18 to 1069·73)	806·48<br>(711·95 to 911·03)	672·61<br>(593·57 to 760·56)	506·74<br>(445·02 to 572·57)	517·39<br>(404·31 to 654·68)
Thurrock	1338·2<br>(967·97 to 1777·73)	1247·7<br>(872·45 to 1722·93)	1214·28<br>(892·01 to 1590·45)	1085·48<br>(917·61 to 1274·66)	902·94<br>(783·78 to 1035·1)	696·1<br>(602·1 to 795·47)	544·17<br>(467·85 to 617·39)	496·31<br>(384·22 to 633·95)
Greater London	1318·48<br>(1074·98 to 1593·86)	1216·95<br>(945·14 to 1518·99)	1178·56<br>(951·3 to 1430·89)	1051·65<br>(934·98 to 1186·05)	903·39<br>(811·72 to 999·92)	738·01<br>(665·84 to 818·89)	534·73<br>(480·42 to 597·88)	488·04<br>(398·19 to 590·61)
Barking and Dagenham	1468·08<br>(1079·41 to 1877·55)	1356·19<br>(976·66 to 1826·71)	1314·67<br>(994·94 to 1718·83)	1165·17<br>(997·17 to 1355·19)	977·19<br>(850·85 to 1116·74)	823·16<br>(719·52 to 938·52)	674·69<br>(583·18 to 768·94)	658·1<br>(503·32 to 841·27)
Barnet	1173·17<br>(858·35 to 1528·82)	1070·77<br>(754·72 to 1450·04)	1017·82<br>(765·56 to 1344·31)	894·13<br>(760·03 to 1036·2)	777·92<br>(676·74 to 891·39)	623·94<br>(548·01 to 714·99)	480·09<br>(417·72 to 553·92)	433·14<br>(337·5 to 549·17)
Bexley	1200·42<br>(909·2 to 1558·93)	1095·23<br>(764·49 to 1472·38)	1051·12<br>(768·33 to 1371·36)	926·38<br>(785·34 to 1077·45)	768·47<br>(662·17 to 878·68)	634·6<br>(551·26 to 721·73)	546·02<br>(469·5 to 629·84)	457·91<br>(354·93 to 584·65)
Brent	1324·39<br>(990·81 to 1749·5)	1213·51<br>(867·32 to 1648·83)	1153·17<br>(865·04 to 1478·39)	1023·22<br>(869·93 to 1187·16)	854·73<br>(746·46 to 986·33)	735·31<br>(643·04 to 839·51)	520·66<br>(452·65 to 598·06)	523·26<br>(404·68 to 676·14)
Bromley	1159·31<br>(865·51 to 1533·6)	1056·26<br>(742·31 to 1457·13)	1007·27<br>(767·11 to 1337·78)	900·29<br>(773·57 to 1046·35)	806·43<br>(701·54 to 925)	629·22<br>(553·48 to 718·58)	452·88<br>(394·07 to 522·13)	424·73<br>(331·14 to 540·19)
Camden	1376·56<br>(1032·4 to 1756·29)	1323·56<br>(939·84 to 1800·59)	1321·79<br>(1012·55 to 1724·47)	1238·36<br>(1064·06 to 1430·22)	957·76<br>(832·46 to 1094·73)	977·56<br>(857·14 to 1109·72)	592·91<br>(512·84 to 677·03)	476·27<br>(365·67 to 601·27)
Croydon	1224·47<br>(893·36 to 1588·7)	1112·31<br>(797·56 to 1546·35)	1054·8<br>(793·99 to 1398·75)	950·58<br>(813·84 to 1107·2)	843·72<br>(734·06 to 974·82)	678·4<br>(592·92 to 781·01)	533·6<br>(464·52 to 618·15)	512·16<br>(399·25 to 648·68)
Ealing	1347·76<br>(1009·68 to 1757·34)	1254·82<br>(886·82 to 1723·18)	1224·41<br>(934·69 to 1595·56)	1089·28<br>(939·5 to 1261·58)	904·18<br>(791·53 to 1035·17)	748·33<br>(655·43 to 847·22)	520·23<br>(453·78 to 595·74)	482·02<br>(374·41 to 611·98)
Enfield	1261·1<br>(946·23 to 1636·96)	1153·32<br>(826·43 to 1561·01)	1102·8<br>(821·94 to 1433·62)	999·43<br>(858·2 to 1155·75)	834·07<br>(722·24 to 948·52)	685·92<br>(597·92 to 778·83)	485·55<br>(424·93 to 563·71)	519·02<br>(398·07 to 655·23)
Greenwich	1386·68<br>(1045·6 to 1796·63)	1273·4<br>(911·51 to 1685·29)	1216·3<br>(923·43 to 1576·43)	1080·56<br>(929·79 to 1259·91)	938·38<br>(816·58 to 1076·53)	827·13<br>(717·51 to 947·21)	601·84<br>(521·05 to 696·15)	533·05<br>(416·63 to 680·85)
Hackney	1401·63<br>(1019·67 to 1834·03)	1320·78<br>(945·93 to 1844·42)	1326·9<br>(1018·72 to 1745·33)	1246·03<br>(1069·07 to 1435·68)	1028·14<br>(894·67 to 1164·95)	802·84<br>(701·46 to 912·1)	600·93<br>(529·31 to 687·84)	602·9<br>(473·63 to 767·41)
Hammersmith and Fulham	1419·4<br>(1038·94 to 1850·83)	1345·58<br>(963·61 to 1824·18)	1328·97<br>(999·15 to 1724·55)	1255·39<br>(1076·1 to 1479·01)	1089·59<br>(948·25 to 1255·76)	873·72<br>(763·19 to 1004·9)	603·13<br>(519·16 to 698·92)	498·08<br>(378·99 to 643·51)
Haringey	1353·44<br>(978·58 to 1759·91)	1261·65<br>(903·13 to 1700·67)	1222·51<br>(926·79 to 1574·79)	1102·48<br>(945·36 to 1275·88)	899·5<br>(785·71 to 1023·64)	837·29<br>(735·76 to 955·01)	634·25<br>(552·03 to 727·21)	501·56<br>(384·21 to 644·66)
Harrow	1167·82<br>(872·08 to 1532·18)	1058·08<br>(750·69 to 1467·24)	1003·05<br>(762·11 to 1348·5)	881·43<br>(749·18 to 1036·45)	769<br>(665·26 to 879·2)	614·73<br>(537·88 to 709·07)	402·75<br>(345·97 to 463·32)	402·26<br>(308·13 to 514·92)
Havering	1209·84<br>(884·81 to 1579·75)	1103·44<br>(774·55 to 1516·82)	1051·01<br>(788·98 to 1392)	944·2<br>(818·74 to 1094·87)	808·77<br>(710·04 to 928·37)	642·5<br>(564·6 to 732·99)	519·77<br>(452·25 to 596·17)	513·99<br>(400·37 to 657·65)
Hillingdon	1223·97<br>(912·51 to 1619·34)	1127·14<br>(805·9 to 1549·52)	1094·77<br>(825·92 to 1434·84)	985·51<br>(840·01 to 1140·62)	816·36<br>(710·71 to 929·49)	720·59<br>(633·28 to 820·01)	527·87<br>(458·82 to 608·31)	468·33<br>(367 to 591·39)
Hounslow	1288·05<br>(967·02 to 1688·15)	1185·95<br>(843·01 to 1613·33)	1140·94<br>(859·77 to 1472·07)	1014·28<br>(869·29 to 1178·42)	897·8<br>(779·65 to 1023·7)	769·22<br>(672·25 to 873·11)	536·43<br>(463·69 to 617·1)	485·26<br>(373·68 to 612·76)
Islington	1397·52<br>(1041·47 to 1781·22)	1307·69<br>(923·3 to 1781·26)	1284·91<br>(957·26 to 1653·23)	1165·61<br>(999·42 to 1353·88)	1028·51<br>(893·85 to 1172·82)	967·91<br>(847·46 to 1103·57)	685·43<br>(591·42 to 794·38)	555·26<br>(422·78 to 699·49)
Kensington and Chelsea	1307·49<br>(975·19 to 1707·04)	1269·83<br>(916·89 to 1705·68)	1263·53<br>(963·57 to 1636·03)	1183·48<br>(1011·23 to 1383·26)	1064·94<br>(929·72 to 1221·07)	675·6<br>(589·25 to 773·34)	463·51<br>(400·37 to 535·49)	437·79<br>(336·25 to 555·37)
Kingston upon Thames	1193·62<br>(878·1 to 1581·05)	1103·56<br>(783·4 to 1524·53)	1063·65<br>(791·27 to 1392·44)	954·57<br>(807·47 to 1116·39)	803·02<br>(702·98 to 914·16)	622·45<br>(544·4 to 715·59)	431·13<br>(375·57 to 501·26)	405·77<br>(309·84 to 522·35)
Lambeth	1519·55<br>(1137·64 to 1938·99)	1432·37<br>(1035·64 to 1929·64)	1413·6<br>(1071·36 to 1804·03)	1196·74<br>(1040·25 to 1390·15)	1034·82<br>(906·8 to 1181·36)	833·59<br>(736·97 to 954·25)	657·1<br>(575·7 to 755·74)	545·63<br>(415·42 to 699)
Lewisham	1389·99<br>(1038·2 to 1829·04)	1274·42<br>(926·37 to 1727·55)	1221·1<br>(902·07 to 1591·78)	1087·88<br>(937·99 to 1265·91)	951·38<br>(828·68 to 1078·61)	851·66<br>(744·25 to 967·42)	611·21<br>(526·88 to 700·07)	578·74<br>(452·08 to 728·34)
Merton	1239·8<br>(918·47 to 1647·41)	1130·77<br>(813·62 to 1525·25)	1078·36<br>(804·73 to 1419·19)	951·49<br>(812·79 to 1127·09)	846·91<br>(733·24 to 968·89)	645·33<br>(566·25 to 732·32)	491·21<br>(427·54 to 565·6)	473·9<br>(362·89 to 615·12)
Newham	1438·94<br>(1065·01 to 1859·49)	1321·08<br>(952 to 1785·57)	1268·84<br>(950·98 to 1609·85)	1133·43<br>(976·59 to 1309·2)	1026·79<br>(895·77 to 1177·95)	918·98<br>(806·2 to 1054·65)	647·74<br>(563·35 to 742·1)	561·82<br>(433·37 to 710·84)
Redbridge	1222·95<br>(895·47 to 1592·6)	1126·68<br>(795·06 to 1517·97)	1080·71<br>(781·2 to 1431·93)	953·45<br>(810·88 to 1110·2)	817·3<br>(709·83 to 932·28)	648·62<br>(566·84 to 740·93)	495·41<br>(426·64 to 570·47)	440·99<br>(342·84 to 559·85)
Richmond upon Thames	1200·18<br>(877·25 to 1556·5)	1126·39<br>(799·74 to 1507·8)	1101·93<br>(830·46 to 1438·18)	1001·19<br>(851·95 to 1176·31)	783·14<br>(680·93 to 897·58)	574·24<br>(497·36 to 657·66)	406·69<br>(352·32 to 471·91)	378·26<br>(296·4 to 487·74)
Southwark	1426·74<br>(1074·81 to 1839·39)	1322·15<br>(931·35 to 1788·49)	1292·85<br>(974·47 to 1659·28)	1137·8<br>(975·81 to 1328·74)	1199·78<br>(1051·57 to 1375·07)	822·11<br>(726·14 to 939·34)	621·94<br>(542·33 to 713·63)	575·31<br>(439·48 to 724·53)
Sutton	1205·74<br>(879·22 to 1597·41)	1081·87<br>(772·46 to 1511·62)	1015·22<br>(758·1 to 1319·28)	884·19<br>(745·78 to 1046·15)	779·52<br>(670·48 to 895·14)	647·37<br>(561·65 to 747·15)	487·78<br>(424·07 to 561·12)	466·36<br>(361·43 to 597·63)

Supplemental Results Table 3b: Age-standardised mortality rates by location, males, 1950, 1960, 1970, 1980, 1990, 2000, 2010, 2019								
Location	1950	1960	1970	1980	1990	2000	2010	2019
Tower Hamlets	1562·45 (1193·39 to 1993·31)	1466·56 (1050·66 to 1950·45)	1483·12 (1131·77 to 1900·66)	1385·3 (1180·12 to 1597·85)	1163·55 (1016·38 to 1323·44)	1000·87 (880·41 to 1140·13)	653·32 (566·09 to 749·39)	567·94 (446·59 to 716·38)
Waltham Forest	1341·61 (985·36 to 1714·33)	1232·14 (889·4 to 1652·16)	1178·75 (895·69 to 1566·14)	1041·07 (894·1 to 1217·93)	869·17 (760·58 to 1006·42)	773·28 (673·51 to 882·58)	581·97 (507·87 to 671·11)	517·01 (396·7 to 655·59)
Wandsworth	1348·42 (1022·91 to 1744·59)	1260·65 (895·14 to 1706·85)	1227·44 (909·58 to 1597·08)	1124·64 (967·9 to 1297·35)	1048·25 (919·28 to 1193·7)	781·91 (680·07 to 897·7)	536·97 (467·88 to 614·6)	437 (335·63 to 566·7)
Westminster	1333·31 (991·86 to 1759·65)	1288·35 (931·01 to 1722·21)	1296·44 (973·22 to 1667·04)	1221·38 (1045·55 to 1411·13)	944·86 (818·01 to 1087·13)	756·21 (660·36 to 861·56)	472·16 (408·92 to 548·87)	459·98 (349·01 to 583·29)
North East England	1506·64 (1224·54 to 1837·23)	1379·48 (1066·93 to 1734·18)	1325·2 (1054·79 to 1635·54)	1170·08 (1034·96 to 1326·26)	1018·58 (913·11 to 1130·37)	820·37 (735·73 to 909·03)	627·43 (562·25 to 697·23)	632·71 (512·31 to 775·88)
County Durham	1501·91 (1132·63 to 1924·44)	1382·44 (983·41 to 1889·37)	1322·54 (1002·97 to 1713·66)	1163·33 (1006·68 to 1350·6)	999·76 (876·6 to 1137·07)	807·44 (709·82 to 916·1)	642·46 (562·36 to 730·9)	617·95 (479·58 to 784·45)
Darlington	1484·39 (1103·81 to 1949·66)	1360·25 (948·7 to 1872·27)	1302·21 (969·69 to 1718·39)	1127·53 (963·17 to 1327·34)	992·62 (868·28 to 1138·72)	820·86 (717·06 to 941·61)	622·67 (537·57 to 713·81)	606·55 (469·89 to 779·78)
Gateshead	1560·18 (1178·93 to 2022·4)	1445·19 (1043·02 to 1980·35)	1406·65 (1065·16 to 1843·4)	1232·33 (1042·67 to 1428·14)	1053·67 (921·98 to 1199·4)	897·68 (773·01 to 1022·05)	623·78 (539·53 to 711·68)	646·33 (499·66 to 815·38)
Hartlepool	1600·92 (1170·93 to 2073·55)	1470·9 (1046·9 to 2000·37)	1409·03 (1054·27 to 1834·87)	1233·93 (1041·14 to 1444·81)	1065·37 (923·4 to 1220·67)	907·55 (785·44 to 1044·06)	667·17 (571·05 to 775·68)	717·27 (547·13 to 929·5)
Middlesbrough	1605·03 (1203·85 to 2113·86)	1475·04 (1044·04 to 1982·11)	1414·62 (1066·97 to 1846·4)	1234·33 (1053·36 to 1435·92)	1048·53 (913·66 to 1199·48)	918·62 (797·41 to 1047·4)	713·38 (616·72 to 821·29)	770·68 (597·87 to 990·6)
Newcastle upon Tyne	1527·8 (1173·73 to 1983·54)	1422·07 (1020·06 to 1892·52)	1375·72 (1033·46 to 1767·03)	1215·17 (1045·49 to 1408·76)	1107·08 (964·7 to 1259·76)	839·22 (731·97 to 956·38)	639·19 (558·97 to 729·47)	624·4 (485·53 to 794·03)
North Tyneside	1457·2 (1082·65 to 1921·4)	1325·57 (956·82 to 1815·27)	1268·03 (942·64 to 1650·92)	1108·94 (957·11 to 1294·72)	981·52 (855·74 to 1124·26)	806·74 (702·16 to 916·35)	627·82 (545·59 to 727·15)	603·97 (456·85 to 770·12)
Northumberland	1420·29 (1075·12 to 1846·84)	1289·51 (895·95 to 1770·7)	1219·36 (924·24 to 1589·2)	1139·23 (978·89 to 1342·27)	917·13 (802·48 to 1050·64)	719·57 (627·14 to 824·12)	555·58 (483·13 to 633·33)	577·38 (451·78 to 742·1)
Redcar and Cleveland	1529·82 (1149·63 to 1988·52)	1406·41 (984·39 to 1956·41)	1344·78 (999·12 to 1756·8)	1170·76 (1009 to 1373)	1035·39 (908·99 to 1187·64)	806·57 (702·55 to 924·09)	595·73 (515·25 to 689·84)	647·41 (502·53 to 837·41)
South Tyneside	1541·64 (1144·13 to 1992·53)	1411·44 (991·2 to 1932·43)	1352·18 (1012·36 to 1768·58)	1182·34 (999·21 to 1385·62)	1045·19 (913 to 1200·01)	863·36 (748·01 to 986·5)	650·99 (556·01 to 750·07)	661·61 (504·56 to 850·99)
Stockton-on-Tees	1460·54 (1059·2 to 1886·16)	1335·46 (945·73 to 1843·67)	1280·7 (949·03 to 1714·65)	1109·84 (943·56 to 1291·64)	1010·16 (883·85 to 1153·01)	762·19 (661·94 to 870·19)	590·73 (512·27 to 675·35)	618·63 (477·19 to 788·12)
Sunderland	1508·45 (1128·12 to 1971·25)	1381·56 (983·44 to 1898·34)	1318·89 (962·53 to 1699·62)	1162·45 (995·48 to 1339·93)	1060·89 (925·27 to 1209·17)	869·44 (762·06 to 986·81)	676·41 (591·1 to 775·09)	687·84 (535·59 to 867·84)
North West England	1483·29 (1221·85 to 1768·25)	1356·2 (1089·66 to 1693·31)	1304·93 (1071·06 to 1584·38)	1150·64 (1036·29 to 1292·31)	998·98 (907·48 to 1117·28)	819·17 (743·07 to 912·55)	632·48 (573·6 to 708·17)	603·65 (502·71 to 730·23)
Blackburn with Darwen	1555·42 (1162·16 to 1975·61)	1423·01 (1050·69 to 1912·84)	1360·55 (1032·36 to 1763·51)	1202·75 (1037·62 to 1402·89)	1097·81 (958·46 to 1252·09)	919·84 (807·47 to 1052·38)	691·76 (607·07 to 804·92)	663·05 (524·84 to 857·39)
Blackpool	1573·97 (1188·63 to 2025·42)	1424·05 (1023·33 to 1895·3)	1353·85 (1033·01 to 1757·2)	1178·87 (1018·18 to 1380·49)	1133·78 (997·74 to 1293·39)	1077·07 (952 to 1238·75)	934·88 (821·79 to 1073·14)	885·62 (693·65 to 1124·45)
Bolton	1530·08 (1146·41 to 1967·97)	1409·1 (1049·38 to 1908·45)	1358·23 (1044·72 to 1733·89)	1184·99 (1013·99 to 1372·99)	1008·76 (898·08 to 1143·82)	850·23 (753·34 to 962·79)	659·4 (583·45 to 753·7)	623·86 (494·95 to 779·15)
Bury	1427·42 (1076·07 to 1848·44)	1310·46 (933·6 to 1788·12)	1255·88 (947·45 to 1633·47)	1096·99 (949·79 to 1272·89)	970·62 (850·24 to 1110·46)	776·11 (681·02 to 894·3)	599·51 (523·53 to 694·5)	559·08 (433·87 to 720·11)
Cheshire East	1299·44 (964·48 to 1689·92)	1193·35 (844·59 to 1637·9)	1150·61 (887·02 to 1485·21)	1018·36 (881·75 to 1172·26)	872·6 (763·87 to 989·43)	706·26 (615·79 to 802·96)	521·07 (456·43 to 595·59)	502·46 (399·52 to 618·13)
Cheshire West and Chester	1330·89 (999·97 to 1731·58)	1224·14 (877·27 to 1679·99)	1182·4 (897·46 to 1552·51)	1063·82 (921·6 to 1231·76)	904·75 (797·41 to 1044·87)	707·53 (623·98 to 806·04)	547·8 (480·9 to 625·79)	533·77 (420·7 to 675·28)
Cumbria	1417·83 (1061·77 to 1800·43)	1291·34 (921·16 to 1706·33)	1235·91 (931·34 to 1608·22)	1090·96 (946·7 to 1261·23)	952·51 (838·1 to 1081·7)	741·39 (652·75 to 841·04)	558·21 (489·01 to 636·17)	592·8 (467·08 to 748·81)
Halton	1484·17 (1101·25 to 1895·66)	1382·66 (1002·6 to 1844·63)	1345·33 (1021·72 to 1737·66)	1197·48 (1022·44 to 1390·68)	1003·39 (869·63 to 1151·3)	881·03 (768·77 to 996·21)	646·97 (555·87 to 745·93)	658·53 (520·31 to 837·52)
Knowsley	1539·67 (1148·24 to 1977·51)	1420·43 (1031·76 to 1934·43)	1377·37 (1052·99 to 1777·43)	1222·86 (1052·67 to 1438·33)	1077·16 (935·96 to 1244·72)	937·27 (814·52 to 1076·94)	716·34 (626·21 to 831·99)	660·2 (517·85 to 845·33)
Lancashire	1450·74 (1116·66 to 1891·83)	1325·58 (987·01 to 1788·61)	1259·75 (947·44 to 1655)	1138·46 (990·06 to 1317·63)	971·84 (860·94 to 1102·52)	787·48 (694·82 to 892·49)	604·6 (532·43 to 686·74)	587·69 (463·04 to 746·74)
Liverpool	1580·4 (1212·91 to 2025·93)	1455·54 (1038·69 to 1969·5)	1409·4 (1054·96 to 1835·63)	1198·46 (1034·42 to 1411·8)	1074·87 (943·43 to 1225·03)	950·91 (831·55 to 1086·43)	737·86 (645·22 to 844·55)	726·68 (578·09 to 922·16)
Manchester	1658·62 (1272·77 to 2144·8)	1532·38 (1096·71 to 2056·58)	1486 (1125·01 to 1900·74)	1297·95 (1120·59 to 1494·72)	1162·74 (1026·61 to 1323·87)	993 (877·42 to 1126·9)	761·03 (671·47 to 867·64)	631·16 (505·45 to 807·16)
Oldham	1550·7 (1194·16 to 1988·98)	1420·91 (1031·52 to 1909·15)	1357·55 (1054·78 to 1750·29)	1202·85 (1037·67 to 1385·36)	1066·22 (939·68 to 1217·46)	857·54 (752·24 to 973·83)	684·17 (595·13 to 786·95)	626·42 (499·67 to 793·24)
Rochdale	1539·99 (1173·68 to 2003·68)	1413 (1005·74 to 1943·17)	1350·67 (1052·13 to 1747·15)	1171·4 (1014·65 to 1362·28)	1038·51 (909·54 to 1185·22)	833·03 (732·94 to 962·82)	696·65 (611·37 to 795·99)	652·46 (504·91 to 824·65)
Salford	1612·79 (1202·28 to 2094·89)	1507·81 (1093·6 to 2057·48)	1479·93 (1126·82 to 1883·49)	1337·73 (1160·22 to 1546·38)	1108·62 (967·98 to 1259·61)	902·5 (792·72 to 1020·83)	732·44 (642·52 to 840·27)	651·7 (508·41 to 818·03)
Sefton	1427·49 (1058·96 to 1876·36)	1297·49 (929·95 to 1766·47)	1245·9 (941·23 to 1632·38)	1097·64 (948·77 to 1277·03)	918·35 (805·76 to 1051·26)	778·04 (683·77 to 890·34)	655·83 (571·73 to 750·62)	630·44 (496·72 to 814·93)
St Helens	1514·8 (1141·58 to 1991·43)	1407·79 (1024·05 to 1905·82)	1373·26 (1040·68 to 1763·18)	1231·61 (1057·97 to 1428·32)	1022·89 (892·59 to 1178·35)	831·04 (727·2 to 955·74)	670·02 (586·81 to 768·19)	677·33 (524·88 to 859·59)
Stockport	1346·69 (1010·28 to 1764·11)	1235·3 (873·49 to 1703·66)	1187·48 (897·55 to 1554·49)	1064·86 (924·1 to 1250·04)	942·03 (826·15 to 1067·98)	710·03 (624·63 to 811·45)	556·21 (483·26 to 640·72)	533·12 (419·97 to 674·53)
Tameside	1514·33 (1168·59 to 1964·54)	1388·25 (1007·78 to 1860·78)	1325·02 (1006·67 to 1703·92)	1163·57 (1005 to 1368·53)	1006·99 (886·51 to 1154·51)	913·16 (802·46 to 1042·38)	688·34 (600·81 to 793·92)	585·77 (458·4 to 748·64)
Trafford	1335·44 (985·1 to 1712·02)	1211·18 (860·47 to 1631·59)	1157·76 (887·61 to 1527·99)	1021·35 (881·68 to 1195·71)	932·67 (813·71 to 1068)	708·19 (619·34 to 807·95)	552·28 (478·29 to 637·83)	492·8 (386·94 to 630·78)
Warrington	1390·99 (1024·23 to 1828·18)	1288·92 (920·34 to 1770·35)	1253·75 (958·13 to 1633·84)	1119·71 (966·99 to 1306·12)	974·04 (850·44 to 1113·85)	760·5 (661·29 to 870·7)	569·16 (494·79 to 653·71)	560·55 (438·39 to 718·58)
Wigan	1497·7 (1143·1 to 1917·36)	1390·77 (1003·53 to 1880·84)	1342·2 (1034·81 to 1739·67)	1202·54 (1044·92 to 1392·17)	1042·8 (911·11 to 1192·16)	860·82 (756·25 to 974·05)	675·97 (592 to 769·3)	626·58 (491·16 to 792·8)
Wirral	1463·82 (1092·29 to 1912·62)	1332·65 (975·41 to 1818·15)	1273·87 (985·3 to 1637·16)	1124·25 (976·21 to 1315·63)	988·27 (873·13 to 1132·92)	808·12 (704·8 to 922·12)	596·88 (523·96 to 686·24)	597·88 (468·48 to 749·89)
South East England	1217·34 (989·85 to 1484·8)	1115·96 (883·83 to 1398·49)	1078·62 (864·18 to 1335·23)	987·64 (887·69 to 1112·48)	824·86 (746·81 to 913·31)	675·34 (610·24 to 744·97)	519·05 (469·24 to 575·65)	495·75 (413·7 to 598·16)
Bracknell Forest	1195·66 (884·88 to 1583·33)	1096·77 (771·75 to 1516·04)	1068·41 (778·5 to 1432·1)	958·93 (815·75 to 1129·21)	821·6 (717·12 to 944·71)	643·28 (558·66 to 740·11)	457·91 (390·12 to 533·69)	454·03 (347·09 to 578·39)
Brighton and Hove	1324·26 (988 to 1742·2)	1218·05 (862·87 to 1669·87)	1167·83 (878·11 to 1538·7)	1035·23 (892·52 to 1215·08)	1024·91 (901·09 to 1175·14)	800·89 (705·76 to 919·32)	628·92 (551·52 to 724·48)	567·21 (441·5 to 710·82)
Buckinghamshire	1118·46 (798·68 to 1463·01)	1033·11 (718·31 to 1420·85)	998·9 (735·54 to 1321·18)	916·29 (791·69 to 1061·54)	797·5 (700·4 to 906·45)	624·05 (551·27 to 706·69)	488·04 (428·83 to 557·96)	448·12 (352·56 to 562·43)
East Sussex	1280·43 (963·54 to 1664·41)	1151·32 (796·26 to 1551·41)	1103·52 (825·85 to 1423·79)	968·18 (833·02 to 1131·88)	776·4 (677·22 to 886·55)	658·63 (577·72 to 751·24)	535·25 (471·25 to 615·71)	529·05 (412·64 to 672·82)
Hampshire	1154·14 (839·77 to 1531·19)	1068·92 (775·87 to 1442·68)	1030·96 (767·88 to 1382·83)	968·47 (839·47 to 1118·43)	801·7 (708·23 to 910·67)	650·16 (571·84 to 734·65)	498·96 (436·9 to 567·61)	476·54 (374·38 to 602·92)
Isle of Wight	1366·82 (1012·93 to 1778·51)	1222·09 (865·08 to 1662·66)	1152·4 (853·94 to 1549·88)	1008·09 (862·73 to 1170·2)	901·29 (789·99 to 1029·74)	712·12 (619·93 to 814·35)	571·14 (497·83 to 658·3)	558·02 (437·38 to 704·31)
Kent	1259·13 (963·02 to 1609·47)	1160·96 (849·77 to 1575·71)	1125·24 (851·57 to 1474·44)	1076·26 (937·85 to 1237·49)	869·54 (773·1 to 977·07)	707·73 (628·87 to 796·51)	543·39 (482·04 to 613·57)	526·18 (413·35 to 665·66)
Medway	1285·47 (934·32 to 1691·22)	1173·94 (826·72 to 1618·31)	1111·83 (829·96 to 1457·35)	999·72 (855·55 to 1170·86)	896·7 (788·6 to 1019·79)	770·99 (678·01 to 875·34)	591·88 (512·91 to 677·53)	583·43 (460·34 to 736·55)
Milton Keynes	1274·74 (924·76 to 1691·53)	1171·96 (848·51 to 1589·36)	1150·73 (834·29 to 1510·35)	1034·66 (882·24 to 1215)	844·5 (740·33 to 962·97)	683·48 (594·5 to 786·25)	530·35 (459·62 to 606·62)	507·07 (393·66 to 633·02)
Oxfordshire	1121·42 (833·46 to 1498·09)	1041·3 (731·04 to 1451·24)	1004·84 (737·55 to 1324·81)	939·16 (814·54 to 1091·19)	781·12 (687·9 to 892·61)	651·91 (574·71 to 747·52)	508·03 (441·22 to 582·77)	457·67 (360·1 to 585·2)
Portsmouth	1373·83 (1021·07 to 1785)	1274·44 (885·59 to 1737·88)	1230·38 (916·55 to 1608·61)	1108·02 (952·43 to 1295·07)	1001·77 (874·01 to 1138·92)	841·2 (733·14 to 959·38)	637·43 (550·27 to 733·42)	569·66 (448·39 to 734·3)
Reading	1279·2 (943·57 to 1677·53)	1198·58 (848·14 to 1616·4)	1187·29 (858·15 to 1575·35)	1080·72 (924·52 to 1280·49)	933·99 (814·84 to 1067·49)	761·78 (663·69 to 869·04)	602·65 (523·69 to 699·5)	601·13 (461·3 to 762·99)
Slough	1308·22 (965·53 to 1719·67)	1206·04 (865·3 to 1645·04)	1170·67 (879·76 to 1552·94)	1053·5 (900·41 to 1228·68)	972·25 (853·06 to 1112·61)	801·33 (694·86 to 916·12)	627·2 (544·05 to 724·65)	586·58 (454·07 to 750·21)
Southampton	1371·76 (990·95 to 1787·63)	1274·74 (914·2 to 1711·15)	1236·3 (911·68 to 1587·68)	1085·65 (937·86 to 1258·45)	948·95 (834·68 to 1078·82)	776·69 (685·93 to 885·61)	582·57 (512·15 to 674·94)	587·66 (459·53 to 749·67)
Surrey	1115·72 (841·54 to 1465·94)	1037·65 (731·63 to 1423·65)	1007·63 (732·56 to 1321·14)	937·28 (818·92 to 1084·43)	772·97 (683·1 to 870·03)	654·38 (576·19 to 738·09)	475·75 (417·71 to 538·42)	450 (354·96 to 569·04)
West Berkshire	1154·82 (839·36 to 1547·13)	1063·63 (751·65 to 1496·78)	1035·21 (761·37 to 1388·64)	933·08 (787·64 to 1097·41)	782·75 (679·22 to 905·76)	620·29 (538·53 to 715·63)	467·59 (403·5 to 539·02)	451·69 (354·44 to 586·33)
West Sussex	1226·62 (929·46 to 1587·1)	1116·67 (784·26 to 1509·91)	1088·43 (808·89 to 1417·9)	980·8 (841·39 to 1129·4)	789·83 (687·11 to 896·45)	651·02 (568·53 to 737·97)	513·84 (447·82 to 585·16)	512·68 (398·45 to 648·17)
Windsor and Maidenhead	1063 (771·8 to 1428·92)	962·27 (672·33 to 1331·25)	907·37 (665·8 to 1217·8)	795·23 (676·44 to 930·34)	750·69 (651·04 to 853·3)	629·3 (547·64 to 721·36)	443·46 (383·32 to 509·8)	426·12 (323·78 to 548·83)
Wokingham	1045·09 (770·91 to 1392·66)	957·97 (673·27 to 1298)	918·36 (658·22 to 1234·23)	815·63 (688·15 to 959·12)	654·76 (571·98 to 745·81)	503·75 (437·5 to 576·73)	390·36 (339·38 to 454·75)	394·2 (305·18 to 503·75)
South West England	1254·28 (1014·6 to 1524·5)	1142·71 (883·79 to 1463·38)	1101·79 (890·48 to 1386·82)	979·47 (873·52 to 1106·51)	829·49 (748·68 to 914·95)	673·09 (607·68 to 744·26)	526·06 (473·83 to 584·34)	513·58 (424·44 to 616·99)
Bath and North East Somerset	1150·7 (859·2 to 1509)	1040·78 (740·7 to 1463·07)	984·61 (718·79 to 1353·6)	855·12 (726·97 to 1018·42)	795·84 (696·34 to 916·12)	632·27 (551·39 to 728·84)	492·54 (423·67 to 564·62)	491·74 (377·36 to 632·46)
Bournemouth	1276·22 (935·91 to 1698·9)	1152·26 (807·93 to 1622·14)	1087·26 (813·02 to 1431·6)	958·7 (811·94 to 1117·55)	927·96 (807·38 to 1057·27)	782·43 (684·6 to 896·9)	624·13 (545·23 to 716·3)	607·68 (473·02 to 767·62)
Bristol, City of	1339·29 (986·48 to 1758·86)	1240·69 (886·22 to 1700·78)	1201·97 (891·61 to 1599·15)	1063·52 (910·92 to 1229·19)	922·47 (805·8 to 1049·94)	764·02 (673·39 to 868·6)	596·63 (516·8 to 687·55)	586·62 (457·62 to 748·11)
Cornwall	1313·72 (985·39 to 1713·05)	1196·88 (840·28 to 1670·41)	1162·19 (878·7 to 1534·95)	1031·36 (883·5 to 1197·83)	844·97 (747·15 to 965·62)	673·08 (590·92 to 766·64)	540·92 (472·71 to 619·51)	536·57 (426·04 to 674·06)
Devon	1235·14 (918·1 to 1626·09)	1131·22 (802·72 to 1526·62)	1096·18 (827·21 to 1453·48)	959·27 (832·37 to 1109·81)	798·62 (702·24 to 900·8)	653·67 (576·49 to 738·76)	508·31 (441·78 to 574·67)	512·25 (403·25 to 639·84)
Dorset	1224·96 (914·29 to 1586·53)	1106 (777·37 to 1508·95)	1054·95 (788·32 to 1427·27)	915·86 (784 to 1074·56)	748·07 (651·31 to 848·32)	612·7 (531·34 to 696·89)	473·31 (413·81 to 538·32)	471·66 (367·37 to 593·31)
Gloucestershire	1201·9 (875·09 to 1580·85)	1105·72 (797·05 to 1520·53)	1064·16 (786·28 to 1400·46)	1005·09 (869·43 to 1169·01)	838·37 (738·11 to 953·75)	668·84 (588·06 to 760·28)	517·92 (455·76 to 588·1)	508·49 (399·42 to 643·64)
North Somerset	1203·08 (886·06 to 1601·16)	1082·41 (759·66 to 1550·12)	1024·26 (755·58 to 1344·42)	892·38 (755·33 to 1042·51)	782·25 (683·03 to 897·61)	648·91 (566·73 to 738·57)	548·94 (476·25 to 638·64)	554·24 (424·36 to 708·68)
Plymouth	1377·47 (997·39 to 1817·38)	1278·24 (910·49 to 1735·97)	1245·13 (962·41 to 1613·13)	1112·79 (961·87 to 1292·68)	922·52 (806·03 to 1038·2)	791·65 (698·4 to 897·81)	606·54 (530·23 to 691·02)	593·1 (471·88 to 737·95)
Poole	1269·66 (935·94 to 1659·81)	1157·94 (811·39 to 1600·61)	1114·86 (833·7 to 1484·99)	993·6 (840·12 to 1182·43)	815·17 (707·51 to 937·25)	639·15 (554·69 to 731·46)	491·31 (424·54 to 564·3)	450·18 (354·63 to 583·46)
Somerset	1229·98 (907·63 to 1624·06)	1121·75 (782·14 to 1562·83)	1082·37 (806·62 to 1431·96)	973·19 (831·68 to 1138·23)	809·16 (707·43 to 918·08)	657·1 (575·22 to 746·96)	521·03 (456·58 to 599·9)	511·9 (399·44 to 651·65)
South Gloucestershire	1164·01 (852 to 1546·9)	1070·49 (751·54 to 1501·07)	1044·91 (766·66 to 1373·29)	923·08 (787·49 to 1082·82)	735·39 (645·65 to 836·74)	634·17 (559·03 to 725·71)	494 (427·6 to 570·45)	426·23 (332·27 to 543·44)
Swindon	1279·14 (952·27 to 1667·09)	1168·63 (813·21 to 1623·48)	1135·54 (858 to 1516·97)	1004·71 (852·02 to 1171·26)	873·37 (763·13 to 998·33)	699·68 (610·48 to 799·95)	521·95 (453·61 to 603·52)	491·83 (385·47 to 625·49)
Torbay	1335·02 (989·82 to 1727·87)	1201·48 (831·01 to 1692·49)	1139·8 (830·93 to 1501·71)	999·3 (854·33 to 1174·15)	893·36 (774·69 to 1020·16)	800·68 (691·23 to 918)	638·59 (552·07 to 736·8)	637·17 (489·18 to 830·58)

Supplemental Results Table 3b: Age-standardised mortality rates by location, males, 1950, 1960, 1970, 1980, 1990, 2000, 2010, 2019

Location	1950	1960	1970	1980	1990	2000	2010	2019
Wiltshire	1194·14 (892·1 to 1597·92)	1092·77 (774·15 to 1512·62)	1040·65 (790·71 to 1361·2)	953·57 (808·26 to 1108·47)	851·42 (740·05 to 971·94)	650·62 (575·45 to 743·18)	499·73 (435·77 to 568·78)	478·24 (369·16 to 611·62)
West Midlands	1394·37 (1130·66 to 1686·29)	1287·43 (1003·04 to 1582)	1243·21 (989·68 to 1516·4)	1121·85 (998·48 to 1260·04)	944·51 (853·23 to 1056·9)	771·35 (702·12 to 858·57)	586·22 (529·7 to 656·68)	569·27 (470·56 to 698·32)
Birmingham	1462·14 (1125·7 to 1866·63)	1348·77 (983·47 to 1778·91)	1295·87 (994·41 to 1702·07)	1190·82 (1032·62 to 1368·17)	1004·22 (887·8 to 1145·61)	835·62 (742·96 to 948·81)	609·57 (536·37 to 695·21)	619·68 (486·48 to 777·81)
Coventry	1430·73 (1061·12 to 1852·23)	1321·75 (939·77 to 1748·37)	1272·07 (960·02 to 1646·9)	1129·29 (974·77 to 1311·38)	981·2 (862·33 to 1131·43)	789·44 (700·08 to 902·34)	641·92 (563·88 to 733·77)	624·25 (490·59 to 792·72)
Dudley	1350·58 (1006·54 to 1790·46)	1239·99 (877·84 to 1692·7)	1180·63 (884·97 to 1543·76)	1074·48 (920·22 to 1254·47)	933·06 (824·42 to 1069·82)	791·96 (698·54 to 912·53)	591·35 (517·07 to 681·07)	551·84 (436·89 to 720·38)
Herefordshire, County of	1300·16 (952·86 to 1736·03)	1177·01 (807·61 to 1605·81)	1119·72 (838·96 to 1495·16)	969·16 (829·96 to 1142·73)	786·28 (679·19 to 906·56)	694·88 (603·35 to 796·44)	528·41 (459·4 to 611·87)	519·34 (403·51 to 676·48)
Sandwell	1525·24 (1135·07 to 1985·34)	1405·24 (1001·99 to 1914·96)	1345·49 (1006·25 to 1749·84)	1181·55 (1029·79 to 1360·53)	1066·11 (934·63 to 1235·49)	883·22 (781·31 to 1020·31)	694·22 (603·87 to 803·83)	602·38 (471·27 to 786·09)
Shropshire	1318·8 (967·27 to 1760·01)	1219·99 (862·8 to 1651·07)	1183·05 (878·27 to 1555·9)	1044·75 (897·14 to 1215·09)	861·87 (759·79 to 987·45)	715·32 (629·46 to 818·45)	525·32 (457·3 to 608·27)	474·84 (364·71 to 610·25)
Solihull	1241·61 (915·99 to 1636·09)	1142·39 (793·86 to 1562·79)	1099·58 (818·29 to 1433·39)	964·77 (830·29 to 1130·41)	785·15 (684·49 to 901·64)	662 (577·13 to 756·24)	475·97 (413·3 to 551·48)	497·25 (382·1 to 649·16)
Staffordshire	1340·78 (988·82 to 1774·91)	1260·64 (890·74 to 1711·87)	1229·12 (905·11 to 1583·58)	1130·52 (977·04 to 1298·42)	949·13 (833·84 to 1079·33)	751·86 (664·37 to 854·01)	558·76 (492·52 to 636·65)	566·9 (451·77 to 715·43)
Stoke-on-Trent	1570·6 (1167·66 to 2057·11)	1464·62 (1028·4 to 1957·75)	1423·2 (1073·32 to 1823·82)	1293·37 (1110·57 to 1493·7)	1075·75 (939·64 to 1237·43)	874·17 (769·92 to 1006·59)	691·58 (606·06 to 798·8)	659·91 (515·78 to 859)
Telford and Wrekin	1415·49 (1054·42 to 1874·95)	1317·71 (930·54 to 1770·69)	1285·64 (951·24 to 1695·95)	1171·5 (1002·04 to 1356·41)	967·75 (848·56 to 1114·33)	746·21 (651·27 to 858·52)	583·53 (504·96 to 670·59)	577·35 (444·54 to 750·45)
Walsall	1471·8 (1091·32 to 1931·53)	1363·12 (957·21 to 1900·7)	1321·62 (998·42 to 1750·06)	1174·34 (1005·71 to 1358·97)	1019·52 (887·97 to 1171·49)	803·28 (704·39 to 920·07)	652·25 (564·25 to 750·75)	611·78 (475·37 to 786·02)
Warwickshire	1291·39 (968·05 to 1681)	1202·84 (841·06 to 1622·55)	1165·34 (862·97 to 1536·95)	1058·42 (908·19 to 1221·05)	869·3 (760·62 to 1004·05)	720·21 (632·99 to 821·58)	551·25 (485·6 to 631·06)	526·97 (413·57 to 673·54)
Wolverhampton	1466·51 (1096·8 to 1915·45)	1357·16 (973·81 to 1851·43)	1310·97 (974·71 to 1711·18)	1156·72 (1003·06 to 1341·91)	987·13 (862·8 to 1138·91)	834·13 (735·6 to 949·02)	673·55 (589·4 to 771·51)	657·24 (512·73 to 832·37)
Worcestershire	1255·39 (922·3 to 1666·38)	1164·6 (816·49 to 1566·65)	1112·19 (838·85 to 1439·2)	1029·31 (888·21 to 1189·62)	876·2 (766·02 to 998·25)	706·53 (622·1 to 801·29)	556·2 (490·07 to 632·3)	546·14 (427·31 to 685·5)
Yorkshire and the Humber	1430·18 (1162·46 to 1747·58)	1312·99 (1025·48 to 1649·06)	1261·7 (1006·24 to 1535·37)	1115·17 (990·93 to 1260·91)	945·19 (852·08 to 1039·14)	755·92 (680·31 to 835·51)	602·32 (542·16 to 669·07)	582·35 (483·08 to 699·2)
Barnsley	1476·07 (1096·84 to 1914·27)	1355·06 (970·38 to 1878·52)	1291·13 (967·3 to 1659·84)	1141·56 (971·69 to 1322·52)	966·11 (835·8 to 1095·15)	788·4 (682·12 to 905·53)	600·76 (523·98 to 680·92)	599·44 (464·51 to 755·82)
Bradford	1519·27 (1119·02 to 1965·5)	1383·18 (997·28 to 1859·61)	1312·27 (986·9 to 1721·47)	1146·43 (996·56 to 1340·03)	996 (868·87 to 1129·36)	822·89 (724·47 to 932·64)	635·15 (558·6 to 719·71)	602·46 (470·52 to 757·25)
Calderdale	1507·71 (1122·36 to 1941·53)	1387·06 (978·46 to 1912·89)	1338·78 (1019·86 to 1728·07)	1183·68 (1020·53 to 1373·64)	1009·7 (881·59 to 1150·84)	758·57 (655·09 to 870·49)	618·24 (536·93 to 710·45)	573·47 (436·74 to 734·84)
Doncaster	1432·59 (1072·88 to 1875·26)	1310·25 (920·33 to 1803·32)	1242·84 (921·1 to 1604·58)	1088·33 (939·12 to 1252·89)	942·93 (826·57 to 1074·18)	804·96 (697·59 to 918·35)	660·63 (576·31 to 752·58)	610·9 (469·9 to 774·52)
East Riding of Yorkshire	1302·89 (957·03 to 1688·25)	1188·97 (834·08 to 1615·85)	1129·37 (850·32 to 1500·61)	988·08 (851·82 to 1145·89)	866·96 (761·98 to 983·44)	709·56 (619·88 to 813·29)	550·49 (482·26 to 626·05)	529·73 (415·59 to 666·01)
Kingston upon Hull, City of	1496·94 (1106·61 to 1921·65)	1369·17 (988·1 to 1840·88)	1289·5 (984·44 to 1696·55)	1131·94 (968·58 to 1313·76)	1001·51 (876·88 to 1131·33)	882·09 (771·09 to 1007·8)	733·97 (641·93 to 840·63)	779·9 (603·55 to 987·24)
Kirklees	1508·56 (1121·46 to 1990·35)	1392·33 (988·95 to 1927·91)	1344·23 (1015·23 to 1741·95)	1207·95 (1038 to 1419·03)	978·63 (862·77 to 1120·61)	769·01 (677·43 to 883·06)	627·05 (547·31 to 726·32)	610·11 (479·76 to 775·98)
Leeds	1428·42 (1067·67 to 1849·67)	1320·11 (934·31 to 1782·55)	1280·43 (940·33 to 1669·19)	1135·95 (988·2 to 1312·02)	928·36 (811·93 to 1050·21)	745·93 (653·04 to 847·28)	612·8 (539·56 to 696·75)	600 (470·52 to 763·2)
North East Lincolnshire	1416·94 (1039·77 to 1859·05)	1279·37 (904·19 to 1775·55)	1195·83 (889·45 to 1558·45)	1037·74 (892·03 to 1212·35)	981·93 (854·95 to 1118·22)	786·98 (683·54 to 903·03)	645·04 (553·79 to 745·81)	631·68 (485·77 to 802·55)
North Lincolnshire	1427·92 (1062·19 to 1859·66)	1320·6 (923·3 to 1836·24)	1273·38 (944·26 to 1646·98)	1124·99 (955·91 to 1312·04)	976·16 (851·32 to 1100·06)	751·64 (650·94 to 861·55)	582·83 (505·3 to 668)	542·46 (421·72 to 696·81)
North Yorkshire	1318·99 (985·38 to 1724·68)	1212·62 (864·86 to 1663·23)	1158·2 (873·03 to 1502·41)	1019·25 (878·41 to 1178·46)	861·64 (753·16 to 975·1)	658·79 (577·27 to 749·63)	520·65 (454·57 to 591·51)	502·67 (391·16 to 632·32)
Rotherham	1465·2 (1108·96 to 1925·86)	1359·28 (963 to 1880·1)	1308·96 (974·46 to 1746·61)	1190·59 (1017·6 to 1382·29)	989·98 (856·84 to 1123·19)	803·53 (700·6 to 911·62)	608·86 (527·84 to 691·62)	644·24 (494·59 to 813·29)
Sheffield	1400·22 (1053·43 to 1822·07)	1302 (910·91 to 1729·96)	1250·64 (932·49 to 1595·73)	1112·37 (955·07 to 1281·57)	957·95 (839·49 to 1081·89)	765·82 (668·72 to 867·53)	603·43 (529·2 to 683·43)	559·33 (439·46 to 695·57)
Wakefield	1506·86 (1127·02 to 1933·02)	1411·52 (1013·36 to 1911·64)	1381·61 (1056·64 to 1781·59)	1238·46 (1078·54 to 1420·56)	996·54 (877·57 to 1127·91)	770·82 (672·66 to 876·42)	643·89 (564·12 to 733·27)	626·61 (492·96 to 787·21)
York	1301·01 (926·65 to 1719·62)	1192·38 (824·28 to 1631·83)	1137·15 (847·79 to 1522·16)	1004·12 (850·38 to 1179·72)	880·11 (765·07 to 1003·95)	667·91 (581·41 to 758·71)	509·76 (442·65 to 581·24)	518·61 (401·83 to 658·32)
Northern Ireland	1263·52 (1176·87 to 1359·31)	1229·41 (1147·37 to 1325·35)	1264·42 (1169·41 to 1369·29)	1247·98 (1154·3 to 1338·45)	1033·74 (956·67 to 1113·34)	804·12 (743·91 to 870·52)	617·39 (571·99 to 665·74)	582·42 (517·93 to 663·27)
Scotland	1609·75 (1498·43 to 1725·6)	1548·21 (1439·03 to 1673)	1423·32 (1315·61 to 1540·12)	1223·06 (1134·62 to 1317·62)	1044·02 (970·52 to 1130·38)	871·97 (809·19 to 943·03)	682·16 (631·66 to 738·4)	660·56 (581·65 to 747·33)
Wales	1480·07 (1362·19 to 1606·62)	1339·37 (1230·48 to 1461·73)	1270·07 (1166·32 to 1386·86)	1112·59 (1032·65 to 1205·8)	926·15 (859·67 to 1000·64)	776·54 (717·25 to 839·26)	606·57 (560·83 to 656·57)	584·39 (518·58 to 663·89)
Latin America and Caribbean	2262·35 (2130·94 to 2400·6)	1830·86 (1744 to 1917·68)	1606·59 (1540·07 to 1676·44)	1277·85 (1228·45 to 1324·98)	1084·1 (1044·96 to 1123·25)	917·53 (884·7 to 949·88)	875·97 (841·52 to 909·75)	763·78 (691·93 to 851·74)
Andean Latin America	2615·27 (2347·17 to 2911·23)	2038·31 (1872·45 to 2217·47)	1913 (1760·33 to 2067·26)	1177·96 (1082·98 to 1279·74)	1024·57 (945·13 to 1107·02)	813·27 (743·03 to 895·46)	747·55 (676·85 to 821·91)	641·23 (524·84 to 767·83)
Bolivia	3335·77 (2956·1 to 3733·98)	2656·26 (2252·61 to 3128·51)	2189·65 (1885·44 to 2512·52)	1757·44 (1533·07 to 2006·24)	1443·16 (1288·43 to 1616·36)	1179·57 (1046·65 to 1328·64)	992·37 (846·57 to 1157·1)	952·79 (748·22 to 1180·93)
Ecuador	1864·23 (1539·18 to 2203·27)	1576·03 (1450·02 to 1726·13)	1411·82 (1280·25 to 1566·9)	1171·73 (1080·93 to 1271·73)	875 (815·61 to 943·06)	778·19 (722·81 to 840·13)	858·95 (761·42 to 961·04)	754·8 (597·01 to 946·9)
Peru	2631·9 (2240·74 to 3090·84)	2045·32 (1805·42 to 2310·76)	2087·04 (1867·7 to 2341·86)	1069·1 (933·71 to 1208·23)	988·57 (863·72 to 1121·07)	734·48 (626·32 to 866·25)	642·04 (544·87 to 751·73)	521·36 (393·31 to 674·23)
Caribbean	1837·58 (1644·39 to 2036·72)	1469·57 (1378·01 to 1568·85)	1369·67 (1296·88 to 1439·32)	1180·87 (1125·86 to 1241·18)	1059·96 (1000·87 to 1134·68)	967·47 (917·97 to 1022·56)	1512·02 (1413·98 to 1627·73)	878·57 (756·72 to 1016·32)
Antigua and Barbuda	2063·36 (1885·62 to 2264·31)	1599·49 (1461·96 to 1748·21)	1322·5 (1217·82 to 1437·84)	1068·77 (920·34 to 1250·81)	1023·99 (940·53 to 1122·64)	851·15 (773·89 to 934·83)	837·39 (757·35 to 926·24)	779·09 (654·55 to 932·3)
The Bahamas	1783·62 (1455·91 to 2142·6)	1529·01 (1275·37 to 1802·13)	1417·17 (1285·49 to 1552·48)	1256·8 (1148·58 to 1374·44)	1143·36 (1043·46 to 1248·46)	1084·86 (985·52 to 1186·31)	944·07 (858·8 to 1041·51)	949·51 (776·75 to 1163·47)
Barbados	1770·26 (1611·49 to 1962·72)	1324·78 (1230·91 to 1428·07)	1301·19 (1202·87 to 1410·05)	1012·65 (935·39 to 1099·12)	914·79 (848·02 to 990·46)	841·6 (778·56 to 916·53)	730·61 (676·88 to 793·01)	760·25 (626·14 to 923·4)
Belize	1919·48 (1713·57 to 2139·94)	1219·69 (1093·07 to 1368·83)	991·47 (901 to 1088·76)	909·89 (829·63 to 1003·9)	810·31 (734·39 to 894·68)	1144·98 (1054·46 to 1241·5)	841·32 (782·18 to 908·6)	842·13 (710·6 to 1011·85)
Bermuda	1630·55 (1470·07 to 1800·45)	1591·36 (1457·93 to 1722·64)	1289·04 (1183·31 to 1392·22)	1136·62 (1051·09 to 1235·69)	1142·91 (1051·64 to 1245·35)	829·39 (762·95 to 903·92)	679·54 (616·79 to 752·85)	628·02 (538·85 to 743·14)
Cuba	1358·81 (1096·16 to 1667·45)	1198·05 (1074·33 to 1338·26)	1159·65 (1085·67 to 1242·78)	947·08 (877·38 to 1026·27)	804·4 (715·83 to 908·48)	720·05 (666·01 to 782·41)	656·89 (606·62 to 713·11)	665·45 (534·85 to 825·45)
Dominica	2341·21 (2119·06 to 2615·75)	1552·11 (1379·66 to 1734·61)	1194·66 (1044·39 to 1360·35)	867·37 (753·48 to 994·42)	1141·38 (1022·23 to 1280·21)	1068·11 (934·71 to 1216·82)	1035·76 (902·9 to 1194·28)	1021·1 (817·65 to 1273·95)
Dominican Republic	1508·62 (1362·1 to 1695·97)	1152·16 (1066·74 to 1246·46)	1135·16 (1018·72 to 1261·07)	960·16 (861·34 to 1068·94)	853·9 (754·84 to 959·94)	858·96 (756·6 to 981·97)	876·72 (725·6 to 1046·9)	927·32 (720·4 to 1196·11)
Grenada	1638·43 (1482·04 to 1808·07)	1365·65 (1215·39 to 1539·87)	1244·25 (1081 to 1439·92)	1089·8 (933·88 to 1279·99)	1307·18 (1186·18 to 1445·42)	1298·92 (1194·17 to 1416·05)	1300·91 (1192·29 to 1429·85)	995·21 (874·35 to 1140·14)
Guyana	2436·43 (2134·6 to 2780·18)	1780·16 (1566·25 to 2017·74)	1670·72 (1451·62 to 1913·73)	1652·21 (1398·57 to 1931·74)	1730·35 (1509·61 to 1966·37)	1524·44 (1310·88 to 1749·44)	1411·34 (1211·03 to 1618·56)	1343·7 (1057·27 to 1696·33)
Haiti	3228·88 (2732·07 to 3736·34)	2703·95 (2202·09 to 3243·05)	2470·02 (2135·05 to 2831·63)	2186·41 (1911·37 to 2483·27)	1891·19 (1625·86 to 2189·29)	1630·89 (1409·48 to 1884·98)	4429·43 (3827·47 to 5097·32)	1324·11 (1015·81 to 1697·18)
Jamaica	1841·1 (1690·52 to 1999·84)	1320·79 (1231·03 to 1421·57)	1045·95 (974·8 to 1122·65)	819·25 (752·02 to 895·56)	735·92 (681·54 to 800·28)	806·88 (746·45 to 872·02)	766·61 (708·99 to 833·93)	727·44 (573·98 to 901·72)
Puerto Rico	1352·03 (1254·59 to 1454·69)	1114·84 (1040·46 to 1194·41)	1173·87 (1090·54 to 1261·66)	964·04 (901·1 to 1029·91)	942·93 (873·62 to 1018·38)	788·67 (735·39 to 850·02)	644·41 (600·54 to 689·62)	585·74 (443·84 to 744·94)
Saint Kitts and Nevis	1527·95 (1263·95 to 1872·09)	1406·01 (1217·34 to 1612·65)	1463·43 (1344·03 to 1595·96)	1411·75 (1284·55 to 1555·7)	1545·25 (1395·83 to 1699·15)	1281·65 (1146·45 to 1420·77)	1128·86 (973·73 to 1294·61)	1121·41 (921·08 to 1390·79)
Saint Lucia	1832·32 (1656·6 to 2037·01)	1505·39 (1371·33 to 1645·53)	1256·36 (1133·19 to 1394·19)	1174·24 (1085·51 to 1268·97)	1263·99 (1164·2 to 1376·88)	1001·9 (919·47 to 1090·03)	850·75 (784·28 to 924)	896·47 (750·2 to 1074·39)
Saint Vincent and the Grenadines	2048·06 (1850·86 to 2273·24)	1441·56 (1307·41 to 1578·18)	1339·73 (1217·53 to 1468·84)	1260·05 (1154·53 to 1382·23)	1112·7 (1016·54 to 1224·15)	1077·52 (989·88 to 1171·34)	927·61 (844·51 to 1017·07)	964·36 (820·28 to 1120·76)
Suriname	1946·81 (1789·19 to 2115·68)	1363·01 (1202·75 to 1532·81)	1305·8 (1180·32 to 1442·34)	1285·5 (1128·42 to 1469·73)	1097·69 (965·38 to 1254·33)	1096·14 (1017·06 to 1186·42)	953·44 (873·93 to 1045·6)	939·53 (767·16 to 1136·69)
Trinidad and Tobago	1865·12 (1731·11 to 2013·48)	1525·56 (1416·45 to 1640·69)	1498·93 (1393·35 to 1617·92)	1385·35 (1296·21 to 1493·66)	1201·62 (1115·41 to 1304·73)	1077·33 (1004·1 to 1164·04)	889·15 (820·13 to 968·88)	836·1 (633·5 to 1081·81)
Virgin Islands	1876·64 (1630·13 to 2146·77)	1751·87 (1576·65 to 1943·63)	1493·64 (1309·94 to 1695·16)	1145·76 (1002·77 to 1299·12)	999·58 (872·47 to 1134·86)	963·5 (851·05 to 1092·53)	1090·68 (992·5 to 1194·82)	1065·66 (897·02 to 1250·32)
Central Latin America	2048·12 (1934·51 to 2168·53)	1635·92 (1563·1 to 1711·97)	1403·46 (1343·97 to 1463·05)	1170·08 (1107·04 to 1234·19)	983·62 (941·64 to 1027·7)	844·62 (808·05 to 882·9)	769·97 (735·77 to 803·89)	748·39 (641·69 to 878·31)
Colombia	1749·43 (1608·19 to 1918·31)	1577·91 (1436·15 to 1746·55)	1107·8 (1015·6 to 1213·64)	1050·77 (889·38 to 1245·42)	953·86 (873·01 to 1043·53)	832·54 (753·11 to 926·24)	666·69 (604·78 to 734·06)	558·99 (435·94 to 706·3)
Costa Rica	1617·64 (1494·89 to 1739·97)	1155·18 (1055·64 to 1269·37)	1114·57 (1022·58 to 1217·98)	815·28 (740·24 to 902·37)	704·76 (634·41 to 782·33)	681·12 (634·81 to 732·2)	620·98 (576·2 to 668·54)	599·3 (457·81 to 764·43)
El Salvador	2275·88 (2044·16 to 2557·93)	1653·78 (1511·81 to 1810·73)	1583·2 (1462·85 to 1717·51)	1273·86 (1101·76 to 1464·26)	1032·19 (950·5 to 1124·87)	949·43 (847·24 to 1063·23)	845·43 (767·28 to 927·5)	844·6 (634·09 to 1094·16)
Guatemala	2503·27 (2256·41 to 2773·69)	2127·95 (1930·78 to 2353·84)	2101·33 (1920·43 to 2301·6)	1593·06 (1467·92 to 1736·96)	1563·62 (1353·1 to 1808·7)	1369·15 (1184·74 to 1557·37)	1151·22 (973·21 to 1341·13)	1021·6 (778·49 to 1312·99)
Honduras	2680·12 (2353·12 to 3049·26)	2126·93 (1900·1 to 2355·16)	1601·91 (1419·59 to 1807·64)	959·27 (837·72 to 1114·99)	1018·53 (872·36 to 1173·28)	935·44 (749·66 to 1181·36)	936·25 (745·39 to 1143·22)	980·99 (864·54 to 1137·36)
Mexico	2200·57 (2009·51 to 2407·44)	1709·72 (1594·92 to 1841·51)	1527·83 (1429·02 to 1639·41)	1230·7 (1151·11 to 1326·16)	979·7 (913·93 to 1054)	808·39 (752·68 to 865·62)	764·22 (714·39 to 818·99)	788·07 (632·25 to 983·32)
Aguascalientes	1973·83 (1664·55 to 2370·74)	1606·79 (1326·79 to 1910·94)	1499·35 (1238·13 to 1778·61)	1187·48 (1072·05 to 1314·36)	956·38 (862·02 to 1054·07)	802·28 (721·96 to 889·52)	703·11 (632·54 to 774·97)	693·4 (532·97 to 909·45)
Baja California	2339·29 (1949·46 to 2750·75)	1830·09 (1531·23 to 2156·1)	1711·18 (1409·69 to 2057·92)	1302·58 (1177·47 to 1442·6)	1233·45 (1115·64 to 1362·46)	1081·5 (973·24 to 1186·9)	897·01 (807·04 to 985·25)	866·47 (662·4 to 1126·61)
Baja California Sur	2178·48 (1814·22 to 2635·87)	1731·59 (1440·16 to 2051·46)	1621·05 (1375·19 to 1903·8)	1233·09 (1113·84 to 1368·98)	962·44 (868·6 to 1066·67)	842·33 (758·83 to 932·04)	749·33 (676·43 to 830·48)	916·61 (697·4 to 1203·78)
Campeche	2045·51 (1692·11 to 2421·53)	1592·18 (1319·13 to 1915·96)	1413·18 (1182·26 to 1673·2)	1141·06 (1025·32 to 1264·62)	908·85 (816·74 to 1006·01)	696·56 (627·07 to 775·23)	688·78 (620·77 to 766·4)	699·12 (532·61 to 910·48)
Chiapas	2210·13 (1858·18 to 2615·28)	1767·49 (1470·7 to 2066·14)	1551·1 (1288·69 to 1858·17)	1231·68 (1107·39 to 1373·96)	938·63 (847·44 to 1046·05)	794·52 (715·12 to 877·24)	723·9 (654·14 to 800·75)	717·86 (545·9 to 942·95)
Chihuahua	2270·46 (1886·1 to 2721·23)	1788·71 (1494·02 to 2113·56)	1612·7 (1339·85 to 1922·41)	1343·31 (1210·58 to 1487·08)	1245·62 (1130·84 to 1381·05)	1044·9 (937·77 to 1154·73)	992·29 (900·85 to 1095·09)	1049·8 (795·36 to 1375·31)
Coahuila	2292·72 (1896·62 to 2719·31)	1803·91 (1528·65 to 2122·14)	1660·33 (1384·12 to 1984·8)	1377·57 (1245·44 to 1513)	1091·85 (983·4 to 1201·83)	870·46 (785·66 to 959·42)	797·74 (722·19 to 879·31)	779·76 (589·23 to 1019·47)
Colima	2697·06 (2273·04 to 3189·19)	2080·88 (1743·03 to 2449·7)	1903·12 (1605·52 to 2242·99)	1463·59 (1315·23 to 1629·22)	1065·48 (959·3 to 1179·22)	801·53 (721·54 to 890·01)	754·81 (680·68 to 831·85)	1011·6 (770·02 to 1322·77)
Durango	2223·03 (1852·3 to 2628·02)	1678·57 (1390·51 to 2022·34)	1452·1 (1193·13 to 1782·96)	1189·76 (1067·82 to 1329·77)	923·65 (829·11 to 1033·02)	738·84 (665·37 to 817·94)	770·78 (696·03 to 854·84)	696·75 (516·69 to 919·09)

Supplemental Results Table 3b: Age-standardised mortality rates by location, males, 1950, 1960, 1970, 1980, 1990, 2000, 2010, 2019

Location	1950	1960	1970	1980	1990	2000	2010	2019
Guanajuato	2334·89 (1949·19 to 2746·54)	1844·43 (1551·95 to 2188·88)	1662·22 (1399·47 to 1970·63)	1219·44 (1105·7 to 1353·65)	1081·24 (984·35 to 1192·46)	839·4 (759·88 to 927·88)	714·8 (648·84 to 787·09)	869·22 (658·02 to 1138·19)
Guerrero	2370·84 (2002·01 to 2814·33)	1964·29 (1650·17 to 2304·51)	1862·44 (1559·34 to 2216·38)	1621·26 (1456·88 to 1803·03)	1162·4 (1056·3 to 1284·37)	1018·24 (926·01 to 1122·99)	1123·15 (1008·32 to 1246·01)	1112·62 (900·42 to 1405·66)
Hidalgo	2408·18 (2011·41 to 2873·14)	1862·6 (1568·04 to 2196·92)	1671·99 (1385·85 to 2017·96)	1422·52 (1285·02 to 1585·95)	907·33 (818·07 to 1002·81)	683·53 (617·39 to 755·87)	673·47 (609·15 to 747·76)	691·29 (525·75 to 913·01)
Jalisco	2046·69 (1709·66 to 2417·92)	1606·45 (1345·37 to 1914·85)	1441·64 (1189·74 to 1732·31)	1110·03 (1009·01 to 1232·88)	995·9 (907·47 to 1107·68)	832·8 (759·26 to 917·8)	763·21 (697·34 to 840·04)	802·38 (610·08 to 1039·12)
México	2102·02 (1752·95 to 2487·53)	1700·8 (1417·25 to 2027·48)	1558·76 (1281·46 to 1867·19)	1292·95 (1095·1 to 1517·99)	1102·38 (1001·62 to 1212·22)	846·92 (770·31 to 927·72)	748·57 (686·14 to 817·42)	767·73 (589·95 to 978·62)
Mexico City	1816·89 (1512·54 to 2175·76)	1416·27 (1174·22 to 1678·96)	1312·1 (1085·5 to 1567·45)	1139·28 (1040·31 to 1252·95)	940·22 (859·4 to 1034·69)	821·01 (749·12 to 903·58)	778·81 (711·93 to 857·34)	772·17 (584·75 to 1018·88)
Michoacán de Ocampo	2271·97 (1897·82 to 2696·99)	1758·91 (1459·82 to 2097·86)	1534·19 (1264·24 to 1873·6)	1250·2 (1127·87 to 1380·85)	883·8 (800·02 to 979·03)	747·14 (672·98 to 826·94)	711·03 (640·71 to 788·48)	771·06 (591·04 to 1008·84)
Morelos	2506·39 (2092·65 to 2939·27)	1895·91 (1588·29 to 2267·08)	1668·6 (1395·19 to 1996·47)	1356·59 (1226·18 to 1511)	968·47 (873·79 to 1073·62)	758·21 (681·87 to 841·72)	713·2 (646·99 to 788·44)	796·06 (607·12 to 1028·43)
Nayarit	2397·72 (1994·49 to 2828·67)	1814·53 (1527·62 to 2163·53)	1533·66 (1271·01 to 1858·08)	1157·09 (1043·58 to 1294·37)	914·51 (821·2 to 1013·74)	691·71 (620·45 to 766·24)	715·54 (645·46 to 788·97)	776·44 (580·92 to 1034·41)
Nuevo León	1803·19 (1495·57 to 2148·96)	1428·64 (1177·94 to 1722·16)	1310·63 (1075·99 to 1592·7)	997·85 (900·97 to 1109·56)	923·18 (839·57 to 1019·21)	786·94 (718·41 to 871·07)	752·97 (688·75 to 825·45)	737·95 (567·3 to 967·18)
Oaxaca	2678·78 (2273·44 to 3147·18)	2118·31 (1792·95 to 2481·53)	1896·79 (1588·84 to 2238·71)	1557·21 (1401·25 to 1715·26)	1051·41 (954·61 to 1162·6)	781·37 (706·41 to 856·91)	724·4 (656·94 to 795·68)	689·34 (525·23 to 902·64)
Puebla	2788 (2365·87 to 3257·94)	2115·26 (1799·26 to 2454·53)	1832·17 (1534·21 to 2175·5)	1469·4 (1333·18 to 1631·91)	1054·49 (959·28 to 1167·81)	867·88 (787·59 to 954·14)	753·61 (686·36 to 832·09)	784·3 (595·21 to 1029·1)
Querétaro	2373·21 (1982·65 to 2858·08)	1855·92 (1565·01 to 2203·34)	1642·03 (1344·37 to 1976·28)	1307·67 (1120·13 to 1513·79)	1082·03 (976·81 to 1191·55)	896·13 (808·68 to 990·03)	743·64 (672·86 to 823·3)	788 (604·37 to 1039·41)
Quintana Roo	1917·21 (1585·06 to 2288·38)	1572·9 (1291·44 to 1885·45)	1432·9 (1184·33 to 1724·68)	1155·53 (1042·95 to 1290·82)	839·82 (757·9 to 934·72)	710·93 (642·74 to 792·66)	745·52 (676·36 to 827·39)	793·46 (605·46 to 1039·84)
San Luis Potosí	2188·63 (1806·37 to 2606·12)	1672·78 (1389·19 to 2000·68)	1490·81 (1231·38 to 1794·49)	976·14 (881·75 to 1076·78)	847·13 (763·05 to 930·27)	697·29 (628·73 to 762·88)	657·9 (596·16 to 724·57)	742·32 (559·56 to 976·66)
Sinaloa	1982·38 (1619·45 to 2353·36)	1563·64 (1282·95 to 1880·37)	1396·97 (1137·9 to 1725·79)	1130·79 (1027·86 to 1249·05)	851·65 (766·77 to 944·21)	729·36 (659·59 to 801·59)	751·46 (680·52 to 831·73)	822·44 (618·74 to 1088·68)
Sonora	1982·5 (1637·89 to 2383·07)	1614·34 (1335·4 to 1941·03)	1520·36 (1227·94 to 1861·56)	1267·48 (1063·26 to 1500·13)	1065·96 (958·76 to 1185·41)	912·33 (819·36 to 1009·1)	860·41 (776·91 to 952·66)	845·76 (638·22 to 1098·34)
Tabasco	2232·74 (1826·43 to 2677·27)	1778·24 (1484·12 to 2114·78)	1627·1 (1367·1 to 1963·18)	1372·49 (1232·56 to 1531·13)	915·4 (825·81 to 1019·97)	763·56 (686·87 to 851·18)	792·44 (717·11 to 875·35)	757·04 (565·89 to 987·45)
Tamaulipas	2035·59 (1702·58 to 2468·45)	1601·46 (1331 to 1894·51)	1432·97 (1182·45 to 1751·73)	1229·39 (1112·29 to 1352·93)	930·3 (844·45 to 1025·27)	735·31 (665·37 to 806·46)	793·28 (720·83 to 870·76)	802·49 (597·56 to 1059·1)
Tlaxcala	2237·39 (1883·1 to 2684·05)	1704·12 (1421·11 to 2033·55)	1482·02 (1216·27 to 1801·2)	1158·99 (988·58 to 1366·88)	918·62 (830·3 to 1025·63)	764·85 (689·66 to 847·7)	671·32 (607·56 to 744·6)	715·52 (543·36 to 948·57)
Veracruz de Ignacio de la Llave	2233·98 (1870·69 to 2670·34)	1704·41 (1434·64 to 1996·29)	1492·28 (1244·31 to 1771·11)	1174·2 (1065·81 to 1301·11)	893·29 (808·25 to 990·49)	776·5 (703·63 to 857·84)	734·05 (666·54 to 811·18)	783·9 (584·44 to 1034·9)
Yucatán	1893·7 (1567·35 to 2265·1)	1599·09 (1350·95 to 1898·54)	1465·32 (1239·04 to 1754·49)	1223·55 (1105·17 to 1352·06)	1010·49 (908·31 to 1115·97)	764·14 (686·15 to 843·12)	743·97 (676·06 to 817·05)	759·27 (566·53 to 982·62)
Zacatecas	1905·71 (1586·1 to 2275·04)	1510·41 (1243·61 to 1813·84)	1352·43 (1104·22 to 1633·61)	1011·91 (915·96 to 1115·77)	806·61 (727·88 to 892·83)	699·29 (628·55 to 774·35)	705·29 (639·27 to 777·26)	783·51 (584·47 to 1025·09)
Nicaragua	2255·14 (2024·59 to 2522·78)	1807·48 (1640·36 to 1999·24)	1673·17 (1506·76 to 1862·17)	1220·44 (1024·55 to 1463·2)	970·1 (878·7 to 1080·78)	906·78 (823·98 to 999·94)	937·86 (843·85 to 1044·24)	913·98 (726·5 to 1123·71)
Panama	1372·83 (1258·93 to 1496·94)	1102·41 (1024·74 to 1189·84)	1052·37 (977·88 to 1139·32)	778·46 (721·34 to 844·91)	691·89 (641·71 to 752·36)	645·71 (600·73 to 696·84)	640·38 (598·13 to 687·94)	565·63 (436·51 to 730·45)
Venezuela	1607·34 (1500·17 to 1727·69)	1277·64 (1185·41 to 1382·09)	1241·69 (1155·55 to 1340·12)	1091·83 (1014·41 to 1173·55)	922·68 (858·36 to 993·12)	903·81 (840·3 to 977·91)	861·27 (800·79 to 925·31)	852·16 (651·43 to 1101·01)
Tropical Latin America	2529·23 (2290·57 to 2802·57)	2109·68 (1927·39 to 2310·26)	1817·88 (1665·76 to 1970·94)	1461·18 (1359·72 to 1568·5)	1219·91 (1134·93 to 1311·61)	1007·02 (939·74 to 1078·53)	886·59 (822·16 to 951·74)	789·78 (698·62 to 901·46)
Brazil	2541·4 (2299·97 to 2817·26)	2141·99 (1957·31 to 2351·24)	1844·27 (1687·03 to 2003·98)	1480·8 (1375·44 to 1591·27)	1235·77 (1146·04 to 1332·27)	1014·61 (944·23 to 1089·42)	890·21 (825·61 to 957·88)	791·45 (699·22 to 905·35)
Acre	2660·46 (2205·82 to 3145·06)	2181·51 (1814·8 to 2582·31)	1835·14 (1526·59 to 2170·2)	1452·22 (1287·17 to 1636·55)	1251·15 (1120·64 to 1407·59)	1009·56 (903·17 to 1118·05)	890·92 (797·41 to 995·25)	885·3 (714·84 to 1084·24)
Alagoas	3143·86 (2664·45 to 3727·44)	2686·19 (2261·93 to 3145·06)	2281·37 (1924·31 to 2679·5)	1811·72 (1614·99 to 2014·32)	1371·86 (1213·42 to 1545·3)	1025·01 (912·23 to 1143·65)	1015·23 (896·47 to 1147·99)	909·13 (712·23 to 1130·37)
Amapá	2789·05 (2285·56 to 3355·58)	2122·52 (1785·06 to 2499·07)	1525·26 (1281·59 to 1808·74)	923·54 (829·29 to 1036·53)	925·31 (829·36 to 1042·27)	979·07 (883·83 to 1084·09)	742·89 (667·81 to 828·97)	772·05 (634·84 to 941·04)
Amazonas	2182·53 (1790·15 to 2672·32)	1787·82 (1464·78 to 2130·99)	1602·34 (1315·87 to 1955·9)	1307·57 (1140·13 to 1489·29)	1014·89 (885·75 to 1156·87)	860·28 (760·51 to 976·79)	762·28 (677·57 to 857·93)	726·63 (584·18 to 893·46)
Bahia	2569·12 (2146·85 to 3027·81)	2163·29 (1809 to 2566·47)	1812·33 (1520·38 to 2161·54)	1345·02 (1168·07 to 1550·47)	1094·1 (947·24 to 1264·19)	928·15 (800·61 to 1068·5)	958·63 (821·8 to 1118·89)	889·7 (694·76 to 1123·31)
Ceará	3428·16 (2912·3 to 4028·99)	2847·77 (2413·1 to 3332·48)	2237·19 (1872·05 to 2622·74)	1563·77 (1298·56 to 1869·9)	1058·89 (890·17 to 1239·31)	926·27 (782·17 to 1080·18)	875·16 (729·03 to 1040·94)	859·93 (648·78 to 1089·86)
Distrito Federal	2326·33 (1931·13 to 2777·89)	2060·44 (1716·52 to 2449·71)	1863·33 (1518·76 to 2242·26)	1674·26 (1456·01 to 1902·24)	1551·62 (1363·06 to 1761·87)	1048·79 (950·02 to 1161·56)	979·55 (882·9 to 1082·62)	795·16 (642·5 to 961·07)
Espírito Santo	2282·81 (1905·53 to 2726·11)	1948·4 (1605·83 to 2366·16)	1702·45 (1414·8 to 2032·42)	1346·51 (1215·39 to 1494·69)	1130·48 (1009·03 to 1264·18)	982·88 (891·82 to 1083·46)	925·79 (829·31 to 1033·43)	846·55 (680·73 to 1039·2)
Goiás	3330·62 (2808·72 to 3953·11)	2811·42 (2352·7 to 3317·3)	2224·52 (1865·37 to 2651·93)	1673·03 (1402·34 to 1984·3)	1339·75 (1156·12 to 1557·52)	970·73 (835·44 to 1123·99)	870·46 (748·2 to 1007·42)	811·09 (634·47 to 1047·32)
Maranhão	3611·93 (3084·88 to 4211·88)	3166·95 (2729·49 to 3660·95)	2758·88 (2343·14 to 3225·55)	2087·4 (1762·59 to 2461·43)	1513·63 (1300·33 to 1785·97)	899·01 (755·35 to 1069·66)	848·39 (704·2 to 1025·75)	990·15 (780·43 to 1239·12)
Mato Grosso	1870·12 (1529·47 to 2255·78)	1666·89 (1353·4 to 2054·34)	1438·54 (1169·53 to 1762·52)	1137·06 (973·25 to 1319·69)	1108·92 (961·85 to 1282·79)	935·01 (808·95 to 1076·99)	824·46 (746·19 to 913·8)	703·49 (568·64 to 864·29)
Mato Grosso do Sul	2234·03 (1830·61 to 2688·59)	1927·84 (1572·04 to 2310·39)	1689·97 (1389·75 to 2050·69)	1392·75 (1241·1 to 1563·3)	1102·56 (979·27 to 1231·09)	992·18 (898·61 to 1095·79)	880·09 (798·16 to 971·87)	731·05 (592·15 to 890·96)
Minas Gerais	2438·55 (2008·46 to 2897·56)	2072·13 (1717·05 to 2462·06)	1790·66 (1477·18 to 2160·67)	1410·92 (1279·65 to 1547·89)	1213·96 (1083·78 to 1345·34)	930·39 (840·55 to 1030·57)	826·77 (742·22 to 917·59)	701·09 (572·92 to 857·17)
Pará	2611·55 (2148·92 to 3138·17)	2116·76 (1755·07 to 2498·21)	1817·83 (1497·26 to 2201·06)	1382·59 (1174·05 to 1622·35)	1132·16 (958·01 to 1315·33)	893·23 (768·55 to 1037·08)	823·19 (699·78 to 960·9)	755·02 (613·51 to 930·4)
Paraíba	2816·11 (2362·04 to 3323·71)	2189·63 (1840·08 to 2609·69)	1719·21 (1429·88 to 2056·88)	1237·1 (1092·86 to 1399·96)	969·67 (855·06 to 1094·85)	856·66 (765·55 to 960·88)	881·86 (775·02 to 992·74)	765·51 (603·49 to 945·3)
Paraná	2132·23 (1748·52 to 2588·74)	1856·5 (1535 to 2214·91)	1658·79 (1378·81 to 2014·13)	1437·44 (1308·14 to 1575·94)	1200·75 (1088·13 to 1320·92)	1011·78 (922·34 to 1109·04)	913·56 (829·69 to 1010·14)	799·47 (640·12 to 989·4)
Pernambuco	3364·98 (2855·3 to 3939·68)	2723·29 (2283·14 to 3195·36)	2138·89 (1827·61 to 2513·05)	1497·69 (1356·17 to 1648·73)	1321·43 (1190·28 to 1466·46)	1091·71 (986·73 to 1208·99)	987·68 (885·95 to 1088·16)	979·54 (794·79 to 1211·51)
Piauí	2672·06 (2237·52 to 3153·87)	2299·22 (1928·67 to 2701·31)	2037·62 (1683·37 to 2409·29)	1483·31 (1268·62 to 1700·37)	1140·39 (1008·38 to 1285·81)	915·53 (815·92 to 1027·11)	757·78 (682·5 to 836·78)	692·43 (565·94 to 847·53)
Rio de Janeiro	2355·58 (1936·43 to 2787·81)	2013·99 (1674·69 to 2397·5)	1855·85 (1526·7 to 2248·1)	1667·27 (1491·4 to 1863·18)	1494·43 (1349·84 to 1663·37)	1178·69 (1073·07 to 1304·03)	998·24 (904·99 to 1099·83)	862·68 (709·96 to 1062·9)
Rio Grande do Norte	2959·86 (2470·26 to 3480·62)	2285·23 (1925·49 to 2717·81)	1776·14 (1461·9 to 2152·02)	1273·59 (1091·76 to 1462·71)	970·31 (837·9 to 1116·72)	816·82 (705·2 to 937·5)	751·91 (640·54 to 872·38)	755·01 (582·07 to 982·24)
Rio Grande do Sul	2136·06 (1755·76 to 2546·6)	1850·36 (1516·44 to 2219·58)	1602·53 (1310·62 to 1940·86)	1335·74 (1198·01 to 1487·9)	1189·32 (1075·32 to 1316·49)	1041·75 (945·24 to 1146·54)	874·11 (793·79 to 961·15)	782·38 (628·94 to 969·09)
Rondônia	3496·25 (2952·82 to 4076·96)	2861·3 (2415·3 to 3352·83)	2354·67 (1993·8 to 2776·49)	1753·86 (1553·48 to 1978·93)	1469·91 (1284·29 to 1665·19)	1105·03 (984·39 to 1235·38)	825·6 (725·73 to 936·42)	785·56 (617·95 to 971·04)
Roraima	3205·07 (2703·56 to 3731·36)	2602·79 (2182·82 to 3098·87)	2150·14 (1833·15 to 2501·33)	1537·33 (1350·59 to 1718·12)	1389·99 (1231·85 to 1563·63)	1057·65 (952·06 to 1175·74)	826·59 (736·79 to 917·68)	834·85 (681·53 to 1012·58)
Santa Catarina	1912·82 (1562·5 to 2327·01)	1674·54 (1361·26 to 2035·87)	1497·83 (1216·39 to 1824·2)	1237·48 (1117·32 to 1367·31)	1210·2 (1078·53 to 1350·05)	1100·47 (988·08 to 1228·77)	859·56 (766·89 to 951)	734·7 (592·08 to 904·54)
São Paulo	2128·45 (1762·24 to 2515·54)	1862·1 (1539·2 to 2228·19)	1712·62 (1401·77 to 2039·09)	1550·21 (1404·03 to 1710·68)	1334·6 (1199·99 to 1488·29)	1122·52 (1031·33 to 1229·21)	888·25 (811·99 to 973·83)	740·9 (609·92 to 904·46)
Sergipe	2507·6 (2057·55 to 2991·31)	2101·77 (1738·42 to 2500·28)	1792·72 (1485·76 to 2160·6)	1480·25 (1303·3 to 1702·87)	1209·7 (1054·07 to 1373·8)	931 (840·39 to 1028·45)	845·59 (736·03 to 981·43)	838·46 (654·8 to 1052·68)
Tocantins	2852·81 (2339·55 to 3403·74)	2414·82 (2001·72 to 2855·59)	1922·82 (1572·84 to 2332·36)	1308·26 (1036·01 to 1632·29)	1149·5 (986·12 to 1322·37)	886·61 (780·74 to 1004·9)	772·24 (663·73 to 891·15)	858·87 (671·06 to 1066·19)
Paraguay	1931·81 (1628·97 to 2272·28)	970·11 (855·09 to 1085·94)	891·96 (788·78 to 996·88)	803·05 (714·41 to 902·58)	704·33 (623·78 to 798·26)	738·72 (649·52 to 837·9)	768·94 (665·84 to 898·19)	756·3 (561·26 to 1004·74)
North Africa and Middle East	2680·81 (2444·31 to 2940)	2217·58 (2035·53 to 2395·82)	1825·47 (1694·43 to 1970·93)	1488·75 (1405·32 to 1585·97)	1260·28 (1191·8 to 1333)	1044·04 (981·63 to 1118·15)	918·8 (859·44 to 982·4)	846·09 (769·46 to 931·51)
North Africa and Middle East	2680·81 (2444·31 to 2940)	2217·58 (2035·53 to 2395·82)	1825·47 (1694·43 to 1970·93)	1488·75 (1405·32 to 1585·97)	1260·28 (1191·8 to 1333)	1044·04 (981·63 to 1118·15)	918·8 (859·44 to 982·4)	846·09 (769·46 to 931·51)
Afghanistan	3066·16 (2334·36 to 4200·81)	2519·46 (2005·14 to 3236·62)	2346·49 (1952·02 to 2869·47)	2816·15 (2248·31 to 3976·53)	1922·15 (1580·51 to 2294·9)	1930·27 (1530·26 to 2475·05)	1561·13 (1140·19 to 2121·57)	1443·24 (1027·46 to 2084·08)
Algeria	1970·76 (1753·63 to 2192·05)	2195·86 (1905·95 to 2505·37)	1869·66 (1605·94 to 2147·61)	1684·24 (1452·14 to 1930·47)	1289·46 (1110·53 to 1492·73)	1049·6 (895·38 to 1237·44)	808·58 (698·26 to 926·51)	729·05 (598·24 to 880·95)
Bahrain	2184·36 (1815·8 to 2616·01)	1938·33 (1633·18 to 2278·7)	1618·3 (1342·04 to 1922·21)	1466·38 (1273·89 to 1681·18)	1389·99 (1235·95 to 1544·7)	1089·07 (957·04 to 1233·51)	957·04 (853·61 to 1065·71)	755·68 (611·32 to 922·97)
Egypt	2674·37 (2387·77 to 2982·71)	2057·54 (1908·74 to 2229·99)	1942·51 (1764·96 to 2135·49)	1592·23 (1451·77 to 1761·04)	1377·82 (1256·69 to 1506·16)	1108·32 (1015·12 to 1211·85)	1075·15 (982·41 to 1184·9)	1016·26 (815·39 to 1261·66)
Iran	3171·7 (2832·33 to 3538·23)	2592·86 (2317·93 to 2903·83)	1884·93 (1690·98 to 2106·21)	1340·69 (1213·71 to 1486·58)	1205·15 (1090·14 to 1331·81)	935·39 (849·67 to 1026·46)	767 (711·3 to 832·24)	658·38 (598·35 to 722·91)
Iraq	1811·38 (1549·91 to 2098·56)	1446·58 (1247·76 to 1656·1)	1287·96 (1088·87 to 1527·93)	1252·35 (1078 to 1437·65)	1235·36 (1038·04 to 1458·05)	1141·41 (899·75 to 1438·62)	1063·95 (785·13 to 1438·89)	985·18 (717·15 to 1334·13)
Jordan	2015·22 (1483·65 to 2715·84)	1753·4 (1402·71 to 2218·65)	1511·55 (1300·32 to 1756·1)	1299·72 (1148·76 to 1469·24)	894·63 (774·36 to 1035·95)	777·63 (644·32 to 926·49)	682·38 (598·38 to 782·29)	597·22 (481·65 to 728·23)
Kuwait	1653·05 (1277·24 to 2089·58)	963·02 (821·53 to 1122·23)	969·2 (900·03 to 1045·38)	871·59 (809·12 to 938·11)	705·44 (631·93 to 778·67)	631·4 (569·05 to 705·12)	528·97 (488·3 to 574·57)	515·67 (418·19 to 640·73)
Lebanon	1785·27 (1457·48 to 2185·87)	1353·34 (1079·11 to 1643·02)	1218·29 (1005·69 to 1445·51)	1167·93 (966·69 to 1423·51)	1181·43 (965·94 to 1475·67)	843·93 (727·34 to 992·53)	877·68 (782·88 to 973·18)	825·71 (690·52 to 982·05)
Libya	2512·7 (2101·03 to 2923·25)	1927·83 (1594·24 to 2303·4)	1143 (1013·67 to 1286·96)	947·94 (841·4 to 1075·42)	844·06 (703·85 to 1006·43)	754·44 (668·33 to 852·97)	693·49 (633·85 to 766·94)	720·81 (581·8 to 923·8)
Morocco	2835·28 (2448·07 to 3258·49)	2471·14 (2139·75 to 2856·99)	1912·13 (1643·73 to 2206·21)	1537·13 (1355·98 to 1750·63)	1182·19 (1030·53 to 1352·02)	1028·78 (875·64 to 1210·8)	865·55 (690·59 to 1056·78)	937·58 (765·03 to 1079·38)
Oman	3579·97 (2485·98 to 5251·92)	2811·04 (2068·16 to 3856·09)	1986·98 (1520·31 to 2600·12)	1649·91 (1293·8 to 2061·95)	1458·47 (1201·79 to 1746·89)	1337·5 (1153·18 to 1551·13)	1233·3 (1149·06 to 1322·62)	1029·8 (905·78 to 1172·16)
Palestine	2203·25 (1706·41 to 2824·35)	1788·71 (1447·44 to 2165·72)	1874·09 (1584·87 to 2207·01)	1344·55 (1055·77 to 1668·97)	1268·22 (1042·75 to 1523·43)	1209·12 (1069·64 to 1366·56)	995·09 (895·69 to 1105·81)	934·08 (813·74 to 1071·9)
Qatar	1470·29 (1018·82 to 2118·49)	1383·68 (1024·21 to 1889·87)	1251·78 (970·21 to 1615·76)	1122·91 (896·93 to 1370·44)	1259·71 (1052·28 to 1511·09)	1076·63 (905·5 to 1289·06)	884·53 (736·92 to 1039·67)	815·12 (664·64 to 1000·89)
Saudi Arabia	1944 (1584·32 to 2371·18)	1932·28 (1562·67 to 2313·64)	1567·79 (1264·54 to 1890·3)	1202·21 (976·38 to 1483·99)	1154·82 (962·01 to 1371·74)	1071·99 (957·79 to 1198·37)	998·98 (906·89 to 1091·8)	821·38 (682·69 to 975·49)
Sudan	2416·27 (2057·55 to 2822·34)	2135·25 (1806·14 to 2526·02)	2039·35 (1744·85 to 2352·55)	1864·22 (1628·38 to 2119·81)	1664·59 (1458·51 to 1887·58)	1413·63 (1161·99 to 1681·34)	1168·45 (935·29 to 1400·24)	1026·75 (839·11 to 1265·55)
Syria	1861·58 (1557·42 to 2210·17)	1412·06 (1169·65 to 1715·51)	1115·6 (961·2 to 1281·97)	923·89 (771·73 to 1114·51)	1082·92 (890·93 to 1294·52)	984·65 (821·28 to 1168·05)	843·82 (687·85 to 1025·66)	911·72 (710·89 to 1174·82)
Tunisia	2879·92 (2454·46 to 3367·06)	2356·87 (2068·56 to 2676·33)	1901·61 (1695·9 to 2139·51)	1393·34 (1243·24 to 1562·66)	935·02 (823·86 to 1057·07)	886·92 (740·21 to 1052·28)	805·84 (614·6 to 1007·92)	725·7 (554·27 to 947·74)

Supplemental Results Table 3b: Age-standardised mortality rates by location, males, 1950, 1960, 1970, 1980, 1990, 2000, 2010, 2019

Location	1950	1960	1970	1980	1990	2000	2010	2019
Turkey	2992·81 (2537·09 to 3487·26)	2282·42 (1936·3 to 2696·27)	1734·06 (1445·51 to 2053·7)	1341·24 (1197·19 to 1507·51)	1125·41 (999·06 to 1254·79)	791·34 (676·35 to 938·22)	739·76 (653·05 to 834·64)	661·97 (531·62 to 803·29)
United Arab Emirates	1690·87 (1283·84 to 2200·16)	1591·78 (1248·15 to 1978·37)	1386·86 (1134·26 to 1703·33)	1248·51 (1027·37 to 1488·1)	1301·42 (1104·97 to 1541·71)	1394·85 (1218·59 to 1602·96)	1025·03 (865·63 to 1197·59)	898·04 (725·81 to 1124·61)
Yemen	4258·27 (2955·45 to 6422·57)	3560·51 (2684·25 to 4957·18)	3037·8 (2398·24 to 3908·29)	2073·68 (1683·96 to 2544·89)	1669·52 (1391·28 to 2001·99)	1416·33 (1159·39 to 1713·98)	1192·63 (972·81 to 1455·93)	1209·06 (959·59 to 1536·18)
South Asia	2783·4 (2422·13 to 3180·97)	2480·26 (2181·15 to 2806·2)	2174·66 (1979·22 to 2386·32)	1816·97 (1694·14 to 1955·52)	1610·26 (1494·5 to 1737·06)	1438·13 (1334·26 to 1552·38)	1187·75 (1097·43 to 1290·67)	993·41 (838·4 to 1152·52)
South Asia	2783·4 (2422·13 to 3180·97)	2480·26 (2181·15 to 2806·2)	2174·66 (1979·22 to 2386·32)	1816·97 (1694·14 to 1955·52)	1610·26 (1494·5 to 1737·06)	1438·13 (1334·26 to 1552·38)	1187·75 (1097·43 to 1290·67)	993·41 (838·4 to 1152·52)
Bangladesh	2946·54 (2502·27 to 3376·41)	2521·57 (2138·78 to 2939·48)	2743·91 (2382·95 to 3178·56)	1796·2 (1573·8 to 2044·35)	1621·76 (1474 to 1798·53)	1243·34 (1096·65 to 1345·4)	1046·55 (940·54 to 1166·68)	774·65 (618·42 to 946·07)
Bhutan	3321·69 (2759·01 to 3904·5)	2760·33 (2208·25 to 3338·88)	2191·86 (1683·12 to 2813·28)	1899·66 (1436·82 to 2542·21)	1435·85 (1145·69 to 1832·99)	1162·14 (989·31 to 1347·72)	956·3 (786·95 to 1120·26)	870·16 (709·46 to 1021·62)
India	2840·7 (2396·41 to 3316·16)	2597·68 (2221·29 to 3021)	2274·56 (2019·06 to 2554·31)	1938·97 (1776·36 to 2130·53)	1655·85 (1504·01 to 1823·94)	1464·44 (1337·49 to 1607·31)	1176·52 (1067·38 to 1302·39)	987·41 (799·3 to 1186·95)
Andhra Pradesh	2587·14 (2028·53 to 3210·49)	2413·47 (1906·84 to 2972·64)	2151 (1710·72 to 2620·24)	1834·69 (1485·69 to 2249·26)	1632·5 (1301·65 to 1984·93)	1476·45 (1155·9 to 1818·47)	1166·98 (905·24 to 1458·93)	877·52 (614·01 to 1202·06)
Arunachal Pradesh	2683·25 (2120·46 to 3326·85)	2516·17 (1997·56 to 3091·29)	2245·46 (1813·22 to 2697·91)	1710·61 (1427·25 to 2168·66)	1556·86 (1254·4 to 1897·16)	1394·92 (1101·09 to 1695·07)	1104·29 (868·62 to 1362·27)	963·55 (707·02 to 1250·54)
Assam	3064·68 (2489·59 to 3727·22)	2802·98 (2262·32 to 3424·52)	2463 (1987·18 to 2989·72)	2047·1 (1668·04 to 2542·33)	1780·47 (1509·16 to 2088·17)	1720·4 (1513·15 to 1931·51)	1404·46 (1240·65 to 1591·97)	1159·33 (889·1 to 1463·43)
Bihar	2507·05 (1990·44 to 3078·29)	2324·19 (1852·7 to 2817·69)	2131·02 (1740·04 to 2550·04)	1883·28 (1555·81 to 2272·35)	1602·09 (1295·86 to 1940)	1439·94 (1203·31 to 1693·55)	1205·42 (1031·18 to 1399·14)	889·28 (680·07 to 1162·81)
Chhattisgarh	2916·26 (2356·61 to 3590·65)	2797·99 (2259·6 to 3418·39)	2492·01 (2055·45 to 2998·81)	2202·66 (1830·9 to 2631·89)	1836·62 (1480·17 to 2214·17)	1573·4 (1336·01 to 1831·81)	1461·84 (1283·7 to 1672·68)	1253·54 (994·21 to 1557·9)
Delhi	2368·94 (1855·67 to 2928·36)	2058·3 (1602·37 to 2548·06)	1803·93 (1442·68 to 2219·13)	1585·11 (1270·6 to 1944·19)	1455·05 (1188·59 to 1755·72)	1359·31 (1178·48 to 1572·47)	1053·38 (927·67 to 1205·32)	802·41 (613·1 to 1039·98)
Goa	1698·35 (1295·35 to 2154·94)	1556·52 (1166·42 to 2048·02)	1416·27 (1095·5 to 1791·46)	1252·35 (989·85 to 1576·31)	1194·53 (920·27 to 1478·31)	1129·62 (882·1 to 1382·08)	896·34 (697·94 to 1109·74)	807·05 (576·79 to 1061·97)
Gujarat	2617·11 (2094·73 to 3230·45)	2397·71 (1919·37 to 3027·14)	2115·75 (1696·1 to 2552·22)	1885·46 (1532·2 to 2298·87)	1650·44 (1383·02 to 1940·89)	1408·98 (1235·41 to 1611·17)	1178·48 (1029·94 to 1340·64)	1060·43 (813·36 to 1357·87)
Haryana	2578·07 (2073·58 to 3213·34)	2412·68 (1884·35 to 3109·69)	2093·74 (1716·96 to 2561·1)	1599·92 (1288·32 to 1929·36)	1309·31 (1084·56 to 1547·9)	1265·11 (1107·92 to 1448·28)	1089·09 (957·95 to 1240·82)	846·78 (644·9 to 1081·88)
Himachal Pradesh	2183·62 (1714·01 to 2714·76)	2044·61 (1563·38 to 2580·21)	1821·11 (1462·41 to 2221·58)	1568·94 (1269·84 to 1932·52)	1378·01 (1147·54 to 1632·02)	1289·32 (1128·31 to 1481·1)	1064·36 (932·26 to 1218·05)	1040·95 (797·57 to 1338·33)
Jammu and Kashmir	1950·78 (1510·64 to 2431·5)	1858·01 (1430·6 to 2353·72)	1650·77 (1291·31 to 2075·65)	1479·71 (1198·15 to 1834·19)	1311·78 (1019·71 to 1631·3)	1171·81 (994·51 to 1368·35)	1010·37 (886·19 to 1151·26)	935·79 (718·45 to 1180·14)
Jharkhand	2622·91 (2099·71 to 3196·08)	2410·7 (1922·16 to 2957·22)	2178·77 (1795·99 to 2625·56)	1987·59 (1620·25 to 2427·38)	1851·9 (1551·31 to 2201·99)	1730·77 (1494·64 to 1978·71)	1247·19 (1100·99 to 1416·39)	669·92 (513·5 to 842·35)
Karnataka	2759·16 (2195·58 to 3364·47)	2543·89 (2043·12 to 3175·37)	2202·37 (1790·92 to 2680·06)	1752·55 (1435·8 to 2146·9)	1473·29 (1230·15 to 1742·98)	1382·85 (1213·92 to 1571·07)	1142·75 (1005·19 to 1305·78)	1041·33 (792·13 to 1350·3)
Kerala	2351·04 (1837·4 to 2916·71)	2008·81 (1572·47 to 2520·25)	1696·25 (1347·81 to 2110·2)	1511·74 (1211·31 to 1843·83)	1366·2 (1136·98 to 1628·36)	1246·25 (1087·3 to 1414·39)	958·05 (837·22 to 1096·35)	791·16 (605·67 to 1023·08)
Madhya Pradesh	3119·55 (2489·98 to 3803·06)	2822·85 (2248·35 to 3485·82)	2474·21 (2035·13 to 2955·24)	2133·26 (1750·23 to 2525·25)	1780·48 (1452·52 to 2140·65)	1523·47 (1307·39 to 1760·65)	1338·23 (1178·2 to 1522·06)	1169·5 (911·71 to 1456·31)
Maharashtra	2633·79 (2117·19 to 3213·43)	2417·23 (1937·44 to 2956·14)	2157·29 (1769·29 to 2596·66)	1805·07 (1478·91 to 2191·73)	1544·02 (1292·26 to 1843·71)	1428·96 (1259·34 to 1609·2)	977·84 (854·89 to 1107·89)	804·98 (604·24 to 1019·22)
Manipur	2290·42 (1794·35 to 2808·52)	2061·18 (1645·41 to 2562·61)	1769·89 (1424·61 to 2217·57)	1519·61 (1209·84 to 1867·63)	1364·37 (1080·64 to 1694·07)	1337·1 (1031·52 to 1657·3)	1131·1 (876·4 to 1378·36)	1000·01 (723·7 to 1301·94)
Meghalaya	1908·34 (1483·84 to 2361·24)	1803·6 (1390·65 to 2275·15)	1705·98 (1366·97 to 2107·81)	1677·96 (1345·17 to 2040·02)	1549·96 (1222·66 to 1905·45)	1404·98 (1117·7 to 1721·21)	1139·63 (904·06 to 1398·53)	1023·16 (759·79 to 1339·01)
Mizoram	1677·26 (1306·45 to 2113·69)	1585·3 (1231·81 to 1999·94)	1474·26 (1141·64 to 1850·12)	1424·92 (1134·1 to 1760·94)	1313·93 (1044·28 to 1632·9)	1265·52 (995·5 to 1547·47)	1103·96 (854·07 to 1375·69)	955·26 (690·82 to 1264·6)
Nagaland	2442·67 (1933·02 to 3033·94)	2178·02 (1712·98 to 2704·57)	1844·65 (1447·39 to 2263·26)	1534·09 (1232·81 to 1900·12)	1368·69 (1090·02 to 1730·85)	1313·34 (1024·8 to 1632·89)	1115·34 (858·52 to 1401·86)	969·83 (696·12 to 1272·18)
Odisha	3217·34 (2543·34 to 3913·64)	2937·52 (2379·84 to 3583·89)	2525·28 (2117·61 to 3098·81)	2125·69 (1756·59 to 2546·53)	1809·32 (1518·29 to 2153·78)	1661·12 (1459·01 to 1898·18)	1241·15 (1082·67 to 1440·5)	979·67 (744·38 to 1264·92)
Punjab	2131·58 (1670·07 to 2646·96)	1978·66 (1463·05 to 2602·85)	1726·83 (1381·73 to 2129·47)	1552·96 (1269·13 to 1888·59)	1398·77 (1163·64 to 1660·51)	1222·71 (1063·28 to 1392·42)	957·19 (836·03 to 1097·36)	754·79 (567·5 to 978·9)
Rajasthan	2378·02 (1890·81 to 2943·62)	2316·37 (1834·67 to 2840·62)	2102·21 (1699·51 to 2523·92)	1903·53 (1575·81 to 2290·47)	1653·71 (1395·32 to 1964·85)	1433·97 (1266·78 to 1634·24)	1160·15 (1025·72 to 1311·45)	1153·45 (898·05 to 1459·16)
Sikkim	2127·14 (1669·04 to 2663·04)	2059·26 (1622·19 to 2561·84)	1879·61 (1493·89 to 2292·16)	1628·68 (1324·41 to 2004·65)	1402·86 (1119·08 to 1743·18)	1299·36 (1026·89 to 1582·32)	1043·42 (836·98 to 1270·09)	870·75 (642·82 to 1142·33)
Tamil Nadu	3180·3 (2562·41 to 3855·78)	2861·39 (2309·85 to 3483·8)	2463·3 (2018·01 to 2931·38)	2106·05 (1740·4 to 2533·75)	1791·37 (1490·39 to 2127·55)	1484·07 (1294·35 to 1689·3)	1257·12 (1088·07 to 1435·98)	993·66 (737·71 to 1291·35)
Telangana	2212·59 (1688·37 to 2812·46)	2075·73 (1581·02 to 2621·17)	1909·04 (1537·73 to 2376·95)	1743·35 (1359·62 to 2175·09)	1638·01 (1258·61 to 2059·74)	1518·91 (1175·16 to 1914·53)	1198·01 (921·41 to 1518·45)	898·13 (636·36 to 1213·63)
Tripura	2884·43 (2280·56 to 3563·48)	2519·79 (2024·04 to 3089·78)	2141·67 (1730·01 to 2605·61)	1749·32 (1410·23 to 2142·84)	1538·97 (1220·89 to 1901·42)	1363·03 (1098·73 to 1665·72)	1109·39 (900·55 to 1353·14)	1002·15 (745·73 to 1312·58)
Union Territories other than Delhi	1948·8 (1524·83 to 2453·81)	1701·45 (1286·97 to 2213·68)	1491·03 (1171·18 to 1858·88)	1298·34 (1018·98 to 1623·37)	1220·57 (950·9 to 1519·73)	1187·89 (945·48 to 1435·97)	979·96 (762·68 to 1216·69)	880·27 (652·13 to 1164·9)
Uttar Pradesh	3633·64 (2969·52 to 4355·52)	3359·06 (2801·02 to 3950·22)	2821·69 (2359·53 to 3308·83)	2296·12 (1946·35 to 2686·05)	1857·58 (1561·35 to 2147·83)	1552·47 (1369·02 to 1772·09)	1345·09 (1198·58 to 1530·19)	1396·32 (1115·81 to 1739·98)
Uttarakhand	2670·46 (2145·57 to 3283·21)	2570·81 (2047·28 to 3174·14)	2341·75 (1867·59 to 2843·4)	2147·72 (1755·14 to 2620·18)	1988·43 (1636·59 to 2365·92)	1823·06 (1524·83 to 2163·25)	1465·39 (1255·97 to 1702·73)	1349·99 (1026·95 to 1739·58)
West Bengal	2684·65 (2160·1 to 3254·81)	2387·63 (1919·03 to 2965·64)	2086·62 (1719·16 to 2549·4)	1829·4 (1514·04 to 2193·87)	1549·59 (1314·93 to 1820·16)	1390·67 (1232·4 to 1560·8)	1019·18 (900·2 to 1154·9)	773·71 (586·08 to 989·4)
Nepal	3095·38 (2613·08 to 3602·08)	2636·41 (2252·77 to 3028·4)	2248·64 (1932·99 to 2614)	2038·87 (1743·86 to 2366·61)	1627·16 (1427·52 to 1889·9)	1260·3 (1125·09 to 1406·63)	1112·92 (1001·34 to 1247·64)	1092·94 (878·86 to 1305·01)
Pakistan	2152·33 (1835·98 to 2541·12)	1750·96 (1475·35 to 2038·42)	1403·69 (1209·77 to 1590·92)	1373·47 (1231·72 to 1539·13)	1401·43 (1253·25 to 1627·53)	1515·32 (1346·25 to 1704·83)	1390·86 (1159·41 to 1666·78)	1214·66 (986·26 to 1476·89)
Southeast Asia, east Asia, and Oceania	2478·26 (2206·2 to 2765·05)	3487·92 (2806·44 to 4468·41)	1650·64 (1436·75 to 1913·19)	1505·47 (1325·51 to 1698·85)	1308·68 (1176·94 to 1429·41)	1166·74 (1080·96 to 1260·74)	1030·28 (953·45 to 1111·95)	873·47 (751·62 to 1008·44)
East Asia	2430·07 (2093·18 to 2800·6)	4011·92 (3063·13 to 5359·6)	1566·01 (1291·54 to 1906·9)	1503·63 (1267·7 to 1759·13)	1317·18 (1148·99 to 1479·73)	1166·04 (1055·27 to 1295·19)	1013·19 (916·69 to 1119·03)	837·23 (685·35 to 1008·6)
China	2359·55 (2017·36 to 2731·06)	4075·49 (3094·69 to 5468·59)	1565·73 (1282·79 to 1920·22)	1512·34 (1267·97 to 1777·04)	1330·76 (1154·46 to 1499·08)	1174·01 (1057·73 to 1306·24)	1032·22 (930·36 to 1142·94)	849·03 (689·22 to 1031·68)
North Korea	7436·48 (5274·73 to 10912·49)	2665·68 (1959·68 to 3670·58)	1945·51 (1493·39 to 2540·96)	1505·83 (1200·54 to 1876·83)	1297·6 (1079·55 to 1550·15)	1802·07 (1304·78 to 2809·62)	1211·81 (1044·66 to 1359)	1062·69 (896·92 to 1273·2)
Taiwan (province of China)	1900·07 (1623·31 to 2206·47)	1537·33 (1520·87 to 1554·13)	1216·91 (1203·67 to 1229·96)	1007·16 (997·11 to 1017·25)	855·37 (846·05 to 864·83)	743·09 (734·83 to 751·15)	624·7 (616·7 to 632·93)	614·55 (491·87 to 776·65)
Oceania	2442·65 (2079·36 to 2893·78)	2056·63 (1793·53 to 2343·92)	1768·56 (1564·16 to 1970·32)	1572·76 (1438·56 to 1717·38)	1569·2 (1370·25 to 1793·65)	1544·9 (1364·9 to 1751·73)	1511·98 (1303·62 to 1748·84)	1413·29 (1188·82 to 1693·75)
American Samoa	1552·87 (1362·24 to 1779·4)	1038·88 (919·26 to 1176·17)	1259·63 (1114·73 to 1424·86)	1240·13 (1090·45 to 1392·78)	1202·22 (1104·64 to 1318·75)	1141·44 (1031·53 to 1257·45)	1080·78 (983·69 to 1186·09)	1027·15 (880·88 to 1210·66)
Cook Islands	2457·44 (2169·2 to 2772·33)	1692·08 (1483·72 to 1916·7)	1420·51 (1248·51 to 1601·24)	1453·78 (1303·11 to 1603·54)	1287·19 (1159·34 to 1420·28)	1055·75 (936·73 to 1184·76)	913·97 (805·94 to 1039·56)	886·56 (765·19 to 1028·93)
Fiji	1650·33 (1521·78 to 1790)	1469·11 (1307·72 to 1635·27)	1561·31 (1394·86 to 1750·61)	1443·61 (1292·23 to 1612·84)	1534·1 (1294·25 to 1798·91)	1714·04 (1558·4 to 1899·17)	1439·5 (1314·13 to 1594)	1419·76 (1153·1 to 1705·18)
Guam	1139·94 (997·23 to 1295·42)	1330·66 (1204·65 to 1456·34)	1400·65 (1298·83 to 1523·54)	1095·87 (1013·73 to 1182·97)	985·88 (889·73 to 1091·83)	773·01 (685·23 to 867·7)	758·12 (683·29 to 841·56)	753·02 (616·93 to 909·59)
Kiribati	3427·02 (3021·15 to 3881·1)	2985·96 (2576·59 to 3452·89)	2702·85 (2333·96 to 3169·23)	2452·26 (2064·41 to 2908·87)	2516·84 (2120·73 to 2911·39)	2375·21 (2023·21 to 2738·93)	2146·23 (1802·83 to 2529·21)	2003·55 (1657·78 to 2382·42)
Marshall Islands	2613·07 (1826·56 to 3779·59)	2248·32 (1670·19 to 2951·48)	2025·49 (1613·61 to 2531·37)	1744·64 (1476·6 to 2035·64)	1862·14 (1684·92 to 2074·34)	1603 (1421·3 to 1807·62)	1523·49 (1288·35 to 1764·73)	1426·93 (1144·15 to 1778·02)
Federated States of Micronesia	2905·09 (2532·13 to 3332·33)	2484·18 (2058·22 to 2998·33)	2216·48 (1731·3 to 2854·2)	1960·8 (1584·49 to 2447·77)	1845·66 (1544·51 to 2177·67)	1750·27 (1548·1 to 1973·09)	1645·6 (1397·1 to 1903·72)	1673·74 (1339·19 to 2034·81)
Nauru	1669·71 (1368·56 to 2010·85)	1540·16 (1294·84 to 1819·24)	1549·06 (1316·91 to 1827·43)	1678·7 (1329·08 to 2092·71)	1853·86 (1533·72 to 2200·91)	2125·1 (1821·99 to 2449·53)	2053·79 (1747·51 to 2391·66)	1767·07 (1394·18 to 2152·77)
Niue	2060·9 (1799·04 to 2361·01)	1547·34 (1317·81 to 1799·48)	1553·36 (1330·97 to 1810·13)	1423·21 (1226·35 to 1636·47)	1621·43 (1168·72 to 1624·45)	1359·57 (1203·24 to 1557·2)	1231·12 (1082·95 to 1397·22)	1179·93 (990·58 to 1390·85)
Northern Mariana Islands	1506·1 (1225·55 to 1809·95)	1434·7 (1169·68 to 1733·34)	1302·68 (1124·18 to 1511·9)	1150 (1006·85 to 1336·74)	1061·07 (951·36 to 1214·23)	1021·55 (910·37 to 1170·09)	1048·4 (932·68 to 1171·26)	1020·69 (869·8 to 1185·36)
Palau	2695·5 (2259·38 to 3219·73)	2311·71 (1952·75 to 2735·48)	1982·95 (1675·23 to 2291·55)	1775·3 (1514·7 to 2089·54)	1586·28 (1320·83 to 1936·91)	1477·24 (1228·64 to 1753·62)	1425·11 (1190·55 to 1696·91)	1378·08 (1126·19 to 1690·71)
Papua New Guinea	2554·83 (2039·52 to 3209·79)	2135·77 (1777·49 to 2555·51)	1735·13 (1467·64 to 2009·84)	1535·41 (1359·44 to 1725·71)	1538·69 (1256·75 to 1857·2)	1508·23 (1255·1 to 1787·2)	1530·98 (1256·5 to 1857·77)	1413·44 (1145·06 to 1747·46)
Samoa	1883·89 (1582·72 to 2245·27)	1417·76 (1222·7 to 1631·89)	1477·36 (1310·56 to 1766·82)	1431·87 (1243 to 1732·84)	1404·43 (1212·44 to 1626·2)	1229·48 (1087·91 to 1439·52)	1117·03 (979·8 to 1277·9)	1054·15 (918·48 to 1257·18)
Solomon Islands	3602·89 (3152·8 to 4116·79)	3169·69 (2753·98 to 3665·77)	2767·72 (2369·24 to 3206·07)	2446·28 (2046·65 to 2916·97)	2346·48 (1973·99 to 2786·74)	2248·88 (1910·95 to 2606·01)	2228·24 (1924·15 to 2575·88)	2070·21 (1688·57 to 2499·32)
Tokelau	2094·3 (1801·62 to 2443·02)	1743·98 (1453·98 to 2087·6)	1468·2 (1251·49 to 1758·49)	1251·98 (1042·48 to 1480·99)	1060·6 (921·73 to 1232·98)	976·96 (836·01 to 1137·95)	888·65 (759·46 to 1054·57)	806 (685·85 to 975·25)
Tonga	1707·98 (1445·64 to 1998·76)	1479·2 (1289·93 to 1690·95)	1336·28 (1164·11 to 1531·85)	1267·69 (1110·66 to 1440·44)	1111·9 (964·14 to 1268·72)	1244·38 (1101·6 to 1418·02)	1108·46 (960·19 to 1260·97)	1048·38 (875·81 to 1249·19)
Tuvalu	3060·79 (2546·44 to 3649·96)	2484·95 (2071·52 to 2939·5)	2171·05 (1754·74 to 2581·46)	2014·85 (1667·06 to 2329·35)	1780·14 (1555·7 to 2023·78)	1744·23 (1563·14 to 1956·82)	1342·04 (1132·3 to 1610·52)	1260·46 (998·83 to 1590·02)
Vanuatu	2684·99 (2284·09 to 3124·75)	2350·6 (1943·77 to 2805·16)	2048·52 (1706·09 to 2423·71)	1726·43 (1476·7 to 2024·64)	1678·62 (1400·88 to 2025·97)	1640·95 (1371·73 to 1976·96)	1582·77 (1333·77 to 1909·57)	1508·08 (1251·94 to 1866)
Southeast Asia	2699·29 (2442·1 to 2971·63)	2081·17 (1891·41 to 2287·24)	1893·97 (1747·84 to 2050·67)	1531·2 (1420·04 to 1651·16)	1328·41 (1251·66 to 1408·03)	1198·78 (1113·17 to 1288·39)	1082·4 (1020·62 to 1149·01)	973·99 (875·12 to 1084·07)
Cambodia	2664·21 (2262·55 to 3167·98)	2317·41 (1937·22 to 2724·86)	2850·83 (2399·61 to 3393·25)	2264·37 (1834·32 to 2875·79)	1852·23 (1617·24 to 2125·42)	1710·16 (1524·52 to 1899·7)	1377·68 (1206·64 to 1553·14)	1230·68 (983·96 to 1461·72)
Indonesia	2933·3 (2550·5 to 3370·81)	2344·4 (2009·16 to 2701·55)	1933·7 (1661·45 to 2233·78)	1536·83 (1354·18 to 1731·44)	1329·16 (1202·89 to 1474·25)	1194·92 (1022·78 to 1388·56)	1168·06 (1026·42 to 1311·91)	1091·31 (887·37 to 1331·11)
Aceh	2303·94 (1843·9 to 2833·36)	1885·17 (1427·63 to 2443·19)	1628·17 (1217·14 to 2076·58)	1332·71 (1063·99 to 1622·68)	1243·99 (1031·28 to 1487·68)	1180·12 (922·28 to 1499·41)	1224·13 (979·88 to 1475·85)	1154·09 (849·35 to 1531·69)
Bali	2556·09 (2061·13 to 3110·45)	2028·97 (1560·93 to 2601·06)	1722·44 (1312·43 to 2202·66)	1431·86 (1155·43 to 1731·04)	1273·51 (1059·44 to 1497·12)	1137·73 (919·55 to 1432·49)	1031·52 (825·87 to 1247·66)	783·28 (560·25 to 1075·9)
Bangka-Belitung Islands	3156·43 (2558·65 to 3853)	2588·28 (2006·24 to 3295·16)	2149·7 (1681·72 to 2702·43)	1695·91 (1372·8 to 2072·78)	1494·3 (1244·6 to 1762·59)	1367·93 (1093·82 to 1677·09)	1306·48 (1077·58 to 1549·4)	1240·78 (924·22 to 1630·87)
Banten	3673·36 (3001·11 to 4393·63)	3137·39 (2503·02 to 3928·26)	2463·93 (1950·59 to 3066·43)	1826·74 (1494·85 to 2239·35)	1456·98 (1218·73 to 1734·3)	1178·2 (942·06 to 1477·12)	1222·81 (992·73 to 1443·32)	1169·79 (853·04 to 1534·05)
Bengkulu	3505·08 (2879·84 to 4186·16)	2767·25 (2189·66 to 3440·43)	2186·87 (1688·93 to 2735·53)	1656·28 (1347·14 to 1981·07)	1345·54 (1128·08 to 1586·73)	1230·36 (965·21 to 1553·64)	1173·04 (949·86 to 1413·01)	1091·18 (804·65 to 1440·88)
Gorontalo	2929·51 (2363·44 to 3602·38)	2435·8 (1922·37 to 3070·2)	1982·46 (1530·54 to 2518·8)	1552·91 (1254·59 to 1901·81)	1249·43 (1039·32 to 1501·21)	1024·59 (803·73 to 1290·23)	994·34 (808·53 to 1225·1)	926·1 (671·75 to 1264·11)
Jakarta	2016·54 (1597·21 to 2489·41)	1696·08 (1295·47 to 2208·31)	1349·98 (1030·84 to 1718·13)	1059·27 (839·45 to 1300·98)	1024·57 (848·52 to 1220·5)	1154·72 (907·77 to 1455·51)	1108·02 (910·67 to 1326·16)	1019·54 (751·99 to 1345·98)
Jambi	3070·71 (2496·03 to 3690·62)	2485 (1944·68 to 3123·36)	2120·75 (1612·32 to 2740·39)	1716·08 (1410·65 to 2057·69)	1459·48 (1228·7 to 1733·87)	1242·28 (986·12 to 1542·21)	1156·49 (952·62 to 1377·65)	1097·99 (816·16 to 1416·33)

Supplemental Results Table 3b: Age-standardised mortality rates by location, males, 1950, 1960, 1970, 1980, 1990, 2000, 2010, 2019

Location	1950	1960	1970	1980	1990	2000	2010	2019
West Java	3386·85 (2826·3 to 3985·8)	2709·24 (2175·36 to 3333·13)	2155·14 (1715·22 to 2683·52)	1637·91 (1358·45 to 1926·58)	1332·43 (1134·9 to 1551·56)	1085·99 (880·11 to 1352·58)	1078·15 (887·77 to 1286·91)	1038·78 (775·6 to 1350·68)
Central Java	2645·1 (2176·82 to 3177·91)	2091·21 (1654·5 to 2625·55)	1728·95 (1347·81 to 2127·53)	1358·9 (1106·45 to 1614·52)	1135·43 (958·99 to 1318·97)	1135·64 (922·19 to 1387·92)	1112·04 (928·84 to 1299·58)	1035·13 (785·97 to 1318·3)
East Java	2726·6 (2261·25 to 3272·03)	2237·28 (1771·82 to 2739·73)	1904·48 (1524·55 to 2395·96)	1582·51 (1330·52 to 1866·13)	1395·15 (1198·81 to 1614·93)	1237·36 (1009·52 to 1523·47)	1209·24 (1012·05 to 1416·55)	1132·73 (868·21 to 1443·92)
West Kalimantan	2661·09 (2158·87 to 3239·21)	2315·09 (1801·5 to 2958·13)	1996·04 (1573·3 to 2518·82)	1594·91 (1299·88 to 1892·73)	1408·43 (1183·55 to 1685·24)	1291·4 (1003·77 to 1609·04)	1189·84 (972·33 to 1412·38)	1067·28 (803·26 to 1400·97)
South Kalimantan	3641·68 (3005·81 to 4359·31)	3066·06 (2447·72 to 3878·5)	2415·17 (1902·92 to 3069·5)	1826·81 (1504·24 to 2171·64)	1672·46 (1415·28 to 1954·89)	1582·69 (1251·65 to 2001·63)	1508·1 (1240·61 to 1793·06)	1357·58 (1004·62 to 1772·09)
Central Kalimantan	2553·64 (2111·2 to 3104·32)	2022·22 (1556·92 to 2595·85)	1733·63 (1328·32 to 2237·46)	1444·51 (1156·42 to 1789·28)	1306·78 (1082·81 to 1558·11)	1131·83 (887·05 to 1416·4)	1188·72 (978·03 to 1411·35)	1151·04 (860 to 1498·46)
East Kalimantan	2196·13 (1754·28 to 2701·53)	1994·61 (1526·74 to 2558·83)	1708·49 (1293·57 to 2208·34)	1356·4 (1097·94 to 1637·59)	1249·69 (1042·66 to 1477·35)	1345·69 (1062·99 to 1677·23)	1241·59 (1021·87 to 1489·44)	1158·86 (852·93 to 1530·9)
North Kalimantan	2198·58 (1724·55 to 2757·51)	2084·92 (1614·91 to 2648·79)	1858·06 (1422·88 to 2341·86)	1417·5 (1153·62 to 1747·52)	1140·48 (911·27 to 1387·49)	922·98 (690·49 to 1180·89)	925·68 (722·35 to 1156·52)	893·45 (640·5 to 1228·68)
Riau Islands	2341·95 (1869·42 to 2874·21)	2044·61 (1557·18 to 2610·31)	1730·18 (1328·94 to 2218·57)	1459·56 (1141·7 to 1808·4)	1295·31 (1056·73 to 1539·24)	927·78 (732·33 to 1177·68)	914·96 (734·72 to 1132·15)	896·65 (638·99 to 1201·92)
Lampung	2792·3 (2251·03 to 3414·9)	2324·53 (1822·73 to 2987·36)	1923·53 (1486·13 to 2444·15)	1531·58 (1240·9 to 1858·62)	1371·63 (1161·96 to 1613·58)	1102·7 (859·79 to 1391·92)	1044·07 (843·35 to 1265·89)	1011·12 (728·18 to 1355·58)
Maluku	3863·31 (3212·89 to 4547·8)	2918·92 (2324·28 to 3639·7)	2363·43 (1871·85 to 2992·4)	1861·81 (1526·97 to 2253·45)	1570·59 (1300·14 to 1856·96)	1338·34 (1044·66 to 1678·68)	1336·96 (1072·27 to 1647·33)	1182·73 (855·61 to 1560·64)
North Maluku	3597·16 (2948·28 to 4352·52)	2925·25 (2293·59 to 3638·5)	2478·42 (1933·74 to 3111·4)	2063·13 (1686·25 to 2496·21)	1496·8 (1239·62 to 1797·38)	1264·48 (992·38 to 1584·79)	1129·44 (915·58 to 1394·75)	1013·13 (730·6 to 1362·81)
West Nusa Tenggara	3854·91 (3203·16 to 4614·3)	3131·36 (2506·29 to 3905·64)	2598 (2047·18 to 3205·1)	1989·75 (1634·26 to 2377·87)	1659·6 (1413·53 to 1958·59)	1411·76 (1119·92 to 1764·72)	1269·32 (1019·43 to 1529·68)	1173·76 (877·22 to 1528·57)
East Nusa Tenggara	3618·69 (2964·23 to 4397·62)	2816·26 (2216·72 to 3553·23)	2308·24 (1798·21 to 2880·41)	1805·57 (1474·4 to 2155·36)	1507·48 (1268·98 to 1755·39)	1318·66 (1041·65 to 1677·66)	1190·91 (968·52 to 1448·39)	1135·59 (824·63 to 1485·19)
Papua	2033·85 (1601·99 to 2552·35)	1900·6 (1459·38 to 2489·17)	1661·4 (1272·81 to 2091·02)	1395·23 (1105·49 to 1720·03)	1350·42 (1121·51 to 1605·42)	1415·75 (1129·56 to 1778·62)	1461·32 (1185·66 to 1750·1)	1338·73 (960·8 to 1772·26)
West Papua	1989·82 (1573·44 to 2448·17)	1802·89 (1379·15 to 2315·41)	1610·46 (1204·37 to 2074·89)	1312·16 (1038·04 to 1621·21)	1156·78 (938·29 to 1402·09)	1074·6 (847·83 to 1377·16)	1145·3 (951·61 to 1372·11)	958·69 (704·58 to 1283·88)
Riau	2525·75 (2021·52 to 3115·21)	2271·2 (1739·23 to 2908·45)	1946·98 (1510·69 to 2458·89)	1537·54 (1243·34 to 1864·19)	1280·48 (1074·3 to 1503·4)	1128·49 (891·72 to 1407·44)	1136·21 (916·84 to 1356·3)	1062·97 (774·44 to 1397·39)
West Sulawesi	4469·82 (3717·13 to 5334·58)	3389·15 (2708·09 to 4222·62)	2749·27 (2148·45 to 3420·25)	2062·16 (1695·88 to 2457·39)	1746·54 (1464·07 to 2045·7)	1513·98 (1170·33 to 1896·53)	1288·05 (1034·49 to 1579·71)	1164·59 (836·64 to 1549·56)
South Sulawesi	3161·32 (2630·54 to 3765·61)	2568·4 (2016·75 to 3219·59)	2128·6 (1667·93 to 2772·87)	1695·24 (1397·86 to 2034·75)	1433·49 (1206·01 to 1692·29)	1230·01 (971·19 to 1534·32)	1194·83 (1002·75 to 1407·47)	1118·66 (830·17 to 1450·22)
Central Sulawesi	3839·32 (3198·07 to 4607·62)	2914·01 (2332·53 to 3649·3)	2382·42 (1883·39 to 3042·12)	1909·73 (1564·18 to 2287·47)	1645·71 (1388·39 to 1948·81)	1481·7 (1183·53 to 1844·56)	1301·78 (1055·86 to 1580·46)	1179·83 (871·2 to 1585·96)
Southeast Sulawesi	3185·24 (2585·76 to 3836·74)	2614·69 (2064·01 to 3265·89)	2175·77 (1713·71 to 2737·06)	1578·16 (1283 to 1941·16)	1296·38 (1071·94 to 1540·1)	1286·55 (1010·52 to 1604·11)	1281·16 (1029·3 to 1545·61)	1206·91 (887·93 to 1594·18)
North Sulawesi	2598·64 (2120·26 to 3211·6)	2149·1 (1654·76 to 2734·18)	1817·67 (1418·84 to 2326·28)	1492·33 (1183·57 to 1811·58)	1421·73 (1206·24 to 1684·1)	1294·93 (1019·91 to 1626·41)	1228·64 (1002·95 to 1464·82)	1161·08 (869·97 to 1514·24)
West Sumatra	3958·85 (3283·86 to 4753·48)	2640·76 (2111·65 to 3348·77)	2029·05 (1584·79 to 2564·1)	1501·54 (1211·35 to 1816·42)	1364·64 (1131·57 to 1593·9)	1326·86 (1045·34 to 1659·64)	1315·54 (1091·19 to 1535·94)	1174·58 (884·68 to 1503·18)
South Sumatra	2859·65 (2356·93 to 3459·86)	2254·48 (1748·7 to 2906·98)	1846·95 (1415·65 to 2419·61)	1397·68 (1142·58 to 1689·97)	1267·95 (1062·51 to 1512·42)	1117·75 (878·36 to 1423·76)	1083·6 (872·68 to 1300·63)	1043·72 (774·61 to 1370·31)
North Sumatra	2664·12 (2151·81 to 3177)	2174·33 (1680·45 to 2754·07)	1843·89 (1427·02 to 2379·37)	1517·8 (1227·39 to 1827·62)	1427·41 (1218·29 to 1689·01)	1393·46 (1109·17 to 1719·94)	1377·52 (1149·89 to 1633·17)	1297·97 (985·87 to 1701·9)
Yogyakarta	1988·4 (1577·74 to 2456·57)	1551·72 (1156·75 to 2045·84)	1345·96 (989·59 to 1753·45)	1136·67 (889·14 to 1422·79)	1066·14 (891·11 to 1272·41)	973·23 (762·79 to 1224·21)	1027·45 (821·42 to 1239·26)	1030·78 (753·56 to 1323·94)
Laos	3470·95 (2494·52 to 4936·9)	3247·85 (2453·04 to 4301·16)	3002·31 (2382·29 to 3707·26)	2526·96 (2098·26 to 3000·6)	2124·46 (1801·87 to 2463·95)	1781·32 (1536·68 to 2030·94)	1411·95 (1195·33 to 1640·71)	1198·12 (976·72 to 1422·47)
Malaysia	2108·78 (1792·87 to 2460·65)	1248·23 (1167·71 to 1339·2)	1260·64 (1178·02 to 1351·73)	1067·05 (958·93 to 1175·86)	992·9 (921·73 to 1077·43)	986·46 (919·74 to 1055·64)	875·07 (806·1 to 947·22)	847·78 (671·06 to 1046·71)
Maldives	3716·55 (3140·5 to 4343·41)	3016·17 (2587·67 to 3472·68)	2220·64 (1958·26 to 2501·27)	1568·55 (1424·76 to 1731·71)	1231·99 (1139·8 to 1326·8)	887·15 (819·19 to 960·53)	638·18 (567·32 to 713·6)	584·09 (494·69 to 686·53)
Mauritius	2332·64 (2163·9 to 2520·36)	1923·34 (1762·73 to 2096·6)	1519·28 (1399·53 to 1656·51)	1472·47 (1344·6 to 1616·32)	1241·01 (1157·51 to 1330·55)	1063·1 (981·87 to 1153·34)	937·67 (863·48 to 1014·54)	832·85 (665·12 to 1026·28)
Myanmar	4254·95 (3569·96 to 5043·67)	3477·79 (2913·63 to 4148·63)	3011·88 (2513·78 to 3578·63)	2381·08 (1978·29 to 2799·38)	2047·52 (1722·48 to 2416·5)	1825·35 (1509·76 to 2196·09)	1486·78 (1288·69 to 1711·88)	1231·05 (1039·32 to 1477·56)
Philippines	1451·84 (1293·92 to 1629·47)	1256·17 (1134·89 to 1387·84)	1348·84 (1228·38 to 1488·97)	1275·72 (1158·71 to 1407·33)	1158·76 (1036·23 to 1284·56)	1089·18 (976·82 to 1213·18)	1110·41 (990·93 to 1231·86)	1032·76 (805·94 to 1293·69)
Seychelles	1685·81 (1513·11 to 1880·39)	1415·51 (1306·05 to 1537·87)	1407·49 (1285·74 to 1541·39)	1083·85 (979·79 to 1203·39)	1319·75 (1195·73 to 1464·15)	1141·99 (1039·9 to 1255·32)	1067·25 (987·01 to 1159·05)	1014·61 (859·53 to 1191·61)
Sri Lanka	1622·05 (1505·09 to 1741·76)	1205·95 (1107·13 to 1321·95)	1252·17 (1161·5 to 1351·09)	1108·34 (1032·05 to 1193·15)	1240·46 (1075·8 to 1435·23)	1140·46 (1000·49 to 1312·71)	987·5 (912·06 to 1066·1)	771·62 (589·42 to 1004·93)
Thailand	1976·81 (1722·7 to 2240·14)	1449·96 (1305·99 to 1616·87)	1355·05 (1205·08 to 1514·03)	1321·93 (1185·08 to 1483·23)	1038·21 (923·25 to 1161·14)	990·19 (849·89 to 1143·18)	710·54 (623·41 to 804)	669·66 (503·19 to 863·23)
Timor-Leste	2829·78 (2273·17 to 3472·21)	2444·35 (2068·81 to 2853·86)	2070·07 (1696·02 to 2479·65)	1941·85 (1694·25 to 2259·02)	1377·07 (1230·81 to 1573·78)	1176·53 (1035·98 to 1355·14)	1041·86 (915·79 to 1172·59)	1088·34 (895·45 to 1249·78)
Vietnam	2661·23 (2207·22 to 3225·44)	1900·78 (1539·9 to 2317·16)	2084 (1773·6 to 2477·17)	1424·87 (1214·17 to 1678·46)	1287·09 (1107·2 to 1495·82)	1156·36 (1037·51 to 1297·16)	1125·12 (1027·32 to 1242·72)	1060·03 (886·27 to 1252·82)
Sub-Saharan Africa	2872·06 (2481·58 to 3275·14)	2600·5 (2324·43 to 2863·97)	2315·7 (2119·56 to 2523·39)	2020·03 (1870·69 to 2178·94)	1942·98 (1839·84 to 2055·3)	2033·12 (1901·74 to 2163·49)	1659·3 (1556·62 to 1776·13)	1355·41 (1243·13 to 1485·49)
Central sub-Saharan Africa	3155·18 (2645·42 to 3712·47)	2735·34 (2321·36 to 3212·87)	2554·09 (2216·42 to 2907·1)	2350·9 (2038·13 to 2691·88)	2166·23 (1899·8 to 2443·24)	2152·04 (1862·29 to 2464·73)	1781·98 (1486·07 to 2127·7)	1467·07 (1161·36 to 1875·23)
Angola	3036·75 (2541·83 to 3576·94)	2823·07 (2385·3 to 3267·26)	2753·81 (2306·25 to 3213·45)	2593·75 (2134·91 to 3115·23)	2447·24 (1940·3 to 3111·33)	2198·49 (1847·69 to 2622·54)	1693·38 (1453·7 to 1963·93)	1421·62 (1191·19 to 1713·46)
Central African Republic	3353·07 (2540·82 to 4349·96)	3081·6 (2474·32 to 3772·02)	2857·74 (2422·31 to 3321·97)	2747·49 (2361·42 to 3128·34)	2838·54 (2509·34 to 3190·74)	3161·06 (2723·76 to 3621·03)	2806·88 (2327·58 to 3355·11)	2517·44 (1995·69 to 3151·66)
Congo (Brazzaville)	3831·12 (2891·74 to 4983·78)	3324·9 (2771·44 to 4023·01)	2976·7 (2554·33 to 3432·99)	2516·85 (2180·85 to 2906·61)	2323·77 (2027·41 to 2657·44)	2177·85 (1897·26 to 2507·78)	1527·24 (1302·09 to 1759·68)	1340·73 (1108·88 to 1619·49)
DR Congo	3141·46 (2589·16 to 3747·85)	2635·85 (2177·03 to 3210·57)	2442·94 (2068·31 to 2831·73)	2252·8 (1916·69 to 2650·35)	2043·16 (1735·83 to 2386·24)	2084·89 (1781·99 to 2521·16)	1780·55 (1385·68 to 2300·37)	1447·69 (1047·03 to 2076·74)
Equatorial Guinea	4619·67 (3904·64 to 5425·67)	3659·04 (3032·14 to 4389·8)	3116·16 (2528·48 to 3766·12)	2607·23 (2179·37 to 3067·12)	2563·27 (2108·82 to 3074·96)	1837·14 (1413·03 to 2347·59)	1258·03 (1000·45 to 1552·1)	1289·6 (1039·99 to 1578·43)
Gabon	3477·45 (2829·99 to 4233·94)	2743·2 (2350·01 to 3163·87)	2462·73 (2036·63 to 2991·55)	2066·36 (1696·01 to 2503·31)	1915·86 (1672·2 to 2194·17)	1843·63 (1571·23 to 2120·5)	1577·36 (1362·4 to 1784·63)	1373·67 (1135·81 to 1600·08)
Eastern sub-Saharan Africa	3137·89 (2711·48 to 3593·23)	2715·23 (2438·35 to 3017·32)	2481·39 (2274·27 to 2701·41)	2189·7 (2048·04 to 2342·95)	2196·36 (2071·24 to 2324·86)	2179·27 (2046·97 to 2329·73)	1635·49 (1533·18 to 1734·64)	1334·23 (1218·54 to 1465·69)
Burundi	3202·32 (2400·57 to 4279·37)	2756·13 (2191·28 to 3408·12)	2650·02 (2226·83 to 3109·17)	2529·51 (2191·52 to 2897·84)	2569·63 (2237·98 to 2938·76)	2869·66 (2473·62 to 3301·5)	1758·39 (1499·71 to 2016·28)	1463·01 (1162·56 to 1831·87)
Comoros	2688·97 (2098·4 to 3380·79)	2444·61 (2018·53 to 2886·89)	2127·65 (1748·26 to 2540·99)	1967·33 (1683·55 to 2292·59)	1717·59 (1466·7 to 1994·28)	1492·74 (1289·42 to 1717)	1224·78 (1033·76 to 1446·22)	1100·51 (889·66 to 1375·17)
Djibouti	2040·01 (1496·93 to 2827·6)	1846·21 (1368·92 to 2452·62)	1680·27 (1283·59 to 2115·34)	1506·72 (1233·96 to 1815·05)	1582·36 (1274·25 to 1925·01)	1634·46 (1276·6 to 2029·96)	1435·43 (1145·27 to 1774·95)	1242·5 (1022·94 to 1570·37)
Eritrea	3691·9 (2703·51 to 5086·92)	3295·09 (2526·15 to 4211·97)	3450·15 (2527·84 to 5704·08)	3281·03 (2899·92 to 3712·88)	3069·13 (2686·07 to 3478·86)	2372·66 (1912·14 to 2873·87)	1949·33 (1470·19 to 2594·25)	1649·97 (1182·69 to 2284·6)
Ethiopia	3722·75 (2754·45 to 5271·46)	3322·79 (2581·88 to 4371·26)	3102·7 (2585·16 to 3767·15)	2875·43 (2516·4 to 3265·57)	2652·95 (2370·41 to 2974·43)	2168·39 (1904·54 to 2470·39)	1383·47 (1243·45 to 1557·83)	1107·62 (922·03 to 1331·42)
Kenya	2425·85 (2081·96 to 2792·58)	1963·82 (1649·08 to 2274·09)	1672·88 (1413·2 to 1980·67)	1406 (1207·71 to 1606·62)	1431·95 (1263·51 to 1612·03)	1952·93 (1704·66 to 2212·82)	1635·67 (1485·12 to 1800·33)	1334·46 (1094·34 to 1592·13)
Baringo	2663·69 (2097·02 to 3318·68)	2357·97 (1758·88 to 3120·96)	2219·78 (1729·09 to 2814·87)	1981·86 (1525·41 to 2504·19)	1918·05 (1488·04 to 2384·62)	2112·96 (1708·7 to 2581·72)	1764·93 (1453·26 to 2152·34)	1379·37 (990·41 to 1853·84)
Bomet	1248·92 (912·79 to 1661·53)	1122·36 (803·34 to 1526·7)	1044·66 (756·59 to 1423·41)	936·48 (685·57 to 1216·6)	1069·44 (851·96 to 1375·74)	1773·91 (1369·63 to 2278·56)	1498·96 (1185·04 to 1873·13)	1215·84 (928·7 to 1605·54)
Bungoma	2806·1 (2186·47 to 3517·58)	2229·61 (1612·9 to 2970·37)	1839·45 (1360·14 to 2392·6)	1553·01 (1178·53 to 2014·15)	1446·7 (1126·09 to 1835·72)	1705·74 (1327·03 to 2162·42)	1271·68 (1010·4 to 1561·8)	1053·68 (760·62 to 1400·6)
Busia	4426·87 (3544·86 to 5456·51)	2978·16 (2257·92 to 3815·44)	2274·64 (1756·18 to 2937·55)	1633·56 (1243·4 to 2077·87)	1669·84 (1268·91 to 2155·91)	2393·1 (1747·35 to 3070·97)	1655·5 (1261·66 to 2114·15)	1272·23 (832·42 to 1771·26)
Elgeyo Marakwet	2054·76 (1558·86 to 2582·29)	1778·22 (1333·46 to 2338·25)	1734·98 (1316·85 to 2230)	1586·69 (1187·82 to 2038·72)	1475·04 (1106·8 to 1913·97)	1736·94 (1334·49 to 2158·14)	1424·18 (1172·41 to 1722·2)	1144·99 (819·75 to 1536·72)
Embu	2667·53 (2099·33 to 3334·04)	1972·16 (1439·34 to 2627·36)	1415·96 (1050·53 to 1865·86)	1062·44 (776·09 to 1389·19)	1054·68 (823·32 to 1364·38)	1332·06 (996·12 to 1799·42)	1321·4 (997·29 to 1674·94)	1184·79 (821·95 to 1652·49)
Garissa	2225·18 (1703·97 to 2889·01)	2035·1 (1486·09 to 2677·01)	1795·76 (1313·2 to 2315·85)	1627·12 (1230·67 to 2119·03)	1559·29 (1185·62 to 2025·83)	1671·68 (1263·24 to 2102·73)	1507 (1176·8 to 1917·59)	1260·65 (889·72 to 1709·73)
Homa Bay	4408·15 (3680·05 to 5197·52)	3247·67 (2575·94 to 4025·11)	2833·08 (2274·44 to 3532·08)	2278·42 (1831·3 to 2801·83)	2300·66 (1797 to 2842·05)	4191·28 (3222·63 to 5282·92)	3077·26 (2408·28 to 3878·97)	2275·02 (1511·83 to 3292·29)
Isiolo	3694·23 (2955·66 to 4514·46)	3278·64 (2508·64 to 4201·99)	2834·63 (2200·1 to 3565·02)	2145·03 (1665·8 to 2716·19)	1872·44 (1452·8 to 2381·85)	2032·05 (1504·01 to 2662·54)	1876·2 (1433·36 to 2403·53)	1537·87 (1061·21 to 2182·36)
Kajiado	1659·81 (1239·31 to 2133·11)	1508·26 (1088·02 to 2013·54)	1431·54 (1039·56 to 1874·98)	1279·73 (947·39 to 1722·3)	1256 (906·27 to 1653·27)	1633·46 (1240·87 to 2030·42)	1385·13 (1116·47 to 1674·4)	1151·37 (826·11 to 1547·27)
Kakamega	2836·73 (2275·78 to 3434·64)	2183·39 (1652·72 to 2798·01)	1840·61 (1430·1 to 2357·83)	1505·17 (1167·37 to 1906·3)	1634·07 (1256·18 to 2067·2)	2332·59 (1764·51 to 2957·74)	1681·94 (1325·64 to 2087·48)	1279·73 (916·22 to 1728·91)
Kericho	1340·24 (975·83 to 1760·46)	1296·82 (906·88 to 1736·4)	1219·06 (881·38 to 1606·06)	1112·36 (819·5 to 1473·74)	1155·34 (858·8 to 1513·62)	1612·23 (1218·44 to 2078·69)	1416·94 (1096·09 to 1770·19)	1170·74 (842·4 to 1593·05)
Kiambu	2101·56 (1626·41 to 2680·73)	1788·68 (1310·95 to 2370·34)	1564 (1167·81 to 2007·95)	1421·3 (1055·87 to 1787·45)	1502·25 (1196·56 to 1820·69)	2083·36 (1701·81 to 2550·11)	1819·26 (1526·86 to 2167·4)	1508·27 (1120·31 to 1977·82)
Kilifi	2853·51 (2272·01 to 3510·86)	2213·48 (1651·41 to 2862·79)	1800·1 (1413·58 to 2324·87)	1427·46 (1108·46 to 1818·43)	1266·83 (979·77 to 1631·78)	1436·77 (1103·29 to 1869·96)	1355·98 (1060·13 to 1670·91)	1142·02 (805·06 to 1561·12)
Kirinyaga	2655·68 (2065·17 to 3348·45)	2045·92 (1479·99 to 2694·54)	1701·28 (1283·88 to 2182·7)	1201·93 (873·7 to 1588·53)	1245·96 (913·65 to 1617·12)	1682·37 (1224·57 to 2166·04)	1537·22 (1201·56 to 1946·42)	1355·68 (940·05 to 1835·98)
Kisii	2326·94 (1810·53 to 2897·66)	2076·74 (1556·16 to 2702·68)	1776·23 (1318·05 to 2303·18)	1548·82 (1150·46 to 2003·76)	1586·65 (1232·34 to 1971·65)	1923·58 (1519·19 to 2377·79)	1415·57 (1151·37 to 1701·33)	1208·33 (855·79 to 1623·96)
Kisumu	2771·76 (2180·88 to 3423·9)	2368·13 (1781·51 to 3005·23)	2133·09 (1606·96 to 2730·07)	1939·42 (1491·77 to 2466·61)	2189·37 (1681·68 to 2753·87)	3778·11 (2962·81 to 4603·06)	2714·16 (2206·95 to 3324·67)	2225·27 (1541·91 to 3067)
Kitui	3109·06 (2466·7 to 3848·19)	2267·82 (1676·94 to 2971·97)	1835·77 (1383·56 to 2365·4)	1455·21 (1103·21 to 1909·02)	1366·05 (1022·34 to 1737·86)	1721·5 (1275·26 to 2221·79)	1615·22 (1316·76 to 1980·06)	1345·51 (958·79 to 1835·31)
Kwale	3163·11 (2501·09 to 3854·6)	2473·67 (1813·85 to 3236·73)	1982·73 (1502·48 to 2580·59)	1566·34 (1191·88 to 1992·47)	1398·38 (1051·94 to 1852·04)	1514·29 (1136·7 to 2000·59)	1625·73 (1295·24 to 1967·87)	1371·32 (951·09 to 1856·04)
Laikipia	1343·53 (977·47 to 1789·73)	1216·71 (858·46 to 1665·33)	1108·95 (789·89 to 1472·19)	977·04 (711·47 to 1325·85)	1121·44 (855·08 to 1475·42)	1693·68 (1260·24 to 2223·08)	1468·15 (1127·9 to 1814·65)	1191·55 (874·53 to 1604·04)
Lamu	3600·09 (2838·82 to 4448·31)	2421·1 (1772·61 to 3194·13)	1778·8 (1330·13 to 2379·6)	1169·39 (872·69 to 1563·72)	1228·39 (931·81 to 1628·22)	1412·68 (1069·89 to 1911·01)	1420·36 (1053·3 to 1905·02)	1199·32 (803·79 to 1694·01)
Machakos	1819·4 (1416·62 to 2314·02)	1535·65 (1122·35 to 2060·39)	1334·77 (984·56 to 1751·2)	1116·8 (830·04 to 1461·3)	1047·58 (804·66 to 1343·34)	1398·96 (1069·39 to 1866·84)	1356·88 (1097·03 to 1664·81)	1196·34 (863·51 to 1610·54)
Makueni	2315·29 (1805·1 to 2899·95)	1714·44 (1251·15 to 2297·76)	1489·12 (1101·94 to 1971·03)	1256·16 (945·4 to 1650·02)	1250·01 (950·48 to 1628·89)	1897·74 (1366·17 to 2568·46)	1674·8 (1314·64 to 2089·18)	1333·42 (998·64 to 1762·74)
Mandera	1993·57 (1509·22 to 2581·25)	1872·87 (1361·64 to 2410·38)	1736·4 (1301·42 to 2244·46)	1620·93 (1199·88 to 2132·42)	1669·4 (1278·17 to 2113·58)	1825·02 (1396·47 to 2330·07)	1717·91 (1362·6 to 2124·52)	1440·31 (1035·7 to 1937·81)

Supplemental Results Table 3b: Age-standardised mortality rates by location, males, 1950, 1960, 1970, 1980, 1990, 2000, 2010, 2019

Location	1950	1960	1970	1980	1990	2000	2010	2019
Marsabit	2119·12 (1600·55 to 2715·09)	1937·69 (1419·17 to 2563·53)	1677·73 (1264·77 to 2176·7)	1409·73 (1051·45 to 1824·22)	1365·42 (1025·91 to 1747·56)	1581·05 (1139·48 to 2136·66)	1613·22 (1227·57 to 2085·15)	1382·12 (932·09 to 1981)
Meru	1768·5 (1346·53 to 2233·75)	1454·36 (1065·61 to 1918·18)	1217·28 (883·63 to 1593·72)	982·59 (729·08 to 1296·65)	1031·76 (790·3 to 1325·84)	1391·44 (1053·44 to 1785·72)	1420·16 (1093·3 to 1773·31)	1253·92 (891·84 to 1712·53)
Migori	3666·81 (2877·9 to 4478·18)	2760·39 (2096·48 to 3541·5)	2340·53 (1781·58 to 2960·86)	2085·84 (1645·51 to 2589·84)	2058·7 (1585·77 to 2594·93)	3038·69 (2209·39 to 3963·45)	2515·54 (2003·9 to 3182·72)	1899·52 (1296·07 to 2699·95)
Mombasa	1875·04 (1428·41 to 2415·38)	1708·55 (1227·91 to 2297·58)	1341·11 (987·41 to 1776·51)	1014·97 (744·3 to 1355·4)	1120·19 (853·64 to 1484·91)	1644·49 (1214·94 to 2200·78)	1503·61 (1147·79 to 1908·14)	1243·79 (881·83 to 1704·45)
Murang'a	2472·46 (1955·72 to 3096·96)	1745·59 (1265·62 to 2257·2)	1444·53 (1079·52 to 1879·14)	1179·94 (884·82 to 1510·3)	1227·57 (955·88 to 1497·79)	1694·59 (1319·22 to 2112·8)	1543·02 (1291·33 to 1860·63)	1227·81 (896·77 to 1630·69)
Nairobi	2257·99 (1762·24 to 2831·99)	1847·9 (1428·16 to 2384·49)	1395·52 (1079·73 to 1767·56)	1127·89 (871·36 to 1437·79)	1244·03 (947·72 to 1572·3)	1845·54 (1422·31 to 2232·26)	1500·63 (1263·06 to 1753·58)	1241·12 (957·13 to 1586·14)
Nakuru	1490·92 (1114·4 to 1934·04)	1396·16 (1005·32 to 1871·33)	1320·73 (991·21 to 1731·63)	1243·2 (936·2 to 1613·29)	1337·25 (1026·79 to 1697·36)	1916·95 (1519 to 2420·6)	1458·99 (1185·82 to 1777·29)	1154·54 (857·67 to 1541·65)
Nandi	2082·13 (1590·57 to 2644·52)	1822·59 (1357·15 to 2407·59)	1696·06 (1281·07 to 2231·23)	1576·46 (1203·56 to 2080·8)	1675·21 (1287·64 to 2147·96)	2264·76 (1790·03 to 2815·82)	1900·34 (1553·56 to 2307·51)	1471·3 (1045·95 to 2000·32)
Narok	1648·83 (1259·64 to 2095·91)	1563·8 (1162·79 to 2095·08)	1586·74 (1169·75 to 2083·12)	1619·35 (1178·16 to 2111·46)	1693·05 (1284·63 to 2146·21)	2051·49 (1623·64 to 2574·33)	1686·39 (1358·08 to 2082·95)	1423·26 (1045·62 to 1911·86)
Nyamira	1945·69 (1501·47 to 2478·04)	1679·81 (1234·63 to 2246·07)	1500·76 (1070·78 to 1989·68)	1378·15 (1004·01 to 1802·58)	1458·19 (1131·06 to 1836·96)	2291·32 (1772·05 to 2934·89)	2058·95 (1624·05 to 2537·11)	1639·65 (1113·5 to 2295·66)
Nyandarua	2384·36 (1804·26 to 3068·86)	2026·94 (1501·67 to 2714·17)	1697·63 (1269·21 to 2203·37)	1443·54 (1089·57 to 1883·52)	1497·66 (1153·6 to 1886·78)	2057·66 (1638·02 to 2557·45)	2040·05 (1679·78 to 2466·85)	1788·49 (1305·16 to 2394·52)
Nyeri	2123·35 (1608·8 to 2653·21)	1596·72 (1155·62 to 2151·3)	1319·53 (968·51 to 1726·51)	1113·4 (804·69 to 1476·61)	1163·89 (884·5 to 1487·2)	1553·57 (1167·04 to 1999·67)	1354·88 (1082·73 to 1686·9)	1188·14 (874·62 to 1644·76)
Samburu	1537·73 (1145·04 to 2030·8)	1409·82 (1006·13 to 1887·04)	1350·41 (990·66 to 1787·05)	1253·67 (909·34 to 1641·99)	1405·93 (1013·7 to 1858·28)	2000·76 (1457·52 to 2625·46)	1795·37 (1369·14 to 2273·56)	1454·06 (983·35 to 2003·12)
Siaya	2917·05 (2318·5 to 3548·89)	2318·24 (1759·3 to 2967·67)	1877·53 (1456·36 to 2356·05)	1605·92 (1242·65 to 2004·42)	1782·52 (1408·77 to 2275·18)	2974·32 (2239·45 to 3817·39)	1957·91 (1512·39 to 2477·82)	1411·29 (1043·66 to 1964·44)
Taita Taveta	2389·72 (1833·46 to 3025·62)	1988·72 (1440·11 to 2625·08)	1773·78 (1343·96 to 2363·53)	1365·68 (1009·35 to 1823·25)	1273·1 (917·02 to 1696·67)	1658·06 (1199·25 to 2261·21)	1656·27 (1256·68 to 2078·86)	1304·07 (894·29 to 1788·06)
Tana River	2689·18 (2058·55 to 3336·17)	2190·74 (1547·85 to 2940·04)	1982·68 (1466·12 to 2642·34)	1782·79 (1349·78 to 2314·76)	1759·91 (1298·17 to 2310·18)	2223·59 (1724·39 to 2833·65)	2144·3 (1694·62 to 2616·63)	1729·85 (1219·82 to 2421·36)
Tharaka Nithi	2568·21 (2030·99 to 3237·98)	1906·02 (1358·99 to 2584·9)	1742·3 (1280·89 to 2278·27)	1534·46 (1146·56 to 2001·06)	1592·9 (1196·02 to 2049·09)	1973·05 (1543·95 to 2443·05)	1779·12 (1452·29 to 2163·95)	1508·33 (1091·32 to 2059·1)
Trans Nzoia	1467·15 (1076·42 to 1978·31)	1349·94 (951·6 to 1825·44)	1225·39 (887·96 to 1634·35)	1100·27 (811·14 to 1455·98)	1145·68 (870·11 to 1500·41)	1644·27 (1195·93 to 2179·09)	1473·57 (1124·46 to 1888·84)	1212·99 (870·39 to 1650·78)
Turkana	1834·53 (1370·75 to 2375·51)	1778·48 (1304·97 to 2332·04)	1800·89 (1344·78 to 2309·13)	1792·63 (1385·69 to 2313·18)	1807·96 (1345·39 to 2367·79)	2655·46 (1861·27 to 3868·3)	2000·44 (1587·18 to 2467·5)	1525·58 (1065·43 to 2085·25)
Uasin Gishu	1241·94 (904·21 to 1638·6)	1149·95 (803·51 to 1552·49)	1058·51 (755 to 1429·93)	974·91 (706·99 to 1309)	1065·02 (813·38 to 1373·09)	1564·74 (1210·17 to 1916·07)	1356·27 (1128·01 to 1608·92)	1095·57 (809·31 to 1443·95)
Vihiga	2663·29 (2090·38 to 3291·11)	1840·62 (1341·42 to 2415·4)	1624·94 (1174·19 to 2104·49)	1437·99 (1082·66 to 1877·83)	1482·13 (1119·18 to 1903·74)	1950·7 (1473·82 to 2488·49)	1551·35 (1196·36 to 1950·23)	1269·15 (853·22 to 1801·14)
Wajir	2714·75 (2131·7 to 3412·48)	2495·9 (1877·22 to 3228·34)	2157·81 (1660·76 to 2755·29)	1870·13 (1439·91 to 2360·77)	1802·64 (1382·39 to 2279·47)	1955·42 (1542·65 to 2380·3)	1753·59 (1380·8 to 2153·78)	1442·94 (993·52 to 1962·41)
West Pokot	1887·75 (1421·54 to 2405·37)	1604·82 (1163·12 to 2139·3)	1506·99 (1120·56 to 1967·59)	1374·16 (1026·48 to 1826·96)	1395·2 (1053·55 to 1804·61)	1575·28 (1174·39 to 2050·88)	1438·6 (1115·26 to 1799·93)	1192·77 (800·98 to 1658·96)
Madagascar	2778·87 (2418·52 to 3213·64)	2317·48 (1995·99 to 2704·27)	2246·55 (1984·87 to 2553·9)	1698·23 (1479·09 to 1953·22)	1796·87 (1585·07 to 2025·27)	1651·18 (1455·14 to 1885·1)	1478·85 (1180·4 to 1839·13)	1305·51 (969·8 to 1755·18)
Malawi	3674·14 (3062·82 to 4359·99)	3178·85 (2670·59 to 3748·61)	2697·03 (2322·54 to 3126·9)	2098·15 (1837·79 to 2374·2)	2207·81 (1966·05 to 2498·54)	2716·56 (2387·1 to 3117·34)	1988·11 (1777·09 to 2238·86)	1493·73 (1302·56 to 1712·78)
Mozambique	3015·34 (2478·2 to 3638·9)	2669·54 (2245·65 to 3121·68)	2370·86 (2038·73 to 2726·86)	2096·6 (1820·94 to 2389·37)	2157·44 (1894·14 to 2449·13)	2206·81 (1970·05 to 2478·28)	2285·34 (2007·61 to 2590·04)	1991·39 (1705·08 to 2321·54)
Rwanda	3920·46 (3305·16 to 4589·14)	2988·84 (2514·64 to 3565·2)	2917·05 (2494·56 to 3385·26)	2459·63 (2149·56 to 2789·98)	2466·31 (2215·81 to 2743·7)	2449·21 (2198·59 to 2717·79)	1392·45 (1247·3 to 1557·55)	1190·59 (1003·63 to 1397·36)
Somalia	2896·5 (2425·63 to 3408·42)	2830·42 (2326·17 to 3406·83)	2673·27 (2208·83 to 3168·23)	2489·65 (2063·41 to 2930·05)	2583·03 (2192·23 to 2967·52)	2438·13 (1990·49 to 3042·2)	2303·12 (1748·3 to 3096·6)	1930·42 (1398·88 to 2756·28)
South Sudan	2318·32 (1826·78 to 2904·33)	2229·92 (1795·1 to 2699·93)	2123·42 (1726·33 to 2583·56)	1881·38 (1578·98 to 2207·28)	1858·73 (1524·14 to 2222·1)	1746·81 (1411·96 to 2130·43)	1517·06 (1233·05 to 1823·18)	1355·03 (1067·68 to 1691·97)
Uganda	2945·67 (2392·45 to 3571·77)	2416·64 (2055·41 to 2803·93)	2184·47 (1871·25 to 2538·66)	2247·57 (1895·27 to 2667·99)	2464·31 (2172·29 to 2847·28)	2510·93 (2261·97 to 2835·1)	1773·39 (1570·92 to 2010·13)	1398·37 (1189·23 to 1624·48)
Tanzania	3104·87 (2555·75 to 3691·55)	2763·71 (2341·61 to 3188·83)	2360·04 (2036·82 to 2702·04)	1839·68 (1601·56 to 2106·1)	1851·92 (1644·06 to 2089·92)	2009·49 (1785·07 to 2265·12)	1508·79 (1347·98 to 1683·78)	1203·79 (1013·63 to 1410·38)
Zambia	2666·76 (2181·74 to 3224)	2316·31 (1911·66 to 2771·83)	1990·51 (1714·33 to 2308·25)	1836·44 (1626·94 to 2076·99)	2130·47 (1905 to 2369·58)	2862·19 (2507·4 to 3209·3)	2014·89 (1805·58 to 2232·46)	1593·12 (1333·66 to 1900·5)
Southern sub-Saharan Africa	2234·01 (2001·87 to 2512·83)	2027·78 (1849·23 to 2225·71)	1829·28 (1676·95 to 1993·88)	1628·19 (1439·99 to 1835·21)	1434·44 (1303·05 to 1573·31)	2240·63 (2038·71 to 2482·31)	2019·57 (1874·88 to 2195·37)	1476·58 (1311·49 to 1656·68)
Botswana	2539·15 (2101·52 to 2995·39)	2406·63 (2007·18 to 2848·75)	2227·85 (1862·87 to 2687·78)	1875·73 (1588·26 to 2188·44)	1841·61 (1563·77 to 2143·95)	3367·92 (2819·95 to 3979·56)	2131·22 (1801·04 to 2517·69)	1729·76 (1420·3 to 2117·53)
eSwatini	3441·2 (2908·75 to 4050·43)	3143·97 (2629·13 to 3714·02)	2624·6 (2225·7 to 3069·8)	2164·78 (1801·28 to 2537·18)	1864·77 (1594·39 to 2143·01)	2930·64 (2441·79 to 3516·45)	3147·49 (2654·47 to 3703·37)	2257·85 (1934·78 to 2715·58)
Lesotho	2731·41 (2331·39 to 3175·75)	2569·66 (2207·03 to 3011·41)	2394·34 (2030·92 to 2835·22)	2056·14 (1728·41 to 2440·87)	1910·37 (1668·94 to 2187·49)	2933·3 (2515·05 to 3416·93)	3143·54 (2740·89 to 3625·86)	2764·36 (2359·12 to 3226·66)
Namibia	2300·06 (1897·27 to 2759·51)	2115·77 (1772·95 to 2507·07)	1945·15 (1647·44 to 2237·65)	1900·43 (1620·13 to 2231·61)	1740·84 (1561·4 to 1926·87)	2312·09 (2032·81 to 2619·44)	1840·21 (1625·63 to 2086·19)	1578·9 (1309·23 to 1894·06)
South Africa	2140·73 (1928·15 to 2410·65)	1952·94 (1766·92 to 2140·12)	1764·23 (1598·14 to 1950·94)	1574·32 (1353·07 to 1816·7)	1355·6 (1203·71 to 1516·34)	2042·2 (1846·66 to 2261·58)	1897·58 (1737·1 to 2096·5)	1367·88 (1186·9 to 1582·25)
Zimbabwe	2466·23 (1809·25 to 3422·22)	2204·95 (1733·85 to 2786·64)	1999·57 (1656·7 to 2409·23)	1792·73 (1592·95 to 2030·69)	1618·92 (1426·56 to 1864·82)	2905·78 (2440·91 to 3481·25)	2445·44 (2183·36 to 2755·57)	1822·89 (1559·97 to 2133·28)
Western sub-Saharan Africa	2753·17 (2263·61 to 3298·18)	2636·8 (2254·94 to 3057·76)	2258·69 (1975·12 to 2577·64)	1904·55 (1705·59 to 2114·54)	1806·49 (1642·62 to 1985·63)	1823·03 (1643·85 to 2021·04)	1557·93 (1403·97 to 1735·01)	1317·84 (1164·23 to 1494·98)
Benin	3060·22 (2228·11 to 4196·17)	2580·41 (2024·2 to 3286·88)	2261·16 (1847·17 to 2702·36)	1962·15 (1720·33 to 2234·71)	1805·53 (1608·48 to 2000·01)	1780·28 (1571·53 to 2025·17)	1576·62 (1347·19 to 1863·64)	1364·17 (1105·73 to 1688·11)
Burkina Faso	3358·8 (2524·02 to 4559·57)	3033·26 (2445·62 to 3789·01)	2593·99 (2220·72 to 3031·36)	2230·37 (1944·84 to 2571·81)	2093·72 (1849·89 to 2396·6)	2026·43 (1779·32 to 2340·17)	1752·02 (1549·4 to 1992·98)	1552·29 (1296·61 to 1877·39)
Cape Verde	2028·55 (1798·55 to 2286·42)	1623·18 (1455·84 to 1816·69)	1444·79 (1297·7 to 1611·64)	1067·1 (975·55 to 1160·92)	1007·79 (927·81 to 1096·82)	1103·86 (940·44 to 1267·77)	848·27 (779·29 to 924·71)	1085·26 (927·54 to 1241·54)
Cameroon	2904·9 (2208·19 to 3827·1)	4720 (3950·23 to 5614·79)	2075·97 (1714·26 to 2450·97)	1788·74 (1534·63 to 2053·36)	1651·92 (1463·89 to 1840·58)	1987·39 (1768·2 to 2266·9)	1768·8 (1519·91 to 2055·71)	1442·27 (1167·12 to 1786·15)
Chad	2849 (1936·55 to 4141·03)	2399·7 (1752·55 to 3353·6)	2293·37 (1777·78 to 3031·31)	2081·44 (1733·37 to 2482·28)	1876·06 (1668·31 to 2135·28)	2068·01 (1779·24 to 2377·68)	1773·67 (1565·25 to 2002·1)	1525·16 (1253·14 to 1843·41)
Côte d'Ivoire	2687·27 (2285·9 to 3126·8)	2502·78 (2065·43 to 2985·32)	2093·63 (1773·14 to 2446·97)	1717·29 (1495·77 to 1975·83)	1920·04 (1705·38 to 2193·31)	2278·98 (2021·1 to 2562·08)	1819·66 (1590·35 to 2088·45)	1399·86 (1191·99 to 1655·77)
The Gambia	2161·44 (1762·7 to 2654·07)	2162·81 (1785·98 to 2600·22)	1937·03 (1623·87 to 2317·84)	1800·84 (1514·91 to 2161·22)	1637·26 (1381·86 to 1944·82)	1595·28 (1373·83 to 1838·88)	1470·02 (1264·95 to 1684·45)	1325·48 (1120·38 to 1549·27)
Ghana	2522·23 (2149·32 to 2911·03)	2161·39 (1844·14 to 2521·47)	1929·42 (1639·75 to 2240·42)	1737·36 (1465·29 to 2064·73)	1632·56 (1389·16 to 1910·41)	1640·83 (1455·39 to 1840·65)	1617·79 (1460·26 to 1793·92)	1367·83 (1164·97 to 1623·49)
Guinea	3059·93 (2521·37 to 3660·28)	2741·5 (2235·43 to 3316·6)	2491·64 (2077·37 to 2971·21)	2152·42 (1848·82 to 2515·71)	1781·92 (1542·56 to 2047·4)	1825·04 (1616·69 to 2052·89)	1711·73 (1471·02 to 1979·4)	1491·52 (1211·39 to 1820·8)
Guinea-Bissau	4515·37 (3925·8 to 5208·39)	3715·69 (3203·81 to 4289·94)	3499·7 (2935·88 to 4165·1)	2829·44 (2412·32 to 3286·89)	2586·03 (2217·4 to 2984·68)	2379·63 (2020·15 to 2797·91)	2051·1 (1787·69 to 2350·94)	1751·14 (1421·78 to 2124·98)
Liberia	3743·83 (2914·18 to 4721·98)	2948·29 (2470·25 to 3429·43)	2496·01 (2140·89 to 2925·19)	1986·19 (1723·26 to 2321·65)	2227·17 (1953·68 to 2544·49)	1739·82 (1502·01 to 2004·12)	1379·49 (1185·53 to 1584·59)	1172·29 (964·21 to 1462·4)
Mali	3473·34 (2858·71 to 4098·1)	3122·71 (2539·93 to 3782·32)	2850·02 (2408·45 to 3381·16)	2332·96 (2053·34 to 2672·01)	1954·6 (1728·96 to 2188·3)	1696·01 (1522·9 to 1901·59)	1476·12 (1304·78 to 1674·01)	1314·64 (1065·32 to 1631·16)
Mauritania	2616·47 (1919·58 to 3543·46)	2241·53 (1758·81 to 2811·72)	1887·31 (1548·12 to 2253·65)	1765·47 (1477·24 to 2050·58)	1578·59 (1388·75 to 1793·64)	1242·25 (1087·16 to 1415·49)	997·13 (861·56 to 1168·5)	901·75 (746·09 to 1088·16)
Niger	3052·48 (2282·04 to 4114·75)	2925·34 (2349·79 to 3685·71)	2611·04 (2222·54 to 3071·73)	2375·16 (2115·33 to 2672·41)	2131·37 (1914·13 to 2373·65)	1847·9 (1653·05 to 2065·59)	1532·48 (1343·98 to 1756·95)	1358·23 (1114·32 to 1686·1)
Nigeria	2590·64 (1843·73 to 3703·59)	2404·84 (1796·71 to 3259·94)	2208·8 (1732·18 to 2876·93)	1815·92 (1478·81 to 2198·81)	1786·57 (1501·37 to 2094·5)	1791·29 (1474·16 to 2172·15)	1483·15 (1213·43 to 1804·98)	1240·23 (1004·07 to 1541·49)
São Tomé and PrÍncipe	3187·55 (2784·12 to 3629·76)	2634·8 (2407·7 to 2870·46)	1827·94 (1670·28 to 1998·03)	1437·55 (1298·34 to 1596·71)	1377·94 (1227·15 to 1554·85)	1262·35 (1122·22 to 1424·88)	1139·04 (1019·49 to 1277·87)	1089·89 (924 to 1262·92)
Senegal	2627·48 (1961·03 to 3535·88)	2468·99 (1991·55 to 3084·19)	2381·69 (2024·24 to 2785·49)	2024·2 (1786·44 to 2283·82)	1676·68 (1497·93 to 1874·56)	1543·26 (1404·28 to 1710·95)	1278·91 (1123·05 to 1448·83)	1132·13 (971·61 to 1331·89)
Sierra Leone	3031·78 (2382·75 to 3880·36)	2726·89 (2254·93 to 3240·52)	2447·23 (2022·22 to 2939·58)	2235·23 (1866·72 to 2661·96)	2065·42 (1747·49 to 2397·62)	1995·97 (1753·08 to 2248)	1748·61 (1502 to 2056·01)	1399·24 (1170·49 to 1705·87)
Togo	2740·61 (2227·27 to 3377·36)	2473·26 (2090·06 to 2871·95)	2064 (1771·78 to 2392·23)	1757·73 (1519·09 to 2027·05)	1688·24 (1449·34 to 1963·42)	1901·33 (1616·83 to 2244·59)	1766·05 (1560·17 to 1997·85)	1437·47 (1185·87 to 1738·56)

Supplemental Results Table 3c: Age-standardised mortality rates by location, females, 1950, 1960, 1970, 1980, 1990, 2000, 2010, 2019

Location	1950	1960	1970	1980	1990	2000	2010	2019
Global	1766·6 (1650·03 to 1884·43)	1864·56 (1689·22 to 2123·01)	1284·82 (1225·96 to 1347·55)	1107·71 (1070·63 to 1152·6)	959·45 (930·78 to 989·75)	872·89 (848·2 to 900·17)	717·66 (695·34 to 741·95)	616·86 (577·93 to 657·36)
Central Europe, eastern Europe, and central Asia	1192·74 (1093·57 to 1299·03)	915·27 (882·88 to 949·64)	860·38 (834·57 to 887·9)	850·71 (822·2 to 879·25)	795·87 (769·89 to 824·29)	852·09 (822·54 to 883·04)	713·78 (691·13 to 737·25)	610·63 (555·01 to 670·63)
Central Asia	1783·3 (1516·39 to 2087·47)	1299·11 (1192·2 to 1406·8)	1025·53 (972·05 to 1085·5)	897·95 (863·74 to 935·4)	840·3 (811·9 to 870·94)	999·85 (954·3 to 1047·54)	938·13 (893·35 to 988·97)	814·49 (745·24 to 898·29)
Armenia	1824·46 (1531·15 to 2189·59)	1158·84 (1012·62 to 1305·08)	941 (853·19 to 1037·38)	799·87 (725·69 to 879·21)	796·52 (736·36 to 858·06)	768 (714·27 to 829·79)	673·27 (621·86 to 730·2)	594·21 (496·9 to 709·85)
Azerbaijan	2482·38 (2105·53 to 2907·94)	1740·69 (1542·15 to 1941·35)	1127·54 (1031·63 to 1237·48)	919·51 (841·37 to 1000·18)	839·11 (768 to 917·39)	983·43 (901·13 to 1082·99)	1046·77 (960·42 to 1143·03)	967·93 (806·31 to 1160·33)
Georgia	1584·74 (1315·28 to 1882·51)	1091·56 (961·44 to 1221·77)	870·84 (789·76 to 957·49)	871·95 (778·72 to 971·23)	886·54 (805·5 to 973·84)	731·05 (654·99 to 813·38)	653·01 (583·18 to 739·25)	596·21 (500·12 to 697·42)
Kazakhstan	1324·92 (1101·59 to 1581·55)	1098·97 (974·74 to 1233·04)	937·94 (857·36 to 1027·44)	821·21 (755·89 to 896·08)	819·65 (757·94 to 890·36)	991·14 (914·4 to 1073·38)	894·65 (869·65 to 923·16)	722·54 (630·33 to 819·41)
Kyrgyzstan	1648·24 (1376·67 to 1987·75)	1302·97 (1144·37 to 1478·02)	1083·38 (978·8 to 1201·69)	902·72 (822·7 to 986·77)	801·47 (733·76 to 873·58)	877·39 (808·96 to 955·25)	815·1 (760·86 to 873·96)	650·15 (563·74 to 762·59)
Mongolia	2886·01 (2406·42 to 3396·16)	2225·07 (1874·95 to 2601·3)	1858·8 (1564·23 to 2160·28)	1427·64 (1207·07 to 1670·76)	1293·97 (1118 to 1493·23)	1425·31 (1258·15 to 1616·45)	1126·86 (970·8 to 1302·25)	918·89 (729·04 to 1138·04)
Tajikistan	2354·08 (1946·95 to 2811·35)	1671·96 (1495·2 to 1852·43)	1162·22 (1054·73 to 1285·68)	972·71 (866·16 to 1087·9)	848·19 (774·74 to 929·84)	1043·48 (955·3 to 1140·13)	1041·97 (930·31 to 1146·29)	988·44 (814·4 to 1203·4)
Turkmenistan	2022·97 (1696·04 to 2403·58)	1448·99 (1305·89 to 1611·82)	1177·31 (1073·85 to 1289·83)	1032·26 (942·94 to 1122·91)	949·99 (878·34 to 1027·95)	919·1 (806·88 to 1069·56)	775·18 (696·27 to 860·56)	729·03 (594·81 to 907·22)
Uzbekistan	1756·44 (1420·91 to 2135·77)	1213·84 (1077·6 to 1356·7)	1005·99 (917·89 to 1100·83)	884·58 (817·27 to 961·98)	786·32 (728·8 to 852·14)	1228·38 (1065·57 to 1418·81)	1430·43 (1182·28 to 1772·8)	1237·28 (942·45 to 1649·19)
Central Europe	1451·16 (1391·88 to 1511·08)	1015·67 (985·9 to 1047·09)	916·15 (888·2 to 943·57)	846·45 (817·39 to 875·58)	788·59 (761·74 to 813·53)	693·91 (670·89 to 717·63)	571·07 (561·07 to 581·68)	506·9 (449·62 to 570·48)
Albania	1876·46 (1668·71 to 2107·9)	1124·25 (1022·68 to 1233·83)	874·47 (731·95 to 1034·05)	704·52 (604·13 to 828·59)	637·49 (586·31 to 689·39)	548·27 (504·7 to 592·46)	487·38 (427·21 to 560·06)	453·17 (355·34 to 563·3)
Bosnia and Herzegovina	2020·59 (1856·35 to 2204·21)	1268·65 (1170·21 to 1374·51)	991·61 (919·94 to 1075·12)	875·91 (810·84 to 947·07)	709·25 (658·62 to 764·1)	578·61 (491·98 to 675·47)	603·45 (549·97 to 663·96)	578·59 (468·41 to 716·24)
Bulgaria	1313·33 (1213·29 to 1416·81)	872·78 (788 to 963·24)	829·29 (748·25 to 918·89)	880·14 (802·7 to 980·45)	916·22 (842·68 to 998·13)	932·09 (858·58 to 1011·67)	764·51 (735·15 to 795·36)	709·26 (579·85 to 854·78)
Croatia	2032·83 (1891·83 to 2193·68)	1320·54 (1227·82 to 1424·89)	970·64 (901·38 to 1047·94)	820·59 (757·68 to 897·24)	737·79 (673·05 to 812·9)	624·68 (572·42 to 682·46)	551·92 (510·66 to 595·43)	466·84 (370·71 to 577·4)
Czech Republic	1217·23 (1135·27 to 1313·73)	908·71 (843·67 to 978·93)	909·38 (848·71 to 975·48)	864·63 (805·86 to 934·95)	772·25 (715·08 to 839·03)	607·26 (595·92 to 618·25)	482·97 (472·44 to 493)	427·41 (356·8 to 506·59)
Hungary	1316·35 (1229·32 to 1412·63)	1039·81 (971·71 to 1116·78)	938·26 (877·47 to 1005·95)	914·57 (854·53 to 977·55)	837·51 (782·01 to 901·54)	708·54 (657·76 to 764·34)	591·61 (549·39 to 639·94)	528·6 (433·53 to 647·61)
Montenegro	977·02 (893·52 to 1064·56)	796·42 (733·68 to 862·88)	633·26 (582·76 to 687·42)	673·14 (622·56 to 728·25)	593·52 (543·55 to 648·32)	679·93 (616·9 to 746·57)	695·02 (638·83 to 758·07)	606·83 (518·01 to 717·12)
North Macedonia	1996·75 (1824·26 to 2187·31)	1499·08 (1382·75 to 1635·6)	1065·21 (981·9 to 1150·69)	827·44 (762·06 to 896·67)	932·97 (843·77 to 1024·7)	936·8 (854·92 to 1021·62)	921·58 (819·53 to 1044·53)	827·72 (655·82 to 1041·18)
Poland	1409·51 (1310·96 to 1515·93)	897·48 (840·16 to 958·91)	811·01 (756·78 to 871·77)	761·13 (701·47 to 829·59)	726·8 (670·95 to 789·41)	613·48 (566·09 to 667·66)	484·12 (478·55 to 489·62)	433·71 (351·75 to 536·23)
Romania	1407·83 (1187·99 to 1659·94)	1068·68 (974·1 to 1176·23)	998·02 (916·22 to 1092·66)	967·41 (886·05 to 1055·93)	883·69 (816·79 to 956·34)	784·15 (722·87 to 852·46)	638·55 (591·04 to 689·33)	556·94 (457·4 to 675·72)
Serbia	1883·9 (1753·54 to 2028·96)	1286·33 (1198·88 to 1394·78)	1113·66 (1027·36 to 1208·74)	861·21 (801·29 to 928·36)	885·81 (822·22 to 961·35)	900·63 (827·94 to 980·24)	808·92 (752·51 to 872·39)	721·42 (594·05 to 882·18)
Slovakia	1194·2 (1170·02 to 1221·85)	861·89 (842·63 to 879·31)	871·89 (855·13 to 891·23)	802·12 (785·12 to 819·05)	736·2 (720·99 to 751·31)	618·54 (602·7 to 634·08)	545·05 (532·12 to 558·97)	491·67 (397·61 to 608·21)
Slovenia	1298·72 (1208·01 to 1401·81)	1173·98 (1084·71 to 1270·04)	962·56 (887·94 to 1044·88)	780·52 (718·24 to 846·66)	645·96 (507·27 to 812·49)	516·54 (498·02 to 536·42)	396·15 (380·28 to 414·15)	334·32 (267·82 to 420·16)
Eastern Europe	943·54 (816·25 to 1086·56)	783·02 (743·16 to 823·73)	781·14 (748·76 to 818·84)	823·96 (785·31 to 869·48)	770·04 (731·99 to 815·12)	885·83 (839·45 to 939·06)	741·01 (703·23 to 783·97)	622·02 (535·71 to 714·97)
Belarus	833·1 (668·04 to 1037·69)	678·56 (621·73 to 742·61)	650·24 (607·41 to 699·76)	708·17 (659·46 to 764·25)	701·12 (652·62 to 758·16)	761·33 (705·78 to 821·85)	669·77 (621·07 to 725·71)	572·52 (449·43 to 740·04)
Estonia	1008·17 (826·08 to 1225·01)	850·88 (823·91 to 878·03)	805·58 (780·87 to 833·36)	795·06 (770·86 to 821·31)	770·43 (744·85 to 794·83)	687·36 (664·66 to 710·48)	478·84 (458·39 to 500·09)	442·06 (357·64 to 538·06)
Latvia	887·24 (710·13 to 1100·45)	796·9 (739·92 to 864·03)	771·02 (713·32 to 830·29)	792·28 (738·09 to 851·43)	762·98 (704·77 to 825·93)	688·83 (639·5 to 742·84)	594·91 (576·26 to 614·52)	520·59 (416·3 to 648·56)
Lithuania	932·81 (735·4 to 1153·44)	770·99 (714·91 to 827·65)	706·5 (657·78 to 759·81)	717·55 (662·71 to 773·68)	681·48 (632 to 739·62)	618·38 (574·69 to 668·2)	572·06 (528·98 to 621·43)	489·34 (406·71 to 587·88)
Moldova	1729·89 (1447·63 to 2049·42)	1525·26 (1367·12 to 1699·47)	1225·37 (1120·26 to 1340·76)	1048·92 (973·43 to 1131·72)	934·67 (868·36 to 1008·97)	859·78 (781·34 to 947·11)	772·85 (693·38 to 866·43)	579·69 (475·81 to 712·16)
Russia	948·65 (775·41 to 1146·75)	794·5 (735·4 to 853·66)	795·04 (742·62 to 852·83)	837·52 (775·52 to 902·57)	775·17 (719·02 to 839·9)	911·3 (840·6 to 988·21)	753·75 (703·85 to 808·86)	608·28 (501·19 to 733·75)
Ukraine	900·58 (736·52 to 1091·67)	736·4 (682·73 to 794·89)	760·35 (712·55 to 810·62)	805·86 (749·98 to 872·35)	766·99 (714·63 to 827·78)	884·11 (824·82 to 950·78)	747·34 (664·12 to 847·64)	709·32 (544·53 to 922·58)
High income	1074·78 (1044·99 to 1104·05)	882·18 (861·24 to 903·99)	779·44 (760·74 to 798·72)	628·76 (613·25 to 645·1)	523·16 (511·34 to 535·62)	450·69 (440·59 to 461·62)	375·78 (367·66 to 384·28)	354·84 (340·47 to 369·86)
Australasia	913·65 (858·67 to 971·4)	800·78 (754·49 to 854·53)	774·3 (727·38 to 827·38)	605·81 (566·75 to 644·91)	511·99 (479·91 to 546·53)	421·19 (394·82 to 449·61)	349·11 (328·24 to 372·48)	327·1 (302·14 to 352·89)
Australia	910·49 (846·37 to 980·24)	796·17 (741·29 to 861·38)	775·77 (721·42 to 839·18)	591·34 (547·45 to 638·53)	500·83 (465·31 to 539·94)	411·77 (382·2 to 444·02)	340·93 (318·59 to 367·69)	320·27 (292·37 to 351·21)
New Zealand	936·14 (872·91 to 1008·38)	821·43 (764·91 to 884·52)	769·34 (715·52 to 829·27)	673·14 (627·33 to 725·88)	568·24 (527·11 to 614·44)	470·17 (437·03 to 507·93)	392·68 (357·82 to 429·71)	364·91 (328·43 to 408·9)
High-income Asia Pacific	1521·48 (1437·56 to 1619·16)	1052·71 (993·71 to 1118·19)	837·2 (788·02 to 892)	636·29 (600·87 to 677·02)	464·38 (438·88 to 493·59)	350·22 (330·87 to 371·93)	281·9 (266·37 to 300·28)	249·38 (230·71 to 271·46)
Brunei	2404·67 (2163·44 to 2648·73)	1844·17 (1665·64 to 2031·98)	1478·46 (1338·64 to 1617·73)	1138·91 (1031·59 to 1268·08)	983·93 (886·94 to 1081·64)	979·09 (898·14 to 1077·57)	793·63 (726·32 to 869·6)	722·99 (629·5 to 834·84)
Japan	1354·4 (1270·37 to 1456·31)	980·16 (919·38 to 1048·76)	765·23 (714·71 to 825·31)	565·62 (529·17 to 606·7)	416·99 (390·76 to 447·86)	320·78 (299·76 to 344·43)	269·57 (251·21 to 291·35)	240·11 (218·26 to 264·9)
Aichi	1325·78 (1082·01 to 1623·35)	978·02 (775·76 to 1208·3)	781·55 (634·87 to 972·53)	592·28 (536·97 to 657·85)	437·42 (398·48 to 486·73)	334·94 (302·64 to 372·41)	273·76 (247·71 to 304·45)	239·75 (200·34 to 285·27)
Akita	1313·05 (1052·49 to 1622·81)	973·78 (775·18 to 1198·07)	797·03 (654·79 to 953·58)	600·12 (542·72 to 662·1)	434·8 (392·85 to 480·06)	331·33 (299·01 to 368·45)	280·1 (252·18 to 310·47)	279·02 (236·92 to 327·95)
Aomori	1397·8 (1148·08 to 1724·89)	1004·64 (807·11 to 1249·08)	771·38 (629·55 to 940·44)	562·75 (510·84 to 620·09)	423·29 (383·49 to 467·36)	335·9 (304·09 to 374·05)	290·49 (261·75 to 323·72)	259·75 (220·15 to 306·34)
Chiba	1368·78 (1099·45 to 1684·83)	984·36 (783·49 to 1212·99)	764·71 (612·6 to 954·49)	572·13 (518·79 to 631·22)	408·54 (369·95 to 450·54)	327·2 (296·24 to 362·29)	276·64 (249·55 to 306·3)	251·72 (213·33 to 296·79)
Ehime	1344·86 (1095·04 to 1625·73)	966·05 (772·03 to 1205·38)	731·03 (594·86 to 892·13)	539·85 (490·81 to 597·19)	407·53 (370·57 to 450·16)	323·87 (293·64 to 358·72)	265·83 (239·67 to 298·61)	247·92 (208·02 to 294·44)
Fukui	1254·36 (1005·52 to 1534·19)	935·95 (748·95 to 1140·92)	713·87 (593·13 to 861·75)	511·65 (461·89 to 569·15)	389·06 (350·23 to 432·61)	293·26 (262·42 to 328·32)	246·39 (220·56 to 274·97)	224·83 (190·97 to 264·89)
Fukuoka	1351·63 (1094·51 to 1637·24)	975·72 (780·5 to 1206·79)	763·88 (617·94 to 945·69)	559·13 (506·02 to 620·75)	412·92 (372·3 to 458·43)	321·13 (290·14 to 354·74)	266·11 (240·65 to 296·82)	242·8 (203·85 to 288·94)
Fukushima	1431·65 (1164·91 to 1746·4)	1010·14 (811·6 to 1231·56)	791·75 (640·44 to 970·18)	556·63 (504·79 to 612·5)	403·06 (368·2 to 442·97)	320·27 (289·38 to 353·13)	274·32 (247·68 to 303·73)	255·38 (215·62 to 302·54)
Gifu	1376·47 (1118 to 1684·87)	1021·25 (813·27 to 1257·33)	825·31 (670·26 to 1009·29)	614·32 (555·39 to 680·02)	433·48 (394·64 to 478·42)	332·88 (300·28 to 367·48)	272·24 (245·07 to 302·17)	243·59 (205·31 to 287·19)
Gunma	1426·94 (1159·52 to 1750·01)	1040·01 (818·89 to 1277·88)	812·64 (655·33 to 991·27)	610·98 (554·8 to 676·39)	431·24 (390·42 to 475·72)	324·71 (293·82 to 358·92)	280·99 (254·64 to 311·11)	249·91 (211·9 to 291·34)
Hiroshima	1391·99 (1136·77 to 1696·17)	988·77 (799·39 to 1221·8)	746·65 (600·34 to 924·51)	553·31 (500·02 to 611·56)	408·91 (368·44 to 451·39)	302·92 (273·61 to 335·25)	255·76 (230·18 to 286·39)	226·56 (187·28 to 270·95)
Hokkaidō	1348·68 (1087·05 to 1655·59)	979·43 (785·22 to 1204·6)	781·41 (637·93 to 959·86)	587·85 (533·87 to 652·75)	419·01 (378·96 to 463·76)	314·48 (283·82 to 349·5)	271·02 (245·02 to 300·67)	244·76 (206·52 to 292·93)
Hyōgo	1333·34 (1072·84 to 1621·59)	974·67 (770·01 to 1213·2)	775·56 (627·29 to 956·61)	586·59 (531·17 to 647·29)	437·98 (395·53 to 483·73)	333·22 (301·92 to 367·37)	273·68 (247·04 to 302·54)	235·92 (197·01 to 279·46)
Ibaraki	1500·09 (1232·37 to 1812·58)	1051·7 (845·4 to 1282·8)	819·17 (660·5 to 1015·33)	591·51 (534·94 to 647·12)	444·77 (401·76 to 490·17)	339·82 (307·61 to 375·03)	290·31 (261·71 to 320·53)	251·89 (211·84 to 295·19)
Ishikawa	1266·36 (1018·9 to 1555·51)	944·36 (752·12 to 1165·97)	732·05 (605·84 to 884·23)	561·88 (507·74 to 621·73)	396·04 (357·74 to 438·73)	312·21 (280·12 to 345·83)	258·53 (232·47 to 286·71)	238·3 (201·44 to 280·95)
Iwate	1415·42 (1161·11 to 1712·28)	1016·29 (817·83 to 1240·85)	797·05 (656·82 to 956·3)	584·01 (525·56 to 644·08)	427·01 (386·16 to 472·91)	320·34 (289·9 to 354·76)	277·45 (248·62 to 310·48)	268·26 (226·93 to 313·77)
Kagawa	1323·1 (1088·54 to 1632·06)	967·66 (777·45 to 1193·58)	732·29 (597·04 to 887·15)	528·76 (480·17 to 585·65)	404·72 (367·63 to 450·63)	324·63 (293·21 to 361·14)	275·65 (248·7 to 305·12)	234·57 (196·27 to 275·32)
Kagoshima	1560·46 (1278·39 to 1885·11)	1087·91 (881·55 to 1326)	844·45 (689·46 to 1025·72)	589·49 (537·29 to 651·72)	421·64 (381·62 to 465·45)	320·21 (289·49 to 353·94)	272·07 (244·84 to 303·82)	235·41 (199·33 to 277·06)
Kanagawa	1237·83 (1001·9 to 1503·55)	899·74 (720·57 to 1127·22)	708·27 (567·64 to 866·91)	542·02 (492·2 to 592·58)	414·11 (376·83 to 454·38)	318·78 (289·63 to 347·86)	268·52 (243·29 to 294·85)	234·91 (197·35 to 277·57)
Kōchi	1481·99 (1188·86 to 1805·85)	1056·55 (863·35 to 1289·64)	807·36 (669·29 to 961·01)	561·79 (509·87 to 622·26)	405·05 (367·83 to 450·13)	322·39 (292·28 to 358·46)	264·64 (237·79 to 295·2)	235·14 (199·02 to 273·26)
Kumamoto	1420·47 (1149·4 to 1712·23)	1014·14 (818·16 to 1244·62)	786·1 (644·41 to 973·56)	552·9 (498·3 to 608·18)	388·71 (352·07 to 429·74)	295·48 (266·8 to 325·61)	252·65 (226·74 to 280·56)	223·13 (189·35 to 262·88)
Kyōto	1292·31 (1044·01 to 1587·77)	938·16 (743·21 to 1157·69)	715·51 (576·64 to 892·04)	522·52 (470 to 579·8)	407·66 (368·29 to 452·58)	308·38 (277·48 to 342·72)	257·88 (231·69 to 285·06)	232·41 (191·85 to 277·36)
Mie	1321·68 (1067 to 1591·74)	968·56 (781·14 to 1191·64)	758·52 (623·32 to 915·28)	566·68 (513·23 to 628·38)	427·49 (388·28 to 473·28)	323·97 (291·11 to 358·34)	272·81 (245·98 to 304·41)	247·32 (208·78 to 291·87)
Miyagi	1361·65 (1109·29 to 1658·7)	972·32 (776·31 to 1206·48)	778·67 (635·98 to 951·34)	589·28 (533·27 to 651·13)	418·42 (378·47 to 461·33)	317·61 (287·03 to 351·6)	264·98 (239·16 to 292·33)	238·91 (202·52 to 281·06)
Miyazaki	1430·01 (1152·53 to 1741·6)	1033·88 (831·14 to 1264·99)	794·6 (648·35 to 949·86)	568·51 (513·67 to 627·88)	404·12 (364·79 to 449·7)	306·74 (276·8 to 340·14)	265·56 (238·58 to 294·46)	244·92 (207·45 to 288·38)
Nagano	1357·1 (1099·44 to 1655·99)	969·83 (776·75 to 1179·29)	749·78 (607·19 to 926·34)	552·19 (497·82 to 608·22)	395·44 (356·79 to 436·76)	299·74 (270·53 to 331·75)	250·02 (225·52 to 276·99)	233·05 (197·55 to 274·02)
Nagasaki	1380·27 (1103·95 to 1684·52)	1001·29 (804·8 to 1236·18)	790·51 (654·65 to 964·19)	583·26 (529·54 to 645·51)	411·42 (373·16 to 457·78)	316·8 (286·84 to 351·14)	268·4 (241·83 to 300·29)	247·4 (206·54 to 294·18)
Nara	1341·95 (1075·8 to 1639·77)	984·83 (795·99 to 1213·84)	791·28 (650·03 to 950·12)	596·07 (536·33 to 659·52)	440·98 (396·02 to 486·13)	322·39 (287·91 to 357·2)	269·79 (243·56 to 299·01)	244·53 (208·56 to 284·21)
Niigata	1307·93 (1054·81 to 1584·99)	950·2 (758·93 to 1166·21)	773·73 (615·36 to 951·67)	586·39 (530·53 to 650·66)	405·2 (367·85 to 449·67)	302·33 (273·4 to 334·87)	250·79 (225·66 to 278·7)	253·49 (211·92 to 302·13)
Ōita	1435·52 (1155·5 to 1745·55)	1037·34 (833·91 to 1286·1)	811·86 (676·96 to 981·33)	576·44 (524·83 to 638·79)	420·34 (379·1 to 466·22)	320·46 (289·16 to 355·01)	255·31 (230·78 to 285·49)	231·54 (195·08 to 274·08)
Okayama	1314·39 (1064·24 to 1602·32)	956·06 (763·78 to 1176·26)	728·31 (592·33 to 889·63)	537·04 (487·7 to 593·08)	393·49 (355·48 to 434·22)	301·45 (272·44 to 332·89)	253·06 (228·77 to 280·59)	218·61 (182·76 to 257·92)
Okinawa	1492·93 (1211·72 to 1839·06)	1014·16 (793 to 1275·65)	765·42 (626·08 to 913·49)	500·93 (454·49 to 552·95)	339·23 (306·99 to 375·83)	281·28 (254·61 to 312·35)	258·22 (233·14 to 288·41)	240·17 (204·21 to 283·35)
Ōsaka	1369·17 (1105·61 to 1665·56)	997·69 (804·46 to 1222·98)	796·28 (650·68 to 988·14)	611·31 (556·74 to 671·83)	460·84 (419·19 to 505·42)	343·52 (311·57 to 380·66)	286·39 (259·8 to 316·83)	244·39 (205·79 to 288·67)
Saga	1394·79 (1140·83 to 1691·69)	992·4 (797·49 to 1230)	753·26 (615·94 to 909·15)	537·42 (484·42 to 597·48)	402·28 (363·76 to 446·54)	309·43 (279·21 to 344·64)	262·06 (234·57 to 291·24)	249·33 (210·81 to 292·82)

Supplemental Results Table 3c: Age-standardised mortality rates by location, females, 1950, 1960, 1970, 1980, 1990, 2000, 2010, 2019

Location	1950	1960	1970	1980	1990	2000	2010	2019
Saitama	1348·56 (1086·27 to 1640·04)	972·58 (774·41 to 1215·03)	769·12 (624·59 to 951·33)	586·97 (531·43 to 646·62)	432·36 (391·82 to 477·6)	333·05 (301·75 to 367·88)	282·84 (254·85 to 312·85)	247·97 (209·54 to 295·37)
Shiga	1357·96 (1108·36 to 1651·73)	1004·76 (823·47 to 1222·95)	800·11 (660·63 to 963·39)	590·89 (532·83 to 652·52)	437·08 (392·88 to 484·37)	307·29 (278·28 to 339·5)	258·66 (231·9 to 288·08)	219·75 (184·5 to 260·35)
Shimane	1330·36 (1082 to 1623·87)	979·01 (791·17 to 1215·47)	746·04 (621·65 to 888·71)	519·71 (468·76 to 579·13)	363·85 (330 to 403·03)	276·38 (249·43 to 307·74)	254·37 (228·44 to 284·42)	229 (194·47 to 270·96)
Shizuoka	1268·33 (1030·37 to 1564·66)	920·75 (728·51 to 1141·13)	723·62 (582·82 to 887·69)	542·64 (491·91 to 595·32)	403·47 (366 to 445·47)	310·43 (281·6 to 341·18)	268·81 (243·22 to 295·73)	234·16 (197·83 to 276·52)
Tochigi	1439·31 (1164·66 to 1754·02)	1038·07 (835·28 to 1262·34)	807·59 (657·31 to 978·8)	613·71 (558·6 to 673·19)	455·73 (414·78 to 502·95)	340·55 (309·53 to 377·33)	291·59 (265·1 to 324·03)	269·58 (227·88 to 317·64)
Tokushima	1432·33 (1149·6 to 1740·49)	1045·28 (839·32 to 1283·66)	817·39 (672·8 to 983·01)	592·35 (534·09 to 658·27)	422·35 (378·9 to 467·89)	319·03 (286·63 to 353·41)	268·59 (240·69 to 300·48)	246·54 (209·16 to 287·34)
Tōkyō	1245·64 (1016·24 to 1537·28)	929·22 (736·87 to 1133)	733·48 (592·53 to 899·35)	549·72 (499·18 to 607·03)	423·33 (385·73 to 465·31)	333·69 (303·05 to 367·39)	268·24 (244·08 to 295·9)	228·67 (192·35 to 268·28)
Tottori	1365·86 (1100·61 to 1680·42)	982·82 (798·53 to 1182·91)	755·43 (628·14 to 907·84)	541·42 (485·21 to 599·06)	408·1 (369·05 to 455·51)	308·08 (276·87 to 341·38)	263·28 (236 to 292·22)	251·27 (212·47 to 294·17)
Toyama	1313·23 (1069·11 to 1595·71)	968·45 (775·14 to 1187·89)	744·23 (615·14 to 891·52)	531·82 (485·72 to 593·04)	384·97 (349·72 to 425·41)	296·88 (268·47 to 332·03)	253·07 (227·85 to 281·55)	217·8 (183·52 to 259·29)
Wakayama	1436·61 (1180·81 to 1745·94)	1045·99 (841·43 to 1284·09)	823·49 (675·25 to 992·53)	600·02 (542·66 to 665·53)	439·38 (396·23 to 487·28)	334·52 (300·53 to 372·39)	287·46 (259·45 to 320·93)	264·19 (223·32 to 313·61)
Yamagata	1358·7 (1100·94 to 1643·48)	997·79 (808·49 to 1229·65)	802·3 (657·36 to 961·95)	554·08 (501·61 to 613·45)	397·76 (359·33 to 441·99)	313·81 (282·8 to 350·86)	263·11 (237·31 to 293·16)	239·69 (203·75 to 281·52)
Yamaguchi	1357·07 (1096·01 to 1643·3)	982·59 (788·74 to 1214·04)	747·62 (611·76 to 909·92)	561·78 (508·75 to 619·23)	402·6 (362·05 to 446·05)	324·63 (294·12 to 359·35)	279 (251·98 to 309·91)	248·63 (210·94 to 294·1)
Yamanashi	1390·04 (1131·32 to 1682·84)	993·96 (799·5 to 1225·76)	753·36 (614·48 to 913·32)	554·33 (501·93 to 614·39)	408·89 (370·42 to 455·91)	317·16 (287·36 to 353·03)	260·56 (235·18 to 290·99)	232·04 (195·79 to 272·87)
South Korea	2242·12 (2077·63 to 2441·53)	1303·05 (1189·77 to 1413·45)	1139·84 (1050·55 to 1239·17)	997·67 (935·36 to 1073·25)	724·36 (681·01 to 779·98)	539·59 (505·46 to 579·36)	354·56 (330·49 to 381·61)	300·65 (272·13 to 331·29)
Singapore	1336·32 (1248·45 to 1435·91)	1039·28 (953·97 to 1140·16)	886·85 (829·37 to 955·35)	762·68 (713·67 to 815·41)	580·17 (541·74 to 621·72)	435·26 (406·18 to 467·15)	322·57 (300·82 to 347·42)	268·91 (250·57 to 291·58)
High-income North America	908·55 (855·86 to 966·12)	803·31 (757·29 to 857·26)	724·28 (680·49 to 771·77)	582·59 (547·08 to 622·84)	520·24 (488·61 to 554·32)	502·27 (472·46 to 535·83)	434·22 (408·58 to 461·77)	423·99 (379·01 to 477·72)
Canada	933·73 (874·48 to 998·95)	780·02 (724·18 to 840·72)	653·46 (608·4 to 704·34)	539·55 (502·63 to 579·42)	475·38 (439·75 to 513·02)	426·24 (398·21 to 459·64)	356·99 (333·44 to 386·92)	340·36 (312·44 to 372·12)
Greenland	1961·69 (1753·03 to 2192·23)	1361·04 (1232·99 to 1507·14)	1176·63 (1070·09 to 1284·05)	1152·27 (1039·31 to 1278·9)	1147·64 (1026·35 to 1280·64)	1198·31 (1093·09 to 1310·95)	772·22 (700·52 to 850·06)	660·5 (551·5 to 791·76)
USA	906 (848·61 to 967·88)	805·52 (755·8 to 865·5)	730·77 (683·43 to 783·83)	586·77 (547·14 to 631·34)	525·18 (490·49 to 564·34)	510·75 (476·83 to 548·98)	443·3 (414·17 to 475·95)	434·18 (382·27 to 495·76)
Alabama	1019·39 (817·06 to 1256·42)	850·37 (770·18 to 941·51)	774·37 (700·5 to 860·96)	615·73 (557·36 to 682·02)	570·9 (514·77 to 631·78)	579·17 (523·36 to 642·27)	567·85 (513·09 to 628·62)	562·16 (433·95 to 716·09)
Alaska	1135·64 (958·97 to 1339·66)	963·06 (869·13 to 1076·01)	773·74 (700·24 to 862·8)	629·06 (565·73 to 701·18)	627·52 (567·22 to 695·23)	605·76 (546·65 to 672·21)	476·48 (429·96 to 527·1)	476·38 (386·25 to 590·32)
Arizona	885·8 (699·92 to 1101·62)	740·78 (668·05 to 822·51)	694·89 (632·07 to 767·59)	572·58 (518·07 to 630·9)	487·27 (442·23 to 538·56)	484·1 (440·23 to 533·92)	409·52 (372·68 to 451·06)	416·02 (315·7 to 542)
Arkansas	879·92 (695·46 to 1103·27)	750·42 (679·65 to 831·98)	689·59 (624·37 to 764·55)	570·88 (513·86 to 630·95)	552 (500·44 to 610·73)	568·98 (514·12 to 627·34)	529 (478·14 to 588·11)	547·64 (422·28 to 701·1)
California	845·63 (681·63 to 1045·85)	746·5 (683·64 to 819·04)	697·93 (637·65 to 765·55)	573·62 (523·17 to 632·2)	512·39 (469·66 to 560·74)	465·85 (425·09 to 512·98)	380·6 (348·24 to 416·74)	358·41 (279·83 to 456·82)
Colorado	847·68 (671·46 to 1047·95)	743·47 (672·19 to 826·85)	658·63 (595·66 to 730·09)	541·26 (489·94 to 602·04)	477·01 (432·21 to 527·02)	483·44 (435·98 to 537·85)	418·61 (378·81 to 460·85)	402·98 (308·31 to 515·98)
Connecticut	865·6 (685·88 to 1094·69)	782·08 (706·34 to 864·29)	674·97 (609·4 to 749·23)	558·06 (503·31 to 620·63)	477·8 (429·27 to 526·97)	457·01 (412·04 to 507·48)	377·56 (339·68 to 420·16)	367·42 (276·9 to 470·46)
Delaware	984·18 (795·14 to 1185·39)	858·18 (777·34 to 949·99)	799·41 (725·49 to 886·66)	627·76 (568·57 to 693·31)	580·29 (525·46 to 642·06)	544·76 (490·93 to 601·52)	476·85 (431·64 to 524·78)	445·2 (355·75 to 554·51)
Washington, DC	1024·52 (825·42 to 1251·37)	940·49 (849·29 to 1044·32)	876·28 (788·79 to 972·53)	696·1 (624·32 to 776·47)	691·79 (622·45 to 764·26)	582·77 (520·54 to 650·63)	597·43 (538·67 to 663·99)	494·36 (389·43 to 623·39)
Florida	858·71 (673·78 to 1060·78)	739·37 (668·09 to 815·42)	681·04 (611·97 to 751·95)	561·84 (505·72 to 624·05)	485·69 (437·82 to 535·15)	471·2 (426·08 to 521·51)	404·95 (364·81 to 446·32)	405·45 (313·46 to 529·64)
Georgia	994·3 (794·56 to 1238)	855·55 (772·2 to 942·53)	779·15 (703·75 to 857·92)	631 (570·49 to 698·43)	581·61 (525·41 to 638·74)	588·52 (532·7 to 649·03)	519·26 (469·46 to 573·35)	492·72 (380·33 to 628·41)
Hawaii	944·79 (766·86 to 1143·89)	873·22 (789·96 to 969·11)	689·17 (623·86 to 760·8)	458·29 (412·05 to 509·13)	423·85 (381·98 to 469·66)	377·44 (338·48 to 418·76)	328·97 (296 to 364·28)	324·43 (258·15 to 403·13)
Idaho	820·37 (649·89 to 1017·4)	706·18 (638·08 to 787)	655·19 (589·56 to 732·19)	521·97 (469·99 to 582·82)	480·88 (433·99 to 533)	486·49 (440·2 to 542·29)	448·1 (405·78 to 499·89)	443·94 (348·76 to 565·44)
Illinois	926·69 (740·49 to 1142·32)	839·75 (761·41 to 930·17)	770·23 (698·75 to 848·96)	603·48 (548·83 to 667·34)	541·99 (491·6 to 598·01)	515·58 (469·11 to 571·67)	433·18 (393·49 to 480·3)	418·64 (320·79 to 541·99)
Indiana	881·41 (703·03 to 1084·47)	806·63 (734·07 to 889·48)	742·24 (672·14 to 818·58)	591·92 (538·31 to 652·52)	536·55 (486·01 to 590·59)	545·24 (492·46 to 599·55)	491·68 (446·99 to 542·44)	496·29 (385·35 to 646·92)
Iowa	768·68 (610·3 to 952·29)	703·71 (635·94 to 776·09)	655·35 (586·87 to 723·15)	498·06 (448·87 to 551·92)	444·67 (401·93 to 490·98)	457·17 (413·01 to 506·35)	419·38 (379·44 to 464·36)	412·38 (310·39 to 550·64)
Kansas	768·01 (604·17 to 955·52)	707·1 (641·24 to 782·68)	637·65 (576·71 to 706·17)	523·02 (472·36 to 579·28)	453·1 (409·5 to 497·35)	487·73 (440 to 541·68)	449·83 (406·33 to 495·68)	440·44 (330·28 to 570·83)
Kentucky	912·51 (735·55 to 1142·39)	807·21 (730·67 to 895·07)	772·25 (694·83 to 857·11)	613·03 (551·48 to 678·84)	559·95 (504·79 to 619·59)	576·04 (524·41 to 637·86)	559·41 (507·46 to 619·8)	575·57 (447·41 to 736·52)
Louisiana	992·73 (793·51 to 1229·74)	868·77 (788·82 to 954·79)	790·67 (712·85 to 874·02)	651·46 (585·36 to 723·26)	602·39 (547 to 661·27)	609·16 (550·28 to 674·61)	539·44 (487·4 to 590·98)	538·38 (414·96 to 694·14)
Maine	914·15 (725·99 to 1131·67)	804·3 (728·98 to 890·89)	732·54 (658·65 to 814·48)	557·82 (504·46 to 621·68)	506·66 (455·77 to 563·76)	506·58 (454·33 to 561·95)	448·41 (403·95 to 495·9)	479·45 (377·11 to 607·56)
Maryland	982·8 (788·06 to 1233·85)	887·52 (804·69 to 982·21)	768·68 (693·88 to 849·26)	614·48 (553·58 to 684·66)	561·12 (507·63 to 618·35)	551·48 (498·17 to 611·02)	442·61 (401·8 to 488·21)	438·24 (336·75 to 568·41)
Massachusetts	883·74 (713·66 to 1099·3)	794·48 (725·96 to 877·09)	688·82 (623·5 to 764·14)	563·16 (510·34 to 624·14)	488·85 (444·18 to 540·03)	472·38 (428·87 to 522·45)	384·34 (348·12 to 423·57)	385·03 (294·98 to 496·03)
Michigan	905·39 (721·93 to 1111·89)	810·16 (735·52 to 897·39)	747·03 (675·23 to 829·63)	595·74 (540·34 to 661·65)	546·36 (491·66 to 605·76)	534·99 (483·46 to 593·42)	475·3 (429·56 to 526·87)	469·84 (356·73 to 606·46)
Minnesota	784·35 (619·64 to 997·5)	726·08 (657·41 to 804·2)	635·46 (575·87 to 702·3)	494·03 (445·77 to 550·66)	452·22 (409·68 to 498)	448·2 (406·62 to 495·14)	381·66 (346·54 to 423·39)	372·52 (281·08 to 481·07)
Mississippi	1035·74 (831·44 to 1290·49)	886·33 (798·04 to 979·43)	802·88 (724·87 to 890·92)	643·71 (580·77 to 711·31)	585·95 (528·01 to 648·01)	623·27 (563·37 to 686·5)	576·79 (521·66 to 635·82)	568·88 (427·25 to 738·34)
Missouri	867·19 (691·24 to 1067·1)	790·75 (721·27 to 873·13)	732·6 (663·26 to 808·9)	587·87 (532·78 to 650·15)	531·96 (483·28 to 587·35)	544·8 (493·9 to 602·25)	490·27 (444·72 to 541·64)	479·96 (363·6 to 615·18)
Montana	871·52 (710·26 to 1060·59)	771·78 (695·84 to 858·46)	702·24 (634·48 to 778·23)	572·85 (515·33 to 639·68)	509·07 (458·75 to 562·13)	495·96 (448·89 to 555·32)	458·72 (414·14 to 510·67)	472·84 (377·66 to 593·83)
Nebraska	755·93 (603·77 to 931·85)	708·47 (641·83 to 789·4)	656·36 (594·41 to 728·55)	521·26 (472·3 to 582·07)	464·4 (420·91 to 516·6)	453·21 (411·77 to 506·27)	416·26 (374·24 to 465·47)	415·28 (326·32 to 520·8)
Nevada	1158·59 (955·83 to 1385·3)	914·04 (828·18 to 1014·83)	858·56 (779·62 to 956·8)	738·52 (664·53 to 824)	678·83 (615·82 to 756·46)	662·14 (599·04 to 734·39)	537·33 (487·37 to 595·48)	496·29 (389·25 to 633·96)
New Hampshire	896·19 (725·65 to 1103·49)	766·42 (688·1 to 855·97)	720·57 (650·35 to 798·87)	567·26 (509·66 to 634·39)	499·12 (448·5 to 554·86)	503·03 (456·24 to 558·47)	415·44 (372·84 to 460·4)	427·13 (337·37 to 539·95)
New Jersey	950·62 (758·96 to 1168·25)	847·97 (769·64 to 937·31)	758·53 (685·87 to 840·48)	608 (550·71 to 674·93)	538·21 (489·17 to 592·3)	498·26 (451·12 to 549·56)	401·25 (362·01 to 443·78)	395·84 (305·08 to 503·39)
New Mexico	906·46 (720·99 to 1119·51)	786·8 (711·86 to 873·54)	716·57 (646·62 to 792·17)	555·61 (499·28 to 615·9)	503·55 (456·52 to 557·43)	491·01 (443·74 to 544·45)	448·11 (407·84 to 496·31)	455·29 (359·63 to 570·01)
New York	956·37 (782·92 to 1169·87)	862·62 (788·66 to 946·79)	771·72 (701·41 to 845·27)	616·06 (563·37 to 676·2)	542·14 (495·76 to 593·63)	475·12 (431·47 to 522·76)	388·23 (353·56 to 426·83)	369·94 (282·13 to 473·1)
North Carolina	946·77 (753·26 to 1179·82)	841·93 (762·68 to 932·33)	757·38 (682·63 to 838·95)	592·7 (534·91 to 655·82)	550·38 (498·18 to 607·94)	562·06 (508·38 to 622·98)	485·03 (437·68 to 535·81)	479·54 (369·26 to 622·31)
North Dakota	814·6 (673·2 to 979·12)	717·19 (644·66 to 794·84)	643·98 (579·34 to 712·39)	512·11 (460·64 to 567·86)	442·45 (400·51 to 490)	436·96 (393·73 to 487·21)	394·91 (355·58 to 438·62)	409·75 (331·88 to 505·9)
Ohio	914·02 (739·67 to 1120·17)	822·25 (749·6 to 900·89)	750·55 (682·36 to 825·72)	617·58 (560·66 to 679·74)	546·28 (497·56 to 599·2)	541·64 (489·83 to 592·49)	491·21 (446·92 to 536·78)	502·09 (389·82 to 644·69)
Oklahoma	815·7 (645·15 to 1026·25)	725·61 (660·91 to 801·56)	682·57 (617·26 to 751·53)	575·4 (522·3 to 634·72)	544·54 (493·71 to 600·43)	584·49 (527·2 to 645·18)	552·56 (498·17 to 611·23)	558·22 (426·12 to 715·82)
Oregon	807·21 (645·59 to 1007·79)	725·5 (656·94 to 801·52)	657·75 (594·49 to 730·43)	538·61 (486·06 to 597·43)	502·53 (454·75 to 559·53)	496·03 (448·16 to 551·16)	439·38 (398·47 to 486·88)	420·12 (316·57 to 544·57)
Pennsylvania	972·52 (787·63 to 1197·96)	862·58 (786·01 to 948·21)	776·69 (705·97 to 856·03)	615·41 (559·23 to 678·88)	541·31 (490·97 to 593·49)	513·89 (468·02 to 564)	448·04 (406·76 to 490·51)	448·48 (352·77 to 557·66)
Rhode Island	902·5 (728·66 to 1102·79)	815·14 (735·93 to 908·59)	732·82 (661·99 to 816·99)	555·34 (498·37 to 616·55)	501·84 (453·52 to 557·96)	474·65 (426·36 to 529·12)	411·44 (371·02 to 456·57)	431·01 (342·05 to 547·52)
South Carolina	1091·91 (865·83 to 1350·39)	903·3 (814·71 to 998·85)	794·31 (711·97 to 870·14)	643·06 (577·05 to 710·15)	585·39 (525·27 to 644·17)	577·11 (518·48 to 637·89)	517·92 (464·86 to 568·06)	506·58 (384·43 to 650·23)
South Dakota	798·29 (647·42 to 982·45)	734·03 (664·37 to 817·53)	667·16 (601·6 to 740·88)	518·31 (465·14 to 579·36)	451·9 (406·21 to 504·35)	447·11 (402·95 to 497·28)	414·02 (372·45 to 461·62)	427·86 (341·76 to 539·6)
Tennessee	935·43 (744·67 to 1156·65)	818·61 (739·1 to 908·64)	755·74 (684·26 to 836·6)	596·65 (538·06 to 662·68)	558·96 (503·24 to 617·7)	578·82 (524·86 to 639·34)	540·62 (489·49 to 596·55)	542·31 (411·52 to 704·78)
Texas	845·84 (661·61 to 1046·73)	738·73 (675·06 to 815·49)	699·96 (636·56 to 773·03)	586·84 (531·22 to 652·29)	523·58 (475·78 to 577·43)	532·65 (485·44 to 586·25)	468·39 (425·46 to 515·01)	445·04 (342·99 to 569·2)
Utah	834·7 (672·04 to 1024·25)	720·7 (652·34 to 798·87)	653·83 (593·32 to 722·18)	546·6 (495·54 to 602·39)	470·03 (426·94 to 520·59)	479·7 (436·1 to 528·96)	433·48 (395·11 to 478·42)	433·29 (345·38 to 548·53)
Vermont	897·54 (731·62 to 1065·99)	793·58 (716·03 to 877·72)	693·95 (624·67 to 776·64)	559·68 (503·11 to 624·87)	509·14 (457·38 to 560·67)	489·29 (442·43 to 538·52)	387·28 (346·85 to 429·66)	406·54 (329 to 502·06)
Virginia	968·19 (765·76 to 1197·53)	848·95 (772·36 to 932·14)	744·25 (676·09 to 819·63)	602 (546·21 to 665·29)	541·31 (491·51 to 599·23)	535·59 (485·97 to 594·21)	447·59 (405·88 to 497·57)	435·08 (331·25 to 561·58)
Washington	817·78 (652·76 to 1014·01)	735·04 (664·84 to 816·33)	686·26 (619·62 to 759·74)	550·56 (497·44 to 606·28)	492·63 (442·02 to 543·78)	477·74 (430·27 to 527·58)	416·19 (375·54 to 458·64)	398·81 (303·46 to 514·95)
West Virginia	922·03 (739·17 to 1150·48)	792·72 (716·58 to 875·56)	817·2 (730·85 to 908·63)	623·54 (562·45 to 695·59)	549·74 (493·36 to 609·49)	590·75 (532·53 to 653·56)	554·87 (499·6 to 613·91)	589·97 (454·17 to 772·96)
Wisconsin	839·05 (663·2 to 1048·46)	775·45 (701·03 to 856·94)	679·58 (612·18 to 754·59)	534·86 (482·4 to 593·33)	491·27 (443·44 to 541·05)	475·79 (428·69 to 526·75)	420·85 (380·33 to 463·81)	428·59 (325·18 to 559·89)
Wyoming	868·15 (712·66 to 1045·39)	771·95 (694·18 to 862·09)	681·17 (615·06 to 758·17)	572·16 (516·64 to 640·87)	513·71 (464·55 to 570·54)	502·94 (450·03 to 560·36)	470·29 (426·04 to 524·34)	456·61 (370·65 to 564·76)
Southern Latin America	1182·43 (1125·13 to 1237·51)	1090·59 (1038·88 to 1146·44)	950·13 (902·78 to 1001·32)	764·96 (728·05 to 805·35)	650·45 (616·05 to 687·31)	561·53 (530·41 to 591·93)	506·37 (479·43 to 533)	484·92 (442 to 530·21)
Argentina	1050·17 (982·12 to 1121·29)	952·96 (892·22 to 1024·66)	891·27 (834·97 to 957·08)	747·08 (700·34 to 799·72)	654·85 (611·63 to 704·75)	580·72 (538·7 to 624·1)	529·74 (495·4 to 564·91)	522·16 (461·04 to 593·3)
Chile	1623·35 (1522·41 to 1743·7)	1613·6 (1507·51 to 1728·78)	1312·52 (1227·44 to 1409·94)	879·98 (810·77 to 958·16)	670·54 (618·77 to 726·42)	514·4 (474·69 to 559·47)	454·83 (408·61 to 510·67)	404·52 (372·94 to 440·27)
Uruguay	999·41 (934·01 to 1075·24)	830·56 (755·58 to 911·99)	822·45 (748·4 to 906·48)	726·77 (658·69 to 805·75)	621·25 (560·8 to 691·09)	553·29 (502·58 to 611·35)	488·44 (453·66 to 527·64)	467·02 (412·68 to 530·38)
Western Europe	1024·4 (988·35 to 1061·86)	864·94 (841·09 to 889·25)	778 (757·87 to 799·24)	643·36 (626·53 to 660·56)	533·88 (523·85 to 544·26)	447·18 (438·87 to 455·82)	366·28 (361·08 to 371·86)	342·56 (330·5 to 354·6)

Supplemental Results Table 3c: Age-standardised mortality rates by location, females, 1950, 1960, 1970, 1980, 1990, 2000, 2010, 2019

Location	1950	1960	1970	1980	1990	2000	2010	2019
Andorra	539·77 (420·34 to 685·05)	538·9 (419·47 to 674·04)	509·45 (401·61 to 620·32)	450·97 (359·84 to 581·92)	399·46 (325·08 to 521·5)	334·03 (268·09 to 439·36)	311·52 (253·62 to 395·73)	322·26 (242·56 to 411·66)
Austria	1114·74 (1045·21 to 1191·92)	913·88 (850·36 to 985·73)	862·84 (801·35 to 930·82)	721·68 (669·82 to 774·93)	567·15 (528·26 to 610·24)	465·83 (434·14 to 501·5)	378·64 (373·14 to 384·3)	340·84 (312·64 to 371·56)
Belgium	1022·93 (959·49 to 1096·04)	885·51 (819·88 to 954·73)	814·62 (762·32 to 872·46)	676·87 (626·74 to 732·43)	541·54 (501·22 to 588·73)	467·5 (433·31 to 504·81)	396·77 (391·75 to 401·86)	363·13 (336·09 to 394·82)
Cyprus	1179·18 (1012·19 to 1360·79)	1105·58 (955·86 to 1256·97)	1050·56 (904·89 to 1246·86)	1030·88 (942·47 to 1125·81)	944·8 (876·51 to 1023·02)	685·13 (635·67 to 740·52)	483·25 (445·69 to 526·35)	453·23 (396·96 to 520·92)
Denmark	957·57 (896·3 to 1031·33)	806·78 (752·95 to 867·11)	694·93 (650·65 to 745·24)	620·83 (611·98 to 629·77)	593·53 (583·96 to 602·64)	532·29 (523·62 to 541·01)	446·4 (439·11 to 453·36)	387·35 (356·75 to 423·23)
Finland	1167·55 (1088·47 to 1255·95)	938·32 (876·23 to 1005·81)	791·87 (781·09 to 802·54)	599·81 (590·14 to 609·01)	545·57 (536·88 to 554·58)	457·66 (450·12 to 466·02)	368·65 (362·16 to 375·76)	332·63 (305·25 to 364·28)
France	982·61 (919·35 to 1055·24)	825·33 (762·61 to 897)	710·37 (656·25 to 768·95)	577·92 (535·77 to 625·77)	458·2 (429·73 to 492·92)	396·02 (368·87 to 426·44)	324·91 (303·99 to 347·88)	295·29 (268·55 to 325·2)
Germany	983·3 (862·96 to 1135·24)	918·9 (856·3 to 988·18)	867·3 (815·25 to 931·83)	703·82 (657·41 to 752·07)	581·57 (576·65 to 586·62)	465·46 (460·81 to 470·12)	395·46 (391·37 to 399·58)	378·56 (346·74 to 413·36)
Greece	854·66 (797·62 to 915·41)	746·9 (697·16 to 806·63)	672·43 (624·03 to 726·55)	627·8 (583·94 to 676·16)	557·05 (517·61 to 601·31)	510·64 (473·36 to 551·5)	419·6 (390·2 to 451·14)	395·01 (352·61 to 449·36)
Iceland	788·79 (748·61 to 834·54)	731·76 (703·72 to 759·1)	677·67 (652·74 to 704·76)	524·58 (504·37 to 546·26)	487·13 (466·07 to 509·57)	419·04 (395·93 to 440·53)	304·99 (285·83 to 325·89)	262·49 (232·98 to 293·97)
Ireland	1180·98 (1105·11 to 1263·95)	961·06 (889 to 1037·48)	860·1 (801·49 to 921·02)	765·76 (707·48 to 829·2)	643·08 (598·13 to 693·64)	554·15 (511·79 to 601·41)	395·29 (364·94 to 429·48)	362·28 (328·69 to 399·23)
Israel	817·71 (763·18 to 877·81)	802·24 (743·13 to 868·37)	842·66 (777·75 to 913·37)	738·75 (678·38 to 803·13)	570·25 (515·58 to 631·5)	492·96 (445·13 to 544·57)	370·77 (341·46 to 404·72)	336·33 (294·31 to 384·65)
Italy	1061·82 (993·48 to 1137·29)	855·92 (795·07 to 929·68)	744·67 (693·11 to 803·63)	637·82 (594·82 to 687·81)	501·56 (469·33 to 539·24)	412·25 (383·67 to 444·75)	339·61 (336·51 to 342·95)	313·35 (288·98 to 340·01)
Luxembourg	1166·65 (1084·99 to 1259·6)	971·84 (902·57 to 1047·2)	840·07 (785·2 to 900·66)	730·04 (672·95 to 790·62)	579·98 (536·12 to 631·71)	485·63 (443·93 to 529·87)	387·58 (368·58 to 406·72)	320·26 (282·97 to 364·24)
Malta	1210·83 (1124·85 to 1312·38)	1002·5 (919·57 to 1095·98)	912·55 (831·46 to 1003·63)	824·79 (741·34 to 916·2)	563·42 (505·16 to 626·2)	477·87 (428·15 to 531·64)	359·15 (327·2 to 394·13)	321·95 (275·39 to 377·15)
Monaco	1067·67 (947·21 to 1218·72)	880·94 (766·38 to 1006·84)	710·19 (620·23 to 825·13)	564·36 (484·9 to 643·27)	465·22 (366·55 to 553·2)	445·75 (339·91 to 541·12)	451·38 (363·51 to 536·87)	415·63 (323·21 to 499·27)
Netherlands	907·61 (897·61 to 918·02)	759·61 (751·87 to 767·7)	686·48 (678·98 to 694·26)	545·7 (539·76 to 551·92)	501·19 (495·59 to 507·04)	485·32 (479·79 to 491·26)	397·07 (391·91 to 401·97)	379·16 (349·2 to 415·97)
Norway	815·87 (761·87 to 876·32)	724·86 (675·68 to 774·96)	639·36 (598·19 to 687·19)	537·56 (500·15 to 573·44)	503·35 (469·13 to 539·72)	447·1 (439·93 to 454·65)	374·73 (368·33 to 381·52)	332·67 (307·53 to 361·28)
Portugal	1313·39 (1222·52 to 1414·29)	1041·18 (969·36 to 1115·45)	970·47 (904·6 to 1041·21)	764·22 (712·12 to 825·02)	643·17 (598·07 to 694·97)	518·03 (481·59 to 557·46)	386·39 (360·97 to 416·29)	341·33 (303·72 to 385·85)
San Marino	681·1 (498·15 to 858·34)	668·9 (534·07 to 809·43)	615·93 (533·1 to 701·79)	524·06 (447·03 to 616·5)	408·13 (336·07 to 497·68)	339·57 (275·84 to 420·43)	358·18 (260·01 to 471·27)	341·4 (237·02 to 484·14)
Spain	1206·09 (1124·85 to 1296·47)	866·46 (808·53 to 926·06)	780·73 (728·46 to 835·41)	585·63 (545·24 to 630·25)	497·36 (461·62 to 532·76)	396·93 (393·11 to 400·57)	319·3 (316·05 to 322·47)	297·92 (273·71 to 326·53)
Sweden	926·36 (864·76 to 994·51)	774·44 (725·19 to 829·88)	638·65 (631·53 to 646·5)	551·06 (544·01 to 557·34)	477·77 (471·64 to 483·92)	419·54 (413·56 to 424·88)	364·43 (359·33 to 369·81)	336·71 (309·03 to 368·53)
Stockholm	936·5 (709·96 to 1177·78)	792·02 (606·34 to 1022·92)	663·4 (525·96 to 825·34)	569·87 (549·58 to 593·02)	501·42 (481·84 to 520·77)	421·84 (406·24 to 437·3)	364·47 (350·17 to 378·19)	332·01 (275·73 to 399·05)
Sweden except Stockholm	924·52 (851·02 to 1003·36)	771·27 (711·11 to 837·96)	633·46 (600·41 to 662·93)	546·97 (538·99 to 554·14)	472·74 (466·06 to 479·23)	418·94 (412·61 to 425·09)	364·53 (358·73 to 370·24)	338·35 (309·35 to 372·74)
Switzerland	968·42 (904·96 to 1038·88)	811·96 (757·52 to 870·66)	703·87 (658·92 to 755·15)	549·55 (512·74 to 586·65)	452·31 (423·3 to 483·53)	389·65 (364·57 to 417·42)	322·55 (317·55 to 327·46)	294·14 (269·01 to 322·83)
UK	988·43 (933·15 to 1046·71)	838·21 (787·23 to 894·37)	759·91 (713·43 to 806·47)	672·13 (633·01 to 717·85)	583·81 (547·94 to 622·31)	506·1 (476·73 to 539·3)	406·24 (381·6 to 433·27)	397·09 (360·52 to 439·42)
England	959·57 (898·42 to 1024·61)	808·98 (753·54 to 870·82)	742·36 (690·59 to 798·26)	658·87 (615·24 to 713·4)	573·12 (531·84 to 618·43)	497·02 (461·8 to 536·37)	397·03 (368·88 to 429·15)	388·84 (346·66 to 441·01)
East Midlands	975·26 (776·66 to 1194·69)	824·79 (639·08 to 1049·88)	756·17 (604·4 to 937·09)	668·74 (597·24 to 751·19)	568·73 (510·41 to 632·44)	500·42 (449·83 to 555·89)	402·44 (360·58 to 447·43)	405·54 (332·81 to 485·56)
Derby	1038·54 (773·25 to 1353·7)	882·13 (625·11 to 1201·13)	808·67 (604·64 to 1091·43)	711·23 (608·39 to 831·26)	607·69 (529·62 to 696·5)	503·99 (434·12 to 578·09)	433·66 (377·13 to 497·17)	411·91 (326·22 to 513·14)
Derbyshire	973·01 (726·45 to 1270·74)	825·65 (597·26 to 1144·81)	757·6 (573·31 to 990·73)	675·43 (588·91 to 782·02)	581·88 (514·67 to 662·93)	502·75 (442·51 to 572·62)	398·78 (349·47 to 453·97)	399·91 (311·12 to 502·4)
Leicester	1071·43 (793·41 to 1397·85)	921·88 (655·09 to 1254·38)	845·34 (634·16 to 1109·78)	747·17 (638·82 to 873·21)	604·44 (522·84 to 691·14)	566·07 (490·72 to 646·65)	441·48 (382·01 to 504·81)	411·89 (322·63 to 515·84)
Leicestershire	888·09 (651·35 to 1168·39)	754·66 (534·74 to 1041·26)	688·43 (514·22 to 897·12)	609·53 (520·57 to 708·92)	520·26 (453·18 to 597·66)	460·58 (401·46 to 528·1)	359·41 (314·81 to 416·13)	361·04 (280·78 to 461·99)
Lincolnshire	957·42 (681·76 to 1255·93)	808·41 (563·81 to 1106·24)	744·21 (565·94 to 978·41)	661·38 (566·86 to 769·17)	558·7 (486·15 to 634·22)	493·65 (433·65 to 561·27)	395·27 (343·9 to 448·55)	401·28 (319·46 to 503·18)
Northamptonshire	945·82 (695·81 to 1247·99)	799·99 (575·64 to 1119·14)	730·22 (545·44 to 947·29)	641·38 (551·94 to 742·9)	559·58 (490·61 to 637·14)	479·16 (421·55 to 547·49)	402·42 (349·83 to 460·66)	430·24 (341·18 to 539·19)
Nottingham	1059·16 (752·49 to 1399·06)	904·4 (629·82 to 1218·45)	826·91 (622·68 to 1073·02)	734·6 (624·34 to 862·34)	616·28 (534·26 to 708·79)	619·05 (538·25 to 708·94)	474·55 (409·99 to 546·58)	433·6 (327·2 to 556·54)
Nottinghamshire	967·88 (720·76 to 1269·44)	816·78 (582·8 to 1115·91)	745·71 (568·85 to 960·65)	659·76 (569·04 to 759·56)	569·85 (501·69 to 647·44)	498·87 (437·26 to 568·84)	412·1 (363·13 to 470·2)	436·5 (344·29 to 544·89)
Rutland	880·36 (639·28 to 1190·03)	739·43 (511·45 to 1038·97)	671·68 (491·93 to 920)	602·93 (486·93 to 724·6)	529·97 (448·29 to 626·46)	424·15 (357·25 to 502·12)	339·27 (280·78 to 405·17)	330·57 (249·45 to 440·29)
East of England	890·56 (714·85 to 1115·15)	753·53 (578·82 to 956·13)	688·74 (551·17 to 857·8)	623·14 (554·86 to 704·46)	540·53 (487·97 to 601·85)	465·69 (419·99 to 518·62)	375·89 (337·99 to 418·37)	370·48 (303·38 to 449·75)
Bedford	908·2 (661 to 1231·38)	766·38 (530·17 to 1058·62)	684·97 (507·87 to 903·41)	599·8 (503 to 708·44)	555·13 (477·86 to 642·04)	480·52 (414·81 to 552·35)	403·14 (347·54 to 467·26)	405·88 (306·58 to 516·61)
Cambridgeshire	830·64 (615·31 to 1133·78)	702·41 (490·72 to 965·81)	631·09 (465·88 to 834·62)	576·07 (493·73 to 676·47)	516 (450·96 to 590·21)	464·1 (406·12 to 535·32)	343·04 (298·92 to 392·42)	342·68 (268 to 438·2)
Central Bedfordshire	934·79 (675·48 to 1246·53)	795·28 (555·61 to 1108·72)	717·12 (528·36 to 960·97)	638·07 (536·97 to 752·76)	569·63 (500·05 to 653·35)	462·29 (400·08 to 539·29)	371·11 (325·16 to 426·57)	347·58 (270·34 to 443·12)
Essex	888·58 (669·35 to 1149·86)	760·49 (545·25 to 1037·3)	703·84 (539·56 to 908·85)	642·88 (558·38 to 744·08)	541·91 (477·54 to 609·95)	457·71 (405 to 518·11)	379·06 (335·63 to 427·04)	394·34 (311·91 to 496·42)
Hertfordshire	862·25 (644·98 to 1132·42)	734·62 (522·67 to 991·2)	680·8 (504·15 to 894·23)	638·58 (555·99 to 739·16)	541·51 (478·9 to 616·88)	476·01 (419·89 to 540·27)	371·56 (328·43 to 423·97)	358·07 (281·14 to 455·13)
Luton	998·61 (742·92 to 1311·24)	861·36 (600·5 to 1208·19)	792·54 (593·45 to 1044·1)	710·54 (603·44 to 830·1)	593·05 (513·98 to 678·53)	523·42 (451·65 to 601·79)	431·76 (375·25 to 502·48)	424·52 (331·32 to 537·89)
Norfolk	882·15 (668·19 to 1164·87)	738·46 (532·63 to 1028·61)	661·13 (498·26 to 864·9)	584 (502·47 to 671·87)	532·2 (469·33 to 602·29)	453·13 (398·74 to 515·4)	375·18 (328·32 to 426·75)	357·75 (285·41 to 452·94)
Peterborough	956·08 (689·54 to 1284·61)	808·15 (568·9 to 1117·11)	730·75 (536·47 to 977·54)	652·79 (556·41 to 769·13)	593·21 (521·49 to 684·98)	517·88 (451·63 to 594·61)	427·6 (371·16 to 497·74)	405·11 (311·95 to 518·46)
Southend-on-Sea	995·56 (746·32 to 1320·23)	837·59 (579·08 to 1158·73)	752·15 (566·99 to 994·69)	661·73 (554·35 to 775·49)	578·73 (498·86 to 663·44)	501·82 (433·08 to 578·52)	414·52 (355·72 to 476·82)	420·41 (321·7 to 553·01)
Suffolk	886·32 (649·65 to 1189·2)	747·64 (516·9 to 1033·79)	680·08 (507·86 to 888·55)	607·85 (524·91 to 703·65)	519·72 (459·09 to 587·76)	451·97 (398·33 to 512·85)	366·14 (320·25 to 413·74)	362·28 (289·34 to 454)
Thurrock	965·9 (702·61 to 1312·62)	832·68 (578·33 to 1123·62)	759·98 (559·65 to 999·93)	681·82 (576·27 to 810·02)	587·82 (509·63 to 675·73)	496·89 (432·02 to 566·76)	405·07 (347·75 to 466·03)	386·08 (299·38 to 487·88)
Greater London	927·31 (755·71 to 1117·45)	791·72 (627·51 to 984·89)	728·2 (582·84 to 885·82)	638·78 (567·31 to 717·91)	561·93 (506·12 to 625·28)	499·38 (449·33 to 554·47)	364·52 (327·51 to 408·73)	350·96 (288·85 to 425·99)
Barking and Dagenham	1027·44 (766·4 to 1325·95)	865·13 (618·24 to 1155·03)	784·4 (587·25 to 1014·15)	675·73 (573·1 to 793·76)	599·01 (521·16 to 687·09)	543·12 (474·51 to 618·59)	431·58 (376·47 to 494·1)	439·76 (341·19 to 562·67)
Barnet	857·3 (627·29 to 1124·69)	726·77 (524·61 to 992·49)	655·47 (485·56 to 856·34)	564·52 (477·81 to 657·71)	508·46 (440·26 to 584·15)	441·1 (382·75 to 505·06)	355·69 (305·59 to 408·84)	311·49 (240·76 to 398·49)
Bexley	863·94 (642·51 to 1147·33)	727·18 (508·41 to 990·12)	655·39 (490·25 to 856·4)	563·68 (472·27 to 659·72)	504·5 (438·39 to 577·14)	442·4 (382·53 to 504·36)	334·09 (289·75 to 383·19)	355·78 (270·35 to 456·08)
Brent	935·69 (706·18 to 1214·78)	801·81 (578·93 to 1078·24)	735·51 (554·8 to 962·17)	642·01 (548·38 to 748·44)	564·27 (488·36 to 645·1)	495·45 (430·15 to 569·24)	320·75 (277·63 to 368·25)	324·86 (252·98 to 416·31)
Bromley	848·94 (633·84 to 1109·19)	717·04 (503·28 to 972·98)	652·8 (497·79 to 843·87)	565·43 (480·78 to 665·16)	488·78 (428·65 to 560·58)	443·65 (387·75 to 510·02)	336·17 (293·61 to 390·37)	314·82 (244·94 to 404·16)
Camden	921·36 (682·96 to 1209·04)	814·94 (588·25 to 1099·02)	768·47 (576·25 to 991·48)	702·39 (598·29 to 811·17)	618·16 (538·81 to 704·76)	540·56 (471·63 to 614·54)	397·94 (342·08 to 455·43)	385·8 (300·64 to 490·09)
Croydon	907·81 (658·77 to 1187·15)	772·83 (548·25 to 1037·61)	714·23 (539·2 to 921·05)	642·86 (547·9 to 746·19)	569·77 (493·34 to 651·96)	486·72 (427·18 to 560·11)	365·67 (320·06 to 425·24)	391·59 (303·05 to 503·45)
Ealing	929·92 (686 to 1221·79)	792·89 (558·25 to 1100·56)	727·29 (539·31 to 941·15)	641·23 (548·62 to 740·79)	561·75 (491·86 to 641·17)	479·7 (419·79 to 547·79)	339·97 (296·18 to 386·09)	324·76 (250·09 to 417·72)
Enfield	891·72 (668·13 to 1190·9)	749·02 (536·57 to 1015·55)	675·38 (505·12 to 882·35)	580·15 (494·95 to 676·92)	514·11 (447·4 to 588·26)	489·94 (425·71 to 560·97)	349·86 (301·48 to 398·55)	327·22 (253·07 to 413·21)
Greenwich	980·07 (739·99 to 1284·7)	823·2 (586·73 to 1100·75)	744·81 (564·76 to 962·04)	646·56 (551·99 to 756·7)	588·46 (514·38 to 674·26)	560·25 (487·42 to 645·97)	399·49 (347·69 to 461·65)	388·8 (299·97 to 494·06)
Hackney	983·58 (737·51 to 1277·13)	851·02 (595·67 to 1170·18)	805·52 (608·54 to 1071·6)	740·79 (639·59 to 856·35)	673·5 (589·52 to 771·88)	586·12 (510·57 to 666·83)	438·71 (381·69 to 504·42)	416·4 (318·99 to 522·97)
Hammersmith and Fulham	961·54 (714·26 to 1249·28)	840·38 (600·77 to 1154·98)	781·39 (589·29 to 1025·14)	716·9 (606 to 848·32)	642·11 (556·02 to 745·44)	555·15 (480·26 to 638·45)	380·08 (329·89 to 442·08)	349·08 (272·79 to 449·22)
Haringey	956·68 (735·79 to 1237·51)	818·5 (591·49 to 1081·52)	747·77 (565·88 to 955·8)	662·26 (565·18 to 772·21)	578·32 (503·8 to 661·51)	527·41 (457·79 to 598·48)	392·43 (338·6 to 453·23)	366·28 (283·82 to 465·19)
Harrow	873·18 (643·21 to 1154·56)	744·44 (532·44 to 1025·07)	684·94 (509·89 to 892·32)	596·63 (509·2 to 704·67)	481·5 (415·45 to 551·27)	429·94 (373·09 to 489·77)	311·73 (268·37 to 363·3)	311·62 (239·6 to 396·07)
Havering	872·01 (654·24 to 1146·27)	739 (523·7 to 1012·76)	676·49 (514·8 to 882·4)	598·57 (508·87 to 692·91)	517·33 (450·14 to 593)	449·99 (396·39 to 510·75)	360·12 (311·77 to 415·65)	334·95 (257·13 to 429·53)
Hillingdon	874·57 (655·61 to 1146·36)	743·08 (520·93 to 1003·69)	680·97 (510·72 to 893·75)	592·88 (505·37 to 695·12)	542·41 (475·99 to 624·95)	493·27 (433·55 to 564·44)	378·19 (328·73 to 435·41)	372·27 (293·35 to 477·06)
Hounslow	930·92 (696·08 to 1223·52)	796·79 (578·83 to 1058·25)	730·77 (541·41 to 959·11)	643·67 (549·76 to 752·07)	569·95 (496·87 to 654·69)	509·65 (446·78 to 583·23)	372·57 (323·88 to 429·55)	330·75 (255·13 to 425·32)
Islington	968·34 (724·61 to 1261·28)	841·41 (600·98 to 1137·01)	789 (598·88 to 1005·36)	720·74 (614·75 to 848·07)	626·19 (544·38 to 713·6)	592·16 (518·24 to 677·38)	415·21 (358·54 to 480·14)	396·64 (305·7 to 500·06)
Kensington and Chelsea	900·31 (655·91 to 1196·57)	815·88 (602·76 to 1113·5)	768·77 (568·9 to 1001·78)	697·92 (592·87 to 809·42)	562 (488·54 to 648·63)	416·78 (363·85 to 477·71)	317·48 (275·88 to 364·45)	334·94 (258·85 to 425·05)
Kingston upon Thames	830·2 (619·03 to 1094·16)	699·84 (480·59 to 952·57)	630·28 (470·15 to 824·65)	548·26 (468 to 645·13)	498·61 (433·25 to 569·86)	446·54 (389·27 to 514·01)	310·77 (270·28 to 360·66)	320·13 (247·6 to 410·05)
Lambeth	1008·68 (758·32 to 1315·96)	867·41 (624·68 to 1166·93)	801·15 (601·44 to 1024·86)	712·54 (607·13 to 829·41)	605·55 (533·87 to 689·93)	591·06 (518·78 to 675·36)	417·56 (361·69 to 481·53)	368·21 (284·23 to 470·46)
Lewisham	968·58 (725·43 to 1257·58)	817·82 (585·11 to 1106·88)	743·1 (557·05 to 963·88)	647·72 (551·62 to 750·66)	601·82 (523·29 to 681·08)	570·35 (498·71 to 649·76)	423·37 (365·94 to 485·02)	383·82 (301·5 to 483·88)
Merton	913·78 (685·37 to 1172·43)	777·64 (556·67 to 1073·54)	708·76 (538·13 to 916·07)	618·57 (527·38 to 724·68)	540·6 (469·17 to 622·38)	470·23 (407·07 to 540·98)	340·94 (295·9 to 395·04)	336·49 (256·44 to 433·6)
Newham	1023·51 (777·28 to 1325·46)	870·52 (620·47 to 1163·89)	802·99 (608·62 to 1044·87)	706·79 (609·1 to 826·13)	599·25 (522·36 to 682·42)	585·16 (511·05 to 668·6)	415·13 (361·69 to 473·68)	394·28 (304·75 to 496·75)
Redbridge	883·65 (662·24 to 1157·36)	748·51 (536·07 to 1022·9)	683·69 (506·83 to 879·66)	577·02 (488·94 to 678·48)	497·46 (432·21 to 566·42)	444·55 (390·27 to 509·69)	335·31 (293·53 to 387·87)	323·52 (249·01 to 412·41)

Supplemental Results Table 3c: Age-standardised mortality rates by location, females, 1950, 1960, 1970, 1980, 1990, 2000, 2010, 2019

Location	1950	1960	1970	1980	1990	2000	2010	2019
Richmond upon Thames	856·25 (641·42 to 1110·22)	734·23 (528·17 to 984·92)	672·97 (506·7 to 872·23)	593·68 (501·22 to 699·25)	500·67 (433·27 to 571·49)	442·03 (383·97 to 508·57)	309·36 (266·78 to 358·04)	309·19 (235·37 to 395·07)
Southwark	976·53 (740·85 to 1264·25)	830·29 (591·33 to 1124·75)	767·33 (579·88 to 1003·85)	687·91 (587·35 to 795·73)	676·59 (589·52 to 773·9)	568·86 (500·7 to 647·91)	421·41 (367·05 to 486·26)	373·65 (288·24 to 473·81)
Sutton	894·16 (667·47 to 1167·9)	750·05 (539·73 to 1014·63)	672·66 (508·3 to 885·27)	580·17 (488·83 to 679·02)	536·24 (465·02 to 614·73)	478·11 (414·16 to 544·19)	337·5 (292·23 to 392·07)	353·06 (266·9 to 450·92)
Tower Hamlets	1032·54 (771·63 to 1321·47)	877·9 (625·68 to 1178·32)	822·81 (615·06 to 1060·45)	735·58 (622·25 to 858·26)	640·52 (554·41 to 731·27)	593·9 (519·37 to 678·77)	415·29 (360·76 to 476·08)	419·29 (324·02 to 535·95)
Waltham Forest	976·28 (738·58 to 1244·83)	826·18 (589·5 to 1119·62)	742·32 (555·56 to 946·34)	640·71 (546·04 to 755·17)	574·92 (500·47 to 661·48)	539·7 (469·91 to 619·11)	382·11 (331·88 to 441·08)	369·25 (283·7 to 475·45)
Wandsworth	967·01 (724·41 to 1252·09)	839·4 (597·87 to 1135·21)	778·95 (598·54 to 1002·73)	681·39 (579·43 to 796·46)	610·58 (534·06 to 694·26)	539·94 (470·92 to 619·03)	367·11 (319·81 to 420·77)	374·26 (285·58 to 478·97)
Westminster	886·14 (665·6 to 1172·46)	798·52 (579 to 1082·52)	752·18 (569·33 to 979·78)	688·4 (584·16 to 805·01)	592·72 (514·31 to 678·7)	477·89 (415·18 to 556·06)	341·03 (292·52 to 394·46)	331·82 (260·38 to 424·95)
North East England	1075·91 (865 to 1312·37)	909·91 (702·52 to 1156·88)	836·75 (662·51 to 1041·8)	734·7 (653·14 to 831·64)	653·62 (586·79 to 724·61)	561·6 (502·03 to 622·46)	455·06 (407·98 to 507·39)	453·43 (373·1 to 552·94)
County Durham	1059·9 (786·67 to 1381·09)	892·07 (644·13 to 1202·61)	823·17 (621·5 to 1076·29)	734·42 (636·08 to 852·82)	660·85 (578·75 to 746·67)	574·56 (503·84 to 653·8)	463·43 (409·7 to 528)	474·22 (369·51 to 596·17)
Darlington	1079·98 (787·01 to 1416·39)	921·25 (642·42 to 1270·03)	849·45 (631·59 to 1128·91)	739·83 (627·75 to 866·45)	656·79 (574·24 to 746·82)	566·89 (490·07 to 651·24)	449·08 (388·96 to 518·31)	435·83 (339·24 to 562·39)
Gateshead	1090·03 (811·1 to 1456·4)	928·1 (654·5 to 1251·12)	851·53 (641·45 to 1098·49)	746·36 (638·43 to 870·75)	634·77 (550·76 to 722·87)	564 (491·38 to 646·49)	449·54 (388·52 to 517·96)	458·78 (360·43 to 592·34)
Hartlepool	1154·06 (855·55 to 1511·44)	983·73 (679·46 to 1371·51)	906·35 (687·18 to 1183·55)	794·18 (667·25 to 934·35)	689·56 (595·67 to 792·21)	603·9 (519·85 to 700·24)	492·8 (424·51 to 570·88)	498·47 (378·02 to 639·6)
Middlesbrough	1186·01 (883·32 to 1533·61)	1014·22 (724·86 to 1408·45)	938·58 (705·01 to 1230·21)	831·14 (712·49 to 963·6)	731·15 (634·68 to 837·33)	635·64 (551·51 to 734·36)	508·69 (437·41 to 587·65)	522·45 (399·95 to 667·17)
Newcastle upon Tyne	1060·6 (791·75 to 1358·24)	904·26 (638·96 to 1229·86)	828·13 (629·95 to 1098·66)	724·55 (619·95 to 848·52)	675·05 (591·48 to 768·58)	571·48 (495·14 to 651·21)	464·51 (403·58 to 532·58)	458·14 (359·69 to 585·57)
North Tyneside	1064·88 (775·93 to 1389·08)	900·02 (655·47 to 1233·82)	823·04 (610·67 to 1084·32)	714·69 (610·95 to 849·72)	647·06 (559·18 to 739·42)	548·45 (476·33 to 626·32)	439·47 (383·52 to 506·26)	416·1 (322·27 to 531·25)
Northumberland	1048·93 (774·69 to 1379·36)	887·74 (636·12 to 1205·87)	814·28 (611·18 to 1080·37)	722·04 (618·53 to 838·35)	615·22 (537·09 to 702·74)	507·45 (441·77 to 582·14)	402·36 (350·74 to 461·12)	390·09 (307·66 to 503·47)
Redcar and Cleveland	1077·63 (804·48 to 1441·68)	917·97 (662·43 to 1261·76)	840·85 (623·76 to 1099·73)	729·07 (622·37 to 855·82)	622·2 (537·72 to 718·47)	557·41 (483·47 to 642·75)	463·74 (401·93 to 536·72)	491·41 (383·02 to 631·52)
South Tyneside	1091·07 (812·66 to 1427·69)	922·5 (647·45 to 1283·63)	839·13 (620·72 to 1092·64)	732·97 (620·89 to 851·46)	664·78 (573·76 to 758·89)	547·58 (472·89 to 626·67)	471·91 (404·63 to 547·35)	447·83 (342·39 to 570·08)
Stockton-on-Tees	1041·64 (766·05 to 1362·46)	881·77 (625·13 to 1201·41)	805·43 (590·85 to 1041·34)	699·13 (590·66 to 818·19)	632·89 (549·74 to 723·11)	529·16 (455·82 to 609·37)	438·06 (379·32 to 504·77)	473·85 (366·16 to 611·9)
Sunderland	1077·07 (808·4 to 1399·87)	913·79 (654·18 to 1247·93)	833·3 (617·35 to 1086·39)	727·21 (623·24 to 847·72)	663 (576·96 to 756·89)	589·47 (510·91 to 671·44)	482·87 (418·27 to 551·18)	466·79 (365·15 to 592·68)
North West England	1050·34 (871·56 to 1254·46)	886·29 (706·09 to 1117·29)	811·43 (651·65 to 1001·37)	719·06 (642·78 to 811·05)	629·26 (571·41 to 700·11)	547·62 (498·6 to 609·62)	450·06 (408·7 to 503·89)	434·41 (359·68 to 522·97)
Blackburn with Darwen	1119·84 (852·64 to 1430·36)	949·47 (687·15 to 1261·42)	861·41 (653·72 to 1115·5)	756·03 (643·63 to 889·76)	671·03 (585·52 to 771·17)	593·97 (520·85 to 680·28)	467·06 (404·66 to 540·46)	462·53 (361·1 to 586·86)
Blackpool	1124·83 (852·67 to 1456·55)	952·26 (695·82 to 1287·61)	864·75 (653·04 to 1143·58)	752·52 (643·41 to 873·86)	660·73 (578·57 to 762·24)	637·56 (561·57 to 732·32)	561·64 (489·46 to 647·22)	546·56 (434·86 to 699·78)
Bolton	1078·05 (813·04 to 1398·13)	911·63 (672·32 to 1229·2)	830·21 (631·94 to 1070·22)	727·11 (627·42 to 852·42)	637·23 (563·33 to 724·88)	562·7 (497·6 to 639·63)	461·83 (407·13 to 527·84)	419·01 (333·57 to 531·53)
Bury	1020·16 (774·89 to 1315·35)	862·77 (609·81 to 1165·28)	771·88 (573·77 to 1025·92)	668·48 (568·03 to 786·37)	610·69 (533·07 to 706·34)	520·22 (452·27 to 601·6)	435·74 (382·95 to 502·13)	438·92 (343·09 to 562·4)
Cheshire East	921·32 (695·71 to 1210·99)	771·37 (544·39 to 1030·06)	693·1 (519·84 to 900·17)	621·45 (534·9 to 715·93)	567·69 (494·52 to 648·99)	466·54 (406·94 to 530·06)	384·33 (335·47 to 437·66)	345·87 (271·98 to 431·97)
Cheshire West and Chester	975·95 (726·5 to 1265·39)	829·46 (603·76 to 1134·22)	765·99 (570·08 to 995·24)	689·73 (596·12 to 809·16)	582·51 (512·88 to 668·37)	482·09 (425·18 to 555·28)	391·3 (344·38 to 449·18)	386·62 (306·13 to 490·35)
Cumbria	1013·49 (767·33 to 1306·06)	857·32 (615·17 to 1145·04)	785·95 (596·4 to 1009·25)	686·81 (593·5 to 797·62)	604·61 (532·59 to 687·9)	502·79 (440·38 to 573·89)	426·33 (372·42 to 486·33)	415·26 (324·38 to 525·07)
Halton	1093·5 (811·43 to 1419·92)	943·13 (680·46 to 1277·62)	869·05 (648·93 to 1148·43)	774·98 (656·84 to 908·66)	664·69 (575·13 to 761·67)	601·48 (524·59 to 684·05)	489·22 (419·69 to 564·17)	449·43 (347·78 to 573·44)
Knowsley	1122·19 (846·95 to 1445)	963·94 (702·49 to 1320·89)	892·03 (668·8 to 1156·14)	796·02 (679·62 to 937·53)	678·91 (591·87 to 787·67)	595·89 (517·56 to 681·32)	484·19 (418·7 to 564·2)	466·79 (358·63 to 601·24)
Lancashire	1007·59 (771·39 to 1274·57)	842·15 (615·6 to 1115·21)	763·3 (572·16 to 987·95)	708·73 (614·84 to 823·15)	620·1 (546·55 to 702·02)	524·17 (464·16 to 595·5)	433·27 (384 to 491·6)	439·86 (350·86 to 547·03)
Liverpool	1116·45 (862·2 to 1430·76)	960·33 (679·37 to 1308·31)	903·77 (693·05 to 1184·15)	761·46 (659·66 to 879·88)	681·48 (600·26 to 779·15)	645·66 (568 to 735·76)	517·58 (454·13 to 598·61)	509·75 (401·83 to 641·72)
Manchester	1119·79 (856·25 to 1445·35)	947·29 (681·96 to 1294·16)	871·82 (670·6 to 1125·42)	785·47 (682·33 to 909·81)	687·48 (602·89 to 781·48)	659·16 (582·61 to 749·94)	535·28 (471·03 to 609·68)	468·73 (372·01 to 596·16)
Oldham	1103·48 (844·37 to 1399·86)	938·77 (685·85 to 1277·25)	851·11 (648·66 to 1119·31)	739·45 (632·95 to 863·25)	648·51 (566·1 to 740·19)	570·09 (497·12 to 650·45)	478·43 (415·5 to 546·67)	460·21 (360·66 to 573·74)
Rochdale	1112·66 (837·97 to 1433·11)	937·87 (684·23 to 1286·93)	844·08 (633·12 to 1100·55)	726·15 (617·39 to 839·48)	698·54 (612·12 to 801·26)	590·97 (518·98 to 673·77)	495·72 (431·55 to 572·2)	500·25 (387·12 to 627·56)
Salford	1098·73 (825·2 to 1416·66)	931·28 (654·14 to 1257·15)	851·01 (638·47 to 1099·48)	750·31 (640·12 to 870·47)	683·82 (597·18 to 774·64)	606·5 (527·48 to 685·18)	485 (423·15 to 555·51)	450·2 (349·58 to 568·59)
Sefton	1027·25 (774·1 to 1324·83)	863·85 (618·23 to 1169·42)	792·99 (597·05 to 1039·69)	700·62 (601·03 to 814·72)	584·46 (514·66 to 669·79)	530·52 (466·15 to 608·83)	438·83 (382·59 to 508·72)	439·37 (343·59 to 557·82)
St Helens	1070·55 (800·78 to 1367·26)	915·43 (662·29 to 1213·15)	834·09 (632·9 to 1072·72)	790·53 (674·32 to 927·65)	644·22 (560·78 to 740·76)	552·28 (483·77 to 631·03)	474·51 (414·85 to 542·65)	472·96 (366·47 to 609·22)
Stockport	954·61 (705·12 to 1263·14)	803·74 (572·29 to 1092)	730·36 (548·06 to 938·94)	664·44 (569·84 to 772·53)	582·25 (512·88 to 663·99)	507·31 (445·38 to 577·62)	410·69 (358·88 to 472·09)	384·91 (300·69 to 487·96)
Tameside	1103·91 (837·73 to 1407·2)	944·94 (682·83 to 1280·23)	863·1 (648·34 to 1136·87)	756·68 (648·76 to 882·27)	664·15 (581·1 to 755·86)	557·35 (482·81 to 643·49)	472·58 (412·27 to 542·48)	445·94 (348·35 to 565·61)
Trafford	1003·08 (748·81 to 1302·92)	856·32 (623·96 to 1149·93)	784·17 (597·6 to 1011·03)	689·48 (586·79 to 809·6)	581·43 (506·23 to 667·62)	514·28 (447·78 to 589·42)	374·68 (326·75 to 429·9)	348·77 (272·79 to 442·34)
Warrington	1025·88 (766·72 to 1336·03)	878·35 (630·42 to 1183·8)	805·31 (611·16 to 1055·68)	718·14 (609·5 to 839·03)	639·48 (560·2 to 735·05)	506·77 (441·41 to 584·14)	427·7 (374·01 to 491·95)	429·01 (335·53 to 545·7)
Wigan	1055·44 (792·47 to 1368·01)	903·13 (649·23 to 1221·83)	822·55 (613·65 to 1068·32)	722·68 (618·84 to 840·51)	637·43 (559·47 to 723·5)	565·81 (499·83 to 645·84)	462·51 (404·66 to 527·98)	451·24 (358·57 to 569·8)
Wirral	1046·04 (786·66 to 1358)	877·32 (620·71 to 1192·54)	800·29 (593·03 to 1043·62)	699·55 (602·11 to 817·15)	610·83 (534·88 to 701·05)	534·72 (470·45 to 611·57)	459·36 (399·58 to 529·57)	450·11 (352·59 to 570·69)
South East England	885·26 (716·16 to 1064·67)	745·79 (583·33 to 940·08)	682·4 (546·03 to 836·08)	610·84 (548·02 to 684·37)	530·67 (481·27 to 584·57)	464·9 (420·59 to 513·61)	370·02 (334·16 to 411·12)	362·13 (302·13 to 442·31)
Bracknell Forest	853·52 (623·86 to 1130·97)	722·28 (511·15 to 987·15)	656·5 (482·24 to 865·43)	585·45 (492 to 697·36)	512·69 (446·66 to 595·78)	439·03 (382·28 to 502·75)	368·58 (316·36 to 425·28)	339·85 (260·65 to 436·62)
Brighton and Hove	958·38 (715·17 to 1247·78)	817·49 (604·11 to 1116·86)	742·44 (562·04 to 959·94)	665·17 (571·27 to 776·19)	567·88 (500·17 to 650·8)	524·99 (461·5 to 604·23)	420·63 (368·97 to 486·52)	397·79 (315·45 to 509·75)
Buckinghamshire	845·95 (619·18 to 1117·63)	715·91 (508·49 to 970·4)	658·19 (479·86 to 848·67)	599·99 (514·27 to 697·4)	526·89 (462·02 to 599·67)	453·8 (397·86 to 514·39)	349·4 (308·42 to 400·82)	327·99 (259·62 to 413·23)
East Sussex	920·73 (672·9 to 1198·46)	767·59 (541·77 to 1037·63)	686·9 (519·71 to 889)	598·27 (512·43 to 696·37)	521·42 (457·75 to 593·89)	453·61 (400·27 to 517·39)	368·94 (321·49 to 420·15)	379·49 (300·03 to 475·58)
Hampshire	853·5 (642·54 to 1114·65)	730·41 (521·6 to 1014·08)	679·04 (506·73 to 892·59)	596·46 (518·6 to 691·09)	520·08 (460·11 to 589·6)	451·15 (396·59 to 512·73)	355·69 (312·59 to 404·93)	348·33 (278·07 to 435·43)
Isle of Wight	956·41 (711·63 to 1245·39)	786·98 (563·75 to 1061·37)	692·42 (519·96 to 892·88)	597·78 (504·55 to 700·02)	534·12 (464·54 to 612·99)	459·38 (398·99 to 525·61)	387·39 (336·16 to 448·49)	375·17 (292·26 to 472·57)
Kent	905·85 (678·96 to 1170·59)	763·88 (554·98 to 1041·07)	704·02 (532·98 to 905·46)	643·19 (564·09 to 733·72)	549·98 (489·49 to 622·79)	494·98 (442·59 to 559·46)	386·14 (343·16 to 435·83)	381·65 (303·64 to 476·33)
Medway	950·08 (693·19 to 1259·74)	807·78 (574·59 to 1126·14)	721·04 (532·45 to 934·66)	634·32 (537·92 to 741·29)	566·95 (495·25 to 645·65)	501·87 (439·9 to 573·53)	429·5 (372·03 to 495·04)	425·78 (334·79 to 561·53)
Milton Keynes	893·09 (657·7 to 1153·53)	753·14 (530·83 to 1015·31)	673·54 (498·69 to 878·46)	597·54 (500·98 to 706·69)	565·39 (494·56 to 651·11)	476·37 (415·66 to 543·61)	403·8 (353·65 to 461·2)	382·68 (298·74 to 486·02)
Oxfordshire	849·35 (624·24 to 1121·15)	726·84 (513·44 to 985·34)	672·62 (503·59 to 887·82)	604·82 (517·71 to 703·18)	509·3 (449·5 to 580·83)	451·24 (395·68 to 514·79)	363·05 (319·64 to 415·79)	356·02 (282·6 to 449·81)
Portsmouth	969·6 (722·06 to 1252·31)	824·03 (582·64 to 1129·73)	740·7 (548·39 to 972·41)	657·56 (556·58 to 767·23)	591·29 (516·17 to 672·04)	563·33 (490·19 to 642·1)	430·19 (374·93 to 498·48)	423·86 (329·92 to 553)
Reading	859·41 (631·49 to 1126·4)	732·08 (508·25 to 1034·3)	655·97 (492·14 to 861·91)	584·56 (496·64 to 687·06)	545·13 (466·9 to 627·46)	501·96 (437·83 to 575·55)	408·04 (351·65 to 473·76)	402·3 (311·12 to 516·17)
Slough	972·05 (729·12 to 1250·28)	832·97 (586·62 to 1170·82)	769·37 (576·36 to 996·69)	700·99 (593·38 to 824·23)	621·98 (539·03 to 714·15)	545·25 (472·65 to 625·89)	399·94 (348·68 to 460·1)	392·46 (311·18 to 496·57)
Southampton	939·55 (689·66 to 1226·39)	801·68 (569·75 to 1085·35)	720·81 (540·78 to 928·84)	636·19 (543·38 to 738·12)	583·72 (511·96 to 668·41)	532·47 (467·36 to 606·04)	430·4 (377·4 to 494·16)	407·9 (323·33 to 520·33)
Surrey	839·15 (626·33 to 1085·72)	709·82 (498·62 to 955·02)	653·38 (496·08 to 850·86)	604·74 (523·98 to 694·99)	515·78 (455·25 to 581·47)	435 (384·51 to 490·68)	337·98 (297·79 to 383·28)	350·17 (278·9 to 441·27)
West Berkshire	811·97 (596·46 to 1076·38)	684·33 (466·3 to 967·11)	611·25 (455·76 to 804·11)	543·58 (451·92 to 644·71)	496·16 (427·33 to 569·49)	450·16 (388·89 to 511·53)	359·67 (308·89 to 415·45)	328·45 (251·13 to 420·59)
West Sussex	877·87 (645·28 to 1141·32)	727·5 (516·99 to 986·47)	662·45 (500·72 to 855·74)	584·28 (499·68 to 679·83)	513·44 (451·58 to 580·03)	450·58 (394·69 to 509·49)	373·1 (324·73 to 425·3)	354·45 (279·04 to 442·24)
Windsor and Maidenhead	848·94 (621·6 to 1124·13)	721·16 (496·44 to 997·47)	658·15 (481·23 to 870·91)	595·03 (503·23 to 695·37)	529·67 (461·3 to 603·09)	406·09 (351·31 to 463·63)	338·48 (293·68 to 390·12)	328·48 (255·14 to 418·26)
Wokingham	786·38 (566·09 to 1053·18)	663·08 (475·12 to 916·26)	593·17 (439·63 to 788·27)	532·02 (447·66 to 631·36)	478·08 (417·35 to 548·32)	397·48 (345·23 to 457·97)	309·78 (268·43 to 357·35)	294·16 (228·57 to 383·34)
South West England	906·28 (727·75 to 1098·79)	758·42 (596·31 to 962·41)	691·77 (551·5 to 869·36)	617·81 (548·34 to 699·45)	528·85 (477·76 to 584·98)	454·6 (410·1 to 505·53)	368·65 (332·45 to 409·19)	366·43 (305·03 to 441·52)
Bath and North East Somerset	842·91 (614·44 to 1104·18)	705·61 (495·29 to 992·38)	633·52 (468·72 to 842·57)	558·74 (476·14 to 659·03)	493·06 (429·96 to 565·75)	433·67 (377·6 to 497)	332·7 (287·39 to 382·59)	338·61 (265·09 to 432·59)
Bournemouth	948·88 (721·04 to 1226·45)	800·48 (572·75 to 1107·96)	725·46 (539·83 to 956·05)	649·76 (551·17 to 771·03)	564·56 (492·94 to 648·53)	505·92 (439·48 to 579·66)	402·78 (349·4 to 460·83)	437·79 (346·14 to 555·14)
Bristol, City of	944·73 (697·53 to 1247·64)	807·04 (568·6 to 1110·82)	744·42 (560·31 to 958·99)	654·92 (562·28 to 762·39)	561·09 (491·58 to 642·16)	504·6 (445·52 to 573·45)	424·51 (369·28 to 485·46)	403·29 (319·59 to 507·6)
Cornwall	931·77 (692·93 to 1217·85)	781·05 (564·22 to 1054·24)	708·47 (531·11 to 914·83)	630·37 (539·34 to 742·74)	539·1 (472·25 to 616·19)	471·54 (415·33 to 535·54)	378·62 (331·15 to 433·04)	383·42 (305·8 to 481·23)
Devon	885·03 (652·79 to 1130·66)	735·13 (534·82 to 992·97)	675·99 (503·18 to 909·36)	614·81 (524·06 to 710·42)	510·22 (447·99 to 577·93)	444·74 (390·83 to 501·36)	351·73 (306·31 to 401·14)	352·54 (277·29 to 439·38)
Dorset	903·55 (683·11 to 1194·82)	752·6 (536·44 to 1029·21)	677·4 (502·6 to 900·71)	599·82 (510·05 to 699·91)	497·83 (432·17 to 566·92)	403 (350·13 to 459·69)	319·52 (277·76 to 366)	335·06 (266·35 to 419·99)
Gloucestershire	883·76 (645·53 to 1158·61)	735·2 (518·51 to 979·03)	669·8 (504·74 to 883·21)	617·95 (531·62 to 720·14)	532·11 (467·68 to 603·08)	454·2 (399·39 to 514·69)	381·77 (334·4 to 434·17)	369·3 (291·67 to 466·06)
North Somerset	887·75 (655·82 to 1168·15)	735·44 (516·1 to 999·65)	655·97 (488·39 to 876·69)	578·16 (492 to 684·79)	511·46 (445·88 to 580·43)	434·64 (377·64 to 501·88)	380·53 (330·96 to 441·22)	375·99 (295·54 to 480·36)
Plymouth	950·08 (702·26 to 1246·42)	804·89 (574·95 to 1106·01)	732·71 (543·84 to 969·55)	655·9 (560·31 to 764·94)	572·68 (502·29 to 647·98)	498·84 (436·4 to 565·31)	420·6 (366·04 to 479·69)	404·6 (321·73 to 501·83)
Poole	894·99 (662·53 to 1171·66)	748 (527·55 to 1060·51)	672·87 (501·57 to 900·18)	600·76 (499·04 to 706·13)	542·63 (470·62 to 629·36)	429·68 (370·83 to 497·28)	359·99 (312·59 to 417·02)	359·13 (281·14 to 458·61)

Supplemental Results Table 3c: Age-standardised mortality rates by location, females, 1950, 1960, 1970, 1980, 1990, 2000, 2010, 2019

Location	1950	1960	1970	1980	1990	2000	2010	2019
Somerset	904·74 (678·62 to 1179·06)	760·17 (526·62 to 1041·99)	695·01 (515·94 to 924·03)	621·35 (538·14 to 724·87)	529·42 (463·92 to 606·17)	460·22 (403·65 to 523·49)	368·05 (322·17 to 417·64)	378·64 (298·31 to 468·37)
South Gloucestershire	847·18 (621·77 to 1109·61)	719·3 (514·88 to 990·8)	654·37 (493·96 to 861·98)	555·44 (472 to 657·52)	510·26 (450·28 to 578·9)	406·44 (357·59 to 466)	318·14 (276·11 to 364·92)	321·09 (250·85 to 405·21)
Swindon	935·4 (691·39 to 1230·26)	793·08 (564·5 to 1089·96)	725·62 (537·47 to 965·94)	656·14 (557·33 to 769·3)	539·54 (473·04 to 618·43)	491·62 (429·88 to 562·19)	386·44 (336·82 to 442·16)	375·68 (296·22 to 475·19)
Torbay	948·56 (693·7 to 1254·31)	793·99 (543·86 to 1115·15)	713·91 (518·09 to 948·38)	629·47 (530·17 to 742·3)	537·03 (466·17 to 618·82)	504·69 (436·65 to 585·19)	431·56 (373·65 to 495·58)	421·89 (326·33 to 536·91)
Wiltshire	884·1 (658·83 to 1156·5)	746·02 (519·77 to 1006·18)	677·49 (512·14 to 879·41)	601·83 (505·63 to 708·83)	535·49 (467·37 to 618·75)	444·03 (389·28 to 508·88)	366·17 (319·89 to 422·61)	348·35 (271·94 to 434·76)
West Midlands	985·43 (796·65 to 1210·59)	836·37 (654·5 to 1067·1)	766·95 (616·92 to 962·22)	684·86 (615·15 to 770·79)	595·44 (535·89 to 669·85)	511·5 (462·26 to 568·05)	408·69 (369·37 to 455·47)	403·66 (334·77 to 492·6)
Birmingham	1008·2 (784·21 to 1285·66)	851·99 (620·68 to 1129·13)	786·34 (590·51 to 1016·42)	712·61 (623·05 to 812·73)	618·66 (545·41 to 704·2)	525·74 (463·99 to 597·88)	410·42 (359·41 to 469·78)	408·67 (323·29 to 513·71)
Coventry	1018·81 (748·85 to 1333·46)	872·64 (615·91 to 1209·5)	802·12 (600·66 to 1054·28)	692·19 (594·22 to 808·54)	608·72 (534·35 to 701·6)	526·7 (461·75 to 601·48)	413·79 (362·49 to 474·77)	416·24 (323·13 to 524·49)
Dudley	939·94 (697·96 to 1255·01)	796·63 (562·9 to 1098·94)	716·28 (533·32 to 954·98)	636 (545·14 to 742)	557·38 (489·83 to 637·87)	499·26 (439·84 to 571·69)	394·27 (346·69 to 453·45)	399·84 (310·54 to 514·45)
Herefordshire, County of	981·38 (724·55 to 1295·1)	832·43 (581·63 to 1131·99)	758·45 (562 to 1004·52)	667·75 (571·2 to 787·19)	538·37 (465·44 to 623·78)	448·29 (390·86 to 514·7)	382·61 (330·73 to 445·18)	390·24 (302·9 to 498·43)
Sandwell	1071·21 (806·1 to 1398·25)	911·49 (659·6 to 1260)	830·68 (627·69 to 1076·39)	723·91 (628·33 to 849·8)	609·62 (531·09 to 704·36)	576·84 (505·08 to 664·6)	482·81 (423·86 to 561·39)	444·89 (349·27 to 564·58)
Shropshire	939·83 (686·09 to 1266·12)	797·34 (565·85 to 1098·62)	719·44 (535·56 to 960·13)	627·82 (539·98 to 733·53)	543·43 (473·24 to 623·03)	455·64 (397·29 to 519·45)	371·73 (323·75 to 429·83)	357·63 (275·57 to 454·41)
Solihull	870·9 (629·65 to 1167·29)	739·28 (519·23 to 1020·19)	663·52 (492·22 to 883·79)	575·92 (491·65 to 678·02)	520·39 (453·26 to 604·47)	470·79 (410·39 to 542·79)	358·42 (312·62 to 410·56)	378·93 (294·36 to 491·9)
Staffordshire	952·8 (691·09 to 1259·85)	814·05 (589·99 to 1124·24)	748·02 (570·17 to 990·29)	684·65 (593·55 to 786·5)	609·01 (532·35 to 695·16)	519·32 (457·67 to 589·92)	406·58 (356·26 to 461·57)	410 (327·5 to 520·08)
Stoke-on-Trent	1110·35 (841·59 to 1443·8)	958·4 (682·97 to 1327·34)	882·63 (669·67 to 1172·49)	787·98 (677·78 to 923·86)	670·67 (585·03 to 773·47)	569·69 (496·61 to 649·15)	499·03 (433·89 to 573·39)	459·57 (354·47 to 588·58)
Telford and Wrekin	985·36 (726·16 to 1304·53)	845·49 (597·52 to 1173·41)	768·71 (570·64 to 1020·35)	679·55 (577·65 to 806·45)	571·07 (496·95 to 661·73)	535·18 (465·15 to 616·36)	428·28 (370·21 to 496·12)	424·79 (329·68 to 548·65)
Walsall	1045·55 (772·33 to 1377·66)	893·9 (639·84 to 1247·78)	814·38 (614·52 to 1092·99)	719·96 (618·64 to 845·74)	645·21 (557·42 to 738·64)	519·41 (453·84 to 595·53)	415·64 (362·94 to 479·22)	430·55 (337·47 to 554·74)
Warwickshire	932·14 (684·91 to 1228·43)	792·76 (553·05 to 1071·36)	728·11 (557·55 to 968·76)	655·06 (564·68 to 762·62)	582·9 (511·52 to 668·55)	478·82 (420·9 to 549·51)	381·23 (333·46 to 439·7)	386·65 (299·04 to 489·22)
Wolverhampton	1029·57 (779·04 to 1360·63)	875·15 (615·63 to 1187·21)	796·72 (592·74 to 1039·99)	704·82 (604·97 to 828·1)	636·47 (557·32 to 731·99)	548·03 (480·87 to 631·7)	466·8 (406·7 to 536·88)	462·11 (364·5 to 588·68)
Worcestershire	910·38 (674·17 to 1179·14)	777·56 (546·03 to 1056·22)	712·03 (535·33 to 937·37)	651·22 (560·2 to 752·14)	561·62 (491·73 to 640·15)	499 (437·31 to 568·72)	394·95 (346·64 to 447·39)	372·27 (291·83 to 467·62)
Yorkshire and the Humber	1009·57 (815·45 to 1235·85)	853·61 (667·98 to 1063·57)	777·24 (625·34 to 941·39)	686·99 (613·01 to 774·75)	600·74 (541·36 to 661·67)	512·05 (461·9 to 565·24)	424·66 (383·31 to 470·29)	419·21 (344·14 to 505·37)
Barnsley	1062·46 (796·28 to 1378·04)	902·61 (652·31 to 1225·77)	813·89 (610·1 to 1056·91)	714·35 (604·36 to 833·4)	622·3 (540·29 to 708·82)	540·32 (470·39 to 621·2)	448·88 (391·48 to 513·9)	436·58 (340·8 to 564·37)
Bradford	1070·3 (799·02 to 1393·9)	906·39 (663·61 to 1213·24)	827·79 (625·18 to 1056·72)	727·59 (629·7 to 843·49)	625·09 (550·03 to 707·87)	540·48 (477·36 to 619·25)	451·32 (396·84 to 514·82)	454·64 (356·98 to 569·03)
Calderdale	1050·92 (783·3 to 1375·92)	888·73 (626·45 to 1202·48)	800·06 (596·09 to 1023·84)	703·5 (599·43 to 827·3)	622·8 (539·25 to 715·9)	515·63 (448·32 to 589·3)	445·99 (388·12 to 515·8)	437·16 (336·84 to 553·98)
Doncaster	1031·29 (747·96 to 1355·59)	869·27 (625·5 to 1174)	780·41 (587·96 to 1012·45)	687·21 (587·53 to 800·23)	656·95 (570·87 to 744·37)	554·03 (481·92 to 627·26)	443·5 (386·28 to 505·52)	472·86 (363·17 to 594·32)
East Riding of Yorkshire	952·78 (702·45 to 1242·91)	802·96 (571·59 to 1094·02)	725·79 (541·93 to 954·29)	642·13 (551·71 to 748·19)	549·73 (481·85 to 626·41)	477·71 (420·22 to 544·27)	389·67 (341·63 to 444·96)	367·58 (288·53 to 462·69)
Kingston upon Hull, City of	1052·41 (778·93 to 1355·25)	892·9 (638·23 to 1202·28)	802·26 (596·42 to 1036·91)	692·46 (590·72 to 819·87)	622 (543·72 to 705·96)	578·29 (505·33 to 660·41)	485·27 (423·01 to 548·94)	484·52 (380·72 to 611·38)
Kirklees	1042·19 (769·99 to 1384·85)	884·94 (625·04 to 1197·03)	804·51 (605·75 to 1043·02)	739·43 (629·62 to 862·57)	625·94 (547·33 to 716·68)	515·52 (451·04 to 593·14)	436·45 (381·36 to 505·7)	416·13 (328·99 to 523·52)
Leeds	990·65 (736·02 to 1305·84)	840·89 (589·34 to 1163·32)	773·22 (588·97 to 988·34)	682·72 (589·29 to 788·43)	586·78 (515·07 to 664·08)	499·79 (437·12 to 571·66)	426·01 (374·39 to 484·45)	419·16 (325·73 to 526·76)
North East Lincolnshire	1036·07 (766·53 to 1364·17)	878·64 (623·21 to 1196·7)	787·38 (588·26 to 1033·26)	690·77 (584·86 to 810·82)	645·09 (561·01 to 734·12)	527·55 (448·93 to 604·92)	432·77 (373·87 to 502·7)	429·43 (327·2 to 543·41)
North Lincolnshire	997·1 (735·92 to 1277·26)	844·49 (601·72 to 1146·16)	757·11 (564·31 to 998·13)	664·27 (557·85 to 770·76)	619·52 (537·89 to 708·84)	527·62 (460·18 to 604·3)	418·95 (362·31 to 483·03)	396·13 (303·73 to 503·92)
North Yorkshire	942·76 (694·87 to 1213·22)	796·67 (569·67 to 1068·71)	726·57 (557·34 to 938·74)	640·94 (550·37 to 745·33)	547·81 (479·19 to 619·1)	473·07 (412·79 to 537)	376·46 (329·72 to 426·88)	372·27 (289·2 to 462·8)
Rotherham	1019·43 (742·19 to 1340·25)	863·91 (605·76 to 1165·9)	773·43 (577·66 to 1014·05)	672·07 (564·08 to 785·19)	606·43 (520·86 to 691·4)	522·36 (450·04 to 594·66)	444·79 (386·6 to 508·98)	448·06 (347·93 to 562·52)
Sheffield	988·45 (734·72 to 1278·28)	839·3 (596·34 to 1136·83)	766·6 (575·24 to 972·58)	688·54 (589·24 to 797·93)	606·04 (532·23 to 688·22)	507·88 (445·58 to 573·76)	430·03 (377·06 to 488·57)	431·79 (339·39 to 541·8)
Wakefield	1056·6 (795·89 to 1381·73)	899·87 (640·79 to 1230·33)	813·12 (592·57 to 1048·98)	713·72 (608·88 to 834·91)	628·96 (546·78 to 714·08)	540·06 (471·83 to 614·26)	442·26 (383·67 to 504·39)	438 (338·86 to 552·58)
York	944·02 (677·36 to 1260·9)	806·51 (573·96 to 1102·9)	728·02 (540·84 to 955·64)	646·25 (547·64 to 758·39)	540·6 (464·78 to 621·8)	438 (376·48 to 500·62)	364·48 (315·68 to 422·75)	390·93 (298·09 to 509·49)
Northern Ireland	1077·17 (1009·13 to 1154·04)	978·61 (911·73 to 1056·8)	836·44 (773·27 to 904·81)	778·97 (719·77 to 837·1)	658·13 (610·32 to 709·38)	539·88 (497·66 to 585·26)	431·86 (402·55 to 465·87)	425·74 (376·97 to 481·47)
Scotland	1207·6 (1123·21 to 1301·15)	1136·68 (1055·7 to 1231·41)	932·94 (863·23 to 1010·68)	770·38 (714·03 to 830·09)	671 (623·31 to 724·41)	575·71 (533·67 to 624·41)	473·22 (440·14 to 510·7)	459·99 (405·72 to 520·39)
Wales	1057·73 (974·11 to 1151·14)	882·17 (807·12 to 961·69)	799·18 (734·4 to 869·03)	685·66 (632·55 to 742·16)	583·05 (541·58 to 629·21)	523·28 (483·7 to 563·92)	429·69 (397·4 to 464·66)	416·69 (372·31 to 474·62)
Latin America and Caribbean	1850·26 (1748·32 to 1968·04)	1455·73 (1391·13 to 1525·01)	1245·32 (1197·75 to 1294·27)	974·65 (936·88 to 1009·1)	788·13 (759·66 to 817)	641·68 (618·66 to 664·11)	596·46 (572·42 to 618·39)	517·64 (470·24 to 571·55)
Andean Latin America	2310·02 (2088·54 to 2557·27)	1810·31 (1673·96 to 1956·51)	1610·35 (1489·78 to 1735·16)	1047·01 (967·22 to 1129·52)	836·44 (775·13 to 898·74)	678·29 (621·11 to 743·52)	599·49 (545·02 to 656·65)	516·77 (432·05 to 608·25)
Bolivia	2840·42 (2526·11 to 3182·24)	2211·26 (1890·15 to 2578·61)	1788·18 (1555·57 to 2035·21)	1471·41 (1300·43 to 1671·75)	1248·67 (1126·16 to 1389·11)	1028·7 (930·51 to 1155·09)	904·13 (791·74 to 1030·99)	852·73 (704·29 to 1033·38)
Ecuador	1749·99 (1473·65 to 2035·28)	1460·55 (1344·56 to 1590·29)	1368·21 (1244·14 to 1513·84)	911·94 (839·7 to 991·61)	745·54 (694·13 to 802·82)	588·86 (545·87 to 635·92)	629·62 (567·9 to 698·83)	569·39 (461·25 to 698·49)
Peru	2341·48 (2014·16 to 2733·09)	1830·34 (1623·79 to 2051)	1695·48 (1519·77 to 1898·27)	988·6 (872·41 to 1107·13)	768·01 (674·55 to 862·32)	619·12 (532·66 to 722·22)	511·35 (437·13 to 589·06)	415·01 (320·82 to 525·11)
Caribbean	1564·34 (1433·19 to 1703·9)	1241·16 (1165·93 to 1325·46)	1099·23 (1043·01 to 1158·28)	945·98 (901·5 to 995·6)	844·16 (797·03 to 901·9)	745·1 (707·65 to 789·98)	1088·15 (1022·93 to 1169·22)	649·83 (565·89 to 743·28)
Antigua and Barbuda	1338·71 (1218·28 to 1471·94)	1075·07 (984·04 to 1170·63)	879·33 (803·64 to 959·23)	700·64 (609·16 to 814·39)	581·2 (524·08 to 644·8)	625·4 (569·78 to 697·82)	583·19 (524·6 to 655·87)	609·27 (524·46 to 708·57)
The Bahamas	1329·47 (1092·88 to 1596·7)	1144·32 (955 to 1346·27)	978·17 (891·84 to 1075·97)	812·61 (741·03 to 889·57)	705·51 (647·74 to 769·98)	696·39 (634·75 to 758·22)	617·71 (566·19 to 675·64)	605·1 (494·92 to 741·08)
Barbados	1394·29 (1274·86 to 1544·36)	955·7 (884·22 to 1036·82)	906·48 (837·62 to 985·87)	742·58 (681·78 to 802·63)	671·7 (620·55 to 727·48)	636·85 (587·17 to 695·84)	614·86 (570·23 to 665·51)	601·64 (499·57 to 727·75)
Belize	1458·76 (1304·86 to 1614)	942·47 (845·69 to 1053·37)	897·29 (821·01 to 979·67)	741·71 (677·66 to 811·14)	705·25 (641·13 to 774·69)	738·74 (681·36 to 801·54)	622·63 (579·47 to 670·93)	556·29 (473·79 to 661·96)
Bermuda	1302·25 (1174·68 to 1439·86)	1013·06 (924·35 to 1106·69)	813·22 (740·68 to 889·03)	618·42 (565·03 to 679·73)	634·3 (581·09 to 693·6)	495·28 (455·05 to 537·92)	333·23 (292·38 to 380·68)	307·94 (258·66 to 379·02)
Cuba	1076·64 (884·68 to 1307·53)	1008·85 (906·35 to 1126·95)	922·66 (864·64 to 987·47)	740·1 (686·03 to 799·76)	622·56 (557·34 to 708·02)	532·07 (491·48 to 577·65)	474·86 (438·23 to 516·23)	457·11 (371·04 to 565·23)
Dominica	1830·56 (1657·26 to 2039·43)	1169·66 (1040·12 to 1308·07)	986·71 (882·03 to 1100·03)	866·64 (783·22 to 956·66)	810·44 (726·52 to 909·56)	728·92 (636·38 to 828·74)	712·01 (617·18 to 821·17)	717·97 (585·44 to 889·67)
Dominican Republic	1422·69 (1288·91 to 1588·16)	1100·98 (1023·21 to 1186·35)	955·64 (858·78 to 1056·21)	812·78 (732·68 to 897·86)	678·7 (604·21 to 764·69)	542·09 (485·15 to 604·73)	611·63 (523·85 to 716·28)	619·97 (494·58 to 785·85)
Grenada	1346·7 (1217·59 to 1482·7)	989·02 (879·05 to 1107·4)	916·66 (805·03 to 1048·2)	805·33 (691·26 to 937·83)	863·37 (780·59 to 961·84)	661·56 (603·26 to 726·02)	692·05 (634·02 to 757·58)	697·38 (607·22 to 804·25)
Guyana	1884·68 (1632·45 to 2163·06)	1412·35 (1244·92 to 1598·47)	1264·48 (1107·7 to 1432·4)	1304·71 (1124·75 to 1507·42)	1256·6 (1098·48 to 1433·38)	1118·96 (973·62 to 1274·28)	1030·54 (903·11 to 1166·31)	945·08 (748·32 to 1186·75)
Haiti	2486·57 (2120·71 to 2883·39)	2125·55 (1733·37 to 2564·47)	1993·87 (1729·91 to 2283·93)	1828·29 (1592·78 to 2080)	1721·56 (1474·57 to 1988·77)	1603·6 (1404·08 to 1829·85)	3285·56 (2864·39 to 3761·39)	1233·59 (953·75 to 1586·73)
Jamaica	1489·81 (1371·55 to 1610·56)	1041·09 (970·05 to 1123·4)	839·91 (781·97 to 901·45)	645·36 (593·4 to 704·57)	612·76 (567·52 to 667·49)	595·65 (549·57 to 644·96)	503·82 (464·86 to 545·94)	560·15 (446·35 to 689·64)
Puerto Rico	1474·18 (1371 to 1583·4)	993·99 (930·02 to 1063·54)	872·63 (812·18 to 937·48)	638·17 (593·86 to 685·25)	558·76 (517·41 to 604·53)	472·32 (440·65 to 508·02)	382·99 (355·83 to 411·77)	356·84 (274·9 to 449·22)
Saint Kitts and Nevis	1220·37 (1007·91 to 1495·67)	1211·55 (1056·83 to 1399·25)	1073·08 (986·08 to 1167·08)	1308·46 (1198·27 to 1429·88)	1231·29 (1105·81 to 1361·22)	855·77 (776·62 to 938·35)	720·21 (625·58 to 831·87)	705·42 (566·98 to 882·45)
Saint Lucia	1763·83 (1605·67 to 1944·34)	1314·53 (1194·49 to 1450·51)	1046·04 (948·64 to 1150·29)	886·49 (820·12 to 960·32)	885·5 (817·01 to 962·01)	667·27 (610·55 to 722·77)	507·21 (465·09 to 553·97)	560·83 (468·44 to 673·11)
Saint Vincent and the Grenadines	1595·1 (1429·65 to 1775·69)	1218·08 (1109·82 to 1337·59)	1051·86 (958·54 to 1150·51)	893·41 (820·23 to 979·44)	894·56 (824·99 to 976·55)	765·3 (700·61 to 833·39)	720·39 (653·85 to 794·42)	727·3 (617·51 to 847·83)
Suriname	1540·82 (1424·15 to 1673)	1176·22 (1045·54 to 1317·18)	944·29 (830·17 to 1058·01)	915·44 (805·42 to 1046·49)	822·66 (727·29 to 938·08)	734·47 (679·73 to 794·45)	631·43 (578·47 to 691·38)	646·2 (535·42 to 771·92)
Trinidad and Tobago	1606·22 (1490·97 to 1733·79)	1118·64 (1038·22 to 1204·05)	1243·23 (1157·91 to 1341·99)	1066·67 (995·29 to 1147·28)	875·32 (811·16 to 945·37)	773·26 (718·1 to 836·22)	597·13 (548·75 to 651·22)	564·36 (433·11 to 724·51)
Virgin Islands	1206·34 (1091·46 to 1368·88)	1216·58 (1100·04 to 1355·1)	930·24 (838·56 to 1055·6)	814·3 (755·08 to 881·48)	698·5 (615·21 to 783·07)	640·8 (587·09 to 703·27)	599·14 (552·42 to 655·03)	557·75 (467·47 to 664·13)
Central Latin America	1900·45 (1801·73 to 2002·91)	1434·01 (1371·42 to 1500·42)	1194·65 (1145·09 to 1247·13)	941·89 (894·15 to 992·55)	736·8 (706·47 to 770·43)	599·19 (572·65 to 625·68)	532·31 (508·85 to 555·11)	508·87 (441·59 to 587·73)
Colombia	1710·25 (1578·48 to 1871·65)	1542·91 (1402·63 to 1707·85)	1069·35 (982·68 to 1172·48)	865·35 (741·47 to 1021·35)	690·21 (630·76 to 755·94)	547·7 (495·37 to 609·91)	436·87 (397·03 to 481·73)	380·89 (305·09 to 472)
Costa Rica	1572·06 (1451·96 to 1701·79)	1077·4 (989·06 to 1184·14)	992·1 (909·71 to 1086·49)	594·52 (539·58 to 661·14)	516·41 (465·18 to 573·26)	477·7 (443·33 to 513·81)	394·41 (365·04 to 425·52)	379·47 (294·43 to 479·14)
El Salvador	2108·35 (1897·42 to 2365·75)	1544·34 (1418·65 to 1684·83)	1326 (1223·09 to 1440·21)	886·75 (767·06 to 1028·39)	661·54 (608·61 to 721·92)	541·3 (482·67 to 602·06)	499·22 (452·06 to 549·1)	489·6 (371·25 to 631·62)
Guatemala	2631·28 (2403·65 to 2879·66)	2365·7 (2150·48 to 2611·45)	1968·37 (1802·1 to 2153·65)	1356·15 (1256·65 to 1468·91)	1326·3 (1163·93 to 1499)	1016·96 (906·73 to 1139·62)	781·29 (661·05 to 907·6)	699·21 (535·38 to 896·95)
Honduras	2459·92 (2183·21 to 2793·27)	1829·7 (1658·49 to 2024·55)	1419·1 (1271·35 to 1586·19)	867·81 (766·13 to 991·15)	764·5 (669·96 to 868·11)	905·15 (748·76 to 1089·38)	901·44 (742·9 to 1081·14)	833·45 (716·5 to 999·34)
Mexico	2022·6 (1854·35 to 2210·07)	1421·98 (1326·64 to 1532·64)	1210·9 (1131·95 to 1300)	1011·39 (946·71 to 1086·77)	773·79 (720·59 to 834·21)	621·09 (577·61 to 666·16)	565·68 (529·17 to 607·18)	552·34 (451·34 to 669·8)
Aguascalientes	1839·46 (1556·31 to 2157·4)	1331·18 (1110·73 to 1573·53)	1172·12 (987·96 to 1375·86)	1024·99 (918·81 to 1140·54)	841·95 (761·43 to 931·63)	662·87 (596·88 to 730·99)	601·2 (542·47 to 664·19)	552·61 (428·83 to 703·52)
Baja California	2045·66 (1704·27 to 2432·63)	1419·87 (1169·13 to 1686·04)	1228·61 (1016·19 to 1467·63)	956·32 (864·61 to 1057·2)	880·66 (790·61 to 970·17)	749·86 (673·22 to 825·95)	622·79 (560·06 to 687·16)	580·62 (441·63 to 744·25)
Baja California Sur	1901·34 (1588·17 to 2251·07)	1282·58 (1084·28 to 1509·89)	1079·65 (915·07 to 1257·05)	855·08 (767·66 to 950·86)	713·6 (638·93 to 795·17)	597·8 (540·41 to 662·18)	563·98 (509·21 to 625·69)	545·5 (422·57 to 700·68)
Campeche	1977·96 (1671·25 to 2318·38)	1399·74 (1170·79 to 1646·75)	1206·71 (1028·29 to 1414·63)	1044·85 (932 to 1166·96)	844·7 (763·07 to 933·78)	610·93 (546·64 to 680·94)	586·57 (526·83 to 655·37)	586·3 (457·68 to 760·94)

Supplemental Results Table 3c: Age-standardised mortality rates by location, females, 1950, 1960, 1970, 1980, 1990, 2000, 2010, 2019

Location	1950	1960	1970	1980	1990	2000	2010	2019
Chiapas	2107·17 (1772·69 to 2504·08)	1535·45 (1299·76 to 1814·41)	1300·65 (1086·62 to 1556·04)	1130·29 (1021·09 to 1255·88)	887·03 (797·69 to 984·76)	677·71 (609·29 to 752·85)	610·39 (548·52 to 673·68)	629·84 (482·64 to 802·23)
Chihuahua	2226·52 (1875·7 to 2610·26)	1591·16 (1339·76 to 1870·56)	1384·53 (1160·33 to 1649·85)	1174·13 (1058·98 to 1305·45)	976·27 (883·06 to 1090·08)	767·73 (689·99 to 848·6)	683·17 (619·65 to 758·13)	692·01 (526·06 to 889·28)
Coahuila	2267·27 (1908·77 to 2695·5)	1587·57 (1329·03 to 1879·38)	1382·48 (1161·63 to 1635·07)	1195·47 (1081·65 to 1316·77)	920·68 (832·01 to 1011·32)	687·93 (619·06 to 757·19)	613·49 (553·77 to 673·57)	604·13 (467·82 to 785·76)
Colima	2297·06 (1937·74 to 2704·17)	1555·46 (1327·71 to 1805·76)	1300·66 (1123·17 to 1522·83)	1063·13 (951·99 to 1186·14)	859·72 (771·09 to 948·45)	650·82 (583·79 to 717·28)	597·2 (537·5 to 656·73)	581·17 (449·83 to 729·51)
Durango	2119·21 (1772·17 to 2499·1)	1455·63 (1218·71 to 1735·51)	1201·73 (1001·93 to 1422·7)	997·72 (895·76 to 1111·54)	715·37 (644·86 to 794·62)	550·42 (497·58 to 612·25)	546·82 (495·68 to 610·96)	501·77 (384·41 to 649·87)
Guanajuato	2077·84 (1786·53 to 2444·42)	1503·44 (1278·48 to 1770·49)	1300·19 (1093·86 to 1528·78)	1042·82 (944·43 to 1153·33)	881·04 (800·32 to 975·62)	670·4 (609·13 to 738·38)	555·5 (505·33 to 611·13)	575·82 (452·39 to 743·98)
Guerrero	2027·23 (1735·71 to 2397·12)	1489·28 (1236·01 to 1778·17)	1343·31 (1137·34 to 1586·87)	1109·01 (1000·15 to 1235·75)	775·05 (701·3 to 857·83)	706·39 (639·24 to 779·95)	523·36 (473·97 to 583·27)	492·98 (377 to 635·18)
Hidalgo	2247·84 (1897·91 to 2672·35)	1582·49 (1304·26 to 1896·77)	1349·97 (1120·28 to 1627·83)	1125·34 (1021·4 to 1250·83)	702·47 (637·21 to 779·43)	508·94 (458·81 to 563·97)	479·82 (437·04 to 533·8)	484·2 (375·07 to 616·1)
Jalisco	1994·27 (1714·92 to 2346·16)	1420·16 (1201·1 to 1654·73)	1220·19 (1029·76 to 1449·58)	1033·05 (940·47 to 1144·21)	874·29 (796·48 to 970·95)	688·8 (628·08 to 759·5)	587·52 (536·56 to 648·03)	553·65 (429·88 to 705·12)
México	1931·7 (1633·55 to 2264·03)	1389·66 (1181 to 1644·41)	1205·65 (1003·78 to 1434·22)	1048·59 (903·05 to 1218·15)	902·22 (819·96 to 994·45)	703·37 (638·39 to 769·87)	593·85 (542·68 to 647·96)	583·98 (460·69 to 738·49)
Mexico City	1673·55 (1427·13 to 1997·73)	1167·15 (974·46 to 1382·18)	1020·07 (856·22 to 1209·08)	911·25 (833·66 to 995·66)	713·7 (649·91 to 786·99)	617·35 (562·6 to 677·44)	582·59 (531·34 to 639·45)	561·84 (438·28 to 717·2)
Michoacán de Ocampo	2007·44 (1685·79 to 2358·82)	1430·46 (1204·9 to 1691·29)	1194·03 (992·14 to 1434·64)	1022·09 (925·41 to 1126·14)	702·34 (630·63 to 782·13)	581·57 (524·58 to 643·64)	514·48 (464·55 to 567·97)	483·92 (375·63 to 632·07)
Morelos	2021·91 (1713·27 to 2376·16)	1398·69 (1174·47 to 1652·9)	1183·99 (990·38 to 1394·52)	971·35 (880·64 to 1076·93)	758·19 (686·88 to 840·05)	598·4 (538·14 to 660·43)	550·05 (498·82 to 606·85)	575·65 (445·33 to 737·09)
Nayarit	2006·64 (1667·99 to 2385·67)	1386·19 (1175·49 to 1646·37)	1136·3 (959·6 to 1341·95)	898·15 (808·25 to 999·62)	701·47 (633·32 to 780·72)	537·64 (483·87 to 597·47)	490·97 (443·56 to 545·63)	481·46 (373·47 to 611·8)
Nuevo León	1849·12 (1563·09 to 2190·32)	1288·24 (1072·84 to 1530·96)	1132·28 (945·03 to 1361·74)	930·43 (843·69 to 1031·52)	770·83 (698·57 to 855·98)	625·08 (563·46 to 694·16)	542·59 (494·81 to 596·74)	533·72 (415·92 to 680·28)
Oaxaca	2429·42 (2041·75 to 2851·8)	1763·65 (1491·87 to 2075·73)	1524·5 (1278·61 to 1812·75)	1295·51 (1171·37 to 1431·12)	923·29 (836·44 to 1019·9)	624·13 (560·86 to 687·09)	523·8 (473·85 to 576·43)	501·86 (385·21 to 639·77)
Puebla	2568·65 (2176·8 to 3029·48)	1802·62 (1525·25 to 2129·37)	1514·76 (1273·32 to 1791·62)	1174·68 (1062·81 to 1304·5)	894·3 (811·34 to 990·09)	659·13 (597·11 to 725·72)	586·5 (534·18 to 647·17)	595·85 (457·04 to 770·93)
Querétaro	2143·39 (1803·19 to 2524·78)	1507·41 (1271·42 to 1774·74)	1271·07 (1051·38 to 1520·73)	1068·77 (936·06 to 1209·48)	855·81 (773·07 to 945·9)	681·37 (615·42 to 754·54)	570·29 (513·29 to 633·38)	580·27 (454 to 734·42)
Quintana Roo	1748·8 (1478·69 to 2075·44)	1290·8 (1080·9 to 1516·66)	1141·51 (974·42 to 1333·09)	918·6 (822·54 to 1025·48)	641·95 (578·75 to 714·48)	633·65 (572·11 to 702·93)	621·1 (563·76 to 685·41)	647·25 (515·23 to 830·57)
San Luis Potosí	2072·96 (1739·9 to 2449·56)	1431·14 (1211·84 to 1705·66)	1198·89 (995·97 to 1420·72)	887·83 (799·79 to 979·08)	713·76 (643·31 to 788·76)	549·58 (494·28 to 603·56)	500·5 (451·42 to 552·26)	512 (392·52 to 660·06)
Sinaloa	1783·87 (1495·46 to 2132·24)	1244·09 (1040·92 to 1482·81)	1052·24 (861·24 to 1272·07)	906·26 (822·12 to 1002·31)	624·62 (565·19 to 691·83)	524·42 (474·8 to 576·18)	482·6 (436·24 to 533·1)	462·51 (353·64 to 583·27)
Sonora	1817·8 (1520·23 to 2159)	1326·99 (1107·45 to 1594·05)	1206·47 (1001·52 to 1456·41)	1082·53 (924·43 to 1264·61)	874·33 (787·29 to 973·98)	699·39 (630·44 to 777·86)	643·99 (580·8 to 714·48)	565·15 (438·41 to 728·85)
Tabasco	2027·77 (1700·02 to 2430·07)	1467·88 (1249·54 to 1753)	1297·84 (1080·73 to 1570·63)	1133·15 (1022·47 to 1263·31)	816·85 (739·56 to 911·44)	668·81 (602·45 to 740·67)	615·61 (560·12 to 682·95)	594·08 (463·61 to 764·04)
Tamaulipas	1882·07 (1588·95 to 2228·58)	1336·94 (1129·88 to 1591·58)	1174·61 (971·4 to 1419·97)	1038·78 (935·82 to 1141·59)	743·06 (670·52 to 824·26)	576·12 (519·8 to 632·64)	524·99 (476·46 to 577·76)	524·63 (404 to 676·28)
Tlaxcala	2116·8 (1773·86 to 2511·75)	1467·87 (1234·58 to 1757·74)	1215·57 (1020·02 to 1456·29)	1009·52 (887·34 to 1161·26)	812·73 (737·32 to 901·17)	636·08 (578·6 to 704·88)	557·82 (506·69 to 619·93)	523·06 (407·45 to 662·94)
Veracruz de Ignacio de la Llave	1958·78 (1627·41 to 2342·81)	1363·16 (1142·88 to 1599·17)	1136·01 (949·31 to 1362·8)	912·54 (830·26 to 1011·75)	689·27 (624·68 to 765·14)	559·1 (506·55 to 618·54)	574·49 (521·35 to 632·48)	570·79 (441·52 to 739·46)
Yucatán	1998·92 (1696·53 to 2336·28)	1526 (1288·08 to 1791·51)	1375·4 (1165·16 to 1629·89)	1236·99 (1114·51 to 1359·03)	892·63 (803·58 to 984·68)	693·57 (623·87 to 764·54)	637·39 (578·11 to 700·33)	606·49 (477·44 to 770·04)
Zacatecas	1792·04 (1512·44 to 2121·89)	1263·12 (1057·41 to 1493·15)	1041·31 (869·01 to 1231·27)	806·59 (728·67 to 889·43)	668·46 (608·09 to 734·74)	566·7 (511·19 to 627·48)	529·41 (480·16 to 581·88)	517·85 (404·42 to 661·04)
Nicaragua	1981·68 (1794·51 to 2191·06)	1537·33 (1393·56 to 1698·24)	1310·66 (1179·34 to 1465·06)	868·65 (738·14 to 1039·76)	539·77 (486·01 to 602·32)	647·43 (586·38 to 713·34)	647·94 (582·71 to 723·4)	584·21 (487·6 to 696·61)
Panama	1198·75 (1103·31 to 1303·5)	966·96 (897·25 to 1043·41)	928·77 (868 to 1009·85)	593·51 (548·97 to 642·59)	509·02 (470·87 to 555·62)	443·21 (410·34 to 478·59)	409·17 (381·77 to 441·36)	382·47 (300·01 to 487·48)
Venezuela	1377·21 (1282·21 to 1481·61)	1078·57 (1001·26 to 1166·45)	1012·49 (944·47 to 1093·57)	816·01 (758·08 to 879·11)	662·36 (616·68 to 713·48)	533·71 (495·11 to 577·38)	478·41 (445·18 to 515·47)	521·27 (404·97 to 670·31)
Tropical Latin America	1757·04 (1596·18 to 1955·64)	1463·05 (1339·3 to 1596·5)	1256·7 (1158·14 to 1358·97)	1011·87 (944·07 to 1082·88)	821·41 (764·79 to 881·93)	657·15 (613·72 to 705·22)	562·72 (523·72 to 603·47)	502·74 (443·6 to 573·68)
Brazil	1779·62 (1618·93 to 1981·72)	1488·46 (1359·83 to 1626·41)	1271·3 (1169·31 to 1378·73)	1021·92 (951·46 to 1095·11)	830·01 (771·67 to 893·24)	660·29 (615·25 to 708·96)	563·67 (523·98 to 605·22)	503·16 (442·85 to 574·51)
Acre	1751·16 (1459·5 to 2072·04)	1418·44 (1188·37 to 1688·05)	1171·28 (995·72 to 1375·85)	946·38 (840·4 to 1060·79)	768·47 (681·62 to 859·41)	717·43 (645·16 to 791·96)	558·19 (497·8 to 623·26)	532·22 (433·64 to 648·44)
Alagoas	2365·7 (1992·55 to 2772·8)	2034·38 (1737·69 to 2354·43)	1725·38 (1474·57 to 2015·72)	1341·59 (1192·88 to 1493·74)	1006·03 (895·68 to 1134·39)	734·85 (654·34 to 822·12)	636·86 (569·27 to 710·31)	598·79 (476·89 to 738·74)
Amapá	2081·8 (1716·92 to 2508·92)	1570·78 (1327·2 to 1856·18)	1162·2 (994·81 to 1354·28)	727·36 (652·98 to 809·66)	600·7 (535·57 to 675·98)	662·04 (598·69 to 730·88)	462·86 (414·89 to 514·74)	483·15 (396·87 to 585·73)
Amazonas	1672·72 (1351·9 to 2068·97)	1349·41 (1134·74 to 1626·25)	1189·57 (984·7 to 1444·36)	929·02 (819·28 to 1049·38)	771·09 (680·73 to 878·31)	630·52 (556·4 to 711·7)	512·41 (456·27 to 573·62)	471·71 (382·54 to 577·63)
Bahia	1841·9 (1567·87 to 2180·31)	1567·34 (1332·35 to 1848·79)	1322·58 (1115·63 to 1575·52)	917·7 (802·36 to 1041·56)	810·42 (709·92 to 922·24)	642·4 (557·99 to 742·18)	544·28 (474·28 to 619·43)	486·6 (386·18 to 613·59)
Ceará	2195·44 (1859·12 to 2568·51)	1804·43 (1549·19 to 2091·3)	1419·13 (1200·82 to 1672·99)	1030·48 (865·51 to 1224·87)	715·69 (618·39 to 833·48)	599·6 (516·07 to 695·08)	546·18 (469·92 to 639·72)	521·3 (403·7 to 666·78)
Distrito Federal	1856·43 (1533·55 to 2216·96)	1536·3 (1292·65 to 1807·16)	1375·15 (1154·77 to 1626·41)	1141·76 (1004·53 to 1292·35)	967·91 (854·78 to 1097·59)	773·06 (700·87 to 856·43)	612·06 (551·21 to 676·27)	535·91 (434·89 to 651·39)
Espírito Santo	1606·31 (1347·42 to 1924·45)	1381·99 (1155·18 to 1638·95)	1217·04 (1021·95 to 1462·98)	972·76 (877·46 to 1075·41)	787·6 (705·64 to 873·83)	621·88 (561·75 to 686·76)	571·6 (509·92 to 636·05)	519·5 (418·11 to 638·72)
Goiás	2373·23 (1974·63 to 2826·86)	1982·64 (1650·83 to 2349·7)	1558·28 (1286·32 to 1859·52)	1210·41 (1015·15 to 1450·89)	947·03 (813·6 to 1097·45)	719·92 (629·18 to 824·5)	603·53 (522·07 to 692·02)	539·43 (423·38 to 685·43)
Maranhão	1963·06 (1652·47 to 2303·52)	1638·75 (1396·81 to 1898·59)	1354·26 (1145·4 to 1594·78)	1003·38 (845·59 to 1196·07)	669·26 (572·19 to 774·93)	575·14 (488·67 to 679·97)	549·19 (468·4 to 641·25)	534·77 (423·72 to 682·61)
Mato Grosso	1480·09 (1220·68 to 1774·26)	1324·62 (1090·59 to 1578·84)	1142·99 (954·62 to 1374·43)	858·07 (750·05 to 975·01)	755·08 (655·91 to 868·42)	635·13 (556·62 to 725·61)	544·19 (490·4 to 606·69)	476·33 (382·33 to 590·15)
Mato Grosso do Sul	1615·42 (1336·88 to 1922·54)	1392·78 (1149·14 to 1670·09)	1206·8 (998·23 to 1443·77)	1012·5 (907·04 to 1130·24)	811·24 (723·73 to 906·94)	661·85 (600·54 to 731·49)	582·56 (526·55 to 645·81)	498·21 (402·3 to 609·48)
Minas Gerais	1748·2 (1467·39 to 2066·22)	1494·95 (1249·56 to 1788·64)	1282·01 (1066·28 to 1533·34)	1037·28 (941·49 to 1140·77)	857·65 (765·72 to 953·57)	629·28 (568·97 to 695·27)	548·64 (492·91 to 608·07)	463·91 (377·37 to 562·38)
Pará	1890·5 (1583·43 to 2229·29)	1515·86 (1285·05 to 1782·22)	1294·07 (1068·44 to 1546·74)	1024·66 (881·91 to 1177·49)	821·11 (710·58 to 944·89)	623·83 (537·99 to 721·96)	529·06 (454·34 to 606·92)	467·22 (379·26 to 579·5)
Paraíba	2163·1 (1849·01 to 2527·16)	1729·56 (1467·39 to 2034·29)	1357·2 (1150·88 to 1608·76)	995·01 (887·48 to 1113·83)	768·64 (685·39 to 864·16)	578·27 (515·87 to 647·05)	537·43 (479·04 to 603·29)	492·12 (397·58 to 614·78)
Paraná	1540·55 (1272·97 to 1848·97)	1351·17 (1114·24 to 1633·73)	1202·21 (996·05 to 1433·09)	1049·7 (952·84 to 1151·07)	880·38 (798·94 to 971·98)	735·16 (669·45 to 806·87)	596·09 (538·75 to 662·02)	524·17 (426·87 to 639·87)
Pernambuco	2527·85 (2177·01 to 2944·19)	2061·26 (1754·1 to 2378·54)	1646·54 (1406·31 to 1932·33)	1144·15 (1038·25 to 1263·48)	961·51 (867·26 to 1064·44)	728·63 (657·09 to 804·27)	637·56 (572·45 to 703·85)	593·82 (481·01 to 719·42)
Piauí	1886·03 (1601·67 to 2209·17)	1602·08 (1345·46 to 1865·37)	1398·45 (1167·99 to 1639·93)	1057·85 (923·93 to 1205·06)	763·79 (670·97 to 858·08)	572·12 (505·87 to 638·46)	494·8 (444·36 to 549·91)	463·16 (375·62 to 565·29)
Rio de Janeiro	1563·17 (1293·34 to 1894·48)	1307·04 (1082 to 1576·7)	1182·64 (979 to 1404·07)	1061·6 (962·66 to 1176·24)	892·67 (805·75 to 992·82)	708·96 (643·72 to 782·29)	612·62 (555·35 to 677·03)	539·89 (443·25 to 657·11)
Rio Grande do Norte	2097·89 (1773·11 to 2487·89)	1633·25 (1387·97 to 1899·64)	1264·23 (1062·63 to 1514·9)	922·1 (800·05 to 1061·38)	681·85 (595·16 to 780·47)	562·15 (490·97 to 649·31)	466·22 (405·04 to 535·9)	443·96 (348·14 to 551·39)
Rio Grande do Sul	1455·27 (1201·05 to 1745·26)	1230·33 (1012·68 to 1477·21)	1051·21 (870·36 to 1253·74)	884·54 (790·09 to 969·89)	753·99 (680·19 to 829·63)	659·14 (596·92 to 722·62)	549·95 (495·82 to 605·96)	507·38 (411·94 to 620·28)
Rondônia	2485·32 (2079·26 to 2937·55)	2023·65 (1722·03 to 2357·07)	1711·02 (1479·11 to 2004·17)	1679·02 (1491·31 to 1871·13)	1126·41 (994·76 to 1256·1)	747·68 (668·3 to 837·18)	573·62 (509·26 to 642·34)	520·5 (419·94 to 633·35)
Roraima	2204·61 (1871·47 to 2592·66)	1771·08 (1509·89 to 2064·01)	1453·94 (1262·16 to 1672·5)	1113·72 (983·72 to 1254·14)	913·13 (813 to 1021·43)	787·97 (710·3 to 878·53)	608·04 (545·94 to 677·57)	588·95 (479·58 to 711·24)
Santa Catarina	1337·81 (1121·6 to 1617·18)	1156·17 (966·29 to 1397·42)	1043·14 (867·85 to 1248·46)	869·59 (784·84 to 960·1)	834·13 (745·04 to 932·73)	717·45 (643·04 to 797·32)	545·29 (486·69 to 609·72)	492·49 (401 to 602·84)
São Paulo	1449·1 (1203·71 to 1706·99)	1264·55 (1046·41 to 1509·76)	1153·8 (959·19 to 1379·57)	1050·49 (956·85 to 1151·68)	859·7 (779·53 to 958·4)	683·49 (626·72 to 746·2)	562·91 (514·84 to 617·25)	494·87 (410·99 to 600·03)
Sergipe	1793·56 (1492·8 to 2140·7)	1525·4 (1290·02 to 1792·65)	1299·16 (1094·84 to 1546·62)	1123·32 (990·96 to 1275·42)	894·08 (785·71 to 1006·01)	668·95 (607·39 to 739·34)	535·69 (466·69 to 612·14)	516·54 (410·33 to 649·39)
Tocantins	2029·23 (1683·98 to 2438·8)	1702·08 (1417·98 to 2009·8)	1339·46 (1130·93 to 1576·79)	943·39 (760·96 to 1148·01)	832·18 (720 to 954·8)	677·7 (598·4 to 765·2)	508·74 (446·09 to 578·47)	486·12 (383·77 to 604·19)
Paraguay	1036·31 (864·42 to 1232·19)	774·76 (690·17 to 864·27)	771·55 (682·98 to 870·03)	673·47 (604·11 to 749·05)	556·09 (493·56 to 627·37)	545·73 (488·28 to 614·89)	536·94 (462·01 to 622·36)	498·1 (373·87 to 654·61)
North Africa and Middle East	2124·6 (1951·13 to 2311·2)	1741·17 (1611·35 to 1873·02)	1457·32 (1364·03 to 1556·12)	1179·46 (1117·17 to 1243·3)	1032·46 (985·95 to 1083·84)	863·68 (815·45 to 918·19)	778·01 (732·19 to 827·63)	701·08 (640·93 to 771·93)
North Africa and Middle East	2124·6 (1951·13 to 2311·2)	1741·17 (1611·35 to 1873·02)	1457·32 (1364·03 to 1556·12)	1179·46 (1117·17 to 1243·3)	1032·46 (985·95 to 1083·84)	863·68 (815·45 to 918·19)	778·01 (732·19 to 827·63)	701·08 (640·93 to 771·93)
Afghanistan	2920·99 (2221·8 to 3990·82)	2386·11 (1912·39 to 3057·96)	2260·5 (1878·93 to 2751·46)	2510·51 (2122·9 to 3091·18)	1859·27 (1534·3 to 2223·28)	1859·24 (1468·91 to 2389·08)	1544·27 (1122·26 to 2102·79)	1503·3 (1059·18 to 2153·11)
Algeria	1556·28 (1398·54 to 1729·12)	1646·82 (1436·64 to 1867·77)	1559·83 (1361·08 to 1768·22)	1589·02 (1408·69 to 1785·38)	1193·7 (1055·59 to 1348·88)	944·53 (818·36 to 1087·47)	842·44 (743·53 to 947·5)	776·66 (651·96 to 919·19)
Bahrain	1729·32 (1428·97 to 2064·11)	1452·3 (1231·92 to 1708·9)	1188·56 (991·37 to 1413·1)	1106·71 (966·88 to 1261·78)	1094·09 (981·39 to 1214·05)	956·47 (853·12 to 1058·63)	869·58 (781·54 to 963·67)	712·76 (592·24 to 854·07)
Egypt	2536·32 (2260·66 to 2826·72)	1999·45 (1854·24 to 2166·73)	1913·2 (1738·33 to 2105·89)	1419·95 (1293·94 to 1572·93)	1301·91 (1186·95 to 1424·35)	1106·11 (1014·49 to 1208·3)	1188·19 (1085·07 to 1311·44)	1115·48 (928·88 to 1339·31)
Iran	2263·43 (2009·85 to 2557·62)	1819·99 (1617·53 to 2062·39)	1325·48 (1186·52 to 1480·9)	933·16 (846·2 to 1038·43)	921·14 (838·12 to 1012·41)	750·81 (681·58 to 822·57)	585·24 (544·74 to 631·6)	534·96 (488·79 to 588·34)
Iraq	1316·31 (1134·84 to 1511·96)	1077·28 (944·51 to 1227·4)	984·81 (843·83 to 1145·72)	951·41 (822·09 to 1090·21)	932·99 (800·68 to 1083)	860·47 (677·7 to 1083·62)	793·68 (591·36 to 1075·1)	697·68 (511·89 to 958·77)
Jordan	1504·26 (1115·78 to 2033·46)	1260·98 (1008·49 to 1569·79)	1080·98 (938·13 to 1242·39)	909·36 (812·18 to 1022·12)	994·68 (870·34 to 1151·03)	938·17 (811·34 to 1085·98)	600·67 (526·51 to 693·23)	552·58 (453·54 to 676·2)
Kuwait	968·27 (777·22 to 1178·66)	748·05 (653·18 to 855·07)	723·59 (671·06 to 775·57)	718·97 (667·46 to 772·65)	633·41 (571·57 to 703·36)	462·88 (418·36 to 514·98)	424·49 (392·57 to 459·77)	327·53 (270·01 to 396·54)
Lebanon	1385·66 (1137·39 to 1673·55)	1021·41 (829·42 to 1226·37)	918·32 (777·44 to 1067·17)	870·09 (727·34 to 1051·51)	833·74 (689·39 to 1024·86)	677·59 (598·61 to 765·15)	594·22 (532·41 to 662·72)	540·18 (456·4 to 636·7)
Libya	2042·03 (1742·29 to 2353·02)	1588·39 (1327 to 1877·54)	1001·01 (890·72 to 1126·09)	811·87 (724·54 to 914·59)	696·9 (592·48 to 814·76)	597·19 (530·41 to 671·49)	584·38 (535·36 to 646·54)	586·65 (477·79 to 748·35)
Morocco	2199·07 (1915·18 to 2509·05)	1891·68 (1656·43 to 2174·8)	1508·44 (1316·48 to 1711·44)	1229·55 (1086·3 to 1388·33)	1004·17 (890·82 to 1126·9)	904·54 (795·51 to 1023·74)	881·62 (746·2 to 1030·99)	774·1 (646·24 to 905·97)
Oman	2776·97 (1909·86 to 4156·11)	2150·97 (1596·64 to 2949·05)	1506·22 (1168·19 to 1935·26)	1269·01 (1014·12 to 1583·83)	1110·88 (916·24 to 1319·11)	999·98 (874·47 to 1143·46)	940·61 (877·29 to 1012·68)	810·77 (718·54 to 908·7)

Supplemental Results Table 3c: Age-standardised mortality rates by location, females, 1950, 1960, 1970, 1980, 1990, 2000, 2010, 2019

Location	1950	1960	1970	1980	1990	2000	2010	2019
Palestine	1537·43 (1202·34 to 1950·97)	1255·97 (1031·59 to 1505·02)	1212·37 (1038·09 to 1417·42)	977·05 (777·17 to 1195·73)	927·29 (760·44 to 1113·64)	822·75 (729·99 to 925·01)	700·86 (631·14 to 778·19)	704·59 (615·34 to 806·25)
Qatar	993·38 (694·55 to 1428·52)	994·25 (744·99 to 1352·02)	920·09 (722·83 to 1163·89)	718·34 (590·1 to 865·1)	1047·59 (886·15 to 1221·23)	1120·54 (961·41 to 1298·43)	1380·68 (1214·74 to 1550·71)	1106·17 (950·76 to 1281·07)
Saudi Arabia	1700·03 (1406·36 to 2047·82)	1571·33 (1299·92 to 1873·38)	1301·07 (1080·99 to 1528·07)	1035·44 (864·53 to 1249·14)	1010·22 (862·01 to 1184·17)	943·58 (837·92 to 1059·58)	805·26 (718·69 to 901·82)	675·34 (537·48 to 845·59)
Sudan	2066·22 (1794·23 to 2364·05)	1915·25 (1650·25 to 2202·72)	1761·17 (1538·31 to 1999·13)	1627·63 (1430·09 to 1834·39)	1352·38 (1197·82 to 1523·52)	1116·77 (944·37 to 1302·81)	941·24 (779·31 to 1106·32)	843·76 (706·5 to 1023·33)
Syria	1534·04 (1290·29 to 1806·84)	1113·53 (939·46 to 1323·52)	864·99 (754·44 to 1002·36)	716·37 (601 to 838·29)	992·82 (822·56 to 1176·91)	1031·83 (897·28 to 1188·83)	877·05 (747·56 to 1026·37)	915·54 (734·24 to 1144·77)
Tunisia	2306·05 (1986·35 to 2670·53)	1903·19 (1681·27 to 2152·3)	1555·78 (1391·11 to 1756·34)	1065·63 (958·3 to 1194·71)	708·03 (631·79 to 798·79)	637·5 (538·1 to 751·03)	566·92 (441·11 to 702·17)	506·25 (395·9 to 644·25)
Turkey	1849·14 (1573·16 to 2144·99)	1578 (1343·62 to 1838·69)	1222·05 (1044 to 1420·72)	935·91 (837·36 to 1040·36)	796·44 (719·12 to 881·54)	562·57 (483·76 to 658·1)	542·96 (485·47 to 610·08)	479·7 (389·8 to 577·57)
United Arab Emirates	1333·51 (1029·86 to 1718·89)	1286·69 (1070·36 to 1557·79)	1135·62 (956·32 to 1365·31)	1102·64 (923·95 to 1301·25)	1217·74 (1045·31 to 1408·89)	1330·37 (1174·62 to 1521·73)	1555·24 (1353·47 to 1789·56)	706·67 (564·85 to 896·73)
Yemen	3446·76 (2380·18 to 5181·9)	2613·68 (1976·27 to 3616·13)	2225·6 (1762·55 to 2864·98)	1580·33 (1304·44 to 1919·7)	1322·15 (1133·97 to 1553·63)	1132·77 (955·9 to 1351·22)	956·89 (809·53 to 1151·13)	951·02 (761·19 to 1204·41)
South Asia	2556·49 (2237·4 to 2897·43)	2336·97 (2065·37 to 2630·93)	2161·87 (1963·57 to 2369·25)	1681·51 (1575·31 to 1813·9)	1471·95 (1376·6 to 1580·01)	1273·97 (1181·45 to 1368·47)	959·09 (892·85 to 1033·89)	846·71 (725·91 to 981·9)
South Asia	2556·49 (2237·4 to 2897·43)	2336·97 (2065·37 to 2630·93)	2161·87 (1963·57 to 2369·25)	1681·51 (1575·31 to 1813·9)	1471·95 (1376·6 to 1580·01)	1273·97 (1181·45 to 1368·47)	959·09 (892·85 to 1033·89)	846·71 (725·91 to 981·9)
Bangladesh	2501·63 (2162·34 to 2840·62)	2172·21 (1874·99 to 2494·21)	2267·72 (1975·41 to 2604·84)	1674·36 (1474·84 to 1901·36)	1376·63 (1244·63 to 1528·45)	1059·74 (960 to 1178·44)	831·5 (747·62 to 921·87)	657·48 (527·87 to 797·05)
Bhutan	2858·32 (2392·11 to 3358·96)	2382·34 (1924 to 2869·8)	1954·3 (1515·19 to 2489·13)	1748·9 (1340·26 to 2325·41)	1376·04 (1114·28 to 1718·07)	1084·63 (937·78 to 1243·57)	840·49 (708·89 to 965·59)	766·91 (643·51 to 873·77)
India	2597·63 (2213·41 to 3001·09)	2441 (2105·3 to 2784·04)	2292·54 (2048·17 to 2563·01)	1783·02 (1641·7 to 1957·3)	1533·45 (1406·74 to 1678·6)	1298·89 (1184·74 to 1418·7)	944·58 (866·31 to 1033·37)	837·3 (693·28 to 997·15)
Andhra Pradesh	2520·54 (2031·1 to 3140·16)	2440·4 (1985·11 to 3001·16)	2336·29 (1916·27 to 2795·44)	1767·14 (1451·96 to 2135·41)	1512·87 (1250·91 to 1827·01)	1233·21 (1017·19 to 1489·41)	938·54 (753·88 to 1143·42)	786·91 (608·61 to 1033·51)
Arunachal Pradesh	2656·64 (2138·08 to 3211·83)	2508·81 (2034·82 to 3064·24)	2380·02 (1958·94 to 2872·22)	1681·02 (1391·25 to 2013·37)	1434·04 (1165·73 to 1732·61)	1152·32 (962·6 to 1389·21)	867·25 (717·67 to 1064·8)	739·86 (569·4 to 953·09)
Assam	2928·48 (2397·84 to 3516·61)	2751·15 (2223·39 to 3343·32)	2592·57 (2142·98 to 3129·67)	1930·24 (1556·17 to 2419·68)	1705·49 (1476·52 to 1992·64)	1696·59 (1503·77 to 1909·13)	1286·02 (1137·44 to 1447·17)	955·08 (763·42 to 1201·21)
Bihar	2359·2 (1911·36 to 2857·18)	2255·71 (1820·8 to 2714·4)	2251·17 (1855·85 to 2699·52)	1815·55 (1535·5 to 2155·27)	1555·27 (1313·39 to 1850·27)	1344·61 (1150·01 to 1581·91)	1025·94 (895·53 to 1165·47)	874·37 (704·84 to 1088·11)
Chhattisgarh	2606·83 (2111·5 to 3155·05)	2531·84 (2031·84 to 3082·3)	2423·86 (1964·6 to 2916·68)	1931·24 (1610·62 to 2322·2)	1676·2 (1371·72 to 2000·36)	1631·01 (1395·6 to 1915·81)	1126·18 (990 to 1284·68)	967·93 (769·86 to 1203·92)
Delhi	2247·57 (1811·06 to 2746·26)	2002·86 (1619·23 to 2484·69)	1860·56 (1513·7 to 2270·61)	1427·19 (1185·4 to 1739·8)	1272·15 (1077·67 to 1520·65)	1122·08 (974·35 to 1281·65)	798·74 (708 to 911·99)	676·66 (541·99 to 833·69)
Goa	1824·71 (1432·14 to 2313·81)	1726·07 (1342·19 to 2221·29)	1659·82 (1335·96 to 2025·8)	1262·59 (1037·72 to 1552·02)	1143·26 (943·88 to 1370·23)	954·48 (777·15 to 1169·3)	695·56 (564·71 to 860·74)	601·36 (448·6 to 787·64)
Gujarat	2320·79 (1892·84 to 2805·56)	2171·04 (1776·34 to 2660·95)	2042·47 (1686·74 to 2484·24)	1673·34 (1399·1 to 1988·86)	1471·89 (1250·72 to 1729·12)	1150·48 (1015·82 to 1300·34)	871·4 (772·41 to 993·82)	843·32 (652·37 to 1075·87)
Haryana	2192·45 (1743·75 to 2680·47)	2095·41 (1645·72 to 2624·02)	1964·45 (1630·45 to 2390·72)	1454·61 (1214·7 to 1743·49)	1265·88 (1086·78 to 1477·48)	1130·56 (1003·75 to 1276·83)	813·54 (714·56 to 925·21)	743·37 (556·75 to 951·08)
Himachal Pradesh	2094·52 (1682·92 to 2604·83)	2026·16 (1567·96 to 2530·86)	1944·92 (1575·84 to 2338·42)	1505·55 (1242·73 to 1792·67)	1339·09 (1133·61 to 1568·27)	1060·99 (942·57 to 1193·85)	744·9 (660·4 to 846·15)	648·24 (508·68 to 804·35)
Jammu and Kashmir	1843·65 (1477·3 to 2276·96)	1805·97 (1431·95 to 2232·19)	1722·87 (1419·2 to 2121·7)	1370·31 (1144·06 to 1685·15)	1220·71 (1013·83 to 1506·35)	1040·17 (902·74 to 1200·82)	849·26 (758·68 to 953·14)	772·73 (614·61 to 982·29)
Jharkhand	2434·98 (1955·89 to 2980·81)	2309·94 (1855·11 to 2829·12)	2235·02 (1805·45 to 2690·74)	1810·04 (1504·11 to 2163·18)	1695·66 (1424·38 to 1989·21)	1426·93 (1248·41 to 1623·05)	1072·81 (949·84 to 1208·98)	853·9 (695·97 to 1046·5)
Karnataka	2434·6 (1961·84 to 2999·04)	2317·94 (1894·37 to 2837·44)	2146·68 (1769·89 to 2573·26)	1549·68 (1289·34 to 1884·9)	1279·6 (1081·62 to 1482·57)	1039·41 (903·66 to 1176·82)	851·01 (749·18 to 969·4)	847·92 (650·89 to 1065·61)
Kerala	1765·64 (1438·43 to 2165·43)	1563·75 (1257·5 to 1933·77)	1422·12 (1163·49 to 1721·4)	1143·97 (937·34 to 1365·28)	1009·48 (847·79 to 1196·03)	888·94 (778·46 to 1007·3)	618·41 (543·53 to 701·44)	493·59 (379·24 to 638·09)
Madhya Pradesh	2819·21 (2278·67 to 3449·5)	2611·54 (2143·37 to 3172·33)	2462·13 (2058·84 to 2926·04)	1940·99 (1640·33 to 2280·15)	1653·15 (1399·38 to 1953·15)	1372·1 (1188·96 to 1569·14)	994·61 (878·44 to 1114·82)	848·83 (684·69 to 1033·97)
Maharashtra	2388·37 (1929·12 to 2920·05)	2250·59 (1828·23 to 2757·45)	2135·86 (1755·52 to 2538·38)	1580·64 (1308·79 to 1899·74)	1344·51 (1133·4 to 1586·2)	1177·86 (1038·11 to 1325·14)	778·75 (689·71 to 886·09)	746·99 (573·9 to 949·91)
Manipur	2256·24 (1818·04 to 2782·46)	2132·6 (1704·11 to 2619·59)	1996·53 (1610·97 to 2448·47)	1495·98 (1240·49 to 1806·37)	1291·72 (1054·86 to 1571·21)	1113·9 (906·6 to 1351·02)	878·6 (695·12 to 1079·72)	754·78 (575·09 to 1006·42)
Meghalaya	1921·89 (1527·38 to 2396·45)	1873·57 (1489·59 to 2317·95)	1869·14 (1536·37 to 2277·15)	1569·98 (1281·11 to 1889·24)	1373·24 (1122·56 to 1659·72)	1106·86 (920·02 to 1324·64)	857·96 (700·08 to 1043·91)	765·72 (582·08 to 985·36)
Mizoram	1735·84 (1376·81 to 2205·09)	1691·12 (1339·33 to 2106·45)	1661·82 (1355·77 to 2069·93)	1370·68 (1112·65 to 1652·69)	1222·61 (1023·26 to 1491·3)	1054·85 (863·38 to 1283·35)	868·7 (688·21 to 1074·97)	747·64 (550·47 to 992·6)
Nagaland	2424·59 (1932·81 to 2997·06)	2266·33 (1819·21 to 2786·12)	2054·38 (1669·48 to 2501·37)	1501·58 (1233·45 to 1804·7)	1281·03 (1053·78 to 1560·35)	1101·36 (909·93 to 1331·14)	897·8 (695·3 to 1121·48)	765·4 (568·3 to 980·19)
Odisha	2992·53 (2415·49 to 3604·65)	2788·7 (2309·26 to 3365·86)	2614·76 (2174·31 to 3084·46)	2019·01 (1692·92 to 2405·65)	1820·67 (1547·33 to 2135·94)	1600·61 (1405·67 to 1818·98)	1138·78 (1002·55 to 1307·16)	858·53 (661·49 to 1099·6)
Punjab	1973·08 (1566·2 to 2441·06)	1876·31 (1467·04 to 2357·31)	1746·91 (1425·96 to 2114·42)	1383·84 (1164·17 to 1674·08)	1239·96 (1064·77 to 1444·44)	1122·14 (993·65 to 1263·81)	828·06 (726·13 to 941·71)	818·17 (639·95 to 1008·33)
Rajasthan	2100·14 (1710·76 to 2568·56)	2093·47 (1692·17 to 2556·05)	2016·88 (1667·9 to 2458·08)	1651·42 (1386·3 to 1948·93)	1432·85 (1218·39 to 1670·76)	1221·6 (1085·21 to 1380·14)	847·77 (754·42 to 956·09)	847·12 (662·98 to 1075·62)
Sikkim	2136·26 (1707·49 to 2659·83)	2119·37 (1691·53 to 2625·11)	2055·7 (1673·73 to 2516·1)	1568·38 (1279 to 1880·17)	1308·45 (1073·27 to 1590·72)	1080·17 (885·28 to 1290·51)	814·52 (657·09 to 995·51)	666·18 (510·6 to 850·56)
Tamil Nadu	2710·71 (2196·2 to 3278·64)	2523·66 (2050·45 to 3075·57)	2344·98 (1932·58 to 2798·45)	1966·82 (1659·56 to 2334·25)	1749·38 (1478·68 to 2046·94)	1488·29 (1314·49 to 1677·47)	1074·51 (943·43 to 1227·14)	879·66 (688·97 to 1108·59)
Telangana	2226·19 (1728·62 to 2783·53)	2165·13 (1713·4 to 2721·46)	2110 (1697·06 to 2608·64)	1688·38 (1375·42 to 2095·83)	1547·46 (1233·63 to 1921·38)	1314·8 (1026·78 to 1653·01)	1027·22 (815·8 to 1292·01)	789·72 (570·49 to 1085·54)
Tripura	2851·9 (2304·51 to 3448·3)	2578·03 (2086·49 to 3148·37)	2337·97 (1911·18 to 2800·74)	1698·13 (1407·67 to 2036·16)	1412·99 (1165·03 to 1713·33)	1124·45 (922·92 to 1348·86)	866·26 (695·5 to 1046·11)	769·29 (577·5 to 1001·67)
Union Territories other than Delhi	2057·21 (1647·39 to 2551·7)	1860·11 (1470·12 to 2342·52)	1737·76 (1414·4 to 2114·79)	1311·79 (1093·27 to 1573·75)	1163·8 (967·44 to 1398·79)	997·09 (812·84 to 1205·91)	761·2 (608·35 to 953·16)	662·87 (512·25 to 868·41)
Uttar Pradesh	3379·21 (2810·3 to 3992·43)	3185·08 (2652·55 to 3768·5)	2906·13 (2482·4 to 3390·68)	2186·34 (1898·41 to 2523·8)	1813·16 (1569·68 to 2085·49)	1462·46 (1316·37 to 1639·53)	1070·61 (956·7 to 1201·33)	1030·31 (832·76 to 1285·02)
Uttarakhand	2220·36 (1741·05 to 2742·69)	2200·11 (1730·05 to 2726·97)	2133·04 (1705·22 to 2626·42)	1707·08 (1393·95 to 2072·05)	1587·13 (1306·64 to 1955·05)	1388·88 (1133·87 to 1679·67)	1040·74 (893·54 to 1227·72)	849·74 (668·45 to 1049·95)
West Bengal	2639·56 (2139·25 to 3173·68)	2406·77 (1957·69 to 2921·39)	2232·89 (1842 to 2683·33)	1757·65 (1464·24 to 2088·39)	1504·53 (1282·4 to 1735·76)	1328·72 (1171·07 to 1487·79)	896·2 (796·81 to 997·89)	772·73 (601·3 to 967·79)
Nepal	2541·55 (2164·56 to 2938·33)	2203·99 (1911·38 to 2517·32)	1915·88 (1676·65 to 2205·99)	1786·72 (1555·73 to 2058·61)	1459·6 (1295·65 to 1676·01)	1054·81 (955·5 to 1163·04)	900·98 (818·27 to 995·99)	849·5 (703·78 to 1001·77)
Pakistan	2192·35 (1914·9 to 2496·36)	1840·39 (1601·93 to 2088·78)	1563·72 (1395·46 to 1767·5)	1320·8 (1192·97 to 1457·01)	1314·1 (1158·57 to 1495·49)	1367·88 (1233·97 to 1508·93)	1222·56 (1041·45 to 1436·67)	1087·8 (918·92 to 1301·34)
Southeast Asia, east Asia, and Oceania	1953·1 (1731·78 to 2196·89)	2858·05 (2279·08 to 3712·34)	1266·5 (1113·81 to 1459·71)	1162·79 (1057·66 to 1304·2)	981·07 (893·56 to 1073·68)	824 (766·48 to 889·52)	659·52 (612·75 to 710·87)	538·1 (462·73 to 620·21)
East Asia	1944·31 (1678·42 to 2253·02)	3325·35 (2538·32 to 4473·19)	1225·83 (1023·8 to 1473·81)	1161·2 (1019·36 to 1352·21)	973·82 (860·9 to 1096·1)	799·39 (727·37 to 884·62)	610·21 (550·95 to 674·32)	484·9 (395·57 to 588·47)
China	1906·91 (1639·12 to 2222·53)	3386·83 (2579·79 to 4560·91)	1227·99 (1020·91 to 1481·86)	1170·48 (1022·9 to 1368·57)	982·06 (862·78 to 1109·59)	795·52 (720·7 to 882·97)	611·26 (549·99 to 678·14)	484·54 (390·54 to 595·07)
North Korea	4223·27 (2981·31 to 6174·07)	1931·49 (1420·98 to 2677·25)	1373·91 (1062·62 to 1798·51)	1060·74 (860·36 to 1310·44)	919·85 (781·63 to 1078·4)	1273·4 (927·54 to 2010·49)	807·69 (699·85 to 922·53)	673·02 (554·98 to 836·46)
Taiwan (province of China)	1531·6 (1337·19 to 1758·47)	1181·61 (1167·34 to 1196·23)	905·64 (895·06 to 916·67)	742·81 (734·05 to 751·87)	616·02 (607·3 to 624·29)	501·69 (494·68 to 509·16)	388·75 (382·69 to 395·23)	364·67 (295·22 to 455·66)
Oceania	1928·97 (1639·57 to 2296·36)	1557·94 (1351·79 to 1787·28)	1305·8 (1153·53 to 1470·23)	1163·58 (1062·04 to 1264·57)	1167·73 (1040·48 to 1327·21)	1171·7 (1038·29 to 1324·47)	1187·81 (1039·23 to 1378·3)	1107·23 (927·62 to 1342·93)
American Samoa	1276·96 (1129·13 to 1452·68)	1100·3 (994·55 to 1222·53)	1043·1 (929·27 to 1176·29)	863·77 (784·07 to 958·21)	778·69 (709·54 to 854·02)	764·93 (696·24 to 840·82)	737·91 (668·3 to 825·61)	764·53 (625·4 to 940·48)
Cook Islands	2391·86 (2122·93 to 2683·49)	1689·07 (1492·32 to 1882·96)	1212·44 (1066·67 to 1377·78)	1071·41 (958·76 to 1211·78)	895·35 (780·17 to 1035·91)	665·7 (584·54 to 752·1)	566·85 (499·88 to 647·66)	557·32 (460·92 to 674·65)
Fiji	1609·26 (1485·31 to 1737·69)	1125·85 (1011·12 to 1246·06)	1025·05 (911·49 to 1148·28)	999·99 (901·13 to 1118·42)	1073·98 (914·05 to 1260·08)	1158·79 (1051·99 to 1280·7)	1061·58 (969·56 to 1170·86)	985·7 (788·55 to 1207·96)
Guam	951·53 (830·17 to 1082·87)	1019·2 (928·21 to 1133·09)	1082·58 (991·29 to 1181·14)	879·61 (810·4 to 959·97)	743·54 (675·45 to 816·47)	568·26 (510·83 to 624·78)	430·89 (390·08 to 478·45)	493·2 (406·8 to 594·13)
Kiribati	2512·61 (2169·95 to 2900·57)	2175·02 (1815·22 to 2568·88)	1964·85 (1635·06 to 2334·87)	1794·31 (1485·57 to 2156·63)	1867·79 (1592·47 to 2169·39)	1759·64 (1517·18 to 2004·07)	1620·32 (1387·79 to 1867·12)	1502·55 (1245·29 to 1780·46)
Marshall Islands	1769·29 (1221·57 to 2594·15)	1472·39 (1078·36 to 1995·42)	1319·24 (1030·68 to 1710·2)	1174·84 (964·24 to 1401·01)	1262·64 (1135·69 to 1409·48)	1423·54 (1268·04 to 1613·49)	1369·16 (1155·49 to 1581·96)	1259·96 (1002·6 to 1561·69)
Federated States of Micronesia	2155·16 (1840·62 to 2514·46)	1790·37 (1444·54 to 2190·87)	1617·36 (1229·68 to 2141·29)	1476·14 (1161·45 to 1858·52)	1421·67 (1167·33 to 1684·7)	1359·22 (1191·99 to 1530·34)	1302·13 (1103·94 to 1503·77)	1307·19 (1049·51 to 1583·16)
Nauru	1761·59 (1460·82 to 2111·53)	1608·14 (1358·57 to 1920·4)	1446·61 (1199·02 to 1719·43)	1349·54 (1081·38 to 1681·27)	1364·59 (1132·95 to 1605·31)	1560·52 (1338·45 to 1811·06)	1522·24 (1300 to 1784·13)	1312·63 (1057·7 to 1608·08)
Niue	1732·15 (1525·32 to 1962·01)	1215·34 (1048·53 to 1403·26)	948·8 (814·32 to 1122·31)	868·95 (734·56 to 1042·33)	921·39 (760·11 to 1125·95)	918·88 (766·82 to 1129·78)	866·13 (716·8 to 1044·2)	808·78 (641·68 to 1034·65)
Northern Mariana Islands	1163·23 (970·22 to 1391·11)	1121·92 (920·55 to 1358·44)	1046·32 (887·85 to 1265·97)	951·19 (785·71 to 1182·64)	912·02 (758 to 1101·87)	780·72 (661·89 to 918·04)	690·66 (589·29 to 808·58)	677·5 (551·39 to 835·48)
Palau	1849·32 (1531·51 to 2243·69)	1560·2 (1309·33 to 1894·8)	1321·42 (1092·76 to 1598·61)	1219·59 (993·66 to 1499·46)	1123·26 (888·19 to 1401·73)	1050·85 (853·24 to 1254·97)	1025·13 (806·87 to 1232·59)	988·04 (773·62 to 1216·85)
Papua New Guinea	2003·71 (1587·63 to 2537·83)	1599 (1325·52 to 1914·57)	1298·79 (1104·89 to 1516·94)	1147·87 (1022·31 to 1274·57)	1153 (969·18 to 1390·84)	1153·94 (967·19 to 1375·52)	1213·5 (1014·66 to 1473·98)	1124·54 (906·29 to 1409·75)
Samoa	1549·42 (1320·8 to 1806·63)	1226·15 (1082·56 to 1386·62)	1074·58 (948·32 to 1238·97)	1003·32 (838·4 to 1186·46)	984·87 (843·79 to 1195·19)	960·87 (799·89 to 1200·09)	941·4 (776·34 to 1143·98)	918·45 (738·66 to 1160·47)
Solomon Islands	2850·48 (2467·71 to 3285·35)	2537·19 (2164·76 to 2958·84)	2238·1 (1892·38 to 2593·81)	2019·08 (1688·44 to 2415·02)	1974·42 (1662·17 to 2338·02)	1926·06 (1648·59 to 2214·37)	1922·05 (1673·2 to 2205·15)	1786·03 (1471·41 to 2141·03)
Tokelau	1800·22 (1528·09 to 2124·31)	1607·28 (1327·66 to 1917·37)	1457·38 (1201·37 to 1769·36)	1337·36 (1130·08 to 1602·89)	1219·71 (1021·69 to 1495·79)	1135·41 (927·41 to 1405·62)	1041·86 (854·95 to 1282·14)	979·17 (785·64 to 1222·94)
Tonga	1315·32 (1122·99 to 1562·22)	1182·65 (1037·59 to 1342·64)	1088·04 (937·79 to 1262·87)	922·31 (800·77 to 1053·19)	752·92 (648·71 to 861·4)	713·21 (625·73 to 816·53)	656·1 (555·21 to 767·92)	649·23 (528·43 to 805·45)
Tuvalu	2220·79 (1833·55 to 2692·91)	1870·59 (1537·16 to 2264·95)	1650·8 (1321·06 to 2010·75)	1554·83 (1265·95 to 1854·73)	1404·13 (1193·87 to 1654·64)	1518·88 (1334·01 to 1725·32)	1143·91 (915·84 to 1390·29)	1069·51 (828·92 to 1347·05)
Vanuatu	2140·77 (1815·91 to 2522·22)	1820·52 (1506·84 to 2217·83)	1498·88 (1228·24 to 1848·24)	1265·97 (1029·46 to 1533·55)	1276·4 (1011·56 to 1558·99)	1266·41 (1003·69 to 1539·19)	1207·91 (955·38 to 1476·1)	1153·01 (913·86 to 1440·91)
Southeast Asia	2010·01 (1826·53 to 2212·18)	1570·72 (1439·45 to 1727·18)	1375·59 (1263·93 to 1491·07)	1146·86 (1074·2 to 1231·46)	991·41 (938·12 to 1048·08)	883·58 (824·86 to 943·71)	771·24 (720·07 to 818·68)	674·78 (603·99 to 747·91)
Cambodia	2198·79 (1836·65 to 2611·31)	1903·49 (1599·48 to 2233·53)	1942·81 (1628·65 to 2316·54)	1656·86 (1346·19 to 2074·66)	1414·64 (1235·29 to 1624·35)	1281·55 (1149·5 to 1419·46)	965·92 (855·12 to 1088·13)	863·46 (710·41 to 1024·15)
Indonesia	2238·18 (1941·67 to 2576)	1842·14 (1599·56 to 2138·84)	1548·44 (1336·15 to 1788·56)	1287·2 (1151·36 to 1439·34)	1110·02 (1010·65 to 1213·07)	1016·88 (882·07 to 1178·6)	935·77 (832·9 to 1035·45)	837·7 (686·77 to 1007·94)
Aceh	2053·85 (1655·38 to 2547·4)	1734·99 (1371·23 to 2183·96)	1526·9 (1199·48 to 1936·43)	1317·24 (1079·4 to 1603·65)	1169·39 (979·28 to 1376·11)	1059·33 (850·04 to 1311·55)	1066·06 (896·06 to 1253·39)	1003·47 (761·72 to 1279·09)

Supplemental Results Table 3c: Age-standardised mortality rates by location, females, 1950, 1960, 1970, 1980, 1990, 2000, 2010, 2019

Location	1950	1960	1970	1980	1990	2000	2010	2019
Bali	2083·32<br>(1671·68 to 2551·26)	1729·54<br>(1358·96 to 2225·18)	1534·41<br>(1175·16 to 1957·87)	1375·63<br>(1111·85 to 1692·46)	1117·23<br>(945·67 to 1303·68)	982·05<br>(795·21 to 1213·78)	852·88<br>(710·69 to 1007·43)	688·28<br>(496·36 to 958·48)
Bangka-Belitung Islands	2698·35<br>(2224·39 to 3266·13)	2248·06<br>(1772·9 to 2853·03)	1920·34<br>(1495·49 to 2414·68)	1610·19<br>(1306·67 to 1970·91)	1446·65<br>(1204·15 to 1720·1)	1336·23<br>(1055·33 to 1650·31)	1212·63<br>(1005·63 to 1430·34)	1086·55<br>(812·53 to 1409·18)
Banten	2727·98<br>(2232·49 to 3300·09)	2401·32<br>(1930·51 to 2988·17)	1916·25<br>(1510·05 to 2377·63)	1487·52<br>(1271·5 to 1779·99)	1156·69<br>(994·65 to 1338·71)	1026·79<br>(823·41 to 1269·22)	983·13<br>(821·96 to 1161·69)	906·24<br>(673·66 to 1166·31)
Bengkulu	3059·11<br>(2525·62 to 3656·95)	2585·89<br>(2023·93 to 3253·4)	2181·77<br>(1697·71 to 2711·33)	1837·73<br>(1507·4 to 2191·76)	1539·71<br>(1307·27 to 1798·06)	1293·13<br>(1036·58 to 1613·46)	1176·28<br>(983·35 to 1390·8)	1053·44<br>(797·71 to 1340·17)
Gorontalo	2570·09<br>(2067·67 to 3104·2)	2291·69<br>(1788·73 to 2916·68)	1991·93<br>(1544·09 to 2527·64)	1728·68<br>(1421·22 to 2108·83)	1603·7<br>(1347·4 to 1877·27)	1616·58<br>(1287·74 to 2013·53)	1572<br>(1293·8 to 1879·24)	1370<br>(1006·16 to 1816·78)
Jakarta	1892·36<br>(1535·14 to 2355·05)	1657·78<br>(1327·04 to 2065·31)	1385·76<br>(1098·09 to 1803·3)	1167·54<br>(998·43 to 1408·66)	1018·47<br>(870·45 to 1192·77)	957·29<br>(764·43 to 1190·26)	873·73<br>(709·18 to 1030·33)	781·5<br>(596·43 to 1012·44)
Jambi	2769·15<br>(2255·41 to 3346·94)	2338·65<br>(1844·97 to 2959·02)	2071·26<br>(1618·4 to 2649·29)	1799·63<br>(1476·66 to 2170·82)	1423·49<br>(1207·87 to 1660·41)	1161·97<br>(928·67 to 1445·13)	1050·31<br>(892·78 to 1233·75)	951·13<br>(719·2 to 1210·4)
West Java	2434·5<br>(2049·02 to 2939·79)	1996·06<br>(1626·39 to 2507·09)	1618·87<br>(1298·71 to 2021·23)	1289·07<br>(1116·15 to 1500·61)	1073·28<br>(942·12 to 1223·02)	958·56<br>(774·44 to 1185·42)	898·11<br>(760·42 to 1055·38)	817·08<br>(629·73 to 1063·83)
Central Java	1957·17<br>(1633·71 to 2356·55)	1592·99<br>(1290·53 to 1980·03)	1332·23<br>(1083·8 to 1646·41)	1095·83<br>(948·03 to 1284·75)	948·52<br>(830·75 to 1068·54)	942·72<br>(763·72 to 1145·13)	878·53<br>(729·23 to 1024·81)	771·01<br>(601·84 to 974)
East Java	1991·68<br>(1684·17 to 2346·32)	1638·45<br>(1337·98 to 1999·4)	1382·19<br>(1137·13 to 1714·78)	1143·71<br>(985·58 to 1322·66)	1006·45<br>(888·33 to 1150·86)	928·65<br>(774·13 to 1130·1)	829·8<br>(701·78 to 969·56)	744·22<br>(576·56 to 967·62)
West Kalimantan	2461·53<br>(1971·96 to 2986·98)	2255·8<br>(1767·32 to 2878·2)	2059·67<br>(1617·58 to 2560·61)	1813·83<br>(1491·18 to 2176·16)	1461·17<br>(1241·86 to 1706·37)	1231·12<br>(975·52 to 1528·08)	1073·17<br>(913·08 to 1260·34)	936·48<br>(694·69 to 1193·87)
South Kalimantan	2767·67<br>(2273·96 to 3350·44)	2432·36<br>(1946·34 to 3040·79)	1994·27<br>(1606·03 to 2482·11)	1625·45<br>(1381·93 to 1943·69)	1420·25<br>(1213·34 to 1657·13)	1296·31<br>(1049·17 to 1614·45)	1139·17<br>(938·38 to 1367·85)	1010·7<br>(778·88 to 1327·75)
Central Kalimantan	2237·33<br>(1811·4 to 2732·17)	1840·46<br>(1429·57 to 2393·34)	1643·88<br>(1243·07 to 2119·55)	1473·54<br>(1190·71 to 1843·54)	1295·65<br>(1097·3 to 1535·97)	1020·68<br>(823·28 to 1265·89)	999·61<br>(838·54 to 1167·42)	925·82<br>(707·22 to 1188·65)
East Kalimantan	1967·05<br>(1588·14 to 2440·51)	1820·53<br>(1430·48 to 2319·57)	1625·14<br>(1239·93 to 2057·25)	1401·17<br>(1145·09 to 1701·5)	1283·79<br>(1098·51 to 1505·66)	1213·99<br>(972·76 to 1513·7)	1082·61<br>(894·9 to 1309·44)	950·01<br>(709·75 to 1214·82)
North Kalimantan	1543·5<br>(1250·5 to 1868·86)	1476·36<br>(1192·45 to 1818·42)	1324·06<br>(1058·68 to 1617·08)	1047·3<br>(862·79 to 1255·19)	812·19<br>(674·21 to 970·8)	685·31<br>(525·79 to 880·83)	617·64<br>(492·54 to 773·73)	582·32<br>(412·76 to 794·81)
Riau Islands	1997·7<br>(1586·29 to 2468·34)	1762·73<br>(1354·36 to 2251·52)	1519·43<br>(1164·57 to 1946·61)	1337·72<br>(1075·33 to 1695·58)	1194·91<br>(1001·53 to 1438·05)	1034·52<br>(814·54 to 1295·63)	1043·43<br>(875·09 to 1205·2)	949·97<br>(713·19 to 1226·5)
Lampung	2142·89<br>(1747·81 to 2639·93)	1825·51<br>(1473·37 to 2300·77)	1501·65<br>(1218·43 to 1858·97)	1207·06<br>(1026·94 to 1425·22)	1007·48<br>(870·95 to 1153·3)	915·84<br>(737·58 to 1133·27)	890·42<br>(748·26 to 1060·92)	823·86<br>(631·46 to 1070·31)
Maluku	3244·35<br>(2705·54 to 3865·22)	2621·26<br>(2093·28 to 3262·48)	2240·98<br>(1765·41 to 2823·31)	1921·15<br>(1578·19 to 2299·73)	1666·01<br>(1410·14 to 1957·14)	1488·81<br>(1179·22 to 1853·19)	1424·91<br>(1174·41 to 1719·45)	1232·61<br>(923·21 to 1588·66)
North Maluku	3224·91<br>(2622·8 to 3931·4)	2747·72<br>(2130·97 to 3522·04)	2412·93<br>(1878·83 to 3103·11)	2129·02<br>(1722·71 to 2557·94)	1851·81<br>(1539·89 to 2185·81)	1753·15<br>(1389·06 to 2182·38)	1683·61<br>(1385·74 to 1977)	1467·85<br>(1103·46 to 1864·16)
West Nusa Tenggara	3162·76<br>(2623·18 to 3802·7)	2721·93<br>(2202·21 to 3397·79)	2347·69<br>(1873·37 to 2934·47)	1955·88<br>(1613·96 to 2329·88)	1570·33<br>(1314·54 to 1849·13)	1314·73<br>(1058·82 to 1631·11)	1112·62<br>(922·72 to 1329·77)	1012·35<br>(776·52 to 1293·9)
East Nusa Tenggara	2701·97<br>(2197·35 to 3255·76)	2234·05<br>(1726·14 to 2785·42)	1912·1<br>(1499·67 to 2425·38)	1610·68<br>(1319·45 to 1913·71)	1413·87<br>(1208·6 to 1642·4)	1229·15<br>(991·44 to 1536·34)	1037·54<br>(877·27 to 1223·7)	963·17<br>(701·1 to 1239·45)
Papua	2081·4<br>(1653·89 to 2541·67)	2030·5<br>(1560·99 to 2612·66)	1880·6<br>(1443·81 to 2394·59)	1718·64<br>(1397·32 to 2076·84)	1586·63<br>(1342·17 to 1867·77)	1454·76<br>(1156·44 to 1804·68)	1386·95<br>(1159·12 to 1670·52)	1220·59<br>(913·28 to 1612·25)
West Papua	2203·03<br>(1765·67 to 2754·08)	2079·67<br>(1593·16 to 2676·11)	1959·4<br>(1502·46 to 2462·01)	1777·24<br>(1420·62 to 2186·48)	1637·32<br>(1334·06 to 1968·43)	1573·47<br>(1260·93 to 1956·64)	1585·75<br>(1336·28 to 1886·58)	1312·59<br>(994·26 to 1720·08)
Riau	1936·75<br>(1565·3 to 2386·35)	1746·15<br>(1383·89 to 2218·08)	1510·22<br>(1189·48 to 1946·43)	1238·08<br>(1045·3 to 1481·01)	1155·56<br>(954·2 to 1350·19)	1044·47<br>(837·22 to 1303·33)	934·2<br>(772·41 to 1095·55)	822·5<br>(606·55 to 1096·7)
West Sulawesi	3810·61<br>(3157·34 to 4536·67)	3245·48<br>(2576·22 to 4014·41)	2802·48<br>(2210·35 to 3500·51)	2319·6<br>(1919·04 to 2790·37)	1908·46<br>(1591·24 to 2230·11)	1584·83<br>(1258·03 to 1995·27)	1369·1<br>(1133·75 to 1650·28)	1221·81<br>(898·14 to 1614·95)
South Sulawesi	2505<br>(2080·7 to 3042·56)	2135·04<br>(1703·94 to 2659·41)	1817·1<br>(1428·05 to 2292·35)	1525·76<br>(1271·27 to 1818·82)	1243·39<br>(1059·35 to 1458·17)	1069·81<br>(859·35 to 1326·96)	984·75<br>(822·12 to 1158·31)	867·56<br>(648 to 1122·09)
Central Sulawesi	3112·45<br>(2559·59 to 3735·48)	2456·77<br>(1968·17 to 3078·32)	2086·86<br>(1635·37 to 2641·3)	1784·83<br>(1461·01 to 2138·81)	1640·94<br>(1391·27 to 1928·8)	1496·11<br>(1186·16 to 1893·69)	1291·69<br>(1064·69 to 1552·48)	1135·02<br>(851·94 to 1470·77)
Southeast Sulawesi	2858·06<br>(2336·27 to 3395·34)	2541·65<br>(1990·74 to 3187·14)	2272·93<br>(1800·97 to 2841·01)	1892·85<br>(1552·26 to 2261·54)	1629·97<br>(1389·68 to 1897·17)	1412·84<br>(1118·22 to 1751·59)	1230·19<br>(1022·35 to 1473·45)	1097·16<br>(812·18 to 1417·9)
North Sulawesi	2300·91<br>(1862·97 to 2823·91)	1952·18<br>(1511·85 to 2489·71)	1714·34<br>(1308·27 to 2237·04)	1502·02<br>(1207·55 to 1855·48)	1331·91<br>(1103·18 to 1589·67)	1167·59<br>(937·04 to 1454·51)	1082·75<br>(916·32 to 1282·38)	1002·52<br>(740·99 to 1296·1)
West Sumatra	3093·18<br>(2562·51 to 3678·68)	2179·6<br>(1744·65 to 2746·47)	1737·25<br>(1365·6 to 2156·71)	1392·88<br>(1180·89 to 1678·51)	1167·95<br>(997·66 to 1357·27)	1045·47<br>(832·93 to 1293·1)	972·28<br>(817·17 to 1140·76)	831·32<br>(632·2 to 1070·55)
South Sumatra	2105·04<br>(1742·14 to 2546·53)	1708·22<br>(1361·77 to 2138·34)	1430·41<br>(1131·45 to 1825·12)	1149·39<br>(980·83 to 1323·81)	1040·75<br>(891·21 to 1191·55)	954·8<br>(774·69 to 1168·3)	901·25<br>(769·74 to 1063·99)	842·47<br>(641·43 to 1085·65)
North Sumatra	2105·26<br>(1729·59 to 2567·25)	1761·76<br>(1399·47 to 2265·56)	1529·38<br>(1169·23 to 1951·32)	1313·99<br>(1078·53 to 1571·7)	1167·24<br>(998·02 to 1350·64)	1099·56<br>(885·22 to 1364·19)	989·1<br>(850·97 to 1167·65)	905·47<br>(682·97 to 1169·09)
Yogyakarta	1717·79<br>(1352·9 to 2126·95)	1404·73<br>(1083·7 to 1813·59)	1268·05<br>(940·23 to 1680·78)	1141·21<br>(895·54 to 1401·07)	1110·82<br>(948·79 to 1295·49)	1028·13<br>(813·15 to 1277·13)	926·45<br>(781·92 to 1103·19)	860·09<br>(661·15 to 1111·39)
Laos	2552·08<br>(1822·32 to 3635·91)	2441·9<br>(1822·02 to 3241·66)	2250·78<br>(1782·09 to 2785·52)	1955·33<br>(1627·02 to 2326·28)	1661·83<br>(1416·99 to 1932·4)	1393·59<br>(1204·37 to 1585·3)	1051·62<br>(896·66 to 1219·34)	897·47<br>(746 to 1063·33)
Malaysia	1537·91<br>(1304·21 to 1792·5)	1008·18<br>(946·57 to 1083·09)	805·44<br>(756·48 to 863·8)	787·03<br>(711·52 to 870·16)	752·72<br>(698·98 to 813·3)	729·56<br>(679·67 to 781·78)	700·47<br>(648·85 to 755·12)	644·13<br>(517·35 to 785·64)
Maldives	3189·61<br>(2709·81 to 3718·51)	2579·98<br>(2211·89 to 2967·68)	1893·9<br>(1660·02 to 2151·41)	1729·22<br>(1582·85 to 1905·65)	1232·62<br>(1142·46 to 1328·87)	886·39<br>(814·89 to 963·28)	545·6<br>(489·21 to 612·95)	482·93<br>(410·43 to 566·74)
Mauritius	2000·37<br>(1852·98 to 2158·78)	1572<br>(1439·06 to 1705·98)	1171·1<br>(1080·04 to 1279·45)	927·98<br>(843·83 to 1025·76)	770·01<br>(712·09 to 833·64)	687·08<br>(634·04 to 744·35)	583·52<br>(537·15 to 632·62)	554·02<br>(444·17 to 680·26)
Myanmar	3061·68<br>(2542·19 to 3655·72)	2444·56<br>(2028·19 to 2937·83)	2100·44<br>(1730·93 to 2515·16)	1687·48<br>(1410·25 to 1997·3)	1476·83<br>(1243·95 to 1745·57)	1332·56<br>(1097·04 to 1609·17)	1012·9<br>(883·11 to 1168·09)	827·47<br>(705·42 to 976·81)
Philippines	1497·65<br>(1348·07 to 1674·21)	1164·38<br>(1064·59 to 1277·63)	1127·14<br>(1031·31 to 1235·46)	1030·89<br>(947·84 to 1128·57)	894·74<br>(818·77 to 977·41)	765·38<br>(694·09 to 844·96)	778·56<br>(705·63 to 857·08)	731·8<br>(594·33 to 897·6)
Seychelles	1238·16<br>(1086·64 to 1393)	1035·13<br>(954·42 to 1124·89)	884·43<br>(812·03 to 962·79)	678·33<br>(614·56 to 754·78)	680·77<br>(612 to 758·48)	656·8<br>(597·08 to 727·7)	646·08<br>(593·89 to 706·71)	625·84<br>(528·64 to 751·54)
Sri Lanka	1644·54<br>(1525·07 to 1769·09)	1266·27<br>(1160·25 to 1383·98)	1099·05<br>(1020·04 to 1186·21)	882·59<br>(821·16 to 953·24)	782·81<br>(683 to 904·3)	669·12<br>(586·55 to 771·07)	612·45<br>(566·7 to 663·29)	503·6<br>(389·58 to 648·11)
Thailand	1571·66<br>(1372·8 to 1771·58)	1062·22<br>(957·66 to 1181·96)	957·21<br>(853·53 to 1055·93)	861·67<br>(775·43 to 962·27)	687·8<br>(618·91 to 767·33)	639·75<br>(564·59 to 720·24)	479·63<br>(427·43 to 532·87)	413·69<br>(316·79 to 523·92)
Timor-Leste	2615·86<br>(2121·04 to 3204·87)	2134·93<br>(1820·43 to 2479·34)	1859·18<br>(1543·93 to 2208·41)	1722·24<br>(1524·86 to 1987·61)	1326·65<br>(1181·64 to 1509·89)	1115·27<br>(994·61 to 1270·06)	902·94<br>(806·78 to 1006·15)	876·06<br>(741·19 to 1008·45)
Vietnam	1630·51<br>(1353·93 to 1988·67)	1192·82<br>(985·27 to 1473·75)	1207·22<br>(1036·47 to 1448·22)	893·07<br>(783·07 to 1042·65)	803·32<br>(699·53 to 918·09)	666·49<br>(600·69 to 756·52)	615·32<br>(546·06 to 695·4)	569·04<br>(466·63 to 680·79)
Sub-Saharan Africa	2285·27<br>(1980·29 to 2590·17)	2089·12<br>(1877·54 to 2310·04)	1856·78<br>(1715·93 to 2009·06)	1642·54<br>(1533·01 to 1756·21)	1551·07<br>(1469·41 to 1638·25)	1662·84<br>(1548·14 to 1791·31)	1326·68<br>(1245·08 to 1416·29)	1052·81<br>(967·04 to 1157·92)
Central sub-Saharan Africa	2171·77<br>(1819·91 to 2575·92)	1915·65<br>(1627·71 to 2247·19)	1797·49<br>(1570·07 to 2050·92)	1678·31<br>(1465·65 to 1916·77)	1622·53<br>(1427·34 to 1827·96)	1672·34<br>(1440·45 to 1914·94)	1404·23<br>(1160·87 to 1690·85)	1111·18<br>(870·1 to 1441·35)
Angola	2129·97<br>(1777·17 to 2528·93)	1980·16<br>(1680·63 to 2312·06)	1911·89<br>(1606·98 to 2233·49)	1820·46<br>(1501·18 to 2205·13)	1776·96<br>(1407·74 to 2254·03)	1640·44<br>(1382·23 to 1969·74)	1254·39<br>(1068·96 to 1472·56)	1043·44<br>(875·96 to 1274·06)
Central African Republic	2289·39<br>(1715·22 to 3034·47)	2092·54<br>(1651·78 to 2619·51)	1937·36<br>(1601·09 to 2302·76)	1866·83<br>(1589·09 to 2158·63)	1968·91<br>(1701·57 to 2269·38)	2530·81<br>(2070·96 to 3030·46)	2133·07<br>(1681·02 to 2674·82)	1779·79<br>(1339·69 to 2346·39)
Congo (Brazzaville)	2603·46<br>(1949·84 to 3452·18)	2224·32<br>(1822·18 to 2717·08)	2002·62<br>(1675·3 to 2383·44)	1695·56<br>(1429·27 to 2028·31)	1766·51<br>(1466·48 to 2076·53)	1939·35<br>(1641·49 to 2276·24)	1478·62<br>(1260·97 to 1730·07)	1196·2<br>(966·57 to 1471·93)
DR Congo	2123·53<br>(1737·72 to 2554·21)	1840·7<br>(1520·88 to 2257·8)	1735·58<br>(1481·06 to 2013·41)	1635·55<br>(1397·89 to 1923·67)	1560·5<br>(1333·95 to 1819·36)	1621·26<br>(1326·14 to 1959·29)	1409·66<br>(1084·94 to 1828·26)	1100·34<br>(790·45 to 1583·44)
Equatorial Guinea	3185·39<br>(2648·54 to 3774·81)	2559·43<br>(2085·59 to 3124·07)	2157·98<br>(1728·04 to 2666·01)	1796·53<br>(1497·77 to 2144·99)	1756·67<br>(1435·32 to 2136·05)	1411·23<br>(1081·78 to 1823·62)	1240·98<br>(936·04 to 1582·54)	1074·32<br>(839·76 to 1385·9)
Gabon	2188·6<br>(1756·24 to 2693·52)	1708·52<br>(1445·02 to 2004·11)	1531·08<br>(1256·45 to 1890·62)	1286·73<br>(1059·11 to 1562·73)	1203·72<br>(1049·08 to 1394·96)	1376·61<br>(1135·81 to 1656·1)	1154·57<br>(980·45 to 1355·01)	908·24<br>(741·88 to 1092·12)
Eastern sub-Saharan Africa	2590·48<br>(2245·39 to 2960·81)	2250·55<br>(2018·39 to 2502·83)	2029·61<br>(1864·19 to 2214·8)	1802·33<br>(1687·43 to 1915·36)	1745·67<br>(1643·06 to 1854·6)	1775·71<br>(1640·66 to 1914·09)	1268·2<br>(1192·25 to 1349·45)	1009·95<br>(924·43 to 1106·61)
Burundi	2695·01<br>(2008·07 to 3590·44)	2307·85<br>(1843·14 to 2879·94)	2186·4<br>(1839·76 to 2563·98)	1979·33<br>(1728·47 to 2269·87)	2008·15<br>(1712·08 to 2372·95)	2338·54<br>(1980·3 to 2751·27)	1402·42<br>(1185·42 to 1622·78)	1124·23<br>(895·76 to 1418·7)
Comoros	2324·2<br>(1830·34 to 2920·69)	2134·8<br>(1773·1 to 2521·58)	1823·8<br>(1526·06 to 2172·01)	1610·94<br>(1394·11 to 1875·7)	1416·94<br>(1224·2 to 1640·42)	1289·72<br>(1128·37 to 1472·17)	1060·91<br>(905·25 to 1242·09)	942·45<br>(766·78 to 1176·51)
Djibouti	1616·37<br>(1203·87 to 2221·2)	1531·96<br>(1163·72 to 2018·26)	1376·23<br>(1080·45 to 1708·61)	1230·27<br>(1031·38 to 1451·38)	1221·74<br>(1006·4 to 1470·92)	1274·63<br>(1008·86 to 1585·51)	1143·68<br>(909·99 to 1413·75)	986·03<br>(809·42 to 1259·66)
Eritrea	2804·64<br>(2022·28 to 3911·92)	2492·81<br>(1884·18 to 3215·99)	2451·41<br>(1864·22 to 3477·28)	2273·48<br>(1994·32 to 2592·06)	2061·18<br>(1776·31 to 2387·52)	1608·18<br>(1239·44 to 2018)	1379·51<br>(996·3 to 1902·64)	1211·76<br>(838·92 to 1756·79)
Ethiopia	3052·39<br>(2208·52 to 4369·34)	2583·24<br>(2016·46 to 3383·26)	2452·3<br>(2039·58 to 3012·79)	2317·86<br>(1999·32 to 2654·62)	2124·86<br>(1882·26 to 2388·78)	1918·15<br>(1683·33 to 2177·54)	1134·07<br>(1024·12 to 1260·28)	887·01<br>(754·57 to 1050·6)
Kenya	1945·86<br>(1693·35 to 2237·44)	1573·98<br>(1357·62 to 1811·48)	1388·06<br>(1201·72 to 1610·96)	1204·3<br>(1063·53 to 1363·97)	1182·53<br>(1060·08 to 1314·21)	1585·68<br>(1342·79 to 1836·75)	1230·96<br>(1118·31 to 1350·73)	982·88<br>(810·51 to 1189·04)
Baringo	1964·97<br>(1556·32 to 2475·27)	1722·25<br>(1295·03 to 2231·69)	1690·6<br>(1332·79 to 2164·59)	1567·41<br>(1237·79 to 1986·77)	1441·18<br>(1140·31 to 1813·1)	1409·16<br>(1108·42 to 1778·82)	1190·3<br>(954·18 to 1456·2)	991·43<br>(738·65 to 1341·02)
Bomet	971·05<br>(746·16 to 1229·62)	835·42<br>(621·53 to 1091·98)	806·29<br>(605 to 1030·03)	749·48<br>(570·79 to 953·99)	828·74<br>(682·49 to 1031·47)	1426·46<br>(1063·39 to 1872·54)	1144·17<br>(926·49 to 1410·13)	957·77<br>(732·79 to 1225·24)
Bungoma	2039·45<br>(1638·48 to 2520·72)	1617·12<br>(1238·16 to 2137·3)	1386·7<br>(1084·74 to 1775·5)	1232·11<br>(959·92 to 1551·89)	1115·02<br>(871·83 to 1407·09)	1263·24<br>(968·09 to 1584·86)	1010·8<br>(813·52 to 1241·97)	867·08<br>(626·37 to 1168·57)
Busia	3220·51<br>(2617·1 to 4002·61)	2224·09<br>(1711·96 to 2926·11)	1752·44<br>(1393·85 to 2219·83)	1290·58<br>(1022·28 to 1640·33)	1241·1<br>(923·14 to 1628·25)	1661·96<br>(1233·44 to 2227·74)	1184·15<br>(885·65 to 1524·03)	916·82<br>(643·2 to 1273·56)
Elgeyo Marakwet	1662·93<br>(1318·94 to 2129·84)	1485·17<br>(1113·22 to 2011·32)	1498·78<br>(1158·86 to 1956·82)	1421·78<br>(1113·96 to 1801·43)	1282·16<br>(988·09 to 1646·56)	1386·49<br>(1056·07 to 1764·72)	1201·78<br>(957·28 to 1499·63)	1050·93<br>(767·91 to 1424·73)
Embu	2473·37<br>(1989·96 to 3036·12)	1835·1<br>(1371·26 to 2382·8)	1355·14<br>(1068·09 to 1730·66)	1038·88<br>(797·42 to 1322·68)	910·65<br>(699·11 to 1163·31)	1089·39<br>(800·08 to 1495·24)	960·48<br>(743·78 to 1252·65)	818·95<br>(614·49 to 1129·12)
Garissa	1952·04<br>(1542·55 to 2533·12)	1784·33<br>(1332·17 to 2379·9)	1589·91<br>(1206·24 to 2099·44)	1478·13<br>(1124·32 to 1946·53)	1326·77<br>(1019·42 to 1699·64)	1330·15<br>(998·1 to 1748·43)	1310·03<br>(1012·43 to 1684·32)	1178·45<br>(832·82 to 1640·31)
Homa Bay	3365·33<br>(2821·01 to 3982·81)	2363·21<br>(1895·41 to 2949·29)	2114·62<br>(1747·67 to 2547·97)	1730·03<br>(1419·42 to 2080·21)	1711·86<br>(1343·7 to 2148·07)	3110·15<br>(2313·95 to 4000·37)	2105·74<br>(1588·39 to 2695·61)	1375·58<br>(955·17 to 2001·13)
Isiolo	3359·14<br>(2719·73 to 4085·05)	2905·84<br>(2255·44 to 3795·08)	2579<br>(2067·47 to 3172·68)	1997·84<br>(1588·4 to 2494·88)	1597·17<br>(1267·48 to 1940·65)	1565·02<br>(1167·7 to 2086·71)	1324·38<br>(967·41 to 1724·75)	1053·25<br>(739·54 to 1465·56)
Kajiado	1522·71<br>(1184·34 to 1967·01)	1382·76<br>(1034·66 to 1823·08)	1356·36<br>(1061·83 to 1802·43)	1266·57<br>(1000·18 to 1648·27)	1226·51<br>(930·08 to 1603·22)	1506·57<br>(1140·26 to 1983·13)	1198·39<br>(942·86 to 1485·76)	1006·79<br>(716·55 to 1359·89)
Kakamega	2153·46<br>(1725·04 to 2646·28)	1746·22<br>(1347·68 to 2263·25)	1491·42<br>(1187·99 to 1879·37)	1257·17<br>(998·55 to 1554·85)	1264·88<br>(993·09 to 1566·21)	1643·89<br>(1239·63 to 2109·07)	1248·05<br>(971·28 to 1550·14)	941·76<br>(689·25 to 1277·33)
Kericho	1179·65<br>(911·53 to 1529·13)	1135·22<br>(839·43 to 1534·66)	1075·38<br>(818·53 to 1406·09)	1002·58<br>(791·96 to 1274·88)	979·41<br>(733·72 to 1237·52)	1272·37<br>(927·16 to 1688·89)	966·32<br>(726·61 to 1287·16)	782·59<br>(588·06 to 1082·64)
Kiambu	1438·58<br>(1128·16 to 1851·85)	1221·81<br>(913·98 to 1615·17)	1114·47<br>(848·39 to 1473·09)	1047·65<br>(808·3 to 1339·35)	1055·89<br>(833·92 to 1322·06)	1443·51<br>(1086·78 to 1840·45)	1074·6<br>(859·99 to 1310·31)	856·1<br>(612·34 to 1154·22)
Kilifi	2811·29<br>(2303·79 to 3435·01)	2189·73<br>(1711·67 to 2766·21)	1873·38<br>(1524·76 to 2304·66)	1564·24<br>(1269·19 to 1958·56)	1428·57<br>(1137·38 to 1781·55)	1639·9<br>(1267·8 to 2106·47)	1199·56<br>(953·57 to 1473·08)	945·28<br>(683·06 to 1312·72)
Kirinyaga	2131·66<br>(1697·25 to 2691·13)	1734·47<br>(1310·79 to 2307·15)	1475·6<br>(1165·79 to 1886·08)	1060·9<br>(817·3 to 1359·32)	963·67<br>(723·86 to 1231·12)	1178·83<br>(886·9 to 1584·06)	969·37<br>(754·12 to 1265·11)	818·73<br>(588·58 to 1127·37)
Kisii	2176·1<br>(1703·33 to 2769·05)	1959·63<br>(1499·28 to 2590·07)	1752·86<br>(1343·05 to 2258·76)	1581·33<br>(1239·05 to 2040·99)	1522·08<br>(1210·11 to 1897·07)	1803·16<br>(1441·34 to 2249·05)	1421·9<br>(1160·51 to 1735·28)	1179·39<br>(844·83 to 1615·66)

Supplemental Results Table 3c: Age-standardised mortality rates by location, females, 1950, 1960, 1970, 1980, 1990, 2000, 2010, 2019

Location	1950	1960	1970	1980	1990	2000	2010	2019
Kisumu	1822·83 (1476·18 to 2248·92)	1457·84 (1119·55 to 1898·5)	1327·15 (1051·98 to 1698·44)	1186·3 (945·63 to 1452·4)	1233·96 (943·84 to 1540·98)	2158·85 (1559·67 to 2868·67)	1571·44 (1181·38 to 1991·97)	1082·47 (742·5 to 1567·55)
Kitui	2183·44 (1764·55 to 2680·97)	1692·81 (1297·15 to 2223·95)	1436·99 (1142·87 to 1843·28)	1193·26 (946·55 to 1498·35)	1103·91 (878·06 to 1368·89)	1313·5 (970·83 to 1717·62)	933·49 (736·96 to 1165·51)	785·81 (591·05 to 1041·84)
Kwale	2569·52 (2034·99 to 3208·56)	2216·18 (1674·93 to 2913·29)	1873·86 (1487·99 to 2370·39)	1578·56 (1238·03 to 2001·63)	1488·33 (1153·39 to 1921·03)	1709·91 (1298·09 to 2192·64)	1279·81 (1029·29 to 1583·71)	1020 (726·56 to 1426·84)
Laikipia	1153·93 (879·8 to 1509·64)	1057·82 (787·19 to 1408·99)	980·4 (736·39 to 1308·66)	885·51 (681·85 to 1109·44)	966·12 (715·88 to 1254·17)	1420·14 (1016·11 to 1884·87)	1131·55 (855·86 to 1476·34)	877·24 (636·51 to 1198·32)
Lamu	3439·09 (2765·43 to 4297·31)	2533·97 (1910·72 to 3306·16)	1906·51 (1509·09 to 2443·68)	1325·45 (1020·15 to 1715·72)	1336·68 (1019·82 to 1759·18)	1485·94 (1053·98 to 2016·82)	1109·59 (823·22 to 1454·12)	915·81 (636·43 to 1288·45)
Machakos	1487·92 (1165·24 to 1874·88)	1287·08 (948·51 to 1712·59)	1165·21 (898·1 to 1494·91)	1002·14 (789·73 to 1271·73)	927·72 (719·21 to 1143·32)	1249·15 (916·69 to 1680·75)	1008·83 (800·42 to 1282·22)	821 (626·26 to 1107·55)
Makueni	1907·75 (1501·35 to 2347·71)	1328·99 (1006·7 to 1688·44)	1175·5 (923·69 to 1461·24)	997·3 (784·8 to 1256·93)	983·25 (757·68 to 1230·04)	1729·54 (1238·25 to 2311·07)	1379·99 (1091·21 to 1744·28)	1047·61 (799·21 to 1390·68)
Mandera	1781·63 (1401·86 to 2268·83)	1613·54 (1204·13 to 2135·59)	1509·89 (1177·36 to 1984·08)	1431·32 (1099 to 1860·04)	1383·54 (1069·96 to 1733·92)	1417 (1047·94 to 1882·29)	1433·31 (1120·71 to 1821·35)	1301·68 (920·02 to 1801·6)
Marsabit	1908·23 (1484·96 to 2427·28)	1741 (1304·19 to 2229·21)	1509·98 (1157·49 to 1956·67)	1275·69 (1009·15 to 1622·96)	1131·45 (864·51 to 1424·2)	1226·09 (921·23 to 1649·54)	1149·23 (848·41 to 1485·67)	976·42 (692·26 to 1347)
Meru	1604·75 (1281 to 1995·54)	1343·92 (1017·05 to 1746·8)	1201·07 (957·84 to 1531·47)	1046·14 (839·34 to 1331·25)	986·33 (792·2 to 1209·2)	1257·72 (917·04 to 1666·32)	1125·2 (880·64 to 1401·67)	951·81 (710·67 to 1270·08)
Migori	2859·13 (2339·12 to 3494·25)	2150·16 (1662·75 to 2784·07)	1887·11 (1510·03 to 2352·33)	1723·41 (1409·56 to 2103·52)	1651·52 (1304·27 to 2054·43)	2407·39 (1778·47 to 3166·45)	1864·02 (1434·82 to 2371·98)	1294·82 (841·16 to 1835·06)
Mombasa	1940·26 (1516·68 to 2472·16)	1727·34 (1324·52 to 2254·46)	1404·03 (1087·04 to 1880·87)	1116·94 (853·47 to 1433·82)	1207·04 (867·52 to 1586·35)	1805·94 (1312·88 to 2425·43)	1220·7 (929·96 to 1553·98)	949·73 (701·37 to 1345·98)
Murang'a	1840·11 (1490·77 to 2235·84)	1332·93 (1008·31 to 1748·29)	1132·7 (893·51 to 1433·62)	934·89 (733·58 to 1176·75)	903·46 (703·73 to 1138·24)	1163·85 (877·49 to 1541·18)	1023·96 (805·36 to 1278·72)	854·86 (627·42 to 1167·39)
Nairobi	2025·77 (1628·71 to 2492·3)	1611·61 (1260·9 to 2047·27)	1231·26 (1000·89 to 1552·04)	1034·38 (842·54 to 1267·26)	1161·98 (904·63 to 1458·43)	1808·51 (1397·73 to 2273·1)	1340·41 (1131·39 to 1575·2)	1054·85 (805·79 to 1366·45)
Nakuru	997·98 (765·45 to 1274·71)	940·43 (683·35 to 1245·15)	916·15 (697·55 to 1205·58)	868·53 (683·59 to 1085·61)	917·76 (701·68 to 1178·77)	1332·61 (993·24 to 1738·59)	1077·41 (843·46 to 1345·77)	857·98 (647·48 to 1166·33)
Nandi	1163·71 (891·6 to 1447·32)	1030·8 (770·84 to 1343·45)	974·36 (753·4 to 1239·83)	910·66 (701·26 to 1140·86)	907·59 (694·34 to 1154·85)	1182·69 (877·85 to 1574·43)	867·65 (683·87 to 1119·2)	747·27 (548·64 to 1028·65)
Narok	1333·95 (1024·4 to 1779·95)	1267·39 (924·2 to 1698·96)	1294·87 (973·99 to 1711·3)	1343·7 (1032·32 to 1799·69)	1364·04 (1034·2 to 1775·86)	1616·03 (1214·32 to 2120·24)	1254·64 (979·91 to 1601·01)	1038·35 (765·59 to 1441)
Nyamira	1693·07 (1338·19 to 2156·04)	1359·91 (1029·62 to 1837·82)	1236·91 (963·98 to 1622·03)	1155·37 (902·91 to 1500·2)	1161·65 (916·69 to 1482·73)	1785·78 (1354·66 to 2321·16)	1730·95 (1374·05 to 2156·88)	1382·18 (930·75 to 1942·04)
Nyandarua	1652·06 (1287·86 to 2118·2)	1378·84 (996·89 to 1868·07)	1187·19 (891·03 to 1580·07)	1047·6 (804·2 to 1372·27)	1029·56 (803·83 to 1311·7)	1420·06 (1037·73 to 1904·03)	1332·04 (1036·88 to 1660·89)	1019·75 (694·64 to 1449·88)
Nyeri	2075·14 (1611·93 to 2631·83)	1540·15 (1149·54 to 2005·79)	1350·52 (1028·61 to 1813·25)	1199·78 (951·61 to 1562·31)	1234·5 (969·38 to 1539·62)	1784·46 (1401·43 to 2279·45)	1380·16 (1089·06 to 1714·64)	1119·57 (762·44 to 1575·19)
Samburu	1348·45 (1043·49 to 1742·97)	1217·11 (904·96 to 1630·56)	1169·47 (910·8 to 1523·72)	1106·64 (875·12 to 1404·34)	1148·28 (846·88 to 1524·29)	1439·78 (1063 to 1928·49)	1210·7 (915·13 to 1588·94)	1006·77 (725·96 to 1386·26)
Siaya	2387·92 (1940·46 to 2921·98)	2031·45 (1587·87 to 2634·21)	1723·03 (1390·09 to 2148·11)	1514·91 (1207·16 to 1857·87)	1609·58 (1279·55 to 1977·38)	2475·73 (1801·31 to 3179·08)	1669·02 (1272·42 to 2113·34)	1115·77 (772·44 to 1563·81)
Taita Taveta	1974·15 (1545·63 to 2476·96)	1626·35 (1202·27 to 2223·26)	1508·52 (1185·19 to 1969·94)	1206·09 (934·12 to 1554·63)	1184·1 (894·3 to 1529·67)	1707·16 (1277·41 to 2237·28)	1514·06 (1171·88 to 1877·66)	1203·93 (811·96 to 1732·08)
Tana River	2079·67 (1628·38 to 2698·05)	1756·65 (1276·63 to 2411·81)	1634·73 (1229·6 to 2237·32)	1537·85 (1154·89 to 2026·44)	1547·94 (1134·36 to 2042·78)	2003·87 (1499·65 to 2614·44)	1701·96 (1315·63 to 2142·59)	1338·14 (920·07 to 1886·16)
Tharaka Nithi	2047·21 (1628·43 to 2547·23)	1445·13 (1066 to 1903·41)	1364·76 (1056·63 to 1804·52)	1232·35 (959·29 to 1595·33)	1215·25 (970·35 to 1518·85)	1515·9 (1147·82 to 1943·81)	1097·81 (878·67 to 1354·21)	937·03 (682·34 to 1256·86)
Trans Nzoia	1299·6 (991·34 to 1676·11)	1192·41 (885·55 to 1577·05)	1099·36 (836·35 to 1420·88)	1013·02 (798·28 to 1262·28)	987·63 (729·25 to 1257·16)	1345·52 (971·93 to 1829·04)	1073·11 (807·05 to 1413·3)	869·33 (644·18 to 1223·27)
Turkana	1442·78 (1123 to 1861·22)	1419·83 (1070·87 to 1902·24)	1412·09 (1114·02 to 1802·07)	1365·52 (1086·22 to 1705·38)	1342·23 (994·03 to 1756·3)	1858·19 (1321·42 to 2512·19)	1420·78 (1041·01 to 1918·68)	1102·04 (762·3 to 1604·92)
Uasin Gishu	1159·69 (896·32 to 1510·07)	1095·63 (815·92 to 1465·12)	1062·04 (807·44 to 1370·53)	1036·35 (819·39 to 1337·94)	1059·33 (817·51 to 1350·88)	1330·65 (983·29 to 1721·37)	1026·02 (804·98 to 1296·81)	844 (623·22 to 1152·02)
Vihiga	2182·09 (1774·93 to 2647·67)	1492·96 (1140·32 to 1942·56)	1364·31 (1065·56 to 1723·25)	1252·47 (989·01 to 1608·91)	1288·69 (1008·33 to 1636·38)	1685·23 (1288·43 to 2166·64)	1382·96 (1091·27 to 1745·41)	1105·29 (762·31 to 1581·59)
Wajir	2533 (2013·27 to 3151·75)	2157·16 (1665·6 to 2860·26)	1883·65 (1487·37 to 2399·32)	1669·07 (1309·45 to 2153·15)	1491·54 (1160·89 to 1882·07)	1443·56 (1126·66 to 1821·48)	1406·19 (1103·06 to 1783·24)	1270·73 (887·02 to 1747·69)
West Pokot	1799·16 (1410·3 to 2311·98)	1630·11 (1213·5 to 2114·67)	1616·23 (1258·78 to 2066·47)	1606·08 (1265·87 to 2065·87)	1507·35 (1182·46 to 1877·73)	1459·86 (1128·96 to 1878·38)	1235·52 (983 to 1537·17)	1025·49 (752·17 to 1396·99)
Madagascar	2706·73 (2385·68 to 3077·64)	2375·88 (2047·81 to 2749·56)	2043·53 (1825·58 to 2313·96)	1501·65 (1322·73 to 1714·08)	1493·54 (1324·29 to 1676·2)	1416·99 (1253·67 to 1615·3)	1265·55 (1017·14 to 1571·76)	1147·02 (851·85 to 1550·64)
Malawi	2916·76 (2440·73 to 3453·7)	2636·98 (2224·91 to 3089·29)	2309·06 (2011·21 to 2647·78)	1866·37 (1662·69 to 2087·53)	1851·23 (1656·83 to 2114·63)	2236·7 (1909·46 to 2607·07)	1417·45 (1252·22 to 1616·7)	991·6 (858·03 to 1138·57)
Mozambique	2398·72 (1993·63 to 2898·16)	2147·62 (1824·58 to 2496·91)	1945·69 (1690·48 to 2225·01)	1790·99 (1581·26 to 2018·6)	1672·84 (1478·44 to 1892·06)	1600·81 (1406·13 to 1828·8)	1544·69 (1329·28 to 1804·33)	1289·76 (1063·76 to 1568·03)
Rwanda	3292·67 (2763·53 to 3872·03)	2446·33 (2052·03 to 2925·64)	2266·47 (1924·04 to 2657·09)	2007·09 (1750·09 to 2287)	1949·45 (1739·22 to 2189·27)	1920·54 (1705·09 to 2169·89)	1059·59 (949·06 to 1183·72)	902·11 (768·17 to 1053·54)
Somalia	2370·51 (1988·9 to 2800·09)	2329·92 (1911·72 to 2819·79)	2169·07 (1792·69 to 2588·31)	1992·65 (1655·44 to 2361·73)	1969·59 (1662·93 to 2321·33)	1818·03 (1462·02 to 2311·93)	1674·34 (1260·33 to 2275·89)	1442·55 (1032·8 to 2123·11)
South Sudan	1858·82 (1509·65 to 2291·36)	1874·81 (1542·53 to 2242·28)	1744·03 (1435·01 to 2098·76)	1559·68 (1333·7 to 1806·33)	1476·77 (1231·01 to 1738·36)	1348·96 (1106·93 to 1626·59)	1188·41 (970·72 to 1418·18)	1059·44 (849·95 to 1299·97)
Uganda	2275·11 (1872·46 to 2737·67)	1864·71 (1601·13 to 2164·74)	1654·37 (1433·29 to 1916·58)	1697·47 (1435·77 to 2009·26)	1849·87 (1576·98 to 2141·45)	1866·07 (1649·82 to 2114·1)	1250·06 (1096·95 to 1421·85)	920·68 (788·88 to 1069·84)
Tanzania	2506·88 (2071·43 to 2982·07)	2270·53 (1917·55 to 2623·13)	1923·94 (1668·08 to 2209·72)	1499·49 (1326·81 to 1693·33)	1505·62 (1341·13 to 1695·55)	1656·92 (1441·4 to 1883·81)	1252·38 (1118·84 to 1392·58)	954·73 (818·92 to 1108·4)
Zambia	2065·17 (1725·81 to 2476·46)	1883·79 (1575·02 to 2231·46)	1633·15 (1432·73 to 1865·6)	1531·94 (1379·06 to 1716·44)	1811·19 (1612·45 to 2023·07)	2383·51 (2035·76 to 2731·23)	1467·88 (1294·25 to 1674·71)	1124·78 (936·26 to 1382·53)
Southern sub-Saharan Africa	1652·68 (1482·29 to 1855·02)	1497·22 (1368·97 to 1637·56)	1354·98 (1249·19 to 1475·34)	1199·02 (1071·05 to 1346·07)	946·63 (860·79 to 1041·34)	1613·77 (1398·8 to 1866·63)	1518·33 (1391·63 to 1672·36)	1035·45 (920·83 to 1168·58)
Botswana	1720·62 (1374·6 to 2088·15)	1601·4 (1294·31 to 1952·4)	1471·49 (1213·68 to 1826·77)	1216·74 (1023·38 to 1474·22)	1164·17 (968·46 to 1449·59)	2315·03 (1680·6 to 2995·67)	1447·74 (1152·44 to 1822·93)	1175 (940·88 to 1522·41)
eSwatini	2134·97 (1770·46 to 2584·77)	1962·62 (1612·81 to 2374·75)	1603·65 (1353·69 to 1932·35)	1301·24 (1081·74 to 1526·63)	1115·91 (952·79 to 1295·41)	2032·3 (1499·63 to 2591·88)	2059·85 (1623·58 to 2579·1)	1284·14 (955·26 to 1654·78)
Lesotho	1592·67 (1340·96 to 1903·72)	1523·27 (1301·27 to 1804)	1421·93 (1197·58 to 1718·86)	1211·31 (1019·97 to 1442·42)	1102·05 (962·59 to 1266·4)	1951·44 (1571·79 to 2427·32)	2124·51 (1779·29 to 2519·57)	1865·21 (1477·73 to 2288·53)
Namibia	1620·16 (1337·32 to 1954·86)	1488·82 (1242·27 to 1770·9)	1376·7 (1170·8 to 1586·92)	1322·15 (1128·35 to 1556·33)	1133·28 (1013·3 to 1262·74)	1718·65 (1436·38 to 2038·85)	1162·58 (966·3 to 1365·16)	974·25 (772·65 to 1209·31)
South Africa	1638·18 (1468·64 to 1822·01)	1482·01 (1346·44 to 1638·66)	1344·09 (1228·58 to 1480·58)	1192·85 (1032·28 to 1379·08)	898·28 (796·99 to 1004·23)	1469·49 (1289·37 to 1669·03)	1440·16 (1301·46 to 1603·46)	967·56 (838·18 to 1119·26)
Zimbabwe	1691·89 (1245·15 to 2341·21)	1519·9 (1194·77 to 1943·93)	1387·82 (1148·89 to 1688·29)	1265·5 (1124·46 to 1439·73)	1132·19 (983·94 to 1337·9)	2111·76 (1593·2 to 2673·18)	1907·1 (1640·46 to 2189·33)	1287·27 (1058·74 to 1594·67)
Western sub-Saharan Africa	2251·17 (1846·11 to 2706·37)	2171 (1846·94 to 2543·8)	1875·53 (1644·88 to 2122·73)	1626·14 (1467·6 to 1796·95)	1543·77 (1428·88 to 1672·08)	1558·87 (1423·12 to 1710·36)	1296 (1182·38 to 1419·33)	1084·27 (966·65 to 1219·99)
Benin	2441·59 (1789·27 to 3322·74)	2129·61 (1685·25 to 2671·55)	1868·84 (1566·74 to 2197·57)	1632·19 (1453·49 to 1834·59)	1439·95 (1295·88 to 1593·43)	1359·18 (1208·13 to 1529·94)	1180·96 (1028·61 to 1379·3)	1042·1 (866·17 to 1273·7)
Burkina Faso	2756·99 (2074·49 to 3744·36)	2445·93 (1985·71 to 3041·67)	2089·62 (1805·54 to 2423·95)	1807·34 (1593·42 to 2068·64)	1731·15 (1532·2 to 1967·17)	1655·39 (1474·9 to 1880·56)	1336·44 (1190·87 to 1513·1)	1154·73 (968·56 to 1388·14)
Cape Verde	1741·69 (1565·02 to 1929·63)	1435·87 (1310·66 to 1584·82)	1317·62 (1194·29 to 1453·44)	880·21 (808·14 to 955·12)	728·8 (665·86 to 795·43)	679·09 (584·16 to 781·76)	566·82 (521·29 to 616·55)	602·55 (508·65 to 700·05)
Cameroon	2357·01 (1781·66 to 3100)	3803·37 (3185·83 to 4533·79)	1799·48 (1529·53 to 2101·63)	1591·72 (1384·41 to 1814·04)	1371·02 (1225·44 to 1520·46)	1615·21 (1425·06 to 1865·41)	1433·1 (1217·08 to 1687·25)	1119·25 (902·22 to 1411·35)
Chad	2298·27 (1587·56 to 3319·31)	1934·24 (1428·06 to 2679·59)	1854·91 (1460·74 to 2444·18)	1729·76 (1453·1 to 2055·45)	1612·2 (1455·76 to 1797·91)	1690·32 (1484·58 to 1941·85)	1465·1 (1302·43 to 1644·02)	1275·48 (1067·03 to 1532·38)
Côte d'Ivoire	2119·8 (1818·75 to 2473·18)	1990·39 (1666·82 to 2363·96)	1692·24 (1459·47 to 1960·06)	1421·83 (1253·56 to 1617·56)	1414·71 (1246·75 to 1661·81)	1782·43 (1512·77 to 2092·59)	1412·42 (1217·44 to 1653·45)	1040·07 (884·32 to 1237·89)
The Gambia	1603·12 (1327·9 to 1962·75)	1565·41 (1313·14 to 1866·04)	1418·93 (1202·59 to 1675·84)	1333·71 (1137·61 to 1589·95)	1228·66 (1059·5 to 1448·75)	1217·38 (1050·64 to 1408·39)	1158·62 (992·54 to 1347·63)	1038·77 (876·05 to 1239·41)
Ghana	1982·96 (1724·62 to 2276·02)	1716·22 (1490·9 to 1979·71)	1552·7 (1346·81 to 1802·25)	1422·45 (1223·89 to 1663·66)	1354·78 (1164·48 to 1571·45)	1343·8 (1191·1 to 1514·58)	1190·39 (1061·52 to 1334·05)	975·3 (828·09 to 1164·52)
Guinea	2525·53 (2117·02 to 2978·05)	2468·65 (2037·5 to 2962·83)	2329·57 (1966·91 to 2747·67)	2093·06 (1835·69 to 2397·93)	1770·92 (1540·89 to 2029·16)	1618·38 (1445·73 to 1815)	1446·65 (1249·08 to 1667·08)	1265·81 (1025·87 to 1549·48)
Guinea-Bissau	3255·59 (2814·08 to 3777·35)	2622·87 (2249·01 to 3043·86)	2367·16 (1956·41 to 2849·62)	1997·42 (1690·78 to 2350·17)	1847·15 (1582·58 to 2143·34)	1758 (1486·69 to 2097·98)	1538·33 (1326·89 to 1791·4)	1280·38 (1028·08 to 1597·02)
Liberia	2756·47 (2152·29 to 3482·14)	2258·34 (1923·21 to 2626)	1994·74 (1727·76 to 2317·2)	1775·16 (1558·72 to 2042·94)	1895·26 (1670·9 to 2151·59)	1601·57 (1388·79 to 1832·81)	1297·31 (1123·92 to 1477·03)	1083·39 (894·09 to 1344·68)
Mali	2848·98 (2375·68 to 3333·83)	2693·56 (2215·34 to 3235·71)	2499·25 (2142·81 to 2934·66)	2147·09 (1903·46 to 2443·13)	1914·93 (1708·21 to 2132·54)	1733·96 (1563·87 to 1928·81)	1406·86 (1261·29 to 1580·91)	1243·72 (1040·03 to 1512·84)
Mauritania	2125·21 (1557·26 to 2871·47)	1912·09 (1522·91 to 2397·09)	1663·71 (1387·48 to 1979·46)	1585·02 (1351·54 to 1834·67)	1477·49 (1308 to 1678·97)	1241·45 (1101·57 to 1397·41)	1012·54 (886·79 to 1174·46)	900·72 (763·78 to 1074·91)
Niger	2378·59 (1803·69 to 3175·01)	2329·93 (1891·81 to 2908·81)	2171·47 (1866·96 to 2508·2)	2068·17 (1858·37 to 2304·75)	1926·69 (1744·1 to 2125·91)	1734·29 (1570·76 to 1917·85)	1339·7 (1195·53 to 1513·05)	1183·8 (990·98 to 1439·63)
Nigeria	2200·27 (1571·57 to 3161·82)	2017·11 (1496·26 to 2760·65)	1832·17 (1467·36 to 2304·15)	1542·08 (1282·52 to 1839·36)	1521·88 (1321·45 to 1746·51)	1544·19 (1293·49 to 1828·48)	1263·11 (1049·08 to 1486·82)	1060·05 (858·89 to 1295·62)
São Tomé and PrÍncipe	2962·76 (2589·38 to 3359·8)	2230·47 (2051·36 to 2420·13)	1431·54 (1317·62 to 1568·03)	1155·93 (1044·38 to 1278·25)	1135·44 (1035·97 to 1253·2)	1156·86 (1037·75 to 1298·88)	982·42 (882·4 to 1095·7)	896·2 (756·74 to 1041·69)
Senegal	2075·31 (1563·87 to 2774·34)	1984·74 (1620·03 to 2465·73)	1926·86 (1653·18 to 2238·68)	1644·01 (1468·41 to 1842·54)	1336·55 (1201·58 to 1488·97)	1249·67 (1139·5 to 1377·38)	1032·58 (923·7 to 1163·41)	926·43 (800·18 to 1090·99)
Sierra Leone	2319·02 (1823·01 to 2958·66)	2148·82 (1792·1 to 2520·6)	1940·75 (1633·92 to 2299·44)	1786·17 (1513·86 to 2085·98)	1655·73 (1415·6 to 1884·93)	1656·24 (1468·68 to 1853·91)	1537·29 (1335·23 to 1793·16)	1236·46 (1047·35 to 1490·1)
Togo	2143·72 (1759·65 to 2625·79)	1951·69 (1674·37 to 2259·96)	1689·31 (1467·78 to 1935·53)	1491·11 (1324·51 to 1682·32)	1382·11 (1197·29 to 1598·18)	1448·75 (1215·43 to 1717·79)	1285·54 (1125·93 to 1475·74)	1015·68 (839·07 to 1234·7)

Supplemental Results Table 4a: Under-5 mortality rates by location, both, 1950, 1960, 1970, 1980, 1990, 2000, 2010, 2019

Location	1950	1960	1970	1980	1990	2000	2010	2019
Global	206·2 (188·5 to 226)	276·1 (234·8 to 335·3)	142 (135·2 to 149·5)	112·3 (107·4 to 117·1)	86·6 (83·5 to 90)	71·2 (68·3 to 74)	53·1 (49·9 to 56·4)	37·1 (33·2 to 41·7)
Central Europe, eastern Europe, and central Asia	104·3 (94·1 to 116·3)	72·8 (68·6 to 77·6)	50·5 (47·6 to 53·8)	38·3 (36·8 to 40)	31·5 (30·2 to 33)	27 (25·7 to 28·4)	16·2 (15·1 to 17·4)	11·5 (10·4 to 12·8)
Central Asia	223·4 (194·2 to 256·2)	155·4 (137·2 to 175·9)	99·7 (89 to 111·1)	72·5 (67·5 to 77·9)	57·3 (53·5 to 61·5)	48·6 (45 to 52·4)	30·8 (27·7 to 34·3)	20·7 (18·3 to 23·7)
Armenia	282·7 (247·6 to 321·8)	172·3 (146·4 to 199·2)	97·2 (83·3 to 112·8)	62·4 (55·7 to 69·5)	40·9 (37·3 to 44·9)	28·9 (25·9 to 32·4)	16·6 (14·3 to 19)	11·4 (9·6 to 13·7)
Azerbaijan	396·5 (354·4 to 441·8)	272·6 (235 to 309·8)	148·6 (128 to 170·8)	90·6 (79·6 to 102·6)	76·5 (68·5 to 85·9)	64·1 (57·1 to 71·6)	38·1 (32·6 to 43·9)	27·6 (23·2 to 33·4)
Georgia	252·4 (219·8 to 289·1)	154·2 (131·2 to 178)	90·4 (77·3 to 105·7)	56·4 (49·6 to 63·4)	41·3 (36·8 to 46·3)	35·1 (30·7 to 39·8)	16·3 (13·7 to 19·2)	10·3 (8·6 to 12·5)
Kazakhstan	122·2 (104 to 143·4)	89·4 (75·4 to 104·5)	56·1 (48·6 to 65·1)	46·1 (41·5 to 51·1)	37·9 (34 to 42)	35·6 (31·3 to 40·1)	21·6 (18·4 to 25·1)	12·1 (10·1 to 14·7)
Kyrgyzstan	222·4 (192·6 to 256·4)	165·7 (141·4 to 191·7)	113·1 (98·3 to 130·2)	80·3 (72 to 88·8)	57·6 (52·6 to 63·3)	44·1 (39·8 to 48·8)	27·5 (26·8 to 28·1)	17·4 (15·9 to 19)
Mongolia	333·9 (295·3 to 376·3)	275·4 (239·1 to 311·4)	192 (169 to 216·7)	130 (117·4 to 143·5)	89·3 (82·1 to 97·6)	57·9 (52·9 to 63·1)	30·1 (26·8 to 33·5)	17 (14·5 to 20·4)
Tajikistan	286·4 (251 to 325·7)	221·6 (192·3 to 252·8)	146·8 (127·2 to 168·5)	105·8 (94·6 to 117·8)	83 (75·6 to 91)	67 (61·1 to 73·1)	42·1 (37·5 to 46·8)	32·1 (27·5 to 37·3)
Turkmenistan	241·4 (209·6 to 277·3)	173·2 (150 to 198·5)	114·9 (97·2 to 136·9)	96·9 (84 to 110·4)	72·3 (64·2 to 81·7)	58 (51·7 to 64·8)	39 (34·1 to 44·2)	25·2 (21·4 to 29·6)
Uzbekistan	162·4 (139·1 to 189·2)	119·3 (100·1 to 139·3)	88·6 (76·8 to 102·9)	67·1 (60·5 to 74·2)	52·8 (47·3 to 58·4)	45·5 (40·3 to 51·9)	31·2 (26·5 to 36·5)	21·2 (17·8 to 25·7)
Central Europe	135·4 (131·4 to 139·9)	76·6 (75·7 to 77·6)	48·8 (48·1 to 49·5)	29·6 (29·3 to 30)	22·6 (22·2 to 22·9)	13·6 (13·3 to 13·9)	7·4 (7·2 to 7·6)	5·1 (4·5 to 5·6)
Albania	218·4 (189·5 to 251·1)	147·9 (126·3 to 170·3)	93·3 (79·2 to 109)	52·3 (45·3 to 59·4)	40·6 (36·5 to 45·1)	27·8 (24·7 to 31·6)	16·2 (13·8 to 18·9)	11·9 (9·9 to 14·4)
Bosnia and Herzegovina	208·8 (206·4 to 211·4)	130·3 (128·5 to 132·4)	75·3 (73·8 to 76·8)	38·7 (37·6 to 39·9)	16·9 (16·2 to 17·6)	10·6 (10 to 11·3)	6·9 (6·4 to 7·5)	5·4 (4·6 to 6·4)
Bulgaria	128·9 (127·2 to 130·5)	57 (55·9 to 58·2)	32·3 (31·5 to 33·2)	25·1 (24·3 to 25·8)	19·4 (18·7 to 20)	17·1 (16·3 to 17·8)	11·1 (10·6 to 11·7)	7·3 (6·2 to 8·6)
Croatia	159 (156·8 to 161·6)	80·7 (79·1 to 82·5)	44·3 (43 to 45·5)	22·5 (21·7 to 23·4)	12·2 (11·6 to 12·8)	8·3 (7·8 to 8·8)	5·2 (4·9 to 5·6)	4 (3·3 to 4·7)
Czech Republic	73·9 (72·9 to 75·1)	24 (23·3 to 24·7)	23·5 (22·8 to 24·2)	18·6 (18 to 19·1)	12·1 (11·6 to 12·6)	5·3 (5 to 5·6)	3·5 (3·2 to 3·7)	2·6 (2·3 to 3·1)
Hungary	104·6 (103·3 to 106·1)	54·8 (53·8 to 55·9)	41·1 (40·2 to 42)	25·3 (24·6 to 25·9)	17·5 (16·9 to 18·1)	10·5 (10 to 11)	5·8 (5·5 to 6·1)	4 (3·5 to 4·6)
Montenegro	80·6 (77 to 84·5)	64·4 (61·5 to 67·7)	44·3 (41·9 to 46·5)	26·6 (25 to 28·4)	16·8 (15·5 to 18·1)	13·8 (12·6 to 15)	7·2 (6·5 to 7·9)	3·7 (3·2 to 4·4)
North Macedonia	229·5 (225·9 to 233·3)	189·6 (186·3 to 193·3)	109·5 (107 to 112·1)	62·8 (61 to 65)	36·7 (35·1 to 38·2)	15·3 (14·4 to 16·3)	9·4 (8·8 to 10·2)	8·5 (7·2 to 10)
Poland	132·1 (131·4 to 133)	64·1 (63·5 to 64·7)	39 (38·5 to 39·4)	24·8 (24·4 to 25·2)	18·6 (18·3 to 18·9)	9·6 (9·3 to 9·8)	5·8 (5·6 to 6)	3·8 (3·3 to 4·5)
Romania	128·6 (110·1 to 150)	89·8 (89 to 90·7)	56·9 (56·3 to 57·6)	35·4 (34·9 to 35·9)	33·6 (33 to 34·2)	22·4 (21·8 to 22·9)	11·5 (11·1 to 11·9)	8 (7 to 9·3)
Serbia	183·7 (181·9 to 185·6)	103·7 (102·3 to 105·1)	69·5 (68·4 to 70·6)	40·1 (39·2 to 41)	28·1 (27·2 to 29)	15·2 (13 to 17·8)	6·9 (6·5 to 7·2)	4·1 (3·5 to 4·9)
Slovakia	127·1 (125 to 129·4)	35·2 (34·1 to 36·3)	30·7 (29·6 to 31·7)	23·4 (22·7 to 24·2)	14·6 (14 to 15·3)	9·8 (9·3 to 10·4)	6·7 (6·4 to 7·2)	5·3 (4·5 to 6·3)
Slovenia	126·5 (123·2 to 129·8)	70·9 (68·9 to 72·9)	37·3 (36 to 38·6)	18·5 (17·7 to 19·3)	10 (9·4 to 10·6)	5·4 (5 to 5·8)	3·3 (3 to 3·6)	2 (1·7 to 2·3)
Eastern Europe	59·9 (50·4 to 71)	44·9 (43·3 to 46·7)	28·8 (27·6 to 30·2)	27 (26 to 28)	20·4 (19·8 to 21·1)	18·3 (18 to 18·5)	9·7 (9·5 to 9·8)	6·9 (6·3 to 7·5)
Belarus	73·8 (62·3 to 87·3)	55·2 (46·5 to 64·5)	34 (28·7 to 39·8)	25 (21·3 to 29·3)	18·7 (15·9 to 21·7)	16·3 (14 to 19)	8·3 (7 to 9·9)	5·3 (4·4 to 6·5)
Estonia	54·1 (45·9 to 63·8)	36·7 (35·1 to 38·2)	22·1 (21·1 to 23·3)	21·7 (20·7 to 22·8)	17·4 (16·6 to 18·3)	11·2 (10·4 to 12)	4·6 (4·3 to 5)	2·6 (2·3 to 3·1)
Latvia	46·8 (39·6 to 55·4)	32·8 (31·4 to 34·4)	22·2 (21·3 to 23·1)	21·6 (20·8 to 22·6)	17·3 (16·6 to 18·1)	13·7 (12·9 to 14·5)	7·8 (7·3 to 8·3)	4·2 (3·6 to 4·9)
Lithuania	69·1 (58·8 to 81·2)	48 (46·8 to 49·4)	23·1 (22·3 to 23·9)	20·1 (19·4 to 20·8)	14·8 (14·2 to 15·4)	11 (10·4 to 11·5)	6 (5·6 to 6·4)	4 (3·5 to 4·6)
Moldova	174·3 (149·7 to 202·6)	122·1 (102·6 to 141·8)	77 (65·4 to 90·2)	52·7 (46 to 60)	32·1 (28·7 to 36)	26·9 (24·1 to 30·6)	16·1 (13·9 to 18·6)	12·2 (10·2 to 14·7)
Russia	58·6 (49·3 to 69·6)	44·3 (44 to 44·5)	28·2 (28 to 28·4)	27·3 (27·1 to 27·5)	21·1 (21 to 21·3)	19·3 (19·1 to 19·6)	9·5 (9·4 to 9·7)	6·5 (5·7 to 7·4)
Ukraine	49·8 (41·8 to 59·2)	37·3 (31·4 to 44)	25·3 (21·3 to 29·9)	24·4 (20·8 to 28·2)	18·1 (15·8 to 20·7)	15·5 (15·2 to 15·8)	10·4 (10·2 to 10·6)	8·8 (7·8 to 9·7)
High income	68·7 (68 to 69·6)	43·5 (43·1 to 44·1)	29 (28·6 to 29·3)	16·8 (16·7 to 17)	11·1 (11 to 11·2)	7·5 (7·5 to 7·6)	6 (5·9 to 6)	4·9 (4·8 to 5·1)
Australasia	31 (30·3 to 31·7)	25·3 (24·8 to 25·7)	21·1 (20·8 to 21·5)	13·5 (13·3 to 13·8)	9·8 (9·6 to 10)	6·6 (6·4 to 6·7)	5·2 (5·1 to 5·3)	3·7 (3·5 to 3·9)
Australia	30·1 (29·5 to 30·7)	24·5 (24·1 to 25)	21·3 (21 to 21·7)	13 (12·7 to 13·3)	9·3 (9·1 to 9·5)	6·3 (6·2 to 6·5)	4·9 (4·8 to 5)	3·6 (3·4 to 3·8)
New Zealand	34·5 (33·3 to 35·8)	27·9 (27·1 to 28·8)	20·3 (19·6 to 21)	15·8 (15·2 to 16·4)	12 (11·5 to 12·5)	7·6 (7·3 to 8)	6·6 (6·3 to 6·9)	4·5 (4·2 to 5)
High-income Asia Pacific	111·4 (108·6 to 115)	57·4 (55 to 59·9)	28·1 (26·9 to 29·5)	15·1 (14·3 to 16)	8·5 (8·1 to 8·9)	5·1 (5·1 to 5·2)	3·4 (3·4 to 3·5)	2·6 (2·5 to 2·7)
Brunei	153·6 (144 to 163)	117·4 (110·4 to 125·6)	53·1 (50·2 to 56·1)	21·7 (19·6 to 23·9)	11·4 (10·5 to 12·5)	9·7 (8·8 to 10·6)	9·3 (8·5 to 10·3)	9·2 (7·7 to 10·9)
Japan	91·7 (91·4 to 92·1)	39·2 (39 to 39·5)	17·2 (17·1 to 17·3)	9·8 (9·7 to 9·9)	6·3 (6·3 to 6·4)	4·4 (4·4 to 4·5)	3·2 (3·1 to 3·2)	2·4 (2·4 to 2·5)
Aichi	82·2 (81·9 to 82·6)	35·5 (32·6 to 38·3)	15·5 (14·8 to 16·2)	9 (8·6 to 9·3)	6 (5·7 to 6·2)	4·2 (4·1 to 4·4)	3·3 (3·1 to 3·4)	2·3 (2·1 to 2·5)
Akita	91·9 (91·6 to 92·3)	40·2 (37·1 to 43·6)	18·8 (17·5 to 20·3)	10·2 (9·6 to 10·8)	6·7 (6·3 to 7·2)	4·3 (4 to 4·7)	3·1 (2·9 to 3·3)	2·5 (2·2 to 2·8)
Aomori	103·7 (103·3 to 104·1)	46 (42·4 to 50)	21·3 (20·1 to 22·4)	11·5 (10·9 to 12)	7·3 (6·9 to 7·7)	5·2 (4·8 to 5·6)	3·5 (3·3 to 3·7)	2·7 (2·5 to 3)
Chiba	92·5 (92·2 to 92·9)	38·4 (34·9 to 42)	15·7 (15·1 to 16·4)	9·2 (8·8 to 9·6)	5·7 (5·5 to 5·9)	4·4 (4·2 to 4·6)	3·3 (3·1 to 3·5)	2·7 (2·5 to 3)
Ehime	94 (93·7 to 94·4)	40·7 (37·6 to 44)	18·5 (17·3 to 19·8)	10·3 (9·7 to 10·9)	6·6 (6·3 to 7·1)	4·3 (4 to 4·6)	2·8 (2·6 to 3)	2·1 (1·9 to 2·3)

Fukui	81·8 (81·5 to 82·2)	36·5 (32·1 to 41·5)	16·5 (14·6 to 18·7)	8·8 (8·1 to 9·5)	6·5 (6 to 7·1)	4·8 (4·4 to 5·2)	3·1 (2·8 to 3·3)	2·4 (2·2 to 2·7)
Fukuoka	87·1 (86·8 to 87·5)	36·4 (33 to 39·8)	16·9 (15·9 to 17·9)	9·3 (9 to 9·6)	6·6 (6·3 to 6·8)	4·6 (4·4 to 4·9)	3·3 (3·1 to 3·5)	2·5 (2·3 to 2·7)
Fukushima	109·1 (108·7 to 109·5)	47·1 (43·5 to 50·8)	22·4 (21·1 to 23·7)	11·7 (11·2 to 12·1)	7·4 (7·1 to 7·8)	4·9 (4·6 to 5·2)	3·2 (3 to 3·5)	2·5 (2·2 to 2·7)
Gifu	88·2 (87·9 to 88·6)	39·3 (36·3 to 42·6)	18·1 (17 to 19·1)	10 (9·6 to 10·5)	5·9 (5·5 to 6·4)	4·4 (4·1 to 4·7)	3·4 (3·2 to 3·6)	2·6 (2·4 to 2·9)
Gunma	95·5 (95·2 to 95·9)	42·4 (39·2 to 45·7)	18·7 (17·7 to 19·8)	10·1 (9·7 to 10·6)	6·4 (6 to 6·8)	4·6 (4·3 to 4·9)	3·2 (3 to 3·4)	2·5 (2·2 to 2·7)
Hiroshima	98·7 (98·3 to 99)	41·1 (36·3 to 46)	18·3 (16·2 to 20·8)	10·2 (9·8 to 10·6)	6·4 (6·1 to 6·7)	4 (3·8 to 4·3)	3·2 (3 to 3·4)	2·5 (2·3 to 2·7)
Hokkaidō	94·2 (93·9 to 94·6)	40·1 (36·7 to 43·4)	18·8 (16·5 to 21·2)	10·4 (10·1 to 10·8)	6·1 (5·9 to 6·4)	4·1 (3·9 to 4·3)	3 (2·8 to 3·2)	2·4 (2·1 to 2·7)
Hyōgo	83·4 (83·1 to 83·7)	35 (32 to 38·3)	15·8 (14·8 to 16·8)	9·3 (8·9 to 9·6)	6·2 (5·9 to 6·5)	4·3 (4·1 to 4·6)	2·9 (2·7 to 3·1)	2·1 (1·9 to 2·3)
Ibaraki	111·9 (111·5 to 112·3)	47·7 (42·2 to 53·3)	21·6 (18·9 to 24·6)	11·6 (11·1 to 12·1)	6·5 (6·2 to 6·8)	4·6 (4·3 to 4·9)	3·4 (3·3 to 3·6)	2·6 (2·4 to 2·8)
Ishikawa	87·5 (87·2 to 87·9)	39·1 (36·1 to 42·2)	17·7 (16·6 to 19·1)	9·9 (9·3 to 10·5)	6·3 (5·8 to 6·8)	4·7 (4·3 to 5·1)	3·2 (3 to 3·4)	2·3 (2·1 to 2·6)
Iwate	107·4 (107 to 107·9)	47·8 (44·2 to 52)	22·9 (21·6 to 24·2)	12·4 (11·8 to 13·1)	6·1 (5·7 to 6·6)	4·3 (4 to 4·7)	3·7 (3·5 to 4)	2·9 (2·6 to 3·2)
Kagawa	90·8 (90·5 to 91·2)	39·2 (36·1 to 42·7)	17·4 (16·2 to 18·8)	9·5 (8·9 to 10·2)	6·3 (5·9 to 6·8)	4·1 (3·7 to 4·4)	3·2 (3 to 3·5)	2·5 (2·2 to 2·7)
Kagoshima	109·8 (109·4 to 110·3)	48·2 (44·5 to 52·2)	23·4 (22 to 24·7)	12·1 (11·6 to 12·7)	6·9 (6·5 to 7·4)	5·1 (4·7 to 5·5)	3·5 (3·3 to 3·7)	2·7 (2·5 to 3)
Kanagawa	78·1 (77·8 to 78·4)	32·1 (29·4 to 34·6)	14 (13·4 to 14·7)	8·8 (8·5 to 9)	6 (5·8 to 6·3)	4·5 (4·3 to 4·7)	3·3 (3·1 to 3·5)	2·6 (2·4 to 2·8)
Kōchi	103 (102·6 to 103·4)	45·1 (41·7 to 48·9)	20·5 (19·1 to 22·1)	11 (10·2 to 11·7)	7·5 (7 to 8·1)	4·9 (4·5 to 5·4)	3·9 (3·5 to 4·2)	2·9 (2·6 to 3·2)
Kumamoto	107·2 (106·8 to 107·7)	47·1 (43·4 to 51)	22 (20·7 to 23·3)	11·5 (10·9 to 12·1)	6·7 (6·3 to 7)	4·6 (4·3 to 4·9)	3·2 (3 to 3·3)	2·3 (2·1 to 2·6)
Kyōto	83·2 (82·9 to 83·5)	35 (32·3 to 37·8)	15·2 (14·3 to 16)	8·7 (8·3 to 9·1)	6·4 (6·1 to 6·8)	4·3 (4·1 to 4·6)	3·1 (3 to 3·3)	2·5 (2·3 to 2·7)
Mie	89·5 (89·1 to 89·8)	39·7 (36·6 to 43)	18·1 (17·1 to 19·1)	10·1 (9·6 to 10·7)	6·6 (6·2 to 7)	4·8 (4·5 to 5·1)	3·5 (3·3 to 3·7)	2·5 (2·2 to 2·7)
Miyagi	99·7 (99·3 to 100·1)	41·7 (38·6 to 45·2)	19·1 (18·1 to 20·2)	10·4 (9·9 to 10·9)	6·3 (5·9 to 6·6)	4·1 (3·8 to 4·4)	3·2 (3 to 3·4)	2·6 (2·3 to 2·8)
Miyazaki	101·4 (101 to 101·8)	44·2 (40·9 to 47·8)	20·4 (19 to 21·9)	11 (10·4 to 11·7)	7·3 (6·9 to 7·8)	4·9 (4·5 to 5·3)	3·3 (3·1 to 3·6)	2·5 (2·3 to 2·8)
Nagano	94·1 (93·7 to 94·4)	40·4 (37·1 to 43·7)	17·5 (16·6 to 18·6)	9·6 (9·2 to 10·1)	6·1 (5·8 to 6·4)	3·6 (3·4 to 3·9)	2·7 (2·5 to 2·8)	2·1 (1·9 to 2·3)
Nagasaki	87 (86·6 to 87·3)	38·2 (35·2 to 41·5)	18·5 (17·5 to 19·5)	9·6 (9·2 to 10·2)	6·3 (5·9 to 6·6)	4·8 (4·4 to 5·1)	3·5 (3·3 to 3·8)	2·6 (2·4 to 2·9)
Nara	88·3 (88 to 88·7)	38·3 (35·3 to 41·5)	17·4 (16·2 to 18·7)	10·2 (9·5 to 10·8)	6·3 (5·9 to 6·8)	4·1 (3·8 to 4·5)	3·4 (3·1 to 3·6)	2·8 (2·5 to 3·1)
Niigata	93 (92·6 to 93·3)	40 (37 to 43·1)	18·2 (17·1 to 19·4)	10·1 (9·7 to 10·6)	5·7 (5·4 to 6)	4·5 (4·2 to 4·8)	2·7 (2·6 to 2·9)	2·1 (1·9 to 2·3)
Ōita	100·3 (99·9 to 100·7)	43·6 (40·2 to 47·4)	20·3 (18·9 to 21·8)	10·9 (10·3 to 11·5)	6·4 (5·9 to 6·8)	4·2 (3·9 to 4·6)	3·3 (3·1 to 3·5)	2·5 (2·3 to 2·8)
Okayama	89·4 (89·1 to 89·8)	38·8 (35·9 to 42)	16·4 (15·5 to 17·3)	9·2 (8·7 to 9·7)	6·6 (6·3 to 7)	4·6 (4·3 to 4·9)	3·1 (2·9 to 3·2)	2·2 (2 to 2·5)
Okinawa	104·2 (103·8 to 104·6)	45·3 (41·8 to 49·3)	22·8 (21·5 to 24·1)	11·1 (10·6 to 11·7)	7·3 (6·9 to 7·8)	5·2 (4·8 to 5·5)	3·3 (3·1 to 3·5)	2·6 (2·4 to 2·9)
Ōsaka	81·7 (81·4 to 82)	34 (31·2 to 36·8)	14·7 (14 to 15·4)	9·2 (8·9 to 9·5)	6·4 (6·2 to 6·7)	4·3 (4·1 to 4·4)	3 (2·8 to 3·2)	2·3 (2·1 to 2·5)
Saga	97·8 (97·5 to 98·2)	42·8 (39·5 to 46·4)	19·9 (18·5 to 21·5)	10·4 (9·7 to 11·2)	6·9 (6·5 to 7·4)	4·8 (4·4 to 5·2)	3 (2·7 to 3·2)	2·2 (2 to 2·4)
Saitama	91 (90·7 to 91·4)	38·2 (35·1 to 41·4)	16 (15·3 to 16·7)	9·4 (9 to 9·7)	6·2 (6 to 6·5)	4·4 (4·2 to 4·6)	3·1 (2·9 to 3·3)	2·3 (2·2 to 2·5)
Shiga	89·4 (89·1 to 89·8)	39·4 (36·3 to 42·7)	17·8 (16·5 to 19·1)	9·4 (8·8 to 10·1)	6·5 (6·1 to 6·9)	4·7 (4·4 to 5·1)	3·3 (3·1 to 3·5)	2·4 (2·2 to 2·7)
Shimane	90·3 (90 to 90·7)	39·4 (35 to 44·7)	18·4 (16·2 to 20·9)	10 (9·3 to 10·8)	6·6 (6·1 to 7·2)	4·6 (4·2 to 5)	3 (2·8 to 3·3)	2·3 (2·1 to 2·6)
Shizuoka	83·5 (83·1 to 83·8)	35 (31 to 39·1)	15·5 (13·6 to 17·7)	8·6 (8·2 to 9)	6·1 (5·9 to 6·4)	4·1 (3·9 to 4·3)	3 (2·8 to 3·1)	2·4 (2·2 to 2·6)
Tochigi	98·2 (97·8 to 98·6)	43·7 (40·5 to 47·4)	20·1 (19 to 21·3)	10·9 (10·3 to 11·4)	7·1 (6·7 to 7·5)	5·3 (4·9 to 5·6)	3·6 (3·4 to 3·8)	2·7 (2·4 to 2·9)
Tokushima	96·7 (96·4 to 97·1)	41·7 (38·6 to 45·4)	19·3 (18 to 20·7)	10·4 (9·6 to 11·1)	6·8 (6·3 to 7·3)	5·2 (4·7 to 5·6)	4·2 (3·8 to 4·5)	3·2 (2·9 to 3·6)
Tōkyō	73·1 (72·8 to 73·4)	31·8 (28·4 to 35·5)	14·8 (13·7 to 16)	8·8 (8·4 to 9·1)	6 (5·7 to 6·3)	4·5 (4·3 to 4·8)	2·9 (2·8 to 3·1)	2·3 (2·1 to 2·5)
Tottori	97·7 (97·4 to 98·1)	42·4 (37·1 to 47·9)	19·8 (17·5 to 22·5)	10·3 (9·6 to 11·1)	6·4 (5·8 to 7)	4·1 (3·8 to 4·6)	3·5 (3·2 to 3·8)	2·8 (2·5 to 3·1)
Toyama	97 (96·7 to 97·4)	43·1 (39·9 to 46·7)	19·7 (18·5 to 21·2)	10·7 (10·2 to 11·4)	7·3 (6·8 to 7·8)	4·7 (4·4 to 5·2)	3·3 (3·1 to 3·5)	2·3 (2·1 to 2·6)
Wakayama	98·7 (98·3 to 99·1)	43·4 (40·1 to 47)	19·8 (18·5 to 21·2)	11·5 (10·8 to 12·2)	6·4 (5·9 to 6·9)	4·3 (3·9 to 4·6)	3·1 (2·9 to 3·4)	2·5 (2·2 to 2·8)
Yamagata	93·8 (93·5 to 94·2)	41·2 (38·1 to 44·7)	19·1 (17·9 to 20·6)	10·2 (9·6 to 10·8)	5·8 (5·4 to 6·3)	4·5 (4·1 to 4·8)	3·5 (3·2 to 3·8)	2·9 (2·6 to 3·2)
Yamaguchi	98·2 (97·9 to 98·6)	42·3 (38·9 to 45·9)	19·1 (18·1 to 20·3)	11 (10·4 to 11·5)	6·8 (6·3 to 7·3)	4·7 (4·3 to 5)	3·3 (3·1 to 3·5)	3 (2·7 to 3·2)
Yamanashi	90·5 (90·2 to 90·9)	39·6 (34·9 to 45)	17·9 (15·8 to 20·3)	9·7 (9 to 10·4)	6·6 (6·2 to 7·1)	4·8 (4·4 to 5·2)	2·9 (2·7 to 3·1)	2·3 (2 to 2·5)
South Korea	176·4 (165 to 190·6)	88·1 (81·8 to 94·9)	50·6 (46·6 to 54·8)	26·2 (23·8 to 28·9)	12·4 (11·3 to 13·6)	6·7 (6·6 to 6·8)	4·1 (4 to 4·2)	3 (2·8 to 3·3)
Singapore	117·7 (115 to 120·4)	48·2 (47 to 49·4)	27·3 (26·4 to 28·3)	15·1 (14·5 to 15·9)	8·2 (7·8 to 8·7)	3·3 (3·1 to 3·5)	2·5 (2·3 to 2·7)	1·8 (1·6 to 2·1)
High-income North America	35·1 (35 to 35·3)	30·4 (30·2 to 30·5)	23·8 (23·6 to 23·9)	14·9 (14·8 to 15)	10·8 (10·7 to 10·9)	8·1 (8·1 to 8·2)	7·1 (7·1 to 7·2)	6·3 (6·2 to 6·5)
Canada	48 (47·4 to 48·7)	32·2 (31·8 to 32·6)	22·3 (22 to 22·7)	12·9 (12·6 to 13·1)	8·3 (8·1 to 8·4)	6·1 (6 to 6·2)	5·7 (5·6 to 5·8)	4·9 (4·7 to 5·1)
Greenland	158·1 (133·3 to 185·9)	87·7 (80·8 to 95)	56·6 (51·2 to 62·1)	40·2 (32·7 to 50·1)	32·7 (28·7 to 36·8)	19·8 (16·9 to 23·2)	12·2 (10 to 14·7)	9·5 (6·9 to 12·9)
USA	33·8 (33·6 to 34)	30·1 (30 to 30·3)	23·9 (23·8 to 24)	15·1 (15 to 15·2)	11·1 (11 to 11·1)	8·3 (8·2 to 8·4)	7·3 (7·2 to 7·3)	6·5 (6·3 to 6·6)

Alabama	42·7 (42·5 to 42·9)	37·6 (36·7 to 38·5)	29·6 (28·5 to 30·6)	17·8 (17·1 to 18·6)	13 (12·4 to 13·7)	11·2 (10·7 to 11·8)	10·1 (9·6 to 10·7)	9·5 (9·1 to 9·9)
Alaska	55·3 (54·1 to 56·5)	46·9 (44·6 to 49·5)	26·6 (25·1 to 28·1)	17·6 (16·6 to 18·7)	12·1 (11·4 to 12·9)	8·9 (8·3 to 9·5)	7·1 (6·6 to 7·7)	7 (5·9 to 8·2)
Arizona	46·9 (46·7 to 47·2)	40·1 (38·6 to 41·6)	24·6 (23·3 to 25·8)	16·2 (15·4 to 17)	11·4 (10·9 to 12)	8·7 (8·2 to 9·1)	7·3 (6·9 to 7·7)	6·3 (6 to 6·5)
Arkansas	38 (37·8 to 38·2)	32·6 (31·3 to 34·2)	26·1 (24·9 to 27·4)	15·2 (14·4 to 16·1)	11·9 (11·1 to 12·7)	10·2 (9·5 to 10·8)	9 (8·4 to 9·5)	8·5 (8·2 to 8·8)
California	32·5 (32·3 to 32·6)	27·4 (27·1 to 27·8)	21·3 (21 to 21·7)	13·9 (13·6 to 14·3)	10·5 (10·3 to 10·7)	6·9 (6·7 to 7·1)	5·8 (5·6 to 5·9)	4·7 (4·5 to 4·9)
Colorado	36·7 (36·5 to 36·9)	31·3 (30·2 to 32·4)	23·5 (22·3 to 24·7)	12·6 (11·9 to 13·3)	10 (9·5 to 10·6)	7·5 (7·1 to 8)	6·9 (6·6 to 7·3)	5·4 (5·2 to 5·7)
Connecticut	26·7 (26·5 to 26·9)	23·6 (22·8 to 24·4)	19·4 (18·7 to 20·2)	12·8 (12·2 to 13·5)	9·1 (8·6 to 9·6)	7 (6·7 to 7·4)	5·9 (5·5 to 6·3)	5·1 (4·3 to 6)
Delaware	32·7 (32 to 33·3)	28·1 (26·5 to 29·7)	22·9 (21·6 to 24·1)	16·3 (15·4 to 17·3)	12·3 (11·6 to 13·1)	10·3 (9·6 to 10·9)	8·8 (8·2 to 9·4)	8·2 (6·9 to 9·7)
Washington, DC	44·8 (43·8 to 45·7)	45 (43·4 to 46·9)	37·9 (36·2 to 39·7)	31·3 (29·8 to 33)	30·4 (28·7 to 32)	16·5 (15·5 to 17·7)	12·4 (11·5 to 13·3)	9 (7·6 to 10·7)
Florida	42·4 (42·2 to 42·6)	36·8 (36 to 37·6)	27·5 (26·6 to 28·4)	18·5 (18 to 19)	12·6 (12·2 to 13)	9 (8·8 to 9·3)	8 (7·8 to 8·3)	7 (6·7 to 7·2)
Georgia	40·7 (40·5 to 40·9)	37·8 (36·9 to 38·7)	27 (26·1 to 27·9)	17·7 (17 to 18·4)	14·2 (13·6 to 14·8)	10·1 (9·7 to 10·5)	7·9 (7·6 to 8·2)	7·9 (7·6 to 8·2)
Hawaii	30·8 (30·2 to 31·4)	26·7 (25·4 to 28·1)	20·7 (19·8 to 21·7)	12·5 (11·8 to 13·3)	9·2 (8·6 to 9·8)	8·4 (7·9 to 9)	7 (6·6 to 7·5)	6·3 (5·3 to 7·4)
Idaho	30·9 (30·3 to 31·5)	27·6 (26·4 to 28·9)	21·7 (20·7 to 22·8)	13·4 (12·7 to 14·1)	10·7 (10·1 to 11·3)	8·1 (7·6 to 8·6)	6·4 (6 to 6·8)	5·8 (4·9 to 6·8)
Illinois	31·5 (31·3 to 31·6)	29·3 (28·8 to 29·8)	25·4 (25 to 25·9)	17·4 (17 to 17·9)	12·5 (12·1 to 12·9)	9·6 (9·3 to 10)	7·9 (7·6 to 8·2)	6·6 (6·3 to 6·8)
Indiana	29·6 (29·5 to 29·8)	27·5 (26·8 to 28·1)	23 (22·2 to 23·8)	14·2 (13·6 to 14·7)	11·1 (10·5 to 11·6)	9·3 (8·8 to 9·7)	8·6 (8·2 to 9)	7·8 (7·5 to 8·1)
Iowa	26 (25·8 to 26·2)	24·6 (23·8 to 25·4)	21·5 (20·7 to 22·3)	12·9 (12·3 to 13·5)	9·1 (8·7 to 9·6)	7·1 (6·7 to 7·5)	5·8 (5·4 to 6·1)	5·8 (4·9 to 6·8)
Kansas	28·2 (27·9 to 28·4)	25·6 (24·7 to 26·5)	21·3 (20·4 to 22·2)	13·4 (12·8 to 14·1)	9·9 (9·4 to 10·4)	8·5 (8·1 to 8·9)	7·6 (7·2 to 8)	6·6 (5·5 to 7·8)
Kentucky	36·8 (36·6 to 37)	33 (32·1 to 33·9)	24·4 (23·4 to 25·5)	15 (14·3 to 15·7)	10·2 (9·7 to 10·9)	8·4 (7·9 to 8·8)	8·2 (7·7 to 8·7)	7·5 (7·2 to 7·8)
Louisiana	42·8 (42·6 to 43)	38 (36·9 to 39·2)	28·5 (27·6 to 29·3)	17·5 (16·8 to 18·3)	13 (12·4 to 13·6)	11·3 (10·7 to 11·9)	9·3 (8·8 to 9·8)	9·3 (8·9 to 9·6)
Maine	35·6 (34·8 to 36·3)	30·4 (29·1 to 31·6)	23·5 (22·6 to 24·6)	11·3 (10·6 to 11·9)	7·9 (7·4 to 8·4)	6·3 (5·8 to 6·7)	6·8 (6·4 to 7·3)	6·7 (5·6 to 7·9)
Maryland	33·9 (33·7 to 34·1)	31·3 (30·5 to 32·2)	22·4 (21·6 to 23·3)	15·8 (15 to 16·5)	11·1 (10·6 to 11·7)	9 (8·6 to 9·4)	7·8 (7·4 to 8·3)	7·3 (7 to 7·5)
Massachusetts	27·7 (27·5 to 27·9)	24·2 (23·4 to 24·9)	19·8 (19 to 20·5)	12·2 (11·6 to 12·7)	8 (7·6 to 8·4)	5·6 (5·2 to 5·9)	5·1 (4·8 to 5·4)	4·5 (3·7 to 5·4)
Michigan	29·3 (29·1 to 29·5)	27·4 (26·7 to 28)	23·7 (23·1 to 24·3)	14·5 (14 to 15)	12 (11·5 to 12·4)	9·3 (8·9 to 9·6)	8 (7·7 to 8·4)	7·2 (6·9 to 7·5)
Minnesota	27·3 (27·2 to 27·5)	25·5 (24·8 to 26·3)	20·7 (19·9 to 21·6)	12 (11·4 to 12·6)	8 (7·6 to 8·5)	6·4 (6 to 6·8)	5·5 (5·1 to 5·8)	5·5 (5·2 to 5·7)
Mississippi	55·3 (55 to 55·6)	47·3 (45·8 to 48·9)	35·1 (33·8 to 36·5)	20·6 (19·6 to 21·5)	14·5 (13·7 to 15·3)	13 (12·3 to 13·8)	11·4 (10·8 to 12·1)	10·1 (8·3 to 12·2)
Missouri	30·9 (30·8 to 31·1)	28 (27·3 to 28·7)	23·4 (22·7 to 24)	15 (14·4 to 15·6)	11·1 (10·6 to 11·6)	8·5 (8·1 to 9)	7·7 (7·3 to 8·1)	7·1 (6·8 to 7·4)
Montana	34·9 (34·2 to 35·6)	31·2 (29·8 to 32·7)	25·4 (24·2 to 26·7)	14·2 (13·4 to 15)	10·1 (9·5 to 10·7)	8·1 (7·6 to 8·6)	7·3 (6·8 to 7·8)	6·6 (5·6 to 7·8)
Nebraska	29·5 (29·2 to 29·7)	26·4 (25·3 to 27·4)	21·4 (20·4 to 22·4)	13·4 (12·8 to 14·2)	9·2 (8·6 to 9·7)	8·1 (7·6 to 8·6)	6·3 (5·9 to 6·7)	6·2 (5·2 to 7·4)
Nevada	51·2 (50·2 to 52·2)	39·5 (37·2 to 41·8)	27·3 (25·8 to 28·6)	14·7 (13·9 to 15·6)	10·3 (9·7 to 10·8)	7·9 (7·5 to 8·4)	6·7 (6·4 to 7·1)	5·8 (5 to 6·8)
New Hampshire	31·3 (30·7 to 31·9)	26·7 (25·3 to 28·1)	20·9 (19·8 to 22)	11·8 (11 to 12·5)	7·9 (7·4 to 8·3)	5·7 (5·3 to 6·1)	5·3 (4·9 to 5·7)	4·5 (3·8 to 5·3)
New Jersey	30·5 (30·4 to 30·7)	27·4 (26·7 to 28·2)	23 (22·4 to 23·7)	14·3 (13·7 to 14·9)	10·6 (10·2 to 11·1)	7·3 (6·9 to 7·6)	5·7 (5·4 to 6)	4·8 (4·6 to 4·9)
New Mexico	49·2 (48·7 to 49·7)	41·3 (39·9 to 42·7)	26·9 (25·7 to 28·1)	15·1 (14·3 to 15·9)	10·8 (10·2 to 11·4)	8 (7·5 to 8·5)	7·2 (6·7 to 7·7)	6·5 (5·4 to 7·7)
New York	31·1 (30·9 to 31·3)	27·7 (27·3 to 28)	23·2 (22·8 to 23·5)	14·8 (14·3 to 15·1)	11·9 (11·5 to 12·2)	7·6 (7·3 to 7·9)	6 (5·8 to 6·3)	4·9 (4·7 to 5·1)
North Carolina	41·5 (41·3 to 41·7)	36·3 (35·5 to 37·1)	28·9 (28 to 29·8)	16·6 (16·1 to 17·2)	12·4 (11·8 to 13)	10·2 (9·7 to 10·6)	8·2 (7·9 to 8·6)	7·7 (7·4 to 7·9)
North Dakota	30·6 (30 to 31·2)	28·5 (27·2 to 29·8)	19·3 (18·2 to 20·5)	13·8 (13 to 14·6)	9·4 (8·8 to 10·2)	8·1 (7·5 to 8·8)	7 (6·4 to 7·6)	6·7 (5·7 to 7·9)
Ohio	29·3 (29·2 to 29·5)	27·4 (26·7 to 28·1)	21·8 (21·4 to 22·2)	14·8 (14·4 to 15·2)	10·9 (10·5 to 11·3)	8·9 (8·5 to 9·2)	8·8 (8·5 to 9·1)	8 (7·7 to 8·3)
Oklahoma	33·4 (33·2 to 33·5)	30·4 (29·5 to 31·5)	25·4 (24·3 to 26·5)	15·2 (14·5 to 15·9)	10·9 (10·3 to 11·5)	9·7 (9·2 to 10·3)	9·3 (8·8 to 9·8)	8·6 (8·2 to 8·9)
Oregon	29·5 (29·3 to 29·8)	26·9 (25·9 to 27·9)	20·7 (19·8 to 21·6)	13·6 (13 to 14·3)	9·6 (9·1 to 10·1)	6·7 (6·4 to 7·1)	5·7 (5·4 to 6·1)	5·2 (4·4 to 6·1)
Pennsylvania	30·4 (30·2 to 30·5)	27·5 (26·9 to 28·2)	23·4 (23 to 23·9)	15 (14·6 to 15·5)	10·9 (10·5 to 11·2)	8·1 (7·8 to 8·5)	8 (7·7 to 8·4)	6·7 (6·4 to 7)
Rhode Island	30 (29·4 to 30·6)	26·3 (25·1 to 27·5)	22·3 (21·2 to 23·4)	13·9 (13·1 to 14·7)	9·4 (8·8 to 10)	7·3 (6·8 to 7·8)	6·9 (6·4 to 7·5)	5·9 (5 to 7)
South Carolina	43·8 (43·6 to 44)	40·2 (39·2 to 41·3)	27·8 (26·6 to 29)	19·1 (18·2 to 19·9)	14 (13·3 to 14·7)	10·4 (9·8 to 11)	8·7 (8·2 to 9·2)	7·7 (7·4 to 8)
South Dakota	33·7 (33 to 34·3)	31·6 (30·2 to 33)	24·3 (23·1 to 25·6)	14·7 (13·8 to 15·6)	11·6 (11 to 12·3)	8·9 (8·3 to 9·5)	8 (7·5 to 8·6)	7·1 (6 to 8·4)
Tennessee	37·3 (37·1 to 37·5)	33·3 (32·4 to 34·1)	25·5 (24·6 to 26·5)	15·8 (15·1 to 16·4)	12·2 (11·6 to 12·9)	10·4 (9·8 to 10·8)	8·8 (8·3 to 9·3)	7·8 (7·5 to 8·1)
Texas	37·9 (37·7 to 38·1)	33·9 (33·3 to 34·5)	25·9 (25·5 to 26·3)	15·5 (15·1 to 16)	10 (9·7 to 10·3)	7·4 (7·2 to 7·6)	7·2 (7 to 7·5)	6·5 (6·2 to 6·7)
Utah	26·3 (26·1 to 26·5)	23·2 (22·1 to 24·2)	18·9 (18 to 19·8)	12·9 (12·3 to 13·5)	8·8 (8·3 to 9·2)	6·5 (6·1 to 6·9)	5·9 (5·5 to 6·2)	5·7 (4·8 to 6·7)
Vermont	33·9 (33·3 to 34·5)	29·1 (27·5 to 30·8)	20·2 (19·1 to 21·5)	12 (11·2 to 12·9)	8·1 (7·4 to 8·7)	6·6 (6 to 7·2)	5·6 (5·1 to 6·2)	4·8 (4 to 5·7)
Virginia	37·6 (37·4 to 37·8)	33·3 (32·5 to 34·1)	24·4 (23·5 to 25·3)	15·6 (15 to 16·3)	11·4 (10·9 to 11·9)	8·2 (7·9 to 8·6)	7·8 (7·5 to 8·2)	6·6 (6·3 to 6·8)
Washington	28·7 (28·5 to 28·8)	26·3 (25·5 to 27·1)	22·1 (21·2 to 23·1)	13·8 (13·1 to 14·4)	9·2 (8·7 to 9·6)	6·3 (6 to 6·7)	5·4 (5·1 to 5·7)	4·8 (4·7 to 5)

West Virginia	34·4 (34·1 to 34·7)	29·7 (28·7 to 30·8)	26·9 (25·8 to 28)	14·8 (14·1 to 15·6)	11 (10·5 to 11·7)	9 (8·4 to 9·6)	8·8 (8·3 to 9·3)	8 (6·7 to 9·5)
Wisconsin	29·1 (29 to 29·3)	26·3 (25·6 to 27)	20 (19·2 to 20·8)	12·2 (11·6 to 12·8)	9·2 (8·8 to 9·7)	7·7 (7·3 to 8·1)	6·7 (6·4 to 7·1)	6·4 (6·1 to 6·7)
Wyoming	37·3 (36·5 to 37·9)	32·4 (30·4 to 34·3)	26·1 (24·7 to 27·6)	14·2 (13·2 to 15·1)	10·5 (9·7 to 11·2)	8·2 (7·5 to 9)	7·5 (6·9 to 8·2)	6·3 (5·3 to 7·5)
Southern Latin America	115 (114·1 to 115·7)	104·3 (103·5 to 105·1)	76·2 (75·7 to 76·7)	39·1 (38·8 to 39·4)	26·1 (25·9 to 26·3)	17·3 (17·2 to 17·5)	12·8 (12·7 to 13)	9·6 (9·1 to 10·2)
Argentina	85·6 (84·8 to 86·4)	77 (76·5 to 77·6)	68·9 (68·3 to 69·5)	39·3 (39 to 39·7)	29·2 (28·9 to 29·4)	19·5 (19·3 to 19·7)	14·2 (14 to 14·3)	10·6 (10·3 to 11)
Chile	191 (189·2 to 192·9)	161·6 (160·3 to 163)	96·2 (95·2 to 97·3)	38·2 (37·5 to 39)	19·5 (19 to 19·9)	11·4 (11·1 to 11·8)	9 (8·7 to 9·3)	6·8 (5·9 to 8)
Uruguay	71·2 (69·1 to 73·4)	56·7 (55·1 to 58·3)	57 (55·4 to 58·6)	41·2 (40 to 42·5)	23·5 (22·6 to 24·4)	16·6 (15·9 to 17·3)	11·7 (10·8 to 12·9)	8·3 (7·3 to 9·5)
Western Europe	63·9 (63·3 to 64·5)	40·4 (40·2 to 40·6)	26·6 (26·4 to 26·7)	14·9 (14·8 to 15)	9·2 (9·2 to 9·3)	5·7 (5·7 to 5·8)	4·3 (4·2 to 4·3)	3·4 (3·3 to 3·6)
Andorra	24·7 (20·7 to 29·6)	17·6 (14·6 to 20·9)	11·7 (9·8 to 14·1)	8·4 (6·9 to 10)	6 (5 to 7·2)	3·5 (2·9 to 4)	2·8 (2·3 to 3·3)	1·8 (1·5 to 2·1)
Austria	77·1 (75·8 to 78·6)	43·7 (42·9 to 44·6)	29·7 (29·1 to 30·4)	17 (16·5 to 17·5)	9·5 (9·2 to 9·8)	5·7 (5·4 to 5·9)	4·5 (4·3 to 4·7)	3·2 (3 to 3·4)
Belgium	59·8 (58·8 to 60·9)	34·3 (33·7 to 34·9)	24·5 (24 to 25·1)	14·8 (14·4 to 15·2)	9·7 (9·5 to 10)	6 (5·7 to 6·2)	4·4 (4·3 to 4·6)	3·5 (3·3 to 3·7)
Cyprus	110·6 (83·1 to 145·1)	84·9 (65·2 to 109·3)	55·4 (45·5 to 66·3)	26·1 (24·4 to 27·6)	12·9 (11·9 to 13·9)	6·8 (6·2 to 7·5)	3·7 (3·2 to 4·1)	3·2 (2·5 to 3·9)
Denmark	35·8 (34·8 to 36·8)	25·6 (24·9 to 26·4)	16·7 (16·1 to 17·3)	10·2 (9·8 to 10·7)	9 (8·6 to 9·4)	5·6 (5·3 to 5·8)	4 (3·8 to 4·3)	3·5 (3·2 to 3·8)
Finland	51·1 (50 to 52·3)	26·2 (25·5 to 27)	16·3 (15·7 to 16·9)	8·8 (8·4 to 9·2)	6·7 (6·4 to 7)	4·3 (4 to 4·5)	3 (2·8 to 3·2)	2·2 (2 to 2·4)
France	56·4 (56 to 56·9)	27·3 (27 to 27·6)	18·1 (17·9 to 18·4)	12·3 (12·1 to 12·5)	8·8 (8·7 to 9)	5·4 (5·3 to 5·5)	4·2 (4·1 to 4·3)	3·6 (3·4 to 3·8)
Germany	65·7 (63·3 to 68·3)	41·5 (41·2 to 41·8)	26·3 (26·1 to 26·5)	15·5 (15·3 to 15·7)	8·7 (8·5 to 8·8)	5·4 (5·3 to 5·4)	4·1 (4·1 to 4·2)	3·3 (3·2 to 3·5)
Greece	64·4 (61·6 to 67·3)	45·1 (44·3 to 46)	32 (31·3 to 32·7)	19·8 (19·2 to 20·3)	10·5 (10·2 to 10·9)	6·3 (6·1 to 6·6)	4·2 (4 to 4·4)	3·9 (3·5 to 4·2)
Iceland	30 (26·4 to 34·1)	20·9 (18·7 to 23·2)	15·6 (14 to 17·5)	9·4 (8·2 to 10·8)	6·7 (5·8 to 7·8)	3·8 (3·2 to 4·6)	2·7 (2·1 to 3·3)	2·3 (1·6 to 3·2)
Ireland	52·3 (50·9 to 53·7)	33·6 (32·7 to 34·5)	22·2 (21·5 to 22·8)	13·8 (13·3 to 14·3)	9·3 (8·9 to 9·7)	6·9 (6·6 to 7·2)	4·3 (4·1 to 4·5)	3·3 (3 to 3·5)
Israel	43·6 (42·6 to 44·7)	38·7 (38 to 39·6)	27 (26·5 to 27·6)	18·4 (18 to 18·8)	11·7 (11·5 to 12)	6·9 (6·7 to 7)	4·5 (4·4 to 4·7)	3·2 (3·1 to 3·3)
Italy	80·6 (80·1 to 81·1)	52·4 (52·1 to 52·8)	33·7 (33·4 to 34)	16·6 (16·4 to 16·8)	9·6 (9·4 to 9·7)	5·5 (5·4 to 5·6)	3·9 (3·8 to 4)	3 (2·9 to 3·1)
Luxembourg	53·1 (48·2 to 58·4)	37·8 (35 to 40·8)	23·5 (21·2 to 25·9)	13·3 (11·8 to 14·9)	9 (8 to 10·3)	4·9 (4·2 to 5·7)	2·8 (2·3 to 3·5)	2·4 (1·7 to 3·4)
Malta	105·6 (100·8 to 110·5)	39·9 (37·7 to 42·3)	27·2 (25 to 29·1)	16 (14·7 to 17·4)	10·8 (9·8 to 11·9)	7·3 (6·5 to 8·3)	6·7 (5·9 to 7·7)	5·7 (4·5 to 7·2)
Monaco	52·9 (45·9 to 60·7)	31·1 (26·5 to 36·2)	15·6 (13·1 to 18·5)	8·7 (7·2 to 10·5)	5·9 (4·9 to 7·1)	4·2 (3·4 to 5)	3·2 (2·7 to 3·9)	2·6 (2·2 to 3·1)
Netherlands	31·5 (30·9 to 32)	21·1 (20·8 to 21·5)	16·1 (15·8 to 16·5)	10·8 (10·6 to 11·1)	8·6 (8·4 to 8·9)	6·2 (6 to 6·4)	4·5 (4·4 to 4·6)	3·7 (3·5 to 3·9)
Norway	33·8 (32·8 to 34·8)	23·2 (22·5 to 23·9)	14·6 (14·1 to 15)	8·9 (8·5 to 9·2)	8·6 (8·3 to 8·9)	4·8 (4·6 to 5·1)	3·3 (3·1 to 3·5)	2·5 (2·3 to 2·7)
Portugal	138·9 (137·5 to 140·3)	106·1 (104·9 to 107·3)	69·9 (68·9 to 70·9)	28·7 (28·1 to 29·3)	14·2 (13·8 to 14·7)	7·3 (7 to 7·6)	3·7 (3·5 to 3·8)	2·8 (2·6 to 3·1)
San Marino	28·9 (24·1 to 35·1)	21·2 (17·8 to 25)	16·6 (14·2 to 19·4)	12·4 (10·6 to 14·4)	8·7 (7·4 to 10·3)	5·7 (4·7 to 6·8)	4·1 (3·4 to 4·9)	3·6 (3 to 4·4)
Spain	99·1 (98·4 to 99·8)	54·2 (53·8 to 54·7)	32·7 (32·4 to 33)	15·3 (15·1 to 15·5)	9·5 (9·3 to 9·6)	5·4 (5·3 to 5·5)	3·9 (3·8 to 4)	3 (2·9 to 3·1)
Sweden	26·5 (25·8 to 27·3)	20·3 (19·7 to 20·9)	13·4 (12·9 to 13·8)	8·1 (7·8 to 8·4)	6·8 (6·5 to 7·1)	3·9 (3·7 to 4·1)	3·1 (2·9 to 3·2)	2·6 (2·4 to 2·8)
Stockholm	24·2 (23·5 to 24·9)	19·4 (17·9 to 20·9)	13·2 (12·1 to 14·4)	8·5 (7·8 to 9·2)	6·5 (6 to 7)	3·8 (3·5 to 4·1)	2·7 (2·4 to 3)	2·2 (2·1 to 2·5)
Sweden except Stockholm	26·9 (26·2 to 27·7)	20·5 (19·9 to 21·1)	13·4 (12·9 to 13·9)	8 (7·7 to 8·4)	6·9 (6·6 to 7·2)	3·9 (3·7 to 4·1)	3·2 (3 to 3·3)	2·7 (2·5 to 2·9)
Switzerland	39·2 (38·2 to 40·2)	27 (26·4 to 27·6)	19 (18·5 to 19·5)	10·9 (10·6 to 11·3)	8·4 (8·1 to 8·7)	5·9 (5·7 to 6·2)	4·5 (4·3 to 4·7)	3·7 (3·5 to 3·9)
UK	34 (33·7 to 34·3)	26·1 (25·8 to 26·3)	21·4 (21·2 to 21·6)	14 (13·8 to 14·2)	9·4 (9·3 to 9·5)	6·5 (6·3 to 6·6)	5·1 (5 to 5·2)	4·1 (4 to 4·2)
England	32·1 (31·6 to 32·4)	25 (24·6 to 25·5)	20·8 (20·5 to 21·1)	13·7 (13·5 to 13·9)	9·4 (9·3 to 9·6)	6·5 (6·4 to 6·6)	5·1 (5·1 to 5·2)	4·2 (4 to 4·3)
East Midlands	31·7 (31·3 to 32·1)	24·8 (23·1 to 26·6)	20·6 (19 to 22·4)	13·5 (12·7 to 14·2)	9·5 (9·2 to 9·8)	6·4 (6·1 to 6·8)	5·2 (4·9 to 5·5)	4·3 (4·2 to 4·4)
Derby	38·2 (37·7 to 38·6)	30 (26·1 to 34·4)	25·5 (22·5 to 28·8)	16·8 (14·6 to 19)	11 (9·9 to 12·2)	7·6 (6·9 to 8·3)	6·6 (6 to 7·3)	5·5 (5·3 to 5·7)
Derbyshire	29·9 (29·5 to 30·3)	23·2 (21·1 to 25·5)	19·5 (17·6 to 21·5)	12·6 (12 to 13·3)	8·1 (7·6 to 8·7)	5·6 (5·2 to 6)	4·3 (4 to 4·6)	3·6 (3·5 to 3·7)
Leicester	39·9 (39·4 to 40·4)	31·5 (27·5 to 36·3)	25·7 (22·9 to 28·7)	16·9 (15 to 18·9)	11·7 (11 to 12·5)	8·6 (7·8 to 9·6)	7·1 (6·5 to 7·7)	5·7 (5·5 to 5·9)
Leicestershire	27·3 (27 to 27·7)	21·2 (19·1 to 23·4)	17·9 (16·1 to 20)	11·9 (10·8 to 13)	8·7 (7·9 to 9·4)	5·4 (5·1 to 5·8)	4·5 (4·2 to 4·9)	3·8 (3·7 to 3·9)
Lincolnshire	30·6 (30·2 to 31)	23·8 (21·6 to 26·2)	19·8 (17·8 to 21·9)	12·8 (12 to 13·6)	9·4 (8·8 to 9·9)	6·5 (6·1 to 7)	5 (4·6 to 5·3)	3·6 (3·5 to 3·8)
Northamptonshire	32·1 (31·7 to 32·5)	24·9 (22·5 to 27·3)	20·7 (18·6 to 23)	13·4 (11·9 to 15)	10·4 (9·7 to 11·1)	6·3 (5·7 to 6·9)	5·2 (4·7 to 5·7)	4·5 (4·4 to 4·6)
Nottingham	37·2 (36·7 to 37·6)	29·5 (25·8 to 33·8)	24·6 (22 to 27·4)	16·1 (13·9 to 18·5)	11·4 (10·7 to 12·2)	9·1 (8·5 to 9·8)	6·8 (6·3 to 7·3)	5·4 (5·2 to 5·5)
Nottinghamshire	29·4 (29 to 29·7)	22·8 (20·3 to 25·5)	19·3 (17·4 to 21·2)	12·6 (11·9 to 13·2)	8·2 (7·8 to 8·7)	5·9 (5·5 to 6·3)	4·7 (4·4 to 5)	4·1 (4 to 4·2)
Rutland	20·3 (20·1 to 20·6)	15·2 (12·5 to 18·3)	12·9 (11·6 to 14·4)	9·5 (8·4 to 10·6)	6·8 (5·8 to 7·9)	4·5 (3·7 to 5·3)	3·1 (2·6 to 3·7)	1·8 (1·7 to 1·9)
East of England	27·6 (27·3 to 28)	21·4 (19·9 to 23)	18 (16·3 to 19·6)	11·9 (11·2 to 12·7)	7·5 (7·1 to 7·8)	5·3 (5·1 to 5·6)	4·5 (4·3 to 4·7)	3·5 (3·4 to 3·6)
Bedford	32 (31·6 to 32·4)	25 (22·4 to 27·8)	21·2 (18·8 to 23·7)	14·3 (12·4 to 16·5)	9·6 (8·5 to 10·8)	6·9 (6·3 to 7·6)	5·7 (5 to 6·4)	4·4 (4 to 4·8)
Cambridgeshire	23·9 (23·6 to 24·2)	18·2 (16·3 to 20·2)	15·3 (13·6 to 17·2)	10·3 (9·2 to 11·5)	6·6 (6·1 to 7·1)	5·3 (4·8 to 5·7)	4·3 (3·9 to 4·7)	3·4 (3·1 to 3·7)

Central Bedfordshire	26·5 (26·1 to 26·8)	20·3 (18·5 to 22·4)	17·1 (15·2 to 19·3)	11·4 (9·8 to 13·3)	7·3 (6·6 to 8)	5·1 (4·5 to 5·7)	4 (3·6 to 4·5)	3·1 (2·9 to 3·4)
Essex	26·7 (26·3 to 27)	20·6 (18·9 to 22·4)	17·2 (15·4 to 19·1)	11·3 (10·2 to 12·5)	7·1 (6·6 to 7·7)	4·9 (4·5 to 5·4)	4·3 (3·9 to 4·7)	3·4 (3 to 3·8)
Hertfordshire	25 (24·7 to 25·3)	19·6 (18 to 21·5)	16·9 (14·6 to 19·4)	11·3 (10·5 to 12·2)	6·9 (6·4 to 7·4)	4·8 (4·4 to 5·2)	4 (3·7 to 4·3)	3 (2·7 to 3·4)
Luton	34·3 (33·8 to 34·7)	27·1 (24·3 to 30·3)	23 (20·4 to 25·8)	15·4 (13·6 to 17·3)	10 (9·2 to 10·9)	8·7 (8 to 9·5)	7·2 (6·6 to 8)	5·7 (5·2 to 6·1)
Norfolk	30·9 (30·5 to 31·3)	23·6 (20·2 to 27·5)	19·5 (17·2 to 22·2)	12·8 (11·8 to 13·8)	7·8 (7·2 to 8·4)	5·5 (5·1 to 5·9)	4·7 (4·3 to 5·1)	3·5 (3·3 to 3·8)
Peterborough	29·7 (29·3 to 30·1)	23·5 (19·9 to 27·3)	20·3 (18·1 to 22·7)	13·7 (12·2 to 15·4)	8·8 (8 to 9·8)	7·5 (6·7 to 8·3)	6 (5·4 to 6·6)	4·6 (4·2 to 5)
Southend-on-Sea	34·3 (33·8 to 34·7)	26·2 (23·5 to 29·1)	21·2 (18·9 to 23·7)	13·6 (11·7 to 15·6)	8·2 (7·2 to 9·2)	5·3 (4·8 to 5·8)	4·4 (3·9 to 4·9)	3·6 (3·3 to 4)
Suffolk	28·7 (28·3 to 29)	21·7 (18·4 to 25·1)	17·8 (15·8 to 20·1)	11·5 (10·1 to 13·2)	7·2 (6·7 to 7·8)	4·9 (4·4 to 5·3)	4·3 (3·9 to 4·7)	3·2 (2·9 to 3·4)
Thurrock	28·8 (28·4 to 29·1)	23·3 (20·9 to 26)	19·7 (17·6 to 22)	12·8 (11·1 to 14·7)	7·9 (7·1 to 8·8)	5·9 (5·2 to 6·6)	4·6 (4·1 to 5·2)	3·6 (3·2 to 3·9)
Greater London	33·7 (33·2 to 34·1)	26·8 (25·9 to 27·9)	22·1 (21·2 to 23)	14·9 (14·3 to 15·5)	10·1 (9·7 to 10·4)	7·1 (6·9 to 7·4)	5·3 (5·1 to 5·6)	3·8 (3·7 to 3·9)
Barking and Dagenham	42·7 (42·2 to 43·2)	35·1 (32·8 to 37·6)	29 (26·9 to 31·2)	18·4 (16·8 to 19·9)	10·6 (9·8 to 11·7)	6·8 (6·1 to 7·5)	5·7 (5·1 to 6·3)	4·2 (4·1 to 4·3)
Barnet	29·1 (28·8 to 29·5)	23·1 (21·7 to 24·6)	19 (17·6 to 20·5)	12·8 (11·9 to 13·9)	7·7 (7 to 8·3)	5·4 (4·9 to 6)	4·2 (3·8 to 4·7)	3 (2·9 to 3·1)
Bexley	24·2 (23·9 to 24·5)	19·5 (18·4 to 20·6)	16·2 (15·2 to 17·2)	10·6 (9·9 to 11·4)	6·9 (6·4 to 7·6)	5·1 (4·7 to 5·6)	4·3 (3·9 to 4·6)	3·2 (3·1 to 3·3)
Brent	36·2 (35·7 to 36·6)	28·8 (26·7 to 31)	23·8 (21·7 to 25·7)	16·2 (14·8 to 17·6)	11·6 (10·5 to 12·6)	8·9 (8·3 to 9·6)	6·1 (5·7 to 6·6)	4·2 (4 to 4·3)
Bromley	25 (24·7 to 25·3)	20·2 (19 to 21·5)	17 (15·6 to 18·4)	11·2 (10·3 to 12·3)	7·8 (7·1 to 8·4)	5·1 (4·7 to 5·6)	3·6 (3·3 to 3·9)	2·8 (2·7 to 2·8)
Camden	34·9 (34·4 to 35·3)	27 (25·3 to 28·8)	21·6 (20·1 to 23·1)	15·5 (14·4 to 16·7)	10·5 (9·7 to 11·4)	6·6 (6·1 to 7·1)	4·6 (4·2 to 5·1)	2·9 (2·8 to 3)
Croydon	30·4 (30 to 30·8)	24·5 (23 to 26·1)	20·3 (18·7 to 21·9)	13·4 (12·1 to 14·8)	10·3 (9·6 to 11)	7·6 (7·1 to 8·2)	5·6 (5·2 to 6·1)	4·1 (4 to 4·2)
Ealing	30·7 (30·3 to 31·1)	24·3 (22·9 to 25·7)	19·9 (18·4 to 21·4)	13·5 (12·5 to 14·6)	9·3 (8·6 to 10·1)	6·4 (6 to 6·9)	4·8 (4·4 to 5·2)	3·3 (3·2 to 3·3)
Enfield	30·8 (30·4 to 31·1)	24·9 (23·1 to 26·8)	20·3 (18·7 to 21·9)	13·5 (12·4 to 14·7)	8·8 (8·1 to 9·5)	6·9 (6·3 to 7·6)	5·9 (5·2 to 6·6)	3·9 (3·8 to 4·1)
Greenwich	41·9 (41·4 to 42·4)	33·9 (31·6 to 36·6)	28·2 (25·9 to 30·5)	18·7 (17·2 to 20·3)	11 (10·2 to 11·8)	7·2 (6·6 to 7·9)	5·8 (5·3 to 6·3)	4·7 (4·6 to 4·9)
Hackney	37·3 (36·8 to 37·7)	30 (27·8 to 32·2)	25·2 (23·2 to 27·2)	17·5 (16·3 to 18·9)	12·7 (11·7 to 13·7)	9·8 (9 to 10·6)	6·8 (6·2 to 7·3)	4·8 (4·7 to 5)
Hammersmith and Fulham	32·6 (32·2 to 33)	25·3 (23·9 to 26·8)	20·1 (18·8 to 21·5)	14·4 (13·4 to 15·5)	11·6 (10·6 to 12·7)	7 (6·4 to 7·6)	4·7 (4·2 to 5·1)	3·3 (3·2 to 3·4)
Haringey	36·4 (35·9 to 36·8)	28·4 (26·2 to 30·6)	23·3 (21·5 to 25·4)	16·1 (15 to 17·3)	11·6 (10·6 to 12·5)	8·8 (8·1 to 9·6)	5·9 (5·5 to 6·3)	3·9 (3·8 to 4)
Harrow	25·9 (25·6 to 26·2)	21 (19·8 to 22·2)	17·1 (16 to 18·3)	11·4 (10·5 to 12·4)	8·7 (7·9 to 9·5)	7·3 (6·8 to 8)	6·5 (5·9 to 7·1)	4·9 (4·7 to 5)
Havering	26·2 (25·8 to 26·5)	21·9 (18·7 to 25·4)	18·8 (17·3 to 20·4)	12·3 (10·7 to 14·1)	7·6 (6·9 to 8·3)	5·2 (4·8 to 5·6)	4·3 (3·9 to 4·7)	2·9 (2·8 to 3)
Hillingdon	28·2 (27·8 to 28·5)	23·1 (21·5 to 24·5)	19·4 (17·9 to 21)	13·1 (12 to 14·3)	8·4 (7·7 to 9·3)	5·8 (5·3 to 6·2)	5 (4·6 to 5·5)	3·5 (3·4 to 3·6)
Hounslow	31·5 (31·1 to 31·9)	25 (21·3 to 29)	20·6 (18·8 to 22·4)	13·7 (11·8 to 15·8)	9·8 (8·9 to 10·6)	6·8 (6·3 to 7·4)	5·8 (5·3 to 6·3)	4·4 (4·3 to 4·6)
Islington	37·9 (37·4 to 38·3)	29·3 (27·6 to 31·2)	24·6 (22·6 to 26·6)	17·3 (15·8 to 18·9)	10·8 (9·9 to 11·8)	7·4 (6·7 to 8)	4·7 (4·3 to 5·1)	3 (2·9 to 3·1)
Kensington and Chelsea	34·6 (34·1 to 35)	26·8 (22·6 to 31·1)	21 (19·6 to 22·4)	15·4 (14 to 16·9)	9·9 (8·9 to 10·9)	6 (5·2 to 6·8)	4·2 (3·6 to 4·8)	3·5 (3·4 to 3·6)
Kingston upon Thames	25·8 (25·4 to 26·1)	20·5 (17·3 to 23·8)	16·6 (15·4 to 17·8)	11 (10·1 to 11·9)	7·6 (6·8 to 8·4)	4·6 (4 to 5·3)	4·3 (3·7 to 4·8)	3·9 (3·8 to 4)
Lambeth	44 (43·4 to 44·5)	34·7 (32·5 to 36·9)	29·3 (27·4 to 31·6)	20·8 (19·3 to 22·6)	12·3 (11·2 to 13·5)	9 (8·2 to 9·7)	6·7 (6·2 to 7·2)	4·6 (4·4 to 4·7)
Lewisham	36·9 (36·4 to 37·3)	29·4 (27·4 to 31·3)	24·7 (23 to 26·6)	16·7 (15·5 to 18·2)	11·3 (10·4 to 12·3)	8·6 (7·9 to 9·4)	5·9 (5·4 to 6·4)	3·9 (3·8 to 4)
Merton	32·8 (32·4 to 33·2)	26·1 (24·6 to 27·5)	21·5 (20·2 to 23)	14·4 (13·3 to 15·6)	9·3 (8·4 to 10·2)	5·7 (5·1 to 6·3)	4·8 (4·3 to 5·4)	3·5 (3·4 to 3·6)
Newham	39·4 (38·9 to 39·8)	31·9 (29·8 to 34)	26·1 (24·1 to 28·2)	17·4 (16·2 to 18·7)	12 (11 to 13·1)	9·4 (8·5 to 10·5)	6·4 (6 to 6·8)	4·2 (4·1 to 4·3)
Redbridge	28·7 (28·3 to 29)	23·2 (19·6 to 26·8)	18·9 (17·3 to 20·7)	12·5 (10·8 to 14·3)	9·1 (8·3 to 10·1)	6·6 (6 to 7·2)	5·2 (4·5 to 5·8)	3·7 (3·6 to 3·8)
Richmond upon Thames	26·8 (26·5 to 27·2)	21·3 (20·1 to 22·6)	17·6 (16·4 to 18·7)	11·9 (11 to 12·7)	7·6 (6·9 to 8·3)	4·6 (4·2 to 5)	3·6 (3·3 to 3·9)	2·7 (2·6 to 2·7)
Southwark	33·3 (32·9 to 33·7)	27·2 (25·3 to 29·2)	22·8 (21 to 24·6)	15·2 (13·8 to 16·8)	12·9 (11·9 to 14·1)	9·9 (9·1 to 10·6)	6·3 (5·9 to 6·8)	4 (3·9 to 4·1)
Sutton	30·3 (29·9 to 30·6)	23·8 (22·6 to 25·2)	19·7 (18·5 to 20·9)	12·9 (11·8 to 14·1)	8·2 (7·4 to 8·9)	5·5 (5 to 5·9)	4·5 (4·1 to 4·9)	3·6 (3·5 to 3·8)
Tower Hamlets	42·3 (41·8 to 42·8)	35 (32·4 to 37·7)	29·8 (27·5 to 32·2)	19·5 (18·1 to 20·9)	10·9 (10 to 12)	7·7 (7·1 to 8·3)	5·8 (5·3 to 6·4)	4·5 (4·4 to 4·6)
Waltham Forest	39·6 (39·1 to 40·1)	31·4 (29·4 to 33·3)	25·2 (23·3 to 27·1)	16·9 (15·6 to 18·3)	11·9 (11 to 13)	8·6 (8 to 9·3)	6·1 (5·7 to 6·6)	3·7 (3·6 to 3·8)
Wandsworth	35·1 (34·6 to 35·5)	27·9 (26·2 to 29·6)	23 (21·2 to 24·8)	16·1 (14·8 to 17·4)	10·7 (9·8 to 11·7)	6·2 (5·6 to 7)	4·5 (4 to 4·9)	3·2 (3·1 to 3·3)
Westminster	41·9 (41·4 to 42·5)	32·7 (27·8 to 38)	26·7 (24·4 to 29·2)	19·8 (17 to 22·7)	12·3 (11·3 to 13·4)	7·5 (6·9 to 8·2)	4·6 (4·3 to 5)	3·2 (3·1 to 3·3)
North East England	30·7 (30·3 to 31)	24·3 (20·2 to 28·8)	20·6 (17·8 to 23·6)	13·2 (12·2 to 14·3)	9·3 (8·9 to 9·8)	6·5 (6·1 to 6·8)	4·5 (4·2 to 4·8)	3·7 (3·6 to 3·8)
County Durham	29·9 (29·5 to 30·2)	23·5 (18·4 to 29·6)	19·9 (16·6 to 23·3)	12·8 (11·3 to 14·5)	8·6 (8·1 to 9·2)	6·6 (6·1 to 7·2)	4·6 (4·2 to 5·1)	4 (3·9 to 4·1)
Darlington	33·8 (33·3 to 34·2)	26·5 (21·2 to 32·4)	22·6 (19·4 to 26·2)	14·9 (13·1 to 16·6)	9·8 (8·7 to 11)	6·7 (5·8 to 7·7)	4·7 (4 to 5·5)	3·5 (3·4 to 3·7)
Gateshead	33·6 (33·2 to 34)	26·6 (22·2 to 31·2)	22·6 (19·4 to 26)	14·8 (13·6 to 16)	10·5 (9·6 to 11·5)	6·5 (5·6 to 7·4)	5 (4·4 to 5·7)	4·1 (4 to 4·3)
Hartlepool	38·7 (38·2 to 39·2)	30·4 (24·1 to 37·7)	26·3 (21·9 to 30·9)	17·2 (14·8 to 19·7)	10·7 (9·4 to 12)	7·2 (6·3 to 8·1)	5 (4·3 to 5·8)	3·8 (3·7 to 4)
Middlesbrough	36·1 (35·6 to 36·5)	29 (22·1 to 36·9)	24·8 (20·7 to 29·1)	15·6 (13·5 to 17·9)	11·1 (10 to 12·3)	7·9 (7·2 to 8·5)	5·6 (5 to 6·1)	4·7 (4·6 to 4·8)

Newcastle upon Tyne	28·6 (28·2 to 29)	22·5 (19 to 26·4)	18·6 (16 to 21·3)	11·8 (10·5 to 13·2)	9·3 (8·5 to 10·2)	6·3 (5·7 to 6·8)	4·5 (4·1 to 4·9)	3·8 (3·7 to 3·9)
North Tyneside	27·2 (26·9 to 27·5)	21·6 (18·1 to 25·4)	18·4 (15·8 to 21·3)	11·7 (10·4 to 13·2)	8·9 (8·3 to 9·5)	5·7 (5·2 to 6·2)	3·8 (3·4 to 4·2)	3 (2·9 to 3)
Northumberland	28·9 (28·6 to 29·3)	22·5 (19 to 26·4)	18·9 (16·3 to 21·9)	12 (10·8 to 13·3)	8·8 (8 to 9·6)	5·9 (5·2 to 6·6)	4 (3·6 to 4·4)	3·1 (3 to 3·2)
Redcar and Cleveland	32·7 (32·3 to 33·1)	25·6 (21·2 to 30·6)	21·8 (18·3 to 25·6)	14·1 (11·7 to 16·9)	9·2 (8·4 to 10)	5·9 (5·2 to 6·6)	4·1 (3·5 to 4·7)	3·3 (3·2 to 3·4)
South Tyneside	26·3 (26 to 26·6)	20·9 (17·5 to 24·7)	18 (15·3 to 20·7)	11·5 (10·1 to 12·9)	7·8 (7·2 to 8·4)	5·8 (5·3 to 6·3)	3·9 (3·5 to 4·3)	2·9 (2·9 to 3)
Stockton-on-Tees	30·6 (30·2 to 30·9)	24·2 (18·5 to 31·1)	20·8 (17·4 to 24·5)	13·5 (11·6 to 15·6)	9·4 (8·7 to 10·1)	6·6 (6·1 to 7·3)	4·8 (4·4 to 5·2)	4·1 (4 to 4·2)
Sunderland	30·8 (30·4 to 31·2)	24·5 (20·8 to 29)	20·7 (18 to 24)	13·1 (11·9 to 14·4)	9·8 (9·2 to 10·5)	6·8 (6·3 to 7·3)	4·4 (4 to 4·8)	3·5 (3·4 to 3·6)
North West England	33·6 (33·1 to 34)	26·2 (25·3 to 27·3)	21·8 (20·9 to 22·7)	14·5 (13·8 to 15·2)	9·7 (9·4 to 10·1)	7·1 (6·7 to 7·4)	5·6 (5·4 to 5·9)	4·9 (4·8 to 5)
Blackburn with Darwen	36·8 (36·3 to 37·2)	29·3 (25·9 to 32·7)	24 (22·2 to 26·1)	15·5 (13·8 to 17·5)	13·2 (12·2 to 14·3)	10·1 (9·3 to 11)	8 (7·4 to 8·7)	6·3 (5·9 to 6·7)
Blackpool	35·6 (35·1 to 36)	28·1 (25·6 to 30·7)	23·1 (21·4 to 24·9)	15·1 (13·7 to 16·6)	10·4 (9·3 to 11·6)	8·4 (7·6 to 9·2)	7 (6·3 to 7·7)	6·1 (5·7 to 6·5)
Bolton	36·9 (36·5 to 37·4)	29·1 (26·5 to 31·9)	24 (22 to 26·2)	15·7 (14·2 to 17·2)	11 (10 to 12)	7·6 (6·8 to 8·4)	5·9 (5·4 to 6·5)	4·6 (4·4 to 4·9)
Bury	35·1 (34·7 to 35·6)	27·2 (25 to 29·7)	22·6 (20·8 to 24·4)	15·3 (13·8 to 16·9)	9·9 (9 to 10·8)	6·1 (5·6 to 6·7)	4·8 (4·4 to 5·4)	4·3 (4 to 4·5)
Cheshire East	29·8 (29·4 to 30·1)	23·2 (21·1 to 25·5)	19·4 (17·9 to 21)	13·2 (12·1 to 14·4)	8·6 (7·9 to 9·2)	5·6 (5·1 to 6)	4·4 (4 to 4·8)	3·9 (3·7 to 4·1)
Cheshire West and Chester	26·3 (25·9 to 26·6)	20·6 (18·9 to 22·3)	17·4 (16 to 18·9)	11·7 (10·8 to 12·7)	7·7 (7·2 to 8·4)	5·5 (4·9 to 6)	4·9 (4·4 to 5·4)	4 (3·8 to 4·2)
Cumbria	34 (33·6 to 34·4)	26·4 (23·4 to 29·2)	22·1 (20·2 to 24·2)	14·7 (13·1 to 16·3)	9·1 (8·5 to 9·7)	5·6 (5·2 to 6·1)	4·4 (4·1 to 4·7)	3·6 (3·4 to 3·8)
Halton	30·5 (30·1 to 30·8)	24·6 (22·3 to 26·9)	20·6 (19·2 to 22·2)	13·5 (12·3 to 14·9)	9·4 (8·5 to 10·5)	7·3 (6·7 to 8)	5·5 (5 to 6·1)	4·3 (4 to 4·6)
Knowsley	33·5 (33·1 to 33·9)	27·1 (23·3 to 30·9)	23·9 (22·1 to 25·6)	15·8 (14·4 to 17·5)	8·8 (8 to 9·5)	6·7 (6 to 7·5)	4·7 (4·1 to 5·4)	4 (3·7 to 4·2)
Lancashire	34 (33·5 to 34·4)	26·2 (23·7 to 28·8)	21·5 (19·5 to 23·6)	14·2 (12·8 to 15·7)	10·3 (9·6 to 11)	7·2 (6·6 to 7·9)	6 (5·4 to 6·6)	5·2 (4·5 to 6)
Liverpool	32·5 (32·1 to 32·9)	25·6 (22·5 to 28·8)	21·1 (19·2 to 23·3)	14 (12·6 to 15·7)	8·6 (8·1 to 9·2)	7·3 (6·8 to 7·9)	5·9 (5·5 to 6·4)	5·6 (5·3 to 5·9)
Manchester	40·2 (39·7 to 40·7)	31·5 (27·1 to 36)	26·2 (23·9 to 28·5)	17·5 (15·7 to 19·6)	12·2 (11·1 to 13·2)	9·8 (9·1 to 10·5)	7·1 (6·6 to 7·7)	6·3 (5·4 to 7·3)
Oldham	38·9 (38·4 to 39·4)	30·7 (27·8 to 33·8)	25·3 (23·3 to 27·3)	16·7 (15·1 to 18·5)	11·3 (10·4 to 12·3)	8·5 (7·8 to 9·2)	7·3 (6·8 to 7·9)	6·3 (6 to 6·6)
Rochdale	39·9 (39·4 to 40·4)	31·8 (28·9 to 35·1)	26·7 (24·7 to 28·8)	17·5 (15·9 to 19·3)	11·3 (10·5 to 12·2)	8·1 (7·5 to 8·8)	5·9 (5·4 to 6·5)	5·5 (5·2 to 5·8)
Salford	37 (36·5 to 37·4)	28·9 (26·5 to 31·5)	24 (22·1 to 25·9)	16 (14·5 to 17·5)	10 (9·3 to 10·7)	7·3 (6·5 to 8·1)	5·9 (5·4 to 6·5)	4·9 (4·7 to 5·2)
Sefton	27·1 (26·8 to 27·5)	21·2 (19·2 to 23·3)	17·8 (16·2 to 19·4)	11·8 (10·7 to 13)	8·4 (7·7 to 9·2)	6·1 (5·7 to 6·6)	5 (4·6 to 5·5)	4·3 (4·1 to 4·6)
St Helens	29 (28·6 to 29·3)	23·1 (20·4 to 25·8)	19·6 (18·1 to 21·2)	13·3 (11·7 to 15·1)	8·9 (8·1 to 9·8)	7·3 (6·5 to 8)	4·7 (4·2 to 5·1)	3·9 (3·6 to 4·1)
Stockport	27·8 (27·4 to 28·1)	21·7 (19·7 to 23·8)	18·2 (16·7 to 19·9)	12·3 (11·1 to 13·5)	8·6 (8 to 9·4)	6 (5·6 to 6·5)	4·8 (4·4 to 5·3)	4·5 (4·2 to 4·7)
Tameside	36·9 (36·4 to 37·3)	29·2 (26·6 to 31·9)	24 (22·3 to 25·7)	15·8 (14·3 to 17·5)	9·3 (8·4 to 10·2)	6·4 (5·9 to 7)	5·1 (4·6 to 5·5)	4·1 (3·9 to 4·4)
Trafford	34·3 (33·9 to 34·7)	26·8 (24·4 to 29·3)	22·9 (21·2 to 24·5)	15·8 (14·3 to 17·6)	9·6 (8·7 to 10·6)	6 (5·5 to 6·5)	4·3 (3·9 to 4·7)	4·2 (3·9 to 4·4)
Warrington	26·9 (26·5 to 27·2)	21·2 (19·3 to 23·2)	17·6 (16·3 to 18·9)	11·9 (10·8 to 13·1)	9·5 (8·8 to 10·4)	6·5 (5·9 to 7·1)	5·1 (4·6 to 5·7)	4·2 (3·9 to 4·4)
Wigan	29·7 (29·3 to 30·1)	23·4 (21·3 to 25·5)	19·1 (17·7 to 20·6)	12·6 (11·5 to 13·9)	8·5 (7·9 to 9·2)	6·6 (6·2 to 7·1)	4·8 (4·4 to 5·2)	4·1 (3·8 to 4·3)
Wirral	33·8 (33·4 to 34·2)	26·3 (24 to 28·5)	21·9 (20·2 to 23·7)	14·4 (13·1 to 15·8)	9·3 (8·4 to 10·3)	6·5 (5·9 to 7)	5·4 (5 to 5·9)	4·9 (4·6 to 5·1)
South East England	31·2 (30·8 to 31·5)	23·2 (22·3 to 24·1)	18·6 (17·8 to 19·4)	12·3 (11·7 to 12·9)	8·5 (8·2 to 8·8)	5·2 (4·9 to 5·4)	4·2 (4·1 to 4·4)	3·7 (3·6 to 3·8)
Bracknell Forest	25·5 (25·2 to 25·8)	19·1 (16·1 to 22·1)	15·8 (14·7 to 17)	10·5 (9·2 to 11·9)	7·3 (6·4 to 8·2)	4·7 (4 to 5·4)	3·7 (3·2 to 4·3)	2·5 (2·3 to 2·6)
Brighton and Hove	36·7 (36·3 to 37·2)	27 (23·7 to 30·1)	21·5 (19·5 to 23·4)	14·5 (12·7 to 16·4)	10·2 (9·3 to 11·1)	6·7 (6 to 7·4)	5·1 (4·7 to 5·6)	4 (3·7 to 4·2)
Buckinghamshire	24·8 (24·5 to 25·1)	18·8 (17·1 to 20·8)	15·2 (14 to 16·5)	10·1 (9·1 to 11·3)	8·5 (7·9 to 9·2)	5·5 (4·9 to 6)	4·8 (4·4 to 5·2)	4·2 (4 to 4·5)
East Sussex	32·3 (31·9 to 32·7)	23·6 (21·5 to 26)	18·3 (16·6 to 20·1)	11·7 (10·6 to 12·9)	8·5 (7·9 to 9·2)	5·6 (5·1 to 6·1)	4·7 (4·3 to 5)	3·9 (3·6 to 4·1)
Hampshire	32·1 (31·7 to 32·5)	23·9 (22 to 25·8)	19·5 (17·9 to 21·2)	13·3 (12·1 to 14·5)	8 (7·4 to 8·6)	4·6 (4·2 to 5·1)	3·8 (3·5 to 4·1)	3·7 (3·2 to 4·3)
Isle of Wight	43 (42·4 to 43·5)	31·2 (26·7 to 36·1)	24·2 (22·4 to 26·1)	15·5 (13·7 to 17·4)	10·4 (9·2 to 11·6)	4·7 (4 to 5·5)	3·2 (2·7 to 3·7)	2·6 (2·4 to 2·8)
Kent	32·9 (32·5 to 33·3)	24·4 (21·8 to 27·1)	19·3 (17·5 to 21·1)	12·4 (11·2 to 13·6)	9 (8·5 to 9·6)	4·9 (4·5 to 5·4)	4 (3·7 to 4·4)	3·8 (3·3 to 4·4)
Medway	35·3 (34·9 to 35·8)	26·9 (24·4 to 29·5)	21·5 (19·7 to 23·7)	14·2 (12·8 to 15·5)	10·1 (9·3 to 10·8)	6·4 (5·9 to 7)	4·5 (4·1 to 5)	3·9 (3·6 to 4·1)
Milton Keynes	32·9 (32·5 to 33·3)	25·1 (22·2 to 28)	20·2 (18·5 to 22·1)	13·2 (11·6 to 14·9)	10·1 (9·3 to 11)	7·2 (6·6 to 7·9)	5·8 (5·2 to 6·4)	5 (4·7 to 5·3)
Oxfordshire	25·1 (24·8 to 25·4)	18·6 (17 to 20·5)	15·1 (13·9 to 16·4)	10·2 (9·3 to 11·2)	7·5 (7 to 8·1)	4·9 (4·5 to 5·4)	4·4 (4·1 to 4·8)	3·7 (3·5 to 3·9)
Portsmouth	38·3 (37·8 to 38·8)	28·5 (25·3 to 32)	22·3 (20·4 to 24·4)	14·3 (12·6 to 16·3)	10·4 (9·6 to 11·4)	6·6 (6 to 7·3)	4·2 (3·8 to 4·7)	3·5 (3·3 to 3·7)
Reading	30·7 (30·3 to 31·1)	23·2 (21 to 25·6)	18·8 (17·4 to 20·4)	12·7 (11·2 to 14·4)	9·5 (8·6 to 10·5)	7·5 (6·8 to 8·3)	5·6 (5·1 to 6·3)	4·2 (3·9 to 4·5)
Slough	33·7 (33·3 to 34·1)	26·9 (24·3 to 29·6)	22·1 (20·6 to 23·9)	14·2 (12·6 to 16)	10·2 (9·2 to 11·3)	6·7 (5·9 to 7·5)	6·1 (5·6 to 6·8)	5·5 (5·2 to 5·9)
Southampton	37·9 (37·4 to 38·3)	28·8 (25·9 to 31·8)	22·7 (21 to 24·5)	14·8 (13·3 to 16·4)	10·2 (9·3 to 11·2)	6·1 (5·6 to 6·7)	4·9 (4·4 to 5·4)	4·2 (3·9 to 4·5)
Surrey	29·5 (29·1 to 29·9)	22·3 (20·7 to 24)	18·4 (17 to 19·8)	12·5 (11·3 to 13·8)	7·7 (7·1 to 8·3)	4·3 (4 to 4·7)	3·5 (3·2 to 3·7)	2·9 (2·8 to 3·1)
West Berkshire	22·6 (22·3 to 22·9)	17·1 (15·6 to 18·7)	13·9 (12·9 to 14·9)	9·1 (8 to 10·3)	7·6 (6·7 to 8·5)	5·1 (4·5 to 5·6)	4 (3·6 to 4·5)	3·5 (3·3 to 3·8)

West Sussex	32·1 (31·7 to 32·5)	23·4 (20·8 to 26·1)	18·6 (16·8 to 20·6)	12 (10·7 to 13·4)	7·9 (7·3 to 8·5)	4·7 (4·3 to 5·1)	4·1 (3·7 to 4·4)	3·3 (3·1 to 3·5)
Windsor and Maidenhead	22·9 (22·6 to 23·2)	17·5 (14·9 to 20·3)	14·3 (13·3 to 15·3)	9·6 (8·6 to 10·9)	7·2 (6·4 to 8·1)	5·2 (4·6 to 5·9)	4 (3·5 to 4·6)	3·6 (3·3 to 3·8)
Wokingham	21·5 (21·2 to 21·7)	16·1 (14·1 to 18·1)	13·2 (12·2 to 14·2)	9·2 (8 to 10·3)	6·8 (6 to 7·6)	4·2 (3·6 to 4·8)	3·8 (3·3 to 4·4)	3·7 (3·4 to 3·9)
South West England	30·7 (30·3 to 31)	23·2 (21·6 to 24·9)	19·1 (17·6 to 20·7)	12·7 (11·9 to 13·4)	8·3 (8 to 8·6)	5·6 (5·3 to 5·9)	4·4 (4·1 to 4·6)	3·4 (3·3 to 3·5)
Bath and North East Somerset	30·1 (29·7 to 30·5)	22·9 (19·1 to 27)	19·2 (17·3 to 21·2)	13 (11·6 to 14·3)	7·6 (6·9 to 8·4)	4·7 (4·2 to 5·4)	3·2 (2·8 to 3·7)	2·5 (2·4 to 2·6)
Bournemouth	29·1 (28·7 to 29·5)	21·5 (18·1 to 25·1)	17·2 (15·5 to 19·1)	11·4 (9·9 to 13·1)	7·5 (6·8 to 8·4)	5·8 (5·2 to 6·6)	3·9 (3·6 to 4·4)	3·2 (3·1 to 3·3)
Bristol, City of	35·1 (34·6 to 35·5)	26·9 (24·3 to 29·8)	22·4 (20·3 to 24·7)	15·2 (13·9 to 16·3)	9·7 (9·1 to 10·4)	6·5 (6 to 6·9)	4·5 (4·1 to 4·8)	3·5 (3·4 to 3·6)
Cornwall	30·3 (29·9 to 30·6)	22·9 (20·7 to 25·4)	18·6 (16·8 to 20·5)	12·1 (10·9 to 13·3)	8·2 (7·6 to 8·8)	5·7 (5·1 to 6·2)	5 (4·6 to 5·4)	3·8 (3·7 to 4)
Devon	31·2 (30·8 to 31·6)	23·3 (20·1 to 26·7)	19 (16·8 to 21·3)	12·5 (11·4 to 13·7)	8·8 (8·1 to 9·6)	5·3 (4·8 to 5·8)	4·4 (3·9 to 5)	3·4 (3·3 to 3·5)
Dorset	29·9 (29·5 to 30·2)	22·3 (20·3 to 24·6)	18·2 (16·4 to 20·2)	12 (10·7 to 13·3)	7·3 (6·8 to 7·9)	5·2 (4·8 to 5·6)	4·3 (3·9 to 4·6)	3·4 (3·3 to 3·5)
Gloucestershire	29 (28·6 to 29·3)	22·2 (19·1 to 25·4)	18·5 (16·5 to 20·7)	12·4 (11 to 13·7)	8·2 (7·6 to 8·8)	5·5 (5·1 to 6)	4·2 (3·9 to 4·5)	3·3 (3·2 to 3·4)
North Somerset	30·5 (30·1 to 30·9)	22·8 (20·4 to 25·3)	18·9 (17 to 20·8)	12·8 (11·3 to 14·2)	7·5 (6·9 to 8·2)	5·8 (5·3 to 6·4)	4·3 (3·9 to 4·8)	3·1 (3 to 3·2)
Plymouth	32·8 (32·4 to 33·2)	25·3 (23·2 to 27·8)	20·9 (18·9 to 23·1)	13·9 (12·6 to 15·1)	8·8 (8·1 to 9·6)	6·6 (6·1 to 7·2)	5·3 (4·8 to 5·7)	3·8 (3·6 to 3·9)
Poole	29·7 (29·3 to 30·1)	22·7 (19 to 26·9)	18·6 (16·8 to 20·5)	12·3 (11 to 13·6)	8 (7·2 to 9)	5·6 (5 to 6·4)	4·1 (3·6 to 4·7)	3 (2·9 to 3·1)
Somerset	27·6 (27·2 to 27·9)	20·9 (19·1 to 22·9)	17·3 (15·4 to 19·3)	11·3 (10·2 to 12·5)	7·1 (6·5 to 7·7)	5·2 (4·8 to 5·6)	4·4 (4 to 4·7)	3·4 (3·3 to 3·5)
South Gloucestershire	25·7 (25·4 to 26·1)	19·8 (16·8 to 22·7)	16·7 (14·9 to 18·6)	11·3 (9·8 to 12·9)	7·8 (7·2 to 8·4)	5 (4·7 to 5·4)	3·5 (3·2 to 3·7)	2·9 (2·8 to 3)
Swindon	37·4 (36·9 to 37·8)	28·8 (26·4 to 31·7)	24·8 (22·4 to 27·5)	16·5 (15 to 18·1)	9·3 (8·5 to 10·2)	6·1 (5·6 to 6·7)	4·3 (3·9 to 4·6)	3·2 (3·1 to 3·3)
Torbay	29·5 (29·1 to 29·8)	22·3 (18·7 to 26·3)	17·7 (16 to 19·5)	11·4 (10·2 to 12·8)	8·4 (7·5 to 9·4)	5·9 (5·1 to 6·8)	5·2 (4·6 to 6)	3·9 (3·7 to 4)
Wiltshire	30·1 (29·7 to 30·5)	22·7 (20·5 to 25·1)	18·6 (16·9 to 20·5)	12·2 (11·2 to 13·2)	8·6 (7·8 to 9·4)	5·3 (4·8 to 5·9)	4·3 (4 to 4·7)	3·3 (3·2 to 3·4)
West Midlands	32·2 (31·8 to 32·6)	26·1 (25·1 to 27·1)	22·4 (21·4 to 23·4)	15 (14·3 to 15·6)	11·4 (10·9 to 11·9)	7·7 (7·4 to 8)	6·5 (6·2 to 6·8)	5·8 (5·7 to 6)
Birmingham	37·2 (36·7 to 37·6)	30 (27·2 to 32·7)	25·8 (24·1 to 27·6)	17·1 (15·9 to 18·5)	14 (13·2 to 14·8)	10·3 (9·5 to 11·2)	8·4 (7·8 to 9·1)	8 (7·1 to 8·9)
Coventry	36·5 (36 to 37)	29·7 (26·7 to 32·9)	26·2 (23·6 to 29)	17·9 (16 to 20)	12·9 (11·8 to 14·1)	8·3 (7·7 to 9)	6·1 (5·6 to 6·6)	5·1 (4·7 to 5·5)
Dudley	27·4 (27·1 to 27·7)	22·1 (19·9 to 24·3)	19 (17·3 to 20·9)	12·5 (11·2 to 14)	10·9 (10 to 11·9)	6·7 (6 to 7·4)	5·5 (4·9 to 6·1)	4·9 (4·5 to 5·3)
Herefordshire, County of	33·3 (32·9 to 33·7)	25·5 (22·6 to 28·4)	22·2 (20·2 to 24·3)	14·9 (13·1 to 16·8)	8·7 (7·8 to 9·7)	4·9 (4·4 to 5·5)	4·3 (3·8 to 4·8)	3·9 (3·6 to 4·3)
Sandwell	40·5 (40 to 41)	33·3 (29·9 to 37·2)	29·2 (26·5 to 32·2)	19·5 (17·5 to 21·7)	12·8 (11·7 to 13·9)	8·7 (8 to 9·5)	7·7 (6·9 to 8·5)	6·5 (6·1 to 7)
Shropshire	27·6 (27·2 to 27·9)	21·5 (19·2 to 24)	18·4 (16·7 to 20·2)	12·4 (11·1 to 13·6)	8·8 (7·9 to 9·7)	5·6 (5·2 to 6)	4·4 (4 to 4·8)	4 (3·6 to 4·3)
Solihull	28·1 (27·7 to 28·4)	22·8 (20·6 to 24·9)	20·4 (18·5 to 22·5)	14·4 (13 to 15·9)	9·4 (8·5 to 10·3)	5·5 (5·1 to 6·1)	4·8 (4·3 to 5·4)	4·7 (4·3 to 5·1)
Staffordshire	29·1 (28·8 to 29·5)	23·1 (21 to 25·4)	20·1 (18·8 to 21·4)	13·6 (12·4 to 14·9)	9·8 (8·8 to 10·7)	6·4 (6 to 6·9)	5·7 (5·3 to 6·2)	5·2 (4·5 to 6)
Stoke-on-Trent	38·7 (38·2 to 39·1)	31·8 (28·8 to 35·1)	27·4 (24·8 to 30)	18·2 (16·6 to 19·9)	12·9 (11·9 to 14)	9·6 (8·8 to 10·3)	7·9 (7·3 to 8·7)	7·3 (6·8 to 7·8)
Telford and Wrekin	32·1 (31·7 to 32·4)	25·6 (22·8 to 28·4)	22·1 (20·2 to 24·1)	15 (13·2 to 17)	11·7 (10·7 to 12·9)	7·5 (6·8 to 8·2)	6 (5·3 to 6·8)	4·9 (4·5 to 5·4)
Walsall	33 (32·6 to 33·4)	27 (24·3 to 29·8)	23·7 (21·6 to 25·8)	16 (14·4 to 17·7)	11·3 (10·4 to 12·3)	8·2 (7·5 to 8·9)	8·2 (7·5 to 9)	6·8 (6·2 to 7·3)
Warwickshire	24·9 (24·6 to 25·2)	19·8 (17·8 to 21·8)	17·1 (15·6 to 18·8)	11·5 (10·5 to 12·6)	9·4 (8·7 to 10·2)	5·6 (5·2 to 6·1)	4·7 (4·4 to 5·1)	4 (3·7 to 4·3)
Wolverhampton	32·1 (31·7 to 32·5)	26·3 (23·7 to 28·9)	23 (21 to 25·3)	15·2 (13·7 to 16·7)	12·5 (11·5 to 13·6)	8·7 (8·1 to 9·4)	7·2 (6·6 to 7·7)	6 (5·6 to 6·5)
Worcestershire	25·7 (25·3 to 26)	20·4 (18·4 to 22·5)	17·4 (15·9 to 19)	11·6 (10·4 to 12·7)	9·5 (8·8 to 10·2)	6·2 (5·7 to 6·7)	4·7 (4·3 to 5·1)	4·1 (3·8 to 4·5)
Yorkshire and the Humber	34·4 (33·9 to 34·8)	27·3 (25·6 to 29·1)	22·8 (21·8 to 23·7)	15 (14·1 to 16)	10·3 (9·9 to 10·6)	7·7 (7·3 to 8)	5·9 (5·6 to 6·2)	4·4 (4·2 to 4·5)
Barnsley	34·4 (34 to 34·8)	27·6 (25·2 to 30·1)	23 (21 to 24·9)	14·7 (13·4 to 16·2)	10·2 (9·4 to 11)	7·1 (6·4 to 7·7)	5 (4·6 to 5·5)	3·4 (3·2 to 3·6)
Bradford	41·9 (41·3 to 42·4)	33·3 (28·7 to 38·7)	27·8 (25·4 to 30·3)	18 (16·1 to 20)	13·2 (12·2 to 14·3)	10·9 (9·9 to 11·9)	8·6 (7·9 to 9·2)	6·3 (5·4 to 7·3)
Calderdale	37·8 (37·3 to 38·3)	29·9 (27·2 to 32·7)	24·8 (22·7 to 27)	16·1 (14·4 to 18)	12·2 (11·1 to 13·4)	8·7 (8 to 9·4)	6·9 (6·3 to 7·6)	5 (4·8 to 5·3)
Doncaster	30·8 (30·4 to 31·2)	25 (22·6 to 27·6)	21·3 (19·4 to 23·2)	13·7 (12·1 to 15·5)	9·7 (9 to 10·5)	7·7 (6·8 to 8·5)	6·1 (5·5 to 6·8)	4·7 (4·5 to 4·9)
East Riding of Yorkshire	27·7 (27·3 to 28)	21·7 (19·4 to 24·3)	18 (16·4 to 19·8)	11·8 (10·6 to 13·2)	8·2 (7·5 to 8·9)	6 (5·5 to 6·5)	4·3 (3·9 to 4·6)	3 (2·8 to 3·1)
Kingston upon Hull, City of	38·4 (37·9 to 38·9)	31·1 (28·4 to 34)	25·6 (23·8 to 27·6)	16·5 (14·8 to 18·5)	10·9 (10 to 11·8)	7·5 (6·7 to 8·5)	5·7 (5·2 to 6·3)	4·5 (4·3 to 4·8)
Kirklees	38·5 (38 to 38·9)	30·2 (26·3 to 34)	25·1 (23 to 27·2)	16·6 (15 to 18·2)	10·9 (10·1 to 11·7)	8·5 (7·9 to 9·2)	6·8 (6·4 to 7·2)	5·2 (5 to 5·4)
Leeds	34·2 (33·7 to 34·6)	27 (24·8 to 29·3)	22·6 (20·8 to 24·4)	15·1 (13·5 to 16·9)	10·4 (9·6 to 11·3)	7·4 (6·9 to 7·9)	5·5 (5·1 to 5·8)	4·1 (3·9 to 4·2)
North East Lincolnshire	31·5 (31·1 to 31·9)	25 (21·6 to 28·6)	21·2 (19·5 to 22·9)	14·1 (12·5 to 15·8)	8·5 (7·7 to 9·4)	7 (6·4 to 7·6)	5·7 (5·1 to 6·2)	4·4 (4·2 to 4·7)
North Lincolnshire	30·3 (29·9 to 30·6)	24·3 (21·6 to 27·3)	20·5 (19·1 to 22)	13·5 (12 to 15·1)	10 (9·1 to 11)	7·2 (6·5 to 7·8)	5 (4·5 to 5·6)	3·8 (3·6 to 4)
North Yorkshire	32 (31·6 to 32·4)	24·3 (21·2 to 27·3)	20·2 (18·6 to 22)	13·6 (12·2 to 15·2)	8·1 (7·5 to 8·7)	5·1 (4·6 to 5·6)	4·1 (3·7 to 4·5)	3 (2·8 to 3·1)
Rotherham	33·1 (32·7 to 33·5)	26·7 (24·4 to 29·3)	22·4 (20·9 to 24·2)	14·6 (13·2 to 16·2)	10·1 (9·4 to 10·9)	7·6 (7 to 8·1)	5·9 (5·4 to 6·3)	4·1 (3·9 to 4·3)
Sheffield	32 (31·5 to 32·3)	25·3 (21·9 to 28·8)	20·8 (18·8 to 22·9)	13·7 (12·3 to 15·1)	10·2 (9·6 to 10·8)	8 (7·4 to 8·5)	5·9 (5·6 to 6·2)	4·5 (4·3 to 4·7)

Wakefield	35·7 (35·2 to 36·1)	28·8 (26·3 to 31·5)	24·4 (22·6 to 26·2)	16·1 (14·6 to 17·8)	9·8 (9 to 10·7)	7·5 (6·9 to 8·1)	5·5 (5·1 to 5·9)	3·8 (3·6 to 4)
York	29·7 (29·3 to 30·1)	23·2 (20·7 to 26)	19·5 (18·2 to 20·9)	13·3 (11·8 to 15)	8·9 (8 to 9·8)	6·7 (6 to 7·4)	4·9 (4·4 to 5·4)	3·6 (3·4 to 3·8)
Northern Ireland	45·1 (43·4 to 46·9)	31·1 (29·9 to 32·3)	26·2 (25·2 to 27·3)	17·6 (16·8 to 18·5)	9·9 (9·4 to 10·5)	6·5 (5·9 to 7)	5·7 (5·3 to 6·1)	4·6 (3·8 to 5·5)
Scotland	42·9 (41·9 to 43·9)	30·5 (29·8 to 31·2)	23·2 (22·7 to 23·9)	14·4 (13·9 to 14·9)	9·3 (9 to 9·7)	6·4 (6·1 to 6·7)	4·5 (4·3 to 4·7)	3·5 (3·1 to 3·9)
Wales	38·3 (37·8 to 38·7)	29·1 (24·5 to 34·5)	23·7 (20·1 to 27·8)	15·2 (14·1 to 16·4)	8·7 (8·3 to 9·2)	6·3 (6 to 6·7)	4·8 (4·5 to 5)	3·7 (3·2 to 4·2)
Latin America and Caribbean	234·6 (211·7 to 262·2)	176·7 (165·7 to 188·3)	132·3 (126·1 to 138·8)	90·1 (86·2 to 94·1)	57·2 (54·9 to 59·6)	37·3 (35·4 to 39·2)	28·8 (26·4 to 31)	19 (16·2 to 22·3)
Andean Latin America	359·7 (334·4 to 387·9)	268·3 (251·4 to 286)	186·1 (176 to 196·4)	116·7 (109·2 to 125·4)	72·9 (68·9 to 77)	44·8 (42·1 to 47·5)	27·7 (25·3 to 30·2)	18·6 (16·5 to 21·1)
Bolivia	392 (354 to 432·8)	318·9 (288·7 to 352·1)	231·6 (213·9 to 249·5)	157·3 (147·7 to 168)	109 (102·3 to 115·7)	70·4 (65·4 to 75·8)	44 (39·2 to 48·8)	29·5 (25·2 to 35·1)
Ecuador	218·3 (190·8 to 248)	163·3 (149·6 to 178·6)	122·6 (112·5 to 132·9)	78·7 (72·3 to 85·3)	43·5 (39·6 to 47·5)	34·7 (30·3 to 39·4)	22·9 (19·4 to 26·7)	15·1 (12·7 to 18·1)
Peru	400 (361·4 to 445·3)	295·4 (268·3 to 321·4)	199·6 (183·9 to 215·7)	119·6 (108·3 to 131·5)	73·1 (66·4 to 80)	39·6 (35·8 to 43·7)	22·9 (20·1 to 25·7)	15·1 (12·6 to 18·1)
Caribbean	154·5 (144·4 to 166·2)	129·1 (123·2 to 135·2)	102·9 (97·9 to 108·5)	87·2 (82·4 to 92·2)	67·9 (64·4 to 71·8)	50·9 (47·6 to 54·3)	75·1 (70·2 to 80·4)	38·8 (33·2 to 45·3)
Antigua and Barbuda	116·5 (99·5 to 138·2)	76·7 (65·7 to 89·4)	39·9 (34 to 46·6)	22·9 (19·2 to 27·1)	14·1 (11·8 to 16·6)	14·3 (12·1 to 16·8)	12 (10·1 to 14·3)	10·3 (8·6 to 12·3)
The Bahamas	117 (99·5 to 137·5)	81·5 (68·5 to 95·5)	48·2 (40·4 to 56·7)	33·4 (28·2 to 38·8)	21·8 (18·1 to 25·7)	15·4 (12·9 to 18·1)	13·2 (11·2 to 15·4)	11·7 (9·9 to 14·1)
Barbados	152·4 (130·9 to 179·6)	86·7 (73·7 to 100·5)	46·2 (39·6 to 54·1)	26 (21·9 to 30·6)	18·2 (15·5 to 21·2)	15 (12·6 to 17·8)	13·7 (11·5 to 16·4)	12·1 (10·2 to 14·5)
Belize	161·8 (139·5 to 187·2)	119·8 (103 to 139·2)	93 (82·6 to 104·3)	63·6 (57·1 to 70·4)	41·5 (37·4 to 45·7)	28 (24·9 to 31·5)	19·9 (17·1 to 22·8)	15·4 (13 to 18·6)
Bermuda	66·5 (60·4 to 73)	42·1 (38·5 to 45·8)	28·3 (25·8 to 31)	17·7 (15·7 to 19·8)	10·6 (9·2 to 12)	5·7 (4·9 to 6·7)	5·3 (4·5 to 6·2)	4·7 (3·9 to 5·5)
Cuba	66·3 (55·9 to 78·6)	47 (46 to 48)	43·3 (42·5 to 44)	24·2 (23·5 to 24·9)	13·7 (13·2 to 14·1)	8·5 (8·2 to 8·9)	5·8 (5·5 to 6·1)	4·7 (4·1 to 5·4)
Dominica	237·3 (206·9 to 274·8)	141·1 (122 to 162·3)	67·8 (58·2 to 78·6)	27·8 (23·6 to 32·8)	20·4 (17·1 to 23·8)	19·5 (16·6 to 22·9)	22·9 (19·3 to 26·9)	26 (21·8 to 31)
Dominican Republic	193·5 (171·2 to 217·7)	166·8 (151·7 to 182·8)	126·5 (116 to 139)	85·9 (77·9 to 94·5)	63·7 (57·7 to 70·4)	41·4 (37·2 to 46·1)	31·6 (27·5 to 36·1)	25·2 (21·1 to 30·2)
Grenada	141 (121·1 to 163·3)	101·3 (86·3 to 116·6)	65·9 (56 to 76·6)	44·6 (37·3 to 53)	25·7 (21·5 to 30·7)	18·1 (15·2 to 21·2)	15·3 (12·8 to 18)	13·8 (11·6 to 16·5)
Guyana	160·5 (143·1 to 179·3)	111 (99·3 to 124·3)	84·4 (76 to 93·8)	61·1 (53·9 to 69·6)	51·8 (47 to 57)	38·7 (34·9 to 42·7)	31·1 (27·1 to 35·3)	23·1 (19·4 to 27·4)
Haiti	289·3 (258·7 to 326·2)	255·9 (236·4 to 276·3)	225·8 (209·1 to 244·4)	189·3 (174·9 to 204·1)	148·3 (137·5 to 159·7)	103·8 (95 to 112·9)	163·5 (152·3 to 175·1)	68·3 (58·7 to 79·5)
Jamaica	128·7 (114·2 to 144·8)	83·5 (76·1 to 91·3)	55·7 (50·3 to 61·3)	37·4 (33·5 to 41·8)	28·7 (25·1 to 32·4)	21·5 (18·5 to 24·8)	18·2 (15·3 to 21·4)	15·7 (13·1 to 18·7)
Puerto Rico	111·5 (109·5 to 113·7)	56·4 (54·8 to 57·9)	32·8 (31·8 to 33·8)	21·4 (20·6 to 22·3)	15·5 (14·8 to 16·2)	11·1 (10·5 to 11·7)	8·8 (8·3 to 9·4)	7·7 (6·5 to 9)
Saint Kitts and Nevis	125·7 (118 to 134)	170·1 (163 to 177·8)	84·5 (79 to 90·4)	61·8 (57·1 to 66·5)	33·9 (30·9 to 37·3)	22·7 (20·3 to 25·5)	17·1 (15 to 19·3)	15·3 (13·1 to 17·9)
Saint Lucia	194·1 (167·9 to 226·7)	142·4 (121·9 to 165·3)	76·9 (65·4 to 90·1)	38·6 (32·8 to 45·2)	23·9 (20·2 to 28·1)	18·8 (15·8 to 22)	17·9 (15 to 21·1)	14·6 (12·2 to 17·4)
Saint Vincent and the Grenadines	188·6 (162·9 to 220·7)	143·9 (123 to 165·8)	93 (78·9 to 107·5)	56·1 (47 to 65·6)	32·5 (27·4 to 38·1)	25·1 (21·4 to 29·5)	19·8 (16·8 to 23·1)	15·2 (12·8 to 18·2)
Suriname	105·8 (90·1 to 125·9)	92·2 (77·9 to 109·2)	75·9 (65·2 to 88·7)	64·3 (56·3 to 72·9)	50·4 (44·4 to 56·3)	42 (36·3 to 48·1)	33 (28·1 to 38·7)	25·7 (21·6 to 30·6)
Trinidad and Tobago	108·9 (94·7 to 124·6)	70·3 (62·9 to 78·4)	51·2 (46 to 56·9)	36·2 (31·8 to 41)	26·4 (23 to 30·2)	26·9 (22·8 to 31·5)	18·1 (15·3 to 21·7)	15·2 (12·7 to 18·2)
Virgin Islands	73·2 (66·6 to 80·4)	48·9 (45·1 to 53)	31·4 (28·3 to 34·7)	26·6 (24·2 to 29·3)	16·9 (15·2 to 18·8)	11·6 (10·3 to 12·9)	8·8 (7·8 to 10·1)	6·8 (5·7 to 8)
Central Latin America	201·8 (180·7 to 227·7)	141·5 (133·3 to 150·2)	103·3 (98·4 to 108·2)	70 (65·8 to 73·9)	43·9 (41·4 to 46·4)	28·9 (27 to 31·1)	20·7 (18·6 to 22·8)	14·8 (12·4 to 17·5)
Colombia	160·7 (142·6 to 183)	120·3 (109·7 to 132·1)	85·2 (78·5 to 92·5)	53·3 (48·5 to 58·2)	33·9 (30·7 to 37)	25·9 (23·4 to 28·8)	18·7 (16·2 to 21·5)	12·6 (10·6 to 15)
Costa Rica	138·2 (134·7 to 142)	95·2 (93·1 to 97·7)	82·4 (80·3 to 84·4)	24 (23·1 to 25)	17·5 (16·8 to 18·2)	12·9 (12·4 to 13·5)	10·7 (10·2 to 11·3)	7·9 (6·8 to 9·3)
El Salvador	274·5 (240·8 to 315·5)	211·3 (186·6 to 237·3)	149·2 (133·7 to 167·2)	94·6 (86·1 to 104·3)	53·4 (48·4 to 58·8)	29·3 (26 to 33·2)	17·3 (15 to 19·8)	10·9 (9·2 to 13·1)
Guatemala	248·9 (217·7 to 287·1)	205·6 (179·8 to 231·6)	170·8 (154·5 to 188)	124·1 (113·1 to 135·2)	78·5 (71·3 to 85·8)	49·4 (43·9 to 54·9)	32·1 (28 to 36·6)	21·7 (18·3 to 25·8)
Honduras	246·2 (216·8 to 282·2)	189·5 (167·7 to 212·4)	136·5 (121·8 to 151·6)	89·1 (80·4 to 98·6)	54·2 (49 to 59·6)	35·7 (31·5 to 40·5)	25·4 (21·7 to 29·4)	17·2 (14·4 to 20·5)
Mexico	220·6 (196·6 to 249·8)	147·8 (134·8 to 161·3)	104·3 (95·5 to 112·5)	72·5 (65·5 to 79·5)	44·6 (40·7 to 48·8)	28 (25·1 to 31·2)	20·2 (17·8 to 23)	14·4 (12·2 to 17·1)
Aguascalientes	188·5 (167·7 to 214)	129·1 (117·4 to 141·8)	94·4 (84·2 to 104·3)	67·6 (60·2 to 75·3)	40·4 (36·2 to 45·1)	25·2 (22·1 to 28·9)	18 (15·5 to 20·8)	12·5 (10·4 to 15)
Baja California	208 (185·2 to 236)	132·2 (119·7 to 145·8)	87·1 (79 to 95·3)	38·9 (34·6 to 43)	35·3 (31·9 to 39)	25·6 (23 to 28·5)	20·7 (18·2 to 23·7)	11·9 (9·9 to 14·3)
Baja California Sur	222·6 (198·5 to 252)	141·5 (127·5 to 156·6)	93·7 (85·8 to 102)	53 (47·3 to 58·8)	26·3 (23·5 to 29·1)	21·2 (18·8 to 23·6)	18·3 (15·7 to 20·9)	12·4 (10 to 15)
Campeche	237·9 (212·3 to 268·9)	164·7 (150·6 to 180·2)	123 (110·1 to 136·8)	92 (82·9 to 101·8)	51 (44·6 to 57·7)	30·2 (26·2 to 34·6)	21·9 (18·8 to 25·4)	15·3 (12·4 to 18·7)
Chiapas	254·8 (227·8 to 287·3)	182·5 (166·7 to 199·9)	139·5 (127·6 to 152·4)	108·9 (98·1 to 120·1)	61 (54·9 to 67·4)	34·6 (30·3 to 39·6)	19·3 (17 to 21·9)	16·7 (13·8 to 20)
Chihuahua	243·2 (217·2 to 274·6)	164·5 (149·6 to 180·2)	119·1 (108·3 to 129·9)	87·2 (78·5 to 96·6)	51·2 (46·4 to 56·4)	35·5 (31·4 to 40·4)	23·9 (20·9 to 27·1)	19·5 (15·9 to 23·6)
Coahuila	243·5 (217·5 to 274·9)	159·2 (145·1 to 174·9)	114·9 (104·6 to 125·6)	80·8 (72·9 to 88·9)	42·6 (38 to 47·1)	27 (23·7 to 30·9)	17·7 (15·4 to 20·1)	15·6 (13 to 18·6)
Colima	272·8 (244·2 to 307)	181·8 (166·7 to 198·8)	126·2 (113·3 to 139·7)	82·3 (69·1 to 96·7)	42 (37·1 to 47·9)	26·4 (22·9 to 30·5)	18·9 (16·1 to 21·8)	11·6 (9·5 to 14·2)
Durango	247·5 (221·1 to 279·2)	167·3 (152·6 to 183)	118 (106·4 to 129·4)	84·7 (76·4 to 93·3)	47·6 (42·8 to 52·9)	31·1 (27·4 to 35·3)	20·8 (18 to 23·7)	15·5 (12·8 to 19)
Guanajuato	247 (220·4 to 279·3)	171·8 (156·1 to 188·9)	125 (111·8 to 138·1)	85·9 (77·2 to 94·5)	53·4 (48·5 to 58·7)	25·5 (22·9 to 28·1)	19 (16·7 to 21·8)	13·4 (11·2 to 16·1)
Guerrero	243·6 (217·6 to 275)	180·4 (147·5 to 213·6)	150 (129·7 to 169·9)	115·1 (96·5 to 133·6)	72·5 (64·2 to 81·3)	48·2 (42·9 to 54)	30·5 (25·2 to 36·5)	16·4 (12·8 to 20·5)

Hidalgo	212·2 (189 to 240·6)	144·3 (130·7 to 158·9)	103·7 (93·8 to 113·9)	64·7 (55·1 to 75·1)	36·6 (31·7 to 41·7)	23·3 (20·3 to 26·9)	16·7 (14·5 to 19·1)	11·7 (9·6 to 14·2)
Jalisco	206·2 (183·7 to 233·9)	138·1 (126·2 to 152·6)	95·5 (84·9 to 106·6)	62·4 (55·7 to 69·7)	37·5 (33·7 to 41·8)	27·3 (23·6 to 31·4)	19·4 (16·9 to 22·1)	13·9 (11·6 to 16·6)
México	199·7 (177·4 to 227·2)	140·6 (127·4 to 155)	99·3 (88·6 to 109·9)	66·4 (59·5 to 72·9)	53·2 (48·5 to 58·4)	30·6 (27·7 to 33·6)	22·7 (19·9 to 25·8)	16·6 (13·8 to 19·7)
Mexico City	151·2 (134 to 172·5)	97·4 (88 to 107·6)	68·2 (62 to 75)	51·4 (46·1 to 56·4)	31·3 (28·5 to 34·3)	22·9 (20·6 to 25·4)	21·4 (18·9 to 24·4)	14·4 (12 to 17·2)
Michoacán de Ocampo	214·1 (190·8 to 242·7)	144·6 (131·7 to 158·9)	93·4 (83·9 to 103·3)	60·9 (54·2 to 67·9)	36·4 (32·6 to 40·4)	23·3 (20·3 to 26·7)	17·6 (15·4 to 20)	11 (9·1 to 13·1)
Morelos	253·4 (226·4 to 286)	169 (153·2 to 186·5)	118·5 (105·4 to 132·3)	78·6 (65 to 92·5)	41·7 (36·6 to 47·4)	26·1 (22·9 to 29·9)	18·6 (16·1 to 21·2)	12·4 (10·3 to 15·3)
Nayarit	255 (227·9 to 287·7)	171·1 (154·7 to 189·1)	113 (102·4 to 124·5)	69·1 (61·3 to 77·5)	41·1 (36·6 to 45·8)	25·6 (22·4 to 29·2)	16·9 (14·5 to 19·4)	13·1 (10·9 to 15·8)
Nuevo León	196·4 (174·7 to 223·1)	122·7 (111·5 to 135·1)	84·4 (75·1 to 94)	53·2 (47 to 60·1)	29·8 (26 to 33·9)	21·6 (18·9 to 24·8)	14·6 (12·7 to 16·8)	12·7 (10·4 to 15·2)
Oaxaca	238·3 (212·7 to 269·1)	167·4 (152·8 to 183·8)	120·8 (109·9 to 132·2)	85·4 (77 to 94·5)	44·9 (40·4 to 49·9)	27·3 (23·7 to 31·5)	16·9 (14·8 to 19·4)	12 (9·8 to 14·6)
Puebla	294·2 (263·9 to 330·5)	204 (187·3 to 222·8)	146 (133·7 to 159·9)	99·2 (89·4 to 109·6)	56·2 (50·8 to 61·9)	35·2 (31 to 40)	24·9 (21·8 to 28·3)	16·9 (13·8 to 20·2)
Querétaro	250·1 (223·5 to 282·5)	172·9 (157·2 to 190·1)	122·2 (110·2 to 134·8)	85·5 (76·7 to 94·5)	46·7 (42·5 to 51·7)	25 (22·4 to 27·6)	18·7 (16·1 to 21·5)	12·8 (10·4 to 15·7)
Quintana Roo	214·8 (191·5 to 243·4)	151·4 (137·7 to 166·7)	114·1 (102·8 to 125·2)	80·2 (71·6 to 89·3)	42·4 (38·1 to 47·3)	27 (23·6 to 30·9)	19·8 (17·2 to 22·7)	13·3 (11·1 to 15·9)
San Luis Potosí	233·7 (208·6 to 264·2)	156·9 (143·3 to 172·5)	109·6 (98·8 to 121·1)	68·8 (61·6 to 77·1)	39·5 (35·3 to 43·8)	25·3 (22·2 to 29)	16·1 (14 to 18·3)	11·5 (9·4 to 14)
Sinaloa	192·3 (171·2 to 218·3)	126·3 (114·3 to 139·3)	84·5 (76·7 to 92·9)	53·2 (46·8 to 60·8)	29 (25·4 to 33)	16·8 (14·5 to 19·4)	13·8 (12 to 15·7)	11·4 (9·4 to 13·7)
Sonora	181·8 (161·4 to 207)	120·2 (109·7 to 132·1)	87·3 (78·4 to 97·2)	62 (51·2 to 73·2)	33·9 (29·6 to 38·5)	26·2 (22·9 to 30)	20·3 (17·7 to 23·2)	13·2 (11 to 15·8)
Tabasco	229·4 (204·7 to 259·6)	161·2 (146·4 to 177·4)	122·9 (112 to 134·1)	90·9 (77·8 to 103·9)	51·8 (45·4 to 59·8)	29·8 (26 to 33·8)	22·1 (19·1 to 25·3)	16·9 (14 to 20·2)
Tamaulipas	200·2 (177·9 to 227·7)	133·3 (120·7 to 146·9)	92·7 (84·6 to 101·3)	64·8 (55·9 to 74·2)	35·8 (31·1 to 41·2)	20·9 (17·8 to 24·3)	18 (15·7 to 20·6)	13·2 (10·7 to 16)
Tlaxcala	256·1 (229 to 288·9)	174·6 (158·7 to 191·9)	122·3 (110·2 to 135)	87·9 (78·7 to 96·8)	53·2 (48·2 to 58·7)	30·8 (27·6 to 34·2)	23 (19·8 to 26·8)	15·4 (12·4 to 18·8)
Veracruz de Ignacio de la Llave	222·9 (198·5 to 252·9)	151 (137 to 166·3)	106·4 (97·4 to 115·8)	71·5 (62 to 82·2)	43·7 (36·2 to 52·2)	25·4 (21·7 to 29·4)	20·4 (17·8 to 23·3)	14·1 (11·7 to 16·8)
Yucatán	195·1 (173·7 to 221·5)	136·5 (123·7 to 150·5)	92·3 (83·9 to 101·4)	65 (57·8 to 71·9)	34·3 (31·1 to 37·8)	21·6 (19·4 to 23·9)	18·1 (15·5 to 21·1)	13·6 (11 to 16·6)
Zacatecas	210·4 (187·5 to 238·4)	146·3 (132·4 to 161·7)	103·3 (92·2 to 115·1)	76·3 (68·4 to 84·5)	42·3 (37·9 to 47·1)	26·8 (23·5 to 30·7)	18·7 (16·1 to 21·3)	13 (10·9 to 15·6)
Nicaragua	287·1 (254·5 to 326·5)	221·4 (197 to 249)	160 (145·8 to 176·2)	101·8 (94·3 to 110·1)	62·4 (57·4 to 67·6)	35·5 (31·8 to 39·5)	21·4 (18·4 to 24·6)	14·4 (12·1 to 17·1)
Panama	110·4 (98·2 to 123·8)	85·3 (77 to 94·2)	57·1 (51·2 to 63·3)	36·5 (32 to 41·4)	27 (23·3 to 30·6)	22·8 (19·8 to 26·3)	18·1 (15·3 to 21·5)	14·5 (12·1 to 17·3)
Venezuela	120·6 (119·2 to 121·9)	75·4 (74·5 to 76·4)	69·1 (68·3 to 69·9)	41·2 (40·6 to 41·8)	32·2 (31·8 to 32·6)	22·7 (22·4 to 23·1)	18·1 (17·8 to 18·5)	14·8 (12·4 to 17·6)
Tropical Latin America	249 (221·7 to 282·2)	199·1 (179·6 to 221·2)	156·9 (143·5 to 170·3)	107·7 (100·2 to 115·5)	67·7 (63·4 to 72·7)	43 (39·3 to 46·8)	28·8 (25·3 to 32·6)	19·8 (16·7 to 23·4)
Brazil	252 (224·5 to 285·5)	201·9 (182 to 224·4)	159·1 (145·5 to 172·8)	109·4 (101·7 to 117·3)	68·9 (64·5 to 74)	43·5 (39·8 to 47·5)	29·2 (25·5 to 33)	20 (16·9 to 23·7)
Acre	294·5 (251·9 to 343·2)	233·9 (197·4 to 272·5)	183·5 (162·5 to 205·8)	136·7 (118·8 to 155·4)	89·9 (81·1 to 99·8)	65·1 (57·4 to 73·8)	45·6 (34·8 to 58·9)	31·2 (24·7 to 39)
Alagoas	384·9 (336 to 439·4)	344·3 (313·6 to 377)	294·7 (268·6 to 322)	241·7 (220 to 263·8)	150·1 (137·7 to 163·9)	79·2 (70·5 to 88·5)	42·5 (36·6 to 49)	23·4 (18·5 to 29·3)
Amapá	352·4 (321·3 to 385·3)	270·9 (238·3 to 305·6)	178·2 (157 to 201·4)	66·6 (60·1 to 73·2)	42·6 (39 to 46·4)	40·1 (37·3 to 43)	32·3 (27·7 to 37·6)	29·4 (26·1 to 33·2)
Amazonas	204·2 (183 to 227·2)	157·6 (134·5 to 185·8)	127·2 (111 to 144)	96·6 (83·2 to 110·3)	61 (53·8 to 68·3)	43·5 (40·6 to 46·3)	26·9 (23·2 to 31·1)	23·3 (20·8 to 25·9)
Bahia	287·4 (247·2 to 333·9)	245·5 (221·5 to 272·3)	198 (178·9 to 218·7)	132 (121·6 to 143·1)	98 (89·2 to 107·3)	61·6 (54·7 to 68·6)	40·7 (35·1 to 47)	25·7 (20·3 to 32·2)
Ceará	433·9 (380·5 to 493)	368·2 (336 to 403·6)	288·6 (262·4 to 315·8)	199·6 (184·5 to 216·2)	116·2 (106·4 to 127·8)	67·5 (60·2 to 75)	38·6 (33·2 to 44·5)	22·1 (17·4 to 27·7)
Distrito Federal	246·4 (222·7 to 271·8)	181·7 (154·8 to 210·8)	127·9 (111·7 to 144·2)	67·9 (62·4 to 74)	36·8 (34·1 to 39·7)	26·3 (24·6 to 28·2)	20·7 (17·8 to 23·8)	16·9 (15 to 19·1)
Espírito Santo	195·5 (163·9 to 233·1)	157·9 (138·8 to 179)	123·3 (108·3 to 139·1)	72·6 (66·8 to 79·2)	46·6 (43·4 to 49·9)	29·2 (26·7 to 31·8)	25·4 (21·7 to 29·5)	18·5 (14·5 to 23·3)
Goiás	291·3 (265·2 to 319)	227·4 (196·4 to 263·7)	146·2 (129·1 to 164·4)	78·8 (68 to 90·4)	46·9 (41·6 to 52·2)	25·4 (23·6 to 27·2)	20·4 (17·6 to 23·6)	18·3 (16·4 to 20·4)
Maranhão	351·5 (303·3 to 406·5)	307 (277·9 to 338·5)	254·6 (231·2 to 279)	177·4 (160·3 to 195·4)	114·8 (104·7 to 125·3)	68·4 (60·6 to 76·2)	41·6 (35·9 to 48)	23·9 (18·9 to 29·9)
Mato Grosso	205 (185 to 226·6)	178·7 (152·2 to 208·6)	141·1 (124·4 to 158·6)	80·8 (72·1 to 89·7)	56 (50·3 to 62·3)	32·6 (30·3 to 34·9)	22·8 (19·5 to 26·4)	18·2 (16·2 to 20·5)
Mato Grosso do Sul	176·6 (156 to 198·9)	142·3 (118·6 to 171)	100 (88·2 to 113·1)	66·1 (60·5 to 71·8)	43·2 (40·2 to 46·4)	37·4 (35·1 to 39·9)	25·3 (21·6 to 29·4)	17·9 (15·9 to 20·3)
Minas Gerais	212·5 (181 to 249·7)	169·8 (151·5 to 190·3)	126·9 (114·2 to 141·6)	80·6 (73·7 to 88·6)	52·9 (47·6 to 58·9)	38·4 (33·7 to 43·3)	26·5 (22·7 to 30·6)	17·8 (14·1 to 22·3)
Pará	280·5 (239·4 to 327·7)	224·6 (201·5 to 249·3)	178·9 (160 to 197·9)	123·9 (111·8 to 135·8)	81·3 (71·9 to 91·2)	52·1 (45·7 to 58·5)	35·4 (30·2 to 40·9)	23·2 (18·3 to 29)
Paraíba	365·9 (318·9 to 418·7)	295·5 (267·6 to 326)	220·1 (199·3 to 242·5)	148·2 (136·8 to 161·2)	89·4 (81·4 to 98·3)	49·2 (43·3 to 55·6)	29·5 (25·3 to 34·1)	17 (13·4 to 21·3)
Paraná	180·9 (152·3 to 214·5)	142·2 (126·3 to 158·9)	105·6 (92·5 to 119·7)	78·5 (72·3 to 85·2)	44·7 (41·8 to 47·9)	30·3 (27·9 to 32·7)	24·4 (20·9 to 28)	17·5 (13·7 to 22)
Pernambuco	413·6 (362·5 to 470·1)	347·5 (316·6 to 380·7)	272 (247·6 to 297·9)	174·7 (161·5 to 189)	110·5 (101·6 to 120·9)	63·7 (57 to 71·3)	37·3 (32·1 to 43·1)	21·7 (17·1 to 27·1)
Piauí	289 (247 to 337·5)	245·3 (221·9 to 271·6)	208·6 (188·1 to 229·7)	136·6 (123·7 to 150·5)	90·3 (82·8 to 99·1)	58·9 (51·5 to 66·1)	37·3 (32·1 to 43)	23 (18·2 to 28·8)
Rio de Janeiro	173·1 (155·6 to 192·2)	131·5 (111·9 to 155·6)	109·3 (96·2 to 123·7)	85·5 (78·7 to 92·8)	51·3 (48 to 54·7)	33·2 (31·2 to 35·3)	23·3 (20·1 to 26·7)	18·8 (17·2 to 20·5)
Rio Grande do Norte	373·4 (325·5 to 427)	293 (265·1 to 322·3)	211·8 (191·3 to 233·6)	137·4 (126·6 to 148·9)	83·3 (75·7 to 91·4)	44·9 (39·6 to 50·5)	26·2 (22·4 to 30·4)	16·3 (12·9 to 20·5)
Rio Grande do Sul	160·9 (144·3 to 179)	123·6 (105·1 to 146·3)	85·6 (74·8 to 96·8)	54·4 (50 to 59·3)	31·7 (29·6 to 34·1)	25·5 (23·9 to 27·1)	18·2 (15·6 to 21)	16·2 (14·7 to 17·7)
Rondônia	314·2 (285·8 to 344·4)	259·2 (224·6 to 297·1)	192·9 (171·3 to 217·9)	101·5 (93·1 to 110·1)	64·8 (60·3 to 69·6)	37 (34·7 to 39·7)	24·8 (21·4 to 28·8)	18·4 (16·4 to 20·9)

Roraima	315·4 (287·7 to 344·7)	252·4 (222·3 to 286·3)	180·1 (160·9 to 202·9)	97·5 (88·6 to 107·2)	55·6 (51·1 to 60·2)	34 (31·6 to 36·5)	26 (22·3 to 30·2)	23·2 (20·7 to 26)
Santa Catarina	138·9 (115·9 to 166·7)	110·1 (97·3 to 124)	90·2 (80·2 to 100·4)	61·2 (55·9 to 67)	43·6 (39 to 48·7)	34·3 (30 to 38·8)	26·6 (22·7 to 30·8)	18·9 (14·9 to 23·7)
São Paulo	148·5 (133·1 to 165·4)	114·9 (97·5 to 136·2)	96 (84·5 to 108·8)	76·4 (70·6 to 82·7)	45·3 (42·4 to 48·5)	29·1 (27·5 to 30·8)	19·8 (17 to 22·8)	16·8 (15·4 to 18·3)
Sergipe	260·9 (223·6 to 304·3)	215·7 (190·2 to 245·4)	170·7 (153·8 to 187·9)	137·8 (119·6 to 158·6)	89·1 (80·6 to 98·1)	56 (49·3 to 62·5)	36·4 (31·3 to 42·1)	23·7 (18·7 to 29·6)
Tocantins	325·9 (293·6 to 360·3)	271 (215·5 to 329·7)	200·5 (178·6 to 223·7)	124·1 (105·9 to 142·8)	81·1 (71 to 92·1)	53·1 (46·5 to 59·8)	34·8 (28·4 to 41·8)	20·9 (17·5 to 25·4)
Paraguay	108·7 (94·4 to 126·8)	81·7 (74·3 to 90·2)	68·4 (62·1 to 74·7)	49·5 (45·1 to 54·7)	34·2 (30·7 to 37·8)	28 (24·6 to 31·3)	20·6 (17·8 to 23·7)	14 (11·8 to 16·6)
North Africa and Middle East	300·7 (277·4 to 327·5)	257·4 (243·2 to 273·3)	191·9 (182·1 to 202·1)	126·5 (120·9 to 132·3)	79·1 (75·7 to 82·7)	54·6 (51·7 to 57·4)	35·9 (33·3 to 38·8)	24·4 (22·3 to 26·7)
North Africa and Middle East	300·7 (277·4 to 327·5)	257·4 (243·2 to 273·3)	191·9 (182·1 to 202·1)	126·5 (120·9 to 132·3)	79·1 (75·7 to 82·7)	54·6 (51·7 to 57·4)	35·9 (33·3 to 38·8)	24·4 (22·3 to 26·7)
Afghanistan	281·3 (246·3 to 320·2)	254·7 (222·4 to 288)	229·2 (197·3 to 265·9)	221·5 (191·8 to 261·7)	151·1 (136·7 to 167·4)	128·5 (117·3 to 140·8)	78·5 (69·7 to 87·7)	55·3 (47·9 to 63·5)
Algeria	152 (138·8 to 166·4)	165·9 (150·1 to 183·3)	158·6 (141·1 to 179)	122·6 (112·2 to 133·9)	59·5 (54·4 to 65·1)	41 (37·3 to 44·9)	27·6 (25·3 to 30·2)	19·5 (17 to 22·4)
Bahrain	224·1 (195·7 to 256·2)	150·3 (132·2 to 170)	74·9 (65·6 to 86·7)	30·1 (28·2 to 32·1)	23·1 (22 to 24·3)	11·9 (11·2 to 12·6)	8·2 (7·6 to 8·7)	6·5 (5·8 to 7·4)
Egypt	370·6 (332·8 to 415·2)	317·6 (288·9 to 347·1)	242·6 (222·4 to 262)	155·3 (141·7 to 169·7)	83·5 (76 to 91·2)	44·4 (39·3 to 49·3)	26·5 (22·9 to 30·5)	15·3 (12·8 to 18·3)
Iran	367 (326·2 to 411·3)	309·6 (271·9 to 351)	206·8 (181·2 to 239·5)	105·6 (93·3 to 119·5)	71·2 (63·6 to 79·1)	41·7 (36·7 to 47·5)	26·5 (23·7 to 29·4)	11·1 (10·2 to 12)
Iraq	176 (151·2 to 204·4)	143·1 (121·2 to 167·5)	115·5 (99·1 to 132·6)	87·4 (80·4 to 94·6)	54·7 (50·1 to 59·8)	42·5 (38·8 to 47·3)	29·1 (25·4 to 32·9)	15·7 (13·2 to 18·9)
Jordan	139 (118·8 to 162·4)	118·2 (102 to 136·8)	84·1 (75·3 to 93·4)	53·1 (48·5 to 58·4)	33·7 (30·8 to 37·1)	25·5 (23 to 28·1)	18 (15·9 to 20·3)	15·3 (13 to 18·3)
Kuwait	91·4 (77·9 to 107·1)	67·7 (59·3 to 77·6)	53·3 (51·5 to 55·3)	34·6 (33·3 to 35·9)	21·2 (19·8 to 22·5)	12·2 (11·5 to 12·9)	11·4 (10·8 to 12)	9·2 (7·8 to 10·8)
Lebanon	142·1 (121·4 to 166·1)	88·9 (74·6 to 104·2)	65·2 (55·9 to 75·2)	45 (39·2 to 51)	31·8 (27·8 to 36)	19 (16·2 to 22·2)	13·1 (11 to 15·5)	9 (7·5 to 11)
Libya	262·9 (229·6 to 303·6)	210·8 (183·6 to 240·4)	118·2 (102·5 to 134·9)	65·4 (57·2 to 74·4)	43·4 (37·7 to 49·9)	25·9 (22·2 to 30)	16·9 (14·3 to 19·9)	13·3 (11·3 to 15·7)
Morocco	290·6 (259·6 to 328)	253·4 (235·9 to 272)	188·5 (175·6 to 201)	125 (116·6 to 134·5)	75·1 (68·3 to 81·9)	48·8 (43 to 54·9)	30·4 (25·6 to 35·8)	17·9 (15 to 21·4)
Oman	306·3 (269·8 to 346·5)	260·3 (226·8 to 294·4)	158·8 (138·6 to 179·9)	86·8 (74·6 to 100·6)	35·3 (30·1 to 42)	15·1 (13·4 to 16·8)	11·3 (10·9 to 11·7)	10·4 (9·4 to 11·4)
Palestine	168·9 (145·6 to 195·2)	130·8 (112·7 to 151·5)	98·4 (85·7 to 111·1)	66·3 (59·8 to 73·6)	43 (39·4 to 47·2)	30·5 (27·8 to 33·4)	21·3 (18·8 to 23·8)	12·4 (10·5 to 14·9)
Qatar	93·8 (79·6 to 110·5)	85 (71·5 to 99·4)	68·1 (57·2 to 80·2)	37·7 (32·1 to 43·9)	26·3 (22·1 to 30·7)	18 (15·3 to 21·1)	11·1 (9·4 to 13·1)	8 (6·7 to 9·7)
Saudi Arabia	206·1 (177·2 to 239·1)	174·8 (150·1 to 200·9)	128·9 (110·5 to 149)	75·9 (65·6 to 86·7)	44·9 (38·2 to 52·2)	24·9 (20·8 to 29·7)	11·4 (9·3 to 13·5)	5·7 (4·8 to 7)
Sudan	260·5 (229·2 to 295·2)	232·2 (209 to 258·3)	213·2 (195·9 to 231·3)	184·8 (170·3 to 200·9)	142·2 (131·8 to 153·7)	99·2 (91·1 to 108·3)	66·3 (59·1 to 73·5)	41·9 (35·7 to 50)
Syria	177·3 (155·2 to 204·9)	124·3 (112·8 to 137·2)	80·9 (72·8 to 89·6)	63·8 (56·8 to 71·7)	49·9 (43·8 to 56·3)	20·7 (20·3 to 21)	14·1 (13·8 to 14·3)	13·6 (11·7 to 15·8)
Tunisia	303 (271·5 to 340·9)	238 (219 to 257·1)	158·3 (146·3 to 170·7)	86·9 (78·8 to 95)	50·7 (45·6 to 56·4)	29 (25·5 to 32·7)	18·8 (16 to 22)	11·3 (9·5 to 13·5)
Turkey	308·1 (274·6 to 348)	263·5 (243·8 to 283·8)	186·3 (172·7 to 199·4)	121·7 (112·2 to 131·4)	70·2 (63·9 to 77·1)	39·9 (35·6 to 44·3)	23·6 (20 to 27·7)	15·4 (12·9 to 18·4)
United Arab Emirates	155·8 (133·8 to 181)	132·9 (113·4 to 154)	86·1 (74·4 to 98·8)	44·8 (38·5 to 51·8)	21·6 (19·2 to 24·3)	11·9 (11·4 to 12·5)	6·8 (6·2 to 7·4)	5 (4·2 to 6)
Yemen	440·6 (399·5 to 488·6)	380 (354·1 to 406·7)	298·6 (278·3 to 320·7)	189 (173·8 to 204·5)	130·6 (120·4 to 140·9)	89·3 (81·5 to 97·1)	58·2 (51 to 66·2)	46·7 (40·2 to 54·4)
South Asia	277·7 (252·3 to 305·7)	244·4 (218·9 to 268·7)	204 (188·1 to 221·2)	156·6 (145·2 to 167·9)	112·6 (105·2 to 120·8)	82·8 (76·9 to 89·6)	61 (55·3 to 66·6)	40·5 (36 to 46)
South Asia	277·7 (252·3 to 305·7)	244·4 (218·9 to 268·7)	204 (188·1 to 221·2)	156·6 (145·2 to 167·9)	112·6 (105·2 to 120·8)	82·8 (76·9 to 89·6)	61 (55·3 to 66·6)	40·5 (36 to 46)
Bangladesh	333·8 (304·9 to 368·1)	279·4 (258·9 to 300·2)	242·4 (226·5 to 259·3)	189 (176 to 203·7)	132·9 (124·8 to 142)	84·8 (78·2 to 92·2)	52·1 (46·4 to 58·1)	29·2 (24·9 to 34·2)
Bhutan	365·5 (324·7 to 409·8)	307·7 (273·2 to 343·4)	240·7 (208·6 to 275·7)	199·6 (175·6 to 224·4)	128·9 (115·4 to 144)	81·8 (74·1 to 90·4)	47·8 (41·5 to 54·4)	31·5 (26·7 to 36·9)
India	265·9 (232·9 to 302·5)	240·3 (209·3 to 271·4)	201·2 (180·4 to 223·6)	152·6 (139·2 to 166·2)	108·5 (99·2 to 118·2)	80·2 (72·6 to 88·7)	58·1 (50·8 to 65·7)	35·8 (30·2 to 43)
Andhra Pradesh	254·2 (222 to 290·5)	232·9 (196·6 to 271·4)	201·2 (177·5 to 228·1)	147·4 (133 to 163·3)	106·1 (95·9 to 117·2)	78·3 (67·6 to 90·4)	53·1 (44·6 to 61·8)	31·9 (25·9 to 40)
Arunachal Pradesh	265·3 (231·8 to 302·7)	243·6 (207·4 to 279·2)	205·2 (181 to 231·8)	131·1 (117·3 to 145·9)	96·7 (87·4 to 107·8)	70·8 (61·4 to 81·3)	41·4 (36·2 to 46·7)	25·7 (21 to 32)
Assam	258·9 (226·2 to 295·6)	234·5 (200 to 268·6)	192·3 (164·4 to 220·6)	135·7 (120·9 to 150·1)	105·8 (96·3 to 116·8)	84·1 (76·2 to 92·7)	68·9 (57 to 81·9)	45 (37 to 55·4)
Bihar	228·7 (198·7 to 262·6)	213·8 (184·1 to 245)	196·8 (169·1 to 224·7)	164·9 (146·3 to 183·3)	119·9 (108·7 to 131·6)	88·9 (80·5 to 97·9)	64·9 (53·8 to 76·7)	40 (32·9 to 49·4)
Chhattisgarh	252·1 (219·5 to 288·6)	234·3 (200·7 to 267·2)	203·2 (171·7 to 238·9)	165 (141·8 to 190·9)	118·9 (106·5 to 133·3)	93 (84 to 102·5)	67·2 (58·1 to 76·7)	38·9 (33·9 to 44·6)
Delhi	240 (209 to 274·9)	182·6 (155 to 213·2)	142·5 (126·3 to 160·7)	103·2 (92·6 to 115·3)	72·1 (65·3 to 80)	56·1 (47·7 to 65)	34·9 (30·7 to 39·2)	22·2 (18·1 to 27·5)
Goa	142·7 (122·1 to 166·5)	120·4 (100·9 to 139·8)	98·9 (87·1 to 111·5)	68·9 (60·9 to 77·1)	47·4 (41 to 54·3)	31·1 (26·2 to 36·6)	21·7 (18·5 to 25·2)	16 (12·9 to 20·1)
Gujarat	230·5 (200·4 to 264·6)	205·6 (175·1 to 237·4)	168·5 (149·8 to 187·7)	131·7 (118·3 to 146·1)	96·6 (87·9 to 106·8)	71·8 (61·3 to 83·2)	50·4 (44·5 to 56·2)	30·7 (25·1 to 38)
Haryana	234·3 (203·8 to 268·6)	213·7 (182·4 to 243·3)	176·3 (157·6 to 196·2)	118·9 (106·9 to 132·5)	86·8 (78·7 to 96·1)	65·3 (57 to 75·6)	47·9 (42·1 to 54)	30·4 (24·9 to 37·8)
Himachal Pradesh	170·4 (146·6 to 197·9)	155 (130·5 to 180·6)	128·1 (113·6 to 144·2)	92·5 (81·9 to 103·4)	64·4 (57·5 to 71·7)	50·8 (43·4 to 58·9)	39·7 (34·9 to 44·6)	26·5 (21·8 to 32·8)
Jammu and Kashmir	161·5 (138·7 to 187·9)	148·1 (124·5 to 171·7)	121·9 (108·4 to 137·6)	101·9 (91·1 to 113·6)	72·3 (65·6 to 80·2)	56·4 (49·3 to 65·2)	44·8 (39·3 to 50·6)	27·5 (22·5 to 34)
Jharkhand	203·3 (175·7 to 234·6)	185·1 (160 to 212·2)	157·9 (131·6 to 188·9)	132 (113·3 to 151·4)	118·4 (106·2 to 131·7)	87·7 (79·3 to 97·1)	60·7 (52·3 to 69·6)	35·3 (30·7 to 40·5)
Karnataka	260·5 (227·8 to 297)	231·6 (198·2 to 265·6)	190·6 (171·1 to 212·4)	130·5 (118·2 to 144·6)	85·8 (77 to 95·1)	60 (51 to 69·8)	42 (36·6 to 47·6)	25·4 (20·8 to 31·6)
Kerala	189·7 (163·7 to 219·4)	142·4 (119·5 to 165·6)	95 (82 to 108·9)	55·1 (46·1 to 65·4)	33·2 (28·2 to 38·9)	21 (18 to 24·5)	14·8 (12·7 to 16·9)	10·1 (8·2 to 12·6)

Madhya Pradesh	286·6 (251·8 to 325·1)	269·9 (234·9 to 306·8)	234·1 (201·6 to 266·8)	192·9 (171·8 to 214·1)	141·9 (128·9 to 155·4)	105·6 (95·6 to 116·5)	76·3 (63·4 to 90·3)	46·5 (38·3 to 57·3)
Maharashtra	240·2 (209·3 to 274·9)	209·8 (178·8 to 242·6)	172·1 (153·5 to 192)	115·1 (103·9 to 127·6)	74·5 (67·7 to 82·4)	51·6 (43·8 to 60·1)	35·1 (31 to 39·5)	21·2 (17·3 to 26·4)
Manipur	183·8 (158·5 to 212·7)	166·6 (139·1 to 194·2)	130·7 (114·3 to 148·2)	92·5 (81·8 to 104·2)	63·7 (57·2 to 70·8)	46·3 (39·5 to 53·7)	33·5 (29·2 to 38)	24·2 (19·7 to 30·2)
Meghalaya	131·2 (112 to 153·5)	120·2 (100·1 to 140)	108·3 (94·6 to 123·8)	109·9 (98·2 to 122·8)	87·2 (76·3 to 99·5)	65·8 (57·2 to 75·9)	47·8 (41·6 to 54·3)	33·6 (27·5 to 41·8)
Mizoram	100·1 (85 to 117·9)	87 (72·6 to 103·6)	71·6 (60·7 to 84·1)	71·1 (62·4 to 80·7)	55·4 (49·9 to 61·7)	45·1 (40·5 to 50·2)	43·2 (35·4 to 51·4)	31 (25·4 to 38·4)
Nagaland	213·4 (185 to 245·6)	187 (162·5 to 214·6)	143·4 (120·1 to 171)	98·5 (86·4 to 112·5)	71·2 (63·3 to 79·9)	56·3 (50·9 to 62·5)	42·8 (35·2 to 51·6)	31·8 (27·5 to 36·7)
Odisha	305·8 (269·5 to 346·1)	284·3 (245·2 to 321·2)	241·2 (216·7 to 266·6)	181·1 (163·9 to 200·8)	131·7 (119·8 to 144·8)	95·6 (83·2 to 110·2)	65·2 (58·1 to 72·7)	41 (33·7 to 50·7)
Punjab	163·7 (140·6 to 190·2)	141·9 (118·8 to 165)	106·5 (94·2 to 119·5)	86·3 (77·3 to 96·5)	64·6 (58·6 to 71·7)	49·7 (43·2 to 57·9)	38·3 (33·6 to 43·4)	24 (19·6 to 29·8)
Rajasthan	219·9 (190·7 to 252·9)	209·2 (182·3 to 237·3)	183·5 (155·9 to 215·8)	155·1 (137·6 to 172·9)	118 (107·5 to 129·8)	88·2 (78·6 to 98·3)	67·7 (58·9 to 76·9)	41·9 (36·5 to 47·9)
Sikkim	175·4 (150·9 to 203·6)	164 (139·4 to 189·6)	142·4 (123·4 to 161·6)	106·1 (92·7 to 120·7)	64·4 (57·3 to 72·6)	45·4 (39·2 to 52·4)	33·4 (29·1 to 38)	20·8 (16·9 to 26)
Tamil Nadu	273·9 (240 to 311·6)	238·5 (205·4 to 274·6)	192·2 (171·8 to 215·3)	133·1 (120·2 to 147·6)	81·3 (73·2 to 90)	49·1 (41·5 to 57·5)	30·6 (26·8 to 34·7)	17·7 (14·4 to 22·1)
Telangana	192·3 (166·1 to 222·2)	179·9 (148·5 to 214·8)	157·6 (141·8 to 174·3)	131·2 (117·4 to 147·3)	99 (89·2 to 109·9)	74·6 (60·8 to 89)	46·3 (39·6 to 53·9)	26·8 (21·8 to 33·6)
Tripura	293·7 (258·3 to 333)	250·9 (215·2 to 286·1)	199·1 (178·5 to 221·7)	136·4 (122·7 to 151·8)	91 (82·4 to 100·9)	60 (52·2 to 69·6)	38·8 (34 to 44)	28·5 (23·2 to 35·6)
Union Territories other than Delhi	167·8 (144 to 195·1)	140·9 (121·8 to 161·1)	108·4 (88·9 to 131·6)	75·4 (62·2 to 90)	52·6 (44·2 to 63)	39·9 (33·2 to 47·6)	30·2 (25·4 to 35·8)	21 (17·9 to 24·5)
Uttar Pradesh	371·8 (331·6 to 415·4)	344·7 (304·1 to 385·5)	286·7 (249·2 to 324)	215·3 (191·8 to 237·8)	147·9 (134·3 to 162·8)	106·6 (97 to 116·8)	79·1 (65·7 to 92·9)	48 (39·6 to 58·9)
Uttarakhand	171·9 (147·7 to 199·6)	153·1 (132·2 to 174·7)	126 (104·2 to 151·4)	100·4 (86·8 to 116·4)	76·3 (66·3 to 87·6)	57·8 (51·8 to 64·7)	44·6 (38·2 to 51·5)	26·4 (22·8 to 30·5)
West Bengal	270 (236·5 to 307·4)	235 (202·6 to 269·4)	194·7 (174·7 to 216·1)	141·4 (126·3 to 158·4)	95·4 (86·2 to 105·2)	63·6 (54·1 to 74·3)	42·7 (37·8 to 47·9)	25·5 (20·8 to 31·7)
Nepal	321 (293·1 to 354·2)	276·7 (255·7 to 297·4)	224·6 (208·4 to 242·5)	197·6 (182·4 to 213·3)	133·4 (123·9 to 143)	81·4 (75 to 88·6)	50·5 (45·1 to 56·2)	29·1 (24·8 to 34·1)
Pakistan	285·1 (258·3 to 317·3)	228·7 (210·4 to 246·6)	176·1 (163·1 to 190·1)	145·2 (132·9 to 158·7)	114·8 (105·1 to 125·4)	93·9 (84·8 to 103·4)	77·8 (68 to 88·6)	63·3 (53·9 to 74·3)
Southeast Asia, east Asia, and Oceania	213·8 (192·1 to 238·1)	430·2 (334·5 to 554·3)	119·7 (110·5 to 130·1)	85·5 (78·6 to 92·3)	60·7 (56·4 to 65·1)	41·7 (39·4 to 44·2)	24·2 (22·4 to 26)	14·8 (13·2 to 16·5)
East Asia	202 (175·6 to 231·9)	505·8 (385·7 to 652·1)	107·7 (95·5 to 121·1)	75·3 (66·7 to 84·2)	54·2 (48·6 to 60·1)	35·9 (32·9 to 39·4)	16·1 (14·8 to 17·4)	8·6 (7·5 to 9·8)
China	198·8 (172·4 to 228·9)	515·8 (393·1 to 664·2)	107·8 (95·6 to 121·1)	75·9 (67·1 to 85·2)	54·7 (49·1 to 60·8)	35·5 (32·5 to 38·7)	16 (14·7 to 17·4)	8·5 (7·5 to 9·7)
North Korea	401·6 (367·5 to 437·5)	246·7 (215·3 to 280·7)	153 (130·9 to 177·5)	92·7 (78·5 to 108·3)	56·1 (47·1 to 65·4)	64·7 (43 to 114·7)	23·2 (19·1 to 27·8)	12·6 (10·5 to 15·4)
Taiwan (province of China)	92·6 (79 to 108·5)	59·9 (59·2 to 60·5)	26·2 (25·7 to 26·6)	15 (14·7 to 15·3)	8·4 (8·2 to 8·7)	7·9 (7·7 to 8·2)	5 (4·8 to 5·2)	4·5 (3·8 to 5·4)
Oceania	178·6 (158·9 to 201·8)	140·6 (124·9 to 157·1)	101·4 (90·4 to 113·3)	77·2 (69 to 86·7)	68·2 (60·9 to 75·4)	61·9 (54·7 to 69·6)	57·8 (48·7 to 67·5)	48·4 (40·7 to 57·5)
American Samoa	83·1 (73·9 to 93·2)	52 (47·6 to 56·8)	37·2 (34 to 41)	22·8 (19·8 to 26·3)	16·8 (15 to 18·7)	14·6 (12·9 to 16·3)	12·3 (10·9 to 13·8)	10·2 (8·7 to 12)
Cook Islands	159·3 (145·2 to 173·6)	98·1 (90 to 106·7)	64·5 (58·5 to 71·5)	44·3 (39·7 to 49·6)	19·9 (17·3 to 23)	11·8 (10·1 to 14)	5·1 (4·4 to 6)	2·5 (2·1 to 2·9)
Fiji	89·5 (79·5 to 100·6)	58·1 (52·6 to 64·1)	43·9 (39·3 to 49·4)	33·9 (29 to 39·1)	25·7 (21·7 to 30·7)	25·8 (21·6 to 30·6)	24·6 (20·7 to 29·2)	21·7 (18·1 to 25·9)
Guam	48 (43·5 to 53·5)	36·4 (33·9 to 39·2)	27·7 (25·7 to 29·7)	16·4 (14·9 to 17·9)	11·8 (10·7 to 13)	11·2 (10·2 to 12·2)	13·4 (12·2 to 14·7)	13·3 (11·6 to 15·3)
Kiribati	199 (173·1 to 227·2)	157·8 (138·2 to 179·2)	116·7 (101·8 to 133·4)	86·2 (73·3 to 102·8)	77·4 (66·7 to 89·4)	61·4 (53·6 to 70·4)	46·1 (39 to 54·4)	33·5 (28·1 to 40)
Marshall Islands	142·2 (121·2 to 166·4)	101·9 (86·1 to 119·8)	76·2 (64·1 to 89·7)	47·8 (41·1 to 55·3)	33·8 (31 to 36·9)	38·4 (34·4 to 42·6)	26·6 (22·8 to 30·6)	19·3 (16·2 to 23·4)
Federated States of Micronesia	146·2 (125 to 173·2)	114·8 (97·6 to 132·6)	81·3 (68·7 to 94·8)	54·5 (46·4 to 64)	41·6 (35·4 to 48·6)	34·8 (29·9 to 40·3)	20·3 (16·8 to 24·2)	14·5 (12·1 to 17·4)
Nauru	94 (79·5 to 110·9)	71·9 (61·1 to 83·8)	53·9 (46·2 to 62·1)	37·6 (31·7 to 44)	32 (27·6 to 36·9)	42·6 (38·1 to 47·4)	38·9 (33·3 to 44·9)	23·2 (19·5 to 28·1)
Niue	137·4 (123 to 152·7)	71·6 (63·2 to 80·6)	46·2 (39·4 to 52·9)	29·2 (24·2 to 34·8)	22·8 (19·2 to 27·7)	25 (20·9 to 29·6)	22·2 (18·4 to 26·4)	18·6 (15·5 to 22·2)
Northern Mariana Islands	67 (56·4 to 79·4)	48·2 (40·4 to 56·8)	31·5 (26·7 to 37·8)	18·6 (15·4 to 22·3)	10·8 (9·1 to 12·8)	7·6 (6·6 to 8·7)	8·3 (7·2 to 9·5)	9·1 (7·7 to 10·7)
Palau	189·7 (163·3 to 219·8)	136·3 (114·8 to 159)	87·2 (73·8 to 102·3)	54·6 (46 to 63·3)	30·9 (26 to 36·2)	19·9 (16·7 to 23·4)	17·2 (14·5 to 20·2)	14 (11·7 to 17)
Papua New Guinea	201·3 (174·9 to 234·2)	161·7 (140·8 to 183·5)	116·1 (101·4 to 131·7)	91·4 (80·6 to 103·5)	82·2 (72·6 to 92·2)	72·1 (63·1 to 82)	65·8 (55·1 to 77·2)	53·8 (45·3 to 63·9)
Samoa	176·5 (154·2 to 200·8)	127·2 (111·3 to 143·7)	91·3 (79 to 105·2)	56·8 (48 to 66·2)	35·6 (29·3 to 43·1)	23·3 (19·2 to 27·6)	18 (15·1 to 21·2)	13·2 (11·1 to 15·9)
Solomon Islands	204·6 (178·1 to 233·4)	160 (139·3 to 181·8)	108 (93 to 124·7)	67·4 (56·7 to 80·1)	51 (43 to 59·8)	40·5 (34·1 to 47·5)	33·7 (28·1 to 39·8)	24·6 (20·6 to 29·4)
Tokelau	86·9 (73·6 to 101·9)	64·2 (53·6 to 75·6)	45·3 (38·3 to 53·4)	37·2 (31·4 to 43·7)	25·5 (21·3 to 30·9)	17·7 (14·7 to 21·1)	11·7 (9·9 to 14)	7·7 (6·4 to 9·3)
Tonga	69·2 (58·4 to 83·3)	49·1 (41·6 to 57·1)	39·9 (33·8 to 46·6)	33·3 (27·9 to 39·3)	25·4 (21·3 to 30)	22 (18·6 to 26)	18·2 (15·4 to 21·6)	13·6 (11·4 to 16·3)
Tuvalu	202·1 (174·8 to 233)	155·6 (132·8 to 181)	117 (99·5 to 136·5)	94·3 (79·8 to 110)	68·6 (60 to 77·7)	45·3 (40·7 to 50·3)	21·9 (18·4 to 25·5)	13·7 (11·4 to 16·7)
Vanuatu	194·5 (169·6 to 222·5)	155·8 (136·1 to 176·2)	99·4 (84·1 to 116)	51·2 (43·2 to 61·2)	36·4 (31·1 to 42·5)	34·1 (29 to 40)	31·2 (26·5 to 36·8)	24·4 (20·5 to 29·2)
Southeast Asia	244·5 (227·5 to 263·6)	191·7 (179·3 to 204·2)	147·2 (139·4 to 155·2)	105·4 (100·8 to 110·7)	73·5 (69·8 to 77·1)	49·5 (46·5 to 52·4)	32·8 (29·8 to 35·8)	22·6 (20·1 to 25·6)
Cambodia	235·4 (204·3 to 270·4)	203·8 (176·9 to 236·8)	191·6 (168·7 to 214·8)	162·6 (147·2 to 179)	120·5 (112·4 to 129)	91·9 (84 to 99·8)	49·1 (43 to 55·2)	31·3 (26·5 to 37·5)
Indonesia	284·8 (259·1 to 315·6)	225·9 (209·9 to 241·8)	170·8 (160·1 to 181·7)	123·3 (116·7 to 130·8)	84·8 (79·6 to 90)	54·4 (50·1 to 59)	36·8 (32·5 to 41·5)	25·5 (21·6 to 30·2)
Aceh	223·1 (198·9 to 251·8)	175·5 (163·4 to 189·2)	139 (128·7 to 150·1)	104·1 (95 to 114·1)	78·2 (70·6 to 86·1)	55·7 (49·9 to 61·9)	39 (34·2 to 44·2)	28·9 (24·2 to 34·5)
Bali	202·4 (180·1 to 228·9)	149·9 (128·7 to 173)	106·9 (95·9 to 117·8)	78·7 (71·4 to 86·5)	43·7 (39·5 to 48)	28·8 (25·1 to 33·3)	18·1 (14·5 to 22·3)	4·7 (3·9 to 5·7)

Bangka-Belitung Islands	301·5 (269·1 to 337·8)	244·2 (224·6 to 264·4)	180·9 (166·5 to 196)	122·3 (109·5 to 136·3)	88·7 (80·8 to 97·4)	51·1 (46 to 56·8)	34·1 (29·7 to 39·5)	28·9 (24·8 to 34·1)
Banten	435·8 (397 to 477·8)	354·1 (330·7 to 381·4)	269·6 (250·5 to 289·5)	191·5 (176·3 to 205·9)	121·3 (111·6 to 130·9)	64·1 (58·6 to 70)	40·5 (35·6 to 46·1)	28·4 (24·4 to 33·3)
Bengkulu	353·3 (320·7 to 390·6)	288·9 (269·2 to 309·9)	212·8 (197·7 to 228·7)	151 (141·8 to 160·7)	99 (92·6 to 105·6)	62·9 (58·2 to 68·5)	41·4 (36·5 to 46·9)	27·3 (22·8 to 32·7)
Gorontalo	323·8 (290·2 to 361·3)	264·5 (243·5 to 290·2)	206·6 (182·9 to 230)	157·3 (141·9 to 174)	116·8 (109·1 to 124·5)	89·3 (79·3 to 100·3)	64·5 (55·7 to 74·1)	45·1 (38·9 to 52·8)
Jakarta	241·7 (216·1 to 271·7)	183·2 (169 to 197·7)	127·8 (115·7 to 140·4)	87·3 (81·4 to 94·1)	55·2 (51·3 to 59)	35·2 (30·9 to 40·5)	25·5 (22·2 to 29·1)	18·8 (15·6 to 22·6)
Jambi	292·8 (263·4 to 326·8)	222·7 (209·6 to 236·2)	170·5 (156·3 to 184·3)	121·9 (110·6 to 132·6)	83·4 (78·1 to 88·7)	53·3 (49·1 to 57·6)	35 (30·8 to 39·8)	24·8 (20·6 to 29·7)
West Java	357 (324·3 to 394·5)	288·7 (268·2 to 309)	214·5 (198·8 to 230·6)	149·1 (139·8 to 159·8)	95·9 (90·3 to 101·9)	57·1 (52·4 to 62·2)	36·7 (32·5 to 41·5)	24·3 (20·3 to 29·1)
Central Java	234·9 (210 to 264·3)	188·8 (173·8 to 203)	138·7 (127·5 to 150·9)	95·6 (88·2 to 103·8)	63·4 (57·8 to 69·4)	39·3 (36·2 to 42·8)	26·7 (23·5 to 30·3)	17·6 (14·7 to 21·2)
East Java	247·2 (221·3 to 277·6)	199·8 (184·4 to 214·9)	153·4 (141·5 to 165·7)	114·9 (107·5 to 123·5)	79 (74·3 to 84·1)	50·1 (45·9 to 54·6)	33 (29·1 to 37·3)	21·8 (18·2 to 26·1)
West Kalimantan	256 (229·2 to 287·3)	224·9 (208·4 to 242·3)	184·3 (171 to 198·4)	140·4 (131·8 to 149·6)	97·1 (90 to 104·4)	61 (55·8 to 66·6)	35·8 (31·6 to 40·5)	20·1 (16·8 to 24·1)
South Kalimantan	385·9 (351·8 to 424·5)	337·1 (314·1 to 359·6)	258·7 (241·3 to 275·5)	193·1 (182·2 to 204·9)	131·8 (121·1 to 143·3)	76·5 (70·6 to 82·7)	48·1 (42·4 to 54·3)	32·9 (27·5 to 39·4)
Central Kalimantan	229 (204·3 to 258·1)	158·5 (148·7 to 168·7)	118·1 (108 to 128)	88·3 (79·8 to 96·3)	70·5 (66·1 to 75·2)	51·7 (47·6 to 55·9)	33·7 (29·7 to 38·3)	25·3 (21·1 to 30·3)
East Kalimantan	221·6 (197·5 to 250·2)	191·6 (165·9 to 218·9)	149·1 (136·8 to 160·9)	103·2 (96·4 to 110·6)	67·3 (62·1 to 72·4)	41·8 (37·5 to 46)	25·5 (20·5 to 31·3)	19·5 (16·2 to 23·5)
North Kalimantan	269·7 (239·3 to 304)	231·8 (212·9 to 251·2)	197·1 (181·9 to 213·8)	134·4 (120 to 149·6)	87·1 (78·6 to 96·5)	61·1 (54·9 to 67·4)	40·1 (34·9 to 46·5)	32·2 (27·6 to 38)
Riau Islands	221·4 (195 to 251·4)	172·6 (158·5 to 188·9)	122·6 (112 to 133·9)	92 (83·5 to 100·5)	65·7 (59·6 to 71·9)	44·1 (39·6 to 48·7)	29·1 (25·4 to 33·6)	21·6 (18·5 to 25·5)
Lampung	297·1 (267·5 to 331·4)	232·3 (213·9 to 250·2)	174·2 (159·8 to 189·2)	123·7 (116·2 to 131·8)	84·9 (79·6 to 90·3)	52·3 (46·6 to 58·1)	36·5 (31·9 to 41·8)	27·7 (23·1 to 33·2)
Maluku	380·8 (346·9 to 419·2)	296 (279·4 to 313)	224·8 (207·5 to 242·2)	168·4 (154·8 to 182·1)	123·4 (113·3 to 133·6)	89·5 (81·2 to 98·2)	62·1 (55 to 69·8)	35·9 (30·1 to 42·9)
North Maluku	318 (284·8 to 355·1)	257·3 (238·2 to 279·2)	197·5 (184·1 to 211·8)	157·7 (143 to 173·9)	114·8 (104·5 to 125)	86·1 (74·3 to 98·9)	61·6 (54·1 to 70·4)	41·7 (36 to 48·7)
West Nusa Tenggara	396·8 (362·3 to 436)	331·3 (308·8 to 353·4)	268·1 (251·7 to 286)	204·8 (192·2 to 218·2)	139·6 (129·4 to 150·4)	85·9 (75·5 to 96·9)	47·3 (41·7 to 53·5)	29·7 (24·8 to 35·6)
East Nusa Tenggara	325·3 (294 to 361·3)	260·9 (245·8 to 276·3)	199·4 (182·7 to 216)	147·4 (135·6 to 159·4)	103·7 (97·5 to 109·8)	76·2 (67·6 to 85·7)	51·4 (45·3 to 57·8)	31·6 (26·4 to 37·7)
Papua	212·4 (189 to 240·1)	184·5 (173·3 to 196·2)	154·1 (143·2 to 165·7)	127·2 (115·1 to 139·7)	105·1 (95·9 to 114·1)	81 (71 to 92)	67·3 (59·4 to 75·9)	54·1 (45·3 to 64·4)
West Papua	174·6 (152·8 to 199·8)	148·3 (134·8 to 162·8)	124·2 (114·8 to 134·8)	91·4 (82·9 to 100·8)	69 (62·4 to 75·4)	57·6 (50·5 to 65·9)	42·8 (37·7 to 49·1)	27·1 (23·3 to 32)
Riau	259·9 (232·9 to 291·5)	222·1 (206·2 to 239·3)	172·3 (160·3 to 185·6)	115·8 (108·6 to 123·5)	80·8 (75·5 to 86·2)	53 (48·8 to 57·3)	36·6 (32·4 to 41·3)	21 (17·5 to 25·2)
West Sulawesi	440·9 (402·9 to 481·8)	366·3 (342·6 to 394·2)	291·9 (270·8 to 312·2)	211·6 (193·8 to 230·3)	142·7 (130·4 to 154·9)	92·2 (83·1 to 101·2)	63·5 (55·8 to 72·8)	43·6 (37·7 to 51·1)
South Sulawesi	300·8 (271·5 to 334·7)	243·1 (224·8 to 261·7)	184 (170·8 to 198·2)	134·8 (126 to 144·2)	89·5 (81·9 to 97·7)	56·3 (51·8 to 61)	36·2 (31·6 to 41·2)	21·8 (18·2 to 26·2)
Central Sulawesi	379·8 (346 to 418·1)	275·1 (255·7 to 296·1)	205 (189·3 to 220·9)	152 (139·3 to 164·8)	107·7 (99·4 to 116·4)	72·7 (66·6 to 78·9)	50·1 (44·4 to 56·5)	35·9 (30·1 to 42·9)
Southeast Sulawesi	323·8 (292·7 to 359·7)	276 (256·8 to 296·5)	225·3 (209·3 to 242·2)	156·7 (143·4 to 169·8)	107 (100·6 to 113·6)	70 (63·3 to 76·8)	49·2 (43·3 to 55·3)	38·1 (31·9 to 45·5)
North Sulawesi	203·9 (181·3 to 230·8)	164·7 (152·7 to 178)	116·9 (108·4 to 125·8)	77·2 (70 to 84·2)	58·5 (53·1 to 64)	44·8 (39·8 to 50·2)	38·3 (33·6 to 43·5)	31·9 (26·7 to 38·1)
West Sumatra	380·6 (346·8 to 419·1)	279·8 (259·9 to 299·9)	197·8 (183·9 to 211·4)	132·4 (123·4 to 142·6)	88·2 (82·7 to 93·6)	59·9 (55·3 to 64·7)	36·5 (32·1 to 41·5)	19·7 (16·4 to 23·7)
South Sumatra	299·2 (269·5 to 333·7)	233·6 (216 to 250·9)	177·8 (163·9 to 191·8)	118·7 (111·5 to 126·3)	85·4 (78·2 to 93·2)	57·9 (53·4 to 62·6)	38·5 (34 to 43·4)	29·6 (24·7 to 35·4)
North Sumatra	229·9 (205·1 to 259·1)	184 (169·6 to 198·1)	139·4 (128·2 to 150·8)	102·3 (96 to 109)	77·2 (70·7 to 84·3)	54·2 (50 to 58·6)	40·7 (36 to 45·9)	34·1 (28·5 to 40·7)
Yogyakarta	132·4 (116·5 to 151·7)	95 (80·9 to 110·7)	68·9 (61·9 to 76)	47·9 (43·1 to 53)	32·1 (28·9 to 35·7)	21 (18·1 to 24·5)	18 (14·4 to 22·3)	16·3 (13·5 to 19·7)
Laos	314·9 (277·9 to 355·6)	295·3 (260·7 to 331·9)	266 (232·4 to 304·5)	223·6 (197 to 251·1)	167·8 (153·7 to 183·8)	117·7 (107·6 to 128·3)	68·7 (61·6 to 75·8)	40·9 (35 to 47·5)
Malaysia	144 (130·3 to 158·7)	100·6 (99·6 to 101·6)	55·5 (54·8 to 56·1)	31·6 (27·9 to 35·7)	17·5 (17·2 to 17·9)	9·1 (8·9 to 9·4)	7·3 (7·1 to 7·5)	6·4 (5·4 to 7·6)
Maldives	438 (394·6 to 484·3)	347·6 (307·5 to 390·3)	239·9 (211·5 to 269·6)	145·2 (129·2 to 162·9)	65·2 (58 to 72·8)	32·5 (28·9 to 36·7)	20·1 (17·5 to 22·8)	16·2 (13·7 to 19·4)
Mauritius	125·3 (120·8 to 129·6)	104·8 (101·7 to 107·9)	79·3 (76·6 to 82·1)	40·9 (39·1 to 42·8)	21·9 (20·6 to 23·2)	17·4 (16·2 to 18·5)	14·3 (13·3 to 15·4)	12·6 (10·8 to 14·7)
Myanmar	362·9 (322·1 to 406·9)	314·7 (279·2 to 352)	259·7 (227·8 to 297·7)	189 (165·9 to 213·6)	142·4 (125·9 to 160·5)	108·5 (96·9 to 120·9)	66·2 (58·2 to 75·2)	40·3 (34·3 to 47·1)
Philippines	198·7 (176·6 to 225·9)	142·6 (130·1 to 156·2)	106·6 (97·5 to 116·2)	81·5 (73·9 to 89·5)	55·4 (50·5 to 60·7)	37·4 (33·7 to 41·6)	29·8 (26·1 to 33·7)	22·6 (19·1 to 26·7)
Seychelles	110·9 (99·8 to 124·6)	95 (89·4 to 100·9)	73·9 (69·6 to 78·3)	36·7 (33·8 to 39·7)	19·1 (17·5 to 20·9)	13·2 (11·8 to 14·7)	13·7 (12·1 to 15·2)	11·5 (9·9 to 13·4)
Sri Lanka	155·7 (154·4 to 157)	95·1 (94·1 to 96·1)	68·5 (67·7 to 69·3)	45·6 (44·9 to 46·2)	27·6 (26·2 to 28·9)	17·2 (16·8 to 17·6)	13·4 (13·1 to 13·8)	7·6 (6·4 to 9·1)
Thailand	159 (142·2 to 177·2)	117·1 (107·5 to 127·4)	75·9 (69·4 to 82·5)	47·9 (43 to 53·7)	28·2 (24·7 to 32·6)	17·5 (15·1 to 20·4)	9·8 (9·7 to 10)	7·6 (6·8 to 8·5)
Timor-Leste	298·6 (262·4 to 338·6)	265·9 (231·7 to 299·8)	221·4 (198·8 to 246·7)	186·9 (173·6 to 202·1)	122·5 (113 to 132·8)	81·4 (73·9 to 90·4)	43·3 (37·8 to 49·1)	32·1 (27·2 to 38·5)
Vietnam	167·8 (145·1 to 193·6)	130·7 (110·9 to 151·5)	106·3 (96·2 to 116·9)	65·2 (59·6 to 71·5)	45·3 (41·8 to 49·7)	27·8 (25 to 30·6)	18·2 (15·7 to 20·7)	12·4 (10·5 to 15)
Sub-Saharan Africa	280·5 (249·8 to 314·3)	257·8 (232·4 to 283·6)	225·6 (211·1 to 241)	194·5 (185·3 to 203·7)	171·2 (164·7 to 178)	147 (141·7 to 152·2)	106·5 (100·6 to 112·7)	74·1 (65·3 to 85·2)
Central sub-Saharan Africa	244·7 (213·1 to 280·1)	228·1 (201·8 to 256·2)	210·5 (190 to 232·1)	189·3 (172·9 to 207·3)	169·2 (157·2 to 182)	153·4 (144·4 to 163·4)	104·7 (95·9 to 113·7)	58·8 (51·7 to 67·5)
Angola	250 (217·5 to 286·6)	244 (213 to 276·5)	229·9 (201·1 to 263·7)	216·8 (193·6 to 242·7)	208 (192·8 to 224·7)	168·3 (155·5 to 181)	97·7 (88·7 to 107)	54·2 (46·4 to 62·9)
Central African Republic	241·5 (210·5 to 276·4)	220·3 (191·4 to 249·8)	194·5 (173·7 to 217)	185·5 (168 to 204·9)	181·4 (166·8 to 197·1)	181·6 (164 to 199·2)	159·1 (139·6 to 179·3)	123·5 (105·4 to 146·5)
Congo (Brazzaville)	259 (226·2 to 295·6)	239·4 (208·6 to 271·7)	200·8 (174·9 to 231·4)	140·4 (125·2 to 157·2)	105·7 (97 to 115·5)	101·3 (92·6 to 110·8)	64·2 (57·4 to 71·4)	39·5 (33·7 to 46·1)

DR Congo	242 (210·7 to 277)	224 (191·5 to 256·2)	208·6 (183·8 to 236)	187·1 (168·9 to 206·2)	163·1 (149·4 to 176·7)	151·6 (139·4 to 164·8)	107·1 (95 to 119·4)	57·9 (49·3 to 69·1)
Equatorial Guinea	281·8 (247 to 320·4)	273·3 (237·9 to 309·1)	225·2 (195·5 to 256·8)	175·9 (155·9 to 197·6)	169·3 (149·4 to 189·8)	121·6 (105·5 to 137·9)	61·8 (51·9 to 72·4)	38·1 (31·9 to 46·3)
Gabon	197·4 (170·3 to 228·2)	185·5 (157·6 to 216)	154·9 (134·3 to 178·7)	111·2 (98·9 to 125)	82·3 (74·2 to 91·3)	67·8 (59·2 to 76·3)	51·5 (44·2 to 59·3)	30·1 (25·3 to 36·4)
Eastern sub-Saharan Africa	282 (250·3 to 317·1)	252·6 (225·5 to 279·4)	223·2 (207·6 to 238·7)	196 (185·6 to 207·1)	170 (162·8 to 177·5)	133·5 (127·4 to 139·7)	87·7 (81 to 94·7)	58·3 (50·5 to 68·1)
Burundi	318·9 (282·3 to 359·2)	279·2 (248·1 to 312·5)	246·9 (222·1 to 272·6)	211·6 (194 to 230·9)	181·8 (167·9 to 197·8)	165·7 (152·3 to 179·7)	98·7 (87·4 to 110·3)	65·4 (55·7 to 77·8)
Comoros	258·6 (225·7 to 295·4)	234·6 (205·3 to 267·3)	193·8 (171·3 to 219·6)	161·6 (145·3 to 178·5)	125 (112·5 to 137·5)	104·3 (93·6 to 116·8)	74·7 (64 to 85·6)	49·8 (42·1 to 59·9)
Djibouti	166·9 (143·3 to 193·9)	153·9 (130·2 to 177·6)	139·2 (119·4 to 160·5)	121·5 (107·2 to 137·1)	114·5 (101·4 to 128·6)	98·3 (86·1 to 112·4)	69·5 (58·5 to 81·3)	47 (39·4 to 56·9)
Eritrea	288·1 (252·5 to 327·6)	261·9 (226·6 to 296·1)	237·4 (204·9 to 301·1)	202·1 (184·9 to 220·5)	162 (148·5 to 175·5)	97·3 (85·7 to 109·9)	70·4 (59·8 to 82·7)	47·5 (39·8 to 57·5)
Ethiopia	304·1 (267·3 to 344·8)	280·5 (247·2 to 313·3)	258·2 (233·1 to 284·5)	230·9 (212·8 to 251·5)	195 (181·1 to 209·4)	145·2 (134·2 to 157·6)	88·1 (78·5 to 98)	52·4 (44·7 to 62·4)
Kenya	234·2 (211 to 262·4)	176·7 (163·4 to 190·2)	142·5 (133·2 to 152·3)	109·2 (101·5 to 117·3)	94·2 (87·7 to 101)	84·3 (77·6 to 91·8)	57·7 (51·1 to 65·1)	40·6 (34·6 to 47·7)
Baringo	211·9 (190·7 to 237·7)	182 (165·3 to 199·1)	179·8 (164·8 to 194·6)	155·3 (142·6 to 168)	133·8 (120·6 to 147·3)	105·5 (95·8 to 116·4)	77·1 (67·2 to 88)	53·5 (45·7 to 62·8)
Bomet	83 (73·8 to 94)	70·1 (60·6 to 80·6)	68·7 (59·1 to 79·2)	61·6 (51·7 to 72·5)	48·9 (41·7 to 56·3)	43·9 (37·2 to 51·8)	37·7 (30·9 to 44·5)	24·2 (20·6 to 28·4)
Bungoma	274·7 (248·2 to 306·8)	216 (197·6 to 234·5)	168·5 (147·7 to 190·5)	132·1 (121·1 to 143·5)	112·6 (102·1 to 123·8)	94·6 (85·7 to 104·7)	59·4 (50·6 to 69·4)	35·3 (30·1 to 41·4)
Busia	401·3 (365·7 to 442·4)	322·4 (292·7 to 351·4)	246·2 (223 to 268·8)	155·3 (142·8 to 169)	133·7 (120·1 to 148·2)	129·9 (115·2 to 145·6)	84·1 (72·5 to 96·2)	53 (45·2 to 62·1)
Elgeyo Marakwet	126·9 (113·4 to 143·2)	111·5 (100·4 to 122·9)	118 (107·7 to 128·5)	111·8 (102·5 to 121·3)	84·9 (76·8 to 93)	57·5 (52 to 63·7)	33·3 (28·9 to 38·1)	21·1 (17·9 to 24·8)
Embu	314 (284·7 to 348·7)	228·9 (202·7 to 255·5)	145·4 (128·4 to 163·8)	88·3 (75 to 102·7)	65·7 (57·3 to 74)	53·7 (48·4 to 59·4)	46·1 (37·9 to 54·4)	33·2 (28·3 to 39)
Garissa	219·2 (197·3 to 245·7)	175·3 (151·9 to 199·8)	146·4 (125·6 to 168·7)	121·3 (110 to 132·9)	102·1 (88·2 to 116·3)	75·9 (64·9 to 88·8)	48·4 (42·4 to 55)	31·5 (26·8 to 37)
Homa Bay	445 (407 to 488·6)	355 (325·4 to 385·2)	322·5 (295 to 352·1)	259·6 (242·2 to 278·4)	212·1 (193·3 to 232·3)	195·3 (176·3 to 217)	107·4 (93·9 to 123)	67·7 (57·9 to 79·1)
Isiolo	498·9 (458·3 to 545·1)	413·1 (371·9 to 453·7)	375·1 (341·5 to 411·7)	294·7 (258 to 333·7)	229·3 (201·7 to 257·4)	170 (155·1 to 187·2)	133·4 (116·1 to 151·5)	92·8 (79·5 to 108·2)
Kajiado	115·2 (102·9 to 130·1)	96·6 (86 to 108·8)	101·4 (92·6 to 110·2)	84·7 (76·2 to 93·2)	60·4 (53·6 to 67·6)	51·8 (46·8 to 57·5)	34·8 (30·1 to 40·1)	20·3 (17·3 to 23·9)
Kakamega	271 (244·8 to 302·3)	217 (198·2 to 236·4)	181·5 (163·7 to 199·7)	146·2 (123·9 to 170·9)	136·1 (125 to 146·8)	132·3 (118·9 to 147)	82·4 (71·2 to 94·3)	49·5 (42·3 to 58)
Kericho	72·7 (64·7 to 82·5)	61·9 (54·7 to 69·8)	67·9 (60·5 to 75·9)	71·7 (65 to 78·7)	52·5 (46·9 to 57·8)	48·5 (42 to 55·6)	37·4 (32·5 to 42·7)	27 (23 to 31·8)
Kiambu	115·4 (103 to 130·3)	82·4 (73·6 to 92·8)	60·7 (54·8 to 67·2)	47·9 (42·5 to 53·5)	45 (40·1 to 50·1)	50·1 (42·9 to 58·5)	41·5 (35·7 to 47·2)	30·5 (26 to 35·9)
Kilifi	369·8 (336·2 to 408·8)	276·5 (252·2 to 300·1)	225·8 (209·6 to 242·1)	169·5 (152·2 to 187·3)	130 (115·5 to 144·8)	101 (92 to 110·9)	65·2 (56·8 to 73·9)	42·4 (36·2 to 49·7)
Kirinyaga	241·6 (217·9 to 270·4)	185·6 (168·7 to 204·4)	155·3 (141·4 to 169·4)	79·8 (68·2 to 92·4)	58·9 (49·4 to 69·6)	58·8 (52·3 to 66·1)	48·4 (41·7 to 55·6)	43 (36·7 to 50·5)
Kisii	230·9 (208 to 258·5)	181·8 (164·3 to 199·3)	150·4 (134·4 to 167·6)	126·3 (112·3 to 141·5)	114·8 (104 to 125·9)	88·7 (80 to 98·6)	47·2 (39·5 to 55·7)	31·1 (26·4 to 36·5)
Kisumu	221·8 (199·9 to 248·5)	176·2 (159·6 to 194·6)	159·1 (141·8 to 177·2)	146·3 (130·2 to 164)	135·7 (122·2 to 149·6)	126·4 (114·4 to 139·6)	64·1 (53·8 to 75·4)	40·9 (34·9 to 48·1)
Kitui	256·9 (231·8 to 287·2)	206·4 (186·6 to 226·6)	156·6 (143·3 to 171·7)	101·9 (92·6 to 111·3)	81 (72 to 91·1)	71·4 (64·5 to 79·2)	45·4 (39·5 to 52)	29 (24·7 to 34·1)
Kwale	354·9 (322·5 to 392·7)	274·3 (249·2 to 299·7)	215·7 (200·7 to 231·7)	159·1 (143·6 to 175·4)	122·7 (110·5 to 136·7)	90·9 (83·3 to 99·5)	68 (59·4 to 77)	44·5 (38 to 52·3)
Laikipia	76·7 (68·3 to 86·9)	60·3 (51·3 to 70·6)	61·9 (54·4 to 70·3)	52·6 (45·6 to 61·1)	47·9 (38·7 to 57·4)	47·9 (41·6 to 54·8)	30·2 (26·1 to 34·8)	17·7 (15 to 20·8)
Lamu	414·5 (378·2 to 456·3)	305·9 (278·8 to 334·8)	232 (198·8 to 266·8)	124·2 (105·5 to 145·3)	126 (103·7 to 151·4)	110·7 (96·5 to 127·6)	87·2 (72 to 104·6)	60·6 (51·8 to 70·9)
Machakos	174 (156·3 to 195·6)	136·3 (122 to 150·5)	115·5 (100·9 to 130·8)	81·6 (69·4 to 94·9)	62·2 (55·3 to 69·5)	55·9 (50·9 to 61·9)	38·3 (31·4 to 45·5)	27 (23 to 31·8)
Makueni	202·5 (182·1 to 227·2)	160·2 (141·8 to 177·9)	133·6 (121·6 to 146·5)	98·8 (84·1 to 115·1)	82·5 (73·1 to 92·9)	63·7 (56·7 to 71·3)	41·3 (36·1 to 47)	24·5 (20·9 to 28·8)
Mandera	162·5 (145·6 to 182·9)	137·4 (119·6 to 157·4)	120·6 (104·7 to 138·3)	112·7 (102·2 to 124)	117·1 (101·8 to 133·6)	94·9 (80·4 to 111·6)	76·7 (67·3 to 86·8)	58·9 (50·3 to 69)
Marsabit	229·1 (206·4 to 256·6)	183 (160·7 to 206·8)	166·2 (152·7 to 179·7)	135·2 (116·8 to 155·2)	112·2 (99·5 to 125·4)	86·7 (77·6 to 96·7)	78 (68·5 to 88·8)	60·2 (51·4 to 70·5)
Meru	153 (136·9 to 172·3)	118 (107 to 129·4)	91·1 (82·1 to 100·9)	60·7 (54·5 to 66·4)	52·4 (46·8 to 58·1)	51·7 (46·5 to 57·7)	51·9 (45·3 to 59)	44·6 (38 to 52·3)
Migori	373·1 (339·5 to 412·3)	309·1 (278·5 to 339·6)	271·2 (249·3 to 295·4)	253·9 (235·9 to 271)	223·1 (202·5 to 244·1)	193·8 (174·9 to 214·4)	109·9 (94·2 to 126·3)	72 (61·6 to 84)
Mombasa	266·3 (240·6 to 297·4)	182·5 (163·7 to 200·8)	135·9 (126 to 146·7)	91·8 (81·9 to 102·1)	81·9 (72·7 to 91·5)	75·4 (68 to 82·8)	53·5 (46·7 to 60·6)	34·9 (29·7 to 41)
Murang'a	195·1 (175·5 to 219·2)	138·2 (124·6 to 152·8)	103 (91·3 to 114·8)	59·9 (51·2 to 69·7)	44·4 (39·4 to 50)	45 (40 to 50·7)	43·4 (37·3 to 49·4)	32·3 (27·5 to 38)
Nairobi	313·9 (284·5 to 349·3)	177·5 (161·9 to 194·3)	106 (96·9 to 115·4)	64·3 (56·6 to 72·8)	66·4 (58·9 to 73·7)	67·2 (60·2 to 74·5)	36·1 (31·7 to 41·1)	23·5 (20 to 27·6)
Nakuru	67·9 (60·4 to 76·9)	53·1 (47·3 to 60·1)	55·6 (50 to 62)	53·2 (47·8 to 58·6)	47·1 (42·5 to 51·8)	50·2 (45·2 to 55·7)	45·9 (39·8 to 52·5)	34·5 (29·4 to 40·5)
Nandi	104 (92·8 to 117·6)	86·5 (76·9 to 96·3)	77 (70·7 to 83·6)	67·5 (55·1 to 80·2)	56·7 (51 to 62·2)	44·5 (40·1 to 49·4)	31·6 (27·3 to 36·4)	21·3 (18·1 to 25·1)
Narok	47·5 (42·2 to 53·9)	46·1 (40·9 to 52·8)	61·4 (54·8 to 68·2)	83·6 (76·3 to 90·9)	72·3 (64·7 to 79·8)	49·9 (45·1 to 55·3)	35 (30·3 to 40·3)	24·7 (21 to 29)
Nyamira	108·2 (96·5 to 122·3)	86 (76·3 to 96·5)	68·5 (62·3 to 75·2)	61·7 (54·8 to 68·3)	49·7 (44·2 to 55·5)	50·6 (44·5 to 57·8)	35·2 (30·4 to 40·7)	24·8 (21·1 to 29·2)
Nyandarua	186 (167·1 to 208·9)	123·8 (111·6 to 137·4)	84·2 (70·1 to 100·2)	48·7 (40·3 to 58·3)	39·3 (31 to 47·9)	43·6 (36·5 to 52·4)	53·4 (43·7 to 64·3)	43 (36·6 to 50·5)
Nyeri	162·4 (145·7 to 182·7)	114·6 (100·7 to 130·2)	80 (71·6 to 88·7)	46·9 (41 to 52·8)	40·8 (34·9 to 47·3)	44·8 (38·2 to 51·9)	40·7 (35·2 to 46·5)	34·5 (29·4 to 40·6)
Samburu	78·2 (69·6 to 88·6)	69·8 (60 to 79·6)	75·5 (63·9 to 88)	81·1 (66·2 to 96·8)	86·3 (69·3 to 104·6)	80·4 (69 to 93·9)	68·8 (57·1 to 81·8)	52·3 (44·6 to 61·3)
Siaya	339·7 (308·4 to 376·4)	277·9 (254·9 to 301·6)	229 (210·4 to 247·9)	199·9 (186·4 to 213·5)	198·8 (180·4 to 218·1)	181·2 (163·5 to 200·8)	99·2 (86·9 to 112·9)	60·6 (51·8 to 70·8)

Taita Taveta	250·4 (226 to 280)	188·5 (171·4 to 206·4)	175·1 (161·3 to 189·6)	111·1 (88·1 to 134·9)	76·2 (68·2 to 85·1)	73·4 (63·4 to 85·5)	49·4 (43 to 56·4)	29·8 (25·3 to 35)
Tana River	193·9 (174·3 to 217·8)	153·8 (140·1 to 168·7)	137·5 (118·2 to 158·6)	118 (95·1 to 143·5)	99·6 (88·1 to 111·8)	102·3 (87 to 120·4)	78·1 (64·5 to 93·2)	51·6 (44·1 to 60·6)
Tharaka Nithi	135·3 (121·1 to 152·5)	109·9 (97·2 to 123·3)	96·8 (84·6 to 110·2)	69·9 (59·8 to 81·3)	72 (63·3 to 82·1)	70·2 (62·8 to 78·9)	44·3 (38·8 to 50·5)	30·6 (26·1 to 36)
Trans Nzoia	128 (114·4 to 144·4)	96·7 (85·9 to 107·6)	94·8 (86·4 to 103·5)	89 (73 to 105·3)	67·8 (60 to 75·2)	56·2 (50·6 to 62·6)	39·3 (34·2 to 45·2)	25·1 (21·4 to 29·5)
Turkana	139·2 (124·5 to 156·9)	121·1 (106·1 to 137)	130·6 (111·5 to 151·7)	141·6 (118·3 to 166·5)	120·1 (105·2 to 134·8)	87·9 (75·5 to 102·8)	60·6 (50·5 to 71·4)	43·6 (37·2 to 51·1)
Uasin Gishu	78·2 (69·6 to 88·5)	58·6 (52·1 to 66·4)	55·1 (49 to 61)	55·4 (50·3 to 60·8)	52·1 (46·9 to 57)	44·6 (40·2 to 49·5)	35·1 (30·4 to 40·3)	25·1 (21·3 to 29·5)
Vihiga	173·4 (155·7 to 194·9)	138·6 (124 to 154·4)	114·2 (98·3 to 132·3)	95·7 (80·7 to 113·3)	96·4 (86·6 to 107·2)	94·1 (81·9 to 107·7)	73·1 (62·2 to 85·5)	48·8 (41·6 to 57·3)
Wajir	309·5 (280·5 to 344·4)	245·7 (216·3 to 278)	203 (175·4 to 231·3)	153 (139·9 to 166·9)	141·5 (122·1 to 161·8)	120·8 (103·6 to 141·3)	89·6 (78·4 to 101·4)	61·8 (52·8 to 72·4)
West Pokot	154 (138 to 173·4)	133·4 (120·4 to 146·6)	141·1 (128·9 to 153·3)	137·6 (126·3 to 149)	123·4 (111·6 to 134·8)	93·1 (84·4 to 102·8)	71·1 (61·9 to 81·5)	51·7 (44·1 to 60·6)
Madagascar	241·6 (210·5 to 276·5)	213·2 (185·2 to 242·5)	182·2 (163·3 to 201·8)	163·5 (151·2 to 176·6)	143·5 (132·7 to 154)	106·3 (97 to 115·7)	79·8 (69·9 to 90)	56·6 (48·1 to 67·7)
Malawi	399·7 (359·1 to 443·3)	363·3 (326·4 to 401·9)	319 (293·7 to 344·5)	254·2 (237·1 to 271·9)	212·7 (200·9 to 225·6)	154·8 (144·9 to 165·4)	94 (85·1 to 103·2)	59·1 (50·6 to 70)
Mozambique	294·4 (258·3 to 334·4)	273·2 (237·1 to 311·2)	246·5 (217·9 to 275)	226·5 (207·3 to 245·7)	209·4 (195·7 to 224·4)	160·1 (148·2 to 172·6)	106·7 (94·9 to 118·5)	69·4 (59 to 82·8)
Rwanda	353·6 (316·7 to 393·5)	283·2 (259·7 to 308·9)	267·5 (249·1 to 285·7)	215 (201 to 229·7)	170·1 (158·4 to 182·4)	148 (137·3 to 158·8)	73·1 (65·3 to 81·9)	46·6 (39·6 to 55·8)
Somalia	239 (207·5 to 274·5)	230·5 (198·7 to 264·3)	217·6 (187·8 to 250·6)	201 (177 to 224·9)	187·1 (168·9 to 206·6)	160·1 (142·5 to 178·5)	137·6 (121·1 to 155·2)	95·4 (80·8 to 114·1)
South Sudan	259·3 (226·4 to 296·1)	246·5 (215·9 to 280·6)	231·1 (200·2 to 268·3)	213·9 (187 to 242·6)	194·4 (175 to 216·3)	149·4 (134·7 to 165·2)	118·2 (103·8 to 133·5)	92·6 (78·9 to 108)
Uganda	243·6 (212·2 to 278·9)	225 (196·3 to 255·4)	198·2 (180·3 to 216·3)	195·8 (182·2 to 209·5)	167·6 (157·7 to 177·4)	134·3 (126·6 to 142·9)	88·1 (80·6 to 96)	58·4 (50·4 to 68·7)
Tanzania	259·8 (227·2 to 296·4)	241·7 (212·1 to 274·7)	209·2 (191·7 to 227)	174·6 (163·4 to 186·7)	153·3 (144·6 to 162·8)	121·8 (113·9 to 130·3)	80·5 (72·1 to 89·2)	57·1 (48·9 to 67·7)
Zambia	248·6 (217 to 283·9)	223·4 (194·7 to 253·7)	186 (170·1 to 202·7)	163·2 (150·9 to 175·4)	159·6 (149·5 to 170·1)	137·9 (127·4 to 149·3)	81·2 (72·3 to 90·6)	51·8 (44 to 61·8)
Southern sub-Saharan Africa	159 (137 to 184·1)	143·9 (124·7 to 164·1)	121·3 (109·5 to 133·9)	101·3 (95·6 to 107·5)	75·3 (71 to 79·8)	77 (72·4 to 82)	59·5 (54·8 to 64·7)	42 (36·3 to 49·3)
Botswana	120·2 (102·4 to 141)	117·9 (100·3 to 137)	104 (93·3 to 116·8)	75·4 (67·9 to 83)	63·3 (57·5 to 69·6)	74·9 (67 to 83·3)	52·8 (45 to 61·2)	41·3 (34·7 to 49·9)
eSwatini	245·6 (213·8 to 281·3)	222·3 (192·9 to 252·9)	168·4 (144·5 to 195)	116·1 (102 to 131·3)	75·1 (68 to 82·4)	89·6 (82·4 to 97·5)	76·5 (68·4 to 84·9)	47·3 (40·3 to 55·3)
Lesotho	160·5 (141·2 to 184·5)	153·2 (140·5 to 166·1)	145 (133·9 to 156·9)	110·4 (100·1 to 120·7)	89·5 (82 to 98)	97·2 (88·9 to 105·9)	85·4 (74·9 to 96·9)	64·4 (54·9 to 75·6)
Namibia	135·5 (115·6 to 158·7)	126 (107 to 146·5)	105·4 (92·7 to 119·5)	93·8 (85·5 to 102·6)	69·4 (63 to 76·3)	66 (59·6 to 73·2)	50·7 (44·1 to 57·8)	35·1 (29·6 to 42·2)
South Africa	162·7 (139·6 to 189·3)	147·6 (127 to 169·8)	125·3 (111·3 to 140·8)	106·5 (99·2 to 114·2)	78·2 (72·6 to 83·8)	76·4 (70·2 to 83·4)	53·3 (46·9 to 60)	36·9 (31·6 to 43·6)
Zimbabwe	141·1 (120·7 to 164·6)	126·9 (108·6 to 147)	103·9 (93 to 115·6)	88·4 (80·9 to 96·5)	67·7 (62·9 to 72·9)	76·7 (70·9 to 83·3)	72·9 (66·3 to 80·2)	52·5 (45 to 62)
Western sub-Saharan Africa	311·5 (279·9 to 345·6)	295·8 (269·3 to 323·6)	255·5 (239·7 to 272)	214 (205·2 to 223)	190·4 (184·1 to 197·3)	168·6 (162·9 to 174)	129·3 (122·3 to 136·5)	95·3 (84·7 to 108·5)
Benin	304·8 (270·9 to 342)	289·6 (264·8 to 315·1)	248·2 (229 to 267·1)	203·7 (188·4 to 217·6)	177·5 (165·1 to 190·1)	141·7 (131·6 to 152·2)	113 (103·4 to 122·9)	85 (73·8 to 99·3)
Burkina Faso	382·4 (341·1 to 427·1)	343·7 (306 to 384·1)	295·9 (270·9 to 321·7)	250 (233·1 to 268·7)	210 (196·5 to 222·7)	179·4 (165·1 to 193·6)	139·5 (123·7 to 156)	108·8 (93 to 129)
Cape Verde	170·4 (153·5 to 188·6)	164·5 (159·1 to 169·9)	166·4 (162·4 to 170·8)	94·2 (90·7 to 98·2)	68·1 (65·1 to 71·3)	51·5 (45 to 59·5)	27·1 (24·9 to 29·5)	17·1 (14·7 to 19·8)
Cameroon	305·5 (275·9 to 340·9)	437·6 (416·1 to 461·3)	204·5 (190·1 to 220·6)	168 (155·8 to 180·9)	138 (127·9 to 148)	131·9 (121·9 to 142·8)	103 (91 to 116)	71·7 (61·2 to 84)
Chad	294·4 (258·2 to 334·5)	270·6 (234·2 to 307·3)	255·6 (225·9 to 284·8)	230·9 (210·9 to 252·5)	198·4 (183·4 to 214·3)	182·9 (168·8 to 198·4)	147·6 (131·9 to 165·2)	112·9 (97 to 133·3)
Côte d'Ivoire	320·2 (285·8 to 361·6)	276·3 (254·6 to 298·2)	220·5 (204·1 to 236·6)	161·8 (150·3 to 175)	147·6 (137·9 to 158·6)	137·6 (127·6 to 147·8)	107·3 (95·8 to 120·2)	73·3 (62·8 to 85·5)
The Gambia	199·7 (172·5 to 230·6)	179·6 (154·4 to 204·7)	153·9 (133·5 to 176·7)	138 (121·4 to 155·4)	111·9 (100·7 to 124·4)	87·2 (78 to 97·2)	59·3 (51·3 to 67·8)	37·8 (31·9 to 45·5)
Ghana	251·4 (223·3 to 285·7)	210·8 (193·7 to 229·3)	176·9 (164·1 to 189·9)	146·2 (136 to 155·9)	121 (113·9 to 128·6)	99 (92·9 to 105·3)	76·5 (69·8 to 83·3)	52·2 (44·9 to 60·7)
Guinea	362·1 (322·4 to 405·1)	331·9 (294·9 to 370·5)	303·1 (272·7 to 338·2)	268·5 (246·6 to 293·1)	224·9 (208·3 to 242·4)	176·2 (162·2 to 190·7)	132·2 (118·3 to 146·4)	97·1 (83·8 to 111·9)
Guinea-Bissau	387·7 (345·2 to 433·7)	339·8 (299·6 to 380·7)	299·2 (264 to 337·9)	252·6 (227·7 to 278·9)	211·6 (192·6 to 232·4)	164·2 (149·2 to 179·7)	106 (93·5 to 118·9)	71·2 (60·7 to 83)
Liberia	368 (328 to 411·4)	337·5 (302 to 375·2)	288·9 (260·9 to 315·6)	245 (225 to 265·3)	223·6 (208·9 to 238·5)	169·1 (157·9 to 180·4)	88·8 (79·6 to 98·5)	60·9 (51·9 to 72·6)
Mali	397·2 (356·2 to 441·3)	368·3 (330·5 to 408)	339 (312·7 to 364·9)	281·5 (262·9 to 299·3)	234·5 (219·2 to 249·9)	196·3 (182·2 to 212·8)	151·5 (137·9 to 165·8)	118·5 (102·6 to 138·4)
Mauritania	228·6 (200·9 to 259·8)	198·3 (181·6 to 215·8)	156·9 (144 to 169·6)	136·5 (125·7 to 148·5)	110·9 (102·1 to 120·1)	91·8 (83·8 to 100·2)	64·8 (58·4 to 71·8)	42·8 (36·6 to 50·8)
Niger	384·9 (343·6 to 429·6)	357·5 (315·8 to 400·4)	328·9 (298·5 to 359·6)	306·7 (284·8 to 328·9)	287 (268·8 to 306·7)	227·8 (208·6 to 247·5)	144·3 (126·5 to 162·6)	110·7 (94·5 to 131·5)
Nigeria	296·7 (261 to 336·2)	276·9 (244·1 to 311·9)	256·7 (234·4 to 280)	213·4 (200·5 to 227·3)	199·8 (190·4 to 210·1)	181·7 (172·8 to 191·1)	143 (133·2 to 153·4)	103·7 (90·6 to 120·3)
São Tomé and PrÍncipe	316·1 (288·1 to 349·5)	265·7 (254·1 to 277·1)	147 (141 to 153)	114 (107·6 to 121·5)	102·1 (93·9 to 109·7)	72·9 (65·9 to 80·4)	39·7 (34·5 to 45·6)	25·4 (21·4 to 30)
Senegal	283·4 (253·7 to 316·1)	263·9 (243·6 to 284·7)	251·4 (234·2 to 267·2)	195·2 (182·8 to 208·9)	138·2 (130 to 146·7)	115·7 (108·8 to 123)	70·9 (65·4 to 76·7)	49·3 (42·8 to 57·7)
Sierra Leone	342·8 (303·7 to 385·5)	314·7 (279·2 to 353·4)	285·9 (252 to 323)	262·6 (235·7 to 290·5)	236·8 (220·6 to 253·7)	209·9 (195·7 to 225·5)	153·3 (139·2 to 167·6)	102·1 (88·6 to 117·1)
Togo	280·6 (246·4 to 318·7)	258 (225·4 to 293·1)	216·3 (195·6 to 238·9)	172·5 (157 to 188·8)	144·3 (132·3 to 157·4)	122·6 (111·1 to 135·7)	93 (82·2 to 104·3)	63·2 (53·9 to 75·3)

Supplemental Results Table 4b: Under-5 mortality rates by location, male, 1950, 1960, 1970, 1980, 1990, 2000, 2010, 2019

Location	1950	1960	1970	1980	1990	2000	2010	2019
Global	212·2 (191·9 to 234·4)	286·2 (241 to 349·8)	145·7 (137·6 to 154·3)	115·2 (109·8 to 120·7)	88·8 (85·2 to 92·7)	73·3 (70 to 76·8)	55 (51·5 to 58·7)	38·8 (34·5 to 44)
Central Europe, eastern Europe, and central Asia	111·7 (99·6 to 126)	78·2 (73·5 to 83·8)	55 (51·6 to 58·8)	42·2 (40·5 to 44·2)	35 (33·5 to 36·7)	30 (28·5 to 31·7)	18 (16·7 to 19·4)	12·8 (11·6 to 14·4)
Central Asia	232·7 (198·7 to 271·5)	163·5 (142·9 to 186·4)	106·6 (94·8 to 119·6)	78·2 (72·4 to 84·5)	62·6 (58·2 to 67·5)	53·5 (49·2 to 58)	34·1 (30·5 to 38)	23·1 (20·4 to 26·5)
Armenia	289·3 (248·8 to 335·1)	178·6 (150·4 to 208·4)	102·4 (86 to 120·6)	66·4 (59·2 to 74·8)	44·4 (40·3 to 48·8)	32·1 (28·7 to 36·1)	18·3 (15·7 to 21·1)	12·5 (10·4 to 15·3)
Azerbaijan	399·7 (351 to 453·2)	275·9 (237 to 316·3)	152·6 (131·4 to 175·2)	93·7 (82·2 to 106·1)	81·7 (73·1 to 91·9)	69 (60·8 to 77·7)	40·7 (34·7 to 47)	29·9 (25·1 to 36·1)
Georgia	269·9 (231 to 314·1)	167 (140 to 195·2)	99 (84 to 116·1)	62·2 (54·4 to 70·3)	46·1 (40·9 to 51·8)	39·1 (33·9 to 44·5)	18·1 (15·1 to 21·2)	11·3 (9·4 to 13·7)
Kazakhstan	131·4 (110 to 156·6)	97 (80·8 to 114·8)	61·7 (53·2 to 72)	51·1 (46·1 to 56·8)	42·4 (38 to 47·1)	40 (35·2 to 45·2)	24·2 (20·6 to 28·1)	13·5 (11·3 to 16·3)
Kyrgyzstan	238·3 (202·9 to 279)	178·8 (151 to 209)	122·6 (106·3 to 142·1)	86·7 (77·6 to 96·1)	63·2 (57·5 to 69·8)	48·7 (43·4 to 54·3)	30·1 (29·3 to 30·9)	19·1 (17·4 to 20·8)
Mongolia	357 (310·1 to 408·8)	294·7 (251·7 to 337·1)	208·3 (181·9 to 236·7)	140·4 (126 to 156)	97 (88·9 to 106·1)	62·7 (57·1 to 68·4)	32·4 (28·7 to 36·3)	18·4 (15·4 to 22·4)
Tajikistan	284·6 (245·3 to 329·1)	222·2 (190·9 to 255·2)	150·1 (128·3 to 175·5)	110·1 (97·7 to 123·4)	88·4 (80·3 to 97·3)	72·7 (66·4 to 79·5)	47·2 (42·1 to 52·6)	36·6 (31·1 to 42·9)
Turkmenistan	253·7 (216·5 to 296·1)	184·3 (158·5 to 213·3)	123·7 (102·9 to 149·9)	104·5 (89·3 to 120·7)	79·3 (69·6 to 90·5)	65 (57·7 to 72·6)	43·8 (38·3 to 49·6)	28·4 (23·8 to 33·6)
Uzbekistan	172·6 (145·3 to 204·2)	128·3 (107·1 to 150·6)	96·6 (83 to 112·7)	73·4 (65·4 to 81·8)	58·3 (52·3 to 64·3)	50·2 (44·4 to 57·5)	34·7 (29·3 to 40·6)	23·6 (19·8 to 28·5)
Central Europe	143·4 (138·7 to 148·8)	81·5 (80·4 to 82·6)	52·6 (51·8 to 53·3)	32·7 (32·2 to 33·1)	25 (24·5 to 25·4)	15 (14·6 to 15·4)	8·1 (7·9 to 8·3)	5·6 (4·9 to 6·3)
Albania	208·9 (181 to 240·9)	142·1 (120·1 to 165·4)	92·7 (78·5 to 108·4)	54·4 (46·7 to 62·4)	42·2 (37·7 to 47)	29·7 (26·1 to 34·1)	17·6 (14·8 to 20·6)	12·9 (10·7 to 15·6)
Bosnia and Herzegovina	210·3 (206·1 to 214·9)	130·9 (128·1 to 134·1)	76·5 (74·6 to 78·5)	41 (39·6 to 42·4)	19 (18·1 to 19·8)	12 (11·3 to 12·8)	7·6 (7 to 8·2)	5·9 (4·9 to 7·1)
Bulgaria	136·8 (134 to 139·6)	61·3 (59·9 to 63)	35·8 (34·8 to 36·8)	28 (27·1 to 28·9)	21·7 (20·8 to 22·5)	18·9 (18·1 to 19·8)	12·4 (11·8 to 13·1)	8·1 (6·8 to 9·6)
Croatia	166 (162·3 to 170)	84·6 (82·2 to 86·9)	46·7 (45·3 to 48·3)	24·2 (23·2 to 25·2)	13·6 (12·9 to 14·4)	9·1 (8·6 to 9·7)	5·6 (5·2 to 6·1)	4·2 (3·5 to 5·1)
Czech Republic	81·6 (80 to 83·3)	27·3 (26·5 to 28·2)	26·9 (26·2 to 27·7)	21·5 (20·8 to 22·1)	13·7 (13·2 to 14·3)	5·9 (5·6 to 6·3)	3·9 (3·7 to 4·2)	3 (2·6 to 3·5)
Hungary	113·7 (111·5 to 116)	60·2 (58·9 to 61·6)	45·2 (44·1 to 46·4)	28·2 (27·5 to 29)	19·4 (18·7 to 20·1)	11·5 (10·9 to 12·1)	6·2 (5·8 to 6·5)	4·3 (3·7 to 5·1)
Montenegro	75·6 (72·2 to 79·5)	60·7 (57·9 to 63·8)	42·6 (40·3 to 44·7)	26·3 (24·6 to 28)	17·5 (16 to 19)	14·7 (13·5 to 16)	7·7 (6·9 to 8·5)	4 (3·4 to 4·8)
North Macedonia	219·1 (215 to 223·9)	179 (175·3 to 182·8)	103·7 (101·2 to 106·5)	60·9 (59 to 63·1)	37·4 (35·7 to 39·1)	16·5 (15·6 to 17·6)	10·1 (9·3 to 11)	9 (7·6 to 10·8)
Poland	143·3 (141·5 to 145·3)	70·7 (69·8 to 71·5)	43·8 (43·2 to 44·5)	28·2 (27·8 to 28·7)	20·8 (20·4 to 21·2)	10·5 (10·2 to 10·8)	6·3 (6·1 to 6·6)	4·2 (3·5 to 5)
Romania	135·2 (113·7 to 160·4)	96 (94·3 to 97·7)	61·2 (60 to 62·3)	38·4 (37·6 to 39·2)	37 (36·3 to 37·8)	24·7 (24·1 to 25·4)	12·7 (12·2 to 13·2)	8·8 (7·6 to 10·4)
Serbia	197·6 (194·1 to 201·3)	112·2 (110·1 to 114·3)	75·5 (73·8 to 77·2)	44·8 (43·7 to 45·9)	32·8 (31·7 to 33·8)	17·7 (15·1 to 20·6)	7·7 (7·3 to 8·1)	4·6 (3·9 to 5·5)
Slovakia	136·7 (133·6 to 140·2)	38·9 (37·7 to 40·2)	34·6 (33·3 to 35·8)	26·8 (25·8 to 27·7)	16·4 (15·7 to 17·1)	10·9 (10·2 to 11·5)	7·4 (7 to 7·9)	5·8 (4·9 to 7)
Slovenia	129·2 (125·2 to 133·2)	73·4 (71 to 76)	39·4 (37·8 to 40·9)	20 (19·1 to 21)	11 (10·4 to 11·7)	6 (5·5 to 6·5)	3·5 (3·1 to 3·8)	2·1 (1·8 to 2·5)
Eastern Europe	66 (54·7 to 79·4)	49·7 (47·7 to 51·8)	32·6 (31·2 to 34·3)	30·6 (29·5 to 31·8)	23·3 (22·6 to 24·2)	20·6 (20·3 to 21)	10·8 (10·6 to 10·9)	7·6 (6·9 to 8·4)
Belarus	82·6 (68·9 to 98·8)	62·2 (52·3 to 73·1)	38·7 (32·5 to 45·5)	28·7 (24·3 to 33·5)	21·4 (18·3 to 25)	18·8 (15·9 to 21·9)	9·4 (7·9 to 11·3)	6 (5 to 7·4)
Estonia	60·5 (50·7 to 72·2)	41·4 (39·6 to 43·2)	25·2 (23·9 to 26·4)	25 (23·8 to 26·2)	20·2 (19·3 to 21·2)	12·5 (11·6 to 13·5)	5 (4·5 to 5·4)	2·8 (2·3 to 3·4)
Latvia	53 (44·2 to 63·4)	37·3 (35·7 to 39·2)	25·5 (24·5 to 26·6)	24·9 (23·8 to 26)	19·6 (18·8 to 20·5)	14·9 (14 to 15·8)	8·3 (7·8 to 8·9)	4·5 (3·8 to 5·4)
Lithuania	76·6 (64·3 to 91·2)	53·6 (52·1 to 55·1)	26·1 (25·2 to 27·1)	22·8 (22 to 23·7)	16·6 (15·8 to 17·3)	12·1 (11·5 to 12·7)	6·6 (6·2 to 7)	4·3 (3·7 to 5)
Moldova	192·4 (162·8 to 226·5)	135·6 (113·7 to 158·4)	86·3 (73·5 to 101·2)	59·2 (51·6 to 67·5)	36·1 (32·3 to 40·5)	29·9 (26·5 to 34)	17·9 (15·3 to 20·7)	13·5 (11·3 to 16·3)
Russia	64·3 (53·2 to 77·4)	48·7 (48·3 to 49·2)	31·9 (31·6 to 32·2)	31 (30·7 to 31·3)	24·2 (24 to 24·4)	21·8 (21·6 to 22·2)	10·6 (10·4 to 10·8)	7·2 (6·3 to 8·3)
Ukraine	55·4 (45·9 to 66·8)	41·8 (35·1 to 49·6)	28·7 (24 to 34·2)	27·6 (23·4 to 31·8)	20·6 (17·8 to 23·5)	17·5 (17·2 to 18)	11·6 (11·3 to 11·9)	9·8 (8·7 to 10·9)
High income	73 (71·6 to 74·6)	47·3 (46·8 to 48)	31·9 (31·5 to 32·3)	18·6 (18·3 to 18·8)	12·3 (12·2 to 12·4)	8·2 (8·2 to 8·3)	6·5 (6·4 to 6·6)	5·4 (5·1 to 5·6)
Australasia	34·3 (33·5 to 35·3)	28·1 (27·6 to 28·6)	23·8 (23·4 to 24·2)	15·2 (14·8 to 15·5)	11 (10·8 to 11·3)	7·3 (7·1 to 7·5)	5·7 (5·6 to 5·9)	4 (3·8 to 4·3)
Australia	33·4 (32·6 to 34·2)	27·3 (26·7 to 27·8)	23·9 (23·5 to 24·4)	14·5 (14·2 to 14·9)	10·4 (10·2 to 10·7)	7 (6·8 to 7·2)	5·4 (5·2 to 5·6)	3·9 (3·6 to 4·1)
New Zealand	38·1 (36·7 to 39·6)	31·3 (30·3 to 32·4)	23 (22·2 to 23·9)	17·8 (17·1 to 18·6)	13·5 (13 to 14·1)	8·5 (8·1 to 8·9)	7·3 (6·9 to 7·6)	4·9 (4·4 to 5·4)
High-income Asia Pacific	113·3 (109·6 to 118·1)	60·5 (58 to 63·2)	30·5 (28·9 to 32·2)	16·2 (15·3 to 17·2)	9·1 (8·6 to 9·6)	5·5 (5·4 to 5·6)	3·7 (3·6 to 3·7)	2·7 (2·6 to 2·9)
Brunei	167·6 (156·9 to 178·1)	128·2 (120·1 to 137·4)	57·8 (54·6 to 61)	23·5 (21·2 to 26·1)	12·4 (11·4 to 13·5)	10·4 (9·5 to 11·4)	10·1 (9·2 to 11·1)	10 (8·2 to 12)
Japan	95·6 (94·2 to 97·1)	42·7 (42·2 to 43·2)	19·3 (19·1 to 19·5)	10·9 (10·7 to 11)	6·9 (6·8 to 7)	4·8 (4·7 to 4·9)	3·4 (3·3 to 3·4)	2·5 (2·4 to 2·6)
Aichi	88·4 (86·9 to 90)	38·9 (35·6 to 42)	17·2 (16·4 to 17·9)	9·8 (9·5 to 10·3)	6·5 (6·3 to 6·8)	4·6 (4·4 to 4·8)	3·5 (3·2 to 3·7)	2·4 (2·2 to 2·6)
Akita	99·8 (98·2 to 101·4)	44·4 (40·7 to 48·1)	20·9 (19·5 to 22·5)	11·3 (10·6 to 11·9)	7·4 (6·9 to 7·9)	4·8 (4·4 to 5·2)	3·4 (3·1 to 3·7)	2·7 (2·4 to 3)
Aomori	114 (112·3 to 115·6)	51·2 (47 to 55·3)	23·9 (22·5 to 25·2)	12·8 (12·1 to 13·4)	7·8 (7·4 to 8·3)	5·4 (5 to 5·8)	3·6 (3·3 to 3·8)	2·8 (2·5 to 3·1)
Chiba	101·9 (100·3 to 103·4)	42·8 (38·7 to 47·2)	17·7 (16·9 to 18·5)	10·3 (9·9 to 10·7)	6·2 (5·9 to 6·4)	4·7 (4·5 to 4·9)	3·5 (3·3 to 3·7)	2·8 (2·6 to 3)
Ehime	99·3 (97·6 to 101·1)	43·8 (40·2 to 47·7)	20·2 (18·9 to 21·6)	11·1 (10·5 to 11·8)	7·1 (6·6 to 7·6)	4·5 (4·2 to 4·9)	2·9 (2·7 to 3·1)	2·2 (2 to 2·5)

Fukui	86·8 (85·2 to 88·4)	39·4 (34·7 to 44·6)	18·1 (15·9 to 20·5)	9·6 (8·8 to 10·4)	7·2 (6·6 to 7·8)	5·3 (4·8 to 5·8)	3·4 (3·1 to 3·6)	2·7 (2·4 to 3)
Fukuoka	92·5 (90·9 to 94·2)	39·4 (35·5 to 43·5)	18·6 (17·4 to 19·7)	10·1 (9·8 to 10·5)	7·2 (6·8 to 7·5)	5 (4·8 to 5·2)	3·5 (3·3 to 3·7)	2·6 (2·4 to 2·8)
Fukushima	118·1 (116·3 to 119·9)	51·8 (47·9 to 55·7)	25 (23·5 to 26·4)	12·9 (12·3 to 13·5)	8·1 (7·7 to 8·5)	5·3 (4·9 to 5·7)	3·5 (3·2 to 3·7)	2·6 (2·3 to 2·9)
Gifu	93 (91·3 to 94·7)	42·2 (39 to 46)	19·7 (18·5 to 20·9)	10·8 (10·3 to 11·4)	6·5 (6 to 6·9)	4·7 (4·4 to 5·1)	3·7 (3·5 to 4)	2·9 (2·6 to 3·2)
Gunma	106·8 (105·3 to 108·1)	47·8 (44·3 to 51·5)	21·4 (20·1 to 22·6)	11·5 (10·9 to 12·1)	7·1 (6·6 to 7·6)	5 (4·7 to 5·3)	3·4 (3·2 to 3·6)	2·6 (2·3 to 2·8)
Hiroshima	106·6 (104·9 to 108·3)	45·2 (39·9 to 50·6)	20·4 (18 to 23·1)	11·2 (10·8 to 11·7)	6·9 (6·6 to 7·3)	4·3 (4 to 4·6)	3·3 (3·1 to 3·5)	2·5 (2·3 to 2·8)
Hokkaidō	101·5 (99·8 to 103·1)	44 (39·9 to 47·8)	20·8 (18·3 to 23·6)	11·5 (11 to 11·9)	6·7 (6·4 to 7)	4·4 (4·2 to 4·7)	3·2 (3 to 3·5)	2·5 (2·2 to 2·9)
Hyōgo	93·3 (92·1 to 94·6)	39·6 (36 to 43·7)	18 (16·9 to 19·1)	10·5 (10·1 to 10·9)	6·9 (6·6 to 7·3)	4·7 (4·5 to 5)	3·1 (2·9 to 3·3)	2·3 (2·1 to 2·4)
Ibaraki	121·2 (119·3 to 123)	52·5 (46·4 to 58·6)	24·1 (21 to 27·5)	12·9 (12·4 to 13·5)	7·2 (6·8 to 7·5)	4·9 (4·6 to 5·2)	3·6 (3·4 to 3·8)	2·7 (2·4 to 2·9)
Ishikawa	92·9 (91·2 to 94·5)	42·1 (38·9 to 45·5)	19·4 (18 to 20·9)	10·7 (10·1 to 11·4)	6·8 (6·2 to 7·3)	5 (4·6 to 5·5)	3·5 (3·2 to 3·7)	2·6 (2·3 to 2·9)
Iwate	115·3 (113·4 to 117·1)	52 (48·4 to 56·2)	25·2 (23·7 to 26·8)	13·7 (12·9 to 14·4)	6·6 (6·1 to 7)	4·5 (4·1 to 4·9)	3·9 (3·6 to 4·1)	2·9 (2·7 to 3·3)
Kagawa	99·3 (97·8 to 100·9)	43·5 (39·8 to 47·4)	19·4 (18·1 to 21)	10·5 (9·8 to 11·3)	6·8 (6·3 to 7·4)	4·3 (3·9 to 4·7)	3·4 (3·1 to 3·7)	2·6 (2·3 to 2·9)
Kagoshima	116·8 (114·8 to 118·7)	52·1 (47·9 to 56·7)	25·7 (24 to 27·2)	13·3 (12·6 to 14)	7·5 (7·1 to 8)	5·5 (5·1 to 5·9)	3·7 (3·5 to 3·9)	2·9 (2·6 to 3·2)
Kanagawa	84·2 (82·7 to 85·7)	35·2 (32 to 38·4)	15·5 (14·8 to 16·2)	9·6 (9·3 to 9·9)	6·5 (6·3 to 6·8)	4·8 (4·6 to 5)	3·5 (3·3 to 3·7)	2·8 (2·6 to 3)
Kōchi	106·1 (104·3 to 108)	47·4 (43·5 to 51·7)	21·9 (20·4 to 23·6)	11·7 (10·8 to 12·5)	8 (7·4 to 8·7)	5·3 (4·8 to 5·8)	4·1 (3·7 to 4·5)	3 (2·7 to 3·4)
Kumamoto	116·4 (114·6 to 118·2)	51·9 (47·6 to 56·7)	24·6 (23·1 to 26·1)	12·8 (12·1 to 13·5)	7·3 (6·9 to 7·8)	5 (4·7 to 5·4)	3·4 (3·1 to 3·6)	2·5 (2·2 to 2·7)
Kyōto	91·5 (90 to 92·9)	39·1 (36·1 to 42·3)	17·1 (16·1 to 18·1)	9·7 (9·2 to 10·2)	7·1 (6·7 to 7·5)	4·6 (4·3 to 4·9)	3·2 (3·1 to 3·4)	2·5 (2·3 to 2·8)
Mie	96 (94·3 to 97·6)	43·3 (39·6 to 47)	19·9 (18·8 to 21·1)	11·1 (10·5 to 11·8)	7·1 (6·7 to 7·6)	5·1 (4·8 to 5·4)	3·7 (3·5 to 4)	2·6 (2·3 to 2·9)
Miyagi	108·2 (106·5 to 109·8)	46 (42·6 to 49·7)	21·3 (20·1 to 22·6)	11·5 (11 to 12·1)	6·8 (6·4 to 7·2)	4·3 (4 to 4·6)	3·3 (3·1 to 3·5)	2·6 (2·4 to 2·9)
Miyazaki	110·1 (108·4 to 111·8)	48·8 (45·2 to 52·7)	22·8 (21·1 to 24·6)	12·3 (11·6 to 13)	8·1 (7·6 to 8·6)	5·3 (4·8 to 5·7)	3·6 (3·4 to 3·9)	2·7 (2·4 to 3)
Nagano	103·4 (101·8 to 104·9)	44·9 (41·1 to 48·8)	19·7 (18·5 to 20·9)	10·7 (10·2 to 11·2)	6·6 (6·2 to 7)	3·8 (3·5 to 4·1)	2·8 (2·6 to 3)	2·1 (1·9 to 2·3)
Nagasaki	95·5 (94 to 96·9)	42·5 (39 to 45·9)	20·8 (19·6 to 22)	10·7 (10·1 to 11·3)	6·8 (6·4 to 7·2)	5·1 (4·7 to 5·5)	3·8 (3·5 to 4)	2·8 (2·5 to 3·1)
Nara	94·9 (93·3 to 96·5)	41·8 (38·6 to 45·1)	19·2 (17·8 to 20·8)	11·2 (10·5 to 11·9)	6·8 (6·4 to 7·3)	4·3 (3·9 to 4·7)	3·5 (3·2 to 3·7)	2·8 (2·6 to 3·1)
Niigata	101·6 (100 to 103·2)	44·3 (41·1 to 47·9)	20·4 (19·2 to 21·7)	11·3 (10·8 to 11·8)	6·2 (5·8 to 6·5)	4·8 (4·5 to 5·2)	2·9 (2·7 to 3·1)	2·2 (2 to 2·5)
Ōita	104·8 (103 to 106·7)	46·4 (42·8 to 50·1)	21·9 (20·4 to 23·6)	11·7 (11·1 to 12·4)	6·8 (6·3 to 7·3)	4·5 (4·1 to 4·8)	3·5 (3·2 to 3·8)	2·7 (2·4 to 3)
Okayama	94·7 (93 to 96·4)	41·7 (38·8 to 45·1)	17·9 (16·9 to 18·9)	10 (9·5 to 10·5)	7·2 (6·8 to 7·6)	5·1 (4·7 to 5·4)	3·4 (3·2 to 3·6)	2·5 (2·2 to 2·7)
Okinawa	112 (110·1 to 113·8)	49·2 (45·5 to 53·1)	25·1 (23·6 to 26·6)	12·1 (11·5 to 12·8)	7·9 (7·4 to 8·3)	5·5 (5·1 to 5·9)	3·5 (3·2 to 3·8)	2·8 (2·5 to 3)
Ōsaka	88 (86·5 to 89·5)	37·3 (34 to 40·4)	16·3 (15·5 to 17·1)	10·1 (9·7 to 10·4)	6·9 (6·6 to 7·2)	4·5 (4·4 to 4·7)	3·2 (3 to 3·4)	2·4 (2·2 to 2·6)
Saga	104·9 (103·1 to 106·6)	46·7 (43·3 to 50·3)	22 (20·4 to 23·8)	11·5 (10·7 to 12·4)	7·5 (7 to 8·1)	5·2 (4·7 to 5·6)	3·1 (2·9 to 3·4)	2·3 (2·1 to 2·6)
Saitama	101·7 (100·3 to 103)	43·2 (39·4 to 47·1)	18·2 (17·4 to 19·1)	10·6 (10·2 to 11)	6·9 (6·6 to 7·2)	4·7 (4·5 to 4·9)	3·3 (3·1 to 3·5)	2·5 (2·3 to 2·6)
Shiga	98·1 (96·6 to 99·6)	43·9 (40·6 to 47·3)	20 (18·6 to 21·5)	10·5 (9·8 to 11·2)	7·1 (6·6 to 7·6)	5·1 (4·7 to 5·4)	3·4 (3·2 to 3·7)	2·5 (2·3 to 2·8)
Shimane	96 (94·3 to 97·7)	42·5 (37·8 to 48·1)	20·1 (17·7 to 22·9)	10·8 (10 to 11·7)	7·1 (6·6 to 7·8)	5 (4·5 to 5·5)	3·2 (2·9 to 3·5)	2·5 (2·2 to 2·8)
Shizuoka	92·1 (90·7 to 93·5)	39·1 (34·6 to 43·8)	17·4 (15·2 to 20)	9·6 (9·2 to 10·1)	6·7 (6·4 to 7)	4·4 (4·2 to 4·7)	3·1 (3 to 3·3)	2·5 (2·2 to 2·7)
Tochigi	105·1 (103·3 to 106·9)	47·6 (43·8 to 51·9)	22·2 (20·8 to 23·7)	12 (11·4 to 12·6)	7·8 (7·3 to 8·2)	5·7 (5·3 to 6)	3·8 (3·6 to 4)	2·8 (2·5 to 3·1)
Tokushima	104·5 (102·8 to 106·2)	45·8 (42·5 to 49·5)	21·5 (20 to 23·2)	11·5 (10·7 to 12·3)	7·5 (6·9 to 8)	5·7 (5·2 to 6·2)	4·6 (4·2 to 4·9)	3·5 (3·1 to 3·9)
Tōkyō	77·8 (76·3 to 79·3)	34·6 (30·6 to 38·8)	16·2 (15·1 to 17·5)	9·5 (9·2 to 9·9)	6·5 (6·2 to 6·8)	5 (4·7 to 5·2)	3·1 (2·9 to 3·3)	2·4 (2·2 to 2·5)
Tottori	103·6 (101·9 to 105·4)	45·6 (40·1 to 51·4)	21·6 (19 to 24·5)	11·1 (10·3 to 12)	6·6 (6 to 7·2)	4·3 (3·8 to 4·7)	3·6 (3·2 to 3·9)	2·9 (2·5 to 3·2)
Toyama	103·1 (101·4 to 104·9)	46·5 (42·9 to 50·7)	21·6 (20·2 to 23·2)	11·7 (11 to 12·4)	7·8 (7·2 to 8·4)	5 (4·6 to 5·4)	3·4 (3·2 to 3·7)	2·4 (2·2 to 2·7)
Wakayama	100·4 (98·7 to 102·2)	44·9 (41·7 to 48·6)	20·8 (19·4 to 22·4)	12 (11·2 to 12·8)	6·6 (6·1 to 7·2)	4·5 (4·1 to 4·9)	3·3 (3 to 3·6)	2·6 (2·3 to 2·9)
Yamagata	100·9 (99·3 to 102·6)	45 (41·7 to 48·7)	21·1 (19·6 to 22·8)	11·1 (10·5 to 11·9)	6·2 (5·8 to 6·6)	4·6 (4·2 to 5)	3·6 (3·3 to 3·8)	3 (2·7 to 3·3)
Yamaguchi	106·7 (105 to 108·3)	46·6 (43·1 to 50·4)	21·3 (20·1 to 22·7)	12·2 (11·5 to 12·9)	7·4 (6·9 to 8)	4·9 (4·6 to 5·4)	3·4 (3·2 to 3·7)	3 (2·7 to 3·3)
Yamanashi	87·6 (86·3 to 89)	39 (34·4 to 44·1)	17·9 (15·7 to 20·4)	9·7 (9 to 10·5)	6·7 (6·2 to 7·2)	4·8 (4·4 to 5·2)	2·9 (2·7 to 3·2)	2·3 (2·1 to 2·6)
South Korea	171 (159·2 to 185·2)	90·5 (83·9 to 97·6)	53·4 (48·6 to 58·4)	27·1 (24·4 to 30·1)	12·9 (11·7 to 14·3)	7·1 (6·9 to 7·2)	4·4 (4·3 to 4·5)	3·2 (3 to 3·5)
Singapore	122·9 (119·3 to 126·4)	52·3 (50·9 to 53·9)	30·4 (29·4 to 31·6)	16·9 (16·1 to 17·8)	8·9 (8·5 to 9·5)	3·6 (3·4 to 3·9)	2·7 (2·5 to 2·9)	1·9 (1·6 to 2·1)
High-income North America	39·3 (39 to 39·6)	34 (33·8 to 34·2)	26·6 (26·4 to 26·7)	16·5 (16·4 to 16·7)	12 (11·9 to 12·1)	8·9 (8·8 to 9)	7·8 (7·7 to 7·9)	6·9 (6·7 to 7·1)
Canada	53·3 (52·4 to 54·2)	35·9 (35·4 to 36·4)	24·9 (24·5 to 25·4)	14·3 (14·1 to 14·6)	9·1 (8·9 to 9·4)	6·6 (6·4 to 6·8)	6·1 (6 to 6·3)	5·2 (4·9 to 5·5)
Greenland	167·2 (138·3 to 199·8)	93·8 (86·4 to 101·8)	61·1 (55 to 67·6)	43·4 (34·8 to 54·5)	34·1 (29·7 to 38·5)	20·2 (17·2 to 23·7)	12·2 (9·9 to 14·8)	9·3 (6·6 to 12·9)
USA	37·8 (37·5 to 38·1)	33·8 (33·5 to 34)	26·7 (26·5 to 26·9)	16·8 (16·6 to 16·9)	12·3 (12·1 to 12·4)	9·1 (9 to 9·2)	8 (7·9 to 8)	7·1 (6·8 to 7·3)

Alabama	46·7 (46 to 47·5)	41·1 (39·9 to 42·3)	32·8 (31·6 to 34·1)	19·7 (18·9 to 20·6)	14·7 (14 to 15·4)	12·6 (12 to 13·2)	11·1 (10·5 to 11·8)	10·5 (10·1 to 10·8)
Alaska	60·6 (59·8 to 61·5)	51·7 (49 to 54·9)	29·5 (27·8 to 31·3)	19·5 (18·3 to 20·7)	13·2 (12·4 to 14·1)	9·7 (9 to 10·4)	7·8 (7·3 to 8·5)	7·7 (6·4 to 9·2)
Arizona	52·2 (51·5 to 52·9)	45 (43·2 to 46·8)	27·2 (25·7 to 28·6)	18·2 (17·3 to 19·1)	12·7 (12·1 to 13·4)	9·6 (9·1 to 10·1)	7·9 (7·5 to 8·4)	6·8 (6·5 to 7)
Arkansas	42·3 (41·7 to 43)	36·5 (34·9 to 38·3)	29·4 (28 to 30·8)	17 (16 to 18)	13·4 (12·6 to 14·3)	11·5 (10·7 to 12·3)	10·1 (9·4 to 10·7)	9·5 (9·2 to 9·8)
California	36·7 (36·2 to 37·2)	31 (30·6 to 31·5)	24·1 (23·7 to 24·5)	15·5 (15·1 to 15·9)	11·6 (11·3 to 11·9)	7·5 (7·2 to 7·7)	6·2 (6·1 to 6·4)	5·1 (4·9 to 5·2)
Colorado	42 (41·5 to 42·5)	36 (34·7 to 37·3)	26·8 (25·5 to 28·3)	14·1 (13·3 to 14·9)	11·3 (10·7 to 12)	8·3 (7·8 to 8·8)	7·7 (7·3 to 8·2)	6 (5·7 to 6·2)
Connecticut	30·6 (30·4 to 30·7)	27 (26·1 to 28)	22 (21·2 to 22·9)	14·4 (13·6 to 15·1)	10·1 (9·6 to 10·6)	7·7 (7·3 to 8·1)	6·5 (6 to 6·9)	5·5 (4·6 to 6·6)
Delaware	35·7 (35·4 to 36)	31·2 (29·5 to 33·1)	25·4 (23·9 to 26·7)	18 (16·9 to 19·2)	13·4 (12·6 to 14·3)	11·1 (10·3 to 11·8)	9·4 (8·8 to 10·1)	8·8 (7·3 to 10·5)
Washington, DC	49·4 (48·9 to 49·8)	50 (48 to 52·1)	42·3 (40·4 to 44·3)	35·2 (33·5 to 37·2)	33·7 (31·8 to 35·6)	18·2 (17 to 19·5)	13·4 (12·3 to 14·5)	9·7 (8·1 to 11·6)
Florida	47·5 (46·9 to 48·2)	41·4 (40·5 to 42·3)	30·7 (29·8 to 31·7)	20·4 (19·8 to 21·1)	13·8 (13·4 to 14·3)	9·8 (9·5 to 10·2)	8·8 (8·5 to 9·1)	7·7 (7·4 to 7·9)
Georgia	45·4 (44·7 to 46·1)	42·3 (41·2 to 43·4)	30 (29 to 31·1)	19·5 (18·8 to 20·3)	15·8 (15·1 to 16·4)	11·3 (10·8 to 11·8)	8·6 (8·3 to 9)	8·6 (8·3 to 8·9)
Hawaii	34·3 (33·9 to 34·6)	30·1 (28·6 to 31·7)	23·3 (22·2 to 24·4)	14·1 (13·4 to 15)	10·4 (9·7 to 11·1)	9·4 (8·8 to 10)	7·9 (7·3 to 8·4)	7·1 (5·9 to 8·4)
Idaho	35 (34·6 to 35·4)	31·6 (30·1 to 33·1)	24·5 (23·4 to 25·8)	15 (14·2 to 15·9)	12 (11·3 to 12·7)	9 (8·5 to 9·6)	7·2 (6·7 to 7·7)	6·4 (5·4 to 7·6)
Illinois	35·3 (34·7 to 35·8)	32·8 (32·2 to 33·5)	28·3 (27·7 to 28·9)	19·3 (18·8 to 19·8)	13·7 (13·2 to 14·2)	10·5 (10·1 to 10·9)	8·6 (8·3 to 9)	7·2 (7 to 7·4)
Indiana	33·2 (32·7 to 33·7)	30·9 (30·1 to 31·6)	25·7 (24·8 to 26·6)	15·9 (15·2 to 16·6)	12·5 (11·9 to 13·1)	10·2 (9·7 to 10·8)	9·4 (8·9 to 9·8)	8·5 (8·2 to 8·7)
Iowa	29·4 (29·2 to 29·6)	27·9 (27 to 28·9)	24·2 (23·3 to 25·3)	14·6 (13·9 to 15·3)	10·3 (9·8 to 10·9)	7·9 (7·5 to 8·4)	6·4 (5·9 to 6·9)	6·3 (5·2 to 7·5)
Kansas	31·8 (31·6 to 32)	29·1 (28 to 30·1)	23·8 (22·8 to 24·9)	14·8 (14·1 to 15·6)	11 (10·5 to 11·6)	9·5 (9·1 to 10)	8·5 (8 to 8·9)	7·3 (6·1 to 8·7)
Kentucky	40·4 (39·7 to 41·1)	36·4 (35·3 to 37·6)	27·3 (26·2 to 28·6)	16·8 (16·1 to 17·6)	11·7 (11 to 12·4)	9·4 (8·9 to 10)	9 (8·5 to 9·6)	8·3 (8 to 8·6)
Louisiana	47 (46·3 to 47·8)	41·9 (40·6 to 43·2)	31·3 (30·4 to 32·3)	19·3 (18·4 to 20·1)	14·2 (13·5 to 14·9)	12·4 (11·7 to 13·1)	10·2 (9·7 to 10·9)	10·2 (9·9 to 10·6)
Maine	40·2 (39·7 to 40·6)	34·6 (33·1 to 36·1)	26·6 (25·5 to 27·9)	12·6 (11·9 to 13·4)	8·7 (8·1 to 9·2)	6·8 (6·3 to 7·3)	7·4 (6·8 to 8)	7·2 (6 to 8·5)
Maryland	37·6 (37 to 38·2)	34·8 (33·7 to 35·8)	24·8 (23·9 to 25·7)	17·2 (16·3 to 18)	12 (11·5 to 12·6)	9·6 (9·2 to 10·1)	8·5 (8 to 8·9)	7·8 (7·5 to 8·1)
Massachusetts	31·7 (31·3 to 32·1)	27·7 (26·9 to 28·6)	22·4 (21·6 to 23·3)	13·6 (13 to 14·3)	8·8 (8·3 to 9·3)	6·1 (5·8 to 6·5)	5·6 (5·3 to 6)	4·9 (4 to 6)
Michigan	32·9 (32·4 to 33·4)	30·8 (29·9 to 31·6)	26·4 (25·7 to 27·2)	16·2 (15·6 to 16·8)	13·2 (12·8 to 13·8)	10·3 (9·9 to 10·7)	8·7 (8·3 to 9·2)	7·9 (7·6 to 8·2)
Minnesota	31 (30·6 to 31·4)	29 (28·2 to 29·9)	23·6 (22·6 to 24·6)	13·7 (13 to 14·4)	9·1 (8·6 to 9·6)	7·3 (6·8 to 7·8)	6·1 (5·8 to 6·6)	6·2 (6 to 6·4)
Mississippi	60·5 (59·6 to 61·4)	51·8 (49·9 to 53·7)	38·7 (37·2 to 40·4)	22·8 (21·7 to 23·9)	15·8 (14·9 to 16·7)	14·4 (13·6 to 15·2)	12·5 (11·8 to 13·2)	10·9 (8·9 to 13·4)
Missouri	34·3 (33·8 to 34·9)	31·2 (30·3 to 32·1)	26 (25·2 to 26·8)	16·6 (15·9 to 17·4)	12·4 (11·8 to 13)	9·4 (9 to 10)	8·5 (8·1 to 9)	8 (7·7 to 8·2)
Montana	39·3 (38·8 to 39·6)	35·3 (33·6 to 37)	28·4 (26·9 to 29·8)	15·7 (14·8 to 16·7)	11 (10·3 to 11·8)	8·7 (8·1 to 9·3)	7·9 (7·3 to 8·5)	7·1 (5·9 to 8·5)
Nebraska	33·3 (33 to 33·5)	29·9 (28·6 to 31·2)	24·3 (23·2 to 25·5)	15·1 (14·3 to 15·9)	10·1 (9·5 to 10·8)	8·9 (8·3 to 9·5)	6·7 (6·3 to 7·2)	6·7 (5·5 to 8)
Nevada	57·5 (56·9 to 58)	44·4 (41·5 to 47·3)	31 (29·4 to 32·6)	16·4 (15·5 to 17·4)	11·4 (10·8 to 12·1)	8·7 (8·2 to 9·2)	7·4 (7 to 7·8)	6·4 (5·4 to 7·5)
New Hampshire	35 (34·7 to 35·4)	30·2 (28·6 to 31·9)	23·5 (22·2 to 24·8)	13·2 (12·4 to 14·1)	8·9 (8·4 to 9·5)	6·4 (5·9 to 6·8)	5·8 (5·3 to 6·3)	4·9 (4·1 to 5·9)
New Jersey	34·1 (33·5 to 34·6)	30·6 (29·7 to 31·5)	25·6 (24·9 to 26·4)	15·7 (15·1 to 16·4)	11·5 (11 to 12·1)	7·9 (7·5 to 8·3)	6·3 (5·9 to 6·6)	5·1 (4·9 to 5·3)
New Mexico	54·1 (53·6 to 54·5)	45·8 (44·1 to 47·6)	30·1 (28·7 to 31·5)	17·2 (16·3 to 18·2)	12·2 (11·5 to 12·9)	8·9 (8·3 to 9·4)	8 (7·4 to 8·5)	7·1 (5·9 to 8·5)
New York	34·8 (34·3 to 35·3)	31 (30·5 to 31·4)	25·8 (25·4 to 26·2)	16·2 (15·7 to 16·6)	13 (12·6 to 13·4)	8·3 (8 to 8·6)	6·5 (6·3 to 6·8)	5·3 (5·1 to 5·4)
North Carolina	45·9 (45·2 to 46·6)	40·3 (39·4 to 41·2)	32 (31 to 33·1)	18·4 (17·8 to 19·1)	13·8 (13·2 to 14·4)	11·2 (10·7 to 11·8)	9·1 (8·7 to 9·5)	8·4 (8·1 to 8·7)
North Dakota	34·4 (34 to 34·8)	32·4 (30·9 to 34)	21·9 (20·6 to 23·2)	15·7 (14·7 to 16·7)	10·3 (9·6 to 11·2)	8·9 (8·1 to 9·7)	7·6 (6·9 to 8·3)	7·3 (6·1 to 8·7)
Ohio	32·8 (32·3 to 33·3)	30·6 (29·8 to 31·5)	24·4 (23·8 to 24·9)	16·5 (16 to 17)	12·1 (11·7 to 12·6)	9·8 (9·4 to 10·2)	9·7 (9·3 to 10·1)	8·8 (8·5 to 9·1)
Oklahoma	37·7 (37·2 to 38·2)	34·5 (33·4 to 35·8)	29·1 (27·8 to 30·4)	17·2 (16·4 to 18·1)	12·2 (11·5 to 13)	10·8 (10·2 to 11·5)	10·3 (9·7 to 10·9)	9·5 (9·2 to 9·9)
Oregon	33·5 (33·3 to 33·7)	30·6 (29·5 to 31·8)	23·2 (22·2 to 24·3)	15·3 (14·6 to 16·1)	10·9 (10·3 to 11·4)	7·4 (7 to 7·8)	6·2 (5·9 to 6·6)	5·6 (4·7 to 6·7)
Pennsylvania	33·9 (33·4 to 34·5)	30·8 (30 to 31·7)	26 (25·5 to 26·6)	16·6 (16 to 17·1)	12·1 (11·6 to 12·5)	8·9 (8·6 to 9·3)	8·8 (8·4 to 9·2)	7·3 (7·1 to 7·5)
Rhode Island	33 (32·7 to 33·2)	29·2 (27·8 to 30·8)	24·7 (23·5 to 26)	15·4 (14·4 to 16·3)	10·6 (9·9 to 11·3)	8·2 (7·6 to 8·8)	7·8 (7·2 to 8·4)	6·6 (5·5 to 7·9)
South Carolina	48·4 (47·7 to 49·2)	44·6 (43·2 to 45·9)	30·6 (29·3 to 32)	20·9 (19·9 to 21·9)	15·4 (14·6 to 16·2)	11·6 (11 to 12·2)	9·7 (9·1 to 10·3)	8·7 (8·4 to 9)
South Dakota	37·7 (37·3 to 38)	35·5 (34 to 37·2)	27·1 (25·7 to 28·4)	16·4 (15·4 to 17·3)	13·2 (12·4 to 14)	9·9 (9·2 to 10·7)	8·9 (8·3 to 9·5)	8 (6·6 to 9·4)
Tennessee	41·4 (40·7 to 42·1)	36·9 (35·9 to 38)	28·4 (27·3 to 29·5)	17·5 (16·8 to 18·3)	13·7 (13 to 14·4)	11·4 (10·8 to 11·9)	9·7 (9·2 to 10·2)	8·6 (8·3 to 8·8)
Texas	42 (41·4 to 42·7)	37·7 (36·9 to 38·4)	29·1 (28·6 to 29·6)	17·3 (16·8 to 17·8)	11·2 (10·8 to 11·6)	8·1 (7·8 to 8·3)	7·9 (7·6 to 8·2)	7·1 (6·8 to 7·3)
Utah	29·8 (29·5 to 30)	26·4 (25·1 to 27·6)	21·7 (20·6 to 22·8)	14·7 (14 to 15·4)	9·9 (9·3 to 10·4)	7·3 (6·9 to 7·7)	6·7 (6·3 to 7·1)	6·5 (5·4 to 7·7)
Vermont	37·7 (37·3 to 38)	32·9 (31 to 35)	22·9 (21·6 to 24·4)	13·4 (12·4 to 14·4)	8·7 (8 to 9·5)	7·1 (6·4 to 7·8)	6·1 (5·5 to 6·7)	5·1 (4·2 to 6·1)
Virginia	41·8 (41·1 to 42·4)	37·1 (36·1 to 38·1)	27·2 (26·3 to 28·3)	17·3 (16·6 to 18·1)	12·6 (12 to 13·2)	9·1 (8·7 to 9·5)	8·5 (8·1 to 8·9)	7·2 (6·9 to 7·4)
Washington	32·8 (32·4 to 33·2)	30·2 (29·3 to 31·2)	25·1 (24·1 to 26·2)	15·6 (14·8 to 16·4)	10·2 (9·7 to 10·7)	6·9 (6·5 to 7·3)	5·9 (5·5 to 6·2)	5·3 (5·1 to 5·5)

West Virginia	38·6 (38·3 to 38·9)	33·5 (32·2 to 34·9)	30·2 (29 to 31·6)	16·4 (15·7 to 17·3)	12·4 (11·7 to 13·1)	10 (9·3 to 10·6)	9·8 (9·2 to 10·4)	8·9 (7·4 to 10·7)
Wisconsin	32·5 (32 to 33)	29·4 (28·6 to 30·3)	22·5 (21·5 to 23·4)	13·6 (12·9 to 14·3)	10·4 (9·9 to 10·9)	8·4 (7·9 to 8·9)	7·4 (6·9 to 7·8)	6·9 (6·7 to 7·2)
Wyoming	41·6 (41·1 to 41·9)	36·7 (34·4 to 39·1)	29·3 (27·7 to 31·1)	15·8 (14·8 to 16·9)	11·6 (10·8 to 12·5)	9·1 (8·2 to 10·1)	8·3 (7·6 to 9)	6·9 (5·7 to 8·3)
Southern Latin America	120·5 (119·3 to 121·7)	110·1 (109 to 111·2)	81·5 (80·7 to 82·3)	42·8 (42·4 to 43·2)	28·8 (28·4 to 29·1)	19·2 (18·9 to 19·5)	14·2 (13·9 to 14·5)	10·6 (9·9 to 11·4)
Argentina	90·4 (89·3 to 91·5)	81·4 (80·5 to 82·3)	73·5 (72·5 to 74·5)	43 (42·6 to 43·5)	32·2 (31·8 to 32·6)	21·7 (21·3 to 22)	15·7 (15·4 to 16·1)	11·7 (11·2 to 12·3)
Chile	197·8 (194·8 to 200·9)	170·1 (167·9 to 172·5)	102·8 (101·2 to 104·4)	41·7 (40·8 to 42·5)	21·4 (20·9 to 21·9)	12·4 (12 to 12·8)	9·7 (9·4 to 10·1)	7·4 (6·2 to 8·7)
Uruguay	77·3 (74·8 to 79·8)	61·5 (59·7 to 63·3)	62·7 (61 to 64·7)	45·5 (44·1 to 47)	26·1 (25·1 to 27·2)	18·5 (17·6 to 19·3)	13 (11·9 to 14·3)	9·1 (7·9 to 10·6)
Western Europe	69·4 (68·3 to 70·4)	44·4 (44·1 to 44·7)	29·6 (29·4 to 29·8)	16·6 (16·4 to 16·8)	10·3 (10·2 to 10·4)	6·3 (6·2 to 6·4)	4·6 (4·6 to 4·7)	3·7 (3·5 to 3·9)
Andorra	29·2 (24·4 to 35)	20·5 (17 to 24·5)	13·1 (10·8 to 15·8)	7·9 (6·6 to 9·6)	5·7 (4·7 to 6·8)	3·2 (2·7 to 3·8)	2·6 (2·1 to 3·1)	1·6 (1·4 to 2)
Austria	85·7 (83·9 to 87·7)	48·9 (47·7 to 49·9)	33·6 (32·8 to 34·4)	19·4 (18·7 to 20)	10·5 (10·2 to 10·9)	6·2 (5·9 to 6·4)	4·9 (4·7 to 5·1)	3·5 (3·2 to 3·8)
Belgium	66·6 (65·1 to 68·1)	38·4 (37·7 to 39·2)	27·7 (27·1 to 28·4)	16·6 (16·1 to 17·1)	11·1 (10·8 to 11·5)	6·7 (6·4 to 6·9)	5 (4·8 to 5·2)	3·9 (3·7 to 4·2)
Cyprus	120·2 (88·8 to 160)	92·5 (70·5 to 119·6)	60·6 (49·9 to 73)	29 (27·1 to 30·8)	14·1 (12·9 to 15·3)	7·1 (6·5 to 7·8)	3·8 (3·3 to 4·2)	3·2 (2·6 to 4·1)
Denmark	40·9 (39·7 to 42·1)	29·2 (28·4 to 30·2)	19·3 (18·7 to 20)	11·7 (11·2 to 12·2)	10·1 (9·6 to 10·6)	6·1 (5·8 to 6·4)	4·3 (4·1 to 4·6)	3·7 (3·4 to 4·2)
Finland	56·8 (55·4 to 58·3)	29·5 (28·6 to 30·4)	18·6 (17·8 to 19·3)	9·9 (9·4 to 10·4)	7·4 (7 to 7·7)	4·7 (4·5 to 5)	3·2 (3 to 3·4)	2·4 (2·1 to 2·6)
France	62·5 (61·7 to 63·4)	30·6 (30·2 to 31)	20·4 (20 to 20·7)	13·9 (13·7 to 14·1)	10·1 (9·9 to 10·3)	6 (5·9 to 6·2)	4·6 (4·5 to 4·7)	3·9 (3·7 to 4·2)
Germany	72·9 (69·2 to 76·7)	46·3 (45·8 to 46·8)	29·6 (29·3 to 29·9)	17·4 (17·1 to 17·6)	9·7 (9·6 to 9·9)	5·9 (5·8 to 6)	4·5 (4·4 to 4·6)	3·6 (3·4 to 3·8)
Greece	65·9 (62·8 to 69·1)	47 (45·8 to 48·1)	34·4 (33·5 to 35·3)	21·8 (21·1 to 22·4)	11·3 (10·9 to 11·8)	6·9 (6·6 to 7·2)	4·6 (4·3 to 4·8)	4·2 (3·8 to 4·7)
Iceland	32·4 (28·5 to 36·6)	22·8 (20·4 to 25·3)	17 (15·3 to 19·1)	10·2 (8·8 to 11·6)	7·2 (6·1 to 8·4)	4·1 (3·4 to 4·9)	2·8 (2·3 to 3·5)	2·5 (1·7 to 3·5)
Ireland	58 (56·3 to 59·9)	37·5 (36·4 to 38·6)	24·6 (23·7 to 25·4)	15·3 (14·7 to 15·9)	10·4 (9·9 to 10·8)	7·7 (7·3 to 8)	4·7 (4·5 to 5)	3·5 (3·2 to 3·9)
Israel	45·1 (43·8 to 46·5)	40 (39 to 41)	29 (28·3 to 29·7)	19·6 (19·1 to 20·1)	12·4 (12 to 12·8)	7·3 (7·1 to 7·6)	4·8 (4·7 to 5)	3·4 (3·2 to 3·6)
Italy	85·1 (83·8 to 86·4)	56·5 (55·7 to 57·2)	37 (36·5 to 37·4)	18·4 (18·1 to 18·7)	10·5 (10·3 to 10·7)	5·9 (5·7 to 6·1)	4·2 (4·1 to 4·3)	3·2 (3·1 to 3·4)
Luxembourg	59·5 (54 to 65·7)	42·5 (39·2 to 45·8)	26·3 (23·7 to 29)	14·8 (13·1 to 16·5)	9·9 (8·8 to 11·4)	5·3 (4·5 to 6·2)	3 (2·5 to 3·7)	2·6 (1·8 to 3·7)
Malta	113·2 (107·8 to 119)	43·7 (41·2 to 46·3)	30 (27·6 to 32·2)	17·8 (16·3 to 19·4)	12·1 (11 to 13·4)	8·2 (7·2 to 9·2)	7·3 (6·3 to 8·3)	6·2 (4·8 to 7·9)
Monaco	50·6 (44 to 58·2)	30·2 (25·5 to 35·6)	15·2 (12·7 to 18·3)	8·4 (6·9 to 10·1)	5·7 (4·7 to 7)	4 (3·3 to 4·8)	3·1 (2·5 to 3·7)	2·5 (2 to 3)
Netherlands	35 (34·2 to 35·7)	23·9 (23·4 to 24·4)	18·3 (17·9 to 18·7)	12·2 (11·9 to 12·5)	9·8 (9·5 to 10·1)	6·9 (6·7 to 7·1)	4·9 (4·8 to 5·1)	4·1 (3·8 to 4·3)
Norway	36·6 (35·4 to 37·9)	25·5 (24·8 to 26·4)	16·5 (15·9 to 17)	9·9 (9·5 to 10·3)	9·4 (9 to 9·8)	5·3 (5·1 to 5·5)	3·5 (3·3 to 3·7)	2·6 (2·4 to 2·9)
Portugal	146·2 (143·4 to 149·4)	112·7 (110·6 to 114·8)	76·2 (74·7 to 77·7)	31·7 (31 to 32·6)	15·9 (15·4 to 16·5)	8·1 (7·8 to 8·4)	4·1 (3·9 to 4·3)	3·2 (2·9 to 3·5)
San Marino	29·5 (24·6 to 35·8)	21·8 (18·2 to 25·8)	17·2 (14·6 to 20·2)	12·8 (10·9 to 14·8)	8·9 (7·4 to 10·6)	5·8 (4·8 to 6·9)	4·1 (3·4 to 5)	3·6 (3 to 4·4)
Spain	105·5 (103·9 to 107·3)	59·6 (58·8 to 60·3)	36·3 (35·8 to 36·8)	17 (16·8 to 17·3)	10·4 (10·2 to 10·6)	5·9 (5·8 to 6·1)	4·2 (4·1 to 4·3)	3·2 (3·1 to 3·4)
Sweden	29·8 (29 to 30·8)	23·1 (22·4 to 23·8)	15·3 (14·7 to 15·8)	9 (8·6 to 9·4)	7·6 (7·2 to 7·9)	4·3 (4·1 to 4·6)	3·3 (3·1 to 3·5)	2·7 (2·5 to 3)
Stockholm	25·8 (24·9 to 26·7)	20·8 (19·2 to 22·5)	14·2 (12·9 to 15·5)	9 (8·3 to 9·9)	6·9 (6·4 to 7·6)	4 (3·7 to 4·3)	2·8 (2·5 to 3·2)	2·3 (2·1 to 2·6)
Sweden except Stockholm	29·5 (28·5 to 30·5)	22·6 (21·9 to 23·4)	14·8 (14·2 to 15·4)	8·8 (8·4 to 9·2)	7·6 (7·3 to 7·9)	4·3 (4·1 to 4·6)	3·5 (3·3 to 3·6)	2·9 (2·7 to 3·2)
Switzerland	43·9 (42·7 to 45·1)	30·5 (29·8 to 31·3)	21·6 (21 to 22·2)	12·4 (12 to 12·9)	9·4 (9·1 to 9·8)	6·6 (6·3 to 6·8)	4·8 (4·6 to 5)	3·9 (3·6 to 4·2)
UK	37·8 (37·3 to 38·3)	29·1 (28·6 to 29·5)	23·9 (23·6 to 24·2)	15·6 (15·4 to 15·9)	10·5 (10·3 to 10·8)	7·2 (7 to 7·3)	5·6 (5·5 to 5·7)	4·5 (4·3 to 4·7)
England	35·9 (35·2 to 36·5)	28·1 (27·5 to 28·8)	23·4 (22·9 to 23·9)	15·5 (15·2 to 15·7)	10·6 (10·4 to 10·7)	7·2 (7 to 7·3)	5·6 (5·5 to 5·7)	4·5 (4·4 to 4·7)
East Midlands	36·1 (35·5 to 36·6)	28·3 (26·4 to 30·5)	23·6 (21·8 to 25·6)	15·4 (14·6 to 16·2)	10·8 (10·4 to 11·1)	7·2 (6·8 to 7·5)	5·7 (5·4 to 6)	4·7 (4·5 to 4·9)
Derby	39·8 (39 to 40·7)	31·6 (27·2 to 36·5)	26·9 (23·7 to 30·5)	17·9 (15·4 to 20·4)	11·6 (10·4 to 13)	7·8 (7·1 to 8·6)	6·7 (6 to 7·4)	5·5 (5·3 to 5·7)
Derbyshire	31 (30·3 to 31·7)	24·2 (22 to 26·5)	20·4 (18·2 to 22·6)	13·1 (12·4 to 13·8)	8·4 (7·8 to 9·1)	5·8 (5·4 to 6·2)	4·4 (4·1 to 4·8)	3·7 (3·5 to 3·8)
Leicester	42·2 (41·4 to 43·1)	33·7 (29·2 to 39·1)	27·6 (24·6 to 30·9)	18·3 (16·2 to 20·4)	12·7 (11·9 to 13·5)	9·3 (8·3 to 10·4)	7·7 (7 to 8·4)	6·2 (6 to 6·4)
Leicestershire	29·6 (29 to 30·2)	23·1 (20·8 to 25·5)	19·6 (17·6 to 21·9)	12·8 (11·7 to 14·1)	9·3 (8·5 to 10·1)	5·7 (5·4 to 6·1)	4·7 (4·4 to 5·1)	4 (3·8 to 4·1)
Lincolnshire	33·8 (33·1 to 34·4)	26·4 (23·9 to 28·9)	22 (19·8 to 24·4)	14·1 (13·3 to 15)	10·1 (9·5 to 10·8)	7 (6·5 to 7·5)	5·3 (5 to 5·7)	3·9 (3·8 to 4·1)
Northamptonshire	35·4 (34·7 to 36)	27·5 (25 to 30·1)	23 (20·5 to 25·6)	14·9 (13·3 to 16·6)	11·5 (10·8 to 12·2)	6·8 (6·2 to 7·5)	5·4 (4·9 to 6)	4·6 (4·4 to 4·7)
Nottingham	39·4 (38·6 to 40·2)	31·5 (27·3 to 36·5)	26·3 (23·6 to 29·4)	17·2 (14·9 to 20)	12·1 (11·2 to 12·9)	9·5 (8·9 to 10·3)	7 (6·4 to 7·6)	5·5 (5·3 to 5·7)
Nottinghamshire	33·3 (32·8 to 33·8)	26 (23·1 to 28·9)	21·9 (19·7 to 24·2)	14·2 (13·4 to 14·9)	9 (8·5 to 9·4)	6·2 (5·7 to 6·7)	4·9 (4·6 to 5·2)	4·3 (4·1 to 4·4)
Rutland	21·9 (21·5 to 22·4)	16·6 (13·6 to 20·1)	14·1 (12·6 to 15·7)	10·3 (9 to 11·6)	7·2 (6·2 to 8·5)	4·7 (3·9 to 5·7)	3·3 (2·8 to 3·9)	1·9 (1·8 to 2)
East of England	31·1 (30·6 to 31·6)	24·2 (22·3 to 26·1)	20·3 (18·5 to 22·2)	13·4 (12·6 to 14·4)	8·4 (8 to 8·7)	6 (5·7 to 6·3)	4·9 (4·7 to 5·2)	3·8 (3·6 to 3·9)
Bedford	35·5 (34·8 to 36·1)	27·7 (24·7 to 31·1)	23·5 (21 to 26·2)	15·9 (13·8 to 18·2)	10·4 (9·2 to 11·7)	7·2 (6·5 to 8)	5·8 (5·2 to 6·5)	4·5 (4·1 to 4·9)
Cambridgeshire	26·7 (26·3 to 27·2)	20·6 (18·4 to 22·9)	17·2 (15·3 to 19·3)	11·5 (10·3 to 12·9)	7·3 (6·7 to 7·8)	5·7 (5·2 to 6·2)	4·6 (4·2 to 5)	3·6 (3·3 to 3·9)

Central Bedfordshire	27·1 (26·6 to 27·8)	21·1 (19 to 23·4)	17·8 (15·8 to 20·1)	11·9 (10·2 to 13·9)	8 (7·2 to 8·7)	5·7 (5 to 6·3)	4·4 (3·9 to 5)	3·5 (3·2 to 3·8)
Essex	29·6 (29 to 30·2)	22·9 (21 to 25)	19·1 (17·2 to 21·3)	12·5 (11·2 to 13·9)	7·7 (7·1 to 8·4)	5·3 (4·8 to 5·8)	4·6 (4·2 to 5)	3·6 (3·2 to 4)
Hertfordshire	27·8 (27·3 to 28·3)	21·9 (20 to 24)	18·8 (16·3 to 21·6)	12·5 (11·5 to 13·5)	7·5 (6·9 to 8)	5 (4·6 to 5·5)	4·1 (3·8 to 4·4)	3·1 (2·8 to 3·5)
Luton	38 (37·3 to 38·6)	30·1 (26·9 to 33·9)	25·6 (22·6 to 28·8)	17 (15·1 to 19·2)	10·8 (9·9 to 11·8)	9·4 (8·6 to 10·2)	7·8 (7·1 to 8·6)	6·1 (5·6 to 6·7)
Norfolk	31·3 (30·6 to 32)	24·3 (20·8 to 28)	20·2 (17·8 to 22·9)	13·3 (12·5 to 14·4)	8·4 (7·7 to 9)	5·8 (5·3 to 6·3)	4·9 (4·5 to 5·3)	3·7 (3·4 to 3·9)
Peterborough	35 (34·5 to 35·4)	27·7 (23·5 to 32·2)	23·9 (21·3 to 26·8)	16·1 (14·3 to 18·2)	10·4 (9·4 to 11·6)	8·6 (7·8 to 9·6)	6·8 (6·1 to 7·5)	5·2 (4·7 to 5·6)
Southend-on-Sea	35·3 (34·6 to 36·1)	27·3 (24·4 to 30·5)	22·1 (19·7 to 24·7)	14·1 (12·1 to 16·4)	8·4 (7·4 to 9·5)	5·4 (4·8 to 5·9)	4·4 (3·9 to 4·9)	3·6 (3·3 to 4)
Suffolk	32·3 (31·7 to 32·8)	24·6 (20·8 to 28·6)	20·2 (17·9 to 22·8)	13 (11·4 to 14·9)	8 (7·4 to 8·7)	5·3 (4·8 to 5·8)	4·6 (4·2 to 5)	3·3 (3 to 3·6)
Thurrock	29·6 (29 to 30·3)	24·2 (21·5 to 27·2)	20·5 (18·4 to 22·8)	13·2 (11·4 to 15·2)	8 (7·2 to 9)	5·9 (5·2 to 6·6)	4·5 (4·1 to 5·1)	3·5 (3·2 to 3·8)
Greater London	37·7 (37 to 38·4)	30·1 (29 to 31·3)	24·9 (23·8 to 25·9)	16·7 (16·1 to 17·4)	11·3 (10·9 to 11·7)	7·9 (7·6 to 8·2)	5·8 (5·5 to 6)	4·1 (3·9 to 4·2)
Barking and Dagenham	44·3 (43·4 to 45·3)	36·7 (34·2 to 39·3)	30·6 (28·3 to 32·9)	19·5 (17·8 to 21·3)	11·5 (10·6 to 12·5)	7·4 (6·6 to 8·1)	6·1 (5·5 to 6·8)	4·5 (4·4 to 4·7)
Barnet	29·1 (28·5 to 29·7)	23·2 (21·7 to 24·7)	19·1 (17·7 to 20·6)	12·9 (11·9 to 13·9)	7·6 (7 to 8·3)	5·3 (4·9 to 5·9)	4·2 (3·7 to 4·6)	2·9 (2·8 to 3)
Bexley	25·2 (24·7 to 25·8)	20·4 (19·2 to 21·7)	16·9 (15·8 to 17·9)	11 (10·2 to 11·9)	7·1 (6·5 to 7·8)	5·2 (4·8 to 5·7)	4·3 (4 to 4·7)	3·3 (3·2 to 3·4)
Brent	40 (39·3 to 40·8)	31·9 (29·6 to 34·6)	26·4 (24·1 to 28·6)	18 (16·4 to 19·4)	12·6 (11·4 to 13·6)	9·5 (8·8 to 10·3)	6·4 (5·9 to 6·9)	4·3 (4·1 to 4·4)
Bromley	27·6 (27 to 28·1)	22·3 (20·8 to 23·8)	18·6 (17 to 20·2)	12·2 (11·2 to 13·4)	8·2 (7·5 to 8·9)	5·3 (4·9 to 5·8)	3·7 (3·4 to 4)	2·8 (2·7 to 2·9)
Camden	38·1 (37·4 to 38·9)	29·4 (27·5 to 31·4)	23·5 (21·9 to 25·2)	16·9 (15·6 to 18·3)	11·3 (10·4 to 12·4)	7 (6·4 to 7·7)	4·9 (4·5 to 5·4)	3 (2·9 to 3·1)
Croydon	33 (32·4 to 33·7)	26·7 (25·1 to 28·4)	22·1 (20·5 to 23·8)	14·5 (13·1 to 16)	11·1 (10·4 to 11·9)	8·3 (7·6 to 8·9)	6·1 (5·7 to 6·6)	4·5 (4·3 to 4·6)
Ealing	31·3 (30·6 to 32)	25 (23·5 to 26·7)	20·6 (18·9 to 22·1)	13·9 (12·9 to 15)	9·8 (9 to 10·6)	6·8 (6·3 to 7·4)	5·1 (4·7 to 5·5)	3·4 (3·3 to 3·5)
Enfield	33·8 (33·1 to 34·4)	27·4 (25·4 to 29·5)	22·4 (20·7 to 24·2)	14·9 (13·7 to 16·3)	9·6 (8·8 to 10·3)	7·5 (6·8 to 8·2)	6·3 (5·6 to 7)	4·1 (4 to 4·3)
Greenwich	45·2 (44·4 to 46·1)	36·9 (34·2 to 39·9)	30·8 (28·3 to 33·3)	20·5 (18·9 to 22·4)	12·1 (11·3 to 13·1)	8·1 (7·3 to 8·8)	6·5 (5·9 to 7)	5·3 (5·1 to 5·4)
Hackney	38 (37·2 to 38·8)	30·9 (28·6 to 33·3)	26·1 (24·1 to 28·3)	18·3 (17 to 19·7)	13·5 (12·5 to 14·6)	10·5 (9·6 to 11·4)	7·2 (6·7 to 7·8)	5·1 (5 to 5·3)
Hammersmith and Fulham	34·4 (33·7 to 35·2)	26·8 (25·1 to 28·5)	21·4 (19·9 to 23)	15·3 (14·2 to 16·5)	12·2 (11·2 to 13·4)	7·3 (6·7 to 8)	4·8 (4·4 to 5·3)	3·4 (3·2 to 3·5)
Haringey	39·5 (38·7 to 40·3)	31 (28·6 to 33·5)	25·6 (23·6 to 27·8)	17·8 (16·5 to 19·2)	13 (11·9 to 14·1)	9·6 (8·8 to 10·5)	6·1 (5·7 to 6·6)	4 (3·8 to 4·1)
Harrow	28·7 (28·1 to 29·2)	23·2 (21·8 to 24·7)	19 (17·8 to 20·3)	12·6 (11·6 to 13·7)	9·5 (8·6 to 10·4)	7·9 (7·2 to 8·6)	6·8 (6·2 to 7·4)	5·1 (4·9 to 5·2)
Havering	28·6 (28·1 to 29·2)	24 (20·6 to 27·6)	20·6 (18·9 to 22·5)	13·5 (11·6 to 15·4)	8·2 (7·4 to 9·1)	5·6 (5·1 to 6·1)	4·6 (4·2 to 5)	3·1 (3 to 3·2)
Hillingdon	29·8 (29·1 to 30·4)	24·5 (22·8 to 26·1)	20·7 (19·1 to 22·3)	13·9 (12·7 to 15·2)	8·9 (8·1 to 9·8)	5·9 (5·4 to 6·4)	5·1 (4·6 to 5·5)	3·4 (3·3 to 3·5)
Hounslow	33·1 (32·4 to 33·8)	26·4 (22·6 to 30·4)	21·8 (19·9 to 23·9)	14·6 (12·6 to 16·7)	10·4 (9·5 to 11·3)	7·1 (6·6 to 7·7)	6 (5·5 to 6·5)	4·6 (4·4 to 4·7)
Islington	38·4 (37·6 to 39·2)	30 (28·2 to 32)	25·3 (23·3 to 27·3)	18 (16·4 to 19·7)	11·4 (10·4 to 12·5)	7·8 (7·1 to 8·6)	4·9 (4·5 to 5·4)	3·1 (3 to 3·2)
Kensington and Chelsea	34 (33·4 to 34·7)	26·6 (22·6 to 30·8)	20·9 (19·6 to 22·4)	15·4 (14 to 16·9)	9·9 (8·9 to 10·9)	6 (5·3 to 6·8)	4·2 (3·7 to 4·9)	3·6 (3·4 to 3·7)
Kingston upon Thames	27·3 (26·8 to 28)	21·9 (18·7 to 25·2)	17·8 (16·5 to 19·1)	11·8 (10·8 to 12·9)	8·4 (7·4 to 9·3)	4·8 (4·1 to 5·5)	4·2 (3·7 to 4·7)	3·7 (3·6 to 3·9)
Lambeth	47·4 (46·5 to 48·3)	37·5 (35 to 39·9)	31·8 (29·6 to 34·1)	22·6 (20·9 to 24·5)	13·2 (12 to 14·4)	9·4 (8·6 to 10·3)	7 (6·5 to 7·5)	4·7 (4·6 to 4·9)
Lewisham	39 (38·2 to 39·8)	31·2 (29 to 33·4)	26·3 (24·5 to 28·3)	17·9 (16·5 to 19·5)	12·1 (11 to 13·1)	9·3 (8·5 to 10·1)	6·4 (5·8 to 6·9)	4·2 (4·1 to 4·4)
Merton	35·6 (34·9 to 36·3)	28·4 (26·6 to 30·2)	23·5 (22 to 25·2)	15·8 (14·5 to 17·1)	10 (9·1 to 11·1)	6 (5·4 to 6·6)	5 (4·4 to 5·6)	3·5 (3·4 to 3·6)
Newham	42·6 (41·8 to 43·4)	34·7 (32·3 to 37·2)	28·5 (26·4 to 30·8)	19 (17·7 to 20·5)	12·9 (11·8 to 14·1)	10·1 (9·1 to 11·2)	6·7 (6·3 to 7·1)	4·4 (4·2 to 4·5)
Redbridge	30 (29·3 to 30·6)	24·4 (20·7 to 28)	20 (18·2 to 21·9)	13·2 (11·4 to 15·1)	9·7 (8·7 to 10·6)	7 (6·4 to 7·7)	5·5 (4·8 to 6·1)	3·9 (3·8 to 4)
Richmond upon Thames	30·5 (30 to 31)	24·2 (22·8 to 25·7)	19·9 (18·6 to 21·2)	13·4 (12·4 to 14·4)	8·5 (7·7 to 9·3)	4·9 (4·5 to 5·3)	3·7 (3·4 to 4)	2·7 (2·6 to 2·8)
Southwark	36·6 (36 to 37·4)	30 (27·9 to 32·2)	25·2 (23·2 to 27·2)	16·8 (15·3 to 18·6)	14·2 (13·2 to 15·5)	10·8 (10·1 to 11·6)	6·9 (6·5 to 7·4)	4·4 (4·3 to 4·5)
Sutton	31·4 (30·7 to 32·1)	24·9 (23·4 to 26·5)	20·6 (19·2 to 21·9)	13·4 (12·2 to 14·7)	8·4 (7·6 to 9·3)	5·6 (5·1 to 6·1)	4·6 (4·1 to 5)	3·7 (3·5 to 3·8)
Tower Hamlets	43·2 (42·3 to 44·1)	36 (33·2 to 38·9)	30·8 (28·6 to 33·1)	20·3 (18·8 to 21·8)	11·3 (10·4 to 12·4)	8 (7·4 to 8·7)	6·2 (5·7 to 6·7)	4·8 (4·6 to 4·9)
Waltham Forest	40·3 (39·5 to 41·2)	32·1 (30·1 to 34·1)	25·9 (24 to 27·9)	17·4 (16·1 to 18·9)	12·4 (11·3 to 13·5)	9·1 (8·4 to 9·8)	6·5 (6 to 7)	3·8 (3·7 to 4)
Wandsworth	35·4 (34·7 to 36·1)	28·4 (26·6 to 30·2)	23·5 (21·7 to 25·3)	16·4 (15·2 to 17·8)	11 (10 to 12·1)	6·6 (5·9 to 7·3)	4·8 (4·3 to 5·3)	3·5 (3·3 to 3·6)
Westminster	43·6 (42·7 to 44·5)	34·2 (29·2 to 39·6)	28·1 (25·6 to 30·8)	20·9 (17·9 to 24)	13·2 (12·1 to 14·3)	8·1 (7·4 to 8·8)	5 (4·6 to 5·4)	3·4 (3·3 to 3·6)
North East England	33·9 (33·3 to 34·5)	27 (22·5 to 32·1)	23 (19·7 to 26·4)	14·7 (13·5 to 16)	10·4 (9·9 to 10·9)	7·1 (6·7 to 7·5)	4·9 (4·6 to 5·2)	4 (3·9 to 4·2)
County Durham	34 (33·5 to 34·6)	26·9 (21·1 to 33·7)	22·8 (18·9 to 26·8)	14·6 (12·9 to 16·5)	9·7 (9·1 to 10·3)	7·2 (6·6 to 7·9)	5 (4·6 to 5·5)	4·4 (4·2 to 4·5)
Darlington	35 (34·3 to 35·8)	27·5 (22 to 33·6)	23·6 (20 to 27·6)	15·4 (13·5 to 17·4)	9·9 (8·7 to 11·3)	6·7 (5·8 to 7·8)	4·7 (4 to 5·4)	3·5 (3·4 to 3·6)
Gateshead	35·7 (35 to 36·5)	28·4 (23·7 to 33·6)	24·3 (20·7 to 27·9)	15·8 (14·5 to 17·2)	11 (10·1 to 12·1)	6·7 (5·8 to 7·6)	5·1 (4·5 to 5·8)	4·2 (4·1 to 4·4)
Hartlepool	39·3 (38·5 to 40·2)	31·1 (24·7 to 38·2)	27·1 (22·5 to 32·2)	17·8 (15·4 to 20·4)	11·3 (9·9 to 12·8)	7·7 (6·6 to 8·8)	5·3 (4·6 to 6·1)	4·1 (3·9 to 4·2)
Middlesbrough	36·8 (36·1 to 37·6)	29·8 (22·8 to 37·7)	25·5 (21 to 30·2)	16·1 (14 to 18·5)	11·1 (10 to 12·3)	7·6 (7 to 8·2)	5·3 (4·7 to 5·8)	4·4 (4·3 to 4·6)

Newcastle upon Tyne	32·9 (32·4 to 33·4)	26·1 (22 to 30·7)	21·5 (18·4 to 24·7)	13·7 (12·1 to 15·3)	10·8 (9·8 to 11·7)	7·2 (6·6 to 7·8)	5·1 (4·7 to 5·6)	4·3 (4·2 to 4·4)
North Tyneside	29·3 (28·7 to 29·9)	23·3 (19·4 to 27·6)	19·9 (16·9 to 22·9)	12·5 (11 to 14·1)	9·2 (8·6 to 10)	5·8 (5·3 to 6·4)	3·9 (3·5 to 4·2)	3 (2·9 to 3·1)
Northumberland	31·8 (31·2 to 32·4)	24·8 (20·8 to 29·1)	20·8 (17·9 to 24·3)	13·2 (11·7 to 14·7)	9·4 (8·5 to 10·4)	6·2 (5·5 to 6·9)	4·2 (3·7 to 4·7)	3·2 (3·1 to 3·3)
Redcar and Cleveland	34·9 (34·2 to 35·7)	27·5 (22·8 to 32·9)	23·4 (19·5 to 27·8)	15·1 (12·5 to 18·1)	9·7 (8·7 to 10·5)	6·2 (5·5 to 7)	4·4 (3·8 to 5)	3·6 (3·5 to 3·7)
South Tyneside	27·1 (26·5 to 27·7)	21·6 (18 to 25·6)	18·6 (15·6 to 21·6)	11·8 (10·2 to 13·4)	7·8 (7·2 to 8·5)	5·8 (5·3 to 6·4)	3·8 (3·5 to 4·2)	2·9 (2·8 to 3)
Stockton-on-Tees	33·7 (33 to 34·3)	26·8 (20·5 to 34·1)	23·1 (19·1 to 27·4)	14·9 (12·8 to 17·1)	10·3 (9·6 to 11·1)	7·2 (6·6 to 7·9)	5·2 (4·8 to 5·6)	4·5 (4·3 to 4·6)
Sunderland	33·3 (32·6 to 34)	26·6 (22·5 to 31·1)	22·5 (19·3 to 26·3)	14·2 (12·8 to 15·6)	10·4 (9·7 to 11·1)	7 (6·5 to 7·6)	4·5 (4·1 to 4·9)	3·6 (3·5 to 3·7)
North West England	37·2 (36·5 to 37·9)	29·3 (28·2 to 30·5)	24·4 (23·4 to 25·4)	16·2 (15·5 to 17·1)	10·9 (10·5 to 11·4)	7·8 (7·5 to 8·2)	6·1 (5·9 to 6·4)	5·3 (5·1 to 5·5)
Blackburn with Darwen	38 (37·2 to 38·8)	30·5 (27·1 to 34)	25·2 (23·2 to 27·3)	16·3 (14·5 to 18·4)	13·9 (12·8 to 15·2)	10·7 (9·8 to 11·6)	8·5 (7·8 to 9·2)	6·7 (6·3 to 7·1)
Blackpool	38·5 (37·8 to 39·3)	30·6 (27·6 to 33·7)	25·3 (23·4 to 27·2)	16·5 (15 to 18·2)	11·2 (9·9 to 12·5)	8·9 (8 to 9·8)	7·3 (6·6 to 8)	6·3 (5·9 to 6·8)
Bolton	40·9 (40·1 to 41·6)	32·4 (29·4 to 35·6)	26·8 (24·4 to 29·1)	17·5 (15·9 to 19·2)	12·1 (11 to 13·3)	8·2 (7·4 to 9)	6·3 (5·7 to 7·1)	4·9 (4·6 to 5·2)
Bury	36·8 (36·1 to 37·6)	28·7 (26·1 to 31·5)	23·8 (21·9 to 25·8)	16·1 (14·5 to 17·8)	10·3 (9·3 to 11·2)	6·4 (5·9 to 7)	5·2 (4·6 to 5·8)	4·6 (4·2 to 4·9)
Cheshire East	31·7 (31·1 to 32·4)	24·9 (22·5 to 27·5)	20·9 (19·3 to 22·6)	14·2 (13 to 15·5)	9·3 (8·6 to 10)	6 (5·5 to 6·4)	4·6 (4·2 to 5·1)	4·2 (3·9 to 4·4)
Cheshire West and Chester	30 (29·5 to 30·4)	23·5 (21·5 to 25·6)	19·8 (18·2 to 21·5)	13·3 (12·2 to 14·4)	8·4 (7·9 to 9·2)	5·8 (5·3 to 6·4)	5·2 (4·7 to 5·8)	4·2 (4 to 4·5)
Cumbria	37 (36·3 to 37·7)	28·8 (25·8 to 31·8)	24·3 (22·1 to 26·6)	16·1 (14·5 to 17·8)	9·8 (9·2 to 10·5)	6 (5·5 to 6·5)	4·6 (4·3 to 5)	3·9 (3·6 to 4·1)
Halton	32 (31·3 to 32·7)	26 (23·5 to 28·6)	21·9 (20·5 to 23·5)	14·4 (13 to 15·9)	10·1 (9 to 11·4)	8 (7·3 to 8·8)	6 (5·4 to 6·6)	4·7 (4·3 to 5)
Knowsley	37 (36·3 to 37·7)	30·1 (26·1 to 34·5)	26·5 (24·6 to 28·5)	17·6 (16 to 19·4)	9·7 (8·9 to 10·6)	7·6 (6·7 to 8·4)	5·3 (4·6 to 6·2)	4·5 (4·2 to 4·8)
Lancashire	37 (36·3 to 37·7)	28·7 (26·1 to 31·6)	23·7 (21·4 to 26·1)	15·6 (14·1 to 17·2)	11·3 (10·5 to 12·1)	8 (7·3 to 8·8)	6·6 (6 to 7·2)	5·8 (5 to 6·6)
Liverpool	35·6 (34·9 to 36·2)	28·2 (25 to 31·5)	23·3 (21·1 to 25·6)	15·4 (13·9 to 17·2)	9·4 (8·8 to 10·1)	8 (7·4 to 8·6)	6·5 (6 to 7)	6·2 (5·9 to 6·5)
Manchester	43·2 (42·4 to 44·1)	34·3 (29·7 to 39)	28·6 (26·1 to 31·1)	19·2 (17·3 to 21·4)	13·3 (12·2 to 14·5)	10·6 (9·9 to 11·5)	7·6 (7·1 to 8·2)	6·7 (5·8 to 7·7)
Oldham	41·1 (40·3 to 42)	32·7 (29·5 to 36·2)	27·1 (24·9 to 29·1)	18 (16·2 to 19·9)	12·3 (11·2 to 13·3)	9·3 (8·5 to 10·1)	8 (7·4 to 8·6)	6·9 (6·5 to 7·3)
Rochdale	40·7 (39·9 to 41·6)	32·7 (29·4 to 36·1)	27·6 (25·5 to 29·8)	18·2 (16·4 to 20·1)	11·5 (10·7 to 12·4)	8·1 (7·5 to 8·8)	5·8 (5·3 to 6·4)	5·4 (5·1 to 5·8)
Salford	40·1 (39·3 to 40·9)	31·6 (28·7 to 34·7)	26·4 (24·3 to 28·3)	17·6 (16 to 19·2)	10·8 (10·1 to 11·7)	7·8 (7 to 8·7)	6·4 (5·8 to 7)	5·3 (5 to 5·7)
Sefton	29·5 (28·9 to 30·1)	23·1 (20·9 to 25·5)	19·4 (17·8 to 21·2)	12·8 (11·6 to 14·1)	9·1 (8·3 to 10)	6·5 (6 to 7)	5·2 (4·7 to 5·7)	4·5 (4·1 to 4·8)
St Helens	30·6 (30 to 31·3)	24·5 (21·8 to 27·3)	20·8 (19·1 to 22·6)	14·1 (12·5 to 15·9)	9·4 (8·4 to 10·3)	7·6 (6·8 to 8·4)	4·8 (4·3 to 5·3)	3·9 (3·7 to 4·2)
Stockport	30·2 (29·6 to 30·8)	23·7 (21·4 to 26·1)	19·9 (18·2 to 21·6)	13·3 (12·1 to 14·7)	9·3 (8·6 to 10·1)	6·4 (5·9 to 6·9)	5 (4·6 to 5·5)	4·7 (4·4 to 5)
Tameside	39 (38·2 to 39·8)	31·1 (28·2 to 34·2)	25·7 (23·8 to 27·7)	17 (15·3 to 18·9)	10 (9 to 11·1)	6·8 (6·2 to 7·4)	5·4 (4·9 to 5·9)	4·4 (4 to 4·7)
Trafford	36·1 (35·4 to 36·9)	28·4 (25·8 to 31·2)	24·4 (22·6 to 26·1)	16·9 (15·3 to 18·9)	10·4 (9·4 to 11·5)	6·5 (5·9 to 7·1)	4·7 (4·3 to 5·2)	4·7 (4·3 to 5)
Warrington	28 (27·4 to 28·7)	22·2 (20 to 24·6)	18·4 (17·1 to 19·8)	12·3 (11·1 to 13·6)	9·7 (8·9 to 10·7)	6·6 (6 to 7·2)	5·2 (4·7 to 5·9)	4·2 (4 to 4·5)
Wigan	32·9 (32·2 to 33·5)	25·9 (23·5 to 28·4)	21·2 (19·7 to 22·8)	13·9 (12·6 to 15·4)	9·3 (8·6 to 10)	7·3 (6·8 to 7·9)	5·3 (4·9 to 5·8)	4·6 (4·3 to 4·9)
Wirral	35·4 (34·7 to 36·2)	27·7 (25·2 to 30·4)	23·1 (21·4 to 24·9)	15·2 (13·8 to 16·8)	9·8 (8·8 to 10·9)	6·7 (6·2 to 7·3)	5·6 (5·2 to 6·1)	5·1 (4·8 to 5·4)
South East England	35·8 (35·3 to 36·3)	26·7 (25·7 to 27·8)	21·4 (20·5 to 22·3)	14·1 (13·5 to 14·9)	9·6 (9·3 to 9·9)	5·8 (5·5 to 6)	4·7 (4·5 to 4·9)	4·1 (3·9 to 4·2)
Bracknell Forest	29·6 (29·2 to 30)	22·2 (18·8 to 25·8)	18·4 (17·1 to 19·8)	12·2 (10·7 to 13·8)	8·4 (7·4 to 9·5)	5·4 (4·7 to 6·3)	4·3 (3·7 to 4·9)	2·8 (2·6 to 3·1)
Brighton and Hove	38 (37·2 to 38·9)	28·2 (25 to 31·2)	22·5 (20·6 to 24·5)	15·2 (13·4 to 17·1)	10·6 (9·7 to 11·6)	7 (6·3 to 7·7)	5·3 (4·9 to 5·8)	4·1 (3·8 to 4·4)
Buckinghamshire	25·8 (25·2 to 26·4)	19·7 (17·8 to 21·8)	15·9 (14·6 to 17·2)	10·4 (9·4 to 11·6)	8·8 (8·1 to 9·6)	5·6 (5 to 6·2)	4·9 (4·5 to 5·3)	4·3 (4 to 4·6)
East Sussex	35·5 (34·8 to 36·2)	25·9 (23·5 to 28·6)	20·1 (18·2 to 22)	12·8 (11·6 to 14)	9·1 (8·4 to 9·8)	5·8 (5·3 to 6·3)	4·7 (4·4 to 5·1)	3·9 (3·6 to 4·2)
Hampshire	34·3 (33·6 to 35)	25·7 (23·6 to 27·9)	21·1 (19·4 to 22·8)	14·3 (13 to 15·8)	8·7 (8 to 9·3)	5 (4·6 to 5·5)	4·1 (3·8 to 4·4)	4 (3·4 to 4·5)
Isle of Wight	45·9 (45 to 46·8)	33·7 (29 to 38·8)	26·2 (24·3 to 28·3)	16·8 (14·8 to 18·9)	10·9 (9·6 to 12·4)	4·8 (4·1 to 5·6)	3·2 (2·7 to 3·7)	2·6 (2·4 to 2·9)
Kent	36·2 (35·5 to 36·9)	26·9 (24·2 to 29·7)	21·3 (19·4 to 23·5)	13·6 (12·4 to 14·9)	9·8 (9·2 to 10·5)	5·4 (4·9 to 5·9)	4·4 (4 to 4·7)	4·1 (3·6 to 4·7)
Medway	36·4 (35·6 to 37·2)	27·9 (25 to 30·8)	22·4 (20·6 to 24·7)	14·8 (13·4 to 16·2)	10·5 (9·7 to 11·3)	6·8 (6·1 to 7·4)	4·8 (4·4 to 5·3)	4·1 (3·8 to 4·4)
Milton Keynes	35 (34·3 to 35·7)	26·8 (23·9 to 29·9)	21·6 (19·8 to 23·6)	14·1 (12·4 to 15·8)	10·7 (9·8 to 11·7)	7·6 (6·9 to 8·3)	6 (5·4 to 6·7)	5·2 (4·9 to 5·5)
Oxfordshire	27·5 (27 to 28·1)	20·7 (18·8 to 22·8)	16·8 (15·4 to 18·3)	11·3 (10·2 to 12·4)	8·3 (7·7 to 8·9)	5·4 (4·9 to 5·9)	4·8 (4·3 to 5·3)	4 (3·7 to 4·2)
Portsmouth	40·1 (39·2 to 40·9)	30·1 (26·8 to 33·7)	23·6 (21·7 to 25·8)	15·2 (13·5 to 17·2)	11·1 (10·2 to 12·1)	7·1 (6·4 to 7·7)	4·5 (4·1 to 5)	3·7 (3·5 to 4)
Reading	30·4 (29·8 to 31·1)	23·2 (20·7 to 25·6)	18·8 (17·4 to 20·3)	12·6 (11·1 to 14·3)	9·3 (8·3 to 10·3)	7·3 (6·5 to 8·1)	5·4 (4·9 to 6)	4·1 (3·8 to 4·3)
Slough	37·4 (36·7 to 38)	30·1 (27 to 33·3)	24·7 (22·9 to 26·5)	15·7 (14 to 17·7)	10·9 (9·8 to 12·2)	7·2 (6·4 to 8·1)	6·8 (6·1 to 7·6)	6·2 (5·8 to 6·6)
Southampton	39·9 (39·1 to 40·7)	30·6 (27·4 to 34·1)	24·4 (22·7 to 26·2)	16 (14·3 to 17·7)	11·2 (10·2 to 12·2)	6·7 (6·1 to 7·3)	5·3 (4·8 to 5·8)	4·6 (4·3 to 4·9)
Surrey	31·5 (30·9 to 32·2)	24 (22·3 to 25·8)	19·9 (18·3 to 21·4)	13·5 (12·1 to 14·9)	8·3 (7·7 to 8·9)	4·6 (4·2 to 5)	3·7 (3·4 to 3·9)	3·1 (2·9 to 3·3)
West Berkshire	25 (24·5 to 25·5)	18·9 (17·1 to 20·8)	15·3 (14·3 to 16·5)	9·9 (8·7 to 11·3)	8·2 (7·2 to 9·2)	5·4 (4·8 to 6)	4·2 (3·7 to 4·8)	3·8 (3·4 to 4·1)

West Sussex	36 (35·4 to 36·6)	26·3 (23·5 to 29·3)	20·9 (18·9 to 23·3)	13·5 (12 to 15·1)	8·8 (8·2 to 9·4)	5·1 (4·7 to 5·5)	4·3 (3·9 to 4·7)	3·5 (3·3 to 3·7)
Windsor and Maidenhead	24·8 (24·3 to 25·3)	19 (15·9 to 22·2)	15·6 (14·4 to 16·7)	10·4 (9·2 to 11·7)	7·6 (6·7 to 8·5)	5·4 (4·7 to 6·1)	4·1 (3·6 to 4·7)	3·6 (3·4 to 3·9)
Wokingham	24·6 (24·2 to 25)	18·5 (16·3 to 20·9)	15·1 (14·1 to 16·3)	10·5 (9·2 to 11·8)	7·7 (6·8 to 8·7)	4·7 (4·1 to 5·4)	4·3 (3·8 to 5)	4·2 (3·9 to 4·5)
South West England	34·7 (34·1 to 35·3)	26·4 (24·4 to 28·4)	21·7 (20 to 23·6)	14·4 (13·5 to 15·2)	9·3 (8·9 to 9·7)	6·1 (5·8 to 6·5)	4·8 (4·5 to 5)	3·7 (3·5 to 3·8)
Bath and North East Somerset	33·5 (32·9 to 34·1)	25·6 (21·4 to 30·3)	21·5 (19·4 to 23·8)	14·5 (13 to 16)	8·5 (7·6 to 9·5)	5·2 (4·6 to 5·9)	3·5 (3 to 4)	2·7 (2·6 to 2·8)
Bournemouth	29·3 (28·7 to 30)	21·9 (18·4 to 25·3)	17·5 (15·7 to 19·4)	11·6 (10 to 13·3)	7·6 (6·8 to 8·6)	5·9 (5·3 to 6·6)	4 (3·6 to 4·4)	3·2 (3·1 to 3·3)
Bristol, City of	36·6 (35·8 to 37·4)	28·3 (25·5 to 31·6)	23·7 (21·5 to 26·2)	16·1 (14·6 to 17·4)	10·3 (9·6 to 11·1)	6·9 (6·4 to 7·5)	4·8 (4·4 to 5·2)	3·7 (3·6 to 3·8)
Cornwall	32·3 (31·6 to 33)	24·6 (22 to 27·5)	19·9 (18 to 22·1)	12·9 (11·6 to 14·2)	8·5 (7·9 to 9·2)	5·7 (5·1 to 6·3)	4·9 (4·6 to 5·3)	3·8 (3·6 to 3·9)
Devon	32·9 (32·2 to 33·6)	24·8 (21·4 to 28·4)	20·3 (17·9 to 22·9)	13·4 (12·2 to 14·7)	9·5 (8·7 to 10·4)	5·6 (5·1 to 6·2)	4·7 (4·1 to 5·3)	3·6 (3·5 to 3·8)
Dorset	31·1 (30·5 to 31·8)	23·5 (21·1 to 25·9)	19·2 (17·3 to 21·4)	12·6 (11·1 to 14)	7·6 (7 to 8·2)	5·4 (5 to 5·9)	4·5 (4·2 to 4·9)	3·6 (3·5 to 3·8)
Gloucestershire	29·4 (28·8 to 30·1)	22·6 (19·5 to 25·8)	18·9 (16·8 to 21·4)	12·5 (11·2 to 13·9)	8·2 (7·7 to 8·8)	5·5 (5·1 to 5·9)	4·2 (3·9 to 4·5)	3·3 (3·2 to 3·4)
North Somerset	30·4 (29·8 to 31·1)	22·9 (20·4 to 25·7)	19 (17·1 to 21)	12·8 (11·3 to 14·3)	7·6 (6·8 to 8·4)	5·9 (5·4 to 6·6)	4·4 (4 to 4·9)	3·1 (3 to 3·3)
Plymouth	36·3 (35·7 to 37)	28·2 (25·7 to 31·2)	23·4 (21·2 to 25·9)	15·5 (14 to 16·9)	9·7 (8·9 to 10·5)	7·2 (6·6 to 7·8)	5·7 (5·2 to 6·1)	4 (3·9 to 4·2)
Poole	34·8 (34·3 to 35·2)	26·4 (22·2 to 31·3)	21·6 (19·5 to 24)	14·3 (12·8 to 15·8)	9·3 (8·2 to 10·4)	6·4 (5·7 to 7·4)	4·6 (4 to 5·3)	3·4 (3·2 to 3·5)
Somerset	30·7 (30·2 to 31·3)	23·4 (21·2 to 25·6)	19·3 (17·2 to 21·5)	12·5 (11·3 to 13·9)	7·7 (7·1 to 8·4)	5·6 (5·1 to 6)	4·6 (4·3 to 5)	3·6 (3·5 to 3·7)
South Gloucestershire	26·7 (26·1 to 27·3)	20·6 (17·6 to 23·6)	17·4 (15·4 to 19·4)	11·7 (10·2 to 13·5)	8·1 (7·5 to 8·7)	5·2 (4·8 to 5·6)	3·6 (3·3 to 3·9)	3 (2·9 to 3·1)
Swindon	40·8 (40 to 41·6)	31·6 (28·7 to 35)	27·3 (24·7 to 30·5)	18·3 (16·5 to 20·1)	10·2 (9·3 to 11·2)	6·6 (6 to 7·2)	4·5 (4·1 to 4·9)	3·3 (3·2 to 3·5)
Torbay	29·8 (29·2 to 30·5)	22·7 (19·1 to 26·6)	18 (16·3 to 19·9)	11·6 (10·2 to 13)	8·5 (7·5 to 9·5)	5·9 (5·1 to 6·8)	5·2 (4·6 to 6)	3·8 (3·7 to 4)
Wiltshire	34·2 (33·6 to 34·7)	25·9 (23·4 to 28·8)	21·3 (19·3 to 23·6)	14 (12·8 to 15·1)	9·7 (8·8 to 10·7)	6 (5·4 to 6·6)	4·8 (4·4 to 5·2)	3·6 (3·5 to 3·7)
West Midlands	35·5 (34·8 to 36·1)	29 (27·9 to 30·1)	25 (23·9 to 26·1)	16·7 (15·9 to 17·5)	12·8 (12·2 to 13·3)	8·6 (8·3 to 9)	7·3 (6·9 to 7·7)	6·6 (6·4 to 6·8)
Birmingham	40·4 (39·6 to 41·2)	32·9 (29·8 to 35·8)	28·3 (26·5 to 30·4)	18·8 (17·5 to 20·3)	15·3 (14·4 to 16·1)	11·2 (10·4 to 12·2)	9·2 (8·5 to 10)	8·9 (8 to 9·8)
Coventry	40·7 (40 to 41·4)	33·2 (30 to 36·7)	29·4 (26·5 to 32·4)	20 (17·9 to 22·4)	13·9 (12·8 to 15·2)	8·8 (8·1 to 9·5)	6·3 (5·8 to 6·8)	5·2 (4·8 to 5·6)
Dudley	29·6 (29 to 30·2)	23·9 (21·4 to 26·6)	20·6 (18·8 to 22·7)	13·6 (12·1 to 15·1)	11·9 (11 to 12·9)	7·3 (6·6 to 8·1)	5·9 (5·3 to 6·6)	5·3 (4·8 to 5·8)
Herefordshire, County of	36·4 (35·7 to 37·1)	28·1 (25·1 to 31·1)	24·5 (22·3 to 26·7)	16·4 (14·5 to 18·5)	9·4 (8·3 to 10·5)	5·1 (4·6 to 5·7)	4·4 (4 to 4·9)	4 (3·7 to 4·4)
Sandwell	42·5 (41·6 to 43·4)	35·3 (31·9 to 39)	31 (28 to 34·2)	20·9 (18·7 to 23·2)	13·6 (12·4 to 14·9)	9·1 (8·4 to 9·9)	7·8 (6·8 to 8·7)	6·5 (5·9 to 7)
Shropshire	30·1 (29·5 to 30·7)	23·5 (20·9 to 26·4)	20·1 (18·3 to 22)	13·5 (12·1 to 14·7)	9·4 (8·5 to 10·3)	6 (5·5 to 6·5)	4·7 (4·3 to 5·2)	4·3 (3·9 to 4·7)
Solihull	32·4 (31·9 to 32·9)	26·1 (23·5 to 28·8)	23·5 (21·3 to 25·8)	16·6 (15 to 18·3)	10·8 (9·7 to 11·8)	6·2 (5·7 to 6·9)	5·3 (4·8 to 5·9)	5·1 (4·7 to 5·6)
Staffordshire	31·9 (31·3 to 32·5)	25·5 (23 to 28)	22·2 (20·6 to 23·7)	15 (13·6 to 16·5)	10·8 (9·7 to 11·8)	7·1 (6·6 to 7·6)	6·2 (5·7 to 6·8)	5·6 (4·8 to 6·5)
Stoke-on-Trent	42·9 (42·1 to 43·6)	35·5 (31·9 to 39·3)	30·6 (27·7 to 33·6)	20·3 (18·5 to 22·2)	14·2 (13 to 15·5)	10·4 (9·6 to 11·3)	8·6 (7·9 to 9·4)	7·9 (7·3 to 8·6)
Telford and Wrekin	33·6 (32·8 to 34·3)	26·9 (24·1 to 29·8)	23·3 (21·3 to 25·6)	15·8 (13·9 to 18)	12·1 (11 to 13·4)	7·6 (6·9 to 8·4)	6 (5·3 to 6·9)	4·9 (4·5 to 5·5)
Walsall	34·6 (33·8 to 35·3)	28·5 (25·5 to 31·6)	25·1 (23·1 to 27·2)	17 (15·2 to 18·8)	12 (11 to 13·1)	8·7 (8 to 9·6)	8·7 (8 to 9·5)	7·2 (6·6 to 7·7)
Warwickshire	26·2 (25·6 to 26·8)	20·9 (18·7 to 23·2)	18·1 (16·6 to 19·8)	12·1 (11·1 to 13·2)	9·9 (9·1 to 10·7)	5·8 (5·4 to 6·3)	4·9 (4·5 to 5·3)	4·2 (3·8 to 4·5)
Wolverhampton	35·4 (34·7 to 36)	29·2 (26·1 to 32·3)	25·6 (23·5 to 28)	16·9 (15·3 to 18·6)	13·8 (12·7 to 15)	9·5 (8·7 to 10·3)	7·6 (6·9 to 8·2)	6·2 (5·7 to 6·8)
Worcestershire	28·1 (27·5 to 28·6)	22·4 (20·1 to 24·7)	19·1 (17·4 to 20·8)	12·6 (11·3 to 13·8)	10·3 (9·5 to 11·2)	6·7 (6·2 to 7·3)	5·1 (4·7 to 5·6)	4·5 (4·2 to 4·9)
Yorkshire and the Humber	38·2 (37·6 to 38·9)	30·5 (28·7 to 32·5)	25·5 (24·4 to 26·6)	16·8 (15·8 to 18)	11·5 (11 to 11·9)	8·4 (8 to 8·7)	6·4 (6 to 6·7)	4·7 (4·5 to 4·8)
Barnsley	37·5 (36·8 to 38·3)	30·2 (27·6 to 33·2)	25·3 (23·1 to 27·3)	16·2 (14·7 to 17·8)	11·1 (10·2 to 12)	7·7 (7 to 8·4)	5·5 (5 to 5·9)	3·8 (3·6 to 4)
Bradford	43·1 (42·3 to 44·1)	34·6 (29·7 to 39·7)	29 (26·5 to 31·5)	18·9 (16·7 to 21·1)	13·8 (12·8 to 15)	11·3 (10·3 to 12·3)	8·7 (8·1 to 9·4)	6·3 (5·5 to 7·3)
Calderdale	37·6 (36·9 to 38·4)	30 (27·1 to 32·8)	25 (22·8 to 27·2)	16·4 (14·6 to 18·4)	12·7 (11·5 to 14·1)	9·1 (8·3 to 9·9)	7·1 (6·5 to 7·8)	5·1 (4·9 to 5·4)
Doncaster	33 (32·4 to 33·7)	27 (24·5 to 29·9)	23 (20·9 to 25·2)	14·8 (13 to 16·8)	10·5 (9·7 to 11·3)	8·2 (7·3 to 9·1)	6·5 (5·8 to 7·3)	5 (4·7 to 5·2)
East Riding of Yorkshire	28·2 (27·6 to 28·9)	22·2 (19·8 to 25)	18·6 (16·8 to 20·3)	12·2 (10·8 to 13·6)	8·6 (7·9 to 9·3)	6·3 (5·8 to 6·9)	4·5 (4·1 to 4·9)	3·1 (2·9 to 3·3)
Kingston upon Hull, City of	42·1 (41·4 to 42·9)	34·2 (31·1 to 37·6)	28·2 (26·2 to 30·4)	18·2 (16·2 to 20·3)	11·7 (10·7 to 12·7)	7·9 (7·1 to 8·9)	6 (5·4 to 6·7)	4·8 (4·6 to 5·1)
Kirklees	42·3 (41·5 to 43·1)	33·4 (29·4 to 37·5)	27·8 (25·5 to 30·2)	18·4 (16·7 to 20·2)	12·1 (11·2 to 13)	9·4 (8·6 to 10·1)	7·4 (7 to 7·8)	5·6 (5·3 to 5·9)
Leeds	36·8 (36·1 to 37·6)	29·3 (26·8 to 31·9)	24·6 (22·7 to 26·6)	16·4 (14·6 to 18·4)	11·2 (10·2 to 12·2)	7·9 (7·4 to 8·5)	5·8 (5·4 to 6·2)	4·3 (4·1 to 4·5)
North East Lincolnshire	33·1 (32·4 to 33·8)	26·4 (23 to 30)	22·4 (20·6 to 24·4)	14·9 (13·2 to 16·8)	8·9 (8·1 to 9·9)	7·3 (6·6 to 8)	5·9 (5·3 to 6·5)	4·6 (4·3 to 4·8)
North Lincolnshire	32·1 (31·4 to 32·8)	25·9 (23 to 29·3)	21·9 (20·5 to 23·4)	14·4 (12·8 to 16·1)	10·6 (9·6 to 11·8)	7·5 (6·8 to 8·3)	5·2 (4·7 to 5·8)	3·9 (3·7 to 4·2)
North Yorkshire	36·2 (35·6 to 36·8)	27·7 (24·3 to 31·1)	23·1 (21·2 to 25·2)	15·5 (14 to 17·3)	9·1 (8·5 to 9·8)	5·5 (4·9 to 6·1)	4·4 (3·9 to 4·8)	3·1 (2·9 to 3·3)
Rotherham	35·5 (34·8 to 36·2)	28·8 (26·2 to 31·7)	24·3 (22·7 to 26)	15·8 (14·2 to 17·4)	10·9 (10·1 to 11·7)	8·1 (7·4 to 8·7)	6·2 (5·7 to 6·7)	4·3 (4 to 4·5)
Sheffield	33·5 (32·8 to 34·3)	26·8 (23·4 to 30·4)	22·2 (20·1 to 24·4)	14·6 (13·2 to 16·2)	10·9 (10·2 to 11·6)	8·4 (7·8 to 9)	6·1 (5·8 to 6·5)	4·6 (4·4 to 4·9)

Wakefield	38·4 (37·6 to 39·1)	31·1 (28·3 to 34·2)	26·4 (24·6 to 28·5)	17·5 (15·8 to 19·2)	10·6 (9·7 to 11·6)	8·1 (7·4 to 8·7)	6 (5·5 to 6·4)	4·1 (3·8 to 4·3)
York	31·6 (30·9 to 32·3)	24·9 (22 to 28·2)	21 (19·5 to 22·4)	14·3 (12·6 to 16·1)	9·6 (8·6 to 10·7)	7·3 (6·6 to 8·1)	5·4 (4·9 to 5·9)	3·9 (3·7 to 4·1)
Northern Ireland	49·4 (47·2 to 51·5)	34 (32·6 to 35·4)	28·6 (27·4 to 29·8)	19·2 (18·3 to 20·3)	10·7 (10·1 to 11·4)	7 (6·4 to 7·6)	6·3 (5·8 to 6·7)	5 (4·1 to 6·1)
Scotland	47·8 (46·5 to 49·2)	34·3 (33·5 to 35·2)	26·2 (25·5 to 26·9)	16·2 (15·7 to 16·8)	10·6 (10·2 to 11·1)	7·2 (6·8 to 7·6)	5 (4·7 to 5·3)	3·8 (3·3 to 4·3)
Wales	42·1 (41·3 to 42·9)	32·3 (27·1 to 38·4)	26·5 (22·5 to 31·3)	17·1 (15·8 to 18·4)	9·8 (9·3 to 10·4)	7·1 (6·7 to 7·5)	5·3 (5 to 5·7)	4·1 (3·6 to 4·7)
Latin America and Caribbean	245·9 (218·8 to 278·6)	187·1 (174·5 to 200·7)	141·5 (134·2 to 149·1)	97·2 (92·4 to 101·8)	62 (59·2 to 64·7)	40·7 (38·6 to 43)	31·4 (28·6 to 34)	20·9 (17·6 to 24·8)
Andean Latin America	366 (335 to 401·7)	274·5 (255·6 to 293·7)	193·1 (181·7 to 204·3)	121·4 (113·2 to 131·1)	75·7 (71·5 to 80·2)	47·6 (44·6 to 50·7)	29·5 (26·8 to 32·2)	19·8 (17·2 to 22·7)
Bolivia	398·9 (359·3 to 440·1)	328·2 (295·9 to 361·4)	243·5 (224·4 to 262·9)	164·1 (153·8 to 175·9)	113·7 (106·8 to 120·8)	73·2 (67·9 to 78·7)	45·6 (40·5 to 50·8)	30·9 (26·4 to 36·7)
Ecuador	221·4 (192·8 to 253·7)	169·2 (154·7 to 184·9)	129 (118·1 to 139·9)	82·7 (75·6 to 89·9)	46·7 (42·7 to 51·1)	37·8 (32·7 to 43·1)	24·9 (21 to 29)	16·4 (13·7 to 19·6)
Peru	406·4 (360·7 to 461·1)	300·5 (272·3 to 329·1)	205 (188 to 221·8)	123·9 (112·2 to 136)	75 (68 to 82)	42·3 (38·3 to 46·6)	24·6 (21·5 to 27·9)	16·2 (13·3 to 19·7)
Caribbean	164·9 (152·7 to 178·8)	137·8 (131·1 to 145·1)	110 (104·3 to 116)	93·7 (88·4 to 99·3)	72·1 (67·8 to 76·5)	55 (51·2 to 59)	79 (73·8 to 84·4)	42·9 (36·6 to 50·4)
Antigua and Barbuda	121·6 (103·6 to 144·3)	80·9 (68·7 to 95)	42·4 (35·7 to 50)	24·4 (20·5 to 28·9)	15 (12·6 to 17·8)	15·3 (12·8 to 18·3)	12·9 (10·7 to 15·4)	11 (9·1 to 13·4)
The Bahamas	126·3 (105·5 to 150·7)	88·1 (73·6 to 103·6)	52·2 (43·6 to 61·5)	36 (30·1 to 41·9)	23·7 (19·5 to 28)	16·6 (13·8 to 19·8)	14·2 (12 to 16·7)	12·6 (10·5 to 15·5)
Barbados	161·3 (136·1 to 193·4)	93·6 (79·3 to 109)	50·9 (43 to 60·3)	28·9 (24·2 to 34·2)	20 (16·9 to 23·4)	15·8 (13 to 19)	14·3 (11·8 to 17·4)	12·6 (10·3 to 15·3)
Belize	174·6 (150·5 to 202·2)	127·9 (109·7 to 148·5)	98·7 (87·3 to 111)	67·8 (60·9 to 75)	45 (40·5 to 49·8)	30·4 (27 to 34·3)	21·6 (18·6 to 24·9)	16·9 (14·2 to 20·3)
Bermuda	72·1 (65·5 to 78·8)	45·6 (41·6 to 49·7)	30·1 (27·4 to 33·1)	18·5 (16·3 to 20·8)	10·9 (9·4 to 12·4)	5·8 (5 to 6·9)	5·4 (4·6 to 6·3)	4·7 (3·9 to 5·7)
Cuba	70·6 (58·4 to 85·2)	50·4 (49·2 to 51·6)	47·3 (46·3 to 48·4)	26·9 (26·1 to 27·8)	15·4 (14·8 to 15·9)	9·5 (9·2 to 9·9)	6·5 (6·2 to 6·9)	5·2 (4·4 to 6)
Dominica	247·2 (211·5 to 291·7)	148·5 (127·8 to 171·2)	72·5 (62 to 84·2)	30·4 (25·7 to 35·8)	22·4 (18·5 to 26·2)	21·2 (18·1 to 25)	24·9 (20·8 to 29·3)	28·3 (23·3 to 34·2)
Dominican Republic	203·6 (177 to 232·7)	177·1 (160·5 to 194·6)	134·1 (122·7 to 147·9)	93·6 (84·8 to 103)	67·8 (61·4 to 74·9)	46·7 (41·9 to 51·9)	35·8 (31·1 to 40·8)	28·9 (24 to 34·8)
Grenada	144·4 (121·9 to 170·1)	105·4 (89·3 to 122·9)	70·3 (58·7 to 83)	48 (39·5 to 57·9)	27·8 (23 to 33·6)	19·5 (16·1 to 23·1)	16·2 (13·4 to 19·5)	14·6 (12·1 to 17·7)
Guyana	170·9 (152·7 to 190·8)	119·4 (106·2 to 133·1)	92·2 (82·8 to 102·5)	67·5 (58·9 to 77·7)	57·5 (52·4 to 63·6)	42·6 (38·4 to 47)	34 (29·5 to 38·7)	25·5 (21·1 to 30·8)
Haiti	312·4 (278·7 to 352·2)	274 (252·9 to 295·8)	240·5 (222·1 to 259·8)	201·4 (184·2 to 217·9)	156·6 (144·8 to 168·7)	110·8 (101·3 to 120·8)	170·4 (158·6 to 183·1)	75·5 (64·8 to 87·9)
Jamaica	137·4 (120·1 to 156·5)	88·6 (80·2 to 97·7)	59·2 (53·3 to 65·2)	40·2 (35·9 to 45)	30·7 (26·9 to 34·8)	22·8 (19·4 to 26·5)	19·3 (16·2 to 23)	16·7 (13·8 to 20·3)
Puerto Rico	117 (114·2 to 120·3)	61·2 (59·3 to 63)	36·5 (35·3 to 37·8)	24 (23·1 to 25)	17·1 (16·4 to 17·9)	12·2 (11·5 to 12·8)	9·7 (9·1 to 10·3)	8·4 (7 to 10)
Saint Kitts and Nevis	129·3 (121·1 to 137·9)	174·5 (166·5 to 183·1)	87·8 (81·8 to 94)	64 (58·7 to 69·3)	32·6 (29·6 to 35·8)	21·2 (19 to 24)	16·8 (14·6 to 19·2)	15·3 (12·9 to 18·2)
Saint Lucia	199·9 (169·8 to 238)	148·1 (124·5 to 174·6)	81·7 (68·3 to 97·3)	42 (35 to 49·5)	25·6 (21·5 to 30·4)	19·6 (16·3 to 23·1)	18·3 (15·1 to 22)	14·8 (12·1 to 18)
Saint Vincent and the Grenadines	187·6 (159·4 to 223·6)	145·8 (123·1 to 167·7)	97 (81·8 to 112·5)	60·2 (50·1 to 71)	35·2 (29·4 to 41·4)	27·2 (22·9 to 32·4)	21·6 (18·1 to 25·4)	16·6 (13·7 to 20·1)
Suriname	113·6 (95·3 to 137·2)	99·8 (83·5 to 119·3)	83 (71·2 to 97)	71·4 (61·9 to 81·5)	56 (49·1 to 62·6)	46·2 (39·9 to 53·1)	36·2 (30·7 to 42·4)	28·2 (23·4 to 34·1)
Trinidad and Tobago	116·6 (101·2 to 134·3)	76·7 (68·4 to 85·8)	56·4 (50·4 to 62·7)	40·2 (35·2 to 45·5)	29·7 (25·8 to 33·9)	29·5 (25 to 34·5)	19·8 (16·7 to 23·7)	16·6 (13·7 to 20·2)
Virgin Islands	77·5 (70·1 to 85·2)	52·4 (48 to 57)	34·4 (31 to 38)	29·8 (27·1 to 32·7)	19·1 (17·1 to 21·4)	13 (11·6 to 14·5)	9·8 (8·6 to 11·3)	7·5 (6·3 to 9)
Central Latin America	207·4 (184 to 234·8)	147·5 (138·2 to 157·8)	108·8 (103 to 114·6)	75·1 (70·5 to 79·6)	47·8 (44·9 to 50·7)	31·6 (29·5 to 34)	22·8 (20·4 to 25·2)	16·3 (13·5 to 19·6)
Colombia	165·4 (146·5 to 188·1)	124·8 (113·4 to 137·6)	88·9 (81·4 to 97·1)	57·4 (52·3 to 62·8)	37·3 (33·7 to 40·8)	28·5 (25·4 to 32)	20·4 (17·7 to 23·6)	13·8 (11·4 to 16·6)
Costa Rica	146·6 (142·3 to 151·4)	100·6 (97·8 to 103·6)	88·3 (85·9 to 91)	26·9 (25·9 to 28)	19·5 (18·7 to 20·3)	14·3 (13·7 to 15)	11·8 (11·2 to 12·4)	8·6 (7·3 to 10·3)
El Salvador	286·2 (246·8 to 334·7)	223 (195·5 to 253·1)	159·4 (141·8 to 179·5)	101·8 (92·5 to 112·3)	57·6 (52·3 to 63·5)	31·6 (27·8 to 35·8)	18·8 (16 to 21·6)	11·8 (9·7 to 14·3)
Guatemala	252·1 (217·1 to 295·8)	209 (182·9 to 235·8)	174·3 (158·1 to 192·4)	128·6 (116·8 to 140·4)	82·6 (74·9 to 90·5)	52·9 (47·1 to 59)	34·7 (30·1 to 39·6)	23·9 (19·8 to 28·7)
Honduras	255·1 (220·8 to 297·6)	196·3 (171·7 to 222·7)	140·4 (124·8 to 157·7)	94·4 (85 to 104·7)	58·4 (52·8 to 64·5)	38·1 (33·5 to 43·2)	27·7 (23·4 to 32·1)	18·7 (15·5 to 22·7)
Mexico	225·9 (200·5 to 255·3)	154·4 (140·7 to 169)	110·2 (100·4 to 119·5)	78·1 (70·6 to 85·9)	48·6 (44·4 to 53·5)	30·9 (27·7 to 34·5)	22·5 (19·6 to 25·6)	16 (13·3 to 19·2)
Aguascalientes	200·3 (177·6 to 227·4)	138·9 (125·4 to 154·2)	102·3 (91·1 to 113·6)	73·9 (65·4 to 82·7)	45·1 (40·2 to 50·5)	28·3 (24·6 to 32·7)	20·2 (17·4 to 23·3)	13·9 (11·6 to 16·7)
Baja California	217·6 (193·3 to 246·7)	140·1 (126·6 to 155·8)	93·2 (84·5 to 102·5)	42·3 (37·6 to 47)	38·7 (35·2 to 42·7)	28·5 (25·5 to 31·6)	23·4 (20·5 to 26·7)	13·4 (11·1 to 16·1)
Baja California Sur	229·8 (204·3 to 260·2)	148·4 (132·2 to 166)	99·2 (89·9 to 108·8)	56·8 (50·2 to 63·3)	28·7 (25·6 to 31·9)	23·3 (20·7 to 26)	20·2 (17·2 to 23·2)	13·6 (11 to 16·5)
Campeche	256·1 (228·4 to 289·4)	179·5 (163 to 198·7)	135·1 (120·8 to 150·2)	101·8 (91·5 to 113·1)	57·1 (49·9 to 64·8)	34·1 (29·6 to 39·3)	24·6 (21·2 to 28·5)	17·2 (13·9 to 21)
Chiapas	276·6 (247·2 to 311·9)	199·8 (182·7 to 218·8)	153·7 (140·3 to 168·6)	120·7 (108·6 to 133·4)	67·6 (60·5 to 75·2)	37·8 (33 to 43·3)	21 (18·3 to 24·1)	18·2 (14·9 to 21·8)
Chihuahua	250·8 (223·5 to 283·4)	171·8 (156·5 to 189)	125·7 (114·7 to 137·6)	93·4 (83·5 to 103·6)	55·5 (50·3 to 61·2)	38·9 (34·2 to 44·4)	26·3 (22·7 to 30)	21·6 (17·7 to 26·1)
Coahuila	251·1 (220·1 to 289·1)	166·7 (151·3 to 183·3)	121·7 (110·8 to 134·1)	87 (78·2 to 96·1)	46·6 (41·6 to 51·7)	29·8 (26·2 to 34·1)	19·6 (16·9 to 22·4)	17·3 (14·2 to 20·8)
Colima	281·7 (252 to 317·4)	189·4 (171·5 to 208·8)	132·9 (119·2 to 147·5)	87·7 (73·4 to 103·3)	45·3 (39·8 to 51·8)	28·7 (24·5 to 33·4)	20·4 (17·3 to 23·6)	12·5 (10·2 to 15·3)
Durango	238·2 (212·6 to 268·9)	162·2 (147·9 to 177·5)	115·3 (103·4 to 126·5)	82·4 (74·3 to 91)	46·3 (41·6 to 51·2)	32·6 (28·6 to 36·9)	22·8 (19·7 to 26)	16·9 (13·9 to 20·7)
Guanajuato	270·6 (241·5 to 305·4)	190 (172·3 to 208·3)	139 (124·2 to 153·8)	96·1 (86·9 to 105·6)	60·3 (54·5 to 66·8)	29·2 (26·2 to 32·1)	21·8 (19 to 24·9)	15·3 (12·7 to 18·3)
Guerrero	254·2 (226·6 to 287·2)	189·4 (155·5 to 225·5)	158·2 (137·1 to 179·1)	122·5 (103 to 142·2)	77·7 (68·6 to 87·4)	51·7 (45·9 to 58)	33 (26·9 to 40)	17·8 (14 to 22·2)

Hidalgo	219·6 (195·1 to 248·9)	151 (136·6 to 168)	109·5 (98·6 to 120·5)	69 (58·6 to 80·5)	39·5 (34·2 to 45)	25·5 (22·2 to 29·3)	18·5 (16·1 to 21·2)	12·9 (10·5 to 15·6)
Jalisco	214·1 (190·1 to 242·8)	145 (131·8 to 159·7)	101·6 (89·6 to 113·7)	67·6 (60 to 76)	41·2 (36·8 to 45·9)	30·1 (26 to 34·6)	21·4 (18·6 to 24·6)	15·4 (12·9 to 18·3)
México	207 (180·9 to 239·5)	147·5 (132·8 to 162·4)	105·7 (94·1 to 117·7)	71·8 (64·7 to 79)	58·1 (52·1 to 64·6)	33·7 (30·3 to 37·1)	25 (21·9 to 28·5)	18·3 (15·1 to 22·1)
Mexico City	157·7 (139·5 to 179·6)	102·9 (92·9 to 113·9)	72·5 (65·7 to 79·8)	55·4 (49·9 to 60·8)	34·5 (31·5 to 37·7)	25·3 (22·9 to 28·1)	23·6 (20·7 to 26·9)	15·8 (13·2 to 18·9)
Michoacán de Ocampo	226·8 (201·6 to 257)	154·6 (140·9 to 170·1)	100·7 (89·8 to 111·6)	66·4 (58·8 to 74·5)	40 (35·7 to 44·6)	25·7 (22·5 to 29·6)	19·3 (16·7 to 22)	11·9 (9·9 to 14·2)
Morelos	295·9 (264·5 to 334·2)	199·3 (180·8 to 220)	140 (124·5 to 156·2)	93·1 (77 to 109·7)	49·2 (43·2 to 55·8)	30·7 (26·8 to 35·2)	21·4 (18·5 to 24·5)	14·2 (11·8 to 17·5)
Nayarit	292·9 (261·8 to 330·7)	197·4 (177·6 to 219·1)	130·8 (118·5 to 144·2)	80·4 (71·1 to 90·1)	47·7 (42·3 to 53·2)	28·9 (25 to 33·1)	18·4 (15·8 to 21·2)	14·1 (11·7 to 16·8)
Nuevo León	206·2 (180·2 to 238·5)	131·7 (119·3 to 145·3)	91·3 (81·4 to 102)	58·1 (50·8 to 66·1)	32·9 (28·8 to 37·5)	24 (21 to 27·5)	16·2 (14 to 18·8)	14·1 (11·4 to 17)
Oaxaca	251 (223·7 to 283·8)	177·8 (162·4 to 195·4)	129·1 (117 to 141)	91·9 (82·2 to 102·4)	48·7 (43·7 to 53·8)	29·7 (25·7 to 34·2)	18·3 (15·9 to 21)	13 (10·6 to 15·7)
Puebla	312·9 (280·4 to 351·5)	218·8 (200·4 to 239·2)	157·6 (143·6 to 173·1)	108·3 (96·7 to 120·5)	62 (55·8 to 68·7)	39·2 (34·6 to 44·5)	27·9 (24·4 to 31·8)	18·9 (15·6 to 22·7)
Querétaro	262·6 (234 to 296·6)	183·7 (166·5 to 202·1)	131 (117·8 to 145·3)	92·5 (82·5 to 102·1)	51·4 (46·7 to 57)	28·1 (25·1 to 31·1)	21·3 (18·3 to 24·6)	14·6 (11·8 to 18)
Quintana Roo	228·8 (203·3 to 259·2)	162·6 (146·4 to 179·8)	123·1 (111·3 to 135·4)	87·2 (77·9 to 96·9)	46·6 (41·8 to 51·9)	29·8 (25·9 to 34·4)	22·1 (19·1 to 25·4)	14·9 (12·4 to 17·8)
San Luis Potosí	244·2 (217·4 to 276·1)	165·5 (150·8 to 181·5)	116·3 (104·1 to 128·7)	73·8 (65·4 to 83·3)	43 (38·4 to 47·9)	27·8 (24·5 to 31·9)	17·7 (15·4 to 20·2)	12·6 (10·3 to 15·3)
Sinaloa	198·1 (175·8 to 224·9)	132·4 (120·3 to 145·9)	89·7 (80·7 to 99·2)	57·3 (49·9 to 65·8)	31·9 (27·9 to 36·2)	19 (16·4 to 21·8)	15·5 (13·5 to 17·7)	12·9 (10·6 to 15·4)
Sonora	198·7 (176·2 to 225·8)	132·6 (120·7 to 146·9)	96·7 (86·8 to 107·3)	69·2 (57 to 82·1)	38·3 (33·4 to 43·6)	29·8 (25·9 to 34·3)	22·9 (19·9 to 26·3)	14·9 (12·4 to 17·8)
Tabasco	246·2 (219·1 to 278·6)	174·6 (158·6 to 191·7)	133·6 (121·4 to 146·6)	99·3 (84·7 to 113·9)	57·2 (50·1 to 65·9)	33·3 (29·2 to 37·8)	24·6 (21·3 to 28)	18·8 (15·6 to 22·6)
Tamaulipas	211·6 (187·9 to 240·1)	141·4 (127·7 to 155·5)	98·8 (89·8 to 108·4)	69·7 (60·2 to 79·3)	39 (33·8 to 45·2)	23·1 (19·7 to 26·8)	19·8 (17·2 to 22·7)	14·5 (11·7 to 17·7)
Tlaxcala	270·7 (241·4 to 305·6)	186·5 (169·1 to 204·6)	131·8 (118·2 to 146)	95·2 (84·7 to 105·4)	58·2 (52·5 to 64·3)	34·3 (30·7 to 38)	25·3 (21·6 to 29·6)	16·8 (13·5 to 20·7)
Veracruz de Ignacio de la Llave	234·4 (208·4 to 265·3)	160·2 (144·8 to 175·9)	113·7 (102·9 to 124·6)	77·1 (66 to 89·2)	47·6 (39·2 to 56·8)	27·9 (23·8 to 32·6)	22·4 (19·5 to 25·5)	15·4 (12·8 to 18·5)
Yucatán	207·3 (184 to 235·4)	146·2 (132·1 to 160·8)	99·4 (90·3 to 109·1)	70·5 (62·4 to 78·3)	37·4 (33·7 to 41·3)	23·4 (21·1 to 25·9)	19·4 (16·5 to 22·6)	14·5 (11·7 to 17·7)
Zacatecas	219·4 (194·9 to 248·6)	154·5 (139·5 to 170·5)	110·1 (98·1 to 122·9)	82·1 (73 to 91·5)	46 (41·2 to 51·5)	29·4 (25·6 to 33·6)	20·4 (17·6 to 23·4)	14·1 (11·8 to 16·9)
Nicaragua	301·2 (262·5 to 348·7)	233·7 (206·6 to 264)	172·5 (156·2 to 190·7)	110·4 (101·6 to 120·8)	68·6 (63 to 74·7)	39 (34·8 to 43·6)	23·6 (20·2 to 27·4)	15·9 (13·2 to 19·1)
Panama	114 (101·3 to 128)	89·9 (81·3 to 99·4)	60·1 (53·8 to 66·5)	38·8 (33·9 to 44·4)	29 (25 to 33·1)	24·6 (21·2 to 28·5)	19·6 (16·5 to 23·3)	15·7 (12·9 to 19)
Venezuela	124·8 (122·2 to 127·5)	79 (77·5 to 80·6)	73·5 (72·1 to 74·9)	44·9 (44 to 45·8)	35·4 (34·8 to 36·1)	24·9 (24·4 to 25·5)	20 (19·5 to 20·4)	16·2 (13·5 to 19·6)
Tropical Latin America	268 (234·5 to 308·9)	215·8 (194 to 241)	171·7 (155·9 to 187)	118·1 (109·1 to 127·4)	74·5 (69·5 to 80)	47·6 (43·3 to 52)	32·1 (28 to 36·5)	22·1 (18·4 to 26·5)
Brazil	271·3 (237·5 to 312·7)	218·9 (196·5 to 244·5)	174·1 (158·1 to 189·8)	119·9 (110·7 to 129·6)	75·9 (70·7 to 81·7)	48·2 (43·9 to 52·7)	32·5 (28·3 to 36·9)	22·4 (18·7 to 26·8)
Acre	322·8 (277·8 to 376·1)	257 (216·2 to 300·6)	202·6 (179·6 to 227·2)	152·2 (131·1 to 174·6)	98·7 (88·8 to 109·3)	70·6 (62 to 80·2)	49·4 (37·9 to 63·7)	34 (26·8 to 42·6)
Alagoas	390·8 (335·7 to 454·2)	349·8 (316·5 to 387·6)	300 (272·2 to 328·9)	256·7 (232·3 to 281·7)	162·7 (149·2 to 178·3)	86·7 (77·1 to 97·4)	46·9 (40·1 to 54·3)	26·2 (20·4 to 33·1)
Amapá	371·5 (332·8 to 412)	286·7 (249·8 to 325·5)	190·3 (167·4 to 217·6)	72·6 (65·3 to 79·8)	47 (43 to 51·4)	43·8 (40·6 to 47·3)	34·7 (29·7 to 40·3)	32·1 (28·5 to 36·2)
Amazonas	204·1 (182·3 to 227·9)	157·6 (134·1 to 186·1)	127·2 (111·3 to 144·1)	103·7 (88·6 to 119·8)	66 (58·1 to 74·4)	47·2 (44·1 to 50·2)	29·4 (25·5 to 34·1)	25·5 (22·6 to 28·6)
Bahia	319·2 (271 to 374·7)	273·3 (245·3 to 305·1)	221·2 (199·6 to 244·6)	148·3 (136·7 to 161·1)	110·1 (100·1 to 120·5)	69 (61·2 to 77)	45·8 (39·4 to 53)	29·2 (22·8 to 36·9)
Ceará	497·5 (434·3 to 567·4)	423·5 (385·2 to 465·3)	332·4 (302·2 to 363·6)	232·5 (215·4 to 252·1)	137·2 (125·5 to 150·9)	79·7 (71·2 to 88·7)	45·7 (39·3 to 52·7)	26·2 (20·7 to 32·9)
Distrito Federal	250·7 (223·4 to 280·4)	186·9 (159 to 217·9)	132·8 (116·6 to 150·6)	71·4 (65·7 to 78·1)	39·3 (36·5 to 42·3)	28·5 (26·6 to 30·5)	22·7 (19·5 to 26·2)	18·5 (16·4 to 20·9)
Espírito Santo	209·9 (176·8 to 250·4)	170·5 (149·4 to 193·9)	133·7 (117·5 to 151·1)	79·9 (73·7 to 87·2)	51·8 (48·3 to 55·4)	32·3 (29·4 to 35·2)	27·9 (23·7 to 32·4)	20·4 (16 to 25·7)
Goiás	316·1 (283·6 to 350·9)	248·1 (214·3 to 289)	161 (142·4 to 181·2)	87·4 (75·4 to 100·6)	52·4 (46·6 to 58·4)	28·7 (26·7 to 30·7)	23·2 (19·9 to 26·7)	20·8 (18·6 to 23·1)
Maranhão	387·6 (330 to 453·1)	339·2 (305·8 to 376·1)	281·9 (255·2 to 310)	197·8 (177·9 to 219·2)	128·9 (117·6 to 140·6)	77·1 (67·9 to 86·4)	47·2 (40·4 to 54·6)	27·3 (21·4 to 34·5)
Mato Grosso	192·4 (171·9 to 214·9)	168·3 (142·5 to 197·4)	133 (116·2 to 150·5)	83·3 (74·1 to 92·6)	63·2 (56·4 to 70·6)	36·6 (34 to 39·2)	25·6 (21·7 to 29·6)	20·5 (18·2 to 22·9)
Mato Grosso do Sul	183·2 (158·7 to 209·7)	148·4 (123·4 to 178)	105·2 (92·7 to 119)	70 (63·7 to 76·3)	46 (42·8 to 49·4)	40·2 (37·7 to 42·7)	27·4 (23·5 to 32)	19·8 (17·5 to 22·2)
Minas Gerais	228·3 (191·1 to 272·6)	183·1 (161·9 to 207)	137·6 (123·3 to 153·5)	88·4 (80·6 to 97·1)	58·4 (52·4 to 65·3)	42·2 (36·9 to 47·7)	29·2 (24·9 to 34)	19·7 (15·3 to 25)
Pará	303 (260·3 to 353·9)	243·6 (216·6 to 272·6)	194·7 (174 to 216·3)	135·5 (121·9 to 149·6)	89·1 (78·9 to 100·3)	57·1 (50·1 to 64·4)	39·1 (33·2 to 45·3)	25·7 (20·2 to 32·2)
Paraíba	373·9 (320·1 to 435·7)	301·8 (271·6 to 335·5)	226 (203·4 to 249·8)	157 (144·3 to 171·1)	96 (87·6 to 105·6)	53·2 (46·6 to 60·3)	32·2 (27·4 to 37·5)	18·7 (14·5 to 23·8)
Paraná	194·7 (164·9 to 231)	153·8 (135·5 to 171·7)	114·8 (100·7 to 130·1)	85·8 (79·2 to 92·8)	49·3 (46·2 to 52·8)	33·6 (30·9 to 36·4)	27 (23 to 31·1)	19·4 (15·1 to 24·3)
Pernambuco	438·9 (378·4 to 506·5)	368·5 (333 to 407·9)	289·1 (262·6 to 317·4)	187·6 (172·7 to 203·4)	119·8 (109·5 to 131·6)	69·5 (61·9 to 78·1)	41 (35·3 to 47·5)	24·1 (18·7 to 30·5)
Piauí	301·9 (253·4 to 359·5)	257 (229·9 to 287·3)	219·3 (197·4 to 241·8)	145·3 (131·3 to 159·7)	96·9 (88·4 to 106·2)	63·3 (55·6 to 71·1)	40·4 (34·7 to 47)	25·2 (19·6 to 32)
Rio de Janeiro	186·4 (166·8 to 207·5)	142·7 (121·3 to 170·6)	118·8 (104·6 to 135·2)	93·9 (86·4 to 102·2)	56·7 (53 to 60·6)	36·6 (34·4 to 38·8)	25·5 (22 to 29·3)	20·7 (18·7 to 22·8)
Rio Grande do Norte	401·3 (352 to 458·2)	315·2 (284 to 347)	229·1 (206 to 253·9)	150 (137·8 to 162·6)	90·8 (82·6 to 99·7)	48·7 (42·8 to 54·9)	28·7 (24·4 to 33·5)	18 (14·1 to 22·5)
Rio Grande do Sul	174·3 (153·8 to 196·5)	133·9 (113·2 to 160·1)	93·2 (80·3 to 106·4)	59·7 (54·6 to 65·4)	34·9 (32·6 to 37·5)	28·1 (26·3 to 30·1)	20·1 (17·1 to 23·3)	17·8 (16·2 to 19·5)
Rondônia	338·2 (302·7 to 375·8)	280 (241·9 to 321·6)	209·6 (183·7 to 238·8)	111·7 (101·5 to 122·3)	72·7 (67·9 to 78·2)	41·7 (38·8 to 44·9)	28·2 (24·2 to 32·8)	21 (18·6 to 23·7)

Roraima	335·8 (301·2 to 372·8)	269·6 (234·6 to 306·6)	193·9 (171·4 to 221)	106·3 (97·2 to 116·5)	61·2 (56 to 66·6)	36·6 (33·9 to 39·7)	28 (23·8 to 32·6)	25·2 (22·5 to 28·3)
Santa Catarina	150 (122·9 to 183·4)	119·9 (105 to 136)	98·6 (87·1 to 110·3)	67·5 (61·6 to 73·8)	48·3 (43·2 to 54)	37·8 (33 to 42·8)	29·2 (24·9 to 34)	20·9 (16·2 to 26·5)
São Paulo	163 (143·8 to 183·8)	126·6 (107·4 to 149·7)	106 (92·2 to 121·2)	84·6 (78·4 to 91·3)	49·9 (46·6 to 53·4)	32 (30·1 to 34)	21·8 (18·7 to 25·3)	18·5 (17 to 20·2)
Sergipe	279 (235·3 to 330·6)	231 (201·7 to 264·6)	183·6 (164·7 to 203)	148·9 (128·4 to 173·1)	97·2 (88·2 to 107·2)	61·4 (53·8 to 69)	40·3 (34·3 to 46·7)	26·4 (20·5 to 33·4)
Tocantins	339·8 (299·9 to 381·4)	283·3 (223·3 to 345·2)	211 (188·2 to 236·2)	132·2 (113·2 to 153)	86·5 (75·3 to 98·7)	56·6 (49·4 to 64·3)	37 (30·3 to 44·4)	22·5 (18·8 to 27·4)
Paraguay	114·8 (99·5 to 134)	87 (78·6 to 96·2)	73·4 (66·7 to 80·4)	52·5 (47·8 to 57·7)	37·1 (33·3 to 41·2)	30·5 (26·8 to 34·2)	22·1 (19·2 to 25·5)	14·8 (12·3 to 17·8)
North Africa and Middle East	309·9 (282·4 to 341·5)	261·1 (245·9 to 278·9)	193·2 (183 to 204·3)	129·6 (123·2 to 136·2)	82·2 (78·1 to 86·5)	57·6 (54·2 to 61·1)	38·2 (35·1 to 41·6)	25·9 (23·4 to 28·6)
North Africa and Middle East	309·9 (282·4 to 341·5)	261·1 (245·9 to 278·9)	193·2 (183 to 204·3)	129·6 (123·2 to 136·2)	82·2 (78·1 to 86·5)	57·6 (54·2 to 61·1)	38·2 (35·1 to 41·6)	25·9 (23·4 to 28·6)
Afghanistan	262·8 (227·8 to 302·3)	239·3 (207·4 to 273·7)	216·4 (186·4 to 251)	209·4 (181·3 to 245·4)	150 (134·3 to 168·5)	130 (117·9 to 142·6)	80·7 (70·7 to 91·1)	57·6 (49·9 to 66·5)
Algeria	158·8 (142·8 to 176·5)	168·9 (152 to 187·1)	161·5 (141·9 to 185·3)	127 (115·1 to 141·2)	64·3 (58·6 to 70·7)	43·7 (39·8 to 48)	29·2 (26·8 to 32·1)	20·7 (17·8 to 24·1)
Bahrain	225·3 (193·6 to 261·7)	152 (132·7 to 173·7)	76·6 (65·8 to 90·4)	31·3 (29 to 33·7)	24 (22·8 to 25·2)	12·4 (11·6 to 13·2)	8·4 (7·8 to 9)	6·6 (5·8 to 7·4)
Egypt	374 (330·2 to 427)	310·1 (282·5 to 338·9)	230·6 (211 to 249·2)	152·1 (138·6 to 166·5)	82·1 (74·8 to 89·8)	46 (40·8 to 51·1)	28 (23·8 to 32·8)	16·2 (13·3 to 19·7)
Iran	377·2 (329·2 to 430·2)	317·4 (278·3 to 360·3)	213 (185·2 to 247·7)	109·9 (96·9 to 125·4)	75·5 (66·6 to 84·5)	45·2 (39·4 to 51·9)	29 (25·9 to 32·2)	12·1 (11·1 to 13·1)
Iraq	191·3 (164·2 to 222·2)	155·5 (130·1 to 183)	125·6 (106·8 to 145·6)	94·7 (87·1 to 102·7)	58·7 (53·6 to 64·3)	45 (40·9 to 49·9)	30 (26·2 to 33·9)	16·2 (13·7 to 19·6)
Jordan	138·1 (116·2 to 164)	117·5 (100·1 to 136·7)	83·7 (74·7 to 93·3)	53·9 (49 to 59·2)	35·1 (32 to 38·5)	27·2 (24·5 to 30·1)	19·6 (17·2 to 22·2)	16·6 (13·9 to 20·2)
Kuwait	95·3 (79·2 to 114·5)	69·7 (60·7 to 79·9)	54·8 (52·7 to 57)	37 (35·5 to 38·5)	21·6 (20·2 to 23·1)	12·7 (12 to 13·4)	12·1 (11·5 to 12·8)	9·9 (8·3 to 11·7)
Lebanon	140·6 (118·2 to 167·2)	88·6 (73·9 to 104·5)	65·3 (55·3 to 76·5)	45·4 (39·5 to 52)	31·7 (27·2 to 36·3)	19·4 (16·2 to 23)	13·5 (11·2 to 16·1)	9·3 (7·6 to 11·5)
Libya	262·6 (229·4 to 303·1)	211·2 (183·7 to 241)	120·6 (104·7 to 137·5)	68·3 (59·4 to 77·6)	45·9 (40 to 52·9)	27·4 (23·6 to 31·7)	17·5 (14·7 to 20·7)	13·5 (11·4 to 15·9)
Morocco	307 (269·5 to 352·7)	265·2 (245·8 to 285·8)	197·4 (183·7 to 212·2)	131·9 (123·1 to 141·9)	79·3 (71·5 to 87·3)	52·5 (45·8 to 59·3)	33·5 (28 to 39·9)	19·9 (16·4 to 24·1)
Oman	298·9 (259·2 to 343·6)	254 (219·7 to 288·8)	156·1 (134·8 to 178·7)	86·1 (73·3 to 100·7)	36·3 (30·6 to 43·5)	15·8 (13·9 to 17·8)	12 (11·5 to 12·5)	11 (10 to 12·2)
Palestine	174 (147·3 to 204·9)	134·4 (114·6 to 157·3)	98 (84·9 to 111·7)	65·9 (59·1 to 73·4)	43·3 (39·6 to 47·5)	31·2 (28·2 to 34·2)	22·7 (19·8 to 25·5)	13·2 (11·2 to 15·9)
Qatar	94·9 (78·9 to 113·9)	85·8 (71·9 to 100·9)	69 (58·2 to 81·3)	38·6 (32·7 to 45)	27·6 (23 to 32·4)	19·3 (16·2 to 22·8)	11·9 (9·9 to 14·1)	8·5 (7·1 to 10·4)
Saudi Arabia	208·3 (176·7 to 244·9)	177·5 (152 to 204·8)	131·5 (112 to 152·6)	78 (67·3 to 89·2)	46·5 (39·4 to 54·4)	26 (21·6 to 31·2)	11·9 (9·6 to 14·2)	5·9 (4·9 to 7·2)
Sudan	270·4 (237·7 to 306·3)	241·7 (217·3 to 268·1)	221·3 (203·3 to 239·9)	191·1 (175·7 to 207·6)	149·1 (137·7 to 161)	106·8 (97·3 to 116·7)	72·5 (64·6 to 80·4)	46·4 (39·4 to 55·3)
Syria	171·7 (148·1 to 201·8)	123 (111·4 to 136)	83·6 (74·3 to 93·8)	67·9 (60·4 to 76·2)	55·4 (48·3 to 63)	22·9 (22·4 to 23·4)	15·5 (15·1 to 15·9)	14·9 (12·7 to 17·6)
Tunisia	324·6 (286 to 371·3)	249·6 (228·8 to 270·7)	162·5 (150·1 to 175·6)	88 (79·5 to 96·6)	53·4 (47·7 to 59·7)	31·6 (27·4 to 35·9)	21 (17·7 to 24·7)	12·6 (10·4 to 15·2)
Turkey	335·5 (298·7 to 379·1)	272·7 (251·1 to 294·4)	189·7 (174·5 to 203·5)	127·7 (117·3 to 138·6)	74·5 (67·2 to 82·8)	43·2 (38·3 to 48)	25·4 (21·6 to 29·8)	16·5 (13·8 to 19·6)
United Arab Emirates	163 (137·5 to 192·7)	139·1 (117·2 to 163·4)	90·8 (78·1 to 104·8)	47·8 (41 to 55·4)	23·4 (20·7 to 26·4)	12·9 (12·3 to 13·6)	7·3 (6·7 to 8)	5·3 (4·5 to 6·4)
Yemen	461·7 (411·9 to 520·3)	397·6 (366·5 to 430·1)	309·1 (286·2 to 334·1)	195·2 (177·5 to 213·2)	132·2 (120·8 to 144·2)	90·9 (82·6 to 99·7)	60 (52·2 to 68·8)	48 (40·4 to 56·8)
South Asia	289·5 (260·7 to 320·2)	253·6 (225·9 to 280)	204·1 (187·7 to 221·5)	154·3 (143 to 166·1)	110·4 (103·3 to 118·3)	80·7 (74·5 to 88·1)	59·5 (53·9 to 64·9)	40 (35·1 to 45·7)
South Asia	289·5 (260·7 to 320·2)	253·6 (225·9 to 280)	204·1 (187·7 to 221·5)	154·3 (143 to 166·1)	110·4 (103·3 to 118·3)	80·7 (74·5 to 88·1)	59·5 (53·9 to 64·9)	40 (35·1 to 45·7)
Bangladesh	348·2 (317·3 to 383·7)	290·5 (268·7 to 312·3)	249·6 (231·8 to 267·5)	188·8 (175·8 to 203·7)	134·6 (126·1 to 144)	86·8 (80 to 94·5)	53·6 (47·5 to 59·9)	30·2 (25·7 to 35·5)
Bhutan	369·4 (322·4 to 421·4)	310·2 (271·4 to 350·2)	241·5 (209 to 278·5)	198·5 (173·7 to 223)	128·5 (115 to 143·8)	82 (73·8 to 91)	48·3 (41·9 to 55·1)	32·1 (27·2 to 37·9)
India	277·6 (242·8 to 315·8)	249·7 (216·2 to 282·8)	200·1 (179 to 222·6)	149·6 (136·6 to 163·5)	105 (96 to 114·5)	76·8 (69·3 to 85·6)	55·3 (48·4 to 62·3)	34·3 (29 to 41·2)
Andhra Pradesh	262·9 (225·3 to 305·9)	237·6 (199·5 to 277)	201·6 (178·2 to 228·4)	146·2 (131·8 to 161·4)	105·6 (95·1 to 116·8)	78·3 (67·4 to 89·7)	52·9 (44·2 to 62)	32 (26·1 to 40)
Arunachal Pradesh	277·8 (238·5 to 322·2)	254·5 (215·7 to 293·8)	213·7 (187·5 to 242·2)	137·2 (121·5 to 154·6)	101·3 (91·1 to 112·6)	75·4 (65·2 to 87)	45·1 (39·2 to 51·2)	28·5 (22·9 to 36)
Assam	279·5 (240·1 to 323·9)	252·6 (214·4 to 290·3)	206·8 (176·2 to 237·2)	147·7 (131 to 164·5)	111·9 (101·4 to 124·1)	85·4 (77·4 to 94·8)	68·4 (56·2 to 82)	44·5 (36·8 to 54·6)
Bihar	249·9 (213·7 to 291)	229·7 (196·6 to 264·4)	202·5 (171·3 to 235·9)	163 (144·7 to 182·1)	114·9 (103·7 to 126·5)	84·3 (76·3 to 93)	61·8 (51 to 73·2)	38·3 (31·6 to 47·3)
Chhattisgarh	274·7 (235·7 to 318·7)	254·5 (217·9 to 291·7)	219·7 (183·9 to 261·5)	177·4 (152·3 to 205·8)	124·5 (110·7 to 140·1)	94·4 (84·8 to 104·2)	67·1 (57·5 to 77·5)	39·1 (33·5 to 45·7)
Delhi	238·1 (203·9 to 277·3)	181·4 (153·8 to 211·1)	141·9 (125·6 to 160·9)	102·7 (91·9 to 114·2)	73 (65·6 to 81·2)	57·8 (48·7 to 67·3)	35·3 (31 to 39·7)	22·5 (18·5 to 27·8)
Goa	155·6 (131 to 184·3)	131·1 (109·3 to 153·4)	105·9 (93 to 119·7)	75·2 (66·5 to 84·1)	51·2 (44·2 to 58·6)	33·7 (28·1 to 39·6)	23·7 (20·1 to 27·6)	17·5 (14·1 to 22)
Gujarat	241·2 (205·9 to 281·7)	213·7 (181·6 to 247·5)	175 (155·8 to 195·8)	135·1 (121·7 to 149·7)	96·9 (87·9 to 107·4)	70·8 (59·7 to 83·1)	50·1 (44·3 to 56·1)	30·9 (25·4 to 38·1)
Haryana	233·7 (199·8 to 272·6)	212 (179·5 to 243·6)	174·1 (155·9 to 194·3)	114·7 (103·1 to 127·6)	84·2 (75·9 to 93·8)	62·2 (54·3 to 72)	45·3 (39·7 to 51·3)	29 (23·8 to 35·9)
Himachal Pradesh	182·7 (154·4 to 215·5)	164·9 (137·3 to 193·4)	134·5 (119 to 152·8)	95 (83·7 to 107·2)	67·6 (60·3 to 75·5)	53·4 (45·2 to 62·4)	41·1 (36 to 46·5)	27·7 (22·4 to 35)
Jammu and Kashmir	170·2 (143·5 to 201·4)	155·5 (130·6 to 180·9)	126·5 (112·5 to 142·6)	106·1 (94·7 to 118·2)	72·9 (65·9 to 81)	56·3 (49 to 65·1)	44·2 (38·7 to 50·1)	27·3 (22·5 to 33·7)
Jharkhand	209·6 (177·9 to 246·3)	190·5 (162·1 to 221·7)	161·4 (133·7 to 194·7)	134·5 (114·9 to 155·8)	121·3 (108·1 to 136·4)	87·8 (79·3 to 97·3)	58·7 (50·4 to 67·8)	34·1 (29·3 to 39·7)
Karnataka	272·1 (233·5 to 315·8)	240·7 (205·8 to 277·1)	197 (176·9 to 219·9)	135·5 (122·1 to 149·2)	88·9 (79·7 to 98·7)	61·6 (52·2 to 72·1)	42·6 (36·9 to 48·4)	25·8 (21·2 to 32)
Kerala	211·7 (180·1 to 247·7)	158·3 (132·9 to 185·5)	105·2 (91·1 to 120·7)	59·4 (49·8 to 70·6)	35·6 (30·1 to 41·9)	22·3 (19 to 26·1)	15·1 (13 to 17·4)	10·1 (8·3 to 12·6)

Madhya Pradesh	283 (244·6 to 326·6)	265·3 (230·8 to 302·7)	228·6 (196·7 to 261·1)	190·4 (169·5 to 211·6)	140 (126·9 to 154·1)	104·6 (94·7 to 115·6)	75·7 (63·2 to 89·4)	46·7 (38·7 to 57·4)
Maharashtra	233·7 (200·5 to 271·8)	205·6 (174·8 to 237·2)	172·4 (154·5 to 192·4)	119·6 (108 to 132·1)	77·6 (70·3 to 86·1)	52·4 (44·1 to 61·5)	35·2 (30·8 to 40)	21·2 (17·3 to 26·3)
Manipur	194·9 (164·9 to 229·5)	176·3 (145·9 to 206·2)	138·5 (120·5 to 157·8)	99·3 (87·3 to 112)	68·8 (61·6 to 76·6)	50·7 (43·1 to 59·1)	37·2 (32·2 to 42·4)	27·1 (21·8 to 34·3)
Meghalaya	141·7 (118·8 to 168·4)	129·6 (108·1 to 151·3)	116·5 (101·4 to 133·5)	116·1 (103·8 to 130·1)	95·4 (83·6 to 108·7)	72·4 (62·4 to 83·8)	51·8 (45 to 59)	36·3 (29·8 to 45)
Mizoram	100·2 (83·6 to 120·1)	87·1 (71·9 to 104·3)	71·5 (60·3 to 84)	70·6 (61·9 to 80·5)	55·5 (50·2 to 61·8)	45·5 (41 to 50·6)	43·5 (35·8 to 51·8)	31·4 (25·8 to 38·8)
Nagaland	219·9 (187 to 257·8)	192·3 (165·4 to 222·2)	147·5 (122·3 to 177)	101·7 (88·9 to 117·3)	74·4 (66·3 to 83·6)	58·4 (52·8 to 64·8)	44·1 (36·2 to 53·1)	32·7 (27·8 to 38·4)
Odisha	323·2 (279·7 to 372)	297·8 (256 to 337·5)	249 (224 to 276)	184 (166·2 to 203·2)	136·4 (123·7 to 150·3)	100·1 (86·7 to 115·8)	68·2 (60·5 to 76·6)	43·1 (35·6 to 53)
Punjab	163·2 (137·8 to 193)	141·2 (118·7 to 164·2)	105·8 (93·1 to 119·1)	83·9 (75 to 93·7)	62·6 (56·6 to 69·9)	49·3 (42·8 to 57·2)	37·5 (32·7 to 42·5)	23·8 (19·6 to 29·5)
Rajasthan	216·9 (185·1 to 253·7)	205·5 (178·1 to 234·3)	179·5 (151·1 to 212·3)	151·3 (133·2 to 170·1)	114·4 (104·2 to 126·3)	84·5 (75·3 to 94·5)	64·3 (55·7 to 73·2)	39·7 (34·3 to 46)
Sikkim	184·7 (155·9 to 218)	171·9 (145·2 to 199·9)	148·7 (127·8 to 170·8)	110·7 (96 to 126·9)	67·2 (59·8 to 76·2)	48·1 (41·3 to 55·7)	35·8 (31 to 41·1)	22·5 (17·9 to 28·6)
Tamil Nadu	288·6 (248·3 to 334·1)	248·1 (213·3 to 285·9)	196·1 (175·7 to 219·8)	132·5 (119·7 to 146·8)	80·1 (71·8 to 88·6)	48·6 (40·8 to 57)	30·7 (26·7 to 34·8)	17·9 (14·6 to 22·2)
Telangana	203·5 (172·6 to 239·3)	189·7 (156 to 228·4)	165·7 (148·5 to 183·4)	137·7 (122·5 to 155)	103·4 (93 to 114·5)	77 (62·5 to 92·6)	47·1 (40 to 55·1)	27·3 (22·3 to 34·1)
Tripura	301·7 (260·4 to 348·2)	257·5 (219·1 to 295·5)	203·5 (180·5 to 227·7)	137·6 (123·5 to 152·7)	98·9 (89·1 to 109·6)	61·4 (53·1 to 71·5)	41·2 (35·8 to 47·1)	30·3 (24·2 to 38·5)
Union Territories other than Delhi	190·2 (161·6 to 223·2)	160 (138·2 to 183·4)	123·4 (101·3 to 149·7)	86 (71 to 102·9)	60·1 (49·9 to 72)	45·7 (37·7 to 54·9)	34·6 (29·1 to 41)	24·1 (20·6 to 28·2)
Uttar Pradesh	373·4 (327·3 to 424·4)	340·8 (300 to 381·2)	277·6 (240·6 to 314·7)	203·7 (181·6 to 225·3)	139·6 (126·7 to 154)	100·5 (91·4 to 110·4)	74·5 (61·6 to 88·2)	45·5 (37·7 to 55·8)
Uttarakhand	180·4 (152·2 to 213·1)	160·7 (137 to 185·5)	131·7 (107·9 to 160·2)	104·9 (90·5 to 122·1)	78·6 (67·9 to 90·4)	61·3 (54·6 to 68·6)	44·1 (37·7 to 51·1)	26 (22·4 to 30·1)
West Bengal	272·1 (234·1 to 315·4)	236·4 (203·5 to 271·1)	195·7 (175·7 to 217·6)	144·3 (128·7 to 161·3)	97·8 (88·2 to 108·6)	65·7 (55·3 to 77·7)	43·2 (38·3 to 48·5)	25·7 (21·1 to 31·9)
Nepal	327·9 (298·6 to 361·6)	280·9 (258·4 to 303·6)	227·4 (210·8 to 245·6)	197·3 (180·4 to 214·2)	133·3 (123 to 143·5)	82·6 (75·7 to 90·2)	51·7 (45·8 to 57·7)	30 (25·5 to 35·2)
Pakistan	294·6 (262·3 to 334)	234·7 (214·5 to 253·8)	176·6 (162·8 to 191·1)	145 (132·1 to 160)	116·6 (106·5 to 127·2)	95·2 (85·8 to 105·3)	79·9 (69·3 to 91)	65·3 (54·6 to 78)
Southeast Asia, east Asia, and Oceania	213·2 (189·6 to 239·6)	441·7 (341 to 573·3)	123·1 (112·7 to 134·5)	88·8 (81·4 to 96·5)	63·3 (58·3 to 68·3)	44·1 (41·5 to 46·9)	26·2 (24·1 to 28·3)	16·2 (14·3 to 18·1)
East Asia	195·2 (167·2 to 227·6)	514·8 (387·3 to 668·8)	107·6 (94·4 to 121·7)	76·2 (66·5 to 85·4)	55·1 (49·1 to 61·5)	37·3 (33·9 to 40·9)	17·4 (15·9 to 18·9)	9·2 (8 to 10·7)
China	192·2 (164·3 to 224·5)	524·8 (394·8 to 680·9)	107·5 (94·7 to 121·9)	76·7 (66·8 to 86·3)	55·6 (49·4 to 62·2)	36·8 (33·5 to 40)	17·3 (15·8 to 18·8)	9·2 (7·9 to 10·6)
North Korea	388·6 (349·7 to 430·7)	250·5 (214·5 to 289·6)	158·3 (133·1 to 186·6)	97 (80·8 to 115·1)	59 (48·6 to 69·7)	68·6 (45·4 to 121·4)	25·1 (20·4 to 30·5)	13·7 (11·2 to 17)
Taiwan (province of China)	91·8 (77 to 109·4)	60 (59 to 61·1)	27·5 (26·8 to 28·1)	16·2 (15·8 to 16·7)	9·1 (8·8 to 9·4)	8·5 (8·1 to 8·9)	5·3 (5·1 to 5·6)	4·9 (4·1 to 5·9)
Oceania	185·4 (165·3 to 209·9)	146·6 (129·7 to 164·2)	106·5 (94·6 to 119·2)	81·7 (72·7 to 91·7)	72 (64·3 to 80·1)	65·1 (56·9 to 74·1)	60·6 (51 to 70·9)	50·8 (42·7 to 60·4)
American Samoa	86·2 (75·3 to 98·2)	54 (49·3 to 59)	38·2 (34·8 to 42·3)	22·9 (19·6 to 27)	16·6 (14·8 to 18·8)	14·4 (12·7 to 16·2)	12·1 (10·7 to 13·6)	10 (8·4 to 11·9)
Cook Islands	161 (146·7 to 176)	100·3 (91·9 to 109·4)	67·1 (60·4 to 74·8)	47·7 (42·5 to 53·6)	21·9 (18·9 to 25·6)	12·9 (11 to 15·5)	5·6 (4·7 to 6·6)	2·7 (2·2 to 3·2)
Fiji	94·9 (83·9 to 106·8)	61·5 (55·3 to 67·9)	46·3 (41·3 to 52·1)	36·1 (30·6 to 42)	27·4 (22·9 to 33·2)	27·4 (22·9 to 32·7)	26·5 (22·1 to 31·5)	23·4 (19·3 to 28·4)
Guam	49·9 (44·4 to 56·9)	38·1 (35·4 to 41)	29 (26·8 to 31·3)	16·9 (15·2 to 18·6)	11·8 (10·6 to 13·1)	11·3 (10·2 to 12·5)	13·7 (12·4 to 15)	13·6 (11·6 to 15·9)
Kiribati	188·3 (163·5 to 215·8)	150·3 (131·1 to 172·3)	111·8 (96·4 to 129·6)	83·1 (70·4 to 99·1)	75·6 (64·3 to 88·2)	60·9 (52·5 to 70·4)	46·5 (39·1 to 54·9)	34·3 (28·7 to 40·9)
Marshall Islands	152·2 (127·7 to 180·8)	110·1 (92 to 131·6)	82·4 (68·6 to 98)	52 (44·4 to 60·8)	36·5 (33·3 to 39·9)	41·3 (36·8 to 46)	28·8 (24·6 to 33·2)	21 (17·3 to 25·9)
Federated States of Micronesia	150·6 (128·6 to 178)	118·8 (100·5 to 138·4)	84·7 (72·1 to 98·8)	57·2 (48·2 to 67·8)	43·8 (36·9 to 51·5)	36·9 (31·6 to 42·9)	21·7 (17·9 to 25·9)	15·5 (12·9 to 18·5)
Nauru	91·8 (76·7 to 109·8)	70·2 (59·2 to 82·2)	53 (45·2 to 61·1)	37·8 (31·3 to 44·9)	32·9 (28·2 to 38)	43·9 (39·2 to 48·8)	40·4 (34·4 to 46·9)	24·5 (20·2 to 30·2)
Niue	146·8 (131 to 163·5)	77 (67·7 to 87·1)	49·3 (41·6 to 57·2)	31 (25·4 to 37·2)	24·2 (20·1 to 29·7)	26·5 (22·1 to 31·5)	23·6 (19·4 to 28·1)	19·6 (16·1 to 23·9)
Northern Mariana Islands	69·3 (57·3 to 83·7)	50·2 (41·8 to 59·8)	33·1 (27·5 to 40·1)	19·7 (16·2 to 24·1)	11·4 (9·6 to 13·7)	8 (6·9 to 9·2)	8·8 (7·5 to 10·2)	9·7 (8·2 to 11·3)
Palau	197·3 (166·8 to 232·6)	142·7 (119·7 to 166·4)	92·1 (77·9 to 108)	58·2 (49 to 67·9)	33·1 (27·8 to 39·1)	21·3 (17·7 to 25·3)	18·2 (15·2 to 21·4)	14·7 (12·2 to 17·9)
Papua New Guinea	207·8 (180·4 to 241·5)	168·7 (146·8 to 191·4)	122·1 (106·5 to 138·9)	96·8 (84·9 to 109·6)	86·9 (76·3 to 97·7)	75·8 (65·4 to 87·5)	68·9 (57·4 to 81·3)	56·4 (47·5 to 67)
Samoa	182·4 (156·5 to 211·1)	132·3 (114 to 151)	95·6 (81·7 to 111·8)	60·6 (50·5 to 71·4)	38·4 (31·2 to 47·4)	25·5 (20·9 to 30·6)	19·8 (16·4 to 23·5)	14·6 (12 to 17·7)
Solomon Islands	208·7 (181·3 to 239·9)	163·9 (142·5 to 187·6)	111·5 (94·6 to 130·4)	70·1 (58·9 to 83·4)	52·7 (43·9 to 62·3)	42 (34·8 to 50)	35·2 (29·3 to 41·7)	25·9 (21·6 to 30·9)
Tokelau	84·9 (71·8 to 100·2)	63 (51·9 to 75·4)	45 (37·3 to 53·6)	36·9 (30·6 to 44)	25·2 (21·1 to 30·6)	17·6 (14·5 to 21)	11·7 (9·8 to 14·1)	7·7 (6·3 to 9·4)
Tonga	70·5 (59·3 to 85)	50·6 (42·2 to 59·6)	41·9 (35·3 to 49·4)	35·7 (29·6 to 42·6)	28 (23·5 to 33·2)	24·6 (20·7 to 29·1)	20·4 (17·1 to 24·5)	15·3 (12·6 to 18·5)
Tuvalu	206·9 (175·4 to 243·5)	159·3 (135·2 to 185·9)	120·6 (102·2 to 141·8)	97·6 (81·4 to 114·3)	70·7 (61·7 to 80·1)	47·1 (42·1 to 52·4)	23·1 (19·4 to 27·2)	14·4 (11·8 to 17·9)
Vanuatu	198·5 (172·9 to 228·2)	159·7 (140 to 181·4)	102·7 (87 to 120·1)	53·5 (45·1 to 63·4)	37·9 (32·2 to 44·6)	35·5 (29·8 to 41·9)	32·6 (27·8 to 38·7)	25·7 (21·5 to 30·7)
Southeast Asia	261 (240 to 285)	206·4 (192·5 to 221·3)	158·8 (149·3 to 168·1)	113·9 (108·3 to 120·1)	79·8 (75·5 to 84·2)	53·8 (50·3 to 57·1)	36·2 (32·6 to 39·8)	25·3 (22·3 to 28·8)
Cambodia	248·9 (212·3 to 290·7)	216·9 (186 to 254·4)	199·8 (173·7 to 226·7)	173·5 (156·2 to 192·3)	129·9 (120·8 to 139·7)	99·5 (91 to 107·8)	53·8 (47 to 60·6)	34·6 (28·8 to 42·1)
Indonesia	302·6 (270·3 to 341·6)	240·6 (223·2 to 258·2)	183·8 (171·5 to 196·2)	131·4 (123·8 to 140·3)	91 (85·4 to 96·8)	58·6 (53·9 to 63·5)	40·9 (35·8 to 46·7)	29·2 (24·4 to 34·8)
Aceh	202·5 (180·6 to 228·5)	160·8 (149·4 to 173·6)	127·7 (118·1 to 138)	96 (87·4 to 105·3)	73·3 (66 to 80·8)	53·5 (47·7 to 59·6)	38·3 (33·4 to 43·6)	28·6 (23·9 to 34·3)
Bali	212·5 (185·4 to 245·2)	158·3 (135·3 to 183·2)	114·5 (102·3 to 126·8)	83·2 (75·1 to 92·1)	46·7 (42·1 to 51·6)	31·2 (27 to 36·2)	19·7 (15·6 to 24·6)	5·1 (4·1 to 6·2)

Bangka-Belitung Islands	303·7 (266·3 to 346·1)	246·8 (225·5 to 269·7)	184·1 (169·4 to 199·6)	125·6 (111·4 to 141·6)	91·7 (82·9 to 101·4)	53·3 (47·7 to 59·4)	36 (31·1 to 41·7)	30·6 (26·3 to 35·9)
Banten	457·4 (409·3 to 510)	371·1 (341·7 to 405·2)	284 (261·1 to 307·3)	204·1 (186·1 to 221·1)	133·8 (123 to 144·5)	70·3 (63·9 to 77·2)	44·7 (39·2 to 51)	31·6 (26·8 to 37·6)
Bengkulu	368·4 (334·1 to 407·3)	301·4 (278·5 to 324·8)	223·3 (206·8 to 240·2)	159·8 (150 to 170·6)	105·9 (98·7 to 113·4)	67·8 (62·3 to 74·1)	45·1 (39·6 to 51·2)	30 (25 to 36·1)
Gorontalo	357·3 (315·1 to 404·4)	290·4 (265 to 319·9)	227·1 (201·1 to 253·2)	173·1 (156·2 to 191·6)	126·8 (118·2 to 135·7)	95·3 (84·4 to 107)	69 (59·5 to 79·5)	48·5 (41·2 to 57·8)
Jakarta	237·5 (208·9 to 272·1)	181 (165·9 to 197)	127·2 (114·2 to 140·1)	89 (82·9 to 95·6)	56·4 (52·3 to 60·3)	36·3 (31·8 to 41·8)	26·5 (22·9 to 30·6)	19·7 (16·1 to 24·1)
Jambi	271·3 (244·4 to 303·4)	207·7 (194·7 to 221·3)	159·5 (145·3 to 173·4)	114·5 (103·8 to 124·4)	78·7 (73·5 to 83·8)	50·6 (46·5 to 54·8)	33·5 (29·3 to 38·2)	23·8 (19·8 to 28·7)
West Java	383·8 (342·4 to 431·6)	310 (287·1 to 331·7)	229·8 (212·8 to 247)	159·5 (149·5 to 170·8)	101·2 (94·8 to 108)	60·7 (55·5 to 66·3)	39·4 (34·4 to 45)	26·3 (21·6 to 32)
Central Java	246·4 (215·9 to 282·9)	198·5 (182·2 to 214)	147·7 (135·6 to 160·8)	102·5 (94·4 to 111·1)	66 (60 to 72·5)	41·7 (38·3 to 45·3)	28·8 (25·1 to 32·8)	19·1 (15·6 to 23·2)
East Java	240·7 (211·9 to 275·3)	196·4 (180·4 to 212·5)	151·8 (140·1 to 163·7)	119·8 (111·6 to 129)	84·9 (79·8 to 90·5)	54·5 (49·8 to 59·5)	36·8 (32·4 to 41·8)	24·6 (20·3 to 29·7)
West Kalimantan	255·6 (229·2 to 287·2)	225·3 (207·2 to 244·1)	185·6 (171·5 to 199·9)	143·3 (133·6 to 153·3)	99·6 (92·3 to 107·4)	62·5 (57·1 to 68·2)	37·2 (32·7 to 42·1)	21·1 (17·5 to 25·5)
South Kalimantan	417·4 (380·5 to 459·2)	363·6 (336·4 to 389·4)	279·6 (261·6 to 298)	207·8 (195·9 to 221·8)	141·5 (129·8 to 154)	85·5 (78·5 to 92·5)	54·2 (47·6 to 61·4)	37·4 (31·1 to 44·7)
Central Kalimantan	233·6 (208·7 to 263·7)	163·1 (150·9 to 175·7)	122·3 (111·9 to 133·2)	92 (83·5 to 100)	74 (69·1 to 78·9)	54·8 (50·3 to 59·6)	36·2 (31·7 to 41·3)	27·4 (22·8 to 33)
East Kalimantan	232·3 (207·3 to 262·6)	201·4 (174·1 to 230·4)	157·6 (144·9 to 170·6)	109·9 (102·3 to 118·5)	71·7 (66·1 to 77·4)	44·4 (39·8 to 49)	27 (21·8 to 33·2)	20·7 (17·1 to 25·1)
North Kalimantan	279·2 (243·5 to 319·9)	240·6 (219·2 to 263·5)	205·4 (187·2 to 225·8)	141·2 (125·9 to 158·4)	92·1 (83·2 to 101·8)	65 (58·3 to 72)	43·1 (37·1 to 50·3)	34·8 (29·4 to 41·8)
Riau Islands	229·6 (198·7 to 265)	180·1 (163·7 to 199·3)	128·9 (116·4 to 142·1)	97·3 (88 to 106·8)	69·8 (63·4 to 76·7)	47·2 (42·3 to 52·5)	31·5 (27·2 to 36·8)	23·5 (19·9 to 28·2)
Lampung	277·3 (249·9 to 309·8)	218·4 (201 to 235·8)	165·3 (151·3 to 180)	121·3 (113·6 to 129·7)	84·6 (79·2 to 90·1)	54 (47·8 to 60·4)	39·1 (33·8 to 44·9)	30·1 (25 to 36·2)
Maluku	414·1 (377·5 to 456·3)	321·8 (300·9 to 343·8)	245·5 (226·1 to 265·3)	184·9 (169·5 to 200·5)	136·8 (125·3 to 148·3)	100·1 (90·6 to 110·1)	70·2 (62·1 to 78·7)	40·9 (34·2 to 48·8)
North Maluku	318·6 (280·2 to 362)	258·6 (236·4 to 284·1)	199·5 (184·7 to 216·5)	160·1 (144·6 to 177·4)	117·3 (106·6 to 128·1)	88·5 (76·2 to 101·9)	63·7 (55·5 to 73·1)	43·5 (37 to 51·8)
West Nusa Tenggara	401·8 (366·5 to 441·6)	335·5 (311·4 to 359·4)	272·3 (255·1 to 291·1)	208·1 (195·2 to 222·5)	148·5 (136·8 to 160·4)	96·8 (85·2 to 109·2)	54·2 (47·8 to 61·2)	34·3 (28·6 to 40·9)
East Nusa Tenggara	367·5 (332·8 to 408·7)	293·4 (274·7 to 312·7)	224·8 (205·9 to 243·6)	166·6 (152·8 to 180·4)	115·3 (108·1 to 122·6)	81·2 (71·7 to 91·3)	54·7 (47·9 to 61·8)	34 (28·3 to 40·7)
Papua	212 (188·9 to 239·9)	184·9 (172 to 198)	154·9 (143 to 166·9)	128·3 (116·2 to 141·4)	106 (96·5 to 115·5)	81·8 (71·5 to 93·1)	68·2 (59·8 to 77·1)	55 (45·9 to 65·9)
West Papua	182·3 (156·7 to 212·1)	155·5 (140·5 to 172·9)	130·8 (119·5 to 143·6)	96·8 (87·6 to 107·1)	73·9 (66·9 to 80·9)	62·3 (54·5 to 71·3)	46·8 (40·9 to 53·9)	30 (25·3 to 35·9)
Riau	275·6 (247·3 to 309·6)	236·4 (216·9 to 256·3)	184·3 (170·4 to 198·6)	124·8 (116·7 to 133·4)	86·7 (80·8 to 92·6)	55·5 (50·9 to 60·3)	38·7 (34 to 43·8)	22·5 (18·7 to 27·1)
West Sulawesi	448·5 (401·9 to 499·9)	371·9 (343·6 to 405·5)	297·5 (273·9 to 322·2)	217·3 (198·1 to 236·5)	150·4 (137·4 to 164)	99·6 (89·7 to 109·6)	69·6 (60·7 to 79·7)	48·2 (41·1 to 57·3)
South Sulawesi	297·5 (263·5 to 337·9)	241·2 (221·7 to 262·4)	183 (169·7 to 197)	133·2 (124·1 to 143·4)	88·6 (80·7 to 96·8)	56·3 (51·8 to 61·3)	36·7 (31·9 to 42·1)	22·4 (18·3 to 27·3)
Central Sulawesi	398·7 (362·8 to 439·1)	288·9 (266·8 to 313)	216·2 (199·2 to 233·5)	161·1 (147 to 174·4)	111·5 (102·7 to 120·7)	75·7 (68·9 to 82·5)	53·3 (46·8 to 60·2)	38·5 (32·1 to 46·1)
Southeast Sulawesi	341·4 (308·6 to 379·4)	290·1 (268·8 to 311·5)	237·1 (219·3 to 255·3)	162·1 (148 to 176)	109·3 (102·4 to 116·4)	73·1 (65·8 to 80·3)	52·3 (46·1 to 59·3)	40·8 (34·1 to 48·9)
North Sulawesi	201·6 (179·5 to 228·4)	164·1 (150·6 to 178·5)	117·3 (108·6 to 126·4)	78·1 (70·6 to 85·8)	59·9 (54·4 to 65·4)	46·2 (41 to 51·6)	39·5 (34·5 to 45)	33·1 (27·6 to 39·8)
West Sumatra	394·9 (359·4 to 434·9)	290·4 (268 to 313·6)	206 (191·7 to 221·1)	134·9 (125·4 to 145·6)	92 (86·1 to 97·9)	63·9 (58·8 to 69·3)	39·8 (34·9 to 45·3)	21·7 (18 to 26·2)
South Sumatra	325·8 (288·6 to 369·3)	254 (233·7 to 274·4)	193·4 (178·5 to 208·3)	127·3 (119·3 to 135·8)	88·2 (80·7 to 96·4)	56·9 (52·1 to 61·6)	37·9 (33·3 to 42·9)	29·2 (24 to 35·4)
North Sumatra	238·1 (212·8 to 268·8)	191·6 (175·9 to 207)	146·2 (134 to 158·2)	108·9 (102·3 to 116·3)	83·1 (76 to 91·2)	58·1 (53·2 to 63·1)	44·2 (39·1 to 50·2)	37·1 (31 to 44·5)
Yogyakarta	135·4 (119·3 to 155·4)	98·1 (83·3 to 114)	72·5 (65·2 to 80)	52·1 (46·6 to 57·9)	34·6 (31 to 38·7)	21 (18 to 24·4)	17·5 (13·9 to 21·7)	15·8 (13 to 19·1)
Laos	326·9 (282·8 to 376·3)	305·4 (268 to 346)	275·7 (237 to 321·2)	233·1 (204·5 to 263·1)	176·7 (161·3 to 193·2)	126·1 (115 to 137·5)	74·3 (66·4 to 82·2)	44·6 (37·6 to 52·5)
Malaysia	156·5 (139·5 to 174·9)	109·9 (108·2 to 111·4)	60·9 (59·9 to 61·9)	34·7 (30·3 to 39·7)	19·2 (18·8 to 19·7)	10 (9·7 to 10·2)	8 (7·7 to 8·2)	7 (5·8 to 8·4)
Maldives	449·2 (397·3 to 505·3)	356·4 (309·1 to 405·3)	247·1 (215·7 to 281·4)	151·1 (133·6 to 170)	68·6 (60·7 to 76·5)	34·4 (30·6 to 38·9)	21·6 (18·8 to 24·5)	17·5 (14·6 to 21·3)
Mauritius	131·8 (126·5 to 137·2)	109·4 (105·8 to 113·1)	84·1 (80·8 to 87·4)	44·7 (42·6 to 46·9)	24·7 (23·2 to 26·2)	19·4 (18·1 to 20·7)	15·8 (14·6 to 17·1)	14·2 (12·1 to 16·7)
Myanmar	395·9 (346·2 to 449·8)	343·5 (301 to 389·6)	283·1 (245·1 to 327·8)	206·9 (180·2 to 235·5)	154·2 (135·2 to 174·7)	117 (104·2 to 130·3)	72·3 (63·5 to 82·4)	44·5 (37·3 to 52·6)
Philippines	212 (185·3 to 245)	153·2 (139·3 to 167·6)	116·1 (106·1 to 126·8)	88·4 (80 to 97·1)	60·9 (55·3 to 66·7)	41·2 (37 to 46)	32·5 (28·4 to 36·9)	24·7 (20·6 to 29·7)
Seychelles	117·1 (103·4 to 134·1)	100·5 (94·4 to 106·9)	78·3 (73·7 to 83·1)	39 (35·7 to 42·3)	19·9 (18·1 to 21·9)	13·2 (11·7 to 14·9)	13·5 (11·9 to 15)	11·2 (9·5 to 13·2)
Sri Lanka	157·2 (154·1 to 160·3)	98·7 (96·9 to 100·7)	72·2 (70·8 to 73·6)	49·4 (48·4 to 50·4)	29·2 (27·7 to 30·8)	18·4 (17·9 to 18·9)	14·1 (13·6 to 14·5)	8·2 (6·8 to 10)
Thailand	168·6 (150·6 to 188·4)	125·9 (115·6 to 137)	81·2 (73·9 to 88·4)	53 (47·3 to 59·5)	31·4 (27·5 to 36·4)	19·1 (16·4 to 22·2)	10·8 (10·5 to 11)	8·4 (7·4 to 9·3)
Timor-Leste	295·4 (255·4 to 340·6)	263·5 (226·4 to 300·7)	220·5 (196·1 to 248·5)	186·4 (170·5 to 204·2)	125·8 (115·4 to 137·4)	85·5 (77·3 to 95·7)	46·9 (40·4 to 53·7)	35·2 (29·3 to 42·8)
Vietnam	188·9 (161·1 to 220·5)	148·3 (125·3 to 172·1)	116·6 (104·2 to 129·4)	74·4 (67·9 to 81·5)	52·3 (48·2 to 57·3)	32·2 (28·9 to 35·6)	21·1 (18·1 to 24·1)	14·4 (12 to 17·5)
Sub-Saharan Africa	291·5 (255·6 to 331·6)	268·3 (239·1 to 297·9)	236·8 (218·8 to 255·2)	205 (194·1 to 215·9)	179·9 (172·2 to 188)	155 (148·6 to 161·7)	113·3 (106·2 to 120·8)	79·4 (69 to 92·7)
Central sub-Saharan Africa	257·1 (219·9 to 299·3)	238·9 (209·1 to 271·8)	220·2 (197·3 to 245·8)	198·3 (179·8 to 218·3)	176·1 (162·3 to 190·6)	161·4 (151·6 to 171·9)	112·1 (101·8 to 122·2)	63·7 (55·4 to 73·9)
Angola	268·7 (229·8 to 312·7)	261·3 (227 to 299·3)	244·7 (210·8 to 285·7)	230·3 (202·2 to 260·4)	217·5 (201 to 235·9)	176·5 (161·3 to 191·4)	105·7 (95·3 to 115·9)	59·4 (50·1 to 69·9)
Central African Republic	244·4 (212·5 to 279·8)	223·5 (193·6 to 253·4)	198·1 (176·1 to 222)	191·1 (172·1 to 211·6)	189·2 (173·4 to 207·1)	191·6 (172·9 to 210·5)	169·4 (148·2 to 191·1)	132·5 (112·9 to 157·1)
Congo (Brazzaville)	280·4 (240·8 to 324·9)	257·1 (223·5 to 292·8)	214·2 (186·1 to 247·8)	149·5 (132·8 to 167·6)	110·3 (100·4 to 121·7)	108·7 (98·9 to 119·5)	71·2 (62·8 to 79·8)	44·4 (37·8 to 52·1)

DR Congo	252 (215 to 294·1)	232·8 (196·2 to 270·6)	217 (188·4 to 248·4)	195 (175·2 to 215·4)	169·2 (153·6 to 185·4)	159·5 (146·3 to 173·4)	114 (100·7 to 127·8)	62·4 (52·1 to 75·8)
Equatorial Guinea	296·9 (255·1 to 343·9)	287 (246·2 to 328·2)	237·3 (203·7 to 272·2)	186·1 (162·8 to 211·4)	178 (156·5 to 201·2)	130·5 (111·8 to 149·7)	67·8 (56·3 to 80·4)	42·2 (34·8 to 52)
Gabon	218·8 (185·8 to 256·5)	204·5 (173·2 to 238·9)	170·8 (146·4 to 197·7)	122·4 (108·5 to 138)	91·2 (82·1 to 101)	76·1 (66·1 to 86)	58·9 (50·3 to 68·3)	34·9 (29·1 to 42·4)
Eastern sub-Saharan Africa	294·8 (257·8 to 336·2)	264·7 (233·9 to 295·8)	236·4 (217·4 to 255·5)	207 (195 to 219·6)	179·8 (171·1 to 188·5)	141·9 (135 to 149)	94·2 (86·5 to 102·4)	63 (53·8 to 74·7)
Burundi	321 (279·1 to 368)	282·2 (248·9 to 318·6)	250·4 (223·9 to 277·9)	216·8 (196·1 to 238·6)	188·6 (173·7 to 206·2)	172·7 (157·4 to 189·3)	105·5 (92·6 to 118·5)	70·4 (58·9 to 85·3)
Comoros	268 (229·6 to 311·8)	243·7 (210·5 to 282·3)	202·5 (176·6 to 233·4)	169·6 (151·9 to 188·8)	131·6 (118·5 to 144·9)	108·7 (96 to 123·6)	77·3 (65·6 to 89·6)	51·8 (43 to 63·5)
Djibouti	181·4 (152·9 to 214·3)	166·3 (139·9 to 193·4)	150·7 (127·4 to 175·1)	131·5 (115·4 to 149·1)	123·3 (108·2 to 139·5)	104·8 (90·9 to 120·6)	74·2 (61·7 to 87·8)	50·4 (41·6 to 62·1)
Eritrea	310 (271·3 to 352·3)	282·7 (244 to 319·5)	254·5 (221·2 to 305)	215·4 (195·1 to 237·7)	171·2 (156·3 to 186)	105·2 (92·7 to 119·1)	76·9 (65·1 to 90·7)	52·1 (43·6 to 63)
Ethiopia	324·7 (281 to 373·3)	301·3 (262·7 to 339·5)	277 (247·4 to 306·4)	247·4 (226·8 to 269·4)	208·2 (191·6 to 225·5)	155·8 (144·1 to 169·3)	96·1 (85·3 to 107·5)	57·7 (48·4 to 69·7)
Kenya	246·5 (218·2 to 281·2)	188·4 (173·1 to 205·1)	150·6 (140·5 to 161·6)	115·9 (107·8 to 124·9)	100·7 (93·1 to 108·6)	90·4 (82·9 to 98·8)	62 (54·3 to 70·5)	43·8 (36·7 to 52·3)
Baringo	229·4 (205·9 to 257·4)	197·5 (178 to 216·8)	194·8 (177·5 to 210·8)	168·8 (154·2 to 183·1)	145 (130·6 to 159·3)	114·4 (103·4 to 126·9)	83·3 (72 to 95·4)	58 (49·5 to 68·3)
Bomet	85·9 (76·3 to 97·5)	73·1 (63·1 to 84·4)	71·6 (61·6 to 82·5)	64·4 (54 to 75·9)	50·9 (43·2 to 59·4)	45·5 (38·5 to 53·5)	39·1 (32·3 to 46·4)	25·4 (21·6 to 29·8)
Bungoma	310·1 (279·9 to 345·7)	244 (223·4 to 265·4)	190·8 (167·4 to 215)	145 (133·2 to 157·1)	120·5 (109·7 to 132·4)	97·6 (88·6 to 107·7)	60·5 (51·7 to 70·8)	36·2 (30·8 to 42·6)
Busia	452·4 (412·8 to 499)	360·5 (326·7 to 392·7)	276 (249·7 to 301·1)	175·3 (161 to 190·8)	148·7 (134 to 164·3)	141·3 (125·6 to 158·6)	92 (78·9 to 105·8)	58·4 (49·7 to 68·5)
Elgeyo Marakwet	134·1 (119·4 to 151·5)	118·4 (106·3 to 131·1)	125·2 (113·8 to 136·4)	118·8 (108·9 to 128·9)	90·8 (81·7 to 99·5)	61·7 (55·5 to 68·9)	36·1 (31·1 to 41·5)	23 (19·6 to 27·1)
Embu	310·5 (281·2 to 345·2)	227·8 (201·8 to 253·5)	146·6 (129·2 to 165)	90·1 (75·6 to 105·3)	71·1 (61·8 to 80·1)	59·6 (53·7 to 66·4)	50·8 (41·6 to 60·2)	36·9 (31·4 to 43·3)
Garissa	215·5 (193·4 to 241·3)	173·3 (149·8 to 197·9)	145·5 (124·5 to 167·3)	120·9 (109·2 to 133·3)	102 (88 to 117)	76·1 (64·6 to 89·1)	49 (42·5 to 55·6)	32·2 (27·3 to 37·8)
Homa Bay	481·3 (440·4 to 528·9)	384·3 (351·1 to 417·1)	348·2 (319·4 to 379·7)	279·6 (260 to 300·1)	224·7 (204·9 to 245·8)	202·6 (183·3 to 223·7)	111·4 (97·2 to 127·8)	70·5 (60·1 to 82·5)
Isiolo	509·2 (468·5 to 556·4)	420·9 (379·7 to 459·7)	382·2 (348·4 to 420·2)	301 (262·4 to 342·7)	237·1 (209·1 to 267·1)	178·8 (163 to 196·7)	143·7 (124·5 to 164·4)	100·7 (86·1 to 117·9)
Kajiado	110·6 (98·6 to 124·9)	93·1 (83 to 105·1)	97·9 (89·2 to 106·3)	81·9 (73·7 to 89·8)	59·8 (52·7 to 67·3)	53·9 (48·6 to 60·1)	38·8 (33·2 to 44·9)	22·9 (19·4 to 27)
Kakamega	294·2 (265·2 to 328·4)	234·5 (213 to 256·3)	195·9 (176·4 to 216·2)	151·2 (127·6 to 177·2)	137·2 (126·1 to 148·4)	132·6 (119·3 to 147·3)	83·7 (72 to 95·9)	50·8 (43·3 to 59·6)
Kericho	78·2 (69·5 to 88·8)	67·2 (59·2 to 76·5)	73·4 (65·1 to 82·6)	77·2 (69·6 to 85·2)	54·1 (48·2 to 59·7)	48·2 (41·3 to 55·4)	37·8 (32·8 to 43·5)	27·6 (23·4 to 32·4)
Kiambu	125·9 (112·1 to 142·1)	90·6 (79·8 to 102·7)	66·9 (60·1 to 74)	52·6 (46·5 to 59·2)	46·3 (41 to 51·8)	49·5 (42·1 to 58·4)	40·7 (34·8 to 46·7)	30·1 (25·6 to 35·6)
Kilifi	338·2 (307·8 to 374·2)	253·4 (231 to 275·9)	207·7 (192·8 to 223)	159·9 (143·5 to 177)	125·2 (111·1 to 139·7)	103·5 (93·3 to 114·5)	69·4 (60·5 to 79·1)	45·8 (39 to 53·7)
Kirinyaga	236·5 (212·6 to 264·6)	182·8 (166 to 202·2)	153·7 (140 to 167·3)	80·1 (68·3 to 92·6)	60·3 (50·8 to 70·8)	61·9 (54·9 to 69·8)	51·6 (44·2 to 59·6)	45·9 (39·1 to 54·3)
Kisii	243·4 (218·3 to 272·1)	192·5 (174·1 to 211·4)	158·9 (142·2 to 176·3)	134 (119·2 to 149·7)	122·8 (111·3 to 134·7)	95 (85·1 to 106·1)	50·3 (42·1 to 59·6)	33·4 (28·4 to 39·2)
Kisumu	234·2 (210 to 262·1)	187·5 (169·4 to 207·4)	169·8 (151·7 to 187·8)	156·6 (138·2 to 177)	145·4 (130·4 to 161·3)	136·3 (123·1 to 150·6)	70·4 (59·2 to 83·1)	45·3 (38·5 to 53·2)
Kitui	294·7 (265·8 to 328·9)	234·8 (212·2 to 258)	178·5 (163·4 to 196)	115·1 (104·7 to 125·8)	86·6 (76·5 to 97·5)	76·5 (68·7 to 85·1)	49·4 (42·9 to 56·8)	31·9 (27·1 to 37·5)
Kwale	369·5 (335·9 to 409·3)	285·3 (259·4 to 311·8)	225·4 (210·1 to 242)	167·1 (150·9 to 184·3)	128·7 (115·6 to 142·7)	95·9 (87·4 to 105·1)	71·9 (62·7 to 81·8)	47·5 (40·4 to 55·8)
Laikipia	86·1 (76·5 to 97·7)	68·6 (58·3 to 80·6)	70·1 (61·5 to 79·5)	59·7 (51·7 to 69·7)	53·6 (43·3 to 64·6)	52·9 (45·5 to 60·9)	32·6 (27·8 to 37·8)	19·2 (16·2 to 22·7)
Lamu	385·8 (352·4 to 425·3)	286·5 (260·2 to 314·6)	217·9 (185·9 to 251·9)	117·8 (100·3 to 138)	119·3 (98·5 to 142·5)	105 (90·8 to 122·1)	82·9 (68·2 to 100·4)	57·9 (49·3 to 68)
Machakos	183·5 (164 to 206·5)	144·1 (129·5 to 159·5)	122·9 (106·4 to 139·8)	89·3 (75·6 to 103·4)	65·1 (57·8 to 73·1)	53·7 (48·6 to 59·4)	36·1 (29·4 to 43)	25·6 (21·7 to 30)
Makueni	185·8 (167·2 to 208·4)	147·4 (130·6 to 163·9)	123·4 (112·5 to 135·5)	91·6 (78·2 to 106·7)	78 (68·8 to 87·9)	63·2 (56·2 to 71·3)	43·3 (37·5 to 49·8)	26·2 (22·3 to 31·1)
Mandera	168·3 (150·3 to 189·6)	143·4 (124·2 to 164·8)	126·5 (109·3 to 144·8)	118·4 (106·8 to 131)	122·8 (106·3 to 139·7)	99·6 (84·2 to 117·1)	80·7 (70·3 to 91·5)	62·2 (53 to 73)
Marsabit	241·4 (216·5 to 270·1)	195·3 (171 to 221·2)	177·8 (163·3 to 192·4)	145·6 (125·7 to 166·2)	122·2 (107·8 to 137·6)	95·1 (84·8 to 106·2)	85·6 (75·1 to 98·1)	66·3 (56·5 to 77·6)
Meru	150·1 (133·8 to 169·2)	116·5 (105·5 to 128·8)	90·7 (81·2 to 100·5)	61·9 (55·2 to 68·3)	55·7 (49·5 to 62)	56·3 (50·6 to 62·8)	56·4 (49·4 to 64·3)	48·6 (41·4 to 57)
Migori	403·6 (367·3 to 446·3)	332·9 (298·6 to 367·2)	292·1 (268·7 to 317·7)	273·2 (253·2 to 292·6)	237·6 (215·7 to 260·1)	202·7 (182·4 to 223·8)	114·2 (97·1 to 131·8)	74·7 (63·7 to 87·5)
Mombasa	269·3 (242·6 to 300·7)	185·4 (165·9 to 205·6)	139·5 (128·9 to 151·1)	95·1 (84·4 to 105·6)	85·6 (76·1 to 96·1)	78 (70·1 to 85·8)	55·5 (48·2 to 63·1)	36·5 (31 to 42·9)
Murang'a	198·4 (177·6 to 222·5)	141·7 (128 to 157)	106·4 (94·4 to 119·2)	64 (54·3 to 74·3)	46·7 (41 to 52·6)	47·8 (42·1 to 54·1)	47·6 (40·8 to 54·2)	35·9 (30·5 to 42·1)
Nairobi	326 (294·7 to 362·7)	187·9 (170·9 to 205·9)	114·3 (104 to 124·9)	69·9 (61·4 to 79·1)	72·5 (64 to 81)	70·5 (62·9 to 79)	37·2 (32·4 to 42·5)	24·4 (20·7 to 28·6)
Nakuru	65·7 (58·3 to 74·5)	51·9 (46 to 58·9)	54·4 (48·8 to 60·6)	53·9 (48·2 to 59·6)	47·6 (42·7 to 52·5)	50·6 (45·1 to 56·8)	46·4 (40·1 to 53·3)	35·1 (29·9 to 41·3)
Nandi	107·9 (96·1 to 122·3)	89·5 (79·3 to 100)	79·9 (72·8 to 87)	70·2 (57·6 to 83·5)	60·9 (54·7 to 67·2)	48·8 (43·7 to 54·2)	35 (30·2 to 40·4)	23·7 (20·2 to 27·9)
Narok	52·1 (46·2 to 59·3)	51·1 (45·3 to 58·5)	67·8 (60·5 to 75·3)	91·7 (83·8 to 100·1)	79·4 (70·7 to 87·9)	55·1 (49·6 to 61·3)	38·9 (33·6 to 44·9)	27·6 (23·5 to 32·5)
Nyamira	99·7 (88·9 to 112·6)	78·9 (70 to 88·7)	63 (57·3 to 69·1)	56·8 (50·5 to 63)	46·2 (40·9 to 51·6)	47·4 (41·7 to 53·9)	33·7 (29·2 to 38·9)	24 (20·4 to 28·2)
Nyandarua	198·4 (177·5 to 222·8)	134·6 (121·1 to 150·2)	92·3 (77·1 to 108·7)	53·9 (44·5 to 64·2)	43·7 (34·4 to 53·5)	48·5 (40·5 to 58·4)	58·6 (47·9 to 70·5)	47·2 (40·2 to 55·5)
Nyeri	154·3 (138 to 173·7)	109·6 (95·8 to 125·1)	77 (68·7 to 85·5)	45·9 (40·1 to 51·8)	41 (35 to 47·6)	46·7 (39·4 to 54·4)	43 (37·1 to 49·2)	36·7 (31·2 to 43·1)
Samburu	82·9 (73·6 to 94)	74·1 (63·8 to 85·1)	80·1 (68·2 to 93·7)	85·8 (69·7 to 102)	91 (72·8 to 110·6)	84·2 (72·3 to 98·3)	72·4 (60·2 to 85·8)	55·3 (47·1 to 64·9)
Siaya	344·5 (312 to 382·2)	282·5 (257·3 to 309·5)	233·9 (216·2 to 253)	206·2 (192·4 to 220·3)	203·9 (183·7 to 225·4)	182·7 (165 to 202·5)	102·2 (89·6 to 115·9)	63 (53·7 to 73·8)

Taita Taveta	258·9 (232·9 to 289·5)	196·1 (177·3 to 215·7)	182·7 (168 to 198)	117·1 (92·9 to 143·6)	80·8 (72·4 to 90·4)	77·7 (66·8 to 90·9)	52·6 (45·4 to 60·1)	32 (27·2 to 37·7)
Tana River	199·7 (179 to 224·2)	158·9 (144·4 to 175·3)	142·6 (123 to 163·7)	122·7 (98·4 to 149·1)	103 (90·8 to 116)	104·6 (89·3 to 122·7)	79·2 (64·8 to 95)	52·5 (44·8 to 61·8)
Tharaka Nithi	139·2 (124·1 to 157·3)	113·9 (100·4 to 127·8)	100·6 (88·3 to 114·7)	73·1 (62 to 85·2)	75·1 (65·2 to 86·4)	73·2 (65·4 to 82)	46·6 (40·6 to 53·3)	32·5 (27·6 to 38·2)
Trans Nzoia	130·9 (116·6 to 147·9)	100 (88·8 to 112·1)	98 (89 to 107·1)	91·9 (75·2 to 109·2)	70·3 (61·8 to 78·3)	58·2 (52·3 to 65·1)	41·2 (35·6 to 47·4)	26·6 (22·6 to 31·3)
Turkana	143 (127·5 to 161·4)	126·1 (110 to 142·4)	135·6 (116·1 to 157)	146·4 (122·6 to 171·8)	124·5 (108·5 to 139·8)	91·5 (78·3 to 107·4)	63·5 (52·8 to 74·8)	46 (39·2 to 54·2)
Uasin Gishu	74·2 (65·9 to 84·2)	56 (49·3 to 64)	52·8 (47 to 58·4)	54·3 (49·4 to 59·4)	52·3 (46·9 to 57·6)	46·2 (41·5 to 51·7)	36·3 (31·5 to 41·7)	26·2 (22·3 to 30·8)
Vihiga	176·4 (157·6 to 198·5)	141·6 (126·1 to 158·4)	117·1 (101·4 to 134·8)	98·6 (83·4 to 116·9)	98·9 (88·8 to 110)	96·1 (83·7 to 110·1)	74·8 (63·2 to 87·7)	50·3 (42·9 to 59·3)
Wajir	313·8 (283·6 to 349·2)	251·3 (220·2 to 285·5)	208·3 (179·1 to 238·7)	158·1 (143·5 to 173·8)	146·3 (126·6 to 167·8)	125·3 (107·2 to 146·5)	93·6 (81·5 to 105·9)	64·9 (55·4 to 76·1)
West Pokot	142·3 (127·3 to 160·3)	124·3 (112·1 to 136·9)	131·4 (120 to 142·6)	128·2 (117·8 to 138·9)	115·1 (103·7 to 125·9)	87·1 (78·9 to 96·6)	66·7 (57·9 to 76·7)	48·7 (41·5 to 57·1)
Madagascar	250·7 (218 to 286·9)	221·3 (192·1 to 251·8)	190 (169·6 to 210·2)	170 (156·9 to 183·9)	150·4 (138·2 to 162·7)	111·8 (101·4 to 122·2)	84 (73·7 to 94·8)	59·9 (50·8 to 71·6)
Malawi	416·4 (367 to 470·4)	378·3 (335·1 to 422·2)	333·2 (304·5 to 361·7)	266·5 (248·6 to 285·5)	222·6 (209·4 to 236·8)	163·7 (153·4 to 174·9)	101·4 (91·5 to 112·1)	64·4 (54·2 to 77·6)
Mozambique	307·6 (265 to 355·5)	285·4 (243·1 to 328·9)	256·9 (224·4 to 290·4)	235·5 (215 to 255·2)	217·1 (202·2 to 232·4)	167·4 (154·1 to 181·7)	112·7 (99·7 to 125·9)	74 (61·8 to 89·8)
Rwanda	333·1 (294·5 to 376)	279 (253·2 to 306·7)	291·7 (271·4 to 314·2)	225 (209·3 to 242·2)	177·8 (164·4 to 191·7)	155·9 (144·2 to 168)	77·8 (68·5 to 87·9)	50·2 (41·9 to 61·1)
Somalia	251·2 (214·3 to 293·4)	242·5 (205·5 to 282·3)	229·2 (195 to 268·1)	212 (184·2 to 239·8)	196·7 (176·5 to 219·1)	168·7 (148·7 to 190·2)	145·3 (125·8 to 165·8)	101·6 (84·5 to 123·7)
South Sudan	275·7 (236 to 320·5)	261·1 (224·9 to 300·5)	244·8 (212·5 to 283·7)	227·2 (197·7 to 258·9)	206·5 (183·8 to 232·7)	158 (142·3 to 174·6)	125·8 (109 to 143·3)	99 (84·2 to 116·2)
Uganda	256·4 (219·2 to 298·7)	234·6 (201·4 to 269·1)	217·5 (196·2 to 239)	207·3 (192·3 to 223·2)	180·2 (168·6 to 190·9)	147 (138·3 to 157·1)	96·4 (87·8 to 105·5)	64·6 (54·8 to 77·1)
Tanzania	264·2 (226·7 to 307)	246·8 (213·4 to 282·8)	214·5 (194·9 to 235·2)	181·3 (169·2 to 195·2)	158·9 (149·4 to 169)	126 (117·3 to 134·9)	84·3 (74·7 to 94·6)	60·2 (50·6 to 72·8)
Zambia	255·2 (218·4 to 297·2)	230·3 (198·9 to 265·2)	194·4 (175·7 to 213·7)	170·2 (156·2 to 184·2)	167·2 (156·3 to 179·1)	146·4 (134 to 159)	87·8 (77·7 to 99·2)	56·5 (47·2 to 68·6)
Southern sub-Saharan Africa	164·6 (139·3 to 194·1)	149·5 (127·9 to 172·5)	127·2 (113·6 to 142·3)	109·4 (102·3 to 116·9)	84·1 (79·1 to 89·4)	86·1 (80·4 to 92·3)	66·1 (60·3 to 72·4)	46·8 (39·9 to 55·7)
Botswana	125·7 (105·1 to 150)	123·3 (104·2 to 143·8)	109 (97·4 to 122·3)	80·4 (72·4 to 89)	67·8 (61·3 to 74·5)	79·8 (70·6 to 89)	56·2 (47·8 to 65)	44·2 (37 to 53·5)
eSwatini	261·6 (223·4 to 305)	236·8 (203·2 to 271·7)	181 (155·3 to 209·5)	126·7 (110·4 to 143·7)	83·3 (74·9 to 92)	98·7 (90·2 to 107·9)	84·1 (74·3 to 94·1)	52·2 (44·4 to 61·3)
Lesotho	175·5 (154·3 to 201·4)	163·8 (150 to 177·8)	153·1 (141·2 to 166)	117 (105·8 to 128·1)	99 (90·3 to 108·5)	108 (98·6 to 117·6)	94·5 (82·6 to 107·4)	71·5 (60·8 to 83·9)
Namibia	141·8 (118·8 to 168·9)	131·9 (111·4 to 154)	110·9 (96·9 to 127)	99·9 (90·1 to 110·3)	75·7 (68·2 to 83·8)	72·5 (65·2 to 80·8)	56·2 (48·4 to 64·8)	39·1 (32·5 to 47·8)
South Africa	167·6 (141·1 to 198·5)	152·8 (129·6 to 177·9)	130·8 (114·3 to 149·3)	114·6 (105·8 to 123·7)	87·9 (81·7 to 94·4)	86·4 (78·7 to 94·8)	60 (52·6 to 68)	41·9 (35·4 to 50·1)
Zimbabwe	146·7 (123·1 to 174·3)	132·2 (110·9 to 154·7)	110·7 (98·2 to 124)	96·8 (88·3 to 106)	74·6 (69 to 80·7)	83·9 (77·7 to 91·2)	79·1 (71·6 to 87)	57 (48·1 to 68·6)
Western sub-Saharan Africa	321 (284·2 to 361·6)	305·6 (275·1 to 337·1)	266·1 (248·1 to 285·5)	225·1 (213·9 to 236·2)	198·6 (191·1 to 206·3)	176 (169·7 to 182·7)	136·3 (128·1 to 144·9)	101·2 (88·5 to 117·1)
Benin	302·4 (264 to 345·6)	289·9 (263·1 to 317·7)	252·3 (231·1 to 272·3)	211 (195·4 to 226·6)	184·2 (170·5 to 198·2)	148·8 (137·8 to 160·9)	120·4 (109 to 132·3)	91·5 (78 to 108·7)
Burkina Faso	387·4 (339·6 to 440)	349·1 (306·5 to 395·3)	304·8 (276·1 to 334·1)	259·2 (240·5 to 279·5)	217·1 (202·2 to 231·1)	184·9 (169 to 200·1)	144·6 (126·5 to 164·2)	113·3 (95 to 136·9)
Cape Verde	178·2 (157·8 to 200·4)	172·2 (166 to 178·4)	173·5 (168·9 to 178·4)	99 (95·2 to 103·4)	72·1 (68·4 to 75·6)	55·4 (47·7 to 64·6)	29·6 (27·1 to 32·2)	18·7 (15·9 to 22·1)
Cameroon	327·2 (290·5 to 371·5)	458·5 (436·5 to 482·1)	217·6 (201·9 to 235)	180·5 (166·7 to 194·6)	147 (135·1 to 158·6)	139·5 (128·7 to 151·5)	109·1 (95·3 to 123·5)	76·2 (63·9 to 90·8)
Chad	314·4 (271·1 to 362·9)	287·3 (244·9 to 330·8)	268·9 (235·1 to 302·5)	239·1 (217·5 to 262)	204 (186·6 to 222·6)	188·6 (173·5 to 204·6)	153·7 (136·3 to 172·3)	118·7 (100·1 to 142·8)
Côte d'Ivoire	338·9 (297·1 to 389·5)	293·8 (270 to 318)	236·9 (218·7 to 254·9)	177·4 (164·5 to 192·1)	156·9 (145·8 to 169)	145·4 (134·5 to 156·4)	114·5 (101·3 to 128·3)	78·7 (66·3 to 93·3)
The Gambia	220·2 (187·7 to 257·3)	199·4 (170·2 to 229·3)	170·8 (146·8 to 197·7)	152·8 (133·5 to 172·9)	121·8 (108·5 to 136·3)	93·4 (83·4 to 104·5)	63·3 (54 to 72·7)	40·6 (33·6 to 49·7)
Ghana	264·3 (234·1 to 300·2)	223 (202·9 to 244·6)	188·6 (174·2 to 203·2)	153·6 (142·7 to 164)	126·7 (119·5 to 134·9)	104·8 (98·1 to 111·8)	81·9 (73·9 to 89·8)	56·1 (48·3 to 65·3)
Guinea	365·8 (319·2 to 417·4)	334·3 (296·1 to 376·9)	305·5 (271·9 to 342·3)	273·2 (249·4 to 300·3)	230·8 (213·2 to 249·3)	184·7 (170·2 to 201)	140·5 (125·7 to 156·1)	103·9 (89·5 to 120·5)
Guinea-Bissau	428·3 (376·5 to 484·3)	375·5 (329 to 423·9)	328·2 (288·9 to 371·8)	278·8 (251·3 to 310·4)	230·9 (208·2 to 255·9)	178·5 (162·3 to 195·3)	115·9 (101·3 to 131·4)	78·2 (66·5 to 91·7)
Liberia	402·5 (358·3 to 449·7)	366 (326 to 407·8)	306·3 (275·2 to 336·4)	257 (235·5 to 279·5)	233·1 (218 to 249)	177·1 (164·5 to 189·2)	93·1 (83·5 to 103·6)	64·1 (54·5 to 76·3)
Mali	406·2 (357·3 to 459·7)	375·5 (332·2 to 421·2)	346·3 (316·9 to 375·1)	288 (268·6 to 307·2)	239 (222·3 to 255)	202·3 (186·9 to 219·8)	160·6 (145·5 to 176·2)	126·8 (107·9 to 150·8)
Mauritania	250·2 (219·6 to 284·2)	210·2 (192·2 to 230·4)	163·8 (150 to 177·8)	143·4 (131·6 to 156·2)	118·2 (108·6 to 127·8)	98·6 (89·7 to 108)	70·1 (62·5 to 78·2)	46·6 (39·8 to 55·4)
Niger	411 (360·4 to 466·3)	378·8 (328·9 to 431·5)	339·5 (305·1 to 374·2)	308·8 (284 to 334·3)	288·9 (269·5 to 309·6)	230·7 (209 to 253·5)	149·8 (129·7 to 170·9)	115·5 (96·8 to 139·9)
Nigeria	301·5 (260·5 to 347·8)	282·7 (245·5 to 322·5)	267·9 (242·4 to 294·5)	228 (212·6 to 244·5)	209·9 (198·1 to 221·3)	190·1 (180·1 to 200·2)	150·7 (140·5 to 162·1)	110·2 (94·5 to 130·2)
São Tomé and PrÍncipe	316·1 (283·7 to 355·4)	263·9 (251·1 to 276·3)	146·7 (139·8 to 152·9)	116·3 (109·1 to 124·2)	105·3 (96·8 to 113)	74·6 (66·9 to 82·7)	41·6 (35·9 to 48·2)	26·9 (22·3 to 32·4)
Senegal	299·4 (263·3 to 339·6)	270·8 (247·4 to 295·3)	256·1 (237·3 to 274·6)	202·4 (188·5 to 217·5)	146·7 (138·1 to 155·9)	122·8 (115·4 to 131)	75·4 (68·9 to 82·2)	52·8 (45 to 62·8)
Sierra Leone	348·6 (303 to 399·4)	319·9 (280·7 to 363·7)	291 (252·6 to 334)	267·5 (236·9 to 300·9)	243·3 (225·1 to 262·1)	218·7 (203 to 236·6)	162·3 (147·2 to 177·8)	109·4 (93·4 to 127·3)
Togo	305·1 (263·8 to 351·2)	279·7 (243·2 to 317·5)	231·3 (208·3 to 256·1)	180·9 (164·3 to 198·7)	153 (138·9 to 168·7)	131·6 (119·1 to 146·1)	100·4 (88·5 to 113·3)	68·8 (57·7 to 83·3)

Supplemental Results Table 4c: Under-5 mortality rates by location, females, 1950, 1960, 1970, 1980, 1990, 2000, 2010, 2019

Location	1950	1960	1970	1980	1990	2000	2010	2019
Global	199·9 (184·5 to 216·5)	265·5 (227·3 to 319·8)	138·1 (131·9 to 144·8)	109·2 (104·8 to 113·5)	84·2 (81·5 to 87·3)	69 (66·5 to 71·6)	51·1 (48·3 to 54)	35·2 (31·9 to 39·2)
Central Europe, eastern Europe, and central Asia	96·4 (88·1 to 105·6)	67·1 (63·4 to 71·1)	45·8 (43·4 to 48·7)	34·3 (33 to 35·6)	27·9 (26·8 to 29·1)	23·8 (22·8 to 24·9)	14·3 (13·4 to 15·3)	10·1 (9·2 to 11·1)
Central Asia	213·4 (189·5 to 239·6)	146·9 (130·9 to 164·2)	92·5 (83·3 to 102·7)	66·6 (62·4 to 71)	51·6 (48·4 to 55·4)	43·3 (40·4 to 46·5)	27·2 (24·6 to 30·1)	18·1 (16·1 to 20·6)
Armenia	275·5 (246·2 to 307·2)	165·7 (142·7 to 190·6)	91·7 (80·2 to 104·6)	58·2 (52·2 to 64·5)	37·2 (33·9 to 41·1)	25·3 (22·7 to 28·3)	14·6 (12·7 to 16·7)	10 (8·6 to 11·9)
Azerbaijan	393·1 (358 to 429·6)	269·1 (233·8 to 305·5)	144·4 (124·4 to 165·5)	87·3 (76·6 to 98·8)	71 (63·7 to 79·6)	58·6 (52·8 to 64·5)	35·1 (30 to 40·5)	25 (20·9 to 30·3)
Georgia	233·6 (207·8 to 262)	140·8 (121·2 to 160·6)	81·4 (69·4 to 95·2)	50·3 (44·5 to 56·5)	36·3 (32·4 to 40·6)	30·7 (27·1 to 34·5)	14·4 (12·2 to 16·9)	9·1 (7·6 to 11·1)
Kazakhstan	112·3 (97·7 to 129·2)	81·4 (69·5 to 94·2)	50·3 (43·6 to 58)	40·9 (36·6 to 45·3)	33·1 (29·8 to 36·7)	30·8 (27·1 to 34·7)	18·8 (16·1 to 21·9)	10·7 (8·9 to 13)
Kyrgyzstan	205·3 (181·7 to 231·8)	152·1 (131·3 to 174·3)	103·3 (90 to 118·5)	73·5 (65·9 to 81·6)	51·8 (47·4 to 56·5)	39·3 (35·8 to 43)	24·7 (24·1 to 25·4)	15·7 (14·3 to 17·1)
Mongolia	310·4 (280·4 to 342·5)	255·3 (226·4 to 283·2)	175 (155·9 to 195·7)	119·2 (108·4 to 131·3)	81·2 (74·6 to 88·5)	52·8 (48·4 to 57·5)	27·6 (24·9 to 30·6)	15·6 (13·5 to 18·3)
Tajikistan	288·8 (257·5 to 322·5)	220·8 (192·7 to 250·8)	143·4 (126·1 to 161·2)	101·2 (91·3 to 112·1)	77·3 (70·7 to 84·6)	60·9 (55·4 to 66·8)	36·6 (32·6 to 40·6)	27·3 (23·7 to 31·3)
Turkmenistan	227 (201·5 to 255·1)	161·7 (141·4 to 184·3)	105·9 (91·5 to 123·3)	89 (78·6 to 99·8)	65·1 (58·5 to 72·5)	50·8 (45·3 to 56·5)	34 (29·8 to 38·7)	21·8 (18·9 to 25·2)
Uzbekistan	151·1 (132·3 to 172·3)	109·9 (93 to 128·1)	80·4 (69·9 to 93·3)	60·7 (55 to 66·6)	47 (42·1 to 52·3)	40·5 (35·9 to 46)	27·5 (23·6 to 32)	18·5 (15·5 to 22·5)
Central Europe	126·9 (123·2 to 131)	71·5 (70·7 to 72·4)	44·7 (44 to 45·5)	26·4 (26·1 to 26·7)	20 (19·8 to 20·3)	12·1 (11·8 to 12·3)	6·6 (6·5 to 6·7)	4·5 (4·1 to 5)
Albania	228·6 (198·8 to 261·2)	154 (132·8 to 175·7)	93·8 (79·7 to 109·8)	49·9 (43·8 to 56·3)	38·9 (35·1 to 43)	25·8 (23·1 to 28·9)	14·7 (12·6 to 17)	10·8 (9 to 13·1)
Bosnia and Herzegovina	207·3 (203 to 212)	129·7 (126·5 to 132·6)	74 (71·9 to 75·9)	36·3 (35·1 to 37·6)	14·7 (14·1 to 15·4)	9·1 (8·5 to 9·7)	6·2 (5·8 to 6·7)	4·9 (4·2 to 5·6)
Bulgaria	120·4 (117·3 to 123)	52·4 (51 to 53·8)	28·6 (27·8 to 29·5)	22 (21·3 to 22·7)	17 (16·3 to 17·6)	15·1 (14·4 to 15·8)	9·7 (9·3 to 10·2)	6·4 (5·6 to 7·4)
Croatia	151·7 (148 to 155·6)	76·5 (74·4 to 78·7)	41·7 (40·3 to 43·1)	20·7 (19·9 to 21·6)	10·7 (10·1 to 11·2)	7·5 (7·1 to 8)	4·8 (4·5 to 5·2)	3·7 (3·2 to 4·2)
Czech Republic	65·8 (64·3 to 67·4)	20·5 (19·8 to 21·1)	19·9 (19·2 to 20·5)	15·5 (15 to 15·9)	10·4 (9·9 to 10·8)	4·6 (4·4 to 4·9)	3 (2·8 to 3·2)	2·2 (2 to 2·6)
Hungary	94·8 (92·8 to 97·1)	49 (47·8 to 50·3)	36·7 (35·7 to 37·7)	22·1 (21·5 to 22·8)	15·5 (14·9 to 16·1)	9·4 (9 to 9·9)	5·4 (5·1 to 5·8)	3·7 (3·2 to 4·2)
Montenegro	85·7 (81·8 to 89·9)	68·3 (65 to 71·8)	46·2 (43·5 to 48·7)	27 (25·4 to 28·9)	16 (14·9 to 17·2)	12·7 (11·6 to 13·8)	6·6 (6·1 to 7·2)	3·5 (3 to 4)
North Macedonia	240·4 (235·5 to 245·1)	200·6 (196·8 to 205)	115·5 (112·6 to 118·7)	64·8 (62·6 to 67·2)	35·9 (34·3 to 37·6)	14·1 (13·2 to 15·1)	8·7 (8·1 to 9·5)	7·9 (6·9 to 9·1)
Poland	120·2 (118·4 to 122·1)	57 (56·3 to 57·9)	33·8 (33·2 to 34·3)	21·2 (20·9 to 21·6)	16·2 (15·9 to 16·6)	8·6 (8·3 to 8·9)	5·3 (5·1 to 5·5)	3·5 (3 to 4)
Romania	121·5 (106·3 to 139)	83·2 (81·7 to 84·8)	52·4 (51·3 to 53·5)	32·2 (31·5 to 32·9)	30 (29·3 to 30·7)	19·9 (19·3 to 20·4)	10·2 (9·8 to 10·6)	7·2 (6·4 to 8·2)
Serbia	168·6 (165 to 172·2)	94·5 (92·2 to 96·6)	63·1 (61·6 to 64·7)	35·1 (34·2 to 36)	23·8 (23 to 24·6)	12·9 (11 to 15)	6 (5·7 to 6·3)	3·6 (3·1 to 4·3)
Slovakia	116·9 (114 to 120)	31·2 (30·1 to 32·3)	26·6 (25·7 to 27·6)	19·9 (19·2 to 20·6)	12·7 (12·2 to 13·3)	8·7 (8·2 to 9·3)	6 (5·6 to 6·4)	4·8 (4·2 to 5·5)
Slovenia	123·5 (119·4 to 127·8)	68·1 (65·8 to 70·5)	35·1 (33·8 to 36·6)	16·8 (16 to 17·7)	8·9 (8·4 to 9·5)	4·8 (4·5 to 5·2)	3 (2·8 to 3·3)	1·9 (1·6 to 2·1)
Eastern Europe	53·4 (45·9 to 62·2)	39·9 (38·6 to 41·3)	24·8 (23·8 to 25·9)	23·2 (22·4 to 24·1)	17·4 (16·9 to 18)	15·8 (15·6 to 16)	8·5 (8·4 to 8·6)	6·1 (5·6 to 6·6)
Belarus	64·3 (55·3 to 75)	47·8 (40·3 to 55·8)	29 (24·7 to 33·8)	21·2 (18·1 to 24·8)	15·7 (13·4 to 18·3)	13·8 (11·9 to 15·9)	7·1 (6 to 8·3)	4·6 (3·9 to 5·5)
Estonia	47·1 (40·7 to 54·7)	31·6 (30·2 to 33·1)	18·9 (18 to 19·9)	18·3 (17·4 to 19·2)	14·5 (13·9 to 15·3)	9·7 (9 to 10·4)	4·3 (3·9 to 4·7)	2·5 (2·2 to 2·9)
Latvia	40·6 (34·9 to 47·4)	28·2 (26·9 to 29·5)	18·7 (18 to 19·5)	18·2 (17·5 to 19)	14·9 (14·2 to 15·6)	12·3 (11·6 to 13·1)	7·2 (6·7 to 7·7)	3·9 (3·4 to 4·5)
Lithuania	61·3 (53·2 to 70·7)	42·3 (41 to 43·7)	20 (19·3 to 20·8)	17·3 (16·6 to 17·9)	13 (12·5 to 13·6)	9·8 (9·3 to 10·3)	5·4 (5·1 to 5·8)	3·7 (3·3 to 4·2)
Moldova	155·5 (136·2 to 177·4)	107·9 (90·8 to 125·2)	67·3 (57·2 to 78·5)	46 (40·1 to 52)	27·8 (24·8 to 31·3)	23·8 (21·4 to 26·8)	14·3 (12·4 to 16·3)	10·8 (9 to 13·1)
Russia	52·5 (45·1 to 61·3)	39·6 (39·2 to 40·1)	24·3 (24 to 24·5)	23·5 (23·2 to 23·7)	17·9 (17·7 to 18·1)	16·7 (16·5 to 16·9)	8·4 (8·3 to 8·5)	5·8 (5·2 to 6·5)
Ukraine	43·8 (37·5 to 51·3)	32·6 (27·5 to 38·4)	21·8 (18·4 to 25·6)	21 (18 to 24·3)	15·6 (13·7 to 17·6)	13·3 (13 to 13·6)	9 (8·8 to 9·3)	7·7 (6·9 to 8·5)
High income	64·2 (63·9 to 64·5)	39·6 (39·1 to 40·1)	25·9 (25·6 to 26·1)	15 (14·9 to 15·2)	9·9 (9·8 to 10)	6·8 (6·7 to 6·8)	5·4 (5·4 to 5·4)	4·5 (4·4 to 4·6)
Australasia	27·4 (26·7 to 28·2)	22·2 (21·8 to 22·7)	18·4 (18 to 18·7)	11·8 (11·5 to 12)	8·6 (8·4 to 8·8)	5·8 (5·7 to 6)	4·6 (4·5 to 4·7)	3·4 (3·3 to 3·5)
Australia	26·6 (26 to 27·3)	21·7 (21·2 to 22·1)	18·6 (18·2 to 19)	11·3 (11·1 to 11·6)	8·2 (7·9 to 8·4)	5·6 (5·5 to 5·8)	4·4 (4·2 to 4·5)	3·3 (3·2 to 3·4)
New Zealand	30·6 (29·5 to 31·8)	24·3 (23·5 to 25·2)	17·4 (16·9 to 18·1)	13·6 (13·1 to 14·2)	10·4 (9·9 to 10·8)	6·8 (6·4 to 7·1)	5·9 (5·6 to 6·2)	4·1 (3·9 to 4·4)
High-income Asia Pacific	109·3 (107·3 to 111·8)	54 (51·6 to 56·6)	25·6 (24·6 to 26·7)	13·9 (13·2 to 14·7)	7·8 (7·4 to 8·2)	4·7 (4·7 to 4·8)	3·2 (3·1 to 3·3)	2·5 (2·4 to 2·6)
Brunei	138·9 (130 to 147·4)	106·3 (99·6 to 114·1)	48·2 (45·4 to 51·3)	19·7 (17·9 to 21·5)	10·4 (9·5 to 11·4)	8·9 (8 to 9·7)	8·5 (7·7 to 9·3)	8·3 (7·2 to 9·7)
Japan	87·7 (86·2 to 89·1)	35·5 (35 to 36)	15 (14·8 to 15·2)	8·6 (8·5 to 8·7)	5·7 (5·6 to 5·8)	4·1 (4 to 4·2)	3 (2·9 to 3)	2·3 (2·3 to 2·4)
Aichi	75·7 (74·2 to 77·3)	31·9 (29·2 to 34·5)	13·7 (13·1 to 14·4)	8 (7·7 to 8·4)	5·3 (5·1 to 5·6)	3·9 (3·7 to 4·1)	3 (2·9 to 3·2)	2·2 (2 to 2·4)
Akita	83·5 (81·9 to 85·3)	35·9 (33·2 to 38·8)	16·5 (15·4 to 17·9)	9 (8·5 to 9·6)	6·1 (5·7 to 6·5)	3·9 (3·6 to 4·2)	2·8 (2·6 to 3)	2·2 (2 to 2·5)
Aomori	92·8 (91 to 94·7)	40·7 (37·2 to 44·4)	18·5 (17·4 to 19·5)	10 (9·5 to 10·6)	6·7 (6·3 to 7·1)	4·9 (4·6 to 5·3)	3·4 (3·3 to 3·6)	2·7 (2·5 to 3)
Chiba	83·1 (81·5 to 84·7)	33·8 (31·1 to 36·6)	13·7 (13 to 14·3)	8·1 (7·7 to 8·5)	5·2 (4·9 to 5·4)	4·1 (3·9 to 4·3)	3·1 (2·9 to 3·4)	2·7 (2·4 to 2·9)
Ehime	88·4 (86·5 to 90·3)	37·5 (34·6 to 40·4)	16·8 (15·6 to 18)	9·4 (8·8 to 10)	6·2 (5·8 to 6·5)	4·1 (3·8 to 4·4)	2·6 (2·5 to 2·8)	2 (1·8 to 2·2)

Fukui	76·7 (75·1 to 78·3)	33·4 (29·3 to 38·1)	14·9 (13·1 to 17)	8 (7·3 to 8·6)	5·8 (5·4 to 6·3)	4·3 (4 to 4·7)	2·8 (2·5 to 3)	2·2 (2 to 2·5)
Fukuoka	81·5 (79·8 to 83·2)	33·2 (30·6 to 36)	15·2 (14·3 to 16·1)	8·4 (8·1 to 8·8)	5·9 (5·7 to 6·2)	4·3 (4·1 to 4·5)	3·1 (2·9 to 3·3)	2·4 (2·2 to 2·7)
Fukushima	99·4 (97·3 to 101·6)	42·1 (38·9 to 45·8)	19·6 (18·5 to 20·8)	10·3 (9·9 to 10·8)	6·7 (6·3 to 7)	4·5 (4·2 to 4·8)	3 (2·8 to 3·2)	2·3 (2·1 to 2·5)
Gifu	83·3 (81·5 to 85)	36·3 (33·4 to 39·5)	16·4 (15·3 to 17·4)	9·1 (8·7 to 9·6)	5·4 (5 to 5·8)	4 (3·7 to 4·3)	3·1 (2·9 to 3·3)	2·4 (2·2 to 2·6)
Gunma	83·6 (82·1 to 85·1)	36·5 (33·6 to 39·6)	16 (15·1 to 16·9)	8·7 (8·3 to 9·1)	5·6 (5·2 to 6)	4·2 (3·9 to 4·5)	3 (2·8 to 3·2)	2·3 (2·1 to 2·5)
Hiroshima	90 (88·2 to 91·9)	36·8 (32·3 to 41·4)	16·1 (14·1 to 18·2)	9 (8·6 to 9·4)	5·8 (5·5 to 6·1)	3·8 (3·5 to 4)	3·1 (2·9 to 3·2)	2·4 (2·2 to 2·6)
Hokkaidō	86·6 (84·8 to 88·4)	36·1 (33·3 to 38·8)	16·6 (14·6 to 18·8)	9·3 (8·9 to 9·7)	5·6 (5·3 to 5·8)	3·8 (3·6 to 4)	2·8 (2·6 to 3)	2·2 (1·9 to 2·6)
Hyōgo	72·9 (71·7 to 74·2)	30·1 (27·6 to 32·7)	13·4 (12·6 to 14·3)	8 (7·7 to 8·3)	5·5 (5·3 to 5·8)	3·9 (3·7 to 4·2)	2·6 (2·5 to 2·8)	2 (1·8 to 2·2)
Ibaraki	101·9 (99·7 to 104·2)	42·7 (37·8 to 48)	18·9 (16·5 to 21·5)	10·2 (9·7 to 10·7)	5·8 (5·6 to 6·1)	4·2 (3·9 to 4·5)	3·3 (3·1 to 3·5)	2·5 (2·3 to 2·7)
Ishikawa	81·8 (80·1 to 83·5)	35·8 (32·8 to 38·8)	16 (14·9 to 17·1)	9 (8·5 to 9·5)	5·8 (5·4 to 6·2)	4·2 (3·9 to 4·6)	2·9 (2·7 to 3·1)	2·1 (1·9 to 2·3)
Iwate	99·1 (96·9 to 101·3)	43·4 (39·7 to 47·7)	20·4 (19·2 to 21·6)	11·1 (10·5 to 11·8)	5·7 (5·3 to 6·1)	4·1 (3·8 to 4·4)	3·6 (3·4 to 3·8)	2·8 (2·5 to 3·1)
Kagawa	81·8 (80·2 to 83·4)	34·8 (32·1 to 37·8)	15·2 (14·1 to 16·5)	8·5 (7·9 to 9·1)	5·8 (5·4 to 6·3)	3·8 (3·5 to 4·1)	3 (2·8 to 3·2)	2·3 (2·1 to 2·6)
Kagoshima	102·5 (100·2 to 104·8)	44·1 (40·8 to 47·8)	21 (19·7 to 22·3)	10·9 (10·4 to 11·5)	6·3 (5·9 to 6·7)	4·7 (4·4 to 5)	3·2 (3·1 to 3·4)	2·6 (2·3 to 2·8)
Kanagawa	71·7 (70·3 to 73·1)	28·7 (26·5 to 30·8)	12·5 (11·9 to 13·1)	7·9 (7·6 to 8·2)	5·5 (5·3 to 5·8)	4·2 (4 to 4·3)	3·1 (2·9 to 3·3)	2·4 (2·2 to 2·7)
Kōchi	99·5 (97·4 to 101·6)	42·8 (39·6 to 46·3)	19 (17·6 to 20·5)	10·2 (9·5 to 11)	7 (6·5 to 7·6)	4·6 (4·2 to 5)	3·6 (3·3 to 3·8)	2·7 (2·4 to 3)
Kumamoto	97·5 (95·5 to 99·6)	42·1 (38·9 to 45·4)	19·3 (18·1 to 20·5)	10·1 (9·5 to 10·6)	5·9 (5·6 to 6·3)	4·2 (3·9 to 4·5)	2·9 (2·8 to 3·1)	2·2 (2 to 2·4)
Kyōto	74·3 (72·9 to 75·7)	30·7 (28·3 to 33·2)	13·1 (12·4 to 13·9)	7·6 (7·3 to 8)	5·8 (5·4 to 6·2)	4 (3·8 to 4·3)	3 (2·9 to 3·2)	2·5 (2·3 to 2·7)
Mie	82·7 (81 to 84·4)	36 (33·2 to 39)	16·1 (15·1 to 17·1)	9·1 (8·6 to 9·6)	6 (5·6 to 6·4)	4·4 (4·1 to 4·7)	3·3 (3·2 to 3·5)	2·4 (2·1 to 2·6)
Miyagi	90·7 (88·8 to 92·6)	37·2 (34·3 to 40·5)	16·7 (15·8 to 17·7)	9·2 (8·8 to 9·6)	5·7 (5·4 to 6)	3·8 (3·5 to 4·1)	3·1 (2·9 to 3·3)	2·5 (2·3 to 2·7)
Miyazaki	92·2 (90·3 to 94·1)	39·5 (36·3 to 43)	17·9 (16·7 to 19·2)	9·7 (9·2 to 10·3)	6·6 (6·2 to 7)	4·4 (4·1 to 4·8)	3 (2·8 to 3·3)	2·3 (2·1 to 2·5)
Nagano	84·1 (82·5 to 85·8)	35·6 (32·9 to 38·4)	15·3 (14·5 to 16·2)	8·5 (8·1 to 8·9)	5·6 (5·3 to 6)	3·4 (3·2 to 3·7)	2·6 (2·4 to 2·7)	2 (1·8 to 2·2)
Nagasaki	78 (76·5 to 79·5)	33·7 (30·8 to 36·9)	16·2 (15·3 to 17)	8·5 (8·1 to 9)	5·7 (5·4 to 6·1)	4·4 (4·1 to 4·8)	3·3 (3·1 to 3·5)	2·5 (2·2 to 2·7)
Nara	81·4 (79·8 to 83·1)	34·6 (31·7 to 37·9)	15·5 (14·4 to 16·5)	9·1 (8·5 to 9·7)	5·8 (5·4 to 6·2)	3·9 (3·6 to 4·2)	3·3 (3 to 3·5)	2·7 (2·5 to 3)
Niigata	83·9 (82·3 to 85·6)	35·4 (32·7 to 38·4)	15·9 (14·9 to 16·9)	8·9 (8·5 to 9·3)	5·1 (4·9 to 5·4)	4·1 (3·8 to 4·4)	2·6 (2·4 to 2·7)	1·9 (1·8 to 2·1)
Ōita	95·5 (93·4 to 97·6)	40·8 (37·3 to 44·6)	18·6 (17·3 to 19·9)	10·1 (9·5 to 10·6)	6 (5·6 to 6·4)	4 (3·7 to 4·3)	3·1 (2·9 to 3·3)	2·4 (2·2 to 2·7)
Okayama	84 (82·2 to 85·7)	35·6 (32·7 to 38·9)	14·8 (13·9 to 15·7)	8·4 (7·9 to 8·8)	6 (5·7 to 6·4)	4·2 (3·9 to 4·5)	2·7 (2·6 to 2·9)	2 (1·8 to 2·2)
Okinawa	96·9 (94·8 to 99)	41·3 (37·7 to 45·3)	20·4 (19·1 to 21·7)	10·1 (9·5 to 10·6)	6·8 (6·4 to 7·2)	4·8 (4·5 to 5·2)	3·1 (2·9 to 3·3)	2·5 (2·3 to 2·7)
Ōsaka	75 (73·5 to 76·5)	30·5 (27·8 to 33·1)	13·1 (12·4 to 13·8)	8·2 (7·9 to 8·6)	5·9 (5·7 to 6·2)	4 (3·8 to 4·1)	2·8 (2·6 to 3)	2·1 (1·9 to 2·4)
Saga	90·4 (88·5 to 92·3)	38·8 (35·3 to 42·4)	17·6 (16·4 to 19)	9·3 (8·7 to 10)	6·2 (5·8 to 6·7)	4·5 (4·1 to 4·9)	2·8 (2·6 to 3)	2·1 (1·8 to 2·3)
Saitama	79·8 (78·4 to 81·2)	32·9 (30·4 to 35·5)	13·6 (13 to 14·3)	8·1 (7·8 to 8·4)	5·5 (5·3 to 5·8)	4 (3·8 to 4·2)	2·9 (2·7 to 3·1)	2·2 (2 to 2·5)
Shiga	80·1 (78·6 to 81·7)	34·7 (31·8 to 38)	15·5 (14·4 to 16·6)	8·3 (7·7 to 8·8)	5·8 (5·4 to 6·2)	4·4 (4·1 to 4·7)	3·1 (2·9 to 3·2)	2·3 (2·1 to 2·5)
Shimane	84·4 (82·6 to 86·2)	36 (32 to 40·9)	16·6 (14·6 to 18·8)	9·1 (8·5 to 9·8)	6·1 (5·6 to 6·6)	4·2 (3·9 to 4·6)	2·8 (2·6 to 3·1)	2·2 (2 to 2·4)
Shizuoka	74·4 (73·1 to 75·8)	30·5 (27·1 to 34·3)	13·4 (11·7 to 15·3)	7·5 (7·1 to 8)	5·5 (5·2 to 5·8)	3·7 (3·5 to 4)	2·8 (2·7 to 3)	2·3 (2·1 to 2·5)
Tochigi	90·9 (88·9 to 92·8)	39·6 (36·8 to 42·7)	17·8 (16·8 to 18·9)	9·7 (9·2 to 10·1)	6·4 (6 to 6·8)	4·9 (4·5 to 5·2)	3·4 (3·2 to 3·6)	2·5 (2·3 to 2·7)
Tokushima	88·3 (86·5 to 90·2)	37·4 (34·3 to 41)	17 (15·8 to 18·2)	9·2 (8·6 to 9·8)	6·1 (5·6 to 6·6)	4·7 (4·3 to 5·1)	3·8 (3·5 to 4·1)	2·9 (2·6 to 3·2)
Tōkyō	68·1 (66·8 to 69·5)	28·9 (25·9 to 32·1)	13·3 (12·3 to 14·4)	7·9 (7·6 to 8·3)	5·4 (5·2 to 5·7)	4·1 (3·9 to 4·3)	2·7 (2·6 to 2·9)	2·1 (1·9 to 2·4)
Tottori	91·7 (89·8 to 93·7)	39·1 (34·2 to 44·5)	18 (15·8 to 20·4)	9·5 (8·8 to 10·3)	6·1 (5·6 to 6·7)	4 (3·7 to 4·5)	3·4 (3·1 to 3·7)	2·8 (2·5 to 3·1)
Toyama	90·6 (88·7 to 92·6)	39·5 (36·6 to 42·7)	17·8 (16·6 to 19·1)	9·8 (9·2 to 10·4)	6·8 (6·2 to 7·3)	4·5 (4·1 to 4·9)	3·2 (3 to 3·4)	2·3 (2 to 2·5)
Wakayama	96·8 (94·7 to 98·7)	41·7 (38·4 to 45·6)	18·7 (17·4 to 20·1)	10·9 (10·3 to 11·6)	6·1 (5·7 to 6·6)	4·1 (3·7 to 4·4)	3 (2·7 to 3·2)	2·4 (2·1 to 2·6)
Yamagata	86·2 (84·4 to 88)	37·2 (34·1 to 40·6)	17 (15·9 to 18·3)	9·2 (8·7 to 9·7)	5·4 (5·1 to 5·9)	4·3 (4 to 4·6)	3·4 (3·2 to 3·7)	2·9 (2·6 to 3·2)
Yamaguchi	89·3 (87·5 to 91·2)	37·8 (34·5 to 41·3)	16·8 (15·9 to 17·8)	9·7 (9·2 to 10·2)	6·2 (5·7 to 6·7)	4·4 (4·1 to 4·7)	3·2 (3 to 3·4)	2·9 (2·6 to 3·2)
Yamanashi	93·6 (92·1 to 94·8)	40·3 (35·4 to 46)	17·8 (15·8 to 20·2)	9·6 (8·9 to 10·4)	6·6 (6·2 to 7·2)	4·7 (4·3 to 5·1)	2·8 (2·6 to 3)	2·2 (2 to 2·4)
South Korea	182·3 (170·3 to 196·3)	85·6 (79·2 to 92·5)	47·5 (44·3 to 51·1)	25·2 (22·9 to 27·8)	11·8 (10·7 to 12·9)	6·2 (6·1 to 6·4)	3·8 (3·7 to 3·9)	2·8 (2·6 to 3)
Singapore	112·2 (109·1 to 115·5)	43·7 (42·4 to 45·1)	23·9 (23 to 24·8)	13·2 (12·5 to 13·8)	7·4 (7 to 7·8)	3 (2·8 to 3·2)	2·3 (2·1 to 2·5)	1·8 (1·6 to 2)
High-income North America	30·8 (30·6 to 31·1)	26·6 (26·4 to 26·8)	20·8 (20·7 to 21)	13·2 (13·1 to 13·3)	9·6 (9·5 to 9·7)	7·3 (7·2 to 7·4)	6·5 (6·4 to 6·5)	5·7 (5·7 to 5·8)
Canada	42·5 (41·7 to 43·2)	28·3 (27·9 to 28·8)	19·6 (19·3 to 19·9)	11·3 (11·1 to 11·5)	7·3 (7·2 to 7·5)	5·6 (5·4 to 5·7)	5·2 (5·1 to 5·4)	4·5 (4·4 to 4·6)
Greenland	148·9 (128·1 to 171·7)	81 (74·5 to 87·9)	51·7 (47 to 56·6)	36·9 (30·4 to 45·2)	31·2 (27·3 to 35·2)	19·5 (16·7 to 22·8)	12·3 (10·2 to 14·7)	9·6 (7·1 to 12·9)
USA	29·6 (29·3 to 29·9)	26·4 (26·2 to 26·6)	20·9 (20·8 to 21·1)	13·4 (13·3 to 13·5)	9·8 (9·7 to 9·9)	7·4 (7·4 to 7·5)	6·6 (6·5 to 6·6)	5·8 (5·8 to 5·9)

Alabama	38·6 (37·9 to 39·3)	33·8 (32·8 to 34·8)	26·2 (25·2 to 27·2)	15·9 (15·1 to 16·6)	11·3 (10·7 to 11·8)	9·8 (9·3 to 10·4)	9 (8·5 to 9·6)	8·5 (8·1 to 9)
Alaska	49·5 (47·6 to 51·3)	42 (39·8 to 44·3)	23·4 (22·1 to 24·9)	15·6 (14·6 to 16·6)	10·9 (10·2 to 11·7)	8·1 (7·6 to 8·6)	6·3 (5·9 to 6·8)	6·1 (5·2 to 7·2)
Arizona	41·4 (40·7 to 42·1)	35 (33·6 to 36·5)	21·8 (20·6 to 23)	14·2 (13·5 to 14·9)	10·1 (9·5 to 10·7)	7·7 (7·3 to 8·1)	6·6 (6·2 to 7)	5·8 (5·5 to 6·1)
Arkansas	33·7 (33·1 to 34·2)	28·6 (27·5 to 30·1)	22·6 (21·5 to 23·9)	13·4 (12·6 to 14·1)	10·3 (9·6 to 10·9)	8·8 (8·3 to 9·4)	7·8 (7·4 to 8·3)	7·5 (7·1 to 7·9)
California	28·1 (27·7 to 28·5)	23·7 (23·3 to 24·1)	18·4 (18·1 to 18·8)	12·3 (11·9 to 12·6)	9·4 (9·1 to 9·6)	6·3 (6·1 to 6·5)	5·2 (5·1 to 5·4)	4·3 (4·1 to 4·5)
Colorado	31·2 (30·8 to 31·5)	26·4 (25·5 to 27·4)	20 (19 to 21·1)	11 (10·4 to 11·7)	8·6 (8·2 to 9·1)	6·7 (6·3 to 7·1)	6·1 (5·7 to 6·4)	4·9 (4·6 to 5·2)
Connecticut	22·7 (22·3 to 23·1)	19·9 (19·2 to 20·7)	16·7 (16·1 to 17·4)	11·2 (10·6 to 11·8)	8 (7·6 to 8·5)	6·3 (6 to 6·7)	5·3 (5 to 5·6)	4·6 (3·9 to 5·4)
Delaware	29·5 (28·5 to 30·5)	24·8 (23·4 to 26·2)	20·3 (19·1 to 21·4)	14·5 (13·6 to 15·5)	11·1 (10·4 to 11·8)	9·4 (8·8 to 10)	8·1 (7·6 to 8·7)	7·6 (6·5 to 8·8)
Washington, DC	40·1 (38·6 to 41·7)	39·9 (38·4 to 41·6)	33·4 (31·9 to 35·1)	27·4 (26 to 28·8)	26·9 (25·4 to 28·5)	14·8 (13·9 to 15·9)	11·3 (10·6 to 12·1)	8·4 (7·2 to 9·8)
Florida	37·1 (36·5 to 37·7)	32 (31·2 to 32·8)	24·1 (23·3 to 24·9)	16·4 (15·9 to 17)	11·3 (10·9 to 11·7)	8·2 (7·9 to 8·5)	7·2 (6·9 to 7·5)	6·2 (5·9 to 6·5)
Georgia	35·8 (35·2 to 36·4)	33·1 (32·1 to 34)	23·8 (23 to 24·7)	15·8 (15·2 to 16·5)	12·6 (12 to 13·1)	8·9 (8·6 to 9·3)	7·2 (6·8 to 7·5)	7·2 (6·9 to 7·6)
Hawaii	27·1 (26·2 to 28)	23·1 (22 to 24·4)	18·1 (17·2 to 19)	10·8 (10·2 to 11·5)	7·9 (7·5 to 8·4)	7·4 (7 to 7·9)	6·2 (5·8 to 6·5)	5·5 (4·7 to 6·5)
Idaho	26·7 (25·8 to 27·5)	23·5 (22·5 to 24·6)	18·7 (17·8 to 19·6)	11·7 (11 to 12·3)	9·3 (8·7 to 9·9)	7·1 (6·7 to 7·6)	5·7 (5·3 to 6)	5·1 (4·4 to 5·9)
Illinois	27·6 (27·2 to 28)	25·6 (25 to 26·2)	22·4 (21·9 to 23)	15·5 (15 to 16)	11·2 (10·9 to 11·6)	8·7 (8·3 to 9)	7·1 (6·8 to 7·5)	5·9 (5·6 to 6·2)
Indiana	26 (25·6 to 26·4)	23·9 (23·2 to 24·6)	20·1 (19·3 to 20·8)	12·4 (11·8 to 12·9)	9·6 (9·1 to 10·1)	8·2 (7·9 to 8·6)	7·7 (7·3 to 8·2)	7·1 (6·7 to 7·4)
Iowa	22·4 (22 to 22·9)	21·1 (20·4 to 21·9)	18·6 (17·9 to 19·4)	11·1 (10·6 to 11·7)	7·8 (7·4 to 8·2)	6·2 (5·9 to 6·6)	5·1 (4·8 to 5·4)	5·2 (4·5 to 6·1)
Kansas	24·3 (23·8 to 24·8)	22 (21·2 to 22·8)	18·7 (17·9 to 19·5)	11·9 (11·2 to 12·4)	8·6 (8·2 to 9·1)	7·5 (7·1 to 7·8)	6·8 (6·4 to 7·2)	5·8 (5 to 6·8)
Kentucky	33·1 (32·5 to 33·7)	29·4 (28·5 to 30·4)	21·3 (20·3 to 22·3)	13 (12·4 to 13·7)	8·7 (8·3 to 9·3)	7·3 (6·8 to 7·7)	7·3 (6·9 to 7·8)	6·8 (6·4 to 7·1)
Louisiana	38·4 (37·7 to 39·1)	34 (32·9 to 35·1)	25·4 (24·6 to 26·4)	15·7 (15 to 16·5)	11·7 (11·1 to 12·2)	10·1 (9·6 to 10·7)	8·2 (7·8 to 8·6)	8·3 (7·8 to 8·7)
Maine	30·6 (29·6 to 31·7)	26 (24·9 to 27·1)	20·3 (19·4 to 21·3)	9·8 (9·2 to 10·5)	7·1 (6·6 to 7·6)	5·7 (5·3 to 6·1)	6·3 (5·9 to 6·6)	6·1 (5·3 to 7·2)
Maryland	30 (29·5 to 30·5)	27·7 (26·9 to 28·6)	19·9 (19·1 to 20·8)	14·3 (13·6 to 15)	10·2 (9·7 to 10·7)	8·3 (7·9 to 8·7)	7·2 (6·8 to 7·6)	6·7 (6·3 to 7·1)
Massachusetts	23·5 (23·2 to 23·7)	20·5 (19·8 to 21·2)	17 (16·3 to 17·7)	10·6 (10·1 to 11·1)	7·2 (6·8 to 7·5)	5 (4·7 to 5·3)	4·6 (4·3 to 4·8)	4 (3·4 to 4·8)
Michigan	25·6 (25·2 to 26)	23·8 (23·1 to 24·5)	20·8 (20·3 to 21·4)	12·8 (12·3 to 13·3)	10·6 (10·2 to 11·1)	8·2 (7·9 to 8·6)	7·2 (6·9 to 7·6)	6·5 (6·1 to 6·8)
Minnesota	23·6 (23·3 to 23·9)	21·9 (21·2 to 22·5)	17·7 (16·9 to 18·5)	10·2 (9·7 to 10·7)	6·9 (6·5 to 7·3)	5·5 (5·2 to 5·8)	4·8 (4·5 to 5)	4·7 (4·5 to 4·9)
Mississippi	50 (49 to 51)	42·6 (41·2 to 44·3)	31·2 (30 to 32·6)	18·2 (17·3 to 19·1)	13·2 (12·4 to 13·9)	11·6 (10·9 to 12·3)	10·3 (9·8 to 11)	9·2 (7·7 to 11)
Missouri	27·4 (27 to 27·9)	24·7 (23·9 to 25·4)	20·6 (20 to 21·3)	13·3 (12·7 to 13·9)	9·7 (9·3 to 10·1)	7·6 (7·2 to 8)	6·8 (6·4 to 7·1)	6·2 (5·9 to 6·5)
Montana	30·5 (29·4 to 31·5)	26·9 (25·7 to 28·3)	22·4 (21·2 to 23·6)	12·6 (11·9 to 13·4)	9·1 (8·6 to 9·8)	7·4 (6·9 to 8)	6·6 (6·2 to 7·1)	6 (5·2 to 7)
Nebraska	25·5 (25 to 26·1)	22·7 (21·7 to 23·6)	18·4 (17·5 to 19·3)	11·7 (11·1 to 12·4)	8·2 (7·7 to 8·7)	7·3 (6·8 to 7·7)	5·8 (5·4 to 6·2)	5·8 (4·9 to 6·8)
Nevada	44·7 (43·2 to 46·2)	34·4 (32·5 to 36·2)	23·4 (22 to 24·6)	12·9 (12·2 to 13·6)	9·1 (8·6 to 9·6)	7·1 (6·7 to 7·5)	6·1 (5·8 to 6·4)	5·3 (4·6 to 6·1)
New Hampshire	27·4 (26·6 to 28·3)	22·9 (21·7 to 24·2)	18·2 (17·2 to 19·2)	10·3 (9·6 to 10·9)	6·8 (6·3 to 7·2)	5 (4·7 to 5·4)	4·8 (4·5 to 5·2)	4·1 (3·5 to 4·8)
New Jersey	26·8 (26·4 to 27·2)	24 (23·3 to 24·8)	20·3 (19·6 to 20·9)	12·8 (12·3 to 13·3)	9·7 (9·2 to 10·1)	6·6 (6·3 to 7)	5·2 (4·9 to 5·5)	4·4 (4·1 to 4·6)
New Mexico	44·1 (42·9 to 45·2)	36·6 (35·2 to 38)	23·7 (22·6 to 24·7)	13 (12·2 to 13·7)	9·4 (8·9 to 9·9)	7·1 (6·6 to 7·5)	6·4 (5·9 to 6·8)	5·8 (5 to 6·8)
New York	27·3 (26·9 to 27·7)	24·2 (23·7 to 24·7)	20·4 (20 to 20·8)	13·2 (12·8 to 13·6)	10·7 (10·4 to 11·1)	6·8 (6·5 to 7·1)	5·5 (5·3 to 5·8)	4·6 (4·3 to 4·8)
North Carolina	36·9 (36·3 to 37·6)	32·1 (31·3 to 33)	25·7 (24·8 to 26·5)	14·8 (14·2 to 15·3)	10·9 (10·4 to 11·5)	9·1 (8·7 to 9·5)	7·4 (7 to 7·7)	6·9 (6·5 to 7·2)
North Dakota	26·6 (25·7 to 27·4)	24·4 (23·3 to 25·6)	16·6 (15·6 to 17·6)	11·8 (11·1 to 12·6)	8·5 (7·9 to 9·1)	7·4 (6·9 to 7·9)	6·3 (5·8 to 6·9)	6·1 (5·2 to 7·2)
Ohio	25·7 (25·4 to 26·1)	23·9 (23·3 to 24·7)	19·1 (18·6 to 19·5)	13 (12·6 to 13·5)	9·6 (9·2 to 9·9)	7·9 (7·6 to 8·2)	7·8 (7·5 to 8·2)	7·1 (6·7 to 7·4)
Oklahoma	28·8 (28·4 to 29·2)	26·2 (25·3 to 27·2)	21·6 (20·6 to 22·5)	13 (12·4 to 13·7)	9·6 (9·1 to 10·1)	8·6 (8·1 to 9·1)	8·3 (7·8 to 8·8)	7·6 (7·2 to 8)
Oregon	25·4 (24·9 to 25·9)	23 (22·1 to 23·8)	18 (17·2 to 18·8)	11·9 (11·3 to 12·5)	8·2 (7·8 to 8·7)	6 (5·7 to 6·4)	5·2 (4·9 to 5·5)	4·7 (4 to 5·5)
Pennsylvania	26·7 (26·3 to 27·1)	24·1 (23·4 to 24·8)	20·7 (20·3 to 21·2)	13·4 (13 to 13·8)	9·6 (9·3 to 10)	7·2 (6·9 to 7·5)	7·2 (6·9 to 7·5)	6·1 (5·8 to 6·4)
Rhode Island	26·8 (25·9 to 27·8)	23·2 (22·1 to 24·3)	19·7 (18·7 to 20·8)	12·4 (11·6 to 13·1)	8·1 (7·6 to 8·6)	6·4 (5·9 to 6·8)	6 (5·6 to 6·5)	5·2 (4·4 to 6·1)
South Carolina	39·1 (38·4 to 39·8)	35·8 (34·6 to 36·9)	24·8 (23·7 to 25·9)	17·2 (16·3 to 18·1)	12·5 (11·9 to 13·2)	9·1 (8·6 to 9·7)	7·6 (7·2 to 8)	6·6 (6·3 to 7)
South Dakota	29·5 (28·5 to 30·6)	27·4 (26·2 to 28·7)	21·5 (20·4 to 22·7)	12·9 (12·2 to 13·7)	10 (9·4 to 10·6)	7·7 (7·3 to 8·2)	7·1 (6·6 to 7·6)	6·3 (5·4 to 7·4)
Tennessee	33·2 (32·6 to 33·7)	29·4 (28·6 to 30·3)	22·6 (21·7 to 23·5)	13·9 (13·3 to 14·5)	10·7 (10·2 to 11·2)	9·3 (8·8 to 9·8)	7·9 (7·4 to 8·3)	7 (6·6 to 7·3)
Texas	33·7 (33·1 to 34·2)	29·9 (29·3 to 30·6)	22·6 (22·1 to 23·1)	13·7 (13·3 to 14·1)	8·8 (8·6 to 9·1)	6·7 (6·4 to 6·9)	6·6 (6·3 to 6·8)	5·9 (5·6 to 6·2)
Utah	22·9 (22·5 to 23·4)	19·8 (18·8 to 20·8)	15·9 (15·1 to 16·7)	11 (10·5 to 11·6)	7·6 (7·2 to 8)	5·6 (5·3 to 6)	5 (4·7 to 5·3)	4·9 (4·2 to 5·7)
Vermont	29·9 (28·9 to 30·8)	25 (23·7 to 26·4)	17·4 (16·4 to 18·5)	10·6 (9·8 to 11·3)	7·3 (6·8 to 8)	6 (5·6 to 6·5)	5·2 (4·7 to 5·7)	4·4 (3·7 to 5·2)
Virginia	33·2 (32·7 to 33·8)	29·3 (28·5 to 30·2)	21·4 (20·6 to 22·2)	13·8 (13·2 to 14·5)	10·1 (9·6 to 10·6)	7·3 (6·9 to 7·6)	7·1 (6·8 to 7·5)	5·9 (5·6 to 6·2)
Washington	24·3 (24 to 24·6)	22·2 (21·4 to 23)	18·9 (18·1 to 19·8)	11·8 (11·2 to 12·4)	8·1 (7·7 to 8·5)	5·7 (5·4 to 6)	4·8 (4·5 to 5·1)	4·4 (4·2 to 4·6)

West Virginia	30 (29·3 to 30·7)	25·8 (24·8 to 26·8)	23·3 (22·3 to 24·3)	13 (12·3 to 13·7)	9·7 (9·1 to 10·3)	8·1 (7·5 to 8·6)	7·8 (7·3 to 8·3)	7 (6 to 8·2)
Wisconsin	25·6 (25·2 to 26)	23 (22·2 to 23·7)	17·4 (16·7 to 18·3)	10·7 (10·1 to 11·3)	8 (7·6 to 8·4)	6·9 (6·5 to 7·3)	6·1 (5·7 to 6·4)	5·9 (5·5 to 6·2)
Wyoming	32·8 (31·7 to 33·8)	27·8 (26·2 to 29·5)	22·7 (21·3 to 24)	12·4 (11·6 to 13·3)	9·2 (8·6 to 10)	7·2 (6·7 to 7·8)	6·7 (6·2 to 7·2)	5·6 (4·8 to 6·6)
Southern Latin America	109·2 (108 to 110·4)	98·4 (97·3 to 99·4)	70·7 (70 to 71·4)	35·3 (35 to 35·7)	23·3 (23·1 to 23·5)	15·4 (15·1 to 15·7)	11·4 (11·2 to 11·6)	8·6 (8·3 to 9)
Argentina	80·6 (79·5 to 81·8)	72·5 (71·7 to 73·3)	64 (63·2 to 64·8)	35·5 (35·1 to 35·9)	26 (25·7 to 26·3)	17·2 (16·9 to 17·6)	12·5 (12·3 to 12·8)	9·4 (9·3 to 9·6)
Chile	184 (180·7 to 187·1)	152·9 (150·7 to 155·1)	89·3 (87·8 to 90·8)	34·7 (33·9 to 35·4)	17·5 (17·1 to 17·9)	10·3 (10 to 10·7)	8·2 (7·9 to 8·5)	6·3 (5·5 to 7·2)
Uruguay	64·9 (62·8 to 67·3)	51·7 (50·1 to 53·4)	50·9 (49·4 to 52·5)	36·6 (35·4 to 37·9)	20·7 (19·9 to 21·6)	14·7 (14 to 15·3)	10·4 (9·6 to 11·4)	7·4 (6·6 to 8·3)
Western Europe	58·1 (57·2 to 58·9)	36·2 (35·9 to 36·4)	23·4 (23·2 to 23·6)	13 (12·9 to 13·1)	8·1 (8 to 8·2)	5·1 (5·1 to 5·2)	3·9 (3·8 to 3·9)	3·1 (3 to 3·2)
Andorra	20·3 (17 to 24·2)	14·4 (12·1 to 17)	10·3 (8·7 to 12·2)	8·8 (7·4 to 10·6)	6·5 (5·4 to 7·6)	3·7 (3·1 to 4·3)	3 (2·5 to 3·5)	1·9 (1·6 to 2·3)
Austria	67·9 (66·3 to 69·5)	38·3 (37·4 to 39·3)	25·7 (25 to 26·3)	14·6 (14·2 to 15)	8·4 (8·1 to 8·8)	5·1 (4·9 to 5·3)	4 (3·9 to 4·2)	2·9 (2·8 to 3)
Belgium	52·6 (51·4 to 53·8)	29·9 (29·2 to 30·6)	21·1 (20·6 to 21·7)	12·8 (12·4 to 13·2)	8·3 (8 to 8·6)	5·2 (5 to 5·4)	3·8 (3·7 to 4)	3 (2·9 to 3·1)
Cyprus	100·4 (77·1 to 129·3)	76·9 (59·5 to 99)	49·8 (41 to 59·1)	23 (21·6 to 24·5)	11·6 (10·8 to 12·6)	6·5 (5·9 to 7·2)	3·6 (3·2 to 4)	3·1 (2·5 to 3·8)
Denmark	30·4 (29·5 to 31·5)	21·7 (21 to 22·4)	14 (13·5 to 14·4)	8·7 (8·3 to 9·1)	7·8 (7·4 to 8·1)	5 (4·8 to 5·2)	3·7 (3·5 to 3·9)	3·2 (3 to 3·4)
Finland	45·2 (43·9 to 46·5)	22·7 (22 to 23·4)	13·9 (13·4 to 14·5)	7·7 (7·3 to 8)	6 (5·7 to 6·4)	3·8 (3·6 to 4·1)	2·7 (2·6 to 2·9)	2 (1·9 to 2·2)
France	50 (49·2 to 50·8)	23·8 (23·5 to 24·2)	15·8 (15·6 to 16)	10·5 (10·4 to 10·7)	7·5 (7·4 to 7·6)	4·7 (4·6 to 4·9)	3·8 (3·7 to 3·9)	3·2 (3·1 to 3·4)
Germany	58·2 (57·1 to 59·4)	36·5 (36 to 37)	22·8 (22·5 to 23·1)	13·5 (13·2 to 13·7)	7·5 (7·4 to 7·7)	4·8 (4·7 to 4·9)	3·7 (3·6 to 3·8)	3·1 (3 to 3·1)
Greece	62·7 (59·8 to 65·8)	43·2 (42·1 to 44·3)	29·4 (28·6 to 30·3)	17·7 (17·1 to 18·2)	9·7 (9·3 to 10)	5·7 (5·4 to 6)	3·9 (3·7 to 4·1)	3·4 (3·2 to 3·7)
Iceland	27·5 (24·2 to 31·3)	18·9 (17 to 21·1)	14·1 (12·6 to 16)	8·7 (7·6 to 9·9)	6·3 (5·4 to 7·3)	3·6 (3 to 4·3)	2·5 (2 to 3·1)	2·1 (1·6 to 2·9)
Ireland	46·2 (44·6 to 47·7)	29·5 (28·6 to 30·5)	19·6 (18·9 to 20·3)	12·3 (11·8 to 12·8)	8·1 (7·7 to 8·5)	6·1 (5·8 to 6·4)	3·9 (3·7 to 4·1)	2·9 (2·8 to 3·1)
Israel	42·1 (40·9 to 43·3)	37·4 (36·4 to 38·4)	25 (24·3 to 25·7)	17·1 (16·6 to 17·6)	11·1 (10·7 to 11·4)	6·4 (6·2 to 6·6)	4·2 (4·1 to 4·4)	2·9 (2·9 to 3)
Italy	75·8 (74·4 to 77·1)	48·2 (47·4 to 48·9)	30·2 (29·8 to 30·7)	14·8 (14·5 to 15)	8·6 (8·5 to 8·8)	5·1 (5 to 5·1)	3·6 (3·5 to 3·7)	2·7 (2·7 to 2·8)
Luxembourg	46·4 (42 to 51·2)	32·9 (30·5 to 35·7)	20·6 (18·7 to 22·7)	11·8 (10·4 to 13·2)	8·1 (7·1 to 9·3)	4·5 (3·8 to 5·2)	2·6 (2·2 to 3·2)	2·2 (1·6 to 3·1)
Malta	97·4 (92·5 to 102·4)	35·9 (33·7 to 38·1)	24·2 (22·2 to 26)	14 (12·9 to 15·4)	9·4 (8·5 to 10·3)	6·4 (5·6 to 7·3)	6·1 (5·4 to 7)	5·3 (4·3 to 6·5)
Monaco	55 (47·7 to 63·1)	32·1 (27·6 to 37)	16 (13·4 to 18·9)	9 (7·5 to 10·9)	6·2 (5·1 to 7·5)	4·4 (3·6 to 5·2)	3·4 (2·8 to 4)	2·7 (2·3 to 3·2)
Netherlands	27·7 (27 to 28·4)	18·2 (17·8 to 18·6)	13·9 (13·6 to 14·2)	9·4 (9·1 to 9·7)	7·4 (7·3 to 7·6)	5·5 (5·3 to 5·7)	4 (3·9 to 4·2)	3·4 (3·3 to 3·5)
Norway	30·9 (29·7 to 32)	20·7 (20 to 21·4)	12·6 (12·2 to 13)	7·8 (7·5 to 8·2)	7·7 (7·4 to 8·1)	4·4 (4·2 to 4·6)	3·1 (2·9 to 3·2)	2·4 (2·3 to 2·5)
Portugal	131 (127·9 to 134)	99 (96·8 to 101·2)	63·2 (61·8 to 64·7)	25·5 (24·8 to 26·1)	12·4 (12 to 12·8)	6·4 (6·1 to 6·7)	3·2 (3 to 3·4)	2·4 (2·3 to 2·6)
San Marino	28·2 (23·5 to 34·2)	20·5 (17·3 to 24·1)	16·1 (13·9 to 18·5)	12 (10·3 to 14)	8·6 (7·3 to 10)	5·6 (4·7 to 6·7)	4·1 (3·4 to 4·9)	3·6 (3 to 4·3)
Spain	92·3 (90·6 to 94)	48·6 (47·8 to 49·4)	28·9 (28·4 to 29·4)	13·4 (13·1 to 13·6)	8·4 (8·2 to 8·6)	4·9 (4·8 to 5·1)	3·6 (3·5 to 3·7)	2·7 (2·7 to 2·8)
Sweden	22·8 (22·1 to 23·6)	17·4 (16·9 to 17·9)	11·3 (10·9 to 11·7)	7·2 (6·9 to 7·5)	6 (5·8 to 6·3)	3·4 (3·2 to 3·6)	2·8 (2·7 to 3)	2·4 (2·3 to 2·6)
Stockholm	22·4 (21·7 to 23·2)	17·9 (16·5 to 19·4)	12·2 (11·1 to 13·2)	7·9 (7·2 to 8·5)	6 (5·5 to 6·5)	3·5 (3·2 to 3·8)	2·6 (2·3 to 2·9)	2·1 (2 to 2·3)
Sweden except Stockholm	24·1 (23·3 to 24·9)	18·2 (17·6 to 18·9)	11·9 (11·4 to 12·4)	7·2 (6·9 to 7·6)	6·1 (5·8 to 6·4)	3·5 (3·4 to 3·7)	2·9 (2·8 to 3·1)	2·5 (2·3 to 2·7)
Switzerland	34·2 (33·2 to 35·3)	23·3 (22·7 to 23·9)	16·2 (15·8 to 16·7)	9·4 (9·1 to 9·7)	7·4 (7·1 to 7·6)	5·3 (5·1 to 5·5)	4·1 (4 to 4·3)	3·4 (3·3 to 3·6)
UK	29·9 (29·4 to 30·4)	22·9 (22·6 to 23·1)	18·7 (18·4 to 19)	12·3 (12·1 to 12·5)	8·2 (8·1 to 8·3)	5·8 (5·6 to 5·9)	4·6 (4·5 to 4·7)	3·7 (3·6 to 3·7)
England	28 (27·5 to 28·6)	21·8 (21·3 to 22·2)	18 (17·6 to 18·4)	11·9 (11·6 to 12·2)	8·2 (8·1 to 8·4)	5·8 (5·7 to 5·9)	4·6 (4·5 to 4·7)	3·8 (3·6 to 3·9)
East Midlands	27 (26·5 to 27·5)	21 (19·5 to 22·5)	17·5 (16·1 to 18·9)	11·4 (10·8 to 12)	8·1 (7·8 to 8·4)	5·7 (5·4 to 6)	4·7 (4·4 to 4·9)	3·9 (3·7 to 4)
Derby	36·3 (35·4 to 37·2)	28·3 (25 to 32·2)	23·9 (21·1 to 26·9)	15·8 (13·7 to 17·7)	10·4 (9·4 to 11·5)	7·3 (6·7 to 8·1)	6·5 (5·9 to 7·2)	5·5 (5·3 to 5·7)
Derbyshire	28·8 (28·1 to 29·5)	22·2 (20 to 24·4)	18·5 (16·9 to 20·4)	12·1 (11·4 to 12·7)	7·8 (7·3 to 8·3)	5·4 (5 to 5·8)	4·1 (3·8 to 4·4)	3·4 (3·3 to 3·6)
Leicester	37·3 (36·4 to 38·3)	29·3 (25·7 to 33·4)	23·7 (21·1 to 26·6)	15·6 (13·8 to 17·3)	10·7 (10 to 11·4)	7·9 (7·1 to 8·7)	6·4 (5·8 to 7·1)	5·2 (5 to 5·4)
Leicestershire	24·9 (24·3 to 25·5)	19·2 (17·2 to 21·2)	16·2 (14·6 to 18)	10·8 (9·8 to 11·9)	8·1 (7·3 to 8·8)	5·1 (4·8 to 5·5)	4·3 (4 to 4·6)	3·6 (3·5 to 3·7)
Lincolnshire	27·2 (26·6 to 27·8)	21·1 (19 to 23·3)	17·5 (15·7 to 19·3)	11·4 (10·7 to 12·1)	8·5 (8·1 to 9·1)	6 (5·7 to 6·4)	4·6 (4·3 to 4·9)	3·4 (3·2 to 3·5)
Northamptonshire	28·8 (28·2 to 29·5)	22 (19·8 to 24·2)	18·3 (16·5 to 20·2)	11·9 (10·6 to 13·2)	9·2 (8·6 to 9·9)	5·8 (5·2 to 6·3)	4·9 (4·4 to 5·5)	4·4 (4·3 to 4·6)
Nottingham	34·6 (33·8 to 35·5)	27·4 (24·1 to 31)	22·7 (20·4 to 25·2)	14·8 (12·9 to 17)	10·8 (10 to 11·5)	8·7 (8·1 to 9·5)	6·6 (6 to 7·1)	5·2 (5 to 5·4)
Nottinghamshire	25·1 (24·7 to 25·6)	19·5 (17·3 to 21·8)	16·5 (14·9 to 18·1)	10·9 (10·3 to 11·4)	7·5 (7 to 7·9)	5·5 (5·1 to 5·9)	4·5 (4·2 to 4·9)	3·9 (3·8 to 4·1)
Rutland	18·3 (17·9 to 18·7)	13·6 (11·1 to 16·4)	11·7 (10·5 to 13)	8·7 (7·8 to 9·6)	6·3 (5·4 to 7·3)	4·2 (3·6 to 4·9)	3 (2·5 to 3·5)	1·7 (1·7 to 1·8)
East of England	24 (23·5 to 24·4)	18·5 (17·2 to 19·8)	15·4 (14 to 16·9)	10·2 (9·6 to 11)	6·5 (6·2 to 6·8)	4·7 (4·5 to 5)	4·1 (3·9 to 4·3)	3·2 (3·1 to 3·3)
Bedford	28·7 (28 to 29·3)	22·1 (19·8 to 24·5)	18·7 (16·5 to 21)	12·7 (10·9 to 14·7)	8·7 (7·7 to 9·8)	6·5 (5·9 to 7·2)	5·6 (4·9 to 6·2)	4·4 (4 to 4·8)
Cambridgeshire	20·4 (20 to 20·8)	15·7 (14 to 17·5)	13·2 (11·7 to 14·8)	9 (8 to 10)	5·9 (5·4 to 6·4)	4·8 (4·4 to 5·2)	4 (3·6 to 4·4)	3·2 (3 to 3·4)

Central Bedfordshire	25·8 (25·2 to 26·4)	19·6 (17·8 to 21·4)	16·3 (14·5 to 18·4)	10·9 (9·4 to 12·7)	6·5 (6 to 7·2)	4·5 (4 to 5)	3·5 (3·2 to 3·9)	2·8 (2·5 to 3)
Essex	23·7 (23·2 to 24·2)	18·2 (16·8 to 19·8)	15·2 (13·6 to 16·8)	10·1 (9·1 to 11·1)	6·5 (5·9 to 7·1)	4·5 (4·2 to 5)	4 (3·7 to 4·3)	3·1 (2·8 to 3·6)
Hertfordshire	22 (21·6 to 22·5)	17·3 (15·9 to 18·9)	14·8 (12·9 to 17·1)	10 (9·4 to 10·8)	6·4 (5·8 to 6·9)	4·6 (4·2 to 5)	3·8 (3·5 to 4·1)	2·9 (2·6 to 3·3)
Luton	30·5 (29·8 to 31·2)	23·9 (21·6 to 26·5)	20·3 (18·1 to 22·7)	13·6 (12 to 15·3)	9·2 (8·4 to 10·1)	8·1 (7·4 to 8·8)	6·7 (6 to 7·4)	5·3 (4·9 to 5·6)
Norfolk	30·5 (29·9 to 31·2)	23 (19·5 to 26·9)	18·7 (16·5 to 21·3)	12·2 (11·1 to 13·3)	7·2 (6·6 to 7·8)	5·1 (4·7 to 5·6)	4·4 (4·1 to 4·8)	3·4 (3·1 to 3·6)
Peterborough	24·2 (23·9 to 24·5)	19·1 (16·2 to 22·2)	16·4 (14·7 to 18·4)	11·1 (9·9 to 12·5)	7·2 (6·5 to 8)	6·3 (5·6 to 7)	5·2 (4·7 to 5·7)	4·1 (3·8 to 4·4)
Southend-on-Sea	33·1 (32·4 to 34)	25·1 (22·4 to 28)	20·2 (18·1 to 22·5)	13 (11·2 to 14·9)	7·9 (7·1 to 8·9)	5·2 (4·7 to 5·8)	4·4 (3·8 to 4·9)	3·6 (3·4 to 3·9)
Suffolk	24·6 (24·2 to 25·1)	18·6 (15·8 to 21·5)	15·3 (13·6 to 17·2)	9·9 (8·7 to 11·3)	6·4 (5·9 to 6·9)	4·4 (4 to 4·8)	4 (3·6 to 4·3)	3 (2·8 to 3·2)
Thurrock	27·9 (27·2 to 28·6)	22·4 (20·3 to 24·7)	18·9 (16·9 to 21·1)	12·3 (10·6 to 14·2)	7·7 (6·9 to 8·5)	5·9 (5·2 to 6·6)	4·7 (4·2 to 5·3)	3·6 (3·3 to 4)
Greater London	29·7 (29·1 to 30·3)	23·4 (22·4 to 24·4)	19·2 (18·3 to 20·1)	13 (12·4 to 13·5)	8·8 (8·5 to 9·2)	6·4 (6·1 to 6·6)	4·9 (4·6 to 5·1)	3·5 (3·3 to 3·6)
Barking and Dagenham	40·9 (39·9 to 42)	33·3 (31 to 35·9)	27·4 (25·2 to 29·7)	17·1 (15·7 to 18·7)	9·7 (8·8 to 10·7)	6·2 (5·6 to 6·8)	5·2 (4·6 to 5·8)	3·9 (3·8 to 4)
Barnet	29·2 (28·6 to 29·9)	23 (21·5 to 24·6)	18·9 (17·4 to 20·5)	12·8 (11·8 to 13·9)	7·8 (7·1 to 8·5)	5·5 (5 to 6·1)	4·3 (3·9 to 4·7)	3·1 (3 to 3·1)
Bexley	23·2 (22·7 to 23·8)	18·6 (17·6 to 19·7)	15·4 (14·4 to 16·5)	10·2 (9·5 to 11)	6·7 (6·2 to 7·3)	5 (4·6 to 5·5)	4·2 (3·8 to 4·6)	3·2 (3·1 to 3·3)
Brent	32·3 (31·6 to 33·1)	25·5 (23·6 to 27·4)	21 (19·1 to 22·8)	14·3 (13·2 to 15·5)	10·5 (9·5 to 11·5)	8·3 (7·8 to 9)	5·9 (5·4 to 6·4)	4 (3·9 to 4·2)
Bromley	22·5 (22 to 23)	18·1 (17 to 19·2)	15·2 (14 to 16·5)	10·2 (9·3 to 11·2)	7·3 (6·7 to 7·9)	4·9 (4·5 to 5·3)	3·4 (3·1 to 3·7)	2·7 (2·6 to 2·8)
Camden	32·3 (31·5 to 33·1)	24·5 (22·9 to 26·1)	19·5 (18 to 20·9)	14·1 (13·1 to 15·2)	9·6 (8·8 to 10·4)	6·1 (5·6 to 6·6)	4·3 (3·9 to 4·8)	2·7 (2·6 to 2·8)
Croydon	27·9 (27·2 to 28·5)	22·2 (20·8 to 23·8)	18·3 (16·8 to 19·9)	12·2 (10·9 to 13·5)	9·4 (8·8 to 10·1)	7 (6·5 to 7·5)	5·1 (4·7 to 5·6)	3·7 (3·6 to 3·9)
Ealing	30 (29·4 to 30·8)	23·6 (22·2 to 25)	19·2 (17·8 to 20·7)	13 (12 to 14·1)	8·8 (8·1 to 9·5)	6 (5·6 to 6·5)	4·6 (4·2 to 5)	3·1 (3 to 3·2)
Enfield	27·7 (27·1 to 28·4)	22·2 (20·5 to 24)	18·1 (16·5 to 19·6)	12·1 (11·1 to 13·2)	7·9 (7·2 to 8·7)	6·4 (5·8 to 7)	5·5 (4·8 to 6·2)	3·7 (3·6 to 3·9)
Greenwich	38·4 (37·5 to 39·4)	30·8 (28·6 to 33·2)	25·4 (23·2 to 27·6)	16·7 (15·3 to 18·2)	9·7 (9 to 10·5)	6·4 (5·8 to 7)	5·1 (4·7 to 5·5)	4·2 (4 to 4·3)
Hackney	36·5 (35·7 to 37·4)	29 (26·9 to 31·2)	24·2 (22·3 to 26·3)	16·7 (15·5 to 18)	11·8 (10·8 to 12·8)	9 (8·3 to 9·8)	6·3 (5·7 to 6·9)	4·5 (4·4 to 4·7)
Hammersmith and Fulham	30·9 (30·2 to 31·7)	23·7 (22·5 to 25·1)	18·8 (17·6 to 20)	13·5 (12·5 to 14·5)	10·9 (10 to 11·9)	6·7 (6·1 to 7·2)	4·5 (4·1 to 5)	3·2 (3·1 to 3·3)
Haringey	33·3 (32·6 to 34·2)	25·6 (23·6 to 27·6)	20·9 (19·3 to 22·7)	14·3 (13·2 to 15·4)	10·1 (9·3 to 11)	8 (7·3 to 8·7)	5·7 (5·2 to 6·1)	3·9 (3·7 to 4)
Harrow	23·1 (22·6 to 23·6)	18·5 (17·4 to 19·6)	15·1 (14·1 to 16·2)	10·2 (9·3 to 11·1)	7·8 (7·1 to 8·5)	6·8 (6·2 to 7·4)	6·1 (5·6 to 6·8)	4·7 (4·5 to 4·8)
Havering	23·6 (23·1 to 24·2)	19·6 (16·5 to 23)	16·8 (15·5 to 18·3)	11·1 (9·6 to 12·7)	7 (6·3 to 7·6)	4·8 (4·4 to 5·1)	4 (3·6 to 4·3)	2·7 (2·6 to 2·8)
Hillingdon	26·5 (25·9 to 27·2)	21·5 (20·1 to 23)	18·1 (16·5 to 19·6)	12·2 (11·1 to 13·4)	8 (7·3 to 8·7)	5·6 (5·1 to 6)	5 (4·6 to 5·5)	3·5 (3·4 to 3·6)
Hounslow	29·9 (29·2 to 30·7)	23·5 (19·8 to 27·3)	19·2 (17·6 to 20·9)	12·8 (11 to 14·8)	9·1 (8·3 to 10)	6·5 (6 to 7·1)	5·6 (5·1 to 6·1)	4·3 (4·1 to 4·4)
Islington	37·4 (36·5 to 38·3)	28·6 (26·7 to 30·6)	23·8 (21·8 to 25·9)	16·6 (15·1 to 18·2)	10·2 (9·3 to 11·2)	6·9 (6·3 to 7·6)	4·4 (4·1 to 4·8)	2·8 (2·7 to 2·9)
Kensington and Chelsea	35 (34·3 to 35·8)	27 (22·6 to 31·6)	21 (19·5 to 22·5)	15·5 (14 to 17)	9·9 (8·9 to 11)	5·9 (5·2 to 6·7)	4·1 (3·5 to 4·7)	3·4 (3·3 to 3·5)
Kingston upon Thames	24·2 (23·6 to 24·8)	19 (16 to 22·3)	15·3 (14·1 to 16·6)	10·2 (9·3 to 11)	6·9 (6·2 to 7·6)	4·5 (3·9 to 5·2)	4·3 (3·8 to 5)	4·1 (3·9 to 4·2)
Lambeth	40·5 (39·5 to 41·6)	31·8 (29·7 to 33·9)	26·8 (24·8 to 29)	18·9 (17·5 to 20·6)	11·4 (10·4 to 12·6)	8·5 (7·7 to 9·2)	6·4 (5·8 to 6·8)	4·4 (4·2 to 4·5)
Lewisham	34·8 (33·9 to 35·7)	27·4 (25·7 to 29·2)	22·9 (21·3 to 24·8)	15·5 (14·3 to 16·9)	10·5 (9·6 to 11·4)	8 (7·3 to 8·7)	5·4 (5 to 5·9)	3·6 (3·4 to 3·7)
Merton	30 (29·3 to 30·7)	23·6 (22·3 to 24·9)	19·4 (18 to 20·7)	13 (12 to 14·1)	8·5 (7·8 to 9·3)	5·4 (4·8 to 5·9)	4·7 (4·2 to 5·2)	3·4 (3·3 to 3·5)
Newham	35·9 (35·1 to 36·8)	28·9 (27 to 31)	23·6 (21·7 to 25·5)	15·7 (14·6 to 16·9)	11 (9·9 to 12·1)	8·8 (7·9 to 9·7)	6 (5·6 to 6·5)	3·9 (3·8 to 4·1)
Redbridge	27·3 (26·7 to 28)	21·9 (18·3 to 25·6)	17·8 (16·3 to 19·5)	11·8 (10·1 to 13·6)	8·6 (7·7 to 9·5)	6·2 (5·7 to 6·8)	4·8 (4·2 to 5·5)	3·4 (3·3 to 3·6)
Richmond upon Thames	23·2 (22·8 to 23·6)	18·3 (17·3 to 19·4)	15 (13·9 to 16·1)	10·2 (9·4 to 11)	6·7 (6·1 to 7·3)	4·3 (3·9 to 4·7)	3·4 (3·1 to 3·8)	2·6 (2·5 to 2·7)
Southwark	30 (29·3 to 30·7)	24·2 (22·5 to 26)	20·2 (18·6 to 21·9)	13·5 (12·3 to 15)	11·6 (10·6 to 12·7)	8·8 (8·2 to 9·6)	5·7 (5·4 to 6·1)	3·6 (3·5 to 3·8)
Sutton	29·2 (28·5 to 29·9)	22·8 (21·6 to 24)	18·7 (17·6 to 20)	12·4 (11·4 to 13·4)	7·9 (7·2 to 8·6)	5·3 (4·9 to 5·8)	4·4 (4 to 4·8)	3·6 (3·5 to 3·7)
Tower Hamlets	41·4 (40·4 to 42·4)	34 (31·5 to 36·6)	28·8 (26·4 to 31·2)	18·6 (17·1 to 20·1)	10·4 (9·5 to 11·5)	7·3 (6·7 to 7·9)	5·5 (4·9 to 6)	4·2 (4 to 4·3)
Waltham Forest	38·9 (38 to 39·9)	30·6 (28·6 to 32·6)	24·4 (22·5 to 26·4)	16·3 (15 to 17·8)	11·5 (10·5 to 12·5)	8·1 (7·6 to 8·8)	5·7 (5·3 to 6·2)	3·5 (3·3 to 3·6)
Wandsworth	34·8 (34 to 35·6)	27·4 (25·8 to 29·2)	22·5 (20·5 to 24·5)	15·7 (14·4 to 17·1)	10·4 (9·5 to 11·4)	5·9 (5·3 to 6·6)	4·1 (3·8 to 4·5)	3 (2·9 to 3·1)
Westminster	40·5 (39·5 to 41·6)	31·2 (26·1 to 36·4)	25·2 (23·1 to 27·5)	18·5 (16 to 21·2)	11·4 (10·4 to 12·4)	6·9 (6·3 to 7·6)	4·3 (3·9 to 4·7)	3 (2·9 to 3·1)
North East England	27·3 (26·7 to 27·9)	21·4 (17·8 to 25·4)	18·1 (15·7 to 20·6)	11·6 (10·7 to 12·5)	8·3 (7·9 to 8·7)	5·8 (5·5 to 6·2)	4·1 (3·8 to 4·4)	3·3 (3·2 to 3·4)
County Durham	25·4 (25 to 25·9)	19·9 (15·5 to 25·2)	16·9 (14·1 to 19·7)	10·9 (9·6 to 12·3)	7·5 (7 to 8)	5·9 (5·5 to 6·4)	4·2 (3·8 to 4·6)	3·6 (3·5 to 3·7)
Darlington	32·7 (31·9 to 33·5)	25·4 (20·2 to 31)	21·6 (18·7 to 24·7)	14·3 (12·7 to 15·8)	9·6 (8·6 to 10·8)	6·7 (5·7 to 7·8)	4·8 (4·1 to 5·6)	3·6 (3·5 to 3·7)
Gateshead	31·5 (30·7 to 32·3)	24·7 (20·8 to 29)	20·9 (18 to 24)	13·7 (12·6 to 14·8)	10 (9 to 11)	6·3 (5·5 to 7·2)	4·9 (4·3 to 5·6)	4·1 (3·9 to 4·2)
Hartlepool	38·1 (37·3 to 39·1)	29·6 (23·3 to 37)	25·5 (21·5 to 29·8)	16·6 (14·2 to 19)	10 (8·9 to 11·2)	6·7 (5·9 to 7·6)	4·7 (4 to 5·5)	3·6 (3·5 to 3·7)
Middlesbrough	35·2 (34·4 to 36·1)	28·2 (21·5 to 36·1)	24 (20·2 to 27·7)	15·1 (13·2 to 17·3)	11·1 (10 to 12·3)	8·1 (7·5 to 8·8)	5·9 (5·3 to 6·4)	4·9 (4·8 to 5·1)

Newcastle upon Tyne	23·9 (23·5 to 24·3)	18·8 (15·9 to 22·1)	15·5 (13·4 to 17·7)	9·9 (8·8 to 11)	7·9 (7·2 to 8·6)	5·3 (4·8 to 5·8)	3·8 (3·5 to 4·2)	3·2 (3·1 to 3·3)
North Tyneside	25·1 (24·6 to 25·7)	19·8 (16·5 to 23·3)	16·9 (14·6 to 19·4)	10·9 (9·6 to 12·2)	8·5 (7·9 to 9·1)	5·6 (5·1 to 6·1)	3·7 (3·4 to 4·1)	2·9 (2·8 to 3)
Northumberland	26·1 (25·5 to 26·7)	20·1 (17 to 23·7)	16·9 (14·7 to 19·3)	10·8 (9·8 to 11·8)	8·1 (7·3 to 8·9)	5·5 (4·8 to 6·2)	3·8 (3·4 to 4·1)	2·9 (2·8 to 3·1)
Redcar and Cleveland	30·4 (29·7 to 31·2)	23·6 (19·7 to 28)	20·1 (17·1 to 23·4)	13·1 (10·9 to 15·6)	8·7 (8 to 9·4)	5·6 (4·9 to 6·2)	3·8 (3·2 to 4·3)	3 (2·9 to 3·1)
South Tyneside	25·6 (25 to 26·2)	20·2 (16·9 to 23·8)	17·3 (15 to 20)	11·2 (9·8 to 12·5)	7·7 (7·1 to 8·3)	5·8 (5·2 to 6·4)	3·9 (3·5 to 4·3)	3 (2·9 to 3·1)
Stockton-on-Tees	27·4 (26·8 to 28)	21·5 (16·4 to 27·6)	18·5 (15·6 to 21·6)	12 (10·3 to 13·9)	8·4 (7·8 to 9·1)	6 (5·5 to 6·6)	4·3 (4 to 4·8)	3·7 (3·6 to 3·9)
Sunderland	28·3 (27·7 to 29)	22·4 (18·9 to 26·6)	18·9 (16·4 to 21·6)	12 (10·8 to 13·2)	9·2 (8·6 to 9·8)	6·5 (6 to 7)	4·3 (3·9 to 4·7)	3·4 (3·3 to 3·5)
North West England	29·7 (29·1 to 30·4)	23 (22 to 24)	19 (18·1 to 19·9)	12·6 (12 to 13·3)	8·5 (8·2 to 8·9)	6·2 (6 to 6·5)	5·1 (4·8 to 5·3)	4·4 (4·3 to 4·6)
Blackburn with Darwen	35·4 (34·5 to 36·3)	28 (24·6 to 31·4)	22·8 (21 to 24·7)	14·7 (13 to 16·7)	12·4 (11·4 to 13·5)	9·5 (8·7 to 10·4)	7·5 (6·9 to 8·2)	5·9 (5·6 to 6·3)
Blackpool	32·6 (31·8 to 33·4)	25·4 (23·3 to 27·7)	20·9 (19·2 to 22·6)	13·6 (12·4 to 15)	9·5 (8·5 to 10·6)	7·8 (7·1 to 8·6)	6·7 (6 to 7·4)	5·9 (5·5 to 6·3)
Bolton	32·7 (32 to 33·5)	25·7 (23·4 to 28)	21·1 (19·2 to 23·2)	13·8 (12·4 to 15·2)	9·9 (9 to 10·7)	6·9 (6·2 to 7·7)	5·5 (5·1 to 6·1)	4·4 (4·1 to 4·6)
Bury	33·5 (32·7 to 34·3)	25·7 (23·7 to 27·9)	21·2 (19·5 to 23)	14·4 (12·9 to 16)	9·4 (8·6 to 10·3)	5·8 (5·3 to 6·4)	4·5 (4·1 to 5)	4 (3·8 to 4·2)
Cheshire East	27·9 (27·2 to 28·6)	21·4 (19·6 to 23·4)	17·9 (16·3 to 19·4)	12·2 (11·1 to 13·3)	7·8 (7·2 to 8·4)	5·1 (4·7 to 5·6)	4·1 (3·7 to 4·5)	3·7 (3·5 to 3·9)
Cheshire West and Chester	22·4 (22·1 to 22·9)	17·5 (16·1 to 19)	14·8 (13·5 to 16·1)	10·1 (9·3 to 11)	6·9 (6·4 to 7·6)	5·1 (4·6 to 5·6)	4·6 (4·1 to 5·1)	3·7 (3·5 to 4)
Cumbria	30·9 (30·2 to 31·6)	23·7 (21 to 26·6)	19·8 (18·1 to 21·7)	13·2 (11·7 to 14·7)	8·3 (7·7 to 8·9)	5·3 (4·9 to 5·7)	4·1 (3·8 to 4·5)	3·4 (3·2 to 3·5)
Halton	28·8 (28·2 to 29·5)	23 (21 to 25·1)	19·3 (17·8 to 20·9)	12·7 (11·5 to 14)	8·7 (7·8 to 9·6)	6·7 (6·1 to 7·3)	5 (4·5 to 5·5)	3·9 (3·6 to 4·2)
Knowsley	29·8 (29·1 to 30·5)	23·9 (20·4 to 27·6)	21 (19·4 to 22·6)	13·9 (12·6 to 15·4)	7·7 (7·1 to 8·4)	5·8 (5·2 to 6·5)	4 (3·5 to 4·7)	3·4 (3·2 to 3·6)
Lancashire	30·8 (30·1 to 31·5)	23·5 (21 to 26)	19·2 (17·3 to 21)	12·6 (11·3 to 14)	9·2 (8·5 to 9·9)	6·5 (5·9 to 7·1)	5·3 (4·8 to 5·9)	4·7 (4 to 5·5)
Liverpool	29·2 (28·5 to 29·9)	22·9 (19·9 to 25·9)	18·8 (17·1 to 20·8)	12·5 (11·2 to 14·1)	7·8 (7·3 to 8·4)	6·6 (6·2 to 7·2)	5·3 (4·9 to 5·7)	5 (4·7 to 5·2)
Manchester	36·8 (35·9 to 37·8)	28·7 (24·5 to 32·9)	23·6 (21·5 to 25·8)	15·7 (14 to 17·7)	10·9 (10 to 11·9)	8·9 (8·2 to 9·6)	6·6 (6·1 to 7·1)	5·9 (5 to 6·9)
Oldham	36·6 (35·7 to 37·5)	28·6 (26·1 to 31·4)	23·4 (21·2 to 25·4)	15·4 (13·9 to 17)	10·4 (9·6 to 11·2)	7·7 (7 to 8·4)	6·6 (6·1 to 7·1)	5·7 (5·4 to 5·9)
Rochdale	39 (38·1 to 40)	30·9 (28·2 to 33·8)	25·8 (23·7 to 28)	16·8 (15·3 to 18·5)	11·1 (10·3 to 11·9)	8·1 (7·5 to 8·8)	6 (5·4 to 6·5)	5·6 (5·3 to 5·8)
Salford	33·5 (32·8 to 34·4)	26 (24 to 28·2)	21·6 (19·8 to 23·4)	14·3 (13 to 15·7)	9·1 (8·4 to 9·8)	6·6 (5·9 to 7·4)	5·4 (4·9 to 6)	4·5 (4·2 to 4·8)
Sefton	24·6 (24·1 to 25·2)	19·1 (17·4 to 20·9)	16 (14·5 to 17·5)	10·7 (9·7 to 11·8)	7·8 (7·2 to 8·4)	5·7 (5·3 to 6·2)	4·8 (4·4 to 5·2)	4·2 (4 to 4·4)
St Helens	27·3 (26·7 to 28)	21·6 (18·8 to 24·3)	18·2 (16·8 to 19·8)	12·5 (10·9 to 14·2)	8·4 (7·6 to 9·2)	6·9 (6·3 to 7·6)	4·5 (4·2 to 4·9)	3·8 (3·5 to 4)
Stockport	25·3 (24·8 to 25·9)	19·6 (17·8 to 21·4)	16·5 (15 to 18)	11·2 (10·1 to 12·4)	8 (7·3 to 8·7)	5·6 (5·2 to 6·1)	4·5 (4·1 to 5)	4·3 (4 to 4·5)
Tameside	34·6 (33·8 to 35·5)	27·1 (24·9 to 29·5)	22·2 (20·4 to 23·9)	14·5 (13·1 to 16·2)	8·6 (7·8 to 9·4)	6 (5·5 to 6·5)	4·7 (4·4 to 5·1)	3·9 (3·7 to 4·1)
Trafford	32·4 (31·7 to 33·3)	25 (22·9 to 27·3)	21·3 (19·6 to 23)	14·7 (13·2 to 16·4)	8·8 (8 to 9·7)	5·4 (4·9 to 5·8)	3·8 (3·5 to 4·1)	3·7 (3·5 to 3·9)
Warrington	25·6 (25 to 26·2)	20·1 (18·4 to 21·9)	16·7 (15·4 to 18·1)	11·4 (10·3 to 12·6)	9·3 (8·6 to 10·1)	6·4 (5·8 to 7)	5 (4·5 to 5·6)	4·1 (3·8 to 4·3)
Wigan	26·4 (25·9 to 27)	20·6 (18·9 to 22·5)	16·9 (15·6 to 18·3)	11·2 (10·2 to 12·5)	7·7 (7·1 to 8·3)	5·9 (5·5 to 6·4)	4·2 (3·9 to 4·5)	3·5 (3·3 to 3·7)
Wirral	32·2 (31·4 to 33)	24·8 (22·9 to 26·7)	20·5 (18·8 to 22·3)	13·5 (12·3 to 14·9)	8·9 (8 to 9·7)	6·2 (5·7 to 6·7)	5·2 (4·8 to 5·7)	4·7 (4·4 to 4·9)
South East England	26·2 (25·8 to 26·7)	19·5 (18·6 to 20·3)	15·6 (14·9 to 16·3)	10·4 (9·9 to 10·9)	7·4 (7·1 to 7·6)	4·5 (4·3 to 4·8)	3·8 (3·6 to 4)	3·3 (3·2 to 3·5)
Bracknell Forest	21 (20·7 to 21·3)	15·7 (13·2 to 18·2)	13 (12·1 to 14)	8·7 (7·6 to 9·9)	6 (5·3 to 6·8)	3·9 (3·4 to 4·5)	3·1 (2·7 to 3·6)	2·1 (1·9 to 2·2)
Brighton and Hove	35·4 (34·5 to 36·3)	25·7 (22·3 to 28·9)	20·3 (18·4 to 22·4)	13·7 (12 to 15·7)	9·7 (8·8 to 10·6)	6·4 (5·8 to 7)	4·9 (4·5 to 5·4)	3·8 (3·5 to 4·1)
Buckinghamshire	23·8 (23·3 to 24·4)	17·9 (16·4 to 19·7)	14·5 (13·3 to 15·8)	9·7 (8·7 to 10·9)	8·3 (7·7 to 8·9)	5·4 (4·8 to 5·9)	4·7 (4·4 to 5·2)	4·2 (4 to 4·4)
East Sussex	29·4 (28·7 to 30·1)	21·1 (19·3 to 23·4)	16·3 (14·7 to 18)	10·6 (9·5 to 11·7)	8 (7·3 to 8·6)	5·4 (5 to 5·9)	4·6 (4·2 to 5)	3·8 (3·7 to 4)
Hampshire	29·9 (29·2 to 30·6)	21·9 (20·2 to 23·6)	17·8 (16·2 to 19·5)	12·1 (11 to 13·2)	7·2 (6·7 to 7·8)	4·2 (3·8 to 4·6)	3·5 (3·2 to 3·8)	3·4 (2·9 to 4)
Isle of Wight	39·6 (38·7 to 40·7)	28·6 (24·2 to 33·4)	22 (20·3 to 23·8)	14·1 (12·5 to 15·9)	9·8 (8·8 to 10·9)	4·6 (4 to 5·3)	3·1 (2·7 to 3·6)	2·6 (2·4 to 2·8)
Kent	29·5 (28·9 to 30·2)	21·7 (19·1 to 24·3)	17·1 (15·5 to 18·7)	11·1 (10 to 12·2)	8·2 (7·6 to 8·7)	4·5 (4·2 to 4·9)	3·7 (3·4 to 4·1)	3·5 (3 to 4·1)
Medway	34·3 (33·5 to 35·2)	25·8 (23·6 to 28·1)	20·6 (18·7 to 22·8)	13·6 (12·2 to 15)	9·6 (8·9 to 10·3)	6 (5·5 to 6·6)	4·2 (3·8 to 4·7)	3·6 (3·4 to 3·9)
Milton Keynes	30·9 (30·1 to 31·6)	23·3 (20·4 to 26)	18·7 (17 to 20·4)	12·2 (10·7 to 13·9)	9·5 (8·7 to 10·4)	6·9 (6·2 to 7·5)	5·6 (5 to 6·2)	4·8 (4·5 to 5·2)
Oxfordshire	22·1 (21·7 to 22·6)	16·4 (15 to 18)	13·3 (12·3 to 14·5)	9 (8·2 to 9·9)	6·7 (6·2 to 7·2)	4·4 (4 to 4·9)	4 (3·7 to 4·4)	3·4 (3·2 to 3·5)
Portsmouth	36·3 (35·4 to 37·2)	26·8 (23·5 to 30·1)	20·8 (19 to 22·8)	13·4 (11·7 to 15·4)	9·8 (8·9 to 10·7)	6·2 (5·6 to 6·8)	3·9 (3·5 to 4·4)	3·3 (3 to 3·5)
Reading	31·1 (30·4 to 31·7)	23·3 (21·2 to 25·6)	18·9 (17·2 to 20·6)	12·8 (11·3 to 14·5)	9·7 (8·7 to 10·8)	7·8 (7 to 8·6)	5·9 (5·3 to 6·5)	4·4 (4·1 to 4·7)
Slough	29·5 (28·9 to 30·2)	23·6 (21·3 to 25·8)	19·4 (17·9 to 21·1)	12·6 (11·1 to 14·2)	9·5 (8·6 to 10·5)	6·1 (5·4 to 6·9)	5·5 (5 to 6)	4·9 (4·5 to 5·2)
Southampton	35·7 (34·9 to 36·7)	26·8 (24·2 to 29·8)	21 (19·2 to 22·9)	13·7 (12·1 to 15·2)	9·2 (8·4 to 10·1)	5·5 (5 to 6·1)	4·4 (3·9 to 4·9)	3·8 (3·6 to 4·1)
Surrey	27·3 (26·7 to 28)	20·5 (18·9 to 22·2)	16·9 (15·5 to 18·3)	11·5 (10·4 to 12·6)	7·1 (6·5 to 7·8)	4·1 (3·8 to 4·4)	3·3 (3 to 3·6)	2·8 (2·6 to 2·9)
West Berkshire	20·2 (19·8 to 20·6)	15·2 (13·9 to 16·5)	12·4 (11·4 to 13·4)	8·2 (7·2 to 9·3)	6·9 (6·2 to 7·7)	4·7 (4·2 to 5·3)	3·8 (3·4 to 4·2)	3·3 (3·1 to 3·5)

West Sussex	28·1 (27·5 to 28·7)	20·2 (17·8 to 22·8)	16·1 (14·5 to 17·9)	10·4 (9·3 to 11·7)	7 (6·5 to 7·6)	4·3 (4 to 4·7)	3·8 (3·5 to 4·1)	3·1 (3 to 3·3)
Windsor and Maidenhead	20·9 (20·4 to 21·4)	15·9 (13·7 to 18·3)	13·1 (12·2 to 14)	8·9 (7·9 to 10)	6·9 (6·1 to 7·7)	5 (4·5 to 5·7)	3·9 (3·4 to 4·4)	3·5 (3·2 to 3·7)
Wokingham	18·1 (17·8 to 18·4)	13·5 (11·8 to 15·3)	11·1 (10·3 to 12)	7·8 (6·8 to 8·8)	5·8 (5·1 to 6·5)	3·6 (3·1 to 4·1)	3·3 (2·9 to 3·8)	3·2 (2·9 to 3·4)
South West England	26·4 (25·9 to 26·9)	19·9 (18·5 to 21·2)	16·3 (15 to 17·7)	10·9 (10·2 to 11·4)	7·2 (6·9 to 7·6)	5 (4·7 to 5·3)	3·9 (3·7 to 4·1)	3 (2·9 to 3·2)
Bath and North East Somerset	26·5 (26 to 27·1)	20 (16·6 to 23·6)	16·7 (15·1 to 18·4)	11·3 (10·2 to 12·5)	6·7 (6·1 to 7·4)	4·3 (3·8 to 4·8)	3 (2·6 to 3·5)	2·3 (2·3 to 2·4)
Bournemouth	29 (28·3 to 29·6)	21·2 (17·8 to 24·8)	16·9 (15·2 to 18·7)	11·3 (9·7 to 13)	7·4 (6·7 to 8·3)	5·8 (5·2 to 6·5)	3·9 (3·6 to 4·3)	3·2 (3 to 3·3)
Bristol, City of	33·4 (32·6 to 34·3)	25·3 (23·1 to 27·9)	21 (19 to 23·2)	14·2 (13·1 to 15·3)	9·1 (8·4 to 9·8)	6 (5·6 to 6·4)	4·1 (3·8 to 4·5)	3·2 (3·1 to 3·4)
Cornwall	28·2 (27·6 to 28·9)	21·2 (19·3 to 23·2)	17·1 (15·5 to 18·8)	11·2 (10·1 to 12·4)	7·8 (7·3 to 8·4)	5·6 (5·1 to 6·1)	5 (4·6 to 5·5)	3·9 (3·8 to 4)
Devon	29·4 (28·7 to 30·1)	21·7 (18·5 to 25·2)	17·6 (15·6 to 19·9)	11·6 (10·6 to 12·8)	8·1 (7·4 to 8·9)	4·9 (4·4 to 5·5)	4·1 (3·7 to 4·6)	3·2 (3·1 to 3·4)
Dorset	28·5 (27·9 to 29·2)	21·1 (19·3 to 23·2)	17·2 (15·4 to 19·2)	11·4 (10·2 to 12·6)	7 (6·5 to 7·6)	4·9 (4·5 to 5·3)	4 (3·7 to 4·4)	3·2 (3·1 to 3·4)
Gloucestershire	28·5 (27·8 to 29·1)	21·7 (18·6 to 25)	18·1 (16·2 to 20·2)	12·2 (10·8 to 13·5)	8·2 (7·6 to 8·9)	5·5 (5·1 to 6)	4·2 (3·9 to 4·5)	3·3 (3·2 to 3·4)
North Somerset	30·7 (30 to 31·3)	22·7 (20·4 to 25·1)	18·7 (16·8 to 20·6)	12·7 (11·3 to 14·2)	7·5 (6·9 to 8·1)	5·7 (5·1 to 6·3)	4·2 (3·8 to 4·6)	3 (2·9 to 3·1)
Plymouth	29 (28·4 to 29·7)	22·3 (20·5 to 24·3)	18·4 (16·6 to 20·2)	12·3 (11·2 to 13·3)	7·9 (7·2 to 8·7)	6·1 (5·5 to 6·6)	4·9 (4·4 to 5·3)	3·5 (3·4 to 3·6)
Poole	24·7 (24·4 to 25·1)	18·7 (15·6 to 22·2)	15·3 (13·8 to 16·9)	10·1 (9·1 to 11·3)	6·8 (6·1 to 7·5)	4·8 (4·3 to 5·5)	3·5 (3 to 4·1)	2·6 (2·5 to 2·7)
Somerset	24·2 (23·7 to 24·7)	18·2 (16·6 to 20)	15·1 (13·4 to 16·9)	9·9 (9 to 11)	6·5 (5·9 to 7·1)	4·9 (4·5 to 5·3)	4·1 (3·7 to 4·5)	3·2 (3·1 to 3·3)
South Gloucestershire	24·8 (24·2 to 25·4)	18·9 (15·9 to 21·9)	15·9 (14·3 to 17·7)	10·9 (9·5 to 12·4)	7·5 (6·9 to 8·2)	4·8 (4·5 to 5·2)	3·3 (3·1 to 3·6)	2·8 (2·7 to 2·9)
Swindon	33·9 (33·1 to 34·8)	25·8 (23·8 to 28·1)	22·1 (20·1 to 24·4)	14·7 (13·3 to 16·1)	8·4 (7·6 to 9·1)	5·7 (5·2 to 6·2)	4 (3·6 to 4·3)	3 (2·9 to 3·1)
Torbay	29·2 (28·5 to 29·8)	21·8 (18·1 to 25·8)	17·3 (15·6 to 19)	11·3 (10·1 to 12·5)	8·4 (7·4 to 9·4)	5·9 (5·1 to 6·8)	5·3 (4·6 to 6)	3·9 (3·8 to 4)
Wiltshire	25·5 (25·1 to 26)	19·2 (17·5 to 21·2)	15·7 (14·3 to 17·3)	10·4 (9·5 to 11·2)	7·4 (6·7 to 8·1)	4·7 (4·2 to 5·2)	3·8 (3·5 to 4·2)	2·9 (2·8 to 3)
West Midlands	28·7 (28·1 to 29·4)	23 (22 to 24·1)	19·7 (18·7 to 20·6)	13·1 (12·6 to 13·6)	9·9 (9·5 to 10·3)	6·7 (6·4 to 7)	5·6 (5·4 to 5·9)	5 (4·9 to 5·2)
Birmingham	33·6 (32·8 to 34·5)	27 (24·4 to 29·5)	23·1 (21·4 to 24·8)	15·3 (14·1 to 16·7)	12·6 (11·8 to 13·5)	9·3 (8·5 to 10·1)	7·5 (6·9 to 8·2)	7·1 (6·2 to 8)
Coventry	31·9 (31·3 to 32·7)	26 (23·1 to 28·9)	23 (20·5 to 25·5)	15·8 (14·1 to 17·6)	11·7 (10·7 to 12·9)	7·9 (7·2 to 8·6)	5·8 (5·4 to 6·3)	4·9 (4·6 to 5·3)
Dudley	25·3 (24·7 to 25·9)	20·1 (18·1 to 22·1)	17·2 (15·7 to 18·9)	11·4 (10·1 to 12·7)	9·8 (9 to 10·7)	6 (5·4 to 6·7)	5 (4·5 to 5·4)	4·5 (4·2 to 4·7)
Herefordshire, County of	29·9 (29·3 to 30·6)	22·8 (20 to 25·6)	19·8 (17·9 to 21·8)	13·3 (11·6 to 15·1)	8 (7·1 to 8·9)	4·7 (4·2 to 5·3)	4·2 (3·7 to 4·7)	3·8 (3·5 to 4·2)
Sandwell	38·2 (37·3 to 39·2)	31·3 (27·8 to 35·2)	27·3 (24·6 to 30)	18·1 (16·2 to 20·3)	11·9 (10·9 to 13)	8·4 (7·7 to 9·2)	7·6 (6·8 to 8·3)	6·6 (6·2 to 7)
Shropshire	25 (24·4 to 25·6)	19·3 (17·4 to 21·5)	16·6 (14·9 to 18·3)	11·3 (10·1 to 12·4)	8·1 (7·4 to 8·9)	5·1 (4·7 to 5·6)	4 (3·7 to 4·4)	3·6 (3·3 to 4)
Solihull	23·9 (23·5 to 24·3)	19·1 (17·4 to 20·9)	17·1 (15·5 to 18·8)	12·1 (10·9 to 13·4)	8 (7·2 to 8·7)	4·8 (4·4 to 5·3)	4·3 (3·9 to 4·8)	4·3 (3·9 to 4·6)
Staffordshire	26·2 (25·7 to 26·8)	20·6 (18·8 to 22·6)	17·9 (16·8 to 18·9)	12·1 (11 to 13·2)	8·7 (7·8 to 9·6)	5·8 (5·3 to 6·3)	5·2 (4·8 to 5·6)	4·8 (4·1 to 5·5)
Stoke-on-Trent	34·1 (33·4 to 34·9)	28 (25·4 to 30·6)	24 (21·7 to 26·4)	15·9 (14·4 to 17·5)	11·5 (10·6 to 12·6)	8·6 (7·9 to 9·4)	7·2 (6·5 to 7·9)	6·7 (6·2 to 7·1)
Telford and Wrekin	30·5 (29·8 to 31·3)	24·2 (21·2 to 27·1)	20·8 (18·9 to 22·8)	14·1 (12·4 to 16)	11·3 (10·3 to 12·4)	7·3 (6·6 to 8·1)	5·9 (5·2 to 6·6)	4·9 (4·6 to 5·3)
Walsall	31·3 (30·6 to 32·1)	25·4 (22·9 to 28)	22·2 (20 to 24·6)	15 (13·4 to 16·6)	10·5 (9·7 to 11·4)	7·6 (7 to 8·3)	7·7 (7 to 8·4)	6·3 (5·8 to 6·8)
Warwickshire	23·6 (23 to 24·1)	18·6 (16·9 to 20·5)	16·1 (14·5 to 17·8)	10·9 (9·8 to 12)	9 (8·2 to 9·7)	5·4 (5 to 5·8)	4·5 (4·1 to 4·9)	3·9 (3·6 to 4·2)
Wolverhampton	28·5 (27·8 to 29·1)	23·2 (21 to 25·5)	20·2 (18·4 to 22·3)	13·3 (12 to 14·7)	11·1 (10·2 to 12·1)	8 (7·4 to 8·6)	6·8 (6·2 to 7·4)	5·8 (5·4 to 6·2)
Worcestershire	23·2 (22·7 to 23·7)	18·3 (16·5 to 20·3)	15·7 (14·2 to 17·1)	10·4 (9·3 to 11·6)	8·6 (7·9 to 9·3)	5·6 (5·2 to 6·1)	4·3 (3·9 to 4·6)	3·8 (3·5 to 4)
Yorkshire and the Humber	30·2 (29·6 to 30·9)	23·9 (22·4 to 25·5)	19·9 (18·9 to 20·7)	13·1 (12·3 to 14)	9 (8·6 to 9·4)	6·9 (6·6 to 7·3)	5·4 (5·1 to 5·7)	4 (3·9 to 4·2)
Barnsley	31·1 (30·4 to 31·8)	24·7 (22·8 to 26·9)	20·6 (18·7 to 22·4)	13·2 (11·9 to 14·6)	9·2 (8·5 to 10)	6·4 (5·8 to 7)	4·5 (4·1 to 4·9)	3·1 (2·9 to 3·2)
Bradford	40·5 (39·5 to 41·6)	32 (27·3 to 37·5)	26·5 (24·1 to 29·1)	17·1 (15·3 to 18·9)	12·5 (11·5 to 13·6)	10·5 (9·5 to 11·5)	8·4 (7·7 to 9·1)	6·3 (5·3 to 7·4)
Calderdale	38·1 (37·3 to 38·9)	29·8 (27·3 to 32·5)	24·6 (22·5 to 26·8)	15·9 (14·2 to 17·8)	11·6 (10·7 to 12·7)	8·3 (7·6 to 9)	6·7 (6·1 to 7·4)	4·9 (4·7 to 5·2)
Doncaster	28·5 (27·8 to 29·2)	22·9 (20·7 to 25·3)	19·5 (17·7 to 21·3)	12·5 (11 to 14·2)	8·9 (8·3 to 9·7)	7·1 (6·3 to 7·9)	5·7 (5·2 to 6·3)	4·4 (4·2 to 4·6)
East Riding of Yorkshire	27·2 (26·5 to 27·8)	21 (18·9 to 23·6)	17·4 (15·8 to 19·2)	11·5 (10·2 to 12·8)	7·7 (7·1 to 8·4)	5·6 (5·1 to 6·1)	4 (3·6 to 4·4)	2·8 (2·6 to 2·9)
Kingston upon Hull, City of	34·5 (33·7 to 35·3)	27·7 (25·4 to 30·3)	22·8 (21·1 to 24·6)	14·7 (13·2 to 16·5)	10·1 (9·3 to 10·9)	7·1 (6·3 to 8)	5·4 (4·9 to 5·9)	4·2 (4 to 4·4)
Kirklees	34·4 (33·6 to 35·2)	26·8 (23·2 to 30·4)	22·1 (20·4 to 24·1)	14·6 (13·2 to 16·2)	9·7 (9 to 10·4)	7·6 (7 to 8·2)	6·2 (5·8 to 6·6)	4·8 (4·6 to 5)
Leeds	31·2 (30·5 to 32)	24·6 (22·6 to 26·7)	20·5 (18·9 to 22·2)	13·8 (12·4 to 15·3)	9·6 (8·8 to 10·4)	6·8 (6·3 to 7·3)	5·1 (4·7 to 5·4)	3·8 (3·7 to 4)
North East Lincolnshire	30 (29·3 to 30·7)	23·6 (20·2 to 27·1)	19·9 (18·2 to 21·5)	13·2 (11·6 to 14·9)	8·1 (7·3 to 8·9)	6·7 (6·1 to 7·2)	5·4 (4·9 to 6)	4·3 (4 to 4·5)
North Lincolnshire	28·2 (27·6 to 28·9)	22·5 (20·2 to 25·2)	18·9 (17·5 to 20·5)	12·5 (11·2 to 14·1)	9·3 (8·5 to 10·2)	6·7 (6·1 to 7·4)	4·7 (4·2 to 5·3)	3·6 (3·4 to 3·8)
North Yorkshire	27·1 (26·6 to 27·6)	20·6 (17·9 to 23·3)	17·1 (15·8 to 18·6)	11·5 (10·4 to 13)	7 (6·5 to 7·6)	4·6 (4·1 to 5·1)	3·9 (3·5 to 4·2)	2·8 (2·7 to 2·9)
Rotherham	30·6 (29·9 to 31·4)	24·5 (22·4 to 26·9)	20·5 (18·9 to 22·2)	13·4 (12 to 14·9)	9·3 (8·7 to 10)	7 (6·5 to 7·6)	5·5 (5·1 to 6)	3·9 (3·8 to 4·1)
Sheffield	30·2 (29·5 to 30·9)	23·6 (20·3 to 27·2)	19·4 (17·3 to 21·3)	12·7 (11·4 to 14)	9·4 (8·8 to 10·1)	7·5 (7 to 8)	5·6 (5·3 to 6)	4·3 (4·2 to 4·5)

Wakefield	32·9 (32·1 to 33·7)	26·3 (24 to 28·7)	22·1 (20·4 to 24)	14·6 (13·2 to 16·1)	9 (8·2 to 9·8)	6·8 (6·3 to 7·4)	5 (4·7 to 5·4)	3·5 (3·3 to 3·6)
York	27·7 (27·1 to 28·4)	21·5 (19·2 to 23·8)	18 (16·7 to 19·3)	12·3 (10·9 to 13·9)	8·1 (7·3 to 9)	6 (5·4 to 6·6)	4·4 (4 to 4·9)	3·2 (3 to 3·4)
Northern Ireland	40·6 (38·8 to 42·4)	27·9 (26·8 to 29·1)	23·6 (22·5 to 24·7)	15·9 (15·1 to 16·8)	9·1 (8·6 to 9·6)	5·9 (5·4 to 6·3)	5·1 (4·7 to 5·5)	4·1 (3·5 to 4·9)
Scotland	37·7 (36·6 to 38·8)	26·4 (25·7 to 27·1)	20·1 (19·5 to 20·7)	12·5 (12 to 13)	7·9 (7·6 to 8·2)	5·6 (5·3 to 5·8)	4 (3·8 to 4·2)	3·1 (2·8 to 3·5)
Wales	34·1 (33·3 to 35)	25·6 (21·7 to 30·2)	20·7 (17·6 to 24·3)	13·2 (12·2 to 14·3)	7·6 (7·2 to 8)	5·5 (5·2 to 5·8)	4·1 (3·9 to 4·4)	3·2 (2·8 to 3·6)
Latin America and Caribbean	222·6 (204·1 to 244·9)	165·7 (156·5 to 175·8)	122·6 (117·4 to 128·1)	82·6 (79·2 to 86)	52·3 (50·2 to 54·3)	33·6 (32 to 35·2)	26·1 (24·1 to 28)	16·9 (14·7 to 19·6)
Andean Latin America	353 (333·4 to 374·1)	261·8 (245·8 to 277·2)	178·9 (169·6 to 188·5)	111·8 (104·6 to 119·9)	70 (66·1 to 74)	41·8 (39·4 to 44·3)	25·9 (23·7 to 28)	17·4 (15·6 to 19·4)
Bolivia	384·7 (347·3 to 424·8)	309·1 (279·6 to 340·8)	219·1 (202·5 to 236·7)	150·2 (141·1 to 160·3)	104·1 (97·3 to 110·9)	67·6 (62·7 to 72·9)	42·2 (37·8 to 46·8)	28·1 (24 to 33·4)
Ecuador	215 (187·6 to 244·8)	157·2 (144·1 to 172)	115·9 (106·4 to 126·1)	74·6 (68·3 to 81·1)	40·2 (36·3 to 43·8)	31·4 (27·6 to 35·5)	20·9 (17·7 to 24·3)	13·8 (11·6 to 16·5)
Peru	393·3 (362 to 428·6)	289·9 (263·8 to 315·8)	193·9 (178·2 to 209·7)	115·2 (104·2 to 126·8)	71·1 (64·6 to 77·7)	36·8 (33·2 to 40·7)	21 (18·7 to 23·4)	14 (12 to 16·4)
Caribbean	143·8 (135·5 to 153·5)	119·9 (114·4 to 125·8)	95·5 (90·8 to 100·6)	80·4 (75·7 to 85·2)	63·6 (60·3 to 67)	46·5 (43·7 to 49·8)	71 (66·4 to 75·8)	34·4 (29·7 to 40)
Antigua and Barbuda	111·1 (94·6 to 131·9)	72·4 (62·2 to 84·2)	37·3 (32·1 to 43·1)	21·3 (17·9 to 25·2)	13·1 (11 to 15·5)	13·2 (11·4 to 15·3)	11·2 (9·4 to 13·1)	9·5 (8·1 to 11·2)
The Bahamas	107·3 (93·2 to 123·6)	74·6 (62·7 to 87·1)	44·1 (37·5 to 51·8)	30·6 (26·1 to 35·5)	20 (16·7 to 23·5)	14·1 (12 to 16·3)	12·2 (10·4 to 14·1)	10·8 (9·3 to 12·8)
Barbados	142·7 (125·2 to 164·5)	79·6 (67·6 to 92)	41·5 (36 to 48)	23 (19·5 to 26·8)	16·4 (14·1 to 19·1)	14·2 (12·2 to 16·6)	13·1 (11·2 to 15·4)	11·7 (10 to 13·7)
Belize	148·4 (128·5 to 171·6)	111·3 (95·7 to 129·2)	87·1 (77·5 to 97·8)	59·2 (53·2 to 65·8)	37·9 (34·1 to 41·6)	25·5 (22·6 to 28·7)	18·1 (15·6 to 20·8)	13·9 (11·7 to 16·8)
Bermuda	60·7 (54·8 to 66·8)	38·5 (35·2 to 42·1)	26·3 (23·9 to 29)	16·9 (15 to 19)	10·3 (9 to 11·6)	5·7 (4·9 to 6·6)	5·3 (4·5 to 6·1)	4·6 (4 to 5·4)
Cuba	61·9 (53·3 to 72)	43·4 (42·2 to 44·6)	39 (38 to 40)	21·3 (20·6 to 21·9)	11·8 (11·4 to 12·3)	7·4 (7·1 to 7·8)	5·1 (4·8 to 5·4)	4·2 (3·8 to 4·8)
Dominica	227·4 (202·2 to 257·6)	133·4 (116·1 to 153)	62·8 (53·6 to 72·7)	25·1 (21·3 to 29·5)	18·5 (15·6 to 21·3)	17·7 (15 to 20·7)	20·9 (17·6 to 24·5)	23·6 (20·3 to 27·6)
Dominican Republic	181·8 (164·2 to 200·4)	155·9 (141·3 to 171·3)	118·5 (108·4 to 130·5)	77·9 (70·3 to 86)	59·4 (53·7 to 66·2)	35·9 (32·2 to 39·9)	27·3 (23·9 to 31·2)	21·4 (18·1 to 25·2)
Grenada	137·4 (120·3 to 156·2)	97·1 (83·4 to 111·7)	61·4 (53·2 to 70·1)	41 (34·9 to 48·1)	23·6 (19·9 to 28)	16·7 (14·3 to 19·2)	14·3 (12·2 to 16·5)	13 (11·1 to 15·1)
Guyana	149·3 (133 to 166·8)	102·1 (91·4 to 114·3)	76·3 (68·5 to 84·9)	54·5 (48·5 to 61·2)	45·8 (41·4 to 50·3)	34·6 (31·1 to 38·3)	28·2 (24·5 to 32·1)	20·5 (17·5 to 24)
Haiti	265·8 (237·1 to 299·4)	237·1 (217·7 to 256·3)	210·6 (194 to 228·5)	176·9 (163·3 to 191·5)	139·7 (129·6 to 150·9)	96·6 (88·4 to 105·3)	156·3 (145·6 to 167·5)	60·9 (52·5 to 70·9)
Jamaica	120·3 (108·4 to 133·1)	78·2 (71·8 to 85)	52 (46·9 to 57·4)	34·6 (31 to 38·6)	26·6 (23·2 to 30)	20·2 (17·6 to 23·1)	16·9 (14·4 to 19·9)	14·6 (12·5 to 17·1)
Puerto Rico	105·5 (102·8 to 108·5)	51·3 (49·7 to 53)	29 (28 to 30)	18·7 (18 to 19·5)	13·8 (13·1 to 14·4)	10 (9·4 to 10·6)	7·8 (7·3 to 8·3)	6·9 (6 to 8)
Saint Kitts and Nevis	121·1 (113·3 to 129·5)	165·5 (157·7 to 173·7)	81 (75·5 to 86·9)	59·5 (55 to 64·2)	35·2 (32 to 38·7)	24·2 (21·7 to 27·2)	17·3 (15·3 to 19·5)	15·4 (13·4 to 17·7)
Saint Lucia	188·5 (166·2 to 215·5)	136·6 (119·3 to 155·5)	71·9 (62·4 to 82·7)	35·2 (30·2 to 40·6)	22·1 (18·9 to 25·7)	18·1 (15·4 to 20·8)	17·4 (14·9 to 20·3)	14·3 (12·2 to 16·8)
Saint Vincent and the Grenadines	189·8 (166·8 to 217·9)	142 (121·6 to 162·8)	88·8 (75·3 to 102·6)	51·8 (43·8 to 60·7)	29·8 (25·3 to 34·9)	23 (19·8 to 26·7)	18 (15·5 to 20·8)	13·8 (11·9 to 16·2)
Suriname	97·7 (84·7 to 114)	84·4 (72·1 to 98·7)	68·6 (58·8 to 80)	57·1 (50·4 to 64·1)	44·6 (39·6 to 49·9)	37·7 (32·7 to 43·2)	29·7 (25·5 to 34·7)	23 (19·7 to 26·9)
Trinidad and Tobago	101·1 (87·8 to 115·7)	63·6 (56·9 to 71·1)	45·9 (41·1 to 50·8)	32 (28 to 36·1)	23 (20·1 to 26·3)	24·2 (20·6 to 28·5)	16·3 (13·8 to 19·7)	13·8 (11·7 to 16·2)
Virgin Islands	68·8 (62·4 to 75·4)	45·3 (41·7 to 49·4)	28·3 (25·5 to 31·3)	23·3 (21·1 to 25·7)	14·7 (13·2 to 16·2)	10·1 (9·1 to 11·2)	7·8 (6·9 to 8·9)	6 (5·2 to 7)
Central Latin America	195·5 (175·8 to 219·8)	135·1 (127·9 to 142·9)	97·6 (93·2 to 101·9)	64·6 (60·9 to 68·3)	40 (37·9 to 42·1)	26·1 (24·4 to 28)	18·5 (16·8 to 20·3)	13·2 (11·3 to 15·4)
Colombia	155·6 (138 to 177·6)	115·6 (105·4 to 126·3)	81·4 (74·9 to 88·3)	49 (44·6 to 53·7)	30·4 (27·6 to 33·3)	23·2 (21·2 to 25·5)	16·8 (14·5 to 19·3)	11·4 (9·8 to 13·3)
Costa Rica	129·5 (125·6 to 133·5)	89·6 (87·2 to 92·3)	76·2 (73·9 to 78·4)	21 (20·1 to 21·9)	15·4 (14·7 to 16·1)	11·4 (10·9 to 12)	9·6 (9·1 to 10·2)	7·2 (6·3 to 8·3)
El Salvador	261·2 (233·8 to 293·9)	199·1 (177·8 to 221·4)	138·6 (125 to 154·7)	87·1 (79·3 to 95·9)	49 (44·4 to 53·9)	26·9 (23·9 to 30·3)	15·8 (13·8 to 17·9)	10·1 (8·6 to 11·8)
Guatemala	245·3 (218·3 to 277·6)	202 (177·3 to 226·8)	167 (151 to 183·3)	119·3 (109·1 to 130·5)	74·2 (67·2 to 81·4)	45·7 (40·7 to 51)	29·4 (25·7 to 33·6)	19·5 (16·8 to 22·8)
Honduras	236·3 (212·2 to 265·1)	182·2 (163·2 to 202·4)	132·2 (118·8 to 146·1)	83·6 (75·3 to 92·3)	49·8 (44·9 to 54·8)	33·1 (29·3 to 37·4)	23·1 (19·8 to 26·6)	15·5 (13·3 to 18·2)
Mexico	214·4 (191·4 to 242·7)	140·7 (128·2 to 153·4)	98·1 (89·7 to 106·6)	66·7 (60·4 to 73·3)	40·5 (36·7 to 44·3)	25 (22·5 to 27·9)	17·9 (15·8 to 20·3)	12·7 (11 to 14·8)
Aguascalientes	174·9 (155·3 to 198·4)	118·6 (108·5 to 129·8)	86·1 (77 to 95·5)	61·1 (54·7 to 67·9)	35·5 (31·9 to 39·4)	22·1 (19·5 to 25·1)	15·7 (13·6 to 18·1)	10·9 (9·1 to 13·1)
Baja California	197·3 (175·6 to 223·7)	123·9 (113·3 to 135·5)	80·7 (73·1 to 88·2)	35·3 (31·5 to 38·9)	31·9 (28·7 to 35·5)	22·8 (20·4 to 25·3)	18 (15·8 to 20·5)	10·4 (8·6 to 12·5)
Baja California Sur	213·5 (189·7 to 241·4)	134·2 (122·1 to 147)	87·9 (80·8 to 95·2)	49·1 (43·8 to 54·4)	23·7 (21·2 to 26·3)	19 (16·9 to 21·1)	16·3 (14·1 to 18·5)	11 (8·9 to 13·4)
Campeche	216·9 (192·9 to 245)	149·1 (137·9 to 161·7)	110·4 (98·4 to 122·6)	81·9 (73·5 to 90·6)	44·8 (39·1 to 51)	26·1 (22·7 to 29·8)	19 (16·4 to 22·1)	13·4 (10·8 to 16·4)
Chiapas	230·6 (205·3 to 260·1)	164 (149·1 to 178·9)	124·5 (113·6 to 135·7)	96·5 (86·9 to 106·3)	54·2 (49 to 59·4)	31·4 (27·3 to 36·1)	17·5 (15·5 to 19·8)	15·2 (12·6 to 18·2)
Chihuahua	233·9 (208 to 264·1)	156·7 (142 to 172·4)	112·1 (101·7 to 122·9)	80·8 (72·8 to 89·3)	46·7 (42·1 to 51·6)	32·1 (28·1 to 36·5)	21·3 (18·8 to 24·1)	17·4 (14·2 to 21·1)
Coahuila	233·5 (212·9 to 257·6)	151·4 (137·3 to 166)	107·8 (97·8 to 118·3)	74·3 (66·7 to 82)	38·4 (34·4 to 42·5)	24·1 (21·1 to 27·6)	15·8 (13·9 to 17·8)	13·9 (11·8 to 16·4)
Colima	263·6 (235 to 296·8)	173·7 (160·9 to 188·4)	119·2 (106·2 to 132·3)	76·6 (64·4 to 90·5)	38·7 (34 to 44·2)	24·2 (21·1 to 27·6)	17·3 (14·9 to 19·8)	10·7 (8·8 to 13·1)
Durango	257·4 (229·6 to 290·6)	172·6 (157·3 to 188·9)	120·9 (109·1 to 132·6)	87 (78·1 to 96·3)	49 (44·1 to 54·5)	29·5 (26 to 33·8)	18·8 (16·4 to 21·4)	13·9 (11·5 to 17·1)
Guanajuato	220·3 (196·5 to 248·7)	152·4 (138·6 to 168·3)	110·1 (98·6 to 121·9)	75·2 (67·2 to 83)	46·2 (42·3 to 50·3)	21·8 (19·6 to 24·1)	16·2 (14·2 to 18·5)	11·4 (9·5 to 13·7)
Guerrero	232·6 (207 to 262·7)	170·8 (140·4 to 202)	141·3 (122 to 161·3)	107·4 (90·6 to 125·5)	67·2 (59·8 to 75·6)	44·7 (39·6 to 50·3)	28 (23·4 to 33·3)	14·9 (11·6 to 18·8)

Hidalgo	203·9 (181·5 to 231)	137·1 (125·4 to 150·3)	97·6 (88·3 to 107·1)	60·3 (51·6 to 69·9)	33·5 (29 to 38·5)	21 (18·3 to 24·2)	14·9 (13 to 17·1)	10·4 (8·5 to 12·7)
Jalisco	198 (175·9 to 224)	130·8 (119 to 145·2)	89·1 (79·2 to 99·8)	57 (51·1 to 63·3)	33·8 (30·3 to 37·8)	24·4 (21·2 to 28·1)	17·2 (15·2 to 19·6)	12·4 (10·4 to 14·9)
México	191 (173·8 to 211·9)	133·4 (121·4 to 147·2)	92·7 (82·4 to 102·8)	60·8 (54 to 67)	48·1 (44·4 to 52·1)	27·4 (24·8 to 30·2)	20·2 (17·8 to 22·9)	14·7 (12·6 to 17·3)
Mexico City	143·9 (127·5 to 163·9)	91·7 (82·9 to 101·4)	63·6 (57·9 to 70·4)	47·3 (41·9 to 52·2)	28 (25·4 to 31)	20·4 (18·3 to 22·6)	19·1 (16·9 to 21·6)	12·8 (10·7 to 15·3)
Michoacán de Ocampo	200·4 (178·1 to 226·8)	134 (122·1 to 147·4)	85·7 (77 to 95·1)	55·2 (49·2 to 61·4)	32·6 (29·2 to 36·4)	20·8 (18·2 to 23·7)	15·8 (13·9 to 17·9)	10 (8·3 to 11·9)
Morelos	203·4 (181·6 to 229·2)	136·6 (123·8 to 150·4)	95·7 (85·5 to 106·8)	63·5 (52·5 to 74·9)	34·1 (30 to 38·8)	21·5 (18·8 to 24·6)	15·6 (13·6 to 17·7)	10·6 (8·7 to 13)
Nayarit	213·5 (190·6 to 240·9)	143 (130·2 to 156·8)	94·3 (85·3 to 103·9)	57·5 (50·9 to 64·5)	34·4 (30·7 to 38·1)	22·2 (19·6 to 25·1)	15·3 (13·2 to 17·6)	12·1 (10·1 to 14·6)
Nuevo León	181·8 (165·3 to 201·7)	113·5 (102·7 to 125·1)	77·2 (68·4 to 86·2)	48·1 (43·1 to 53·6)	26·6 (23·1 to 30·4)	19·2 (16·8 to 22)	13 (11·3 to 14·8)	11·3 (9·4 to 13·4)
Oaxaca	224·8 (199·9 to 254)	156·6 (142·6 to 171·3)	112·1 (101·8 to 123·2)	78·6 (71·2 to 86·9)	41·1 (36·9 to 45·9)	24·9 (21·6 to 28·7)	15·5 (13·6 to 17·8)	11·1 (9 to 13·4)
Puebla	272·9 (243·4 to 306·7)	188·3 (172·3 to 205)	133·9 (122 to 146·5)	89·7 (81·7 to 98·4)	50·2 (45·4 to 55·3)	31 (27·3 to 35·3)	21·7 (19·1 to 24·6)	14·7 (12 to 17·6)
Querétaro	235·7 (209·8 to 266)	161·6 (147·1 to 177·7)	112·9 (101·5 to 124·4)	78·3 (70 to 86·7)	41·8 (38·2 to 46·3)	21·9 (19·5 to 24·3)	15·9 (13·8 to 18·3)	10·9 (8·8 to 13·4)
Quintana Roo	199·4 (177·2 to 225·7)	139·6 (128·2 to 152·3)	104·6 (93·6 to 115·2)	73 (65·3 to 81·5)	38·2 (34·3 to 42·7)	24 (21·2 to 27·3)	17·4 (15·1 to 19·9)	11·7 (9·7 to 14)
San Luis Potosí	222·1 (197·4 to 251)	147·8 (134·6 to 162·7)	102·5 (92·4 to 113·6)	63·6 (57·4 to 70·7)	35·9 (32·2 to 40)	22·8 (19·9 to 26·1)	14·4 (12·5 to 16·4)	10·4 (8·4 to 12·6)
Sinaloa	185·1 (164·4 to 210)	119·9 (108·4 to 132·3)	79 (72·3 to 85·6)	49 (43·3 to 55)	26 (22·8 to 29·8)	14·6 (12·6 to 16·8)	11·9 (10·5 to 13·7)	9·9 (8·2 to 11·9)
Sonora	163·9 (145·5 to 186·1)	107·3 (98·2 to 117·4)	77·5 (69·1 to 86·5)	54·5 (45 to 64·2)	29·4 (25·7 to 33·2)	22·5 (19·9 to 25·6)	17·5 (15·3 to 20·1)	11·4 (9·5 to 13·7)
Tabasco	211 (187·9 to 238·4)	147 (133·4 to 162·5)	111·7 (101·9 to 121·5)	82·2 (70·9 to 93·9)	46·3 (40·5 to 53·3)	26·1 (22·7 to 29·7)	19·5 (16·9 to 22·2)	14·8 (12·2 to 17·8)
Tamaulipas	190·7 (169·5 to 216·1)	124·9 (113·2 to 138·3)	86·3 (78·8 to 94·5)	59·9 (50·9 to 69·1)	32·4 (28·5 to 37)	18·7 (15·9 to 21·8)	16·1 (14·2 to 18·5)	11·8 (9·7 to 14·2)
Tlaxcala	240·8 (214·4 to 271·6)	162·1 (147·2 to 178·6)	112·4 (101·3 to 123)	80·4 (72·3 to 88·2)	48 (43·5 to 52·9)	27·3 (24·4 to 30·3)	20·6 (17·9 to 23·9)	13·8 (11·2 to 17·1)
Veracruz de Ignacio de la Llave	210·5 (187·5 to 238)	141·2 (128·6 to 156·1)	98·7 (91·2 to 106·7)	65·7 (57·2 to 74·7)	39·8 (32·9 to 47·6)	22·7 (19·4 to 26·2)	18·4 (16 to 20·9)	12·7 (10·5 to 15·2)
Yucatán	181·1 (160·9 to 205·4)	126·2 (114·7 to 139·6)	84·9 (77 to 93·4)	59·3 (53·3 to 65·2)	31·1 (28·1 to 34·3)	19·8 (17·7 to 22)	16·7 (14·3 to 19·5)	12·6 (10·2 to 15·6)
Zacatecas	199·7 (177·5 to 225·9)	137·9 (124·1 to 152·9)	96·3 (86 to 107·7)	70·3 (63·5 to 77·5)	38·6 (34·6 to 42·9)	24·2 (21·1 to 27·6)	16·8 (14·6 to 19·2)	11·9 (9·9 to 14·3)
Nicaragua	271·8 (245·9 to 302·4)	208·3 (186·5 to 232·5)	146·9 (132·8 to 161·8)	92·7 (86·6 to 99·9)	55·8 (51·5 to 60·6)	31·8 (28·6 to 35·4)	19 (16·4 to 21·8)	12·8 (11 to 15)
Panama	106·7 (94·8 to 119·9)	80·5 (72·4 to 89·1)	54 (48·6 to 59·8)	34·1 (29·9 to 38·6)	24·8 (21·6 to 28·2)	20·9 (18·2 to 24)	16·6 (14 to 19·5)	13·3 (11·3 to 15·6)
Venezuela	116·3 (113·7 to 118·9)	71·7 (70·2 to 73·1)	64·5 (63·1 to 65·8)	37·4 (36·5 to 38·3)	28·8 (28·2 to 29·4)	20·3 (19·8 to 20·8)	16·2 (15·8 to 16·6)	13·2 (11·3 to 15·4)
Tropical Latin America	229·3 (208·5 to 254·1)	181·8 (164·8 to 200·4)	141·6 (130·2 to 153·4)	96·9 (90·8 to 103·3)	60·6 (56·7 to 64·9)	38·2 (34·8 to 41·5)	25·4 (22·4 to 28·7)	17·4 (15 to 20·2)
Brazil	232 (211·1 to 256·9)	184·3 (167·1 to 203·2)	143·5 (131·9 to 155·7)	98·3 (92 to 105)	61·7 (57·6 to 66·1)	38·6 (35·2 to 42·1)	25·7 (22·6 to 29)	17·6 (15·1 to 20·4)
Acre	265·1 (224·8 to 310·3)	209·5 (177·6 to 243·2)	163·4 (144·8 to 182·5)	120·4 (106·3 to 135·2)	80·8 (72·9 to 89·8)	59·2 (52·4 to 67·1)	41·7 (31·6 to 54)	28·2 (22·2 to 34·9)
Alagoas	379·4 (337·1 to 423·7)	338·5 (310 to 368·1)	289 (263·7 to 315·5)	225·9 (207·4 to 245·7)	136·7 (125·2 to 149·7)	71·3 (63·2 to 79·6)	37·8 (32·8 to 43·4)	20·5 (16·5 to 25·2)
Amapá	332 (309·6 to 355)	254·3 (224·3 to 285·8)	165·5 (146·3 to 185·5)	60·3 (54·3 to 66·3)	38 (34·7 to 41·5)	36·1 (33·7 to 38·9)	29·7 (25·6 to 34·7)	26·6 (23·5 to 30·1)
Amazonas	206·5 (185·4 to 229·1)	157·4 (134·3 to 185·5)	126·9 (110·5 to 144·3)	89 (77·7 to 100·8)	55·7 (49·1 to 62·4)	39·7 (36·7 to 42·7)	24·2 (20·9 to 28)	20·9 (18·8 to 23·1)
Bahia	253·9 (222·2 to 289·2)	216·5 (196·6 to 237·8)	173·6 (157·2 to 191·6)	115 (105·5 to 125·2)	85·3 (77·5 to 93·7)	53·9 (47·4 to 60·3)	35·4 (30·6 to 40·6)	22·1 (17·8 to 27·3)
Ceará	361·2 (321·4 to 404·8)	308·3 (282·7 to 336)	241·7 (220·3 to 264·6)	164·8 (152·2 to 178·6)	94·1 (86·1 to 103·5)	54·4 (48·6 to 60·5)	31·1 (26·8 to 35·8)	17·8 (14·1 to 22·2)
Distrito Federal	240·3 (222·3 to 258·9)	176·3 (151·3 to 204·4)	122·8 (107·3 to 138·8)	64·2 (58·6 to 70·3)	34·1 (31·6 to 36·9)	24 (22·4 to 25·9)	18·5 (16 to 21·4)	15·1 (13·4 to 17·1)
Espírito Santo	181·1 (150·1 to 217·2)	144·9 (127·8 to 164·4)	112·4 (98·4 to 127)	64·9 (59·3 to 71)	41·3 (38·3 to 44·4)	26 (23·8 to 28·3)	22·8 (19·6 to 26·4)	16·5 (12·9 to 20·8)
Goiás	264·4 (246·2 to 283·6)	205·7 (177·6 to 239·8)	130·7 (115·1 to 147·5)	69·7 (60·4 to 79·6)	41·1 (36·4 to 45·7)	21·9 (20·4 to 23·5)	17·6 (15·2 to 20·4)	15·8 (14·1 to 17·7)
Maranhão	311·5 (273·7 to 352·9)	272·4 (249·2 to 298)	225·4 (204·5 to 246·5)	155·8 (141·3 to 170·5)	99·9 (91 to 109·2)	59·2 (52·4 to 65·9)	35·8 (30·9 to 41·1)	20·3 (16·3 to 25·1)
Mato Grosso	217·7 (198·4 to 237·8)	189·3 (162·4 to 220·7)	149·5 (132·8 to 166·8)	78·2 (69·9 to 86·3)	48·6 (43·8 to 53·8)	28·4 (26·5 to 30·5)	20 (17·1 to 23)	15·9 (14·1 to 17·9)
Mato Grosso do Sul	170 (153·2 to 188·1)	135·9 (113·8 to 163·3)	94·7 (83·6 to 106·5)	62 (57·1 to 67·1)	40·2 (37·3 to 43·4)	34·5 (32·1 to 37·2)	23·1 (19·8 to 26·8)	16·1 (14·2 to 18·2)
Minas Gerais	196·9 (170·8 to 226·3)	156·1 (140·2 to 173·2)	115·8 (103·9 to 128·7)	72·4 (66·1 to 79·6)	47·1 (42·4 to 52·4)	34·4 (30·2 to 38·7)	23·8 (20·4 to 27·4)	15·8 (12·8 to 19·5)
Pará	257·5 (217·8 to 302·2)	205·1 (183·8 to 227·1)	162·5 (144·6 to 180·7)	111·9 (101·2 to 122·4)	73·2 (65·1 to 82·6)	46·8 (41·3 to 52·6)	31·6 (27·1 to 36·3)	20·4 (16·1 to 25·6)
Paraíba	359·3 (318·5 to 402·4)	289 (263·3 to 316·4)	214 (193·6 to 235·4)	139·1 (128·5 to 151·1)	82·4 (74·7 to 90·9)	45 (39·7 to 50·5)	26·6 (23 to 30·6)	15·2 (12·2 to 18·7)
Paraná	166·8 (139·1 to 198·9)	130·1 (116·3 to 145)	96 (83·4 to 108·6)	70·9 (64·8 to 78·2)	39·9 (37·1 to 42·9)	26·8 (24·7 to 29)	21·7 (18·5 to 24·9)	15·5 (12·1 to 19·5)
Pernambuco	387·1 (346 to 429·9)	325·8 (298·6 to 354·1)	254·4 (232 to 278·5)	161·3 (148·7 to 174·8)	100·9 (92·3 to 110)	57·7 (51·8 to 64·2)	33·4 (28·9 to 38·3)	19·1 (15·4 to 23·6)
Piauí	275·8 (240·1 to 315·1)	233·4 (212·4 to 256·9)	197·5 (177·6 to 217·1)	127·5 (114·7 to 140·7)	83·5 (76·2 to 91·2)	54·2 (47·2 to 60·7)	34 (29·4 to 39)	20·7 (16·7 to 25·4)
Rio de Janeiro	159·6 (144 to 176·6)	120·2 (102·9 to 141·5)	99·5 (87·2 to 112·7)	76·8 (70·4 to 83·6)	45·7 (42·7 to 49)	29·6 (27·7 to 31·7)	20·9 (18 to 24·1)	16·8 (15·5 to 18·1)
Rio Grande do Norte	344·9 (298·2 to 396·5)	269·8 (245·2 to 297·6)	193·7 (175·7 to 212·5)	124·1 (113·9 to 134·9)	75·5 (68·7 to 83)	40·9 (36·1 to 46)	23·6 (20·2 to 27·1)	14·6 (11·5 to 18·4)
Rio Grande do Sul	148·4 (136·1 to 161·6)	112·8 (96·7 to 132·7)	77·7 (69 to 86·6)	48·9 (45 to 53·2)	28·3 (26·4 to 30·5)	22·8 (21·4 to 24·1)	16·2 (14 to 18·6)	14·4 (13·1 to 15·8)
Rondônia	288·6 (268·5 to 309·6)	237·4 (206·3 to 272·5)	175·4 (158·6 to 193·9)	90·8 (83·9 to 97·9)	56·6 (52·5 to 61)	32·1 (30·2 to 34·2)	21·4 (18·5 to 24·7)	15·8 (14 to 17·9)

Roraima	294·3 (274·5 to 314·9)	234·3 (207·4 to 264)	165·7 (149·2 to 183·9)	88·3 (79·5 to 97·9)	49·7 (45·7 to 54)	31·1 (29 to 33·3)	23·9 (20·5 to 27·6)	21·1 (18·8 to 23·6)
Santa Catarina	126·6 (107·7 to 149)	100 (88·8 to 111·6)	81·6 (72·7 to 90·7)	54·6 (49·8 to 59·9)	38·6 (34·6 to 43·4)	30·6 (26·8 to 34·6)	23·8 (20·5 to 27·4)	16·8 (13·5 to 20·7)
São Paulo	133·6 (122·2 to 145·8)	102·8 (87·3 to 121·7)	85·6 (76·3 to 95·6)	67·8 (62 to 74·2)	40·6 (37·9 to 43·5)	26·1 (24·5 to 27·6)	17·6 (15·3 to 20·2)	15 (13·7 to 16·3)
Sergipe	242·9 (212·3 to 276·9)	199·7 (177·2 to 224·4)	157·3 (141·9 to 173)	126 (110·2 to 143·8)	80·7 (73·1 to 89·1)	50·3 (44·6 to 55·6)	32·4 (28·1 to 37·2)	20·8 (16·7 to 25·6)
Tocantins	311·7 (286·5 to 337·3)	258·3 (206 to 313·3)	189·6 (168 to 210·1)	115·6 (98·8 to 133·5)	75·5 (66·6 to 85·2)	49·5 (43·1 to 55·6)	32·4 (26·4 to 39)	19·3 (16·1 to 23·4)
Paraguay	102·2 (88·5 to 119·4)	76 (69·1 to 83·7)	63 (57·1 to 68·9)	46·3 (42·1 to 51·1)	31 (27·8 to 34·4)	25·3 (22·2 to 28·3)	18·9 (16·3 to 21·7)	13·1 (11·3 to 15·3)
North Africa and Middle East	290·8 (271·5 to 312·4)	253·4 (240·3 to 267·2)	190·4 (181·5 to 200·2)	123·3 (118·3 to 128·4)	75·8 (72·9 to 78·9)	51·4 (49·1 to 53·8)	33·6 (31·4 to 35·8)	22·8 (21 to 24·7)
North Africa and Middle East	290·8 (271·5 to 312·4)	253·4 (240·3 to 267·2)	190·4 (181·5 to 200·2)	123·3 (118·3 to 128·4)	75·8 (72·9 to 78·9)	51·4 (49·1 to 53·8)	33·6 (31·4 to 35·8)	22·8 (21 to 24·7)
Afghanistan	300·4 (265·3 to 338·7)	271 (238·9 to 304·2)	242·6 (209·5 to 280·8)	234·2 (202·5 to 279·2)	152·4 (139 to 167·3)	127 (115·5 to 139·2)	76·2 (68·5 to 84·2)	52·8 (45·6 to 60·8)
Algeria	144·4 (134·2 to 155·2)	162·7 (147·9 to 179·6)	155·6 (140·5 to 172·6)	117·9 (109·4 to 127·6)	54·4 (49·7 to 59·4)	38 (34·5 to 41·5)	25·9 (23·7 to 28·2)	18·2 (16·2 to 20·5)
Bahrain	222·5 (198·3 to 249·1)	148·5 (131 to 167·4)	73·1 (65·2 to 83)	28·8 (27·2 to 30·6)	22·2 (21·1 to 23·3)	11·4 (10·7 to 12·1)	8 (7·5 to 8·6)	6·5 (5·7 to 7·3)
Egypt	367 (335·7 to 402·6)	325·2 (295·5 to 356·5)	254·9 (233·5 to 276·2)	158·7 (144·5 to 173·5)	84·9 (77·4 to 93·1)	42·7 (37·9 to 47·5)	24·8 (21·9 to 28·1)	14·3 (12·2 to 16·8)
Iran	356·2 (323·1 to 390·9)	301·1 (265·7 to 340·4)	200·3 (175·2 to 230·7)	100·9 (89·7 to 113·9)	66·8 (59·9 to 73·9)	38 (33·8 to 42·8)	23·8 (21·4 to 26·4)	10·1 (9·2 to 10·9)
Iraq	159·6 (137·7 to 185·2)	129·8 (110·6 to 150·2)	104·6 (90·5 to 119·6)	79·7 (73·1 to 86·5)	50·5 (46·2 to 55·1)	39·9 (36·4 to 44·4)	28·2 (24·7 to 32)	15 (12·7 to 18·1)
Jordan	140·2 (122 to 160·7)	118·8 (102·5 to 137·6)	84·4 (75·8 to 93·4)	52·3 (47·6 to 57·6)	32·3 (29·5 to 35·6)	23·8 (21·4 to 26·2)	16·4 (14·4 to 18·3)	13·9 (12 to 16·2)
Kuwait	90·5 (78·2 to 104·7)	65·6 (57·4 to 75)	51·7 (49·7 to 53·8)	32·2 (30·9 to 33·5)	20·7 (19·4 to 22·1)	11·7 (11 to 12·4)	10·6 (10 to 11·2)	8·4 (7·1 to 10)
Lebanon	143·8 (124·9 to 165·2)	89·2 (76 to 103·9)	65 (56·4 to 74·4)	44·6 (39 to 50·4)	31·9 (28·2 to 35·9)	18·6 (16 to 21·4)	12·7 (10·7 to 14·9)	8·7 (7·4 to 10·4)
Libya	263·4 (229·6 to 304·1)	210·4 (183·9 to 240·2)	115·6 (100·3 to 132·3)	62·5 (54·8 to 71·3)	40·8 (35·4 to 46·9)	24·3 (20·8 to 28·2)	16·3 (13·6 to 19·2)	13·1 (11·3 to 15·4)
Morocco	273 (249·1 to 301)	241 (223·9 to 258·8)	179·1 (166·4 to 191·7)	117·7 (109·5 to 127)	70·7 (64·7 to 76·6)	44·9 (39·7 to 50·3)	27·1 (23·1 to 31·5)	15·8 (13·5 to 18·6)
Oman	314·7 (281·3 to 350·5)	267·5 (235·5 to 302·5)	162 (143·1 to 181·8)	87·4 (75·6 to 100·6)	34·2 (29·5 to 40·1)	14·3 (12·9 to 15·7)	10·7 (10·2 to 11·1)	9·7 (8·8 to 10·7)
Palestine	163·6 (144 to 185·4)	126·9 (111·2 to 145)	98·9 (86·8 to 111·3)	66·8 (60·4 to 74)	42·7 (39·2 to 47·1)	29·8 (27·2 to 32·5)	19·8 (17·8 to 21·9)	11·5 (9·7 to 13·8)
Qatar	93·5 (80·7 to 108·3)	84·3 (70·8 to 98·2)	67·3 (56·6 to 78·8)	36·7 (31·4 to 42·7)	24·9 (21·1 to 28·9)	16·6 (14·3 to 19·3)	10·3 (8·8 to 12)	7·5 (6·2 to 9·1)
Saudi Arabia	202·1 (178 to 228·9)	171·9 (147·9 to 196·9)	126·2 (108·6 to 144·8)	73·6 (63·5 to 84·2)	43·4 (36·9 to 50·3)	23·7 (19·9 to 28·1)	10·9 (9 to 12·8)	5·5 (4·6 to 6·8)
Sudan	247 (217·8 to 279·8)	219·8 (197·3 to 244·5)	203·8 (186·8 to 221·1)	177·6 (163·2 to 193·4)	134·7 (124·7 to 145·7)	91 (83·2 to 99·6)	59·7 (53·2 to 66·2)	37·1 (31·5 to 44·2)
Syria	183·2 (162·5 to 208·2)	125·6 (114·1 to 139)	78 (71·1 to 85·5)	59·4 (52·6 to 66·7)	44 (39 to 49·1)	18·3 (17·9 to 18·8)	12·5 (12·2 to 12·9)	12·2 (10·7 to 13·9)
Tunisia	279·7 (256 to 307·5)	225·7 (208·2 to 243·3)	153·8 (141·1 to 166·2)	85·8 (78 to 93·9)	47·8 (43·2 to 52·9)	26·3 (23·3 to 29·3)	16·6 (14·2 to 19·1)	9·9 (8·5 to 11·6)
Turkey	283·5 (252·3 to 320·1)	253·7 (233·6 to 274·5)	182·7 (169·5 to 195·7)	115·3 (106·7 to 123·9)	65·6 (60·4 to 71·1)	36·4 (32·4 to 40·6)	21·7 (18·3 to 25·4)	14·2 (12 to 17)
United Arab Emirates	148·3 (130·1 to 168·7)	126·3 (110·1 to 143·8)	81·3 (70·4 to 92·9)	41·8 (35·6 to 48·4)	19·7 (17·4 to 22·2)	10·9 (10·4 to 11·5)	6·3 (5·7 to 6·9)	4·6 (3·9 to 5·6)
Yemen	411·2 (381·6 to 444·4)	361·5 (338·8 to 384·3)	287·5 (268·3 to 307·4)	182·6 (170 to 195·3)	128·8 (119·7 to 137·6)	87·5 (80 to 95·3)	56·4 (49·8 to 63·6)	45·4 (39·9 to 51·8)
South Asia	265·1 (241·9 to 290·8)	234·8 (212·6 to 256·5)	203·9 (187·9 to 221·2)	159 (147·7 to 170·3)	115 (107·4 to 123·5)	84·9 (79·2 to 91·4)	62·7 (56·8 to 68·8)	41·1 (36·7 to 46·4)
South Asia	265·1 (241·9 to 290·8)	234·8 (212·6 to 256·5)	203·9 (187·9 to 221·2)	159 (147·7 to 170·3)	115 (107·4 to 123·5)	84·9 (79·2 to 91·4)	62·7 (56·8 to 68·8)	41·1 (36·7 to 46·4)
Bangladesh	319·1 (290·4 to 351·7)	267·7 (247·2 to 288·5)	234·8 (220 to 250·1)	189·2 (175·7 to 203·6)	131·3 (122·8 to 140·5)	82·8 (76·4 to 89·7)	50·6 (45·2 to 56·4)	28 (24 to 32·9)
Bhutan	361·1 (327·1 to 396·8)	305 (273·1 to 338·5)	239·9 (207·5 to 276)	200·9 (176·1 to 225·7)	129·3 (115·6 to 143·8)	81·5 (73·5 to 90·3)	47·2 (41·1 to 53·7)	30·8 (26·1 to 36·1)
India	253·5 (222·9 to 288·4)	230·5 (203·1 to 258·9)	202·3 (181·3 to 224·2)	155·7 (142·3 to 169·6)	112·3 (102·8 to 122·5)	83·8 (76·2 to 92·1)	61·2 (53·5 to 69)	37·4 (31·5 to 44·8)
Andhra Pradesh	245·4 (218·6 to 274·7)	228·1 (193·7 to 266·1)	200·7 (175·8 to 227·8)	148·6 (133·8 to 165·7)	106·5 (96·4 to 117·7)	78·3 (67·6 to 90·1)	53·2 (45·1 to 61·8)	31·8 (25·9 to 39·7)
Arunachal Pradesh	251·2 (224·4 to 280·5)	232·1 (200·7 to 263·8)	196·3 (173·9 to 221·1)	124·6 (112·5 to 137·9)	91·8 (83·2 to 101·7)	65·8 (57·4 to 75·2)	37·3 (33 to 42)	22·6 (18·8 to 27·7)
Assam	236·6 (211·2 to 264·6)	215·5 (185·2 to 246·1)	177·1 (151·7 to 202·1)	122·9 (110·5 to 135·5)	99·1 (89·9 to 109·3)	82·7 (75·3 to 90·9)	69·5 (58 to 81·8)	45·5 (37·7 to 55·9)
Bihar	205·9 (182·7 to 231·9)	196·8 (170 to 224·6)	190·8 (167·4 to 214·4)	166·8 (148·6 to 185·6)	125·3 (113·7 to 137·7)	93·9 (85 to 103·3)	68·4 (57 to 80·7)	41·9 (34·6 to 51·7)
Chhattisgarh	226·4 (201·5 to 254)	212·7 (182·6 to 243·2)	185·6 (158·4 to 215·3)	151·8 (131 to 174·9)	112·8 (101·8 to 125·5)	91·5 (82·8 to 101·1)	67·2 (58·7 to 76)	38·7 (34·2 to 43·6)
Delhi	242·7 (215 to 273)	184·1 (156·3 to 215·4)	143·2 (126·6 to 161·3)	103·8 (92·9 to 116·2)	71·2 (64·6 to 78·6)	54·2 (46·7 to 62·5)	34·4 (30·2 to 38·7)	21·8 (18 to 27)
Goa	129 (112·7 to 147·7)	109·2 (92·3 to 126·4)	91·5 (80·7 to 102·6)	62·2 (54·8 to 69·8)	43·4 (37·4 to 49·8)	28·2 (23·8 to 33·2)	19·5 (16·8 to 22·5)	14·4 (11·8 to 17·9)
Gujarat	219 (194·5 to 246·1)	197·1 (168·6 to 228·3)	161·8 (144·1 to 180·3)	128·1 (114·6 to 143·4)	96·3 (87·4 to 106·3)	72·8 (62·7 to 83·4)	50·8 (44·9 to 56·7)	30·4 (25·1 to 37·6)
Haryana	234·8 (207·9 to 264·2)	215·5 (184·5 to 245·4)	178·7 (159·9 to 198·3)	123·5 (110·7 to 137·8)	89·9 (81·5 to 99·3)	68·9 (60·2 to 79·6)	50·9 (44·8 to 57·4)	32 (26·3 to 39·6)
Himachal Pradesh	157·3 (138·2 to 178·9)	144·6 (123 to 168·1)	121·4 (108 to 135·8)	89·9 (80·1 to 100)	60·9 (54·5 to 67·8)	48·1 (41·2 to 55·4)	38·2 (33·7 to 42·8)	25·2 (21·2 to 30·4)
Jammu and Kashmir	152·5 (133·7 to 173·7)	140·4 (119·2 to 162·5)	117·1 (103·5 to 131·9)	97·4 (86·3 to 109·3)	71·6 (64·9 to 79·2)	56·4 (49·2 to 65·1)	45·5 (40 to 51·5)	27·7 (22·8 to 34·1)
Jharkhand	196·6 (173·5 to 222·3)	179·5 (158 to 202·8)	154·2 (129·4 to 182·6)	129·5 (111·2 to 148)	115·2 (104·1 to 127·2)	87·5 (79 to 97·1)	62·8 (54·3 to 71·6)	36·6 (32·2 to 41·4)
Karnataka	247·9 (221·3 to 276·9)	221·9 (189·7 to 255·1)	183·9 (163·9 to 205·5)	125·1 (112·8 to 139·6)	82·3 (74 to 91·5)	58·3 (49·6 to 67·6)	41·4 (36·3 to 46·7)	25 (20·5 to 31)
Kerala	166·9 (146·8 to 189·8)	125·9 (105·9 to 146·8)	84·3 (72·5 to 97·1)	50·4 (42·3 to 60·1)	30·6 (26·1 to 35·7)	19·6 (16·9 to 22·7)	14·4 (12·4 to 16·5)	10·1 (8·2 to 12·5)

Madhya Pradesh	290·3 (259·2 to 323·6)	274·6 (239·4 to 310·4)	239·9 (207·1 to 273·5)	195·5 (173·3 to 217·3)	144 (131·2 to 158·5)	106·7 (96·7 to 117·1)	77·1 (64·1 to 91·1)	46·3 (38·4 to 56·9)
Maharashtra	246·5 (217·9 to 277·7)	214 (183·7 to 247·4)	171·8 (153 to 192·5)	110·4 (98·9 to 123·1)	71·1 (64·4 to 78·7)	50·6 (43·1 to 58·8)	34·9 (31·1 to 39·2)	21·2 (17·3 to 26·3)
Manipur	172·7 (152·1 to 195·9)	156·4 (132·5 to 181)	122·4 (107·8 to 138·2)	85·3 (75·9 to 95·8)	58·2 (52·2 to 64·7)	41·5 (35·4 to 48·3)	29·5 (25·9 to 33·2)	21 (17·4 to 25·8)
Meghalaya	120·5 (105·1 to 138·2)	110·5 (92·5 to 128·6)	99·8 (87·3 to 113·9)	103·3 (91·9 to 115·5)	78·5 (68·4 to 89·8)	58·7 (51·3 to 67·5)	43·4 (38·1 to 49·2)	30·7 (25·2 to 38)
Mizoram	100 (86·4 to 115·5)	87 (73 to 103)	71·8 (60·4 to 84)	71·7 (62·9 to 81·4)	55·3 (49·7 to 61·7)	44·7 (40 to 49·9)	42·8 (35·1 to 51·3)	30·5 (25·1 to 37·8)
Nagaland	207·5 (183·5 to 234·2)	181·7 (158·9 to 207·5)	139·1 (117·1 to 164·4)	95·1 (83·4 to 108)	67·9 (60·3 to 76·6)	53·9 (48·5 to 60·1)	41·4 (34 to 49·8)	30·8 (27·2 to 34·9)
Odisha	287·1 (258·5 to 318·1)	270·1 (234·6 to 306·4)	232·9 (209·1 to 258·2)	177·9 (159·7 to 197·5)	126·6 (115·2 to 138·9)	90·6 (79·2 to 103·9)	61·9 (55·3 to 69)	38·8 (32 to 47·8)
Punjab	164·2 (143·5 to 187·3)	142·6 (120·2 to 165·9)	107·4 (95·3 to 120·6)	88·9 (79·5 to 99·5)	67 (61·1 to 73·9)	50·3 (43·7 to 58·3)	39·3 (34·5 to 44·3)	24·2 (19·9 to 30)
Rajasthan	222·8 (196·5 to 251·8)	213·4 (187 to 241·3)	188 (160·3 to 220·3)	159·3 (142·3 to 176·2)	122 (111 to 134)	92·2 (82·4 to 102·6)	71·5 (62·3 to 80·8)	44·2 (39 to 50)
Sikkim	165·8 (145·7 to 188·4)	155·9 (134 to 179·3)	135·8 (119 to 152·8)	101·2 (89 to 115)	61·4 (54·5 to 69·3)	42·5 (36·9 to 48·8)	30·9 (27 to 35)	19 (15·8 to 23·2)
Tamil Nadu	258·7 (231·6 to 288·3)	228·6 (196·9 to 262·4)	188·1 (167·3 to 209·3)	133·7 (120·1 to 149·5)	82·6 (74·3 to 91·6)	49·7 (42·4 to 57·8)	30·6 (26·7 to 34·7)	17·5 (14·3 to 21·7)
Telangana	180·4 (159·2 to 204·3)	169·4 (141·2 to 200·7)	149 (134·7 to 165·3)	124·2 (110·9 to 139·7)	94·2 (84·7 to 104·7)	71·9 (58·7 to 85·4)	45·5 (39·1 to 52·4)	26·2 (21·4 to 32·8)
Tripura	284·9 (255·6 to 316·5)	243·9 (210·6 to 277·5)	194·4 (175·4 to 216·4)	135 (120·8 to 149·9)	82·6 (74·3 to 91·8)	58·4 (50·8 to 67·4)	36·2 (31·8 to 40·8)	26·5 (22·1 to 32·4)
Union Territories other than Delhi	143·6 (125·2 to 164·6)	120·4 (104·8 to 137·3)	92·5 (75·7 to 112·1)	64 (53 to 76·2)	44·5 (37·2 to 53·3)	33·6 (28·3 to 39·6)	25·4 (21·4 to 30)	17·5 (15 to 20·4)
Uttar Pradesh	370·2 (336·4 to 405·6)	348·9 (308·1 to 390·1)	296·7 (258·3 to 335·3)	227·9 (203·7 to 251·4)	156·9 (142·6 to 172·6)	113·3 (103 to 124)	84·3 (70·1 to 98·8)	50·7 (42·1 to 62·2)
Uttarakhand	162·8 (143 to 185·2)	145 (126·9 to 163·4)	120 (99·5 to 142·4)	95·5 (82·6 to 109·8)	73·7 (64·3 to 84·8)	54 (48·3 to 60·5)	45·2 (38·4 to 52·2)	26·9 (23·3 to 30·9)
West Bengal	268 (239·1 to 299·2)	233·5 (202 to 268·7)	193·6 (173·2 to 214·6)	138·4 (122·6 to 156·1)	92·9 (84 to 102·5)	61·2 (52·8 to 70·6)	42·2 (37·3 to 47·1)	25·2 (20·7 to 31·3)
Nepal	314·7 (286·4 to 347·1)	272·2 (251·4 to 293·2)	221·5 (204·8 to 239·2)	197·8 (183·3 to 212·1)	133·4 (124·3 to 142·8)	80·1 (73·8 to 87·4)	49·2 (44 to 54·9)	28·1 (24·1 to 33)
Pakistan	273·8 (253·5 to 297·5)	222·4 (204 to 240·2)	175·6 (162·3 to 189·7)	145·4 (133·7 to 157·4)	113 (103 to 123·9)	92·6 (83·3 to 101·7)	75·5 (66·5 to 85·1)	61·2 (53·2 to 70·4)
Southeast Asia, east Asia, and Oceania	214·6 (194·7 to 235·9)	417·8 (329·3 to 533·5)	116·1 (107·7 to 124·9)	81·9 (75·7 to 88·5)	57·8 (54·2 to 61·4)	39·1 (37 to 41·2)	21·9 (20·5 to 23·4)	13·3 (12 to 14·6)
East Asia	209·6 (184·7 to 236·9)	495·9 (384·3 to 633·7)	107·8 (96·6 to 119·6)	74·4 (66·2 to 82·9)	53·1 (48·2 to 58·4)	34·4 (31·5 to 37·7)	14·6 (13·4 to 15·7)	7·8 (7 to 8·7)
China	206·2 (181·3 to 233·8)	505·9 (391·8 to 645·8)	108·1 (96·8 to 120)	75·1 (66·6 to 83·9)	53·8 (48·7 to 59·2)	33·9 (31·1 to 37)	14·5 (13·3 to 15·7)	7·7 (6·9 to 8·7)
North Korea	414·7 (385·6 to 444·1)	242·7 (215·5 to 271)	147·5 (128·4 to 167·7)	88 (76 to 100·9)	53 (45·6 to 60·8)	60·6 (40·3 to 107·6)	21·1 (17·9 to 24·8)	11·5 (9·7 to 13·7)
Taiwan (province of China)	93·6 (81·2 to 107·7)	59·7 (58·6 to 60·8)	24·8 (24·2 to 25·4)	13·7 (13·3 to 14·1)	7·7 (7·4 to 8)	7·3 (7·1 to 7·5)	4·6 (4·4 to 4·8)	4·1 (3·6 to 4·8)
Oceania	170 (151·7 to 192·8)	134·2 (119·3 to 150·2)	96 (85·5 to 107·1)	72·4 (64·8 to 81·3)	64 (57·6 to 70·8)	58·5 (52·6 to 64·9)	54·8 (46·3 to 63·7)	45·8 (38·7 to 54·3)
American Samoa	79·3 (72 to 87·1)	50 (45·7 to 54·6)	36·2 (33 to 39·7)	22·8 (20·1 to 25·7)	16·9 (15·2 to 18·8)	14·8 (13·2 to 16·4)	12·4 (11·1 to 14)	10·4 (9 to 12)
Cook Islands	156·8 (143 to 171·2)	95·6 (87·4 to 104·3)	61·6 (56·2 to 67·9)	40·6 (36·4 to 45·4)	17·8 (15·7 to 20·3)	10·5 (9·1 to 12·3)	4·7 (4 to 5·4)	2·2 (1·9 to 2·6)
Fiji	84·5 (74·8 to 94·7)	54·6 (49·4 to 60·4)	41·5 (37·1 to 46·3)	31·5 (27·3 to 36)	23·9 (20·5 to 28)	24·1 (20·3 to 28·4)	22·6 (19 to 26·6)	19·8 (17 to 23·2)
Guam	46·3 (42·8 to 50·6)	34·6 (32·2 to 37·3)	26·3 (24·4 to 28·4)	15·9 (14·6 to 17·3)	11·7 (10·8 to 12·8)	11·1 (10·2 to 12)	13·2 (12 to 14·3)	13 (11·6 to 14·7)
Kiribati	209·4 (182·4 to 239·4)	165·5 (145·9 to 186·4)	121·8 (106·5 to 138·3)	89·4 (76·2 to 106·7)	79·4 (69·3 to 90·8)	61·9 (54·4 to 70·3)	45·6 (38·6 to 53·3)	32·7 (27·6 to 39)
Marshall Islands	130 (113·4 to 149)	93·2 (80·4 to 107)	69·5 (58·9 to 80·8)	43·4 (37·4 to 49·7)	30·8 (28·3 to 33·7)	35·4 (31·8 to 39·4)	24·3 (20·7 to 27·9)	17·5 (15 to 20·8)
Federated States of Micronesia	141·6 (120·8 to 167·6)	110·5 (94·1 to 127·5)	77·7 (65·5 to 90·7)	51·7 (44·4 to 60)	39·3 (33·7 to 45·5)	32·5 (28·2 to 37·6)	18·9 (15·6 to 22·4)	13·4 (11·3 to 16·1)
Nauru	97·2 (83·6 to 112·8)	73·8 (62·9 to 85·9)	54·9 (47·1 to 63·3)	37·4 (32·2 to 43·1)	31 (26·9 to 35·8)	41·2 (36·9 to 45·9)	37·2 (32 to 42·8)	21·8 (18·7 to 25·8)
Niue	127·4 (114·2 to 142·4)	65·6 (58·2 to 73·8)	42·6 (37·1 to 48)	27·1 (22·8 to 31·8)	21·3 (18·2 to 25·4)	23·6 (19·9 to 27·9)	21 (17·5 to 25·1)	17·5 (15 to 20·5)
Northern Mariana Islands	64·3 (55·3 to 74·7)	46 (39 to 53·7)	29·5 (25·4 to 34·8)	17·2 (14·6 to 20·4)	10·1 (8·5 to 11·9)	7·1 (6·2 to 8·2)	7·8 (6·8 to 8·7)	8·5 (7·2 to 10)
Palau	181·6 (159·7 to 206·1)	129·4 (110 to 150·2)	81·9 (69·1 to 96·2)	50·8 (42·5 to 59·3)	28·5 (24·1 to 33·1)	18·4 (15·6 to 21·4)	16·1 (13·5 to 18·9)	13·2 (11 to 16·2)
Papua New Guinea	192·1 (166·5 to 223·4)	154·2 (133·8 to 176)	109·7 (95·5 to 124·6)	85·6 (75·5 to 97·1)	77·1 (68·4 to 86·4)	68·2 (60·4 to 76·6)	62·5 (52·1 to 73·1)	51 (43·1 to 60·5)
Samoa	170 (151·7 to 189·7)	121·8 (107·9 to 136·3)	86·9 (75·9 to 99·3)	52·7 (45·1 to 60·8)	32·5 (27·3 to 38·6)	20·9 (17·5 to 24·5)	16 (13·7 to 18·7)	11·8 (10·1 to 13·9)
Solomon Islands	200·6 (174·3 to 229·4)	155·9 (136·7 to 176·5)	104·3 (91·4 to 118·5)	64·6 (54·6 to 76·8)	49·1 (41·8 to 57·1)	38·8 (33·3 to 44·7)	32·1 (26·9 to 37·8)	23·3 (19·5 to 27·8)
Tokelau	88·1 (74·6 to 103·2)	64·8 (54·7 to 75·5)	45·6 (39·2 to 53·1)	37·1 (31·8 to 43)	25 (20·7 to 30·3)	17·4 (14·5 to 20·7)	11·7 (9·9 to 13·8)	7·8 (6·6 to 9·2)
Tonga	67·9 (57·2 to 81·7)	47·5 (41 to 54·6)	37·7 (32 to 44·1)	30·8 (25·9 to 36·1)	22·7 (19 to 26·7)	19·3 (16·2 to 22·7)	15·8 (13·5 to 18·7)	11·8 (10 to 13·9)
Tuvalu	198·4 (174·7 to 224·7)	151·4 (129·6 to 175·6)	113·3 (97·3 to 131·7)	90·9 (77·9 to 104·5)	66·4 (58·1 to 75·1)	43·4 (38·9 to 48·2)	20·6 (17·3 to 24)	12·9 (11 to 15·4)
Vanuatu	190·3 (165·4 to 217·4)	151·7 (132 to 171·9)	95·8 (81·2 to 112·3)	48·8 (40·8 to 58·3)	34·7 (29·9 to 40·4)	32·6 (27·9 to 37·6)	29·7 (25·1 to 34·9)	23·1 (19·4 to 27·6)
Southeast Asia	227·6 (214·9 to 241·8)	176·3 (166·2 to 186·9)	134·9 (128·7 to 141·7)	96·4 (92·4 to 100·8)	66·8 (63·8 to 69·7)	45 (42·3 to 47·7)	29·2 (26·8 to 31·6)	19·8 (17·8 to 22)
Cambodia	220·4 (195·6 to 247·9)	190·1 (166·7 to 217·7)	183 (163·3 to 202·6)	151 (137·9 to 165·5)	110·6 (102·8 to 118·2)	83·9 (76·5 to 91)	44·2 (39 to 49·6)	27·8 (24 to 32·8)
Indonesia	267·6 (248·3 to 290·1)	210·4 (194·8 to 226·3)	157·2 (147·4 to 168)	114·7 (108·3 to 121·4)	78·1 (73·3 to 83·3)	50 (46 to 54·4)	32·4 (29 to 36·1)	21·7 (18·6 to 25·3)
Aceh	241·7 (215·4 to 273·2)	190·9 (178·1 to 205·7)	150·7 (139·6 to 162·7)	112·6 (102·5 to 123·4)	83·3 (75·5 to 91·7)	58 (52·1 to 64·3)	39·8 (34·9 to 44·9)	29·1 (24·2 to 34·9)
Bali	192·3 (174·6 to 212·9)	141·1 (121·6 to 162·4)	99·1 (89 to 109·1)	74 (67·4 to 80·9)	40·5 (36·6 to 44·6)	26·4 (23·2 to 30·3)	16·4 (13·2 to 20)	4·4 (3·7 to 5·2)

Bangka-Belitung Islands	299·1 (271·6 to 328·7)	241·4 (222·9 to 259·7)	177·6 (163 to 192·9)	118·8 (107·2 to 130·7)	85·6 (78 to 93·2)	48·8 (43·7 to 54·1)	32·1 (28 to 37·1)	27·1 (23·3 to 31·8)
Banten	413·8 (384 to 444·1)	336 (314·4 to 357·9)	254·4 (237·8 to 270·9)	178·1 (165·2 to 191·6)	107·9 (99·4 to 116·9)	57·4 (52·6 to 62·8)	36 (31·7 to 40·8)	25 (21·9 to 28·8)
Bengkulu	338·8 (305·1 to 375·7)	275·8 (256·9 to 295·7)	201·8 (186·8 to 217·5)	141·7 (132·7 to 151·4)	91·8 (85·8 to 97·7)	57·7 (53·4 to 62·5)	37·5 (33·2 to 42·4)	24·5 (20·3 to 29·4)
Gorontalo	292·8 (267·1 to 320·7)	237·6 (219·3 to 258·4)	185·1 (163·8 to 206)	140·5 (126·7 to 155·8)	106·2 (98·7 to 113·8)	83 (73·3 to 93·6)	59·8 (51·5 to 69)	41·5 (36·6 to 47·5)
Jakarta	245·9 (223·3 to 271·5)	185·6 (171·8 to 199·9)	128·4 (116·5 to 141·4)	85·4 (79·7 to 92)	53·8 (50·3 to 57·8)	34·1 (29·7 to 39·3)	24·3 (21·5 to 27·7)	17·9 (15·2 to 21·1)
Jambi	313·4 (281·4 to 350·2)	238·6 (225·3 to 252)	181·9 (167·1 to 196·4)	129·7 (118·1 to 140·9)	88·4 (82·6 to 94)	56·2 (51·7 to 61)	36·6 (32·1 to 41·7)	25·7 (21·3 to 31)
West Java	330·4 (306·4 to 357·3)	266·3 (246·3 to 285·9)	198·5 (183·3 to 214)	138·1 (129 to 148)	90·2 (85·1 to 96)	53·3 (49 to 58·3)	33·9 (30·1 to 38)	22·2 (18·9 to 26·1)
Central Java	223·8 (204·1 to 246·5)	178·6 (163·4 to 193·3)	129·4 (118·5 to 140·8)	88·2 (81·1 to 96·2)	60·6 (54·9 to 66·4)	36·8 (33·8 to 40·2)	24·6 (21·7 to 27·8)	16·1 (13·7 to 19)
East Java	252·9 (229·6 to 279·4)	203·4 (188·2 to 217·9)	155·1 (143 to 168·1)	109·8 (102·3 to 118·1)	72·7 (68·4 to 77·7)	45·4 (41·6 to 49·5)	28·9 (25·7 to 32·6)	18·9 (16 to 22·3)
West Kalimantan	256·3 (228 to 288)	224·4 (207·8 to 242·6)	183 (169·5 to 197·7)	137·2 (128·6 to 146·1)	94·4 (87·4 to 101·8)	59·3 (54·2 to 64·8)	34·4 (30·4 to 38·9)	19·1 (15·8 to 23)
South Kalimantan	355 (321·2 to 391·5)	309·2 (287·9 to 330·4)	236·7 (220 to 252·8)	177·6 (167·2 to 189·4)	121·5 (111·6 to 132·9)	66·9 (61·7 to 72·4)	41·6 (36·7 to 46·9)	28·2 (23·4 to 33·9)
Central Kalimantan	224·7 (199·1 to 253·6)	153·7 (145·5 to 161·8)	113·7 (103·7 to 123·4)	84·4 (76·2 to 92·5)	66·8 (62·4 to 71·4)	48·4 (44·5 to 52·4)	31·1 (27·4 to 35·3)	23·1 (19·2 to 27·8)
East Kalimantan	210·8 (186·4 to 238·3)	181·2 (156·6 to 207·3)	140·1 (128·3 to 151·8)	96·1 (89·4 to 103·2)	62·7 (57·7 to 67·5)	39 (35·1 to 43)	23·9 (19·1 to 29·4)	18·2 (15 to 22)
North Kalimantan	259·8 (235·2 to 286·7)	222·5 (206 to 239·2)	188·3 (175 to 202)	127·2 (113·7 to 141·5)	81·8 (73·6 to 90·8)	57 (51·3 to 63·1)	36·9 (32·3 to 42·4)	29·4 (25·7 to 33·9)
Riau Islands	212·8 (191·3 to 236·7)	164·7 (152 to 178·6)	116 (106·3 to 126·2)	86·5 (78·4 to 94·8)	61·3 (55·5 to 67·1)	40·8 (36·6 to 44·9)	26·5 (23·4 to 30·3)	19·6 (17·1 to 22·6)
Lampung	316·4 (284·1 to 352·8)	247 (227·9 to 265·7)	183·4 (168·7 to 198·8)	126·2 (118·5 to 134·5)	85·3 (79·7 to 90·8)	50·6 (45 to 56·3)	33·7 (29·6 to 38·5)	25·3 (20·9 to 30·4)
Maluku	347·1 (313·9 to 383·2)	268·4 (255·3 to 281·7)	202·7 (186·9 to 217·9)	150·6 (139·2 to 163)	109 (99·7 to 117·9)	78·2 (71·1 to 86·1)	53·6 (47·5 to 60·1)	30·6 (25·6 to 36·6)
North Maluku	317·5 (288·9 to 348·1)	256·1 (237·8 to 275·7)	195·5 (183 to 208·9)	155·2 (141 to 171·2)	112·1 (101·6 to 122·3)	83·7 (72 to 95·9)	59·3 (52·6 to 67·2)	39·7 (35·1 to 45·3)
West Nusa Tenggara	391·8 (355·2 to 431·6)	326·9 (304·9 to 351)	263·6 (246·1 to 281·5)	201·2 (188·3 to 215·7)	130·2 (119·9 to 140·1)	74·3 (65·3 to 83·8)	40·1 (35·3 to 45·1)	24·9 (20·7 to 29·9)
East Nusa Tenggara	284·2 (255·6 to 315·9)	226·2 (214·8 to 237·7)	172·4 (158·7 to 186·9)	126·9 (116·5 to 137·5)	91·2 (85·3 to 96·7)	71 (63 to 80·3)	47·8 (42·4 to 53·8)	29·1 (24·2 to 34·9)
Papua	212·7 (188·2 to 240·8)	184·2 (173·5 to 195)	153·3 (142·7 to 164·5)	126·1 (113·7 to 139·1)	104·2 (94·8 to 113·4)	80·2 (70·4 to 91·2)	66·3 (58·4 to 75)	53·1 (44·2 to 63·5)
West Papua	166·6 (148·7 to 186·8)	140·7 (128·6 to 154·3)	117·3 (108·8 to 126·1)	85·6 (77·6 to 94·4)	63·6 (57·4 to 70·1)	52·6 (45·8 to 60·3)	38·7 (34·1 to 44·1)	24·1 (21 to 27·8)
Riau	243·3 (216·3 to 273·7)	207·1 (193 to 221·6)	159·8 (148·2 to 172·3)	106·3 (99·5 to 113·5)	74·6 (69·5 to 80)	50·4 (46·3 to 54·7)	34·4 (30·4 to 38·9)	19·5 (16·1 to 23·5)
West Sulawesi	434 (402·5 to 465·7)	360·4 (336·9 to 384·9)	286 (267 to 304·3)	205·6 (188·5 to 223·6)	134·6 (122·9 to 146·9)	84·2 (76·2 to 92·4)	57·1 (50·5 to 64·7)	38·8 (34·1 to 44·5)
South Sulawesi	303·1 (277·7 to 331·5)	244·8 (227 to 264)	185·1 (171·2 to 199·4)	136·5 (128·2 to 145·6)	90·5 (82·5 to 99·3)	56·3 (51·8 to 61·2)	35·7 (31·5 to 40·5)	21·2 (18 to 25)
Central Sulawesi	360·6 (325·9 to 398·1)	260·4 (241·8 to 278·3)	193·2 (177·3 to 209·7)	142·4 (130·8 to 154·5)	103·6 (95·4 to 112·5)	69·4 (64·2 to 75·1)	46·8 (41·5 to 52·7)	33·2 (27·6 to 39·7)
Southeast Sulawesi	307·1 (275·4 to 342·3)	261·2 (242·8 to 282·9)	212·9 (197·9 to 229·6)	151·1 (138·1 to 164·6)	104·5 (97·8 to 111·2)	66·7 (60·2 to 73·5)	45·9 (40·4 to 51·7)	35·3 (29·3 to 42·2)
North Sulawesi	205·7 (181·9 to 233·2)	165·5 (153·7 to 176·7)	116·4 (107·9 to 125·8)	76·2 (69·4 to 83·3)	57·1 (51·6 to 62·7)	43·4 (38·6 to 48·3)	37 (32·5 to 41·9)	30·6 (25·4 to 36·8)
West Sumatra	366·8 (331·6 to 404·8)	268·8 (250·9 to 288·3)	189·2 (175·6 to 203)	129·7 (120·5 to 139·3)	84·1 (78·8 to 89·9)	55·7 (51·2 to 60·2)	33 (29 to 37·6)	17·5 (14·5 to 21·2)
South Sumatra	272·5 (250·7 to 297·6)	212 (196·6 to 226·7)	161·3 (148·4 to 174·8)	109·6 (102·6 to 117·4)	82·5 (75·5 to 90·5)	59 (54·6 to 63·8)	39·1 (34·7 to 44·1)	30 (25·5 to 35·3)
North Sumatra	221·5 (196·2 to 250·1)	176 (162·7 to 190·1)	132·2 (121·2 to 143·8)	95·4 (89·4 to 101·9)	71 (65·1 to 77·7)	50 (46·1 to 54·1)	37 (32·7 to 41·5)	30·8 (25·6 to 36·9)
Yogyakarta	129·2 (113 to 148·3)	91·8 (78·2 to 107·3)	65·1 (58·5 to 72·4)	43·4 (39·2 to 48·1)	29·4 (26·7 to 32·7)	21 (18·1 to 24·5)	18·6 (14·8 to 23)	16·9 (13·9 to 20·4)
Laos	304·9 (274·3 to 337·7)	284·5 (252·2 to 317·9)	255·8 (226·5 to 287·9)	213·5 (189·6 to 238·6)	158·4 (144·9 to 173·4)	108·9 (99·7 to 118·9)	62·8 (56·5 to 69·2)	37 (32·4 to 42·2)
Malaysia	132·3 (122 to 143·3)	90·9 (89·4 to 92·4)	49·7 (48·8 to 50·7)	28·3 (25·5 to 31·6)	15·8 (15·3 to 16·2)	8·3 (8 to 8·5)	6·6 (6·4 to 6·8)	5·8 (5 to 6·8)
Maldives	424·7 (390·4 to 459·8)	338·3 (305·2 to 373·2)	232·4 (207·3 to 258)	139·1 (123·6 to 156)	61·6 (54·9 to 68·9)	30·5 (27 to 34·4)	18·5 (16·2 to 21)	14·9 (12·9 to 17·4)
Mauritius	118·6 (114 to 123·3)	100 (96·6 to 103·4)	74·3 (71·3 to 77·2)	37·1 (35·4 to 38·8)	19 (17·8 to 20·2)	15·3 (14·3 to 16·4)	12·8 (11·8 to 13·8)	10·9 (9·6 to 12·5)
Myanmar	326·2 (296 to 358)	284·1 (256·1 to 313·9)	235 (208·5 to 264·7)	170·2 (149·6 to 191·5)	130 (115·8 to 145·8)	99·5 (88·8 to 110·9)	59·8 (52·8 to 67·9)	35·9 (31·1 to 41·2)
Philippines	184 (166·9 to 204·6)	131·4 (119·9 to 143·7)	96·4 (88·1 to 105·1)	74·2 (67·1 to 81·3)	49·5 (45·2 to 54·2)	33·2 (29·9 to 37·1)	26·8 (23·6 to 30·1)	20·2 (17·4 to 23·5)
Seychelles	104·5 (96 to 114·8)	89·3 (83·8 to 95·3)	69·4 (65·1 to 74)	34·3 (31·6 to 37·3)	18·3 (16·8 to 20)	13·1 (11·8 to 14·6)	13·9 (12·3 to 15·4)	11·8 (10·3 to 13·5)
Sri Lanka	153·8 (150·9 to 156·9)	91·4 (89·5 to 93·2)	64·7 (63·3 to 66·1)	41·6 (40·7 to 42·5)	25·8 (24·6 to 27·3)	15·9 (15·5 to 16·4)	12·8 (12·4 to 13·2)	7 (6 to 8·2)
Thailand	148·3 (132·5 to 165·2)	107·8 (98·9 to 117·2)	70·4 (64·4 to 76·4)	42·6 (38·4 to 47·8)	24·8 (21·8 to 28·8)	15·9 (13·7 to 18·4)	8·8 (8·6 to 9·1)	6·9 (6·1 to 7·6)
Timor-Leste	303·9 (271·5 to 338·7)	268·5 (236·7 to 299·6)	221·8 (200·8 to 244·4)	187·4 (175·6 to 200·1)	119·1 (110·2 to 128·3)	77·2 (70·1 to 84·8)	39·6 (35 to 44·5)	29 (25 to 34)
Vietnam	147 (129·4 to 166·9)	112·2 (95·6 to 129·8)	95·4 (87·6 to 104)	55·4 (50·6 to 61)	38 (34·9 to 41·6)	23·1 (20·8 to 25·4)	15·1 (13·1 to 17·1)	10·4 (8·8 to 12·4)
Sub-Saharan Africa	269·1 (243·9 to 296·3)	246·9 (224·6 to 268·9)	214·1 (202·1 to 226·7)	183·5 (176 to 191·3)	162·1 (156·3 to 168·4)	138·6 (134 to 143·4)	99·4 (94·7 to 104·6)	68·6 (61·5 to 77·4)
Central sub-Saharan Africa	232·8 (206·7 to 261·5)	217 (193·9 to 241·4)	200·5 (182·4 to 219·3)	180 (165 to 196·4)	162 (151·6 to 173·2)	145·1 (136·2 to 154·6)	97·2 (89·9 to 105·1)	53·8 (48 to 60·8)
Angola	230 (204·5 to 258·2)	225·3 (198 to 252·2)	213·7 (189·6 to 240·3)	202·4 (182·1 to 224·1)	198·2 (185 to 214·5)	159·8 (148·7 to 171·4)	89·4 (81·2 to 98)	48·8 (42·6 to 55·7)
Central African Republic	238·5 (208·5 to 272·9)	216·8 (187·9 to 246·7)	190·7 (170·7 to 213·5)	179·8 (163·5 to 197·7)	173·2 (158·8 to 188·9)	171·2 (153·9 to 188)	148·3 (130·1 to 167·6)	114·1 (97·2 to 135·3)
Congo (Brazzaville)	236·8 (211·3 to 265)	221·4 (193·9 to 250·6)	187 (163·1 to 216·2)	131·2 (116·6 to 147·3)	101·1 (93·3 to 109·6)	93·7 (85·8 to 102·5)	57·1 (51·7 to 62·8)	34·6 (29·4 to 40·3)

DR Congo	233·2 (207 to 261·9)	215 (186·4 to 243·2)	200·1 (177·9 to 223·3)	179 (161·8 to 198·2)	156·7 (144·8 to 168·2)	143·5 (131·6 to 156·5)	99·9 (89·2 to 111·5)	53·3 (46·3 to 62·2)
Equatorial Guinea	268·3 (239·9 to 299·1)	259·2 (228·1 to 289·7)	212·7 (186·2 to 241·5)	165·3 (147·4 to 184·5)	160 (141·5 to 179·1)	111·8 (98·4 to 125·3)	55·4 (47·2 to 64·3)	33·7 (28·7 to 40·1)
Gabon	177·1 (155·8 to 201·2)	166·2 (141·8 to 191·4)	138·6 (121·5 to 157·8)	99·8 (88·8 to 111·9)	73·2 (66 to 81·2)	59·4 (52·3 to 66·3)	43·9 (38·1 to 50·3)	25·3 (21·5 to 30·2)
Eastern sub-Saharan Africa	268·3 (242·3 to 296·6)	240 (216·8 to 262·4)	209·5 (197·2 to 222)	184·4 (175·1 to 194·6)	159·9 (153·9 to 166·4)	124·7 (119·3 to 130)	81 (75·6 to 86·8)	53·4 (47·1 to 61·3)
Burundi	316·5 (285·3 to 349·6)	276·1 (246·5 to 306·4)	243·2 (220·5 to 266·9)	206 (190·4 to 222·8)	174·9 (161·4 to 190·2)	158·6 (146·3 to 171·1)	91·7 (81·7 to 102·4)	60·2 (52·4 to 70·2)
Comoros	249·1 (221·9 to 278·8)	225·2 (199·9 to 255·2)	184·8 (165·6 to 206)	153·3 (138·9 to 167·8)	118·1 (106·9 to 129·7)	99·6 (90·5 to 110·2)	71·9 (62·4 to 81·7)	47·7 (41·2 to 56·1)
Djibouti	154·1 (135 to 175·7)	140·8 (119·5 to 162·4)	126·8 (110·6 to 144·3)	110·6 (98 to 124·6)	105 (93·8 to 117·1)	91·3 (81 to 103·6)	64·4 (54·9 to 74·6)	43·3 (37·1 to 51·2)
Eritrea	264·6 (232·6 to 300·8)	240 (207·5 to 271·5)	219·2 (188 to 295·1)	188·1 (174 to 202·2)	152·5 (139·8 to 165·6)	89 (78·1 to 100·7)	63·7 (54·3 to 74·3)	42·7 (35·7 to 51·7)
Ethiopia	278 (250·1 to 308·3)	258·3 (230·1 to 285)	237·8 (217·2 to 259·8)	212·9 (195·8 to 232·1)	181 (169·3 to 193·6)	133·8 (123·5 to 145·2)	79·6 (71·3 to 88·5)	46·8 (40·7 to 54·6)
Kenya	220·9 (203·2 to 241·9)	164·5 (153·1 to 175·7)	134 (124·9 to 143·4)	102·2 (94·7 to 109·8)	87·4 (81·2 to 93·5)	77·9 (71·6 to 84·8)	53·3 (47·9 to 59·4)	37·2 (32·4 to 42·8)
Baringo	195·1 (175 to 218·6)	166·5 (151·7 to 182·5)	163·8 (150·6 to 177·7)	141 (129·4 to 152·6)	122·1 (109·6 to 134·1)	96·2 (87·3 to 106·3)	70·7 (62·3 to 80·1)	48·8 (41·8 to 56·9)
Bomet	79·1 (70·6 to 89·4)	66·9 (58 to 76·3)	65·6 (56·3 to 76·2)	58·8 (49·1 to 69·6)	46·8 (40·2 to 53·5)	42·2 (35·6 to 50)	36·2 (29·6 to 42·7)	22·9 (19·6 to 26·9)
Bungoma	236·1 (213 to 263·3)	185·9 (170·3 to 201·9)	145·5 (127·4 to 165·8)	119 (108·8 to 130)	104·4 (94·3 to 115·4)	91·5 (82·4 to 101·7)	58·3 (49·7 to 67·8)	34·4 (29·4 to 40·4)
Busia	353·4 (320·5 to 390·3)	281·7 (255·5 to 307·7)	214·8 (195 to 234·7)	134·8 (123·7 to 147·4)	118·6 (106·3 to 132·1)	118·1 (104·4 to 132·3)	75·8 (65·9 to 86·2)	47·4 (40·6 to 55·6)
Elgeyo Marakwet	119·1 (106·5 to 134·4)	104·3 (94·4 to 114·8)	110·6 (101·2 to 120·8)	104·6 (95·3 to 114·1)	78·9 (71·2 to 86·2)	53·1 (48·1 to 59)	30·3 (26·4 to 34·8)	19 (16·2 to 22·3)
Embu	317·6 (286·9 to 353·1)	229·8 (203·5 to 258)	144·2 (127 to 162·4)	86·4 (73·8 to 100·3)	60·3 (52·6 to 68)	47·6 (43·2 to 52·3)	41·2 (33·9 to 48·3)	29·4 (25·2 to 34·5)
Garissa	223 (200·6 to 249·9)	177·6 (153·7 to 201·5)	147·8 (126·8 to 170·8)	121·9 (110·5 to 133·3)	102·5 (88·6 to 116·6)	75·9 (65 to 88·9)	48 (42 to 54·7)	30·9 (26·4 to 36·3)
Homa Bay	401·9 (364·5 to 442·7)	324·2 (296·8 to 353)	295·7 (270·1 to 322·3)	238·9 (222·6 to 257·1)	199·1 (180·9 to 217·6)	187·8 (168·5 to 209·7)	103·2 (89·6 to 118·2)	64·9 (55·6 to 75·9)
Isiolo	486·3 (442·5 to 534·3)	404·7 (364·8 to 446·9)	367·4 (332·3 to 405·6)	288·1 (251·3 to 325·7)	221·2 (194·5 to 248·4)	160·8 (146·5 to 177·4)	122·6 (107·8 to 138·1)	84·5 (72·7 to 98·2)
Kajiado	120 (107·3 to 135·3)	100·3 (89·5 to 113·1)	105·3 (96·2 to 114·8)	87·6 (78·7 to 96·6)	60·9 (54·1 to 68·1)	49·6 (44·9 to 55·1)	30·8 (26·9 to 35·2)	17·6 (15 to 20·6)
Kakamega	249·9 (225 to 278·7)	198·9 (181·3 to 215·9)	166·5 (150·8 to 183·4)	141·1 (119·5 to 164·9)	134·8 (123·2 to 146·3)	131·9 (118·1 to 146·8)	81·1 (70·3 to 92·3)	48·2 (41·3 to 56·6)
Kericho	65·6 (58·5 to 74·2)	56·4 (49·6 to 63·5)	62·2 (55·6 to 69·6)	66·1 (60·1 to 72·4)	51 (45·6 to 56·4)	48·9 (42·4 to 56)	37 (32·1 to 42·4)	26·4 (22·6 to 31·1)
Kiambu	104·2 (93·1 to 117·5)	74 (66·9 to 82·3)	54·2 (48·9 to 59·9)	43 (38·2 to 47·8)	43·7 (39 to 48·8)	50·8 (43·7 to 58·9)	42·3 (36·5 to 48)	30·9 (26·4 to 36·1)
Kilifi	401·9 (365·4 to 444·7)	299·8 (274 to 324·7)	244·2 (226·8 to 261·7)	179·4 (161 to 198·6)	134·9 (120·2 to 150·1)	98·4 (90·2 to 106·9)	60·8 (53 to 69·1)	38·9 (33·3 to 45·7)
Kirinyaga	246·3 (221·9 to 275·6)	188·2 (171·5 to 206·6)	156·9 (143 to 171·4)	79·4 (67·7 to 92)	57·4 (48·1 to 68·2)	55·7 (49·5 to 62·4)	45·2 (39·2 to 51·5)	40 (34·3 to 46·7)
Kisii	218·3 (196·4 to 244·4)	170·7 (152·9 to 188·5)	141·6 (125·3 to 158·3)	118·5 (105·1 to 132·8)	106·7 (96·3 to 116·6)	82·2 (74·2 to 91·4)	43·9 (36·8 to 51·6)	28·7 (24·5 to 33·7)
Kisumu	208·4 (187·4 to 233·4)	164·6 (148·9 to 181·2)	148·1 (131·6 to 165·3)	135·6 (121·6 to 151·1)	125·6 (113·1 to 138·3)	116·1 (104·7 to 128·3)	57·6 (48·6 to 67·5)	36·4 (31·2 to 42·8)
Kitui	222 (200·1 to 247·9)	176·6 (159·8 to 193·8)	133·6 (121·5 to 146·7)	88 (80·1 to 96·3)	75·2 (66·8 to 84·4)	66·1 (59·7 to 73·5)	41·1 (35·7 to 47·2)	26 (22·2 to 30·5)
Kwale	342·5 (309·1 to 380·1)	262·6 (237·3 to 288·6)	205·4 (189·2 to 222·2)	150·8 (135·6 to 166·8)	116·4 (104·4 to 130)	85·8 (78·6 to 93·6)	63·9 (55·6 to 72·6)	41·5 (35·5 to 48·7)
Laikipia	65·9 (58·8 to 74·7)	52·1 (44·6 to 60·9)	53·3 (46·8 to 60·5)	45·2 (39·4 to 52·7)	41·9 (33·9 to 50·3)	42·7 (37·3 to 48·9)	27·7 (24·2 to 31·7)	16·2 (13·8 to 18·9)
Lamu	439·7 (400·5 to 483·6)	326·1 (297·1 to 356)	246·5 (212·7 to 281·7)	130·9 (111·6 to 152·6)	132·9 (108·9 to 161·1)	116·6 (102·2 to 132·9)	91·6 (76 to 109·8)	63·4 (54·3 to 73·8)
Machakos	165·6 (148·4 to 186·2)	127·9 (114·4 to 140·9)	107·6 (94·7 to 121·4)	73·6 (62·3 to 85·6)	59·1 (52·6 to 66·3)	58·3 (53·1 to 64·2)	40·6 (33·3 to 48·1)	28·6 (24·4 to 33·4)
Makueni	219·3 (197·3 to 246·2)	173 (152·8 to 192)	144 (131·1 to 158·1)	106·1 (90·6 to 123·4)	87·1 (77·2 to 97·9)	64·1 (56·8 to 71·9)	39·1 (34·5 to 44·4)	22·7 (19·4 to 26·6)
Mandera	154·6 (138·5 to 174·1)	130·3 (113 to 148·6)	114·2 (98·9 to 131·1)	106·6 (96·8 to 117)	111·2 (96·3 to 127·2)	90·3 (76·7 to 105·9)	72·8 (63·7 to 83)	55·6 (47·6 to 65·1)
Marsabit	213·4 (191·9 to 238·9)	170·5 (149·9 to 192·5)	154·1 (141 to 167·2)	124·6 (107·5 to 143·7)	101·7 (90·6 to 113·3)	77·9 (69·8 to 86·9)	70 (61·2 to 79·6)	53·8 (46·1 to 63)
Meru	156·5 (140·2 to 176)	119·5 (108 to 131·5)	91·6 (82·1 to 101·8)	59·4 (53·6 to 65)	48·9 (43·8 to 54·4)	46·9 (41·9 to 52·6)	47·1 (40·9 to 53·8)	40·4 (34·6 to 47·4)
Migori	344·4 (311·5 to 381·4)	283·9 (255·9 to 312·5)	249·2 (228·2 to 271·4)	233·8 (217·9 to 250·2)	208·3 (188·8 to 228·4)	184·6 (165·8 to 205)	105·5 (90·6 to 121·1)	69·2 (59·2 to 80·9)
Mombasa	264·7 (238·2 to 295·5)	178·7 (161 to 195·6)	132·1 (121·7 to 142·8)	88·5 (79·3 to 98·4)	78·1 (69·2 to 87·1)	72·6 (65·4 to 80)	51·4 (45 to 58·6)	33·2 (28·4 to 39)
Murang'a	192·4 (172·7 to 216)	134·6 (121 to 149)	99·4 (87·4 to 111·5)	55·7 (47·7 to 64·6)	42·2 (37·2 to 47·5)	42·2 (37·8 to 47·1)	39·1 (33·6 to 44·5)	28·6 (24·5 to 33·6)
Nairobi	296·1 (266·9 to 329·2)	165·7 (151·3 to 181·3)	98·1 (89·6 to 106·6)	58·7 (51·2 to 66·6)	60·1 (53·3 to 66·9)	63·8 (57·6 to 70·3)	35 (30·6 to 40)	22·6 (19·4 to 26·6)
Nakuru	69·7 (62·2 to 79·1)	54·5 (48·7 to 61·5)	57 (51·2 to 63·8)	52·5 (47·4 to 57·7)	46·5 (41·9 to 51·2)	49·8 (45·3 to 54·6)	45·3 (39·4 to 51·8)	33·8 (28·9 to 39·7)
Nandi	101·3 (90·5 to 114·4)	83·2 (74·1 to 92·7)	73·9 (67·9 to 80·3)	64·6 (52·6 to 76·8)	52·3 (46·9 to 57·8)	40 (36·1 to 44·4)	28·1 (24·4 to 32·4)	18·8 (16·1 to 22·1)
Narok	41·8 (37·3 to 47·5)	41 (36·3 to 47)	54·9 (49 to 61)	75·2 (68·7 to 82·1)	65 (58·3 to 71·6)	44·6 (40·5 to 49·6)	31 (26·9 to 35·6)	21·6 (18·5 to 25·4)
Nyamira	118·5 (105·8 to 133·9)	93·3 (82·9 to 104·7)	74·1 (67·3 to 81·5)	66·6 (59·3 to 73·8)	53·4 (47·5 to 59·4)	53·9 (47·3 to 61·9)	36·8 (31·9 to 42·7)	25·7 (21·9 to 30)
Nyandarua	169·5 (152 to 190·4)	112·4 (101·8 to 124·3)	75·8 (62·9 to 91·1)	43·4 (35·8 to 51·7)	34·7 (27·8 to 42·3)	38·7 (32·4 to 46·4)	48 (39·4 to 57·6)	38·6 (33 to 45)
Nyeri	171·3 (153·7 to 192·6)	119·8 (105·3 to 136·1)	83·1 (74·4 to 91·6)	47·8 (42 to 53·8)	40·5 (34·9 to 46·9)	42·8 (36·8 to 49·3)	38·3 (33·3 to 43·7)	32·3 (27·6 to 37·9)
Samburu	73·6 (65·7 to 83·3)	65·5 (56·3 to 74·8)	70·9 (59·7 to 83·1)	76·3 (62·3 to 91·3)	81·7 (65·6 to 99)	76·6 (65·5 to 89·6)	65·1 (53·8 to 77·1)	49·1 (42·1 to 57·6)
Siaya	335·5 (303·3 to 372·1)	273 (249·8 to 295·5)	223·9 (203·8 to 243·4)	193·3 (179·3 to 207)	193·5 (175·8 to 211·6)	179·7 (162·2 to 199·5)	96 (83·6 to 110)	58·1 (49·7 to 68)

Taita Taveta	241·7 (217·3 to 270·1)	180·3 (164·5 to 197·3)	167·1 (153·6 to 180·3)	104·8 (82·9 to 127·4)	71·5 (63·7 to 80·1)	69 (59·5 to 80·3)	46·1 (40·2 to 52·6)	27·4 (23·4 to 32·1)
Tana River	188·7 (169·3 to 211·7)	148·2 (135·1 to 162·4)	132·4 (113 to 153·8)	113 (90·9 to 137·3)	96 (84·4 to 107·8)	99·9 (84·4 to 118)	76·9 (63·8 to 92·1)	50·7 (43·5 to 59·1)
Tharaka Nithi	130·8 (117 to 147·3)	105·8 (93·3 to 119·2)	92·9 (80·9 to 105·6)	66·7 (57·2 to 77·4)	68·7 (60·9 to 77·7)	67·2 (60 to 75·3)	41·9 (36·7 to 47·9)	28·7 (24·5 to 33·7)
Trans Nzoia	124·2 (111 to 140)	93·5 (82·8 to 104·1)	91·5 (83·5 to 99·9)	85·9 (71·2 to 101·5)	65·4 (58·2 to 72·5)	54·1 (49·2 to 60)	37·4 (32·4 to 42·7)	23·5 (20·1 to 27·7)
Turkana	133·4 (119·4 to 150·4)	116·8 (102·5 to 132·9)	126·2 (106·9 to 147·8)	136·8 (113·7 to 161)	115·6 (101·8 to 130)	84·2 (71·9 to 98·6)	57·6 (48·1 to 67·5)	41 (35·1 to 47·9)
Uasin Gishu	82·2 (73·3 to 93·1)	61·3 (54·6 to 68·9)	57·3 (51 to 63·6)	56·5 (51 to 62·1)	51·7 (46·7 to 56·7)	42·9 (38·6 to 47·5)	33·8 (29·4 to 39)	23·9 (20·4 to 28·1)
Vihiga	170·8 (153·1 to 192·1)	135·4 (121·2 to 150·4)	111·1 (95 to 129)	92·8 (78·2 to 109·5)	94·1 (84·5 to 105·2)	92 (79·8 to 105·5)	71·4 (60·8 to 83)	47·2 (40·5 to 55·1)
Wajir	302·8 (273·4 to 336·8)	240·3 (211·1 to 271·1)	197·5 (171·4 to 225·6)	147·9 (135·5 to 160·2)	136·5 (118·2 to 155·2)	116·3 (99·4 to 136·2)	85·5 (75·2 to 97·4)	58·6 (50·2 to 68·7)
West Pokot	163·8 (146·9 to 184·4)	142·7 (129·1 to 156·6)	150·8 (138 to 163·5)	147·1 (134·9 to 159·6)	131·7 (119·2 to 143·7)	99·2 (89·9 to 109·6)	75·6 (65·9 to 86·8)	54·8 (46·9 to 64·1)
Madagascar	232·6 (203·2 to 266)	204·6 (176·9 to 234·7)	174 (155·5 to 193·2)	156·5 (144·4 to 170)	136·2 (126·5 to 146)	100·4 (91·6 to 109·7)	75·4 (65·9 to 85·2)	53·2 (45·1 to 63·6)
Malawi	383·8 (351·8 to 417·2)	348 (317·2 to 378·8)	304·5 (281·6 to 327·9)	241·5 (224·7 to 258·4)	202·7 (190·1 to 215·3)	145·7 (136·2 to 155·9)	86·4 (78·7 to 94·6)	53·7 (46·9 to 62·4)
Mozambique	280·5 (251·4 to 312·1)	260·5 (229·4 to 292·6)	235·7 (211·2 to 261·1)	217·3 (198·1 to 237·2)	201·5 (187·8 to 216·5)	152·6 (141·7 to 163·7)	100·5 (90·1 to 111·3)	64·7 (56·2 to 75·5)
Rwanda	370·3 (334·8 to 407·7)	287·4 (266·3 to 309·8)	242·4 (225·2 to 259·5)	204·6 (191·6 to 218·3)	162·2 (151·6 to 173)	139·9 (129·9 to 150·4)	68·4 (61·5 to 75·7)	43 (37·3 to 50·3)
Somalia	225·7 (200·3 to 253·6)	217·3 (190·9 to 244·5)	204·6 (180·2 to 231·3)	188·8 (168·7 to 208·6)	176·6 (160·3 to 194·4)	150·9 (135·5 to 166·5)	129·4 (115·3 to 143·9)	88·8 (76·9 to 103·8)
South Sudan	244·9 (218·1 to 274·2)	231·1 (205·4 to 257·5)	216·5 (187·3 to 250·2)	199·8 (175·1 to 226·5)	181·5 (165·6 to 199·5)	140·3 (127·2 to 154·4)	110 (98·1 to 122·2)	85·8 (73·1 to 100)
Uganda	230·4 (204·9 to 258·4)	215 (190·2 to 240·9)	178·3 (163 to 193·7)	183·9 (171·3 to 196·3)	154·6 (145·8 to 164·2)	121·1 (113·8 to 129)	79·6 (72·9 to 86·6)	52 (45·7 to 60)
Tanzania	255·6 (227·6 to 285·9)	236·5 (210·1 to 265·3)	203·8 (187·7 to 219·6)	167·9 (157·2 to 179·4)	147·5 (138·8 to 156·8)	117·4 (109·8 to 125·8)	76·5 (69·4 to 83·9)	53·9 (47·1 to 62·5)
Zambia	242 (215·2 to 271·2)	216·2 (191·2 to 242·5)	177·3 (163·3 to 191·9)	156·1 (145·1 to 167·3)	151·8 (141·6 to 161·7)	129·3 (119·7 to 140)	74·5 (67 to 82·7)	46·9 (40·7 to 54·9)
Southern sub-Saharan Africa	152·9 (134·5 to 173·5)	138·1 (121·5 to 155·8)	115·1 (105·8 to 125·7)	93·1 (88·4 to 98)	66·4 (62·7 to 70·1)	67·7 (64·2 to 71·5)	52·8 (48·7 to 57)	37 (32·6 to 42·7)
Botswana	114·1 (99·2 to 131·1)	112·3 (96·1 to 130)	98·9 (88·4 to 110·6)	70·3 (63·2 to 77·3)	58·6 (53·1 to 64·7)	69·9 (62·9 to 77·6)	49·3 (42·1 to 57·3)	38·2 (32·1 to 46·3)
eSwatini	230·5 (204·8 to 258·8)	207·4 (182·4 to 233)	155·7 (134·1 to 180·4)	105·4 (92·9 to 119)	66·8 (61·2 to 72·8)	80·1 (73·7 to 87·3)	68·6 (62·2 to 75·2)	42·2 (36 to 49·3)
Lesotho	145·9 (128 to 167·6)	142·4 (130·1 to 154·2)	136·7 (125·9 to 147·6)	103·6 (93·6 to 114·1)	79·7 (72·8 to 87·4)	86·1 (78·5 to 94·1)	76 (66·9 to 86·2)	57·2 (48·9 to 67·1)
Namibia	128·8 (112·3 to 147·7)	119·9 (102·7 to 137·9)	99·6 (88·9 to 111·8)	87·5 (79·9 to 95·4)	63 (57·6 to 68·5)	59·4 (53·7 to 65·5)	45·2 (39·6 to 51·1)	30·9 (26·6 to 36·6)
South Africa	157·2 (137·6 to 179·1)	142·2 (124·2 to 161·4)	119·7 (107·8 to 133)	98·3 (92·4 to 104·7)	68·3 (63·5 to 73·2)	66 (61·1 to 71·5)	46·3 (40·9 to 51·8)	31·8 (27·6 to 37)
Zimbabwe	135·7 (118·5 to 155·2)	121·4 (105 to 139·1)	96·9 (87·5 to 107)	79·7 (73 to 86·9)	60·7 (56·2 to 65·4)	69·3 (63·9 to 75·1)	66·5 (60·6 to 73·2)	47·8 (41·8 to 55·3)
Western sub-Saharan Africa	301·9 (275·9 to 329·1)	285·7 (262·4 to 309·3)	244·4 (230·8 to 258·9)	202·6 (195·6 to 210)	181·8 (175·8 to 188·3)	160·8 (155·2 to 166)	122 (116·3 to 128·2)	89·2 (80·6 to 99·6)
Benin	307 (277·2 to 338·8)	289·2 (266·8 to 311·9)	243·8 (225 to 262·4)	195·9 (181·8 to 209·5)	170·4 (158·6 to 182·6)	134·3 (124·8 to 144·4)	105·2 (96·9 to 113·8)	78·3 (69·3 to 89·4)
Burkina Faso	375·7 (341·9 to 410·9)	338 (304·5 to 371·5)	286·6 (264·7 to 309·3)	240·4 (223·3 to 258·3)	202·5 (188·9 to 215·4)	173·5 (160·5 to 186·6)	134·1 (120·9 to 148)	104·1 (90·9 to 120·7)
Cape Verde	162·6 (149·2 to 176·7)	156·4 (150·7 to 162·2)	158·9 (154·6 to 163·6)	89·2 (85·7 to 93·4)	63·9 (61·1 to 67)	47·5 (42·2 to 53·7)	24·5 (22·6 to 26·6)	15·3 (13·4 to 17·5)
Cameroon	283·1 (261·1 to 308·8)	415·4 (394·2 to 438·2)	190·8 (176·9 to 206)	154·8 (143 to 166·7)	128·5 (119·7 to 137·2)	123·9 (114·5 to 134·3)	96·5 (85·8 to 108·3)	67 (58·4 to 76·9)
[truncated: 555,476 more chars]
