# Supplementary material for: Global age-sex-specific fertility, mortality, healthy life expectancy (HALE), and population estimates in 204 countries and territories, 1950–2019: a comprehensive demographic analysis for the Global Burden of Disease Study 2019
Source: Lancet. 2020 Oct 17;396(10258):1160–203. doi: 10.1016/S0140-6736(20)30977-6 (PMC7566045; doi:10.1016/S0140-6736(20)30977-6)
Supplement: Supplementary appendix 1 [file mmc1.pdf]

THE LANCET

Supplementary appendix 1

This appendix formed part of the original submission and has been peer reviewed. We post it as supplied by the authors.

Supplement to: GBD 2019 Demographics Collaborators. Global age-sex-specific fertility, mortality, healthy life expectancy (HALE), and population estimates in 204 countries and territories, 1950–2019: a comprehensive demographic analysis for the Global Burden of Disease Study 2019. *Lancet* 2020; **396:** 1160–203.

Appendix 1: methods appendix to "Global age-sex-specific fertility, mortality, healthy life expectancy (HALE), and population estimates for 204 countries and territories, 1950–2019: a comprehensive demographic analysis for the Global Burden of Disease Study 2019"

This appendix provides further methodological detail for "Global age-sex-specific fertility, mortality, healthy life expectancy (HALE), and population estimates for 204 countries and territories, 1950–2019: a comprehensive demographic analysis for the Global Burden of Disease Study 2019."

Preamble

This appendix provides further methodological detail for "Global age-sex-specific fertility, mortality, healthy life expectancy (HALE), and population estimates for 204 countries and territories, 1950–2019: a comprehensive demographic analysis for the Global Burden of Disease Study 2019." This study complies with the Guidelines for Accurate and Transparent Health Estimates Reporting (GATHER) recommendations.[1] It includes detailed tables and information on data in an effort to maximise transparency in our estimation processes and provide a comprehensive description of analytical steps. We intend this appendix to be a living document, to be updated with each iteration of the Global Burden of Disease Study.

Portions of this appendix have been reproduced or adapted from appendices for Wang,[2] Wang,[3] Dicker,[4] Murray,[5] and Kyu.[6] References are provided for reproduced or adapted sections.

Authors contributions

Managing the estimation process

Haidong Wang, Tahiya Alam, Charlton SKH Callender, Katherine Paulson, and Christopher J L Murray.

Writing the first draft of the manuscript

Haidong Wang, Greg Bertolacci, Charlton SKH Callender, Haley Comfort, Andrew T Leever, Claudia I Mastrogiacomo, Erin A May, Molly R Nixon, Katherine Paulson, Spencer Pease, David Shaw, Muming Tang, Heather Taylor, and Christopher J L Murray.

Providing data or critical feedback on data sources

Haidong Wang, Hassan Abolhassani, Lucas Abreu, Michael Abrigo, Abdelrahman Abushouk, Maryam Adabi, Tim Adair, Oladimeji Adebayo, Isaac Adedeji, Victor Adekanmbi, Olatunji Adetokunboh, Shailesh Advani, Mohammad Aghaali, Bali Ahmed, Muktar Ahmed, Ziyad Al-Aly, Khurshid Alam, Jacqueline Alcalde-Rabanal, Muhammad Ali, Cyrus Alinia, Vahid Alipour, Peter Allebeck, Amir Almasi-Hashiani, Arwa Alumran, Nelson Alvis-Guzman, Sofia Androudi, Fereshteh Ansari, Davood Anvari, Faraj Anwar, Seth Christopher Yaw Appiah, Jalal Arabloo, Morteza Arab-Zozani, Ziapour Arash, Olatunde Aremu, Johan Ärnlöv, Fomenkov Artem, Krishna Aryal, Darwesh Aso, Madhu Atteraya, Beatriz Paulina Ayala Quintanilla, Eleni Bagli, Maciej Banach, Palash Banik, Till Bärnighausen, Akbar Barzegar, Sanjay Basu, Adam Berman, Reshmi Bhageerathy, Ali Bijani, Antonio Biondi, Antonio Borzì, Michael Brauer, Charlton Callender, Juan Carrero, Joao Mauricio Castaldelli-Maia, Franz Castro, Ferrán Catalá-López, Vijay Kumar Chattu, Dinh-Toi Chu, Massimo Cirillo, Herteliu Claudiu, Vera Costa, Matthew Cunningham, Tat Nguyen Cuong, Giovanni Damiani, Ahmad Daryani, Rajat Das Gupta, José Das Neves, Moosavi Delaram, Nikolaos Dervenis, Rupak Desai, Govinda Dhungana, Diana Dias Da Silva, Fariba Dorostkar, Leila Doshmangir, Kerrie Doyle, David Edvardsson, Andem Effiong, Firooz Esmaeilzadeh, Mohammad Farahmand, Mohammad Fareed, Andre Faro, Nazir Fattahi, Valery Feigin, Seyed-Mohammad Fereshtehnejad, Carsten Flohr, Morenike Folayan, Artem Fomenkov, Takeshi Fukumoto, João Furtado, Mohamed Gad, Farhad Ghamari, Syed Amir Gilani, Alessandra Goulart, Yuming Guo, Rajeev Gupta, Quang Pham Hai, Arvin Haj-Mirzaian, Kanaan Hamagharib Abdullah, Komaki Hamidreza, Chieh Han, Hamidreza Haririan, Josep Haro, Soheil Hassanipour, Hadi Hassankhani, Simon Hay, Nathaniel Henry, Thi Do Hoa, Praveen Hoogar, Mehdi Hosseinzadeh, Mowafa Househ, Guoqing Hu, Lan Nguyen Huong, Filip Irina, Md.Mohaimenul Islam, Olusanya Jacob, Kathryn Jacobsen, Deepa Jahagirdar, Nader Jahanmehr, Spencer James, Ravi Jha, Vivekanand Jha, John Ji, Morgado-Da-Costa Joana, Jost Jonas, Farahnaz Joukar, Jacek Jozwiak, Mikk Jürisson, Zubair Kabir, Leila Kalankesh, Tanuj Kanchan, André Karch, Salah Eddin Karimi, Nicholas J Kassebaum, Srinivasa Katikireddi, Gbenga Kayode, Yousef Khader, Nauman Khalid, Ejaz Khan, Young-Ho Khang, Amir Khater, Mahalaqua Nazli Khatib, Salman Khazaei, Mohammad Taghi Khodayari, Jagdish Khubchandani, Cho-Il Kim, Young-Eun Kim, Yun Jin Kim, Soewarta Kosen, Michael Kravchenko, Kewal Krishan, Bhattacharya Krittika, Kris Krohn, Manasi Kumar, Pushpendra Kumar, Vivek Kumar, Dian Kusuma, Carlo La Vecchia, Dharmesh Lal, Savita Lasrado, Shaun Lee, Kate Legrand, Lee-Ling Lim, Alton Lu, Cámera Luis, Bohluli Mahdi, Abdullah Mamun, Navid Manafi, Fariborz Mansour-Ghanaei, Borhan Mansouri, Mohammad Ali Mansournia, Ausloos Marcel, Vacante Marco, Titova Maria, Francisco Rogerlândio Martins-Melo, Manu Mathur, Colm Mcalinden, Walter Mendoza, Ritesh Menezes, Endalkachew Mengesha, Atte Meretoja, Tomislav Mestrovic, George Milne, Anjomshoa Mina, Erkin Mirrakhimov, Babak Moazen, Masoud Moghadaszadeh, Dara Mohammad, Abdollah Mohammadian-Hafshejani, Shafiu Mohammed, Farnam Mohebi, Ali Mokdad, Natalie Momen, Lorenzo Monasta, Ghobad Moradi, Masoud Moradi, Mohammad Moradi-Joo, Ilais Moreno Velásquez, Seyyed Meysam Mousavi, Ulrich Mueller, Kamarul Imran Musa, Mohsen Naghavi, Duduzile Ndwandwe, Kianipour Neda, Ionut Negoi, Josephine Ngunjiri, Huong Nguyen, Yeshambel Nigatu, Amin Reza Nikpoor, Shuhei Nomura, Jean Jacques Noubiap, Christoph Nowak, Andrew Olagunju, Bolajoko Olusanya, Kanyin Ong, Obinna Onwujekwe, Alberto Ortiz, Adrian Oțoiu, Nikita Otstavnov, Simon Øverland, Mayowa Owolabi, Mahesh P A, Jagadish Rao. Padubidri, Keyvan Pakshir, Adrian Pana, Deepak Kumar Pasupula, Sangram Patel, Veincent Christian Pepito, Alexandre Pereira, David Pigott, Khem Pokhrel, Maarten Postma, Hadi Pourjafar, Reza Pourmirza Kalhori, Anna Poznańska, Sergio Prada, Zahiruddin Quazi Syed, Amir Radfar, Alireza Rafiei, Ali Rajabpour-Sanati, Chhabi Ranabhat, Sowmya J Rao, Priya Rathi, Lal Rawal, Vishnu Renjith, Andre Renzaho, Nima Rezaei, Mohammad Sadegh Rezai, Jennifer Rickard, Ancuceanu Robert Viorel, Leonardo Roever, Luca Ronfani, Gholamreza Roshandel, Enrico Rubagotti, Negoi Ruxandra Irina, Godfrey Rwegerera, Basema Saddik, Ehsan Sadeghi, Yahya Safari, Saeid Safiri,

Rajesh Sagar, S Mohammad Sajadi, Joseph Salama, Payman Salamati, Inbal Salz, Zainab Samad, Abdallah Samy, Damian Santomauro, Milena Santric-Milicevic, Sivan Saraswathy, Benn Sartorius, Arash Sarveazad, Brijesh Sathian, David Schwebel, Mario Sekerija, Edson Serván-Mori, Masood Shaikh, Mehran Shams-Beyranvand, Mohammed Shannawaz, Li Shanshan, David Shaw, Jae Il Shin, Soraya Siabani, Jasvinder Singh, Eirini Skiadaresi, Valentin Skryabin, Amin Soheili, Panda-Jonas Songhomitra, Reed Sorensen, Joan Soriano, Chandrashekhar Sreeramareddy, Otstavnov Stanislav, Leo Stockfelt, Mark Stokes, Agus Sudaryanto, Rafael Tabarés-Seisdedos, Karen Tabb, Amir Taherkhani, Nuno Taveira, Nihal Thomas, Roman Topor-Madry, Marcos Roberto Tovani-Palone, Bach Tran, Huyen Nguyen Trang, Ravensara S Travillian, Sergi Trias-Llimós, Thomas Truelsen, Bhaskaran Unnikrishnan, Era Upadhyay, Pascual Valdez, Tommi Vasankari, Yasser Vasseghian, Narayanaswamy Venketasubramanian, Vasily Vlassov, Bay Vo, Theo Vos, Yasir Waheed, Stefanie Watson, Jordan Weiss, Girmay Weldesamuel, Ronny Westerman, Harvey Whiteford, Taweewat Wiangkham, Tissa Wijeratne, Charles Wiysonge, Bogdan Wojtyniak, Ai-Min Wu, Gelin Xu, Sanni Yaya, Jamal Yearwood, Christopher Yilgwan, Naohiro Yonemoto, Seok-Jun Yoon, Mustafa Younis, Theodore Younker, Abdilahi Yousuf, Josefina Zakzuk, Mohammad Zamani, Zhi-Jiang Zhang, Maigeng Zhou, and Christopher J L Murray.

Developing methods or computational machinery

Haidong Wang, Amir Almasi-Hashiani, Davood Anvari, Ziapour Arash, Aleksandr Y Aravkin, Greg Bertolacci, Charlton Callender, Matthew Cunningham, Ahmad Daryani, Rajat Das Gupta, Ilse Dippenaar, Firooz Esmaeilzadeh, Ali Akbar Fazaeli, Chieh Han, Mowafa Househ, Deepa Jahagirdar, Spencer James, Young-Eun Kim, Pushpendra Kumar, Andrew Leever, Alton Lu, Borhan Mansouri, Claudia Mastrogiacomo, Erin May, Fereshteh Mehri, Masoud Moghadaszadeh, Reza Mohammadpourhodki, Ali Mokdad, Mohsen Naghavi, Kianipour Neda, Katherine Paulson, Spencer Pease, Reza Pourmirza Kalhori, Liliana Preotescu, Zahiruddin Quazi Syed, Chhabi Ranabhat, Enrico Rubagotti, Yahya Safari, Mohammad Salahshoor, Zainab Samad, Abdallah Samy, Damian Santomauro, David Shaw, Yasser Vasseghian, Narayanaswamy Venketasubramanian, Theo Vos, Magdalene Walters, Seok-Jun Yoon, Hunter York, Theodore Younker, Peng Zheng, and Christopher J L Murray.

Providing critical feedback on methods or results

Haidong Wang, Kaja Abbas, Foad Abd-Allah, Ahmed Abdelalim, Hassan Abolhassani, Lucas Abreu, Michael Abrigo, Abdelrahman Abushouk, Tim Adair, Oladimeji Adebayo, Isaac Adedeji, Victor Adekanmbi, Olatunji Adetokunboh, Shailesh Advani, Ashkan Afshin, Mohammad Aghaali, Anurag Agrawal, Keivan Ahmadi, Bali Ahmed, Muktar Ahmed, Ziyad Al-Aly, Khurshid Alam, Fahad Alanezi, Turki Alanzi, Jacqueline Alcalde-Rabanal, Gianfranco Alicandro, Mehran Alijanzadeh, Vahid Alipour, Vakilian Alireza, Syed Aljunid, Peter Allebeck, Amir Almasi-Hashiani, Hesham Al-Mekhlafi, Khalid Altirkawi, Arwa Alumran, Nelson Alvis-Guzman, Arianna Maever Amit, Briko Andrey, Colin Angus, Fereshteh Ansari, Iman Ansari, Alireza Ansari-Moghaddam, Carl Abelardo Antonio, Davood Anvari, Faraj Anwar, Seth Christopher Yaw Appiah, Jalal Arabloo, Morteza Arab-Zozani, Ziapour Arash, Olatunde Aremu, Johan Ärnlöv, Fomenkov Artem, Krishna Aryal, Badiye Ashish, Darwesh Aso, Madhu Atteraya, Euripide Avokpaho, Beatriz Paulina Ayala Quintanilla, Getinet Ayano, Martin Ayanore, Ghasem Azarian, Ebrahim Babaee, Ashish Badiye, Eleni Bagli, Ahad Bakhtiari, Maciej Banach, Palash Banik, Suzanne Barker-Collo, Till Bärnighausen, Sanjay Basu, Bernhard Baune, Mohsen Bayati, Gholamreza Bazmandegan, Neeraj Bedi, Michellr Bell, Derrick Bennett, Isabela Bensenor, Kidanemaryam Berhe, Adam Berman, Reshmi Bhageerathy, Zulfiqar Bhutta, Antonio Biondi, Donal Bisanzio, Tone Bjørge, Kassawmar Bogale, Antonio Borzì, Oliver Brady, Dejana Braithwaite, Nikolay Briko, Zahid Butt, Florentino Luciano Caetano Dos Santos, Charlton Callender, Ismael Campos-Nonato, Rosario Cárdenas, Juan Carrero, Joao Mauricio Castaldelli-Maia, Franz Castro, Ferrán Catalá-López, Christopher Cederroth, Ester Cerin, Vijay Kumar Chattu, Ken Chin, Dinh-Toi Chu, Liliana Ciobanu, Herteliu Claudiu, Vera Costa, Richard Cowden, Elizabeth Cromwell, Andrew Croneberger, Matthew Cunningham, Tat Nguyen Cuong, Giovanni Damiani, Emanuele D'amico, Paul Dargan, Ahmad Daryani, Rajat Das Gupta, José Das Neves, Sattin Davide, Kairat Davletov, Diego De Leo, Moosavi Delaram, Kebede Deribe, Nikolaos Dervenis, Rupak Desai, Diana Dias Da Silva, Daniel Diaz, David Doku, Fariba Dorostkar, Chirag Doshi, Leila Doshmangir, Kerrie Doyle, Andre Duraes, David Edvardsson, Andem Effiong, Glushkova Ekaterina, Iman El Sayed, Maha El Tantawi, Sharareh Eskandarieh, Firooz Esmaeilzadeh, Mohammad Farahmand, Mohammad Fareed, Andre Faro, Farshad Farzadfar, Valery Feigin, Seyed-Mohammad Fereshtehnejad, Manuela Ferreira, Florian Fischer, Carsten Flohr, Nataliya Foigt, Morenike Folayan, Artem Fomenkov, Marisa Freitas, Takeshi Fukumoto, Mohamed Gad, Silvano Gallus, Ketema Gebremedhin, Farhad Ghamari, Ahmad Ghashghaee, Sameer Gopalani, Yuming Guo,

Rajeev Gupta, Subodh Gupta, Quang Pham Hai, Arya Haj-Mirzaian, Randah Hamadeh, Kanaan Hamagharib Abdullah, Ahmadieh Hamid, Komaki Hamidreza, Chieh Han, Demelash Woldeyohannes Handiso, Soheil Hassanipour, Hadi Hassankhani, Simon Hay, Reza Heidari-Soureshjani, Nathaniel Henry, Fatemeh Heydarpour, Thi Do Hoa, Michael Hole, Praveen Hoogar, H Dean Hosgood, Naznin Hossain, Mehdi Hosseinzadeh, Mihaela Hostiuc, Sorin Hostiuc, Mowafa Househ, Guoqing Hu, Lan Nguyen Huong, Segun Ibitoye, Olayinka Ilesanmi, Irena Ilic, Milena Ilic, Mohammad Hasan Imani-Nasab, Filip Irina, Md.Mohaimenul Islam, Chinwe Iwu, Jalil Jaafari, Olusanya Jacob, Kathryn Jacobsen, Nader Jahanmehr, Amir Jalali, Farzad Jalilian, Spencer James, Hosna Janjani, Ravi Jha, Vivekanand Jha, John Ji, Morgado-Da-Costa Joana, Jost Jonas, Farahnaz Joukar, Jacek Jozwiak, Mikk Jürisson, Zubair Kabir, Leila Kalankesh, Tanuj Kanchan, Neeti Kapoor, André Karch, Salah Eddin Karimi, Seyed Asaad Karimi, Nicholas J Kassebaum, Srinivasa Katikireddi, Norito Kawakami, Gbenga Kayode, Peter Keiyoro, Yousef Khader, Nauman Khalid, Ejaz Khan, Maseer Khan, Mona Khater, Mahalaqua Nazli Khatib, Mohammad Taghi Khodayari, Jagdish Khubchandani, Cho-Il Kim, Young-Eun Kim, Yun Jin Kim, Yohannes Kinfu, Katarzyna Kissimova-Skarbek, Mika Kivimäki, Jacek Kopec, Parvaiz Koul, Ai Koyanagi, Kewal Krishan, Bhattacharya Krittika, Kris Krohn, Manasi Kumar, Pushpendra Kumar, Vivek Kumar, Dian Kusuma, Hmwe Kyu, Carlo La Vecchia, Dharmesh Lal, Ratilal Lalloo, Anders Larsson, Savita Lasrado, Zohra Lassi, Shaun Lee, Andrew Leever, Kate Legrand, Shanshan Li, Lee-Ling Lim, Shai Linn, Rakesh Lodha, Giancarlo Logroscino, Alan Lopez, Paulo Lotufo, Rafael Lozano, Alton Lu, Cámera Luis, Raimundas Lunevicius, Hassan Magdy Abd El Razek, Muhammed Magdy Abd El Razek, Phetole Mahasha, Mokhtar Mahdavi, Bohluli Mahdi, Abdullah Mamun, Navid Manafi, Fariborz Mansour-Ghanaei, Borhan Mansouri, Mohammad Ali Mansournia, Chabila Christopher Mapoma, Ausloos Marcel, Vacante Marco, Titova Maria, Santi Martini, Francisco Rogerlândio Martins-Melo, Anthony Masaka, Manu Mathur, Leonardi Matilde, Colm Mcalinden, Kala Mehta, Wahengbam Bigyananda Meitei, Peter Memiah, Walter Mendoza, Ritesh Menezes, Endalkachew Mengesha, Tuomo Meretoja, Irmina Maria Michalek, Kebadnew Mihretie, Ted Miller, Edward Mills, George Milne, Erkin Mirrakhimov, Hamed Mirzaei, Maryam Mirzaei, Mehdi Mirzaei-Alavijeh, Abbasifard Mitra, Babak Moazen, Masoud Moghadaszadeh, Efat Mohamadi, Dara Mohammad, Rabiee Mohammad, Yousef Mohammad, Sahraian Mohammad Ali, Abolfazl Mohammadbeigi, Abdollah Mohammadian-Hafshejani, Reza Mohammadpourhodki, Shafiu Mohammed, Farnam Mohebi, Amin Mokari, Ali Mokdad, Natalie Momen, Ghobad Moradi, Mohammad Moradi-Joo, Maziar Moradi-Lakeh, Rahmatollah Moradzadeh, Ilais Moreno Velásquez, Shane Morrison, Jonathan Mosser, Simin Mouodi, Amin Mousavi Khaneghah, Ulrich Mueller, Kamarul Imran Musa, Saravanan Muthupandian, Mehdi Naderi, Ahamarshan Nagarajan, Mohsen Naghavi, Gurudatta Naik, Farid Najafi, Vinay Nangia, Jobert Richie Nansseu, Duduzile Ndwandwe, Kianipour Neda, Kapoor Neeti, Ionut Negoi, Josephine Ngunjiri, Huong Nguyen, Yeshambel Nigatu, Rajan Nikbakhsh, Amin Reza Nikpoor, Molly Nixon, Chukwudi Nnaji, Shuhei Nomura, Jean Jacques Noubiap, Soraya Nouraei Motlagh, Christoph Nowak, In-Hwan Oh, Andrew Olagunju, Bolajoko Olusanya, Kanyin Ong, Obinna Onwujekwe, Alberto Ortiz, Adrian Oțoiu, Nikita Otstavnov, Simon Øverland, Mayowa Owolabi, Mahesh P A, Jagadish Rao. Padubidri, Raffaele Palladino, Adrian Pana, James Park, Jenil Patel, Sangram Patel, George Patton, Katherine Paulson, Hamidreza Pazoki Toroudi, Amy Peden, Veincent Christian Pepito, Emmanuel Peprah, Alexandre Pereira, David Pereira, David Pigott, Meghdad Pirsaheb, Lopukhov Platon, Khem Pokhrel, Maarten Postma, Hadi Pourjafar, Farshad Pourmalek, Reza Pourmirza Kalhori, Akram Pourshams, Sergio Prada, Sanjay Prakash, Liliana Preotescu, Zahiruddin Quazi Syed, Navid Rabiee, Amir Radfar, Alireza Rafiei, Alberto Raggi, Muhammad Aziz Rahman, Ali Rajabpour-Sanati, Pradhum Ram, Chhabi Ranabhat, Sowmya J Rao, Vahid Rashedi, Prateek Rastogi, Priya Rathi, Lal Rawal, Vishnu Renjith, Andre Renzaho, Serge Resnikoff, Malekzadeh Reza, Nima Rezaei, Mohammad Sadegh Rezai, Aziz Rezapour, Jennifer Rickard, Ancuceanu Robert Viorel, Leonardo Roever, Luca Ronfani, Gholamreza Roshandel, Enrico Rubagotti, Negoi Ruxandra Irina, Godfrey Rwegerera, Siamak Sabour, Basema Saddik, Ehsan Sadeghi, Yahya Safari, Sare Safi, Rajesh Sagar, S Mohammad Sajadi, Payman Salamati, Marwa Salem, Yahya Salimi, Joshua Salomon, Inbal Salz, Zainab Samad, Abdallah Samy, Juan Sanabria, Damian Santomauro, Milena Santric-Milicevic, Sivan Saraswathy, Benn Sartorius, Arash Sarveazad, Brijesh Sathian, Thirunavukkarasu Sathish, Mete Saylan, Lauren Schaeffer, David Schwebel, Falk Schwendicke, Anbissa Senbeta, Subramanian Senthilkumaran, Sadaf Sepanlou, Edson Serván-Mori, Irandoost Seyed Fahim , Mahsima Shabani, Saeed Shahabi, Mohammad Shahbaz, Amira Shaheen, Masood Shaikh, Ali Shalash, Mehran Shams-Beyranvand, Morteza Shamsizadeh, Mohammed Shannawaz, Li Shanshan, Kiomars Sharafi, David Shaw, Aziz Sheikh, Jae Il Shin, Rahman Shiri, Mark Shrime, Kerem Shuval, Soraya Siabani, Sabour Siamak, Diego Silva, Schiavolin Silvia, Biagio Simonetti, Kyle Simpson, Jasvinder Singh, Eirini Skiadaresi, Valentin Skryabin, Amin Soheili, Panda-Jonas Songhomitra, Reed Sorensen,

Joan Soriano, Muluken Sorrie, Ireneous Soyiri, Emma Spurlock, Chandrashekhar Sreeramareddy, Otstavnov Stanislav, Leo Stockfelt, Mark Stokes, Jacob Stubbs, Agus Sudaryanto, Mu'awiyyah Sufiyan, Rizwan Suliankatchi Abdulkader, Bryan Sykes, Rafael Tabarés-Seisdedos, Karen Tabb, Santosh Tadakamadla, Nuno Taveira, Arash Tehrani-Banihashemi, Berhane Teklehaimanot, Zemenu Tessema, Kavumpurathu Thankappan, Nihal Thomas, Amanda Thrift, Hamid Reza Tohidinik, Marcello Tonelli, Fotis Topouzis, Marcos Roberto Tovani-Palone, Eugenio Traini, Bach Tran, Huyen Nguyen Trang, Ravensara S Travillian, Sergi Trias-Llimós, Thomas Truelsen, Lorainne Tudor Car, Bhaskaran Unnikrishnan, Era Upadhyay, Pascual Valdez, Alessandro Valli, Constantine Vardavas, Ana Maria Vasconcelos, Yasser Vasseghian, Yousef Veisani, Narayanaswamy Venketasubramanian, Francesco Violante, Bay Vo, Stein Emil Vollset, Theo Vos, Isidora Vujcic, Ana Vukovic, Rade Vukovic, Yasir Waheed, Mitchell Wallin, Yuan-Pang Wang, Stefanie Watson, Jingkai Wei, Jordan Weiss, Girmay Weldesamuel, Ronny Westerman, Taweewat Wiangkham, Kirsten Wiens, Tissa Wijeratne, Charles Wiysonge, Bogdan Wojtyniak, Charles Wolfe, Adam Wondmieneh, Ai-Min Wu, Gelin Xu, Tomohide Yamada, Yuichiro Yano, Sanni Yaya, Vahid Yazdi-Feyzabadi, Christopher Yilgwan, Paul Yip, Naohiro Yonemoto, Seok-Jun Yoon, Javad Yoosefi Lebni, Mustafa Younis, Taraneh Yousefinezhadi, Abdilahi Yousuf, Guo Yuming, Hasan Yusefzadeh, Telma Zahirian Moghadam, Kamiab Zahra, Josefina Zakzuk, Sojib Bin Zaman, Maryam Zamanian, Hamed Zandian, Zhi-Jiang Zhang, Maigeng Zhou, and Christopher J L Murray.

Drafting the work or revising is critically for important intellectual content

Haidong Wang, Kaja Abbas, Foad Abd-Allah, Ahmed Abdelalim, Hassan Abolhassani, Lucas Abreu, Abdelrahman Abushouk, Oladimeji Adebayo, Isaac Adedeji, Victor Adekanmbi, Abiodun Adeoye, Olatunji Adetokunboh, Shailesh Advani, Mohammad Aghaali, Bali Ahmed, Muktar Ahmed, Khurshid Alam, Tahiya Alam, Jacqueline Alcalde-Rabanal, Muhammad Ali, Cyrus Alinia, Peter Allebeck, Majid Almadi, Amir Almasi-Hashiani, Hesham Al-Mekhlafi, Arwa Alumran, Nelson Alvis-Guzman, Mostafa Amini-Rarani, Arya Aminorroaya, Briko Andrey, Colin Angus, Iman Ansari, Carl Abelardo Antonio, Jalal Arabloo, Morteza Arab-Zozani, Olatunde Aremu, Johan Ärnlöv, Ali Asadi-Pooya, Badiye Ashish, Leticia Avila-Burgos, Beatriz Paulina Ayala Quintanilla, Martin Ayanore, Ashish Badiye, Eleni Bagli, Maciej Banach, Suzanne Barker-Collo, Till Bärnighausen, Sanjay Basu, Bernhard Baune, Neeraj Bedi, Michellr Bell, Derrick Bennett, Isabela Bensenor, Kidanemaryam Berhe, Reshmi Bhageerathy, Antonio Biondi, Catherine Bisignano, Raaj Kishore Biswas, Tone Bjørge, Srinivasa Rao Bolla, Antonio Borzì, Oliver Brady, Dejana Braithwaite, Nikolay Briko, Sharath Burugina Nagaraja, Zahid Butt, Florentino Luciano Caetano Dos Santos, Charlton Callender, Ismael Campos-Nonato, Giulia Carreras, Juan Carrero, Felix Carvalho, Joao Mauricio Castaldelli-Maia, Giulio Castelpietra, Franz Castro, Christopher Cederroth, Ester Cerin, Ken Chin, Liliana Ciobanu, Massimo Cirillo, Herteliu Claudiu, Vera Costa, Richard Cowden, Matthew Cunningham, Tat Nguyen Cuong, Giovanni Damiani, Emanuele D'amico, Paul Dargan, José Das Neves, Sattin Davide, Moosavi Delaram, Edgar Denova-Gutiérrez, Kebede Deribe, Nikolaos Dervenis, Rupak Desai, Govinda Dhungana, Diana Dias Da Silva, Daniel Diaz, Klara Dokova, Eleonora Dubljanin, Fernandes Eduarda, David Edvardsson, Andem Effiong, Glushkova Ekaterina, Iman El Sayed, Maha El Tantawi, Iffat Elbarazi, Shaimaa El-Jaafary, Sharareh Eskandarieh, Firooz Esmaeilzadeh, Andre Faro, Mehdi Fazlzadeh, Valery Feigin, Seyed-Mohammad Fereshtehnejad, Eduarda Fernandes, Manuela Ferreira, Florian Fischer, Carsten Flohr, Nataliya Foigt, Morenike Folayan, Marisa Freitas, Takeshi Fukumoto, João Furtado, Mohamed Gad, Silvano Gallus, Farhad Ghamari, Ahmad Ghashghaee, Asadollah Gholamian, Mojgan Gitimoghaddam, Sameer Gopalani, Alessandra Goulart, Rajeev Gupta, Juanita Haagsma, Quang Pham Hai, Arya Haj-Mirzaian, Iman Halvaei, Kanaan Hamagharib Abdullah, Komaki Hamidreza, Pazoki-Toroudi Hamidreza , Demelash Woldeyohannes Handiso, Graeme Hankey, Josep Haro, Ahmed Hasaballah, Simon Hay, Behzad Heibati, Reza Heidari-Soureshjani, Thi Do Hoa, Michael Hole, Praveen Hoogar, Naznin Hossain, Mihaela Hostiuc, Sorin Hostiuc, Mowafa Househ, Lan Nguyen Huong, Segun Ibitoye, Olayinka Ilesanmi, Irena Ilic, Milena Ilic, Mohammad Hasan Imani-Nasab, Filip Irina, Hiroyasu Iso, Olusanya Jacob, Kathryn Jacobsen, Ensiyeh Jenabi, Ravi Jha, Vivekanand Jha, John Ji, Morgado-Da-Costa Joana, Jost Jonas, Jacek Jozwiak, Mikk Jürisson, Hamed Kalani, Neeti Kapoor, André Karch, Salah Eddin Karimi, Seyed Asaad Karimi, Nicholas J Kassebaum, Srinivasa Katikireddi, Gbenga Kayode, Nauman Khalid, Ejaz Khan, Maseer Khan, Young-Ho Khang, Mona Khater, Mahalaqua Nazli Khatib, Jagdish Khubchandani, Young-Eun Kim, Yun Jin Kim, Mika Kivimäki, Parvaiz Koul, Ai Koyanagi, Michael Kravchenko, Kewal Krishan, Bhattacharya Krittika, Kris Krohn, Pushpendra Kumar, Vivek Kumar, Dian Kusuma, Carlo La Vecchia, Ben Lacey, Ratilal Lalloo, Faris Lami, Sonia Lansky, Anders Larsson, Jeffrey Lazarus, Paul Lee, Shaun Lee, Andrew Leever, Kate Legrand, Shanshan Li, Lee-Ling Lim,

Alton Lu, Cámera Luis, Raimundas Lunevicius, Mohammed Madadin, Hassan Magdy Abd El Razek, Muhammed Magdy Abd El Razek, Abdullah Mamun, Navid Manafi, Borhan Mansouri, Mohammad Ali Mansournia, Ausloos Marcel, Vacante Marco, Santi Martini, Francisco Rogerlândio Martins-Melo, Claudia Mastrogiacomo, Manu Mathur, Leonardi Matilde, Erin May, Colm Mcalinden, John Mcgrath, Martin Mckee, Kala Mehta, Wahengbam Bigyananda Meitei, Walter Mendoza, Ritesh Menezes, Endalkachew Mengesha, George Mensah, Atte Meretoja, Tuomo Meretoja, Tomislav Mestrovic, Irmina Maria Michalek, Ted Miller, Edward Mills, Anjomshoa Mina, Masoud Moghadaszadeh, Dara Mohammad, Rabiee Mohammad, Yousef Mohammad, Sahraian Mohammad Ali, Abdollah Mohammadian-Hafshejani, Hussen Mohammed, Shafiu Mohammed, Farnam Mohebi, Ali Mokdad, Natalie Momen, Lorenzo Monasta, Maziar Moradi-Lakeh, Paula Moraga, Shane Morrison, Jonathan Mosser, Seyyed Meysam Mousavi, Amin Mousavi Khaneghah, Ulrich Mueller, Kamarul Imran Musa, Saravanan Muthupandian, Behnam Nabavizadeh, Ahamarshan Nagarajan, Behshad Naghshtabrizi, Vinay Nangia, Jobert Richie Nansseu, Kapoor Neeti, Ionut Negoi, Josephine Ngunjiri, Huong Nguyen, Yeshambel Nigatu, Rajan Nikbakhsh, Amin Reza Nikpoor, Molly Nixon, Jean Jacques Noubiap, Christoph Nowak, In-Hwan Oh, Morteza Oladnabi, Andrew Olagunju, Bolajoko Olusanya, Obinna Onwujekwe, Alberto Ortiz, Adrian Oțoiu, Simon Øverland, Mayowa Owolabi, Mahesh P A, Jagadish Rao. Padubidri, Raffaele Palladino, Adrian Pana, James Park, Jenil Patel, Sangram Patel, Katherine Paulson, Spencer Pease, Amy Peden, Veincent Christian Pepito, Emmanuel Peprah, Alexandre Pereira, David Pereira, Norberto Perico, Thomas Pilgrim, Lopukhov Platon, Maarten Postma, Farshad Pourmalek, Sergio Prada, Zahiruddin Quazi Syed, Navid Rabiee, Amir Radfar, Alberto Raggi, Muhammad Aziz Rahman, Ali Rajabpour-Sanati, Pradhum Ram, Chhabi Ranabhat, Sowmya J Rao, Davide Rasella, Vahid Rashedi, Prateek Rastogi, Lal Rawal, Vishnu Renjith, Malekzadeh Reza, Nima Rezaei, Jennifer Rickard, Ancuceanu Robert Viorel, Leonardo Roever, Luca Ronfani, Gholamreza Roshandel, Morteza Rostamian, Negoi Ruxandra Irina, Godfrey Rwegerera, Basema Saddik, Masoumeh Sadeghi, Yahya Safari, Saeid Safiri, Rajesh Sagar, Amirhossein Sahebkar, Valadan Tahbaz Sahel, Joseph Salama, Marwa Salem, Joshua Salomon, Zainab Samad, Abdallah Samy, Juan Sanabria, Damian Santomauro, Milena Santric-Milicevic, Arash Sarveazad, David Schwebel, Falk Schwendicke, Sadaf Sepanlou, Mahsima Shabani, Mohammad Shahbaz, Masood Shaikh, Ali Shalash, Mehran Shams-Beyranvand, Mohammadbagher Shamsi, Morteza Shamsizadeh, Zeinab Sharafi, Rajesh Sharma, David Shaw, Mark Shrime, Kerem Shuval, Diego Silva, Schiavolin Silvia, Kyle Simpson, Jasvinder Singh, Eirini Skiadaresi, Anton Sokhan, Panda-Jonas Songhomitra, Joan Soriano, Muluken Sorrie, Ireneous Soyiri, Chandrashekhar Sreeramareddy, Leo Stockfelt, Mark Stokes, Jacob Stubbs, Mu'awiyyah Sufiyan, Bryan Sykes, Rafael Tabarés-Seisdedos, Karen Tabb, Santosh Tadakamadla, Muming Tang, Nuno Taveira, Zemenu Tessema, Nihal Thomas, Amanda Thrift, Hamid Reza Tohidinik, Marcello Tonelli, Roman Topor-Madry, Fotis Topouzis, Marcos Roberto Tovani-Palone, Bach Tran, Huyen Nguyen Trang, Ravensara S Travillian, Thomas Truelsen, Era Upadhyay, Pascual Valdez, Constantine Vardavas, Tommi Vasankari, Ana Maria Vasconcelos, Yasser Vasseghian, Narayanaswamy Venketasubramanian, Simone Vidale, Francesco Violante, Vasily Vlassov, Bay Vo, Stein Emil Vollset, Ana Vukovic, Rade Vukovic, Yasir Waheed, Mitchell Wallin, Yuan-Pang Wang, Ronny Westerman, Kirsten Wiens, Tissa Wijeratne, Charles Wiysonge, Eve Wool, Ai-Min Wu, Seyed Hossein Yahyazadeh Jabbari, Kazumasa Yamagishi, Sanni Yaya, Vahid Yazdi-Feyzabadi, Tomas Yeheyis, Christopher Yilgwan, Seok-Jun Yoon, Zabihollah Yousefi, Guo Yuming, Sojib Bin Zaman, Mohammad Zamani, Maryam Zamanian, and Christopher J L Murray.

Extracting, cleaning, or cataloging data; designing or coding figures and tables

Olatunji Adetokunboh, Muktar Ahmed, Tahiya Alam, Greg Bertolacci, Charlton Callender, Franz Castro, Haley Comfort, Matthew Cunningham, Ahmad Daryani, Moosavi Delaram, Ilse Dippenaar, Firooz Esmaeilzadeh, Takeshi Fukumoto, John Fuller, Farhad Ghamari, Chieh Han, Kiana Henny, Kevin Ikuta, Spencer James, Cathleen Keller, Maseer Khan, Mahalaqua Nazli Khatib, Pushpendra Kumar, Samantha Larson, Andrew Leever, Alton Lu, Emilie Maddison, Hassan Magdy Abd El Razek, Muhammed Magdy Abd EL Razek, Navid Manafi, Borhan Mansouri, Claudia Mastrogiacomo, Erin May, Masoud Moghadaszadeh, Reza Mohammadpourhodki, Ali Mokdad, Mohsen Naghavi, Amin Reza Nikpoor, Spencer Pease, David Pigott, Enrico Rubagotti, Yahya Safari, Joseph Salama, Zainab Samad, Abdallah Samy, Damian Santomauro, Fablina Sharara, David Shaw, Kyle Simpson, Emma Spurlock, Muming Tang, Whitney Teagle, Yasser Vasseghian, Magdalene Walters, Tissa Wijeratne, Junjie Wu, Theodore Younker.

Managing the overall research enterprise

Haidong Wang, Ashkan Afshin, Tahiya Alam, Peter Allebeck, Michael Brauer, Zulfiqar Bhutta, Elizabeth Cromwell, Lalit Dandona, Rakhi Dandona, Simon I Hay, Spencer James, Nicholas J Kassebaum, Yohannes Kinfu, Alan Lopez, Rafael Lozano, George Mensah, Ali Mokdad, Molly Nixon, Christopher Odell, Simon Øverland, George Patton, Joshua Salomon, Benn Sartorius, Roman Topor-Madry, Stein Emil Vollset, Andrea Werdecker, Harvey Whiteford, Eve Wool, and Christopher J L Murray.

Table of contents

Preamble 2
Authors contributions 3
List of appendix figures and tables 10
Appendix figures 10
Appendix tables 10
Section 1: GBD overview 11
Section 1.1: Global Burden of Diseases, Injuries, and Risk Factors Study 2019 11
Section 1.2: Harmonising estimation of population, fertility, and mortality 11
Section 1.3: Geographical locations of the analysis 11
Section 1.4: Time period of the analysis 11
Section 1.5: Statement of GATHER compliance 11
Section 1.6: List of abbreviations 11
Section 1.7: GBD results overview 14
Section 1.8: Data input sources overview 15
Section 1.9: Funding sources 15
Section 2: All-cause mortality, HIV mortality, and fatal discontinuities[4,5] 16
Section 2.1: Overview 16
Section 2.2: Child mortality 16
Section 2.3: Estimation of adult mortality rate 26
Section 2.4: Model life table system 37
Section 2.5: HIV/AIDS estimation 42
Section 2.6: Age-specific mortality estimation for all GBD age groups: with and without HIV ... 45
Section 2.7: Fatal discontinuities 46
Section 3: Fertility[4,5] 51
Section 3.1: Overview 51
Section 3.2: Data sources 51
Section 3.3: Modelling strategy 52
Section 3.4: Sex ratio at birth 56
Section 4: Estimating healthy life expectancy (HALE)[6] 57
Section 5: Population[4,5] 58
Section 5.1: Overview 58
Section 5.2: Data sources and processing 58
Section 5.3: Model inputs 61
Section 5.4: Modelling strategy 62
Section 6: Socio-demographic Index (SDI) analysis[6] 66
Section 6.1: Overview 66
Section 6.2: SDI calculation 66
Section 7: Additional methods 68
Section 7.1: Correlation estimation 68
Section 7.2: Annualised rates of change 68
Section 7.3: Gross domestic product (GDP) and lag-distributed income 68
Section 7.4: Educational attainment covariate 68
Section 8: References 70
Section 9: Appendix table and figures 74

List of appendix figures and tables

Appendix figures

Appendix Figure 1. Analytical flowchart for the estimation of all-cause mortality by age and sex, and HIV/AIDS incidence, prevalence, and mortality for GBD 2019

Appendix Figure 2. Estimated completeness of death registration 1950−2019

Appendix Figure 3. Analytical flowchart for the GBD 2019 fertility estimation process

Appendix Figure 4. Analytical flowchart for the GBD 2019 population estimation process

Appendix Figure 5. Census and registry availability by location and year

Appendix tables

Appendix Table 1. GBD location hierarchy with levels

Appendix Table 2. GATHER checklist

Appendix Table 3. Number of all-cause mortality data sources by type and location, 1950–2019

Appendix Table 4. Number of all-cause mortality data sources by type and year, 1950–2019

Appendix Table 5. Under-5 mortality reference sources by source date and location, 1950–2019

Appendix Table 6. New data sources added for GBD 2019 mortality estimation

Appendix Table 7. Number of outliered datapoints for the age/sex model by location, source type, and age group

Appendix Table 8. Distribution of empirical life tables by decade and GBD super-region, 1950–2019

Appendix Table 9. Locations by HIV estimation group

Appendix Table 10. Number of sources used for the analysis of age-specific fertility (VR, CBH-survey, SBH-survey, SBH-census) for each location

Appendix Table 11. Number of sources used for the analysis of age-specific fertility by year (VR, CBH-survey, SBH-survey, SBH-census)

Appendix Table 12. List of all confirmed censuses by location and year

Appendix Table 13. GBD world population age standard

Appendix Table 14. Socio-demographic Index values for all estimated GBD 2019 locations, 1950–1969

Appendix Table 15. Socio-demographic Index values for all estimated GBD 2019 locations, 1970–1989

Appendix Table 16. Socio-demographic Index values for all estimated GBD 2019 locations, 1990–2019

Section 1: GBD overview

Section 1.1: Global Burden of Diseases, Injuries, and Risk Factors Study 2019

The Global Burden of Diseases, Injuries, and Risk Factors Study (GBD) is a collaborative research effort aimed at estimating worldwide population, fertility, morbidity, and mortality. GBD draws on the expertise of an extensive collaborator network from around the world. During the last research cycle, GBD 2017, the project undertook in-house estimation of population and fertility for the first time. In this paper, we estimated fertility, mortality, and population by age, sex, and location from 1950 to 2019. We estimated healthy life expectancy (HALE) from 1990 to 2019.

Section 1.2: Harmonising estimation of population, fertility, and mortality

The GDB demographic analytical framework is unique in that mortality, fertility, and population estimates are generated using a closely interconnected modelling process. For GBD 2019, we first generated age-specific fertility and mortality separately using the analytical approaches described in the sections that follow. In these steps, population was used as an input for the calculation of age-specific mortality rates and fertility rates from vital and civil registration systems. The outputs from these two processes were used to generate age-specific and sex-specific population by location and year using a Bayesian hierarchical cohort component model for population projection. Once the new population numbers were estimated, they were used as inputs to the same fertility and mortality estimation models that were being run again in the second loop, which allowed us to update our fertility and mortality estimates using new population input data. The population estimation was then run again to produce the final population estimates for GBD 2019. To maintain consistency between mortality, fertility, and population in count space, we updated death number and birth number estimates using the rates estimated in the second loop and the final population estimates.

Section 1.3: Geographical locations of the analysis

We produced estimates for 204 countries and territories that were grouped into 21 regions and seven super-regions. For GBD 2019, nine countries and territories (Cook Islands, Monaco, San Marino, Nauru, Niue, Palau, Saint Kitts and Nevis, Tokelau, and Tuvalu) were added, such that the GBD location hierarchy now includes all WHO member states. This year, GBD includes subnational analyses for Italy, Nigeria, Pakistan, the Philippines, Poland, and 16 countries previously estimated at subnational levels (Brazil, China, Ethiopia, India, Indonesia, Iran, Japan, Kenya, Mexico, New Zealand, Norway, Russia, South Africa, Sweden, the UK, and the USA). All analyses are at the first level of administrative organisation within each country except for New Zealand (by Māori ethnicity), Sweden (by Stockholm and non-Stockholm), the UK (by local government authorities), Kenya (by district and province) and the Philippines (by provinces). For the demographic analyses, we seek to make the most of rich demographic data, more readily available and robust at aggregate level, and increase the precision of estimates at the aggregate level by running the modelling process at both the most detailed level and at the aggregate level (whether national, subnational, or both national and subnational). In this publication we present subnational estimates for Brazil, India, Indonesia, Japan, Kenya, Mexico, Sweden, the UK, and the USA); given space constraints, these results are presented in appendix 2 instead of the main text.

Section 1.4: Time period of the analysis

We estimated numbers and rates of all-cause mortality, fertility, and population for the years 1950–2019. We also generated estimates of observed life expectancy in comparison to life expectancy based on the Socio-demographic Index (SDI) from 1950 to 2019. HALE was estimated for 1990-2019.

Section 1.5: Statement of GATHER compliance

This study complies with the Guidelines for Accurate and Transparent Health Estimates Reporting (GATHER) recommendations.[1] We have documented the steps involved in our analytical procedures and detailed the data sources used. See appendix table 2 (section 9) for the GATHER checklist.

The GATHER recommendations can be found here: http://gather-statement.org/

Section 1.6: List of abbreviations

Abbreviation	Full phrase
$_1q_0$	probability of death from birth to age 1 year

${}_{45}m_{15}$	mortality rate between age 15 years and 60 years
${}_{45}q_{15}$	probability of death between age 15 years and 60 years
${}_{4}q_{1}$	probability of death between age 1 year and 5 years
${}_{5}m_{0}$	mortality rate between birth and age 5 years
${}_{5}q_{0}$	probability of death between birth and age 5 years, also commonly known as under-5 mortality rate (U5MR)
ACLED	Armed Conflict Location and Event Database
ART	antiretroviral therapy
ASFR	age-specific fertility rate
BCCMP	Bayesian hierarchical cohort component model for population projection
CBH	complete birth history
CCF50	cohort completed fertility by age 50
CCMPP	cohort component method for population projection
CD4	CD4+ T lymphocyte; an indicator of immune function
CEB	children ever born
CIBA	cohort incidence bias adjustment
CIBA-Spectrum	cohort incidence bias adjusted spectrum output
CoD	causes of death
CR	civil registration
CODEm	Cause of Death Ensemble model
DALYs	disability-adjusted life-years
DDM	death distribution methods
DHS	Demographic and Health Surveys
DSP	Disease Surveillance Points System
DYB	Demographic Yearbook
EDU15+	Mean years of education for those aged 15 and older
EM-DAT	Centre for Research on the Epidemiology of Disasters' International Disaster Database
enn	early neonatal
EPP	Estimation and Projection Package
EU	European Union
EPP-ASM	EPP-Age Sex Model

GATHER	Guidelines for Accurate and Transparent Health Estimates Reporting
GBD	Global Burden of Diseases, Injuries, and Risk Factors Study
GGB	generalised growth balance method
GGB-SEG	combined generalised growth balance and synthetic extinct generations
GHDx	Global Health Data Exchange
GIDEON	Global Infectious Diseases and Epidemiology Network
GK	Gakidou-King
GLMM	Gulf Labour Markets, Migration, and Population
GPR	Gaussian process regression
GTD	Global Terrorism Database
HALE	healthy life expectancy
HDI	human development index
HFC	Human Fertility Collection
HIV	human immunodeficiency virus
HIV CDR	crude death rate due to HIV/AIDS
HMD	Human Mortality Database
IBC	Iraq Body Count
IISS	International Institute for Strategic Studies
IPUMS	Integrated Public Use Microdata Series
LDI	lag-distributed income per capita
lnn	late neonatal
MAD	median absolute deviation
MAPE	mean average percentage error
MCCD	Medical Certification of Causes of Death
MICS	Multiple Indicator Cluster Surveys
MPIDR	Max Planck Institute for Demographic Research
MR-BRT	meta-regression—Bayesian, regularised, trimmed
${}_{n}m_{x}$	mortality rate between age x and x+n
PES	post-enumeration survey
pnn	post neonatal
PRIO	Peace Research Institute Oslo

${}_nq_x$	probability of death between age x and x+n
RHS	Reproductive Health Surveys
SBH	summary birth history
SDGs	United Nations Sustainable Development Goals
SDI	Socio-demographic Index
SEG	synthetic extinct generations
SRS	Sample Registration System
ST-GPR	spatiotemporal Gaussian process regression
TFO30	total fertility over age 30
TFR	total fertility rate
TFU25	total fertility under age 25
U5MR	under-5 mortality rate
UCDP	Uppsala Conflict Data Program
UI	uncertainty interval
UN	United Nations
UNAIDS	Joint United Nations Programme on HIV/AIDS
UNDYB	United Nations
UNHCR	United Nations High Commissioner for Refugees
UNICEF	United Nations International Children's Emergency Fund
UNMLT	United Nations Model Life Tables
UNSD	United Nations Statistics Division
USAID	United States Agency for International Development
VR	vital registration
WFS	World Fertility Survey
WHO	World Health Organization
WPP	World Population Prospects
YLDs	years lived with disability
YLLs	years of life lost

Section 1.7: GBD results overview

Results are available through an interactive data downloading tool on the Global Health Data exchange (GHDx)\. The GHDx is the world's most comprehensive catalogue of surveys, censuses, vital statistics, and other health-related data.

The latest version of the data download tool, available here: http://ghdx.healthdata.org/gbd-results-tool, contains core summary results for GBD 2019. These results include deaths, years of life lost (YLLs), years lived with disability (YLDs), disability-adjusted life-years (DALYs), prevalence, incidence, and rate of change. The GHDx includes data for causes, risks, cause-risk attribution, aetiologies, and impairments.

Data above a certain size cannot be viewed online but can be downloaded. Depending on the size of the download, users may need to enter an email address; a download location will be sent to them when the files are prepared.

All GBD 2019 online data visualisations are available at http://vizhub.healthdata.org/gbd-compare, which provides results for all GBD health metrics.

Section 1.8: Data input sources overview

GBD 2019 synthesises a large and growing number of data input sources including surveys, censuses, vital statistics, and other health-related data sources. The data from these sources is used to estimate mortality and morbidity; causes of death, illness, and injury; and attributable risk for 204 countries and territories from 1990 to 2019. The input sources are accessible through an interactive citation tool available in the GHDx. While we reviewed and extracted all input sources included in this tool, some sources were not included in our models as a result of data deduplication in the modelling process.

Citations for specific GBD components, causes and risks, and locations can be found through the Data Input Sources Tool in GHDx: http://ghdx.healthdata.org/gbd-2019/data-input-sources. This tool allows users to view and access GHDx records for input sources and export a CSV file that includes metadata, citations, and information about where the data were used in GBD. As required by GATHER, additional metadata for input sources are available through the citation tool as well.

Section 1.9: Funding sources

Research reported in this publication was supported by the Bill & Melinda Gates Foundation, the University of Melbourne, Public Health England, the Norwegian Institute of Public Health, the National Institute on Aging of the National Institutes of Health (award P30AG047845), and the National Institute of Mental Health of the National Institutes of Health (award R01MH110163). The content is solely the responsibility of the authors and does not necessarily represent the official views of the funders. The funders of the study had no role in study design, data collection, data analysis, data interpretation, or writing of the report. All authors had full access to all data in the study and had final responsibility for the decision to submit for publication.

Section 2: All-cause mortality, HIV mortality, and fatal discontinuities[4,5]

Section 2.1: Overview

We aimed to generate the most accurate estimates for all-cause mortality estimates for all GBD age groups, by sex, for all 1062 locations in the GBD 2019 location hierarchy, for a 70-year time series from 1950 to 2019.

One methodological challenge we faced was variability in the completeness and quality of data. Calculating all-cause mortality for different age groups in different locations required drawing on a diverse set of data sources (appendix tables 3 and 4 [section 9]). More generally, estimating all-cause mortality was complicated by the fact that not all countries and territories have complete vital registration (VR) systems recording the event of death and periodic censuses. Several methods were used to estimate completeness and adjust VR accordingly.

Here, we provide a detailed description of the methodology we used to estimate all-cause mortality. Due to the interdependence between HIV mortality estimation and all-cause mortality estimation, this appendix includes a partial overview of the epidemiological modelling of HIV. In what follows, we describe five major methodological tasks: estimating the probability of death between birth and age 5 years (${}_5q_0$); estimating the probability of death between age 15 years and 60 years (${}_{45}q_{15}$); estimating a complete set of age-specific mortality rates; estimating HIV mortality; and producing final estimates of age-specific mortality, including HIV mortality and fatal discontinuities. We describe primary input data and analytical steps undertaken in relation to each step. The appendix figure 1 (section 9) flowchart shows the entire modelling process. We ran the full model twice in order to update the input mortality data with the updated population estimates to improve internal consistency among different model components in not only our mortality estimation model, but also fertility and population estimation models. Inputs that were updated for the second iteration of the model are shown in appendix figure 1 using patterned boxes.

Section 2.2: Child mortality

Section 2.2.1: Data sources

Overview

For the estimation of child mortality, we used data from vital registration (VR) systems, sample registration systems (SRS), and disease surveillance point systems, household surveys (complete birth histories [CBH], summary birth histories [SBH]), censuses (SBHs, CBH in rare occasions), and Demographic Surveillance Sites (DSS). It was important that data were fully representative of the given age-sex group and location-year.

VRs, sample registration systems, and DSPs from other sources

We tried to incorporate all available data from VR systems in our all-cause mortality estimation process. These included multi-country VR sources such as the WHO Mortality Database, United Nations Demographic Yearbooks, Human Mortality Database, country statistics offices, and the Organisation for Economic Co-operation and Development (OECD) databases. We updated the data from these sources in our systems as new data were updated. Wherever possible, we also catalogued all data sources from each system for ongoing national VR systems (eg, the USA National Vital Statistics System).

When vital registration data were not available, we also extracted data from the Sample Registration System from India and the Disease Surveillance Points System (DSP) from China. Data from the Health and Demographic Surveillance System were also used.

Under-5 populations and livebirths

Livebirths and population in the under-5 age groups were produced as part of the fertility and population estimation, described in sections 3 and 5.

CBH microdata

Where VR data were unavailable or unreliable, complete birth histories (CBHs) were the preferred source for child mortality data. CBHs are surveys conducted with mothers about all livebirths they have ever had by the time of the survey regardless of their survival status, including birthdate, survival status, and date of death if deceased. Since CBH data include the age at which mothers gave birth to each child, we could calculate period and age-specific mortality rates in the time period before the survey, assuming no survivor, migration-related, or recall bias. Many surveys, including the Demographic and Health Surveys (DHS), World Fertility Surveys (WFS), Multiple Indicator

Cluster Surveys (MICS), and many national surveys include CBH modules. When available, we calculated mortality rates using micro-level data from surveys instead of the reported values for three five-year periods that are typically reported by surveys.

CBH tabulated data

Some reports release tabulated results before they release microdata, so we also included these datapoints in our probability of death from birth to age 5 years ($_5q_0$) database. Once we were able to obtain microdata from the same surveys, however, we replaced tabulated report estimates in our database with the microdata.

SBH microdata

Summary birth history (SBH) questionnaires are shorter and less detailed than CBHs and lack information on dates of birth and death of children. They simply ask mothers the number of livebirths they have ever had and how many of those children have died, as well as the age of the mother at the time of the interview. For this study, we collected all available SBH data that had microdata, or those that reported data on the proportion of a mother's children who died, by maternal age group. GBD has developed its own summary birth history method to generate under-5 mortality rate (U5MR) using the aforementioned SBH data.

Under-5 age-sex patterns for VR/SRS/DSP

In high-income countries, under-5 mortality estimates were derived primarily from VR system data. Often, these data were divided into age groups: early neonatal (0-6 days), late neonatal (7-27 days), post-neonatal (28-364 days), and 1 to 4 years. Some country-years of data had less specific age groups, with early and late neonatal or all under-1 groups combined. Age patterns of mortality in the under-5 age groups were also available from sample registration system and disease surveillance point systems.

Under-5 age-sex patterns from CBH

Aside from VR/SRS/DSP, complete birth histories (CBHs) from surveys provide another important source on the distribution of mortality among under-5 age groups. These data reflect age patterns primarily from low-income countries and countries with higher levels of under-5 mortality. We only used datapoints from the 15 years leading up to the survey, so children who were born to mothers more than 15 years before they were surveyed were not included in our analysis. We divided observations into 5-year time periods in order to produce more robust input point estimates of the probability of death for each under-5 age-sex group.

Section 2.2.2: VR prioritisation

As we worked with a variety of sources of vital registration data, we developed a rank ordered list of preferred VR data sources. For a given location-year, our first preference was to use WHO data from GBD cause-specific mortality estimation, followed by unadjusted WHO data, then Human Mortality Database (HMD) data, and then UN Demographic Yearbook data. We made exceptions in some cases, however. We assessed single-country VR data sources according to whether there were inconsistencies with other data sources or VR system documentation. We used HMD VR data for Germany, Taiwan (province of China), and Spain, for instance, because WHO data produced mortality rates inconsistent with established trends.

Section 2.2.3: VR under-enumeration for bias correction

For under-5 mortality, VR system completeness was estimated in the same way as in previous iterations of GBD, and like GBD 2017, most VR bias was corrected using the mixed effects non-linear model described in section 2.2.6.

Section 2.2.4: Computation of 5q0 from CBH

We used CBH microdata from unpooled surveys to calculate biennial under-5 mortality rate. We analysed each survey separately (eg, Kenya DHS 2014; Bangladesh MICS 2012–2013) instead of pooling all DHS or MICS CBH surveys from a single country (eg, Nigeria MICS surveys from 2007, 2011, and 2016–2017) so that we could better understand and address data quality issues in specific surveys. By grouping observation points into two-year intervals to generate biennial estimates, we overcame the issues of smaller sample sizes and lower stability that came from having unpooled data. We only included biennial estimates that contained more than 10 000 person-months of data.

Processing of tabular CBH

Microdata were not available for all CBH surveys. We still added results from these surveys to our database if survey reports provided $_5q_0$ datapoint estimates based on CBH.

Section 2.2.5: SBH method

We used an SBH method developed and updated by Rajaratnam and colleagues to estimate $_5q_0$ from SBH data from censuses and surveys.[7] We found this method to be more accurate and provide more timely estimates than previous methods.

In cases where the only data available were tabular data of number of livebirths ever born and number of children who have died, aggregated by the mother's age, we applied Rajaratnam and colleagues' Maternal Age Cohort model.[7]

Section 2.2.6: $_5q_0$ data synthesis

Data synthesis using ST-GPR

We used a spatiotemporal Gaussian process regression (ST-GPR) to estimate under-5 mortality rate.[8] We also incorporated data bias adjustment into the model based on the under-5 mortality synthesis model used in previous GBD iterations.[2–4,8–10] To adjust for bias, we ran the first-stage non-linear effects model for GBD 2019 standard locations only and then took predicted fixed effect coefficients from the non-linear mixed effect model and applied them to all locations. A set of fixed effects were estimated for each data source type used in our analysis. The list of source types can be found table below. We also used a random effect for each data source within a location. The combination of source-type fixed effect and source-specific random effect was the basis for bias adjustment. When a reference source was selected, all other sources were adjusted based on the difference between the bias value from the reference source and the source of interest. We did not adjust reference sources when more than one source was used as the reference source. In this case, all other sources were adjusted to the average bias values of all selected reference sources. Removing systematic differences in sources meant that we avoided estimating false trends caused by overlapping sources that had different levels of non-sampling variance. We then synthesised the bias-adjusted child mortality data using a combination of a non-linear effects model, spatiotemporal regression, and Gaussian process regression (GPR). This process produced consistent time series estimates of mortality for location, with 95% uncertainty intervals (UIs).

Source types used in child mortality bias correction

1. CBH: AIDS Indicator Survey and Malaria Indicator Survey
2. CBH: Census
3. CBH: Demographic and Health Survey
4. CBH: Multiple Indicator Cluster Survey
5. CBH: World Fertility Survey
6. CBH: Other survey series
7. Household Death Recall: Census
8. Household Death Recall: Other survey series
9. Household Death Recall: Incomplete VR/Sample Registration/Surveillance
10. SBH: AIDS Indicator Survey and Malaria Indicator Survey
11. SBH: Census
12. SBH: Demographic and Health Survey
13. SBH: Multiple Indicator Cluster Survey
14. SBH: World Fertility Survey
15. SBH: Other survey series
16. VR/Sample Registration/Surveillance: complete
17. VR/Sample Registration/Surveillance: incomplete

A list of under-5 mortality reference sources is provided in appendix table 5 (section 9).

Non-linear mixed-effects model
To estimate data bias and provide first stage estimates for $_5m_0$, we used a non-linear mixed effects regression model:

$$_5m_{0_{ctsi}} = \exp[\alpha_k + \gamma_{0c} + \gamma_{0cs} + (\beta_1 + \gamma_{1c}) * \log(LDI_{ct}) + (\beta_2 + \gamma_{2c}) * education_{ct}] + \beta_3 * HIV_{ct} + \varepsilon_{cysi}$$

$\gamma_{0c} \sim N\left(0, \sigma_{\gamma_0}^2\right)$
$\gamma_{1c} \sim N\left(0, \sigma_{\gamma_1}^2\right)$
$\gamma_{2c} \sim N\left(0, \sigma_{\gamma_2}^2\right)$
$\gamma_{0cs} \sim N\left(0, \sigma_{\gamma_{0s}}^2\right)$
$\varepsilon_{cts} \sim N(0, \sigma_\varepsilon^2)$

Where

Index c is location, t is time, s is source, k is source type of datapoint i

$_5m_0$ is under-5 mortality rate

LDI is lag-distributed income per capita

education is mean years of education for women of reproductive age (15-49 years)

HIV is crude death rate due to HIV in age groups 0-4

γ_{0c} is a country random intercept

γ_{0cs} is a country-source random intercept

γ_{1c} is a country random slope for *LDI*

γ_{2c} is a country random slope for *education*

α is a fixed intercept for source

β_i is a fixed covariate coefficient

ε is the residual

$\gamma_{0c}, \gamma_{0cs}, \gamma_{1c}, \gamma_{2c}$ are all pairwise independent

Sources were categorised into one of the 17 source types (k) listed above. See sections 7.3 and 7.4 for details on how LDI and education were estimated. This regression used HIV mortality rates estimated as part of the GBD 2019 cycle (section 2.5).

We turned to country experts to identify data sources in each location that were likely to be least biased, of higher data quality to be used as reference source or sources. When it was believed that a VR system was complete, we used it as the reference source. When data from complete VR systems were not available, we used DHS estimates from CBHs as the reference source if available. Specific source or sources were selected for a location by location basis when neither complete VR nor CBH from DHS or other surveys were available.

The bias adjustment was done using the equation below:

$$adjusted_5m_{0,cts} = \exp[\alpha_t + \gamma_{0c} + \gamma_{0ref,c+}(\beta_1 + \gamma_{1c}) * \log(LDI_{ct}) + (\beta_2 + \gamma_{2c}) * education_{cy}] + \beta_3 * HIV_{ct} + \varepsilon_{cts}$$

For incomplete VR data, we calculated an upward adjustment using a five-year rolling mean of the difference between incomplete VR data and a Loess regression of the already-adjusted survey data.

Spatiotemporal smoothing
To smooth the residuals between the predicted $_5q_0$ time series using the non-linear mixed effect model and the adjusted raw data, we applied a combination of spatiotemporal smoothing functions. This stage smoothed the residuals over time and across locations within each GBD region. First-stage prediction of $_5q_0$ is generated using the equation below:

$$predicted_5m_{0,ct} = \exp[\alpha_t + \beta_1 * \log(LDI_{ct}) + \beta_2 * education_{ct}] + \beta_3 * HIV_{ct}$$

Here, the prediction only used the fixed effects from the non-linear mixed effects model where source type fixed effects is set to zero, the value of the absorbed source, complete vital registration system.

After calculating the predicted time series, we calculated the residuals between the prediction and the adjusted data. Next, we applied the smoothing functions to the residuals. We weighted residuals in each region for each location-year based on how close the data were to this location-year in space and time. 99% of the weight went to in-location residuals, with the remaining 1% going to out-of-location but within GBD region residuals. The weight for time is defined using the equation below:

$$w_t = \left(1 - \left(\frac{|r_t - r_{est}|}{1 + argmax_t|r_t - r_{est}|}\right)^{\lambda}\right)^3$$

Where

r_t is the year of interest

r_{est} is the year of the residual being weighted

$argmax_t|r_t - r_{est}|$ is the maximum distance between the year of interest and a residual within the region

λ is the weighting function that dictates how quickly the weights fall off as the distance in time increases

When λ is larger, the assigned weights diminish more slowly over time compared to a smaller λ. Next, we used a linear fit to the weighted data (akin to a Loess fit) to generate a single estimate of the smoothed residuals. We also generated a second estimate of the smoothed residuals using the weighted average of the data.

We then pooled the two estimates of the smoothed residuals into one final estimate. We assigned more weight to the local linear fit in countries and territories with high quality and completeness of data and more weight to the weighted average in countries and territories with sparse data using the following equation:

$$final\ smoothed\ residual = k * linear\ estimate + (1 - k) * weighted\ average$$

Where

$$k = \frac{number\ of\ in\ country\ datapoints}{number\ of\ in\ country\ datapoints\ +\ number\ of\ country\ years\ with\ no\ data}$$

The pooling of two estimates of the smoothed residuals is developed to account for artificial trend in smoothed residuals from either method especially when the year of interest is further away from the last year where there is empirical data. Such a method also helps improve comparability among estimates for different locations that otherwise might need to employ different method for smoothing the residuals.

Last, we incorporated the smoothed residuals back into the above predictions to produce a smoothed approximation of the adjusted data. We used this estimate as the prior for the GPR explained in the next section.
To avoid producing space-time smoothed U5MR that is over 1 or under zero, both space-time smoothing and the Gaussian process regression described below are done using U5MR in logit space.

GPR

We used Gaussian process regression (GPR) to generate a final time series of point estimates as well as UIs.
The GPR model was as follows:

$$\mu_{c,t} = f_c(t) + S_{c,t}$$

$$f_c(t) \sim GP(M, C)$$

Where

t is time, c is location

μ_t is the true $\log_{10}({}_5q_0)$

$f(t)$ is the baseline mortality risk

S_t is excess mortality due to fatal discontinuities estimated independently of $f(t)$

M is the mean function for the Gaussian process

C is the covariance function for the Gaussian process

The mean function (M) is an initial guess at our unknown function f_c. We choose this to be our second-stage predictions.

The covariance function (C) defines the covariance between any two points t_i and t_j. It controls the smoothness of realizations from the Gaussian process, and the amount of deviation from the mean that is tolerated. There are some conventions regarding good choices for the covariance function in Gaussian process regression, but no single function that must be used. We select the Matérn covariance function:

$$C_{matern}(x_i, x_j \mid a, s, \nu) = a^2 \frac{1}{\Gamma(\nu) 2^{(\nu-1)}} \left[\sqrt{2\nu} \frac{|x_i - x_j|}{s}\right]^{\nu} K_\nu\left(\sqrt{2\nu} \frac{|x_i - x_j|}{s}\right)$$

Where a is amplitude, s is scale, ν is degree of differentiability, Γ is the gamma function, and K_ν is the modified Bessel function of the second kind and order ν. To select the *amplitude* parameter, we first calculated the variance of logit-difference between the first-stage and second-stage predictions in high data-density years separately by location. Then, this location-specific variance was used as amplitude for countries with complete VR and the mean of these variances was used as amplitude for countries with incomplete VR. *Scale* is fixed by location based on data density (see table A below), and degree of differentiability is fixed at 1.

In addition to our Gaussian process prior for f, we observe the value of f for some set of years o_i – this is our data. We assume the observation error is normally distributed and calculate the data variance as described in the next section. Then, the posterior distribution for f given the observations and the prior is another Gaussian process with a closed form solution for posterior $\widetilde{M}$ and $\tilde{C}$. Let V be a matrix with observation variances $[v_0, \ldots, v_{n-1}]$ on its diagonal and o be the vector of years for which we've observed $f(o)$. Then the posterior distribution, $\tilde{f}$, is:

$$\tilde{f}_c \mid data \sim GP(\widetilde{M}, \tilde{C})$$

$$\widetilde{M}(t) = M(t) + C(t,o)[C(o,o) + V]^{-1}\big(f(o) - M(o)\big)$$
$$\tilde{C}(t_i, t_j) = C(t_i, t_j) - C(t_i, o)[C(o,o) + V]^{-1} C(o, t_j)$$

From this Gaussian process posterior distribution, we take realizations, where one realization is a complete function from our domain of years to the outcome of interest. 1000 realizations are draws that can be collapsed to get a mean and 95% uncertainty interval for every location and year separately.

GPR was implemented using the PyMC package in Python.

Data variance

We calculated data variance for each $_5q_0$ input datapoint, which reflects sampling errors. Additional non-sampling errors were added when the datapoint was from a non-reference data source. Specifically, we calculated data variance using the following methods, depending on the type of data:

1. **Complete VR data:** we assumed there was only sampling error, not non-sampling error, as complete VR is assumed to be unbiased. We calculated this error using the binomial distribution $p(1-p)/N$, where N is the population aged 0 to 5 years for the location and p is the under-5 mortality rate, $_5m_0$. We then used the delta method[11] to transform the variance of $_5m_0$ to the variance of $\log_{10}(_5q_0)$.
2. **Incomplete VR data:** we included both sampling error and non-sampling error from uncertainty in our estimate of data completeness. We calculated the total data variance as the sum of the variances from sampling error (see calculation method for complete VR data) and non-sampling error based on the completeness estimate.
3. **CBH data:** f draws of $_5q_0$, transformed these draws into $\log_{10}$ space, and calculated the sampling variance from the transformed draws.
4. **SBH data:** we used the standard error from the mean residuals.
5. **Other:** we used the maximum standard error from non-VR points in a country or territory if we could not produce data variance for a source. When no other source was available, we set the data variance to be the maximum standard error from non-VR data sources within the GBD region the location belongs to.

Finally, for all data types other than complete VR data, we calculated the within-source-type variance of the source-specific random effect for each source type. We then converted it to $\log_{10}$ space and added it to the variance already calculated using one of the methods above.

Hyperparameter selection for under-5 mortality rate ST-GPR

We selected hyperparameters based on a data density score for each location, first used in GBD 2017.[4] We calculated data density scores based on the number of deaths from VR sources plus the number of unique CBH and SBH sources available for the given location, using the following computational methods for each type of data source:

1. **Complete VR data density score:** calculated based on the number of deaths reported from unbiased VR sources in a single location. We capped the number of deaths for each source-year at 500 and then divided that number by 500 to generate a score between 0 and 1 where 1 represents a complete VR system with at least 500 registered deaths and a 0 represents a complete VR system with 0 registered deaths. Final complete VR scores ranged from 0 to 68.
2. **Incomplete VR data density score:** calculated the same way as with complete VR data, using biased VR sources rather than unbiased VR sources.
3. **Total CBH sources:** the number of unique complete birth histories for a single location.
4. **Total SBH sources:** the number of unique summary birth histories for a single location.

After making these calculations, we used the following formula to compute the final data density for each location:

$$data\_density = complete\_vr\_deaths + (0.5 \times incomplete\_vr\_deaths) + (2 \times cbh\_sources) + (0.25 * sbh\_sources)$$

We then assigned hyperparameters based on final data densities, as designated in table A. Zeta determines the magnitude of space weights in the spatiotemporal smoothing. Lambda determines the magnitude of time weights. Scale is the scale parameter of the Matérn covariance function, which controls correlation over time.

Table A

Data density	Zeta	Lambda	Scale
0 to 9	0·7	0·7	15
10 to 19	0·7	0·5	15
20 to 29	0·8	0·4	15

30 to 49	0·9	0·3	10
50 plus	0·99	0·2	5

Difference between GBD's application of ST-GPR and GPR used in geospatial models

It is important to point out that the ST-GPR model described above and implemented in GBD is different from how it is utilised in other research fields such as model-based geostatistics. In model-based geospatial research, for example, it requires longitude and latitude for the input dataset to produce a continuous surface of metrics of interest over latitude and longitude. However, that is not how it is implemented in GBD estimation. In GBD, we estimate at the national or subnational level, in contrast to the pixel level. While we are interested in geospatial analyses, we routinely rake the geospatial analyses to the GBD results at the national or admin 1 level as we have much more confidence in the rigor of the GBD analysis at the national level than we do in the full geospatial analysis integrated to the national level. The reason is that at the national level we can detect and correct for non-sampling error in the models using source random effects. We also fully take into account the random sampling nature of the national surveys or the full coverage of vital registration data. In contrast, our and others' geospatial models struggle to distinguish local spatial variation from non-sampling error.

While our ST-GPR model is definitely not a geospatial model, it is a model that borrows strength from countries within a GBD region through the smoothing of residuals in time and space.

Section 2.2.7: Identify and remove outliers

We carefully reviewed child mortality data and estimates to ensure quality. First, we outliered datapoints from years in which fatal discontinuities occurred, unless cause of death information was available for the VR datapoint such that deaths from specific causes of death identified as fatal discontinuity could be removed. We excluded fatal discontinuity data because our objective was to identify the underlying mortality risk, which would be warped by mortality data from large stochastic events. We added back fatal discontinuity data later in the estimation process (see section 2.7). Second, we outliered data sources whose data quality we were concerned about, such as the Afghanistan DHS from 2010. Collaborators from our extensive network reviewed sources in which they had expert knowledge and identified those with known quality issues.

Section 2.2.8: Rake subnational estimates to national level (excluding South Africa)

One strength of GBD is that we ensure consistency throughout our location hierarchy, meaning that subnational mortality estimates always add up to national mortality estimates. The estimation process for $_5q_0$ described above does not enforce this consistency, so the following adjustments in locations with subnational estimates after the aforementioned estimation process are made. We first population-weighted the subnational estimates to form an estimate at the national level. A scalar was then calculated by year by dividing the separately estimated national value by this weighted average of subnational estimates. We then multiplied the scalars from this calculation by all the subnational estimates to get scaled estimates for each subnational location. We chose this strategy for subnational scaling because national-level estimates are generally more reliable as they are more likely to be based on more empirical data and the data themselves are less likely to be affected by the small number issue. The effect of scaling was minimal in locations where VR data were high-quality.

South Africa was the one country where we did not use this method. We instead aggregated provincial-level estimates from the GPR to the national level. This was to ensure consistency between our estimates at the subnational level and the observed deaths from the vital registration system in South Africa at the national level.

Section 2.2.9: Review estimates for quality

We compared our $_5q_0$ estimates with GBD 2017 and the UN International Children's Emergency Fund (UNICEF) estimates from 2017 and 2019.[4,12] We identified all differences in results and determined whether they were caused by updates to the available data, changes to the hyperparameters we set, or modifications to input covariates. We made revisions to our estimates as part of this review process and through consultation with collaborators in the GBD mortality network.

Section 2.2.10: Under-5 age and sex pattern model estimation

To disaggregate under-5 mortality into age- and sex-specific groups, we used a multi-stage modelling process similar to the method first published in GBD 2016.[3] The result of this process is sex-specific mortality for the early neonatal (0-6 days), late neonatal (7-27 days), post-neonatal (28-364 days), and childhood (1-4 years) age groups.

First, we estimated the ratio of male to female $_5q_0$, and second, we estimated the sex-specific age distributions of under-5 mortality. Both the sex and age components have the following stages: (1) generalised linear model, (2) spatiotemporal smoothing, and (3) Gaussian process regression. Finally, the components are scaled and synthesised into age- and sex-specific mortality.

Data from VR, sample registration system, and CBH sources were converted to mortality risks for each age group. Some sources included age-specific data for all four age groups, while others only had infant (early, late, and post-neonatal) and child distinctions.

The sex model first estimated the ratio of male to female $_5q_0$ for each location i in region j in year y, in rescaled logit space. We excluded observed ratios below 0·8 or above 1·5, then rescaled the ratio data from 0·8–1·5 to 0–1 to place our data within the domain of the logit function. Data from non-standard locations were also excluded in the first-stage model fitting.

With these data, we fit the following sex-ratio model:

$$logit\ \left(\frac{Male\ _5q_0\ scaled}{Female\ _5q_0\ \ scaled}\right)_{ijt} = \beta_0 + \delta_j + \gamma_i + S_{_5q_0} + \varepsilon_{ijt}$$

$$\delta_j \sim N\left(0, \sigma^2_{\delta_j}\right)$$

$$\gamma_i \sim N\left(0, \sigma^2_{\gamma_i}\right)$$

$$\varepsilon_{ijt} \sim N(0, \sigma^2_\varepsilon)$$

Where

i is location, j is region,

t is time

β_0 is an intercept term

$S_{_5q_0}$ is a natural spline on both-sex $_5q_0$

δ_j is the region random intercept

γ_i is the location random intercept (nested in region)

ε is the residual

We then used the parameter estimates from this model, and our estimated $_5q_0$, to predict first-stage sex-ratio of under-5 mortality for all GBD location-years. Spatiotemporal smoothing and GPR were used to produce final sex-ratio estimates. We chose ST-GPR hyperparameters using the same method in section 2.2.6.

Next, we estimated $_5q_0$ for males and females using the equation:

$$_5q_0 = \left(\frac{1}{1 + r_{birth}}\right) * (female\,_5q_0) + \left(\frac{r_{birth}}{1 + r_{birth}}\right) * (male\,_5q_0)$$

where r_{birth} is the sex-ratio at birth, $_5q_0$ is the direct output from the estimation process described in section 2.2.1–2.2.10, and male $_5q_0$ can be replaced with female $_5q_0$ times the predicted $_5q_0$ sex-ratio to create a system with one unknown.

We then fit a separate age-specific model for each sex-specific age group, resulting in five models for each sex: early neonatal, late neonatal, post-neonatal, infant (neonatal, late neonatal, and post-neonatal combined), and child. The outcome modelled was the log-proportion of sex-specific under-5 deaths occurring in age group x, in other words, the probability of death in age group x conditional on death before age 5. This outcome facilitates scaling to our estimates for ${}_5q_0$.

The first-stage model for estimating this age distribution is:

$$log(Pr(death\ at\ age\ x|u5\ death)_{jits}) = \beta_0 + \gamma_{js} + \gamma_{is} + a_{1x}\beta_{1s} * HIV_{its} + a_{2x}\beta_{2s} * Mat.Ed._{it} +$$

$$a_{3x}\beta_{3s} * Completeness_{it} + S_{{}_5q_0} + \varepsilon_{jits}$$
$$\gamma_{js} \sim N(0,\sigma^2)$$
$$\gamma_{is} \sim N(0,\tau^2)$$

Where

x is age, s is sex, i is location, j is region, t is time

γ_i is a country random intercept (nested in region)

$\gamma_{j[i]}$ is a region random intercept

$S_{{}_5q_0}$ is a natural spline on ${}_5q_0$

β_i is a fixed covariate coefficient

a_{1x} is an indicator for HIV covariate inclusion: 0 for enn, 1 otherwise

a_{2x} is an indicator for maternal education covariate inclusion: 1 for child, 0 otherwise

a_{3x} is an indicator for source completeness covariate inclusion: 1 for child, 0 otherwise

ε is the residual

The literature on HIV mortality in different under-5 age groups is limited, and there is no clear guidance on how to age-split under-1 deaths due to HIV.[13,14] Prior research has, however, suggested that HIV mortality risk differs between different under-5 age groups,[14,15] with essentially all HIV deaths that occur in the first year of life occurring in the post-neonatal stage. We therefore included under-5 crude death rates from HIV from the GBD 2019 model (see section 2.5) in the model for post-neonatal, infant, and child, but not the neonatal age groups. Including this covariate improved the fit and predictive validity of the model in countries with high HIV prevalence. In locations where HIV/AIDS contributes a large burden to under-5 mortality, using ${}_5q_0$ as the only predictor in this model typically leads to an overestimation of neonatal mortality.[16]

We also included a maternal education covariate and a source-specific child completeness covariate for the 1 to 4 age group only. We calculated child completeness by dividing the source-specific ${}_5q_0$ point estimate by the final GPR ${}_5q_0$ estimate. In the prediction of this model, we used a completeness value of 1, to predict from a hypothetically complete source.

After prediction of the first-stage model for all GBD location-years, ST-GPR was used to generate stage-three results by age and sex. We used the same ST-GPR process as described previously for the overall under-5 mortality rate.

Finally, we ensured consistency in probability of death between the separate and collective under-5 age groups by scaling. We first scaled the infant and child conditional probabilities to add to 1 and then scaled the early neonatal, late neonatal, and post-neonatal conditional probabilities to add to the infant conditional probability. Next, we calculated the probability of early neonatal death by multiplying the rescaled conditional probability of early neonatal death given under-5 death by ${}_5q_0$ using the equation below:

$$q_{enn} = \Pr(death\ in\ enn \mid u5\ death) * 5q0$$

where enn is early neonatal.

To calculate the probability of late neonatal death, we multiplied the rescaled conditional probability of late neonatal death given under-5 death by ${}_5q_0$ and then divided that by the probability of survival to the start of the age group (7 days), using the following equation:

$$q_{lnn} = \Pr(death\ in\ lnn \mid u5\ death) * 5q0/(1 - q_{enn})$$

where lnn refers to late neonatal.

We calculated probability of death for the post-neonatal and 1–4-year age groups in the same way.

Section 2.2.11: Identify and remove outliers from age and sex models

We removed outliers from the under-5 age-sex pattern model in several instances.

For the sex model, we removed non-VR datapoints from locations with high-quality VR data (based on the GBD VR quality rating system) to make sure we were only using the highest-quality data.

For the age model, we outliered the following:

1. VR data that were incomplete. We compared the nine-year rolling average of the ${}_5q_0$ value from the VR data to the nine-year rolling average of the ${}_5q_0$ estimates and then the raw data value of ${}_5q_0$ to the final ${}_5q_0$ estimate for each data-year. We outliered data for which the second comparison yielded a ratio of 0·85 or less, unless the ratio of the nine-year rolling average was above 0·85.
2. Data that were identified as outliers earlier in the ${}_5q_0$ analysis.
3. CBH datapoints from more than 15 years before the survey was administered.
4. CBH data in locations with both VR and CBH data, but only if the data conflicted.
5. Other datapoints that were manually outliered for various reasons. For instance, the definition of a livebirth included a minimum weight requirement in some eastern European countries before the 1990s, which led to inconsistencies in livebirth data over time. We therefore outliered age group data in ages that would include childbirth deaths when and where the definition of livebirth included a minimum weight.

Section 2.2.12: Under-5 death number estimation

We estimated the number of under-5 deaths by ageing birth cohorts through our estimated age-sex-specific probabilities of death. To do this, we divided the number of births for each location-year into weekly birth cohorts and moved these cohorts through our mortality estimates in weekly increments. This process produced estimates of the number of person-years and deaths in each under-5 age group.

Section 2.3: Estimation of adult mortality rate

Section 2.3.1: Data sources

Adult population estimates

We used multiple data types to estimate adult mortality rate, including VR systems; surveys and censuses, from which we extracted household death recall data; the Sample Registration System (SRS) in India; and the DSP in China.

VR/SRS/DSP

Refer to section 2.2.1 for details on how we identified and prioritised VR, SRS, and DSP data.

Household recall of deaths

Household death recall modules can be found in censuses and surveys, where the information on the number of deaths that occurred in each household over a specific recall period is collected. Information on the number of usual residents in the household for the same recall period is collected as well. Such information, combined, can be used to adjust data deemed incomplete in the recall of household deaths to generate sex-specific and age-specific mortality rates.

Sibling survival histories

We also acquired sibling survival history data from surveys where participants are asked about the status of their siblings (alive or dead). Usable surveys needed to ask participants to provide a full account of all siblings (children born to the same mother), including the year of death and age at death (if applicable), year of birth, and sex.

Section 2.3.2: Completeness assessment: death distribution methods and completeness estimates synthesis

As with under-5 mortality VR data, we had to evaluate the completeness of available adult mortality VR data. For this study, we used five death distribution methods (DDMs) as well as our findings about under-5 VR data completeness to make this assessment.[17] We used the three DDMs most common in demography: generalised growth balance method (GGB), synthetic extinct generations (SEG), and a combined approach (GGB-SEG),[18–22] which estimate completeness by comparing the age distribution of the population between two censuses with the age distribution of deaths between those same censuses. For GBD 2019, we added two additional methods that utilise the GBD Bayesian Population Model.

To test performance of the traditional DDM methods and newer methods, we simulated census and VR data while varying the level of VR completeness and factors that may bias completeness estimates, including total population size and age-structure, census completeness, net migration, and extent of age-misreporting in census and VR data. We tested two new methods that used the Cohort Component Method of Population Projection (CCMPP) in the GBD Bayesian Population Model to estimate completeness of VR in between censuses.[17] These two methods age a population forward in time using CCMPP with the input mortality data and estimates of completeness in order to match the population at the second time point. These two methods differ only in whether the proportion of net migrants is also estimated in addition to completeness. For each of the five methods (new and traditional) we tested 105 age trims ranging from a starting age of 5 and an end age of 95. Table B shows the top ten age trims by method.

Table B

Rank	CCMP fixed migration		CCMP not fixed migration		GGB		GGBSEG		SEG	
	Start age trim	End age trim	Start age trim	End age trim	Start age trim	End age trim	Start age trim	End age trim	Start age trim	End age trim
1	55	95	25	95	5	70	5	70	45	85
2	65	95	25	90	15	65	10	70	40	85
3	55	90	35	95	10	65	5	75	50	95
4	65	90	30	95	20	65	15	65	45	95
5	45	95	20	95	5	85	20	65	55	85
6	60	95	15	95	5	60	15	70	50	85
7	50	95	35	90	10	85	10	60	40	90
8	45	90	45	95	10	70	10	65	60	85
9	50	90	30	90	15	85	15	60	40	95
10	60	90	20	90	10	60	10	75	35	85

From the simulation testing, we found that no single method performed significantly better than the other methods, but certain age trims for each method performed significantly better than other age trims. To account for this when producing point estimates of completeness for each pair of censuses and VR data in between censuses, we excluded the highest and lowest point estimates across the five methods and only kept the three middle estimates. For each method, we only kept the top-performing age trim as ranked in our simulation testing. These three-point estimates of

completeness for each census pair were then combined to form a full time series of completeness using a spatiotemporal regression model, as described next.

Our assessment used a two-stage model. We first used estimated child completeness to predict adult completeness, followed by a spatiotemporal regression model to incorporate estimates of adult completeness from the DDMs to generate a series of estimates from 1950 to 2019 of source- and country-specific adult death registration completeness. We assume that the completeness of a system changes gradually, so within the GBD standard locations, we used DDM estimates from neighbouring years to help assess the completeness of data from any given year in the study period. We also assumed that completeness was likely to be similar between countries in the same region, so we factored in completeness estimates from nearby countries as well.

We calculated child completeness as the ratio of observed child mortality to estimated child mortality in a given source, country, and year, using the estimation process described in section 2.2. While data were only available from certain years for an individual country-source, we made a complete time series of child completeness estimates using a smoothing process. When a country-source had no more than three years of data, we assumed a constant level of child completeness that matched the mean of the available years' completeness. When a country-source had more than three years of data, we used a Loess regression to fill in gaps in the time series. In cases where we had to make out-of-sample estimates (country-sources without data from 1950 or 2019), we held child completeness constant before the first and after the last observation instead of using the Loess regression to predict completeness levels. When a country-source had a gap of more than five years between data, we filled in the gap with a linear interpolation of the two closest observations instead of using the Loess regression.

After estimating child completeness, we conducted our first-stage regression using simple linear regression of calculated adult completeness on child completeness, and predicted adult completeness using the obtained coefficients for SRS, VR, DSP, Medical Certification of Causes of Death (MCCD), and civil registration (CR) sources in log space, using the following formula:

$$\hat{\mu}(\log(Adult\ completeness)) = \hat{\beta}_0 + \hat{\beta}_1 * \log(Child\ completeness)$$

For other sources, we assumed completeness for the first stage due to lack of child mortality data. In our second-stage model, we took the residuals from the first-stage regression and applied spatiotemporal smoothing to the residuals, in order to borrow strength across the full time series and nearby locations. We then produced draw level estimates of completeness using the calculated prediction from stage 1 as mean, and standard deviation calculated the smoothed residuals for stage 2 assuming a normal distribution. After setting logged completeness to 0 for estimates greater than 0, we produced final estimates and uncertainty from the anti-logged individual truncated draws.

For VR, SRS, and DSP sources, we considered country-years that had completeness estimates of 95% or higher to be 100% complete. We then scaled completeness values between 90% and 95% up to 100% as follows:

$$scaled\ completeness = 0.9 + 2\ (estimated\ completeness - 0.9).$$

We also assumed that if a country-source was complete across the full time-series, its subnational locations were complete as well. Our exceptions to this assumption were Brazil and Iran, where we found that level of completeness differed across subnational locations, despite a complete and accurate time-series at the national level.

Section 2.3.3: Sibling survival method

While vital registration systems are the preferred sources for adult mortality rates, in countries with few to no data from a working vital registration system, adult mortality rates can be derived from sibling survival modules, which provide much-needed information on the level and trends of adult mortality. We define a sibship as a group of offspring who have the same biological parents. Without necessary adjustment, adult mortality estimates from sibling survival modules are biased in four primary ways:[23]

1. Selection bias (under-representation of siblings from sibships with high mortality)

2. Zero reporter bias (sibships not represented in the survey due to sex composition and/or absence of any alive sibling of a sibship by the time of the interview)
3. Sparse data
4. Recall bias (under-reporting of deaths of siblings living in different places, having died in the distant past, or for any other reason that the respondent could not recall the death).

The technique we used to estimate adult mortality from sibling survival data and minimise bias was based on methods in Obermeyer and colleagues,[23] with the following changes to their methods:

1. Incorporated appropriate survival weights that accounted for the study design
2. Implemented a zero-survivor correction that accounted for mortality in families who were underrepresented in the data because none of the siblings were alive to participate in the survey
3. Refined recall bias adjustment.

The correction to account for mortality in sibships with high mortality rates, proposed by Gakidou and King,[24] incorporates a sibship-level weight:

$$W_j = \frac{B_j}{S_j}$$

Where

j is a given sibship

B_j is the original size of sibship j

S_j is the number of siblings in sibship j who survive to the time of the survey

We used the Gakidou-King (GK) weight on observations being analysed at the sibship level to compute the weighted average of the proportions of siblings who had died, as reported by each respondent. The weight corrected for underrepresentation of high-mortality sibships.

Since we analyse data at the sibling level (with one observation for each sibling instead of for each sibship), we also used the following sibling-level weight:

$$W_i = \frac{1}{S_j}$$

for sibling i in sibship j.[25,26]

Previously, we applied the GK sibship-level weight to data that had been disaggregated to the sibling level, which led to inaccuracies in the estimates. Our sibling-level weight overcomes this challenge.

Eligibility criteria for different surveys also needed to be factored into the correction.[25] In GK's survivorship correction, $\frac{S_j}{B_j}$ refers to the probability that a sibling in sibship j survived and was eligible to be selected as a survey participant. For instance, only women between the ages of 15 and 49 are eligible to participate in DHSs, so for DHS data, S_j refers to the number of surviving women in sibship j who were aged 15-49 at the time of the survey. For this study, we made the S_j value consistent with the eligibility criteria for each survey.

As previously discussed, the sampled population in sibling histories did not include sibships that had no eligible siblings to participate in the surveys. As a result, these sibships are not present in the data. To account for this, we

estimated the number of deaths among missing sibships by age and sibship size using a zero-survivor correction. We then added these estimated siblings to the observed sample before calculating age-specific mortality rates.

We applied the zero-survivor correction to sibships with one or two female siblings. It factored in the true number of sibships with one (or two) females as it related to the cumulative probability of dying before the time of the survey. The result of the correction was an estimate of the number of missing sibling deaths. For one-sibling sibships, we used the following formula:

$$K_{obs}^{1} = K_{true}^{1} * (1 - {}_{a}q_{0}^{1})$$

$$K_{miss}^{1} = K_{true}^{1} * {}_{a}q_{0}^{1}$$

Where

K_{obs}^{1} is the number of sibships with one sister that were observed in the sample

K_{true}^{1} is the true number of sibships with one sister in the sample

K_{miss}^{1} is the number of sibships with one sister that were not represented in the sample due to no surviving sibling

${}_{a}q_{0}^{1}$ is the probability of death between birth and age a

$(1 - {}_{a}q_{0}^{1})$ is the probability that the sister has survived to the time of the survey

We were able to determine that the number of sibships with only one sister that were not represented in the sampled population as a result of zero-survivor bias was:

$$K_{miss}^{1} = \frac{K_{obs}^{1}}{1 - {}_{a}q_{0}^{1}} \times {}_{a}q_{0}^{1}$$

We then multiplied the estimated number of missing sibships (K_{miss}^{1}) by the number of females in the sibship (one, in this instance) to estimate the number of females missing from each age group because they had died. We made each missing female one observation point, assigned birth and death dates based on the distribution in the sampled population, and added them to the dataset. We conducted the same process with families with two sisters using the formula below:

$$K_{obs}^{2} = K_{true}^{2} * (1 - {}_{a}q_{0}^{1} * {}_{a}q_{0}^{2})$$

$$K_{miss}^{2} = K_{true}^{2} * {}_{a}q_{0}^{1} * {}_{a}q_{0}^{2}$$

$$\therefore K_{miss}^{2} = \frac{K_{obs}^{2}}{1 - {}_{a}q_{0}^{1} * {}_{a}q_{0}^{2}} * {}_{a}q_{0}^{1} * {}_{a}q_{0}^{2}$$

Where

K_{obs}^{2} is the number of sibships with two sisters that were observed in the sampled population

K_{true}^{2} is the true number of sibships with two sisters in the population

K_{miss}^{2} is the number of sibships with two sisters that were not represented in the sampled population due to zero-survivor bias

$_{a}q_{0}^{1}$ is the probability of death for the first sister between birth and age a

$_{a}q_{0}^{2}$ is the probability of death for the second sister between birth and age a.

In two-sister sibships with only one 15- to 49-year-old sister, we used the following equation to account for the second sister not contributing to the probability of the sibship being observed in the sample:

$$K_{obs}^{2} = K_{true}^{2} * (1 - {_{a}q_{0}^{1}})$$

$$K_{miss}^{2} = K_{true}^{2} * {_{a}q_{0}^{1}} * {_{a}q_{0}^{2}} + K_{true}^{2} * {_{a}q_{0}^{1}} * (1 - {_{a}q_{0}^{2}})$$

$$\therefore K_{miss}^{2} = \frac{K_{obs}^{2}}{1 - {_{a}q_{0}^{1}}} * {_{a}q_{0}^{1}} * {_{a}q_{0}^{2}} * \frac{K_{obs}^{2}}{1 - {_{a}q_{0}^{1}}} * {_{a}q_{0}^{1}} * (1 - {_{a}q_{0}^{2}})$$

The original GK and Obermeyer and colleagues methods used a logistic regression to account for bias in the time period before the survey.[23,24] For this study, however, we used an updated method to adjust for recall bias. Once we had estimated the probability of death between 15 and 60 years ($_{45}q_{15}$) from each survey, we paired up estimates that overlap the same time period. Overlaps occurred in countries and territories where at least two surveys were conducted within 15 years of one another, since we included 15 years of recall. For each year with overlapping surveys, we computed the difference in the years of recall as the number of years between surveys. We quantified the relationship between years of recall and level of mortality for each sex of sibling using the following linear regression model:

$$\Delta({_{45}q_{15}})_{i,j} = \beta \times \Delta(survey\ date)_{i,j} + \xi$$

Where

$\Delta({_{45}q_{15}})_{i,j}$ is the difference in $_{45}q_{15}$

$\Delta(survey\ date)_{i,j}$ is the difference in survey date

j is the survey pair

i is the country

We also calculated 95% UIs.

We adjusted the $_{45}q_{15}$ estimates to account for recall bias using the period coefficient.

Section 2.3.4: $_{45}q_{15}$ data synthesis using non-linear mixed effects model and ST-GPR

Overview of adult ($_{45}q_{15}$) mortality estimation

We produced a time series of $_{45}q_{15}$ estimates for each location in the GBD hierarchy. For each location, we modelled the underlying mortality risk and then separately modelled excess mortality due to fatal discontinuities. We used a three-stage process to model underlying mortality risk. Through this process, we incorporated all available data for each country and territory that had not been outliered or excluded for conflict or natural disaster. Broadly, we did the following in each stage of the model:

1. Applied a nonlinear mixed effects model that attempted to explain the variation in mortality rate in the 15 to 59-year age group ($_{45}m_{15}$) using covariates.
2. Smoothed residuals between input data and the first-stage prediction. We then added the smoothed residuals back into the first-stage regression predictions. Through this, we generated a complete time series of prior $_{45}q_{15}$ for each location.
3. Final estimates were generated using the prior generated from the first two steps and input data on adult mortality rates.

After completing these three stages, we modelled shocks to mortality by estimating the excess risk of mortality in country-years in which a natural disaster or conflict occurred. We then added the estimated excess mortality risk to the underlying mortality risk to generate final time series estimates of ${}_{45}q_{15}$. A full description of the stages from this part of the model are found below.

First-stage nonlinear mixed effects regression

The first stage of the adult mortality model was a nonlinear mixed effects regression. This regression produced adult mortality rate estimates for each location, year, and sex. We incorporated the following covariates: lag-distributed income per capita, mean years of education in the 15 to 59-year age group, crude death rate due to HIV/AIDS in the 15 to 59-year age group, and estimated ${}_{5}q_{0}$ for the location from our study. We also included a fixed effect on sex, which allowed us to fit the model using males and females together. The equation for the nonlinear mixed effects regression was as follows:

$$ {}_{45}m_{15}^{observed}{}_{s,c,t,i} = \exp(\alpha_s + \gamma_c + \beta_1 \cdot Edu_{s,c,t} + \beta_2 \cdot ln\left(LDI_{c,t}\right) + \beta_3 \; {}_{5}q_{0_{c,t}}) + \beta_5 \cdot HIV + \mathcal{E}_{s,c,t,i} $$

$$ \gamma_c \sim N(0, \sigma_c^2) $$

Where

s is sex, c is location, t is time for datapoint i

Edu is the mean years of education for the age group 15 to 59

LDI is lag-distributed income

${}_{5}q_{0}$ is probability of death from birth to age 5 years

α_s is a fixed intercept for sex

γ_c is a country- or territory-level random effect

HIV is the crude mortality rate from HIV for ages 15 to 59

The predictions generated through stage one of the model were based on predictions from the above equation, but without the location-level random effect. We did this to enable the modelling of spatial mortality trends in the next stage. After predictions were generated, we converted them from mortality rates to probabilities so they could be used in the second stage. When converting from mortality rate (${}_{n}m_{x}$) to probability of death (${}_{n}q_{x}$), we assumed a constant mortality rate within the 45-year wide age group.

Second stage spatiotemporal smoothing of residuals

We used the second stage of the regression model in part to capture more fully the pattern of variation in ${}_{45}q_{15}$ that the covariates could not account for. To do this, we smoothed residuals across time and among locations within the same GBD region.

We fit the local regressions for each of the 21 GBD regions. These local regressions included two variations, both of which used the same weighting scheme for incorporating spatial and temporal correlations. Both variations included conducting weighted linear regressions for each location-year of interest. A description of the weighting scheme that was used in both variations is described below, followed by a description of the two variations.

For each location-year regression, we weighted all the residuals with respect to that location and year. We first weighted residuals with respect to time using the following weighting function, which was similar to the one used in the U5MR estimation process:

$$w_t = \left(1 - \left(\frac{|r_t - r_{est}|}{1 + argmax_t|r_t - r_{est}|}\right)^{\lambda}\right)^3$$

Where

r_t is the year of interest

r_{est} is the year of the residual being weighted

$argmax_t|r_t - r_{est}|$ is the maximum distance between the year of interest and a residual within the region

The λ parameter determined how quickly the weights diminished over time, with a smaller λ meaning a more rapidly diminishing weight over time than a larger λ.

Second, we weighted residuals with respect to space using the weighting function below, which was applied to the modified time weights. We multiplied the weight of residuals for each country of interest by a factor of:

$$\frac{\zeta \sum_{i \notin c_{est}} w_i}{(1-\zeta) \sum_{i \in c_{est}} w_i}$$

Where

c_{est} is the location of interest

w_i is the time weight

Applying this formula meant that 100·ζ% of the weight was placed on residuals in a single country or territory, with the remaining 100·(1-ζ)% placed on residuals from other countries or territories in the same region. We applied a factor of 0 to countries and territories that had no residuals (ie, those with no data), which meant they were not re-weighted. For those locations, all the weight was consequently applied to the other countries in the region. We set λ and ζ based on each location's data density.

The first local regression variation, or the linear local regression, was a weighted linear regression of the residuals by country-year. It was an indicator of the residual from the country or territory being assessed and was estimated using the following equation:

$$r_{est} = \beta_0 + \beta_1 t + \beta_2 c_{est} + \mathcal{E}$$

We dropped the indicator if it could not be estimated due to the country or territory not having any residuals.

The second variation was a simple weighted average of the residuals in the country or territory being assessed.

We combined the estimates from both variations. The local linear regression included covariates such as year, but in locations with sparse data, this led to implausible out-of-time sample predictions because the extrapolation relied so heavily on the covariate information. The variation did not include an explicit time trend, so it did not have the same problem with out-of-time sampling, but it was less equipped to fit the data in data-rich countries and territories.

Using the equation below, we then calculated the data density (d_c) in each country and territory of interest (c_{est}) and from that, the weighted average of the predictions from the two local regression variations, with d_c% of the weight assigned to the linear local regression and the remainder to the fixed effects local regression. This gave appropriate weight to each of the variations based on completeness of VR data in each location.

$$d_c = 100 \times \left(\frac{\#\, VR\ points\ in\ c_{est}}{Maximum\ \#\, VR\ points\ in\ any\ country\ in\ the\ region}\right)$$

We logit-transformed the residuals before smoothing. We used the smoothed residuals to adjust the logit-transformed predictions from the first-stage regression, before reverse-logit transforming this estimate. Because we conducted the first two stages in logit-space, our predictions' domain was restricted to the range between 0 and 1. We called these the second-stage predictions.

Model

We used a GPR model for the third stage of our prediction model. The equation was as follows:

$$\mu_{t,c} = f_c(t) + S_{t,c}$$

$$f_c(t) \sim GP(M, C)$$

Where

t is time, c is location

μ_t is the true $\log_{10}({}_{45}q_{15})$

$f(t)$ is the baseline mortality risk

S_t captures excess mortality due to war and disasters and is estimated independently of $f(t)$

M is the Gaussian process mean function

C is the Gaussian process covariance function

Because the Dominican Republic, Peru, Madagascar, and Morocco had very different estimates from sibling histories versus VR systems and we could not determine the direction of the bias in each source, we used a slightly different model from the one above. For these countries, we instead used a model that included a bias term for each source (β_s):

$$\mu_{t,c} = f_c(t) + \beta_s + S_{t,s}$$

$$\beta_s \sim Normal(0, 0.01^2)$$

$$f_c(t) \sim GP(M, C)$$

As with ${}_5q_0$ (see section 2.2.6), the prior distribution of $f(t)$ was based on second-stage predictions as the mean prior (M) and a Matérn covariance function as the covariance prior (C). The covariance function included three parameters: scale, amplitude, and degree of differentiability. Respectively, these parameters controlled the distance over which the function was correlated; controlled the amount by which estimates from the Gaussian process distribution could deviate from the mean function; and influenced sample smoothness from the Gaussian process. The selection of these and other hyperparameters is explained in the next section. For more details about Gaussian process regression and the Matérn covariance function, see section 2.2.6.

Hyperparameter and parameter selection for adult mortality rate ST-GPR

The following hyperparameters and parameters are used:

- Stage 2 (Space-time smoothing)
    - Zeta

    - Lambda
- Stage 3 (Gaussian process)
    - Scale
    - Amplitude
    - Degree of differentiability

Similar to selecting parameters for U5MR, we selected zeta, lambda, and scale hyperparameters for adult mortality based on a data density score. We calculated the data density score for each location and sex based on the number of sibling histories and number of deaths, following the steps outlined below:

1. Calculated the death number score based on the number of deaths calculated during the DDM part of the model, including unadjusted complete deaths and adjusted deaths from incomplete sources.
2. Assigned a score of 15 for each sibling history for a single location, year, and sex.
3. Added the death counts and sibling history scores together for each location, year, and sex; capped the resulting number at 1000 and then divided the number by 1000. The result was a score of 0 to 1 for each location, year, and sex.
4. Added the score for each year in the full time series to calculate the final complete VR score for each location and sex, over the study period.
5. Assigned the following parameters based on the data density for each location.

Table C

Data density	Zeta	Lambda	Scale
0 to 9	0·6	0·7	25
10 to 19	0·7	0·5	20
20 to 29	0·8	0·4	15
30 to 49	0·9	0·3	10
50 plus	0·99	0·2	5

To develop the data density score system, we conducted iterative testing that accounted for the assumption that sources such as complete birth histories and complete VR systems are more susceptible to non-sampling measurement error than other sources. We tested different cutoffs for VR deaths to account for sample size. We also conducted iterative testing and refinement for weights.

To get the *amplitude* parameter for GPR, we first calculated the variance of logit-difference between the first-stage and second-stage predictions in high data-density years separately by location. Then, this location-specific variance was used as amplitude for countries with complete VR and the mean of these variances was used as amplitude for countries with incomplete VR.

The *degree of differentiability* parameter for GPR was selected as 1.

Data variance

We calculated data variance for each ${}_{45}q_{15}$ empirical observation using different methods depending on the type of data.

For sampling variance of ${}_{45}m_{15}$, we used the following binomial distribution:

$$\frac{p(1-p)}{N}$$

Where

N is the national population aged 15 to 59 years

p is the mortality rate, ${}_{45}m_{15}$

We then used the delta method to transform variance into $\log_{10}({}_{45}q_{15})$ space. For estimates that came from a combination of complete and incomplete VR data, we added the sampling variance (calculated for complete VR data) and the variance based on the level of estimated completeness from DDM synthesis (calculated as in section 2.2.6) together to get the total data variance.

For estimates that came from survey, sibling history, or census data, we calculated the median absolute deviation (MAD) estimator of the variance by source type using the second-stage predictions. We employed the following equation:

$$\theta_s^2 = (1.4826 \cdot MAD_s)^2$$
$$= 1.4826\ x\ median\Big(\Big|\Big(log_{10}\big({}_{45}q_{15}^{observed}\big) - log_{10}\big({}_{45}q_{15}^{predicted}\big)\Big)$$
$$- median\ \Big(log_{10}\big({}_{45}q_{15}^{observed}\big) - log_{10}\big({}_{45}q_{15}^{predicted}\big)\Big)\Big|\Big)^2$$

Where s is the source type (sibling history, census, or survey).

Section 2.3.5: Identify and remove outliers

We excluded certain implausible outliers from the ST-GPR regression. We generally outliered raw datapoints that met one of the following criteria:

1. Raw input ${}_{45}q_{15}$ datapoints from years affected by natural disasters, wars, and other fatal discontinuities defined elsewhere[3] from the analysis explained in section 2.7.
2. Raw datasets with poor quality data, as determined by examining the dataset. Examples include Afghanistan 2010 Mortality Survey, which was not nationally representative, and VR data from Serbia that excluded deaths from Kosovo.
3. Raw input data and time series estimates of ${}_{45}q_{15}$ that were unexplainably different from adjacent points from a similar source, as determined by visual inspection. Unexplained differences indicated a data reporting issue and compilation error in the direct sources.
4. Subnational-level single-year ${}_{45}q_{15}$ estimates from sibling survival methods that had implausibly high or low estimates due to small sample sizes.

Section 2.3.6: Scale subnational estimates to national level (excluding South Africa)

As explained in section 2.2.8, a strength of GBD is that we ensure consistency throughout our location hierarchy, meaning that subnational mortality estimates always add up to national mortality estimates. National-level data were typically more robust, included more data sources, and spanned longer time periods. To eliminate any correlation between subnational and national draws that was introduced earlier in the model, we randomised the order of the 1000 draws at the subnational and national levels. Then, to ensure consistency between the aggregated subnational-level and separately estimated national-level estimates, we raked subnational ${}_{45}q_{15}$ to the national level using the following formula:

$${}_{45}q_{15}^{s'} = 1 - e^{-45 \times {}_{45}M_{15}^{S} \times r}$$

And

$$r = \frac{\dfrac{ln(1 - {}_{45}q_{15}^{N})}{-45}}{\sum_{s=1}^{n} \dfrac{ln(1 - {}_{45}q_{15}^{N})}{-45} \times \dfrac{p_s}{p_N}}$$

Where

s is subnational locations within country N

p is population in age group 15 to 59

$_{45}q_{15}$ is the estimate of adult mortality rate from the ST-GPR process

$_{45}q_{15}^{s'}$ is the post-scaling $_{45}q_{15}$ for subnational locations

As with $_5q_0$, South Africa was the one country where we did not use this method, because HIV/AIDS is such a large contributor to mortality. We instead aggregated provincial-level estimates to the national level using population for the corresponding age group as weights.

Section 2.4: Model life table system

Section 2.4.1: Overview

We used model life tables to produce estimates of age-specific mortality using summary mortality indices of $_5q_0$ and $_{45}q_{15}$. In particular, we relied on this method for location-years without VR systems, including recent years for locations with high-quality VR systems because VR data often takes several years to publish after being collected.

Ideally, a model life table system has the following attributes. First, it should only require several entry parameters to generate a full life table. Second, it should accurately reflect the age patterns of mortality observed in real populations. Third, it should make reasonable estimates of age-specific mortality in countries and territories that have high levels of mortality, particularly those countries with large HIV/AIDS epidemics. Fourth, it should produce realistic age-specific mortality time trends. Fifth, age-specific mortality should be positively related to entry parameters such as $_5q_0$ and $_{45}q_{15}$.

For this and earlier GBD iterations, we used a relational model life table system akin to the one proposed by Brass and Coale.[19] They posited that the logit-transformed survival curves from two life tables can be linearly related to each other. Murray and colleagues found, however, that as mortality levels in a population moved further from the reference standard, the linearity in logit *lx* space no longer held true.[27] They instead proposed a set of bend factors to account for the deviation from linearity. This modified logit life table system also gives users the option to predict $_{45}q_{15}$ from $_5q_0$. It has been used broadly by WHO since the early 2000s.[27]

Murray and colleagues' modified logit life table system does, however, have two important limitations. First, in location-years where adult mortality was much higher than child mortality, such as with the HIV/AIDS epidemic, the mortality age patterns generated by the system did not fit well to the observed mortality age patterns. Second, when the system was applied to the $_5q_0$ and $_{45}q_{15}$ time series, a contradictory pattern emerged with adult and child mortality both declining but age-specific mortality rates increasing in certain age groups.

We extended the modified logit life table system in four distinct steps, summarised below. A full description of the modifications can be found elsewhere.[2,8]

Section 2.4.2: Building an empirical model life table database: data sources and quality review

Relational model life table systems are heavily dependent on the empirical database used to produce the standard life tables and test their predictive validity. For the latest GBD analyses of all-cause and cause-specific mortality, we compiled 60 124 abridged empirical life tables prior to outliering or smoothing using data from full VR systems and sample VR systems such as the Disease Surveillance Points system in China and the SRS in India. We adjusted each source by estimated completeness. For ages under 1 and 1 to 4, we applied under-5 completeness; for ages 15 and above, we applied adult completeness; and for ages 5 to 9 and 10 to 14, we applied a combination of under-5 and adult completeness.

For each life table in each location, we sorted by year and conducted smoothing using moving averages of widths of 3, 5, and 7 years. The smoothing helped minimise drops or jumps in age-specific mortality in locations where low mortality rates contributed to high variability in mortality patterns across ages.

As with previous iterations of GBD, we had two sets of life tables that met the inclusion criteria for this study. First, we had a universal set of life tables that were used to identify matches for all locations. Second, we had a location-specific set of life tables that were used only for a certain location or locations. Life tables that had jumps in age-specific mortality or other implausible trends due to too-low populations or too-low mortality contributing to inconsistent estimates, old-age misreporting or heaping, or data extraction or reporting issues were excluded.

In order to categorise life tables, a neural network machine vision model (based in Python, using Keras from the TensorFlow package) was trained and deployed to separate likely outliers from likely non-outliers. All life tables were formatted and saved as .jpg plots of log $_nq_x$ over age. A random set of 6000 plots were reviewed manually for implausible age patterns to create the training set. We looked for a general age pattern of mortality among the human population, which starts with relatively high probability of death in the first year of life, followed by a drop into late childhood, and then roughly log-linear increase through most of adulthood and after assuming there is no major epidemic of any kind. Life tables with too much noise were excluded from the universal set. A two-person review system was used to verify or modify these machine vision designations. Therefore, the machine vision was not the arbiter of inclusion, but rather a tool to facilitate speedy manual review.

Life tables that were outliered for $_5q_0$ or $_{45}q_{15}$ estimation, or had estimated completeness below 50%, were also excluded from the empirical life table database. Life tables with estimated completeness between 50% and 85% were included only in the location-specific category, not in the universal set.

For some locations, life tables that did not qualify upon initial review were included as location-specific life tables, in order to improve modelled envelope fit to the vital registration data.

Our database of life tables that met the inclusion criteria now includes 11 139 universal and 24 267 location-specific life tables, for a total of 35 406 life tables.

Section 2.4.3: Extending age-specific mortality

We extrapolated age-specific mortality up to age 105-109 using the recursive model below, fit to high-quality VR data from the Human Mortality Database:

$$logit\left({}_5q_{x+5}^{j,t,g}\right) - logit\left({}_5q_x^{j,t,g}\right) = \beta_0^g + \gamma^{j,g} + \beta^g\left[logit\left({}_5q_x^{j,t,g}\right) - logit\left({}_5q_{x-5}^{j,t,g}\right)\right]$$
$$\gamma^{j,g} \sim N(0,\sigma_\gamma^2)$$

Where

j refers to country

g refers to sex

t refers to time

β is a fixed covariate coefficient

γ is a random intercept

This was a new approach for GBD 2019, where a difference in $_nq_x$ is used to predict a difference in $_nq_x$. Previously, we used the last (oldest age) observed level of $_nq_x$ to predict the difference to the next $_nq_x$ value. However, we conducted an analysis of these two extension methods and found that the new method was a better predictor out of sample. Additionally, using the last slope to predict the next slope removed cases of $_nq_x$ kinks (sharp changes in slope) that were produced by the old method.

After empirical life tables were extended to age 105-109, an iterative scaling algorithm was applied to match $_5q_{95}$, $_5q_{100}$, and $_5q_{105}$ to the observed $_nm_x$ implied by vital registration deaths over GBD-estimated population.

Another modification for GBD 2019 was intended to reduce the frequency of cases where female mortality rate becomes greater than male mortality rate in the extended ages. Life tables with male-female crossover in late adulthood were designated to the location-specific category rather than the universal life table group. Another change was made to address this issue, which is covered in section 2.4.4.

Section 2.4.4: GBD relational model life table system with a flexible standard selection mechanism

We based our relational model life table system on the idea that to capture the very high levels of young adult mortality that occur in locations with high HIV prevalence, we had to first develop a model life table for a counterfactual population without HIV and then add the effects of HIV by age and sex into that life table.

There were three steps to our system. First, we estimated counterfactual levels of ${}_5q_0$ and ${}_{45}q_{15}$ without HIV. Second, we generated a full set of age-specific mortality rates using the counterfactual model life table system. Third, we estimated the increase in mortality when HIV was included back in, for each age group.

Model for populations free of HIV/AIDS

Our relational model life table system was different from previous systems in several ways. First, we modelled ${}_nq_x$ rather than l_x in logit space. Modelling ${}_nq_x$ allowed us to better capture the impacts of changes to ${}_5q_0$ and ${}_{45}q_{15}$ on different age groups. We used the following equation to generate life tables for populations not affected by HIV/AIDS:

$$logit({}_nq_x^c) = logit({}_nq_x^s) + \beta_x^1 \cdot \left(logit({}_5q_0^c) - logit({}_5q_0^s)\right) + \beta_x^2 \cdot \left(logit({}_{45}q_{15}^c) - logit({}_{45}q_{15}^s)\right)$$

Where

$logit({}_{45}q_{15}^s)$ is the logit transformation of ${}_{45}q_{15}$ in the standard life table

$logit({}_{45}q_{15}^c)$ is the logit transformation of ${}_{45}q_{15}$ value for a country without HIV or the counterfactual level of ${}_{45}q_{15}$ in the absence of HIV in a country affected by HIV/AIDS

$logit({}_5q_0^s)$ is the logit transformation of ${}_5q_0$ in the standard population

$logit({}_5q_0^c)$ is the logit transformation of ${}_5q_0$ for a country without HIV or the counterfactual level of ${}_5q_0$ in the absence of HIV in a country affected by HIV/AIDS

$logit({}_nq_x^s)$ is the logit of the probability of death in the standard population from age x to $x + n$

$logit({}_nq_x^c)$ is the logit transformation of the probability of death from age x to $x + n$ in a country or territory without HIV or the counterfactual level of ${}_nq_x$ in the absence of HIV in a country or territory affected by HIV/AIDS

β_x^1 and β_x^2 are coefficients that vary by age x and that measure the impact of differences in child and adult mortality rates between a target life table and the standard life table on the estimated age pattern of mortality. These coefficients determined how much the estimated age pattern of mortality deviated from the standard by age and from linearity.

The design of this equation was based on our assumption that the logit-transformed age-specific probability of death in a target life table (c) could be represented as a function of (1) the corresponding logit-transformed age-specific probability of death in a standard life table (s), (2) the difference in ${}_5q_0$ in logit scale between life tables c and s, and (3) the difference in ${}_{45}q_{15}$ in logit scale between life tables c and s. Life table c was our life table without HIV, either with counterfactual levels of probability of death without HIV/AIDS for countries and territories affected by HIV/AIDS, or as a complete life table for countries and territories not affected by HIV/AIDS. The design of the model was informed by previous observations that in the absence of an HIV/AIDS epidemic, differences in age-specific probability of death in logit scale are correlated strongly with differences in ${}_5q_0$ or ${}_{45}q_{15}$ in logit scale.

We estimated the β_x^1 and β_x^2 coefficients using the equation below:

$$logit\ {}_nq_x^c - logit\ {}_nq_x^s = \beta_x^1 \times (logit\ {}_5q_0^c - logit\ {}_5q_0^s) + \beta_x^2 \times (logit_{45}q_{15}^c - logit_{45}q_{15}^s)$$

To estimate the parameters, we used location-time-specific standards for each of the life tables in our database that were from country-years not affected by HIV/AIDS. We used country/territory-time-specific standard life tables any time we had in our database an observed life table from the same country within a 15-year time frame. We produced region-specific standard life tables by collapsing all the life tables in our dataset without HIV from the same GBD region by sex. Next we paired the life tables in our dataset without HIV with the region-specific life tables we had produced.

We estimated $\hat{\beta}_1$ and $\hat{\beta}_2$, the results from which are found in the table below. We restricted the effects of ${}_5q_0$ and ${}_{45}q_{15}$ to specific age groups so as to prevent improbable outputs in instances where ${}_5q_0$ and ${}_{45}q_{15}$ changed in opposite directions in the same population. We generated full life tables for locations without HIV/AIDS epidemics using the values in the table below. We used the ${}_5q_0$ and ${}_{45}q_{15}$ values as our entry parameters for this model life table system.

Table D. Model life table coefficients

Age group	Difference in ${}_5q_0$ (logit scale)		Difference in ${}_{45}q_{15}$ (logit scale)	
	Male	Female	Male	Female
0	1·013	1·011	--	--
1-4	0·922	0·947	--	--
5-9	0·728	0·676	--	--
10-14	0·424	0·335	0·454	0·465
15-19	0·194	0·238	0·740	0·521
20-24	--	--	0·726	0·921
25-29	--	--	0·574	1·038
30-34	--	--	0·597	0·989
35-39	--	--	0·720	0·978
40-44	--	--	0·848	0·949
45-49	--	--	0·970	0·954
50-54	--	--	1·001	0·952
55-59	--	--	1·014	0·932
60-64	--	--	0·954	0·857
65-69	--	--	0·918	0·778
70-74	--	--	0·899	0·762
75-79	--	--	0·866	0·756
80-84	--	--	0·819	0·718
85-89	--	--	0·702	0·801
90-94	--	--	0·710	0·775
95-99	--	--	0·712	0·755
100-104	--	--	0·712	0·732
105-109	--	--	0·711	0·708

The process we used to compute standard life tables considered observed relationships between variances in geography, time, and mortality age patterns. To produce a standard life table, we computed the Mahalanobis distance between the target life table and all the observed life tables in our database without HIV based on ${}_5q_0$ and ${}_{45}q_{15}$ in logit scale. We used Mahalanobis distance rather than Euclidean distance because Mahalanobis distance includes the relationship between ${}_5q_0$ and ${}_{45}q_{15}$ in logit space in its calculation of distances between life table pairs. We expressed the Mahalanobis distance between two sets of ${}_5q_0$ and ${}_{45}q_{15}$ as:

$$D_M^i(Q^i) = \sqrt{(Q^i - O)^{\tau} S^{-1} (Q^i - O)}$$

Where

O is a multivariate vector representing entry parameters ${}_5q_0$ and ${}_{45}q_{15}$ in logit scale

$Q^i = (logit(\ {}_5q_0^i), logit({}_{45}q_{15}^i))$ is a multivariate vector that corresponds to an empirical life table i in our life table database.

$_5q_0$ and $_{45}q_{15}$ were highly coordinated in logit space. We kept the 100 life tables that were most similar to the target life table, as determined by the Mahalanobis distance, as well as all the life tables in our database from the location, within 30 years. After combining all these life tables, we kept the 100 that were closest based on geography and distance in years.

For GBD 2017, this process was done separately by location, year, and sex. For GBD 2019, we selected life tables by location-year, but in pairs by sex. This was done to reduce unsubstantiated crossover of male and female mortality in the elderly. The result is that the 100 life tables selected for males for a given location-year are from the same location-years as the 100 life tables selected for females for a given location-year.

We then calculated the weighted average of the selected life tables by applying empirical weights to each table.

Figure A. Empirical weights by lag in time and geographic region for males

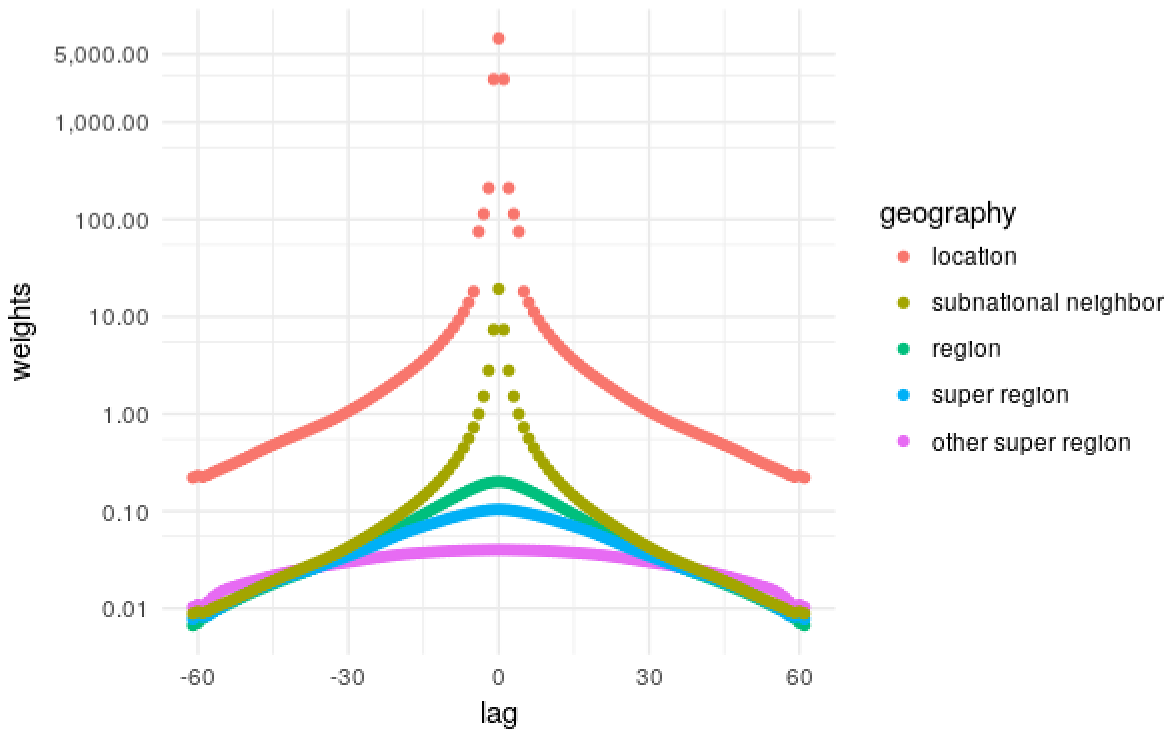

Estimating AIDS

Unlike other existing model life table systems, our system provides a solution to the challenge of incorporating deaths from HIV/AIDS. Since GBD 2016, we have incorporated an updated process to estimate the HIV counterfactual mortality age pattern (see section 2.6) using HIV counterfactual $_5q_0$ and $_{45}q_{15}$ as entry parameters. We then added excess mortality due to HIV/AIDS to the under-5 and 15–59 summary age groups as well as specific age groups by location, year, and sex to the age-specific mortality without HIV. For the age-specific step, we used the Spectrum HIV model to extract the number of deaths due to HIV/AIDS and calculated the relative risk (RR). We defined RR as the ratio of HIV-specific mortality between a specific age group and the 40 to 44 age group, using the following equation:

$$R_i = \frac{{}_nM_x^{HIV}}{{}_5M_{40}^{HIV}}$$

Where i indicates different age groups 0, 1–4, 4–9, 10–14, etc.

We ran the Estimation and Projection Package Age Sex Model (EPP-ASM) at the 1000-draw level, for draws of the RR of death from HIV/AIDS by location, sex, and time

Estimating all-cause mortality for South Africa

We followed the steps for incorporating HIV/AIDS into all-cause mortality described in the previous section for all locations except South Africa. For estimating all-cause mortality in South Africa, we instead followed the steps in reverse order. South Africa has a near-complete VR system, so we estimated all-cause mortality with HIV using observed VR and then removed excess mortality due to HIV using the RR of death from HIV estimated in the Spectrum model.

This method allowed us to match the completeness-adjusted VR data by age, and the age pattern of HIV-specific mortality matched the age pattern from the EPP-ASM epidemiological model.

Uncertainty in the estimated life tables

One strength of our model was that we included uncertainty in each step of the mortality estimation process. We followed the method described in King and colleagues[28] and incorporated uncertainty from the entry parameters ($_5q_0$ and $_{45}q_{15}$) and the coefficients of each model. In short, we repeated the model life table process described in the previous sections 1000 times based on randomly paired entry parameters (ie, different standard life tables) and model coefficients. The result was wide UIs for mortality estimates in countries and territories where uncertainties in the estimated entry parameters were high.

Section 2.4.5: Rake subnational life tables to national level (excluding South Africa)

As explained in previous sections, for all countries and territories but South Africa, we raked subnational-level estimates to separately estimated national-level estimates. We did this because we needed subnational mortality estimates to add up to national estimates and data at the national level were generally more robust than at the subnational level.

For South Africa, we instead aggregated subnational-level estimates from our EPP-Spectrum model as our national-level estimates. As explained briefly in previous sections, we did this because the substantial heterogeneity in HIV/AIDS mortality rates at the subnational level in South Africa made it difficult to accurately estimate all-cause and HIV cause-specific mortality at the national level. We used all subnational child and adult mortality data available. The number of data sources was similar at the national and provincial levels.

Section 2.5: HIV/AIDS estimation

Section 2.5.1: Age-specific mortality (with and without HIV)

We used age-specific mortality rates with-HIV and without-HIV from the model life table process as inputs to the HIV/AIDS estimation described in this section.

Section 2.5.2: HIV-free survival rates (for Spectrum)

Our inputs for Spectrum were single-year HIV-free mortality based on five-year abridged life tables, generated from the age-specific HIV-free survival probabilities produced through the process described above.

Section 2.5.3: EPP and Spectrum

For our HIV analysis, we modified two tools developed by the Joint United Nations Programme on HIV/AIDS (UNAIDS):

1. EPP and EPP-Age Sex Model (EPP-ASM):
    a. These tools generate HIV burden estimates in line with observed prevalence data. They incorporate many of the same assumptions as Spectrum. EPP fits a simpler model to HIV prevalence data from representative surveys and surveillance sites to estimate prevalence and incidence for the 15–49 year age groups. EPP-ASM incorporates the full population project of Spectrum and produces age-sex-specific prevalence, incidence, and mortality.
    b. We modified EPP-ASM by building a paediatric module that mirrored that of Spectrum. Perinatal and breastfeeding transmission was calculated as a function of prevalence among pregnant women and maternal-to-child transmission programme data. We additionally improved the fit to

prevalence data by allowing flexibility in the age distribution of incidence over time. We parameterised the ratio of incidence among ages 15–24:25+ as a constant before year 2000 and a linear regression thereafter. This allowed for the shifts in the age distribution of incidence observed over the course of the HIV epidemic to be reflected in our results.

2. Spectrum natural history model:
   a. This model generates HIV incidence, prevalence, and death by age, sex, and year using time series estimates of HIV incidence, demographic inputs (such as HIV-free mortality and population), assumptions about CD4 progression rates, and assumptions about on-antiretroviral therapy (ART) and off-ART HIV mortality rates by age, sex, and CD4 rate. We used treatment coverage time series from UNAIDS.
   b. We made several modifications after creating a replica of Spectrum in Python (starting with GBD 2013). First, we sex-split incidence based on a model that was fit to the sex ratio of observed prevalence in countries and territories with nationally representative surveys. Second, our child model included CD4 progression and CD4-specific mortality rates that came from a model fit to survival data from the International epidemiology Databases to Evaluate AIDS (IeDEA), as well as ART distribution data from IeDEA. Additionally, we scaled all input values by a uniformly sampled factor between 0·9 and 1·1 to generate estimates with realistic ranges of uncertainty.

Section 2.5.4: HIV mortality reckoning

We used the reckoning process to resolve and merge separate estimates of HIV mortality produced by two different estimation processes in the GBD framework. The separate estimates were those from the model life table system and from the natural history model of EPP-Spectrum. We also used space-time GPR-smoothed VR data on HIV-specific mortality in countries and territories with high-quality VR systems instead of mortality estimates from Spectrum.

We assigned all GBD 2019 locations to a modelling strategy group (appendix table 9 [section 9]) based on the level of HIV in the country or territory and the completeness of HIV and VR data. The group a location was assigned to determine which sources we used for HIV-specific mortality data and how we calculated final HIV and all-cause mortality estimates. The groups were as follows:

- Group 1A: locations with greater than 0·25% adult HIV prevalence and available HIV prevalence survey data and/or antenatal care clinic data. For all Group 1A locations, we ran the EPP model using these available data from each location. The demographic assessments for Group 1 locations relied heavily on sibling history data analyses, which had large UIs and for which there were often varied subnational response biases.
- Group 1B: India, Sudan, and Somalia. These locations constituted their own group because they had available surveillance data but fairly low adult HIV prevalence – less than 0·5%. We used the model life table system to get all-cause mortality estimates in these locations. We used EPP-Spectrum to get HIV-specific deaths. For India, we used SRS data to adjust age-specific incidence in Spectrum and generate the mortality age pattern. The adjusted result better matched observed deaths.
- Group 2A: locations with high-quality (4 or 5 star) VR systems, based on the GBD VR quality rating system.[29] All-cause mortality estimates in these locations used national data such as the VR system.
- Group 2B: locations with lower-quality VR systems.
- Group 2C: locations without any VR data.

For HIV-specific mortality estimates, Group 1A and 1B locations used Spectrum output, Group 2A locations used mortality output straight from the ST-GPR process, and Group 2B and 2C locations used output cohort incidence bias adjusted (CIBA) deaths caused by HIV/AIDS from the Spectrum model.[30]

The outputs from the model included the under-1 age group but not early-, late-, and post-neonatal age groups. We assumed that all HIV deaths that occurred in the first year of life occurred in the post-neonatal stage. Previous research is limited but suggests higher mortality in the post-neonatal stage.[13,14] There was no definitive guidance on how better to age-split under-1 HIV deaths.

Section 2.5.5: Envelope calculation

We produced the all-cause and without-HIV envelopes largely by combining the results from the with-HIV and HIV-free life tables, as well as particular HIV mortality from ST-GPR, Spectrum output, or CIBA-Spectrum output. We determined the implied HIV mortality from the model life table system.

We conducted a separate calculation for the under-5 age group using results from the under-5 age-sex process described in section 2.2.10. For Group 1A and 1B locations, we produced a scalar from HIV-specific and non-HIV deaths Spectrum results, which we then used to produce an HIV-deleted envelope from the all-cause results in the envelope. For locations in other groups, we capped HIV mortality, which was taken directly from ST-GPR or Spectrum, at 90% of the all-cause envelope, and then subtracted it from the all-cause envelope to produce an HIV-deleted envelope. We assumed that all under-1 HIV-specific deaths happened in the post-neonatal stage.

For all locations in groups other than Group 1A, we followed the same procedure described above and capped HIV mortality for ages 6-14, which was taken directly from ST-GPR or Spectrum, at 90% of the all-cause envelope, and then subtracted it from the all-cause envelope to produce an HIV-deleted envelope for that age group. For Group 1A locations, we added HIV mortality from Spectrum to the HIV-free mortality taken directly from our estimates to produce the all-cause with-HIV envelope.

For the age groups above 15 and below 95, we used one approach for Group 1A and 1B locations and another approach for Group 2A, 2B, and 2C locations. For Group 1A and 1B locations, we used HIV mortality estimated from an ensemble model. For this, we calculated the average of the implied HIV mortality from the model life table process and HIV mortality from Spectrum, which were related to one another through the age pattern of draw-level HIV-free mortality. For Group 2A, 2B, and 2C locations, we used HIV mortality taken directly from ST-GPR and CIBA-Spectrum. For all locations in groups other than Group 1A, we subtracted HIV mortality, calculated using one of the approaches just described, from the all-cause envelope to produce HIV-deleted envelope deaths. For Group 1A locations, we instead added HIV mortality, again calculated using the approach just described, to the HIV-free mortality that had been estimated through the model life table process, which produced all-cause mortality. This process allowed us to adjust all-cause mortality based on ensemble HIV estimates in Group 1A locations and was a response to the inherent uncertainty in all-cause mortality estimates that are based primarily on sibling survival data (as was the case in Group 1A locations) and the assumptions about mortality and data that are necessary in EPP and Spectrum.

For the 95+ age group, we approximated the over-95 all-cause mortality rate by using our life table output and dividing l_x by T_x from the 95-99 age group values in the country-specific life tables (T_x is the life table parameter from the total person-years lived after age x). We also calculated the 95+ mortality all-cause mortality rate using the envelope deaths/populations. Next, we developed a scalar from the approximated 95+ all-cause mortality to the 95+ all-cause mortality. We rescaled the with-HIV and HIV-free life tables and the implied HIV mortality rate to death counts space using this scalar. Finally, we calculated HIV-deleted and all-cause mortality using the approach described for other age groups above 15 years old.

Section 2.5.6: Life table calculation

For the most part, we calculated life tables using the same approach as for calculating the envelope. There were, however, several differences in the calculation that had to do with sex and location aggregation.

For all locations in groups other than Group 1A, we divided the all-cause life table results by the ratio of HIV-free mortality to with-HIV mortality to get HIV-free life table results. For Group 1A locations, we instead multiplied the HIV-free life table results by the same ratio to get all-cause life table results. This process aligned with how we produced envelopes for different location groups.

For aggregate locations (ie, regions, super-regions, and global), we produced aggregate ${}_nm_x$ and ${}_na_x$ (defined as the average number of years lived in the age interval, from among those who died in that age interval) by weighting ${}_nm_x$ by population and ${}_na_x$ by deaths:

$${}_nm_x \times population$$

We used these death- and population-weighted ${}_nm_x$ and ${}_na_x$ values to produce life tables at the regional level.

We calculated the life tables for the under-5 age groups using a similar approach. We did, however, use national-level under-5 results instead of the aggregated ${}_nm_x$ and ${}_na_x$ values when calculating the all-cause life table for countries with subnational locations. We used this approach to preserve the under-5 mortality estimates produced through the processes described above. We did this because those estimates were not always the same as the estimates produced by the aggregated ${}_nm_x$ and ${}_na_x$ values from the model life table process. We used the standard weights for HIV-deleted life tables to aggregate subnational units.

Section 2.6: Age-specific mortality estimation for all GBD age groups: with and without HIV

Section 2.6.1: HIV-deleted age-specific mortality

We used HIV-deleted age-specific mortality in our CoD analyses to prevent the spillover effect of HIV mortality into other causes, which was a potential problem especially in locations with a high HIV burden. We calculated this as the difference between the HIV-specific mortality from the HIV reckoning process (section 2.5.4) and the with-HIV age-specific mortality without fatal discontinuities (section 2.6.3).

Section 2.6.2: Age-specific mortality without discontinuities (with HIV/AIDS)

We produced age-specific mortality rates without fatal discontinuities using the model life table system (section 2.4), the age-sex model (section 2.2.11), and the HIV reckoning process (section 2.5.4), with some methods specific to certain location groups.

For all locations, we used our age-sex model, which split U5MR into early mortality for the early neonatal (enn), late neonatal (lnn), post neonatal (pnn), and 1 to 4 years age groups, to calculate under-5 age-specific with-HIV mortality. For the age groups over 5 years, we used the GBD model life table system to calculate age-specific with-HIV mortality for locations not in Group 1A. For Group 1A locations, we instead calculated age-specific with-HIV mortality as the sum of the HIV-specific mortality calculated in the ensemble model (section 2.5) and the HIV-free mortality calculated in the first step of the model life table system (section 2.4).

Section 2.6.3: Add fatal discontinuities

We excluded data from years with fatal discontinuities from our mortality estimation process because we did not want sudden and temporary jumps in mortality to warp long-term mortality trends in that country or territory. The following section explains how we added deaths caused by fatal discontinuities to the all-cause mortality envelope and life tables. Section 2.7 provides more information about how we estimated the fatal discontinuities mortality. To add fatal discontinuities mortality into the mortality envelope, we added 1000 draws of deaths due to fatal discontinuities pairwise to 1000 draws of the with-HIV mortality envelope for each location, sex, and age group. We calculated 95% UIs in the same way as for other estimates, using the 2·5th and 97·5th percentile of the summed draws.

We then had to add fatal discontinuity deaths into the life table. To do this, we first produced full single-year with-HIV life tables, to which the fatal discontinuity deaths would later be added. We then calculated fatal discontinuity mortality rates for each single-year age by assuming the same mortality rate for each year in an age group. This kept the five-year mortality rates consistent between the abridged and full life tables. We then added the fatal discontinuity mortality rates to the with-HIV mortality rates in the full life tables, creating full and abridged life tables that had both fatal discontinuities and HIV included. To calculate the probability of death in the under-1 age group (${}_1q_0$), we used results from the under-5 age-sex model, calculated the with-fatal-discontinuities mortality rate for each of the under-1 age groups, back-calculated ${}_nq_x$ for the same age groups, and aggregated to ${}_1q_0$. We also used the probability of death between ages 1 and 4 (${}_4q_1$) that were produced by the age-sex model.

While using the same mortality rate for fatal discontinuities did, for the most part, lead to a corresponding increase in the mortality rate in the abridged life tables, there were instances where the difference in abridged mortality rate between the with- and without-fatal-discontinuities life tables did not quite align with the fatal discontinuity

mortality rate that was input. There were very few instances where this occurred, and they were primarily in age groups such as 90 to 94, where without-fatal-discontinuities mortality was very high and varied within the age group, and where fatal discontinuities mortality was fairly low. As a result, the difference between the mortality rate from the final abridged life tables and those calculated using the mortality envelopes and population results was very minor.

Section 2.6.4: Ages-specific deaths with discontinuities and HIV/AIDS

We used the results from section 2.6.3 to produce location-, sex-, year-, and age-specific mortality numbers that included fatal discontinuities (appendix 2 figure 7).

Section 2.6.5: Life tables with HIV/AIDS and fatal discontinuities

We used the results from section 2.6.4 to produce location-, sex-, and year-specific ${}_5q_0$, ${}_{45}q_{15}$, and life expectancy at birth (appendix 2 tables 2-4).

Section 2.7: Fatal discontinuities

Figure B

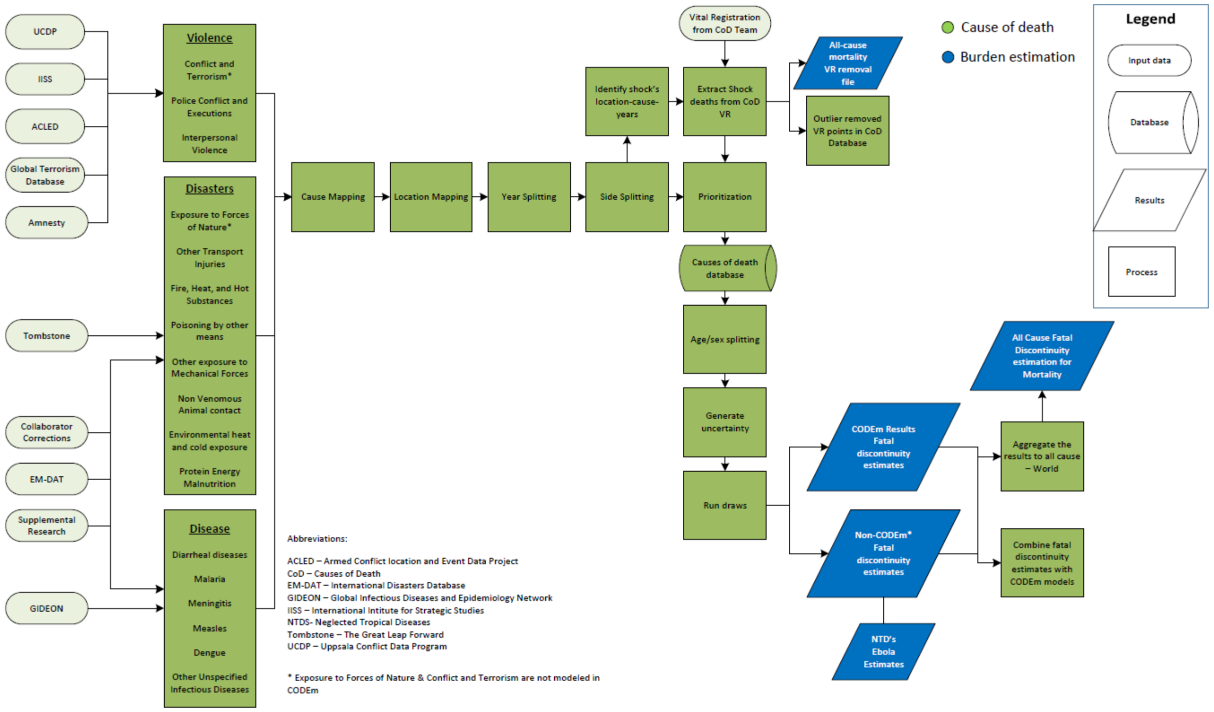

Fatal discontinuities are defined as events that are stochastic in nature and cannot be modelled because they do not have a predictable time trend. Some causes have both fatal discontinuities as well as a continuous background mortality that has a smooth time trend and can be modelled. These include police violence and executions; interpersonal violence; other transport injuries; fire, heat, and hot substances; poisoning by other means; other exposure to mechanical forces; non-venomous animal contact; environmental heat and cold exposure; protein-energy malnutrition; diarrhoeal disease; malaria; meningitis; measles; dengue; and other unspecified infectious diseases. Causes without a continuous background mortality and that are exclusively estimated using the fatal discontinuity method are conflict and terrorism and exposure to forces of nature. Any other causes are not captured in fatal discontinuities.

Section 2.7.1: Input data

Overall

We collected data on fatal discontinuities from a range of sources, namely from country-level VR systems and international databases that reported several cause-specific fatal discontinuities. We also collected supplemental data that had known issues related to quality, representativeness, or time lags in reporting. We used a Twitter scrape in place of a systematic literature review as a way to identify supplemental input data for missing fatal discontinuities. We describe the different fatal discontinuity data sources, presented by fatal discontinuity sub-cause, below.

Discontinuities only (non-CODEm)

For causes not modelled in CODEm, all deaths captured in VR were considered to be fatal discontinuities. Deaths that were extracted from cause-specific VR were then subtracted from the all-cause VR data used in the all-cause mortality estimation process.

Conflict and terrorism

In GBD 2019, war is defined as "a state of armed conflict between states, governments, societies, and paramilitary groups."[31] It is "generally characterised by extreme violence, aggression, destruction, and mortality, and the use of regular or irregular military forces."[31] Terrorism is defined as "the unlawful use or threatened use of force or violence against individuals or property in an attempt to coerce or intimidate governments or societies to achieve political, religious, or ideological objectives."[32] We used conflict and terrorism data from the Uppsala Conflict Data Program (UCDP), International Institute for Strategic Studies (IISS), Armed Conflict Location & Event Data Project (ACLED), Global Terrorism Database (GTD), and vital registration systems and other supplemental data sources. Case were assigned for each event using the source's cause coding and any description from available notes.

Table E. Conflict and terrorism data sources

Data source name	Date accessed	Years of data downloaded	Type of data included
**Uppsala Conflict Data Program[33]**			
Georeferenced Event Dataset, Version 19.1	6/10/2019	1989-2018	UCDP battles, non-state, and one-sided conflict deaths with the most disaggregated location information available
PRIO Battles Deaths Dataset, Version 3.1	1/16/2018	1946-2008	Armed conflict (civil wars, etc.)
**International Institute for Strategic Studies**			
Armed Conflict Dataset	11/17/2016	1997-2016	Insurgency, Inter-state, Intra-state conflict deaths
**Robert S. Strauss Center for International Security And Law**			
Armed Conflict Location and Event Dataset (ACLED)	2/5/2019	1997-2019	Actions of opposition groups, governments, and militias in selected locations in Africa, Asia, and the Middle East specifying the exact location and date of battle events, transfers of military control, headquarter establishment, civilian violence, and rioting
**University of Maryland, Global Terrorism Database**			
Global Terrorism Database (GTD)	6/10/2019	1970-2017	Attacks aimed at attaining political, economic, religious, or social goal, includes evidence of intention to coerce, action was outside precepts of International Humanitarian Law.
**University of Chicago, Chicago Project on Security and Threats**			
Suicide Attack Database (CPOST SAD)	11/26/2018	1982-2018	Attacks in which an attacker kills him/herself in a deliberate attempt to kill others, includes only attacks perpetrated by non-state actors

Amnesty International			
Amnesty	6/20/2019	1991-2018	Police conflict and executions

Four major conflicts were identified that were not represented in these databases: genocide in Bangladesh in 1971,[34] genocide in Burundi in 1972 and 1993[35], and civil conflict in Albania in 1997.[36] In these cases, literature sources were used to account for these fatal discontinuities.

Exposure to forces of nature

In GBD 2019, exposure to forces of nature is defined as a force that is beyond human control.[37] The Centre for Research on the Epidemiology of Disasters' International Disaster Database (EM-DAT)[38] served as the primary non-VR source of fatal discontinuities due to exposure to forces of nature (ie, natural disasters, lightning, earthquakes, volcanic eruptions, avalanches, storms, and floods). Data from EM-DAT were last accessed June 20, 2019. Supplemental online research was conducted for events where EM-DAT and VR were not up-to-date.

Partial discontinuity (CODEm)

For causes modelled in CODEm with fatal discontinuities hiding in the time trend, a process was established to avoid duplication of fatal discontinuity deaths in CODEm and the fatal discontinuity estimates. First, location-cause-years were identified through outside non-VR sources. If these location-cause-years also had VR death estimates that were greater than the average of the immediate surrounding years, the difference between the identified year and the average of the surrounding years was included in the relevant cause for the fatal discontinuities database. The extracted deaths for all fatal discontinuity causes from VR are then subtracted from the all-cause VR data used in the all-cause mortality estimation process.

Executions and policy conflict

In GBD 2019, executions and police conflict is defined as "the lawful use or threatened use of force or violence against individuals or groups of people or property in an attempt to achieve political or socioeconomic objectives for a state."[39] Data for executions and police conflict came primarily from Amnesty International, but other sources such as UCDP, ACLED, and VR that reported deaths due to legal intervention were also cause-mapped to executions and police conflict.

Homicide

In GBD 2019, homicide is defined as "the use of violence against an individual or group of people in an attempt to achieve non-political, religious, or ideological objectives."[39] Data for homicide came from VR, IISS, GED, ACLED, and other supplements. Events were mapped to homicide where the notes found in the raw data indicated gang violence. Deaths from IISS, GED, and ACLED were then split among three homicide sub-types; physical violence by firearms, physical violence by sharp object, and physical violence by other means, based on the rates calculated from VR by country and territory if available, and by region if country VR was unavailable.

Protein-energy malnutrition (PEM)

In GBD 2019, protein-energy malnutrition is defined as a lack of dietary protein and/or energy and covers famines as well as severe droughts.[40] The primary source for PEM data, other than VR, was EM-DAT. The Tombstone report was used to estimate deaths attributed to the famine during the Great Leap Forward in China in the 1960s.[41]

Other injury causes

Other injury causes include other transport injuries (ie, plane, train, and boat accidents); poisonings; fire, heat, and hot substances; and other exposure to mechanical forces (eg, building collapse). The primary data source for these events other than VR was EM-DAT. Supplemental online research was conducted for events where EM-DAT and VR were not up to date.

Meningococcal meningitis and other diseases

In GBD 2019, fatal discontinuities due to a subset of infectious diseases were estimated, including meningococcal meningitis (or meningococcal infection), diarrhoeal disease caused by cholera, dengue, and malaria. These

infectious diseases were first included on the fatal discontinuity cause list for GBD 2016 because (1) their current modelling strategies in the Cause of Death Ensemble model (CODEm) does not optimally capture the potentially highly variable—or epidemic—mortality levels and trends characteristic of these causes; and (2) they can contribute to significant total fatalities in a given location-year. Other infectious diseases for which the latter is true—high death rates in the presence of an outbreak or epidemic—are currently modelled with alternative cause of death methods (eg, natural history models for measles and yellow fever), which allow for greater variation year-over-year if or when outbreaks occur.

The primary data sources for collating cholera and meningococcal meningitis or meningococcal infection death reports were The Global Infectious Diseases and Epidemiology Network (GIDEON) and EM-DAT.[42,43] For any year that cholera or meningococcal meningitis deaths were recorded in a country or territory covered by GBD, reported deaths were directly extracted from 1950 to 2019. If GIDEON or EM-DAT had reporting gaps in cholera or meningococcal meningitis deaths, and WHO reports had coverage for those years, the WHO reports were used. For the Yemen Cholera outbreak in 2016 and 2017, estimates from local collaborators were used in the absence of other data sources.

Section 2.7.2: Location mapping

Every event in the fatal discontinuities database was mapped to a GBD location using a four-step process that includes the following steps in succession: (1) manual mapping, (2) string matching, (3) GPS overlay, and (4) geocoding. If an event was manually mapped, the location was assigned without the use of any other map types. In manual mapping, events are manually assigned to locations by matching the location provided in the raw data to a GBD location. During string matching, an event's location strings were directly compared to the GBD ASCII location names. During GPS overlay, events that have GPS coordinates provided were overlaid onto a map of GBD locations. If the event was placed over a GBD most-detailed location, the event was assigned to that location. During geocoding, the event's location string was entered into Open Street Maps, which returns GPS coordinates. These coordinates were processed using GPS overlay to return GBD locations. This hierarchy provides results where the results of manual mappings are considered the most reliable, followed successively by string matching, GPS coordinates, and then geocoding.

Section 2.7.3: Side splitting

Many fatal discontinuities, such as war, have deaths that are reported across multiple locations. In these instances, deaths were split between the population from both locations, unless estimates by side were provided. If the resulting locations were at the most detailed GBD location level, no further splitting was needed. If a location was not most detailed, the deaths were distributed among the child locations by population.

Section 2.7.4: Prioritisation

Where multiple sources reported shock deaths for the same location-year-cause, a cause-specific prioritisation scheme was followed that reflected the available detail in the cause-specific datasets. For example, the Georeferenced Event Dataset from UCDP was prioritised above all other non-VR sources because it included detail on how deaths were distributed between multiple actors and locations in each conflict event. In most cases, VR from 4-star or 5-star locations was used where available. In some cases, VR from 4-star or 5-star locations was not chosen if there were well-known data quality issues or discrepancies in the cause of death data reporting related to a particular event (eg, supplemental death data for Louisiana were used for Hurricane Katrina because of established data reporting issues).

Section 2.7.5: Age-sex splitting

We ran all compiled data through the causes of death age-sex splitting process, except in cases where we had substantial and reliable information about the age distribution of specific events with high mortality, such as United States mortality during the Vietnam war and Iran mortality during the Iran-Iraq conflict in the early 1980s.[29]

Section 2.7.6: Assigning uncertainty and generating draws

Uncertainty analysis

We generated UIs for deaths caused by conflict and terrorism using UCDP high and low mortality estimates, except for deaths in Iraq from 2003 to 2016. During this time period, deaths due to conflict and terrorism were estimated using a combination of supplemental sources. We used death counts from the Iraq Body Count (IBC),[44] which had the lowest number of deaths from among the sources we found, as the lower bound of the UI from 2003 to 2016. We used estimates from the Iraq Mortality Study (IMS) by Hagopian and colleagues[45] from 2003 to 2006, the deadliest years of the war, to scale deaths to produce the upper UI limits using the formula below:

$$deaths_{GBD\ 2017,\ high} = deaths_{IBC} \cdot \overline{\left[\frac{deaths_{IMS}}{deaths_{IBC}}\right]}_{2003-2006}$$

GBD 2019 used the average ratio between IMS and IBC reported deaths between 2003 and 2006, multiplied by the number of deaths reported by the IBC. This high estimate was carried forward through 2017 under the assumption that IBC similarly undercounts the number of deaths due to the ongoing civil war in Iraq. The final, best estimate for conflict and terrorism deaths in Iraq from 2003 to 2016 is the midpoint of the high and low estimates given above.

When high and low estimates were not included in the existing data, we applied the regional average UI to the available mortality estimate, for all fatal discontinuity causes.

We assumed a log-normal distribution using mean mortality rates and standard error based on high and low estimates. When the standard error was less than 10e-8, we set the draws equal to the mean rate. We then sampled 1000 draws from the log-normal distribution and converted the draws back to counts space. We used the count space draws to calculate final means and UIs.

Section 2.7.7: Changes from GBD 2017

In GBD 2019, all events were assigned a unique identifier that was derived from the source's internal tracking system. This unique identifier is consistent over time and improved versioning of changes made during cause and location mapping.

In GBD 2017, the location matching process only retained location detail from one phase of location mapping at a time. In GBD 2019, each location mapping phase retains the detail that was provided by the previous phases. For instance, if string matching provides national location information, the following phases will only map subnational locations that correspond with that national location.

In past GBD rounds, if an event spanned multiple years, and no detail on the distribution of deaths across years was provided in the raw data, deaths were split evenly across the time span. In GBD 2019, months were used when distributing deaths over time, to improve accuracy. Year distributions were calculated by taking the months of a year an event occurred over and dividing by 12. These weights were then normalised to sum to one. For example, for an event that started in September and lasted until June, the weight for year one would be $\frac{4}{12}$ and the weight for year two would be $\frac{6}{12}$. The fractions were then multiplied by the inverse of the sum of both fractions so that they summed to 1 and could be used to distribute deaths.

Section 3: Fertility[4,5]

Section 3.1: Overview

Prior to GBD 2016, we used United Nations World Population Prospects fertility estimates for all countries and territories.[46] Starting with GBD 2016, we estimated fertility rates within the GBD framework. We based our estimate of total fertility rate (TFR) on a systematic synthesis of all data available for all GBD 2016 locations, using the age-specific fertility pattern from World Population Prospects.[47] For GBD 2017 and 2019, we estimated age-specific fertility rates for ages 10–54 years based on a systematic synthesis of all available data for all GBD locations. We calculated TFR as a function of the age-specific fertility rates.

Section 3.2: Data sources

Fertility estimates were based on three main types of data sources: (1) the number of livebirths reported in vital registration (VR) systems; (2) complete birth histories (CBH); and (3) summary birth histories (SBH). We compiled a total of 37 286 unique location-source-years of data for women aged 10 to 54 for the period between 1950 and 2019. Appendix table 10 and appendix table 11 (section 9) portray the number of sources by location and year.

Section 3.2.1: Fertility data source types

We sought to use accurate and complete accounts of livebirths reported by the age of mothers. Complete livebirth registration reports are designed to account for all births in a single country, territory, or subnational location in a single year, which makes them the gold standard for fertility data. Most high-income countries and territories had high-quality VR systems with information on dates and locations for all births as well as demographic characteristics of each mother. Many lower-income countries and territories, however, had birth registries with incomplete data coverage or interrupted and/or delayed reporting. In these locations, we used household surveys with birth history information for women aged 15 to 49 at the time of the survey, but had to use birth registries for females aged 10 to 14 and 50 to 54 since most household surveys do not collect birth histories from those age groups.

For triangulating the level and age-pattern of fertility, we had to rely on other types of data sources, primarily household surveys and censuses, where birth registration data quality and completeness were low. Household surveys and censuses had two primary types of fertility information: CBHs and SBHs. See section 2.2.1 for more information about CBHs and SBHs.

Section 3.2.2: Fertility data identification and synthesis

We got VR data from the UN Demographic Yearbook (DYB) from the UN Statistics Division (UNSD),[48] the Human Fertility Collection (HFC) from the Max Planck Institute for Demographic Research (MPIDR),[49] the WHO mortality database, official publications, online data portals of national statistical offices, and international collaborators. The HFC and DYB are both compilations of registry-based fertility data from national statistical offices and research institutes. We obtained DYB data on live births by age of mother for every year available from 1948 to 2019. We obtained the complete set of age-specific HFC empirical data up to 2018 but excluded country-year-ages already accounted for by the DYB. In addition to DYB and HFC data, we obtained data from SRSs where available, primarily in South Asian countries such as India, Pakistan, and Bangladesh. In total, we had 8046 unique country-source-years of VR data, with 2577 from before 1970 and 1864 from after 2000. We also had 32 country-source-years of data from SRS.

We identified fertility data from censuses and household surveys using the Global Health Data Exchange (GHDx) by searching for “complete birth history,” “summary birth history,” and “fertility” from among the records categorised as “survey” and “census.” Research team members reviewed these data to verify whether they included the necessary information for GBD analysis. We then conducted additional research to identify and fill gaps in data, filling gaps primarily through country statistical office websites and surveys such as DHS, MICS, WFS, and Reproductive Health Surveys (RHS). In-country collaborators also assisted in acquiring data that were not publicly available. For low-income locations (especially in sub-Saharan Africa), we sought out colonial censuses from the 1950s and 1960s with SBH data. For sources that contained microdata, we computed period age-specific fertility rates (ASFR) every three years over a 15-year recall using CBH data and calculated the average number of children ever born (CEB) for each year of mother’s age, which would later be split by cohort age patterns from the first

modelling stage (see section 3.3.5), using SBH data. For sources that did not contain microdata, we extracted period ASFR or average CEB by mother's age from reports or other publications. In total, we used 439 CBH and 628 SBH sources from surveys and 349 censuses. We were occasionally unable to identify whether a survey that had tabulated period ASFRs was a CBH or SBH survey, but these data only accounted for 156 country-source-years from 45 sources. We have provided details on the nature and quantity of identified sources in appendix tables 10 and 11.

Section 3.3: Modelling strategy

Section 3.3.1: Age-specific fertility rate estimation

We used the data described in section 3.2 to estimate ASFRs for ages 10 to 54 years, separated into five-year age groups. We first estimated ASFRs for age groups in the 15 to 49 year range using ST-GPR. We then estimated fertility rates for the 10 to 14 year age group as a function of estimated fertility in the 15 to 19 year age group and for the 50 to 54 year age group as a function of the estimated fertility in the 45 to 49 year group. We provide more information about the age-specific fertility estimation process below. After estimating ASFRs, we computed summary measures of fertility including TFR, total fertility under age 25 (TFU25), and total fertility over age 30 (TFO30).

Section 3.3.2: Age-specific fertility rate estimation for 15 to 49 years

As stated above, we used ST-GPR to estimate ASFR for age groups 15–19, 20–24, 25–29, 30–34, 35–39, 40–44, and 45–49. The methods for this process have been described in full elsewhere[5], but in short, we did the following: (1) estimated ASFR for the 20–24 age group using age-specific data from CBH and VR sources, using mean years of education in that age group as a covariate; (2) estimated ASFR for the other age groups using age-specific data from CBH and VR sources as well as age-specific mean years of education and the 20–24 age group ASFR; (3) split SBH and other total births data by age and period using estimated location, time, and ASFR for each age group; (4) re-estimated ASFR for the 20–24 age group using CBH, VR, and period-age-split SBH data; and (5) re-estimated ASFR for the other age groups using CBH, VR, and the period-age-split SBH data.

We implemented the ST-GPR models for ASFR as explained below. The first stage of our mixed effect regression was fit in bounded logit space:

$$Logit\left(\frac{ASFR\ data - lower\ bound_{age}}{upper\ bound_{age} - lower\ bound_{age}}\right)$$

We set the lower bound as the minimum fertility by age across time and location and the upper bound, after dropping implausibly high ASFRs over 0·5, as the 99·3 percentile of fertility by age across time and location. The upper bound set an implied maximum TFR of 9·35.

We used the following formula for our mixed effects regression:

$$logit_{bound}(ASFR_{20-24})_{c,t,s,i} = \beta_0 + \beta_1 * female\ education_{c,t} + \gamma_{cs} + \varepsilon_{c,t,s,i}$$

$$logit_{bound}(ASFR_{n-n+4})_{c,t,s,i} = \beta_0 + \beta_1 * fem\ edu_{c,t} + spline(ASFR_{20-24,c,t}) + \gamma_{cs} + \varepsilon_{c,t,s,i}$$
$$\gamma_{cs} \sim N(0, \sigma^2_{\gamma_{cs}})$$
$$\varepsilon_{c,t,s,i} \sim N(0, \sigma^2_{\varepsilon})$$

Where

c is location, t is time, s is source of datapoint i

n is between 15 and 45

β_0 is the intercept

β_1 is the coefficient on female education

γ_{cs} is a location-source random intercept

ε is the residual

Female education and the 20-24 age group ASFR estimates were specific to each country or territory and year.

We only used female education as a covariate in high-income locations for the 20-24 age group, not for the other age groups. We fit separate models for high-income, sub-Saharan Africa, central Europe, eastern Europe, and central Asia to factor in the differences in the relationships between 20-24 age group ASFR and the ASFR of other age groups. We selected the knots in the linear spline (in logit space) by super-region and age group, as outlined in the table below.

Table F. Knots on ASFR 20–24

Region	Age	Knot
Central Europe, eastern Europe, and central Asia	15	NA
Central Europe, eastern Europe, and central Asia	25	-1·5
Central Europe, eastern Europe, and central Asia	30	-2
Central Europe, eastern Europe, and central Asia	35	-1·75
Central Europe, eastern Europe, and central Asia	40	-1·75
Central Europe, eastern Europe, and central Asia	45	-2
High-income	15	NA
High-income	25	NA
High-income	30	-2·25
High-income	35	-2
High-income	40	-2·25
High-income	45	-2·25
Others	15	NA
Others	25	-1·5
Others	30	-1·3
Others	35	-1·3
Others	40	-2
Others	45	-2·5
Sub-Saharan Africa	15	NA
Sub-Saharan Africa	25	-1·75
Sub-Saharan Africa	30	-1·25
Sub-Saharan Africa	35	-1·3
Sub-Saharan Africa	40	-1·5
Sub-Saharan Africa	45	-1·75

We outliered data that reported improbably high ASFR (ie, ASFR over 0·5); had 0 values as a result of sampling error, particularly in the 45 to 49 year age group; reflected an undercounting of births, when we could not adjust the data using other sources; or reported implausibly high mortality levels or trends compared to complete VR data or other more reliable sources.

Section 3.3.3: Data source adjustment

After running the mixed effects model, we adjusted data to a reference source using the random intercept on the concatenation of location and source. To get the adjustment factor, we did the following using the equation below: (1) calculated the difference between the fixed and random effects of the reference source, (2) calculated the difference between the fixed and random effects of the datapoint for the specific source, (3) added the two differences together. We then added this adjustment factor to the data to get an adjusted value.

$$Adjustment\ Factor = (Location\ Source\ RE_{ref} - Location\ Source\ RE_{data\ point})$$

where RE represents a random intercept of either a reference source or a datapoint-specific location-source.

When we had more than one reference source for a single location, we averaged the values of the location source random effects from all the reference sources and used that for the $Location\ Source\ RE_{ref}$ part of the equation.

We primarily chose reference sources as those that met one of the following criteria: (1) complete VR for locations with complete VR, (2) an average of complete birth history sources for locations with one or more complete birth histories, and (3) an average of all the sources for each location for locations without complete VR or complete birth histories. We considered VR to be complete if the median completeness of child death registrations in the location over all available years was over 95%.[4] We also chose reference sources for some locations using expert judgement. For example, the 1950s and 1960s censuses in sub-Saharan Africa are widely viewed as an accurate reflection of depressed fertility in that region at that time, so we used those as reference sources.

Section 3.3.4: Hyperparameter selection

We used the outputs of the previous processes to implement residual smoothing and GPR. We chose hyperparameters for these steps based on a location- and age-specific data density score. We calculated data density scores based on the years for which VR sources were available plus the number of unique CBH and SBH sources available for the given location, using the following computational methods for each type of data source:

1. **Complete VR sources:** calculated as the number of years for which VR data were available. If the number of births in the age group was below 100, this part of the score was down-weighted by the difference between the number of births and 100.
2. **Incomplete VR sources:** calculated the same way as with complete VR data, but down-weighted by 0·5.
3. **Total CBH sources:** the number of unique complete birth histories for a single location.
4. **Total SBH sources:** the number of unique summary birth histories for a single location.

We calculated the data density score using the following equation:

$$\begin{aligned} DD\ Score_{loc,age} = \ & Complete\ VR\ years_{loc,age} + \left(2 * Number\ CBH\ Sources_{loc,age}\right) \\ & + \left(0.25 * Number\ SBH\ sources_{loc,age}\right) + \left(0.5 * Incomplete\ VR\ years_{loc,age}\right) \\ & + Number\ Other\ Sources_{loc,age} \end{aligned}$$

Where

DD is the data density

CBH is complete birth history

SBH is summary birth history

All elements of the equation integrate year and age.

We then assigned hyperparameters α and β for a beta density function, generally based on final data densities, as shown in Table G. However, there were exceptions where we manually assigned a different set of hyperparameters.

Table G. Hyperparameter values by data density

Data density	Alpha	Beta	Zeta	Scale
Over 50	40	40	0·99	5
Between 30 and 50	30	30	0·9	10
Between 20 and 29	20	20	0·8	15
Between 10 and 19	15	15	0·7	15
Under 10	10	10	0·6	15

In cases of incomplete VR sources, we defined data variance as the difference between the spatiotemporal prediction and the unadjusted data. Some location-ages had very little data, resulting in implausible variance. As such, for location-ages with fewer than five datapoints, we used the maximum data variance in the location's GBD region. For complete VR sources, we assumed that non-sampling variance was 0. We calculated sampling variance for these sources using the following binomial equation:

$$Sampling\ Variance = \frac{ASFR * (1 - ASFR)}{Births}$$

We then calculated amplitude and applied it to all locations other than high-income VR-only national locations with 40+ years of VR data. For these locations, we took the mean of the location-specific standard deviation of the difference between the mixed effect regression and the spatiotemporal smoothing. However, we only included national locations with a data density score of more than 50 from 1990 to 2019. For the high-income VR-only national locations, we used their location-specific value instead of the mean, which is based on the difference between the unadjusted data and the spatiotemporal smoothing.

Section 3.3.5: SBH methods

For the earlier years of the study period, CEB data were more readily available from SBHs than from CBHs. While there are numerous methods for calculating period- and age-specific fertility from SBH information, the Brass Parity/Fertility ratio method is used most often. However, this method assumes a constant ASFR over time. We wanted a dynamic measure of cohort age patterns over time, so we instead used ASFR estimates from CBH and VR data from the first run-through of ST-GPR described above to split SBH into period ASFR. We used these estimates to compute implied annualised fertility for all the five-year birth cohorts represented in a given SBH from age 10 through whichever came sooner: age 54 or the year of the survey. To account for births that occurred in years when part of the initial age group had moved into the next age group, we calculated the weighted average of estimated ASFRs in the age groups on either side of the selected age group and took that average as the fertility experienced by that hypothetical cohort in that year, assuming a uniform age distribution within the age group. For example, for a cohort of women aged 20 to 24 in 1984 with ASFR F, we would compute the ASFR experienced by this cohort as women aged 21 to 25 in 1985 as:

$$.8 *_5 F_{20}^{1984} + .2 *_5 F_{25}^{1989}$$

since 20% of the cohort had aged into the 25 to 29 age group by the following year.
We then used the implied annualised cohort ASFR to calculate cumulative cohort fertility up to the age of each cohort at the time of the survey. We compared implied cumulative fertility to observed cumulative fertility (average CEB from SBHs) by each cohort to get a scaling factor. We then applied this scaling factor to the original implied cohort age pattern, which distributed CEB back across time and age. This method only covered birth cohorts between 1940 (who began to experience ASFR 10-14 in 1950, the first year of our study period) and 2009 (who began to experience ASFR 10-14 in 2019, at the end of our study period).

Splitting of total birth and historic location aggregate data

We could only get a large portion of the data aggregated by age and/or location (eg, total live births instead of births by mother's age; former USSR prior to its dissolution). For these data, we split just the CBH and VR data using the age and location proportions designated in the first ST-GPR run-through. After splitting the data, we reran the estimation process describe above using all CBH, VR, and period and age-split data as well as location and age-split miscellany. This improved the availability of past data and gave us more information about aggregate levels of fertility over time.

Section 3.3.6: Age-specific fertility rate estimation for 10- to 14-year-olds and 50- to 54-year-olds

We estimated ASFR for the 10 to 14 and 50 to 54 year age groups separately because data for these age groups in locations without VR systems were scarce. We used the relationship between ASFR in sequential age groups to estimate both age groups. For the 10 to 14 age group, we ran a mixed effects regression on the log of the ratio of ASFR 10-14 over ASFR 15-19, and used ASFR 15-19 and nested random intercepts by super-region, region, and location as predictors, as defined by the equation below:

$$\log\left(\frac{ASFR\ 10-14}{ASFR\ 15-19}\right) = \beta_0 + \beta_1 \log(\text{ASFR } 15-19) + \gamma_{k[j]} + \gamma_{jk[i]} + \gamma_{ijk}$$
$$\gamma_{k[j]} \sim N\left(0, \sigma^2_{\gamma_{k[j]}}\right)$$
$$\gamma_{jk[i]} \sim N\left(0, \sigma^2_{\gamma_{jk[i]}}\right)$$
$$\gamma_{ijk} \sim N(0, \sigma^2_{\gamma_{ijk}})$$

Where

i is location, j is region, k is super-region

β_i is a fixed covariate coefficient

$\gamma_{k[j]}$, $\gamma_{jk[i]}$, and γ_{ijk} are nested super-region, region, and location random intercepts

For ASFR in the 50 to 54 age group, we instead estimated a regression on the log ratio of ASFR 50–54 over ASFR 45–49 with a constant. We did this because there was not a clear relationship between this ratio and ASFR 45–49. We produced 1000 draws from the variance-covariance matrix to generate uncertainty using methods described previously.

Section 3.3.7: Fertility metrics

We calculated TFR as the time-weighted sum of the ASFRs from each age group. To do this, we added ASFR for each five-year age group and multiplied that by the five years spent in each age group. We calculated TFU25 and TFO30 in the same way, but with only the relevant age groups included. We defined livebirths as the sum of ASFR from all age groups multiplied by the 10- to 54-year-old female population from the population model described in section 4.

Section 3.4: Sex ratio at birth

Section 3.4.1: Overview

Another component of population structures and reproductive capacity is the sex ratio at birth (SRB). For GBD analysis, we defined SRB as the ratio of total male to total female livebirths in each location in a given calendar year. The naturally occurring SRB is generally approximately 1·05 males per female, with some location-specific variations.[50] Since the introduction of ultrasound technologies and the ability to conduct sex-selective abortions, previously stable SRBs have shifted in some locations as a result of systematic sex preferences for children. SRBs in recent years are particularly skewed in the Caucasus, south Asia, and east Asia. To reflect historic equilibria and recent shifts in SRB, we developed a model to estimate SRB in all GBD countries and territories, Hong Kong, and Macau from 1950 to 2019.

Section 3.4.2: Modelling approach

For GBD 2019, we used GBD 2017 SRB estimates. We did not update the model or re-run it, instead using our results from the previous iteration. Details of the model are published elsewhere.[5]

Section 4: Estimating healthy life expectancy (HALE)[6]

We calculated healthy life expectancy (HALE) for every location-age-sex-year population group from 1990 to 2019. Key inputs from GBD 2019 were YLDs per capita for every location-age-sex-year population group from 1990 to 2019. Details on calculating these inputs and on COMO are available elsewhere.[51]

We used Sullivan's method to incorporate average health values into life tables. We defined *Tx* as person-years lived above age x. We defined *nLx* as total person-years lived in an age interval between age *x* and age *x*+*n*.

We calculated health-adjusted person years by calculating 1 minus YLDs per capita. We calculated an adjusted *nLx* by multiplying the column with *nLx* by the average health value for that age group.

$$nLx_{adjusted} = nLx * (1 - YLDs)$$

We used the adjusted *nLx* values to recalculate the rest of the life table. We calculated health-adjusted person-years lived in and above a certain age interval (adjusted *Tx*) for each age group by summing the health-adjusted person-years, *nLx*, for all age intervals above that age group. For each age group *i* in $[1..t]$ where *t* is the terminal age group, we calculated:

$$Tx_{adjusted,i} = \sum_{i}^{t} nLx_{adjusted}$$

We calculated HALE by dividing adjusted *Tx* for each age group by lx:

$$HALE = \frac{Tx_{adjusted}}{lx}$$

Finally, we calculated summaries (the mean, upper UI, and lower UI). We also calculated percent change from 1990 to 2019, 2010 to 2019, and 1990 to 2010.

Section 5: Population[4,5]

Section 5.1: Overview

The demographic balancing equation expresses how population (N) changes between two points in time.

$$N(T) = N(0) + B[0,T] - D[0,T] + I[0,T] - O[0,T]$$

Increases in population counts can only be attributed to births (B) or immigration (I) and decreases to deaths (D) or emigration (O). In this analysis, immigration and emigration are collapsed into net migration (G), where net immigration is indicated by a positive value and net emigration is indicated by a negative value.

$$N(T) = N(0) + B[0,T] - D[0,T] + G[0,T]$$

To estimate population size over a period of time, we need to know the initial population and how many people are entering and leaving the population through births, deaths, and migration. To summarise, the Bayesian population model used in this study reconciles data on population size obtained through population censuses and registries with GBD fertility and mortality estimates. First, we will describe each data source used as an input to the model and then we will describe the modelling processes used to estimate migration and population. The calculated GBD world population standard is shown in appendix table 13.

Section 5.2: Data sources and processing

Section 5.2.1: Census and registry lists

Population censuses, which are conducted in most countries in about ten-year intervals, are the main source of data for population size by age and sex. In certain countries, continuous population registries are also maintained. We synthesised population census data by first compiling a list of censuses as documented by UNSD,[52] UN Population Division,[53] UN DYB,[48] the Integrated Public Use Microdata Series (IPUMS),[54] and the Population Research Center at The University of Texas at Austin.[55] This list can be found in appendix table 12 (section 9); all censuses and registries can be found for each country in appendix figure 5.

Section 5.2.2: Data extraction

We extracted age-sex-specific population census data and registry counts from the UN DYB,[48] IPUMS,[54] national statistic websites, and by searching the WorldCat catalog.[56] Occasionally, multiple sources of population counts for a given census or registry-year were available. In these instances, preference was given to registry data rather than census data, data where population counts were reported as de facto (when people are counted based on place of enumeration) over de jure (when people are counted based on place of usual residence), and data reporting population by more granular age and sex groups. In certain cases, only a portion of the total census population was reported with more granular detail for age and sex (with a sample ranging from 1% to 20% of the total census). In these instances, we assumed that the age and sex structure of the sub-sample was identical to that of the total census. Then, we scaled up the sub-sample counts using the total census population counts.

In total, data were extracted for 1250 censuses out of 1296 censuses known to have occurred. Of the censuses extracted, 668 were de facto, 517 were de jure, and in 64 instances we were unable to determine whether the data were de facto or de jure. The 46 un-extracted censuses include those that were never or not yet released by the coordinating organisation. In total, 747 location-years of registry data was extracted for 28 locations.

Any instance of published limitations of the census was also noted, including both limitations identified by media and independent experts in addition to limitations provided by the country's statistical division upon publication. In total, data were outliered from 88 censuses because they were representative of only a subset of the population, utilised questionable methods, or were inconsistent with adjacent data. Some censuses were excluded because countries may have artificially inflated population counts or undercounted minorities for political reasons. Others were excluded because they failed to enumerate the non-white population (for example, Rwanda in 1953 and Burundi in both 1952 and 1958).

Section 5.2.3: Data processing

Population data could be inaccurate for a number of reasons including age misreporting, representativeness of the de facto population, and under/over-enumeration. While identifying censuses, we preferentially utilised raw census and registry population counts. Then, we corrected for the issues mentioned by applying a standardised set of methods.

First, we distributed counts of individuals where age and/or sex were unknown. The age-sex structure of the remaining data from that particular census was used to distribute the counts of this group of individuals.

The next step was adjusting for age heaping that occurs when, instead of reporting their exact age, individuals report their age as a round number (usually ending in 0 or 5). Population totals were aggregated first to the largest age interval length originally in the data, so each population count was in standardised age groups and the censuses with unusually wide age groups were collapsed to total population by sex only. For each census, the age ratio score (for males and females), sex ratio score, and joint score (JS) were calculated. These measures are explained in detail in the US Census Bureau Population Analysis System documentation.[57]

We use a combination of three previously developed age-heaping methods for this analysis. One of these, the Feeney correction, which is applicable only in census counts with single-year age groups, redistributes people reporting their age as multiples of five to the eight adjacent single-year age groups, resulting in corrected counts with proportional adjacent age groups that then form a linear progression. The corrected counts are reported in five-year age groups. The Arriaga correction takes input census counts in five-year age groups and smooths them by combining the three nearest ten-year age groups with a second-degree polynomial. The advantage of using this technique instead of other similar methods is that it also smooths the youngest and oldest age groups. The Arriaga strong method averages three consecutive ten-year age groups, which addresses cases with severe age misreporting. These three methods were used in countries outside of the high-income and central Europe, eastern Europe, and central Asia super-regions according to the age group length of the population counts and the joint score, as seen below.

Table H. Age heaping corrections

Age group length	Joint score (JS)	Age heaping correction
1	JS <= 20	None
1	JS > 20	Feeney
5	JS <= 20	None
5	20 < JS >= 40	Arriaga
5	JS > 40	Arriaga strong
10	JS <= 20	None
10	JS > 20	Arriaga strong
Total population	Not available	None

After performing a visual inspection, 33 censuses were identified as being incorrectly scored or poor-quality, and these data were not corrected for age heaping. This was predominantly due to sex ratio scores that were higher than normal, but still thought to be a real phenomenon, occurring among the Persian Gulf states, for example. 21 censuses were also identified as exhibiting age heaping but were not corrected due to being in either the high-income or the central Europe, eastern Europe, and central Asia super-regions. After applying age heaping corrections, we aggregated the single-year age group population counts conducted outside of the high-income and the central Europe, eastern Europe, and central Asia super-regions into five-year age groups.

For the third step, we corrected data in cases that were not representative of the de facto population of interest, either due to the coverage of geographical area, subpopulations, or a combination of both. A number of countries that exist in 2019 are combinations of two or more countries that existed in the past, like Germany. Others have had semi-autonomous territories conduct separate censuses, like Transnistria, and others still have contemporary states that are part of historical aggregates, like states that were previously part of Yugoslavia. Censuses for fragmented or semi-autonomous areas often occurred in different years and collected information about different age groups.

The population of the complete modern geographic unit for which we had a time series of separate censuses from all constituent historical or disputed territories was estimated. All available censuses for the constituent parts were collapsed to the most granular set of common age groups. Age-and-sex-specific populations were interpolated after standardisation using the age-and-sex-specific annual rate of change to generate annual time series spanning the available census data for each constituent. Finally, for all original census years corresponding to the largest constituent, where there was also overlap with the estimated populations from the constituents that were smaller, the

populations of all the constituents were summed at the age-sex level to produce a census estimate that was representative of the entire modern geography. This method was utilised in the combination of censuses from East and West Germany, Moldova and Transnistria, Cyprus and the Turkish Republic of Northern Cyprus, Serbia and Kosovo, and Malaysia Peninsular, Sarawak, and Sabah. For modern countries formerly part of Yugoslavia, only historical census data that were reported according to ethnic (rather than geographical) affiliation were able to be extracted. In these cases, the estimated age-sex proportions of the total historical aggregate population from the previous iteration of the Bayesian demographic balancing model were applied to create modern geographies from the historic census data. These split data were used only for Serbia, as census data for the five other modern countries that were formerly part of Yugoslavia were obtained.

Singapore censuses only contained granular age and sex population counts for residents, but non-residents accounted for a significant portion of the de facto population, while registry data for Singapore residents and non-residents were only available in broad age groups (0–14, 15–64, 65+). Using available empirical death counts for both residents and non-residents, and assuming that both subpopulations have similar mortality rates, the relative age-pattern in the death counts was utilised to scale the resident population from the census data to the total population. These age- and sex-specific population counts were then scaled to the broad age group data from the population registries in Singapore.

A fourth step was to correct for improper enumeration. Many censuses suffer from under-enumeration, since it is logistically very difficult to reach every single member of the population except in the smallest and most navigable countries. In order to address this issue, national statistical offices will often conduct post-enumeration surveys (PES) shortly after the completion of censuses to assess the degree to which individuals were either missed or double-counted. The PES quantifies the overall bias of a given census, and population totals can be adjusted using those results. We searched for PES using the UNSD division's list of known or planned PES efforts in the 2000 and 2010 census rounds. We looked for PES corresponding to any known census by looking through country statistical websites, academic publications, the WorldCat catalog, and data presented by countries during UN Stats symposia on the topic of PES as well. 161 PES were identified that reported a net underenumeration percentage, of which 36 reported underenumeration percentage by age and/or sex. As most extracted census data were unadjusted and a corresponding PES was unable to be identified for many cases, a simple linear regression was used to predict percentage adjustment for all ages and both sexes on the basis of the Socio-demographic Index (SDI) (appendix section 6).

$$totalPctAdjust = \beta_0 + \beta_1 SDI + \varepsilon$$

We used the variance-covariance matrix from this model to simulate 1000 draws of the predicted total percentage adjustment ($totalPctAdjust$) for every possible value of SDI. We incorporated the variance of the 1000 draws simulated from the model into the overall calculation of population uncertainty (See appendix section 2.3.2)

Seeing as how over- and underenumeration varies significantly depending on age and sex, the 36 PES that reported underenumeration by age and/or sex were used to obtain an underenumeration global age pattern. In order to address the issue of PES reporting inconsistent age groups, we used DisMod-MR, an age-integrating Bayesian meta-regression tool widely used in GBD processes[58,59] and included SDI as a predictor in the model. The age-sex pattern was then shifted up or down to equal the predicted mean total percentage adjustment and was applied to all census counts where prior adjustment was not identified.

The Bayesian demographic balancing model and the cohort component method of population projection (CCMPP) requires a starting population as an input from which it can project populations forward in time, but most countries did not conduct a census in 1950. The last step of data processing is to produce a prior estimate of the population in 1950 in each location in single-year age groups for each sex. In instances where post-processed censuses were available for 1950 with continuous single-year age groups up to the age group 95+, the census was used directly. In all other cases, aggregate age groups were split in the oldest census available with person years lived (${}_nL_x$) in single-year age intervals from the estimated life table for that location in 1950.[4] Then, this age pattern was smoothed using a local first-degree polynomial with bandwidth equal to 2 if the original age groups were in age interval lengths of 1 or 5 and bandwidth equal to 5 if in larger age groups.[60] If this oldest census was not from 1950, we projected the census backward using an inverse version of CCMPP (assuming zero migration). A description of standard CCMPP can be found in section 2.3.2; backward CCMPP solves for the population at the previous time point rather than the projecting to the next time point.

$$ {}_1N_{x-1}(t) = {}_1N_x\ (t+1) \cdot \frac{{}_1L_{x-1}(t)}{{}_1L_x\ (t)} $$

The equation for the open-ended age group is indeterminate and leaves what we call the upper missing triangle as the backward projection continues further back in time. The missing age groups are estimated using the 1950 ${}_nL_x$ age pattern found in the estimated life tables. In certain locations, as the census counts are projected backward, age misreporting in the older age groups becomes extremely evident and leads to implausible age patterns; we addressed this by collapsing the original census to a lower open-ended age group ignoring the oldest age groups in the original census. 34 countries used annualised rate of change between the oldest two censuses to back-project the total population, then scaled the back-projected age-specific population to the back-projected total population. In 36 countries, the assumption of zero migration used in backward CCMPP is not appropriate, or the oldest census was not close enough temporally to 1950 to back-project a reasonable age pattern; in these countries we instead used previous GBD population estimates in 1950 as the baseline population. The 1950 populations were used as the prior for the Bayesian demographic balancing model but were input with considerable uncertainty to account for the fact that a census did not occur in 1950 in most countries.

Section 5.3: Model inputs

Section 5.3.1: Migration

The demographic balancing equation has four main components: population, births, deaths, and migration. Except in high-income countries, migration was not measured as well as the other components, thus impacting CCMPP which requires either the net migration proportion or net number of migrations by age, sex, and year. In locations with frequent censuses, we set the prior for net migration to zero in the Bayesian population model (further described below). However, we recognised the model was not able to estimate migration accurately when large migration occurred between censuses or in the years after the last census was conducted. Therefore, we describe our process of replacing the zero prior in these specific types of locations by identifying and extracting migration data below.

For migration, we utilised a refugee stock dataset from the United Nations High Commissioner for Refugees (UNHCR)[61] which included end-of-year (EOY) counts of refugees residing in each country of destination (D), organised by country of origin (O), to calculate net flow of refugees in or out of countries. For example, change in the number of refugees residing in Rwanda in 1994 was calculated by taking the number of refugees in the country at the EOY in 1994 $D_{RWA}(EOY\ 1994)$ and subtracting the number of refugees at the EOY in 1993 $D_{RWA}(EOY\ 1993)$.

$$ \Delta D_{RWA}[1994] = D_{RWA}(EOY\ 1994) - D_{RWA}(EOY\ 1993) $$

Likewise, change in the number of refugees originating from Rwanda who had relocated elsewhere in 1994 was calculated by taking the number of refugees originating from Rwanda at the EOY in 1994 $O_{RWA}\ (EOY\ 1994)$ and substracting the number of refugees originating from Rwanda at the EOY in 1993 $O_{RWA}(EOY\ 1993)$.

$$ \Delta O_{RWA}[1994] = O_{RWA}(EOY\ 1994) - O_{RWA}(EOY\ 1993) $$

Net refugee migration for Rwanda in 1994 was calculated as:

$$ \text{Net refugee migration}[1994] = (\Delta D_{RWA}[1994] - \Delta O_{RWA}[1994]) $$

We converted net refugee migration to mid-year migration totals by averaging totals for adjacent years. While the UNHCR dataset also reported totals for internally displaced persons, these types of individuals were excluded from our analysis. In location-years of refugee crises, the total number of migrants were primarily made up of the large influxes or outfluxes of people, replacing the zero prior with the UNHCR data.

There are certain countries, such as Germany, the Czech Republic, Romania, the United Arab Emirates, and Bahrain, where large non-refugee migrations have taken place too recently to have been captured by the last census so they were not accounted for in the Bayesian population model. In locations where this was the case, migration data was taken from EUROSTAT,[62] the Gulf Labour Markets, Migration and Population (GLMM) programme,[63] or national statistics websites.

EUROSTAT was the only source which provided age-and-sex-specific migration data; the other sources reported total number of migrants over specific time periods. Since age-and-sex-specific migration is required as an input to CCMPP, we scaled age-sex patterns of migration to the input net migrant totals. The aggregated EUROSTAT age and sex pattern was used for most locations, but we used the Bayesian demographic balancing model's age and sex pattern for Qatar in places where the majority of migration has occurred among young adult male temporary workers (Saudi Arabia, Bahrain, United Arab Emirates, Oman, and Kuwait). Qatar was chosen because its most recent census was conducted in 2015 and captured this recent uptick in migration. Lastly, given the genocide in Rwanda in 1994, we assumed the migrant age pattern was similar to neither the typical migrant age pattern reported in the EU nor the age pattern of migratory workers in Qatar, so we used an age pattern that was uniform across age and sex for the country.

Section 5.3.2: Mortality

The GBD mortality model produced yearly complete period life tables with single-year age groups up to 95+, as described in section 2.

Section 5.3.3: Fertility

The GBD fertility model produced single-year age group fertility rates between age 10 and 54 as described in section 3.

Section 5.3.4: Sex ratio at birth

The GBD sex ratio at birth (SRB) model produces yearly sex ratio estimates, which we used to split total livebirths into sex-specific livebirths during CCMPP. See section 3.4 for more information.

Section 5.4: Modelling strategy

Section 5.4.1: CCMPP

CCMPP allows projection of population forward in discrete time intervals by age and sex by applying mortality, fertility, and migration. Since more information about this method is provided in Preston,[20] we only present the fundamental equation here.

$$ {}_1N_x\ (t+1) = \left[\left({}_1N_{x-1}(t) + \frac{{}_1G_{x-1}[t,t+1]}{2}\right)\cdot\frac{{}_1L_x(t)}{{}_1L_{x-1}(t)}\right] + \frac{{}_1G_{x-1}[t,t+1]}{2} $$

The survivorship ratio, $\frac{{}_1L_x}{{}_1L_{x-1}}$, which is derived from the GBD period life tables, is used to survive any age group (the youngest and oldest age groups are slightly different) forward by one time interval. One key assumption made was that the number of migrants is evenly split, with half migrating during the beginning of the time interval and the other half migrating at the end of the time interval. Although G was defined by Preston as the net flow of migrants during the projection period in the age interval x - 1 to x,[20] we defined ${}_1G_{x-1}$ as the net flow of migrants during the projection period for the cohort initially between age x – 1 and x at the beginning of the projection period because we used the GBD Bayesian Demographic Balancing Model (described below) to estimate migration. Net migration proportions were estimated by this model instead of counts. We defined these proportions as the ratio of net migrants in a cohort to the population at the beginning of the projection period.

$$ {}_1g_x = \frac{{}_1G_x}{{}_1N_x} $$

Section 5.4.2: Bayesian demographic balancing model

A Bayesian hierarchical model (popReconstruct) which reconstructs population by age and sex back in time was previously developed by Wheldon and colleagues.[64] Baseline population counts, mortality, fertility, migration, and sex ratio at birth are simultaneously estimated by the popReconstruct model to reconcile CCMPP's population projections with recent census data.

In Wheldon's version, bias-reduced input values reported by the United Nations were used for the necessary components of population change which included net migration. In our application of the model, we used initial GBD estimates of fertility and mortality which were derived from replicable methods. Since GBD did not produce net migration estimates, attributable to a lack of migration data and difficulties in directly modelling the variable, we modified Wheldon's popReconstruct model to estimate net migration indirectly consistent with available census data and the input demographic estimates.

Section 5.4.3: Model description

In both popReconstruct and our Demographic Balancing Model, CCMPP is embedded into a Bayesian hierarchical model. To keep the variables consistent with the popReconstruct model, n, g, s, f, and srb were used to symbolise population, the net migration proportion, survivorship ratio, age-specific fertility rate, and sex-ratio at birth, respectively. We indexed the variables by sex (l), single-year age groups (a) and single calendar years (t). Initial parameter values are indicated by an asterisk ($*$).

Level 1: The percentage difference between non-baseline sex-age-year-specific census counts ($n^*_{l,a,t}$) and the corresponding projected sex-age-year-specific populations counts ($n_{l,a,t}$) from CCMPP in Level 2 was modelled. We modelled percentage difference to ensure the variance ($\sigma^2_{n^*_{l,a,t}}$) associated with each census datapoint was estimated on a consistent scale across age groups and locations for a variety of population magnitudes.

$$Level\ 1: \frac{n^*_{l,a,t} - n_{l,a,t}}{n_{l,a,t}} \sim Normal(0, \sigma^2_{n^*_{l,a,t}})$$

Level 2: The model inputs were transformed into projected sex-age-year-specific population counts ($n_{l,a,t}$).

$$Level\ 2: n_{l,a,t} = CCMPP(n_{l,a,t_0}, g_{l,a,t}, s^*_{l,a,t}, f^*_{l,a,t}, srb^*_t)$$

Level 3: The net migration proportion ($g_{l,a,t}$) and baseline population in 1950 (n_{l,a,t_0}) were modelled. Like in level 1, the baseline population was modelled as the percentage difference between the estimated baseline population in 1950 and the input values. To avoid major discontinuities in the age pattern and time series, net migration proportion was modelled as an autoregressive process over age and time. Contrary to the popReconstruct model, we did not also estimate ASFR ($f^*_{l,a,t}$), survival ratios ($s^*_{l,a,t}$) and sex ratio at birth (srb^*_t) because the zero prior for migration makes it difficult to jointly estimate all components.

$$Level\ 3: \frac{n_{l,a,t_0} - n^*_{l,a,t_0}}{n^*_{l,a,t_0}} \sim Normal\left(0, \sigma^2_{n^*_{l,a,t_0}}\right)$$

$$g_{l,a,t} - g^*_{l,a,t} \sim AR1: AR1(\sigma_g, \rho_{g_a}, \rho_{g_t})$$

Level 4: Hyper-priors were defined for the prior distribution on the net migration proportion. We specified greater correlation over time than age for the net migration proportion correlation.

$$Level\ 4: \log(\sigma_g) \sim Normal(-5, 9)$$

$$logit(\rho_{g_a}) \sim Normal(-3, 0.01\ )$$

$$logit(\rho_{g_t}) \sim Normal(3, 0.01)$$

Rather than estimating one variance term for all census counts, the initial standard deviation for every age-sex-year-specific census datapoint ($\sigma_{n^*_{l,a,t}}$) was set to 0·01 for locations in the high-income super-region, 0·03 for locations in the central Europe, eastern Europe, and central Asia super-region, and 0·05 for locations in the other super-regions. Several issues were addressed by multiplying the standard deviation by scalars in order to adjust the weight of a datapoint. These issues included: 1) We doubled the standard deviation of the 1950 baseline counts so that the estimated baseline count could change more from the input 1950 counts from census processing. 2) Our model was constructed to compare projected population counts to the available census age groups, whether that was single-year age groups or just total population. When a census only had less granular age groups, there were fewer datapoints included, which gave less weight to the census year as a whole. To resolve this, the standard deviation for each

census datapoint was divided by the age interval's width, resulting in all censuses being assigned similar weights. 3) Age misreporting among older ages was accounted for by multiplying the standard deviation by a scalar which increased linearly from 1 among 50-year-olds to 3 among 95+-year-olds in the terminal age group. 4) Under-enumeration in the under-5 age group was addressed by multiplying standard deviation of population counts by a linear scalar that increased from 1 for age 5 to 3 for age 0. For the locations not included in the high-income or central Europe, eastern Europe, and central Asia super-regions, non-baseline under-5 population counts were not input for all censuses, making under-5 populations dependent on the fertility estimates, under-5 mortality estimates, and census counts from older age groups.

Older age misreporting seemed to be quite common, demonstrated by the unrealistically large immigration values produced by the model to compensate for the high counts of individuals among the older age groups. This problem was partly addressed by the methods we described above which gave less weight to census counts among the older age groups. In addition, we ran the model multiple times for each location with each version excluding data above a certain maximum age between 55 and 95. Upon completion of each model version for a location, we calculated: 1) mean average percentage error (MAPE) to compare input census data to the estimated population for each datapoint that was included in the model and 2) the weighted mean of the absolute value of the net migration proportions for five-year age groups between 55 and 95 by assigning a weight equal to 1 for age group 55, 2 for age group 60, and so on, capping at a weight of 9 assigned to age group 95+. Then, we selected the version with the highest maximum age that was within 5% of the minimum MAPE value from the various model versions and within 0·005 of the minimum weighted mean net migration proportion for older age groups. By using this method, we were able to balance decreasing migration among the oldest age groups and ensure a good fit to the census data we included in our model.

For each location we separately fit the Bayesian Demographic Balancing model from 1950 to 2019 with single-year age groups up to the terminal age group of 95+. CCMPP was the used to derive final population estimates using GBD estimates of mortality, fertility, and sex ratio at birth as well as posterior estimates of baseline population and the net migration proportion.

Section 5.4.4: Population uncertainty

Population uncertainty was estimated using a combination of out-of-sample predictive validity the uncertainty in the adjustment for census completeness (appendix section 2.3.1) after estimating a full time series of age-and-sex-specific population with CCMPP.

A major cause of uncertainty was related to the PES results and the census completeness model, leading to additional uncertainty in population estimates for all years, since censuses are used to inform population size in both census and non-census years. Using our census completeness model, we calculated the variance of the percentage adjustment for completeness ($Var_{completeness}$) that was associated with each location-year and dependent on the SDI value.

There was additional uncertainty in our population estimates for non-census years which was related to the amount of time to the closest census and GBD estimates of mortality, fertility, and migration. To measure this, we withheld census data in the 143 national locations where there are a minimum of five censuses with age-and-sex-specific population totals after undergoing post-census processing (see appendix section 2.3.1). For each location, the model was fit 20 times using a random subset of one to five of the location's censuses. Percentage error between the held-out census data and the projected posterior population estimates were compiled across locations by the absolute number of years from the closest census. A linear regression on the root mean squared percentage error ($RMSPE$) linear dependent on the number of years from the closest census ($YearsToCensus$) was fit.

$$RMSPE = \beta_0 + \beta_1 YearsToCensus + \varepsilon_i$$

This gave us the predicted variance of the percentage adjustment for completeness ($Var_{completeness}$) for each location-year based on the specific year's SDI and the predicted out-of-sample percentage error we calculated based on the number of years from the closest census ($RMSPE$). We calculated annual total uncertainty in variance space by combining the different types of uncertainty we identified.

$$Var_{pop} = Var_{completeness} + RMSPE^2$$

We used the total variance for population to take simulated draws of the population percentage error ($PopPctError$) by location-year.

$$PopPctError \sim Normal(0, Var_{pop})$$

Finally, we used the percentage errors to calculate new population draws which incorporate both PES adjustment uncertainty and also out-of-sample variance associated with estimation errors in the GBD fertility, mortality, and migration estimates and the amount of time to the closest census.

$$Pop_{draw} = Pop_{mean} + (Pop_{mean} * PopPctError_{draw})$$

Section 5.4.5: GBD world population age standard

The GBD world population age standard was used to calculate age-standardised rates presented throughout GBD. In previous GBD years, 2013, 2015, and 2016, specifically, a standard population age structure was generated by taking the non-weighted mean of the 2010 to 2035 age-specific proportional distributions for national locations reported by the UN Population Division World Population Prospects 2012 revision. In GBD 2017 and 2019, we used the non-weighted mean of the GBD year's age-specific proportional distributions for national locations with populations greater than 5 million in the GBD year to update the world population age standard. The final values used for the age standard can be found in appendix table 13 (section 9).

Section 6: Socio-demographic Index (SDI) analysis[6]

Section 6.1: Overview

The Socio-demographic Index (SDI) is a summary indicator created to get at the background social and economic conditions that shape health outcomes in a given location. Introduced in GBD 2015, it represents an update to the Human Development Index (HDI), while also providing an alternative to outdated development language.

SDI includes three components. It includes an economic indicator: lag-distributed income (LDI) per capita. It also includes two demographic indicators – total fertility rate under the age of 25 (TFU25) and mean educational attainment for those aged 15 and older (EDU15+). TFU25 is intended to serve as an indicator of the status of women. Beginning in GBD 2017, we began using TFU25 instead of total fertility rate (TFR), because we found it to be a better proxy for women's status.

To create the index, we rescaled each component to obtain a value between 0·005 to 1. We set this scale using selected health indicators. At the low end of this scale, 0·005 represents the minimum level of each covariate input past which selected health outcomes do not get worse. At the high end of this scale, 1 represents the maximum level of each covariate input past which selected health outcomes do not improve. Finally, we calculated SDI by taking the geometric mean of those rescaled values.

Section 6.2: SDI calculation

GBD originally used the Human Development Index (HDI) methodology in developing SDI for the 2015 cycle. At the core of this method was the use of three covariate inputs: TFR in ages 15 to 49, EDU15+, and LDI per capita. The scale of the index was from 0 to 1. The observed minimum for each covariate over the estimation period determined the lower boundary of this range, whereas the observed maximum for each covariate over the estimation period determined the upper end of this range.[29]

Further refinements to the method for calculating SDI have been implemented since that time. Beginning in GBD 2017, we decided to use TFU25 instead of the TFR component. The rationale for this was to attempt to better capture women's social status, given that it covers ages when women tend to enter the workforce and pursue further educational opportunities. It is also important that there has been a consistent decline in TFU25 over time in highly developed countries. In contrast, there have been rebounds in TFR driven by increasing fertility in older ages. The concordance correlation coefficient was 0·981 between SDI using the GBD 2016 method and the updated GBD 2017 method.

In order to improve the stability of the interpretation of SDI over time, we switched from relative index scales to absolute index scales during GBD 2016 when we noticed the introduction of subnational units led to stretched empirical minima and maxima.[7] The minima and maxima of the scales were selected by looking at the relationships between each of the inputs and life expectancy at birth and under-5 mortality then identifying points of limiting returns at both high and low values, if they occurred prior to theoretical limits (eg, a EDU15+ of 0).

An index score of 0 therefore represents the point at which decreasing each covariate does not worsen selected health outcomes and an index score of 1 represents the level at which increasing the level of each covariate does not improve selected health outcomes. The means that a location with an SDI of 1 would have the theoretical maximum level of development relevant to these selected health outcomes. A location with an SDI approaching 0, on the other hand, would have the level of development relevant to these selected health outcomes approaching the theoretical minimum. However, an index score of exact 0 is not technically possible; for each component, we set a floor of 0.005 so that a single component doesn't pull the index score to 0

Using scales which define the upper and lower bound for each input, we computed the index scores underlying SDI as follows:

$$I_{Cly} = max(\frac{C_{ly} - C_{low}}{C_{high} - C_{low}}, 0.005)$$

Where I_{cly} – the index for covariate *C*, location *l*, and year *y* – is equal to the quotient of the difference between the value of that covariate in that location-year and the lower bound of the covariate and the difference between the upper and lower bounds for that covariate. If the values of input covariates fell above the upper bounds or below the lower bounds, they were set equal to the respective upper or lower bounds. The index value for TFU25 was computed as $1 - I_{TFU25ly}$, to account for the negative relationship between TFU25 and development, and thus between TFU25 and index score. For GBD 2019, we expanded the computation of SDI to 1062 national and subnational locations spanning the time period 1950–2019.

The composite SDI was calculated as the geometric mean of these three indices for a given location-year. The cutoff values used to determine quintiles for analysis were then computed using country-level estimates of SDI for the year 2019, excluding countries with populations less than 1 million. For subnational locations with a parent population of less than 200 million, we set the subnational quintile to the parent quintile.

For reporting, final SDI values were multiplied by 100 to facilitate interpretation and engagement.

Section 7: Additional methods

Section 7.1: Correlation estimation

To estimate correlation, we used the Pearson correlation coefficient in Stata 14, defined by the function below:[65]

$$\hat{\rho} = \frac{\sum_{i=1}^{n} w_i (x_i - \bar{x})(y_i - \bar{y})}{\sqrt{\sum_{i-1}^{n} w_i (x_i - \bar{x})^2}\sqrt{\sum_{i-1}^{n} w_i (y_i - \bar{y})^2}}$$

Where

w_i are the weights, if specified, or $w_i = 1$ if weights are not specified

$\bar{x} = (\sum w_i x_i)/(\sum w_i)$ is the mean of x

$\bar{y}$ is similarly defined

Section 7.2: Annualised rates of change

We calculated annualised rates of change by taking the log of ${}_5q_0$ in the target year $(y + t)$, dividing that by the ${}_5q_0$ at the baseline year y, and dividing that by the difference in years between the target and baseline years (t), using the following equation:

$$aroc_{{}_5q_0} = \frac{ln\left(\frac{{}_5q_{0\ y+t}}{{}_5q_{0\ y}}\right)}{t}$$

Section 7.3: Gross domestic product (GDP) and lag-distributed income

We used four gross domestic product (GDP) per capita series to calculate GDP per capita: World Bank World Development Indicators, International Monetary Fund World Economic Outlook report, Angus Maddison's research homepage at the University of Groningen Department of Economics, and the University of Pennsylvania (Penn) Center for International Comparisons of Production, Income, and Prices. We imputed each series separately using growth regressions and then averaged the four series to make a unified GDP per capita series for GBD. We used mixed effects regression with region-specific random effects to interpolate or extrapolate any country data that were missing from all four series. These methods are described in full in James and colleagues.[66]

Lag-distributed income (LDI) per capita is a moving average transformation of GDP per capita. We calculated LDI, a ten-year lagged average of GDP, with the formula below:

$$LDI_{pc,t} = \frac{1}{5.5}\left(GDPpc_t + \sum_{i=1}^{9} GDPpc_{t-i} \cdot \left(1 - \frac{i}{10}\right)\right)$$

where LDI at time t is a function of current GDP (at t) and the previous nine years of GDP with an inverse moving weight, and a normalisation factor (the sum of all weights).

Section 7.4: Educational attainment covariate

We based our estimates of average years of education on a collection of 3675 censuses and household surveys. The approach we used to estimate educational attainment, which we describe below, was based on previously published methods.[20,67] Each data source we used had information about the distribution of educational attainment by country or territory, year, sex, and five-year or ten-year age group. For sources that only provided education data for multi-year bins (, the percentage of the population with between two and five years of completed schooling), we split data into single-year distributions from 0 to 18 years based on a database of 1226 sources that did report single years of educational attainment, using the average of the 12 single-year distributions closest to the source in terms of geographical proximity and year. From the single-year distributions of each data source, we then calculated the mean years of education by age and sex.

In the next step, we used an age-cohort modelling process to carry educational attainment in observed cohorts forward through time, since education levels are relatively constant after age 25. For datapoints from cohorts aged 25 or older, we extrapolated the data forward and backward in time so all year-age combinations in that cohort contained that data (eg, a datapoint for a cohort aged 40–44 in 1995 was projected forward for 45–49-year-olds in 2000, 50–54-year-olds in 2005, etc. and backward for 35–39-year-olds in 1990, 30–34-year-olds in 1985, etc.).

Following cohort imputation, we fit age-period models to all data (i.e. the original input data and the imputed cohort data), allowing us to estimate a complete single-year series of educational attainment from 1950 through 2019 by age, sex, and location.

For each sex and GBD location, we calculated the mean level of educational attainment of the country-age-year-specific population, ($Edu_{c,a,\,s,t}$) using the following formula:

$$logit\left(\frac{Edu_{c,a,s,t}}{Edu_{max\,a}}\right) = \beta_{s,r} + \delta_{s,r}\,Year + I_{s,r} + \propto_{c,a,s}$$

$$\alpha_{c,a,s} \sim N(0, \sigma_{\alpha}^{2})$$

where:

c is location, a is age, s is sex, t is time

$Edu_{max\,a}$ is the maximum mean educational attainment for each age group, defined as 3 for ages 5-9, 8 for ages 10-14, 13 for ages 15-19, and 18 for all age groups 20-24 and up

$\beta_{s,r}$is a sex- and region-specific intercept

$\delta_{s,r}$ captures the linear secular trend for each sex and region

$I_{s,r}$is a natural spline on age to capture the non-linear age pattern by sex and region, with knots at 45 and 65 years of age

$\propto_{c,s}$ is a country-sex-specific random intercept.

Last, we used GPR to smooth the age-period model residuals. This step allowed us to account for uncertainty in each datapoint and combine data and model uncertainty to estimate UIs. These methods are described in more detail elsewhere.[68]

Section 8: References

1 Stevens G, Alkema L, Black R, et al. Guidelines for Accurate and Transparent Health Estimates Reporting: the GATHER statement. *The Lancet* 2016; **388**: 19–23.

2 Wang H, Dwyer-Lindgren L, Lofgren K, et al. Age-specific and sex-specific mortality in 187 countries, 1970–2010: a systematic analysis for the Global Burden of Disease Study 2010. *The Lancet* 2012; **380**: 2071–94.

3 Wang H, Abajobir A, Abate K, et al. Global, regional, and national under-5 mortality, adult mortality, age-specific mortality, and life expectancy, 1970–2016: a systematic analysis for the Global Burden of Disease Study 2016. *The Lancet* 2017; **390**: 1084–150.

4 Dicker D, Nguyen G, Abate D, et al. Global, regional, and national age-sex-specific mortality and life expectancy, 1950–2017: a systematic analysis for the Global Burden of Disease Study 2017. *The Lancet* 2018; **392**: 1684–735.

5 Murray CJL, Callender CSKH, Kulikoff XR. Population and fertility by age and sex for 195 countries and territories, 1950–2017: a systematic analysis for the Global Burden of Disease Study 2017. *The Lancet* 2018; **392**: 1995–2051.

6 Kyu HH, Abate D, Abate KH, *et al*. Global, regional, and national disability-adjusted life-years (DALYs) for 359 diseases and injuries and healthy life expectancy (HALE) for 195 countries and territories, 1990–2017: a systematic analysis for the Global Burden of Disease Study 2017. *The Lancet* 2018; **392**: 1859–922.

7 Rajaratnam J, Tran L, Lopez A, Murray C. Measuring under-five mortality: validation of new low-cost methods. *PLOS Med* 2010; **7**: e1000253.

8 Wang H, Liddell C, Coates M, et al. Global, regional, and national levels of neonatal, infant, and under-5 mortality during 1990–2013: a systematic analysis for the Global Burden of Disease Study 2013. *The Lancet* 2014; **384**: 957–79.

9 Murray C, Ezzati M, Flaxman A, et al. GBD 2010: design, definitions, and metrics. *The Lancet* 2012; **380**: 2063–6.

10 Wang H, Bhutta Z, Coates M, et al. Global, regional, national, and selected subnational levels of stillbirths, neonatal, infant, and under-5 mortality, 1980–2015: a systematic analysis for the Global Burden of Disease Study 2015. *The Lancet* 2016; **388**: 1725–74.

11 Gompertz B. Gompertz B. On one uniform law of mortality from birth to extreme old age, and on the law of sickness. J Inst Actuar Assur Mag 1871; 16: 329–44. *J Inst Actruar Assur Mag* 1871; **16**: 329–44.

12 The UN inter-agency group for child mortality estimation. Child mortality estimates. 2019. http://www.childmortality.org/ (accessed Jan 28, 2019).

13 Brocklehurst P, French R. The association between maternal HIV infection and perinatal outcome: a systematic review of the literature and meta-analysis. *Br J Obstet Gynaecol* 1998; **105**: 836–48.

14 Kim H-Y, Kasonde P, Mwiya M, et al. Pregnancy loss and role of infant HIV status on perinatal mortality among HIV-infected women. *BMC Pediatr* 2012; **12**: 138.

15 Kerber K, Lawn J, Johnson L, et al. South African child deaths 1990-2011: have HIV services reversed the trend enough to meet Millennium Development Goal 4? *AIDS Lond Engl* 2013; **27**: 2637–48.

16 Bradshaw D, Dorrington R. Child mortality in South Africa - we have lost touch. *Afr Med J* 2007; **97**: 582.

17 Murray C, Rajaratnam J, Marcus J, Laakso T, Lopez A. What can we conclude from death registration? *PLOS Med* 2010; **7**: e1000262.

18 Hill K. Estimating census and death registration completeness. *Asian Pac Popul Forum* 1987; **1**: 8–13, 23–4.

19 Brass W, Coale A. Methods of analysis and estimation in mathematical demography. In: Smith D, Keyfitz N, eds. Mathematical Demography. Biomathematics, vol 6. Springer, Berlin, Heidelberg, 1977: 307–13.

20 Preston S. The changing relation between mortality and level of economic development. *Popul Stud* 1975; **29**: 231–48.

21 Preston S, Coale A, Trussell J, Weinstein M. Estimating the completeness of reporting of adult deaths in populations that are approximately stable. *Popul Index* 1980; **46**: 179–202.

22 Bennett N, Horiuchi S. Estimating the completeness of death registration in a closed population. *Popul Index* 1981; **47**: 207–11.

23 Obermeyer Z, Rajaratnam J, Park C, et al. Measuring adult mortality using sibling survival: a new analytical method and new results for 44 countries, 1974–2006. *PLOS Med* 2010; **7**: e1000260.

24 Gakidou E, King G. Death by survey: estimating adult mortality without selection bias from sibling survival data. *Demography* 2006; **43**: 569–85.

25 Masquelier B. Adult mortality from sibling survival data: a reappraisal of selection biases. *Demography* 2013; **50**: 207–28.

26 Rogers R, Crimmins E, editors. International handbook of adult mortality. Springer Netherlands, 2011.

27 Murray C, Ferguson B, Lopez A, Guillot M, Salomon J, Ahmad O. Modified logit life table system: principles, empirical validation, and application. *Popul Stud* 2003; **57**: 165–82.

28 King G, Tomz M, Wittenberg J. Making the most of statistical analyses: improving interpretation and presentation. *Am J Polit Sci* 2000; **44**: 341–55.

29 Roth G, Abate D, Abate K, et al. Global, regional, and national age-sex-specific mortality for 282 causes of death in 195 countries and territories, 1980–2017: a systematic analysis for the Global Burden of Disease Study 2017. *The Lancet* 2018; **392**: 1736–88.

30 Wang H, Wolock T, Carter A, et al. Estimates of global, regional, and national incidence, prevalence, and mortality of HIV, 1980–2015: the Global Burden of Disease Study 2015. *Lancet HIV* 2016; **3**: e361-87.

31 War. Wikepedia. 2020; published online March 19. https://en.wikiquote.org/wiki/War (accessed March 25, 2020).

32 NATO. NATO's military concept for defence against terrorism. NATO. http://www.nato.int/cps/en/natohq/topics_69482.htm (accessed March 25, 2020).

33 Themnér L, Uppsala Conflict Data Program (UCDP). UCDP/PRIO Armed Conflict Dataset codebook version 18.1. Uppsala Conflict Data Program (UCDP) and Center for the Study of Civil Wars - International Peace Research Institute (PRIO), 2012.

34 Obermeyer Z, Murray C, Gakidou E. Fifty years of violent war deaths from Vietnam to Bosnia: analysis of data from the world health survey programme. *BMJ* 2008; **336**: 1482–6.

35 Leitenberg M. Rwanda, 1994: international incompetence produces genocide. *Peacekeeping Int Relat* 1994; **23**.

36 Jarvis C. The rise and fall of Albania's pyramid schemes. *Finance Dev* 2000; **37**.

37 Act of God. Wikipedia. 2019; published online Nov 16. https://en.wikipedia.org/w/index.php?title=Act_of_God&oldid=926475660 (accessed March 25, 2020).

38 Centre for Research on the Epidemiology of Disaster (CRED). EM-DAT: The OFDA/CRED International Disaster Database. Brussels: Catholic University of Leuven.

39 Schmid A. Terrorism - The Definitional Problem. *Case West Reserve J Int Law JIL* 2004; **36**: 375–419.

40 TMF Health Quality Institute, the Medicare Quality Improvement Organization for Texas. Protein-Energy Malnutrition Definition. http://healthinsight.org/Internal/assets/Nursing%20Home/PRU%20-%20Protein%20Energy%20and%20Malnutrition.pdf.

41 Jisheng Y, Friedman E, Guo J, Mosher S. Tombstone: the great Chinese famine, 1958-1962. New York: Farrar, Straus and Giroux (Macmillan), 2012.

42 Inc G, Berger D. Bacterial meningitis: global status: 2017 edition. GIDEON Informatics Inc, 2017.

43 Inc G, Berger D. Cholera: global status: 2017 edition. GIDEON Informatics Inc, 2017.

44 Documented civilian deaths from violence. Iraq Body Count. 2003; published online 2020. https://www.iraqbodycount.org/database/ (accessed May 8, 2017).

45 Hagopian A, Flaxman A, Takaro T, et al. Mortality in Iraq associated with the 2003–2011 war and occupation: findings from a national cluster sample survey by the University Collaborative Iraq Mortality Study. *PLOS Med* 2013; **10**: e1001533.

46 Wang H, Naghavi M, Allen C, *et al*. Global, regional, and national life expectancy, all-cause mortality, and cause-specific mortality for 249 causes of death, 1980–2015: a systematic analysis for the Global Burden of Disease Study 2015. *The Lancet* 2016; **388**: 1459–544.

47 Wang H, Abajobir AA, Abate KH, *et al*. Global, regional, and national under-5 mortality, adult mortality, age-specific mortality, and life expectancy, 1970–2016: a systematic analysis for the Global Burden of Disease Study 2016. *The Lancet* 2017; **390**: 1084–150.

48 United Nations Department of Economic and Social Affairs. United Nations demographic yearbook. New York: United Nations, 2019.

49 Max Planck Institute for Demographic Research (Germany), Vienna Institute of Demography (Austria). Human fertility collection. 2018. www.fertilitydata.org.

50 Preston S, Heuveline P, Guillot M. Demography: measuring and modeling population processes, 1 edition. Wiley-Blackwell, 2000.

51 GBD 2019 Diseases, Injuries, and Impairments Collaborators. Global burden of 369 diseases and injuries in 204 countries and territories, 1990–2019: a systematic analysis for the Global Burden of Disease Study 2019. *The Lancet* in press.

52 United Nations Department of Economic and Social Affairs Statistics Division. Welcome to UNSD. 2020. https://unstats.un.org/home/ (accessed April 7, 2018).

53 United Nations Department of Economic and Social Affairs. Population. http://www.un.org/en/development/desa/population/ (accessed April 7, 2018).

54 IPUMS USA. U.S. census data for social, economic, and health research. 2019. https://usa.ipums.org/usa/ (accessed April 7, 2018).

55 The University of Texas at Austin College of Liberal Arts. Welcome to the Population Research Center (PRC). 2020. https://liberalarts.utexas.edu/prc/.

56 WorldCat. WorldCat. https://www.worldcat.org/.

57 Arriaga EE, Johnson PD, Jamison E. Population analysis with microcomputers - Volume 1 Presentation of Techniques. 1994; published online Nov. https://www2.census.gov/software/pas/documentation/pamvi-archive.pdf.

58 Flaxman AD, Vos T, Murray CJL, Kiyono P, editors. An integrative metaregression framework for descriptive epidemiology, 1 edition. Seattle: University of Washington Press, 2015.

59 James SL, Abate D, Abate KH, *et al*. Global, regional, and national incidence, prevalence, and years lived with disability for 354 diseases and injuries for 195 countries and territories, 1990–2017: a systematic analysis for the Global Burden of Disease Study 2017. *The Lancet* 2018; **392**: 1789–858.

60 Wand M, Ripley B. KernSmooth: functions for Kernel Smoothing supporting Wand & Jones (1995). 2015 https://CRAN.R-project.org/package=KernSmooth (accessed July 10, 2018).

61 UNCHR. UNHCR - The UN Refugee Agency. 2001; published online 2020. http://www.unhcr.org/en-us/ (accessed April 7, 2018).

62 European Commission. eurostat: your key to European statistics. 2020. http://ec.europa.eu/eurostat (accessed April 8, 2018).

63 European University Institute and Gulf Research Center. Gulf Labour Markets, Migration, and Population (GLMM) Programme. GLMM. 2020. http://gulfmigration.eu/ (accessed April 7, 2018).

64 Wheldon M, Raftery A, Clark S, Gerland P. Reconstructing past populations with uncertainty from fragmentary data. *J Am Stat Assoc* 2013; **108**: 96–110.

65 Stata:Corp. Stata: release 13. College Station, TX: StataCorp LP, 2013.

66 James S, Gubbins P, Murray C, Gakidou E. Developing a comprehensive time series of GDP per capita for 210 countries from 1950 to 2015. *Popul Health Metr* 2012; **10**: 12.

67 Gakidou E, Cowling K, Lozano R, Murray C. Increased educational attainment and its effect on child mortality in 175 countries between 1970 and 2009: a systematic analysis. *Lancet Lond Eng* 2010; **376**: 959–74.

68 Friedman J, York H, Graetz N, *et al.* Measuring and forecasting progress towards the education-related SDG targets. *Nature* 2020; **580**: 636–9.

Section 9: Appendix table and figures

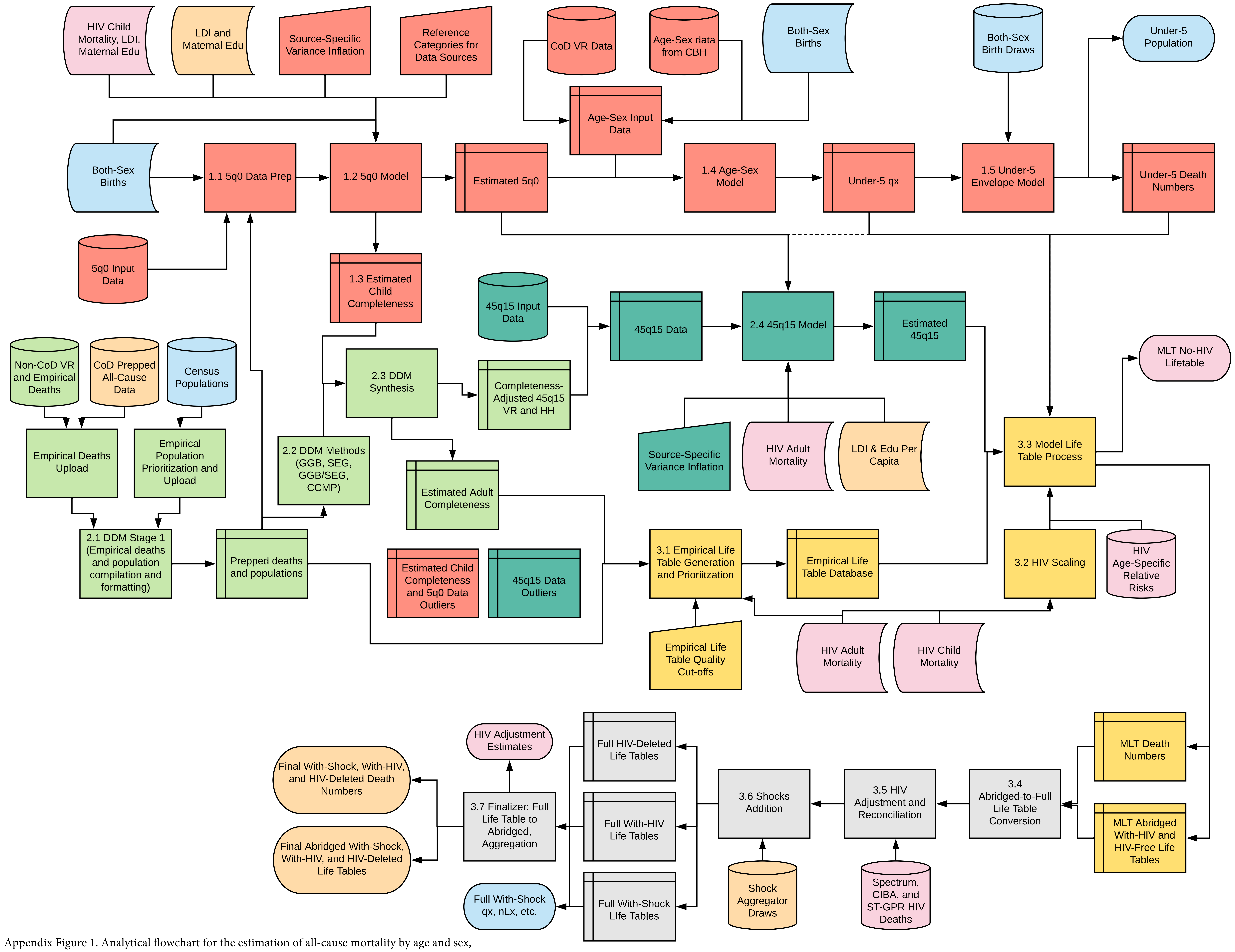

Appendix Figure 1. Analytical flowchart for the estimation of all-cause mortality by age and sex, and HIV/AIDS incidence, prevalence, and mortality for GBD 2019

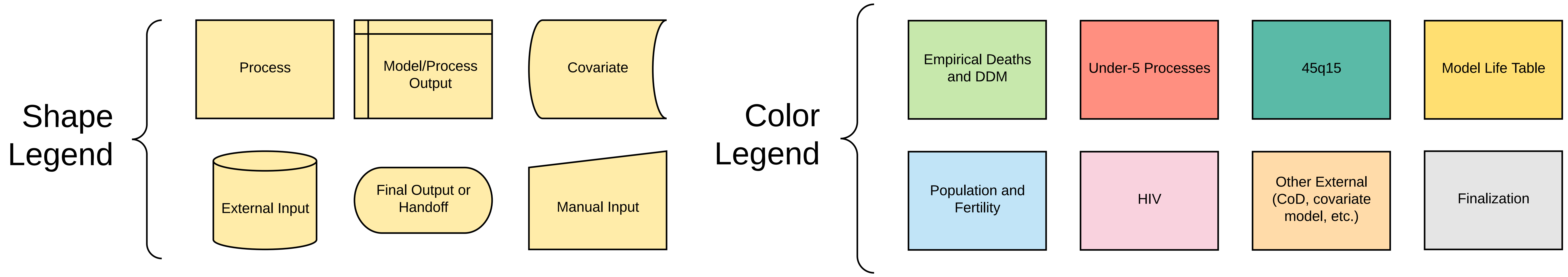

Appendix Figure 2. Estimated completeness of under−5 death registration, 1950−2019

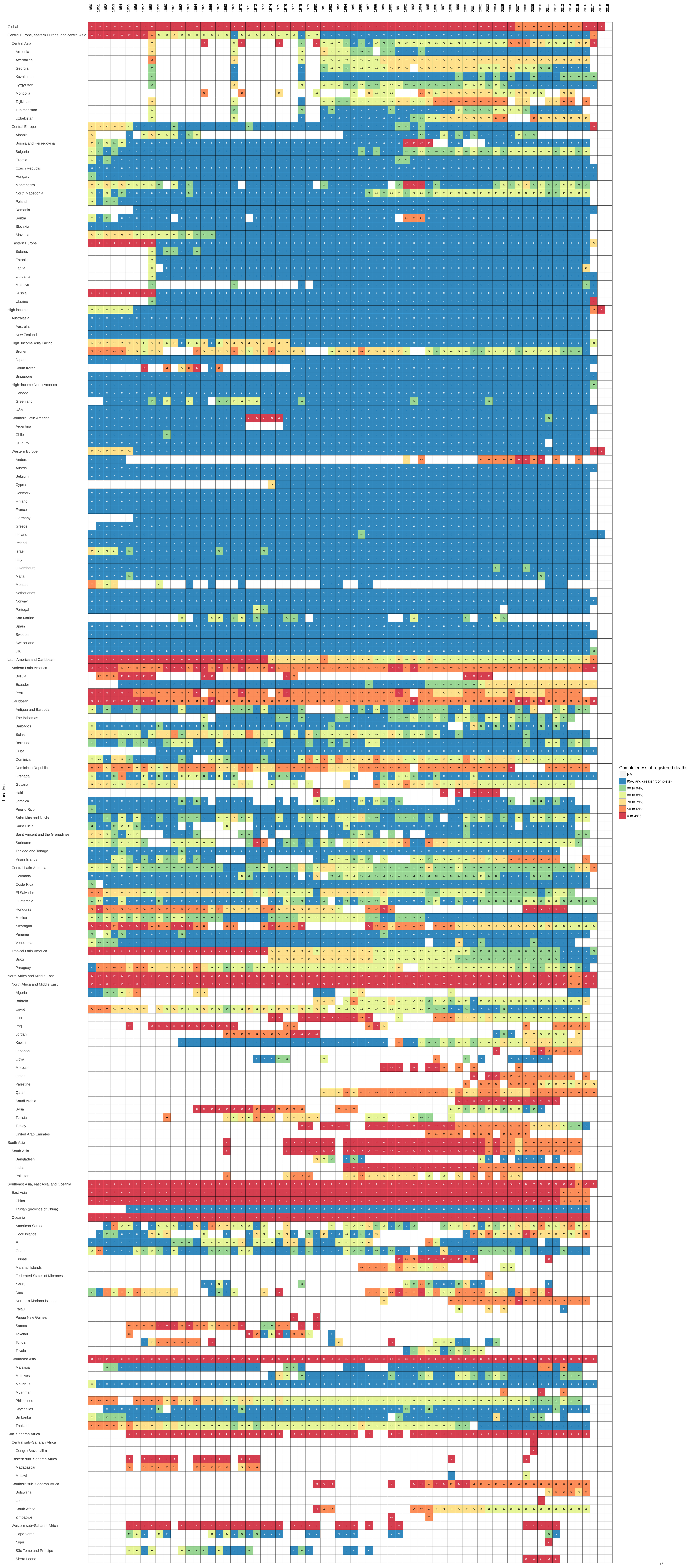

Appendix Figure 3. Analytical flowchart for the GBD 2019 fertility estimation process

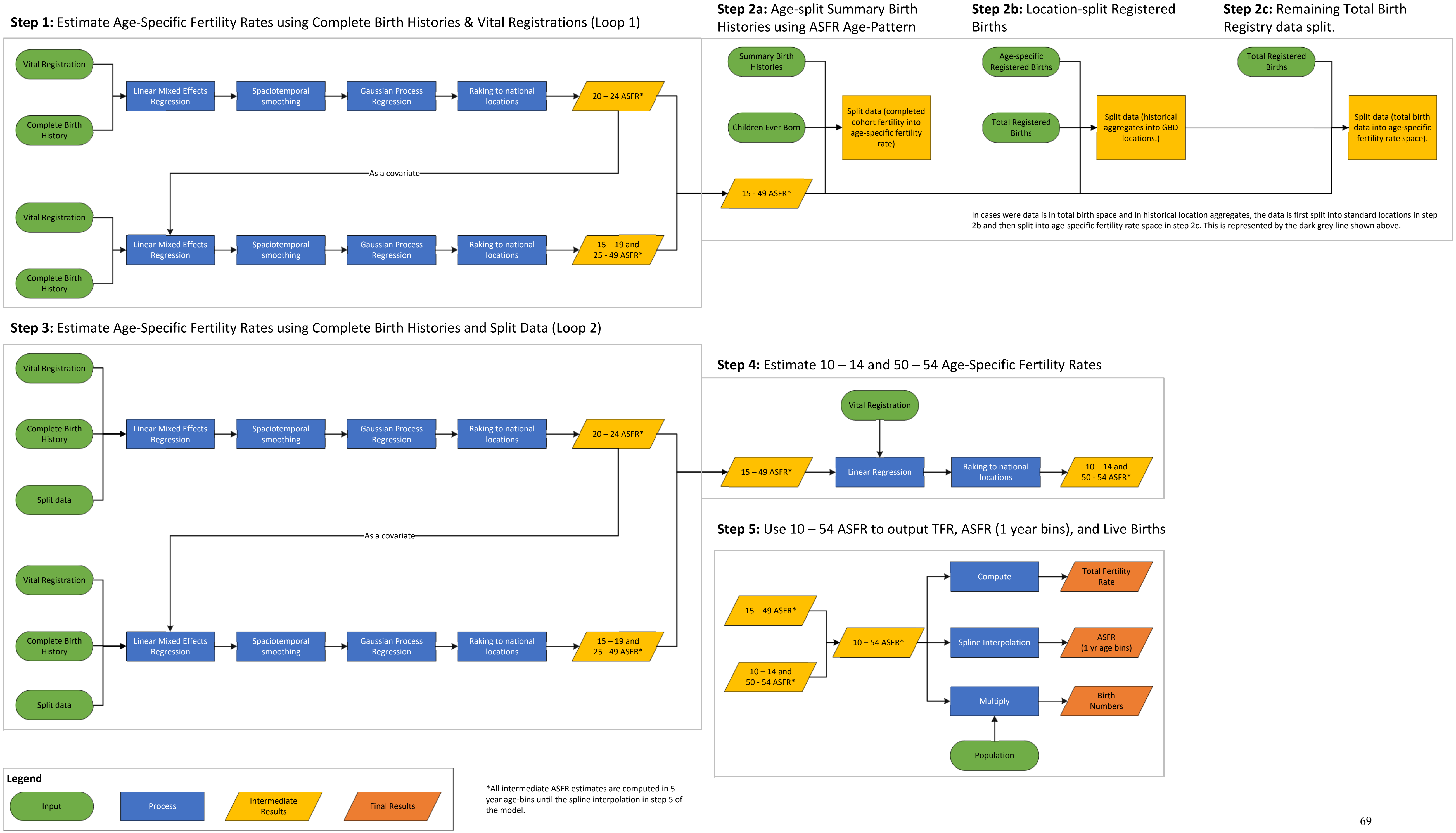

*All intermediate ASFR estimates are computed in 5 year age-bins until the spline interpolation in step 5 of the model.

Appendix Figure 4. Analytical flowchart for the GBD 2019 population estimation process

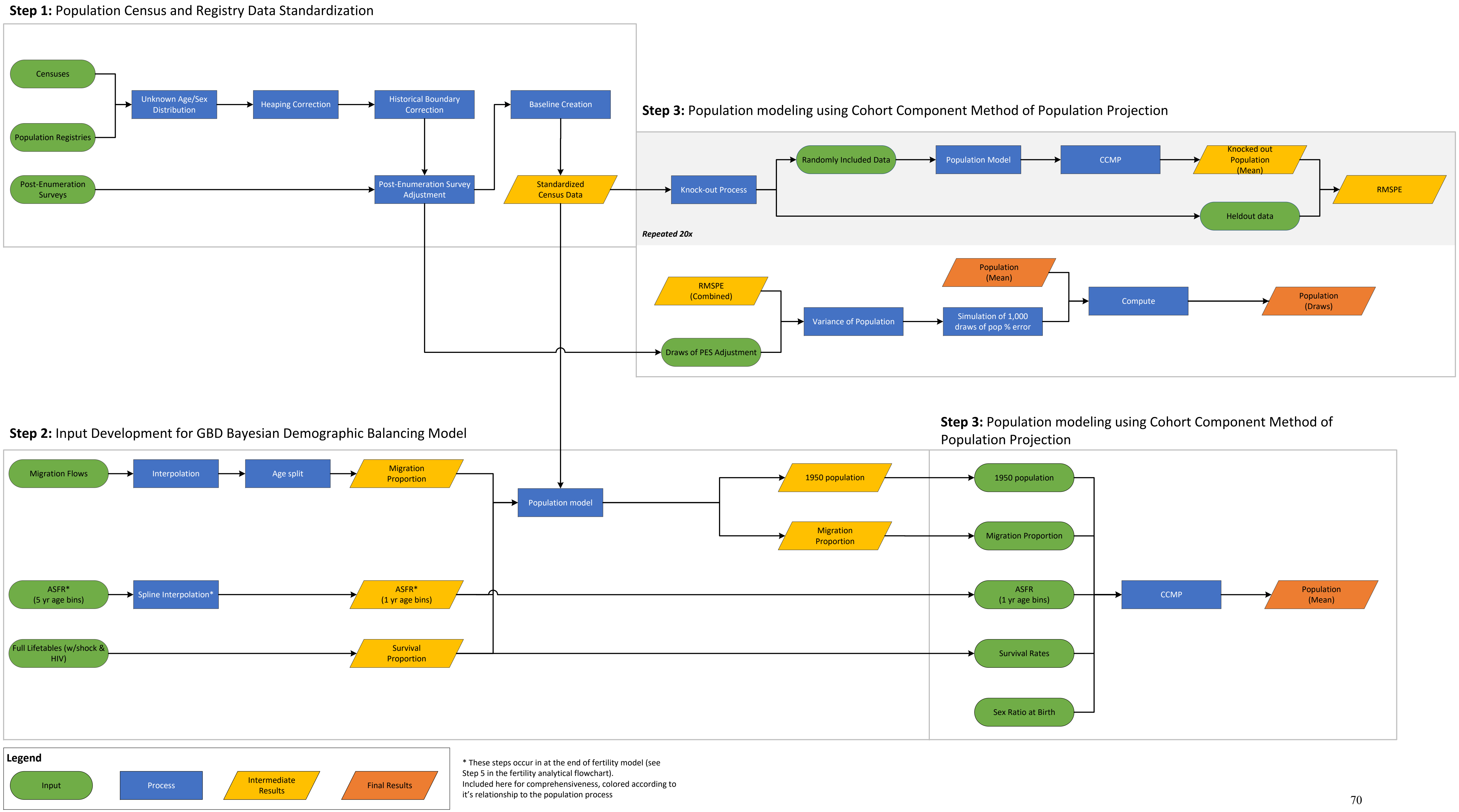

* These steps occur in at the end of fertility model (see Step 5 in the fertility analytical flowchart).
Included here for comprehensiveness, colored according to it's relationship to the population process

Appendix Figure 5. Census and registry availability by location and year

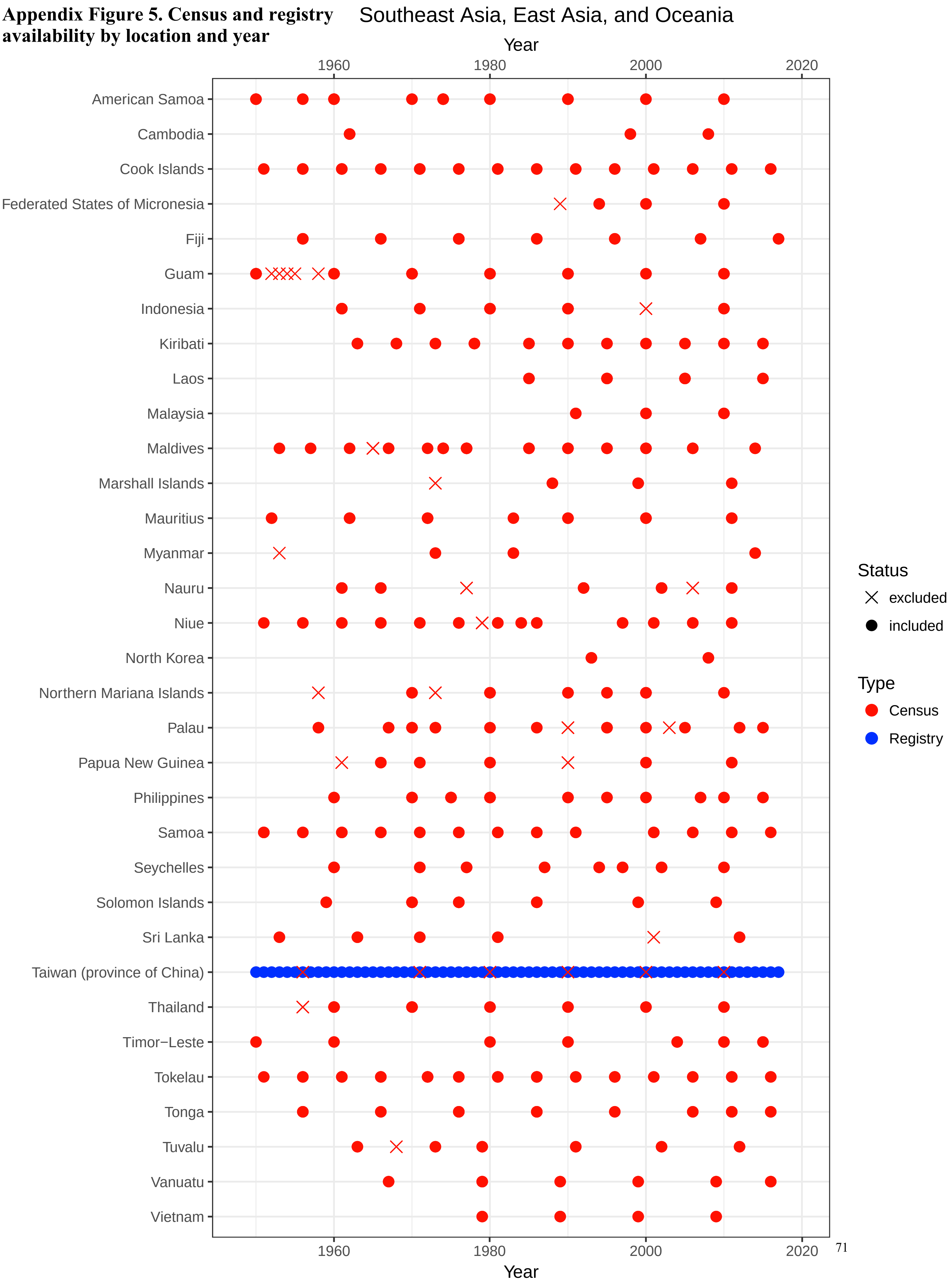

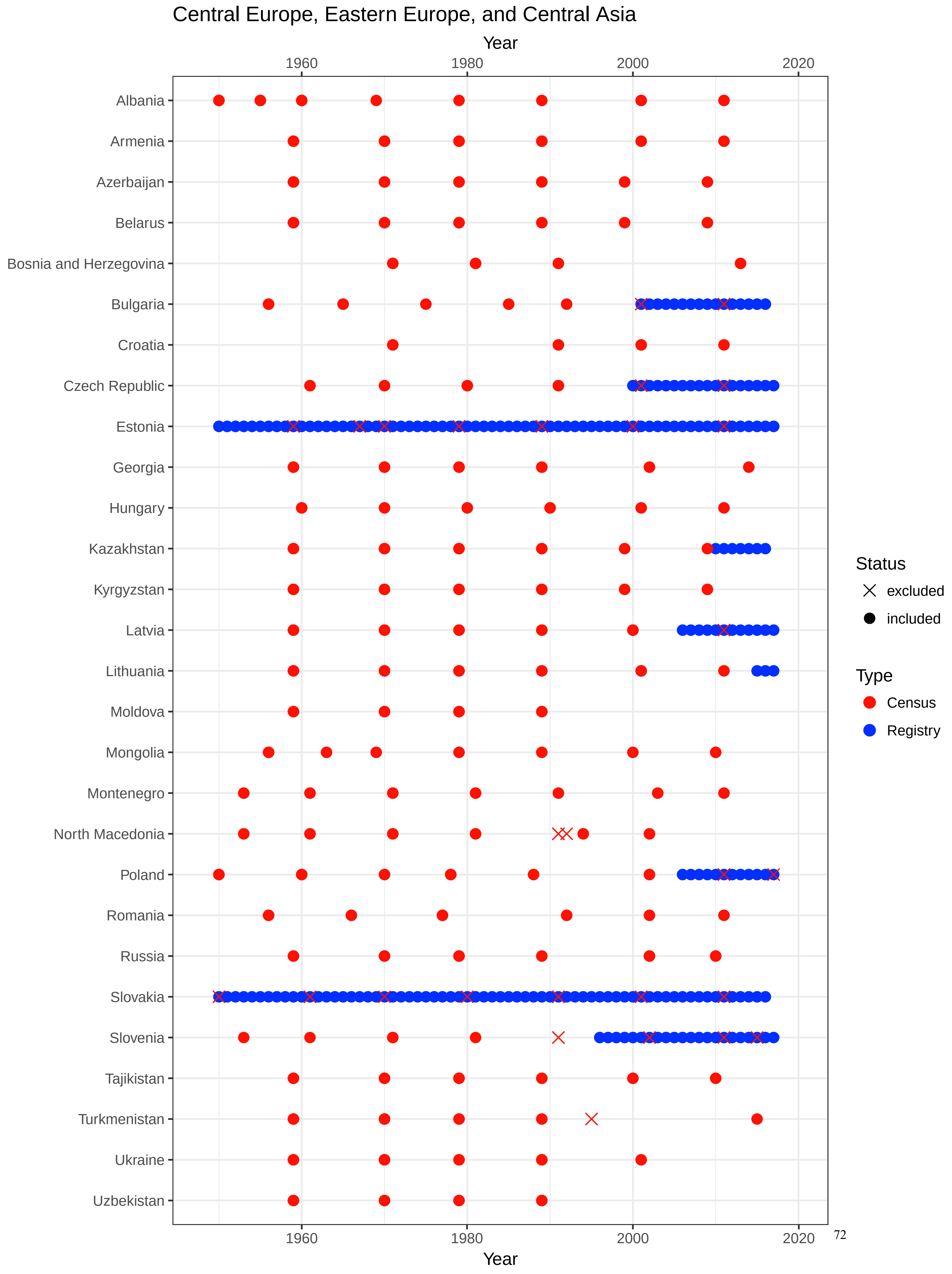
Central Europe, Eastern Europe, and Central Asia
Year
1960
1980
2000
2020
Albania
Armenia
Azerbaijan
Belarus
Bosnia and Herzegovina
Bulgaria
Croatia
Czech Republic
Estonia
Georgia
Hungary
Kazakhstan
Kyrgyzstan
Latvia
Lithuania
Moldova
Mongolia
Montenegro
North Macedonia
Poland
Romania
Russia
Slovakia
Slovenia
Tajikistan
Turkmenistan
Ukraine
Uzbekistan
Status
excluded
included
Type
Census
Registry

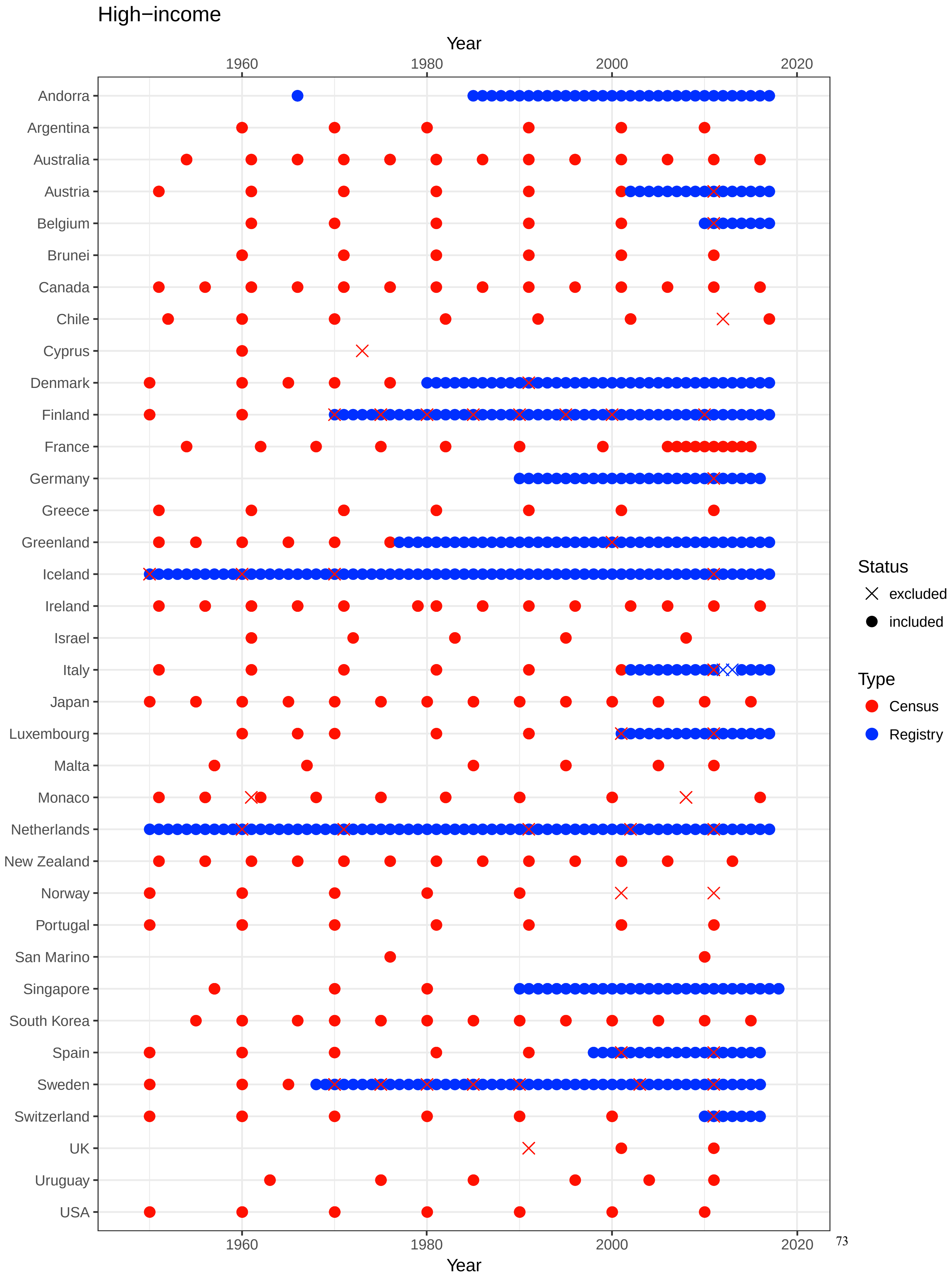

High−income
Year
1960
1980
2000
2020
Andorra
Argentina
Australia
Austria
Belgium
Brunei
Canada
Chile
Cyprus
Denmark
Finland
France
Germany
Greece
Greenland
Iceland
Ireland
Israel
Italy
Japan
Luxembourg
Malta
Monaco
Netherlands
New Zealand
Norway
Portugal
San Marino
Singapore
South Korea
Spain
Sweden
Switzerland
UK
Uruguay
USA
Status
excluded
included
Type
Census
Registry

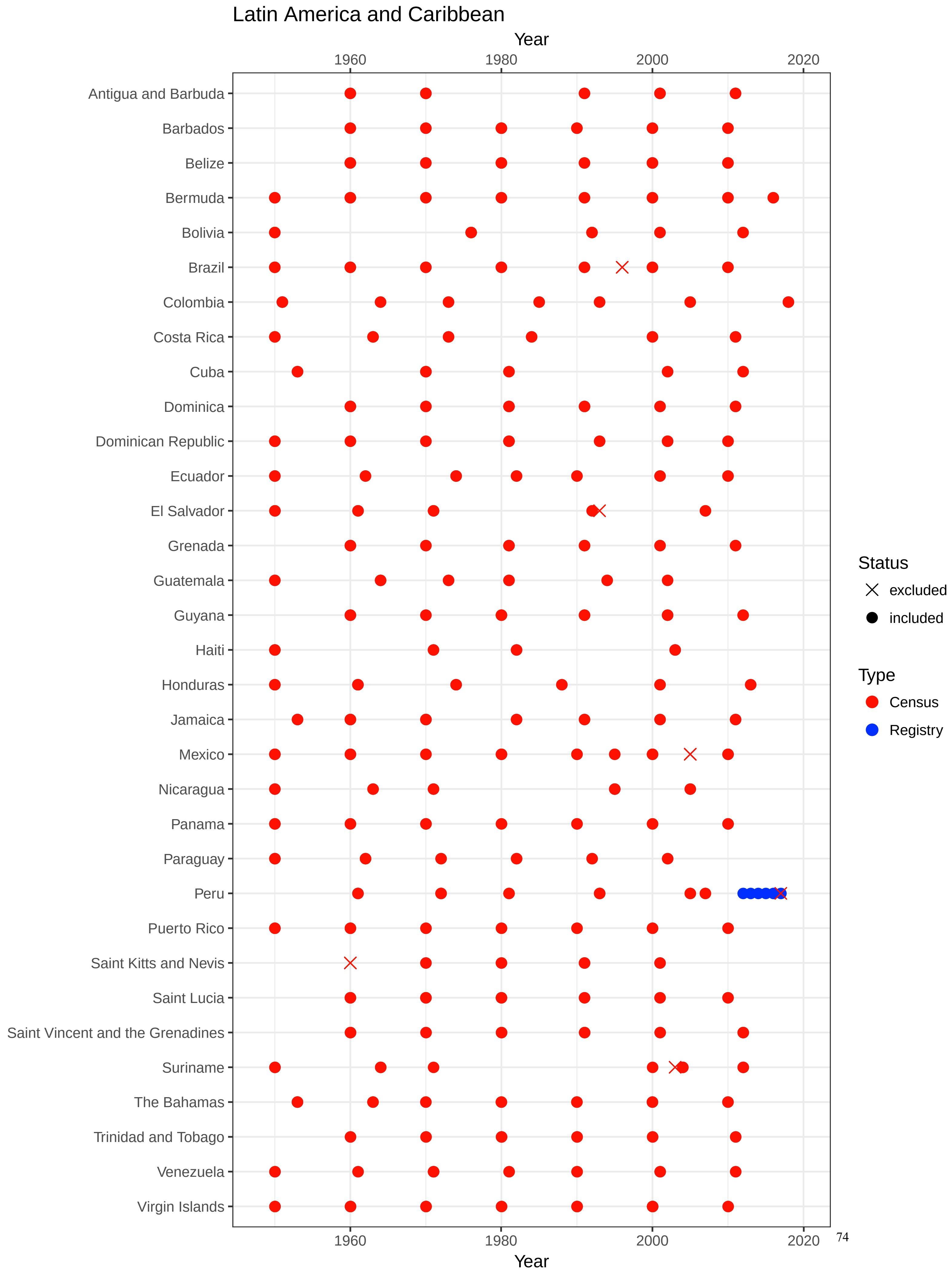

Latin America and Caribbean
Year
1960
1980
2000
2020
Antigua and Barbuda
Barbados
Belize
Bermuda
Bolivia
Brazil
Colombia
Costa Rica
Cuba
Dominica
Dominican Republic
Ecuador
El Salvador
Grenada
Guatemala
Guyana
Haiti
Honduras
Jamaica
Mexico
Nicaragua
Panama
Paraguay
Peru
Puerto Rico
Saint Kitts and Nevis
Saint Lucia
Saint Vincent and the Grenadines
Suriname
The Bahamas
Trinidad and Tobago
Venezuela
Virgin Islands
1960
1980
2000
2020
Year
Status
excluded
included
Type
Census
Registry

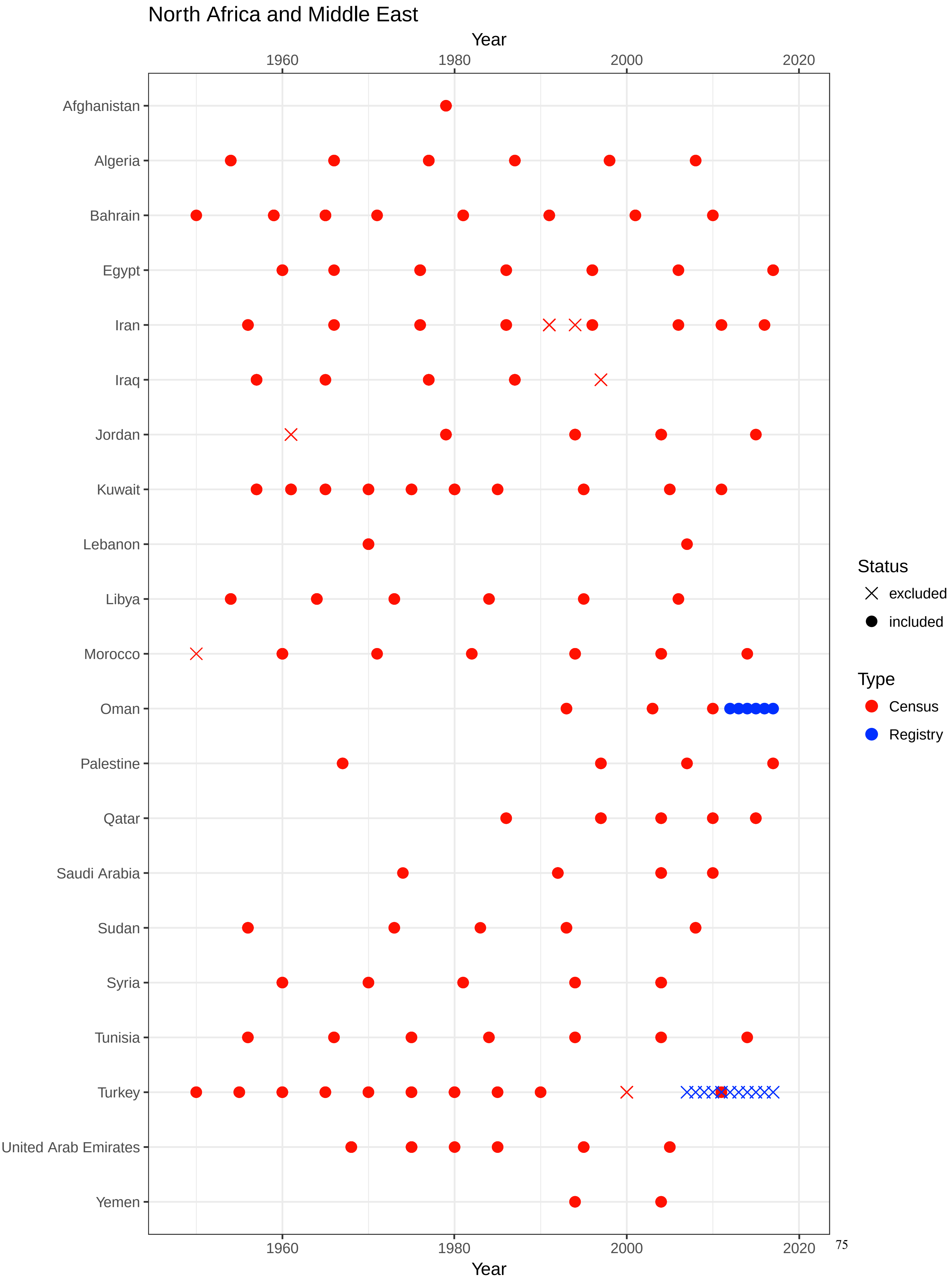
North Africa and Middle East
Year
1960
1980
2000
2020
Afghanistan
Algeria
Bahrain
Egypt
Iran
Iraq
Jordan
Kuwait
Lebanon
Libya
Morocco
Oman
Palestine
Qatar
Saudi Arabia
Sudan
Syria
Tunisia
Turkey
United Arab Emirates
Yemen
Status
excluded
included
Type
Census
Registry

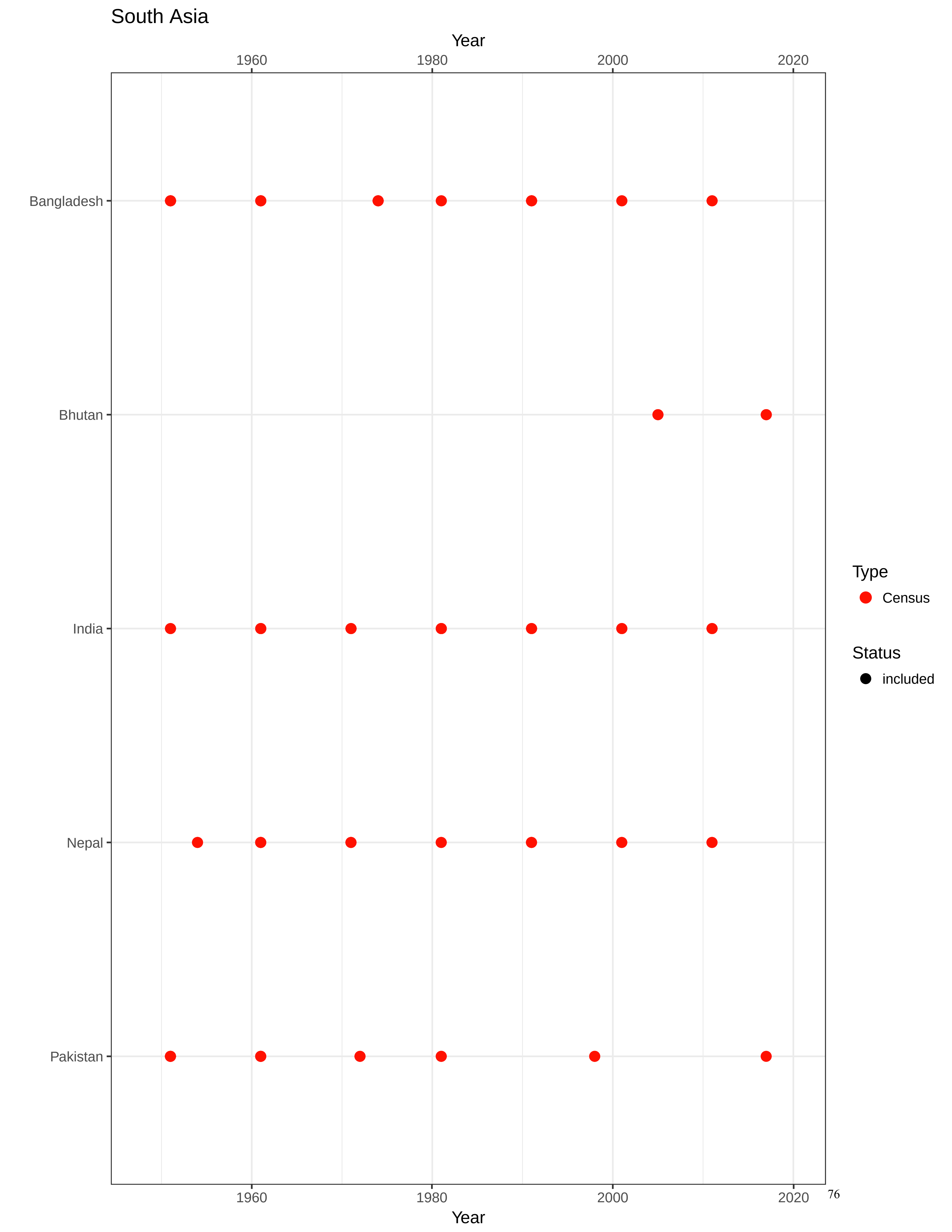
South Asia
Year
1960
1980
2000
2020
Bangladesh
Bhutan
India
Nepal
Pakistan
Type
Census
Status
included
Year

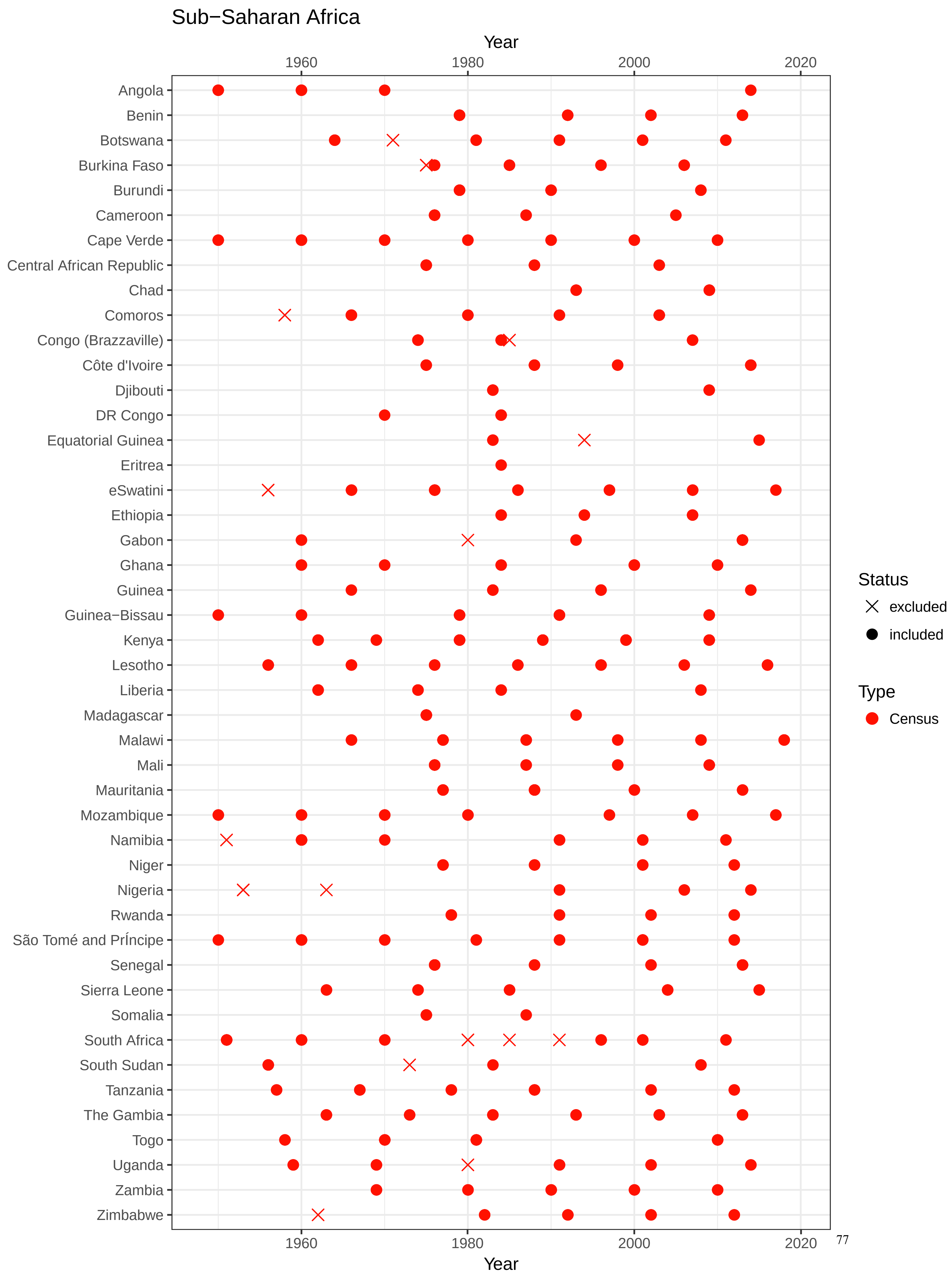

Sub−Saharan Africa
Year
1960
1980
2000
2020
Angola
Benin
Botswana
Burkina Faso
Burundi
Cameroon
Cape Verde
Central African Republic
Chad
Comoros
Congo (Brazzaville)
Côte d'Ivoire
Djibouti
DR Congo
Equatorial Guinea
Eritrea
eSwatini
Ethiopia
Gabon
Ghana
Guinea
Guinea−Bissau
Kenya
Lesotho
Liberia
Madagascar
Malawi
Mali
Mauritania
Mozambique
Namibia
Niger
Nigeria
Rwanda
São Tomé and PrÍncipe
Senegal
Sierra Leone
Somalia
South Africa
South Sudan
Tanzania
The Gambia
Togo
Uganda
Zambia
Zimbabwe
Status
excluded
included
Type
Census

Appendix Table 1. GBD location hierarchy with levels	
**Geography**	**Level**
Global	0
Low SDI	1
Low-middle SDI	1
Middle SDI	1
High-middle SDI	1
High SDI	1
Central Europe, Eastern Europe, and Central Asia	1
Central Asia	2
Armenia	3
Azerbaijan	3
Georgia	3
Kazakhstan	3
Kyrgyzstan	3
Mongolia	3
Tajikistan	3
Turkmenistan	3
Uzbekistan	3
Central Europe	2
Albania	3
Bosnia and Herzegovina	3
Bulgaria	3
Croatia	3
Czech Republic	3

Hungary	3
Montenegro	3
Macedonia	3
Poland	3
Romania	3
Serbia	3
Slovakia	3
Slovenia	3
Eastern Europe	2
Belarus	3
Estonia	3
Latvia	3
Lithuania	3
Moldova	3
Russian Federation	3
Ukraine	3
High-income	1
Australasia	2
Australia	3
New Zealand	3
High-income Asia-Pacific	2
Brunei	3
Japan	3
Aichi	4
Akita	4

Aomori	4
Chiba	4
Ehime	4
Fukui	4
Fukuoka	4
Fukushima	4
Gifu	4
Gunma	4
Hiroshima	4
Hokkaidō	4
Hyōgo	4
Ibaraki	4
Ishikawa	4
Iwate	4
Kagawa	4
Kagoshima	4
Kanagawa	4
Kōchi	4
Kumamoto	4
Kyōto	4
Mie	4
Miyagi	4
Miyazaki	4
Nagano	4
Nagasaki	4

Nara	4
Niigata	4
Ōita	4
Okayama	4
Okinawa	4
Ōsaka	4
Saga	4
Saitama	4
Shiga	4
Shimane	4
Shizuoka	4
Tochigi	4
Tokushima	4
Tōkyō	4
Tottori	4
Toyama	4
Wakayama	4
Yamagata	4
Yamaguchi	4
Yamanashi	4
South Korea	3
Singapore	3
High-income North America	2
Canada	3
Greenland	3

USA	3
Alabama	4
Alaska	4
Arizona	4
Arkansas	4
California	4
Colorado	4
Connecticut	4
Delaware	4
Washington, DC	4
Florida	4
Georgia	4
Hawaii	4
Idaho	4
Illinois	4
Indiana	4
Iowa	4
Kansas	4
Kentucky	4
Louisiana	4
Maine	4
Maryland	4
Massachusetts	4
Michigan	4
Minnesota	4

Mississippi	4
Missouri	4
Montana	4
Nebraska	4
Nevada	4
New Hampshire	4
New Jersey	4
New Mexico	4
New York	4
North Carolina	4
North Dakota	4
Ohio	4
Oklahoma	4
Oregon	4
Pennsylvania	4
Rhode Island	4
South Carolina	4
South Dakota	4
Tennessee	4
Texas	4
Utah	4
Vermont	4
Virginia	4
Washington	4
West Virginia	4

Wisconsin	4
Wyoming	4
Southern Latin America	2
Argentina	3
Chile	3
Uruguay	3
Western Europe	2
Andorra	3
Austria	3
Belgium	3
Cyprus	3
Denmark	3
Finland	3
France	3
Germany	3
Greece	3
Iceland	3
Ireland	3
Israel	3
Italy	3
Luxembourg	3
Malta	3
Netherlands	3
Norway	3
Portugal	3

Principality of Monaco	3
Republic of San Marino	3
Spain	3
Sweden	3
Stockholm	4
Sweden except Stockholm	4
Switzerland	3
United Kingdom	3
England	4
East Midlands	5
Derby	6
Derbyshire	6
Leicester	6
Leicestershire	6
Lincolnshire	6
Northamptonshire	6
Nottingham	6
Nottinghamshire	6
Rutland	6
East of England	5
Bedford	6
Cambridgeshire	6
Central Bedfordshire	6
Essex	6
Hertfordshire	6

Luton	6
Norfolk	6
Peterborough	6
Southend-on-Sea	6
Suffolk	6
Thurrock	6
Greater London	5
Barking and Dagenham	6
Barnet	6
Bexley	6
Brent	6
Bromley	6
Camden	6
Croydon	6
Ealing	6
Enfield	6
Greenwich	6
Hackney	6
Hammersmith and Fulham	6
Haringey	6
Harrow	6
Havering	6
Hillingdon	6
Hounslow	6
Islington	6

Kensington and Chelsea	6
Kingston upon Thames	6
Lambeth	6
Lewisham	6
Merton	6
Newham	6
Redbridge	6
Richmond upon Thames	6
Southwark	6
Sutton	6
Tower Hamlets	6
Waltham Forest	6
Wandsworth	6
Westminster	6
North East England	5
County Durham	6
Darlington	6
Gateshead	6
Hartlepool	6
Middlesbrough	6
Newcastle upon Tyne	6
North Tyneside	6
Northumberland	6
Redcar and Cleveland	6
South Tyneside	6

Stockton-on-Tees	6
Sunderland	6
North West England	5
Blackburn with Darwen	6
Blackpool	6
Bolton	6
Bury	6
Cheshire East	6
Cheshire West and Chester	6
Cumbria	6
Halton	6
Knowsley	6
Lancashire	6
Liverpool	6
Manchester	6
Oldham	6
Rochdale	6
Salford	6
Sefton	6
St Helens	6
Stockport	6
Tameside	6
Trafford	6
Warrington	6
Wigan	6

Wirral	6
South East England	5
Bracknell Forest	6
Brighton and Hove	6
Buckinghamshire	6
East Sussex	6
Hampshire	6
Isle of Wight	6
Kent	6
Medway	6
Milton Keynes	6
Oxfordshire	6
Portsmouth	6
Reading	6
Slough	6
Southampton	6
Surrey	6
West Berkshire	6
West Sussex	6
Windsor and Maidenhead	6
Wokingham	6
South West England	5
Bath and North East Somerset	6
Bournemouth	6
Bristol, City of	6

Cornwall	6
Devon	6
Dorset	6
Gloucestershire	6
North Somerset	6
Plymouth	6
Poole	6
Somerset	6
South Gloucestershire	6
Swindon	6
Torbay	6
Wiltshire	6
West Midlands	5
Birmingham	6
Coventry	6
Dudley	6
Herefordshire, County of	6
Sandwell	6
Shropshire	6
Solihull	6
Staffordshire	6
Stoke-on-Trent	6
Telford and Wrekin	6
Walsall	6
Warwickshire	6

Wolverhampton	6
Worcestershire	6
Yorkshire and the Humber	5
Barnsley	6
Bradford	6
Calderdale	6
Doncaster	6
East Riding of Yorkshire	6
Kingston upon Hull, City of	6
Kirklees	6
Leeds	6
North East Lincolnshire	6
North Lincolnshire	6
North Yorkshire	6
Rotherham	6
Sheffield	6
Wakefield	6
York	6
Northern Ireland	4
Scotland	4
Wales	4
Latin America and Caribbean	1
Andean Latin America	2
Ecuador	3
Peru	3

Bolivia	3
Caribbean	2
Antigua and Barbuda	3
Barbados	3
Belize	3
Bermuda	3
The Bahamas	3
Cuba	3
Dominica	3
Dominican Republic	3
Grenada	3
Guyana	3
Haiti	3
Jamaica	3
Puerto Rico	3
Saint Kitts and Nevis	3
Saint Lucia	3
Saint Vincent and the Grenadines	3
Suriname	3
Trinidad and Tobago	3
Virgin Islands	3
Central Latin America	2
Venezuela	3
Colombia	3
Costa Rica	3

El Salvador	3
Guatemala	3
Honduras	3
Mexico	3
Aguascalientes	4
Baja California	4
Baja California Sur	4
Campeche	4
Chiapas	4
Chihuahua	4
Coahuila	4
Colima	4
Durango	4
Guanajuato	4
Guerrero	4
Hidalgo	4
Jalisco	4
México	4
Mexico City	4
Michoacán de Ocampo	4
Morelos	4
Nayarit	4
Nuevo León	4
Oaxaca	4
Puebla	4

Querétaro	4
Quintana Roo	4
San Luis Potosí	4
Sinaloa	4
Sonora	4
Tabasco	4
Tamaulipas	4
Tlaxcala	4
Veracruz de Ignacio de la Llave	4
Yucatán	4
Zacatecas	4
Nicaragua	3
Panama	3
Tropical Latin America	2
Brazil	3
Acre	4
Alagoas	4
Amapá	4
Amazonas	4
Bahia	4
Ceará	4
Distrito Federal	4
Espírito Santo	4
Goiás	4
Maranhão	4

Mato Grosso	4
Mato Grosso do Sul	4
Minas Gerais	4
Pará	4
Paraíba	4
Paraná	4
Pernambuco	4
Piauí	4
Rio de Janeiro	4
Rio Grande do Norte	4
Rio Grande do Sul	4
Rondônia	4
Roraima	4
Santa Catarina	4
São Paulo	4
Sergipe	4
Tocantins	4
Paraguay	3
North Africa and Middle East	1
North Africa and Middle East	2
Afghanistan	3
Algeria	3
Bahrain	3
Egypt	3
Iraq	3

Iran	3
Jordan	3
Kuwait	3
Lebanon	3
Libya	3
Morocco	3
Palestine	3
Oman	3
Qatar	3
Saudi Arabia	3
Sudan	3
Syria	3
Tunisia	3
Turkey	3
United Arab Emirates	3
Yemen	3
South Asia	1
South Asia	2
Bangladesh	3
Bhutan	3
India	3
Andhra Pradesh	4
Arunachal Pradesh	4
Assam	4
Bihar	4

Chhattisgarh	4
Delhi	4
Goa	4
Gujarat	4
Haryana	4
Himachal Pradesh	4
Jammu and Kashmir	4
Jharkhand	4
Karnataka	4
Kerala	4
Madhya Pradesh	4
Maharashtra	4
Manipur	4
Meghalaya	4
Mizoram	4
Nagaland	4
Odisha	4
Punjab	4
Rajasthan	4
Sikkim	4
Tamil Nadu	4
Telangana	4
Tripura	4
Union Territories other than Delhi	4
Uttar Pradesh	4

Uttarakhand	4
West Bengal	4
Nepal	3
Pakistan	3
Southeast Asia, East Asia, and Oceania	1
East Asia	2
China	3
North Korea	3
Taiwan (Province of China)	3
Oceania	2
American Samoa	3
Cook Islands	3
Federated States of Micronesia	3
Fiji	3
Guam	3
Kiribati	3
Marshall Islands	3
Northern Mariana Islands	3
Papua New Guinea	3
Republic of Nauru	3
Republic of Niue	3
Republic of Palau	3
Samoa	3
Solomon Islands	3
Tokelau	3

Tonga	3
Tuvalu	3
Vanuatu	3
Southeast Asia	2
Cambodia	3
Indonesia	3
Aceh	4
Bali	4
Bangka-Belitung Islands	4
Banten	4
Bengkulu	4
Gorontalo	4
Jakarta	4
Jambi	4
West Java	4
Central Java	4
East Java	4
West Kalimantan	4
South Kalimantan	4
Central Kalimantan	4
East Kalimantan	4
North Kalimantan	4
Riau Islands	4
Lampung	4
Maluku	4

North Maluku	4
West Nusa Tenggara	4
East Nusa Tenggara	4
Papua	4
West Papua	4
Riau	4
West Sulawesi	4
South Sulawesi	4
Central Sulawesi	4
Southeast Sulawesi	4
North Sulawesi	4
West Sumatra	4
South Sumatra	4
North Sumatra	4
Yogyakarta	4
Laos	3
Malaysia	3
Maldives	3
Mauritius	3
Myanmar	3
Philippines	3
Sri Lanka	3
Seychelles	3
Vietnam	3
Thailand	3

Timor-Leste	3
Sub-Saharan Africa	1
Central sub-Saharan Africa	2
Angola	3
Central African Republic	3
Congo (Brazzaville)	3
DR Congo	3
Equatorial Guinea	3
Gabon	3
Eastern sub-Saharan Africa	2
Burundi	3
Comoros	3
Djibouti	3
Eritrea	3
Ethiopia	3
Kenya	3
Baringo	4
Bomet	4
Bungoma	4
Busia	4
Elgeyo Marakwet	4
Embu	4
Garissa	4
Homa Bay	4
Isiolo	4

Kajiado	4
Kakamega	4
Kericho	4
Kiambu	4
Kilifi	4
Kirinyaga	4
Kisii	4
Kisumu	4
Kitui	4
Kwale	4
Laikipia	4
Lamu	4
Machakos	4
Makueni	4
Mandera	4
Marsabit	4
Meru	4
Migori	4
Mombasa	4
Murang'a	4
Nairobi	4
Nakuru	4
Nandi	4
Narok	4
Nyamira	4

Nyandarua	4
Nyeri	4
Samburu	4
Siaya	4
Taita Taveta	4
Tana River	4
Tharaka Nithi	4
Trans Nzoia	4
Turkana	4
Uasin Gishu	4
Vihiga	4
Wajir	4
West Pokot	4
Madagascar	3
Malawi	3
Mozambique	3
Rwanda	3
Somalia	3
South Sudan	3
Tanzania	3
Uganda	3
Zambia	3
Southern sub-Saharan Africa	2
Botswana	3
Lesotho	3

Namibia	3
South Africa	3
eSwatini	3
Zimbabwe	3
Western sub-Saharan Africa	2
Benin	3
Burkina Faso	3
Cameroon	3
Chad	3
Ghana	3
Guinea	3
Guinea-Bissau	3
Liberia	3
Mali	3
Mauritania	3
Niger	3
Nigeria	3
Cape Verde	3
Cote d'Ivoire	3
The Gambia	3
Sao Tome and Principe	3
Senegal	3
Sierra Leone	3
Togo	3

Appendix Table 2. GATHER checklist of information that should be included in reports of global health estimates, with description of compliance and location of information for GBD 2019

#	GATHER checklist item	Description of compliance	Reference
**Objectives and funding**			
1	Define the indicators, populations, and time periods for which estimates were made.	Narrative provided in paper and methods appendix describing indicators, definitions, and populations	Main text (Methods—Overview, Geographical units and time periods) and methods appendix
2	List the funding sources for the work.	Funding sources listed in paper	Main text (Summary)
**Data Inputs**			
For all data inputs from multiple sources that are synthesized as part of the study:			
3	Describe how the data were identified and how the data were accessed.	Narrative provided in paper and methods appendix describing data seeking methods	Main text (Methods) and methods appendix
4	Specify the inclusion and exclusion criteria. Identify all ad-hoc exclusions.	Narrative provided in paper and methods appendix describing inclusion and exclusion criteria by data type	Main text (Methods) and methods appendix
5	Provide information on all included data sources and their main characteristics. For each data source used, report reference information or contact name/institution, population represented, data collection method, year(s) of data collection, sex and age range, diagnostic criteria or measurement method, and sample size, as relevant.	Metadata for data sources by component, geography, cause, risk, or impairment is available through an interactive, online data source tool	Online data citation tools, link to the ghdx to be provided upon publication.
6	Identify and describe any categories of input data that have potentially important biases (e.g., based on characteristics listed in item 5).	Summary of known biases included in methods appendix	Methods appendix
For data inputs that contribute to the analysis but were not synthesized as part of the study:			
7	Describe and give sources for any other data inputs.	Included in online data source tool	Online data citation tools, link to the ghdx to be provided upon publication.
For all data inputs:			
8	Provide all data inputs in a file format from which data can be efficiently extracted (e.g., a spreadsheet as opposed to a PDF), including all relevant meta-data listed in item 5. For any data inputs that cannot be shared due to ethical or legal reasons, such as third-party ownership, provide a contact	Downloads of input data available through online tools, including data visualization tools	Online data visualization tools, data query tools, and the Global Health Data Exchange,

	name or the name of the institution that retains the right to the data.	and data query tools; input data not available in tools will be made available upon request	http://ghdx.healthdata.org
**Data analysis**			
9	Provide a conceptual overview of the data analysis method. A diagram may be helpful.	Flow diagrams of the overall methodological processes, as well as cause-specific modelling processes, have been provided	Main text (Methods) and methods appendix
10	Provide a detailed description of all steps of the analysis, including mathematical formulae. This description should cover, as relevant, data cleaning, data pre-processing, data adjustments and weighting of data sources, and mathematical or statistical model(s).	Flow diagrams and corresponding methodological write-ups have been provided	Main text (Methods) and methods appendix
11	Describe how candidate models were evaluated and how the final model(s) were selected.	Details on evaluation of model performance have been provided	Methods appendix
12	Provide the results of an evaluation of model performance, if done, as well as the results of any relevant sensitivity analysis.	Details on evaluation of model performance have been provided	Methods appendix
13	Describe methods for calculating uncertainty of the estimates. State which sources of uncertainty were, and were not, accounted for in the uncertainty analysis.	Details on uncertainty calculations have been provided	Methods appendix
14	State how analytic or statistical source code used to generate estimates can be accessed.	Access statement provided	Code is provided in an online repository, link to the ghdx to be provided upon publication.
**Results and Discussion**			
15	Provide published estimates in a file format from which data can be efficiently extracted.	Results are available through online data visualization tools, the Global Health Data Exchange, and the online data query tool	Online data tools (data visualization tools, data query tools, and the Global Health Data Exchange, link to the ghdx to be provided upon publication.)
16	Report a quantitative measure of the uncertainty of the estimates (e.g. uncertainty intervals).	Uncertainty intervals are provided with all results	Main text, methods appendix, and online data tools (data visualization tools, data query tools, and the Global Health Data Exchange, link to the ghdx to be provided upon publication.)

17	Interpret results in light of existing evidence. If updating a previous set of estimates, describe the reasons for changes in estimates.	Discussion of methodological differences between GBD estimates and other available evidence provided in the paper and methods appendix	Main text (Methods and Discussion) and methods appendix
18	Discuss limitations of the estimates. Include a discussion of any modelling assumptions or data limitations that affect interpretation of the estimates.	Discussion of limitations provided was provided	Main text (Limitations) and methods appendix

Appendix Table 3. Number of all-cause mortality data sources by type and location, 1950-2019						
Geography	SBH	CBH	HH	SIBS	VR/SRS/DSP	DSS
Armenia	6	4	0	0	38	0
Azerbaijan	4	1	2	0	39	0
Georgia	3	3	1	0	38	0
Kazakhstan	8	2	0	0	40	0
Kyrgyzstan	7	4	0	0	40	0
Mongolia	8	3	1	0	29	0
Tajikistan	8	2	1	0	37	0
Turkmenistan	3	1	1	0	38	0
Uzbekistan	4	2	0	0	39	0
Albania	6	2	0	0	37	0
Bosnia and Herzegovina	0	0	0	0	61	0
Bulgaria	4	0	0	0	71	0
Croatia	0	0	0	0	67	0
Czech Republic	0	0	0	0	68	0
Hungary	1	0	0	0	82	0
Macedonia	2	0	0	0	67	0
Montenegro	1	0	0	0	62	0
Poland	1	0	0	0	110	0
Romania	4	1	1	0	62	0
Serbia	3	0	4	0	61	0
Slovakia	0	0	0	0	67	0
Slovenia	0	0	0	0	67	0
Belarus	3	0	0	0	59	0
Estonia	1	0	0	0	58	0
Latvia	1	0	0	0	57	0
Lithuania	0	0	0	0	59	0
Moldova	4	2	0	0	41	0
Russian Federation	1	0	0	0	63	0
Ukraine	6	2	0	0	59	0
Australia	0	0	0	0	85	0
New Zealand	0	0	0	0	95	0
Brunei	1	0	0	0	58	0
Japan	0	0	0	0	119	0
Aichi	0	0	0	0	39	0
Akita	0	0	0	0	39	0
Aomori	0	0	0	0	39	0
Chiba	0	0	0	0	39	0
Ehime	0	0	0	0	39	0
Fukui	0	0	0	0	39	0
Fukuoka	0	0	0	0	39	0
Fukushima	0	0	0	0	39	0
Gifu	0	0	0	0	39	0
Gunma	0	0	0	0	39	0
Hiroshima	0	0	0	0	39	0
Hokkaidō	0	0	0	0	39	0
Hyōgo	0	0	0	0	38	0
Ibaraki	0	0	0	0	39	0
Ishikawa	0	0	0	0	39	0
Iwate	0	0	0	0	38	0
Kagawa	0	0	0	0	39	0
Kagoshima	0	0	0	0	39	0
Kanagawa	0	0	0	0	39	0
Kōchi	0	0	0	0	39	0

Appendix Table 3. Number of all-cause mortality data sources by type and location, 1950-2019						
Geography	SBH	CBH	HH	SIBS	VR/SRS/DSP	DSS
Kumamoto	0	0	0	0	39	0
Kyōto	0	0	0	0	39	0
Mie	0	0	0	0	39	0
Miyagi	0	0	0	0	38	0
Miyazaki	0	0	0	0	39	0
Nagano	0	0	0	0	39	0
Nagasaki	0	0	0	0	39	0
Nara	0	0	0	0	39	0
Niigata	0	0	0	0	39	0
Ōita	0	0	0	0	39	0
Okayama	0	0	0	0	39	0
Okinawa	0	0	0	0	39	0
Ōsaka	0	0	0	0	39	0
Saga	0	0	0	0	39	0
Saitama	0	0	0	0	39	0
Shiga	0	0	0	0	39	0
Shimane	0	0	0	0	39	0
Shizuoka	0	0	0	0	39	0
Tochigi	0	0	0	0	39	0
Tokushima	0	0	0	0	39	0
Tōkyō	0	0	0	0	39	0
Tottori	0	0	0	0	39	0
Toyama	0	0	0	0	39	0
Wakayama	0	0	0	0	39	0
Yamagata	0	0	0	0	39	0
Yamaguchi	0	0	0	0	39	0
Yamanashi	0	0	0	0	39	0
South Korea	6	1	0	0	41	0
Singapore	0	0	0	0	103	0
Canada	0	0	0	0	86	0
Greenland	0	0	0	0	55	0
USA	0	0	0	0	112	0
Alabama	0	0	0	0	58	0
Alaska	0	0	0	0	58	0
Arizona	0	0	0	0	58	0
Arkansas	0	0	0	0	58	0
California	0	0	0	0	58	0
Colorado	0	0	0	0	58	0
Connecticut	0	0	0	0	58	0
Delaware	0	0	0	0	58	0
Washington, DC	0	0	0	0	58	0
Florida	0	0	0	0	58	0
Georgia	0	0	0	0	58	0
Hawaii	0	0	0	0	58	0
Idaho	0	0	0	0	58	0
Illinois	0	0	0	0	58	0
Indiana	0	0	0	0	58	0
Iowa	0	0	0	0	58	0
Kansas	0	0	0	0	58	0
Kentucky	0	0	0	0	58	0
Louisiana	0	0	0	0	58	0
Maine	0	0	0	0	58	0
Maryland	0	0	0	0	58	0

Appendix Table 3. Number of all-cause mortality data sources by type and location, 1950-2019						
**Geography**	**SBH**	**CBH**	**HH**	**SIBS**	**VR/SRS/DSP**	**DSS**
Massachusetts	0	0	0	0	58	0
Michigan	0	0	0	0	58	0
Minnesota	0	0	0	0	58	0
Mississippi	0	0	0	0	58	0
Missouri	0	0	0	0	58	0
Montana	0	0	0	0	58	0
Nebraska	0	0	0	0	58	0
Nevada	0	0	0	0	58	0
New Hampshire	0	0	0	0	58	0
New Jersey	0	0	0	0	58	0
New Mexico	0	0	0	0	58	0
New York	0	0	0	0	58	0
North Carolina	0	0	0	0	58	0
North Dakota	0	0	0	0	58	0
Ohio	0	0	0	0	58	0
Oklahoma	0	0	0	0	58	0
Oregon	0	0	0	0	58	0
Pennsylvania	0	0	0	0	58	0
Rhode Island	0	0	0	0	58	0
South Carolina	0	0	0	0	58	0
South Dakota	0	0	0	0	58	0
Tennessee	0	0	0	0	58	0
Texas	0	0	0	0	58	0
Utah	0	0	0	0	58	0
Vermont	0	0	0	0	58	0
Virginia	0	0	0	0	58	0
Washington	0	0	0	0	58	0
West Virginia	0	0	0	0	58	0
Wisconsin	0	0	0	0	58	0
Wyoming	0	0	0	0	58	0
Argentina	3	0	0	0	63	0
Chile	4	0	0	0	87	0
Uruguay	6	0	0	0	77	0
Andorra	0	0	0	0	13	0
Austria	0	0	0	0	82	0
Belgium	2	0	0	0	81	0
Cyprus	3	0	0	0	43	0
Denmark	0	0	0	0	85	0
Finland	0	0	0	0	84	0
France	0	0	0	0	84	0
Germany	0	0	0	0	61	0
Greece	0	0	0	0	73	0
Iceland	0	0	0	0	89	0
Ireland	0	0	1	0	85	0
Israel	0	0	0	0	70	0
Italy	0	0	0	0	126	0
Luxembourg	0	0	0	0	71	0
Malta	0	0	0	0	69	0
Netherlands	0	0	0	0	86	0
Norway	0	0	0	0	67	0
Portugal	1	1	0	0	86	0
Spain	0	0	0	0	67	0
Sweden	0	0	0	0	115	0

Appendix Table 3. Number of all-cause mortality data sources by type and location, 1950-2019						
**Geography**	**SBH**	**CBH**	**HH**	**SIBS**	**VR/SRS/DSP**	**DSS**
Stockholm	0	0	0	0	35	0
Sweden except Stockholm	0	0	0	0	35	0
Switzerland	1	0	0	0	85	0
United Kingdom	0	0	0	0	67	0
England	0	0	0	0	37	0
East Midlands	0	0	0	0	37	0
Derby	0	0	0	0	37	0
Derbyshire	0	0	0	0	37	0
Leicester	0	0	0	0	37	0
Leicestershire	0	0	0	0	37	0
Lincolnshire	0	0	0	0	37	0
Northamptonshire	0	0	0	0	37	0
Nottingham	0	0	0	0	37	0
Nottinghamshire	0	0	0	0	37	0
Rutland	0	0	0	0	37	0
East of England	0	0	0	0	37	0
Bedford	0	0	0	0	37	0
Cambridgeshire	0	0	0	0	37	0
Central Bedfordshire	0	0	0	0	37	0
Essex	0	0	0	0	37	0
Hertfordshire	0	0	0	0	37	0
Luton	0	0	0	0	37	0
Norfolk	0	0	0	0	37	0
Peterborough	0	0	0	0	37	0
Southend-on-Sea	0	0	0	0	37	0
Suffolk	0	0	0	0	37	0
Thurrock	0	0	0	0	37	0
Greater London	0	0	0	0	37	0
Barking and Dagenham	0	0	0	0	37	0
Barnet	0	0	0	0	37	0
Bexley	0	0	0	0	37	0
Brent	0	0	0	0	37	0
Bromley	0	0	0	0	37	0
Camden	0	0	0	0	37	0
Croydon	0	0	0	0	37	0
Ealing	0	0	0	0	37	0
Enfield	0	0	0	0	37	0
Greenwich	0	0	0	0	37	0
Hackney	0	0	0	0	37	0
Hammersmith and Fulham	0	0	0	0	37	0
Haringey	0	0	0	0	37	0
Harrow	0	0	0	0	37	0
Havering	0	0	0	0	37	0
Hillingdon	0	0	0	0	37	0
Hounslow	0	0	0	0	37	0
Islington	0	0	0	0	37	0
Kensington and Chelsea	0	0	0	0	37	0
Kingston upon Thames	0	0	0	0	37	0
Lambeth	0	0	0	0	37	0
Lewisham	0	0	0	0	37	0
Merton	0	0	0	0	37	0
Newham	0	0	0	0	37	0
Redbridge	0	0	0	0	37	0

Appendix Table 3. Number of all-cause mortality data sources by type and location, 1950-2019

Geography	SBH	CBH	HH	SIBS	VR/SRS/DSP	DSS
Richmond upon Thames	0	0	0	0	37	0
Southwark	0	0	0	0	37	0
Sutton	0	0	0	0	37	0
Tower Hamlets	0	0	0	0	37	0
Waltham Forest	0	0	0	0	37	0
Wandsworth	0	0	0	0	37	0
Westminster	0	0	0	0	37	0
North East England	0	0	0	0	37	0
County Durham	0	0	0	0	37	0
Darlington	0	0	0	0	37	0
Gateshead	0	0	0	0	37	0
Hartlepool	0	0	0	0	37	0
Middlesbrough	0	0	0	0	37	0
Newcastle upon Tyne	0	0	0	0	37	0
North Tyneside	0	0	0	0	37	0
Northumberland	0	0	0	0	37	0
Redcar and Cleveland	0	0	0	0	37	0
South Tyneside	0	0	0	0	37	0
Stockton-on-Tees	0	0	0	0	37	0
Sunderland	0	0	0	0	37	0
North West England	0	0	0	0	37	0
Blackburn with Darwen	0	0	0	0	37	0
Blackpool	0	0	0	0	37	0
Bolton	0	0	0	0	37	0
Bury	0	0	0	0	37	0
Cheshire East	0	0	0	0	37	0
Cheshire West and Chester	0	0	0	0	37	0
Cumbria	0	0	0	0	37	0
Halton	0	0	0	0	37	0
Knowsley	0	0	0	0	37	0
Lancashire	0	0	0	0	37	0
Liverpool	0	0	0	0	37	0
Manchester	0	0	0	0	37	0
Oldham	0	0	0	0	37	0
Rochdale	0	0	0	0	37	0
Salford	0	0	0	0	37	0
Sefton	0	0	0	0	37	0
St Helens	0	0	0	0	37	0
Stockport	0	0	0	0	37	0
Tameside	0	0	0	0	37	0
Trafford	0	0	0	0	37	0
Warrington	0	0	0	0	37	0
Wigan	0	0	0	0	37	0
Wirral	0	0	0	0	37	0
South East England	0	0	0	0	37	0
Bracknell Forest	0	0	0	0	37	0
Brighton and Hove	0	0	0	0	37	0
Buckinghamshire	0	0	0	0	37	0
East Sussex	0	0	0	0	37	0
Hampshire	0	0	0	0	37	0
Isle of Wight	0	0	0	0	37	0
Kent	0	0	0	0	37	0
Medway	0	0	0	0	37	0

Appendix Table 3. Number of all-cause mortality data sources by type and location, 1950-2019						
**Geography**	**SBH**	**CBH**	**HH**	**SIBS**	**VR/SRS/DSP**	**DSS**
Milton Keynes	0	0	0	0	37	0
Oxfordshire	0	0	0	0	37	0
Portsmouth	0	0	0	0	37	0
Reading	0	0	0	0	37	0
Slough	0	0	0	0	37	0
Southampton	0	0	0	0	37	0
Surrey	0	0	0	0	37	0
West Berkshire	0	0	0	0	37	0
West Sussex	0	0	0	0	37	0
Windsor and Maidenhead	0	0	0	0	37	0
Wokingham	0	0	0	0	37	0
South West England	0	0	0	0	37	0
Bath and North East Somerset	0	0	0	0	37	0
Bournemouth	0	0	0	0	37	0
Bristol, City of	0	0	0	0	37	0
Cornwall	0	0	0	0	37	0
Devon	0	0	0	0	37	0
Dorset	0	0	0	0	37	0
Gloucestershire	0	0	0	0	37	0
North Somerset	0	0	0	0	37	0
Plymouth	0	0	0	0	37	0
Poole	0	0	0	0	37	0
Somerset	0	0	0	0	37	0
South Gloucestershire	0	0	0	0	37	0
Swindon	0	0	0	0	37	0
Torbay	0	0	0	0	37	0
Wiltshire	0	0	0	0	37	0
West Midlands	0	0	0	0	37	0
Birmingham	0	0	0	0	37	0
Coventry	0	0	0	0	37	0
Dudley	0	0	0	0	37	0
Herefordshire, County of	0	0	0	0	37	0
Sandwell	0	0	0	0	37	0
Shropshire	0	0	0	0	37	0
Solihull	0	0	0	0	37	0
Staffordshire	0	0	0	0	37	0
Stoke-on-Trent	0	0	0	0	37	0
Telford and Wrekin	0	0	0	0	37	0
Walsall	0	0	0	0	37	0
Warwickshire	0	0	0	0	37	0
Wolverhampton	0	0	0	0	37	0
Worcestershire	0	0	0	0	37	0
Yorkshire and the Humber	0	0	0	0	37	0
Barnsley	0	0	0	0	37	0
Bradford	0	0	0	0	37	0
Calderdale	0	0	0	0	37	0
Doncaster	0	0	0	0	37	0
East Riding of Yorkshire	0	0	0	0	37	0
Kingston upon Hull, City of	0	0	0	0	37	0
Kirklees	0	0	0	0	37	0
Leeds	0	0	0	0	37	0
North East Lincolnshire	0	0	0	0	37	0
North Lincolnshire	0	0	0	0	37	0

Appendix Table 3. Number of all-cause mortality data sources by type and location, 1950-2019						
**Geography**	**SBH**	**CBH**	**HH**	**SIBS**	**VR/SRS/DSP**	**DSS**
North Yorkshire	0	0	0	0	37	0
Rotherham	0	0	0	0	37	0
Sheffield	0	0	0	0	37	0
Wakefield	0	0	0	0	37	0
York	0	0	0	0	37	0
Northern Ireland	0	0	0	0	67	0
Scotland	0	0	0	0	67	0
Wales	0	0	0	0	37	0
Bolivia	25	6	1	3	12	0
Ecuador	17	7	1	2	70	0
Peru	21	14	0	4	67	0
Antigua and Barbuda	0	0	0	0	63	0
The Bahamas	0	0	0	0	49	0
Barbados	0	0	0	0	69	0
Belize	6	3	0	0	71	0
Bermuda	1	0	0	0	74	0
Cuba	1	0	0	0	60	0
Dominica	0	0	0	0	68	0
Dominican Republic	15	13	0	2	69	0
Grenada	0	0	0	0	56	0
Guyana	7	4	0	0	46	0
Haiti	9	7	2	2	1	0
Jamaica	5	1	1	0	54	0
Puerto Rico	1	1	0	0	73	0
Saint Lucia	0	0	0	0	56	0
Saint Vincent and the Grenadines	0	0	0	0	51	0
Suriname	2	0	0	0	61	0
Trinidad and Tobago	5	2	0	0	78	0
Virgin Islands	0	0	0	0	53	0
Colombia	13	8	0	0	90	0
Costa Rica	7	3	0	0	73	0
El Salvador	10	6	2	3	83	0
Guatemala	10	6	0	0	72	0
Honduras	12	5	2	1	44	0
Mexico	25	2	2	0	108	0
Aguascalientes	16	1	1	0	39	0
Baja California	16	1	0	0	39	0
Baja California Sur	16	1	0	0	39	0
Campeche	16	1	1	0	39	0
Chiapas	16	1	1	0	39	0
Chihuahua	16	1	1	0	39	0
Coahuila	16	1	1	0	39	0
Colima	16	1	0	0	39	0
Durango	16	1	1	0	39	0
Guanajuato	16	1	1	0	39	0
Guerrero	16	1	1	0	39	0
Hidalgo	14	1	1	0	39	0
Jalisco	16	1	1	0	39	0
México	16	1	0	0	39	0
Mexico City	16	1	1	0	39	0
Michoacán de Ocampo	16	1	1	0	39	0
Morelos	16	1	1	0	39	0
Nayarit	16	1	1	0	39	0

Appendix Table 3. Number of all-cause mortality data sources by type and location, 1950-2019						
Geography	SBH	CBH	HH	SIBS	VR/SRS/DSP	DSS
Nuevo León	16	1	1	0	39	0
Oaxaca	16	1	1	0	39	0
Puebla	16	1	1	0	39	0
Querétaro	16	1	1	0	39	0
Quintana Roo	16	1	1	0	39	0
San Luis Potosí	16	1	1	0	39	0
Sinaloa	16	1	1	0	39	0
Sonora	16	1	1	0	39	0
Tabasco	16	1	0	0	39	0
Tamaulipas	16	1	1	0	39	0
Tlaxcala	16	1	0	0	39	0
Veracruz de Ignacio de la Llave	16	1	1	0	39	0
Yucatán	16	1	1	0	39	0
Zacatecas	16	1	1	0	39	0
Nicaragua	12	5	0	0	54	0
Panama	9	1	0	0	79	0
Venezuela	4	1	1	0	77	0
Brazil	27	2	0	0	47	0
Acre	8	1	0	0	39	0
Alagoas	20	2	0	0	39	0
Amapá	8	1	0	0	39	0
Amazonas	9	1	0	0	39	0
Bahia	20	2	0	0	39	0
Ceará	20	2	0	0	39	0
Distrito Federal	20	1	0	0	39	0
Espírito Santo	20	1	0	0	39	0
Goiás	20	1	0	0	39	0
Maranhão	20	2	0	0	39	0
Mato Grosso	20	1	0	0	39	0
Mato Grosso do Sul	20	1	0	0	39	0
Minas Gerais	20	1	0	0	39	0
Pará	9	1	0	0	39	0
Paraíba	20	2	0	0	38	0
Paraná	20	1	0	0	39	0
Pernambuco	20	2	0	0	39	0
Piauí	20	2	0	0	39	0
Rio de Janeiro	20	1	0	0	39	0
Rio Grande do Norte	20	2	0	0	39	0
Rio Grande do Sul	20	1	0	0	39	0
Rondônia	8	1	0	0	39	0
Roraima	8	1	0	0	39	0
Santa Catarina	20	1	0	0	39	0
São Paulo	19	1	0	0	39	0
Sergipe	20	2	0	0	39	0
Tocantins	8	1	0	0	29	0
Paraguay	15	7	0	0	71	0
Afghanistan	6	1	1	0	0	0
Algeria	5	3	0	0	25	0
Bahrain	4	0	2	0	36	0
Egypt	13	11	2	0	67	0
Iran	5	1	9	0	32	0
Iraq	7	4	4	0	24	1
Jordan	12	7	1	1	23	0

Appendix Table 3. Number of all-cause mortality data sources by type and location, 1950-2019						
**Geography**	**SBH**	**CBH**	**HH**	**SIBS**	**VR/SRS/DSP**	**DSS**
Kuwait	3	0	1	0	58	0
Lebanon	4	2	1	1	6	0
Libya	4	1	0	0	15	0
Morocco	9	6	2	2	10	0
Palestine	8	4	3	0	19	0
Oman	4	0	3	0	12	0
Qatar	1	0	2	0	42	0
Saudi Arabia	2	0	4	0	11	0
Sudan	9	5	3	1	0	0
Syria	6	4	1	0	36	0
Tunisia	7	5	1	0	20	0
Turkey	14	5	1	0	36	0
United Arab Emirates	2	0	6	0	12	0
Yemen	7	5	2	0	0	0
Bangladesh	13	9	1	3	42	2
Bhutan	4	0	1	1	0	0
India	12	4	0	0	60	0
Nepal	12	7	3	2	0	0
Pakistan	14	8	2	0	24	0
China	5	0	30	0	23	0
North Korea	0	0	1	0	0	0
Taiwan (Province of China)	0	0	0	0	68	0
American Samoa	1	0	0	0	54	0
Federated States of Micronesia	3	0	0	0	1	0
Fiji	7	1	0	0	55	0
Guam	0	0	0	0	66	0
Kiribati	7	0	1	0	12	0
Marshall Islands	2	0	1	0	14	0
Northern Mariana Islands	0	0	0	0	20	0
Papua New Guinea	8	1	2	0	2	0
Samoa	5	0	4	0	22	0
Solomon Islands	4	0	3	0	0	0
Tonga	3	0	2	0	18	0
Vanuatu	4	0	1	0	0	0
Cambodia	11	5	1	4	0	0
Indonesia	46	15	1	5	0	0
Aceh	32	4	2	4	0	0
Bali	40	6	6	5	0	0
Bangka-Belitung Islands	25	5	5	4	0	0
Banten	33	5	4	4	0	0
Bengkulu	36	5	5	5	0	0
Gorontalo	22	4	5	5	0	0
Jakarta	40	6	4	5	0	0
Jambi	34	4	5	5	0	0
West Java	40	6	6	5	0	0
Central Java	40	6	6	5	0	0
East Java	40	6	6	5	0	0
West Kalimantan	36	5	6	5	0	0
South Kalimantan	40	6	5	5	0	0
Central Kalimantan	36	5	4	5	0	0
East Kalimantan	34	6	6	5	0	0
North Kalimantan	17	2	3	0	0	0
Riau Islands	29	4	3	5	0	0

Appendix Table 3. Number of all-cause mortality data sources by type and location, 1950-2019

Geography	SBH	CBH	HH	SIBS	VR/SRS/DSP	DSS
Lampung	39	6	4	5	0	0
Maluku	33	4	0	4	0	0
North Maluku	24	4	4	3	0	0
West Nusa Tenggara	40	6	4	5	0	0
East Nusa Tenggara	34	5	6	5	0	0
Papua	30	3	3	4	0	0
West Papua	29	3	4	4	0	0
Riau	37	6	6	5	0	0
West Sulawesi	30	4	5	3	0	0
South Sulawesi	40	6	6	5	0	0
Central Sulawesi	36	5	5	5	0	0
Southeast Sulawesi	35	6	4	5	0	0
North Sulawesi	36	5	5	5	0	0
West Sumatra	40	6	4	5	0	0
South Sumatra	40	6	6	5	0	0
North Sumatra	40	6	3	5	0	0
Yogyakarta	40	6	6	5	0	0
Laos	4	2	1	1	0	0
Malaysia	3	1	0	0	53	0
Maldives	7	2	1	0	39	0
Mauritius	0	0	0	0	81	0
Myanmar	6	1	2	1	1	0
Philippines	13	6	0	0	75	0
Sri Lanka	5	2	4	0	82	0
Seychelles	2	0	1	0	65	0
Thailand	11	2	0	0	66	0
Timor-Leste	9	6	0	4	0	0
Vietnam	16	3	3	0	0	2
Angola	4	2	0	1	0	0
Central African Republic	5	1	1	1	0	0
Congo (Brazzaville)	5	4	1	2	0	0
DR Congo	5	2	0	2	0	0
Equatorial Guinea	1	0	1	0	0	0
Gabon	2	2	0	2	0	0
Burundi	8	3	2	1	0	0
Comoros	4	2	2	1	0	0
Djibouti	3	1	0	0	0	0
Eritrea	3	2	2	1	0	0
Ethiopia	8	4	1	4	0	5
Kenya	19	7	0	4	0	4
Madagascar	10	4	1	4	14	0
Malawi	19	8	6	6	2	1
Mozambique	9	4	1	3	0	1
Rwanda	13	7	1	4	0	0
Somalia	2	1	0	0	0	0
South Sudan	2	1	0	0	0	0
Tanzania	14	7	3	4	0	3
Uganda	17	7	1	5	0	1
Zambia	11	5	7	5	0	0
Botswana	9	2	3	0	7	0
Lesotho	10	4	0	3	1	0
Namibia	5	4	1	4	0	0
South Africa	10	2	5	1	31	3

Appendix Table 3. Number of all-cause mortality data sources by type and location, 1950-2019						
**Geography**	**SBH**	**CBH**	**HH**	**SIBS**	**VR/SRS/DSP**	**DSS**
Swaziland	4	3	1	1	0	0
Zimbabwe	9	8	3	6	2	0
Benin	9	7	2	2	0	0
Burkina Faso	12	4	5	3	0	2
Cameroon	9	6	1	3	0	0
Cape Verde	3	1	1	0	25	0
Chad	5	3	0	3	0	0
Cote d'Ivoire	6	10	1	3	0	1
The Gambia	6	1	1	0	0	1
Ghana	20	9	1	1	0	1
Guinea	4	4	3	3	0	0
Guinea-Bissau	4	1	0	1	0	0
Liberia	7	5	1	2	0	0
Mali	10	6	3	4	0	0
Mauritania	8	5	1	2	0	0
Niger	6	4	0	3	0	0
Nigeria	22	6	1	0	0	0
Sao Tome and Principe	6	2	1	2	20	0
Senegal	13	13	1	3	0	3
Sierra Leone	6	3	1	2	5	0
Togo	8	3	1	2	0	0

Appendix Table 4. Number of all-cause mortality data sources by type and year, 1950-2019						
**Year**	**SBH**	**CBH**	**HH**	**SIBS**	**VR/SRS/DSP**	**DSS**
1950	0	0	0	0	92	0
1951	0	0	0	0	98	0
1952	0	0	0	0	106	0
1953	0	0	1	0	105	0
1954	0	0	0	0	107	0
1955	0	1	0	0	123	0
1956	2	0	0	0	122	0
1957	0	1	0	0	122	0
1958	1	0	1	0	131	0
1959	0	1	1	0	178	0
1960	23	0	1	0	180	0
1961	3	1	1	0	187	0
1962	0	0	0	0	185	0
1963	1	1	0	0	190	0
1964	1	2	0	0	195	0
1965	1	1	1	0	251	0
1966	4	4	0	0	198	0
1967	2	2	1	0	197	0
1968	1	7	0	0	238	0
1969	1	3	0	0	268	0
1970	39	10	1	0	254	0
1971	39	3	3	0	251	0
1972	3	13	0	0	251	0
1973	9	4	1	0	251	1
1974	8	22	2	0	259	0
1975	13	13	0	0	193	0
1976	14	24	1	0	266	0
1977	8	11	1	0	267	0
1978	10	29	0	0	275	0
1979	7	10	1	0	363	0
1980	113	28	1	0	341	0
1981	17	6	2	0	507	0
1982	38	26	7	0	510	0
1983	5	7	1	0	508	0
1984	8	28	21	0	513	0
1985	45	11	1	0	497	0
1986	33	44	1	0	522	0
1987	38	42	7	0	527	0
1988	48	58	4	1	502	0
1989	55	17	33	1	597	0
1990	148	44	2	1	603	4
1991	114	45	9	6	604	4
1992	148	165	14	2	603	4
1993	168	35	8	5	602	6

Appendix Table 4. Number of all-cause mortality data sources by type and year, 1950-2019						
**Year**	**SBH**	**CBH**	**HH**	**SIBS**	**VR/SRS/DSP**	**DSS**
1994	134	46	5	33	606	7
1995	106	52	15	6	650	7
1996	121	48	49	4	684	8
1997	83	74	45	48	691	9
1998	121	108	73	3	695	9
1999	335	59	66	11	705	10
2000	292	91	51	14	711	11
2001	180	56	38	2	738	11
2002	75	167	39	34	742	11
2003	242	77	7	7	712	14
2004	117	46	39	10	759	16
2005	232	109	69	7	759	18
2006	306	81	24	5	845	21
2007	221	69	14	38	845	21
2008	287	157	38	4	847	23
2009	260	44	14	10	899	26
2010	349	20	50	6	894	30
2011	389	132	53	7	831	30
2012	192	71	36	39	844	29
2013	150	72	34	42	879	24
2014	117	88	36	13	848	21
2015	193	50	32	3	859	2
2016	134	83	32	11	678	0
2017	110	50	36	0	462	0
2018	3	0	2	0	4	0

Appendix Table 5. Number of 5q0 data sources by type and location, 1950-2019					
**Geography**	**SBH**	**CBH**	**HH**	**VR/SRS/DSP**	**DSS**
Armenia	6	4	0	38	0
Azerbaijan	4	1	2	39	0
Georgia	3	3	1	38	0
Kazakhstan	8	2	0	40	0
Kyrgyzstan	7	4	0	40	0
Mongolia	8	3	1	28	0
Tajikistan	8	2	1	37	0
Turkmenistan	3	1	1	25	0
Uzbekistan	4	2	0	38	0
Albania	6	2	0	37	0
Bosnia and Herzegovina	0	0	0	61	0
Bulgaria	4	0	0	67	0
Croatia	0	0	0	66	0
Czech Republic	0	0	0	68	0
Hungary	1	0	0	68	0
Macedonia	2	0	0	67	0
Montenegro	1	0	0	57	0
Poland	1	0	0	67	0
Romania	4	1	1	62	0
Serbia	3	0	4	50	0
Slovakia	0	0	0	67	0
Slovenia	0	0	0	67	0
Belarus	3	0	0	58	0
Estonia	1	0	0	58	0
Latvia	1	0	0	57	0
Lithuania	0	0	0	59	0
Moldova	4	2	0	41	0
Russian Federation	1	0	0	59	0
Ukraine	6	2	0	58	0
Australia	0	0	0	67	0
New Zealand	0	0	0	67	0
Brunei	1	0	0	58	0
Japan	0	0	0	68	0
Aichi	0	0	0	39	0
Akita	0	0	0	39	0
Aomori	0	0	0	39	0
Chiba	0	0	0	39	0
Ehime	0	0	0	39	0
Fukui	0	0	0	39	0
Fukuoka	0	0	0	39	0
Fukushima	0	0	0	39	0
Gifu	0	0	0	39	0
Gunma	0	0	0	39	0
Hiroshima	0	0	0	39	0
Hokkaidō	0	0	0	39	0
Hyōgo	0	0	0	38	0

Appendix Table 5. Number of 5q0 data sources by type and location, 1950-2019

Geography	SBH	CBH	HH	VR/SRS/DSP	DSS
Ibaraki	0	0	0	39	0
Ishikawa	0	0	0	39	0
Iwate	0	0	0	38	0
Kagawa	0	0	0	39	0
Kagoshima	0	0	0	39	0
Kanagawa	0	0	0	39	0
Kōchi	0	0	0	39	0
Kumamoto	0	0	0	39	0
Kyōto	0	0	0	39	0
Mie	0	0	0	39	0
Miyagi	0	0	0	38	0
Miyazaki	0	0	0	39	0
Nagano	0	0	0	39	0
Nagasaki	0	0	0	39	0
Nara	0	0	0	39	0
Niigata	0	0	0	39	0
Ōita	0	0	0	39	0
Okayama	0	0	0	39	0
Okinawa	0	0	0	39	0
Ōsaka	0	0	0	39	0
Saga	0	0	0	39	0
Saitama	0	0	0	39	0
Shiga	0	0	0	39	0
Shimane	0	0	0	39	0
Shizuoka	0	0	0	39	0
Tochigi	0	0	0	39	0
Tokushima	0	0	0	39	0
Tōkyō	0	0	0	39	0
Tottori	0	0	0	39	0
Toyama	0	0	0	39	0
Wakayama	0	0	0	39	0
Yamagata	0	0	0	39	0
Yamaguchi	0	0	0	39	0
Yamanashi	0	0	0	39	0
South Korea	6	1	0	40	0
Singapore	0	0	0	68	0
Canada	0	0	0	67	0
Greenland	0	0	0	55	0
USA	0	0	0	68	0
Alabama	0	0	0	58	0
Alaska	0	0	0	58	0
Arizona	0	0	0	58	0
Arkansas	0	0	0	58	0
California	0	0	0	58	0
Colorado	0	0	0	58	0
Connecticut	0	0	0	58	0

Appendix Table 5. Number of 5q0 data sources by type and location, 1950-2019					
**Geography**	**SBH**	**CBH**	**HH**	**VR/SRS/DSP**	**DSS**
Delaware	0	0	0	58	0
Washington, DC	0	0	0	58	0
Florida	0	0	0	58	0
Georgia	0	0	0	58	0
Hawaii	0	0	0	58	0
Idaho	0	0	0	58	0
Illinois	0	0	0	58	0
Indiana	0	0	0	58	0
Iowa	0	0	0	58	0
Kansas	0	0	0	58	0
Kentucky	0	0	0	58	0
Louisiana	0	0	0	58	0
Maine	0	0	0	58	0
Maryland	0	0	0	58	0
Massachusetts	0	0	0	58	0
Michigan	0	0	0	58	0
Minnesota	0	0	0	58	0
Mississippi	0	0	0	58	0
Missouri	0	0	0	58	0
Montana	0	0	0	58	0
Nebraska	0	0	0	58	0
Nevada	0	0	0	58	0
New Hampshire	0	0	0	58	0
New Jersey	0	0	0	58	0
New Mexico	0	0	0	58	0
New York	0	0	0	58	0
North Carolina	0	0	0	58	0
North Dakota	0	0	0	58	0
Ohio	0	0	0	58	0
Oklahoma	0	0	0	58	0
Oregon	0	0	0	58	0
Pennsylvania	0	0	0	58	0
Rhode Island	0	0	0	58	0
South Carolina	0	0	0	58	0
South Dakota	0	0	0	58	0
Tennessee	0	0	0	58	0
Texas	0	0	0	58	0
Utah	0	0	0	58	0
Vermont	0	0	0	58	0
Virginia	0	0	0	58	0
Washington	0	0	0	58	0
West Virginia	0	0	0	58	0
Wisconsin	0	0	0	58	0
Wyoming	0	0	0	58	0
Argentina	3	0	0	61	0
Chile	4	0	0	67	0

Appendix Table 5. Number of 5q0 data sources by type and location, 1950-2019

Geography	SBH	CBH	HH	VR/SRS/DSP	DSS
Uruguay	6	0	0	65	0
Andorra	0	0	0	13	0
Austria	0	0	0	68	0
Belgium	2	0	0	67	0
Cyprus	3	0	0	43	0
Denmark	0	0	0	67	0
Finland	0	0	0	67	0
France	0	0	0	67	0
Germany	0	0	0	61	0
Greece	0	0	0	66	0
Iceland	0	0	0	68	0
Ireland	0	0	0	67	0
Israel	0	0	0	67	0
Italy	0	0	0	67	0
Luxembourg	0	0	0	67	0
Malta	0	0	0	67	0
Netherlands	0	0	0	67	0
Norway	0	0	0	67	0
Portugal	1	1	0	64	0
Spain	0	0	0	67	0
Sweden	0	0	0	68	0
Stockholm	0	0	0	34	0
Sweden except Stockholm	0	0	0	34	0
Switzerland	1	0	0	67	0
United Kingdom	0	0	0	66	0
England	0	0	0	37	0
East Midlands	0	0	0	37	0
Derby	0	0	0	37	0
Derbyshire	0	0	0	37	0
Leicester	0	0	0	37	0
Leicestershire	0	0	0	37	0
Lincolnshire	0	0	0	37	0
Northamptonshire	0	0	0	37	0
Nottingham	0	0	0	37	0
Nottinghamshire	0	0	0	37	0
Rutland	0	0	0	37	0
East of England	0	0	0	37	0
Bedford	0	0	0	37	0
Cambridgeshire	0	0	0	37	0
Central Bedfordshire	0	0	0	37	0
Essex	0	0	0	37	0
Hertfordshire	0	0	0	37	0
Luton	0	0	0	37	0
Norfolk	0	0	0	37	0
Peterborough	0	0	0	37	0
Southend-on-Sea	0	0	0	37	0

Appendix Table 5. Number of 5q0 data sources by type and location, 1950-2019					
**Geography**	**SBH**	**CBH**	**HH**	**VR/SRS/DSP**	**DSS**
Suffolk	0	0	0	37	0
Thurrock	0	0	0	37	0
Greater London	0	0	0	37	0
Barking and Dagenham	0	0	0	37	0
Barnet	0	0	0	37	0
Bexley	0	0	0	37	0
Brent	0	0	0	37	0
Bromley	0	0	0	37	0
Camden	0	0	0	37	0
Croydon	0	0	0	37	0
Ealing	0	0	0	37	0
Enfield	0	0	0	37	0
Greenwich	0	0	0	37	0
Hackney	0	0	0	37	0
Hammersmith and Fulham	0	0	0	37	0
Haringey	0	0	0	37	0
Harrow	0	0	0	37	0
Havering	0	0	0	37	0
Hillingdon	0	0	0	37	0
Hounslow	0	0	0	37	0
Islington	0	0	0	37	0
Kensington and Chelsea	0	0	0	37	0
Kingston upon Thames	0	0	0	37	0
Lambeth	0	0	0	37	0
Lewisham	0	0	0	37	0
Merton	0	0	0	37	0
Newham	0	0	0	37	0
Redbridge	0	0	0	37	0
Richmond upon Thames	0	0	0	37	0
Southwark	0	0	0	37	0
Sutton	0	0	0	37	0
Tower Hamlets	0	0	0	37	0
Waltham Forest	0	0	0	37	0
Wandsworth	0	0	0	37	0
Westminster	0	0	0	37	0
North East England	0	0	0	37	0
County Durham	0	0	0	37	0
Darlington	0	0	0	37	0
Gateshead	0	0	0	37	0
Hartlepool	0	0	0	37	0
Middlesbrough	0	0	0	37	0
Newcastle upon Tyne	0	0	0	37	0
North Tyneside	0	0	0	37	0
Northumberland	0	0	0	37	0
Redcar and Cleveland	0	0	0	37	0
South Tyneside	0	0	0	37	0

Appendix Table 5. Number of 5q0 data sources by type and location, 1950-2019					
**Geography**	**SBH**	**CBH**	**HH**	**VR/SRS/DSP**	**DSS**
Stockton-on-Tees	0	0	0	37	0
Sunderland	0	0	0	37	0
North West England	0	0	0	37	0
Blackburn with Darwen	0	0	0	37	0
Blackpool	0	0	0	37	0
Bolton	0	0	0	37	0
Bury	0	0	0	37	0
Cheshire East	0	0	0	37	0
Cheshire West and Chester	0	0	0	37	0
Cumbria	0	0	0	37	0
Halton	0	0	0	37	0
Knowsley	0	0	0	37	0
Lancashire	0	0	0	37	0
Liverpool	0	0	0	37	0
Manchester	0	0	0	37	0
Oldham	0	0	0	37	0
Rochdale	0	0	0	37	0
Salford	0	0	0	37	0
Sefton	0	0	0	37	0
St Helens	0	0	0	37	0
Stockport	0	0	0	37	0
Tameside	0	0	0	37	0
Trafford	0	0	0	37	0
Warrington	0	0	0	37	0
Wigan	0	0	0	37	0
Wirral	0	0	0	37	0
South East England	0	0	0	37	0
Bracknell Forest	0	0	0	37	0
Brighton and Hove	0	0	0	37	0
Buckinghamshire	0	0	0	37	0
East Sussex	0	0	0	37	0
Hampshire	0	0	0	37	0
Isle of Wight	0	0	0	37	0
Kent	0	0	0	37	0
Medway	0	0	0	37	0
Milton Keynes	0	0	0	37	0
Oxfordshire	0	0	0	37	0
Portsmouth	0	0	0	37	0
Reading	0	0	0	37	0
Slough	0	0	0	37	0
Southampton	0	0	0	37	0
Surrey	0	0	0	37	0
West Berkshire	0	0	0	37	0
West Sussex	0	0	0	37	0
Windsor and Maidenhead	0	0	0	37	0
Wokingham	0	0	0	37	0

Appendix Table 5. Number of 5q0 data sources by type and location, 1950-2019					
**Geography**	**SBH**	**CBH**	**HH**	**VR/SRS/DSP**	**DSS**
South West England	0	0	0	37	0
Bath and North East Somerset	0	0	0	37	0
Bournemouth	0	0	0	37	0
Bristol, City of	0	0	0	37	0
Cornwall	0	0	0	37	0
Devon	0	0	0	37	0
Dorset	0	0	0	37	0
Gloucestershire	0	0	0	37	0
North Somerset	0	0	0	37	0
Plymouth	0	0	0	37	0
Poole	0	0	0	37	0
Somerset	0	0	0	37	0
South Gloucestershire	0	0	0	37	0
Swindon	0	0	0	37	0
Torbay	0	0	0	37	0
Wiltshire	0	0	0	37	0
West Midlands	0	0	0	37	0
Birmingham	0	0	0	37	0
Coventry	0	0	0	37	0
Dudley	0	0	0	37	0
Herefordshire, County of	0	0	0	37	0
Sandwell	0	0	0	37	0
Shropshire	0	0	0	37	0
Solihull	0	0	0	37	0
Staffordshire	0	0	0	37	0
Stoke-on-Trent	0	0	0	37	0
Telford and Wrekin	0	0	0	37	0
Walsall	0	0	0	37	0
Warwickshire	0	0	0	37	0
Wolverhampton	0	0	0	37	0
Worcestershire	0	0	0	37	0
Yorkshire and the Humber	0	0	0	37	0
Barnsley	0	0	0	37	0
Bradford	0	0	0	37	0
Calderdale	0	0	0	37	0
Doncaster	0	0	0	37	0
East Riding of Yorkshire	0	0	0	37	0
Kingston upon Hull, City of	0	0	0	37	0
Kirklees	0	0	0	37	0
Leeds	0	0	0	37	0
North East Lincolnshire	0	0	0	37	0
North Lincolnshire	0	0	0	37	0
North Yorkshire	0	0	0	37	0
Rotherham	0	0	0	37	0
Sheffield	0	0	0	37	0
Wakefield	0	0	0	37	0

Appendix Table 5. Number of 5q0 data sources by type and location, 1950-2019					
**Geography**	**SBH**	**CBH**	**HH**	**VR/SRS/DSP**	**DSS**
York	0	0	0	37	0
Northern Ireland	0	0	0	67	0
Scotland	0	0	0	67	0
Wales	0	0	0	37	0
Bolivia	25	6	1	12	0
Ecuador	17	7	0	64	0
Peru	21	14	0	63	0
Antigua and Barbuda	0	0	0	58	0
The Bahamas	0	0	0	49	0
Barbados	0	0	0	62	0
Belize	6	3	0	67	0
Bermuda	1	0	0	67	0
Cuba	1	0	0	59	0
Dominica	0	0	0	64	0
Dominican Republic	15	13	0	60	0
Grenada	0	0	0	56	0
Guyana	7	4	0	46	0
Haiti	9	7	1	0	0
Jamaica	5	1	1	49	0
Puerto Rico	1	1	0	67	0
Saint Lucia	0	0	0	56	0
Saint Vincent and the Grenadines	0	0	0	51	0
Suriname	2	0	0	57	0
Trinidad and Tobago	5	2	0	63	0
Virgin Islands	0	0	0	50	0
Colombia	13	8	0	65	0
Costa Rica	7	3	0	66	0
El Salvador	10	6	0	65	0
Guatemala	10	6	0	66	0
Honduras	12	5	1	44	0
Mexico	25	2	2	93	0
Aguascalientes	16	1	1	39	0
Baja California	16	1	0	39	0
Baja California Sur	16	1	0	39	0
Campeche	16	1	1	39	0
Chiapas	16	1	1	39	0
Chihuahua	16	1	1	39	0
Coahuila	16	1	1	39	0
Colima	16	1	0	39	0
Durango	16	1	1	39	0
Guanajuato	16	1	1	39	0
Guerrero	16	1	1	39	0
Hidalgo	14	1	1	39	0
Jalisco	16	1	1	39	0
México	16	1	0	39	0
Mexico City	16	1	1	38	0

Appendix Table 5. Number of 5q0 data sources by type and location, 1950-2019					
**Geography**	**SBH**	**CBH**	**HH**	**VR/SRS/DSP**	**DSS**
Michoacán de Ocampo	16	1	1	39	0
Morelos	16	1	1	39	0
Nayarit	16	1	1	39	0
Nuevo León	16	1	1	39	0
Oaxaca	16	1	1	39	0
Puebla	16	1	1	39	0
Querétaro	16	1	1	39	0
Quintana Roo	16	1	1	39	0
San Luis Potosí	16	1	1	39	0
Sinaloa	16	1	1	39	0
Sonora	16	1	1	39	0
Tabasco	16	1	0	39	0
Tamaulipas	16	1	1	39	0
Tlaxcala	16	1	0	39	0
Veracruz de Ignacio de la Llave	16	1	1	39	0
Yucatán	16	1	1	39	0
Zacatecas	16	1	1	39	0
Nicaragua	12	5	0	29	0
Panama	9	1	0	66	0
Venezuela	4	1	1	63	0
Brazil	27	2	0	44	0
Acre	8	1	0	39	0
Alagoas	20	2	0	39	0
Amapá	8	1	0	39	0
Amazonas	9	1	0	39	0
Bahia	20	2	0	39	0
Ceará	20	2	0	39	0
Distrito Federal	20	1	0	39	0
Espírito Santo	20	1	0	39	0
Goiás	20	1	0	39	0
Maranhão	20	2	0	39	0
Mato Grosso	20	1	0	39	0
Mato Grosso do Sul	20	1	0	39	0
Minas Gerais	20	1	0	39	0
Pará	9	1	0	39	0
Paraíba	20	2	0	38	0
Paraná	20	1	0	39	0
Pernambuco	20	2	0	39	0
Piauí	20	2	0	39	0
Rio de Janeiro	20	1	0	39	0
Rio Grande do Norte	20	2	0	39	0
Rio Grande do Sul	20	1	0	39	0
Rondônia	8	1	0	39	0
Roraima	8	1	0	39	0
Santa Catarina	20	1	0	39	0
São Paulo	19	1	0	39	0

Appendix Table 5. Number of 5q0 data sources by type and location, 1950-2019					
Geography	SBH	CBH	HH	VR/SRS/DSP	DSS
Sergipe	20	2	0	39	0
Tocantins	8	1	0	28	0
Paraguay	15	7	0	65	0
Afghanistan	6	1	0	0	0
Algeria	5	3	0	25	0
Bahrain	4	0	2	35	0
Egypt	13	11	2	64	0
Iran	5	1	3	2	0
Iraq	7	4	3	11	1
Jordan	12	7	0	23	0
Kuwait	3	0	1	53	0
Lebanon	4	2	0	6	0
Libya	4	1	0	15	0
Morocco	9	6	1	10	0
Palestine	8	4	3	10	0
Oman	4	0	3	12	0
Qatar	1	0	2	37	0
Saudi Arabia	2	0	4	0	0
Sudan	9	5	3	0	0
Syria	6	4	0	31	0
Tunisia	7	5	0	20	0
Turkey	14	5	1	36	0
United Arab Emirates	2	0	6	12	0
Yemen	7	5	2	0	0
Bangladesh	13	9	0	42	0
Bhutan	4	0	1	0	0
India	12	4	0	54	0
Nepal	12	7	2	0	0
Pakistan	14	8	2	24	0
China	5	0	29	23	0
Taiwan (Province of China)	0	0	0	62	0
American Samoa	1	0	0	53	0
Federated States of Micronesia	3	0	0	1	0
Fiji	7	1	0	55	0
Guam	0	0	0	66	0
Kiribati	7	0	1	12	0
Marshall Islands	2	0	1	14	0
Northern Mariana Islands	0	0	0	19	0
Papua New Guinea	8	1	2	2	0
Samoa	5	0	2	22	0
Solomon Islands	4	0	2	0	0
Tonga	3	0	2	16	0
Vanuatu	4	0	1	0	0
Cambodia	11	5	0	0	0
Indonesia	46	15	1	0	0
Aceh	32	4	0	0	0

Appendix Table 5. Number of 5q0 data sources by type and location, 1950-2019					
**Geography**	**SBH**	**CBH**	**HH**	**VR/SRS/DSP**	**DSS**
Bali	40	6	0	0	0
Bangka-Belitung Islands	25	5	2	0	0
Banten	33	5	0	0	0
Bengkulu	36	5	0	0	0
Gorontalo	22	4	1	0	0
Jakarta	40	6	0	0	0
Jambi	34	4	0	0	0
West Java	40	6	0	0	0
Central Java	40	6	0	0	0
East Java	40	6	0	0	0
West Kalimantan	36	5	0	0	0
South Kalimantan	40	6	0	0	0
Central Kalimantan	36	5	0	0	0
East Kalimantan	34	6	0	0	0
North Kalimantan	17	2	0	0	0
Riau Islands	29	4	0	0	0
Lampung	39	6	0	0	0
Maluku	33	4	0	0	0
North Maluku	24	4	0	0	0
West Nusa Tenggara	40	6	0	0	0
East Nusa Tenggara	34	5	0	0	0
Papua	30	3	1	0	0
West Papua	29	3	1	0	0
Riau	37	6	0	0	0
West Sulawesi	30	4	0	0	0
South Sulawesi	40	6	0	0	0
Central Sulawesi	36	5	0	0	0
Southeast Sulawesi	35	6	0	0	0
North Sulawesi	36	5	0	0	0
West Sumatra	40	6	0	0	0
South Sumatra	40	6	0	0	0
North Sumatra	40	6	0	0	0
Yogyakarta	40	6	2	0	0
Laos	4	2	1	0	0
Malaysia	3	1	0	53	0
Maldives	7	2	1	39	0
Mauritius	0	0	0	68	0
Myanmar	6	1	2	1	0
Philippines	13	6	0	63	0
Sri Lanka	5	2	4	59	0
Seychelles	2	0	0	64	0
Thailand	11	2	0	66	0
Timor-Leste	9	6	0	0	0
Vietnam	16	3	1	0	0
Angola	4	2	0	0	0
Central African Republic	5	1	0	0	0

Appendix Table 5. Number of 5q0 data sources by type and location, 1950-2019

Geography	SBH	CBH	HH	VR/SRS/DSP	DSS
Congo (Brazzaville)	5	4	0	0	0
DR Congo	5	2	0	0	0
Equatorial Guinea	1	0	1	0	0
Gabon	2	2	0	0	0
Burundi	8	3	0	0	0
Comoros	4	2	1	0	0
Djibouti	3	1	0	0	0
Eritrea	3	2	1	0	0
Ethiopia	8	4	0	0	0
Kenya	19	7	0	0	0
Madagascar	10	4	0	14	0
Malawi	19	8	0	0	0
Mozambique	9	4	0	0	0
Rwanda	13	7	0	0	0
Somalia	2	1	0	0	0
South Sudan	2	1	0	0	0
Tanzania	14	7	1	0	0
Uganda	17	7	0	0	0
Zambia	11	5	2	0	0
Botswana	9	2	1	0	0
Lesotho	10	4	0	1	0
Namibia	5	4	0	0	0
South Africa	10	2	4	31	0
Swaziland	4	3	0	0	0
Zimbabwe	9	8	1	2	0
Benin	9	7	2	0	0
Burkina Faso	12	4	3	0	0
Cameroon	9	6	0	0	0
Cape Verde	3	1	1	24	0
Chad	5	3	0	0	0
Cote d'Ivoire	6	10	0	0	0
The Gambia	6	1	1	0	0
Ghana	20	9	0	0	0
Guinea	4	4	1	0	0
Guinea-Bissau	4	1	0	0	0
Liberia	7	5	0	0	0
Mali	10	6	0	0	0
Mauritania	8	5	0	0	0
Niger	6	4	0	0	0
Nigeria	22	6	1	0	0
Sao Tome and Principe	6	2	0	20	0
Senegal	13	13	0	0	0
Sierra Leone	6	3	0	5	0
Togo	8	3	1	0	0

Appendix Table 6. New data sources added for GBD 2019 mortality estimation

Location	Year	Sources
Afghanistan	1981-2010	Afghanistan Special Demographic and Health Survey 2010
Afghanistan	2015-2015	Afghanistan Health Survey 2015
Albania	1990-2017	Albania Demographic and Health Survey 2017-2018
Andorra	2004-2008	Andorra Statistical Yearbook 1999-2008
Angola	1979-1995	Angola Multiple Indicator Cluster Survey 1996
Angola	2008-2008	Angola Integrated Inquiry into People's Well-Being 2008-2009
Argentina	2009-2009	Argentina Births and Deaths by Place of Residence 2009
Argentina	2010-2010	Argentina Births and Deaths by Place of Residence 2010
Argentina	2015-2015	Argentina Vital Statistics 2015
Argentina	2016-2016	Argentina Vital Statistics 2016
Bahrain	2000-2000	Bahrain Health Statistics 2000
Bahrain	2001-2001	Bahrain Health Statistics 2001
Bahrain	2002-2002	Bahrain Health Statistics 2002
Bahrain	2003-2003	Bahrain Health Statistics 2003
Bahrain	2004-2004	Bahrain Health Statistics 2004
Bahrain	2005-2005	Bahrain Health Statistics 2005
Bahrain	2006-2006	Bahrain Health Statistics 2006
Bahrain	2007-2007	Bahrain Health Statistics 2007
Bahrain	2008-2008	Bahrain Health Statistics 2008
Bahrain	2009-2009	Bahrain Health Statistics 2009
Bahrain	2010-2010	Bahrain Health Statistics 2010
Bahrain	2011-2011	Bahrain Health Statistics 2011
Bahrain	2012-2012	Bahrain Health Statistics 2012
Bahrain	2013-2013	Bahrain Health Statistics 2013
Bahrain	2014-2014	Bahrain Health Statistics 2014
Bahrain	2015-2015	Bahrain Health Statistics 2015
Bahrain	2016-2016	Bahrain Health Statistics 2016
Bangladesh	1984-2008	Bangladesh Multiple Indicator Cluster Survey 2009
Bangladesh	2012-2016	Bangladesh Sample Vital Registration System 2016
Bangladesh	2015-2015	Bangladesh Sample Vital Registration System 2015
Belarus	1978-1998	Belarus Population Census 1999 - IPUMS
Belize	1999-2003	Health Statistics of Belize 1999-2003
Belize	2004-2007	Health Statistics of Belize 2003-2007
Belize	2008-2010	Health Statistics of Belize 2006-2010
Benin	1988-2017	Benin Demographic and Health Survey 2017-2018
Benin	1989-2013	Benin Population and Housing Census 2013 - IPUMS
Benin	2002-2002	Benin Population and Housing Census 2002
Benin	2013-2013	Benin Population and Housing Census 2013
Bolivia	1965-1989	Bolivia Integrated Household Survey February 1989
Bolivia	1975-1999	Bolivia Household Survey 1999
Bolivia	1982-2006	Bolivia Household Survey 2006
Bolivia	1983-2007	Bolivia Household Survey 2007
Bolivia	1984-2008	Bolivia Household Survey 2008
Bolivia	1985-2009	Bolivia Household Survey 2009
Bolivia	1987-2011	Bolivia Household Survey 2011

Bolivia	1989-2013	Bolivia Household Survey 2013
Bolivia	1990-2014	Bolivia Household Survey 2014
Bolivia	1991-2015	Bolivia Household Survey 2015
Bolivia	1992-2016	Bolivia Household Survey 2016
Bolivia	1993-2017	Bolivia Household Survey 2017
Bolivia	2012-2012	Bolivia Population and Housing Census 2012
Bosnia and Herzegovina	1991-2010	Bosnia and Herzegovina Multiple Indicator Cluster Survey 2011-2012
Bosnia and Herzegovina	2005-2014	Bosnia and Herzegovina Demography and Social Statistics 2015
Botswana	1967-1991	Botswana Population and Housing Census 1991 - IPUMS
Botswana	1971-2017	Botswana Demographic Survey 2017
Botswana	1977-2001	Botswana Population and Housing Census 2001 - IPUMS
Botswana	2012-2012	Botswana Vital Statistics Report 2012
Botswana	2016-2016	Botswana Vital Statistics Report 2016
Brazil	1972-1996	Brazil Living Standards Measurement Survey 1996-1997
Brazil	1989-2013	Brazil National Health Survey 2013
Brazil	2016-2016	Brazil Civil Registry Statistics - Deaths, by Year of Occurrence, Type of Death, Sex, Age, Place of Occurrence, and Place of Residence of the Deceased
Brazil	2017-2017	Brazil Mortality Information System - Deaths 2017
Burkina Faso	1983-2007	Burkina Faso Global Fund Household Health Coverage Survey 2008
Burkina Faso	1986-2010	Burkina Faso Food Consumption and Iron Status Survey in Two Rural Provinces 2010
Cameroon	1977-2001	Cameroon Household Survey 2001
Canada	2010-2011	Canada CANSIM Database - Annual Deaths by Age, Sex, Provinces and Territories
Canada	2012-2016	Canada CANSIM Database - Deaths, by Age and Sex, Canada, Provinces and Territories, Annual
Chile	1994-1994	Chile Vital Statistics - Deaths 1994
Chile	1995-1995	Chile Vital Statistics - Deaths 1995
Chile	1996-1996	Chile Vital Statistics - Deaths 1996
China	1982-2006	China Health and Nutrition Survey 2006
China	2009-2009	Macao Demographics Statistics 2009
China	2010-2010	Macao Demographics Statistics 2010
China	2011-2011	Macao Demographics Statistics 2011
China	2012-2012	Macao Demographics Statistics 2012
Colombia	2012-2012	Colombia Vital Statistics - Deaths 2012
Colombia	2013-2013	Colombia Vital Statistics - Deaths 2013
Colombia	2014-2014	Colombia Vital Statistics - Deaths 2014
Colombia	2015-2015	Colombia Vital Statistics - Deaths 2015
Colombia	2016-2016	Colombia Vital Statistics - Deaths 2016
Comoros	1991-1991	Comoros Population and Housing Census 1991
Cook Islands	1989-2009	Cook Islands Population and Housing Census 2011
Costa Rica	1991-2009	Costa Rica Census 2011
Costa Rica	2015-2015	Costa Rica Registered Deaths 2015
Costa Rica	2016-2016	Costa Rica Death Statistics. Preliminary Data 2016
Costa Rica	2016-2016	Costa Rica Registered Deaths 2016
Côte d'Ivoire	1993-2017	Cote d'Ivoire Performance Monitoring and Accountability 2020 Survey, Round 1 2017
Croatia	2015-2015	Croatia Deaths by Single Age and Sex
Cuba	1967-1985	Cuba National Fertility Survey 1987
Cuba	1986-2010	Cuba Multiple Indicator Cluster Survey 2010-2011
Cuba	1989-2013	Cuba Multiple Indicator Cluster Survey 2014
Cuba	2010-2010	Cuba Statistical Yearbook 2010

Cuba	2011-2011	Cuba Statistical Yearbook 2011
Cuba	2012-2012	Cuba Statistical Yearbook 2012
Cuba	2013-2013	Cuba Statistical Yearbook 2013
Cuba	2013-2013	Cuba Health Statistics Yearbook 2013
Cuba	2014-2014	Cuba Statistical Yearbook 2014
Cuba	2014-2014	Cuba Health Statistics Yearbook 2014
Cuba	2015-2015	Cuba Statistical Yearbook 2015
Cuba	2015-2015	Cuba Health Statistics Yearbook 2015
Cuba	2016-2016	Cuba Health Statistics Yearbook 2016
Cuba	2017-2017	Cuba Statistical Yearbook 2017
Czech Republic	1971-1992	Czech Republic Reproductive Health Survey 1993
Czech Republic	1982-1989	Czechoslovakia Population and Housing Census 1991
Dominican Republic	1992-2013	Dominican Republic Special Demographic and Health Survey 2013
Ecuador	2013-2013	Ecuador Statistics of Births and Deaths (General and Fetal) 2013
Ecuador	2014-2014	Ecuador Vital Statistics Yearbook: Births and Deaths 2014
Ecuador	2017-2017	Ecuador General Deaths 2017
Egypt	1972-1996	Egypt In Depth Demographic and Health Survey 1996-1997
eSwatini	2007-2007	Swaziland Population and Housing Census 2007
Ethiopia	1994-2017	Ethiopia Mini Demographic and Health Survey 2014
Ghana	1984-2008	Ghana Child Verbal Autopsy Study 2008
Ghana	1988-2017	Ghana Special Demographic and Health Survey 2017
Ghana	1993-2017	Ghana Performance Monitoring and Accountability 2020 Survey, Round 6 2017
Ghana	2010-2010	Ghana Population and Housing Census 2010
Greece	1969-1987	Greece Population and Housing Census 1991 - IPUMS
Greenland	1979-2014	Greenland Deaths and Mean Population in Municipalities
Greenland	1988-2016	Greenland Deaths by District, Residence, Place of Birth, Age and Gender
Guam	2004-2004	Guam Statistical Yearbook 2004
Guatemala	1986-2010	Guatemala National Survey of Living Conditions 2011
Guatemala	2017-2017	Guatemala Vital Statistics 2017
Guyana	2014-2016	Guyana Statistical Bulletin June 2017
Honduras	1950-1974	Honduras Population and Housing Census 1974 - IPUMS
Honduras	1964-1988	Honduras Population and Housing Census 1988 - IPUMS
Honduras	1977-2001	Honduras Population and Housing Census 2001 - IPUMS
Hungary	2016-2016	Hungary Deaths by District 2016
Iceland	1981-2017	Iceland Deaths by Municipalities, Sex and Age
Iceland	2018-2018	Iceland Causes of Death Register 2018
India	1992-2016	India - Rajasthan Performance Monitoring and Accountability 2020 Survey, Round 2 2017
India	2007-2007	IHME GBD India Medical Certification of Cause of Death Estimates by State 2007
India	2017-2017	India SRS Statistical Report 2017
Indonesia	1986-2017	Indonesia Demographic and Health Survey 2017
Indonesia	1987-2011	Indonesia - West Papua Multiple Indicator Cluster Survey 2011
Indonesia	1987-2011	Indonesia - Papua Multiple Indicator Cluster Survey 2011
Indonesia	1992-2016	Indonesia Performance Monitoring and Accountability 2020 Survey, Round 2 2016-2017
Ireland	2011-2011	Ireland Census 2011
Italy	1969-1969	Italy Vital Registration - Deaths 1969
Italy	1970-1970	Italy Vital Registration - Deaths 1970
Italy	1971-1971	Italy Vital Registration - Deaths 1971

Italy	1972-1972	Italy Vital Registration - Deaths 1972
Italy	1973-1973	Italy Vital Registration - Deaths 1973
Italy	1974-1974	Italy Vital Registration - Deaths 1974
Italy	1975-1975	Italy Vital Registration - Deaths 1975
Italy	1976-1976	Italy Vital Registration - Deaths 1976
Italy	1977-1977	Italy Vital Registration - Deaths 1977
Italy	1978-1978	Italy Vital Registration - Deaths 1978
Italy	1979-1979	Italy Vital Registration - Deaths 1979
Italy	1980-1980	Italy Vital Registration - Deaths 1980
Italy	1981-1981	Italy Vital Registration - Deaths 1981
Italy	1982-1982	Italy Vital Registration - Deaths 1982
Italy	1983-1983	Italy Vital Registration - Deaths 1983
Italy	1984-1984	Italy Vital Registration - Deaths 1984
Italy	1985-1985	Italy Vital Registration - Deaths 1985
Italy	1986-1986	Italy Vital Registration - Deaths 1986
Italy	1987-1987	Italy Vital Registration - Deaths 1987
Italy	1988-1988	Italy Vital Registration - Deaths 1988
Italy	1989-1989	Italy Vital Registration - Deaths 1989
Italy	1990-1990	Italy Vital Registration - Deaths 1990
Italy	1991-1991	Italy Vital Registration - Deaths 1991
Italy	1992-1992	Italy Vital Registration - Deaths 1992
Italy	1993-1993	Italy Vital Registration - Deaths 1993
Italy	1994-1994	Italy Vital Registration - Deaths 1994
Italy	1995-1995	Italy Vital Registration - Deaths 1995
Italy	1996-1996	Italy Vital Registration - Deaths 1996
Italy	1997-1997	Italy Vital Registration - Deaths 1997
Italy	1998-1998	Italy Vital Registration - Deaths 1998
Italy	1999-1999	Italy Vital Registration - Deaths 1999
Italy	2000-2000	Italy Vital Registration - Deaths 2000
Italy	2001-2001	Italy Vital Registration - Deaths 2001
Italy	2002-2002	Italy Vital Registration - Deaths 2002
Italy	2003-2003	Italy Vital Registration - Deaths 2003
Italy	2004-2004	Italy Vital Registration - Deaths 2004
Italy	2005-2005	Italy Vital Registration - Deaths 2005
Italy	2006-2006	Italy Vital Registration - Deaths 2006
Italy	2007-2007	Italy Vital Registration - Deaths 2007
Italy	2008-2008	Italy Vital Registration - Deaths 2008
Italy	2009-2009	Italy Vital Registration - Deaths 2009
Italy	2010-2010	Italy Vital Registration - Deaths 2010
Italy	2011-2011	Italy Vital Registration - Deaths 2011
Italy	2012-2012	Italy Vital Registration - Deaths 2012
Italy	2014-2014	Italy Vital Registration - Deaths 2014
Italy	2015-2015	Italy Vital Registration - Deaths 2015
Italy	2016-2016	Italy Vital Registration - Deaths 2016
Jamaica	2011-2011	Jamaica Population and Housing Census 2011
Jamaica	2012-2012	Jamaica Vital Registration - Deaths 2012
Jamaica	2013-2013	Jamaica Vital Registration - Deaths 2013

Jamaica	2014-2014	Jamaica Vital Registration - Deaths 2014
Japan	2016-2016	Japan Vital Registration - Deaths 2016
Japan	2017-2017	Japan Vital Registration - Deaths 2017
Jordan	1988-2017	Jordan Demographic and Health Survey 2017-2018
Kazakhstan	1991-2015	Kazakhstan Multiple Indicator Cluster Survey 2015
Kazakhstan	2009-2016	Kazakhstan Demographic Yearbook 2017
Kyrgyzstan	2017-2017	Kyrgyzstan Vital Registration - Deaths 2017
Laos	1987-2011	Laos Demographic and Health Survey 2011-2012
Laos	1988-2017	Laos Multiple Indicator Cluster Survey 2017
Latvia	2016-2016	Latvia Dead by Gender and Age Group in Statistical Regions, Republic Cities and Districts
Lesotho	1972-1996	Lesotho Population and Housing Census 1996 - IPUMS
Lesotho	1982-2006	Lesotho Population and Housing Census 2006 - IPUMS
Lesotho	2006-2006	Lesotho Population and Housing Census 2006
Lithuania	2010-2010	Lithuania Causes of Death 2010
Lithuania	2011-2011	Lithuania Causes of Death 2011
Lithuania	2012-2012	Lithuania Causes of Death 2012
Madagascar	1984-1995	Madagascar - Antananorivo Mortality Report 1984-1995
Madagascar	1991-2015	Madagascar Malaria Indicator Survey 2016
Malawi	1998-1998	Malawi Statistical Yearbook 2001
Malawi	2008-2008	Malawi Statistical Yearbook 2009
Malaysia	1980-1980	Malaysia - Peninsular Vital Statistics 1980
Malaysia	1981-1981	Malaysia - Peninsular Vital Statistics 1981
Malaysia	1982-1982	Malaysia - Peninsular Vital Statistics 1982
Maldives	1988-2017	Maldives Demographic and Health Survey 2016-2017
Maldives	2005-2014	Maldives Health Statistics 2014
Maldives	2015-2015	Maldives Statistical Archive
Maldives	2016-2016	Maldives Statistical Yearbook 2016
Mali	1981-1984	Mali - Twelve Years of Urban Mortality in the Sahel. Levels, Trends, Seasons, and Causes of Mortality in Bamako, 1974-1985
Mali	2009-2009	Mali Population and Housing Census 2009
Mexico	1959-1983	Mexico Household Income and Expenditure Survey 1983-1984
Mexico	1980-2004	Mexico Household Income and Expenditure Survey 2004
Mexico	1981-2005	Mexico Household Income and Expenditure Survey 2005
Mexico	1982-2006	Mexico Household Income and Expenditure Survey 2006
Mexico	1990-2014	Mexico National Survey of Demographic Dynamics 2014
Mexico	1992-2016	Mexico Household Income and Expenditure Survey 2016
Mexico	1993-2017	Mexico Vital Registration - Registered Births 2017
Mexico	1994-2017	Mexico National Survey of Demographic Dynamics 2018
Mexico	2017-2017	Mexico Vital Registration - Deaths 2017
Moldova	2016-2017	Moldova Statistical Databank - Deceased by Age Group and Gender, in Territorial Profile
Mongolia	1992-2003	Tracking Maternal Mortality Declines in Mongolia Between 1992 and 2007: The Importance of Collaboration
Mongolia	1993-2016	Mongolia - Khuvsgul Multiple Indicator Cluster Survey 2016
Montenegro	1989-2010	Montenegro Multiple Indicator Cluster Survey 2013
Morocco	1962-2010	Morocco National Multiround Demographic Survey 2009-2010
Mozambique	1991-2015	Mozambique AIDS Indicator Survey 2015
Mozambique	2001-2001	Mozambique Main Causes of Reported Death Study 2001
Myanmar	1990-1990	Myanmar Population Change and Fertility Survey 1991
Myanmar	1995-1995	Myanmar Multiple Indicator Cluster Survey 1995

Myanmar	2003-2003	Myanmar Overall and Cause Specific Under-Five Mortality Survey 2002-2003
Nauru	1987-2006	Nauru Demographic and Health Survey 2007
Nauru	1990-2011	Nauru Population and Housing Census 2011
Nauru	1992-2002	Nauru Vital Statistics Report 2008-2013
Nauru	2002-2002	Nauru Population and Housing Census 2002
Nepal	2001-2011	Nepal Population and Housing Census 2011
New Zealand	2015-2015	New Zealand Mortality Collection 2015
Niger	1992-2016	Niger - Niamey Performance Monitoring and Accountability 2020 Survey, Round 3 2016
Niger	1993-2017	Niger Performance Monitoring and Accountability 2020 Survey, Round 4 2017
Nigeria	1983-2007	Nigeria General Household Survey 2008
Nigeria	1983-2007	Nigeria National HIV/AIDS and Reproductive Health Survey 2007
Nigeria	1987-1997	Nigeria Demographic and Health Survey 1999
Nigeria	1988-2012	Nigeria National HIV/AIDS and Reproductive Health Survey 2012
Nigeria	1991-2015	Nigeria Performance Monitoring and Accountability 2020 Survey, Round 3 2016
Nigeria	1992-2016	Nigeria Performance Monitoring and Accountability 2020 Survey, Round 4 2017
Nigeria	2006-2016	Nigeria Demographic and Health Survey 2018
Niue	1987-2011	Niue Vital Statistics Report 1987-2011
North Korea	2008-2008	North Korea Population Census 2008
Norway	1951-1951	Norway Cause of Death Registry 1951
Norway	1952-1952	Norway Cause of Death Registry 1952
Norway	1953-1953	Norway Cause of Death Registry 1953
Norway	1954-1954	Norway Cause of Death Registry 1954
Norway	1955-1955	Norway Cause of Death Registry 1955
Norway	1956-1956	Norway Cause of Death Registry 1956
Norway	1957-1957	Norway Cause of Death Registry 1957
Norway	1958-1958	Norway Cause of Death Registry 1958
Norway	1959-1959	Norway Cause of Death Registry 1959
Norway	1960-1960	Norway Cause of Death Registry 1960
Norway	1961-1961	Norway Cause of Death Registry 1961
Norway	1962-1962	Norway Cause of Death Registry 1962
Norway	1963-1963	Norway Cause of Death Registry 1963
Norway	1964-1964	Norway Cause of Death Registry 1964
Norway	1965-1965	Norway Cause of Death Registry 1965
Norway	1966-1966	Norway Cause of Death Registry 1966
Norway	1967-1967	Norway Cause of Death Registry 1967
Norway	1968-1968	Norway Cause of Death Registry 1968
Norway	2016-2016	Norway Cause of Death Registry 2016
Norway	2017-2017	Norway Cause of Death Registry 2017
Pakistan	1985-2009	Pakistan - Balochistan Multiple Indicator Cluster Survey 2010
Pakistan	1987-2011	Pakistan - Punjab Multiple Indicator Cluster Survey 2011
Pakistan	1989-2013	Pakistan - Sindh Multiple Indicator Cluster Survey 2014
Pakistan	1990-2014	Pakistan - Punjab Multiple Indicator Cluster Survey 2014
Pakistan	1993-2017	Pakistan Demographic and Health Survey 2017-2018
Pakistan	2017-2017	Pakistan - Punjab Multiple Indicator Cluster Survey 2017-2018
Palau	1974-1994	Palau Population and Housing Census 1995
Palau	1978-1999	Palau Population and Housing Census 2000
Palestine	1953-1965	Palestine Population and Housing Census 1967

Palestine	1976-2000	Palestine - West Bank and Gaza Strip Multiple Indicator Cluster Survey 2000 - UNICEF
Palestine	2012-2012	Palestine - Gaza Strip and West Bank Mortality by Sex, Age, and Cause 2012
Palestine	2013-2013	Palestine - Gaza Strip and West Bank Mortality by Sex, Age, and Cause 2013
Palestine	2014-2014	Palestine - Gaza Strip and West Bank Mortality by Sex, Age, and Cause 2014
Palestine	2017-2017	Palestine - Gaza Strip and West Bank Mortality by Sex, Age, and Cause 2017
Panama	1984-2008	Panama Living Standard Measurement Survey 2008
Papua New Guinea	1959-1979	Papua New Guinea Population and Housing Census 1980 - IPUMS
Papua New Guinea	1981-1998	Papua New Guinea Population and Housing Census 2000 - IPUMS
Peru	1976-2000	Peru National Household Survey, Second Quarter 2000
Peru	1993-2017	Peru Demographic and Family Health Survey 2017
Peru	1999-2013	Peru Continuous Demographic and Health Survey 2014
Philippines	1965-1965	Philippines Vital Statistics Report 1965
Philippines	1968-1968	Philippines Vital Statistics Report 1968
Philippines	1969-1969	Philippines Vital Statistics Report 1969
Philippines	1970-1970	Philippines Vital Statistics Report 1970
Philippines	1971-1971	Philippines Vital Statistics Report 1971
Philippines	1972-1972	Philippines Vital Statistics Report 1972
Philippines	1973-1973	Philippines Vital Statistics Report 1973
Philippines	1974-1974	Philippines Vital Statistics Report 1974
Philippines	1976-1976	Philippines Vital Statistics Report 1976
Philippines	1977-1977	Philippines Vital Statistics Report 1977
Philippines	1978-1978	Philippines Vital Statistics Report 1978
Philippines	1979-1979	Philippines Vital Statistics Report 1979
Philippines	1986-2010	Philippines Population and Housing Census 2010 - IPUMS
Philippines	1992-2017	Philippines Demographic and Health Survey 2017
Philippines	2006-2006	Philippines Vital Registration - Deaths 2006
Philippines	2007-2007	Philippines Vital Registration - Deaths 2007
Philippines	2008-2008	Philippines Vital Registration - Deaths 2008
Philippines	2009-2009	Philippines Vital Registration - Deaths 2009
Philippines	2010-2010	Philippines Vital Registration - Deaths 2010
Philippines	2011-2011	Philippines Vital Registration - Deaths 2011
Poland	1980-1980	Poland Vital Registration - Death Data 1980
Poland	1981-1981	Poland Vital Registration - Death Data 1981
Poland	1982-1982	Poland Vital Registration - Death Data 1982
Poland	1983-1983	Poland Vital Registration - Death Data 1983
Poland	1984-1984	Poland Vital Registration - Death Data 1984
Poland	1985-1985	Poland Vital Registration - Death Data 1985
Poland	1986-1986	Poland Vital Registration - Death Data 1986
Poland	1987-1987	Poland Vital Registration - Death Data 1987
Poland	1988-1988	Poland Vital Registration - Death Data 1988
Poland	1989-1989	Poland Vital Registration - Death Data 1989
Poland	1990-1990	Poland Vital Registration - Death Data 1990
Poland	1991-1991	Poland Vital Registration - Death Data 1991
Poland	1992-1992	Poland Vital Registration - Death Data 1992
Poland	1993-1993	Poland Vital Registration - Death Data 1993
Poland	1994-1994	Poland Vital Registration - Death Data 1994
Poland	1995-1995	Poland Vital Registration - Death Data 1995

Poland	1996-1996	Poland Vital Registration - Death Data 1996
Poland	1997-1997	Poland Vital Registration - Death Data 1997
Poland	1998-1998	Poland Vital Registration - Death Data 1998
Poland	1999-1999	Poland Vital Registration - Causes of Death Data 1999
Poland	2000-2000	Poland Vital Registration - Causes of Death Data 2000
Poland	2001-2001	Poland Vital Registration - Causes of Death Data 2001
Poland	2002-2002	Poland Vital Registration - Causes of Death Data 2002
Poland	2003-2003	Poland Vital Registration - Causes of Death Data 2003
Poland	2004-2004	Poland Vital Registration - Causes of Death Data 2004
Poland	2005-2005	Poland Vital Registration - Causes of Death Data 2005
Poland	2006-2006	Poland Vital Registration - Causes of Death Data 2006
Poland	2007-2007	Poland Vital Registration - Causes of Death Data 2007
Poland	2008-2008	Poland Vital Registration - Causes of Death Data 2008
Poland	2010-2010	Poland Vital Registration - Causes of Death Data 2010
Poland	2011-2011	Poland Vital Registration - Causes of Death Data 2011
Poland	2012-2012	Poland Vital Registration - Causes of Death Data 2012
Poland	2013-2013	Poland Vital Registration - Causes of Death Data 2013
Poland	2014-2014	Poland Vital Registration - Causes of Death Data 2014
Poland	2015-2015	Poland Vital Registration - Causes of Death Data 2015
Poland	2016-2016	Poland Vital Registration - Causes of Death Data 2016
Portugal	1980-1980	Portugal Vital Registration - Deaths 1980
Portugal	1981-1981	Portugal Vital Registration - Deaths 1981
Portugal	1982-1982	Portugal Vital Registration - Deaths 1982
Portugal	1983-1983	Portugal Vital Registration - Deaths 1983
Russia	1950-2016	Russia - Udmurtiya Deaths by Sex and Age 1939-2016
Russia	1989-1998	Russia Mortality Rates by Region, Age, Sex, and Cause of Death 1989-1998
Russia	1999-2005	Russia Mortality Rates by Region, Age, Sex, and Cause of Death 1999-2005
Russia	2006-2010	Russia Mortality Rates by Region, Age, Sex, and Cause of Death 2006-2012
Russia	2011-2014	Russia Mortality Rates by Region, Cause of Death, 5-Year Age Groups, and Sex, 2011-2014
Russia	2015-2016	Russia Mortality Rates by Region, 1-Year Age Groups, and Sex
Russia	2017-2017	Russia Mortality Rates by Region, 1-Year Age Groups 2015-2017
Russia	2017-2017	Russia Mortality by Region, Age, Sex, and Cause of Death 2017
Rwanda	1988-2012	Rwanda World Poll 2013
Rwanda	1988-2012	Rwanda Population and Housing Census 2012 - IPUMS
Rwanda	1993-2017	Rwanda Malaria Indicator Survey 2017
Samoa	2011-2011	Samoa Population and Housing Census 2011
São Tomé and PrÍncipe	2012-2012	Sao Tome and Principe Population and Housing Census 2012
Senegal	1990-2015	Senegal - Dakar Urban Multiple Indicator Cluster Survey 2015-2016
Senegal	1992-1999	Senegal Multiple Indicator Cluster Survey 2000
Serbia	1953-1961	UNSD Demographic Statistics - Deaths by Age, Sex, and Urban/Rural Residence
Serbia	1981-2003	Serbia and Montenegro - Serbia Multiple Indicator Cluster Survey 2005-2006
Serbia	1994-2012	Kosovo Multiple Indicator Cluster Survey 2013-2014
Sierra Leone	1988-2017	Sierra Leone Multiple Indicator Cluster Survey 2017
Sierra Leone	1992-2016	Sierra Leone Malaria Indicator Survey 2016
South Africa	1979-2003	South Africa Demographic and Health Survey 2003-2004
South Africa	1990-2014	South Africa National Income Dynamics Study - Wave 4 2014-2015
South Africa	1993-2017	Southern Africa Labour and Development Research Unit. National Income Dynamics Study 2017

South Africa	2010-2010	South Africa Mortality and Causes of Death 2010
South Africa	2016-2016	South Africa Vital Registration - Causes of Death 2016
South Korea	1983-2012	South Korea KOSIS Database - Deaths and Death Rates by Sex and Age Group
Sri Lanka	1992-1992	Sri Lanka Vital Statistics - Deaths 1992
Sri Lanka	1994-1994	Sri Lanka Vital Statistics - Deaths 1994
Sri Lanka	1995-1995	Sri Lanka Vital Statistics - Deaths 1995
Sri Lanka	1996-1996	Sri Lanka Vital Statistics - Deaths 1996
Sri Lanka	1997-1997	Sri Lanka Vital Statistics - Deaths 1997
Sri Lanka	1998-1998	Sri Lanka Vital Statistics - Deaths 1998
Sri Lanka	1999-1999	Sri Lanka Vital Statistics - Deaths 1999
Sri Lanka	2000-2000	Sri Lanka Vital Statistics - Deaths 2000
Sri Lanka	2001-2001	Sri Lanka Vital Statistics - Deaths 2001
Sri Lanka	2002-2002	Sri Lanka Vital Statistics - Deaths 2002
Sri Lanka	2003-2003	Sri Lanka Vital Statistics - Deaths 2003
Sri Lanka	2004-2004	Sri Lanka Vital Statistics - Deaths 2004
Sri Lanka	2005-2005	Sri Lanka Vital Statistics - Deaths 2005
Sri Lanka	2006-2006	Sri Lanka Vital Statistics - Deaths 2006
Sri Lanka	2009-2009	Sri Lanka Vital Statistics - Deaths 2009
Sri Lanka	2010-2010	Sri Lanka Vital Statistics - Deaths 2010
Sweden	1980-1980	Sweden Cause of Death Register 1980
Sweden	1981-1981	Sweden Cause of Death Register 1981
Sweden	1982-1982	Sweden Cause of Death Register 1982
Sweden	1983-1983	Sweden Cause of Death Register 1983
Sweden	1984-1984	Sweden Cause of Death Register 1984
Sweden	1985-1985	Sweden Cause of Death Register 1985
Sweden	1986-1986	Sweden Cause of Death Register 1986
Sweden	1987-1987	Sweden Cause of Death Register 1987
Sweden	1988-1988	Sweden Cause of Death Register 1988
Sweden	1989-1989	Sweden Cause of Death Register 1989
Sweden	1990-1990	Sweden Cause of Death Register 1990
Sweden	1991-1991	Sweden Cause of Death Register 1991
Sweden	1992-1992	Sweden Cause of Death Register 1992
Sweden	1993-1993	Sweden Cause of Death Register 1993
Sweden	1994-1994	Sweden Cause of Death Register 1994
Sweden	1995-1995	Sweden Cause of Death Register 1995
Sweden	1996-1996	Sweden Cause of Death Register 1996
Sweden	1997-1997	Sweden Cause of Death Register 1997
Sweden	1998-1998	Sweden Cause of Death Register 1998
Sweden	1999-1999	Sweden Cause of Death Register 1999
Sweden	2000-2000	Sweden Cause of Death Register 2000
Sweden	2001-2001	Sweden Cause of Death Register 2001
Sweden	2002-2002	Sweden Cause of Death Register 2002
Sweden	2003-2003	Sweden Cause of Death Register 2003
Sweden	2004-2004	Sweden Cause of Death Register 2004
Sweden	2005-2005	Sweden Cause of Death Register 2005
Sweden	2006-2006	Sweden Cause of Death Register 2006
Sweden	2007-2007	Sweden Cause of Death Register 2007

Sweden	2008-2008	Sweden Cause of Death Register 2008
Sweden	2009-2009	Sweden Cause of Death Register 2009
Sweden	2010-2010	Sweden Cause of Death Register 2010
Sweden	2011-2011	Sweden Cause of Death Register 2011
Sweden	2017-2017	Sweden Cause of Death Register 2017
Syria	2000-2000	Syria Multiple Indicator Cluster Survey 2000
Taiwan (province of China)	2007-2012	Taiwan Deaths by Sex, 5-Year Age Group, Counties, and Cities 2007-2012 - Department of Household Registration
Taiwan (province of China)	2011-2011	Taiwan Vital Registration - Deaths 2011
Taiwan (province of China)	2012-2012	Taiwan Vital Registration - Deaths 2012
Taiwan (province of China)	2013-2013	Taiwan Vital Registration - Deaths 2013
Taiwan (province of China)	2014-2014	Taiwan Vital Registration - Deaths 2014
Tanzania	1993-2017	Tanzania Malaria Indicator Survey 2017
Tanzania	2007-2007	Tanzania - Kigoma Reproductive Health Survey 2014
Thailand	1991-2015	Thailand - Bangkok Small Community Multiple Indicator Cluster Survey 2016
Thailand	1992-2016	Thailand Multiple Indicator Cluster Survey 2015-2016
Timor-Leste	1983-2007	Timor-Leste Living Standards and Measurement Survey 2007-2008
Togo	1993-2017	Togo Malaria Indicator Survey 2017
Togo	1996-1996	Togo Multiple Indicator Cluster Survey 1996
Tokelau	1980-1980	Mortality Trends in Pacific Island States
Trinidad and Tobago	1987-2011	Trinidad and Tobago Multiple Indicator Cluster Survey 2011
Turkey	2012-2012	Turkey Death Statistics 2010-2012
Turkey	2013-2013	Turkey Death Statistics 2011-2013
Turkey	2014-2014	Turkey Death Statistics 2014
Tuvalu	1986-2006	Tuvalu Demographic and Health Survey 2007
Tuvalu	1992-2002	Tuvalu Population and Housing Census 2002
Uganda	1987-2011	Uganda Living Standards Measurement Survey - Integrated Survey on Agriculture 2011-2012
Uganda	1990-2014	Uganda Performance Monitoring and Accountability 2020 Survey, Round 2 2015
Uganda	1992-2016	Uganda Performance Monitoring and Accountability 2020 Survey, Round 5 2017
UK	2017-2017	United Kingdom - England and Wales Mortality Statistics 2017
Ukraine	1989-2016	Ukraine Distribution of Deaths by Gender and Age Groups
Ukraine	2015-2015	Ukraine Vital Registration Deaths 2015
Ukraine	2015-2016	Russia Mortality Rates by Region, 1-Year Age Groups, and Sex
Ukraine	2016-2016	Ukraine Vital Registration Deaths 2016
Ukraine	2017-2017	Ukraine Mortality by Region, Age, Sex, and Cause of Death 2017
United Arab Emirates	2008-2008	United Arab Emirates Health Statistics 2008
United Arab Emirates	2009-2012	United Arab Emirates in Figures 2013
Uruguay	1989-2013	Uruguay Continuous Household Survey 2013
Uruguay	1992-2016	Uruguay Continuous Household Survey 2016
Uruguay	1993-2017	Uruguay Continuous Household Survey 2017
USA	1968-1978	United States CDC Wonder Compressed Mortality File 1968-1978
USA	1979-1979	United States CDC Wonder Compressed Mortality File 1979-1998
USA	2015-2015	United States NVSS Mortality Data 2015
USA	2017-2017	United States Deaths: Final Data for 2017
Vanuatu	1989-1989	Vanuatu Population and Housing Census 1989
Vanuatu	1999-1999	Vanuatu Population and Housing Census 1999
Vietnam	2008-2008	Vietnam Health Statistics Yearbook 2008
Zambia	2006-2016	Zambia Demographic and Health Survey 2018-2019

Zambia	2010-2010	Zambia Census of Population and Housing 2010
Zimbabwe	1988-2012	Zimbabwe Population Census 2012 - IPUMS
Zimbabwe	1992-1997	Zimbabwe Intercensal Demographic Survey 1997
Zimbabwe	2007-2007	Zimbabwe Mortality Report 2007

Appendix Table 7. Number of outliers for the age-sex model by location, source type, and age group

Location	Source	Outliers Early Neonatal	Outliers Late Neonatal	Outliers Post Neonatal	Outliers Infant	Outliers Child
Afghanistan	DHS	33	33	33	33	33
Albania	VR	108	45	45	45	45
Albania	LSMS	15	15	15	15	15
Albania	DHS	36	21	18	18	20
Albania	RHS	12	4	3	3	3
Algeria	VR	9	9	9	9	9
Algeria	Other	12	12	12	12	12
Algeria	PAPFAM	9	9	9	9	9
Algeria	MICS	9	9	9	9	9
American Samoa	VR	12	25	13	9	20
Andorra	VR	27	27	27	34	43
Angola	Other	9	9	9	9	9
Angola	DHS	9	9	9	9	9
Antigua and Barbuda	VR	54	63	52	51	56
Antigua and Barbuda	Other	2	2	2	2	2
Argentina	VR	3	3	3	3	3
Argentina	Other	20	20	20	20	20
Armenia	VR	114	114	114	114	114
Armenia	DHS	36	39	36	36	37
Azerbaijan	VR	117	117	117	117	117
Azerbaijan	DHS	9	9	9	9	9
Bahrain	VR	6	6	6	6	6
Bangladesh	WFS	6	6	6	6	6
Bangladesh	DHS	90	90	90	90	90
Bangladesh	Other	24	24	24	24	24
Barbados	VR	0	0	0	0	9
Barbados	Other	6	6	6	6	6
Belarus	VR	75	75	75	75	75
Belgium	Other	4	4	4	4	4
Belize	RHS	15	18	15	15	15
Belize	MICS	9	12	9	9	9
Belize	Other	3	3	3	3	3
Benin	WFS	9	9	9	9	9
Benin	DHS	45	45	45	45	45
Benin	MICS	9	9	9	9	9
Bermuda	VR	48	70	63	48	78
Bermuda	Other	2	2	2	2	2
Bolivia	VR	36	36	36	36	36
Bolivia	DHS	54	54	45	45	45
Bolivia	Other	18	18	18	18	18
Botswana	DHS	15	6	6	6	6
Botswana	Other	23	21	14	14	14
Botswana	VR	21	21	21	21	21
Brazil	DHS	24	24	24	24	24
Brazil	VR	45	45	45	45	45
Brazil	Other	29	29	29	29	29
Brazil - Acre	VR	54	54	54	54	54
Brazil - Acre	DHS	9	9	9	9	9
Brazil - Acre	Other	21	21	21	21	21
Brazil - Alagoas	DHS	17	21	12	12	14
Brazil - Alagoas	VR	21	21	21	21	21
Brazil - Alagoas	Other	6	6	6	6	6
Brazil - Amapá	VR	6	6	6	6	6
Brazil - Amapá	DHS	9	9	9	9	9
Brazil - Amapá	Other	23	23	23	23	23
Brazil - Amazonas	DHS	12	14	12	9	11
Brazil - Amazonas	Other	12	12	12	12	12
Brazil - Bahia	DHS	21	22	21	21	21
Brazil - Bahia	VR	3	3	3	3	3
Brazil - Ceará	DHS	15	16	15	15	17
Brazil - Distrito Federal	DHS	18	18	18	18	18
Brazil - Distrito Federal	VR	30	30	30	30	30
Brazil - Distrito Federal	Other	19	19	19	19	19
Brazil - Espírito Santo	DHS	21	21	21	21	21
Brazil - Espírito Santo	Other	28	28	28	28	28
Brazil - Goiás	DHS	21	21	21	21	21
Brazil - Goiás	VR	3	3	3	3	3
Brazil - Goiás	Other	9	9	9	9	9
Brazil - Maranhão	DHS	19	17	12	12	12
Brazil - Maranhão	VR	9	9	9	9	9
Brazil - Mato Grosso	DHS	13	11	10	9	10
Brazil - Mato Grosso	VR	9	9	9	9	9
Brazil - Mato Grosso	Other	18	18	18	18	18
Brazil - Mato Grosso do Sul	DHS	9	13	10	9	14
Brazil - Mato Grosso do Sul	VR	15	15	15	15	15
Brazil - Mato Grosso do Sul	Other	23	23	23	23	23
Brazil - Minas Gerais	DHS	12	14	13	12	14
Brazil - Minas Gerais	Other	23	23	23	23	23
Brazil - Paraná	DHS	21	21	21	21	21
Brazil - Paraná	VR	9	9	9	9	9
Brazil - Paraná	Other	36	36	36	36	36

Appendix Table 7. Number of outliers for the age-sex model by location, source type, and age group

Location	Source	Outliers Early Neonatal	Outliers Late Neonatal	Outliers Post Neonatal	Outliers Infant	Outliers Child
Brazil - Paraíba	DHS	11	14	9	9	14
Brazil - Paraíba	VR	3	3	3	3	3
Brazil - Paraíba	Other	4	4	4	4	4
Brazil - Pará	DHS	13	13	9	9	10
Brazil - Pará	VR	15	15	15	15	15
Brazil - Pará	Other	5	5	5	5	5
Brazil - Pernambuco	DHS	19	18	18	18	19
Brazil - Pernambuco	Other	27	27	27	27	27
Brazil - Piauí	DHS	21	25	21	21	21
Brazil - Piauí	VR	3	3	3	3	3
Brazil - Piauí	Other	12	12	12	12	12
Brazil - Rio Grande do Norte	DHS	18	17	12	12	14
Brazil - Rio Grande do Norte	Other	6	6	6	6	6
Brazil - Rio Grande do Sul	DHS	13	16	13	12	19
Brazil - Rio Grande do Sul	VR	30	30	30	30	30
Brazil - Rio Grande do Sul	Other	23	23	23	23	23
Brazil - Rio de Janeiro	DHS	13	15	13	12	17
Brazil - Rio de Janeiro	Other	33	33	33	33	33
Brazil - Rondônia	DHS	12	12	12	12	12
Brazil - Roraima	VR	63	63	63	63	63
Brazil - Roraima	DHS	9	9	9	9	9
Brazil - Roraima	Other	10	10	10	10	10
Brazil - Santa Catarina	DHS	18	18	18	18	18
Brazil - Santa Catarina	Other	35	35	35	35	35
Brazil - Sergipe	DHS	12	15	10	9	15
Brazil - Sergipe	VR	6	6	6	6	6
Brazil - Sergipe	Other	27	27	27	27	27
Brazil - São Paulo	DHS	15	16	15	15	19
Brazil - São Paulo	VR	24	24	24	24	24
Brazil - São Paulo	Other	24	24	24	24	24
Brazil - Tocantins	DHS	9	9	9	9	9
Brazil - Tocantins	VR	9	9	9	9	9
Brazil - Tocantins	Other	10	10	10	10	10
Burkina Faso	DHS	36	36	36	36	36
Burkina Faso	Other	3	3	3	3	3
Burundi	DHS	24	24	24	24	24
Cambodia	DHS	45	45	45	45	45
Cameroon	WFS	9	9	9	9	9
Cameroon	DHS	39	39	39	39	39
Cameroon	MICS	9	9	9	9	9
Canada	Other	3	3	3	3	3
Cape Verde	RHS	6	6	6	6	6
Central African Republic	DHS	9	9	9	9	9
Chad	DHS	30	30	30	30	30
Chile	Other	4	4	4	4	4
Colombia	VR	51	51	51	51	51
Colombia	WFS	6	6	6	6	6
Colombia	DHS	69	69	69	69	69
Colombia	Other	19	19	19	19	19
Comoros	DHS	18	18	18	18	18
Congo (Brazzaville)	DHS	48	48	48	48	48
Congo (Brazzaville)	MICS	9	9	9	9	9
Cook Islands	VR	36	36	36	36	51
Costa Rica	WFS	6	6	6	6	6
Costa Rica	RHS	5	5	5	5	5
Croatia	VR	3	3	3	3	3
Cyprus	VR	12	12	12	12	12
Czech Republic	RHS	15	15	15	15	15
Côte d'Ivoire	WFS	9	9	9	9	9
Côte d'Ivoire	DHS	24	24	24	24	24
Côte d'Ivoire	LSMS	3	3	3	3	3
Côte d'Ivoire	Other	9	9	9	9	9
Côte d'Ivoire	MICS	9	9	9	9	9
DR Congo	DHS	21	21	21	21	21
Djibouti	PAPFAM	15	15	15	15	15
Dominica	VR	189	189	189	189	189
Dominican Republic	VR	144	144	144	144	144
Dominican Republic	WFS	33	33	33	33	33
Dominican Republic	DHS	78	82	78	78	78
Dominican Republic	MICS	12	12	12	12	12
Ecuador	WFS	18	18	18	18	18
Ecuador	VR	180	72	72	72	72
Ecuador	DHS	15	6	6	6	6
Ecuador	RHS	45	27	27	27	27
Egypt	VR	171	171	171	171	171
Egypt	WFS	9	9	9	9	9
Egypt	Other	12	12	12	12	12
Egypt	DHS	81	81	81	81	81
El Salvador	VR	195	195	195	195	195
El Salvador	RHS	30	30	30	30	30
El Salvador	MICS	9	9	9	9	9

Appendix Table 7. Number of outliers for the age-sex model by location, source type, and age group

Location	Source	Outliers Early Neonatal	Outliers Late Neonatal	Outliers Post Neonatal	Outliers Infant	Outliers Child
England - Barking and Dagenham	VR	18	21	5	3	18
England - Barnet	VR	15	19	2	0	12
England - Barnsley	VR	27	25	11	6	23
England - Bath and North East Somerset	VR	39	43	31	20	45
England - Bedford	VR	26	29	21	10	37
England - Bexley	VR	25	29	11	6	36
England - Birmingham	VR	15	15	0	0	0
England - Blackburn with Darwen	VR	18	20	1	0	13
England - Blackpool	VR	18	21	7	0	36
England - Bolton	VR	19	20	4	3	16
England - Bournemouth	VR	25	27	19	9	52
England - Bracknell Forest	VR	33	35	37	12	49
England - Bradford	VR	15	16	0	0	0
England - Brent	VR	18	19	3	3	13
England - Brighton and Hove	VR	16	17	10	0	25
England - Bristol, City of	VR	15	15	1	0	5
England - Bromley	VR	16	22	9	0	14
England - Buckinghamshire	VR	15	17	0	0	2
England - Bury	VR	16	19	7	0	24
England - Calderdale	VR	22	26	8	6	20
England - Cambridgeshire	VR	15	17	1	0	3
England - Camden	VR	27	28	15	10	29
England - Central Bedfordshire	VR	21	24	24	6	20
England - Cheshire East	VR	15	18	5	0	14
England - Cheshire West and Chester	VR	18	19	10	3	10
England - Cornwall	VR	15	18	2	0	8
England - County Durham	VR	18	19	3	3	16
England - Coventry	VR	15	15	1	0	10
England - Croydon	VR	16	16	1	0	6
England - Cumbria	VR	15	21	1	0	8
England - Darlington	VR	30	34	24	13	61
England - Derby	VR	15	18	1	0	15
England - Derbyshire	VR	15	17	1	0	0
England - Devon	VR	15	15	0	0	2
England - Doncaster	VR	15	15	1	0	10
England - Dorset	VR	16	18	9	0	17
England - Dudley	VR	15	16	2	0	10
England - Ealing	VR	18	24	7	3	17
England - East Midlands	VR	15	15	0	0	0
England - East Riding of Yorkshire	VR	21	23	17	6	17
England - East Sussex	VR	15	21	2	0	3
England - East of England	VR	15	15	0	0	0
England - Enfield	VR	18	20	4	3	15
England - England	VR	15	15	0	0	0
England - Essex	VR	15	15	0	0	0
England - Gateshead	VR	20	24	13	4	28
England - Gloucestershire	VR	15	17	0	0	8
England - Greater London	VR	15	15	0	0	0
England - Greenwich	VR	15	18	3	0	13
England - Hackney	VR	15	16	0	0	6
England - Halton	VR	28	30	23	10	44
England - Hammersmith and Fulham	VR	21	26	9	6	34
England - Hampshire	VR	15	15	0	0	0
England - Haringey	VR	16	20	2	0	15
England - Harrow	VR	21	24	9	6	27
England - Hartlepool	VR	41	45	34	18	52
England - Havering	VR	21	24	18	3	27
England - Herefordshire, County of	VR	23	23	18	3	40
England - Hertfordshire	VR	15	15	0	0	1
England - Hillingdon	VR	22	19	5	3	21
England - Hounslow	VR	21	24	6	6	23
England - Isle of Wight	VR	47	51	48	27	65
England - Islington	VR	19	24	7	3	19
England - Kensington and Chelsea	VR	36	41	22	16	46
England - Kent	VR	15	15	0	0	0
England - Kingston upon Hull, City of	VR	15	17	0	0	18
England - Kingston upon Thames	VR	37	42	38	21	59
England - Kirklees	VR	15	17	0	0	3
England - Knowsley	VR	20	23	13	4	30
England - Lambeth	VR	15	17	1	0	13
England - Lancashire	VR	15	15	0	0	0
England - Leeds	VR	13	14	0	0	3
England - Leicester	VR	15	17	0	0	9
England - Leicestershire	VR	15	15	2	0	8
England - Lewisham	VR	15	18	2	0	12
England - Lincolnshire	VR	15	15	0	0	4
England - Liverpool	VR	15	15	0	0	7
England - Luton	VR	18	22	3	3	16
England - Manchester	VR	15	16	0	0	2
England - Medway	VR	15	18	5	0	20
England - Merton	VR	22	31	12	6	31

Appendix Table 7. Number of outliers for the age-sex model by location, source type, and age group						
**Location**	**Source**	**Outliers Early Neonatal**	**Outliers Late Neonatal**	**Outliers Post Neonatal**	**Outliers Infant**	**Outliers Child**
England - Middlesbrough	VR	17	18	9	0	20
England - Milton Keynes	VR	18	19	5	3	14
England - Newcastle upon Tyne	VR	19	28	14	3	15
England - Newham	VR	15	16	0	0	5
England - Norfolk	VR	15	16	0	0	2
England - North East England	VR	15	15	0	0	0
England - North East Lincolnshire	VR	24	25	16	6	26
England - North Lincolnshire	VR	22	28	19	6	47
England - North Somerset	VR	26	32	22	10	35
England - North Tyneside	VR	26	28	24	9	40
England - North West England	VR	15	15	0	0	0
England - North Yorkshire	VR	13	17	4	0	3
England - Northamptonshire	VR	15	15	0	0	2
England - Northumberland	VR	23	26	16	7	26
England - Nottingham	VR	15	17	0	0	11
England - Nottinghamshire	VR	15	16	0	0	3
England - Oldham	VR	15	17	2	0	12
England - Oxfordshire	VR	15	17	0	0	1
England - Peterborough	VR	16	20	0	0	13
England - Plymouth	VR	18	21	5	3	26
England - Poole	VR	35	38	24	8	48
England - Portsmouth	VR	19	25	9	3	37
England - Reading	VR	18	28	11	3	45
England - Redbridge	VR	16	17	2	0	12
England - Redcar and Cleveland	VR	41	43	40	22	59
England - Richmond upon Thames	VR	26	30	21	6	44
England - Rochdale	VR	15	23	1	0	14
England - Rotherham	VR	18	21	4	3	17
England - Rutland	VR	89	89	92	79	109
England - Salford	VR	16	16	4	0	17
England - Sandwell	VR	15	17	0	0	9
England - Sefton	VR	21	23	13	6	36
England - Sheffield	VR	15	15	1	0	5
England - Shropshire	VR	18	22	11	3	24
England - Slough	VR	18	25	5	3	29
England - Solihull	VR	30	35	25	15	45
England - Somerset	VR	16	19	2	0	7
England - South East England	VR	15	15	0	0	0
England - South Gloucestershire	VR	24	30	18	9	29
England - South Tyneside	VR	32	35	26	13	54
England - South West England	VR	15	15	0	0	0
England - Southampton	VR	19	24	7	3	33
England - Southend-on-Sea	VR	21	27	12	3	44
England - Southwark	VR	15	16	3	0	15
England - St Helens	VR	32	35	23	15	42
England - Staffordshire	VR	15	16	1	0	2
England - Stockport	VR	16	17	6	0	24
England - Stockton-on-Tees	VR	21	27	17	6	35
England - Stoke-on-Trent	VR	15	20	1	0	16
England - Suffolk	VR	15	15	1	0	3
England - Sunderland	VR	23	25	12	6	21
England - Surrey	VR	15	15	0	0	2
England - Sutton	VR	20	24	13	4	40
England - Swindon	VR	16	16	10	0	32
England - Tameside	VR	18	23	4	1	18
England - Telford and Wrekin	VR	19	22	10	3	23
England - Thurrock	VR	16	22	13	0	24
England - Torbay	VR	32	37	35	10	61
England - Tower Hamlets	VR	16	20	1	0	14
England - Trafford	VR	19	22	14	3	20
England - Wakefield	VR	16	18	4	0	14
England - Walsall	VR	15	16	0	0	15
England - Waltham Forest	VR	21	21	7	6	12
England - Wandsworth	VR	15	16	2	0	13
England - Warrington	VR	16	15	8	0	26
England - Warwickshire	VR	13	16	3	0	8
England - West Berkshire	VR	30	34	31	12	52
England - West Midlands	VR	15	15	0	0	0
England - West Sussex	VR	15	16	0	0	2
England - Westminster	VR	20	23	8	4	19
England - Wigan	VR	15	19	3	0	9
England - Wiltshire	VR	15	17	0	0	14
England - Windsor and Maidenhead	VR	37	37	32	19	53
England - Wirral	VR	21	24	8	6	11
England - Wokingham	VR	40	50	47	21	53
England - Wolverhampton	VR	15	16	0	0	21
England - Worcestershire	VR	15	16	1	0	6
England - York	VR	28	26	21	10	34
England - Yorkshire and the Humber	VR	15	15	0	0	0
Eritrea	DHS	18	18	18	18	18
Ethiopia	DHS	57	57	57	57	57

Appendix Table 7. Number of outliers for the age-sex model by location, source type, and age group

Location	Source	Outliers Early Neonatal	Outliers Late Neonatal	Outliers Post Neonatal	Outliers Infant	Outliers Child
Federated States of Micronesia	VR	3	3	3	3	3
Fiji	VR	165	165	165	39	42
Fiji	WFS	15	15	15	6	6
Gabon	DHS	18	20	18	18	18
Georgia	VR	69	69	69	69	69
Georgia	RHS	29	29	29	29	29
Germany	Other	1	1	1	1	1
Ghana	WFS	9	9	9	9	9
Ghana	DHS	75	84	75	75	75
Ghana	MICS	18	18	18	18	18
Grenada	VR	18	30	18	18	22
Grenada	Other	3	3	3	3	3
Guam	VR	0	8	0	0	3
Guatemala	VR	3	6	3	3	3
Guatemala	DHS	36	36	36	36	36
Guatemala	RHS	18	18	18	18	18
Guinea	DHS	27	27	27	27	27
Guinea	MICS	9	9	9	9	9
Guinea-Bissau	MICS	9	9	9	9	9
Guyana	VR	129	129	129	129	129
Guyana	WFS	6	6	6	6	6
Guyana	Other	9	10	9	9	10
Guyana	DHS	9	9	9	9	10
Guyana	MICS	9	9	9	9	9
Haiti	WFS	6	6	6	6	6
Haiti	DHS	45	45	45	45	45
Haiti	VR	15	15	15	15	15
Honduras	VR	132	132	132	132	132
Honduras	RHS	18	18	18	18	18
Honduras	DHS	21	21	21	21	21
Iceland	VR	3	6	3	3	4
India	VR	45	45	45	45	45
India	DHS	48	48	48	48	48
India	Other	33	33	33	33	33
India - Andhra Pradesh	DHS	54	54	54	54	54
India - Andhra Pradesh	Other	90	90	90	90	90
India - Andhra Pradesh	VR	9	9	9	9	9
India - Arunachal Pradesh	DHS	12	13	12	12	12
India - Arunachal Pradesh	Other	9	9	9	9	9
India - Arunachal Pradesh	VR	9	9	9	9	9
India - Assam	DHS	24	24	24	24	24
India - Assam	Other	75	75	75	75	75
India - Assam	VR	3	3	3	3	3
India - Bihar	DHS	27	27	27	27	27
India - Bihar	Other	68	68	68	68	68
India - Bihar	VR	3	3	3	3	3
India - Chhattisgarh	DHS	9	9	9	9	9
India - Chhattisgarh	Other	68	68	68	68	68
India - Chhattisgarh	VR	6	6	6	6	6
India - Delhi	DHS	21	21	21	21	21
India - Delhi	Other	77	77	77	77	77
India - Goa	DHS	21	24	21	21	21
India - Goa	Other	9	9	9	9	9
India - Goa	VR	21	21	21	21	21
India - Gujarat	DHS	24	24	24	24	24
India - Gujarat	Other	89	89	89	89	89
India - Gujarat	VR	15	15	15	15	15
India - Haryana	DHS	21	21	21	21	21
India - Haryana	Other	89	89	89	89	89
India - Himachal Pradesh	DHS	24	24	24	24	24
India - Himachal Pradesh	Other	89	89	89	89	90
India - Himachal Pradesh	VR	15	15	15	15	15
India - Jammu and Kashmir	DHS	24	24	24	24	24
India - Jammu and Kashmir	Other	56	56	56	56	57
India - Jammu and Kashmir	VR	15	15	15	15	15
India - Jharkhand	DHS	6	6	6	6	6
India - Jharkhand	Other	65	65	65	65	65
India - Karnataka	DHS	27	27	27	27	27
India - Karnataka	Other	91	91	91	91	91
India - Karnataka	VR	21	21	21	21	21
India - Kerala	DHS	27	31	27	27	29
India - Kerala	Other	102	102	102	102	102
India - Kerala	VR	21	21	21	21	21
India - Madhya Pradesh	DHS	27	27	27	27	27
India - Madhya Pradesh	Other	66	66	66	66	66
India - Madhya Pradesh	VR	21	21	21	21	21
India - Maharashtra	DHS	27	27	27	27	27
India - Maharashtra	Other	105	105	105	105	105
India - Maharashtra	VR	9	9	9	9	9
India - Manipur	DHS	21	21	21	21	21
India - Manipur	Other	9	9	9	9	9

Appendix Table 7. Number of outliers for the age-sex model by location, source type, and age group						
**Location**	**Source**	**Outliers Early Neonatal**	**Outliers Late Neonatal**	**Outliers Post Neonatal**	**Outliers Infant**	**Outliers Child**
India - Manipur	VR	15	15	15	15	15
India - Meghalaya	DHS	15	15	15	15	15
India - Meghalaya	Other	9	9	9	9	9
India - Mizoram	DHS	15	23	15	15	15
India - Mizoram	Other	9	9	9	9	9
India - Nagaland	DHS	18	17	17	15	19
India - Nagaland	Other	6	6	6	6	6
India - Nagaland	VR	15	15	15	15	15
India - Odisha	DHS	27	27	27	27	27
India - Odisha	Other	90	90	90	90	90
India - Odisha	VR	21	21	21	21	21
India - Punjab	DHS	18	18	18	18	18
India - Punjab	Other	89	89	89	89	89
India - Punjab	VR	21	21	21	21	21
India - Rajasthan	DHS	27	27	27	27	27
India - Rajasthan	Other	90	90	90	90	90
India - Rajasthan	VR	18	18	18	18	18
India - Sikkim	DHS	9	9	9	9	10
India - Sikkim	Other	9	9	9	9	9
India - Sikkim	VR	15	15	15	15	15
India - Tamil Nadu	DHS	27	27	27	27	27
India - Tamil Nadu	Other	89	89	89	89	89
India - Tamil Nadu	VR	21	21	21	21	21
India - Telangana	Other	72	72	72	72	73
India - Telangana	VR	6	6	6	6	6
India - Tripura	DHS	18	20	18	18	18
India - Tripura	Other	9	9	9	9	9
India - Tripura	VR	9	9	9	9	9
India - Union Territories other than Delhi	Other	9	9	9	9	9
India - Union Territories other than Delhi	VR	15	15	15	15	15
India - Uttar Pradesh	DHS	27	27	27	27	27
India - Uttar Pradesh	Other	80	80	80	80	80
India - Uttarakhand	DHS	6	6	6	6	6
India - Uttarakhand	Other	29	29	29	29	29
India - West Bengal	DHS	27	27	27	27	27
India - West Bengal	Other	89	89	89	89	89
India - West Bengal	VR	3	3	3	3	3
Indonesia	Other	45	45	45	45	45
Indonesia	WFS	6	6	6	6	6
Indonesia	DHS	96	96	96	96	96
Indonesia - Aceh	DHS	24	24	24	24	24
Indonesia - Bali	DHS	30	30	30	30	30
Indonesia - Bangka-Belitung Islands	DHS	15	15	15	15	15
Indonesia - Banten	DHS	18	18	18	18	18
Indonesia - Bengkulu	DHS	24	24	24	24	24
Indonesia - Central Java	DHS	30	30	30	30	30
Indonesia - Central Kalimantan	DHS	12	12	12	12	12
Indonesia - Central Sulawesi	DHS	21	21	21	21	21
Indonesia - East Java	DHS	30	30	30	30	30
Indonesia - East Kalimantan	DHS	15	15	15	15	15
Indonesia - East Nusa Tenggara	DHS	12	12	12	12	12
Indonesia - Gorontalo	DHS	12	12	12	12	12
Indonesia - Jakarta	DHS	30	30	30	30	30
Indonesia - Jambi	DHS	15	15	15	15	15
Indonesia - Lampung	DHS	27	27	27	27	27
Indonesia - Maluku	DHS	12	12	12	12	12
Indonesia - North Maluku	DHS	12	12	12	12	12
Indonesia - North Sulawesi	DHS	24	24	24	24	24
Indonesia - North Sumatra	DHS	30	30	30	30	30
Indonesia - Papua	DHS	12	12	12	12	12
Indonesia - Riau	DHS	27	27	27	27	27
Indonesia - Riau Islands	DHS	12	12	12	12	12
Indonesia - South Kalimantan	DHS	24	24	24	24	24
Indonesia - South Sulawesi	DHS	27	27	27	27	27
Indonesia - South Sumatra	DHS	27	27	27	27	27
Indonesia - Southeast Sulawesi	DHS	21	21	21	21	21
Indonesia - West Java	DHS	36	36	36	36	36
Indonesia - West Kalimantan	DHS	24	24	24	24	24
Indonesia - West Nusa Tenggara	DHS	24	24	24	24	24
Indonesia - West Papua	DHS	12	12	12	12	12
Indonesia - West Sulawesi	DHS	12	12	12	12	12
Indonesia - West Sumatra	DHS	27	27	27	27	27
Indonesia - Yogyakarta	DHS	30	30	30	30	30
Iran	Other	12	12	12	12	12
Iran	VR	78	78	78	78	78
Iraq	VR	60	60	60	60	60
Iraq	MICS	30	30	30	30	30
Iraq	Other	3	3	3	3	3
Jamaica	VR	42	42	42	42	42
Jamaica	WFS	6	6	6	6	6
Jamaica	Other	9	9	9	9	9

Appendix Table 7. Number of outliers for the age-sex model by location, source type, and age group

Location	Source	Outliers Early Neonatal	Outliers Late Neonatal	Outliers Post Neonatal	Outliers Infant	Outliers Child
Japan	VR	3	3	3	3	3
Japan - Akita	VR	1	15	2	1	1
Japan - Aomori	VR	0	5	0	0	1
Japan - Ehime	VR	0	10	0	0	0
Japan - Fukui	VR	1	13	1	0	3
Japan - Fukuoka	VR	0	2	0	0	0
Japan - Fukushima	VR	0	1	0	0	0
Japan - Gunma	VR	0	1	0	0	0
Japan - Hiroshima	VR	0	1	0	0	0
Japan - Hyōgo	VR	3	4	3	3	3
Japan - Ibaraki	VR	0	4	0	0	0
Japan - Ishikawa	VR	1	6	0	0	1
Japan - Iwate	VR	4	8	3	3	4
Japan - Kagawa	VR	1	11	1	1	0
Japan - Kagoshima	VR	0	2	0	0	0
Japan - Kyōto	VR	0	7	0	0	0
Japan - Kōchi	VR	0	13	1	0	3
Japan - Mie	VR	0	1	0	0	0
Japan - Miyagi	VR	3	5	3	3	3
Japan - Miyazaki	VR	0	1	0	0	0
Japan - Nagano	VR	0	9	0	0	0
Japan - Nara	VR	1	5	1	0	0
Japan - Niigata	VR	0	1	0	0	0
Japan - Okayama	VR	0	2	0	0	0
Japan - Saga	VR	2	14	1	0	0
Japan - Shiga	VR	0	1	0	0	1
Japan - Shimane	VR	2	13	1	0	2
Japan - Shizuoka	VR	0	2	0	0	0
Japan - Tochigi	VR	0	1	0	0	0
Japan - Tokushima	VR	1	5	1	0	1
Japan - Tottori	VR	8	20	6	3	4
Japan - Toyama	VR	0	5	0	0	1
Japan - Wakayama	VR	0	9	0	0	3
Japan - Yamagata	VR	0	4	0	0	2
Japan - Yamaguchi	VR	0	11	0	0	0
Japan - Yamanashi	VR	5	8	1	0	5
Japan - Ōita	VR	1	7	0	0	0
Jordan	DHS	63	63	63	63	63
Jordan	VR	66	66	66	66	66
Kazakhstan	VR	39	39	39	39	39
Kazakhstan	DHS	21	23	21	21	21
Kenya	WFS	9	9	9	9	9
Kenya	DHS	57	57	57	57	57
Kenya	Other	3	3	3	3	3
Kenya - Baringo	DHS	3	4	4	3	4
Kenya - Bomet	DHS	13	19	14	12	13
Kenya - Bungoma	DHS	14	17	7	6	6
Kenya - Bungoma	MICS	6	6	6	6	6
Kenya - Busia	DHS	35	44	34	33	37
Kenya - Elgeyo Marakwet	DHS	37	46	38	36	44
Kenya - Embu	DHS	49	55	48	48	49
Kenya - Embu	MICS	6	6	6	6	6
Kenya - Garissa	DHS	1	10	4	0	2
Kenya - Homa Bay	MICS	6	6	6	6	6
Kenya - Homa Bay	DHS	8	13	6	6	6
Kenya - Isiolo	DHS	21	22	21	21	23
Kenya - Isiolo	MICS	3	4	3	3	3
Kenya - Kajiado	DHS	44	52	44	40	45
Kenya - Kakamega	DHS	14	23	9	9	9
Kenya - Kakamega	MICS	6	8	6	6	6
Kenya - Kericho	DHS	20	34	19	18	23
Kenya - Kiambu	DHS	33	44	32	30	38
Kenya - Kilifi	DHS	19	32	19	18	20
Kenya - Kirinyaga	DHS	32	46	39	32	34
Kenya - Kisii	DHS	17	29	19	16	16
Kenya - Kisii	MICS	6	7	6	6	6
Kenya - Kisumu	DHS	11	23	3	3	4
Kenya - Kisumu	MICS	6	9	6	6	6
Kenya - Kitui	MICS	12	12	12	12	12
Kenya - Kitui	DHS	22	46	20	16	25
Kenya - Kwale	DHS	12	34	11	5	13
Kenya - Laikipia	DHS	48	48	49	48	51
Kenya - Lamu	DHS	24	27	23	23	24
Kenya - Machakos	DHS	30	37	32	30	34
Kenya - Machakos	MICS	6	9	7	6	6
Kenya - Makueni	DHS	25	27	25	24	25
Kenya - Makueni	MICS	6	6	6	6	6
Kenya - Mandera	DHS	11	17	5	3	7
Kenya - Marsabit	MICS	21	21	21	21	21
Kenya - Marsabit	DHS	21	24	22	21	22
Kenya - Meru	DHS	7	37	15	6	14

Appendix Table 7. Number of outliers for the age-sex model by location, source type, and age group

Location	Source	Outliers Early Neonatal	Outliers Late Neonatal	Outliers Post Neonatal	Outliers Infant	Outliers Child
Kenya - Meru	MICS	9	9	9	9	9
Kenya - Migori	DHS	5	13	3	3	3
Kenya - Migori	MICS	3	4	3	3	3
Kenya - Mombasa	DHS	3	30	9	0	8
Kenya - Mombasa	MICS	3	5	3	3	3
Kenya - Murang'a	DHS	32	52	34	30	41
Kenya - Nairobi	DHS	11	25	10	6	9
Kenya - Nakuru	DHS	23	49	22	18	32
Kenya - Nandi	DHS	37	51	38	36	37
Kenya - Narok	DHS	37	47	35	33	37
Kenya - Nyamira	DHS	25	26	26	24	29
Kenya - Nyamira	MICS	6	8	6	6	6
Kenya - Nyandarua	DHS	49	45	44	43	46
Kenya - Nyeri	DHS	35	46	38	33	44
Kenya - Samburu	DHS	18	21	18	18	19
Kenya - Siaya	DHS	16	36	12	12	12
Kenya - Siaya	MICS	6	6	6	6	6
Kenya - Taita Taveta	DHS	36	43	39	34	38
Kenya - Tana River	DHS	27	29	27	27	27
Kenya - Tharaka Nithi	MICS	6	10	6	6	6
Kenya - Tharaka Nithi	DHS	14	17	17	14	15
Kenya - Trans Nzoia	DHS	40	47	33	31	36
Kenya - Turkana	DHS	14	24	13	12	12
Kenya - Turkana	MICS	3	5	3	3	3
Kenya - Uasin Gishu	DHS	27	35	27	24	26
Kenya - Vihiga	DHS	14	20	9	7	9
Kenya - Wajir	DHS	5	11	5	5	4
Kenya - West Pokot	DHS	37	42	37	36	41
Kiribati	VR	12	12	12	12	12
Kyrgyzstan	VR	48	48	48	48	48
Kyrgyzstan	DHS	15	15	15	15	15
Kyrgyzstan	LSMS	6	6	6	6	6
Kyrgyzstan	MICS	9	9	9	9	9
Kyrgyzstan	Other	1	1	1	1	1
Laos	MICS	18	18	18	18	18
Latvia	VR	0	0	0	0	3
Lebanon	Other	12	12	12	12	13
Lebanon	PAPFAM	9	9	9	9	12
Lebanon	VR	24	24	24	24	24
Lesotho	WFS	6	6	6	6	6
Lesotho	DHS	27	27	27	27	27
Lesotho	VR	3	3	3	3	3
Liberia	DHS	39	39	39	39	39
Liberia	Other	9	9	9	9	9
Libya	Other	12	12	12	12	12
Libya	VR	12	12	12	12	12
Luxembourg	VR	2	15	3	0	7
Madagascar	VR	42	42	42	42	42
Madagascar	DHS	39	39	39	39	39
Malawi	DHS	51	51	51	51	51
Malawi	MICS	21	21	21	21	21
Malaysia	WFS	6	6	6	6	6
Maldives	VR	63	63	63	63	63
Maldives	DHS	18	18	18	18	18
Mali	DHS	45	45	45	45	45
Mali	MICS	9	9	9	9	9
Mali	Other	3	3	3	3	3
Malta	VR	0	6	0	0	4
Mauritania	WFS	9	9	9	9	9
Mauritania	Other	9	9	9	9	9
Mauritania	DHS	9	9	9	9	9
Mauritania	MICS	21	21	21	21	21
Mexico	VR	54	54	54	54	54
Mexico	WFS	6	6	6	6	6
Mexico	DHS	9	9	9	9	9
Mexico	Other	39	39	39	39	39
Mexico - Aguascalientes	Other	57	57	48	48	48
Mexico - Aguascalientes	DHS	9	9	9	9	9
Mexico - Aguascalientes	VR	36	36	36	36	36
Mexico - Baja California	Other	66	66	57	57	58
Mexico - Baja California	DHS	9	9	9	9	9
Mexico - Baja California	VR	9	9	9	9	9
Mexico - Baja California Sur	Other	47	47	38	38	39
Mexico - Baja California Sur	VR	54	54	54	54	54
Mexico - Campeche	Other	54	54	45	45	45
Mexico - Campeche	DHS	9	9	9	9	9
Mexico - Campeche	VR	51	51	51	51	51
Mexico - Chiapas	DHS	12	12	12	12	12
Mexico - Chiapas	Other	72	72	63	63	63
Mexico - Chihuahua	Other	69	69	60	60	60
Mexico - Chihuahua	DHS	9	9	9	9	9

Appendix Table 7. Number of outliers for the age-sex model by location, source type, and age group						
**Location**	**Source**	**Outliers Early Neonatal**	**Outliers Late Neonatal**	**Outliers Post Neonatal**	**Outliers Infant**	**Outliers Child**
Mexico - Coahuila	Other	69	69	60	60	60
Mexico - Coahuila	DHS	9	9	9	9	9
Mexico - Colima	Other	56	56	47	47	47
Mexico - Colima	DHS	9	9	9	9	9
Mexico - Colima	VR	36	36	36	36	36
Mexico - Durango	Other	67	67	58	58	58
Mexico - Durango	DHS	9	9	9	9	9
Mexico - Durango	VR	6	6	6	6	6
Mexico - Guanajuato	Other	68	68	59	59	59
Mexico - Guanajuato	DHS	9	9	9	9	9
Mexico - Guanajuato	VR	3	3	3	3	3
Mexico - Guerrero	DHS	12	12	12	12	12
Mexico - Guerrero	Other	65	65	56	56	56
Mexico - Guerrero	VR	12	12	12	12	12
Mexico - Hidalgo	Other	65	65	56	56	56
Mexico - Hidalgo	DHS	9	9	9	9	9
Mexico - Hidalgo	VR	21	21	21	21	21
Mexico - Jalisco	DHS	12	12	12	12	12
Mexico - Jalisco	Other	69	69	60	60	60
Mexico - Mexico City	DHS	12	12	12	12	12
Mexico - Mexico City	Other	69	69	60	60	60
Mexico - Michoacán de Ocampo	Other	68	68	59	59	59
Mexico - Michoacán de Ocampo	DHS	9	9	9	9	9
Mexico - Michoacán de Ocampo	VR	12	12	12	12	12
Mexico - Morelos	Other	65	65	56	56	56
Mexico - Morelos	DHS	9	9	9	9	9
Mexico - Morelos	VR	12	12	12	12	12
Mexico - México	Other	69	69	60	60	60
Mexico - México	DHS	9	9	9	9	9
Mexico - Nayarit	Other	62	62	53	53	53
Mexico - Nayarit	DHS	9	9	9	9	9
Mexico - Nayarit	VR	15	15	15	15	15
Mexico - Nuevo León	DHS	12	12	12	12	12
Mexico - Nuevo León	Other	67	67	58	58	59
Mexico - Nuevo León	VR	6	6	6	6	6
Mexico - Oaxaca	Other	67	67	58	58	58
Mexico - Oaxaca	DHS	9	9	9	9	9
Mexico - Oaxaca	VR	6	6	6	6	6
Mexico - Puebla	Other	70	70	61	61	61
Mexico - Puebla	DHS	9	9	9	9	9
Mexico - Puebla	VR	6	6	6	6	6
Mexico - Querétaro	DHS	12	12	12	12	12
Mexico - Querétaro	Other	63	63	54	54	54
Mexico - Querétaro	VR	9	9	9	9	9
Mexico - Quintana Roo	Other	59	59	50	50	50
Mexico - Quintana Roo	VR	27	27	27	27	27
Mexico - San Luis Potosí	Other	70	70	61	61	61
Mexico - San Luis Potosí	DHS	9	9	9	9	9
Mexico - San Luis Potosí	VR	6	6	6	6	6
Mexico - Sinaloa	Other	60	60	51	51	52
Mexico - Sinaloa	DHS	9	9	9	9	9
Mexico - Sinaloa	VR	30	30	30	30	30
Mexico - Sonora	Other	59	59	50	50	51
Mexico - Sonora	DHS	9	9	9	9	9
Mexico - Sonora	VR	30	30	30	30	30
Mexico - Tabasco	Other	66	66	57	57	57
Mexico - Tabasco	DHS	9	9	9	9	9
Mexico - Tabasco	VR	21	21	21	21	21
Mexico - Tamaulipas	Other	68	68	59	59	59
Mexico - Tamaulipas	DHS	9	9	9	9	9
Mexico - Tamaulipas	VR	3	3	3	3	3
Mexico - Tlaxcala	Other	60	60	51	51	51
Mexico - Tlaxcala	DHS	9	9	9	9	9
Mexico - Tlaxcala	VR	12	12	12	12	12
Mexico - Veracruz de Ignacio de la Llave	DHS	12	12	12	12	12
Mexico - Veracruz de Ignacio de la Llave	Other	64	64	55	55	55
Mexico - Veracruz de Ignacio de la Llave	VR	15	15	15	15	15
Mexico - Yucatán	Other	62	62	53	53	53
Mexico - Yucatán	DHS	9	9	9	9	9
Mexico - Yucatán	VR	18	18	18	18	18
Mexico - Zacatecas	Other	61	61	52	52	53
Mexico - Zacatecas	DHS	9	9	9	9	9
Mexico - Zacatecas	VR	21	21	21	21	21
Moldova	VR	75	75	75	75	75
Moldova	DHS	9	13	9	9	9
Moldova	MICS	9	13	9	9	12
Monaco	VR	33	33	31	33	43
Mongolia	VR	66	66	66	66	66
Mongolia	RHS	21	21	21	21	21
Mongolia	MICS	15	15	15	15	16
Montenegro	VR	9	9	9	9	10

Appendix Table 7. Number of outliers for the age-sex model by location, source type, and age group						
**Location**	**Source**	**Outliers Early Neonatal**	**Outliers Late Neonatal**	**Outliers Post Neonatal**	**Outliers Infant**	**Outliers Child**
Morocco	WFS	9	9	9	9	9
Morocco	DHS	51	51	51	51	51
Morocco	Other	9	9	9	9	9
Morocco	PAPFAM	24	24	24	24	24
Morocco	VR	30	30	30	30	30
Mozambique	DHS	45	45	45	45	45
Mozambique	MICS	12	12	12	12	12
Mozambique	RHS	6	6	6	6	6
Myanmar	DHS	9	9	9	9	9
Myanmar	VR	3	3	3	3	3
Namibia	DHS	36	36	36	36	36
Nauru	VR	9	9	9	9	19
Nepal	WFS	6	6	6	6	6
Nepal	DHS	45	45	45	45	45
Nepal	MICS	9	9	9	9	9
Nicaragua	VR	108	108	108	108	108
Nicaragua	RHS	18	18	18	18	18
Nicaragua	DHS	39	39	30	30	30
Nicaragua	Other	18	18	18	18	18
Niger	DHS	36	36	36	36	36
Nigeria	DHS	81	78	78	78	78
Nigeria	Other	12	12	12	12	12
Nigeria	MICS	12	12	12	12	12
Niue	VR	72	72	72	83	91
Northern Mariana Islands	VR	22	36	19	17	31
Norway	VR	48	48	48	48	48
Pakistan	WFS	6	6	6	6	6
Pakistan	DHS	51	51	51	51	51
Pakistan	Other	78	78	69	69	69
Pakistan	LSMS	18	18	12	12	12
Palau	VR	3	3	3	3	3
Palestine	Other	12	12	12	12	12
Palestine	DHS	18	18	9	9	9
Palestine	MICS	21	21	21	21	21
Palestine	VR	48	48	48	48	48
Panama	WFS	6	6	6	6	6
Papua New Guinea	VR	6	6	6	6	6
Papua New Guinea	DHS	18	18	18	18	18
Paraguay	VR	144	144	144	144	144
Paraguay	WFS	18	18	18	18	18
Paraguay	DHS	9	9	9	9	9
Paraguay	RHS	27	28	27	27	27
Paraguay	MICS	9	10	9	9	9
Peru	VR	180	180	180	180	180
Peru	WFS	9	9	9	9	9
Peru	DHS	186	186	186	186	186
Peru	Other	66	66	66	66	66
Philippines	VR	189	189	189	189	189
Philippines	WFS	9	9	9	9	9
Philippines	DHS	63	63	63	63	63
Portugal	VR	6	6	6	6	6
Portugal	WFS	9	9	9	9	9
Portugal	Other	2	2	2	2	2
Puerto Rico	RHS	9	9	9	9	12
Qatar	VR	108	108	108	108	108
Romania	LSMS	20	20	20	20	20
Rwanda	WFS	9	9	9	9	9
Rwanda	DHS	105	105	105	105	105
Saint Kitts and Nevis	VR	0	24	19	0	10
Saint Lucia	VR	3	5	4	3	5
Saint Lucia	Other	7	7	7	7	7
Saint Vincent and the Grenadines	VR	15	18	15	15	19
Saint Vincent and the Grenadines	Other	3	3	3	3	3
Samoa	VR	63	63	63	63	63
San Marino	VR	42	48	40	44	93
Saudi Arabia	VR	30	30	30	30	30
Senegal	WFS	9	9	9	9	9
Senegal	DHS	90	90	90	90	90
Senegal	Other	9	9	9	9	9
Senegal	MICS	9	9	9	9	9
Serbia	VR	30	30	30	30	30
Seychelles	VR	12	15	13	12	25
Sierra Leone	DHS	21	21	21	21	21
Sierra Leone	MICS	9	9	9	9	9
Sierra Leone	VR	15	15	15	15	15
Singapore	Other	26	26	26	26	26
Somalia	MICS	6	6	6	6	6
South Africa	DHS	18	21	18	18	18
South Africa	Other	32	32	32	32	32
South Africa	VR	21	21	21	21	21
South Korea	WFS	6	6	6	6	6

Appendix Table 7. Number of outliers for the age-sex model by location, source type, and age group

Location	Source	Outliers Early Neonatal	Outliers Late Neonatal	Outliers Post Neonatal	Outliers Infant	Outliers Child
South Korea	VR	87	87	87	87	87
South Sudan	MICS	9	9	9	9	9
Sri Lanka	VR	6	6	6	6	6
Sri Lanka	WFS	6	6	6	6	6
Sri Lanka	DHS	9	9	9	9	9
Sudan	WFS	9	9	9	9	9
Sudan	Other	12	12	12	12	12
Sudan	DHS	9	9	9	9	9
Sudan	MICS	18	18	18	18	18
Suriname	VR	138	138	138	138	138
Sweden	VR	3	3	3	3	3
Sweden	Other	3	3	3	3	3
Sweden - Stockholm	VR	3	4	3	3	3
Sweden - Sweden except Stockholm	VR	3	3	3	3	3
Sweden - Sweden except Stockholm	Other	3	3	3	3	3
Syria	WFS	9	9	9	9	9
Syria	VR	60	60	60	60	60
Syria	Other	9	9	9	9	9
Syria	PAPFAM	30	30	30	30	30
São Tomé and PrÍncipe	DHS	9	9	9	9	9
São Tomé and PrÍncipe	MICS	9	9	9	9	9
Tajikistan	VR	81	81	81	81	81
Tajikistan	LSMS	20	20	20	20	20
Tajikistan	DHS	18	18	18	18	18
Tanzania	DHS	54	54	54	54	54
Tanzania	Other	9	9	9	9	9
Thailand	VR	174	174	174	174	174
Thailand	WFS	6	6	6	6	6
Thailand	DHS	9	9	9	9	9
Thailand	Other	1	1	1	1	1
The Bahamas	VR	9	147	11	9	9
The Gambia	DHS	9	9	9	9	9
Timor-Leste	DHS	21	23	21	21	21
Timor-Leste	Other	6	6	6	6	6
Togo	DHS	24	24	24	24	24
Tokelau	VR	27	27	27	30	29
Tonga	VR	48	48	48	48	48
Trinidad and Tobago	WFS	9	9	9	9	9
Trinidad and Tobago	VR	102	102	105	102	102
Trinidad and Tobago	DHS	9	10	9	9	9
Trinidad and Tobago	Other	2	2	2	2	2
Tunisia	WFS	9	9	9	9	9
Tunisia	VR	60	60	60	60	60
Tunisia	DHS	9	9	9	9	9
Tunisia	Other	12	12	12	12	12
Tunisia	PAPFAM	9	9	9	9	9
Tunisia	MICS	9	9	9	9	9
Turkey	WFS	9	9	9	9	9
Turkey	DHS	36	36	36	36	36
Turkey	VR	108	108	108	108	108
Turkmenistan	VR	51	51	51	51	51
Turkmenistan	MICS	6	7	6	6	6
Tuvalu	VR	0	0	0	1	13
UK	VR	30	30	30	12	12
USA - Montana	VR	0	1	0	0	0
USA - Nevada	VR	0	5	0	0	0
USA - New Hampshire	VR	0	0	0	0	3
USA - North Dakota	VR	0	4	0	0	1
USA - South Dakota	VR	0	1	0	0	0
USA - Vermont	VR	0	3	1	0	4
USA - Wyoming	VR	0	2	0	0	0
Uganda	DHS	57	57	57	57	57
Uganda	Other	9	9	9	9	9
Ukraine	VR	0	174	0	0	0
Ukraine	RHS	18	18	9	9	11
Ukraine	DHS	20	36	18	18	18
Uruguay	Other	24	24	24	24	24
Uzbekistan	VR	63	63	63	63	63
Uzbekistan	DHS	15	15	15	15	15
Venezuela	WFS	15	15	15	15	15
Vietnam	DHS	15	15	15	15	15
Vietnam	MICS	9	10	9	9	9
Virgin Islands	VR	12	18	14	12	26
Yemen	WFS	6	6	6	6	6
Yemen	Other	21	21	21	21	21
Yemen	DHS	24	24	24	24	24
Yemen	PAPFAM	9	9	9	9	9
Yemen	MICS	9	9	9	9	9
Zambia	DHS	48	48	48	48	48
Zimbabwe	DHS	54	54	54	54	54
Zimbabwe	MICS	18	18	18	18	18

Appendix Table 7. Number of outliers for the age-sex model by location, source type, and age group

Location	Source	Outliers Early Neonatal	Outliers Late Neonatal	Outliers Post Neonatal	Outliers Infant	Outliers Child
Zimbabwe	VR	3	3	3	3	3
eSwatini	DHS	9	9	9	9	9
eSwatini	MICS	18	18	18	18	18

Appendix Table 8. Distribution of empirical life tables by decade and GBD super-region, 1950-2019

Location	Life table type	1950-1959	1960-1969	1970-1979	1980-1989	1990-1999	2000-2009	2010-2019	All years
Central Europe, eastern Europe, and central Asia	Total	249	367	345	485	490	502	349	2787
	Location specific	201	182	208	395	377	397	291	2051
	Universal	48	185	137	90	113	105	58	736
High income	Total	681	1593	1643	3427	3489	3249	1991	16073
	Location specific	490	1240	1237	1591	1305	1681	1259	8803
	Universal	191	353	406	1836	2184	1568	732	7270
Latin America and Caribbean	Total	232	299	425	1200	1420	1322	930	5828
	Location specific	164	260	381	1125	1369	1281	895	5475
	Universal	68	39	44	75	51	41	35	353
North Africa and Middle East	Total	2	12	44	49	78	117	96	398
	Location specific	2	12	41	49	78	109	89	380
	Universal	0	0	3	0	0	8	7	18
South Asia	Total	0	0	16	16	46	125	136	339
	Location specific	0	0	16	16	46	125	136	339
	Universal	0	0	0	0	0	0	0	0
Southeast Asia, east Asia, and Oceania	Total	59	126	134	140	130	150	71	810
	Location specific	42	107	102	114	126	144	69	704
	Universal	17	19	32	26	4	6	2	106
Sub-Saharan Africa	Total	4	4	2	20	18	20	16	84
	Location specific	4	4	2	20	18	20	16	84
	Universal	0	0	0	0	0	0	0	0
All locations	Total	1227	2401	2609	5337	5671	5485	3589	26319
	Location specific	903	1805	1987	3310	3319	3757	2755	17836
	Universal	324	596	622	2027	2352	1728	834	8483

Appendix Table 9. Locations by HIV estimation group			
**Group 1**	**Group 2A**	**Group 2B**	**Group 2C**
Angola	Antigua and Barbuda	Albania	Afghanistan
Benin	Argentina	American Samoa	Algeria
Botswana	Armenia	Azerbaijan	Andorra
Burkina Faso	Australia	Bahrain	Bangladesh
Burundi	Austria	Bosnia and Herzegovina	Bhutan
Cambodia	Barbados	Brunei	Bolivia
Cameroon	Belarus	China	Comoros
Cape Verde	Belgium	Cyprus	Cook Islands
Central African Republic	Belize	Egypt	Federated States of Micronesia
Chad	Bermuda	El Salvador	Laos
Congo (Brazzaville)	Brazil	Fiji	Lebanon
Cote d'Ivoire	Bulgaria	Greenland	Libya
Djibouti	Canada	Guam	Marshall Islands
Dominican Republic	Chile	Honduras	Mauritania
DR Congo	Colombia	Indonesia	Morocco
Equatorial Guinea	Costa Rica	Iran	Nepal
Eritrea	Croatia	Iraq	North Korea
Ethiopia	Cuba	Jordan	Pakistan
Gabon	Czech Republic	Kiribati	Principality of Monaco
Ghana	Denmark	Macedonia	Republic of Nauru
Guinea	Dominica	Malaysia	Republic of Niue
Guinea-Bissau	Ecuador	Maldives	Republic of Palau
Haiti	Estonia	Mongolia	Republic of San Marino
Kenya	Finland	Montenegro	Saint Kitts and Nevis
Lesotho	France	Nicaragua	Samoa
Liberia	Georgia	Northern Mariana Islands	Solomon Islands
Madagascar	Germany	Oman	Timor-Leste
Malawi	Greece	Palestine	Tokelau
Mali	Grenada	Paraguay	Tunisia
Mozambique	Guatemala	Peru	Tuvalu
Myanmar	Guyana	Qatar	United Arab Emirates
Namibia	Hungary	Sao Tome and Principe	Vanuatu
Niger	Iceland	Saudi Arabia	Yemen
Nigeria	Ireland	Serbia	
Papua New Guinea	Israel	Seychelles	
Rwanda	Italy	Slovakia	
Senegal	Jamaica	Sri Lanka	
Sierra Leone	Japan	Syria	
South Africa	Kazakhstan	Tajikistan	
South Sudan	Kuwait	Thailand	
Swaziland	Kyrgyzstan	Tonga	
Tanzania	Latvia	Turkey	
The Gambia	Lithuania	Vietnam	
Togo	Luxembourg		
Uganda	Malta		
Zambia	Mauritius		
Zimbabwe	Mexico		
India (1B)	Moldova		
Somalia (1B)	Netherlands		
Sudan (1B)	New Zealand		
	Norway		
	Panama		
	Philippines		
	Poland		
	Portugal		
	Puerto Rico		
	Romania		

Appendix Table 9. Locations by HIV estimation group

Group 1	Group 2A	Group 2B	Group 2C
	Russian Federation		
	Saint Lucia		
	Saint Vincent and the Grenadines		
	Singapore		
	Slovenia		
	South Korea		
	Spain		
	Suriname		
	Sweden		
	Switzerland		
	Taiwan (Province of China)		
	The Bahamas		
	Trinidad and Tobago		
	Turkmenistan		
	Ukraine		
	United Kingdom		
	Uruguay		
	USA		
	Uzbekistan		
	Venezuela		
	Virgin Islands		

Appendix Table 10. Number of sources used for the analysis of age-specific fertility (VR, CBH-survey, SBH-survey, SBH-census) for each location

Location	Vital Registrations	Complete Birth Histories	Summary Birth Histories	Censuses	Other	Sample Registrations
Afghanistan	1	2	5	1	1	0
Albania	64	3	5	2	0	0
Algeria	61	2	3	0	0	0
American Samoa	60	0	0	1	0	0
Andorra	48	0	0	0	0	0
Angola	24	3	4	0	0	0
Antigua and Barbuda	52	0	0	0	1	0
Argentina	68	0	0	4	0	0
Armenia	57	4	1	2	0	0
Australia	70	0	0	1	0	0
Austria	70	0	0	1	0	0
Azerbaijan	63	1	3	2	0	0
Bahrain	45	0	1	2	0	0
Bangladesh	38	10	6	2	4	0
Barbados	65	0	1	1	2	0
Belarus	67	0	2	2	0	0
Belgium	69	0	0	1	0	0
Belize	66	0	5	3	1	0
Benin	2	6	3	2	0	0
Bermuda	60	0	0	2	0	0
Bhutan	1	0	2	2	0	0
Bolivia	50	5	15	4	1	0
Bosnia and Herzegovina	29	1	1	0	1	0
Botswana	18	1	6	4	7	0
Brazil	57	4	16	3	0	0
Brunei	61	0	0	1	0	0
Bulgaria	71	0	2	1	0	0
Burkina Faso	0	5	5	4	0	0
Burundi	5	2	8	1	0	0
Cambodia	1	5	5	2	0	0
Cameroon	0	6	4	2	0	0
Canada	70	0	0	1	24	0
Cape Verde	44	0	2	1	0	0
Central African Republic	0	1	4	2	0	0
Chad	0	3	3	0	0	0
Chile	69	0	0	4	0	0
Colombia	65	8	4	4	0	0
Comoros	10	2	2	1	0	0
Congo (Brazzaville)	0	3	4	0	0	0
Cook Islands	0	0	0	2	0	0
Costa Rica	66	1	3	3	0	0
Côte d'Ivoire	1	8	6	1	0	1
Croatia	68	0	0	0	0	0
Cuba	67	0	2	1	0	0
Cyprus	69	0	0	1	39	0
Czech Republic	68	0	0	0	0	0
Denmark	70	0	0	0	16	0
Djibouti	28	1	4	0	0	0
Dominica	52	0	0	2	1	0
Dominican Republic	54	12	4	4	0	0

DR Congo	0	2	7	0	0	0
Ecuador	60	6	9	5	0	0
Egypt	64	9	8	2	0	0
El Salvador	60	6	3	3	0	0
Equatorial Guinea	19	1	1	0	0	0
Eritrea	0	2	2	0	0	0
Estonia	68	0	0	0	0	0
eSwatini	3	3	3	4	0	0
Ethiopia	0	6	4	2	0	0
Federated States of Micronesia	8	0	0	3	0	0
Fiji	61	1	2	5	0	0
Finland	70	0	0	0	0	0
France	70	0	0	1	0	0
Gabon	0	2	1	1	0	0
Georgia	57	1	3	0	26	0
Germany	69	0	0	0	0	0
Ghana	30	10	13	3	0	0
Greece	68	0	0	1	4	0
Greenland	71	0	0	0	0	0
Grenada	46	0	0	1	1	0
Guam	69	0	0	1	0	0
Guatemala	60	6	2	1	0	0
Guinea	2	4	2	2	0	1
Guinea-Bissau	23	1	4	0	0	0
Guyana	36	6	7	1	1	0
Haiti	0	6	3	2	3	0
Honduras	38	4	7	3	0	0
Hungary	70	0	0	2	13	0
Iceland	70	0	0	1	0	0
India	64	6	14	5	0	25
Indonesia	15	9	14	5	1	0
Iran	61	0	3	2	0	0
Iraq	40	4	6	2	0	0
Ireland	69	0	0	2	0	0
Israel	68	0	0	1	0	0
Italy	70	0	0	0	0	0
Jamaica	58	1	3	4	0	0
Japan	70	0	0	1	0	0
Jordan	48	7	7	1	0	0
Kazakhstan	53	2	5	2	0	0
Kenya	49	8	9	4	0	0
Kiribati	20	0	1	3	0	0
Kuwait	58	0	0	1	2	0
Kyrgyzstan	54	3	5	2	0	0
Laos	0	3	4	1	0	0
Latvia	68	0	0	0	0	0
Lebanon	29	1	4	0	0	0
Lesotho	2	3	3	3	1	0
Liberia	5	4	4	4	2	0
Libya	47	0	2	1	0	0
Lithuania	68	0	0	0	0	0

Luxembourg	70	0	0	1	0	0
Madagascar	26	6	5	0	0	0
Malawi	4	7	9	5	2	0
Malaysia	62	0	1	1	0	0
Maldives	49	2	3	4	0	0
Mali	3	6	3	3	0	0
Malta	70	0	0	1	0	0
Marshall Islands	26	0	0	2	0	0
Mauritania	1	2	6	1	0	0
Mauritius	70	0	0	0	0	0
Mexico	70	1	15	6	0	0
Moldova	52	2	4	0	0	0
Monaco	7	0	0	0	0	0
Mongolia	45	3	9	0	0	0
Montenegro	26	0	1	0	0	0
Morocco	18	5	5	2	0	0
Mozambique	30	4	4	4	0	0
Myanmar	6	2	3	2	0	0
Namibia	2	4	1	2	0	0
Nauru	1	0	0	2	0	0
Nepal	0	8	5	2	0	0
Netherlands	69	0	0	1	0	0
New Zealand	70	0	0	1	0	0
Nicaragua	55	5	6	4	0	0
Niger	0	4	4	1	0	0
Nigeria	2	7	15	3	0	0
Niue	0	0	0	3	0	0
North Korea	2	1	0	1	2	0
North Macedonia	32	0	2	1	1	0
Northern Mariana Islands	8	0	0	0	0	0
Norway	70	0	0	1	0	0
Oman	11	0	0	1	9	0
Pakistan	17	7	14	9	2	1
Palau	0	0	0	1	0	0
Palestine	20	3	6	2	0	0
Panama	70	1	3	5	0	0
Papua New Guinea	4	0	3	3	0	0
Paraguay	42	6	6	4	0	0
Peru	61	14	11	2	0	0
Philippines	68	7	4	5	3	0
Poland	70	0	0	1	6	0
Portugal	69	1	0	1	0	0
Puerto Rico	61	0	0	1	0	0
Qatar	48	0	0	1	2	0
Romania	69	0	0	1	0	0
Russia	67	0	0	0	0	0
Rwanda	19	8	7	5	0	0
Saint Kitts and Nevis	12	0	0	2	1	0
Saint Lucia	55	0	0	3	1	0
Saint Vincent and the Grenadines	62	0	0	2	1	0
Samoa	40	0	0	1	0	3

São Tomé and PrÍncipe	43	1	4	2	0	0
Saudi Arabia	8	0	2	0	1	0
Senegal	3	14	4	3	0	0
Serbia	67	0	3	0	0	0
Seychelles	71	0	0	2	0	0
Sierra Leone	12	3	5	1	0	0
Singapore	71	0	0	1	0	0
Slovakia	68	0	0	0	0	0
Slovenia	65	0	0	0	1	0
Solomon Islands	1	1	0	3	0	0
Somalia	0	1	3	0	0	0
South Africa	28	4	9	1	0	0
South Korea	43	1	1	3	22	0
South Sudan	0	0	1	1	0	0
Spain	70	0	0	1	0	0
Sri Lanka	68	3	1	2	6	0
Sudan	4	5	5	3	0	0
Suriname	53	0	2	1	0	0
Sweden	71	0	0	0	0	0
Switzerland	70	0	0	1	0	0
Syria	59	2	5	2	0	0
Taiwan (province of China)	68	0	0	0	0	0
Tajikistan	44	2	6	1	0	0
Tanzania	1	7	7	1	1	0
Thailand	69	1	7	5	6	0
The Bahamas	70	0	0	1	1	0
The Gambia	0	1	5	1	0	0
Timor-Leste	24	5	13	0	0	0
Togo	1	4	5	2	1	0
Tokelau	0	0	0	1	0	0
Tonga	48	0	1	2	0	0
Trinidad and Tobago	66	2	6	2	0	0
Tunisia	69	4	8	1	0	0
Turkey	26	4	8	4	4	0
Turkmenistan	35	1	3	0	0	0
Tuvalu	1	0	0	1	0	0
Uganda	3	8	9	2	1	1
UK	69	0	0	0	0	0
Ukraine	67	1	4	1	0	0
United Arab Emirates	42	0	3	1	1	0
Uruguay	59	0	3	5	0	0
USA	70	0	0	1	0	0
Uzbekistan	46	2	4	0	0	0
Vanuatu	1	0	2	3	0	0
Venezuela	67	1	0	3	2	0
Vietnam	5	3	14	4	5	0
Virgin Islands	49	0	0	1	0	0
Yemen	4	3	6	1	2	0
Zambia	3	5	5	4	0	0
Zimbabwe	1	8	3	2	2	0

Appendix Table 11. Number of sources used for the analysis of age-specific fertility for each year (VR, CBH-survey, SBH-survey, SBH-census)

Year	Vital Registrations	Complete Birth Histories	Summary Birth Histories	Censuses	Other	Sample Registrations
1948	93	0	0	1	0	0
1949	96	0	0	0	0	0
1950	118	0	0	0	1	0
1951	119	1	0	0	1	0
1952	117	0	0	0	0	0
1953	118	0	0	0	0	0
1954	117	0	0	2	0	0
1955	120	0	0	0	1	0
1956	120	0	0	1	0	0
1957	120	0	0	0	0	0
1958	122	1	0	1	2	0
1959	122	0	0	0	0	0
1960	127	0	0	11	3	0
1961	123	0	0	2	3	0
1962	121	0	0	0	3	0
1963	124	0	0	1	3	0
1964	124	0	0	2	4	0
1965	119	0	0	0	4	0
1966	115	0	0	2	4	0
1967	113	0	0	2	4	0
1968	114	0	0	0	2	0
1969	115	0	0	0	2	0
1970	123	0	0	11	7	0
1971	121	0	3	8	5	1
1972	113	1	0	1	6	0
1973	111	3	0	6	2	0
1974	113	8	4	4	4	0
1975	112	3	4	4	4	0
1976	116	8	5	6	4	1
1977	112	4	4	4	2	0
1978	114	5	8	2	2	0
1979	121	0	5	3	2	0
1980	124	0	4	32	5	0
1981	123	0	7	17	3	1
1982	120	1	0	6	3	0
1983	119	1	4	4	3	0
1984	122	4	4	5	2	0
1985	124	7	4	4	3	0
1986	125	4	1	6	4	1
1987	125	10	7	6	6	0
1988	120	6	9	7	2	0
1989	121	4	5	5	4	0
1990	125	10	7	14	13	0
1991	124	10	11	12	7	1
1992	125	9	5	8	5	0
1993	117	9	13	5	5	0
1994	114	10	6	5	6	0
1995	109	9	12	2	5	0
1996	110	16	10	7	6	1
1997	96	8	10	5	5	0
1998	98	11	16	3	3	1
1999	101	13	18	8	4	1
2000	107	6	47	18	4	1
2001	101	15	14	18	4	1
2002	112	12	10	8	4	1
2003	110	13	15	3	4	1
2004	107	19	15	1	3	1
2005	110	24	27	6	4	2
2006	109	14	39	5	5	1
2007	113	16	20	6	4	1
2008	113	12	21	8	5	1
2009	110	19	22	7	3	2
2010	105	20	33	9	4	1
2011	110	15	28	11	7	1
2012	135	23	38	5	4	1
2013	132	17	22	1	4	1
2014	126	15	28	2	4	2
2015	112	7	19	1	4	1
2016	92	8	16	1	1	4
2017	59	8	27	2	1	1
2018	8	0	1	2	0	0

Appendix Table 12. List of all confirmed censuses by location and year

Location	Year	Type	Age detail	Enumeration method	Included or excluded from estimation
Afghanistan	1979	Census	5-year age groups	De facto	Included
Albania	1950	Census	5-year age groups	De facto	Included
Albania	1955	Census	5-year age groups	De facto	Included
Albania	1960	Census	non-std age groups	De facto	Included
Albania	1969	Census	all ages only	De facto	Included
Albania	1979	Census	all ages only	De jure	Included
Albania	1989	Census	all ages only	De jure	Included
Albania	2001	Census	5-year age groups	De facto	Included
Albania	2011	Census	5-year age groups	De jure	Included
Algeria	1954	Census	5-year age groups	Unknown	Included
Algeria	1966	Census	5-year age groups	De jure	Included
Algeria	1977	Census	5-year age groups	De jure	Included
Algeria	1987	Census	5-year age groups	De jure	Included
Algeria	1998	Census	5-year age groups	De jure	Included
Algeria	2008	Census	1-year age groups	De jure	Included
American Samoa	1950	Census	5-year age groups	De jure	Included
American Samoa	1956	Census	1-year age groups	De jure	Included
American Samoa	1960	Census	5-year age groups	De jure	Included
American Samoa	1970	Census	5-year age groups	De jure	Included
American Samoa	1974	Census	5-year age groups	De jure	Included
American Samoa	1980	Census	1-year age groups	De jure	Included
American Samoa	1990	Census	5-year age groups	De jure	Included
American Samoa	2000	Census	5-year age groups	De jure	Included
American Samoa	2010	Census	5-year age groups	De jure	Included
Andorra	1966	Registry	non-std age groups	NA	Included
Andorra	1985	Registry	1-year age groups	NA	Included
Andorra	1986	Registry	1-year age groups	NA	Included
Andorra	1987	Registry	1-year age groups	NA	Included
Andorra	1988	Registry	1-year age groups	NA	Included
Andorra	1989	Registry	1-year age groups	NA	Included
Andorra	1990	Registry	1-year age groups	NA	Included
Andorra	1991	Registry	1-year age groups	NA	Included
Andorra	1992	Registry	1-year age groups	NA	Included
Andorra	1993	Registry	1-year age groups	NA	Included
Andorra	1994	Registry	1-year age groups	NA	Included
Andorra	1995	Registry	1-year age groups	NA	Included
Andorra	1996	Registry	1-year age groups	NA	Included
Andorra	1997	Registry	1-year age groups	NA	Included
Andorra	1998	Registry	1-year age groups	NA	Included
Andorra	1999	Registry	1-year age groups	NA	Included
Andorra	2000	Registry	1-year age groups	NA	Included
Andorra	2001	Registry	1-year age groups	NA	Included
Andorra	2002	Registry	1-year age groups	NA	Included
Andorra	2003	Registry	1-year age groups	NA	Included
Andorra	2004	Registry	1-year age groups	NA	Included
Andorra	2005	Registry	1-year age groups	NA	Included
Andorra	2006	Registry	1-year age groups	NA	Included
Andorra	2007	Registry	1-year age groups	NA	Included
Andorra	2008	Registry	1-year age groups	NA	Included

Andorra	2009	Registry	1-year age groups	NA	Included
Andorra	2010	Registry	1-year age groups	NA	Included
Andorra	2011	Registry	1-year age groups	NA	Included
Andorra	2012	Registry	1-year age groups	NA	Included
Andorra	2013	Registry	1-year age groups	NA	Included
Andorra	2014	Registry	1-year age groups	NA	Included
Andorra	2015	Registry	1-year age groups	NA	Included
Andorra	2016	Registry	1-year age groups	NA	Included
Andorra	2017	Registry	1-year age groups	NA	Included
Angola	1950	Census	5-year age groups	De facto	Included
Angola	1960	Census	5-year age groups	De jure	Included
Angola	1970	Census	all ages only	De facto	Included
Angola	2014	Census	1-year age groups	De facto	Included
Antigua and Barbuda	1960	Census	1-year age groups	De facto	Included
Antigua and Barbuda	1970	Census	5-year age groups	De jure	Included
Antigua and Barbuda	1991	Census	1-year age groups	De jure	Included
Antigua and Barbuda	2001	Census	1-year age groups	De facto	Included
Antigua and Barbuda	2011	Census	5-year age groups	De jure	Included
Argentina	1960	Census	1-year age groups	De facto	Included
Argentina	1970	Census	1-year age groups	De facto	Included
Argentina	1980	Census	1-year age groups	De facto	Included
Argentina	1991	Census	1-year age groups	De facto	Included
Argentina	2001	Census	1-year age groups	De facto	Included
Argentina	2010	Census	1-year age groups	De facto	Included
Armenia	1959	Census	5-year age groups	De facto	Included
Armenia	1970	Census	5-year age groups	De facto	Included
Armenia	1979	Census	5-year age groups	De facto	Included
Armenia	1989	Census	5-year age groups	De jure	Included
Armenia	2001	Census	1-year age groups	De jure	Included
Armenia	2011	Census	1-year age groups	De jure	Included
Australia	1954	Census	5-year age groups	De facto	Included
Australia	1961	Census	1-year age groups	De facto	Included
Australia	1966	Census	5-year age groups	De facto	Included
Australia	1971	Census	1-year age groups	De facto	Included
Australia	1976	Census	5-year age groups	De facto	Included
Australia	1981	Census	5-year age groups	De facto	Included
Australia	1986	Census	1-year age groups	De facto	Included
Australia	1991	Census	1-year age groups	De facto	Included
Australia	1996	Census	1-year age groups	De facto	Included
Australia	2001	Census	1-year age groups	De jure	Included
Australia	2006	Census	1-year age groups	De jure	Included
Australia	2011	Census	1-year age groups	De jure	Included
Australia	2016	Census	5-year age groups	De jure	Excluded
Austria	1951	Census	1-year age groups	De jure	Included
Austria	1961	Census	1-year age groups	De jure	Included
Austria	1971	Census	1-year age groups	De jure	Included
Austria	1981	Census	1-year age groups	De jure	Included
Austria	1991	Census	1-year age groups	De jure	Included
Austria	2001	Census	1-year age groups	De jure	Included
Austria	2002	Registry	5-year age groups	NA	Included
Austria	2003	Registry	5-year age groups	NA	Included

Austria	2004	Registry	5-year age groups	NA	Included
Austria	2005	Registry	5-year age groups	NA	Included
Austria	2006	Registry	5-year age groups	NA	Included
Austria	2007	Registry	5-year age groups	NA	Included
Austria	2008	Registry	5-year age groups	NA	Included
Austria	2009	Registry	5-year age groups	NA	Included
Austria	2010	Registry	5-year age groups	NA	Included
Austria	2011	Registry	5-year age groups	NA	Included
Austria	2012	Registry	5-year age groups	NA	Included
Austria	2013	Registry	5-year age groups	NA	Included
Austria	2014	Registry	5-year age groups	NA	Included
Austria	2015	Registry	5-year age groups	NA	Included
Austria	2016	Registry	5-year age groups	NA	Included
Austria	2017	Registry	5-year age groups	NA	Included
Azerbaijan	1959	Census	5-year age groups	De facto	Included
Azerbaijan	1970	Census	5-year age groups	De facto	Included
Azerbaijan	1979	Census	5-year age groups	De facto	Included
Azerbaijan	1989	Census	5-year age groups	De jure	Included
Azerbaijan	1999	Census	1-year age groups	De jure	Included
Azerbaijan	2009	Census	1-year age groups	De jure	Included
Bahrain	1950	Census	all ages only	De facto	Included
Bahrain	1959	Census	non-std age groups	De facto	Included
Bahrain	1965	Census	non-std age groups	De facto	Included
Bahrain	1971	Census	1-year age groups	De facto	Included
Bahrain	1981	Census	5-year age groups	De facto	Included
Bahrain	1991	Census	5-year age groups	De jure	Excluded
Bahrain	2001	Census	1-year age groups	De jure	Included
Bahrain	2010	Census	1-year age groups	De jure	Included
Bangladesh	1951	Census	non-std age groups	De jure	Included
Bangladesh	1961	Census	5-year age groups	De jure	Included
Bangladesh	1974	Census	5-year age groups	De facto	Included
Bangladesh	1981	Census	5-year age groups	De facto	Included
Bangladesh	1991	Census	1-year age groups	De facto	Included
Bangladesh	2001	Census	1-year age groups	De facto	Included
Bangladesh	2011	Census	5-year age groups	De facto	Included
Barbados	1960	Census	1-year age groups	De facto	Included
Barbados	1970	Census	1-year age groups	De facto	Included
Barbados	1980	Census	1-year age groups	De facto	Included
Barbados	1990	Census	5-year age groups	De jure	Included
Barbados	2000	Census	1-year age groups	De facto	Included
Barbados	2010	Census	5-year age groups	De jure	Included
Belarus	1959	Census	5-year age groups	De facto	Included
Belarus	1970	Census	5-year age groups	De facto	Included
Belarus	1979	Census	5-year age groups	De facto	Included
Belarus	1989	Census	1-year age groups	De jure	Included
Belarus	1999	Census	1-year age groups	De jure	Included
Belarus	2009	Census	1-year age groups	De jure	Included
Belgium	1961	Census	1-year age groups	De jure	Included
Belgium	1970	Census	1-year age groups	De jure	Included
Belgium	1981	Census	1-year age groups	De jure	Included
Belgium	1991	Census	5-year age groups	De jure	Included

Belgium	2001	Census	1-year age groups	De jure	Included
Belgium	2010	Registry	1-year age groups	NA	Included
Belgium	2011	Census	1-year age groups	De jure	Excluded
Belgium	2012	Registry	1-year age groups	NA	Included
Belgium	2013	Registry	1-year age groups	NA	Included
Belgium	2014	Registry	1-year age groups	NA	Included
Belgium	2015	Registry	1-year age groups	NA	Included
Belgium	2016	Registry	1-year age groups	NA	Included
Belgium	2017	Registry	1-year age groups	NA	Included
Belize	1960	Census	5-year age groups	De facto	Included
Belize	1970	Census	1-year age groups	De facto	Included
Belize	1980	Census	1-year age groups	De facto	Included
Belize	1991	Census	1-year age groups	De facto	Included
Belize	2000	Census	non-std age groups	De jure	Included
Belize	2010	Census	5-year age groups	De jure	Included
Benin	1979	Census	5-year age groups	De facto	Included
Benin	1992	Census	1-year age groups	De facto	Included
Benin	2002	Census	1-year age groups	De jure	Included
Benin	2013	Census	5-year age groups	De jure	Included
Bermuda	1950	Census	1-year age groups	De jure	Included
Bermuda	1960	Census	1-year age groups	De jure	Included
Bermuda	1970	Census	1-year age groups	De jure	Included
Bermuda	1980	Census	1-year age groups	De jure	Included
Bermuda	1991	Census	1-year age groups	De jure	Included
Bermuda	2000	Census	1-year age groups	De jure	Included
Bermuda	2010	Census	1-year age groups	De jure	Included
Bermuda	2016	Census	1-year age groups	De jure	Excluded
Bhutan	2005	Census	1-year age groups	De facto	Included
Bhutan	2017	Census	1-year age groups	De facto	Included
Bolivia	1950	Census	5-year age groups	De facto	Excluded
Bolivia	1976	Census	5-year age groups	De facto	Included
Bolivia	1992	Census	1-year age groups	De facto	Included
Bolivia	2001	Census	1-year age groups	De facto	Included
Bolivia	2012	Census	1-year age groups	De facto	Included
Bosnia and Herzegovina	1971	Census	5-year age groups	De jure	Excluded
Bosnia and Herzegovina	1981	Census	5-year age groups	De jure	Included
Bosnia and Herzegovina	1991	Census	1-year age groups	De jure	Included
Bosnia and Herzegovina	2013	Census	1-year age groups	De jure	Included
Botswana	1964	Census	1-year age groups	De facto	Included
Botswana	1971	Census	1-year age groups	De facto	Excluded
Botswana	1981	Census	1-year age groups	De facto	Included
Botswana	1991	Census	1-year age groups	De facto	Included
Botswana	2001	Census	5-year age groups	De facto	Included
Botswana	2011	Census	1-year age groups	De facto	Included
Brazil	1950	Census	1-year age groups	De facto	Included
Brazil	1960	Census	1-year age groups	De facto	Included
Brazil	1970	Census	1-year age groups	De facto	Included
Brazil	1980	Census	1-year age groups	De jure	Included
Brazil	1991	Census	1-year age groups	De jure	Included
Brazil	1996	Census	5-year age groups	De jure	Excluded
Brazil	2000	Census	1-year age groups	De jure	Included

Brazil	2010	Census	1-year age groups	De jure	Included
Brunei	1960	Census	5-year age groups	De facto	Included
Brunei	1971	Census	1-year age groups	De facto	Included
Brunei	1981	Census	1-year age groups	De facto	Included
Brunei	1991	Census	1-year age groups	De facto	Included
Brunei	2001	Census	5-year age groups	De facto	Included
Brunei	2011	Census	1-year age groups	De jure	Included
Bulgaria	1956	Census	5-year age groups	De facto	Included
Bulgaria	1965	Census	5-year age groups	De facto	Included
Bulgaria	1975	Census	5-year age groups	De facto	Included
Bulgaria	1985	Census	5-year age groups	De jure	Included
Bulgaria	1992	Census	5-year age groups	De jure	Included
Bulgaria	2001	Census	5-year age groups	De jure	Excluded
Bulgaria	2002	Registry	5-year age groups	NA	Included
Bulgaria	2003	Registry	5-year age groups	NA	Included
Bulgaria	2004	Registry	5-year age groups	NA	Included
Bulgaria	2005	Registry	5-year age groups	NA	Included
Bulgaria	2006	Registry	5-year age groups	NA	Included
Bulgaria	2007	Registry	5-year age groups	NA	Included
Bulgaria	2008	Registry	5-year age groups	NA	Included
Bulgaria	2009	Registry	5-year age groups	NA	Included
Bulgaria	2010	Registry	5-year age groups	NA	Included
Bulgaria	2011	Registry	5-year age groups	NA	Included
Bulgaria	2012	Registry	5-year age groups	NA	Included
Bulgaria	2013	Registry	5-year age groups	NA	Included
Bulgaria	2014	Registry	5-year age groups	NA	Included
Bulgaria	2015	Registry	5-year age groups	NA	Included
Bulgaria	2016	Registry	5-year age groups	NA	Included
Burkina Faso	1975	Census	5-year age groups	De jure	Excluded
Burkina Faso	1976	Census	non-std age groups	Unknown	Included
Burkina Faso	1985	Census	1-year age groups	De jure	Included
Burkina Faso	1996	Census	1-year age groups	De jure	Included
Burkina Faso	2006	Census	1-year age groups	De jure	Included
Burundi	1979	Census	5-year age groups	De jure	Included
Burundi	1990	Census	1-year age groups	De jure	Included
Burundi	2008	Census	1-year age groups	De jure	Included
Cambodia	1962	Census	1-year age groups	De facto	Included
Cambodia	1998	Census	1-year age groups	De facto	Included
Cambodia	2008	Census	1-year age groups	De facto	Included
Cameroon	1976	Census	5-year age groups	De jure	Included
Cameroon	1987	Census	1-year age groups	Unknown	Included
Cameroon	2005	Census	1-year age groups	De jure	Included
Canada	1951	Census	5-year age groups	De jure	Included
Canada	1956	Census	1-year age groups	De jure	Included
Canada	1961	Census	5-year age groups	De jure	Included
Canada	1966	Census	5-year age groups	De jure	Included
Canada	1971	Census	5-year age groups	De jure	Included
Canada	1976	Census	5-year age groups	De jure	Included
Canada	1981	Census	1-year age groups	De jure	Included
Canada	1986	Census	5-year age groups	De jure	Included
Canada	1991	Census	1-year age groups	De jure	Included

Canada	1996	Census	1-year age groups	De jure	Included
Canada	2001	Census	1-year age groups	De jure	Included
Canada	2006	Census	1-year age groups	De jure	Included
Canada	2011	Census	1-year age groups	De jure	Included
Canada	2016	Census	1-year age groups	De jure	Included
Cape Verde	1950	Census	non-std age groups	De facto	Included
Cape Verde	1960	Census	non-std age groups	De facto	Included
Cape Verde	1970	Census	5-year age groups	De facto	Included
Cape Verde	1980	Census	5-year age groups	De jure	Included
Cape Verde	1990	Census	1-year age groups	De facto	Included
Cape Verde	2000	Census	5-year age groups	De facto	Included
Cape Verde	2010	Census	1-year age groups	De jure	Included
Central African Republic	1975	Census	5-year age groups	De facto	Included
Central African Republic	1988	Census	1-year age groups	De facto	Included
Central African Republic	2003	Census	5-year age groups	De facto	Included
Chad	1993	Census	5-year age groups	De jure	Included
Chad	2009	Census	5-year age groups	De jure	Included
Chile	1952	Census	5-year age groups	De facto	Included
Chile	1960	Census	5-year age groups	De facto	Included
Chile	1970	Census	1-year age groups	De facto	Included
Chile	1982	Census	1-year age groups	De facto	Included
Chile	1992	Census	1-year age groups	De facto	Included
Chile	2002	Census	5-year age groups	De facto	Included
Chile	2012	Census	1-year age groups	De jure	Excluded
Chile	2017	Census	5-year age groups	De facto	Included
Colombia	1951	Census	5-year age groups	De facto	Included
Colombia	1964	Census	1-year age groups	De facto	Included
Colombia	1973	Census	1-year age groups	De facto	Included
Colombia	1985	Census	1-year age groups	De facto	Included
Colombia	1993	Census	1-year age groups	De facto	Included
Colombia	2005	Census	1-year age groups	De facto	Included
Colombia	2018	Census	5-year age groups	De jure	Included
Comoros	1958	Census	5-year age groups	De facto	Excluded
Comoros	1966	Census	non-std age groups	Unknown	Included
Comoros	1980	Census	1-year age groups	De facto	Included
Comoros	1991	Census	5-year age groups	De jure	Included
Comoros	2003	Census	non-std age groups	De jure	Included
Congo (Brazzaville)	1974	Census	5-year age groups	De jure	Included
Congo (Brazzaville)	1984	Census	1-year age groups	De jure	Included
Congo (Brazzaville)	1985	Census	1-year age groups	De jure	Excluded
Congo (Brazzaville)	2007	Census	1-year age groups	De facto	Included
Cook Islands	1951	Census	5-year age groups	De jure	Excluded
Cook Islands	1956	Census	5-year age groups	De facto	Included
Cook Islands	1961	Census	5-year age groups	De facto	Included
Cook Islands	1966	Census	5-year age groups	De facto	Excluded
Cook Islands	1971	Census	5-year age groups	Unknown	Included
Cook Islands	1976	Census	5-year age groups	De facto	Excluded
Cook Islands	1981	Census	1-year age groups	De facto	Included
Cook Islands	1986	Census	5-year age groups	De facto	Included
Cook Islands	1991	Census	5-year age groups	De facto	Included
Cook Islands	1996	Census	5-year age groups	De facto	Included

Cook Islands	2001	Census	5-year age groups	De facto	Included
Cook Islands	2006	Census	5-year age groups	De facto	Included
Cook Islands	2011	Census	5-year age groups	De facto	Included
Cook Islands	2016	Census	5-year age groups	De facto	Included
Costa Rica	1950	Census	1-year age groups	De jure	Included
Costa Rica	1963	Census	1-year age groups	De jure	Included
Costa Rica	1973	Census	1-year age groups	De jure	Included
Costa Rica	1984	Census	1-year age groups	De jure	Included
Costa Rica	2000	Census	5-year age groups	De jure	Included
Costa Rica	2011	Census	1-year age groups	De jure	Included
Côte d'Ivoire	1975	Census	5-year age groups	De facto	Included
Côte d'Ivoire	1988	Census	1-year age groups	De facto	Included
Côte d'Ivoire	1998	Census	5-year age groups	Unknown	Included
Côte d'Ivoire	2014	Census	1-year age groups	De jure	Included
Croatia	1971	Census	1-year age groups	De facto	Included
Croatia	1991	Census	5-year age groups	Unknown	Excluded
Croatia	2001	Census	1-year age groups	De jure	Included
Croatia	2011	Census	1-year age groups	De jure	Included
Cuba	1953	Census	5-year age groups	De jure	Included
Cuba	1970	Census	5-year age groups	De jure	Included
Cuba	1981	Census	1-year age groups	De jure	Included
Cuba	2002	Census	1-year age groups	De jure	Included
Cuba	2012	Census	1-year age groups	De jure	Included
Cyprus	1960	Census	5-year age groups	De jure	Included
Cyprus	1973	Census	5-year age groups	De jure	Excluded
Czech Republic	1961	Census	1-year age groups	De facto	Included
Czech Republic	1970	Census	1-year age groups	De jure	Included
Czech Republic	1980	Census	1-year age groups	De jure	Included
Czech Republic	1991	Census	1-year age groups	De jure	Included
Czech Republic	2000	Registry	1-year age groups	NA	Included
Czech Republic	2001	Census	5-year age groups	De jure	Excluded
Czech Republic	2002	Registry	1-year age groups	NA	Included
Czech Republic	2003	Registry	1-year age groups	NA	Included
Czech Republic	2004	Registry	1-year age groups	NA	Included
Czech Republic	2005	Registry	1-year age groups	NA	Included
Czech Republic	2006	Registry	1-year age groups	NA	Included
Czech Republic	2007	Registry	1-year age groups	NA	Included
Czech Republic	2008	Registry	1-year age groups	NA	Included
Czech Republic	2009	Registry	1-year age groups	NA	Included
Czech Republic	2010	Registry	1-year age groups	NA	Included
Czech Republic	2011	Census	1-year age groups	De jure	Excluded
Czech Republic	2012	Registry	1-year age groups	NA	Included
Czech Republic	2013	Registry	1-year age groups	NA	Included
Czech Republic	2014	Registry	1-year age groups	NA	Included
Czech Republic	2015	Registry	1-year age groups	NA	Included
Czech Republic	2016	Registry	1-year age groups	NA	Included
Czech Republic	2017	Registry	1-year age groups	NA	Included
Denmark	1950	Census	1-year age groups	De jure	Included
Denmark	1960	Census	1-year age groups	De jure	Included
Denmark	1965	Census	5-year age groups	De jure	Included
Denmark	1970	Census	1-year age groups	De jure	Included

Denmark	1976	Census	1-year age groups	De jure	Included
Denmark	1980	Registry	1-year age groups	NA	Included
Denmark	1981	Registry	1-year age groups	NA	Included
Denmark	1982	Registry	1-year age groups	NA	Included
Denmark	1983	Registry	1-year age groups	NA	Included
Denmark	1984	Registry	1-year age groups	NA	Included
Denmark	1985	Registry	1-year age groups	NA	Included
Denmark	1986	Registry	1-year age groups	NA	Included
Denmark	1987	Registry	1-year age groups	NA	Included
Denmark	1988	Registry	1-year age groups	NA	Included
Denmark	1989	Registry	1-year age groups	NA	Included
Denmark	1990	Registry	1-year age groups	NA	Included
Denmark	1991	Census	1-year age groups	De jure	Excluded
Denmark	1992	Registry	1-year age groups	NA	Included
Denmark	1993	Registry	1-year age groups	NA	Included
Denmark	1994	Registry	1-year age groups	NA	Included
Denmark	1995	Registry	1-year age groups	NA	Included
Denmark	1996	Registry	1-year age groups	NA	Included
Denmark	1997	Registry	1-year age groups	NA	Included
Denmark	1998	Registry	1-year age groups	NA	Included
Denmark	1999	Registry	1-year age groups	NA	Included
Denmark	2000	Registry	1-year age groups	NA	Included
Denmark	2001	Registry	1-year age groups	NA	Included
Denmark	2002	Registry	1-year age groups	NA	Included
Denmark	2003	Registry	1-year age groups	NA	Included
Denmark	2004	Registry	1-year age groups	NA	Included
Denmark	2005	Registry	1-year age groups	NA	Included
Denmark	2006	Registry	1-year age groups	NA	Included
Denmark	2007	Registry	1-year age groups	NA	Included
Denmark	2008	Registry	1-year age groups	NA	Included
Denmark	2009	Registry	1-year age groups	NA	Included
Denmark	2010	Registry	1-year age groups	NA	Included
Denmark	2011	Registry	1-year age groups	NA	Included
Denmark	2012	Registry	1-year age groups	NA	Included
Denmark	2013	Registry	1-year age groups	NA	Included
Denmark	2014	Registry	1-year age groups	NA	Included
Denmark	2015	Registry	1-year age groups	NA	Included
Denmark	2016	Registry	1-year age groups	NA	Included
Denmark	2017	Registry	1-year age groups	NA	Included
Djibouti	1983	Census	5-year age groups	De jure	Included
Djibouti	2009	Census	5-year age groups	De jure	Included
Dominica	1960	Census	5-year age groups	De facto	Included
Dominica	1970	Census	5-year age groups	De facto	Included
Dominica	1981	Census	5-year age groups	De facto	Excluded
Dominica	1991	Census	5-year age groups	De facto	Included
Dominica	2001	Census	5-year age groups	De facto	Included
Dominica	2011	Census	5-year age groups	De facto	Included
Dominican Republic	1950	Census	1-year age groups	De jure	Included
Dominican Republic	1960	Census	1-year age groups	De jure	Included
Dominican Republic	1970	Census	1-year age groups	De jure	Included
Dominican Republic	1981	Census	5-year age groups	De jure	Included

Dominican Republic	1993	Census	1-year age groups	De jure	Included
Dominican Republic	2002	Census	1-year age groups	De jure	Included
Dominican Republic	2010	Census	1-year age groups	De jure	Included
DR Congo	1970	Census	all ages only	De jure	Included
DR Congo	1984	Census	1-year age groups	Unknown	Included
Ecuador	1950	Census	1-year age groups	De facto	Included
Ecuador	1962	Census	1-year age groups	De facto	Included
Ecuador	1974	Census	5-year age groups	De facto	Included
Ecuador	1982	Census	1-year age groups	De facto	Included
Ecuador	1990	Census	1-year age groups	De facto	Included
Ecuador	2001	Census	1-year age groups	De facto	Included
Ecuador	2010	Census	1-year age groups	De facto	Included
Egypt	1960	Census	5-year age groups	De facto	Included
Egypt	1966	Census	all ages only	De facto	Included
Egypt	1976	Census	5-year age groups	De facto	Included
Egypt	1986	Census	5-year age groups	De facto	Included
Egypt	1996	Census	5-year age groups	De facto	Included
Egypt	2006	Census	1-year age groups	De facto	Included
Egypt	2017	Census	5-year age groups	De facto	Included
El Salvador	1950	Census	1-year age groups	De facto	Included
El Salvador	1961	Census	1-year age groups	De facto	Included
El Salvador	1971	Census	1-year age groups	De facto	Included
El Salvador	1992	Census	1-year age groups	De facto	Included
El Salvador	1993	Census	5-year age groups	Unknown	Excluded
El Salvador	2007	Census	1-year age groups	De jure	Included
Equatorial Guinea	1983	Census	5-year age groups	De facto	Included
Equatorial Guinea	1994	Census	1-year age groups	Unknown	Excluded
Equatorial Guinea	2015	Census	all ages only	Unknown	Included
Eritrea	1984	Census	all ages only	De jure	Included
Estonia	1950	Registry	5-year age groups	NA	Included
Estonia	1951	Registry	5-year age groups	NA	Included
Estonia	1952	Registry	5-year age groups	NA	Included
Estonia	1953	Registry	5-year age groups	NA	Included
Estonia	1954	Registry	5-year age groups	NA	Included
Estonia	1955	Registry	5-year age groups	NA	Included
Estonia	1956	Registry	5-year age groups	NA	Included
Estonia	1957	Registry	5-year age groups	NA	Included
Estonia	1958	Registry	5-year age groups	NA	Included
Estonia	1959	Census	5-year age groups	De facto	Excluded
Estonia	1960	Registry	5-year age groups	NA	Included
Estonia	1961	Registry	5-year age groups	NA	Included
Estonia	1962	Registry	5-year age groups	NA	Included
Estonia	1963	Registry	5-year age groups	NA	Included
Estonia	1964	Registry	5-year age groups	NA	Included
Estonia	1965	Registry	5-year age groups	NA	Included
Estonia	1966	Registry	5-year age groups	NA	Included
Estonia	1967	Census	5-year age groups	De facto	Excluded
Estonia	1968	Registry	5-year age groups	NA	Included
Estonia	1969	Registry	5-year age groups	NA	Included
Estonia	1970	Census	5-year age groups	De facto	Excluded
Estonia	1971	Registry	5-year age groups	NA	Included

Estonia	1972	Registry	5-year age groups	NA	Included
Estonia	1973	Registry	5-year age groups	NA	Included
Estonia	1974	Registry	5-year age groups	NA	Included
Estonia	1975	Registry	5-year age groups	NA	Included
Estonia	1976	Registry	5-year age groups	NA	Included
Estonia	1977	Registry	5-year age groups	NA	Included
Estonia	1978	Registry	5-year age groups	NA	Included
Estonia	1979	Census	5-year age groups	De facto	Excluded
Estonia	1980	Registry	5-year age groups	NA	Included
Estonia	1981	Registry	5-year age groups	NA	Included
Estonia	1982	Registry	5-year age groups	NA	Included
Estonia	1983	Registry	5-year age groups	NA	Included
Estonia	1984	Registry	5-year age groups	NA	Included
Estonia	1985	Registry	5-year age groups	NA	Included
Estonia	1986	Registry	5-year age groups	NA	Included
Estonia	1987	Registry	5-year age groups	NA	Included
Estonia	1988	Registry	5-year age groups	NA	Included
Estonia	1989	Census	5-year age groups	De facto	Excluded
Estonia	1990	Registry	5-year age groups	NA	Included
Estonia	1991	Registry	5-year age groups	NA	Included
Estonia	1992	Registry	5-year age groups	NA	Included
Estonia	1993	Registry	5-year age groups	NA	Included
Estonia	1994	Registry	5-year age groups	NA	Included
Estonia	1995	Registry	5-year age groups	NA	Included
Estonia	1996	Registry	5-year age groups	NA	Included
Estonia	1997	Registry	5-year age groups	NA	Included
Estonia	1998	Registry	5-year age groups	NA	Included
Estonia	1999	Registry	5-year age groups	NA	Included
Estonia	2000	Registry	5-year age groups	NA	Included
Estonia	2001	Registry	5-year age groups	NA	Included
Estonia	2002	Registry	5-year age groups	NA	Included
Estonia	2003	Registry	5-year age groups	NA	Included
Estonia	2004	Registry	5-year age groups	NA	Included
Estonia	2005	Registry	5-year age groups	NA	Included
Estonia	2006	Registry	5-year age groups	NA	Included
Estonia	2007	Registry	5-year age groups	NA	Included
Estonia	2008	Registry	5-year age groups	NA	Included
Estonia	2009	Registry	5-year age groups	NA	Included
Estonia	2010	Registry	5-year age groups	NA	Included
Estonia	2011	Registry	5-year age groups	NA	Included
Estonia	2012	Registry	5-year age groups	NA	Included
Estonia	2013	Registry	5-year age groups	NA	Included
Estonia	2014	Registry	5-year age groups	NA	Included
Estonia	2015	Registry	5-year age groups	NA	Included
Estonia	2016	Registry	5-year age groups	NA	Included
Estonia	2017	Registry	5-year age groups	NA	Included
eSwatini	1956	Census	5-year age groups	De facto	Excluded
eSwatini	1966	Census	1-year age groups	De facto	Included
eSwatini	1976	Census	5-year age groups	De facto	Included
eSwatini	1986	Census	1-year age groups	De facto	Included
eSwatini	1997	Census	1-year age groups	De facto	Included

eSwatini	2007	Census	5-year age groups	De facto	Excluded
eSwatini	2017	Census	all ages only	Unknown	Included
Ethiopia	1984	Census	1-year age groups	De facto	Included
Ethiopia	1994	Census	1-year age groups	De facto	Included
Ethiopia	2007	Census	1-year age groups	De facto	Included
Federated States of Micronesia	1989	Census	all ages only	De jure	Excluded
Federated States of Micronesia	1994	Census	5-year age groups	De jure	Included
Federated States of Micronesia	2000	Census	1-year age groups	De jure	Included
Federated States of Micronesia	2010	Census	5-year age groups	De facto	Included
Fiji	1956	Census	5-year age groups	De facto	Included
Fiji	1966	Census	5-year age groups	De facto	Included
Fiji	1976	Census	5-year age groups	De facto	Included
Fiji	1986	Census	1-year age groups	De facto	Included
Fiji	1996	Census	1-year age groups	De facto	Included
Fiji	2007	Census	5-year age groups	De facto	Included
Fiji	2017	Census	5-year age groups	De jure	Included
Finland	1950	Census	1-year age groups	De jure	Included
Finland	1960	Census	5-year age groups	De jure	Excluded
Finland	1970	Census	1-year age groups	De jure	Excluded
Finland	1971	Registry	1-year age groups	NA	Included
Finland	1972	Registry	1-year age groups	NA	Included
Finland	1973	Registry	1-year age groups	NA	Included
Finland	1974	Registry	1-year age groups	NA	Included
Finland	1975	Census	5-year age groups	De jure	Excluded
Finland	1976	Registry	1-year age groups	NA	Included
Finland	1977	Registry	1-year age groups	NA	Included
Finland	1978	Registry	1-year age groups	NA	Included
Finland	1979	Registry	1-year age groups	NA	Included
Finland	1980	Registry	1-year age groups	NA	Included
Finland	1981	Registry	1-year age groups	NA	Included
Finland	1982	Registry	1-year age groups	NA	Included
Finland	1983	Registry	1-year age groups	NA	Included
Finland	1984	Registry	1-year age groups	NA	Included
Finland	1985	Census	1-year age groups	De jure	Excluded
Finland	1986	Registry	1-year age groups	NA	Included
Finland	1987	Registry	1-year age groups	NA	Included
Finland	1988	Registry	1-year age groups	NA	Included
Finland	1989	Registry	1-year age groups	NA	Included
Finland	1990	Census	1-year age groups	De jure	Excluded
Finland	1991	Registry	1-year age groups	NA	Included
Finland	1992	Registry	1-year age groups	NA	Included
Finland	1993	Registry	1-year age groups	NA	Included
Finland	1994	Registry	1-year age groups	NA	Included
Finland	1995	Registry	1-year age groups	NA	Included
Finland	1996	Registry	1-year age groups	NA	Included
Finland	1997	Registry	1-year age groups	NA	Included
Finland	1998	Registry	1-year age groups	NA	Included
Finland	1999	Registry	1-year age groups	NA	Included
Finland	2000	Census	1-year age groups	De jure	Excluded
Finland	2001	Registry	1-year age groups	NA	Included
Finland	2002	Registry	1-year age groups	NA	Included

Finland	2003	Registry	1-year age groups	NA	Included
Finland	2004	Registry	1-year age groups	NA	Included
Finland	2005	Registry	1-year age groups	NA	Included
Finland	2006	Registry	1-year age groups	NA	Included
Finland	2007	Registry	1-year age groups	NA	Included
Finland	2008	Registry	1-year age groups	NA	Included
Finland	2009	Registry	1-year age groups	NA	Included
Finland	2010	Census	1-year age groups	De jure	Excluded
Finland	2011	Registry	1-year age groups	NA	Included
Finland	2012	Registry	1-year age groups	NA	Included
Finland	2013	Registry	1-year age groups	NA	Included
Finland	2014	Registry	1-year age groups	NA	Included
Finland	2015	Registry	1-year age groups	NA	Included
Finland	2016	Registry	1-year age groups	NA	Included
Finland	2017	Registry	1-year age groups	NA	Included
France	1954	Census	5-year age groups	De jure	Included
France	1962	Census	5-year age groups	De jure	Included
France	1968	Census	5-year age groups	De jure	Included
France	1975	Census	5-year age groups	De jure	Included
France	1982	Census	5-year age groups	De jure	Included
France	1990	Census	5-year age groups	De jure	Included
France	1999	Census	5-year age groups	De jure	Included
France	2006	Census	1-year age groups	De jure	Included
France	2007	Census	1-year age groups	De jure	Included
France	2008	Census	1-year age groups	De jure	Included
France	2009	Census	1-year age groups	De jure	Included
France	2010	Census	1-year age groups	De jure	Included
France	2011	Census	1-year age groups	De jure	Included
France	2012	Census	1-year age groups	De jure	Included
France	2013	Census	1-year age groups	De jure	Included
France	2014	Census	1-year age groups	De jure	Included
France	2015	Census	5-year age groups	Unknown	Included
Gabon	1960	Census	5-year age groups	De facto	Included
Gabon	1980	Census	5-year age groups	De jure	Excluded
Gabon	1993	Census	1-year age groups	De facto	Included
Gabon	2013	Census	non-std age groups	De jure	Included
Georgia	1959	Census	5-year age groups	De facto	Included
Georgia	1970	Census	5-year age groups	De facto	Included
Georgia	1979	Census	5-year age groups	De facto	Included
Georgia	1989	Census	5-year age groups	De jure	Included
Georgia	2002	Census	1-year age groups	De jure	Included
Georgia	2014	Census	1-year age groups	De jure	Included
Germany	1990	Registry	1-year age groups	NA	Included
Germany	1991	Registry	1-year age groups	NA	Included
Germany	1992	Registry	1-year age groups	NA	Included
Germany	1993	Registry	1-year age groups	NA	Included
Germany	1994	Registry	1-year age groups	NA	Included
Germany	1995	Registry	1-year age groups	NA	Included
Germany	1996	Registry	1-year age groups	NA	Included
Germany	1997	Registry	1-year age groups	NA	Included
Germany	1998	Registry	1-year age groups	NA	Included

Germany	1999	Registry	1-year age groups	NA	Included
Germany	2000	Registry	1-year age groups	NA	Included
Germany	2001	Registry	1-year age groups	NA	Included
Germany	2002	Registry	1-year age groups	NA	Included
Germany	2003	Registry	1-year age groups	NA	Included
Germany	2004	Registry	1-year age groups	NA	Included
Germany	2005	Registry	1-year age groups	NA	Included
Germany	2006	Registry	1-year age groups	NA	Included
Germany	2007	Registry	1-year age groups	NA	Included
Germany	2008	Registry	1-year age groups	NA	Included
Germany	2009	Registry	1-year age groups	NA	Included
Germany	2010	Registry	1-year age groups	NA	Included
Germany	2011	Census	1-year age groups	De jure	Excluded
Germany	2012	Registry	1-year age groups	NA	Included
Germany	2013	Registry	1-year age groups	NA	Included
Germany	2014	Registry	1-year age groups	NA	Included
Germany	2015	Registry	1-year age groups	NA	Included
Germany	2016	Registry	1-year age groups	NA	Included
Ghana	1960	Census	5-year age groups	De facto	Excluded
Ghana	1970	Census	1-year age groups	De facto	Included
Ghana	1984	Census	1-year age groups	De facto	Included
Ghana	2000	Census	5-year age groups	De facto	Included
Ghana	2010	Census	1-year age groups	De facto	Included
Greece	1951	Census	5-year age groups	De facto	Included
Greece	1961	Census	5-year age groups	De facto	Included
Greece	1971	Census	5-year age groups	De facto	Included
Greece	1981	Census	1-year age groups	De facto	Included
Greece	1991	Census	1-year age groups	De facto	Included
Greece	2001	Census	1-year age groups	De jure	Included
Greece	2011	Census	1-year age groups	De facto	Included
Greenland	1951	Census	5-year age groups	De jure	Included
Greenland	1955	Census	5-year age groups	De jure	Included
Greenland	1960	Census	non-std age groups	De jure	Included
Greenland	1965	Census	1-year age groups	De jure	Included
Greenland	1970	Census	1-year age groups	De jure	Included
Greenland	1976	Census	5-year age groups	De jure	Included
Greenland	1977	Registry	1-year age groups	NA	Included
Greenland	1978	Registry	1-year age groups	NA	Included
Greenland	1979	Registry	1-year age groups	NA	Included
Greenland	1980	Registry	1-year age groups	NA	Included
Greenland	1981	Registry	1-year age groups	NA	Included
Greenland	1982	Registry	1-year age groups	NA	Included
Greenland	1983	Registry	1-year age groups	NA	Included
Greenland	1984	Registry	1-year age groups	NA	Included
Greenland	1985	Registry	1-year age groups	NA	Included
Greenland	1986	Registry	1-year age groups	NA	Included
Greenland	1987	Registry	1-year age groups	NA	Included
Greenland	1988	Registry	1-year age groups	NA	Included
Greenland	1989	Registry	1-year age groups	NA	Included
Greenland	1990	Registry	1-year age groups	NA	Included
Greenland	1991	Registry	1-year age groups	NA	Included

Greenland	1992	Registry	1-year age groups	NA	Included
Greenland	1993	Registry	1-year age groups	NA	Included
Greenland	1994	Registry	1-year age groups	NA	Included
Greenland	1995	Registry	1-year age groups	NA	Included
Greenland	1996	Registry	1-year age groups	NA	Included
Greenland	1997	Registry	1-year age groups	NA	Included
Greenland	1998	Registry	1-year age groups	NA	Included
Greenland	1999	Registry	1-year age groups	NA	Included
Greenland	2000	Census	5-year age groups	De jure	Excluded
Greenland	2001	Registry	1-year age groups	NA	Included
Greenland	2002	Registry	1-year age groups	NA	Included
Greenland	2003	Registry	1-year age groups	NA	Included
Greenland	2004	Registry	1-year age groups	NA	Included
Greenland	2005	Registry	1-year age groups	NA	Included
Greenland	2006	Registry	1-year age groups	NA	Included
Greenland	2007	Registry	1-year age groups	NA	Included
Greenland	2008	Registry	1-year age groups	NA	Included
Greenland	2009	Registry	1-year age groups	NA	Included
Greenland	2010	Registry	1-year age groups	NA	Included
Greenland	2011	Registry	1-year age groups	NA	Included
Greenland	2012	Registry	1-year age groups	NA	Included
Greenland	2013	Registry	1-year age groups	NA	Included
Greenland	2014	Registry	1-year age groups	NA	Included
Greenland	2015	Registry	1-year age groups	NA	Included
Greenland	2016	Registry	1-year age groups	NA	Included
Greenland	2017	Registry	1-year age groups	NA	Included
Grenada	1960	Census	5-year age groups	De facto	Included
Grenada	1970	Census	5-year age groups	De facto	Included
Grenada	1981	Census	5-year age groups	De facto	Excluded
Grenada	1991	Census	5-year age groups	De facto	Included
Grenada	2001	Census	5-year age groups	De facto	Included
Grenada	2011	Census	5-year age groups	De jure	Included
Guam	1950	Census	5-year age groups	De jure	Included
Guam	1952	Census	all ages only	Unknown	Excluded
Guam	1953	Census	all ages only	Unknown	Excluded
Guam	1954	Census	all ages only	Unknown	Excluded
Guam	1955	Census	all ages only	Unknown	Excluded
Guam	1958	Census	all ages only	Unknown	Excluded
Guam	1960	Census	non-std age groups	De jure	Included
Guam	1970	Census	5-year age groups	De jure	Included
Guam	1980	Census	1-year age groups	De jure	Included
Guam	1990	Census	1-year age groups	De jure	Included
Guam	2000	Census	1-year age groups	De jure	Included
Guam	2010	Census	5-year age groups	De jure	Included
Guatemala	1950	Census	1-year age groups	De jure	Included
Guatemala	1964	Census	1-year age groups	De jure	Included
Guatemala	1973	Census	1-year age groups	De jure	Included
Guatemala	1981	Census	1-year age groups	De jure	Included
Guatemala	1994	Census	1-year age groups	De jure	Included
Guatemala	2002	Census	all ages only	De jure	Included
Guinea	1966	Census	all ages only	Unknown	Included

Guinea	1983	Census	1-year age groups	Unknown	Included
Guinea	1996	Census	5-year age groups	De facto	Included
Guinea	2014	Census	1-year age groups	De jure	Included
Guinea-Bissau	1950	Census	5-year age groups	De facto	Included
Guinea-Bissau	1960	Census	5-year age groups	De facto	Included
Guinea-Bissau	1979	Census	5-year age groups	De jure	Included
Guinea-Bissau	1991	Census	non-std age groups	De facto	Included
Guinea-Bissau	2009	Census	1-year age groups	De jure	Included
Guyana	1960	Census	5-year age groups	De facto	Included
Guyana	1970	Census	1-year age groups	De facto	Included
Guyana	1980	Census	5-year age groups	Unknown	Excluded
Guyana	1991	Census	5-year age groups	De facto	Included
Guyana	2002	Census	5-year age groups	De facto	Included
Guyana	2012	Census	5-year age groups	De jure	Included
Haiti	1950	Census	1-year age groups	De jure	Included
Haiti	1971	Census	1-year age groups	De jure	Included
Haiti	1982	Census	1-year age groups	De jure	Included
Haiti	2003	Census	1-year age groups	Unknown	Included
Honduras	1950	Census	5-year age groups	De facto	Included
Honduras	1961	Census	5-year age groups	De facto	Included
Honduras	1974	Census	5-year age groups	De facto	Included
Honduras	1988	Census	1-year age groups	De facto	Included
Honduras	2001	Census	1-year age groups	De jure	Included
Honduras	2013	Census	1-year age groups	De facto	Included
Hungary	1960	Census	1-year age groups	De facto	Included
Hungary	1970	Census	1-year age groups	De facto	Included
Hungary	1980	Census	1-year age groups	De facto	Included
Hungary	1990	Census	1-year age groups	De facto	Included
Hungary	2001	Census	1-year age groups	Unknown	Included
Hungary	2011	Census	1-year age groups	De facto	Included
Iceland	1950	Census	5-year age groups	De jure	Excluded
Iceland	1951	Registry	1-year age groups	NA	Included
Iceland	1952	Registry	1-year age groups	NA	Included
Iceland	1953	Registry	1-year age groups	NA	Included
Iceland	1954	Registry	1-year age groups	NA	Included
Iceland	1955	Registry	1-year age groups	NA	Included
Iceland	1956	Registry	1-year age groups	NA	Included
Iceland	1957	Registry	1-year age groups	NA	Included
Iceland	1958	Registry	1-year age groups	NA	Included
Iceland	1959	Registry	1-year age groups	NA	Included
Iceland	1960	Census	1-year age groups	De jure	Excluded
Iceland	1961	Registry	1-year age groups	NA	Included
Iceland	1962	Registry	1-year age groups	NA	Included
Iceland	1963	Registry	1-year age groups	NA	Included
Iceland	1964	Registry	1-year age groups	NA	Included
Iceland	1965	Registry	1-year age groups	NA	Included
Iceland	1966	Registry	1-year age groups	NA	Included
Iceland	1967	Registry	1-year age groups	NA	Included
Iceland	1968	Registry	1-year age groups	NA	Included
Iceland	1969	Registry	1-year age groups	NA	Included
Iceland	1970	Census	5-year age groups	De jure	Excluded

Iceland	1971	Registry	1-year age groups	NA	Included
Iceland	1972	Registry	1-year age groups	NA	Included
Iceland	1973	Registry	1-year age groups	NA	Included
Iceland	1974	Registry	1-year age groups	NA	Included
Iceland	1975	Registry	1-year age groups	NA	Included
Iceland	1976	Registry	1-year age groups	NA	Included
Iceland	1977	Registry	1-year age groups	NA	Included
Iceland	1978	Registry	1-year age groups	NA	Included
Iceland	1979	Registry	1-year age groups	NA	Included
Iceland	1980	Registry	1-year age groups	NA	Included
Iceland	1981	Registry	1-year age groups	NA	Included
Iceland	1982	Registry	1-year age groups	NA	Included
Iceland	1983	Registry	1-year age groups	NA	Included
Iceland	1984	Registry	1-year age groups	NA	Included
Iceland	1985	Registry	1-year age groups	NA	Included
Iceland	1986	Registry	1-year age groups	NA	Included
Iceland	1987	Registry	1-year age groups	NA	Included
Iceland	1988	Registry	1-year age groups	NA	Included
Iceland	1989	Registry	1-year age groups	NA	Included
Iceland	1990	Registry	1-year age groups	NA	Included
Iceland	1991	Registry	1-year age groups	NA	Included
Iceland	1992	Registry	1-year age groups	NA	Included
Iceland	1993	Registry	1-year age groups	NA	Included
Iceland	1994	Registry	1-year age groups	NA	Included
Iceland	1995	Registry	1-year age groups	NA	Included
Iceland	1996	Registry	1-year age groups	NA	Included
Iceland	1997	Registry	1-year age groups	NA	Included
Iceland	1998	Registry	1-year age groups	NA	Included
Iceland	1999	Registry	1-year age groups	NA	Included
Iceland	2000	Registry	1-year age groups	NA	Included
Iceland	2001	Registry	1-year age groups	NA	Included
Iceland	2002	Registry	1-year age groups	NA	Included
Iceland	2003	Registry	1-year age groups	NA	Included
Iceland	2004	Registry	1-year age groups	NA	Included
Iceland	2005	Registry	1-year age groups	NA	Included
Iceland	2006	Registry	1-year age groups	NA	Included
Iceland	2007	Registry	1-year age groups	NA	Included
Iceland	2008	Registry	1-year age groups	NA	Included
Iceland	2009	Registry	1-year age groups	NA	Included
Iceland	2010	Registry	1-year age groups	NA	Included
Iceland	2011	Census	5-year age groups	De jure	Excluded
Iceland	2012	Registry	1-year age groups	NA	Included
Iceland	2013	Registry	1-year age groups	NA	Included
Iceland	2014	Registry	1-year age groups	NA	Included
Iceland	2015	Registry	1-year age groups	NA	Included
Iceland	2016	Registry	1-year age groups	NA	Included
Iceland	2017	Registry	1-year age groups	NA	Included
India	1951	Census	1-year age groups	De facto	Included
India	1961	Census	5-year age groups	De facto	Included
India	1971	Census	1-year age groups	De facto	Included
India	1981	Census	5-year age groups	De facto	Included

India	1991	Census	1-year age groups	De facto	Included
India	2001	Census	1-year age groups	De facto	Included
India	2011	Census	1-year age groups	De facto	Included
Indonesia	1961	Census	non-std age groups	De facto	Included
Indonesia	1971	Census	5-year age groups	De jure	Excluded
Indonesia	1980	Census	1-year age groups	De facto	Included
Indonesia	1990	Census	5-year age groups	De facto	Included
Indonesia	2000	Census	1-year age groups	De jure	Excluded
Indonesia	2010	Census	1-year age groups	De jure	Included
Iran	1956	Census	5-year age groups	De facto	Included
Iran	1966	Census	5-year age groups	De jure	Excluded
Iran	1976	Census	5-year age groups	De facto	Included
Iran	1986	Census	5-year age groups	De facto	Included
Iran	1991	Census	5-year age groups	De jure	Excluded
Iran	1994	Census	1-year age groups	De jure	Excluded
Iran	1996	Census	5-year age groups	De jure	Excluded
Iran	2006	Census	1-year age groups	De jure	Included
Iran	2011	Census	1-year age groups	De jure	Included
Iran	2016	Census	5-year age groups	De jure	Excluded
Iraq	1957	Census	5-year age groups	De facto	Included
Iraq	1965	Census	1-year age groups	De facto	Included
Iraq	1977	Census	5-year age groups	De facto	Included
Iraq	1987	Census	5-year age groups	De facto	Included
Iraq	1997	Census	5-year age groups	De facto	Excluded
Ireland	1951	Census	1-year age groups	De facto	Included
Ireland	1956	Census	5-year age groups	De jure	Included
Ireland	1961	Census	5-year age groups	De facto	Included
Ireland	1966	Census	5-year age groups	De facto	Included
Ireland	1971	Census	1-year age groups	De facto	Included
Ireland	1979	Census	5-year age groups	De facto	Included
Ireland	1981	Census	5-year age groups	De facto	Included
Ireland	1986	Census	5-year age groups	De facto	Included
Ireland	1991	Census	1-year age groups	De facto	Included
Ireland	1996	Census	1-year age groups	De facto	Included
Ireland	2002	Census	1-year age groups	De facto	Included
Ireland	2006	Census	1-year age groups	De facto	Included
Ireland	2011	Census	1-year age groups	De facto	Included
Ireland	2016	Census	1-year age groups	De jure	Included
Israel	1961	Census	1-year age groups	De jure	Included
Israel	1972	Census	5-year age groups	De jure	Included
Israel	1983	Census	5-year age groups	De jure	Included
Israel	1995	Census	1-year age groups	De facto	Included
Israel	2008	Census	1-year age groups	De facto	Included
Italy	1951	Census	5-year age groups	De jure	Included
Italy	1961	Census	1-year age groups	De jure	Included
Italy	1971	Census	1-year age groups	De jure	Included
Italy	1981	Census	1-year age groups	De facto	Included
Italy	1991	Census	1-year age groups	De facto	Included
Italy	2001	Census	1-year age groups	De jure	Included
Italy	2002	Registry	1-year age groups	NA	Included
Italy	2003	Registry	1-year age groups	NA	Included

Italy	2004	Registry	1-year age groups	NA	Included
Italy	2005	Registry	1-year age groups	NA	Included
Italy	2006	Registry	1-year age groups	NA	Included
Italy	2007	Registry	1-year age groups	NA	Included
Italy	2008	Registry	1-year age groups	NA	Included
Italy	2009	Registry	1-year age groups	NA	Included
Italy	2010	Registry	1-year age groups	NA	Included
Italy	2011	Census	5-year age groups	De jure	Excluded
Italy	2012	Registry	1-year age groups	NA	Excluded
Italy	2013	Registry	1-year age groups	NA	Excluded
Italy	2014	Registry	1-year age groups	NA	Included
Italy	2015	Registry	1-year age groups	NA	Included
Italy	2016	Registry	1-year age groups	NA	Included
Italy	2017	Registry	1-year age groups	NA	Included
Jamaica	1953	Census	5-year age groups	De jure	Included
Jamaica	1960	Census	5-year age groups	De jure	Included
Jamaica	1970	Census	5-year age groups	De jure	Included
Jamaica	1982	Census	1-year age groups	De jure	Included
Jamaica	1991	Census	1-year age groups	De jure	Included
Jamaica	2001	Census	1-year age groups	De jure	Included
Jamaica	2011	Census	1-year age groups	De jure	Included
Japan	1950	Census	1-year age groups	De facto	Included
Japan	1955	Census	5-year age groups	De facto	Included
Japan	1960	Census	5-year age groups	De facto	Included
Japan	1965	Census	1-year age groups	De facto	Included
Japan	1970	Census	1-year age groups	De facto	Included
Japan	1975	Census	5-year age groups	De facto	Included
Japan	1980	Census	1-year age groups	De facto	Included
Japan	1985	Census	5-year age groups	De facto	Included
Japan	1990	Census	1-year age groups	De facto	Included
Japan	1995	Census	1-year age groups	De facto	Included
Japan	2000	Census	1-year age groups	De jure	Included
Japan	2005	Census	1-year age groups	De jure	Included
Japan	2010	Census	1-year age groups	De jure	Included
Japan	2015	Census	5-year age groups	De jure	Excluded
Jordan	1961	Census	non-std age groups	De facto	Excluded
Jordan	1979	Census	5-year age groups	De facto	Included
Jordan	1994	Census	5-year age groups	De facto	Included
Jordan	2004	Census	5-year age groups	De facto	Included
Jordan	2015	Census	1-year age groups	De facto	Included
Kazakhstan	1959	Census	non-std age groups	De facto	Included
Kazakhstan	1970	Census	non-std age groups	De facto	Included
Kazakhstan	1979	Census	5-year age groups	De facto	Included
Kazakhstan	1989	Census	1-year age groups	De facto	Included
Kazakhstan	1999	Census	1-year age groups	De jure	Included
Kazakhstan	2009	Census	5-year age groups	De facto	Included
Kazakhstan	2010	Registry	all ages only	NA	Included
Kazakhstan	2011	Registry	all ages only	NA	Included
Kazakhstan	2012	Registry	all ages only	NA	Included
Kazakhstan	2013	Registry	all ages only	NA	Included
Kazakhstan	2014	Registry	all ages only	NA	Included

Kazakhstan	2015	Registry	all ages only	NA	Included
Kazakhstan	2016	Registry	all ages only	NA	Included
Kenya	1962	Census	5-year age groups	Unknown	Excluded
Kenya	1969	Census	1-year age groups	De facto	Included
Kenya	1979	Census	5-year age groups	De facto	Included
Kenya	1989	Census	1-year age groups	De jure	Included
Kenya	1999	Census	5-year age groups	De facto	Included
Kenya	2009	Census	1-year age groups	De facto	Included
Kiribati	1963	Census	5-year age groups	De facto	Included
Kiribati	1968	Census	5-year age groups	De facto	Included
Kiribati	1973	Census	5-year age groups	De facto	Included
Kiribati	1978	Census	5-year age groups	De facto	Included
Kiribati	1985	Census	5-year age groups	De facto	Included
Kiribati	1990	Census	5-year age groups	De facto	Included
Kiribati	1995	Census	5-year age groups	De facto	Included
Kiribati	2000	Census	5-year age groups	De facto	Included
Kiribati	2005	Census	5-year age groups	De facto	Included
Kiribati	2010	Census	1-year age groups	De facto	Included
Kiribati	2015	Census	5-year age groups	De jure	Included
Kuwait	1957	Census	5-year age groups	De facto	Included
Kuwait	1961	Census	non-std age groups	De facto	Included
Kuwait	1965	Census	1-year age groups	De facto	Included
Kuwait	1970	Census	1-year age groups	De facto	Included
Kuwait	1975	Census	5-year age groups	De facto	Included
Kuwait	1980	Census	5-year age groups	De facto	Included
Kuwait	1985	Census	1-year age groups	De facto	Included
Kuwait	1995	Census	5-year age groups	De facto	Included
Kuwait	2005	Census	5-year age groups	De facto	Included
Kuwait	2011	Census	1-year age groups	De facto	Included
Kyrgyzstan	1959	Census	non-std age groups	De facto	Included
Kyrgyzstan	1970	Census	non-std age groups	De facto	Included
Kyrgyzstan	1979	Census	5-year age groups	De facto	Included
Kyrgyzstan	1989	Census	1-year age groups	De jure	Included
Kyrgyzstan	1999	Census	1-year age groups	De jure	Included
Kyrgyzstan	2009	Census	1-year age groups	De jure	Included
Laos	1985	Census	5-year age groups	De jure	Included
Laos	1995	Census	5-year age groups	De facto	Included
Laos	2005	Census	1-year age groups	De jure	Included
Laos	2015	Census	1-year age groups	De jure	Included
Latvia	1959	Census	non-std age groups	De facto	Included
Latvia	1970	Census	non-std age groups	De facto	Included
Latvia	1979	Census	5-year age groups	De jure	Included
Latvia	1989	Census	5-year age groups	De jure	Included
Latvia	2000	Census	5-year age groups	De jure	Included
Latvia	2006	Registry	1-year age groups	NA	Included
Latvia	2007	Registry	1-year age groups	NA	Included
Latvia	2008	Registry	1-year age groups	NA	Included
Latvia	2009	Registry	1-year age groups	NA	Included
Latvia	2010	Registry	1-year age groups	NA	Included
Latvia	2011	Census	1-year age groups	De jure	Excluded
Latvia	2012	Registry	1-year age groups	NA	Included

Latvia	2013	Registry	1-year age groups	NA	Included
Latvia	2014	Registry	1-year age groups	NA	Included
Latvia	2015	Registry	1-year age groups	NA	Included
Latvia	2016	Registry	1-year age groups	NA	Included
Latvia	2017	Registry	1-year age groups	NA	Included
Lebanon	1970	Survey	5-year age groups	De facto	Included
Lebanon	2007	Census	5-year age groups	De facto	Included
Lesotho	1956	Census	non-std age groups	De facto	Included
Lesotho	1966	Census	1-year age groups	De facto	Included
Lesotho	1976	Census	5-year age groups	De jure	Included
Lesotho	1986	Census	5-year age groups	De jure	Included
Lesotho	1996	Census	5-year age groups	De jure	Included
Lesotho	2006	Census	1-year age groups	De jure	Included
Lesotho	2016	Census	5-year age groups	De jure	Included
Liberia	1962	Census	5-year age groups	De facto	Included
Liberia	1974	Census	5-year age groups	De facto	Included
Liberia	1984	Census	1-year age groups	De facto	Included
Liberia	2008	Census	1-year age groups	De facto	Included
Libya	1954	Census	5-year age groups	De facto	Included
Libya	1964	Census	non-std age groups	De facto	Included
Libya	1973	Census	1-year age groups	De jure	Included
Libya	1984	Census	1-year age groups	De facto	Included
Libya	1995	Census	all ages only	Unknown	Included
Libya	2006	Census	5-year age groups	De facto	Included
Lithuania	1959	Census	non-std age groups	De facto	Included
Lithuania	1970	Census	non-std age groups	De facto	Included
Lithuania	1979	Census	5-year age groups	De facto	Included
Lithuania	1989	Census	1-year age groups	De jure	Included
Lithuania	2001	Census	5-year age groups	De jure	Included
Lithuania	2011	Census	1-year age groups	De jure	Included
Lithuania	2015	Registry	1-year age groups	NA	Included
Lithuania	2016	Registry	1-year age groups	NA	Included
Lithuania	2017	Registry	1-year age groups	NA	Included
Luxembourg	1960	Census	1-year age groups	De jure	Included
Luxembourg	1966	Census	5-year age groups	De jure	Included
Luxembourg	1970	Census	1-year age groups	De jure	Included
Luxembourg	1981	Census	1-year age groups	De jure	Included
Luxembourg	1991	Census	5-year age groups	De jure	Included
Luxembourg	2001	Registry	5-year age groups	NA	Included
Luxembourg	2002	Registry	5-year age groups	NA	Included
Luxembourg	2003	Registry	5-year age groups	NA	Included
Luxembourg	2004	Registry	5-year age groups	NA	Included
Luxembourg	2005	Registry	5-year age groups	NA	Included
Luxembourg	2006	Registry	5-year age groups	NA	Included
Luxembourg	2007	Registry	5-year age groups	NA	Included
Luxembourg	2008	Registry	5-year age groups	NA	Included
Luxembourg	2009	Registry	5-year age groups	NA	Included
Luxembourg	2010	Registry	5-year age groups	NA	Included
Luxembourg	2011	Registry	5-year age groups	NA	Included
Luxembourg	2012	Registry	5-year age groups	NA	Included
Luxembourg	2013	Registry	5-year age groups	NA	Included

Luxembourg	2014	Registry	5-year age groups	NA	Included
Luxembourg	2015	Registry	5-year age groups	NA	Included
Luxembourg	2016	Registry	5-year age groups	NA	Included
Luxembourg	2017	Registry	5-year age groups	NA	Included
Madagascar	1975	Census	5-year age groups	De facto	Included
Madagascar	1993	Census	5-year age groups	De facto	Included
Malawi	1966	Census	5-year age groups	De facto	Included
Malawi	1977	Census	5-year age groups	De facto	Included
Malawi	1987	Census	1-year age groups	De facto	Included
Malawi	1998	Census	1-year age groups	De facto	Included
Malawi	2008	Census	1-year age groups	De facto	Included
Malawi	2018	Census	5-year age groups	De jure	Included
Malaysia	1991	Census	1-year age groups	De facto	Included
Malaysia	2000	Census	5-year age groups	De jure	Included
Malaysia	2010	Census	1-year age groups	De jure	Included
Maldives	1953	Census	all ages only	De jure	Included
Maldives	1957	Census	all ages only	De jure	Included
Maldives	1962	Census	all ages only	De jure	Included
Maldives	1965	Census	non-std age groups	De facto	Excluded
Maldives	1967	Census	1-year age groups	De facto	Included
Maldives	1972	Census	all ages only	De jure	Included
Maldives	1974	Census	5-year age groups	De facto	Included
Maldives	1977	Census	5-year age groups	De facto	Included
Maldives	1985	Census	1-year age groups	De facto	Included
Maldives	1990	Census	1-year age groups	De facto	Included
Maldives	1995	Census	1-year age groups	De facto	Included
Maldives	2000	Census	1-year age groups	De facto	Included
Maldives	2006	Census	1-year age groups	De facto	Included
Maldives	2014	Census	1-year age groups	De facto	Included
Mali	1976	Census	5-year age groups	De facto	Included
Mali	1987	Census	1-year age groups	De jure	Included
Mali	1998	Census	5-year age groups	De jure	Included
Mali	2009	Census	1-year age groups	De facto	Included
Malta	1957	Census	5-year age groups	De facto	Included
Malta	1967	Census	5-year age groups	De facto	Included
Malta	1985	Census	5-year age groups	De facto	Included
Malta	1995	Census	1-year age groups	De jure	Included
Malta	2005	Census	1-year age groups	De jure	Included
Malta	2011	Census	1-year age groups	De facto	Included
Marshall Islands	1973	Census	5-year age groups	De facto	Excluded
Marshall Islands	1988	Census	1-year age groups	De facto	Included
Marshall Islands	1999	Census	1-year age groups	De facto	Included
Marshall Islands	2011	Census	all ages only	De jure	Included
Mauritania	1977	Census	5-year age groups	De facto	Included
Mauritania	1988	Census	5-year age groups	De facto	Included
Mauritania	2000	Census	5-year age groups	De facto	Included
Mauritania	2013	Census	5-year age groups	De jure	Included
Mauritius	1952	Census	1-year age groups	De jure	Included
Mauritius	1962	Census	1-year age groups	De facto	Included
Mauritius	1972	Census	1-year age groups	De jure	Included
Mauritius	1983	Census	all ages only	De jure	Included

Mauritius	1990	Census	1-year age groups	De facto	Included
Mauritius	2000	Census	1-year age groups	De jure	Included
Mauritius	2011	Census	1-year age groups	De jure	Included
Mexico	1950	Census	1-year age groups	De jure	Included
Mexico	1960	Census	5-year age groups	De jure	Excluded
Mexico	1970	Census	1-year age groups	De jure	Included
Mexico	1980	Census	1-year age groups	De jure	Included
Mexico	1990	Census	1-year age groups	De jure	Included
Mexico	1995	Census	5-year age groups	Unknown	Included
Mexico	2000	Census	1-year age groups	De jure	Included
Mexico	2005	Census	1-year age groups	De jure	Excluded
Mexico	2010	Census	1-year age groups	De facto	Included
Moldova	1959	Census	non-std age groups	De facto	Included
Moldova	1970	Census	non-std age groups	De facto	Included
Moldova	1979	Census	5-year age groups	De facto	Included
Moldova	1989	Census	1-year age groups	De jure	Included
Monaco	1951	Census	5-year age groups	De jure	Included
Monaco	1956	Census	5-year age groups	De jure	Included
Monaco	1961	Census	1-year age groups	De jure	Excluded
Monaco	1962	Census	5-year age groups	De jure	Included
Monaco	1968	Census	5-year age groups	De jure	Included
Monaco	1975	Census	5-year age groups	De jure	Excluded
Monaco	1982	Census	5-year age groups	De jure	Excluded
Monaco	1990	Census	non-std age groups	De jure	Excluded
Monaco	2000	Census	5-year age groups	Unknown	Included
Monaco	2008	Census	5-year age groups	De jure	Excluded
Monaco	2016	Census	1-year age groups	Unknown	Included
Mongolia	1956	Census	non-std age groups	De facto	Included
Mongolia	1963	Census	5-year age groups	De jure	Included
Mongolia	1969	Census	5-year age groups	De jure	Included
Mongolia	1979	Census	5-year age groups	De jure	Included
Mongolia	1989	Census	5-year age groups	De facto	Included
Mongolia	2000	Census	1-year age groups	De facto	Included
Mongolia	2010	Census	5-year age groups	De facto	Included
Montenegro	1953	Census	5-year age groups	De jure	Included
Montenegro	1961	Census	5-year age groups	De jure	Included
Montenegro	1971	Census	5-year age groups	De jure	Included
Montenegro	1981	Census	5-year age groups	De jure	Included
Montenegro	1991	Census	5-year age groups	De facto	Included
Montenegro	2003	Census	1-year age groups	De jure	Included
Montenegro	2011	Census	1-year age groups	De jure	Included
Morocco	1950	Census	all ages only	De jure	Excluded
Morocco	1960	Census	5-year age groups	De jure	Included
Morocco	1971	Census	1-year age groups	De jure	Included
Morocco	1982	Census	5-year age groups	De facto	Included
Morocco	1994	Census	5-year age groups	De facto	Included
Morocco	2004	Census	1-year age groups	De facto	Included
Morocco	2014	Census	5-year age groups	De jure	Included
Mozambique	1950	Census	5-year age groups	De facto	Included
Mozambique	1960	Census	5-year age groups	De jure	Included
Mozambique	1970	Census	5-year age groups	De facto	Included

Mozambique	1980	Census	1-year age groups	De facto	Included
Mozambique	1997	Census	1-year age groups	De jure	Included
Mozambique	2007	Census	1-year age groups	De facto	Included
Mozambique	2017	Census	1-year age groups	Unknown	Included
Myanmar	1953	Census	all ages only	Unknown	Excluded
Myanmar	1973	Census	5-year age groups	De jure	Included
Myanmar	1983	Census	1-year age groups	De facto	Included
Myanmar	2014	Census	1-year age groups	De facto	Included
Namibia	1951	Census	5-year age groups	De facto	Excluded
Namibia	1960	Census	5-year age groups	De facto	Included
Namibia	1970	Census	all ages only	De jure	Included
Namibia	1991	Census	1-year age groups	De facto	Included
Namibia	2001	Census	1-year age groups	De facto	Included
Namibia	2011	Census	1-year age groups	De facto	Included
Nauru	1961	Census	5-year age groups	De facto	Excluded
Nauru	1966	Census	5-year age groups	De facto	Excluded
Nauru	1977	Census	5-year age groups	De facto	Excluded
Nauru	1992	Census	5-year age groups	De facto	Excluded
Nauru	2002	Census	5-year age groups	De facto	Excluded
Nauru	2006	Census	5-year age groups	Unknown	Excluded
Nauru	2011	Census	5-year age groups	Unknown	Included
Nepal	1954	Census	5-year age groups	De facto	Included
Nepal	1961	Census	5-year age groups	De facto	Included
Nepal	1971	Census	non-std age groups	De jure	Included
Nepal	1981	Census	1-year age groups	De jure	Included
Nepal	1991	Census	1-year age groups	De jure	Included
Nepal	2001	Census	1-year age groups	De jure	Included
Nepal	2011	Census	1-year age groups	De jure	Included
Netherlands	1950	Registry	1-year age groups	NA	Included
Netherlands	1951	Registry	1-year age groups	NA	Included
Netherlands	1952	Registry	1-year age groups	NA	Included
Netherlands	1953	Registry	1-year age groups	NA	Included
Netherlands	1954	Registry	1-year age groups	NA	Included
Netherlands	1955	Registry	1-year age groups	NA	Included
Netherlands	1956	Registry	1-year age groups	NA	Included
Netherlands	1957	Registry	1-year age groups	NA	Included
Netherlands	1958	Registry	1-year age groups	NA	Included
Netherlands	1959	Registry	1-year age groups	NA	Included
Netherlands	1960	Census	1-year age groups	De jure	Excluded
Netherlands	1961	Registry	1-year age groups	NA	Included
Netherlands	1962	Registry	1-year age groups	NA	Included
Netherlands	1963	Registry	1-year age groups	NA	Included
Netherlands	1964	Registry	1-year age groups	NA	Included
Netherlands	1965	Registry	1-year age groups	NA	Included
Netherlands	1966	Registry	1-year age groups	NA	Included
Netherlands	1967	Registry	1-year age groups	NA	Included
Netherlands	1968	Registry	1-year age groups	NA	Included
Netherlands	1969	Registry	1-year age groups	NA	Included
Netherlands	1970	Registry	1-year age groups	NA	Included
Netherlands	1971	Registry	1-year age groups	NA	Included
Netherlands	1971	Census	1-year age groups	Unknown	Excluded

Netherlands	1972	Registry	1-year age groups	NA	Included
Netherlands	1973	Registry	1-year age groups	NA	Included
Netherlands	1974	Registry	1-year age groups	NA	Included
Netherlands	1975	Registry	1-year age groups	NA	Included
Netherlands	1976	Registry	1-year age groups	NA	Included
Netherlands	1977	Registry	1-year age groups	NA	Included
Netherlands	1978	Registry	1-year age groups	NA	Included
Netherlands	1979	Registry	1-year age groups	NA	Included
Netherlands	1980	Registry	1-year age groups	NA	Included
Netherlands	1981	Registry	1-year age groups	NA	Included
Netherlands	1982	Registry	1-year age groups	NA	Included
Netherlands	1983	Registry	1-year age groups	NA	Included
Netherlands	1984	Registry	1-year age groups	NA	Included
Netherlands	1985	Registry	1-year age groups	NA	Included
Netherlands	1986	Registry	1-year age groups	NA	Included
Netherlands	1987	Registry	1-year age groups	NA	Included
Netherlands	1988	Registry	1-year age groups	NA	Included
Netherlands	1989	Registry	1-year age groups	NA	Included
Netherlands	1990	Registry	1-year age groups	NA	Included
Netherlands	1991	Census	5-year age groups	De jure	Excluded
Netherlands	1992	Registry	1-year age groups	NA	Included
Netherlands	1993	Registry	1-year age groups	NA	Included
Netherlands	1994	Registry	1-year age groups	NA	Included
Netherlands	1995	Registry	1-year age groups	NA	Included
Netherlands	1996	Registry	1-year age groups	NA	Included
Netherlands	1997	Registry	1-year age groups	NA	Included
Netherlands	1998	Registry	1-year age groups	NA	Included
Netherlands	1999	Registry	1-year age groups	NA	Included
Netherlands	2000	Registry	1-year age groups	NA	Included
Netherlands	2001	Registry	1-year age groups	NA	Included
Netherlands	2002	Census	1-year age groups	De jure	Excluded
Netherlands	2003	Registry	1-year age groups	NA	Included
Netherlands	2004	Registry	1-year age groups	NA	Included
Netherlands	2005	Registry	1-year age groups	NA	Included
Netherlands	2006	Registry	1-year age groups	NA	Included
Netherlands	2007	Registry	1-year age groups	NA	Included
Netherlands	2008	Registry	1-year age groups	NA	Included
Netherlands	2009	Registry	1-year age groups	NA	Included
Netherlands	2010	Registry	1-year age groups	NA	Included
Netherlands	2011	Census	1-year age groups	De jure	Excluded
Netherlands	2012	Registry	1-year age groups	NA	Included
Netherlands	2013	Registry	1-year age groups	NA	Included
Netherlands	2014	Registry	1-year age groups	NA	Included
Netherlands	2015	Registry	1-year age groups	NA	Included
Netherlands	2016	Registry	1-year age groups	NA	Included
Netherlands	2017	Registry	1-year age groups	NA	Included
New Zealand	1951	Census	1-year age groups	De facto	Included
New Zealand	1956	Census	5-year age groups	De jure	Included
New Zealand	1961	Census	1-year age groups	De jure	Included
New Zealand	1966	Census	5-year age groups	De facto	Included
New Zealand	1971	Census	5-year age groups	De facto	Included

New Zealand	1976	Census	5-year age groups	De facto	Included
New Zealand	1981	Census	1-year age groups	De jure	Included
New Zealand	1986	Census	1-year age groups	De jure	Included
New Zealand	1991	Census	1-year age groups	De facto	Included
New Zealand	1996	Census	1-year age groups	De jure	Included
New Zealand	2001	Census	5-year age groups	De jure	Included
New Zealand	2006	Census	1-year age groups	De jure	Included
New Zealand	2013	Census	1-year age groups	De jure	Included
Nicaragua	1950	Census	1-year age groups	De jure	Included
Nicaragua	1963	Census	1-year age groups	De jure	Included
Nicaragua	1971	Census	1-year age groups	De jure	Included
Nicaragua	1995	Census	1-year age groups	Unknown	Included
Nicaragua	2005	Census	1-year age groups	De jure	Included
Niger	1977	Census	5-year age groups	De jure	Included
Niger	1988	Census	5-year age groups	De facto	Included
Niger	2001	Census	1-year age groups	De jure	Included
Niger	2012	Census	1-year age groups	De jure	Included
Nigeria	1953	Census	non-std age groups	De facto	Excluded
Nigeria	1963	Census	1-year age groups	De facto	Excluded
Nigeria	1991	Census	1-year age groups	De facto	Included
Nigeria	2006	Census	5-year age groups	De facto	Included
Nigeria	2014	Census	5-year age groups	Micro-census	Included
Niue	1951	Census	5-year age groups	De facto	Excluded
Niue	1956	Census	1-year age groups	De facto	Included
Niue	1961	Census	1-year age groups	De facto	Included
Niue	1966	Census	5-year age groups	De jure	Included
Niue	1971	Census	1-year age groups	De facto	Excluded
Niue	1976	Census	5-year age groups	De facto	Excluded
Niue	1979	Census	5-year age groups	De facto	Excluded
Niue	1981	Census	5-year age groups	De facto	Included
Niue	1984	Census	1-year age groups	De facto	Included
Niue	1986	Census	1-year age groups	De facto	Included
Niue	1997	Census	5-year age groups	De facto	Excluded
Niue	2001	Census	5-year age groups	De facto	Excluded
Niue	2006	Census	5-year age groups	De jure	Included
Niue	2011	Census	5-year age groups	De facto	Included
North Korea	1993	Census	1-year age groups	De jure	Included
North Korea	2008	Census	1-year age groups	De jure	Included
North Macedonia	1953	Census	5-year age groups	De jure	Included
North Macedonia	1961	Census	5-year age groups	De jure	Included
North Macedonia	1971	Census	5-year age groups	De jure	Included
North Macedonia	1981	Census	5-year age groups	De jure	Included
North Macedonia	1991	Census	5-year age groups	De jure	Excluded
North Macedonia	1992	Census	5-year age groups	Unknown	Excluded
North Macedonia	1994	Census	5-year age groups	De jure	Included
North Macedonia	2002	Census	5-year age groups	De jure	Excluded
Northern Mariana Islands	1958	Census	5-year age groups	De jure	Excluded
Northern Mariana Islands	1970	Census	non-std age groups	De jure	Included
Northern Mariana Islands	1973	Census	1-year age groups	De facto	Excluded
Northern Mariana Islands	1980	Census	5-year age groups	De facto	Included
Northern Mariana Islands	1990	Census	1-year age groups	De facto	Included

Northern Mariana Islands	1995	Census	5-year age groups	De facto	Included
Northern Mariana Islands	2000	Census	non-std age groups	De jure	Included
Northern Mariana Islands	2010	Census	5-year age groups	De facto	Included
Norway	1950	Census	5-year age groups	De jure	Excluded
Norway	1960	Census	5-year age groups	De jure	Excluded
Norway	1970	Census	1-year age groups	De jure	Included
Norway	1980	Census	1-year age groups	De jure	Included
Norway	1990	Census	5-year age groups	De jure	Included
Norway	2001	Census	1-year age groups	De jure	Excluded
Norway	2011	Census	1-year age groups	De jure	Excluded
Oman	1993	Census	5-year age groups	De facto	Included
Oman	2003	Census	1-year age groups	De facto	Included
Oman	2010	Census	5-year age groups	De facto	Included
Oman	2012	Registry	5-year age groups	NA	Included
Oman	2013	Registry	5-year age groups	NA	Included
Oman	2014	Registry	5-year age groups	NA	Included
Oman	2015	Registry	5-year age groups	NA	Included
Oman	2016	Registry	5-year age groups	NA	Included
Oman	2017	Registry	5-year age groups	NA	Included
Pakistan	1951	Census	non-std age groups	De jure	Included
Pakistan	1961	Census	non-std age groups	De jure	Included
Pakistan	1972	Census	5-year age groups	De facto	Excluded
Pakistan	1981	Census	1-year age groups	De facto	Included
Pakistan	1998	Census	5-year age groups	De facto	Included
Pakistan	2017	Census	all ages only	Unknown	Included
Palau	1958	Census	5-year age groups	De facto	Included
Palau	1967	Census	5-year age groups	De facto	Included
Palau	1970	Census	5-year age groups	De jure	Excluded
Palau	1973	Census	5-year age groups	De facto	Excluded
Palau	1980	Census	5-year age groups	De facto	Excluded
Palau	1986	Census	5-year age groups	De facto	Excluded
Palau	1990	Census	5-year age groups	De jure	Excluded
Palau	1995	Census	5-year age groups	De facto	Included
Palau	2000	Census	1-year age groups	De jure	Excluded
Palau	2003	Census	non-std age groups	Unknown	Excluded
Palau	2005	Census	5-year age groups	De jure	Included
Palau	2012	Census	5-year age groups	De jure	Excluded
Palau	2015	Census	5-year age groups	De jure	Excluded
Palestine	1967	Census	5-year age groups	De jure	Included
Palestine	1997	Census	1-year age groups	De facto	Included
Palestine	2007	Census	1-year age groups	De facto	Included
Palestine	2017	Census	1-year age groups	De facto	Included
Panama	1950	Census	1-year age groups	De facto	Included
Panama	1960	Census	1-year age groups	De facto	Included
Panama	1970	Census	5-year age groups	De facto	Included
Panama	1980	Census	1-year age groups	De facto	Included
Panama	1990	Census	1-year age groups	De facto	Included
Panama	2000	Census	1-year age groups	De facto	Included
Panama	2010	Census	5-year age groups	De facto	Included
Papua New Guinea	1961	Census	1-year age groups	De facto	Excluded
Papua New Guinea	1966	Census	5-year age groups	De facto	Included

Papua New Guinea	1971	Census	1-year age groups	De facto	Included
Papua New Guinea	1980	Census	1-year age groups	De facto	Included
Papua New Guinea	1990	Census	5-year age groups	De facto	Excluded
Papua New Guinea	2000	Census	1-year age groups	De facto	Included
Papua New Guinea	2011	Census	5-year age groups	De facto	Included
Paraguay	1950	Census	1-year age groups	De facto	Included
Paraguay	1962	Census	1-year age groups	De facto	Included
Paraguay	1972	Census	1-year age groups	De facto	Included
Paraguay	1982	Census	1-year age groups	De facto	Included
Paraguay	1992	Census	1-year age groups	De facto	Included
Paraguay	2002	Census	1-year age groups	De facto	Included
Peru	1961	Census	1-year age groups	De facto	Included
Peru	1972	Census	1-year age groups	De facto	Included
Peru	1981	Census	1-year age groups	De facto	Included
Peru	1993	Census	1-year age groups	Unknown	Included
Peru	2005	Census	1-year age groups	De facto	Included
Peru	2007	Census	1-year age groups	De facto	Included
Peru	2012	Registry	all ages only	NA	Included
Peru	2013	Registry	all ages only	NA	Included
Peru	2014	Registry	all ages only	NA	Included
Peru	2015	Registry	all ages only	NA	Included
Peru	2016	Registry	all ages only	NA	Included
Peru	2017	Registry	all ages only	NA	Included
Peru	2017	Census	1-year age groups	Unknown	Excluded
Philippines	1960	Census	1-year age groups	De jure	Included
Philippines	1970	Census	1-year age groups	De jure	Included
Philippines	1975	Census	5-year age groups	De jure	Included
Philippines	1980	Census	5-year age groups	De jure	Included
Philippines	1990	Census	1-year age groups	De jure	Included
Philippines	1995	Census	1-year age groups	De jure	Included
Philippines	2000	Census	1-year age groups	De jure	Included
Philippines	2007	Census	1-year age groups	De jure	Included
Philippines	2010	Census	5-year age groups	De jure	Included
Philippines	2015	Census	1-year age groups	De jure	Included
Poland	1950	Census	5-year age groups	De facto	Included
Poland	1960	Census	5-year age groups	De facto	Included
Poland	1970	Census	1-year age groups	De facto	Included
Poland	1978	Census	5-year age groups	De facto	Included
Poland	1988	Census	1-year age groups	De facto	Included
Poland	2002	Census	1-year age groups	De jure	Included
Poland	2006	Registry	1-year age groups	NA	Included
Poland	2007	Registry	1-year age groups	NA	Included
Poland	2008	Registry	1-year age groups	NA	Included
Poland	2009	Registry	1-year age groups	NA	Included
Poland	2010	Registry	1-year age groups	NA	Included
Poland	2011	Census	1-year age groups	De jure	Excluded
Poland	2012	Registry	1-year age groups	NA	Included
Poland	2013	Registry	1-year age groups	NA	Included
Poland	2014	Registry	1-year age groups	NA	Included
Poland	2015	Registry	1-year age groups	NA	Included
Poland	2016	Registry	1-year age groups	NA	Included

Poland	2017	Registry	1-year age groups	NA	Included
Poland	2017	Census	1-year age groups	Unknown	Excluded
Portugal	1950	Census	1-year age groups	De facto	Included
Portugal	1960	Census	5-year age groups	De facto	Included
Portugal	1970	Census	1-year age groups	De facto	Included
Portugal	1981	Census	1-year age groups	De facto	Included
Portugal	1991	Census	1-year age groups	De facto	Included
Portugal	2001	Census	5-year age groups	De facto	Included
Portugal	2011	Census	1-year age groups	De jure	Included
Puerto Rico	1950	Census	1-year age groups	De jure	Included
Puerto Rico	1960	Census	5-year age groups	De jure	Included
Puerto Rico	1970	Census	1-year age groups	De jure	Included
Puerto Rico	1980	Census	1-year age groups	De jure	Included
Puerto Rico	1990	Census	1-year age groups	De jure	Included
Puerto Rico	2000	Census	1-year age groups	De jure	Included
Puerto Rico	2010	Census	1-year age groups	De jure	Included
Qatar	1986	Census	1-year age groups	De facto	Included
Qatar	1997	Census	5-year age groups	De facto	Included
Qatar	2004	Census	5-year age groups	De facto	Included
Qatar	2010	Census	5-year age groups	De facto	Included
Qatar	2015	Census	5-year age groups	De facto	Included
Romania	1956	Census	5-year age groups	De jure	Included
Romania	1966	Census	5-year age groups	De jure	Excluded
Romania	1977	Census	5-year age groups	De facto	Included
Romania	1992	Census	1-year age groups	De jure	Included
Romania	2002	Census	1-year age groups	De jure	Included
Romania	2011	Census	1-year age groups	De jure	Included
Russia	1959	Census	5-year age groups	De facto	Included
Russia	1970	Census	5-year age groups	De facto	Included
Russia	1979	Census	5-year age groups	De jure	Included
Russia	1989	Census	1-year age groups	De jure	Included
Russia	2002	Census	5-year age groups	De jure	Included
Russia	2010	Census	1-year age groups	De jure	Included
Rwanda	1978	Census	5-year age groups	De facto	Included
Rwanda	1991	Census	5-year age groups	De facto	Included
Rwanda	2002	Census	1-year age groups	De jure	Included
Rwanda	2012	Census	1-year age groups	De jure	Included
Saint Kitts and Nevis	1960	Census	1-year age groups	De facto	Excluded
Saint Kitts and Nevis	1970	Census	1-year age groups	De facto	Included
Saint Kitts and Nevis	1980	Census	5-year age groups	Unknown	Included
Saint Kitts and Nevis	1991	Census	5-year age groups	De facto	Excluded
Saint Kitts and Nevis	2001	Census	5-year age groups	Unknown	Included
Saint Lucia	1960	Census	5-year age groups	De facto	Included
Saint Lucia	1970	Census	5-year age groups	De facto	Included
Saint Lucia	1980	Census	1-year age groups	De facto	Included
Saint Lucia	1991	Census	1-year age groups	De facto	Included
Saint Lucia	2001	Census	1-year age groups	De facto	Included
Saint Lucia	2010	Census	5-year age groups	De jure	Included
Saint Vincent and the Grenadines	1960	Census	5-year age groups	De facto	Included
Saint Vincent and the Grenadines	1970	Census	5-year age groups	De facto	Included
Saint Vincent and the Grenadines	1980	Census	1-year age groups	De facto	Included

Saint Vincent and the Grenadines	1991	Census	1-year age groups	De facto	Included
Saint Vincent and the Grenadines	2001	Census	1-year age groups	De facto	Included
Saint Vincent and the Grenadines	2012	Census	5-year age groups	De jure	Included
Samoa	1951	Census	5-year age groups	De facto	Included
Samoa	1956	Census	1-year age groups	De facto	Included
Samoa	1961	Census	5-year age groups	De facto	Included
Samoa	1966	Census	5-year age groups	De facto	Included
Samoa	1971	Census	1-year age groups	De facto	Included
Samoa	1976	Census	5-year age groups	De facto	Included
Samoa	1981	Census	5-year age groups	De facto	Included
Samoa	1986	Census	5-year age groups	De facto	Included
Samoa	1991	Census	5-year age groups	De facto	Included
Samoa	2001	Census	1-year age groups	De facto	Included
Samoa	2006	Census	5-year age groups	De facto	Included
Samoa	2011	Census	5-year age groups	De facto	Included
Samoa	2016	Census	all ages only	Unknown	Included
San Marino	1976	Census	5-year age groups	De facto	Excluded
San Marino	2010	Census	non-std age groups	De facto	Included
São Tomé and PrÍncipe	1950	Census	all ages only	Unknown	Included
São Tomé and PrÍncipe	1960	Census	5-year age groups	De jure	Included
São Tomé and PrÍncipe	1970	Census	5-year age groups	De facto	Included
São Tomé and PrÍncipe	1981	Census	1-year age groups	De facto	Included
São Tomé and PrÍncipe	1991	Census	1-year age groups	De facto	Included
São Tomé and PrÍncipe	2001	Census	5-year age groups	De facto	Included
São Tomé and PrÍncipe	2012	Census	1-year age groups	De jure	Included
Saudi Arabia	1974	Census	all ages only	Unknown	Included
Saudi Arabia	1992	Census	5-year age groups	De facto	Included
Saudi Arabia	2004	Census	5-year age groups	De facto	Included
Saudi Arabia	2010	Census	5-year age groups	De facto	Included
Senegal	1976	Census	5-year age groups	De jure	Included
Senegal	1988	Census	5-year age groups	De jure	Included
Senegal	2002	Census	5-year age groups	De jure	Included
Senegal	2013	Census	5-year age groups	De facto	Included
Seychelles	1960	Census	5-year age groups	De facto	Included
Seychelles	1971	Census	1-year age groups	De jure	Included
Seychelles	1977	Census	5-year age groups	De facto	Included
Seychelles	1987	Census	1-year age groups	De facto	Included
Seychelles	1994	Census	5-year age groups	De facto	Included
Seychelles	1997	Census	1-year age groups	De facto	Included
Seychelles	2002	Census	1-year age groups	De jure	Included
Seychelles	2010	Census	1-year age groups	De facto	Included
Sierra Leone	1963	Census	5-year age groups	De facto	Included
Sierra Leone	1974	Census	5-year age groups	De facto	Included
Sierra Leone	1985	Census	5-year age groups	De facto	Included
Sierra Leone	2004	Census	1-year age groups	Unknown	Included
Sierra Leone	2015	Census	5-year age groups	De facto	Included
Singapore	1957	Census	5-year age groups	De facto	Included
Singapore	1970	Census	1-year age groups	De facto	Included
Singapore	1980	Census	1-year age groups	De facto	Included
Singapore (Residents Only)	1990	Registry	non-std age groups	NA	Included
Singapore (Residents Only)	1991	Registry	non-std age groups	NA	Included

Singapore (Residents Only)	1992	Registry	non-std age groups	NA	Included
Singapore (Residents Only)	1993	Registry	non-std age groups	NA	Included
Singapore (Residents Only)	1994	Registry	non-std age groups	NA	Included
Singapore (Residents Only)	1995	Registry	non-std age groups	NA	Included
Singapore (Residents Only)	1996	Registry	non-std age groups	NA	Included
Singapore (Residents Only)	1997	Registry	non-std age groups	NA	Included
Singapore (Residents Only)	1998	Registry	non-std age groups	NA	Included
Singapore (Residents Only)	1999	Registry	non-std age groups	NA	Included
Singapore (Residents Only)	2000	Registry	non-std age groups	NA	Included
Singapore (Residents Only)	2001	Registry	non-std age groups	NA	Included
Singapore (Residents Only)	2002	Registry	non-std age groups	NA	Included
Singapore (Residents Only)	2003	Registry	non-std age groups	NA	Included
Singapore (Residents Only)	2004	Registry	non-std age groups	NA	Included
Singapore (Residents Only)	2005	Registry	non-std age groups	NA	Included
Singapore (Residents Only)	2006	Registry	non-std age groups	NA	Included
Singapore (Residents Only)	2007	Registry	non-std age groups	NA	Included
Singapore (Residents Only)	2008	Registry	non-std age groups	NA	Included
Singapore (Residents Only)	2009	Registry	non-std age groups	NA	Included
Singapore (Residents Only)	2010	Registry	non-std age groups	NA	Included
Singapore (Residents Only)	2011	Registry	non-std age groups	NA	Included
Singapore (Residents Only)	2012	Registry	non-std age groups	NA	Included
Singapore (Residents Only)	2013	Registry	non-std age groups	NA	Included
Singapore (Residents Only)	2014	Registry	non-std age groups	NA	Included
Singapore (Residents Only)	2015	Registry	non-std age groups	NA	Included
Singapore (Residents Only)	2016	Registry	non-std age groups	NA	Included
Singapore (Residents Only)	2017	Registry	non-std age groups	NA	Included
Singapore (Residents Only)	2018	Registry	non-std age groups	NA	Included
Slovakia	1950	Census	5-year age groups	De jure	Excluded
Slovakia	1951	Registry	1-year age groups	NA	Included
Slovakia	1952	Registry	1-year age groups	NA	Included
Slovakia	1953	Registry	1-year age groups	NA	Included
Slovakia	1954	Registry	1-year age groups	NA	Included
Slovakia	1955	Registry	1-year age groups	NA	Included
Slovakia	1956	Registry	1-year age groups	NA	Included
Slovakia	1957	Registry	1-year age groups	NA	Included
Slovakia	1958	Registry	1-year age groups	NA	Included
Slovakia	1959	Registry	1-year age groups	NA	Included
Slovakia	1960	Registry	1-year age groups	NA	Included
Slovakia	1961	Census	1-year age groups	De jure	Excluded
Slovakia	1962	Registry	1-year age groups	NA	Included
Slovakia	1963	Registry	1-year age groups	NA	Included
Slovakia	1964	Registry	1-year age groups	NA	Included
Slovakia	1965	Registry	1-year age groups	NA	Included
Slovakia	1966	Registry	1-year age groups	NA	Included
Slovakia	1967	Registry	1-year age groups	NA	Included
Slovakia	1968	Registry	1-year age groups	NA	Included
Slovakia	1969	Registry	1-year age groups	NA	Included
Slovakia	1970	Registry	1-year age groups	NA	Included
Slovakia	1970	Census	all ages only	Unknown	Excluded
Slovakia	1971	Registry	1-year age groups	NA	Included
Slovakia	1972	Registry	1-year age groups	NA	Included

Slovakia	1973	Registry	1-year age groups	NA	Included
Slovakia	1974	Registry	1-year age groups	NA	Included
Slovakia	1975	Registry	1-year age groups	NA	Included
Slovakia	1976	Registry	1-year age groups	NA	Included
Slovakia	1977	Registry	1-year age groups	NA	Included
Slovakia	1978	Registry	1-year age groups	NA	Included
Slovakia	1979	Registry	1-year age groups	NA	Included
Slovakia	1980	Census	5-year age groups	De jure	Excluded
Slovakia	1981	Registry	1-year age groups	NA	Included
Slovakia	1982	Registry	1-year age groups	NA	Included
Slovakia	1983	Registry	1-year age groups	NA	Included
Slovakia	1984	Registry	1-year age groups	NA	Included
Slovakia	1985	Registry	1-year age groups	NA	Included
Slovakia	1986	Registry	1-year age groups	NA	Included
Slovakia	1987	Registry	1-year age groups	NA	Included
Slovakia	1988	Registry	1-year age groups	NA	Included
Slovakia	1989	Registry	1-year age groups	NA	Included
Slovakia	1990	Registry	1-year age groups	NA	Included
Slovakia	1991	Census	5-year age groups	De jure	Excluded
Slovakia	1992	Registry	1-year age groups	NA	Included
Slovakia	1993	Registry	1-year age groups	NA	Included
Slovakia	1994	Registry	1-year age groups	NA	Included
Slovakia	1995	Registry	1-year age groups	NA	Included
Slovakia	1996	Registry	1-year age groups	NA	Included
Slovakia	1997	Registry	1-year age groups	NA	Included
Slovakia	1998	Registry	1-year age groups	NA	Included
Slovakia	1999	Registry	1-year age groups	NA	Included
Slovakia	2000	Registry	1-year age groups	NA	Included
Slovakia	2001	Census	1-year age groups	De jure	Excluded
Slovakia	2002	Registry	1-year age groups	NA	Included
Slovakia	2003	Registry	1-year age groups	NA	Included
Slovakia	2004	Registry	1-year age groups	NA	Included
Slovakia	2005	Registry	1-year age groups	NA	Included
Slovakia	2006	Registry	1-year age groups	NA	Included
Slovakia	2007	Registry	1-year age groups	NA	Included
Slovakia	2008	Registry	1-year age groups	NA	Included
Slovakia	2009	Registry	1-year age groups	NA	Included
Slovakia	2010	Registry	1-year age groups	NA	Included
Slovakia	2011	Census	1-year age groups	De jure	Excluded
Slovakia	2012	Registry	1-year age groups	NA	Included
Slovakia	2013	Registry	1-year age groups	NA	Included
Slovakia	2014	Registry	1-year age groups	NA	Included
Slovakia	2015	Registry	1-year age groups	NA	Included
Slovakia	2016	Registry	1-year age groups	NA	Included
Slovenia	1953	Census	all ages only	Unknown	Included
Slovenia	1961	Census	all ages only	Unknown	Included
Slovenia	1971	Census	1-year age groups	De facto	Included
Slovenia	1981	Census	all ages only	Unknown	Included
Slovenia	1991	Census	1-year age groups	De jure	Excluded
Slovenia	1996	Registry	1-year age groups	NA	Included
Slovenia	1997	Registry	1-year age groups	NA	Included

Slovenia	1998	Registry	1-year age groups	NA	Included
Slovenia	1999	Registry	1-year age groups	NA	Included
Slovenia	2000	Registry	1-year age groups	NA	Included
Slovenia	2001	Registry	1-year age groups	NA	Included
Slovenia	2002	Census	1-year age groups	De jure	Excluded
Slovenia	2003	Registry	1-year age groups	NA	Included
Slovenia	2004	Registry	1-year age groups	NA	Included
Slovenia	2005	Registry	1-year age groups	NA	Included
Slovenia	2006	Registry	1-year age groups	NA	Included
Slovenia	2007	Registry	1-year age groups	NA	Included
Slovenia	2008	Registry	1-year age groups	NA	Included
Slovenia	2009	Registry	1-year age groups	NA	Included
Slovenia	2010	Registry	1-year age groups	NA	Included
Slovenia	2011	Census	1-year age groups	De jure	Excluded
Slovenia	2012	Registry	1-year age groups	NA	Included
Slovenia	2013	Registry	1-year age groups	NA	Included
Slovenia	2014	Registry	1-year age groups	NA	Included
Slovenia	2015	Census	1-year age groups	De jure	Excluded
Slovenia	2016	Registry	1-year age groups	NA	Included
Slovenia	2017	Registry	1-year age groups	NA	Included
Solomon Islands	1959	Census	5-year age groups	De facto	Included
Solomon Islands	1970	Census	5-year age groups	De facto	Included
Solomon Islands	1976	Census	5-year age groups	De facto	Included
Solomon Islands	1986	Census	5-year age groups	De facto	Included
Solomon Islands	1999	Census	5-year age groups	De facto	Included
Solomon Islands	2009	Census	5-year age groups	De facto	Included
Somalia	1975	Census	5-year age groups	De jure	Included
Somalia	1987	Census	all ages only	Unknown	Included
South Africa	1951	Census	5-year age groups	De facto	Included
South Africa	1960	Census	1-year age groups	De facto	Included
South Africa	1970	Census	1-year age groups	De facto	Included
South Africa	1980	Census	5-year age groups	De facto	Excluded
South Africa	1985	Census	1-year age groups	De facto	Excluded
South Africa	1991	Census	1-year age groups	De facto	Excluded
South Africa	1996	Census	1-year age groups	De facto	Included
South Africa	2001	Census	5-year age groups	De facto	Included
South Africa	2011	Census	5-year age groups	De facto	Included
South Korea	1955	Census	5-year age groups	De facto	Included
South Korea	1960	Census	5-year age groups	De facto	Included
South Korea	1966	Census	1-year age groups	De facto	Included
South Korea	1970	Census	1-year age groups	De facto	Included
South Korea	1975	Census	5-year age groups	De facto	Included
South Korea	1980	Census	1-year age groups	De facto	Included
South Korea	1985	Census	1-year age groups	De facto	Included
South Korea	1990	Census	1-year age groups	De facto	Included
South Korea	1995	Census	1-year age groups	De jure	Included
South Korea	2000	Census	1-year age groups	De jure	Included
South Korea	2005	Census	1-year age groups	De jure	Included
South Korea	2010	Census	5-year age groups	De jure	Included
South Korea	2015	Census	1-year age groups	De jure	Included
South Sudan	1956	Census	non-std age groups	De jure	Included

South Sudan	1973	Census	5-year age groups	De facto	Excluded
South Sudan	1983	Census	all ages only	De jure	Included
South Sudan	2008	Census	1-year age groups	De facto	Included
Spain	1950	Census	1-year age groups	De facto	Included
Spain	1960	Census	1-year age groups	De jure	Included
Spain	1970	Census	5-year age groups	De jure	Included
Spain	1981	Census	5-year age groups	De jure	Included
Spain	1991	Census	1-year age groups	De jure	Included
Spain	1998	Registry	1-year age groups	NA	Included
Spain	1999	Registry	1-year age groups	NA	Included
Spain	2000	Registry	1-year age groups	NA	Included
Spain	2001	Census	1-year age groups	De facto	Excluded
Spain	2002	Registry	1-year age groups	NA	Included
Spain	2003	Registry	1-year age groups	NA	Included
Spain	2004	Registry	1-year age groups	NA	Included
Spain	2005	Registry	1-year age groups	NA	Included
Spain	2006	Registry	1-year age groups	NA	Included
Spain	2007	Registry	1-year age groups	NA	Included
Spain	2008	Registry	1-year age groups	NA	Included
Spain	2009	Registry	1-year age groups	NA	Included
Spain	2010	Registry	1-year age groups	NA	Included
Spain	2011	Census	1-year age groups	De jure	Excluded
Spain	2012	Registry	1-year age groups	NA	Included
Spain	2013	Registry	1-year age groups	NA	Included
Spain	2014	Registry	1-year age groups	NA	Included
Spain	2015	Registry	1-year age groups	NA	Included
Spain	2016	Registry	1-year age groups	NA	Included
Sri Lanka	1953	Census	5-year age groups	De facto	Included
Sri Lanka	1963	Census	1-year age groups	De facto	Included
Sri Lanka	1971	Census	1-year age groups	De facto	Included
Sri Lanka	1981	Census	1-year age groups	De facto	Included
Sri Lanka	2001	Census	1-year age groups	De facto	Excluded
Sri Lanka	2012	Census	1-year age groups	De jure	Included
Sudan	1956	Census	non-std age groups	De jure	Included
Sudan	1973	Census	5-year age groups	De facto	Included
Sudan	1983	Census	all ages only	De jure	Included
Sudan	1993	Census	5-year age groups	De jure	Included
Sudan	2008	Census	1-year age groups	Unknown	Included
Suriname	1950	Census	1-year age groups	De facto	Included
Suriname	1964	Census	5-year age groups	De facto	Included
Suriname	1971	Census	all ages only	Unknown	Included
Suriname	2000	Census	all ages only	Unknown	Included
Suriname	2003	Census	5-year age groups	De jure	Excluded
Suriname	2004	Census	1-year age groups	De jure	Included
Suriname	2012	Census	1-year age groups	De jure	Included
Sweden	1950	Census	1-year age groups	De jure	Included
Sweden	1960	Census	5-year age groups	De jure	Included
Sweden	1965	Census	5-year age groups	De jure	Included
Sweden	1968	Registry	1-year age groups	NA	Included
Sweden	1969	Registry	1-year age groups	NA	Included
Sweden	1970	Census	5-year age groups	De jure	Excluded

Sweden	1971	Registry	1-year age groups	NA	Included
Sweden	1972	Registry	1-year age groups	NA	Included
Sweden	1973	Registry	1-year age groups	NA	Included
Sweden	1974	Registry	1-year age groups	NA	Included
Sweden	1975	Census	5-year age groups	De jure	Excluded
Sweden	1976	Registry	1-year age groups	NA	Included
Sweden	1977	Registry	1-year age groups	NA	Included
Sweden	1978	Registry	1-year age groups	NA	Included
Sweden	1979	Registry	1-year age groups	NA	Included
Sweden	1980	Census	1-year age groups	De jure	Excluded
Sweden	1981	Registry	1-year age groups	NA	Included
Sweden	1982	Registry	1-year age groups	NA	Included
Sweden	1983	Registry	1-year age groups	NA	Included
Sweden	1984	Registry	1-year age groups	NA	Included
Sweden	1985	Census	5-year age groups	De jure	Excluded
Sweden	1986	Registry	1-year age groups	NA	Included
Sweden	1987	Registry	1-year age groups	NA	Included
Sweden	1988	Registry	1-year age groups	NA	Included
Sweden	1989	Registry	1-year age groups	NA	Included
Sweden	1990	Census	1-year age groups	De jure	Excluded
Sweden	1991	Registry	1-year age groups	NA	Included
Sweden	1992	Registry	1-year age groups	NA	Included
Sweden	1993	Registry	1-year age groups	NA	Included
Sweden	1994	Registry	1-year age groups	NA	Included
Sweden	1995	Registry	1-year age groups	NA	Included
Sweden	1996	Registry	1-year age groups	NA	Included
Sweden	1997	Registry	1-year age groups	NA	Included
Sweden	1998	Registry	1-year age groups	NA	Included
Sweden	1999	Registry	1-year age groups	NA	Included
Sweden	2000	Registry	1-year age groups	NA	Included
Sweden	2001	Registry	1-year age groups	NA	Included
Sweden	2002	Registry	1-year age groups	NA	Included
Sweden	2003	Census	1-year age groups	De jure	Excluded
Sweden	2004	Registry	1-year age groups	NA	Included
Sweden	2005	Registry	1-year age groups	NA	Included
Sweden	2006	Registry	1-year age groups	NA	Included
Sweden	2007	Registry	1-year age groups	NA	Included
Sweden	2008	Registry	1-year age groups	NA	Included
Sweden	2009	Registry	1-year age groups	NA	Included
Sweden	2010	Registry	1-year age groups	NA	Included
Sweden	2011	Census	1-year age groups	De jure	Excluded
Sweden	2012	Registry	1-year age groups	NA	Included
Sweden	2013	Registry	1-year age groups	NA	Included
Sweden	2014	Registry	1-year age groups	NA	Included
Sweden	2015	Registry	1-year age groups	NA	Included
Sweden	2016	Registry	1-year age groups	NA	Included
Switzerland	1950	Census	1-year age groups	De jure	Included
Switzerland	1960	Census	5-year age groups	De jure	Included
Switzerland	1970	Census	1-year age groups	De jure	Included
Switzerland	1980	Census	1-year age groups	De jure	Included
Switzerland	1990	Census	1-year age groups	De jure	Included

Switzerland	2000	Census	5-year age groups	De facto	Included
Switzerland	2010	Registry	1-year age groups	NA	Included
Switzerland	2011	Census	1-year age groups	De jure	Excluded
Switzerland	2012	Registry	1-year age groups	NA	Included
Switzerland	2013	Registry	1-year age groups	NA	Included
Switzerland	2014	Registry	1-year age groups	NA	Included
Switzerland	2015	Registry	1-year age groups	NA	Included
Switzerland	2016	Registry	1-year age groups	NA	Included
Syria	1960	Census	5-year age groups	De facto	Included
Syria	1970	Census	1-year age groups	De facto	Included
Syria	1981	Census	1-year age groups	De facto	Included
Syria	1994	Census	1-year age groups	De facto	Included
Syria	2004	Census	5-year age groups	De facto	Included
Taiwan (province of China)	1950	Registry	1-year age groups	NA	Included
Taiwan (province of China)	1951	Registry	1-year age groups	NA	Included
Taiwan (province of China)	1952	Registry	1-year age groups	NA	Included
Taiwan (province of China)	1953	Registry	1-year age groups	NA	Included
Taiwan (province of China)	1954	Registry	1-year age groups	NA	Included
Taiwan (province of China)	1955	Registry	1-year age groups	NA	Included
Taiwan (province of China)	1956	Census	5-year age groups	De facto	Excluded
Taiwan (province of China)	1957	Registry	1-year age groups	NA	Included
Taiwan (province of China)	1958	Registry	1-year age groups	NA	Included
Taiwan (province of China)	1959	Registry	1-year age groups	NA	Included
Taiwan (province of China)	1960	Registry	1-year age groups	NA	Included
Taiwan (province of China)	1961	Registry	1-year age groups	NA	Included
Taiwan (province of China)	1962	Registry	1-year age groups	NA	Included
Taiwan (province of China)	1963	Registry	1-year age groups	NA	Included
Taiwan (province of China)	1964	Registry	1-year age groups	NA	Included
Taiwan (province of China)	1965	Registry	5-year age groups	NA	Included
Taiwan (province of China)	1966	Registry	5-year age groups	NA	Included
Taiwan (province of China)	1967	Registry	5-year age groups	NA	Included
Taiwan (province of China)	1968	Registry	5-year age groups	NA	Included
Taiwan (province of China)	1969	Registry	5-year age groups	NA	Included
Taiwan (province of China)	1970	Registry	1-year age groups	NA	Included
Taiwan (province of China)	1971	Census	5-year age groups	De jure	Excluded
Taiwan (province of China)	1972	Registry	1-year age groups	NA	Included
Taiwan (province of China)	1973	Registry	1-year age groups	NA	Included
Taiwan (province of China)	1974	Registry	1-year age groups	NA	Included
Taiwan (province of China)	1975	Registry	1-year age groups	NA	Included
Taiwan (province of China)	1976	Registry	1-year age groups	NA	Included
Taiwan (province of China)	1977	Registry	1-year age groups	NA	Included
Taiwan (province of China)	1978	Registry	1-year age groups	NA	Included
Taiwan (province of China)	1979	Registry	1-year age groups	NA	Included
Taiwan (province of China)	1980	Census	5-year age groups	Unknown	Excluded
Taiwan (province of China)	1981	Registry	1-year age groups	NA	Included
Taiwan (province of China)	1982	Registry	1-year age groups	NA	Included
Taiwan (province of China)	1983	Registry	1-year age groups	NA	Included
Taiwan (province of China)	1984	Registry	1-year age groups	NA	Included
Taiwan (province of China)	1985	Registry	1-year age groups	NA	Included
Taiwan (province of China)	1986	Registry	1-year age groups	NA	Included
Taiwan (province of China)	1987	Registry	1-year age groups	NA	Included

Taiwan (province of China)	1988	Registry	1-year age groups	NA	Included
Taiwan (province of China)	1989	Registry	1-year age groups	NA	Included
Taiwan (province of China)	1990	Census	1-year age groups	De facto	Excluded
Taiwan (province of China)	1991	Registry	1-year age groups	NA	Included
Taiwan (province of China)	1992	Registry	1-year age groups	NA	Included
Taiwan (province of China)	1993	Registry	1-year age groups	NA	Included
Taiwan (province of China)	1994	Registry	1-year age groups	NA	Included
Taiwan (province of China)	1995	Registry	1-year age groups	NA	Included
Taiwan (province of China)	1996	Registry	1-year age groups	NA	Included
Taiwan (province of China)	1997	Registry	1-year age groups	NA	Included
Taiwan (province of China)	1998	Registry	1-year age groups	NA	Included
Taiwan (province of China)	1999	Registry	1-year age groups	NA	Included
Taiwan (province of China)	2000	Census	1-year age groups	De facto	Excluded
Taiwan (province of China)	2001	Registry	1-year age groups	NA	Included
Taiwan (province of China)	2002	Registry	1-year age groups	NA	Included
Taiwan (province of China)	2003	Registry	1-year age groups	NA	Included
Taiwan (province of China)	2004	Registry	1-year age groups	NA	Included
Taiwan (province of China)	2005	Registry	1-year age groups	NA	Included
Taiwan (province of China)	2006	Registry	1-year age groups	NA	Included
Taiwan (province of China)	2007	Registry	1-year age groups	NA	Included
Taiwan (province of China)	2008	Registry	1-year age groups	NA	Included
Taiwan (province of China)	2009	Registry	1-year age groups	NA	Included
Taiwan (province of China)	2010	Census	5-year age groups	De facto	Excluded
Taiwan (province of China)	2011	Registry	1-year age groups	NA	Included
Taiwan (province of China)	2012	Registry	1-year age groups	NA	Included
Taiwan (province of China)	2013	Registry	1-year age groups	NA	Included
Taiwan (province of China)	2014	Registry	1-year age groups	NA	Included
Taiwan (province of China)	2015	Registry	1-year age groups	NA	Included
Taiwan (province of China)	2016	Registry	1-year age groups	NA	Included
Taiwan (province of China)	2017	Registry	1-year age groups	NA	Included
Tajikistan	1959	Census	non-std age groups	De facto	Included
Tajikistan	1970	Census	non-std age groups	De facto	Included
Tajikistan	1979	Census	5-year age groups	De facto	Included
Tajikistan	1989	Census	1-year age groups	De jure	Included
Tajikistan	2000	Census	1-year age groups	De facto	Included
Tajikistan	2010	Census	1-year age groups	De facto	Included
Tanzania	1957	Census	5-year age groups	De jure	Included
Tanzania	1967	Census	1-year age groups	De facto	Included
Tanzania	1978	Census	5-year age groups	De facto	Included
Tanzania	1988	Census	1-year age groups	De facto	Included
Tanzania	2002	Census	5-year age groups	De facto	Included
Tanzania	2012	Census	1-year age groups	De facto	Included
Thailand	1956	Census	5-year age groups	De jure	Excluded
Thailand	1960	Census	5-year age groups	De jure	Included
Thailand	1970	Census	5-year age groups	De jure	Included
Thailand	1980	Census	1-year age groups	De jure	Included
Thailand	1990	Census	5-year age groups	De jure	Included
Thailand	2000	Census	1-year age groups	De jure	Included
Thailand	2010	Census	1-year age groups	De jure	Included
The Bahamas	1953	Census	5-year age groups	De facto	Included
The Bahamas	1963	Census	non-std age groups	De facto	Included

The Bahamas	1970	Census	1-year age groups	De facto	Included
The Bahamas	1980	Census	1-year age groups	De facto	Included
The Bahamas	1990	Census	1-year age groups	De facto	Included
The Bahamas	2000	Census	1-year age groups	De facto	Included
The Bahamas	2010	Census	1-year age groups	De jure	Included
The Gambia	1963	Census	non-std age groups	De facto	Included
The Gambia	1973	Census	1-year age groups	De facto	Included
The Gambia	1983	Census	1-year age groups	De facto	Included
The Gambia	1993	Census	1-year age groups	De facto	Included
The Gambia	2003	Census	5-year age groups	De facto	Included
The Gambia	2013	Census	5-year age groups	De facto	Included
Timor-Leste	1950	Census	all ages only	Unknown	Included
Timor-Leste	1960	Census	all ages only	Unknown	Included
Timor-Leste	1980	Census	all ages only	Unknown	Included
Timor-Leste	1990	Census	1-year age groups	De jure	Included
Timor-Leste	2004	Census	5-year age groups	De facto	Included
Timor-Leste	2010	Census	1-year age groups	De facto	Included
Timor-Leste	2015	Census	5-year age groups	De facto	Included
Togo	1958	Census	1-year age groups	De facto	Included
Togo	1970	Census	5-year age groups	De jure	Included
Togo	1981	Census	5-year age groups	De facto	Included
Togo	2010	Census	5-year age groups	De jure	Included
Tokelau	1951	Census	5-year age groups	De facto	Excluded
Tokelau	1956	Census	1-year age groups	De facto	Included
Tokelau	1961	Census	5-year age groups	Unknown	Included
Tokelau	1966	Census	5-year age groups	Unknown	Included
Tokelau	1972	Census	5-year age groups	Unknown	Included
Tokelau	1976	Census	5-year age groups	De facto	Excluded
Tokelau	1981	Census	1-year age groups	Unknown	Included
Tokelau	1986	Census	5-year age groups	Unknown	Included
Tokelau	1991	Census	5-year age groups	De facto	Included
Tokelau	1996	Census	5-year age groups	De facto	Included
Tokelau	2001	Census	5-year age groups	De facto	Included
Tokelau	2006	Census	5-year age groups	De facto	Included
Tokelau	2011	Census	5-year age groups	De facto	Included
Tokelau	2016	Census	5-year age groups	De facto	Included
Tonga	1956	Census	5-year age groups	De facto	Included
Tonga	1966	Census	5-year age groups	De facto	Included
Tonga	1976	Census	5-year age groups	De jure	Included
Tonga	1986	Census	5-year age groups	De facto	Included
Tonga	1996	Census	5-year age groups	De facto	Included
Tonga	2006	Census	1-year age groups	De jure	Included
Tonga	2011	Census	non-std age groups	De jure	Included
Tonga	2016	Census	1-year age groups	De facto	Included
Trinidad and Tobago	1960	Census	1-year age groups	De jure	Included
Trinidad and Tobago	1970	Census	1-year age groups	De facto	Included
Trinidad and Tobago	1980	Census	1-year age groups	De facto	Included
Trinidad and Tobago	1990	Census	5-year age groups	De facto	Included
Trinidad and Tobago	2000	Census	5-year age groups	De facto	Included
Trinidad and Tobago	2011	Census	1-year age groups	De jure	Included
Tunisia	1956	Census	non-std age groups	De facto	Included

Tunisia	1966	Census	1-year age groups	De facto	Included
Tunisia	1975	Census	5-year age groups	De facto	Included
Tunisia	1984	Census	1-year age groups	De facto	Included
Tunisia	1994	Census	1-year age groups	De facto	Included
Tunisia	2004	Census	5-year age groups	De facto	Included
Tunisia	2014	Census	5-year age groups	De facto	Included
Turkey	1950	Census	5-year age groups	De facto	Included
Turkey	1955	Census	5-year age groups	De facto	Included
Turkey	1960	Census	5-year age groups	De facto	Included
Turkey	1965	Census	5-year age groups	De facto	Included
Turkey	1970	Census	5-year age groups	De facto	Excluded
Turkey	1975	Census	5-year age groups	De facto	Included
Turkey	1980	Census	5-year age groups	De facto	Included
Turkey	1985	Census	5-year age groups	De facto	Excluded
Turkey	1990	Census	5-year age groups	De facto	Excluded
Turkey	2000	Census	5-year age groups	De facto	Excluded
Turkey	2007	Registry	5-year age groups	NA	Excluded
Turkey	2008	Registry	5-year age groups	NA	Excluded
Turkey	2009	Registry	5-year age groups	NA	Excluded
Turkey	2010	Registry	5-year age groups	NA	Excluded
Turkey	2011	Registry	5-year age groups	NA	Excluded
Turkey	2012	Registry	5-year age groups	NA	Excluded
Turkey	2013	Registry	5-year age groups	NA	Excluded
Turkey	2014	Registry	5-year age groups	NA	Excluded
Turkey	2015	Registry	5-year age groups	NA	Excluded
Turkey	2016	Registry	5-year age groups	NA	Excluded
Turkey	2017	Registry	5-year age groups	NA	Excluded
Turkmenistan	1959	Census	non-std age groups	De facto	Included
Turkmenistan	1970	Census	non-std age groups	De facto	Included
Turkmenistan	1979	Census	5-year age groups	De facto	Included
Turkmenistan	1989	Census	5-year age groups	De facto	Included
Turkmenistan	1995	Census	5-year age groups	De facto	Excluded
Turkmenistan	2015	Census	all ages only	Unknown	Included
Tuvalu	1963	Census	5-year age groups	De facto	Included
Tuvalu	1968	Census	5-year age groups	De facto	Excluded
Tuvalu	1973	Census	5-year age groups	De facto	Included
Tuvalu	1979	Census	5-year age groups	De facto	Included
Tuvalu	1991	Census	5-year age groups	De facto	Included
Tuvalu	2002	Census	5-year age groups	De facto	Included
Tuvalu	2012	Census	5-year age groups	De facto	Included
Uganda	1959	Census	5-year age groups	De jure	Included
Uganda	1969	Census	1-year age groups	De facto	Included
Uganda	1980	Census	5-year age groups	De facto	Excluded
Uganda	1991	Census	1-year age groups	De facto	Included
Uganda	2002	Census	1-year age groups	De facto	Included
Uganda	2014	Census	1-year age groups	De facto	Included
UK	1991	Census	1-year age groups	De facto	Excluded
UK	2001	Census	1-year age groups	De facto	Included
UK	2011	Census	1-year age groups	De jure	Included
Ukraine	1959	Census	non-std age groups	De facto	Included
Ukraine	1970	Census	non-std age groups	De facto	Included

Ukraine	1979	Census	5-year age groups	De facto	Included
Ukraine	1989	Census	5-year age groups	De jure	Included
Ukraine	2001	Census	1-year age groups	De facto	Included
United Arab Emirates	1968	Census	non-std age groups	De facto	Included
United Arab Emirates	1975	Census	5-year age groups	De facto	Included
United Arab Emirates	1980	Census	5-year age groups	De jure	Included
United Arab Emirates	1985	Census	5-year age groups	De facto	Included
United Arab Emirates	1995	Census	5-year age groups	De facto	Included
United Arab Emirates	2005	Census	5-year age groups	De facto	Included
Uruguay	1963	Census	1-year age groups	De facto	Included
Uruguay	1975	Census	5-year age groups	De facto	Included
Uruguay	1985	Census	1-year age groups	De facto	Included
Uruguay	1996	Census	1-year age groups	De facto	Included
Uruguay	2004	Census	1-year age groups	De facto	Included
Uruguay	2011	Census	1-year age groups	De jure	Included
United States	1950	Census	1-year age groups	De jure	Included
United States	1960	Census	5-year age groups	De jure	Included
United States	1970	Census	5-year age groups	De jure	Included
United States	1980	Census	1-year age groups	De jure	Included
United States	1990	Census	5-year age groups	De jure	Included
United States	2000	Census	1-year age groups	De jure	Included
United States	2010	Census	1-year age groups	De jure	Included
Uzbekistan	1959	Census	non-std age groups	De facto	Included
Uzbekistan	1970	Census	non-std age groups	De facto	Included
Uzbekistan	1979	Census	5-year age groups	De facto	Included
Uzbekistan	1989	Census	5-year age groups	De jure	Included
Vanuatu	1967	Census	5-year age groups	De facto	Included
Vanuatu	1979	Census	5-year age groups	De facto	Included
Vanuatu	1989	Census	1-year age groups	De jure	Included
Vanuatu	1999	Census	5-year age groups	De facto	Included
Vanuatu	2009	Census	1-year age groups	De facto	Included
Vanuatu	2016	Census	1-year age groups	De jure	Included
Venezuela	1950	Census	1-year age groups	De facto	Included
Venezuela	1961	Census	1-year age groups	De facto	Included
Venezuela	1971	Census	5-year age groups	De facto	Included
Venezuela	1981	Census	1-year age groups	De facto	Included
Venezuela	1990	Census	1-year age groups	De facto	Included
Venezuela	2001	Census	non-std age groups	De facto	Included
Venezuela	2011	Census	1-year age groups	De jure	Included
Vietnam	1979	Census	5-year age groups	De facto	Included
Vietnam	1989	Census	1-year age groups	De jure	Included
Vietnam	1999	Census	1-year age groups	De jure	Included
Vietnam	2009	Census	1-year age groups	De jure	Included
Virgin Islands, U.S.	1950	Census	5-year age groups	De jure	Included
Virgin Islands, U.S.	1960	Census	5-year age groups	De jure	Included
Virgin Islands, U.S.	1970	Census	5-year age groups	De jure	Included
Virgin Islands, U.S.	1980	Census	1-year age groups	De jure	Included
Virgin Islands, U.S.	1990	Census	5-year age groups	De jure	Included
Virgin Islands, U.S.	2000	Census	5-year age groups	De jure	Included
Virgin Islands, U.S.	2010	Census	5-year age groups	De jure	Included
Yemen	1994	Census	1-year age groups	De facto	Included

Yemen	2004	Census	5-year age groups	De facto	Included
Zambia	1969	Census	1-year age groups	De facto	Included
Zambia	1980	Census	1-year age groups	De facto	Included
Zambia	1990	Census	1-year age groups	De facto	Included
Zambia	2000	Census	1-year age groups	De facto	Included
Zambia	2010	Census	1-year age groups	De jure	Included
Zimbabwe	1962	Census	non-std age groups	De jure	Excluded
Zimbabwe	1982	Census	1-year age groups	De facto	Included
Zimbabwe	1992	Census	1-year age groups	De facto	Included
Zimbabwe	2002	Census	1-year age groups	De facto	Included
Zimbabwe	2012	Census	1-year age groups	De facto	Included

Appendix Table 13. GBD world population age standard		
**Age Group**	**Percent of Population**	**Rounded**
Early Neonatal	0.039706188	0.04
Late Neonatal	0.118021789	0.12
Post Neonatal	1.868264909	1.87
<1	2.025992886	2.03
1 to 4	7.909875913	7.91
5 to 9	9.568418272	9.57
10 to 14	8.990277942	8.99
15 to 19	8.324362192	8.32
20 to 24	7.866450176	7.87
25 to 29	7.632917343	7.63
30 to 34	7.331511124	7.33
35 to 39	6.811055	6.81
40 to 44	6.136798184	6.14
45 to 49	5.509495973	5.51
50 to 54	4.921822565	4.92
55 to 59	4.345633072	4.35
60 to 64	3.684473754	3.68
65 to 69	2.991239718	2.99
70 to 74	2.272487547	2.27
75 to 79	1.607371655	1.61
80 to 84	1.113034599	1.11
85 to 89	0.61707008	0.62
90 to 94	0.255008068	0.26
95 plus	0.084703935	0.08

Appendix Table 14. Socio-demographic Index values for all estimated GBD 2019 locations, 1950–1969																				
Location	1950	1951	1952	1953	1954	1955	1956	1957	1958	1959	1960	1961	1962	1963	1964	1965	1966	1967	1968	1969
Global	0·354	0·356	0·359	0·361	0·363	0·365	0·367	0·369	0·371	0·374	0·377	0·381	0·385	0·389	0·393	0·398	0·402	0·406	0·411	0·416
Central Europe, eastern Europe, and central Asia	0·511	0·514	0·521	0·525	0·526	0·53	0·532	0·534	0·536	0·539	0·54	0·548	0·559	0·562	0·568	0·573	0·573	0·574	0·578	0·581
Central Asia	0·393	0·395	0·398	0·401	0·404	0·407	0·411	0·414	0·418	0·421	0·425	0·43	0·435	0·439	0·443	0·448	0·452	0·457	0·462	0·468
Armenia	0·373	0·374	0·378	0·382	0·385	0·39	0·396	0·401	0·407	0·411	0·418	0·424	0·431	0·436	0·442	0·448	0·454	0·459	0·464	0·468
Azerbaijan	0·352	0·353	0·357	0·361	0·364	0·369	0·375	0·38	0·386	0·391	0·397	0·404	0·411	0·416	0·424	0·432	0·44	0·448	0·457	0·465
Georgia	0·493	0·496	0·501	0·506	0·51	0·516	0·522	0·528	0·533	0·537	0·543	0·548	0·553	0·556	0·56	0·565	0·569	0·573	0·576	0·579
Kazakhstan	0·428	0·43	0·433	0·435	0·437	0·439	0·442	0·444	0·447	0·449	0·453	0·458	0·463	0·468	0·474	0·481	0·487	0·493	0·499	0·505
Kyrgyzstan	0·404	0·407	0·41	0·413	0·416	0·419	0·423	0·426	0·429	0·431	0·434	0·437	0·441	0·444	0·448	0·452	0·455	0·458	0·46	0·461
Mongolia	0·303	0·306	0·309	0·312	0·315	0·317	0·32	0·322	0·324	0·326	0·327	0·329	0·33	0·332	0·333	0·334	0·336	0·338	0·34	0·342
Tajikistan	0·276	0·276	0·278	0·28	0·283	0·285	0·289	0·291	0·295	0·297	0·301	0·304	0·307	0·31	0·314	0·318	0·322	0·326	0·33	0·334
Turkmenistan	0·368	0·37	0·373	0·377	0·381	0·385	0·391	0·395	0·4	0·403	0·408	0·413	0·418	0·421	0·427	0·432	0·437	0·442	0·447	0·451
Uzbekistan	0·324	0·325	0·328	0·332	0·335	0·339	0·343	0·347	0·352	0·356	0·36	0·365	0·369	0·373	0·378	0·382	0·387	0·392	0·396	0·401
Central Europe	0·476	0·481	0·483	0·485	0·487	0·49	0·493	0·497	0·501	0·506	0·51	0·517	0·523	0·527	0·533	0·54	0·539	0·537	0·544	0·551
Albania	0·339	0·342	0·344	0·345	0·346	0·348	0·35	0·353	0·356	0·36	0·364	0·369	0·374	0·38	0·387	0·393	0·4	0·406	0·413	0·42
Bosnia and Herzegovina	0·31	0·314	0·319	0·322	0·324	0·33	0·333	0·337	0·342	0·349	0·356	0·363	0·368	0·375	0·382	0·388	0·395	0·401	0·407	0·413
Bulgaria	0·441	0·456	0·457	0·463	0·465	0·465	0·467	0·471	0·474	0·474	0·479	0·487	0·495	0·499	0·506	0·517	0·524	0·52	0·518	0·527
Croatia	0·473	0·477	0·479	0·482	0·484	0·49	0·497	0·507	0·514	0·52	0·526	0·533	0·541	0·548	0·55	0·549	0·554	0·56	0·565	0·572
Czech Republic	0·557	0·56	0·566	0·571	0·574	0·575	0·576	0·581	0·588	0·592	0·594	0·595	0·593	0·591	0·599	0·609	0·617	0·624	0·627	0·628
Hungary	0·498	0·505	0·506	0·5	0·499	0·505	0·512	0·519	0·525	0·532	0·537	0·546	0·555	0·559	0·563	0·565	0·563	0·562	0·566	0·572
Montenegro	0·486	0·491	0·495	0·497	0·5	0·507	0·512	0·518	0·523	0·529	0·535	0·544	0·551	0·559	0·568	0·576	0·583	0·59	0·597	0·603
North Macedonia	0·422	0·426	0·43	0·433	0·437	0·443	0·449	0·455	0·461	0·467	0·473	0·479	0·484	0·49	0·499	0·505	0·512	0·518	0·523	0·529
Poland	0·485	0·487	0·488	0·489	0·489	0·489	0·49	0·491	0·496	0·502	0·509	0·516	0·523	0·529	0·536	0·543	0·551	0·558	0·563	0·567
Romania	0·397	0·408	0·412	0·418	0·422	0·43	0·434	0·439	0·444	0·45	0·457	0·465	0·473	0·479	0·485	0·497	0·474	0·44	0·461	0·48
Serbia	0·486	0·487	0·491	0·493	0·495	0·503	0·511	0·514	0·514	0·512	0·509	0·513	0·517	0·52	0·52	0·522	0·528	0·533	0·536	0·543
Slovakia	0·507	0·509	0·514	0·519	0·521	0·521	0·522	0·527	0·532	0·534	0·537	0·543	0·548	0·552	0·56	0·567	0·576	0·584	0·588	0·59
Slovenia	0·535	0·54	0·545	0·549	0·555	0·559	0·564	0·569	0·574	0·578	0·583	0·587	0·589	0·592	0·594	0·597	0·601	0·608	0·614	0·618
Eastern Europe	0·54	0·544	0·553	0·559	0·561	0·565	0·567	0·568	0·569	0·571	0·571	0·581	0·595	0·6	0·607	0·612	0·613	0·616	0·617	0·616
Belarus	0·426	0·43	0·439	0·445	0·447	0·45	0·455	0·458	0·461	0·466	0·472	0·479	0·487	0·493	0·499	0·504	0·506	0·508	0·51	0·511
Estonia	0·519	0·524	0·531	0·537	0·542	0·549	0·556	0·56	0·564	0·568	0·572	0·577	0·585	0·589	0·594	0·599	0·603	0·603	0·601	0·601
Latvia	0·532	0·537	0·544	0·549	0·551	0·554	0·558	0·562	0·567	0·571	0·575	0·58	0·588	0·594	0·602	0·608	0·61	0·612	0·613	0·614
Lithuania	0·493	0·499	0·506	0·513	0·52	0·525	0·53	0·533	0·536	0·542	0·546	0·551	0·559	0·566	0·572	0·576	0·58	0·582	0·583	0·586
Moldova	0·421	0·424	0·429	0·434	0·439	0·445	0·451	0·456	0·462	0·467	0·473	0·479	0·485	0·49	0·496	0·501	0·507	0·512	0·517	0·52
Russia	0·552	0·555	0·567	0·572	0·574	0·578	0·579	0·579	0·579	0·581	0·579	0·592	0·61	0·614	0·623	0·627	0·628	0·63	0·632	0·629
Ukraine	0·525	0·529	0·534	0·538	0·542	0·546	0·549	0·551	0·554	0·556	0·56	0·565	0·57	0·576	0·581	0·586	0·59	0·592	0·594	0·596
High income	0·58	0·584	0·586	0·589	0·591	0·592	0·593	0·596	0·597	0·599	0·601	0·604	0·609	0·613	0·618	0·626	0·632	0·638	0·643	0·648
Australasia	0·577	0·573	0·569	0·569	0·568	0·566	0·565	0·566	0·567	0·568	0·568	0·571	0·578	0·588	0·598	0·606	0·611	0·613	0·615	0·619
Australia	0·571	0·566	0·562	0·562	0·561	0·558	0·556	0·557	0·558	0·56	0·56	0·564	0·572	0·582	0·593	0·601	0·606	0·608	0·61	0·614
New Zealand	0·592	0·59	0·589	0·589	0·591	0·594	0·597	0·597	0·596	0·594	0·594	0·596	0·599	0·603	0·61	0·618	0·623	0·626	0·629	0·634
High-income Asia Pacific	0·483	0·494	0·503	0·511	0·517	0·524	0·531	0·537	0·541	0·547	0·556	0·564	0·572	0·579	0·584	0·594	0·601	0·606	0·618	0·627
Brunei	0·36	0·361	0·362	0·361	0·361	0·361	0·362	0·363	0·366	0·371	0·378	0·387	0·396	0·406	0·415	0·426	0·437	0·449	0·462	0·475
Japan	0·512	0·525	0·535	0·544	0·551	0·558	0·567	0·573	0·577	0·584	0·593	0·603	0·611	0·618	0·622	0·633	0·641	0·646	0·659	0·667
Aichi	0·524	0·537	0·547	0·556	0·563	0·569	0·578	0·584	0·588	0·594	0·603	0·612	0·62	0·627	0·63	0·64	0·648	0·652	0·665	0·674
Akita	0·483	0·495	0·504	0·513	0·519	0·525	0·533	0·538	0·541	0·547	0·555	0·563	0·57	0·577	0·579	0·59	0·598	0·603	0·615	0·624
Aomori	0·46	0·473	0·482	0·491	0·497	0·504	0·512	0·518	0·521	0·527	0·535	0·543	0·55	0·557	0·559	0·57	0·578	0·582	0·595	0·604
Chiba	0·492	0·505	0·515	0·524	0·53	0·537	0·545	0·551	0·556	0·562	0·572	0·583	0·592	0·602	0·608	0·621	0·631	0·638	0·653	0·664
Ehime	0·491	0·504	0·513	0·522	0·529	0·535	0·543	0·549	0·552	0·558	0·566	0·575	0·582	0·589	0·592	0·602	0·61	0·615	0·628	0·637
Fukui	0·489	0·502	0·511	0·52	0·526	0·532	0·54	0·545	0·548	0·553	0·561	0·569	0·576	0·582	0·584	0·594	0·601	0·605	0·618	0·627
Fukuoka	0·519	0·532	0·542	0·551	0·558	0·564	0·572	0·578	0·582	0·588	0·596	0·604	0·611	0·617	0·62	0·629	0·637	0·641	0·654	0·663
Fukushima	0·472	0·485	0·495	0·503	0·51	0·516	0·524	0·529	0·532	0·538	0·546	0·554	0·561	0·567	0·57	0·581	0·589	0·593	0·607	0·616
Gifu	0·495	0·508	0·517	0·526	0·533	0·539	0·547	0·553	0·556	0·562	0·571	0·58	0·587	0·594	0·597	0·607	0·615	0·619	0·632	0·641
Gunma	0·486	0·498	0·508	0·516	0·523	0·529	0·537	0·542	0·546	0·553	0·561	0·571	0·579	0·588	0·593	0·604	0·614	0·62	0·634	0·644
Hiroshima	0·511	0·524	0·534	0·543	0·55	0·556	0·564	0·57	0·574	0·58	0·589	0·598	0·606	0·613	0·616	0·627	0·635	0·64	0·654	0·663
Hokkaidō	0·508	0·521	0·531	0·54	0·547	0·553	0·561	0·567	0·57	0·576	0·584	0·592	0·599	0·605	0·607	0·617	0·624	0·627	0·639	0·647
Hyōgo	0·534	0·547	0·557	0·566	0·573	0·579	0·587	0·593	0·596	0·602	0·61	0·619	0·626	0·632	0·635	0·645	0·652	0·656	0·669	0·677
Ibaraki	0·479	0·492	0·502	0·511	0·518	0·524	0·532	0·538	0·542	0·549	0·558	0·567	0·575	0·583	0·587	0·597	0·605	0·61	0·624	0·634
Ishikawa	0·494	0·507	0·516	0·525	0·531	0·538	0·545	0·551	0·554	0·56	0·569	0·578	0·585	0·592	0·594	0·605	0·612	0·616	0·63	0·639
Iwate	0·463	0·476	0·486	0·494	0·501	0·507	0·515	0·52	0·523	0·53	0·538	0·546	0·554	0·561	0·564	0·575	0·582	0·586	0·599	0·608
Kagawa	0·497	0·51	0·52	0·529	0·536	0·542	0·55	0·555	0·558	0·564	0·572	0·58	0·587	0·594	0·596	0·607	0·615	0·62	0·635	0·644
Kagoshima	0·463	0·475	0·484	0·492	0·498	0·505	0·512	0·518	0·521	0·527	0·535	0·543	0·55	0·557	0·56	0·57	0·578	0·583	0·596	0·605
Kanagawa	0·56	0·574	0·583	0·592	0·599	0·606	0·614	0·62	0·625	0·632	0·641	0·651	0·66	0·669	0·673	0·684	0·692	0·696	0·708	0·716
Kōchi	0·473	0·487	0·496	0·505	0·512	0·518	0·526	0·531	0·534	0·54	0·548	0·557	0·564	0·571	0·575	0·585	0·593	0·598	0·611	0·619
Kumamoto	0·469	0·481	0·491	0·5	0·506	0·513	0·52	0·526	0·529	0·535	0·543	0·551	0·559	0·566	0·569	0·581	0·589	0·594	0·608	0·617
Kyōto	0·531	0·543	0·553	0·561	0·568	0·574	0·582	0·588	0·592	0·599	0·607	0·617	0·625	0·632	0·637	0·647	0·655	0·661	0·673	0·683
Mie	0·495	0·508	0·518	0·526	0·533	0·54	0·548	0·553	0·556	0·562	0·571	0·58	0·587	0·595	0·597	0·608	0·615	0·619	0·632	0·641
Miyagi	0·491	0·503	0·513	0·521	0·528	0·534	0·542	0·548	0·551	0·557	0·566	0·575	0·583	0·59	0·594	0·605	0·613	0·619	0·632	0·641

Appendix Table 14. Socio-demographic Index values for all estimated GBD 2019 locations, 1950–1969																				
**Location**	**1950**	**1951**	**1952**	**1953**	**1954**	**1955**	**1956**	**1957**	**1958**	**1959**	**1960**	**1961**	**1962**	**1963**	**1964**	**1965**	**1966**	**1967**	**1968**	**1969**
Miyazaki	0·464	0·477	0·487	0·495	0·502	0·509	0·517	0·522	0·525	0·531	0·538	0·547	0·554	0·561	0·564	0·575	0·583	0·588	0·601	0·61
Nagano	0·495	0·507	0·516	0·524	0·53	0·537	0·544	0·55	0·555	0·562	0·571	0·581	0·589	0·597	0·603	0·614	0·623	0·629	0·642	0·652
Nagasaki	0·475	0·488	0·497	0·506	0·512	0·519	0·527	0·532	0·536	0·541	0·549	0·556	0·561	0·567	0·569	0·578	0·586	0·59	0·602	0·611
Nara	0·516	0·528	0·538	0·546	0·553	0·56	0·568	0·574	0·578	0·584	0·592	0·601	0·608	0·615	0·619	0·63	0·638	0·643	0·656	0·665
Niigata	0·485	0·498	0·507	0·515	0·521	0·528	0·536	0·541	0·545	0·551	0·559	0·567	0·575	0·582	0·586	0·597	0·605	0·61	0·622	0·631
Ōita	0·485	0·497	0·507	0·516	0·522	0·529	0·537	0·543	0·547	0·553	0·561	0·569	0·576	0·583	0·586	0·597	0·605	0·611	0·624	0·633
Okayama	0·495	0·508	0·518	0·527	0·533	0·54	0·548	0·553	0·557	0·563	0·571	0·58	0·588	0·596	0·599	0·611	0·62	0·625	0·64	0·65
Okinawa	0·43	0·445	0·456	0·466	0·474	0·481	0·49	0·497	0·5	0·507	0·516	0·525	0·532	0·539	0·541	0·551	0·558	0·561	0·574	0·582
Ōsaka	0·546	0·559	0·569	0·578	0·585	0·592	0·6	0·606	0·61	0·617	0·626	0·635	0·644	0·651	0·655	0·666	0·673	0·678	0·69	0·698
Saga	0·478	0·491	0·5	0·508	0·515	0·521	0·529	0·534	0·538	0·544	0·552	0·56	0·567	0·574	0·577	0·588	0·596	0·601	0·614	0·623
Saitama	0·493	0·506	0·516	0·524	0·531	0·538	0·546	0·553	0·557	0·564	0·574	0·585	0·594	0·603	0·609	0·621	0·63	0·636	0·65	0·66
Shiga	0·509	0·522	0·531	0·54	0·547	0·553	0·562	0·567	0·571	0·577	0·585	0·593	0·601	0·607	0·61	0·62	0·628	0·633	0·646	0·655
Shimane	0·47	0·482	0·492	0·5	0·507	0·513	0·521	0·527	0·53	0·536	0·544	0·553	0·559	0·566	0·568	0·579	0·586	0·591	0·603	0·612
Shizuoka	0·507	0·52	0·53	0·539	0·545	0·552	0·56	0·566	0·57	0·576	0·585	0·594	0·602	0·609	0·611	0·622	0·629	0·633	0·646	0·654
Tochigi	0·492	0·505	0·514	0·523	0·53	0·536	0·544	0·549	0·553	0·559	0·567	0·576	0·583	0·591	0·594	0·606	0·614	0·619	0·633	0·642
Tokushima	0·476	0·489	0·499	0·507	0·514	0·521	0·529	0·535	0·538	0·545	0·554	0·563	0·57	0·577	0·58	0·591	0·599	0·604	0·617	0·626
Tōkyō	0·611	0·623	0·632	0·64	0·646	0·653	0·66	0·666	0·67	0·675	0·683	0·691	0·698	0·705	0·708	0·717	0·724	0·729	0·74	0·748
Tottori	0·485	0·498	0·508	0·516	0·523	0·529	0·537	0·542	0·545	0·551	0·558	0·566	0·573	0·579	0·581	0·592	0·599	0·604	0·617	0·626
Toyama	0·504	0·517	0·526	0·535	0·541	0·547	0·555	0·56	0·563	0·569	0·578	0·587	0·594	0·601	0·602	0·613	0·62	0·624	0·637	0·646
Wakayama	0·501	0·514	0·524	0·533	0·54	0·546	0·554	0·559	0·562	0·567	0·575	0·583	0·591	0·598	0·6	0·611	0·618	0·622	0·634	0·642
Yamagata	0·474	0·486	0·495	0·503	0·509	0·515	0·523	0·529	0·532	0·539	0·547	0·556	0·563	0·57	0·573	0·584	0·592	0·597	0·61	0·619
Yamaguchi	0·507	0·519	0·529	0·538	0·545	0·551	0·559	0·564	0·567	0·574	0·582	0·591	0·598	0·605	0·608	0·619	0·627	0·631	0·644	0·653
Yamanashi	0·489	0·5	0·509	0·517	0·524	0·53	0·538	0·544	0·549	0·556	0·565	0·574	0·583	0·591	0·596	0·606	0·615	0·621	0·633	0·643
South Korea	0·327	0·326	0·33	0·339	0·347	0·354	0·36	0·367	0·373	0·38	0·385	0·391	0·396	0·401	0·407	0·412	0·418	0·424	0·431	0·44
Singapore	0·356	0·349	0·337	0·332	0·337	0·341	0·346	0·35	0·354	0·367	0·381	0·389	0·396	0·405	0·416	0·427	0·438	0·457	0·475	0·489
High-income North America	0·614	0·609	0·608	0·602	0·598	0·593	0·585	0·585	0·586	0·587	0·59	0·597	0·61	0·622	0·639	0·654	0·664	0·676	0·683	0·685
Canada	0·629	0·625	0·62	0·616	0·614	0·614	0·611	0·608	0·608	0·609	0·613	0·618	0·625	0·634	0·649	0·664	0·674	0·683	0·69	0·696
Greenland	0·487	0·49	0·489	0·486	0·481	0·477	0·475	0·472	0·467	0·459	0·451	0·445	0·439	0·434	0·434	0·442	0·46	0·483	0·506	0·532
USA	0·612	0·607	0·606	0·6	0·596	0·591	0·581	0·582	0·583	0·584	0·587	0·594	0·608	0·621	0·638	0·653	0·663	0·675	0·682	0·684
Alabama	0·563	0·558	0·557	0·551	0·547	0·541	0·532	0·532	0·534	0·533	0·535	0·542	0·557	0·569	0·586	0·602	0·613	0·627	0·636	0·639
Alaska	0·595	0·584	0·581	0·568	0·557	0·544	0·522	0·516	0·514	0·507	0·503	0·51	0·531	0·549	0·575	0·598	0·615	0·636	0·651	0·658
Arizona	0·596	0·589	0·588	0·58	0·573	0·566	0·553	0·552	0·554	0·554	0·556	0·565	0·583	0·598	0·618	0·636	0·647	0·66	0·667	0·666
Arkansas	0·547	0·54	0·538	0·531	0·525	0·517	0·505	0·503	0·504	0·502	0·503	0·51	0·527	0·541	0·561	0·579	0·592	0·607	0·616	0·619
California	0·633	0·628	0·627	0·622	0·618	0·613	0·604	0·605	0·608	0·609	0·612	0·62	0·635	0·648	0·664	0·678	0·687	0·698	0·705	0·707
Colorado	0·625	0·619	0·618	0·612	0·607	0·602	0·592	0·592	0·593	0·594	0·596	0·604	0·619	0·632	0·65	0·666	0·677	0·689	0·698	0·701
Connecticut	0·652	0·648	0·648	0·643	0·639	0·635	0·627	0·627	0·627	0·627	0·629	0·635	0·647	0·658	0·674	0·689	0·699	0·712	0·721	0·727
Delaware	0·62	0·613	0·611	0·603	0·596	0·589	0·576	0·574	0·574	0·571	0·571	0·577	0·592	0·606	0·625	0·644	0·657	0·674	0·686	0·693
Washington, DC	0·643	0·637	0·634	0·626	0·619	0·611	0·597	0·594	0·594	0·591	0·59	0·597	0·614	0·628	0·646	0·662	0·676	0·692	0·706	0·713
Florida	0·613	0·608	0·607	0·602	0·597	0·592	0·583	0·583	0·584	0·584	0·585	0·592	0·605	0·617	0·632	0·647	0·657	0·669	0·677	0·679
Georgia	0·567	0·561	0·559	0·552	0·546	0·539	0·526	0·524	0·524	0·521	0·52	0·526	0·541	0·553	0·572	0·59	0·602	0·618	0·628	0·632
Hawaii	0·61	0·604	0·603	0·596	0·591	0·584	0·573	0·572	0·573	0·572	0·574	0·582	0·598	0·612	0·632	0·65	0·664	0·679	0·69	0·695
Idaho	0·581	0·571	0·568	0·557	0·548	0·537	0·52	0·516	0·515	0·511	0·51	0·518	0·537	0·553	0·578	0·601	0·616	0·634	0·645	0·649
Illinois	0·623	0·618	0·618	0·613	0·609	0·605	0·598	0·6	0·603	0·605	0·61	0·619	0·634	0·646	0·662	0·676	0·684	0·693	0·696	0·694
Indiana	0·604	0·598	0·596	0·588	0·582	0·574	0·561	0·557	0·555	0·551	0·548	0·552	0·565	0·576	0·595	0·612	0·625	0·641	0·653	0·66
Iowa	0·613	0·607	0·605	0·598	0·592	0·585	0·575	0·573	0·573	0·572	0·574	0·581	0·595	0·608	0·627	0·644	0·655	0·669	0·678	0·682
Kansas	0·617	0·611	0·609	0·602	0·596	0·589	0·577	0·575	0·574	0·571	0·57	0·574	0·588	0·6	0·619	0·636	0·649	0·665	0·678	0·685
Kentucky	0·569	0·565	0·564	0·559	0·554	0·549	0·54	0·54	0·542	0·542	0·544	0·55	0·564	0·576	0·593	0·607	0·618	0·631	0·64	0·642
Louisiana	0·561	0·554	0·553	0·545	0·539	0·532	0·519	0·517	0·516	0·514	0·513	0·519	0·533	0·546	0·565	0·583	0·596	0·612	0·623	0·628
Maine	0·595	0·588	0·586	0·579	0·572	0·565	0·552	0·55	0·549	0·546	0·545	0·55	0·564	0·577	0·597	0·615	0·627	0·643	0·653	0·658
Maryland	0·628	0·624	0·623	0·618	0·614	0·609	0·6	0·6	0·602	0·602	0·604	0·612	0·626	0·638	0·655	0·67	0·681	0·694	0·702	0·706
Massachusetts	0·659	0·656	0·656	0·653	0·65	0·648	0·643	0·645	0·647	0·649	0·654	0·661	0·672	0·683	0·697	0·709	0·716	0·725	0·73	0·731
Michigan	0·611	0·605	0·605	0·599	0·594	0·589	0·58	0·58	0·583	0·584	0·587	0·595	0·611	0·624	0·641	0·656	0·666	0·676	0·681	0·681
Minnesota	0·625	0·619	0·618	0·612	0·607	0·602	0·593	0·591	0·591	0·59	0·592	0·598	0·611	0·623	0·641	0·658	0·67	0·685	0·696	0·703
Mississippi	0·516	0·508	0·505	0·496	0·489	0·48	0·465	0·464	0·466	0·466	0·469	0·479	0·5	0·518	0·541	0·561	0·574	0·589	0·598	0·597
Missouri	0·596	0·59	0·588	0·581	0·576	0·569	0·558	0·556	0·556	0·554	0·554	0·56	0·575	0·588	0·607	0·624	0·637	0·652	0·664	0·669
Montana	0·61	0·603	0·601	0·593	0·586	0·578	0·566	0·564	0·564	0·562	0·563	0·57	0·586	0·599	0·62	0·638	0·65	0·665	0·674	0·676
Nebraska	0·617	0·61	0·609	0·602	0·596	0·589	0·578	0·577	0·578	0·577	0·578	0·585	0·599	0·612	0·631	0·648	0·66	0·674	0·683	0·687
Nevada	0·638	0·634	0·634	0·629	0·625	0·62	0·61	0·611	0·613	0·612	0·614	0·62	0·635	0·647	0·663	0·677	0·687	0·7	0·709	0·711
New Hampshire	0·613	0·607	0·605	0·598	0·593	0·586	0·575	0·574	0·574	0·573	0·574	0·581	0·596	0·609	0·629	0·647	0·659	0·675	0·685	0·69
New Jersey	0·642	0·638	0·638	0·634	0·631	0·627	0·621	0·622	0·625	0·627	0·631	0·639	0·651	0·663	0·678	0·691	0·7	0·709	0·714	0·714
New Mexico	0·58	0·573	0·571	0·563	0·556	0·548	0·534	0·533	0·533	0·531	0·531	0·539	0·555	0·569	0·59	0·608	0·621	0·636	0·646	0·649
New York	0·642	0·638	0·638	0·634	0·631	0·629	0·623	0·626	0·629	0·632	0·637	0·646	0·659	0·67	0·685	0·697	0·704	0·712	0·715	0·714
North Carolina	0·582	0·578	0·577	0·571	0·567	0·563	0·554	0·554	0·556	0·556	0·558	0·564	0·578	0·59	0·606	0·62	0·631	0·644	0·653	0·656
North Dakota	0·592	0·584	0·581	0·573	0·565	0·557	0·545	0·542	0·541	0·539	0·54	0·546	0·561	0·576	0·597	0·617	0·632	0·649	0·661	0·668
Ohio	0·614	0·609	0·609	0·604	0·601	0·597	0·59	0·592	0·596	0·599	0·604	0·613	0·628	0·64	0·656	0·669	0·676	0·683	0·685	0·681

Appendix Table 14. Socio-demographic Index values for all estimated GBD 2019 locations, 1950–1969

Location	1950	1951	1952	1953	1954	1955	1956	1957	1958	1959	1960	1961	1962	1963	1964	1965	1966	1967	1968	1969
Oklahoma	0·594	0·589	0·588	0·582	0·578	0·572	0·563	0·563	0·564	0·564	0·566	0·573	0·587	0·6	0·617	0·632	0·643	0·656	0·665	0·667
Oregon	0·627	0·621	0·619	0·612	0·606	0·599	0·587	0·586	0·585	0·583	0·584	0·59	0·605	0·618	0·637	0·655	0·668	0·683	0·695	0·702
Pennsylvania	0·628	0·624	0·624	0·621	0·618	0·616	0·611	0·613	0·617	0·62	0·626	0·634	0·647	0·658	0·673	0·684	0·691	0·698	0·699	0·696
Rhode Island	0·625	0·619	0·618	0·613	0·608	0·603	0·594	0·593	0·594	0·594	0·596	0·602	0·615	0·627	0·644	0·66	0·671	0·684	0·693	0·697
South Carolina	0·566	0·561	0·56	0·554	0·548	0·542	0·531	0·53	0·531	0·53	0·531	0·538	0·553	0·566	0·584	0·6	0·612	0·627	0·636	0·639
South Dakota	0·595	0·588	0·585	0·577	0·571	0·563	0·551	0·549	0·548	0·546	0·547	0·555	0·57	0·584	0·605	0·624	0·637	0·652	0·663	0·668
Tennessee	0·571	0·565	0·564	0·558	0·552	0·546	0·535	0·533	0·533	0·531	0·53	0·535	0·549	0·56	0·578	0·594	0·606	0·621	0·632	0·638
Texas	0·583	0·576	0·574	0·566	0·559	0·551	0·537	0·534	0·533	0·529	0·528	0·532	0·547	0·56	0·579	0·598	0·61	0·626	0·638	0·643
Utah	0·613	0·606	0·604	0·596	0·589	0·58	0·568	0·566	0·566	0·565	0·566	0·574	0·591	0·605	0·626	0·645	0·657	0·67	0·678	0·678
Vermont	0·612	0·606	0·604	0·598	0·592	0·585	0·575	0·574	0·574	0·573	0·574	0·581	0·595	0·608	0·627	0·644	0·657	0·672	0·682	0·687
Virginia	0·601	0·596	0·596	0·59	0·585	0·58	0·571	0·571	0·573	0·574	0·577	0·585	0·6	0·613	0·631	0·647	0·658	0·671	0·68	0·683
Washington	0·628	0·622	0·62	0·613	0·607	0·6	0·589	0·587	0·587	0·586	0·586	0·593	0·608	0·62	0·639	0·656	0·669	0·684	0·695	0·703
West Virginia	0·581	0·576	0·575	0·569	0·564	0·559	0·55	0·549	0·55	0·55	0·551	0·558	0·571	0·582	0·598	0·613	0·624	0·636	0·644	0·646
Wisconsin	0·621	0·615	0·614	0·609	0·603	0·598	0·589	0·588	0·588	0·588	0·59	0·596	0·61	0·622	0·64	0·656	0·668	0·682	0·691	0·696
Wyoming	0·598	0·588	0·584	0·573	0·564	0·553	0·535	0·531	0·53	0·526	0·525	0·532	0·55	0·566	0·59	0·612	0·628	0·646	0·659	0·665
Southern Latin America	0·465	0·469	0·47	0·471	0·473	0·475	0·476	0·479	0·483	0·486	0·49	0·492	0·493	0·496	0·5	0·506	0·512	0·517	0·516	0·516
Argentina	0·471	0·474	0·475	0·476	0·478	0·481	0·483	0·487	0·492	0·496	0·501	0·503	0·505	0·508	0·512	0·518	0·524	0·528	0·523	0·522
Chile	0·446	0·45	0·452	0·454	0·455	0·455	0·454	0·456	0·459	0·459	0·46	0·464	0·465	0·468	0·472	0·477	0·483	0·491	0·5	0·503
Uruguay	0·472	0·477	0·478	0·478	0·478	0·478	0·479	0·48	0·481	0·481	0·481	0·481	0·484	0·486	0·489	0·493	0·499	0·503	0·505	0·507
Western Europe	0·583	0·586	0·588	0·591	0·594	0·596	0·598	0·6	0·601	0·603	0·605	0·606	0·607	0·608	0·611	0·615	0·62	0·626	0·629	0·635
Andorra	0·69	0·694	0·699	0·703	0·707	0·711	0·715	0·719	0·723	0·726	0·73	0·734	0·737	0·741	0·746	0·75	0·755	0·759	0·764	0·768
Austria	0·593	0·596	0·597	0·599	0·601	0·6	0·599	0·599	0·596	0·598	0·6	0·6	0·603	0·608	0·614	0·616	0·615	0·617	0·621	0·629
Belgium	0·577	0·58	0·581	0·582	0·582	0·582	0·582	0·582	0·582	0·584	0·585	0·586	0·587	0·588	0·592	0·597	0·602	0·609	0·617	0·621
Cyprus	0·477	0·481	0·486	0·491	0·493	0·494	0·496	0·497	0·499	0·501	0·504	0·507	0·512	0·517	0·52	0·524	0·528	0·534	0·543	0·554
Denmark	0·625	0·625	0·625	0·625	0·625	0·626	0·627	0·63	0·633	0·636	0·639	0·641	0·643	0·644	0·646	0·65	0·659	0·672	0·685	0·694
Finland	0·559	0·561	0·562	0·563	0·564	0·566	0·568	0·574	0·579	0·579	0·584	0·588	0·588	0·592	0·601	0·607	0·612	0·618	0·628	0·638
France	0·542	0·547	0·551	0·554	0·556	0·558	0·562	0·565	0·566	0·567	0·568	0·57	0·571	0·57	0·575	0·581	0·588	0·597	0·603	0·609
Germany	0·643	0·646	0·647	0·656	0·664	0·667	0·669	0·672	0·673	0·674	0·676	0·676	0·678	0·681	0·685	0·688	0·692	0·701	0·699	0·707
Greece	0·526	0·531	0·534	0·537	0·538	0·542	0·548	0·551	0·553	0·554	0·559	0·562	0·562	0·563	0·564	0·564	0·563	0·563	0·564	0·568
Iceland	0·559	0·558	0·555	0·55	0·544	0·541	0·542	0·545	0·546	0·548	0·554	0·56	0·563	0·566	0·572	0·579	0·591	0·605	0·615	0·622
Ireland	0·578	0·58	0·584	0·587	0·59	0·593	0·595	0·596	0·597	0·598	0·599	0·599	0·599	0·601	0·603	0·606	0·609	0·612	0·614	0·615
Israel	0·505	0·501	0·499	0·507	0·509	0·511	0·519	0·526	0·541	0·546	0·547	0·556	0·566	0·574	0·582	0·589	0·596	0·602	0·606	0·607
Italy	0·534	0·538	0·541	0·544	0·546	0·549	0·552	0·555	0·558	0·561	0·564	0·567	0·568	0·568	0·568	0·571	0·576	0·58	0·584	0·588
Luxembourg	0·673	0·672	0·673	0·673	0·674	0·674	0·675	0·677	0·678	0·679	0·681	0·683	0·685	0·685	0·686	0·688	0·691	0·697	0·705	0·712
Malta	0·421	0·428	0·435	0·441	0·446	0·45	0·454	0·457	0·458	0·46	0·467	0·476	0·485	0·496	0·509	0·519	0·525	0·53	0·536	0·541
Monaco	0·689	0·693	0·697	0·701	0·704	0·708	0·712	0·716	0·719	0·723	0·727	0·73	0·734	0·738	0·741	0·745	0·749	0·752	0·756	0·76
Netherlands	0·638	0·638	0·641	0·645	0·647	0·649	0·65	0·651	0·65	0·652	0·653	0·653	0·653	0·654	0·656	0·658	0·66	0·666	0·669	0·673
Norway	0·661	0·66	0·657	0·655	0·652	0·65	0·649	0·65	0·651	0·654	0·653	0·649	0·651	0·653	0·655	0·658	0·663	0·666	0·668	0·673
Portugal	0·442	0·445	0·448	0·453	0·457	0·46	0·46	0·459	0·459	0·459	0·461	0·463	0·466	0·469	0·472	0·476	0·479	0·483	0·489	0·494
San Marino	0·653	0·657	0·661	0·665	0·669	0·673	0·677	0·681	0·685	0·689	0·693	0·697	0·702	0·706	0·71	0·714	0·718	0·723	0·727	0·731
Spain	0·469	0·473	0·475	0·477	0·478	0·479	0·48	0·481	0·485	0·488	0·491	0·494	0·498	0·5	0·504	0·509	0·513	0·516	0·517	0·523
Sweden	0·624	0·626	0·626	0·629	0·631	0·631	0·634	0·638	0·642	0·647	0·65	0·651	0·652	0·651	0·652	0·655	0·659	0·669	0·681	0·687
Stockholm	0·683	0·686	0·687	0·69	0·692	0·693	0·696	0·701	0·705	0·709	0·713	0·715	0·717	0·718	0·719	0·722	0·725	0·73	0·737	0·739
Sweden except Stockholm	0·612	0·614	0·614	0·617	0·619	0·618	0·621	0·625	0·629	0·634	0·637	0·637	0·638	0·637	0·636	0·64	0·644	0·654	0·667	0·674
Switzerland	0·747	0·75	0·752	0·754	0·755	0·756	0·756	0·756	0·759	0·76	0·76	0·758	0·755	0·754	0·757	0·763	0·767	0·771	0·776	0·781
UK	0·579	0·582	0·584	0·586	0·587	0·588	0·588	0·588	0·588	0·587	0·587	0·588	0·589	0·591	0·596	0·601	0·608	0·614	0·622	0·629
England	0·581	0·584	0·586	0·588	0·59	0·591	0·592	0·594	0·595	0·596	0·596	0·597	0·599	0·601	0·606	0·611	0·618	0·625	0·632	0·639
East Midlands	0·549	0·552	0·554	0·556	0·557	0·559	0·56	0·562	0·563	0·563	0·564	0·565	0·566	0·569	0·574	0·58	0·587	0·594	0·601	0·609
Derby	0·563	0·566	0·568	0·57	0·571	0·572	0·573	0·574	0·574	0·574	0·574	0·574	0·575	0·577	0·582	0·587	0·594	0·602	0·609	0·617
Derbyshire	0·55	0·553	0·555	0·556	0·558	0·559	0·56	0·561	0·562	0·562	0·562	0·562	0·563	0·566	0·57	0·576	0·582	0·59	0·597	0·604
Leicester	0·539	0·542	0·544	0·546	0·548	0·549	0·55	0·552	0·553	0·554	0·555	0·556	0·558	0·561	0·566	0·572	0·579	0·587	0·594	0·602
Leicestershire	0·587	0·591	0·593	0·595	0·597	0·598	0·6	0·601	0·602	0·603	0·604	0·606	0·608	0·611	0·615	0·621	0·627	0·634	0·641	0·647
Lincolnshire	0·551	0·554	0·556	0·558	0·559	0·56	0·561	0·563	0·564	0·564	0·564	0·565	0·566	0·569	0·573	0·579	0·585	0·593	0·6	0·607
Northamptonshire	0·563	0·566	0·567	0·569	0·57	0·571	0·572	0·574	0·574	0·574	0·574	0·575	0·576	0·578	0·583	0·588	0·595	0·603	0·61	0·618
Nottingham	0·575	0·578	0·58	0·583	0·584	0·586	0·588	0·59	0·591	0·592	0·593	0·595	0·597	0·601	0·606	0·611	0·618	0·625	0·632	0·639
Nottinghamshire	0·551	0·554	0·556	0·558	0·559	0·56	0·561	0·563	0·564	0·564	0·564	0·565	0·566	0·568	0·573	0·578	0·585	0·592	0·599	0·606
Rutland	0·592	0·595	0·598	0·6	0·601	0·602	0·604	0·605	0·606	0·606	0·607	0·607	0·608	0·611	0·616	0·621	0·628	0·636	0·643	0·65
East of England	0·573	0·577	0·579	0·581	0·583	0·584	0·585	0·587	0·588	0·589	0·59	0·591	0·592	0·595	0·6	0·605	0·612	0·619	0·626	0·633
Bedford	0·581	0·585	0·587	0·589	0·59	0·592	0·593	0·595	0·596	0·596	0·597	0·598	0·599	0·602	0·606	0·612	0·619	0·626	0·633	0·64
Cambridgeshire	0·616	0·619	0·621	0·624	0·625	0·627	0·629	0·631	0·632	0·634	0·635	0·637	0·639	0·642	0·647	0·652	0·659	0·665	0·672	0·679
Central Bedfordshire	0·578	0·581	0·583	0·586	0·587	0·588	0·59	0·591	0·592	0·593	0·593	0·594	0·595	0·598	0·602	0·608	0·615	0·622	0·629	0·637
Essex	0·566	0·569	0·572	0·574	0·575	0·577	0·578	0·58	0·581	0·582	0·583	0·584	0·586	0·588	0·593	0·598	0·605	0·612	0·619	0·626
Hertfordshire	0·618	0·621	0·624	0·626	0·628	0·629	0·631	0·632	0·634	0·635	0·635	0·637	0·638	0·641	0·645	0·651	0·657	0·664	0·67	0·677
Luton	0·558	0·561	0·563	0·565	0·566	0·567	0·568	0·569	0·569	0·57	0·569	0·57	0·571	0·573	0·577	0·583	0·589	0·597	0·604	0·611
Norfolk	0·558	0·561	0·563	0·565	0·566	0·568	0·569	0·57	0·571	0·572	0·572	0·573	0·575	0·577	0·582	0·587	0·594	0·601	0·608	0·615

Appendix Table 14. Socio-demographic Index values for all estimated GBD 2019 locations, 1950–1969

Location	1950	1951	1952	1953	1954	1955	1956	1957	1958	1959	1960	1961	1962	1963	1964	1965	1966	1967	1968	1969
Peterborough	0·553	0·556	0·558	0·559	0·56	0·561	0·561	0·562	0·562	0·562	0·561	0·561	0·561	0·563	0·567	0·573	0·58	0·588	0·595	0·603
Southend-on-Sea	0·539	0·542	0·544	0·545	0·547	0·548	0·549	0·55	0·551	0·551	0·552	0·552	0·554	0·557	0·561	0·567	0·574	0·581	0·588	0·596
Suffolk	0·56	0·562	0·564	0·566	0·567	0·568	0·569	0·57	0·571	0·571	0·571	0·572	0·573	0·575	0·579	0·585	0·592	0·6	0·607	0·614
Thurrock	0·553	0·556	0·558	0·56	0·561	0·562	0·563	0·564	0·565	0·565	0·565	0·566	0·567	0·569	0·574	0·579	0·586	0·593	0·601	0·608
Greater London	0·636	0·639	0·641	0·643	0·645	0·647	0·648	0·65	0·652	0·653	0·654	0·656	0·658	0·661	0·665	0·671	0·677	0·683	0·69	0·696
Barking and Dagenham	0·524	0·527	0·528	0·529	0·53	0·531	0·531	0·532	0·533	0·533	0·533	0·533	0·534	0·536	0·54	0·546	0·552	0·559	0·567	0·574
Barnet	0·615	0·618	0·62	0·622	0·623	0·625	0·626	0·628	0·629	0·63	0·631	0·633	0·635	0·638	0·642	0·648	0·654	0·66	0·667	0·674
Bexley	0·56	0·563	0·565	0·567	0·568	0·57	0·571	0·572	0·573	0·574	0·575	0·576	0·578	0·58	0·584	0·59	0·596	0·603	0·61	0·616
Brent	0·585	0·588	0·59	0·592	0·594	0·595	0·597	0·598	0·599	0·6	0·601	0·602	0·604	0·607	0·611	0·616	0·623	0·63	0·636	0·643
Bromley	0·602	0·604	0·606	0·608	0·61	0·612	0·613	0·614	0·616	0·617	0·618	0·619	0·621	0·624	0·628	0·633	0·639	0·646	0·653	0·659
Camden	0·723	0·727	0·729	0·732	0·735	0·738	0·741	0·743	0·746	0·749	0·751	0·754	0·757	0·761	0·765	0·77	0·776	0·782	0·788	0·794
Croydon	0·578	0·581	0·583	0·585	0·586	0·588	0·589	0·59	0·591	0·591	0·592	0·593	0·594	0·597	0·601	0·607	0·613	0·621	0·628	0·635
Ealing	0·602	0·605	0·608	0·61	0·611	0·613	0·614	0·616	0·617	0·618	0·619	0·62	0·622	0·625	0·629	0·635	0·641	0·648	0·655	0·662
Enfield	0·567	0·569	0·571	0·573	0·574	0·575	0·576	0·578	0·579	0·579	0·58	0·581	0·582	0·585	0·589	0·595	0·601	0·608	0·615	0·623
Greenwich	0·558	0·56	0·562	0·564	0·565	0·566	0·567	0·568	0·569	0·57	0·57	0·571	0·573	0·576	0·58	0·585	0·592	0·599	0·606	0·613
Hackney	0·642	0·645	0·647	0·647	0·648	0·647	0·647	0·647	0·647	0·646	0·644	0·643	0·643	0·644	0·647	0·652	0·658	0·665	0·672	0·68
Hammersmith and Fulham	0·674	0·678	0·68	0·683	0·685	0·688	0·69	0·693	0·695	0·698	0·7	0·703	0·706	0·709	0·714	0·719	0·725	0·731	0·738	0·744
Haringey	0·589	0·592	0·594	0·596	0·598	0·6	0·602	0·604	0·605	0·606	0·607	0·609	0·611	0·614	0·618	0·624	0·63	0·637	0·644	0·651
Harrow	0·602	0·604	0·606	0·608	0·61	0·611	0·612	0·614	0·615	0·616	0·617	0·618	0·62	0·622	0·627	0·632	0·638	0·645	0·651	0·658
Havering	0·56	0·562	0·564	0·566	0·567	0·569	0·57	0·571	0·572	0·573	0·574	0·575	0·576	0·579	0·583	0·589	0·595	0·601	0·608	0·615
Hillingdon	0·621	0·624	0·626	0·628	0·63	0·631	0·632	0·634	0·635	0·636	0·636	0·638	0·639	0·642	0·646	0·652	0·658	0·664	0·671	0·678
Hounslow	0·607	0·61	0·612	0·614	0·616	0·617	0·618	0·62	0·621	0·622	0·623	0·624	0·625	0·628	0·632	0·638	0·644	0·651	0·659	0·666
Islington	0·671	0·675	0·677	0·68	0·682	0·685	0·687	0·689	0·691	0·693	0·695	0·697	0·699	0·703	0·707	0·712	0·718	0·725	0·731	0·738
Kensington and Chelsea	0·713	0·716	0·719	0·722	0·725	0·728	0·731	0·734	0·736	0·739	0·742	0·745	0·748	0·752	0·757	0·762	0·768	0·774	0·781	0·788
Kingston upon Thames	0·648	0·651	0·653	0·655	0·657	0·659	0·661	0·663	0·665	0·667	0·669	0·671	0·673	0·676	0·681	0·686	0·691	0·697	0·703	0·709
Lambeth	0·617	0·62	0·622	0·625	0·627	0·629	0·63	0·632	0·634	0·635	0·637	0·638	0·64	0·643	0·648	0·653	0·659	0·666	0·673	0·679
Lewisham	0·568	0·571	0·573	0·575	0·577	0·579	0·58	0·582	0·583	0·584	0·585	0·587	0·589	0·592	0·596	0·601	0·607	0·614	0·621	0·627
Merton	0·604	0·607	0·609	0·611	0·612	0·614	0·615	0·616	0·618	0·618	0·619	0·621	0·622	0·625	0·629	0·635	0·641	0·648	0·654	0·661
Newham	0·54	0·542	0·544	0·545	0·547	0·548	0·548	0·549	0·55	0·55	0·55	0·551	0·552	0·554	0·559	0·564	0·571	0·578	0·586	0·593
Redbridge	0·581	0·583	0·585	0·587	0·588	0·59	0·591	0·593	0·594	0·595	0·596	0·597	0·599	0·602	0·606	0·612	0·618	0·624	0·631	0·638
Richmond upon Thames	0·671	0·674	0·676	0·678	0·68	0·682	0·684	0·686	0·688	0·689	0·691	0·693	0·695	0·698	0·703	0·707	0·713	0·719	0·726	0·732
Southwark	0·629	0·632	0·634	0·636	0·638	0·64	0·641	0·643	0·644	0·645	0·646	0·647	0·649	0·652	0·656	0·662	0·668	0·675	0·681	0·688
Sutton	0·576	0·578	0·58	0·582	0·584	0·585	0·586	0·588	0·589	0·59	0·59	0·592	0·593	0·596	0·6	0·605	0·612	0·619	0·626	0·633
Tower Hamlets	0·617	0·62	0·621	0·623	0·624	0·624	0·625	0·626	0·626	0·626	0·626	0·626	0·627	0·629	0·633	0·638	0·646	0·653	0·661	0·668
Waltham Forest	0·545	0·547	0·549	0·551	0·552	0·553	0·555	0·556	0·557	0·558	0·558	0·559	0·561	0·564	0·568	0·573	0·58	0·587	0·594	0·601
Wandsworth	0·649	0·652	0·654	0·657	0·659	0·661	0·663	0·666	0·668	0·67	0·671	0·674	0·676	0·679	0·684	0·689	0·695	0·701	0·708	0·714
Westminster	0·741	0·743	0·746	0·748	0·75	0·751	0·753	0·754	0·756	0·757	0·759	0·76	0·762	0·764	0·768	0·771	0·776	0·781	0·786	0·791
North East England	0·527	0·53	0·531	0·533	0·534	0·534	0·535	0·536	0·536	0·535	0·534	0·534	0·534	0·537	0·541	0·547	0·554	0·562	0·57	0·578
County Durham	0·535	0·537	0·539	0·541	0·541	0·542	0·542	0·543	0·543	0·542	0·542	0·541	0·542	0·544	0·548	0·553	0·56	0·568	0·575	0·583
Darlington	0·552	0·554	0·556	0·557	0·558	0·558	0·558	0·558	0·557	0·556	0·554	0·553	0·553	0·554	0·558	0·563	0·57	0·578	0·586	0·594
Gateshead	0·543	0·545	0·547	0·548	0·549	0·549	0·549	0·549	0·549	0·548	0·547	0·546	0·546	0·548	0·551	0·557	0·564	0·572	0·579	0·587
Hartlepool	0·512	0·515	0·516	0·517	0·518	0·517	0·517	0·517	0·516	0·515	0·513	0·511	0·51	0·511	0·515	0·52	0·528	0·536	0·544	0·552
Middlesbrough	0·515	0·518	0·519	0·52	0·521	0·52	0·52	0·52	0·519	0·518	0·516	0·514	0·514	0·515	0·519	0·525	0·532	0·541	0·549	0·557
Newcastle upon Tyne	0·585	0·588	0·59	0·592	0·594	0·595	0·597	0·598	0·599	0·6	0·601	0·602	0·603	0·606	0·611	0·617	0·623	0·63	0·637	0·644
North Tyneside	0·547	0·549	0·551	0·552	0·553	0·553	0·554	0·554	0·554	0·553	0·552	0·551	0·551	0·553	0·557	0·562	0·569	0·577	0·584	0·592
Northumberland	0·542	0·544	0·546	0·547	0·548	0·548	0·548	0·548	0·548	0·547	0·545	0·545	0·544	0·546	0·55	0·555	0·562	0·57	0·578	0·586
Redcar and Cleveland	0·518	0·521	0·522	0·523	0·524	0·524	0·524	0·524	0·523	0·522	0·52	0·518	0·518	0·519	0·523	0·528	0·536	0·544	0·552	0·56
South Tyneside	0·513	0·516	0·517	0·518	0·519	0·519	0·519	0·519	0·519	0·518	0·516	0·515	0·515	0·516	0·52	0·525	0·533	0·54	0·548	0·556
Stockton-on-Tees	0·551	0·554	0·556	0·557	0·557	0·557	0·557	0·557	0·556	0·555	0·553	0·552	0·551	0·552	0·556	0·561	0·569	0·577	0·585	0·593
Sunderland	0·524	0·526	0·528	0·529	0·529	0·529	0·529	0·529	0·529	0·527	0·526	0·524	0·524	0·525	0·529	0·535	0·542	0·55	0·558	0·566
North West England	0·555	0·558	0·561	0·562	0·564	0·565	0·566	0·568	0·569	0·569	0·569	0·57	0·571	0·574	0·579	0·585	0·592	0·599	0·607	0·614
Blackburn with Darwen	0·518	0·521	0·522	0·523	0·524	0·524	0·524	0·525	0·524	0·523	0·522	0·522	0·522	0·524	0·528	0·534	0·541	0·55	0·557	0·565
Blackpool	0·518	0·52	0·522	0·523	0·524	0·524	0·525	0·526	0·527	0·526	0·526	0·526	0·527	0·529	0·533	0·539	0·546	0·553	0·56	0·568
Bolton	0·536	0·539	0·541	0·543	0·544	0·544	0·545	0·545	0·546	0·545	0·545	0·545	0·545	0·548	0·552	0·558	0·565	0·573	0·58	0·588
Bury	0·556	0·559	0·561	0·563	0·564	0·565	0·566	0·568	0·568	0·568	0·568	0·569	0·57	0·572	0·577	0·582	0·589	0·597	0·604	0·612
Cheshire East	0·61	0·613	0·615	0·617	0·619	0·62	0·621	0·622	0·623	0·623	0·624	0·624	0·625	0·628	0·632	0·638	0·645	0·652	0·659	0·666
Cheshire West and Chester	0·599	0·603	0·605	0·607	0·609	0·61	0·611	0·613	0·613	0·614	0·614	0·615	0·616	0·619	0·623	0·629	0·636	0·643	0·65	0·658
Cumbria	0·562	0·565	0·567	0·569	0·57	0·571	0·572	0·574	0·574	0·575	0·575	0·575	0·576	0·579	0·583	0·589	0·595	0·603	0·61	0·617
Halton	0·545	0·548	0·55	0·552	0·552	0·553	0·553	0·554	0·554	0·554	0·553	0·552	0·553	0·555	0·559	0·564	0·572	0·58	0·587	0·595
Knowsley	0·524	0·527	0·529	0·53	0·532	0·532	0·533	0·534	0·534	0·534	0·533	0·533	0·534	0·536	0·54	0·545	0·552	0·56	0·567	0·574
Lancashire	0·566	0·569	0·571	0·573	0·574	0·575	0·576	0·577	0·578	0·578	0·578	0·578	0·579	0·582	0·586	0·592	0·599	0·606	0·614	0·621
Liverpool	0·56	0·563	0·566	0·568	0·57	0·571	0·573	0·575	0·576	0·578	0·579	0·58	0·583	0·586	0·591	0·597	0·604	0·611	0·618	0·625
Manchester	0·586	0·589	0·592	0·594	0·596	0·597	0·599	0·601	0·602	0·604	0·605	0·606	0·608	0·612	0·616	0·622	0·629	0·636	0·643	0·65
Oldham	0·519	0·522	0·524	0·525	0·526	0·526	0·527	0·528	0·528	0·527	0·526	0·526	0·527	0·529	0·533	0·539	0·546	0·554	0·562	0·569
Rochdale	0·523	0·526	0·528	0·529	0·53	0·531	0·531	0·532	0·532	0·532	0·531	0·531	0·531	0·533	0·537	0·543	0·55	0·558	0·566	0·574

Appendix Table 14. Socio-demographic Index values for all estimated GBD 2019 locations, 1950–1969

Location	1950	1951	1952	1953	1954	1955	1956	1957	1958	1959	1960	1961	1962	1963	1964	1965	1966	1967	1968	1969
Salford	0·552	0·555	0·557	0·558	0·559	0·56	0·561	0·562	0·563	0·563	0·563	0·563	0·564	0·566	0·571	0·576	0·583	0·591	0·598	0·606
Sefton	0·555	0·558	0·56	0·562	0·564	0·565	0·567	0·568	0·569	0·57	0·57	0·571	0·573	0·576	0·58	0·586	0·592	0·6	0·607	0·614
St Helens	0·534	0·537	0·538	0·54	0·541	0·542	0·543	0·544	0·545	0·545	0·545	0·545	0·546	0·548	0·552	0·558	0·565	0·572	0·58	0·587
Stockport	0·586	0·589	0·591	0·593	0·595	0·596	0·597	0·598	0·599	0·6	0·6	0·6	0·601	0·604	0·608	0·614	0·621	0·628	0·635	0·642
Tameside	0·53	0·532	0·534	0·536	0·537	0·538	0·538	0·539	0·54	0·54	0·539	0·539	0·54	0·543	0·547	0·552	0·559	0·567	0·575	0·582
Trafford	0·617	0·62	0·622	0·624	0·626	0·627	0·628	0·629	0·63	0·631	0·631	0·632	0·633	0·635	0·64	0·645	0·652	0·659	0·666	0·673
Warrington	0·593	0·596	0·598	0·6	0·601	0·603	0·604	0·605	0·605	0·605	0·605	0·606	0·607	0·609	0·614	0·619	0·626	0·634	0·642	0·649
Wigan	0·53	0·533	0·535	0·537	0·538	0·539	0·54	0·541	0·542	0·542	0·542	0·542	0·543	0·545	0·55	0·555	0·562	0·57	0·578	0·585
Wirral	0·545	0·548	0·55	0·551	0·553	0·553	0·554	0·555	0·556	0·556	0·556	0·556	0·557	0·559	0·563	0·569	0·576	0·583	0·591	0·598
South East England	0·611	0·614	0·616	0·618	0·62	0·622	0·624	0·626	0·627	0·628	0·63	0·631	0·633	0·636	0·64	0·646	0·652	0·659	0·666	0·672
Bracknell Forest	0·624	0·627	0·63	0·632	0·634	0·636	0·637	0·639	0·641	0·641	0·642	0·643	0·645	0·648	0·652	0·658	0·664	0·671	0·678	0·685
Brighton and Hove	0·63	0·633	0·635	0·638	0·64	0·642	0·645	0·647	0·649	0·651	0·654	0·656	0·659	0·663	0·668	0·673	0·678	0·684	0·69	0·696
Buckinghamshire	0·635	0·639	0·641	0·644	0·646	0·647	0·649	0·651	0·652	0·654	0·655	0·656	0·658	0·661	0·665	0·671	0·677	0·684	0·69	0·697
East Sussex	0·563	0·566	0·568	0·569	0·571	0·572	0·573	0·574	0·575	0·576	0·577	0·578	0·579	0·582	0·586	0·592	0·598	0·606	0·613	0·62
Hampshire	0·606	0·609	0·612	0·614	0·616	0·617	0·619	0·621	0·622	0·623	0·624	0·625	0·627	0·63	0·634	0·64	0·646	0·654	0·661	0·668
Isle of Wight	0·546	0·549	0·551	0·552	0·553	0·554	0·555	0·556	0·557	0·557	0·557	0·558	0·559	0·561	0·565	0·571	0·578	0·585	0·592	0·599
Kent	0·576	0·579	0·581	0·583	0·585	0·586	0·588	0·589	0·59	0·591	0·592	0·593	0·595	0·598	0·602	0·608	0·614	0·622	0·629	0·636
Medway	0·549	0·552	0·554	0·556	0·558	0·559	0·56	0·562	0·563	0·563	0·564	0·565	0·566	0·569	0·573	0·579	0·586	0·594	0·601	0·608
Milton Keynes	0·601	0·604	0·606	0·607	0·608	0·609	0·61	0·611	0·611	0·611	0·611	0·611	0·612	0·614	0·619	0·625	0·632	0·64	0·648	0·656
Oxfordshire	0·641	0·645	0·647	0·65	0·652	0·654	0·656	0·659	0·661	0·662	0·664	0·666	0·668	0·672	0·676	0·682	0·688	0·694	0·7	0·707
Portsmouth	0·593	0·596	0·598	0·6	0·602	0·604	0·606	0·608	0·609	0·611	0·612	0·614	0·616	0·62	0·625	0·631	0·637	0·644	0·651	0·659
Reading	0·647	0·65	0·652	0·655	0·657	0·659	0·661	0·663	0·665	0·666	0·668	0·669	0·672	0·675	0·68	0·685	0·692	0·698	0·705	0·711
Slough	0·612	0·615	0·617	0·618	0·619	0·62	0·621	0·622	0·622	0·622	0·622	0·622	0·622	0·625	0·629	0·635	0·641	0·649	0·656	0·664
Southampton	0·605	0·608	0·611	0·613	0·615	0·617	0·619	0·621	0·623	0·624	0·626	0·628	0·631	0·634	0·639	0·645	0·651	0·657	0·664	0·67
Surrey	0·652	0·655	0·657	0·66	0·662	0·664	0·666	0·668	0·669	0·671	0·672	0·674	0·676	0·679	0·684	0·689	0·695	0·701	0·707	0·713
West Berkshire	0·637	0·64	0·642	0·644	0·646	0·647	0·648	0·65	0·651	0·651	0·651	0·652	0·653	0·656	0·66	0·666	0·673	0·68	0·688	0·695
West Sussex	0·594	0·597	0·599	0·601	0·603	0·604	0·606	0·607	0·608	0·609	0·61	0·611	0·613	0·616	0·621	0·626	0·633	0·64	0·647	0·653
Windsor and Maidenhead	0·657	0·66	0·663	0·665	0·667	0·669	0·671	0·673	0·675	0·676	0·677	0·679	0·681	0·684	0·688	0·693	0·7	0·706	0·713	0·719
Wokingham	0·666	0·67	0·672	0·674	0·677	0·679	0·681	0·683	0·684	0·686	0·687	0·689	0·691	0·694	0·699	0·704	0·71	0·717	0·724	0·73
South West England	0·581	0·584	0·586	0·588	0·59	0·591	0·592	0·594	0·595	0·596	0·597	0·598	0·599	0·602	0·607	0·612	0·619	0·626	0·633	0·64
Bath and North East Somerset	0·635	0·638	0·64	0·643	0·645	0·647	0·649	0·652	0·654	0·655	0·657	0·66	0·662	0·665	0·67	0·675	0·68	0·686	0·692	0·697
Bournemouth	0·59	0·593	0·595	0·597	0·599	0·6	0·602	0·604	0·606	0·608	0·609	0·612	0·614	0·618	0·622	0·627	0·634	0·64	0·646	0·652
Bristol, City of	0·621	0·624	0·627	0·629	0·631	0·633	0·635	0·637	0·638	0·64	0·641	0·643	0·645	0·648	0·653	0·659	0·665	0·671	0·678	0·684
Cornwall	0·562	0·565	0·567	0·568	0·569	0·57	0·571	0·572	0·573	0·573	0·573	0·573	0·574	0·576	0·58	0·586	0·593	0·6	0·607	0·615
Devon	0·582	0·585	0·587	0·589	0·59	0·592	0·593	0·595	0·596	0·596	0·597	0·598	0·6	0·603	0·607	0·612	0·619	0·625	0·632	0·639
Dorset	0·579	0·582	0·584	0·586	0·587	0·588	0·589	0·591	0·591	0·592	0·592	0·592	0·594	0·596	0·6	0·606	0·612	0·619	0·626	0·633
Gloucestershire	0·598	0·602	0·604	0·606	0·607	0·609	0·61	0·611	0·612	0·613	0·613	0·614	0·616	0·618	0·623	0·628	0·635	0·642	0·648	0·655
North Somerset	0·576	0·579	0·581	0·583	0·585	0·586	0·587	0·588	0·589	0·589	0·59	0·591	0·592	0·594	0·599	0·604	0·611	0·618	0·625	0·632
Plymouth	0·571	0·574	0·576	0·578	0·58	0·581	0·583	0·584	0·586	0·586	0·587	0·588	0·59	0·593	0·598	0·603	0·61	0·617	0·624	0·631
Poole	0·588	0·591	0·593	0·595	0·596	0·597	0·598	0·599	0·6	0·6	0·6	0·601	0·602	0·605	0·609	0·614	0·621	0·628	0·636	0·643
Somerset	0·568	0·571	0·573	0·575	0·576	0·577	0·578	0·579	0·58	0·58	0·58	0·58	0·581	0·584	0·588	0·593	0·6	0·607	0·615	0·622
South Gloucestershire	0·615	0·618	0·62	0·622	0·624	0·626	0·627	0·629	0·63	0·63	0·631	0·632	0·634	0·636	0·641	0·646	0·653	0·659	0·666	0·673
Swindon	0·596	0·599	0·601	0·603	0·605	0·605	0·606	0·607	0·608	0·608	0·607	0·607	0·608	0·61	0·614	0·62	0·627	0·634	0·641	0·649
Torbay	0·543	0·545	0·547	0·548	0·549	0·55	0·551	0·551	0·552	0·552	0·552	0·552	0·553	0·556	0·56	0·565	0·572	0·579	0·586	0·593
Wiltshire	0·585	0·588	0·591	0·592	0·594	0·595	0·596	0·597	0·598	0·598	0·598	0·598	0·599	0·601	0·605	0·611	0·618	0·625	0·633	0·64
West Midlands	0·542	0·545	0·547	0·548	0·55	0·55	0·551	0·552	0·553	0·553	0·553	0·553	0·554	0·556	0·56	0·566	0·573	0·581	0·589	0·596
Birmingham	0·544	0·546	0·548	0·55	0·551	0·551	0·552	0·553	0·553	0·553	0·552	0·552	0·553	0·556	0·56	0·566	0·573	0·581	0·588	0·596
Coventry	0·569	0·572	0·574	0·576	0·577	0·578	0·579	0·58	0·581	0·581	0·581	0·581	0·582	0·584	0·589	0·594	0·601	0·609	0·616	0·623
Dudley	0·534	0·537	0·539	0·54	0·542	0·542	0·543	0·544	0·544	0·544	0·543	0·543	0·544	0·546	0·55	0·555	0·562	0·57	0·577	0·585
Herefordshire, County of	0·555	0·558	0·56	0·562	0·563	0·563	0·564	0·565	0·565	0·565	0·565	0·564	0·565	0·567	0·571	0·577	0·584	0·592	0·599	0·607
Sandwell	0·511	0·514	0·515	0·516	0·517	0·517	0·517	0·517	0·517	0·516	0·515	0·514	0·514	0·516	0·519	0·525	0·532	0·54	0·548	0·555
Shropshire	0·561	0·564	0·566	0·568	0·569	0·57	0·571	0·572	0·572	0·572	0·572	0·572	0·573	0·576	0·58	0·585	0·592	0·6	0·608	0·615
Solihull	0·598	0·601	0·603	0·605	0·606	0·607	0·608	0·609	0·609	0·609	0·609	0·609	0·609	0·611	0·615	0·621	0·628	0·635	0·643	0·65
Staffordshire	0·557	0·56	0·562	0·564	0·565	0·566	0·567	0·568	0·569	0·569	0·569	0·569	0·57	0·572	0·576	0·582	0·589	0·597	0·604	0·612
Stoke-on-Trent	0·516	0·519	0·52	0·522	0·523	0·523	0·523	0·524	0·524	0·524	0·523	0·523	0·523	0·526	0·53	0·535	0·542	0·55	0·558	0·565
Telford and Wrekin	0·548	0·551	0·553	0·554	0·555	0·555	0·556	0·556	0·556	0·555	0·554	0·553	0·553	0·555	0·559	0·565	0·572	0·58	0·588	0·596
Walsall	0·517	0·52	0·522	0·523	0·524	0·525	0·525	0·526	0·526	0·525	0·524	0·524	0·524	0·526	0·53	0·535	0·542	0·55	0·557	0·565
Warwickshire	0·587	0·59	0·592	0·594	0·596	0·597	0·598	0·599	0·6	0·6	0·6	0·6	0·602	0·604	0·608	0·614	0·621	0·628	0·636	0·643
Wolverhampton	0·529	0·532	0·534	0·535	0·536	0·536	0·537	0·537	0·537	0·537	0·536	0·535	0·536	0·538	0·541	0·547	0·554	0·562	0·569	0·577
Worcestershire	0·563	0·566	0·568	0·57	0·571	0·572	0·573	0·574	0·575	0·575	0·575	0·575	0·576	0·579	0·583	0·588	0·595	0·603	0·61	0·618
Yorkshire and the Humber	0·545	0·548	0·55	0·552	0·553	0·554	0·555	0·557	0·557	0·558	0·558	0·559	0·56	0·563	0·568	0·573	0·581	0·588	0·596	0·603
Barnsley	0·511	0·513	0·515	0·517	0·518	0·518	0·519	0·52	0·52	0·52	0·519	0·519	0·52	0·522	0·526	0·532	0·539	0·546	0·554	0·562
Bradford	0·521	0·524	0·525	0·527	0·527	0·528	0·528	0·529	0·528	0·527	0·526	0·526	0·526	0·528	0·532	0·538	0·546	0·554	0·562	0·571
Calderdale	0·545	0·548	0·549	0·551	0·552	0·552	0·553	0·553	0·553	0·553	0·552	0·552	0·552	0·555	0·559	0·565	0·572	0·58	0·589	0·597
Doncaster	0·512	0·515	0·517	0·518	0·519	0·519	0·52	0·52	0·52	0·52	0·519	0·518	0·518	0·52	0·524	0·53	0·537	0·545	0·553	0·561

Appendix Table 14. Socio-demographic Index values for all estimated GBD 2019 locations, 1950–1969

Location	1950	1951	1952	1953	1954	1955	1956	1957	1958	1959	1960	1961	1962	1963	1964	1965	1966	1967	1968	1969
East Riding of Yorkshire	0·564	0·567	0·569	0·571	0·572	0·574	0·575	0·576	0·577	0·577	0·577	0·578	0·579	0·582	0·586	0·592	0·599	0·607	0·614	0·622
Kingston upon Hull, City of	0·518	0·521	0·523	0·524	0·525	0·526	0·527	0·528	0·528	0·528	0·527	0·527	0·528	0·531	0·535	0·541	0·549	0·557	0·564	0·573
Kirklees	0·538	0·541	0·543	0·545	0·546	0·547	0·548	0·549	0·549	0·549	0·549	0·549	0·55	0·552	0·557	0·563	0·57	0·578	0·586	0·594
Leeds	0·588	0·591	0·593	0·596	0·597	0·599	0·601	0·602	0·604	0·605	0·606	0·607	0·609	0·612	0·617	0·623	0·63	0·637	0·644	0·651
North East Lincolnshire	0·514	0·517	0·518	0·52	0·52	0·52	0·521	0·521	0·521	0·52	0·518	0·517	0·517	0·519	0·523	0·529	0·536	0·545	0·553	0·562
North Lincolnshire	0·557	0·56	0·561	0·563	0·564	0·564	0·565	0·565	0·565	0·564	0·563	0·563	0·563	0·565	0·569	0·575	0·582	0·59	0·598	0·607
North Yorkshire	0·577	0·58	0·582	0·584	0·585	0·586	0·587	0·588	0·589	0·589	0·589	0·59	0·591	0·594	0·598	0·604	0·611	0·619	0·626	0·634
Rotherham	0·52	0·523	0·525	0·526	0·527	0·528	0·529	0·529	0·529	0·529	0·528	0·528	0·529	0·531	0·535	0·54	0·548	0·556	0·563	0·571
Sheffield	0·579	0·582	0·585	0·587	0·589	0·591	0·592	0·594	0·596	0·597	0·598	0·6	0·602	0·606	0·61	0·616	0·622	0·629	0·636	0·642
Wakefield	0·526	0·529	0·531	0·532	0·533	0·534	0·534	0·535	0·535	0·535	0·534	0·534	0·535	0·537	0·541	0·547	0·554	0·562	0·57	0·578
York	0·626	0·629	0·632	0·634	0·636	0·638	0·64	0·642	0·644	0·646	0·647	0·649	0·652	0·655	0·66	0·665	0·671	0·678	0·684	0·691
Northern Ireland	0·521	0·522	0·521	0·52	0·518	0·515	0·511	0·506	0·501	0·494	0·485	0·476	0·468	0·463	0·464	0·47	0·479	0·489	0·499	0·512
Scotland	0·6	0·603	0·605	0·607	0·608	0·606	0·597	0·584	0·573	0·566	0·563	0·56	0·559	0·561	0·563	0·567	0·569	0·572	0·582	0·591
Wales	0·522	0·525	0·527	0·529	0·53	0·532	0·533	0·534	0·535	0·535	0·536	0·536	0·538	0·54	0·545	0·551	0·558	0·566	0·573	0·581
Latin America and Caribbean	0·278	0·281	0·284	0·287	0·29	0·293	0·296	0·3	0·303	0·306	0·31	0·314	0·318	0·323	0·328	0·334	0·34	0·346	0·352	0·357
Andean Latin America	0·274	0·277	0·28	0·282	0·285	0·288	0·291	0·293	0·295	0·298	0·301	0·305	0·309	0·313	0·318	0·323	0·328	0·334	0·34	0·346
Bolivia	0·234	0·239	0·244	0·248	0·252	0·257	0·261	0·264	0·266	0·269	0·271	0·274	0·278	0·282	0·288	0·293	0·3	0·307	0·313	0·32
Ecuador	0·291	0·292	0·294	0·295	0·295	0·296	0·296	0·296	0·297	0·298	0·3	0·302	0·305	0·309	0·313	0·318	0·324	0·33	0·337	0·344
Peru	0·279	0·283	0·286	0·289	0·292	0·295	0·298	0·301	0·304	0·307	0·31	0·314	0·318	0·323	0·327	0·332	0·337	0·342	0·347	0·352
Caribbean	0·332	0·335	0·337	0·339	0·341	0·342	0·345	0·347	0·348	0·347	0·347	0·346	0·347	0·35	0·355	0·363	0·375	0·387	0·391	0·395
Antigua and Barbuda	0·318	0·322	0·324	0·327	0·329	0·332	0·336	0·34	0·344	0·35	0·356	0·362	0·369	0·376	0·384	0·391	0·4	0·409	0·418	0·428
The Bahamas	0·472	0·474	0·476	0·478	0·484	0·492	0·5	0·508	0·513	0·516	0·516	0·514	0·51	0·508	0·512	0·521	0·534	0·547	0·557	0·566
Barbados	0·408	0·407	0·403	0·4	0·399	0·403	0·408	0·415	0·421	0·428	0·436	0·446	0·455	0·463	0·469	0·476	0·484	0·494	0·503	0·512
Belize	0·255	0·257	0·258	0·259	0·26	0·261	0·261	0·262	0·262	0·263	0·263	0·264	0·265	0·267	0·269	0·273	0·277	0·281	0·286	0·291
Bermuda	0·414	0·419	0·423	0·427	0·43	0·433	0·436	0·439	0·442	0·445	0·449	0·456	0·464	0·474	0·485	0·496	0·508	0·519	0·529	0·537
Cuba	0·397	0·399	0·4	0·401	0·402	0·405	0·406	0·406	0·402	0·395	0·386	0·377	0·37	0·368	0·372	0·383	0·403	0·421	0·421	0·421
Dominica	0·347	0·351	0·354	0·354	0·354	0·353	0·353	0·354	0·354	0·355	0·355	0·355	0·357	0·359	0·361	0·363	0·366	0·371	0·377	0·385
Dominican Republic	0·198	0·202	0·206	0·208	0·211	0·214	0·218	0·223	0·227	0·231	0·236	0·24	0·245	0·25	0·256	0·261	0·267	0·273	0·28	0·287
Grenada	0·194	0·196	0·198	0·201	0·203	0·206	0·209	0·214	0·219	0·226	0·233	0·242	0·25	0·259	0·268	0·277	0·286	0·295	0·303	0·312
Guyana	0·246	0·248	0·25	0·252	0·255	0·257	0·261	0·264	0·268	0·273	0·279	0·285	0·292	0·299	0·307	0·315	0·324	0·333	0·342	0·35
Haiti	0·198	0·2	0·203	0·205	0·208	0·21	0·213	0·215	0·218	0·221	0·224	0·226	0·229	0·232	0·234	0·237	0·24	0·243	0·246	0·248
Jamaica	0·36	0·363	0·365	0·367	0·368	0·368	0·368	0·368	0·367	0·365	0·364	0·363	0·362	0·361	0·361	0·362	0·365	0·369	0·374	0·381
Puerto Rico	0·394	0·403	0·414	0·421	0·419	0·416	0·421	0·43	0·437	0·445	0·453	0·461	0·467	0·473	0·481	0·496	0·517	0·535	0·548	0·556
Saint Kitts and Nevis	0·283	0·287	0·292	0·297	0·302	0·307	0·312	0·318	0·323	0·329	0·336	0·343	0·35	0·357	0·364	0·371	0·379	0·387	0·395	0·403
Saint Lucia	0·268	0·269	0·27	0·271	0·271	0·271	0·271	0·271	0·272	0·272	0·273	0·274	0·275	0·276	0·277	0·278	0·28	0·283	0·286	0·291
Saint Vincent and the Grenadines	0·202	0·202	0·202	0·201	0·199	0·197	0·195	0·193	0·192	0·193	0·194	0·197	0·2	0·205	0·21	0·216	0·222	0·229	0·237	0·246
Suriname	0·29	0·29	0·29	0·29	0·291	0·291	0·292	0·292	0·291	0·292	0·293	0·296	0·301	0·309	0·318	0·326	0·335	0·343	0·351	0·361
Trinidad and Tobago	0·369	0·373	0·375	0·376	0·377	0·379	0·382	0·385	0·389	0·394	0·4	0·408	0·416	0·426	0·437	0·448	0·46	0·472	0·484	0·496
Virgin Islands	0·408	0·41	0·412	0·413	0·413	0·414	0·414	0·414	0·415	0·417	0·419	0·422	0·426	0·43	0·436	0·443	0·452	0·462	0·473	0·485
Central Latin America	0·275	0·278	0·281	0·283	0·286	0·289	0·293	0·295	0·298	0·301	0·304	0·308	0·313	0·318	0·323	0·329	0·335	0·34	0·346	0·352
Colombia	0·266	0·269	0·272	0·275	0·278	0·281	0·284	0·286	0·288	0·291	0·294	0·298	0·303	0·308	0·314	0·32	0·327	0·334	0·341	0·348
Costa Rica	0·31	0·312	0·315	0·317	0·316	0·316	0·315	0·314	0·311	0·309	0·313	0·319	0·329	0·34	0·353	0·366	0·38	0·393	0·406	0·418
El Salvador	0·206	0·208	0·21	0·212	0·214	0·217	0·219	0·222	0·225	0·228	0·231	0·234	0·238	0·242	0·247	0·252	0·258	0·263	0·269	0·274
Guatemala	0·205	0·209	0·212	0·216	0·22	0·223	0·226	0·228	0·229	0·23	0·231	0·234	0·236	0·24	0·244	0·249	0·255	0·26	0·264	0·267
Honduras	0·181	0·184	0·186	0·189	0·191	0·193	0·196	0·199	0·202	0·204	0·207	0·21	0·212	0·215	0·218	0·222	0·225	0·229	0·232	0·236
Mexico	0·285	0·289	0·292	0·295	0·298	0·301	0·305	0·308	0·312	0·316	0·32	0·324	0·328	0·332	0·336	0·341	0·345	0·35	0·354	0·359
Aguascalientes	0·298	0·302	0·305	0·308	0·311	0·315	0·318	0·322	0·326	0·33	0·334	0·338	0·342	0·347	0·351	0·356	0·361	0·366	0·371	0·376
Baja California	0·314	0·318	0·322	0·325	0·328	0·331	0·335	0·338	0·342	0·346	0·35	0·354	0·359	0·363	0·368	0·373	0·378	0·384	0·389	0·395
Baja California Sur	0·278	0·281	0·285	0·288	0·291	0·294	0·297	0·3	0·304	0·308	0·312	0·316	0·32	0·324	0·329	0·334	0·338	0·343	0·348	0·353
Campeche	0·275	0·278	0·28	0·282	0·285	0·287	0·29	0·293	0·296	0·298	0·301	0·304	0·307	0·31	0·313	0·317	0·32	0·324	0·327	0·331
Chiapas	0·242	0·245	0·247	0·249	0·251	0·254	0·256	0·259	0·262	0·265	0·269	0·272	0·275	0·279	0·282	0·286	0·29	0·294	0·298	0·301
Chihuahua	0·29	0·294	0·297	0·3	0·303	0·306	0·309	0·312	0·316	0·319	0·322	0·326	0·33	0·333	0·338	0·342	0·346	0·35	0·355	0·359
Coahuila	0·303	0·307	0·31	0·313	0·316	0·319	0·322	0·325	0·329	0·332	0·336	0·34	0·343	0·347	0·351	0·355	0·359	0·363	0·367	0·371
Colima	0·285	0·288	0·292	0·295	0·298	0·302	0·305	0·309	0·313	0·317	0·321	0·325	0·329	0·334	0·338	0·343	0·348	0·352	0·357	0·362
Durango	0·232	0·235	0·237	0·239	0·241	0·243	0·245	0·248	0·25	0·253	0·256	0·259	0·262	0·266	0·269	0·273	0·277	0·281	0·285	0·289
Guanajuato	0·252	0·255	0·258	0·26	0·263	0·266	0·269	0·273	0·276	0·279	0·283	0·287	0·29	0·294	0·299	0·303	0·307	0·311	0·315	0·319
Guerrero	0·232	0·234	0·236	0·238	0·24	0·242	0·245	0·248	0·25	0·253	0·256	0·26	0·263	0·266	0·27	0·273	0·277	0·281	0·284	0·288
Hidalgo	0·213	0·216	0·219	0·22	0·222	0·225	0·227	0·23	0·233	0·236	0·239	0·243	0·246	0·25	0·254	0·257	0·261	0·265	0·269	0·273
Jalisco	0·297	0·301	0·305	0·308	0·311	0·315	0·318	0·322	0·326	0·33	0·335	0·339	0·343	0·347	0·352	0·357	0·361	0·366	0·371	0·375
México	0·282	0·285	0·288	0·291	0·294	0·297	0·3	0·304	0·308	0·312	0·316	0·32	0·325	0·329	0·334	0·339	0·344	0·349	0·354	0·36
Mexico City	0·357	0·361	0·365	0·368	0·371	0·374	0·378	0·382	0·386	0·389	0·393	0·398	0·402	0·406	0·411	0·416	0·421	0·426	0·431	0·436
Michoacán de Ocampo	0·258	0·261	0·264	0·266	0·269	0·272	0·275	0·279	0·282	0·285	0·289	0·292	0·296	0·3	0·304	0·308	0·312	0·315	0·319	0·323
Morelos	0·286	0·29	0·293	0·296	0·299	0·302	0·306	0·309	0·313	0·317	0·321	0·325	0·329	0·333	0·337	0·341	0·346	0·35	0·355	0·359
Nayarit	0·261	0·264	0·266	0·269	0·271	0·274	0·277	0·28	0·284	0·287	0·29	0·294	0·298	0·302	0·306	0·31	0·315	0·319	0·324	0·329
Nuevo León	0·311	0·315	0·319	0·322	0·325	0·329	0·333	0·337	0·341	0·345	0·349	0·353	0·358	0·362	0·367	0·373	0·378	0·383	0·389	0·394

Appendix Table 14. Socio-demographic Index values for all estimated GBD 2019 locations, 1950–1969

Location	1950	1951	1952	1953	1954	1955	1956	1957	1958	1959	1960	1961	1962	1963	1964	1965	1966	1967	1968	1969
Oaxaca	0·242	0·244	0·247	0·248	0·25	0·253	0·255	0·258	0·261	0·263	0·266	0·269	0·273	0·276	0·28	0·283	0·287	0·291	0·294	0·298
Puebla	0·265	0·268	0·271	0·273	0·276	0·279	0·282	0·285	0·288	0·291	0·295	0·298	0·302	0·305	0·31	0·314	0·318	0·322	0·326	0·33
Querétaro	0·269	0·272	0·275	0·278	0·281	0·285	0·288	0·292	0·296	0·3	0·304	0·309	0·313	0·318	0·323	0·328	0·333	0·338	0·344	0·349
Quintana Roo	0·26	0·263	0·266	0·268	0·27	0·273	0·275	0·278	0·281	0·284	0·287	0·29	0·293	0·296	0·3	0·304	0·307	0·311	0·315	0·319
San Luis Potosí	0·258	0·261	0·264	0·266	0·269	0·272	0·274	0·278	0·281	0·284	0·288	0·291	0·295	0·299	0·303	0·307	0·311	0·315	0·319	0·323
Sinaloa	0·28	0·284	0·287	0·289	0·292	0·295	0·299	0·302	0·305	0·309	0·312	0·316	0·32	0·323	0·327	0·331	0·335	0·339	0·344	0·348
Sonora	0·3	0·304	0·307	0·31	0·313	0·317	0·32	0·324	0·328	0·332	0·336	0·341	0·345	0·35	0·355	0·36	0·365	0·37	0·375	0·38
Tabasco	0·262	0·265	0·268	0·27	0·273	0·276	0·278	0·281	0·284	0·287	0·291	0·294	0·297	0·3	0·304	0·308	0·312	0·316	0·32	0·324
Tamaulipas	0·302	0·306	0·309	0·312	0·315	0·319	0·322	0·326	0·33	0·334	0·338	0·342	0·346	0·351	0·356	0·361	0·365	0·37	0·375	0·38
Tlaxcala	0·249	0·252	0·255	0·257	0·26	0·263	0·266	0·27	0·273	0·276	0·28	0·284	0·288	0·292	0·296	0·3	0·304	0·309	0·313	0·318
Veracruz de Ignacio de la Llave	0·272	0·275	0·278	0·28	0·283	0·286	0·289	0·292	0·295	0·298	0·302	0·305	0·308	0·312	0·316	0·319	0·323	0·327	0·331	0·334
Yucatán	0·289	0·293	0·296	0·298	0·301	0·304	0·307	0·31	0·313	0·316	0·319	0·321	0·324	0·327	0·33	0·333	0·336	0·339	0·343	0·346
Zacatecas	0·272	0·276	0·278	0·281	0·284	0·287	0·29	0·293	0·296	0·299	0·303	0·306	0·31	0·313	0·317	0·321	0·325	0·329	0·334	0·338
Nicaragua	0·119	0·122	0·126	0·129	0·133	0·136	0·14	0·144	0·148	0·152	0·156	0·161	0·166	0·171	0·177	0·183	0·188	0·194	0·2	0·205
Panama	0·337	0·331	0·325	0·321	0·32	0·323	0·326	0·329	0·332	0·334	0·337	0·341	0·345	0·35	0·355	0·359	0·363	0·368	0·373	0·381
Venezuela	0·319	0·32	0·321	0·321	0·323	0·326	0·328	0·328	0·325	0·323	0·324	0·329	0·337	0·345	0·354	0·363	0·372	0·379	0·387	0·397
Tropical Latin America	0·258	0·261	0·265	0·268	0·272	0·276	0·28	0·284	0·288	0·293	0·298	0·304	0·309	0·315	0·32	0·325	0·33	0·336	0·341	0·347
Brazil	0·257	0·26	0·264	0·267	0·271	0·275	0·279	0·283	0·288	0·292	0·298	0·303	0·309	0·314	0·319	0·325	0·33	0·335	0·341	0·347
Acre	0·172	0·174	0·176	0·178	0·18	0·182	0·185	0·187	0·19	0·194	0·198	0·203	0·208	0·212	0·217	0·221	0·225	0·229	0·233	0·238
Alagoas	0·154	0·155	0·157	0·159	0·161	0·163	0·165	0·167	0·17	0·174	0·178	0·182	0·187	0·191	0·195	0·199	0·203	0·207	0·211	0·215
Amapá	0·247	0·25	0·253	0·257	0·26	0·264	0·268	0·272	0·276	0·281	0·287	0·293	0·299	0·304	0·31	0·316	0·321	0·326	0·332	0·338
Amazonas	0·193	0·195	0·198	0·2	0·203	0·205	0·208	0·211	0·215	0·219	0·224	0·229	0·235	0·239	0·244	0·249	0·253	0·258	0·262	0·267
Bahia	0·179	0·181	0·183	0·185	0·187	0·19	0·192	0·195	0·198	0·202	0·206	0·21	0·214	0·218	0·222	0·226	0·23	0·234	0·239	0·243
Ceará	0·186	0·188	0·19	0·193	0·195	0·198	0·201	0·204	0·208	0·212	0·216	0·221	0·226	0·231	0·236	0·241	0·245	0·249	0·253	0·256
Distrito Federal	0·369	0·373	0·378	0·383	0·388	0·393	0·398	0·403	0·409	0·414	0·42	0·426	0·432	0·438	0·444	0·449	0·454	0·459	0·464	0·469
Espírito Santo	0·231	0·233	0·236	0·239	0·242	0·246	0·249	0·253	0·257	0·262	0·267	0·273	0·279	0·285	0·291	0·297	0·303	0·31	0·316	0·323
Goiás	0·185	0·187	0·19	0·193	0·196	0·199	0·202	0·206	0·21	0·215	0·22	0·225	0·232	0·237	0·243	0·249	0·254	0·259	0·266	0·275
Maranhão	0·118	0·119	0·12	0·122	0·123	0·125	0·126	0·128	0·131	0·133	0·137	0·14	0·144	0·148	0·152	0·155	0·158	0·161	0·164	0·167
Mato Grosso	0·243	0·245	0·248	0·251	0·254	0·257	0·261	0·264	0·268	0·273	0·278	0·284	0·289	0·295	0·3	0·305	0·31	0·315	0·319	0·324
Mato Grosso do Sul	0·229	0·232	0·235	0·238	0·241	0·245	0·249	0·252	0·257	0·262	0·267	0·273	0·28	0·286	0·292	0·297	0·302	0·308	0·314	0·32
Minas Gerais	0·253	0·256	0·259	0·263	0·266	0·27	0·274	0·278	0·282	0·287	0·292	0·297	0·302	0·307	0·312	0·317	0·321	0·326	0·331	0·337
Pará	0·21	0·212	0·215	0·217	0·22	0·223	0·227	0·23	0·234	0·238	0·243	0·248	0·254	0·258	0·263	0·268	0·272	0·276	0·28	0·284
Paraíba	0·181	0·183	0·185	0·187	0·19	0·192	0·195	0·198	0·201	0·205	0·21	0·214	0·219	0·223	0·228	0·232	0·235	0·238	0·241	0·244
Paraná	0·263	0·266	0·27	0·273	0·277	0·281	0·284	0·288	0·293	0·297	0·302	0·306	0·311	0·315	0·319	0·323	0·326	0·33	0·334	0·338
Pernambuco	0·191	0·193	0·195	0·198	0·2	0·203	0·206	0·209	0·212	0·216	0·22	0·225	0·23	0·235	0·24	0·245	0·25	0·254	0·259	0·263
Piauí	0·145	0·147	0·149	0·15	0·153	0·155	0·158	0·16	0·164	0·167	0·171	0·176	0·181	0·186	0·19	0·194	0·198	0·202	0·206	0·209
Rio de Janeiro	0·341	0·345	0·35	0·355	0·359	0·365	0·37	0·375	0·38	0·386	0·392	0·399	0·405	0·411	0·417	0·422	0·428	0·434	0·44	0·447
Rio Grande do Norte	0·187	0·189	0·191	0·194	0·196	0·199	0·202	0·205	0·208	0·212	0·216	0·221	0·226	0·231	0·235	0·239	0·244	0·247	0·251	0·254
Rio Grande do Sul	0·314	0·318	0·322	0·326	0·33	0·335	0·34	0·344	0·349	0·354	0·36	0·366	0·372	0·378	0·384	0·39	0·396	0·403	0·409	0·416
Rondônia	0·214	0·216	0·219	0·221	0·224	0·227	0·23	0·233	0·236	0·241	0·245	0·25	0·255	0·26	0·266	0·271	0·276	0·281	0·286	0·292
Roraima	0·207	0·209	0·211	0·214	0·217	0·22	0·223	0·226	0·23	0·234	0·24	0·245	0·251	0·257	0·263	0·268	0·274	0·279	0·284	0·29
Santa Catarina	0·278	0·282	0·285	0·289	0·292	0·297	0·301	0·305	0·31	0·315	0·321	0·327	0·334	0·339	0·345	0·35	0·355	0·361	0·366	0·373
São Paulo	0·321	0·325	0·329	0·334	0·338	0·343	0·347	0·352	0·357	0·363	0·369	0·375	0·381	0·387	0·393	0·399	0·405	0·411	0·417	0·424
Sergipe	0·186	0·188	0·19	0·192	0·195	0·198	0·2	0·203	0·207	0·21	0·215	0·219	0·225	0·229	0·234	0·238	0·242	0·247	0·251	0·255
Tocantins	0·154	0·156	0·158	0·159	0·162	0·164	0·166	0·168	0·171	0·175	0·178	0·183	0·188	0·192	0·197	0·201	0·204	0·208	0·213	0·219
Paraguay	0·277	0·281	0·283	0·286	0·289	0·293	0·296	0·3	0·304	0·307	0·311	0·315	0·319	0·324	0·328	0·333	0·337	0·342	0·347	0·352
North Africa and Middle East	0·157	0·16	0·164	0·168	0·172	0·176	0·18	0·183	0·187	0·191	0·196	0·2	0·205	0·21	0·215	0·221	0·227	0·233	0·239	0·246
North Africa and Middle East	0·157	0·16	0·164	0·168	0·172	0·176	0·18	0·183	0·187	0·191	0·196	0·2	0·205	0·21	0·215	0·221	0·227	0·233	0·239	0·246
Afghanistan	0·0947	0·0966	0·0986	0·101	0·103	0·105	0·107	0·109	0·112	0·114	0·117	0·119	0·121	0·124	0·126	0·128	0·131	0·134	0·136	0·139
Algeria	0·162	0·165	0·168	0·172	0·175	0·178	0·182	0·185	0·189	0·192	0·196	0·199	0·202	0·206	0·21	0·215	0·219	0·224	0·229	0·235
Bahrain	0·22	0·225	0·229	0·235	0·24	0·246	0·251	0·258	0·264	0·271	0·279	0·286	0·294	0·303	0·311	0·32	0·328	0·337	0·346	0·355
Egypt	0·164	0·166	0·168	0·171	0·175	0·178	0·182	0·185	0·189	0·193	0·197	0·202	0·206	0·211	0·216	0·221	0·226	0·232	0·237	0·243
Iran	0·153	0·156	0·159	0·163	0·167	0·171	0·175	0·178	0·181	0·185	0·19	0·196	0·202	0·208	0·213	0·22	0·227	0·233	0·24	0·246
Iraq	0·138	0·141	0·144	0·149	0·154	0·158	0·162	0·166	0·171	0·175	0·18	0·186	0·191	0·196	0·201	0·207	0·213	0·219	0·225	0·231
Jordan	0·0486	0·0498	0·051	0·0522	0·0534	0·0546	0·0561	0·0576	0·0591	0·0607	0·0622	0·064	0·0657	0·0674	0·0692	0·0858	0·13	0·16	0·183	0·203
Kuwait	0·274	0·278	0·282	0·285	0·289	0·292	0·298	0·304	0·306	0·301	0·297	0·295	0·294	0·294	0·299	0·306	0·313	0·315	0·326	0·347
Lebanon	0·193	0·201	0·208	0·215	0·222	0·229	0·235	0·242	0·247	0·253	0·258	0·264	0·27	0·276	0·281	0·288	0·294	0·3	0·306	0·313
Libya	0·118	0·12	0·122	0·123	0·125	0·127	0·13	0·132	0·135	0·137	0·141	0·144	0·148	0·152	0·158	0·164	0·169	0·175	0·181	0·187
Morocco	0·103	0·105	0·108	0·111	0·114	0·117	0·12	0·123	0·126	0·129	0·133	0·136	0·141	0·145	0·15	0·155	0·16	0·166	0·172	0·178
Oman	0·114	0·118	0·121	0·124	0·128	0·132	0·136	0·14	0·144	0·148	0·153	0·158	0·163	0·168	0·173	0·178	0·184	0·193	0·204	0·216
Palestine	0·0899	0·0927	0·0956	0·0986	0·102	0·105	0·109	0·112	0·116	0·12	0·124	0·129	0·133	0·138	0·143	0·148	0·154	0·159	0·164	0·169
Qatar	0·221	0·227	0·233	0·24	0·247	0·254	0·261	0·269	0·277	0·285	0·294	0·302	0·311	0·32	0·329	0·338	0·347	0·356	0·366	0·375
Saudi Arabia	0·158	0·162	0·167	0·171	0·176	0·181	0·186	0·191	0·196	0·202	0·208	0·214	0·221	0·227	0·234	0·242	0·249	0·257	0·265	0·274
Sudan	0·0907	0·0932	0·0958	0·0984	0·101	0·104	0·106	0·109	0·111	0·114	0·116	0·119	0·121	0·123	0·125	0·127	0·129	0·13	0·132	0·134
Syria	0·123	0·125	0·129	0·133	0·137	0·14	0·144	0·148	0·151	0·154	0·157	0·161	0·165	0·169	0·173	0·178	0·182	0·186	0·19	0·196

Appendix Table 14. Socio-demographic Index values for all estimated GBD 2019 locations, 1950–1969

Location	1950	1951	1952	1953	1954	1955	1956	1957	1958	1959	1960	1961	1962	1963	1964	1965	1966	1967	1968	1969
Tunisia	0·141	0·144	0·148	0·152	0·156	0·16	0·164	0·167	0·172	0·176	0·181	0·186	0·191	0·196	0·202	0·207	0·213	0·219	0·225	0·231
Turkey	0·177	0·182	0·187	0·191	0·195	0·199	0·202	0·206	0·211	0·215	0·22	0·225	0·231	0·237	0·244	0·25	0·258	0·265	0·273	0·281
United Arab Emirates	0·209	0·215	0·22	0·226	0·231	0·237	0·243	0·25	0·256	0·263	0·27	0·277	0·285	0·293	0·301	0·31	0·318	0·327	0·337	0·348
Yemen	0·0645	0·0657	0·067	0·0684	0·0698	0·0711	0·0725	0·074	0·0753	0·0767	0·0782	0·0796	0·081	0·0824	0·0837	0·0851	0·0865	0·0879	0·0894	0·0909
South Asia	0·169	0·172	0·174	0·177	0·18	0·183	0·186	0·188	0·191	0·194	0·198	0·201	0·204	0·208	0·211	0·214	0·218	0·221	0·225	0·229
South Asia	0·169	0·172	0·174	0·177	0·18	0·183	0·186	0·188	0·191	0·194	0·198	0·201	0·204	0·208	0·211	0·214	0·218	0·221	0·225	0·229
Bangladesh	0·045	0·0454	0·046	0·0464	0·0469	0·0472	0·0477	0·0481	0·0485	0·0489	0·0494	0·0499	0·0504	0·0519	0·0695	0·0834	0·0959	0·107	0·119	0·129
Bhutan	0·0673	0·0697	0·072	0·0745	0·0771	0·0798	0·0826	0·0854	0·0883	0·0914	0·0947	0·0981	0·101	0·105	0·109	0·112	0·116	0·12	0·124	0·128
India	0·179	0·182	0·184	0·187	0·19	0·193	0·196	0·199	0·203	0·206	0·21	0·213	0·217	0·221	0·225	0·228	0·232	0·236	0·239	0·244
Andhra Pradesh	0·139	0·141	0·143	0·145	0·147	0·15	0·152	0·154	0·157	0·159	0·162	0·165	0·168	0·171	0·175	0·178	0·18	0·184	0·187	0·191
Arunachal Pradesh	0·132	0·134	0·136	0·139	0·141	0·143	0·146	0·148	0·151	0·154	0·157	0·161	0·164	0·168	0·172	0·175	0·179	0·183	0·188	0·192
Assam	0·174	0·176	0·179	0·183	0·186	0·189	0·193	0·196	0·199	0·203	0·207	0·211	0·215	0·219	0·223	0·227	0·231	0·235	0·239	0·243
Bihar	0·151	0·153	0·154	0·157	0·159	0·161	0·163	0·165	0·167	0·169	0·172	0·175	0·177	0·18	0·183	0·185	0·188	0·19	0·193	0·196
Chhattisgarh	0·135	0·137	0·14	0·142	0·145	0·148	0·151	0·154	0·157	0·16	0·163	0·167	0·17	0·174	0·178	0·181	0·184	0·188	0·192	0·196
Delhi	0·33	0·334	0·339	0·344	0·349	0·355	0·36	0·365	0·371	0·376	0·382	0·388	0·393	0·399	0·405	0·41	0·415	0·42	0·426	0·431
Goa	0·262	0·266	0·271	0·276	0·281	0·286	0·291	0·296	0·301	0·307	0·312	0·318	0·324	0·33	0·336	0·341	0·347	0·353	0·359	0·365
Gujarat	0·205	0·207	0·21	0·213	0·217	0·22	0·223	0·226	0·23	0·233	0·237	0·241	0·244	0·248	0·253	0·256	0·26	0·264	0·268	0·272
Haryana	0·189	0·192	0·194	0·197	0·201	0·204	0·208	0·211	0·215	0·219	0·224	0·228	0·233	0·238	0·243	0·248	0·253	0·258	0·263	0·269
Himachal Pradesh	0·148	0·15	0·153	0·156	0·16	0·163	0·167	0·171	0·175	0·179	0·184	0·189	0·194	0·199	0·204	0·209	0·214	0·219	0·224	0·23
Jammu and Kashmir	0·187	0·19	0·192	0·195	0·198	0·201	0·204	0·207	0·211	0·214	0·218	0·222	0·226	0·23	0·235	0·239	0·243	0·247	0·252	0·257
Jharkhand	0·116	0·118	0·12	0·123	0·126	0·128	0·131	0·134	0·137	0·14	0·143	0·147	0·15	0·153	0·157	0·16	0·164	0·167	0·171	0·175
Karnataka	0·195	0·198	0·2	0·203	0·206	0·209	0·213	0·215	0·219	0·222	0·226	0·229	0·233	0·237	0·241	0·244	0·247	0·25	0·254	0·258
Kerala	0·24	0·244	0·248	0·253	0·257	0·262	0·266	0·271	0·276	0·281	0·286	0·291	0·297	0·302	0·308	0·314	0·319	0·325	0·33	0·337
Madhya Pradesh	0·147	0·15	0·152	0·154	0·157	0·16	0·162	0·165	0·168	0·171	0·174	0·178	0·181	0·185	0·189	0·192	0·196	0·2	0·204	0·209
Maharashtra	0·23	0·232	0·235	0·238	0·242	0·245	0·248	0·251	0·255	0·258	0·262	0·265	0·269	0·273	0·277	0·28	0·284	0·288	0·291	0·296
Manipur	0·163	0·166	0·17	0·173	0·177	0·181	0·185	0·189	0·193	0·198	0·203	0·208	0·213	0·218	0·224	0·229	0·235	0·241	0·246	0·253
Meghalaya	0·144	0·147	0·15	0·153	0·157	0·16	0·164	0·167	0·171	0·175	0·18	0·184	0·188	0·193	0·198	0·202	0·206	0·211	0·216	0·221
Mizoram	0·191	0·194	0·197	0·201	0·205	0·209	0·213	0·217	0·222	0·226	0·231	0·236	0·241	0·246	0·251	0·256	0·261	0·266	0·271	0·277
Nagaland	0·159	0·162	0·165	0·169	0·174	0·178	0·182	0·187	0·192	0·196	0·202	0·207	0·213	0·219	0·225	0·231	0·237	0·243	0·249	0·256
Odisha	0·126	0·128	0·13	0·133	0·136	0·139	0·142	0·144	0·148	0·151	0·154	0·158	0·162	0·166	0·17	0·174	0·178	0·182	0·186	0·191
Punjab	0·221	0·224	0·228	0·231	0·235	0·239	0·243	0·247	0·252	0·256	0·261	0·266	0·272	0·277	0·283	0·288	0·294	0·299	0·305	0·311
Rajasthan	0·15	0·152	0·154	0·156	0·158	0·16	0·163	0·165	0·167	0·169	0·172	0·175	0·177	0·18	0·183	0·185	0·188	0·191	0·193	0·196
Sikkim	0·143	0·146	0·149	0·152	0·155	0·158	0·161	0·164	0·167	0·171	0·175	0·178	0·182	0·186	0·19	0·194	0·198	0·202	0·207	0·211
Tamil Nadu	0·21	0·213	0·216	0·219	0·223	0·226	0·23	0·233	0·237	0·24	0·244	0·248	0·252	0·257	0·262	0·266	0·27	0·274	0·279	0·283
Telangana	0·149	0·151	0·154	0·156	0·159	0·161	0·164	0·166	0·169	0·172	0·175	0·179	0·182	0·185	0·189	0·192	0·195	0·199	0·203	0·207
Tripura	0·156	0·159	0·162	0·165	0·168	0·172	0·175	0·179	0·183	0·187	0·191	0·195	0·2	0·204	0·209	0·214	0·218	0·223	0·227	0·233
Union Territories other than Delhi	0·197	0·2	0·204	0·208	0·212	0·216	0·221	0·225	0·229	0·234	0·239	0·244	0·249	0·255	0·26	0·266	0·271	0·277	0·283	0·29
Uttar Pradesh	0·156	0·159	0·161	0·163	0·166	0·168	0·171	0·173	0·176	0·179	0·182	0·185	0·188	0·191	0·194	0·197	0·199	0·202	0·205	0·209
Uttarakhand	0·151	0·153	0·156	0·159	0·162	0·165	0·168	0·171	0·174	0·178	0·181	0·185	0·189	0·193	0·197	0·2	0·204	0·208	0·212	0·216
West Bengal	0·184	0·187	0·19	0·193	0·196	0·199	0·203	0·206	0·209	0·213	0·216	0·22	0·224	0·227	0·231	0·235	0·238	0·242	0·245	0·25
Nepal	0·111	0·112	0·114	0·116	0·118	0·12	0·122	0·124	0·126	0·129	0·131	0·133	0·135	0·137	0·139	0·141	0·143	0·144	0·146	0·147
Pakistan	0·126	0·127	0·128	0·129	0·131	0·132	0·134	0·135	0·137	0·139	0·14	0·142	0·145	0·147	0·15	0·152	0·155	0·158	0·161	0·164
Southeast Asia, east Asia, and Oceania	0·192	0·197	0·203	0·208	0·212	0·216	0·22	0·225	0·23	0·235	0·239	0·243	0·247	0·251	0·257	0·262	0·268	0·274	0·28	0·286
East Asia	0·17	0·179	0·186	0·193	0·197	0·202	0·207	0·213	0·218	0·223	0·228	0·23	0·233	0·236	0·242	0·248	0·254	0·26	0·266	0·273
China	0·163	0·173	0·181	0·188	0·193	0·197	0·202	0·208	0·213	0·218	0·223	0·224	0·226	0·229	0·234	0·239	0·246	0·251	0·256	0·262
North Korea	0·185	0·179	0·181	0·189	0·196	0·202	0·207	0·213	0·219	0·224	0·229	0·235	0·24	0·246	0·252	0·258	0·265	0·272	0·28	0·289
Taiwan (province of China)	0·298	0·299	0·313	0·32	0·323	0·325	0·335	0·344	0·345	0·346	0·352	0·355	0·361	0·372	0·377	0·379	0·386	0·395	0·408	0·424
Oceania	0·232	0·235	0·238	0·24	0·243	0·246	0·249	0·252	0·256	0·259	0·263	0·266	0·27	0·275	0·279	0·284	0·288	0·293	0·297	0·302
American Samoa	0·438	0·442	0·446	0·451	0·455	0·459	0·462	0·465	0·468	0·47	0·471	0·473	0·474	0·475	0·477	0·48	0·485	0·49	0·496	0·503
Cook Islands	0·401	0·407	0·412	0·418	0·424	0·43	0·436	0·442	0·448	0·454	0·46	0·466	0·471	0·477	0·483	0·49	0·496	0·503	0·51	0·516
Fiji	0·299	0·303	0·308	0·312	0·316	0·321	0·326	0·331	0·336	0·341	0·347	0·354	0·36	0·367	0·375	0·382	0·388	0·394	0·401	0·408
Guam	0·539	0·535	0·53	0·523	0·512	0·5	0·488	0·481	0·48	0·482	0·488	0·496	0·509	0·523	0·538	0·552	0·565	0·575	0·579	0·579
Kiribati	0·278	0·281	0·284	0·287	0·29	0·294	0·297	0·301	0·305	0·309	0·314	0·318	0·323	0·328	0·333	0·338	0·344	0·349	0·354	0·359
Marshall Islands	0·219	0·222	0·225	0·229	0·232	0·235	0·238	0·242	0·245	0·248	0·252	0·255	0·258	0·261	0·265	0·269	0·272	0·276	0·28	0·284
Federated States of Micronesia	0·243	0·246	0·249	0·252	0·256	0·259	0·262	0·266	0·269	0·273	0·277	0·281	0·285	0·289	0·293	0·298	0·303	0·308	0·313	0·319
Nauru	0·338	0·341	0·344	0·347	0·35	0·354	0·357	0·36	0·364	0·367	0·371	0·374	0·378	0·382	0·386	0·39	0·394	0·399	0·403	0·408
Niue	0·343	0·347	0·352	0·356	0·361	0·366	0·371	0·376	0·381	0·386	0·391	0·397	0·402	0·408	0·414	0·42	0·426	0·432	0·439	0·445
Northern Mariana Islands	0·444	0·449	0·454	0·458	0·462	0·467	0·471	0·475	0·48	0·485	0·49	0·495	0·501	0·506	0·512	0·517	0·523	0·53	0·537	0·543
Palau	0·383	0·388	0·393	0·398	0·404	0·409	0·414	0·42	0·425	0·431	0·436	0·441	0·447	0·453	0·459	0·465	0·471	0·477	0·484	0·492
Papua New Guinea	0·158	0·16	0·162	0·164	0·166	0·168	0·171	0·173	0·175	0·177	0·18	0·183	0·187	0·19	0·193	0·197	0·201	0·205	0·209	0·214
Samoa	0·317	0·321	0·324	0·327	0·33	0·334	0·337	0·341	0·344	0·348	0·353	0·357	0·362	0·367	0·372	0·378	0·383	0·389	0·396	0·402
Solomon Islands	0·155	0·157	0·159	0·16	0·162	0·165	0·167	0·169	0·171	0·174	0·176	0·179	0·181	0·184	0·187	0·189	0·193	0·195	0·198	0·202
Tokelau	0·211	0·215	0·218	0·222	0·226	0·23	0·234	0·239	0·243	0·248	0·253	0·258	0·263	0·268	0·273	0·277	0·282	0·286	0·291	0·296
Tonga	0·269	0·273	0·278	0·283	0·289	0·294	0·299	0·305	0·31	0·316	0·321	0·327	0·332	0·337	0·343	0·348	0·353	0·358	0·362	0·367
Tuvalu	0·265	0·268	0·271	0·275	0·279	0·282	0·286	0·29	0·294	0·298	0·302	0·306	0·311	0·315	0·319	0·323	0·328	0·332	0·337	0·342

Appendix Table 14. Socio-demographic Index values for all estimated GBD 2019 locations, 1950–1969

Location	1950	1951	1952	1953	1954	1955	1956	1957	1958	1959	1960	1961	1962	1963	1964	1965	1966	1967	1968	1969
Vanuatu	0·209	0·212	0·215	0·217	0·22	0·223	0·227	0·23	0·233	0·236	0·24	0·243	0·247	0·25	0·254	0·258	0·262	0·266	0·27	0·274
Southeast Asia	0·219	0·223	0·226	0·23	0·233	0·237	0·24	0·244	0·247	0·251	0·255	0·259	0·264	0·269	0·274	0·278	0·283	0·288	0·294	0·299
Cambodia	0·161	0·163	0·165	0·167	0·169	0·171	0·174	0·177	0·18	0·184	0·187	0·19	0·193	0·197	0·2	0·203	0·206	0·209	0·212	0·215
Indonesia	0·204	0·208	0·211	0·215	0·218	0·221	0·224	0·227	0·23	0·233	0·236	0·239	0·243	0·246	0·249	0·252	0·255	0·259	0·263	0·268
Aceh	0·24	0·244	0·248	0·251	0·255	0·258	0·261	0·265	0·268	0·271	0·274	0·277	0·281	0·284	0·287	0·291	0·294	0·298	0·302	0·307
Bali	0·185	0·188	0·191	0·194	0·196	0·199	0·201	0·204	0·206	0·208	0·211	0·213	0·216	0·218	0·221	0·223	0·226	0·229	0·233	0·238
Bangka-Belitung Islands	0·221	0·225	0·229	0·232	0·236	0·239	0·242	0·246	0·249	0·252	0·256	0·26	0·263	0·267	0·27	0·274	0·277	0·281	0·285	0·29
Banten	0·219	0·224	0·227	0·23	0·234	0·237	0·24	0·244	0·247	0·25	0·253	0·257	0·26	0·263	0·266	0·269	0·272	0·275	0·279	0·284
Bengkulu	0·186	0·19	0·194	0·197	0·201	0·204	0·207	0·21	0·213	0·215	0·218	0·222	0·225	0·227	0·23	0·232	0·235	0·237	0·24	0·244
Gorontalo	0·153	0·158	0·161	0·165	0·169	0·172	0·176	0·179	0·182	0·185	0·189	0·192	0·196	0·199	0·201	0·204	0·207	0·209	0·212	0·216
Jakarta	0·272	0·276	0·28	0·284	0·289	0·293	0·296	0·3	0·304	0·307	0·311	0·315	0·32	0·323	0·327	0·331	0·336	0·34	0·345	0·351
Jambi	0·184	0·188	0·191	0·194	0·198	0·201	0·203	0·207	0·209	0·212	0·214	0·218	0·221	0·223	0·226	0·228	0·231	0·233	0·237	0·241
West Java	0·203	0·207	0·21	0·213	0·216	0·219	0·222	0·225	0·228	0·23	0·233	0·236	0·239	0·242	0·245	0·248	0·251	0·254	0·258	0·262
Central Java	0·183	0·186	0·189	0·192	0·195	0·198	0·2	0·203	0·205	0·207	0·21	0·212	0·215	0·218	0·22	0·223	0·226	0·229	0·233	0·237
East Java	0·187	0·191	0·194	0·196	0·199	0·202	0·205	0·208	0·21	0·212	0·215	0·218	0·221	0·224	0·226	0·229	0·233	0·236	0·24	0·245
West Kalimantan	0·173	0·176	0·179	0·181	0·184	0·187	0·189	0·192	0·194	0·197	0·199	0·202	0·205	0·207	0·209	0·212	0·214	0·217	0·22	0·224
South Kalimantan	0·194	0·198	0·201	0·204	0·208	0·211	0·214	0·217	0·22	0·223	0·226	0·229	0·232	0·235	0·238	0·242	0·245	0·248	0·252	0·257
Central Kalimantan	0·215	0·219	0·223	0·226	0·23	0·233	0·236	0·24	0·243	0·246	0·249	0·252	0·256	0·259	0·262	0·265	0·268	0·271	0·275	0·28
East Kalimantan	0·27	0·274	0·278	0·281	0·285	0·288	0·291	0·295	0·298	0·301	0·305	0·309	0·313	0·316	0·32	0·324	0·328	0·333	0·338	0·344
North Kalimantan	0·27	0·273	0·276	0·279	0·282	0·284	0·287	0·29	0·293	0·296	0·299	0·303	0·307	0·31	0·314	0·317	0·321	0·325	0·33	0·335
Riau Islands	0·298	0·303	0·307	0·312	0·316	0·32	0·324	0·329	0·333	0·337	0·341	0·345	0·35	0·354	0·358	0·362	0·366	0·371	0·376	0·381
Lampung	0·175	0·179	0·182	0·185	0·189	0·192	0·195	0·198	0·2	0·203	0·206	0·209	0·212	0·214	0·217	0·219	0·221	0·223	0·227	0·231
Maluku	0·237	0·241	0·246	0·25	0·254	0·258	0·262	0·267	0·27	0·274	0·278	0·282	0·287	0·29	0·294	0·298	0·302	0·306	0·31	0·316
North Maluku	0·148	0·153	0·157	0·161	0·166	0·17	0·173	0·177	0·18	0·183	0·187	0·191	0·194	0·197	0·2	0·203	0·205	0·207	0·211	0·215
West Nusa Tenggara	0·136	0·14	0·143	0·147	0·15	0·153	0·156	0·159	0·162	0·164	0·167	0·17	0·173	0·175	0·178	0·18	0·182	0·184	0·187	0·19
East Nusa Tenggara	0·144	0·149	0·153	0·157	0·162	0·166	0·169	0·173	0·176	0·179	0·183	0·187	0·191	0·193	0·196	0·199	0·201	0·203	0·207	0·211
Papua	0·246	0·25	0·253	0·256	0·26	0·263	0·266	0·27	0·272	0·275	0·278	0·281	0·285	0·287	0·29	0·294	0·296	0·299	0·303	0·308
West Papua	0·227	0·231	0·234	0·237	0·24	0·243	0·246	0·249	0·252	0·255	0·258	0·261	0·264	0·267	0·27	0·273	0·276	0·279	0·283	0·288
Riau	0·266	0·27	0·274	0·277	0·281	0·284	0·288	0·292	0·295	0·298	0·302	0·305	0·309	0·313	0·316	0·32	0·324	0·328	0·333	0·338
West Sulawesi	0·163	0·167	0·17	0·173	0·176	0·179	0·182	0·185	0·187	0·19	0·193	0·196	0·199	0·201	0·204	0·206	0·209	0·211	0·214	0·218
South Sulawesi	0·18	0·184	0·187	0·19	0·194	0·197	0·2	0·204	0·206	0·209	0·212	0·216	0·219	0·222	0·225	0·228	0·231	0·234	0·238	0·242
Central Sulawesi	0·173	0·178	0·181	0·185	0·188	0·192	0·195	0·199	0·201	0·204	0·208	0·211	0·214	0·217	0·22	0·222	0·225	0·227	0·23	0·234
Southeast Sulawesi	0·155	0·159	0·162	0·164	0·167	0·17	0·173	0·175	0·177	0·18	0·182	0·185	0·187	0·19	0·192	0·194	0·196	0·199	0·202	0·206
North Sulawesi	0·212	0·216	0·22	0·224	0·228	0·232	0·235	0·239	0·242	0·246	0·249	0·253	0·257	0·26	0·263	0·266	0·269	0·272	0·276	0·281
West Sumatra	0·22	0·225	0·229	0·233	0·237	0·241	0·245	0·249	0·252	0·256	0·26	0·264	0·268	0·271	0·275	0·278	0·282	0·285	0·289	0·294
South Sumatra	0·222	0·226	0·23	0·233	0·237	0·24	0·244	0·247	0·25	0·253	0·256	0·26	0·263	0·267	0·27	0·273	0·276	0·28	0·284	0·288
North Sumatra	0·228	0·232	0·236	0·24	0·245	0·248	0·252	0·256	0·26	0·263	0·267	0·271	0·275	0·279	0·283	0·286	0·29	0·294	0·298	0·303
Yogyakarta	0·216	0·221	0·225	0·229	0·233	0·237	0·241	0·245	0·248	0·251	0·255	0·259	0·262	0·266	0·269	0·272	0·275	0·278	0·282	0·287
Laos	0·14	0·141	0·143	0·144	0·146	0·148	0·149	0·151	0·153	0·155	0·157	0·159	0·162	0·164	0·166	0·169	0·171	0·174	0·177	0·179
Malaysia	0·259	0·261	0·265	0·268	0·271	0·274	0·277	0·281	0·287	0·296	0·299	0·291	0·305	0·317	0·328	0·338	0·339	0·351	0·363	0·372
Maldives	0·162	0·165	0·167	0·17	0·173	0·175	0·177	0·179	0·181	0·183	0·184	0·186	0·187	0·188	0·189	0·19	0·191	0·193	0·194	0·196
Mauritius	0·278	0·28	0·281	0·282	0·281	0·279	0·279	0·282	0·287	0·292	0·295	0·299	0·306	0·316	0·328	0·341	0·356	0·37	0·381	0·392
Myanmar	0·0947	0·104	0·107	0·11	0·11	0·113	0·117	0·121	0·124	0·129	0·134	0·138	0·143	0·148	0·152	0·156	0·159	0·161	0·164	0·168
Philippines	0·284	0·288	0·292	0·297	0·301	0·306	0·311	0·315	0·32	0·325	0·329	0·334	0·339	0·343	0·348	0·352	0·357	0·362	0·366	0·371
Seychelles	0·346	0·35	0·352	0·354	0·355	0·355	0·356	0·356	0·356	0·356	0·357	0·356	0·357	0·358	0·36	0·362	0·365	0·368	0·372	0·376
Sri Lanka	0·304	0·309	0·312	0·315	0·319	0·324	0·328	0·332	0·336	0·34	0·345	0·35	0·355	0·36	0·365	0·371	0·376	0·381	0·386	0·392
Thailand	0·241	0·245	0·248	0·252	0·255	0·258	0·262	0·265	0·269	0·273	0·278	0·282	0·288	0·293	0·299	0·307	0·315	0·323	0·33	0·337
Timor-Leste	0·122	0·124	0·127	0·129	0·132	0·134	0·137	0·139	0·142	0·144	0·149	0·152	0·155	0·157	0·16	0·166	0·17	0·175	0·179	0·184
Vietnam	0·196	0·199	0·203	0·206	0·21	0·214	0·218	0·222	0·226	0·23	0·235	0·239	0·245	0·25	0·255	0·26	0·265	0·268	0·27	0·273
Sub-Saharan Africa	0·149	0·151	0·154	0·156	0·158	0·161	0·163	0·166	0·168	0·171	0·173	0·176	0·179	0·182	0·185	0·188	0·192	0·195	0·198	0·202
Central sub-Saharan Africa	0·128	0·131	0·133	0·136	0·138	0·141	0·144	0·146	0·149	0·151	0·154	0·156	0·158	0·161	0·164	0·166	0·169	0·172	0·174	0·178
Angola	0·111	0·113	0·114	0·115	0·117	0·118	0·12	0·121	0·123	0·124	0·126	0·128	0·13	0·131	0·134	0·136	0·139	0·141	0·144	0·147
Central African Republic	0·0876	0·089	0·0906	0·0921	0·0937	0·0954	0·097	0·0987	0·1	0·102	0·104	0·106	0·108	0·11	0·111	0·113	0·115	0·116	0·118	0·12
Congo (Brazzaville)	0·132	0·134	0·136	0·138	0·14	0·143	0·145	0·147	0·15	0·152	0·155	0·158	0·161	0·164	0·167	0·17	0·174	0·177	0·181	0·185
DR Congo	0·129	0·133	0·136	0·139	0·142	0·145	0·148	0·151	0·154	0·156	0·159	0·161	0·163	0·166	0·168	0·171	0·174	0·176	0·179	0·182
Equatorial Guinea	0·0609	0·0625	0·064	0·0655	0·0671	0·0687	0·0704	0·0722	0·074	0·076	0·0786	0·0815	0·0846	0·0885	0·0926	0·0967	0·101	0·104	0·108	0·112
Gabon	0·147	0·149	0·151	0·154	0·156	0·159	0·161	0·164	0·167	0·17	0·174	0·177	0·181	0·185	0·189	0·193	0·197	0·202	0·206	0·211
Eastern sub-Saharan Africa	0·11	0·112	0·114	0·116	0·118	0·12	0·122	0·124	0·126	0·128	0·131	0·133	0·136	0·138	0·141	0·144	0·147	0·15	0·154	0·157
Burundi	0·0811	0·0833	0·0851	0·0871	0·0893	0·0915	0·0937	0·0961	0·0985	0·101	0·104	0·105	0·107	0·109	0·111	0·113	0·115	0·118	0·121	0·123
Comoros	0·129	0·131	0·133	0·134	0·135	0·137	0·138	0·139	0·139	0·14	0·141	0·142	0·144	0·146	0·148	0·149	0·151	0·154	0·156	0·158
Djibouti	0·129	0·131	0·133	0·135	0·137	0·14	0·142	0·144	0·146	0·149	0·151	0·153	0·156	0·158	0·16	0·162	0·165	0·168	0·17	0·173
Eritrea	0·0556	0·0575	0·0595	0·0616	0·064	0·0665	0·0691	0·0718	0·0745	0·0773	0·0804	0·0836	0·0867	0·0898	0·0931	0·0965	0·1	0·104	0·107	0·111
Ethiopia	0·045	0·0481	0·05	0·0518	0·0533	0·0546	0·0562	0·0577	0·059	0·0608	0·0628	0·0647	0·0667	0·0686	0·0709	0·0731	0·0755	0·0777	0·08	0·0825
Kenya	0·112	0·116	0·118	0·12	0·123	0·126	0·129	0·132	0·135	0·138	0·142	0·145	0·149	0·153	0·157	0·161	0·166	0·171	0·176	0·182
Baringo	0·0912	0·0939	0·0948	0·0956	0·0969	0·0985	0·1	0·102	0·103	0·105	0·107	0·109	0·111	0·113	0·115	0·117	0·12	0·123	0·126	0·128

Appendix Table 14. Socio-demographic Index values for all estimated GBD 2019 locations, 1950–1969

Location	1950	1951	1952	1953	1954	1955	1956	1957	1958	1959	1960	1961	1962	1963	1964	1965	1966	1967	1968	1969
Bomet	0·0857	0·0884	0·0898	0·0912	0·0931	0·095	0·0969	0·0988	0·101	0·102	0·105	0·107	0·109	0·111	0·113	0·115	0·118	0·121	0·125	0·128
Bungoma	0·0774	0·081	0·083	0·0848	0·0873	0·0901	0·0928	0·0957	0·0986	0·102	0·105	0·108	0·112	0·116	0·12	0·124	0·13	0·135	0·14	0·146
Busia	0·0496	0·0548	0·0586	0·0622	0·0658	0·0694	0·0729	0·0764	0·0798	0·0833	0·087	0·0908	0·0948	0·0991	0·104	0·108	0·114	0·12	0·126	0·132
Elgeyo Marakwet	0·0871	0·0906	0·0918	0·093	0·0949	0·097	0·0991	0·101	0·103	0·106	0·108	0·11	0·113	0·116	0·119	0·122	0·126	0·13	0·135	0·139
Embu	0·132	0·137	0·139	0·141	0·144	0·148	0·151	0·155	0·158	0·162	0·166	0·17	0·175	0·179	0·184	0·189	0·195	0·201	0·206	0·212
Garissa	0·0815	0·0841	0·085	0·0858	0·0872	0·0889	0·0907	0·0926	0·0946	0·0966	0·0987	0·101	0·103	0·105	0·108	0·111	0·114	0·117	0·12	0·124
Homa Bay	0·0919	0·0952	0·0967	0·0981	0·1	0·102	0·104	0·106	0·109	0·111	0·113	0·115	0·117	0·12	0·122	0·125	0·128	0·131	0·135	0·138
Isiolo	0·0909	0·0941	0·0951	0·096	0·0976	0·0995	0·102	0·104	0·106	0·108	0·11	0·113	0·115	0·118	0·121	0·125	0·129	0·132	0·136	0·14
Kajiado	0·137	0·141	0·143	0·145	0·147	0·15	0·153	0·157	0·16	0·163	0·167	0·17	0·173	0·177	0·18	0·184	0·188	0·193	0·197	0·202
Kakamega	0·0723	0·076	0·0785	0·0809	0·0837	0·0867	0·0896	0·0925	0·0954	0·0985	0·102	0·105	0·109	0·113	0·117	0·122	0·127	0·132	0·138	0·144
Kericho	0·0419	0·0434	0·0439	0·0443	0·045	0·0458	0·0467	0·0475	0·0483	0·0491	0·0499	0·0507	0·0515	0·0524	0·0534	0·0544	0·0556	0·0567	0·0579	0·059
Kiambu	0·138	0·142	0·145	0·147	0·15	0·154	0·158	0·162	0·166	0·17	0·175	0·179	0·184	0·189	0·194	0·199	0·205	0·211	0·218	0·224
Kilifi	0·106	0·109	0·11	0·111	0·112	0·114	0·116	0·118	0·12	0·122	0·124	0·126	0·129	0·132	0·135	0·138	0·142	0·147	0·152	0·157
Kirinyaga	0·0992	0·103	0·104	0·106	0·108	0·111	0·113	0·116	0·119	0·122	0·125	0·128	0·131	0·135	0·14	0·144	0·149	0·156	0·162	0·169
Kisii	0·0869	0·0915	0·0943	0·0971	0·1	0·104	0·108	0·112	0·115	0·119	0·124	0·128	0·132	0·137	0·143	0·148	0·154	0·16	0·167	0·173
Kisumu	0·105	0·11	0·112	0·114	0·117	0·12	0·123	0·126	0·129	0·133	0·136	0·14	0·144	0·148	0·152	0·157	0·162	0·168	0·173	0·179
Kitui	0·0864	0·0894	0·0907	0·0919	0·0937	0·0958	0·098	0·1	0·103	0·105	0·108	0·111	0·114	0·117	0·121	0·124	0·129	0·133	0·138	0·143
Kwale	0·0987	0·102	0·103	0·103	0·105	0·107	0·108	0·11	0·112	0·114	0·116	0·118	0·121	0·124	0·127	0·13	0·135	0·14	0·145	0·15
Laikipia	0·102	0·106	0·109	0·112	0·115	0·118	0·122	0·125	0·128	0·132	0·136	0·139	0·143	0·147	0·151	0·155	0·16	0·166	0·171	0·176
Lamu	0·0958	0·0987	0·0999	0·101	0·102	0·104	0·106	0·108	0·11	0·112	0·115	0·118	0·121	0·124	0·128	0·131	0·137	0·142	0·148	0·154
Machakos	0·129	0·133	0·134	0·136	0·139	0·142	0·145	0·148	0·151	0·154	0·158	0·161	0·165	0·169	0·174	0·178	0·184	0·189	0·195	0·2
Makueni	0·096	0·0994	0·101	0·102	0·104	0·106	0·109	0·111	0·114	0·116	0·119	0·121	0·124	0·127	0·13	0·134	0·138	0·142	0·146	0·151
Mandera	0·0763	0·0791	0·0801	0·081	0·0826	0·0844	0·0863	0·0883	0·0905	0·0928	0·0952	0·0975	0·1	0·103	0·106	0·109	0·112	0·115	0·118	0·12
Marsabit	0·084	0·0866	0·0875	0·0884	0·0899	0·0915	0·0933	0·0952	0·0971	0·0991	0·101	0·103	0·105	0·107	0·11	0·112	0·115	0·118	0·12	0·123
Meru	0·109	0·113	0·115	0·117	0·119	0·122	0·125	0·128	0·131	0·134	0·138	0·141	0·145	0·149	0·154	0·158	0·163	0·169	0·175	0·181
Migori	0·0946	0·0975	0·0987	0·0999	0·102	0·104	0·106	0·108	0·11	0·112	0·114	0·116	0·119	0·121	0·124	0·126	0·13	0·133	0·137	0·14
Mombasa	0·167	0·173	0·176	0·179	0·183	0·187	0·191	0·195	0·199	0·204	0·208	0·213	0·217	0·222	0·228	0·234	0·241	0·248	0·255	0·262
Murang'a	0·112	0·115	0·117	0·119	0·121	0·124	0·126	0·129	0·132	0·135	0·138	0·141	0·145	0·148	0·152	0·155	0·16	0·165	0·17	0·175
Nairobi	0·227	0·235	0·239	0·244	0·25	0·257	0·263	0·27	0·277	0·284	0·292	0·299	0·307	0·315	0·323	0·331	0·341	0·35	0·359	0·368
Nakuru	0·125	0·13	0·133	0·135	0·138	0·141	0·145	0·149	0·152	0·156	0·16	0·163	0·167	0·171	0·175	0·179	0·184	0·189	0·194	0·2
Nandi	0·0891	0·0919	0·0933	0·0947	0·0966	0·0987	0·101	0·103	0·105	0·108	0·11	0·113	0·115	0·118	0·122	0·124	0·129	0·133	0·137	0·141
Narok	0·076	0·0774	0·0774	0·0774	0·0777	0·078	0·0783	0·0785	0·0786	0·0787	0·0789	0·0788	0·0788	0·0786	0·0784	0·0775	0·0777	0·0778	0·0779	0·0782
Nyamira	0·109	0·113	0·116	0·119	0·122	0·125	0·129	0·132	0·136	0·14	0·144	0·148	0·152	0·157	0·161	0·166	0·172	0·178	0·184	0·19
Nyandarua	0·123	0·127	0·129	0·131	0·134	0·136	0·139	0·143	0·146	0·149	0·152	0·155	0·159	0·163	0·167	0·17	0·175	0·18	0·184	0·189
Nyeri	0·105	0·11	0·112	0·115	0·118	0·121	0·125	0·128	0·132	0·136	0·14	0·145	0·149	0·154	0·16	0·165	0·171	0·178	0·184	0·191
Samburu	0·0534	0·0555	0·0562	0·0568	0·0579	0·059	0·0602	0·0613	0·0625	0·0637	0·065	0·0663	0·0678	0·0692	0·0709	0·0722	0·0744	0·0765	0·0787	0·081
Siaya	0·0827	0·0866	0·088	0·0893	0·0913	0·0935	0·0958	0·0981	0·1	0·103	0·105	0·107	0·11	0·113	0·116	0·119	0·123	0·126	0·13	0·134
Taita Taveta	0·126	0·13	0·132	0·133	0·135	0·138	0·14	0·143	0·145	0·148	0·151	0·154	0·157	0·16	0·164	0·168	0·173	0·179	0·184	0·19
Tana River	0·0677	0·0702	0·071	0·0717	0·073	0·0744	0·0759	0·0774	0·079	0·0806	0·0824	0·0842	0·0863	0·0887	0·0916	0·0941	0·098	0·102	0·106	0·111
Tharaka Nithi	0·135	0·139	0·141	0·143	0·145	0·148	0·151	0·154	0·157	0·16	0·163	0·166	0·17	0·173	0·177	0·181	0·186	0·191	0·197	0·202
Trans Nzoia	0·126	0·131	0·133	0·135	0·138	0·141	0·144	0·147	0·15	0·153	0·156	0·159	0·162	0·165	0·169	0·172	0·176	0·18	0·184	0·188
Turkana	0·0749	0·0772	0·0779	0·0784	0·0795	0·0808	0·0821	0·0834	0·0848	0·0861	0·0877	0·0892	0·0908	0·0927	0·0947	0·0967	0·0992	0·102	0·104	0·107
Uasin Gishu	0·104	0·108	0·111	0·113	0·116	0·119	0·123	0·126	0·129	0·133	0·136	0·14	0·144	0·148	0·152	0·156	0·161	0·167	0·172	0·178
Vihiga	0·0867	0·0907	0·0926	0·0944	0·097	0·0997	0·102	0·105	0·108	0·11	0·113	0·116	0·119	0·122	0·126	0·129	0·133	0·137	0·141	0·146
Wajir	0·0746	0·0775	0·0784	0·0793	0·0808	0·0825	0·0843	0·0862	0·0882	0·0902	0·0923	0·0942	0·0964	0·0987	0·101	0·104	0·106	0·108	0·11	0·112
West Pokot	0·0682	0·0704	0·0711	0·0718	0·0729	0·0741	0·0752	0·0764	0·0775	0·0786	0·0799	0·0811	0·0824	0·0838	0·0853	0·0864	0·0886	0·0906	0·0927	0·0949
Madagascar	0·144	0·146	0·148	0·15	0·152	0·153	0·155	0·156	0·158	0·16	0·161	0·162	0·163	0·164	0·165	0·166	0·167	0·168	0·17	0·173
Malawi	0·0943	0·0965	0·0988	0·101	0·104	0·106	0·109	0·112	0·115	0·118	0·12	0·123	0·126	0·128	0·13	0·133	0·137	0·141	0·144	0·148
Mozambique	0·0569	0·0583	0·0598	0·0614	0·0627	0·0646	0·0661	0·0675	0·0692	0·0712	0·0732	0·0751	0·0774	0·0793	0·0814	0·0832	0·085	0·0873	0·0901	0·0932
Rwanda	0·0965	0·0989	0·101	0·103	0·106	0·108	0·111	0·114	0·116	0·119	0·122	0·125	0·128	0·131	0·133	0·135	0·137	0·14	0·143	0·148
Somalia	0·0593	0·06	0·0605	0·0612	0·062	0·0628	0·0636	0·0643	0·0648	0·0651	0·0656	0·0661	0·0665	0·0669	0·0676	0·0684	0·0691	0·0698	0·0705	0·0714
South Sudan	0·142	0·144	0·146	0·148	0·151	0·153	0·155	0·157	0·16	0·162	0·164	0·167	0·169	0·171	0·174	0·176	0·179	0·181	0·184	0·187
Uganda	0·0882	0·0901	0·0915	0·0924	0·0935	0·0947	0·0961	0·0978	0·0994	0·101	0·103	0·105	0·106	0·109	0·111	0·113	0·116	0·118	0·121	0·124
Tanzania	0·115	0·118	0·12	0·121	0·123	0·124	0·126	0·128	0·129	0·131	0·133	0·135	0·138	0·141	0·144	0·147	0·15	0·154	0·158	0·162
Zambia	0·122	0·122	0·122	0·122	0·122	0·122	0·122	0·123	0·123	0·125	0·126	0·128	0·131	0·133	0·137	0·141	0·146	0·152	0·158	0·164
Southern sub-Saharan Africa	0·285	0·289	0·292	0·296	0·3	0·304	0·309	0·313	0·317	0·322	0·326	0·331	0·337	0·342	0·348	0·354	0·36	0·366	0·372	0·378
Botswana	0·155	0·157	0·159	0·162	0·164	0·167	0·169	0·172	0·175	0·178	0·181	0·184	0·188	0·192	0·196	0·2	0·204	0·21	0·216	0·222
eSwatini	0·148	0·15	0·153	0·155	0·158	0·161	0·163	0·166	0·169	0·172	0·176	0·181	0·187	0·193	0·199	0·206	0·213	0·219	0·224	0·23
Lesotho	0·15	0·154	0·156	0·159	0·163	0·166	0·169	0·173	0·177	0·18	0·184	0·188	0·193	0·2	0·206	0·212	0·217	0·223	0·227	0·231
Namibia	0·229	0·232	0·235	0·238	0·242	0·245	0·249	0·253	0·257	0·261	0·266	0·271	0·276	0·282	0·288	0·294	0·301	0·307	0·313	0·319
South Africa	0·31	0·315	0·318	0·323	0·328	0·332	0·337	0·342	0·347	0·352	0·357	0·363	0·368	0·374	0·38	0·387	0·393	0·4	0·407	0·414
Zimbabwe	0·174	0·177	0·18	0·183	0·185	0·188	0·192	0·195	0·198	0·202	0·205	0·209	0·213	0·216	0·219	0·222	0·225	0·229	0·232	0·236
Western sub-Saharan Africa	0·136	0·139	0·141	0·144	0·146	0·149	0·151	0·153	0·156	0·158	0·161	0·164	0·166	0·169	0·172	0·175	0·178	0·18	0·182	0·185
Benin	0·097	0·0984	0·0997	0·101	0·102	0·104	0·105	0·107	0·108	0·11	0·112	0·114	0·115	0·117	0·119	0·121	0·124	0·126	0·128	0·131
Burkina Faso	0·0524	0·0535	0·0547	0·056	0·0573	0·0586	0·06	0·0614	0·0629	0·0646	0·0664	0·0681	0·07	0·0716	0·0731	0·0746	0·0761	0·0778	0·0794	0·081

Appendix Table 14. Socio-demographic Index values for all estimated GBD 2019 locations, 1950–1969

Location	1950	1951	1952	1953	1954	1955	1956	1957	1958	1959	1960	1961	1962	1963	1964	1965	1966	1967	1968	1969
Cape Verde	0·132	0·134	0·135	0·136	0·137	0·138	0·139	0·14	0·141	0·143	0·145	0·148	0·15	0·153	0·155	0·158	0·163	0·168	0·174	0·181
Cameroon	0·143	0·146	0·148	0·151	0·154	0·158	0·161	0·164	0·167	0·17	0·174	0·177	0·18	0·183	0·186	0·189	0·192	0·194	0·197	0·199
Chad	0·061	0·0618	0·0627	0·0637	0·0649	0·0659	0·0669	0·0679	0·069	0·0701	0·0714	0·0726	0·074	0·0751	0·0761	0·077	0·0782	0·0792	0·0802	0·0812
Côte d'Ivoire	0·124	0·126	0·128	0·13	0·132	0·135	0·137	0·139	0·141	0·144	0·146	0·149	0·152	0·155	0·158	0·161	0·164	0·167	0·171	0·174
The Gambia	0·123	0·125	0·126	0·128	0·13	0·131	0·133	0·135	0·136	0·138	0·14	0·142	0·143	0·145	0·147	0·149	0·151	0·153	0·155	0·157
Ghana	0·202	0·205	0·206	0·209	0·212	0·214	0·215	0·217	0·219	0·221	0·223	0·226	0·228	0·23	0·233	0·235	0·237	0·239	0·241	0·244
Guinea	0·0631	0·065	0·0669	0·069	0·0715	0·074	0·0768	0·0798	0·0829	0·0861	0·0896	0·0931	0·0966	0·1	0·103	0·106	0·109	0·112	0·115	0·117
Guinea-Bissau	0·064	0·0664	0·069	0·0716	0·0755	0·0784	0·0819	0·0855	0·089	0·0925	0·0961	0·0998	0·103	0·107	0·111	0·115	0·119	0·123	0·127	0·131
Liberia	0·129	0·131	0·134	0·136	0·138	0·14	0·142	0·145	0·147	0·15	0·152	0·154	0·157	0·159	0·161	0·163	0·166	0·168	0·171	0·174
Mali	0·0596	0·0606	0·0616	0·0627	0·0638	0·0649	0·0661	0·0673	0·0686	0·0699	0·0713	0·0726	0·0737	0·075	0·0761	0·0773	0·0785	0·0798	0·0813	0·0827
Mauritania	0·168	0·171	0·175	0·177	0·18	0·183	0·187	0·19	0·193	0·197	0·201	0·204	0·207	0·21	0·214	0·219	0·223	0·226	0·23	0·233
Niger	0·0526	0·0523	0·053	0·0535	0·054	0·0543	0·0547	0·0554	0·0561	0·0567	0·0573	0·058	0·0584	0·0591	0·0597	0·0605	0·0608	0·0609	0·0609	0·0609
Nigeria	0·153	0·156	0·16	0·162	0·166	0·169	0·171	0·174	0·177	0·18	0·183	0·187	0·19	0·193	0·197	0·201	0·204	0·207	0·209	0·213
São Tomé and PrÍncipe	0·153	0·155	0·157	0·159	0·16	0·16	0·161	0·162	0·163	0·163	0·165	0·166	0·168	0·171	0·173	0·176	0·18	0·184	0·188	0·193
Senegal	0·102	0·104	0·105	0·107	0·109	0·111	0·113	0·115	0·117	0·119	0·12	0·122	0·124	0·125	0·127	0·128	0·13	0·132	0·134	0·136
Sierra Leone	0·109	0·111	0·113	0·115	0·117	0·12	0·122	0·124	0·126	0·129	0·131	0·134	0·136	0·138	0·141	0·144	0·147	0·149	0·152	0·154
Togo	0·109	0·111	0·113	0·115	0·117	0·119	0·121	0·123	0·126	0·128	0·131	0·134	0·136	0·139	0·142	0·147	0·151	0·155	0·159	0·164

Appendix Table 15. Socio-demographic Index values for all estimated GBD 2019 locations, 1970–1989

Location	1970	1971	1972	1973	1974	1975	1976	1977	1978	1979	1980	1981	1982	1983	1984	1985	1986	1987	1988	1989
Global	0·421	0·426	0·431	0·435	0·438	0·442	0·446	0·451	0·455	0·46	0·465	0·469	0·474	0·478	0·483	0·488	0·492	0·497	0·502	0·507
Central Europe, eastern Europe, and central Asia	0·584	0·587	0·592	0·595	0·597	0·601	0·604	0·608	0·611	0·614	0·618	0·621	0·622	0·623	0·626	0·628	0·63	0·633	0·637	0·642
Central Asia	0·473	0·478	0·481	0·486	0·49	0·494	0·499	0·504	0·509	0·513	0·517	0·52	0·524	0·527	0·53	0·533	0·536	0·54	0·544	0·548
Armenia	0·473	0·477	0·481	0·486	0·49	0·494	0·498	0·502	0·506	0·51	0·512	0·514	0·515	0·517	0·519	0·521	0·523	0·525	0·528	0·532
Azerbaijan	0·473	0·481	0·489	0·498	0·506	0·513	0·52	0·528	0·534	0·54	0·545	0·549	0·553	0·557	0·56	0·562	0·565	0·568	0·571	0·574
Georgia	0·582	0·586	0·589	0·592	0·596	0·6	0·603	0·607	0·611	0·615	0·617	0·62	0·623	0·625	0·628	0·631	0·635	0·638	0·644	0·649
Kazakhstan	0·51	0·516	0·522	0·528	0·534	0·54	0·546	0·552	0·558	0·563	0·567	0·57	0·573	0·576	0·579	0·582	0·585	0·588	0·592	0·597
Kyrgyzstan	0·462	0·463	0·464	0·466	0·468	0·471	0·474	0·477	0·481	0·484	0·488	0·491	0·495	0·499	0·504	0·508	0·513	0·517	0·522	0·527
Mongolia	0·345	0·349	0·352	0·356	0·36	0·365	0·369	0·375	0·38	0·386	0·393	0·399	0·407	0·414	0·421	0·429	0·436	0·443	0·45	0·457
Tajikistan	0·339	0·343	0·348	0·354	0·36	0·365	0·371	0·378	0·385	0·392	0·398	0·405	0·412	0·419	0·427	0·434	0·442	0·448	0·456	0·462
Turkmenistan	0·455	0·46	0·463	0·467	0·472	0·476	0·481	0·486	0·491	0·496	0·5	0·504	0·509	0·514	0·518	0·523	0·528	0·532	0·538	0·543
Uzbekistan	0·405	0·41	0·414	0·419	0·424	0·428	0·434	0·439	0·445	0·45	0·455	0·459	0·463	0·467	0·471	0·475	0·478	0·481	0·484	0·487
Central Europe	0·557	0·562	0·566	0·567	0·568	0·573	0·578	0·583	0·587	0·592	0·598	0·601	0·605	0·608	0·61	0·614	0·618	0·622	0·627	0·634
Albania	0·427	0·436	0·444	0·453	0·462	0·471	0·481	0·49	0·498	0·506	0·513	0·519	0·525	0·53	0·535	0·538	0·54	0·54	0·54	0·54
Bosnia and Herzegovina	0·419	0·426	0·432	0·439	0·446	0·452	0·458	0·464	0·471	0·478	0·485	0·492	0·498	0·503	0·509	0·514	0·519	0·523	0·527	0·531
Bulgaria	0·534	0·541	0·543	0·538	0·54	0·546	0·548	0·554	0·558	0·563	0·571	0·575	0·579	0·585	0·591	0·595	0·598	0·602	0·608	0·62
Croatia	0·576	0·578	0·584	0·591	0·599	0·607	0·613	0·618	0·623	0·628	0·634	0·638	0·642	0·645	0·649	0·655	0·66	0·664	0·669	0·674
Czech Republic	0·631	0·631	0·627	0·618	0·615	0·618	0·623	0·627	0·63	0·64	0·652	0·656	0·658	0·661	0·663	0·668	0·674	0·677	0·681	0·686
Hungary	0·577	0·58	0·584	0·578	0·569	0·573	0·58	0·588	0·594	0·603	0·612	0·62	0·629	0·634	0·635	0·637	0·643	0·648	0·653	0·657
Montenegro	0·61	0·617	0·623	0·628	0·634	0·639	0·644	0·65	0·656	0·661	0·667	0·673	0·678	0·682	0·686	0·69	0·693	0·696	0·698	0·7
North Macedonia	0·535	0·541	0·547	0·552	0·558	0·563	0·568	0·572	0·577	0·582	0·586	0·589	0·592	0·595	0·598	0·601	0·604	0·608	0·611	0·615
Poland	0·571	0·575	0·579	0·582	0·585	0·588	0·592	0·596	0·599	0·602	0·603	0·602	0·601	0·602	0·604	0·609	0·615	0·619	0·624	0·628
Romania	0·494	0·504	0·514	0·515	0·514	0·523	0·528	0·533	0·54	0·545	0·552	0·561	0·573	0·578	0·577	0·577	0·578	0·584	0·593	0·609
Serbia	0·549	0·552	0·556	0·56	0·563	0·566	0·57	0·575	0·579	0·581	0·589	0·593	0·593	0·595	0·598	0·604	0·608	0·609	0·615	0·622
Slovakia	0·595	0·598	0·598	0·597	0·599	0·602	0·605	0·608	0·609	0·615	0·623	0·626	0·627	0·629	0·63	0·634	0·641	0·646	0·651	0·655
Slovenia	0·621	0·625	0·629	0·633	0·637	0·64	0·643	0·647	0·651	0·657	0·664	0·671	0·678	0·685	0·691	0·696	0·701	0·706	0·713	0·72
Eastern Europe	0·617	0·62	0·626	0·631	0·635	0·638	0·642	0·646	0·648	0·651	0·654	0·657	0·657	0·658	0·661	0·663	0·664	0·666	0·67	0·674
Belarus	0·512	0·516	0·523	0·529	0·535	0·54	0·544	0·548	0·552	0·555	0·559	0·563	0·565	0·567	0·57	0·573	0·576	0·578	0·581	0·586
Estonia	0·603	0·607	0·614	0·618	0·621	0·624	0·627	0·63	0·633	0·634	0·636	0·639	0·641	0·643	0·646	0·649	0·648	0·647	0·649	0·656
Latvia	0·616	0·62	0·626	0·631	0·635	0·639	0·644	0·647	0·649	0·65	0·652	0·654	0·652	0·652	0·655	0·656	0·657	0·66	0·664	0·669
Lithuania	0·588	0·592	0·601	0·609	0·616	0·62	0·623	0·629	0·633	0·638	0·648	0·656	0·659	0·662	0·665	0·667	0·67	0·67	0·67	0·67
Moldova	0·524	0·528	0·531	0·534	0·538	0·542	0·546	0·549	0·553	0·556	0·559	0·561	0·563	0·566	0·567	0·569	0·572	0·574	0·577	0·581
Russia	0·629	0·633	0·64	0·646	0·65	0·653	0·657	0·661	0·663	0·666	0·669	0·672	0·671	0·671	0·675	0·678	0·677	0·68	0·684	0·688
Ukraine	0·597	0·599	0·602	0·605	0·608	0·612	0·615	0·618	0·621	0·623	0·625	0·627	0·629	0·631	0·633	0·635	0·638	0·641	0·644	0·649
High income	0·653	0·66	0·668	0·674	0·68	0·686	0·691	0·696	0·701	0·705	0·71	0·715	0·72	0·726	0·731	0·735	0·74	0·744	0·748	0·751
Australasia	0·618	0·621	0·634	0·646	0·658	0·668	0·675	0·683	0·69	0·695	0·698	0·702	0·706	0·712	0·717	0·721	0·727	0·731	0·736	0·739
Australia	0·613	0·615	0·629	0·641	0·652	0·661	0·669	0·676	0·683	0·689	0·692	0·695	0·699	0·705	0·711	0·715	0·722	0·727	0·732	0·735
New Zealand	0·635	0·639	0·65	0·664	0·678	0·689	0·697	0·706	0·712	0·717	0·722	0·728	0·735	0·74	0·744	0·747	0·748	0·749	0·751	0·753
High-income Asia Pacific	0·632	0·636	0·641	0·648	0·657	0·665	0·673	0·681	0·688	0·695	0·702	0·708	0·713	0·719	0·726	0·733	0·739	0·746	0·753	0·76
Brunei	0·489	0·504	0·517	0·53	0·542	0·553	0·564	0·574	0·583	0·593	0·602	0·61	0·618	0·627	0·634	0·642	0·649	0·656	0·663	0·67
Japan	0·672	0·674	0·679	0·686	0·695	0·704	0·712	0·72	0·726	0·733	0·739	0·744	0·748	0·752	0·758	0·763	0·768	0·774	0·78	0·786
Aichi	0·678	0·679	0·683	0·689	0·698	0·708	0·717	0·725	0·732	0·739	0·745	0·75	0·754	0·758	0·765	0·771	0·776	0·782	0·789	0·796
Akita	0·627	0·628	0·631	0·636	0·643	0·651	0·659	0·668	0·676	0·684	0·691	0·697	0·702	0·707	0·713	0·719	0·725	0·731	0·736	0·741
Aomori	0·608	0·609	0·612	0·618	0·627	0·637	0·646	0·655	0·664	0·672	0·679	0·685	0·689	0·694	0·701	0·707	0·712	0·719	0·725	0·731
Chiba	0·67	0·673	0·679	0·686	0·695	0·704	0·713	0·72	0·727	0·733	0·739	0·743	0·746	0·75	0·756	0·761	0·766	0·771	0·778	0·784
Ehime	0·641	0·643	0·647	0·653	0·662	0·671	0·68	0·687	0·694	0·701	0·707	0·712	0·717	0·721	0·726	0·731	0·735	0·741	0·746	0·752
Fukui	0·63	0·631	0·635	0·641	0·651	0·662	0·671	0·68	0·688	0·696	0·704	0·711	0·716	0·721	0·729	0·735	0·741	0·747	0·754	0·761
Fukuoka	0·668	0·672	0·677	0·685	0·694	0·703	0·711	0·719	0·725	0·732	0·738	0·742	0·745	0·749	0·754	0·758	0·763	0·768	0·773	0·778
Fukushima	0·621	0·623	0·627	0·634	0·643	0·652	0·66	0·67	0·678	0·686	0·693	0·7	0·704	0·708	0·715	0·721	0·727	0·734	0·74	0·746
Gifu	0·646	0·648	0·652	0·659	0·668	0·678	0·686	0·694	0·701	0·708	0·714	0·72	0·725	0·729	0·736	0·741	0·747	0·753	0·759	0·766
Gunma	0·649	0·653	0·658	0·665	0·674	0·683	0·692	0·699	0·706	0·713	0·719	0·725	0·729	0·733	0·74	0·745	0·751	0·756	0·762	0·768
Hiroshima	0·667	0·669	0·673	0·681	0·692	0·703	0·713	0·721	0·728	0·735	0·74	0·745	0·748	0·752	0·758	0·762	0·767	0·772	0·778	0·784
Hokkaidō	0·65	0·652	0·656	0·663	0·672	0·682	0·691	0·699	0·707	0·714	0·72	0·726	0·73	0·733	0·739	0·744	0·748	0·753	0·758	0·764
Hyōgo	0·681	0·683	0·688	0·694	0·702	0·711	0·719	0·727	0·734	0·74	0·746	0·751	0·754	0·757	0·763	0·767	0·772	0·777	0·783	0·789
Ibaraki	0·639	0·643	0·648	0·656	0·666	0·676	0·686	0·694	0·702	0·709	0·715	0·721	0·725	0·73	0·736	0·743	0·748	0·755	0·761	0·768
Ishikawa	0·642	0·642	0·645	0·651	0·66	0·67	0·679	0·688	0·696	0·703	0·71	0·717	0·722	0·727	0·734	0·74	0·746	0·753	0·76	0·767
Iwate	0·611	0·612	0·616	0·622	0·632	0·642	0·651	0·66	0·668	0·675	0·682	0·688	0·692	0·697	0·704	0·71	0·716	0·722	0·728	0·733
Kagawa	0·649	0·651	0·655	0·662	0·672	0·683	0·692	0·701	0·708	0·714	0·72	0·725	0·729	0·733	0·739	0·744	0·749	0·754	0·76	0·766
Kagoshima	0·61	0·614	0·619	0·628	0·637	0·647	0·657	0·665	0·672	0·68	0·687	0·693	0·698	0·704	0·711	0·717	0·722	0·727	0·733	0·739
Kanagawa	0·719	0·72	0·724	0·729	0·736	0·744	0·751	0·758	0·764	0·77	0·774	0·779	0·782	0·786	0·791	0·795	0·8	0·804	0·81	0·815
Kōchi	0·624	0·626	0·63	0·636	0·645	0·654	0·663	0·672	0·68	0·688	0·694	0·699	0·703	0·707	0·713	0·719	0·724	0·729	0·735	0·74
Kumamoto	0·622	0·626	0·631	0·639	0·649	0·659	0·668	0·677	0·684	0·692	0·699	0·705	0·709	0·713	0·72	0·726	0·732	0·738	0·744	0·749
Kyōto	0·689	0·693	0·698	0·705	0·713	0·721	0·728	0·735	0·742	0·748	0·753	0·758	0·762	0·765	0·77	0·774	0·779	0·784	0·79	0·795
Mie	0·644	0·645	0·649	0·655	0·664	0·673	0·682	0·689	0·696	0·703	0·709	0·714	0·718	0·722	0·727	0·733	0·738	0·744	0·751	0·758
Miyagi	0·647	0·65	0·655	0·662	0·671	0·68	0·688	0·696	0·704	0·711	0·717	0·723	0·728	0·732	0·739	0·745	0·75	0·757	0·763	0·769

Appendix Table 15. Socio-demographic Index values for all estimated GBD 2019 locations, 1970–1989																				
**Location**	**1970**	**1971**	**1972**	**1973**	**1974**	**1975**	**1976**	**1977**	**1978**	**1979**	**1980**	**1981**	**1982**	**1983**	**1984**	**1985**	**1986**	**1987**	**1988**	**1989**
Miyazaki	0·614	0·616	0·62	0·627	0·635	0·645	0·654	0·662	0·67	0·678	0·684	0·69	0·695	0·7	0·706	0·712	0·717	0·724	0·73	0·736
Nagano	0·658	0·662	0·668	0·676	0·684	0·692	0·7	0·708	0·715	0·722	0·728	0·734	0·739	0·742	0·747	0·752	0·756	0·762	0·767	0·773
Nagasaki	0·616	0·62	0·626	0·634	0·644	0·654	0·663	0·671	0·678	0·685	0·691	0·697	0·701	0·706	0·712	0·717	0·722	0·727	0·733	0·738
Nara	0·67	0·673	0·678	0·684	0·692	0·7	0·708	0·715	0·721	0·727	0·731	0·735	0·738	0·742	0·747	0·752	0·756	0·762	0·767	0·773
Niigata	0·635	0·637	0·642	0·648	0·656	0·665	0·673	0·682	0·689	0·697	0·704	0·71	0·714	0·718	0·725	0·731	0·736	0·742	0·747	0·753
Ōita	0·638	0·641	0·647	0·654	0·663	0·672	0·68	0·688	0·696	0·703	0·71	0·716	0·721	0·725	0·732	0·737	0·742	0·748	0·753	0·759
Okayama	0·654	0·656	0·66	0·667	0·677	0·687	0·697	0·705	0·712	0·718	0·723	0·728	0·732	0·736	0·743	0·748	0·753	0·76	0·766	0·772
Okinawa	0·586	0·587	0·592	0·6	0·612	0·624	0·635	0·645	0·654	0·662	0·67	0·678	0·684	0·689	0·696	0·703	0·709	0·717	0·724	0·731
Ōsaka	0·702	0·704	0·708	0·714	0·723	0·731	0·739	0·746	0·752	0·758	0·762	0·766	0·769	0·772	0·776	0·781	0·784	0·789	0·795	0·801
Saga	0·628	0·631	0·637	0·644	0·653	0·663	0·671	0·679	0·686	0·693	0·7	0·706	0·71	0·714	0·72	0·725	0·731	0·736	0·742	0·747
Saitama	0·665	0·668	0·673	0·68	0·688	0·698	0·706	0·714	0·721	0·727	0·733	0·737	0·741	0·745	0·751	0·756	0·761	0·766	0·772	0·778
Shiga	0·661	0·665	0·671	0·679	0·689	0·699	0·707	0·715	0·722	0·73	0·737	0·742	0·746	0·75	0·755	0·761	0·766	0·773	0·78	0·787
Shimane	0·617	0·619	0·624	0·632	0·641	0·65	0·659	0·667	0·673	0·68	0·687	0·693	0·697	0·702	0·708	0·713	0·718	0·724	0·729	0·735
Shizuoka	0·658	0·659	0·663	0·67	0·679	0·688	0·697	0·706	0·713	0·72	0·726	0·731	0·735	0·739	0·745	0·751	0·756	0·762	0·769	0·775
Tochigi	0·648	0·651	0·656	0·664	0·673	0·682	0·691	0·699	0·706	0·714	0·72	0·726	0·73	0·733	0·739	0·744	0·749	0·755	0·761	0·768
Tokushima	0·631	0·634	0·639	0·646	0·656	0·666	0·675	0·683	0·69	0·696	0·702	0·707	0·71	0·714	0·721	0·726	0·731	0·737	0·743	0·749
Tōkyō	0·753	0·757	0·762	0·77	0·779	0·787	0·795	0·801	0·807	0·813	0·817	0·822	0·826	0·83	0·835	0·841	0·845	0·851	0·856	0·862
Tottori	0·631	0·634	0·639	0·646	0·656	0·666	0·675	0·684	0·691	0·698	0·704	0·71	0·714	0·718	0·724	0·73	0·736	0·742	0·747	0·753
Toyama	0·649	0·649	0·652	0·659	0·667	0·677	0·686	0·695	0·703	0·711	0·719	0·725	0·73	0·734	0·74	0·746	0·751	0·758	0·764	0·772
Wakayama	0·644	0·645	0·648	0·655	0·666	0·678	0·688	0·696	0·701	0·703	0·704	0·705	0·707	0·712	0·72	0·727	0·733	0·739	0·744	0·75
Yamagata	0·624	0·626	0·631	0·637	0·645	0·653	0·661	0·669	0·677	0·685	0·693	0·7	0·705	0·709	0·715	0·72	0·725	0·732	0·737	0·743
Yamaguchi	0·657	0·658	0·663	0·669	0·678	0·688	0·696	0·703	0·709	0·716	0·721	0·726	0·729	0·733	0·738	0·744	0·748	0·754	0·76	0·766
Yamanashi	0·649	0·654	0·66	0·668	0·677	0·686	0·694	0·702	0·708	0·715	0·722	0·728	0·733	0·737	0·743	0·749	0·754	0·759	0·765	0·771
South Korea	0·449	0·458	0·469	0·482	0·495	0·509	0·522	0·535	0·549	0·561	0·572	0·582	0·592	0·603	0·615	0·626	0·638	0·649	0·662	0·674
Singapore	0·5	0·51	0·52	0·533	0·55	0·56	0·571	0·584	0·592	0·6	0·607	0·616	0·625	0·635	0·643	0·651	0·659	0·665	0·671	0·68
High-income North America	0·692	0·708	0·722	0·729	0·733	0·739	0·742	0·745	0·749	0·75	0·753	0·756	0·759	0·764	0·766	0·768	0·772	0·773	0·772	0·77
Canada	0·702	0·713	0·722	0·728	0·732	0·736	0·741	0·747	0·753	0·757	0·761	0·764	0·767	0·771	0·775	0·78	0·784	0·787	0·788	0·789
Greenland	0·558	0·581	0·6	0·616	0·625	0·631	0·635	0·64	0·644	0·647	0·651	0·655	0·66	0·665	0·666	0·666	0·665	0·663	0·66	0·658
USA	0·691	0·708	0·721	0·729	0·733	0·74	0·742	0·745	0·749	0·749	0·751	0·755	0·758	0·763	0·765	0·767	0·77	0·771	0·77	0·768
Alabama	0·643	0·654	0·665	0·673	0·679	0·687	0·692	0·697	0·703	0·705	0·71	0·716	0·72	0·726	0·729	0·731	0·734	0·735	0·733	0·731
Alaska	0·669	0·689	0·707	0·717	0·725	0·734	0·737	0·739	0·744	0·744	0·748	0·75	0·752	0·756	0·756	0·757	0·76	0·76	0·756	0·75
Arizona	0·668	0·68	0·693	0·701	0·709	0·721	0·728	0·732	0·735	0·734	0·735	0·736	0·736	0·738	0·738	0·739	0·743	0·744	0·742	0·74
Arkansas	0·623	0·637	0·65	0·657	0·663	0·672	0·678	0·682	0·689	0·69	0·695	0·7	0·704	0·709	0·711	0·712	0·715	0·715	0·712	0·71
California	0·719	0·738	0·751	0·754	0·755	0·759	0·76	0·762	0·765	0·763	0·764	0·765	0·767	0·77	0·771	0·771	0·773	0·771	0·767	0·761
Colorado	0·709	0·725	0·74	0·748	0·755	0·763	0·767	0·769	0·772	0·772	0·773	0·775	0·777	0·78	0·782	0·784	0·787	0·789	0·789	0·788
Connecticut	0·738	0·756	0·77	0·778	0·783	0·788	0·791	0·793	0·796	0·797	0·801	0·804	0·808	0·812	0·815	0·818	0·822	0·825	0·826	0·827
Delaware	0·704	0·722	0·738	0·748	0·754	0·76	0·762	0·762	0·765	0·765	0·768	0·771	0·774	0·777	0·779	0·781	0·784	0·785	0·786	0·785
Washington, DC	0·722	0·737	0·751	0·761	0·768	0·776	0·779	0·781	0·785	0·786	0·788	0·79	0·79	0·791	0·791	0·79	0·791	0·791	0·788	0·785
Florida	0·682	0·692	0·704	0·713	0·721	0·733	0·74	0·745	0·749	0·749	0·751	0·753	0·754	0·757	0·758	0·759	0·762	0·762	0·76	0·758
Georgia	0·64	0·658	0·674	0·684	0·693	0·703	0·708	0·713	0·718	0·718	0·721	0·725	0·729	0·734	0·736	0·738	0·742	0·743	0·742	0·74
Hawaii	0·702	0·715	0·727	0·734	0·74	0·748	0·751	0·754	0·759	0·76	0·763	0·765	0·766	0·768	0·768	0·769	0·772	0·773	0·772	0·77
Idaho	0·657	0·673	0·686	0·692	0·696	0·702	0·701	0·702	0·707	0·709	0·716	0·724	0·73	0·738	0·74	0·742	0·746	0·747	0·745	0·743
Illinois	0·698	0·716	0·731	0·738	0·742	0·748	0·75	0·754	0·757	0·754	0·756	0·76	0·764	0·77	0·773	0·776	0·779	0·78	0·778	0·776
Indiana	0·671	0·69	0·705	0·712	0·716	0·723	0·726	0·73	0·734	0·733	0·736	0·741	0·745	0·751	0·754	0·757	0·76	0·76	0·759	0·757
Iowa	0·692	0·711	0·726	0·733	0·736	0·741	0·742	0·744	0·747	0·748	0·752	0·758	0·763	0·768	0·772	0·774	0·778	0·78	0·78	0·78
Kansas	0·696	0·713	0·727	0·734	0·737	0·741	0·741	0·742	0·745	0·745	0·748	0·751	0·754	0·759	0·761	0·764	0·768	0·77	0·77	0·769
Kentucky	0·646	0·656	0·667	0·675	0·68	0·686	0·688	0·691	0·697	0·7	0·705	0·712	0·717	0·723	0·726	0·729	0·732	0·733	0·731	0·729
Louisiana	0·636	0·653	0·668	0·678	0·684	0·69	0·691	0·692	0·697	0·697	0·701	0·706	0·711	0·717	0·72	0·723	0·727	0·728	0·727	0·725
Maine	0·666	0·683	0·699	0·709	0·716	0·723	0·725	0·728	0·735	0·738	0·743	0·748	0·752	0·756	0·758	0·761	0·764	0·767	0·768	0·77
Maryland	0·715	0·734	0·75	0·76	0·767	0·772	0·775	0·777	0·781	0·782	0·785	0·787	0·789	0·792	0·793	0·795	0·798	0·799	0·799	0·799
Massachusetts	0·738	0·754	0·767	0·774	0·78	0·786	0·789	0·792	0·795	0·797	0·8	0·804	0·807	0·811	0·814	0·817	0·821	0·824	0·826	0·827
Michigan	0·688	0·707	0·722	0·73	0·736	0·744	0·748	0·75	0·752	0·75	0·752	0·756	0·76	0·766	0·77	0·773	0·776	0·777	0·776	0·774
Minnesota	0·714	0·73	0·743	0·75	0·754	0·759	0·76	0·762	0·765	0·766	0·769	0·774	0·778	0·784	0·787	0·791	0·796	0·799	0·801	0·802
Mississippi	0·6	0·616	0·633	0·644	0·652	0·663	0·668	0·673	0·679	0·681	0·687	0·693	0·698	0·704	0·707	0·709	0·712	0·712	0·71	0·707
Missouri	0·678	0·694	0·708	0·716	0·72	0·727	0·729	0·731	0·734	0·732	0·735	0·739	0·743	0·748	0·751	0·754	0·758	0·759	0·759	0·757
Montana	0·68	0·693	0·705	0·713	0·719	0·727	0·73	0·733	0·738	0·74	0·745	0·75	0·754	0·759	0·76	0·762	0·765	0·765	0·764	0·763
Nebraska	0·695	0·71	0·723	0·73	0·734	0·741	0·744	0·746	0·751	0·752	0·756	0·76	0·764	0·769	0·771	0·774	0·777	0·779	0·78	0·78
Nevada	0·714	0·724	0·733	0·74	0·747	0·755	0·758	0·759	0·76	0·757	0·758	0·76	0·761	0·764	0·764	0·764	0·765	0·763	0·758	0·752
New Hampshire	0·699	0·716	0·731	0·741	0·749	0·757	0·761	0·765	0·77	0·773	0·777	0·781	0·784	0·789	0·791	0·794	0·799	0·803	0·806	0·809
New Jersey	0·721	0·738	0·753	0·761	0·767	0·773	0·777	0·78	0·783	0·784	0·788	0·792	0·796	0·801	0·804	0·807	0·811	0·813	0·814	0·814
New Mexico	0·654	0·668	0·68	0·687	0·695	0·704	0·707	0·708	0·71	0·707	0·71	0·713	0·716	0·722	0·724	0·726	0·73	0·73	0·727	0·723
New York	0·722	0·74	0·754	0·76	0·762	0·767	0·769	0·773	0·778	0·78	0·784	0·787	0·79	0·793	0·795	0·797	0·799	0·801	0·801	0·8
North Carolina	0·66	0·67	0·681	0·69	0·698	0·707	0·712	0·717	0·724	0·728	0·733	0·738	0·741	0·744	0·745	0·746	0·748	0·748	0·747	0·746
North Dakota	0·677	0·692	0·706	0·714	0·721	0·728	0·729	0·731	0·735	0·736	0·741	0·746	0·751	0·757	0·76	0·764	0·769	0·773	0·774	0·776
Ohio	0·685	0·703	0·719	0·726	0·73	0·736	0·739	0·742	0·744	0·744	0·746	0·75	0·753	0·758	0·761	0·763	0·767	0·768	0·767	0·765

Appendix Table 15. Socio-demographic Index values for all estimated GBD 2019 locations, 1970–1989

Location	1970	1971	1972	1973	1974	1975	1976	1977	1978	1979	1980	1981	1982	1983	1984	1985	1986	1987	1988	1989
Oklahoma	0·67	0·681	0·691	0·698	0·704	0·711	0·714	0·715	0·718	0·716	0·718	0·722	0·726	0·732	0·735	0·738	0·743	0·744	0·742	0·739
Oregon	0·712	0·728	0·741	0·747	0·751	0·755	0·756	0·756	0·758	0·757	0·759	0·763	0·766	0·77	0·772	0·773	0·775	0·776	0·774	0·773
Pennsylvania	0·7	0·716	0·731	0·738	0·743	0·749	0·752	0·755	0·759	0·76	0·763	0·766	0·77	0·774	0·777	0·779	0·782	0·784	0·785	0·785
Rhode Island	0·705	0·721	0·736	0·746	0·753	0·76	0·762	0·765	0·769	0·772	0·775	0·779	0·781	0·784	0·786	0·788	0·792	0·794	0·796	0·797
South Carolina	0·642	0·653	0·665	0·675	0·684	0·695	0·701	0·706	0·712	0·715	0·721	0·726	0·729	0·733	0·735	0·736	0·738	0·738	0·736	0·735
South Dakota	0·674	0·687	0·698	0·705	0·71	0·716	0·718	0·719	0·723	0·723	0·727	0·732	0·737	0·743	0·745	0·748	0·752	0·755	0·755	0·754
Tennessee	0·646	0·663	0·678	0·686	0·693	0·701	0·705	0·708	0·713	0·715	0·719	0·724	0·728	0·733	0·734	0·736	0·738	0·739	0·737	0·734
Texas	0·653	0·672	0·689	0·698	0·705	0·713	0·717	0·718	0·72	0·717	0·718	0·72	0·722	0·727	0·729	0·732	0·737	0·738	0·737	0·733
Utah	0·682	0·695	0·706	0·709	0·708	0·709	0·705	0·701	0·702	0·7	0·705	0·713	0·721	0·73	0·735	0·739	0·745	0·748	0·748	0·748
Vermont	0·696	0·712	0·726	0·736	0·744	0·752	0·756	0·759	0·763	0·765	0·769	0·773	0·776	0·779	0·781	0·784	0·789	0·792	0·795	0·797
Virginia	0·691	0·707	0·722	0·73	0·735	0·743	0·747	0·753	0·76	0·762	0·767	0·771	0·774	0·778	0·78	0·782	0·785	0·787	0·787	0·787
Washington	0·717	0·739	0·755	0·76	0·763	0·766	0·767	0·768	0·77	0·768	0·769	0·771	0·773	0·777	0·779	0·781	0·784	0·785	0·785	0·783
West Virginia	0·649	0·66	0·671	0·679	0·685	0·691	0·693	0·695	0·7	0·703	0·709	0·716	0·721	0·727	0·73	0·733	0·735	0·736	0·735	0·733
Wisconsin	0·705	0·722	0·735	0·74	0·742	0·748	0·752	0·755	0·758	0·757	0·759	0·763	0·766	0·772	0·775	0·779	0·783	0·786	0·787	0·787
Wyoming	0·675	0·693	0·707	0·715	0·721	0·727	0·727	0·724	0·724	0·72	0·722	0·726	0·73	0·737	0·74	0·744	0·751	0·755	0·756	0·756
Southern Latin America	0·521	0·524	0·525	0·528	0·53	0·532	0·536	0·541	0·543	0·547	0·552	0·557	0·563	0·568	0·571	0·572	0·573	0·576	0·578	0·582
Argentina	0·528	0·531	0·532	0·533	0·531	0·53	0·532	0·535	0·537	0·541	0·548	0·554	0·56	0·565	0·568	0·567	0·569	0·572	0·576	0·581
Chile	0·505	0·509	0·512	0·519	0·529	0·537	0·545	0·55	0·553	0·556	0·556	0·559	0·567	0·571	0·575	0·579	0·58	0·581	0·582	0·586
Uruguay	0·508	0·51	0·511	0·514	0·517	0·52	0·525	0·529	0·535	0·541	0·547	0·554	0·558	0·562	0·565	0·567	0·57	0·572	0·575	0·577
Western Europe	0·641	0·646	0·653	0·66	0·666	0·672	0·677	0·682	0·688	0·692	0·698	0·704	0·71	0·716	0·722	0·726	0·731	0·736	0·741	0·745
Andorra	0·772	0·776	0·78	0·784	0·787	0·791	0·795	0·799	0·802	0·805	0·809	0·812	0·815	0·818	0·822	0·825	0·827	0·828	0·83	0·831
Austria	0·639	0·646	0·654	0·66	0·666	0·677	0·686	0·693	0·698	0·7	0·702	0·705	0·712	0·721	0·727	0·733	0·738	0·742	0·746	0·751
Belgium	0·624	0·631	0·639	0·649	0·658	0·665	0·671	0·677	0·682	0·687	0·692	0·698	0·705	0·712	0·718	0·724	0·729	0·733	0·738	0·742
Cyprus	0·564	0·573	0·58	0·587	0·589	0·589	0·59	0·592	0·593	0·595	0·598	0·602	0·606	0·612	0·619	0·625	0·632	0·639	0·646	0·654
Denmark	0·7	0·705	0·71	0·715	0·72	0·726	0·734	0·741	0·748	0·755	0·762	0·768	0·775	0·78	0·784	0·788	0·793	0·797	0·8	0·803
Finland	0·646	0·655	0·664	0·67	0·672	0·677	0·683	0·69	0·697	0·702	0·708	0·712	0·716	0·723	0·729	0·736	0·743	0·747	0·75	0·754
France	0·612	0·617	0·624	0·635	0·646	0·656	0·661	0·665	0·672	0·675	0·68	0·686	0·693	0·7	0·705	0·711	0·717	0·723	0·728	0·733
Germany	0·721	0·726	0·739	0·746	0·751	0·757	0·76	0·765	0·771	0·774	0·777	0·782	0·788	0·794	0·8	0·8	0·803	0·809	0·813	0·813
Greece	0·576	0·581	0·587	0·589	0·589	0·591	0·594	0·597	0·599	0·601	0·608	0·616	0·623	0·63	0·639	0·649	0·656	0·662	0·67	0·677
Iceland	0·626	0·626	0·63	0·641	0·653	0·664	0·674	0·682	0·686	0·691	0·698	0·706	0·715	0·725	0·735	0·743	0·748	0·751	0·754	0·758
Ireland	0·616	0·617	0·62	0·625	0·631	0·638	0·644	0·65	0·655	0·66	0·666	0·673	0·681	0·689	0·696	0·702	0·708	0·714	0·72	0·725
Israel	0·608	0·615	0·621	0·624	0·625	0·626	0·632	0·643	0·65	0·655	0·662	0·666	0·67	0·676	0·681	0·69	0·698	0·703	0·708	0·713
Italy	0·591	0·594	0·597	0·6	0·604	0·61	0·618	0·626	0·634	0·642	0·651	0·657	0·663	0·67	0·676	0·683	0·69	0·695	0·701	0·707
Luxembourg	0·716	0·724	0·734	0·741	0·748	0·752	0·756	0·76	0·767	0·772	0·774	0·777	0·783	0·788	0·792	0·796	0·801	0·804	0·806	0·81
Malta	0·546	0·552	0·559	0·562	0·565	0·572	0·584	0·593	0·601	0·609	0·618	0·625	0·63	0·636	0·642	0·647	0·65	0·653	0·658	0·662
Monaco	0·764	0·767	0·771	0·775	0·779	0·782	0·786	0·79	0·793	0·797	0·8	0·803	0·807	0·81	0·813	0·817	0·82	0·824	0·827	0·83
Netherlands	0·682	0·691	0·701	0·71	0·719	0·726	0·732	0·739	0·744	0·748	0·752	0·758	0·763	0·767	0·771	0·775	0·78	0·784	0·788	0·792
Norway	0·678	0·683	0·692	0·703	0·712	0·723	0·734	0·743	0·749	0·755	0·762	0·767	0·774	0·781	0·786	0·79	0·794	0·797	0·799	0·802
Portugal	0·5	0·506	0·511	0·515	0·516	0·514	0·514	0·519	0·527	0·535	0·542	0·549	0·554	0·56	0·566	0·573	0·579	0·586	0·593	0·6
San Marino	0·736	0·74	0·744	0·749	0·753	0·757	0·762	0·766	0·77	0·774	0·778	0·782	0·785	0·789	0·792	0·796	0·799	0·803	0·806	0·81
Spain	0·526	0·527	0·531	0·536	0·538	0·541	0·545	0·551	0·559	0·566	0·575	0·583	0·592	0·599	0·605	0·613	0·619	0·625	0·633	0·64
Sweden	0·687	0·689	0·696	0·7	0·706	0·716	0·724	0·73	0·736	0·739	0·743	0·749	0·754	0·759	0·763	0·764	0·767	0·768	0·767	0·767
Stockholm	0·738	0·739	0·745	0·751	0·758	0·768	0·774	0·779	0·783	0·786	0·79	0·796	0·801	0·806	0·808	0·809	0·81	0·81	0·81	0·811
Sweden except Stockholm	0·675	0·677	0·684	0·688	0·693	0·703	0·711	0·718	0·724	0·727	0·731	0·737	0·742	0·747	0·751	0·753	0·755	0·756	0·756	0·755
Switzerland	0·786	0·792	0·799	0·805	0·812	0·82	0·826	0·831	0·834	0·836	0·837	0·84	0·843	0·847	0·851	0·856	0·859	0·862	0·864	0·866
UK	0·637	0·645	0·654	0·663	0·673	0·682	0·69	0·694	0·696	0·697	0·703	0·71	0·716	0·72	0·723	0·726	0·729	0·732	0·736	0·741
England	0·647	0·654	0·662	0·671	0·681	0·688	0·696	0·7	0·702	0·704	0·709	0·716	0·722	0·726	0·729	0·732	0·735	0·738	0·742	0·746
East Midlands	0·617	0·624	0·633	0·642	0·652	0·66	0·668	0·672	0·674	0·676	0·682	0·689	0·695	0·699	0·702	0·706	0·709	0·712	0·716	0·721
Derby	0·625	0·633	0·641	0·651	0·661	0·67	0·677	0·681	0·682	0·684	0·689	0·697	0·704	0·709	0·712	0·716	0·72	0·723	0·728	0·732
Derbyshire	0·612	0·62	0·628	0·637	0·647	0·656	0·663	0·668	0·67	0·672	0·677	0·685	0·69	0·694	0·697	0·7	0·703	0·705	0·709	0·713
Leicester	0·61	0·617	0·625	0·634	0·644	0·652	0·659	0·664	0·665	0·667	0·673	0·68	0·687	0·692	0·696	0·7	0·705	0·709	0·714	0·719
Leicestershire	0·655	0·662	0·669	0·678	0·687	0·695	0·702	0·706	0·708	0·711	0·716	0·722	0·727	0·731	0·734	0·736	0·739	0·741	0·745	0·75
Lincolnshire	0·615	0·623	0·631	0·641	0·651	0·659	0·667	0·671	0·673	0·675	0·68	0·687	0·693	0·696	0·699	0·701	0·704	0·706	0·709	0·714
Northamptonshire	0·626	0·634	0·643	0·653	0·663	0·672	0·68	0·685	0·686	0·688	0·693	0·701	0·707	0·711	0·714	0·716	0·719	0·721	0·725	0·73
Nottingham	0·646	0·653	0·661	0·669	0·678	0·685	0·692	0·696	0·698	0·701	0·706	0·713	0·719	0·724	0·728	0·732	0·736	0·74	0·745	0·75
Nottinghamshire	0·614	0·622	0·63	0·64	0·649	0·658	0·665	0·669	0·671	0·673	0·678	0·685	0·691	0·695	0·697	0·7	0·703	0·706	0·71	0·714
Rutland	0·658	0·666	0·674	0·683	0·693	0·701	0·707	0·711	0·712	0·713	0·718	0·725	0·731	0·735	0·739	0·743	0·747	0·75	0·755	0·76
East of England	0·641	0·648	0·656	0·665	0·675	0·682	0·689	0·694	0·696	0·698	0·703	0·71	0·715	0·719	0·722	0·726	0·729	0·731	0·736	0·74
Bedford	0·648	0·655	0·663	0·672	0·682	0·689	0·696	0·701	0·702	0·704	0·709	0·717	0·722	0·727	0·731	0·734	0·738	0·741	0·745	0·75
Cambridgeshire	0·686	0·693	0·7	0·708	0·717	0·724	0·73	0·734	0·736	0·739	0·744	0·75	0·755	0·759	0·762	0·765	0·768	0·771	0·775	0·78
Central Bedfordshire	0·644	0·652	0·66	0·67	0·68	0·688	0·695	0·699	0·701	0·702	0·707	0·715	0·72	0·724	0·727	0·731	0·734	0·737	0·742	0·747
Essex	0·634	0·641	0·649	0·658	0·667	0·675	0·682	0·687	0·688	0·69	0·696	0·703	0·708	0·712	0·715	0·717	0·72	0·723	0·727	0·731
Hertfordshire	0·684	0·691	0·699	0·708	0·716	0·724	0·73	0·735	0·736	0·738	0·743	0·75	0·755	0·759	0·762	0·765	0·768	0·771	0·775	0·779
Luton	0·619	0·627	0·635	0·645	0·655	0·663	0·671	0·675	0·676	0·678	0·683	0·692	0·698	0·702	0·706	0·709	0·713	0·715	0·72	0·725
Norfolk	0·623	0·631	0·638	0·648	0·657	0·665	0·672	0·677	0·679	0·681	0·686	0·693	0·698	0·702	0·705	0·708	0·711	0·714	0·718	0·722

Appendix Table 15. Socio-demographic Index values for all estimated GBD 2019 locations, 1970–1989

Location	1970	1971	1972	1973	1974	1975	1976	1977	1978	1979	1980	1981	1982	1983	1984	1985	1986	1987	1988	1989
Peterborough	0·611	0·62	0·628	0·639	0·65	0·66	0·668	0·672	0·673	0·674	0·68	0·689	0·695	0·7	0·703	0·706	0·71	0·712	0·716	0·72
Southend-on-Sea	0·604	0·611	0·619	0·629	0·639	0·647	0·654	0·659	0·661	0·663	0·669	0·677	0·682	0·687	0·69	0·694	0·698	0·701	0·705	0·71
Suffolk	0·623	0·631	0·639	0·649	0·659	0·668	0·675	0·68	0·681	0·682	0·688	0·695	0·701	0·705	0·709	0·712	0·715	0·718	0·722	0·727
Thurrock	0·616	0·624	0·632	0·641	0·651	0·659	0·667	0·671	0·672	0·674	0·679	0·686	0·691	0·695	0·698	0·7	0·704	0·706	0·71	0·714
Greater London	0·703	0·71	0·717	0·725	0·733	0·74	0·747	0·751	0·753	0·756	0·761	0·768	0·773	0·777	0·781	0·784	0·788	0·791	0·795	0·8
Barking and Dagenham	0·582	0·59	0·598	0·608	0·618	0·627	0·634	0·639	0·641	0·644	0·649	0·657	0·663	0·667	0·67	0·673	0·676	0·678	0·682	0·686
Barnet	0·681	0·688	0·696	0·705	0·713	0·721	0·727	0·732	0·734	0·737	0·742	0·749	0·755	0·759	0·762	0·766	0·77	0·773	0·778	0·782
Bexley	0·624	0·632	0·639	0·649	0·658	0·666	0·673	0·678	0·68	0·683	0·689	0·696	0·701	0·705	0·708	0·711	0·714	0·717	0·72	0·725
Brent	0·651	0·658	0·666	0·675	0·685	0·693	0·7	0·705	0·707	0·71	0·716	0·723	0·73	0·735	0·739	0·743	0·747	0·751	0·755	0·76
Bromley	0·667	0·674	0·682	0·691	0·7	0·708	0·714	0·719	0·722	0·725	0·73	0·737	0·742	0·746	0·749	0·752	0·755	0·757	0·761	0·766
Camden	0·801	0·807	0·812	0·817	0·823	0·828	0·832	0·835	0·837	0·839	0·843	0·848	0·852	0·856	0·858	0·861	0·863	0·865	0·867	0·87
Croydon	0·643	0·651	0·659	0·669	0·679	0·688	0·695	0·7	0·702	0·705	0·711	0·719	0·724	0·729	0·732	0·735	0·739	0·741	0·745	0·749
Ealing	0·67	0·678	0·685	0·695	0·704	0·712	0·719	0·723	0·725	0·728	0·733	0·741	0·747	0·752	0·756	0·761	0·765	0·769	0·774	0·779
Enfield	0·63	0·638	0·646	0·656	0·666	0·674	0·681	0·686	0·689	0·692	0·697	0·705	0·71	0·714	0·718	0·721	0·725	0·728	0·732	0·737
Greenwich	0·621	0·629	0·637	0·647	0·657	0·665	0·672	0·677	0·679	0·682	0·688	0·695	0·701	0·705	0·708	0·712	0·715	0·718	0·722	0·727
Hackney	0·687	0·695	0·704	0·713	0·724	0·733	0·741	0·745	0·745	0·745	0·75	0·759	0·765	0·769	0·771	0·773	0·776	0·777	0·78	0·784
Hammersmith and Fulham	0·751	0·757	0·763	0·77	0·777	0·783	0·788	0·793	0·795	0·799	0·804	0·81	0·815	0·82	0·825	0·829	0·834	0·839	0·844	0·85
Haringey	0·659	0·666	0·674	0·683	0·693	0·701	0·708	0·713	0·715	0·718	0·724	0·731	0·737	0·742	0·746	0·75	0·754	0·757	0·761	0·766
Harrow	0·666	0·673	0·681	0·69	0·699	0·707	0·714	0·718	0·721	0·724	0·729	0·736	0·741	0·745	0·748	0·751	0·755	0·758	0·762	0·768
Havering	0·622	0·629	0·637	0·646	0·655	0·663	0·669	0·674	0·677	0·68	0·685	0·691	0·696	0·7	0·703	0·705	0·708	0·71	0·714	0·718
Hillingdon	0·685	0·693	0·7	0·71	0·719	0·727	0·733	0·738	0·741	0·744	0·749	0·756	0·762	0·766	0·77	0·773	0·776	0·779	0·783	0·787
Hounslow	0·673	0·681	0·689	0·699	0·708	0·717	0·724	0·729	0·731	0·734	0·739	0·747	0·752	0·757	0·76	0·764	0·768	0·771	0·775	0·78
Islington	0·745	0·751	0·758	0·766	0·774	0·781	0·787	0·792	0·795	0·798	0·803	0·81	0·815	0·82	0·823	0·826	0·83	0·832	0·835	0·839
Kensington and Chelsea	0·795	0·8	0·806	0·812	0·818	0·824	0·828	0·832	0·835	0·838	0·842	0·848	0·852	0·856	0·859	0·862	0·866	0·869	0·873	0·878
Kingston upon Thames	0·716	0·722	0·729	0·737	0·744	0·751	0·756	0·761	0·764	0·767	0·772	0·778	0·782	0·786	0·79	0·793	0·798	0·801	0·806	0·811
Lambeth	0·687	0·694	0·701	0·71	0·718	0·725	0·732	0·736	0·738	0·741	0·747	0·754	0·76	0·765	0·77	0·774	0·779	0·783	0·789	0·794
Lewisham	0·635	0·642	0·649	0·658	0·667	0·675	0·681	0·686	0·688	0·691	0·697	0·704	0·711	0·716	0·72	0·725	0·729	0·733	0·738	0·743
Merton	0·668	0·676	0·684	0·693	0·702	0·71	0·716	0·721	0·723	0·726	0·731	0·738	0·743	0·748	0·751	0·755	0·759	0·763	0·768	0·773
Newham	0·601	0·609	0·617	0·627	0·638	0·647	0·654	0·659	0·661	0·663	0·669	0·677	0·684	0·689	0·693	0·697	0·702	0·705	0·709	0·714
Redbridge	0·645	0·652	0·66	0·669	0·678	0·685	0·692	0·697	0·699	0·702	0·708	0·715	0·72	0·724	0·728	0·731	0·734	0·737	0·74	0·744
Richmond upon Thames	0·739	0·745	0·752	0·76	0·768	0·774	0·78	0·784	0·787	0·79	0·795	0·8	0·805	0·809	0·812	0·816	0·819	0·823	0·827	0·832
Southwark	0·695	0·703	0·71	0·719	0·728	0·735	0·742	0·746	0·749	0·752	0·757	0·765	0·771	0·776	0·781	0·785	0·79	0·794	0·799	0·805
Sutton	0·64	0·648	0·656	0·666	0·675	0·684	0·691	0·696	0·698	0·701	0·707	0·714	0·719	0·723	0·726	0·729	0·733	0·735	0·739	0·744
Tower Hamlets	0·677	0·685	0·694	0·704	0·715	0·724	0·732	0·736	0·736	0·736	0·741	0·749	0·756	0·76	0·764	0·768	0·772	0·774	0·778	0·783
Waltham Forest	0·609	0·617	0·625	0·635	0·644	0·653	0·66	0·664	0·667	0·669	0·675	0·683	0·689	0·694	0·698	0·702	0·706	0·71	0·715	0·72
Wandsworth	0·721	0·727	0·734	0·741	0·749	0·755	0·761	0·765	0·767	0·77	0·775	0·782	0·787	0·792	0·797	0·802	0·807	0·812	0·818	0·824
Westminster	0·796	0·801	0·806	0·811	0·817	0·823	0·827	0·831	0·833	0·835	0·838	0·843	0·847	0·85	0·853	0·855	0·857	0·859	0·862	0·864
North East England	0·587	0·596	0·605	0·616	0·628	0·637	0·645	0·651	0·652	0·654	0·66	0·669	0·675	0·68	0·683	0·687	0·691	0·694	0·699	0·704
County Durham	0·591	0·599	0·608	0·619	0·629	0·639	0·647	0·651	0·653	0·655	0·661	0·669	0·676	0·68	0·684	0·687	0·691	0·693	0·697	0·702
Darlington	0·604	0·613	0·622	0·634	0·646	0·656	0·665	0·67	0·671	0·672	0·679	0·687	0·694	0·698	0·701	0·704	0·707	0·708	0·711	0·715
Gateshead	0·596	0·605	0·614	0·625	0·636	0·646	0·654	0·659	0·66	0·661	0·667	0·676	0·682	0·686	0·689	0·693	0·696	0·698	0·703	0·708
Hartlepool	0·561	0·57	0·58	0·592	0·604	0·614	0·623	0·628	0·629	0·63	0·636	0·645	0·652	0·656	0·659	0·661	0·664	0·666	0·67	0·675
Middlesbrough	0·567	0·576	0·586	0·597	0·61	0·62	0·629	0·634	0·635	0·636	0·642	0·651	0·657	0·662	0·666	0·669	0·673	0·676	0·681	0·687
Newcastle upon Tyne	0·652	0·66	0·667	0·676	0·685	0·693	0·7	0·705	0·707	0·71	0·715	0·722	0·727	0·732	0·735	0·739	0·743	0·747	0·752	0·758
North Tyneside	0·601	0·609	0·618	0·629	0·64	0·65	0·658	0·663	0·664	0·666	0·672	0·68	0·685	0·689	0·692	0·695	0·698	0·7	0·704	0·709
Northumberland	0·595	0·604	0·613	0·624	0·636	0·646	0·654	0·659	0·66	0·662	0·668	0·677	0·683	0·688	0·692	0·695	0·699	0·701	0·705	0·71
Redcar and Cleveland	0·569	0·578	0·588	0·599	0·611	0·621	0·63	0·635	0·635	0·637	0·642	0·651	0·658	0·662	0·665	0·668	0·671	0·674	0·678	0·683
South Tyneside	0·565	0·573	0·583	0·594	0·605	0·615	0·623	0·628	0·63	0·631	0·637	0·645	0·65	0·654	0·656	0·658	0·661	0·663	0·668	0·673
Stockton-on-Tees	0·602	0·612	0·621	0·633	0·645	0·655	0·664	0·669	0·67	0·671	0·677	0·686	0·692	0·696	0·699	0·702	0·705	0·707	0·711	0·716
Sunderland	0·576	0·585	0·594	0·605	0·618	0·628	0·636	0·641	0·642	0·643	0·649	0·658	0·665	0·67	0·673	0·677	0·681	0·684	0·689	0·694
North West England	0·622	0·63	0·638	0·648	0·658	0·667	0·674	0·679	0·681	0·683	0·688	0·696	0·701	0·705	0·708	0·712	0·715	0·717	0·722	0·726
Blackburn with Darwen	0·574	0·583	0·592	0·603	0·615	0·625	0·633	0·638	0·639	0·64	0·646	0·655	0·662	0·667	0·67	0·674	0·677	0·679	0·683	0·687
Blackpool	0·576	0·584	0·592	0·603	0·613	0·622	0·63	0·635	0·637	0·64	0·646	0·653	0·659	0·663	0·665	0·667	0·669	0·67	0·674	0·678
Bolton	0·597	0·605	0·614	0·624	0·635	0·645	0·652	0·657	0·658	0·659	0·665	0·673	0·679	0·683	0·686	0·69	0·693	0·695	0·7	0·704
Bury	0·62	0·628	0·636	0·646	0·656	0·665	0·672	0·677	0·678	0·68	0·685	0·692	0·697	0·701	0·703	0·706	0·709	0·71	0·714	0·719
Cheshire East	0·674	0·682	0·69	0·7	0·71	0·718	0·725	0·73	0·731	0·733	0·738	0·745	0·75	0·754	0·756	0·758	0·761	0·763	0·767	0·771
Cheshire West and Chester	0·665	0·673	0·681	0·69	0·7	0·708	0·715	0·719	0·721	0·722	0·727	0·734	0·739	0·743	0·745	0·748	0·751	0·753	0·757	0·762
Cumbria	0·625	0·633	0·642	0·651	0·661	0·67	0·677	0·682	0·683	0·685	0·69	0·698	0·703	0·708	0·711	0·714	0·717	0·719	0·722	0·726
Halton	0·604	0·613	0·622	0·633	0·644	0·653	0·661	0·666	0·667	0·668	0·673	0·681	0·687	0·69	0·692	0·694	0·697	0·698	0·702	0·707
Knowsley	0·582	0·59	0·599	0·609	0·619	0·628	0·636	0·64	0·641	0·643	0·648	0·656	0·663	0·667	0·671	0·674	0·677	0·679	0·683	0·687
Lancashire	0·629	0·637	0·646	0·656	0·666	0·675	0·682	0·687	0·688	0·69	0·695	0·703	0·708	0·712	0·715	0·718	0·721	0·723	0·727	0·732
Liverpool	0·632	0·64	0·647	0·656	0·665	0·672	0·679	0·683	0·685	0·687	0·692	0·699	0·704	0·709	0·712	0·716	0·72	0·723	0·728	0·733
Manchester	0·657	0·664	0·672	0·68	0·689	0·697	0·704	0·708	0·71	0·713	0·718	0·725	0·731	0·736	0·739	0·743	0·747	0·75	0·755	0·76
Oldham	0·578	0·586	0·595	0·606	0·617	0·626	0·634	0·639	0·64	0·641	0·647	0·655	0·661	0·665	0·668	0·671	0·674	0·676	0·679	0·683
Rochdale	0·582	0·591	0·599	0·61	0·621	0·631	0·639	0·644	0·645	0·646	0·652	0·66	0·666	0·67	0·672	0·675	0·677	0·679	0·682	0·686

Appendix Table 15. Socio-demographic Index values for all estimated GBD 2019 locations, 1970–1989

Location	1970	1971	1972	1973	1974	1975	1976	1977	1978	1979	1980	1981	1982	1983	1984	1985	1986	1987	1988	1989
Salford	0·614	0·622	0·63	0·64	0·65	0·658	0·666	0·67	0·671	0·673	0·678	0·686	0·692	0·696	0·699	0·702	0·705	0·707	0·711	0·715
Sefton	0·622	0·629	0·637	0·647	0·656	0·664	0·672	0·676	0·678	0·68	0·685	0·692	0·697	0·701	0·704	0·706	0·709	0·711	0·715	0·72
St Helens	0·595	0·603	0·611	0·621	0·631	0·64	0·647	0·652	0·653	0·655	0·66	0·668	0·674	0·678	0·681	0·684	0·687	0·689	0·693	0·697
Stockport	0·65	0·658	0·666	0·676	0·686	0·694	0·701	0·706	0·707	0·709	0·714	0·721	0·726	0·73	0·733	0·735	0·738	0·74	0·745	0·75
Tameside	0·591	0·599	0·607	0·617	0·628	0·637	0·645	0·649	0·651	0·652	0·658	0·666	0·671	0·676	0·679	0·681	0·684	0·686	0·69	0·694
Trafford	0·681	0·689	0·697	0·706	0·716	0·724	0·731	0·736	0·737	0·739	0·744	0·751	0·756	0·76	0·762	0·765	0·768	0·77	0·774	0·779
Warrington	0·657	0·665	0·674	0·684	0·694	0·702	0·71	0·714	0·715	0·716	0·722	0·729	0·735	0·738	0·741	0·744	0·747	0·749	0·753	0·758
Wigan	0·593	0·601	0·609	0·619	0·63	0·638	0·646	0·65	0·651	0·653	0·658	0·666	0·672	0·676	0·68	0·683	0·686	0·689	0·693	0·697
Wirral	0·607	0·615	0·623	0·633	0·644	0·652	0·66	0·665	0·666	0·668	0·673	0·681	0·686	0·69	0·693	0·695	0·698	0·7	0·703	0·708
South East England	0·68	0·687	0·694	0·703	0·711	0·719	0·725	0·73	0·731	0·734	0·738	0·745	0·75	0·754	0·757	0·76	0·763	0·766	0·77	0·774
Bracknell Forest	0·692	0·699	0·706	0·715	0·724	0·731	0·738	0·742	0·743	0·745	0·75	0·757	0·762	0·767	0·77	0·774	0·777	0·779	0·783	0·787
Brighton and Hove	0·702	0·708	0·714	0·721	0·728	0·734	0·739	0·744	0·746	0·75	0·754	0·76	0·765	0·769	0·772	0·776	0·781	0·785	0·79	0·795
Buckinghamshire	0·704	0·711	0·718	0·727	0·735	0·742	0·749	0·753	0·755	0·757	0·762	0·768	0·773	0·777	0·78	0·784	0·787	0·789	0·792	0·796
East Sussex	0·627	0·635	0·643	0·652	0·662	0·67	0·677	0·682	0·684	0·686	0·692	0·699	0·704	0·708	0·711	0·714	0·717	0·72	0·724	0·729
Hampshire	0·675	0·682	0·69	0·699	0·708	0·716	0·722	0·727	0·728	0·73	0·735	0·742	0·747	0·751	0·754	0·757	0·76	0·763	0·766	0·771
Isle of Wight	0·607	0·615	0·623	0·633	0·643	0·651	0·658	0·663	0·665	0·667	0·672	0·68	0·685	0·689	0·691	0·694	0·697	0·7	0·704	0·708
Kent	0·643	0·651	0·659	0·668	0·677	0·685	0·692	0·697	0·699	0·701	0·707	0·713	0·719	0·722	0·725	0·728	0·73	0·733	0·736	0·74
Medway	0·616	0·624	0·632	0·641	0·651	0·659	0·667	0·671	0·672	0·674	0·679	0·687	0·692	0·696	0·699	0·702	0·705	0·707	0·712	0·716
Milton Keynes	0·665	0·674	0·683	0·694	0·705	0·714	0·722	0·727	0·728	0·73	0·735	0·743	0·749	0·752	0·755	0·758	0·762	0·764	0·768	0·773
Oxfordshire	0·713	0·72	0·726	0·734	0·742	0·748	0·754	0·758	0·76	0·762	0·767	0·773	0·778	0·782	0·785	0·788	0·792	0·794	0·798	0·802
Portsmouth	0·666	0·674	0·681	0·69	0·699	0·707	0·714	0·719	0·721	0·724	0·73	0·736	0·742	0·745	0·749	0·752	0·756	0·759	0·763	0·768
Reading	0·718	0·725	0·732	0·74	0·749	0·755	0·762	0·766	0·768	0·77	0·774	0·781	0·786	0·79	0·793	0·797	0·801	0·805	0·81	0·815
Slough	0·672	0·68	0·688	0·698	0·708	0·716	0·724	0·728	0·729	0·73	0·735	0·743	0·749	0·753	0·757	0·76	0·764	0·767	0·772	0·776
Southampton	0·677	0·684	0·691	0·699	0·706	0·713	0·719	0·724	0·726	0·728	0·733	0·739	0·744	0·748	0·751	0·755	0·758	0·761	0·765	0·769
Surrey	0·72	0·726	0·733	0·741	0·749	0·755	0·761	0·766	0·768	0·77	0·775	0·781	0·785	0·789	0·792	0·795	0·798	0·801	0·804	0·809
West Berkshire	0·703	0·711	0·719	0·729	0·739	0·747	0·754	0·759	0·76	0·762	0·767	0·774	0·779	0·783	0·786	0·789	0·792	0·794	0·799	0·803
West Sussex	0·661	0·668	0·676	0·685	0·694	0·702	0·709	0·714	0·716	0·718	0·724	0·731	0·736	0·74	0·744	0·747	0·75	0·753	0·756	0·761
Windsor and Maidenhead	0·726	0·733	0·74	0·749	0·757	0·764	0·77	0·774	0·776	0·778	0·782	0·789	0·793	0·797	0·8	0·803	0·807	0·809	0·813	0·817
Wokingham	0·737	0·744	0·75	0·758	0·766	0·773	0·778	0·782	0·784	0·786	0·79	0·796	0·8	0·803	0·805	0·808	0·811	0·814	0·818	0·823
South West England	0·647	0·655	0·663	0·672	0·681	0·689	0·696	0·7	0·702	0·704	0·709	0·716	0·722	0·726	0·729	0·732	0·735	0·738	0·742	0·747
Bath and North East Somerset	0·703	0·709	0·716	0·723	0·73	0·736	0·741	0·745	0·748	0·751	0·755	0·76	0·765	0·768	0·771	0·774	0·777	0·78	0·784	0·789
Bournemouth	0·659	0·665	0·672	0·68	0·687	0·694	0·7	0·704	0·707	0·71	0·715	0·721	0·726	0·731	0·735	0·739	0·743	0·747	0·752	0·757
Bristol, City of	0·691	0·698	0·705	0·713	0·721	0·728	0·734	0·739	0·741	0·743	0·748	0·755	0·76	0·764	0·767	0·771	0·774	0·778	0·783	0·787
Cornwall	0·623	0·63	0·639	0·648	0·658	0·667	0·674	0·679	0·68	0·682	0·687	0·694	0·699	0·703	0·706	0·709	0·712	0·714	0·718	0·723
Devon	0·646	0·654	0·661	0·67	0·679	0·687	0·693	0·698	0·7	0·702	0·708	0·714	0·72	0·724	0·727	0·729	0·732	0·735	0·739	0·743
Dorset	0·641	0·649	0·657	0·666	0·676	0·684	0·691	0·696	0·697	0·699	0·704	0·711	0·717	0·72	0·723	0·725	0·728	0·73	0·734	0·738
Gloucestershire	0·663	0·67	0·677	0·687	0·696	0·704	0·71	0·715	0·716	0·718	0·723	0·731	0·736	0·74	0·743	0·746	0·75	0·752	0·756	0·76
North Somerset	0·64	0·648	0·656	0·665	0·675	0·683	0·69	0·695	0·696	0·698	0·703	0·71	0·716	0·72	0·723	0·726	0·729	0·732	0·736	0·741
Plymouth	0·638	0·646	0·653	0·662	0·671	0·679	0·686	0·691	0·692	0·694	0·7	0·707	0·713	0·718	0·721	0·725	0·73	0·733	0·737	0·742
Poole	0·651	0·658	0·667	0·676	0·686	0·694	0·702	0·706	0·708	0·71	0·715	0·722	0·727	0·731	0·734	0·737	0·74	0·743	0·747	0·751
Somerset	0·63	0·638	0·646	0·656	0·666	0·674	0·681	0·686	0·687	0·689	0·695	0·702	0·708	0·712	0·715	0·717	0·72	0·721	0·725	0·729
South Gloucestershire	0·68	0·687	0·694	0·703	0·712	0·72	0·726	0·731	0·732	0·734	0·739	0·745	0·75	0·754	0·757	0·759	0·762	0·765	0·769	0·773
Swindon	0·657	0·665	0·673	0·683	0·693	0·702	0·709	0·714	0·715	0·716	0·722	0·73	0·735	0·74	0·743	0·746	0·749	0·752	0·756	0·761
Torbay	0·601	0·609	0·617	0·627	0·637	0·645	0·653	0·658	0·66	0·662	0·668	0·675	0·681	0·684	0·687	0·691	0·694	0·697	0·701	0·706
Wiltshire	0·648	0·656	0·664	0·673	0·683	0·692	0·699	0·703	0·704	0·705	0·71	0·718	0·724	0·728	0·732	0·735	0·738	0·74	0·744	0·748
West Midlands	0·605	0·613	0·621	0·632	0·642	0·651	0·659	0·664	0·665	0·667	0·672	0·68	0·686	0·691	0·694	0·697	0·7	0·703	0·707	0·712
Birmingham	0·604	0·612	0·621	0·631	0·641	0·65	0·658	0·662	0·663	0·665	0·67	0·679	0·685	0·69	0·693	0·697	0·702	0·705	0·71	0·715
Coventry	0·631	0·639	0·647	0·656	0·666	0·675	0·682	0·687	0·688	0·689	0·694	0·702	0·707	0·711	0·714	0·717	0·721	0·724	0·728	0·734
Dudley	0·593	0·601	0·61	0·62	0·63	0·639	0·647	0·651	0·652	0·654	0·659	0·667	0·673	0·677	0·68	0·683	0·686	0·688	0·692	0·696
Herefordshire, County of	0·615	0·624	0·633	0·643	0·654	0·663	0·671	0·676	0·677	0·679	0·684	0·692	0·698	0·702	0·705	0·707	0·709	0·711	0·714	0·718
Sandwell	0·564	0·572	0·581	0·591	0·602	0·612	0·62	0·624	0·624	0·625	0·631	0·64	0·647	0·651	0·655	0·658	0·662	0·664	0·669	0·674
Shropshire	0·624	0·632	0·64	0·651	0·661	0·67	0·678	0·682	0·684	0·686	0·691	0·698	0·704	0·707	0·71	0·712	0·714	0·716	0·72	0·724
Solihull	0·658	0·666	0·675	0·685	0·695	0·703	0·71	0·715	0·716	0·717	0·722	0·73	0·735	0·738	0·739	0·741	0·743	0·745	0·748	0·753
Staffordshire	0·62	0·628	0·636	0·646	0·657	0·665	0·673	0·678	0·679	0·681	0·686	0·694	0·699	0·703	0·705	0·708	0·71	0·712	0·716	0·721
Stoke-on-Trent	0·574	0·582	0·59	0·601	0·611	0·62	0·628	0·633	0·633	0·635	0·64	0·649	0·655	0·66	0·664	0·667	0·671	0·674	0·678	0·683
Telford and Wrekin	0·606	0·614	0·623	0·635	0·646	0·656	0·664	0·669	0·669	0·67	0·676	0·684	0·691	0·695	0·698	0·701	0·705	0·706	0·71	0·715
Walsall	0·573	0·581	0·59	0·6	0·611	0·62	0·628	0·632	0·633	0·634	0·64	0·648	0·654	0·659	0·662	0·665	0·668	0·67	0·674	0·678
Warwickshire	0·651	0·659	0·667	0·677	0·687	0·695	0·703	0·707	0·709	0·71	0·715	0·723	0·728	0·732	0·734	0·737	0·74	0·742	0·745	0·75
Wolverhampton	0·585	0·594	0·602	0·612	0·623	0·632	0·64	0·645	0·646	0·647	0·653	0·661	0·668	0·672	0·675	0·678	0·682	0·684	0·687	0·692
Worcestershire	0·626	0·634	0·643	0·653	0·664	0·672	0·68	0·685	0·686	0·688	0·693	0·701	0·706	0·71	0·713	0·715	0·718	0·72	0·723	0·727
Yorkshire and the Humber	0·611	0·619	0·628	0·638	0·648	0·657	0·664	0·669	0·671	0·673	0·678	0·686	0·692	0·696	0·699	0·702	0·706	0·708	0·713	0·718
Barnsley	0·57	0·579	0·587	0·597	0·608	0·617	0·625	0·629	0·63	0·632	0·638	0·646	0·652	0·656	0·659	0·662	0·665	0·667	0·671	0·675
Bradford	0·58	0·589	0·598	0·609	0·621	0·631	0·639	0·644	0·644	0·645	0·651	0·66	0·667	0·671	0·675	0·679	0·683	0·685	0·69	0·695
Calderdale	0·606	0·614	0·624	0·635	0·646	0·655	0·663	0·668	0·669	0·671	0·676	0·685	0·691	0·695	0·698	0·7	0·704	0·706	0·71	0·715
Doncaster	0·57	0·578	0·587	0·598	0·609	0·618	0·626	0·631	0·632	0·633	0·639	0·647	0·653	0·657	0·66	0·662	0·665	0·667	0·67	0·674

Appendix Table 15. Socio-demographic Index values for all estimated GBD 2019 locations, 1970–1989

Location	1970	1971	1972	1973	1974	1975	1976	1977	1978	1979	1980	1981	1982	1983	1984	1985	1986	1987	1988	1989
East Riding of Yorkshire	0·63	0·638	0·647	0·656	0·666	0·675	0·682	0·687	0·689	0·691	0·696	0·703	0·709	0·712	0·715	0·717	0·719	0·721	0·724	0·728
Kingston upon Hull, City of	0·581	0·59	0·599	0·609	0·62	0·629	0·637	0·641	0·643	0·644	0·65	0·658	0·664	0·668	0·671	0·674	0·677	0·679	0·683	0·688
Kirklees	0·602	0·611	0·62	0·63	0·641	0·65	0·657	0·662	0·663	0·665	0·67	0·678	0·684	0·689	0·692	0·696	0·699	0·702	0·706	0·711
Leeds	0·658	0·666	0·673	0·682	0·691	0·698	0·705	0·709	0·711	0·713	0·718	0·725	0·731	0·735	0·738	0·741	0·745	0·748	0·753	0·758
North East Lincolnshire	0·571	0·58	0·589	0·601	0·613	0·623	0·631	0·636	0·637	0·638	0·643	0·652	0·658	0·661	0·663	0·665	0·668	0·669	0·673	0·678
North Lincolnshire	0·615	0·624	0·633	0·644	0·655	0·665	0·673	0·678	0·678	0·679	0·685	0·693	0·699	0·703	0·705	0·708	0·71	0·712	0·715	0·719
North Yorkshire	0·642	0·65	0·659	0·669	0·679	0·687	0·695	0·7	0·702	0·704	0·709	0·717	0·722	0·726	0·728	0·731	0·733	0·735	0·738	0·742
Rotherham	0·58	0·588	0·597	0·608	0·618	0·627	0·635	0·64	0·641	0·642	0·648	0·656	0·662	0·666	0·668	0·671	0·674	0·675	0·679	0·683
Sheffield	0·65	0·657	0·664	0·672	0·681	0·688	0·694	0·699	0·701	0·704	0·709	0·715	0·72	0·724	0·727	0·73	0·733	0·736	0·74	0·745
Wakefield	0·586	0·595	0·604	0·614	0·625	0·634	0·642	0·647	0·648	0·65	0·655	0·664	0·669	0·674	0·676	0·679	0·682	0·684	0·687	0·691
York	0·698	0·705	0·711	0·719	0·727	0·734	0·74	0·744	0·746	0·749	0·753	0·759	0·764	0·768	0·771	0·774	0·778	0·781	0·786	0·79
Northern Ireland	0·529	0·55	0·576	0·602	0·622	0·633	0·641	0·646	0·643	0·642	0·648	0·659	0·666	0·67	0·671	0·674	0·677	0·68	0·688	0·696
Scotland	0·594	0·606	0·622	0·634	0·646	0·66	0·672	0·678	0·678	0·677	0·681	0·69	0·698	0·703	0·705	0·708	0·711	0·712	0·717	0·722
Wales	0·589	0·597	0·606	0·616	0·627	0·636	0·643	0·649	0·65	0·652	0·658	0·666	0·672	0·677	0·68	0·684	0·688	0·691	0·696	0·701
Latin America and Caribbean	0·363	0·369	0·375	0·382	0·389	0·396	0·403	0·41	0·417	0·424	0·431	0·438	0·445	0·452	0·458	0·464	0·47	0·476	0·481	0·486
Andean Latin America	0·353	0·359	0·366	0·373	0·38	0·387	0·395	0·402	0·41	0·418	0·426	0·435	0·443	0·45	0·457	0·463	0·47	0·476	0·481	0·485
Bolivia	0·326	0·332	0·338	0·343	0·346	0·348	0·35	0·352	0·356	0·36	0·365	0·37	0·377	0·382	0·387	0·391	0·395	0·399	0·403	0·407
Ecuador	0·352	0·36	0·368	0·377	0·387	0·396	0·405	0·415	0·423	0·433	0·442	0·451	0·459	0·466	0·473	0·48	0·486	0·49	0·495	0·499
Peru	0·358	0·364	0·37	0·377	0·385	0·393	0·401	0·409	0·418	0·426	0·435	0·444	0·453	0·46	0·467	0·474	0·481	0·488	0·494	0·498
Caribbean	0·398	0·398	0·403	0·412	0·422	0·432	0·441	0·452	0·461	0·468	0·475	0·478	0·48	0·487	0·491	0·497	0·503	0·505	0·507	0·511
Antigua and Barbuda	0·439	0·448	0·457	0·464	0·467	0·469	0·469	0·471	0·475	0·482	0·491	0·5	0·51	0·52	0·529	0·538	0·547	0·556	0·565	0·572
The Bahamas	0·574	0·582	0·592	0·6	0·603	0·6	0·599	0·605	0·61	0·612	0·615	0·621	0·629	0·638	0·65	0·666	0·679	0·685	0·688	0·692
Barbados	0·521	0·53	0·539	0·549	0·559	0·57	0·578	0·585	0·591	0·596	0·6	0·603	0·607	0·612	0·62	0·627	0·635	0·64	0·644	0·646
Belize	0·296	0·301	0·307	0·312	0·318	0·323	0·328	0·334	0·34	0·347	0·354	0·361	0·367	0·373	0·38	0·386	0·393	0·401	0·409	0·418
Bermuda	0·545	0·555	0·565	0·576	0·586	0·595	0·603	0·61	0·617	0·624	0·63	0·636	0·641	0·646	0·652	0·658	0·664	0·669	0·675	0·68
Cuba	0·414	0·4	0·4	0·414	0·433	0·453	0·473	0·496	0·512	0·523	0·532	0·53	0·528	0·536	0·539	0·549	0·559	0·56	0·562	0·568
Dominica	0·396	0·408	0·422	0·437	0·452	0·467	0·48	0·492	0·504	0·511	0·518	0·526	0·532	0·538	0·543	0·548	0·554	0·56	0·566	0·572
Dominican Republic	0·294	0·302	0·31	0·319	0·328	0·337	0·345	0·352	0·36	0·367	0·374	0·381	0·387	0·395	0·401	0·407	0·411	0·414	0·418	0·422
Grenada	0·321	0·329	0·338	0·346	0·354	0·362	0·37	0·377	0·384	0·391	0·397	0·402	0·407	0·412	0·417	0·422	0·428	0·436	0·444	0·453
Guyana	0·359	0·367	0·375	0·382	0·389	0·396	0·402	0·408	0·413	0·417	0·421	0·425	0·428	0·431	0·433	0·436	0·439	0·442	0·445	0·448
Haiti	0·251	0·255	0·257	0·26	0·264	0·267	0·27	0·273	0·276	0·279	0·282	0·284	0·287	0·289	0·291	0·293	0·296	0·298	0·301	0·304
Jamaica	0·391	0·402	0·414	0·427	0·439	0·45	0·461	0·47	0·479	0·486	0·492	0·498	0·503	0·508	0·513	0·517	0·521	0·526	0·531	0·536
Puerto Rico	0·56	0·565	0·573	0·579	0·586	0·591	0·589	0·592	0·602	0·611	0·619	0·628	0·638	0·646	0·653	0·656	0·659	0·663	0·665	0·665
Saint Kitts and Nevis	0·411	0·42	0·429	0·437	0·446	0·455	0·464	0·472	0·481	0·489	0·498	0·507	0·515	0·522	0·53	0·538	0·547	0·556	0·565	0·574
Saint Lucia	0·296	0·303	0·31	0·318	0·326	0·335	0·344	0·354	0·364	0·374	0·383	0·391	0·4	0·409	0·418	0·428	0·438	0·449	0·46	0·472
Saint Vincent and the Grenadines	0·256	0·267	0·279	0·29	0·301	0·311	0·322	0·333	0·343	0·353	0·364	0·374	0·384	0·394	0·405	0·416	0·426	0·435	0·445	0·454
Suriname	0·372	0·383	0·394	0·402	0·408	0·412	0·414	0·418	0·422	0·425	0·427	0·429	0·432	0·438	0·446	0·457	0·468	0·476	0·484	0·491
Trinidad and Tobago	0·506	0·515	0·524	0·532	0·539	0·546	0·552	0·558	0·563	0·568	0·573	0·578	0·583	0·587	0·591	0·596	0·6	0·605	0·61	0·614
Virgin Islands	0·497	0·51	0·524	0·537	0·55	0·561	0·57	0·579	0·586	0·594	0·601	0·607	0·613	0·618	0·624	0·629	0·634	0·64	0·645	0·65
Central Latin America	0·358	0·364	0·37	0·375	0·381	0·387	0·393	0·399	0·406	0·413	0·421	0·43	0·438	0·446	0·453	0·46	0·466	0·471	0·476	0·48
Colombia	0·355	0·362	0·37	0·378	0·385	0·393	0·4	0·407	0·413	0·42	0·427	0·434	0·441	0·448	0·455	0·461	0·466	0·47	0·473	0·476
Costa Rica	0·429	0·438	0·445	0·451	0·455	0·458	0·461	0·465	0·47	0·476	0·482	0·488	0·493	0·495	0·497	0·5	0·506	0·512	0·519	0·525
El Salvador	0·279	0·284	0·288	0·293	0·298	0·303	0·309	0·317	0·325	0·335	0·343	0·352	0·359	0·366	0·371	0·376	0·38	0·383	0·385	0·388
Guatemala	0·27	0·271	0·272	0·272	0·271	0·27	0·267	0·265	0·264	0·264	0·266	0·271	0·277	0·285	0·292	0·3	0·305	0·308	0·31	0·312
Honduras	0·24	0·243	0·247	0·25	0·254	0·258	0·262	0·267	0·273	0·279	0·284	0·29	0·295	0·3	0·305	0·309	0·313	0·317	0·322	0·326
Mexico	0·363	0·368	0·372	0·377	0·383	0·388	0·394	0·401	0·409	0·417	0·427	0·437	0·446	0·455	0·464	0·472	0·48	0·488	0·494	0·501
Aguascalientes	0·381	0·386	0·392	0·398	0·404	0·41	0·417	0·424	0·432	0·441	0·45	0·46	0·469	0·478	0·487	0·496	0·503	0·511	0·518	0·525
Baja California	0·401	0·406	0·412	0·419	0·426	0·433	0·441	0·449	0·459	0·469	0·48	0·492	0·503	0·513	0·523	0·532	0·54	0·548	0·554	0·56
Baja California Sur	0·359	0·364	0·37	0·376	0·383	0·391	0·4	0·409	0·419	0·431	0·443	0·456	0·469	0·481	0·492	0·503	0·513	0·522	0·531	0·539
Campeche	0·335	0·339	0·343	0·347	0·352	0·358	0·364	0·37	0·378	0·387	0·396	0·407	0·416	0·426	0·435	0·444	0·452	0·459	0·467	0·474
Chiapas	0·305	0·309	0·313	0·316	0·32	0·325	0·329	0·334	0·34	0·346	0·353	0·361	0·368	0·375	0·382	0·388	0·393	0·398	0·403	0·408
Chihuahua	0·364	0·369	0·374	0·38	0·386	0·393	0·4	0·408	0·417	0·428	0·439	0·451	0·462	0·473	0·483	0·493	0·501	0·509	0·517	0·523
Coahuila	0·375	0·379	0·383	0·388	0·392	0·398	0·404	0·41	0·418	0·427	0·437	0·447	0·458	0·468	0·478	0·488	0·497	0·506	0·514	0·522
Colima	0·367	0·372	0·377	0·383	0·389	0·395	0·402	0·409	0·417	0·426	0·436	0·446	0·456	0·466	0·476	0·486	0·496	0·506	0·516	0·526
Durango	0·293	0·296	0·301	0·306	0·311	0·317	0·324	0·332	0·342	0·352	0·364	0·375	0·387	0·398	0·408	0·417	0·426	0·433	0·44	0·447
Guanajuato	0·323	0·327	0·33	0·334	0·338	0·342	0·347	0·352	0·358	0·365	0·372	0·381	0·389	0·398	0·406	0·414	0·422	0·429	0·437	0·444
Guerrero	0·292	0·295	0·298	0·302	0·306	0·31	0·314	0·319	0·324	0·331	0·338	0·346	0·354	0·362	0·369	0·376	0·383	0·389	0·396	0·402
Hidalgo	0·276	0·28	0·283	0·287	0·292	0·297	0·302	0·308	0·316	0·324	0·334	0·345	0·356	0·366	0·376	0·386	0·395	0·404	0·413	0·421
Jalisco	0·38	0·384	0·388	0·393	0·397	0·403	0·408	0·414	0·421	0·429	0·438	0·448	0·458	0·466	0·475	0·484	0·492	0·5	0·508	0·516
México	0·365	0·37	0·376	0·382	0·389	0·396	0·404	0·412	0·421	0·431	0·443	0·454	0·466	0·476	0·486	0·496	0·505	0·514	0·521	0·529
Mexico City	0·441	0·446	0·452	0·458	0·464	0·47	0·477	0·485	0·494	0·503	0·514	0·525	0·535	0·545	0·554	0·563	0·571	0·579	0·587	0·594
Michoacán de Ocampo	0·326	0·329	0·332	0·336	0·339	0·343	0·347	0·351	0·356	0·363	0·371	0·379	0·388	0·397	0·405	0·415	0·423	0·433	0·442	0·451
Morelos	0·363	0·368	0·372	0·377	0·382	0·387	0·393	0·4	0·407	0·416	0·425	0·436	0·446	0·456	0·466	0·476	0·486	0·496	0·505	0·514
Nayarit	0·334	0·339	0·344	0·35	0·356	0·363	0·37	0·378	0·386	0·396	0·406	0·416	0·426	0·435	0·444	0·453	0·461	0·468	0·475	0·482
Nuevo León	0·4	0·406	0·412	0·419	0·426	0·434	0·443	0·451	0·461	0·472	0·484	0·496	0·508	0·519	0·529	0·539	0·548	0·556	0·563	0·57

Appendix Table 15. Socio-demographic Index values for all estimated GBD 2019 locations, 1970–1989																				
**Location**	**1970**	**1971**	**1972**	**1973**	**1974**	**1975**	**1976**	**1977**	**1978**	**1979**	**1980**	**1981**	**1982**	**1983**	**1984**	**1985**	**1986**	**1987**	**1988**	**1989**
Oaxaca	0·302	0·306	0·31	0·314	0·318	0·323	0·328	0·333	0·34	0·347	0·356	0·365	0·373	0·382	0·39	0·398	0·405	0·411	0·417	0·421
Puebla	0·334	0·337	0·341	0·345	0·349	0·353	0·358	0·363	0·369	0·376	0·384	0·392	0·401	0·409	0·417	0·425	0·432	0·439	0·446	0·453
Querétaro	0·354	0·359	0·365	0·37	0·376	0·382	0·389	0·395	0·403	0·411	0·421	0·431	0·44	0·449	0·458	0·467	0·475	0·484	0·492	0·5
Quintana Roo	0·322	0·326	0·33	0·335	0·34	0·345	0·352	0·359	0·368	0·377	0·388	0·4	0·411	0·422	0·433	0·444	0·454	0·464	0·474	0·484
San Luis Potosí	0·327	0·331	0·335	0·339	0·343	0·348	0·353	0·358	0·364	0·372	0·38	0·388	0·397	0·405	0·413	0·421	0·428	0·434	0·44	0·446
Sinaloa	0·353	0·358	0·363	0·369	0·375	0·383	0·39	0·399	0·408	0·419	0·43	0·442	0·453	0·463	0·473	0·483	0·491	0·499	0·506	0·513
Sonora	0·385	0·39	0·395	0·401	0·407	0·413	0·42	0·427	0·435	0·445	0·455	0·466	0·477	0·487	0·497	0·507	0·516	0·524	0·532	0·539
Tabasco	0·329	0·333	0·338	0·343	0·348	0·354	0·361	0·368	0·377	0·386	0·397	0·408	0·418	0·428	0·438	0·447	0·455	0·463	0·471	0·478
Tamaulipas	0·385	0·389	0·393	0·398	0·403	0·408	0·413	0·419	0·425	0·433	0·442	0·451	0·461	0·469	0·478	0·486	0·495	0·504	0·513	0·521
Tlaxcala	0·322	0·327	0·331	0·336	0·341	0·346	0·352	0·357	0·364	0·372	0·38	0·389	0·398	0·407	0·416	0·425	0·434	0·444	0·454	0·464
Veracruz de Ignacio de la Llave	0·338	0·342	0·346	0·35	0·355	0·36	0·365	0·372	0·379	0·387	0·396	0·406	0·416	0·425	0·433	0·442	0·449	0·455	0·461	0·466
Yucatán	0·35	0·353	0·357	0·361	0·365	0·37	0·376	0·382	0·388	0·396	0·405	0·414	0·423	0·431	0·44	0·448	0·455	0·461	0·467	0·472
Zacatecas	0·342	0·347	0·352	0·357	0·363	0·369	0·376	0·383	0·391	0·4	0·41	0·42	0·43	0·439	0·448	0·457	0·465	0·473	0·48	0·487
Nicaragua	0·211	0·216	0·221	0·225	0·23	0·234	0·238	0·243	0·248	0·253	0·258	0·265	0·272	0·28	0·288	0·297	0·305	0·314	0·322	0·33
Panama	0·389	0·399	0·41	0·42	0·431	0·442	0·452	0·461	0·47	0·478	0·485	0·492	0·499	0·504	0·511	0·517	0·524	0·53	0·535	0·539
Venezuela	0·409	0·42	0·428	0·436	0·442	0·448	0·453	0·459	0·465	0·471	0·478	0·485	0·492	0·498	0·502	0·505	0·506	0·506	0·507	0·507
Tropical Latin America	0·354	0·361	0·368	0·376	0·384	0·391	0·398	0·406	0·412	0·419	0·426	0·432	0·438	0·444	0·449	0·456	0·462	0·469	0·475	0·481
Brazil	0·353	0·36	0·368	0·375	0·383	0·391	0·398	0·405	0·412	0·419	0·426	0·432	0·438	0·443	0·449	0·456	0·462	0·469	0·475	0·481
Acre	0·242	0·247	0·251	0·255	0·259	0·262	0·266	0·27	0·275	0·281	0·287	0·294	0·3	0·307	0·314	0·322	0·331	0·339	0·348	0·356
Alagoas	0·219	0·223	0·228	0·234	0·24	0·245	0·252	0·258	0·264	0·27	0·277	0·284	0·292	0·299	0·308	0·317	0·326	0·334	0·342	0·349
Amapá	0·344	0·35	0·356	0·361	0·366	0·37	0·375	0·381	0·388	0·395	0·403	0·411	0·419	0·428	0·437	0·447	0·457	0·467	0·476	0·486
Amazonas	0·273	0·28	0·287	0·295	0·303	0·31	0·319	0·327	0·336	0·345	0·354	0·364	0·374	0·384	0·395	0·406	0·418	0·429	0·44	0·45
Bahia	0·248	0·254	0·26	0·266	0·272	0·279	0·285	0·291	0·298	0·305	0·313	0·32	0·328	0·336	0·345	0·355	0·364	0·373	0·382	0·39
Ceará	0·259	0·263	0·267	0·271	0·275	0·28	0·285	0·29	0·297	0·304	0·311	0·319	0·326	0·334	0·342	0·35	0·358	0·366	0·373	0·38
Distrito Federal	0·474	0·48	0·485	0·491	0·497	0·502	0·509	0·515	0·521	0·528	0·535	0·542	0·547	0·552	0·556	0·561	0·566	0·57	0·576	0·581
Espírito Santo	0·33	0·338	0·346	0·354	0·362	0·369	0·377	0·385	0·393	0·402	0·41	0·417	0·424	0·431	0·438	0·445	0·452	0·459	0·466	0·473
Goiás	0·285	0·294	0·304	0·314	0·324	0·333	0·342	0·351	0·359	0·367	0·375	0·382	0·388	0·394	0·4	0·407	0·414	0·421	0·428	0·435
Maranhão	0·17	0·173	0·178	0·182	0·187	0·192	0·196	0·201	0·206	0·211	0·216	0·221	0·225	0·23	0·235	0·241	0·248	0·255	0·261	0·268
Mato Grosso	0·329	0·334	0·34	0·345	0·35	0·354	0·359	0·364	0·369	0·374	0·38	0·385	0·391	0·397	0·403	0·41	0·418	0·426	0·433	0·441
Mato Grosso do Sul	0·327	0·334	0·343	0·353	0·363	0·372	0·38	0·388	0·395	0·402	0·409	0·415	0·421	0·427	0·433	0·439	0·446	0·453	0·459	0·465
Minas Gerais	0·342	0·348	0·355	0·363	0·371	0·379	0·387	0·395	0·403	0·411	0·419	0·427	0·434	0·44	0·447	0·454	0·461	0·468	0·474	0·481
Pará	0·288	0·292	0·297	0·303	0·308	0·314	0·32	0·326	0·333	0·34	0·348	0·355	0·362	0·368	0·376	0·383	0·392	0·4	0·409	0·417
Paraíba	0·246	0·249	0·253	0·258	0·263	0·268	0·275	0·281	0·287	0·294	0·301	0·308	0·315	0·322	0·33	0·338	0·347	0·355	0·363	0·371
Paraná	0·343	0·349	0·356	0·365	0·374	0·384	0·394	0·404	0·414	0·423	0·432	0·44	0·448	0·455	0·462	0·468	0·475	0·482	0·488	0·494
Pernambuco	0·268	0·274	0·28	0·286	0·292	0·298	0·305	0·311	0·318	0·324	0·331	0·338	0·345	0·352	0·36	0·368	0·377	0·385	0·393	0·401
Piauí	0·213	0·217	0·222	0·227	0·233	0·238	0·244	0·25	0·256	0·262	0·269	0·275	0·281	0·287	0·293	0·3	0·308	0·315	0·322	0·328
Rio de Janeiro	0·454	0·461	0·469	0·478	0·486	0·494	0·501	0·508	0·515	0·521	0·527	0·532	0·536	0·54	0·544	0·548	0·553	0·557	0·561	0·566
Rio Grande do Norte	0·258	0·262	0·266	0·272	0·279	0·285	0·292	0·3	0·308	0·316	0·324	0·332	0·341	0·348	0·357	0·365	0·374	0·382	0·389	0·396
Rio Grande do Sul	0·423	0·43	0·438	0·446	0·454	0·461	0·468	0·475	0·481	0·487	0·493	0·498	0·504	0·508	0·514	0·519	0·526	0·532	0·538	0·544
Rondônia	0·297	0·303	0·309	0·315	0·319	0·324	0·329	0·334	0·339	0·345	0·351	0·357	0·364	0·371	0·379	0·388	0·397	0·406	0·415	0·423
Roraima	0·295	0·301	0·306	0·312	0·318	0·323	0·328	0·333	0·338	0·343	0·349	0·355	0·361	0·368	0·375	0·383	0·392	0·401	0·41	0·418
Santa Catarina	0·379	0·386	0·394	0·402	0·411	0·419	0·428	0·437	0·446	0·456	0·465	0·473	0·481	0·489	0·496	0·503	0·511	0·518	0·525	0·532
São Paulo	0·431	0·439	0·446	0·454	0·461	0·468	0·474	0·481	0·487	0·493	0·499	0·504	0·509	0·514	0·519	0·525	0·531	0·537	0·543	0·549
Sergipe	0·26	0·265	0·27	0·277	0·283	0·29	0·297	0·304	0·311	0·319	0·328	0·336	0·346	0·357	0·368	0·379	0·39	0·4	0·409	0·417
Tocantins	0·226	0·234	0·242	0·25	0·258	0·265	0·272	0·279	0·286	0·292	0·299	0·305	0·312	0·318	0·324	0·331	0·339	0·345	0·351	0·357
Paraguay	0·358	0·363	0·368	0·373	0·379	0·384	0·389	0·395	0·401	0·407	0·413	0·42	0·425	0·43	0·434	0·439	0·443	0·448	0·453	0·459
North Africa and Middle East	0·252	0·259	0·266	0·272	0·279	0·286	0·292	0·298	0·304	0·311	0·319	0·327	0·336	0·345	0·354	0·365	0·374	0·384	0·394	0·404
North Africa and Middle East	0·252	0·259	0·266	0·272	0·279	0·286	0·292	0·298	0·304	0·311	0·319	0·327	0·336	0·345	0·354	0·365	0·374	0·384	0·394	0·404
Afghanistan	0·142	0·145	0·147	0·149	0·152	0·154	0·157	0·159	0·161	0·161	0·16	0·16	0·164	0·168	0·172	0·175	0·178	0·181	0·183	0·185
Algeria	0·241	0·247	0·254	0·261	0·269	0·277	0·286	0·295	0·305	0·315	0·326	0·337	0·348	0·36	0·371	0·383	0·395	0·405	0·416	0·426
Bahrain	0·365	0·375	0·386	0·398	0·41	0·422	0·434	0·446	0·458	0·468	0·479	0·488	0·497	0·505	0·513	0·52	0·527	0·534	0·54	0·547
Egypt	0·249	0·254	0·259	0·263	0·267	0·271	0·277	0·283	0·289	0·295	0·302	0·31	0·318	0·326	0·335	0·345	0·355	0·367	0·379	0·391
Iran	0·252	0·258	0·263	0·268	0·272	0·273	0·273	0·268	0·263	0·259	0·26	0·265	0·275	0·29	0·309	0·328	0·345	0·36	0·375	0·389
Iraq	0·237	0·244	0·25	0·257	0·264	0·272	0·281	0·289	0·299	0·308	0·318	0·326	0·335	0·342	0·35	0·358	0·365	0·373	0·38	0·386
Jordan	0·22	0·236	0·25	0·264	0·278	0·291	0·306	0·321	0·337	0·353	0·371	0·39	0·407	0·424	0·441	0·457	0·472	0·486	0·5	0·511
Kuwait	0·369	0·382	0·394	0·407	0·417	0·429	0·444	0·462	0·481	0·5	0·517	0·533	0·551	0·568	0·583	0·597	0·608	0·618	0·628	0·642
Lebanon	0·319	0·326	0·335	0·344	0·353	0·36	0·366	0·371	0·376	0·381	0·387	0·392	0·396	0·402	0·411	0·422	0·432	0·443	0·451	0·456
Libya	0·192	0·198	0·205	0·211	0·22	0·228	0·238	0·249	0·26	0·272	0·285	0·296	0·308	0·319	0·331	0·343	0·355	0·366	0·378	0·391
Morocco	0·185	0·191	0·198	0·205	0·213	0·221	0·229	0·238	0·246	0·255	0·264	0·272	0·281	0·289	0·298	0·306	0·315	0·323	0·331	0·339
Oman	0·226	0·235	0·245	0·253	0·261	0·27	0·28	0·29	0·299	0·308	0·318	0·328	0·339	0·351	0·363	0·376	0·388	0·401	0·414	0·428
Palestine	0·175	0·181	0·188	0·195	0·202	0·209	0·217	0·224	0·232	0·239	0·247	0·254	0·261	0·267	0·274	0·28	0·287	0·294	0·3	0·306
Qatar	0·385	0·395	0·405	0·416	0·427	0·436	0·446	0·456	0·465	0·475	0·484	0·493	0·502	0·511	0·52	0·529	0·539	0·55	0·561	0·573
Saudi Arabia	0·283	0·292	0·302	0·313	0·324	0·335	0·344	0·354	0·363	0·373	0·383	0·394	0·404	0·413	0·422	0·431	0·441	0·45	0·459	0·469
Sudan	0·136	0·138	0·14	0·143	0·145	0·149	0·153	0·158	0·163	0·168	0·172	0·177	0·182	0·188	0·193	0·198	0·203	0·208	0·214	0·22
Syria	0·201	0·207	0·213	0·22	0·228	0·236	0·245	0·254	0·262	0·271	0·279	0·289	0·298	0·307	0·315	0·324	0·332	0·341	0·35	0·358

Appendix Table 15. Socio-demographic Index values for all estimated GBD 2019 locations, 1970–1989

Location	1970	1971	1972	1973	1974	1975	1976	1977	1978	1979	1980	1981	1982	1983	1984	1985	1986	1987	1988	1989
Tunisia	0·238	0·245	0·253	0·261	0·269	0·278	0·286	0·296	0·305	0·315	0·325	0·336	0·346	0·357	0·368	0·379	0·39	0·401	0·412	0·423
Turkey	0·29	0·298	0·307	0·316	0·325	0·334	0·344	0·353	0·362	0·371	0·38	0·389	0·398	0·407	0·416	0·425	0·434	0·444	0·454	0·463
United Arab Emirates	0·358	0·37	0·383	0·396	0·409	0·422	0·435	0·448	0·461	0·474	0·488	0·502	0·515	0·527	0·539	0·551	0·563	0·576	0·591	0·606
Yemen	0·0932	0·0959	0·0988	0·102	0·105	0·108	0·112	0·116	0·119	0·123	0·127	0·131	0·135	0·139	0·143	0·148	0·153	0·158	0·163	0·169
South Asia	0·233	0·237	0·24	0·244	0·246	0·248	0·251	0·253	0·256	0·258	0·262	0·265	0·269	0·273	0·278	0·283	0·288	0·294	0·3	0·307
South Asia	0·233	0·237	0·24	0·244	0·246	0·248	0·251	0·253	0·256	0·258	0·262	0·265	0·269	0·273	0·278	0·283	0·288	0·294	0·3	0·307
Bangladesh	0·14	0·15	0·159	0·167	0·175	0·181	0·188	0·194	0·2	0·206	0·211	0·216	0·22	0·225	0·23	0·235	0·24	0·246	0·253	0·26
Bhutan	0·132	0·136	0·14	0·144	0·147	0·151	0·154	0·157	0·161	0·165	0·169	0·174	0·179	0·185	0·191	0·196	0·202	0·209	0·215	0·222
India	0·248	0·252	0·255	0·259	0·261	0·263	0·265	0·267	0·27	0·272	0·275	0·278	0·282	0·286	0·291	0·296	0·301	0·307	0·314	0·32
Andhra Pradesh	0·196	0·2	0·204	0·208	0·211	0·214	0·217	0·219	0·222	0·224	0·227	0·231	0·235	0·239	0·244	0·25	0·257	0·264	0·271	0·279
Arunachal Pradesh	0·197	0·201	0·205	0·21	0·213	0·217	0·22	0·224	0·228	0·232	0·237	0·242	0·248	0·255	0·263	0·271	0·279	0·287	0·296	0·305
Assam	0·248	0·252	0·255	0·258	0·261	0·263	0·265	0·267	0·269	0·271	0·273	0·277	0·281	0·286	0·291	0·297	0·304	0·31	0·317	0·324
Bihar	0·199	0·202	0·205	0·207	0·208	0·21	0·211	0·212	0·214	0·215	0·217	0·22	0·223	0·227	0·232	0·236	0·241	0·246	0·252	0·257
Chhattisgarh	0·2	0·204	0·207	0·211	0·213	0·216	0·218	0·22	0·223	0·226	0·229	0·233	0·237	0·242	0·248	0·254	0·26	0·266	0·273	0·281
Delhi	0·437	0·442	0·447	0·451	0·454	0·456	0·458	0·46	0·462	0·463	0·465	0·467	0·468	0·47	0·472	0·475	0·479	0·482	0·487	0·491
Goa	0·372	0·378	0·384	0·39	0·395	0·401	0·407	0·413	0·419	0·425	0·431	0·438	0·445	0·452	0·459	0·466	0·473	0·48	0·487	0·495
Gujarat	0·276	0·279	0·283	0·285	0·287	0·289	0·29	0·293	0·295	0·298	0·301	0·304	0·308	0·313	0·319	0·325	0·333	0·341	0·349	0·357
Haryana	0·274	0·279	0·284	0·288	0·292	0·295	0·298	0·302	0·305	0·308	0·311	0·314	0·317	0·32	0·324	0·328	0·333	0·338	0·343	0·349
Himachal Pradesh	0·236	0·241	0·246	0·251	0·254	0·258	0·261	0·265	0·27	0·274	0·279	0·285	0·29	0·296	0·302	0·309	0·315	0·322	0·33	0·338
Jammu and Kashmir	0·262	0·266	0·271	0·275	0·278	0·282	0·286	0·29	0·294	0·297	0·301	0·305	0·309	0·313	0·317	0·321	0·325	0·329	0·333	0·338
Jharkhand	0·179	0·183	0·186	0·189	0·191	0·193	0·195	0·197	0·199	0·201	0·204	0·207	0·211	0·216	0·221	0·227	0·234	0·24	0·248	0·255
Karnataka	0·261	0·265	0·267	0·27	0·271	0·273	0·274	0·275	0·277	0·278	0·279	0·282	0·285	0·288	0·293	0·298	0·304	0·311	0·319	0·327
Kerala	0·343	0·349	0·354	0·359	0·362	0·366	0·368	0·371	0·375	0·377	0·38	0·384	0·387	0·391	0·395	0·4	0·405	0·41	0·416	0·423
Madhya Pradesh	0·213	0·217	0·221	0·224	0·227	0·229	0·232	0·234	0·237	0·24	0·243	0·247	0·251	0·256	0·261	0·266	0·272	0·278	0·284	0·29
Maharashtra	0·299	0·303	0·306	0·309	0·31	0·312	0·313	0·314	0·316	0·317	0·318	0·321	0·324	0·327	0·332	0·337	0·343	0·35	0·357	0·365
Manipur	0·259	0·265	0·271	0·277	0·282	0·287	0·292	0·298	0·304	0·309	0·315	0·322	0·328	0·335	0·342	0·35	0·357	0·365	0·374	0·382
Meghalaya	0·226	0·231	0·236	0·24	0·243	0·247	0·251	0·254	0·258	0·262	0·267	0·271	0·276	0·282	0·288	0·295	0·301	0·308	0·316	0·324
Mizoram	0·282	0·288	0·293	0·297	0·301	0·305	0·309	0·314	0·318	0·322	0·327	0·332	0·338	0·344	0·352	0·361	0·37	0·379	0·388	0·397
Nagaland	0·263	0·269	0·275	0·281	0·286	0·291	0·296	0·302	0·308	0·313	0·319	0·325	0·332	0·339	0·346	0·354	0·361	0·369	0·378	0·386
Odisha	0·197	0·201	0·206	0·21	0·214	0·218	0·221	0·225	0·228	0·232	0·236	0·24	0·245	0·25	0·255	0·261	0·268	0·274	0·282	0·289
Punjab	0·317	0·323	0·328	0·332	0·336	0·339	0·343	0·346	0·35	0·353	0·356	0·359	0·363	0·366	0·37	0·374	0·378	0·383	0·388	0·393
Rajasthan	0·199	0·202	0·205	0·207	0·208	0·21	0·211	0·212	0·214	0·215	0·217	0·219	0·222	0·225	0·229	0·233	0·237	0·242	0·248	0·255
Sikkim	0·216	0·221	0·225	0·23	0·234	0·238	0·241	0·246	0·25	0·254	0·259	0·264	0·27	0·277	0·284	0·292	0·301	0·31	0·319	0·328
Tamil Nadu	0·288	0·292	0·296	0·3	0·303	0·306	0·309	0·312	0·316	0·319	0·322	0·326	0·33	0·334	0·339	0·344	0·35	0·355	0·362	0·368
Telangana	0·21	0·214	0·218	0·221	0·223	0·226	0·228	0·231	0·233	0·236	0·239	0·243	0·247	0·252	0·257	0·263	0·269	0·275	0·282	0·29
Tripura	0·238	0·244	0·248	0·253	0·257	0·261	0·265	0·268	0·273	0·276	0·281	0·286	0·291	0·296	0·302	0·308	0·315	0·322	0·329	0·337
Union Territories other than Delhi	0·296	0·302	0·308	0·313	0·318	0·322	0·326	0·33	0·335	0·341	0·347	0·353	0·36	0·367	0·375	0·383	0·391	0·399	0·407	0·415
Uttar Pradesh	0·212	0·215	0·217	0·219	0·22	0·221	0·222	0·223	0·225	0·226	0·228	0·23	0·233	0·237	0·241	0·245	0·25	0·256	0·262	0·268
Uttarakhand	0·221	0·225	0·228	0·232	0·234	0·236	0·238	0·24	0·243	0·246	0·25	0·254	0·259	0·265	0·271	0·277	0·284	0·29	0·296	0·303
West Bengal	0·254	0·258	0·262	0·265	0·268	0·271	0·273	0·276	0·279	0·282	0·286	0·29	0·295	0·3	0·306	0·311	0·318	0·324	0·33	0·336
Nepal	0·148	0·15	0·151	0·152	0·153	0·155	0·157	0·158	0·16	0·162	0·164	0·167	0·169	0·171	0·174	0·177	0·181	0·184	0·188	0·193
Pakistan	0·167	0·17	0·173	0·176	0·179	0·182	0·185	0·189	0·192	0·195	0·199	0·203	0·207	0·211	0·216	0·22	0·226	0·231	0·236	0·242
Southeast Asia, east Asia, and Oceania	0·294	0·301	0·308	0·316	0·323	0·33	0·337	0·344	0·353	0·361	0·369	0·377	0·386	0·394	0·402	0·41	0·419	0·427	0·436	0·444
East Asia	0·281	0·289	0·297	0·305	0·312	0·32	0·327	0·334	0·342	0·351	0·359	0·367	0·375	0·383	0·392	0·402	0·411	0·421	0·43	0·439
China	0·27	0·279	0·286	0·293	0·3	0·307	0·313	0·32	0·328	0·336	0·344	0·351	0·359	0·368	0·377	0·387	0·396	0·406	0·416	0·425
North Korea	0·297	0·306	0·314	0·323	0·331	0·338	0·346	0·353	0·359	0·366	0·372	0·378	0·384	0·39	0·396	0·402	0·408	0·414	0·42	0·426
Taiwan (province of China)	0·438	0·453	0·467	0·478	0·49	0·491	0·496	0·512	0·519	0·531	0·545	0·556	0·57	0·584	0·598	0·615	0·628	0·636	0·648	0·658
Oceania	0·307	0·311	0·316	0·321	0·326	0·33	0·335	0·339	0·343	0·347	0·351	0·355	0·358	0·361	0·365	0·368	0·371	0·374	0·377	0·38
American Samoa	0·51	0·518	0·527	0·536	0·544	0·551	0·558	0·564	0·569	0·574	0·578	0·581	0·584	0·586	0·588	0·591	0·593	0·596	0·599	0·602
Cook Islands	0·522	0·527	0·53	0·534	0·538	0·542	0·546	0·55	0·554	0·559	0·564	0·569	0·574	0·58	0·586	0·593	0·6	0·607	0·613	0·619
Fiji	0·415	0·422	0·429	0·437	0·444	0·452	0·459	0·465	0·471	0·477	0·483	0·488	0·492	0·496	0·5	0·504	0·508	0·512	0·516	0·521
Guam	0·575	0·571	0·575	0·589	0·612	0·637	0·66	0·677	0·688	0·693	0·696	0·698	0·701	0·703	0·705	0·704	0·704	0·704	0·702	0·698
Kiribati	0·364	0·369	0·374	0·381	0·391	0·401	0·407	0·411	0·416	0·419	0·419	0·419	0·419	0·419	0·418	0·418	0·419	0·419	0·421	0·423
Marshall Islands	0·288	0·293	0·297	0·304	0·31	0·317	0·322	0·328	0·334	0·339	0·343	0·347	0·351	0·356	0·361	0·365	0·371	0·378	0·385	0·391
Federated States of Micronesia	0·324	0·33	0·337	0·343	0·35	0·356	0·363	0·37	0·377	0·383	0·39	0·396	0·402	0·408	0·414	0·419	0·425	0·43	0·436	0·441
Nauru	0·412	0·417	0·422	0·427	0·432	0·437	0·442	0·447	0·451	0·456	0·46	0·464	0·468	0·472	0·476	0·48	0·484	0·488	0·491	0·495
Niue	0·452	0·458	0·464	0·47	0·476	0·482	0·488	0·494	0·5	0·507	0·513	0·519	0·524	0·53	0·535	0·54	0·545	0·55	0·555	0·561
Northern Mariana Islands	0·55	0·556	0·563	0·569	0·577	0·586	0·595	0·603	0·611	0·618	0·625	0·631	0·638	0·646	0·654	0·661	0·668	0·675	0·681	0·687
Palau	0·499	0·505	0·51	0·516	0·522	0·528	0·534	0·539	0·545	0·552	0·558	0·564	0·57	0·575	0·58	0·586	0·593	0·6	0·607	0·614
Papua New Guinea	0·218	0·223	0·228	0·232	0·236	0·241	0·244	0·248	0·252	0·255	0·259	0·262	0·266	0·269	0·273	0·276	0·279	0·283	0·287	0·29
Samoa	0·408	0·416	0·423	0·431	0·437	0·443	0·45	0·456	0·463	0·47	0·477	0·482	0·488	0·493	0·498	0·503	0·509	0·515	0·52	0·526
Solomon Islands	0·205	0·208	0·211	0·215	0·219	0·222	0·226	0·23	0·235	0·241	0·245	0·25	0·253	0·257	0·26	0·263	0·266	0·269	0·272	0·275
Tokelau	0·302	0·309	0·315	0·321	0·327	0·332	0·338	0·345	0·352	0·359	0·366	0·373	0·379	0·385	0·391	0·398	0·404	0·409	0·415	0·421
Tonga	0·372	0·377	0·381	0·385	0·39	0·394	0·398	0·403	0·407	0·412	0·418	0·426	0·435	0·444	0·456	0·468	0·479	0·489	0·497	0·504
Tuvalu	0·347	0·351	0·355	0·358	0·361	0·365	0·368	0·37	0·373	0·375	0·377	0·38	0·381	0·382	0·385	0·388	0·393	0·4	0·409	0·416

Appendix Table 15. Socio-demographic Index values for all estimated GBD 2019 locations, 1970–1989																				
Location	1970	1971	1972	1973	1974	1975	1976	1977	1978	1979	1980	1981	1982	1983	1984	1985	1986	1987	1988	1989
Vanuatu	0·278	0·282	0·286	0·291	0·298	0·303	0·308	0·313	0·318	0·324	0·327	0·33	0·334	0·337	0·341	0·345	0·348	0·351	0·354	0·357
Southeast Asia	0·305	0·311	0·318	0·325	0·332	0·339	0·346	0·354	0·362	0·37	0·378	0·385	0·393	0·401	0·409	0·417	0·424	0·431	0·439	0·447
Cambodia	0·217	0·218	0·219	0·22	0·221	0·22	0·22	0·22	0·221	0·223	0·225	0·227	0·23	0·233	0·237	0·242	0·246	0·251	0·256	0·261
Indonesia	0·274	0·28	0·287	0·296	0·304	0·312	0·321	0·33	0·339	0·348	0·357	0·367	0·376	0·386	0·395	0·405	0·414	0·424	0·433	0·443
Aceh	0·312	0·318	0·324	0·332	0·339	0·347	0·354	0·362	0·37	0·378	0·386	0·395	0·404	0·413	0·422	0·431	0·441	0·451	0·461	0·472
Bali	0·243	0·25	0·257	0·265	0·275	0·284	0·293	0·303	0·313	0·323	0·334	0·345	0·356	0·367	0·378	0·389	0·4	0·41	0·421	0·432
Bangka-Belitung Islands	0·295	0·301	0·308	0·315	0·323	0·33	0·337	0·344	0·351	0·358	0·366	0·373	0·38	0·387	0·394	0·401	0·409	0·417	0·425	0·433
Banten	0·289	0·295	0·302	0·31	0·318	0·326	0·333	0·341	0·349	0·357	0·366	0·374	0·382	0·391	0·399	0·408	0·416	0·425	0·434	0·443
Bengkulu	0·249	0·254	0·26	0·266	0·274	0·28	0·287	0·294	0·301	0·308	0·316	0·323	0·331	0·339	0·348	0·356	0·365	0·375	0·384	0·395
Gorontalo	0·221	0·227	0·233	0·241	0·249	0·256	0·264	0·271	0·278	0·286	0·294	0·301	0·308	0·315	0·322	0·329	0·336	0·343	0·351	0·359
Jakarta	0·359	0·366	0·375	0·385	0·396	0·406	0·417	0·428	0·44	0·452	0·464	0·476	0·488	0·5	0·513	0·525	0·536	0·548	0·559	0·57
Jambi	0·246	0·252	0·259	0·267	0·275	0·283	0·291	0·299	0·307	0·316	0·325	0·335	0·344	0·353	0·363	0·373	0·382	0·392	0·402	0·412
West Java	0·268	0·274	0·28	0·288	0·296	0·304	0·312	0·32	0·329	0·338	0·347	0·357	0·366	0·376	0·386	0·396	0·406	0·416	0·425	0·435
Central Java	0·243	0·249	0·256	0·264	0·273	0·281	0·29	0·299	0·307	0·316	0·326	0·335	0·344	0·352	0·362	0·37	0·379	0·388	0·397	0·405
East Java	0·251	0·258	0·265	0·274	0·283	0·292	0·301	0·31	0·32	0·329	0·34	0·35	0·36	0·37	0·38	0·389	0·399	0·408	0·417	0·426
West Kalimantan	0·229	0·234	0·24	0·247	0·254	0·261	0·268	0·276	0·283	0·291	0·299	0·308	0·316	0·325	0·333	0·342	0·351	0·36	0·37	0·379
South Kalimantan	0·263	0·269	0·276	0·283	0·292	0·3	0·308	0·316	0·325	0·334	0·343	0·353	0·362	0·371	0·381	0·39	0·4	0·409	0·418	0·428
Central Kalimantan	0·285	0·291	0·298	0·306	0·314	0·321	0·329	0·337	0·345	0·353	0·362	0·37	0·379	0·387	0·396	0·405	0·414	0·423	0·432	0·442
East Kalimantan	0·35	0·358	0·366	0·375	0·384	0·394	0·404	0·414	0·425	0·436	0·448	0·46	0·472	0·484	0·497	0·509	0·52	0·532	0·542	0·553
North Kalimantan	0·341	0·347	0·354	0·363	0·371	0·379	0·388	0·397	0·405	0·414	0·423	0·432	0·441	0·45	0·459	0·467	0·476	0·485	0·494	0·504
Riau Islands	0·387	0·394	0·402	0·41	0·419	0·427	0·436	0·444	0·452	0·461	0·47	0·479	0·487	0·496	0·505	0·514	0·523	0·532	0·542	0·551
Lampung	0·235	0·241	0·247	0·254	0·262	0·269	0·277	0·285	0·292	0·3	0·309	0·317	0·325	0·333	0·342	0·35	0·359	0·367	0·376	0·385
Maluku	0·322	0·328	0·335	0·344	0·352	0·36	0·369	0·377	0·385	0·393	0·402	0·41	0·418	0·425	0·433	0·441	0·448	0·456	0·463	0·471
North Maluku	0·221	0·227	0·235	0·244	0·253	0·261	0·27	0·278	0·287	0·295	0·304	0·312	0·319	0·326	0·333	0·34	0·347	0·354	0·361	0·368
West Nusa Tenggara	0·195	0·2	0·206	0·213	0·22	0·227	0·234	0·241	0·247	0·254	0·261	0·269	0·276	0·283	0·291	0·298	0·306	0·314	0·322	0·331
East Nusa Tenggara	0·217	0·223	0·231	0·24	0·25	0·259	0·267	0·276	0·285	0·293	0·301	0·31	0·317	0·323	0·329	0·335	0·341	0·347	0·353	0·36
Papua	0·313	0·318	0·324	0·331	0·338	0·345	0·352	0·359	0·365	0·372	0·38	0·387	0·394	0·401	0·408	0·415	0·423	0·43	0·437	0·445
West Papua	0·293	0·298	0·305	0·312	0·319	0·326	0·334	0·341	0·348	0·356	0·364	0·372	0·379	0·387	0·395	0·402	0·41	0·418	0·426	0·434
Riau	0·344	0·35	0·357	0·365	0·373	0·38	0·388	0·396	0·405	0·413	0·422	0·431	0·44	0·45	0·46	0·47	0·481	0·491	0·502	0·513
West Sulawesi	0·223	0·228	0·234	0·241	0·249	0·256	0·263	0·27	0·277	0·285	0·293	0·301	0·308	0·315	0·323	0·331	0·338	0·346	0·354	0·362
South Sulawesi	0·248	0·253	0·26	0·268	0·276	0·284	0·292	0·3	0·308	0·316	0·325	0·333	0·342	0·35	0·358	0·367	0·375	0·383	0·392	0·4
Central Sulawesi	0·24	0·245	0·252	0·26	0·268	0·275	0·283	0·291	0·299	0·308	0·317	0·326	0·334	0·343	0·352	0·361	0·369	0·378	0·387	0·396
Southeast Sulawesi	0·21	0·216	0·222	0·229	0·237	0·244	0·251	0·259	0·267	0·275	0·284	0·292	0·301	0·309	0·318	0·327	0·335	0·344	0·354	0·363
North Sulawesi	0·286	0·293	0·3	0·308	0·318	0·326	0·336	0·345	0·355	0·365	0·376	0·387	0·397	0·408	0·418	0·428	0·437	0·446	0·455	0·464
West Sumatra	0·299	0·305	0·312	0·32	0·328	0·336	0·344	0·352	0·359	0·367	0·376	0·385	0·393	0·401	0·41	0·419	0·427	0·436	0·444	0·453
South Sumatra	0·294	0·299	0·306	0·313	0·321	0·328	0·335	0·343	0·35	0·358	0·366	0·375	0·383	0·391	0·4	0·409	0·418	0·427	0·436	0·445
North Sumatra	0·309	0·315	0·322	0·33	0·338	0·346	0·354	0·362	0·369	0·377	0·386	0·394	0·402	0·41	0·419	0·427	0·435	0·443	0·451	0·46
Yogyakarta	0·293	0·299	0·307	0·315	0·324	0·333	0·341	0·35	0·359	0·369	0·378	0·388	0·397	0·406	0·415	0·423	0·432	0·441	0·449	0·458
Laos	0·182	0·185	0·188	0·192	0·195	0·199	0·203	0·206	0·21	0·213	0·217	0·222	0·227	0·232	0·237	0·243	0·248	0·253	0·258	0·263
Malaysia	0·376	0·385	0·397	0·405	0·413	0·421	0·43	0·441	0·449	0·463	0·475	0·481	0·491	0·499	0·506	0·515	0·52	0·522	0·531	0·538
Maldives	0·199	0·201	0·204	0·206	0·209	0·211	0·213	0·215	0·218	0·222	0·227	0·232	0·238	0·244	0·251	0·258	0·266	0·275	0·284	0·293
Mauritius	0·4	0·407	0·415	0·422	0·429	0·435	0·442	0·447	0·451	0·458	0·467	0·476	0·487	0·496	0·503	0·509	0·512	0·514	0·517	0·521
Myanmar	0·176	0·183	0·19	0·196	0·201	0·207	0·213	0·218	0·224	0·229	0·235	0·241	0·247	0·253	0·26	0·266	0·271	0·276	0·278	0·281
Philippines	0·377	0·382	0·387	0·393	0·399	0·405	0·412	0·419	0·426	0·433	0·44	0·447	0·455	0·462	0·467	0·472	0·477	0·482	0·487	0·492
Seychelles	0·382	0·389	0·396	0·405	0·414	0·423	0·434	0·445	0·457	0·47	0·481	0·491	0·5	0·509	0·518	0·527	0·535	0·543	0·551	0·559
Sri Lanka	0·398	0·403	0·408	0·414	0·419	0·424	0·428	0·431	0·434	0·437	0·44	0·444	0·448	0·454	0·46	0·468	0·476	0·484	0·491	0·498
Thailand	0·345	0·353	0·36	0·368	0·375	0·382	0·39	0·399	0·407	0·415	0·421	0·428	0·436	0·444	0·452	0·461	0·47	0·479	0·489	0·498
Timor-Leste	0·188	0·192	0·197	0·203	0·209	0·21	0·215	0·219	0·223	0·227	0·231	0·234	0·238	0·242	0·246	0·25	0·254	0·259	0·264	0·269
Vietnam	0·276	0·279	0·283	0·287	0·292	0·295	0·301	0·308	0·314	0·321	0·326	0·332	0·338	0·345	0·352	0·359	0·365	0·371	0·377	0·383
Sub-Saharan Africa	0·205	0·209	0·213	0·218	0·222	0·226	0·231	0·235	0·24	0·244	0·249	0·253	0·257	0·262	0·266	0·271	0·275	0·279	0·283	0·287
Central sub-Saharan Africa	0·181	0·184	0·188	0·192	0·196	0·2	0·204	0·208	0·212	0·216	0·22	0·225	0·229	0·234	0·239	0·244	0·249	0·254	0·259	0·264
Angola	0·15	0·154	0·158	0·162	0·166	0·17	0·174	0·178	0·183	0·187	0·192	0·196	0·2	0·205	0·209	0·214	0·218	0·223	0·228	0·233
Central African Republic	0·123	0·125	0·127	0·13	0·132	0·135	0·138	0·141	0·144	0·148	0·15	0·154	0·158	0·161	0·164	0·168	0·172	0·175	0·179	0·183
Congo (Brazzaville)	0·19	0·196	0·201	0·208	0·214	0·22	0·228	0·235	0·242	0·25	0·26	0·269	0·28	0·291	0·303	0·314	0·324	0·334	0·344	0·354
DR Congo	0·185	0·188	0·191	0·195	0·199	0·201	0·204	0·207	0·21	0·214	0·217	0·221	0·225	0·229	0·233	0·238	0·243	0·247	0·252	0·256
Equatorial Guinea	0·114	0·117	0·12	0·124	0·128	0·133	0·139	0·145	0·15	0·155	0·159	0·164	0·168	0·172	0·176	0·181	0·185	0·19	0·196	0·202
Gabon	0·216	0·221	0·227	0·234	0·241	0·248	0·258	0·267	0·275	0·284	0·293	0·302	0·31	0·319	0·329	0·339	0·348	0·357	0·367	0·377
Eastern sub-Saharan Africa	0·161	0·164	0·168	0·171	0·175	0·178	0·182	0·186	0·189	0·193	0·197	0·2	0·204	0·208	0·212	0·216	0·22	0·223	0·227	0·231
Burundi	0·127	0·131	0·134	0·137	0·14	0·143	0·146	0·151	0·155	0·159	0·162	0·166	0·169	0·173	0·176	0·18	0·183	0·187	0·191	0·194
Comoros	0·161	0·164	0·166	0·17	0·173	0·178	0·182	0·186	0·191	0·196	0·202	0·208	0·214	0·221	0·228	0·236	0·243	0·251	0·259	0·266
Djibouti	0·176	0·18	0·183	0·187	0·191	0·195	0·2	0·205	0·21	0·215	0·22	0·223	0·227	0·231	0·236	0·241	0·247	0·253	0·261	0·269
Eritrea	0·115	0·119	0·123	0·127	0·132	0·136	0·14	0·144	0·148	0·152	0·156	0·16	0·164	0·168	0·172	0·176	0·18	0·184	0·189	0·193
Ethiopia	0·0847	0·0868	0·0895	0·092	0·0941	0·0967	0·099	0·101	0·103	0·106	0·109	0·112	0·116	0·12	0·124	0·127	0·13	0·134	0·137	0·14
Kenya	0·187	0·193	0·199	0·205	0·212	0·218	0·224	0·231	0·239	0·246	0·254	0·261	0·269	0·277	0·285	0·292	0·3	0·309	0·317	0·325
Baringo	0·131	0·133	0·136	0·139	0·142	0·145	0·148	0·151	0·155	0·16	0·165	0·171	0·177	0·184	0·192	0·2	0·209	0·218	0·228	0·238

Appendix Table 15. Socio-demographic Index values for all estimated GBD 2019 locations, 1970–1989																				
**Location**	**1970**	**1971**	**1972**	**1973**	**1974**	**1975**	**1976**	**1977**	**1978**	**1979**	**1980**	**1981**	**1982**	**1983**	**1984**	**1985**	**1986**	**1987**	**1988**	**1989**
Bomet	0·131	0·135	0·138	0·142	0·146	0·151	0·154	0·159	0·164	0·17	0·177	0·184	0·192	0·201	0·21	0·219	0·229	0·239	0·249	0·26
Bungoma	0·151	0·156	0·161	0·167	0·172	0·178	0·183	0·188	0·194	0·2	0·206	0·212	0·219	0·226	0·233	0·241	0·249	0·257	0·265	0·273
Busia	0·137	0·142	0·148	0·153	0·159	0·164	0·169	0·174	0·179	0·185	0·19	0·196	0·202	0·208	0·214	0·22	0·227	0·234	0·241	0·248
Elgeyo Marakwet	0·143	0·147	0·152	0·156	0·161	0·166	0·17	0·174	0·179	0·184	0·19	0·195	0·201	0·208	0·215	0·222	0·23	0·238	0·247	0·255
Embu	0·217	0·223	0·23	0·236	0·242	0·249	0·255	0·262	0·269	0·278	0·287	0·296	0·305	0·314	0·324	0·333	0·343	0·352	0·361	0·369
Garissa	0·127	0·13	0·134	0·137	0·141	0·144	0·147	0·15	0·153	0·156	0·158	0·161	0·163	0·165	0·168	0·17	0·173	0·176	0·18	0·183
Homa Bay	0·141	0·144	0·147	0·15	0·152	0·154	0·156	0·158	0·16	0·162	0·164	0·167	0·169	0·173	0·176	0·18	0·184	0·19	0·196	0·202
Isiolo	0·144	0·148	0·153	0·157	0·161	0·165	0·169	0·173	0·178	0·182	0·187	0·192	0·197	0·202	0·207	0·212	0·217	0·223	0·229	0·235
Kajiado	0·206	0·211	0·215	0·221	0·226	0·232	0·237	0·243	0·25	0·258	0·266	0·274	0·283	0·292	0·301	0·311	0·321	0·33	0·34	0·35
Kakamega	0·149	0·155	0·16	0·166	0·171	0·177	0·182	0·188	0·193	0·2	0·206	0·213	0·22	0·227	0·234	0·242	0·25	0·257	0·266	0·273
Kericho	0·0601	0·0628	0·0684	0·0746	0·0811	0·0909	0·0976	0·105	0·114	0·123	0·133	0·144	0·155	0·166	0·178	0·189	0·2	0·211	0·222	0·233
Kiambu	0·23	0·237	0·245	0·252	0·26	0·268	0·276	0·285	0·295	0·305	0·316	0·328	0·339	0·351	0·362	0·373	0·385	0·395	0·406	0·416
Kilifi	0·162	0·167	0·172	0·177	0·182	0·188	0·193	0·199	0·205	0·211	0·217	0·224	0·231	0·238	0·245	0·252	0·26	0·268	0·276	0·284
Kirinyaga	0·175	0·182	0·19	0·198	0·206	0·214	0·223	0·232	0·241	0·251	0·262	0·273	0·284	0·296	0·307	0·319	0·33	0·341	0·352	0·362
Kisii	0·179	0·185	0·191	0·198	0·204	0·211	0·217	0·224	0·231	0·239	0·247	0·255	0·264	0·274	0·283	0·293	0·303	0·313	0·323	0·334
Kisumu	0·184	0·189	0·195	0·2	0·205	0·211	0·216	0·221	0·227	0·234	0·242	0·25	0·258	0·267	0·276	0·285	0·295	0·304	0·314	0·323
Kitui	0·147	0·152	0·156	0·161	0·166	0·171	0·175	0·18	0·185	0·19	0·197	0·203	0·209	0·216	0·222	0·229	0·237	0·244	0·251	0·259
Kwale	0·155	0·161	0·167	0·173	0·179	0·185	0·19	0·196	0·202	0·208	0·214	0·221	0·227	0·234	0·24	0·247	0·254	0·262	0·269	0·277
Laikipia	0·181	0·186	0·192	0·198	0·204	0·21	0·215	0·222	0·229	0·237	0·246	0·255	0·265	0·275	0·286	0·296	0·307	0·317	0·327	0·336
Lamu	0·161	0·167	0·174	0·181	0·188	0·196	0·203	0·21	0·217	0·225	0·233	0·241	0·249	0·257	0·265	0·272	0·28	0·288	0·295	0·303
Machakos	0·206	0·212	0·218	0·224	0·23	0·236	0·242	0·248	0·255	0·262	0·269	0·277	0·285	0·293	0·301	0·309	0·317	0·326	0·334	0·343
Makueni	0·155	0·159	0·164	0·168	0·173	0·177	0·181	0·186	0·191	0·196	0·201	0·206	0·212	0·217	0·223	0·229	0·235	0·242	0·248	0·255
Mandera	0·123	0·125	0·128	0·13	0·133	0·135	0·137	0·139	0·141	0·143	0·145	0·147	0·15	0·152	0·154	0·156	0·159	0·162	0·165	0·169
Marsabit	0·126	0·129	0·132	0·135	0·138	0·142	0·144	0·148	0·151	0·155	0·159	0·163	0·167	0·171	0·175	0·179	0·184	0·188	0·193	0·197
Meru	0·187	0·193	0·199	0·206	0·212	0·218	0·224	0·23	0·236	0·243	0·25	0·258	0·265	0·273	0·28	0·288	0·296	0·305	0·313	0·322
Migori	0·143	0·146	0·149	0·151	0·154	0·156	0·158	0·16	0·162	0·164	0·167	0·17	0·174	0·178	0·182	0·186	0·192	0·197	0·203	0·21
Mombasa	0·27	0·278	0·287	0·296	0·305	0·313	0·32	0·328	0·336	0·344	0·352	0·36	0·368	0·375	0·382	0·388	0·395	0·402	0·409	0·415
Murang'a	0·179	0·185	0·19	0·196	0·202	0·209	0·215	0·223	0·231	0·241	0·251	0·261	0·272	0·283	0·295	0·306	0·317	0·327	0·338	0·348
Nairobi	0·377	0·387	0·397	0·407	0·416	0·424	0·432	0·441	0·45	0·46	0·469	0·478	0·487	0·495	0·502	0·509	0·516	0·522	0·527	0·532
Nakuru	0·204	0·21	0·215	0·221	0·226	0·232	0·237	0·243	0·25	0·256	0·264	0·272	0·28	0·288	0·296	0·305	0·314	0·323	0·332	0·34
Nandi	0·145	0·15	0·154	0·159	0·164	0·17	0·175	0·181	0·188	0·195	0·204	0·213	0·223	0·234	0·245	0·256	0·268	0·28	0·291	0·303
Narok	0·0776	0·0775	0·0776	0·078	0·0785	0·0819	0·0836	0·0864	0·0904	0·0957	0·103	0·111	0·119	0·129	0·139	0·149	0·16	0·171	0·181	0·192
Nyamira	0·196	0·202	0·209	0·215	0·221	0·228	0·233	0·239	0·245	0·252	0·259	0·267	0·274	0·282	0·29	0·298	0·307	0·316	0·325	0·334
Nyandarua	0·193	0·198	0·203	0·207	0·212	0·218	0·222	0·227	0·233	0·24	0·247	0·255	0·263	0·272	0·281	0·29	0·299	0·308	0·317	0·326
Nyeri	0·198	0·205	0·212	0·219	0·227	0·234	0·242	0·25	0·259	0·268	0·279	0·29	0·301	0·312	0·324	0·336	0·347	0·358	0·369	0·379
Samburu	0·0828	0·0849	0·0871	0·0893	0·0916	0·0947	0·0972	0·1	0·104	0·108	0·113	0·118	0·123	0·129	0·136	0·142	0·149	0·156	0·163	0·17
Siaya	0·138	0·141	0·145	0·148	0·151	0·154	0·156	0·159	0·162	0·165	0·169	0·173	0·177	0·182	0·187	0·192	0·198	0·204	0·211	0·218
Taita Taveta	0·196	0·202	0·209	0·216	0·223	0·23	0·236	0·243	0·251	0·258	0·267	0·275	0·283	0·292	0·3	0·309	0·317	0·326	0·334	0·343
Tana River	0·115	0·12	0·125	0·13	0·136	0·141	0·146	0·151	0·157	0·162	0·168	0·173	0·179	0·184	0·189	0·195	0·201	0·207	0·213	0·219
Tharaka Nithi	0·207	0·213	0·219	0·225	0·231	0·237	0·243	0·249	0·256	0·262	0·269	0·276	0·283	0·289	0·297	0·304	0·311	0·318	0·326	0·333
Trans Nzoia	0·191	0·195	0·199	0·203	0·207	0·212	0·215	0·219	0·224	0·23	0·236	0·243	0·25	0·258	0·267	0·275	0·284	0·293	0·303	0·312
Turkana	0·11	0·112	0·115	0·118	0·121	0·124	0·127	0·13	0·133	0·136	0·139	0·143	0·147	0·15	0·154	0·159	0·163	0·168	0·173	0·179
Uasin Gishu	0·183	0·19	0·196	0·203	0·21	0·217	0·224	0·231	0·24	0·249	0·259	0·27	0·281	0·293	0·305	0·316	0·328	0·34	0·351	0·361
Vihiga	0·15	0·155	0·16	0·165	0·169	0·173	0·178	0·183	0·189	0·194	0·2	0·206	0·213	0·22	0·227	0·234	0·242	0·25	0·258	0·267
Wajir	0·114	0·116	0·118	0·119	0·121	0·122	0·123	0·124	0·125	0·126	0·128	0·129	0·13	0·131	0·132	0·134	0·136	0·138	0·14	0·143
West Pokot	0·0965	0·0984	0·1	0·102	0·104	0·107	0·109	0·112	0·114	0·118	0·122	0·126	0·132	0·137	0·143	0·15	0·157	0·165	0·173	0·181
Madagascar	0·175	0·179	0·182	0·186	0·19	0·194	0·199	0·203	0·208	0·213	0·219	0·224	0·229	0·234	0·239	0·244	0·249	0·253	0·257	0·261
Malawi	0·152	0·156	0·161	0·165	0·169	0·172	0·176	0·179	0·183	0·186	0·189	0·192	0·194	0·197	0·2	0·204	0·207	0·209	0·21	0·212
Mozambique	0·0961	0·0991	0·102	0·105	0·107	0·109	0·11	0·112	0·113	0·114	0·115	0·117	0·118	0·118	0·118	0·117	0·116	0·115	0·116	0·118
Rwanda	0·152	0·157	0·161	0·166	0·171	0·175	0·181	0·187	0·192	0·199	0·205	0·212	0·218	0·224	0·23	0·235	0·241	0·246	0·25	0·254
Somalia	0·0725	0·074	0·0788	0·08	0·0806	0·0828	0·0823	0·0822	0·081	0·0752	0·0655	0·0533	0·0461	0·0468	0·0475	0·0481	0·0488	0·0494	0·0499	0·0504
South Sudan	0·189	0·192	0·195	0·198	0·201	0·204	0·207	0·21	0·213	0·216	0·219	0·222	0·224	0·227	0·23	0·233	0·236	0·239	0·242	0·245
Uganda	0·126	0·129	0·132	0·134	0·137	0·139	0·141	0·144	0·145	0·147	0·148	0·149	0·15	0·152	0·154	0·156	0·157	0·159	0·161	0·164
Tanzania	0·166	0·17	0·175	0·179	0·184	0·189	0·194	0·2	0·205	0·21	0·215	0·22	0·224	0·229	0·234	0·239	0·243	0·247	0·251	0·255
Zambia	0·17	0·177	0·184	0·191	0·198	0·206	0·213	0·22	0·227	0·234	0·241	0·248	0·254	0·261	0·267	0·273	0·278	0·284	0·29	0·295
Southern sub-Saharan Africa	0·385	0·391	0·397	0·404	0·411	0·417	0·424	0·431	0·437	0·444	0·451	0·458	0·465	0·471	0·478	0·484	0·49	0·496	0·502	0·508
Botswana	0·23	0·239	0·249	0·26	0·27	0·28	0·29	0·299	0·308	0·317	0·327	0·337	0·347	0·356	0·366	0·377	0·387	0·397	0·408	0·42
eSwatini	0·236	0·242	0·249	0·255	0·262	0·27	0·279	0·286	0·294	0·301	0·308	0·315	0·323	0·33	0·338	0·345	0·354	0·363	0·373	0·382
Lesotho	0·235	0·237	0·24	0·246	0·251	0·255	0·259	0·265	0·273	0·278	0·282	0·285	0·288	0·291	0·295	0·298	0·302	0·305	0·31	0·315
Namibia	0·325	0·331	0·338	0·344	0·351	0·358	0·364	0·371	0·378	0·385	0·392	0·399	0·406	0·412	0·418	0·424	0·43	0·436	0·442	0·448
South Africa	0·421	0·428	0·435	0·442	0·45	0·457	0·464	0·471	0·478	0·485	0·493	0·5	0·507	0·513	0·52	0·526	0·531	0·537	0·542	0·547
Zimbabwe	0·241	0·247	0·252	0·258	0·264	0·27	0·276	0·281	0·287	0·293	0·3	0·308	0·317	0·327	0·336	0·347	0·357	0·367	0·376	0·386
Western sub-Saharan Africa	0·189	0·193	0·197	0·2	0·205	0·209	0·214	0·218	0·223	0·227	0·231	0·235	0·239	0·243	0·247	0·251	0·254	0·257	0·261	0·264
Benin	0·134	0·136	0·139	0·142	0·146	0·149	0·152	0·155	0·158	0·162	0·166	0·17	0·174	0·178	0·183	0·187	0·192	0·196	0·2	0·204
Burkina Faso	0·0824	0·0838	0·0855	0·0869	0·0883	0·0896	0·091	0·0925	0·0946	0·0968	0·0989	0·101	0·104	0·106	0·108	0·11	0·113	0·116	0·119	0·122

Appendix Table 15. Socio-demographic Index values for all estimated GBD 2019 locations, 1970–1989

Location	1970	1971	1972	1973	1974	1975	1976	1977	1978	1979	1980	1981	1982	1983	1984	1985	1986	1987	1988	1989
Cape Verde	0·187	0·194	0·2	0·206	0·212	0·216	0·22	0·223	0·227	0·231	0·235	0·238	0·242	0·247	0·252	0·257	0·264	0·27	0·277	0·285
Cameroon	0·202	0·205	0·208	0·211	0·214	0·218	0·221	0·225	0·23	0·235	0·24	0·246	0·253	0·26	0·267	0·276	0·284	0·292	0·299	0·307
Chad	0·0823	0·0834	0·0844	0·0852	0·0861	0·0874	0·0886	0·0897	0·0908	0·091	0·0911	0·091	0·0911	0·092	0·0932	0·095	0·097	0·0993	0·102	0·105
Côte d'Ivoire	0·177	0·181	0·184	0·188	0·191	0·194	0·198	0·202	0·205	0·209	0·213	0·216	0·22	0·224	0·227	0·232	0·236	0·241	0·246	0·251
The Gambia	0·16	0·161	0·163	0·165	0·167	0·169	0·172	0·174	0·176	0·179	0·181	0·184	0·187	0·19	0·194	0·197	0·201	0·205	0·209	0·214
Ghana	0·248	0·252	0·255	0·259	0·264	0·269	0·274	0·279	0·285	0·29	0·296	0·301	0·306	0·311	0·316	0·322	0·328	0·334	0·341	0·348
Guinea	0·119	0·12	0·122	0·125	0·127	0·13	0·135	0·138	0·141	0·144	0·147	0·149	0·152	0·154	0·157	0·159	0·162	0·164	0·167	0·171
Guinea-Bissau	0·135	0·139	0·143	0·146	0·15	0·153	0·157	0·16	0·163	0·167	0·169	0·172	0·174	0·176	0·179	0·182	0·184	0·188	0·192	0·196
Liberia	0·177	0·18	0·182	0·186	0·188	0·19	0·193	0·196	0·198	0·201	0·204	0·206	0·207	0·209	0·212	0·214	0·217	0·22	0·222	0·222
Mali	0·0843	0·0859	0·0875	0·089	0·0902	0·092	0·0943	0·0968	0·099	0·102	0·104	0·106	0·108	0·11	0·112	0·114	0·116	0·118	0·12	0·123
Mauritania	0·236	0·239	0·241	0·243	0·245	0·247	0·25	0·253	0·255	0·258	0·261	0·265	0·268	0·272	0·276	0·281	0·285	0·291	0·296	0·302
Niger	0·0608	0·0608	0·0607	0·0602	0·0599	0·0593	0·059	0·0588	0·059	0·0595	0·0601	0·0609	0·0618	0·0629	0·0638	0·0649	0·0663	0·0678	0·0694	0·0711
Nigeria	0·217	0·223	0·228	0·233	0·239	0·244	0·25	0·255	0·261	0·266	0·27	0·275	0·279	0·283	0·287	0·292	0·294	0·296	0·299	0·301
São Tomé and PrÍncipe	0·199	0·205	0·211	0·217	0·223	0·229	0·235	0·242	0·248	0·255	0·261	0·265	0·27	0·274	0·278	0·282	0·286	0·289	0·292	0·295
Senegal	0·138	0·14	0·143	0·145	0·148	0·15	0·154	0·157	0·161	0·165	0·169	0·173	0·179	0·184	0·189	0·195	0·201	0·207	0·214	0·22
Sierra Leone	0·158	0·161	0·164	0·167	0·17	0·172	0·175	0·177	0·179	0·181	0·184	0·186	0·188	0·19	0·193	0·195	0·197	0·2	0·202	0·205
Togo	0·168	0·172	0·176	0·18	0·185	0·19	0·194	0·199	0·205	0·211	0·217	0·222	0·227	0·232	0·236	0·241	0·246	0·25	0·255	0·261

Appendix Table 16. Socio-demographic Index values for all estimated GBD 2019 locations, 1990–2019

Location	1990	1991	1992	1993	1994	1995	1996	1997	1998	1999	2000	2001	2002	2003	2004	2005	2006	2007	2008	2009	2010	2011	2012	2013	2014	2015	2016	2017	2018	2019
Global	0·511	0·516	0·521	0·525	0·529	0·534	0·538	0·542	0·547	0·551	0·556	0·561	0·566	0·571	0·576	0·581	0·586	0·591	0·596	0·601	0·607	0·612	0·616	0·621	0·626	0·631	0·635	0·641	0·647	0·651
Central Europe, eastern Europe, and central Asia	0·648	0·654	0·662	0·666	0·669	0·672	0·675	0·678	0·681	0·684	0·687	0·69	0·694	0·698	0·704	0·71	0·714	0·719	0·724	0·728	0·732	0·735	0·738	0·742	0·745	0·748	0·751	0·754	0·758	0·76
Central Asia	0·551	0·555	0·557	0·558	0·559	0·559	0·56	0·56	0·561	0·563	0·566	0·569	0·574	0·579	0·585	0·591	0·598	0·605	0·611	0·617	0·622	0·627	0·632	0·637	0·642	0·647	0·651	0·655	0·659	0·663
Armenia	0·536	0·541	0·541	0·542	0·544	0·546	0·55	0·554	0·559	0·564	0·57	0·577	0·586	0·596	0·606	0·616	0·626	0·637	0·647	0·652	0·658	0·662	0·666	0·67	0·673	0·676	0·679	0·682	0·686	0·689
Azerbaijan	0·576	0·578	0·579	0·578	0·576	0·573	0·569	0·565	0·561	0·559	0·559	0·561	0·564	0·569	0·575	0·583	0·594	0·607	0·619	0·628	0·637	0·645	0·652	0·658	0·664	0·669	0·673	0·677	0·68	0·683
Georgia	0·654	0·658	0·657	0·654	0·65	0·644	0·638	0·633	0·628	0·625	0·624	0·626	0·63	0·636	0·641	0·646	0·651	0·656	0·66	0·663	0·665	0·668	0·672	0·676	0·68	0·684	0·688	0·693	0·697	0·702
Kazakhstan	0·602	0·606	0·611	0·615	0·619	0·622	0·625	0·628	0·63	0·632	0·635	0·639	0·644	0·649	0·655	0·661	0·667	0·674	0·679	0·683	0·688	0·692	0·696	0·7	0·704	0·708	0·712	0·716	0·72	0·723
Kyrgyzstan	0·532	0·537	0·541	0·543	0·542	0·541	0·539	0·537	0·536	0·534	0·534	0·535	0·537	0·54	0·544	0·546	0·549	0·552	0·555	0·558	0·56	0·563	0·565	0·569	0·574	0·578	0·583	0·588	0·592	0·596
Mongolia	0·465	0·47	0·475	0·48	0·484	0·49	0·495	0·501	0·506	0·512	0·517	0·523	0·528	0·534	0·539	0·545	0·55	0·555	0·56	0·563	0·566	0·57	0·575	0·579	0·584	0·588	0·592	0·597	0·601	0·606
Tajikistan	0·468	0·473	0·474	0·474	0·472	0·468	0·462	0·457	0·451	0·445	0·441	0·44	0·443	0·448	0·456	0·463	0·47	0·477	0·483	0·489	0·495	0·5	0·505	0·511	0·516	0·521	0·526	0·531	0·535	0·539
Turkmenistan	0·548	0·551	0·554	0·557	0·557	0·558	0·558	0·557	0·556	0·557	0·561	0·565	0·57	0·576	0·582	0·588	0·595	0·601	0·606	0·611	0·616	0·622	0·628	0·635	0·642	0·648	0·654	0·66	0·666	0·67
Uzbekistan	0·49	0·492	0·494	0·496	0·498	0·501	0·505	0·51	0·515	0·52	0·525	0·531	0·536	0·541	0·546	0·551	0·556	0·561	0·567	0·572	0·578	0·584	0·59	0·597	0·603	0·609	0·616	0·622	0·627	0·631
Central Europe	0·641	0·647	0·652	0·658	0·665	0·672	0·678	0·683	0·689	0·695	0·702	0·709	0·715	0·72	0·726	0·731	0·736	0·74	0·745	0·75	0·756	0·76	0·764	0·768	0·771	0·775	0·778	0·781	0·785	0·788
Albania	0·54	0·537	0·534	0·533	0·535	0·538	0·544	0·549	0·555	0·561	0·569	0·577	0·585	0·593	0·601	0·608	0·615	0·621	0·627	0·631	0·636	0·64	0·645	0·651	0·658	0·664	0·669	0·674	0·678	0·681
Bosnia and Herzegovina	0·533	0·534	0·532	0·529	0·527	0·528	0·54	0·558	0·576	0·591	0·604	0·616	0·626	0·636	0·644	0·651	0·658	0·665	0·671	0·677	0·682	0·686	0·691	0·695	0·698	0·702	0·706	0·71	0·714	0·718
Bulgaria	0·631	0·641	0·648	0·656	0·666	0·671	0·676	0·681	0·677	0·675	0·68	0·688	0·693	0·697	0·701	0·706	0·71	0·715	0·718	0·724	0·733	0·737	0·74	0·743	0·746	0·75	0·752	0·755	0·76	0·764
Croatia	0·68	0·688	0·692	0·692	0·692	0·691	0·691	0·697	0·703	0·707	0·713	0·719	0·725	0·73	0·734	0·739	0·745	0·748	0·753	0·758	0·763	0·767	0·77	0·774	0·777	0·781	0·784	0·788	0·791	0·794
Czech Republic	0·688	0·696	0·705	0·718	0·736	0·748	0·755	0·76	0·765	0·771	0·776	0·782	0·786	0·79	0·794	0·798	0·801	0·804	0·807	0·81	0·813	0·816	0·818	0·819	0·82	0·82	0·82	0·822	0·825	0·828
Hungary	0·659	0·663	0·671	0·678	0·685	0·693	0·7	0·707	0·713	0·718	0·724	0·73	0·735	0·741	0·746	0·751	0·756	0·76	0·763	0·768	0·772	0·773	0·774	0·774	0·775	0·778	0·781	0·784	0·788	0·791
Montenegro	0·701	0·701	0·699	0·695	0·69	0·687	0·686	0·687	0·69	0·692	0·696	0·701	0·706	0·712	0·717	0·723	0·729	0·736	0·743	0·749	0·754	0·759	0·764	0·768	0·773	0·777	0·78	0·784	0·788	0·791
North Macedonia	0·618	0·62	0·623	0·625	0·627	0·631	0·635	0·64	0·646	0·651	0·656	0·662	0·668	0·674	0·679	0·684	0·689	0·694	0·7	0·704	0·709	0·713	0·717	0·722	0·726	0·73	0·734	0·738	0·741	0·744
Poland	0·632	0·637	0·644	0·653	0·661	0·67	0·677	0·685	0·693	0·701	0·709	0·717	0·724	0·73	0·735	0·74	0·743	0·747	0·752	0·757	0·763	0·77	0·775	0·78	0·784	0·788	0·791	0·795	0·798	0·802
Romania	0·625	0·632	0·635	0·638	0·643	0·649	0·653	0·655	0·659	0·664	0·669	0·677	0·682	0·686	0·693	0·698	0·702	0·707	0·711	0·718	0·726	0·729	0·734	0·74	0·741	0·744	0·747	0·752	0·756	0·76
Serbia	0·626	0·635	0·639	0·639	0·64	0·64	0·644	0·647	0·651	0·657	0·661	0·665	0·67	0·676	0·685	0·694	0·702	0·709	0·716	0·723	0·729	0·735	0·739	0·744	0·748	0·753	0·756	0·76	0·763	0·767
Slovakia	0·656	0·662	0·668	0·679	0·693	0·702	0·709	0·716	0·724	0·731	0·739	0·746	0·752	0·756	0·76	0·766	0·772	0·777	0·781	0·784	0·789	0·794	0·798	0·801	0·803	0·803	0·804	0·805	0·808	0·812
Slovenia	0·726	0·731	0·736	0·741	0·746	0·751	0·756	0·762	0·768	0·774	0·78	0·787	0·793	0·797	0·802	0·807	0·811	0·814	0·818	0·82	0·822	0·824	0·825	0·827	0·829	0·831	0·833	0·835	0·838	0·84
Eastern Europe	0·68	0·687	0·697	0·702	0·702	0·705	0·707	0·708	0·709	0·711	0·711	0·713	0·716	0·72	0·727	0·734	0·74	0·745	0·751	0·757	0·762	0·765	0·768	0·772	0·777	0·781	0·785	0·788	0·791	0·793
Belarus	0·591	0·595	0·6	0·606	0·611	0·614	0·618	0·62	0·621	0·624	0·629	0·635	0·642	0·65	0·658	0·665	0·671	0·678	0·687	0·695	0·703	0·709	0·713	0·719	0·725	0·73	0·734	0·738	0·742	0·745
Estonia	0·665	0·676	0·687	0·696	0·7	0·705	0·711	0·717	0·721	0·726	0·733	0·741	0·748	0·753	0·759	0·765	0·771	0·777	0·785	0·792	0·798	0·804	0·809	0·813	0·817	0·821	0·825	0·829	0·833	0·835
Latvia	0·675	0·682	0·691	0·7	0·708	0·713	0·716	0·719	0·721	0·723	0·727	0·733	0·739	0·745	0·753	0·76	0·766	0·774	0·784	0·793	0·797	0·798	0·801	0·803	0·804	0·805	0·809	0·813	0·817	0·82
Lithuania	0·67	0·672	0·682	0·691	0·694	0·696	0·7	0·705	0·709	0·714	0·723	0·73	0·736	0·743	0·752	0·76	0·765	0·771	0·782	0·792	0·797	0·801	0·808	0·813	0·817	0·822	0·829	0·835	0·839	0·843
Moldova	0·585	0·589	0·591	0·594	0·594	0·595	0·594	0·593	0·591	0·587	0·585	0·585	0·588	0·593	0·6	0·607	0·615	0·622	0·63	0·637	0·644	0·651	0·658	0·665	0·672	0·677	0·683	0·688	0·693	0·696
Russia	0·695	0·703	0·716	0·72	0·719	0·722	0·724	0·725	0·726	0·728	0·728	0·728	0·73	0·734	0·741	0·749	0·754	0·759	0·764	0·77	0·775	0·777	0·779	0·784	0·788	0·793	0·797	0·801	0·803	0·805
Ukraine	0·653	0·657	0·661	0·665	0·666	0·667	0·667	0·667	0·666	0·665	0·664	0·665	0·668	0·672	0·679	0·685	0·692	0·699	0·706	0·71	0·713	0·718	0·721	0·725	0·727	0·729	0·73	0·732	0·734	0·736
High income	0·755	0·76	0·765	0·769	0·773	0·777	0·78	0·783	0·786	0·788	0·791	0·795	0·799	0·801	0·804	0·806	0·807	0·809	0·812	0·816	0·82	0·823	0·826	0·829	0·832	0·835	0·839	0·842	0·845	0·847
Australasia	0·742	0·746	0·749	0·753	0·757	0·761	0·765	0·769	0·773	0·777	0·781	0·785	0·789	0·793	0·797	0·799	0·799	0·8	0·803	0·807	0·81	0·812	0·816	0·821	0·825	0·828	0·832	0·835	0·837	0·84
Australia	0·738	0·741	0·745	0·749	0·753	0·757	0·761	0·766	0·77	0·774	0·778	0·782	0·787	0·791	0·795	0·797	0·798	0·799	0·802	0·806	0·809	0·812	0·815	0·82	0·824	0·828	0·832	0·834	0·837	0·839
New Zealand	0·757	0·762	0·765	0·769	0·772	0·774	0·778	0·782	0·785	0·787	0·79	0·794	0·796	0·798	0·802	0·803	0·8	0·8	0·803	0·807	0·809	0·812	0·816	0·821	0·825	0·828	0·832	0·835	0·838	0·84
High-income Asia Pacific	0·767	0·773	0·779	0·785	0·79	0·796	0·801	0·805	0·809	0·813	0·816	0·819	0·823	0·826	0·83	0·833	0·836	0·839	0·842	0·844	0·847	0·85	0·853	0·856	0·859	0·862	0·865	0·868	0·871	0·873
Brunei	0·676	0·682	0·688	0·694	0·7	0·706	0·712	0·717	0·723	0·729	0·735	0·741	0·747	0·753	0·758	0·764	0·769	0·774	0·779	0·784	0·789	0·793	0·797	0·801	0·806	0·809	0·813	0·817	0·82	0·823
Japan	0·791	0·796	0·801	0·805	0·809	0·813	0·817	0·82	0·822	0·824	0·826	0·828	0·83	0·833	0·836	0·838	0·84	0·842	0·844	0·846	0·848	0·85	0·853	0·855	0·857	0·86	0·862	0·865	0·867	0·87
Aichi	0·801	0·806	0·811	0·816	0·82	0·825	0·828	0·831	0·833	0·835	0·837	0·838	0·841	0·843	0·846	0·848	0·85	0·852	0·854	0·856	0·859	0·861	0·864	0·866	0·869	0·871	0·874	0·876	0·879	0·881
Akita	0·746	0·75	0·754	0·759	0·763	0·768	0·772	0·776	0·779	0·781	0·784	0·786	0·789	0·791	0·794	0·796	0·798	0·8	0·802	0·803	0·806	0·808	0·811	0·813	0·816	0·819	0·822	0·824	0·827	0·83
Aomori	0·736	0·74	0·745	0·749	0·754	0·758	0·762	0·766	0·768	0·771	0·773	0·775	0·779	0·782	0·786	0·789	0·791	0·794	0·796	0·798	0·8	0·803	0·806	0·809	0·811	0·814	0·817	0·82	0·823	0·826
Chiba	0·79	0·794	0·8	0·804	0·808	0·813	0·817	0·82	0·822	0·824	0·825	0·826	0·827	0·829	0·832	0·834	0·835	0·837	0·838	0·84	0·842	0·844	0·846	0·848	0·85	0·852	0·855	0·857	0·859	0·861
Ehime	0·757	0·761	0·766	0·771	0·775	0·78	0·784	0·788	0·791	0·795	0·797	0·8	0·802	0·805	0·807	0·809	0·811	0·812	0·814	0·816	0·818	0·82	0·822	0·824	0·827	0·83	0·833	0·836	0·838	0·841
Fukui	0·767	0·772	0·778	0·782	0·787	0·791	0·795	0·799	0·802	0·806	0·808	0·811	0·813	0·816	0·818	0·821	0·823	0·826	0·828	0·831	0·833	0·836	0·838	0·84	0·842	0·844	0·846	0·849	0·851	0·854
Fukuoka	0·783	0·786	0·791	0·795	0·799	0·803	0·807	0·81	0·813	0·815	0·818	0·82	0·822	0·825	0·828	0·829	0·831	0·832	0·833	0·835	0·837	0·84	0·842	0·844	0·846	0·848	0·85	0·853	0·855	0·858
Fukushima	0·751	0·755	0·76	0·764	0·768	0·773	0·776	0·779	0·781	0·784	0·785	0·787	0·79	0·794	0·797	0·8	0·803	0·805	0·807	0·809	0·812	0·814	0·816	0·818	0·821	0·823	0·826	0·83	0·833	0·836
Gifu	0·771	0·776	0·781	0·786	0·79	0·795	0·798	0·802	0·804	0·806	0·808	0·81	0·813	0·815	0·818	0·821	0·823	0·825	0·826	0·828	0·831	0·833	0·836	0·838	0·84	0·842	0·845	0·847	0·849	0·852
Gunma	0·774	0·778	0·783	0·788	0·792	0·796	0·8	0·803	0·805	0·807	0·808	0·81	0·813	0·816	0·819	0·821	0·823	0·825	0·828	0·83	0·833	0·836	0·838	0·841	0·844	0·847	0·85	0·852	0·855	0·858
Hiroshima	0·788	0·793	0·798	0·802	0·807	0·811	0·815	0·818	0·821	0·823	0·825	0·827	0·829	0·832	0·834	0·836	0·837	0·839	0·84	0·841	0·843	0·846	0·849	0·851	0·854	0·857	0·859	0·862	0·865	0·868
Hokkaidō	0·768	0·772	0·777	0·782	0·786	0·791	0·794	0·797	0·799	0·802	0·803	0·805	0·808	0·81	0·813	0·815	0·817	0·818	0·82	0·821	0·823	0·825	0·827	0·829	0·831	0·833	0·836	0·838	0·841	0·843
Hyōgo	0·794	0·798	0·804	0·808	0·812	0·817	0·82	0·823	0·826	0·828	0·83	0·832	0·833	0·835	0·837	0·838	0·839	0·841	0·842	0·844	0·845	0·848	0·85	0·852	0·854	0·857	0·859	0·862	0·864	0·866
Ibaraki	0·773	0·777	0·782	0·787	0·791	0·796	0·8	0·803	0·805	0·807	0·808	0·81	0·812	0·815	0·818	0·821	0·823	0·826	0·828	0·831	0·834	0·837	0·839	0·842	0·844	0·847	0·85	0·852	0·855	0·858
Ishikawa	0·774	0·78	0·785	0·79	0·795	0·799	0·803	0·807	0·81	0·813	0·816	0·818	0·821	0·824	0·826	0·829	0·83	0·832	0·833	0·834	0·835	0·837	0·839	0·841	0·843	0·846	0·848	0·851	0·854	0·856
Iwate	0·738	0·742	0·747	0·752	0·757	0·762	0·766	0·769	0·772	0·775	0·777	0·779	0·782	0·785	0·789	0·791	0·793	0·796	0·798	0·8	0·803	0·806	0·808	0·811	0·814	0·818	0·821	0·824	0·828	0·831
Kagawa	0·772	0·777	0·783	0·788	0·792	0·796	0·8	0·803	0·805	0·807	0·809	0·812	0·814	0·817	0·82	0·823	0·824	0·826	0·827	0·829	0·831	0·834	0·837	0·839	0·842	0·844	0·847	0·85	0·853	0·855
Kagoshima	0·744	0·748	0·753	0·757	0·761	0·766	0·77	0·773	0·776	0·779	0·781	0·784	0·787	0·79	0·793	0·795	0·797	0·799	0·801	0·803	0·806	0·808	0·811	0·813	0·816	0·819	0·822	0·825	0·828	0·831
Kanagawa	0·82	0·824	0·828	0·832	0·836	0·84	0·843	0·846	0·848	0·849	0·85	0·851	0·853	0·855	0·857	0·859	0·86	0·862	0·864	0·865	0·867	0·869	0·87	0·872	0·873	0·875	0·877	0·878	0·88	0·882
Kōchi	0·744	0·748	0·752	0·757	0·761	0·766	0·77	0·774	0·777	0·781	0·784	0·787	0·79	0·793	0·796	0·798	0·799	0·801	0·803	0·805	0·807	0·81	0·812	0·815	0·817	0·82	0·823	0·826	0·829	0·832
Kumamoto	0·754	0·758	0·763	0·767	0·771	0·775	0·778	0·78	0·782	0·784	0·786	0·788	0·791	0·794	0·797	0·8	0·801	0·803	0·804	0·806	0·808	0·811	0·813	0·816	0·819	0·822	0·825	0·828	0·831	0·834
Kyōto	0·801	0·805	0·81	0·814	0·818	0·822	0·825	0·828	0·831	0·833	0·836	0·838	0·84	0·842	0·845	0·847	0·849	0·85	0·852	0·854	0·855	0·857	0·859	0·861	0·863	0·865	0·868	0·87	0·872	0·875
Mie	0·765	0·77	0·776	0·781	0·785	0·79	0·794	0·797	0·8	0·802	0·805	0·808	0·811	0·814	0·818	0·821	0·823	0·825	0·828	0·83	0·832	0·835	0·838	0·841	0·844	0·847	0·85	0·853	0·856	0·859
Miyagi	0·774	0·779	0·784	0·789	0·793	0·798	0·801	0·803	0·805	0·807	0·808	0·81	0·813	0·816	0·819	0·822	0·823	0·826	0·827	0·829	0·831	0·834	0·837	0·839	0·842	0·845	0·848	0·851	0·854	0·857
Miyazaki	0·741	0·744	0·749	0·752	0·755	0·759	0·763	0·767	0·771	0·774	0·777	0·779	0·782	0·785	0·788	0·791	0·792	0·794	0·795	0·797	0·799	0·802	0·805	0·807	0·81	0·814	0·817	0·82	0·823	0·826
Nagano	0·778	0·782	0·787	0·791	0·795	0·799	0·803	0·806	0·808	0·811	0·813	0·815	0·817	0·82	0·822	0·825	0·827	0·829	0·831	0·833	0·835	0·837	0·839	0·841	0·843	0·845	0·848	0·851	0·853	0·856
Nagasaki	0·743	0·747	0·752	0·756	0·761	0·765	0·769	0·772	0·775	0·778	0·78	0·783	0·785	0·787	0·79	0·792	0·794	0·796	0·797	0·799	0·801	0·804	0·807	0·81	0·813	0·815	0·818	0·821	0·824	0·827
Nara	0·779	0·784	0·789	0·794	0·799	0·803	0·807	0·81	0·812	0·815	0·817	0·819	0·822	0·824	0·826	0·827	0·829	0·83	0·831	0·833	0·834	0·836	0·837	0·839	0·841	0·843	0·845	0·847	0·849	0·851
Niigata	0·758	0·762	0·767	0·772	0·776	0·78	0·784	0·788	0·791	0·794	0·796	0·799	0·803	0·805	0·808	0·811	0·813	0·815	0·817	0·819	0·821	0·823	0·826	0·829	0·832	0·834	0·837	0·84	0·842	0·845
Ōita	0·765	0·769	0·774	0·779	0·783	0·787	0·791	0·795	0·798	0·8	0·803	0·805	0·808	0·811	0·814	0·817	0·818	0·82	0·821	0·823	0·825	0·827	0·83	0·832	0·834	0·836	0·839	0·841	0·844	0·847
Okayama	0·778	0·783	0·788	0·792	0·796	0·8	0·804	0·807	0·809	0·812	0·814	0·816	0·819	0·822	0·825	0·827	0·829	0·831	0·833	0·836	0·838	0·841	0·843	0·845	0·848	0·85	0·853	0·855	0·858	0·861
Okinawa	0·736	0·74	0·745	0·749	0·753	0·757	0·76	0·763	0·764	0·766	0·768	0·77	0·774	0·778	0·782	0·785	0·787	0·789	0·791	0·793	0·795	0·798	0·8	0·802	0·804	0·807	0·81	0·813	0·816	0·819
Ōsaka	0·806	0·81	0·816	0·82	0·824	0·828	0·832	0·834	0·837	0·839	0·841	0·842	0·844	0·846	0·848	0·849	0·85	0·852	0·853	0·854	0·856	0·858	0·86	0·862	0·864	0·866	0·869	0·871	0·873	0·875
Saga	0·752	0·756	0·761	0·765	0·77	0·774	0·778	0·781	0·783	0·786	0·788	0·79	0·794	0·797	0·8	0·802	0·804	0·806	0·808	0·81	0·812	0·814	0·816	0·818	0·82	0·822	0·825	0·828	0·831	0·834
Saitama	0·783	0·788	0·793	0·798	0·802	0·806	0·81	0·813	0·816	0·817	0·818	0·819	0·821	0·823	0·826	0·828	0·829	0·831	0·833	0·835	0·837	0·839	0·841	0·843	0·845	0·847	0·849	0·851	0·854	0·856
Shiga	0·794	0·799	0·805	0·81	0·814	0·819	0·823	0·826	0·829	0·831	0·833	0·835	0·837	0·84	0·843	0·845	0·847	0·849	0·85	0·852	0·853	0·855	0·858	0·86	0·862	0·864	0·867	0·869	0·872	0·874
Shimane	0·74	0·745	0·75	0·754	0·759	0·764	0·768	0·773	0·776	0·78	0·783	0·785	0·788	0·792	0·795	0·798	0·801	0·803	0·805	0·807	0·809	0·812	0·815	0·818	0·821	0·824	0·827	0·83	0·833	0·836
Shizuoka	0·781	0·785	0·79	0·794	0·799	0·803	0·807	0·81	0·812	0·814	0·816	0·817	0·82	0·823	0·827	0·83	0·831	0·834	0·836	0·838	0·84	0·843	0·845	0·848	0·851	0·853	0·856	0·858	0·861	0·864
Tochigi	0·773	0·778	0·783	0·788	0·793	0·797	0·8	0·803	0·805	0·807	0·809	0·811	0·813	0·816	0·819	0·822	0·824	0·827	0·829	0·831	0·834	0·836	0·839	0·841	0·844	0·847	0·851	0·854	0·857	0·859
Tokushima	0·755	0·76	0·766	0·771	0·775	0·779	0·783	0·787	0·79	0·794	0·797	0·8	0·804	0·808	0·812	0·816	0·818	0·821	0·823	0·825	0·828	0·831	0·833	0·836	0·839	0·842	0·845	0·848	0·851	0·854
Tōkyō	0·867	0·872	0·877	0·881	0·884	0·888	0·891	0·894	0·896	0·897	0·898	0·899	0·9	0·902	0·904	0·906	0·907	0·908	0·91	0·912	0·913	0·915	0·917	0·918	0·92	0·921	0·923	0·924	0·925	0·927
Tottori	0·758	0·762	0·767	0·772	0·776	0·781	0·785	0·788	0·791	0·794	0·796	0·798	0·8	0·802	0·805	0·807	0·808	0·81	0·812	0·813	0·815	0·816	0·818	0·82	0·822	0·825	0·827	0·83	0·832	0·835
Toyama	0·778	0·783	0·789	0·793	0·797	0·801	0·805	0·808	0·811	0·814	0·817	0·819	0·822	0·825	0·829	0·831	0·833	0·836	0·838	0·839	0·842	0·844	0·846	0·848	0·85	0·853	0·855	0·858	0·861	0·863
Wakayama	0·755	0·759	0·764	0·769	0·772	0·777	0·78	0·783	0·786	0·789	0·792	0·794	0·798	0·801	0·804	0·807	0·81	0·812	0·814	0·816	0·819	0·822	0·825	0·828	0·832	0·835	0·838	0·842	0·845	0·848
Yamagata	0·748	0·752	0·757	0·761	0·765	0·769	0·772	0·775	0·778	0·78	0·782	0·784	0·787	0·79	0·794	0·797	0·799	0·802	0·805	0·807	0·81	0·813	0·816	0·818	0·821	0·824	0·827	0·83	0·833	0·835
Yamaguchi	0·771	0·775	0·78	0·783	0·787	0·791	0·795	0·798	0·801	0·804	0·806	0·808	0·811	0·814	0·817	0·82	0·821	0·824	0·825	0·828	0·83	0·833	0·836	0·839	0·841	0·844	0·847	0·849	0·852	0·855
Yamanashi	0·777	0·782	0·787	0·791	0·795	0·799	0·803	0·806	0·808	0·81	0·812	0·814	0·816	0·818	0·821	0·824	0·827	0·83	0·832	0·834	0·836	0·839	0·841	0·843	0·846	0·848	0·851	0·853	0·856	0·858
South Korea	0·686	0·697	0·708	0·719	0·73	0·74	0·75	0·76	0·767	0·776	0·784	0·791	0·799	0·805	0·811	0·817	0·823	0·828	0·833	0·837	0·842	0·846	0·851	0·855	0·859	0·863	0·867	0·871	0·875	0·878
Singapore	0·688	0·697	0·705	0·714	0·723	0·731	0·74	0·749	0·756	0·762	0·769	0·776	0·783	0·789	0·794	0·801	0·808	0·814	0·822	0·828	0·835	0·839	0·843	0·847	0·85	0·852	0·855	0·858	0·86	0·861
High-income North America	0·771	0·773	0·777	0·78	0·784	0·787	0·79	0·792	0·794	0·797	0·8	0·805	0·809	0·811	0·814	0·815	0·814	0·817	0·822	0·828	0·834	0·837	0·841	0·844	0·847	0·85	0·854	0·857	0·859	0·86
Canada	0·79	0·792	0·795	0·797	0·8	0·804	0·809	0·812	0·815	0·819	0·824	0·828	0·832	0·835	0·838	0·84	0·842	0·843	0·845	0·848	0·851	0·853	0·856	0·859	0·861	0·864	0·867	0·869	0·871	0·873
Greenland	0·655	0·652	0·651	0·651	0·653	0·653	0·653	0·655	0·661	0·664	0·667	0·671	0·678	0·684	0·689	0·696	0·704	0·71	0·715	0·721	0·728	0·734	0·737	0·74	0·743	0·747	0·751	0·756	0·759	0·761

Appendix Table 16. Socio-demographic Index values for all estimated GBD 2019 locations, 1990–2019

Location	1990	1991	1992	1993	1994	1995	1996	1997	1998	1999	2000	2001	2002	2003	2004	2005	2006	2007	2008	2009	2010	2011	2012	2013	2014	2015	2016	2017	2018	2019
USA	0·768	0·771	0·775	0·778	0·782	0·785	0·788	0·789	0·791	0·794	0·797	0·802	0·806	0·809	0·811	0·812	0·811	0·814	0·819	0·826	0·832	0·835	0·839	0·842	0·845	0·849	0·853	0·856	0·858	0·859
Alabama	0·732	0·736	0·741	0·745	0·749	0·751	0·753	0·753	0·754	0·757	0·761	0·767	0·772	0·776	0·779	0·779	0·777	0·78	0·786	0·794	0·801	0·805	0·809	0·811	0·812	0·814	0·816	0·818	0·819	0·819
Alaska	0·748	0·75	0·754	0·76	0·766	0·771	0·776	0·779	0·782	0·784	0·787	0·793	0·796	0·799	0·801	0·801	0·798	0·8	0·804	0·811	0·817	0·821	0·826	0·83	0·834	0·839	0·844	0·848	0·85	0·851
Arizona	0·739	0·742	0·745	0·747	0·75	0·752	0·754	0·755	0·757	0·758	0·761	0·766	0·769	0·771	0·774	0·775	0·775	0·782	0·791	0·801	0·81	0·814	0·818	0·822	0·825	0·829	0·834	0·838	0·84	0·842
Arkansas	0·711	0·716	0·723	0·727	0·731	0·734	0·735	0·736	0·737	0·739	0·742	0·748	0·752	0·755	0·758	0·757	0·756	0·76	0·766	0·774	0·783	0·787	0·79	0·794	0·796	0·799	0·803	0·807	0·809	0·811
California	0·757	0·757	0·76	0·762	0·767	0·772	0·778	0·783	0·788	0·793	0·798	0·804	0·809	0·811	0·814	0·815	0·816	0·82	0·825	0·831	0·838	0·841	0·845	0·848	0·852	0·856	0·861	0·865	0·868	0·87
Colorado	0·788	0·791	0·794	0·798	0·801	0·803	0·805	0·806	0·807	0·808	0·81	0·815	0·818	0·821	0·824	0·825	0·825	0·829	0·834	0·84	0·847	0·851	0·855	0·859	0·863	0·867	0·871	0·874	0·876	0·877
Connecticut	0·829	0·832	0·835	0·838	0·841	0·843	0·845	0·847	0·849	0·852	0·855	0·86	0·864	0·865	0·867	0·868	0·868	0·87	0·873	0·877	0·881	0·884	0·887	0·89	0·893	0·896	0·899	0·901	0·902	0·902
Delaware	0·787	0·791	0·796	0·798	0·801	0·803	0·805	0·805	0·806	0·808	0·81	0·815	0·818	0·82	0·822	0·822	0·822	0·826	0·831	0·838	0·843	0·847	0·85	0·852	0·854	0·857	0·859	0·861	0·862	0·863
Washington, DC	0·784	0·788	0·793	0·798	0·805	0·812	0·82	0·826	0·833	0·839	0·846	0·853	0·859	0·862	0·865	0·865	0·865	0·867	0·869	0·872	0·876	0·877	0·879	0·88	0·881	0·883	0·884	0·886	0·886	0·886
Florida	0·759	0·763	0·768	0·773	0·777	0·78	0·783	0·784	0·786	0·789	0·792	0·798	0·802	0·804	0·807	0·807	0·807	0·812	0·818	0·826	0·832	0·836	0·839	0·842	0·844	0·846	0·85	0·852	0·854	0·856
Georgia	0·742	0·747	0·752	0·757	0·761	0·764	0·767	0·768	0·768	0·769	0·772	0·777	0·78	0·783	0·785	0·785	0·784	0·789	0·796	0·804	0·812	0·817	0·821	0·825	0·828	0·832	0·835	0·838	0·84	0·841
Hawaii	0·769	0·771	0·775	0·78	0·785	0·791	0·796	0·799	0·802	0·804	0·806	0·811	0·814	0·817	0·82	0·819	0·818	0·821	0·825	0·83	0·836	0·84	0·843	0·847	0·85	0·853	0·856	0·858	0·859	0·86
Idaho	0·744	0·748	0·753	0·758	0·762	0·765	0·767	0·768	0·769	0·77	0·772	0·777	0·78	0·781	0·784	0·783	0·782	0·786	0·792	0·8	0·807	0·81	0·813	0·815	0·817	0·82	0·823	0·825	0·827	0·827
Illinois	0·775	0·777	0·781	0·784	0·788	0·792	0·796	0·799	0·802	0·805	0·809	0·815	0·819	0·823	0·826	0·828	0·828	0·832	0·837	0·842	0·848	0·851	0·854	0·858	0·86	0·863	0·867	0·869	0·871	0·872
Indiana	0·758	0·761	0·766	0·769	0·772	0·774	0·775	0·776	0·777	0·779	0·782	0·787	0·791	0·793	0·795	0·794	0·792	0·795	0·8	0·806	0·812	0·816	0·819	0·823	0·826	0·829	0·833	0·835	0·837	0·838
Iowa	0·781	0·784	0·788	0·791	0·794	0·796	0·798	0·8	0·802	0·803	0·806	0·81	0·813	0·815	0·817	0·816	0·816	0·818	0·822	0·828	0·833	0·836	0·84	0·843	0·847	0·851	0·856	0·859	0·862	0·864
Kansas	0·771	0·774	0·778	0·781	0·784	0·786	0·788	0·789	0·79	0·792	0·794	0·799	0·803	0·805	0·807	0·806	0·804	0·806	0·81	0·815	0·822	0·826	0·83	0·835	0·84	0·845	0·85	0·854	0·857	0·858
Kentucky	0·729	0·733	0·738	0·743	0·747	0·749	0·752	0·753	0·754	0·756	0·76	0·766	0·77	0·773	0·776	0·775	0·772	0·774	0·778	0·784	0·79	0·794	0·797	0·8	0·803	0·806	0·81	0·812	0·814	0·815
Louisiana	0·725	0·728	0·733	0·738	0·742	0·745	0·747	0·748	0·75	0·752	0·755	0·762	0·768	0·771	0·775	0·775	0·773	0·776	0·781	0·788	0·794	0·798	0·801	0·805	0·808	0·812	0·817	0·82	0·822	0·823
Maine	0·774	0·78	0·787	0·792	0·796	0·8	0·802	0·803	0·805	0·807	0·81	0·814	0·818	0·82	0·823	0·824	0·824	0·827	0·831	0·835	0·84	0·842	0·845	0·848	0·85	0·853	0·856	0·858	0·86	0·862
Maryland	0·801	0·805	0·809	0·813	0·817	0·82	0·823	0·824	0·826	0·828	0·831	0·836	0·84	0·844	0·847	0·848	0·849	0·852	0·856	0·861	0·867	0·87	0·873	0·876	0·878	0·881	0·884	0·885	0·886	0·887
Massachusetts	0·83	0·834	0·837	0·84	0·843	0·846	0·849	0·852	0·854	0·858	0·861	0·866	0·869	0·872	0·874	0·876	0·877	0·879	0·882	0·886	0·889	0·892	0·894	0·897	0·899	0·902	0·904	0·906	0·907	0·907
Michigan	0·775	0·778	0·783	0·787	0·792	0·795	0·798	0·8	0·802	0·805	0·808	0·814	0·817	0·82	0·823	0·823	0·822	0·825	0·828	0·832	0·836	0·838	0·84	0·843	0·845	0·849	0·854	0·858	0·861	0·863
Minnesota	0·804	0·808	0·812	0·815	0·818	0·82	0·822	0·824	0·826	0·828	0·83	0·834	0·837	0·84	0·842	0·843	0·843	0·846	0·85	0·855	0·86	0·863	0·866	0·87	0·873	0·876	0·879	0·882	0·884	0·886
Mississippi	0·708	0·712	0·717	0·721	0·725	0·729	0·731	0·732	0·733	0·735	0·738	0·743	0·747	0·748	0·75	0·748	0·746	0·749	0·756	0·765	0·774	0·779	0·784	0·789	0·792	0·796	0·8	0·803	0·804	0·805
Missouri	0·758	0·761	0·766	0·77	0·774	0·777	0·779	0·78	0·782	0·784	0·787	0·792	0·796	0·797	0·799	0·799	0·797	0·8	0·805	0·811	0·818	0·821	0·825	0·828	0·831	0·834	0·838	0·841	0·843	0·844
Montana	0·764	0·767	0·773	0·778	0·783	0·787	0·79	0·792	0·793	0·794	0·796	0·8	0·803	0·805	0·808	0·808	0·807	0·809	0·813	0·818	0·823	0·826	0·829	0·833	0·837	0·842	0·847	0·851	0·854	0·856
Nebraska	0·781	0·785	0·789	0·793	0·796	0·799	0·801	0·802	0·803	0·804	0·806	0·809	0·811	0·813	0·816	0·816	0·815	0·817	0·821	0·827	0·833	0·836	0·84	0·845	0·849	0·853	0·857	0·86	0·862	0·862
Nevada	0·749	0·75	0·752	0·755	0·758	0·762	0·765	0·768	0·771	0·775	0·779	0·785	0·789	0·791	0·793	0·793	0·793	0·799	0·806	0·814	0·822	0·825	0·827	0·829	0·829	0·83	0·832	0·834	0·834	0·835
New Hampshire	0·813	0·818	0·823	0·826	0·828	0·83	0·832	0·834	0·837	0·841	0·845	0·849	0·853	0·856	0·859	0·86	0·86	0·863	0·866	0·87	0·874	0·877	0·88	0·883	0·886	0·89	0·893	0·895	0·897	0·898
New Jersey	0·816	0·819	0·823	0·826	0·83	0·833	0·835	0·837	0·838	0·84	0·843	0·847	0·85	0·853	0·856	0·857	0·858	0·86	0·863	0·868	0·872	0·875	0·878	0·88	0·883	0·886	0·888	0·89	0·892	0·892
New Mexico	0·721	0·724	0·729	0·733	0·739	0·743	0·747	0·748	0·75	0·751	0·753	0·759	0·762	0·765	0·767	0·767	0·765	0·769	0·775	0·784	0·793	0·798	0·803	0·807	0·811	0·815	0·819	0·823	0·825	0·826
New York	0·802	0·805	0·808	0·811	0·815	0·818	0·821	0·823	0·826	0·828	0·832	0·837	0·84	0·843	0·846	0·847	0·847	0·85	0·854	0·858	0·863	0·866	0·869	0·872	0·875	0·878	0·881	0·883	0·884	0·884
North Carolina	0·747	0·752	0·757	0·761	0·764	0·767	0·768	0·768	0·769	0·771	0·774	0·78	0·784	0·787	0·79	0·79	0·789	0·792	0·798	0·806	0·813	0·817	0·822	0·826	0·829	0·832	0·835	0·836	0·837	0·838
North Dakota	0·778	0·782	0·785	0·788	0·791	0·795	0·798	0·802	0·806	0·809	0·813	0·816	0·818	0·819	0·82	0·819	0·818	0·82	0·823	0·827	0·832	0·835	0·84	0·846	0·853	0·861	0·868	0·873	0·876	0·876
Ohio	0·766	0·769	0·773	0·777	0·78	0·783	0·785	0·786	0·788	0·79	0·793	0·798	0·802	0·804	0·807	0·807	0·806	0·808	0·812	0·817	0·823	0·826	0·829	0·832	0·835	0·838	0·841	0·843	0·845	0·846
Oklahoma	0·738	0·74	0·745	0·748	0·751	0·753	0·754	0·753	0·753	0·754	0·757	0·763	0·767	0·77	0·773	0·772	0·77	0·772	0·778	0·785	0·793	0·797	0·801	0·805	0·808	0·813	0·818	0·823	0·826	0·827
Oregon	0·773	0·776	0·78	0·784	0·787	0·79	0·793	0·795	0·797	0·8	0·804	0·81	0·814	0·817	0·82	0·82	0·82	0·823	0·827	0·833	0·838	0·842	0·845	0·848	0·852	0·856	0·86	0·864	0·866	0·868
Pennsylvania	0·787	0·791	0·795	0·8	0·803	0·807	0·809	0·811	0·813	0·815	0·818	0·822	0·826	0·828	0·83	0·831	0·831	0·833	0·837	0·841	0·846	0·849	0·852	0·855	0·857	0·861	0·864	0·866	0·868	0·87
Rhode Island	0·8	0·804	0·808	0·811	0·814	0·816	0·818	0·82	0·822	0·825	0·828	0·832	0·836	0·838	0·841	0·842	0·843	0·846	0·85	0·855	0·86	0·863	0·866	0·869	0·872	0·875	0·877	0·879	0·881	0·882
South Carolina	0·736	0·742	0·749	0·755	0·759	0·763	0·765	0·766	0·766	0·768	0·77	0·776	0·78	0·782	0·785	0·784	0·783	0·786	0·792	0·799	0·807	0·811	0·816	0·82	0·823	0·826	0·828	0·83	0·831	0·832
South Dakota	0·756	0·76	0·766	0·771	0·776	0·78	0·784	0·786	0·789	0·79	0·793	0·796	0·798	0·799	0·8	0·799	0·798	0·8	0·804	0·81	0·817	0·82	0·824	0·828	0·833	0·838	0·843	0·847	0·849	0·85
Tennessee	0·735	0·739	0·744	0·748	0·752	0·756	0·758	0·76	0·761	0·763	0·766	0·77	0·772	0·773	0·774	0·773	0·771	0·774	0·78	0·788	0·795	0·8	0·805	0·81	0·815	0·82	0·823	0·826	0·827	0·827
Texas	0·732	0·734	0·737	0·739	0·742	0·744	0·747	0·748	0·749	0·751	0·754	0·76	0·763	0·766	0·769	0·768	0·767	0·771	0·777	0·786	0·794	0·799	0·803	0·808	0·812	0·818	0·824	0·83	0·833	0·835
Utah	0·751	0·755	0·76	0·764	0·767	0·77	0·772	0·774	0·776	0·779	0·782	0·787	0·79	0·793	0·795	0·794	0·793	0·797	0·803	0·81	0·818	0·822	0·827	0·831	0·834	0·839	0·843	0·847	0·849	0·851
Vermont	0·801	0·806	0·81	0·814	0·817	0·82	0·822	0·824	0·827	0·829	0·833	0·838	0·841	0·845	0·848	0·85	0·851	0·853	0·856	0·86	0·864	0·866	0·869	0·872	0·875	0·879	0·883	0·885	0·887	0·889
Virginia	0·788	0·792	0·796	0·8	0·803	0·806	0·809	0·81	0·812	0·813	0·816	0·821	0·825	0·828	0·831	0·833	0·834	0·837	0·843	0·849	0·854	0·858	0·861	0·864	0·867	0·869	0·872	0·874	0·876	0·877
Washington	0·784	0·787	0·791	0·795	0·799	0·802	0·806	0·809	0·812	0·815	0·82	0·825	0·829	0·831	0·834	0·834	0·833	0·835	0·839	0·844	0·85	0·853	0·856	0·86	0·863	0·866	0·87	0·873	0·875	0·876
West Virginia	0·734	0·738	0·743	0·748	0·752	0·755	0·757	0·758	0·759	0·76	0·762	0·767	0·77	0·771	0·772	0·771	0·768	0·769	0·772	0·777	0·783	0·786	0·789	0·793	0·797	0·802	0·806	0·809	0·811	0·812
Wisconsin	0·79	0·793	0·797	0·801	0·804	0·807	0·81	0·811	0·813	0·815	0·818	0·822	0·825	0·827	0·83	0·83	0·829	0·831	0·835	0·839	0·844	0·848	0·851	0·855	0·858	0·861	0·864	0·866	0·868	0·869
Wyoming	0·758	0·763	0·769	0·774	0·779	0·782	0·785	0·786	0·788	0·789	0·792	0·796	0·799	0·802	0·805	0·805	0·805	0·809	0·815	0·823	0·829	0·833	0·837	0·84	0·843	0·847	0·85	0·853	0·855	0·856
Southern Latin America	0·584	0·589	0·597	0·602	0·608	0·614	0·62	0·625	0·63	0·634	0·64	0·644	0·648	0·651	0·653	0·658	0·663	0·664	0·667	0·671	0·676	0·681	0·686	0·689	0·692	0·701	0·71	0·716	0·719	0·721
Argentina	0·581	0·585	0·593	0·599	0·605	0·611	0·617	0·622	0·625	0·628	0·634	0·637	0·64	0·641	0·642	0·649	0·653	0·655	0·657	0·661	0·665	0·67	0·674	0·677	0·679	0·687	0·696	0·702	0·706	0·708
Chile	0·592	0·6	0·606	0·611	0·617	0·624	0·63	0·637	0·644	0·651	0·657	0·663	0·671	0·678	0·683	0·686	0·689	0·692	0·695	0·7	0·706	0·712	0·719	0·724	0·728	0·738	0·747	0·753	0·756	0·759
Uruguay	0·581	0·584	0·588	0·591	0·594	0·597	0·6	0·606	0·612	0·618	0·622	0·626	0·628	0·631	0·633	0·636	0·639	0·642	0·645	0·649	0·653	0·658	0·663	0·668	0·673	0·678	0·684	0·688	0·693	0·697
Western Europe	0·75	0·756	0·762	0·767	0·772	0·775	0·779	0·782	0·784	0·787	0·79	0·794	0·797	0·8	0·802	0·805	0·807	0·81	0·812	0·815	0·817	0·821	0·824	0·827	0·83	0·832	0·835	0·838	0·841	0·843
Andorra	0·834	0·838	0·84	0·841	0·841	0·841	0·843	0·845	0·847	0·849	0·851	0·854	0·855	0·859	0·862	0·865	0·867	0·869	0·872	0·874	0·876	0·879	0·881	0·883	0·885	0·887	0·889	0·891	0·892	0·894
Austria	0·753	0·754	0·757	0·761	0·768	0·773	0·778	0·783	0·787	0·791	0·795	0·799	0·803	0·805	0·808	0·811	0·815	0·818	0·821	0·824	0·826	0·83	0·833	0·835	0·838	0·839	0·841	0·844	0·847	0·849
Belgium	0·746	0·75	0·756	0·762	0·767	0·771	0·775	0·779	0·782	0·784	0·787	0·792	0·796	0·799	0·802	0·805	0·808	0·81	0·813	0·816	0·82	0·824	0·829	0·834	0·837	0·841	0·843	0·846	0·849	0·851
Cyprus	0·662	0·67	0·68	0·691	0·702	0·713	0·723	0·732	0·741	0·75	0·758	0·767	0·774	0·78	0·786	0·791	0·797	0·804	0·81	0·816	0·82	0·824	0·827	0·829	0·831	0·832	0·834	0·836	0·838	0·841
Denmark	0·806	0·809	0·813	0·816	0·82	0·824	0·828	0·833	0·836	0·84	0·844	0·848	0·852	0·855	0·858	0·86	0·862	0·864	0·865	0·867	0·87	0·873	0·875	0·878	0·88	0·882	0·884	0·886	0·888	0·89
Finland	0·757	0·759	0·762	0·765	0·769	0·773	0·777	0·782	0·785	0·788	0·792	0·797	0·802	0·805	0·808	0·812	0·815	0·818	0·821	0·824	0·828	0·831	0·834	0·837	0·84	0·844	0·848	0·851	0·853	0·856
France	0·738	0·743	0·75	0·755	0·759	0·763	0·767	0·77	0·773	0·775	0·777	0·781	0·785	0·787	0·79	0·793	0·796	0·799	0·801	0·803	0·806	0·809	0·812	0·815	0·819	0·822	0·826	0·829	0·832	0·834
Germany	0·819	0·83	0·834	0·838	0·841	0·843	0·844	0·844	0·844	0·844	0·847	0·853	0·856	0·858	0·861	0·863	0·866	0·869	0·873	0·875	0·878	0·881	0·883	0·886	0·888	0·89	0·892	0·894	0·896	0·898
Greece	0·682	0·688	0·695	0·702	0·707	0·713	0·717	0·723	0·729	0·735	0·74	0·743	0·748	0·753	0·758	0·761	0·765	0·768	0·771	0·775	0·779	0·782	0·785	0·786	0·786	0·787	0·788	0·79	0·792	0·794
Iceland	0·764	0·77	0·774	0·778	0·782	0·785	0·788	0·79	0·794	0·799	0·806	0·813	0·818	0·822	0·824	0·827	0·83	0·834	0·838	0·842	0·846	0·847	0·847	0·848	0·85	0·854	0·858	0·863	0·866	0·869
Ireland	0·73	0·735	0·741	0·747	0·753	0·758	0·763	0·768	0·774	0·78	0·786	0·793	0·799	0·806	0·812	0·816	0·819	0·821	0·824	0·827	0·831	0·835	0·839	0·842	0·845	0·85	0·854	0·859	0·864	0·867
Israel	0·717	0·721	0·726	0·73	0·734	0·738	0·742	0·745	0·749	0·752	0·756	0·76	0·762	0·765	0·769	0·773	0·776	0·778	0·778	0·779	0·781	0·784	0·787	0·79	0·792	0·794	0·796	0·798	0·8	0·803
Italy	0·712	0·717	0·722	0·727	0·732	0·737	0·74	0·744	0·747	0·75	0·753	0·757	0·761	0·763	0·766	0·768	0·771	0·773	0·775	0·777	0·78	0·782	0·784	0·787	0·789	0·791	0·794	0·796	0·798	0·801
Luxembourg	0·815	0·818	0·82	0·823	0·828	0·833	0·836	0·839	0·842	0·844	0·847	0·85	0·853	0·855	0·857	0·858	0·862	0·866	0·869	0·871	0·872	0·874	0·877	0·88	0·883	0·886	0·889	0·892	0·894	0·895
Malta	0·666	0·67	0·675	0·682	0·69	0·695	0·696	0·7	0·708	0·715	0·722	0·729	0·733	0·737	0·741	0·745	0·749	0·753	0·757	0·761	0·764	0·768	0·772	0·775	0·779	0·784	0·788	0·793	0·797	0·801
Monaco	0·834	0·837	0·84	0·843	0·846	0·849	0·852	0·855	0·857	0·86	0·862	0·865	0·867	0·87	0·872	0·875	0·877	0·879	0·881	0·883	0·886	0·888	0·89	0·892	0·893	0·895	0·897	0·899	0·901	0·902
Netherlands	0·796	0·801	0·806	0·81	0·814	0·818	0·821	0·824	0·827	0·83	0·832	0·836	0·839	0·842	0·845	0·848	0·851	0·853	0·856	0·858	0·861	0·864	0·866	0·869	0·871	0·874	0·876	0·878	0·881	0·883
Norway	0·807	0·812	0·818	0·823	0·828	0·832	0·837	0·843	0·847	0·851	0·856	0·861	0·866	0·869	0·872	0·874	0·876	0·877	0·879	0·881	0·885	0·889	0·893	0·896	0·9	0·903	0·907	0·91	0·912	0·913
Portugal	0·607	0·615	0·622	0·629	0·636	0·641	0·647	0·651	0·656	0·661	0·666	0·671	0·676	0·681	0·686	0·69	0·694	0·698	0·701	0·705	0·709	0·714	0·718	0·722	0·726	0·729	0·732	0·736	0·739	0·743
San Marino	0·814	0·817	0·82	0·824	0·828	0·832	0·837	0·841	0·846	0·85	0·852	0·854	0·856	0·859	0·861	0·862	0·864	0·866	0·868	0·87	0·872	0·874	0·876	0·877	0·879	0·881	0·882	0·882	0·883	0·884
Spain	0·647	0·655	0·662	0·669	0·675	0·681	0·686	0·691	0·696	0·7	0·705	0·709	0·713	0·717	0·721	0·725	0·728	0·73	0·734	0·739	0·743	0·746	0·749	0·752	0·754	0·756	0·759	0·761	0·764	0·767
Sweden	0·769	0·775	0·782	0·788	0·794	0·801	0·806	0·811	0·815	0·819	0·823	0·826	0·829	0·832	0·835	0·838	0·84	0·842	0·844	0·846	0·849	0·852	0·855	0·858	0·86	0·863	0·865	0·868	0·87	0·872
Stockholm	0·813	0·818	0·824	0·829	0·834	0·839	0·844	0·848	0·852	0·855	0·859	0·862	0·865	0·867	0·87	0·873	0·875	0·878	0·88	0·883	0·886	0·889	0·891	0·893	0·895	0·897	0·899	0·901	0·903	0·904
Sweden except Stockholm	0·758	0·764	0·771	0·777	0·784	0·79	0·796	0·801	0·805	0·809	0·813	0·816	0·819	0·822	0·825	0·827	0·83	0·832	0·834	0·835	0·838	0·841	0·844	0·846	0·849	0·851	0·853	0·856	0·858	0·86
Switzerland	0·868	0·869	0·873	0·876	0·878	0·88	0·881	0·883	0·884	0·886	0·889	0·893	0·894	0·896	0·898	0·9	0·902	0·905	0·908	0·909	0·912	0·914	0·917	0·919	0·921	0·922	0·924	0·926	0·928	0·929
UK	0·745	0·749	0·755	0·761	0·766	0·769	0·771	0·774	0·778	0·784	0·789	0·793	0·797	0·799	0·802	0·804	0·806	0·808	0·811	0·813	0·816	0·82	0·825	0·83	0·834	0·837	0·839	0·842	0·845	0·847
England	0·75	0·755	0·76	0·765	0·77	0·774	0·776	0·779	0·783	0·788	0·793	0·797	0·801	0·803	0·805	0·807	0·81	0·812	0·815	0·817	0·82	0·823	0·828	0·833	0·837	0·84	0·842	0·845	0·848	0·85
East Midlands	0·725	0·73	0·735	0·74	0·745	0·749	0·751	0·754	0·758	0·764	0·769	0·773	0·776	0·779	0·781	0·784	0·786	0·788	0·791	0·793	0·796	0·8	0·804	0·809	0·813	0·816	0·819	0·821	0·824	0·826
Derby	0·736	0·741	0·747	0·753	0·759	0·762	0·764	0·767	0·771	0·776	0·781	0·785	0·789	0·791	0·793	0·795	0·797	0·798	0·8	0·802	0·805	0·809	0·814	0·821	0·825	0·829	0·832	0·835	0·838	0·841
Derbyshire	0·716	0·72	0·725	0·73	0·735	0·738	0·74	0·743	0·746	0·751	0·756	0·759	0·762	0·764	0·766	0·768	0·77	0·772	0·774	0·776	0·779	0·783	0·789	0·795	0·799	0·803	0·806	0·809	0·811	0·814
Leicester	0·724	0·729	0·734	0·74	0·745	0·748	0·751	0·754	0·758	0·763	0·767	0·771	0·775	0·777	0·78	0·783	0·786	0·789	0·792	0·794	0·797	0·801	0·805	0·809	0·812	0·815	0·817	0·819	0·821	0·823
Leicestershire	0·754	0·758	0·763	0·768	0·773	0·776	0·778	0·781	0·784	0·789	0·793	0·797	0·8	0·802	0·805	0·807	0·81	0·812	0·815	0·817	0·819	0·822	0·826	0·83	0·833	0·835	0·838	0·84	0·842	0·845
Lincolnshire	0·717	0·721	0·726	0·731	0·736	0·739	0·741	0·743	0·747	0·753	0·757	0·761	0·764	0·765	0·767	0·769	0·77	0·771	0·773	0·774	0·777	0·781	0·786	0·791	0·795	0·799	0·802	0·804	0·807	0·81
Northamptonshire	0·734	0·739	0·745	0·75	0·756	0·758	0·76	0·762	0·765	0·77	0·774	0·777	0·779	0·781	0·782	0·784	0·786	0·788	0·79	0·792	0·795	0·799	0·804	0·81	0·814	0·817	0·82	0·822	0·825	0·827
Nottingham	0·754	0·759	0·764	0·769	0·774	0·777	0·781	0·784	0·789	0·794	0·799	0·804	0·807	0·81	0·813	0·815	0·818	0·82	0·823	0·826	0·828	0·832	0·836	0·84	0·842	0·844	0·846	0·848	0·85	0·852
Nottinghamshire	0·718	0·722	0·727	0·732	0·737	0·74	0·742	0·745	0·749	0·754	0·759	0·763	0·766	0·768	0·77	0·772	0·774	0·775	0·777	0·779	0·781	0·784	0·789	0·794	0·797	0·8	0·803	0·806	0·809	0·811

Appendix Table 16. Socio-demographic Index values for all estimated GBD 2019 locations, 1990–2019

Location	1990	1991	1992	1993	1994	1995	1996	1997	1998	1999	2000	2001	2002	2003	2004	2005	2006	2007	2008	2009	2010	2011	2012	2013	2014	2015	2016	2017	2018	2019
Rutland	0·764	0·769	0·773	0·778	0·782	0·784	0·786	0·788	0·79	0·794	0·797	0·8	0·802	0·803	0·806	0·808	0·81	0·813	0·816	0·818	0·821	0·824	0·827	0·831	0·834	0·836	0·838	0·84	0·842	0·844
East of England	0·744	0·749	0·754	0·759	0·764	0·767	0·77	0·773	0·777	0·782	0·787	0·791	0·794	0·797	0·799	0·801	0·804	0·806	0·809	0·811	0·814	0·817	0·822	0·827	0·83	0·833	0·836	0·839	0·841	0·844
Bedford	0·754	0·759	0·763	0·768	0·773	0·776	0·778	0·781	0·785	0·79	0·795	0·798	0·801	0·803	0·806	0·808	0·81	0·811	0·814	0·815	0·818	0·821	0·825	0·83	0·834	0·836	0·839	0·842	0·844	0·847
Cambridgeshire	0·784	0·789	0·794	0·798	0·803	0·806	0·809	0·812	0·816	0·82	0·825	0·829	0·832	0·835	0·837	0·839	0·842	0·844	0·846	0·848	0·851	0·854	0·858	0·862	0·865	0·868	0·87	0·872	0·875	0·877
Central Bedfordshire	0·751	0·755	0·76	0·765	0·77	0·773	0·775	0·778	0·781	0·785	0·79	0·793	0·796	0·798	0·8	0·802	0·804	0·805	0·808	0·809	0·811	0·815	0·819	0·824	0·827	0·83	0·833	0·836	0·839	0·841
Essex	0·735	0·74	0·745	0·751	0·756	0·759	0·762	0·765	0·768	0·773	0·778	0·782	0·786	0·788	0·791	0·794	0·796	0·798	0·801	0·803	0·805	0·809	0·813	0·817	0·821	0·823	0·825	0·828	0·83	0·833
Hertfordshire	0·783	0·788	0·793	0·798	0·802	0·806	0·808	0·811	0·815	0·82	0·826	0·83	0·834	0·837	0·839	0·842	0·844	0·846	0·848	0·849	0·851	0·854	0·857	0·862	0·865	0·867	0·87	0·872	0·875	0·877
Luton	0·729	0·734	0·739	0·745	0·75	0·753	0·756	0·759	0·762	0·767	0·772	0·775	0·778	0·78	0·783	0·786	0·789	0·793	0·797	0·8	0·803	0·807	0·812	0·817	0·82	0·823	0·825	0·828	0·83	0·832
Norfolk	0·726	0·73	0·735	0·74	0·745	0·749	0·751	0·754	0·758	0·763	0·768	0·772	0·776	0·778	0·78	0·782	0·784	0·786	0·788	0·79	0·793	0·796	0·801	0·806	0·81	0·813	0·815	0·818	0·821	0·823
Peterborough	0·722	0·726	0·73	0·735	0·74	0·743	0·745	0·748	0·752	0·757	0·761	0·765	0·767	0·768	0·769	0·77	0·772	0·773	0·776	0·777	0·78	0·785	0·791	0·798	0·802	0·806	0·809	0·813	0·816	0·818
Southend-on-Sea	0·714	0·718	0·722	0·727	0·732	0·735	0·737	0·74	0·745	0·751	0·756	0·761	0·765	0·768	0·77	0·772	0·774	0·776	0·778	0·78	0·783	0·787	0·792	0·797	0·8	0·803	0·805	0·807	0·81	0·812
Suffolk	0·731	0·736	0·741	0·747	0·751	0·754	0·756	0·758	0·762	0·767	0·771	0·775	0·777	0·779	0·781	0·782	0·785	0·786	0·789	0·791	0·794	0·799	0·804	0·81	0·813	0·816	0·819	0·822	0·824	0·827
Thurrock	0·717	0·722	0·727	0·733	0·738	0·741	0·744	0·747	0·751	0·756	0·759	0·762	0·764	0·765	0·766	0·767	0·768	0·77	0·772	0·774	0·776	0·78	0·784	0·789	0·792	0·794	0·796	0·799	0·801	0·804
Greater London	0·804	0·808	0·813	0·818	0·823	0·826	0·829	0·832	0·836	0·841	0·845	0·849	0·852	0·854	0·857	0·859	0·861	0·863	0·866	0·868	0·871	0·874	0·878	0·883	0·886	0·888	0·89	0·892	0·894	0·895
Barking and Dagenham	0·689	0·693	0·699	0·704	0·71	0·714	0·717	0·721	0·726	0·732	0·736	0·74	0·743	0·743	0·744	0·745	0·745	0·746	0·747	0·749	0·751	0·756	0·761	0·768	0·773	0·777	0·78	0·783	0·787	0·79
Barnet	0·786	0·791	0·796	0·8	0·805	0·808	0·811	0·815	0·819	0·824	0·829	0·833	0·837	0·839	0·841	0·843	0·844	0·846	0·848	0·849	0·852	0·855	0·859	0·863	0·866	0·869	0·872	0·874	0·876	0·878
Bexley	0·729	0·734	0·74	0·745	0·75	0·753	0·755	0·758	0·762	0·767	0·772	0·777	0·78	0·783	0·786	0·788	0·79	0·792	0·795	0·797	0·799	0·803	0·807	0·812	0·817	0·82	0·824	0·827	0·83	0·833
Brent	0·764	0·769	0·773	0·778	0·783	0·787	0·789	0·792	0·796	0·802	0·806	0·81	0·813	0·814	0·816	0·817	0·819	0·82	0·821	0·822	0·824	0·827	0·832	0·837	0·84	0·842	0·844	0·846	0·848	0·851
Bromley	0·77	0·774	0·779	0·784	0·788	0·792	0·794	0·797	0·802	0·807	0·812	0·816	0·82	0·822	0·825	0·827	0·829	0·83	0·832	0·833	0·835	0·837	0·841	0·844	0·847	0·849	0·852	0·854	0·856	0·858
Camden	0·872	0·875	0·879	0·882	0·885	0·888	0·89	0·892	0·895	0·898	0·9	0·903	0·904	0·906	0·907	0·909	0·911	0·912	0·914	0·915	0·917	0·92	0·922	0·925	0·926	0·928	0·929	0·93	0·931	0·931
Croydon	0·753	0·758	0·764	0·769	0·774	0·777	0·78	0·782	0·786	0·792	0·797	0·801	0·804	0·806	0·808	0·809	0·811	0·812	0·814	0·815	0·816	0·818	0·822	0·826	0·829	0·831	0·833	0·835	0·838	0·84
Ealing	0·783	0·787	0·792	0·797	0·802	0·806	0·809	0·812	0·816	0·821	0·826	0·83	0·833	0·835	0·836	0·837	0·838	0·839	0·84	0·841	0·842	0·845	0·849	0·854	0·858	0·862	0·865	0·868	0·87	0·872
Enfield	0·74	0·745	0·75	0·756	0·761	0·764	0·766	0·77	0·774	0·779	0·784	0·788	0·791	0·793	0·795	0·796	0·798	0·8	0·802	0·804	0·806	0·81	0·815	0·82	0·823	0·826	0·829	0·831	0·834	0·836
Greenwich	0·732	0·737	0·743	0·749	0·755	0·759	0·762	0·765	0·769	0·775	0·78	0·784	0·787	0·788	0·791	0·793	0·795	0·797	0·799	0·802	0·805	0·808	0·813	0·818	0·822	0·825	0·827	0·83	0·832	0·834
Hackney	0·787	0·793	0·799	0·805	0·809	0·812	0·812	0·813	0·815	0·819	0·823	0·826	0·828	0·83	0·832	0·835	0·838	0·841	0·845	0·849	0·853	0·858	0·864	0·87	0·873	0·876	0·877	0·879	0·881	0·882
Hammersmith and Fulham	0·854	0·859	0·864	0·869	0·873	0·876	0·879	0·882	0·886	0·89	0·893	0·895	0·897	0·898	0·9	0·902	0·903	0·905	0·907	0·909	0·912	0·914	0·917	0·92	0·922	0·924	0·925	0·927	0·928	0·929
Haringey	0·77	0·775	0·779	0·784	0·788	0·791	0·793	0·795	0·799	0·803	0·808	0·812	0·815	0·817	0·82	0·822	0·824	0·826	0·828	0·829	0·832	0·835	0·84	0·845	0·848	0·851	0·854	0·856	0·858	0·86
Harrow	0·772	0·777	0·782	0·787	0·791	0·794	0·797	0·8	0·804	0·81	0·814	0·819	0·822	0·824	0·826	0·828	0·83	0·831	0·832	0·833	0·834	0·836	0·839	0·843	0·845	0·847	0·848	0·85	0·852	0·854
Havering	0·722	0·727	0·732	0·737	0·741	0·744	0·747	0·75	0·754	0·759	0·764	0·769	0·772	0·775	0·778	0·781	0·784	0·786	0·788	0·79	0·793	0·796	0·8	0·805	0·808	0·811	0·814	0·817	0·82	0·823
Hillingdon	0·791	0·796	0·801	0·806	0·811	0·814	0·817	0·82	0·824	0·83	0·835	0·839	0·843	0·845	0·846	0·848	0·849	0·85	0·851	0·853	0·856	0·859	0·863	0·868	0·871	0·874	0·877	0·879	0·881	0·882
Hounslow	0·784	0·789	0·794	0·799	0·804	0·807	0·809	0·812	0·816	0·822	0·827	0·832	0·835	0·837	0·839	0·841	0·843	0·844	0·846	0·847	0·85	0·854	0·858	0·864	0·869	0·873	0·876	0·88	0·883	0·885
Islington	0·842	0·846	0·85	0·855	0·859	0·863	0·865	0·868	0·871	0·875	0·878	0·881	0·883	0·885	0·887	0·889	0·891	0·893	0·895	0·898	0·9	0·903	0·906	0·909	0·911	0·913	0·914	0·916	0·917	0·918
Kensington and Chelsea	0·882	0·886	0·89	0·894	0·897	0·9	0·903	0·905	0·907	0·909	0·91	0·912	0·914	0·915	0·916	0·917	0·919	0·921	0·923	0·924	0·926	0·929	0·931	0·934	0·935	0·937	0·938	0·939	0·94	0·941
Kingston upon Thames	0·816	0·821	0·826	0·83	0·835	0·838	0·841	0·845	0·849	0·854	0·859	0·863	0·866	0·869	0·872	0·874	0·877	0·879	0·881	0·882	0·884	0·886	0·888	0·891	0·893	0·895	0·896	0·898	0·9	0·902
Lambeth	0·799	0·805	0·81	0·815	0·82	0·824	0·827	0·83	0·834	0·839	0·844	0·848	0·851	0·854	0·857	0·861	0·864	0·867	0·871	0·874	0·878	0·881	0·886	0·89	0·894	0·897	0·899	0·902	0·904	0·906
Lewisham	0·747	0·753	0·758	0·763	0·768	0·772	0·775	0·778	0·782	0·787	0·792	0·797	0·8	0·802	0·804	0·806	0·808	0·81	0·812	0·814	0·816	0·82	0·824	0·829	0·833	0·836	0·839	0·842	0·844	0·846
Merton	0·777	0·783	0·788	0·793	0·798	0·802	0·805	0·809	0·814	0·819	0·824	0·828	0·831	0·833	0·835	0·836	0·837	0·839	0·841	0·842	0·845	0·848	0·853	0·858	0·862	0·866	0·869	0·872	0·874	0·876
Newham	0·717	0·722	0·727	0·733	0·738	0·741	0·743	0·746	0·751	0·757	0·763	0·768	0·773	0·776	0·779	0·783	0·786	0·788	0·792	0·794	0·798	0·803	0·808	0·814	0·818	0·822	0·824	0·827	0·829	0·831
Redbridge	0·748	0·752	0·757	0·762	0·767	0·77	0·773	0·776	0·78	0·785	0·79	0·794	0·797	0·8	0·802	0·804	0·806	0·808	0·81	0·811	0·813	0·816	0·82	0·825	0·828	0·831	0·834	0·836	0·839	0·841
Richmond upon Thames	0·836	0·841	0·845	0·85	0·854	0·857	0·86	0·863	0·867	0·872	0·876	0·88	0·883	0·886	0·888	0·89	0·892	0·894	0·895	0·897	0·899	0·901	0·904	0·908	0·911	0·914	0·917	0·92	0·922	0·924
Southwark	0·81	0·815	0·821	0·826	0·831	0·835	0·839	0·842	0·847	0·853	0·858	0·863	0·867	0·87	0·872	0·874	0·876	0·878	0·881	0·884	0·887	0·891	0·895	0·899	0·902	0·904	0·906	0·908	0·909	0·91
Sutton	0·748	0·753	0·758	0·763	0·769	0·773	0·776	0·78	0·784	0·79	0·795	0·8	0·803	0·806	0·808	0·81	0·812	0·813	0·815	0·816	0·819	0·822	0·826	0·831	0·835	0·838	0·841	0·844	0·847	0·849
Tower Hamlets	0·787	0·792	0·798	0·804	0·81	0·814	0·818	0·821	0·826	0·832	0·837	0·841	0·845	0·848	0·852	0·856	0·859	0·863	0·866	0·869	0·873	0·877	0·881	0·885	0·888	0·89	0·892	0·893	0·895	0·896
Waltham Forest	0·724	0·729	0·734	0·74	0·745	0·749	0·752	0·756	0·76	0·765	0·77	0·774	0·777	0·779	0·781	0·783	0·785	0·786	0·788	0·79	0·792	0·796	0·801	0·807	0·811	0·815	0·818	0·822	0·825	0·827
Wandsworth	0·828	0·833	0·838	0·843	0·848	0·851	0·855	0·858	0·863	0·868	0·872	0·877	0·88	0·883	0·886	0·888	0·891	0·893	0·895	0·897	0·899	0·901	0·904	0·907	0·909	0·911	0·913	0·915	0·916	0·918
Westminster	0·867	0·87	0·874	0·878	0·881	0·884	0·886	0·888	0·891	0·894	0·896	0·899	0·9	0·902	0·903	0·905	0·907	0·908	0·91	0·911	0·914	0·916	0·919	0·922	0·924	0·925	0·927	0·928	0·929	0·93
North East England	0·709	0·714	0·72	0·726	0·732	0·736	0·738	0·742	0·747	0·753	0·758	0·763	0·767	0·769	0·772	0·775	0·778	0·78	0·783	0·785	0·788	0·792	0·797	0·802	0·805	0·808	0·811	0·814	0·817	0·819
County Durham	0·705	0·71	0·715	0·721	0·726	0·729	0·732	0·735	0·739	0·744	0·749	0·754	0·757	0·759	0·762	0·764	0·767	0·769	0·772	0·774	0·777	0·78	0·785	0·79	0·793	0·796	0·798	0·801	0·803	0·805
Darlington	0·718	0·723	0·728	0·734	0·74	0·743	0·745	0·747	0·751	0·756	0·761	0·766	0·769	0·772	0·775	0·778	0·78	0·783	0·786	0·788	0·791	0·796	0·801	0·807	0·812	0·815	0·818	0·821	0·824	0·826
Gateshead	0·712	0·717	0·723	0·728	0·733	0·736	0·738	0·741	0·745	0·751	0·756	0·761	0·765	0·768	0·772	0·775	0·778	0·781	0·784	0·786	0·79	0·794	0·799	0·805	0·809	0·812	0·815	0·817	0·82	0·822
Hartlepool	0·678	0·684	0·689	0·695	0·7	0·704	0·705	0·708	0·712	0·717	0·722	0·726	0·729	0·73	0·732	0·735	0·737	0·739	0·742	0·745	0·749	0·753	0·759	0·766	0·771	0·774	0·778	0·781	0·784	0·786
Middlesbrough	0·692	0·698	0·703	0·709	0·714	0·718	0·72	0·723	0·727	0·733	0·738	0·742	0·745	0·748	0·751	0·753	0·756	0·758	0·761	0·763	0·765	0·769	0·774	0·78	0·783	0·786	0·788	0·791	0·793	0·796
Newcastle upon Tyne	0·763	0·768	0·773	0·778	0·783	0·787	0·79	0·794	0·798	0·803	0·808	0·813	0·818	0·822	0·826	0·83	0·834	0·838	0·841	0·844	0·846	0·849	0·853	0·856	0·858	0·86	0·861	0·863	0·865	0·866
North Tyneside	0·713	0·719	0·725	0·731	0·736	0·739	0·742	0·744	0·748	0·754	0·759	0·764	0·767	0·77	0·774	0·777	0·781	0·783	0·787	0·789	0·793	0·797	0·802	0·808	0·812	0·815	0·818	0·821	0·824	0·826
Northumberland	0·713	0·718	0·723	0·728	0·733	0·737	0·739	0·742	0·746	0·751	0·756	0·76	0·764	0·766	0·769	0·771	0·774	0·776	0·779	0·781	0·784	0·787	0·792	0·797	0·801	0·804	0·807	0·809	0·812	0·814
Redcar and Cleveland	0·686	0·691	0·697	0·703	0·708	0·712	0·714	0·717	0·721	0·726	0·731	0·735	0·737	0·739	0·741	0·744	0·746	0·748	0·751	0·753	0·756	0·76	0·765	0·772	0·776	0·78	0·783	0·786	0·789	0·791
South Tyneside	0·678	0·683	0·689	0·695	0·7	0·704	0·706	0·709	0·714	0·72	0·725	0·73	0·734	0·736	0·74	0·743	0·746	0·748	0·751	0·753	0·756	0·76	0·766	0·772	0·776	0·78	0·783	0·786	0·789	0·792
Stockton-on-Tees	0·721	0·727	0·733	0·74	0·746	0·75	0·752	0·755	0·76	0·765	0·769	0·773	0·775	0·777	0·779	0·781	0·783	0·784	0·787	0·788	0·791	0·795	0·8	0·805	0·809	0·812	0·815	0·817	0·82	0·822
Sunderland	0·699	0·705	0·712	0·718	0·724	0·728	0·731	0·734	0·738	0·744	0·749	0·753	0·757	0·759	0·762	0·765	0·768	0·77	0·772	0·775	0·777	0·781	0·786	0·792	0·796	0·799	0·802	0·805	0·807	0·81
North West England	0·73	0·735	0·74	0·745	0·75	0·754	0·756	0·759	0·763	0·768	0·773	0·777	0·781	0·783	0·785	0·788	0·79	0·792	0·795	0·797	0·8	0·804	0·809	0·815	0·819	0·822	0·825	0·827	0·83	0·832
Blackburn with Darwen	0·69	0·694	0·698	0·703	0·707	0·709	0·711	0·713	0·717	0·723	0·728	0·732	0·735	0·736	0·739	0·741	0·744	0·746	0·749	0·752	0·755	0·761	0·767	0·775	0·78	0·785	0·789	0·792	0·795	0·798
Blackpool	0·681	0·685	0·69	0·695	0·699	0·702	0·703	0·705	0·709	0·715	0·719	0·722	0·725	0·725	0·727	0·729	0·73	0·731	0·733	0·735	0·737	0·741	0·746	0·753	0·757	0·76	0·763	0·766	0·769	0·772
Bolton	0·708	0·713	0·718	0·723	0·728	0·731	0·732	0·734	0·737	0·741	0·746	0·749	0·751	0·752	0·754	0·756	0·757	0·759	0·761	0·763	0·766	0·77	0·775	0·782	0·786	0·789	0·792	0·795	0·797	0·8
Bury	0·722	0·727	0·733	0·738	0·743	0·746	0·748	0·75	0·754	0·759	0·763	0·766	0·769	0·77	0·772	0·774	0·776	0·778	0·78	0·781	0·784	0·788	0·793	0·8	0·804	0·808	0·811	0·814	0·817	0·819
Cheshire East	0·774	0·779	0·784	0·789	0·794	0·797	0·799	0·801	0·805	0·81	0·815	0·819	0·822	0·824	0·826	0·828	0·83	0·831	0·833	0·835	0·838	0·842	0·847	0·852	0·856	0·86	0·863	0·865	0·868	0·87
Cheshire West and Chester	0·766	0·771	0·776	0·781	0·786	0·789	0·791	0·794	0·798	0·802	0·807	0·811	0·814	0·816	0·819	0·821	0·824	0·826	0·829	0·831	0·834	0·837	0·842	0·847	0·85	0·853	0·855	0·858	0·86	0·862
Cumbria	0·729	0·733	0·737	0·742	0·747	0·75	0·752	0·754	0·758	0·763	0·767	0·771	0·773	0·775	0·777	0·779	0·781	0·783	0·786	0·789	0·793	0·797	0·803	0·81	0·815	0·819	0·822	0·825	0·828	0·83
Halton	0·711	0·716	0·722	0·727	0·733	0·736	0·737	0·739	0·742	0·747	0·751	0·755	0·758	0·759	0·761	0·763	0·766	0·768	0·771	0·774	0·778	0·783	0·789	0·797	0·802	0·806	0·81	0·813	0·817	0·819
Knowsley	0·69	0·694	0·699	0·704	0·708	0·711	0·713	0·715	0·72	0·725	0·731	0·736	0·739	0·742	0·744	0·747	0·75	0·752	0·755	0·758	0·762	0·766	0·772	0·779	0·783	0·787	0·79	0·794	0·797	0·8
Lancashire	0·736	0·741	0·746	0·752	0·757	0·76	0·762	0·765	0·769	0·773	0·778	0·781	0·784	0·786	0·788	0·79	0·792	0·794	0·796	0·799	0·801	0·805	0·81	0·815	0·819	0·822	0·824	0·827	0·829	0·831
Liverpool	0·738	0·742	0·747	0·752	0·757	0·76	0·763	0·766	0·771	0·777	0·782	0·787	0·792	0·796	0·8	0·804	0·808	0·811	0·815	0·818	0·821	0·824	0·827	0·831	0·833	0·835	0·836	0·838	0·84	0·842
Manchester	0·764	0·768	0·773	0·778	0·783	0·787	0·79	0·794	0·799	0·804	0·81	0·815	0·819	0·823	0·827	0·83	0·834	0·838	0·842	0·845	0·848	0·852	0·856	0·86	0·863	0·865	0·867	0·869	0·872	0·873
Oldham	0·686	0·691	0·696	0·701	0·705	0·707	0·707	0·708	0·711	0·715	0·719	0·722	0·725	0·726	0·728	0·73	0·733	0·735	0·738	0·741	0·744	0·749	0·755	0·762	0·766	0·77	0·773	0·776	0·779	0·781
Rochdale	0·689	0·693	0·698	0·704	0·709	0·712	0·713	0·716	0·719	0·725	0·729	0·733	0·736	0·737	0·739	0·741	0·743	0·745	0·747	0·749	0·753	0·757	0·763	0·77	0·774	0·778	0·781	0·784	0·787	0·79
Salford	0·719	0·723	0·728	0·734	0·739	0·742	0·744	0·747	0·752	0·758	0·763	0·769	0·773	0·775	0·778	0·781	0·784	0·786	0·788	0·79	0·793	0·798	0·803	0·81	0·814	0·817	0·82	0·822	0·825	0·827
Sefton	0·724	0·729	0·735	0·74	0·745	0·749	0·751	0·754	0·758	0·762	0·767	0·771	0·774	0·777	0·779	0·782	0·784	0·785	0·787	0·788	0·789	0·792	0·795	0·799	0·802	0·804	0·806	0·808	0·81	0·812
St Helens	0·7	0·704	0·709	0·714	0·719	0·721	0·723	0·725	0·729	0·734	0·739	0·743	0·746	0·748	0·75	0·752	0·755	0·757	0·759	0·761	0·765	0·769	0·774	0·78	0·784	0·787	0·789	0·792	0·794	0·797
Stockport	0·754	0·759	0·764	0·77	0·775	0·777	0·779	0·782	0·786	0·791	0·796	0·8	0·803	0·805	0·808	0·81	0·812	0·814	0·816	0·818	0·82	0·824	0·828	0·834	0·837	0·84	0·843	0·845	0·848	0·85
Tameside	0·697	0·702	0·707	0·712	0·717	0·721	0·723	0·725	0·729	0·735	0·739	0·743	0·746	0·747	0·749	0·75	0·751	0·752	0·753	0·754	0·757	0·76	0·766	0·772	0·776	0·779	0·782	0·785	0·788	0·79
Trafford	0·783	0·787	0·793	0·798	0·802	0·805	0·807	0·81	0·813	0·818	0·823	0·827	0·831	0·834	0·837	0·839	0·842	0·844	0·846	0·848	0·851	0·854	0·859	0·864	0·867	0·871	0·874	0·876	0·879	0·881
Warrington	0·762	0·767	0·772	0·777	0·781	0·784	0·785	0·787	0·791	0·796	0·801	0·806	0·81	0·812	0·815	0·818	0·821	0·823	0·825	0·827	0·83	0·834	0·84	0·846	0·85	0·854	0·858	0·861	0·864	0·866
Wigan	0·7	0·705	0·709	0·714	0·719	0·722	0·723	0·726	0·729	0·734	0·738	0·741	0·744	0·745	0·747	0·749	0·751	0·752	0·754	0·756	0·759	0·763	0·769	0·775	0·779	0·782	0·785	0·788	0·791	0·793
Wirral	0·711	0·716	0·721	0·726	0·731	0·734	0·736	0·739	0·742	0·747	0·752	0·755	0·758	0·76	0·762	0·764	0·766	0·767	0·769	0·771	0·774	0·777	0·783	0·788	0·792	0·796	0·798	0·801	0·804	0·806
South East England	0·777	0·782	0·786	0·791	0·795	0·798	0·8	0·803	0·806	0·811	0·815	0·819	0·822	0·823	0·826	0·828	0·83	0·832	0·834	0·836	0·838	0·841	0·845	0·85	0·853	0·855	0·858	0·86	0·862	0·864
Bracknell Forest	0·79	0·794	0·798	0·802	0·805	0·807	0·809	0·812	0·815	0·821	0·826	0·83	0·833	0·836	0·838	0·84	0·842	0·843	0·845	0·847	0·849	0·852	0·857	0·861	0·865	0·868	0·871	0·873	0·876	0·878
Brighton and Hove	0·799	0·804	0·808	0·813	0·817	0·821	0·824	0·828	0·832	0·836	0·841	0·845	0·849	0·852	0·856	0·859	0·862	0·865	0·867	0·869	0·871	0·873	0·876	0·879	0·881	0·883	0·885	0·887	0·889	0·891
Buckinghamshire	0·799	0·803	0·807	0·811	0·816	0·819	0·821	0·823	0·827	0·831	0·835	0·839	0·841	0·843	0·845	0·846	0·848	0·849	0·851	0·852	0·853	0·856	0·86	0·864	0·866	0·869	0·871	0·873	0·875	0·877
East Sussex	0·732	0·737	0·742	0·747	0·752	0·755	0·757	0·76	0·763	0·768	0·773	0·777	0·78	0·782	0·784	0·786	0·788	0·789	0·791	0·793	0·795	0·799	0·803	0·809	0·812	0·816	0·819	0·822	0·825	0·827
Hampshire	0·774	0·779	0·783	0·787	0·792	0·794	0·796	0·799	0·802	0·807	0·811	0·815	0·818	0·819	0·821	0·823	0·825	0·827	0·829	0·83	0·833	0·836	0·841	0·846	0·85	0·853	0·855	0·858	0·86	0·862
Isle of Wight	0·712	0·716	0·721	0·727	0·732	0·735	0·738	0·741	0·745	0·751	0·755	0·76	0·763	0·764	0·766	0·768	0·771	0·772	0·775	0·776	0·778	0·781	0·785	0·79	0·793	0·796	0·798	0·8	0·803	0·805
Kent	0·744	0·748	0·753	0·757	0·762	0·765	0·767	0·769	0·773	0·777	0·781	0·785	0·788	0·79	0·792	0·794	0·796	0·798	0·8	0·802	0·805	0·808	0·813	0·818	0·821	0·824	0·826	0·828	0·831	0·833
Medway	0·72	0·725	0·73	0·735	0·74	0·743	0·745	0·747	0·75	0·754	0·758	0·762	0·764	0·766	0·768	0·77	0·772	0·774	0·776	0·778	0·78	0·783	0·788	0·792	0·796	0·798	0·8	0·802	0·805	0·807
Milton Keynes	0·777	0·782	0·787	0·792	0·797	0·8	0·801	0·803	0·806	0·81	0·814	0·817	0·82	0·821	0·823	0·824	0·826	0·827	0·829	0·831	0·834	0·838	0·844	0·85	0·855	0·859	0·862	0·866	0·869	0·87

Appendix Table 16. Socio-demographic Index values for all estimated GBD 2019 locations, 1990–2019

Location	1990	1991	1992	1993	1994	1995	1996	1997	1998	1999	2000	2001	2002	2003	2004	2005	2006	2007	2008	2009	2010	2011	2012	2013	2014	2015	2016	2017	2018	2019
Oxfordshire	0·805	0·809	0·813	0·817	0·822	0·824	0·827	0·829	0·832	0·837	0·841	0·844	0·847	0·849	0·852	0·854	0·856	0·858	0·86	0·862	0·864	0·867	0·871	0·875	0·878	0·881	0·883	0·886	0·888	0·89
Portsmouth	0·772	0·776	0·781	0·786	0·79	0·794	0·797	0·8	0·804	0·808	0·813	0·816	0·819	0·822	0·824	0·826	0·828	0·83	0·832	0·834	0·836	0·839	0·842	0·846	0·849	0·85	0·852	0·854	0·856	0·858
Reading	0·819	0·823	0·827	0·832	0·836	0·839	0·841	0·845	0·848	0·853	0·858	0·862	0·865	0·868	0·871	0·872	0·873	0·874	0·875	0·876	0·878	0·88	0·883	0·886	0·889	0·89	0·891	0·893	0·894	0·896
Slough	0·78	0·785	0·79	0·795	0·8	0·804	0·806	0·809	0·813	0·816	0·819	0·821	0·823	0·823	0·823	0·824	0·824	0·824	0·826	0·828	0·831	0·835	0·841	0·847	0·851	0·854	0·857	0·86	0·862	0·864
Southampton	0·772	0·777	0·782	0·787	0·791	0·795	0·799	0·803	0·807	0·812	0·816	0·82	0·823	0·826	0·828	0·83	0·831	0·833	0·834	0·835	0·837	0·839	0·841	0·844	0·846	0·848	0·849	0·851	0·853	0·855
Surrey	0·812	0·816	0·82	0·824	0·828	0·831	0·833	0·836	0·84	0·845	0·849	0·853	0·856	0·859	0·861	0·863	0·865	0·866	0·868	0·87	0·871	0·874	0·877	0·881	0·884	0·886	0·889	0·891	0·893	0·895
West Berkshire	0·807	0·812	0·817	0·822	0·826	0·829	0·831	0·833	0·836	0·841	0·845	0·849	0·851	0·851	0·851	0·851	0·852	0·852	0·853	0·855	0·857	0·861	0·865	0·87	0·874	0·876	0·878	0·88	0·882	0·883
West Sussex	0·765	0·769	0·774	0·779	0·783	0·786	0·788	0·79	0·794	0·798	0·803	0·807	0·81	0·811	0·814	0·815	0·817	0·819	0·821	0·823	0·825	0·829	0·833	0·838	0·841	0·844	0·847	0·849	0·852	0·854
Windsor and Maidenhead	0·82	0·823	0·827	0·831	0·834	0·837	0·839	0·841	0·845	0·849	0·854	0·858	0·861	0·863	0·866	0·868	0·87	0·872	0·874	0·875	0·877	0·88	0·884	0·889	0·892	0·895	0·898	0·9	0·903	0·905
Wokingham	0·826	0·831	0·835	0·839	0·843	0·846	0·848	0·851	0·854	0·859	0·863	0·866	0·869	0·871	0·874	0·876	0·878	0·879	0·881	0·881	0·883	0·885	0·887	0·891	0·893	0·896	0·898	0·9	0·902	0·904
South West England	0·751	0·755	0·76	0·765	0·77	0·773	0·776	0·779	0·783	0·788	0·793	0·797	0·8	0·802	0·805	0·807	0·81	0·812	0·814	0·816	0·819	0·823	0·827	0·832	0·835	0·838	0·841	0·843	0·846	0·848
Bath and North East Somerset	0·793	0·797	0·801	0·805	0·809	0·812	0·815	0·818	0·822	0·827	0·831	0·836	0·84	0·844	0·848	0·851	0·855	0·858	0·861	0·863	0·865	0·868	0·871	0·874	0·876	0·878	0·88	0·882	0·884	0·886
Bournemouth	0·761	0·766	0·771	0·776	0·781	0·785	0·788	0·792	0·796	0·801	0·806	0·811	0·815	0·818	0·822	0·825	0·828	0·831	0·833	0·835	0·838	0·84	0·844	0·847	0·85	0·852	0·854	0·856	0·858	0·861
Bristol, City of	0·792	0·796	0·801	0·806	0·811	0·814	0·817	0·821	0·825	0·829	0·834	0·837	0·841	0·843	0·845	0·848	0·85	0·853	0·856	0·858	0·861	0·864	0·868	0·872	0·875	0·877	0·88	0·882	0·884	0·886
Cornwall	0·727	0·732	0·738	0·743	0·748	0·752	0·754	0·756	0·76	0·765	0·77	0·774	0·777	0·779	0·782	0·784	0·787	0·789	0·791	0·793	0·796	0·799	0·804	0·809	0·812	0·815	0·818	0·82	0·823	0·825
Devon	0·747	0·752	0·757	0·762	0·766	0·769	0·771	0·774	0·778	0·783	0·788	0·792	0·796	0·798	0·802	0·805	0·807	0·81	0·812	0·814	0·817	0·821	0·825	0·829	0·832	0·835	0·837	0·839	0·841	0·843
Dorset	0·741	0·746	0·751	0·755	0·76	0·763	0·765	0·768	0·772	0·777	0·782	0·785	0·788	0·79	0·792	0·794	0·795	0·797	0·8	0·802	0·805	0·808	0·813	0·818	0·822	0·825	0·827	0·83	0·833	0·835
Gloucestershire	0·764	0·768	0·773	0·778	0·783	0·786	0·788	0·79	0·794	0·799	0·803	0·808	0·811	0·814	0·816	0·819	0·821	0·823	0·825	0·827	0·829	0·833	0·837	0·842	0·846	0·849	0·852	0·855	0·857	0·859
North Somerset	0·744	0·749	0·754	0·759	0·764	0·766	0·768	0·77	0·773	0·778	0·782	0·786	0·789	0·791	0·794	0·796	0·799	0·801	0·804	0·806	0·81	0·814	0·819	0·825	0·829	0·833	0·836	0·838	0·841	0·843
Plymouth	0·746	0·751	0·755	0·76	0·765	0·768	0·77	0·773	0·776	0·781	0·785	0·789	0·791	0·793	0·796	0·798	0·8	0·802	0·805	0·807	0·809	0·812	0·816	0·821	0·824	0·826	0·828	0·83	0·832	0·834
Poole	0·755	0·76	0·765	0·77	0·775	0·778	0·78	0·783	0·787	0·792	0·797	0·801	0·803	0·805	0·807	0·809	0·811	0·813	0·815	0·817	0·819	0·823	0·828	0·833	0·837	0·84	0·843	0·846	0·848	0·85
Somerset	0·732	0·736	0·742	0·747	0·752	0·755	0·757	0·759	0·763	0·768	0·772	0·775	0·778	0·78	0·782	0·784	0·786	0·787	0·79	0·791	0·794	0·798	0·802	0·808	0·812	0·815	0·818	0·821	0·824	0·826
South Gloucestershire	0·776	0·78	0·784	0·788	0·792	0·795	0·797	0·801	0·805	0·81	0·815	0·819	0·823	0·825	0·828	0·831	0·833	0·836	0·839	0·841	0·843	0·847	0·851	0·856	0·86	0·863	0·865	0·868	0·871	0·873
Swindon	0·765	0·77	0·775	0·781	0·785	0·788	0·789	0·791	0·794	0·798	0·802	0·806	0·808	0·809	0·81	0·812	0·813	0·814	0·817	0·818	0·821	0·825	0·83	0·835	0·839	0·842	0·844	0·846	0·849	0·85
Torbay	0·71	0·715	0·72	0·725	0·73	0·733	0·735	0·738	0·741	0·746	0·75	0·753	0·755	0·755	0·757	0·758	0·76	0·761	0·762	0·763	0·765	0·768	0·772	0·777	0·78	0·782	0·785	0·787	0·79	0·793
Wiltshire	0·751	0·755	0·759	0·764	0·769	0·771	0·773	0·776	0·779	0·784	0·789	0·793	0·796	0·798	0·8	0·802	0·804	0·805	0·807	0·809	0·811	0·815	0·82	0·825	0·829	0·833	0·836	0·839	0·841	0·844
West Midlands	0·716	0·721	0·727	0·733	0·738	0·742	0·744	0·747	0·752	0·757	0·763	0·767	0·771	0·773	0·776	0·778	0·781	0·783	0·786	0·789	0·792	0·796	0·801	0·807	0·811	0·814	0·817	0·82	0·823	0·825
Birmingham	0·719	0·724	0·73	0·736	0·741	0·744	0·746	0·749	0·754	0·759	0·764	0·769	0·773	0·775	0·778	0·781	0·784	0·787	0·79	0·793	0·797	0·801	0·806	0·811	0·815	0·818	0·82	0·823	0·825	0·827
Coventry	0·738	0·744	0·75	0·756	0·761	0·765	0·769	0·772	0·777	0·782	0·787	0·791	0·794	0·796	0·799	0·801	0·802	0·804	0·806	0·807	0·81	0·813	0·818	0·823	0·826	0·829	0·832	0·835	0·837	0·84
Dudley	0·699	0·704	0·708	0·714	0·718	0·721	0·723	0·726	0·73	0·735	0·74	0·744	0·747	0·748	0·751	0·752	0·754	0·756	0·757	0·758	0·76	0·763	0·768	0·773	0·777	0·779	0·782	0·785	0·788	0·79
Herefordshire, County of	0·722	0·727	0·733	0·739	0·745	0·748	0·751	0·755	0·759	0·765	0·771	0·775	0·779	0·781	0·783	0·786	0·788	0·789	0·792	0·793	0·796	0·8	0·805	0·811	0·816	0·819	0·823	0·826	0·829	0·832
Sandwell	0·677	0·682	0·687	0·692	0·698	0·701	0·702	0·705	0·709	0·714	0·719	0·723	0·726	0·728	0·73	0·731	0·733	0·734	0·737	0·738	0·741	0·746	0·752	0·759	0·764	0·768	0·772	0·775	0·778	0·781
Shropshire	0·728	0·733	0·738	0·744	0·749	0·752	0·754	0·757	0·762	0·768	0·773	0·778	0·781	0·784	0·787	0·79	0·792	0·795	0·797	0·799	0·802	0·806	0·81	0·815	0·819	0·822	0·825	0·827	0·83	0·832
Solihull	0·756	0·762	0·767	0·773	0·778	0·782	0·785	0·788	0·792	0·798	0·803	0·808	0·811	0·813	0·816	0·818	0·82	0·822	0·823	0·825	0·827	0·83	0·834	0·839	0·842	0·846	0·849	0·852	0·855	0·858
Staffordshire	0·725	0·73	0·736	0·742	0·747	0·75	0·752	0·755	0·759	0·765	0·769	0·773	0·776	0·778	0·78	0·782	0·784	0·786	0·788	0·79	0·793	0·797	0·801	0·805	0·808	0·811	0·813	0·815	0·817	0·819
Stoke-on-Trent	0·687	0·692	0·698	0·703	0·709	0·712	0·714	0·717	0·72	0·725	0·729	0·732	0·734	0·734	0·735	0·736	0·737	0·737	0·74	0·742	0·745	0·75	0·756	0·764	0·769	0·773	0·776	0·78	0·783	0·786
Telford and Wrekin	0·718	0·723	0·729	0·735	0·741	0·744	0·747	0·75	0·754	0·759	0·764	0·767	0·769	0·769	0·77	0·771	0·772	0·773	0·774	0·775	0·778	0·782	0·787	0·794	0·798	0·801	0·804	0·807	0·81	0·813
Walsall	0·681	0·685	0·689	0·694	0·699	0·701	0·702	0·703	0·706	0·711	0·716	0·72	0·723	0·724	0·726	0·728	0·73	0·732	0·735	0·737	0·74	0·745	0·751	0·758	0·762	0·766	0·77	0·773	0·776	0·779
Warwickshire	0·754	0·76	0·765	0·771	0·776	0·779	0·782	0·785	0·789	0·795	0·799	0·803	0·807	0·808	0·811	0·813	0·815	0·817	0·82	0·822	0·825	0·829	0·834	0·839	0·843	0·846	0·849	0·852	0·855	0·857
Wolverhampton	0·695	0·7	0·706	0·712	0·717	0·721	0·723	0·726	0·73	0·736	0·741	0·745	0·748	0·75	0·752	0·754	0·756	0·758	0·76	0·761	0·764	0·768	0·774	0·78	0·784	0·787	0·79	0·793	0·796	0·799
Worcestershire	0·731	0·736	0·741	0·747	0·752	0·755	0·757	0·761	0·765	0·772	0·777	0·782	0·785	0·788	0·79	0·792	0·794	0·796	0·798	0·799	0·802	0·805	0·809	0·814	0·818	0·82	0·823	0·826	0·828	0·831
Yorkshire and the Humber	0·721	0·726	0·732	0·737	0·742	0·745	0·748	0·751	0·755	0·76	0·765	0·77	0·773	0·775	0·778	0·78	0·783	0·785	0·788	0·79	0·793	0·797	0·802	0·808	0·812	0·815	0·818	0·821	0·823	0·826
Barnsley	0·677	0·681	0·685	0·69	0·695	0·697	0·699	0·702	0·706	0·711	0·716	0·719	0·722	0·723	0·725	0·727	0·729	0·73	0·732	0·734	0·737	0·741	0·746	0·753	0·758	0·761	0·765	0·768	0·771	0·774
Bradford	0·698	0·703	0·708	0·713	0·718	0·72	0·721	0·722	0·725	0·729	0·733	0·737	0·74	0·741	0·744	0·746	0·749	0·751	0·754	0·757	0·761	0·766	0·773	0·78	0·784	0·788	0·791	0·794	0·797	0·8
Calderdale	0·719	0·724	0·73	0·736	0·741	0·743	0·745	0·747	0·75	0·755	0·76	0·763	0·766	0·767	0·769	0·771	0·773	0·775	0·777	0·78	0·783	0·788	0·794	0·802	0·806	0·81	0·813	0·816	0·819	0·821
Doncaster	0·677	0·681	0·686	0·691	0·696	0·698	0·699	0·701	0·705	0·71	0·715	0·719	0·722	0·724	0·727	0·729	0·732	0·734	0·737	0·739	0·743	0·748	0·754	0·761	0·766	0·77	0·774	0·777	0·781	0·783
East Riding of Yorkshire	0·732	0·737	0·742	0·748	0·753	0·756	0·758	0·76	0·764	0·769	0·773	0·777	0·78	0·781	0·784	0·786	0·788	0·789	0·791	0·792	0·794	0·798	0·802	0·806	0·81	0·812	0·814	0·817	0·819	0·821
Kingston upon Hull, City of	0·692	0·696	0·702	0·707	0·712	0·716	0·718	0·722	0·727	0·733	0·738	0·743	0·747	0·749	0·751	0·754	0·756	0·758	0·76	0·762	0·764	0·768	0·772	0·778	0·781	0·783	0·785	0·787	0·79	0·792
Kirklees	0·715	0·72	0·726	0·731	0·736	0·738	0·74	0·742	0·745	0·75	0·754	0·758	0·761	0·762	0·764	0·767	0·769	0·77	0·773	0·775	0·777	0·782	0·787	0·793	0·797	0·8	0·803	0·805	0·808	0·81
Leeds	0·762	0·767	0·773	0·778	0·783	0·787	0·79	0·793	0·798	0·803	0·808	0·812	0·816	0·819	0·822	0·826	0·829	0·831	0·834	0·836	0·838	0·842	0·845	0·849	0·853	0·855	0·858	0·86	0·862	0·864
North East Lincolnshire	0·682	0·688	0·694	0·7	0·705	0·708	0·709	0·711	0·714	0·719	0·723	0·726	0·729	0·729	0·731	0·733	0·735	0·737	0·741	0·743	0·746	0·751	0·757	0·764	0·768	0·771	0·774	0·778	0·781	0·784
North Lincolnshire	0·722	0·727	0·732	0·737	0·742	0·745	0·746	0·748	0·752	0·756	0·76	0·762	0·764	0·765	0·767	0·768	0·77	0·772	0·775	0·777	0·781	0·785	0·79	0·796	0·799	0·8	0·801	0·803	0·805	0·806
North Yorkshire	0·746	0·75	0·755	0·76	0·765	0·767	0·769	0·772	0·775	0·781	0·786	0·79	0·793	0·796	0·798	0·801	0·803	0·804	0·807	0·808	0·811	0·815	0·819	0·824	0·827	0·83	0·832	0·834	0·836	0·838
Rotherham	0·686	0·69	0·695	0·701	0·706	0·708	0·71	0·712	0·715	0·72	0·725	0·729	0·732	0·734	0·737	0·739	0·741	0·743	0·745	0·747	0·75	0·754	0·76	0·766	0·77	0·773	0·775	0·778	0·781	0·784
Sheffield	0·749	0·753	0·758	0·762	0·767	0·77	0·773	0·776	0·78	0·785	0·789	0·794	0·798	0·801	0·804	0·807	0·811	0·814	0·817	0·819	0·822	0·826	0·83	0·834	0·837	0·84	0·842	0·844	0·847	0·848
Wakefield	0·694	0·699	0·704	0·71	0·715	0·718	0·72	0·722	0·726	0·731	0·735	0·739	0·742	0·743	0·745	0·746	0·748	0·749	0·751	0·753	0·755	0·76	0·765	0·772	0·777	0·78	0·783	0·786	0·789	0·792
York	0·795	0·799	0·804	0·809	0·813	0·816	0·819	0·822	0·826	0·83	0·834	0·839	0·842	0·845	0·849	0·852	0·855	0·857	0·859	0·861	0·863	0·865	0·868	0·871	0·873	0·875	0·877	0·879	0·881	0·883
Northern Ireland	0·701	0·707	0·716	0·726	0·733	0·737	0·738	0·74	0·744	0·751	0·759	0·765	0·77	0·774	0·777	0·779	0·781	0·783	0·786	0·789	0·792	0·796	0·802	0·808	0·811	0·815	0·818	0·821	0·823	0·825
Scotland	0·724	0·728	0·735	0·743	0·75	0·754	0·754	0·756	0·76	0·768	0·774	0·78	0·784	0·787	0·789	0·791	0·792	0·794	0·797	0·801	0·805	0·809	0·814	0·819	0·823	0·826	0·828	0·831	0·833	0·834
Wales	0·706	0·711	0·717	0·723	0·729	0·733	0·736	0·74	0·745	0·751	0·757	0·762	0·766	0·769	0·772	0·775	0·778	0·78	0·783	0·785	0·788	0·792	0·797	0·802	0·806	0·809	0·812	0·815	0·818	0·82
Latin America and Caribbean	0·491	0·496	0·501	0·506	0·511	0·516	0·521	0·526	0·531	0·536	0·541	0·546	0·55	0·554	0·558	0·563	0·568	0·573	0·578	0·583	0·589	0·594	0·6	0·605	0·611	0·616	0·62	0·625	0·629	0·633
Andean Latin America	0·489	0·492	0·496	0·5	0·504	0·509	0·514	0·519	0·524	0·529	0·534	0·538	0·543	0·548	0·553	0·558	0·564	0·569	0·575	0·58	0·585	0·591	0·596	0·602	0·608	0·613	0·618	0·624	0·628	0·632
Bolivia	0·412	0·417	0·422	0·428	0·434	0·441	0·448	0·455	0·462	0·469	0·474	0·48	0·485	0·491	0·496	0·501	0·506	0·51	0·514	0·518	0·521	0·525	0·528	0·533	0·538	0·544	0·55	0·556	0·562	0·566
Ecuador	0·503	0·506	0·51	0·514	0·517	0·521	0·524	0·528	0·532	0·535	0·539	0·544	0·549	0·554	0·559	0·565	0·57	0·575	0·581	0·586	0·591	0·597	0·603	0·609	0·615	0·621	0·626	0·632	0·636	0·64
Peru	0·501	0·505	0·508	0·511	0·516	0·521	0·526	0·532	0·537	0·543	0·548	0·552	0·557	0·562	0·566	0·571	0·577	0·583	0·589	0·595	0·601	0·608	0·614	0·62	0·625	0·63	0·635	0·64	0·645	0·648
Caribbean	0·517	0·522	0·525	0·528	0·531	0·533	0·536	0·539	0·542	0·547	0·552	0·557	0·563	0·569	0·573	0·579	0·585	0·588	0·591	0·594	0·598	0·601	0·605	0·609	0·612	0·616	0·621	0·625	0·628	0·631
Antigua and Barbuda	0·579	0·586	0·592	0·598	0·604	0·61	0·616	0·623	0·631	0·638	0·645	0·652	0·659	0·665	0·672	0·679	0·686	0·694	0·7	0·705	0·709	0·713	0·716	0·719	0·723	0·727	0·731	0·735	0·739	0·743
The Bahamas	0·692	0·684	0·676	0·679	0·686	0·692	0·697	0·702	0·71	0·721	0·731	0·735	0·737	0·739	0·742	0·749	0·755	0·759	0·762	0·767	0·774	0·779	0·78	0·782	0·784	0·786	0·789	0·791	0·794	0·796
Barbados	0·649	0·653	0·658	0·663	0·667	0·67	0·672	0·674	0·676	0·677	0·68	0·683	0·687	0·691	0·695	0·699	0·703	0·707	0·711	0·714	0·718	0·721	0·725	0·728	0·73	0·733	0·735	0·737	0·74	0·742
Belize	0·428	0·437	0·447	0·457	0·466	0·475	0·482	0·488	0·494	0·499	0·505	0·51	0·515	0·521	0·528	0·534	0·541	0·547	0·553	0·559	0·564	0·569	0·574	0·579	0·583	0·588	0·592	0·596	0·6	0·603
Bermuda	0·685	0·689	0·693	0·697	0·701	0·704	0·707	0·71	0·714	0·719	0·724	0·731	0·737	0·743	0·749	0·755	0·76	0·766	0·773	0·779	0·785	0·79	0·795	0·799	0·802	0·805	0·807	0·809	0·811	0·813
Cuba	0·578	0·586	0·589	0·588	0·586	0·583	0·583	0·582	0·579	0·581	0·586	0·588	0·595	0·602	0·605	0·611	0·618	0·618	0·616	0·618	0·62	0·624	0·631	0·636	0·64	0·645	0·653	0·66	0·665	0·668
Dominica	0·579	0·585	0·591	0·597	0·604	0·61	0·617	0·623	0·63	0·637	0·643	0·649	0·655	0·66	0·666	0·672	0·677	0·683	0·689	0·695	0·7	0·705	0·709	0·713	0·717	0·721	0·724	0·727	0·728	0·729
Dominican Republic	0·425	0·427	0·43	0·434	0·437	0·442	0·446	0·452	0·458	0·465	0·471	0·478	0·485	0·492	0·499	0·508	0·516	0·524	0·532	0·538	0·544	0·549	0·554	0·559	0·563	0·569	0·574	0·58	0·587	0·592
Grenada	0·463	0·474	0·485	0·495	0·505	0·515	0·524	0·533	0·543	0·553	0·562	0·57	0·577	0·585	0·592	0·6	0·606	0·613	0·619	0·624	0·628	0·633	0·637	0·641	0·645	0·65	0·654	0·659	0·664	0·669
Guyana	0·452	0·456	0·461	0·467	0·474	0·481	0·488	0·496	0·503	0·51	0·516	0·522	0·527	0·532	0·536	0·54	0·544	0·549	0·554	0·559	0·565	0·571	0·577	0·583	0·59	0·596	0·602	0·608	0·614	0·618
Haiti	0·307	0·311	0·314	0·317	0·32	0·323	0·328	0·333	0·338	0·344	0·35	0·356	0·361	0·366	0·371	0·375	0·379	0·383	0·387	0·392	0·395	0·399	0·403	0·407	0·412	0·416	0·42	0·424	0·428	0·432
Jamaica	0·542	0·547	0·553	0·56	0·566	0·573	0·58	0·586	0·592	0·598	0·603	0·609	0·614	0·62	0·625	0·63	0·635	0·64	0·645	0·649	0·653	0·657	0·661	0·664	0·668	0·671	0·675	0·678	0·681	0·684
Puerto Rico	0·67	0·676	0·679	0·684	0·689	0·693	0·699	0·705	0·711	0·714	0·72	0·731	0·738	0·742	0·744	0·748	0·752	0·756	0·76	0·764	0·769	0·774	0·779	0·785	0·793	0·802	0·808	0·811	0·813	0·814
Saint Kitts and Nevis	0·583	0·592	0·599	0·607	0·614	0·62	0·626	0·633	0·638	0·644	0·65	0·657	0·663	0·668	0·673	0·679	0·685	0·69	0·696	0·701	0·706	0·71	0·714	0·719	0·724	0·728	0·733	0·738	0·742	0·746
Saint Lucia	0·483	0·493	0·504	0·514	0·524	0·534	0·543	0·551	0·56	0·568	0·575	0·581	0·587	0·593	0·6	0·606	0·612	0·617	0·623	0·629	0·634	0·639	0·643	0·648	0·652	0·656	0·659	0·663	0·667	0·67
Saint Vincent and the Grenadines	0·462	0·471	0·479	0·487	0·495	0·502	0·509	0·515	0·522	0·528	0·534	0·539	0·545	0·551	0·557	0·562	0·568	0·574	0·58	0·585	0·589	0·593	0·598	0·602	0·606	0·61	0·615	0·619	0·623	0·627
Suriname	0·498	0·503	0·507	0·511	0·513	0·515	0·518	0·523	0·528	0·533	0·538	0·545	0·552	0·56	0·568	0·574	0·579	0·584	0·588	0·593	0·598	0·602	0·607	0·611	0·616	0·62	0·625	0·629	0·633	0·636
Trinidad and Tobago	0·618	0·622	0·626	0·63	0·634	0·639	0·644	0·65	0·656	0·662	0·669	0·675	0·682	0·689	0·696	0·704	0·711	0·718	0·724	0·728	0·732	0·736	0·739	0·742	0·745	0·748	0·751	0·753	0·755	0·757
Virgin Islands	0·667	0·68	0·689	0·697	0·704	0·71	0·715	0·72	0·724	0·728	0·731	0·734	0·742	0·749	0·756	0·762	0·768	0·773	0·778	0·782	0·785	0·788	0·79	0·791	0·792	0·794	0·795	0·796	0·798	0·799
Central Latin America	0·485	0·49	0·495	0·501	0·507	0·512	0·517	0·523	0·528	0·533	0·538	0·543	0·547	0·551	0·554	0·559	0·564	0·569	0·575	0·579	0·584	0·589	0·594	0·599	0·604	0·609	0·614	0·618	0·623	0·626
Colombia	0·478	0·48	0·482	0·486	0·492	0·498	0·503	0·509	0·514	0·518	0·522	0·526	0·529	0·533	0·536	0·54	0·546	0·552	0·559	0·567	0·574	0·582	0·589	0·596	0·603	0·609	0·616	0·622	0·628	0·633
Costa Rica	0·532	0·538	0·544	0·55	0·555	0·56	0·565	0·571	0·577	0·584	0·592	0·598	0·603	0·607	0·611	0·615	0·619	0·623	0·627	0·632	0·637	0·642	0·647	0·652	0·657	0·662	0·667	0·672	0·676	0·68
El Salvador	0·39	0·393	0·397	0·402	0·408	0·415	0·423	0·431	0·441	0·45	0·46	0·469	0·478	0·486	0·493	0·499	0·505	0·511	0·517	0·521	0·526	0·531	0·536	0·542	0·547	0·552	0·558	0·563	0·568	0·573
Guatemala	0·315	0·32	0·328	0·336	0·345	0·352	0·36	0·369	0·378	0·387	0·395	0·403	0·412	0·421	0·43	0·439	0·446	0·453	0·459	0·465	0·472	0·478	0·485	0·491	0·498	0·504	0·51	0·516	0·522	0·526
Honduras	0·33	0·335	0·34	0·345	0·351	0·357	0·363	0·37	0·377	0·384	0·391	0·397	0·404	0·411	0·417	0·424	0·43	0·437	0·443	0·448	0·454	0·459	0·464	0·469	0·473	0·478	0·482	0·487	0·492	0·496
Mexico	0·507	0·514	0·52	0·526	0·532	0·537	0·542	0·547	0·553	0·558	0·563	0·569	0·574	0·578	0·583	0·588	0·592	0·597	0·601	0·605	0·608	0·613	0·617	0·621	0·626	0·631	0·636	0·64	0·645	0·649
Aguascalientes	0·532	0·538	0·544	0·549	0·554	0·559	0·564	0·569	0·574	0·58	0·585	0·591	0·597	0·602	0·607	0·611	0·616	0·621	0·625	0·629	0·633	0·637	0·641	0·646	0·65	0·655	0·66	0·664	0·669	0·673

Appendix Table 16. Socio-demographic Index values for all estimated GBD 2019 locations, 1990–2019

Location	1990	1991	1992	1993	1994	1995	1996	1997	1998	1999	2000	2001	2002	2003	2004	2005	2006	2007	2008	2009	2010	2011	2012	2013	2014	2015	2016	2017	2018	2019
Baja California	0·565	0·57	0·575	0·58	0·585	0·589	0·593	0·598	0·604	0·609	0·615	0·619	0·624	0·628	0·632	0·636	0·64	0·644	0·647	0·65	0·653	0·656	0·66	0·663	0·667	0·671	0·675	0·68	0·685	0·688
Baja California Sur	0·546	0·553	0·559	0·565	0·571	0·576	0·582	0·588	0·594	0·599	0·604	0·609	0·614	0·619	0·625	0·63	0·636	0·641	0·646	0·649	0·653	0·658	0·662	0·666	0·67	0·675	0·68	0·684	0·689	0·693
Campeche	0·481	0·488	0·495	0·502	0·509	0·515	0·522	0·529	0·536	0·542	0·548	0·554	0·56	0·565	0·571	0·576	0·581	0·586	0·591	0·595	0·6	0·606	0·611	0·617	0·623	0·629	0·634	0·639	0·644	0·648
Chiapas	0·413	0·419	0·424	0·43	0·437	0·442	0·446	0·451	0·456	0·461	0·466	0·47	0·475	0·48	0·486	0·491	0·497	0·503	0·508	0·512	0·517	0·522	0·527	0·532	0·536	0·541	0·545	0·55	0·554	0·557
Chihuahua	0·529	0·534	0·538	0·542	0·546	0·549	0·553	0·558	0·563	0·568	0·573	0·578	0·584	0·588	0·593	0·598	0·602	0·607	0·611	0·614	0·617	0·62	0·623	0·627	0·632	0·637	0·643	0·648	0·654	0·659
Coahuila	0·529	0·536	0·543	0·549	0·556	0·561	0·566	0·571	0·576	0·581	0·586	0·591	0·595	0·6	0·604	0·609	0·613	0·617	0·62	0·623	0·625	0·628	0·631	0·634	0·639	0·644	0·648	0·653	0·658	0·663
Colima	0·535	0·543	0·551	0·557	0·563	0·568	0·574	0·58	0·585	0·59	0·596	0·601	0·606	0·611	0·615	0·62	0·625	0·629	0·634	0·638	0·642	0·646	0·65	0·654	0·659	0·664	0·669	0·673	0·678	0·682
Durango	0·453	0·46	0·466	0·472	0·478	0·483	0·489	0·496	0·504	0·511	0·517	0·524	0·53	0·537	0·543	0·548	0·553	0·558	0·563	0·566	0·571	0·575	0·58	0·585	0·591	0·596	0·602	0·608	0·614	0·618
Guanajuato	0·452	0·459	0·467	0·474	0·481	0·488	0·495	0·502	0·51	0·517	0·525	0·532	0·538	0·544	0·55	0·556	0·562	0·567	0·572	0·577	0·582	0·588	0·594	0·6	0·606	0·612	0·618	0·624	0·63	0·634
Guerrero	0·408	0·414	0·419	0·424	0·429	0·434	0·44	0·446	0·452	0·457	0·464	0·47	0·476	0·481	0·487	0·493	0·498	0·504	0·51	0·516	0·522	0·529	0·535	0·541	0·548	0·553	0·558	0·563	0·568	0·572
Hidalgo	0·43	0·438	0·445	0·453	0·46	0·467	0·474	0·481	0·488	0·494	0·501	0·507	0·513	0·518	0·524	0·53	0·536	0·542	0·548	0·553	0·559	0·565	0·571	0·577	0·584	0·59	0·596	0·601	0·606	0·611
Jalisco	0·523	0·53	0·537	0·544	0·551	0·556	0·561	0·566	0·572	0·577	0·582	0·587	0·591	0·596	0·6	0·604	0·608	0·613	0·617	0·621	0·625	0·629	0·634	0·638	0·643	0·647	0·652	0·657	0·661	0·665
México	0·536	0·544	0·551	0·558	0·565	0·569	0·573	0·577	0·58	0·583	0·587	0·591	0·595	0·599	0·604	0·608	0·612	0·616	0·62	0·622	0·625	0·629	0·632	0·636	0·64	0·644	0·648	0·652	0·657	0·66
Mexico City	0·601	0·607	0·614	0·621	0·627	0·633	0·638	0·644	0·65	0·656	0·662	0·667	0·671	0·675	0·679	0·683	0·687	0·69	0·693	0·695	0·697	0·7	0·704	0·708	0·712	0·716	0·72	0·724	0·729	0·732
Michoacán de Ocampo	0·459	0·467	0·475	0·482	0·487	0·492	0·497	0·503	0·508	0·514	0·519	0·524	0·529	0·534	0·54	0·545	0·55	0·555	0·56	0·564	0·568	0·572	0·576	0·581	0·585	0·59	0·594	0·598	0·602	0·606
Morelos	0·522	0·53	0·536	0·542	0·548	0·553	0·559	0·564	0·57	0·575	0·581	0·586	0·59	0·594	0·597	0·601	0·604	0·608	0·611	0·614	0·617	0·621	0·624	0·628	0·632	0·637	0·641	0·646	0·65	0·654
Nayarit	0·489	0·495	0·501	0·506	0·511	0·516	0·522	0·529	0·535	0·541	0·548	0·555	0·561	0·567	0·574	0·58	0·586	0·591	0·595	0·599	0·603	0·607	0·612	0·617	0·621	0·626	0·63	0·635	0·639	0·643
Nuevo León	0·577	0·583	0·589	0·594	0·599	0·602	0·606	0·611	0·615	0·62	0·625	0·629	0·634	0·638	0·642	0·645	0·649	0·652	0·656	0·658	0·661	0·665	0·669	0·673	0·677	0·682	0·686	0·691	0·696	0·699
Oaxaca	0·426	0·429	0·433	0·437	0·441	0·445	0·449	0·453	0·458	0·462	0·467	0·472	0·477	0·483	0·49	0·496	0·502	0·509	0·516	0·522	0·528	0·534	0·54	0·545	0·55	0·555	0·561	0·566	0·571	0·574
Puebla	0·46	0·467	0·473	0·48	0·486	0·49	0·495	0·5	0·504	0·509	0·514	0·52	0·525	0·53	0·536	0·541	0·546	0·552	0·557	0·561	0·567	0·572	0·577	0·583	0·588	0·593	0·598	0·603	0·608	0·612
Querétaro	0·507	0·514	0·52	0·526	0·533	0·539	0·545	0·552	0·559	0·567	0·575	0·584	0·591	0·597	0·603	0·608	0·613	0·618	0·622	0·626	0·63	0·634	0·639	0·644	0·649	0·653	0·658	0·663	0·667	0·671
Quintana Roo	0·494	0·504	0·513	0·522	0·531	0·54	0·549	0·557	0·565	0·572	0·579	0·585	0·591	0·596	0·601	0·606	0·61	0·615	0·62	0·624	0·629	0·634	0·638	0·643	0·647	0·652	0·657	0·662	0·666	0·67
San Luis Potosí	0·451	0·457	0·464	0·471	0·478	0·486	0·493	0·501	0·508	0·516	0·523	0·53	0·536	0·543	0·55	0·557	0·564	0·571	0·578	0·584	0·59	0·596	0·602	0·607	0·612	0·617	0·622	0·626	0·631	0·635
Sinaloa	0·519	0·525	0·53	0·536	0·542	0·547	0·553	0·559	0·565	0·57	0·576	0·58	0·585	0·589	0·594	0·599	0·604	0·608	0·613	0·617	0·621	0·626	0·631	0·636	0·641	0·647	0·652	0·657	0·662	0·667
Sonora	0·546	0·552	0·558	0·564	0·569	0·574	0·579	0·584	0·59	0·596	0·602	0·607	0·612	0·616	0·621	0·626	0·631	0·635	0·639	0·642	0·645	0·649	0·653	0·658	0·662	0·668	0·673	0·678	0·683	0·687
Tabasco	0·485	0·492	0·499	0·505	0·511	0·516	0·523	0·529	0·536	0·542	0·548	0·553	0·558	0·562	0·566	0·571	0·575	0·58	0·584	0·588	0·592	0·597	0·602	0·608	0·613	0·619	0·624	0·629	0·634	0·638
Tamaulipas	0·529	0·535	0·54	0·546	0·551	0·557	0·563	0·57	0·576	0·581	0·585	0·589	0·594	0·598	0·602	0·606	0·61	0·614	0·618	0·621	0·624	0·628	0·632	0·636	0·641	0·647	0·652	0·657	0·662	0·666
Tlaxcala	0·474	0·483	0·492	0·501	0·509	0·516	0·523	0·529	0·536	0·542	0·549	0·555	0·561	0·566	0·572	0·577	0·582	0·587	0·592	0·596	0·601	0·605	0·61	0·614	0·619	0·623	0·628	0·632	0·636	0·64
Veracruz de Ignacio de la Llave	0·471	0·475	0·48	0·485	0·489	0·493	0·497	0·502	0·507	0·512	0·517	0·522	0·527	0·532	0·537	0·542	0·548	0·553	0·558	0·562	0·567	0·572	0·578	0·582	0·587	0·592	0·596	0·601	0·605	0·609
Yucatán	0·478	0·484	0·49	0·497	0·504	0·511	0·517	0·524	0·53	0·535	0·54	0·545	0·549	0·553	0·559	0·564	0·571	0·577	0·583	0·588	0·594	0·599	0·605	0·611	0·616	0·622	0·627	0·631	0·636	0·64
Zacatecas	0·493	0·499	0·505	0·511	0·516	0·52	0·525	0·529	0·534	0·539	0·544	0·55	0·555	0·56	0·564	0·568	0·572	0·576	0·581	0·584	0·587	0·591	0·595	0·599	0·604	0·609	0·615	0·62	0·625	0·63
Nicaragua	0·338	0·345	0·353	0·36	0·368	0·376	0·385	0·393	0·402	0·411	0·419	0·426	0·433	0·438	0·444	0·449	0·453	0·458	0·462	0·466	0·47	0·474	0·479	0·484	0·489	0·495	0·5	0·506	0·512	0·517
Panama	0·544	0·549	0·555	0·559	0·562	0·565	0·568	0·573	0·579	0·586	0·592	0·597	0·602	0·605	0·607	0·61	0·613	0·616	0·62	0·623	0·627	0·63	0·635	0·642	0·65	0·658	0·666	0·674	0·68	0·686
Venezuela	0·509	0·514	0·522	0·53	0·537	0·542	0·546	0·551	0·554	0·557	0·559	0·56	0·558	0·552	0·548	0·55	0·557	0·566	0·575	0·582	0·586	0·591	0·596	0·6	0·604	0·607	0·608	0·608	0·608	0·607
Tropical Latin America	0·487	0·492	0·497	0·503	0·508	0·513	0·519	0·524	0·529	0·533	0·538	0·543	0·547	0·552	0·556	0·561	0·566	0·572	0·578	0·584	0·59	0·597	0·604	0·61	0·617	0·622	0·627	0·632	0·636	0·64
Brazil	0·487	0·492	0·498	0·503	0·508	0·513	0·519	0·524	0·529	0·533	0·538	0·543	0·547	0·551	0·556	0·561	0·566	0·572	0·577	0·583	0·59	0·597	0·603	0·61	0·616	0·622	0·627	0·632	0·636	0·64
Acre	0·364	0·371	0·379	0·386	0·394	0·402	0·41	0·417	0·423	0·429	0·435	0·441	0·446	0·452	0·458	0·464	0·47	0·477	0·485	0·493	0·501	0·509	0·518	0·526	0·533	0·54	0·547	0·552	0·558	0·562
Alagoas	0·356	0·362	0·367	0·372	0·377	0·382	0·387	0·391	0·396	0·4	0·404	0·408	0·413	0·417	0·422	0·427	0·433	0·439	0·446	0·453	0·461	0·469	0·477	0·485	0·492	0·498	0·504	0·509	0·514	0·518
Amapá	0·494	0·501	0·508	0·515	0·521	0·528	0·534	0·54	0·545	0·549	0·553	0·557	0·56	0·563	0·566	0·569	0·573	0·578	0·583	0·588	0·594	0·6	0·606	0·612	0·618	0·624	0·629	0·633	0·637	0·641
Amazonas	0·459	0·467	0·474	0·481	0·486	0·491	0·495	0·498	0·501	0·503	0·505	0·507	0·51	0·512	0·516	0·52	0·524	0·53	0·536	0·541	0·548	0·555	0·562	0·569	0·576	0·582	0·588	0·593	0·598	0·602
Bahia	0·397	0·404	0·409	0·415	0·421	0·426	0·431	0·435	0·44	0·444	0·448	0·452	0·456	0·461	0·466	0·471	0·477	0·483	0·491	0·498	0·505	0·514	0·521	0·529	0·536	0·542	0·548	0·553	0·558	0·562
Ceará	0·386	0·392	0·398	0·404	0·41	0·416	0·422	0·428	0·433	0·437	0·442	0·447	0·451	0·456	0·461	0·466	0·472	0·479	0·486	0·493	0·501	0·51	0·518	0·525	0·533	0·539	0·544	0·549	0·554	0·558
Distrito Federal	0·587	0·594	0·602	0·61	0·619	0·63	0·641	0·652	0·661	0·669	0·676	0·683	0·689	0·694	0·699	0·704	0·709	0·715	0·72	0·726	0·732	0·739	0·745	0·751	0·756	0·761	0·766	0·77	0·774	0·777
Espírito Santo	0·479	0·485	0·491	0·498	0·504	0·511	0·518	0·525	0·531	0·537	0·543	0·548	0·554	0·56	0·566	0·572	0·579	0·586	0·593	0·599	0·607	0·614	0·622	0·629	0·636	0·642	0·647	0·652	0·657	0·66
Goiás	0·442	0·449	0·455	0·462	0·469	0·476	0·483	0·49	0·496	0·502	0·508	0·514	0·52	0·526	0·532	0·538	0·545	0·551	0·558	0·565	0·573	0·581	0·588	0·596	0·603	0·609	0·614	0·619	0·624	0·628
Maranhão	0·274	0·281	0·287	0·293	0·299	0·305	0·311	0·316	0·32	0·324	0·327	0·33	0·333	0·336	0·34	0·344	0·349	0·355	0·361	0·368	0·376	0·385	0·394	0·403	0·412	0·42	0·427	0·433	0·439	0·444
Mato Grosso	0·448	0·455	0·462	0·469	0·477	0·484	0·491	0·497	0·503	0·509	0·516	0·522	0·528	0·535	0·543	0·55	0·557	0·564	0·572	0·579	0·587	0·595	0·603	0·61	0·617	0·623	0·629	0·634	0·638	0·642
Mato Grosso do Sul	0·471	0·477	0·483	0·488	0·494	0·5	0·505	0·511	0·516	0·521	0·526	0·531	0·536	0·542	0·547	0·553	0·558	0·565	0·571	0·578	0·585	0·593	0·601	0·608	0·615	0·621	0·626	0·631	0·636	0·639
Minas Gerais	0·487	0·493	0·498	0·504	0·51	0·515	0·52	0·525	0·529	0·534	0·538	0·542	0·547	0·552	0·558	0·563	0·569	0·576	0·582	0·589	0·596	0·603	0·61	0·616	0·622	0·627	0·632	0·636	0·64	0·643
Pará	0·425	0·432	0·438	0·444	0·449	0·454	0·457	0·46	0·462	0·464	0·465	0·467	0·469	0·472	0·475	0·479	0·484	0·489	0·496	0·502	0·51	0·518	0·526	0·534	0·542	0·549	0·554	0·56	0·565	0·569
Paraíba	0·378	0·385	0·391	0·396	0·402	0·408	0·413	0·418	0·422	0·426	0·431	0·435	0·439	0·444	0·449	0·454	0·46	0·467	0·474	0·482	0·49	0·499	0·507	0·515	0·522	0·528	0·534	0·539	0·544	0·548
Paraná	0·499	0·504	0·509	0·514	0·519	0·525	0·531	0·537	0·543	0·549	0·555	0·561	0·567	0·573	0·579	0·584	0·59	0·596	0·603	0·609	0·615	0·621	0·628	0·634	0·64	0·645	0·65	0·654	0·659	0·662
Pernambuco	0·407	0·414	0·419	0·424	0·429	0·434	0·438	0·442	0·446	0·45	0·453	0·457	0·461	0·465	0·47	0·475	0·481	0·488	0·495	0·502	0·51	0·519	0·527	0·536	0·543	0·55	0·556	0·561	0·567	0·571
Piauí	0·334	0·34	0·345	0·351	0·357	0·363	0·369	0·375	0·38	0·384	0·388	0·392	0·396	0·4	0·405	0·41	0·416	0·423	0·431	0·439	0·448	0·457	0·466	0·474	0·482	0·489	0·494	0·5	0·505	0·509
Rio de Janeiro	0·569	0·574	0·578	0·582	0·586	0·59	0·595	0·6	0·604	0·609	0·613	0·618	0·622	0·626	0·63	0·634	0·639	0·643	0·648	0·653	0·658	0·664	0·67	0·675	0·681	0·686	0·69	0·694	0·698	0·702
Rio Grande do Norte	0·402	0·408	0·413	0·419	0·424	0·429	0·434	0·44	0·444	0·449	0·454	0·459	0·464	0·469	0·475	0·481	0·488	0·495	0·503	0·51	0·519	0·527	0·535	0·543	0·551	0·557	0·562	0·567	0·572	0·576
Rio Grande do Sul	0·549	0·554	0·558	0·563	0·567	0·572	0·576	0·58	0·584	0·588	0·593	0·597	0·602	0·606	0·611	0·616	0·62	0·626	0·631	0·636	0·642	0·648	0·653	0·659	0·664	0·669	0·673	0·677	0·681	0·684
Rondônia	0·43	0·436	0·441	0·446	0·451	0·455	0·46	0·465	0·47	0·474	0·48	0·485	0·49	0·496	0·503	0·509	0·516	0·524	0·531	0·539	0·547	0·556	0·565	0·573	0·58	0·587	0·592	0·598	0·603	0·606
Roraima	0·425	0·431	0·437	0·441	0·446	0·452	0·458	0·465	0·471	0·476	0·482	0·488	0·494	0·5	0·506	0·512	0·519	0·526	0·534	0·541	0·55	0·558	0·566	0·575	0·582	0·589	0·595	0·601	0·606	0·61
Santa Catarina	0·539	0·544	0·549	0·554	0·559	0·564	0·569	0·574	0·579	0·584	0·589	0·594	0·6	0·605	0·611	0·616	0·622	0·628	0·634	0·64	0·646	0·652	0·659	0·665	0·67	0·676	0·68	0·684	0·688	0·691
São Paulo	0·554	0·559	0·564	0·569	0·575	0·581	0·587	0·593	0·599	0·604	0·61	0·615	0·62	0·624	0·629	0·633	0·638	0·643	0·648	0·652	0·658	0·663	0·669	0·675	0·68	0·686	0·69	0·694	0·698	0·702
Sergipe	0·424	0·43	0·436	0·441	0·446	0·451	0·456	0·46	0·465	0·469	0·473	0·478	0·483	0·488	0·493	0·498	0·504	0·511	0·518	0·524	0·532	0·539	0·546	0·553	0·56	0·566	0·57	0·575	0·579	0·583
Tocantins	0·361	0·366	0·37	0·374	0·38	0·386	0·393	0·4	0·408	0·415	0·422	0·43	0·438	0·447	0·456	0·465	0·474	0·484	0·494	0·504	0·514	0·524	0·534	0·544	0·552	0·56	0·567	0·573	0·579	0·583
Paraguay	0·465	0·471	0·477	0·484	0·491	0·498	0·505	0·512	0·519	0·524	0·529	0·534	0·539	0·544	0·549	0·554	0·559	0·564	0·57	0·575	0·582	0·588	0·594	0·601	0·608	0·615	0·621	0·627	0·633	0·638
North Africa and Middle East	0·414	0·423	0·432	0·441	0·45	0·46	0·469	0·478	0·487	0·497	0·506	0·515	0·524	0·532	0·541	0·55	0·56	0·569	0·578	0·587	0·595	0·603	0·611	0·619	0·626	0·633	0·64	0·647	0·654	0·66
North Africa and Middle East	0·414	0·423	0·432	0·441	0·45	0·46	0·469	0·478	0·487	0·497	0·506	0·515	0·524	0·532	0·541	0·55	0·56	0·569	0·578	0·587	0·595	0·603	0·611	0·619	0·626	0·633	0·64	0·647	0·654	0·66
Afghanistan	0·187	0·191	0·195	0·196	0·194	0·194	0·193	0·192	0·19	0·189	0·188	0·188	0·194	0·202	0·209	0·216	0·224	0·234	0·243	0·253	0·264	0·274	0·285	0·295	0·304	0·313	0·321	0·329	0·337	0·343
Algeria	0·436	0·446	0·456	0·465	0·474	0·483	0·492	0·5	0·509	0·518	0·526	0·534	0·542	0·55	0·558	0·566	0·573	0·58	0·587	0·593	0·599	0·605	0·611	0·617	0·623	0·628	0·634	0·64	0·646	0·652
Bahrain	0·553	0·56	0·566	0·573	0·58	0·587	0·595	0·602	0·61	0·618	0·626	0·634	0·643	0·653	0·662	0·672	0·68	0·688	0·697	0·705	0·711	0·715	0·72	0·726	0·731	0·735	0·739	0·743	0·747	0·751
Egypt	0·403	0·415	0·426	0·437	0·448	0·459	0·469	0·478	0·487	0·496	0·504	0·512	0·52	0·527	0·535	0·542	0·549	0·557	0·565	0·573	0·582	0·591	0·6	0·609	0·617	0·626	0·635	0·643	0·651	0·658
Iran	0·404	0·419	0·433	0·446	0·457	0·468	0·477	0·486	0·496	0·506	0·517	0·529	0·541	0·552	0·562	0·571	0·581	0·593	0·603	0·613	0·622	0·63	0·635	0·64	0·645	0·649	0·654	0·659	0·665	0·67
Iraq	0·392	0·397	0·402	0·407	0·412	0·417	0·422	0·429	0·439	0·451	0·462	0·475	0·485	0·493	0·504	0·514	0·525	0·535	0·547	0·558	0·57	0·583	0·597	0·61	0·622	0·632	0·644	0·654	0·663	0·671
Jordan	0·52	0·529	0·537	0·546	0·554	0·562	0·57	0·577	0·585	0·592	0·6	0·607	0·614	0·621	0·63	0·639	0·648	0·656	0·664	0·673	0·681	0·688	0·695	0·702	0·707	0·713	0·718	0·723	0·727	0·731
Kuwait	0·655	0·659	0·662	0·667	0·673	0·68	0·689	0·699	0·709	0·717	0·724	0·729	0·735	0·742	0·75	0·76	0·769	0·777	0·785	0·793	0·801	0·808	0·815	0·822	0·828	0·834	0·839	0·844	0·848	0·851
Lebanon	0·462	0·47	0·477	0·485	0·493	0·502	0·511	0·52	0·53	0·54	0·548	0·557	0·565	0·574	0·582	0·591	0·6	0·609	0·618	0·628	0·639	0·649	0·66	0·67	0·677	0·685	0·691	0·698	0·704	0·708
Libya	0·405	0·422	0·438	0·455	0·472	0·489	0·506	0·522	0·538	0·552	0·566	0·58	0·593	0·606	0·619	0·632	0·645	0·658	0·67	0·681	0·691	0·695	0·703	0·707	0·707	0·707	0·705	0·705	0·707	0·709
Morocco	0·347	0·354	0·361	0·367	0·374	0·38	0·386	0·392	0·398	0·403	0·409	0·414	0·42	0·426	0·432	0·439	0·445	0·452	0·46	0·467	0·475	0·483	0·491	0·499	0·508	0·516	0·524	0·533	0·541	0·548
Oman	0·441	0·455	0·469	0·485	0·5	0·514	0·528	0·543	0·558	0·573	0·588	0·601	0·615	0·628	0·641	0·653	0·664	0·678	0·692	0·704	0·715	0·726	0·737	0·747	0·754	0·76	0·767	0·773	0·778	0·783
Palestine	0·314	0·32	0·329	0·337	0·347	0·356	0·365	0·375	0·386	0·397	0·407	0·415	0·422	0·43	0·439	0·449	0·458	0·467	0·476	0·486	0·497	0·509	0·521	0·533	0·543	0·553	0·564	0·573	0·582	0·588
Qatar	0·585	0·598	0·61	0·621	0·632	0·643	0·654	0·666	0·676	0·685	0·694	0·703	0·711	0·719	0·727	0·735	0·743	0·75	0·757	0·764	0·772	0·779	0·786	0·793	0·799	0·806	0·812	0·818	0·825	0·83
Saudi Arabia	0·48	0·491	0·504	0·516	0·529	0·541	0·554	0·566	0·578	0·59	0·602	0·614	0·625	0·637	0·65	0·664	0·677	0·69	0·703	0·715	0·726	0·738	0·75	0·76	0·769	0·778	0·786	0·793	0·8	0·805
Sudan	0·227	0·234	0·241	0·248	0·256	0·263	0·271	0·28	0·289	0·298	0·308	0·318	0·327	0·337	0·348	0·358	0·37	0·381	0·393	0·404	0·416	0·428	0·44	0·451	0·462	0·474	0·485	0·497	0·507	0·515
Syria	0·367	0·376	0·387	0·398	0·409	0·421	0·432	0·443	0·454	0·465	0·476	0·486	0·497	0·508	0·521	0·535	0·548	0·56	0·572	0·583	0·594	0·601	0·605	0·606	0·607	0·608	0·61	0·613	0·616	0·619
Tunisia	0·434	0·444	0·455	0·466	0·476	0·487	0·498	0·508	0·518	0·528	0·538	0·548	0·556	0·565	0·574	0·582	0·591	0·599	0·607	0·614	0·622	0·628	0·634	0·64	0·646	0·651	0·657	0·662	0·667	0·672
Turkey	0·473	0·483	0·493	0·504	0·513	0·523	0·534	0·545	0·556	0·566	0·577	0·587	0·597	0·607	0·619	0·63	0·641	0·652	0·663	0·671	0·68	0·689	0·698	0·707	0·715	0·723	0·729	0·736	0·743	0·748
United Arab Emirates	0·621	0·637	0·653	0·669	0·685	0·7	0·714	0·727	0·74	0·751	0·762	0·773	0·783	0·792	0·801	0·81	0·819	0·829	0·838	0·846	0·853	0·859	0·863	0·868	0·87	0·872	0·874	0·876	0·879	0·88
Yemen	0·176	0·183	0·191	0·198	0·207	0·215	0·224	0·234	0·243	0·253	0·263	0·273	0·283	0·293	0·303	0·314	0·325	0·335	0·346	0·356	0·366	0·375	0·384	0·393	0·402	0·407	0·41	0·412	0·413	0·412
South Asia	0·313	0·319	0·325	0·331	0·337	0·344	0·351	0·357	0·364	0·371	0·378	0·385	0·391	0·398	0·405	0·413	0·421	0·43	0·438	0·447	0·456	0·465	0·475	0·485	0·495	0·505	0·515	0·525	0·535	0·543
South Asia	0·313	0·319	0·325	0·331	0·337	0·344	0·351	0·357	0·364	0·371	0·378	0·385	0·391	0·398	0·405	0·413	0·421	0·43	0·438	0·447	0·456	0·465	0·475	0·485	0·495	0·505	0·515	0·525	0·535	0·543
Bangladesh	0·267	0·275	0·283	0·29	0·297	0·304	0·311	0·317	0·324	0·33	0·336	0·343	0·349	0·355	0·362	0·369	0·376	0·384	0·392	0·4	0·408	0·416	0·425	0·433	0·441	0·449	0·457	0·466	0·475	0·483
Bhutan	0·228	0·232	0·237	0·243	0·251	0·258	0·266	0·275	0·282	0·29	0·298	0·306	0·314	0·322	0·33	0·338	0·347	0·356	0·365	0·375	0·384	0·394	0·403	0·411	0·419	0·426	0·434	0·442	0·449	0·455
India	0·327	0·333	0·339	0·345	0·351	0·358	0·364	0·371	0·378	0·386	0·393	0·4	0·407	0·414	0·421	0·429	0·437	0·446	0·455	0·463	0·473	0·483	0·493	0·504	0·515	0·526	0·537	0·547	0·558	0·566
Andhra Pradesh	0·287	0·294	0·301	0·309	0·316	0·324	0·332	0·34	0·348	0·356	0·364	0·373	0·381	0·388	0·395	0·404	0·413	0·422	0·431	0·441	0·451	0·461	0·471	0·481	0·492	0·504	0·515	0·527	0·537	0·546

Appendix Table 16. Socio-demographic Index values for all estimated GBD 2019 locations, 1990–2019

Location	1990	1991	1992	1993	1994	1995	1996	1997	1998	1999	2000	2001	2002	2003	2004	2005	2006	2007	2008	2009	2010	2011	2012	2013	2014	2015	2016	2017	2018	2019
Arunachal Pradesh	0·314	0·322	0·329	0·337	0·344	0·351	0·358	0·364	0·37	0·376	0·382	0·389	0·395	0·403	0·411	0·419	0·428	0·438	0·448	0·458	0·469	0·481	0·492	0·503	0·515	0·526	0·537	0·548	0·558	0·566
Assam	0·331	0·337	0·342	0·348	0·354	0·36	0·366	0·373	0·38	0·388	0·396	0·404	0·411	0·418	0·425	0·432	0·439	0·446	0·453	0·46	0·467	0·476	0·484	0·493	0·502	0·512	0·522	0·532	0·542	0·551
Bihar	0·261	0·264	0·266	0·267	0·267	0·267	0·267	0·267	0·269	0·272	0·276	0·28	0·285	0·291	0·296	0·303	0·31	0·318	0·326	0·335	0·346	0·356	0·367	0·378	0·389	0·4	0·412	0·423	0·434	0·444
Chhattisgarh	0·287	0·294	0·3	0·306	0·312	0·317	0·323	0·329	0·335	0·342	0·349	0·355	0·362	0·37	0·378	0·386	0·396	0·406	0·415	0·425	0·436	0·447	0·458	0·469	0·48	0·492	0·503	0·514	0·524	0·533
Delhi	0·497	0·503	0·51	0·517	0·524	0·53	0·536	0·542	0·547	0·553	0·56	0·566	0·573	0·579	0·586	0·593	0·6	0·608	0·617	0·625	0·634	0·643	0·652	0·661	0·671	0·682	0·692	0·701	0·71	0·717
Goa	0·502	0·509	0·516	0·524	0·531	0·539	0·547	0·555	0·563	0·571	0·579	0·586	0·592	0·599	0·607	0·614	0·622	0·631	0·639	0·647	0·655	0·663	0·669	0·676	0·682	0·689	0·697	0·704	0·711	0·717
Gujarat	0·364	0·369	0·375	0·382	0·389	0·397	0·405	0·414	0·423	0·431	0·438	0·445	0·45	0·456	0·462	0·47	0·478	0·487	0·496	0·505	0·515	0·524	0·534	0·545	0·557	0·569	0·58	0·591	0·601	0·609
Haryana	0·355	0·36	0·366	0·372	0·378	0·384	0·391	0·398	0·405	0·412	0·419	0·426	0·433	0·439	0·447	0·455	0·463	0·473	0·483	0·494	0·506	0·518	0·53	0·542	0·555	0·567	0·579	0·59	0·601	0·609
Himachal Pradesh	0·346	0·354	0·362	0·371	0·381	0·391	0·402	0·412	0·423	0·435	0·446	0·457	0·466	0·476	0·485	0·495	0·505	0·514	0·524	0·534	0·544	0·555	0·566	0·577	0·588	0·599	0·61	0·62	0·63	0·638
Jammu and Kashmir	0·343	0·348	0·353	0·36	0·366	0·373	0·381	0·389	0·398	0·408	0·418	0·429	0·439	0·449	0·459	0·469	0·479	0·489	0·498	0·508	0·518	0·529	0·539	0·549	0·558	0·568	0·578	0·587	0·596	0·605
Jharkhand	0·263	0·269	0·276	0·282	0·288	0·294	0·3	0·307	0·313	0·32	0·327	0·334	0·341	0·348	0·356	0·365	0·374	0·384	0·394	0·404	0·415	0·426	0·437	0·448	0·46	0·47	0·481	0·491	0·501	0·51
Karnataka	0·335	0·343	0·351	0·359	0·367	0·375	0·383	0·391	0·399	0·407	0·415	0·422	0·429	0·436	0·443	0·451	0·459	0·468	0·477	0·486	0·496	0·506	0·518	0·529	0·541	0·554	0·566	0·578	0·589	0·598
Kerala	0·43	0·437	0·444	0·451	0·458	0·465	0·472	0·48	0·489	0·498	0·507	0·515	0·522	0·53	0·539	0·547	0·556	0·564	0·571	0·579	0·587	0·595	0·604	0·614	0·625	0·636	0·646	0·655	0·664	0·671
Madhya Pradesh	0·296	0·301	0·305	0·309	0·313	0·317	0·321	0·326	0·33	0·336	0·342	0·348	0·353	0·359	0·364	0·37	0·376	0·382	0·389	0·397	0·405	0·415	0·425	0·436	0·448	0·46	0·472	0·484	0·495	0·505
Maharashtra	0·373	0·381	0·388	0·396	0·404	0·412	0·42	0·428	0·436	0·444	0·451	0·459	0·466	0·474	0·482	0·49	0·499	0·509	0·517	0·526	0·536	0·545	0·555	0·566	0·576	0·587	0·599	0·609	0·619	0·628
Manipur	0·389	0·396	0·403	0·409	0·416	0·422	0·429	0·436	0·444	0·452	0·459	0·467	0·474	0·48	0·487	0·494	0·501	0·508	0·514	0·521	0·527	0·534	0·541	0·548	0·556	0·564	0·572	0·58	0·588	0·595
Meghalaya	0·332	0·339	0·346	0·353	0·36	0·367	0·374	0·382	0·389	0·397	0·405	0·413	0·421	0·428	0·436	0·444	0·453	0·462	0·47	0·479	0·488	0·497	0·506	0·515	0·523	0·532	0·54	0·549	0·557	0·564
Mizoram	0·405	0·412	0·419	0·426	0·432	0·439	0·445	0·451	0·458	0·464	0·471	0·478	0·484	0·49	0·495	0·501	0·507	0·514	0·52	0·527	0·535	0·543	0·552	0·561	0·571	0·582	0·592	0·603	0·612	0·621
Nagaland	0·394	0·402	0·409	0·417	0·425	0·432	0·44	0·447	0·453	0·46	0·467	0·474	0·481	0·489	0·496	0·504	0·512	0·52	0·528	0·536	0·544	0·551	0·559	0·567	0·576	0·584	0·593	0·602	0·61	0·618
Odisha	0·295	0·301	0·307	0·312	0·318	0·325	0·332	0·339	0·346	0·355	0·362	0·37	0·377	0·385	0·394	0·403	0·413	0·423	0·433	0·442	0·452	0·462	0·472	0·482	0·493	0·504	0·514	0·524	0·534	0·542
Punjab	0·397	0·402	0·408	0·414	0·421	0·428	0·436	0·443	0·45	0·458	0·465	0·472	0·479	0·486	0·493	0·501	0·51	0·518	0·527	0·535	0·544	0·553	0·561	0·57	0·58	0·589	0·598	0·607	0·615	0·623
Rajasthan	0·261	0·267	0·273	0·279	0·286	0·293	0·3	0·307	0·314	0·322	0·33	0·337	0·344	0·351	0·358	0·366	0·374	0·384	0·393	0·403	0·415	0·427	0·439	0·451	0·464	0·476	0·489	0·501	0·512	0·521
Sikkim	0·336	0·344	0·352	0·36	0·369	0·378	0·387	0·396	0·406	0·416	0·425	0·435	0·445	0·454	0·463	0·473	0·484	0·495	0·507	0·52	0·535	0·55	0·564	0·577	0·589	0·601	0·612	0·622	0·632	0·64
Tamil Nadu	0·374	0·38	0·386	0·392	0·399	0·406	0·414	0·421	0·429	0·437	0·445	0·453	0·46	0·468	0·476	0·485	0·493	0·502	0·511	0·519	0·528	0·538	0·548	0·558	0·57	0·582	0·593	0·603	0·613	0·621
Telangana	0·297	0·304	0·311	0·319	0·326	0·334	0·342	0·35	0·358	0·366	0·374	0·383	0·39	0·398	0·407	0·415	0·425	0·435	0·445	0·456	0·467	0·479	0·491	0·503	0·516	0·528	0·54	0·552	0·563	0·572
Tripura	0·344	0·35	0·356	0·363	0·369	0·377	0·384	0·392	0·4	0·409	0·417	0·425	0·433	0·44	0·446	0·453	0·46	0·466	0·473	0·479	0·486	0·493	0·499	0·507	0·515	0·523	0·532	0·541	0·549	0·557
Union Territories other than Delhi	0·423	0·43	0·437	0·444	0·451	0·459	0·467	0·476	0·485	0·495	0·504	0·514	0·523	0·532	0·541	0·55	0·559	0·568	0·577	0·585	0·593	0·601	0·609	0·617	0·625	0·633	0·641	0·649	0·657	0·664
Uttar Pradesh	0·274	0·279	0·284	0·29	0·295	0·301	0·307	0·314	0·32	0·327	0·334	0·341	0·348	0·355	0·362	0·37	0·379	0·388	0·397	0·407	0·417	0·428	0·439	0·45	0·461	0·472	0·483	0·494	0·505	0·513
Uttarakhand	0·309	0·315	0·321	0·328	0·334	0·341	0·349	0·357	0·365	0·375	0·385	0·396	0·408	0·419	0·431	0·444	0·458	0·472	0·487	0·501	0·516	0·531	0·545	0·559	0·572	0·585	0·597	0·608	0·619	0·628
West Bengal	0·342	0·347	0·352	0·358	0·363	0·369	0·375	0·382	0·388	0·395	0·402	0·409	0·415	0·421	0·427	0·434	0·441	0·448	0·455	0·462	0·469	0·476	0·484	0·491	0·5	0·509	0·518	0·528	0·537	0·545
Nepal	0·198	0·203	0·208	0·215	0·221	0·228	0·236	0·244	0·251	0·259	0·267	0·276	0·284	0·291	0·299	0·307	0·315	0·322	0·33	0·339	0·347	0·356	0·365	0·373	0·382	0·391	0·399	0·408	0·416	0·422
Pakistan	0·247	0·253	0·259	0·265	0·271	0·277	0·283	0·289	0·295	0·301	0·307	0·313	0·32	0·326	0·333	0·34	0·347	0·355	0·363	0·371	0·379	0·387	0·394	0·402	0·41	0·418	0·426	0·434	0·442	0·449
Southeast Asia, east Asia, and Oceania	0·452	0·46	0·468	0·476	0·485	0·494	0·503	0·511	0·519	0·526	0·533	0·541	0·548	0·556	0·563	0·571	0·58	0·589	0·597	0·605	0·614	0·622	0·629	0·636	0·643	0·647	0·651	0·659	0·667	0·673
East Asia	0·447	0·456	0·464	0·473	0·483	0·492	0·502	0·511	0·52	0·528	0·537	0·545	0·554	0·562	0·571	0·58	0·59	0·6	0·609	0·618	0·628	0·637	0·644	0·652	0·66	0·662	0·665	0·675	0·684	0·691
China	0·433	0·441	0·45	0·459	0·469	0·479	0·489	0·499	0·508	0·516	0·525	0·534	0·543	0·552	0·561	0·571	0·581	0·591	0·601	0·611	0·621	0·631	0·638	0·646	0·654	0·657	0·659	0·669	0·679	0·686
North Korea	0·431	0·436	0·439	0·442	0·445	0·447	0·45	0·451	0·452	0·455	0·458	0·463	0·467	0·473	0·478	0·485	0·49	0·496	0·502	0·507	0·513	0·518	0·523	0·528	0·534	0·538	0·543	0·548	0·553	0·558
Taiwan (province of China)	0·667	0·678	0·685	0·694	0·702	0·711	0·719	0·731	0·743	0·747	0·754	0·763	0·772	0·779	0·787	0·795	0·802	0·81	0·817	0·824	0·83	0·833	0·838	0·843	0·848	0·852	0·856	0·86	0·865	0·868
Oceania	0·383	0·385	0·388	0·391	0·394	0·397	0·4	0·403	0·405	0·408	0·41	0·412	0·413	0·414	0·416	0·417	0·419	0·421	0·422	0·424	0·426	0·428	0·431	0·433	0·436	0·44	0·443	0·446	0·449	0·452
American Samoa	0·606	0·609	0·613	0·616	0·619	0·623	0·627	0·63	0·634	0·637	0·641	0·645	0·649	0·652	0·656	0·66	0·663	0·667	0·671	0·674	0·678	0·682	0·686	0·69	0·694	0·698	0·702	0·706	0·709	0·712
Cook Islands	0·625	0·631	0·637	0·643	0·649	0·654	0·659	0·663	0·667	0·671	0·676	0·682	0·687	0·692	0·697	0·701	0·706	0·711	0·715	0·719	0·722	0·726	0·73	0·734	0·739	0·744	0·75	0·755	0·76	0·764
Fiji	0·527	0·532	0·538	0·544	0·55	0·555	0·561	0·567	0·572	0·577	0·582	0·587	0·592	0·596	0·601	0·605	0·609	0·612	0·616	0·619	0·622	0·626	0·63	0·635	0·639	0·644	0·649	0·654	0·659	0·664
Guam	0·693	0·688	0·684	0·684	0·688	0·695	0·704	0·715	0·728	0·741	0·753	0·762	0·767	0·768	0·77	0·771	0·773	0·775	0·778	0·782	0·785	0·789	0·792	0·795	0·797	0·799	0·803	0·807	0·81	0·813
Kiribati	0·425	0·427	0·429	0·432	0·435	0·438	0·441	0·444	0·447	0·451	0·455	0·459	0·463	0·468	0·472	0·476	0·48	0·484	0·487	0·49	0·492	0·495	0·497	0·501	0·504	0·509	0·514	0·518	0·523	0·527
Marshall Islands	0·398	0·404	0·41	0·416	0·423	0·43	0·435	0·439	0·442	0·446	0·45	0·454	0·458	0·463	0·467	0·472	0·477	0·482	0·487	0·493	0·498	0·504	0·509	0·515	0·52	0·525	0·531	0·536	0·541	0·544
Federated States of Micronesia	0·447	0·453	0·459	0·465	0·471	0·478	0·483	0·488	0·492	0·497	0·502	0·507	0·511	0·516	0·521	0·525	0·53	0·534	0·538	0·542	0·546	0·55	0·554	0·558	0·561	0·565	0·569	0·573	0·577	0·58
Nauru	0·499	0·501	0·503	0·504	0·504	0·505	0·505	0·504	0·504	0·503	0·503	0·503	0·503	0·503	0·504	0·506	0·509	0·51	0·515	0·521	0·529	0·538	0·547	0·559	0·573	0·585	0·595	0·605	0·613	0·618
Niue	0·566	0·571	0·576	0·581	0·586	0·591	0·595	0·6	0·605	0·609	0·614	0·618	0·622	0·626	0·631	0·638	0·645	0·651	0·657	0·663	0·669	0·675	0·681	0·685	0·69	0·695	0·699	0·703	0·707	0·711
Northern Mariana Islands	0·692	0·698	0·704	0·709	0·714	0·718	0·722	0·725	0·727	0·731	0·738	0·744	0·748	0·75	0·751	0·752	0·752	0·753	0·753	0·752	0·751	0·75	0·75	0·75	0·75	0·751	0·756	0·761	0·767	0·771
Palau	0·621	0·629	0·636	0·642	0·647	0·652	0·658	0·663	0·668	0·672	0·676	0·68	0·683	0·686	0·69	0·693	0·697	0·7	0·703	0·705	0·707	0·71	0·714	0·717	0·72	0·725	0·729	0·732	0·735	0·738
Papua New Guinea	0·292	0·295	0·299	0·304	0·309	0·313	0·317	0·32	0·323	0·326	0·329	0·331	0·333	0·335	0·337	0·339	0·342	0·345	0·348	0·352	0·356	0·36	0·363	0·367	0·372	0·377	0·382	0·386	0·391	0·394
Samoa	0·531	0·535	0·539	0·543	0·546	0·55	0·555	0·559	0·563	0·567	0·571	0·576	0·581	0·586	0·59	0·595	0·599	0·603	0·607	0·61	0·612	0·615	0·618	0·62	0·623	0·626	0·629	0·633	0·637	0·641
Solomon Islands	0·279	0·283	0·288	0·294	0·3	0·306	0·312	0·317	0·321	0·326	0·328	0·33	0·331	0·332	0·333	0·336	0·339	0·343	0·348	0·353	0·358	0·364	0·371	0·377	0·382	0·387	0·393	0·398	0·403	0·407
Tokelau	0·427	0·432	0·438	0·444	0·45	0·456	0·463	0·47	0·477	0·484	0·491	0·498	0·504	0·511	0·519	0·526	0·534	0·542	0·55	0·557	0·565	0·573	0·58	0·588	0·595	0·602	0·608	0·615	0·621	0·626
Tonga	0·51	0·517	0·522	0·527	0·533	0·538	0·543	0·547	0·551	0·555	0·559	0·563	0·568	0·572	0·576	0·58	0·583	0·587	0·59	0·594	0·598	0·602	0·606	0·61	0·614	0·618	0·622	0·627	0·632	0·636
Tuvalu	0·426	0·434	0·442	0·45	0·458	0·465	0·47	0·476	0·483	0·49	0·496	0·502	0·509	0·514	0·519	0·523	0·527	0·531	0·537	0·541	0·545	0·549	0·553	0·558	0·562	0·567	0·573	0·579	0·584	0·589
Vanuatu	0·361	0·365	0·369	0·373	0·377	0·381	0·386	0·39	0·395	0·399	0·403	0·407	0·41	0·413	0·417	0·42	0·425	0·43	0·435	0·44	0·446	0·451	0·455	0·46	0·464	0·468	0·473	0·477	0·481	0·485
Southeast Asia	0·455	0·463	0·471	0·479	0·488	0·496	0·504	0·512	0·517	0·523	0·528	0·533	0·538	0·543	0·548	0·554	0·559	0·566	0·572	0·578	0·585	0·592	0·599	0·606	0·612	0·619	0·626	0·632	0·639	0·644
Cambodia	0·266	0·272	0·277	0·282	0·286	0·291	0·296	0·301	0·307	0·313	0·321	0·328	0·337	0·345	0·354	0·363	0·373	0·382	0·391	0·399	0·406	0·413	0·421	0·428	0·435	0·442	0·449	0·456	0·463	0·469
Indonesia	0·452	0·462	0·472	0·481	0·49	0·499	0·508	0·516	0·522	0·527	0·533	0·537	0·542	0·547	0·552	0·558	0·564	0·571	0·578	0·585	0·593	0·601	0·609	0·617	0·625	0·633	0·64	0·647	0·654	0·66
Aceh	0·482	0·493	0·502	0·512	0·521	0·529	0·537	0·544	0·55	0·554	0·559	0·563	0·567	0·572	0·577	0·582	0·587	0·592	0·598	0·603	0·608	0·614	0·62	0·626	0·633	0·64	0·646	0·653	0·66	0·666
Bali	0·443	0·454	0·465	0·475	0·485	0·495	0·505	0·513	0·519	0·524	0·528	0·532	0·537	0·541	0·545	0·55	0·555	0·561	0·567	0·574	0·582	0·59	0·598	0·606	0·613	0·621	0·628	0·635	0·642	0·648
Bangka-Belitung Islands	0·442	0·452	0·461	0·471	0·48	0·489	0·498	0·506	0·512	0·517	0·522	0·527	0·532	0·537	0·542	0·547	0·553	0·56	0·567	0·574	0·581	0·589	0·597	0·604	0·612	0·619	0·627	0·634	0·641	0·647
Banten	0·452	0·462	0·471	0·481	0·49	0·499	0·508	0·516	0·521	0·526	0·529	0·533	0·536	0·539	0·542	0·546	0·551	0·556	0·562	0·568	0·575	0·583	0·591	0·599	0·606	0·614	0·621	0·629	0·636	0·642
Bengkulu	0·405	0·416	0·427	0·437	0·448	0·458	0·468	0·477	0·483	0·488	0·492	0·496	0·5	0·505	0·509	0·515	0·52	0·527	0·534	0·542	0·55	0·558	0·567	0·575	0·583	0·591	0·598	0·606	0·613	0·619
Gorontalo	0·367	0·376	0·385	0·393	0·402	0·411	0·419	0·427	0·432	0·436	0·439	0·442	0·446	0·449	0·453	0·458	0·464	0·47	0·478	0·487	0·496	0·505	0·514	0·523	0·532	0·54	0·548	0·555	0·563	0·569
Jakarta	0·581	0·591	0·602	0·612	0·623	0·633	0·643	0·652	0·659	0·665	0·671	0·677	0·683	0·69	0·696	0·703	0·709	0·716	0·724	0·732	0·74	0·748	0·756	0·764	0·771	0·778	0·785	0·791	0·797	0·802
Jambi	0·422	0·432	0·442	0·452	0·462	0·471	0·481	0·489	0·495	0·5	0·505	0·51	0·515	0·52	0·525	0·531	0·538	0·545	0·554	0·562	0·571	0·581	0·59	0·598	0·606	0·614	0·621	0·628	0·635	0·641
West Java	0·446	0·456	0·466	0·475	0·485	0·494	0·503	0·511	0·517	0·521	0·526	0·53	0·534	0·538	0·542	0·547	0·553	0·559	0·567	0·574	0·582	0·59	0·598	0·606	0·614	0·621	0·629	0·636	0·643	0·648
Central Java	0·415	0·424	0·433	0·442	0·45	0·459	0·467	0·474	0·48	0·484	0·488	0·492	0·496	0·501	0·506	0·511	0·518	0·524	0·532	0·539	0·547	0·555	0·563	0·571	0·579	0·586	0·593	0·6	0·607	0·613
East Java	0·436	0·445	0·454	0·463	0·472	0·481	0·49	0·498	0·504	0·51	0·515	0·521	0·526	0·532	0·538	0·544	0·55	0·557	0·565	0·572	0·58	0·589	0·597	0·604	0·612	0·619	0·626	0·633	0·64	0·646
West Kalimantan	0·389	0·4	0·41	0·421	0·431	0·441	0·452	0·461	0·468	0·473	0·479	0·483	0·488	0·492	0·497	0·502	0·507	0·513	0·519	0·525	0·532	0·54	0·548	0·555	0·563	0·57	0·578	0·585	0·592	0·598
South Kalimantan	0·438	0·447	0·457	0·467	0·477	0·486	0·495	0·504	0·51	0·515	0·519	0·524	0·528	0·532	0·537	0·541	0·546	0·552	0·558	0·564	0·571	0·579	0·586	0·594	0·601	0·609	0·616	0·623	0·63	0·636
Central Kalimantan	0·452	0·462	0·472	0·483	0·495	0·505	0·516	0·525	0·531	0·536	0·54	0·544	0·548	0·551	0·555	0·559	0·564	0·569	0·575	0·582	0·589	0·596	0·604	0·611	0·619	0·626	0·633	0·64	0·647	0·653
East Kalimantan	0·563	0·573	0·582	0·59	0·598	0·606	0·614	0·621	0·627	0·632	0·637	0·642	0·646	0·651	0·656	0·662	0·667	0·674	0·681	0·688	0·695	0·703	0·711	0·719	0·727	0·734	0·742	0·749	0·756	0·762
North Kalimantan	0·513	0·523	0·533	0·542	0·551	0·561	0·57	0·58	0·588	0·595	0·603	0·611	0·619	0·627	0·635	0·644	0·653	0·662	0·671	0·68	0·689	0·698	0·707	0·715	0·723	0·731	0·738	0·745	0·752	0·758
Riau Islands	0·561	0·57	0·58	0·589	0·598	0·607	0·615	0·623	0·629	0·634	0·638	0·641	0·644	0·646	0·649	0·652	0·656	0·661	0·667	0·673	0·68	0·687	0·695	0·702	0·709	0·717	0·724	0·731	0·737	0·742
Lampung	0·394	0·404	0·414	0·423	0·433	0·444	0·454	0·463	0·47	0·476	0·482	0·487	0·492	0·497	0·502	0·507	0·513	0·52	0·528	0·537	0·546	0·556	0·565	0·574	0·582	0·59	0·598	0·605	0·612	0·617
Maluku	0·479	0·487	0·495	0·503	0·51	0·516	0·522	0·526	0·527	0·526	0·523	0·519	0·514	0·509	0·503	0·498	0·494	0·493	0·494	0·498	0·503	0·511	0·519	0·527	0·535	0·544	0·552	0·56	0·568	0·575
North Maluku	0·376	0·385	0·394	0·403	0·412	0·422	0·432	0·441	0·446	0·45	0·454	0·457	0·459	0·461	0·462	0·463	0·465	0·468	0·473	0·479	0·487	0·495	0·504	0·513	0·522	0·531	0·539	0·547	0·555	0·562
West Nusa Tenggara	0·341	0·351	0·361	0·372	0·383	0·394	0·405	0·415	0·423	0·43	0·437	0·445	0·452	0·459	0·467	0·474	0·482	0·489	0·497	0·505	0·513	0·522	0·53	0·538	0·546	0·554	0·561	0·568	0·575	0·582
East Nusa Tenggara	0·367	0·374	0·382	0·39	0·398	0·406	0·414	0·421	0·424	0·427	0·429	0·432	0·435	0·437	0·441	0·444	0·448	0·453	0·459	0·465	0·472	0·48	0·488	0·497	0·505	0·513	0·521	0·529	0·537	0·543
Papua	0·453	0·461	0·47	0·478	0·486	0·495	0·503	0·51	0·516	0·52	0·524	0·527	0·529	0·531	0·533	0·536	0·539	0·542	0·547	0·553	0·559	0·567	0·575	0·583	0·591	0·599	0·607	0·615	0·623	0·629
West Papua	0·443	0·452	0·461	0·47	0·478	0·487	0·495	0·503	0·508	0·512	0·516	0·52	0·524	0·528	0·532	0·537	0·543	0·55	0·559	0·569	0·58	0·593	0·604	0·615	0·625	0·634	0·643	0·652	0·66	0·666
Riau	0·524	0·535	0·545	0·555	0·564	0·572	0·58	0·588	0·594	0·599	0·605	0·611	0·617	0·622	0·627	0·632	0·637	0·643	0·649	0·656	0·663	0·67	0·678	0·685	0·693	0·7	0·707	0·714	0·721	0·727
West Sulawesi	0·371	0·38	0·388	0·397	0·405	0·413	0·421	0·428	0·433	0·437	0·44	0·444	0·447	0·45	0·454	0·458	0·463	0·47	0·477	0·486	0·495	0·505	0·515	0·524	0·533	0·542	0·55	0·558	0·566	0·573
South Sulawesi	0·409	0·419	0·428	0·437	0·446	0·455	0·465	0·473	0·479	0·485	0·49	0·495	0·501	0·506	0·511	0·517	0·523	0·53	0·537	0·545	0·554	0·564	0·573	0·581	0·59	0·598	0·605	0·613	0·62	0·626
Central Sulawesi	0·406	0·416	0·425	0·435	0·445	0·455	0·465	0·475	0·482	0·488	0·494	0·499	0·505	0·51	0·516	0·521	0·527	0·534	0·541	0·549	0·557	0·566	0·575	0·583	0·592	0·6	0·608	0·615	0·622	0·628
Southeast Sulawesi	0·373	0·384	0·394	0·404	0·414	0·423	0·433	0·443	0·45	0·456	0·462	0·468	0·473	0·479	0·486	0·493	0·5	0·508	0·517	0·526	0·536	0·546	0·556	0·565	0·574	0·582	0·59	0·598	0·606	0·612
North Sulawesi	0·473	0·483	0·492	0·501	0·51	0·52	0·529	0·538	0·543	0·548	0·551	0·555	0·558	0·561	0·565	0·568	0·573	0·578	0·585	0·592	0·6	0·608	0·615	0·623	0·631	0·638	0·645	0·652	0·658	0·664
West Sumatra	0·462	0·47	0·479	0·488	0·497	0·506	0·515	0·523	0·529	0·534	0·539	0·543	0·548	0·553	0·558	0·563	0·57	0·576	0·584	0·592	0·6	0·609	0·617	0·625	0·633	0·641	0·648	0·655	0·662	0·668
South Sumatra	0·455	0·464	0·473	0·482	0·49	0·498	0·507	0·514	0·52	0·525	0·529	0·534	0·538	0·543	0·548	0·554	0·56	0·567	0·574	0·582	0·59	0·598	0·607	0·615	0·622	0·63	0·637	0·644	0·651	0·657
North Sumatra	0·468	0·477	0·486	0·495	0·504	0·512	0·521	0·529	0·534	0·539	0·544	0·548	0·553	0·558	0·563	0·569	0·575	0·582	0·589	0·597	0·605	0·614	0·623	0·631	0·639	0·647	0·654	0·662	0·669	0·675
Yogyakarta	0·467	0·477	0·487	0·498	0·509	0·52	0·53	0·539	0·545	0·55	0·555	0·559	0·563	0·567	0·571	0·576	0·582	0·587	0·594	0·601	0·608	0·616	0·624	0·631	0·639	0·646	0·652	0·659	0·665	0·671

Appendix Table 16. Socio-demographic Index values for all estimated GBD 2019 locations, 1990–2019

Location	1990	1991	1992	1993	1994	1995	1996	1997	1998	1999	2000	2001	2002	2003	2004	2005	2006	2007	2008	2009	2010	2011	2012	2013	2014	2015	2016	2017	2018	2019
Laos	0·268	0·274	0·279	0·285	0·29	0·296	0·302	0·309	0·315	0·322	0·329	0·336	0·344	0·351	0·359	0·367	0·376	0·385	0·394	0·403	0·413	0·422	0·431	0·441	0·45	0·458	0·467	0·475	0·483	0·49
Malaysia	0·542	0·548	0·554	0·562	0·572	0·581	0·59	0·6	0·611	0·622	0·63	0·638	0·646	0·652	0·659	0·665	0·671	0·677	0·681	0·687	0·693	0·698	0·704	0·71	0·716	0·722	0·726	0·728	0·732	0·737
Maldives	0·303	0·314	0·324	0·336	0·347	0·359	0·37	0·382	0·394	0·406	0·417	0·427	0·437	0·447	0·456	0·464	0·473	0·481	0·49	0·497	0·504	0·511	0·518	0·525	0·532	0·538	0·544	0·551	0·557	0·562
Mauritius	0·527	0·532	0·535	0·543	0·556	0·565	0·57	0·576	0·583	0·588	0·593	0·598	0·603	0·608	0·614	0·621	0·627	0·633	0·64	0·646	0·652	0·658	0·665	0·673	0·68	0·686	0·69	0·695	0·7	0·705
Myanmar	0·284	0·287	0·29	0·295	0·3	0·306	0·313	0·32	0·327	0·335	0·344	0·353	0·363	0·373	0·384	0·395	0·406	0·417	0·427	0·437	0·446	0·455	0·464	0·473	0·482	0·49	0·498	0·506	0·514	0·521
Philippines	0·497	0·501	0·505	0·509	0·513	0·518	0·522	0·526	0·529	0·532	0·534	0·537	0·54	0·542	0·545	0·547	0·55	0·554	0·558	0·562	0·567	0·572	0·577	0·583	0·589	0·596	0·603	0·61	0·617	0·623
Seychelles	0·567	0·576	0·584	0·592	0·6	0·607	0·614	0·621	0·629	0·636	0·642	0·647	0·652	0·656	0·659	0·662	0·666	0·67	0·673	0·676	0·679	0·683	0·687	0·691	0·696	0·702	0·707	0·713	0·719	0·724
Sri Lanka	0·504	0·511	0·518	0·525	0·532	0·539	0·547	0·554	0·561	0·567	0·573	0·578	0·582	0·587	0·592	0·597	0·602	0·609	0·615	0·621	0·628	0·636	0·644	0·651	0·658	0·666	0·672	0·678	0·684	0·69
Thailand	0·508	0·518	0·527	0·536	0·544	0·553	0·562	0·569	0·574	0·578	0·583	0·589	0·594	0·599	0·605	0·61	0·616	0·623	0·629	0·633	0·638	0·643	0·649	0·655	0·66	0·666	0·671	0·676	0·682	0·687
Timor-Leste	0·274	0·28	0·286	0·293	0·3	0·308	0·317	0·326	0·334	0·339	0·345	0·352	0·358	0·364	0·374	0·388	0·406	0·421	0·436	0·448	0·458	0·469	0·48	0·488	0·493	0·498	0·503	0·508	0·511	0·514
Vietnam	0·39	0·397	0·404	0·412	0·42	0·429	0·438	0·447	0·455	0·463	0·471	0·478	0·486	0·493	0·501	0·509	0·517	0·525	0·533	0·541	0·549	0·558	0·566	0·573	0·581	0·589	0·596	0·604	0·611	0·617
Sub-Saharan Africa	0·291	0·295	0·299	0·302	0·306	0·309	0·314	0·318	0·322	0·327	0·331	0·336	0·341	0·346	0·352	0·359	0·365	0·372	0·38	0·386	0·394	0·401	0·409	0·416	0·423	0·431	0·438	0·445	0·452	0·456
Central sub-Saharan Africa	0·269	0·273	0·276	0·279	0·281	0·283	0·286	0·289	0·292	0·295	0·298	0·302	0·308	0·313	0·32	0·328	0·336	0·346	0·357	0·367	0·378	0·389	0·4	0·412	0·423	0·434	0·445	0·454	0·463	0·47
Angola	0·238	0·243	0·247	0·25	0·253	0·257	0·262	0·267	0·273	0·278	0·283	0·289	0·296	0·303	0·311	0·319	0·33	0·341	0·353	0·364	0·376	0·387	0·398	0·41	0·421	0·432	0·443	0·454	0·463	0·47
Central African Republic	0·186	0·19	0·193	0·196	0·199	0·202	0·205	0·208	0·211	0·215	0·218	0·221	0·225	0·227	0·23	0·233	0·236	0·24	0·244	0·248	0·253	0·258	0·263	0·263	0·263	0·264	0·266	0·268	0·271	0·274
Congo (Brazzaville)	0·364	0·373	0·381	0·389	0·396	0·403	0·41	0·416	0·421	0·426	0·431	0·437	0·442	0·447	0·452	0·458	0·464	0·469	0·475	0·482	0·491	0·5	0·509	0·519	0·528	0·538	0·547	0·556	0·563	0·568
DR Congo	0·26	0·262	0·263	0·263	0·262	0·261	0·259	0·256	0·253	0·25	0·246	0·242	0·239	0·238	0·239	0·242	0·244	0·247	0·251	0·257	0·266	0·277	0·289	0·305	0·321	0·336	0·35	0·362	0·374	0·382
Equatorial Guinea	0·208	0·214	0·222	0·23	0·24	0·252	0·268	0·295	0·318	0·341	0·364	0·391	0·415	0·438	0·46	0·482	0·502	0·522	0·543	0·561	0·578	0·594	0·611	0·626	0·64	0·652	0·663	0·673	0·681	0·685
Gabon	0·388	0·399	0·409	0·42	0·431	0·442	0·453	0·463	0·474	0·483	0·493	0·502	0·51	0·519	0·528	0·537	0·546	0·554	0·563	0·57	0·579	0·587	0·596	0·605	0·614	0·623	0·632	0·641	0·649	0·656
Eastern sub-Saharan Africa	0·235	0·239	0·242	0·245	0·249	0·252	0·257	0·261	0·265	0·27	0·275	0·28	0·285	0·29	0·295	0·301	0·307	0·314	0·321	0·328	0·336	0·343	0·351	0·359	0·367	0·375	0·383	0·391	0·399	0·405
Burundi	0·198	0·201	0·204	0·207	0·209	0·21	0·21	0·211	0·212	0·213	0·213	0·214	0·216	0·218	0·22	0·223	0·226	0·23	0·234	0·238	0·243	0·248	0·254	0·26	0·266	0·27	0·274	0·278	0·282	0·284
Comoros	0·274	0·281	0·288	0·296	0·302	0·309	0·315	0·322	0·328	0·334	0·34	0·346	0·353	0·359	0·365	0·371	0·378	0·384	0·39	0·396	0·401	0·407	0·413	0·419	0·426	0·432	0·438	0·444	0·45	0·455
Djibouti	0·275	0·277	0·28	0·285	0·289	0·293	0·297	0·302	0·306	0·311	0·317	0·322	0·327	0·333	0·339	0·345	0·353	0·36	0·368	0·376	0·384	0·392	0·4	0·408	0·416	0·425	0·434	0·443	0·452	0·459
Eritrea	0·198	0·203	0·21	0·218	0·228	0·238	0·248	0·258	0·267	0·275	0·283	0·289	0·296	0·302	0·307	0·312	0·317	0·322	0·326	0·33	0·334	0·34	0·346	0·353	0·36	0·367	0·375	0·382	0·39	0·396
Ethiopia	0·144	0·146	0·147	0·149	0·151	0·153	0·157	0·161	0·164	0·167	0·171	0·176	0·18	0·183	0·189	0·195	0·203	0·213	0·223	0·233	0·244	0·256	0·268	0·279	0·291	0·302	0·313	0·324	0·334	0·343
Kenya	0·333	0·341	0·348	0·354	0·36	0·366	0·372	0·378	0·383	0·388	0·392	0·397	0·401	0·404	0·409	0·413	0·418	0·424	0·429	0·435	0·441	0·448	0·455	0·463	0·47	0·478	0·486	0·494	0·502	0·508
Baringo	0·248	0·257	0·266	0·274	0·283	0·291	0·299	0·307	0·315	0·322	0·329	0·335	0·341	0·348	0·353	0·359	0·365	0·372	0·378	0·384	0·392	0·4	0·408	0·417	0·426	0·436	0·447	0·457	0·468	0·476
Bomet	0·27	0·28	0·289	0·298	0·307	0·315	0·324	0·332	0·34	0·347	0·355	0·362	0·369	0·376	0·383	0·39	0·398	0·407	0·415	0·424	0·433	0·443	0·454	0·465	0·476	0·487	0·499	0·511	0·522	0·531
Bungoma	0·281	0·289	0·296	0·302	0·308	0·314	0·32	0·325	0·329	0·334	0·338	0·342	0·346	0·35	0·355	0·36	0·366	0·373	0·38	0·387	0·395	0·404	0·413	0·423	0·433	0·444	0·454	0·465	0·475	0·483
Busia	0·256	0·263	0·269	0·275	0·282	0·288	0·294	0·299	0·305	0·31	0·315	0·32	0·325	0·329	0·334	0·339	0·345	0·351	0·356	0·363	0·37	0·378	0·387	0·396	0·405	0·415	0·425	0·435	0·445	0·453
Elgeyo Marakwet	0·264	0·273	0·28	0·288	0·295	0·302	0·308	0·315	0·321	0·327	0·333	0·339	0·344	0·35	0·356	0·363	0·371	0·379	0·387	0·396	0·406	0·416	0·426	0·437	0·449	0·46	0·471	0·483	0·494	0·503
Embu	0·377	0·385	0·392	0·398	0·404	0·409	0·415	0·419	0·424	0·429	0·433	0·437	0·44	0·444	0·447	0·451	0·456	0·461	0·466	0·471	0·478	0·485	0·492	0·5	0·508	0·516	0·525	0·534	0·542	0·55
Garissa	0·187	0·192	0·195	0·199	0·203	0·207	0·211	0·215	0·218	0·221	0·224	0·227	0·23	0·233	0·235	0·239	0·242	0·246	0·25	0·255	0·259	0·264	0·27	0·275	0·281	0·287	0·293	0·299	0·305	0·31
Homa Bay	0·209	0·215	0·222	0·229	0·236	0·243	0·25	0·257	0·264	0·271	0·277	0·283	0·289	0·295	0·301	0·307	0·314	0·322	0·33	0·338	0·348	0·358	0·369	0·381	0·393	0·405	0·418	0·431	0·443	0·452
Isiolo	0·241	0·246	0·252	0·257	0·263	0·268	0·273	0·278	0·283	0·289	0·293	0·298	0·303	0·307	0·311	0·315	0·32	0·324	0·329	0·333	0·339	0·346	0·353	0·361	0·369	0·378	0·387	0·396	0·405	0·413
Kajiado	0·359	0·367	0·374	0·382	0·388	0·395	0·401	0·407	0·413	0·419	0·423	0·428	0·433	0·437	0·441	0·446	0·452	0·459	0·465	0·472	0·48	0·489	0·498	0·507	0·516	0·526	0·536	0·545	0·555	0·563
Kakamega	0·281	0·288	0·295	0·301	0·307	0·313	0·319	0·325	0·33	0·336	0·341	0·347	0·351	0·356	0·361	0·367	0·373	0·379	0·386	0·393	0·401	0·41	0·419	0·429	0·439	0·449	0·46	0·47	0·481	0·489
Kericho	0·243	0·252	0·261	0·269	0·278	0·286	0·294	0·302	0·31	0·318	0·326	0·333	0·341	0·348	0·356	0·363	0·372	0·381	0·389	0·398	0·408	0·419	0·43	0·441	0·453	0·465	0·477	0·489	0·501	0·509
Kiambu	0·425	0·434	0·441	0·447	0·453	0·459	0·465	0·47	0·474	0·479	0·483	0·488	0·492	0·496	0·5	0·505	0·511	0·518	0·524	0·531	0·538	0·546	0·554	0·562	0·57	0·578	0·586	0·594	0·602	0·609
Kilifi	0·292	0·299	0·306	0·312	0·319	0·325	0·331	0·337	0·343	0·348	0·353	0·357	0·362	0·366	0·37	0·375	0·38	0·386	0·392	0·399	0·407	0·415	0·424	0·433	0·443	0·453	0·463	0·474	0·484	0·492
Kirinyaga	0·372	0·38	0·387	0·393	0·399	0·404	0·41	0·414	0·419	0·423	0·428	0·432	0·436	0·44	0·444	0·449	0·454	0·46	0·465	0·471	0·477	0·484	0·491	0·499	0·506	0·514	0·523	0·531	0·539	0·546
Kisii	0·343	0·353	0·362	0·369	0·377	0·384	0·391	0·397	0·402	0·407	0·412	0·416	0·42	0·424	0·428	0·433	0·438	0·445	0·452	0·459	0·468	0·477	0·487	0·497	0·507	0·518	0·528	0·539	0·549	0·557
Kisumu	0·333	0·341	0·349	0·356	0·363	0·37	0·376	0·382	0·388	0·394	0·4	0·405	0·41	0·415	0·42	0·426	0·432	0·44	0·448	0·456	0·465	0·475	0·485	0·496	0·507	0·518	0·529	0·54	0·551	0·559
Kitui	0·266	0·273	0·28	0·286	0·293	0·299	0·305	0·311	0·317	0·322	0·327	0·333	0·338	0·342	0·348	0·353	0·359	0·366	0·372	0·38	0·388	0·396	0·406	0·415	0·425	0·435	0·446	0·456	0·466	0·474
Kwale	0·284	0·292	0·298	0·305	0·311	0·318	0·324	0·329	0·334	0·339	0·343	0·347	0·351	0·354	0·358	0·362	0·367	0·372	0·377	0·383	0·39	0·397	0·405	0·414	0·423	0·432	0·442	0·452	0·461	0·469
Laikipia	0·344	0·352	0·358	0·364	0·37	0·376	0·382	0·387	0·393	0·399	0·404	0·41	0·416	0·423	0·43	0·437	0·445	0·453	0·462	0·47	0·479	0·489	0·498	0·508	0·518	0·528	0·538	0·549	0·559	0·567
Lamu	0·31	0·317	0·323	0·329	0·335	0·341	0·347	0·352	0·356	0·361	0·365	0·369	0·373	0·377	0·382	0·386	0·391	0·397	0·402	0·408	0·414	0·421	0·429	0·436	0·444	0·453	0·462	0·471	0·48	0·488
Machakos	0·351	0·359	0·367	0·373	0·38	0·386	0·393	0·398	0·403	0·409	0·413	0·418	0·423	0·427	0·432	0·437	0·443	0·45	0·457	0·464	0·472	0·48	0·489	0·498	0·508	0·517	0·527	0·536	0·546	0·553
Makueni	0·262	0·268	0·274	0·28	0·286	0·293	0·299	0·305	0·311	0·317	0·322	0·328	0·333	0·338	0·343	0·348	0·354	0·361	0·367	0·374	0·381	0·389	0·398	0·406	0·416	0·425	0·435	0·445	0·455	0·462
Mandera	0·172	0·176	0·179	0·183	0·186	0·19	0·193	0·197	0·201	0·204	0·207	0·211	0·214	0·217	0·22	0·223	0·227	0·231	0·235	0·24	0·245	0·251	0·257	0·264	0·27	0·277	0·283	0·29	0·296	0·302
Marsabit	0·202	0·207	0·212	0·217	0·222	0·227	0·232	0·238	0·243	0·249	0·254	0·26	0·265	0·27	0·275	0·281	0·287	0·293	0·299	0·305	0·313	0·32	0·328	0·337	0·346	0·354	0·363	0·372	0·381	0·388
Meru	0·33	0·338	0·345	0·352	0·358	0·365	0·371	0·377	0·383	0·388	0·393	0·398	0·403	0·407	0·412	0·417	0·422	0·428	0·434	0·44	0·448	0·456	0·464	0·472	0·481	0·49	0·5	0·509	0·518	0·525
Migori	0·217	0·223	0·23	0·236	0·243	0·25	0·256	0·262	0·268	0·274	0·28	0·285	0·291	0·296	0·301	0·307	0·314	0·321	0·328	0·336	0·345	0·354	0·364	0·375	0·386	0·397	0·409	0·421	0·433	0·442
Mombasa	0·422	0·428	0·433	0·438	0·443	0·447	0·452	0·456	0·459	0·463	0·465	0·468	0·471	0·473	0·476	0·479	0·483	0·489	0·494	0·5	0·507	0·514	0·522	0·53	0·539	0·548	0·557	0·566	0·575	0·582
Murang'a	0·357	0·365	0·373	0·379	0·386	0·392	0·398	0·404	0·409	0·415	0·42	0·426	0·431	0·436	0·441	0·447	0·453	0·459	0·465	0·471	0·478	0·486	0·493	0·501	0·509	0·518	0·526	0·535	0·543	0·55
Nairobi	0·536	0·54	0·543	0·545	0·547	0·549	0·551	0·553	0·556	0·558	0·561	0·564	0·567	0·57	0·573	0·577	0·582	0·587	0·593	0·598	0·604	0·61	0·617	0·624	0·63	0·637	0·645	0·652	0·66	0·665
Nakuru	0·349	0·357	0·364	0·37	0·377	0·383	0·389	0·394	0·398	0·403	0·407	0·41	0·414	0·417	0·421	0·426	0·431	0·438	0·445	0·452	0·461	0·47	0·479	0·489	0·499	0·509	0·52	0·53	0·54	0·548
Nandi	0·314	0·324	0·333	0·341	0·349	0·357	0·365	0·372	0·379	0·385	0·392	0·397	0·403	0·408	0·413	0·419	0·425	0·432	0·438	0·445	0·453	0·462	0·471	0·481	0·491	0·502	0·513	0·524	0·534	0·543
Narok	0·202	0·212	0·22	0·229	0·236	0·244	0·251	0·257	0·263	0·269	0·274	0·279	0·284	0·289	0·293	0·299	0·304	0·31	0·316	0·322	0·33	0·338	0·347	0·356	0·366	0·376	0·387	0·398	0·409	0·418
Nyamira	0·343	0·352	0·361	0·369	0·377	0·385	0·392	0·399	0·406	0·413	0·419	0·426	0·432	0·437	0·443	0·45	0·456	0·464	0·471	0·478	0·487	0·496	0·506	0·517	0·527	0·538	0·55	0·561	0·571	0·579
Nyandarua	0·334	0·341	0·348	0·354	0·361	0·367	0·373	0·379	0·385	0·391	0·398	0·404	0·41	0·417	0·423	0·43	0·437	0·445	0·452	0·459	0·467	0·475	0·483	0·492	0·501	0·51	0·519	0·529	0·538	0·546
Nyeri	0·388	0·396	0·404	0·41	0·417	0·423	0·428	0·433	0·438	0·442	0·446	0·45	0·454	0·457	0·461	0·466	0·471	0·477	0·483	0·489	0·496	0·504	0·512	0·52	0·528	0·536	0·545	0·553	0·561	0·568
Samburu	0·177	0·183	0·188	0·193	0·199	0·204	0·209	0·214	0·218	0·223	0·228	0·232	0·236	0·241	0·245	0·25	0·255	0·261	0·267	0·274	0·281	0·289	0·297	0·306	0·314	0·324	0·333	0·343	0·353	0·361
Siaya	0·225	0·232	0·238	0·244	0·25	0·257	0·263	0·269	0·274	0·28	0·285	0·289	0·294	0·299	0·303	0·309	0·315	0·323	0·33	0·338	0·347	0·358	0·368	0·38	0·392	0·404	0·416	0·428	0·44	0·449
Taita Taveta	0·351	0·359	0·366	0·373	0·379	0·386	0·392	0·397	0·402	0·407	0·411	0·416	0·419	0·423	0·427	0·431	0·437	0·443	0·449	0·455	0·462	0·47	0·478	0·486	0·495	0·504	0·513	0·522	0·531	0·539
Tana River	0·226	0·232	0·238	0·243	0·249	0·254	0·259	0·264	0·268	0·272	0·275	0·279	0·281	0·284	0·287	0·29	0·295	0·3	0·305	0·31	0·317	0·324	0·331	0·34	0·348	0·357	0·366	0·375	0·384	0·391
Tharaka Nithi	0·341	0·348	0·355	0·362	0·369	0·376	0·383	0·39	0·396	0·403	0·41	0·416	0·423	0·429	0·436	0·442	0·449	0·457	0·464	0·471	0·479	0·488	0·497	0·506	0·516	0·526	0·536	0·546	0·556	0·564
Trans Nzoia	0·321	0·329	0·337	0·344	0·351	0·357	0·364	0·371	0·377	0·383	0·388	0·394	0·4	0·405	0·411	0·416	0·423	0·429	0·436	0·443	0·451	0·459	0·468	0·478	0·488	0·498	0·509	0·52	0·53	0·539
Turkana	0·185	0·191	0·196	0·202	0·208	0·214	0·22	0·226	0·232	0·237	0·242	0·247	0·252	0·256	0·26	0·263	0·268	0·272	0·276	0·28	0·286	0·292	0·299	0·306	0·314	0·322	0·33	0·339	0·347	0·355
Uasin Gishu	0·371	0·38	0·388	0·395	0·402	0·408	0·414	0·42	0·425	0·43	0·435	0·441	0·445	0·45	0·455	0·461	0·467	0·474	0·48	0·488	0·496	0·505	0·514	0·524	0·535	0·545	0·556	0·566	0·577	0·585
Vihiga	0·276	0·284	0·292	0·3	0·307	0·315	0·322	0·329	0·335	0·341	0·346	0·351	0·355	0·359	0·363	0·367	0·371	0·376	0·38	0·385	0·391	0·398	0·405	0·413	0·421	0·429	0·438	0·447	0·457	0·464
Wajir	0·146	0·15	0·154	0·157	0·161	0·165	0·169	0·173	0·176	0·18	0·183	0·185	0·188	0·19	0·192	0·194	0·197	0·2	0·203	0·206	0·21	0·215	0·22	0·225	0·231	0·236	0·242	0·248	0·253	0·259
West Pokot	0·189	0·196	0·203	0·21	0·217	0·224	0·23	0·237	0·243	0·249	0·255	0·261	0·267	0·273	0·28	0·286	0·293	0·301	0·309	0·317	0·326	0·336	0·346	0·357	0·368	0·379	0·39	0·402	0·413	0·422
Madagascar	0·265	0·268	0·27	0·273	0·275	0·277	0·28	0·283	0·286	0·29	0·294	0·299	0·302	0·307	0·311	0·316	0·32	0·324	0·328	0·332	0·336	0·342	0·348	0·355	0·361	0·369	0·376	0·383	0·391	0·396
Malawi	0·213	0·215	0·215	0·217	0·218	0·22	0·225	0·23	0·235	0·24	0·245	0·249	0·254	0·26	0·267	0·274	0·281	0·29	0·299	0·308	0·317	0·326	0·335	0·342	0·35	0·358	0·365	0·372	0·379	0·384
Mozambique	0·12	0·122	0·123	0·126	0·129	0·131	0·137	0·144	0·152	0·159	0·165	0·172	0·179	0·186	0·194	0·201	0·208	0·215	0·223	0·23	0·237	0·244	0·252	0·26	0·268	0·277	0·285	0·294	0·301	0·307
Rwanda	0·257	0·259	0·262	0·265	0·261	0·261	0·262	0·265	0·269	0·272	0·277	0·283	0·291	0·298	0·307	0·315	0·325	0·333	0·342	0·351	0·359	0·368	0·376	0·384	0·391	0·399	0·407	0·415	0·422	0·429
Somalia	0·0508	0·0514	0·0522	0·053	0·0539	0·0547	0·0556	0·0565	0·0574	0·0584	0·0593	0·0603	0·0613	0·0622	0·0632	0·0641	0·0651	0·0661	0·0671	0·0682	0·0692	0·0703	0·0716	0·0728	0·0742	0·0756	0·077	0·0785	0·0799	0·081
South Sudan	0·248	0·251	0·253	0·256	0·259	0·261	0·264	0·267	0·271	0·274	0·277	0·281	0·284	0·288	0·292	0·296	0·301	0·305	0·31	0·315	0·32	0·325	0·33	0·334	0·339	0·342	0·345	0·351	0·358	0·363
Uganda	0·167	0·169	0·172	0·176	0·181	0·187	0·194	0·201	0·208	0·216	0·225	0·234	0·243	0·253	0·262	0·272	0·282	0·293	0·303	0·314	0·325	0·335	0·345	0·355	0·364	0·373	0·382	0·391	0·399	0·404
Tanzania	0·26	0·265	0·269	0·273	0·276	0·28	0·284	0·288	0·292	0·296	0·301	0·305	0·31	0·315	0·32	0·326	0·331	0·337	0·342	0·348	0·354	0·361	0·368	0·375	0·383	0·391	0·399	0·408	0·416	0·423
Zambia	0·299	0·304	0·307	0·311	0·314	0·316	0·319	0·323	0·326	0·33	0·334	0·34	0·345	0·352	0·359	0·367	0·376	0·386	0·396	0·407	0·418	0·429	0·44	0·451	0·462	0·472	0·481	0·491	0·499	0·505
Southern sub-Saharan Africa	0·513	0·517	0·522	0·526	0·53	0·535	0·539	0·544	0·548	0·553	0·557	0·562	0·567	0·571	0·576	0·581	0·586	0·591	0·596	0·601	0·605	0·61	0·614	0·619	0·623	0·628	0·632	0·636	0·639	0·642
Botswana	0·431	0·441	0·451	0·459	0·467	0·475	0·483	0·491	0·498	0·506	0·514	0·521	0·528	0·535	0·541	0·548	0·555	0·562	0·569	0·575	0·581	0·587	0·593	0·6	0·606	0·612	0·618	0·624	0·63	0·634
eSwatini	0·392	0·401	0·41	0·419	0·427	0·435	0·443	0·45	0·456	0·462	0·468	0·474	0·479	0·484	0·49	0·495	0·501	0·507	0·513	0·519	0·526	0·532	0·538	0·545	0·551	0·557	0·563	0·569	0·574	0·577
Lesotho	0·321	0·327	0·333	0·34	0·346	0·353	0·36	0·367	0·373	0·38	0·387	0·393	0·399	0·405	0·411	0·417	0·423	0·429	0·435	0·441	0·448	0·455	0·462	0·469	0·476	0·483	0·489	0·496	0·502	0·507
Namibia	0·454	0·459	0·465	0·47	0·475	0·48	0·486	0·491	0·495	0·5	0·505	0·509	0·514	0·518	0·523	0·529	0·534	0·54	0·546	0·552	0·558	0·564	0·571	0·577	0·584	0·591	0·597	0·603	0·608	0·612
South Africa	0·552	0·556	0·56	0·564	0·567	0·572	0·576	0·58	0·584	0·588	0·593	0·597	0·602	0·607	0·611	0·616	0·622	0·627	0·632	0·637	0·642	0·647	0·651	0·656	0·66	0·664	0·668	0·672	0·676	0·678
Zimbabwe	0·394	0·403	0·409	0·415	0·421	0·426	0·432	0·437	0·441	0·444	0·446	0·448	0·448	0·447	0·444	0·441	0·437	0·433	0·428	0·425	0·425	0·427	0·432	0·438	0·445	0·452	0·459	0·465	0·471	0·476
Western sub-Saharan Africa	0·268	0·272	0·276	0·28	0·284	0·288	0·292	0·297	0·301	0·306	0·311	0·316	0·322	0·328	0·335	0·343	0·351	0·359	0·367	0·375	0·383	0·391	0·399	0·407	0·415	0·422	0·43	0·437	0·443	0·448
Benin	0·209	0·213	0·218	0·222	0·227	0·231	0·236	0·241	0·245	0·25	0·254	0·259	0·263	0·267	0·271	0·275	0·279	0·283	0·288	0·292	0·297	0·301	0·306	0·312	0·318	0·324	0·331	0·338	0·346	0·352
Burkina Faso	0·125	0·128	0·131	0·134	0·137	0·14	0·144	0·147	0·152	0·156	0·161	0·166	0·17	0·175	0·18	0·186	0·191	0·196	0·2	0·205	0·21	0·215	0·22	0·226	0·231	0·236	0·241	0·247	0·252	0·257
Cape Verde	0·292	0·298	0·305	0·311	0·318	0·326	0·333	0·342	0·35	0·36	0·37	0·379	0·388	0·397	0·407	0·415	0·424	0·434	0·443	0·452	0·461	0·469	0·477	0·484	0·491	0·498	0·505	0·512	0·519	0·525

Appendix Table 16. Socio-demographic Index values for all estimated GBD 2019 locations, 1990–2019

Location	1990	1991	1992	1993	1994	1995	1996	1997	1998	1999	2000	2001	2002	2003	2004	2005	2006	2007	2008	2009	2010	2011	2012	2013	2014	2015	2016	2017	2018	2019
Cameroon	0·313	0·32	0·325	0·33	0·334	0·338	0·342	0·346	0·349	0·353	0·357	0·36	0·364	0·368	0·373	0·378	0·384	0·39	0·397	0·404	0·412	0·42	0·428	0·436	0·445	0·455	0·464	0·474	0·483	0·49
Chad	0·108	0·112	0·115	0·118	0·12	0·123	0·125	0·128	0·13	0·132	0·135	0·137	0·14	0·144	0·149	0·156	0·162	0·167	0·173	0·179	0·185	0·191	0·197	0·203	0·21	0·216	0·223	0·228	0·234	0·238
Côte d'Ivoire	0·256	0·26	0·265	0·27	0·275	0·279	0·285	0·29	0·295	0·301	0·305	0·31	0·314	0·318	0·322	0·325	0·329	0·333	0·337	0·341	0·346	0·35	0·355	0·362	0·369	0·376	0·384	0·393	0·401	0·408
The Gambia	0·218	0·223	0·227	0·232	0·237	0·241	0·246	0·251	0·256	0·262	0·268	0·274	0·28	0·286	0·293	0·3	0·306	0·313	0·319	0·327	0·334	0·341	0·348	0·356	0·363	0·37	0·378	0·385	0·393	0·399
Ghana	0·355	0·362	0·368	0·374	0·38	0·385	0·391	0·396	0·401	0·406	0·411	0·415	0·42	0·426	0·431	0·437	0·444	0·45	0·458	0·466	0·474	0·484	0·494	0·504	0·514	0·523	0·531	0·541	0·549	0·557
Guinea	0·175	0·178	0·182	0·186	0·19	0·194	0·199	0·204	0·209	0·214	0·219	0·224	0·229	0·235	0·239	0·244	0·249	0·253	0·258	0·263	0·267	0·272	0·278	0·284	0·29	0·296	0·303	0·31	0·318	0·325
Guinea-Bissau	0·2	0·205	0·209	0·214	0·219	0·224	0·23	0·236	0·239	0·244	0·249	0·253	0·257	0·261	0·266	0·271	0·275	0·28	0·286	0·291	0·297	0·304	0·31	0·316	0·322	0·328	0·335	0·342	0·349	0·355
Liberia	0·221	0·222	0·219	0·214	0·206	0·196	0·183	0·176	0·175	0·184	0·203	0·22	0·238	0·245	0·252	0·258	0·265	0·272	0·279	0·287	0·296	0·305	0·314	0·325	0·335	0·344	0·351	0·358	0·365	0·37
Mali	0·126	0·129	0·132	0·136	0·139	0·143	0·147	0·151	0·155	0·159	0·163	0·168	0·173	0·178	0·183	0·188	0·193	0·198	0·203	0·209	0·214	0·22	0·225	0·23	0·235	0·241	0·247	0·253	0·259	0·263
Mauritania	0·308	0·314	0·319	0·326	0·332	0·338	0·344	0·349	0·355	0·36	0·365	0·369	0·374	0·379	0·384	0·39	0·398	0·406	0·413	0·42	0·427	0·435	0·443	0·45	0·459	0·467	0·474	0·482	0·49	0·496
Niger	0·0728	0·0746	0·0761	0·0777	0·0793	0·0808	0·0822	0·0836	0·0853	0·0871	0·0887	0·0907	0·093	0·0956	0·0982	0·101	0·104	0·108	0·111	0·115	0·119	0·123	0·128	0·133	0·138	0·143	0·148	0·153	0·158	0·162
Nigeria	0·305	0·308	0·312	0·315	0·319	0·324	0·329	0·334	0·339	0·344	0·35	0·356	0·363	0·371	0·381	0·392	0·402	0·412	0·422	0·432	0·442	0·451	0·46	0·469	0·478	0·487	0·495	0·503	0·51	0·515
São Tomé and PrÍncipe	0·299	0·302	0·306	0·309	0·313	0·317	0·322	0·327	0·332	0·338	0·344	0·351	0·358	0·365	0·373	0·381	0·39	0·398	0·407	0·416	0·424	0·433	0·443	0·452	0·461	0·47	0·478	0·487	0·495	0·502
Senegal	0·227	0·233	0·239	0·245	0·251	0·257	0·262	0·267	0·272	0·277	0·282	0·286	0·29	0·295	0·299	0·304	0·308	0·313	0·318	0·324	0·33	0·336	0·342	0·348	0·354	0·361	0·368	0·375	0·382	0·389
Sierra Leone	0·207	0·209	0·21	0·212	0·215	0·218	0·218	0·218	0·219	0·218	0·219	0·221	0·224	0·229	0·234	0·239	0·245	0·252	0·26	0·267	0·275	0·283	0·292	0·304	0·314	0·321	0·328	0·335	0·342	0·347
Togo	0·266	0·272	0·278	0·281	0·286	0·291	0·296	0·302	0·306	0·31	0·313	0·317	0·32	0·323	0·327	0·33	0·334	0·338	0·342	0·347	0·352	0·358	0·364	0·371	0·379	0·386	0·394	0·402	0·411	0·417
